# Uncovering a Dual Regulatory Role for Caspases During Endoplasmic Reticulum Stress-induced Cell Death[Fn FN1]

**DOI:** 10.1074/mcp.M115.055376

**Published:** 2016-04-28

**Authors:** Veronica G. Anania, Kebing Yu, Florian Gnad, Rebecca R. Pferdehirt, Han Li, Taylur P. Ma, Diana Jeon, Nikolaus Fortelny, William Forrest, Avi Ashkenazi, Christopher M. Overall, Jennie R. Lill

**Affiliations:** From the Departments of ‡Protein Chemistry,; §Bioinformatics and Computational Biology,; ¶Molecular Oncology,; **Biostatistics, Genentech, Inc., South San Francisco, CA,; ‖Departments of Oral Biological and Medical Sciences, and University of British Columbia, Vancouver, British Columbia, Canada

## Abstract

Many diseases are associated with endoplasmic reticulum (ER) stress, which results from an accumulation of misfolded proteins. This triggers an adaptive response called the “unfolded protein response” (UPR), and prolonged exposure to ER stress leads to cell death. Caspases are reported to play a critical role in ER stress-induced cell death but the underlying mechanisms by which they exert their effect continue to remain elusive. To understand the role caspases play during ER stress, a systems level approach integrating analysis of the transcriptome, proteome, and proteolytic substrate profile was employed. This quantitative analysis revealed transcriptional profiles for most human genes, provided information on protein abundance for 4476 proteins, and identified 445 caspase substrates. Based on these data sets many caspase substrates were shown to be downregulated at the protein level during ER stress suggesting caspase activity inhibits their cellular function. Additionally, RNA sequencing revealed a role for caspases in regulation of ER stress-induced transcriptional pathways and gene set enrichment analysis showed expression of multiple gene targets of essential transcription factors to be upregulated during ER stress upon inhibition of caspases. Furthermore, these transcription factors were degraded in a caspase-dependent manner during ER stress. These results indicate that caspases play a dual role in regulating the cellular response to ER stress through both post-translational and transcriptional regulatory mechanisms. Moreover, this study provides unique insight into progression of the unfolded protein response into cell death, which may help identify therapeutic strategies to treat ER stress-related diseases.

Endoplasmic reticulum (ER)^1^ stress results from the accumulation of misfolded proteins in the lumen of the ER, subsequently activating a coordinated adaptive program, termed the “unfolded protein response” (UPR). The ability of a cell to detect and circumvent ER stress during times of elevated physiological demand for protein folding is key to maintaining cellular homeostasis. ER stress has been linked to many diseases including cancer, neurodegeneration, and diabetes (Reviewed in (1)); therefore, understanding the underlying molecular mechanisms of this pathway are important for the development of novel therapeutic strategies for a wide range of disease pathologies.

Mammalian cells have three key mediators of the ER stress response: double-stranded RNA-dependent protein kinase (PKR)-like ER kinase (PERK), Inositol-Requiring Enzyme 1α (IRE1α), and Activating Transcription Factor 6 (ATF6). Once misfolded proteins are detected, PERK, IRE1α, and ATF6 activate transcription factors that are released to the nucleus, where they initiate a transcriptional response primarily involved in cellular adaptation and survival (Reviewed in (2, 3)). Additionally, the kinase domain of PERK phosphorylates elongation initiation factor 2α (eIF2α) suppressing general translation, whereas the endo-ribonuclease domain of IRE1α degrades nascent mRNA. These cellular adaptations promote cell survival by preventing an influx of additional nascent polypeptides into the ER and provide the cell with an opportunity to degrade or fold proteins that have already accumulated. If the underlying cause of stress is not properly resolved, apoptotic cell death can occur, however the mechanisms that regulate induction of cell death in response to ER stress are unknown.

Caspases are a family of cysteine aspartate-specific proteases involved in cell death and inflammation (Reviewed in (4)). There are three main classes of caspases: those involved in inflammation, apoptotic initiators, and apoptotic executioners. Apoptotic initiators such as caspase-8 and -9 exist as inactive monomers that upon dimerization undergo auto-proteolysis between the large and small subunits to create a stable, active dimer. Executioner caspases including caspase-3 or -7 exist as inactive dimers that subsequently become activated by proteolytic cleavage between the large and small subunit by active initiator caspases. Interestingly, introduction of an active executioner caspase into any mammalian cell induces cell death, however, the underlying mechanisms are not well characterized. Several studies have investigated the role of specific caspases known to become activated during ER stress, however, the exact process by which caspases induce cell death in response to ER stress, remain elusive (5–17).

In this study, a comprehensive systems level approach was used to profile the biological response to ER stress and resultant data were interrogated to better understand the role of caspases during ER stress. Taken together, these data reveal a dual regulatory role for caspases in ER stress-induced cell death: a post-translational regulatory role controlling protein expression of direct caspase substrates and a transcriptional regulatory role that controls expression of genes involved in cell fate decisions in response to ER stress. Furthermore, this study provides unique insight into the progression of the UPR at the transcript, protein, and post-translational level providing a valuable resource for further investigation into the cellular response to ER stress.

## Materials and Methods

### 

#### 

##### Cell Culture and Reagents

HeLa cells and HCT116 cells maintained in DMEM supplemented with 10% fetal bovine serum (FBS) and 100 μg/ml penicillin/streptomycin. Cells were treated with 5 μg/ml tunicamycin (Sigma, St. Louis, MO) or 500 nM thapsigargin (Sigma). To block caspase activity, cells were pre-treated for 1 h with 20 μm z-VAD-FMK (Enzo Life Sciences, Farmingdale, NY) prior to treatment with the ER stress inducing reagent. In fresh z-VAD-FMK treatment conditions, new z-VAD-FMK was added every 24 h. MS grade trypsin and LysC was purchased from Wako Chemicals Inc. (Richmond, VA), and MS grade chymotrypsin was purchased from Pierce Biotechnology (Rockford, IL). Annexin V and Propidium Iodide staining were performed using the BD Biosciences Annexin V: FITC Apoptosis Detection kit II (San Jose, CA) according to the manufacturer's instructions. The samples were analyzed by flow-cytometry using a BD FACSCanto II (BD Biosciences).

##### Western Blots, Immunoprecipitations, Global Protein Profiling, and Tandem Mass Tag (TMT) Labeling

Antibodies against caspase-2, 3, 4, 7, 8, 9, 10, BiP, IRE1, IRE1-phospho, PERK, eIF2α, t-JNK, p-JNK, GAPDH, FXR1, ATP Citrate Lyase, Phosphorylated Glycogen Synthase, Glycogen Synthase, MAP3K7, Kinectin 1, eIF4G1, phosphorylated eIF4G1, DIDO1, caspase 2/3/7 motif, caspase 6/8 motif, SRF, STAT6, CREB1, and anti-Rabbit-HRP were all purchased from Cell Signaling Technology (Danvers, MA) and used according to the manufacturer's suggestions for Western blot analysis. Anti-β-actin was purchased from Abcam (Cambridge, MA) and used according to manufacturer's instructions. FLAG-Apo2L was cross-linked with anti-FLAG M2 (Sigma) and cells were treated with 1 μg/ml for 4 h. Western blots were quantified by densitometry using ImageJ Gel Analyzer. Densitometry values were all normalized to β-actin or GAPDH loading controls.

Cells were subjected to immunoaffinity enrichment of caspase-cleaved peptides using the PTMScan® protocol (Cell Signaling Technology), as previously described (18). Briefly, cells were lysed in urea lysis buffer, lysates were reduced with DTT at 60**°**C, iced for 10 min, alkylated with IAA at room temperature and 10 mg of material was digested with trypsin (1:50 enzyme/protein ratio) or chymotrypsin (1:25 enzyme/protein ratio) overnight at room temperature. Resultant peptides were desalted and dried. Peptides were resuspended in IAP buffer (Cell Signaling Technology) and C-terminal Asp motif peptides were immunoprecipitated using the caspase motif antibodies conjugated to agarose beads for 2 h at 4 °C. Peptides were eluted using 0.15% TFA and dried under vacuum. Peptides were labeled with TMT according to the manufacturer's instruction (Pierce Biotechnology). Briefly, each sample was individually dissolved in 10 μl of 20 mm HEPES (pH 8) and mixed with one of the TMT6plex labeling reagents (10% of one unit was used for each sample.). The reaction was carried out for 1 h at room temperature and the addition of hydroxylamine immediately quenched extra reagent. Samples were combined, desalted with a C18 STAGE tip (Proxeon, Thermo Fisher Scientific, Waltham, MA), and labeling efficiency was analyzed by LC-MS/MS using an HCD-MS2 method.

For global protein profiling, cells were lysed in urea lysis buffer, lysates were reduced with DTT at 60**°**C, iced for 10 min, alkylated with IAA at room temperature, and 1 mg of material was digested with LysC (1:25 enzyme/protein ratio) overnight at room temperature. 100 μg of peptide from each time point was labeled with TMT according to the manufacturer's instructions. Samples were combined, dried down, and fractionated using a high pH reverse phase column on an Agilent 1100 series offline HPLC system. Lyophilized peptides were re-suspended in HPLC grade water at 10 μg/μl. 400 μg was loaded onto a Agilent Zorbax 300 Extend C18 analytical column with 2.1 × 150 mm dimensions and 3.5 μm particle size. Solvent A consisted of 25 mM Ammonium Formate (pH 9.7) whereas solvent B was 100% ACN. A gradient of 15% to 60% B was applied over 65 min and fractions were collected in 45 s intervals. 96 fractions were collected and every 16^th^ fraction was combined to form a final set of 16 distinct groups, for example, fraction 1, 17, 33, 49, 65, 81 were combined into one fraction. Samples were lyophilized, STAGE tipped and injected for LC-MS/MS analysis using a CID-MS2/HCD-MS3 method.

##### LC-MS/MS and Clustering

Peptide mixtures were injected *via* an auto-sampler and loaded onto a Symmetry® C18 column (1.7 μm BEH-130, 0.1 × 100 mm) at a flow rate of 1.5 μl/min using a NanoAcquity UPLC system (Waters, Milford, MA) as previously described (19). Peptides were separated at 1 μl/min using a gradient of 2% Solvent B to 25% Solvent B (where Solvent A is 0.1% Formic acid/2% ACN/water and Solvent B is 0.1% formic acid/2% water/ACN) applied over 90 min with a total analysis time of 120 min. Peptides were eluted directly into an Orbitrap Elite mass spectrometer (ThermoFisher, San Jose, CA) at a spray voltage of 1.2 kV using an Advance CaptiveSpray ionization source (Michrom BioResources/Bruker, Auburn, CA). For the HCD-MS2 method, precursor ions were analyzed in the orbitrap at 30,000 resolution. MS/MS was performed by isolating the 10 most abundant precursor ions with a 2-Da window, fragmented using HCD at 40% normalized collision energy, and analyzed in the orbitrap at 30,000 resolution. For the CID-MS2/HCD-MS3 method, precursor ions were analyzed in the orbitrap at 60,000 resolution. MS/MS was performed by isolating the 10 most abundant precursor ions with a 1.8-Da window, fragmented using CID at 35% normalized collision energy, and analyzed in the ion trap. Multiple fragment ions were further subjected to HCD fragmentation at a normalized collision energy of 50 and acquired at 30,000 resolution in the Orbitrap for tandem mass tag (TMT) quantitation (20).

For peptide identification, ReAdW (v.4.3.1) was used to generate peaks and MS/MS spectra were searched using Mascot (v.2.3.02) against a concatenated target-decoy database comprised of human protein sequences (UniProt Dec. 2011, 119,247 total entries) and known contaminants. MS/MS spectra were searched by Mascot using trypsin or chymotrypsin plus C-terminal aspartic acid cleavage with up to four missed cleavages total. For example, the trypsin plus C-terminal aspartic acid mascot search allowed for N-terminal cleavage at K or R and C-terminal cleavage at K, R, or D with a total of four potential missed cleavages. Searches also included a 50 ppm precursor ion tolerance, and 0.8 Da fragment ion tolerance. Variable modifications were permitted for methionine oxidation (+15.9949 Da), carbamidomethylation (+57.0215) on cysteine residues and fixed TMT modification of the N terminus and lysine residues (+229.1269). TMT reporter ion quantification was performed by selecting the highest peak intensity within 20 ppm of the theoretical TMT reporter ion *m*/*z* using in-house software called Mojave as previously described (21).

Given that single protein analysis offers limited numbers of peptide spectral matches, peptide assignments were initially filtered to a 5% peptide-spectra-matching level false discovery rate (FDR) using linear discriminant analysis (22). Global protein profiling (GPP) samples were further processed to a 2% protein level FDR using Protein Sieve (22). Protein level data was determined by averaging peptide data; peptide level data are determined by summing peptide spectral match level TMT intensity and fed downstream as the relative abundance to the maximal intensity. Peptide spectral match level data were fed downstream as raw abundance. Only peptide spectral matches with a combined reporter ion intensity greater than 2000 were included in further analyses. All mass spectrometry raw data files can be accessed in MassIVE, Accession # MSV000079302 (http://massive.ucsd.edu).

To identify novel caspase cleavage sites after comparison with existing repositories, caspase cleavage sites of all human caspases were downloaded from TopFIND (23) and CASBAH (24). CASBAH data were parsed using an in-house Ruby script. UniProt IDs and accession codes of human SwissProt entries were downloaded from UniProt (25) to cross-map entries between the databases. Position of cleavage sites from this study and CASBAH were mapped to protein sequences using the TopFIND Explorer software (23). Overlaps were calculated based on cleavage sites using an in-house R script. Caspase motif logos were generated using WebLogo v3.4. Peptide and flanking sequences from the identified caspase substrate peptides were extracted from the cleavage sites +6 to −5 residues. A logo was constructed using residue probability as units.

Clusters were generated using a fuzzy c-means algorithm as implemented in mfuzz in R (26, 27). For the GPP data set, proteins were standardized so that each protein has an average of 0 and standard deviation of 1 across conditions. Cluster size was set to 6 and fuzzifier (m) was set to 1.5. Relative peptide intensity from the caspase substrate profiling (CSP) and transcripts quantified in the RNA sequencing data set were clustered in a similar way, except that cluster number was set to 5 for caspase substrates and 9 for transcripts. GPP cluster #5 and #6 were overlaid on CSP clusters based on matched protein names. Transcripts that map to these proteins were also overlaid. Pearson correlations among the GPP, CSP and transcripts were calculated by an internal function cor.test() in R.

For the scatter plot showing correlation between transcript level and protein abundance of caspase substrates, a linear regression was performed. Protein and transcript data obtained from global protein profiling and RNA sequencing were standardized to have a mean value of zero and a standard deviation of one. A Pearson correlation was used to calculate correlation between caspase substrate protein expression and their transcript expression level (R stats package, version 3.2.2).

##### RNA Sequencing Data and Transcription Factor Gene Set Enrichment Analysis

RNA was isolated from HeLa cell samples (three biological replicates per condition) using the RNeasy kit from Qiagen (Valencia, CA) and sequenced using the Illumina High-Throughput Sequencing System. RNAseq reads were first aligned to ribosomal RNA sequences to remove ribosomal reads. Remaining reads were aligned to the human reference genome (NCBI Build 37) using GSNAP (28) version “2013–10^−10^”, allowing maximum of 2 mismatches per 75 base sequence (parameters: “-M 2 -n 10 -B 2 -i 1 -N 1 -w 200000 -E 1 -pairmax-rna = 200000”). Gene expression levels were quantified with RPKM (reads per kilobase of transcript per million mapped reads) values derived from the number of reads mapped to each RefSeq gene. Using the limma R package (29) we applied linear model analysis via the “voom” function (30) to measure differential gene expression between various conditions, reported as fold change and associated adjusted *p* values. Genes that did not show read counts in any sample were excluded from the analysis. DESeq was applied to derive variance stabilized count data, which were used to cluster samples based on the 500 most variable genes (31). Ward clustering was applied using Euclidean distance in R (http://www.R-project.org).

To reveal transcription factor target genes that are higher ranked relative to other genes in terms of differential expression, we applied gene set enrichment analyses. Using 615 transcription factor target gene sets (a subset of the “c3” motif gene sets) from the Molecular Signatures Database (v4.0) (32), we applied camera to the voom-generated differential expression results at default parameters.

## Results

### 

#### 

##### Caspases are Required for Inducing Cell Death in Response to ER Stress

**Fig. 1.**
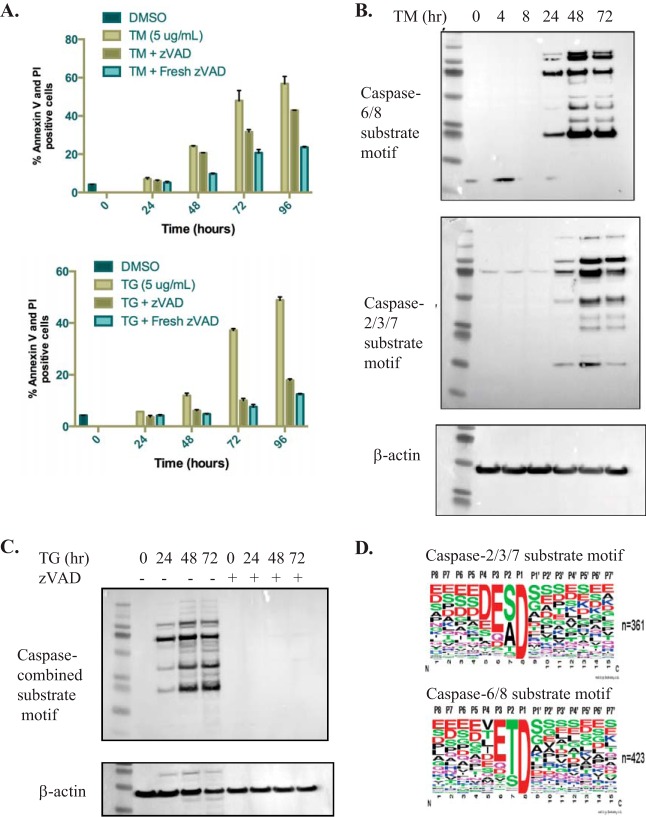
**Caspase activity is required for ER stress-induced cell death.** *A*, ER stress was induced using tunicamycin (top panel) or thapsigargin (bottom panel). HeLa cells were treated for 0 to 96 h with ER stress inducer alone or ER stress inducer plus the pan caspase inhibitor z-VAD-FMK. Samples labeled Fresh zVAD had fresh z-VAD-FMK added every 24 h during ER stress treatment. Error bars represent the standard deviation between two biological replicates. *B*, Caspase activation levels were analyzed using antibodies that recognize a motif similar to the caspase-6 and -8 consensus motif or that recognize a motif similar to the caspase-2, -3, and -7 consensus motif. β-actin was included as a loading control. *C*, Caspase activation levels were analyzed in response to thapsigargin treatment alone or with the pan caspase inhibitor z-VAD-FMK over a 72 h time course. A 50/50 combination the caspase-2, -3, and -7 substrate motif antibody and the caspase-6 and -8 substrate motif antibody was used to probe Western blots. *D*, Reported logo recognition motifs for both caspase substrate antibody clones (reprinted with permission from Cell Signaling Technology, more information available at www.cellsignal.com).

Caspases are known to become activated in response to ER stress (Reviewed in (33, 34)), however, whether they are required to induce cell death in response to ER stress is uncertain. ER stress can be induced by a variety of chemical perturbations including thapsigargin, a calcium channel inhibitor, or tunicamycin, an *N*-linked glycosylation inhibitor. To determine whether ER stress-induced cell death requires the activity of caspases, HeLa cells were treated for 0 to 96 h with thapsigargin or tunicamycin in the presence or absence of the pan caspase inhibitor z-VAD-FMK. At multiple time points during treatment, cells were stained for cell death markers Annexin V and propidium iodide and the percentage of cells staining positive was determined by flow-cytometry (Fig. 1*A*). It was observed that inhibition of caspase activity resulted in a significant reduction in cell death in both tunicamycin or thapsigargin treated cells. For example, at the 72 h time point, 48% of tunicamycin treated cells and 40% of cells treated with thapsigargin stained positive for cell death markers. In contrast, in the absence of caspase activity, only 21% and 9% of cells were positive, respectively. These data confirm that caspases play an important role in inducing cell death in response to ER stress.

In concordance, several reports have indicated that caspases may be contributing to cell death in response to ER stress (5–14, 17, 35–38). The exact role of caspases in this process, however, remains unclear. To investigate the involvement of caspases in ER stress-induced cell death, a study was conducted to monitor the kinetics of caspase substrate cleavage during ER stress. HeLa cells were treated for 0 to 72 h with tunicamycin and lysed in a urea-based lysis buffer. To determine which caspases become activated in response to ER stress, Western blot analysis was used to examine proteolytic cleavage and activation of caspases-2, 3, 4, 7, 8, 9, and 10 during ER stress (Supplemental Fig. 1A). Interestingly, all caspases examined have some level of proteolytic activation during the ER stress time course with the exception of caspase-4, which is thought to be required for activation of the inflammasome (39). Western blot analysis of key mediators of ER stress was also performed to monitor levels of ER stress induction over the tunicamycin treatment window. Levels of PERK and the ER chaperone BiP increased after 4 h of tunicamycin treatment and levels of IRE1α increased after 24 h of ER stress. Phosphorylated IRE1α levels increase after 24 h of tunicamycin treatment and sustained IRE1α activation also results in phosphorylation of c-Jun N-terminal kinase (JNK) beginning at this time point. Treatment of cells with a FLAG-tagged, cross-linked version of the extrinsic apoptosis inducer Apo2L (Apo2L-XL) was used as a positive control for apoptosis induction, and GAPDH was provided as a loading control (supplemental Fig. S1*A*, S1*B*).

In this study, antibodies that recognize a caspase-2, -3 and -7 P1 motif and a caspase-6, and -8 P1 motif were used for enrichment of caspase-cleaved substrates. Using these two antibodies in Western blot analysis of the tunicamycin time course revealed accumulation of caspase-cleaved proteins beginning before 24 h of ER stress (Fig. 1*B*). Notably, these bands are not present if cells are pre-treated with a pan caspase inhibitor (Fig. 1*C*). Motif logo analysis on peptides captured with these antibodies was used by the manufacturer to determine the specificity of these reagents (Cell Signaling Technologies, more information available at www.cellsignal.com) (amino acid positions P8-P7′) and the results are depicted in Fig. 1*D*.

##### Identification of Caspase Substrates During ER Stress

**Fig. 2.**
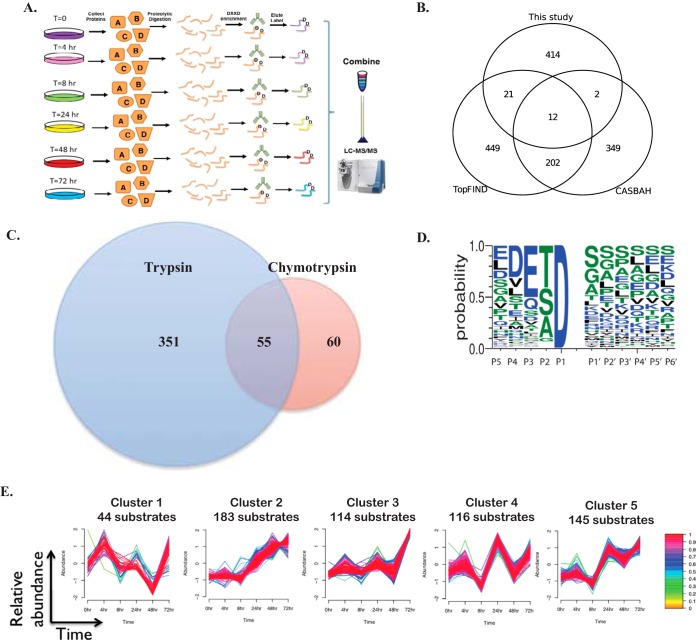
**Kinetic analysis of caspase substrates during ER stress.** *A*, Experimental design for peptide level immunoaffinity caspase motif enrichment and identification. HeLa cells were treated with tunicamycin for 0 to 72 h, lysed, and digested with trypsin or chymotrypsin. Immunoaffinity enrichment was used to capture peptides resulting from caspase cleavage events, and peptides were eluted and differentially labeled with TMT. Samples were combined, desalted, and analyzed by LC-MS/MS. *B*, Venn diagram comparing caspase substrate cleavage sites identified in this study to those reported in CASBAH or TopFIND. *C*, Venn diagram showing the number of capase cleavage sites identified from tryptic digestion *versus* chymotryptic digestion prior to immunoaffinity enrichment of caspase-cleaved peptides. *D*, Logo motif analysis for enriched caspase substrate peptides identified in this study. *E*, Fuzzy c-means clustering was used to group peptides with similar expression profiles over the tunicamycin treatment time course. Each line represents a caspase-cleaved peptide and the color of the line indicates how closely the peptide profile correlates with the mean of the cluster.

Peptide level immunoaffinity-based approaches targeting post-translational modifications have proven to be powerful tools for the dissection of intracellular signaling pathways (18, 40, 41). This technique has been successfully applied to characterize caspase substrates in response to pan-kinase inhibition and DNA damage (42). Here we utilize this immunoaffinity-based approach in combination with multiplexed tandem mass tag (TMT) labeling to identify caspase substrates and to determine the kinetics of caspase substrate cleavage in response to ER stress. HeLa cells were treated with tunicamycin for 0, 4, 8, 24, 48, or 72 h and peptide-level immunoprecipitations were performed to enrich for peptides containing a C-terminal aspartic acid motif. A 50:50 combination of both caspase motif antibodies (caspase 2/3/7 combined with caspase 6/8 substrate motifs) was used to capture caspase substrates. Enriched peptides were eluted and labeled with TMT reagents, combined together, and analyzed by mass spectrometry (Fig. 2*A*).

Using this method, 625 unique C-terminal aspartic acid containing peptides were identified with less than 4% peptide-level FDR. TMT reporter ion quantification was employed, allowing the kinetics of caspase cleavage for 445 protein substrates (602 unique peptides) to be monitored, including 449 total cleavage sites (Fig. 2*B*, for mass spectra of all identified peptides see supplemental Fig. S2). Because this method requires capture of the neo C-terminal peptide (or P1 peptide) created as a result of caspase cleavage, two complementary proteolytic enzymes, trypsin and chymotrypsin, were employed to provide increased likelihood of detecting a wider range of substrates and provide greater coverage in the mass spectrometry results (Fig. 2*C*). Motif logo analysis was performed on the cleaved peptide cleavage sites (positions P5-P6′) and is shown in Fig. 2*D*.

Caspase-mediated deactivation of many of the caspase substrates identified in this study likely impact how cells respond to the accumulation of misfolded proteins. For instance, some substrates identified here are known to play key roles in the ER stress response and ER trafficking (Hsp105, Hsp74L, Hsp7C, CopA, Sec24D, Sec16A, Sec62, and Sequestosome/p62) (supplemental Fig. S2). Additionally, YAP and Rb, two proteins shown to have an important role in cancer and tumor metastasis (reviewed in (43, 44)) are both shown to be cleaved and degraded in a caspase dependent manner in response to ER stress (supplemental Figs. S2 and S3).

To further characterize the caspase substrate cleavage profile, the expression level of caspase-cleaved peptides over time was clustered to determine the kinetic substrate specificity of caspases in response to ER stress (26, 27). The resulting five clusters of caspase substrates were categorized into four main groups of i) substrates cleaved before 24 h of tunicamycin treatment (cluster 1, 44 substrates), ii) substrates that accumulate after 24 h of tunicamycin treatment (cluster 2, 183 substrates), iii) substrates that begin to accumulate by 72 h of tunicamycin treatment (cluster 3, 114 substrates), and iv) two populations of substrates with bimodal expression patterns (cluster 4, 116 substrates, and cluster 5, 145 substrates) (Fig. 2*E*, See supplemental Table S1 for a full list of all cluster members and peptide intensities). Each line in Fig. 2*E* represents a cleaved peptide and the color of the line indicates how closely the expression level profile correlates with the mean of the cluster. Overall, these results demonstrate that there is a preference for certain substrates earlier in the ER stress response, which may subsequently impact cell fate decisions.

##### Global Protein Profiling Reveals Caspase Substrates That Are Downregulated In Response to ER Stress

Caspases are thought to potentially have thousands of substrates, and most likely, many of them are not required for caspase-dependent cell death. Additionally, with the exception of a few substrates known to be activated by caspase activity (*e.g.* interleukin-1β (45–47)), most substrates are thought to be deactivated by caspase cleavage (4). We postulated that identification of caspase substrates where caspase activity leads to loss of protein expression may help identify which caspase substrates are likely responsible for caspase-dependent cell death. To better understand the effect of ER stress on caspase substrates at the protein level, global protein profiling was performed. Whole cell lysate samples from each of the six time points described in the caspase substrate profiling experiment were proteolytically digested, chemically labeled with TMT and chromatographically fractionated using a high pH reverse phase gradient. Fractions were analyzed by mass spectrometry and protein levels at each time point were inferred by the cumulative abundance of reporter ions from all peptides from a given protein.

**Fig. 3.**
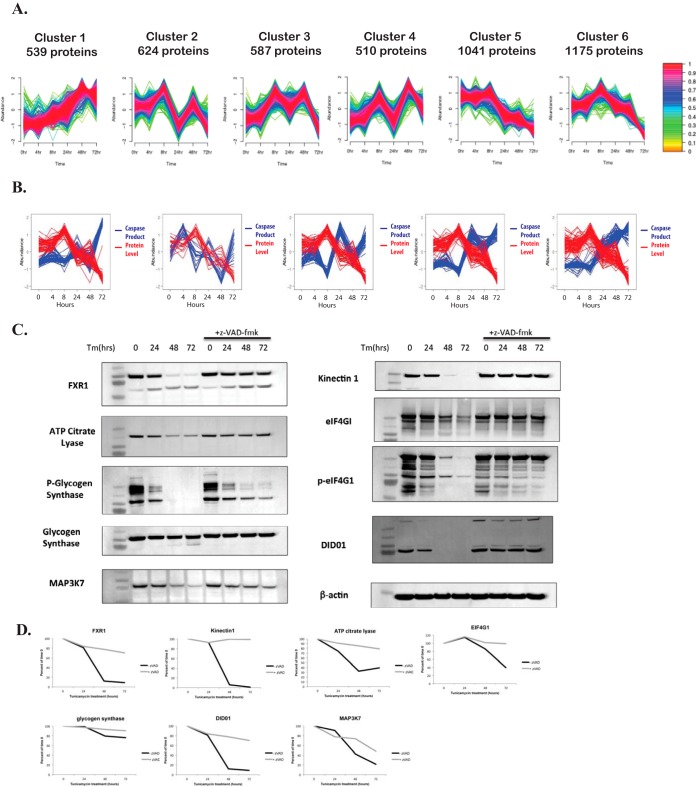
**Global protein profiling reveals caspase substrates that are downregulated in response to ER stress.** *A*, Fuzzy c-means clustering was used to group proteins with similar expression profiles during tunicamycin treatment. Each line represents a protein and the color of the line indicates how closely the protein expression profile correlates with the mean of the cluster. *B*, Overlay graphs were created by plotting the abundance of proteins identified to be downregulated at the protein level (clusters GPP 5 and GPP 6) (red line) and the abundance of caspase cleavage products (blue lines) over time. The five overlay graphs are divided into the five unique caspase cleavage product clusters from Fig. 2*E. C*, A subset of proteins identified as being downregulated at the protein level and cleaved by caspases was examined by Western blot analysis. HeLa cells were treated with tunicamycin or tunicamycin plus the pan caspase inhibitor z-VAD-FMK for 0 to 72 h. Western blot analysis was performed against FXR1, ATP Citrate Lyase, phospho-glycogen synthase, glycogen synthase, MAP3K7, Kinectin 1, eiF4G1, phospho-eIF4G1, DIDO1, and β-actin (loading control). *D*, Densitometry to quantify the abundance of bands for FXR1, ATP Citrate Lyase, glycogen synthase, MAP3K7, Kinectin 1, eiF4G1, and DIDO1. Quantified bands were normalized to the β-actin loading control and graphed as % of time point 0. Black lines represent cells treated with tunicamycin alone and gray lines represent cells treated with tunicamycin plus z-VAD-FMK.

This method allowed relative kinetic quantification of the expression levels for 4476 proteins (from 39,044 unique peptides) in response to ER stress with 2% protein-level FDR. Fuzzy c-means clustering yielded six distinct clusters (Fig. 3*A*). Two clusters, GPP 5 (1041 proteins) and GPP 6 (1175 proteins), contain proteins that are downregulated at the protein level in response to ER stress (See supplemental Table S2 for a full list of all cluster members). Next we investigated which of these downregulated proteins were also caspase substrates in response to ER stress. Fig. 3*B* shows the overlay plots illustrating protein expression and caspase substrate cleavage accumulation over time. Several proteins identified in this comparison were evaluated by Western blot analysis to determine if these proteins are degraded in a caspase-dependent manner. Fig. 3*C* shows Western blots analysis for FXR1, ATP Citrate Lyase, Glycogen synthase (both phosphorylated and un-phosphorylated), MAP3K7, Kinectin-1, eIF4G1 (both phosphorylated and un-phosphorylated) and DIDO1 in response to tunicamycin treatment in the presence or absence of the pan caspase inhibitor z-VAD-FMK. Densitometry was performed to quantify abundance of these substrates (Fig. 3*D*), and importantly, all substrates tested were confirmed to be degraded in a caspase-dependent manner. These results demonstrate that use of an integrated approach involving clustering of the global proteome in combination with quantification of caspase substrate cleavage products enables identification of many substrates that are downregulated at the protein level in a caspase-dependent manner.

##### Characterization of the Transcriptional Response to ER Stress

**Fig. 4.**
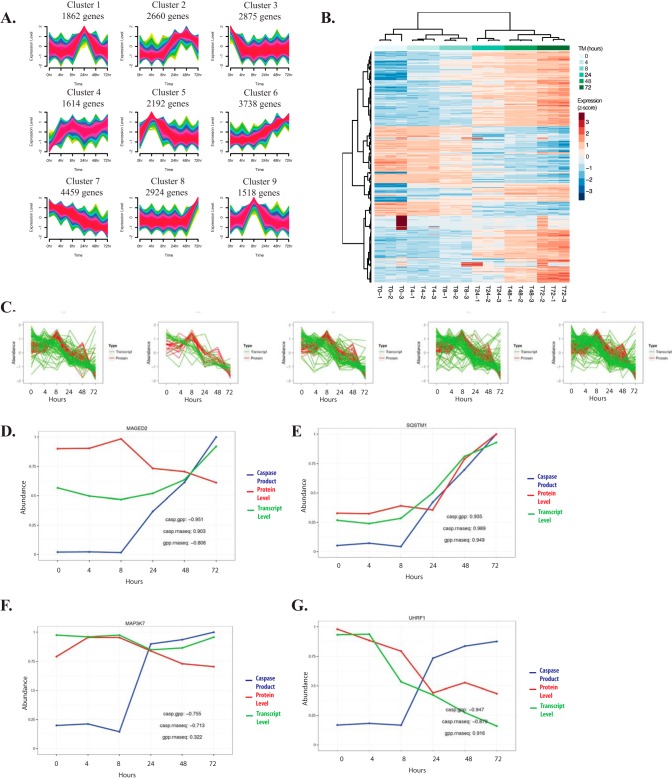
**Characterization of the transcriptional response to ER stress.** *A*, Fuzzy c-means clustering was used to group transcripts with similar expression profiles from HeLa cells treated with tunicamycin. Each line represents the average gene transcript level from three biological replicates and the color of the line indicates how closely the transcriptional profile correlates with the mean of the cluster. 9 unique clusters with distinct expression patterns were identified. *B*, Heat map of the most significant changes during the ER stress response. *C*, Overlay graphs were created by plotting the abundance of caspase cleaved substrates, protein abundance (red lines), and transcript abundance (green lines). The 5 overlay graphs are divided into the 5 unique caspase cleavage product clusters from Fig. 2*E*. Correlation plots are provided for MAGED2 (*D*), Sequestosome (SQSTM1) (*E*), MAP3K7 (*F*), and UHRF1 (*G*). Red lines indicate protein level, green lines indicate transcript level, and blue lines indicate the level of caspase cleavage product.

Several studies have looked into the transcriptional response to ER stress (48, 49), however none of these previous reports extended characterization of the transcriptional response beyond 24 h of exposure to cellular stress. To characterize the prolonged transcriptional response to tunicamycin, RNA sequencing analysis at the same time points discussed in the caspase substrate capture and global protein profiling experiments were performed. Biological triplicates were sampled at each time point, sequenced using the Illumina High-Throughput Sequencing System, and raw reads were mapped to the human genome by GSNAP-based transcriptome analysis pipeline (28, 50). Transcript levels were quantified with reads per kilobase per million and clustered using fuzzy c-means clustering. Nine unique clusters were identified representing expression levels for 23,842 genes (Fig. 4*A*, supplemental Tables S3 and S4). Fig. 4*B* illustrates a heat map depicting the top 500 most variable genes in our time course analysis. Sample clustering made clear that expression levels correlated well between biological replicates. Additionally, this analysis revealed that there are transcriptional fluctuations occurring much later in the ER stress response. For example, Cluster 8 contains 2924 genes that are upregulated after 72 h of treatment. These previously unreported, delayed transcriptional changes might play a role in the ability of the cell to induce cell death in response to ER stress.

This data set was further interrogated to identify transcriptional changes in genes encoding caspase substrates identified in this study in response to ER stress. Because transcript level can impact protein abundance during prolonged exposure to ER stress, the correlation between protein level and transcript level for caspase substrates was examined and determined to be high (Pearson correlation = 0.57, *p* value <2.2e-16, supplemental Fig. S4). Most caspase substrates are downregulated at both the transcript and protein level (supplemental Fig. S4 and Fig. *4**C*). Using this approach, substrates that were post-transcriptionally downregulated and therefore likely degraded in a caspase-dependent manner were identified. For example, Fig. 4*D* shows data for MAGED2 which has transcript level and caspase substrate product abundance inversely correlated with protein level. It is transcriptionally upregulated, downregulated at the protein level, and cleaved by caspases after 24 h of treatment with tunicamycin. This pattern strongly suggests that expression levels of MAGED2 are downregulated most likely as a result of proteolytic cleavage by caspases leading to increased protein turnover during ER stress. Fig. 4*E* shows the correlation plot for Sequestosome/p62, an example of a substrate where all three data sets are highly correlated. Sequestosome is upregulated both at the transcriptional and at the protein level and therefore it is uncertain whether caspase cleavage of this protein would impact the ability of Sequestosome to carry out cellular processes. MAP3K7 is an example of a caspase substrate that is downregulated at the protein level but transcript levels remain flat throughout the treatment window (Fig. 4*F* and Fig. 3*C*). Because transcript levels are largely unaffected, downregulation of MAP3K7 at the protein level is likely a result of direct caspase activity. Last, caspase substrates like the E3 ubiquitin ligase UHRF1 have transcript levels highly correlated with protein level data. These substrates are downregulated both transcriptionally and at the protein level, and although loss of function may contribute to cell death in response to ER stress, it is unclear whether these proteins are downregulated in a caspase-dependent manner (Fig. 4*G*) (See all 346 correlation plots in supplemental Fig. S5).

##### Caspase-dependent Transcriptional Regulation Occurs During ER Stress

**Fig. 5.**
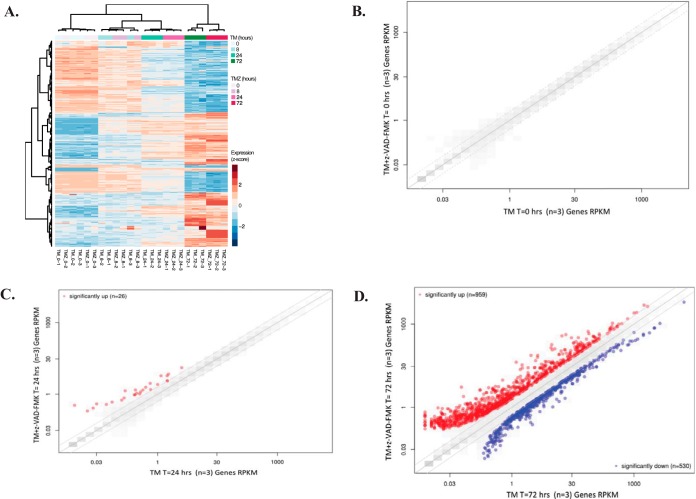
**Caspase inhibition leads to upregulation of genes.** *A*, Heat map of the top 1,000 most significant changes during the ER stress response in HeLa cells in the presence or absence of caspase activity. Transcript levels between samples with active caspases and samples where caspases are inhibited with z-VAD-FMK were compared at time point 0 (*B*), 24 h of tunicamycin treatment (*C*), or 72 h of tunicamycin treatment (*D*).

Based on the observation that many caspase substrates appeared to be transcriptionally downregulated, the impact of caspases on the transcriptional response to ER stress was investigated. Transcription factors are known to be caspase substrates in response to other cellular perturbations; however, it remains unknown whether caspase cleavage of these proteins impacts the transcriptional response to ER stress. To address this, RNA sequencing was performed to determine the impact of ER stress on transcription in the presence or absence of caspase activity. HeLa cells were treated with tunicamycin for 0, 8, 24, or 72 h alone or in combination with the pan caspase inhibitor z-VAD-FMK, and transcript levels of three biological replicates for each time point were compared. The majority of transcriptional changes occurring across the samples were driven by time point rather than presence or absence of caspase activity (Fig. 5*A*). However, when caspase activity was inhibited, differential expression analysis (see Materials and Methods section 3.4) showed that 26 and 959 genes were significantly upregulated (adjusted *p* value < 0.05, fold change > 2) at the 24 and 72 h time points, respectively (Fig. 5*B*–5*D*, supplemental Tables S5 and S6). Hierarchical clustering of the samples also illustrated that the most apparent differences between z-VAD-FMK treated and untreated samples occurred 24 h post treatment (Fig. 5*A*). These results suggest that caspase activity specifically prevents expression of these genes during the ER stress response.

**Fig. 6.**
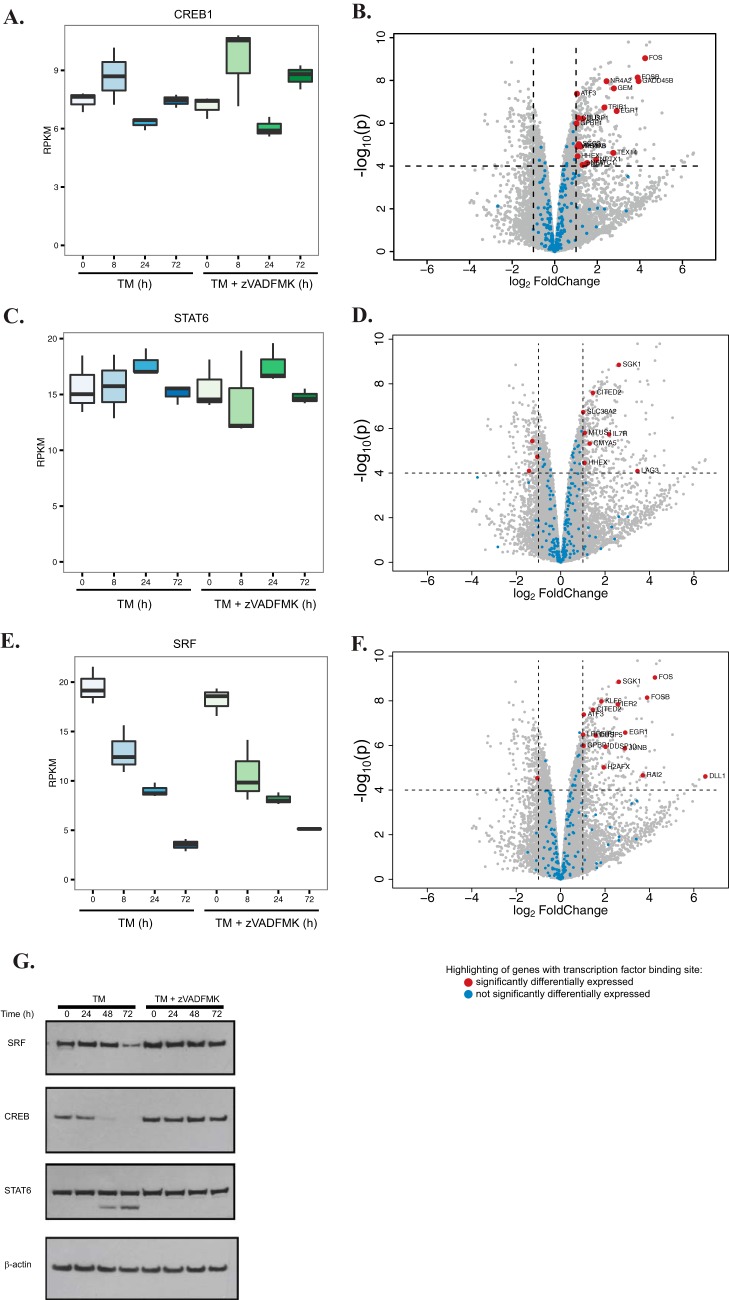
**Caspases impact the transcriptional profile during the ER stress response.** Box plots provided indicate transcript levels for CREB1 (*A*), STAT6 (*C*), or SRF (E) during tunicamycin treatment in the presence or absence of caspase activity. Volcano plots for CREB1 (*B*), STAT6 (*D*), or SRF (*F*) plot the log-scaled mean fold change and adjusted *p* values for all human genes resulting from the differential expression analysis between caspase inhibited (TMZ) and caspase active (TM) samples at 72 h. Using a more stringent cutoff for regulation (adjusted *p* value < 0.0001, fold change > 2), the most significantly differentially expressed transcription factor target genes are highlighted in red. Blue dots indicate gene targets that are not significantly changed. *G*, Western blot analysis of SRF, CREB1, and STAT6 after treatment with tunicamycin alone or in combination with z-VAD-FMK to block caspase activity. β-actin is included as a loading control.

To identify potential transcription factors that may be driving transcriptional changes present when caspases are inhibited, a gene set enrichment analysis was performed (see Materials and Methods). The analysis was based on the gene set collection “c3” from the MSigDB (51), which contains the putative target genes for 615 transcription factors. This analysis revealed that target genes for 81 transcription factor sets were upregulated in response to caspase inhibition during ER stress (supplemental Table S7). Strikingly, we uncovered several transcription factors whose gene expression levels were not substantially affected by caspase activity (fold change < 2, supplemental Fig. S5) and displayed increased expression of multiple target genes in response to caspase inhibition. For example, transcript levels of cyclic AMP-Responsive Element-Binding protein 1 (CREB1) and Signal Transducer and Activator of Transcription 6 (STAT6) were not significantly affected by altered caspase activity (1.17- and 0.98-fold change in transcript levels, respectively), whereas multiple target genes were upregulated in response to pan caspase inhibitor treatment 72 h post-ER stress induction (Figs. 6*A*–6*D*). We also found a similar trend for Serum Response Factor (SRF), although SRF transcript levels were slightly elevated upon caspase inhibition during ER stress (1.47-fold increase in transcript levels) (Figs. 6*E* and 6*F*). It was therefore hypothesized that these transcription factors may be cleaved by caspases in response to ER stress resulting in inhibition of transcription of their downstream target genes. Indeed Western blot analysis showed that CREB1, STAT6, and SRF are substantially degraded in a caspase-dependent manner (Fig. 6*G*). These results suggest that caspases impact the transcriptional response to ER stress and therefore act as both transcriptional and post-translational cellular regulators.

##### Discussion and Conclusions

The results of this study indicate that caspases can influence cellular signaling pathways both by directly cleaving substrates and by deactivating transcription factors required for upregulation of genes involved in cell fate decisions. Using immunoaffinity enrichment of caspase substrates combined with global proteome profiling and RNA sequencing, we were able to identify caspase substrates that are post-transcriptionally downregulated at the protein level. This analysis allowed identification of proteins that are deactivated by caspases and thereby potentially contributing to cell death. Unexpectedly, transcription levels of many caspase substrates were greatly downregulated during ER stress. Based on this observation we investigated the role of caspases in transcriptional regulation in response to ER stress. Our findings show that activity of caspases on transcription factors CREB1, STAT6, and SRF resulted in a loss of expression of these three proteins thereby preventing expression of a subset of their target genes. This previously unappreciated role for caspases in transcriptional regulation is likely playing a role in cell fate decisions during chronic ER stress by altering the balance of cell survival and cell death.

##### Caspases Directly Regulate Protein Expression Through Proteolytic Cleavage of Substrates

Proteolytic cleavage of substrates by caspases could result in a number of outcomes: (1) loss of function of the protein, (2) the cleavage product could act as a dominant negative and inhibit normal cellular function of the uncleaved protein, (3) activation of a novel function of that protein, or (4) have no effect at all. Furthermore, newly formed proteolysis products may have varying stabilities, with some neo-peptides either stabilized or destabilized in comparison to their uncleaved precursor. This adds additional caveats when interpreting protein level data, as this is determined through averaging peptide level data. However, in general, averaging peptide abundance to determine the protein level is still the most feasible way to quantify protein levels on a global scale. With these considerations in mind, we believe our data show that the majority of caspase substrates identified have reduced protein levels during ER stress (346/445) indicating that caspase activity most likely results in loss of function for these proteins. It has long been thought that caspases induce cell death through deactivation of many proteins and that it is the cumulative effect of cleaving many proteins that ultimately results in the cell's demise. Further investigation into these caspase substrates that are downregulated at the protein level will be required to fully appreciate whether loss of expression of substrates identified in this study are sufficient for cell death.

Interestingly several clusters appear to show a decrease in caspase substrate abundance at the 48 h time point (clusters 1, 3, 4, and 5). We see this same pattern in peptides immunoprecipitated from both trypsin and chymotrypsin digested cell lysates (which were performed separately), suggesting this variation is likely biological rather than technical (see Rb example, supplemental Fig. S3). More studies are required to further characterize this observation and to determine whether fluctuations in caspase activity, protein synthesis, protein turnover or all of the above are playing a role in this decrease in caspase cleavage product late in the ER stress response.

Many substrates identified in this study are known to play key roles in the ER stress response. For example, calreticulin, Ero1A, and several heat shock proteins (Hsp105, Hsp74L, and Hsp7C) that serve as protein folding chaperones were all identified as caspase substrates in this analysis. These proteins are known to play an important role in protein folding and thus it is tempting to speculate that caspases may actively inhibit this process. Moreover, proteins involved in ER trafficking and ER-associated degradation (CopA, Sec24D, Sec16A, Sec62, and Sequestosome/p62) are also revealed to be caspase substrates in this study. These results strongly suggest that one function of caspase activation may be to counteract the adaptive phase of the UPR by preventing proper protein folding and degradation, thereby driving cells into apoptosis.

Some of the substrates identified also play an important role in cancer and tumor metastasis. As many of these proteins are important for cellular growth and proliferation, it is not entirely unexpected that these would be caspase substrates if the ultimate aim of caspase activation is cell death. For example, YAP and Rb are both cleaved by caspases and degraded in a caspase dependent manner. These substrates are particularly interesting because of their well-known role in cellular proliferation and cell cycle control, respectively. Caspase-mediated inhibition of these proteins is likely to impact cell survival and indicates that caspases not only actively induce cell death but also prevent cell survival.

##### Caspases Indirectly Regulate Protein Expression Through Proteolytic Cleavage of Transcription Factors

Using gene set enrichment analysis, we identified transcription factors for which a significant number of transcriptional target genes were upregulated in response to caspase inhibition. Analogous to their target genes, several of these transcription factors displayed greater than 2-fold change in gene expression in response to caspase inhibition and were therefore excluded from further analyses (for example, EGR1 and ATF3, supplemental Table S7). However, some transcription factors including CREB1, STAT6, and SRF, exhibited only minor gene expression changes compared with their target genes in response to caspase inhibition. Interestingly, in contrast to their gene expression profile, these transcription factors displayed drastic changes in protein level expression suggesting caspases could be playing a role in regulating expression of these proteins during ER stress. Furthermore, Western blot analyses confirmed that during ER stress, CREB1, STAT6, and SRF are all degraded in a caspase dependent manner.

CREB1 is a member of the leucine zipper family of CRE sequence binding proteins that has been shown to play an important role in cellular growth, proliferation, and may contribute to the development of cancer. CREB1 has been previously shown to play a role in protection against tunicamycin treatment in rat cells potentially through competition for CRE binding sites with ATF4 and ATF6 that would otherwise upregulate a pro-apoptotic gene expression profile (52). Additionally, CREB1 has also been shown to be a caspase substrate in response to treatment with the pan-kinase inhibitor staurosporine (53). Combining these observations with the results of this study, one could hypothesize that CREB1 initially plays a pro-survival role in the early stages of ER stress, however following caspase activation, CREB1 is degraded in a caspase-dependent manner in order to allow for expression of pro-apoptotic genes.

Similarly, SRF has previously been described as a caspase substrate in response to extrinsic apoptosis inducers and cleavage of SRF results in a loss of c-Fos expression (54, 55). SRF is a member of the MADS family of transcription factors that binds to Serum Response Elements (SRE) and leads to stimulation of cellular proliferation and differentiation. In conditions where caspases are inhibited, for instance in viral infection or chemical perturbation, both CREB1 and SRF induce expression of genes that are pro-survival. Caspase regulation of these transcription factors may aim to extinguish expression of a subset of prosurvival genes normally regulated by these two proteins.

In contrast to SRF and CREB1, STAT6 has never been previously shown to be a caspase substrate prior to this study. STAT6 is a STAT family transcription factor found to upregulate expression of pro-survival Bcl-2 family members in response to IL-4 treatment of B cells (56, 57). During ER stress, STAT6 is cleaved and unlike CREB1 and SRF, the protein is not degraded but rather is present as a stable, lower molecular weight fragment. Based on antibody epitope and molecular weight shift by Western blot we have determined that cleavage of STAT6 results in a loss of the C terminus including the LXXLL motif known to be important for dimerization with transcriptional co-regulatory proteins (58, 59). This truncated STAT6 may potentially act as a dominant negative because the protein homodimerizes during transcriptional activation. One gene upregulated by STAT6 during ER stress in the absence of caspase activity is CITED2 (Cbp/p300-interacting transactivator 2), which plays a role in cellular differentiation and loss of expression of this gene is associated with congenital cardiovascular defects. Furthermore, STAT6 activation also leads to upregulation of Sgk1, which is known to phosphorylate and activate CREB1. Although further experiments are required to fully assess the relationship between STAT6 and CREB1, it is possible that STAT6 plays a role in CREB1 activation in response to ER stress.

Overall in this study, quantitative proteomic approaches combined with gene expression profiling and transcription factor gene set analysis were employed to identify caspase substrates that are deactivated during chronic exposure to ER stress. The identities of proteins responsible for caspase-dependent cell death have long remained elusive. Our study has revealed the identity of 346 caspase substrates downregulated at the protein level during ER stress, likely resulting in cell death. Only through integration of multiple data sets could we determine a transcriptional regulatory role for caspases during ER stress. These data sets can inform future systems-level studies as well as studies focused on a particular protein of interest. Moreover, these results can be further queried to identify unique signatures of ER stress that may lead to the identification of relevant, measurable biomarkers to be used in studies focusing on ER stress-related disease pathologies.

## Supplementary Material

Supplemental Data

## Footnotes

^1^ The abbreviations used are:

ER: endoplasmic reticulum
UPR: unfolded protein response
PERK: PKR-like ER response
IRE1α: inositol-requiring enzyme 1 α
TMT: tandem mass tag.
