# Supplementary material for: Uncovering a Dual Regulatory Role for Caspases During Endoplasmic Reticulum Stress-induced Cell Death
Source: Mol Cell Proteomics. 2016 Apr 28;15(7):2293–307. doi: 10.1074/mcp.M115.055376 (PMC4937505; doi:10.1074/mcp.M115.055376)
Supplement: Supplemental Data [file supp_15_7_2293__index.html]

Uncovering a dual regulatory role for caspases during endoplasmic reticulum stress-induced cell death — Uncovering a Dual Regulatory Role for Caspases During Endoplasmic Reticulum Stress-induced Cell Death — Caspases and ER Stress-induced cell death — Supplemental Data 

# Uncovering a Dual Regulatory Role for Caspases During Endoplasmic Reticulum Stress-induced Cell Death

## Supplemental Data

- Supplemental Figure 1 (.pdf, 1.9 MB) - Supplemental Figure 1
- Supplemental Figure 2 (.pdf, 10.6 MB) - Supplemental Figure 2
- Supplemental Figure 3 (.pdf, 2.1 MB) - Supplemental Figure 3
- Supplemental Figure 4 (.pdf, 391 KB) - Supplemental Figure 4
- Supplemental Figure 5 (.pdf, 512 KB) - Supplemental Figure 5
- Supplemental Figure 6 (.pdf, 481 KB) - Supplemental Figure 6
- Supplemental Figure 7 (.pdf, 2.0 MB) - Supplemental Figure 7
- Supplemental Table 1 (.xls, 310 KB) - Supplemental Table 1
- Supplemental Table 2 (.xls, 2.2 MB) - Supplemental Table 2
- Supplemental Table 3 (.xls, 4.1 MB) - Supplemental Table 3
- Supplemental Table 4 (.xlsx, 4.2 MB) - Supplemental Table 4
- Supplemental Table 5 (.xlsx, 5.3 MB) - Supplemental Table 5
- Supplemental Table 6 (.xlsx, 176 KB) - Supplemental Table 6
- Supplemental Table 7 (.xls, 39 KB) - Supplemental Table 7
- Supplemental Table 8 (.xls, 56 KB) - Supplemental Table 8
- Supplemental Table & Figure Legend (.pdf, 59 KB) - Supplemental Table & Figure Legend
