# Supplementary figures and images for: Uncovering a Dual Regulatory Role for Caspases During Endoplasmic Reticulum Stress-induced Cell Death
Source: Mol Cell Proteomics. 2016 Apr 28;15(7):2293–307. doi: 10.1074/mcp.M115.055376 (PMC4937505; doi:10.1074/mcp.M115.055376)

Supplemental Figure 1

A.

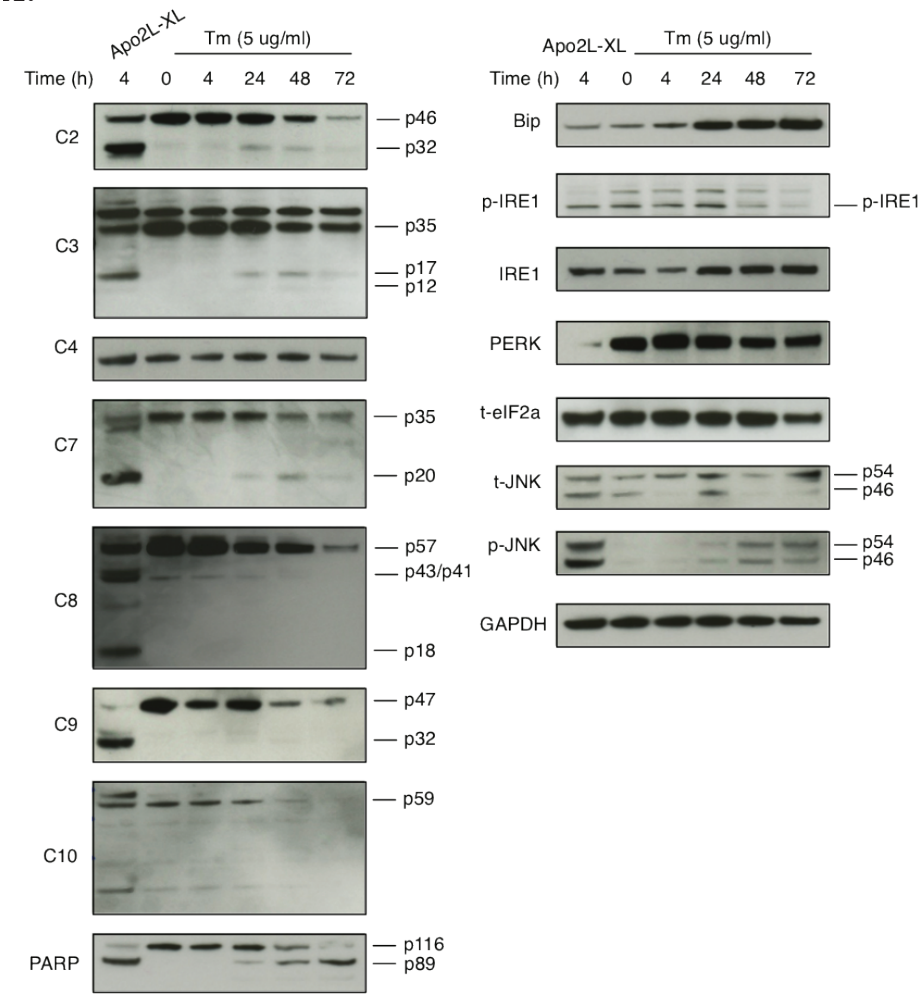

B.

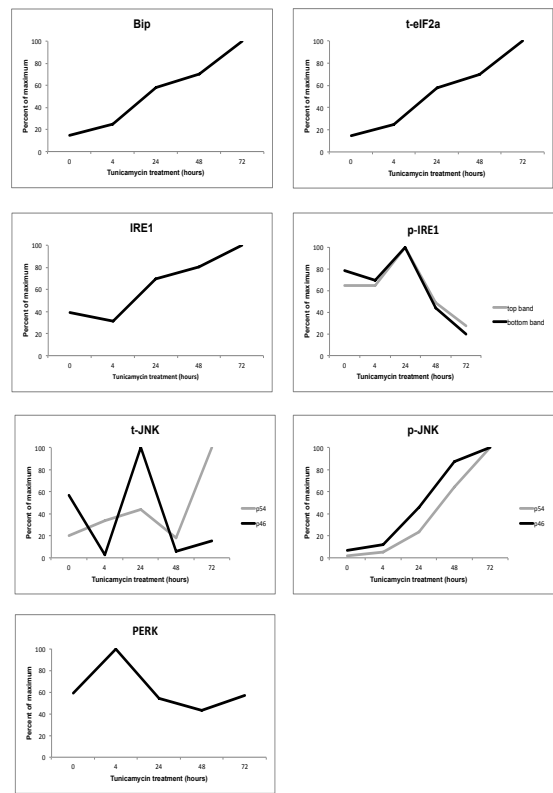

Supplemental Figure 3

A.

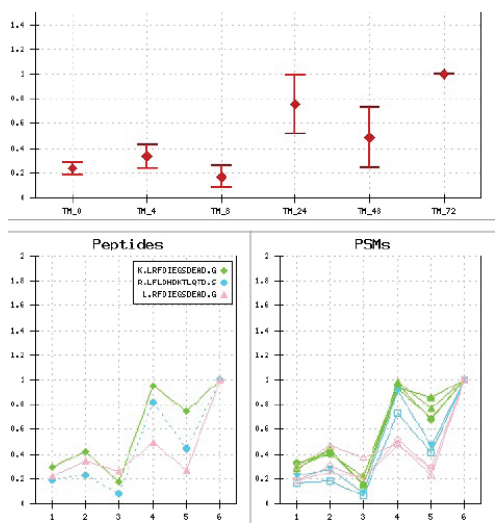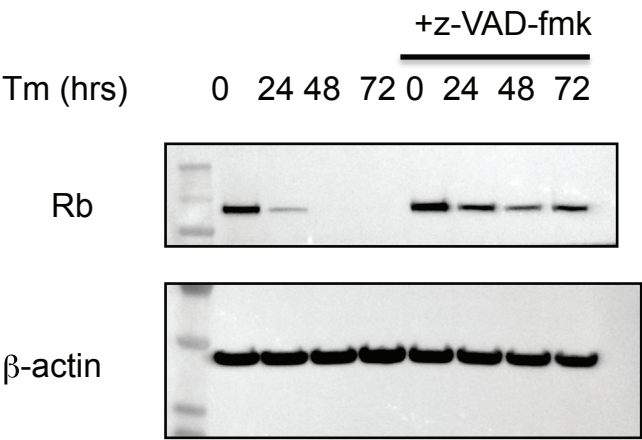

B.

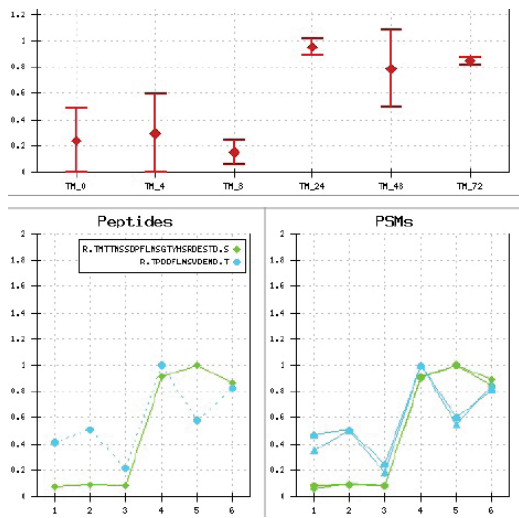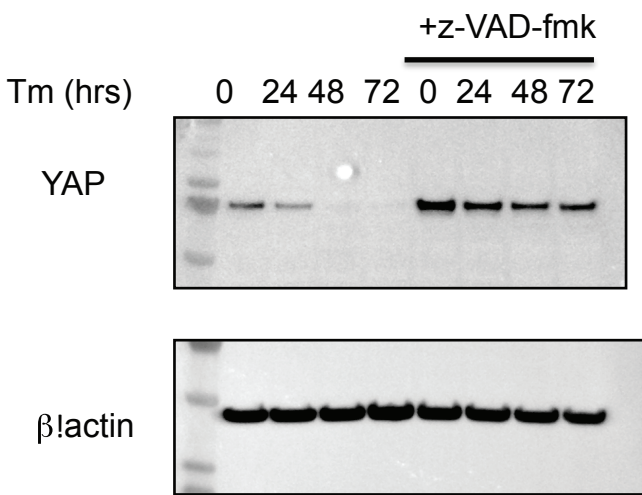

Supplemental Figure 4

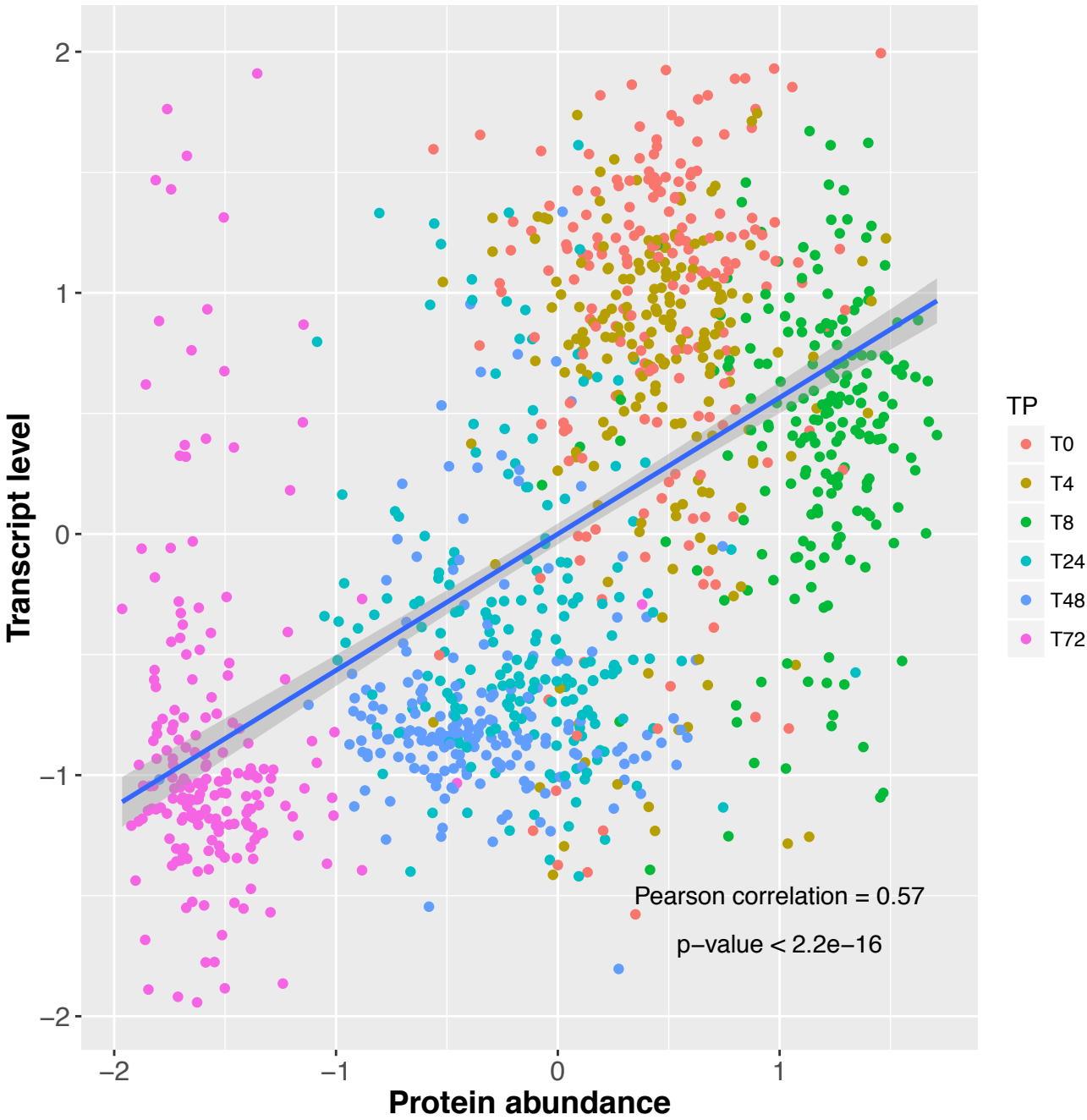

Supplemental Figure 6

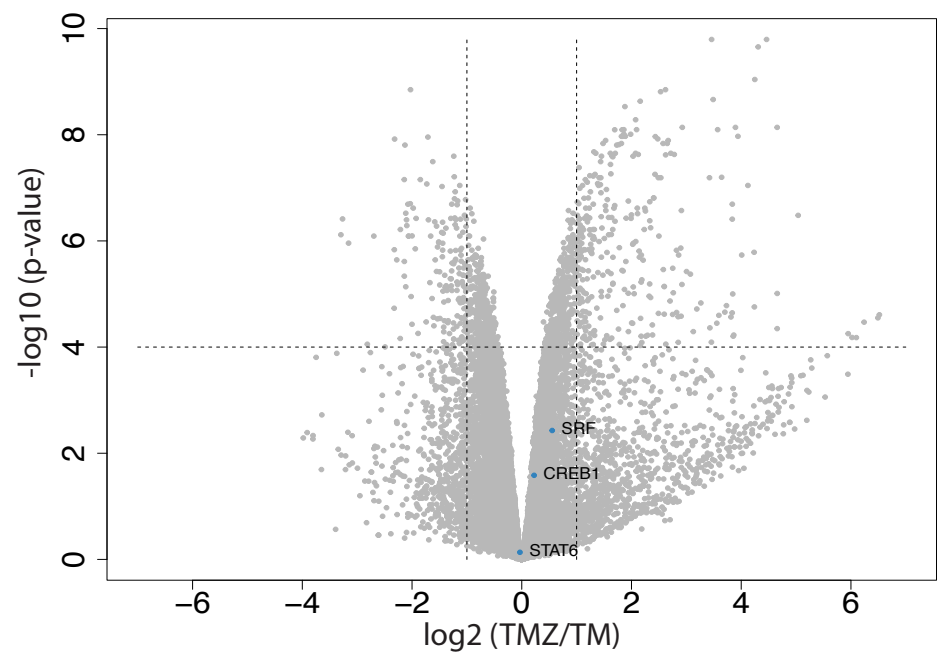

Supplement: Supplemental Data [file 10.1074_M115.055376_mcp.M115.055376-1.pdf]

Supplemental Figure 3

A.

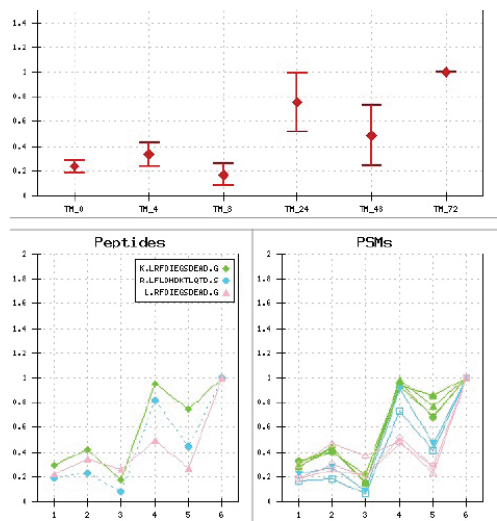

B.

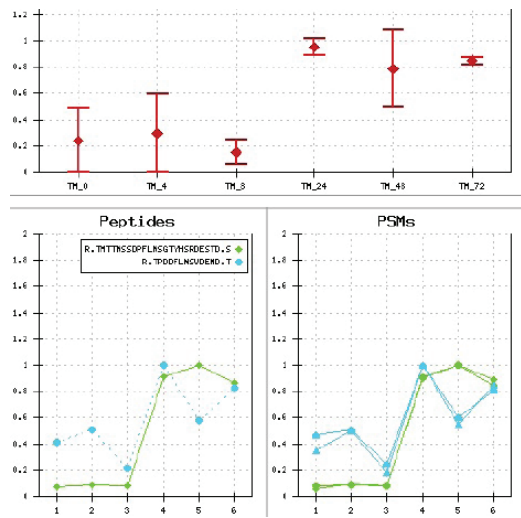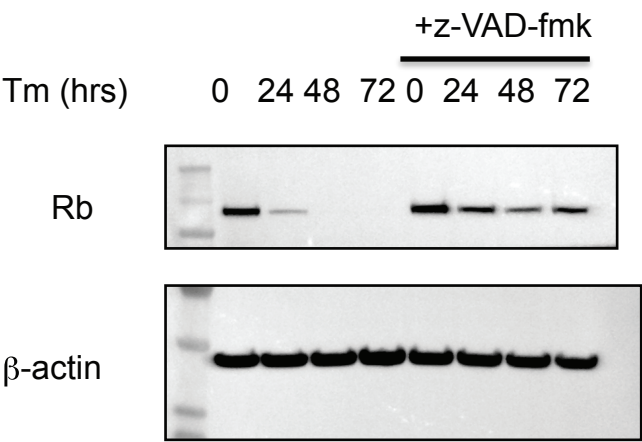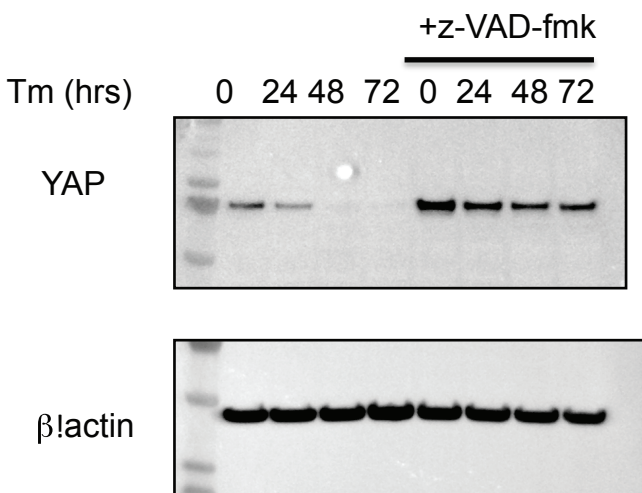

Supplement: Supplemental Data [file 10.1074_M115.055376_mcp.M115.055376-3.pdf]

Supplemental Figure 4

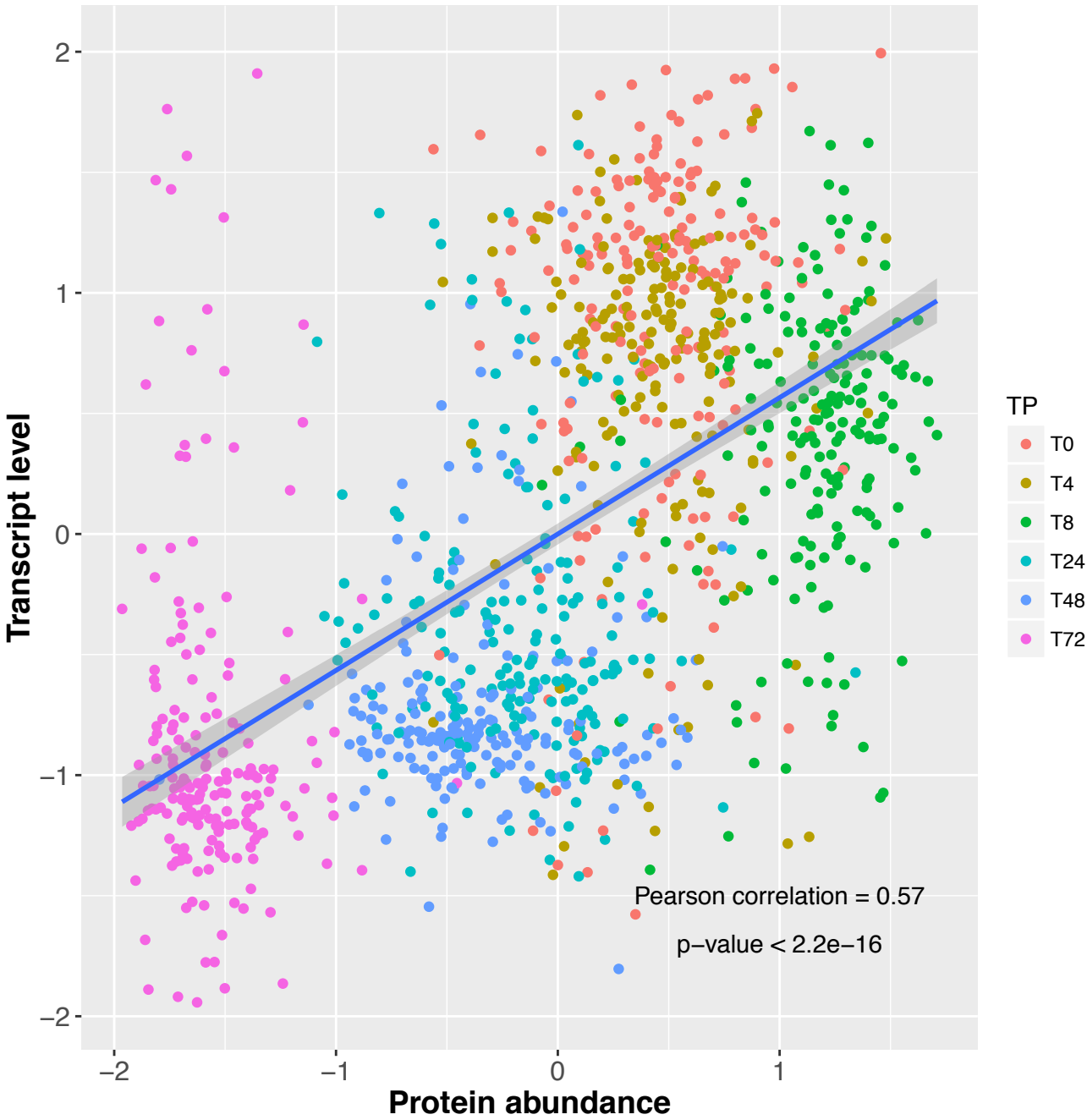

Supplement: Supplemental Data [file 10.1074_M115.055376_mcp.M115.055376-4.pdf]

Supplemental Figure 6

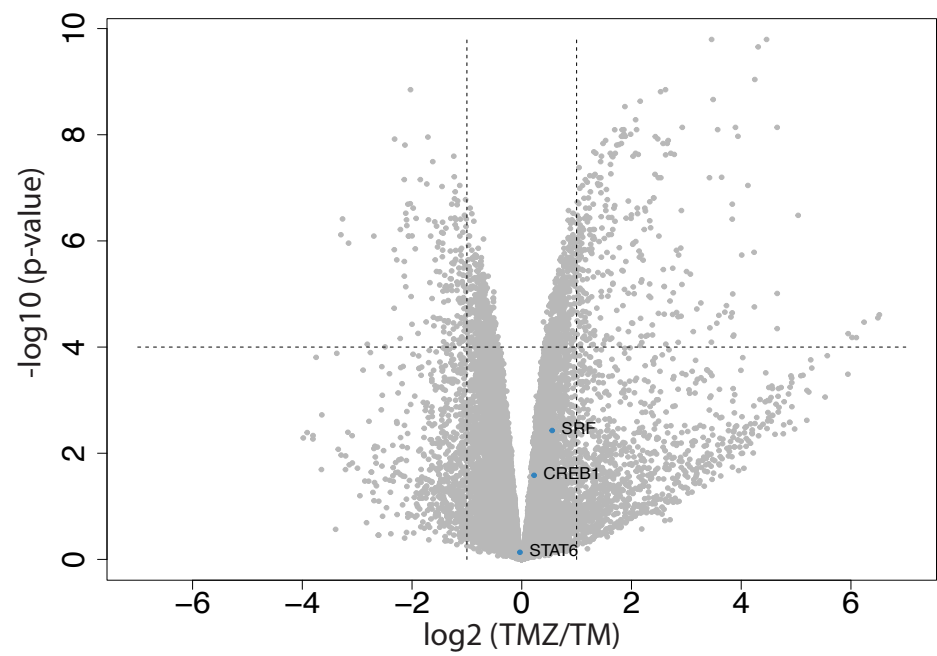

Supplement: Supplemental Data [file 10.1074_M115.055376_mcp.M115.055376-6.pdf]
