# Supplementary material for: Uncovering a Dual Regulatory Role for Caspases During Endoplasmic Reticulum Stress-induced Cell Death
Source: Mol Cell Proteomics. 2016 Apr 28;15(7):2293–307. doi: 10.1074/mcp.M115.055376 (PMC4937505; doi:10.1074/mcp.M115.055376)
Supplement: Supplemental Data [file 10.1074_M115.055376_mcp.M115.055376-5.pdf]

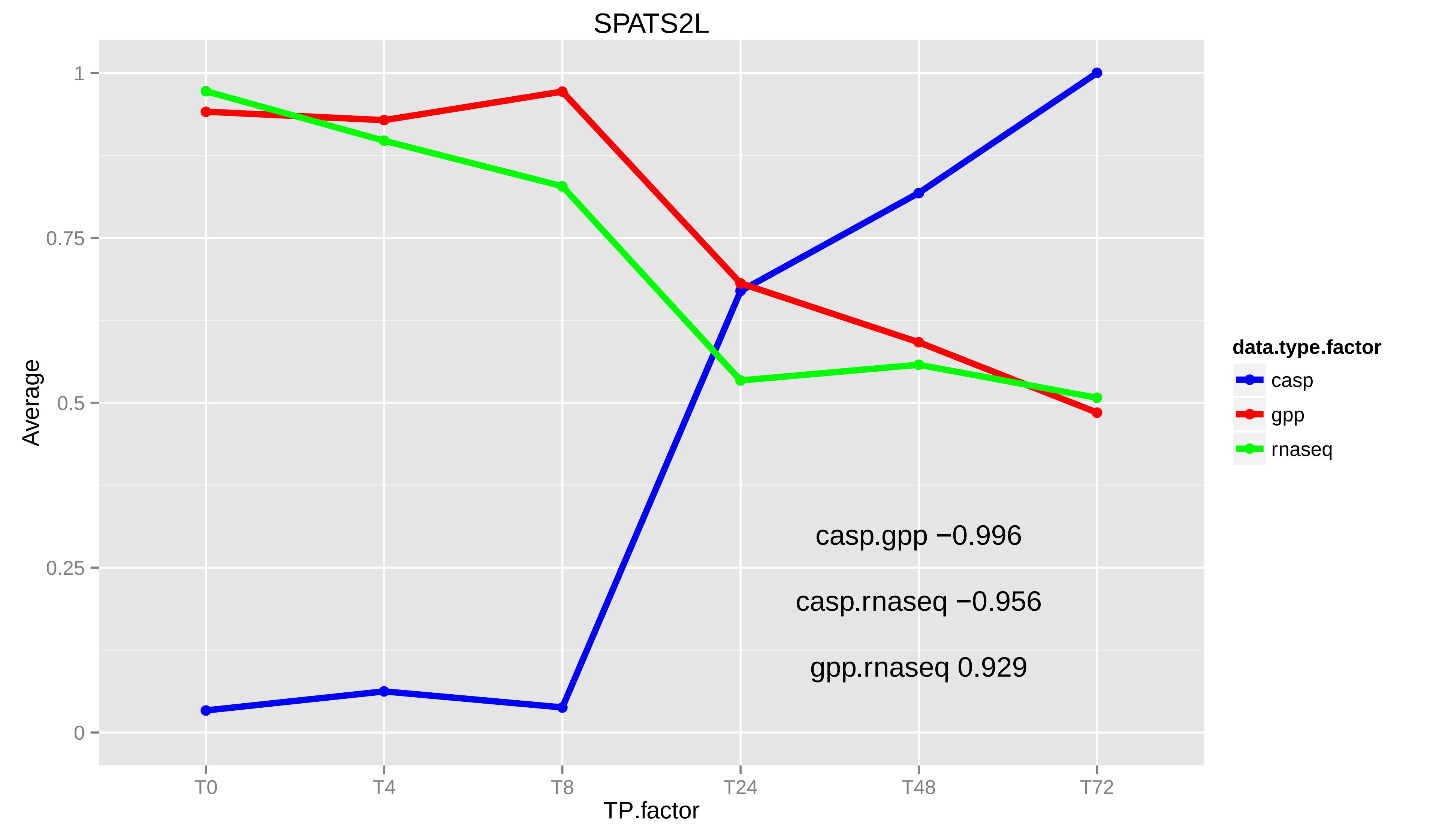
SPATS2L
Average
1
0.75
0.5
0.25
0
T0
T4
T8
T24
T48
T72
TP.factor
data.type.factor
casp
gpp
rnaseq
casp.gpp −0.996
casp.rnaseq −0.956
gpp.rnaseq 0.929

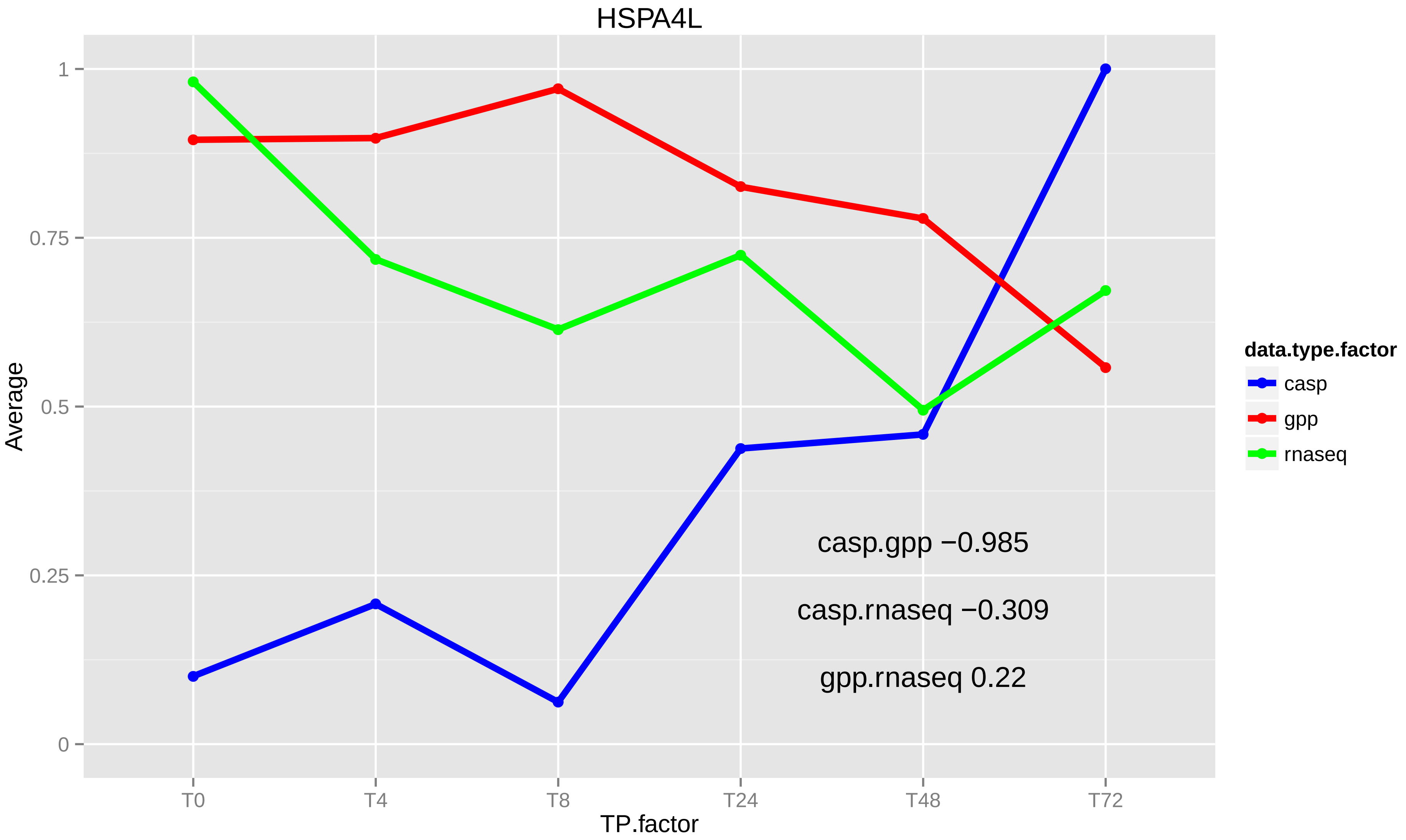

HSPA4L
Average
1
0.75
0.5
0.25
0
T0
T4
T8
T24
T48
T72
TP.factor
data.type.factor
casp
gpp
rnaseq
casp.gpp −0.985
casp.rnaseq −0.309
gpp.rnaseq 0.22

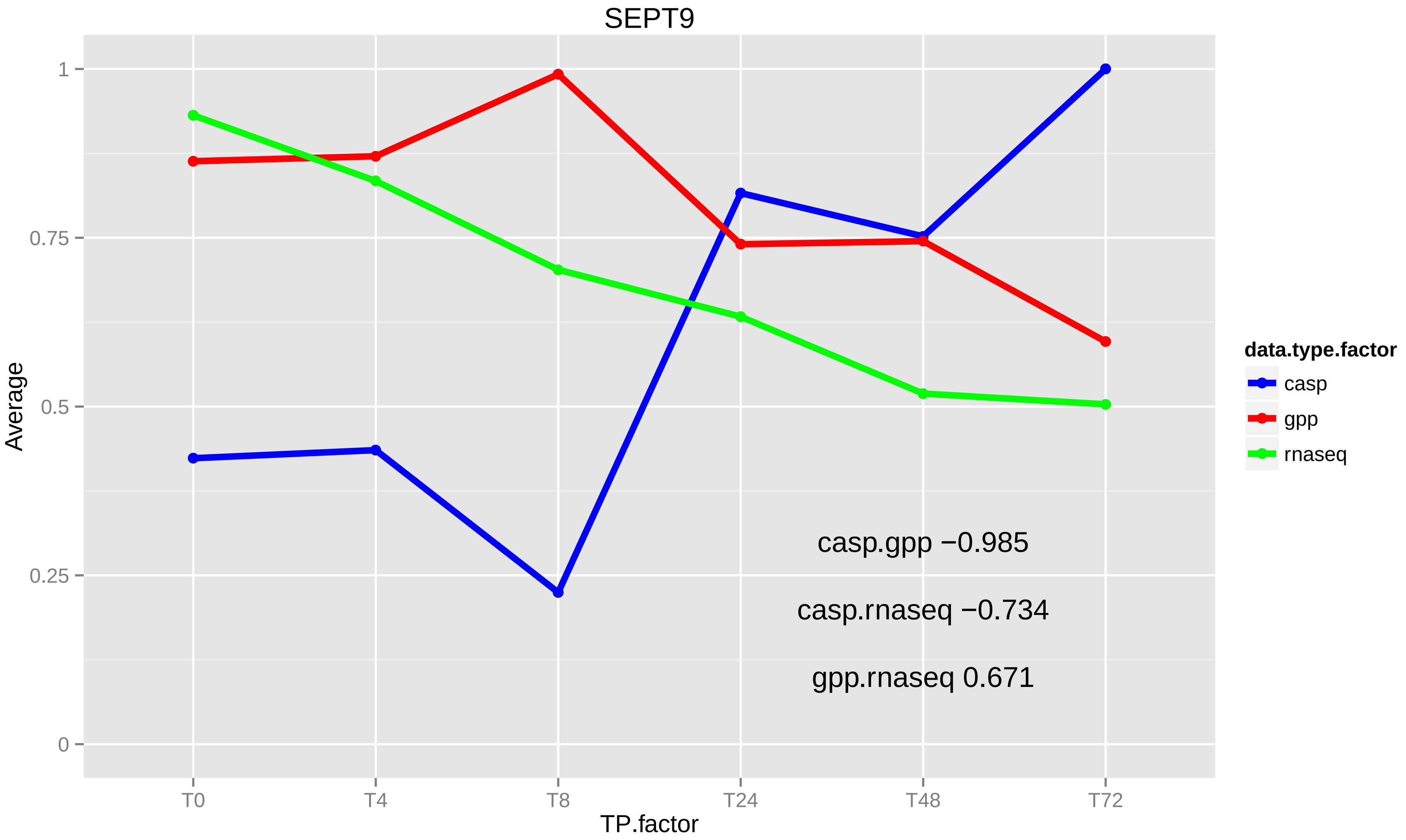

SEPT9
1
0.75
0.5
0.25
0
Average
T0
T4
T8
T24
T48
T72
TP.factor
data.type.factor
casp
gpp
rnaseq
casp.gpp −0.985
casp.rnaseq −0.734
gpp.rnaseq 0.671

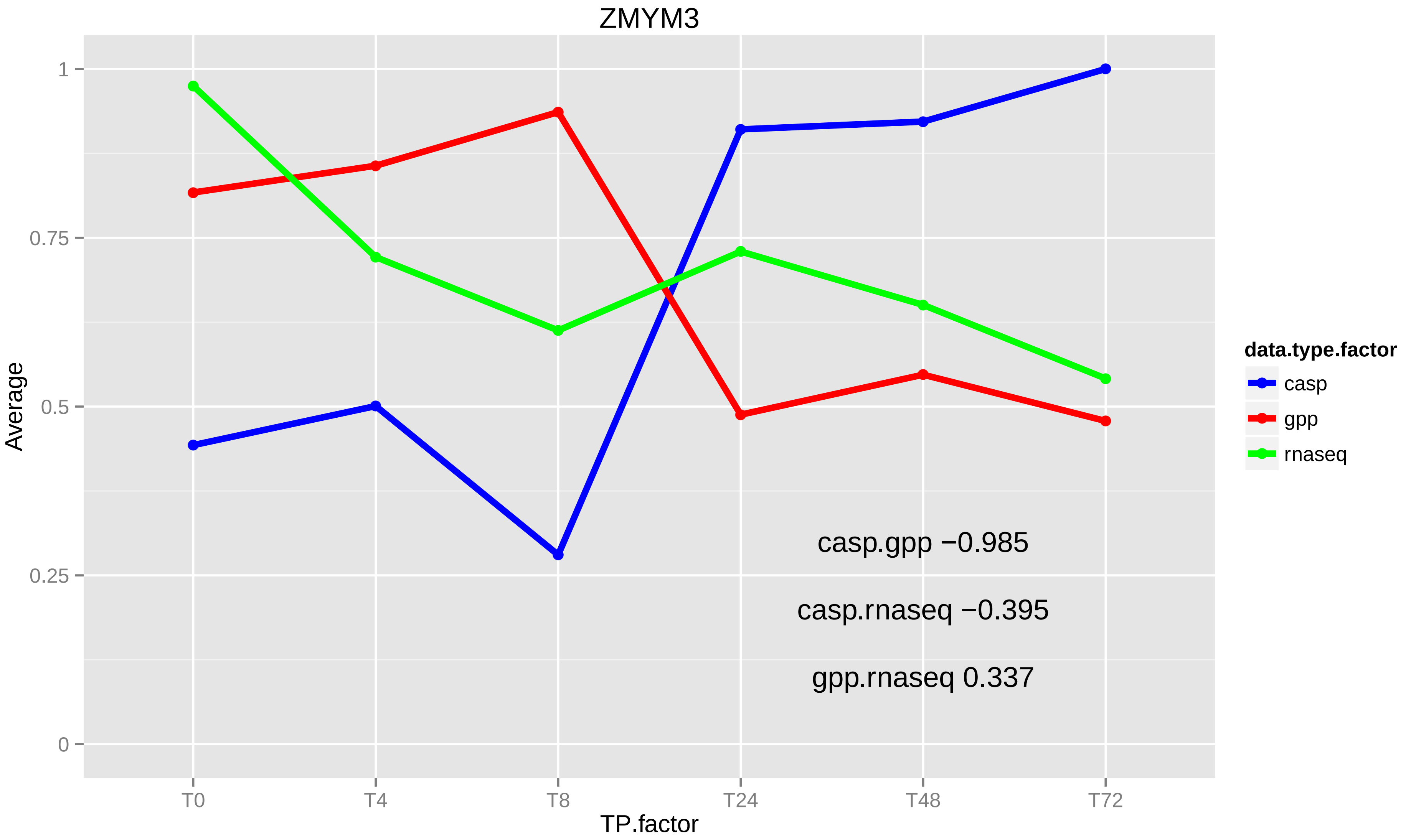
ZMYM3
Average
1
0.75
0.5
0.25
0
T0
T4
T8
T24
T48
T72
TP.factor
data.type.factor
casp
gpp
rnaseq
casp.gpp −0.985
casp.rnaseq −0.395
gpp.rnaseq 0.337

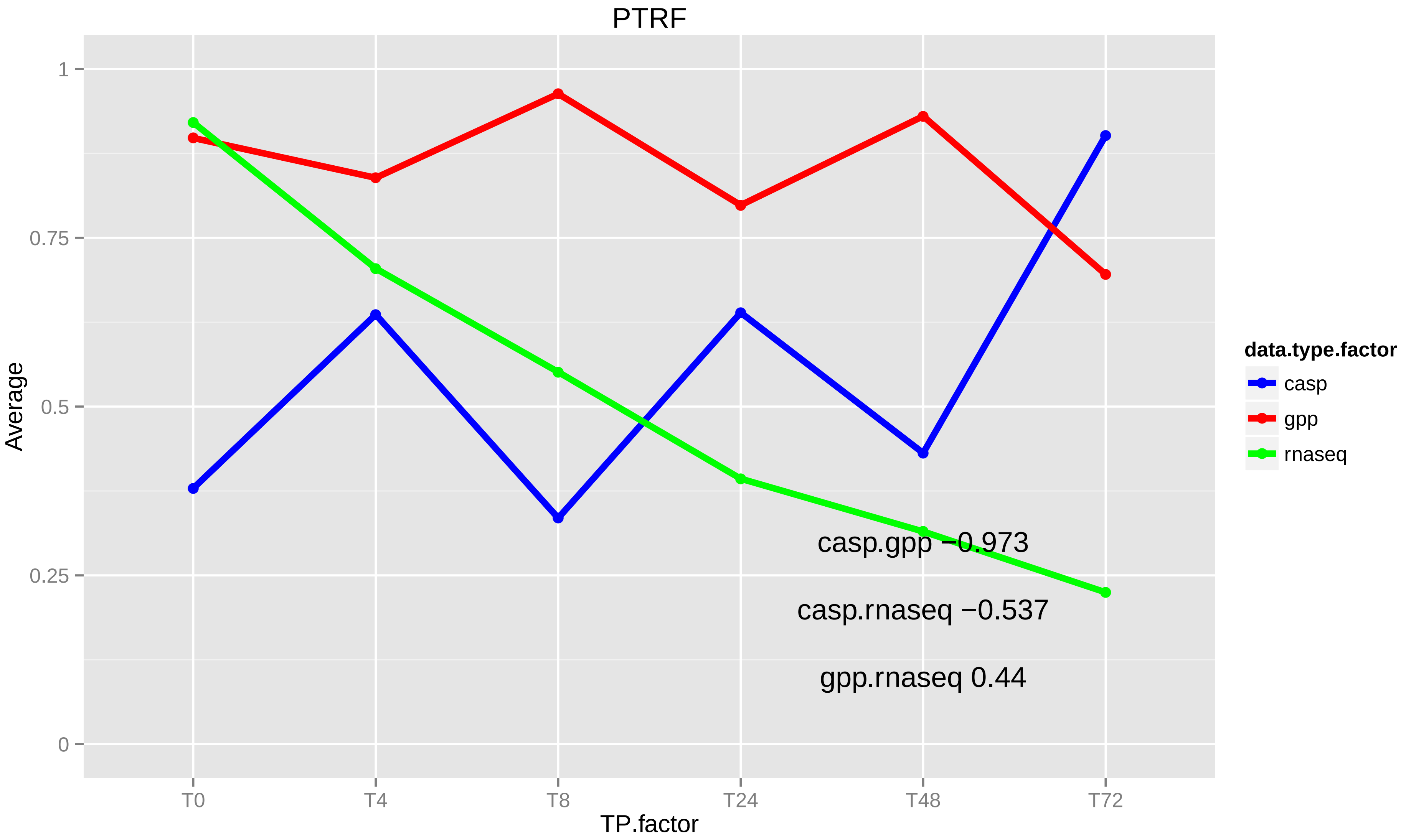

PTRF
Average
TP.factor
T0
T4
T8
T24
T48
T72
0
0.25
0.5
0.75
1
casp.gpp −0.973
casp.rnaseq −0.537
gpp.rnaseq 0.44
data.type.factor
casp
gpp
rnaseq

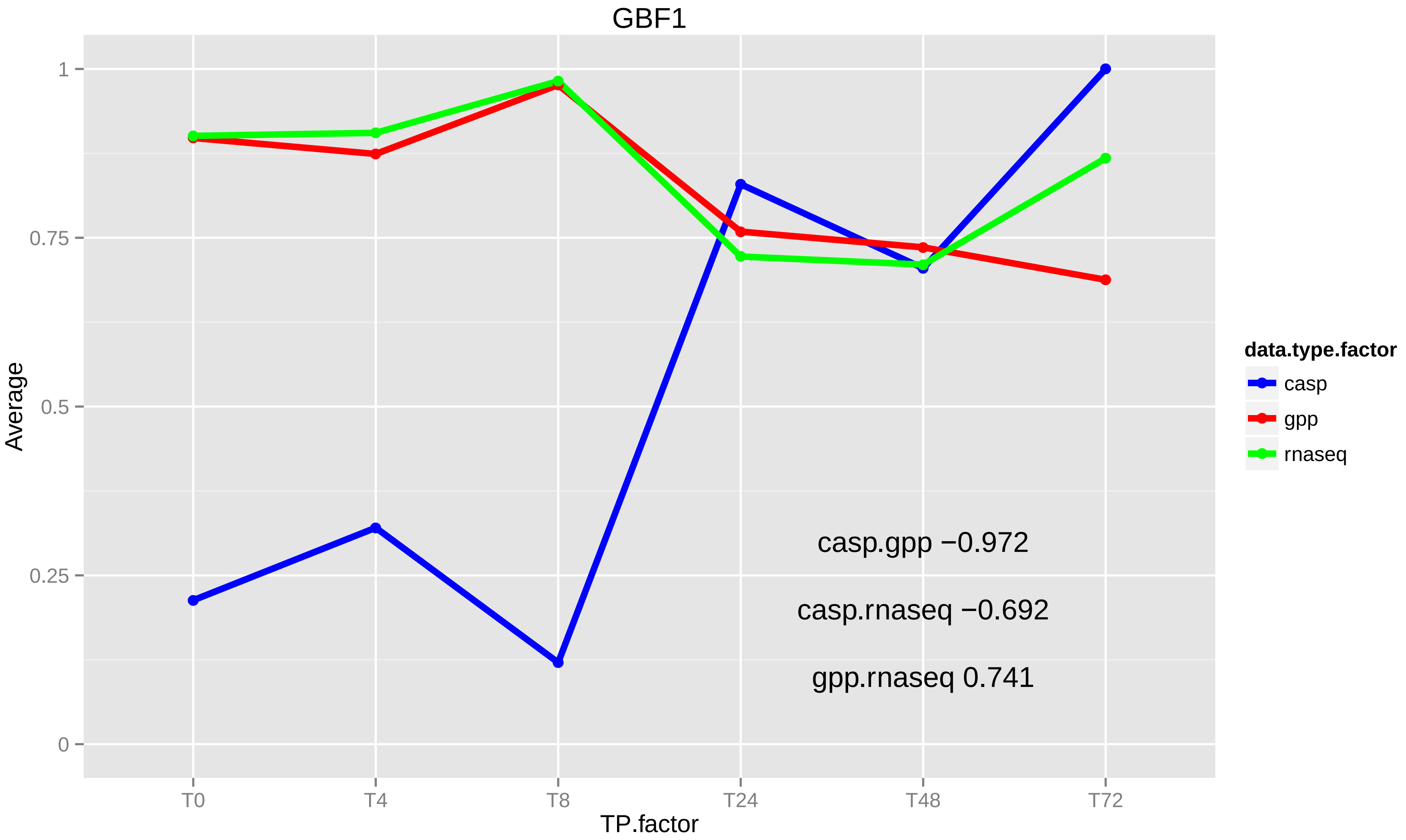

GBF1
1
0.75
0.5
0.25
0
Average
T0
T4
T8
T24
T48
T72
TP.factor
data.type.factor
casp
gpp
rnaseq
casp.gpp −0.972
casp.rnaseq −0.692
gpp.rnaseq 0.741

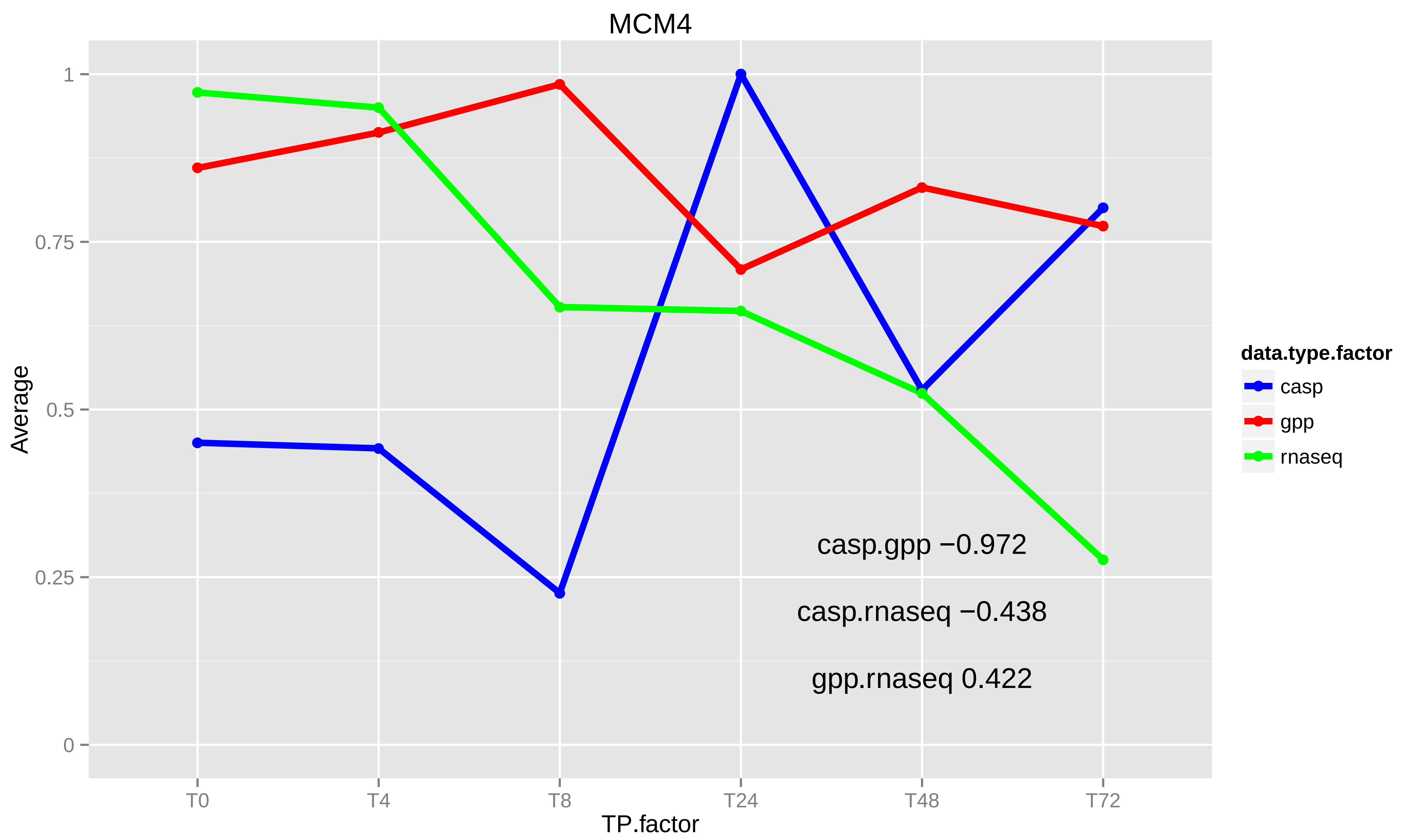
MCM4
Average
1
0.75
0.5
0.25
0
T0
T4
T8
T24
T48
T72
TP.factor
data.type.factor
casp
gpp
rnaseq
casp.gpp −0.972
casp.rnaseq −0.438
gpp.rnaseq 0.422

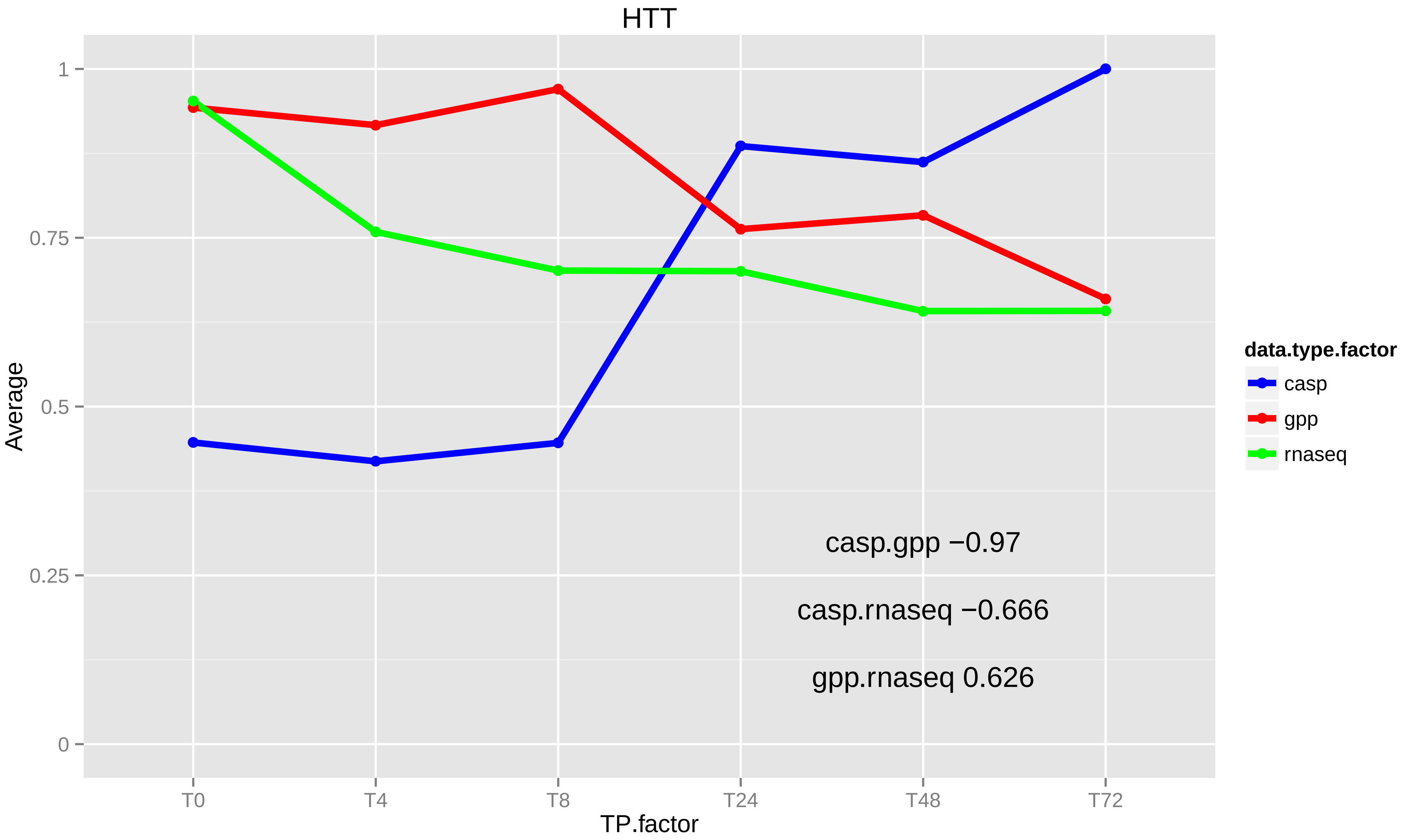

HTT
1
0.75
0.5
0.25
0
Average
T0
T4
T8
T24
T48
T72
TP.factor
data.type.factor
casp
gpp
rnaseq
casp.gpp −0.97
casp.rnaseq −0.666
gpp.rnaseq 0.626

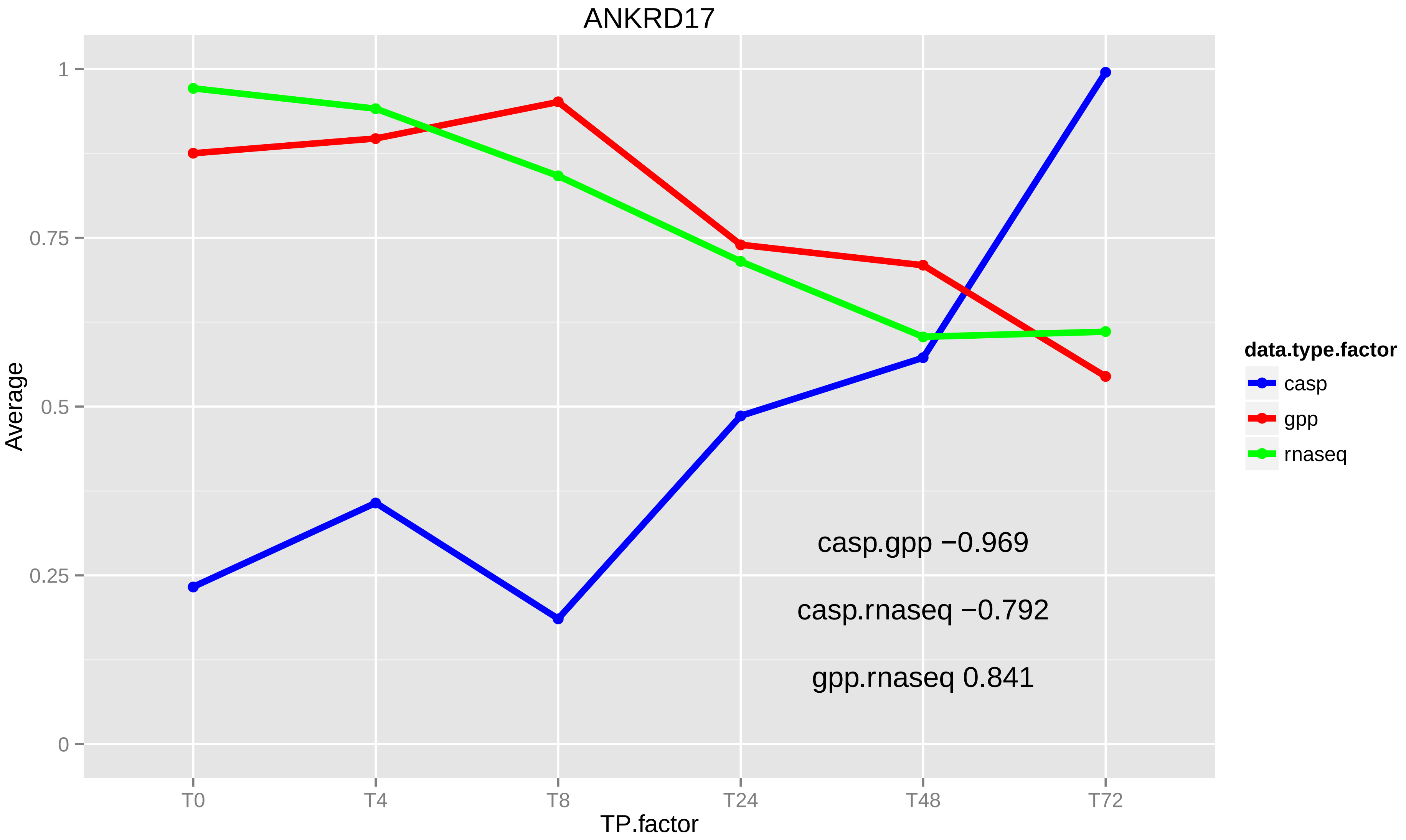

ANKRD17
Average
1
0.75
0.5
0.25
0
T0
T4
T8
T24
T48
T72
TP.factor
data.type.factor
casp
gpp
rnaseq
casp.gpp −0.969
casp.rnaseq −0.792
gpp.rnaseq 0.841

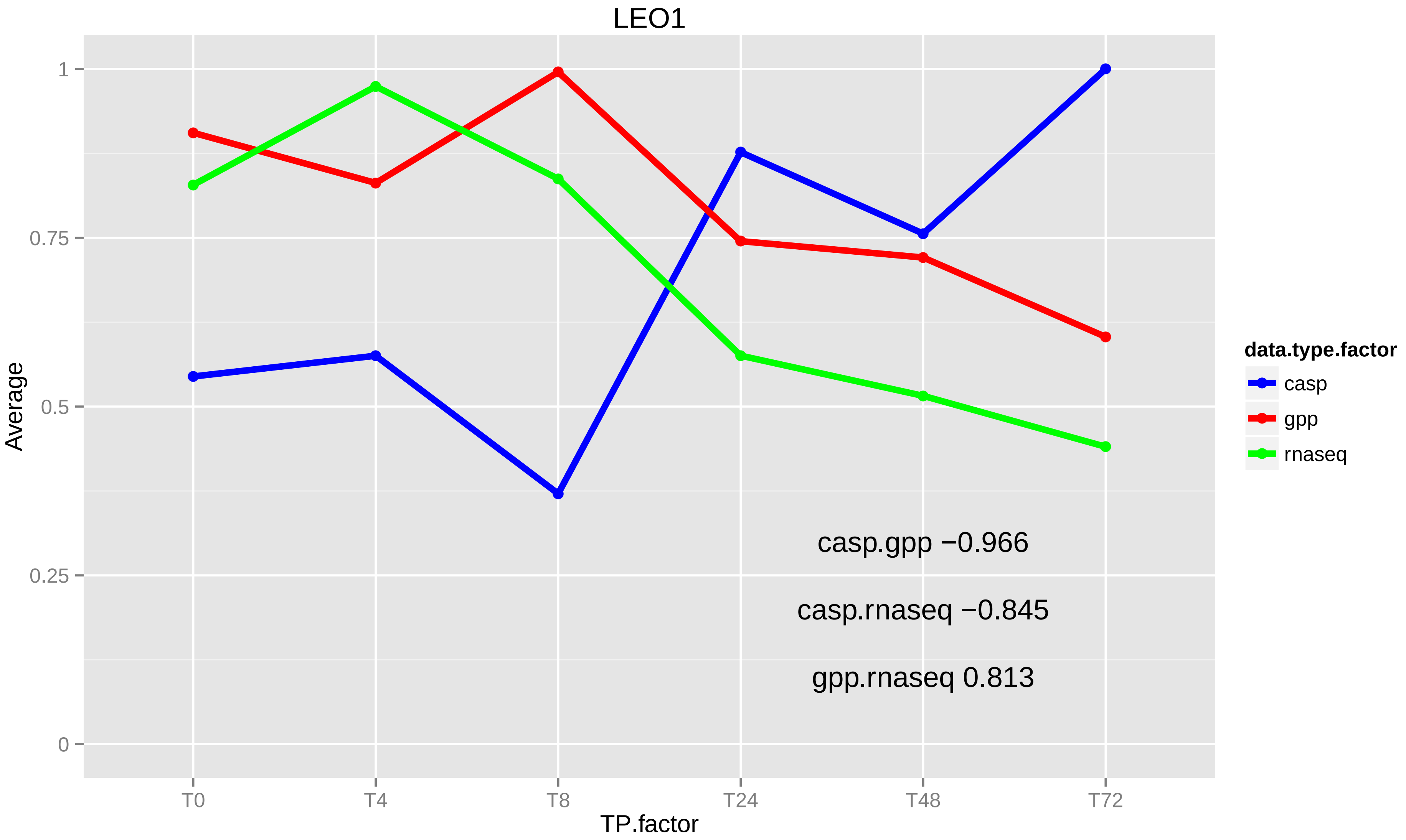
LEO1
Average
1
0.75
0.5
0.25
0
T0
T4
T8
T24
T48
T72
TP.factor
data.type.factor
casp
gpp
rnaseq
casp.gpp −0.966
casp.rnaseq −0.845
gpp.rnaseq 0.813

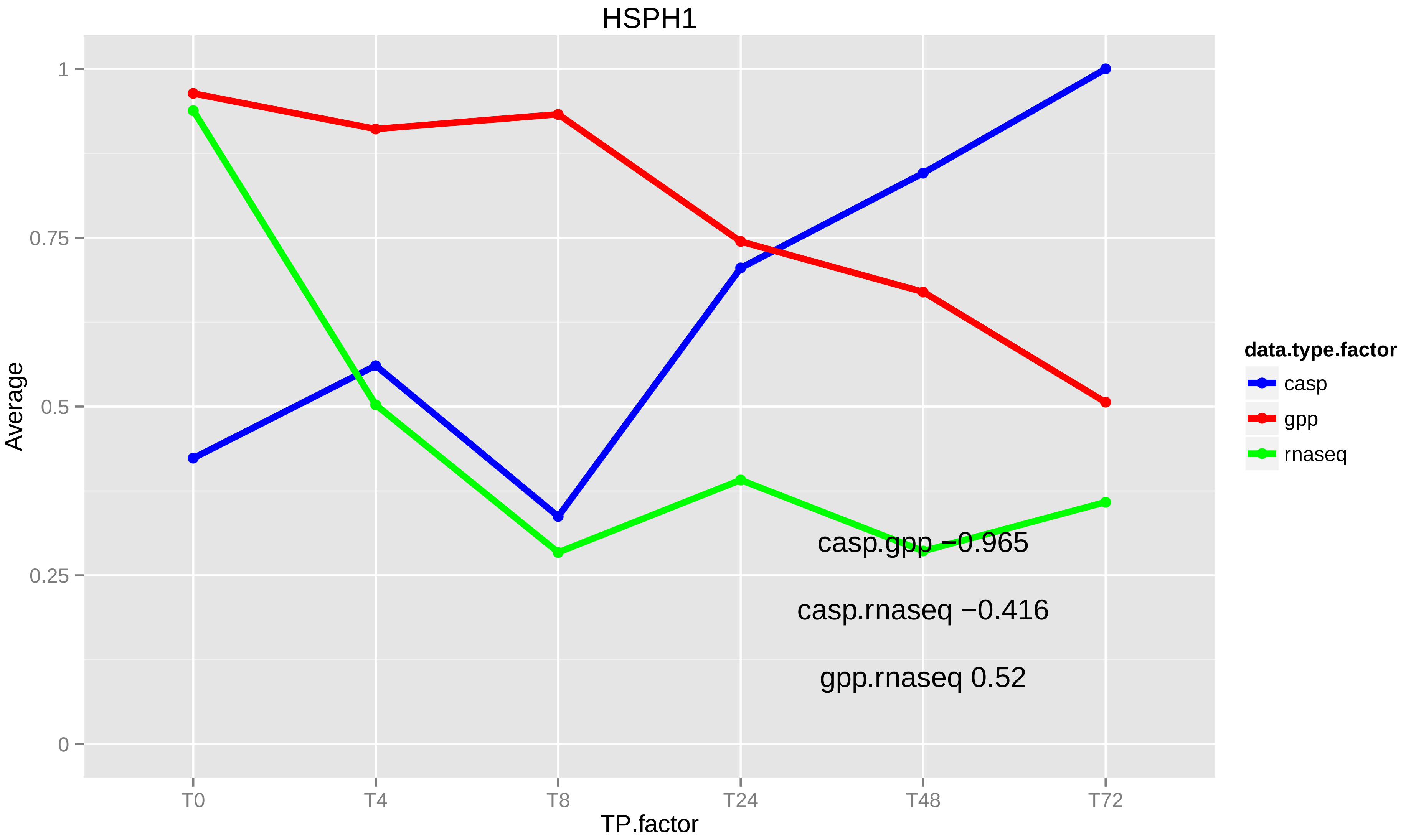
HSPH1
1
0.75
0.5
0.25
0
Average
T0
T4
T8
T24
T48
T72
TP.factor
data.type.factor
casp
gpp
rnaseq
casp.gpp −0.965
casp.rnaseq −0.416
gpp.rnaseq 0.52

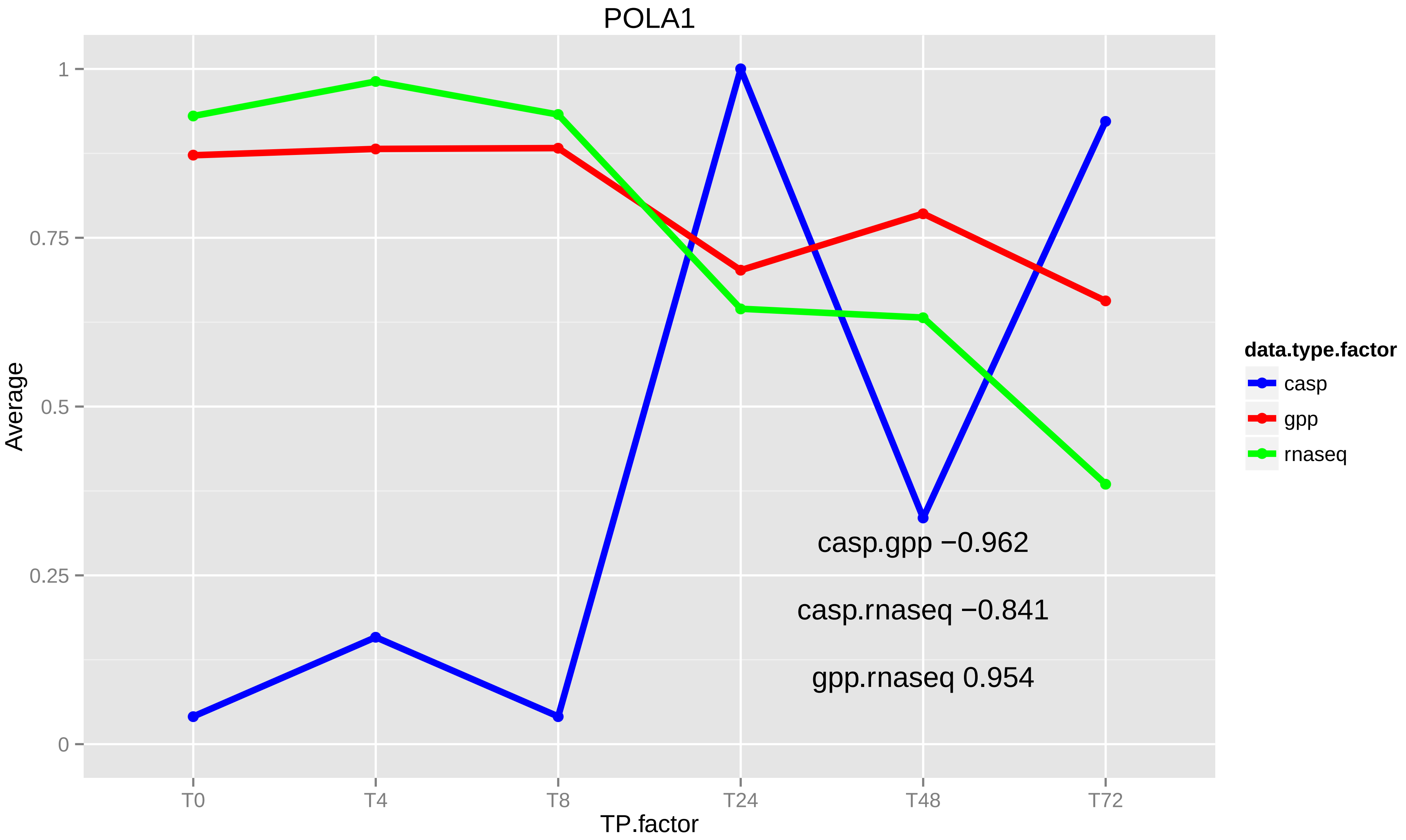
POLA1
Average
1
0.75
0.5
0.25
0
T0
T4
T8
T24
T48
T72
TP.factor
data.type.factor
casp
gpp
rnaseq
casp.gpp −0.962
casp.rnaseq −0.841
gpp.rnaseq 0.954

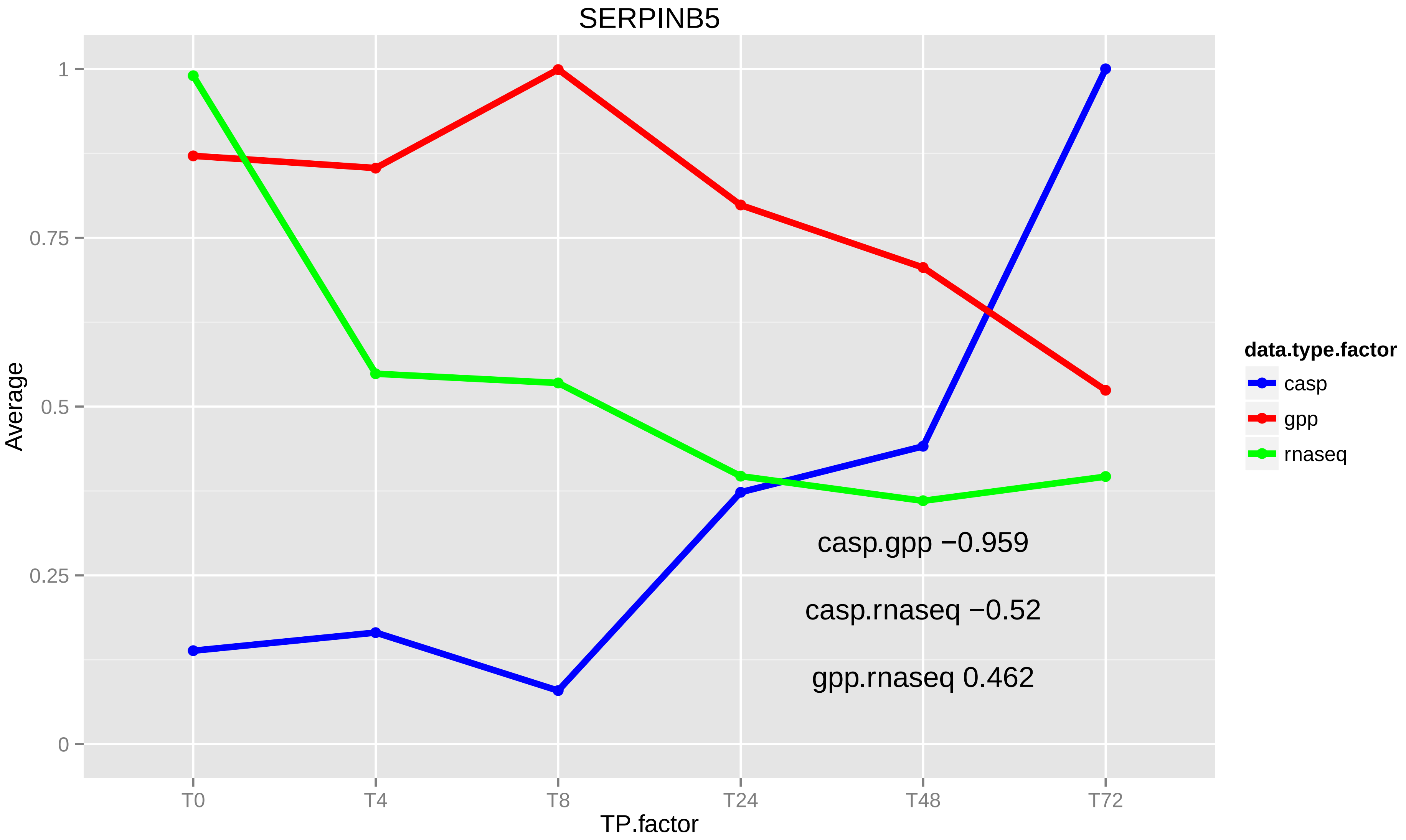
SERPINB5
Average
1
0.75
0.5
0.25
0
T0
T4
T8
T24
T48
T72
TP.factor
data.type.factor
casp
gpp
rnaseq
casp.gpp −0.959
casp.rnaseq −0.52
gpp.rnaseq 0.462

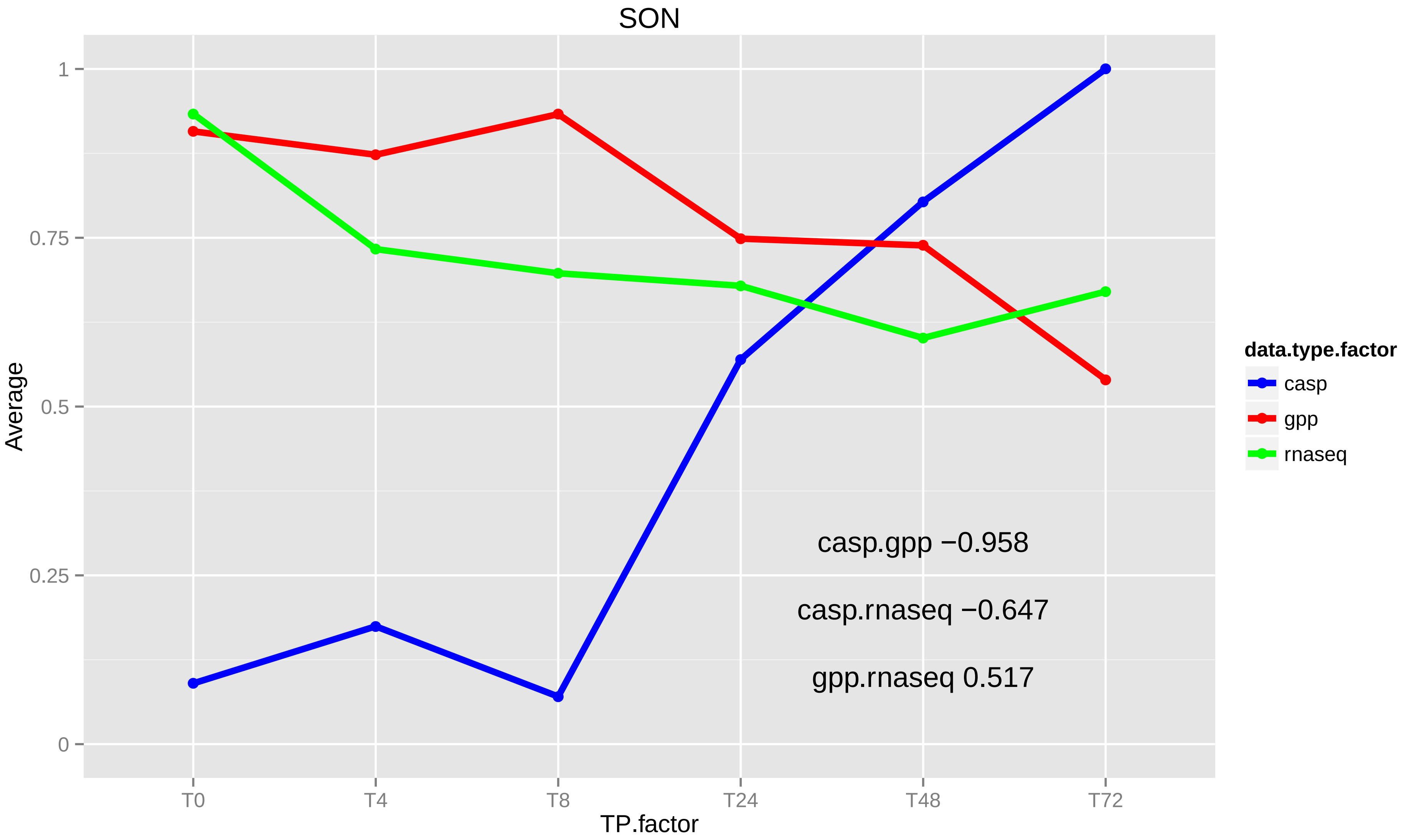

SON
1
0.75
0.5
0.25
0
Average
T0
T4
T8
T24
T48
T72
TP.factor
data.type.factor
casp
gpp
rnaseq
casp.gpp −0.958
casp.rnaseq −0.647
gpp.rnaseq 0.517

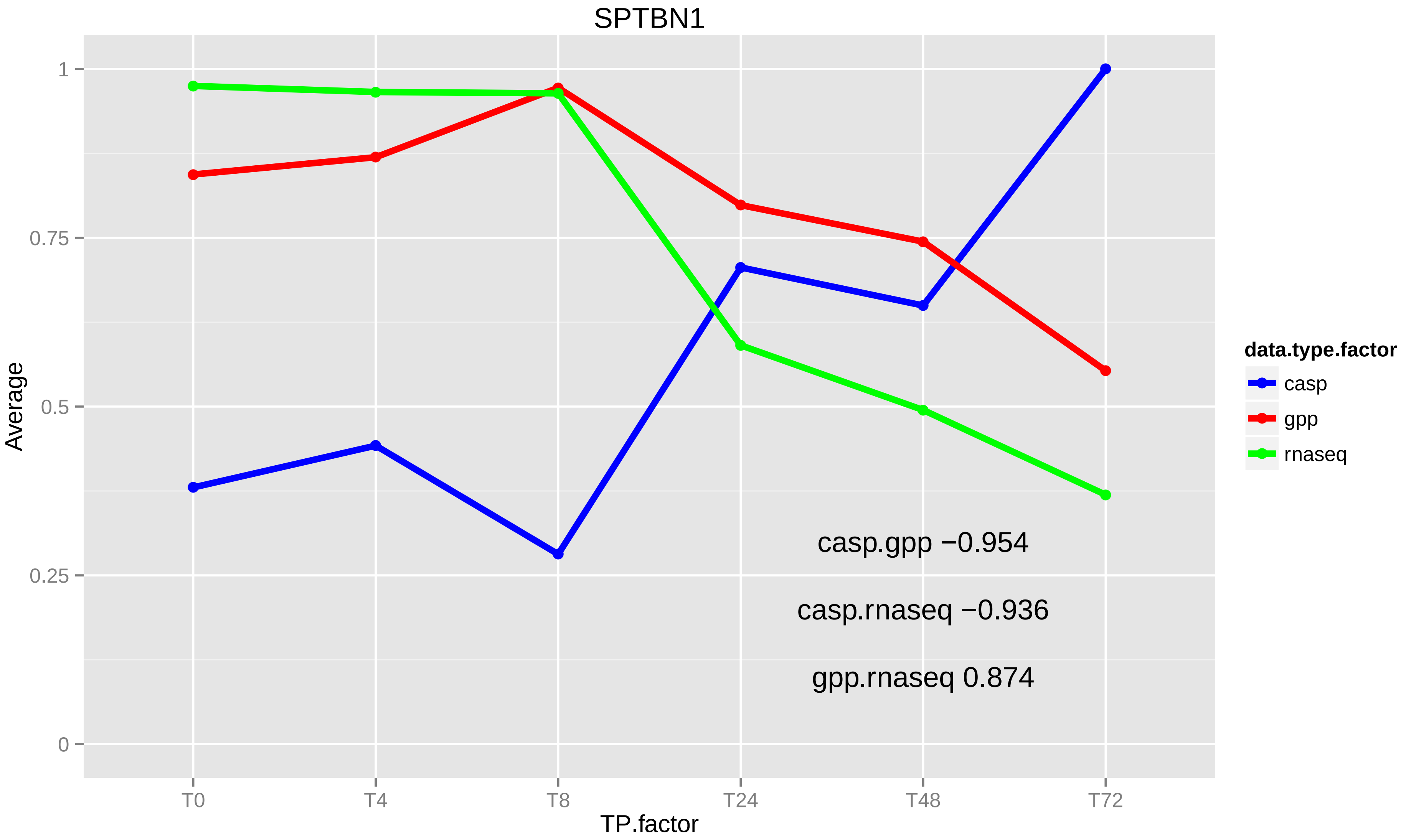
SPTBN1
Average
1
0.75
0.5
0.25
0
T0
T4
T8
T24
T48
T72
TP.factor
data.type.factor
casp
gpp
rnaseq
casp.gpp −0.954
casp.rnaseq −0.936
gpp.rnaseq 0.874

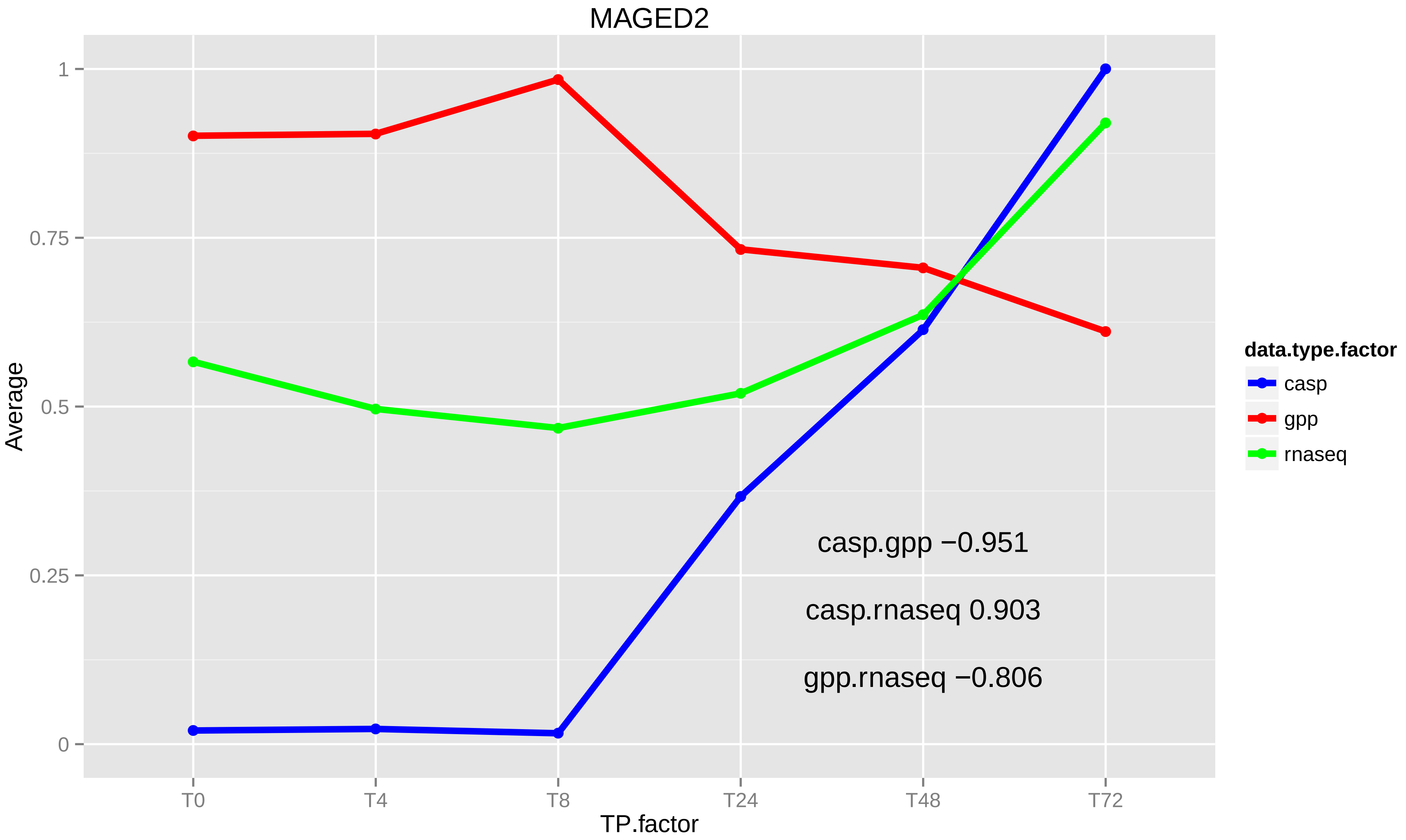
MAGED2
Average
1
0.75
0.5
0.25
0
T0
T4
T8
T24
T48
T72
TP.factor
data.type.factor
casp
gpp
rnaseq
casp.gpp −0.951
casp.rnaseq 0.903
gpp.rnaseq −0.806

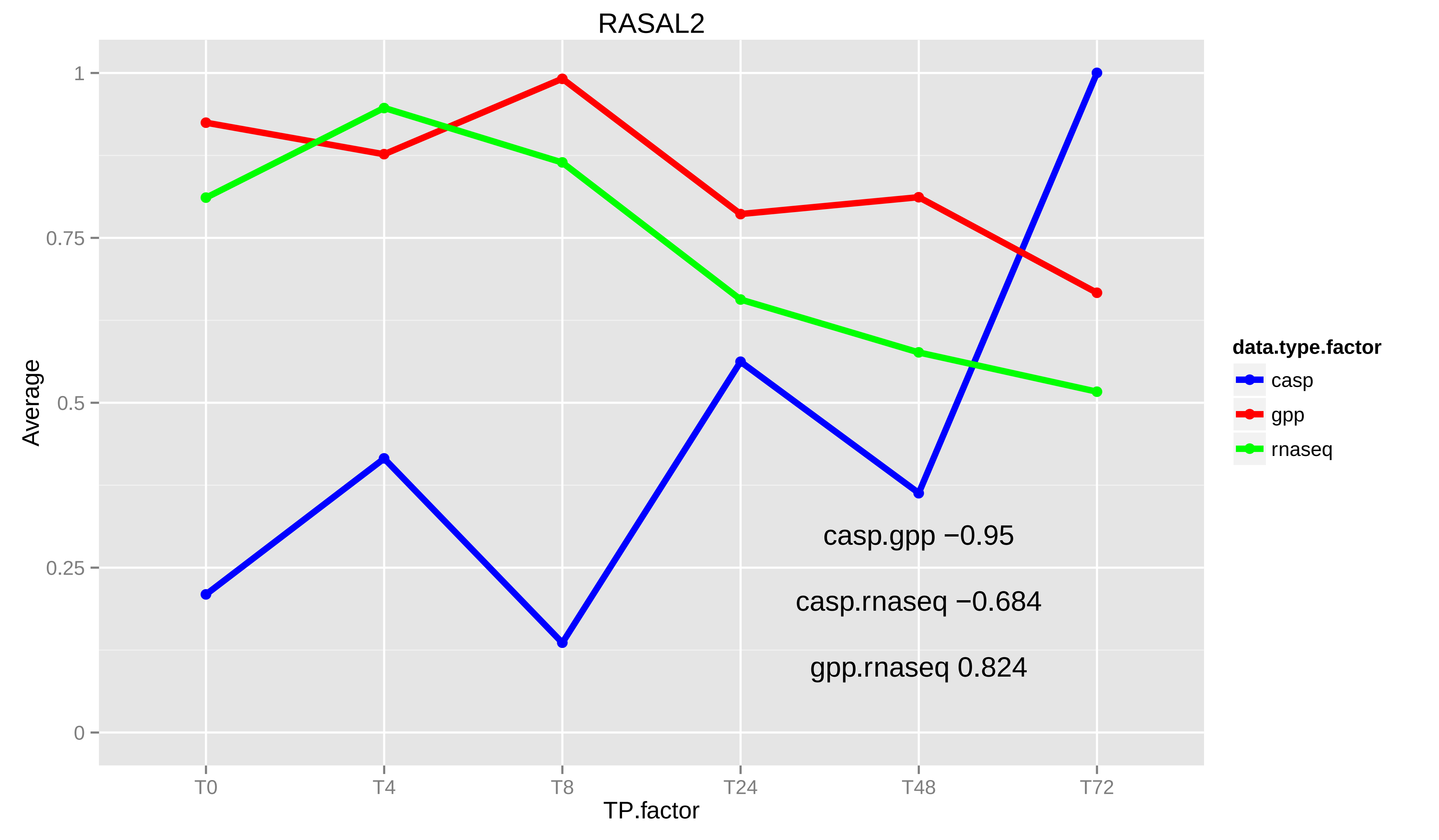
RASAL2
Average
1
0.75
0.5
0.25
0
T0
T4
T8
T24
T48
T72
TP.factor
data.type.factor
casp
gpp
rnaseq
casp.gpp −0.95
casp.rnaseq −0.684
gpp.rnaseq 0.824

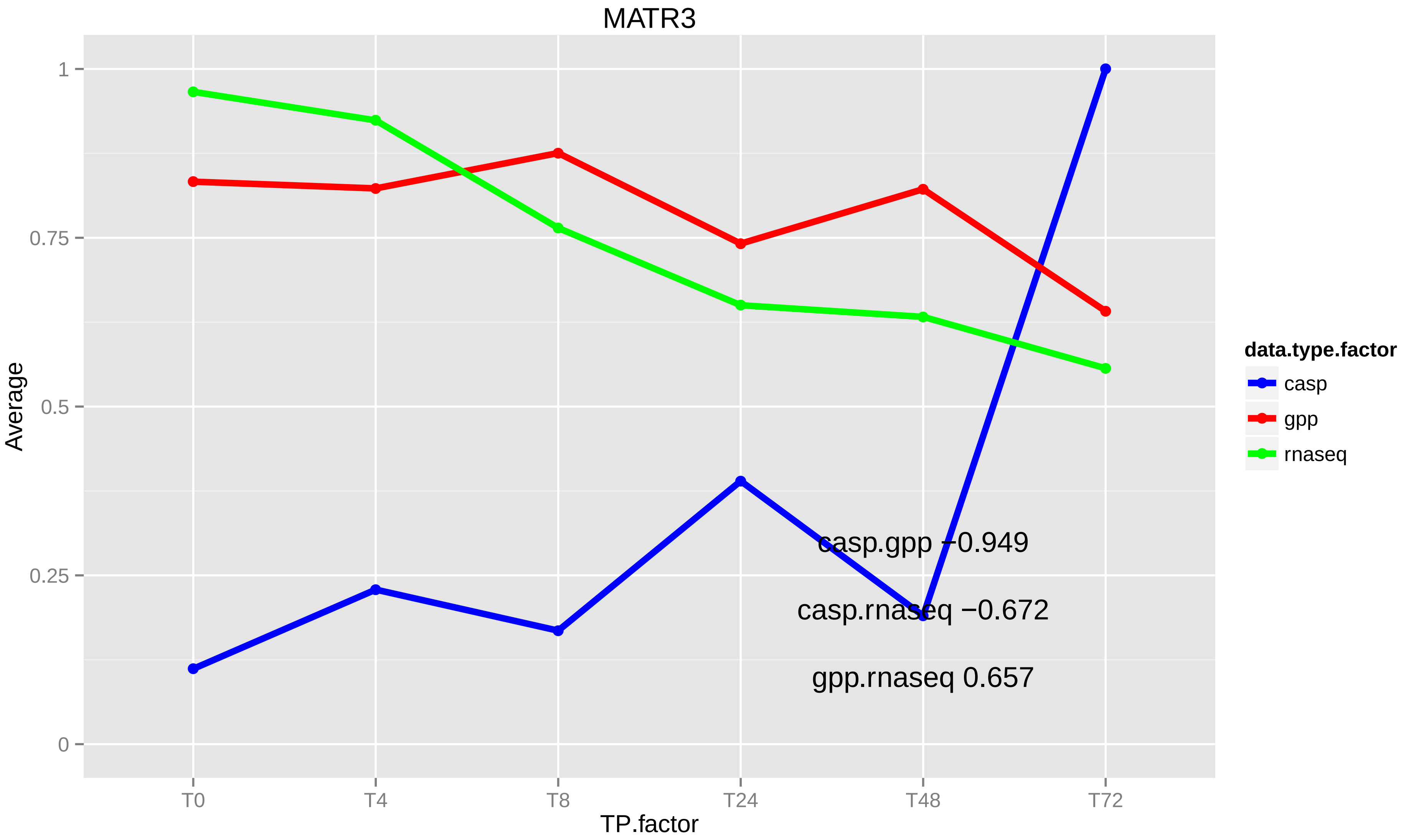
MATR3
Average
1
0.75
0.5
0.25
0
T0
T4
T8
T24
T48
T72
TP.factor
casp.gpp −0.949
casp.rnaseq −0.672
gpp.rnaseq 0.657
data.type.factor
casp
gpp
rnaseq

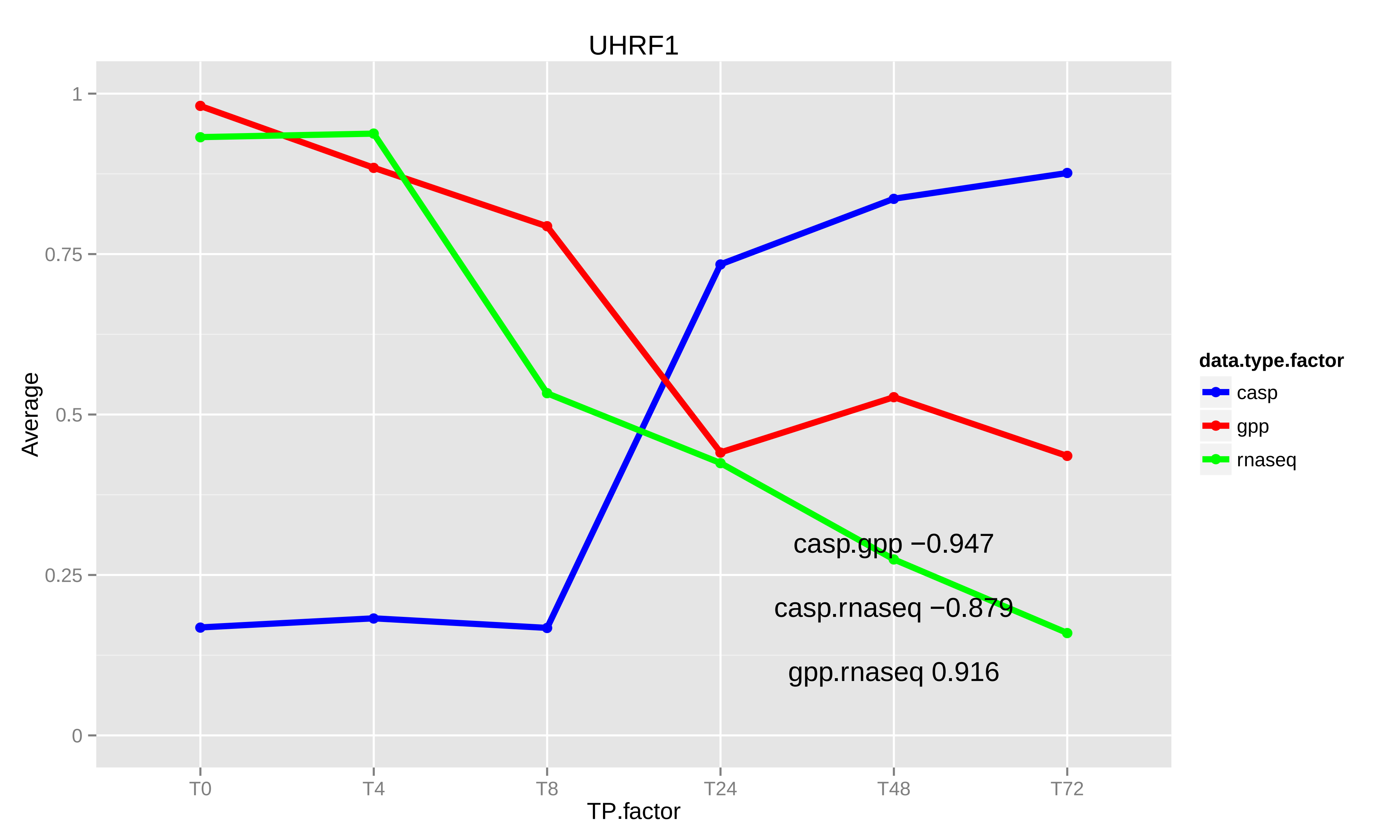

UHRF1
Average
1
0.75
0.5
0.25
0
T0
T4
T8
T24
T48
T72
TP.factor
data.type.factor
casp
gpp
rnaseq
casp.gpp −0.947
casp.rnaseq −0.879
gpp.rnaseq 0.916

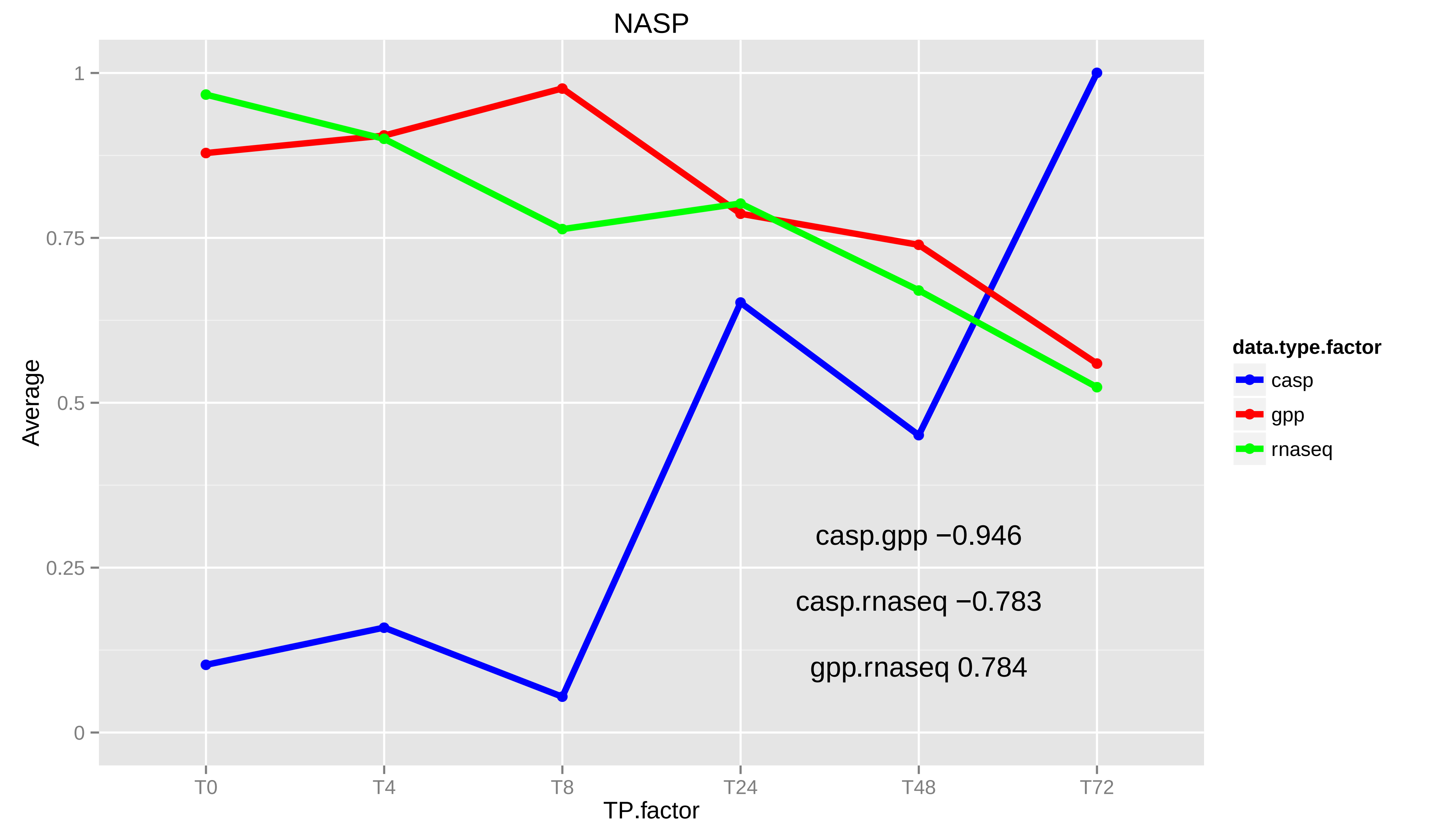
NASP
Average
1
0.75
0.5
0.25
0
T0
T4
T8
T24
T48
T72
TP.factor
data.type.factor
casp
gpp
rnaseq
casp.gpp −0.946
casp.rnaseq −0.783
gpp.rnaseq 0.784

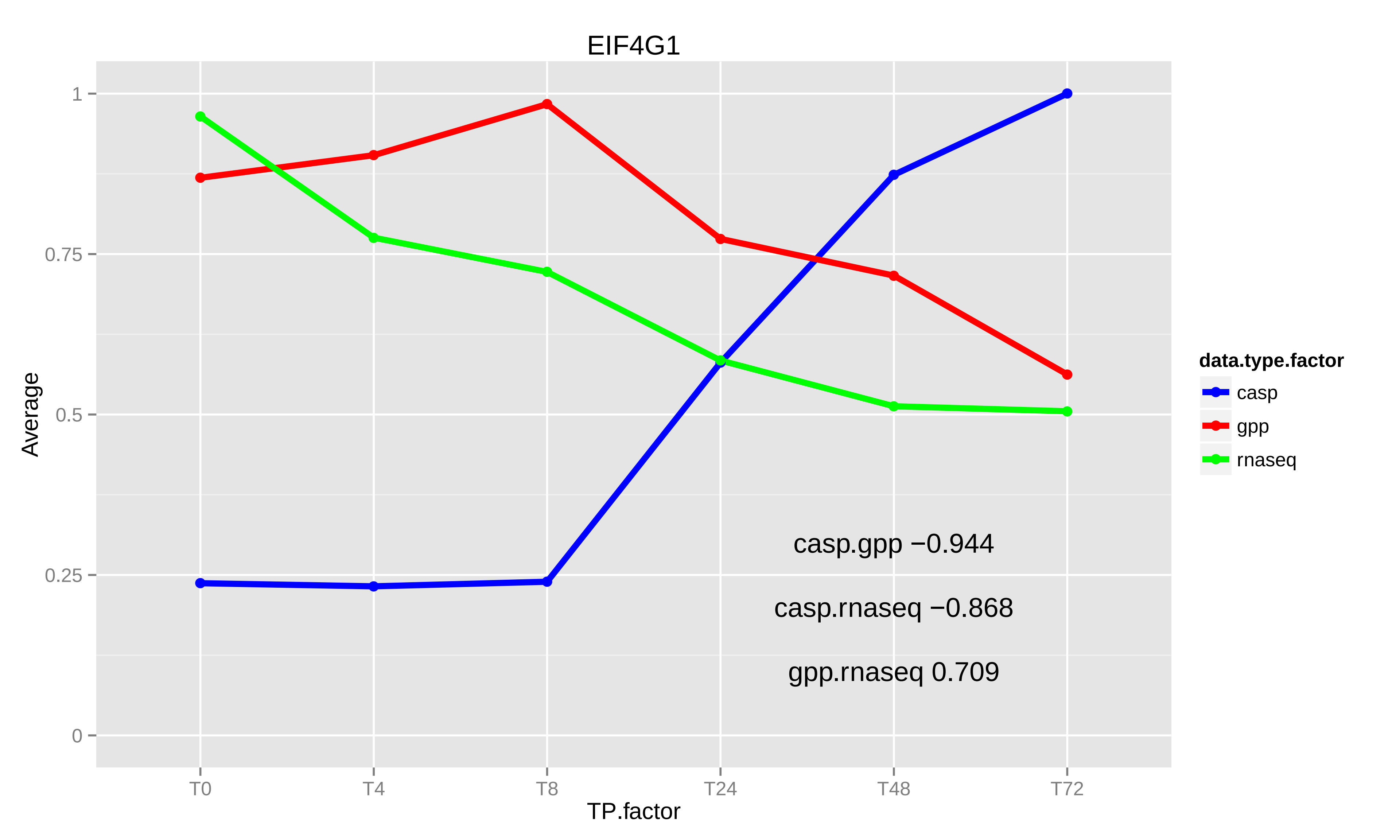

EIF4G1
Average
1
0.75
0.5
0.25
0
T0
T4
T8
T24
T48
T72
TP.factor
data.type.factor
casp
gpp
rnaseq
casp.gpp −0.944
casp.rnaseq −0.868
gpp.rnaseq 0.709

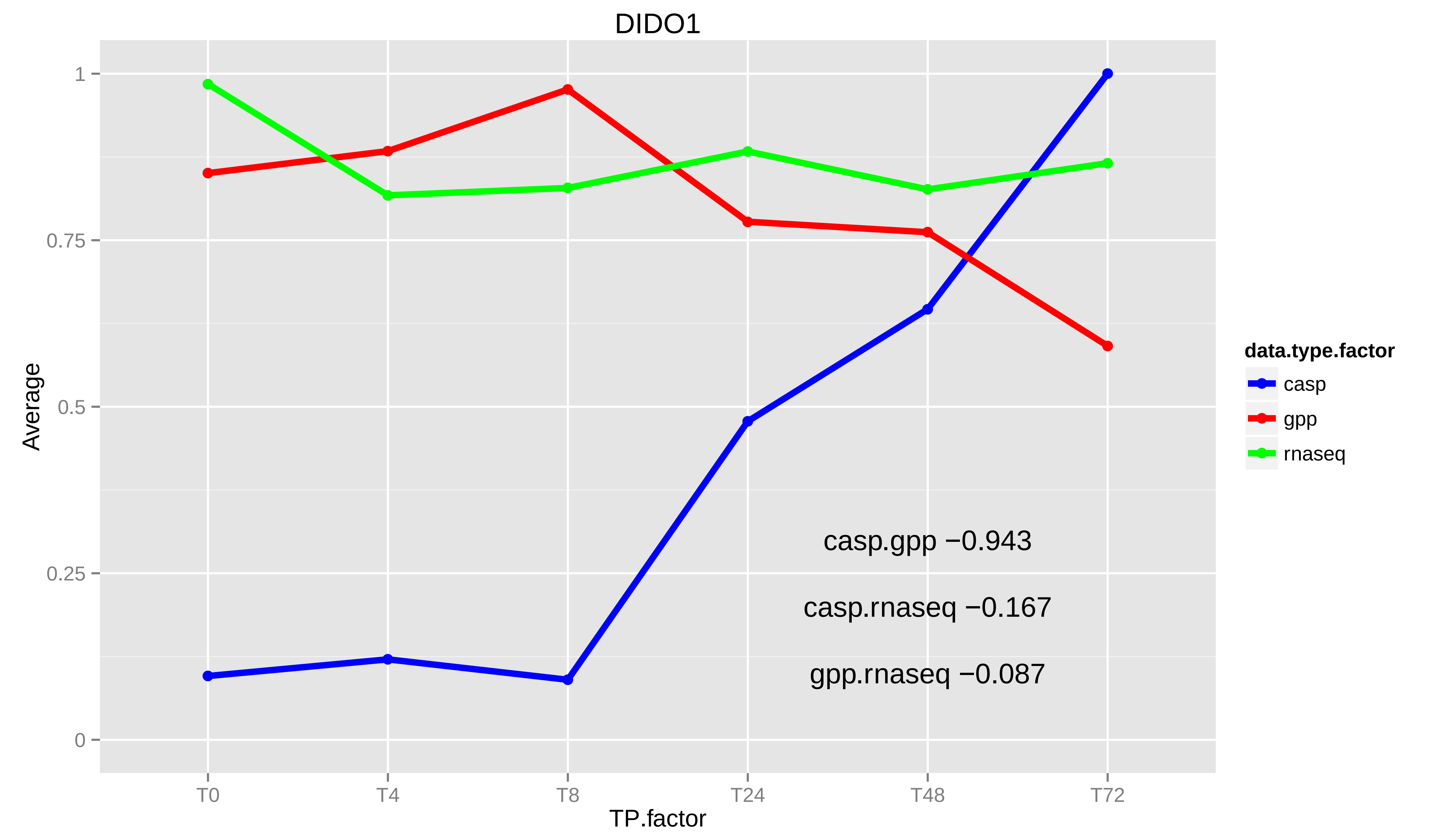

DIDO1
1
0.75
0.5
0.25
0
Average
T0
T4
T8
T24
T48
T72
TP.factor
data.type.factor
casp
gpp
rnaseq
casp.gpp −0.943
casp.rnaseq −0.167
gpp.rnaseq −0.087

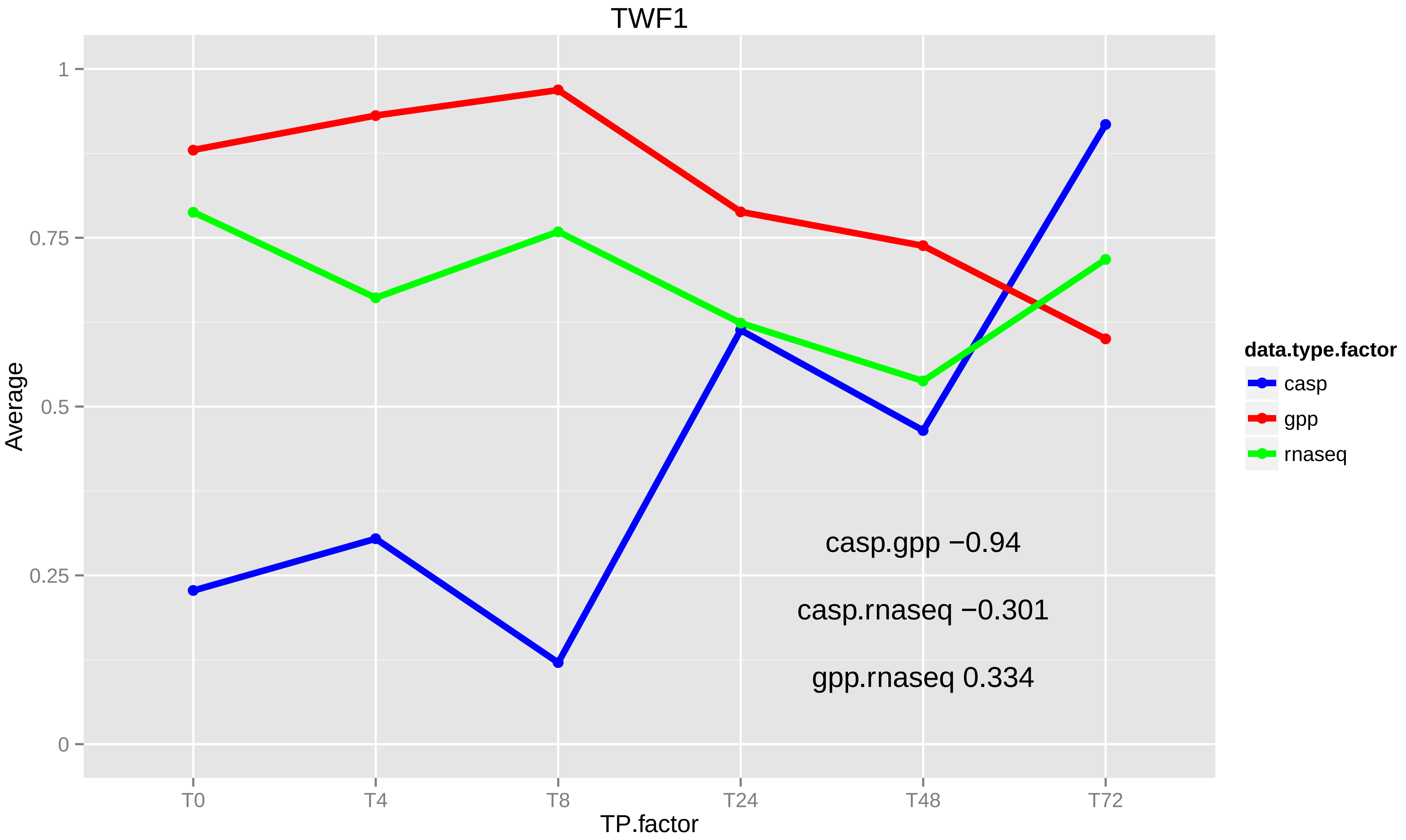
TWF1
Average
1
0.75
0.5
0.25
0
T0
T4
T8
T24
T48
T72
TP.factor
data.type.factor
casp
gpp
rnaseq
casp.gpp −0.94
casp.rnaseq −0.301
gpp.rnaseq 0.334

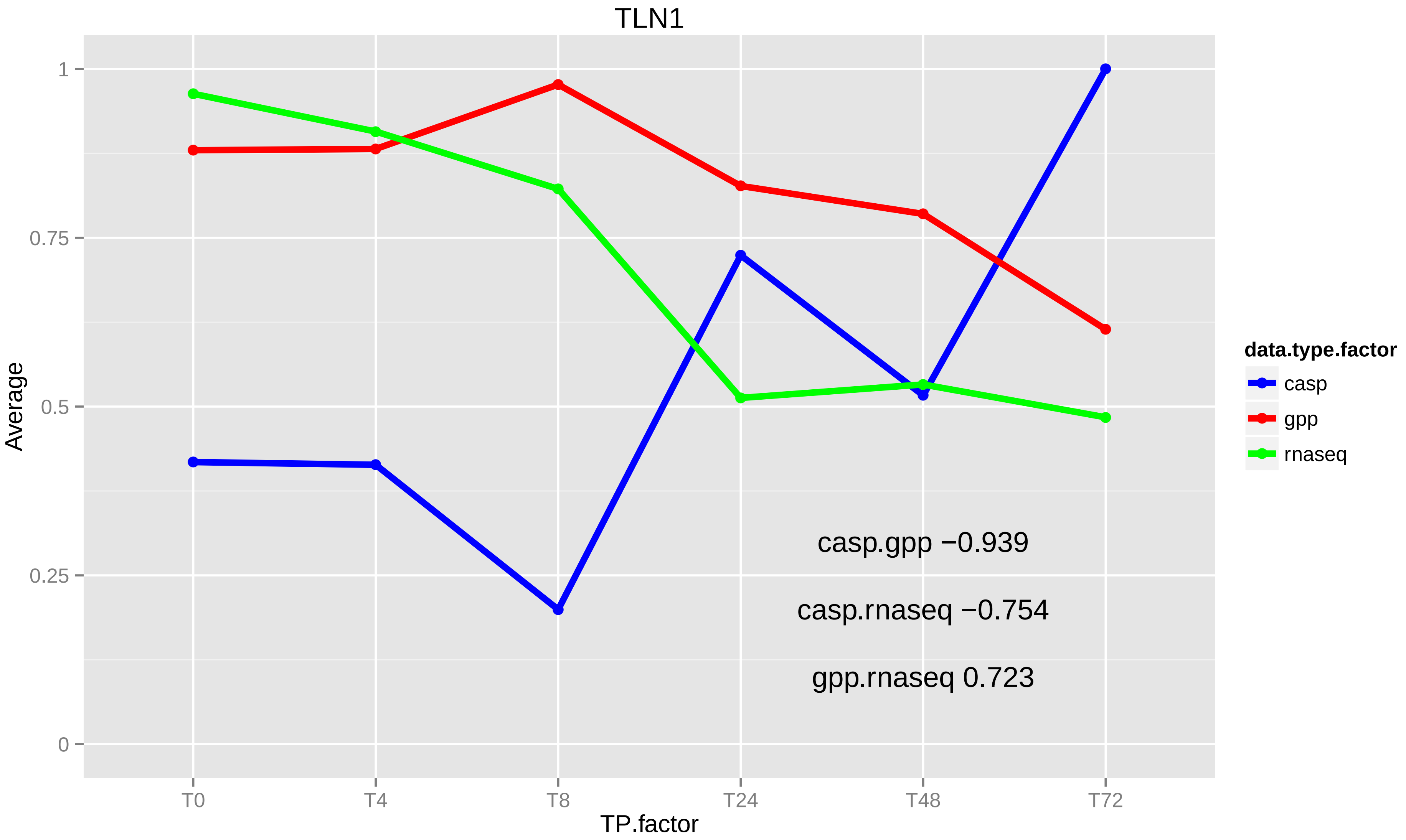

TLN1
1
0.75
0.5
0.25
0
Average
T0
T4
T8
T24
T48
T72
TP.factor
data.type.factor
casp
gpp
rnaseq
casp.gpp −0.939
casp.rnaseq −0.754
gpp.rnaseq 0.723

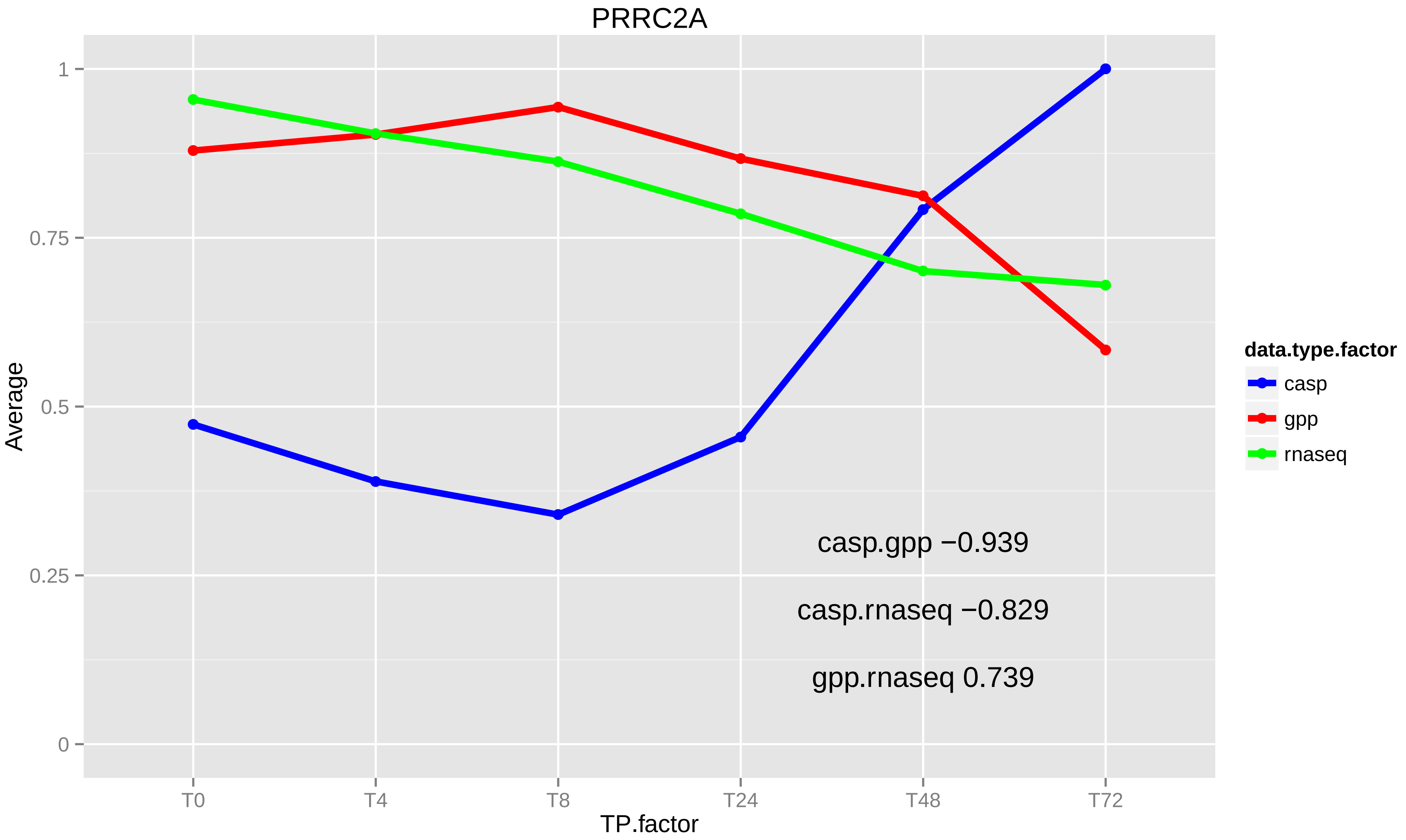

PRRC2A
1
0.75
0.5
0.25
0
Average
T0
T4
T8
T24
T48
T72
TP.factor
data.type.factor
casp
gpp
rnaseq
casp.gpp −0.939
casp.rnaseq −0.829
gpp.rnaseq 0.739

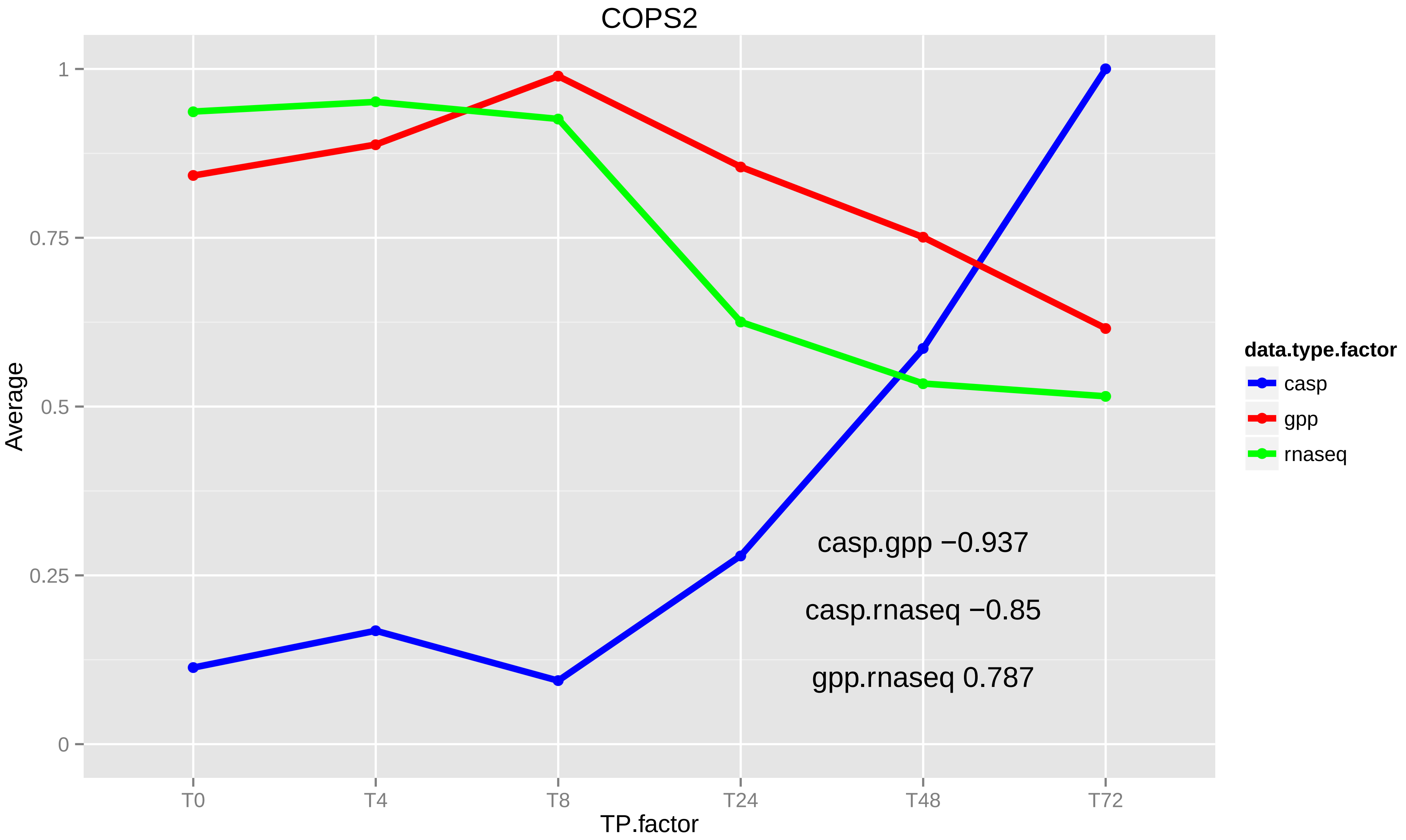

COPS2
Average
1
0.75
0.5
0.25
0
T0
T4
T8
T24
T48
T72
TP.factor
casp.gpp −0.937
casp.rnaseq −0.85
gpp.rnaseq 0.787
data.type.factor
casp
gpp
rnaseq

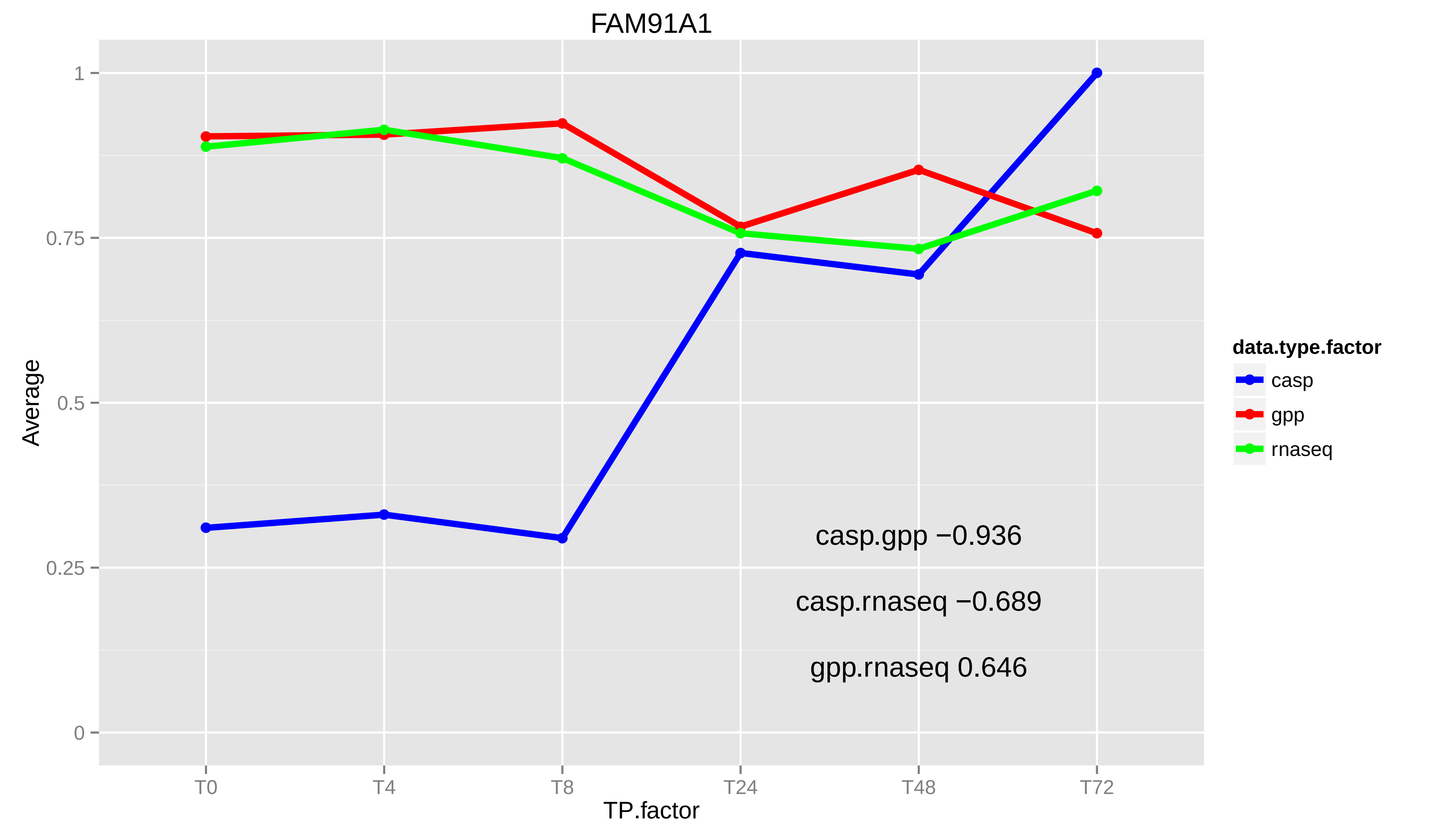

FAM91A1
Average
TP.factor
T0
T4
T8
T24
T48
T72
0
0.25
0.5
0.75
1
data.type.factor
casp
gpp
rnaseq
casp.gpp −0.936
casp.rnaseq −0.689
gpp.rnaseq 0.646

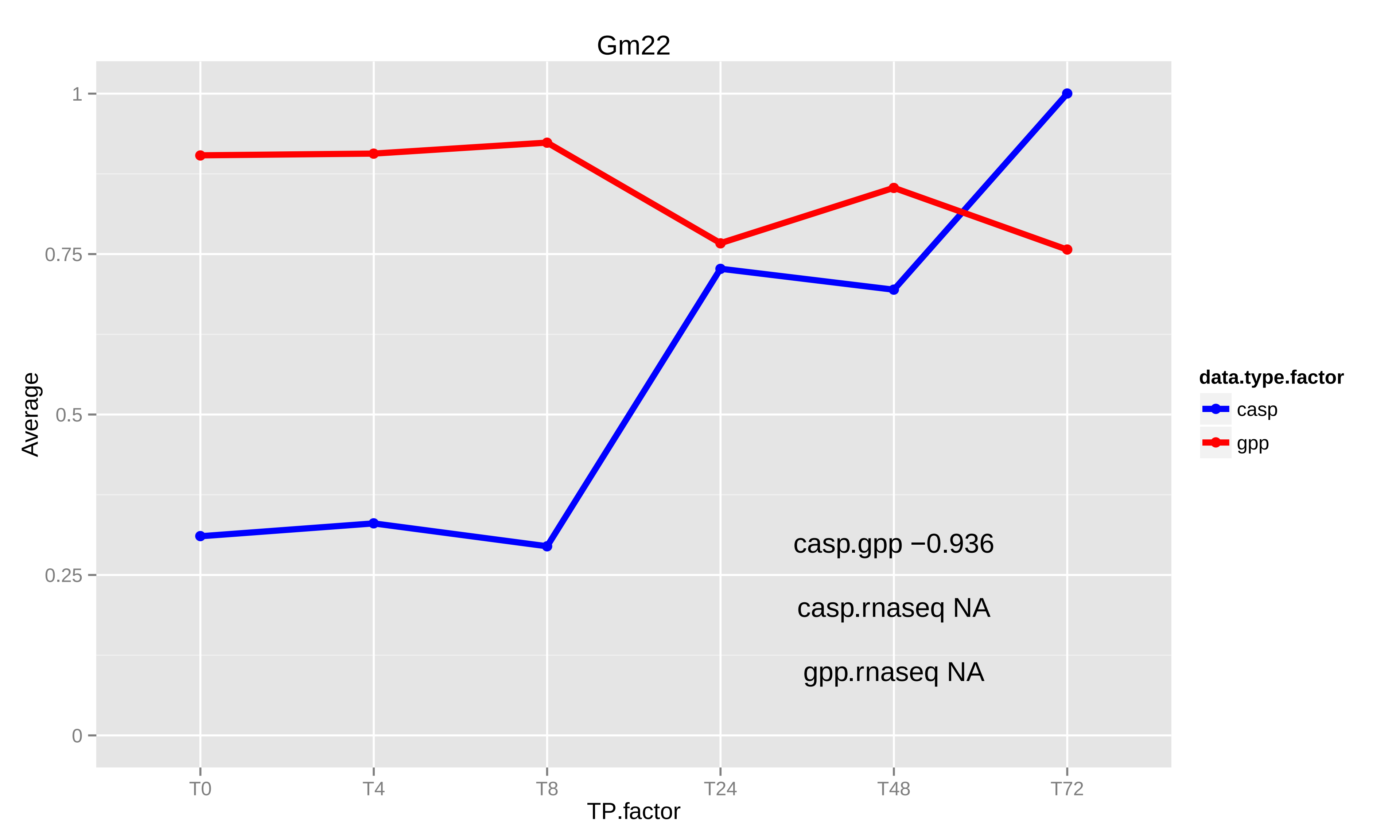

Gm22
Average
1
0.75
0.5
0.25
0
T0
T4
T8
T24
T48
T72
TP.factor
data.type.factor
casp
gpp
casp.gpp −0.936
casp.rnaseq NA
gpp.rnaseq NA

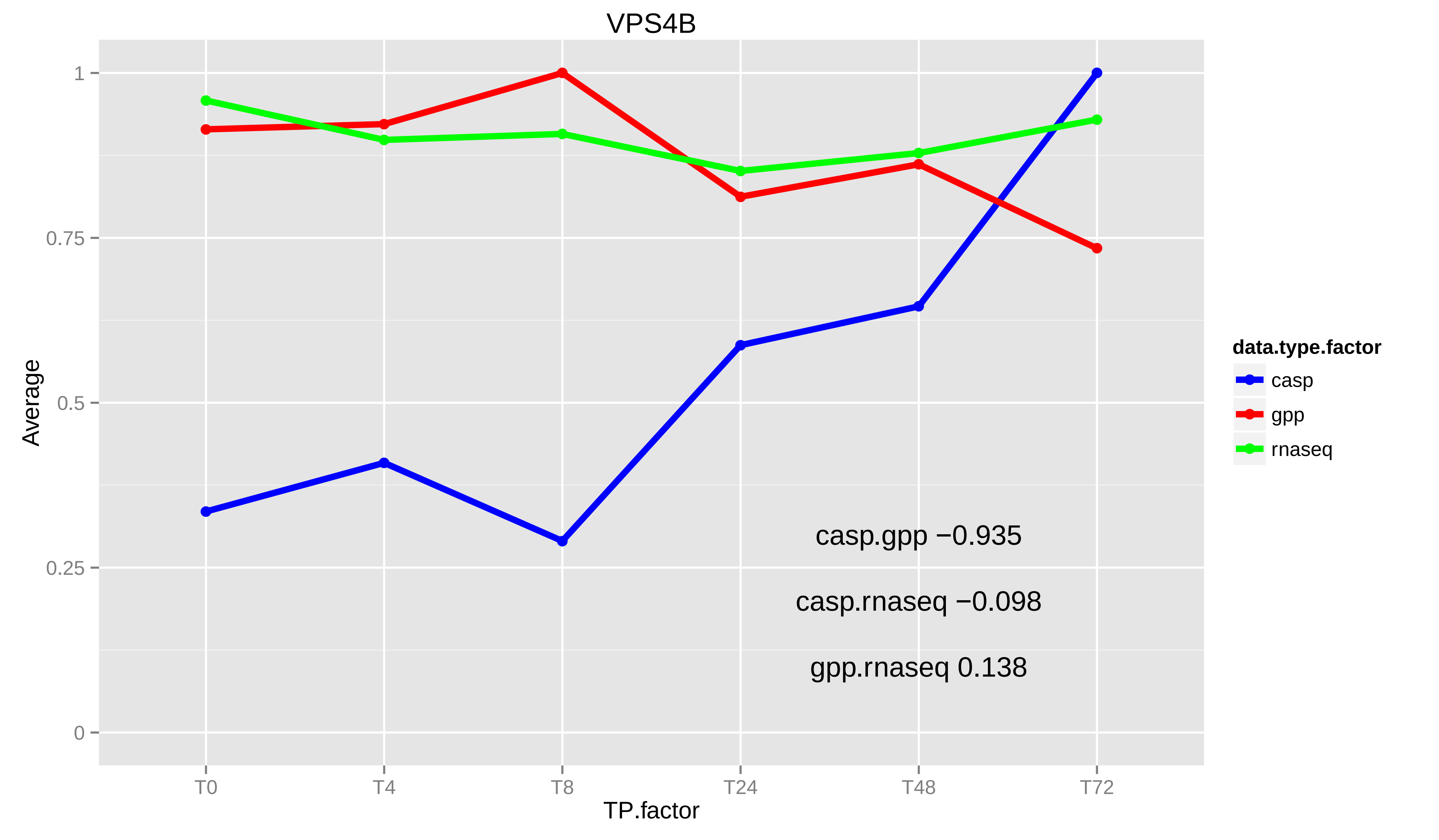

VPS4B
Average
1
0.75
0.5
0.25
0
T0
T4
T8
T24
T48
T72
TP.factor
data.type.factor
casp
gpp
rnaseq
casp.gpp −0.935
casp.rnaseq −0.098
gpp.rnaseq 0.138

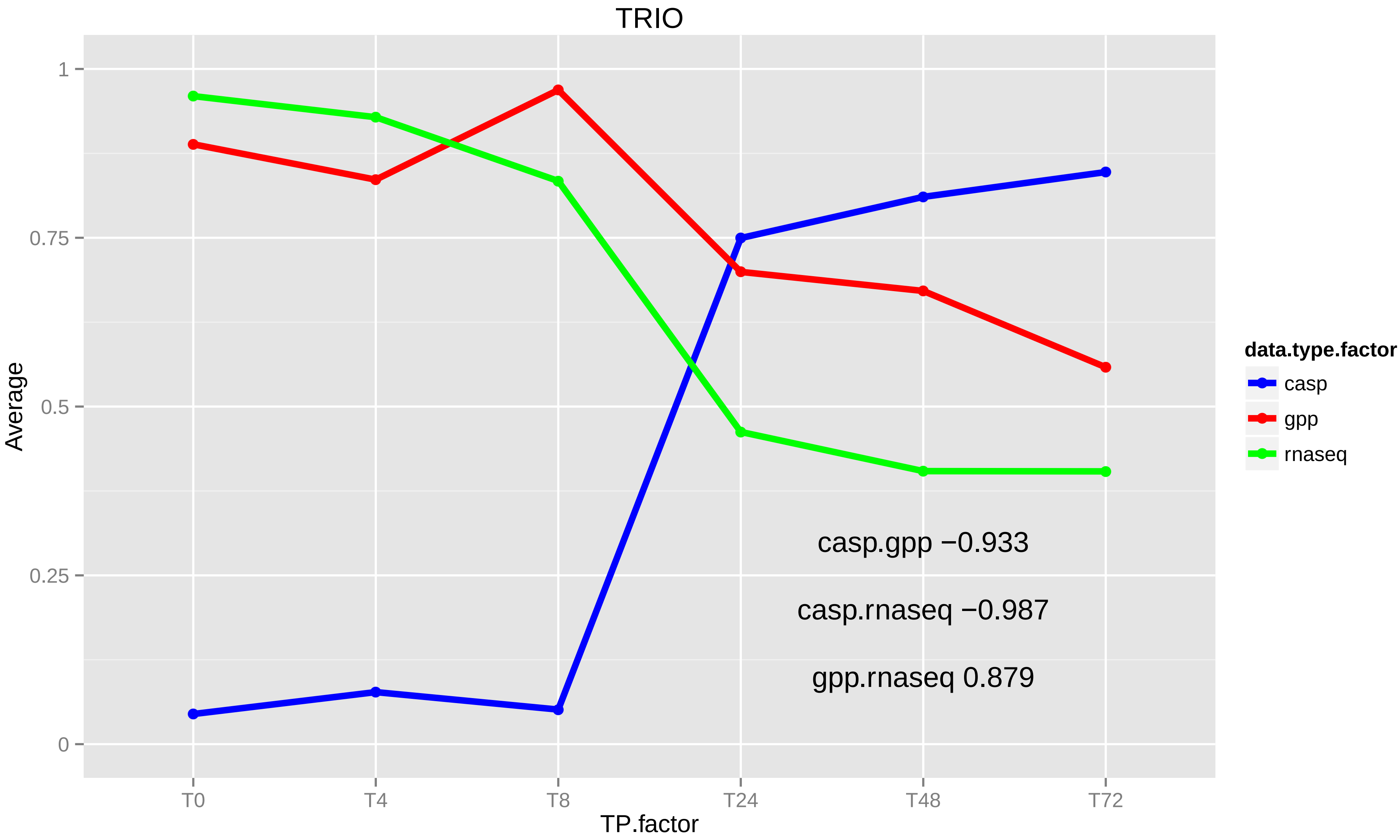
TRIO
Average
1
0.75
0.5
0.25
0
T0
T4
T8
T24
T48
T72
TP.factor
data.type.factor
casp
gpp
rnaseq
casp.gpp −0.933
casp.rnaseq −0.987
gpp.rnaseq 0.879

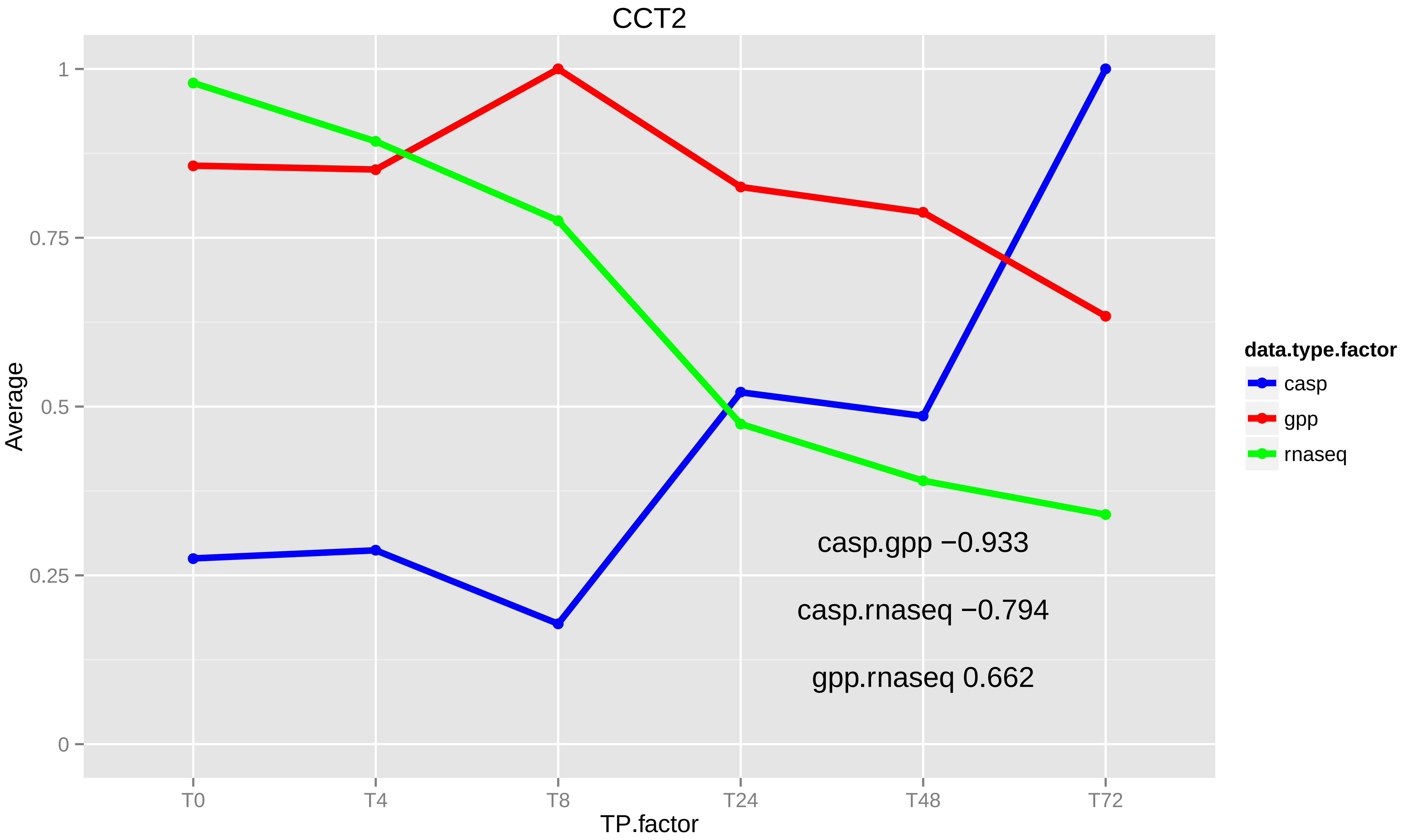

CCT2
1
0.75
0.5
0.25
0
Average
T0
T4
T8
T24
T48
T72
TP.factor
data.type.factor
casp
gpp
rnaseq
casp.gpp −0.933
casp.rnaseq −0.794
gpp.rnaseq 0.662

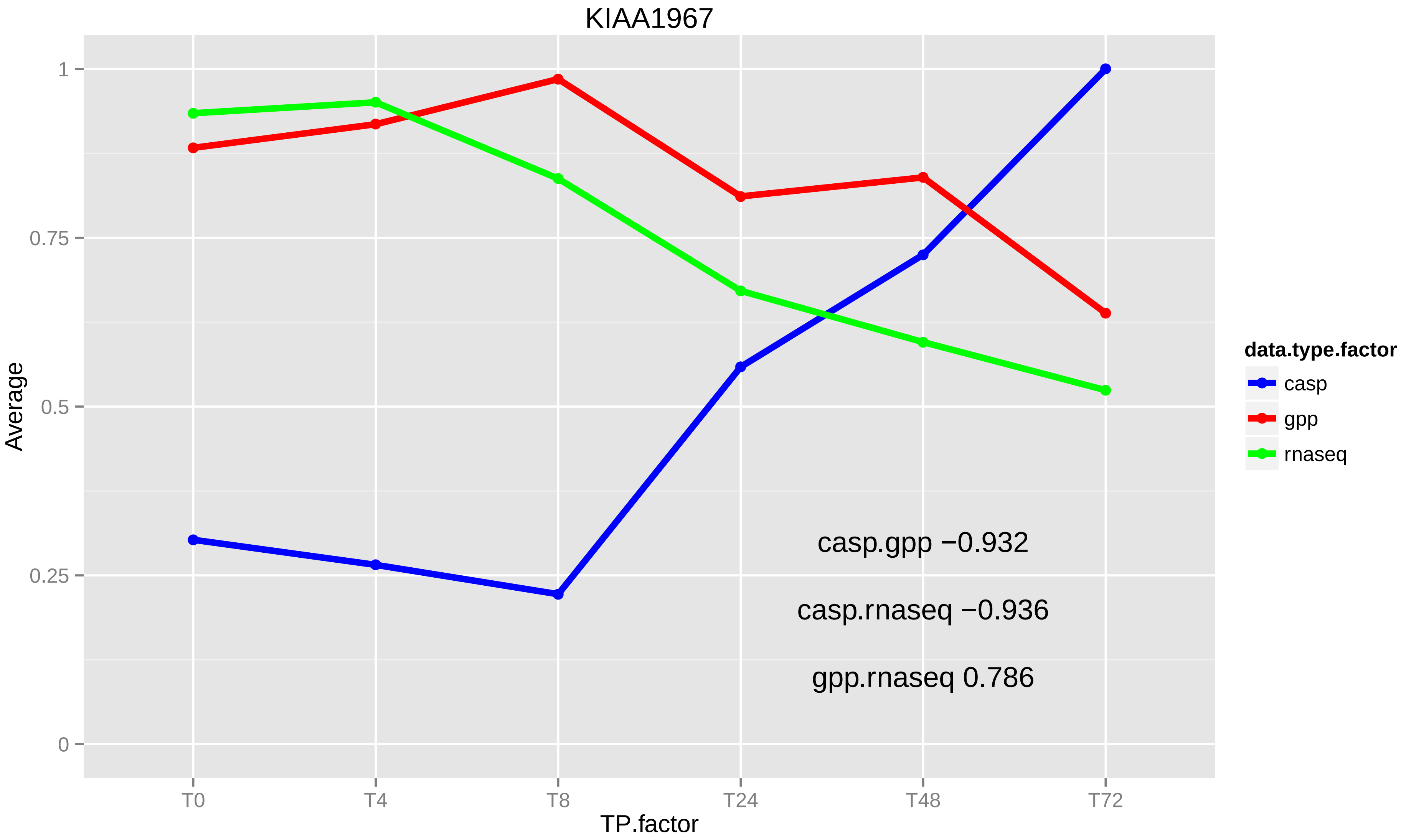
KIAA1967
Average
1
0.75
0.5
0.25
0
T0
T4
T8
T24
T48
T72
TP.factor
data.type.factor
casp
gpp
rnaseq
casp.gpp −0.932
casp.rnaseq −0.936
gpp.rnaseq 0.786

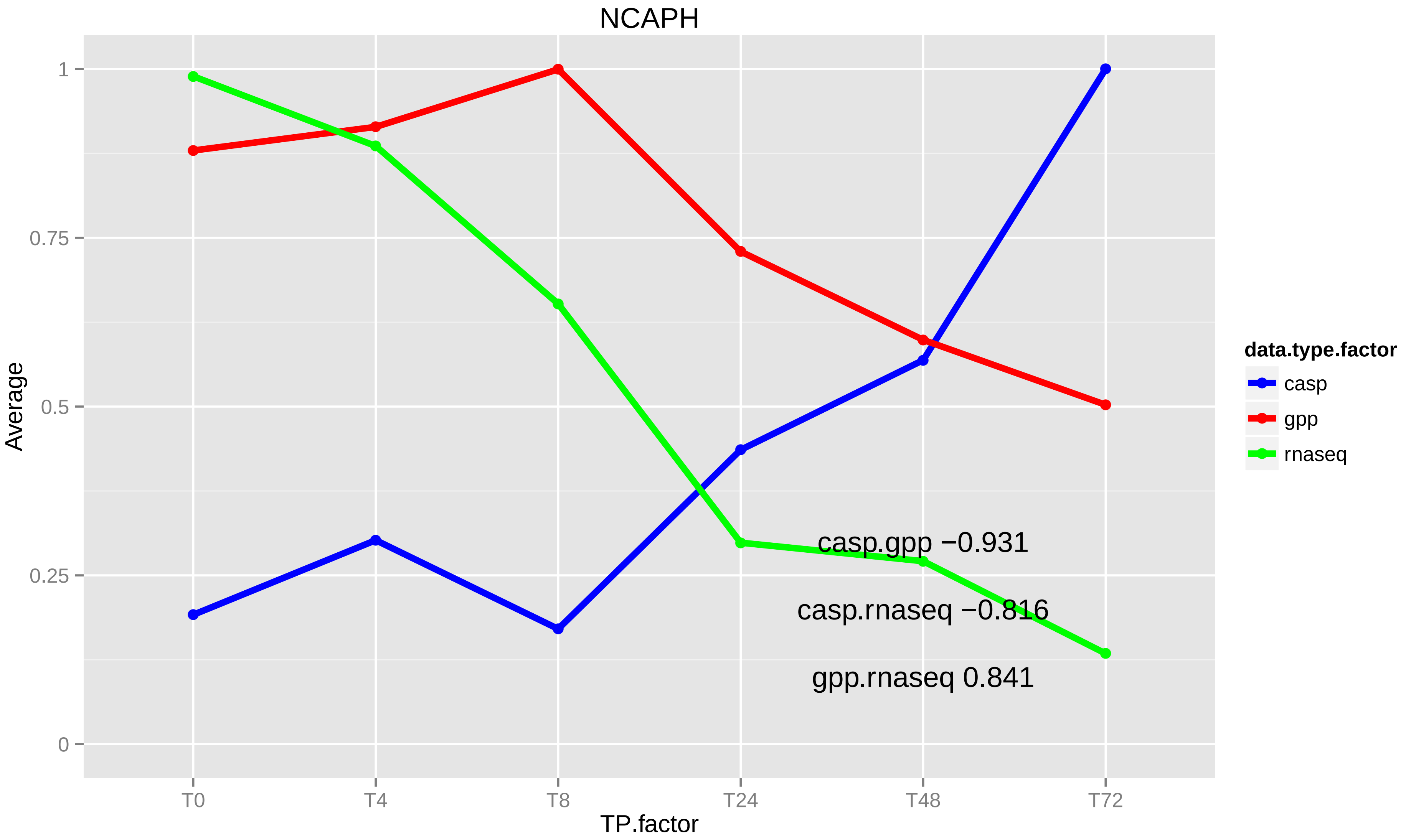
NCAPH
Average
1
0.75
0.5
0.25
0
T0
T4
T8
T24
T48
T72
TP.factor
data.type.factor
casp
gpp
rnaseq
casp.gpp −0.931
casp.rnaseq −0.816
gpp.rnaseq 0.841

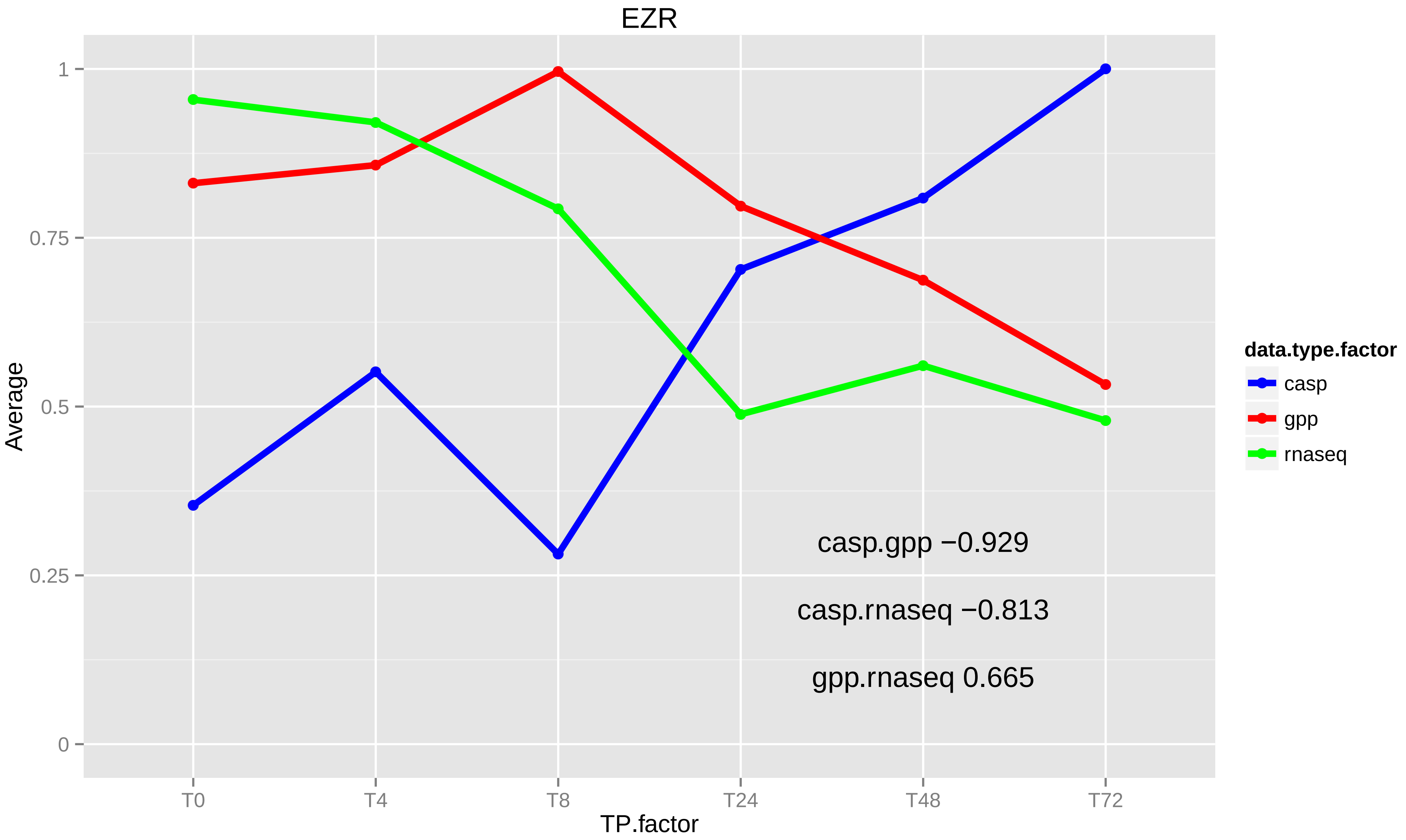

EZR
1
0.75
0.5
0.25
0
Average
T0
T4
T8
T24
T48
T72
TP.factor
data.type.factor
casp
gpp
rnaseq
casp.gpp −0.929
casp.rnaseq −0.813
gpp.rnaseq 0.665

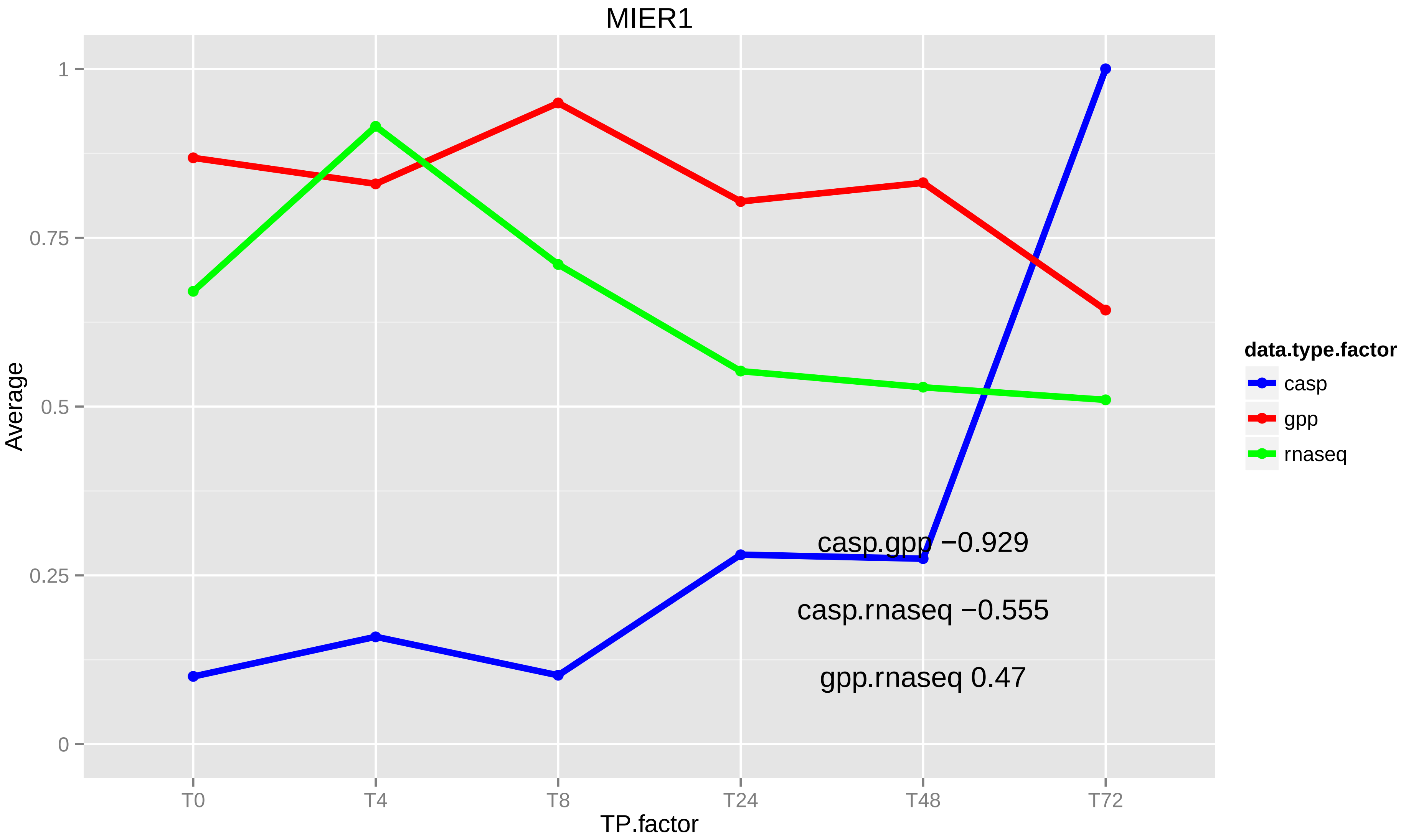
MIER1
1
0.75
0.5
0.25
0
Average
T0
T4
T8
T24
T48
T72
TP.factor
data.type.factor
casp
gpp
rnaseq
casp.gpp −0.929
casp.rnaseq −0.555
gpp.rnaseq 0.47

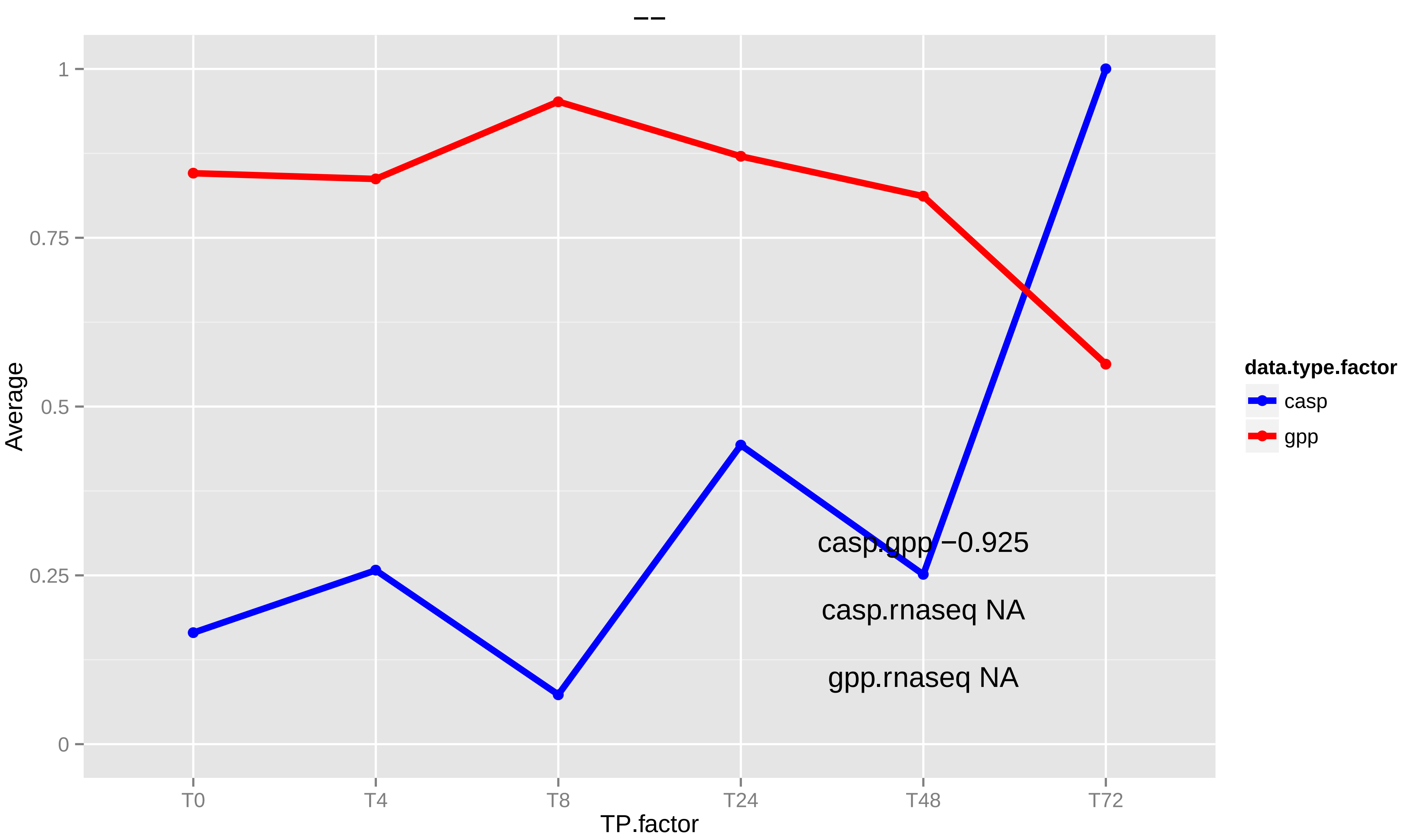

--
Average
1
0.75
0.5
0.25
0
T0
T4
T8
T24
T48
T72
TP.factor
data.type.factor
casp
gpp
casp.gpp −0.925
casp.rnaseq NA
gpp.rnaseq NA

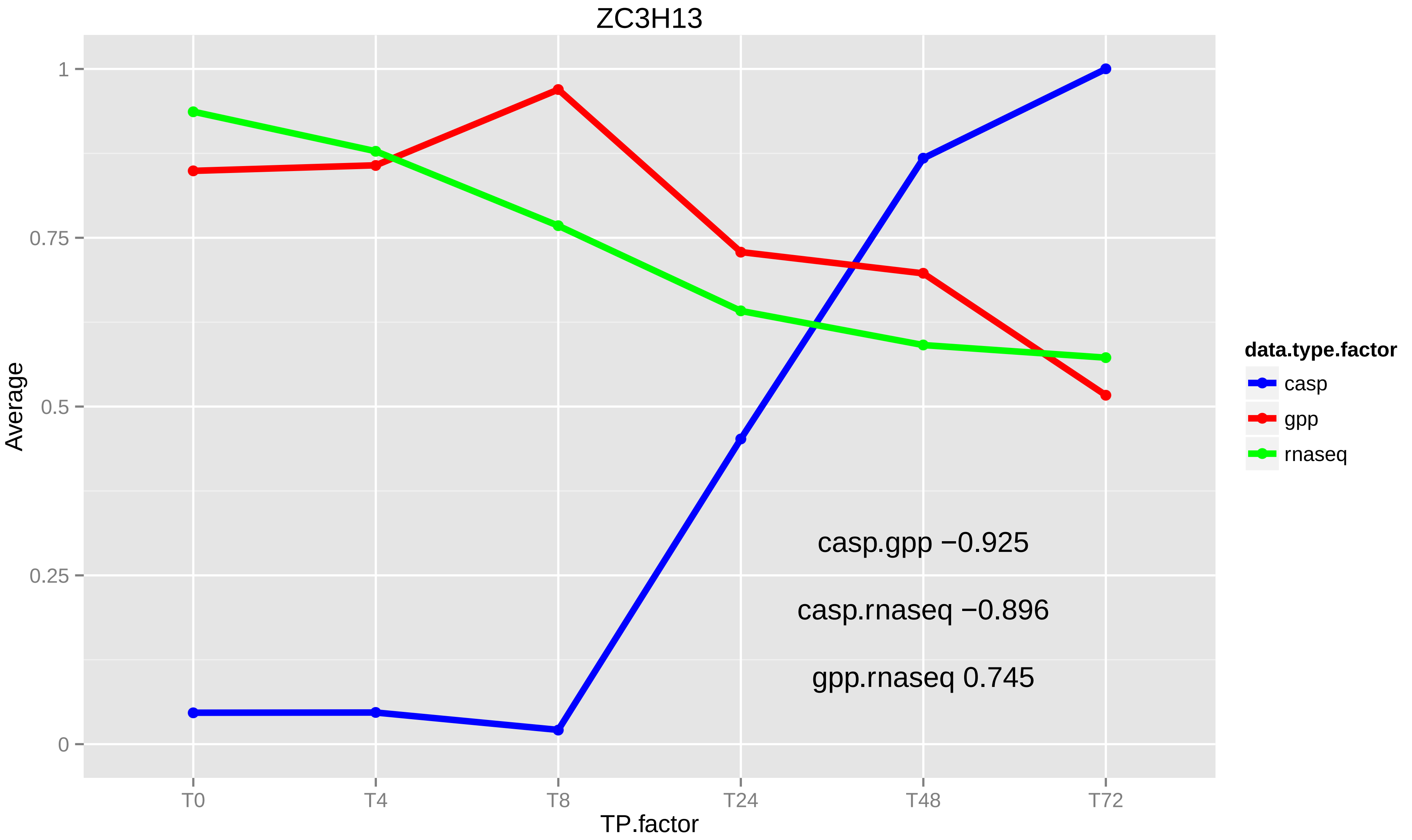
ZC3H13
Average
1
0.75
0.5
0.25
0
T0
T4
T8
T24
T48
T72
TP.factor
data.type.factor
casp
gpp
rnaseq
casp.gpp −0.925
casp.rnaseq −0.896
gpp.rnaseq 0.745

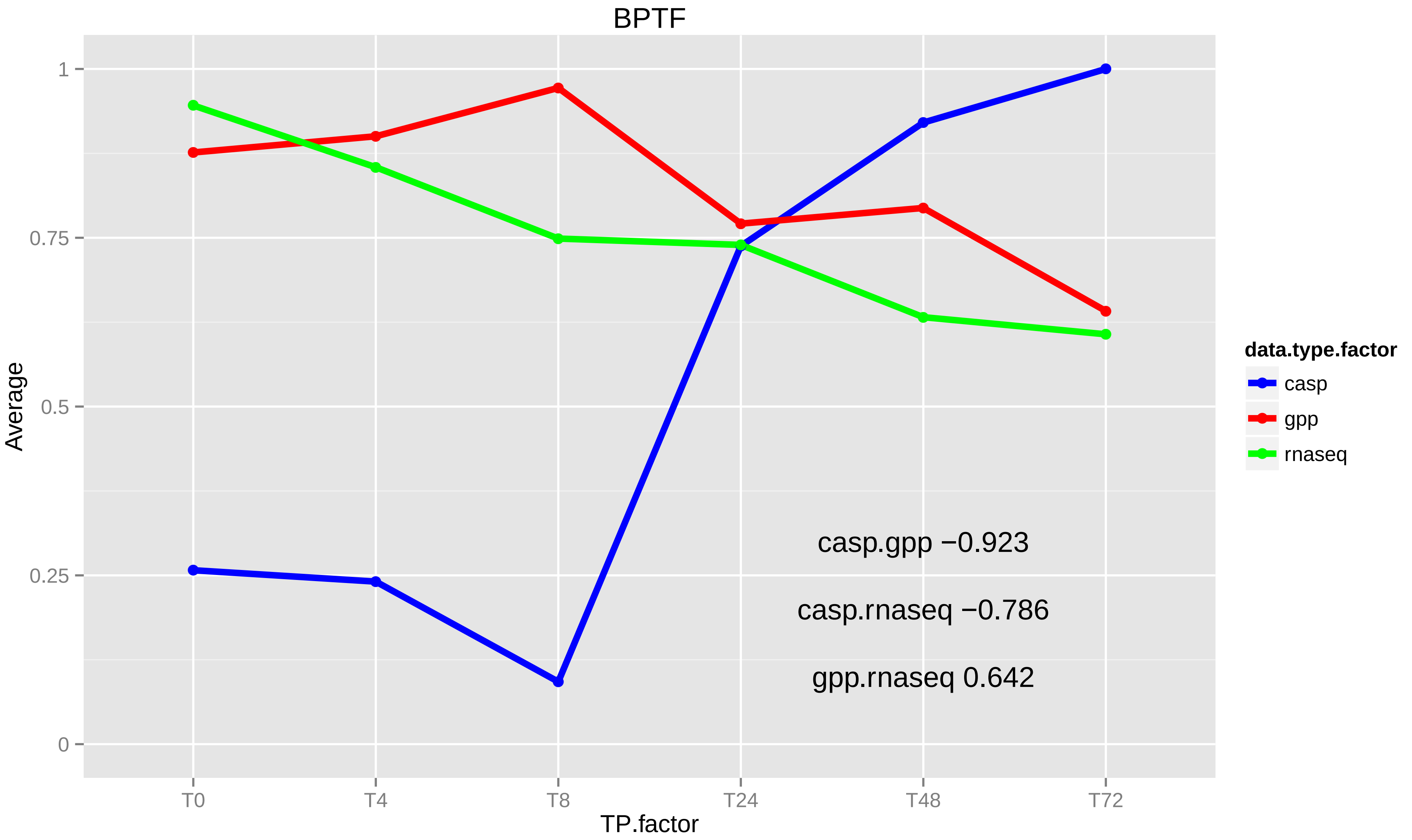

BPTF
Average
1
0.75
0.5
0.25
0
T0
T4
T8
T24
T48
T72
TP.factor
data.type.factor
casp
gpp
rnaseq
casp.gpp −0.923
casp.rnaseq −0.786
gpp.rnaseq 0.642

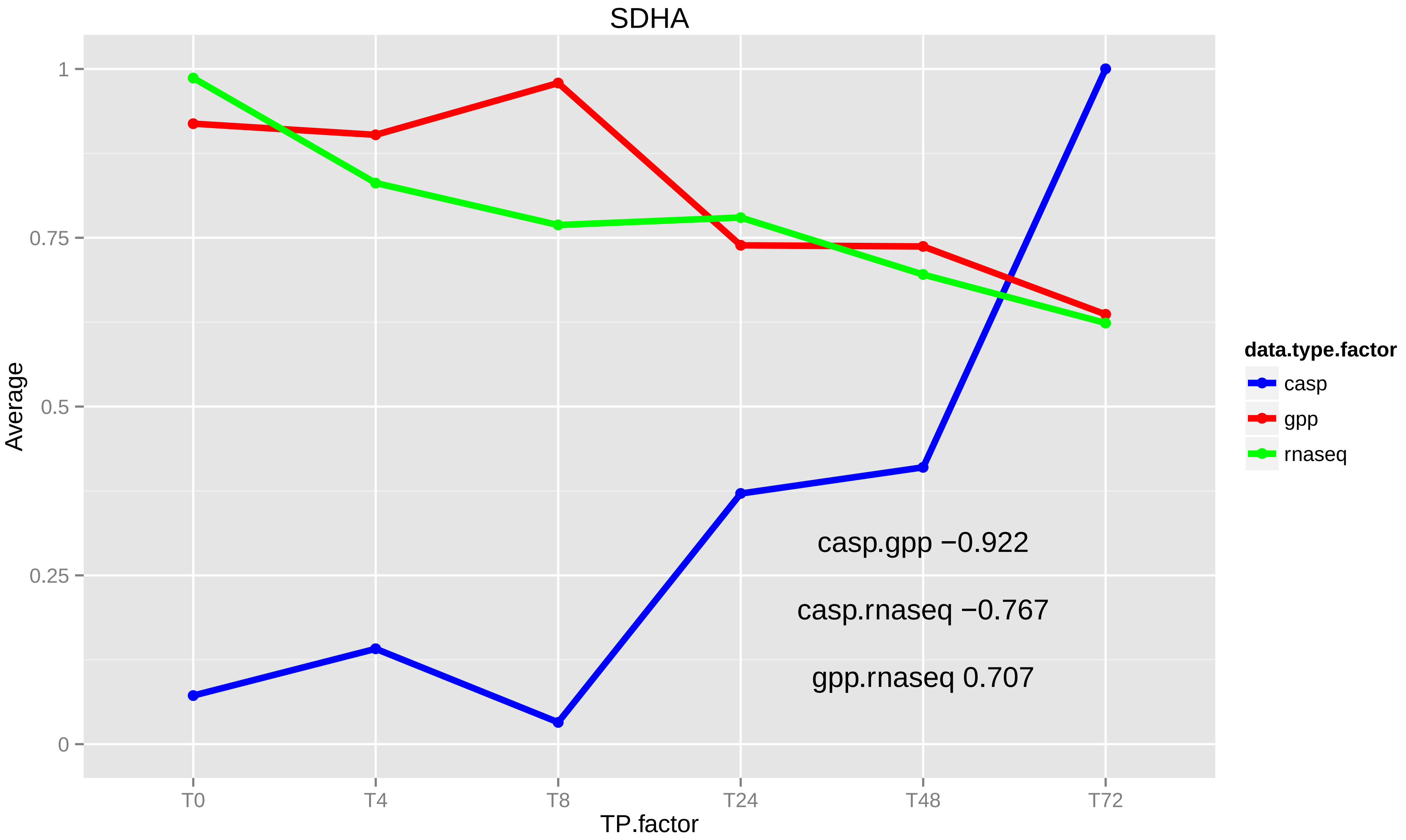
SDHA
1
0.75
0.5
0.25
0
Average
T0
T4
T8
T24
T48
T72
TP.factor
data.type.factor
casp
gpp
rnaseq
casp.gpp −0.922
casp.rnaseq −0.767
gpp.rnaseq 0.707

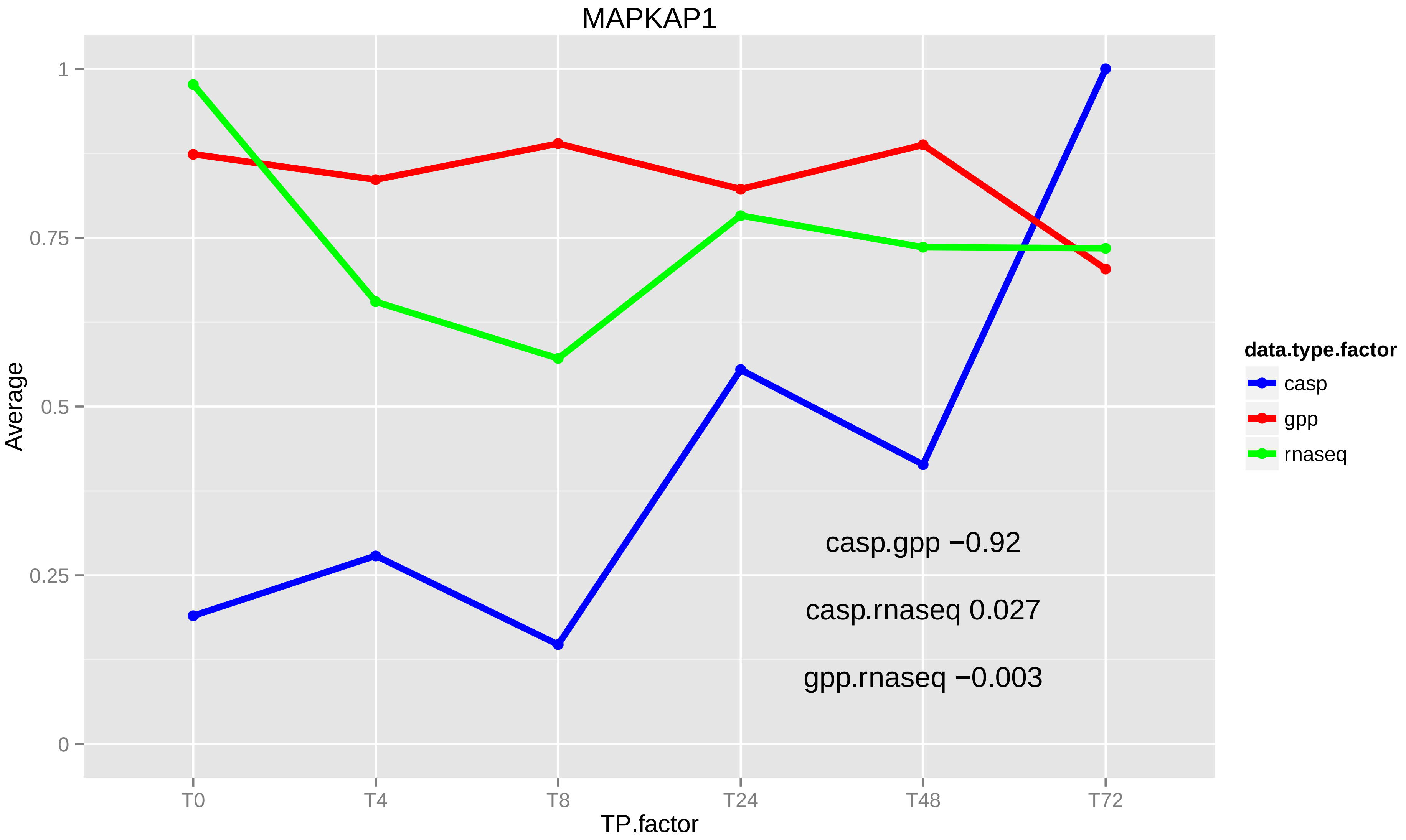

MAPKAP1
Average
1
0.75
0.5
0.25
0
T0
T4
T8
T24
T48
T72
TP.factor
data.type.factor
casp
gpp
rnaseq
casp.gpp −0.92
casp.rnaseq 0.027
gpp.rnaseq −0.003

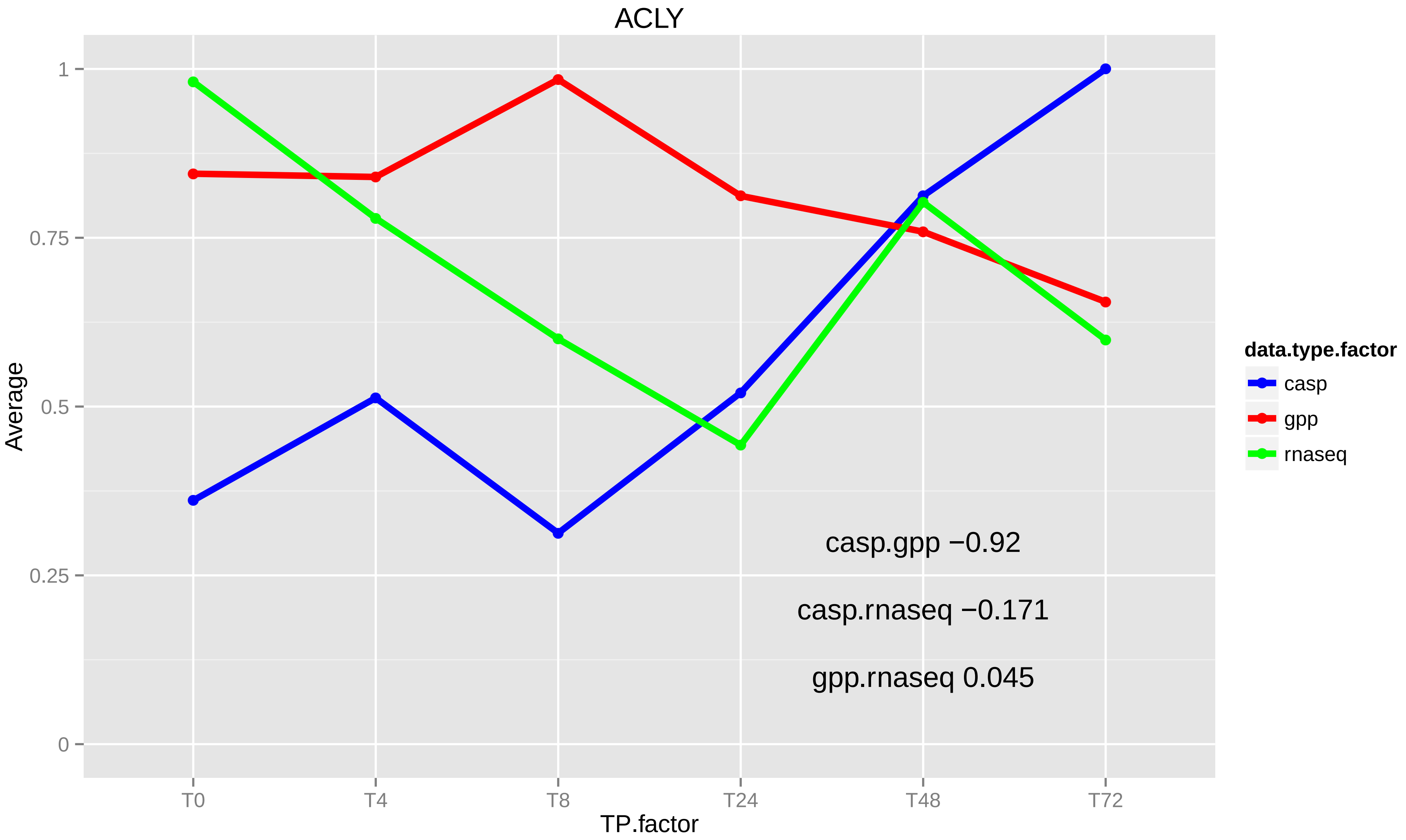
ACLY
Average
1
0.75
0.5
0.25
0
T0
T4
T8
T24
T48
T72
TP.factor
data.type.factor
casp
gpp
rnaseq
casp.gpp −0.92
casp.rnaseq −0.171
gpp.rnaseq 0.045

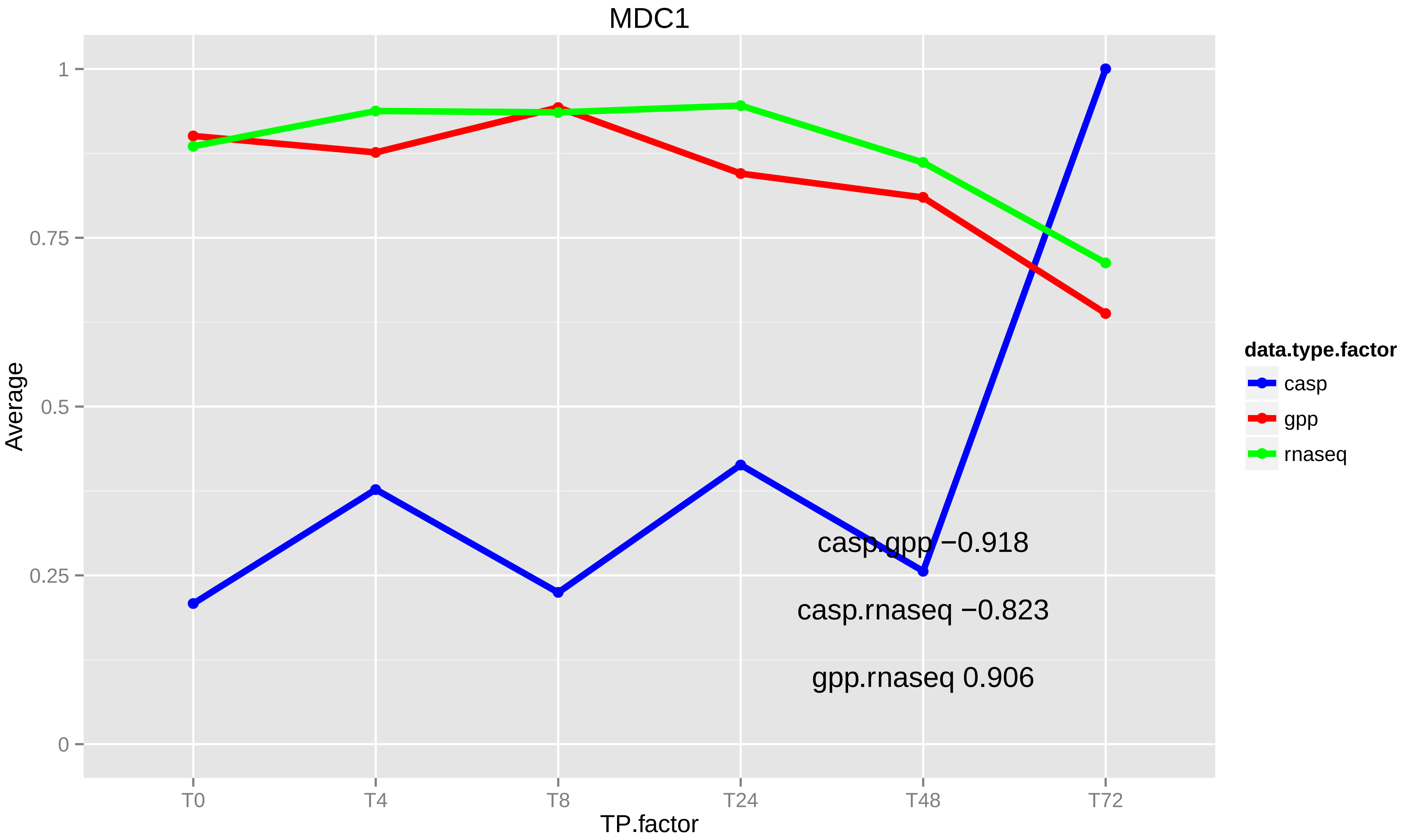

MDC1
1
0.75
0.5
0.25
0
Average
T0
T4
T8
T24
T48
T72
TP.factor
data.type.factor
casp
gpp
rnaseq
casp.gpp −0.918
casp.rnaseq −0.823
gpp.rnaseq 0.906

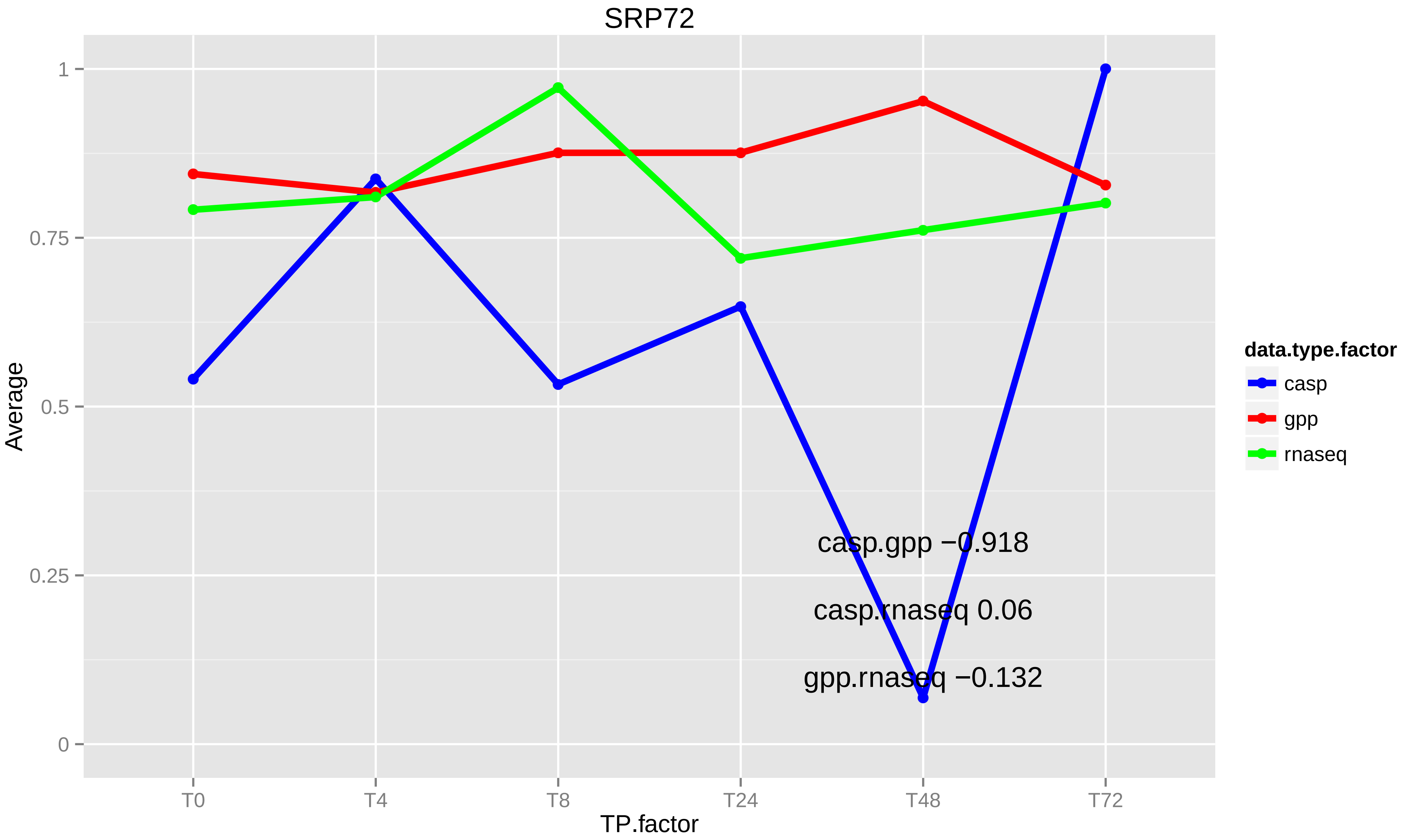

SRP72
Average
1
0.75
0.5
0.25
0
T0
T4
T8
T24
T48
T72
TP.factor
data.type.factor
casp
gpp
rnaseq
casp.gpp −0.918
casp.rnaseq 0.06
gpp.rnaseq −0.132

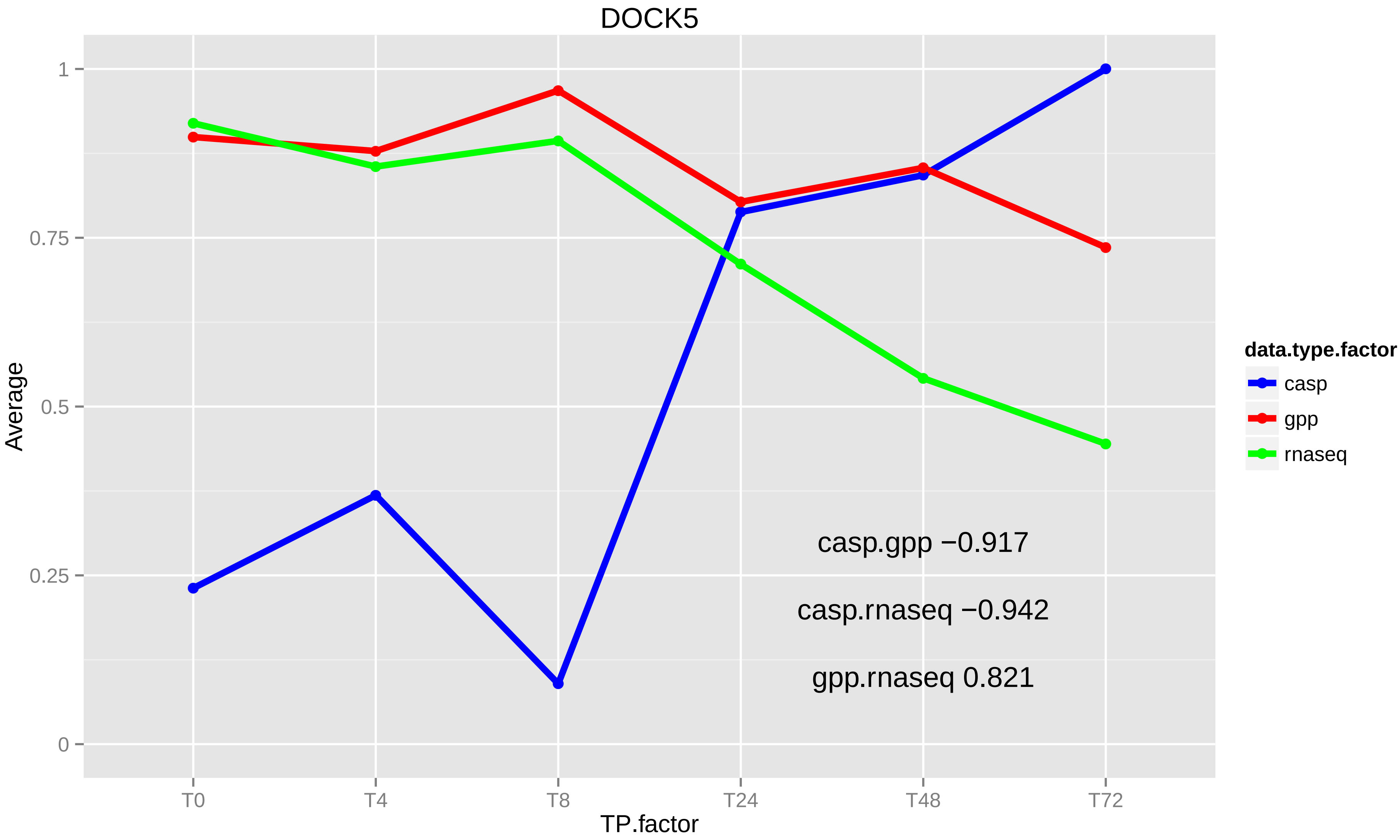
DOCK5
1
0.75
0.5
0.25
0
Average
T0
T4
T8
T24
T48
T72
TP.factor
data.type.factor
casp
gpp
rnaseq
casp.gpp −0.917
casp.rnaseq −0.942
gpp.rnaseq 0.821

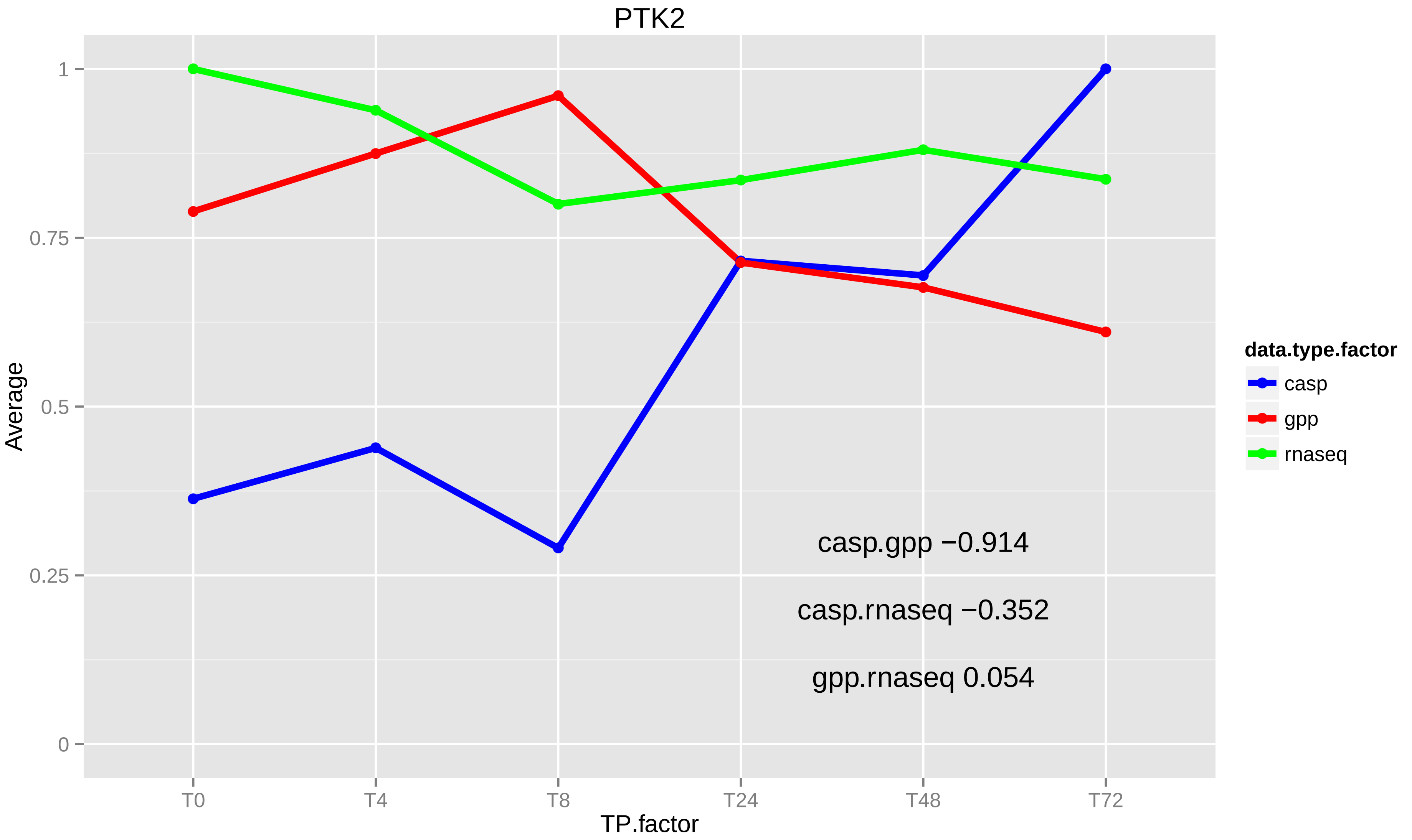
PTK2
Average
1
0.75
0.5
0.25
0
T0
T4
T8
T24
T48
T72
TP.factor
data.type.factor
casp
gpp
rnaseq
casp.gpp −0.914
casp.rnaseq −0.352
gpp.rnaseq 0.054

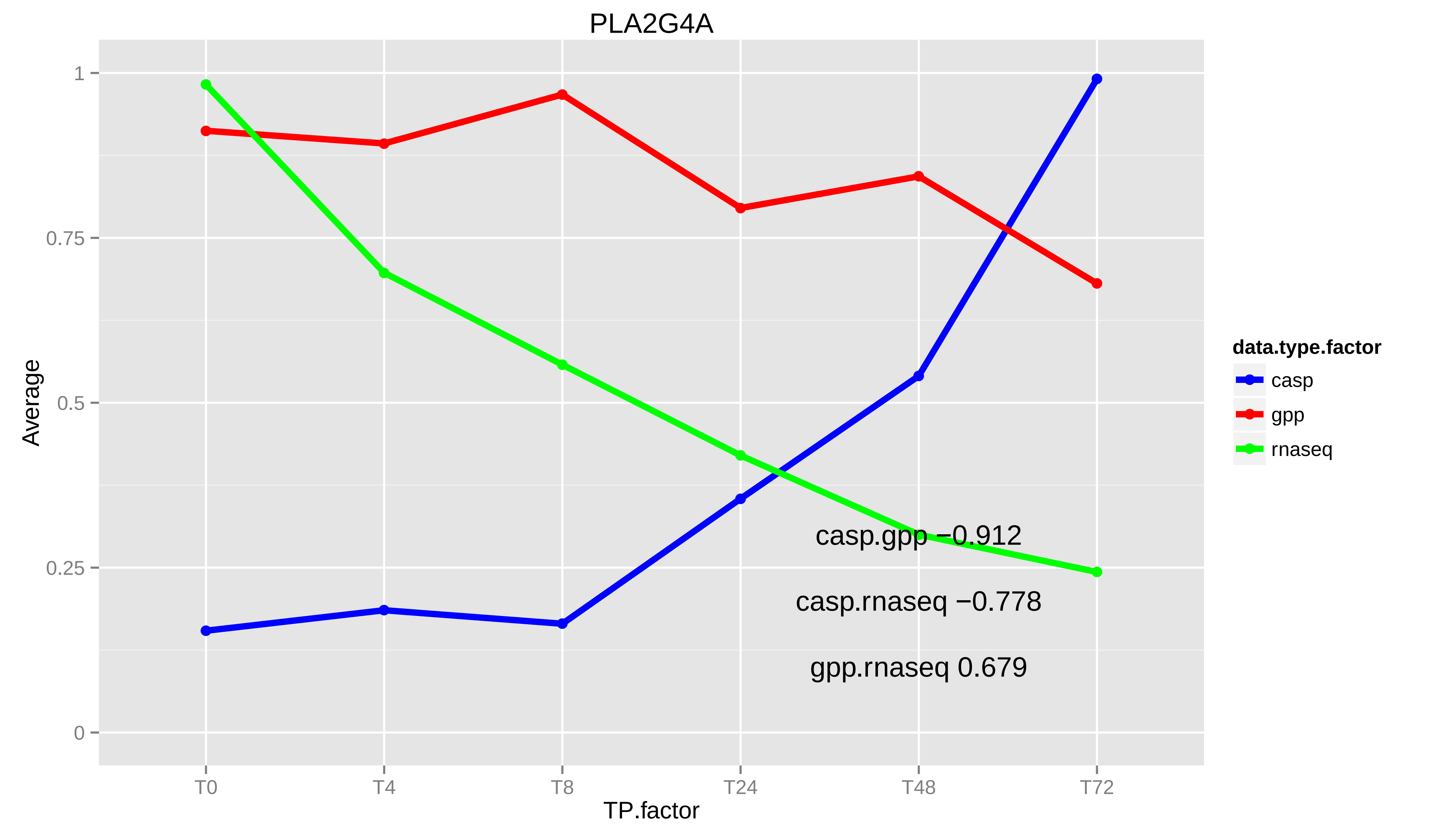

PLA2G4A
Average
1
0.75
0.5
0.25
0
T0
T4
T8
T24
T48
T72
TP.factor
data.type.factor
casp
gpp
rnaseq
casp.gpp −0.912
casp.rnaseq −0.778
gpp.rnaseq 0.679

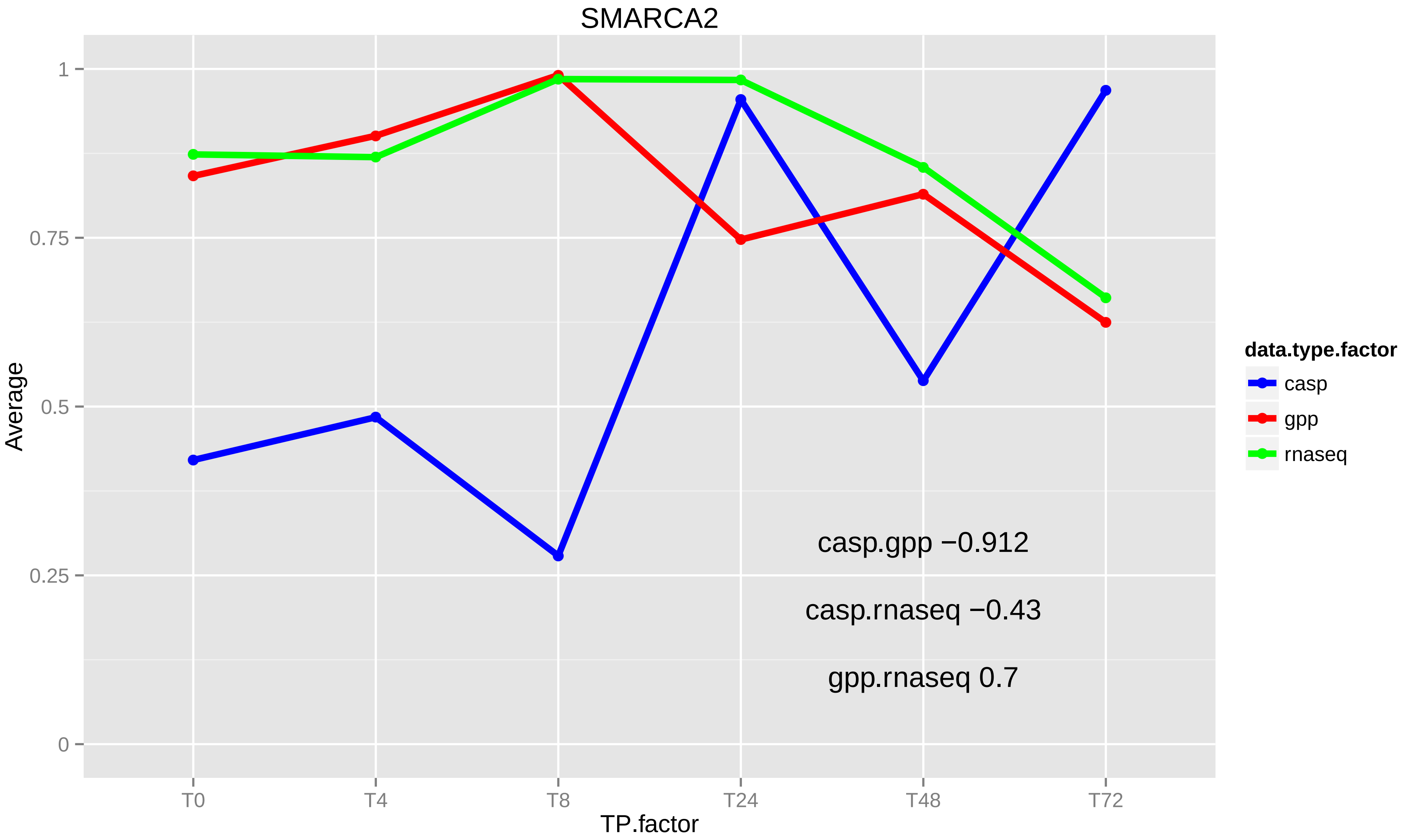
SMARCA2
1
0.75
0.5
0.25
0
Average
T0
T4
T8
T24
T48
T72
TP.factor
data.type.factor
casp
gpp
rnaseq
casp.gpp −0.912
casp.rnaseq −0.43
gpp.rnaseq 0.7

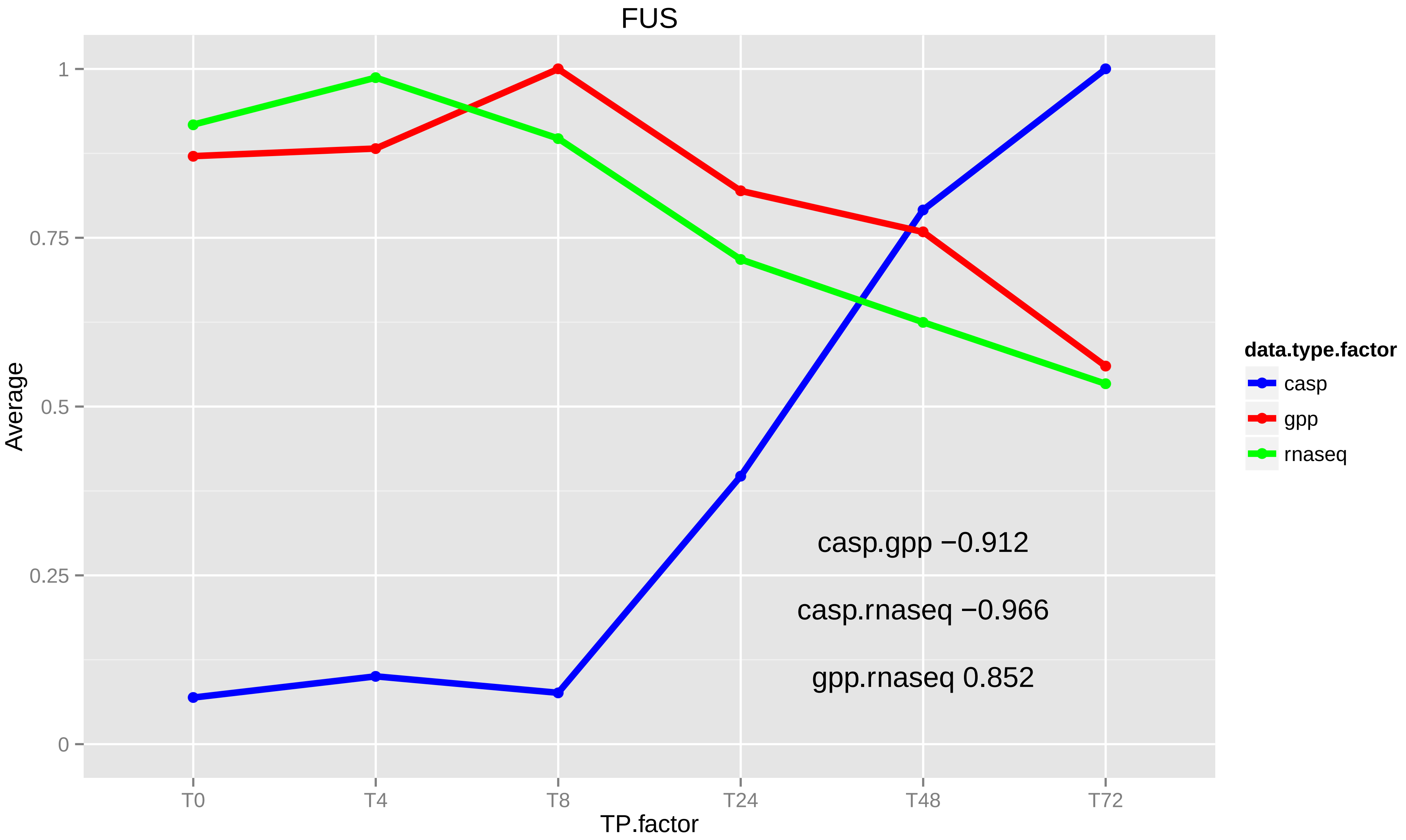

FUS
1
0.75
0.5
0.25
0
Average
T0
T4
T8
T24
T48
T72
TP.factor
data.type.factor
casp
gpp
rnaseq
casp.gpp −0.912
casp.rnaseq −0.966
gpp.rnaseq 0.852

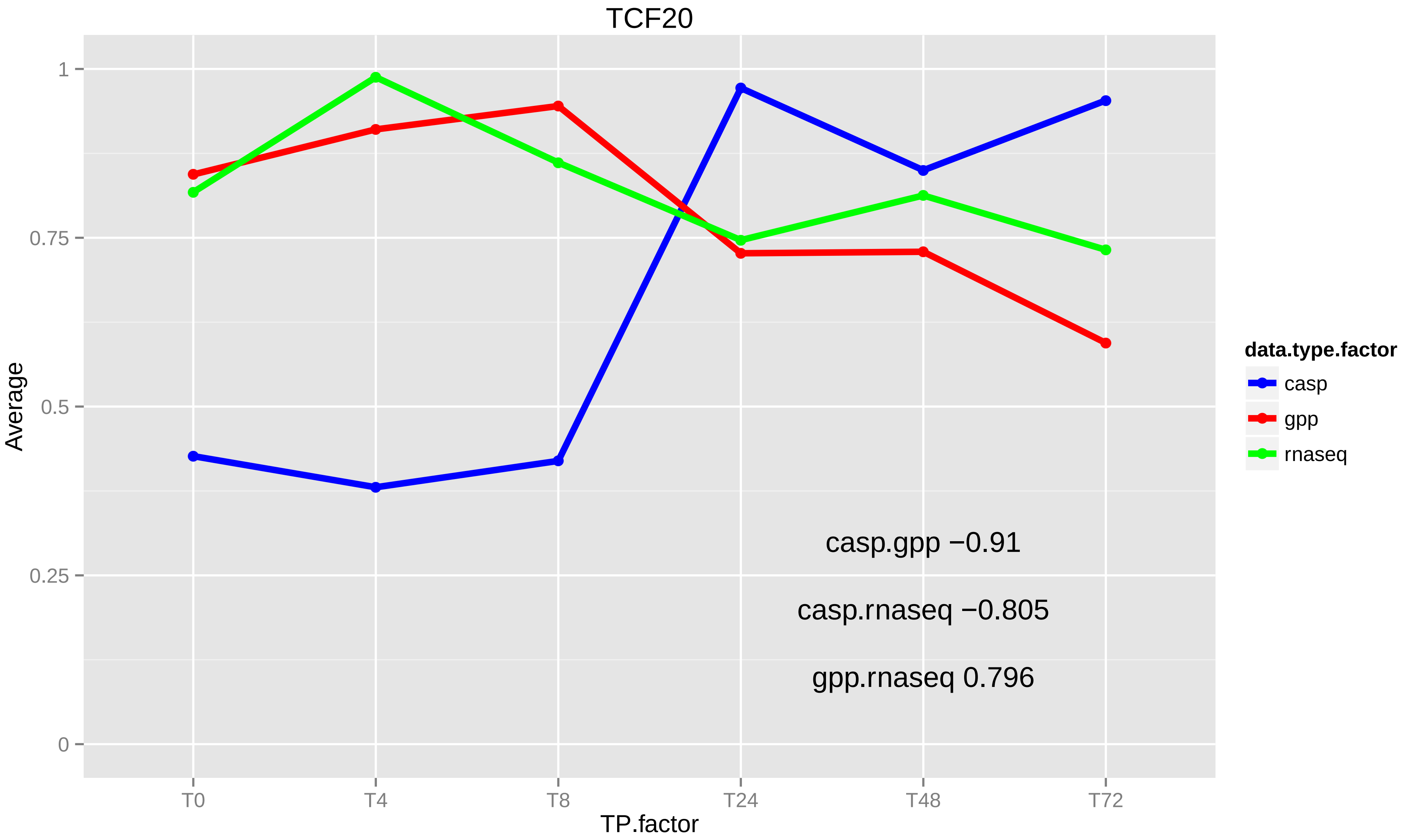
TCF20
1
0.75
0.5
0.25
0
Average
T0
T4
T8
T24
T48
T72
TP.factor
data.type.factor
casp
gpp
rnaseq
casp.gpp −0.91
casp.rnaseq −0.805
gpp.rnaseq 0.796

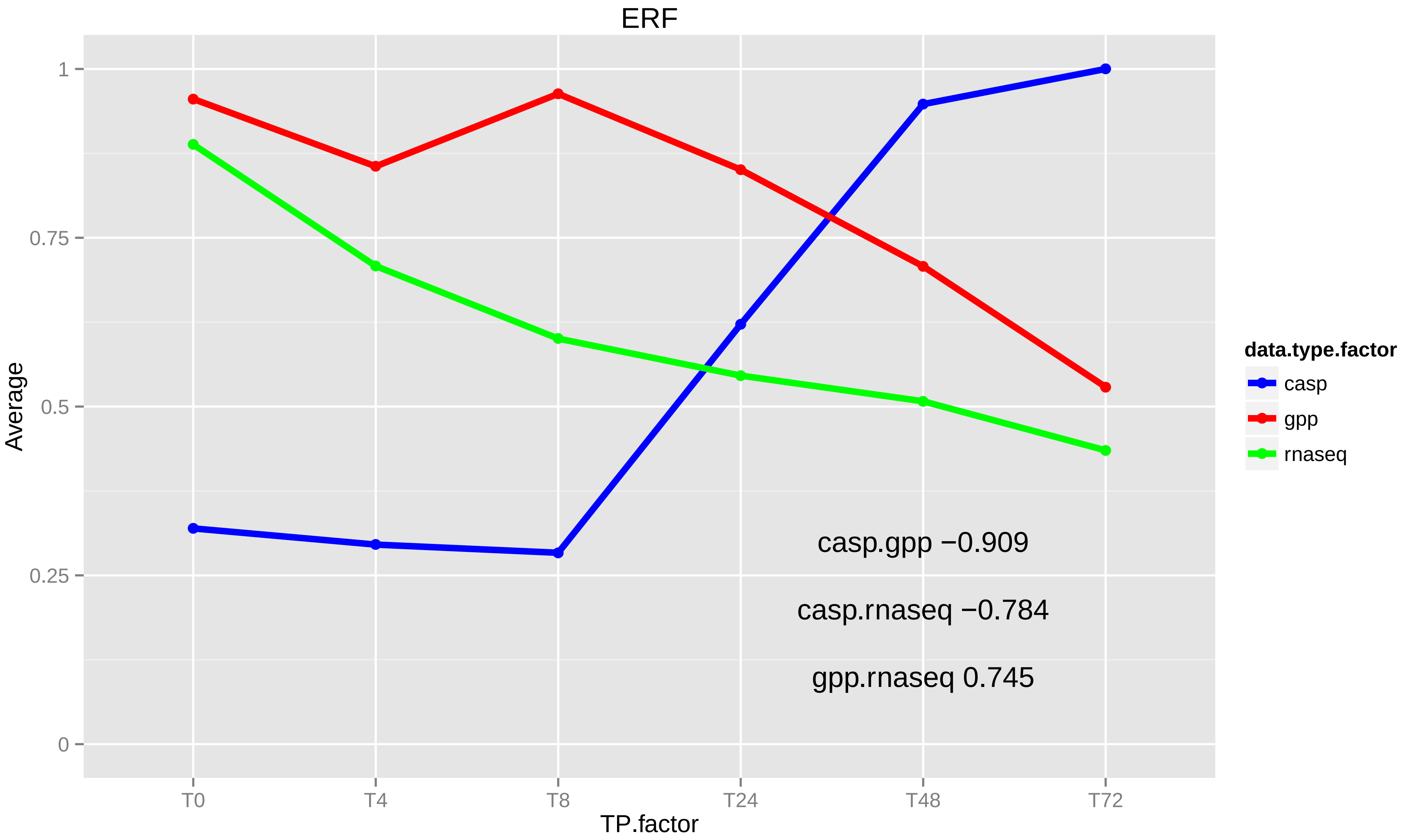
ERF
Average
1
0.75
0.5
0.25
0
T0
T4
T8
T24
T48
T72
TP.factor
data.type.factor
casp
gpp
rnaseq
casp.gpp −0.909
casp.rnaseq −0.784
gpp.rnaseq 0.745

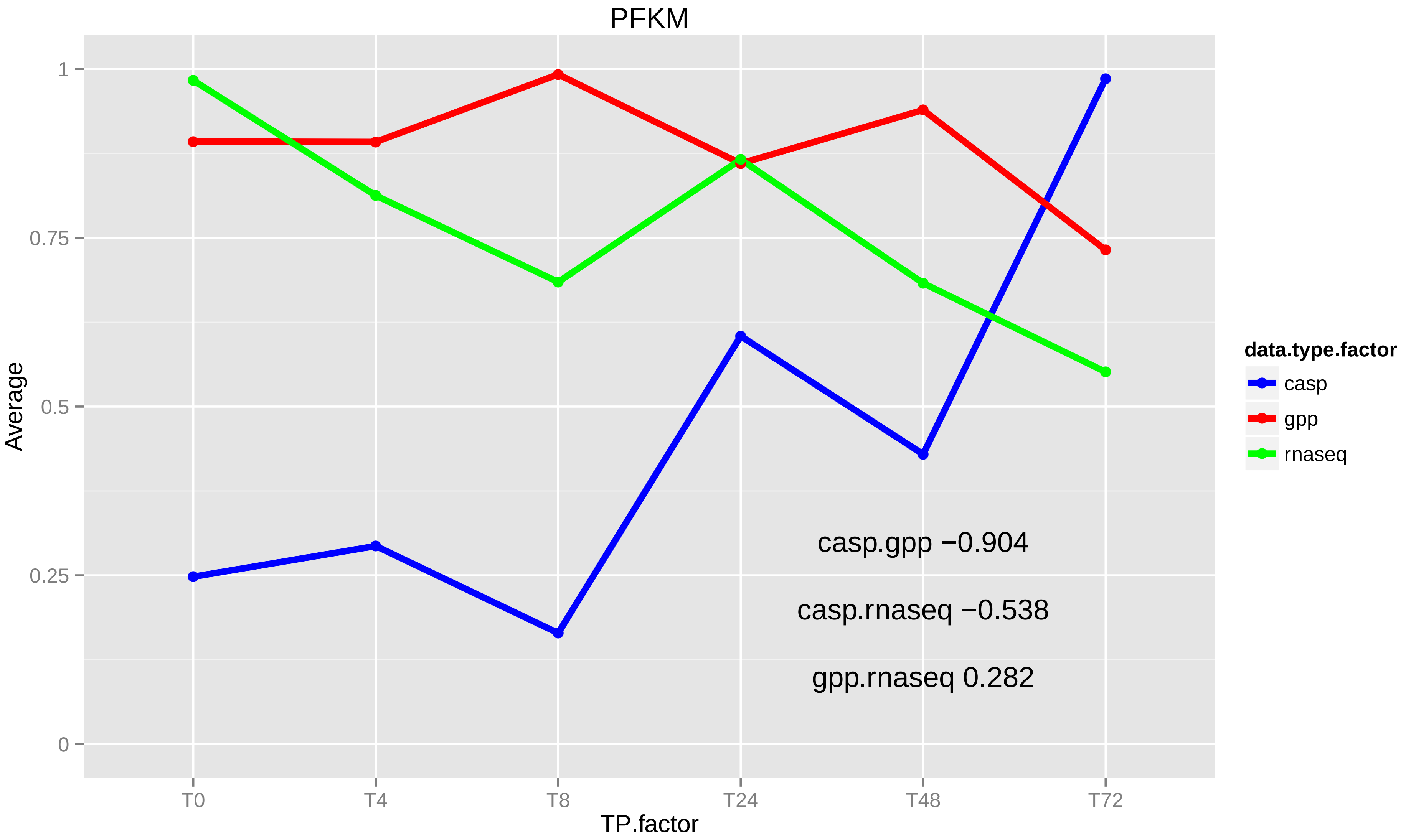
PFKM
Average
1
0.75
0.5
0.25
0
T0
T4
T8
T24
T48
T72
TP.factor
data.type.factor
casp
gpp
rnaseq
casp.gpp −0.904
casp.rnaseq −0.538
gpp.rnaseq 0.282

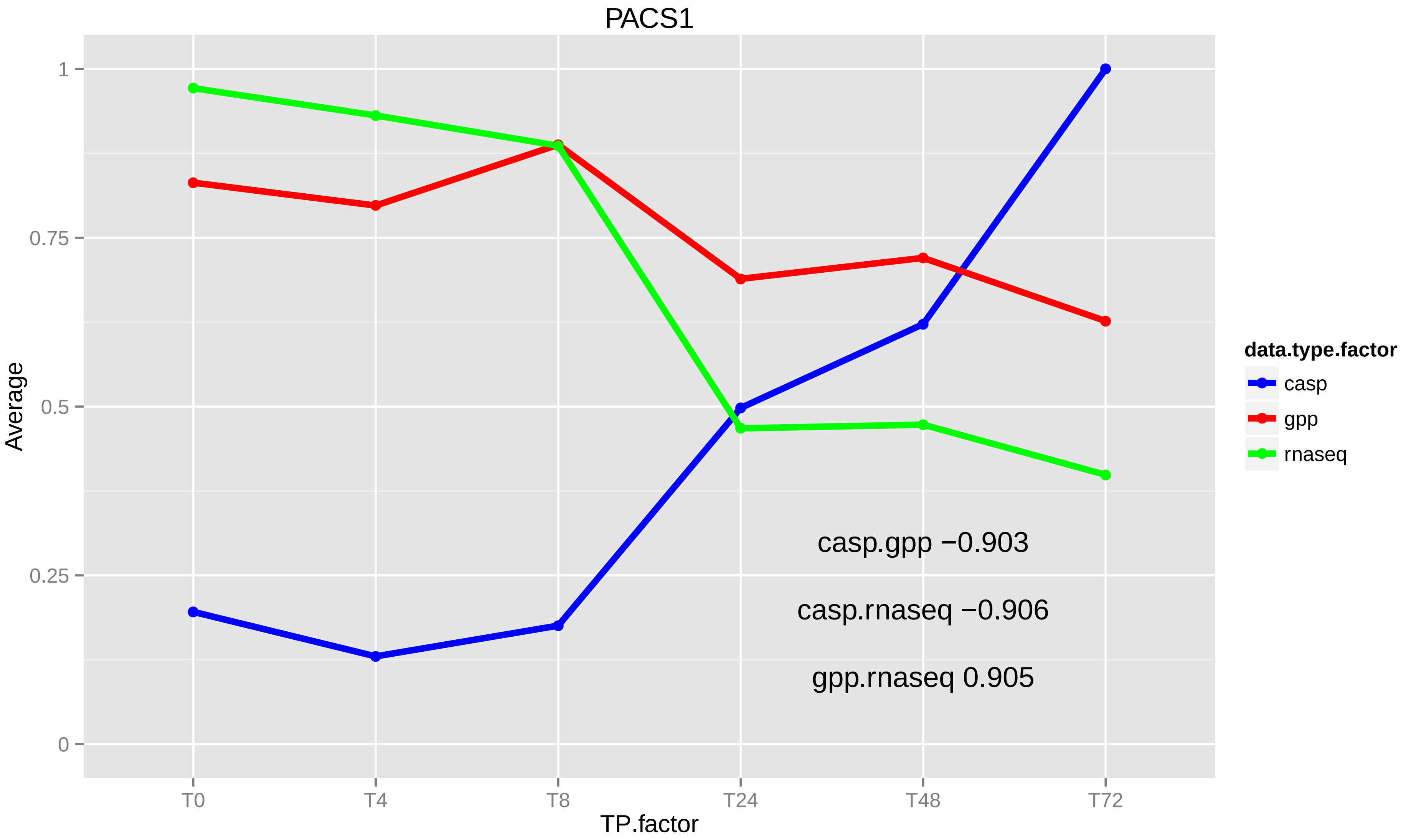
PACS1
Average
1
0.75
0.5
0.25
0
T0
T4
T8
T24
T48
T72
TP.factor
data.type.factor
casp
gpp
rnaseq
casp.gpp −0.903
casp.rnaseq −0.906
gpp.rnaseq 0.905

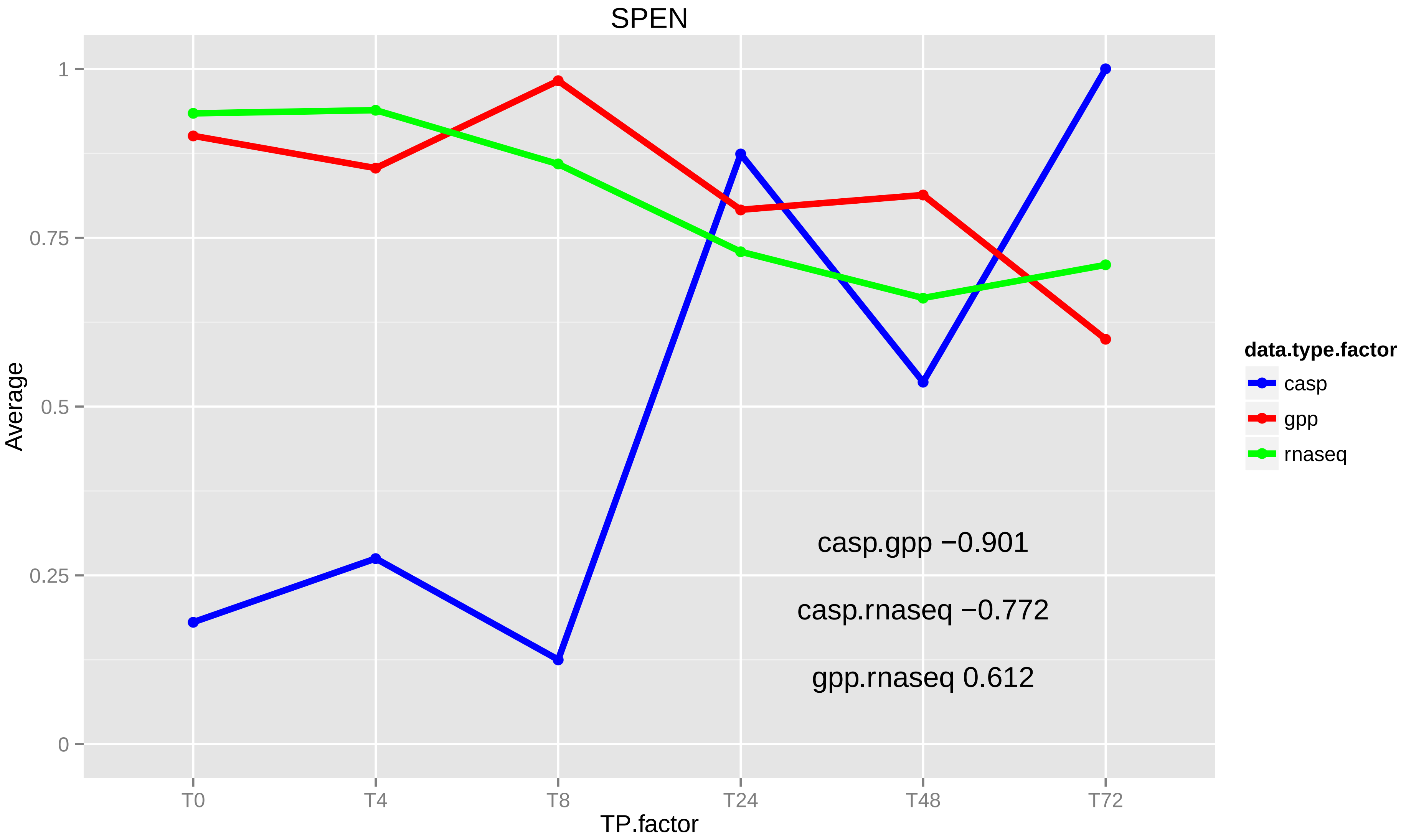

SPEN
Average
1
0.75
0.5
0.25
0
T0
T4
T8
T24
T48
T72
TP.factor
data.type.factor
casp
gpp
rnaseq
casp.gpp −0.901
casp.rnaseq −0.772
gpp.rnaseq 0.612

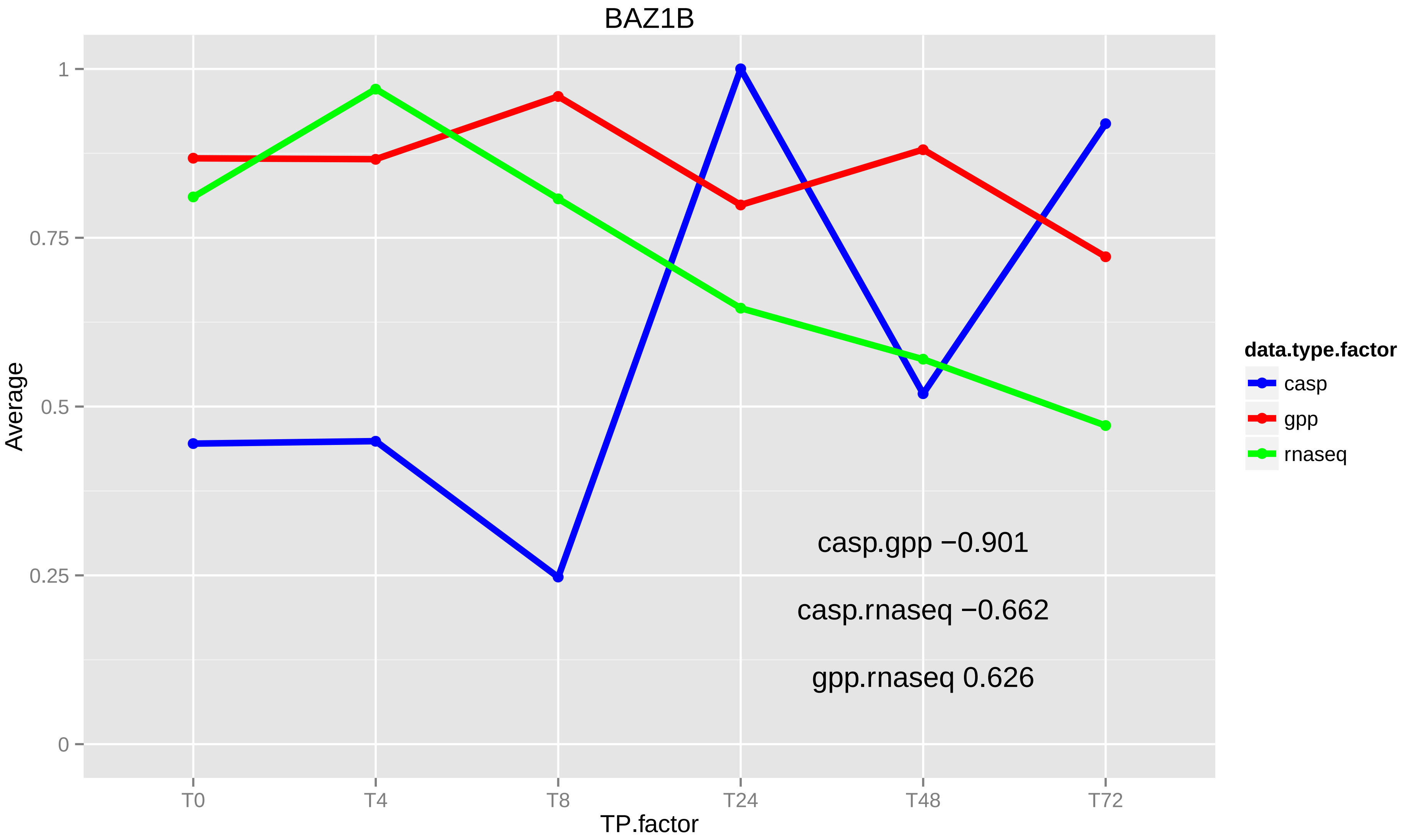

BAZ1B
Average
1
0.75
0.5
0.25
0
T0
T4
T8
T24
T48
T72
TP.factor
data.type.factor
casp
gpp
rnaseq
casp.gpp −0.901
casp.rnaseq −0.662
gpp.rnaseq 0.626

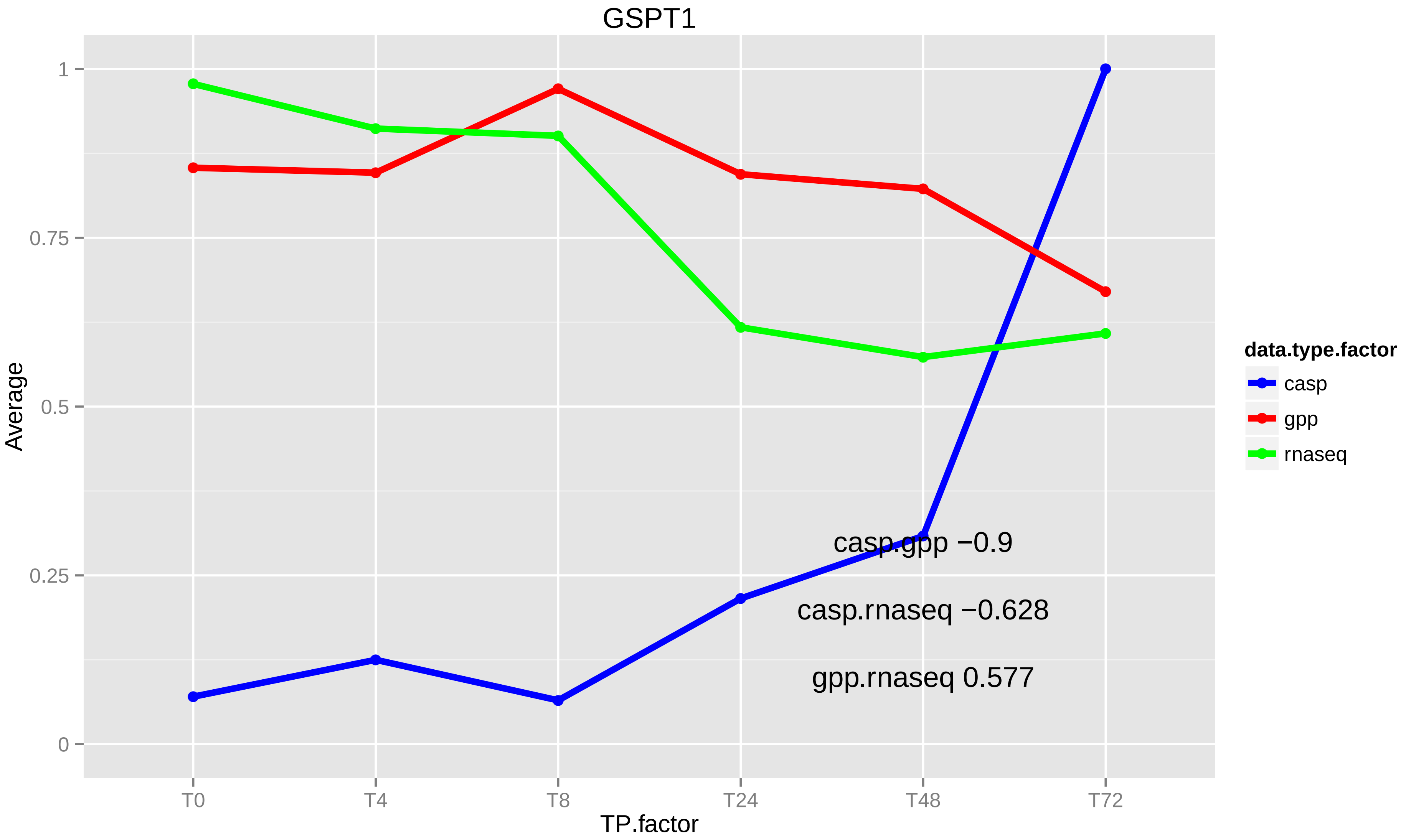

GSPT1
Average
1
0.75
0.5
0.25
0
T0
T4
T8
T24
T48
T72
TP.factor
casp.gpp −0.9
casp.rnaseq −0.628
gpp.rnaseq 0.577
data.type.factor
casp
gpp
rnaseq

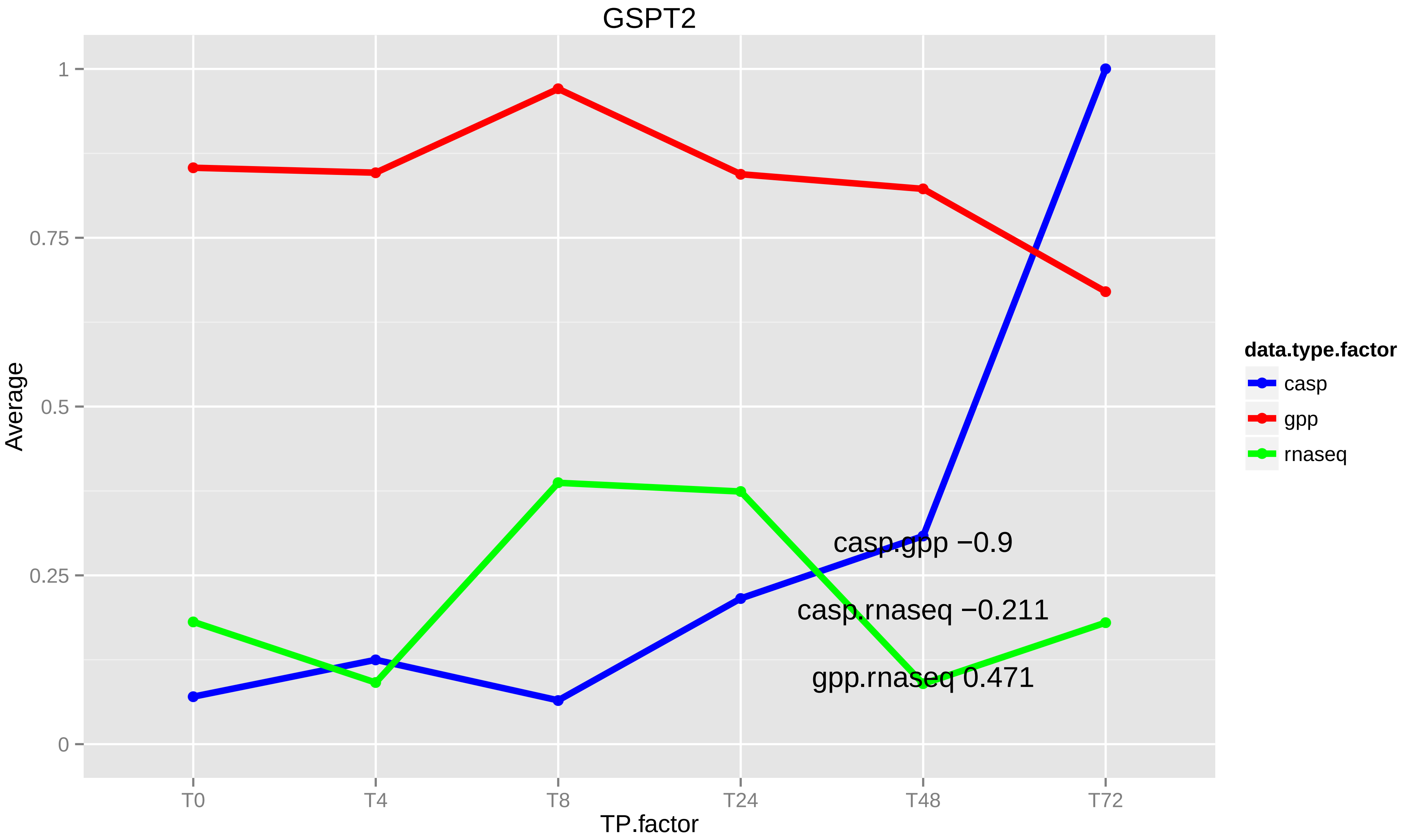
GSPT2
Average
1
0.75
0.5
0.25
0
T0
T4
T8
T24
T48
T72
TP.factor
casp.gpp −0.9
casp.rnaseq −0.211
gpp.rnaseq 0.471
data.type.factor
casp
gpp
rnaseq

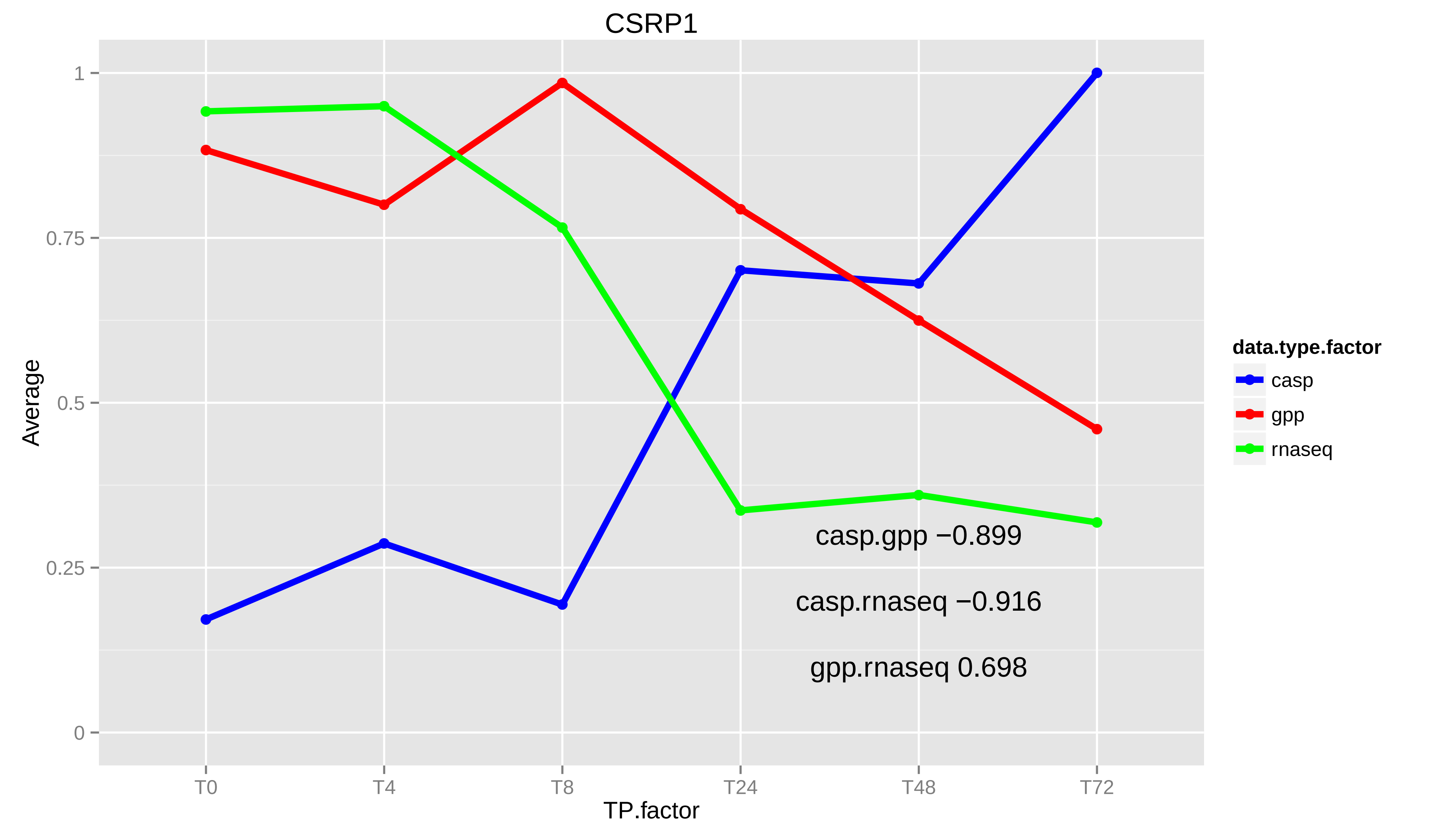

CSRP1
Average
1
0.75
0.5
0.25
0
T0
T4
T8
T24
T48
T72
TP.factor
data.type.factor
casp
gpp
rnaseq
casp.gpp −0.899
casp.rnaseq −0.916
gpp.rnaseq 0.698

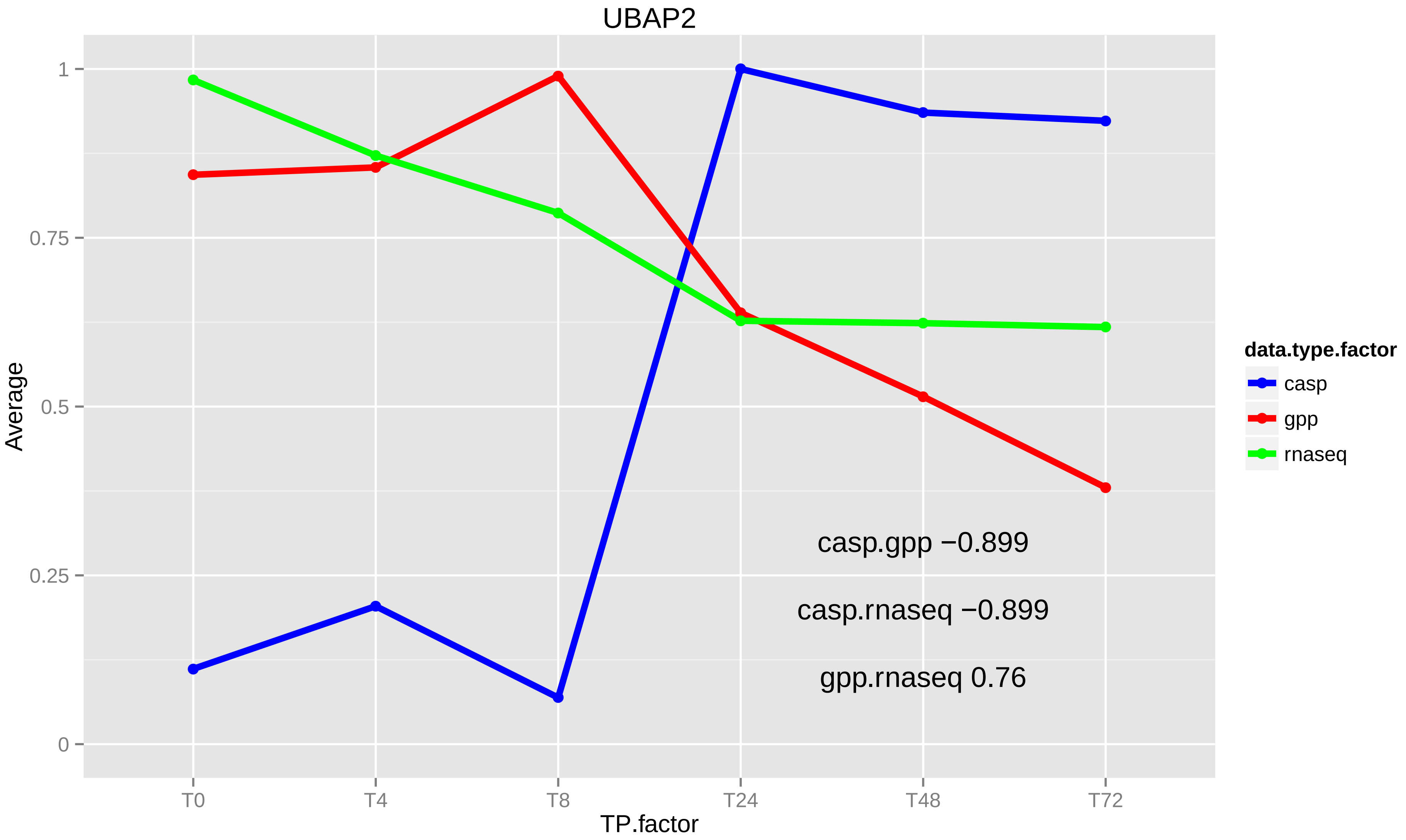

UBAP2
1
0.75
0.5
0.25
0
Average
T0
T4
T8
T24
T48
T72
TP.factor
data.type.factor
casp
gpp
rnaseq
casp.gpp −0.899
casp.rnaseq −0.899
gpp.rnaseq 0.76

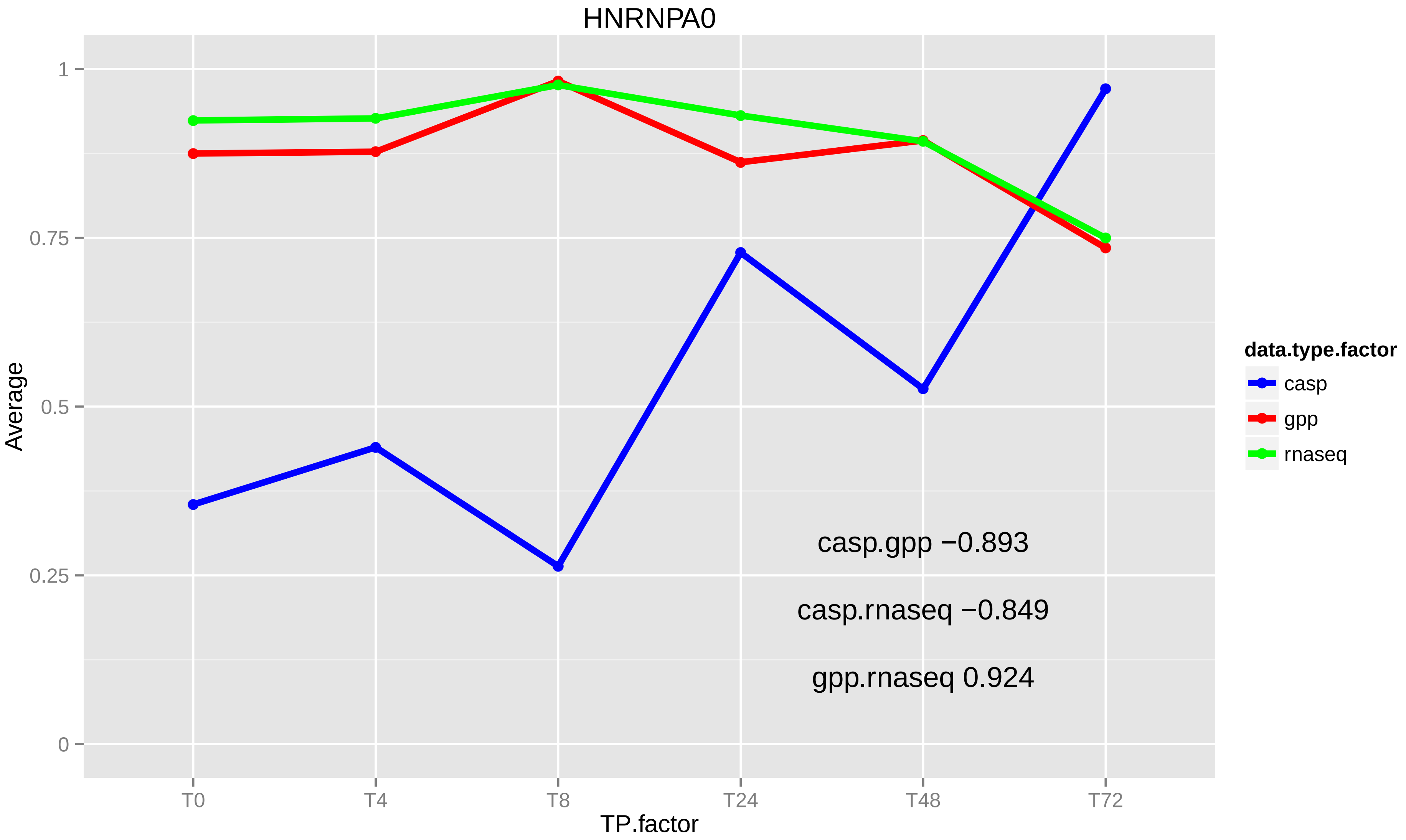
HNRNPA0
Average
1
0.75
0.5
0.25
0
T0
T4
T8
T24
T48
T72
TP.factor
data.type.factor
casp
gpp
rnaseq
casp.gpp −0.893
casp.rnaseq −0.849
gpp.rnaseq 0.924

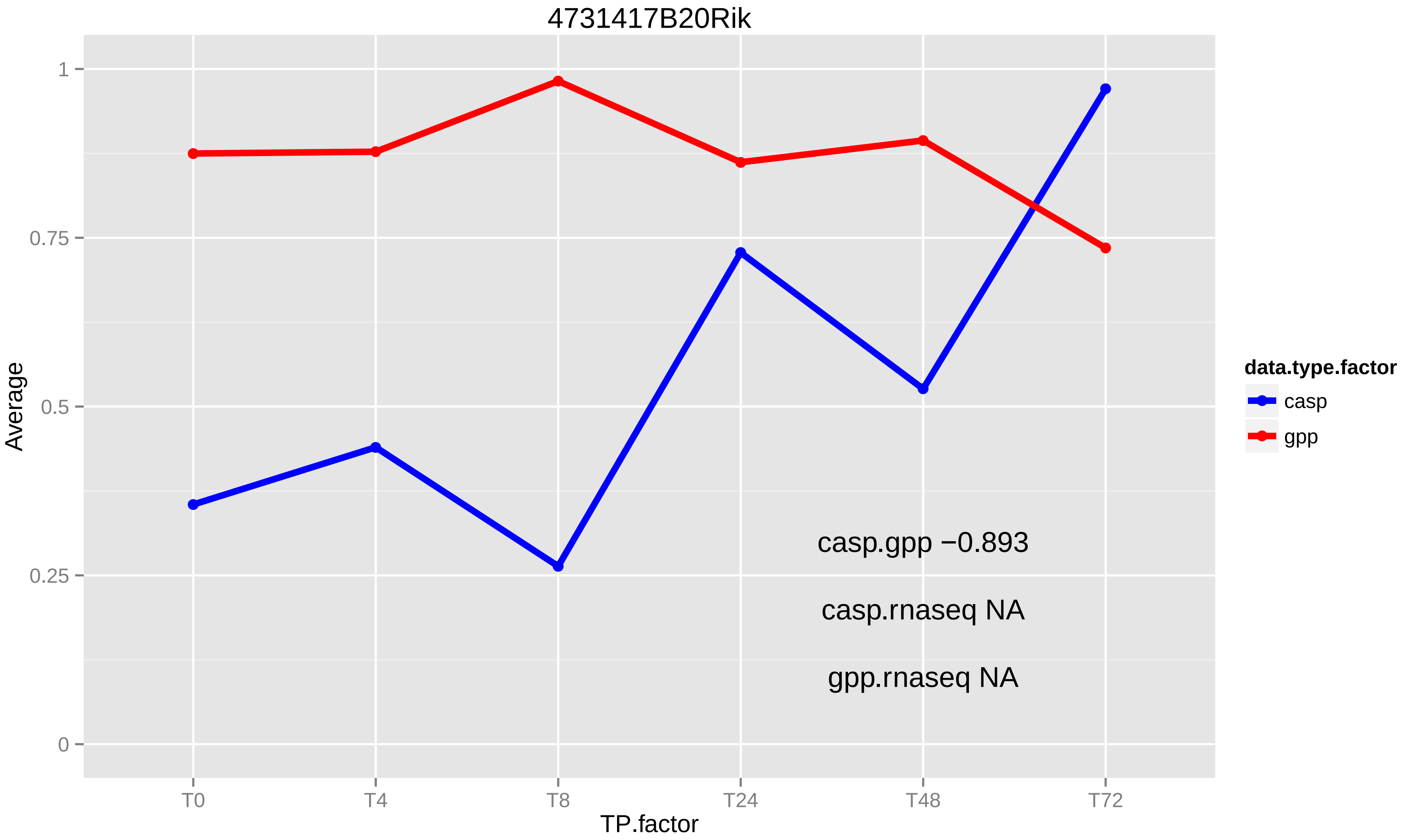
4731417B20Rik
Average
1
0.75
0.5
0.25
0
T0
T4
T8
T24
T48
T72
TP.factor
data.type.factor
casp
gpp
casp.gpp −0.893
casp.rnaseq NA
gpp.rnaseq NA

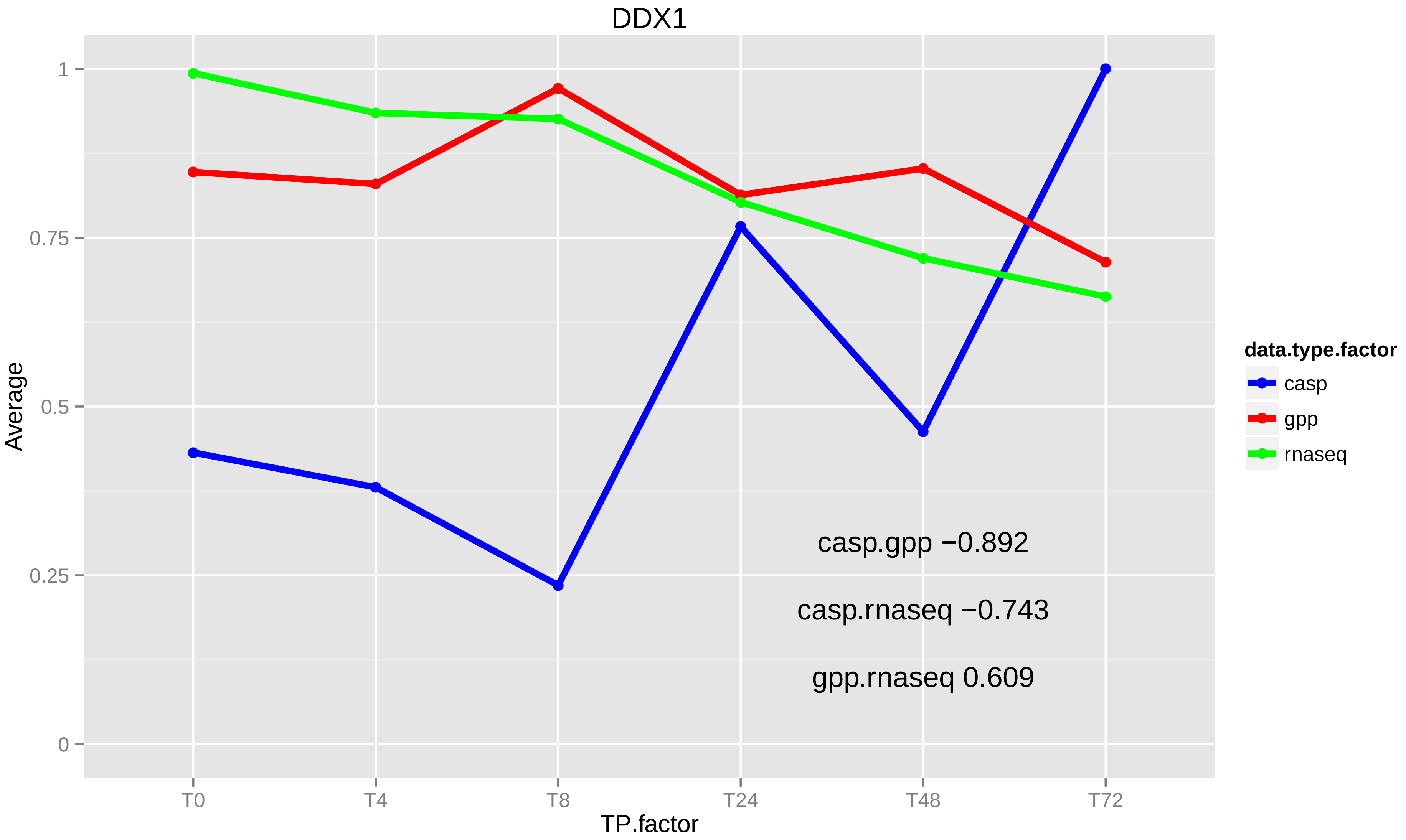
DDX1
1
0.75
0.5
0.25
0
Average
T0
T4
T8
T24
T48
T72
TP.factor
data.type.factor
casp
gpp
rnaseq
casp.gpp −0.892
casp.rnaseq −0.743
gpp.rnaseq 0.609

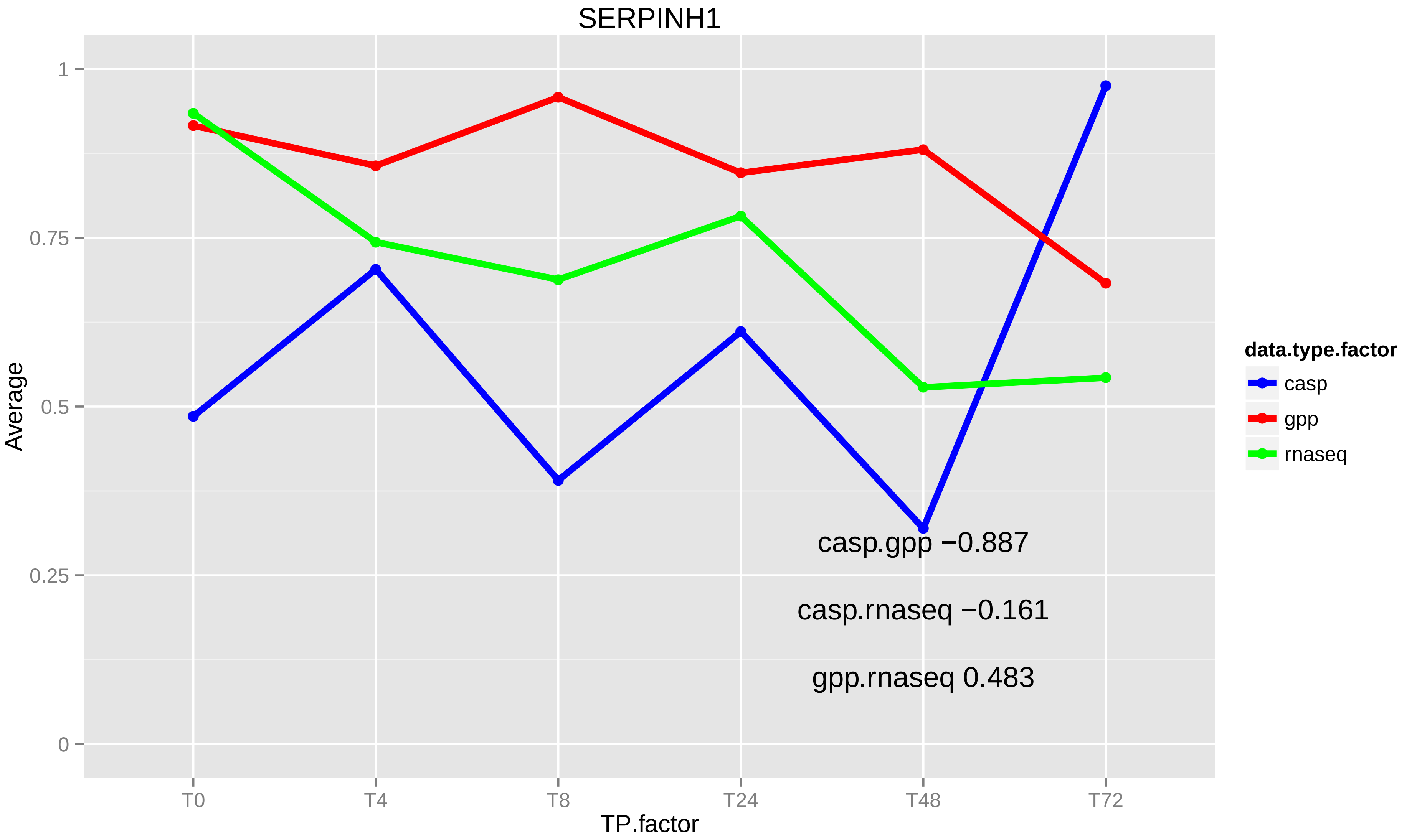
SERPINH1
Average
1
0.75
0.5
0.25
0
T0
T4
T8
T24
T48
T72
TP.factor
data.type.factor
casp
gpp
rnaseq
casp.gpp −0.887
casp.rnaseq −0.161
gpp.rnaseq 0.483

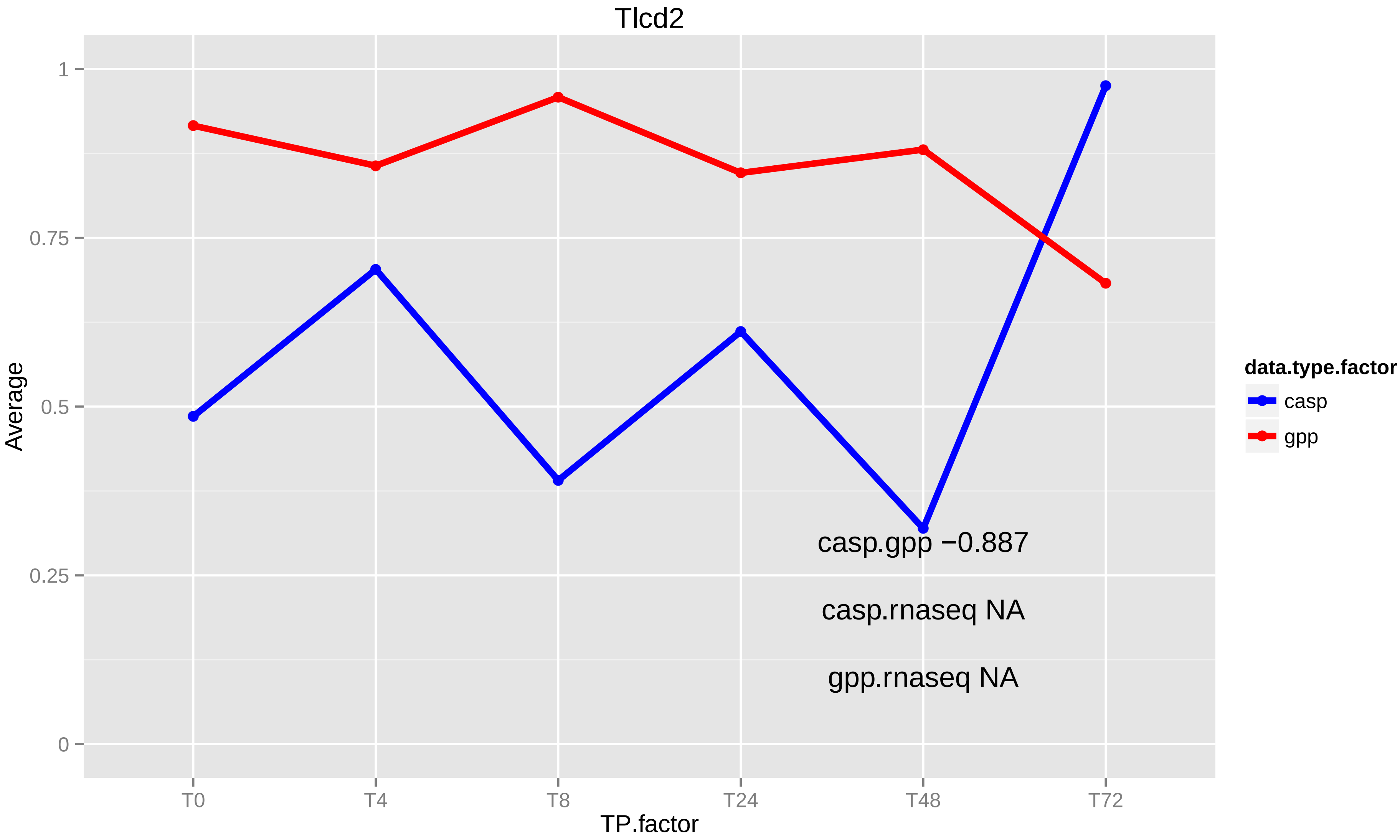
Tlcd2
Average
1
0.75
0.5
0.25
0
T0
T4
T8
T24
T48
T72
TP.factor
data.type.factor
casp
gpp
casp.gpp −0.887
casp.rnaseq NA
gpp.rnaseq NA

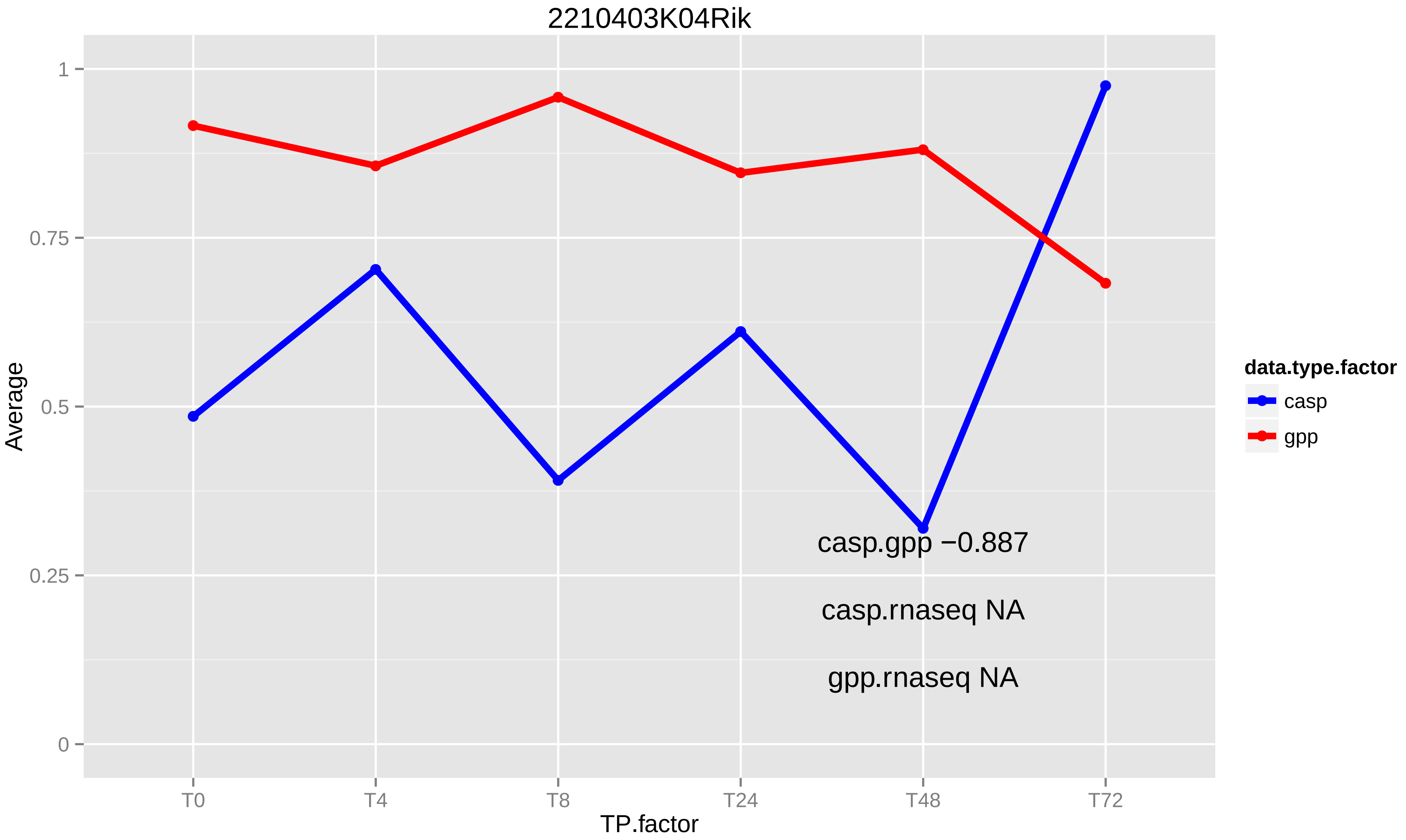
2210403K04Rik
1
0.75
0.5
0.25
0
Average
T0
T4
T8
T24
T48
T72
TP.factor
data.type.factor
casp
gpp
casp.gpp −0.887
casp.rnaseq NA
gpp.rnaseq NA

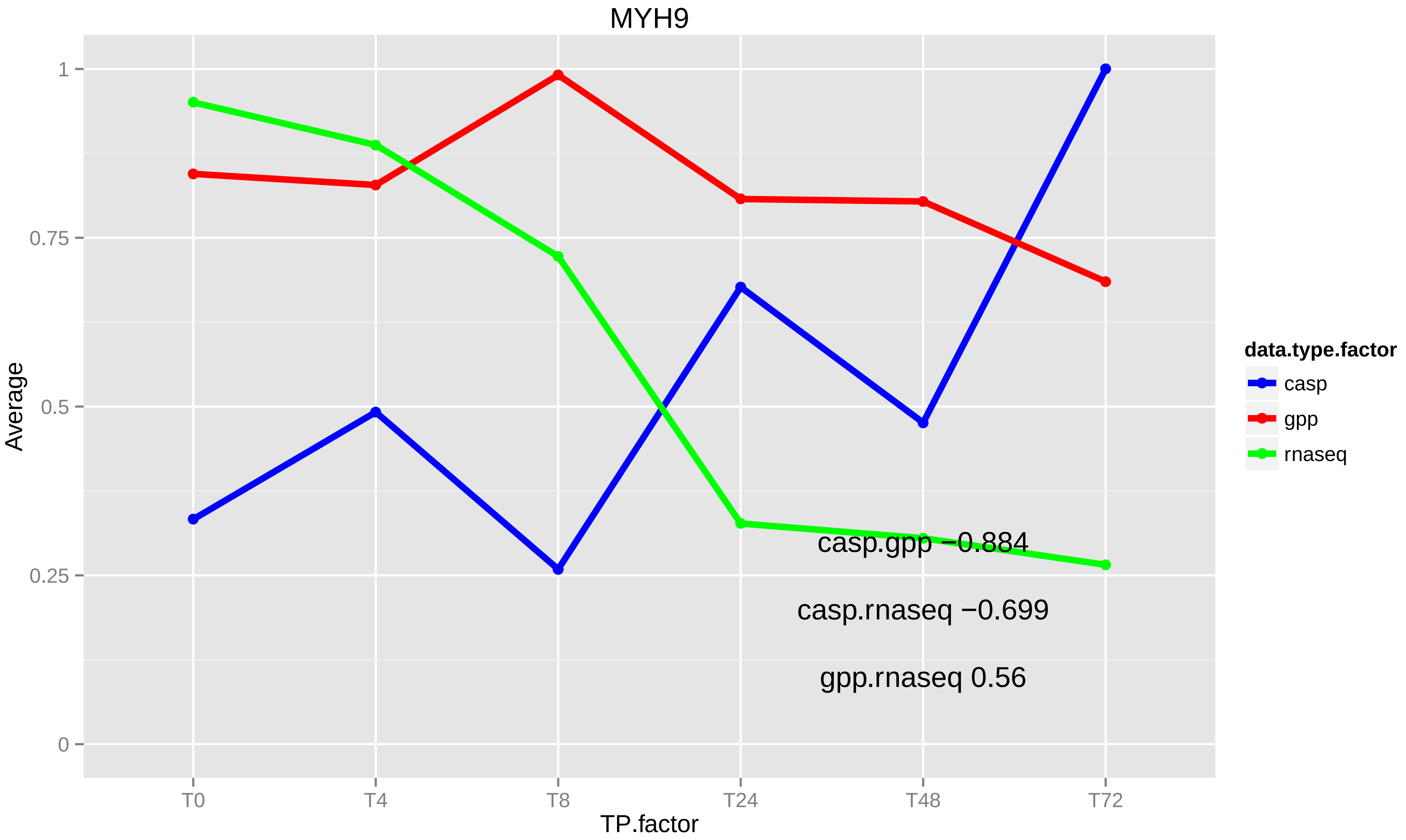
MYH9
Average
1
0.75
0.5
0.25
0
T0
T4
T8
T24
T48
T72
TP.factor
data.type.factor
casp
gpp
rnaseq
casp.gpp −0.884
casp.rnaseq −0.699
gpp.rnaseq 0.56

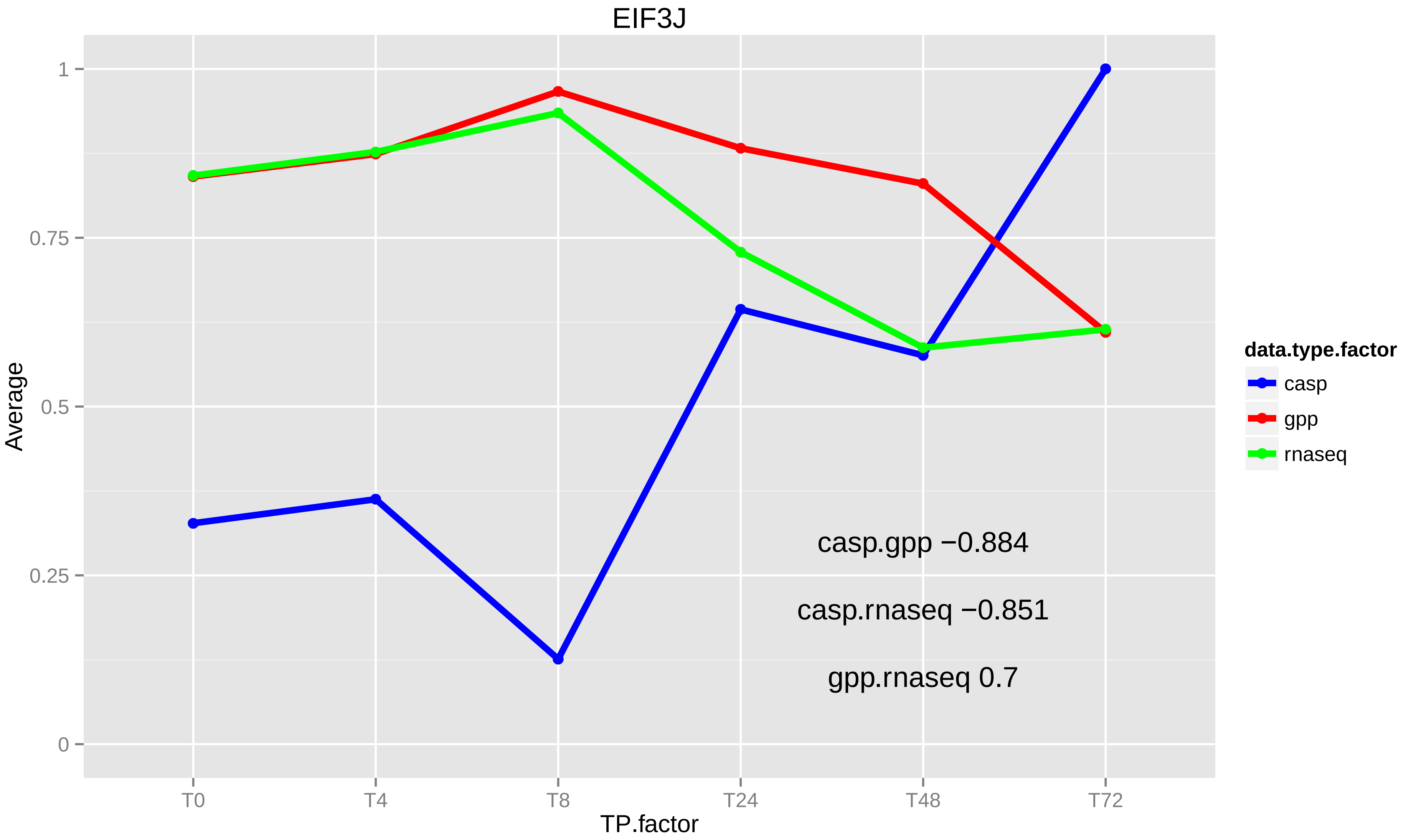

EIF3J
1
0.75
0.5
0.25
0
Average
T0
T4
T8
T24
T48
T72
TP.factor
data.type.factor
casp
gpp
rnaseq
casp.gpp −0.884
casp.rnaseq −0.851
gpp.rnaseq 0.7

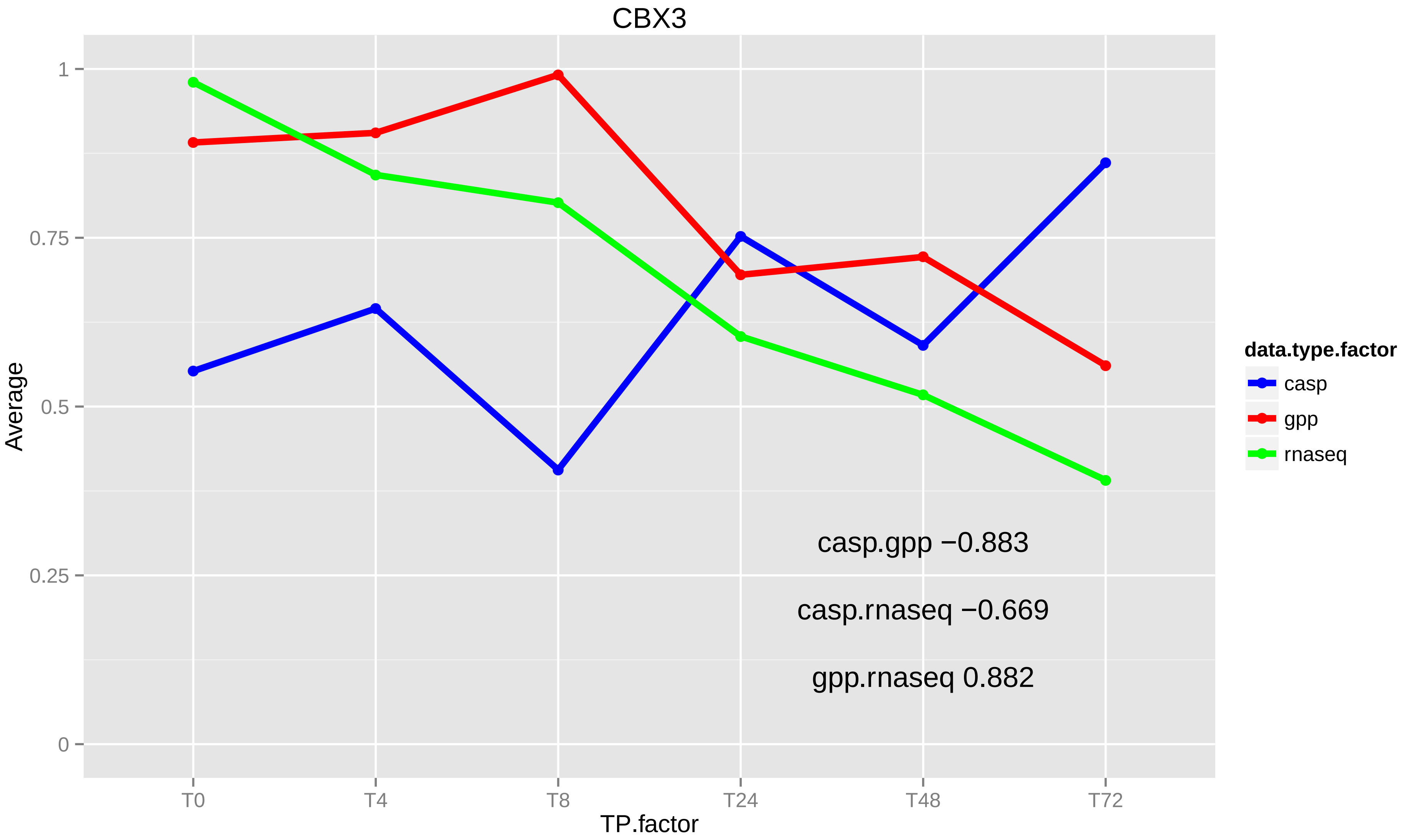
CBX3
1
0.75
0.5
0.25
0
Average
T0
T4
T8
T24
T48
T72
TP.factor
data.type.factor
casp
gpp
rnaseq
casp.gpp −0.883
casp.rnaseq −0.669
gpp.rnaseq 0.882

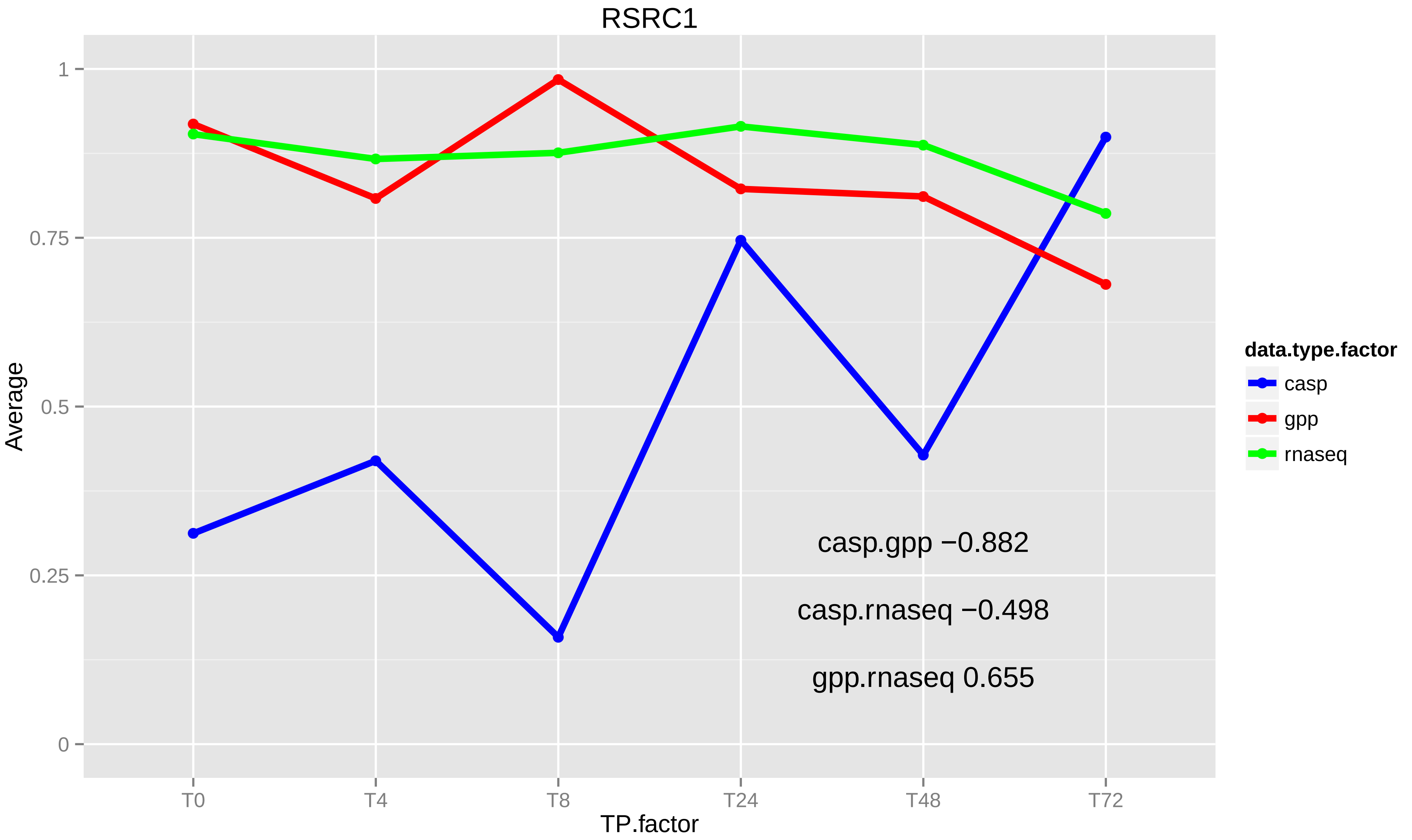

RSRC1
1
0.75
0.5
0.25
0
Average
T0
T4
T8
T24
T48
T72
TP.factor
data.type.factor
casp
gpp
rnaseq
casp.gpp −0.882
casp.rnaseq −0.498
gpp.rnaseq 0.655

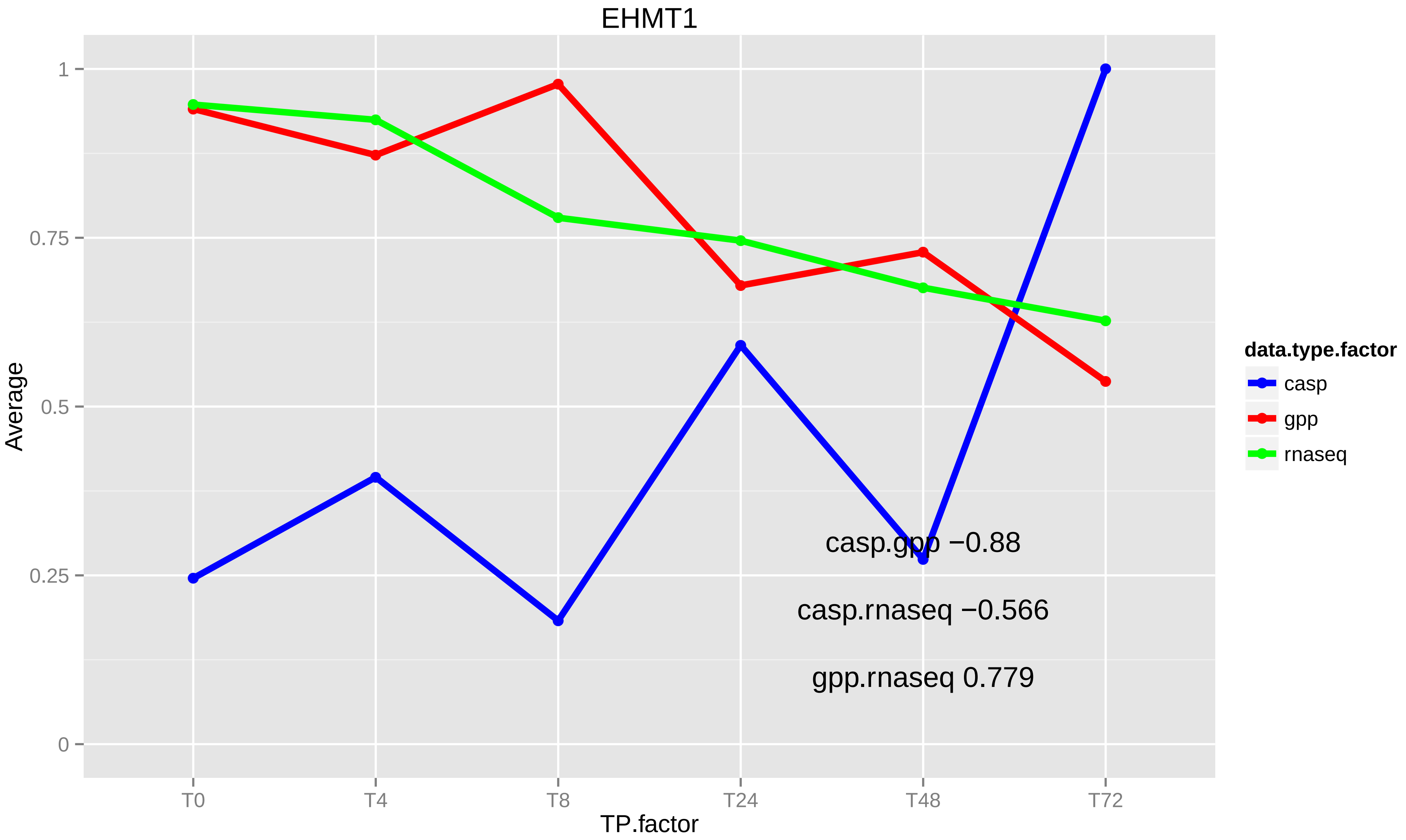

EHMT1
Average
1
0.75
0.5
0.25
0
T0
T4
T8
T24
T48
T72
TP.factor
data.type.factor
casp
gpp
rnaseq
casp.gpp −0.88
casp.rnaseq −0.566
gpp.rnaseq 0.779

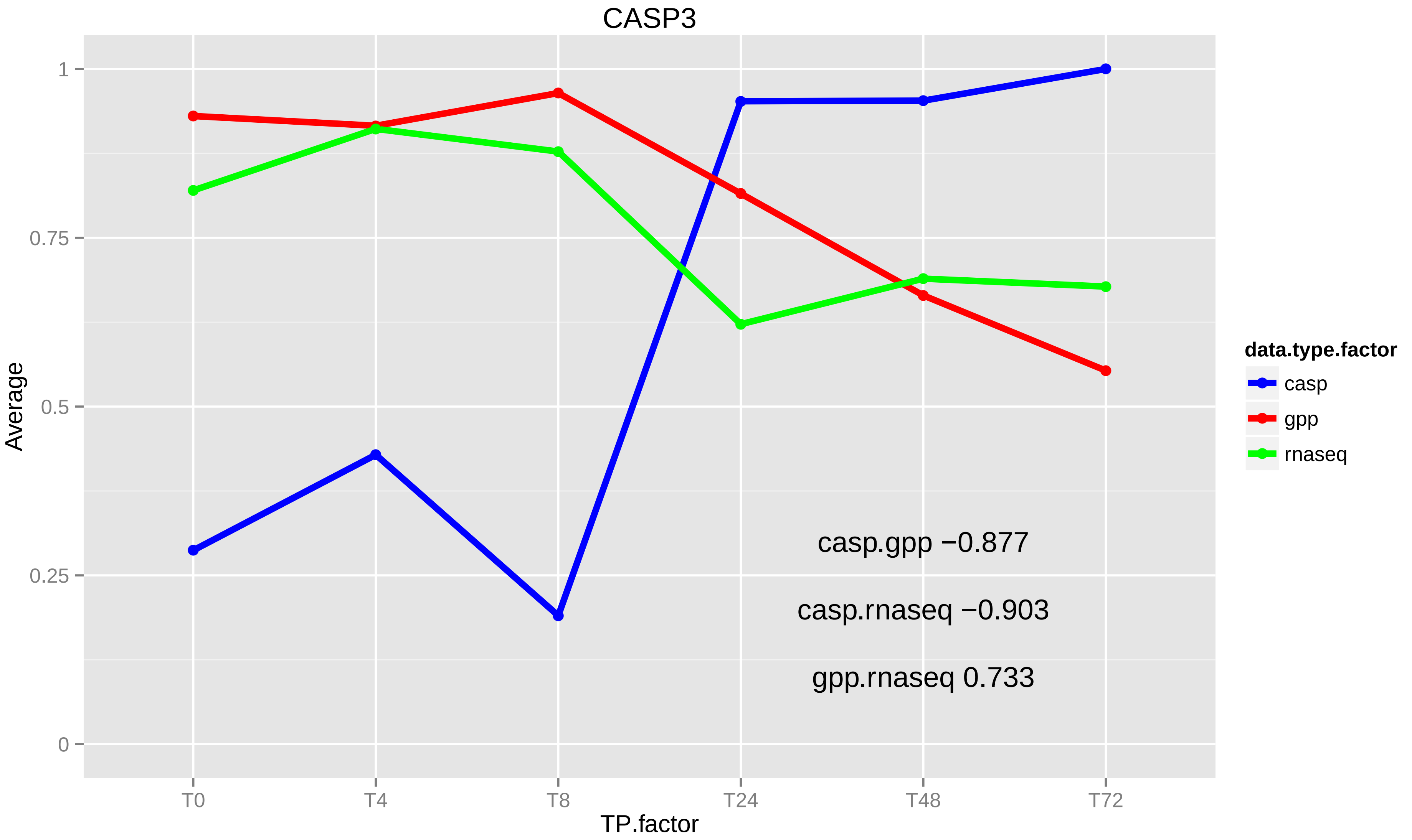

CASP3
Average
1
0.75
0.5
0.25
0
T0
T4
T8
T24
T48
T72
TP.factor
data.type.factor
casp
gpp
rnaseq
casp.gpp −0.877
casp.rnaseq −0.903
gpp.rnaseq 0.733

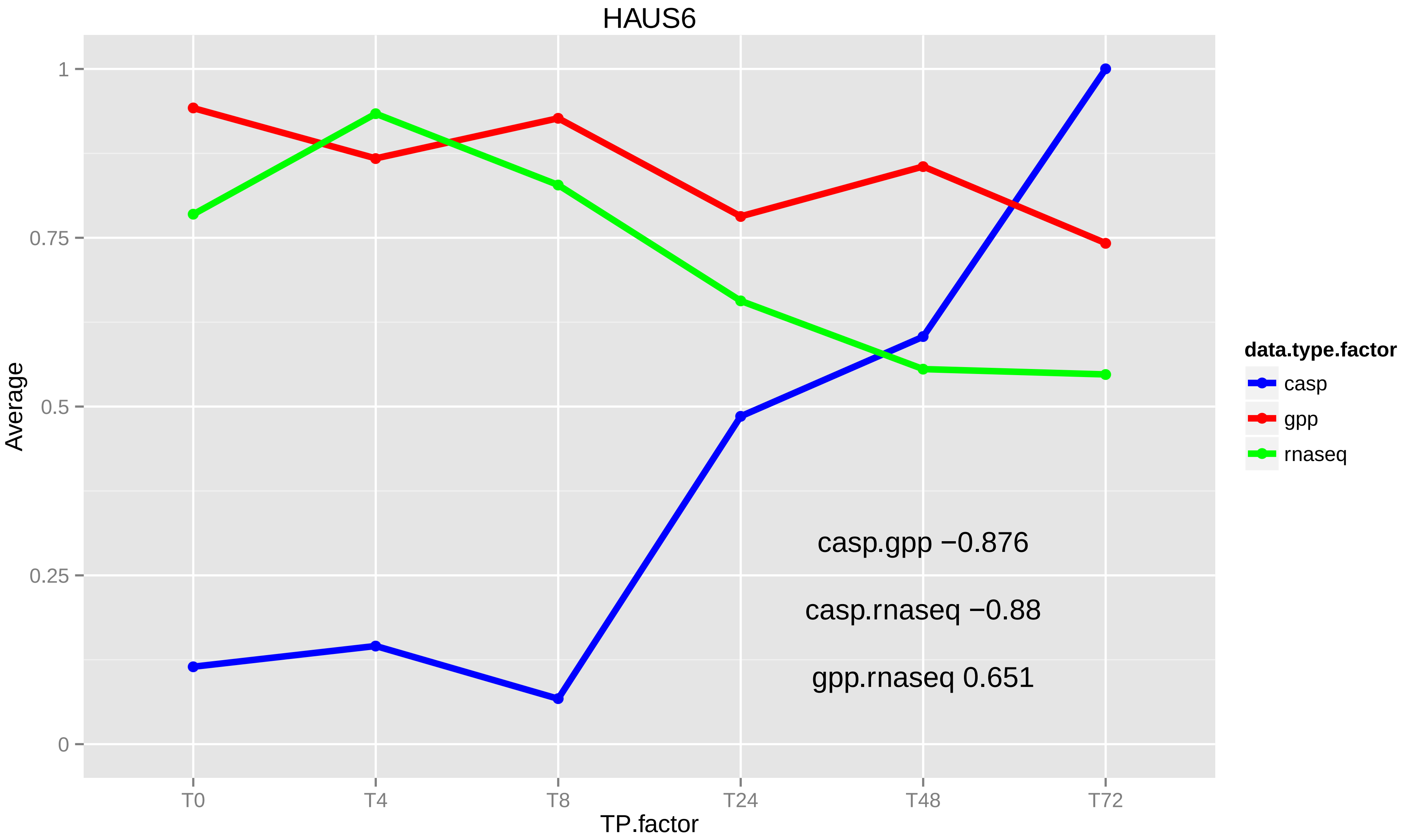
HAUS6
Average
1
0.75
0.5
0.25
0
T0
T4
T8
T24
T48
T72
TP.factor
data.type.factor
casp
gpp
rnaseq
casp.gpp −0.876
casp.rnaseq −0.88
gpp.rnaseq 0.651

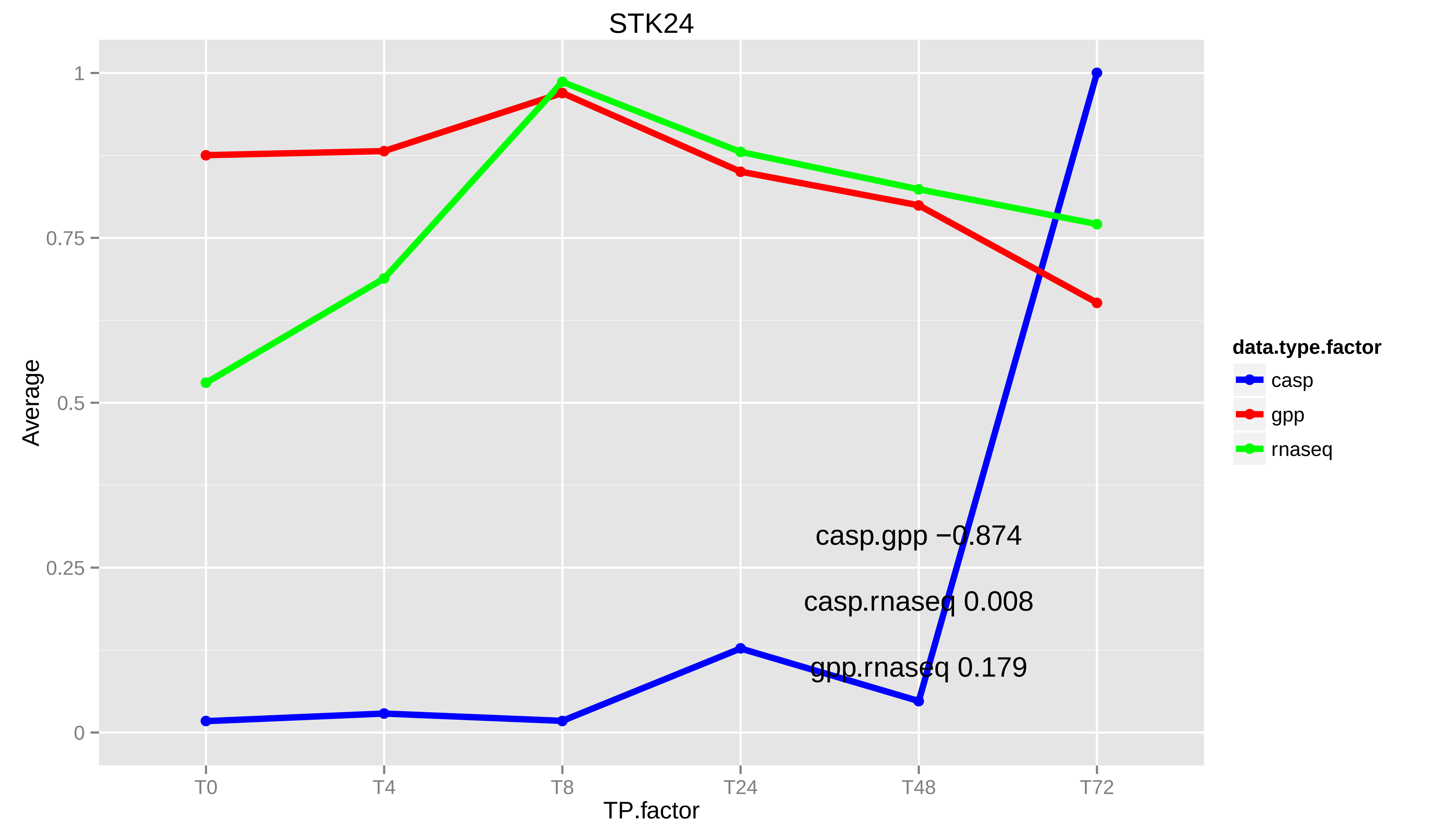
STK24
1
0.75
0.5
0.25
0
Average
T0
T4
T8
T24
T48
T72
TP.factor
data.type.factor
casp
gpp
rnaseq
casp.gpp −0.874
casp.rnaseq 0.008
gpp.rnaseq 0.179

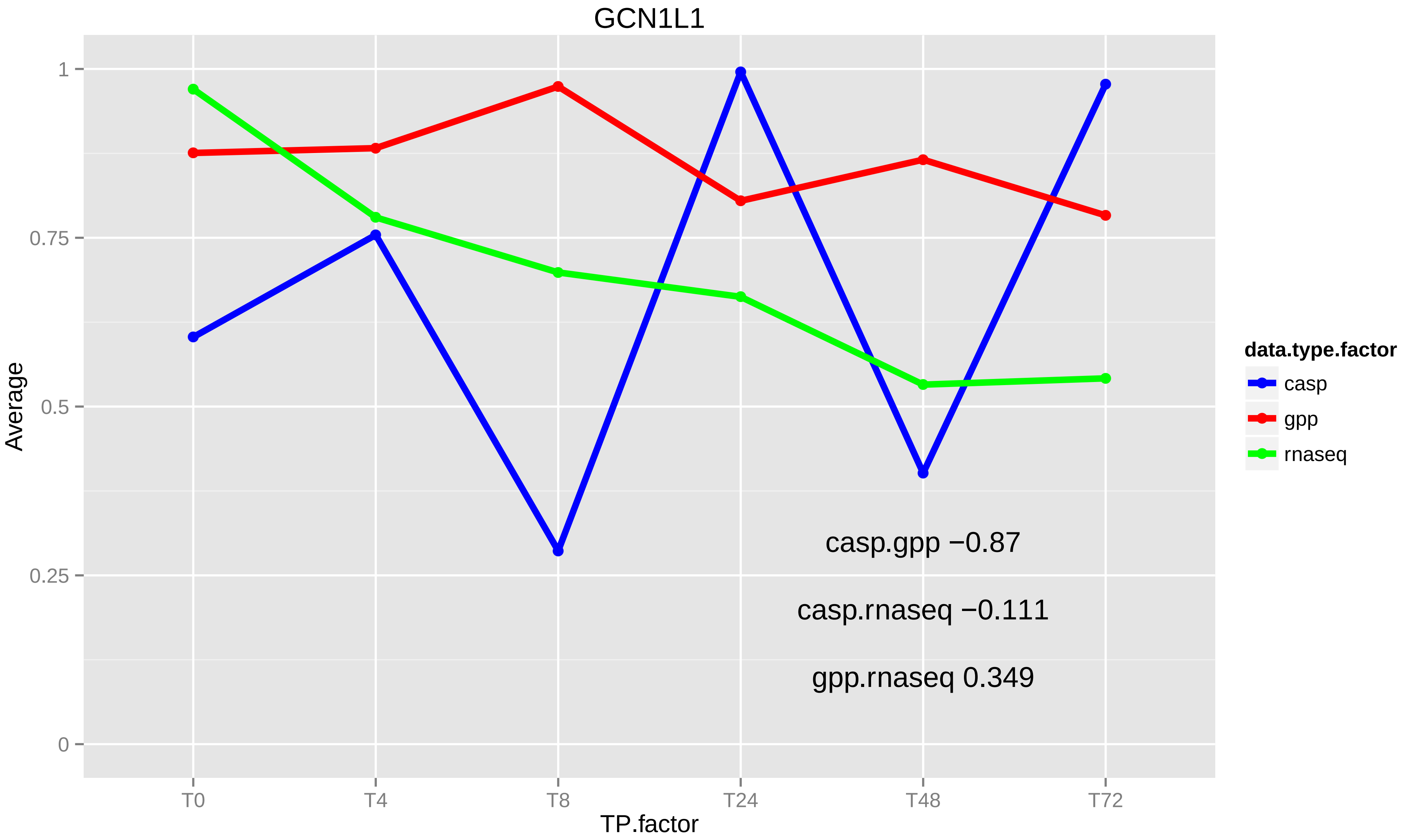
GCN1L1
Average
1
0.75
0.5
0.25
0
T0
T4
T8
T24
T48
T72
TP.factor
data.type.factor
casp
gpp
rnaseq
casp.gpp −0.87
casp.rnaseq −0.111
gpp.rnaseq 0.349

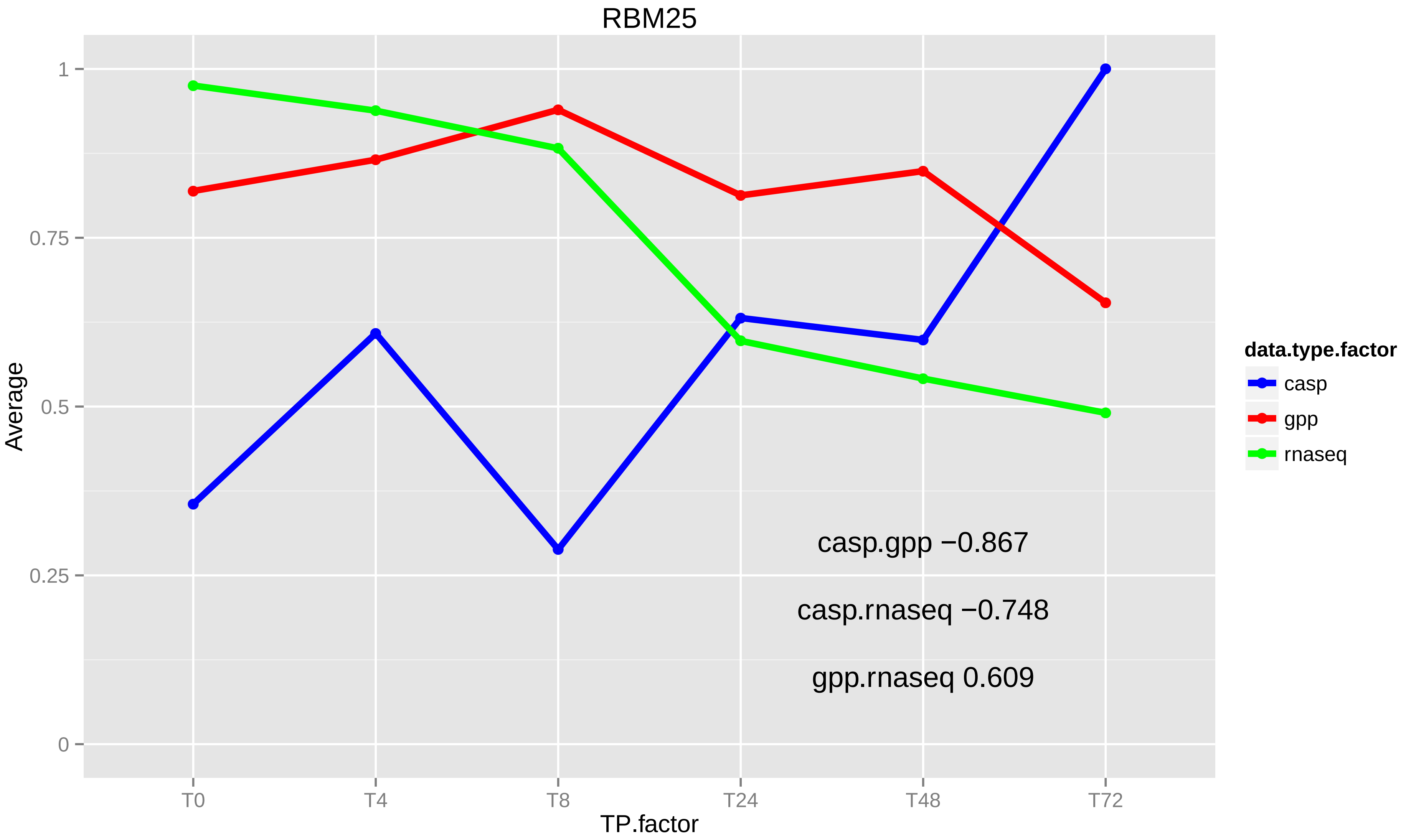
RBM25
Average
1
0.75
0.5
0.25
0
T0
T4
T8
T24
T48
T72
TP.factor
data.type.factor
casp
gpp
rnaseq
casp.gpp −0.867
casp.rnaseq −0.748
gpp.rnaseq 0.609

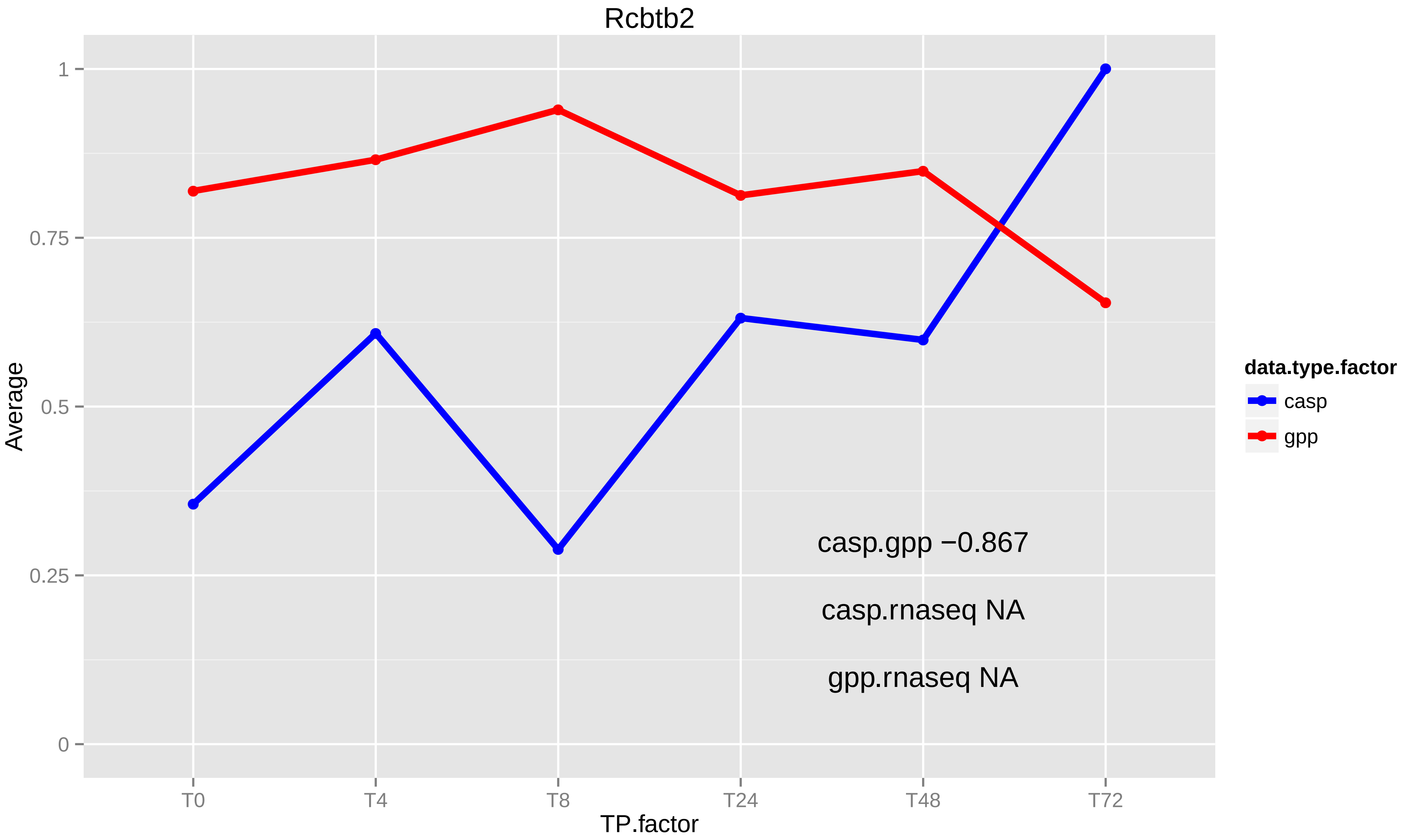
Rcbtb2
Average
TP.factor
1
0.75
0.5
0.25
0
T0
T4
T8
T24
T48
T72
data.type.factor
casp
gpp
casp.gpp −0.867
casp.rnaseq NA
gpp.rnaseq NA

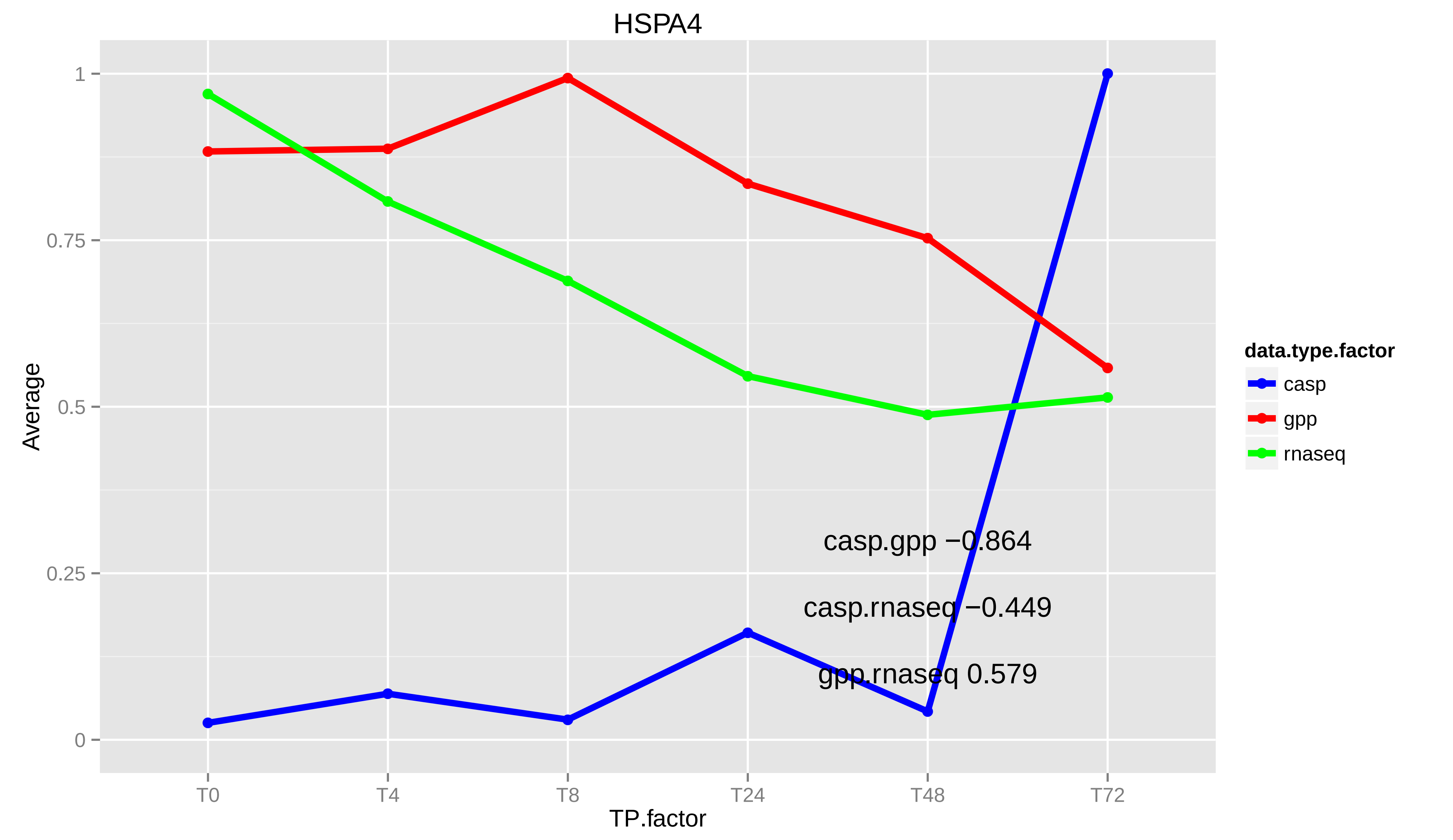

HSPA4
Average
1
0.75
0.5
0.25
0
T0
T4
T8
T24
T48
T72
TP.factor
data.type.factor
casp
gpp
rnaseq
casp.gpp −0.864
casp.rnaseq −0.449
gpp.rnaseq 0.579

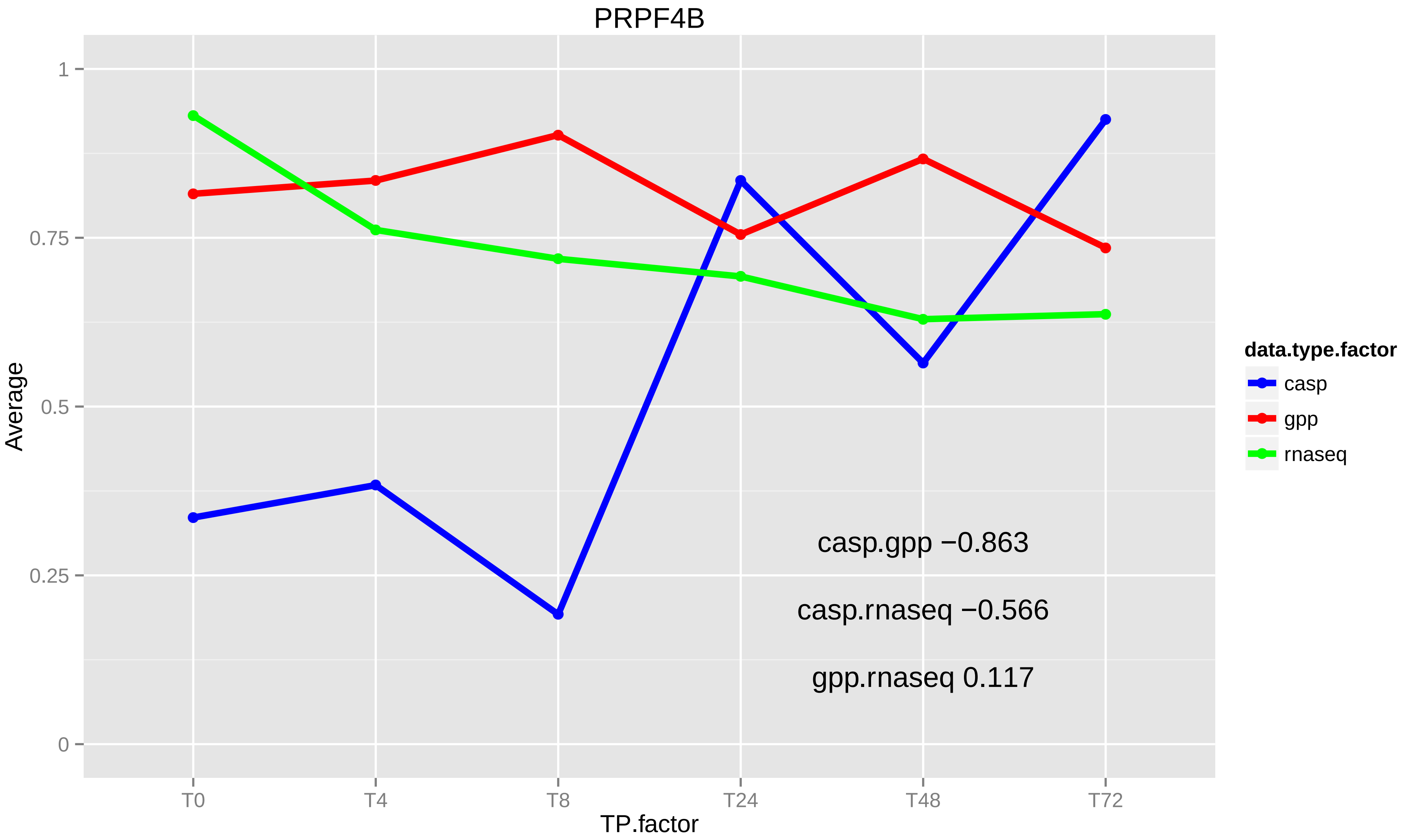
PRPF4B
Average
1
0.75
0.5
0.25
0
T0
T4
T8
T24
T48
T72
TP.factor
data.type.factor
casp
gpp
rnaseq
casp.gpp −0.863
casp.rnaseq −0.566
gpp.rnaseq 0.117

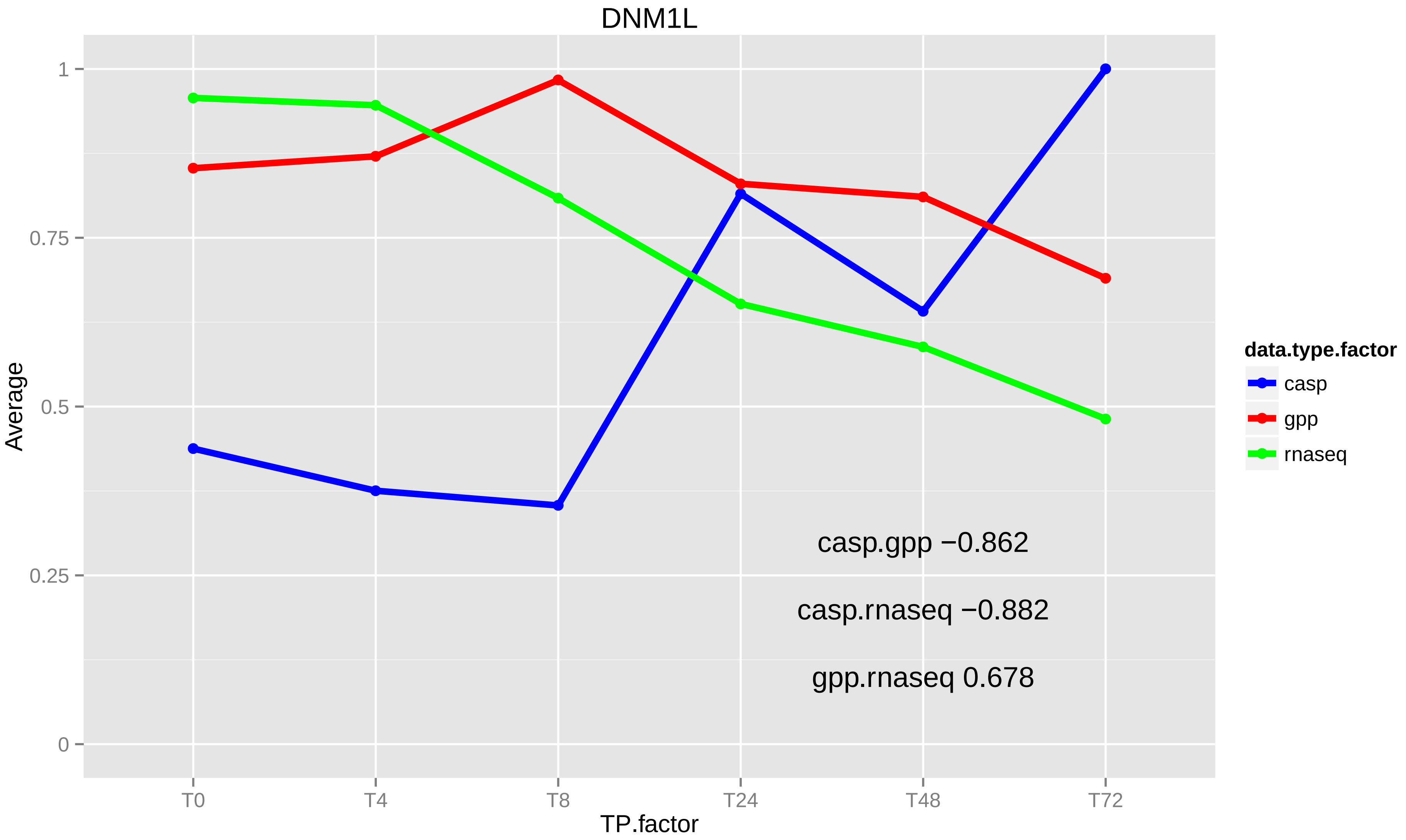
DNM1L
Average
1
0.75
0.5
0.25
0
T0
T4
T8
T24
T48
T72
TP.factor
data.type.factor
casp
gpp
rnaseq
casp.gpp −0.862
casp.rnaseq −0.882
gpp.rnaseq 0.678

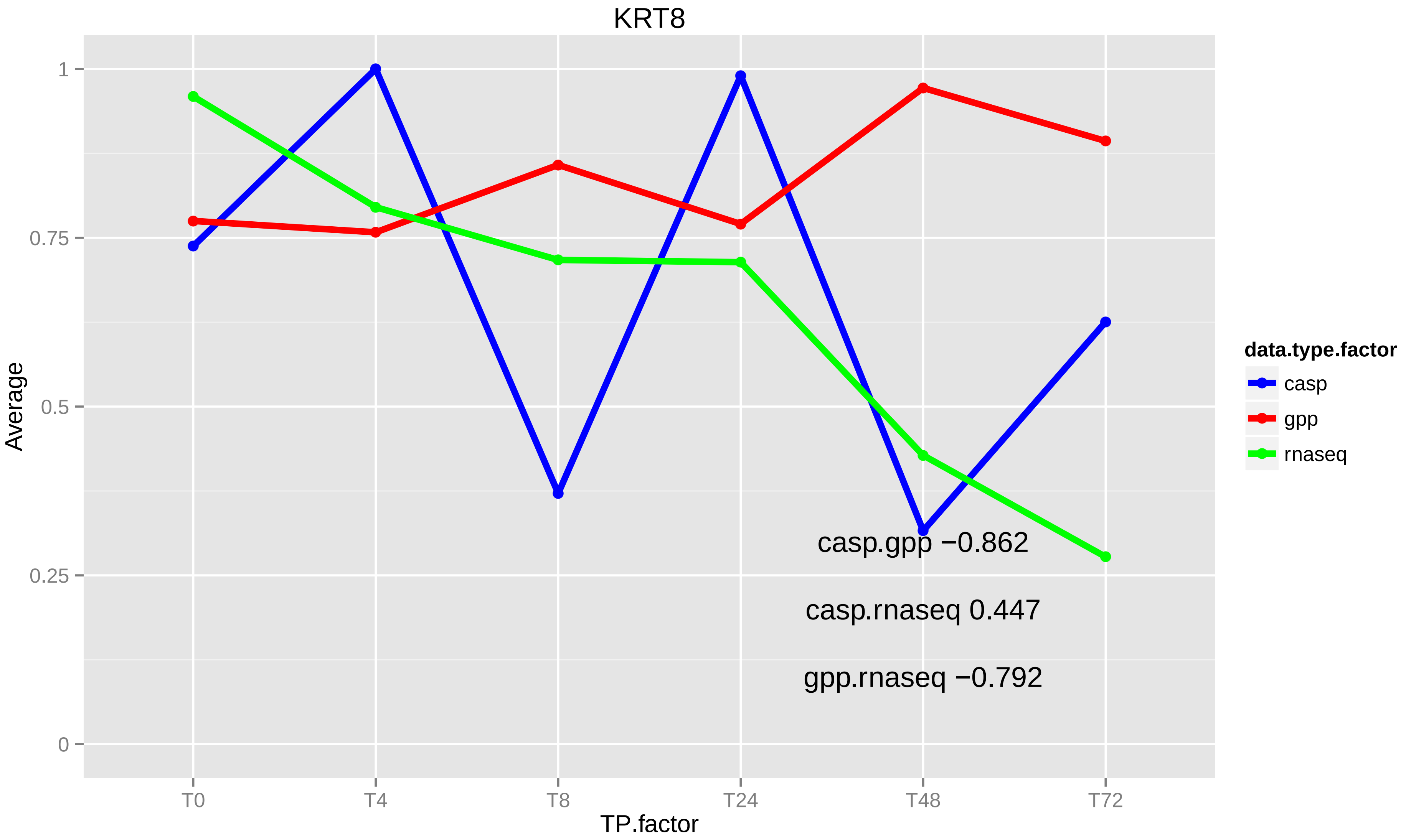

KRT8
Average
1
0.75
0.5
0.25
0
T0
T4
T8
T24
T48
T72
TP.factor
data.type.factor
casp
gpp
rnaseq
casp.gpp −0.862
casp.rnaseq 0.447
gpp.rnaseq −0.792

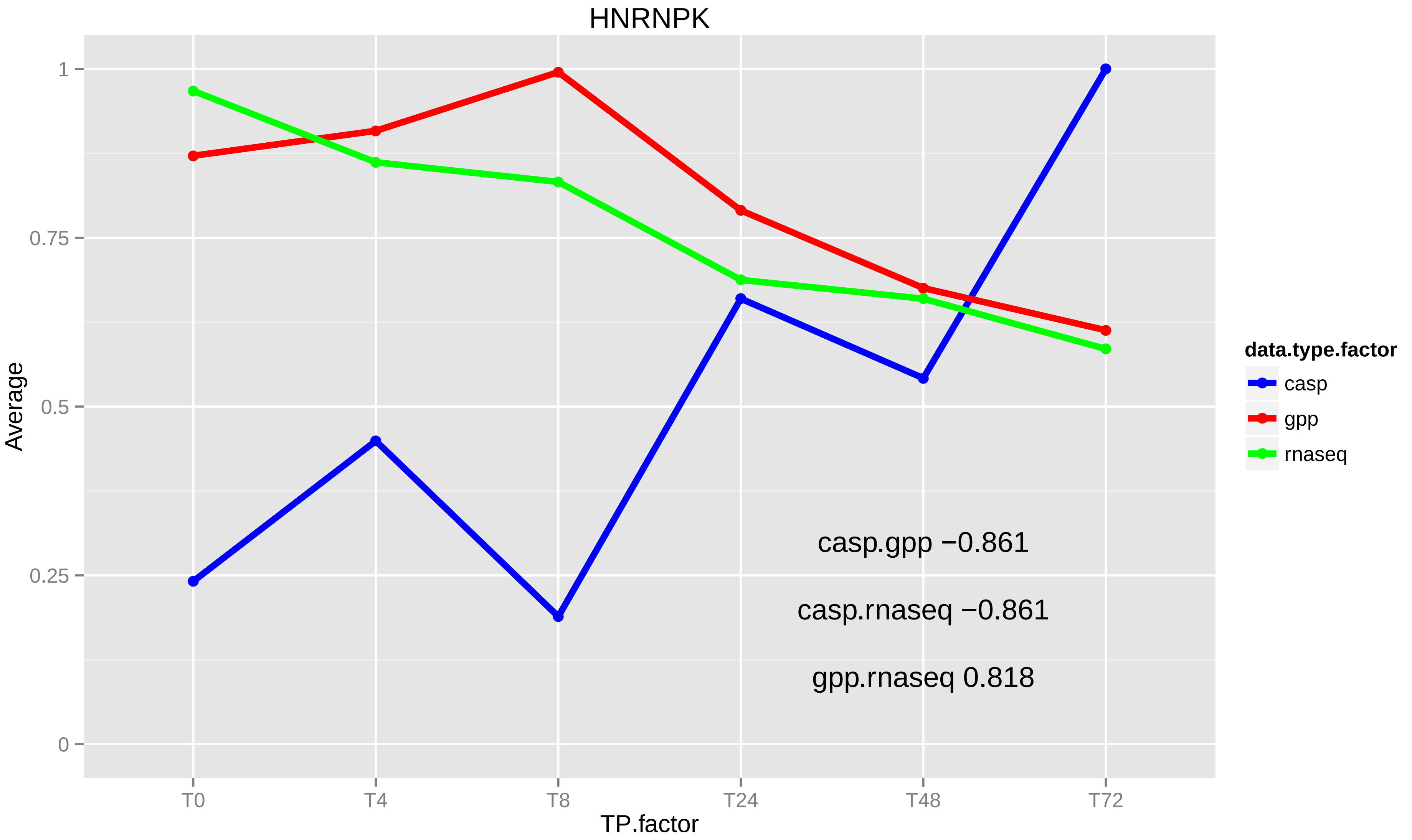
HNRNPK
Average
1
0.75
0.5
0.25
0
T0
T4
T8
T24
T48
T72
TP.factor
data.type.factor
casp
gpp
rnaseq
casp.gpp −0.861
casp.rnaseq −0.861
gpp.rnaseq 0.818

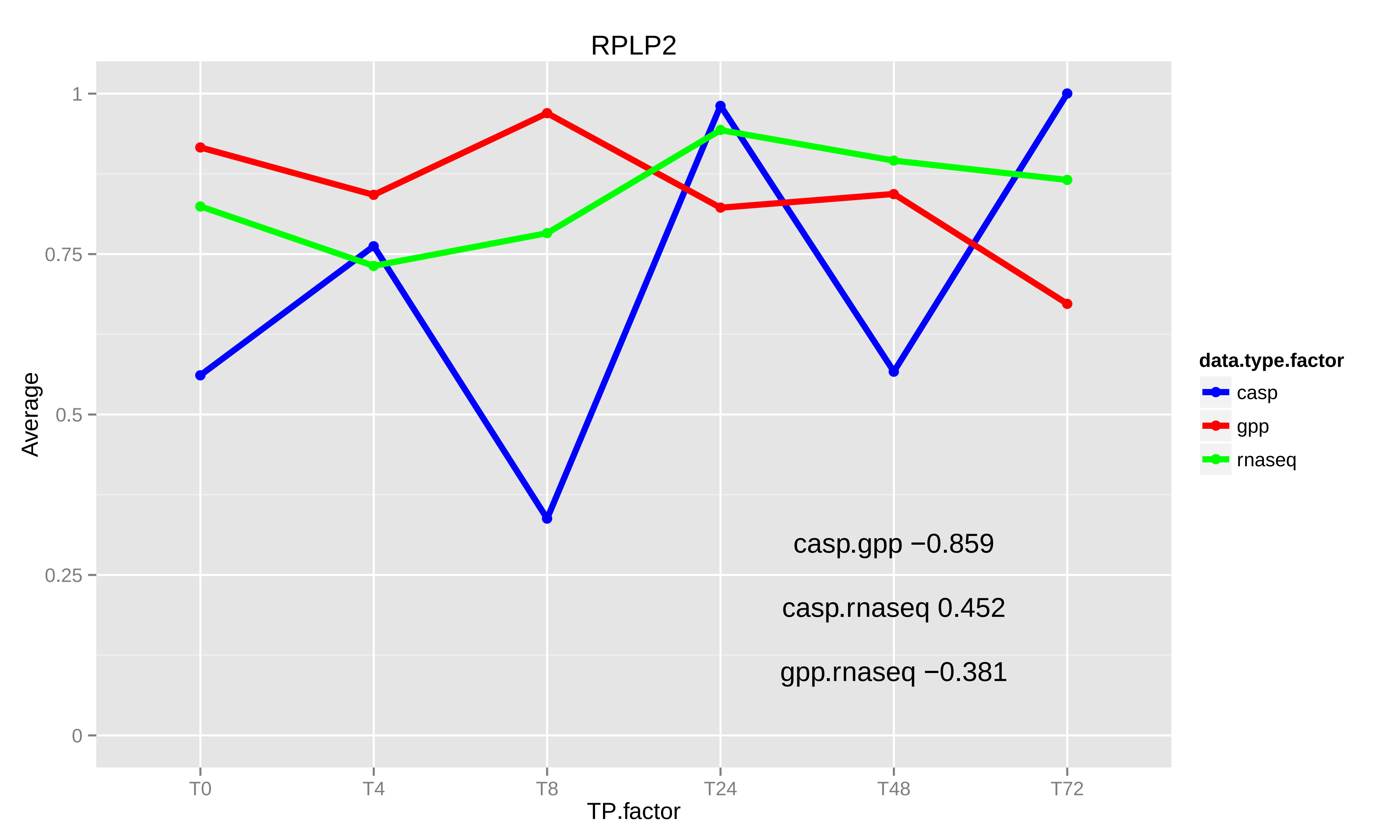
RPLP2
Average
1
0.75
0.5
0.25
0
T0
T4
T8
T24
T48
T72
TP.factor
data.type.factor
casp
gpp
rnaseq
casp.gpp −0.859
casp.rnaseq 0.452
gpp.rnaseq −0.381

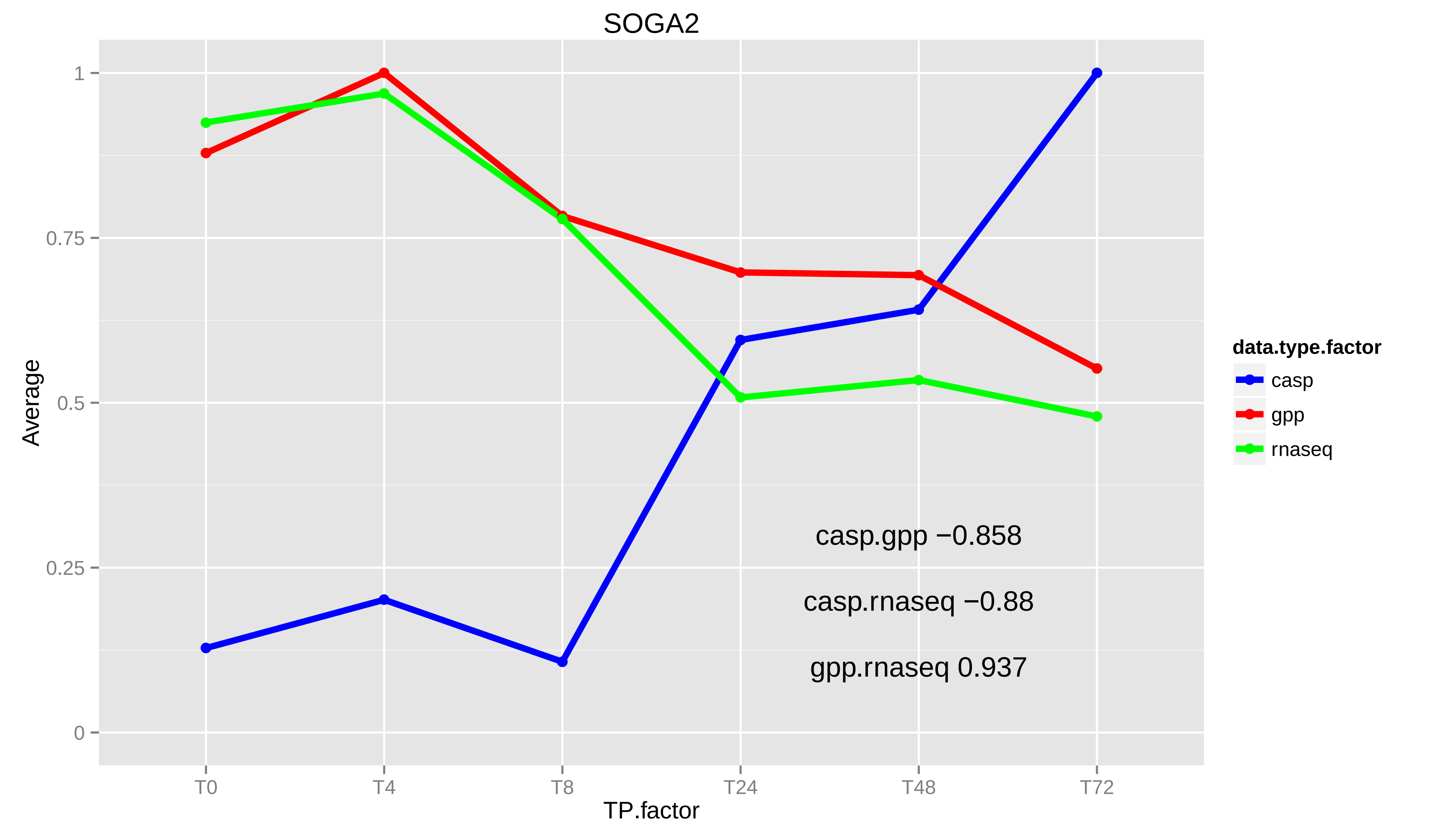

SOGA2
Average
1
0.75
0.5
0.25
0
T0
T4
T8
T24
T48
T72
TP.factor
data.type.factor
casp
gpp
rnaseq
casp.gpp −0.858
casp.rnaseq −0.88
gpp.rnaseq 0.937

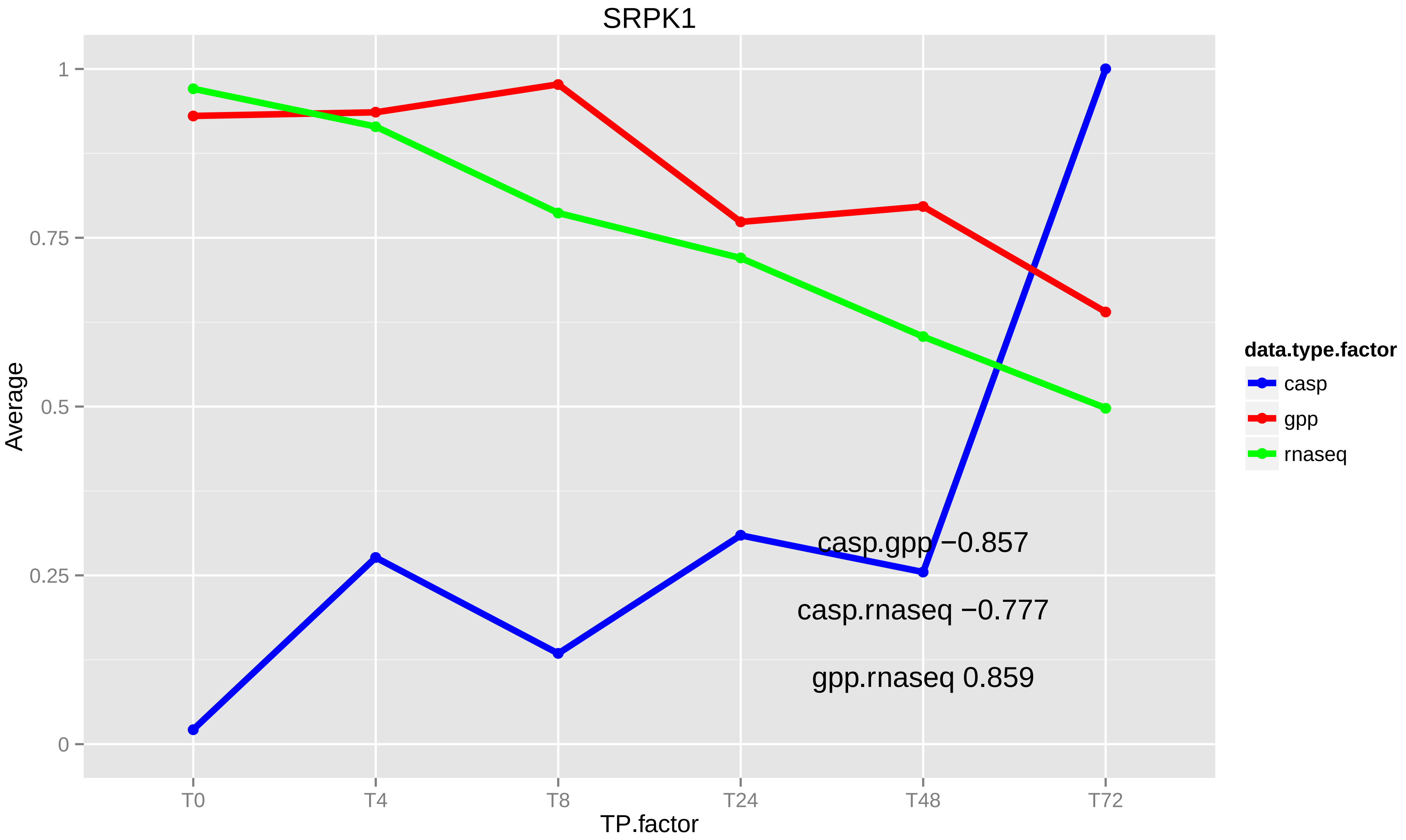
SRPK1
1
0.75
0.5
0.25
0
Average
T0
T4
T8
T24
T48
T72
TP.factor
data.type.factor
casp
gpp
rnaseq
casp.gpp −0.857
casp.rnaseq −0.777
gpp.rnaseq 0.859

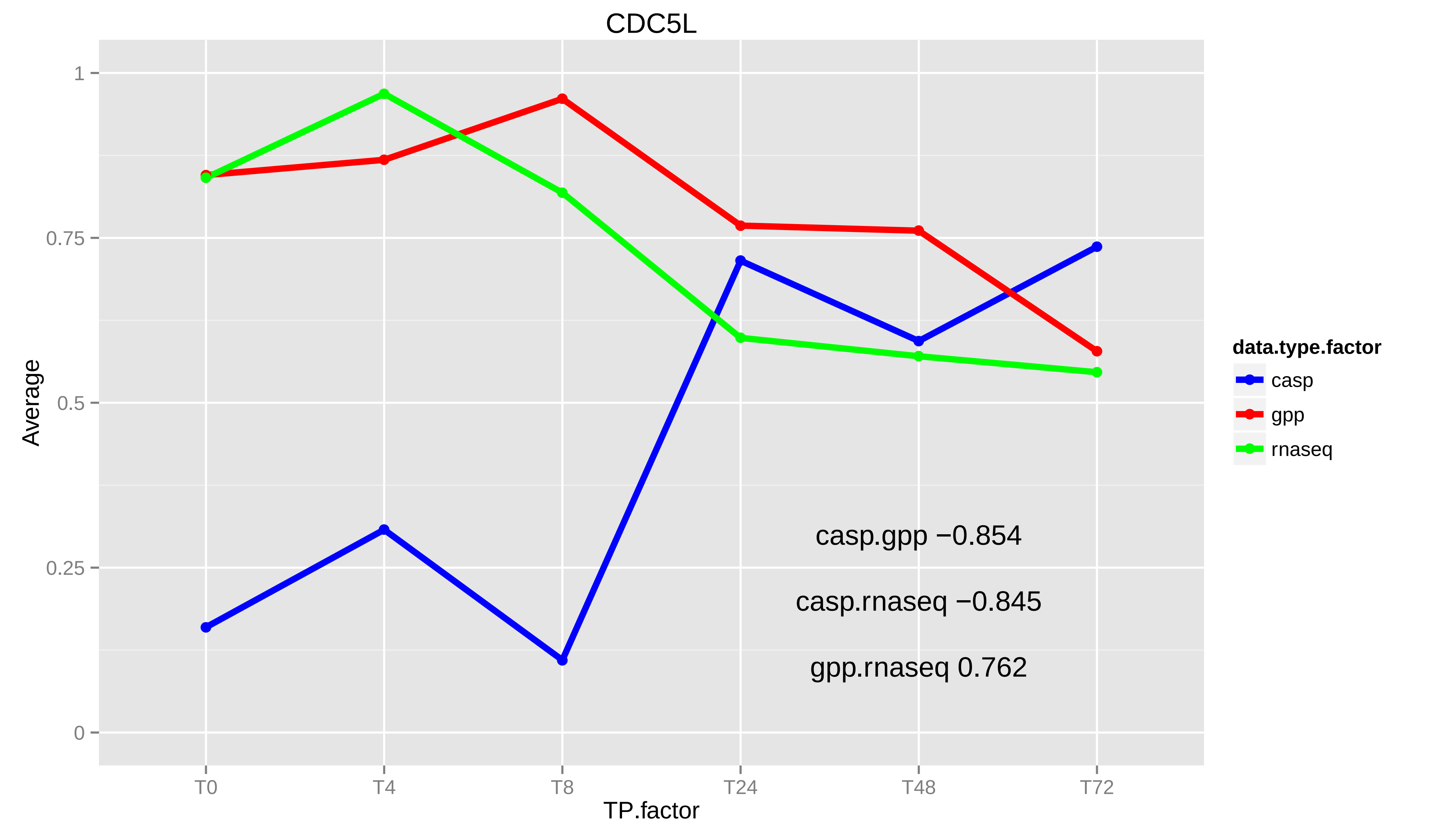

CDC5L
Average
1
0.75
0.5
0.25
0
T0
T4
T8
T24
T48
T72
TP.factor
data.type.factor
casp
gpp
rnaseq
casp.gpp −0.854
casp.rnaseq −0.845
gpp.rnaseq 0.762

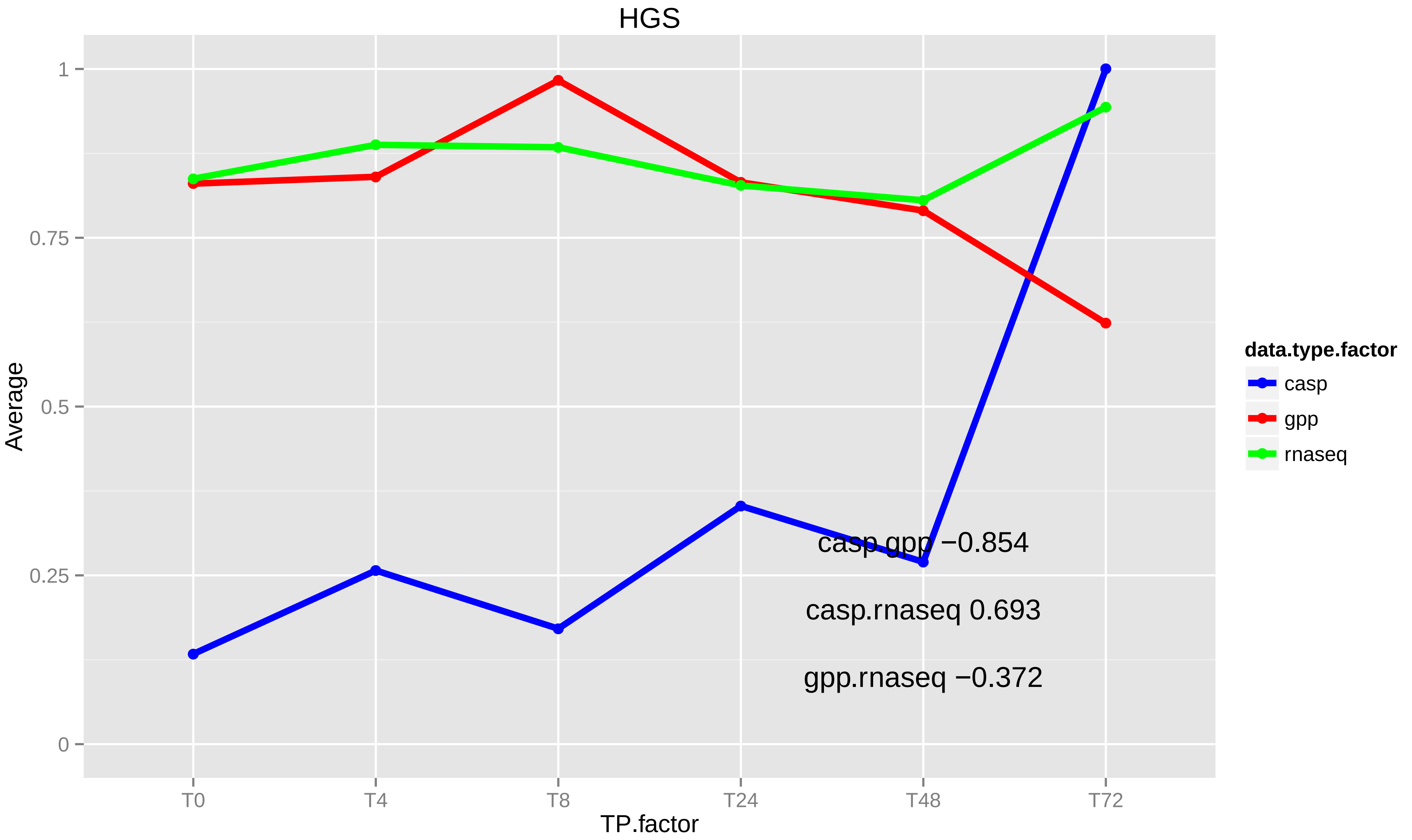
HGS
1
0.75
0.5
0.25
0
Average
T0
T4
T8
T24
T48
T72
TP.factor
casp.gpp −0.854
casp.rnaseq 0.693
gpp.rnaseq −0.372
data.type.factor
casp
gpp
rnaseq

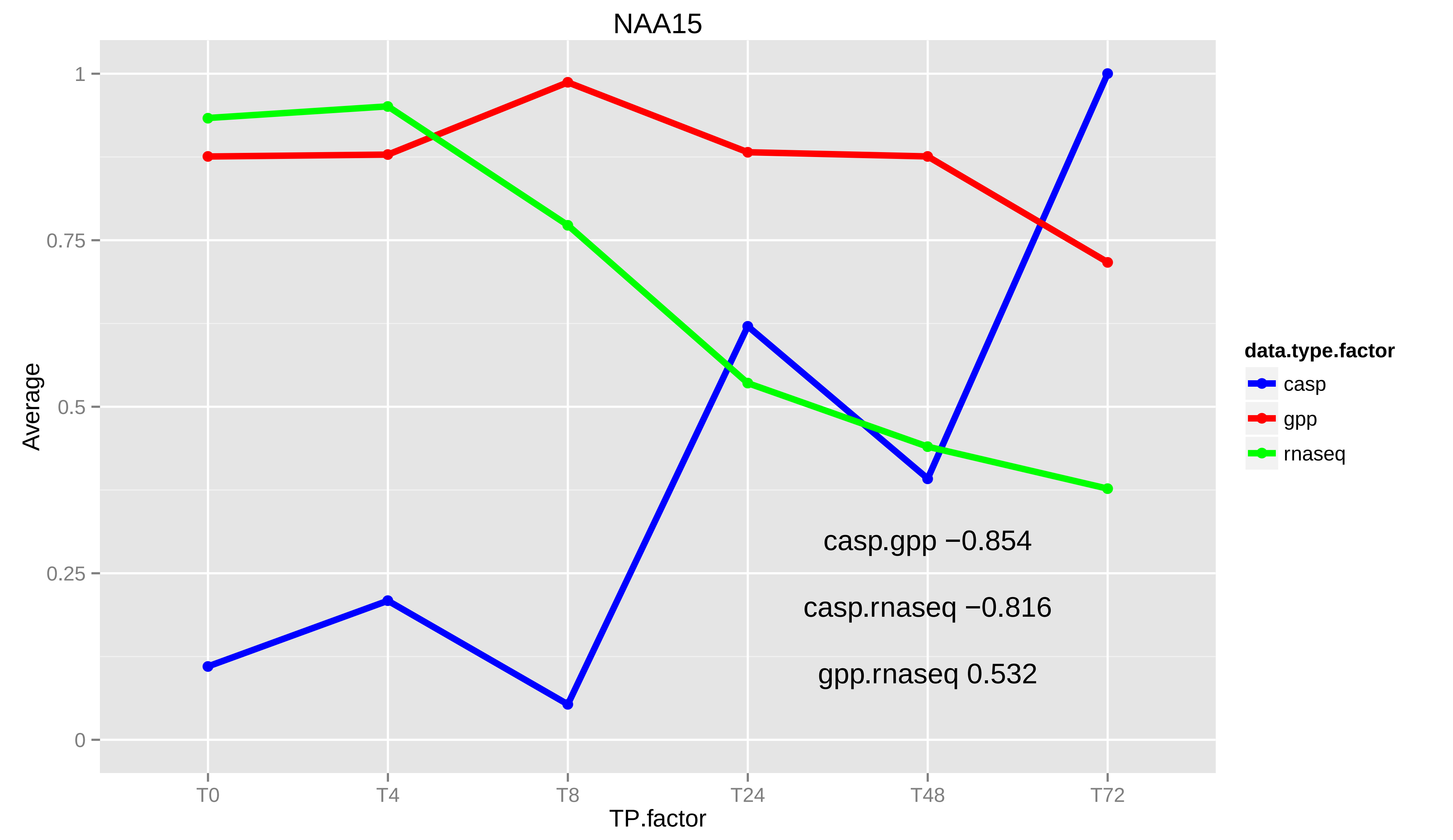
NAA15
Average
1
0.75
0.5
0.25
0
T0
T4
T8
T24
T48
T72
TP.factor
data.type.factor
casp
gpp
rnaseq
casp.gpp −0.854
casp.rnaseq −0.816
gpp.rnaseq 0.532

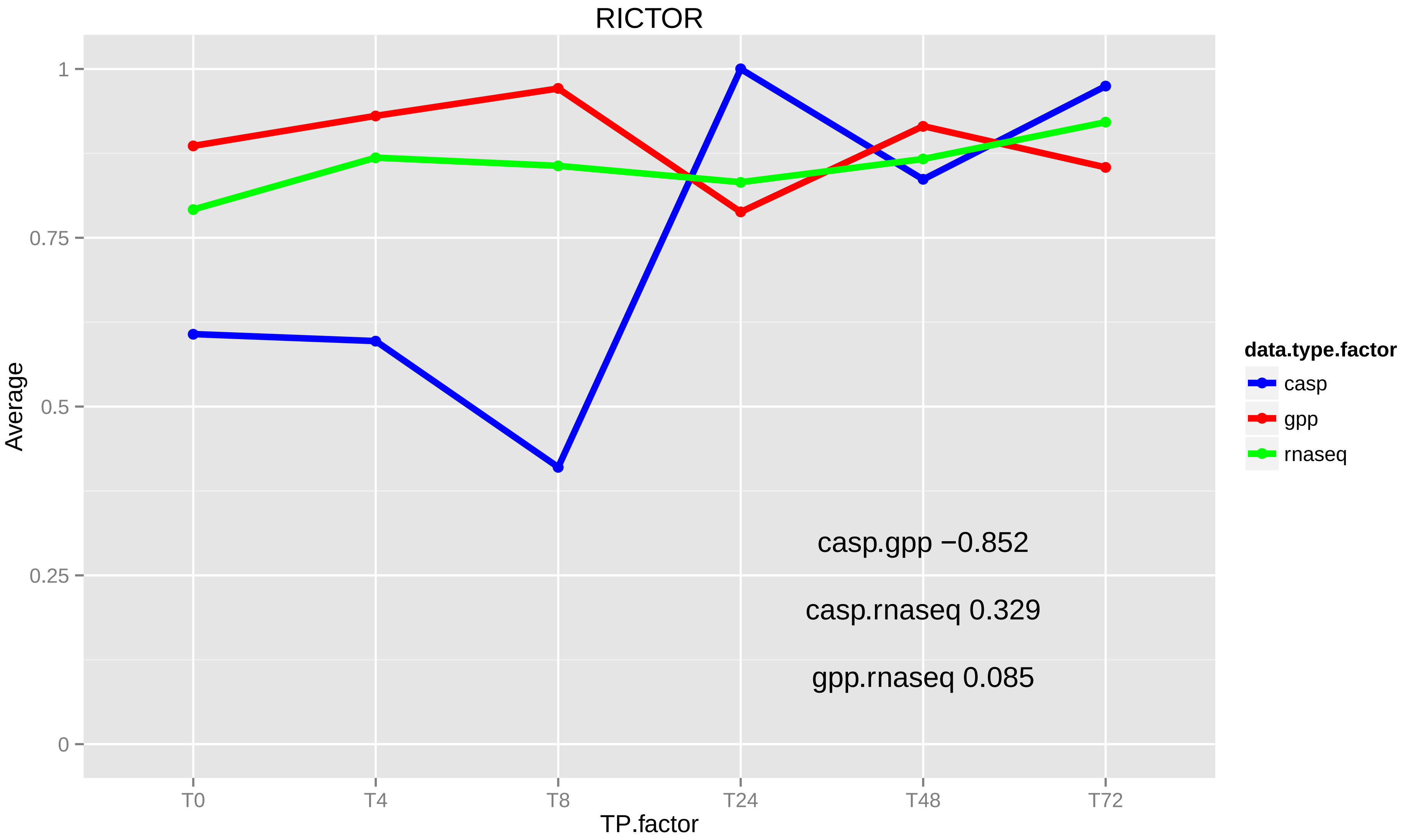
RICTOR
1
0.75
0.5
0.25
0
Average
T0
T4
T8
T24
T48
T72
TP.factor
data.type.factor
casp
gpp
rnaseq
casp.gpp −0.852
casp.rnaseq 0.329
gpp.rnaseq 0.085

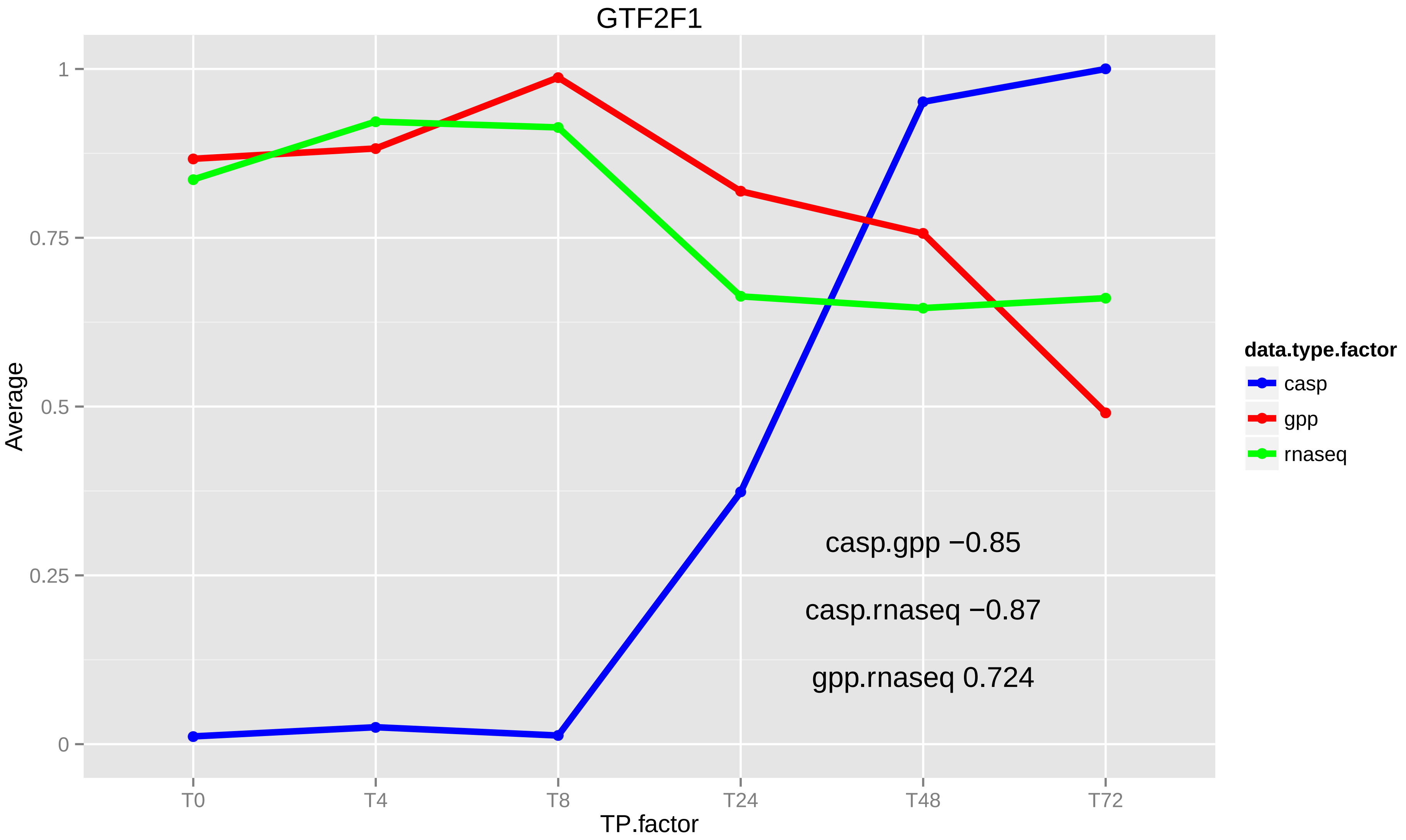
GTF2F1
Average
1
0.75
0.5
0.25
0
T0
T4
T8
T24
T48
T72
TP.factor
data.type.factor
casp
gpp
rnaseq
casp.gpp −0.85
casp.rnaseq −0.87
gpp.rnaseq 0.724

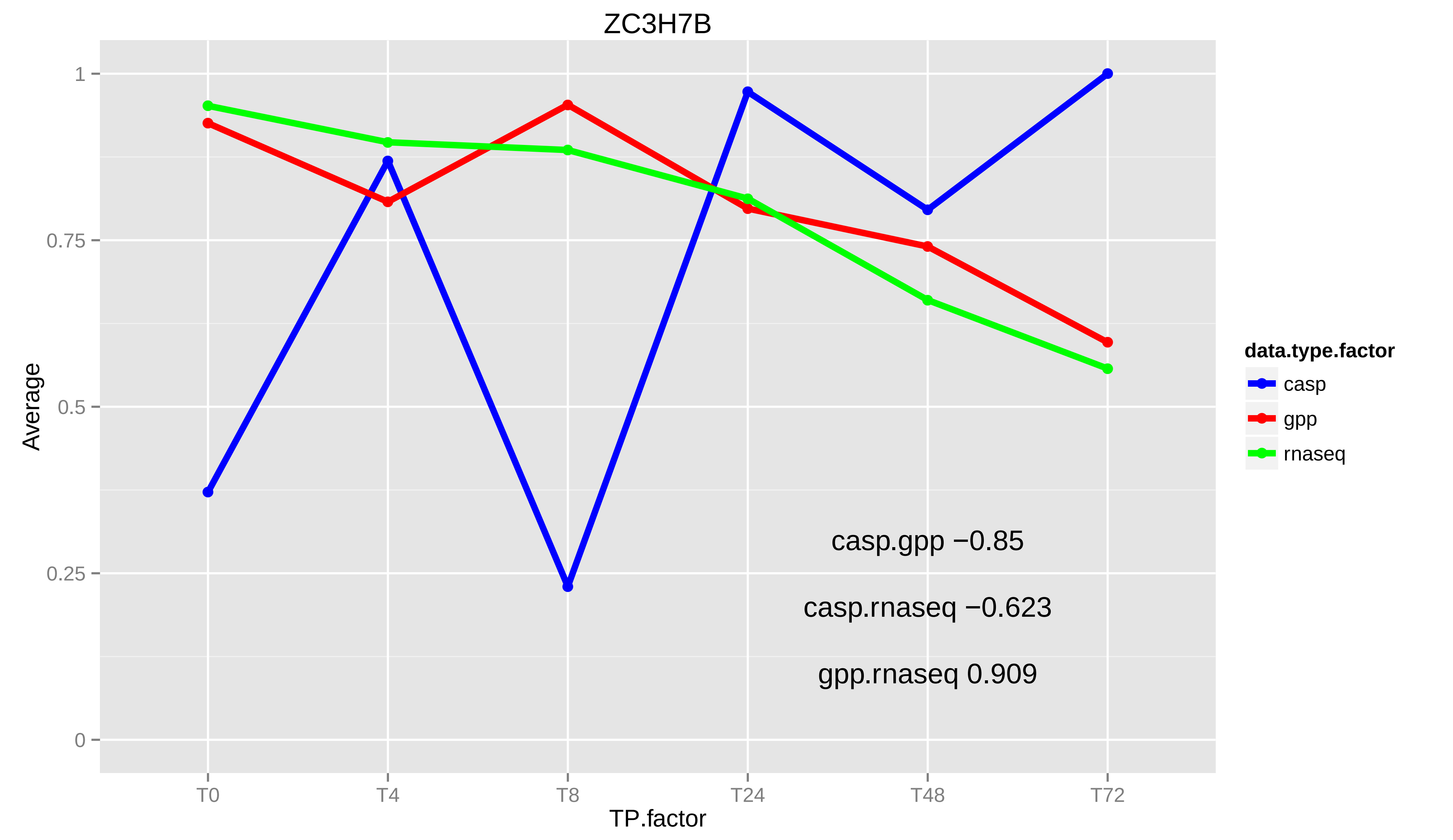
ZC3H7B
Average
1
0.75
0.5
0.25
0
T0
T4
T8
T24
T48
T72
TP.factor
data.type.factor
casp
gpp
rnaseq
casp.gpp −0.85
casp.rnaseq −0.623
gpp.rnaseq 0.909

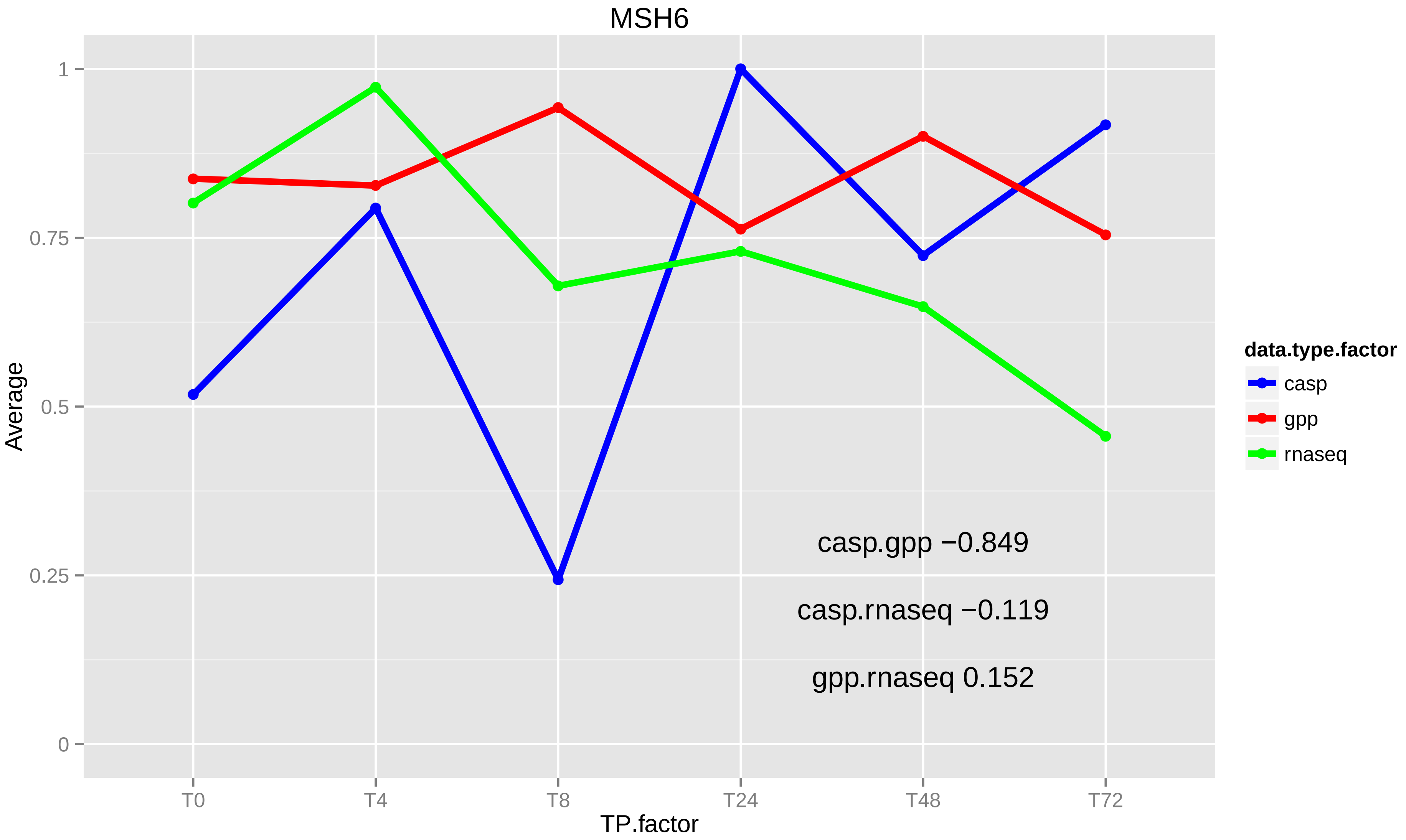
MSH6
Average
1
0.75
0.5
0.25
0
T0
T4
T8
T24
T48
T72
TP.factor
data.type.factor
casp
gpp
rnaseq
casp.gpp −0.849
casp.rnaseq −0.119
gpp.rnaseq 0.152

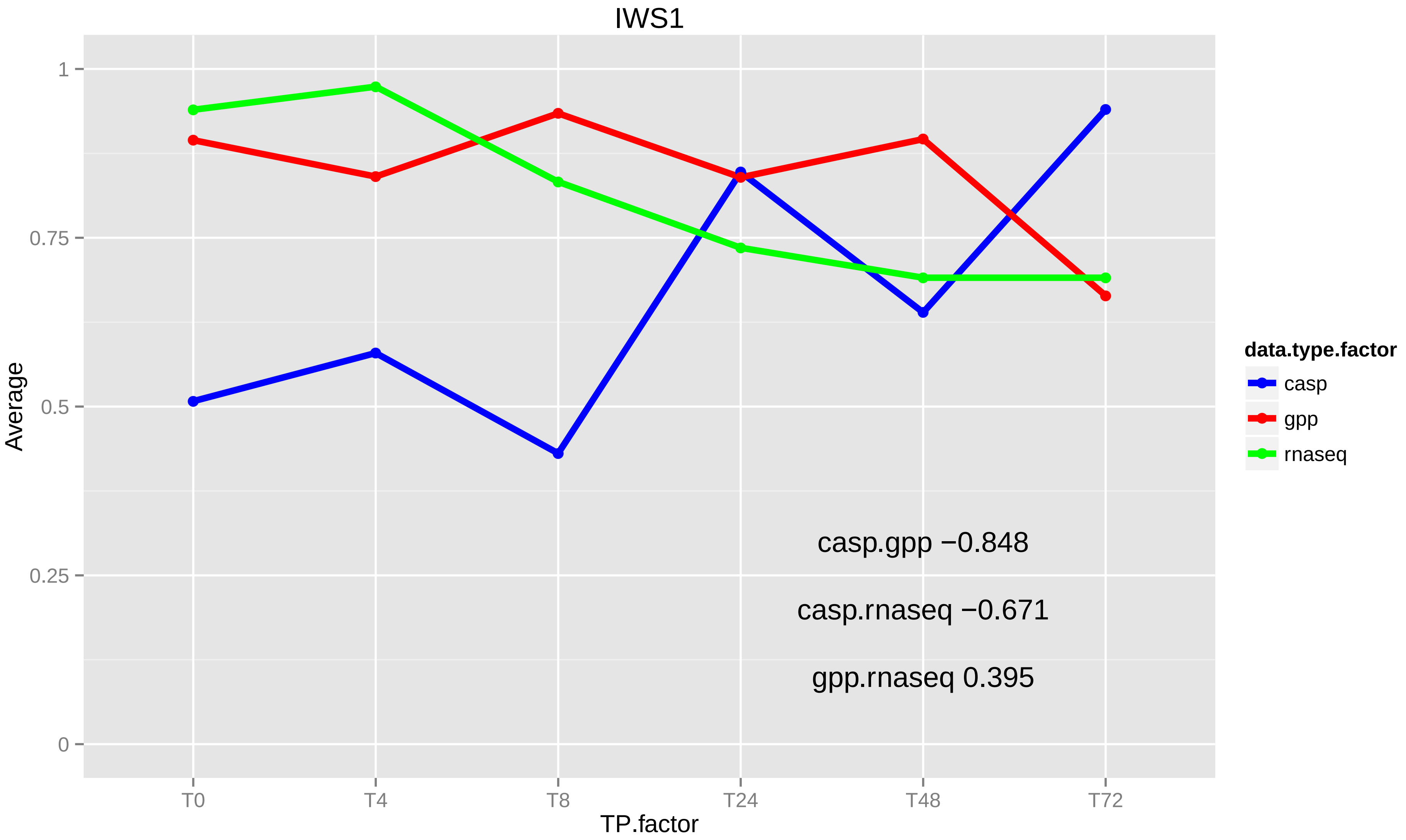
IWS1
Average
1
0.75
0.5
0.25
0
T0
T4
T8
T24
T48
T72
TP.factor
data.type.factor
casp
gpp
rnaseq
casp.gpp −0.848
casp.rnaseq −0.671
gpp.rnaseq 0.395

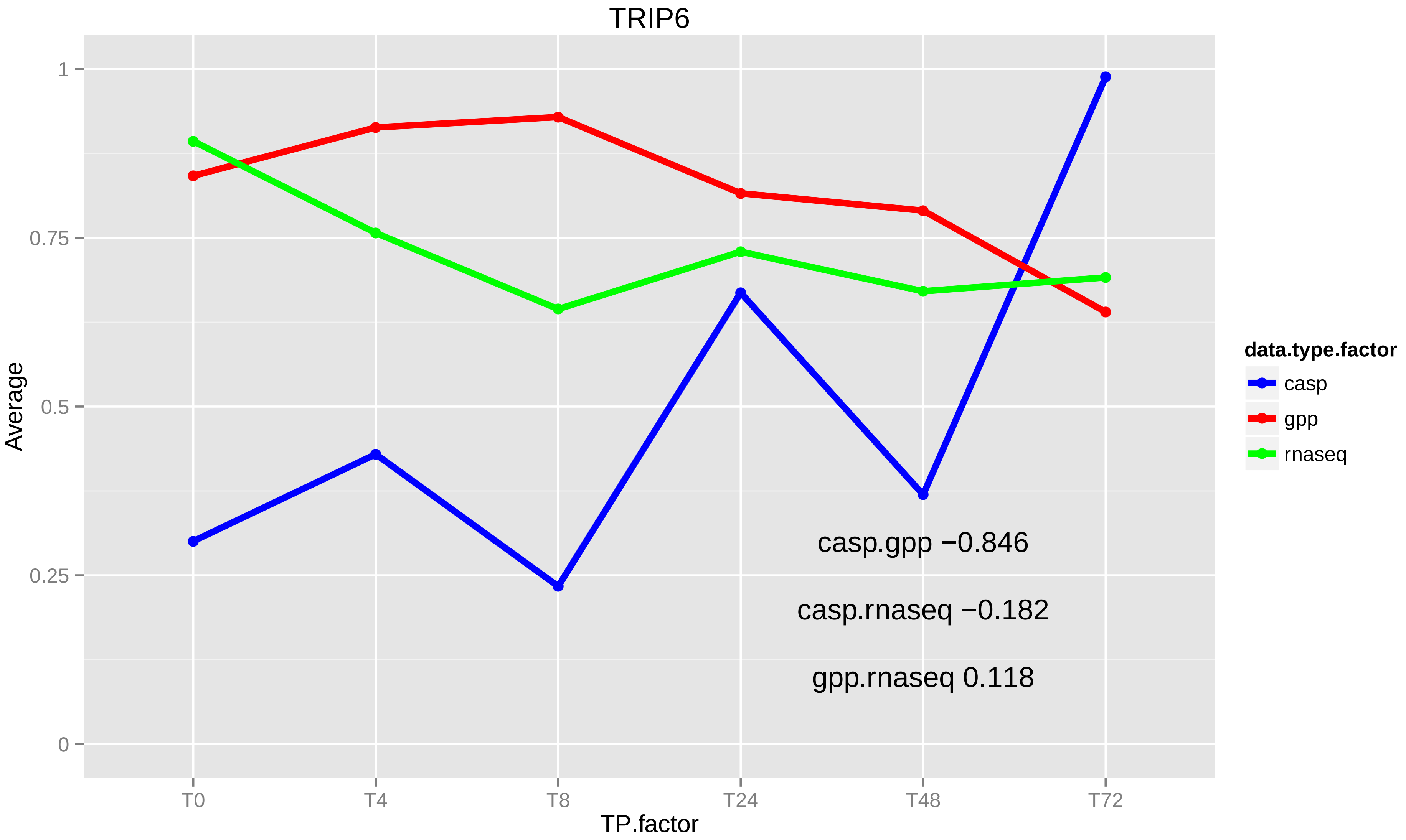
TRIP6
Average
1
0.75
0.5
0.25
0
T0
T4
T8
T24
T48
T72
TP.factor
data.type.factor
casp
gpp
rnaseq
casp.gpp −0.846
casp.rnaseq −0.182
gpp.rnaseq 0.118

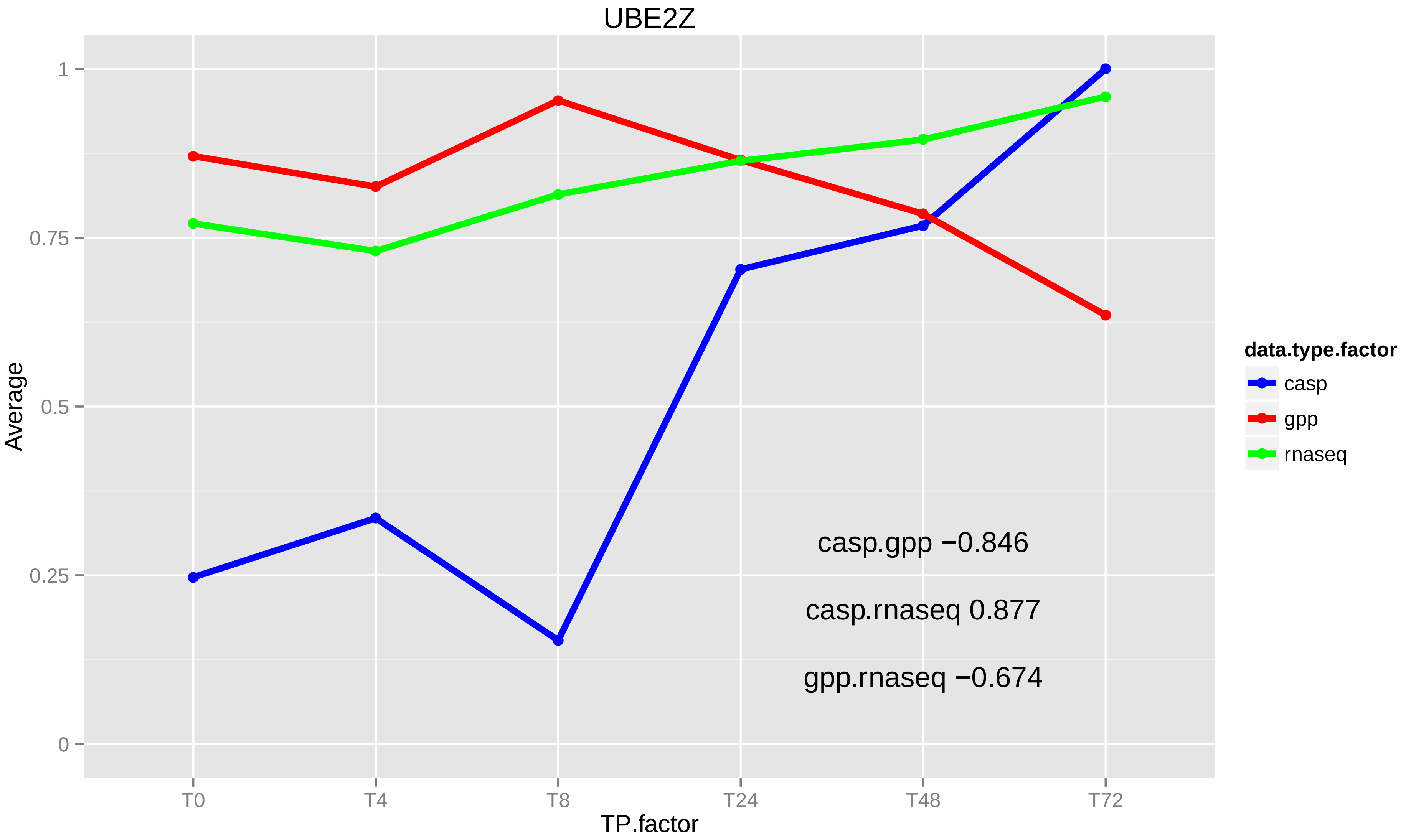

UBE2Z
1
0.75
0.5
0.25
0
Average
T0
T4
T8
T24
T48
T72
TP.factor
data.type.factor
casp
gpp
rnaseq
casp.gpp −0.846
casp.rnaseq 0.877
gpp.rnaseq −0.674

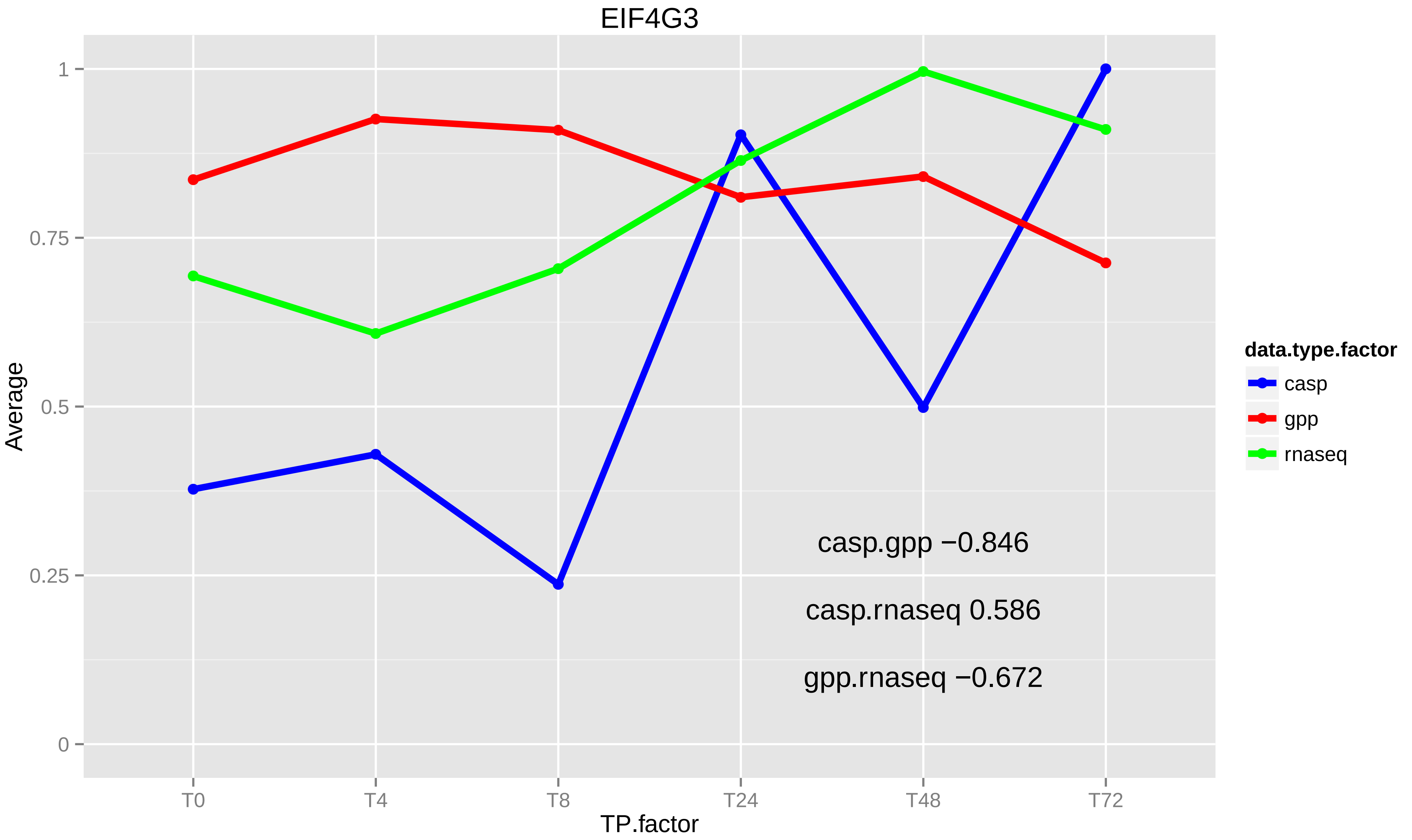
EIF4G3
1
0.75
0.5
0.25
0
Average
T0
T4
T8
T24
T48
T72
TP.factor
data.type.factor
casp
gpp
rnaseq
casp.gpp −0.846
casp.rnaseq 0.586
gpp.rnaseq −0.672

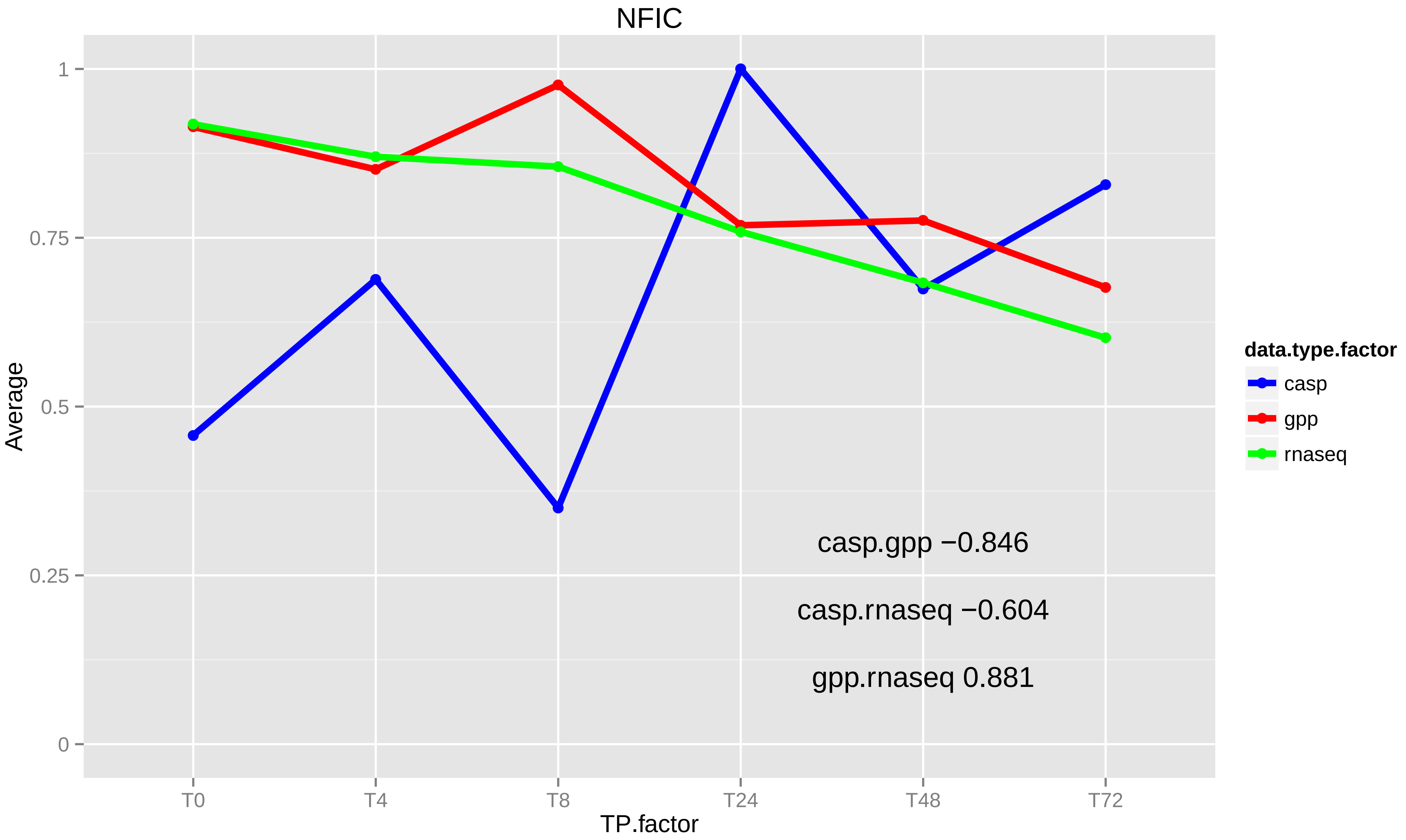

NFIC
1
0.75
0.5
0.25
0
Average
T0
T4
T8
T24
T48
T72
TP.factor
data.type.factor
casp
gpp
rnaseq
casp.gpp −0.846
casp.rnaseq −0.604
gpp.rnaseq 0.881

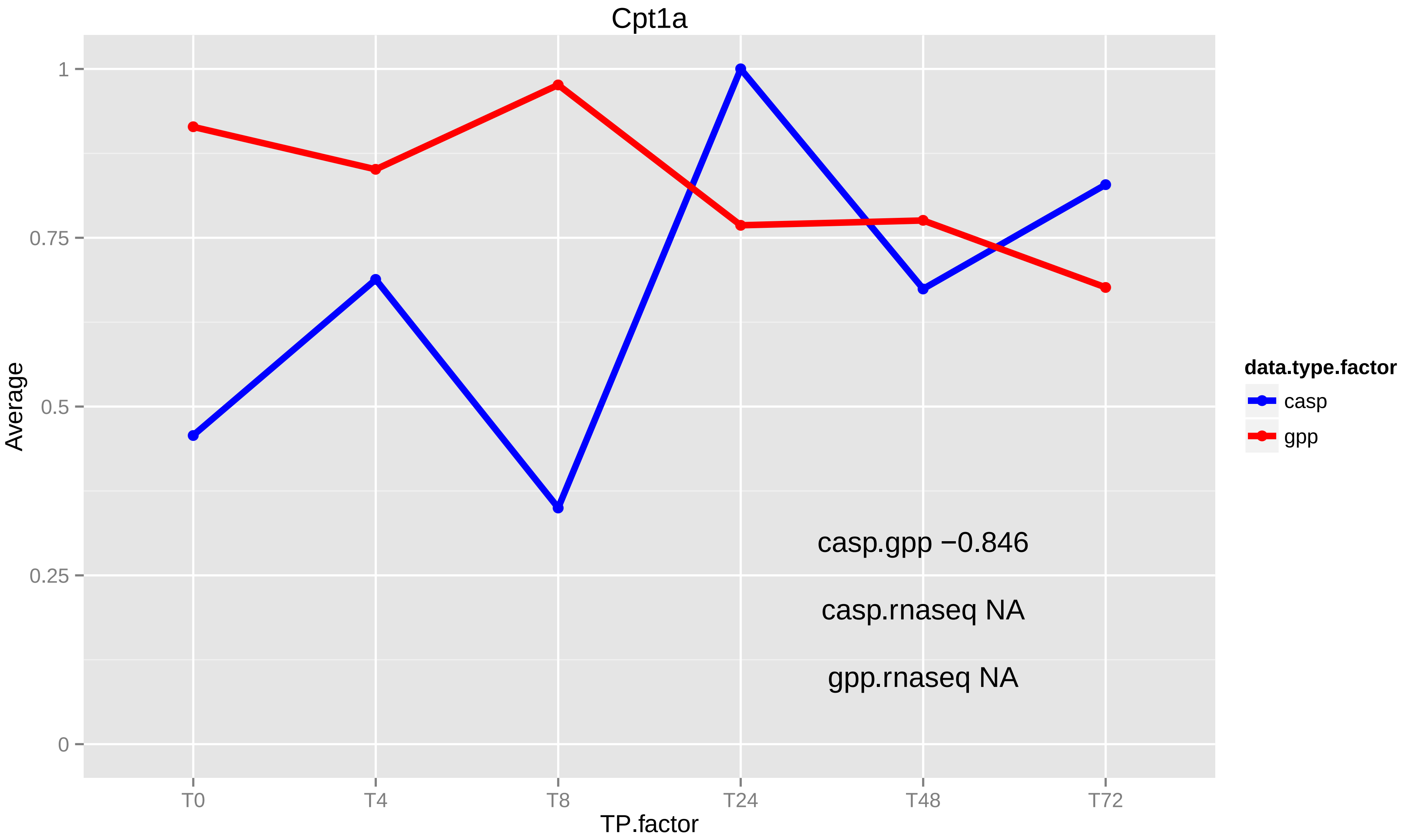
Cpt1a
1
0.75
0.5
0.25
0
Average
T0
T4
T8
T24
T48
T72
TP.factor
data.type.factor
casp
gpp
casp.gpp −0.846
casp.rnaseq NA
gpp.rnaseq NA

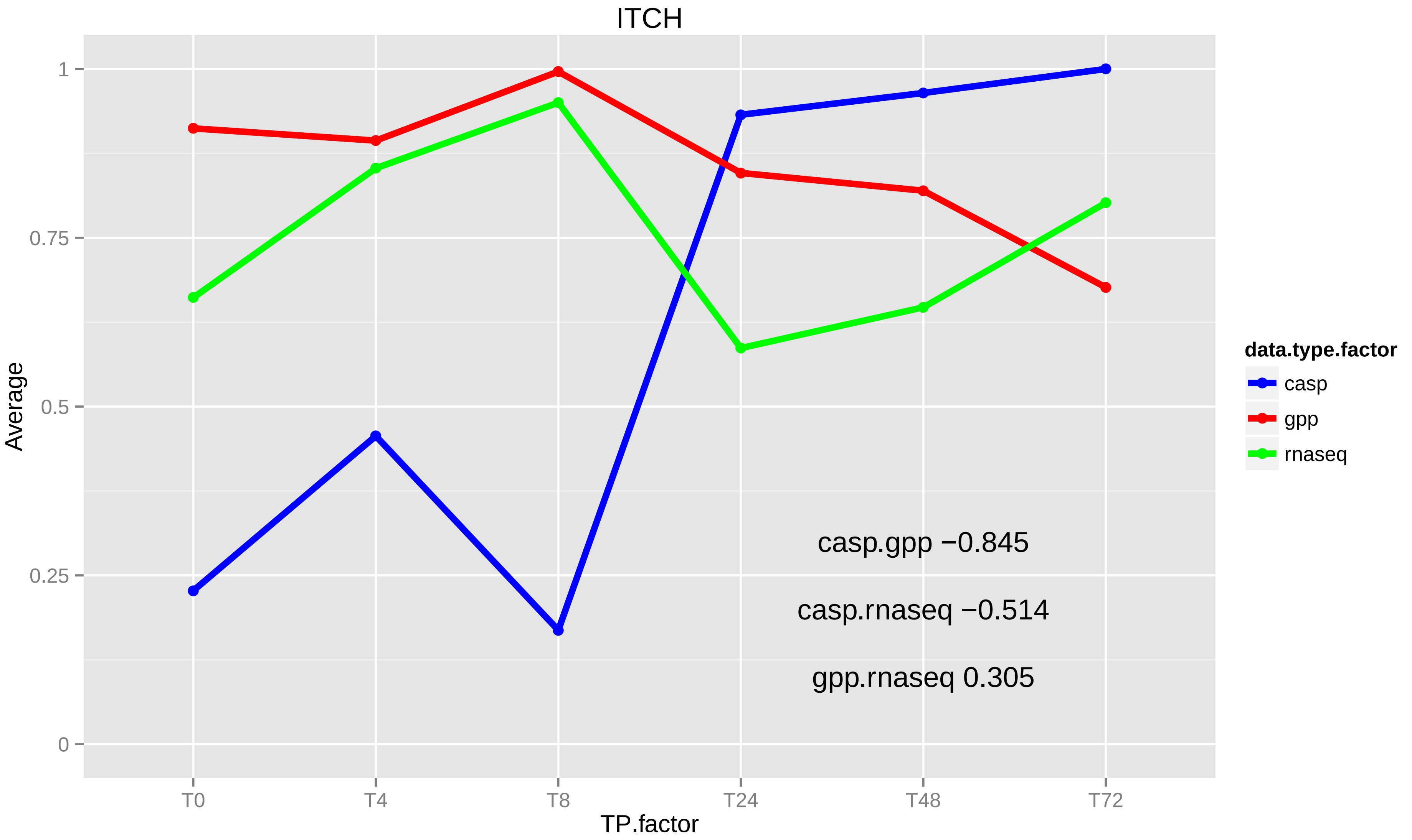
ITCH
Average
1
0.75
0.5
0.25
0
T0
T4
T8
T24
T48
T72
TP.factor
data.type.factor
casp
gpp
rnaseq
casp.gpp −0.845
casp.rnaseq −0.514
gpp.rnaseq 0.305

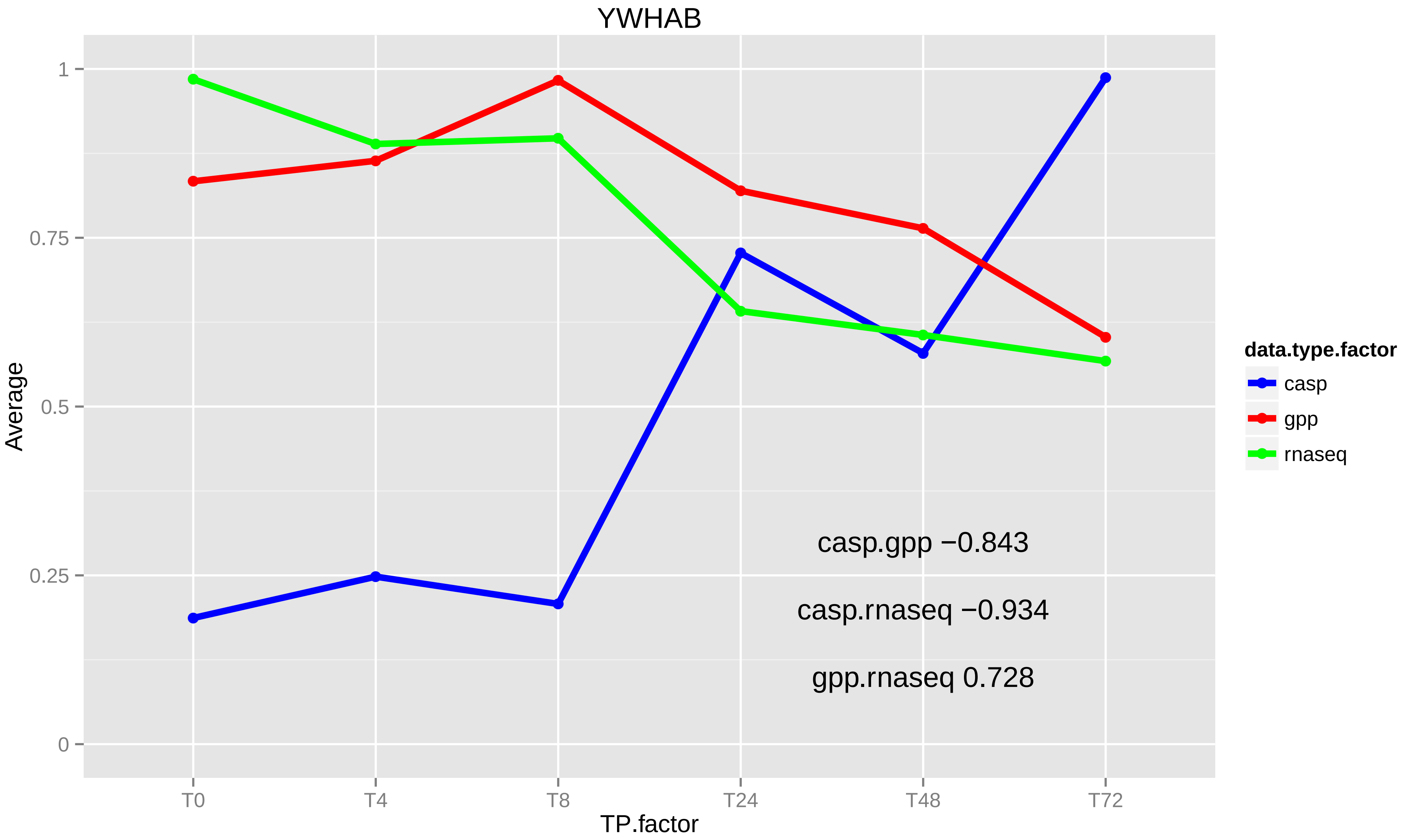
YWHAB
Average
1
0.75
0.5
0.25
0
T0
T4
T8
T24
T48
T72
TP.factor
data.type.factor
casp
gpp
rnaseq
casp.gpp −0.843
casp.rnaseq −0.934
gpp.rnaseq 0.728

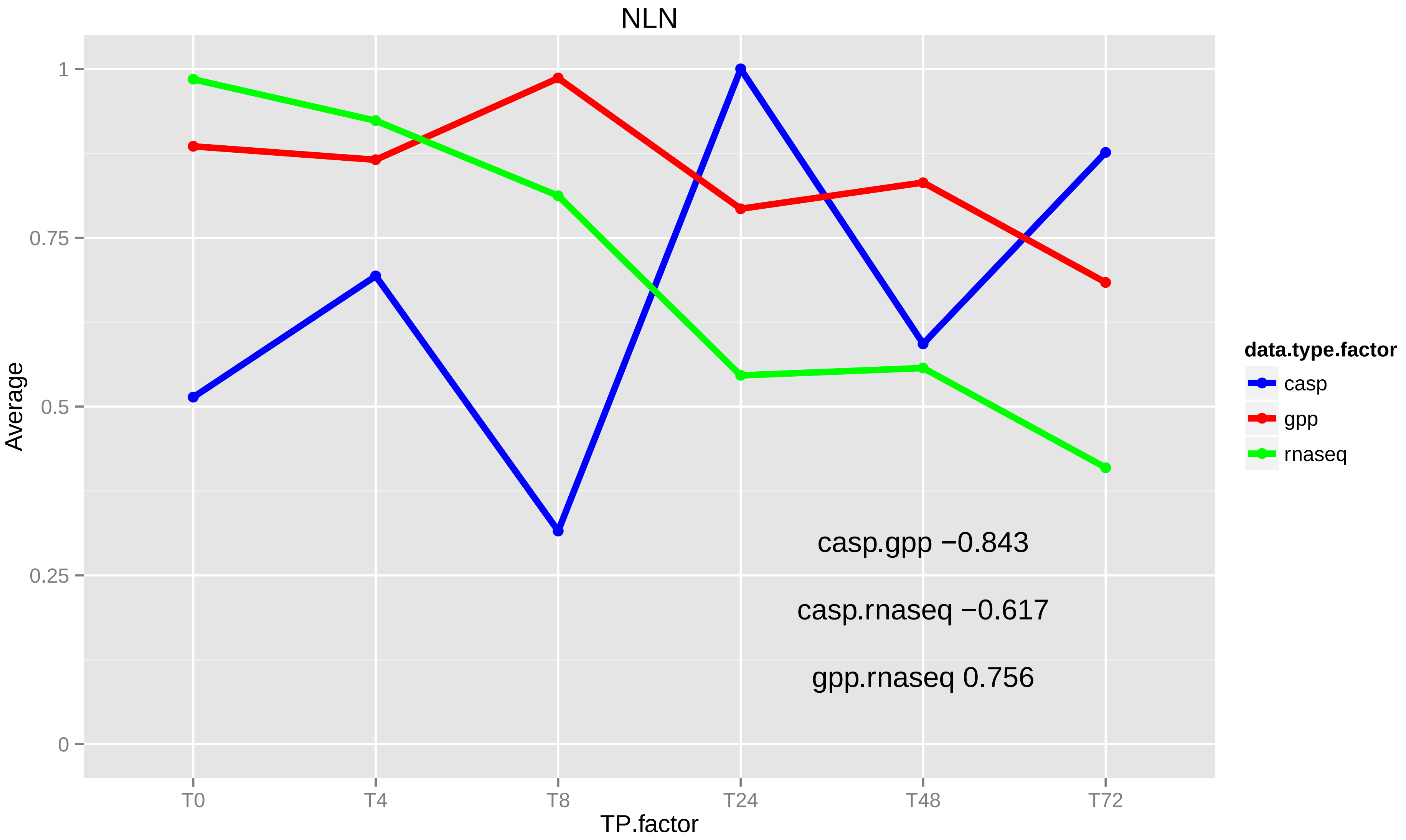

NLN
1
0.75
0.5
0.25
0
Average
T0
T4
T8
T24
T48
T72
TP.factor
data.type.factor
casp
gpp
rnaseq
casp.gpp −0.843
casp.rnaseq −0.617
gpp.rnaseq 0.756

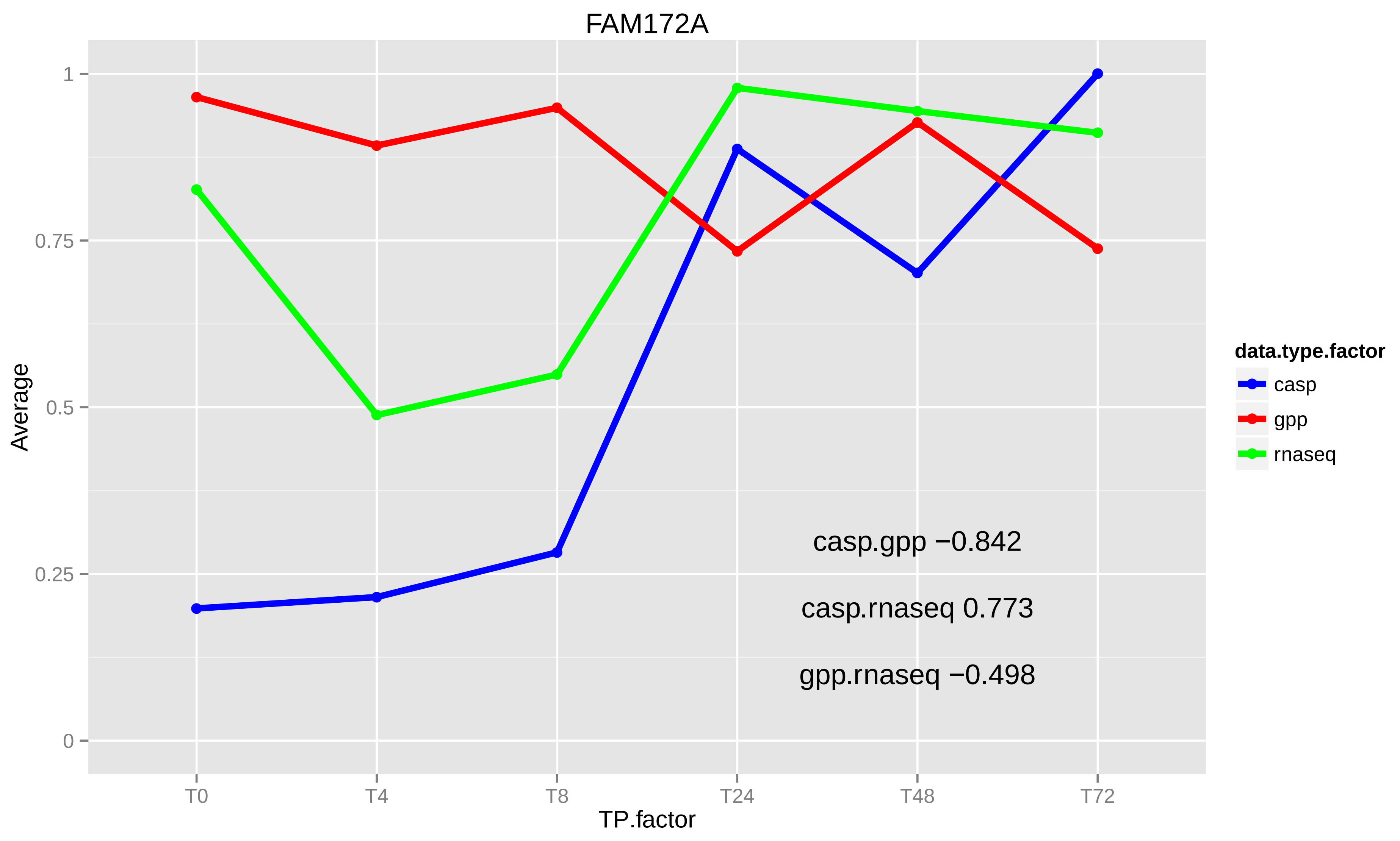

FAM172A
Average
1
0.75
0.5
0.25
0
T0
T4
T8
T24
T48
T72
TP.factor
data.type.factor
casp
gpp
rnaseq
casp.gpp −0.842
casp.rnaseq 0.773
gpp.rnaseq −0.498

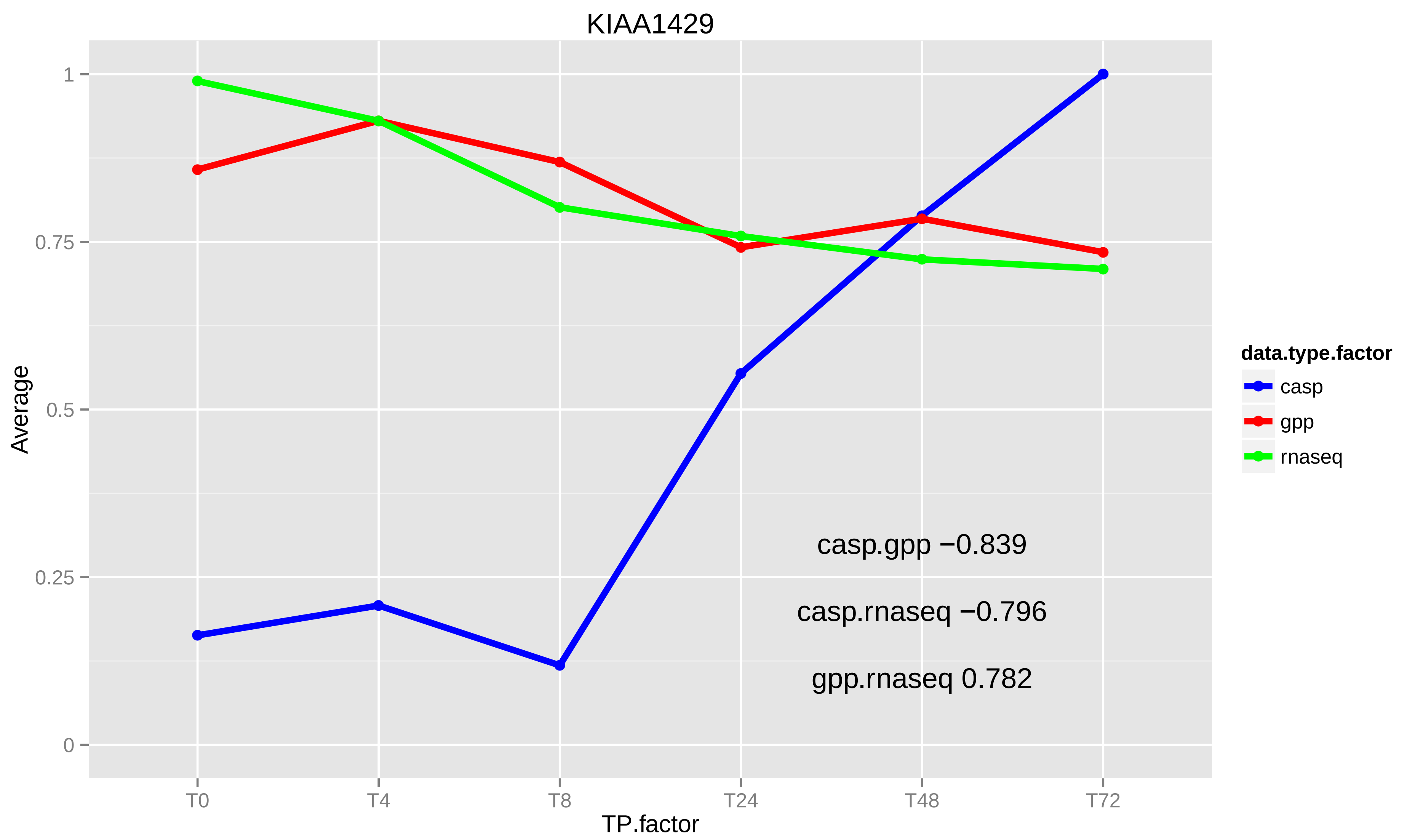

KIAA1429
1
0.75
0.5
0.25
0
Average
T0
T4
T8
T24
T48
T72
TP.factor
casp.gpp −0.839
casp.rnaseq −0.796
gpp.rnaseq 0.782
data.type.factor
casp
gpp
rnaseq

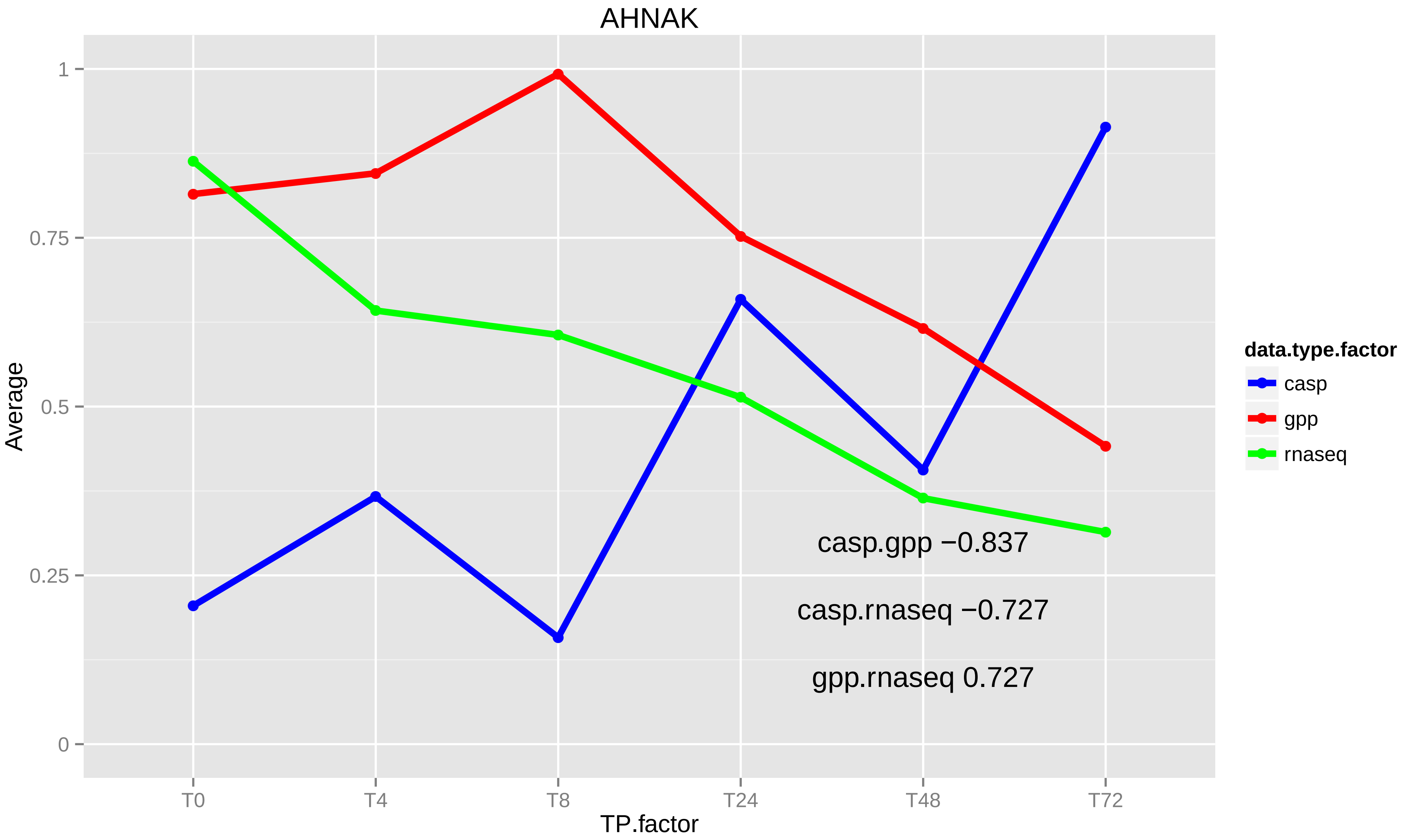
AHNAK
Average
1
0.75
0.5
0.25
0
T0
T4
T8
T24
T48
T72
TP.factor
data.type.factor
casp
gpp
rnaseq
casp.gpp −0.837
casp.rnaseq −0.727
gpp.rnaseq 0.727

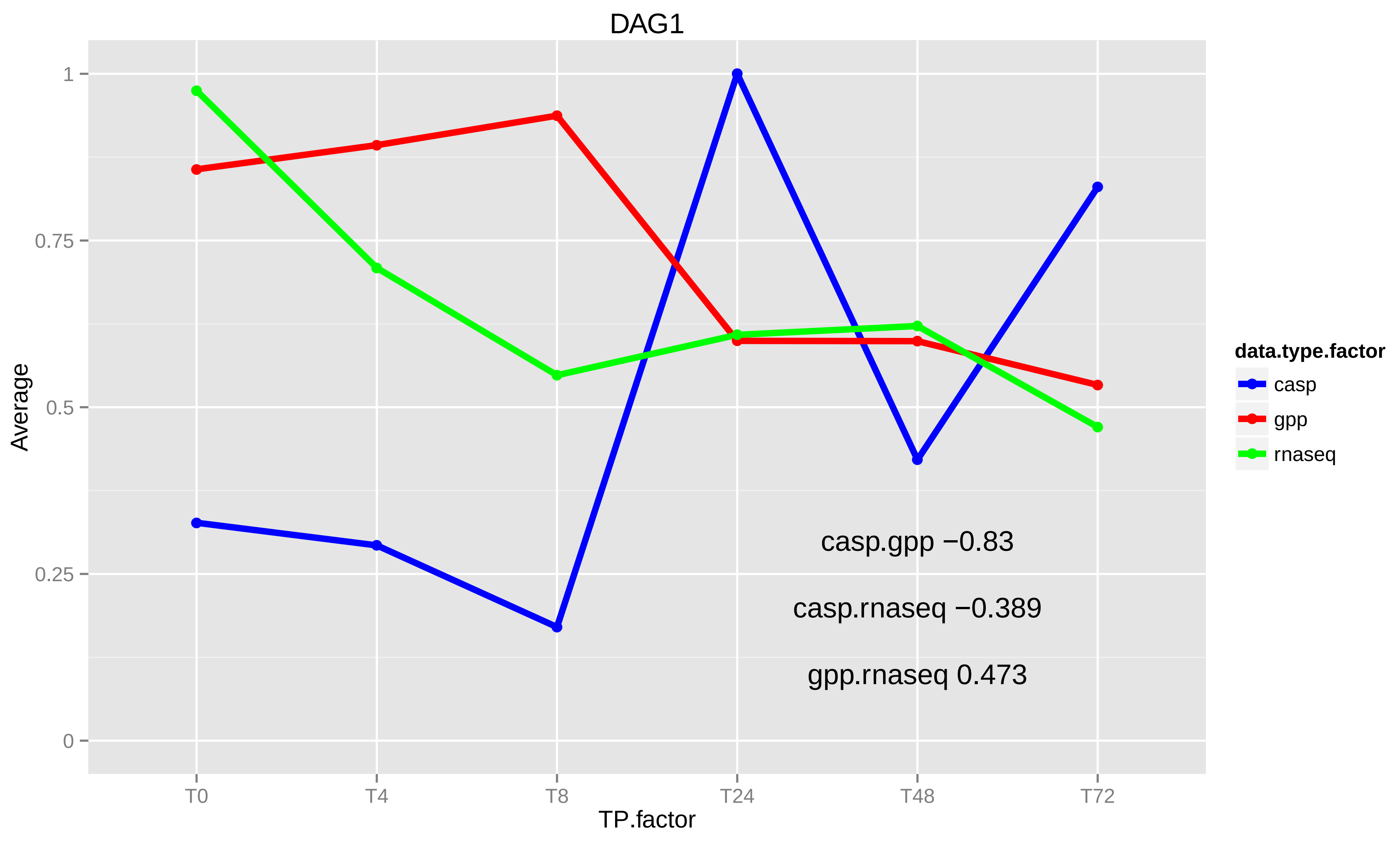

DAG1
Average
1
0.75
0.5
0.25
0
T0
T4
T8
T24
T48
T72
TP.factor
data.type.factor
casp
gpp
rnaseq
casp.gpp −0.83
casp.rnaseq −0.389
gpp.rnaseq 0.473

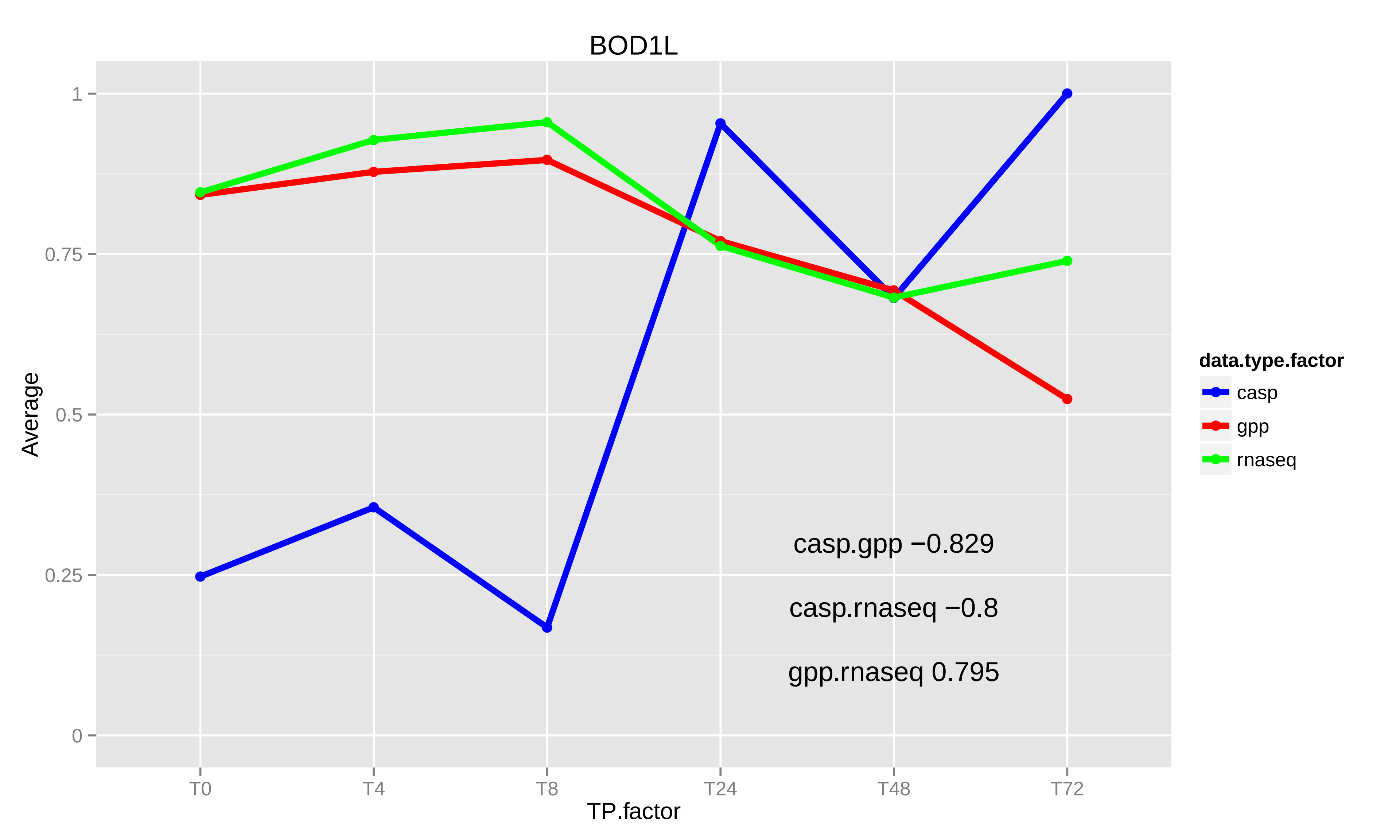
BOD1L
Average
1
0.75
0.5
0.25
0
T0
T4
T8
T24
T48
T72
TP.factor
data.type.factor
casp
gpp
rnaseq
casp.gpp −0.829
casp.rnaseq −0.8
gpp.rnaseq 0.795

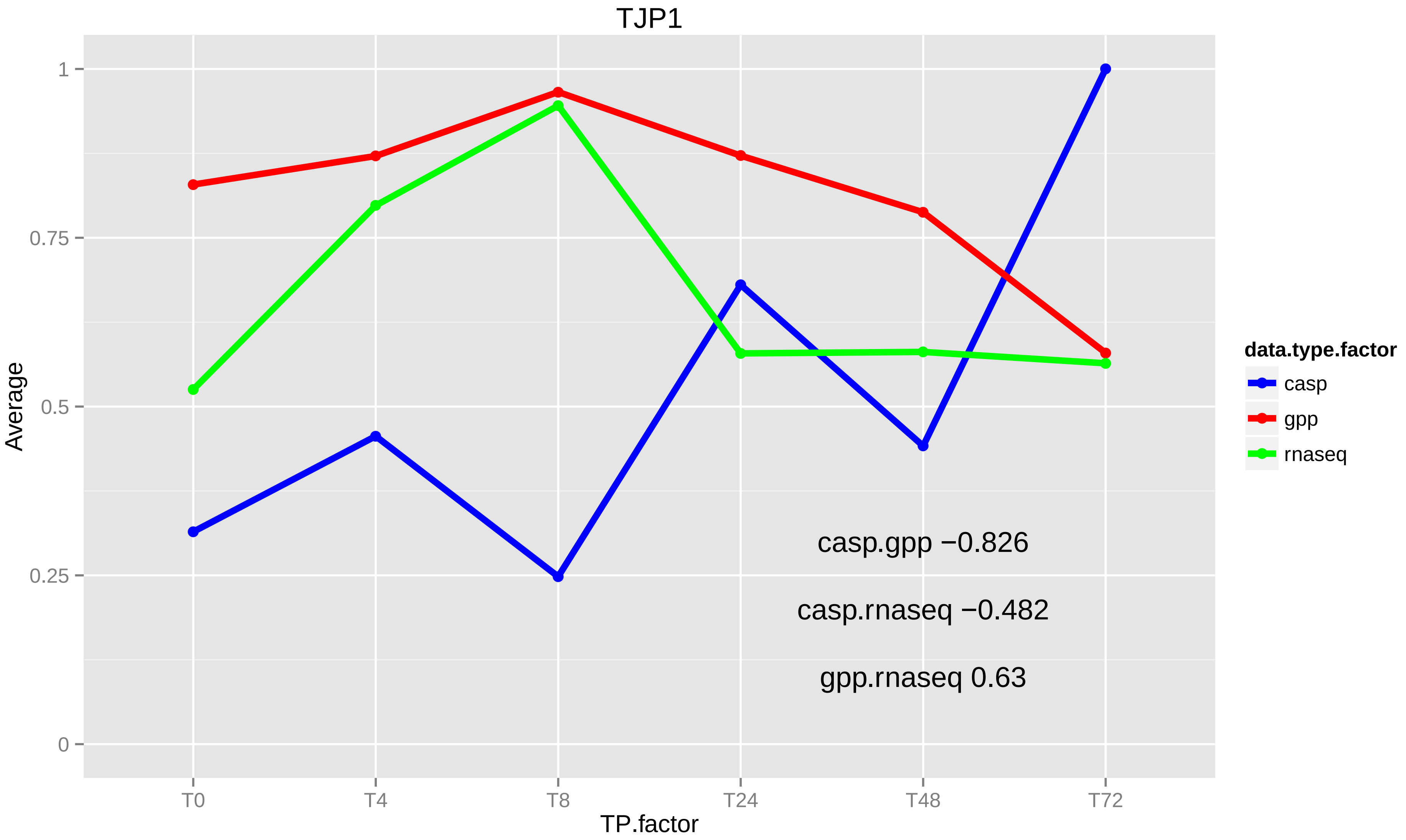

TJP1
Average
1
0.75
0.5
0.25
0
T0
T4
T8
T24
T48
T72
TP.factor
data.type.factor
casp
gpp
rnaseq
casp.gpp −0.826
casp.rnaseq −0.482
gpp.rnaseq 0.63

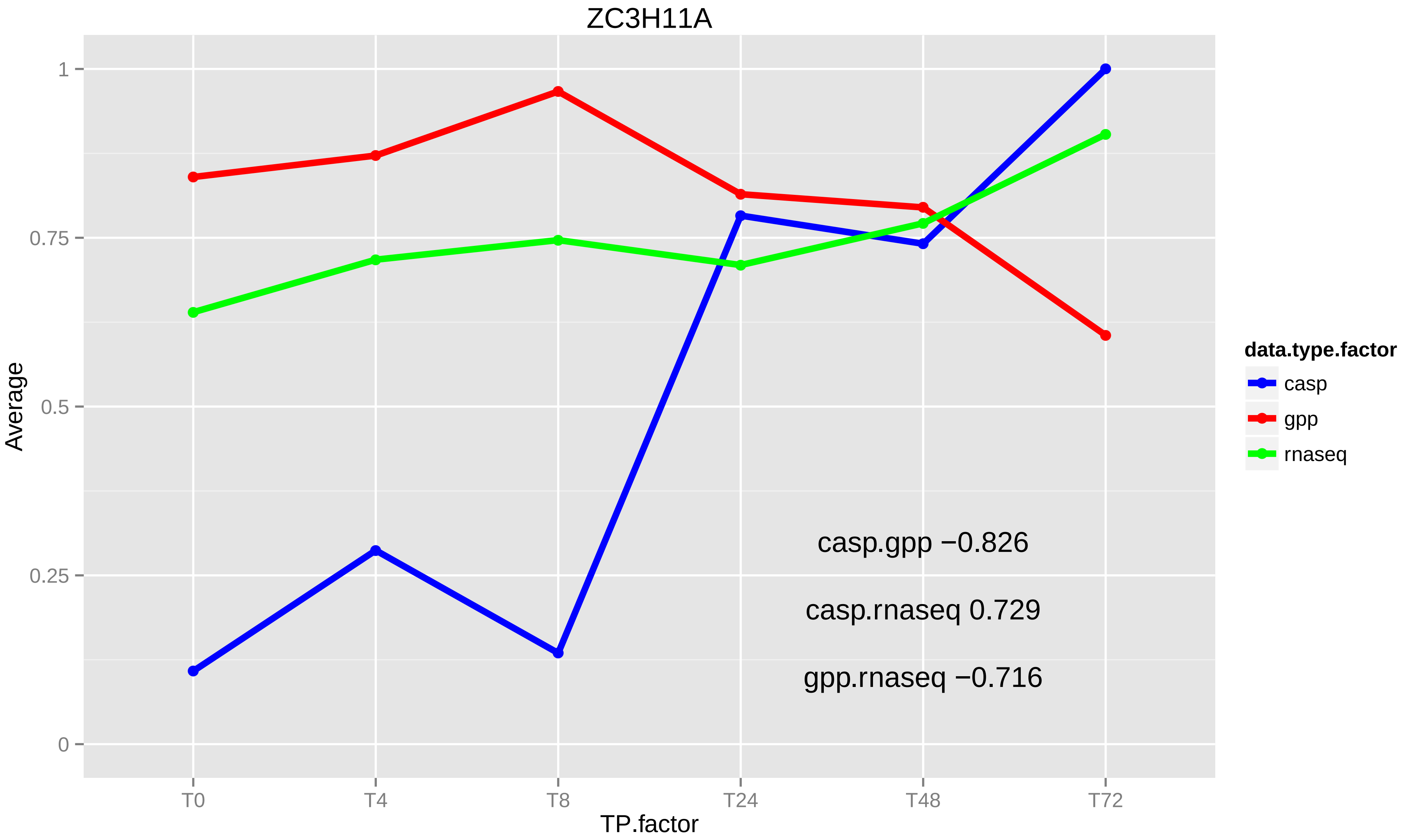
ZC3H11A
Average
TP.factor
T0
T4
T8
T24
T48
T72
0
0.25
0.5
0.75
1
data.type.factor
casp
gpp
rnaseq
casp.gpp −0.826
casp.rnaseq 0.729
gpp.rnaseq −0.716

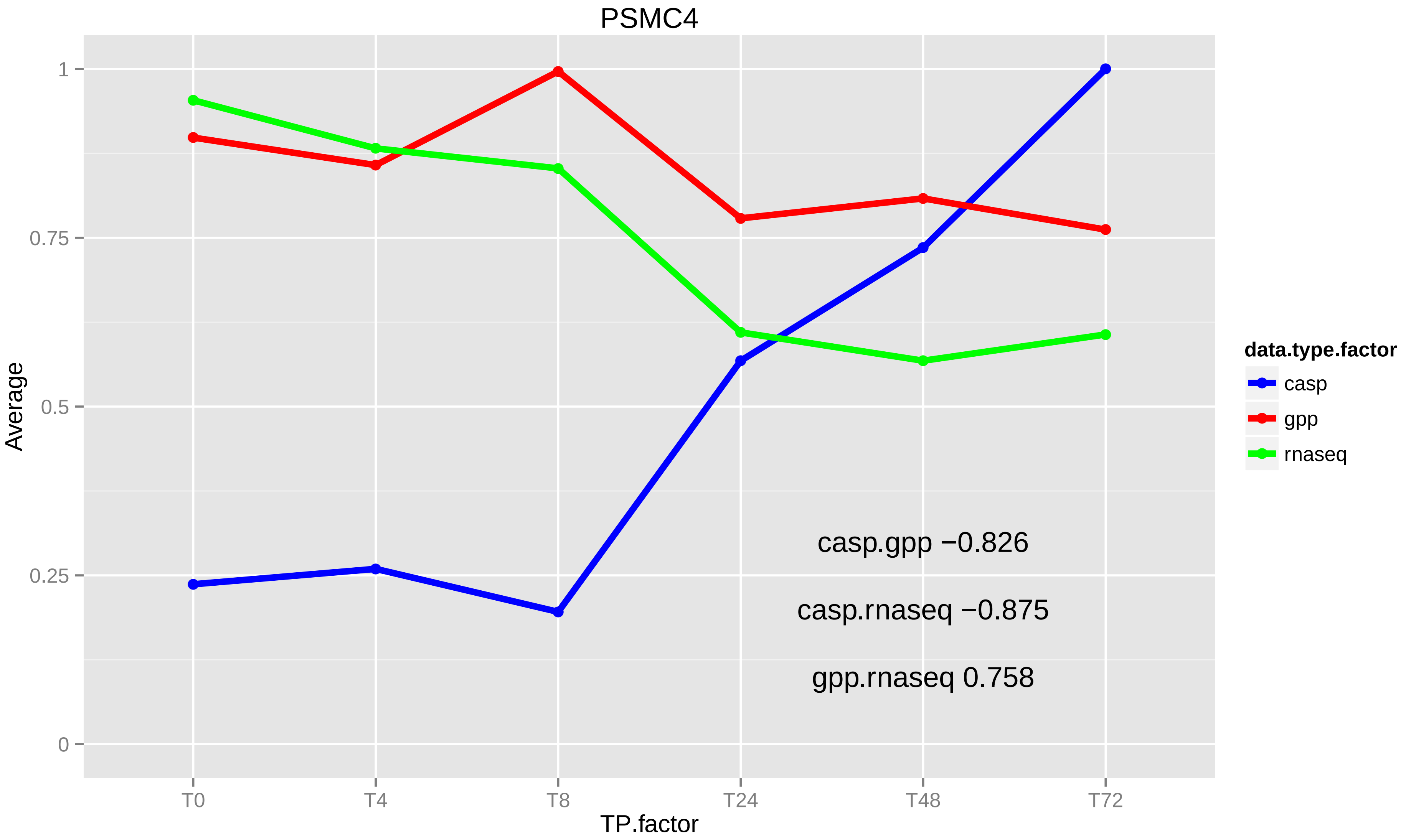

PSMC4
Average
1
0.75
0.5
0.25
0
T0
T4
T8
T24
T48
T72
TP.factor
data.type.factor
casp
gpp
rnaseq
casp.gpp −0.826
casp.rnaseq −0.875
gpp.rnaseq 0.758

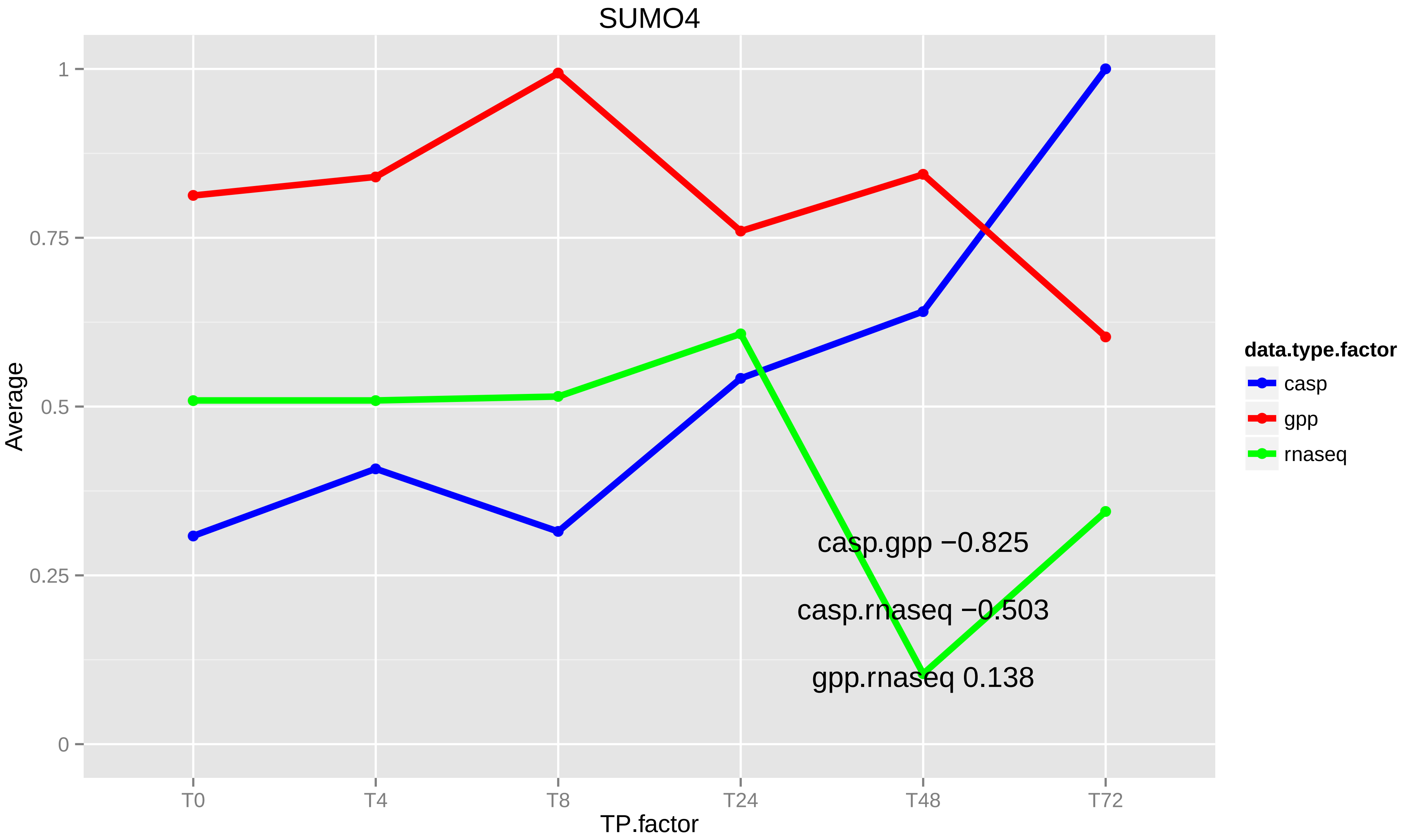

SUMO4
Average
1
0.75
0.5
0.25
0
T0
T4
T8
T24
T48
T72
TP.factor
data.type.factor
casp
gpp
rnaseq
casp.gpp −0.825
casp.rnaseq −0.503
gpp.rnaseq 0.138

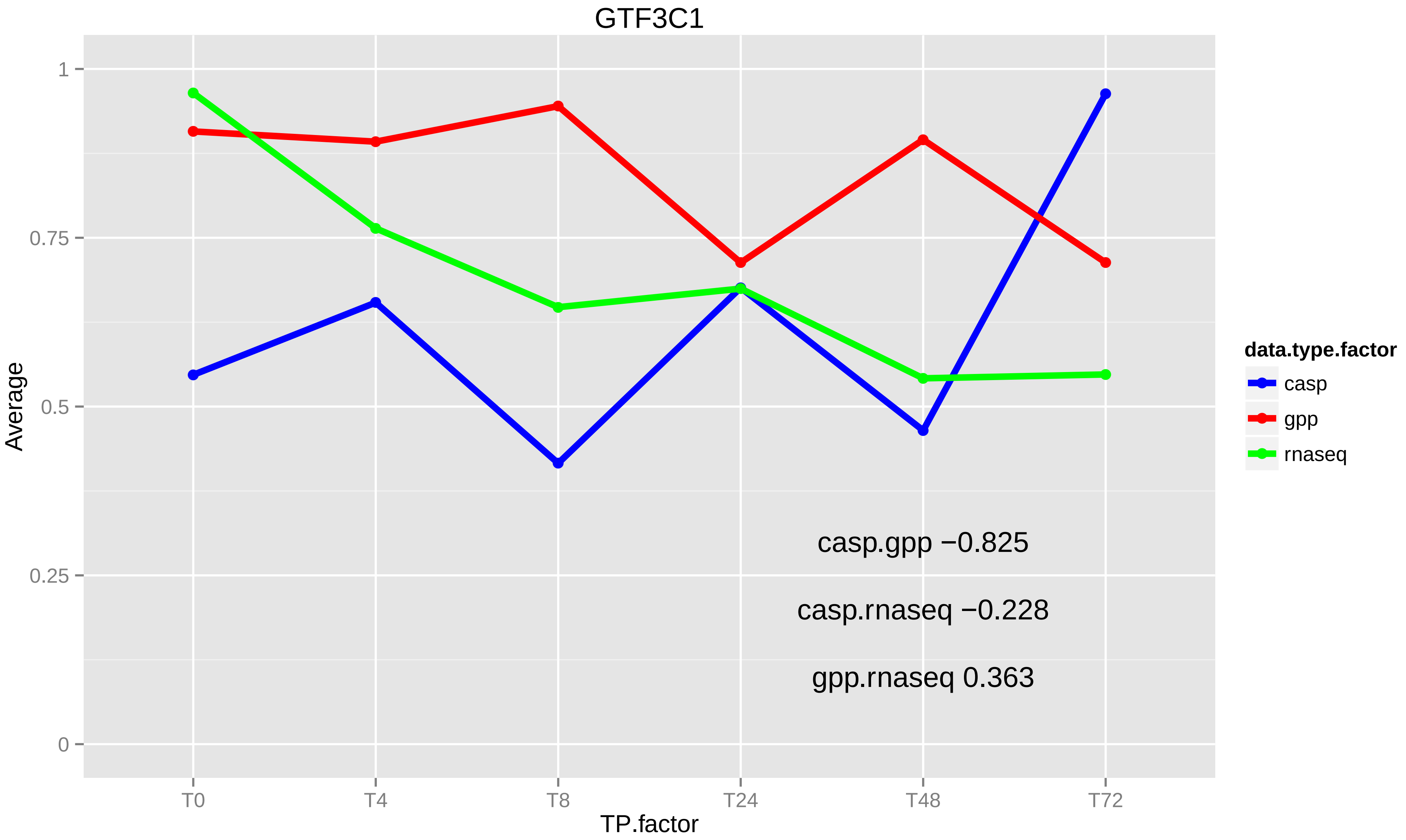
GTF3C1
Average
1
0.75
0.5
0.25
0
T0
T4
T8
T24
T48
T72
TP.factor
data.type.factor
casp
gpp
rnaseq
casp.gpp −0.825
casp.rnaseq −0.228
gpp.rnaseq 0.363

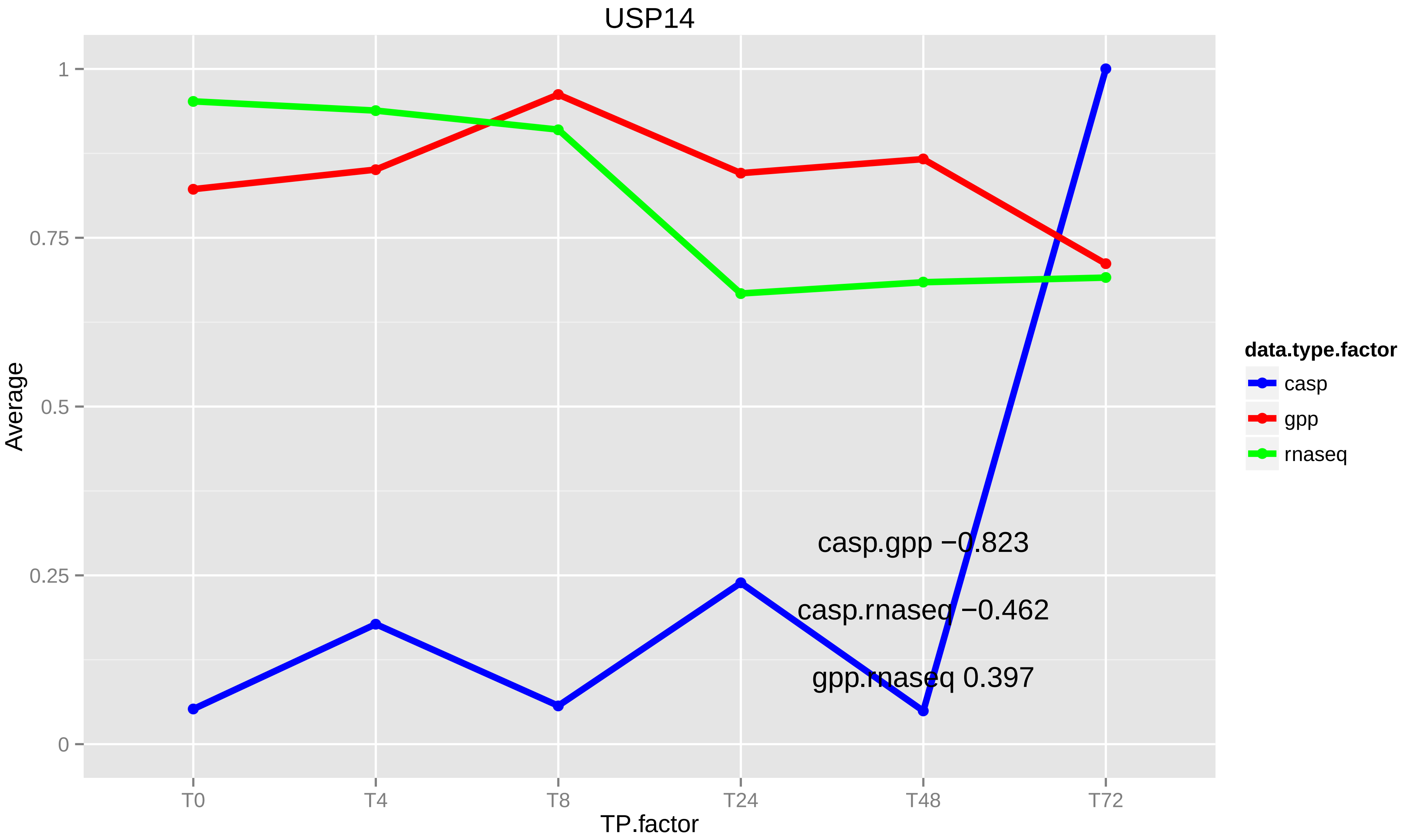
USP14
1
0.75
0.5
0.25
0
Average
T0
T4
T8
T24
T48
T72
TP.factor
data.type.factor
casp
gpp
rnaseq
casp.gpp −0.823
casp.rnaseq −0.462
gpp.rnaseq 0.397

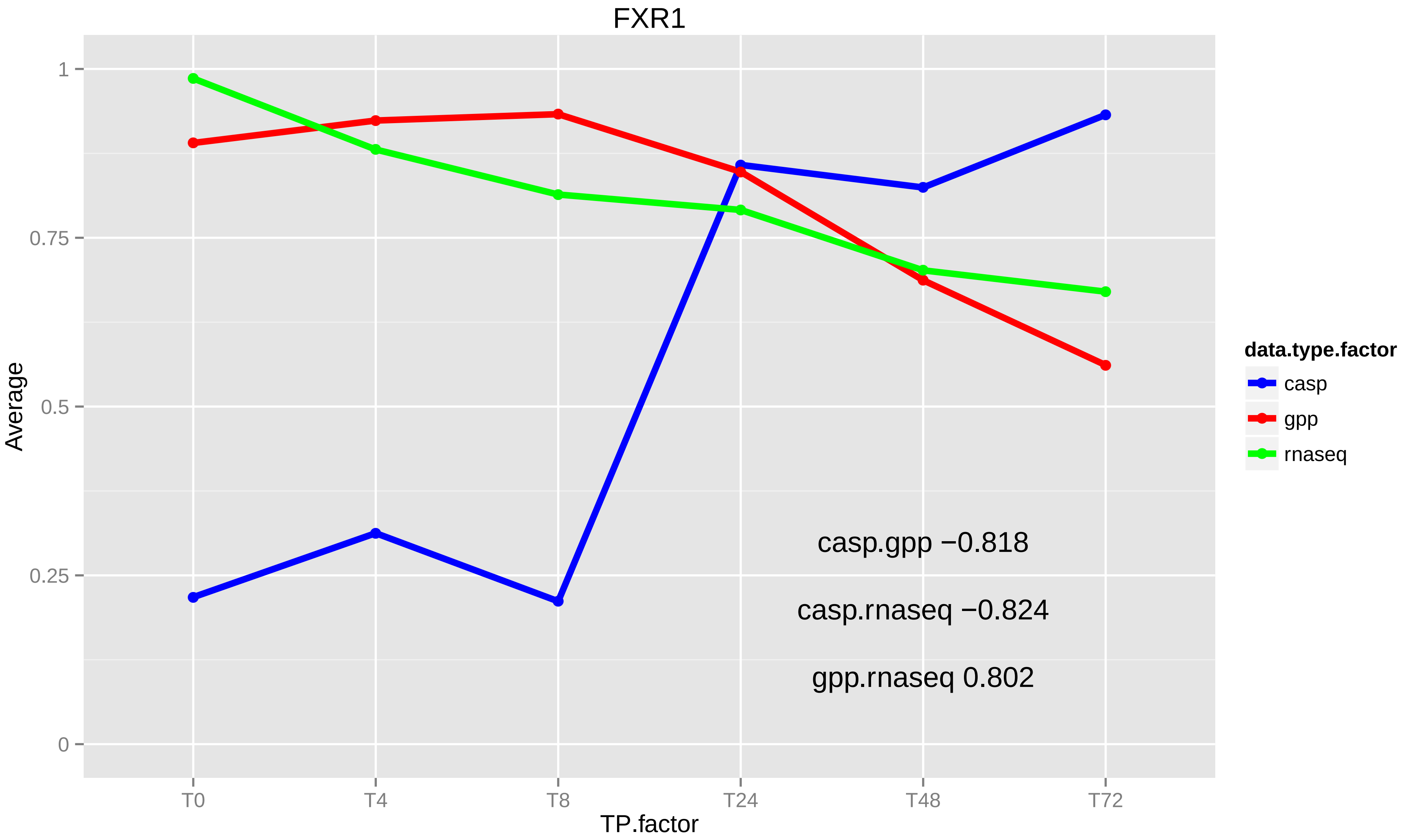

FXR1
Average
1
0.75
0.5
0.25
0
T0
T4
T8
T24
T48
T72
TP.factor
data.type.factor
casp
gpp
rnaseq
casp.gpp −0.818
casp.rnaseq −0.824
gpp.rnaseq 0.802

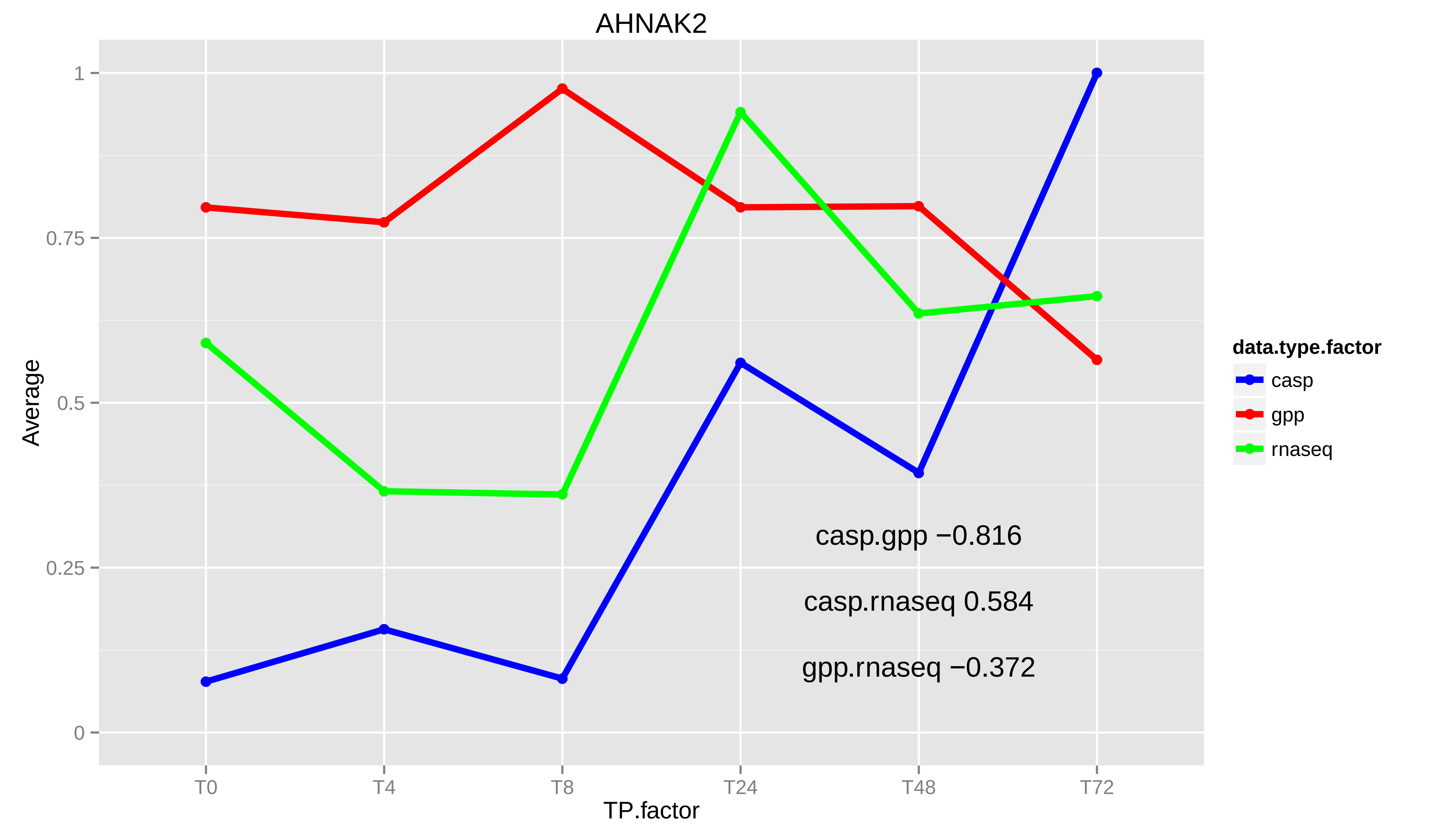

AHNAK2
1
0.75
0.5
0.25
0
Average
T0
T4
T8
T24
T48
T72
TP.factor
data.type.factor
casp
gpp
rnaseq
casp.gpp −0.816
casp.rnaseq 0.584
gpp.rnaseq −0.372

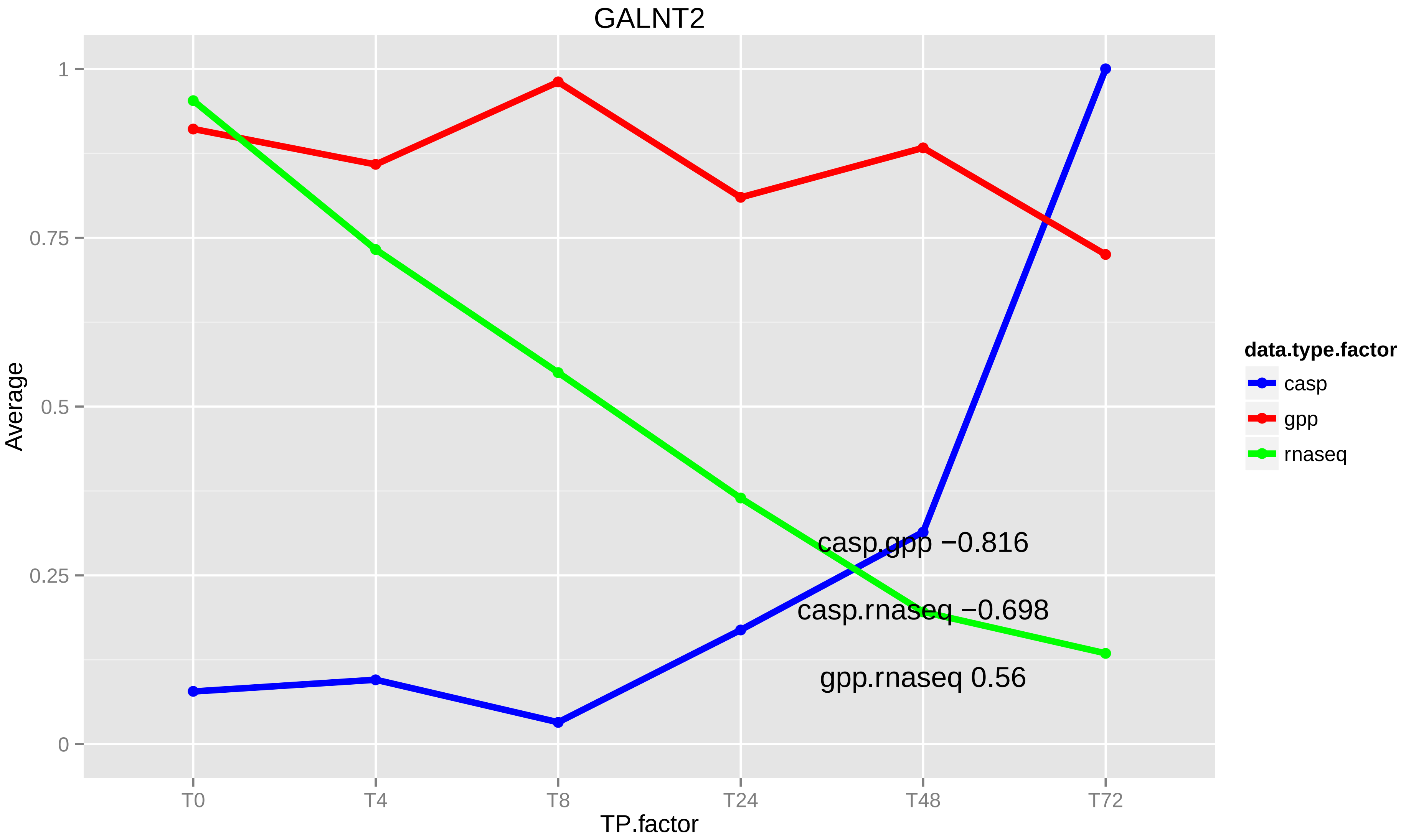
GALNT2
Average
1
0.75
0.5
0.25
0
T0
T4
T8
T24
T48
T72
TP.factor
data.type.factor
casp
gpp
rnaseq
casp.gpp −0.816
casp.rnaseq −0.698
gpp.rnaseq 0.56

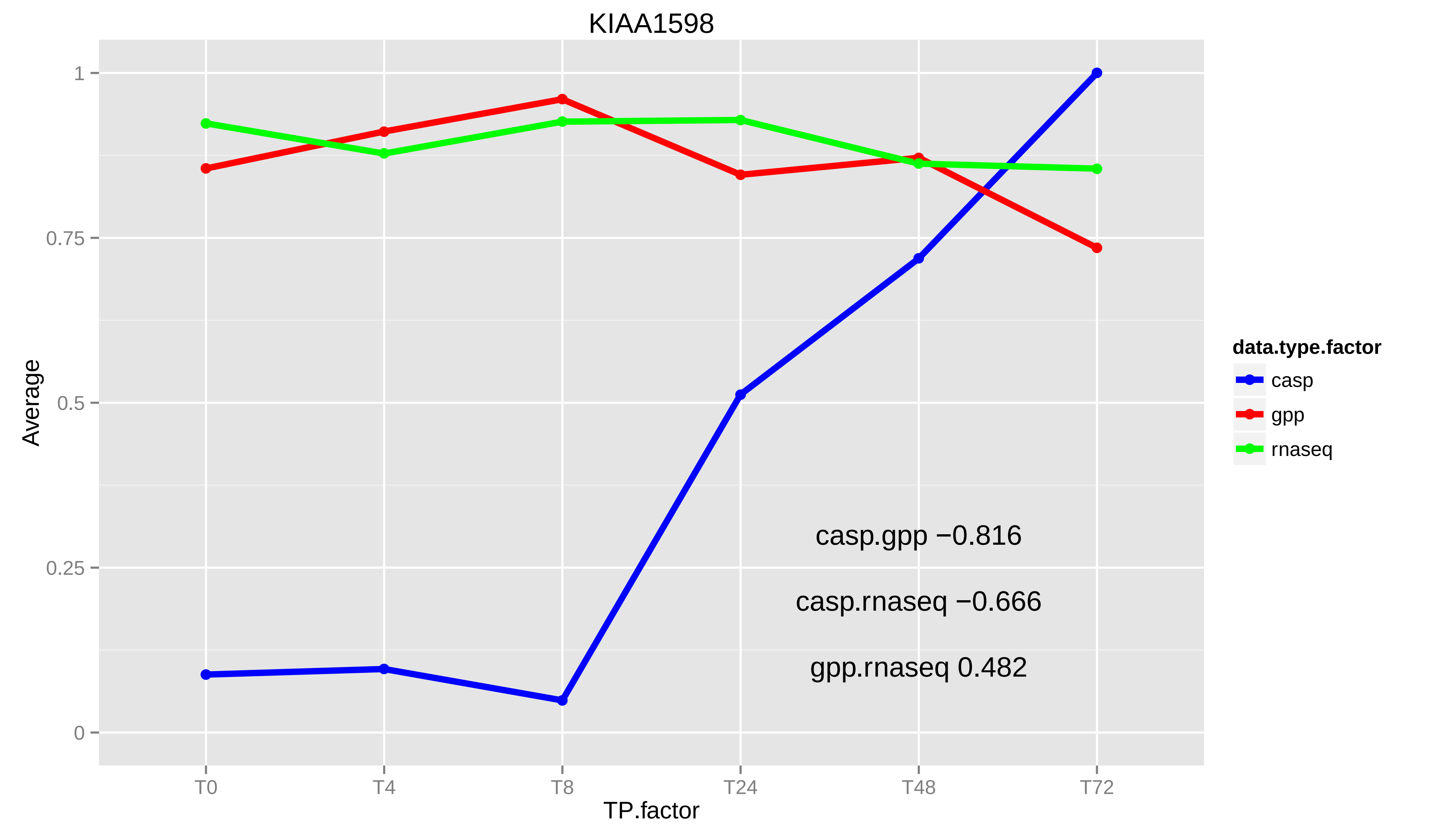

KIAA1598
Average
1
0.75
0.5
0.25
0
T0
T4
T8
T24
T48
T72
TP.factor
data.type.factor
casp
gpp
rnaseq
casp.gpp −0.816
casp.rnaseq −0.666
gpp.rnaseq 0.482

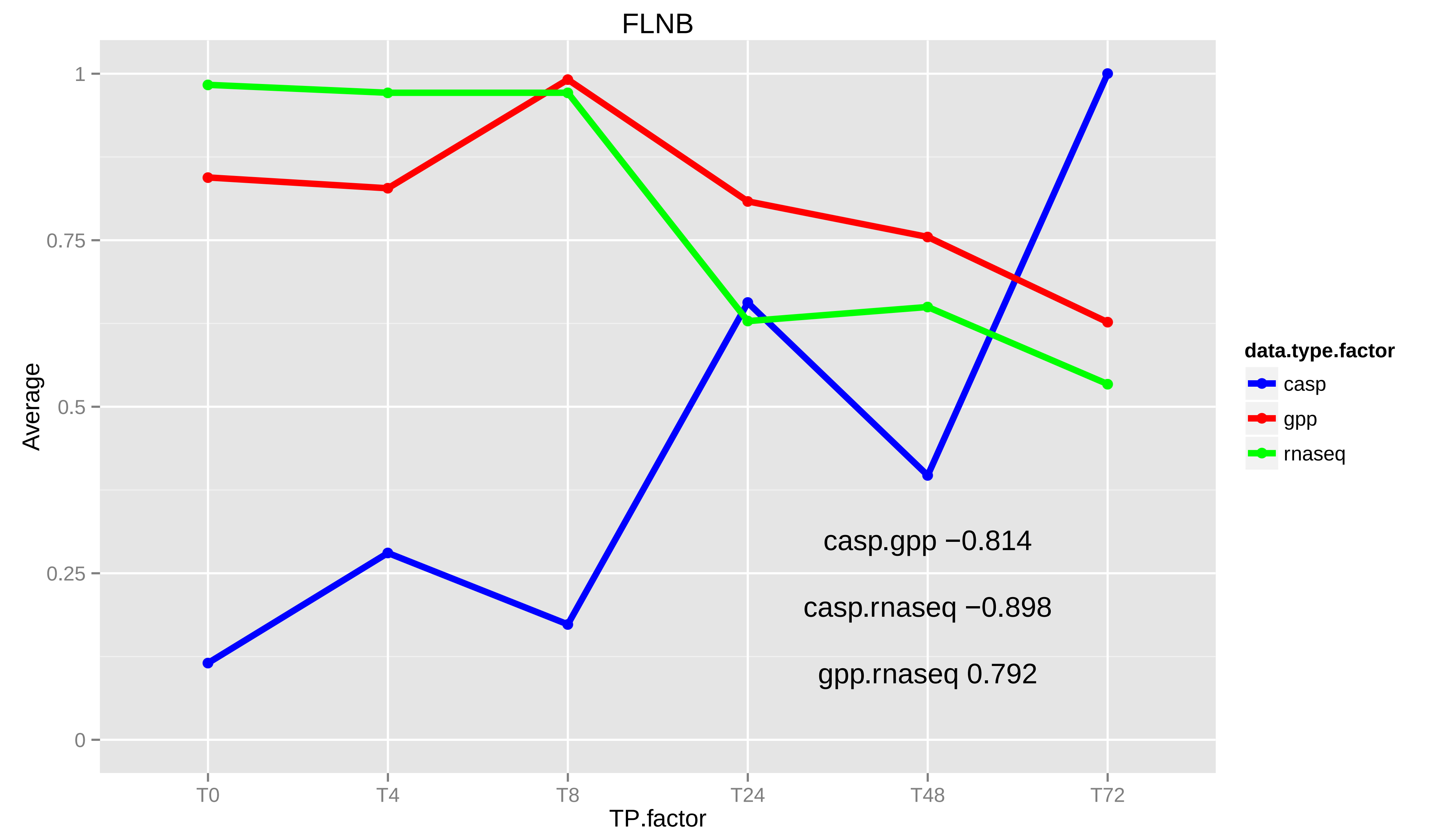

FLNB
Average
1
0.75
0.5
0.25
0
T0
T4
T8
T24
T48
T72
TP.factor
data.type.factor
casp
gpp
rnaseq
casp.gpp −0.814
casp.rnaseq −0.898
gpp.rnaseq 0.792

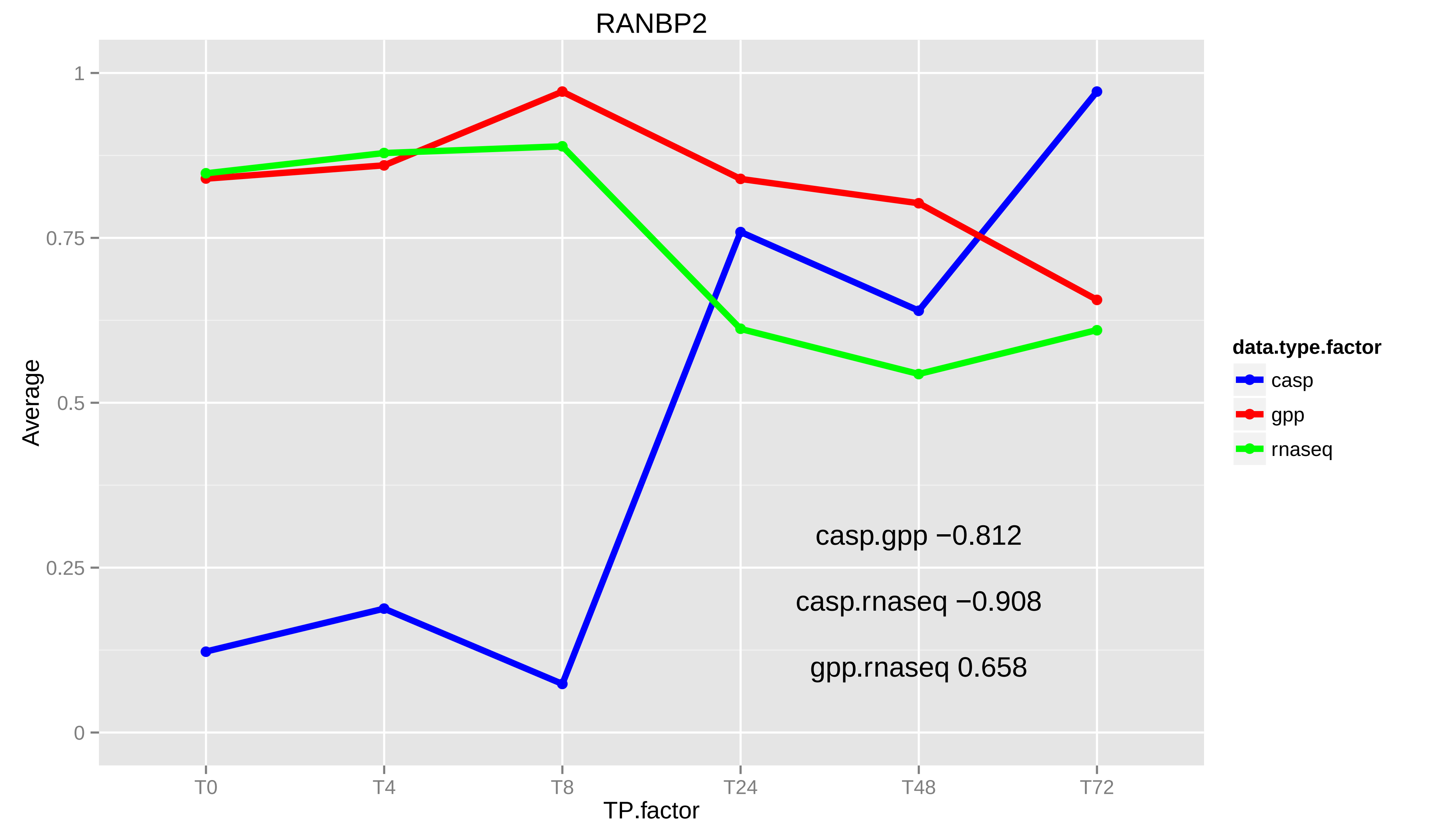
RANBP2
Average
TP.factor
T0
T4
T8
T24
T48
T72
0
0.25
0.5
0.75
1
data.type.factor
casp
gpp
rnaseq
casp.gpp −0.812
casp.rnaseq −0.908
gpp.rnaseq 0.658

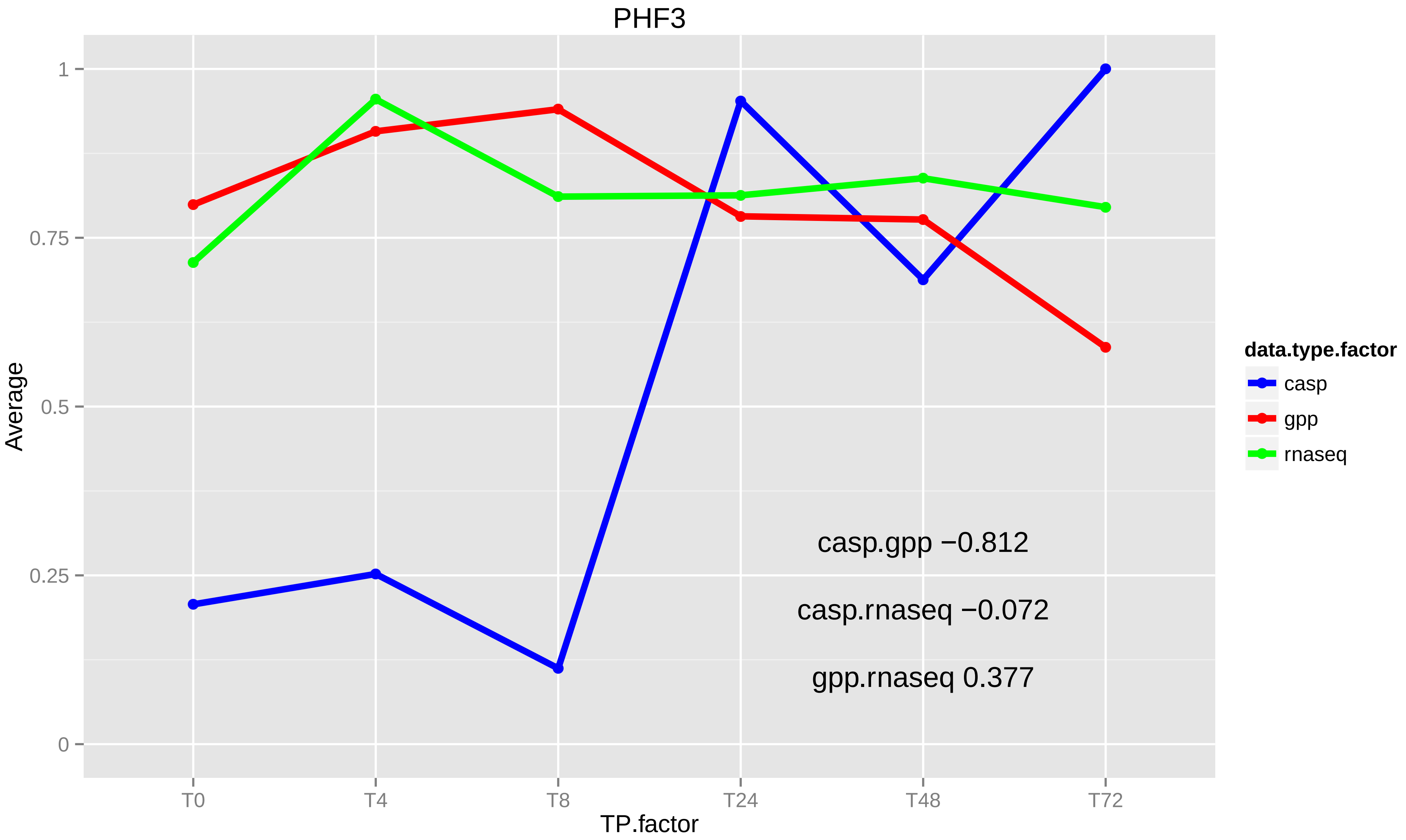

PHF3
1
0.75
0.5
0.25
0
Average
T0
T4
T8
T24
T48
T72
TP.factor
data.type.factor
casp
gpp
rnaseq
casp.gpp −0.812
casp.rnaseq −0.072
gpp.rnaseq 0.377

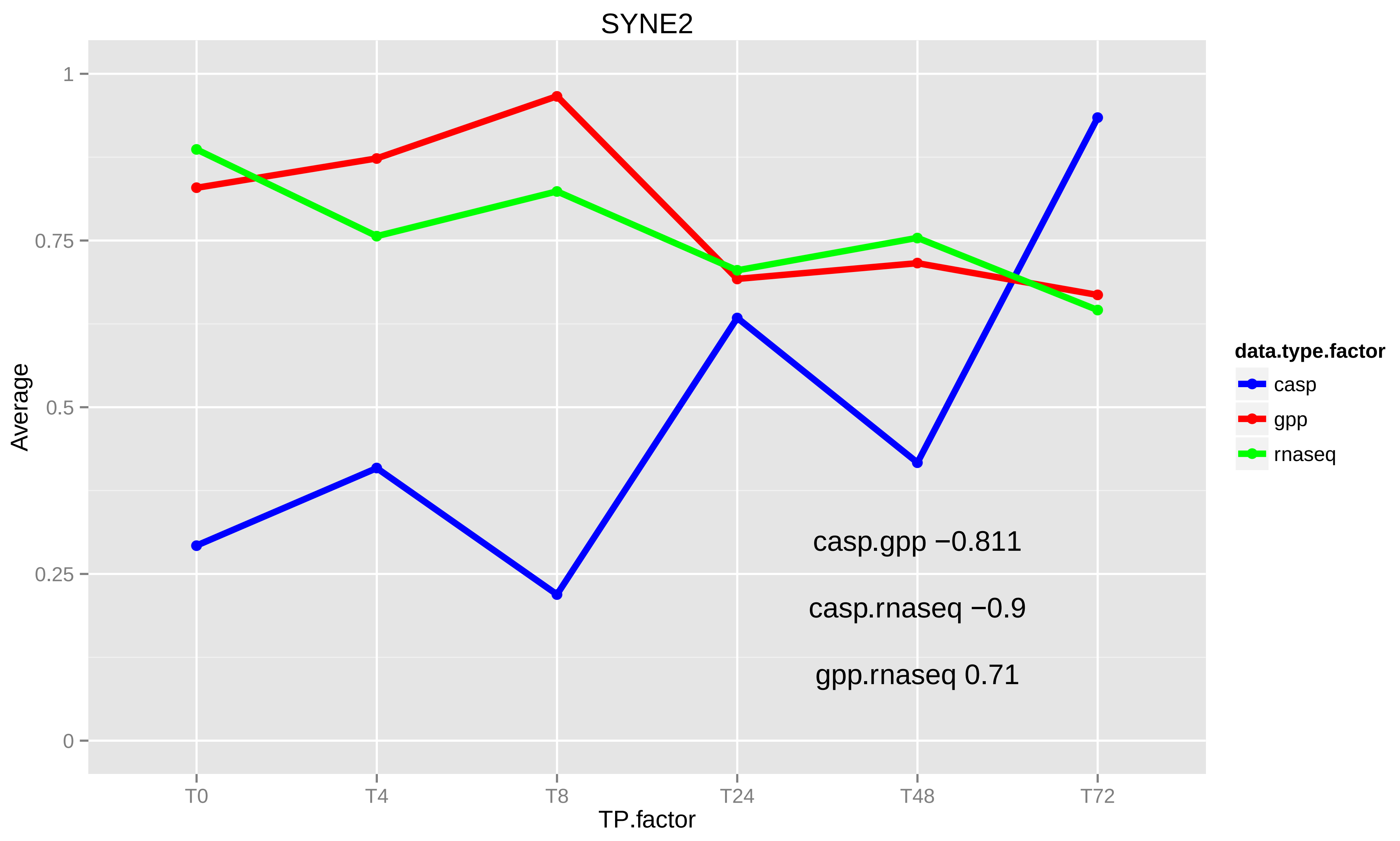

SYNE2
1
0.75
0.5
0.25
0
Average
T0
T4
T8
T24
T48
T72
TP.factor
data.type.factor
casp
gpp
rnaseq
casp.gpp −0.811
casp.rnaseq −0.9
gpp.rnaseq 0.71

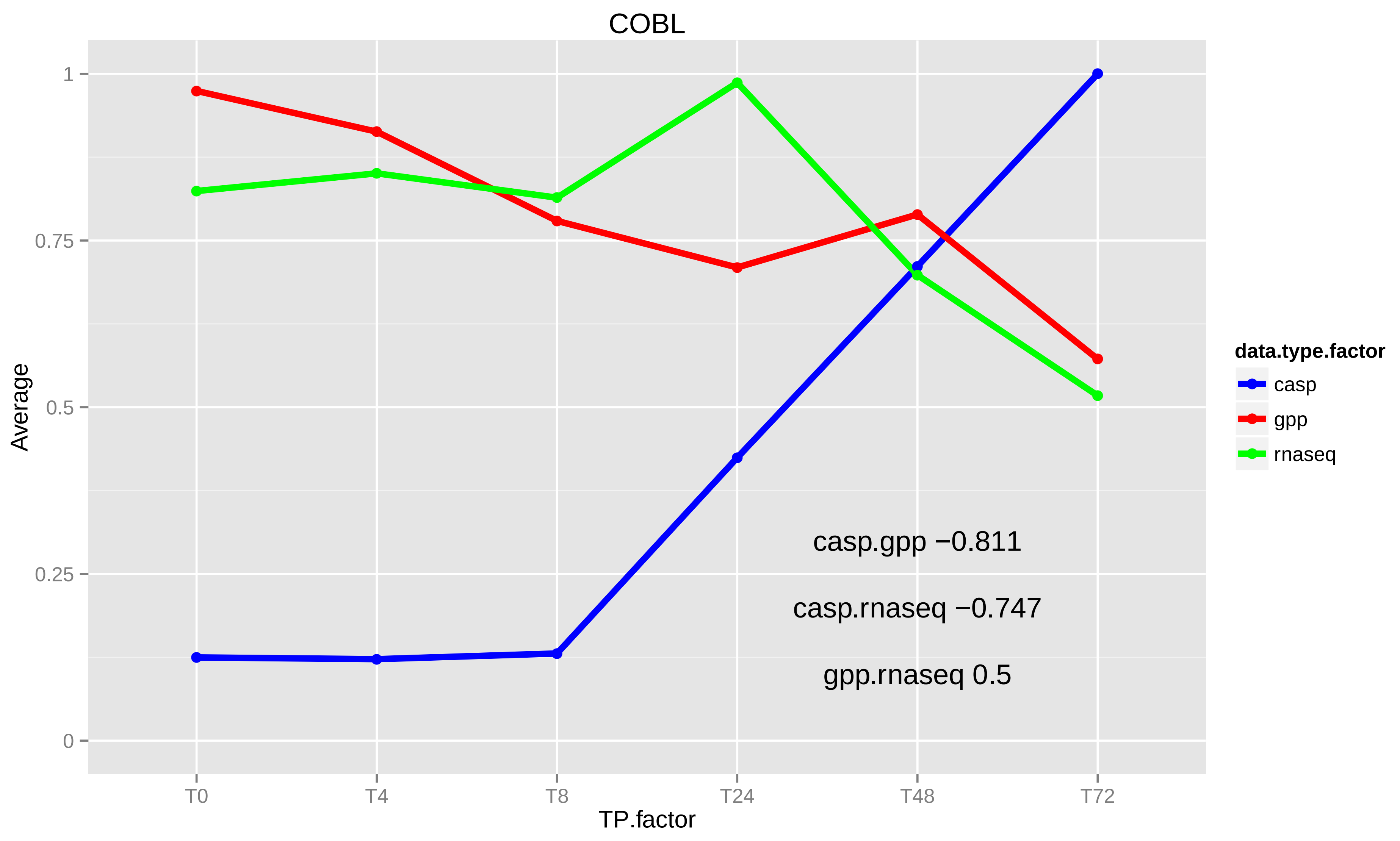
COBL
Average
1
0.75
0.5
0.25
0
T0
T4
T8
T24
T48
T72
TP.factor
data.type.factor
casp
gpp
rnaseq
casp.gpp −0.811
casp.rnaseq −0.747
gpp.rnaseq 0.5

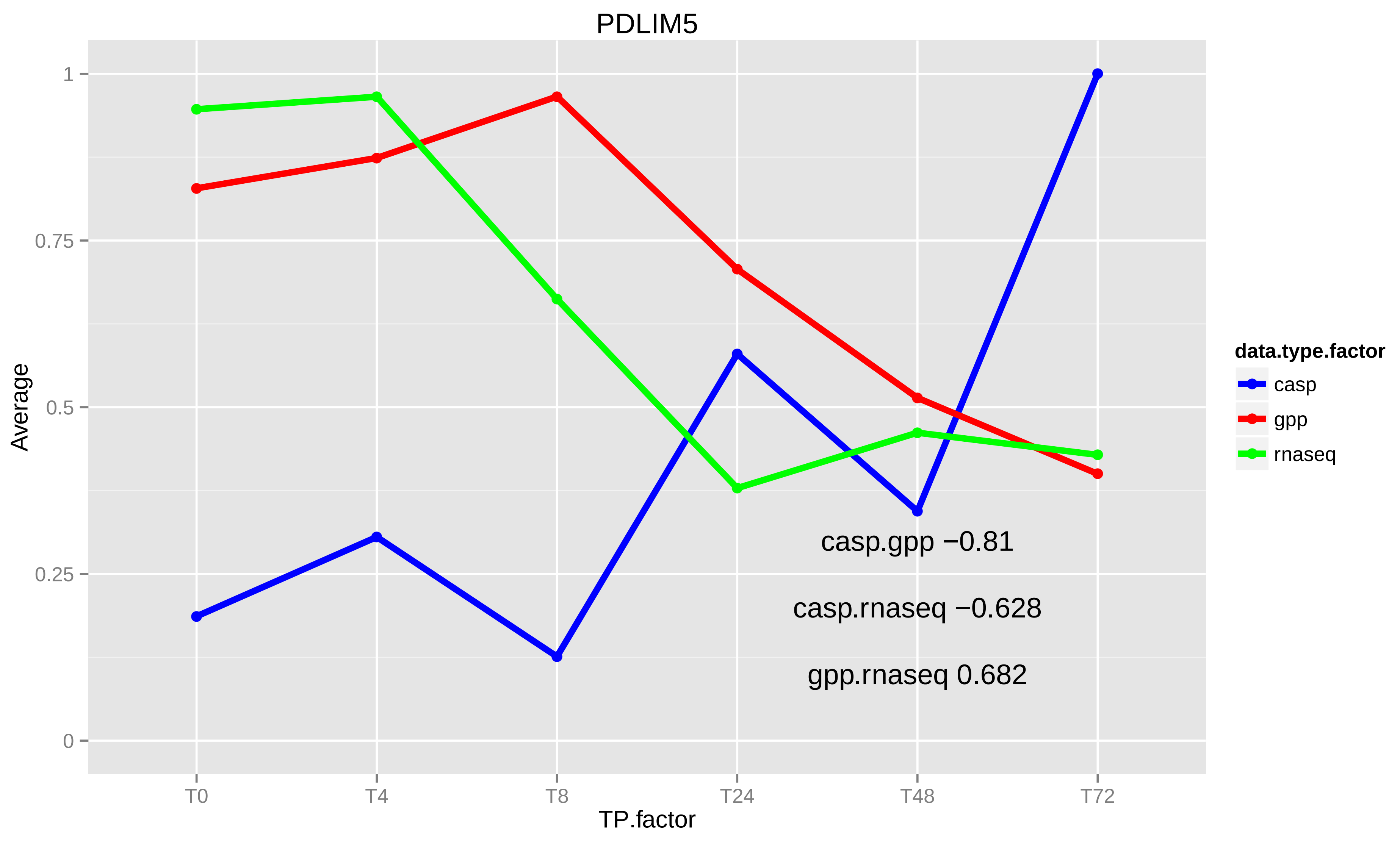
PDLIM5
Average
1
0.75
0.5
0.25
0
T0
T4
T8
T24
T48
T72
TP.factor
data.type.factor
casp
gpp
rnaseq
casp.gpp −0.81
casp.rnaseq −0.628
gpp.rnaseq 0.682

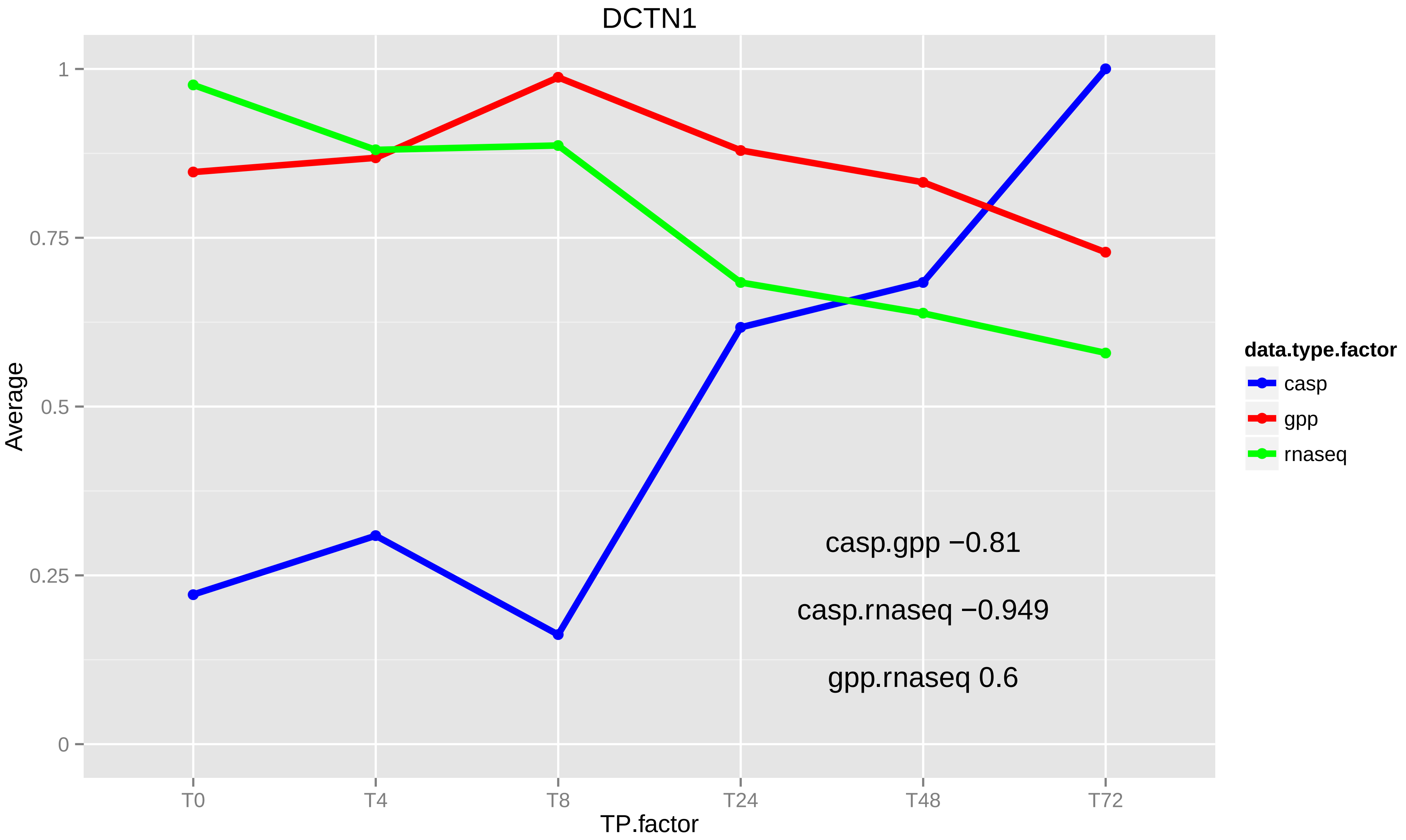
DCTN1
Average
1
0.75
0.5
0.25
0
T0
T4
T8
T24
T48
T72
TP.factor
data.type.factor
casp
gpp
rnaseq
casp.gpp −0.81
casp.rnaseq −0.949
gpp.rnaseq 0.6

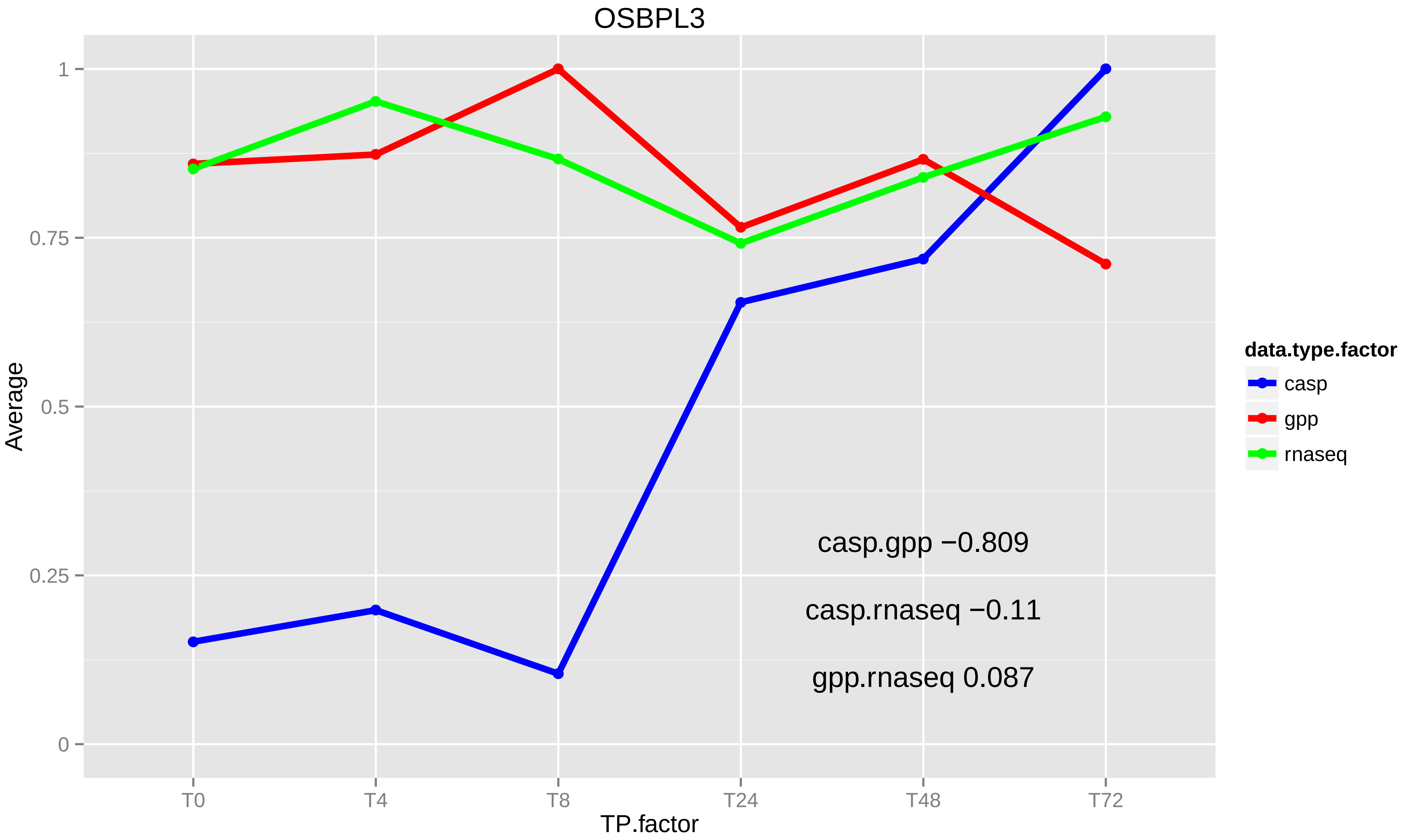
OSBPL3
Average
1
0.75
0.5
0.25
0
T0
T4
T8
T24
T48
T72
TP.factor
data.type.factor
casp
gpp
rnaseq
casp.gpp −0.809
casp.rnaseq −0.11
gpp.rnaseq 0.087

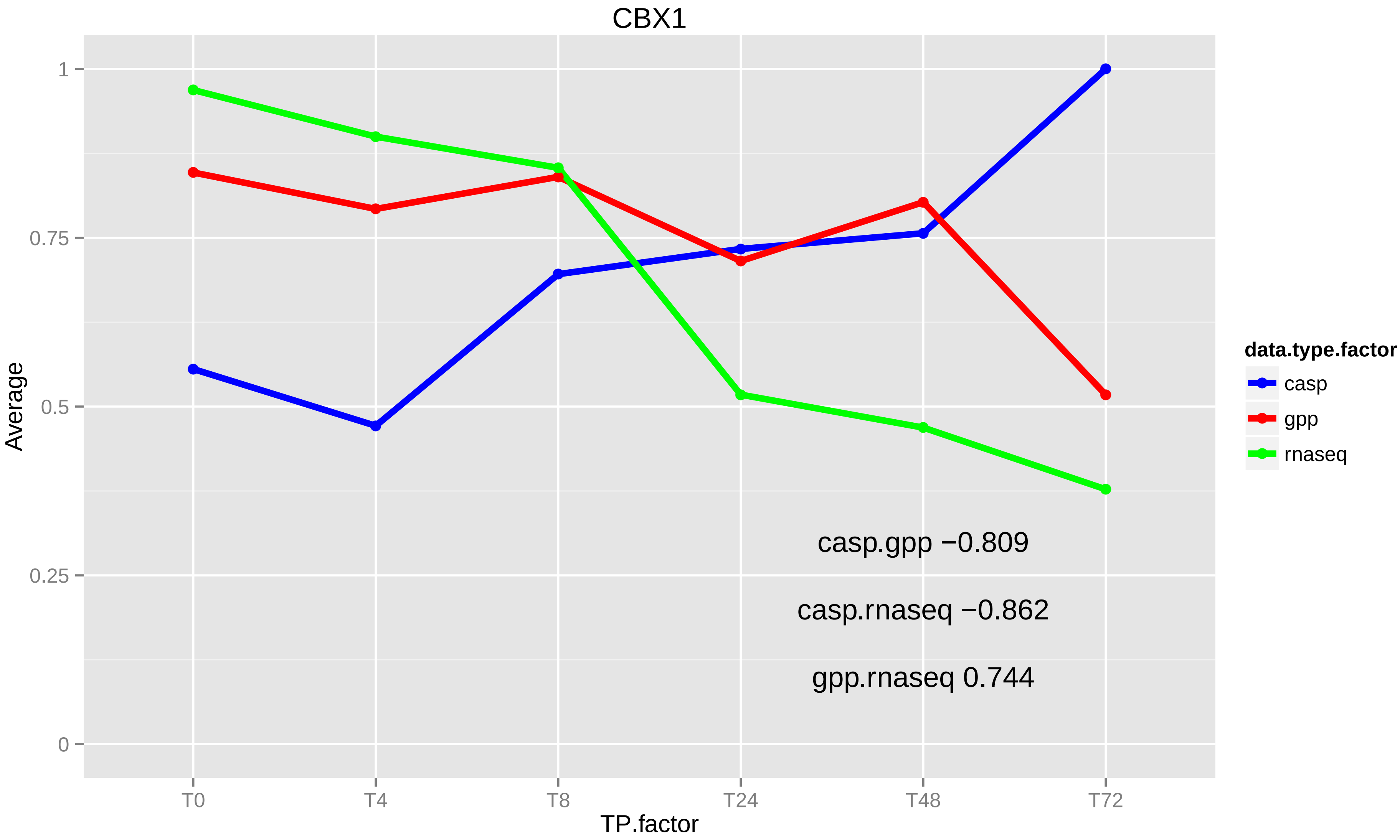
CBX1
1
0.75
0.5
0.25
0
Average
T0
T4
T8
T24
T48
T72
TP.factor
data.type.factor
casp
gpp
rnaseq
casp.gpp −0.809
casp.rnaseq −0.862
gpp.rnaseq 0.744

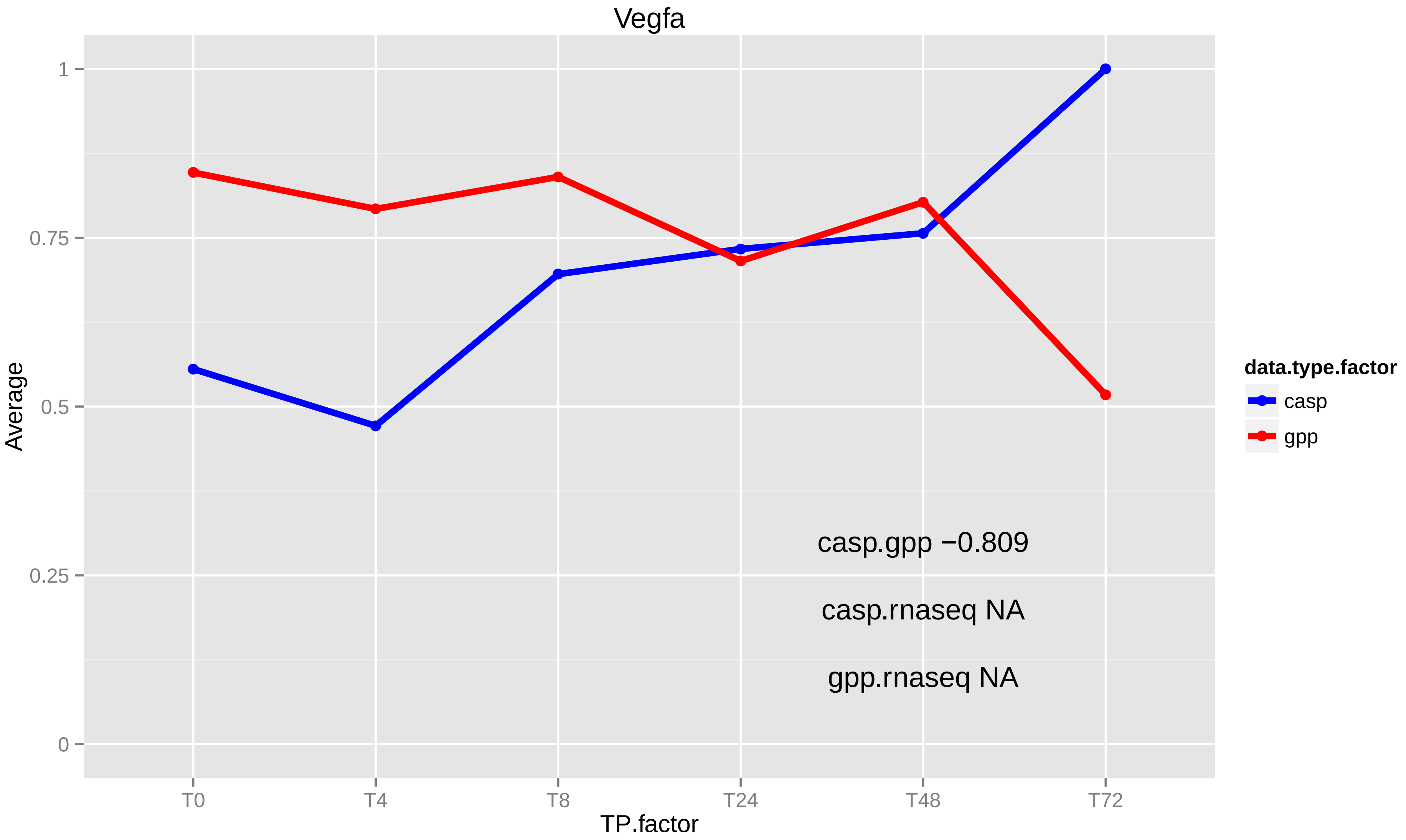

Vegfa
1
0.75
0.5
0.25
0
Average
T0
T4
T8
T24
T48
T72
TP.factor
data.type.factor
casp
gpp
casp.gpp −0.809
casp.rnaseq NA
gpp.rnaseq NA

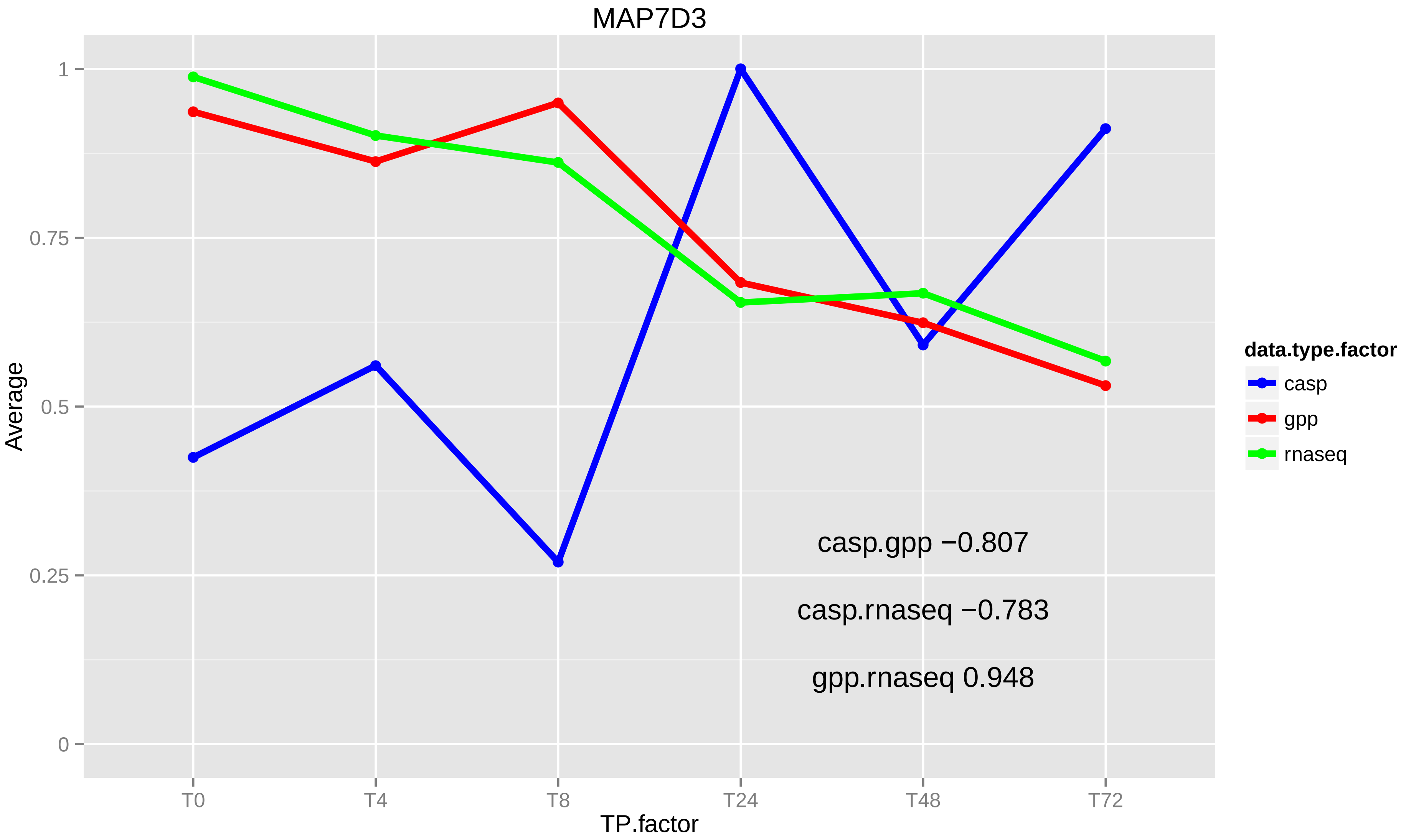

MAP7D3
Average
1
0.75
0.5
0.25
0
T0
T4
T8
T24
T48
T72
TP.factor
data.type.factor
casp
gpp
rnaseq
casp.gpp −0.807
casp.rnaseq −0.783
gpp.rnaseq 0.948

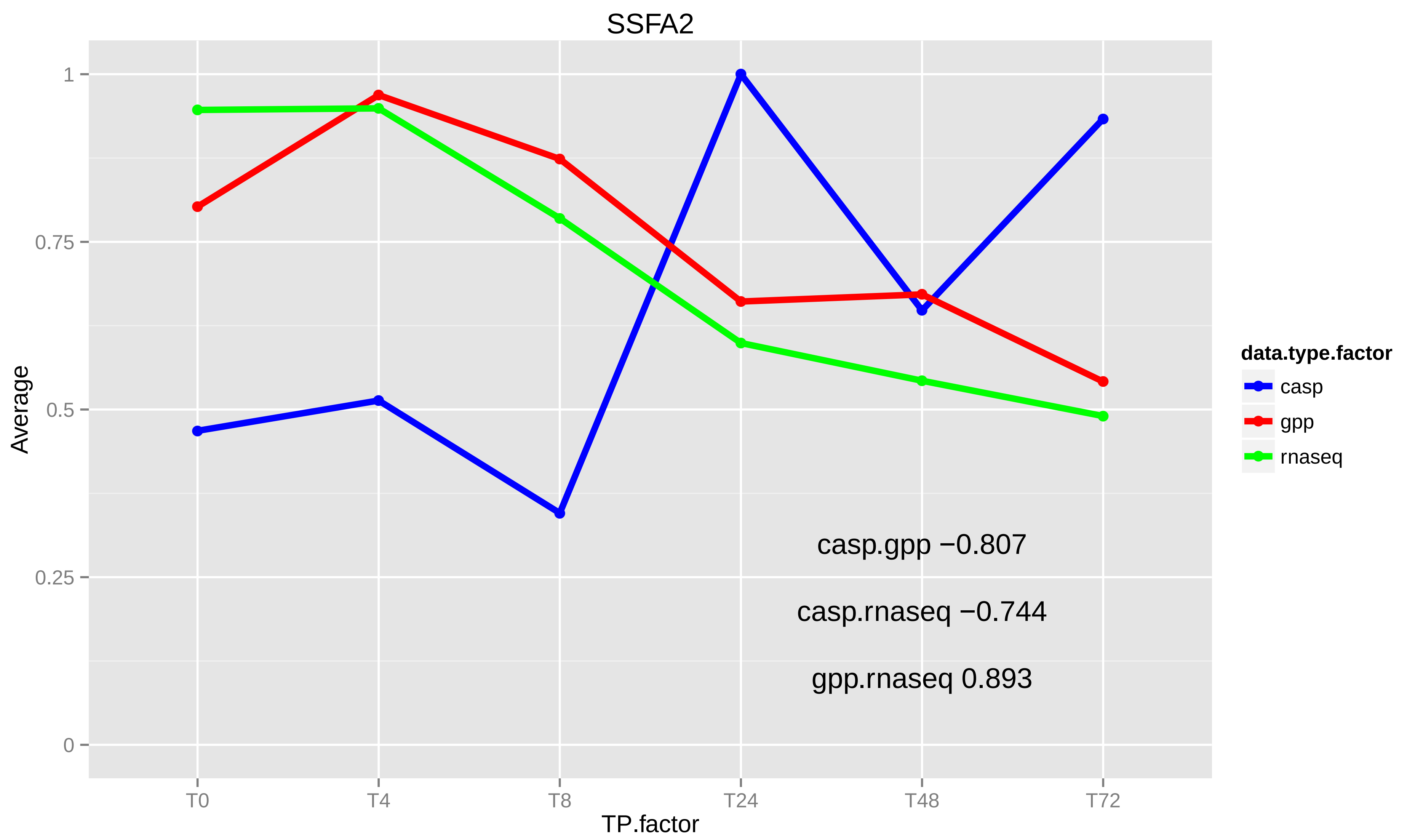
SSFA2
Average
TP.factor
T0
T4
T8
T24
T48
T72
0
0.25
0.5
0.75
1
data.type.factor
casp
gpp
rnaseq
casp.gpp −0.807
casp.rnaseq −0.744
gpp.rnaseq 0.893

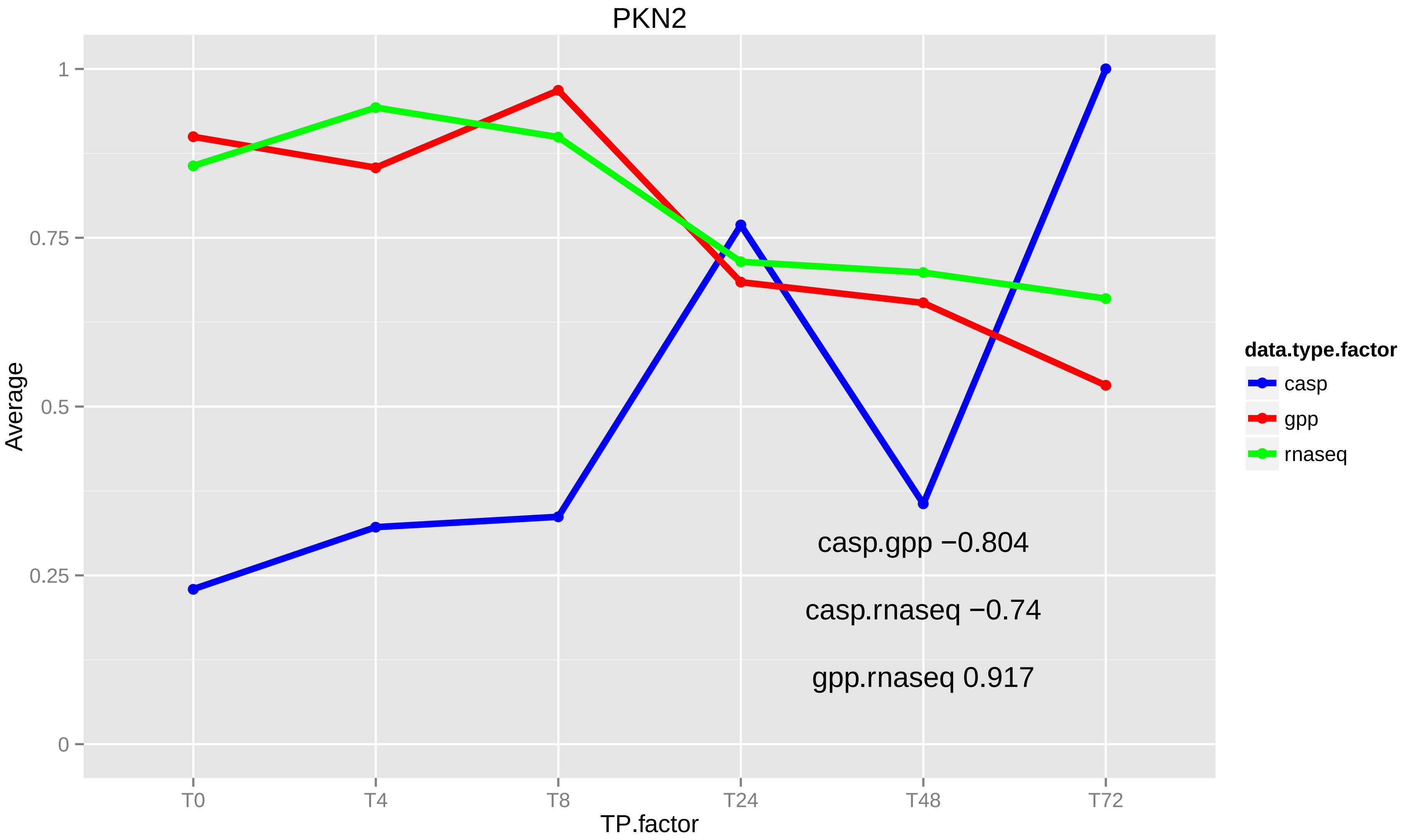

PKN2
1
0.75
0.5
0.25
0
Average
T0
T4
T8
T24
T48
T72
TP.factor
data.type.factor
casp
gpp
rnaseq
casp.gpp −0.804
casp.rnaseq −0.74
gpp.rnaseq 0.917

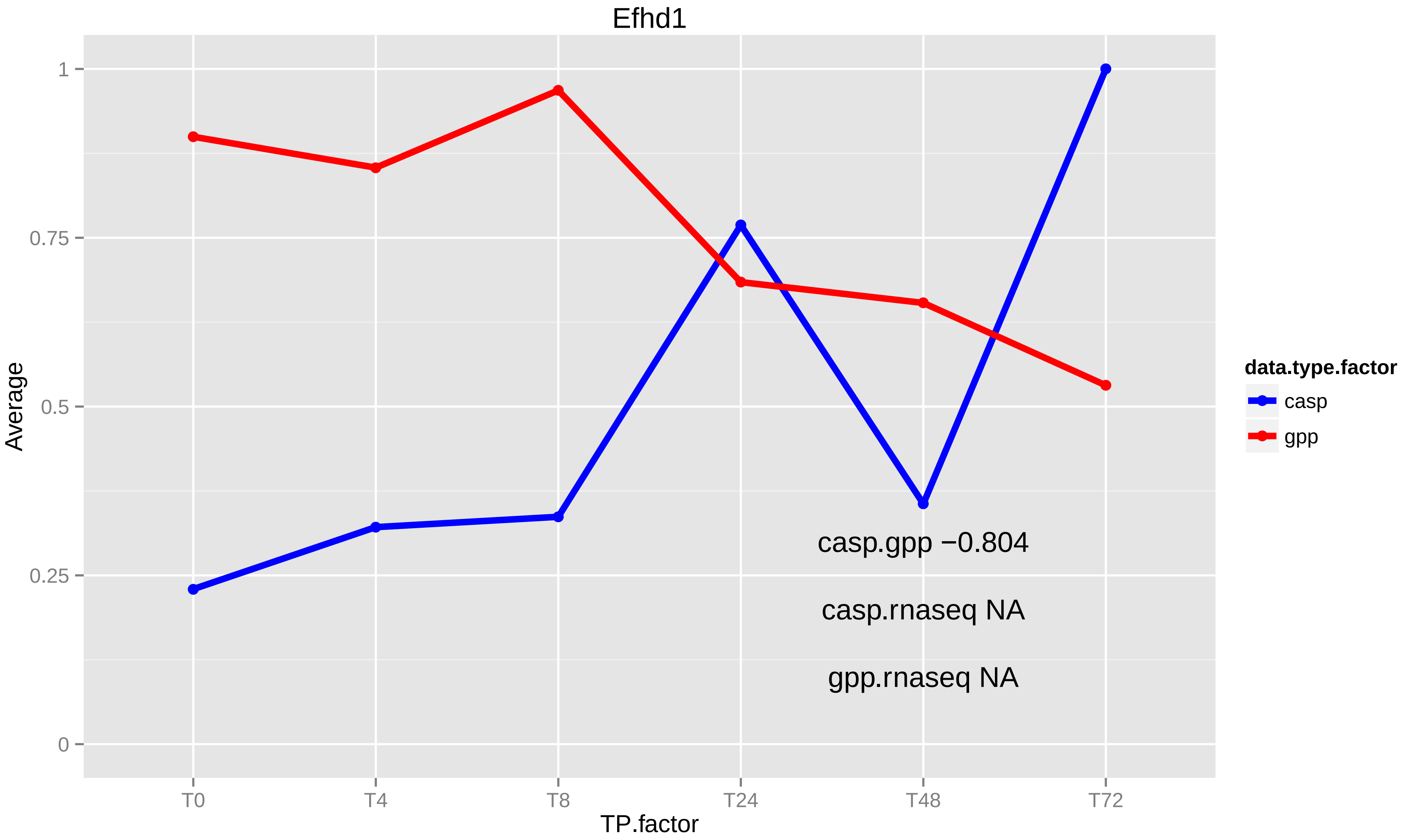

Efhd1
Average
1
0.75
0.5
0.25
0
T0
T4
T8
T24
T48
T72
TP.factor
data.type.factor
casp
gpp
casp.gpp −0.804
casp.rnaseq NA
gpp.rnaseq NA

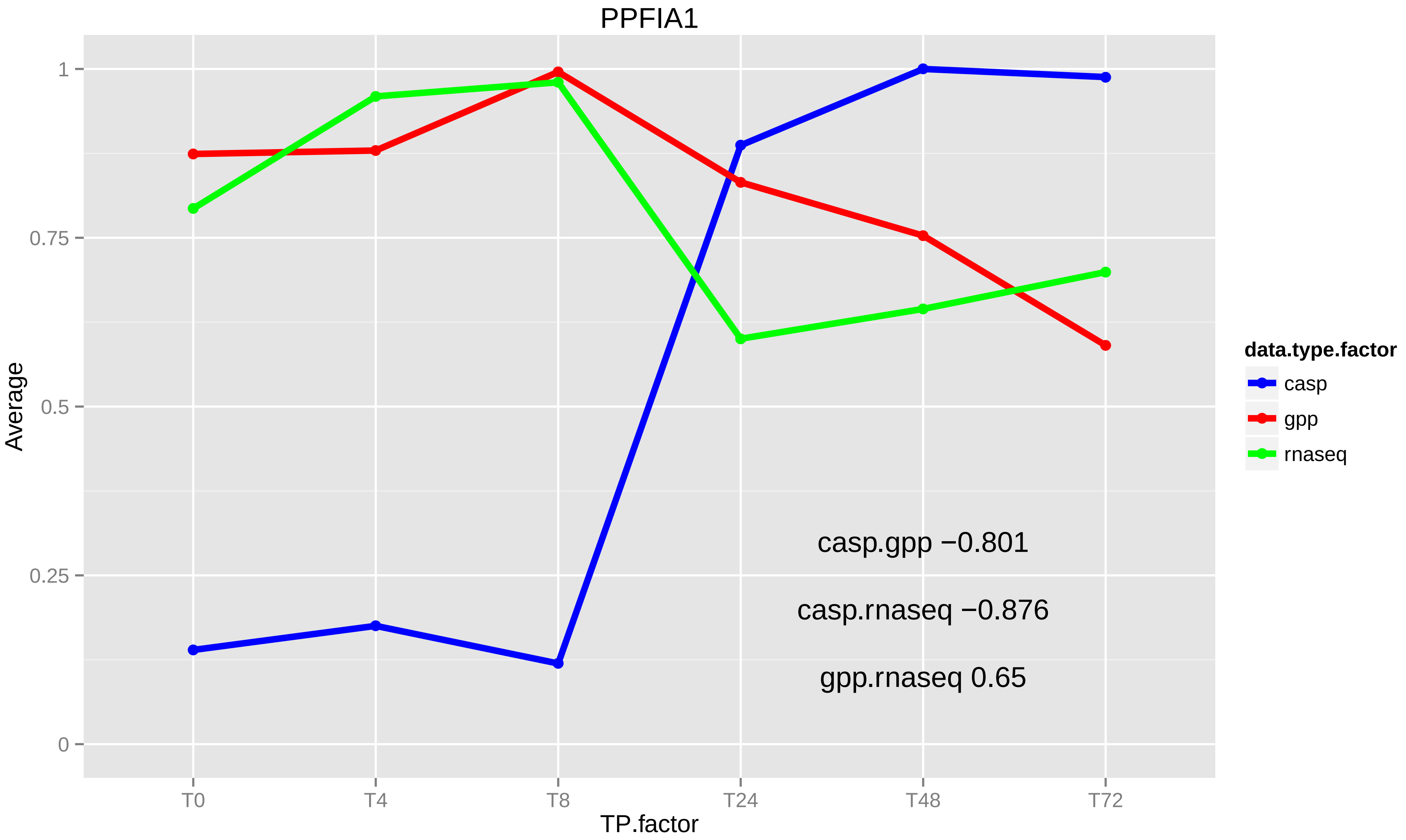
PPFIA1
Average
1
0.75
0.5
0.25
0
T0
T4
T8
T24
T48
T72
TP.factor
data.type.factor
casp
gpp
rnaseq
casp.gpp −0.801
casp.rnaseq −0.876
gpp.rnaseq 0.65

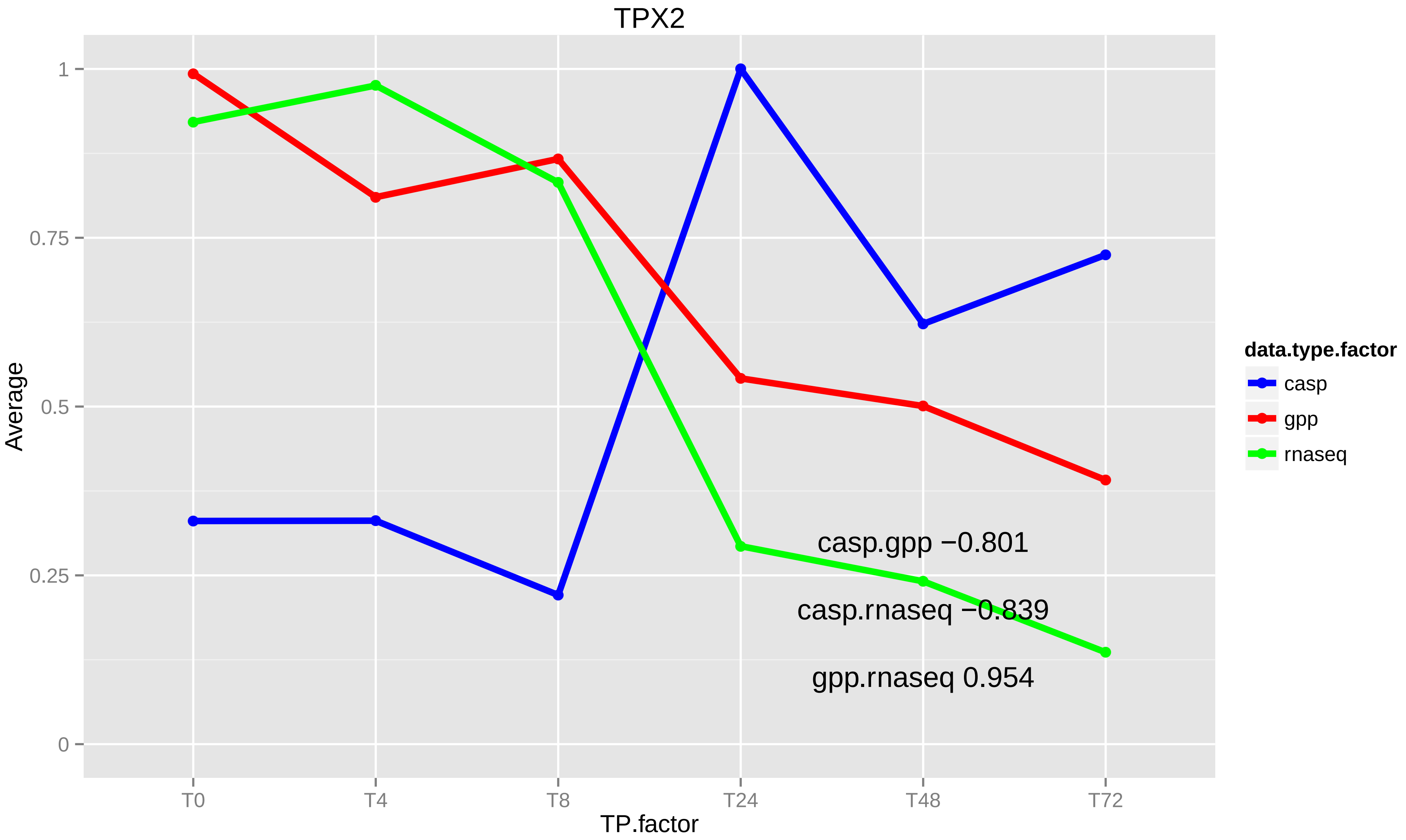

TPX2
Average
1
0.75
0.5
0.25
0
T0
T4
T8
T24
T48
T72
TP.factor
data.type.factor
casp
gpp
rnaseq
casp.gpp −0.801
casp.rnaseq −0.839
gpp.rnaseq 0.954

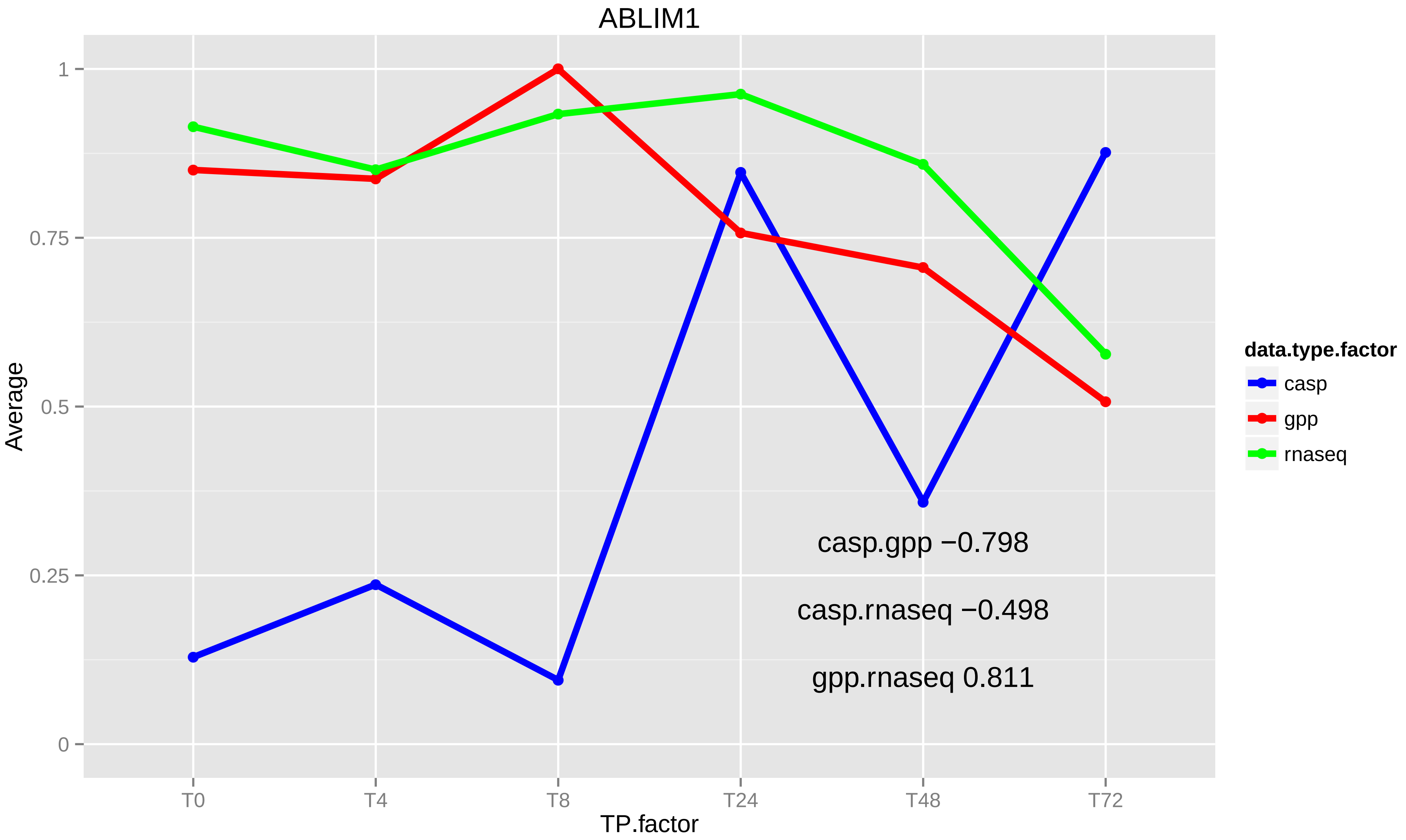
ABLIM1
Average
1
0.75
0.5
0.25
0
T0
T4
T8
T24
T48
T72
TP.factor
data.type.factor
casp
gpp
rnaseq
casp.gpp −0.798
casp.rnaseq −0.498
gpp.rnaseq 0.811

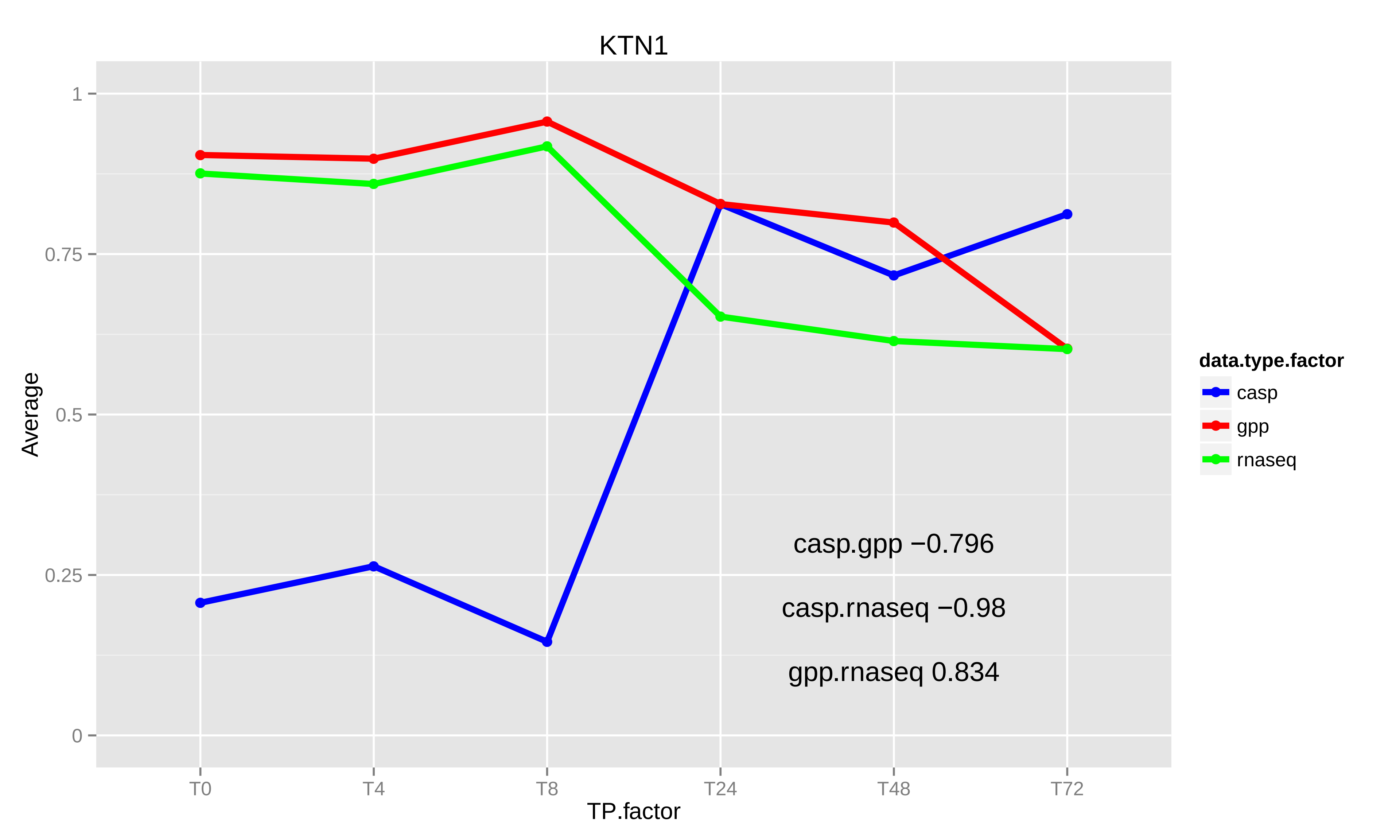
KTN1
Average
1
0.75
0.5
0.25
0
T0
T4
T8
T24
T48
T72
TP.factor
data.type.factor
casp
gpp
rnaseq
casp.gpp −0.796
casp.rnaseq −0.98
gpp.rnaseq 0.834

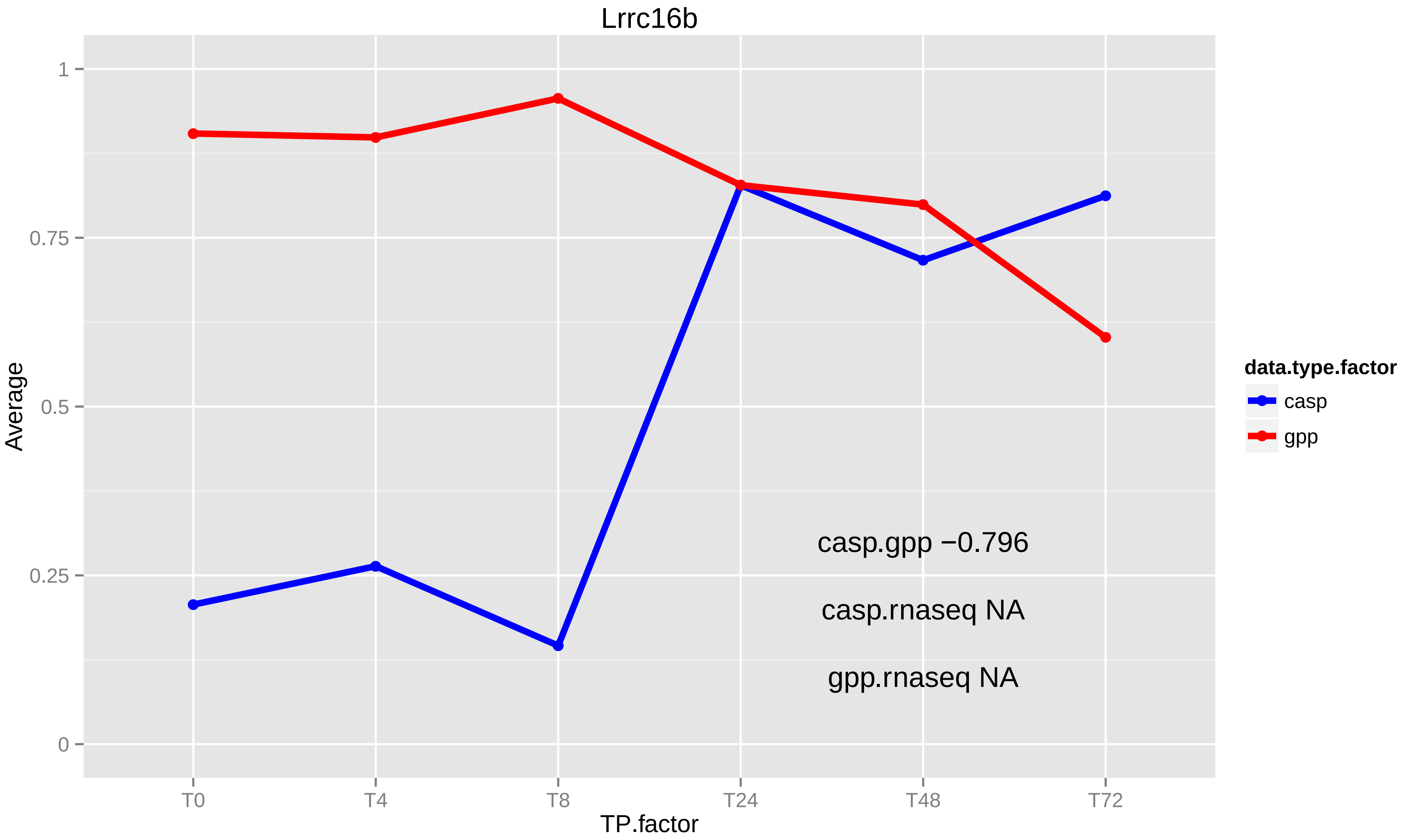

Lrrc16b
Average
1
0.75
0.5
0.25
0
T0
T4
T8
T24
T48
T72
TP.factor
data.type.factor
casp
gpp
casp.gpp −0.796
casp.rnaseq NA
gpp.rnaseq NA

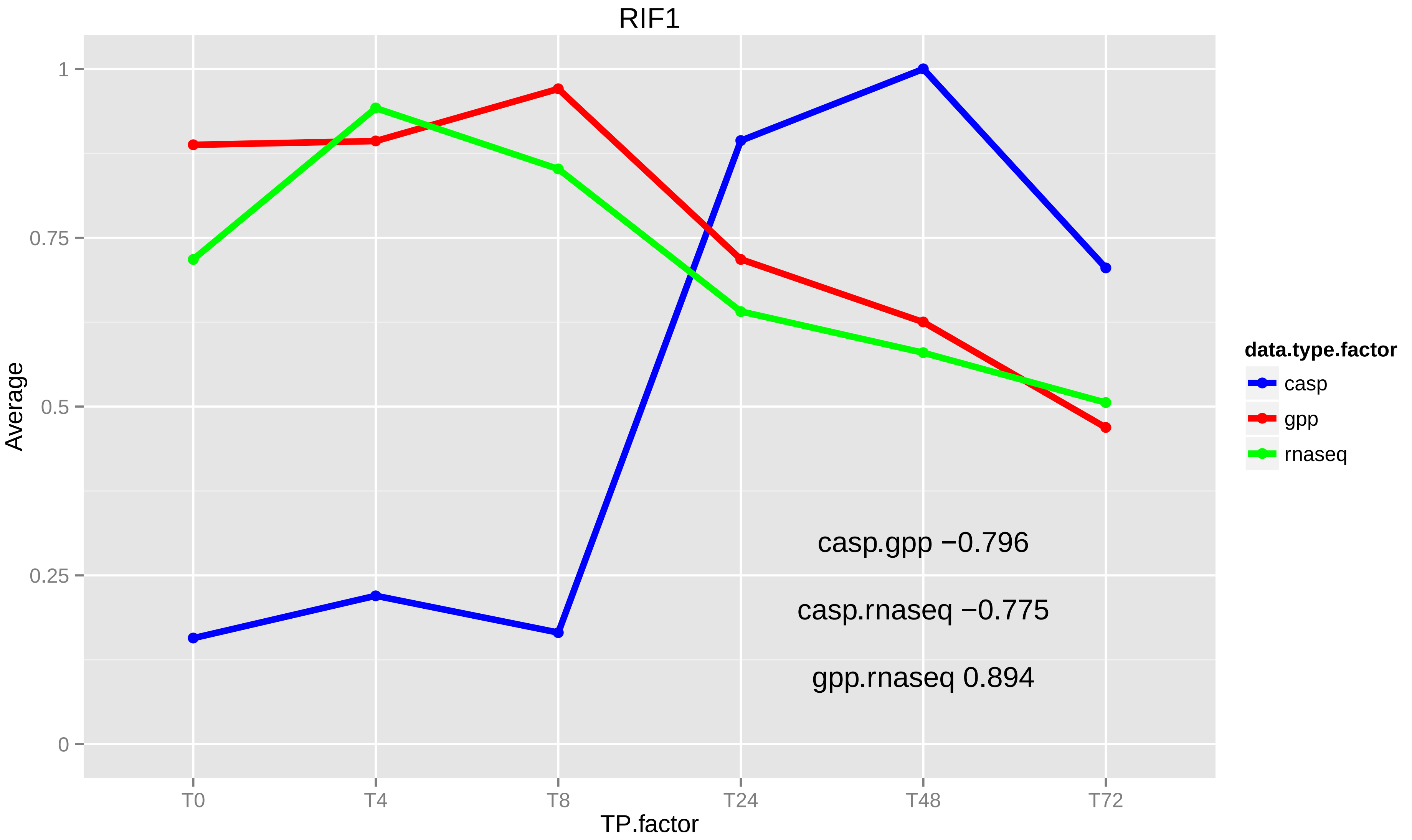
RIF1
Average
1
0.75
0.5
0.25
0
T0
T4
T8
T24
T48
T72
TP.factor
data.type.factor
casp
gpp
rnaseq
casp.gpp −0.796
casp.rnaseq −0.775
gpp.rnaseq 0.894

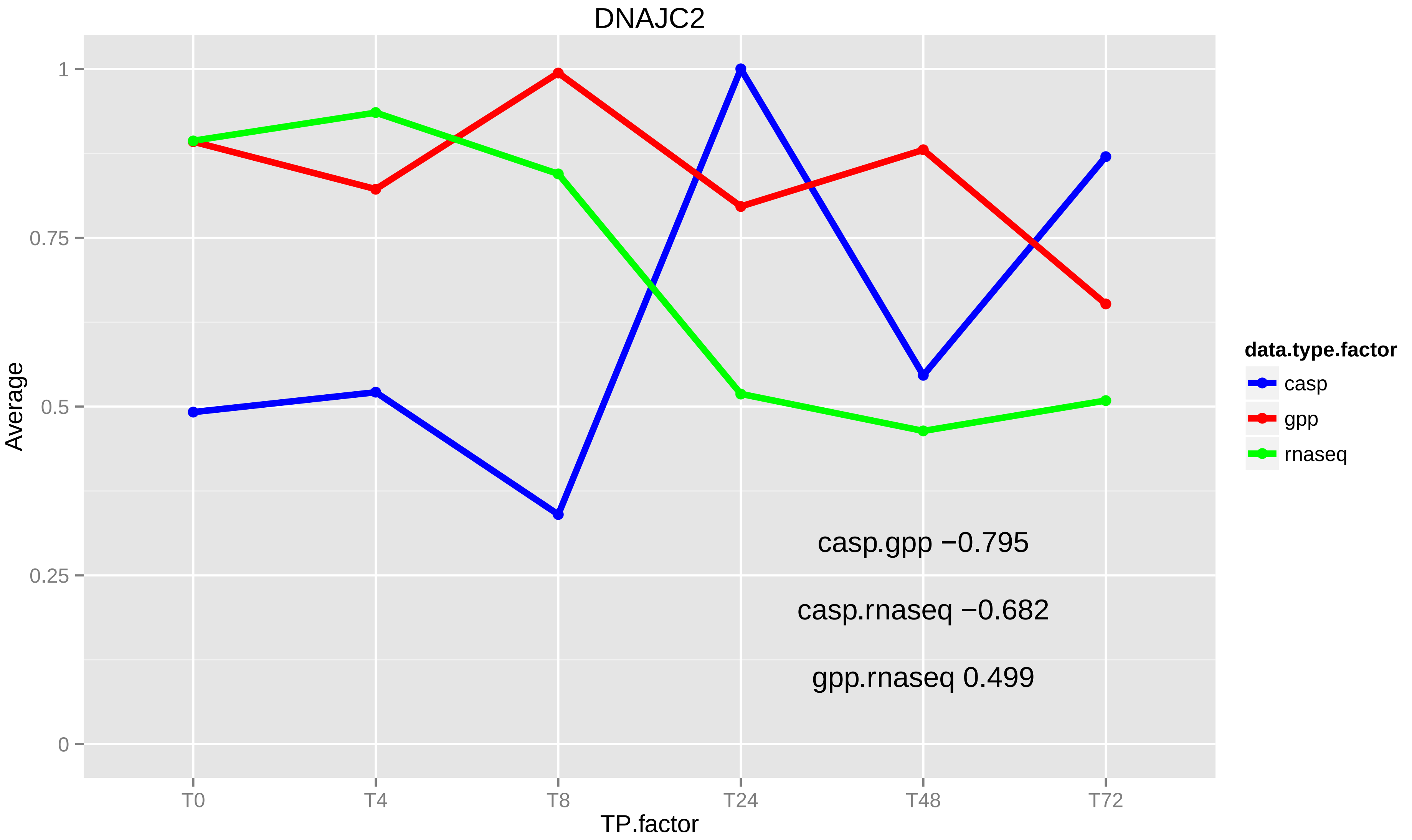
DNAJC2
1
0.75
0.5
0.25
0
Average
T0
T4
T8
T24
T48
T72
TP.factor
data.type.factor
casp
gpp
rnaseq
casp.gpp −0.795
casp.rnaseq −0.682
gpp.rnaseq 0.499

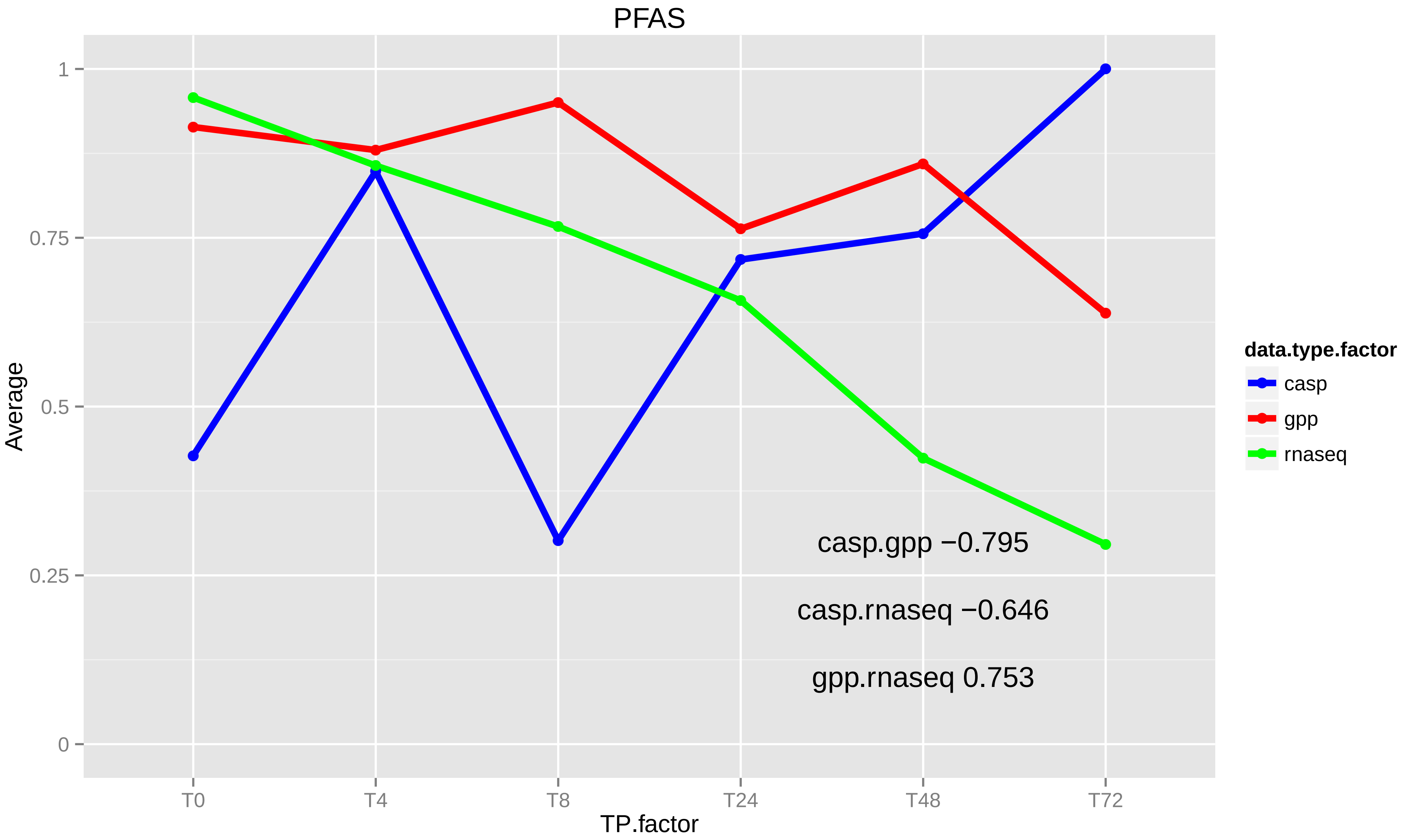

PFAS
1
0.75
0.5
0.25
0
Average
T0
T4
T8
T24
T48
T72
TP.factor
data.type.factor
casp
gpp
rnaseq
casp.gpp −0.795
casp.rnaseq −0.646
gpp.rnaseq 0.753

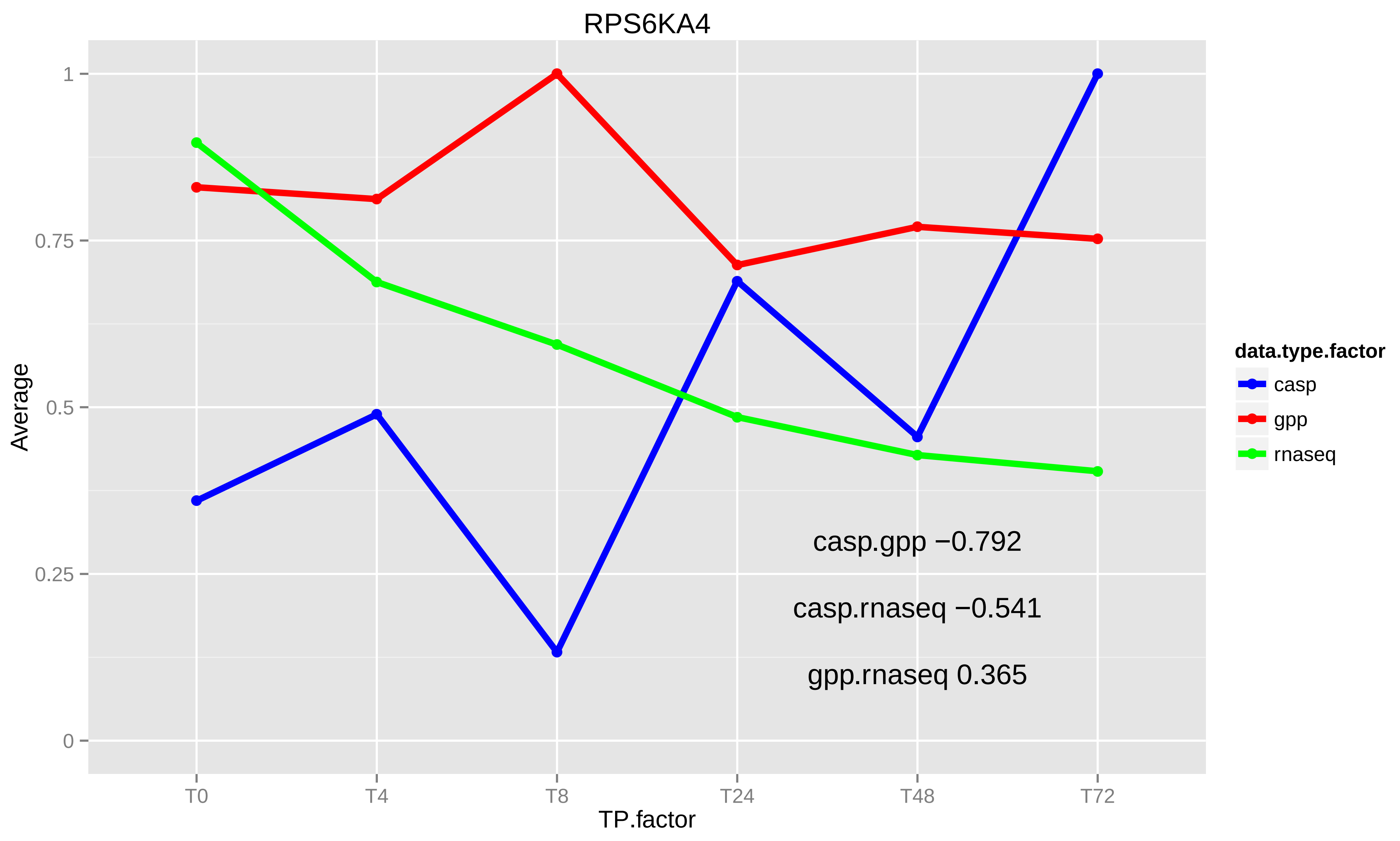
RPS6KA4
1
0.75
0.5
0.25
0
Average
T0
T4
T8
T24
T48
T72
TP.factor
data.type.factor
casp
gpp
rnaseq
casp.gpp −0.792
casp.rnaseq −0.541
gpp.rnaseq 0.365

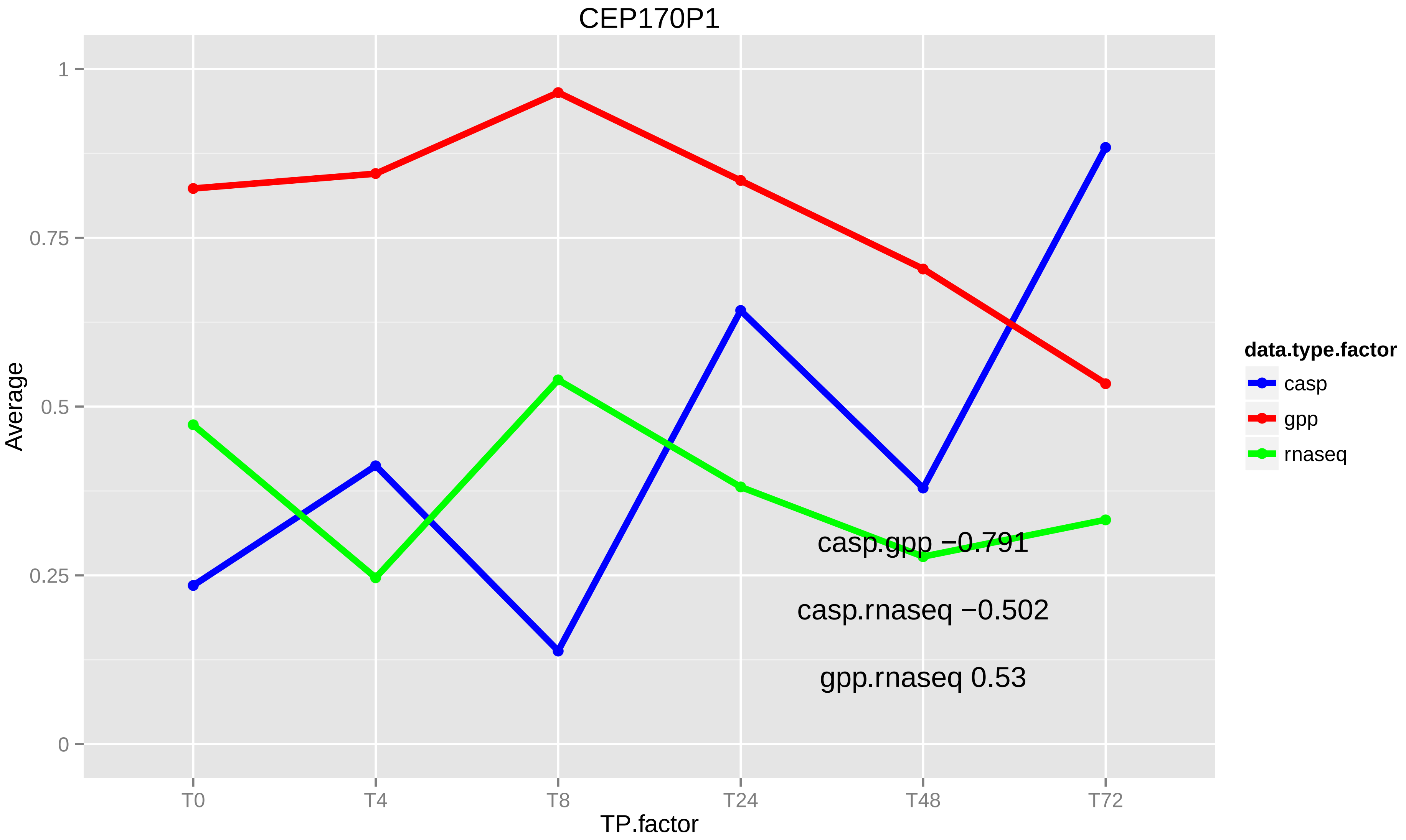

CEP170P1
Average
1
0.75
0.5
0.25
0
T0
T4
T8
T24
T48
T72
TP.factor
data.type.factor
casp
gpp
rnaseq
casp.gpp −0.791
casp.rnaseq −0.502
gpp.rnaseq 0.53

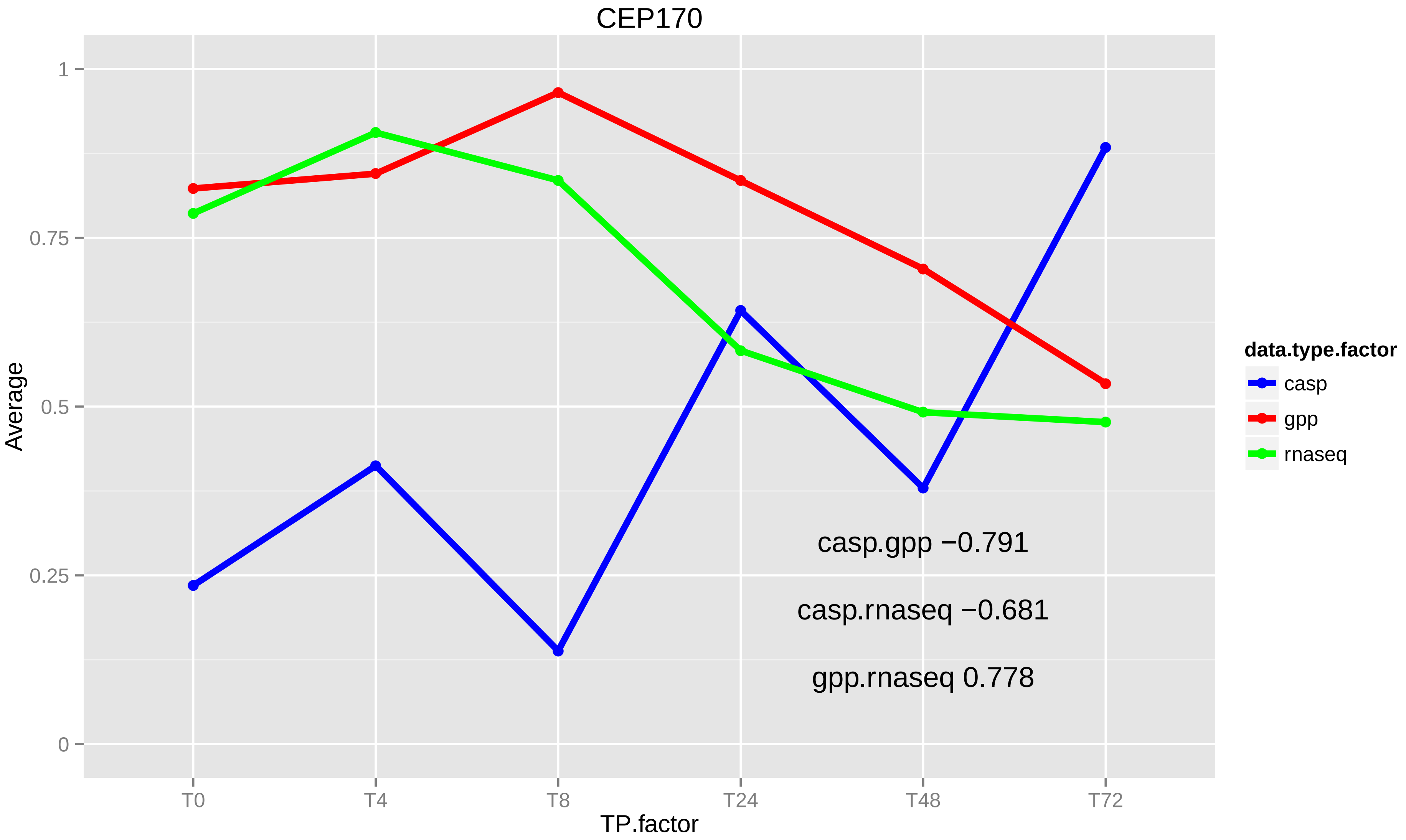
CEP170
Average
1
0.75
0.5
0.25
0
T0
T4
T8
T24
T48
T72
TP.factor
data.type.factor
casp
gpp
rnaseq
casp.gpp −0.791
casp.rnaseq −0.681
gpp.rnaseq 0.778

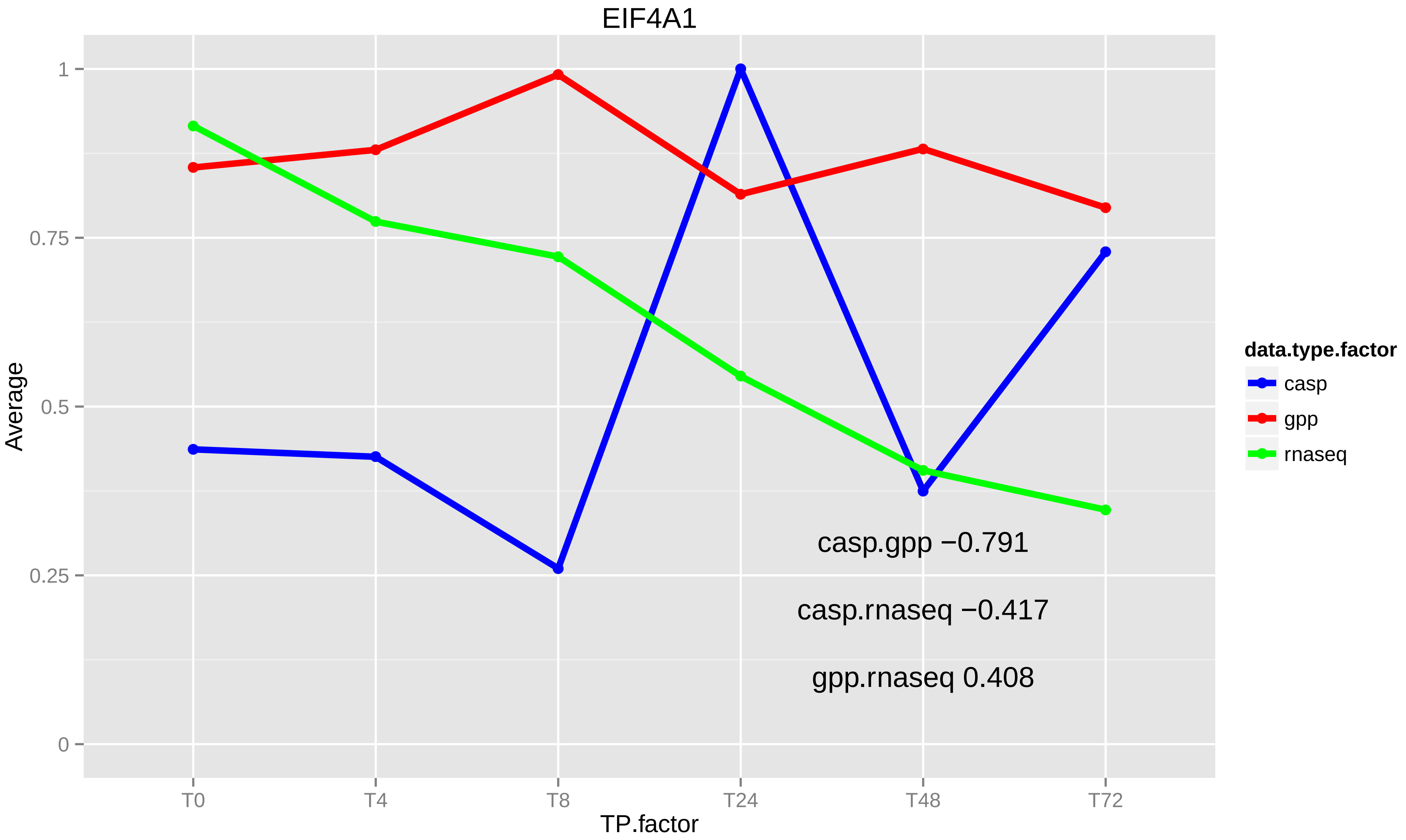
EIF4A1
Average
TP.factor
1
0.75
0.5
0.25
0
T0
T4
T8
T24
T48
T72
data.type.factor
casp
gpp
rnaseq
casp.gpp −0.791
casp.rnaseq −0.417
gpp.rnaseq 0.408

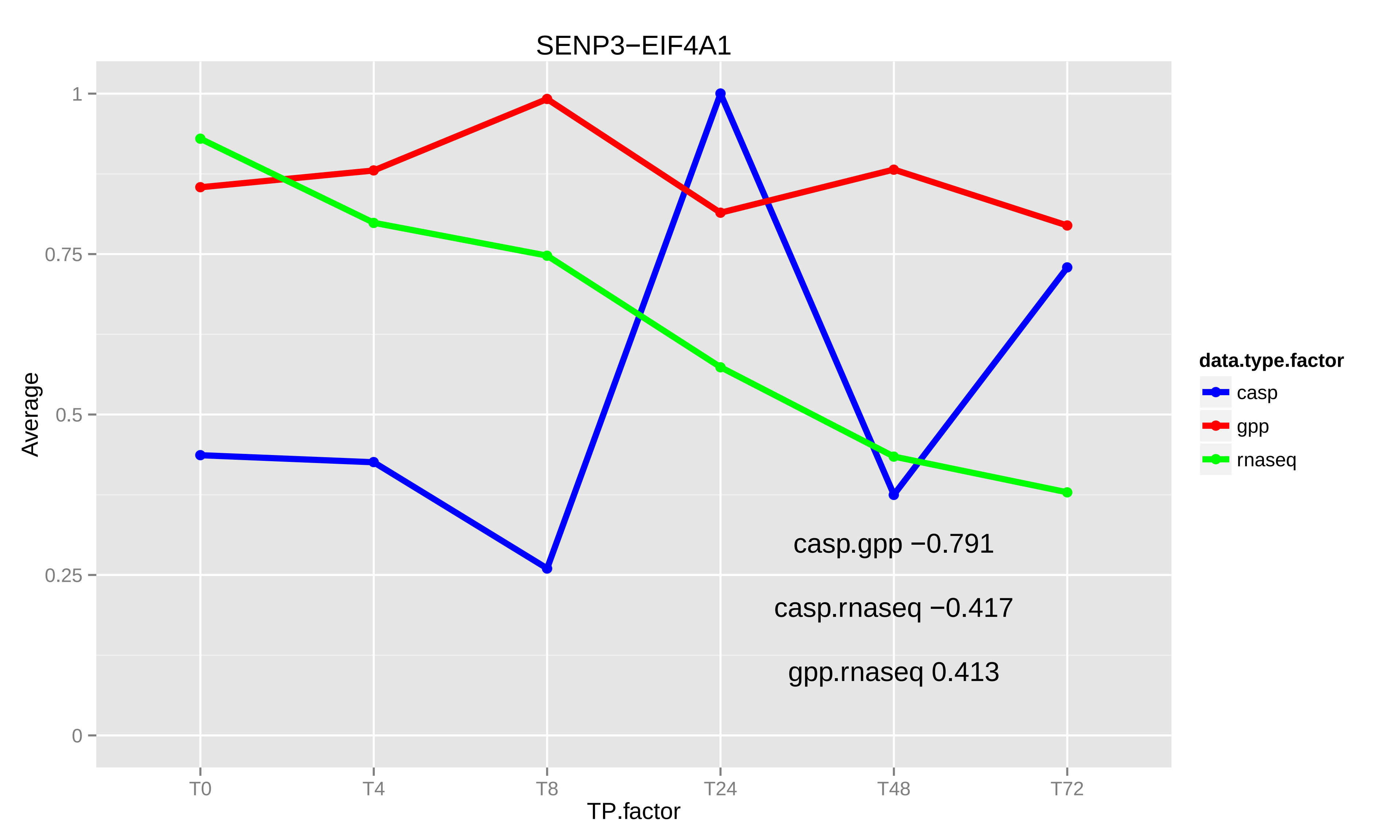

SENP3−EIF4A1
Average
1
0.75
0.5
0.25
0
T0
T4
T8
T24
T48
T72
TP.factor
data.type.factor
casp
gpp
rnaseq
casp.gpp −0.791
casp.rnaseq −0.417
gpp.rnaseq 0.413

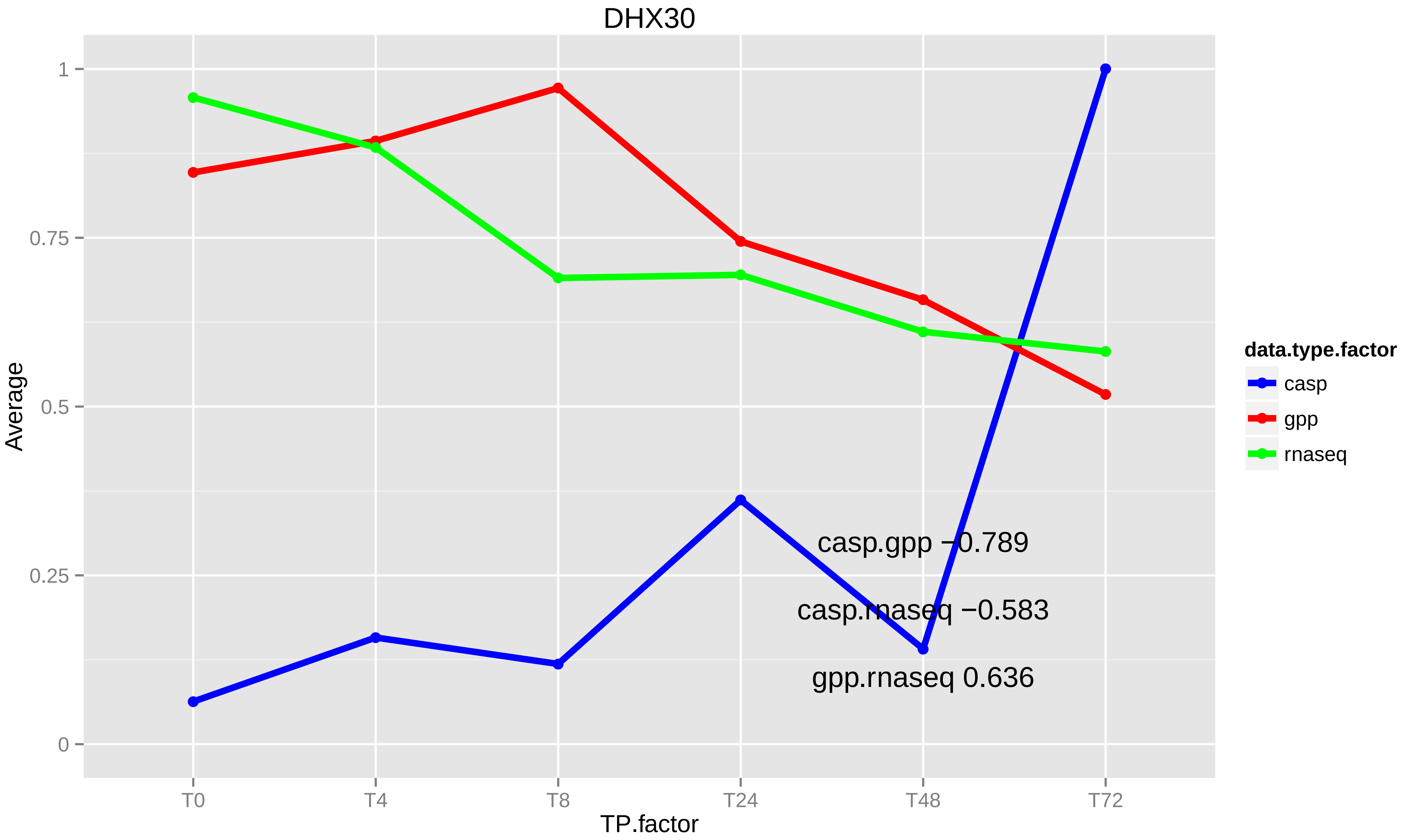
DHX30
Average
1
0.75
0.5
0.25
0
T0
T4
T8
T24
T48
T72
TP.factor
data.type.factor
casp
gpp
rnaseq
casp.gpp −0.789
casp.rnaseq −0.583
gpp.rnaseq 0.636

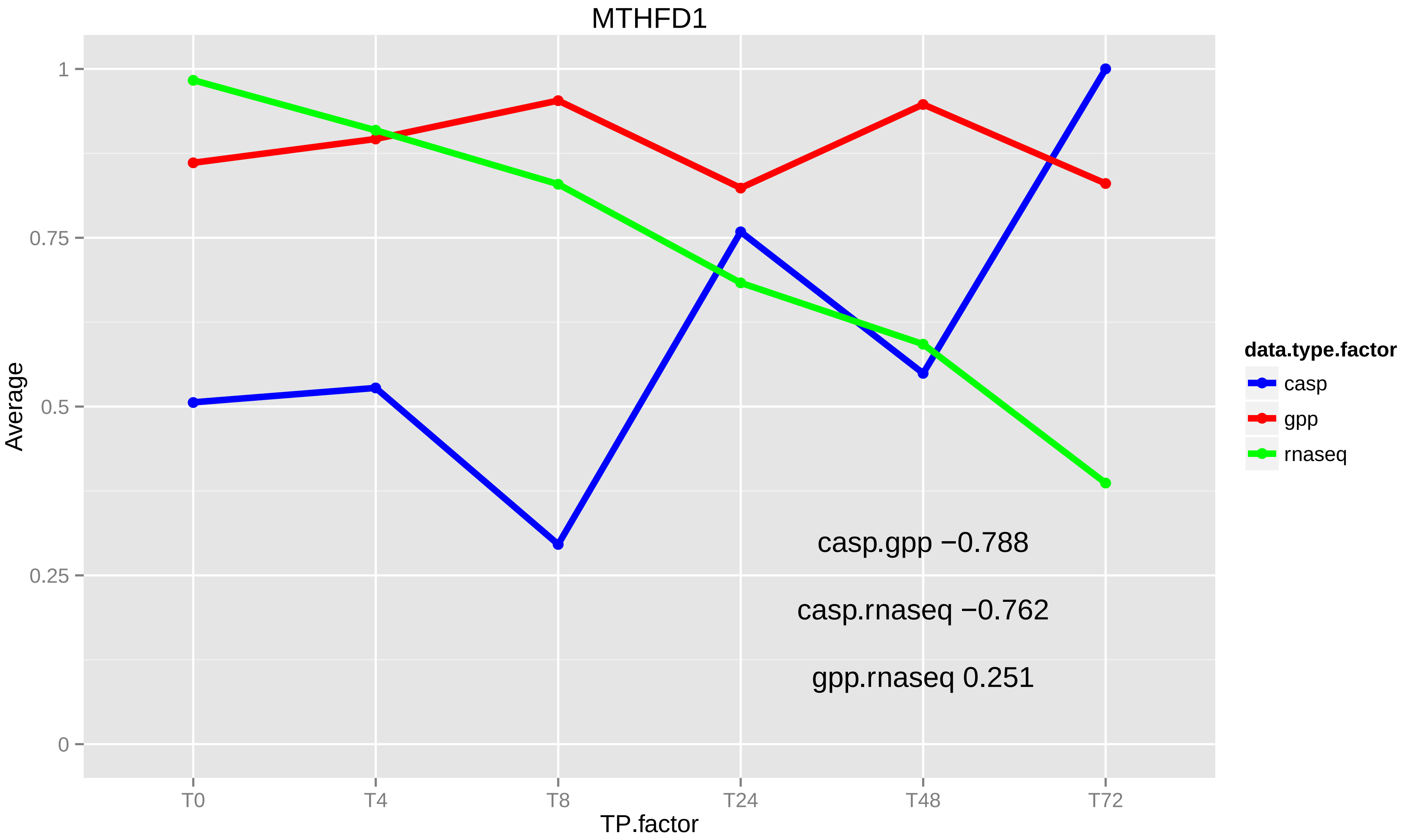
MTHFD1
Average
1
0.75
0.5
0.25
0
T0
T4
T8
T24
T48
T72
TP.factor
data.type.factor
casp
gpp
rnaseq
casp.gpp −0.788
casp.rnaseq −0.762
gpp.rnaseq 0.251

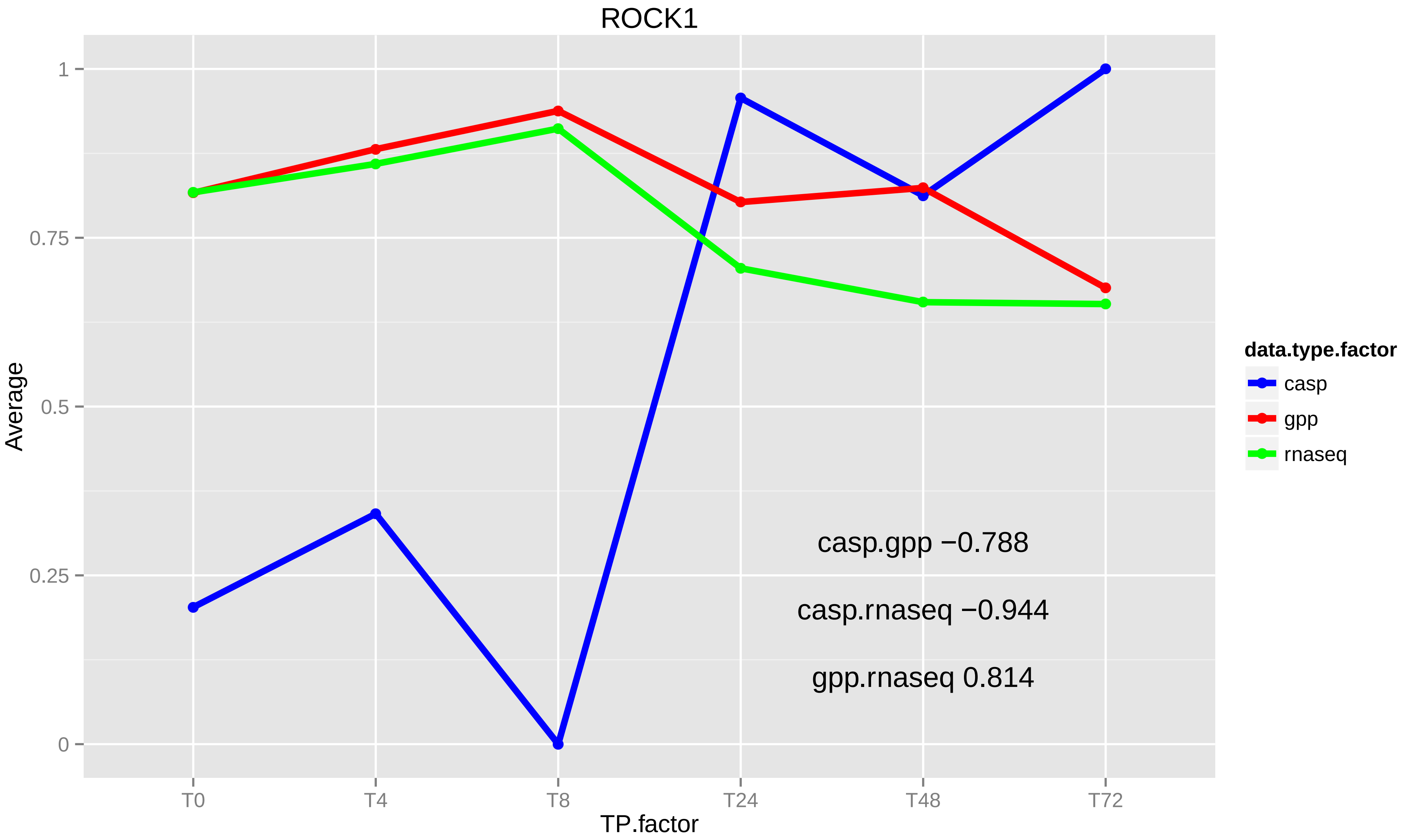
ROCK1
Average
1
0.75
0.5
0.25
0
T0
T4
T8
T24
T48
T72
TP.factor
data.type.factor
casp
gpp
rnaseq
casp.gpp −0.788
casp.rnaseq −0.944
gpp.rnaseq 0.814

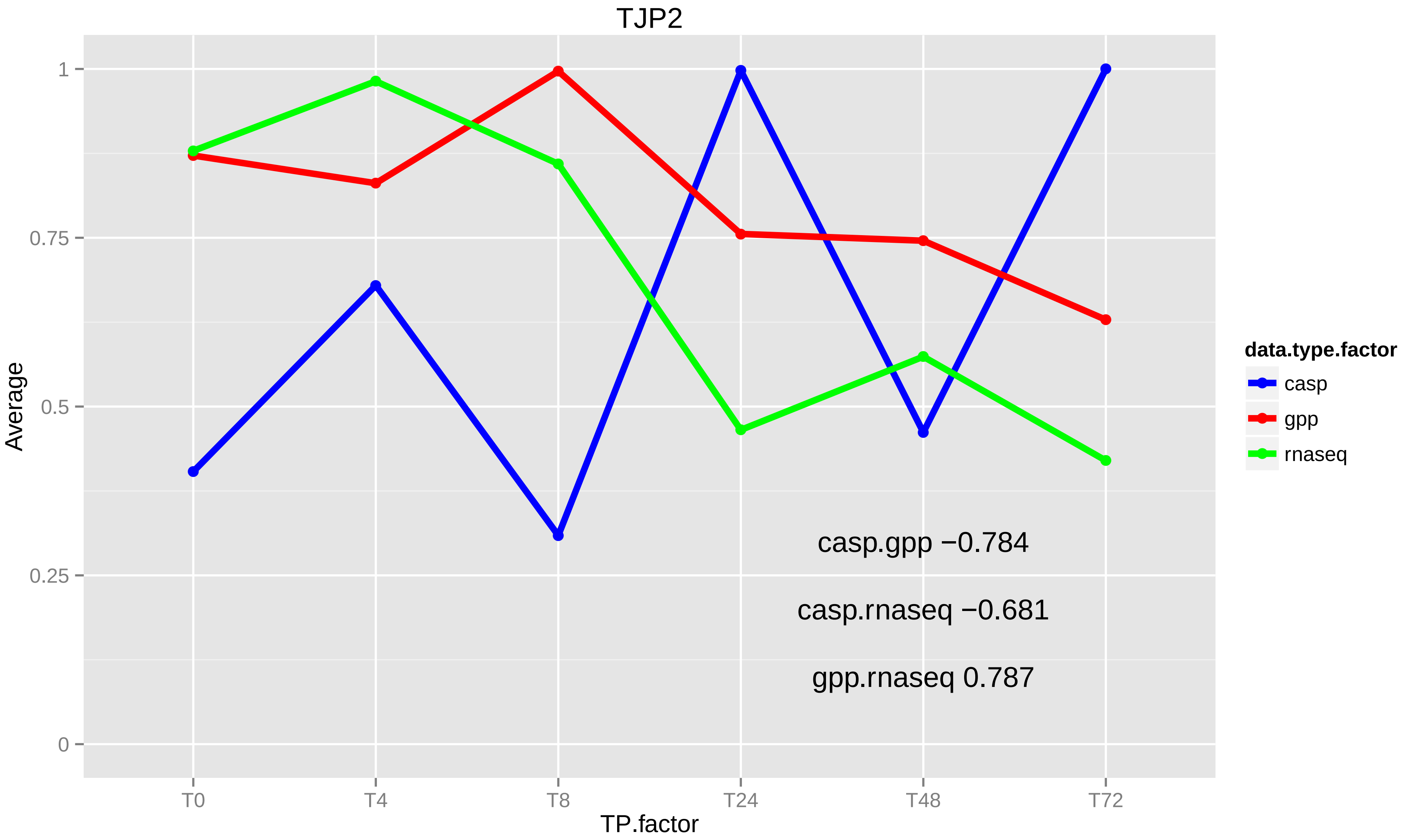
TJP2
Average
1
0.75
0.5
0.25
0
T0
T4
T8
T24
T48
T72
TP.factor
data.type.factor
casp
gpp
rnaseq
casp.gpp −0.784
casp.rnaseq −0.681
gpp.rnaseq 0.787

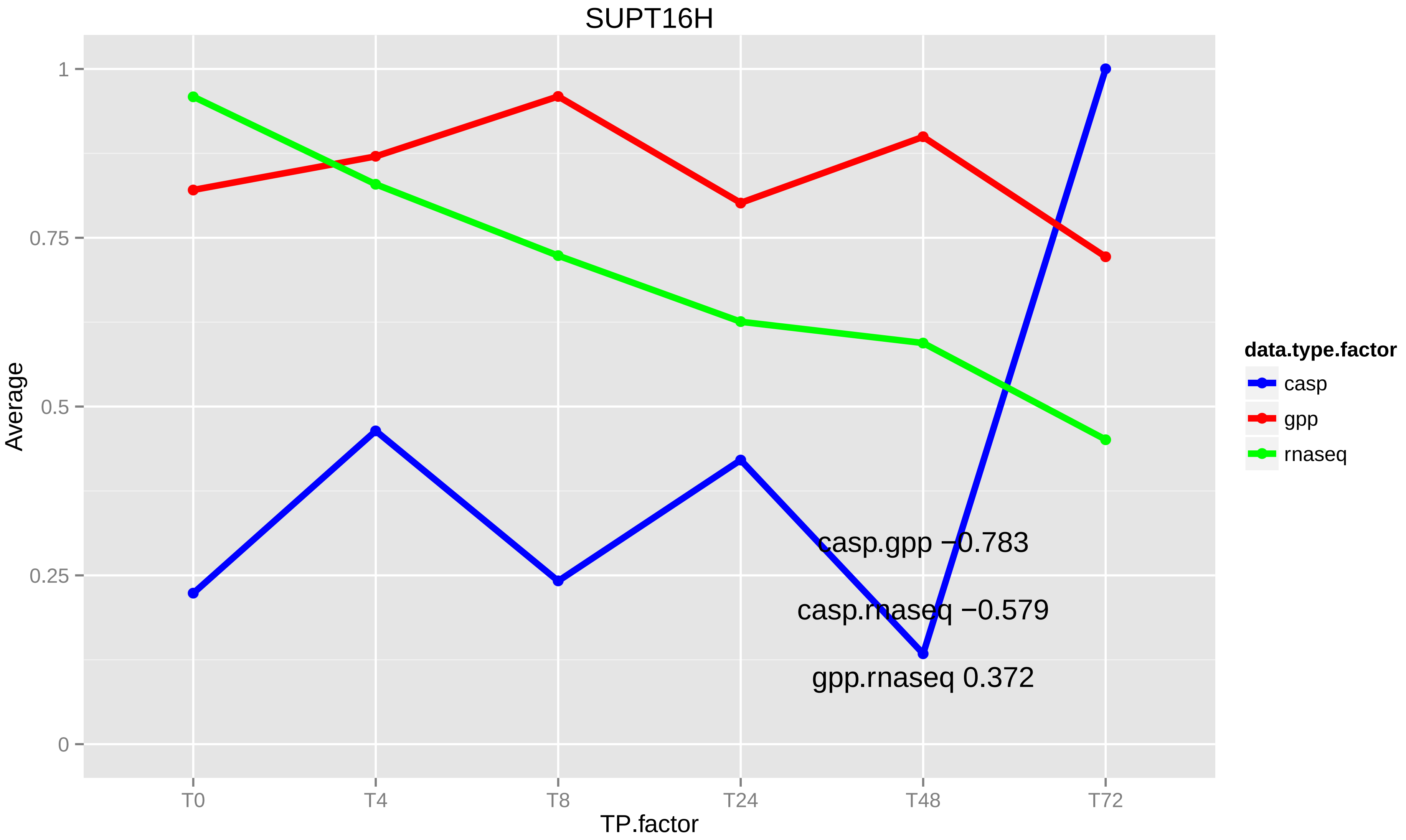
SUPT16H
Average
1
0.75
0.5
0.25
0
T0
T4
T8
T24
T48
T72
TP.factor
data.type.factor
casp
gpp
rnaseq
casp.gpp −0.783
casp.rnaseq −0.579
gpp.rnaseq 0.372

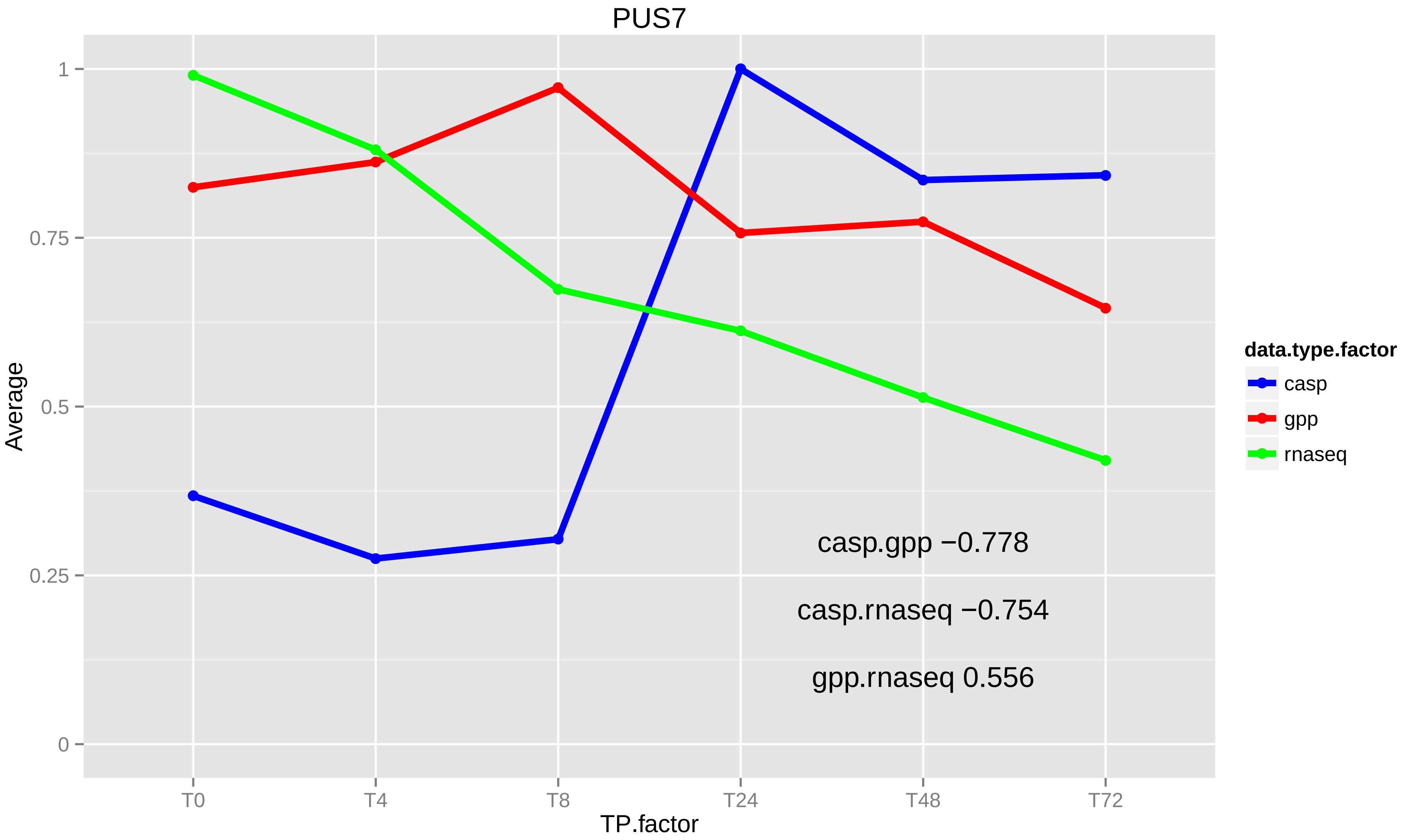
PUS7
Average
1
0.75
0.5
0.25
0
T0
T4
T8
T24
T48
T72
TP.factor
data.type.factor
casp
gpp
rnaseq
casp.gpp −0.778
casp.rnaseq −0.754
gpp.rnaseq 0.556

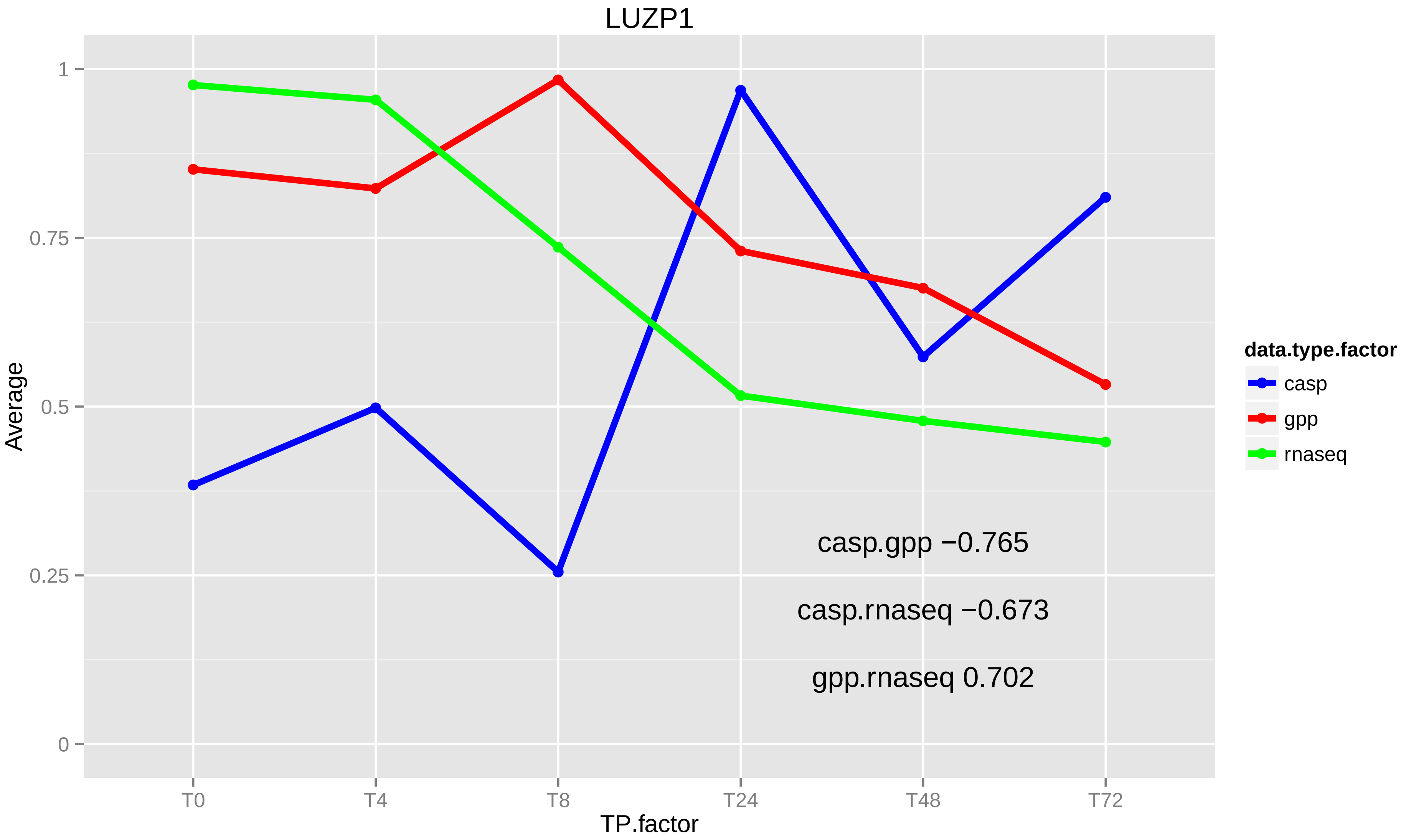

LUZP1
1
0.75
0.5
0.25
0
Average
T0
T4
T8
T24
T48
T72
TP.factor
data.type.factor
casp
gpp
rnaseq
casp.gpp −0.765
casp.rnaseq −0.673
gpp.rnaseq 0.702

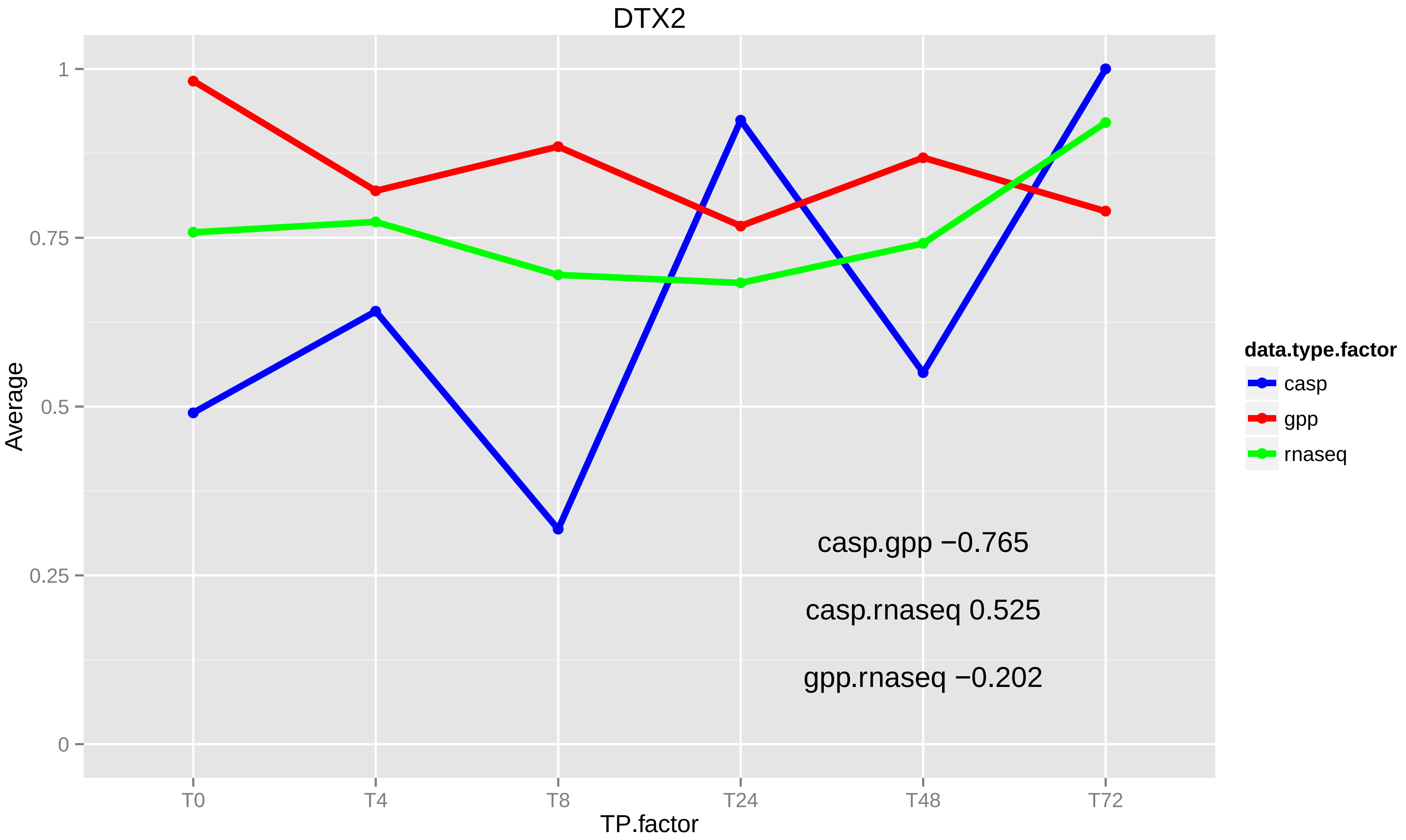

DTX2
1
0.75
0.5
0.25
0
Average
T0
T4
T8
T24
T48
T72
TP.factor
data.type.factor
casp
gpp
rnaseq
casp.gpp −0.765
casp.rnaseq 0.525
gpp.rnaseq −0.202

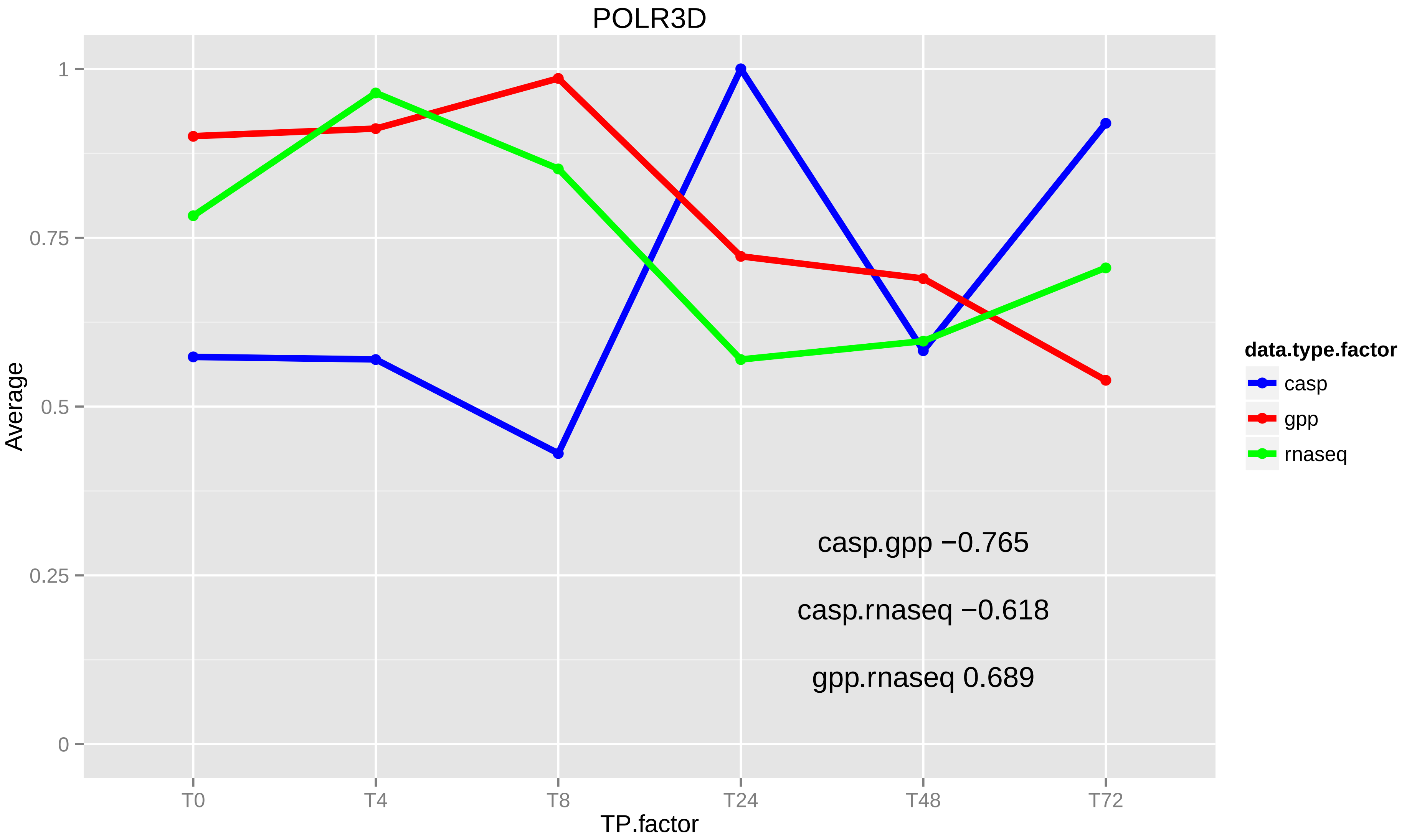

POLR3D
Average
1
0.75
0.5
0.25
0
T0
T4
T8
T24
T48
T72
TP.factor
data.type.factor
casp
gpp
rnaseq
casp.gpp −0.765
casp.rnaseq −0.618
gpp.rnaseq 0.689

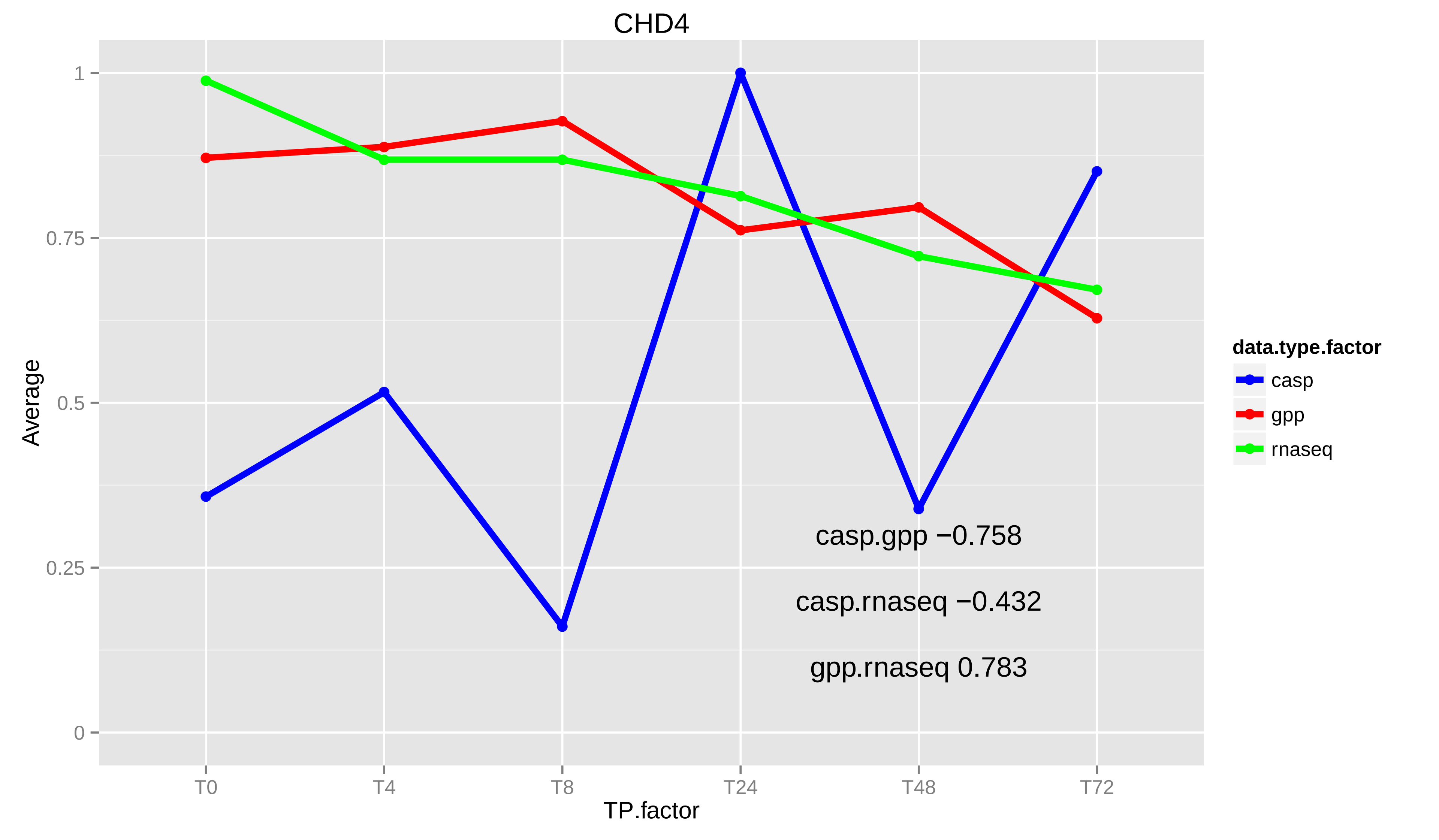

CHD4
1
0.75
0.5
0.25
0
Average
T0
T4
T8
T24
T48
T72
TP.factor
data.type.factor
casp
gpp
rnaseq
casp.gpp −0.758
casp.rnaseq −0.432
gpp.rnaseq 0.783

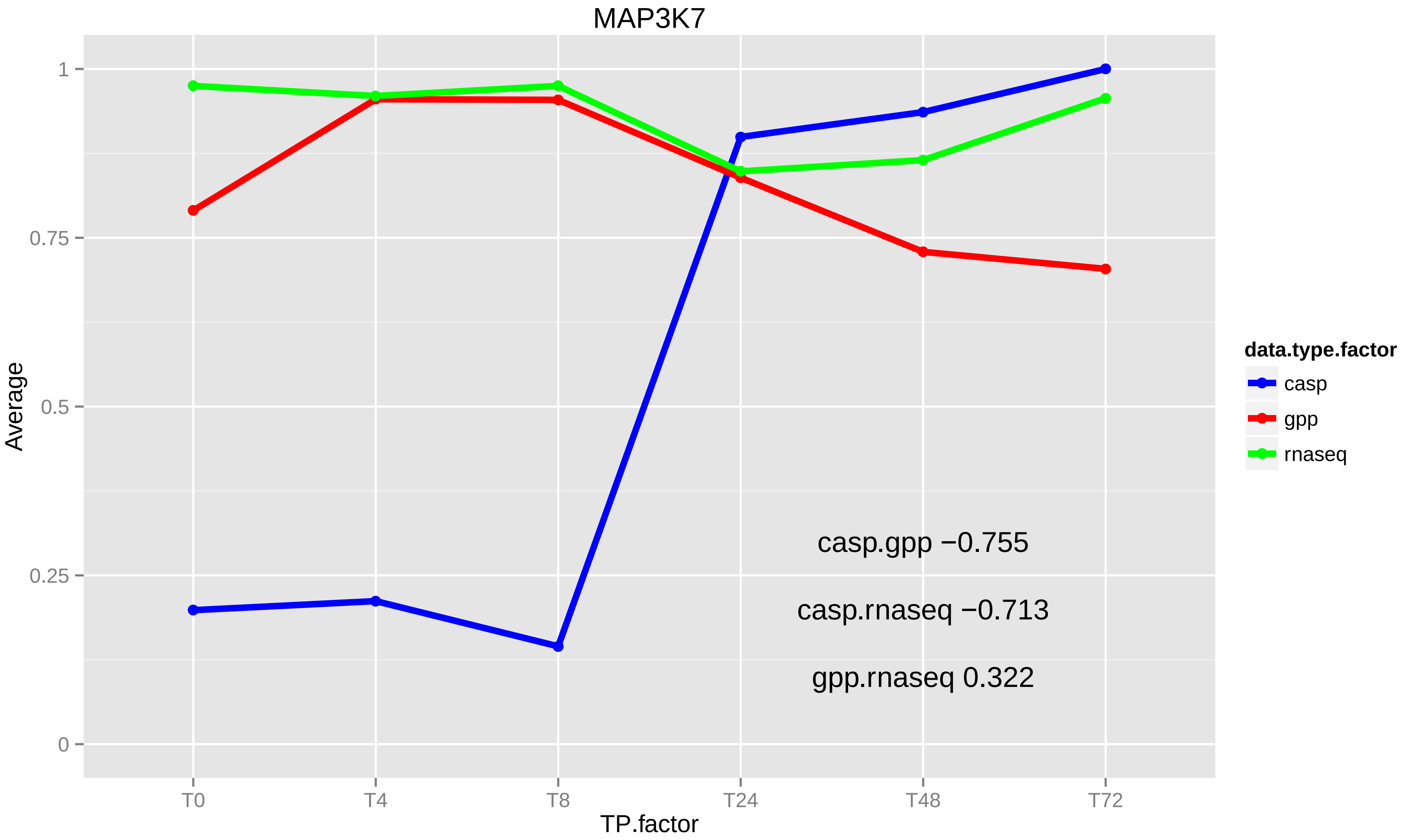
MAP3K7
Average
1
0.75
0.5
0.25
0
T0
T4
T8
T24
T48
T72
TP.factor
data.type.factor
casp
gpp
rnaseq
casp.gpp −0.755
casp.rnaseq −0.713
gpp.rnaseq 0.322

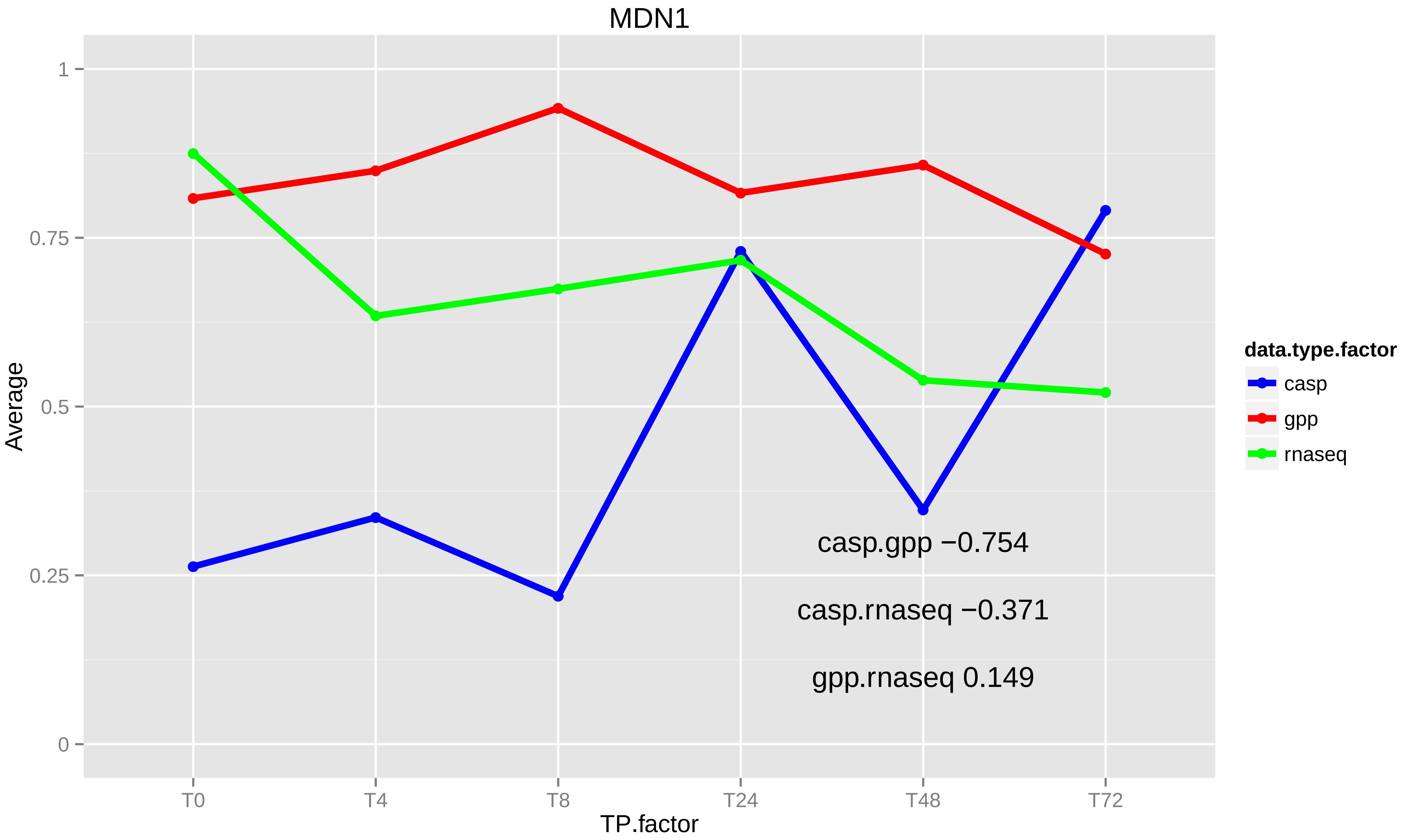

MDN1
Average
1
0.75
0.5
0.25
0
T0
T4
T8
T24
T48
T72
TP.factor
data.type.factor
casp
gpp
rnaseq
casp.gpp −0.754
casp.rnaseq −0.371
gpp.rnaseq 0.149

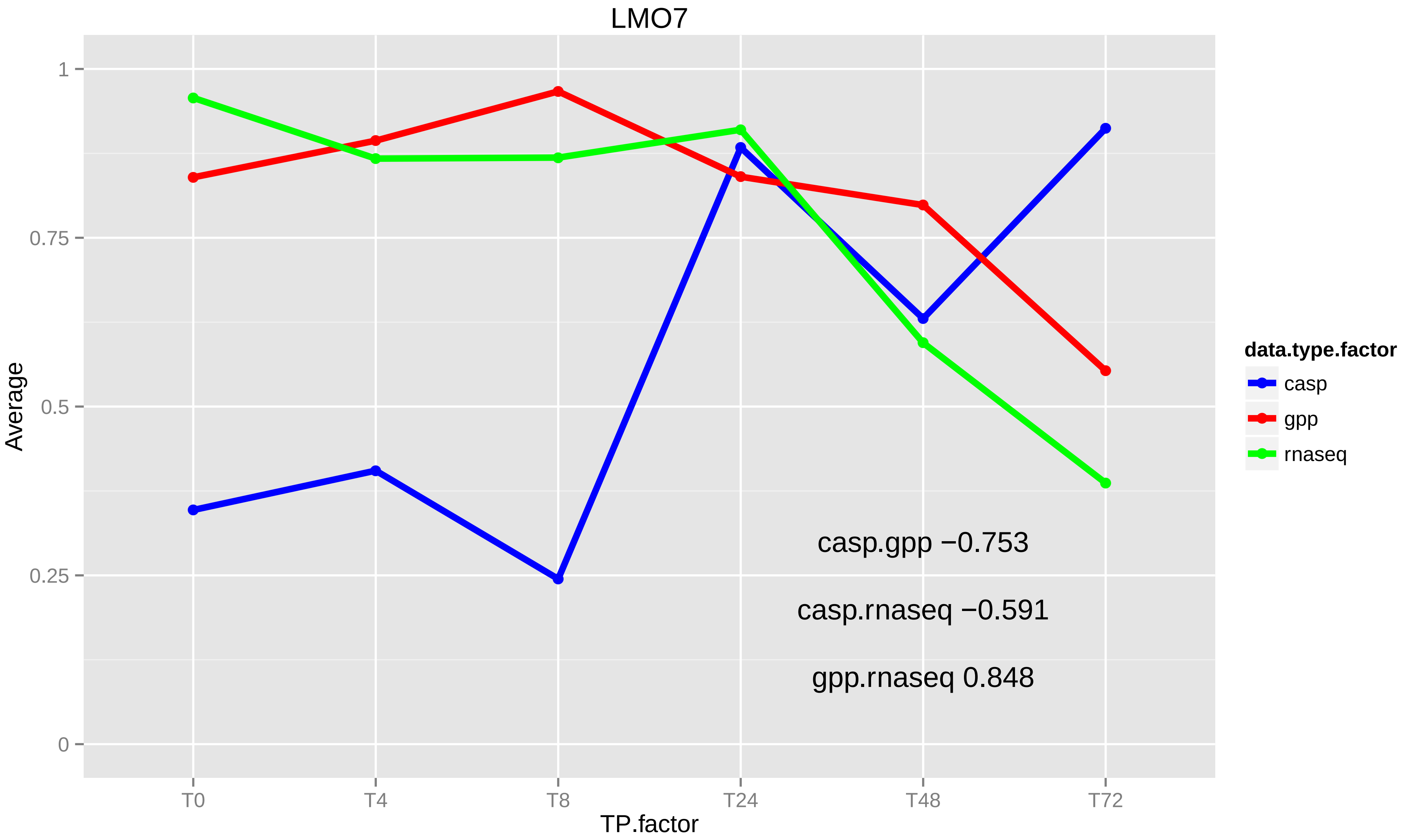

LMO7
1
0.75
0.5
0.25
0
Average
T0
T4
T8
T24
T48
T72
TP.factor
data.type.factor
casp
gpp
rnaseq
casp.gpp −0.753
casp.rnaseq −0.591
gpp.rnaseq 0.848

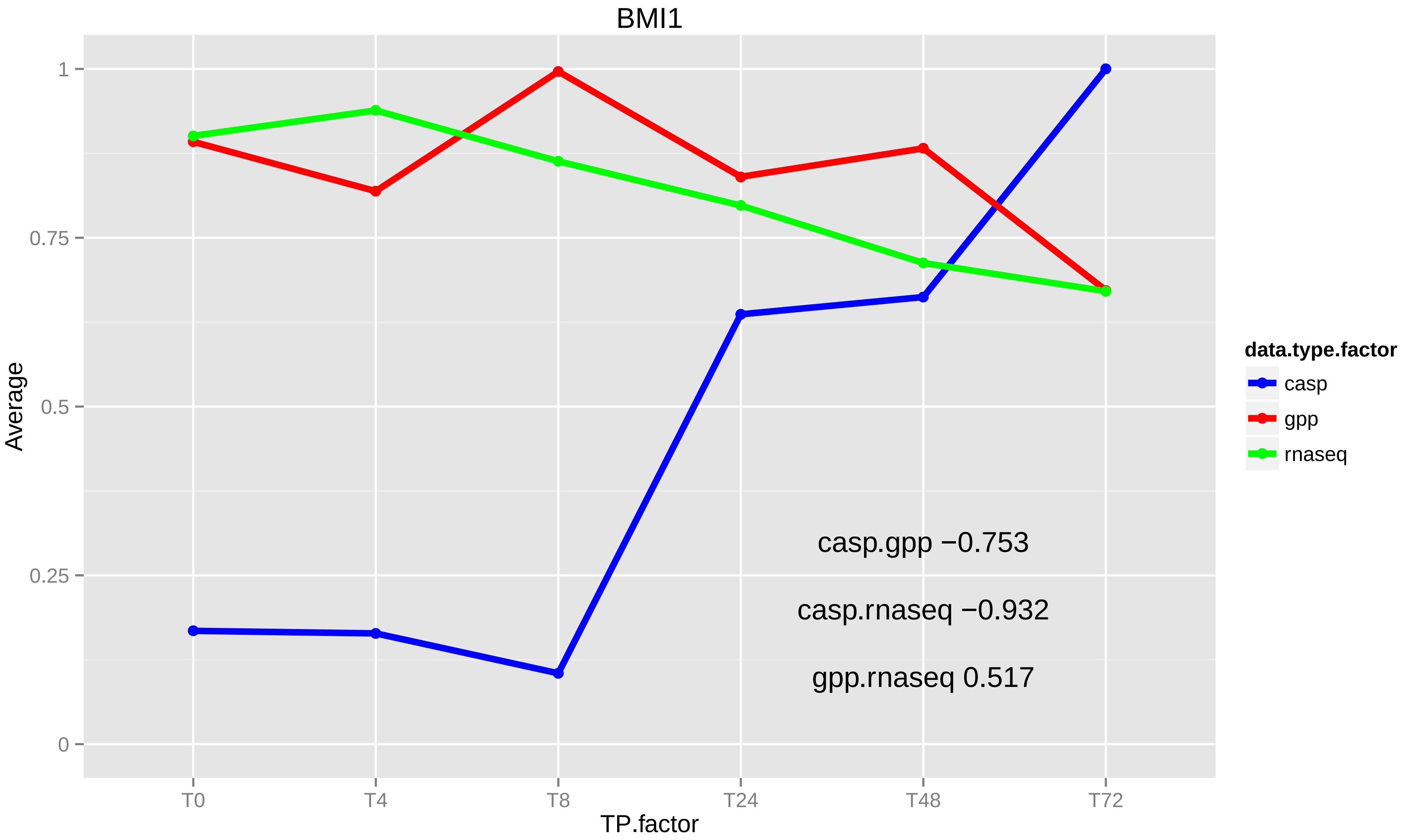

BMI1
Average
1
0.75
0.5
0.25
0
T0
T4
T8
T24
T48
T72
TP.factor
data.type.factor
casp
gpp
rnaseq
casp.gpp −0.753
casp.rnaseq −0.932
gpp.rnaseq 0.517

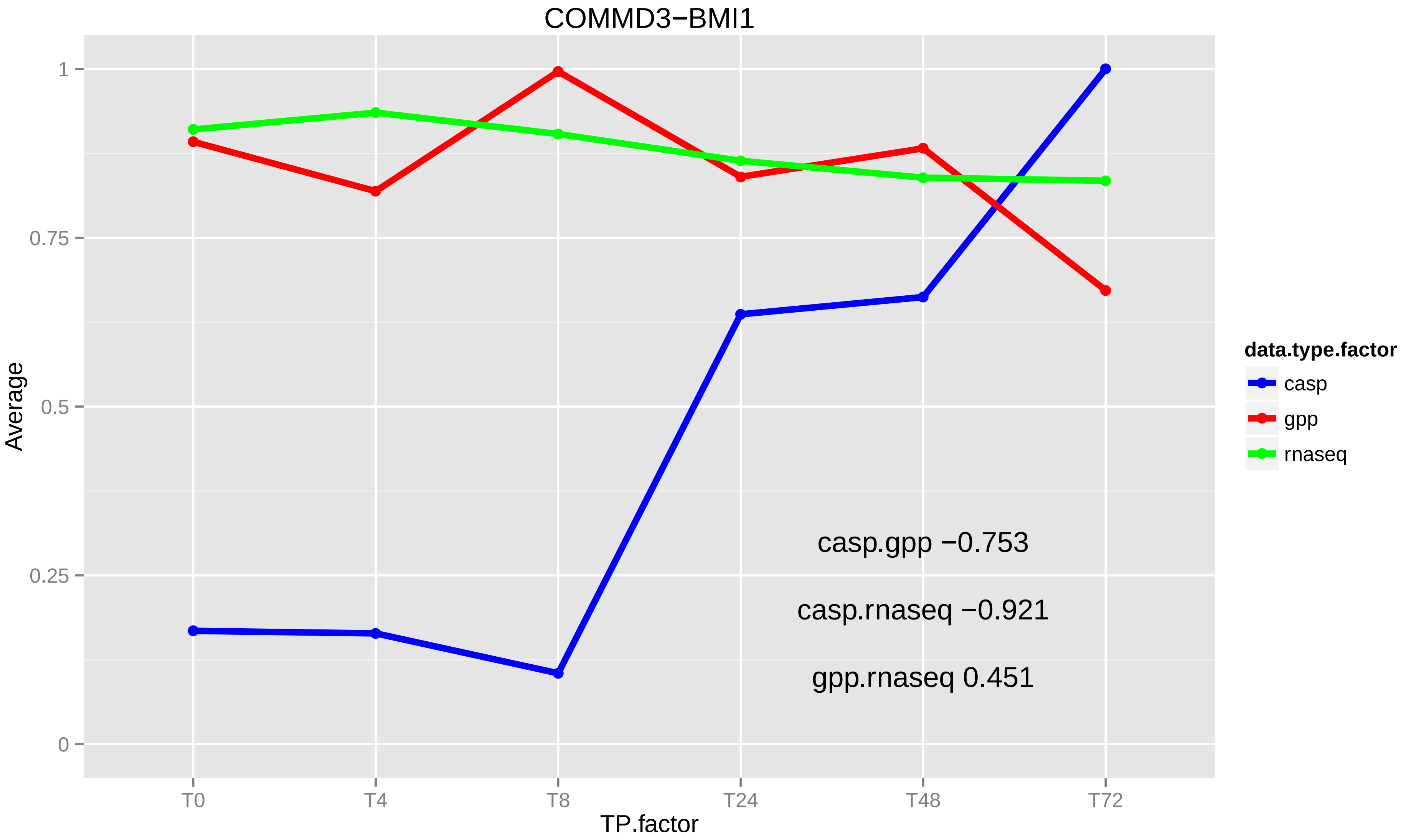

COMMD3−BMI1
Average
1
0.75
0.5
0.25
0
T0
T4
T8
T24
T48
T72
TP.factor
data.type.factor
casp
gpp
rnaseq
casp.gpp −0.753
casp.rnaseq −0.921
gpp.rnaseq 0.451

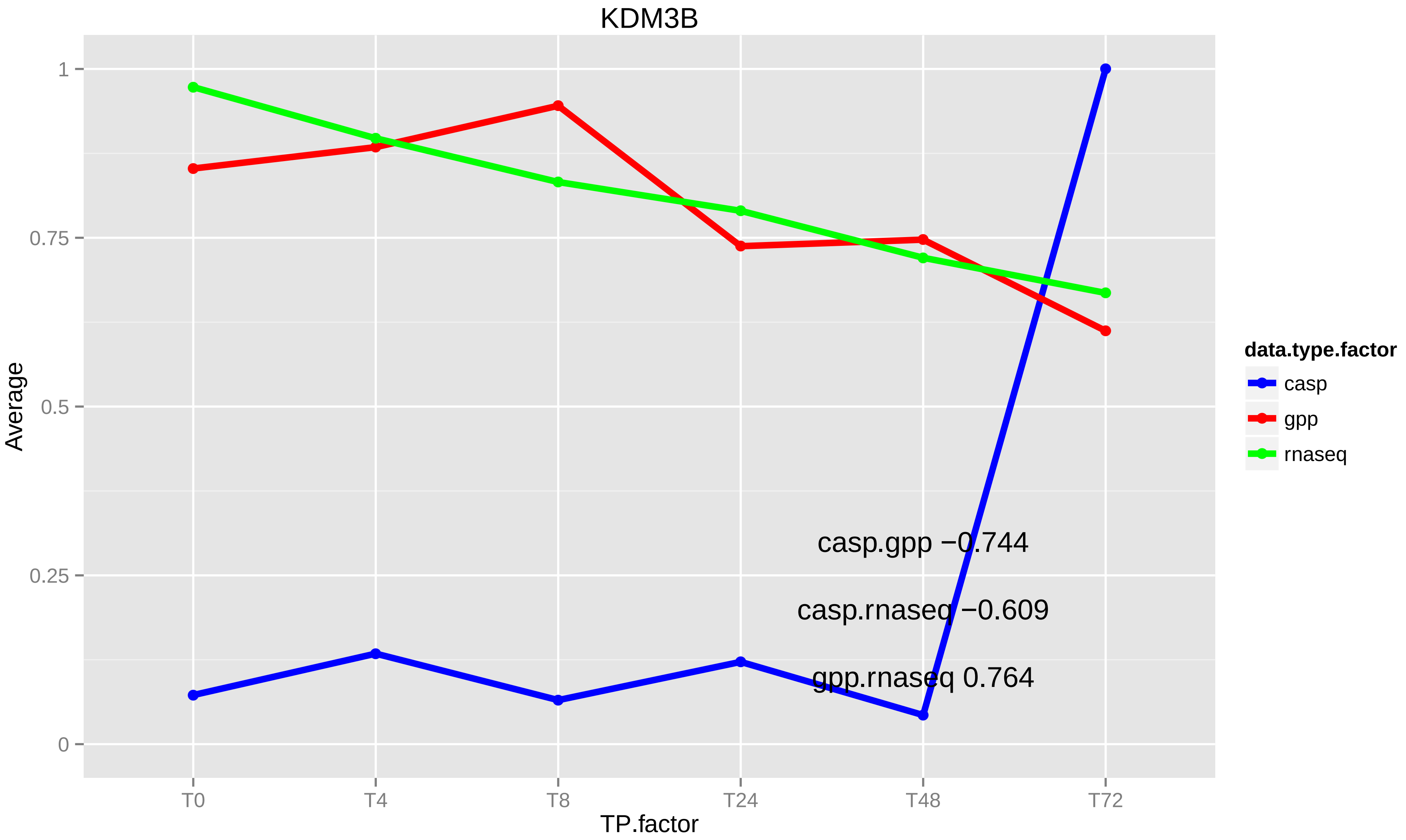

KDM3B
Average
1
0.75
0.5
0.25
0
T0
T4
T8
T24
T48
T72
TP.factor
data.type.factor
casp
gpp
rnaseq
casp.gpp −0.744
casp.rnaseq −0.609
gpp.rnaseq 0.764

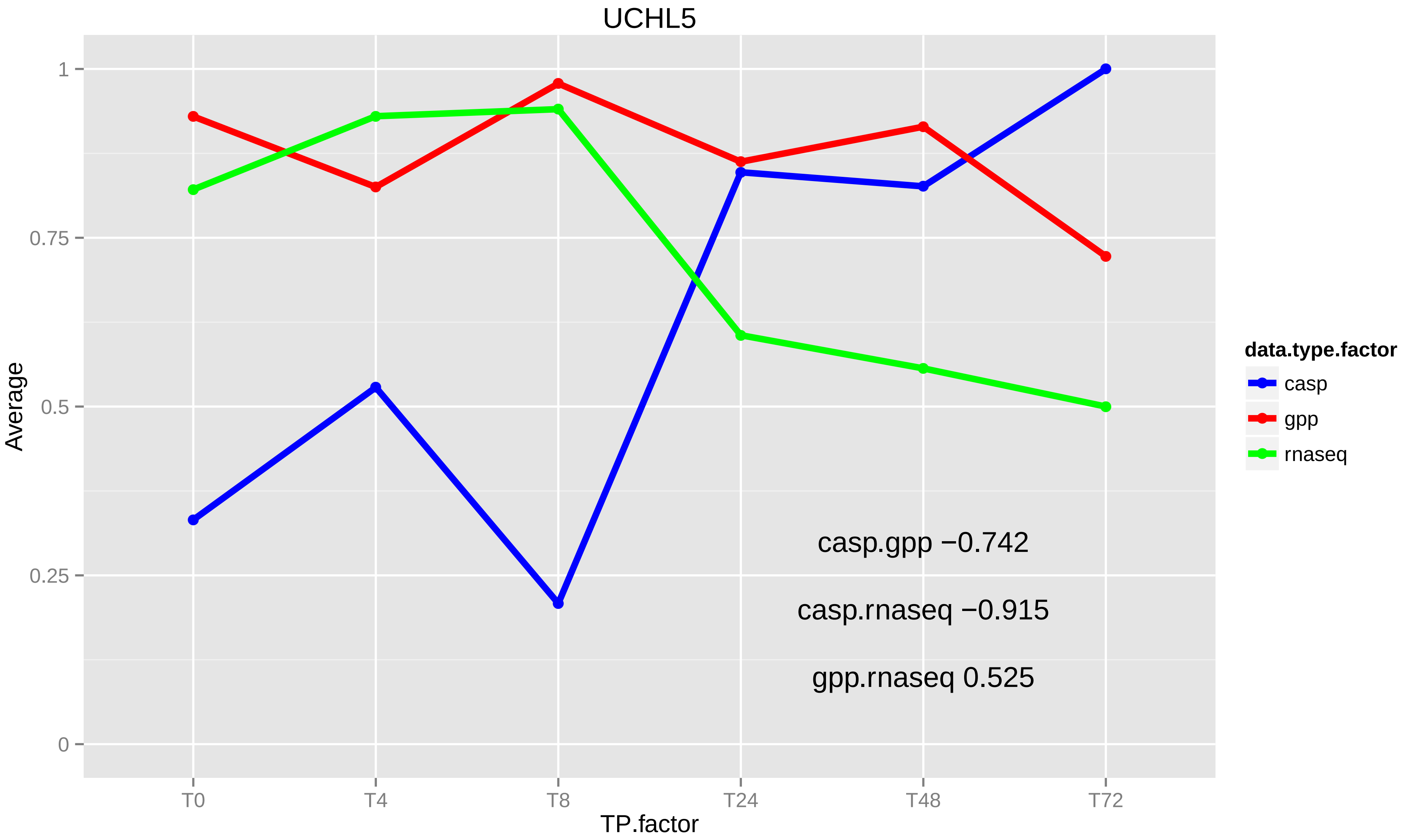

UCHL5
Average
1
0.75
0.5
0.25
0
T0
T4
T8
T24
T48
T72
TP.factor
data.type.factor
casp
gpp
rnaseq
casp.gpp −0.742
casp.rnaseq −0.915
gpp.rnaseq 0.525

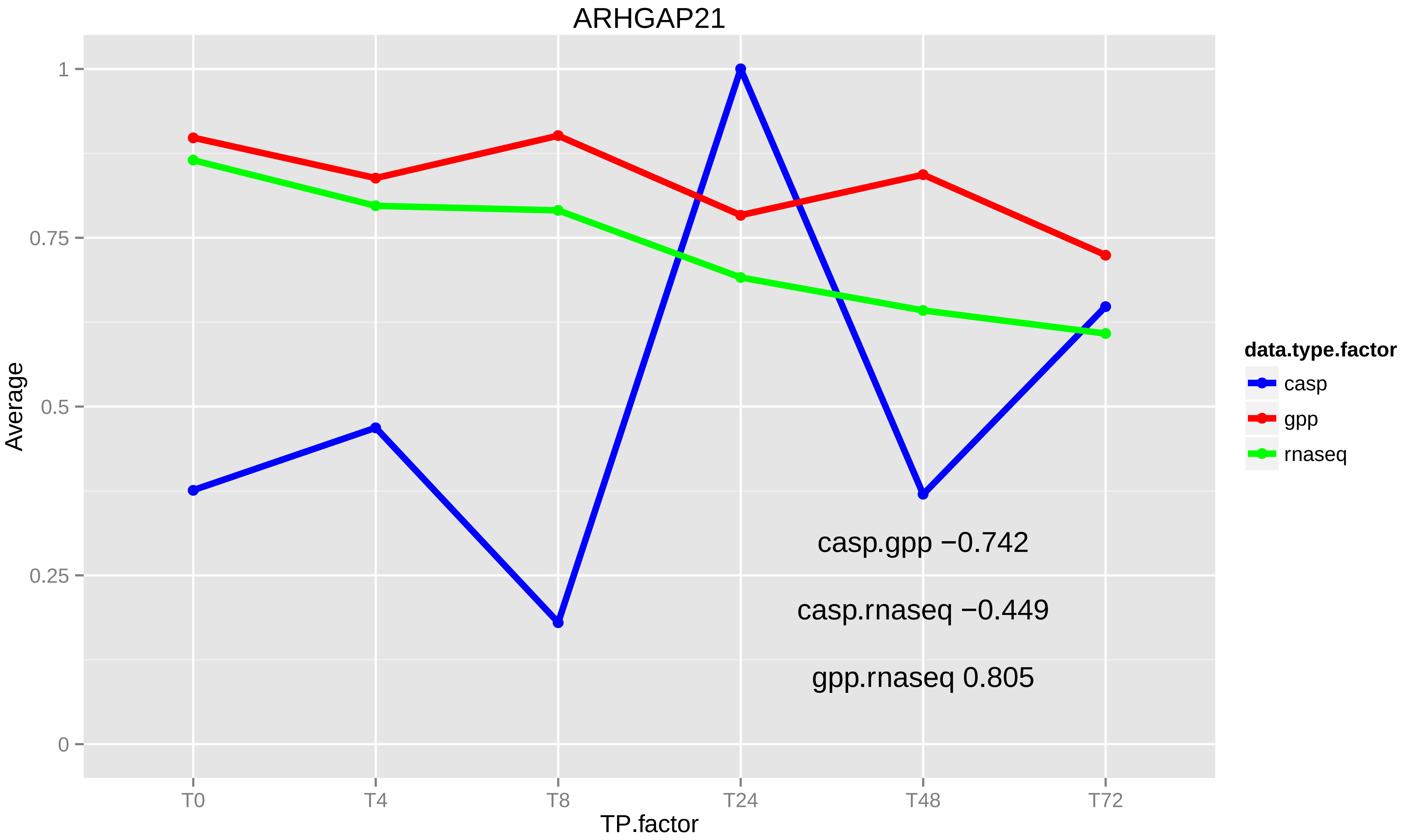
ARHGAP21
Average
1
0.75
0.5
0.25
0
T0
T4
T8
T24
T48
T72
TP.factor
data.type.factor
casp
gpp
rnaseq
casp.gpp −0.742
casp.rnaseq −0.449
gpp.rnaseq 0.805

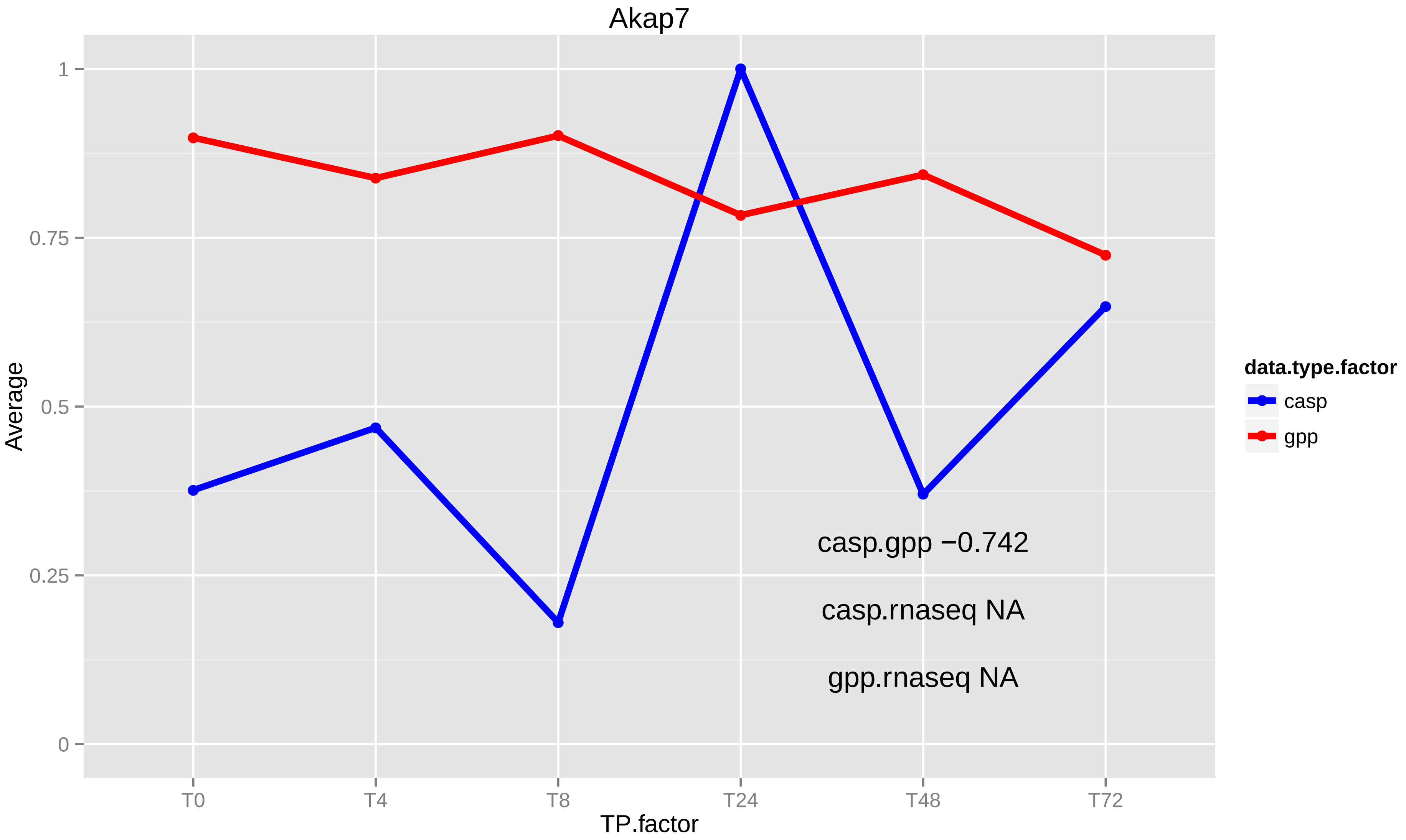

Akap7
1
0.75
0.5
0.25
0
Average
T0
T4
T8
T24
T48
T72
TP.factor
data.type.factor
casp
gpp
casp.gpp −0.742
casp.rnaseq NA
gpp.rnaseq NA

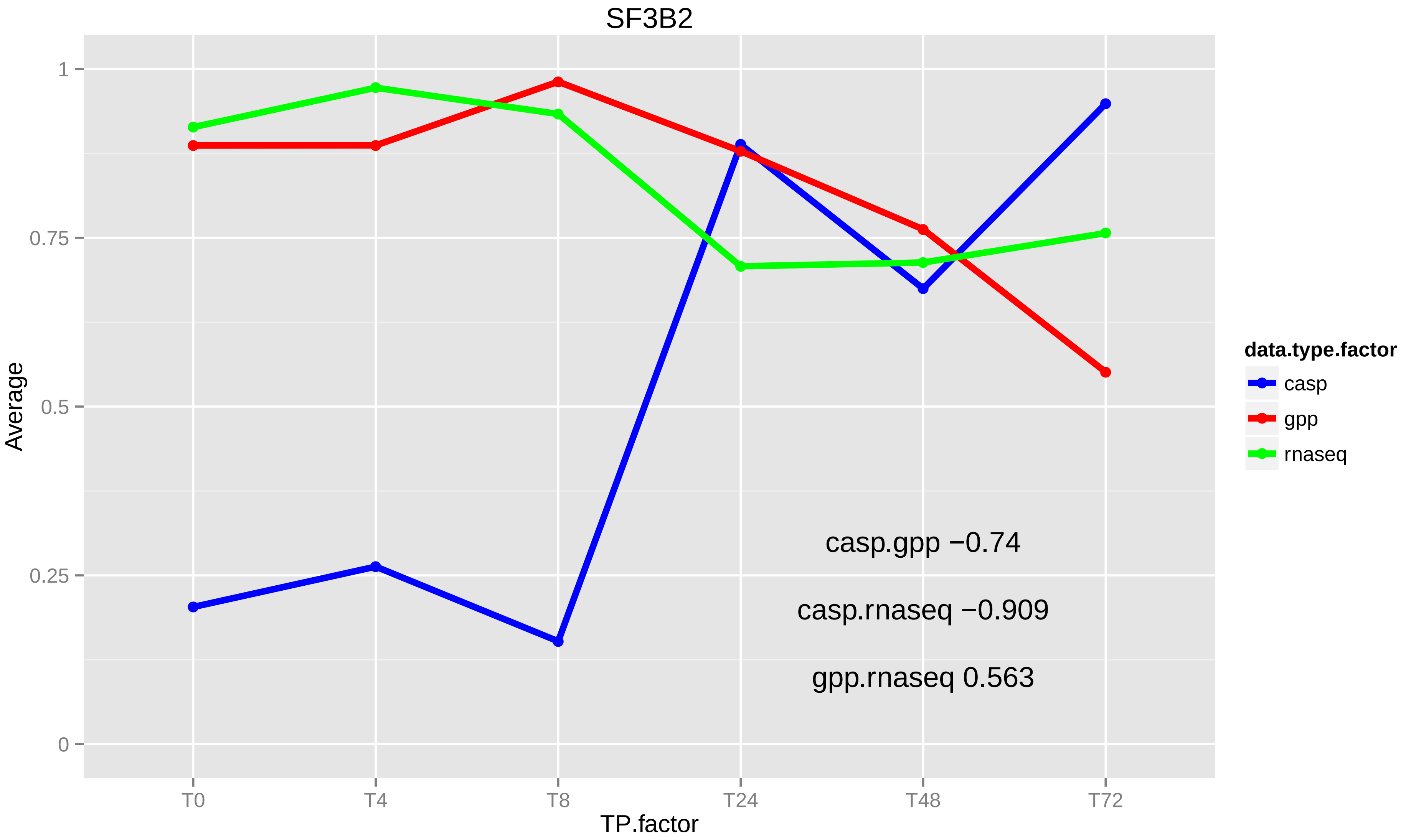

SF3B2
Average
1
0.75
0.5
0.25
0
T0
T4
T8
T24
T48
T72
TP.factor
data.type.factor
casp
gpp
rnaseq
casp.gpp −0.74
casp.rnaseq −0.909
gpp.rnaseq 0.563

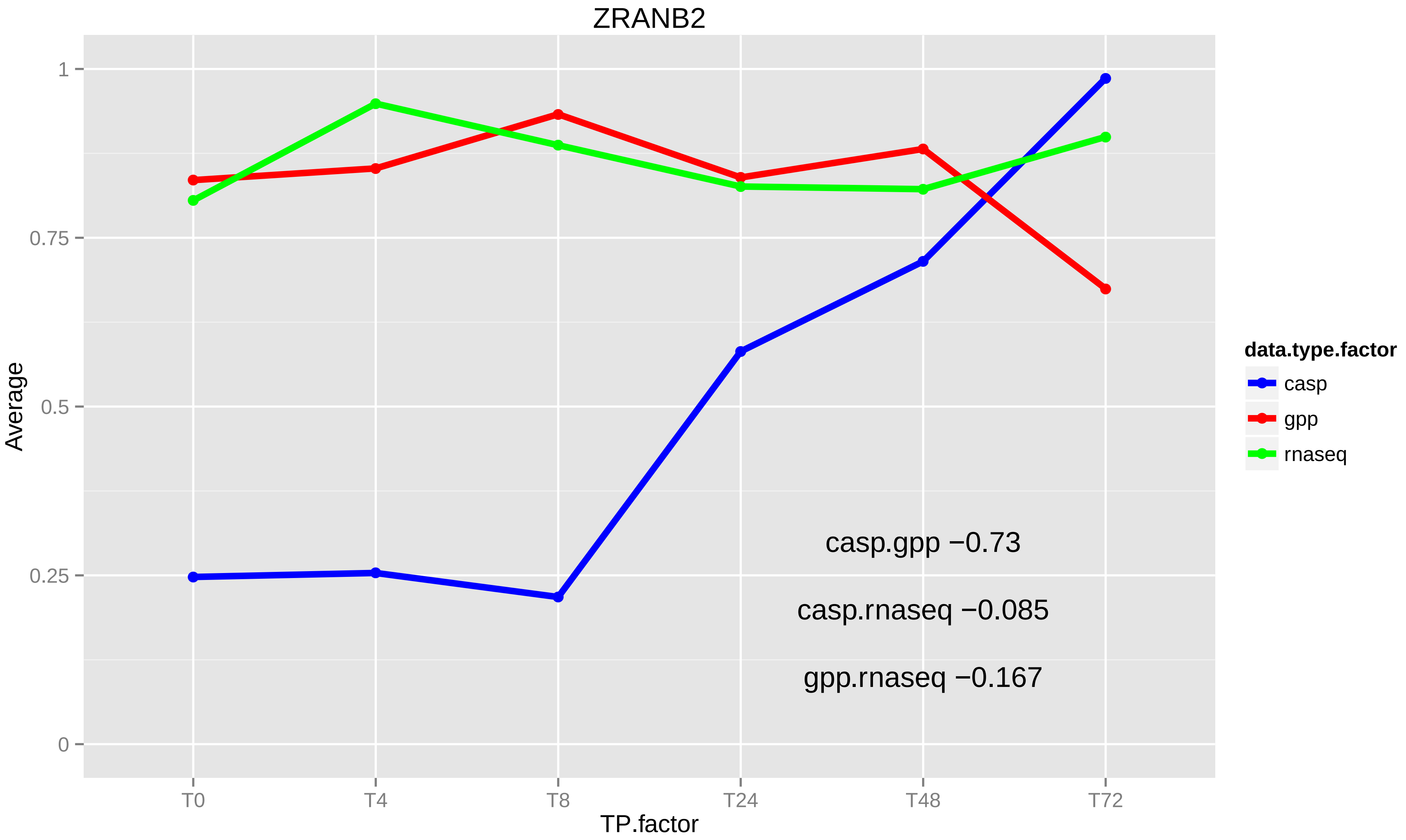

ZRANB2
Average
1
0.75
0.5
0.25
0
T0
T4
T8
T24
T48
T72
TP.factor
data.type.factor
casp
gpp
rnaseq
casp.gpp −0.73
casp.rnaseq −0.085
gpp.rnaseq −0.167

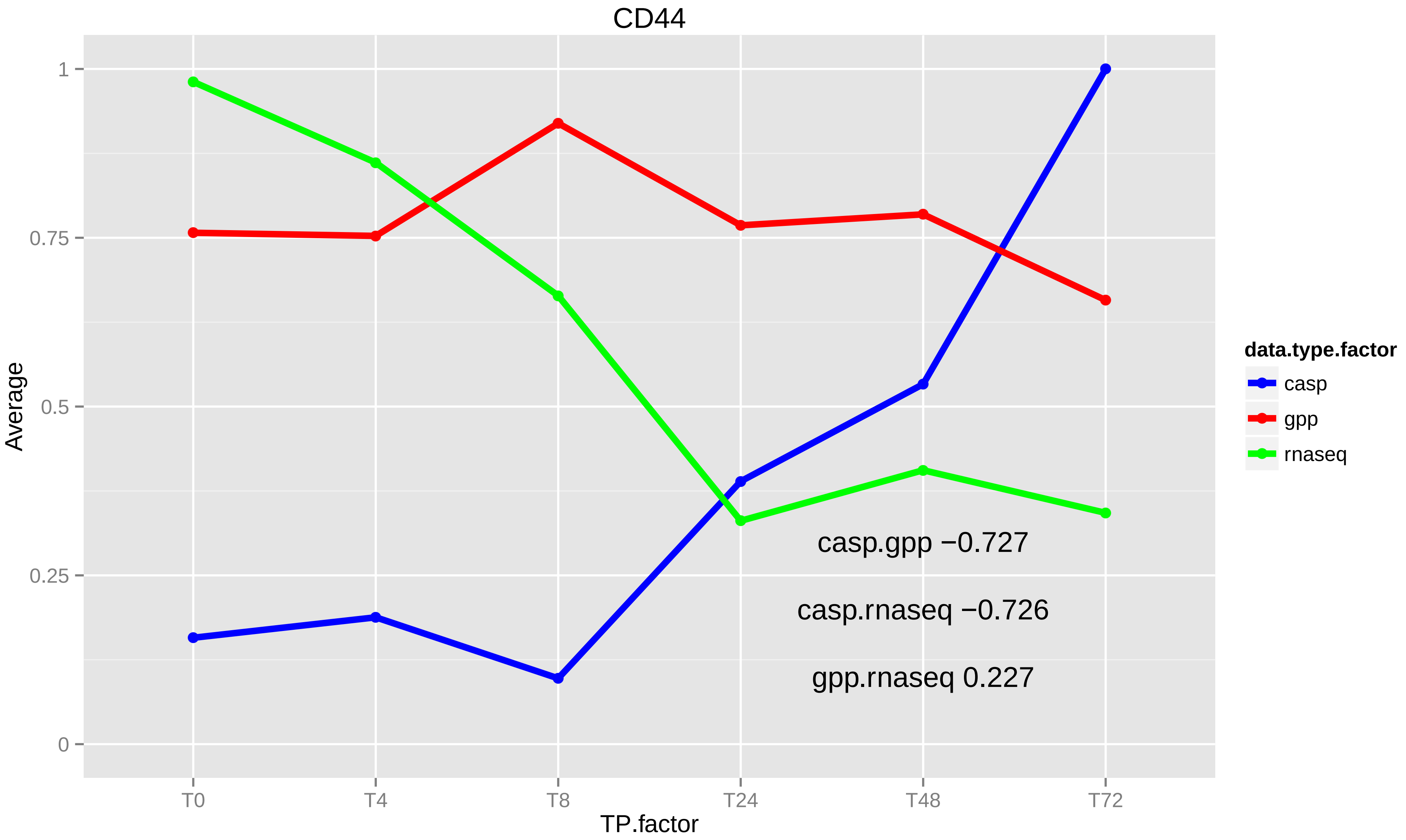

CD44
1
0.75
0.5
0.25
0
Average
T0
T4
T8
T24
T48
T72
TP.factor
data.type.factor
casp
gpp
rnaseq
casp.gpp −0.727
casp.rnaseq −0.726
gpp.rnaseq 0.227

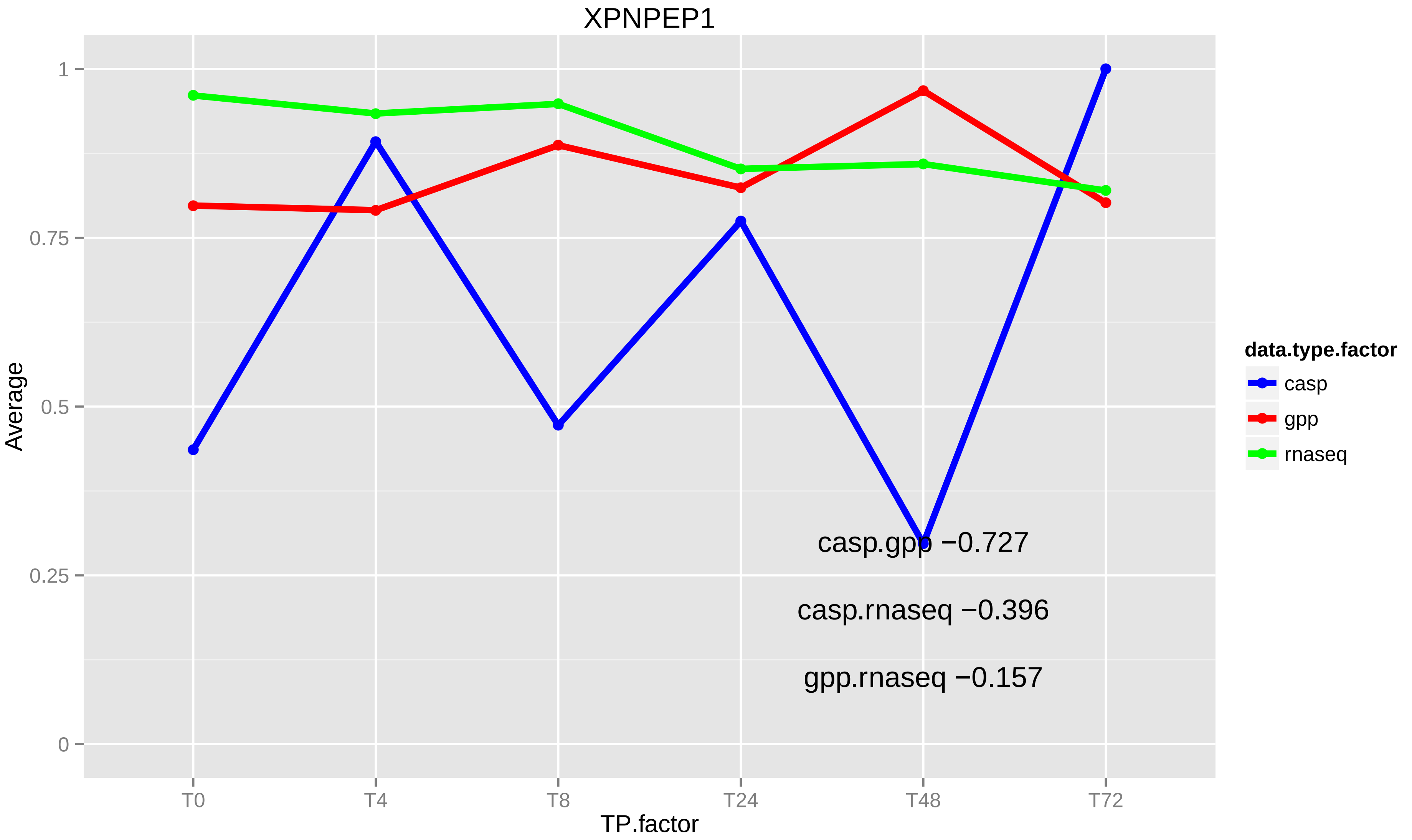
XPNPEP1
Average
1
0.75
0.5
0.25
0
T0
T4
T8
T24
T48
T72
TP.factor
data.type.factor
casp
gpp
rnaseq
casp.gpp −0.727
casp.rnaseq −0.396
gpp.rnaseq −0.157

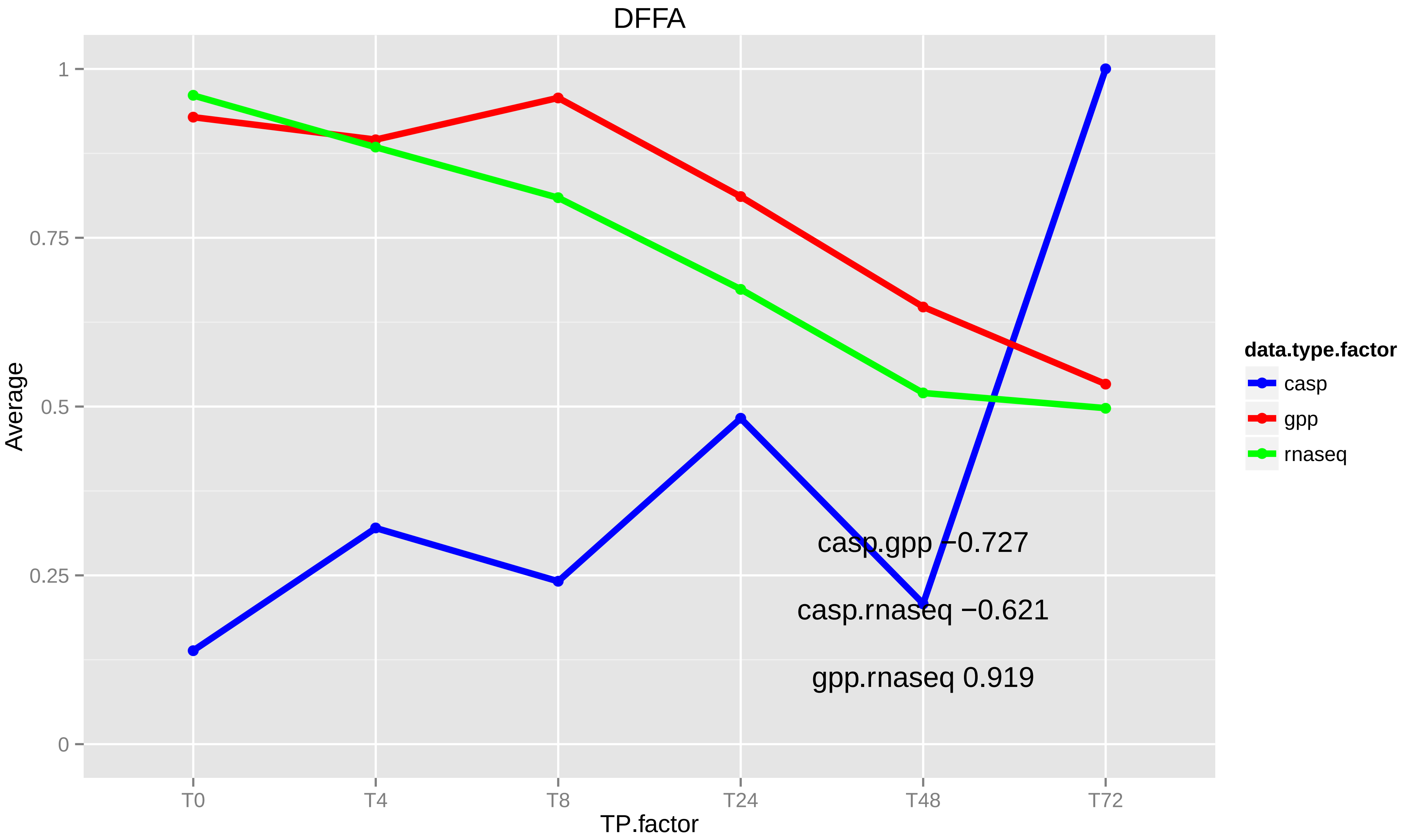

DFFA
1
0.75
0.5
0.25
0
Average
T0
T4
T8
T24
T48
T72
TP.factor
data.type.factor
casp
gpp
rnaseq
casp.gpp −0.727
casp.rnaseq −0.621
gpp.rnaseq 0.919

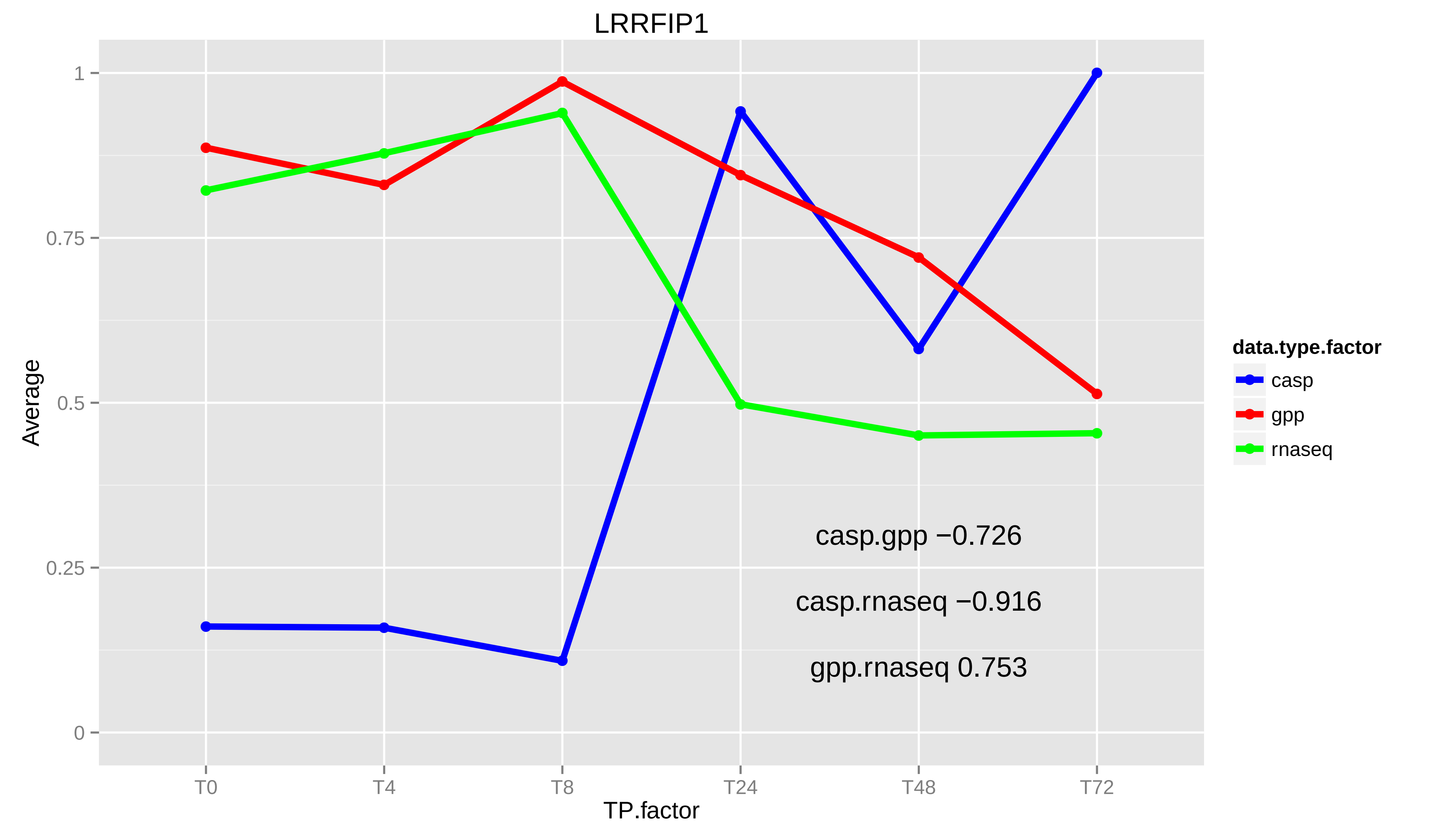
LRRFIP1
Average
1
0.75
0.5
0.25
0
T0
T4
T8
T24
T48
T72
TP.factor
data.type.factor
casp
gpp
rnaseq
casp.gpp −0.726
casp.rnaseq −0.916
gpp.rnaseq 0.753

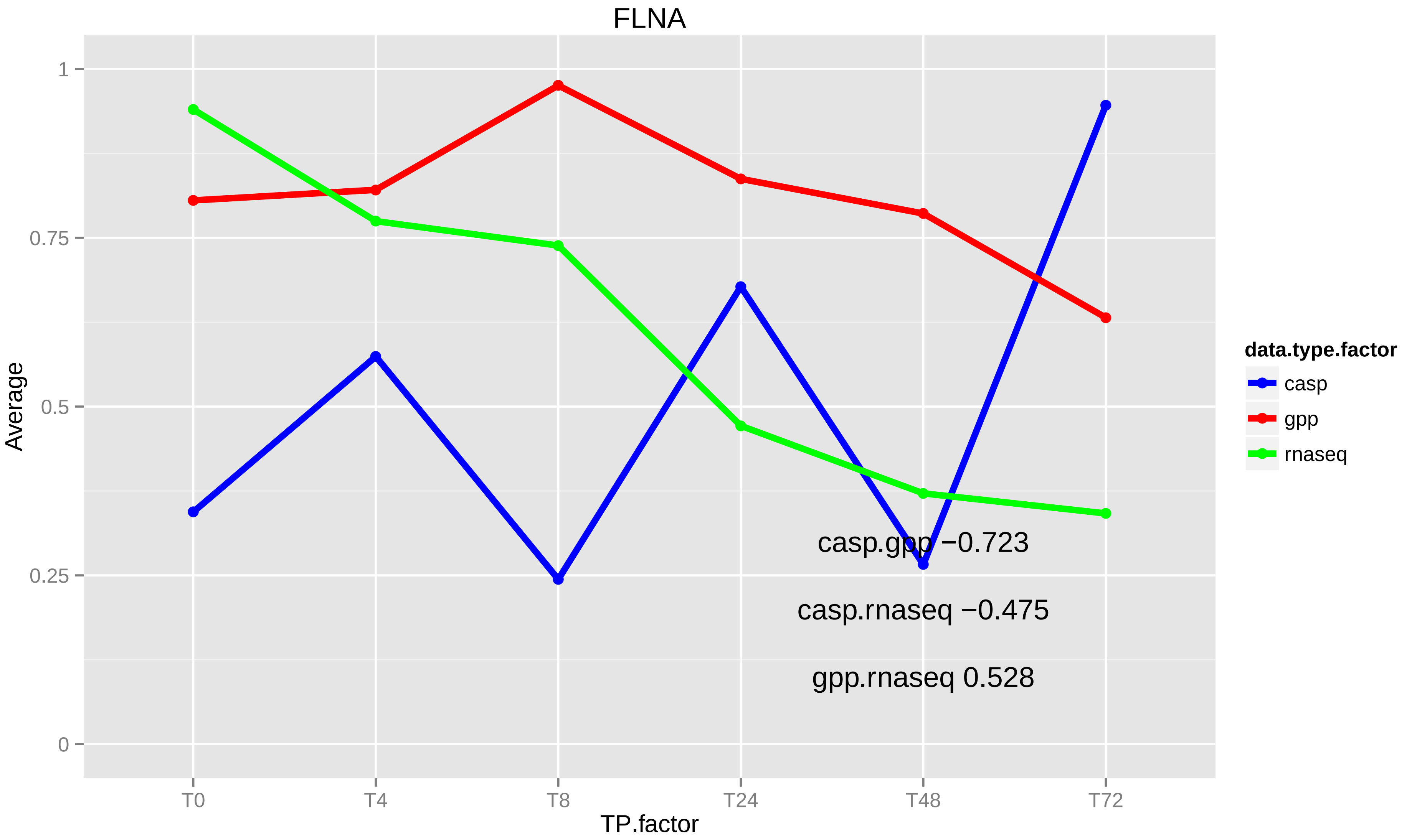
FLNA
Average
1
0.75
0.5
0.25
0
T0
T4
T8
T24
T48
T72
TP.factor
data.type.factor
casp
gpp
rnaseq
casp.gpp −0.723
casp.rnaseq −0.475
gpp.rnaseq 0.528

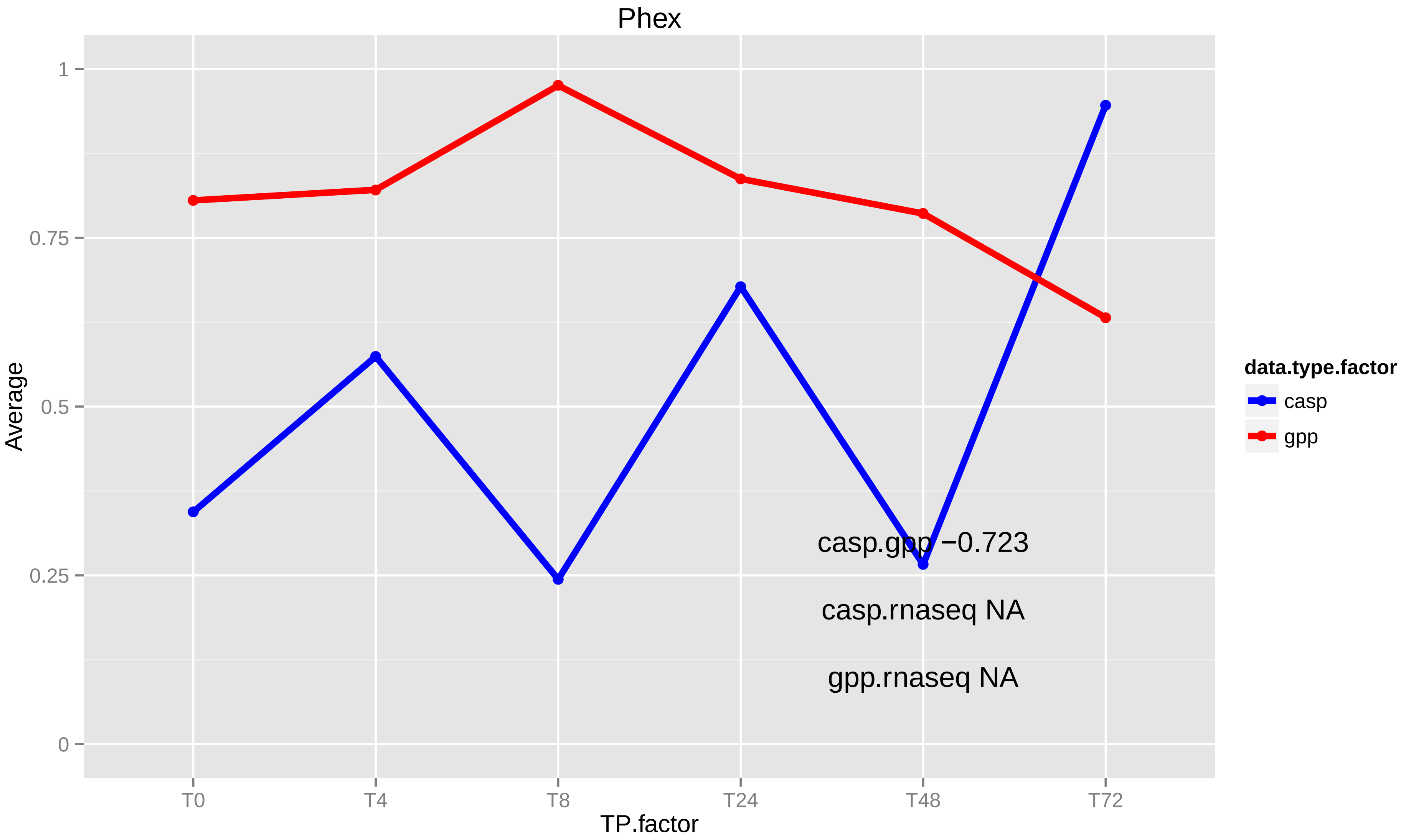

Phex
1
0.75
0.5
0.25
0
Average
T0
T4
T8
T24
T48
T72
TP.factor
data.type.factor
casp
gpp
casp.gpp −0.723
casp.rnaseq NA
gpp.rnaseq NA

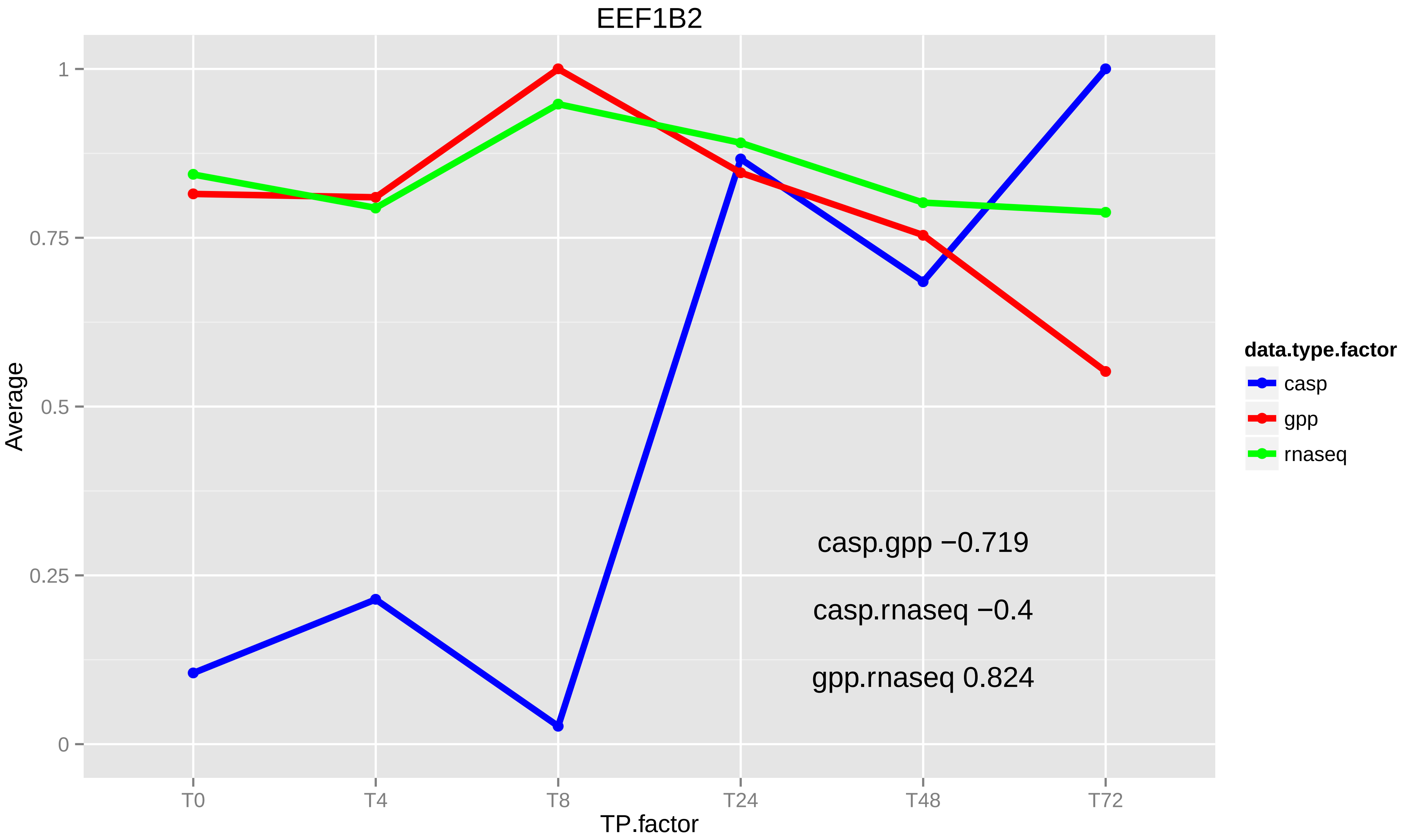

EEF1B2
1
0.75
0.5
0.25
0
Average
T0
T4
T8
T24
T48
T72
TP.factor
data.type.factor
casp
gpp
rnaseq
casp.gpp −0.719
casp.rnaseq −0.4
gpp.rnaseq 0.824

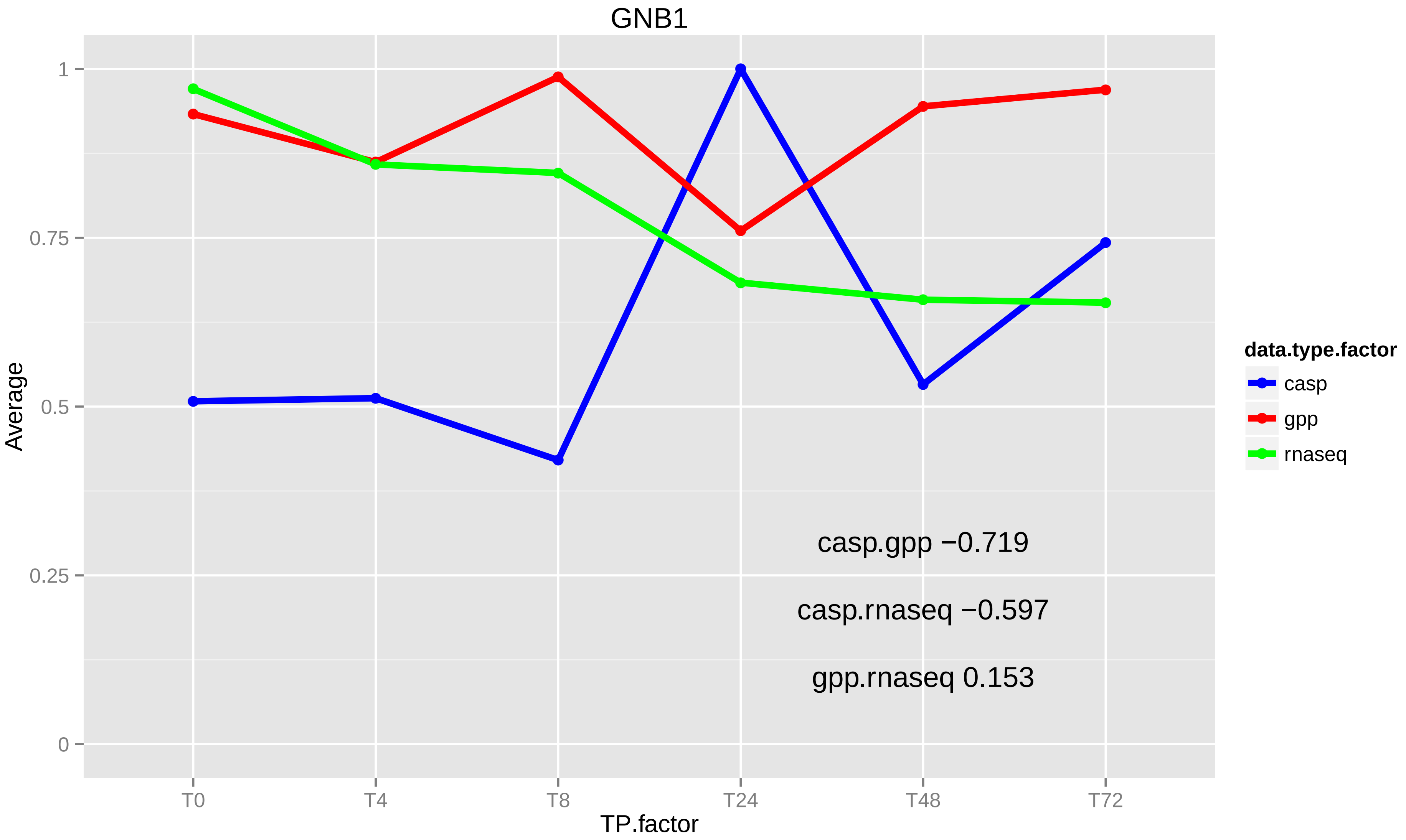
GNB1
1
0.75
0.5
0.25
0
Average
T0
T4
T8
T24
T48
T72
TP.factor
data.type.factor
casp
gpp
rnaseq
casp.gpp −0.719
casp.rnaseq −0.597
gpp.rnaseq 0.153

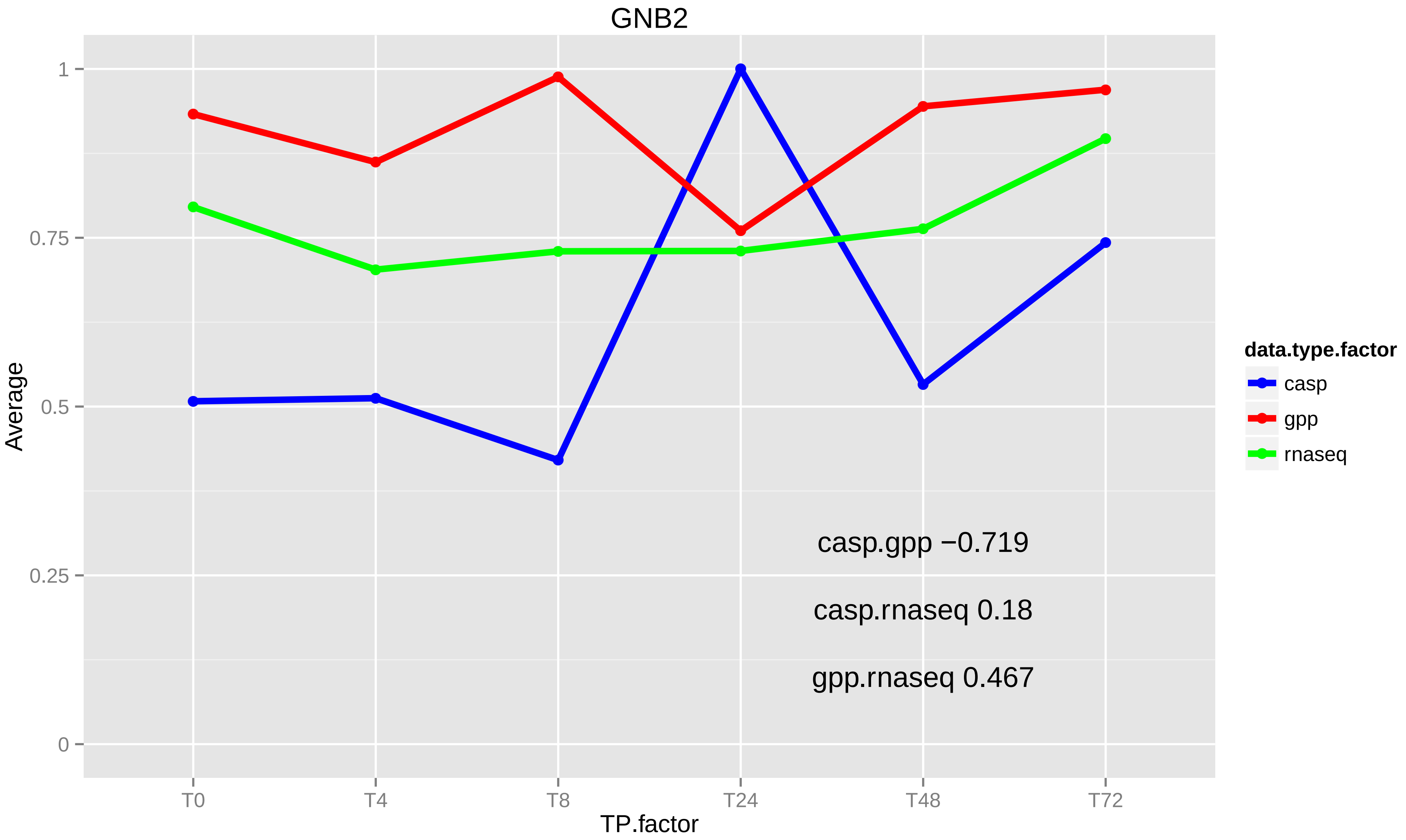
GNB2
Average
1
0.75
0.5
0.25
0
T0
T4
T8
T24
T48
T72
TP.factor
data.type.factor
casp
gpp
rnaseq
casp.gpp −0.719
casp.rnaseq 0.18
gpp.rnaseq 0.467

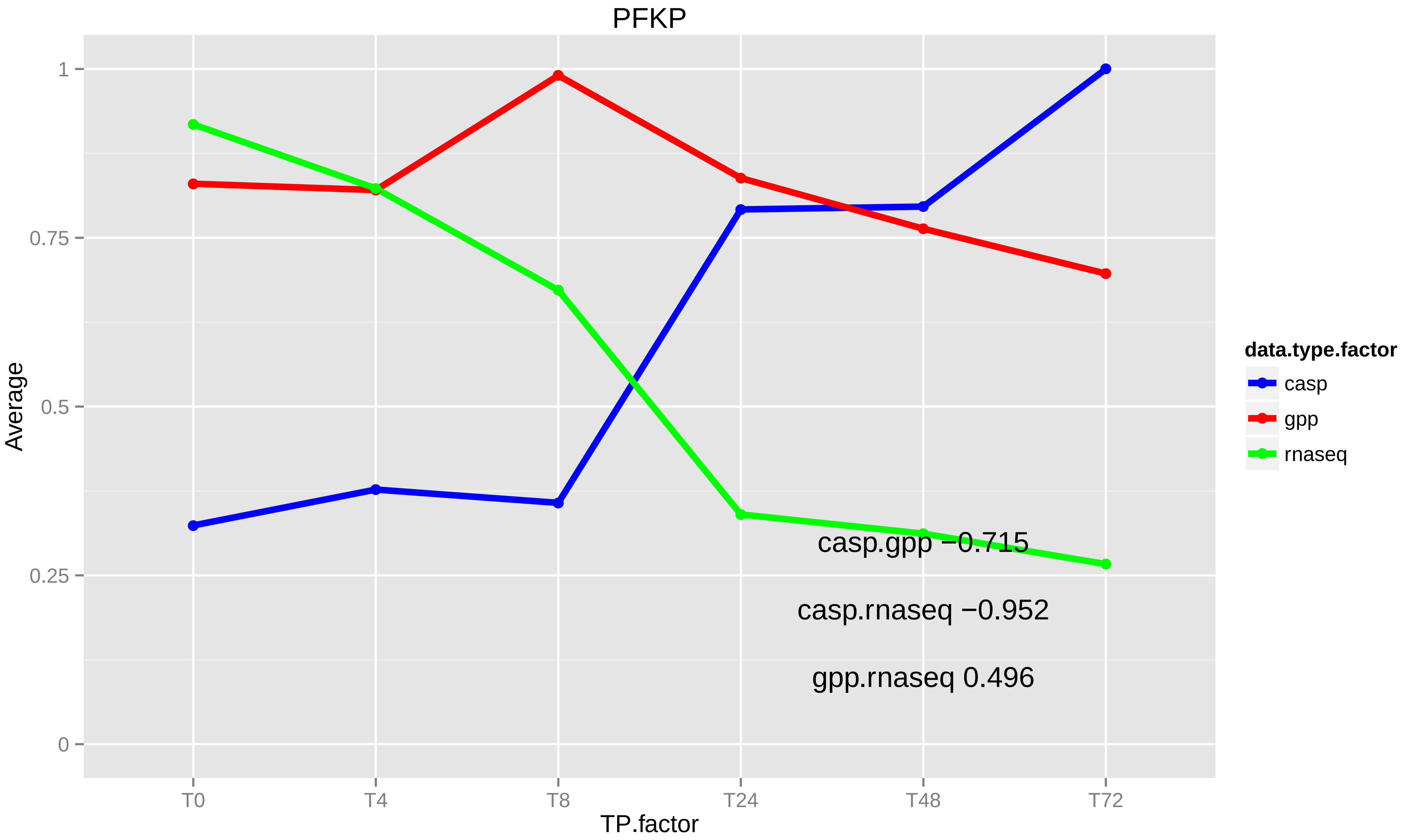
PFKP
Average
1
0.75
0.5
0.25
0
T0
T4
T8
T24
T48
T72
TP.factor
data.type.factor
casp
gpp
rnaseq
casp.gpp −0.715
casp.rnaseq −0.952
gpp.rnaseq 0.496

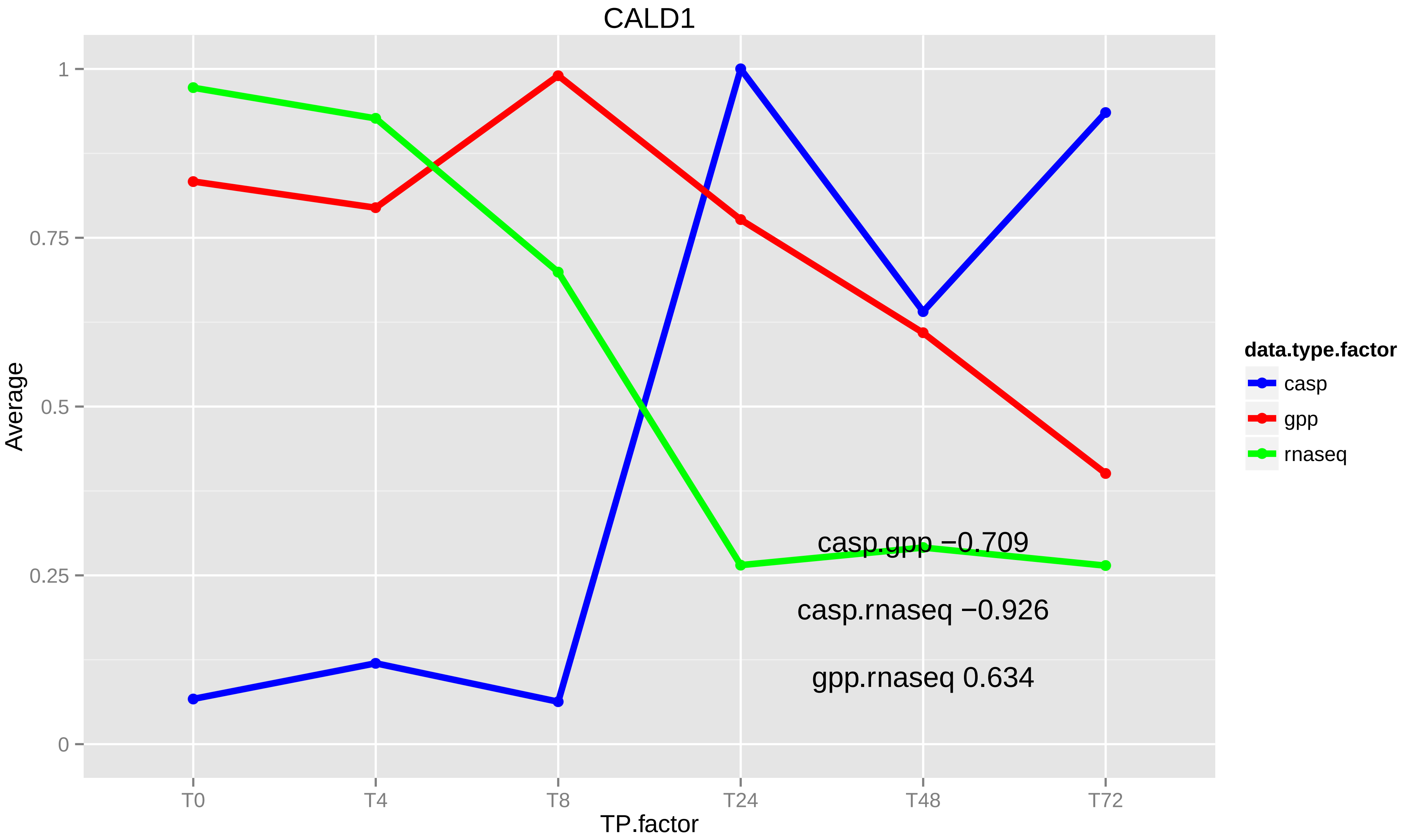
CALD1
1
0.75
0.5
0.25
0
Average
T0
T4
T8
T24
T48
T72
TP.factor
data.type.factor
casp
gpp
rnaseq
casp.gpp −0.709
casp.rnaseq −0.926
gpp.rnaseq 0.634

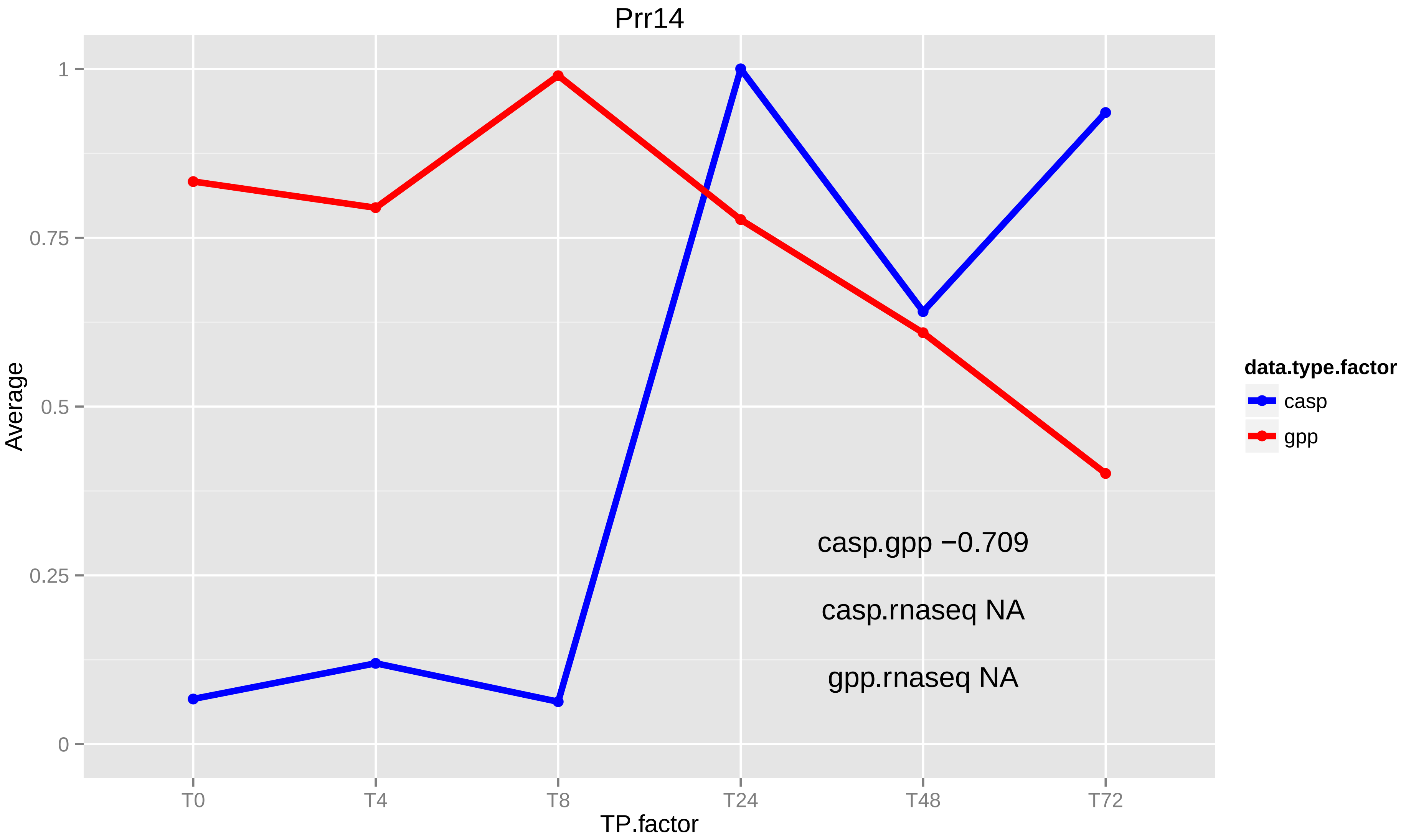

Prr14
Average
1
0.75
0.5
0.25
0
T0
T4
T8
T24
T48
T72
TP.factor
data.type.factor
casp
gpp
casp.gpp −0.709
casp.rnaseq NA
gpp.rnaseq NA

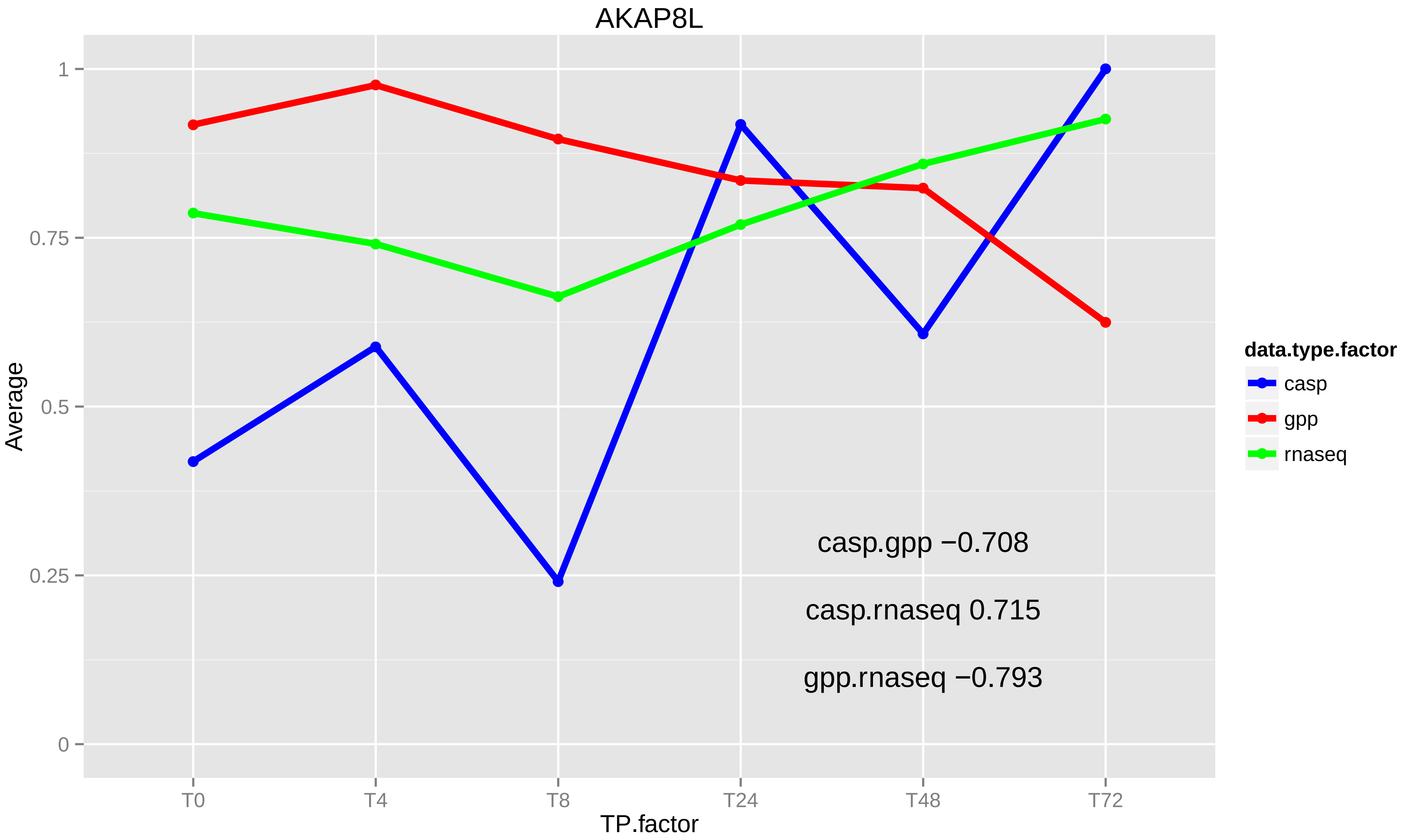
AKAP8L
Average
1
0.75
0.5
0.25
0
T0
T4
T8
T24
T48
T72
TP.factor
data.type.factor
casp
gpp
rnaseq
casp.gpp −0.708
casp.rnaseq 0.715
gpp.rnaseq −0.793

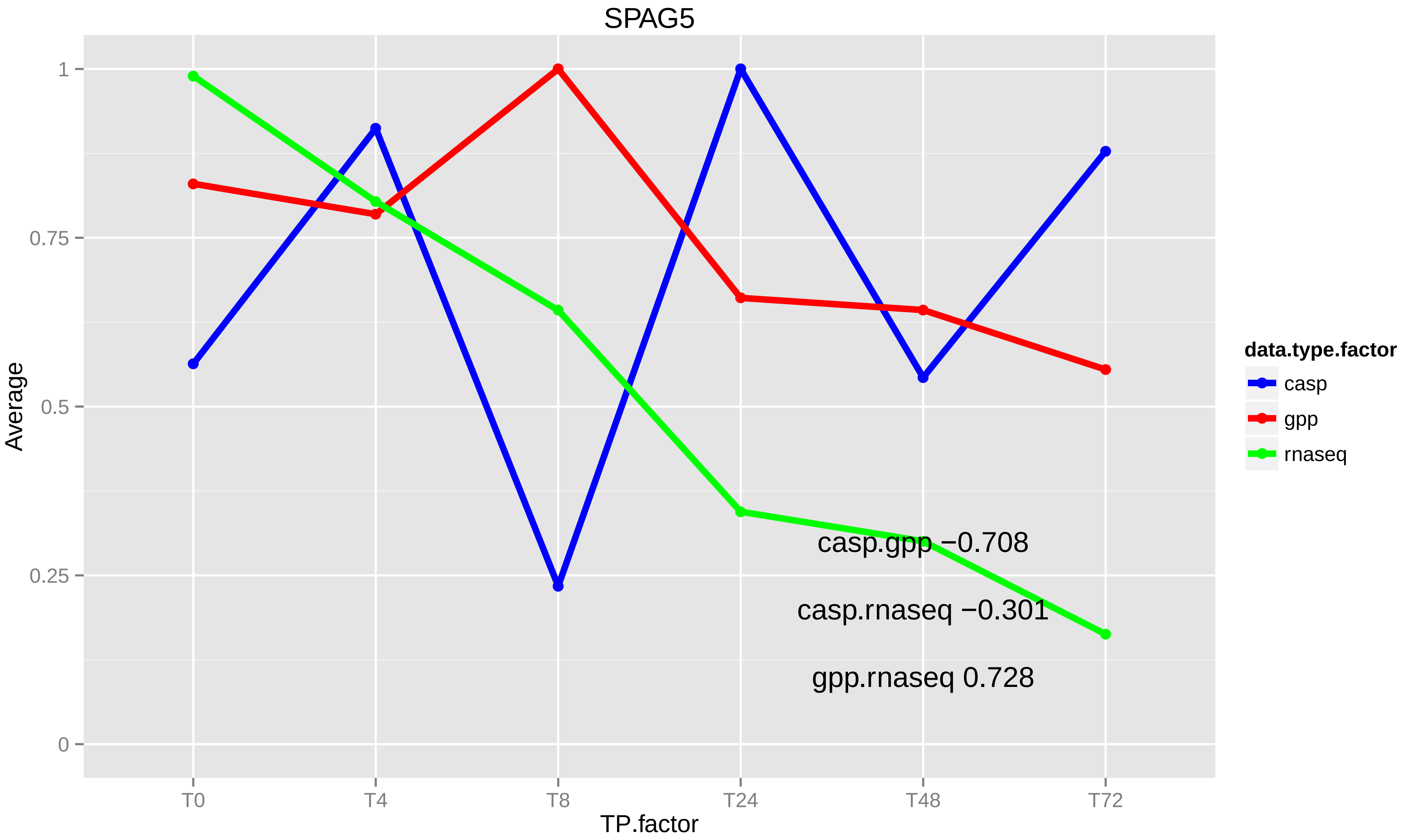

SPAG5
Average
1
0.75
0.5
0.25
0
T0
T4
T8
T24
T48
T72
TP.factor
data.type.factor
casp
gpp
rnaseq
casp.gpp −0.708
casp.rnaseq −0.301
gpp.rnaseq 0.728

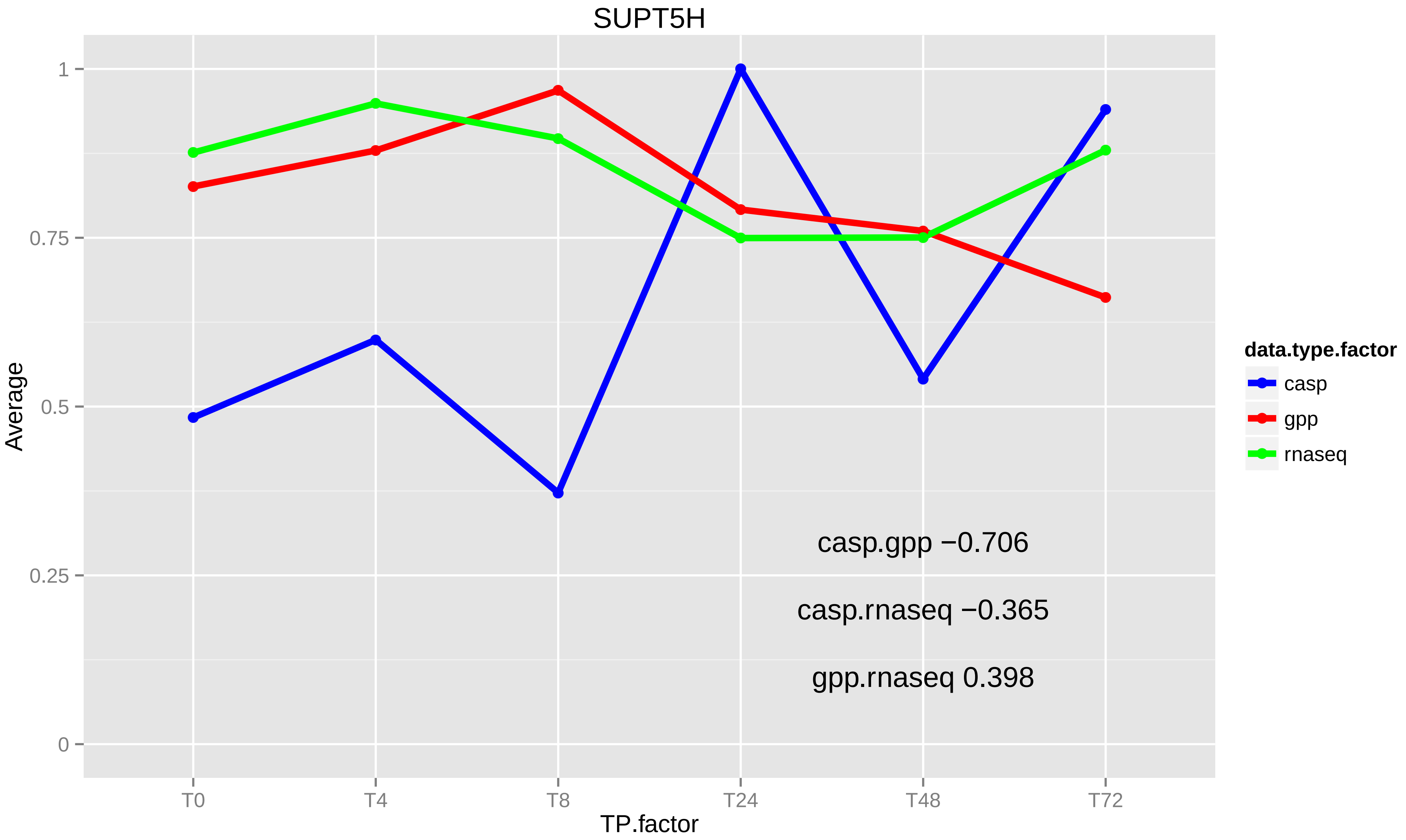

SUPT5H
Average
1
0.75
0.5
0.25
0
T0
T4
T8
T24
T48
T72
TP.factor
data.type.factor
casp
gpp
rnaseq
casp.gpp −0.706
casp.rnaseq −0.365
gpp.rnaseq 0.398

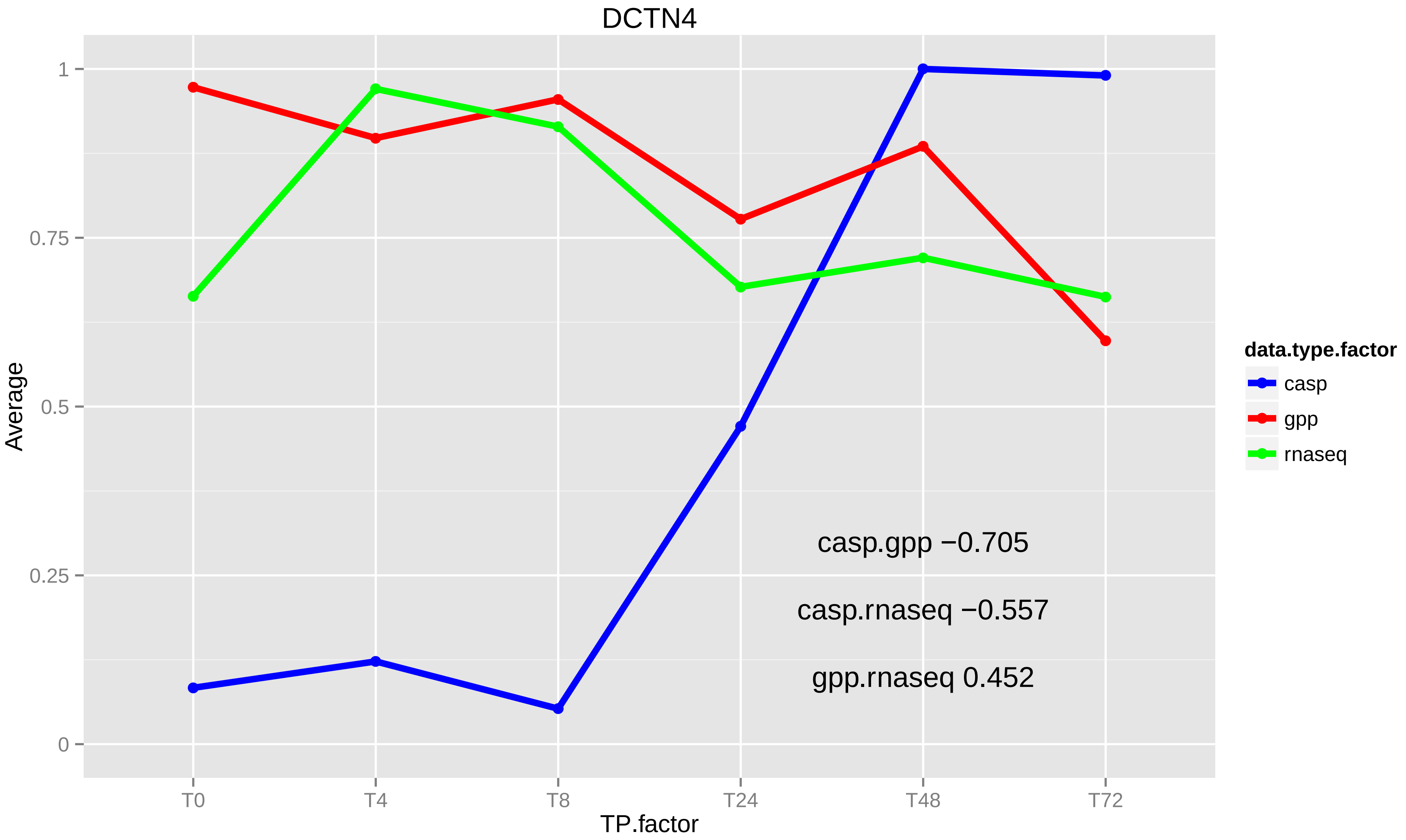

DCTN4
Average
1
0.75
0.5
0.25
0
T0
T4
T8
T24
T48
T72
TP.factor
data.type.factor
casp
gpp
rnaseq
casp.gpp −0.705
casp.rnaseq −0.557
gpp.rnaseq 0.452

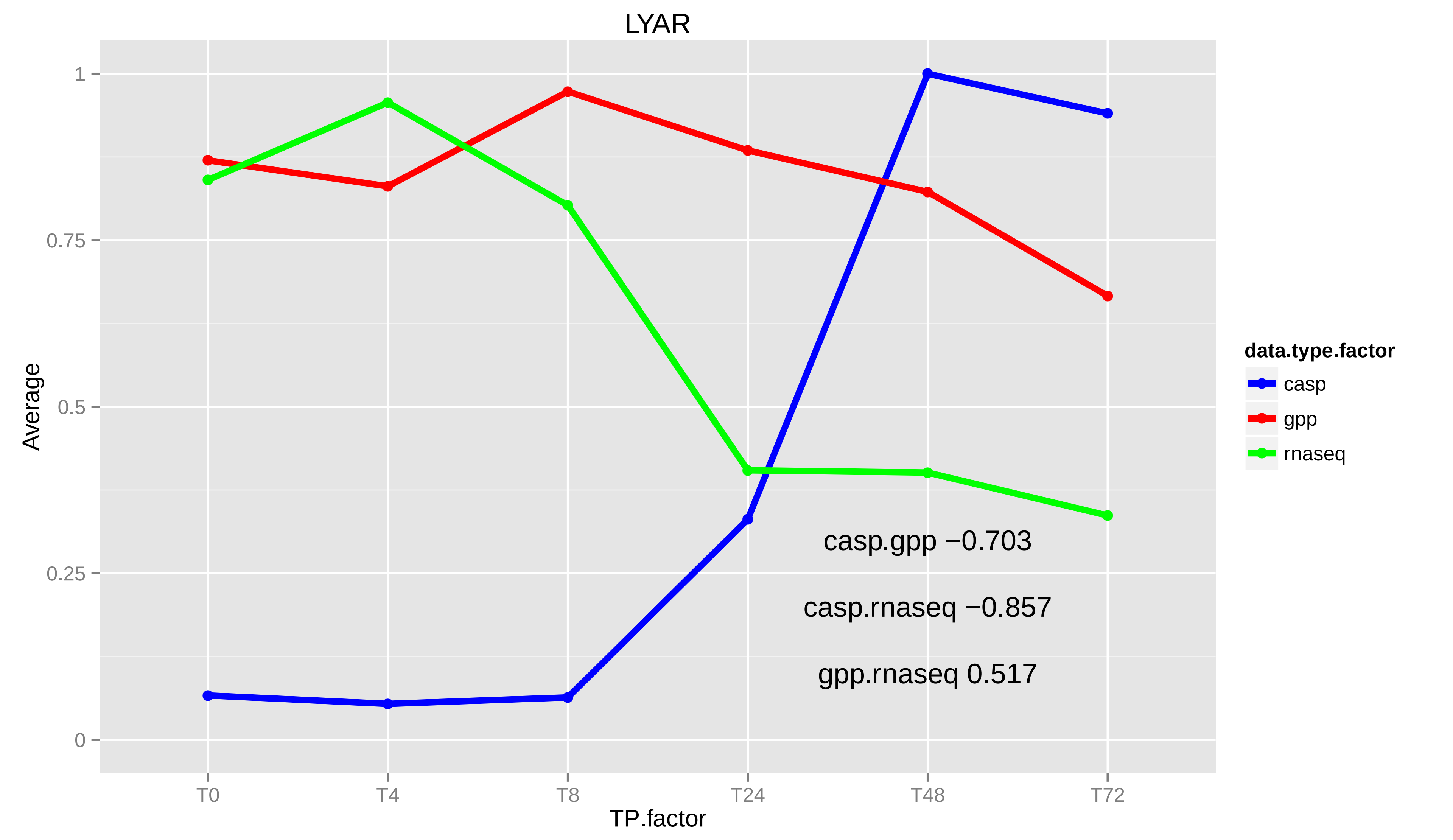

LYAR
1
0.75
0.5
0.25
0
Average
T0
T4
T8
T24
T48
T72
TP.factor
data.type.factor
casp
gpp
rnaseq
casp.gpp −0.703
casp.rnaseq −0.857
gpp.rnaseq 0.517

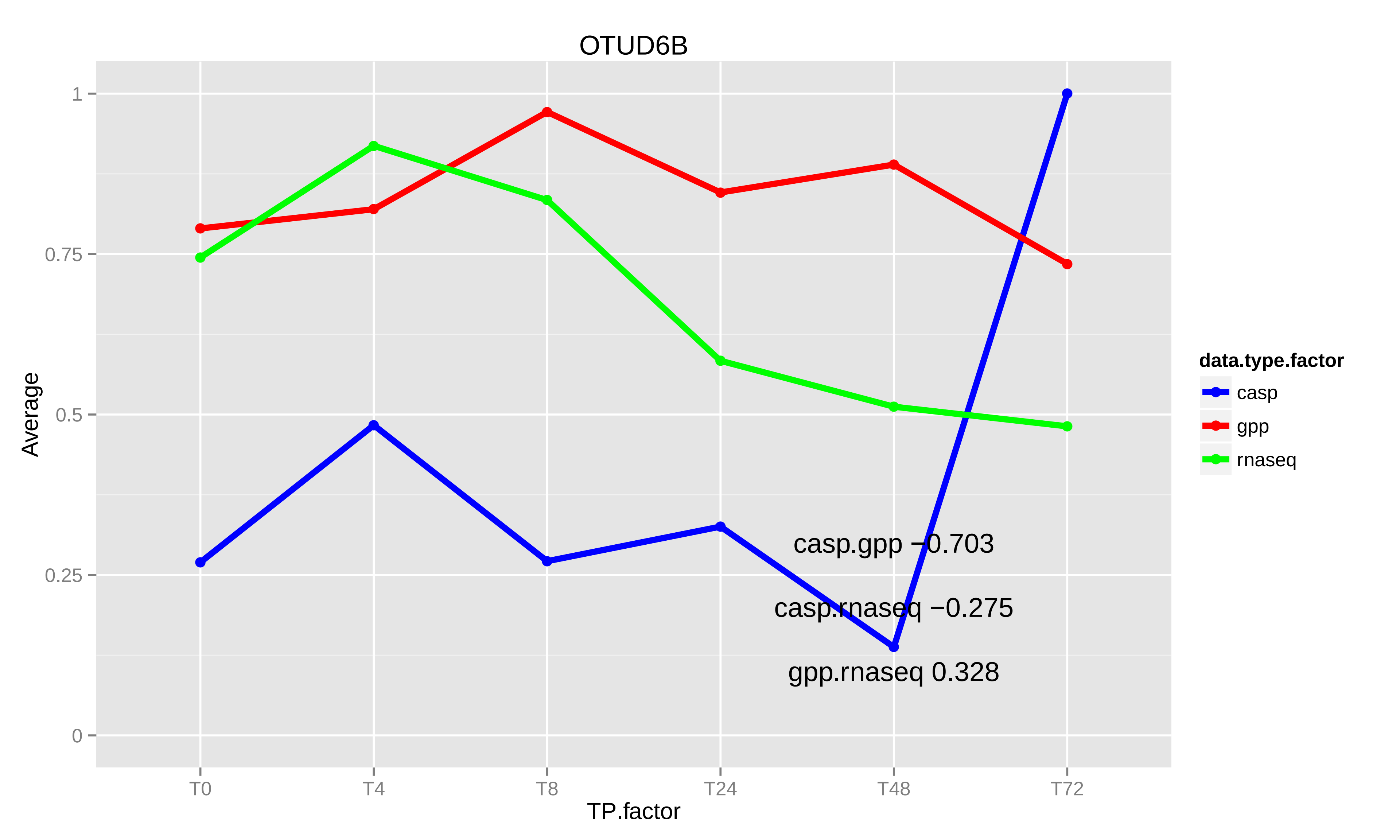
OTUD6B
Average
1
0.75
0.5
0.25
0
T0
T4
T8
T24
T48
T72
TP.factor
data.type.factor
casp
gpp
rnaseq
casp.gpp −0.703
casp.rnaseq −0.275
gpp.rnaseq 0.328

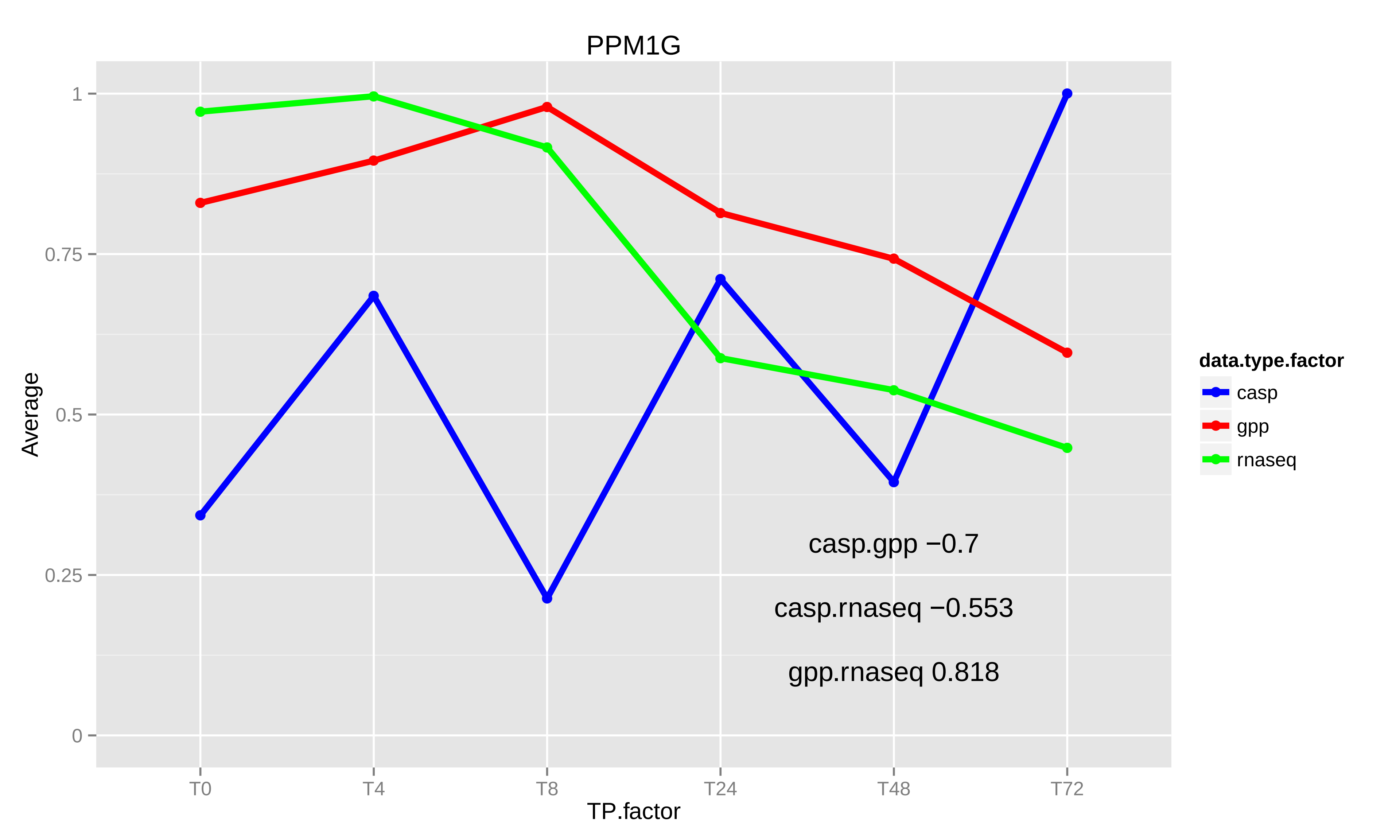

PPM1G
Average
1
0.75
0.5
0.25
0
T0
T4
T8
T24
T48
T72
TP.factor
data.type.factor
casp
gpp
rnaseq
casp.gpp −0.7
casp.rnaseq −0.553
gpp.rnaseq 0.818

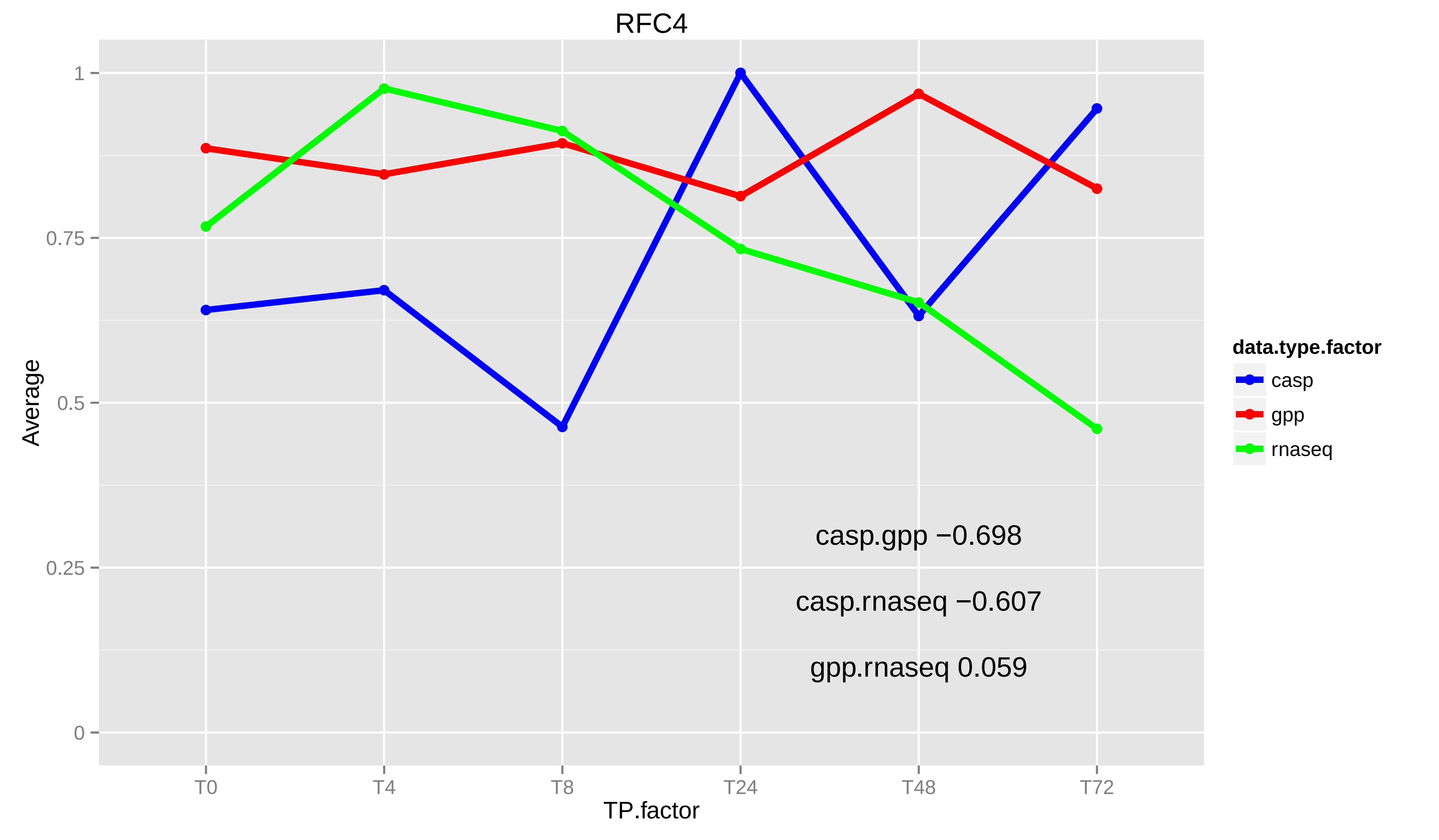
RFC4
1
0.75
0.5
0.25
0
Average
T0
T4
T8
T24
T48
T72
TP.factor
data.type.factor
casp
gpp
rnaseq
casp.gpp −0.698
casp.rnaseq −0.607
gpp.rnaseq 0.059

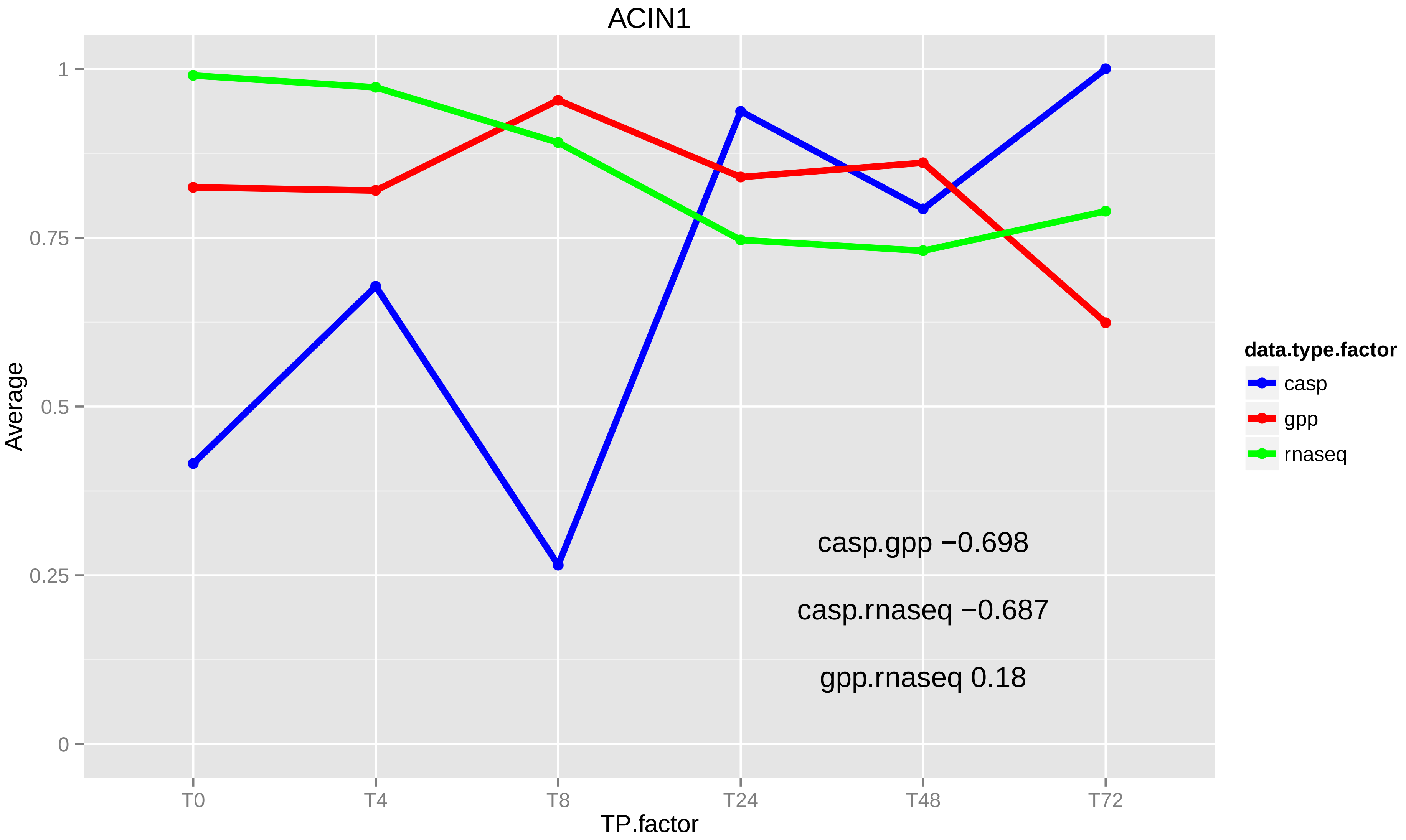

ACIN1
Average
1
0.75
0.5
0.25
0
T0
T4
T8
T24
T48
T72
TP.factor
data.type.factor
casp
gpp
rnaseq
casp.gpp −0.698
casp.rnaseq −0.687
gpp.rnaseq 0.18

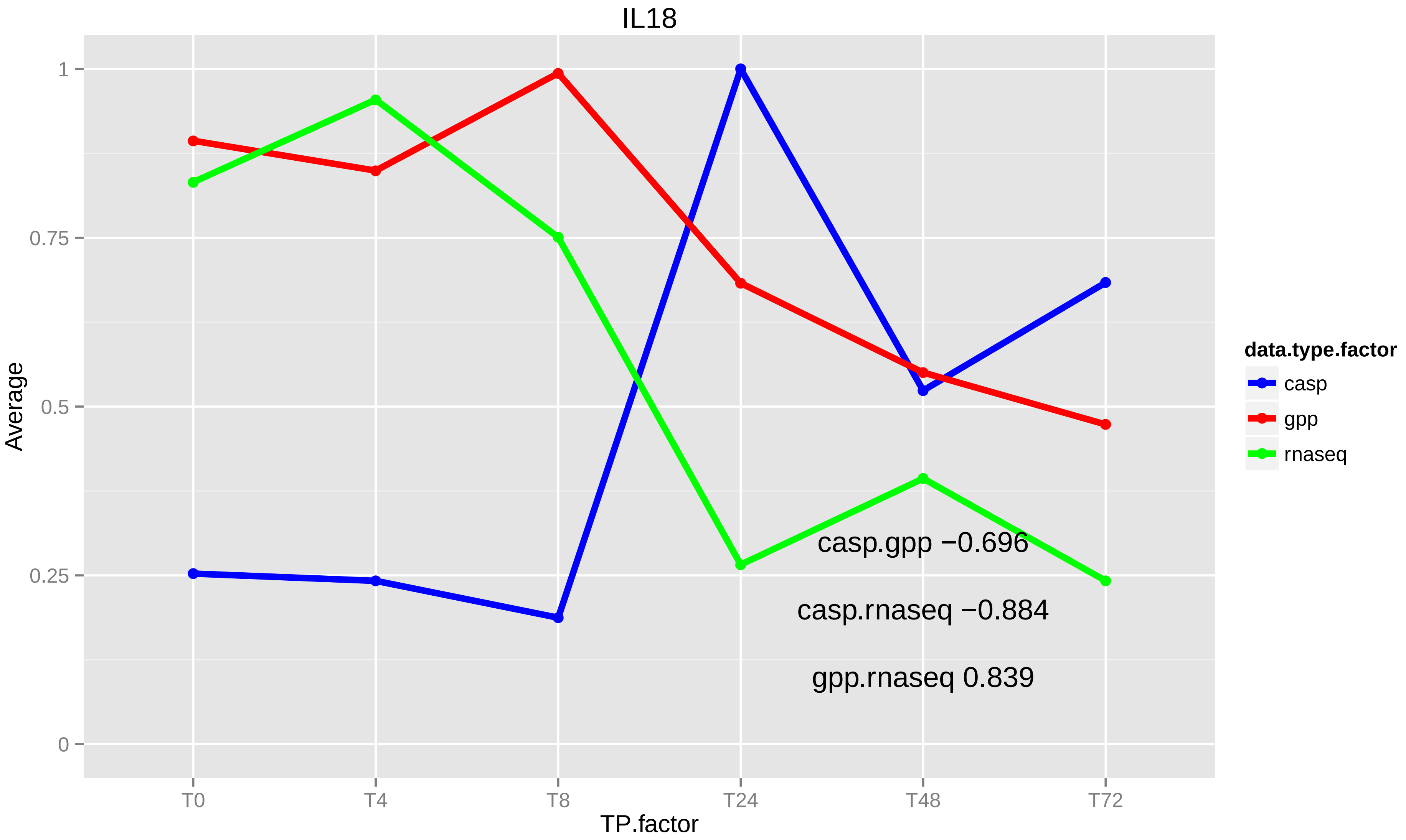
IL18
Average
1
0.75
0.5
0.25
0
T0
T4
T8
T24
T48
T72
TP.factor
data.type.factor
casp
gpp
rnaseq
casp.gpp −0.696
casp.rnaseq −0.884
gpp.rnaseq 0.839

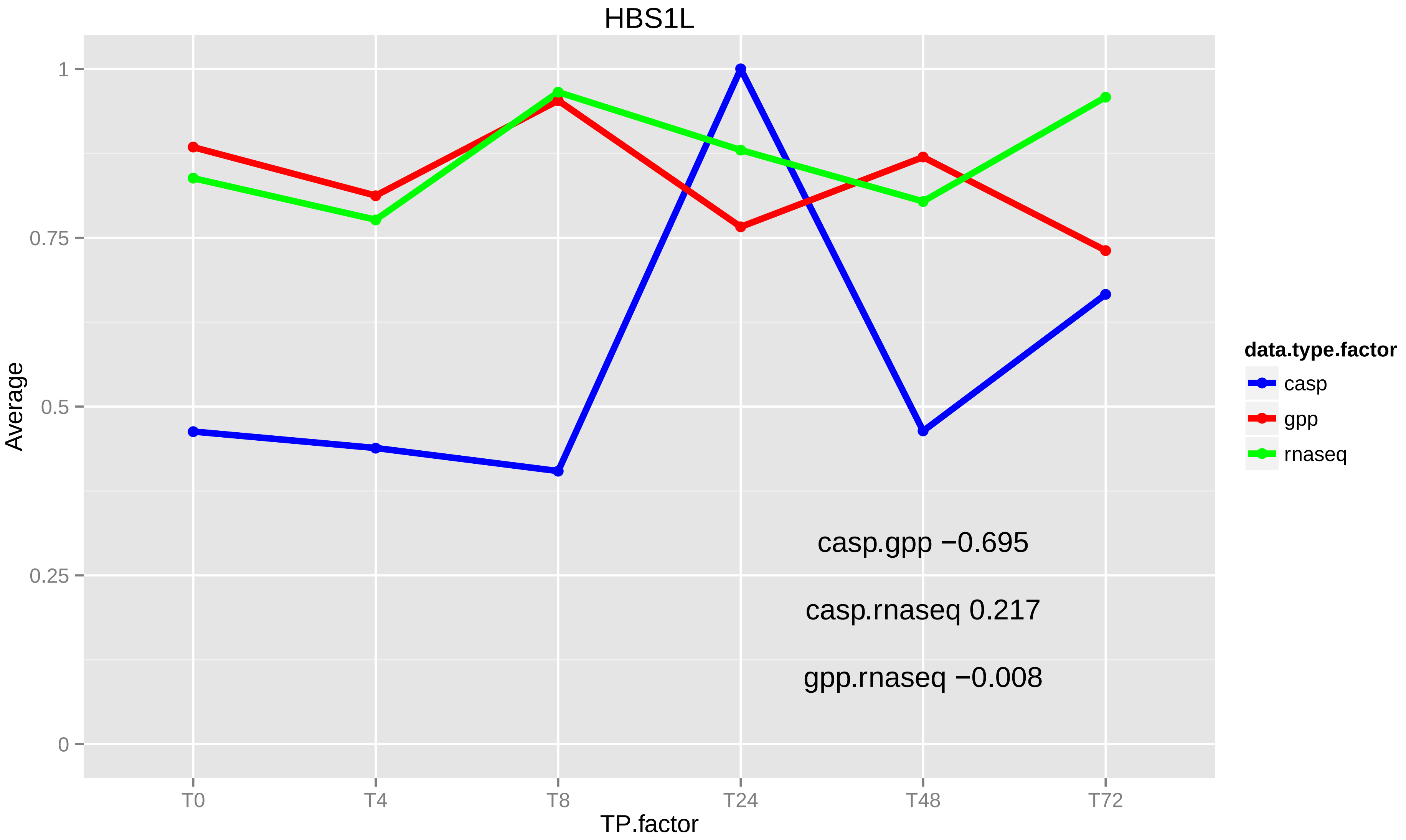
HBS1L
1
0.75
0.5
0.25
0
Average
T0
T4
T8
T24
T48
T72
TP.factor
data.type.factor
casp
gpp
rnaseq
casp.gpp −0.695
casp.rnaseq 0.217
gpp.rnaseq −0.008

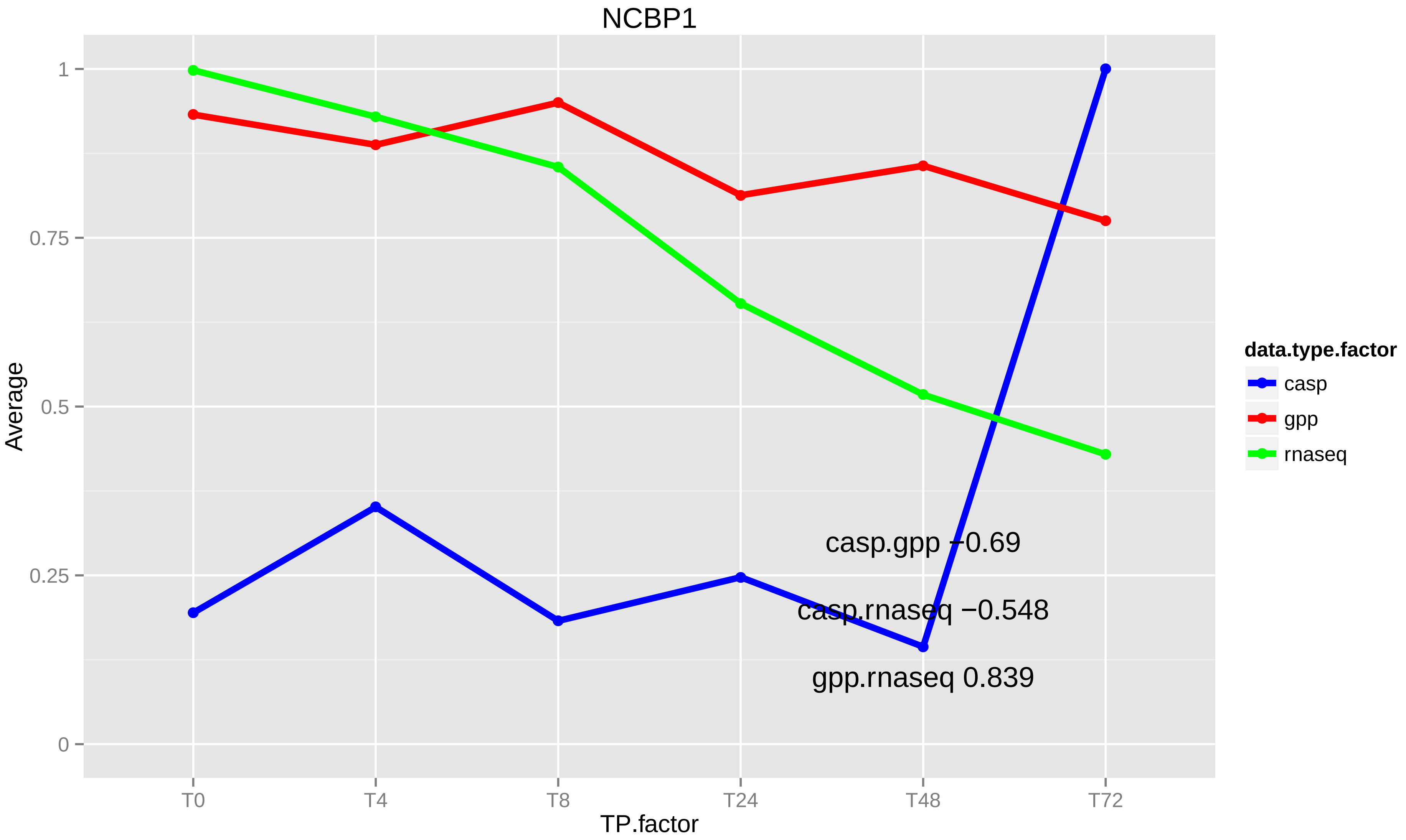
NCBP1
1
0.75
0.5
0.25
0
Average
T0
T4
T8
T24
T48
T72
TP.factor
data.type.factor
casp
gpp
rnaseq
casp.gpp −0.69
casp.rnaseq −0.548
gpp.rnaseq 0.839

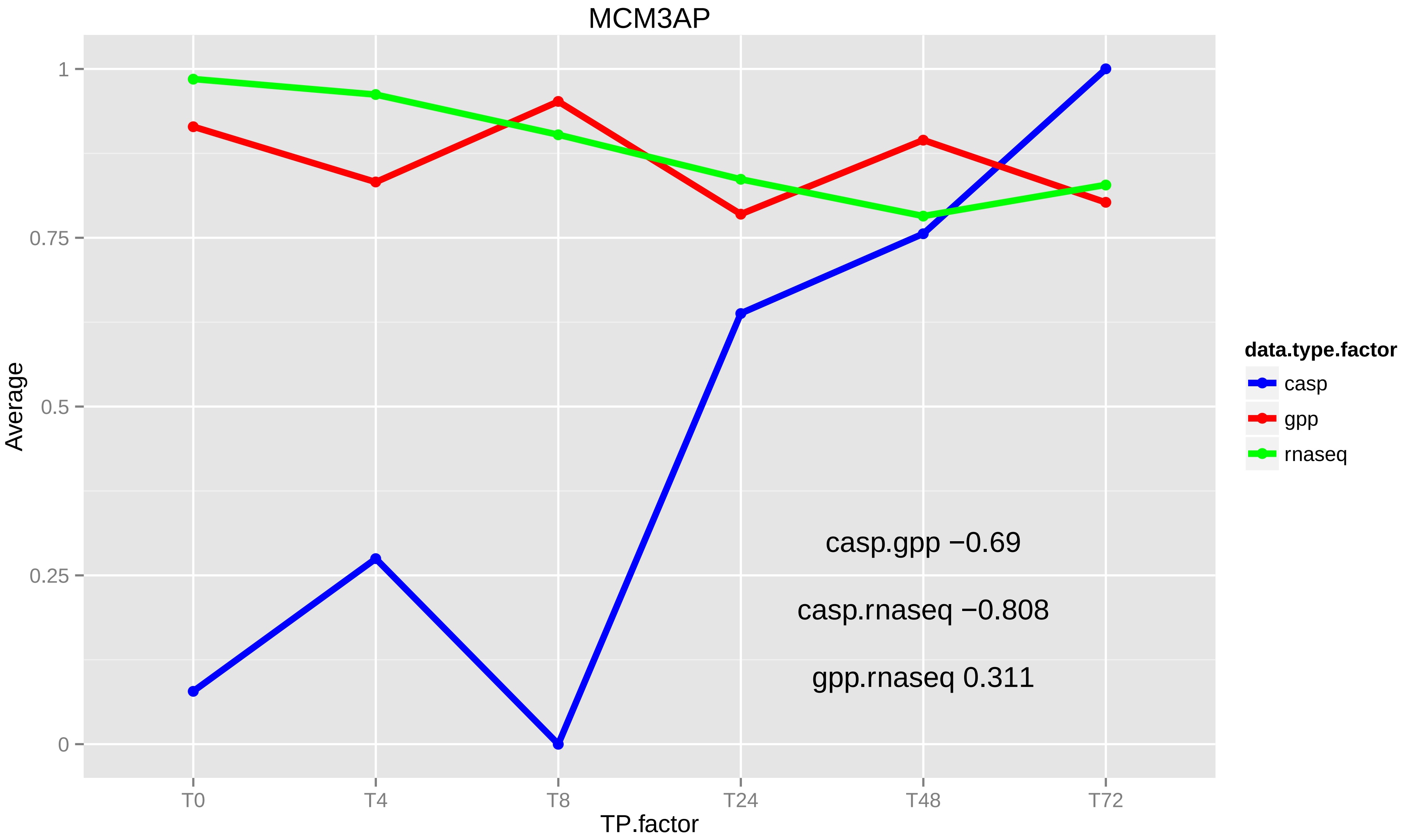
MCM3AP
Average
1
0.75
0.5
0.25
0
T0
T4
T8
T24
T48
T72
TP.factor
data.type.factor
casp
gpp
rnaseq
casp.gpp −0.69
casp.rnaseq −0.808
gpp.rnaseq 0.311

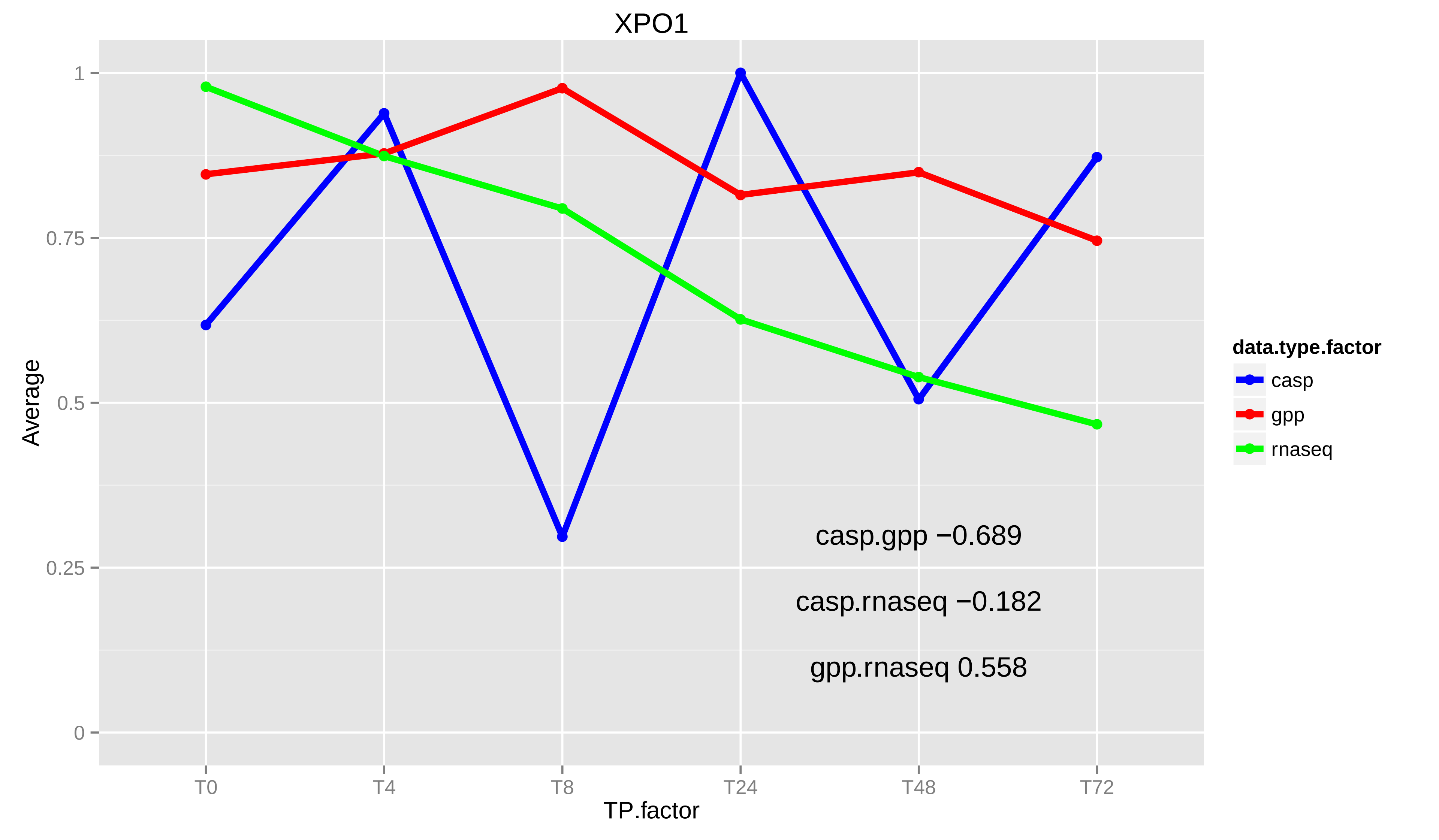

XPO1
Average
1
0.75
0.5
0.25
0
T0
T4
T8
T24
T48
T72
TP.factor
data.type.factor
casp
gpp
rnaseq
casp.gpp −0.689
casp.rnaseq −0.182
gpp.rnaseq 0.558

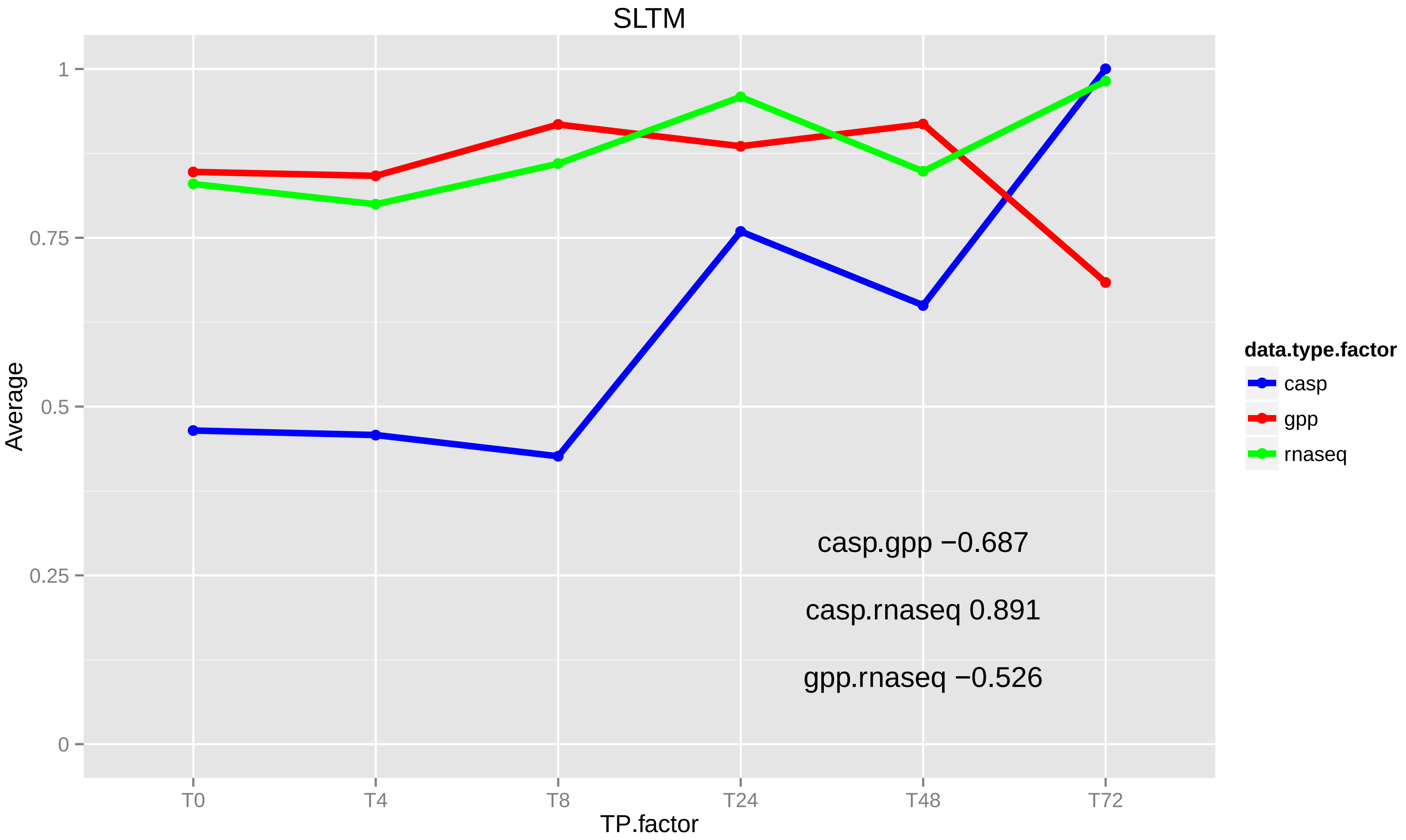

SLTM
Average
1
0.75
0.5
0.25
0
T0
T4
T8
T24
T48
T72
TP.factor
data.type.factor
casp
gpp
rnaseq
casp.gpp −0.687
casp.rnaseq 0.891
gpp.rnaseq −0.526

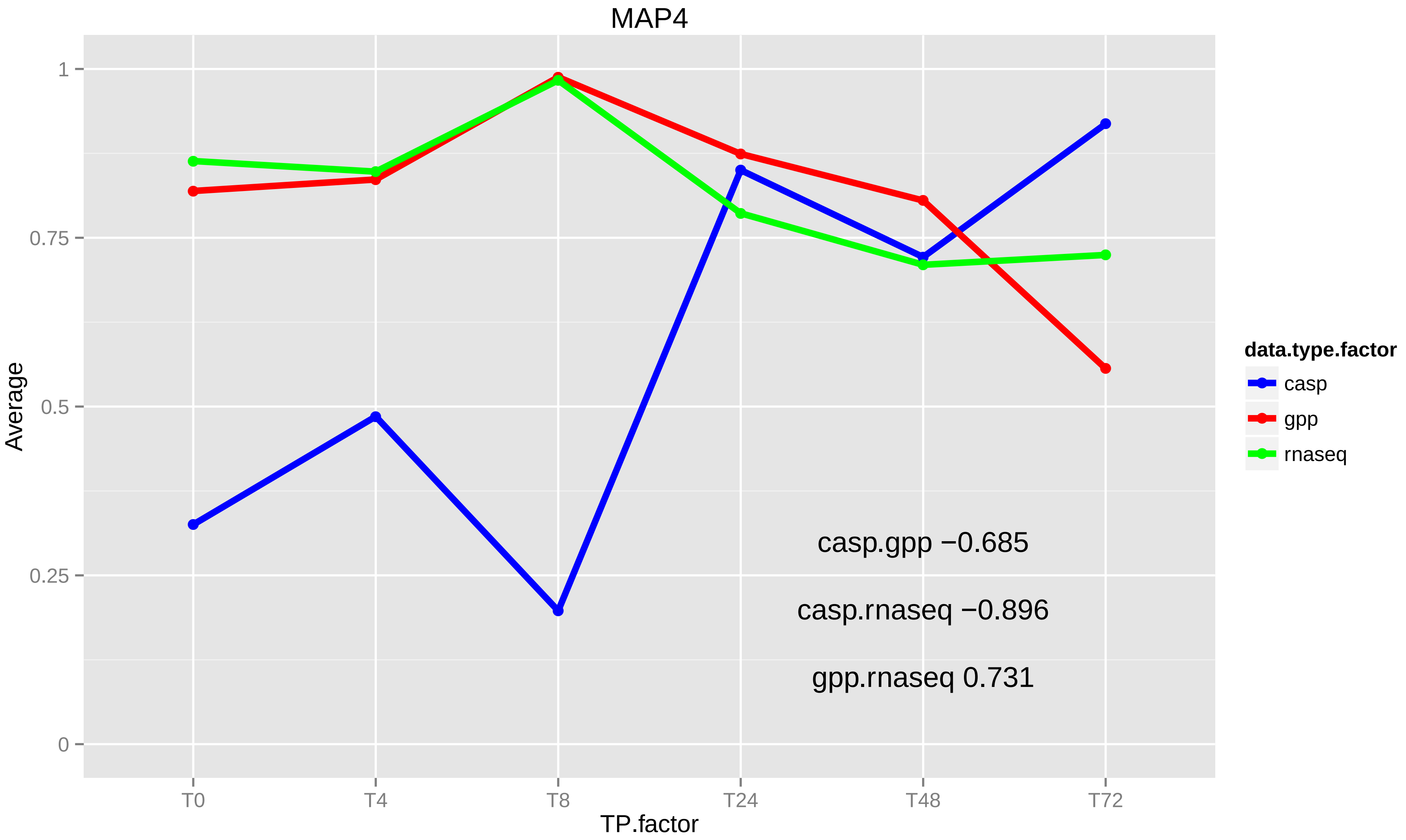

MAP4
1
0.75
0.5
0.25
0
Average
T0
T4
T8
T24
T48
T72
TP.factor
data.type.factor
casp
gpp
rnaseq
casp.gpp −0.685
casp.rnaseq −0.896
gpp.rnaseq 0.731

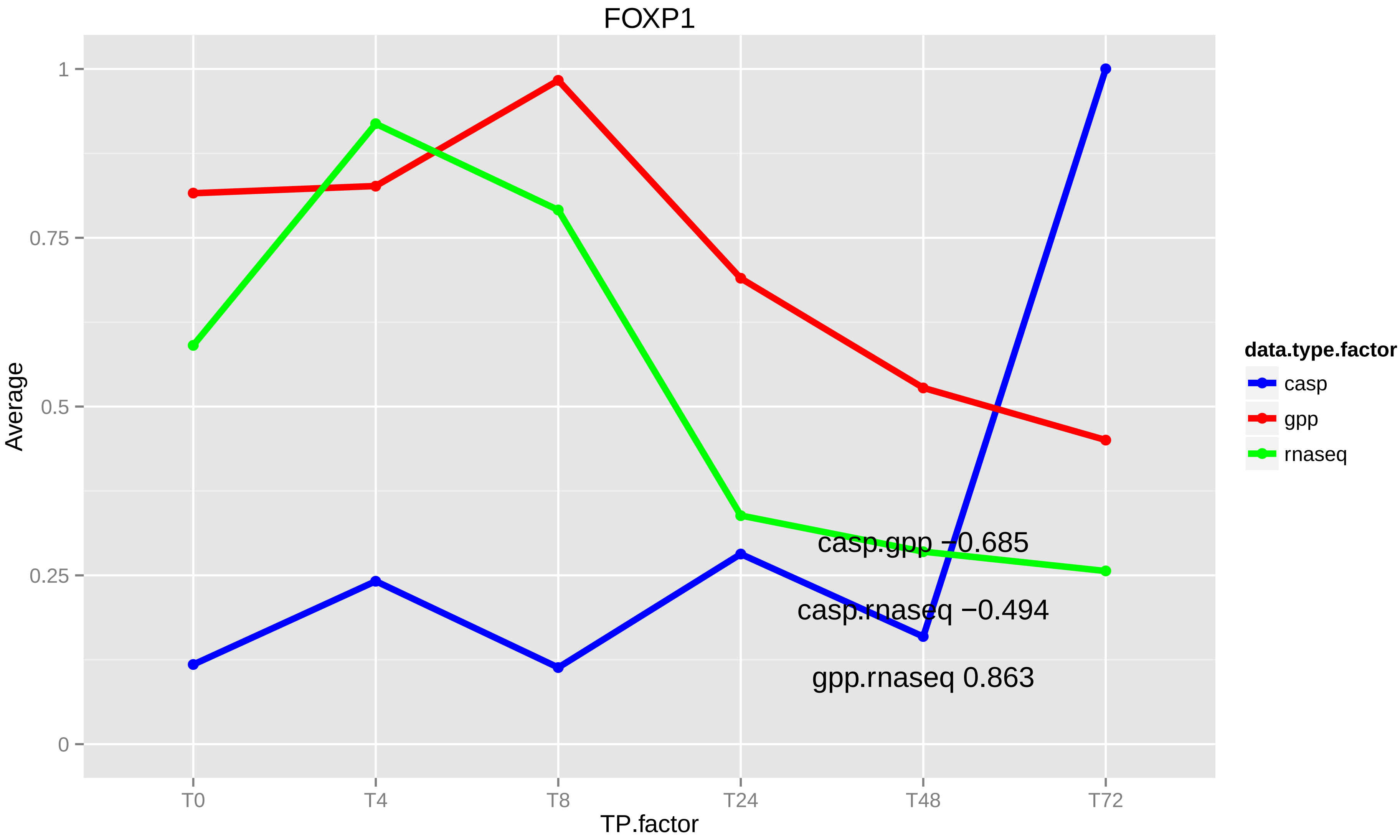
FOXP1
Average
1
0.75
0.5
0.25
0
T0
T4
T8
T24
T48
T72
TP.factor
data.type.factor
casp
gpp
rnaseq
casp.gpp −0.685
casp.rnaseq −0.494
gpp.rnaseq 0.863

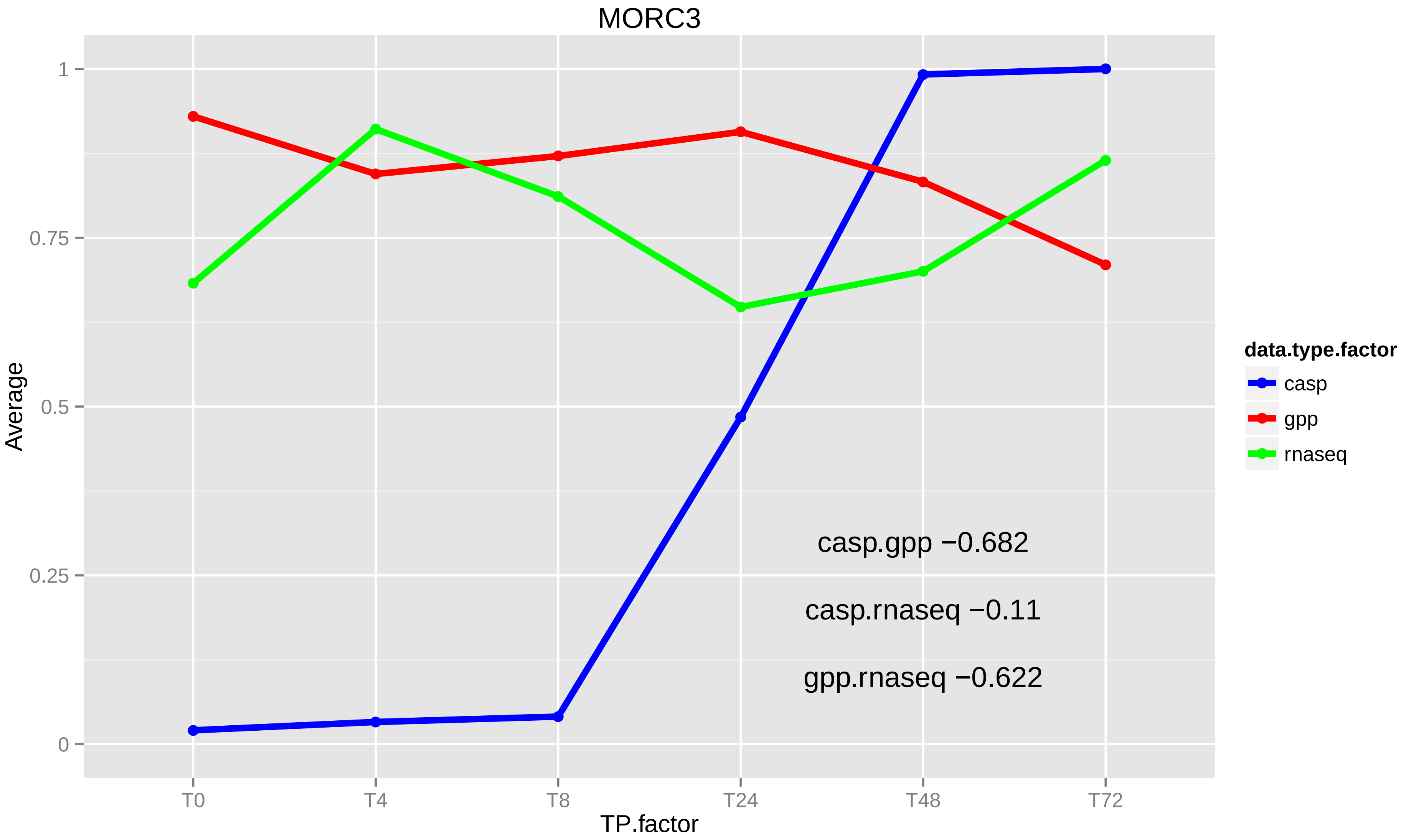

MORC3
1
0.75
0.5
0.25
0
Average
T0
T4
T8
T24
T48
T72
TP.factor
data.type.factor
casp
gpp
rnaseq
casp.gpp −0.682
casp.rnaseq −0.11
gpp.rnaseq −0.622

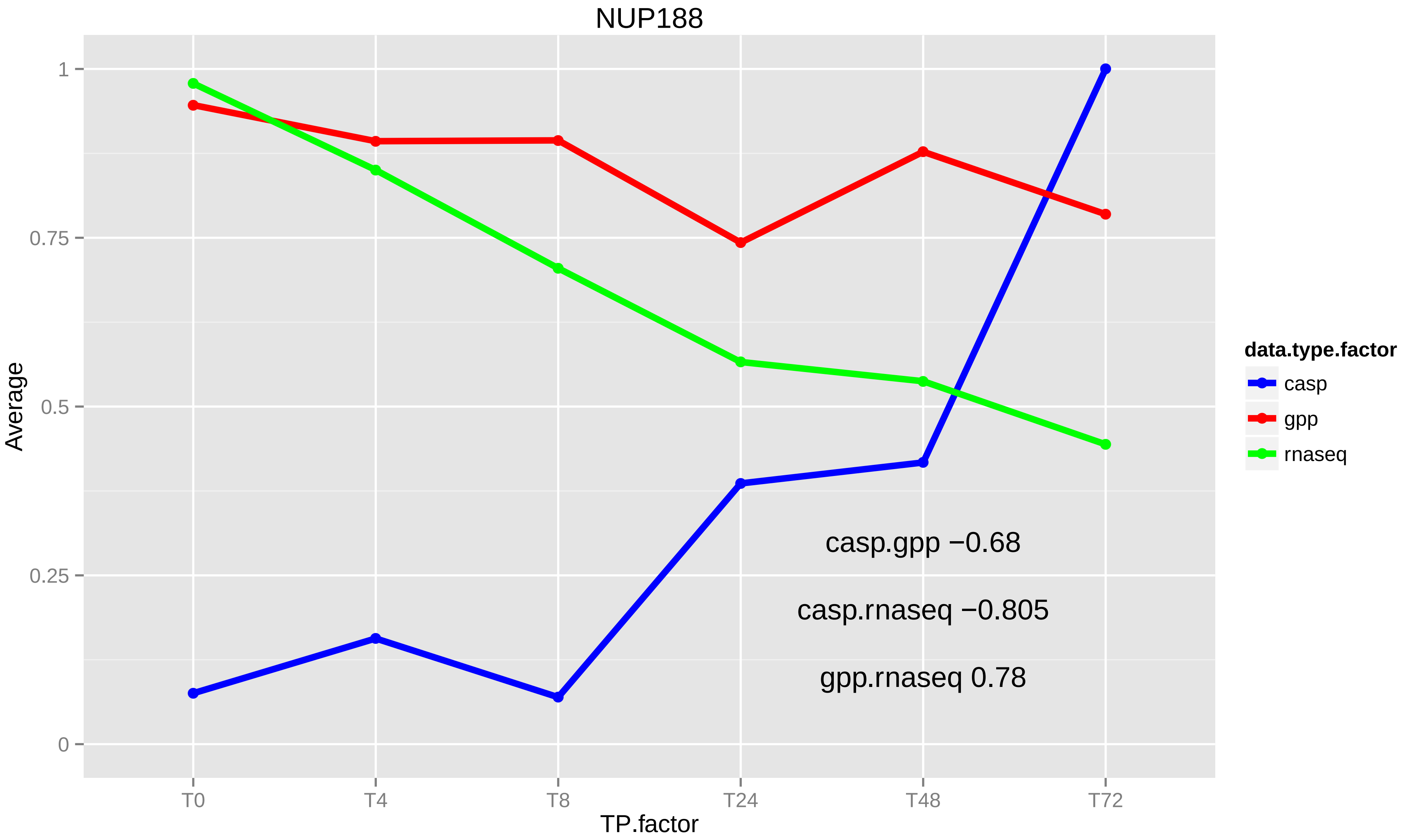

NUP188
Average
1
0.75
0.5
0.25
0
T0
T4
T8
T24
T48
T72
TP.factor
data.type.factor
casp
gpp
rnaseq
casp.gpp −0.68
casp.rnaseq −0.805
gpp.rnaseq 0.78

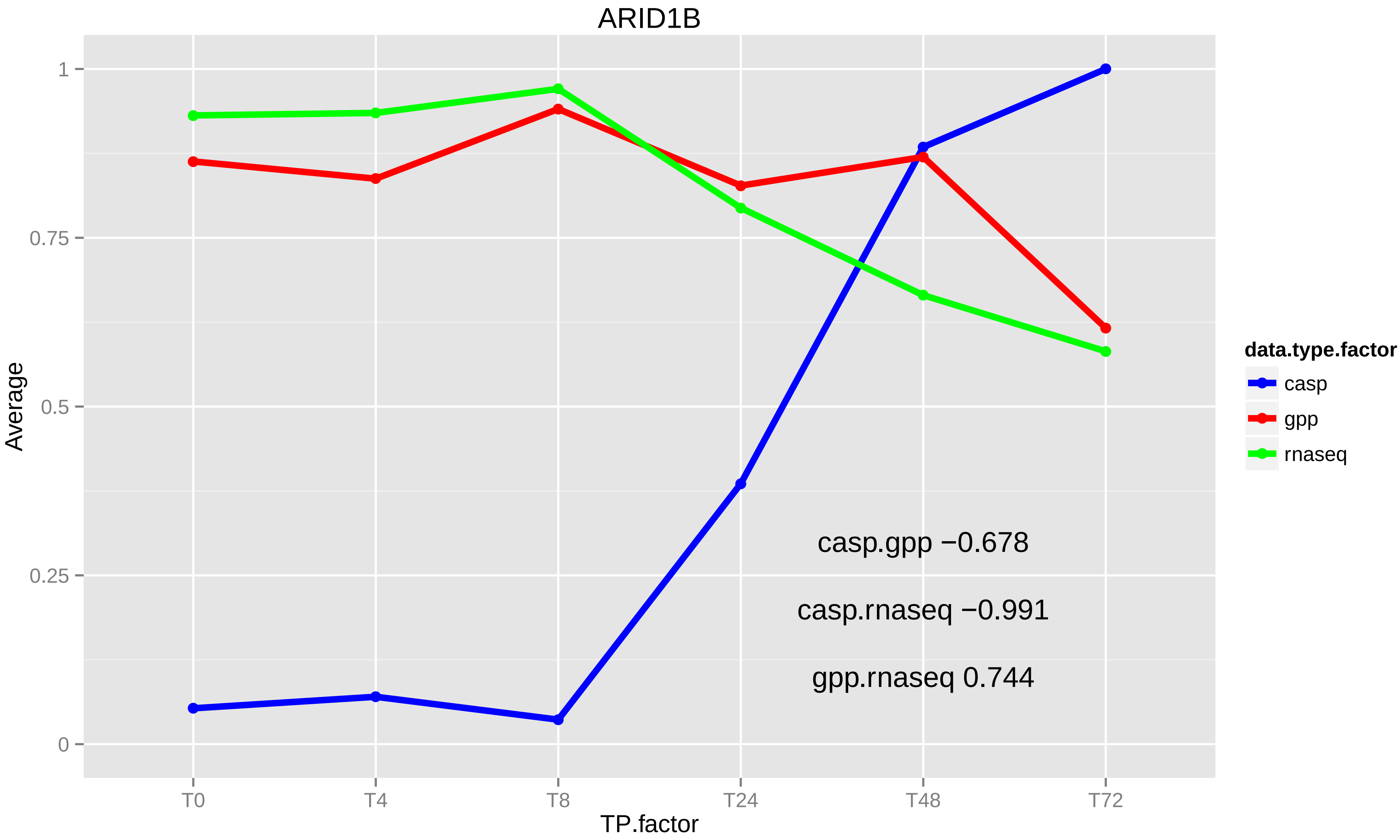

ARID1B
1
0.75
0.5
0.25
0
Average
T0
T4
T8
T24
T48
T72
TP.factor
data.type.factor
casp
gpp
rnaseq
casp.gpp −0.678
casp.rnaseq −0.991
gpp.rnaseq 0.744

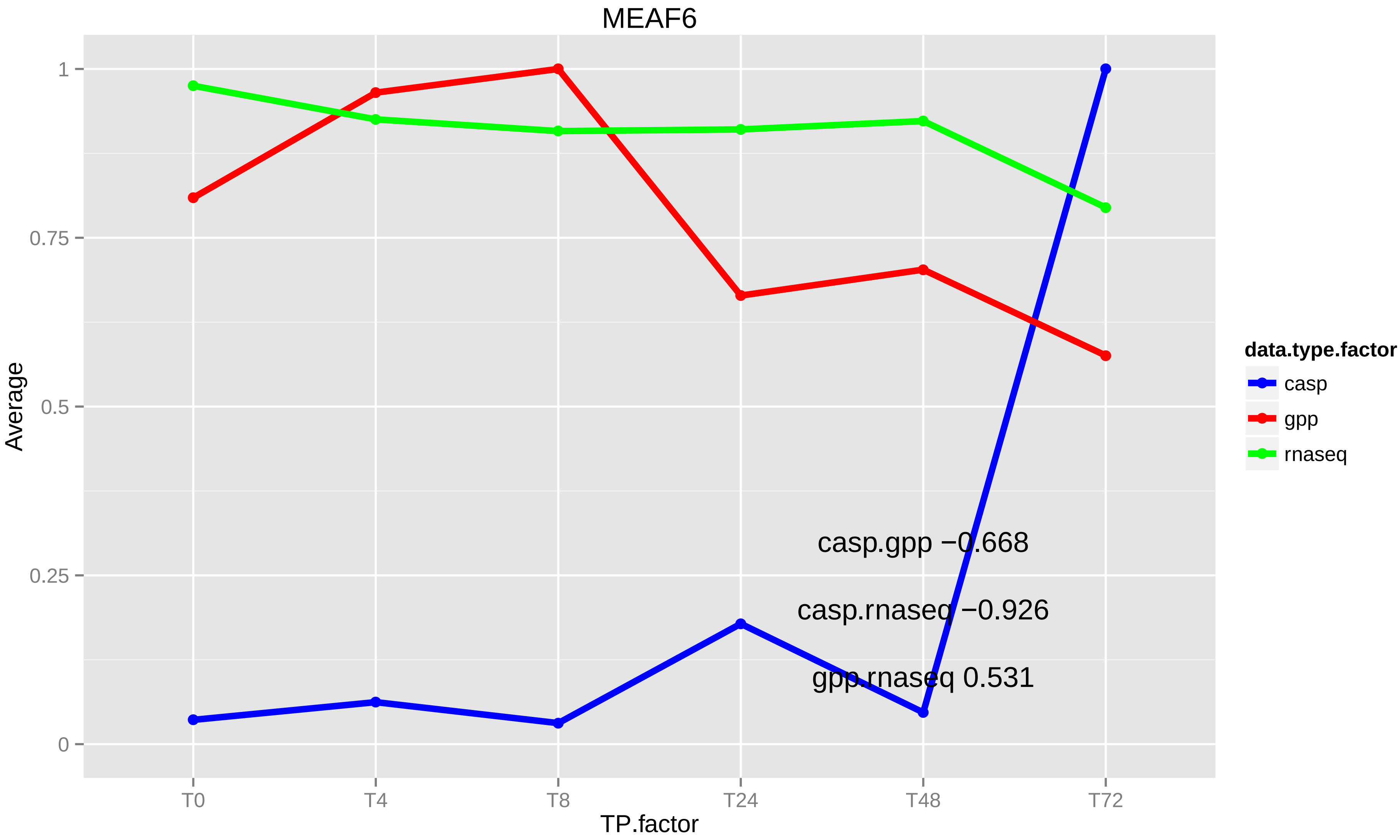
MEAF6
Average
1
0.75
0.5
0.25
0
T0
T4
T8
T24
T48
T72
TP.factor
data.type.factor
casp
gpp
rnaseq
casp.gpp −0.668
casp.rnaseq −0.926
gpp.rnaseq 0.531

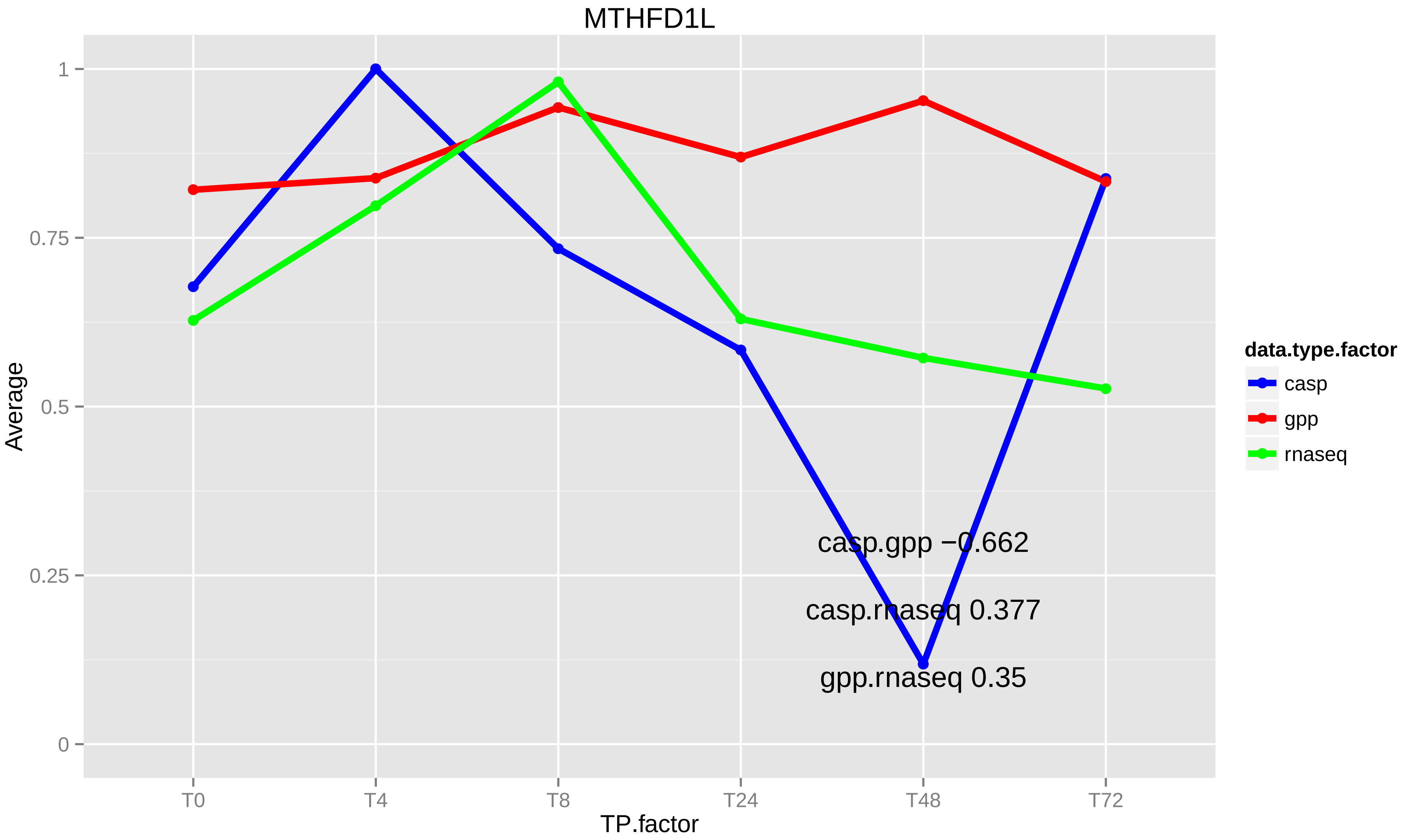
MTHFD1L
Average
1
0.75
0.5
0.25
0
TP.factor
T0
T4
T8
T24
T48
T72
data.type.factor
casp
gpp
rnaseq
casp.gpp −0.662
casp.rnaseq 0.377
gpp.rnaseq 0.35

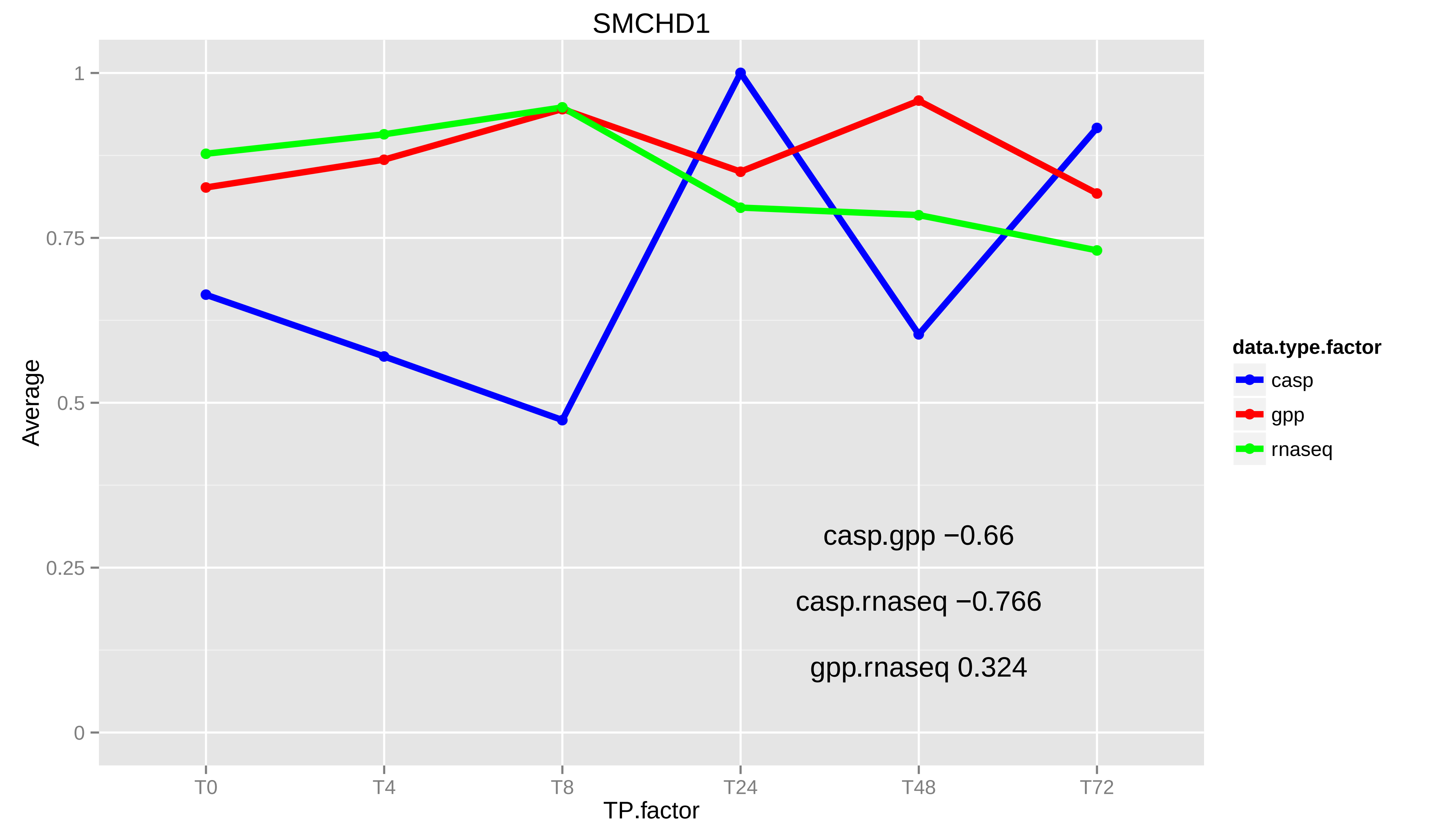

SMCHD1
1
0.75
0.5
0.25
0
Average
T0
T4
T8
T24
T48
T72
TP.factor
data.type.factor
casp
gpp
rnaseq
casp.gpp −0.66
casp.rnaseq −0.766
gpp.rnaseq 0.324

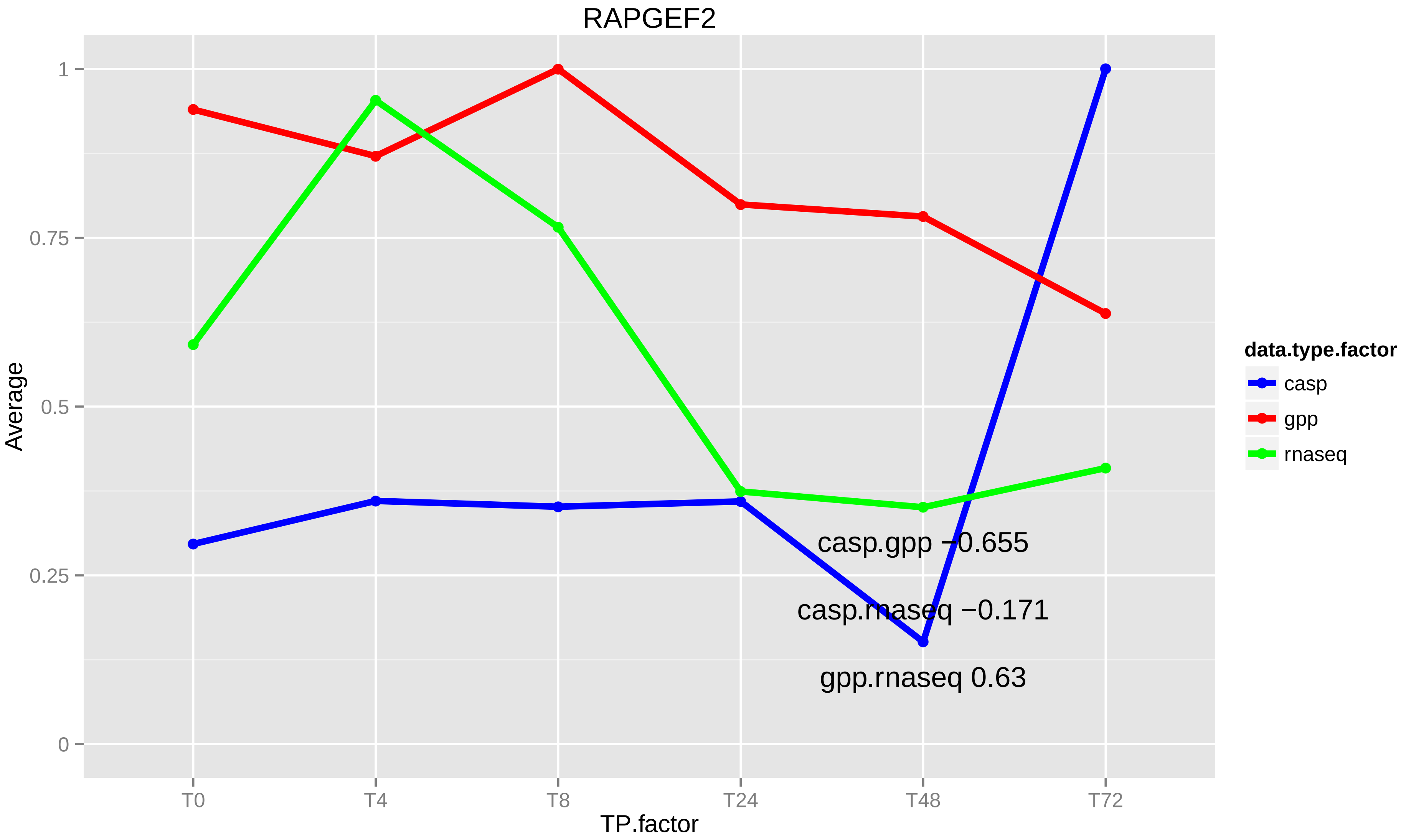
RAPGEF2
Average
1
0.75
0.5
0.25
0
T0
T4
T8
T24
T48
T72
TP.factor
data.type.factor
casp
gpp
rnaseq
casp.gpp −0.655
casp.rnaseq −0.171
gpp.rnaseq 0.63

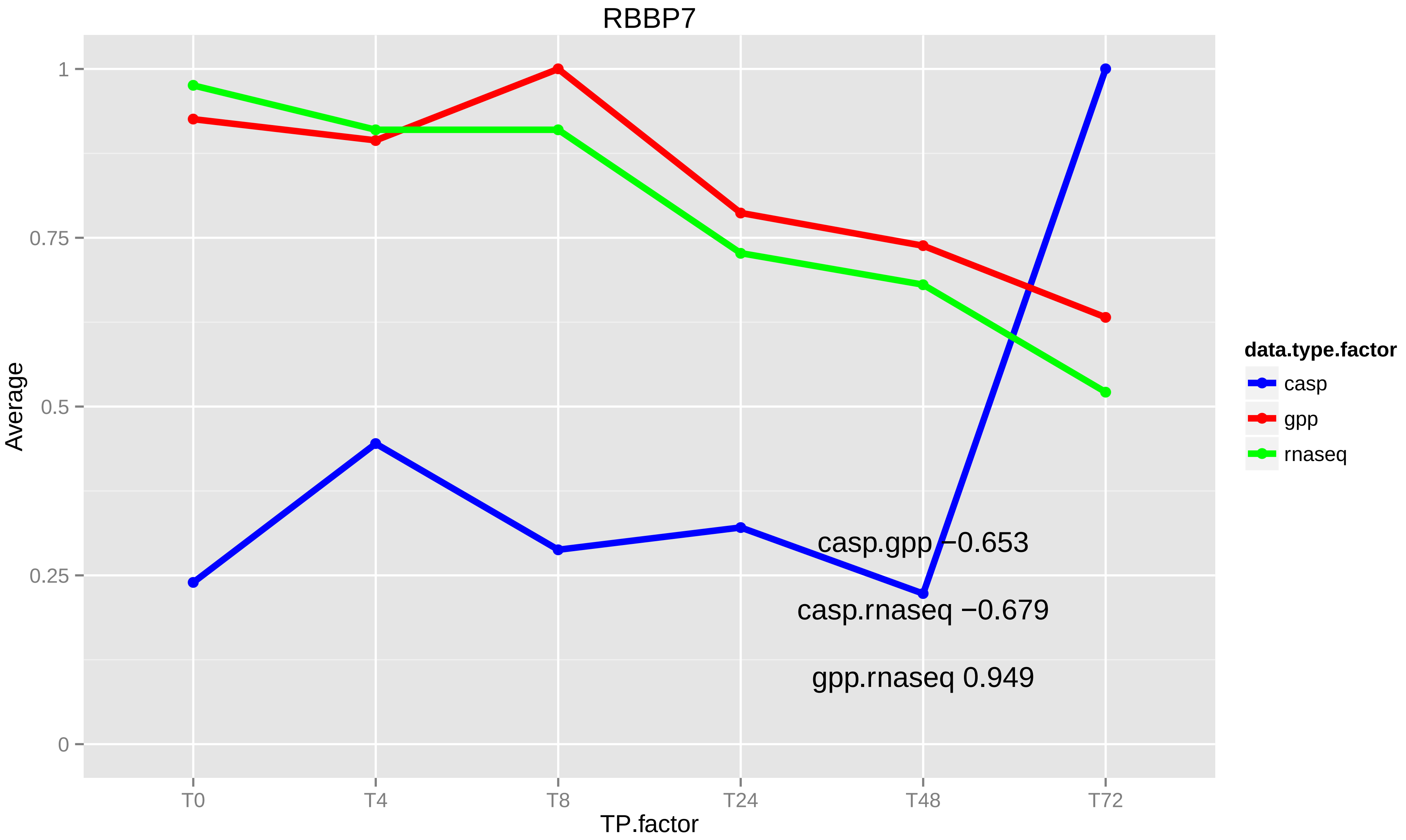

RBBP7
Average
1
0.75
0.5
0.25
0
T0
T4
T8
T24
T48
T72
TP.factor
data.type.factor
casp
gpp
rnaseq
casp.gpp −0.653
casp.rnaseq −0.679
gpp.rnaseq 0.949

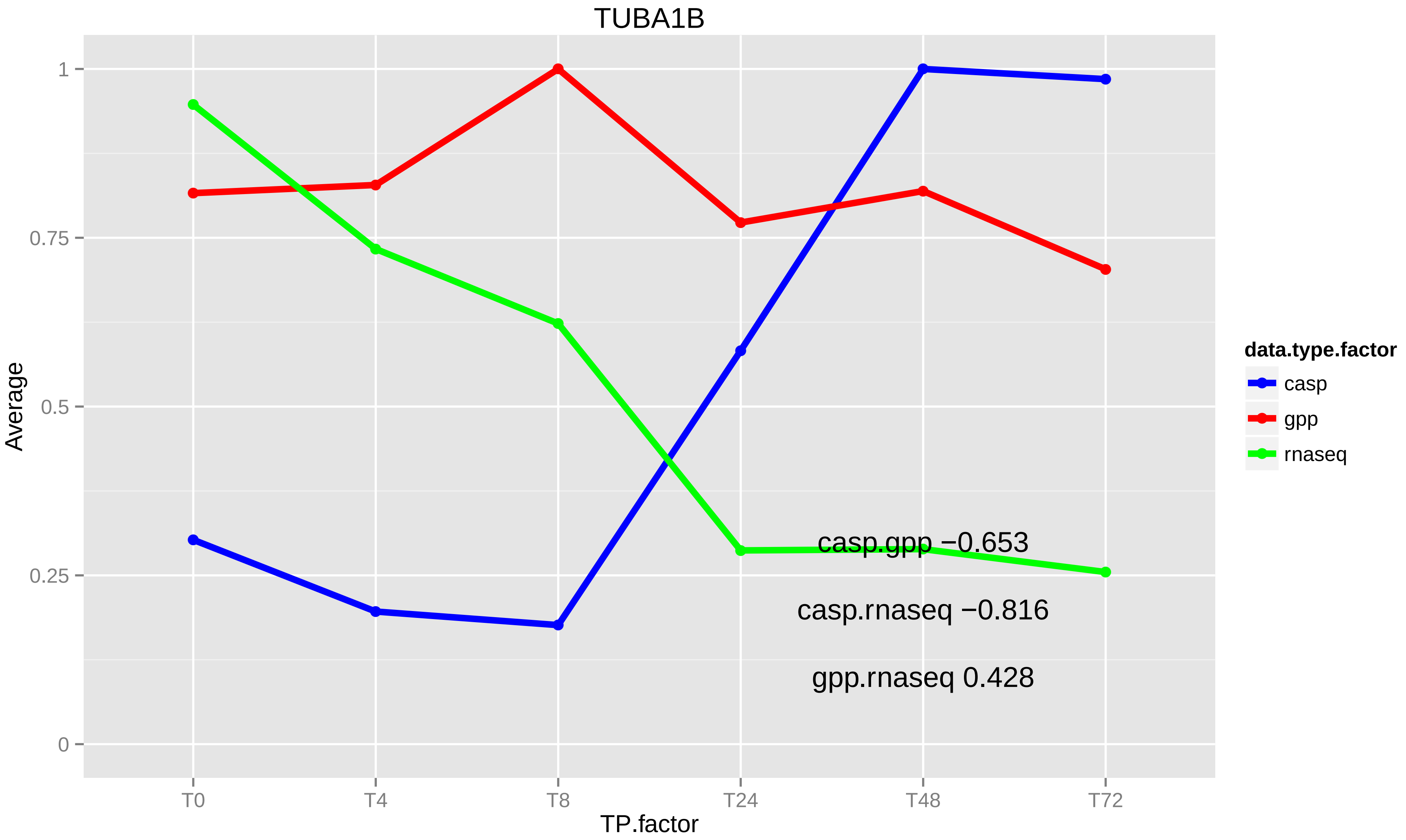
TUBA1B
Average
1
0.75
0.5
0.25
0
T0
T4
T8
T24
T48
T72
TP.factor
data.type.factor
casp
gpp
rnaseq
casp.gpp −0.653
casp.rnaseq −0.816
gpp.rnaseq 0.428

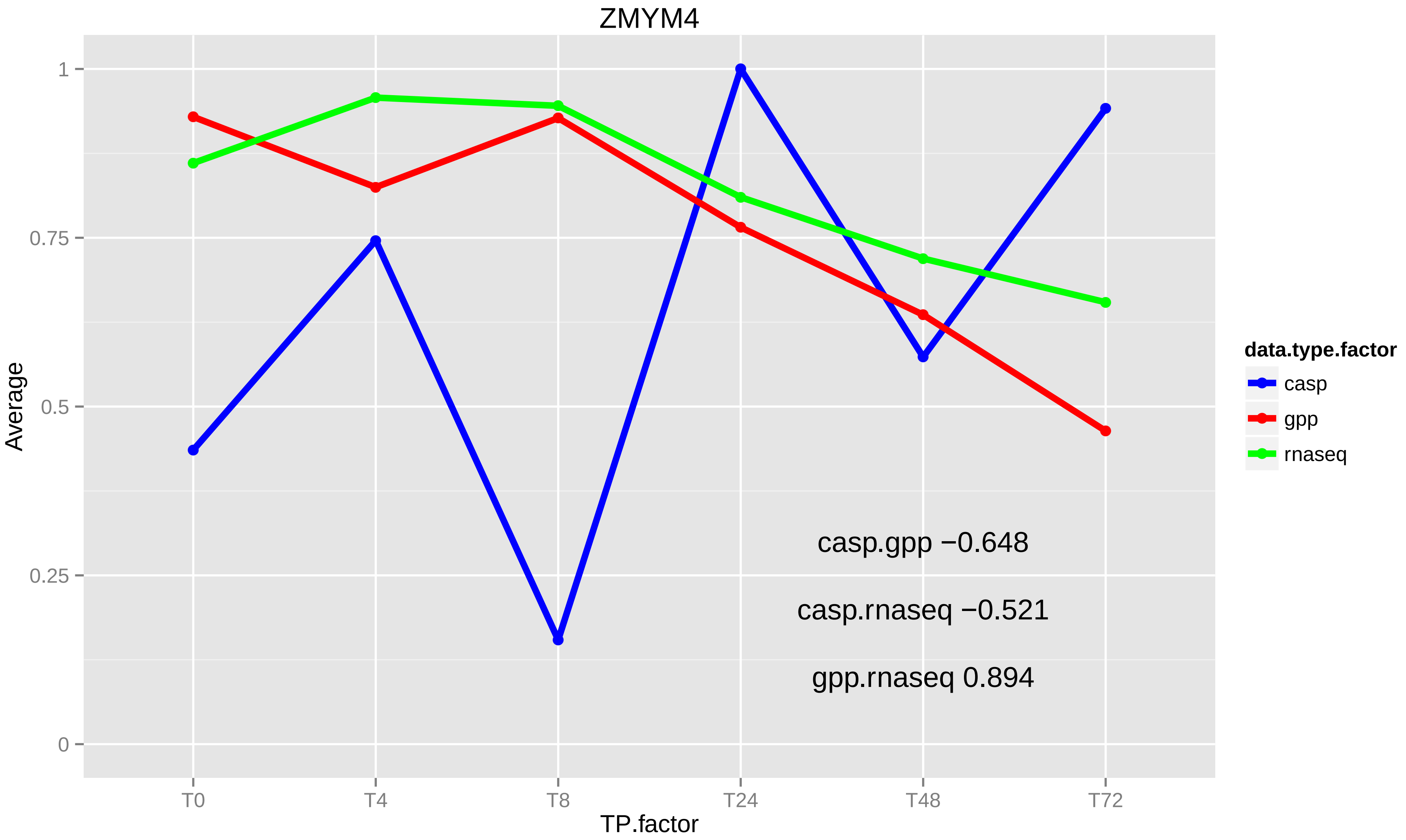

ZMYM4
Average
1
0.75
0.5
0.25
0
T0
T4
T8
T24
T48
T72
TP.factor
data.type.factor
casp
gpp
rnaseq
casp.gpp −0.648
casp.rnaseq −0.521
gpp.rnaseq 0.894

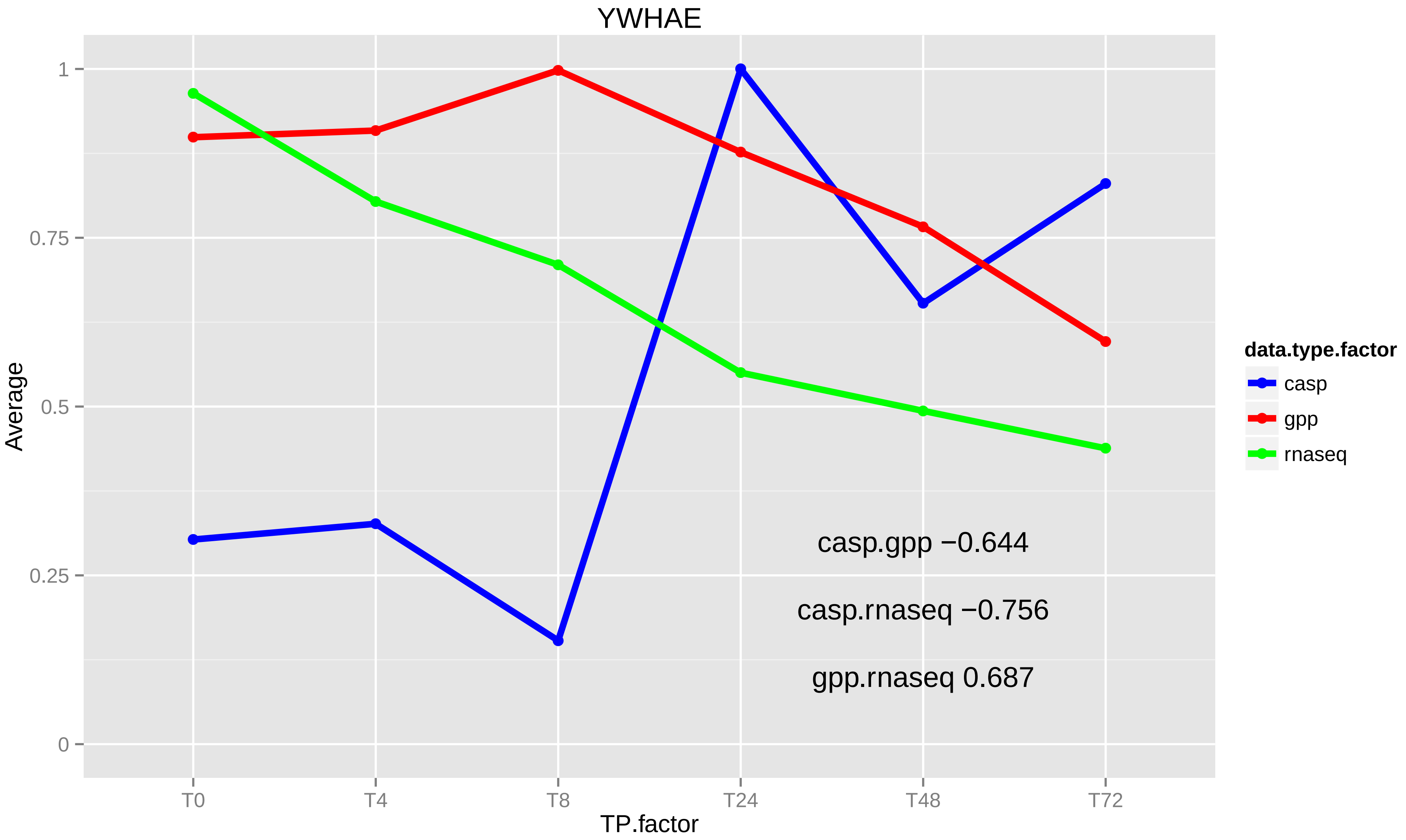
YWHAE
Average
1
0.75
0.5
0.25
0
T0
T4
T8
T24
T48
T72
TP.factor
data.type.factor
casp
gpp
rnaseq
casp.gpp −0.644
casp.rnaseq −0.756
gpp.rnaseq 0.687

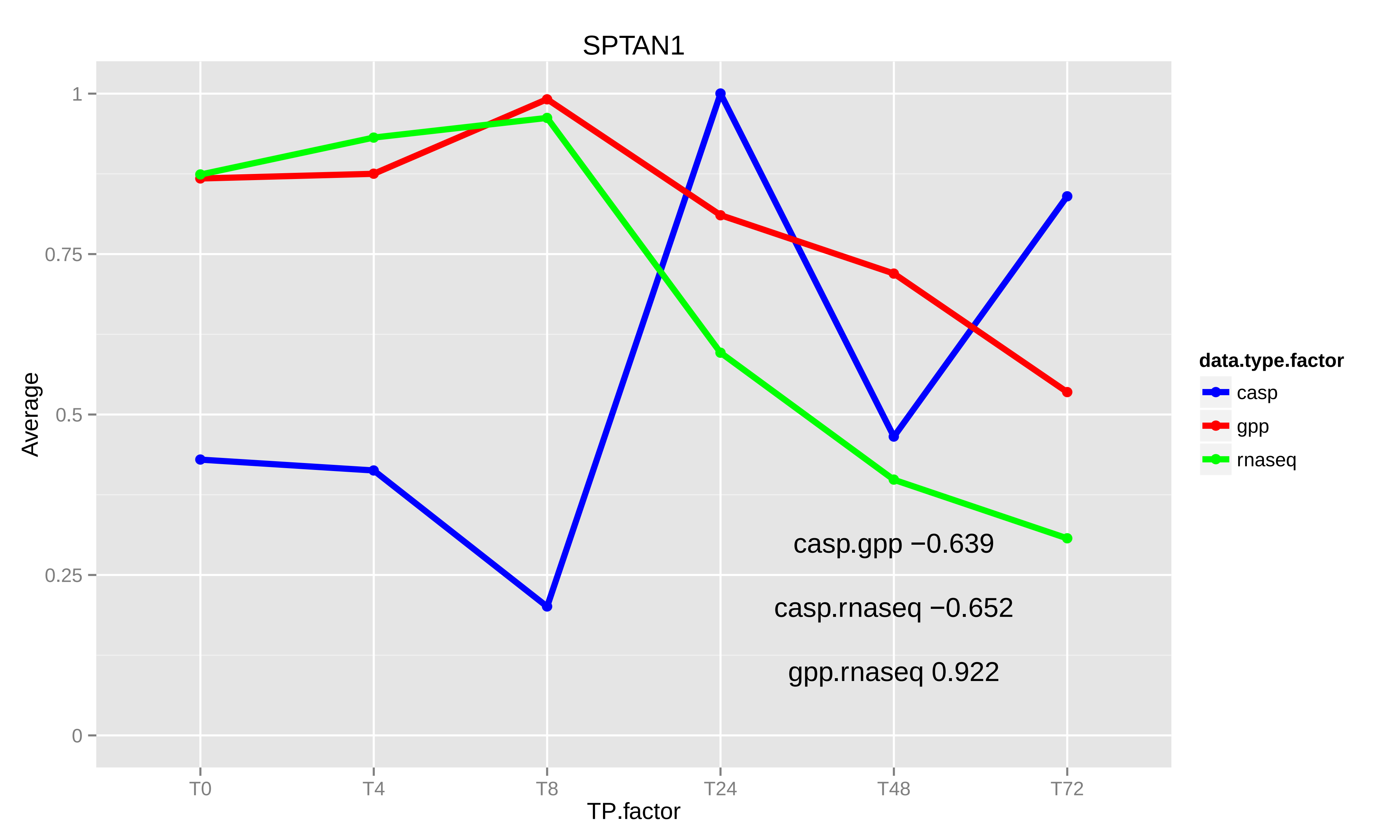
SPTAN1
Average
1
0.75
0.5
0.25
0
T0
T4
T8
T24
T48
T72
TP.factor
data.type.factor
casp
gpp
rnaseq
casp.gpp −0.639
casp.rnaseq −0.652
gpp.rnaseq 0.922

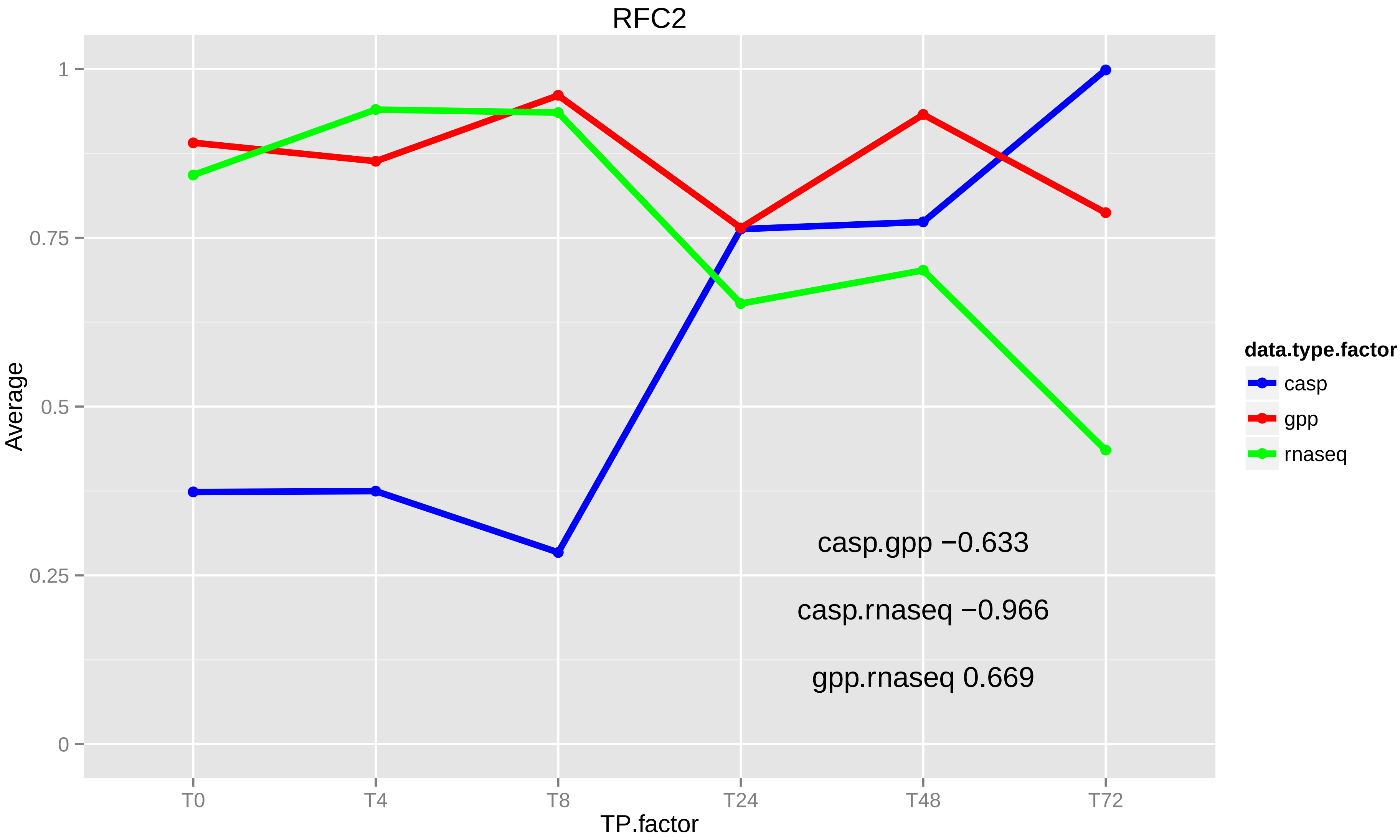
RFC2
1
0.75
0.5
0.25
0
Average
T0
T4
T8
T24
T48
T72
TP.factor
data.type.factor
casp
gpp
rnaseq
casp.gpp −0.633
casp.rnaseq −0.966
gpp.rnaseq 0.669

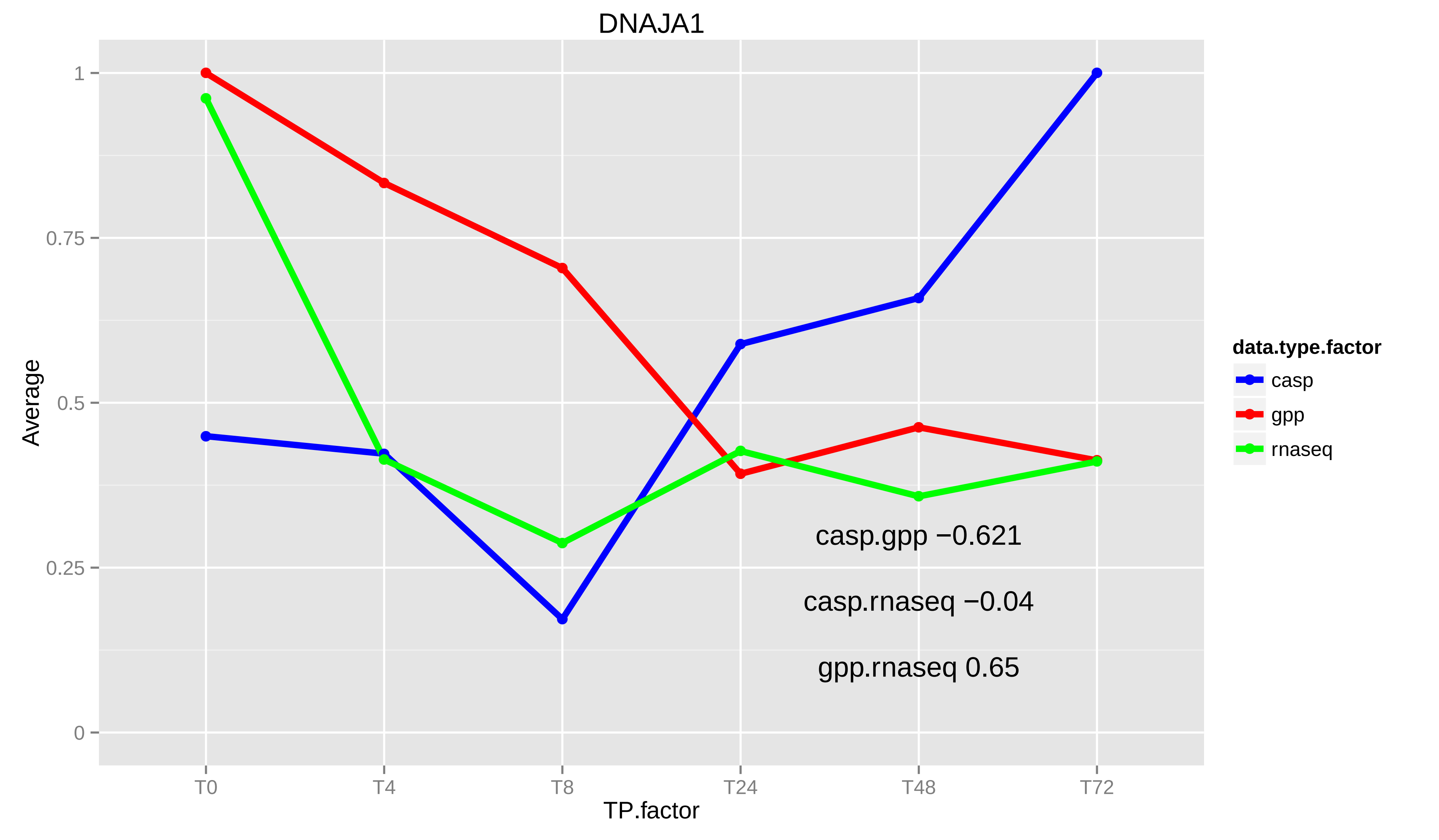
DNAJA1
1
0.75
0.5
0.25
0
Average
T0
T4
T8
T24
T48
T72
TP.factor
data.type.factor
casp
gpp
rnaseq
casp.gpp −0.621
casp.rnaseq −0.04
gpp.rnaseq 0.65

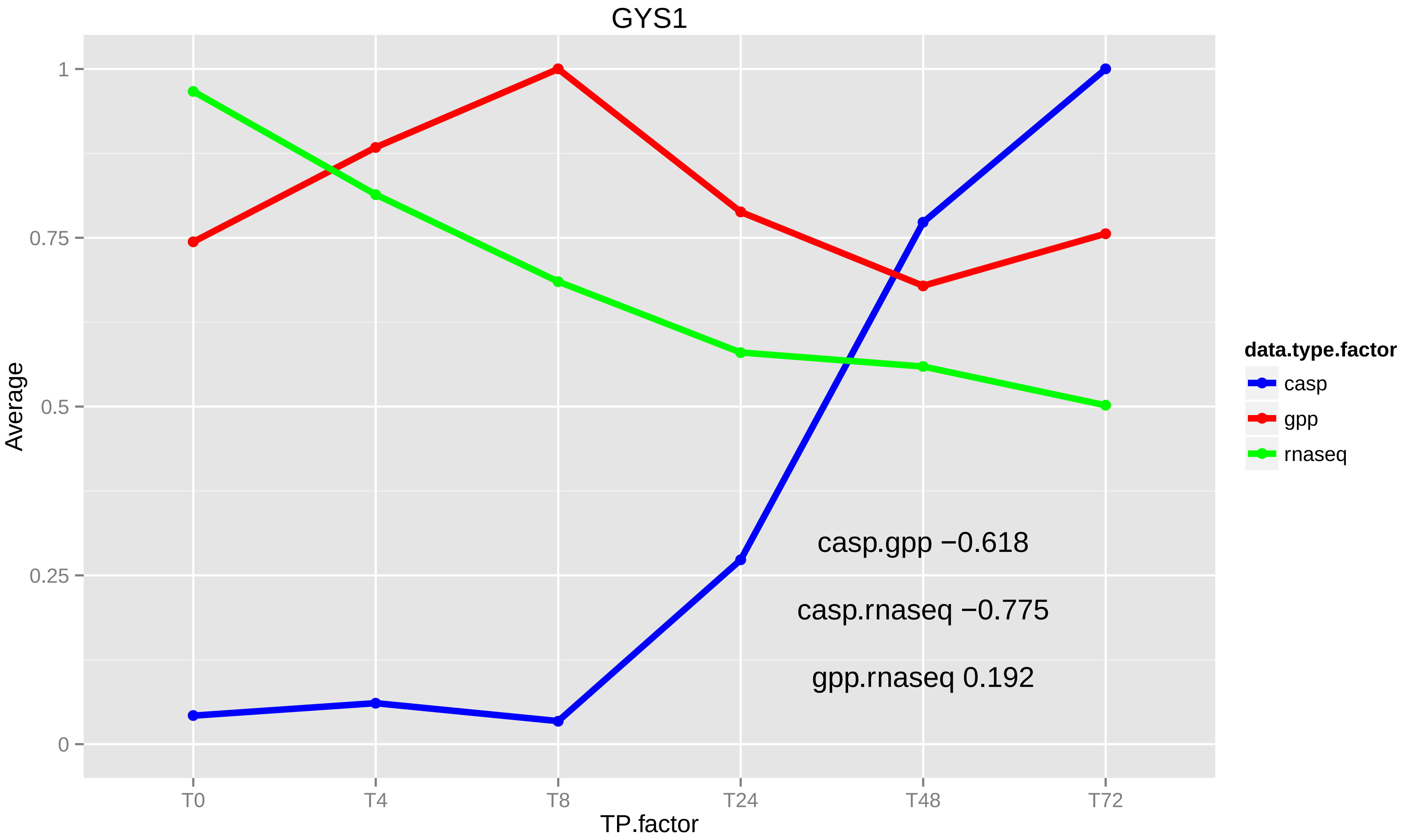

GYS1
Average
1
0.75
0.5
0.25
0
T0
T4
T8
T24
T48
T72
TP.factor
data.type.factor
casp
gpp
rnaseq
casp.gpp −0.618
casp.rnaseq −0.775
gpp.rnaseq 0.192

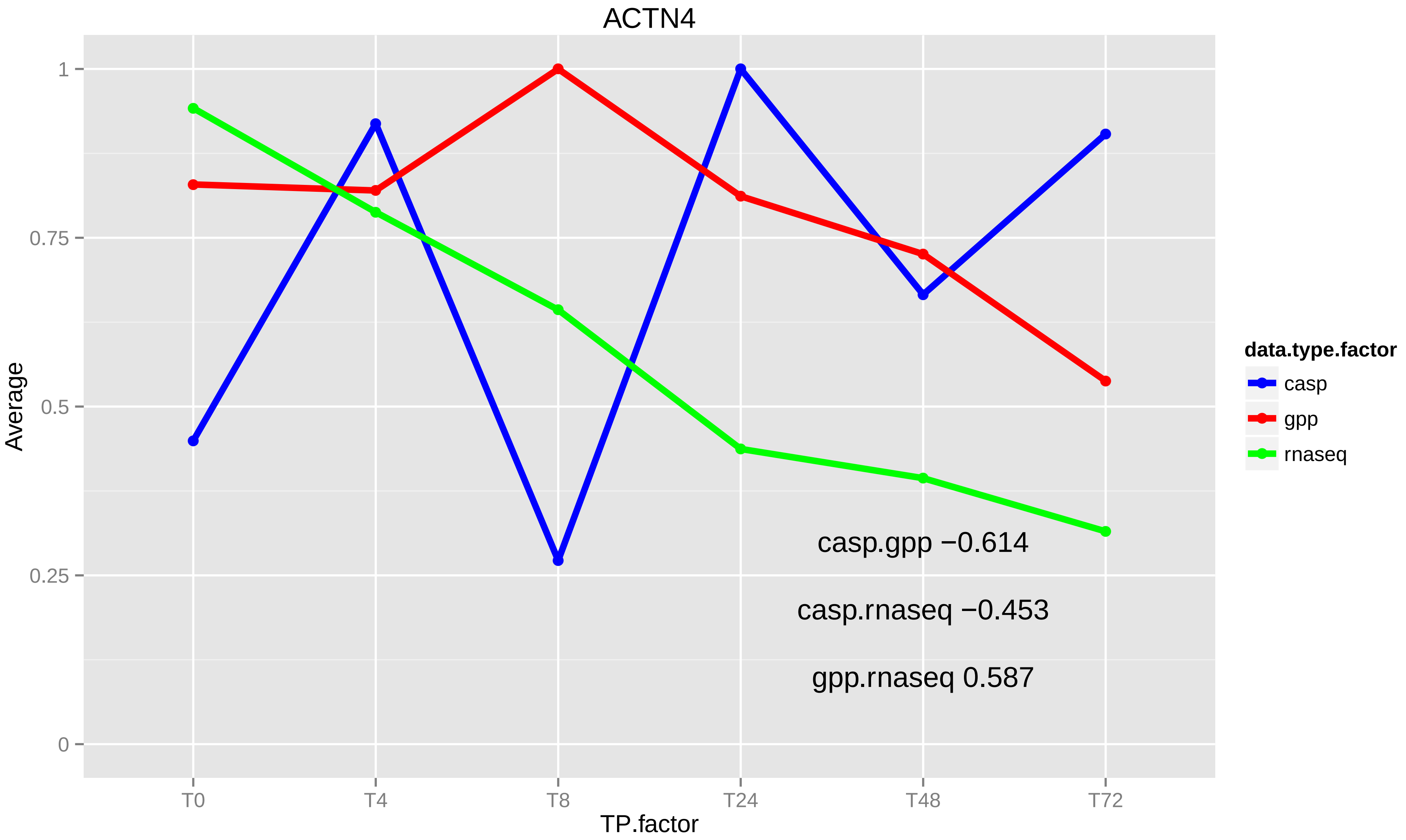
ACTN4
1
0.75
0.5
0.25
0
Average
T0
T4
T8
T24
T48
T72
TP.factor
data.type.factor
casp
gpp
rnaseq
casp.gpp −0.614
casp.rnaseq −0.453
gpp.rnaseq 0.587

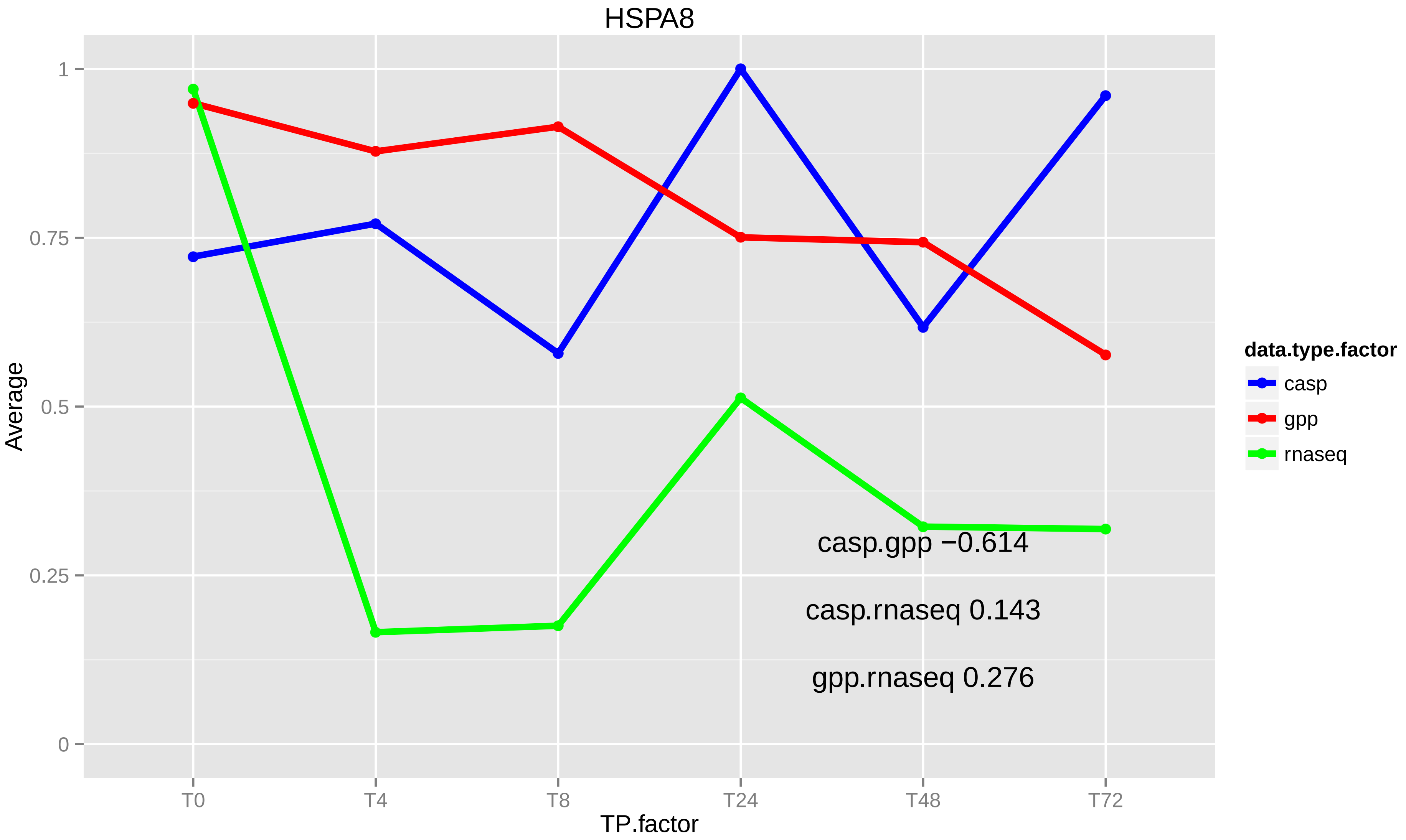

HSPA8
Average
1
0.75
0.5
0.25
0
T0
T4
T8
T24
T48
T72
TP.factor
data.type.factor
casp
gpp
rnaseq
casp.gpp −0.614
casp.rnaseq 0.143
gpp.rnaseq 0.276

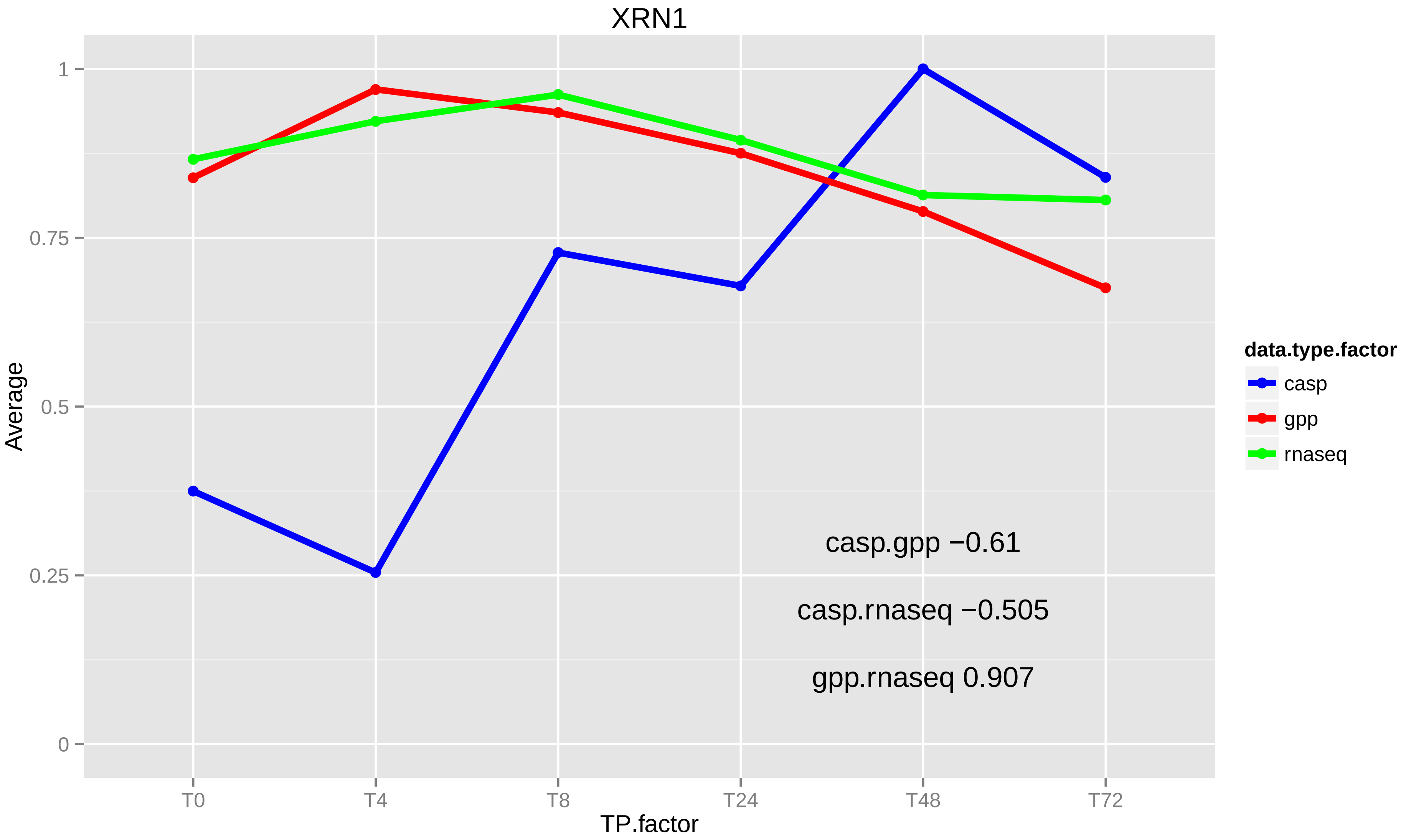
XRN1
Average
1
0.75
0.5
0.25
0
T0
T4
T8
T24
T48
T72
TP.factor
data.type.factor
casp
gpp
rnaseq
casp.gpp −0.61
casp.rnaseq −0.505
gpp.rnaseq 0.907

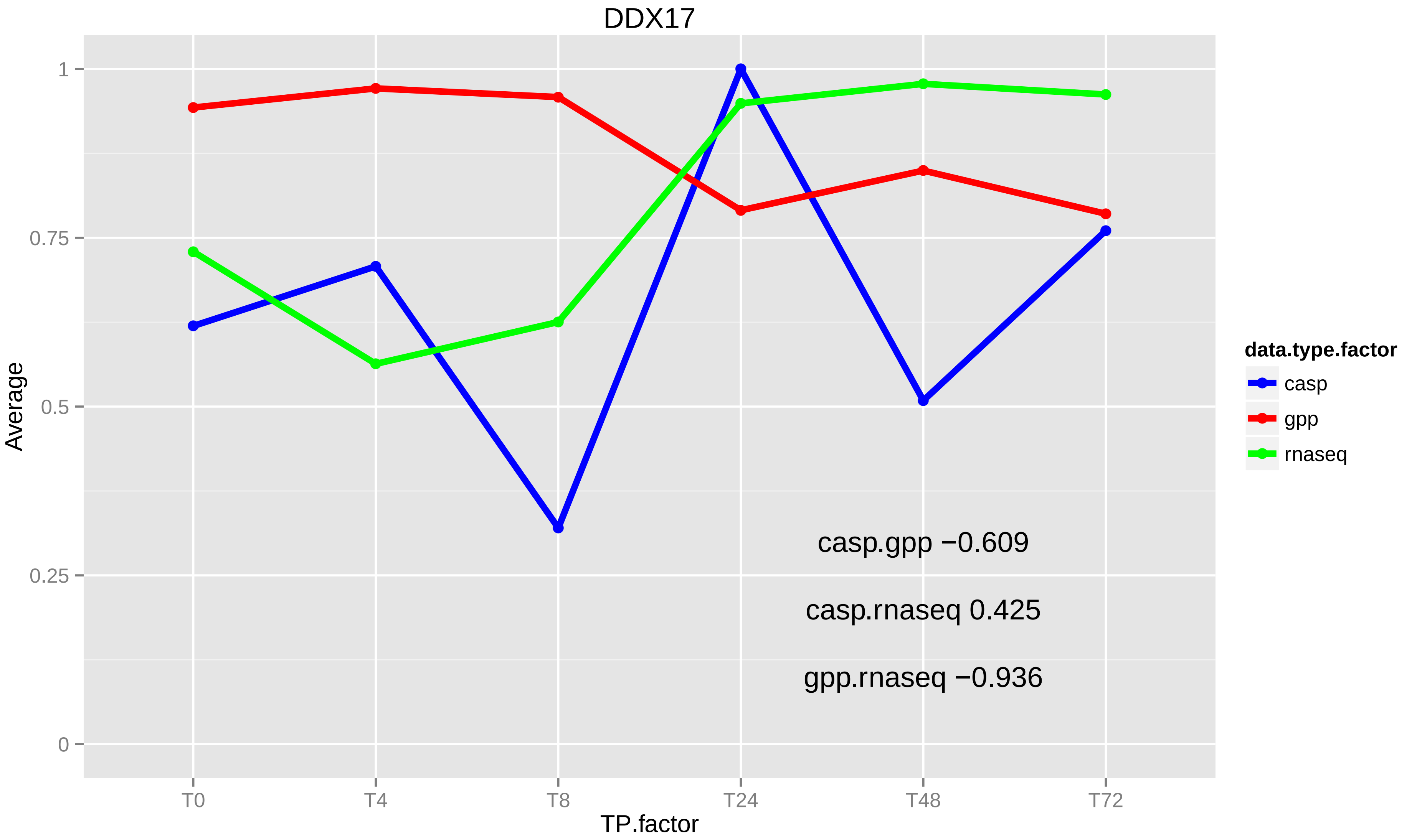

DDX17
1
0.75
0.5
0.25
0
Average
T0
T4
T8
T24
T48
T72
TP.factor
data.type.factor
casp
gpp
rnaseq
casp.gpp −0.609
casp.rnaseq 0.425
gpp.rnaseq −0.936

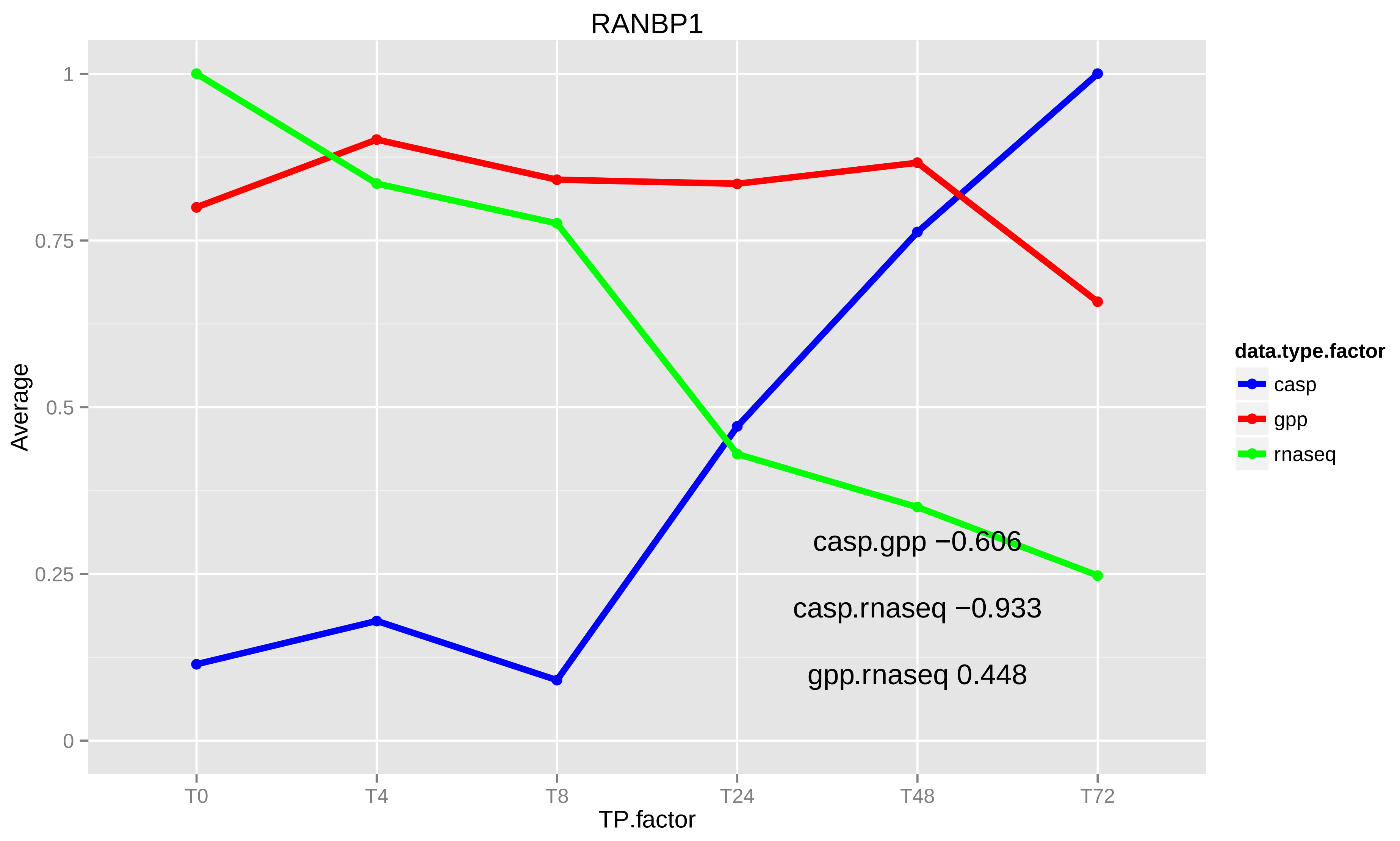

RANBP1
Average
1
0.75
0.5
0.25
0
T0
T4
T8
T24
T48
T72
TP.factor
data.type.factor
casp
gpp
rnaseq
casp.gpp −0.606
casp.rnaseq −0.933
gpp.rnaseq 0.448

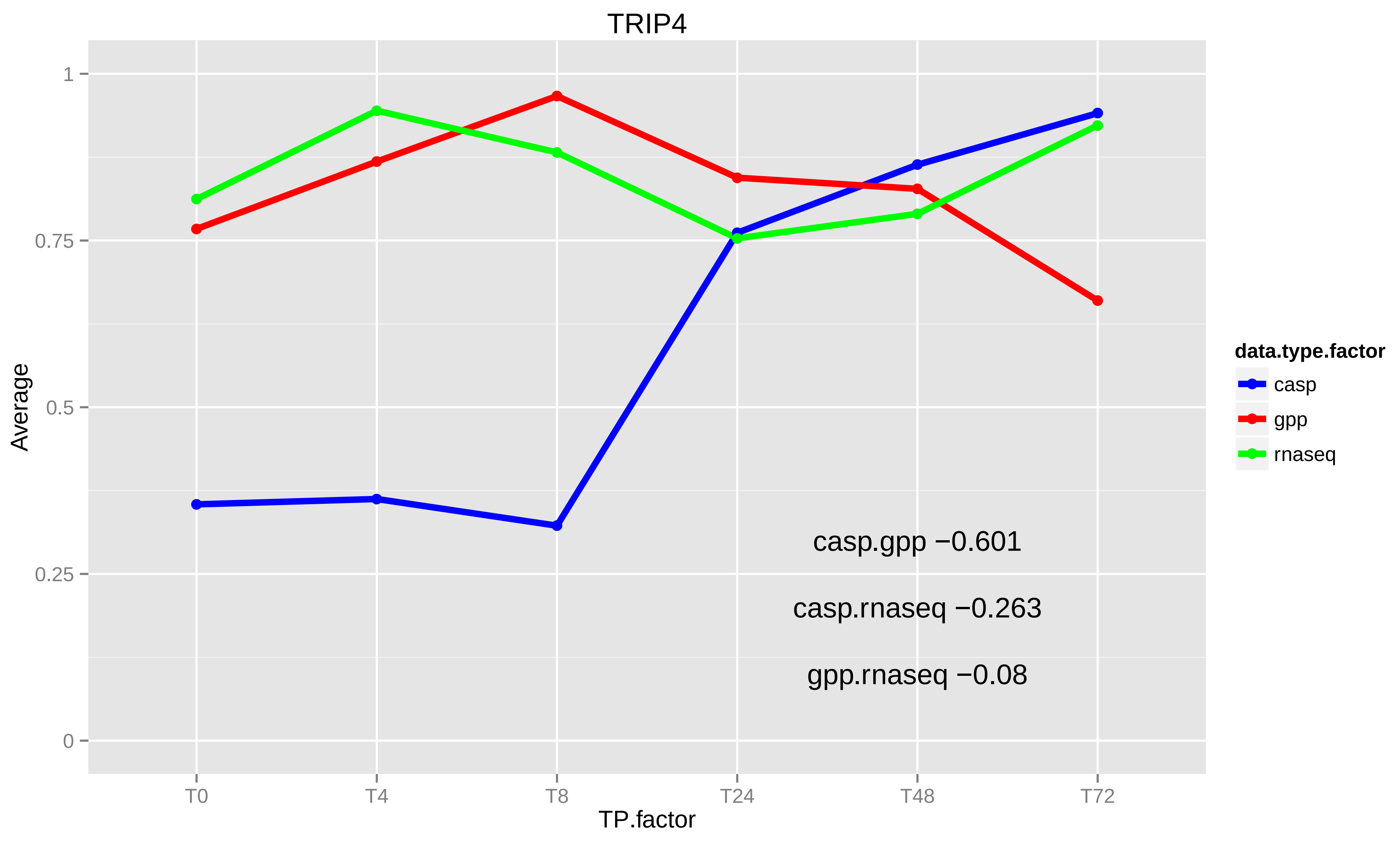

TRIP4
1
0.75
0.5
0.25
0
Average
T0
T4
T8
T24
T48
T72
TP.factor
data.type.factor
casp
gpp
rnaseq
casp.gpp −0.601
casp.rnaseq −0.263
gpp.rnaseq −0.08

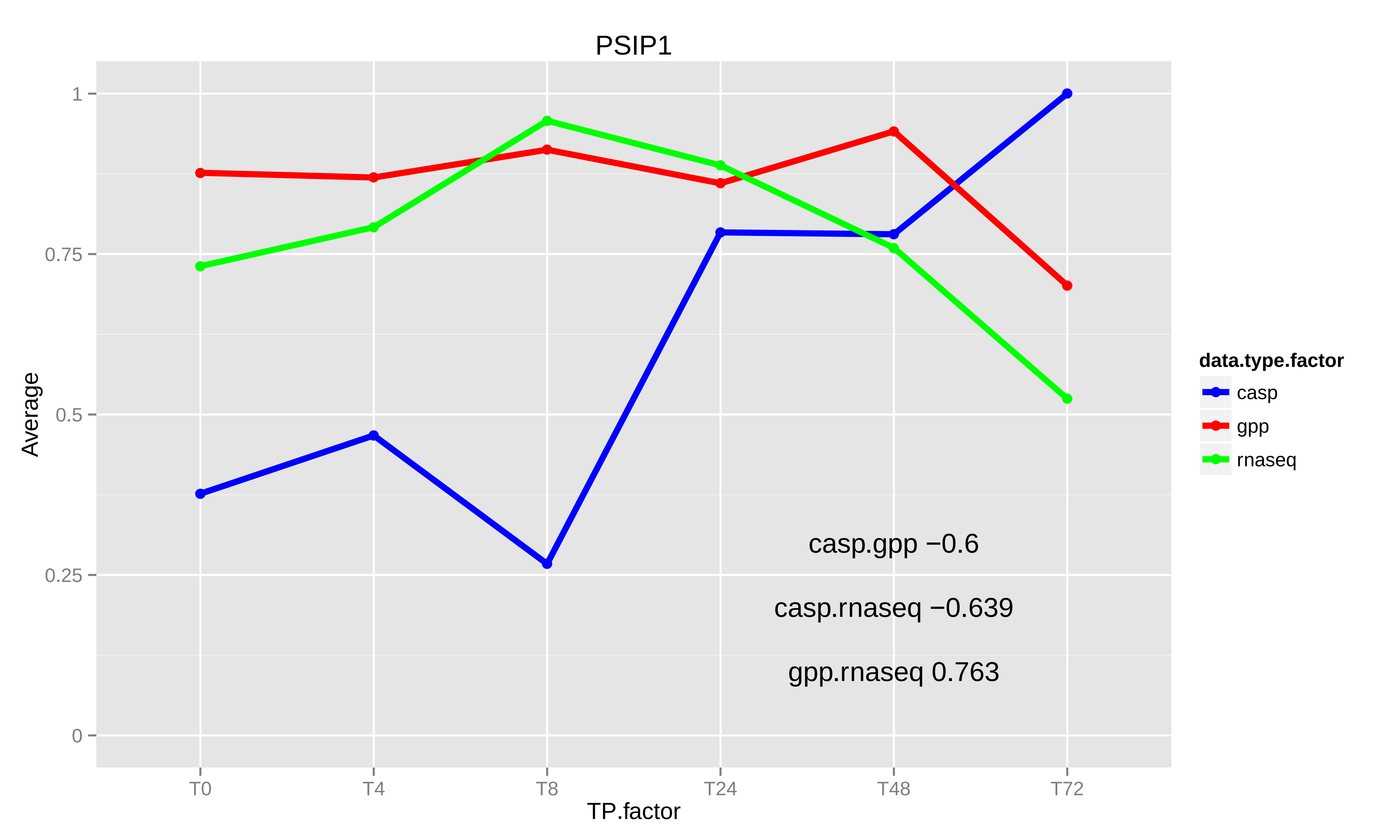

PSIP1
1
0.75
0.5
0.25
0
Average
T0
T4
T8
T24
T48
T72
TP.factor
data.type.factor
casp
gpp
rnaseq
casp.gpp −0.6
casp.rnaseq −0.639
gpp.rnaseq 0.763

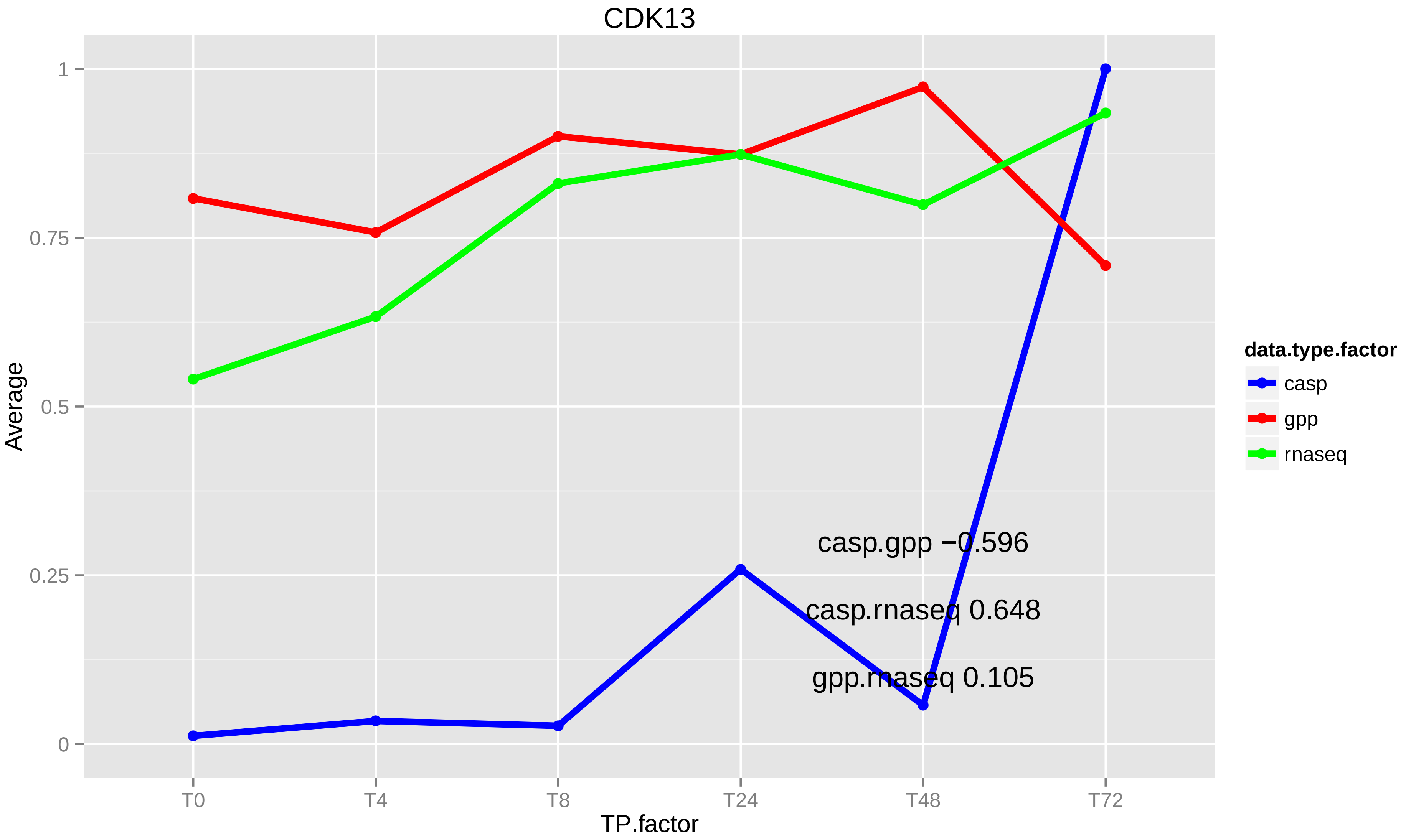
CDK13
Average
1
0.75
0.5
0.25
0
T0
T4
T8
T24
T48
T72
TP.factor
data.type.factor
casp
gpp
rnaseq
casp.gpp −0.596
casp.rnaseq 0.648
gpp.rnaseq 0.105

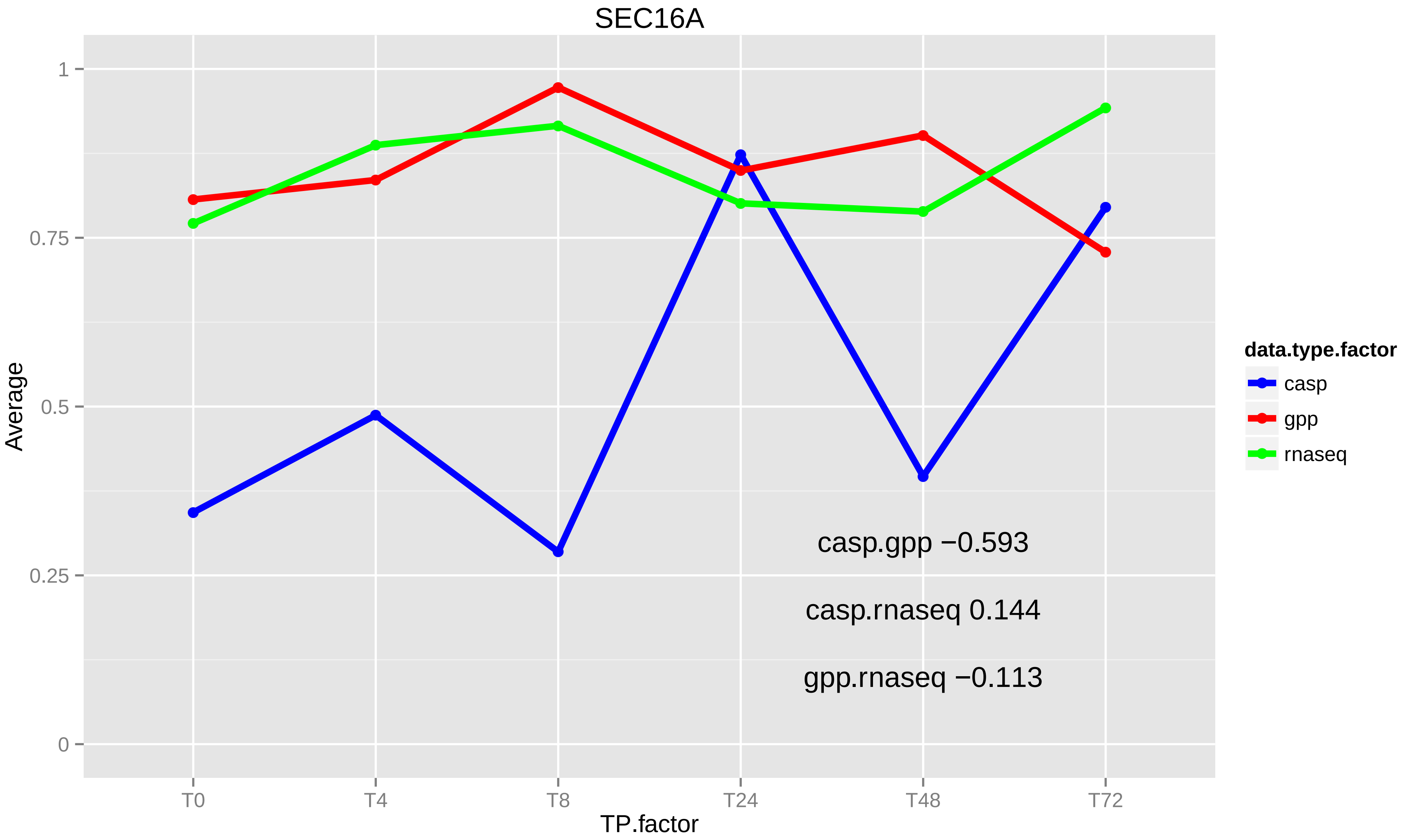
SEC16A
Average
1
0.75
0.5
0.25
0
T0
T4
T8
T24
T48
T72
TP.factor
data.type.factor
casp
gpp
rnaseq
casp.gpp −0.593
casp.rnaseq 0.144
gpp.rnaseq −0.113

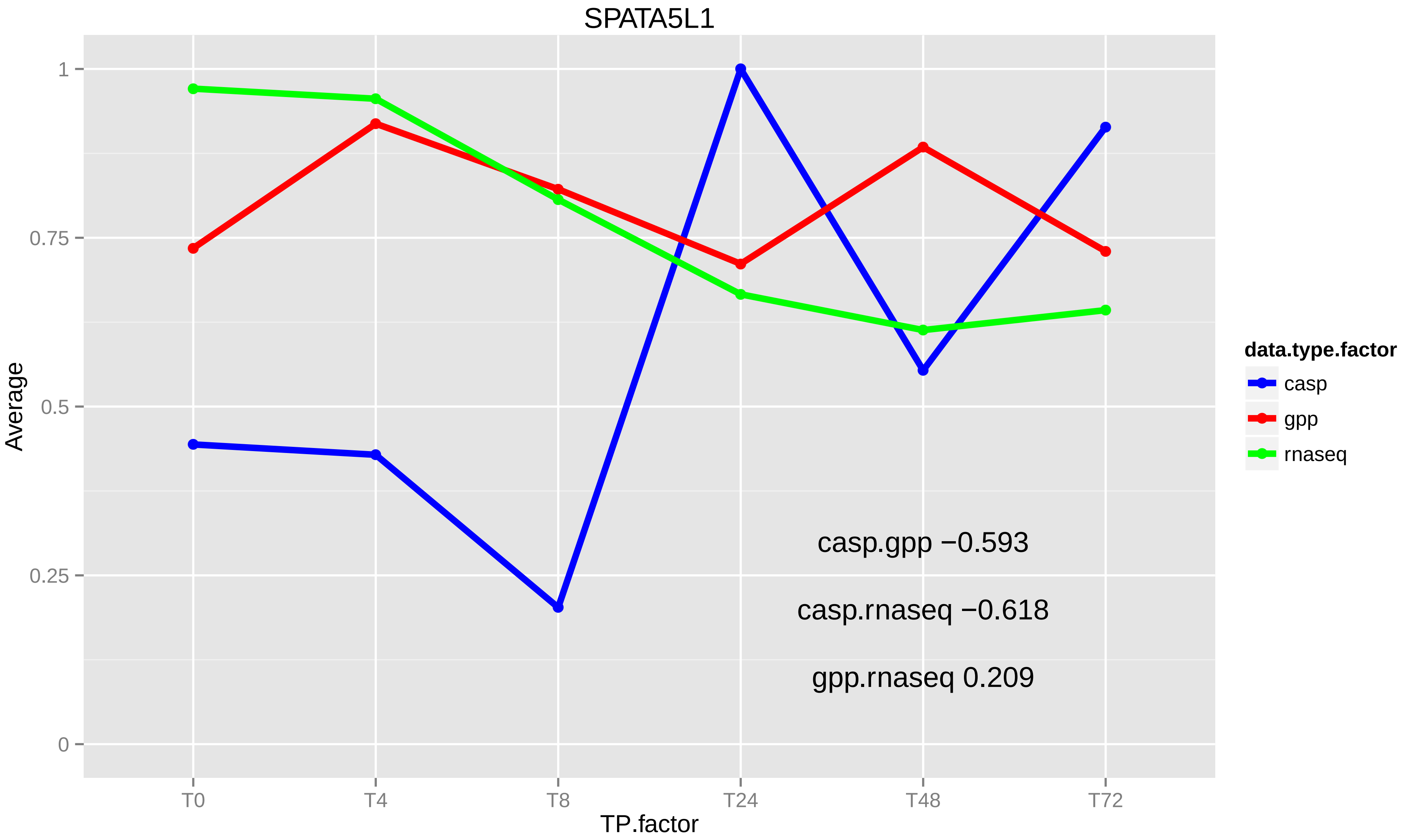
SPATA5L1
Average
1
0.75
0.5
0.25
0
T0
T4
T8
T24
T48
T72
TP.factor
data.type.factor
casp
gpp
rnaseq
casp.gpp −0.593
casp.rnaseq −0.618
gpp.rnaseq 0.209

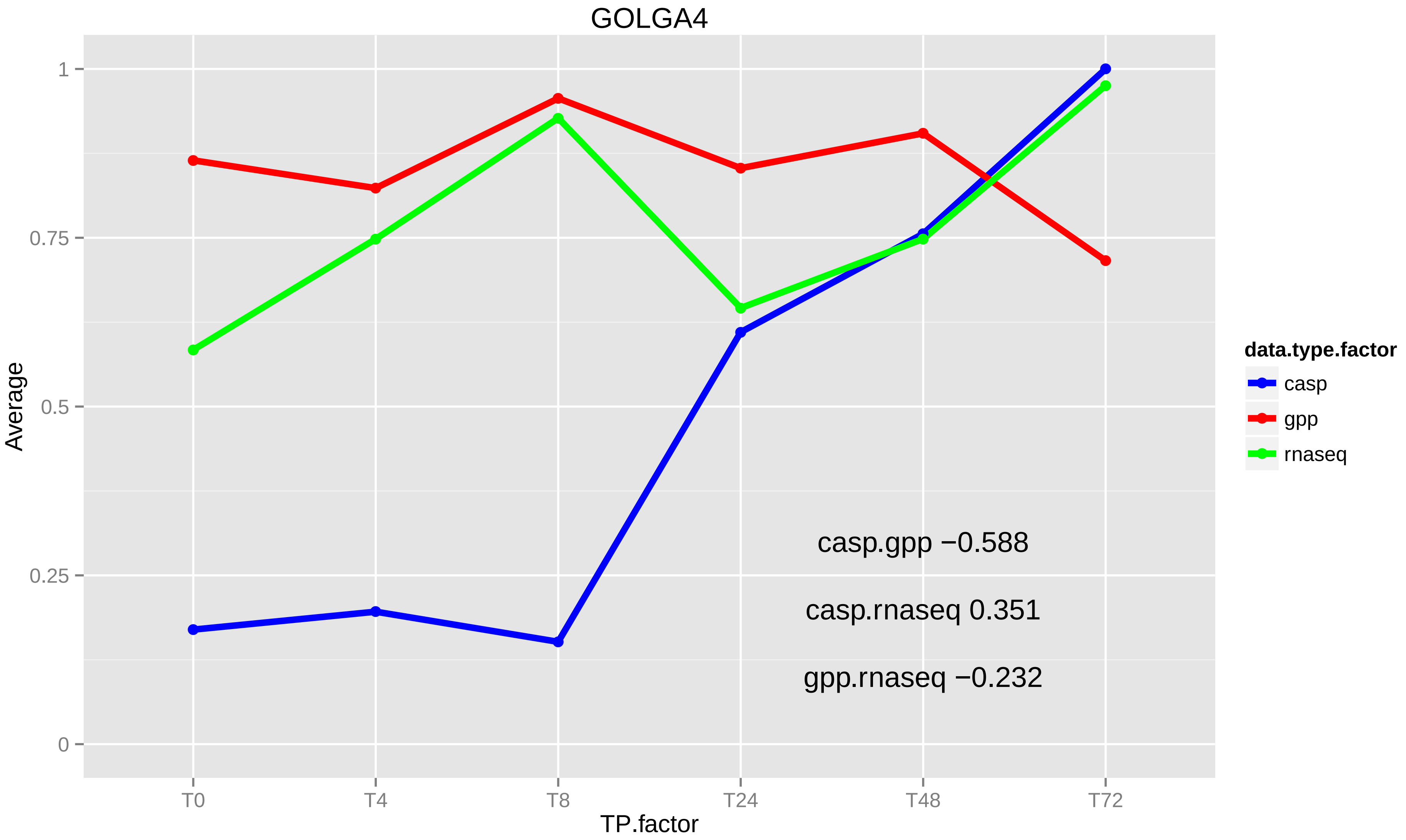

GOLGA4
Average
1
0.75
0.5
0.25
0
T0
T4
T8
T24
T48
T72
TP.factor
data.type.factor
casp
gpp
rnaseq
casp.gpp −0.588
casp.rnaseq 0.351
gpp.rnaseq −0.232

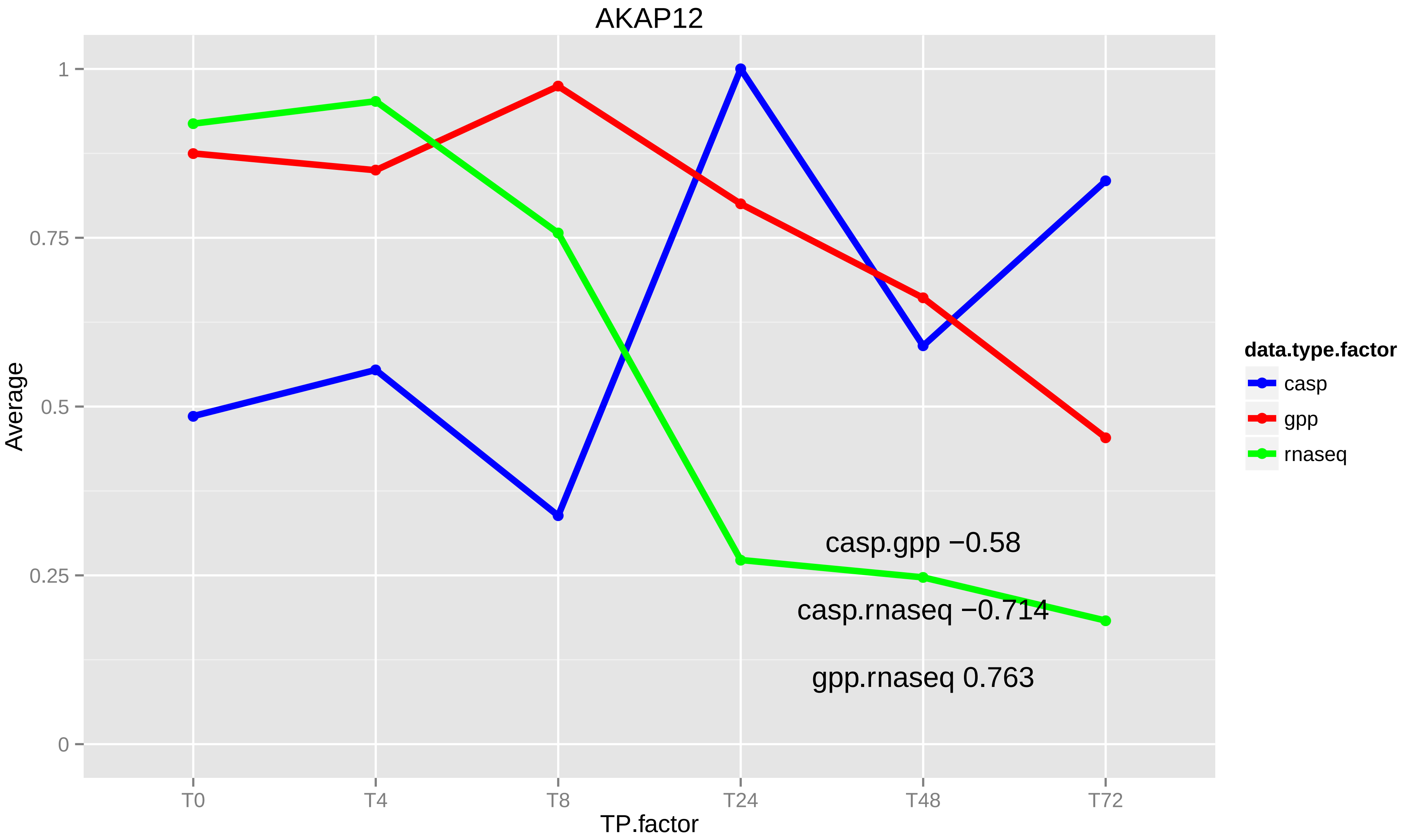
AKAP12
Average
1
0.75
0.5
0.25
0
T0
T4
T8
T24
T48
T72
TP.factor
data.type.factor
casp
gpp
rnaseq
casp.gpp −0.58
casp.rnaseq −0.714
gpp.rnaseq 0.763

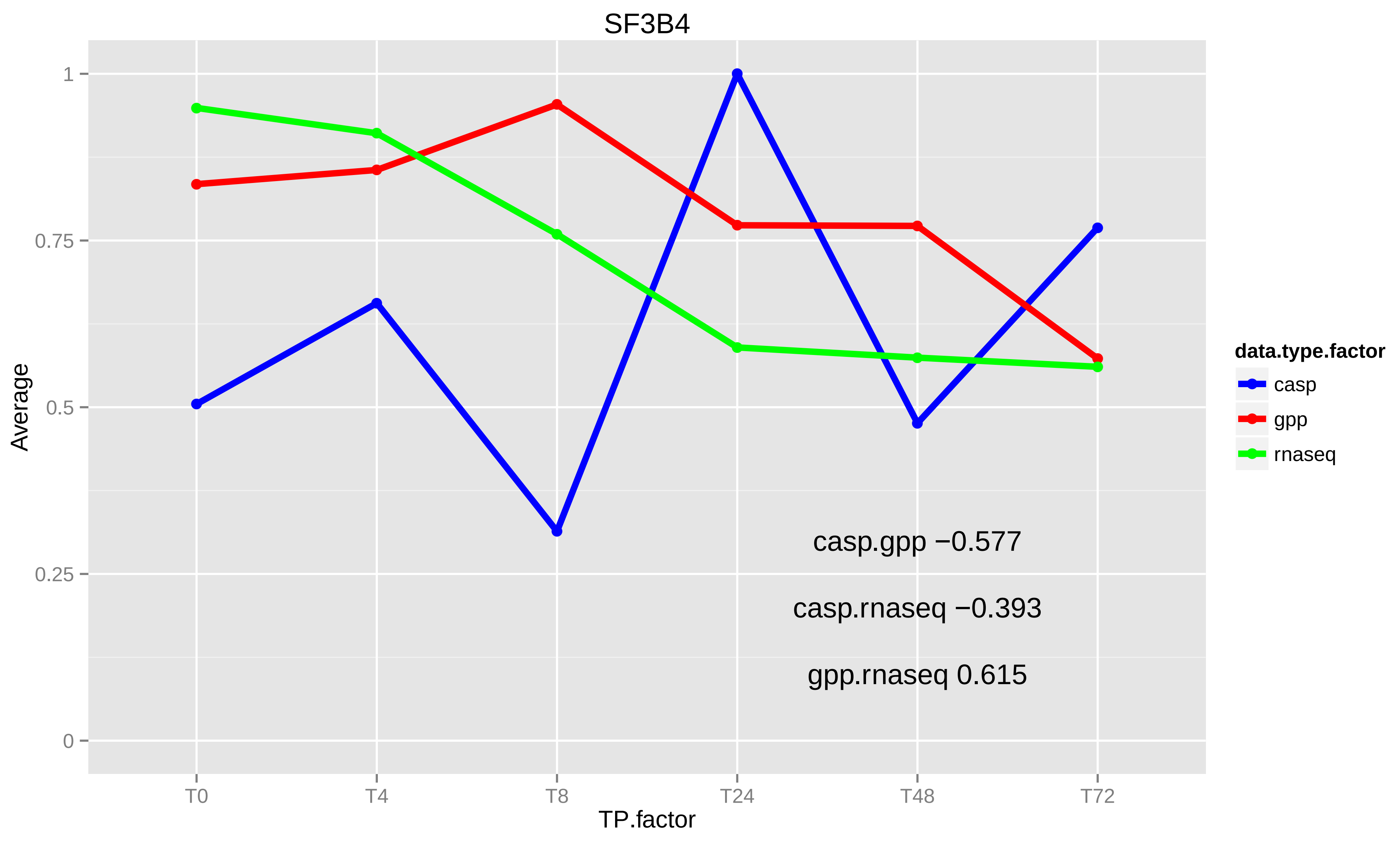
SF3B4
Average
1
0.75
0.5
0.25
0
T0
T4
T8
T24
T48
T72
TP.factor
data.type.factor
casp
gpp
rnaseq
casp.gpp −0.577
casp.rnaseq −0.393
gpp.rnaseq 0.615

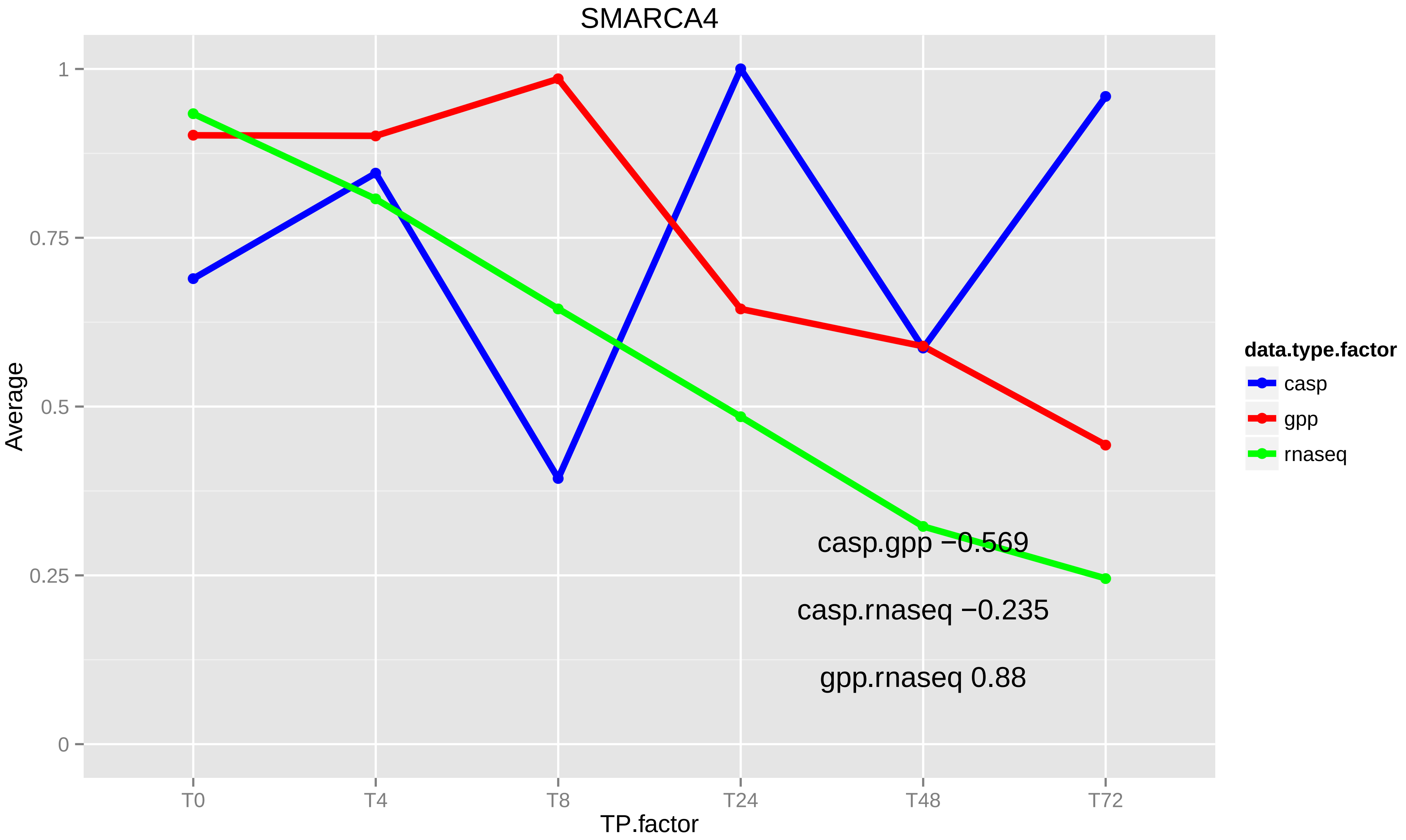
SMARCA4
Average
1
0.75
0.5
0.25
0
T0
T4
T8
T24
T48
T72
TP.factor
data.type.factor
casp
gpp
rnaseq
casp.gpp −0.569
casp.rnaseq −0.235
gpp.rnaseq 0.88

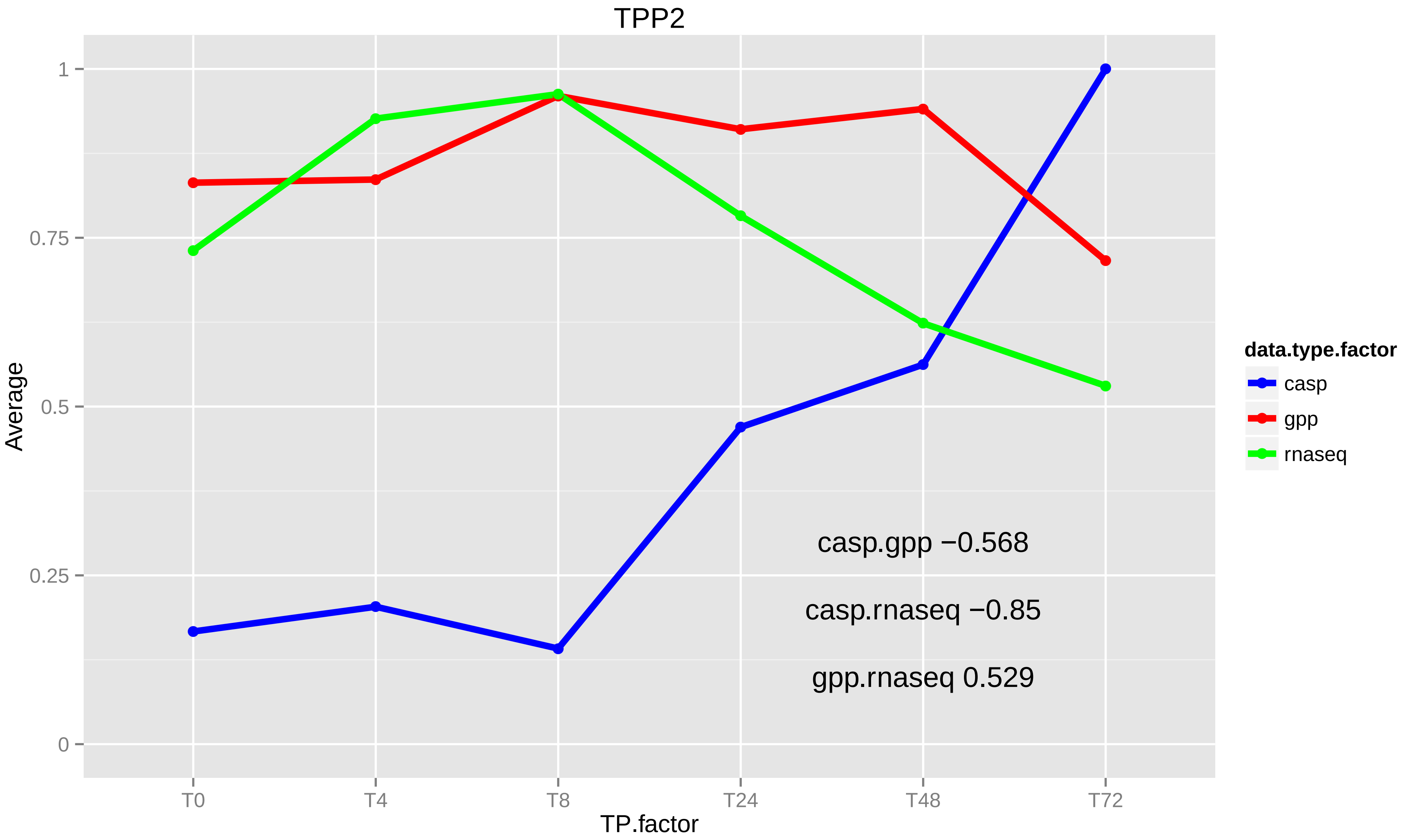

TPP2
1
0.75
0.5
0.25
0
Average
T0
T4
T8
T24
T48
T72
TP.factor
data.type.factor
casp
gpp
rnaseq
casp.gpp −0.568
casp.rnaseq −0.85
gpp.rnaseq 0.529

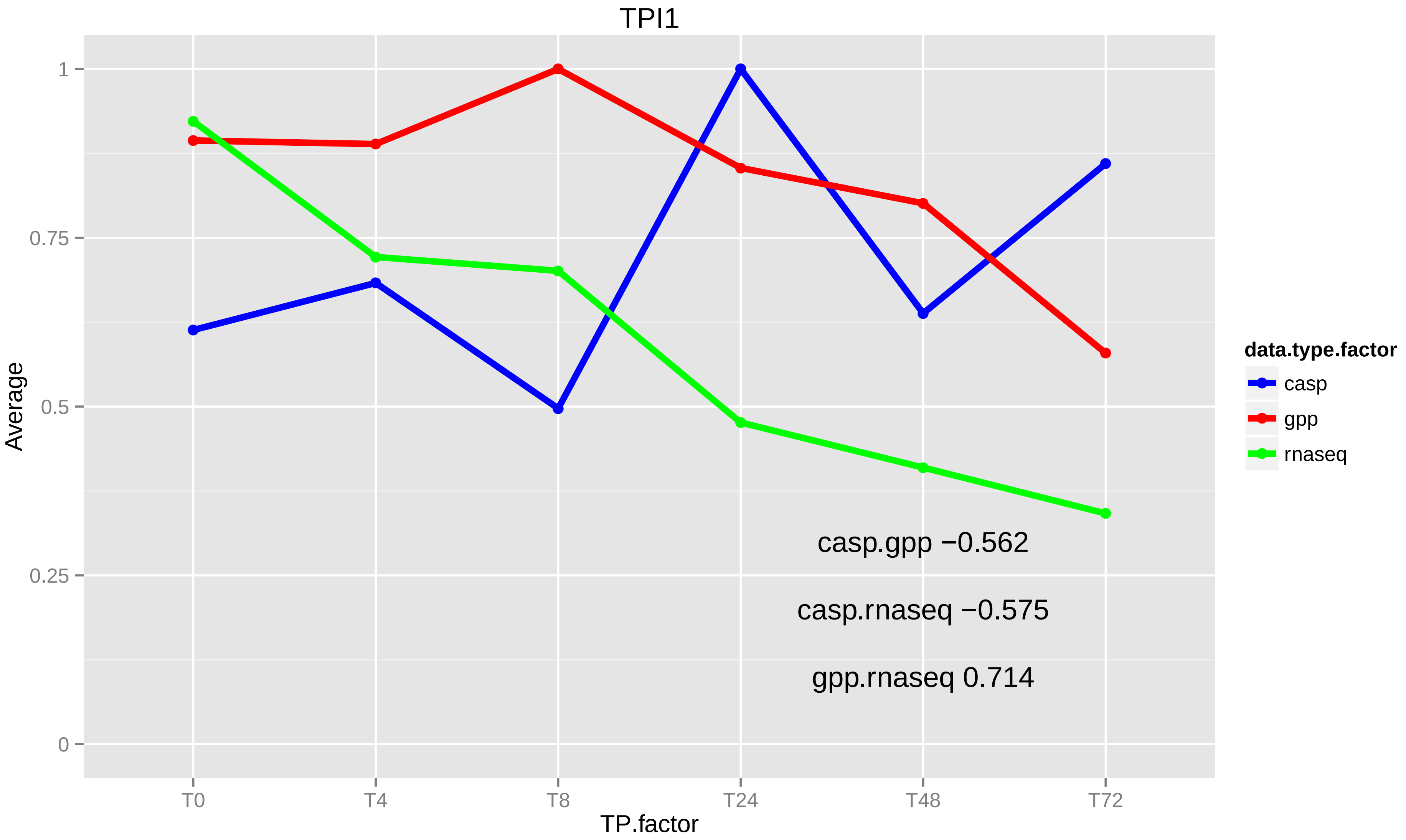

TPI1
1
0.75
0.5
0.25
0
Average
T0
T4
T8
T24
T48
T72
TP.factor
data.type.factor
casp
gpp
rnaseq
casp.gpp −0.562
casp.rnaseq −0.575
gpp.rnaseq 0.714

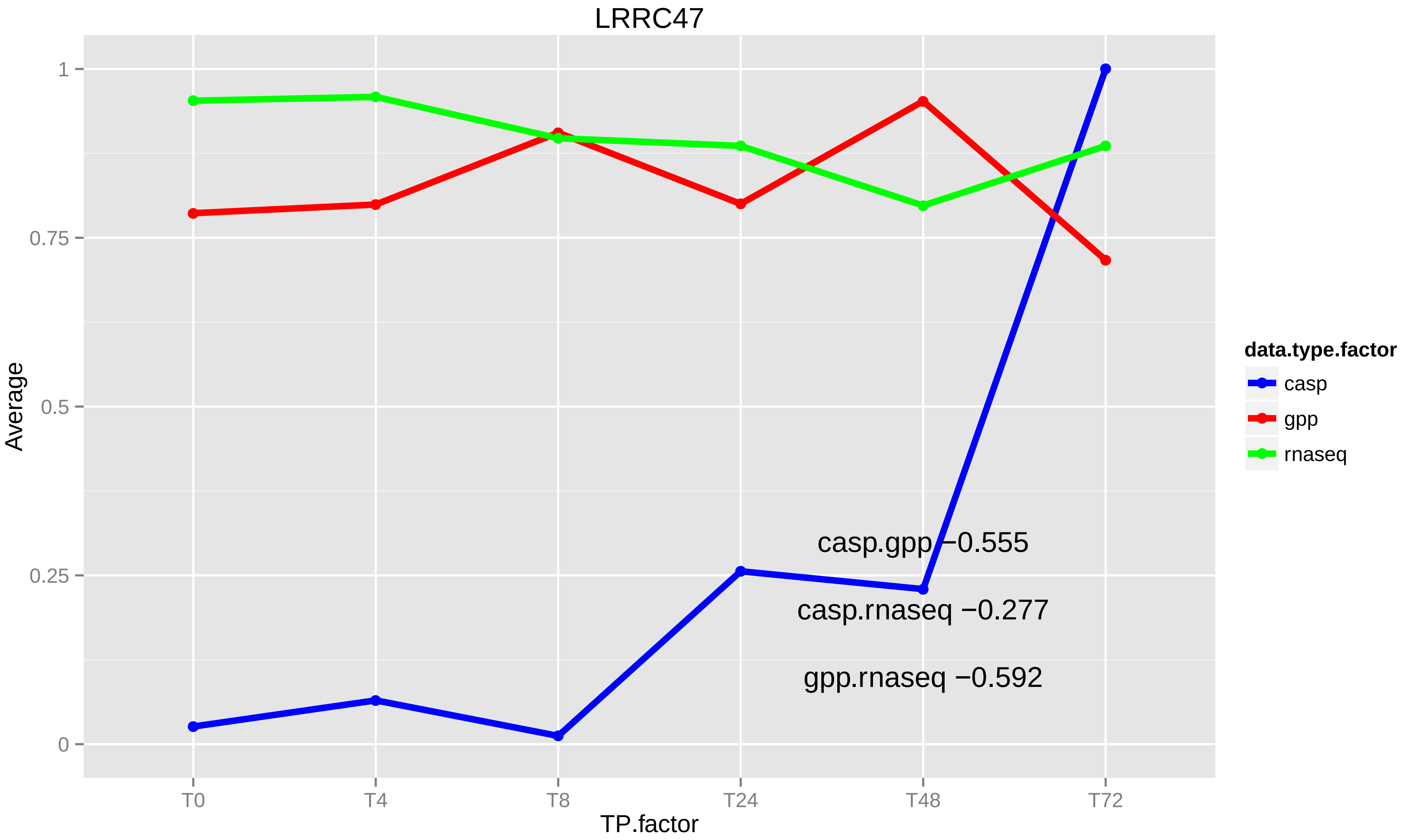
LRRC47
Average
TP.factor
1
0.75
0.5
0.25
0
T0
T4
T8
T24
T48
T72
data.type.factor
casp
gpp
rnaseq
casp.gpp −0.555
casp.rnaseq −0.277
gpp.rnaseq −0.592

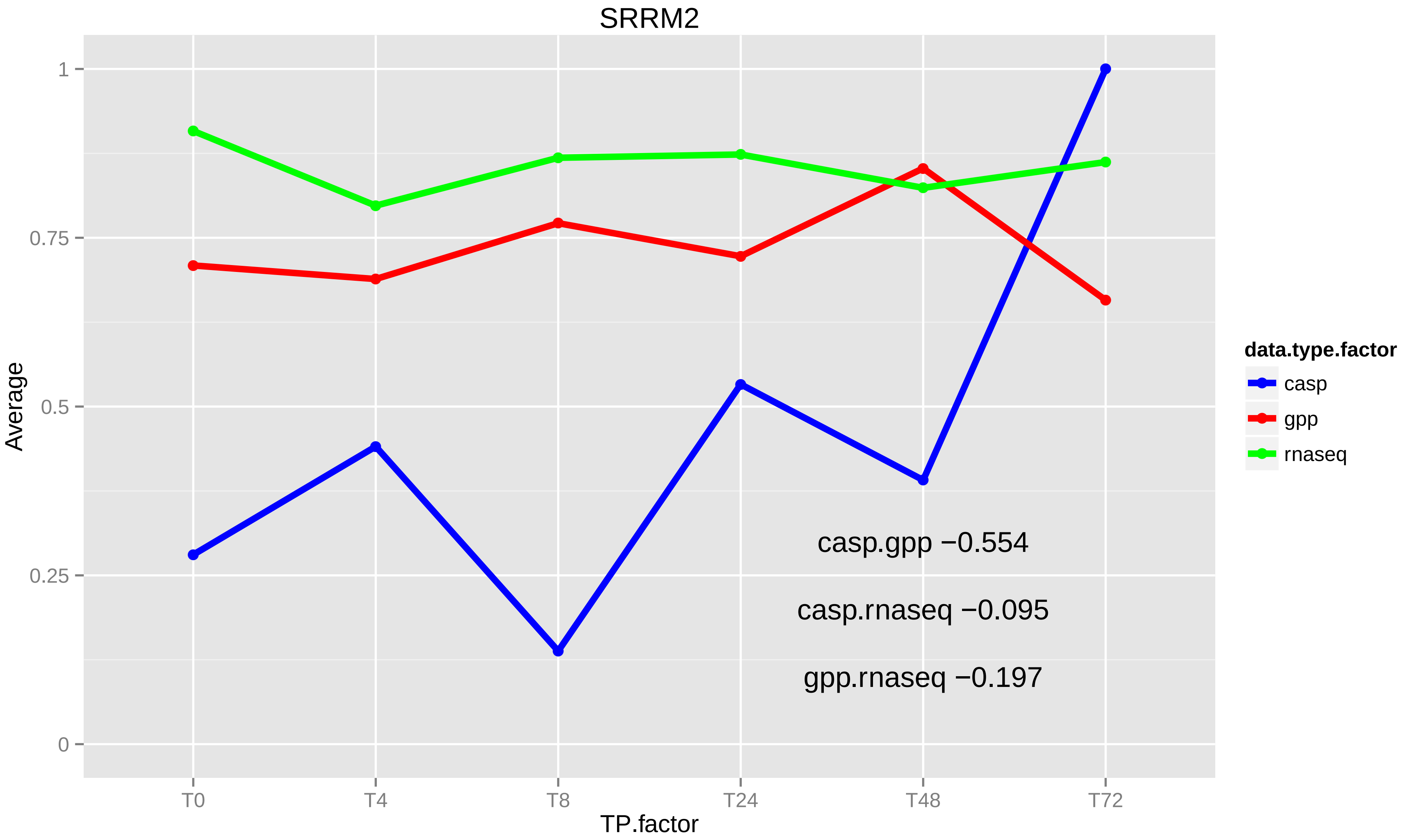

SRRM2
Average
1
0.75
0.5
0.25
0
T0
T4
T8
T24
T48
T72
TP.factor
data.type.factor
casp
gpp
rnaseq
casp.gpp −0.554
casp.rnaseq −0.095
gpp.rnaseq −0.197

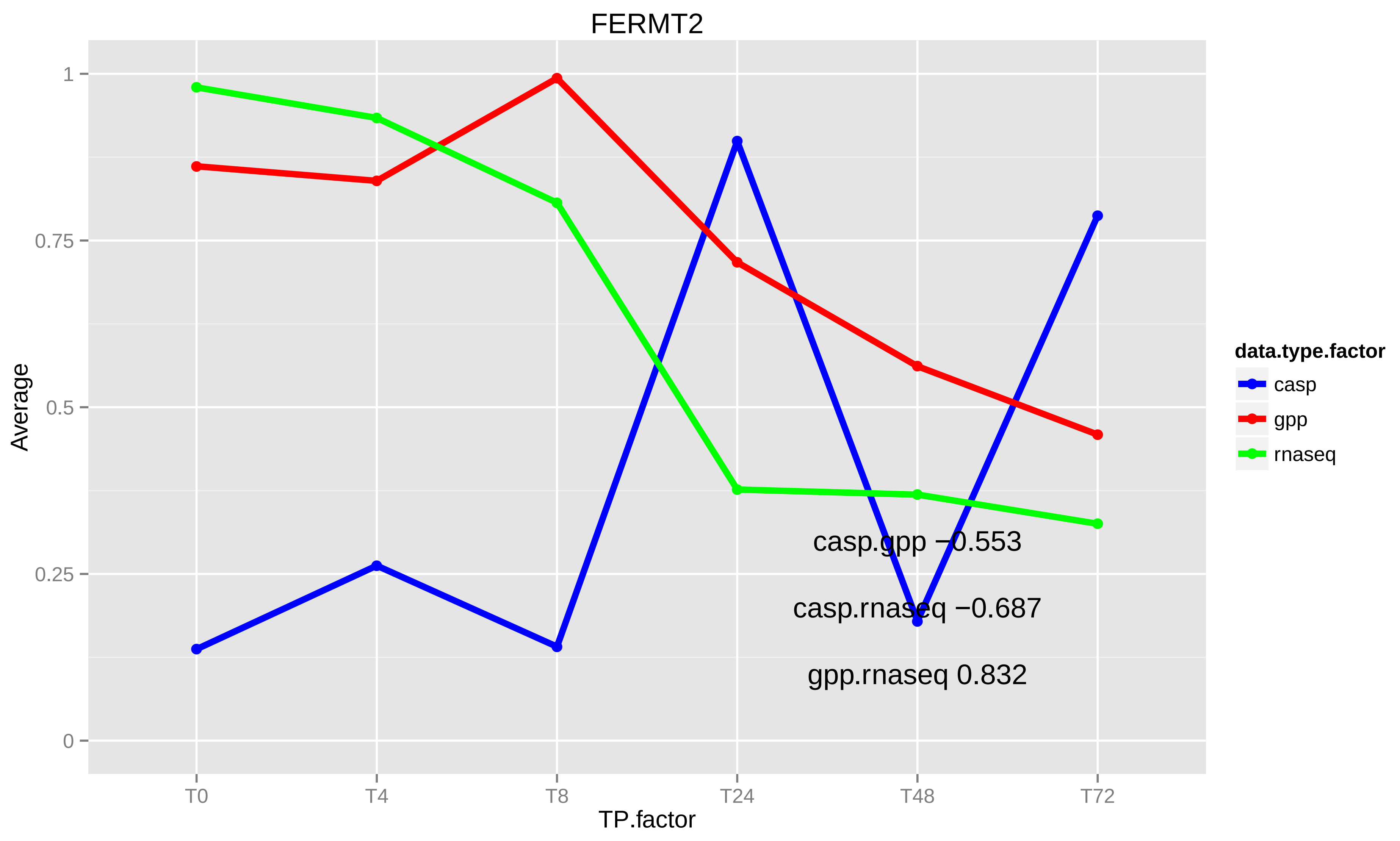

FERMT2
Average
1
0.75
0.5
0.25
0
T0
T4
T8
T24
T48
T72
TP.factor
data.type.factor
casp
gpp
rnaseq
casp.gpp −0.553
casp.rnaseq −0.687
gpp.rnaseq 0.832

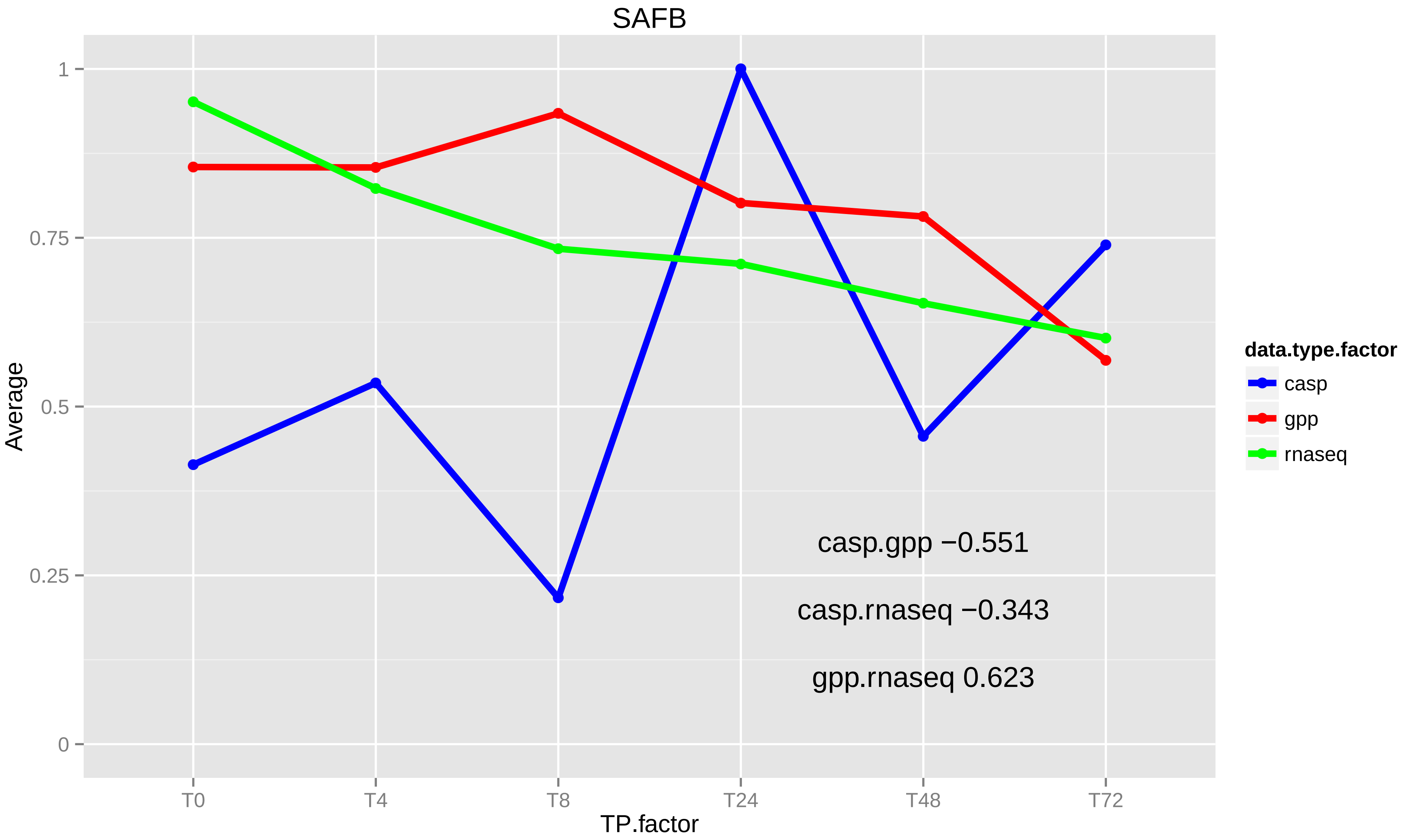

SAFB
1
0.75
0.5
0.25
0
Average
T0
T4
T8
T24
T48
T72
TP.factor
data.type.factor
casp
gpp
rnaseq
casp.gpp −0.551
casp.rnaseq −0.343
gpp.rnaseq 0.623

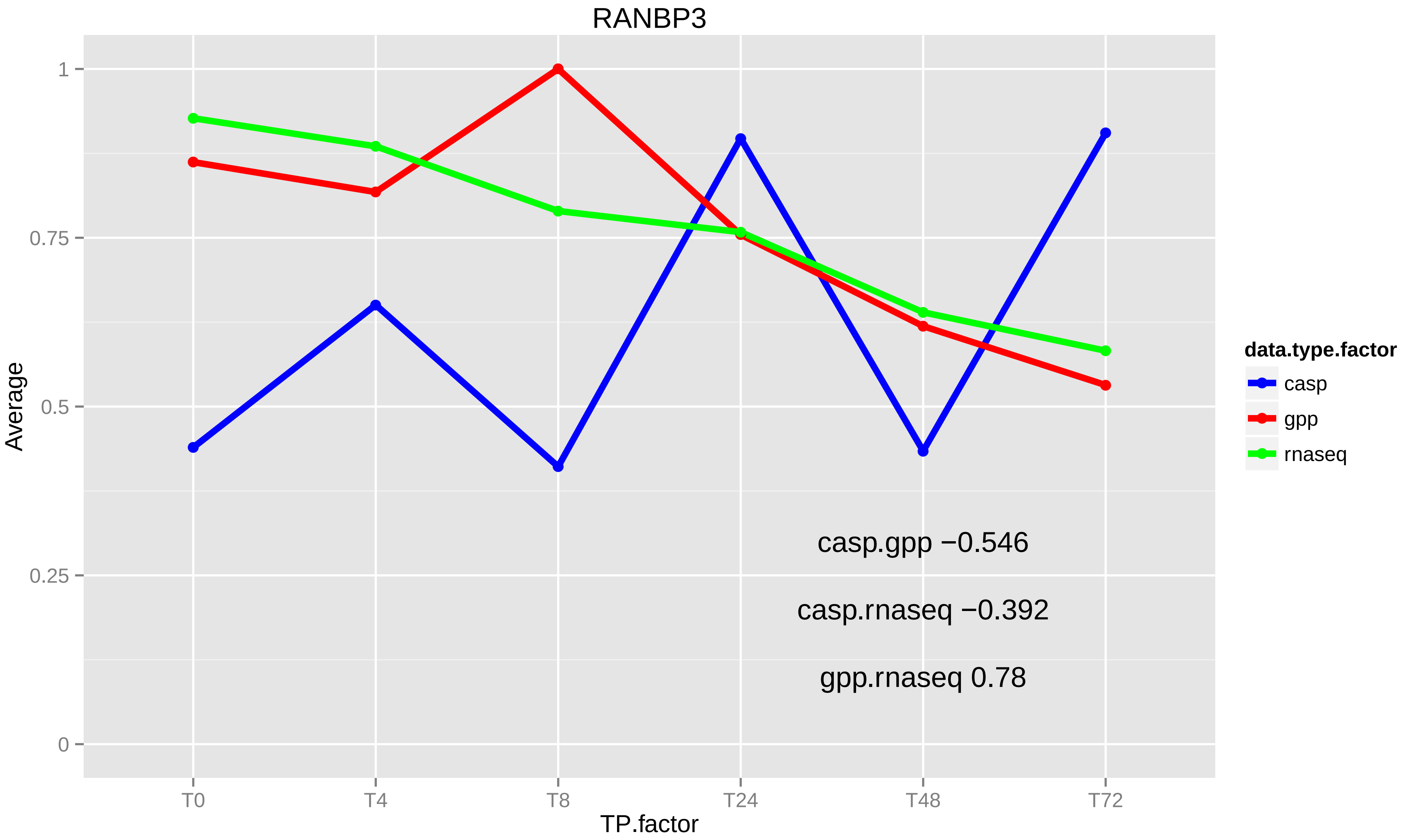

RANBP3
Average
1
0.75
0.5
0.25
0
T0
T4
T8
T24
T48
T72
TP.factor
data.type.factor
casp
gpp
rnaseq
casp.gpp −0.546
casp.rnaseq −0.392
gpp.rnaseq 0.78

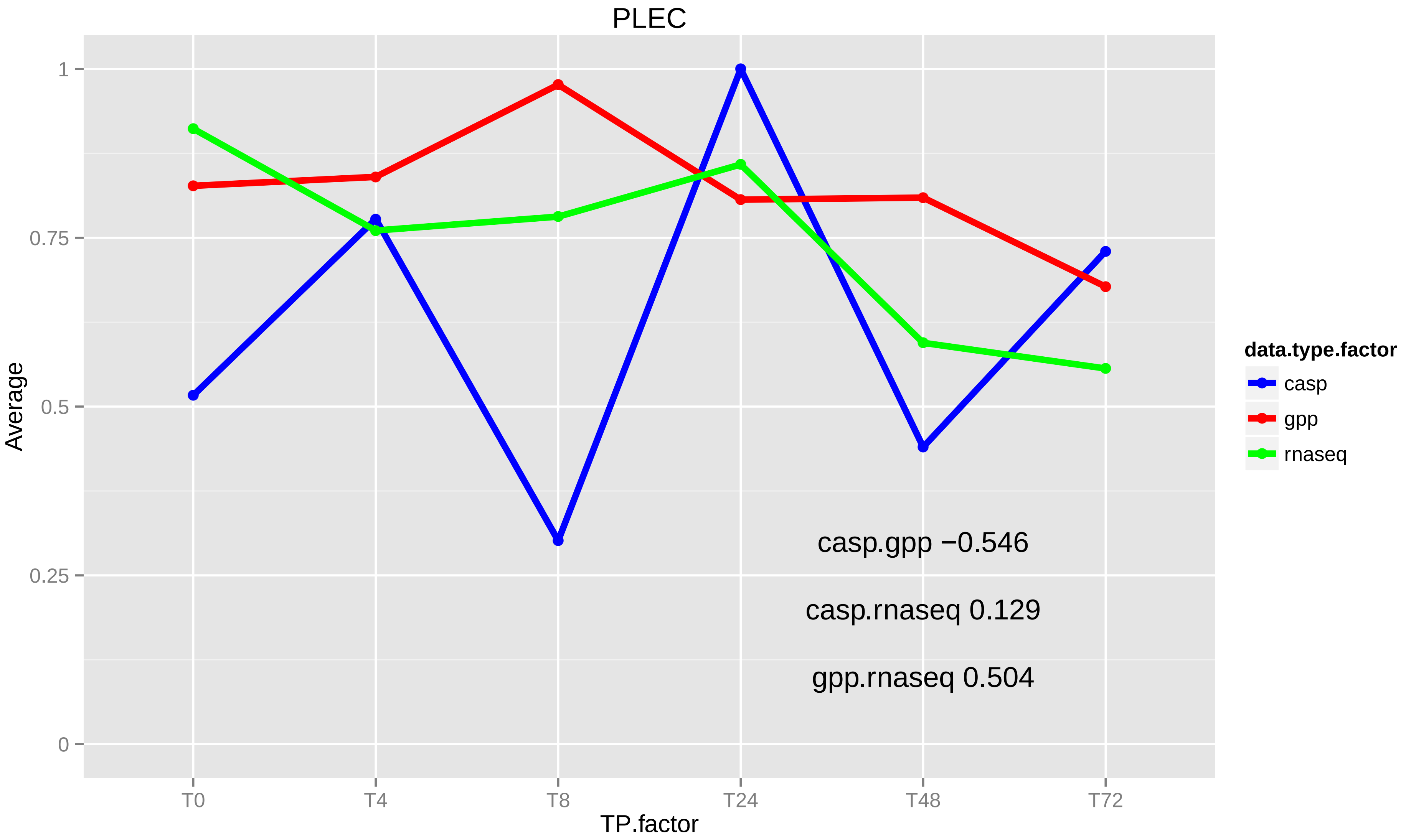

PLEC
Average
1
0.75
0.5
0.25
0
T0
T4
T8
T24
T48
T72
TP.factor
data.type.factor
casp
gpp
rnaseq
casp.gpp −0.546
casp.rnaseq 0.129
gpp.rnaseq 0.504

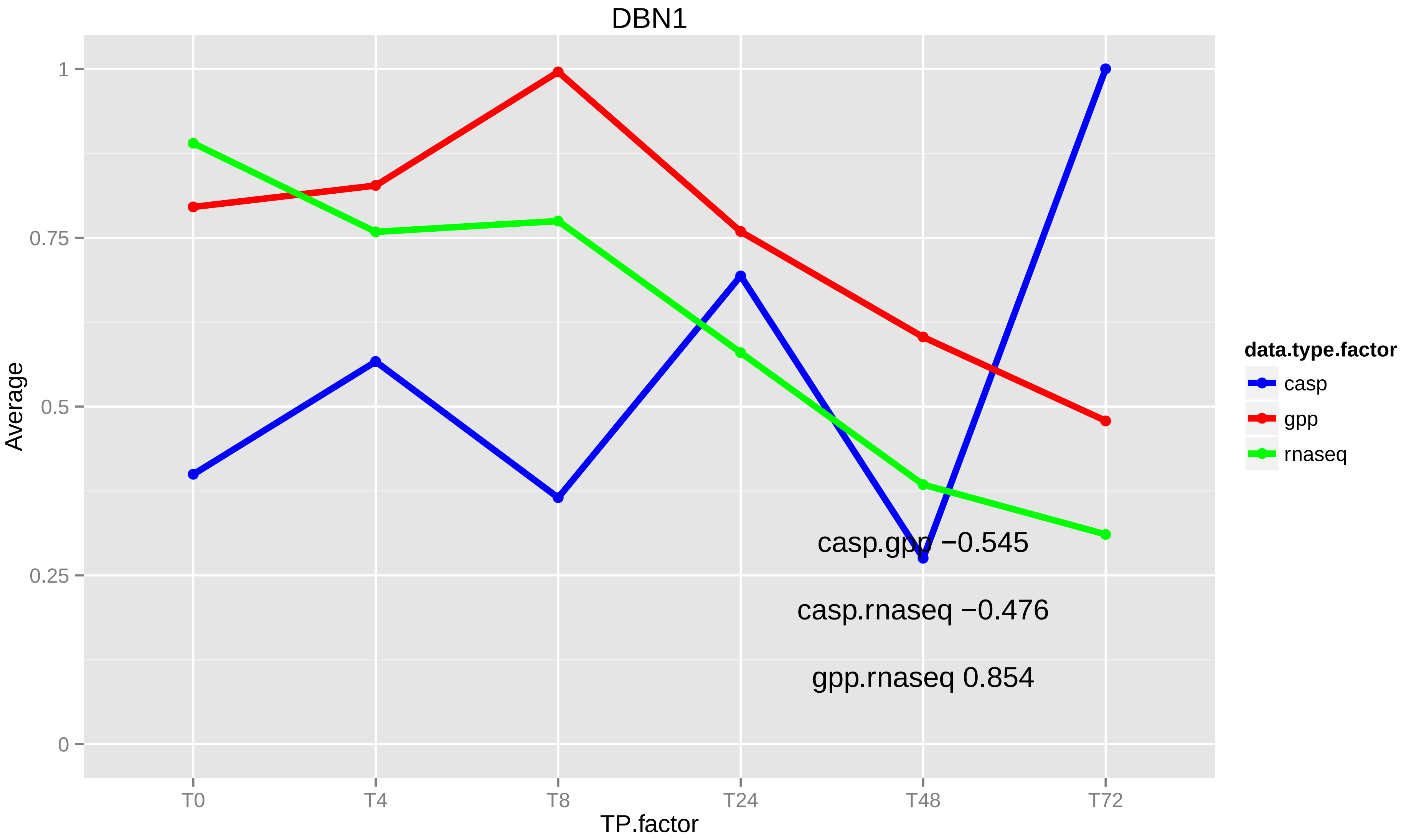

DBN1
Average
1
0.75
0.5
0.25
0
T0
T4
T8
T24
T48
T72
TP.factor
data.type.factor
casp
gpp
rnaseq
casp.gpp −0.545
casp.rnaseq −0.476
gpp.rnaseq 0.854

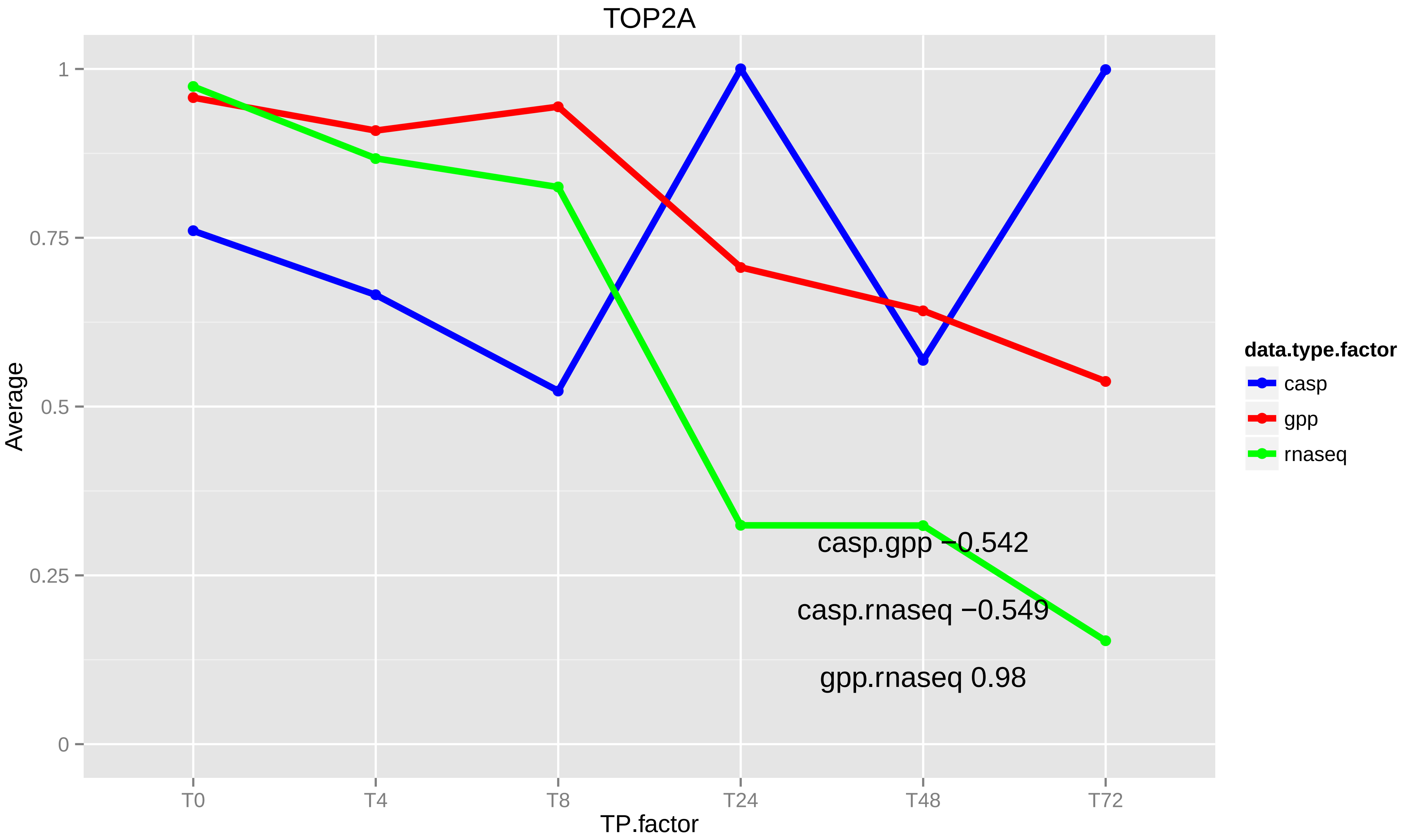
TOP2A
Average
1
0.75
0.5
0.25
0
T0
T4
T8
T24
T48
T72
TP.factor
data.type.factor
casp
gpp
rnaseq
casp.gpp −0.542
casp.rnaseq −0.549
gpp.rnaseq 0.98

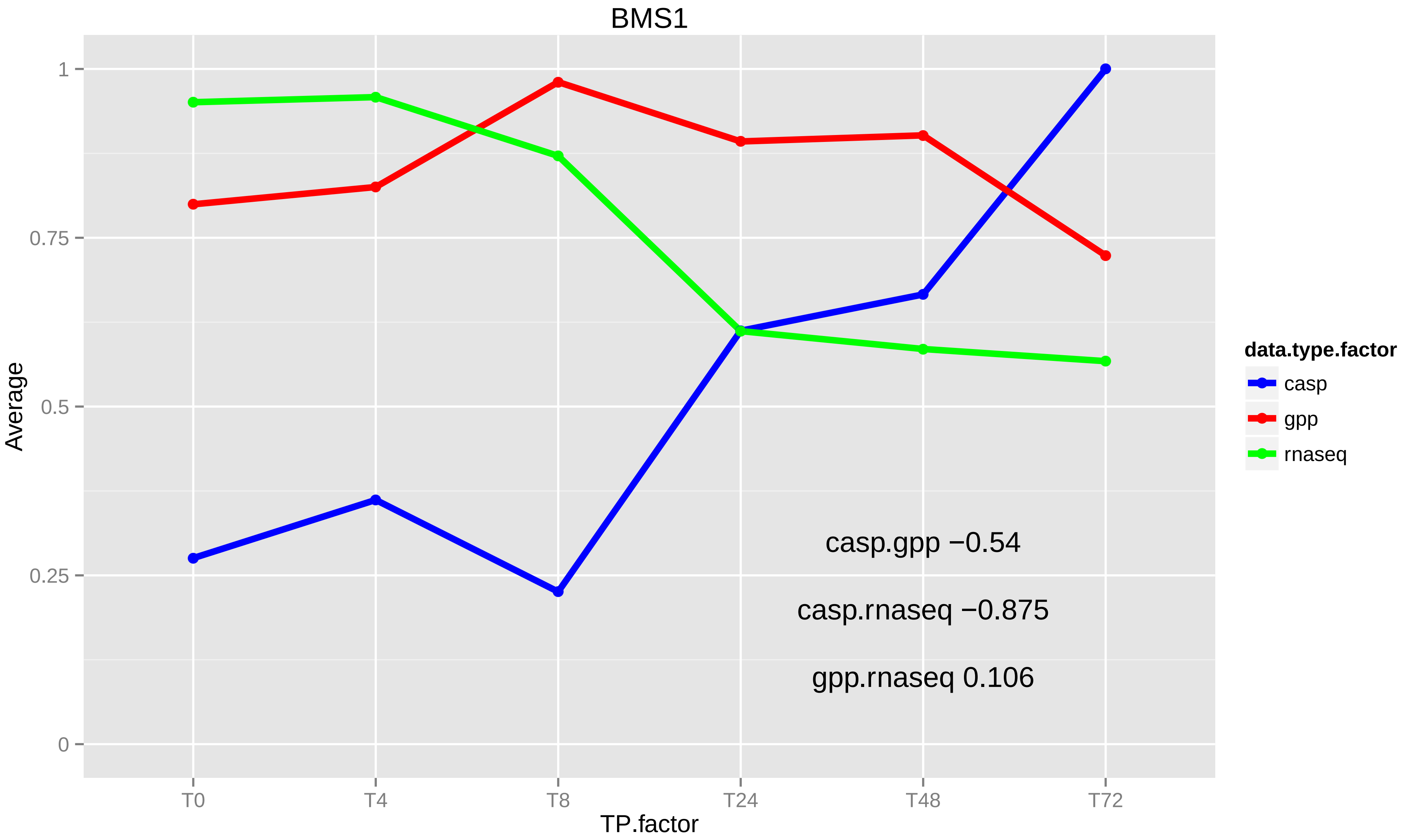
BMS1
1
0.75
0.5
0.25
0
Average
T0
T4
T8
T24
T48
T72
TP.factor
data.type.factor
casp
gpp
rnaseq
casp.gpp −0.54
casp.rnaseq −0.875
gpp.rnaseq 0.106

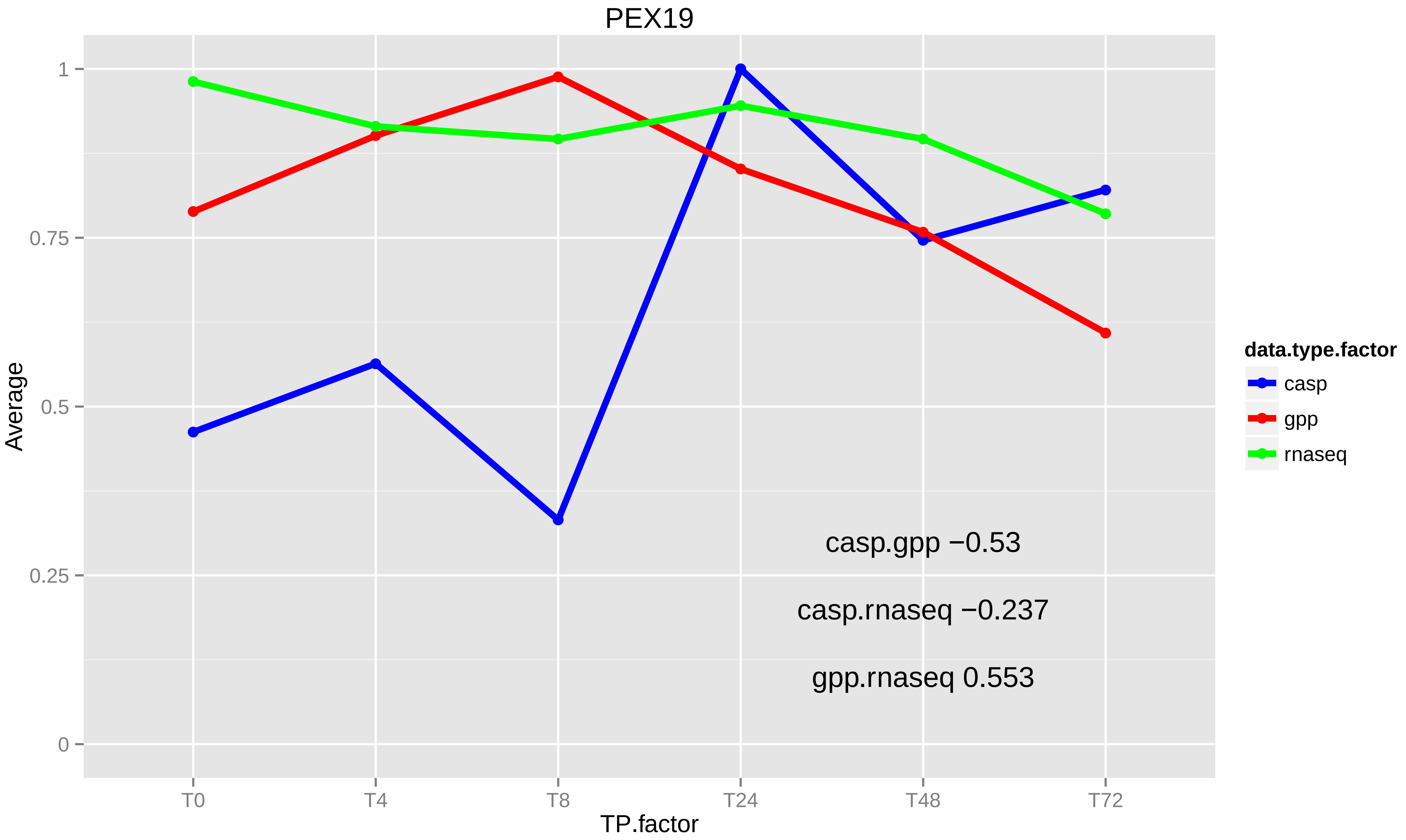
PEX19
Average
1
0.75
0.5
0.25
0
T0
T4
T8
T24
T48
T72
TP.factor
data.type.factor
casp
gpp
rnaseq
casp.gpp −0.53
casp.rnaseq −0.237
gpp.rnaseq 0.553

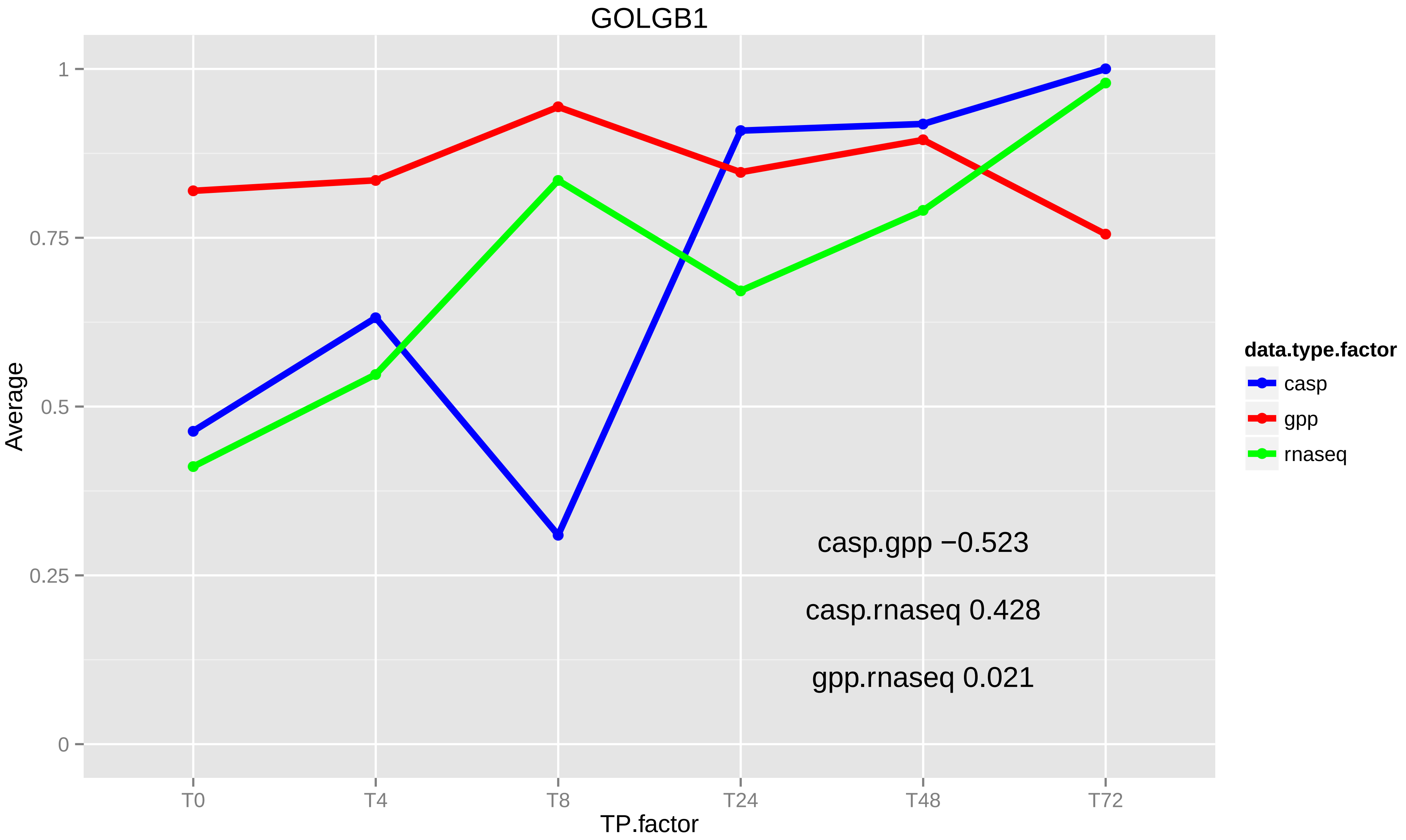

GOLGB1
Average
TP.factor
1
0.75
0.5
0.25
0
T0
T4
T8
T24
T48
T72
data.type.factor
casp
gpp
rnaseq
casp.gpp −0.523
casp.rnaseq 0.428
gpp.rnaseq 0.021

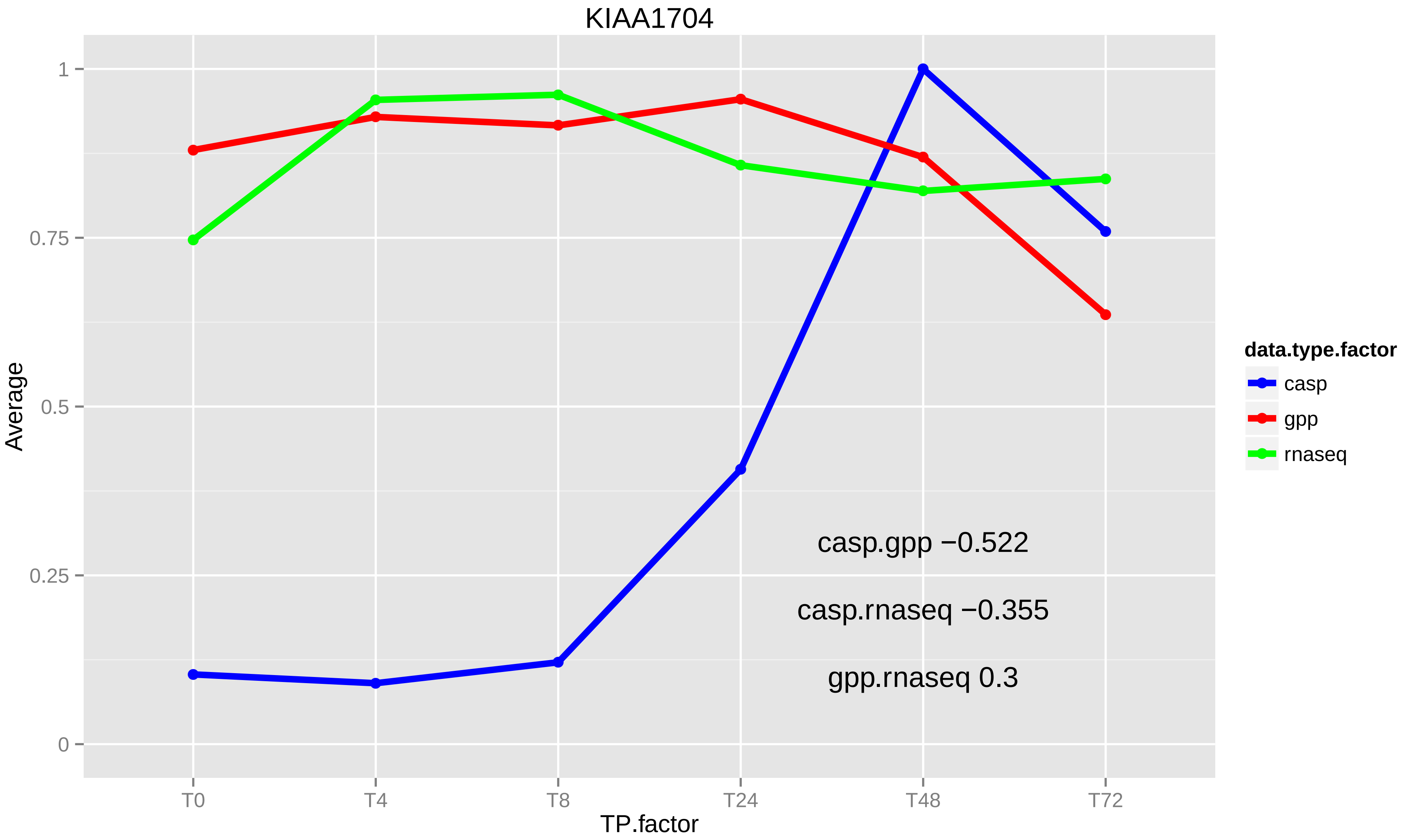
KIAA1704
Average
1
0.75
0.5
0.25
0
T0
T4
T8
T24
T48
T72
TP.factor
data.type.factor
casp
gpp
rnaseq
casp.gpp −0.522
casp.rnaseq −0.355
gpp.rnaseq 0.3

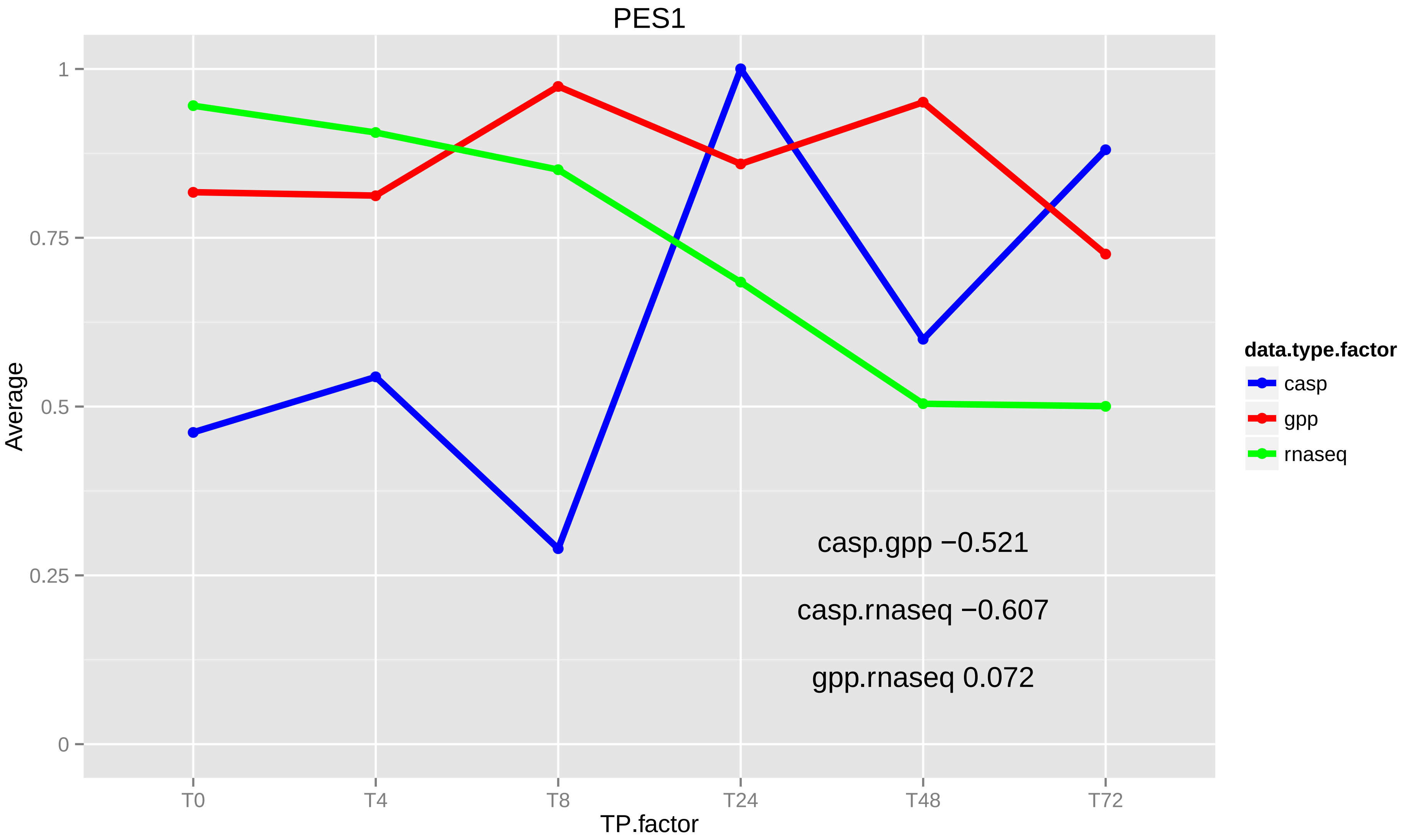

PES1
Average
1
0.75
0.5
0.25
0
T0
T4
T8
T24
T48
T72
TP.factor
data.type.factor
casp
gpp
rnaseq
casp.gpp −0.521
casp.rnaseq −0.607
gpp.rnaseq 0.072

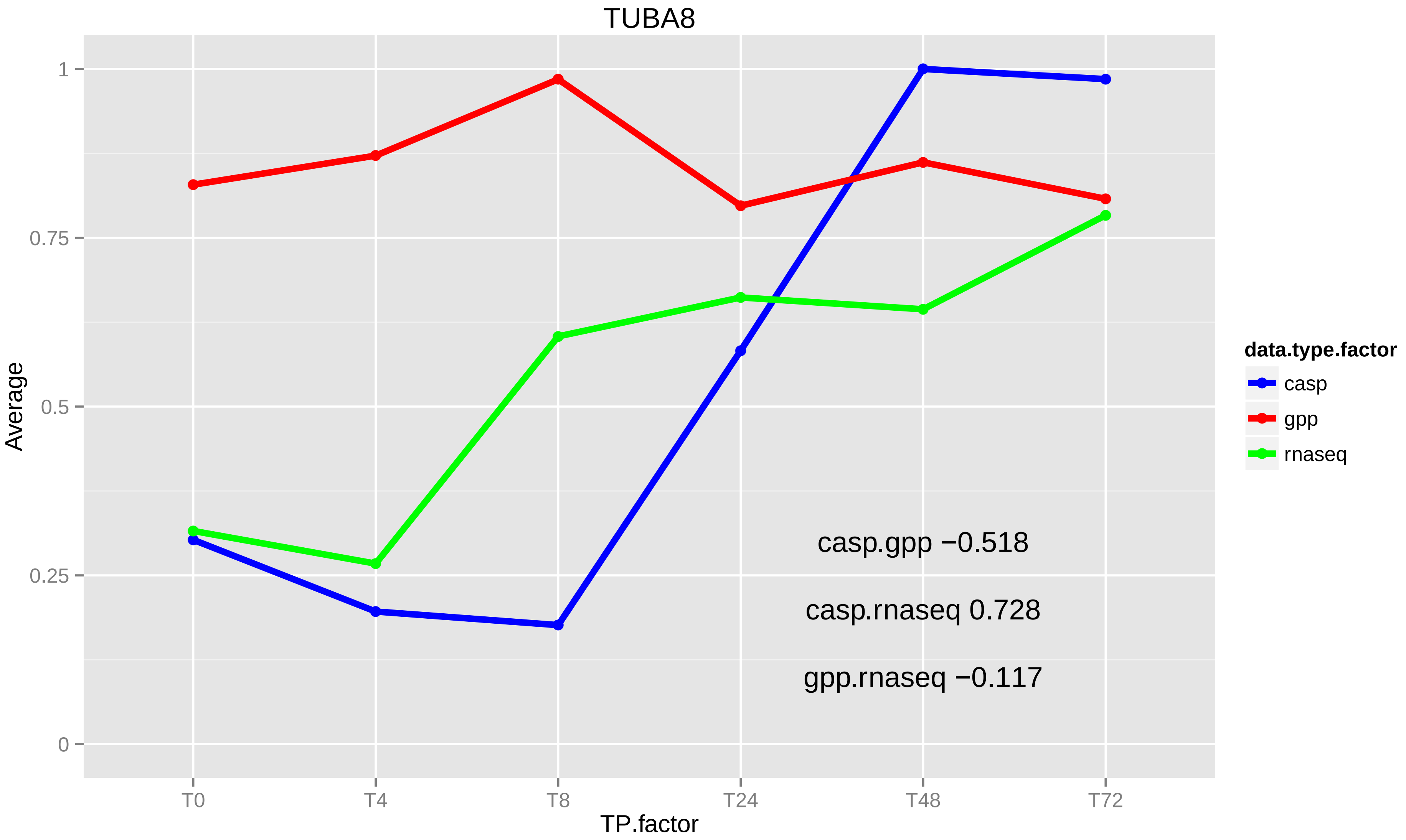

TUBA8
1
0.75
0.5
0.25
0
Average
T0
T4
T8
T24
T48
T72
TP.factor
data.type.factor
casp
gpp
rnaseq
casp.gpp −0.518
casp.rnaseq 0.728
gpp.rnaseq −0.117

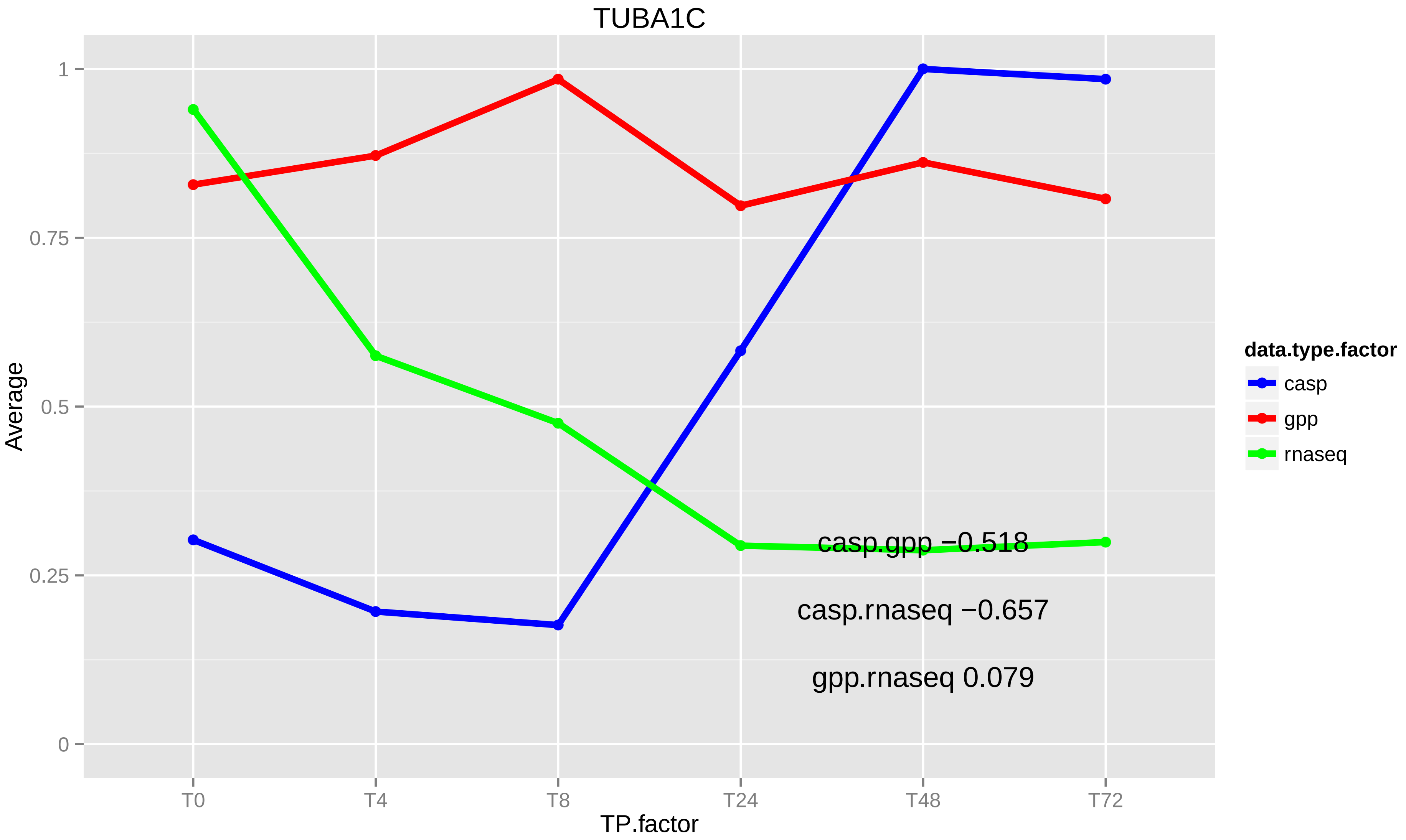
TUBA1C
1
0.75
0.5
0.25
0
Average
T0
T4
T8
T24
T48
T72
TP.factor
data.type.factor
casp
gpp
rnaseq
casp.gpp −0.518
casp.rnaseq −0.657
gpp.rnaseq 0.079

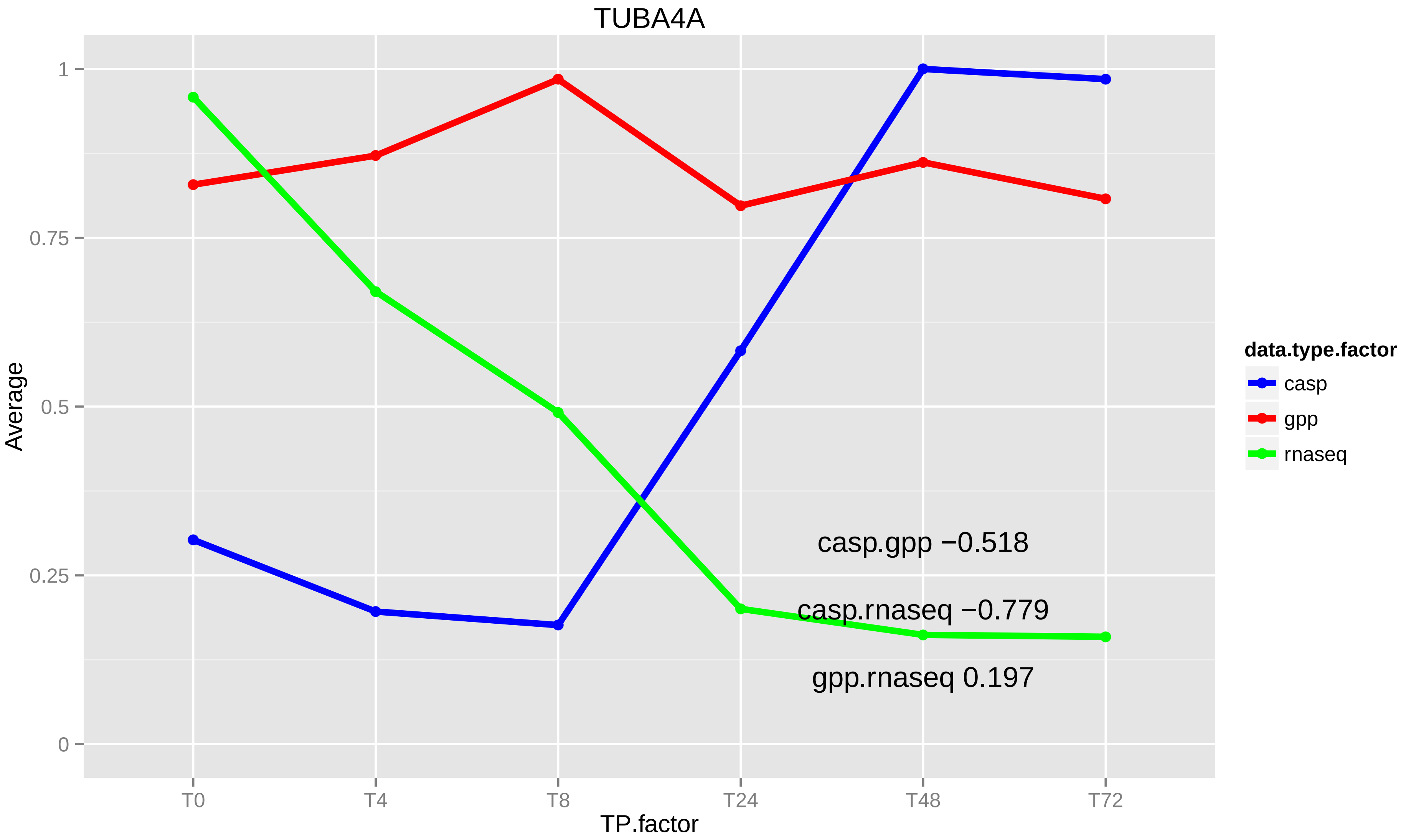
TUBA4A
Average
1
0.75
0.5
0.25
0
T0
T4
T8
T24
T48
T72
TP.factor
data.type.factor
casp
gpp
rnaseq
casp.gpp −0.518
casp.rnaseq −0.779
gpp.rnaseq 0.197

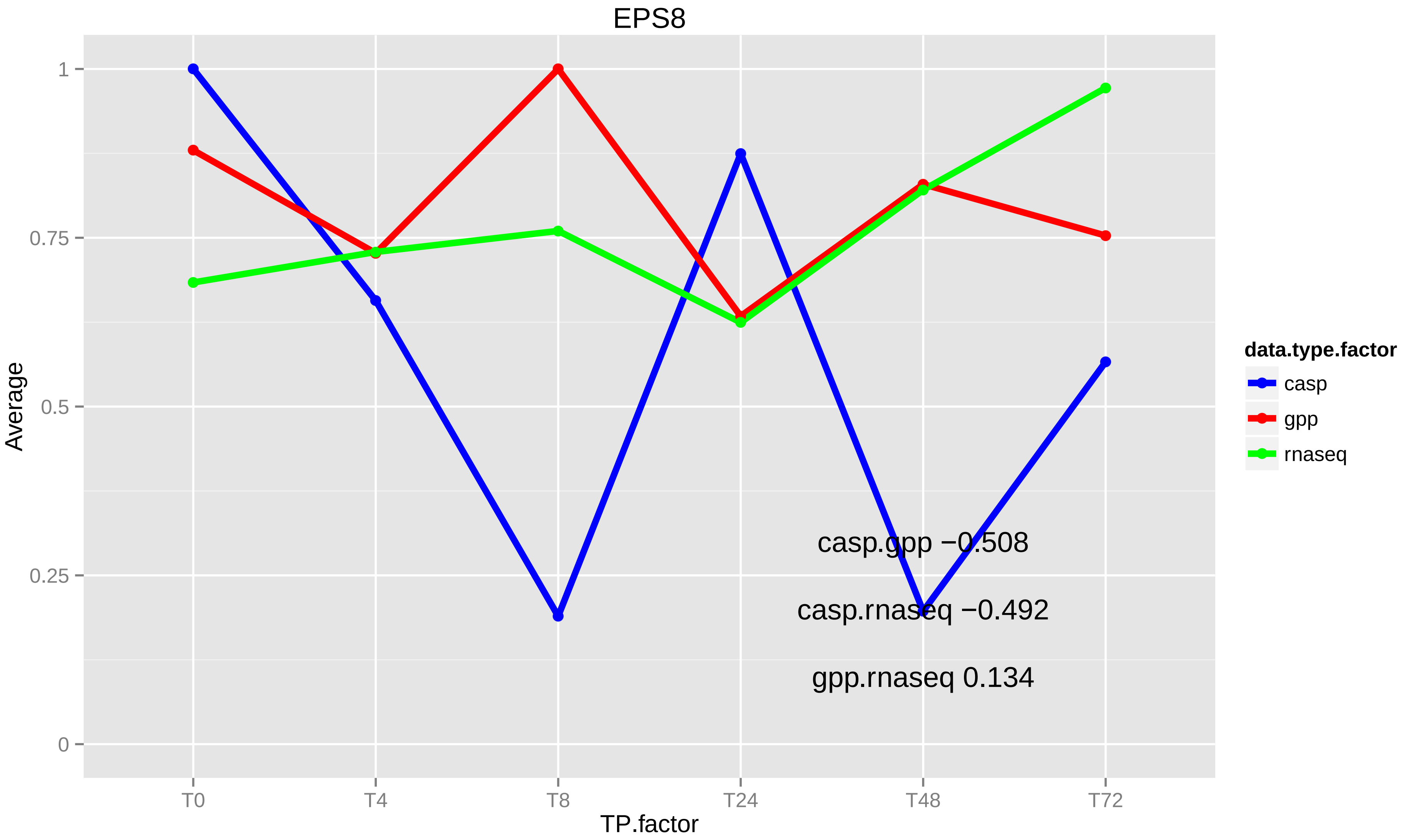
EPS8
1
0.75
0.5
0.25
0
Average
T0
T4
T8
T24
T48
T72
TP.factor
data.type.factor
casp
gpp
rnaseq
casp.gpp −0.508
casp.rnaseq −0.492
gpp.rnaseq 0.134

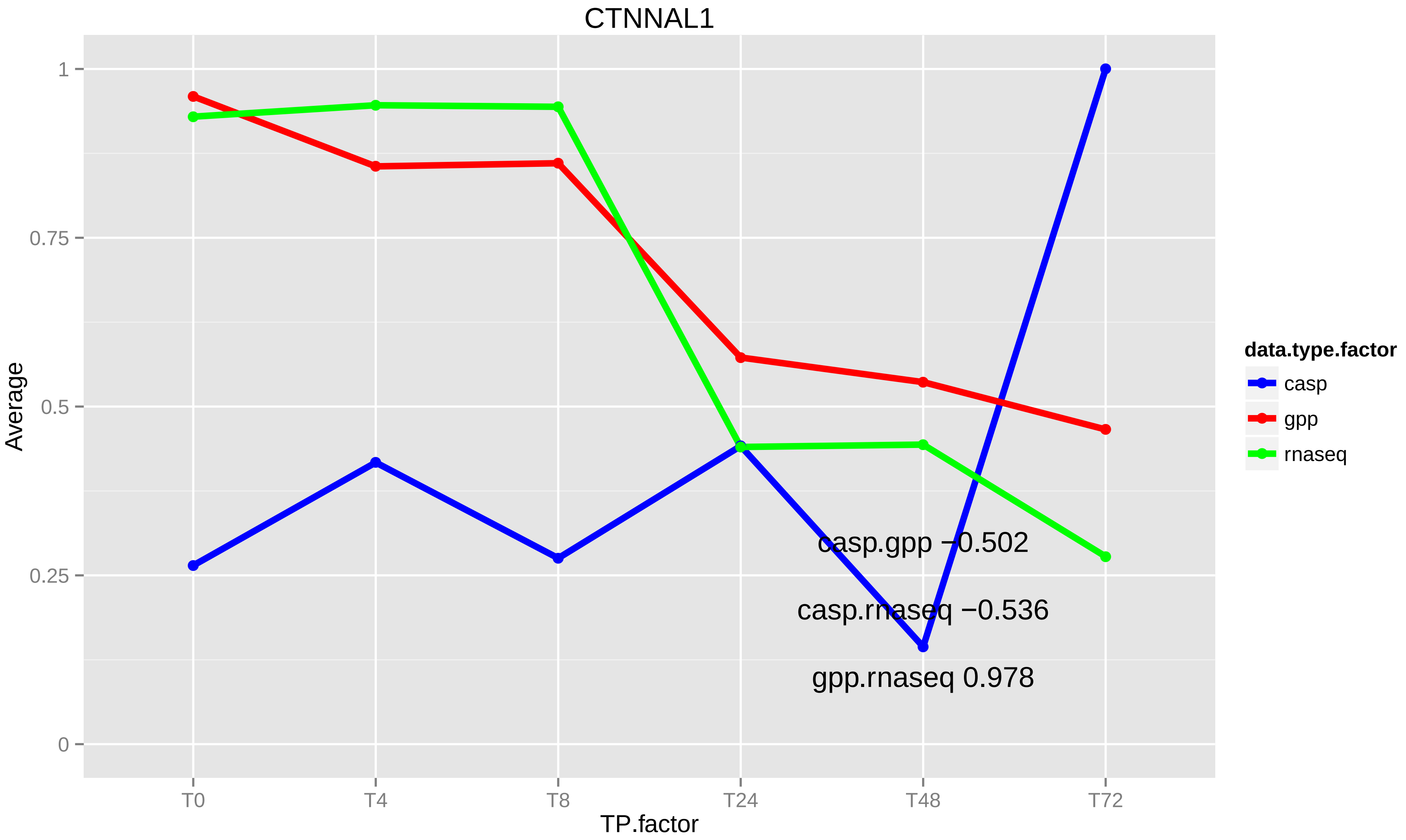

CTNNAL1
Average
1
0.75
0.5
0.25
0
T0
T4
T8
T24
T48
T72
TP.factor
casp.gpp −0.502
casp.rnaseq −0.536
gpp.rnaseq 0.978
data.type.factor
casp
gpp
rnaseq

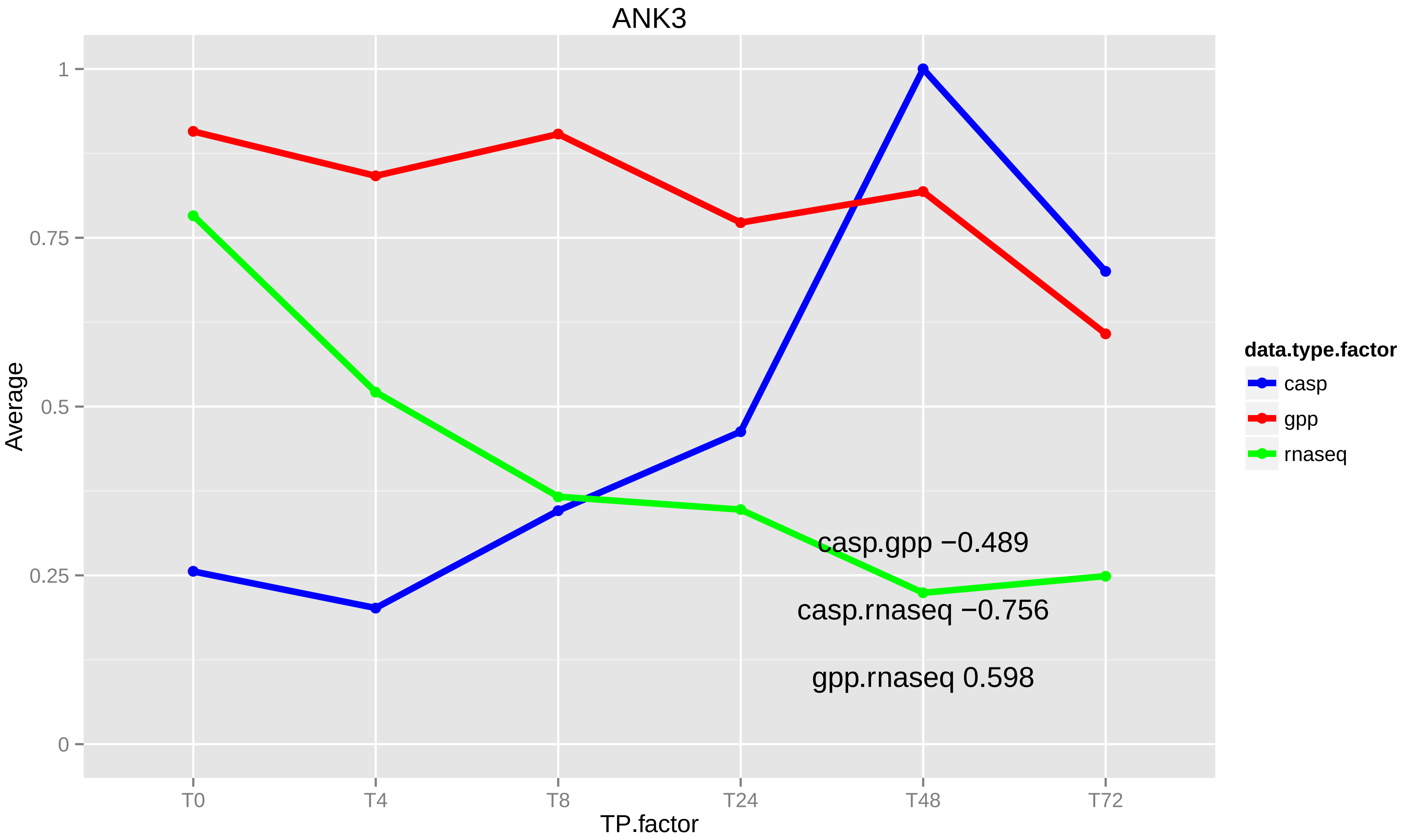
ANK3
Average
1
0.75
0.5
0.25
0
T0
T4
T8
T24
T48
T72
TP.factor
data.type.factor
casp
gpp
rnaseq
casp.gpp −0.489
casp.rnaseq −0.756
gpp.rnaseq 0.598

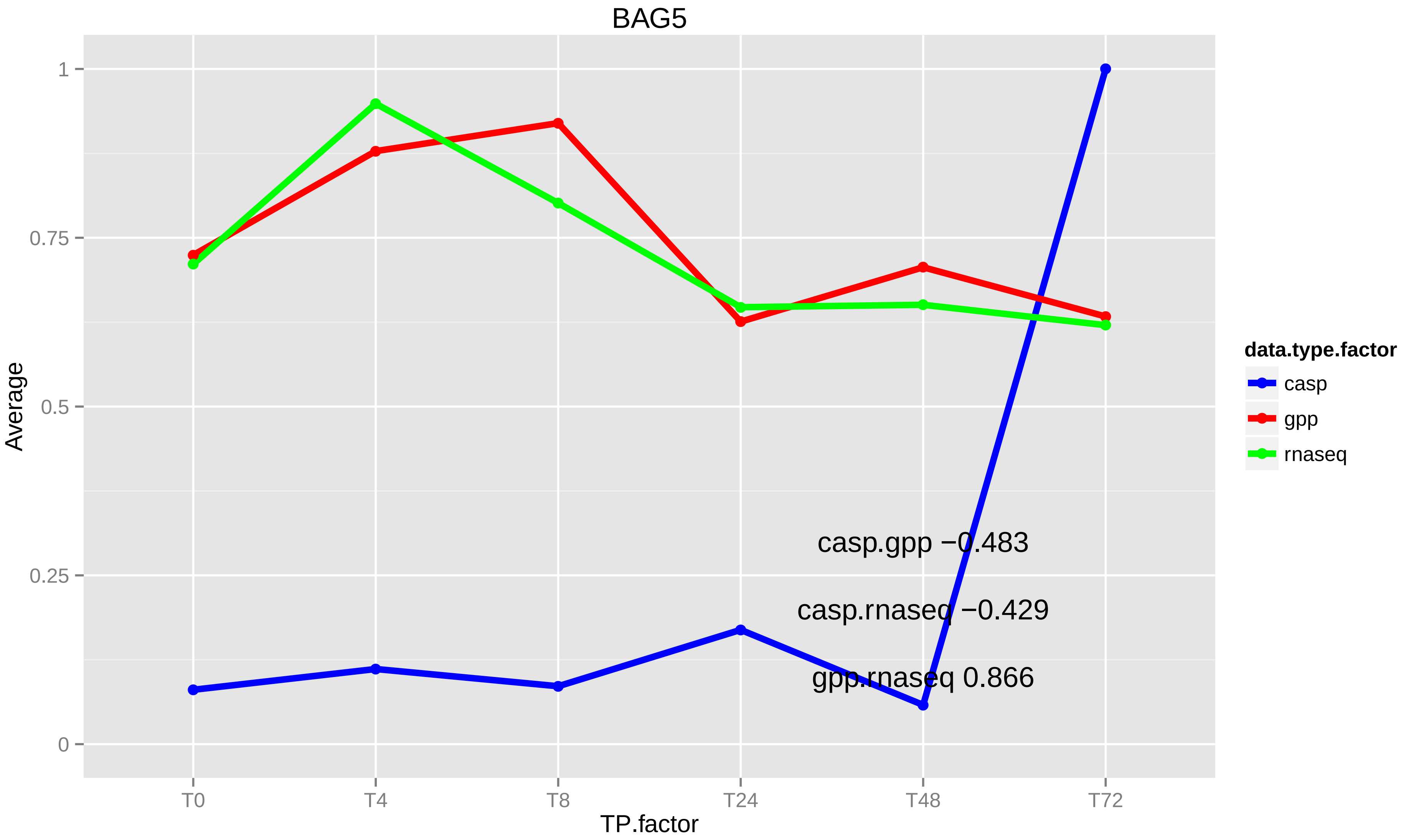
BAG5
Average
1
0.75
0.5
0.25
0
T0
T4
T8
T24
T48
T72
TP.factor
data.type.factor
casp
gpp
rnaseq
casp.gpp −0.483
casp.rnaseq −0.429
gpp.rnaseq 0.866

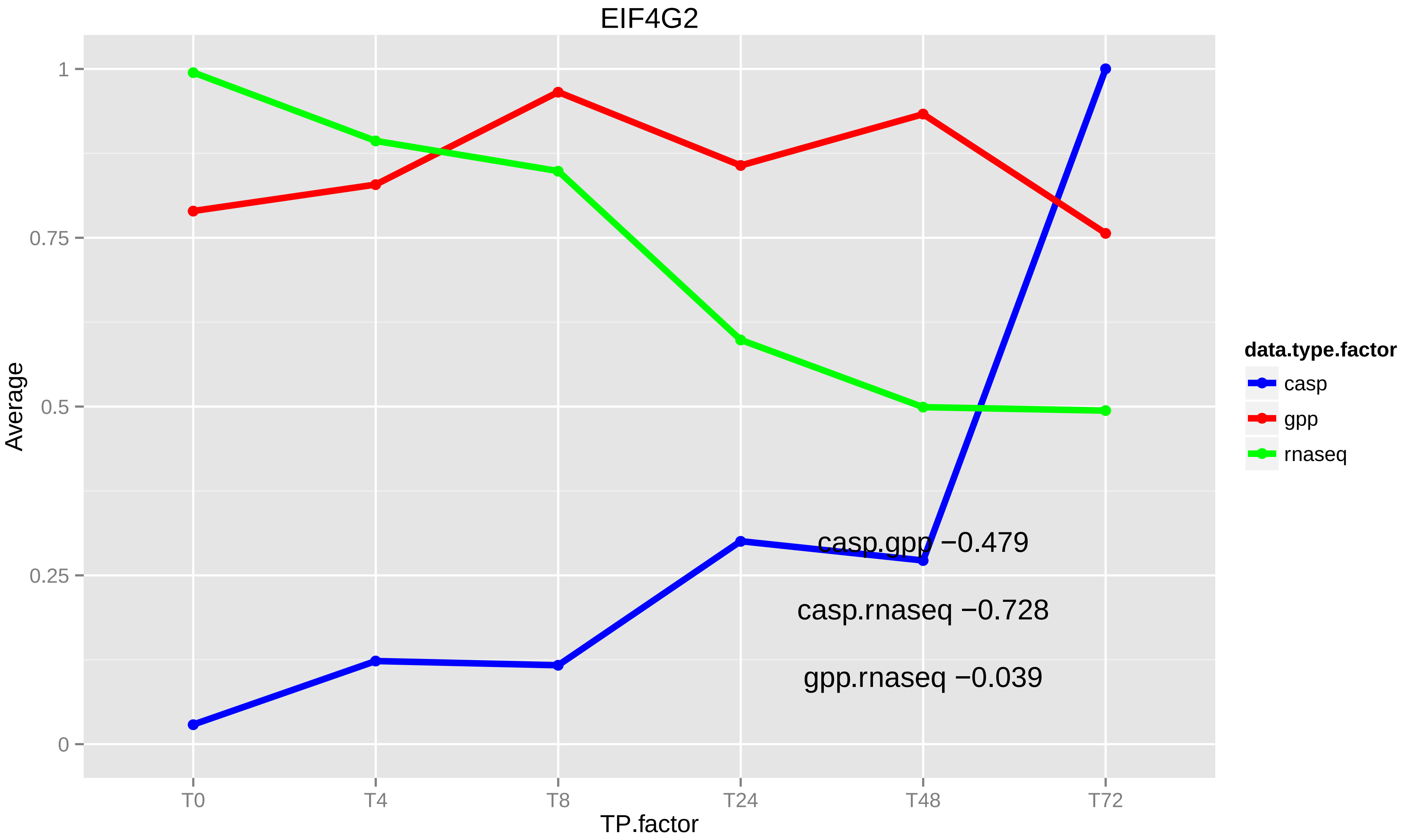

EIF4G2
Average
1
0.75
0.5
0.25
0
T0
T4
T8
T24
T48
T72
TP.factor
data.type.factor
casp
gpp
rnaseq
casp.gpp −0.479
casp.rnaseq −0.728
gpp.rnaseq −0.039

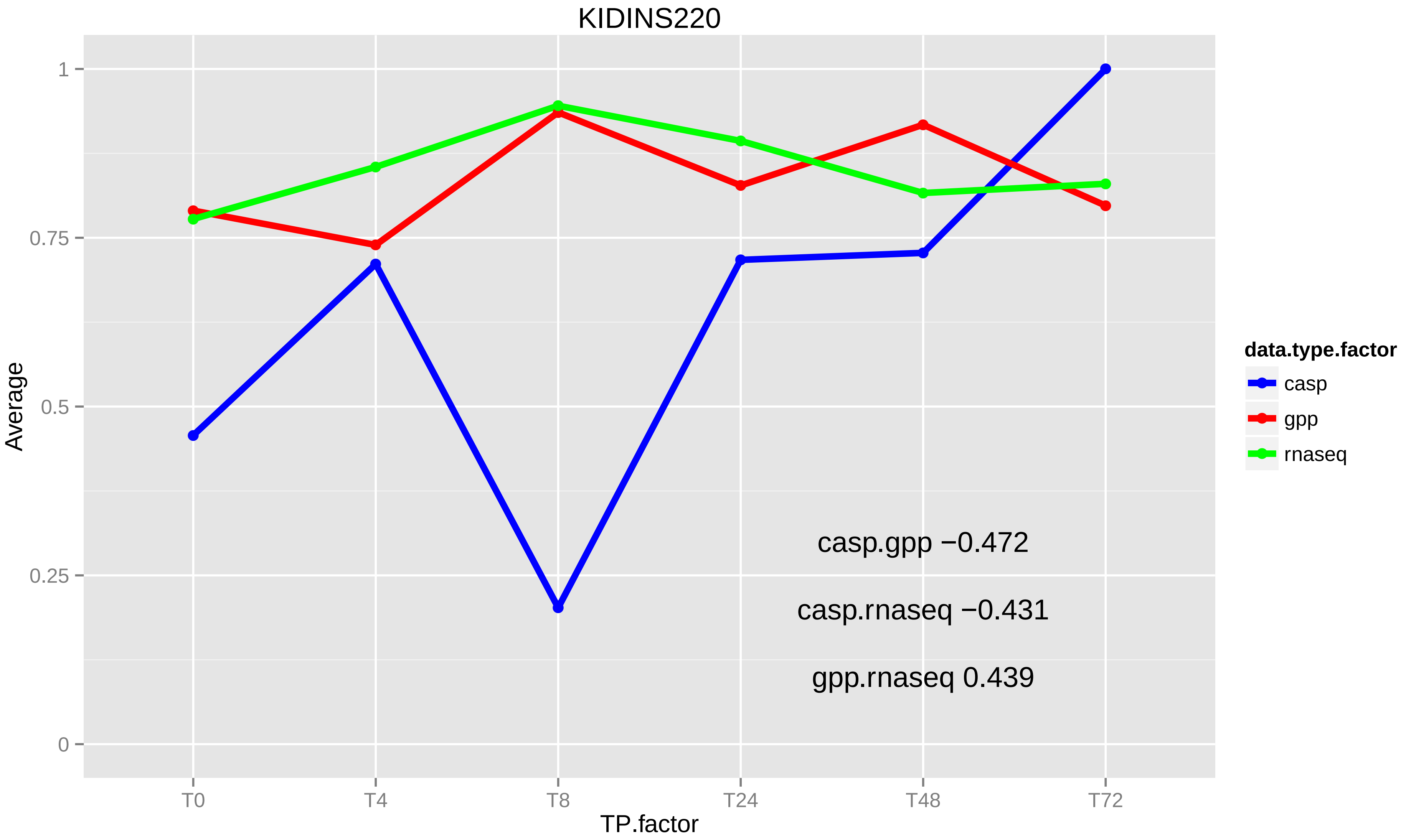
KIDINS220
Average
TP.factor
T0
T4
T8
T24
T48
T72
0
0.25
0.5
0.75
1
data.type.factor
casp
gpp
rnaseq
casp.gpp −0.472
casp.rnaseq −0.431
gpp.rnaseq 0.439

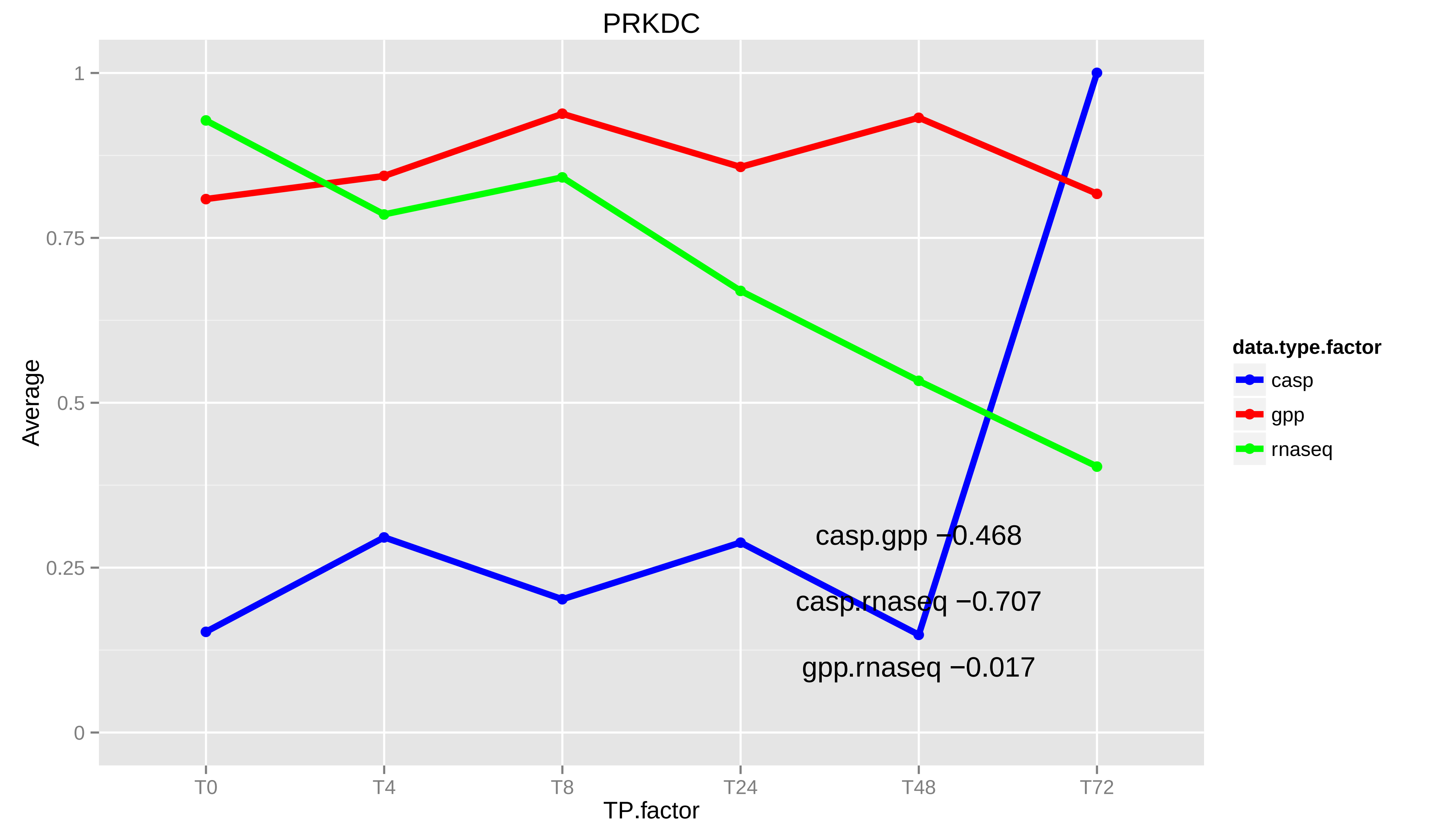
PRKDC
Average
1
0.75
0.5
0.25
0
T0
T4
T8
T24
T48
T72
TP.factor
data.type.factor
casp
gpp
rnaseq
casp.gpp −0.468
casp.rnaseq −0.707
gpp.rnaseq −0.017

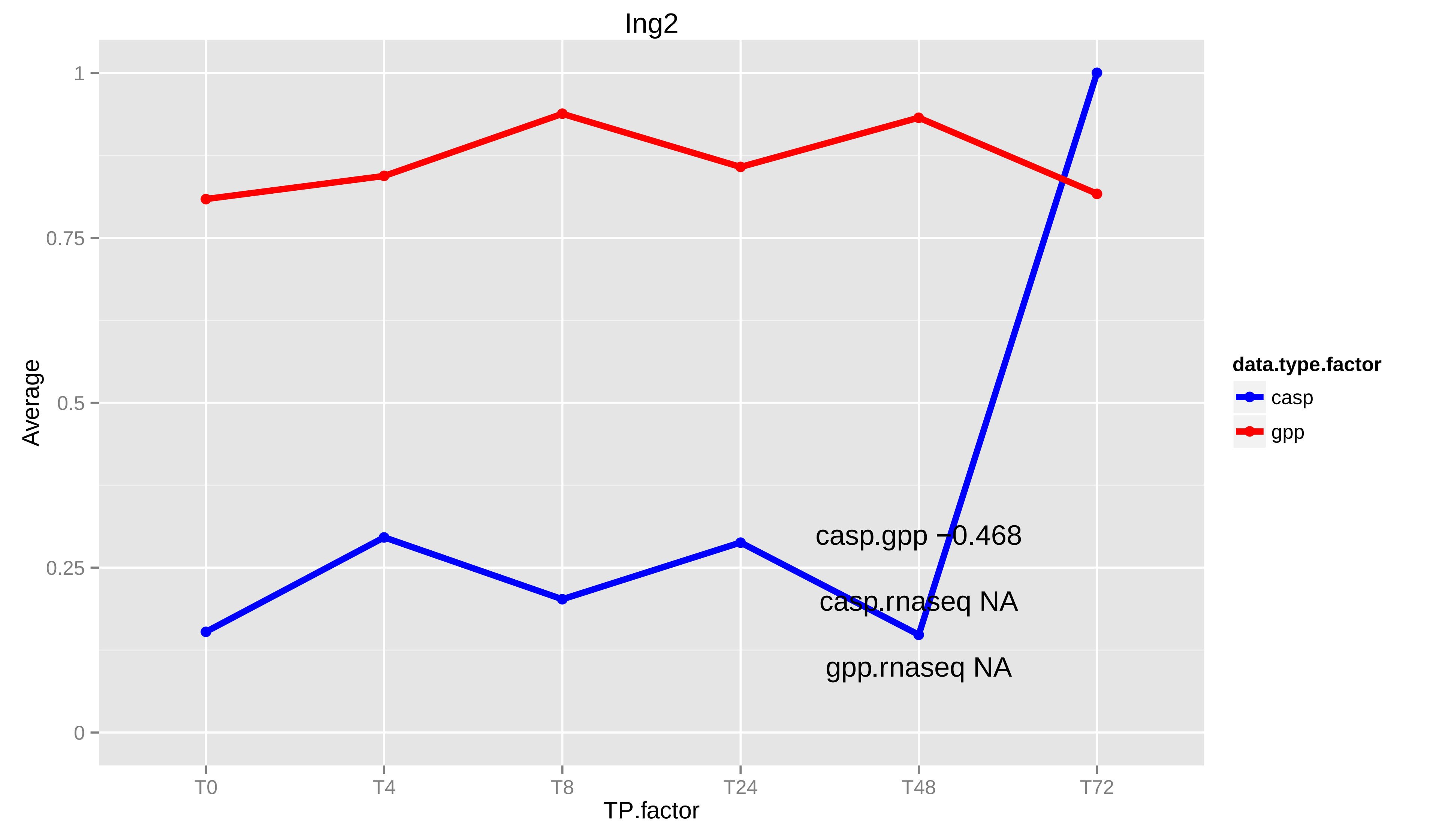

Ing2
1
0.75
0.5
0.25
0
Average
T0
T4
T8
T24
T48
T72
TP.factor
data.type.factor
casp
gpp
casp.gpp −0.468
casp.rnaseq NA
gpp.rnaseq NA

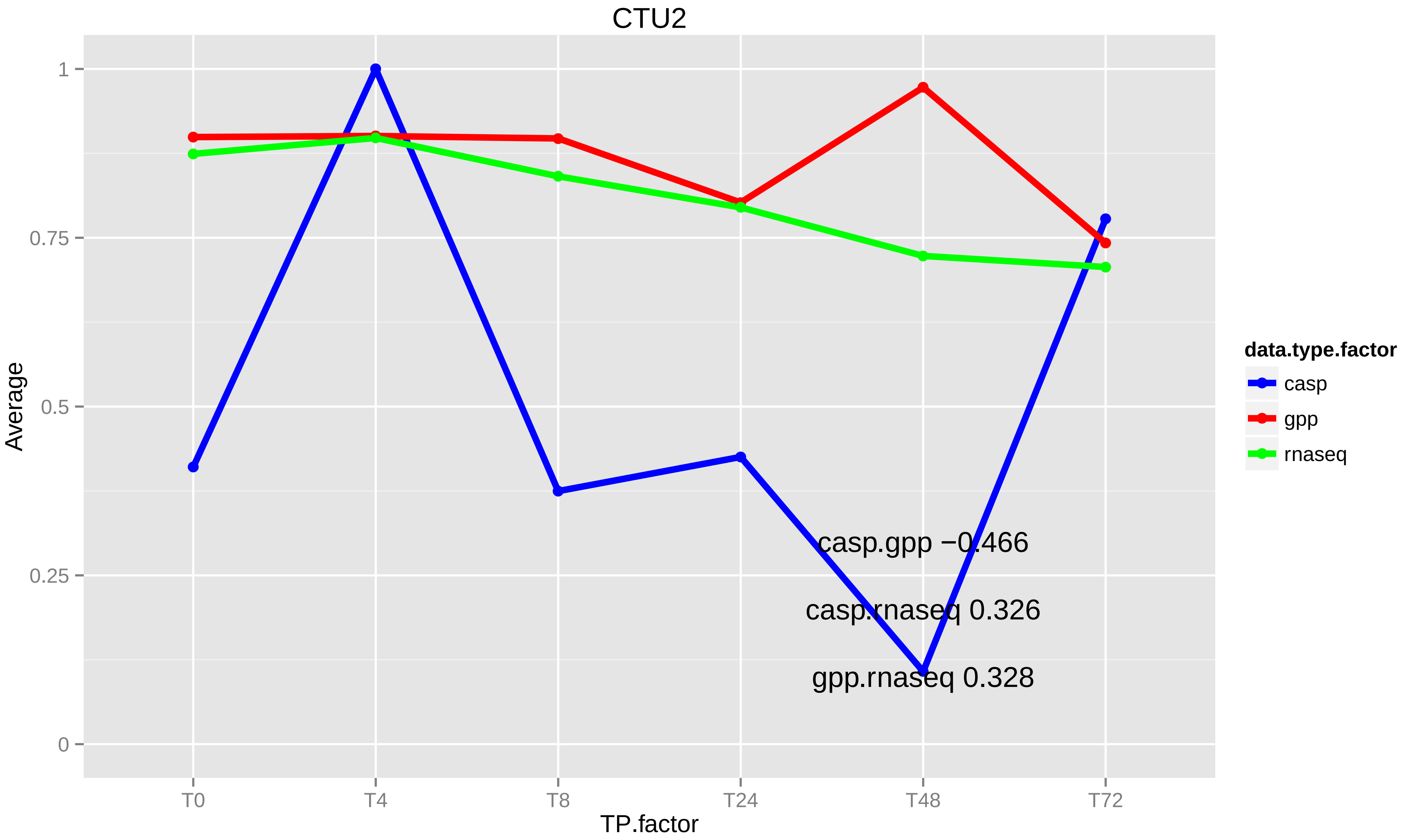
CTU2
Average
1
0.75
0.5
0.25
0
T0
T4
T8
T24
T48
T72
TP.factor
data.type.factor
casp
gpp
rnaseq
casp.gpp −0.466
casp.rnaseq 0.326
gpp.rnaseq 0.328

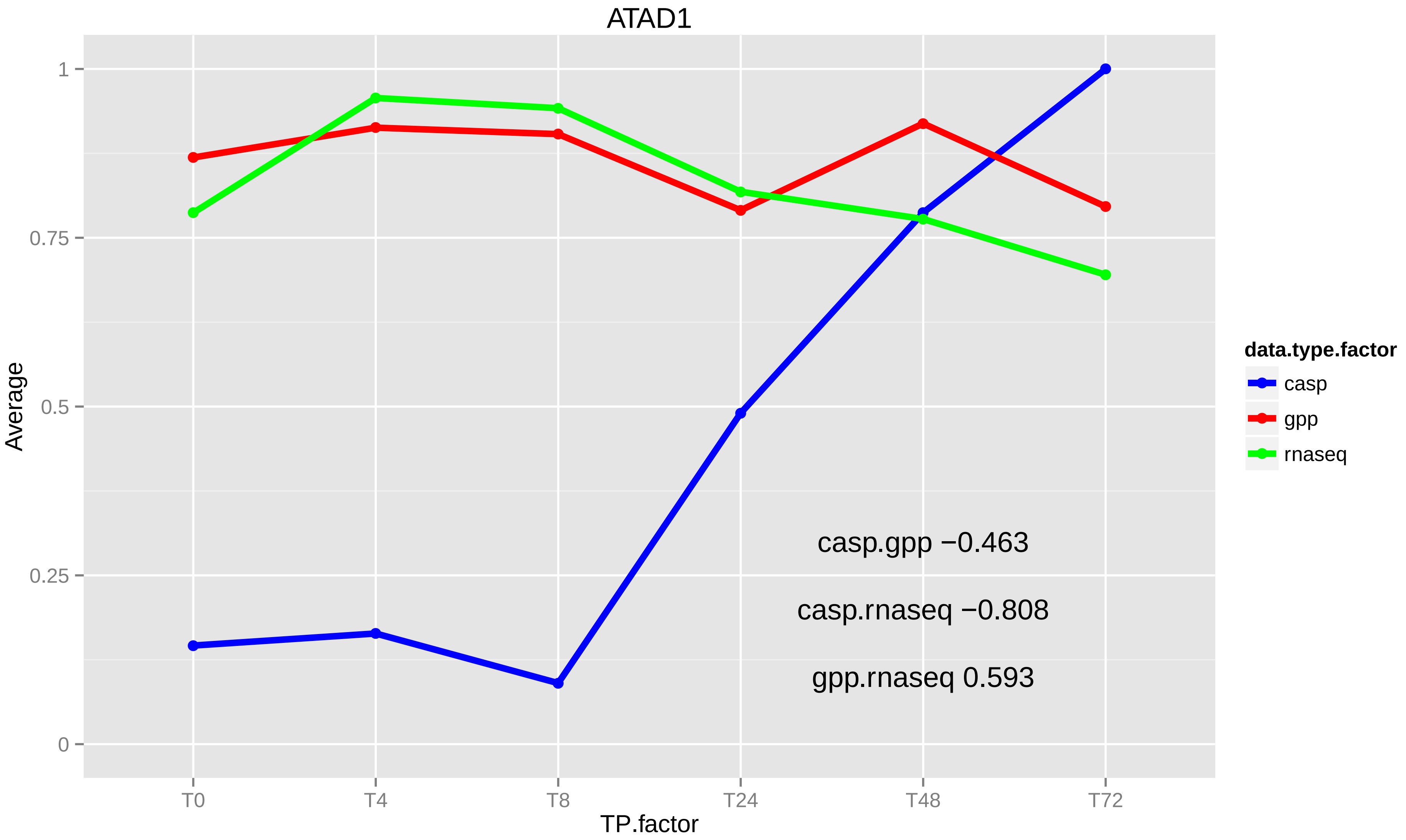
ATAD1
Average
1
0.75
0.5
0.25
0
T0
T4
T8
T24
T48
T72
TP.factor
data.type.factor
casp
gpp
rnaseq
casp.gpp −0.463
casp.rnaseq −0.808
gpp.rnaseq 0.593

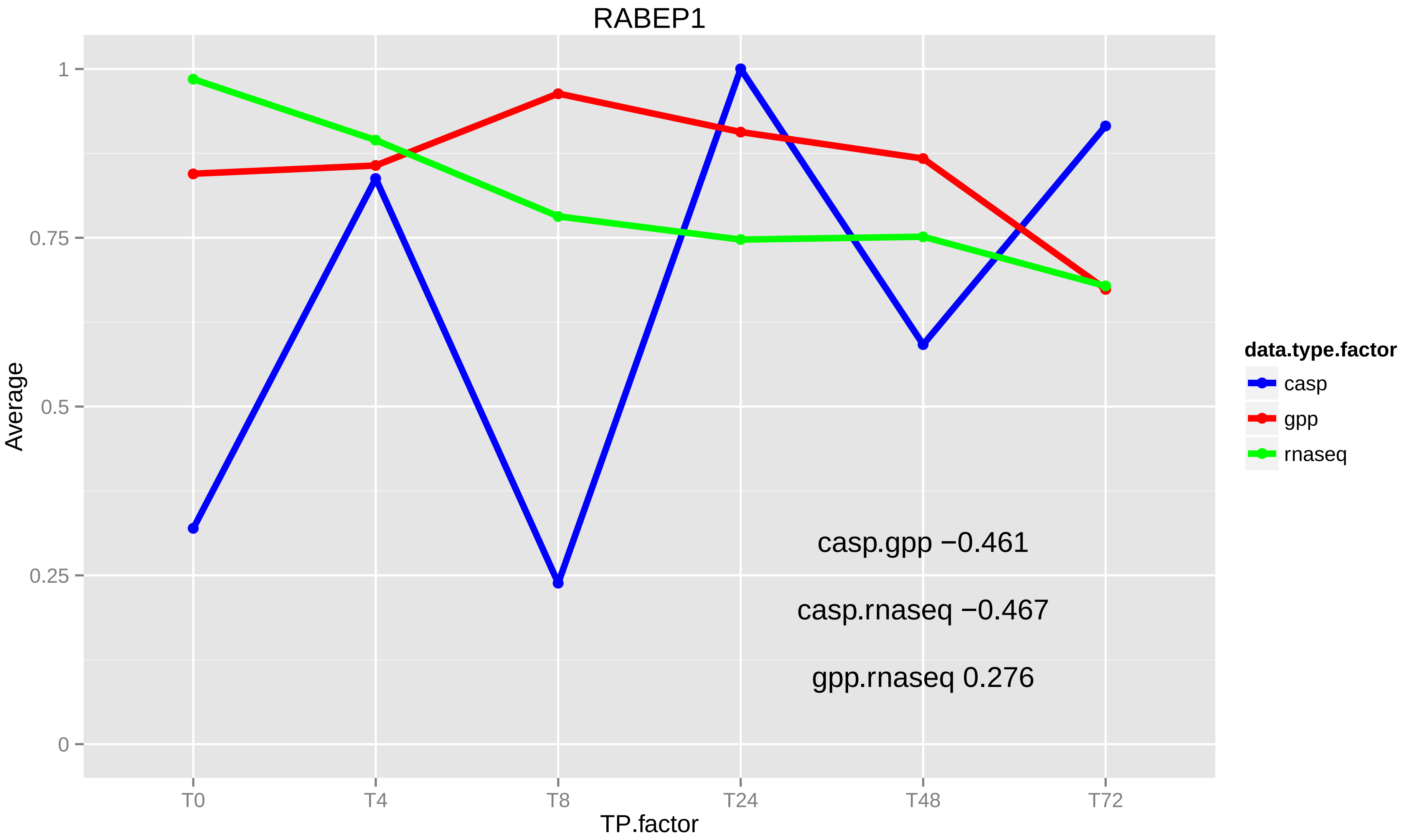
RABEP1
Average
1
0.75
0.5
0.25
0
T0
T4
T8
T24
T48
T72
TP.factor
data.type.factor
casp
gpp
rnaseq
casp.gpp −0.461
casp.rnaseq −0.467
gpp.rnaseq 0.276

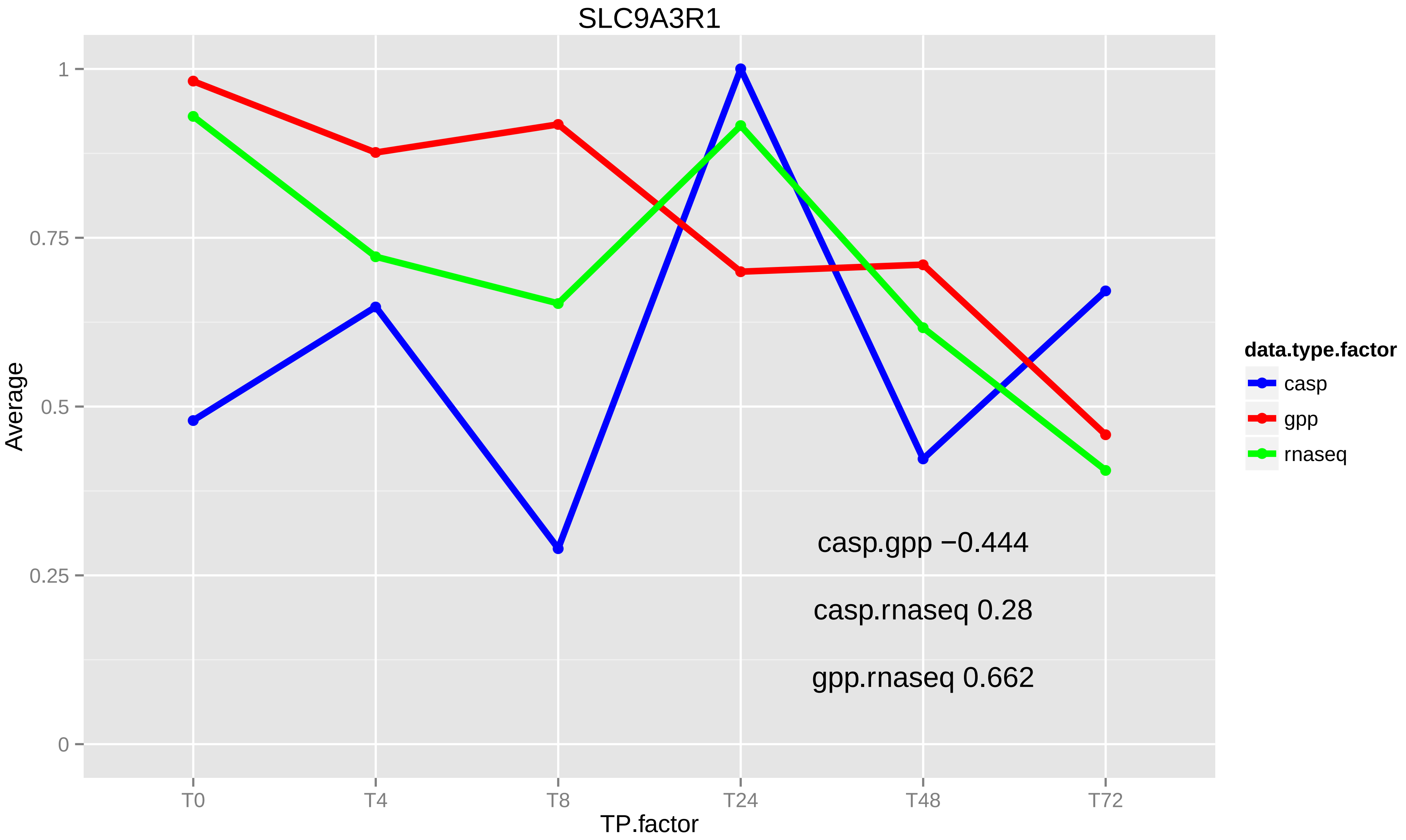

SLC9A3R1
Average
1
0.75
0.5
0.25
0
T0
T4
T8
T24
T48
T72
TP.factor
data.type.factor
casp
gpp
rnaseq
casp.gpp −0.444
casp.rnaseq 0.28
gpp.rnaseq 0.662

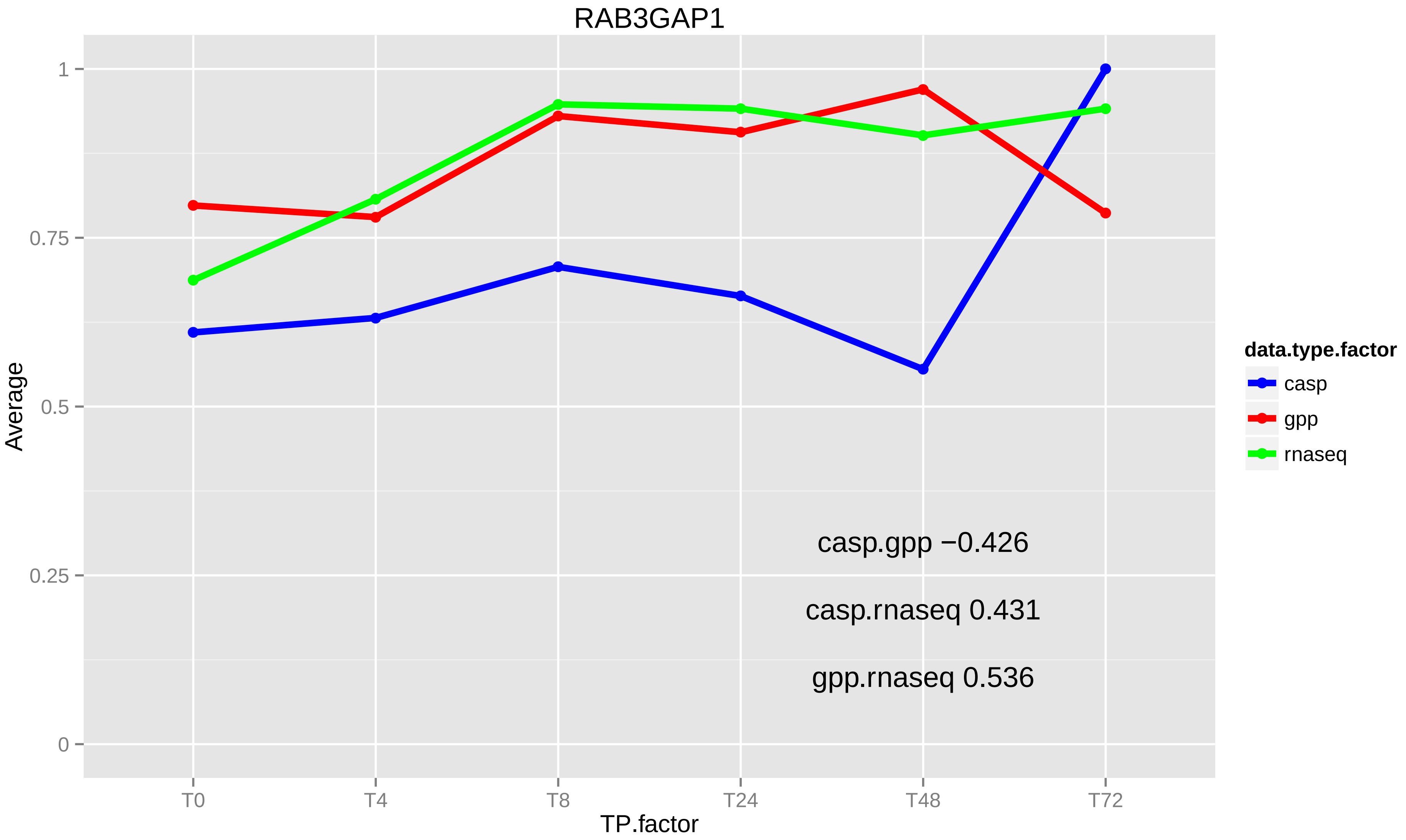

RAB3GAP1
1
0.75
0.5
0.25
0
Average
T0
T4
T8
T24
T48
T72
TP.factor
data.type.factor
casp
gpp
rnaseq
casp.gpp −0.426
casp.rnaseq 0.431
gpp.rnaseq 0.536

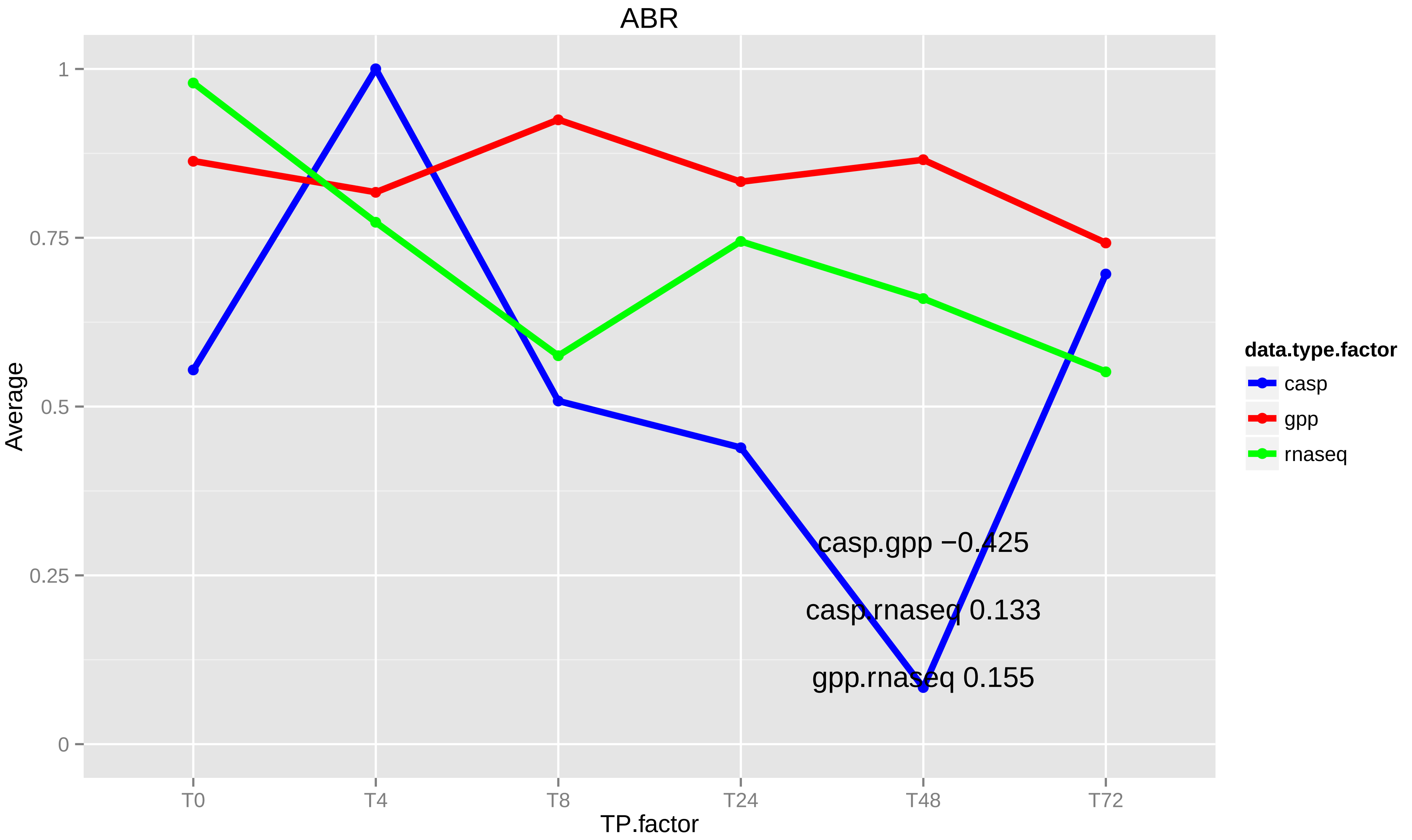

ABR
Average
1
0.75
0.5
0.25
0
T0
T4
T8
T24
T48
T72
TP.factor
data.type.factor
casp
gpp
rnaseq
casp.gpp −0.425
casp.rnaseq 0.133
gpp.rnaseq 0.155

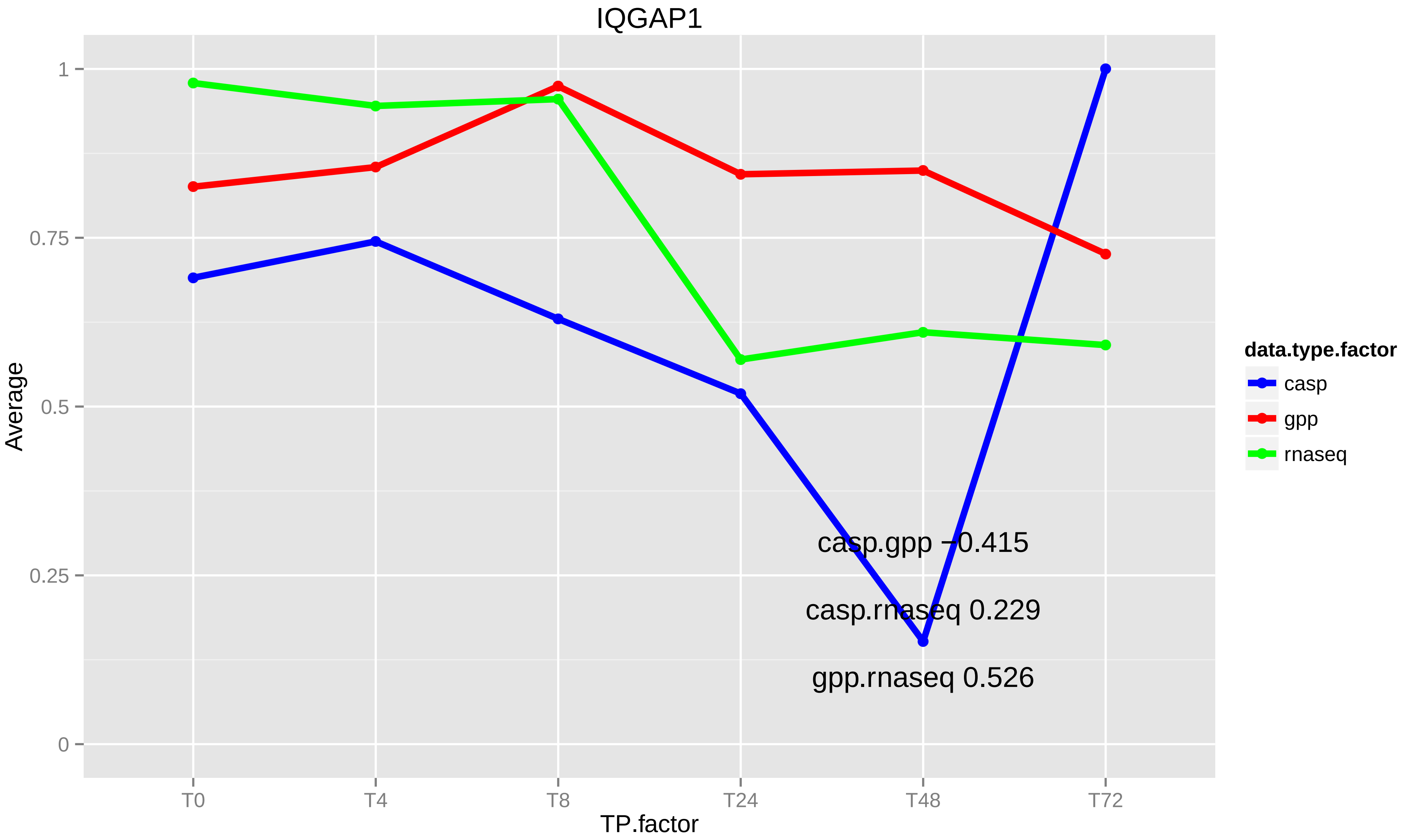
IQGAP1
Average
1
0.75
0.5
0.25
0
T0
T4
T8
T24
T48
T72
TP.factor
data.type.factor
casp
gpp
rnaseq
casp.gpp −0.415
casp.rnaseq 0.229
gpp.rnaseq 0.526

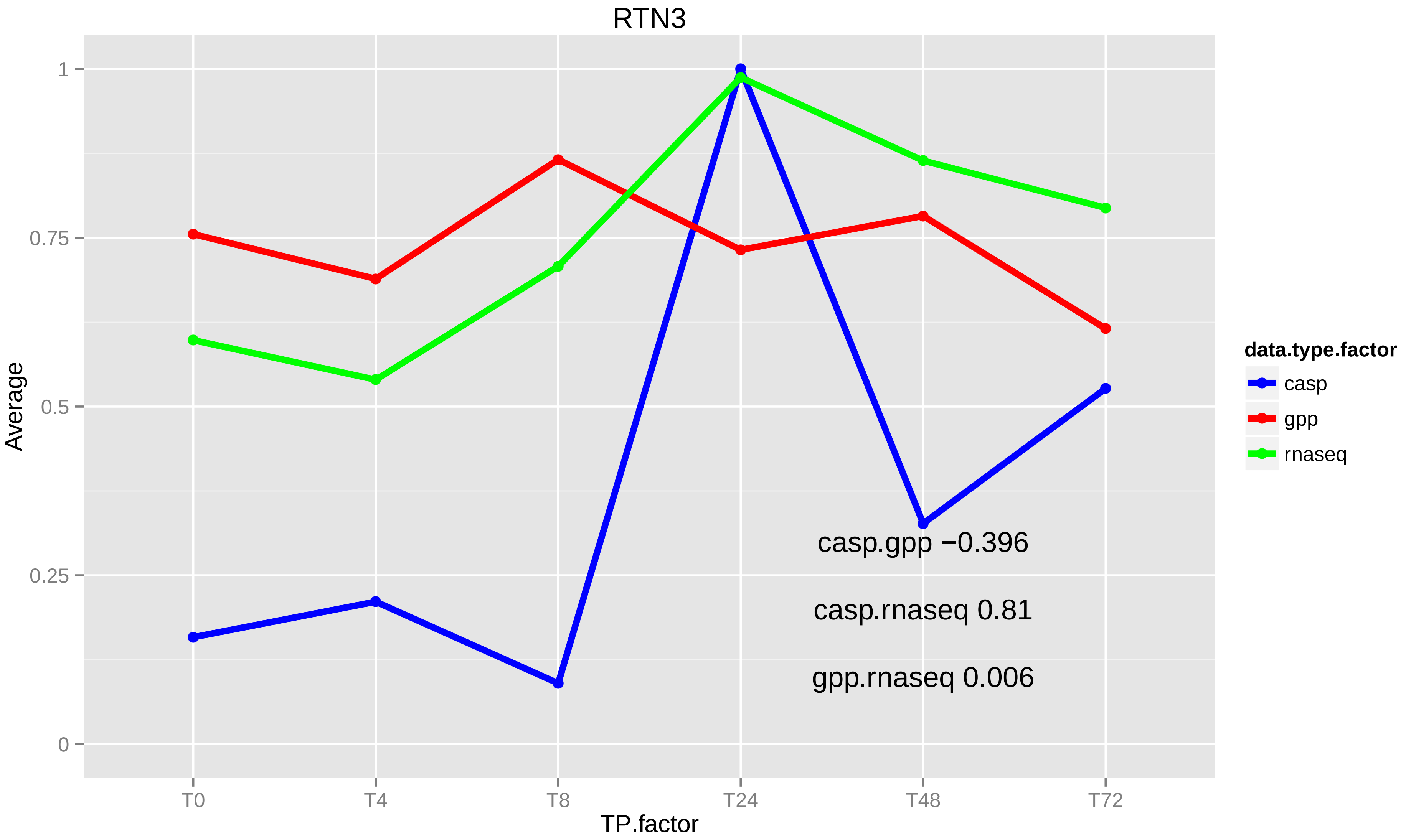
RTN3
Average
1
0.75
0.5
0.25
0
T0
T4
T8
T24
T48
T72
TP.factor
data.type.factor
casp
gpp
rnaseq
casp.gpp −0.396
casp.rnaseq 0.81
gpp.rnaseq 0.006

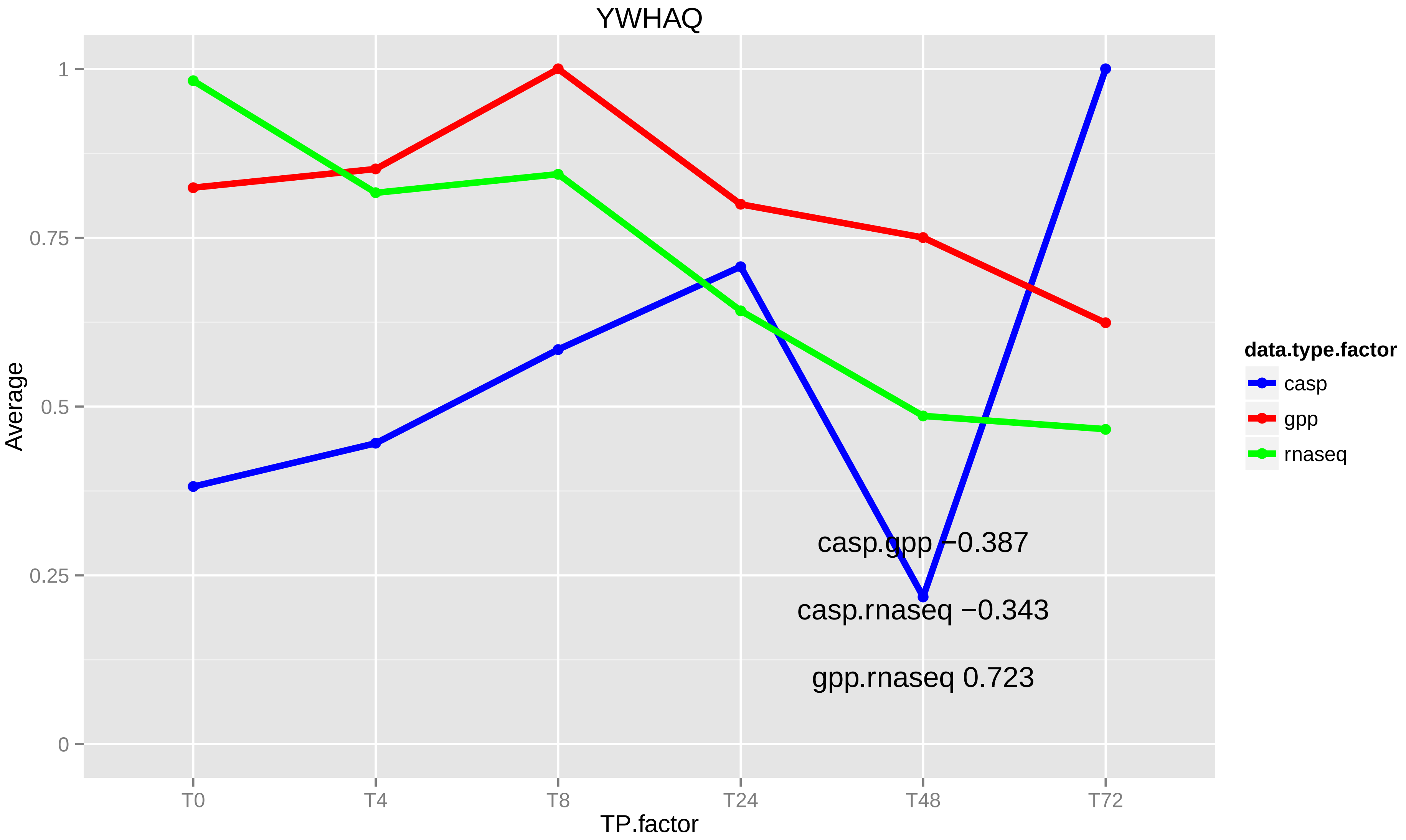
YWHAQ
1
0.75
0.5
0.25
0
Average
T0
T4
T8
T24
T48
T72
TP.factor
data.type.factor
casp
gpp
rnaseq
casp.gpp −0.387
casp.rnaseq −0.343
gpp.rnaseq 0.723

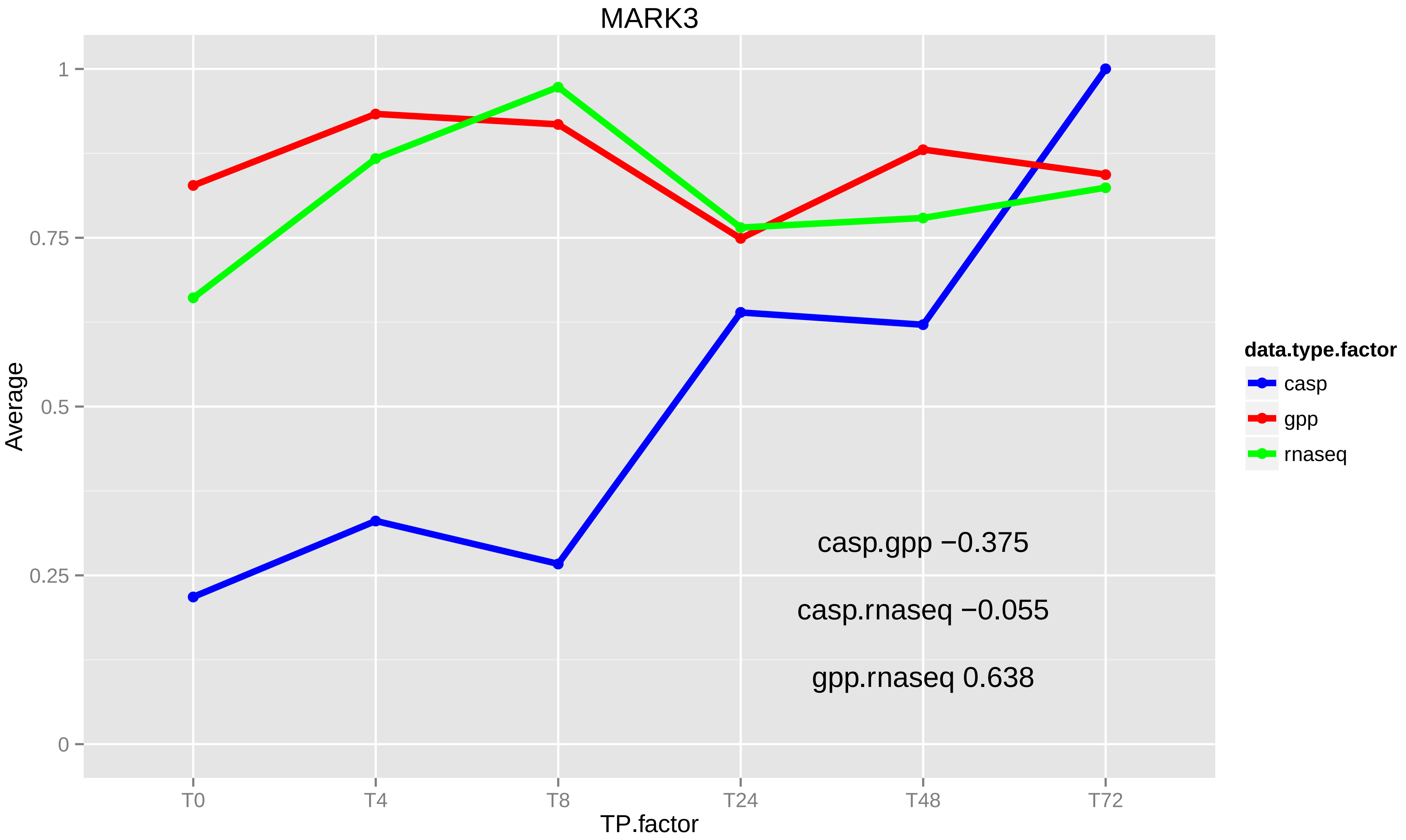
MARK3
Average
1
0.75
0.5
0.25
0
T0
T4
T8
T24
T48
T72
TP.factor
data.type.factor
casp
gpp
rnaseq
casp.gpp −0.375
casp.rnaseq −0.055
gpp.rnaseq 0.638

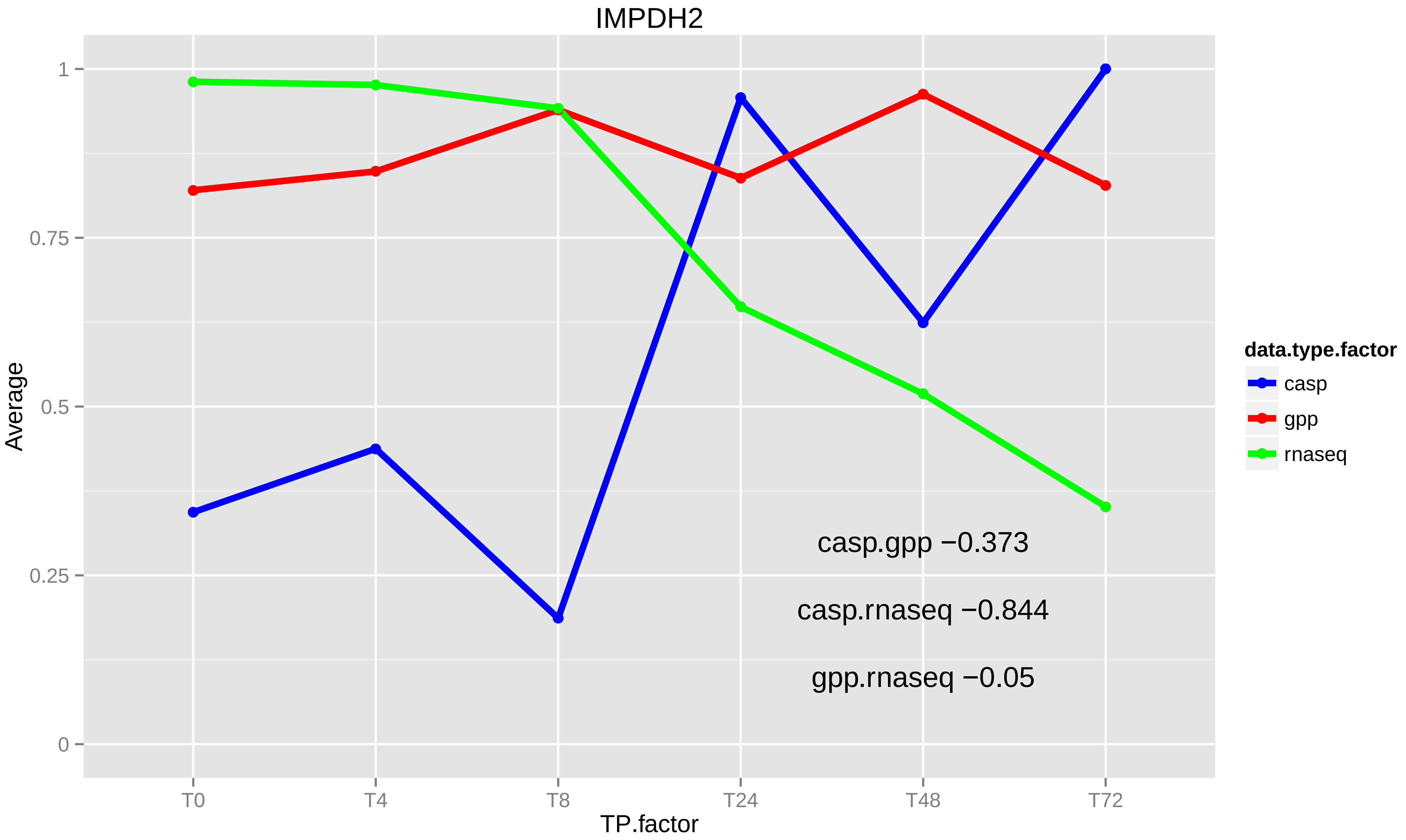
IMPDH2
Average
1
0.75
0.5
0.25
0
T0
T4
T8
T24
T48
T72
TP.factor
data.type.factor
casp
gpp
rnaseq
casp.gpp −0.373
casp.rnaseq −0.844
gpp.rnaseq −0.05

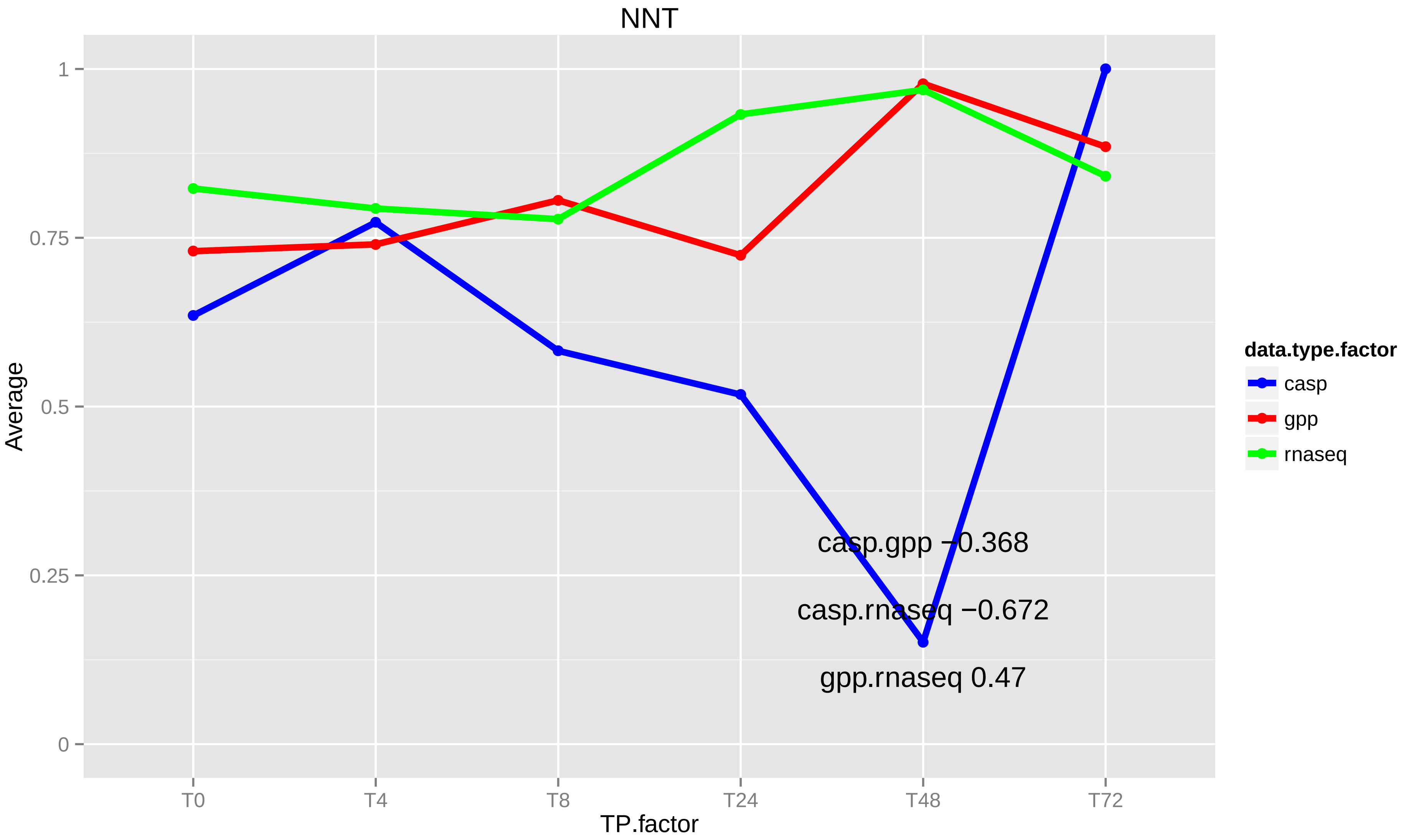

NNT
1
0.75
0.5
0.25
0
Average
T0
T4
T8
T24
T48
T72
TP.factor
casp.gpp −0.368
casp.rnaseq −0.672
gpp.rnaseq 0.47
data.type.factor
casp
gpp
rnaseq

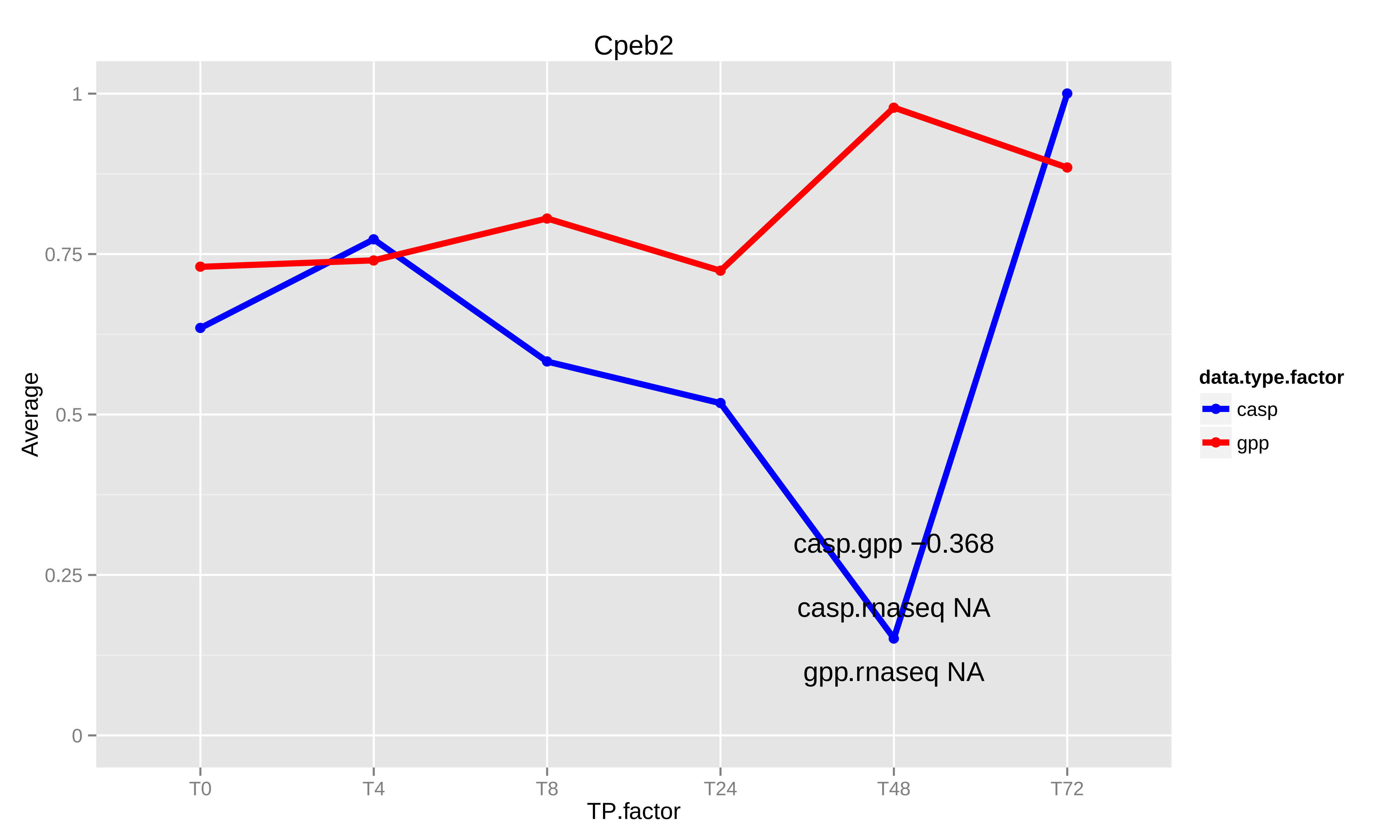

Cpeb2
1
0.75
0.5
0.25
0
Average
T0
T4
T8
T24
T48
T72
TP.factor
data.type.factor
casp
gpp
casp.gpp −0.368
casp.rnaseq NA
gpp.rnaseq NA

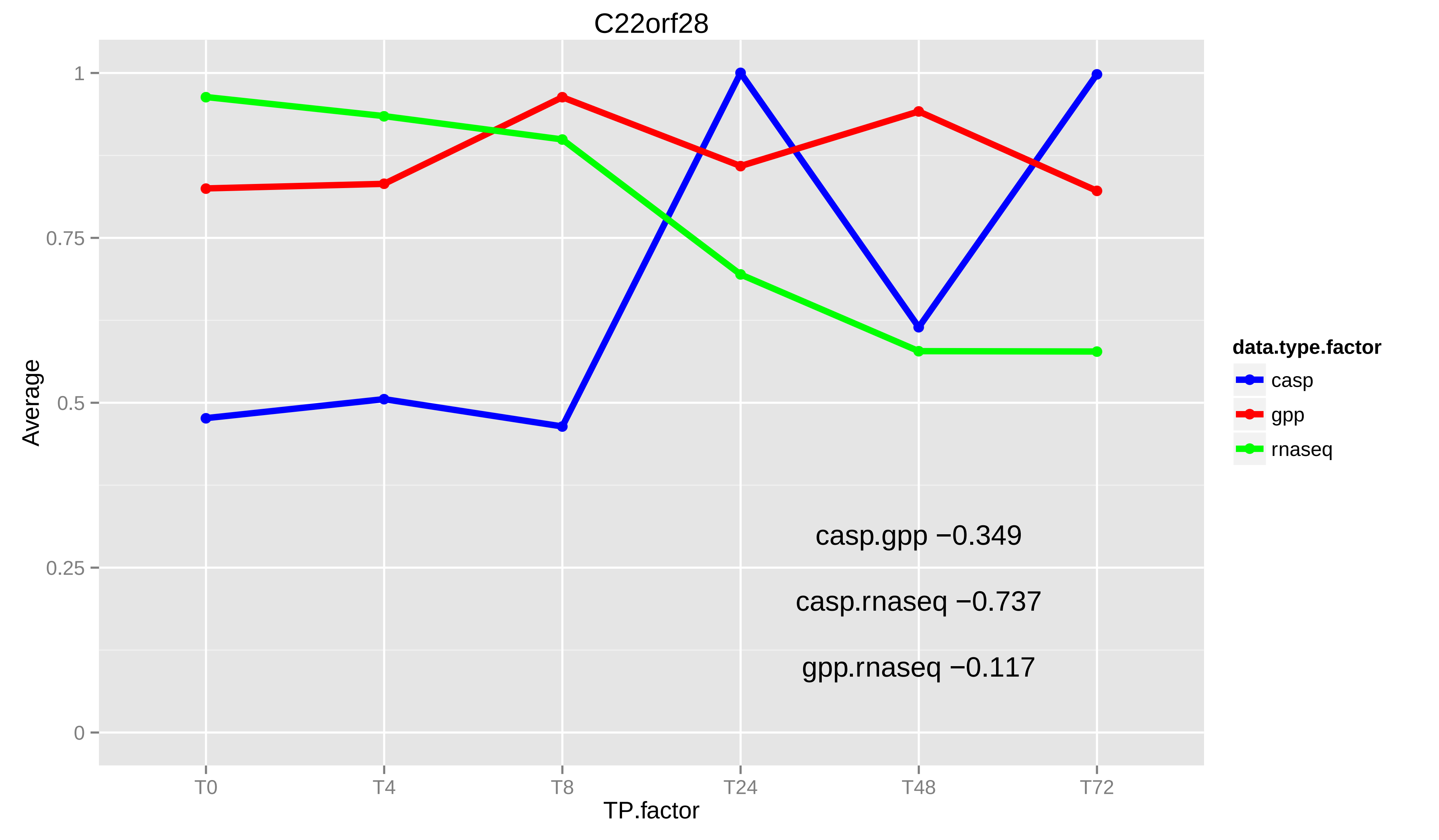

C22orf28
1
0.75
0.5
0.25
0
Average
T0
T4
T8
T24
T48
T72
TP.factor
data.type.factor
casp
gpp
rnaseq
casp.gpp −0.349
casp.rnaseq −0.737
gpp.rnaseq −0.117

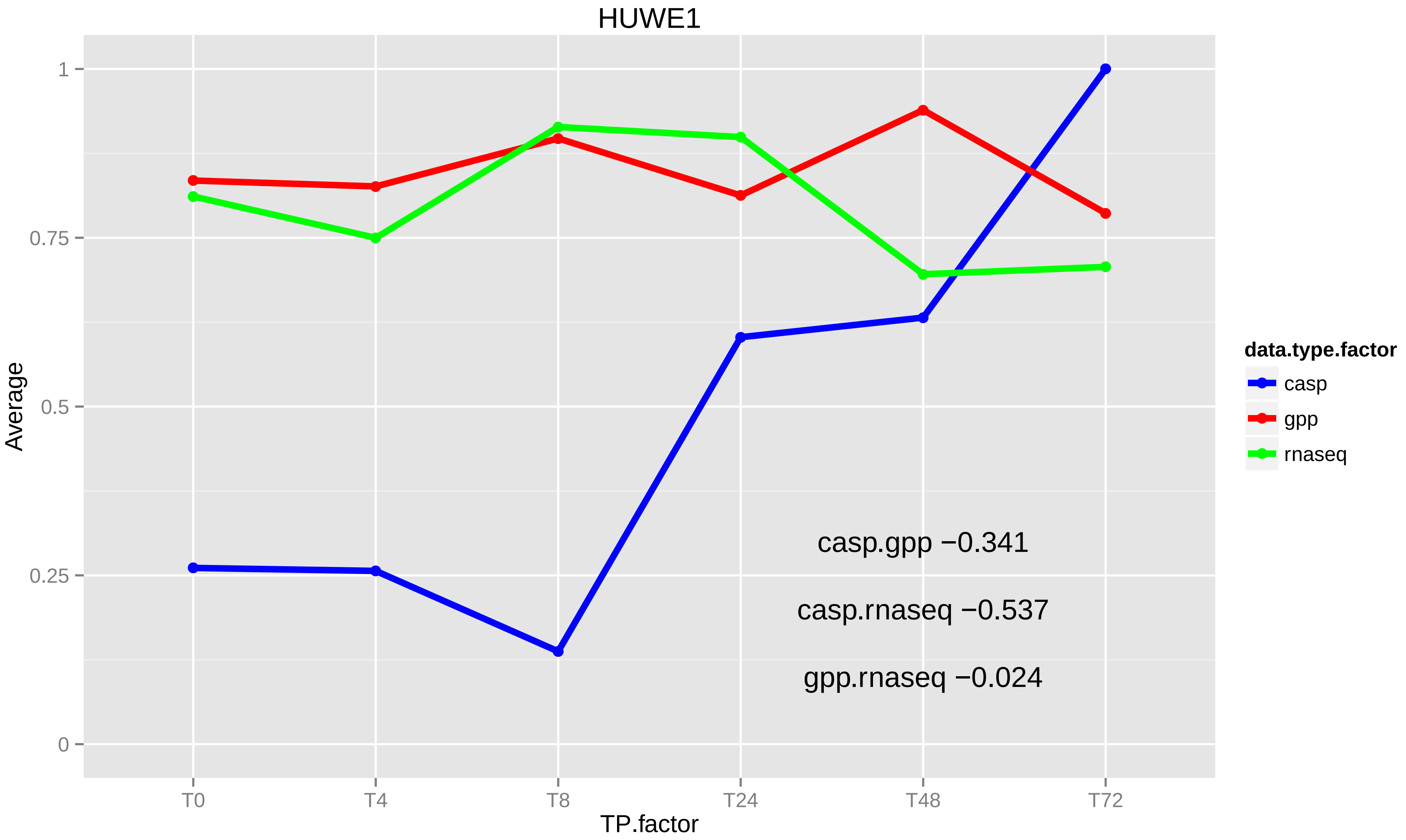
HUWE1
Average
1
0.75
0.5
0.25
0
T0
T4
T8
T24
T48
T72
TP.factor
data.type.factor
casp
gpp
rnaseq
casp.gpp −0.341
casp.rnaseq −0.537
gpp.rnaseq −0.024

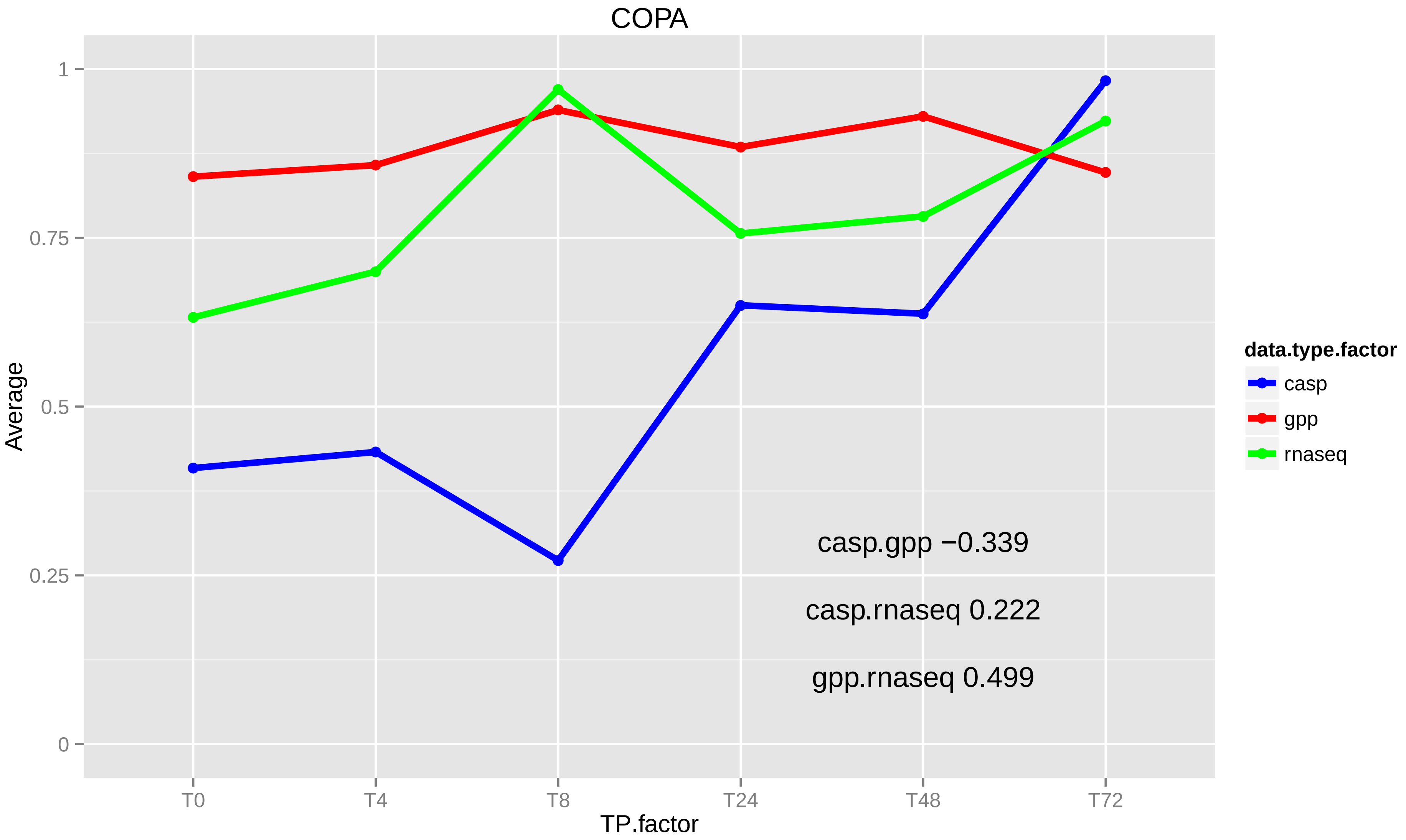
COPA
Average
1
0.75
0.5
0.25
0
T0
T4
T8
T24
T48
T72
TP.factor
data.type.factor
casp
gpp
rnaseq
casp.gpp −0.339
casp.rnaseq 0.222
gpp.rnaseq 0.499

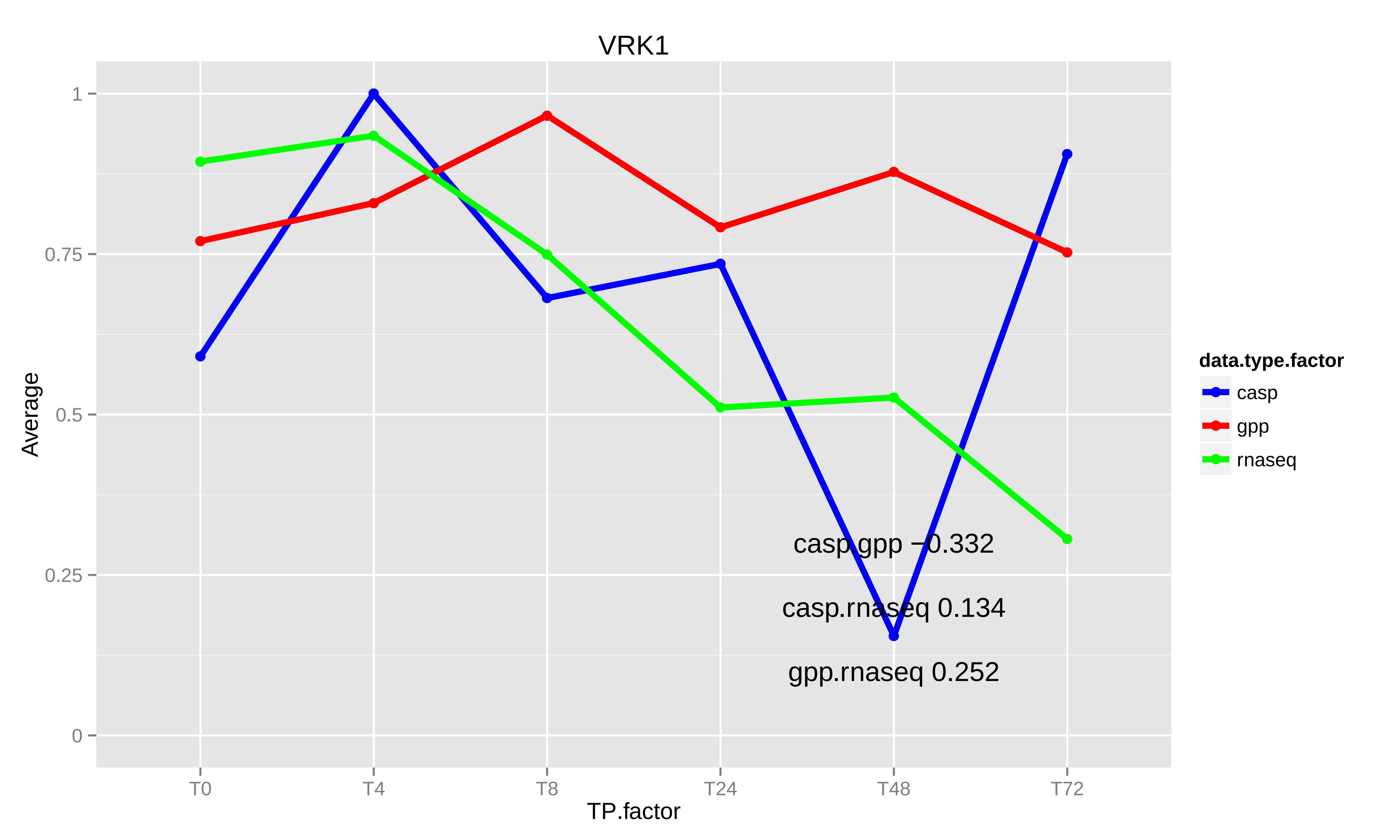
VRK1
Average
1
0.75
0.5
0.25
0
T0
T4
T8
T24
T48
T72
TP.factor
data.type.factor
casp
gpp
rnaseq
casp.gpp −0.332
casp.rnaseq 0.134
gpp.rnaseq 0.252

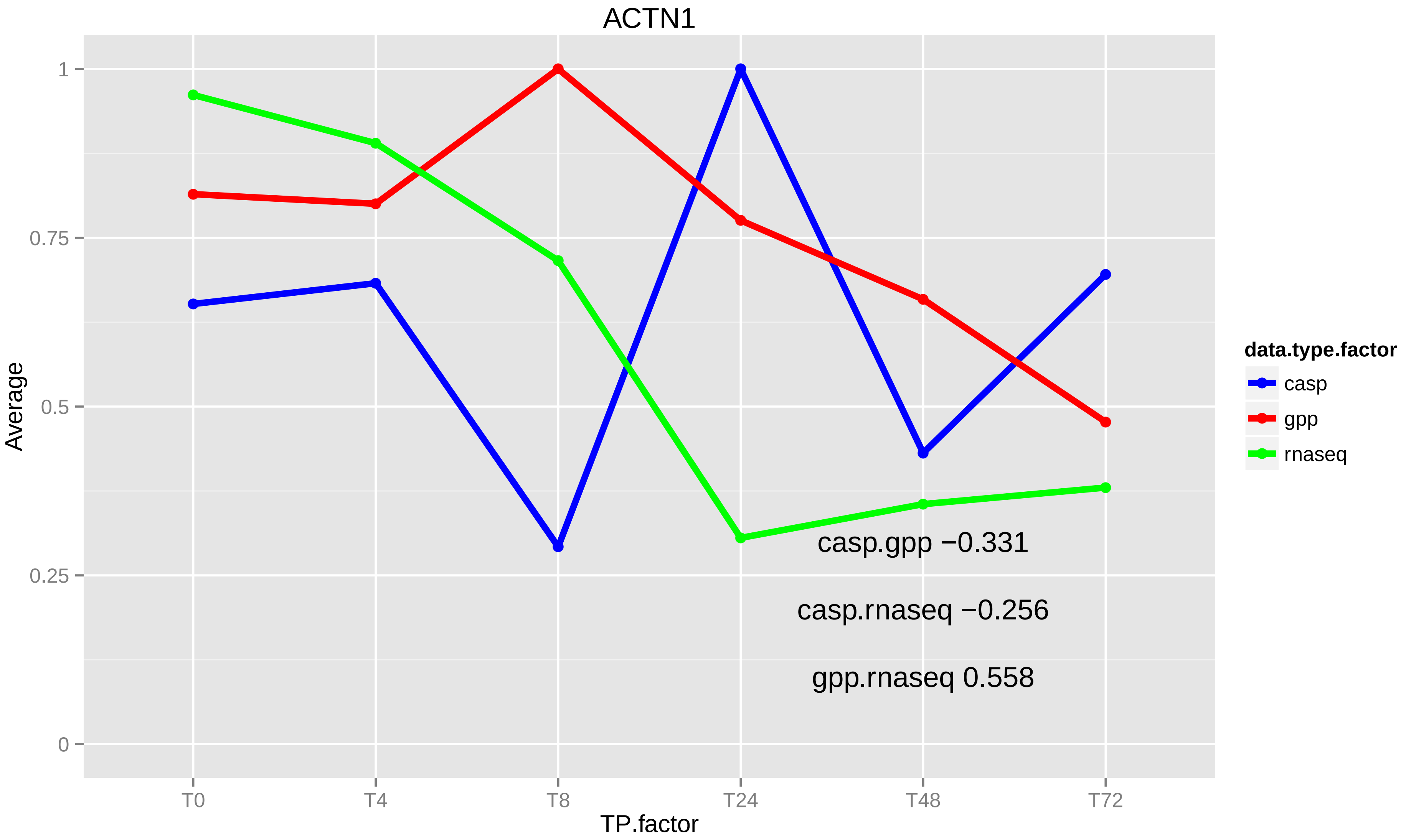

ACTN1
Average
1
0.75
0.5
0.25
0
T0
T4
T8
T24
T48
T72
TP.factor
data.type.factor
casp
gpp
rnaseq
casp.gpp −0.331
casp.rnaseq −0.256
gpp.rnaseq 0.558

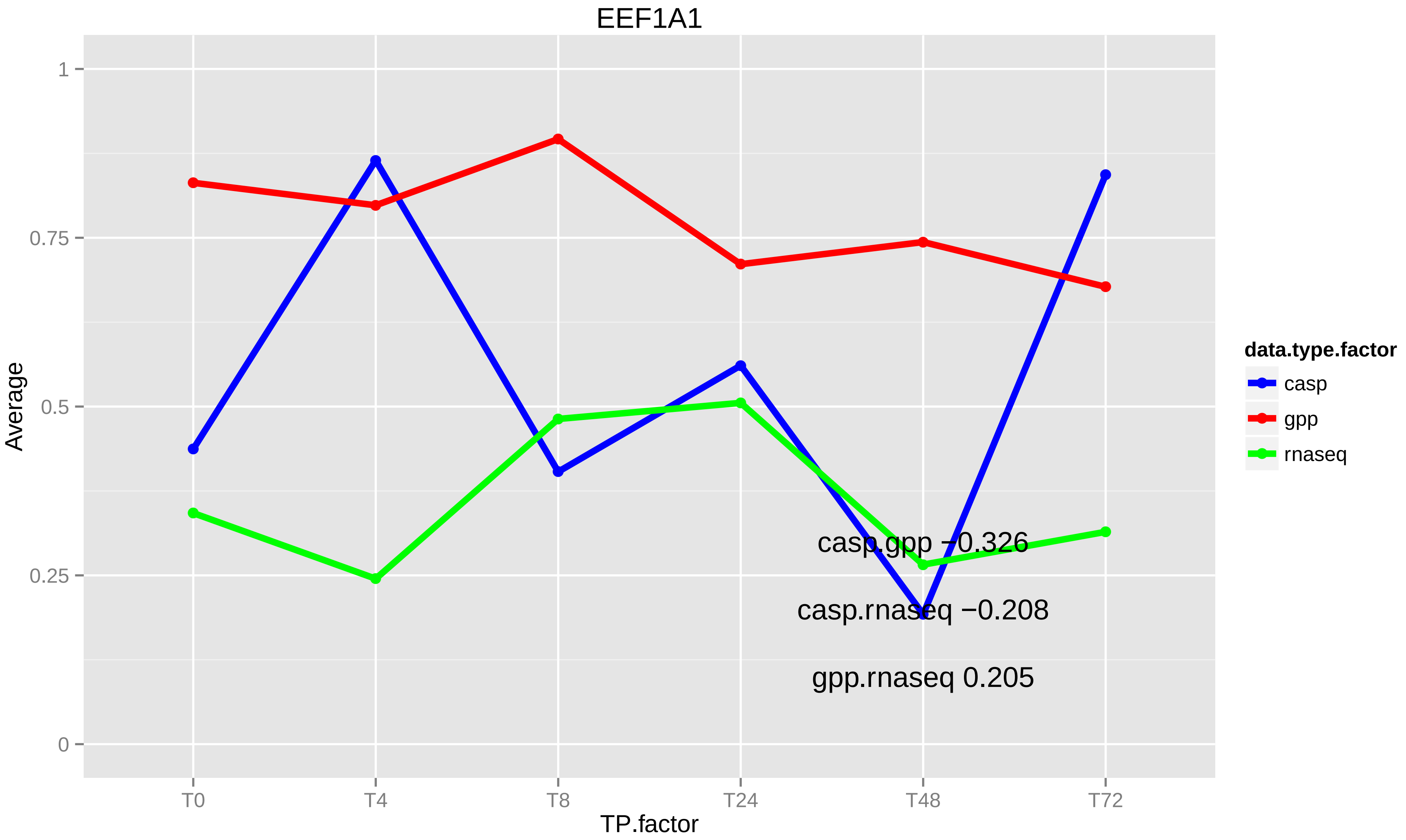
EEF1A1
Average
1
0.75
0.5
0.25
0
T0
T4
T8
T24
T48
T72
TP.factor
data.type.factor
casp
gpp
rnaseq
casp.gpp −0.326
casp.rnaseq −0.208
gpp.rnaseq 0.205

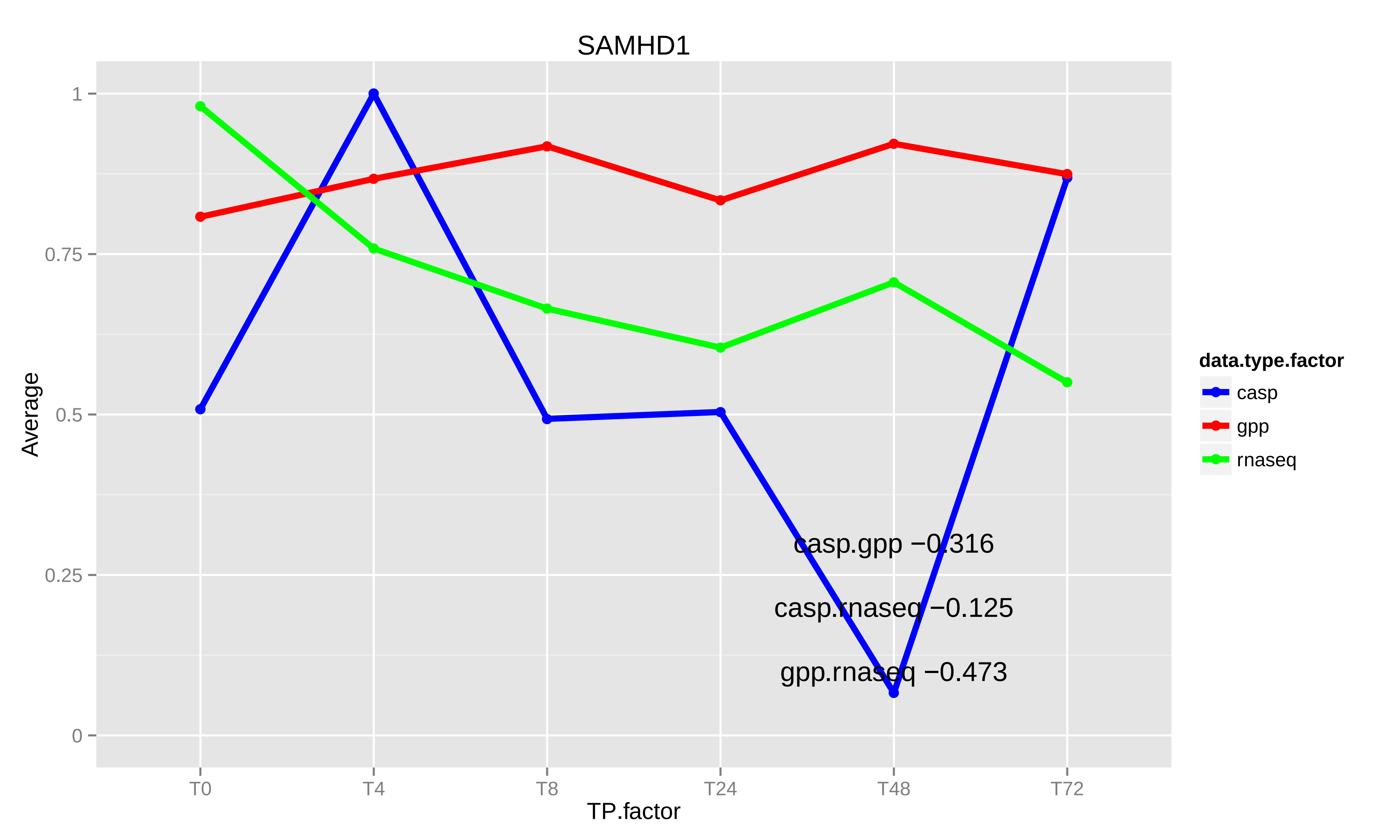
SAMHD1
Average
1
0.75
0.5
0.25
0
T0
T4
T8
T24
T48
T72
TP.factor
data.type.factor
casp
gpp
rnaseq
casp.gpp −0.316
casp.rnaseq −0.125
gpp.rnaseq −0.473

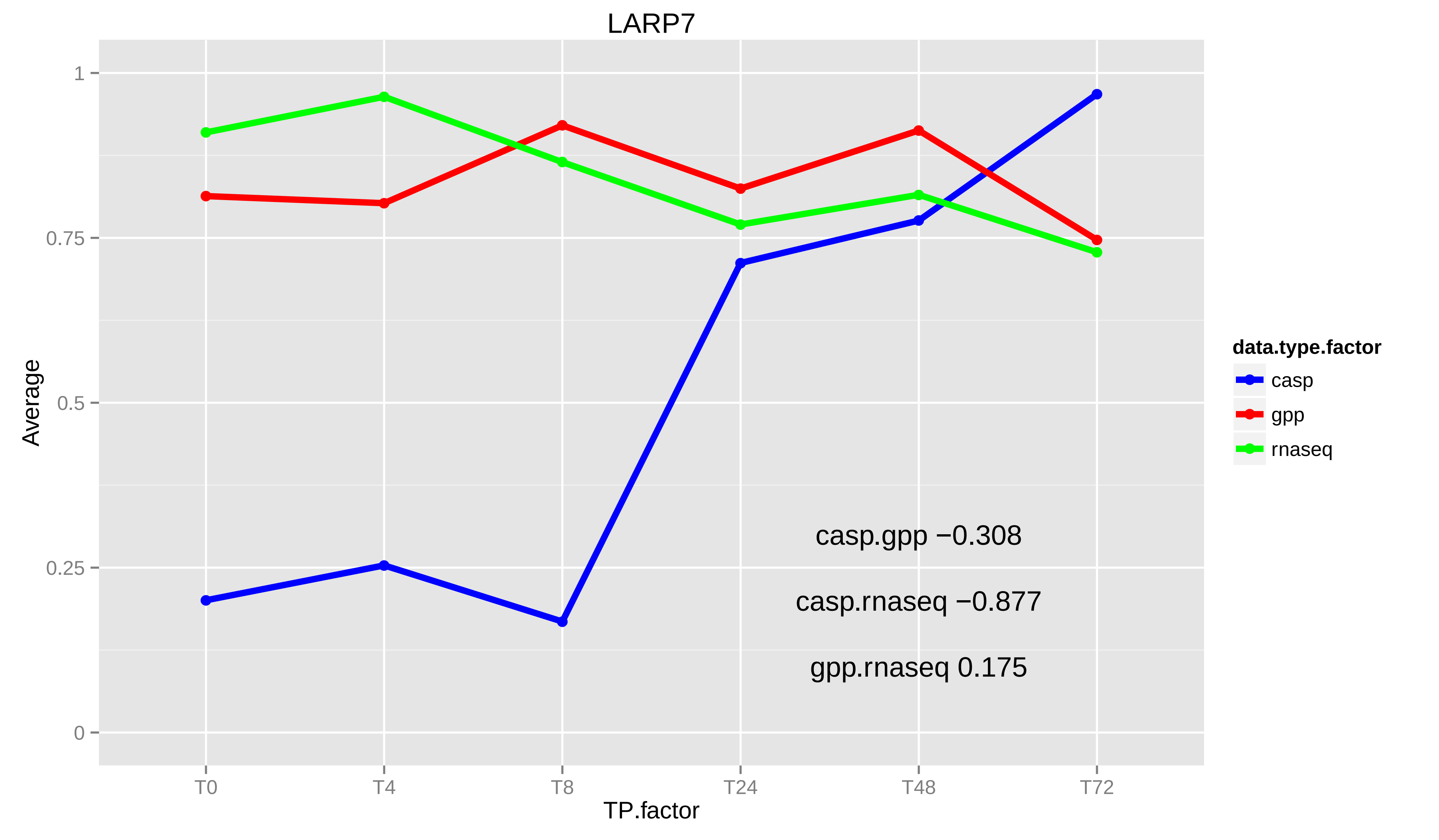

LARP7
Average
1
0.75
0.5
0.25
0
T0
T4
T8
T24
T48
T72
TP.factor
data.type.factor
casp
gpp
rnaseq
casp.gpp −0.308
casp.rnaseq −0.877
gpp.rnaseq 0.175

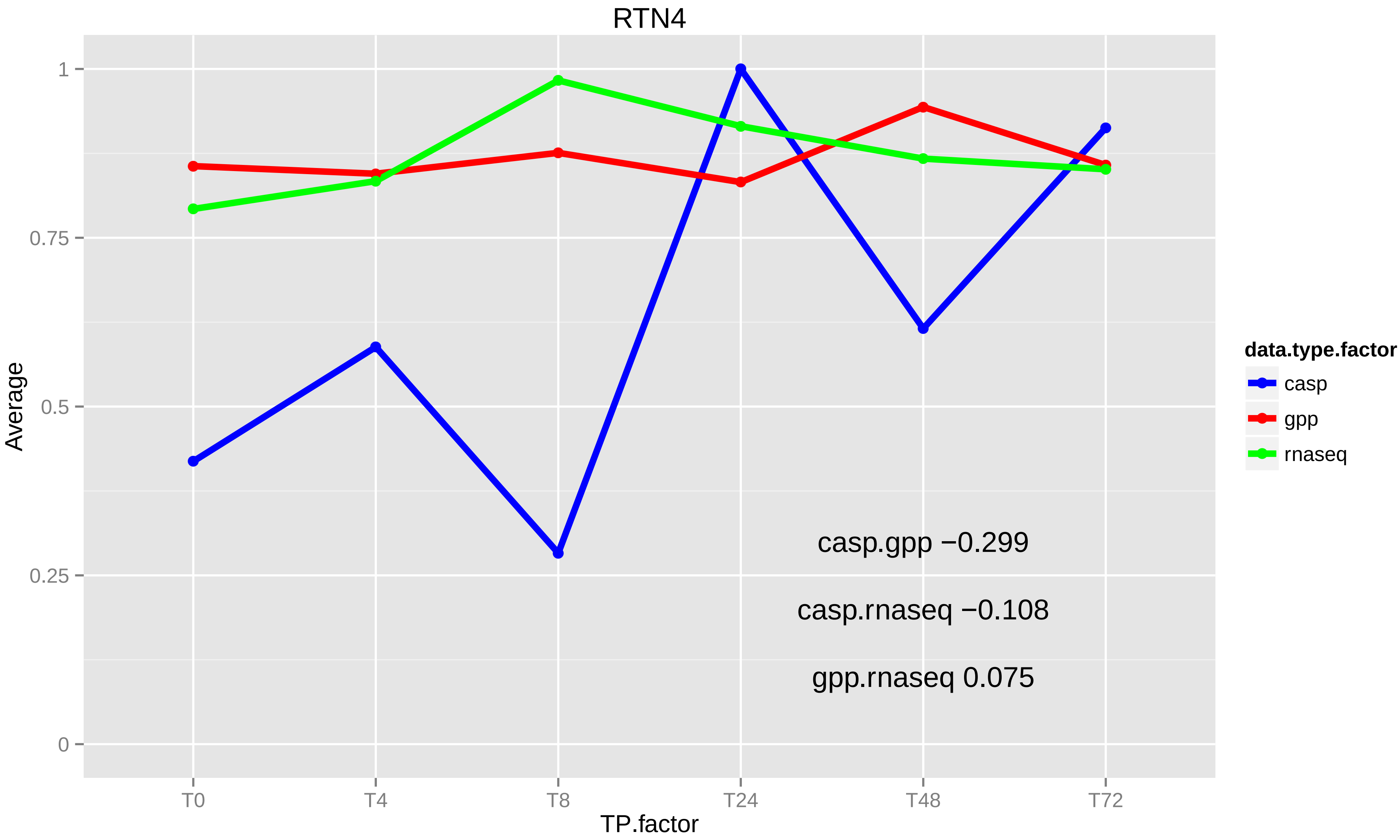

RTN4
1
0.75
0.5
0.25
0
Average
T0
T4
T8
T24
T48
T72
TP.factor
data.type.factor
casp
gpp
rnaseq
casp.gpp −0.299
casp.rnaseq −0.108
gpp.rnaseq 0.075

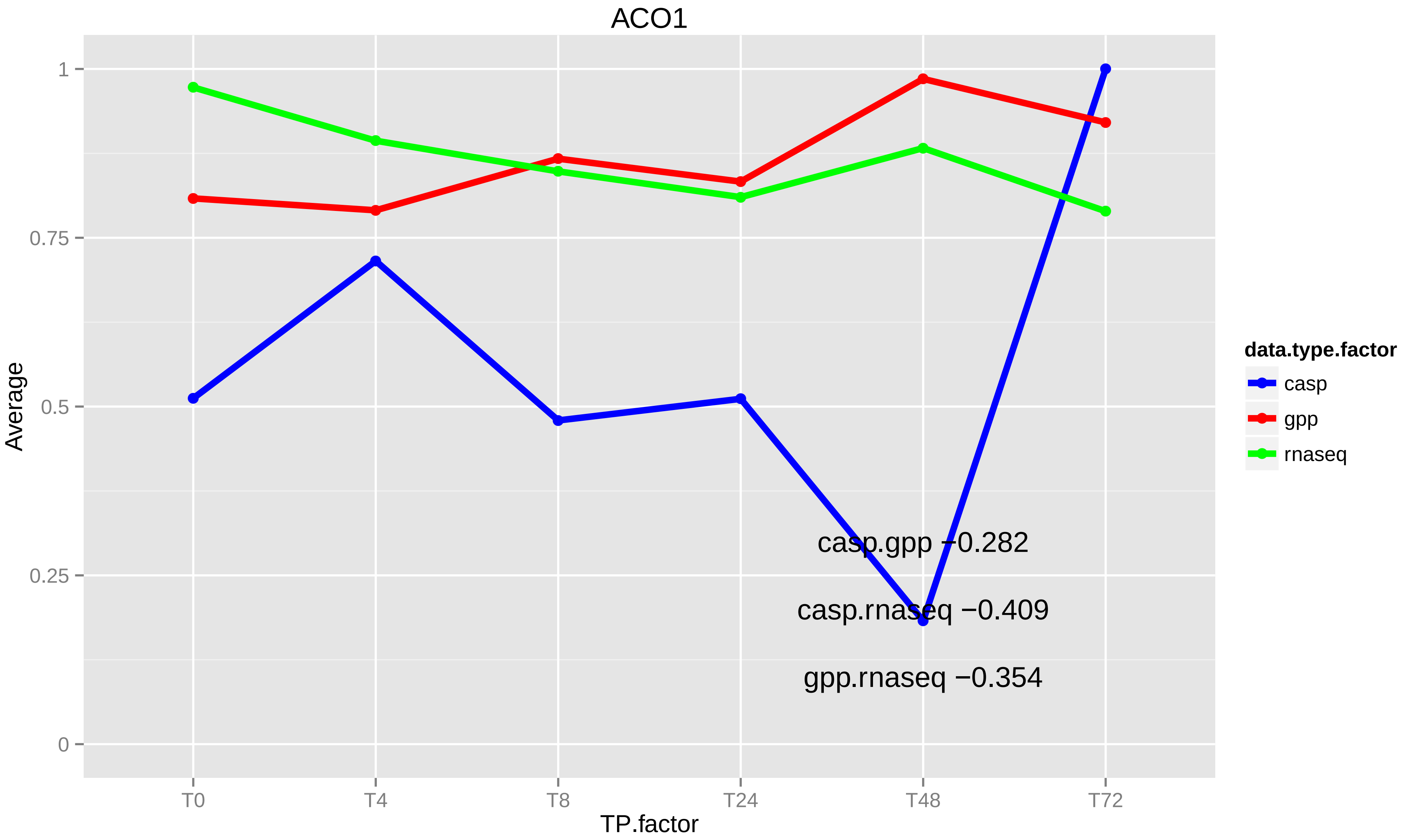

ACO1
1
0.75
0.5
0.25
0
Average
T0
T4
T8
T24
T48
T72
TP.factor
data.type.factor
casp
gpp
rnaseq
casp.gpp −0.282
casp.rnaseq −0.409
gpp.rnaseq −0.354

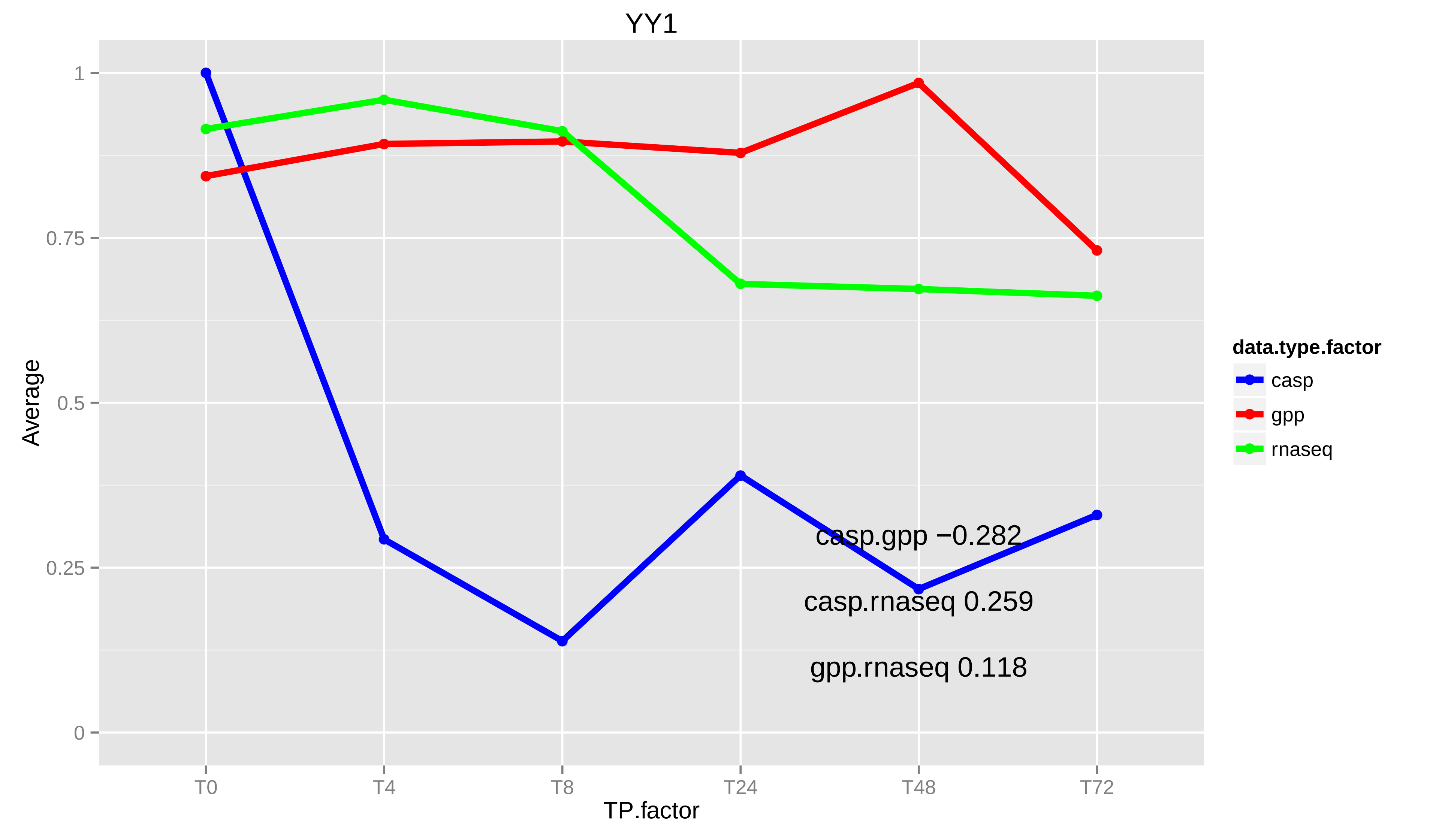

YY1
Average
1
0.75
0.5
0.25
0
T0
T4
T8
T24
T48
T72
TP.factor
data.type.factor
casp
gpp
rnaseq
casp.gpp −0.282
casp.rnaseq 0.259
gpp.rnaseq 0.118

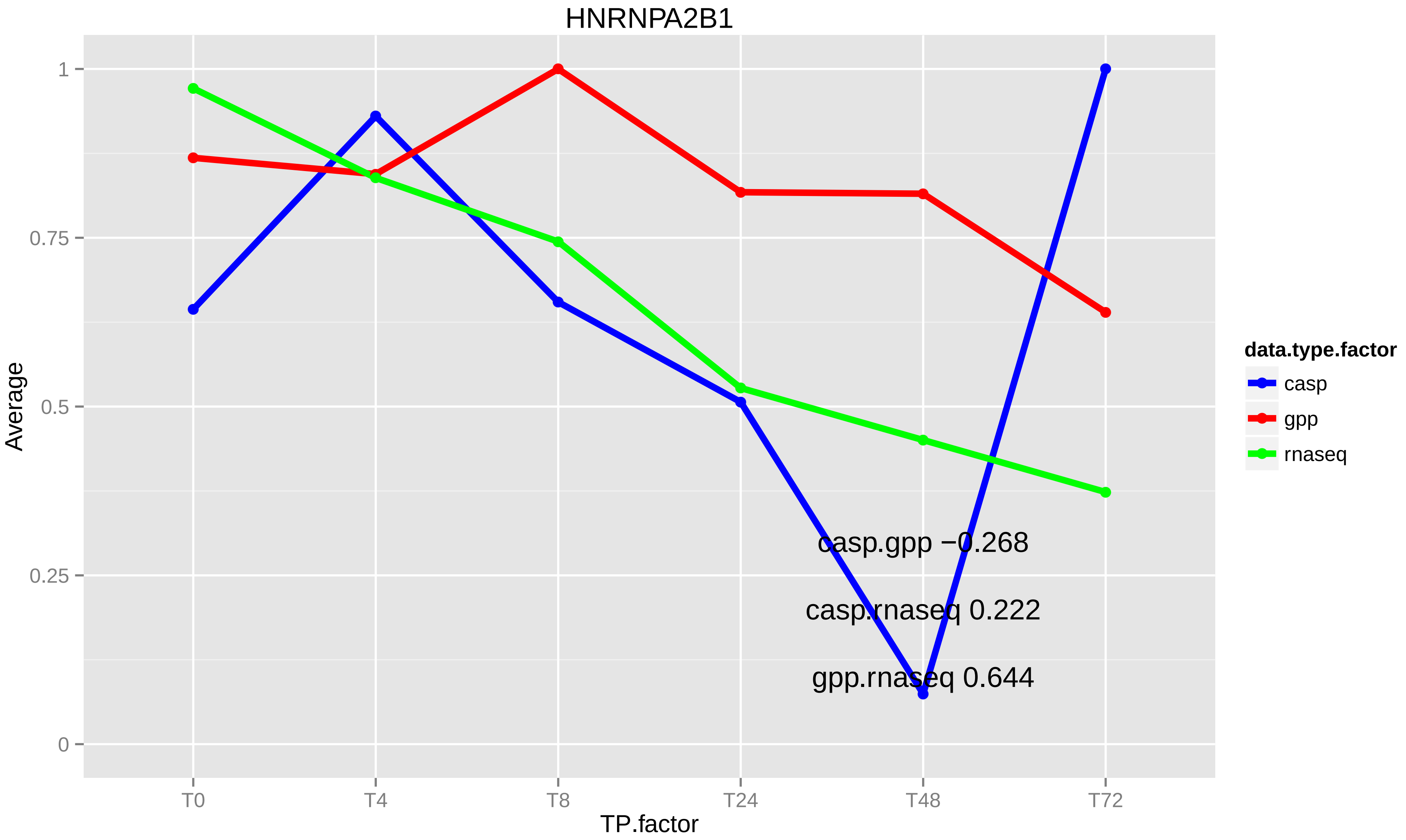

HNRNPA2B1
Average
1
0.75
0.5
0.25
0
T0
T4
T8
T24
T48
T72
TP.factor
data.type.factor
casp
gpp
rnaseq
casp.gpp −0.268
casp.rnaseq 0.222
gpp.rnaseq 0.644

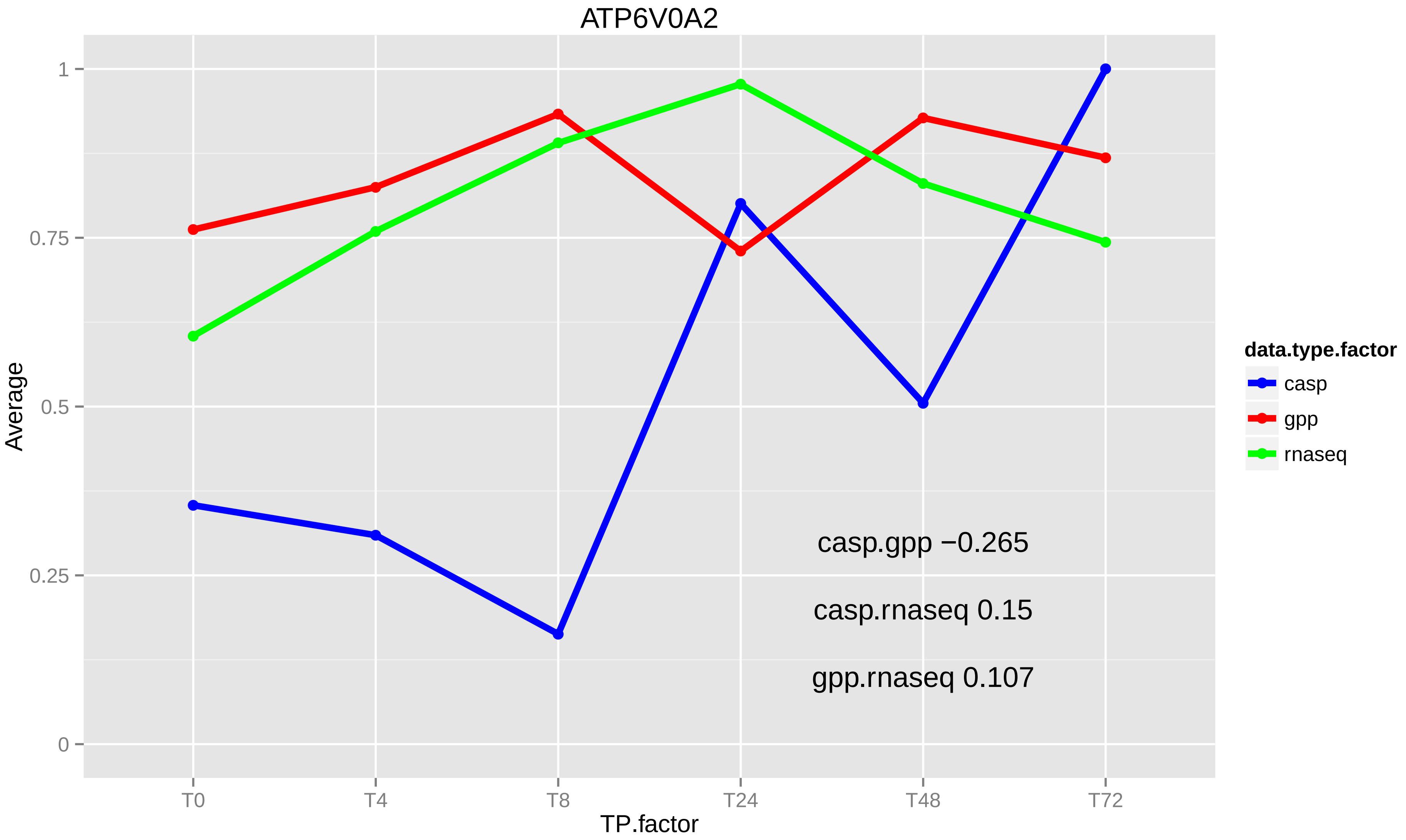
ATP6V0A2
Average
1
0.75
0.5
0.25
0
T0
T4
T8
T24
T48
T72
TP.factor
data.type.factor
casp
gpp
rnaseq
casp.gpp −0.265
casp.rnaseq 0.15
gpp.rnaseq 0.107

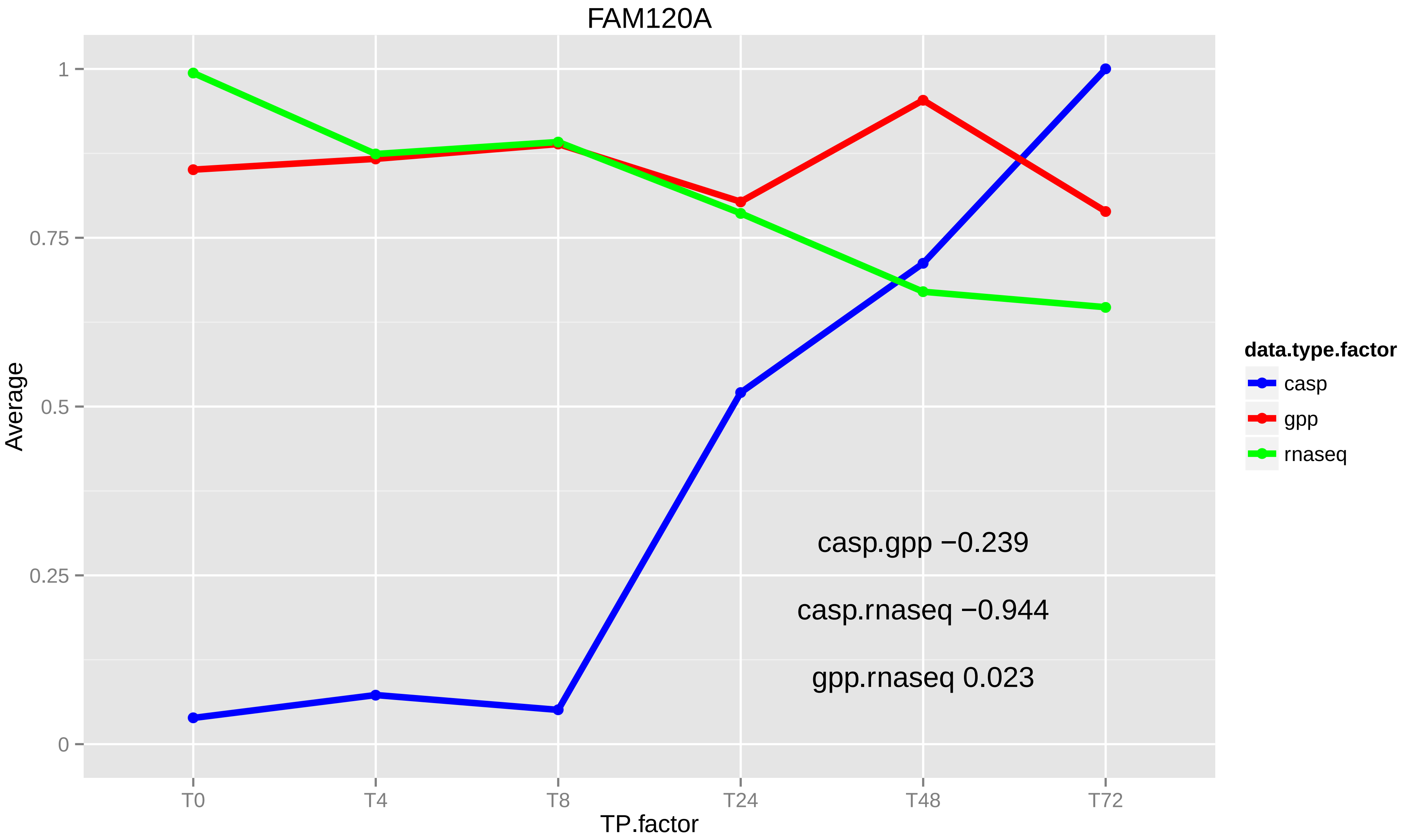
FAM120A
Average
1
0.75
0.5
0.25
0
T0
T4
T8
T24
T48
T72
TP.factor
data.type.factor
casp
gpp
rnaseq
casp.gpp −0.239
casp.rnaseq −0.944
gpp.rnaseq 0.023

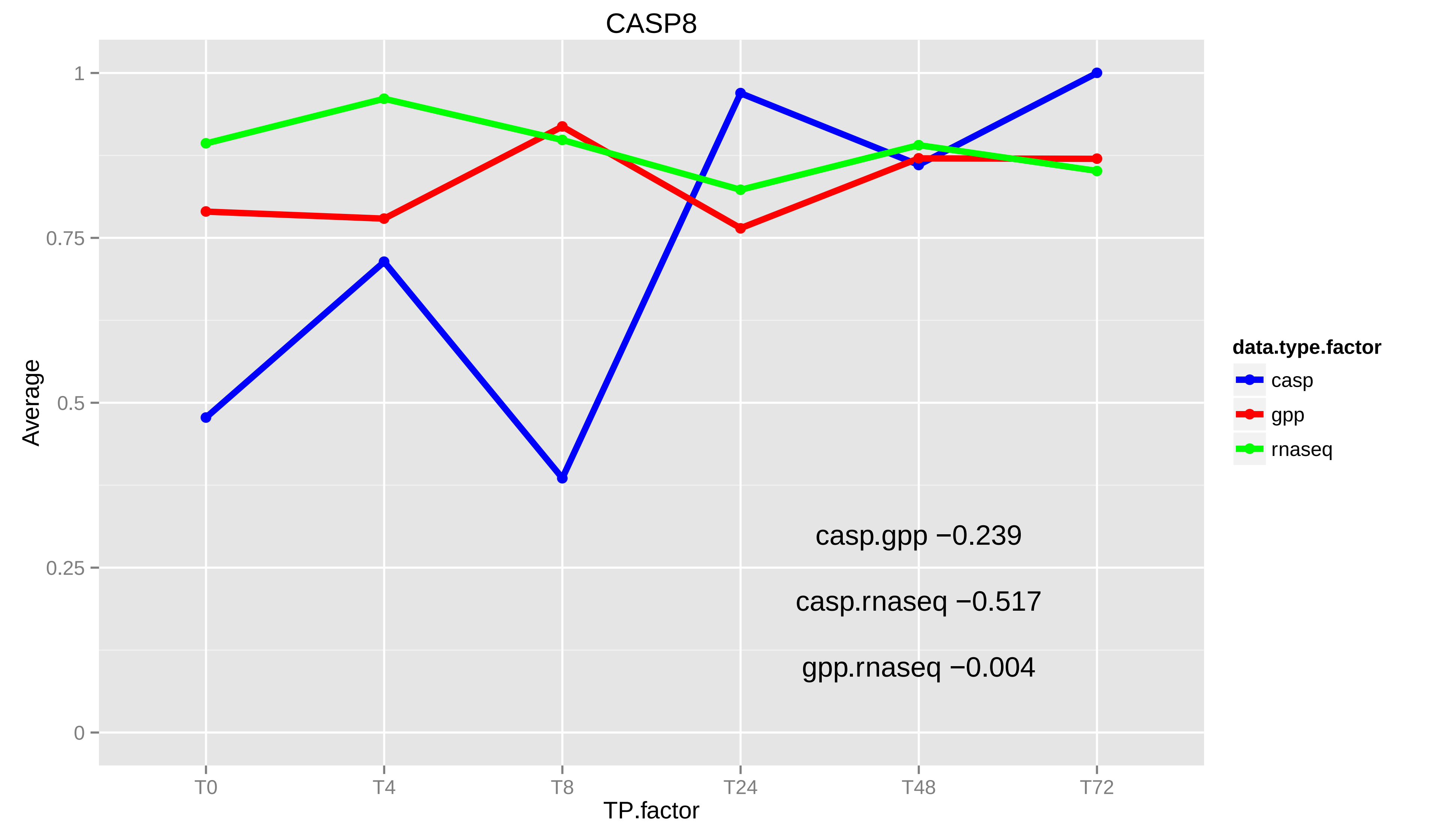
CASP8
Average
1
0.75
0.5
0.25
0
T0
T4
T8
T24
T48
T72
TP.factor
data.type.factor
casp
gpp
rnaseq
casp.gpp −0.239
casp.rnaseq −0.517
gpp.rnaseq −0.004

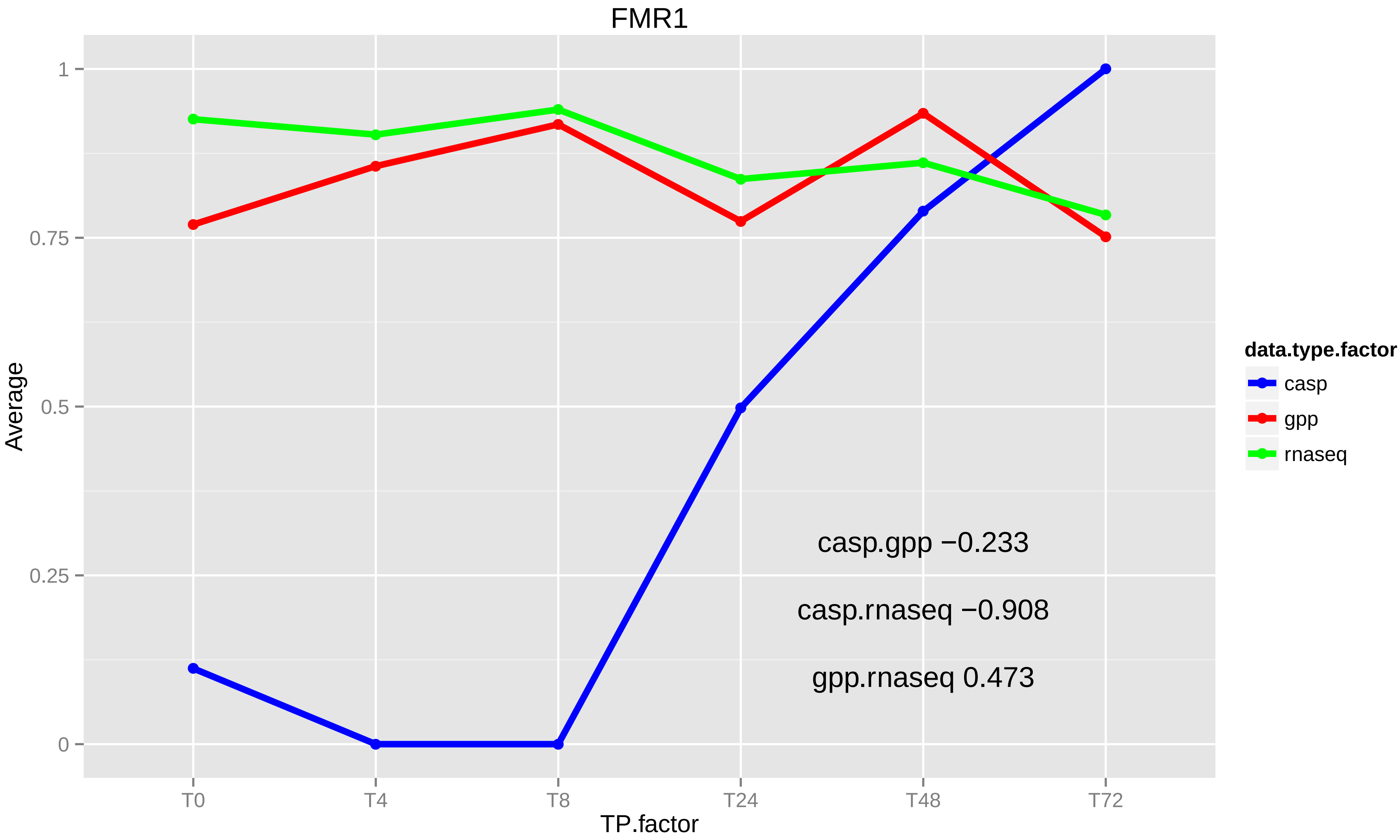
FMR1
1
0.75
0.5
0.25
0
Average
T0
T4
T8
T24
T48
T72
TP.factor
data.type.factor
casp
gpp
rnaseq
casp.gpp −0.233
casp.rnaseq −0.908
gpp.rnaseq 0.473

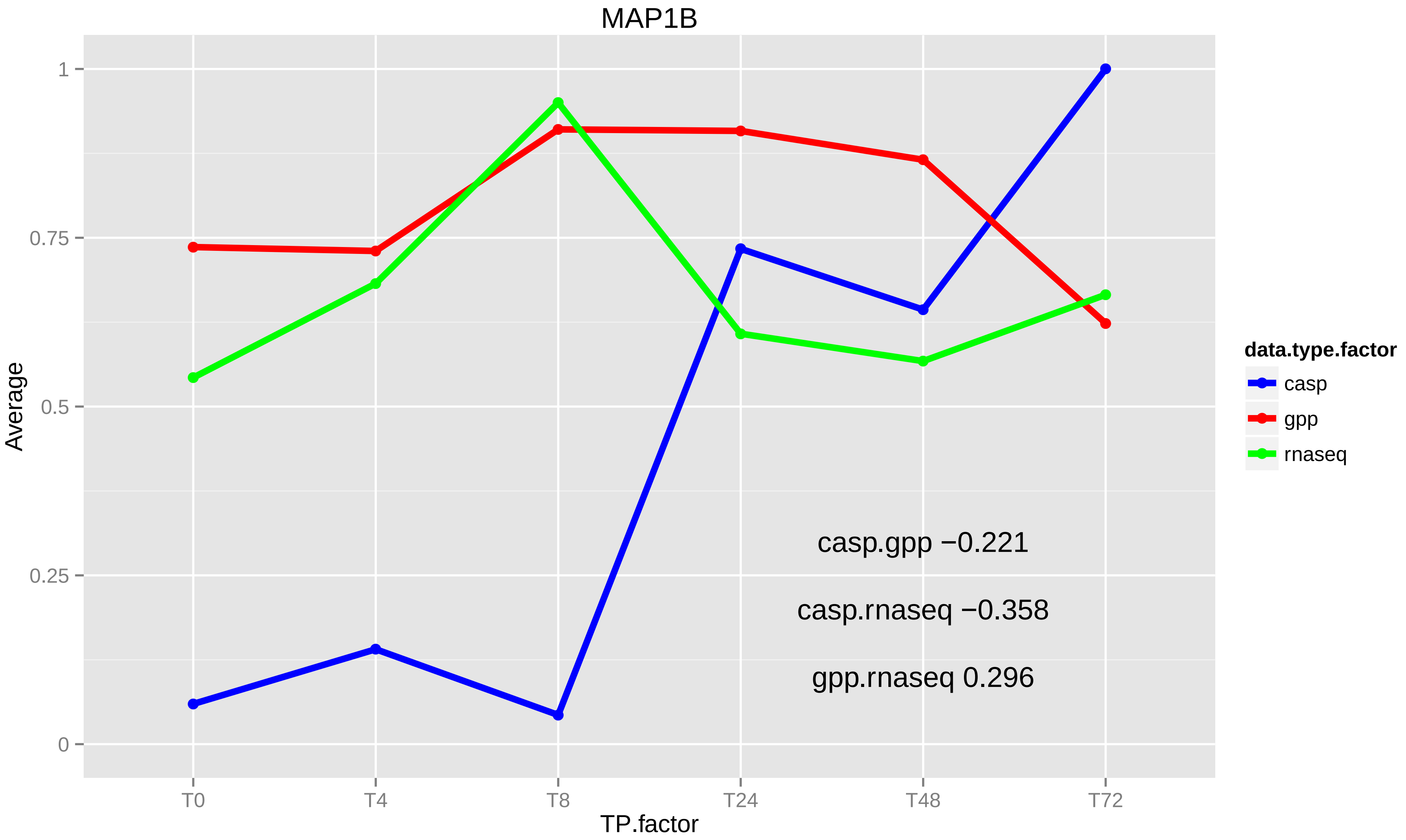

MAP1B
1
0.75
0.5
0.25
0
Average
T0
T4
T8
T24
T48
T72
TP.factor
data.type.factor
casp
gpp
rnaseq
casp.gpp −0.221
casp.rnaseq −0.358
gpp.rnaseq 0.296

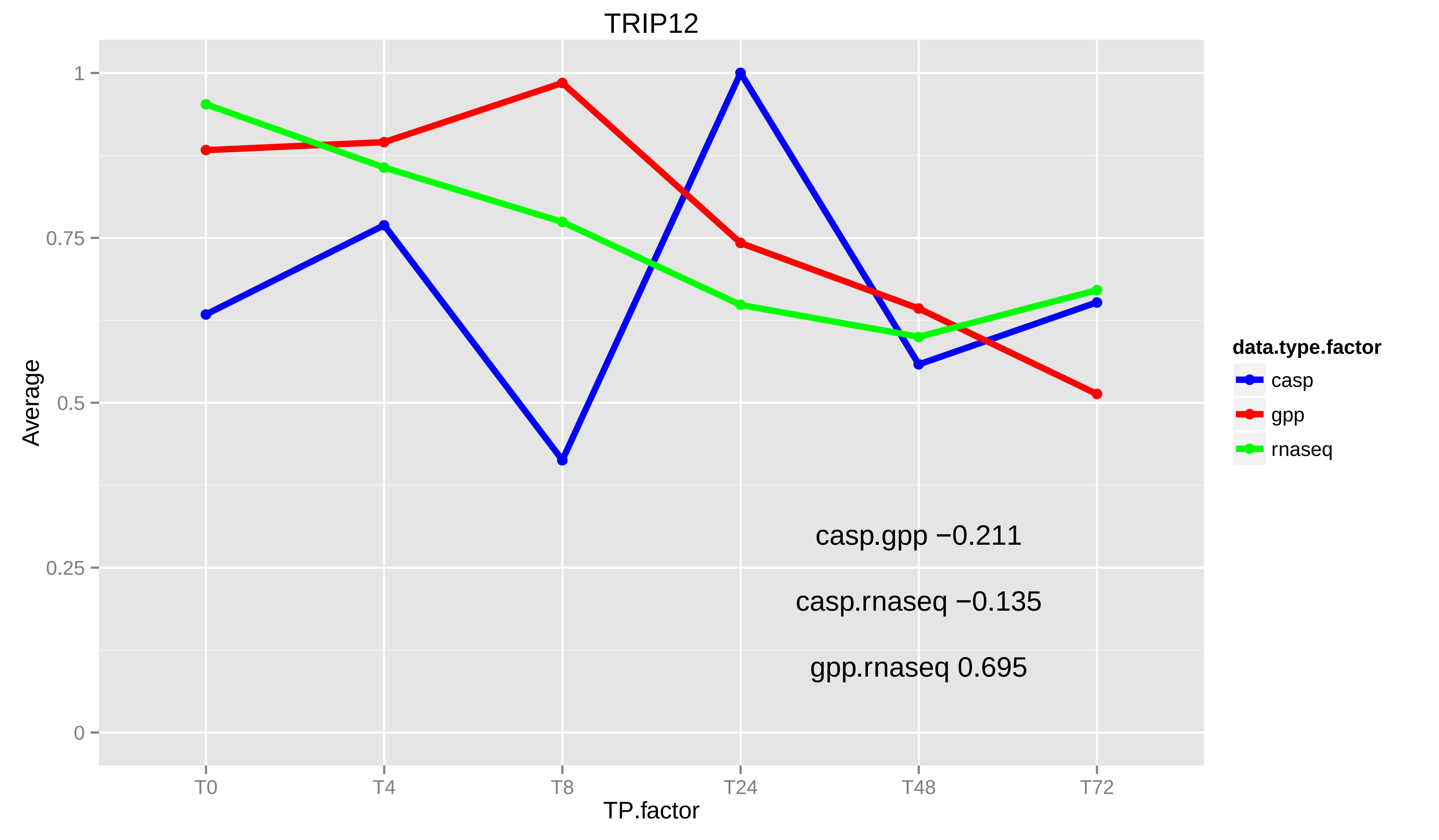
TRIP12
1
0.75
0.5
0.25
0
Average
T0
T4
T8
T24
T48
T72
TP.factor
data.type.factor
casp
gpp
rnaseq
casp.gpp −0.211
casp.rnaseq −0.135
gpp.rnaseq 0.695

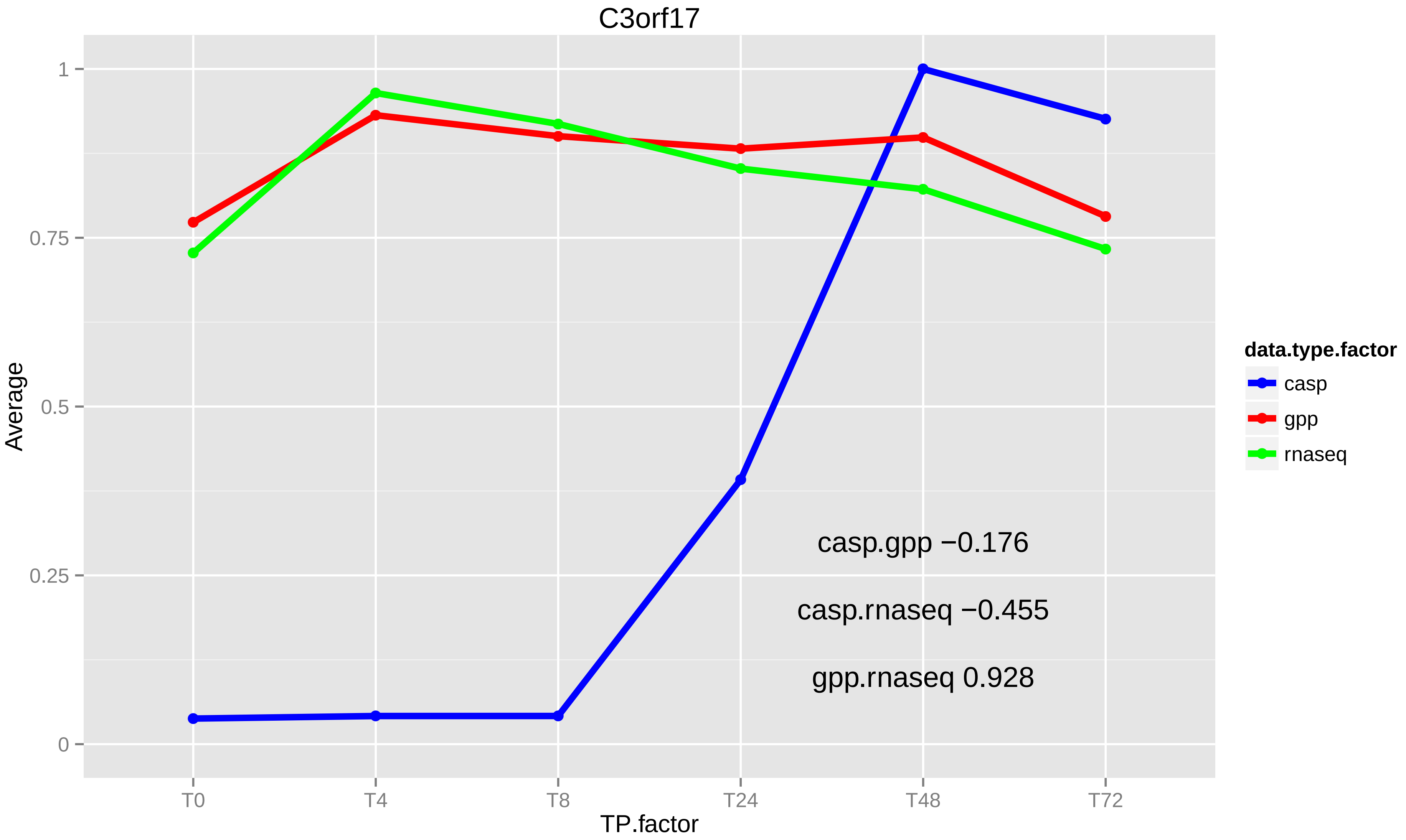
C3orf17
Average
1
0.75
0.5
0.25
0
T0
T4
T8
T24
T48
T72
TP.factor
data.type.factor
casp
gpp
rnaseq
casp.gpp −0.176
casp.rnaseq −0.455
gpp.rnaseq 0.928

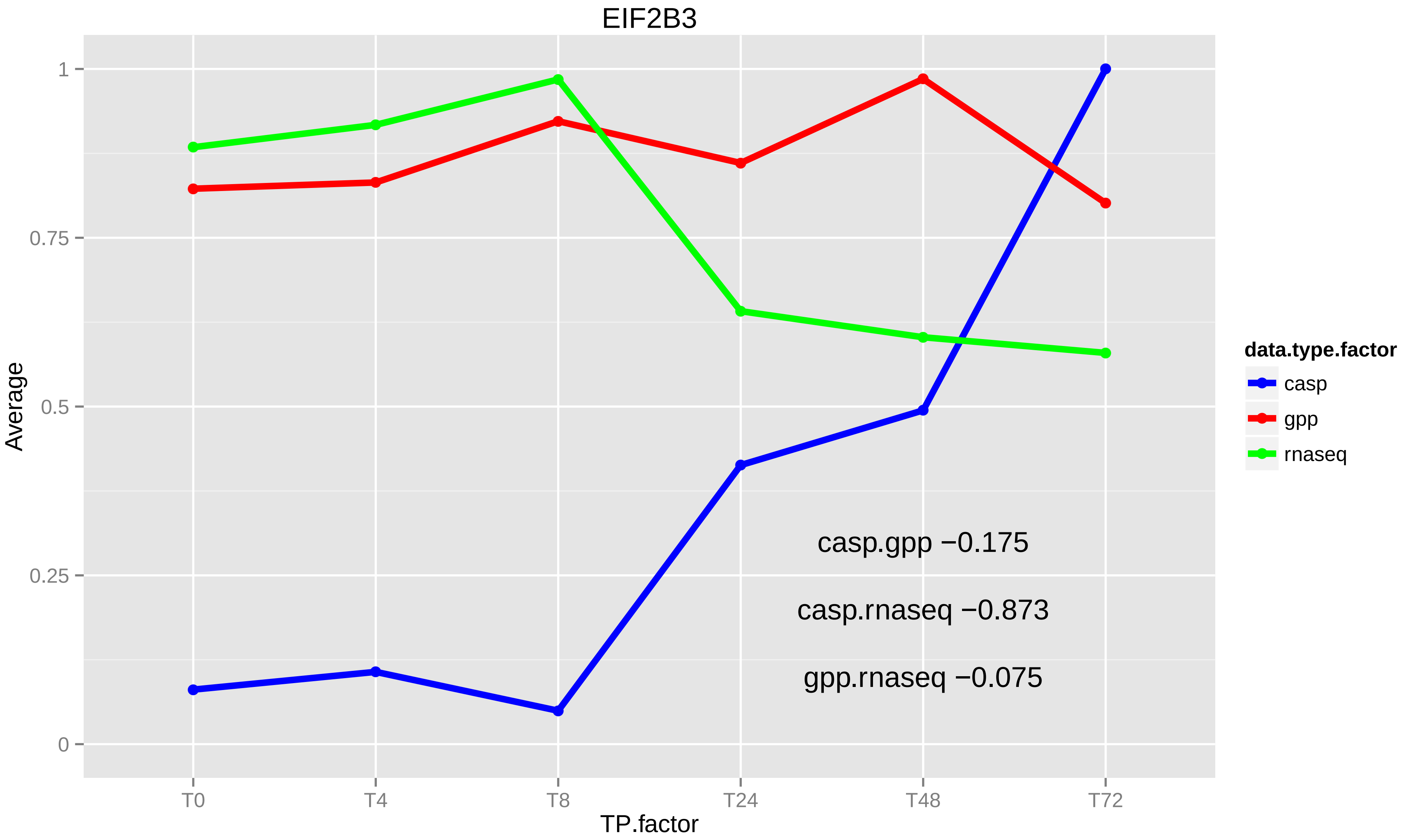

EIF2B3
Average
1
0.75
0.5
0.25
0
T0
T4
T8
T24
T48
T72
TP.factor
data.type.factor
casp
gpp
rnaseq
casp.gpp −0.175
casp.rnaseq −0.873
gpp.rnaseq −0.075

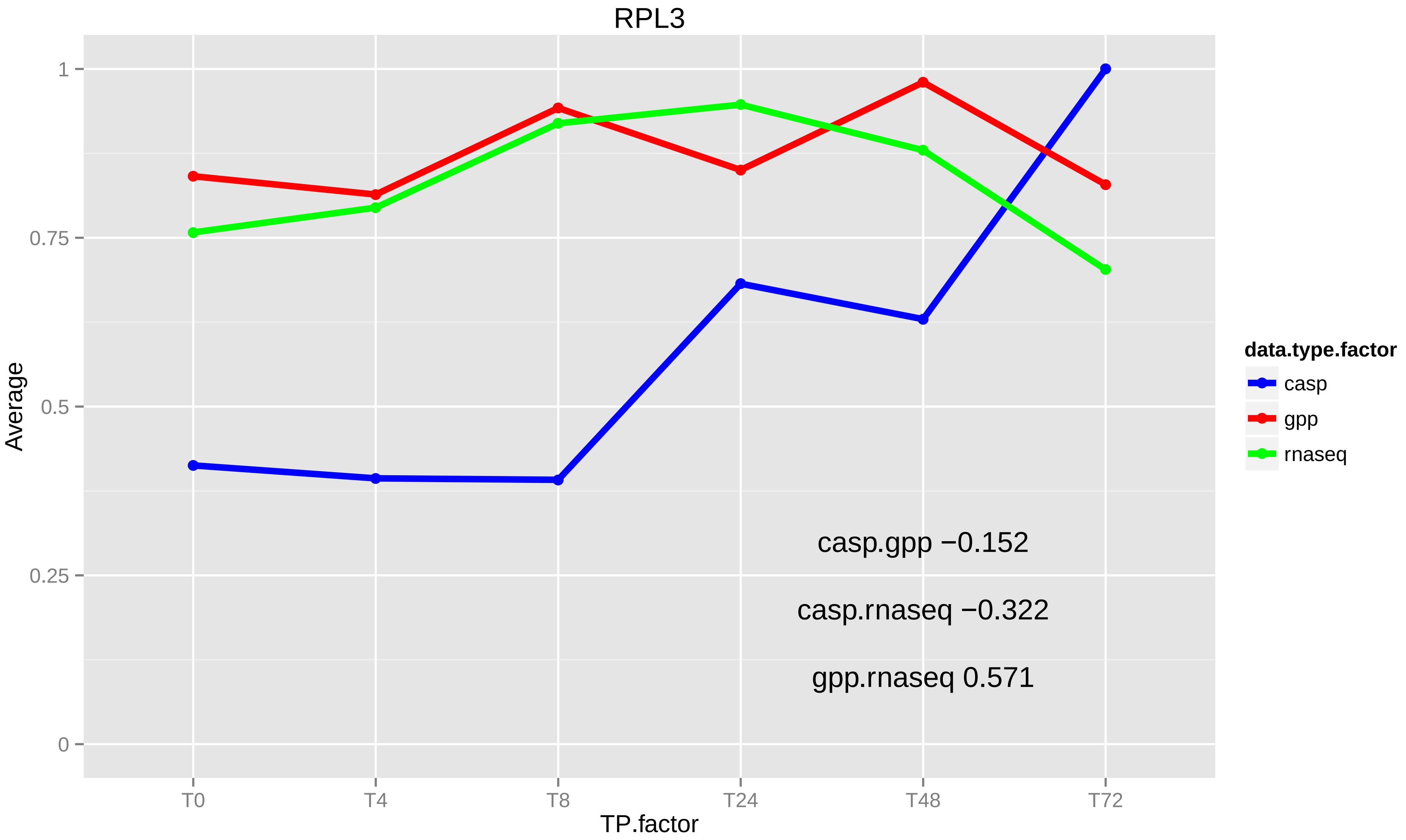
RPL3
Average
1
0.75
0.5
0.25
0
T0
T4
T8
T24
T48
T72
TP.factor
data.type.factor
casp
gpp
rnaseq
casp.gpp −0.152
casp.rnaseq −0.322
gpp.rnaseq 0.571

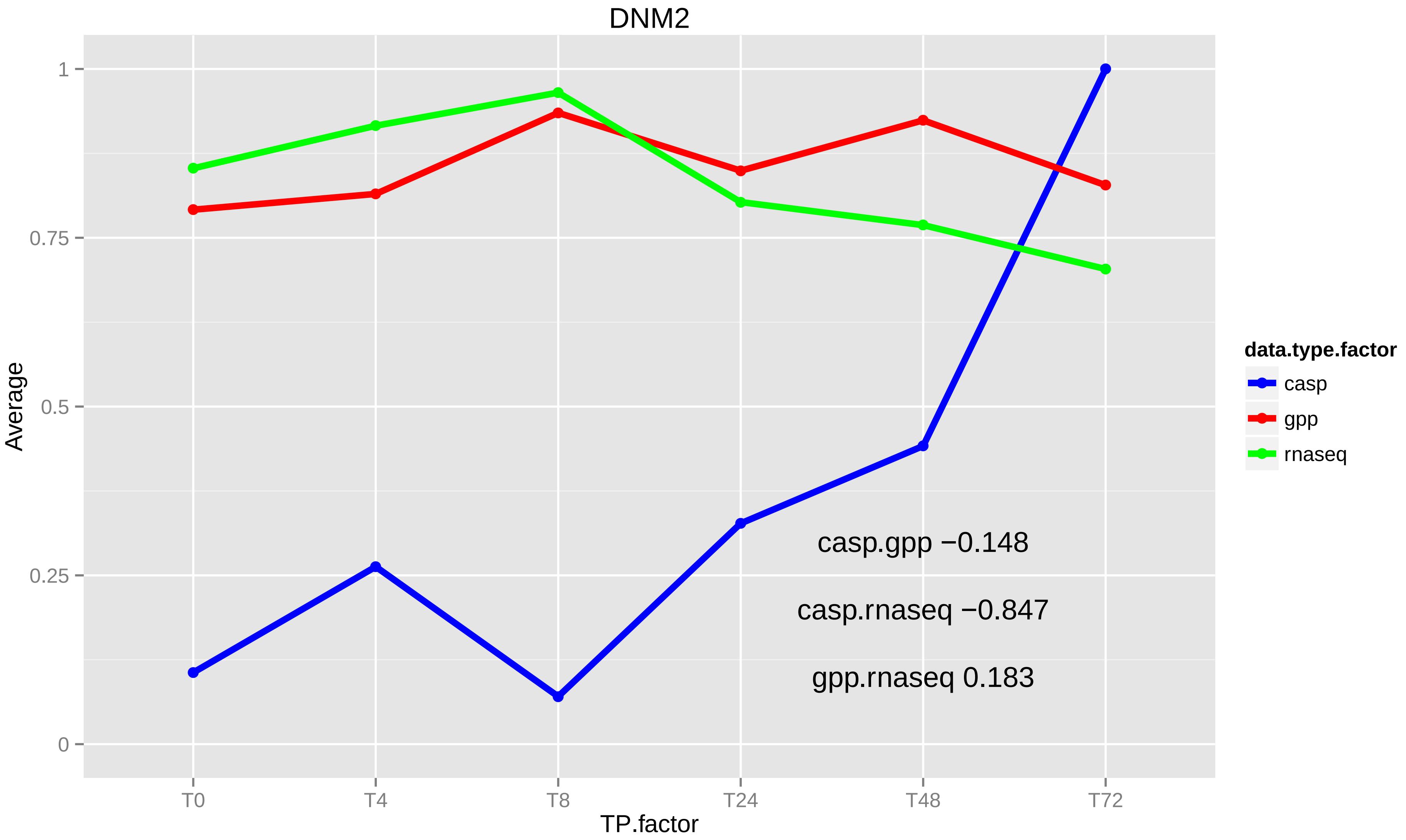
DNM2
1
0.75
0.5
0.25
0
Average
T0
T4
T8
T24
T48
T72
TP.factor
casp.gpp −0.148
casp.rnaseq −0.847
gpp.rnaseq 0.183
data.type.factor
casp
gpp
rnaseq

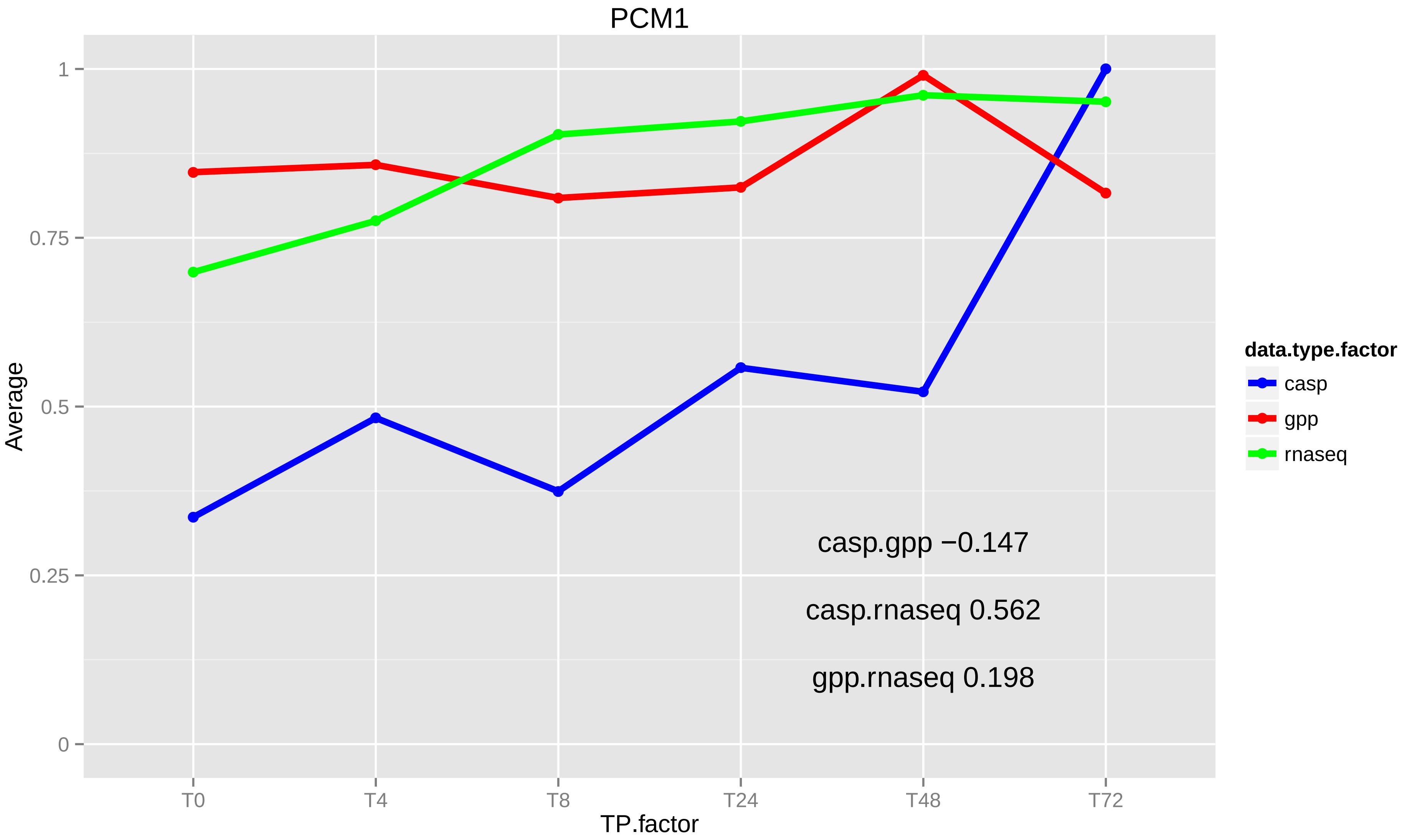
PCM1
Average
1
0.75
0.5
0.25
0
T0
T4
T8
T24
T48
T72
TP.factor
data.type.factor
casp
gpp
rnaseq
casp.gpp −0.147
casp.rnaseq 0.562
gpp.rnaseq 0.198

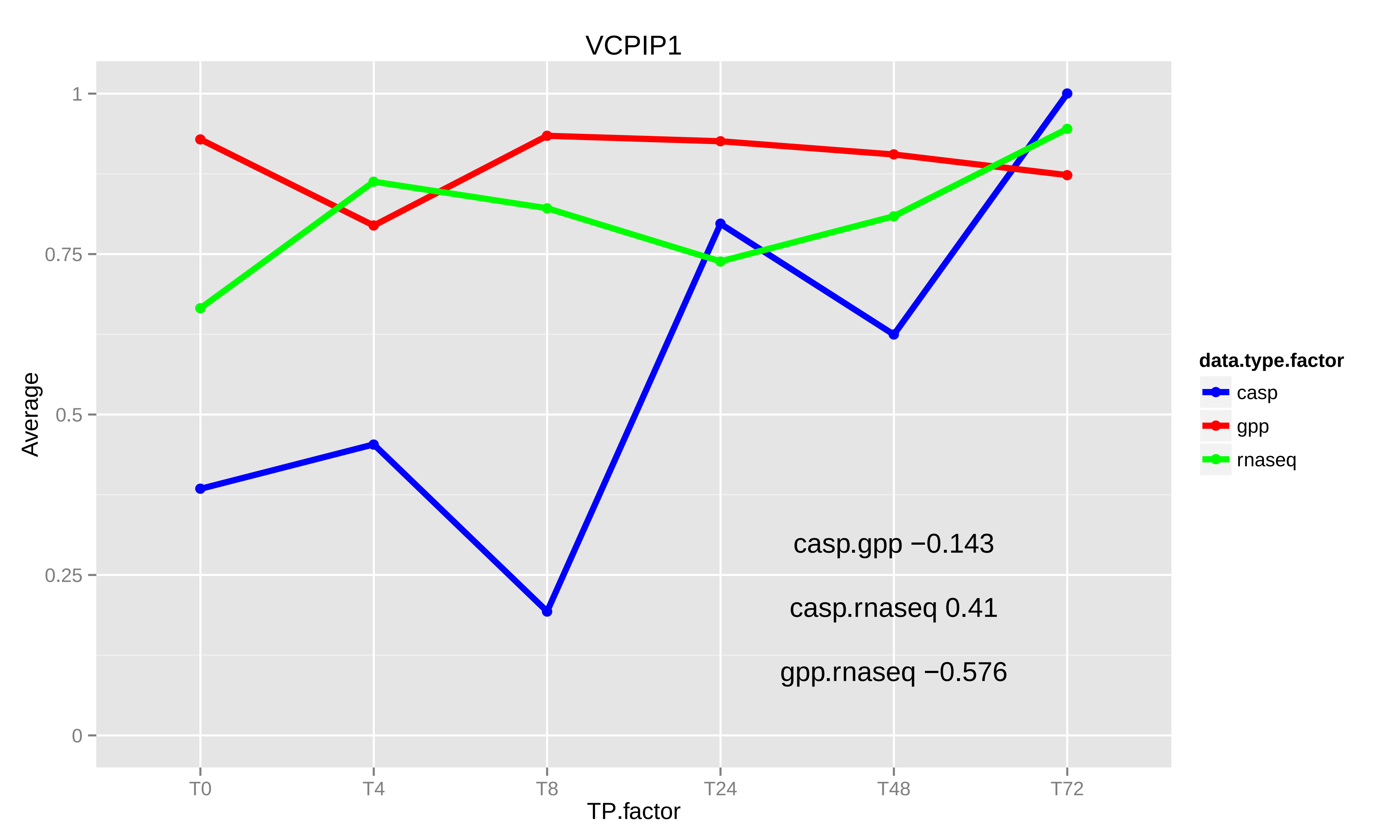

VCPIP1
Average
1
0.75
0.5
0.25
0
T0
T4
T8
T24
T48
T72
TP.factor
data.type.factor
casp
gpp
rnaseq
casp.gpp −0.143
casp.rnaseq 0.41
gpp.rnaseq −0.576

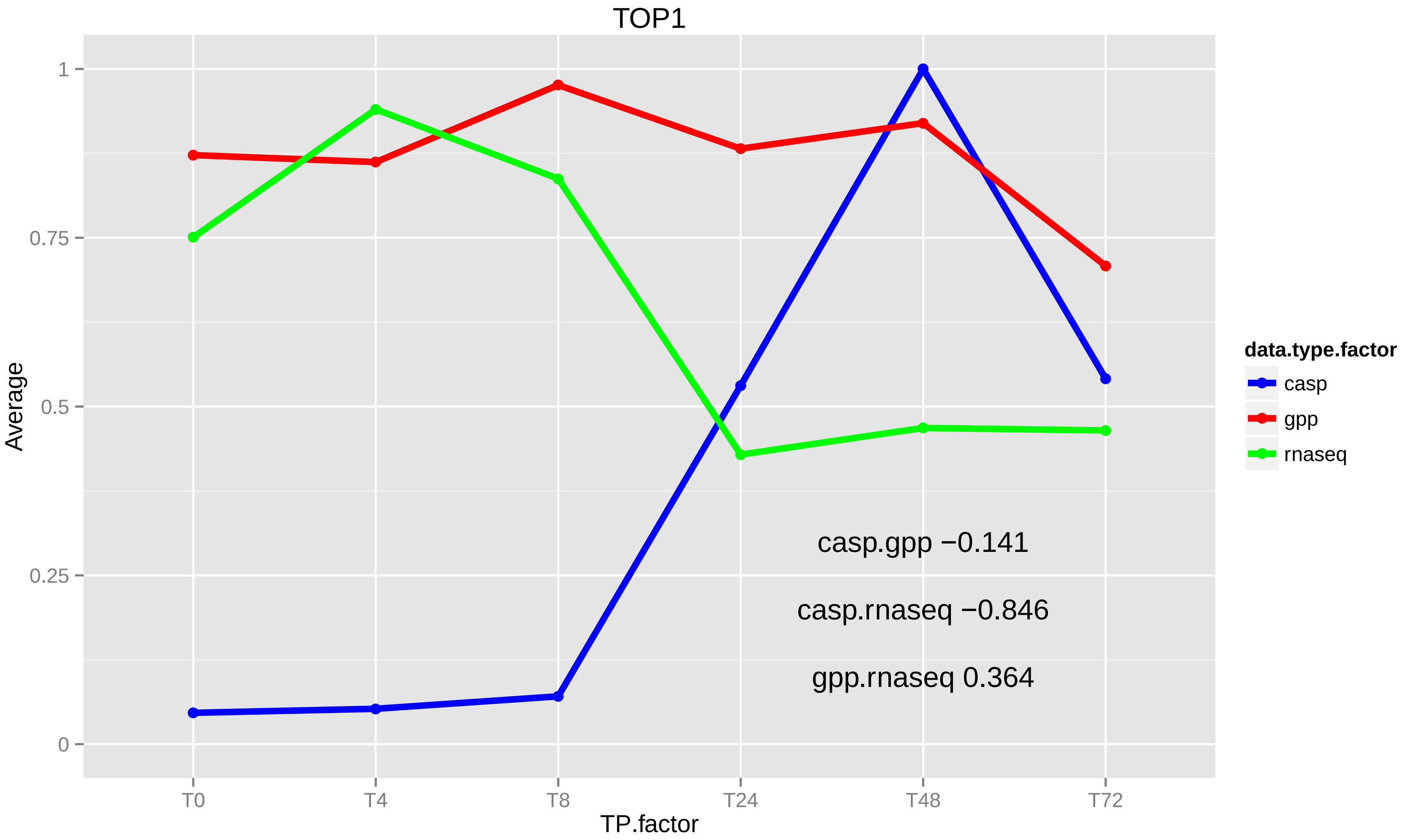
TOP1
Average
1
0.75
0.5
0.25
0
T0
T4
T8
T24
T48
T72
TP.factor
data.type.factor
casp
gpp
rnaseq
casp.gpp −0.141
casp.rnaseq −0.846
gpp.rnaseq 0.364

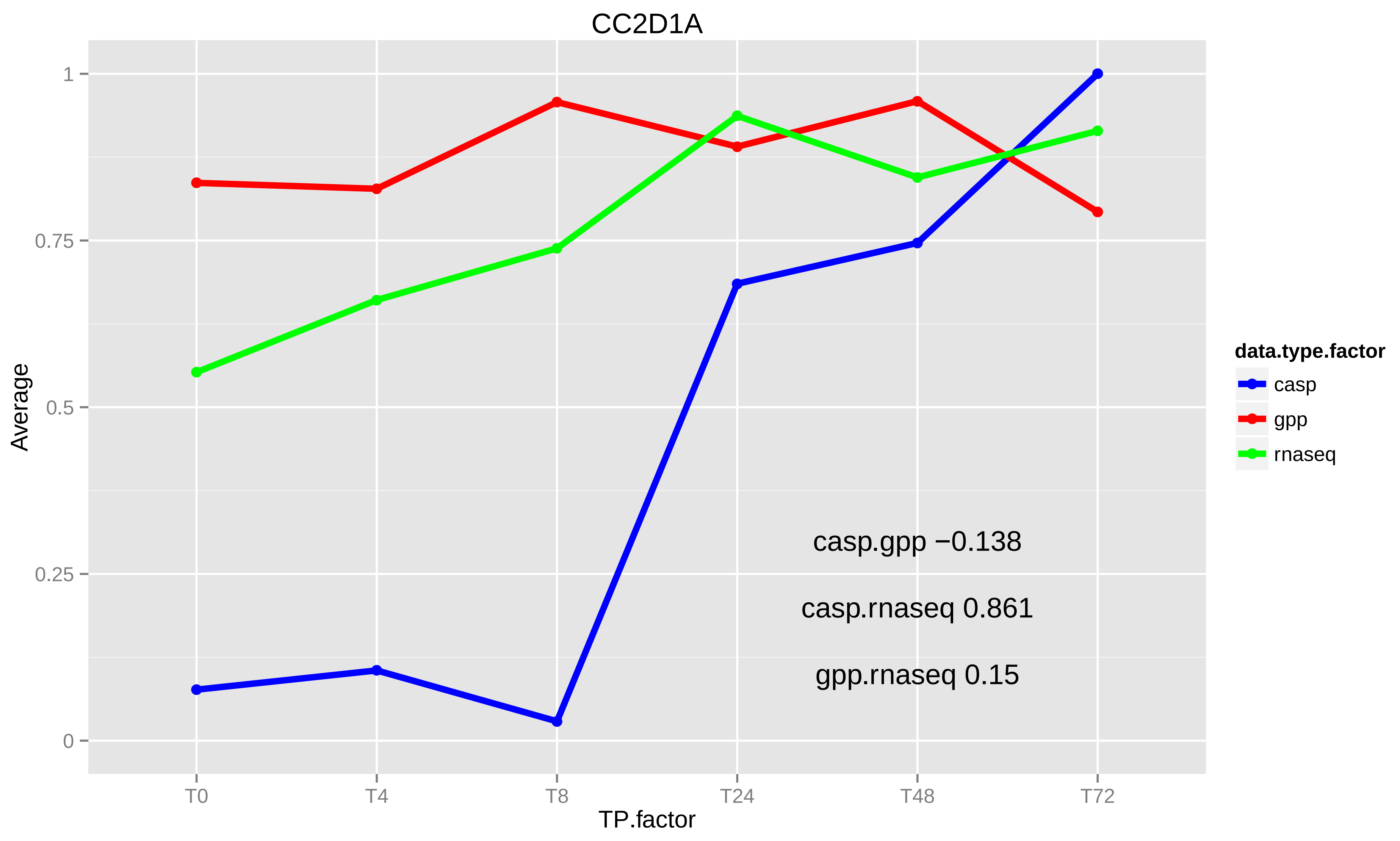
CC2D1A
Average
TP.factor
1
0.75
0.5
0.25
0
T0
T4
T8
T24
T48
T72
data.type.factor
casp
gpp
rnaseq
casp.gpp −0.138
casp.rnaseq 0.861
gpp.rnaseq 0.15

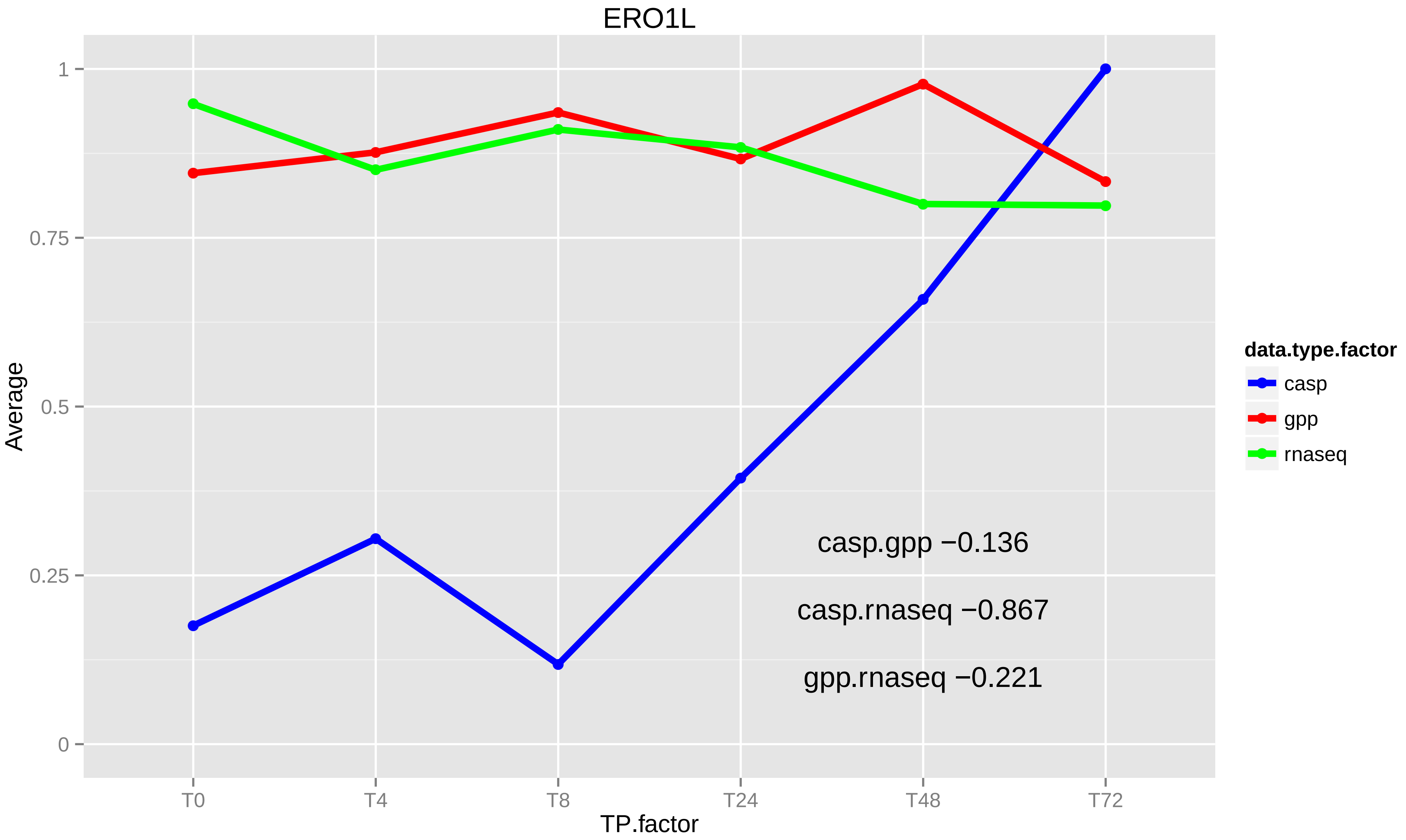

ERO1L
1
0.75
0.5
0.25
0
Average
T0
T4
T8
T24
T48
T72
TP.factor
data.type.factor
casp
gpp
rnaseq
casp.gpp −0.136
casp.rnaseq −0.867
gpp.rnaseq −0.221

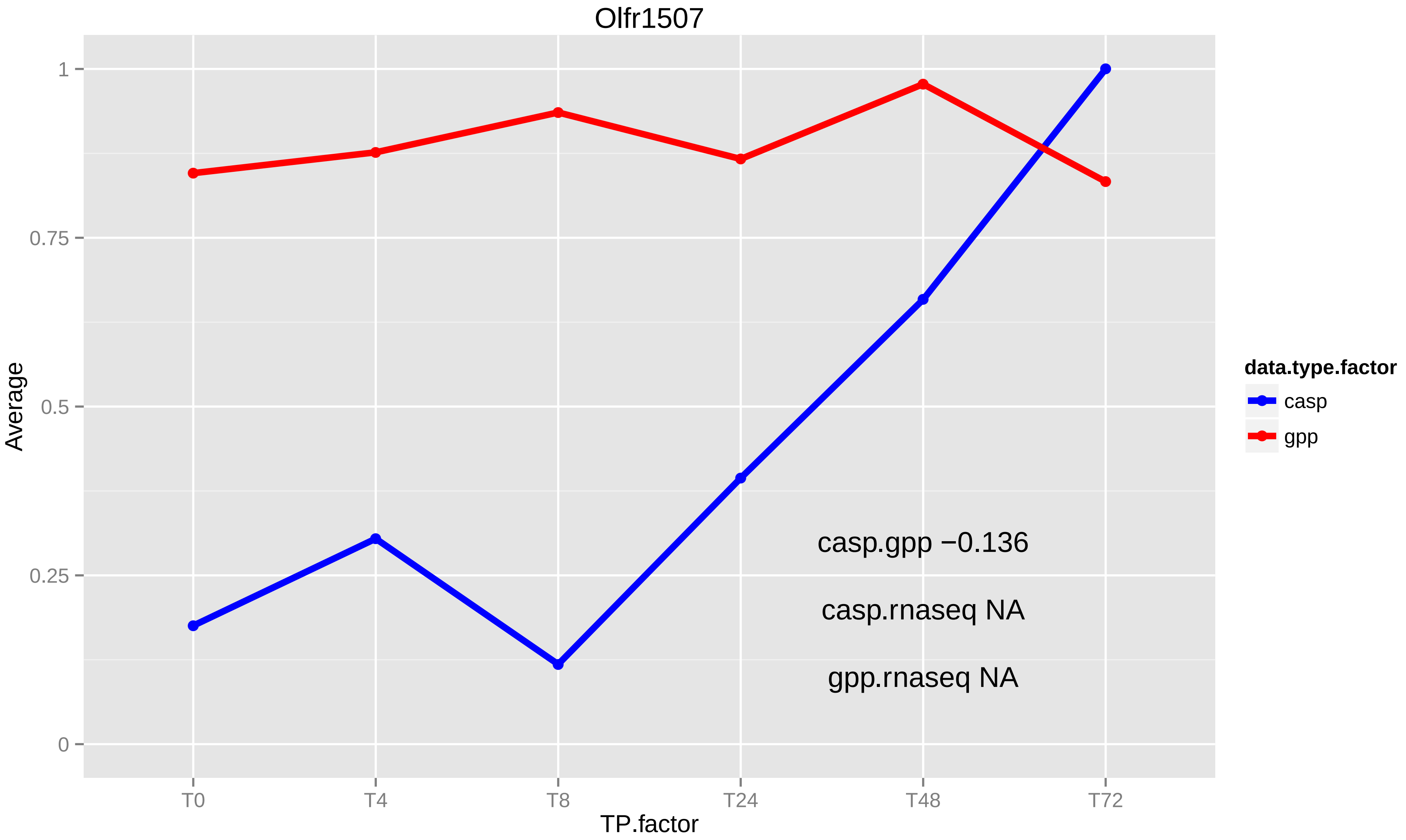

Olfr1507
1
0.75
0.5
0.25
0
Average
T0
T4
T8
T24
T48
T72
TP.factor
data.type.factor
casp
gpp
casp.gpp −0.136
casp.rnaseq NA
gpp.rnaseq NA

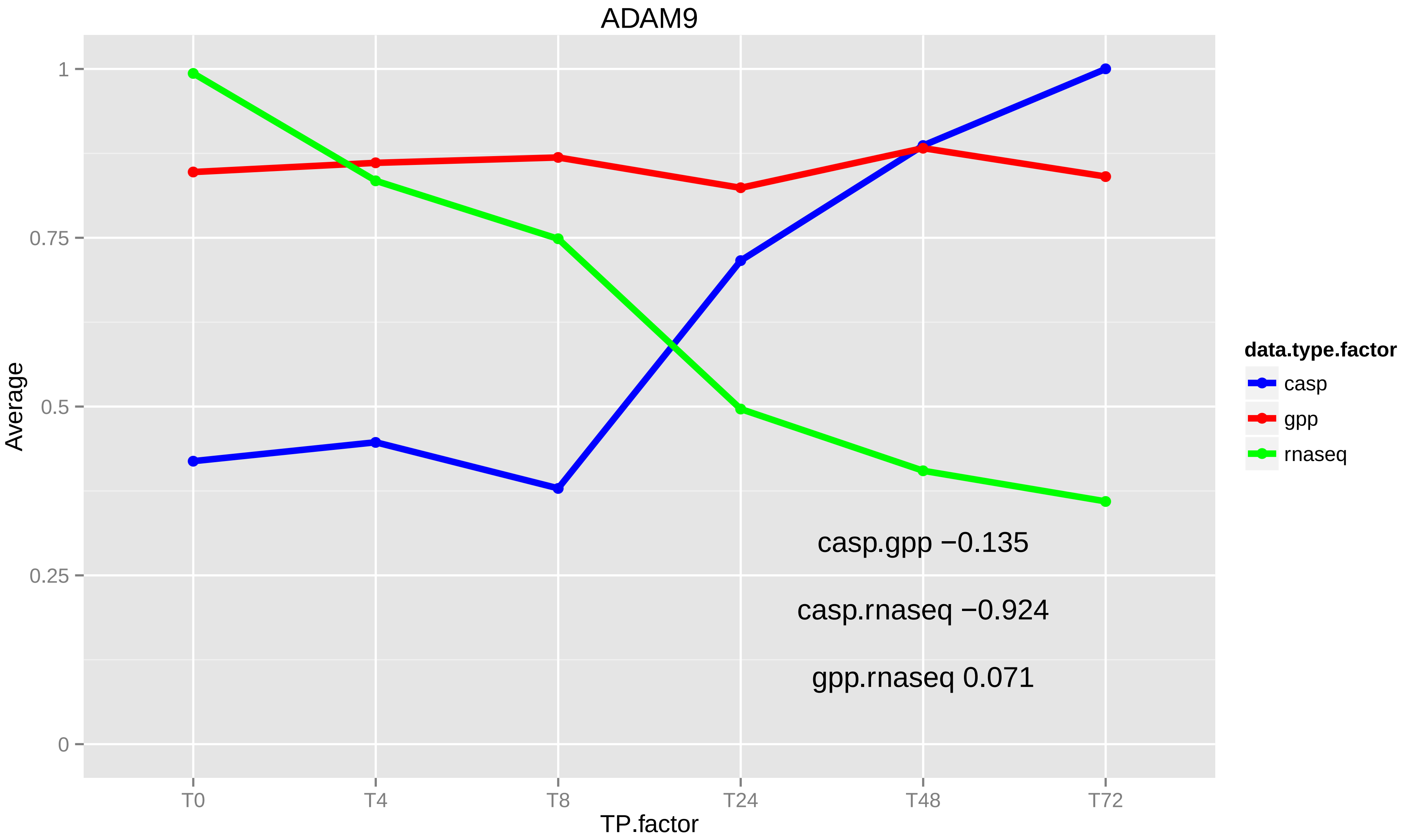
ADAM9
Average
1
0.75
0.5
0.25
0
T0
T4
T8
T24
T48
T72
TP.factor
data.type.factor
casp
gpp
rnaseq
casp.gpp −0.135
casp.rnaseq −0.924
gpp.rnaseq 0.071

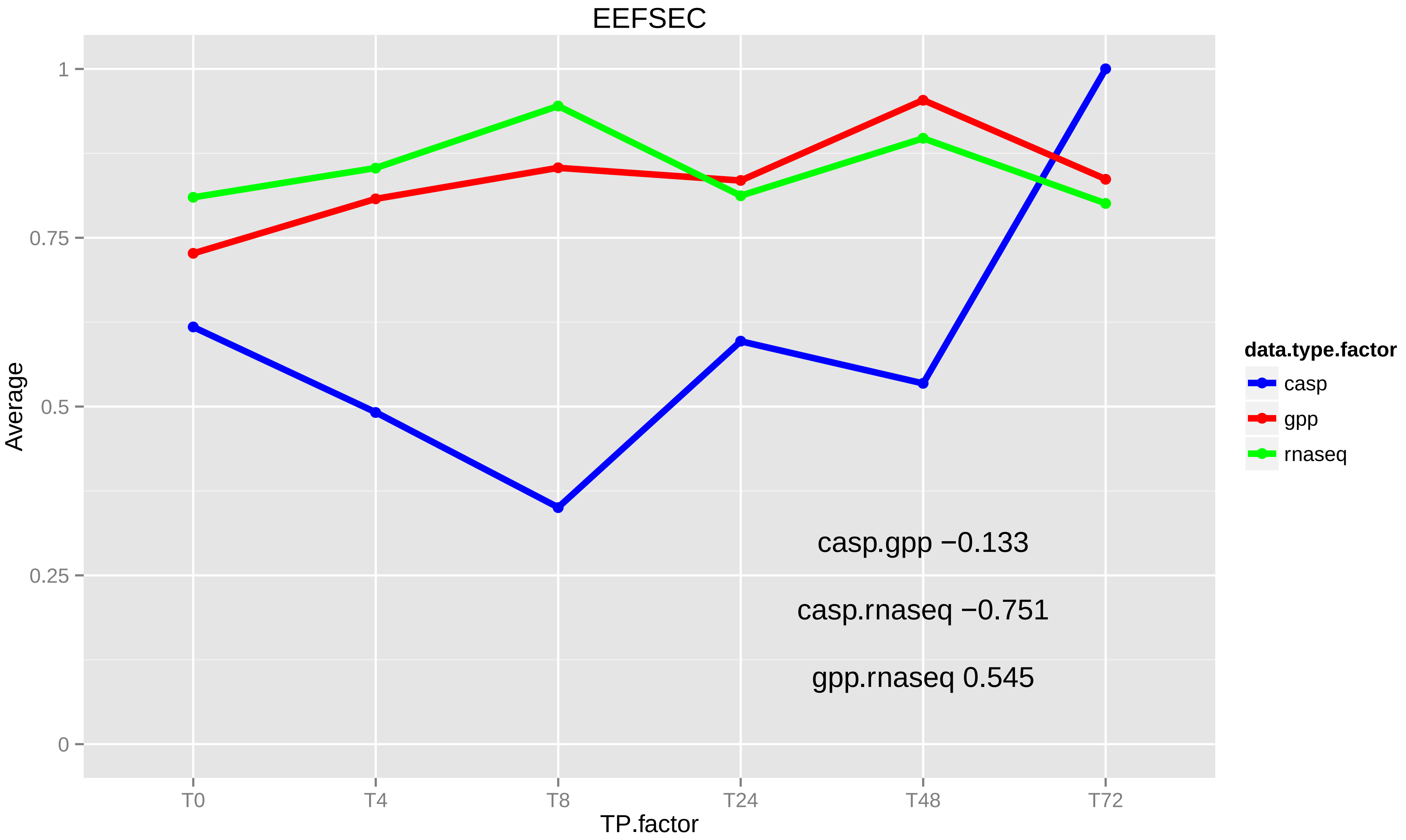
EEFSEC
1
0.75
0.5
0.25
0
Average
T0
T4
T8
T24
T48
T72
TP.factor
data.type.factor
casp
gpp
rnaseq
casp.gpp −0.133
casp.rnaseq −0.751
gpp.rnaseq 0.545

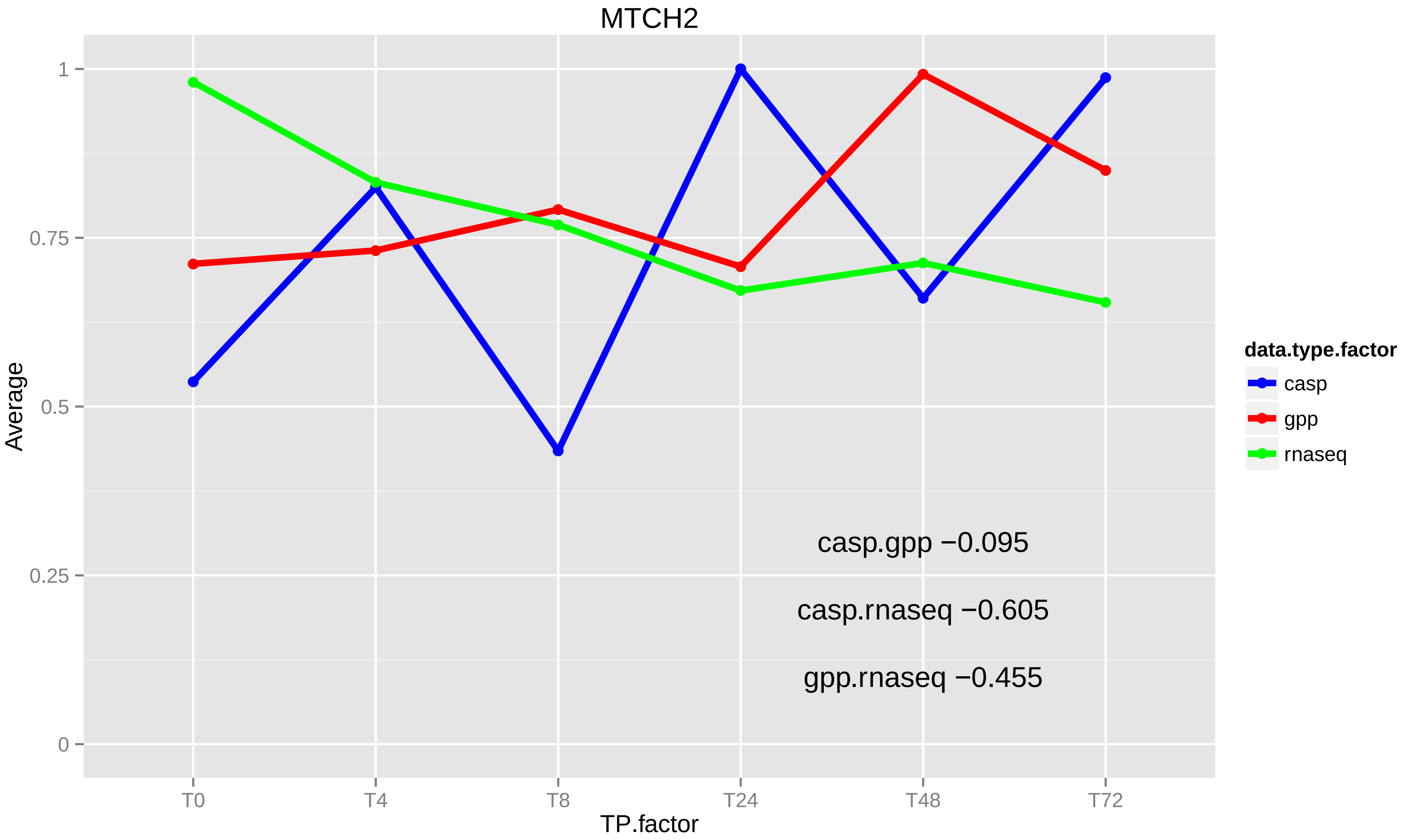

MTCH2
1
0.75
0.5
0.25
0
Average
T0
T4
T8
T24
T48
T72
TP.factor
data.type.factor
casp
gpp
rnaseq
casp.gpp −0.095
casp.rnaseq −0.605
gpp.rnaseq −0.455

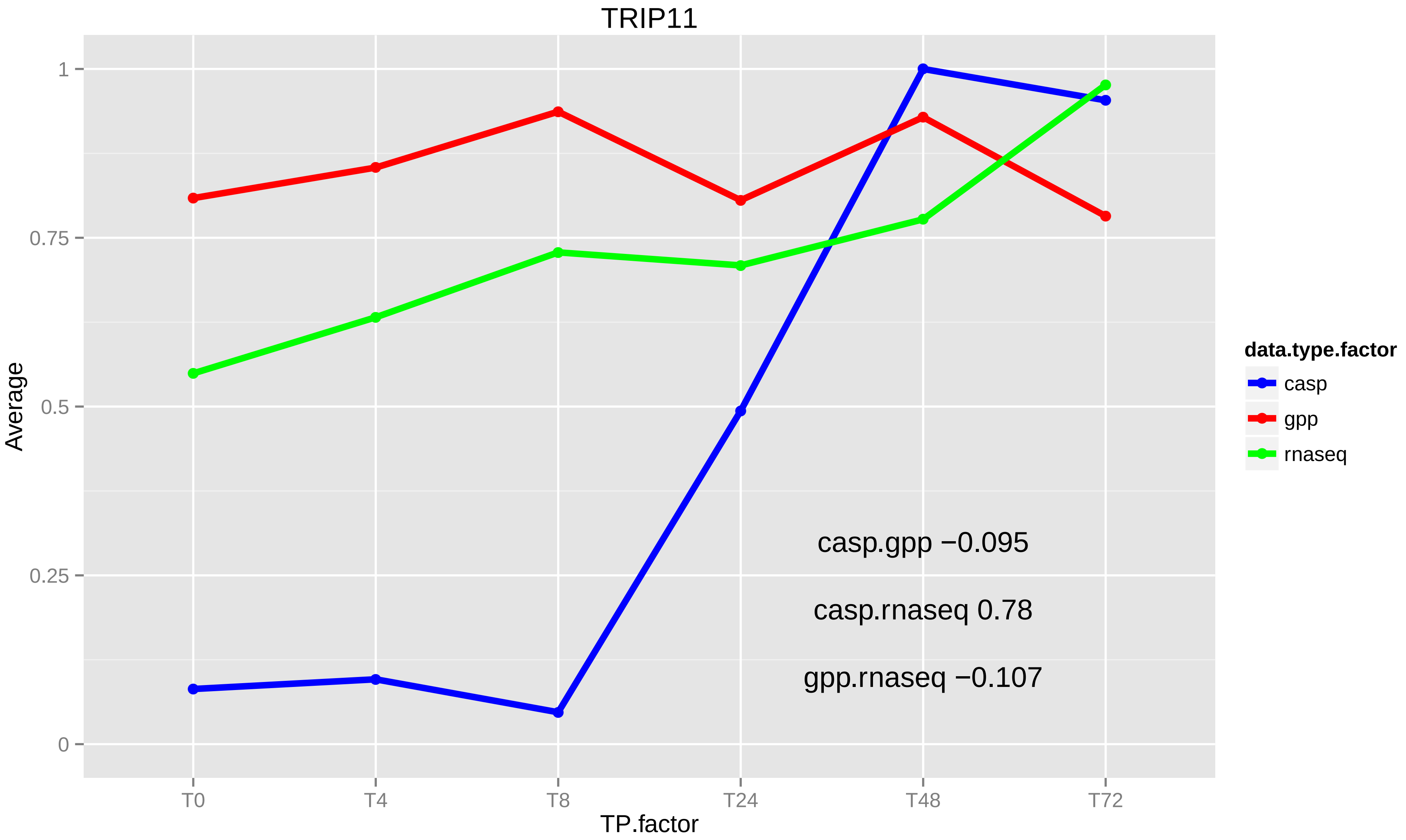

TRIP11
Average
1
0.75
0.5
0.25
0
T0
T4
T8
T24
T48
T72
TP.factor
data.type.factor
casp
gpp
rnaseq
casp.gpp −0.095
casp.rnaseq 0.78
gpp.rnaseq −0.107

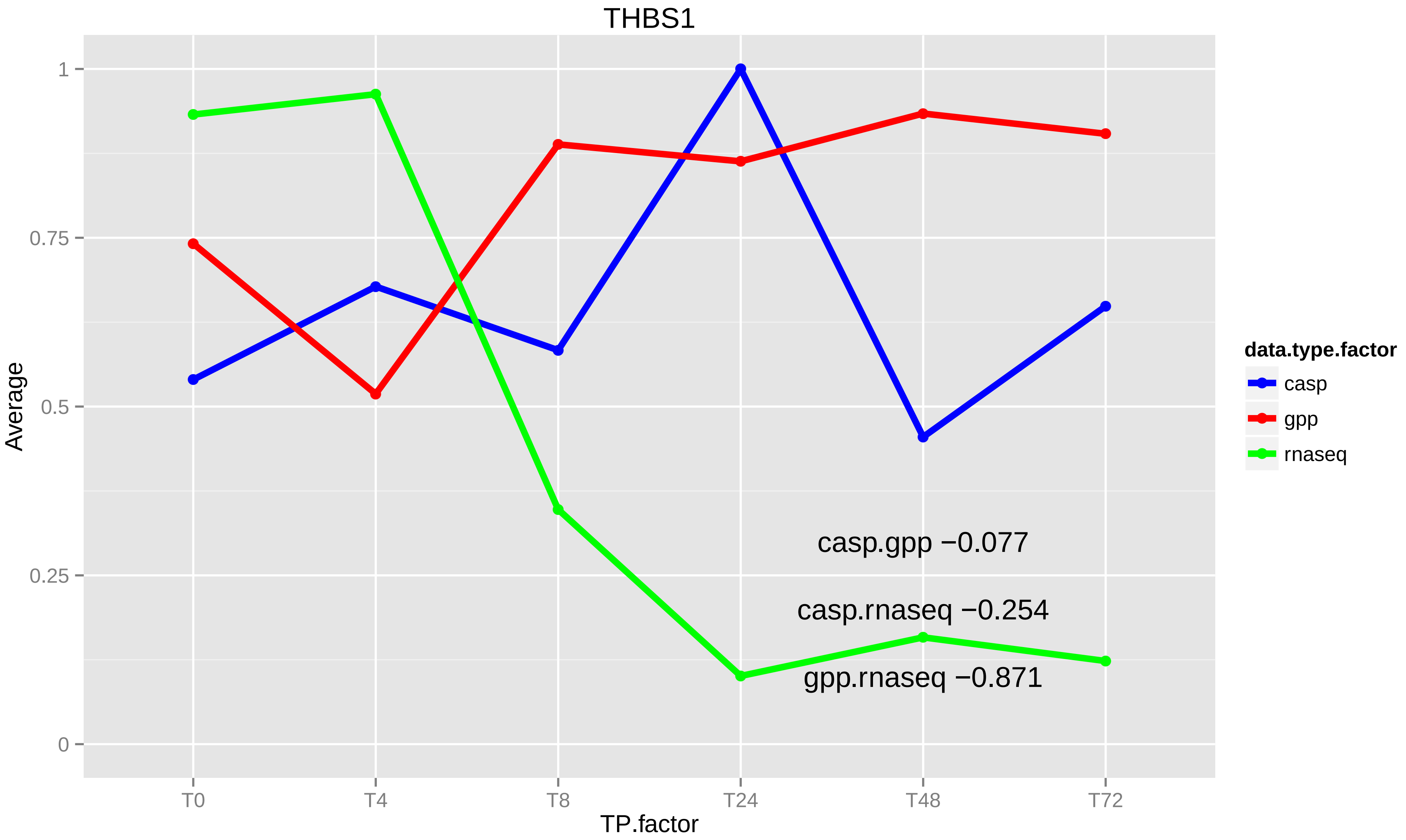
THBS1
Average
1
0.75
0.5
0.25
0
T0
T4
T8
T24
T48
T72
TP.factor
data.type.factor
casp
gpp
rnaseq
casp.gpp −0.077
casp.rnaseq −0.254
gpp.rnaseq −0.871

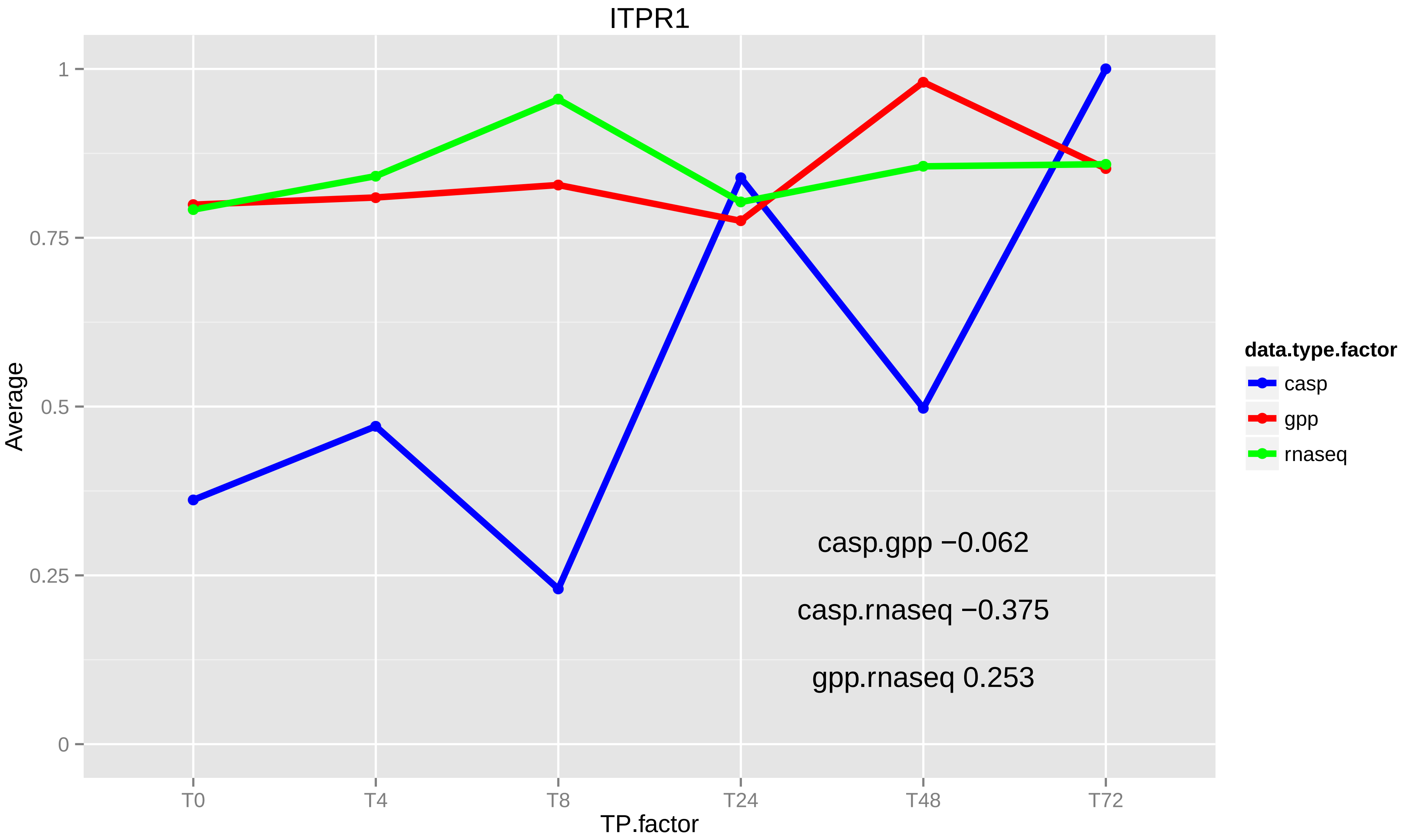

ITPR1
Average
1
0.75
0.5
0.25
0
T0
T4
T8
T24
T48
T72
TP.factor
data.type.factor
casp
gpp
rnaseq
casp.gpp −0.062
casp.rnaseq −0.375
gpp.rnaseq 0.253

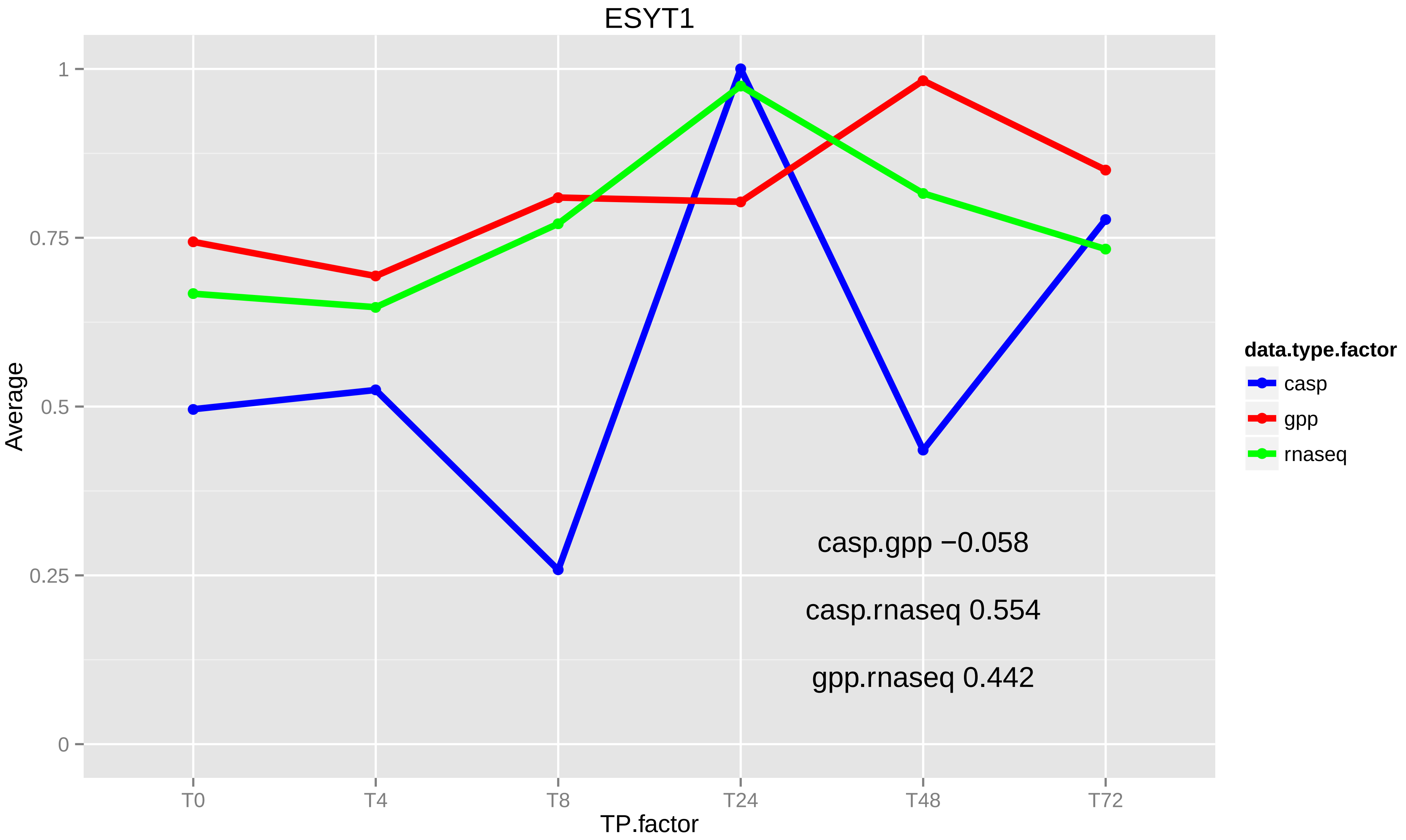
ESYT1
Average
1
0.75
0.5
0.25
0
T0
T4
T8
T24
T48
T72
TP.factor
data.type.factor
casp
gpp
rnaseq
casp.gpp −0.058
casp.rnaseq 0.554
gpp.rnaseq 0.442

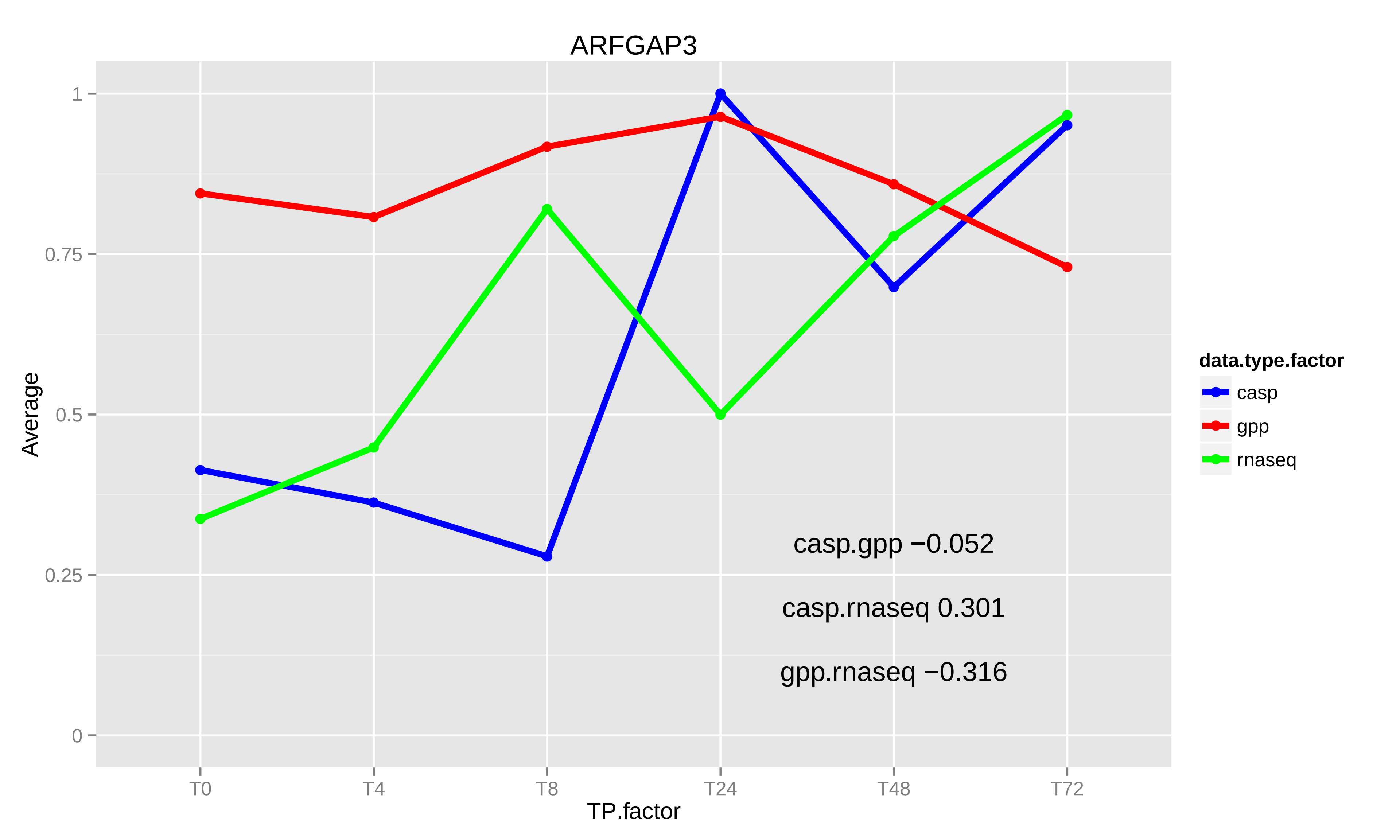
ARFGAP3
Average
1
0.75
0.5
0.25
0
T0
T4
T8
T24
T48
T72
TP.factor
data.type.factor
casp
gpp
rnaseq
casp.gpp −0.052
casp.rnaseq 0.301
gpp.rnaseq −0.316

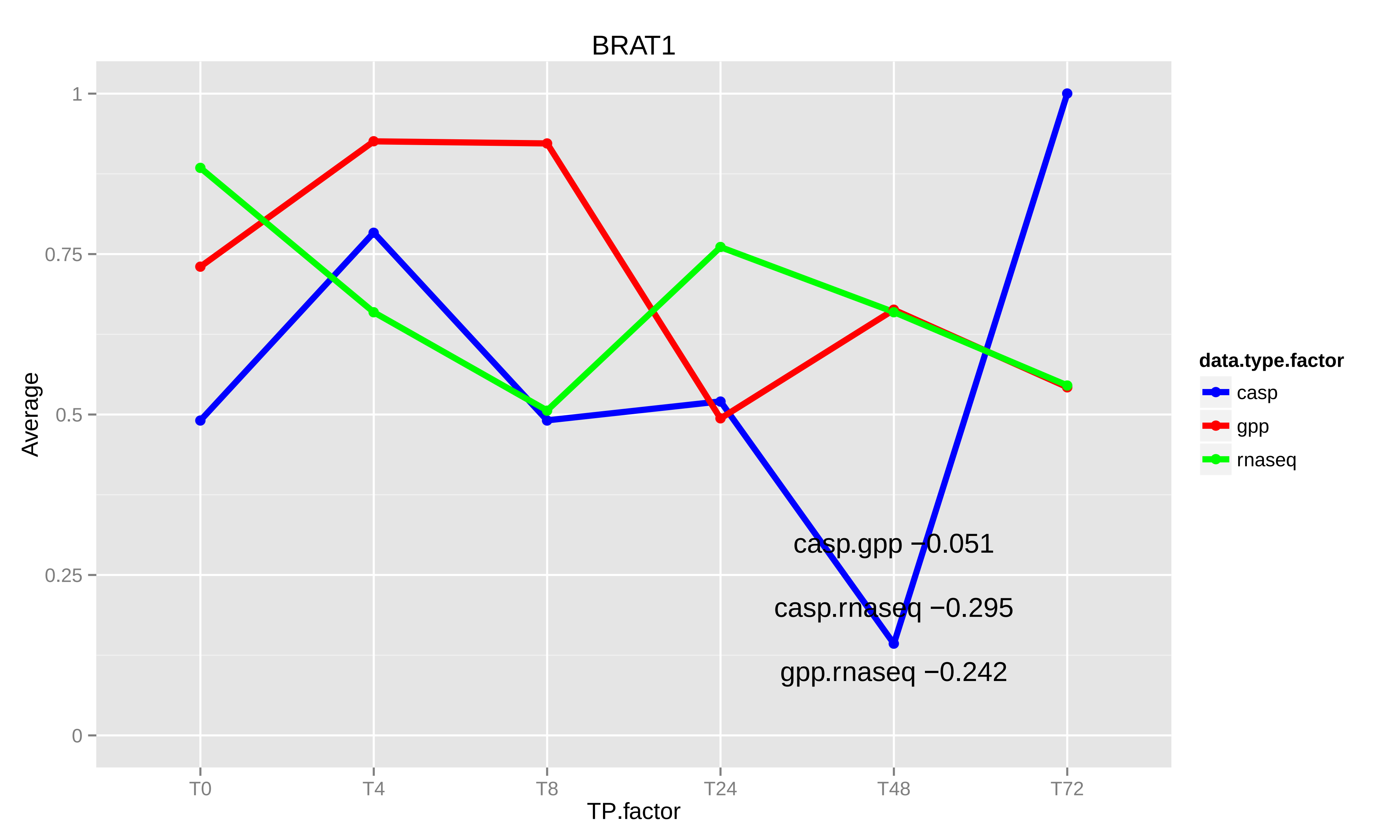

BRAT1
Average
1
0.75
0.5
0.25
0
T0
T4
T8
T24
T48
T72
TP.factor
data.type.factor
casp
gpp
rnaseq
casp.gpp −0.051
casp.rnaseq −0.295
gpp.rnaseq −0.242

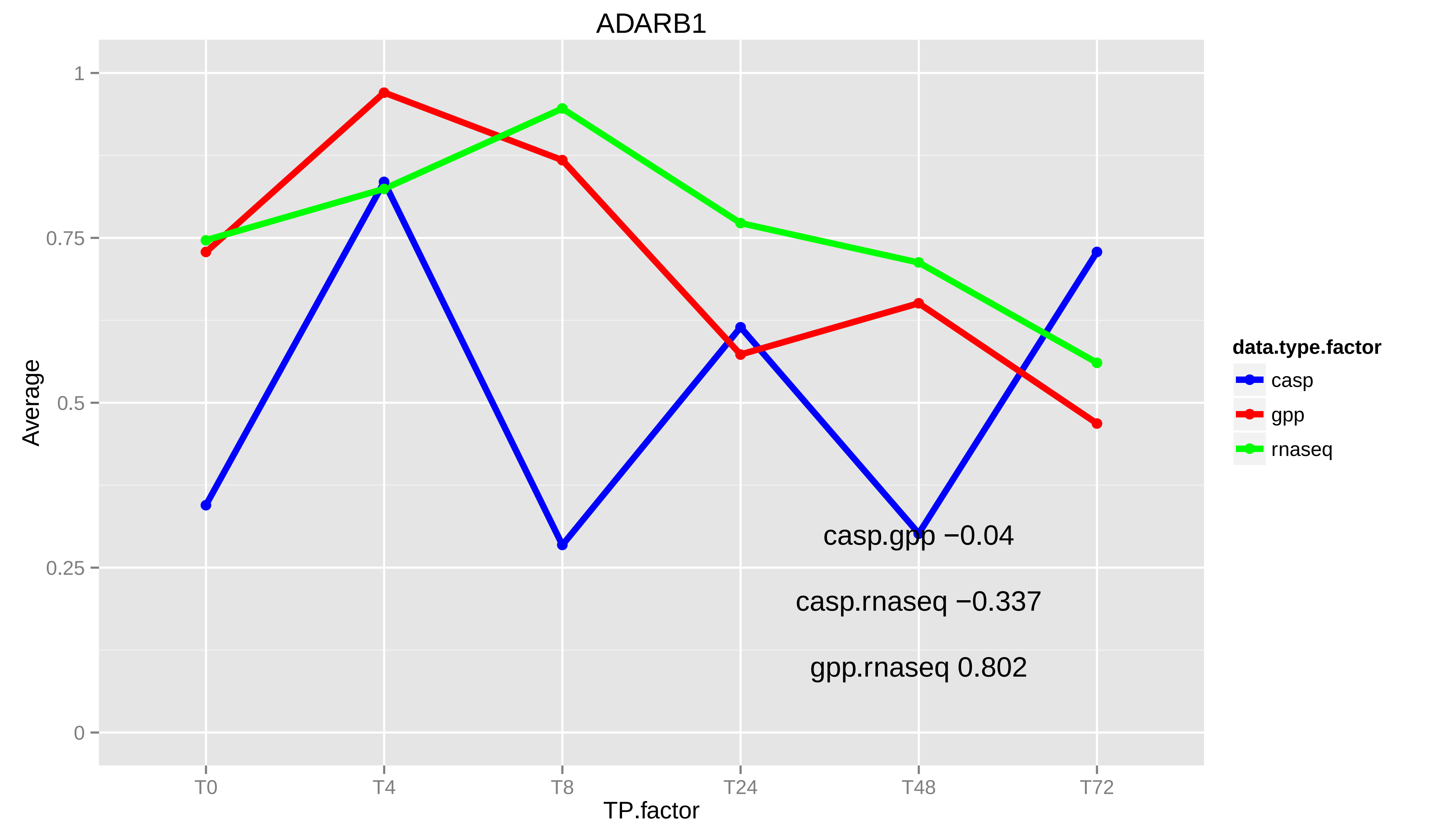

ADARB1
Average
1
0.75
0.5
0.25
0
T0
T4
T8
T24
T48
T72
TP.factor
data.type.factor
casp
gpp
rnaseq
casp.gpp −0.04
casp.rnaseq −0.337
gpp.rnaseq 0.802

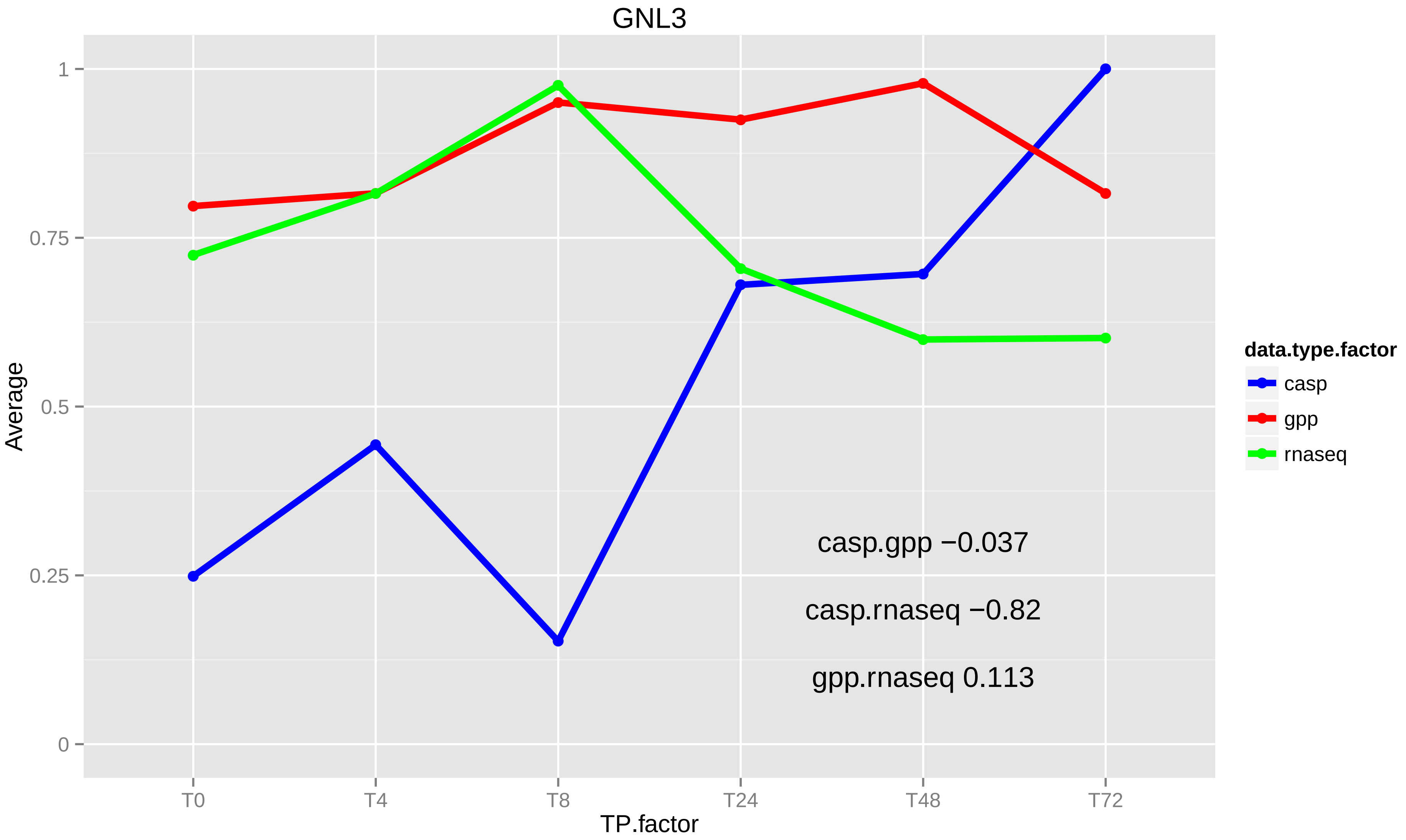
GNL3
Average
1
0.75
0.5
0.25
0
T0
T4
T8
T24
T48
T72
TP.factor
data.type.factor
casp
gpp
rnaseq
casp.gpp −0.037
casp.rnaseq −0.82
gpp.rnaseq 0.113

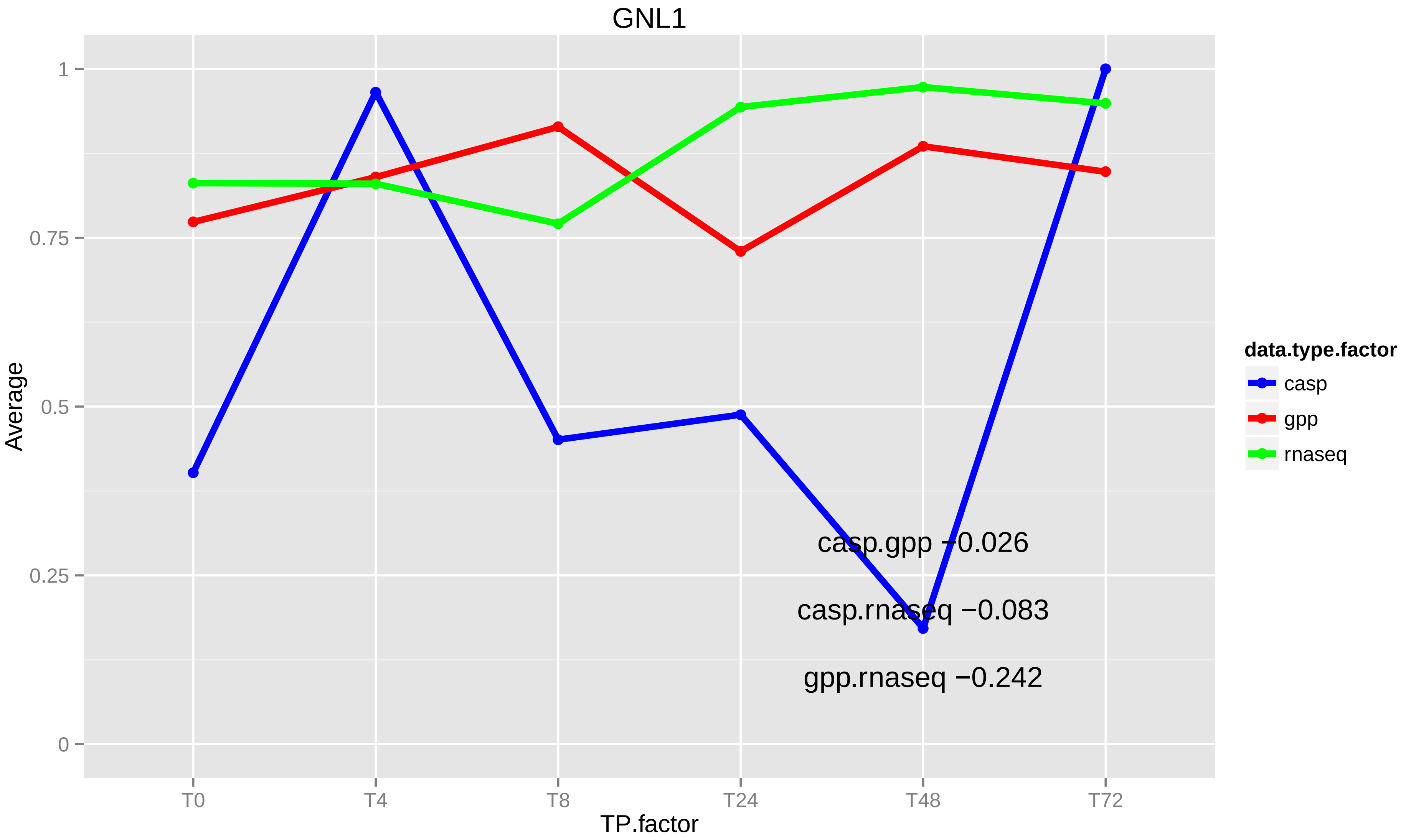
GNL1
Average
1
0.75
0.5
0.25
0
T0
T4
T8
T24
T48
T72
TP.factor
data.type.factor
casp
gpp
rnaseq
casp.gpp −0.026
casp.rnaseq −0.083
gpp.rnaseq −0.242

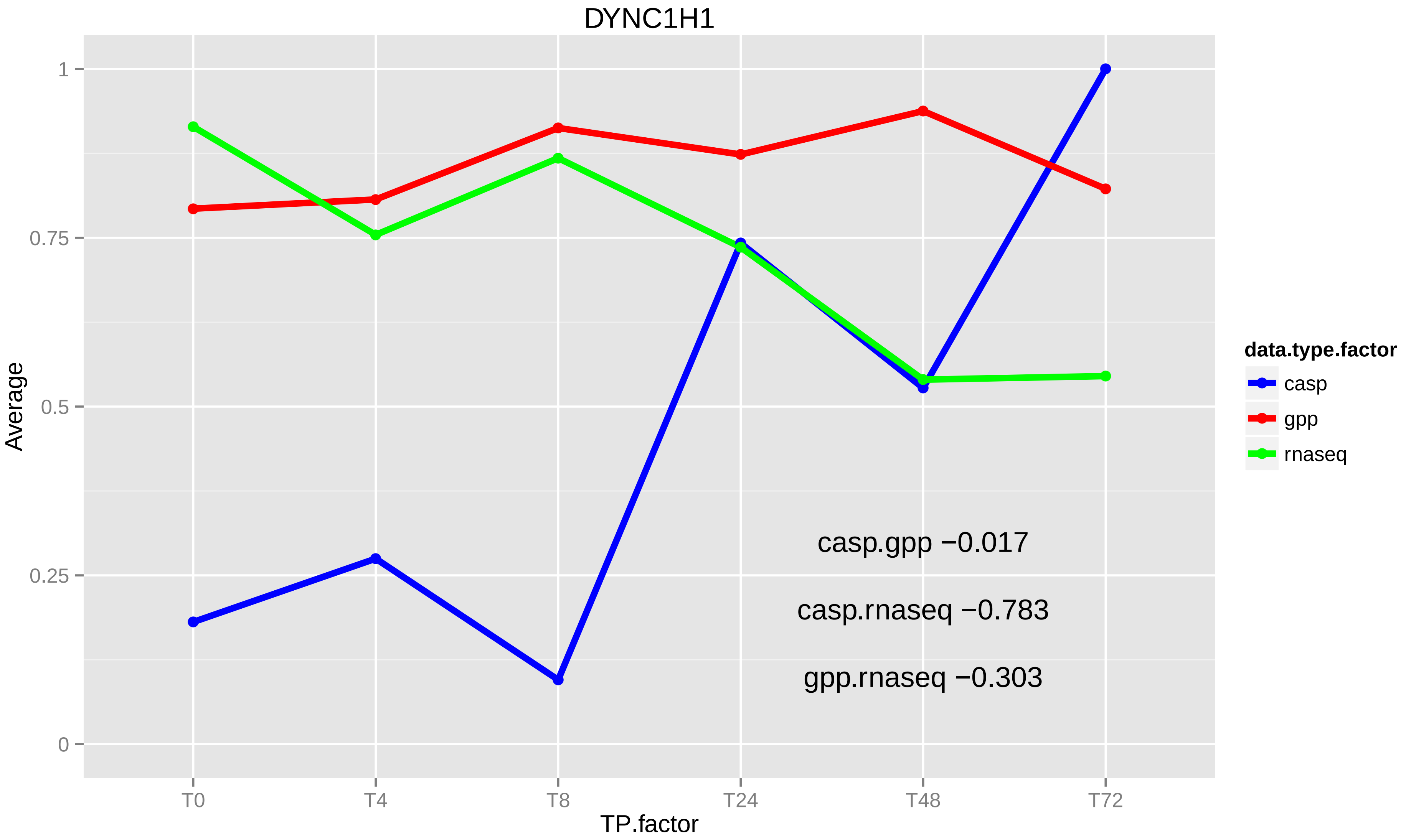
DYNC1H1
1
0.75
0.5
0.25
0
Average
T0
T4
T8
T24
T48
T72
TP.factor
data.type.factor
casp
gpp
rnaseq
casp.gpp −0.017
casp.rnaseq −0.783
gpp.rnaseq −0.303

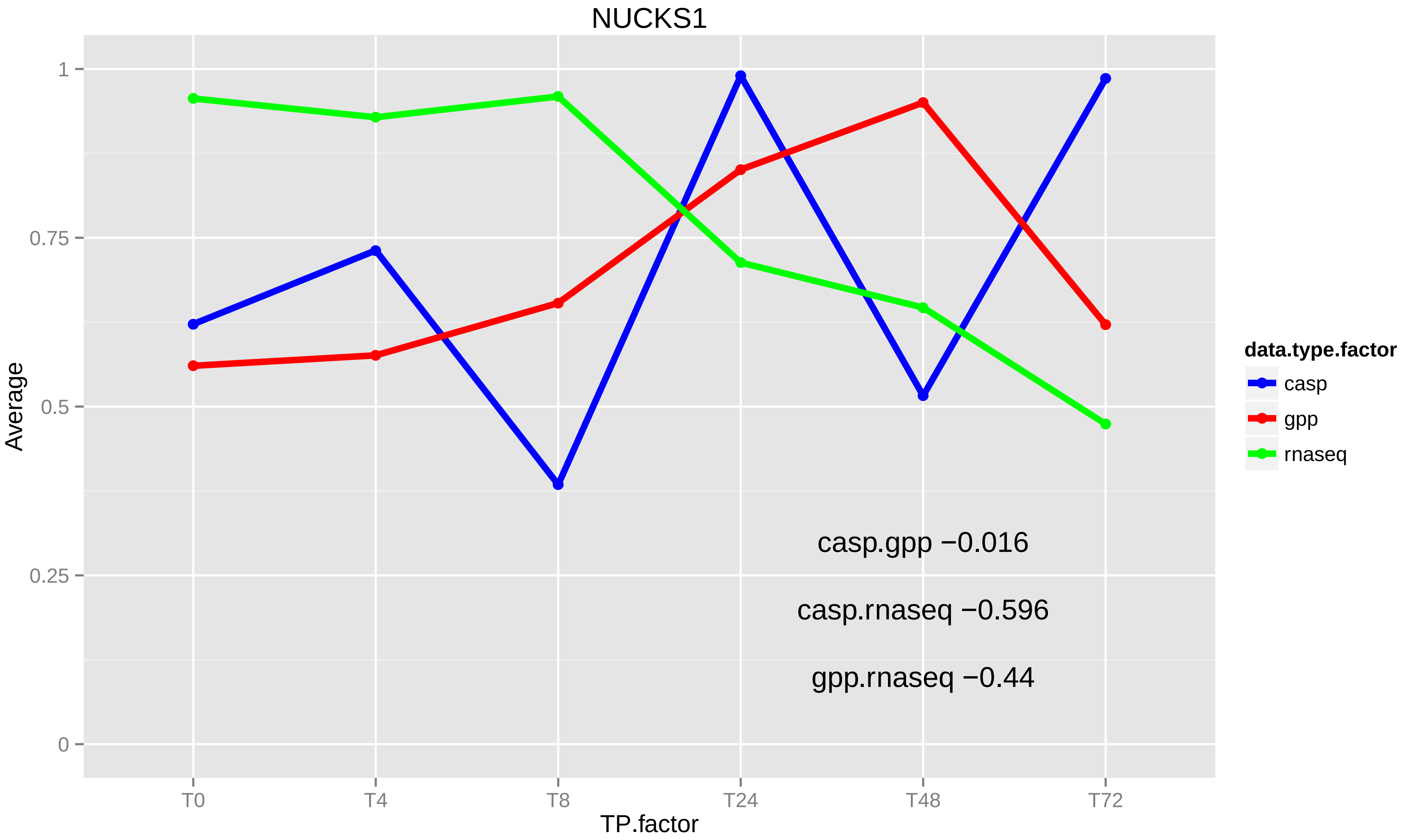

NUCKS1
1
0.75
0.5
0.25
0
Average
T0
T4
T8
T24
T48
T72
TP.factor
data.type.factor
casp
gpp
rnaseq
casp.gpp −0.016
casp.rnaseq −0.596
gpp.rnaseq −0.44

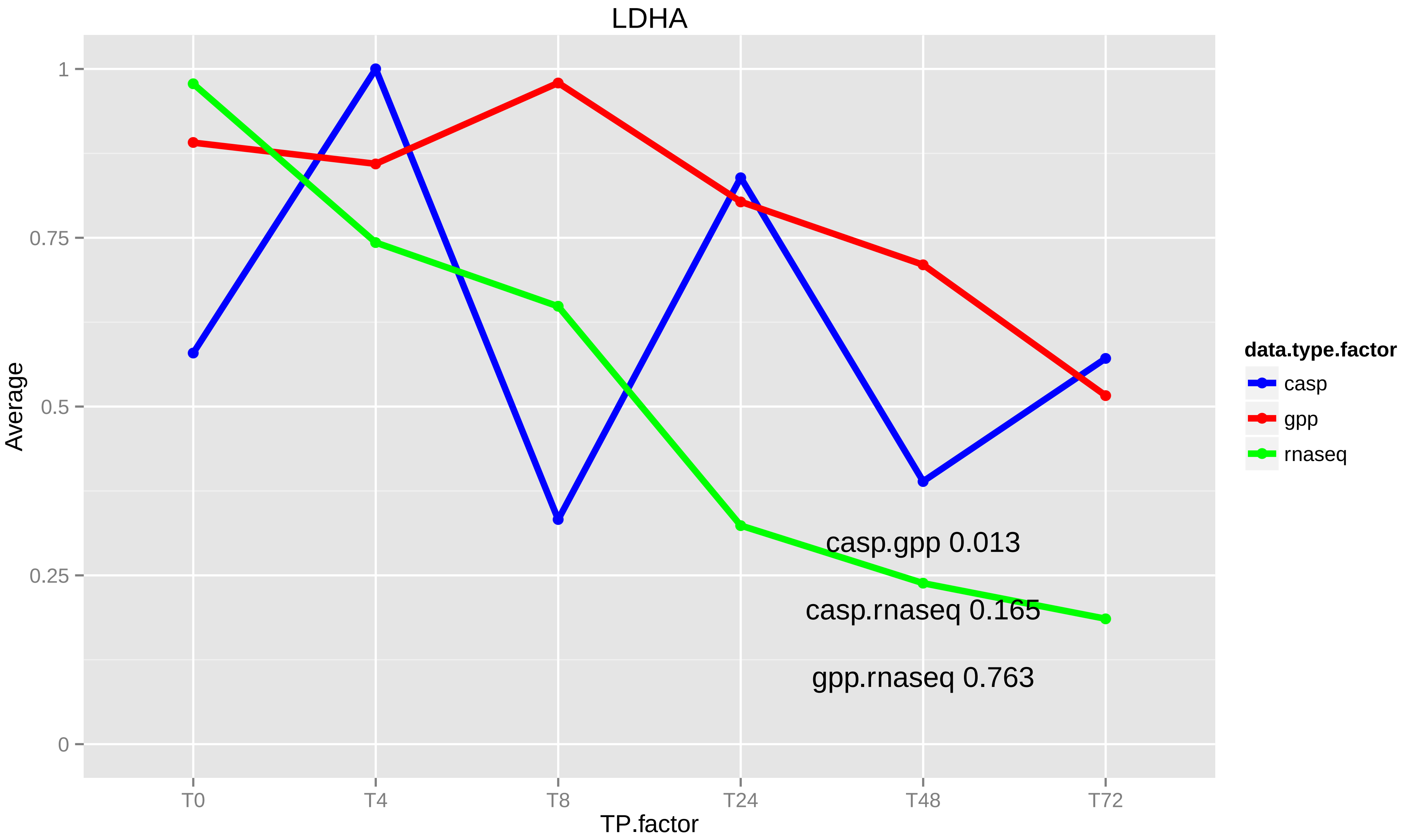

LDHA
1
0.75
0.5
0.25
0
Average
T0
T4
T8
T24
T48
T72
TP.factor
data.type.factor
casp
gpp
rnaseq
casp.gpp 0.013
casp.rnaseq 0.165
gpp.rnaseq 0.763

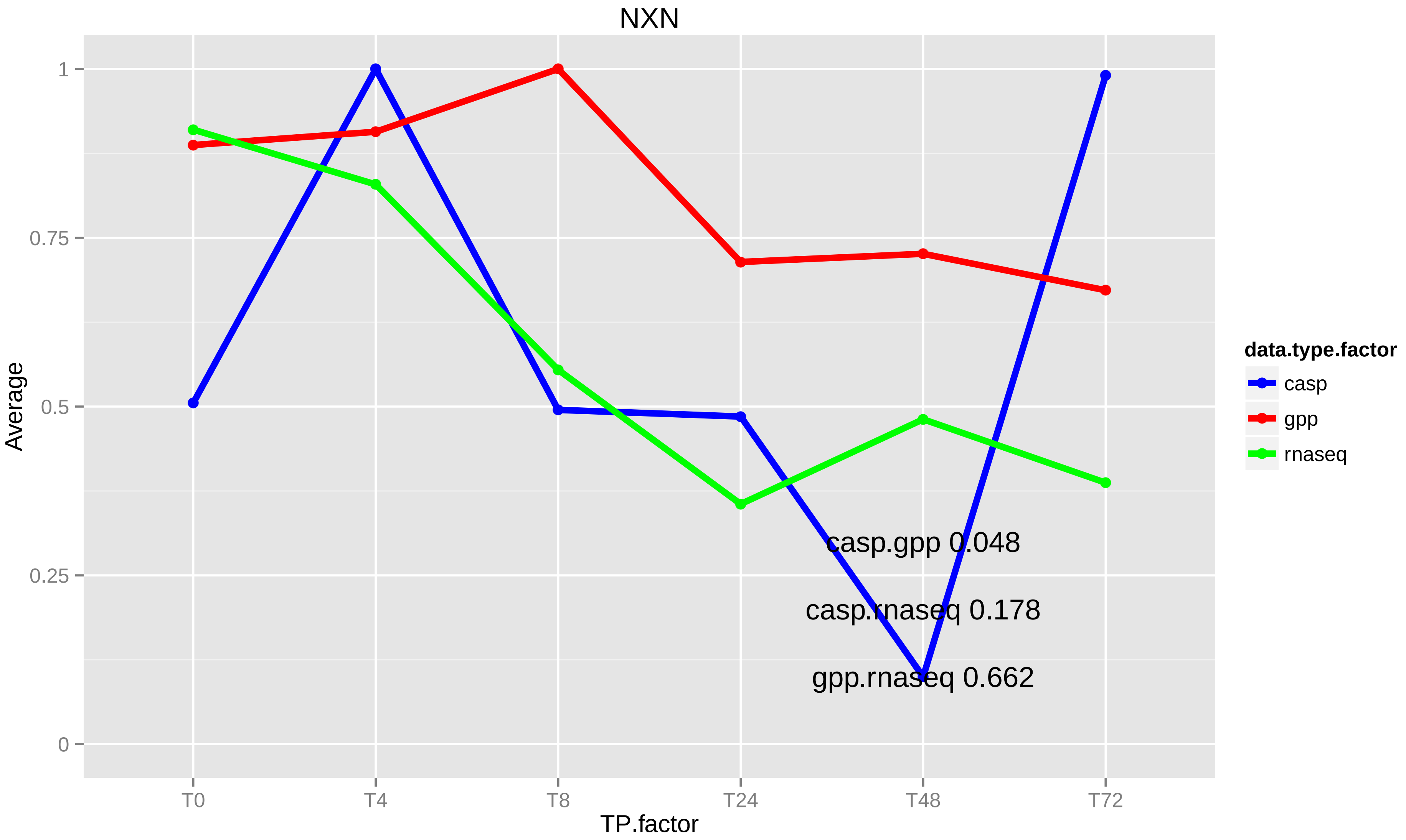
NXN
1
0.75
0.5
0.25
0
Average
T0
T4
T8
T24
T48
T72
TP.factor
casp.gpp 0.048
casp.rnaseq 0.178
gpp.rnaseq 0.662
data.type.factor
casp
gpp
rnaseq

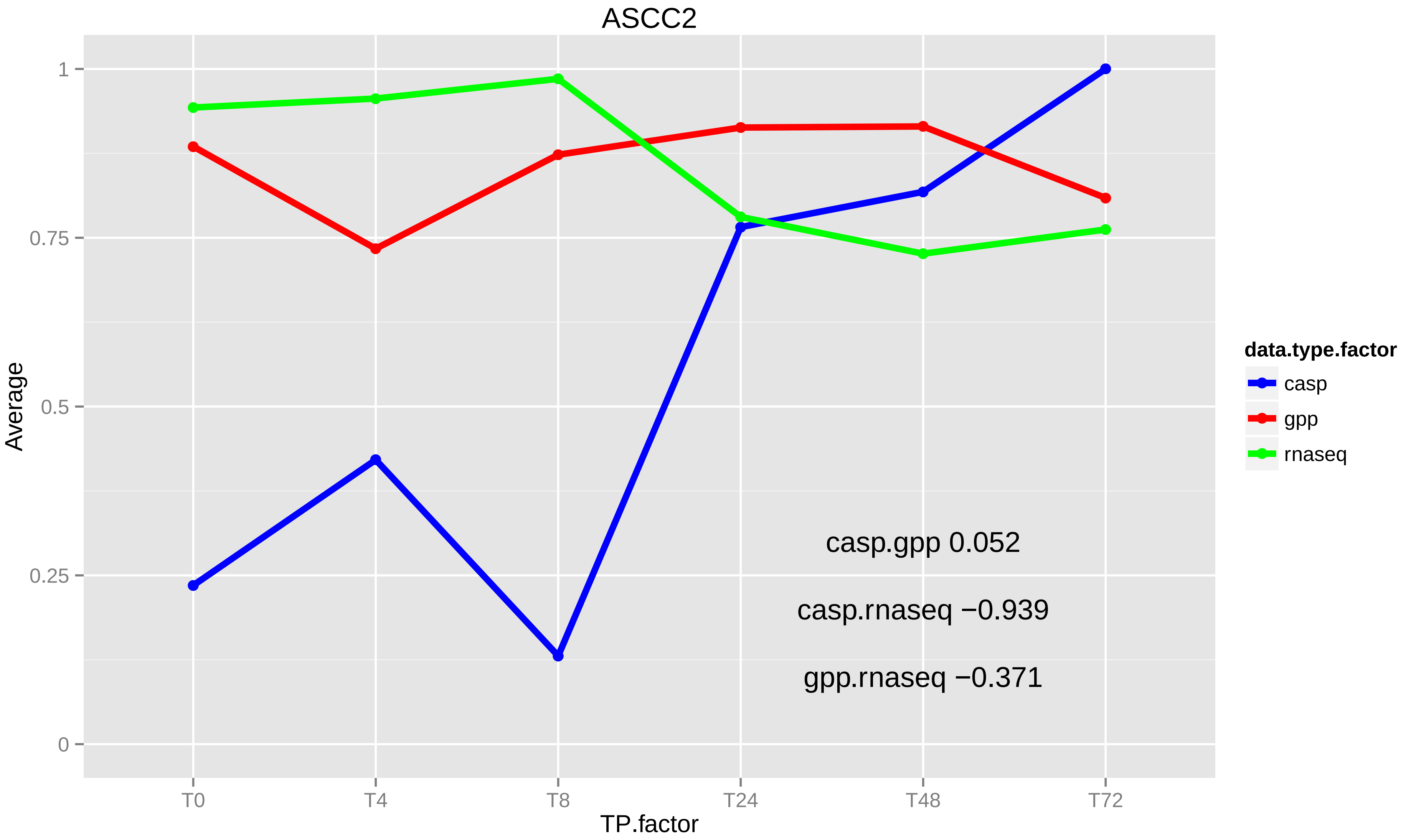
ASCC2
Average
1
0.75
0.5
0.25
0
T0
T4
T8
T24
T48
T72
TP.factor
data.type.factor
casp
gpp
rnaseq
casp.gpp 0.052
casp.rnaseq −0.939
gpp.rnaseq −0.371

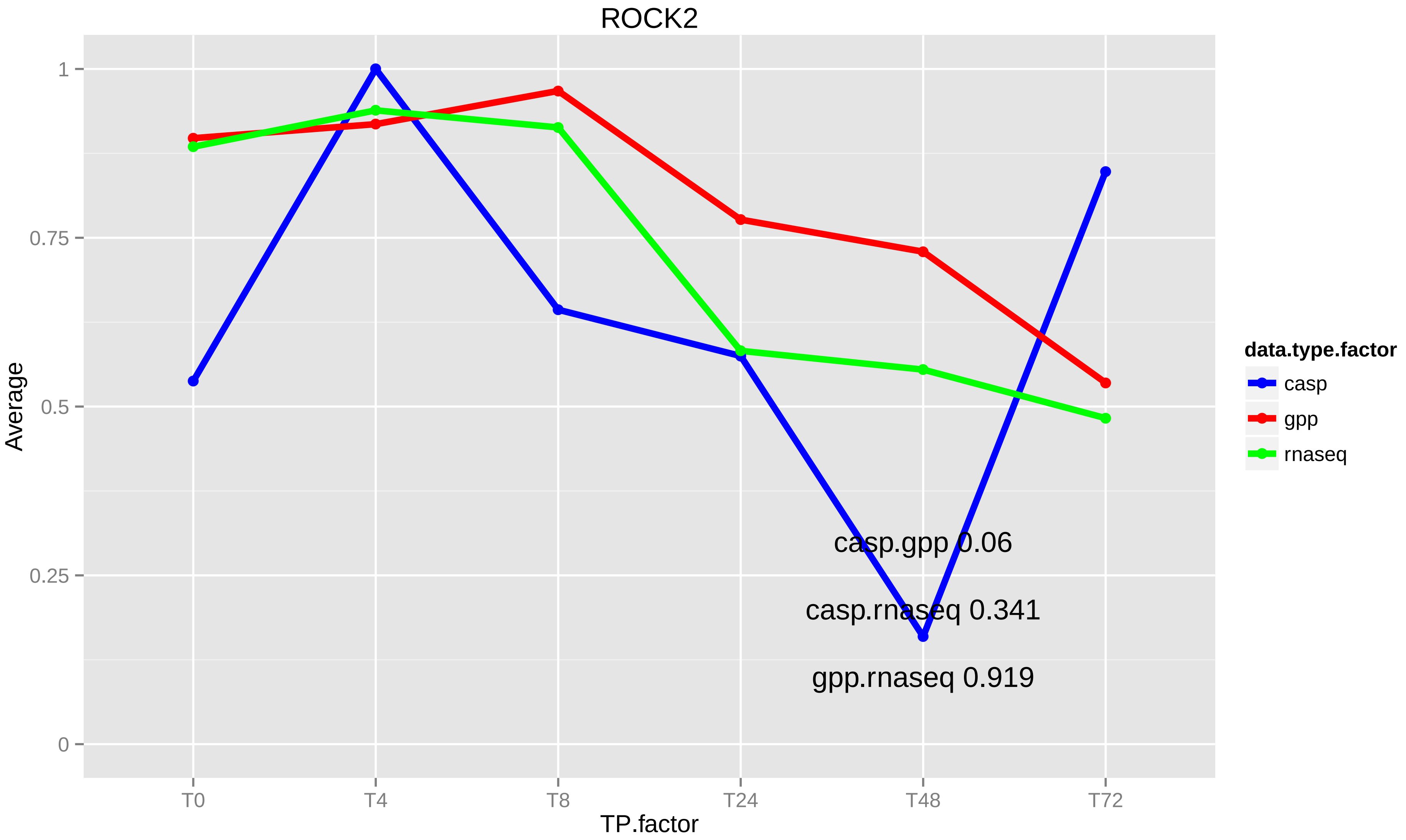
ROCK2
Average
1
0.75
0.5
0.25
0
T0
T4
T8
T24
T48
T72
TP.factor
data.type.factor
casp
gpp
rnaseq
casp.gpp 0.06
casp.rnaseq 0.341
gpp.rnaseq 0.919

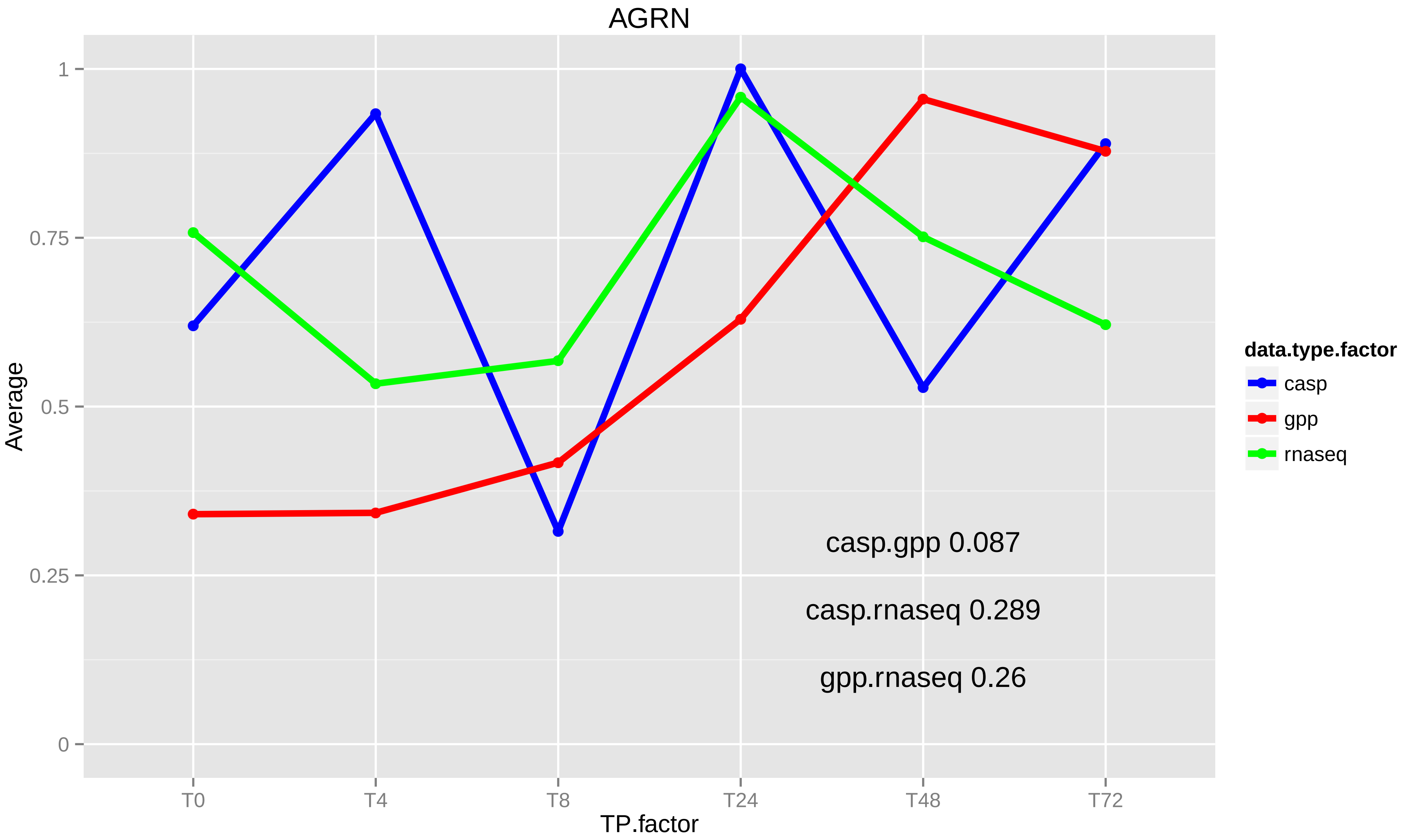
AGRN
Average
1
0.75
0.5
0.25
0
T0
T4
T8
T24
T48
T72
TP.factor
data.type.factor
casp
gpp
rnaseq
casp.gpp 0.087
casp.rnaseq 0.289
gpp.rnaseq 0.26

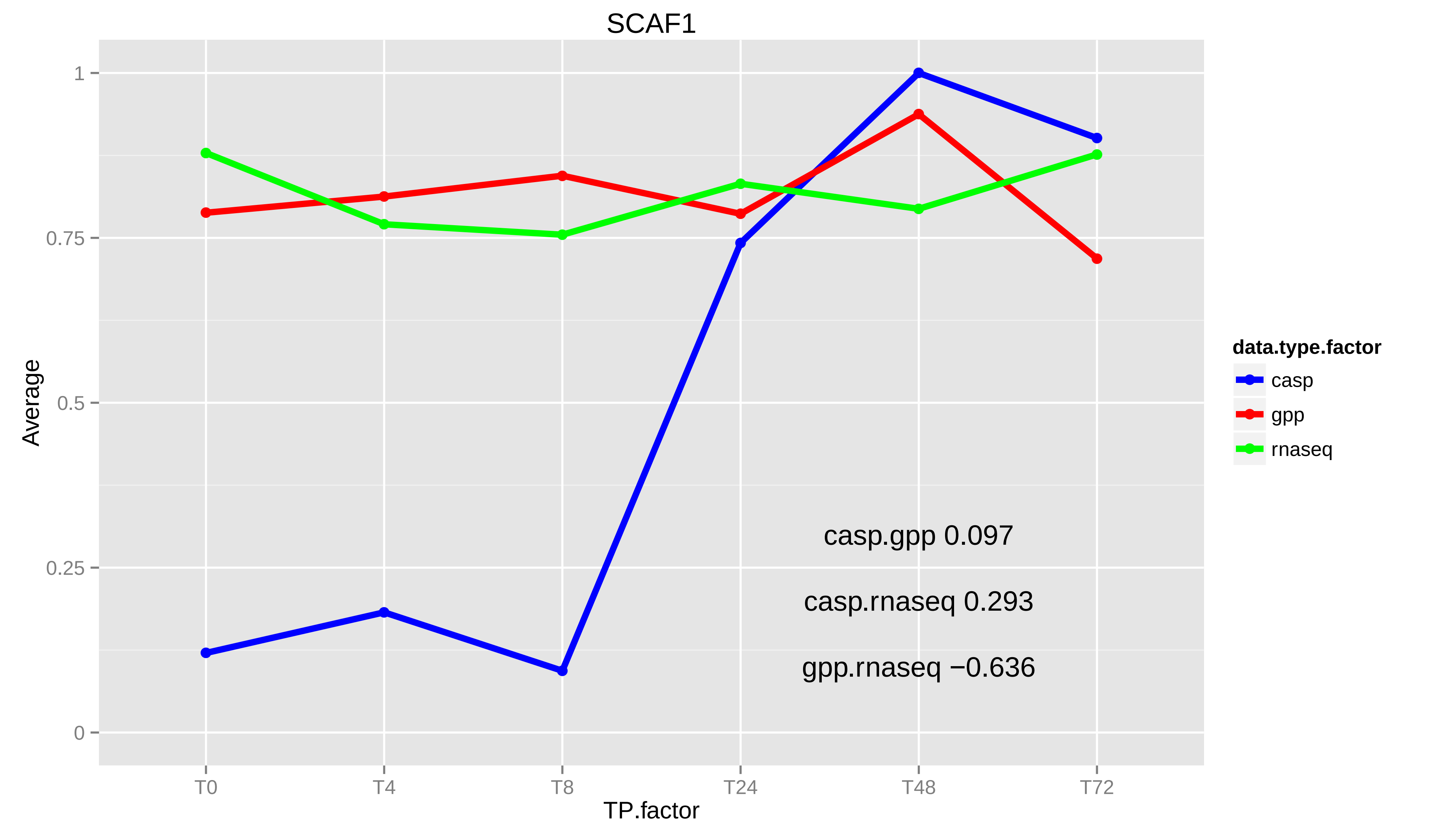

SCAF1
Average
1
0.75
0.5
0.25
0
T0
T4
T8
T24
T48
T72
TP.factor
data.type.factor
casp
gpp
rnaseq
casp.gpp 0.097
casp.rnaseq 0.293
gpp.rnaseq −0.636

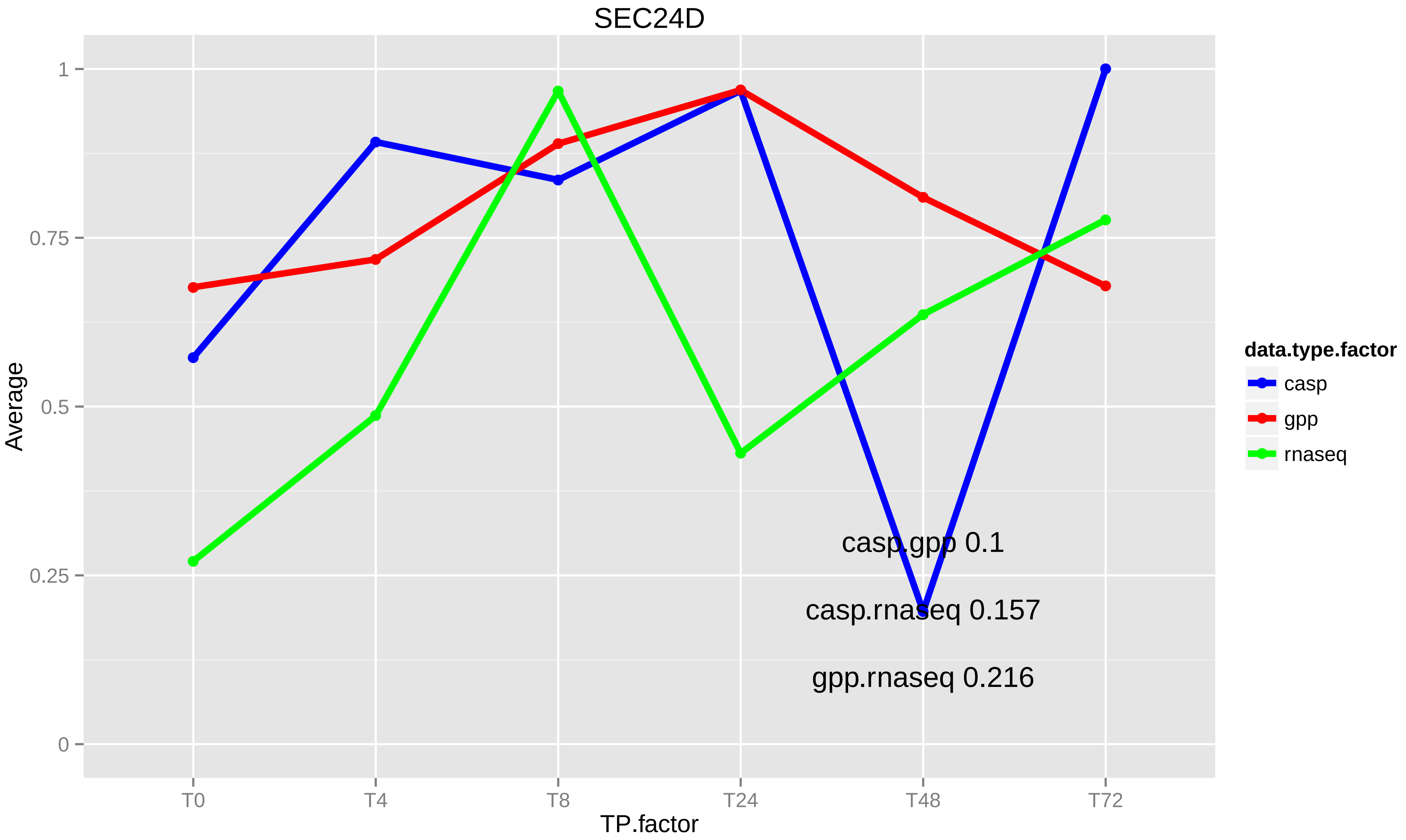
SEC24D
Average
1
0.75
0.5
0.25
0
T0
T4
T8
T24
T48
T72
TP.factor
data.type.factor
casp
gpp
rnaseq
casp.gpp 0.1
casp.rnaseq 0.157
gpp.rnaseq 0.216

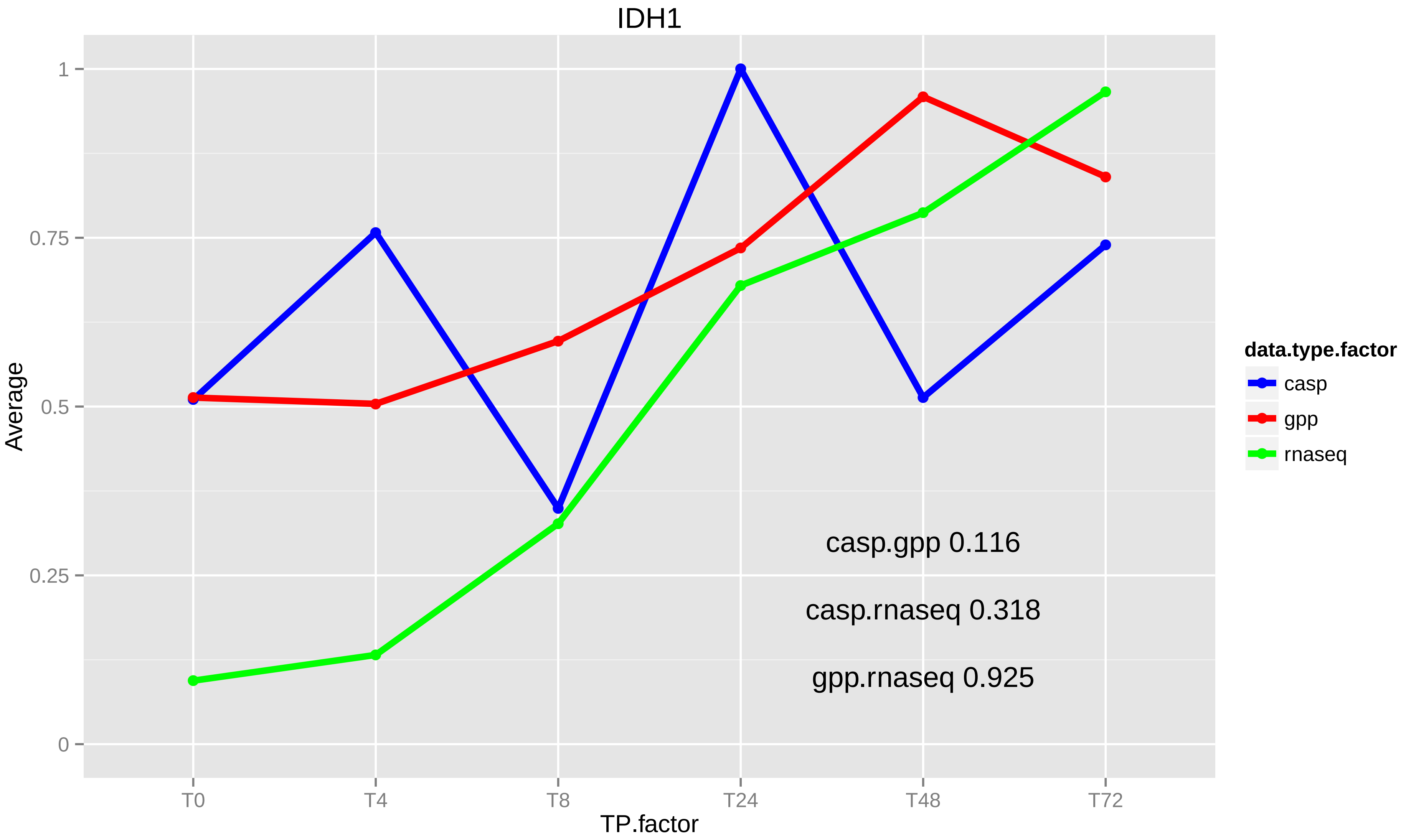

IDH1
1
0.75
0.5
0.25
0
Average
T0
T4
T8
T24
T48
T72
TP.factor
data.type.factor
casp
gpp
rnaseq
casp.gpp 0.116
casp.rnaseq 0.318
gpp.rnaseq 0.925

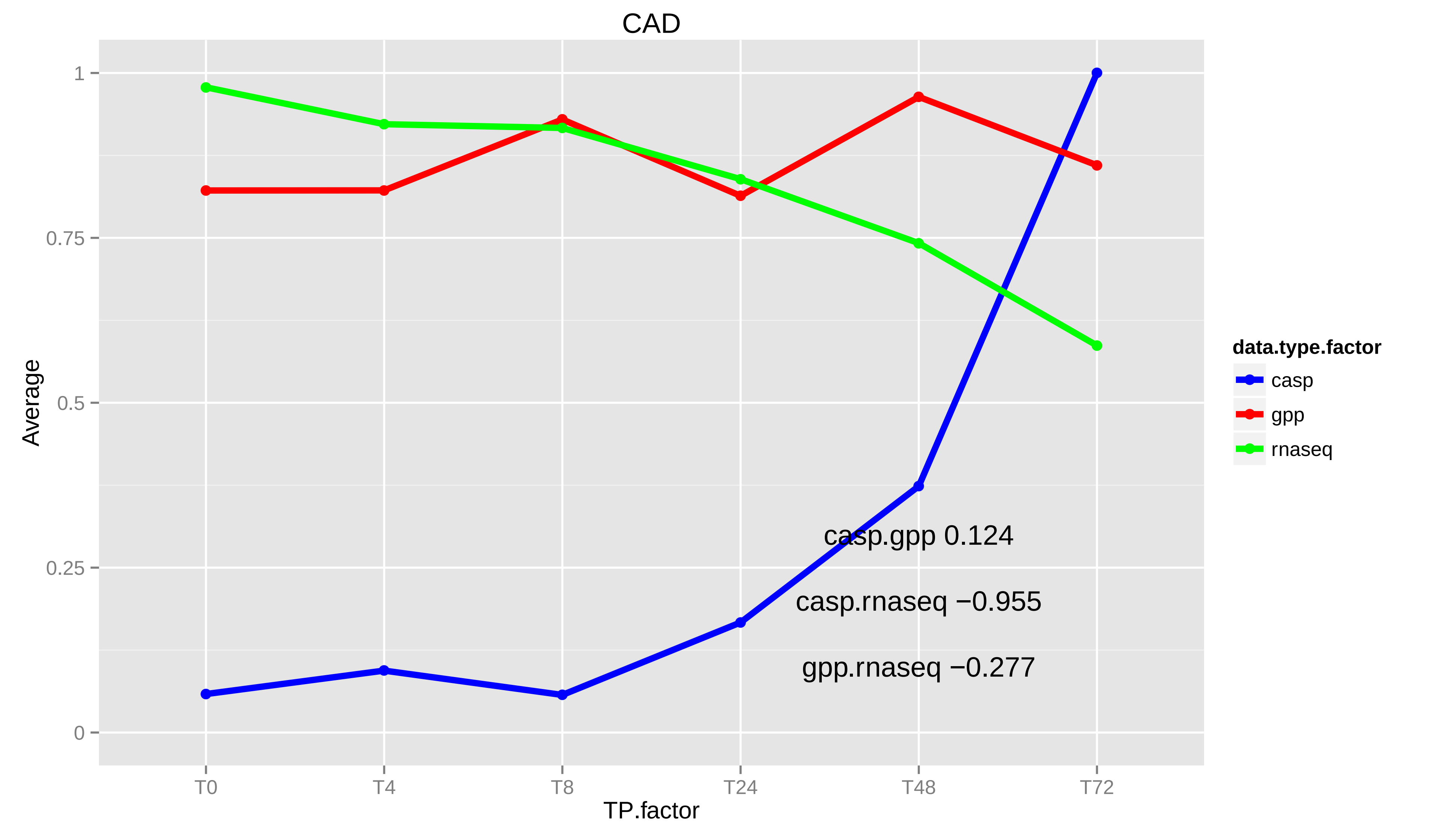
CAD
Average
1
0.75
0.5
0.25
0
T0
T4
T8
T24
T48
T72
TP.factor
data.type.factor
casp
gpp
rnaseq
casp.gpp 0.124
casp.rnaseq −0.955
gpp.rnaseq −0.277

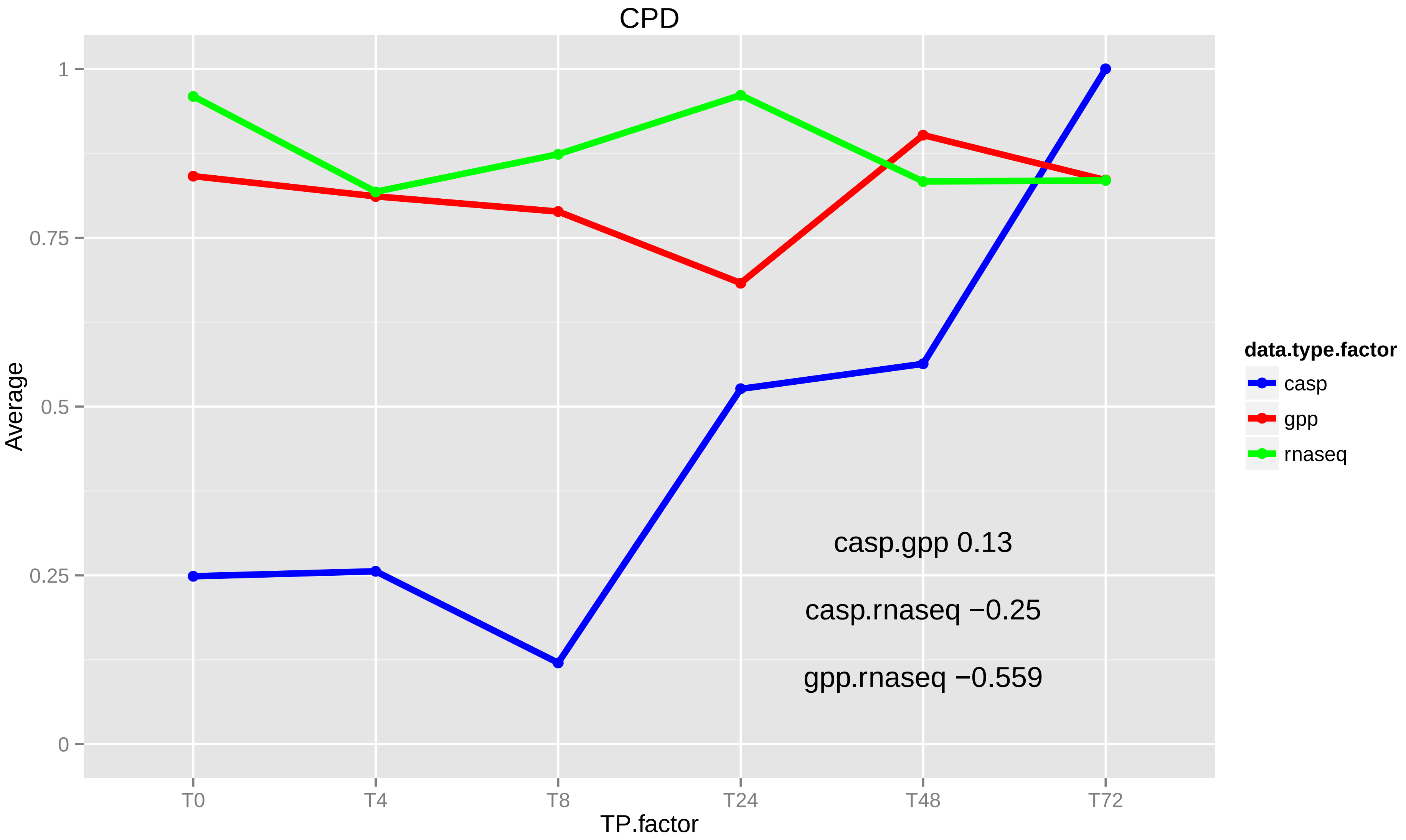
CPD
Average
1
0.75
0.5
0.25
0
T0
T4
T8
T24
T48
T72
TP.factor
data.type.factor
casp
gpp
rnaseq
casp.gpp 0.13
casp.rnaseq −0.25
gpp.rnaseq −0.559

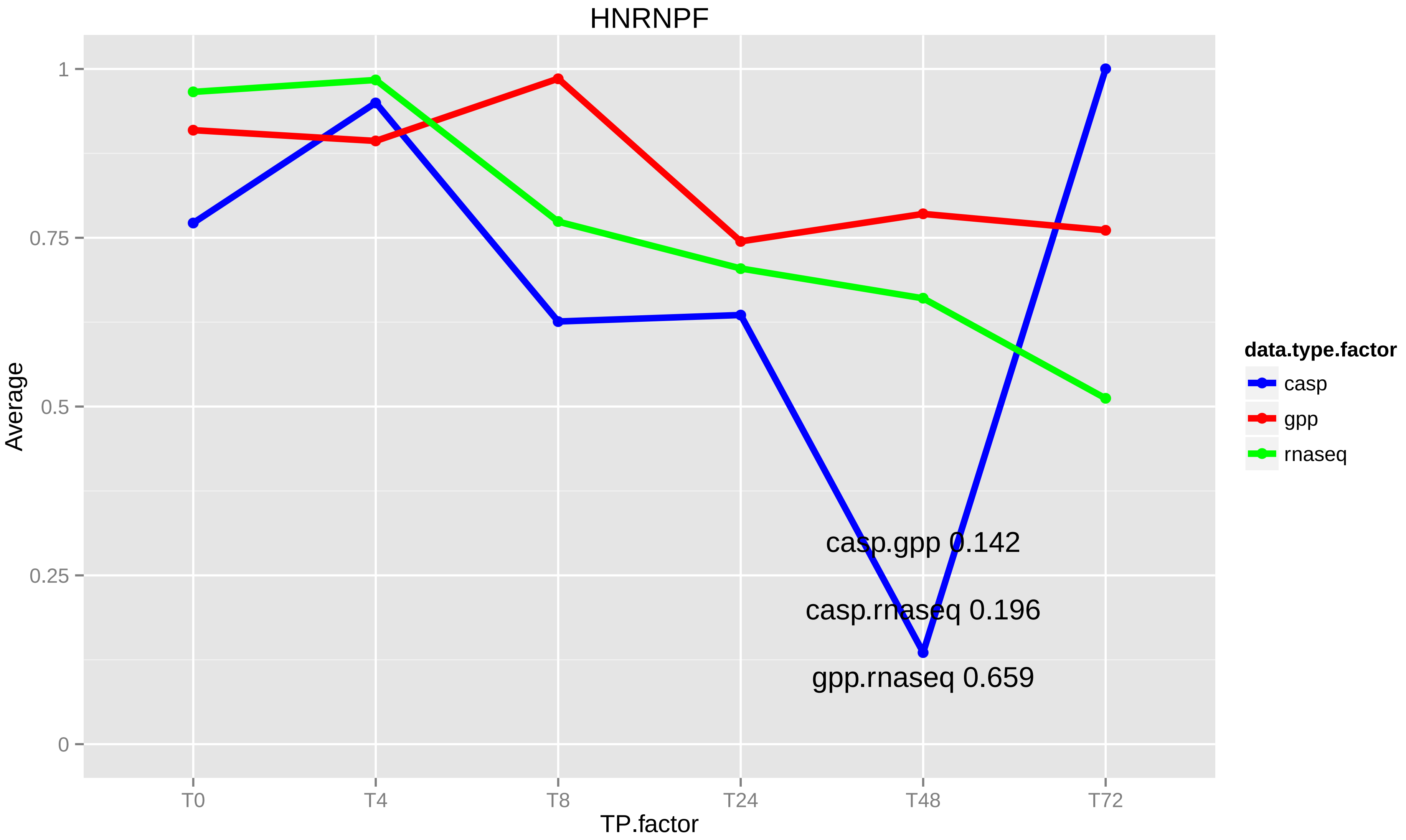
HNRNPF
Average
1
0.75
0.5
0.25
0
T0
T4
T8
T24
T48
T72
TP.factor
data.type.factor
casp
gpp
rnaseq
casp.gpp 0.142
casp.rnaseq 0.196
gpp.rnaseq 0.659

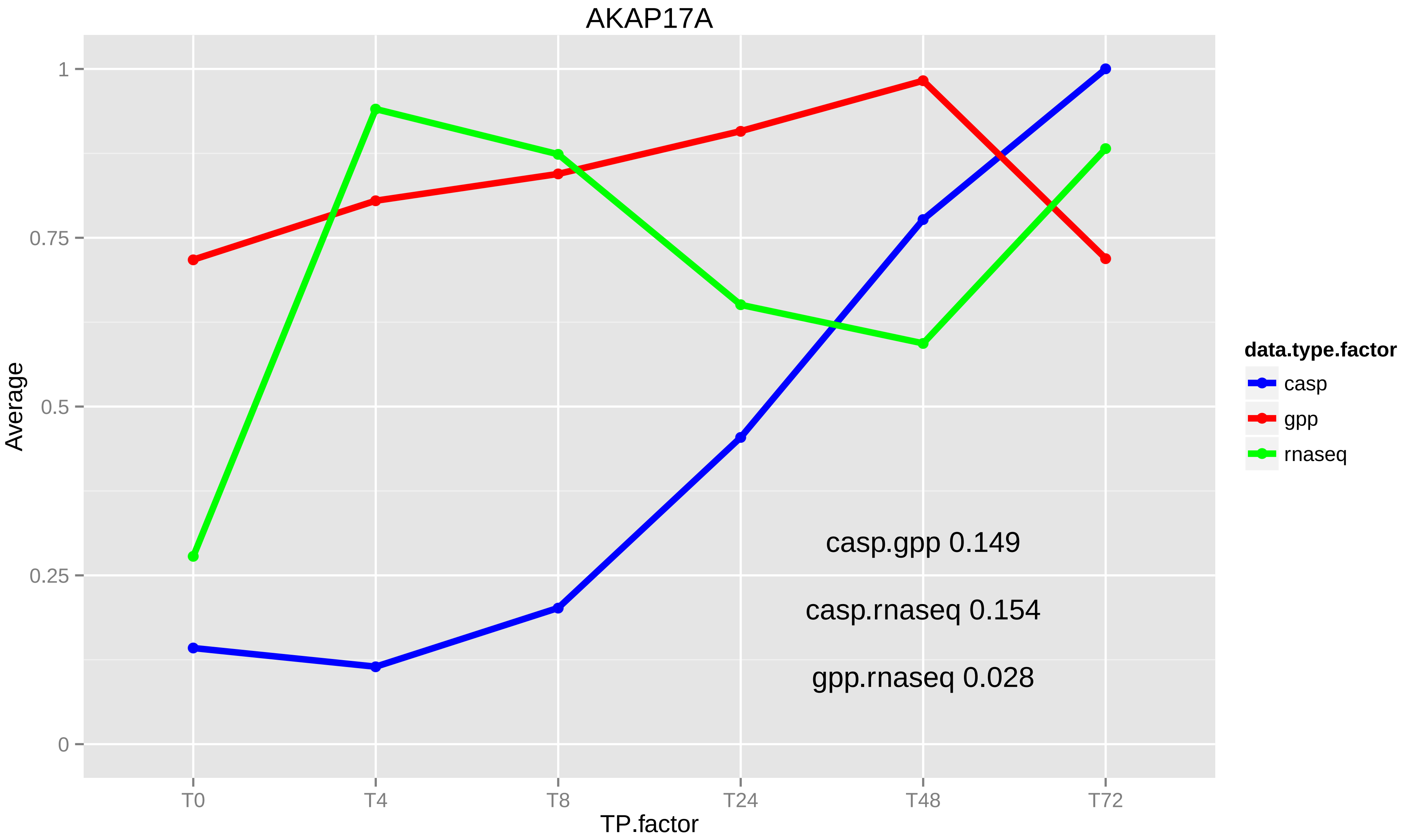
AKAP17A
Average
1
0.75
0.5
0.25
0
T0
T4
T8
T24
T48
T72
TP.factor
data.type.factor
casp
gpp
rnaseq
casp.gpp 0.149
casp.rnaseq 0.154
gpp.rnaseq 0.028

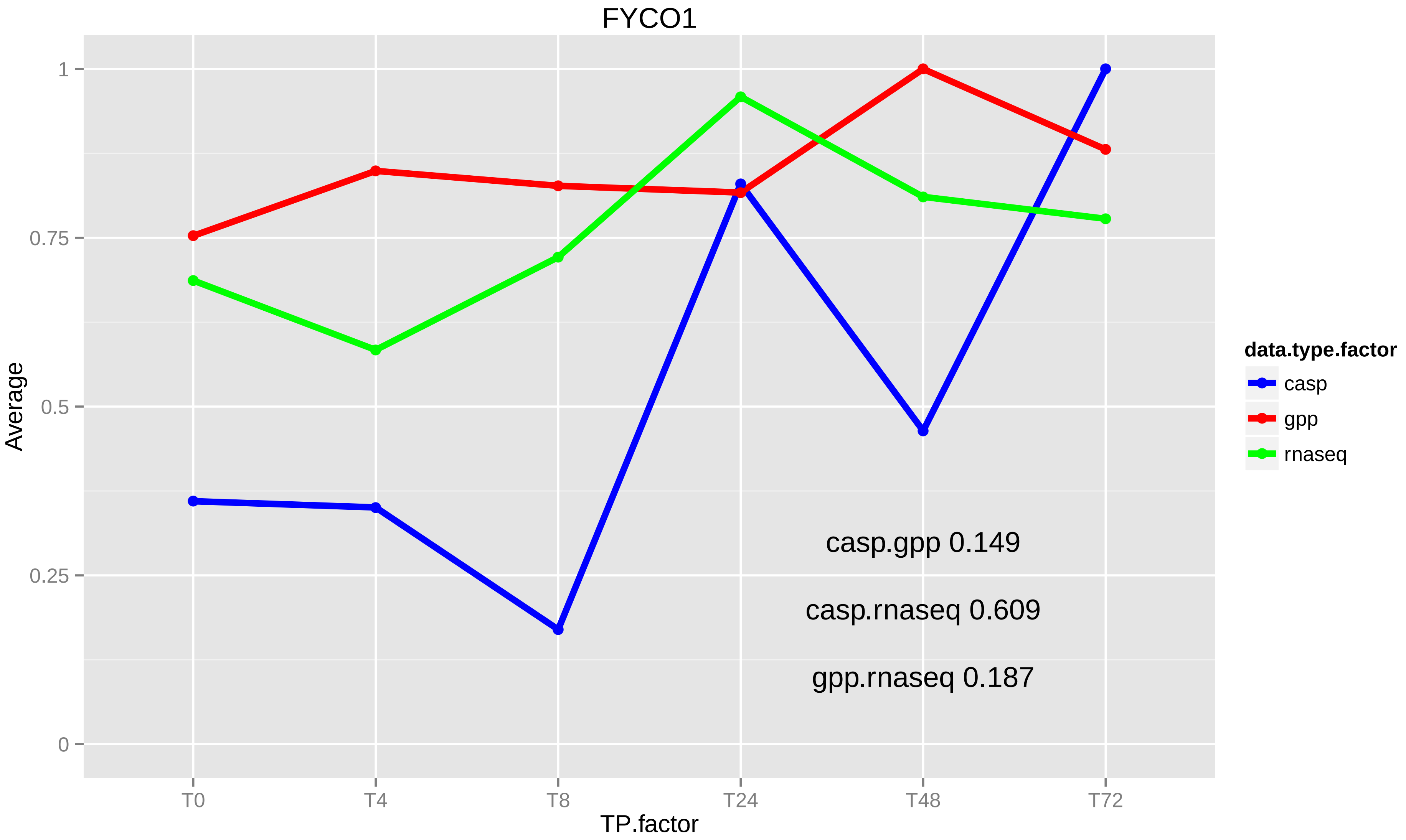
FYCO1
Average
1
0.75
0.5
0.25
0
T0
T4
T8
T24
T48
T72
TP.factor
data.type.factor
casp
gpp
rnaseq
casp.gpp 0.149
casp.rnaseq 0.609
gpp.rnaseq 0.187

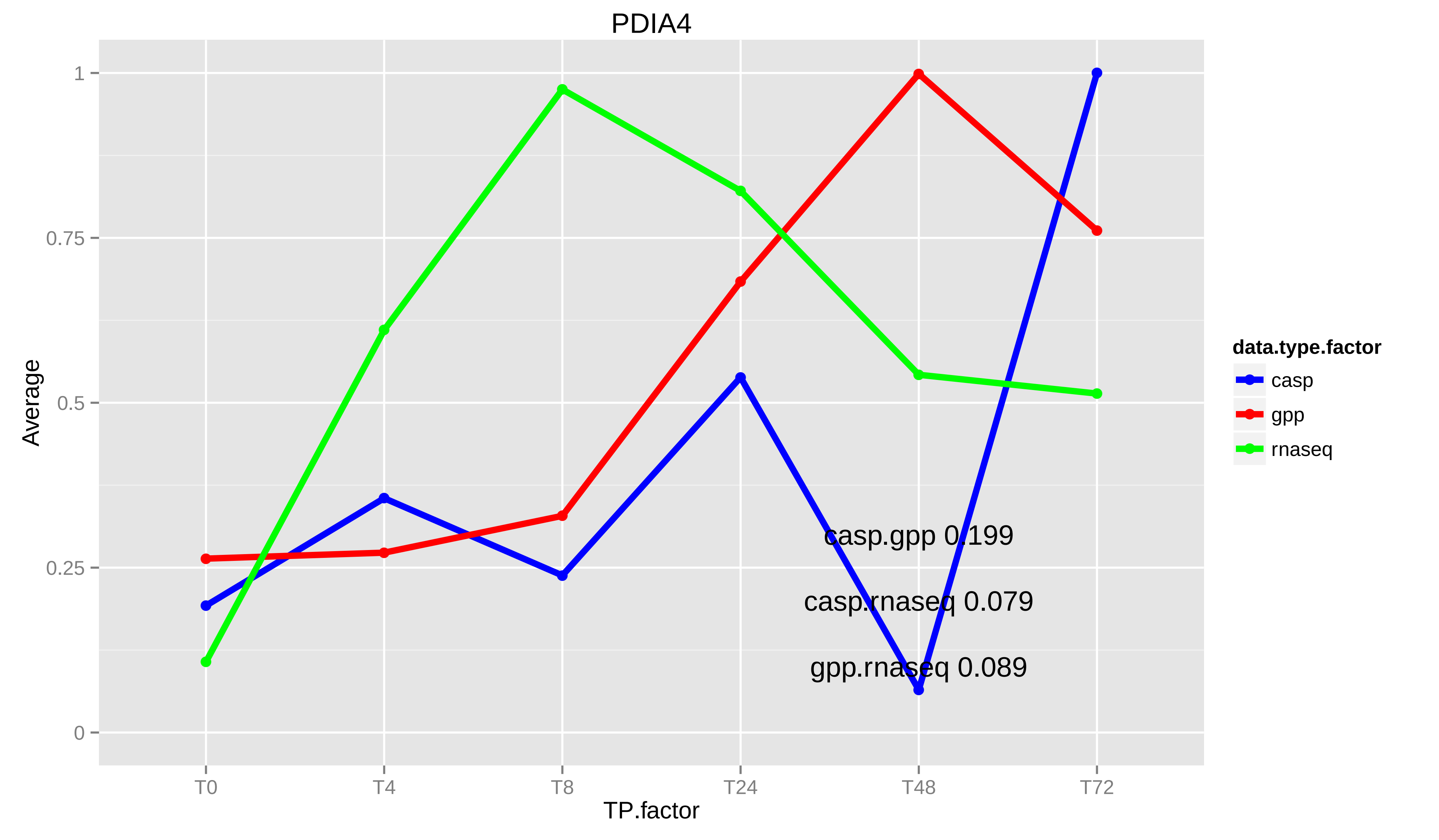

PDIA4
1
0.75
0.5
0.25
0
Average
T0
T4
T8
T24
T48
T72
TP.factor
data.type.factor
casp
gpp
rnaseq
casp.gpp 0.199
casp.rnaseq 0.079
gpp.rnaseq 0.089

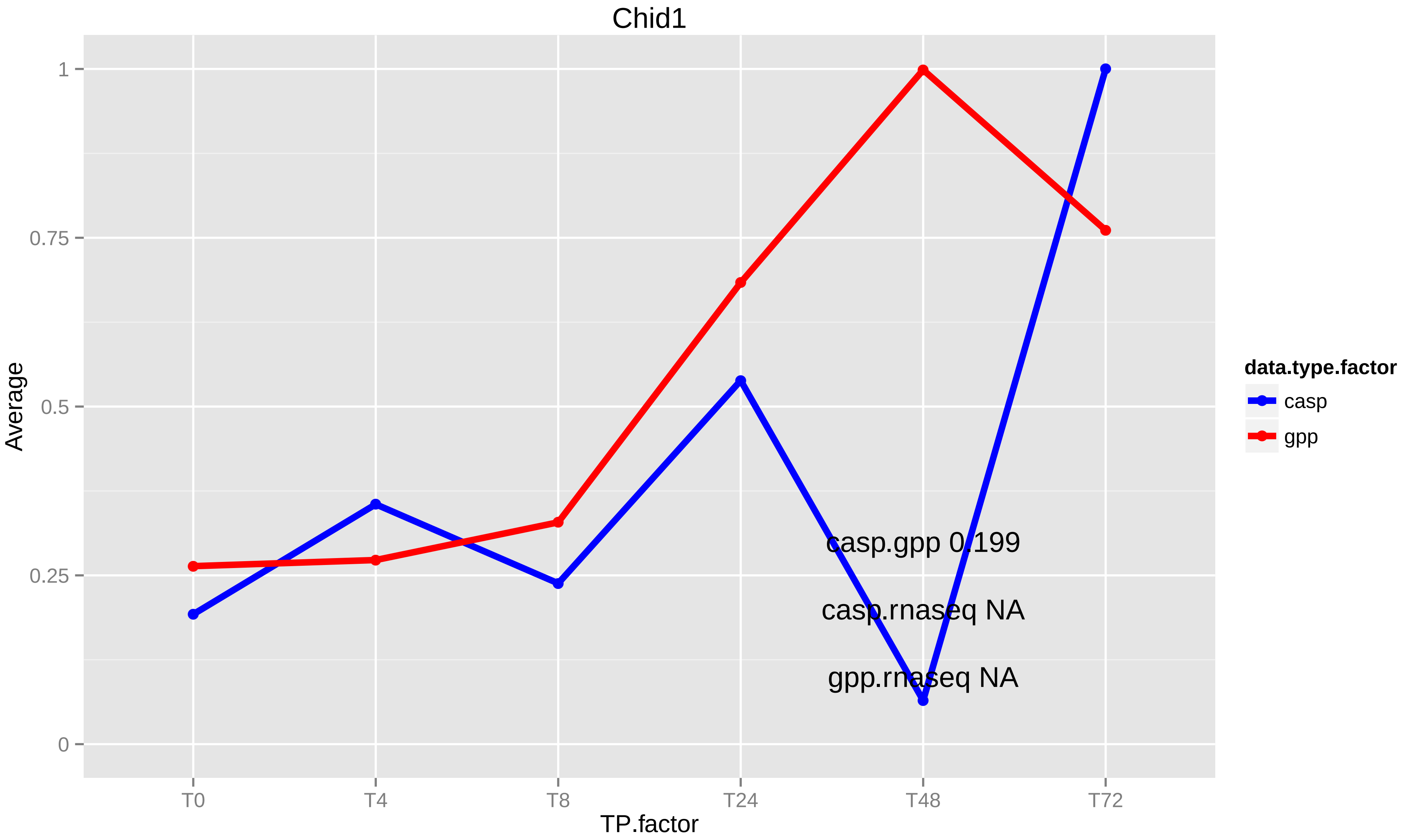

Chid1
1
0.75
0.5
0.25
0
Average
T0
T4
T8
T24
T48
T72
TP.factor
data.type.factor
casp
gpp
casp.gpp 0.199
casp.rnaseq NA
gpp.rnaseq NA

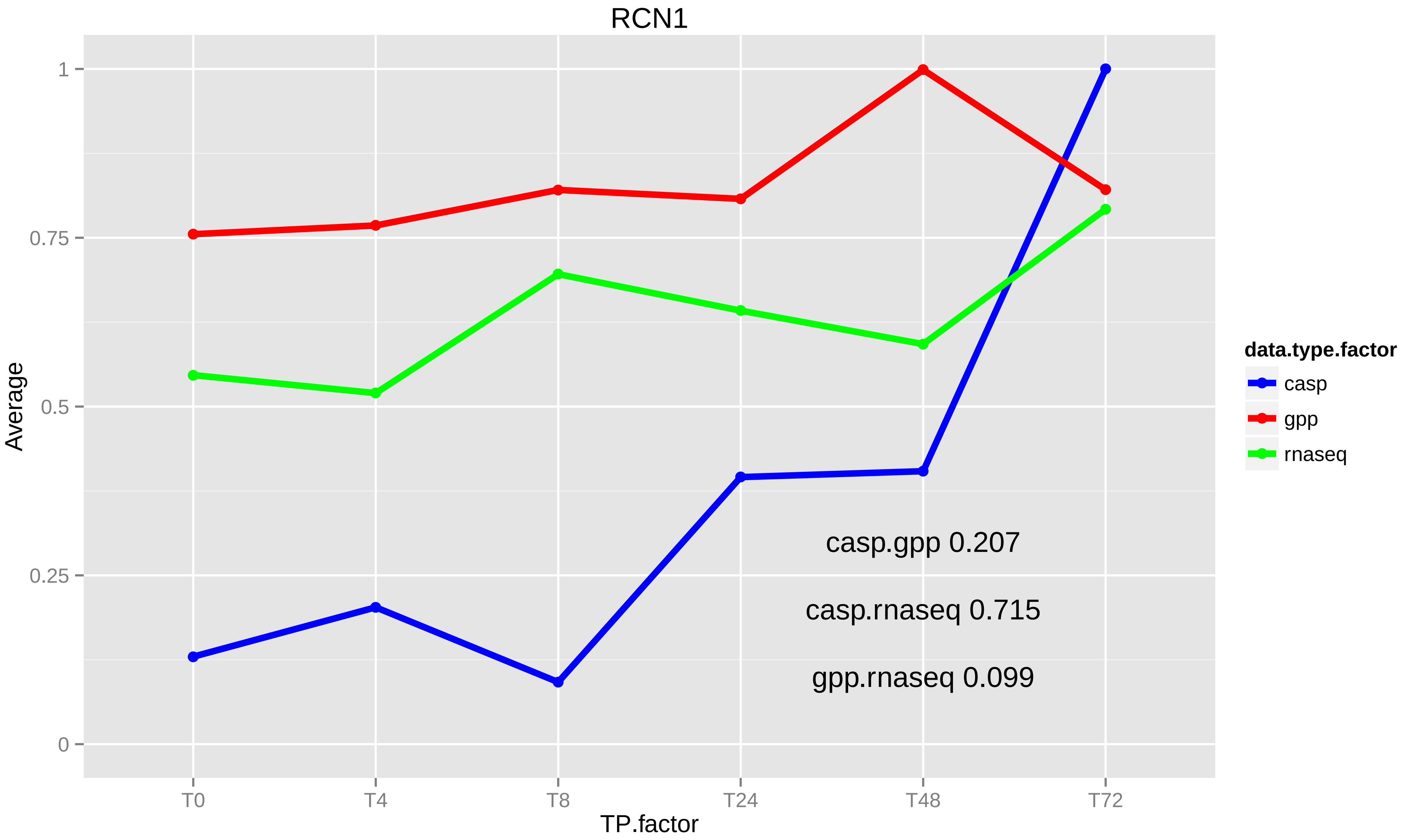

RCN1
1
0.75
0.5
0.25
0
Average
casp.gpp 0.207
casp.rnaseq 0.715
gpp.rnaseq 0.099
T0
T4
T8
T24
T48
T72
TP.factor
data.type.factor
casp
gpp
rnaseq

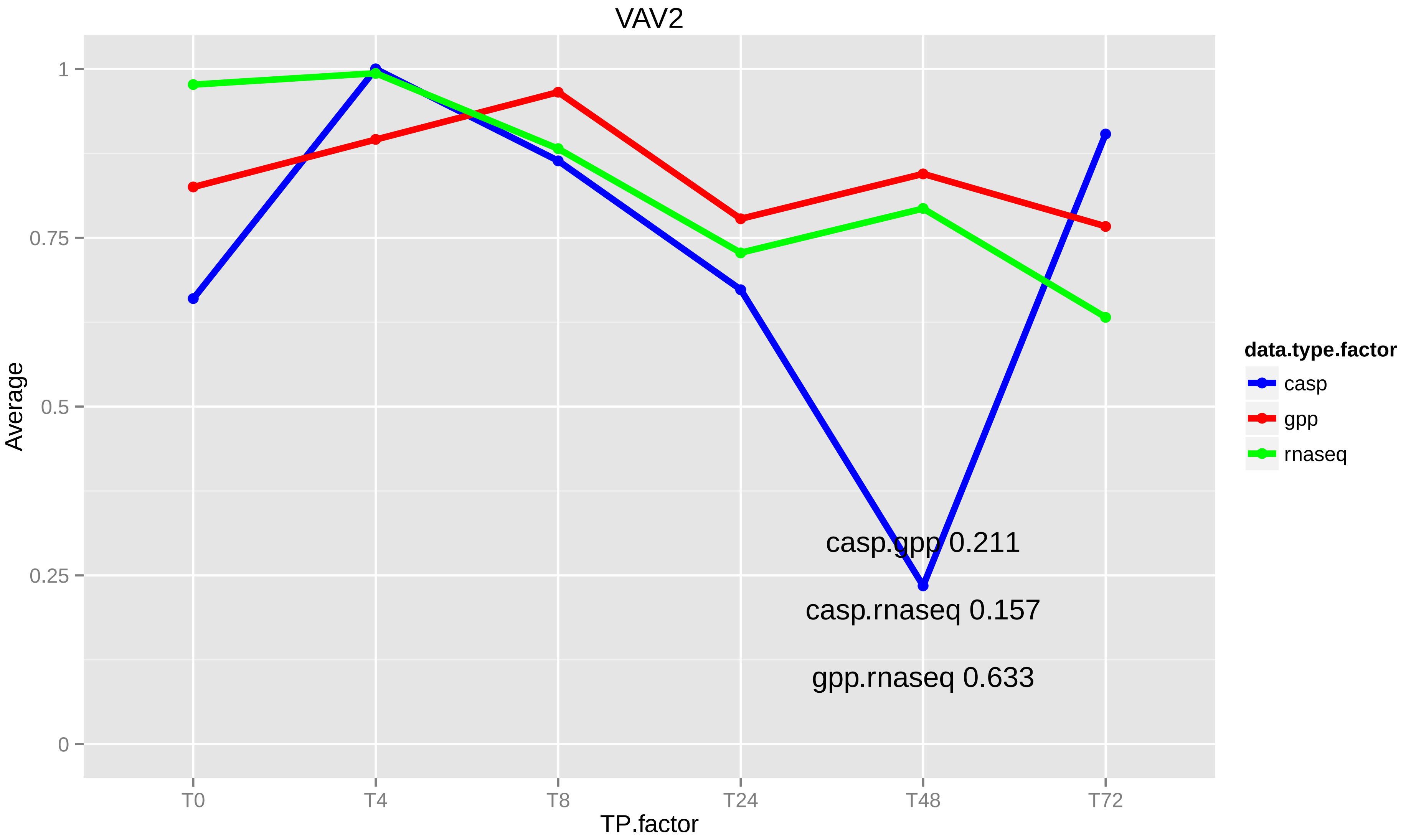
VAV2
Average
1
0.75
0.5
0.25
0
T0
T4
T8
T24
T48
T72
TP.factor
data.type.factor
casp
gpp
rnaseq
casp.gpp 0.211
casp.rnaseq 0.157
gpp.rnaseq 0.633

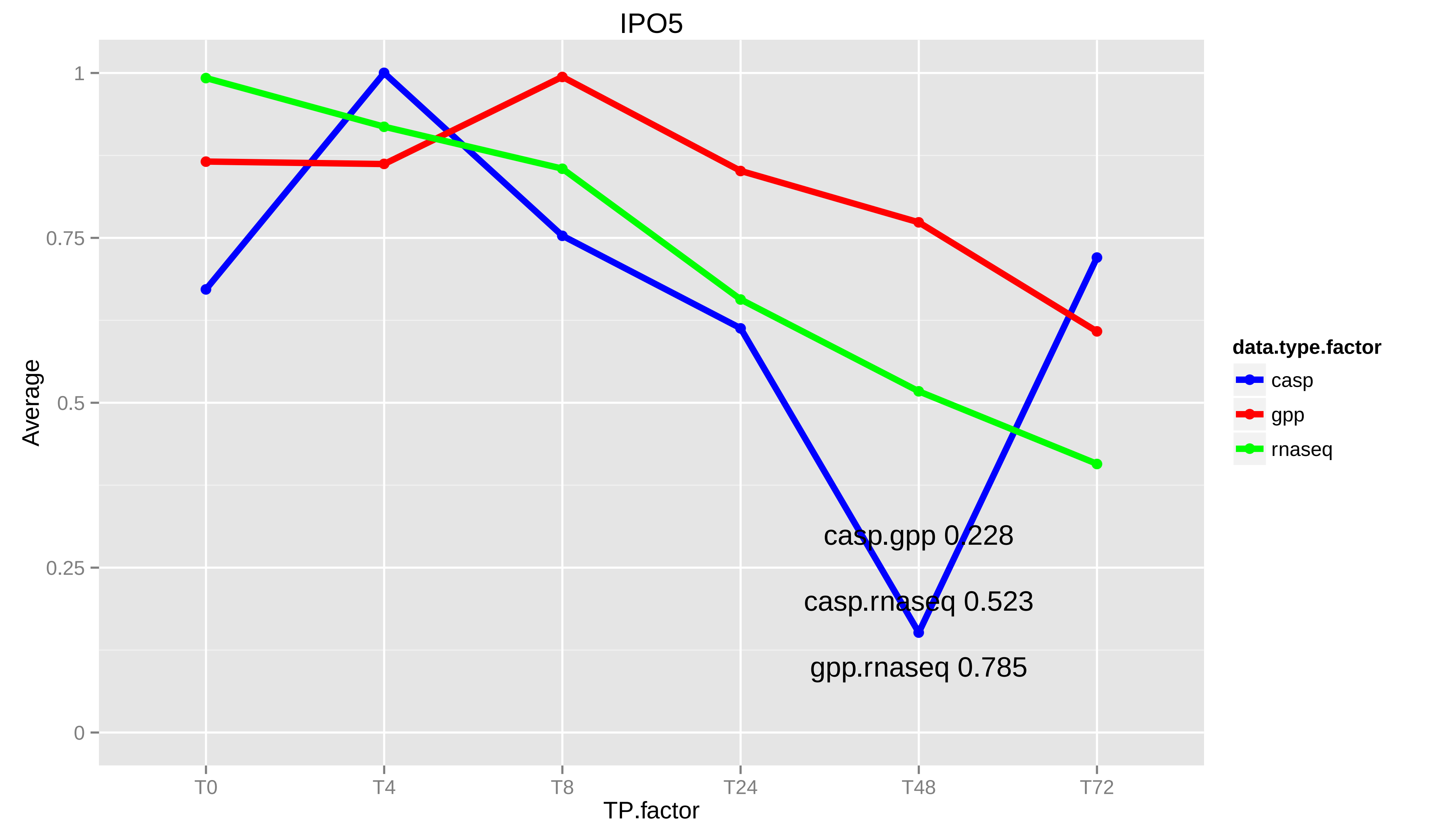

IPO5
1
0.75
0.5
0.25
0
Average
T0
T4
T8
T24
T48
T72
TP.factor
data.type.factor
casp
gpp
rnaseq
casp.gpp 0.228
casp.rnaseq 0.523
gpp.rnaseq 0.785

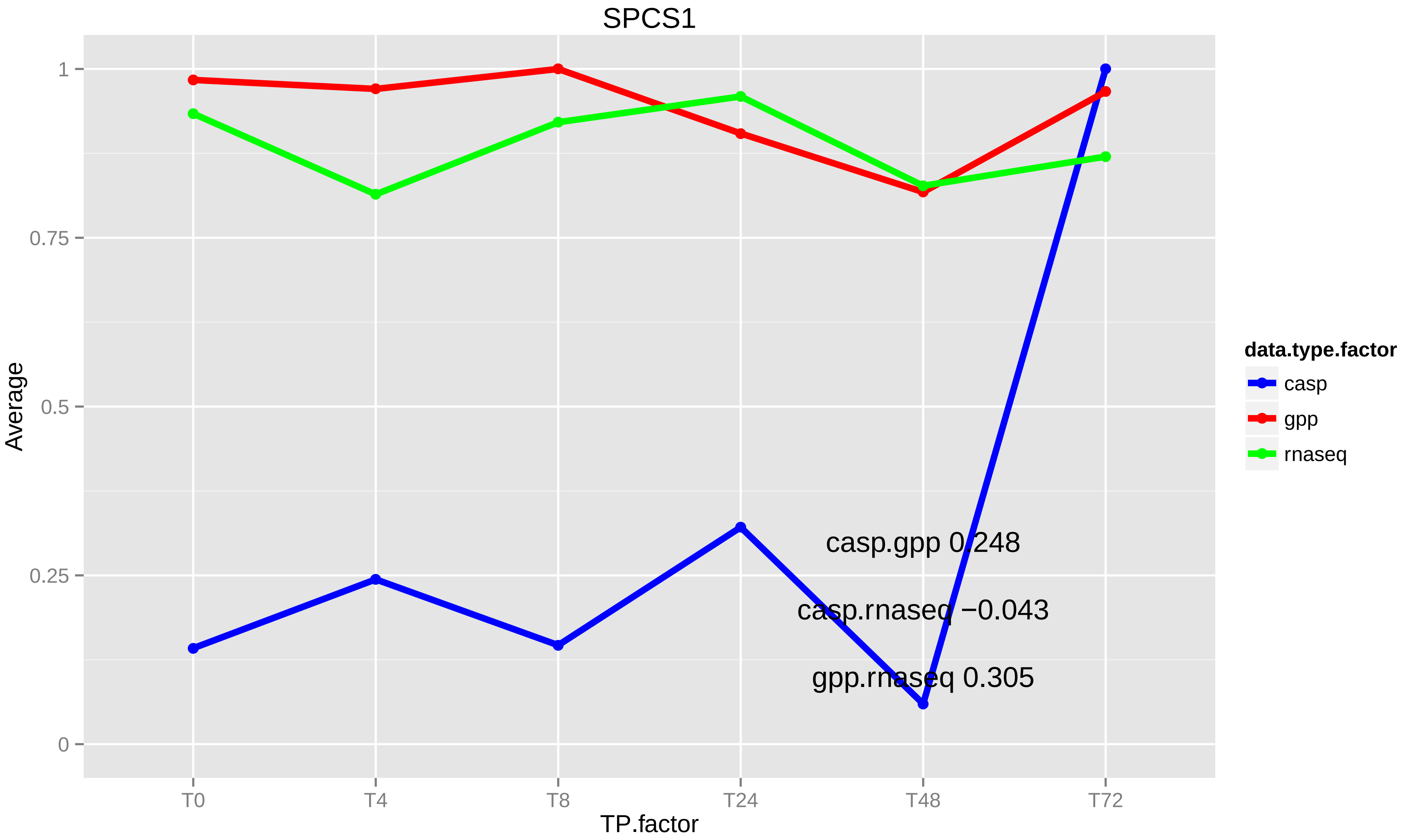
SPCS1
Average
1
0.75
0.5
0.25
0
T0
T4
T8
T24
T48
T72
TP.factor
data.type.factor
casp
gpp
rnaseq
casp.gpp 0.248
casp.rnaseq −0.043
gpp.rnaseq 0.305

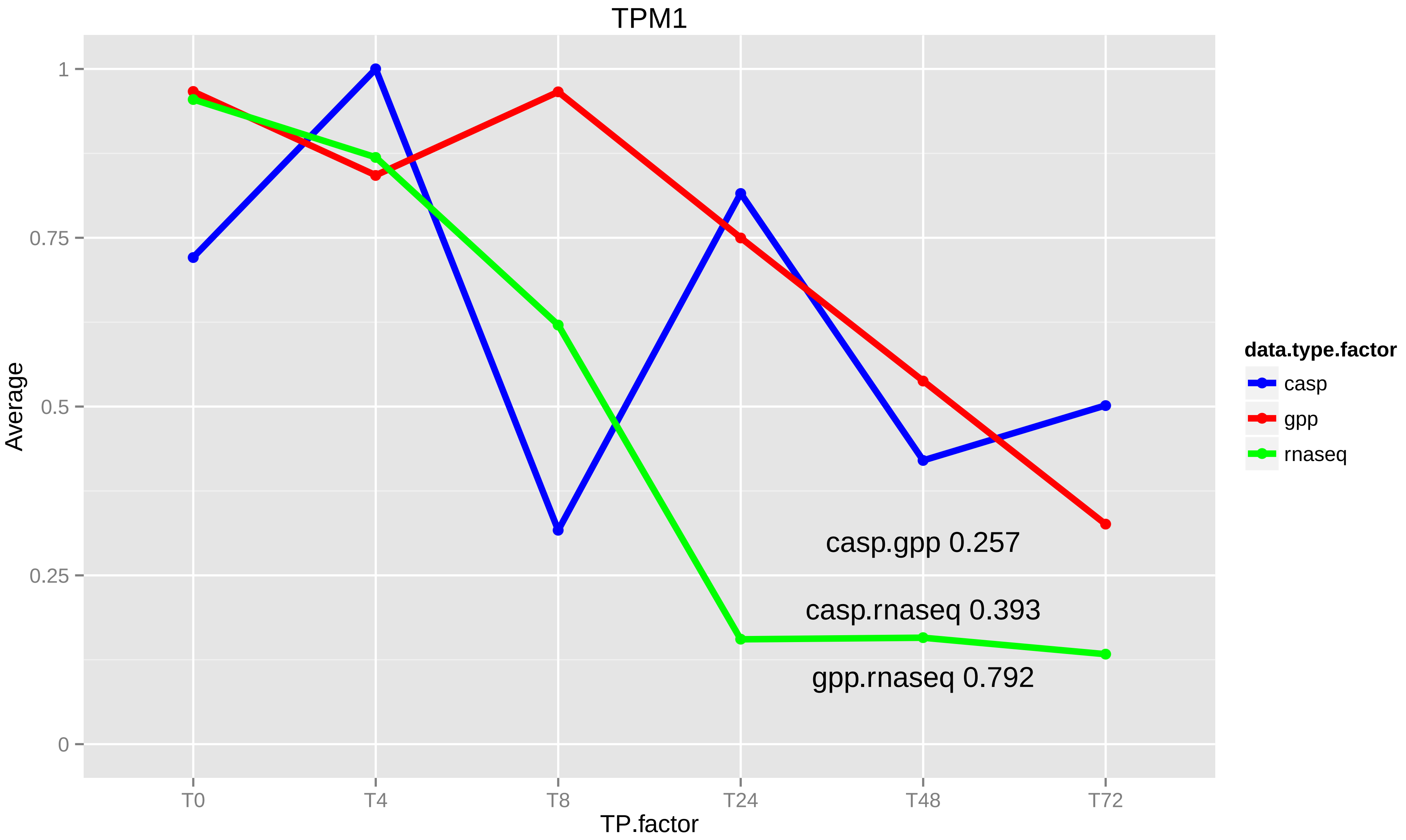
TPM1
1
0.75
0.5
0.25
0
Average
T0
T4
T8
T24
T48
T72
TP.factor
casp.gpp 0.257
casp.rnaseq 0.393
gpp.rnaseq 0.792
data.type.factor
casp
gpp
rnaseq

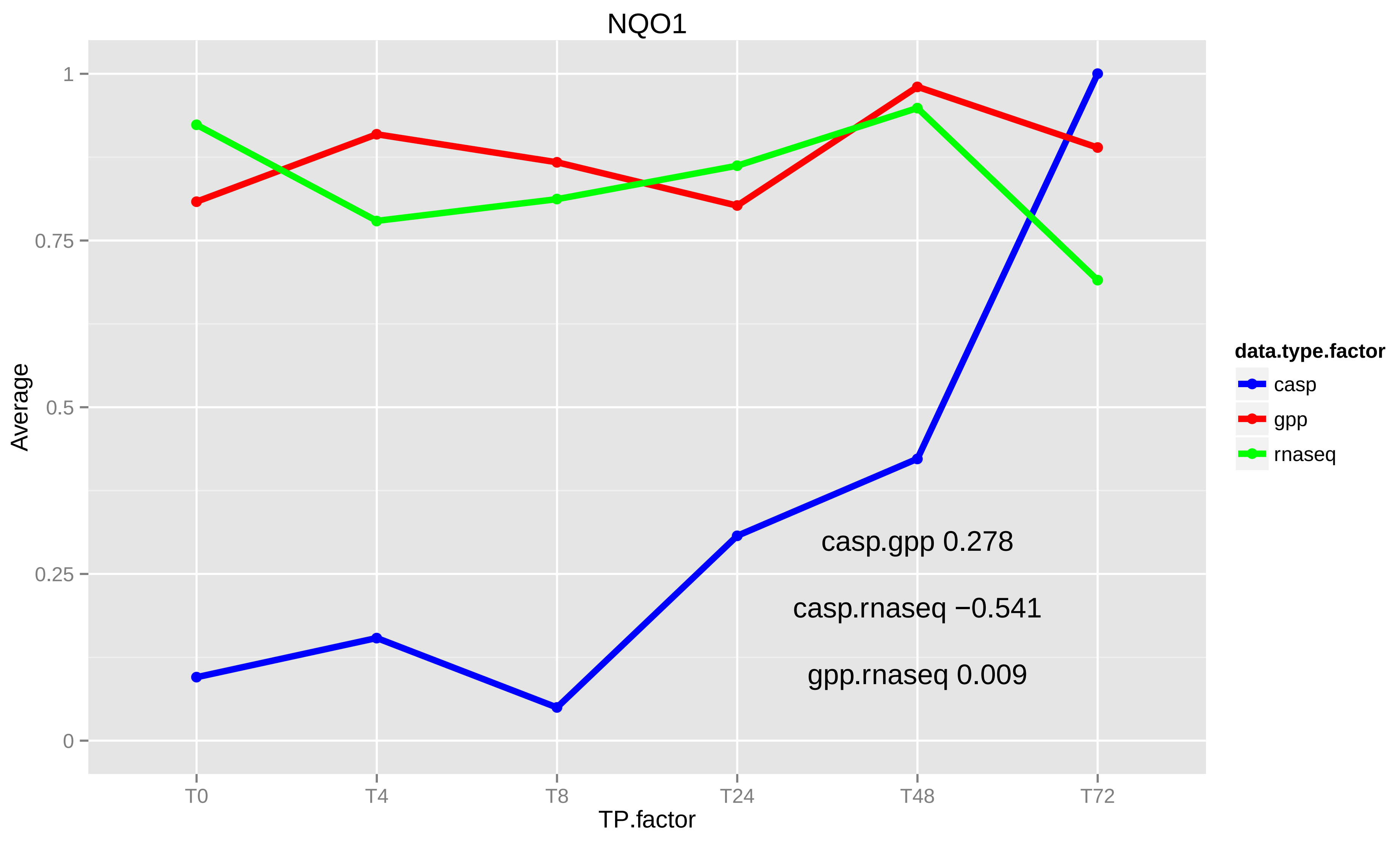

NQO1
Average
1
0.75
0.5
0.25
0
T0
T4
T8
T24
T48
T72
TP.factor
data.type.factor
casp
gpp
rnaseq
casp.gpp 0.278
casp.rnaseq −0.541
gpp.rnaseq 0.009

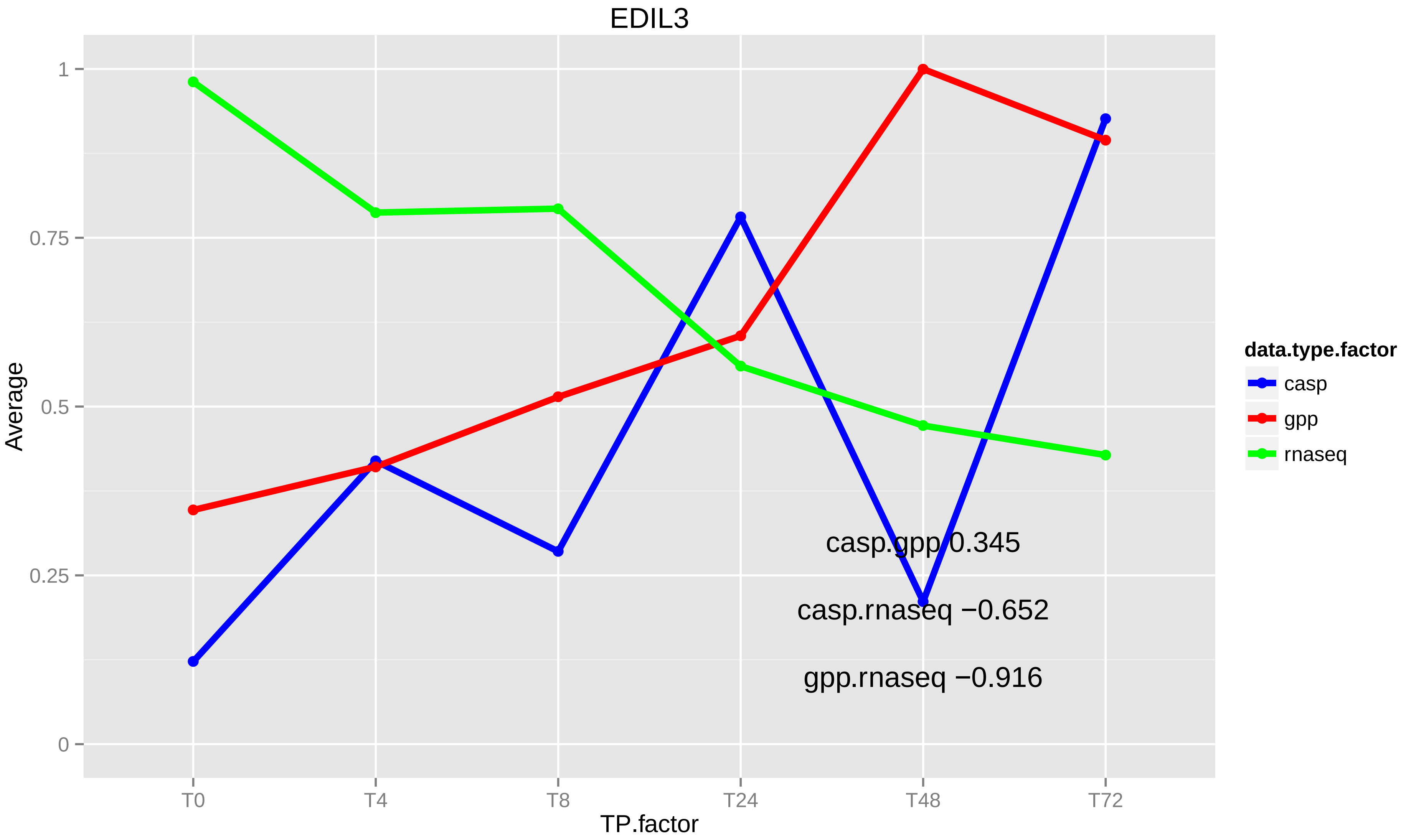

EDIL3
Average
1
0.75
0.5
0.25
0
T0
T4
T8
T24
T48
T72
TP.factor
data.type.factor
casp
gpp
rnaseq
casp.gpp 0.345
casp.rnaseq −0.652
gpp.rnaseq −0.916

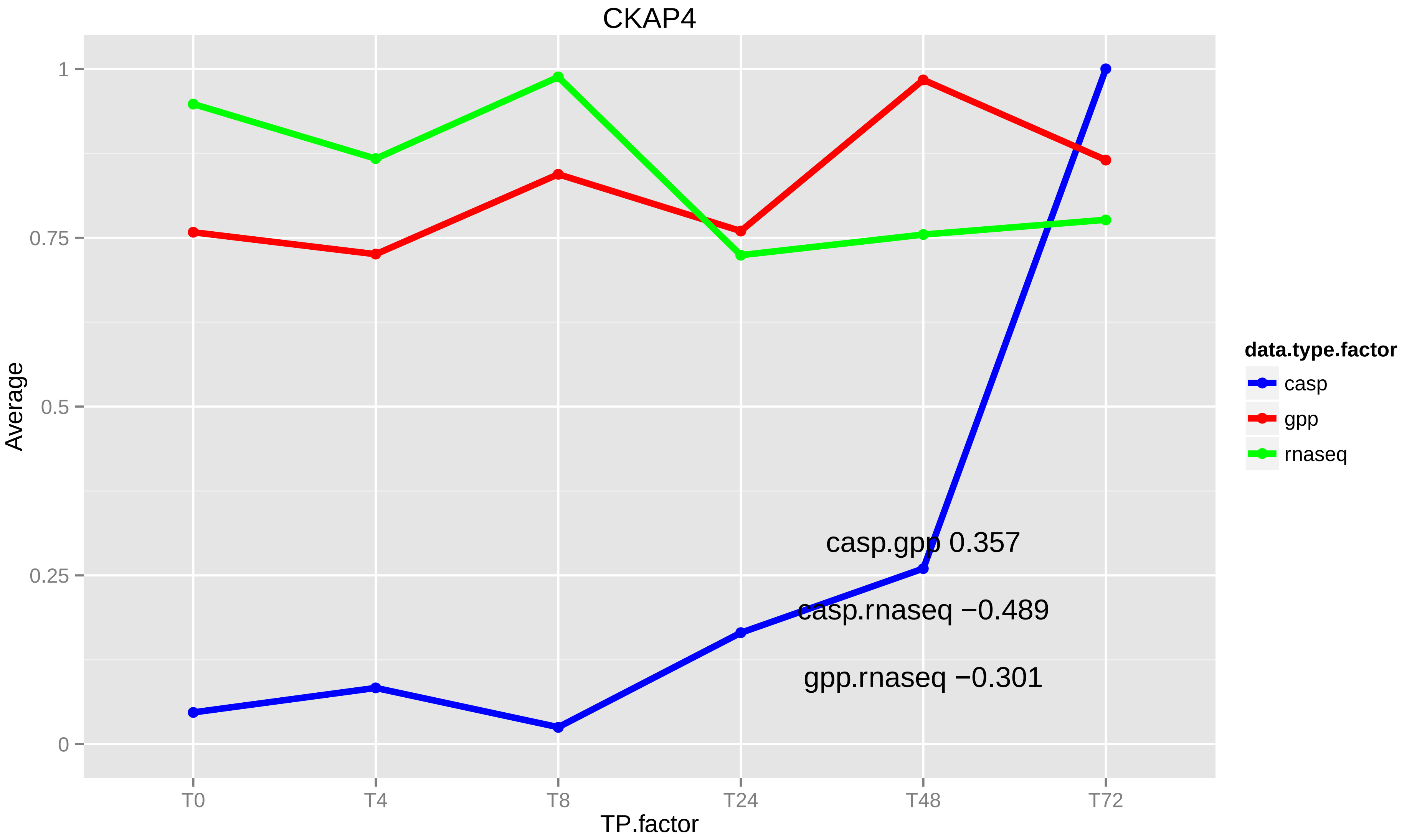
CKAP4
Average
1
0.75
0.5
0.25
0
T0
T4
T8
T24
T48
T72
TP.factor
data.type.factor
casp
gpp
rnaseq
casp.gpp 0.357
casp.rnaseq −0.489
gpp.rnaseq −0.301

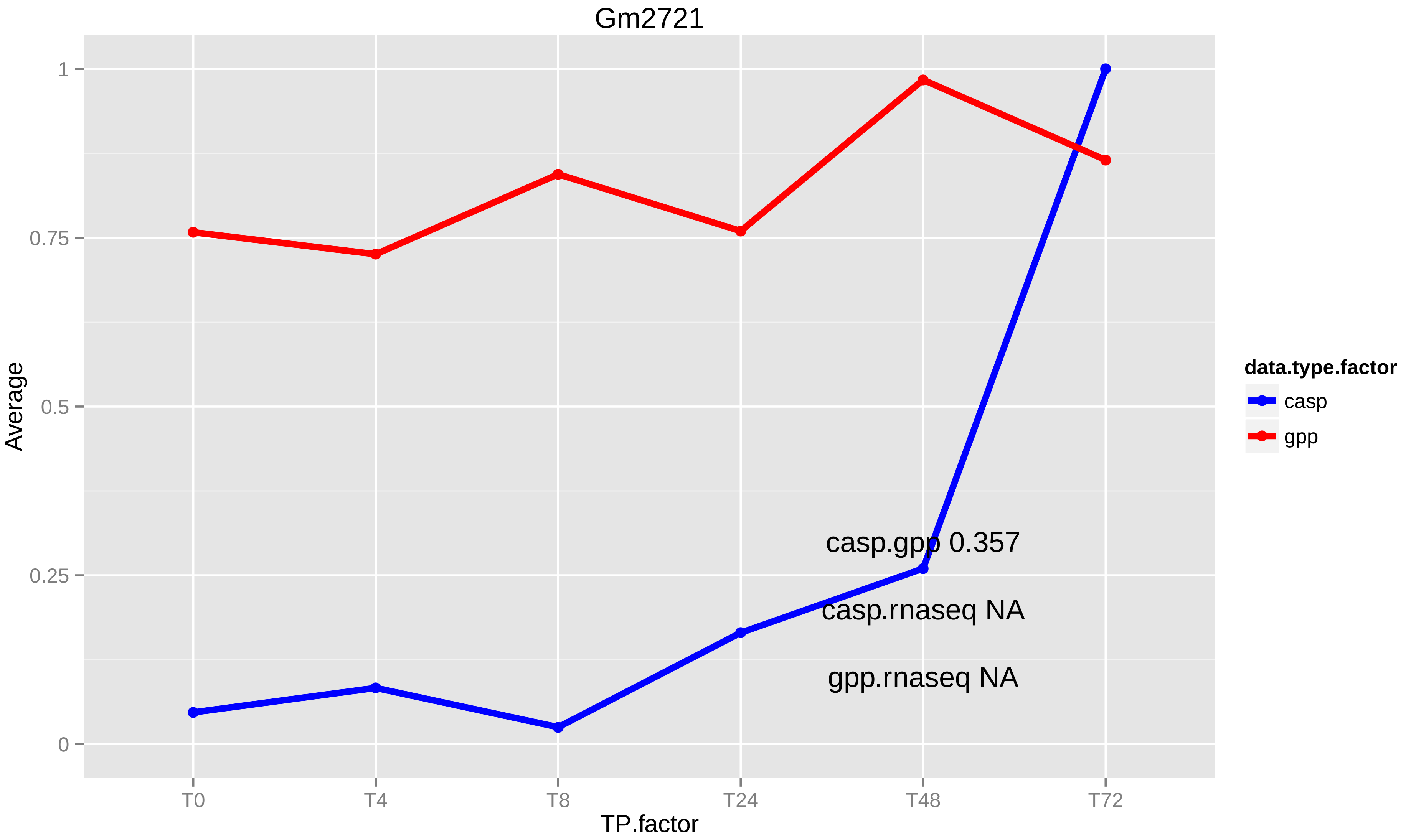

Gm2721
Average
1
0.75
0.5
0.25
0
T0
T4
T8
T24
T48
T72
TP.factor
casp.gpp 0.357
casp.rnaseq NA
gpp.rnaseq NA
data.type.factor
casp
gpp

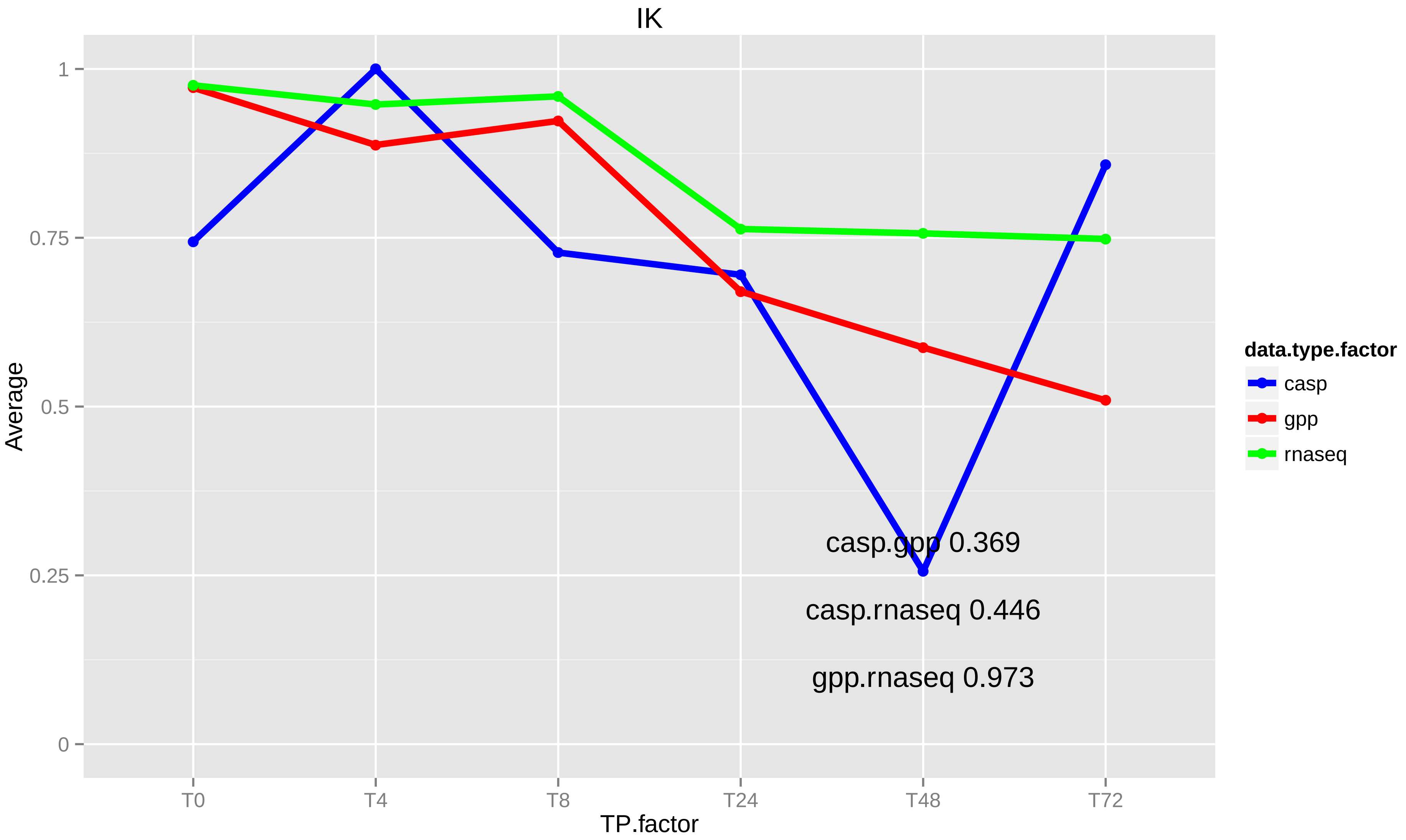
IK
Average
1
0.75
0.5
0.25
0
T0
T4
T8
T24
T48
T72
TP.factor
data.type.factor
casp
gpp
rnaseq
casp.gpp 0.369
casp.rnaseq 0.446
gpp.rnaseq 0.973

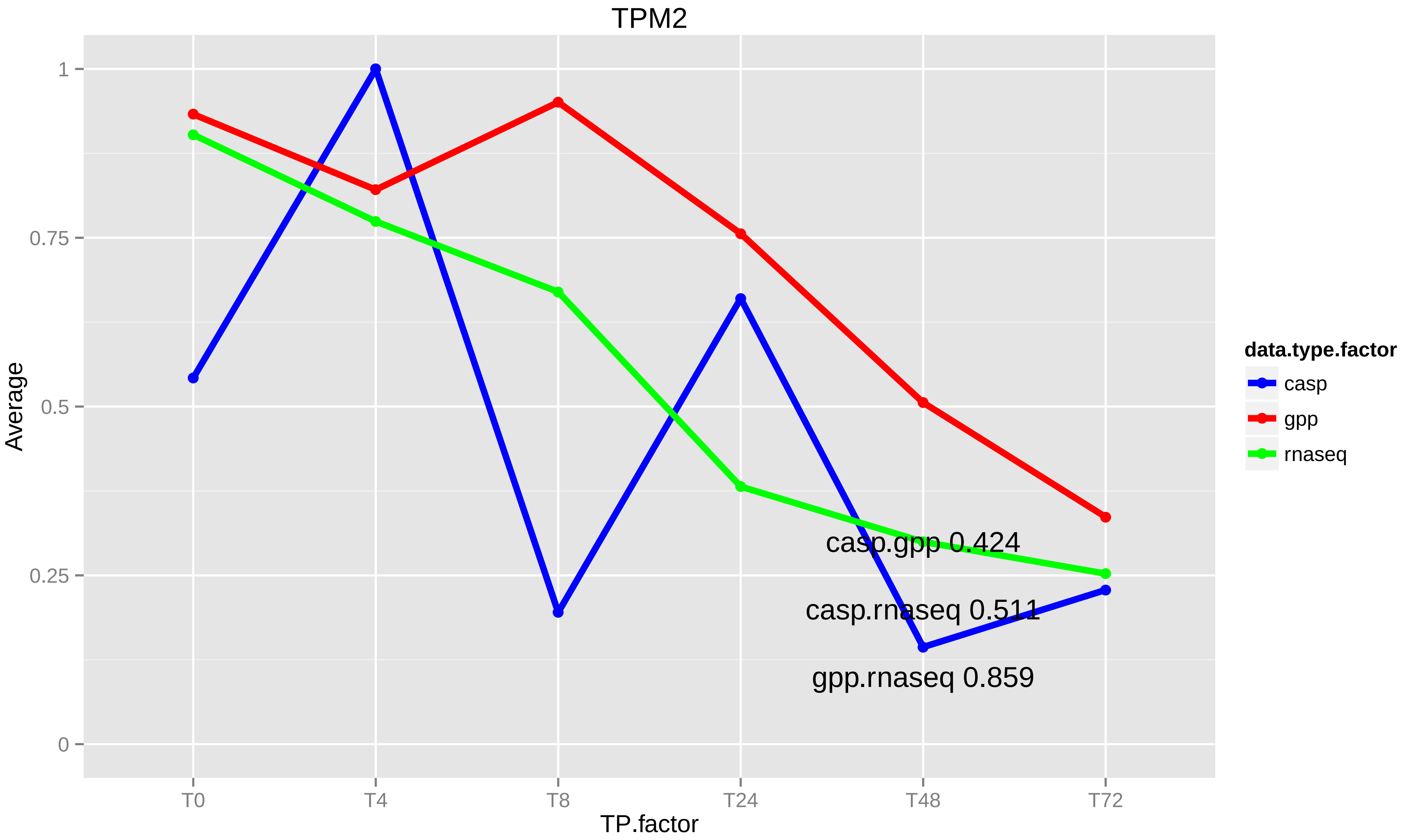

TPM2
1
0.75
0.5
0.25
0
Average
T0
T4
T8
T24
T48
T72
TP.factor
data.type.factor
casp
gpp
rnaseq
casp.gpp 0.424
casp.rnaseq 0.511
gpp.rnaseq 0.859

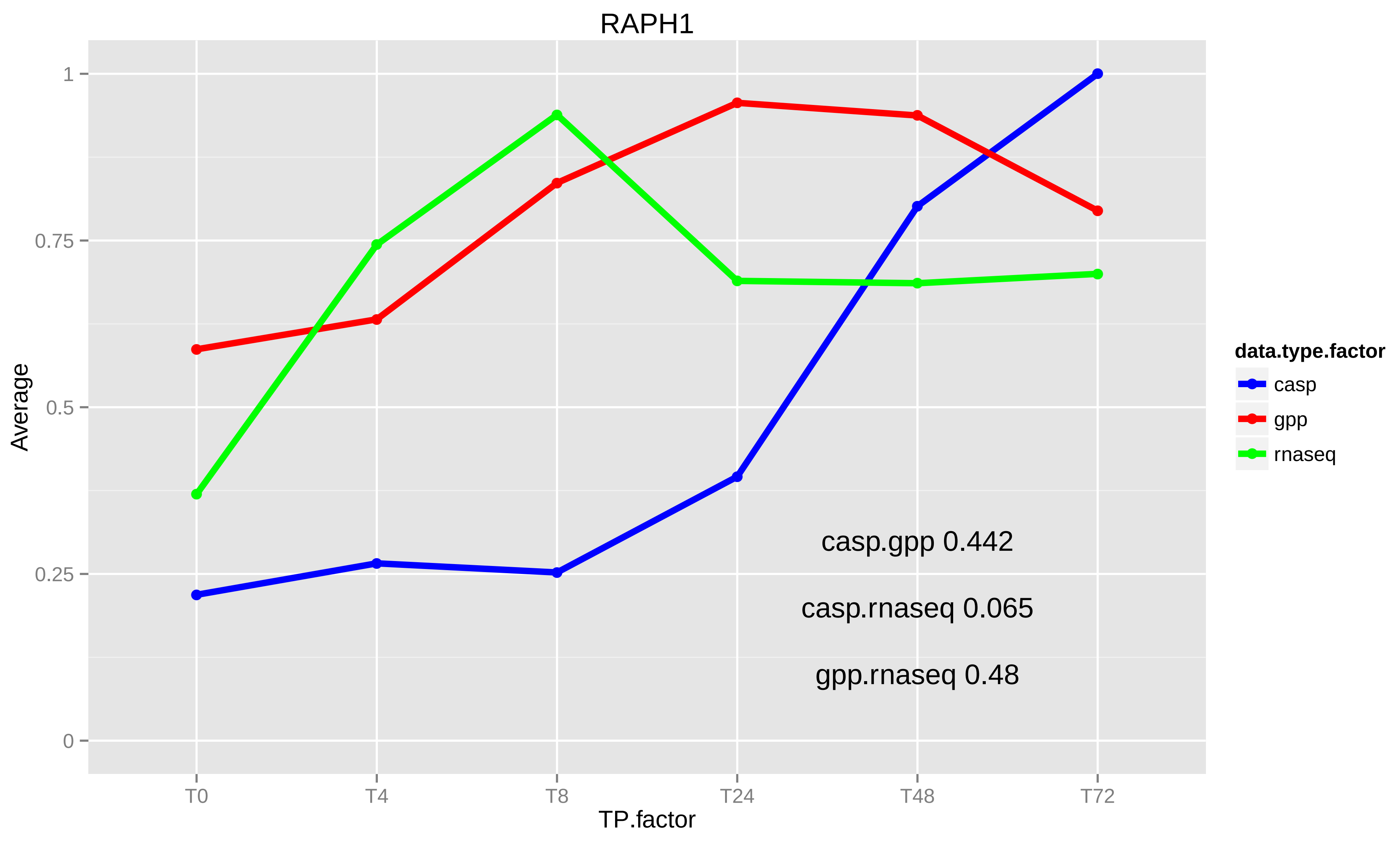

RAPH1
1
0.75
0.5
0.25
0
Average
T0
T4
T8
T24
T48
T72
TP.factor
data.type.factor
casp
gpp
rnaseq
casp.gpp 0.442
casp.rnaseq 0.065
gpp.rnaseq 0.48

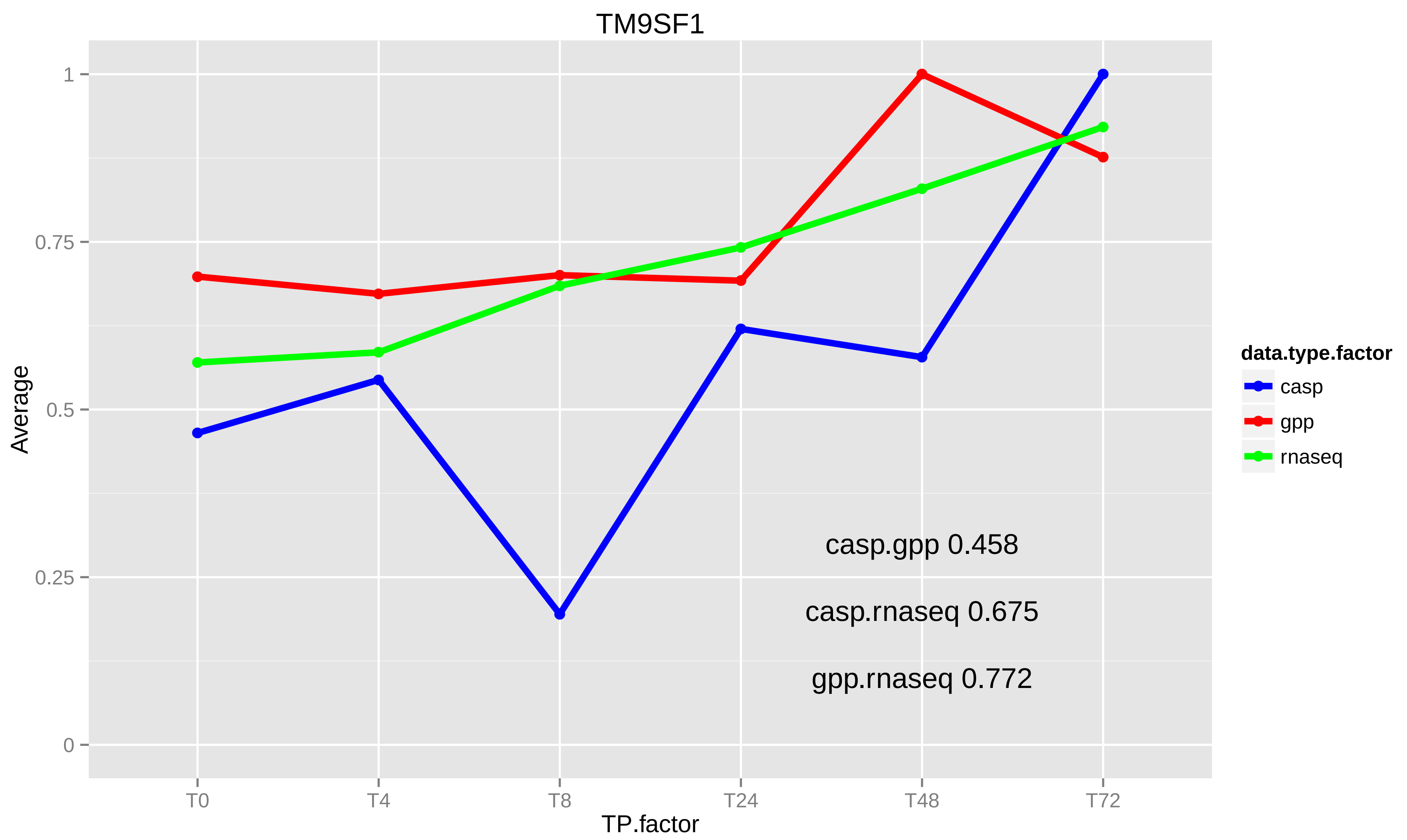
TM9SF1
1
0.75
0.5
0.25
0
Average
T0
T4
T8
T24
T48
T72
TP.factor
data.type.factor
casp
gpp
rnaseq
casp.gpp 0.458
casp.rnaseq 0.675
gpp.rnaseq 0.772

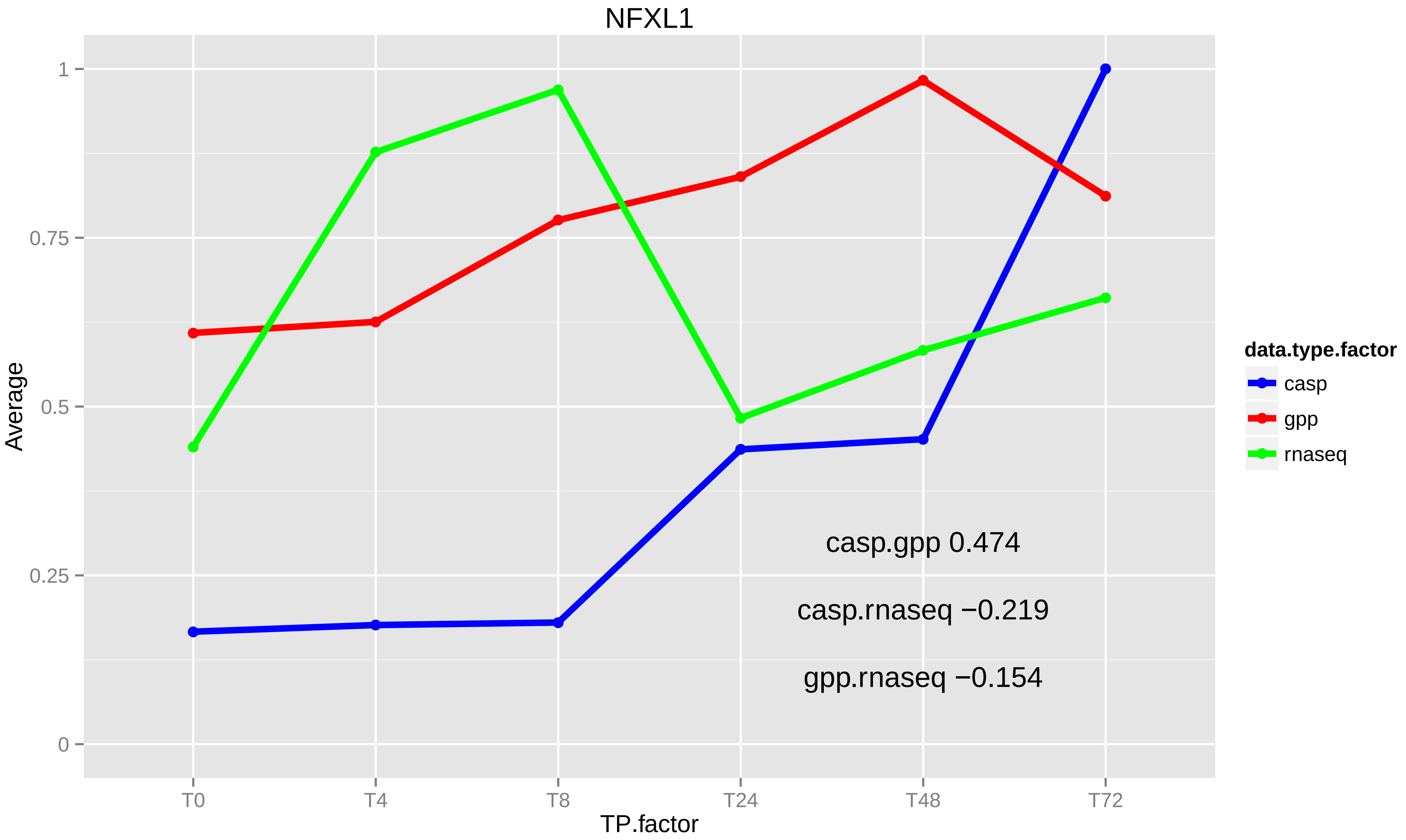
NFXL1
1
0.75
0.5
0.25
0
Average
T0
T4
T8
T24
T48
T72
TP.factor
data.type.factor
casp
gpp
rnaseq
casp.gpp 0.474
casp.rnaseq −0.219
gpp.rnaseq −0.154

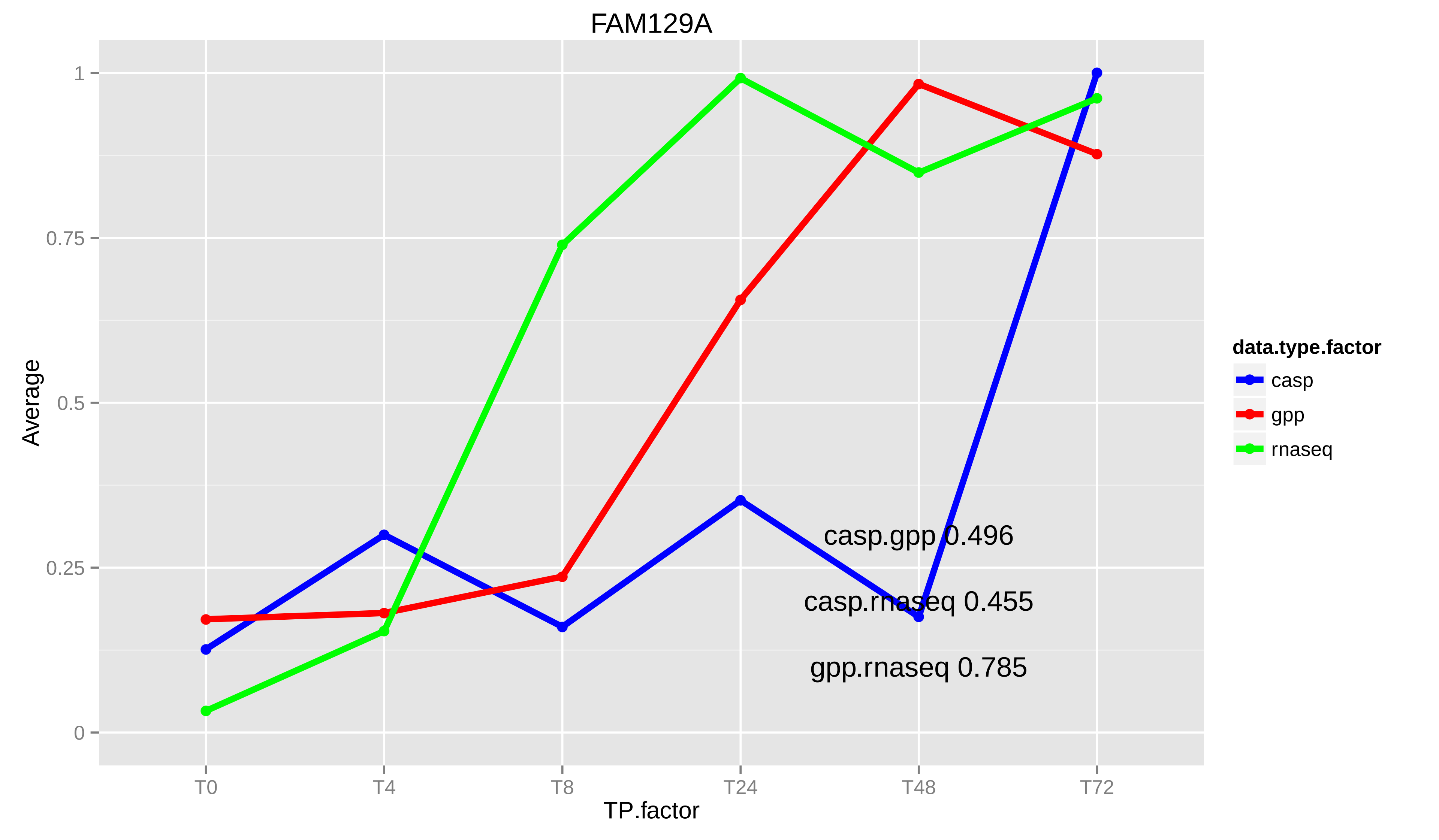

FAM129A
Average
1
0.75
0.5
0.25
0
T0
T4
T8
T24
T48
T72
TP.factor
data.type.factor
casp
gpp
rnaseq
casp.gpp 0.496
casp.rnaseq 0.455
gpp.rnaseq 0.785

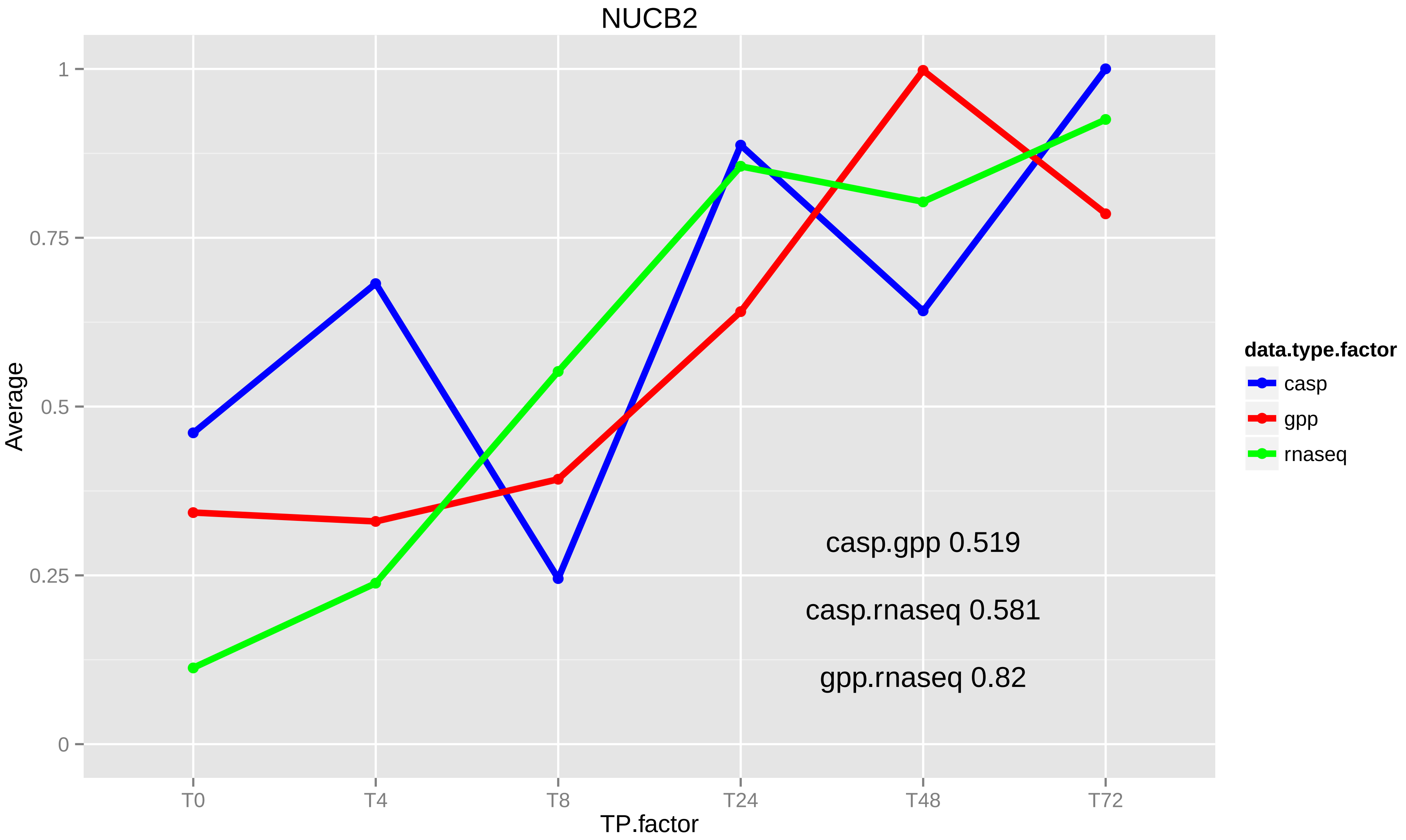
NUCB2
1
0.75
0.5
0.25
0
Average
T0
T4
T8
T24
T48
T72
TP.factor
data.type.factor
casp
gpp
rnaseq
casp.gpp 0.519
casp.rnaseq 0.581
gpp.rnaseq 0.82

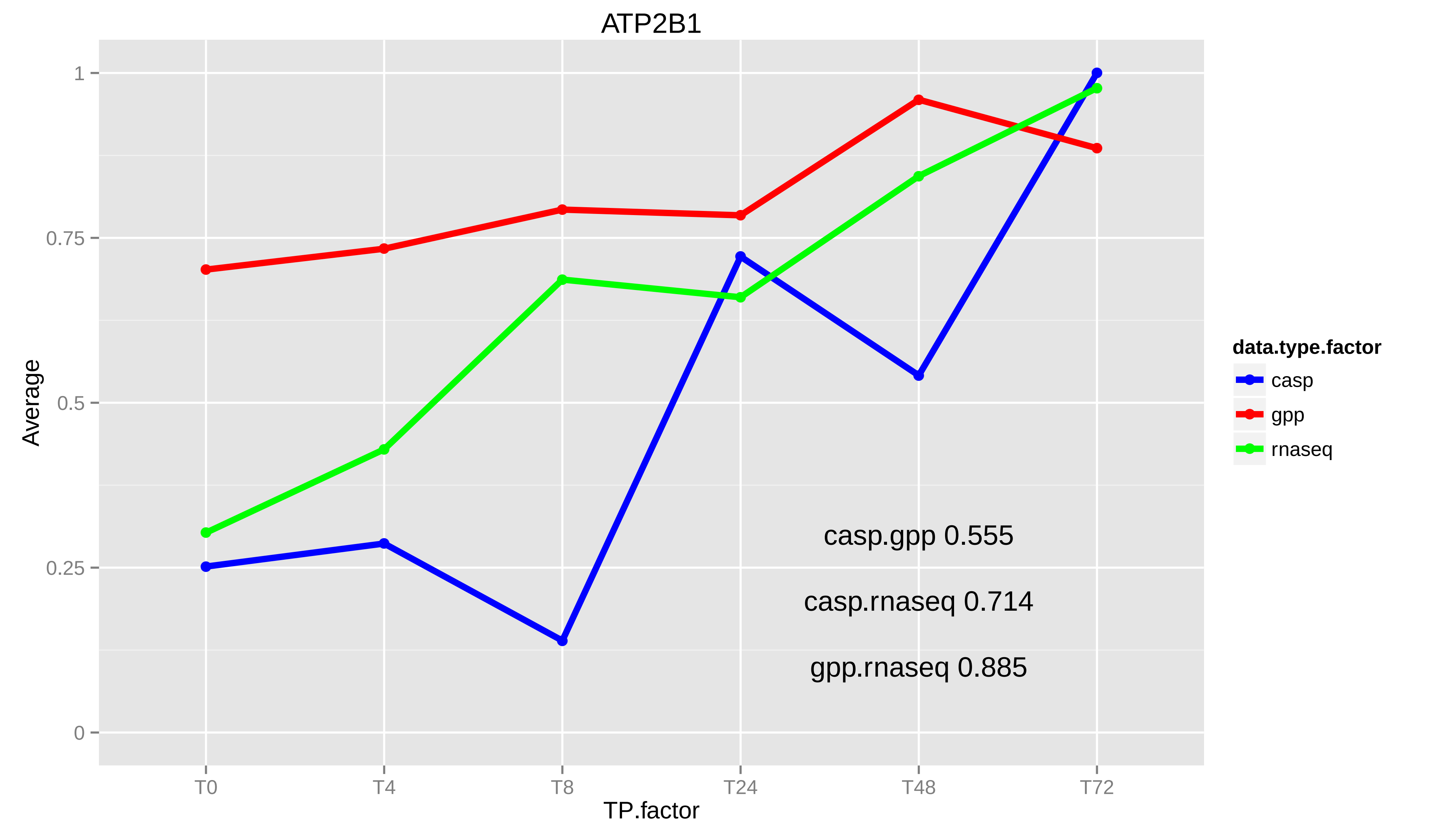

ATP2B1
Average
1
0.75
0.5
0.25
0
T0
T4
T8
T24
T48
T72
TP.factor
data.type.factor
casp
gpp
rnaseq
casp.gpp 0.555
casp.rnaseq 0.714
gpp.rnaseq 0.885

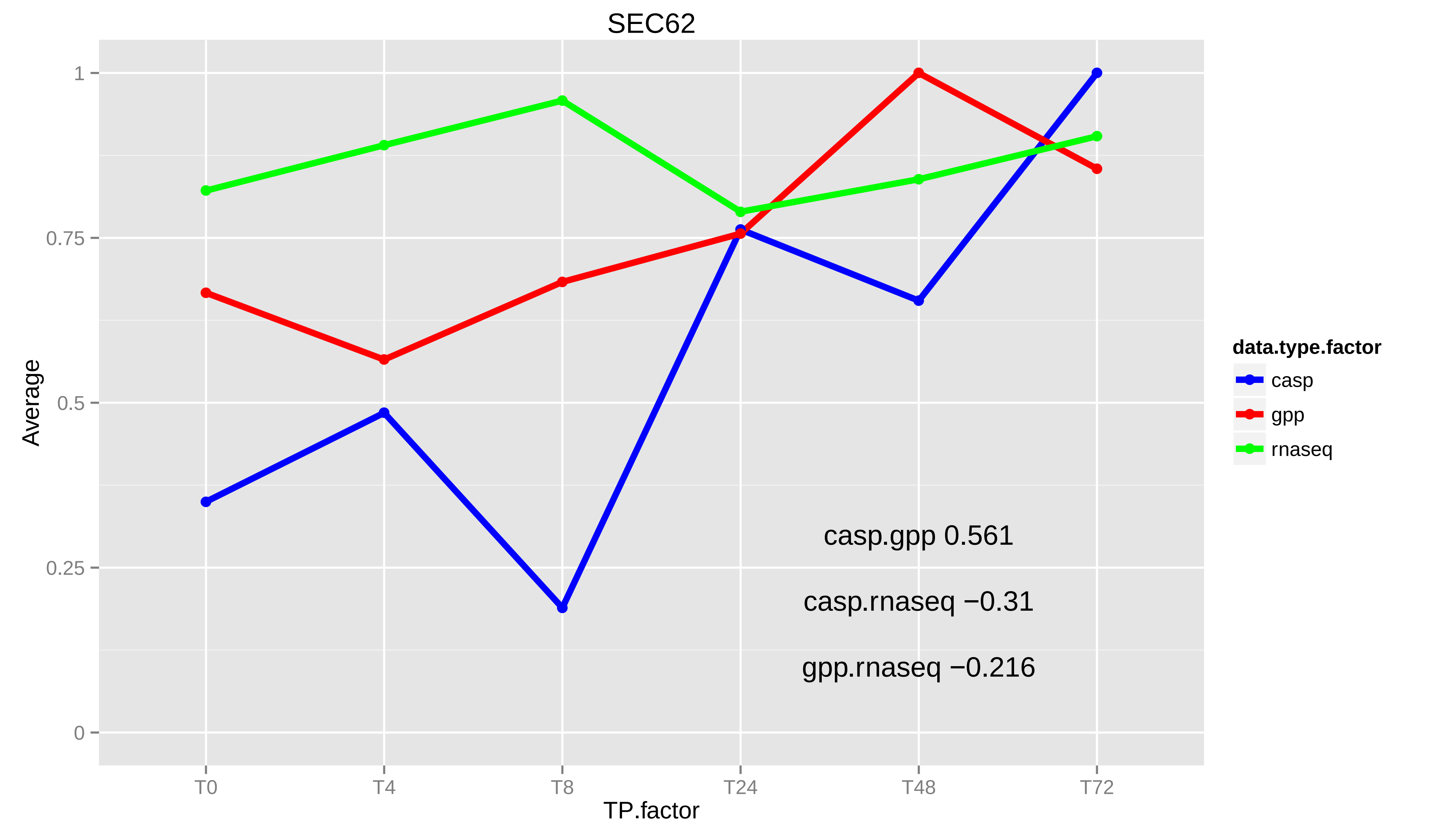

SEC62
Average
1
0.75
0.5
0.25
0
T0
T4
T8
T24
T48
T72
TP.factor
data.type.factor
casp
gpp
rnaseq
casp.gpp 0.561
casp.rnaseq −0.31
gpp.rnaseq −0.216

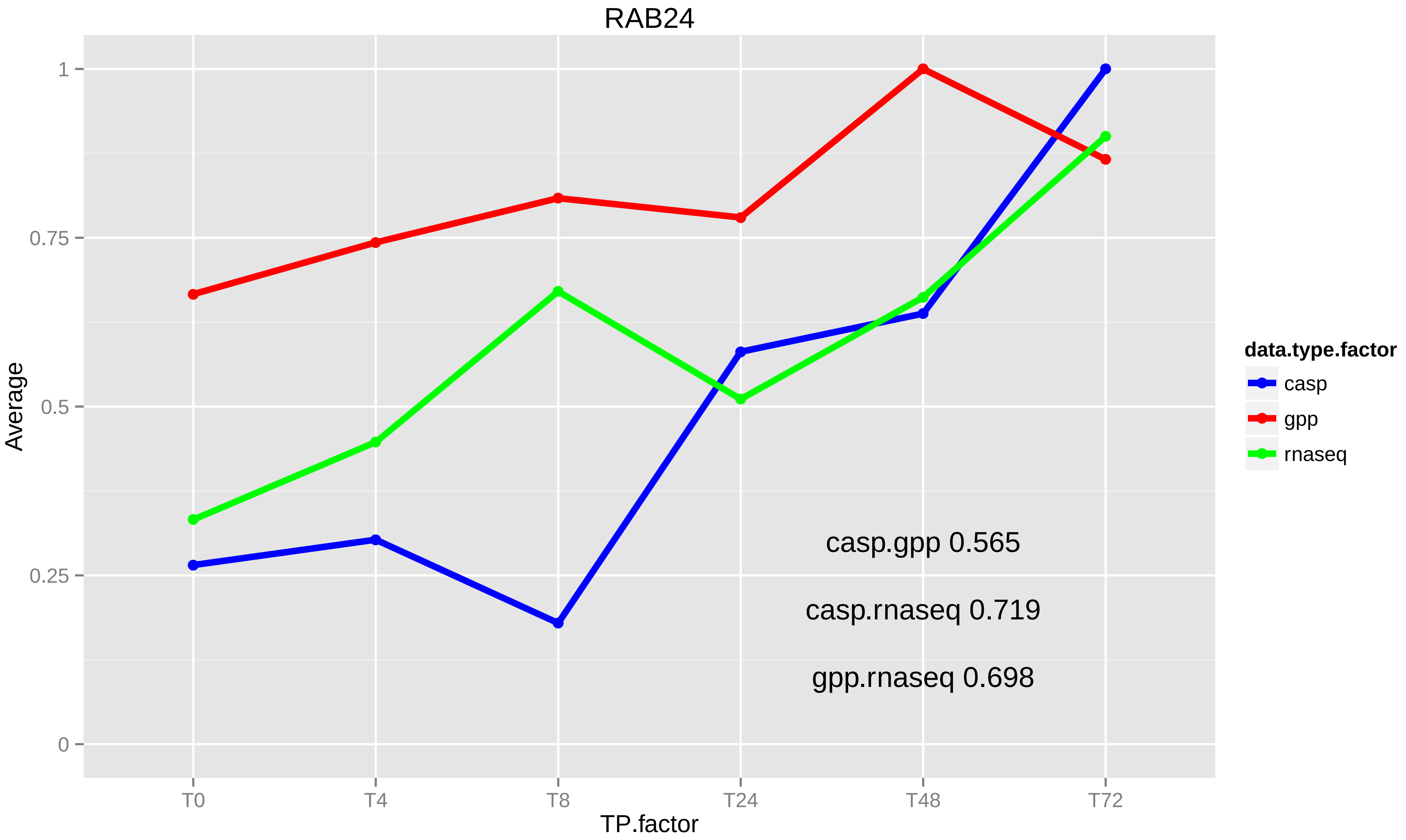

RAB24
Average
1
0.75
0.5
0.25
0
T0
T4
T8
T24
T48
T72
TP.factor
data.type.factor
casp
gpp
rnaseq
casp.gpp 0.565
casp.rnaseq 0.719
gpp.rnaseq 0.698

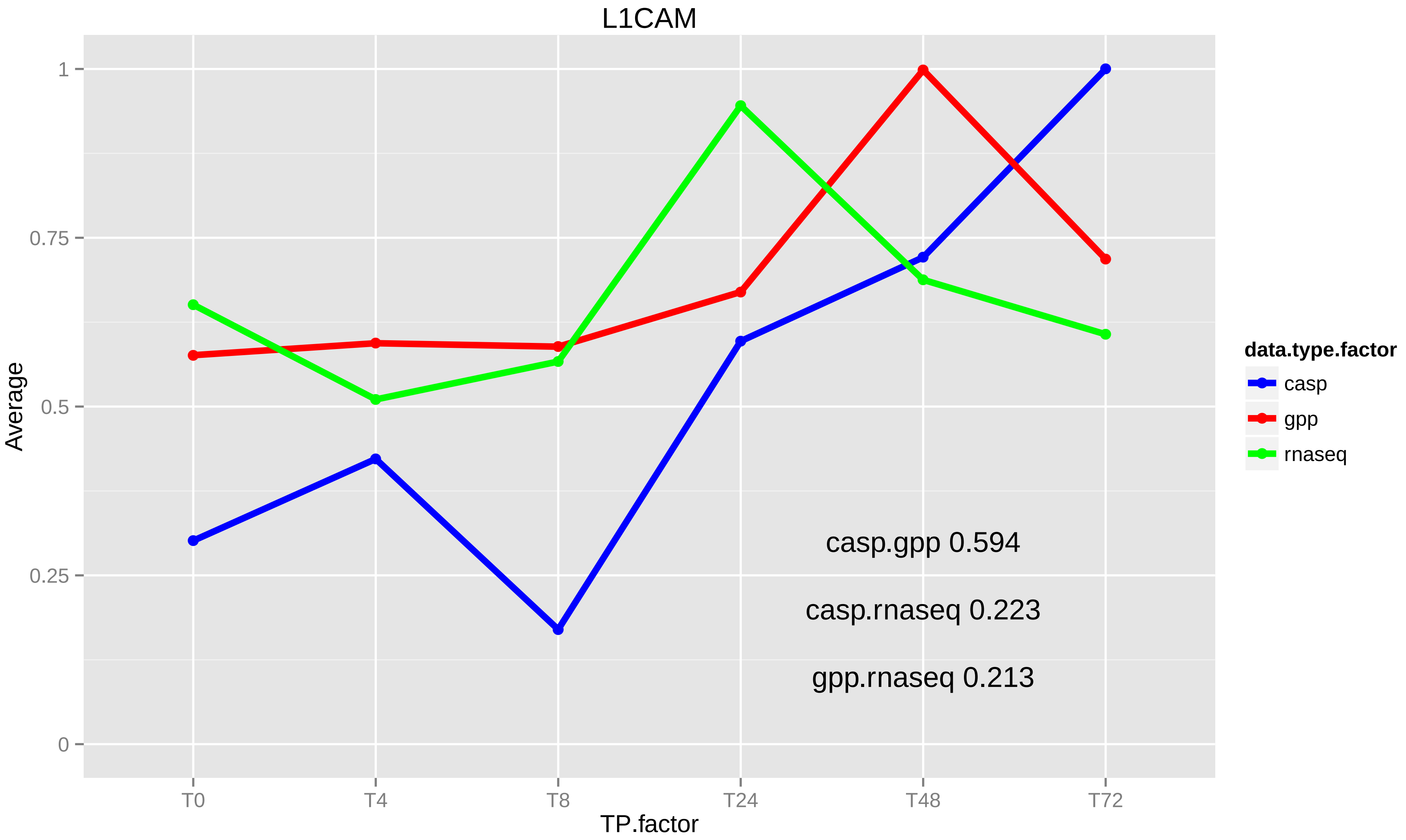

L1CAM
1
0.75
0.5
0.25
0
Average
T0
T4
T8
T24
T48
T72
TP.factor
data.type.factor
casp
gpp
rnaseq
casp.gpp 0.594
casp.rnaseq 0.223
gpp.rnaseq 0.213

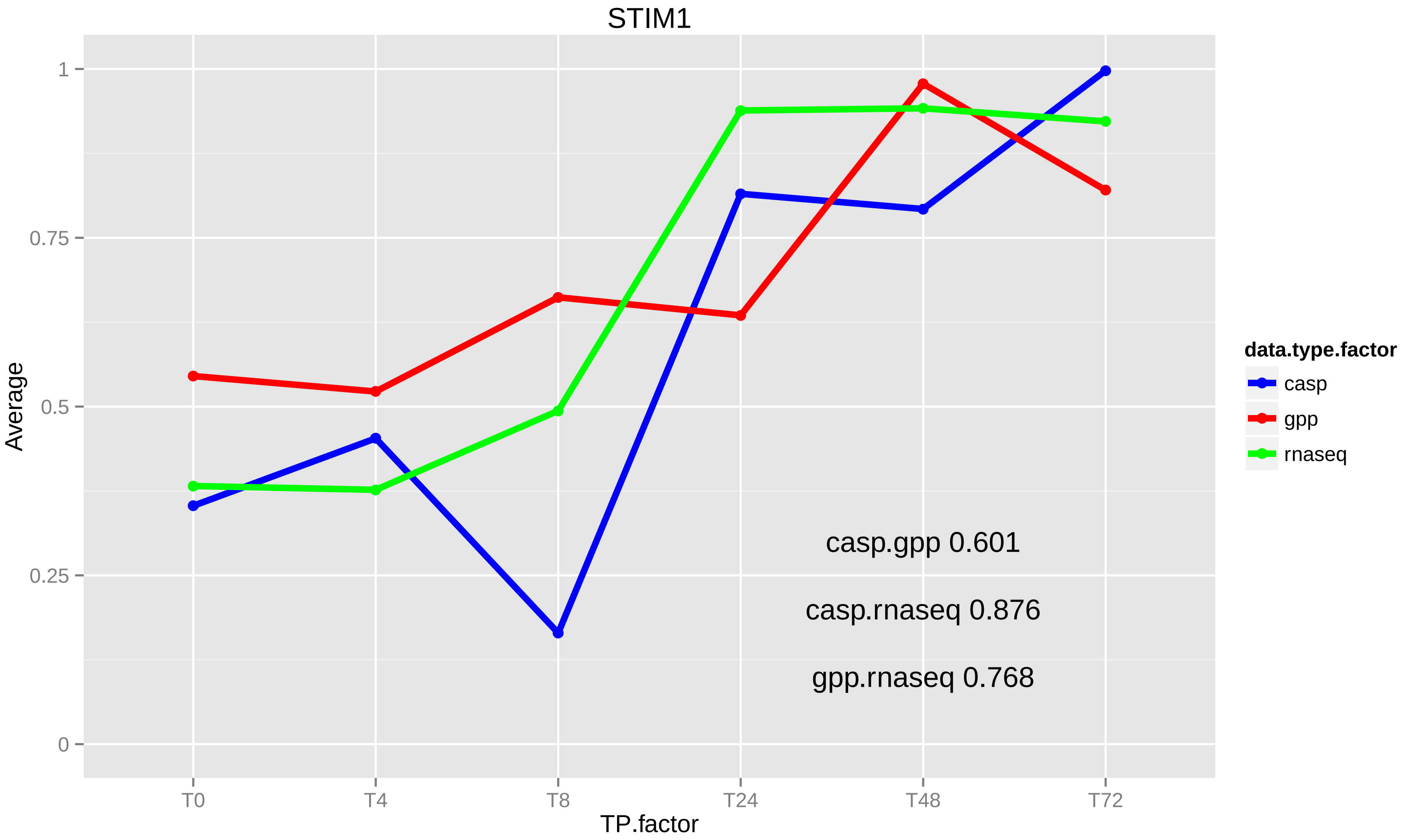

STIM1
1
0.75
0.5
0.25
0
Average
T0
T4
T8
T24
T48
T72
TP.factor
data.type.factor
casp
gpp
rnaseq
casp.gpp 0.601
casp.rnaseq 0.876
gpp.rnaseq 0.768

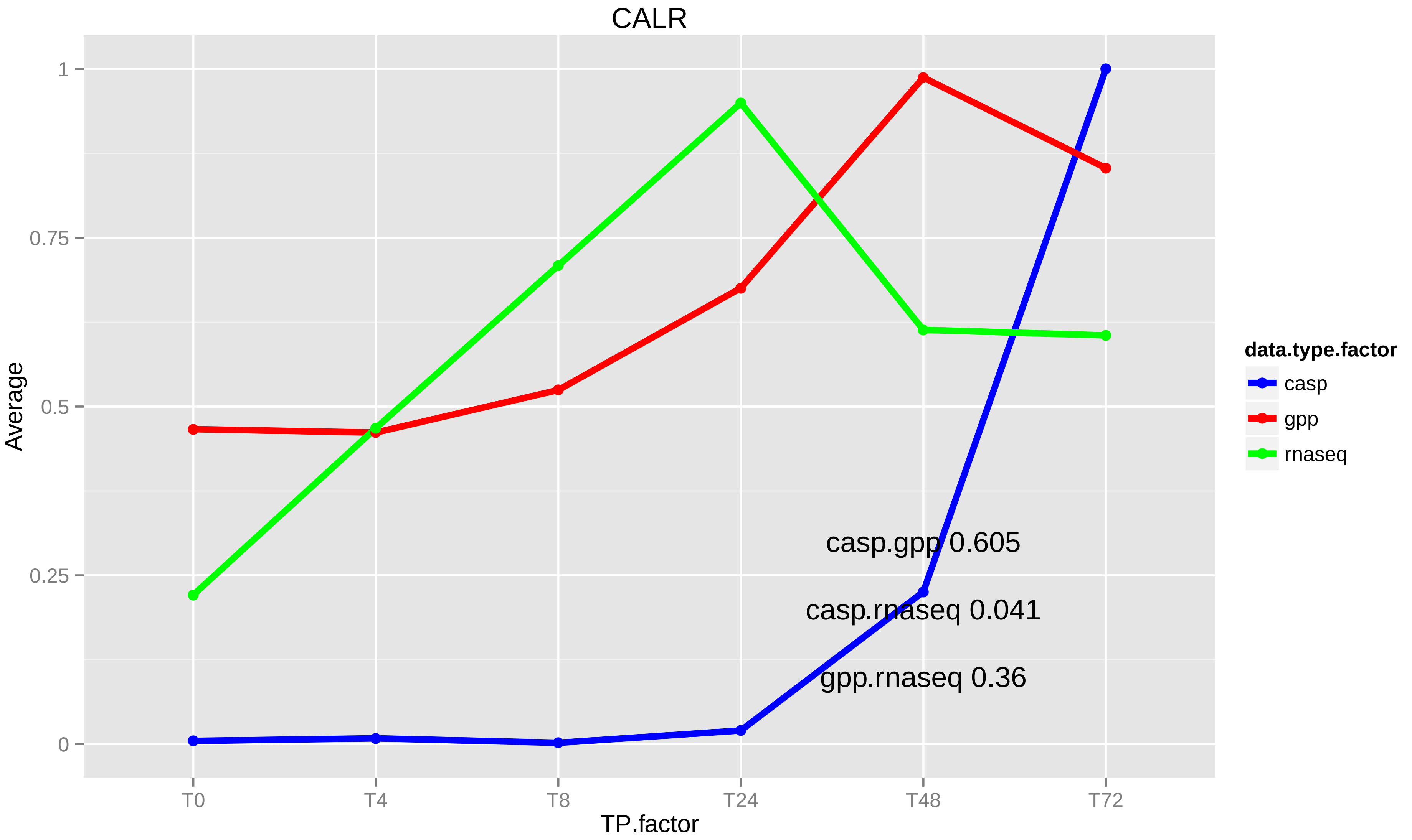

CALR
1
0.75
0.5
0.25
0
Average
T0
T4
T8
T24
T48
T72
TP.factor
data.type.factor
casp
gpp
rnaseq
casp.gpp 0.605
casp.rnaseq 0.041
gpp.rnaseq 0.36

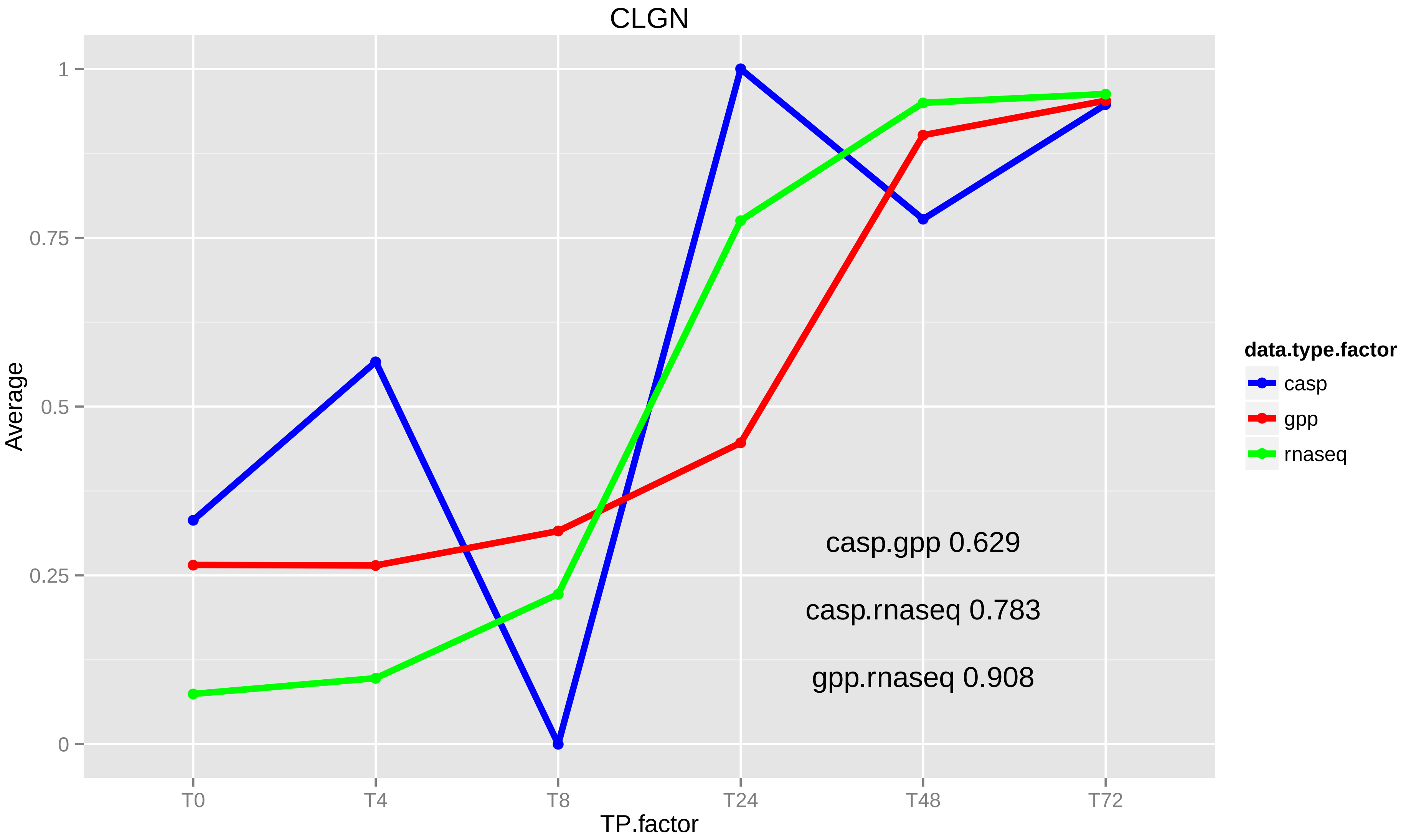
CLGN
Average
1
0.75
0.5
0.25
0
T0
T4
T8
T24
T48
T72
TP.factor
data.type.factor
casp
gpp
rnaseq
casp.gpp 0.629
casp.rnaseq 0.783
gpp.rnaseq 0.908

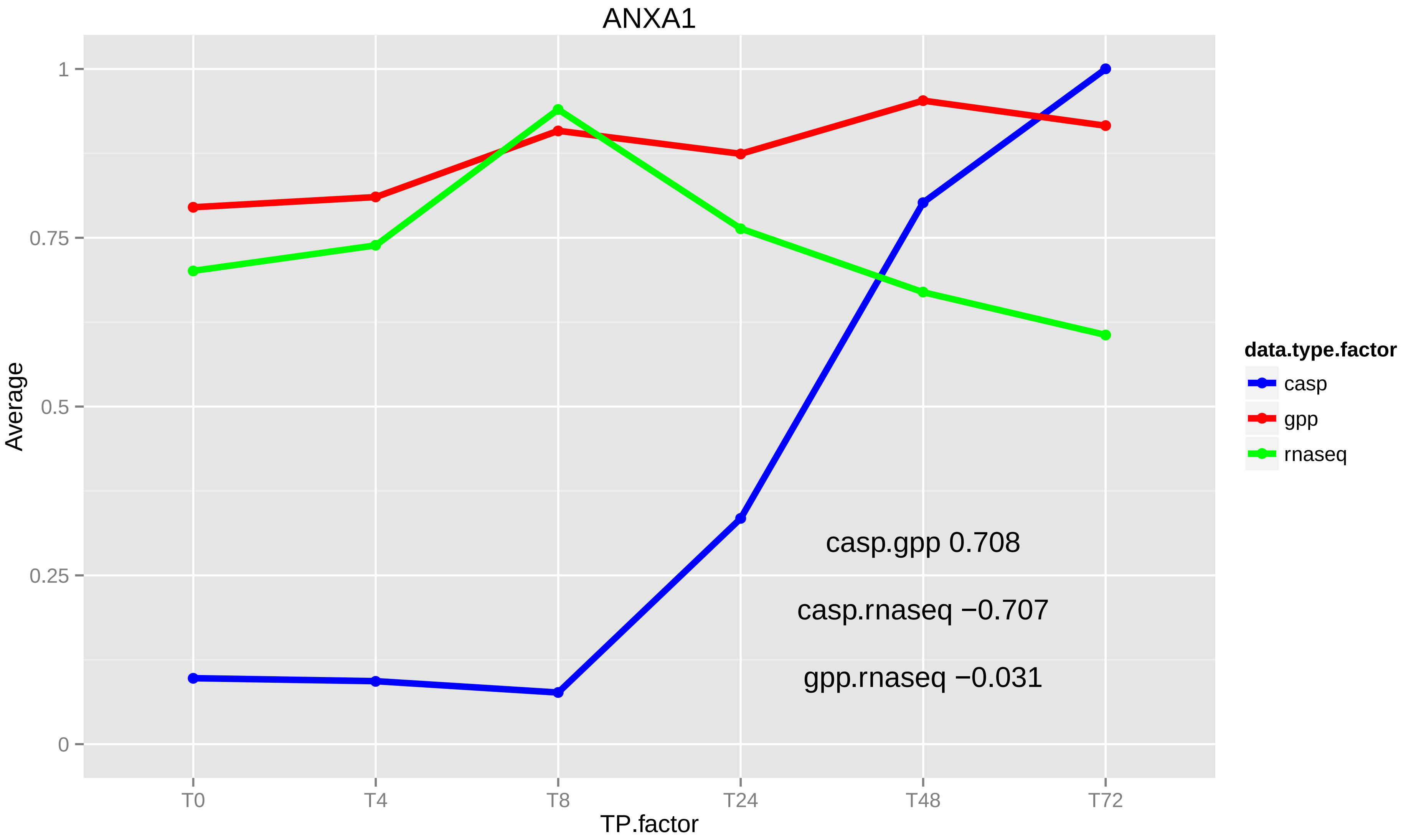

ANXA1
Average
TP.factor
1
0.75
0.5
0.25
0
T0
T4
T8
T24
T48
T72
data.type.factor
casp
gpp
rnaseq
casp.gpp 0.708
casp.rnaseq −0.707
gpp.rnaseq −0.031

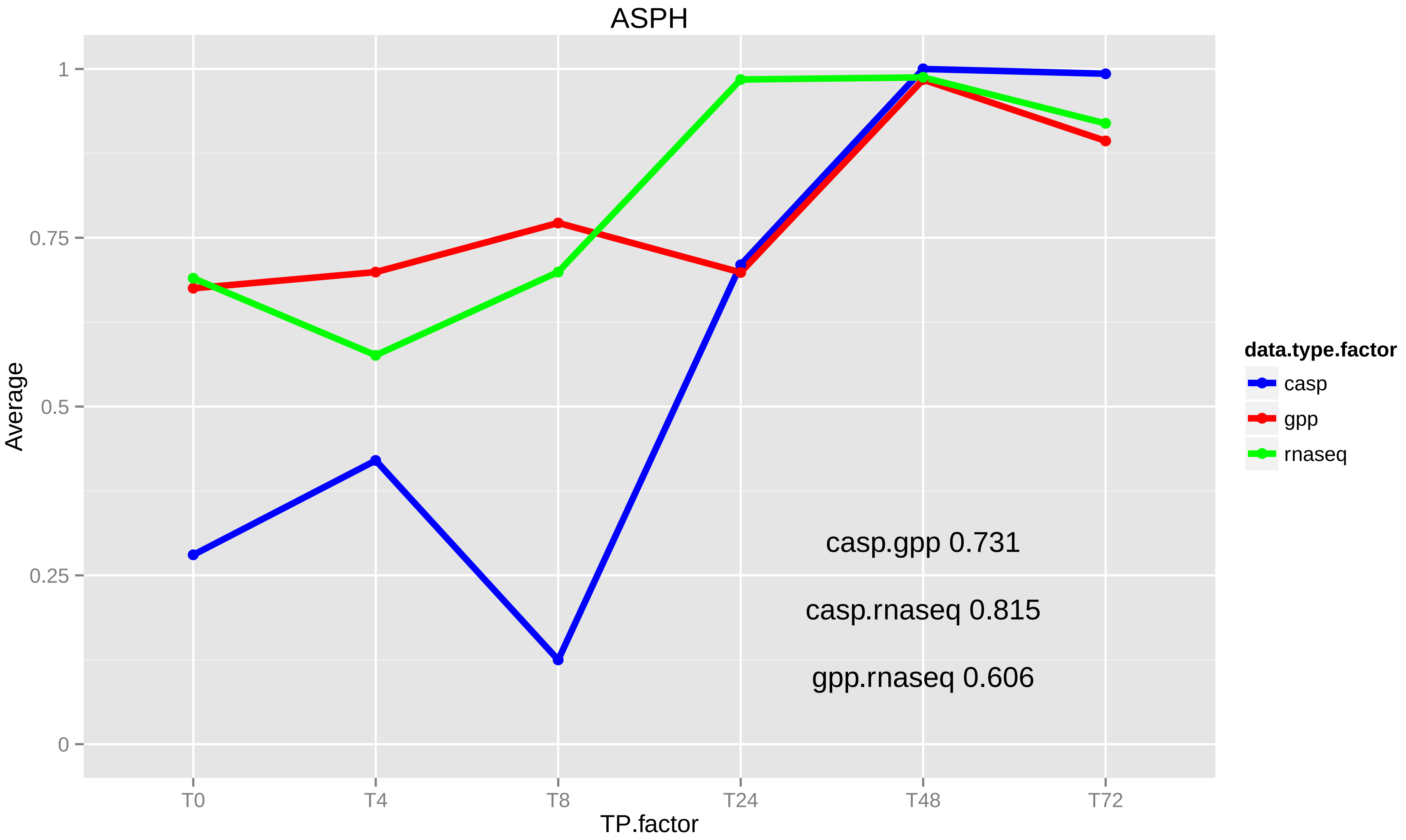
ASPH
1
0.75
0.5
0.25
0
Average
T0
T4
T8
T24
T48
T72
TP.factor
casp.gpp 0.731
casp.rnaseq 0.815
gpp.rnaseq 0.606
data.type.factor
casp
gpp
rnaseq

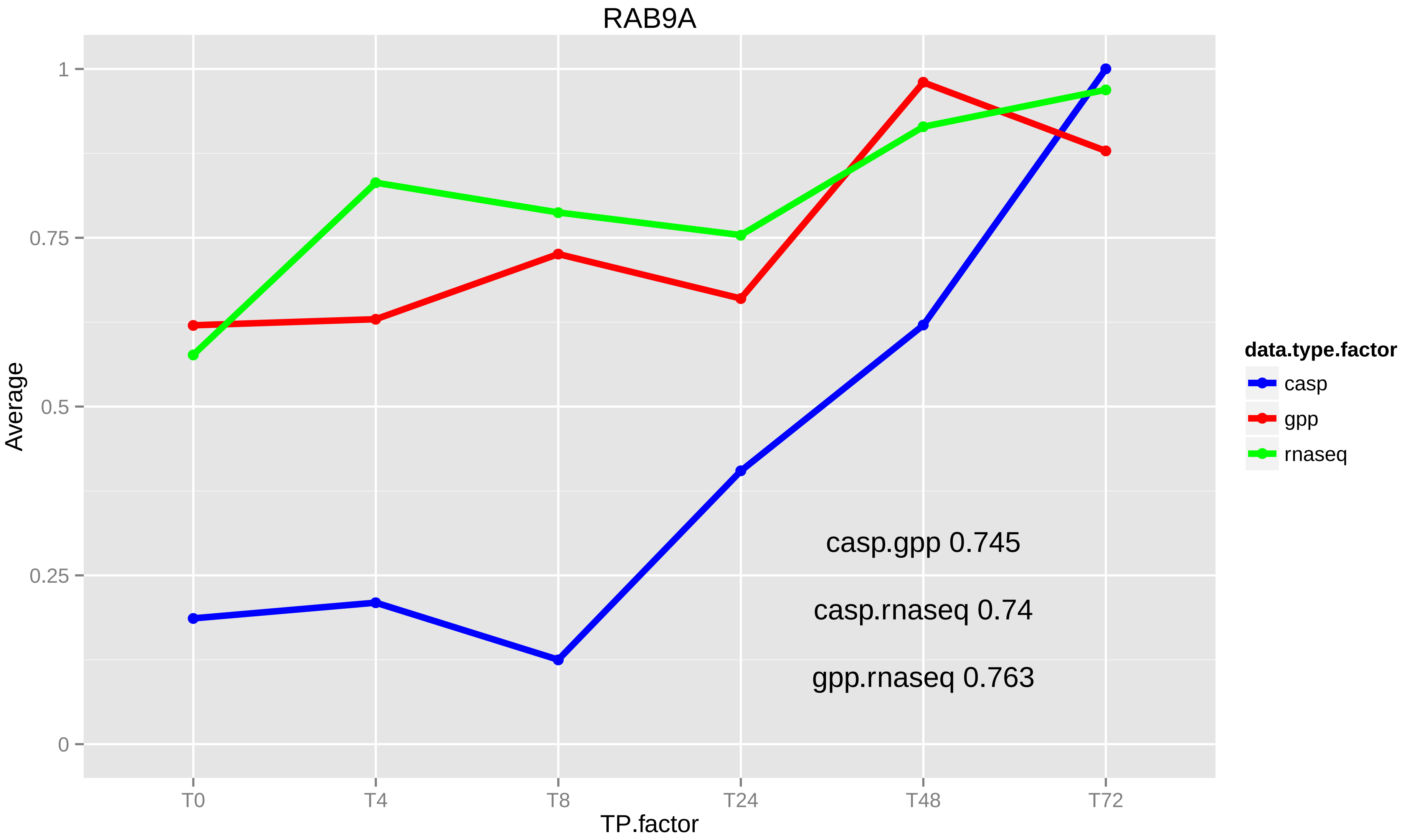
RAB9A
Average
1
0.75
0.5
0.25
0
T0
T4
T8
T24
T48
T72
TP.factor
data.type.factor
casp
gpp
rnaseq
casp.gpp 0.745
casp.rnaseq 0.74
gpp.rnaseq 0.763

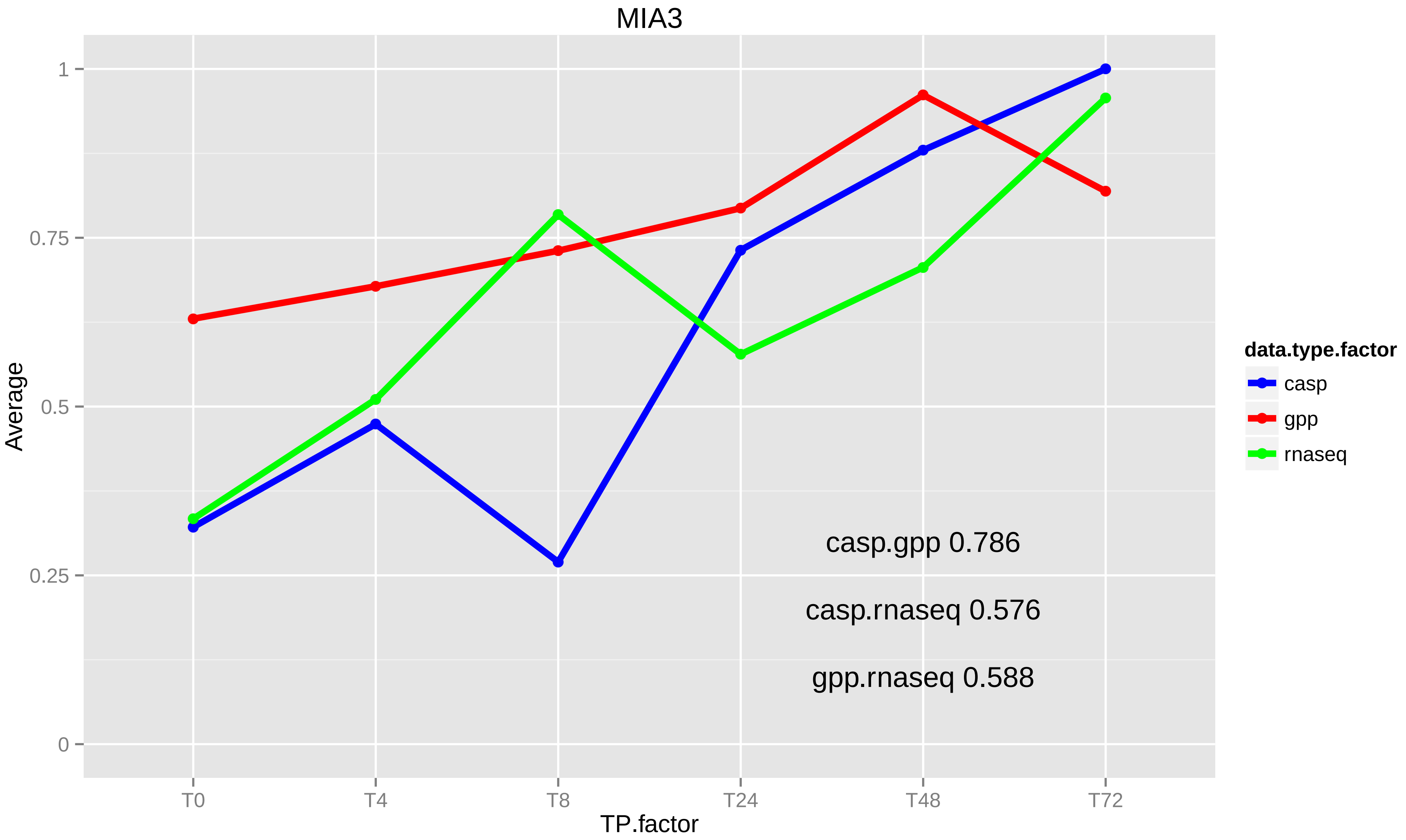

MIA3
1
0.75
0.5
0.25
0
Average
T0
T4
T8
T24
T48
T72
TP.factor
data.type.factor
casp
gpp
rnaseq
casp.gpp 0.786
casp.rnaseq 0.576
gpp.rnaseq 0.588

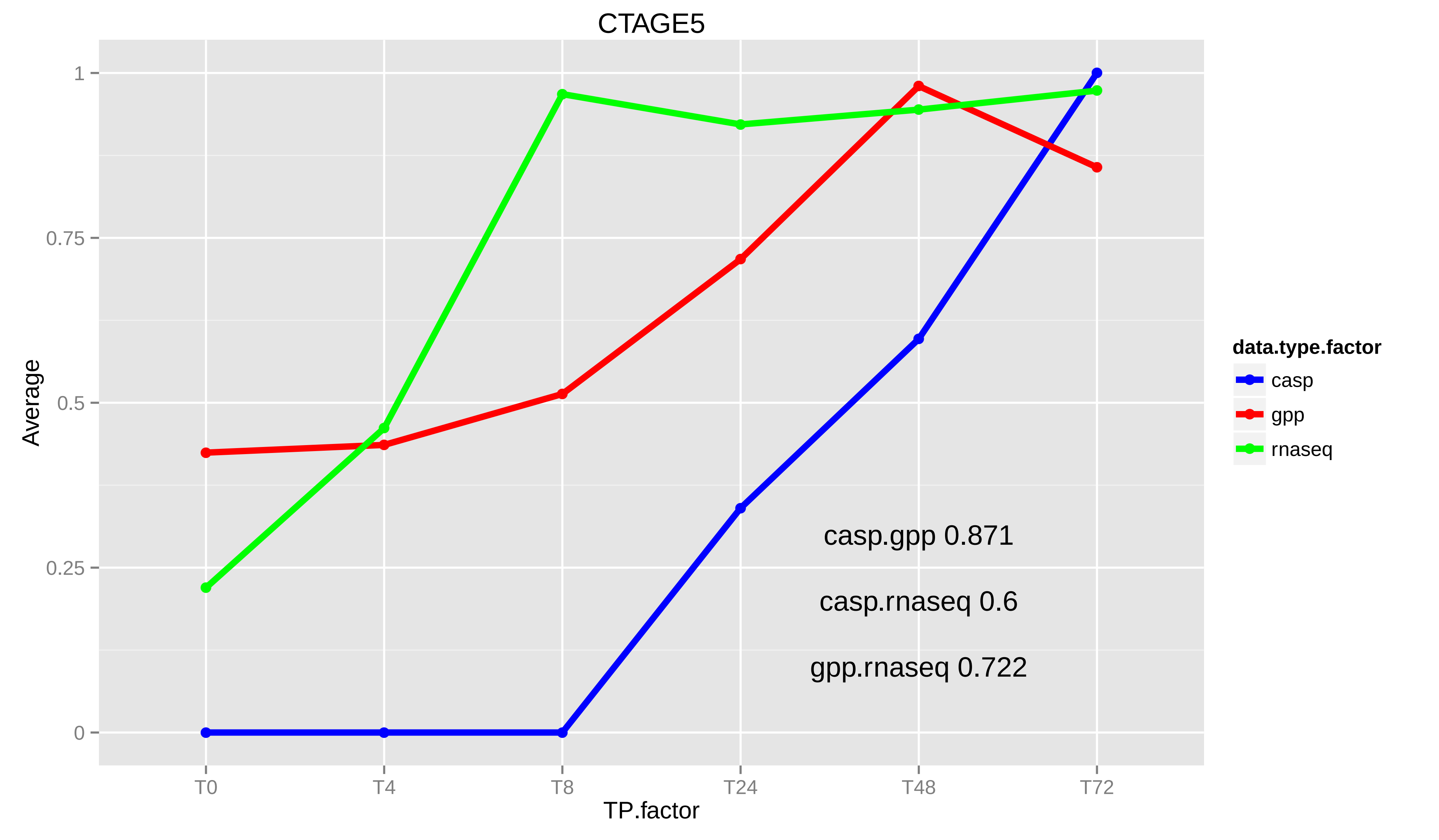

CTAGE5
Average
1
0.75
0.5
0.25
0
T0
T4
T8
T24
T48
T72
TP.factor
casp.gpp 0.871
casp.rnaseq 0.6
gpp.rnaseq 0.722
data.type.factor
casp
gpp
rnaseq

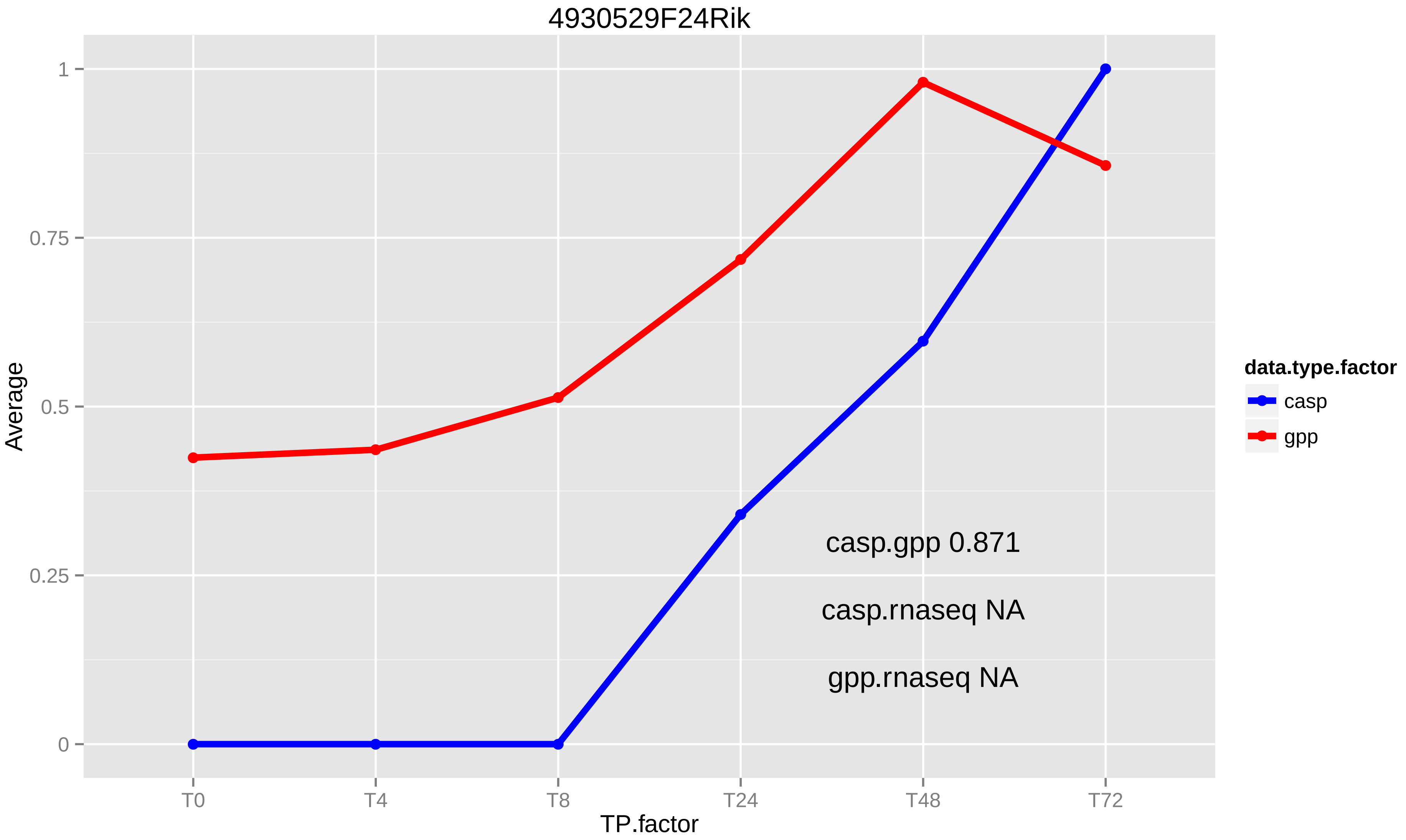
4930529F24Rik
Average
TP.factor
T0
T4
T8
T24
T48
T72
0
0.25
0.5
0.75
1
data.type.factor
casp
gpp
casp.gpp 0.871
casp.rnaseq NA
gpp.rnaseq NA

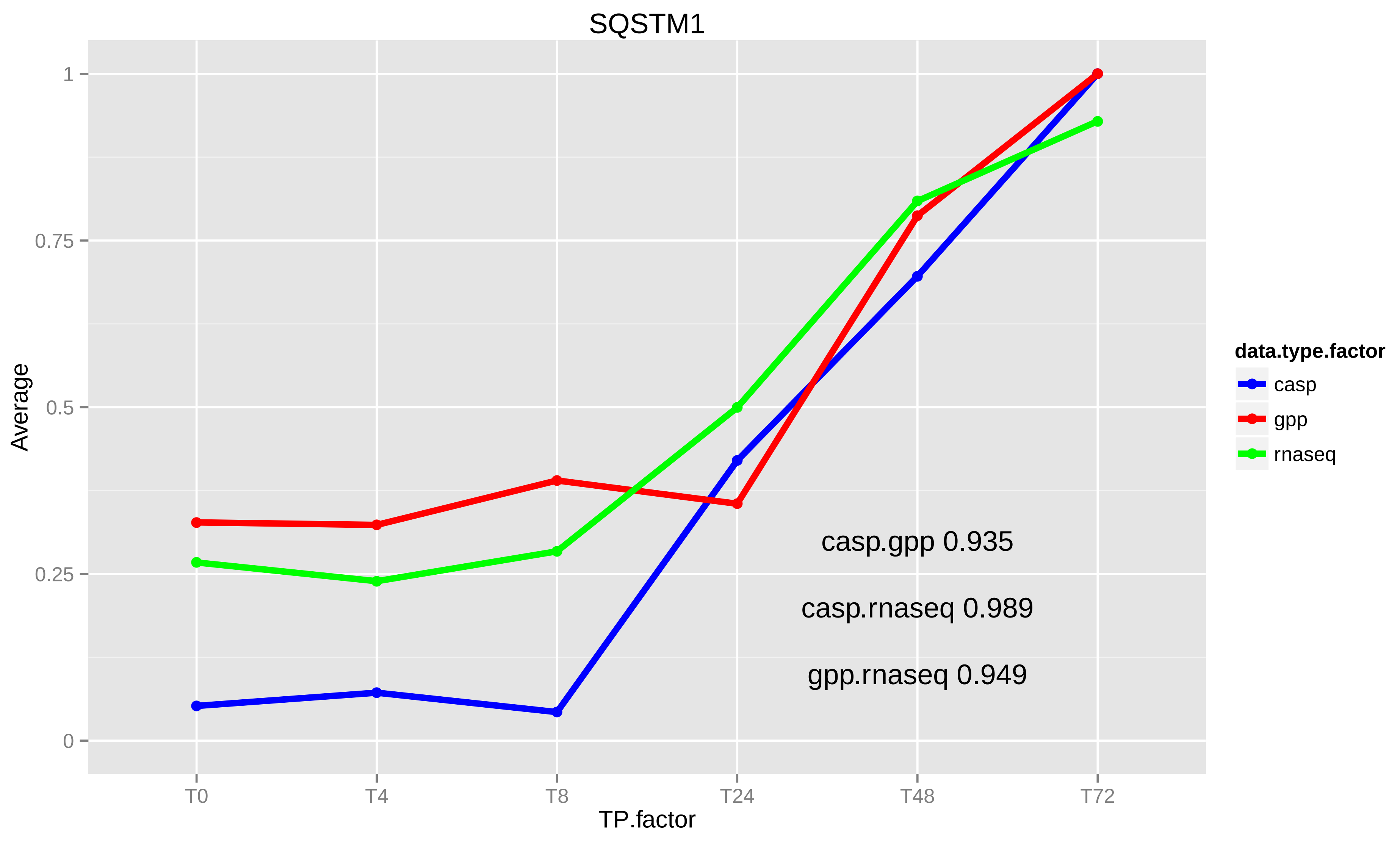
SQSTM1
1
0.75
0.5
0.25
0
Average
T0
T4
T8
T24
T48
T72
TP.factor
data.type.factor
casp
gpp
rnaseq
casp.gpp 0.935
casp.rnaseq 0.989
gpp.rnaseq 0.949
