# Supplementary material for: Uncovering a Dual Regulatory Role for Caspases During Endoplasmic Reticulum Stress-induced Cell Death
Source: Mol Cell Proteomics. 2016 Apr 28;15(7):2293–307. doi: 10.1074/mcp.M115.055376 (PMC4937505; doi:10.1074/mcp.M115.055376)
Supplement: Supplemental Data [file 10.1074_M115.055376_mcp.M115.055376-7.pdf]

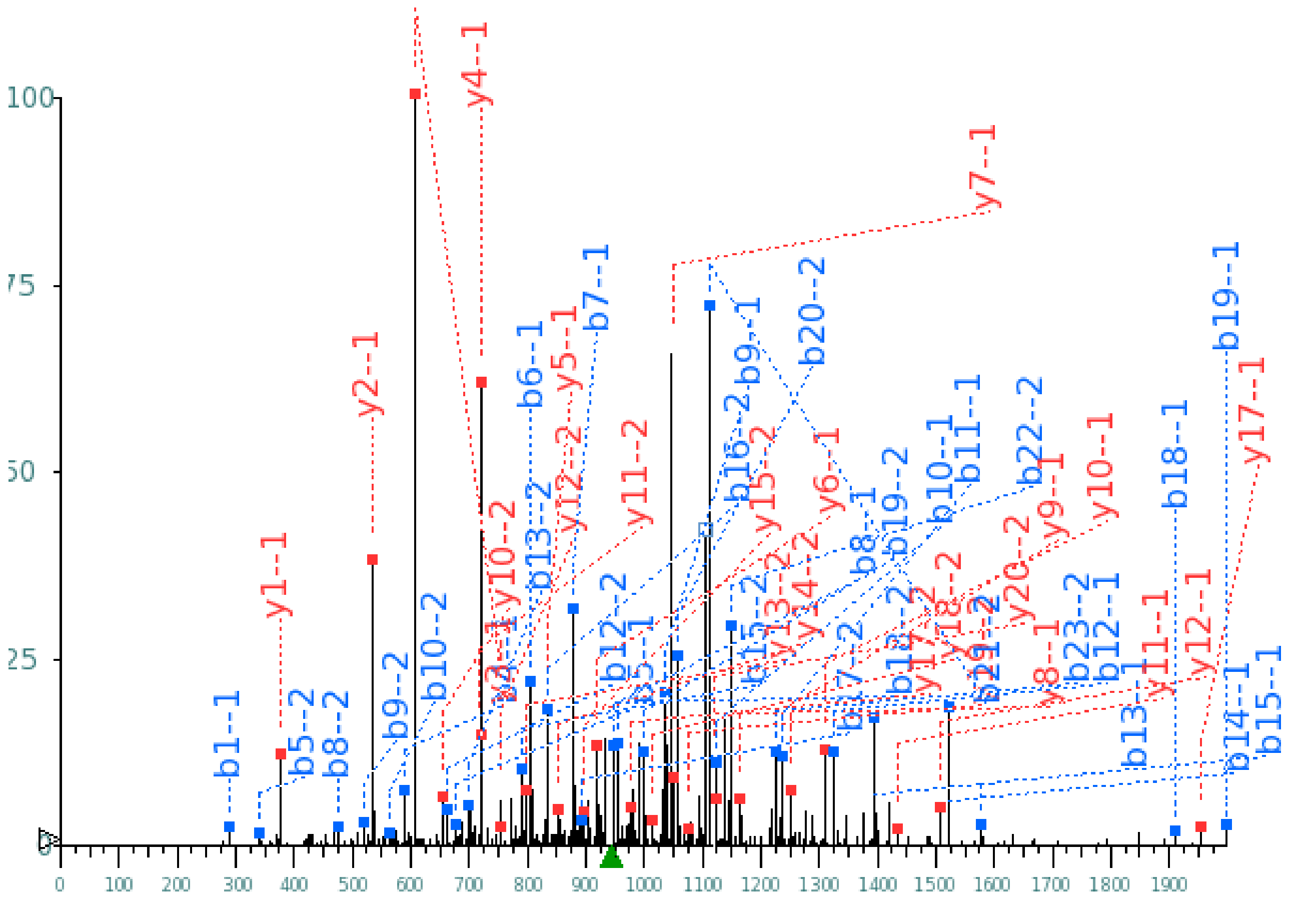

GGSC*SQAASSNSAQGSDESLIAC*K (+3)

Primary Reference: 1C03_HUMAN
Search ID: 41865
Search Name: 20130330_ananiav_TMT_GPP_10percent_fraction1_lysC_2MC_IAA
Scan#: 11121
Observed Mass: 944.1165 (0.1 ppm)
PSM Score: 97.78

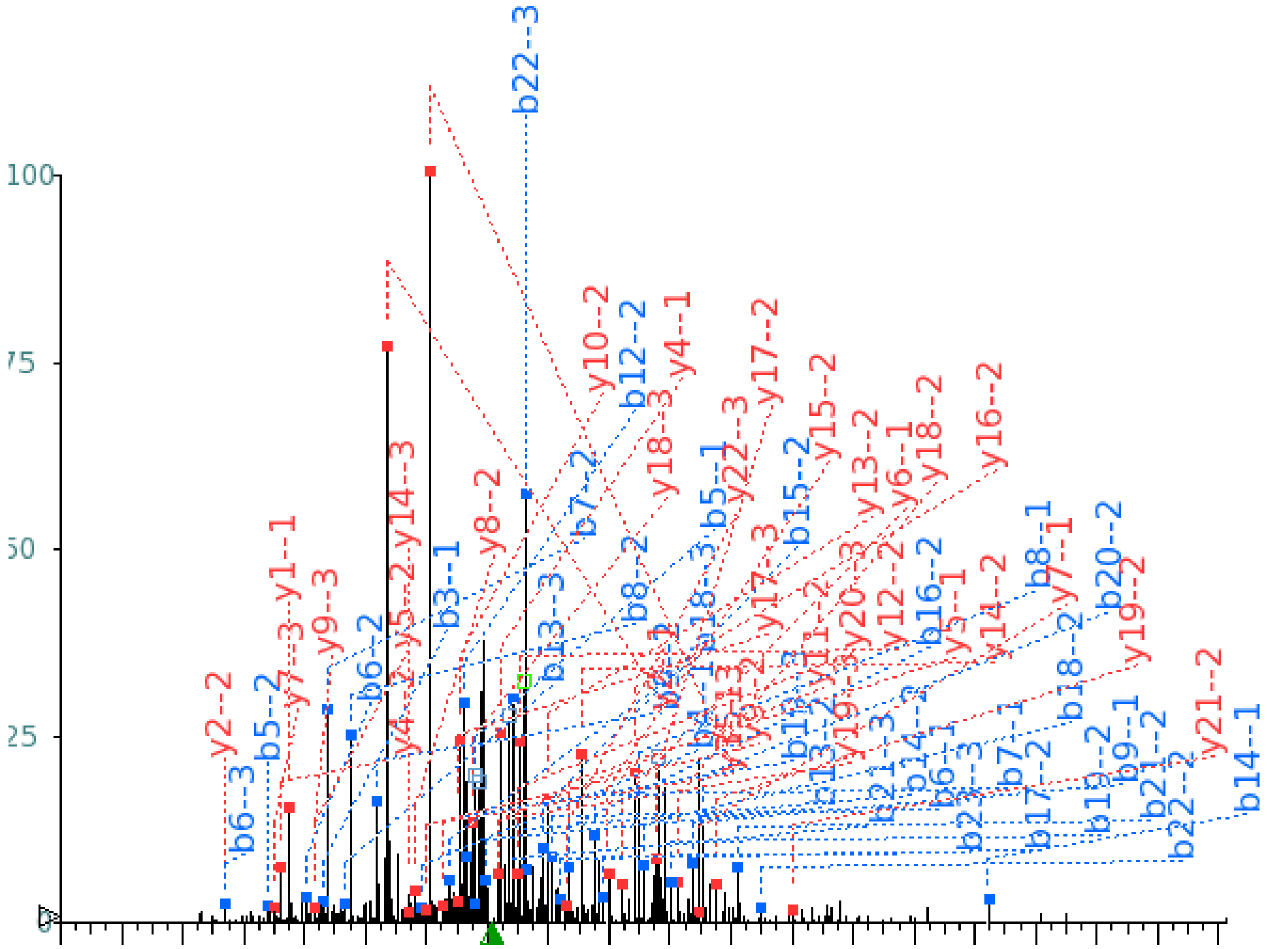

GGSC*SQAASSNSAQGSDESLIAC*K (+4)

Primary Reference: 1C03_HUMAN
Search ID: 41865
Search Name: 20130330_ananiav_TMT_GPP_10percent_fraction1_lysC_2MC_IAA
Scan#: 11133
Observed Mass: 708.3392 (0.1 ppm)
PSM Score: 61.02

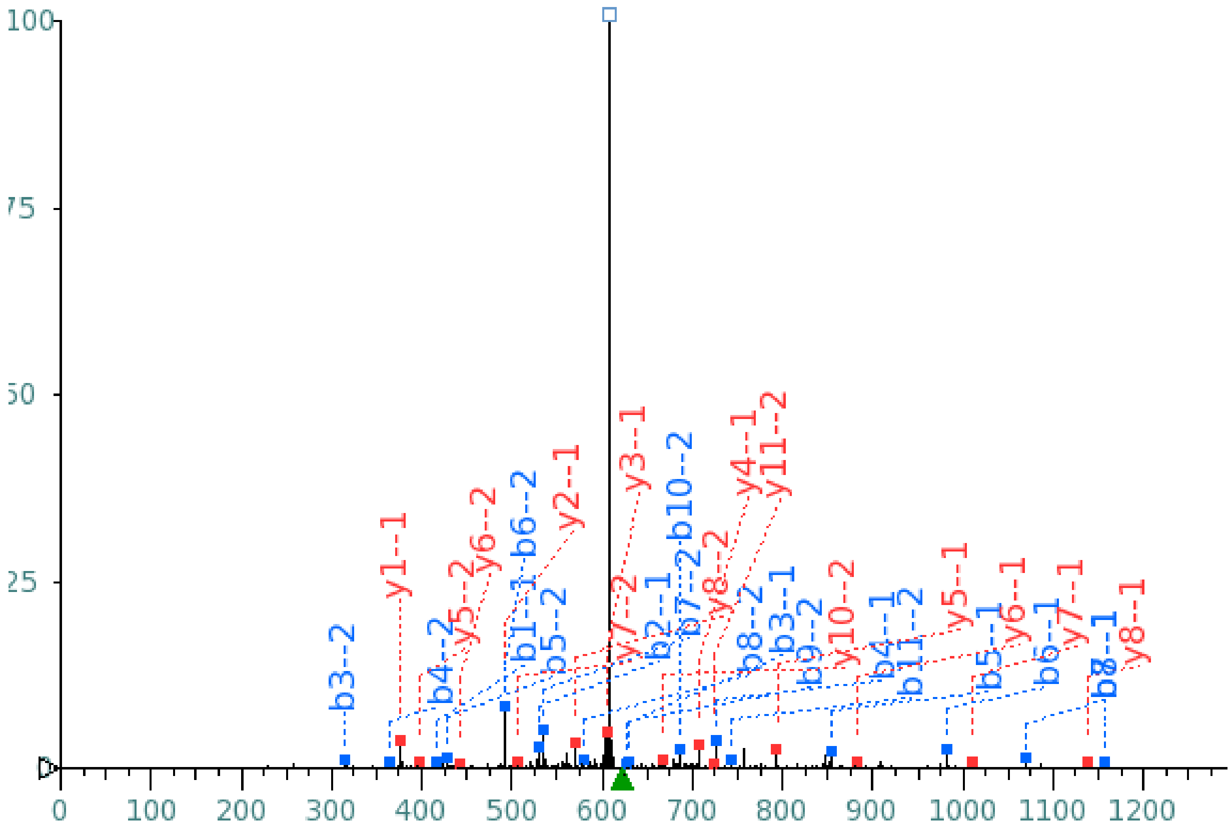

WLPVQESSTDDK (+3)

Primary Reference: SPCS1_HUMAN
Search ID: 41865
Search Name: 20130330_ananiav_TMT_GPP_10percent_fraction1_lysC_2MC_IAA
Scan#: 15890
Observed Mass: 621.6692 (1.7 ppm)
PSM Score: 36.87

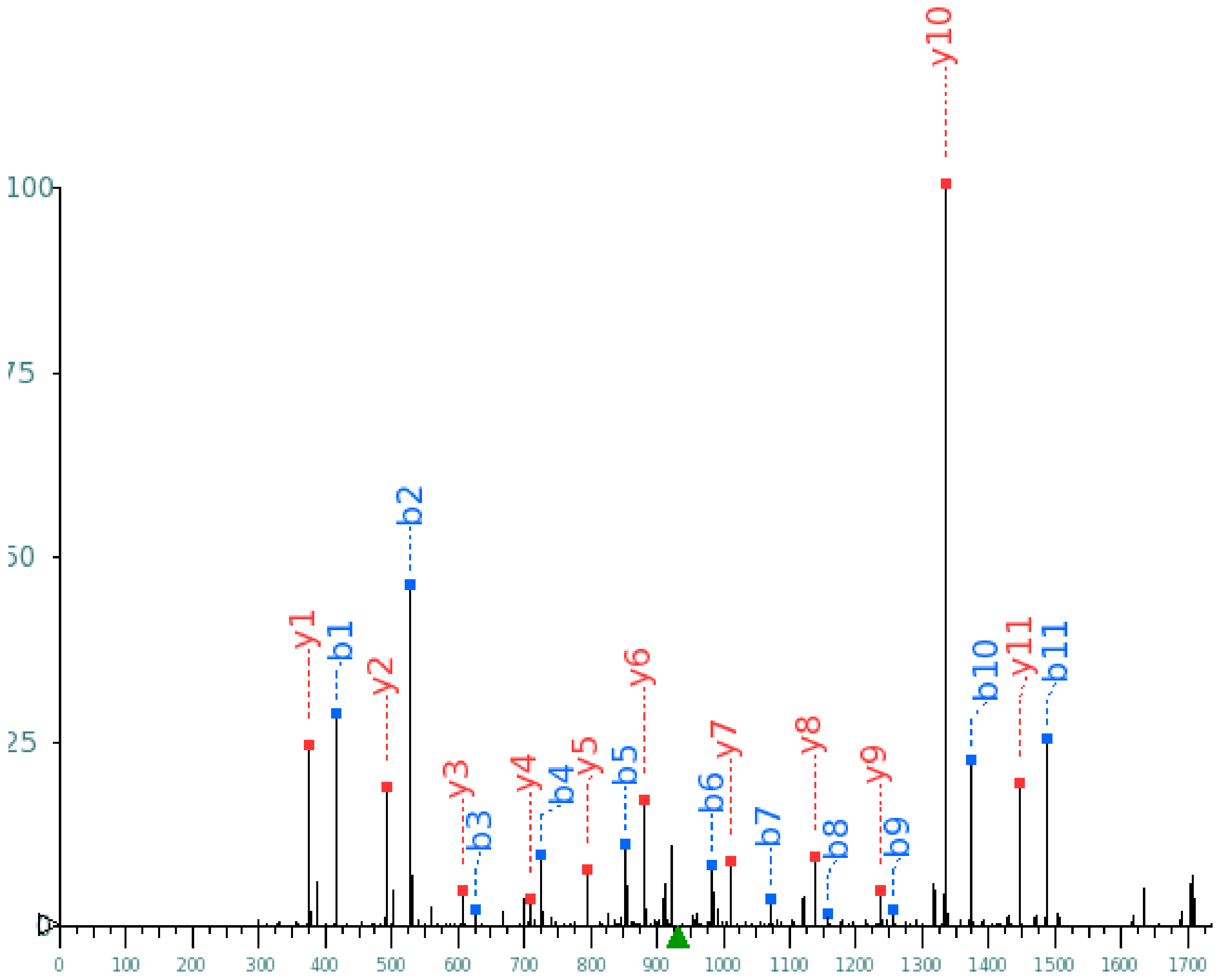

WLPVQESSTDDK (+2)

Primary Reference: SPCS1_HUMAN
Search ID: 41865
Search Name: 20130330_ananiav_TMT_GPP_10percent_fraction1_lysC_2MC_IAA
Scan#: 15911
Observed Mass: 931.9991 (0.5 ppm)
PSM Score: 73.97

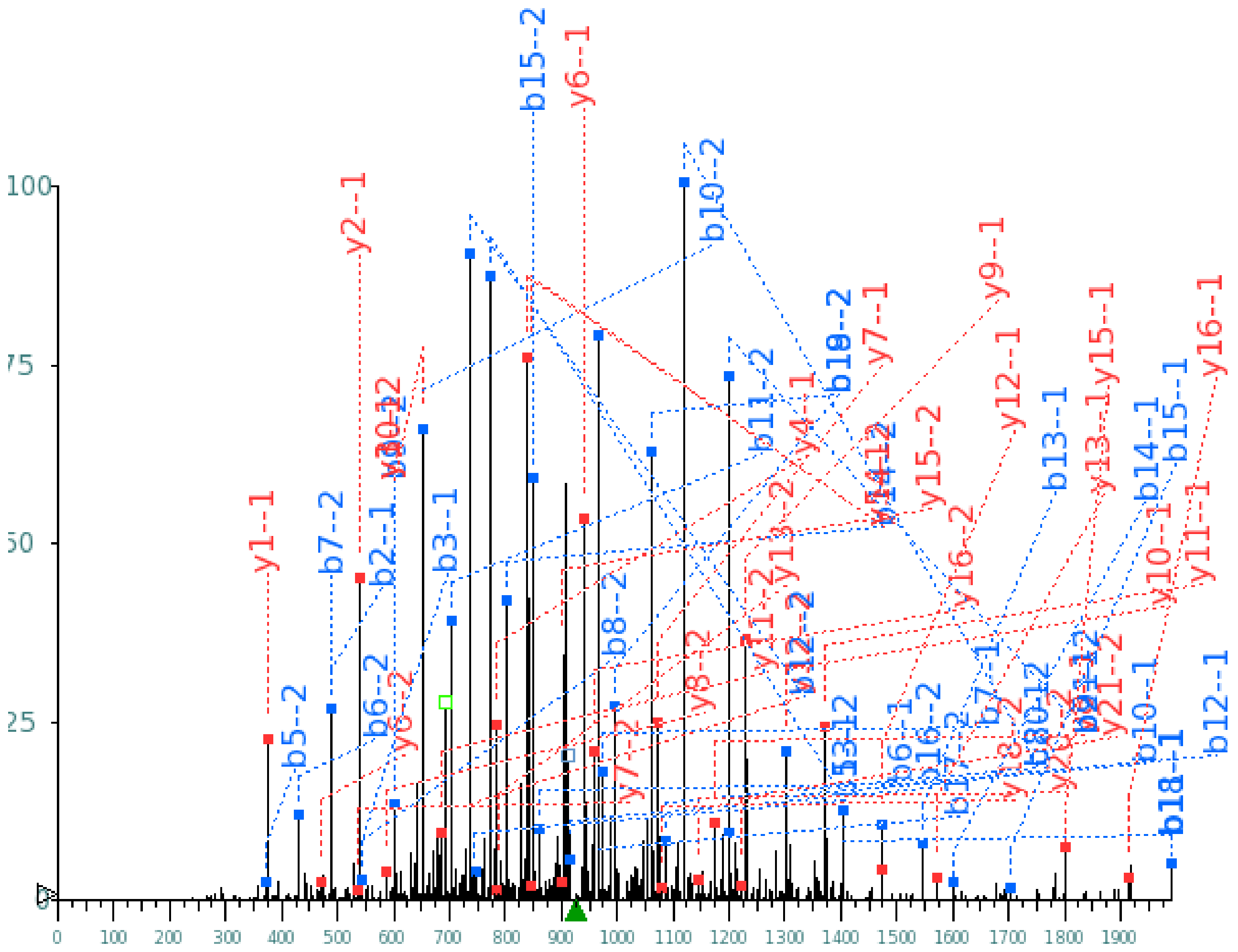

TRGAEDDLNTVAAGTMTGMLYK (+3)

Primary Reference: TI23B_HUMAN
Search ID: 41865
Search Name: 20130330_ananiav_TMT_GPP_10percent_fraction1_lysC_2MC_IAA
Scan#: 20156
Observed Mass: 925.147 (0 ppm)
PSM Score: 100.52

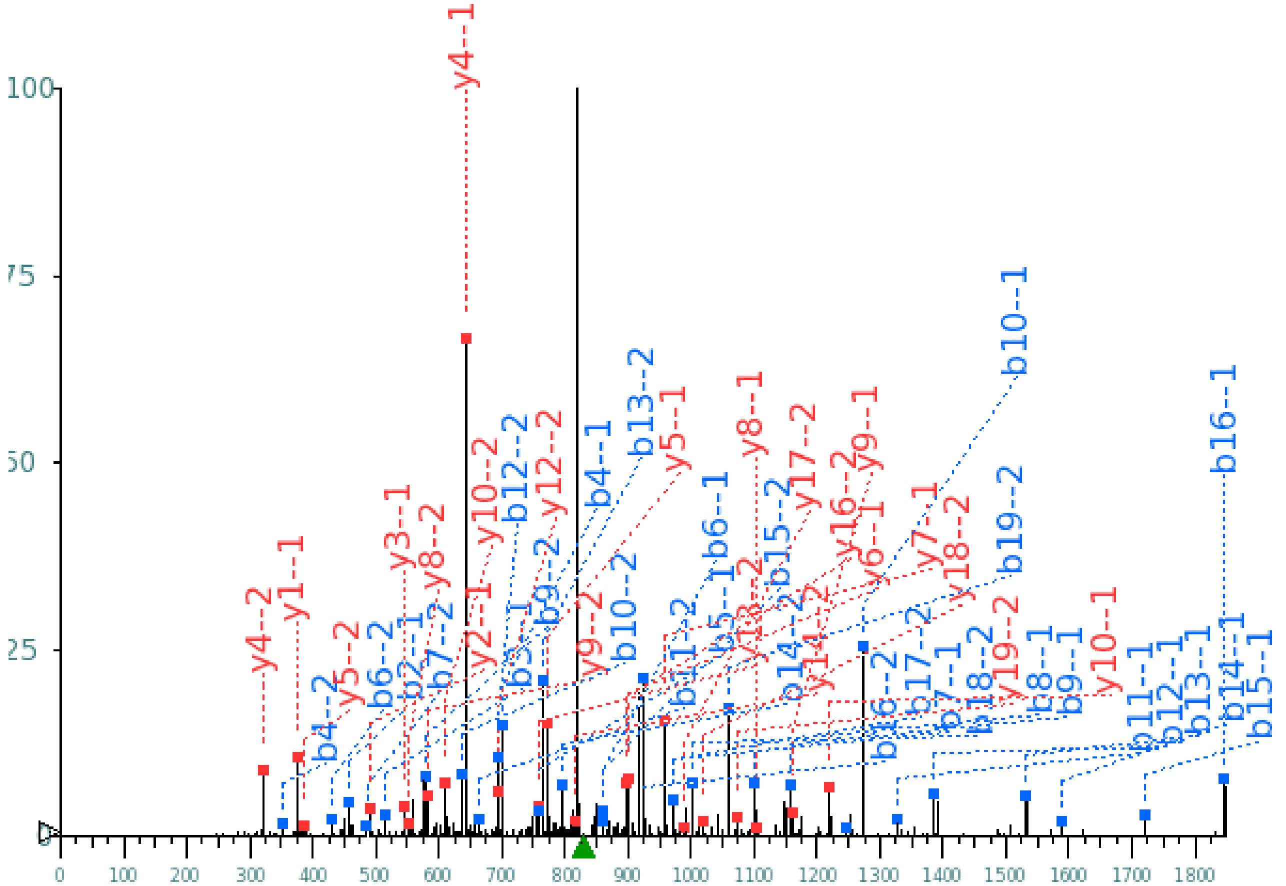

NIGWGTDQGIGGFGEEPGIK (+3)

Primary Reference: IR3IP_HUMAN
Search ID: 41865
Search Name: 20130330_ananiav_TMT_GPP_10percent_fraction1_lysC_2MC_IAA
Scan#: 20653
Observed Mass: 830.7734 (1.1 ppm)
PSM Score: 88.15

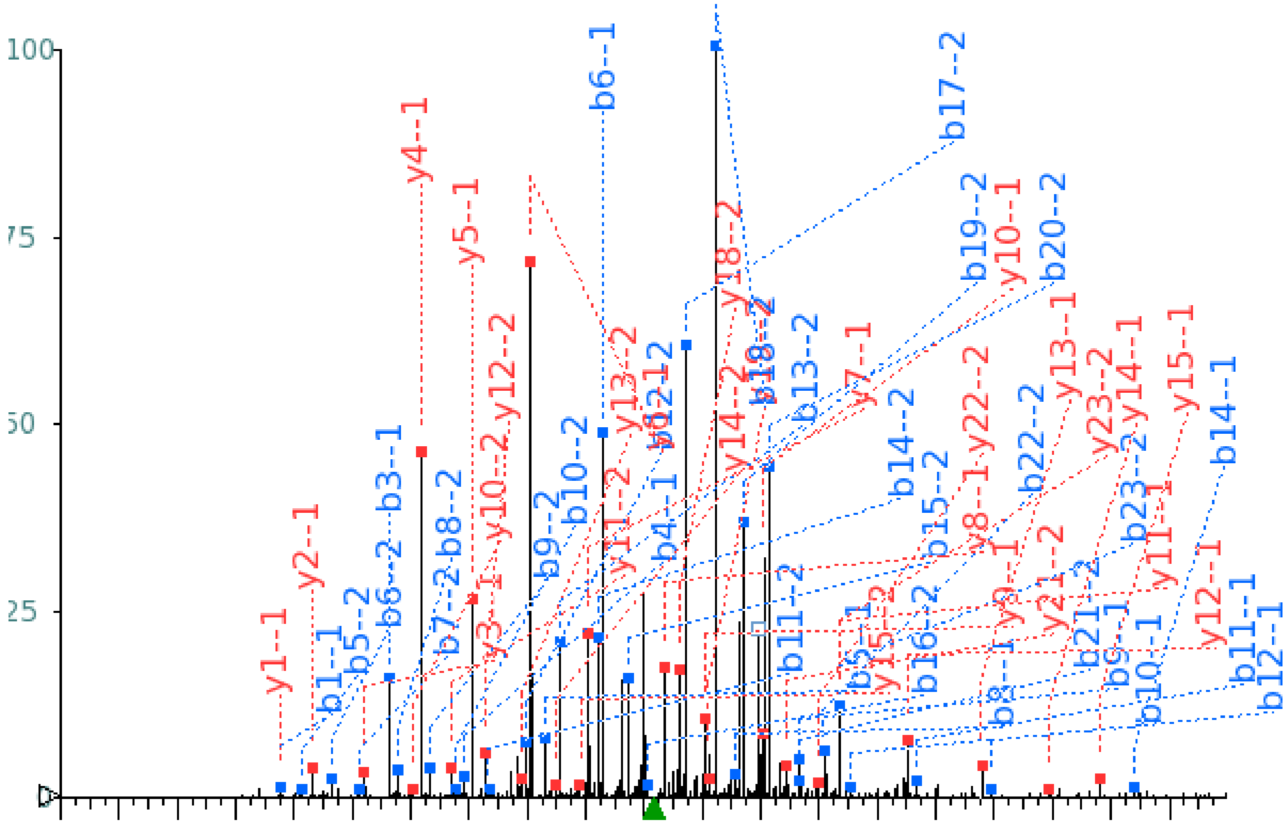

FADQHVPEISIQDMTAQVTSPSGK (+3)

Primary Reference: Q5HY90_HUMAN
Search ID: 41865
Search Name: 20130330_ananiav_TMT_GPP_10percent_fraction1_lysC_2MC_IAA
Scan#: 21502
Observed Mass: 1015.5318 (1.5 ppm)
PSM Score: 87.35

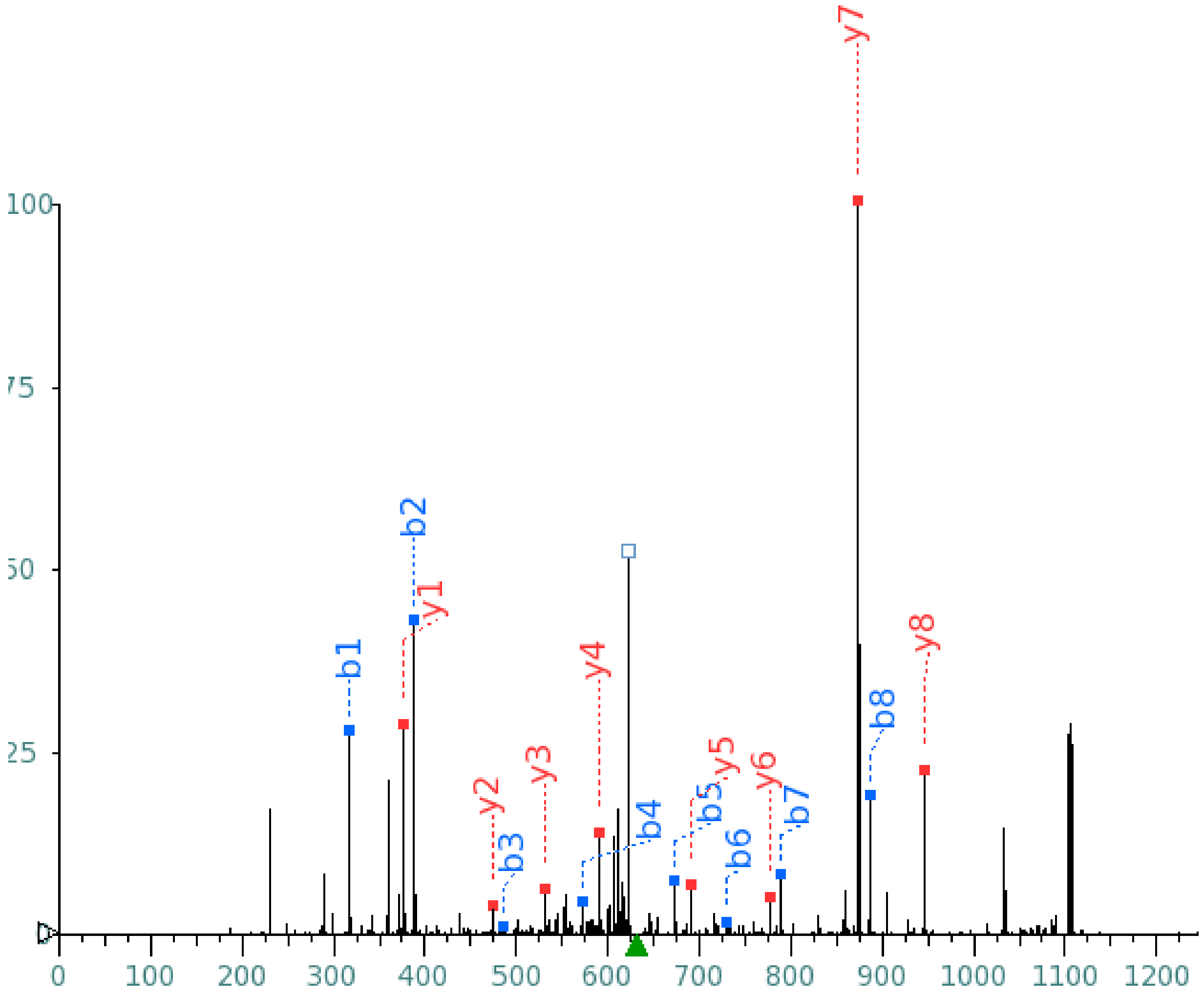

SAPSTGGVK (+2)

Primary Reference: H33_HUMAN
Search ID: 41866
Search Name: 20130330_ananiav_TMT_GPP_10percent_fraction2_lysC_2MC_IAA
Scan#: 6229
Observed Mass: 631.3798 (0.6 ppm)
PSM Score: 49.27

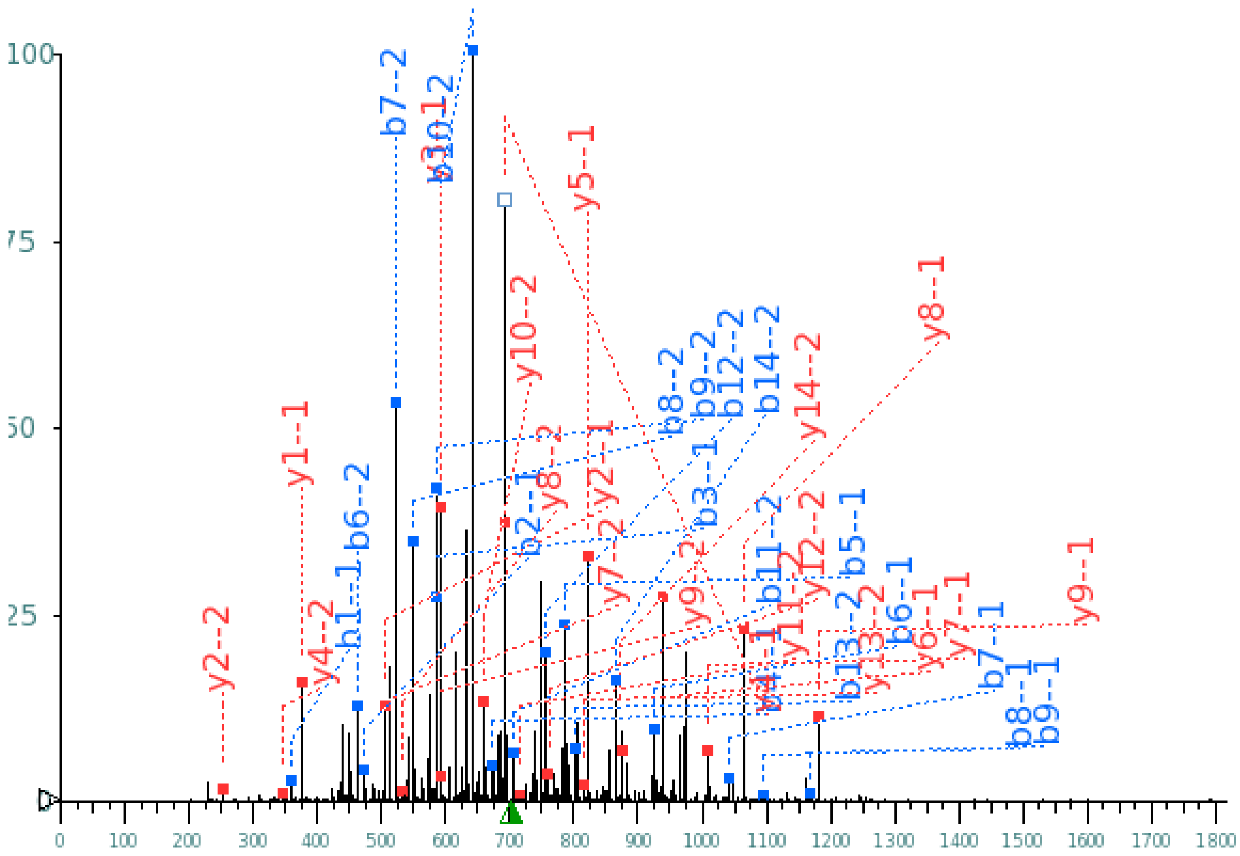

ELNSNHDGADETSEK (+3)

Primary Reference: B2RCX0_HUMAN
Search ID: 41866
Search Name: 20130330_ananiav_TMT_GPP_10percent_fraction2_lysC_2MC_IAA
Scan#: 6243
Observed Mass: 702.0116 (0.4 ppm)
PSM Score: 34.1

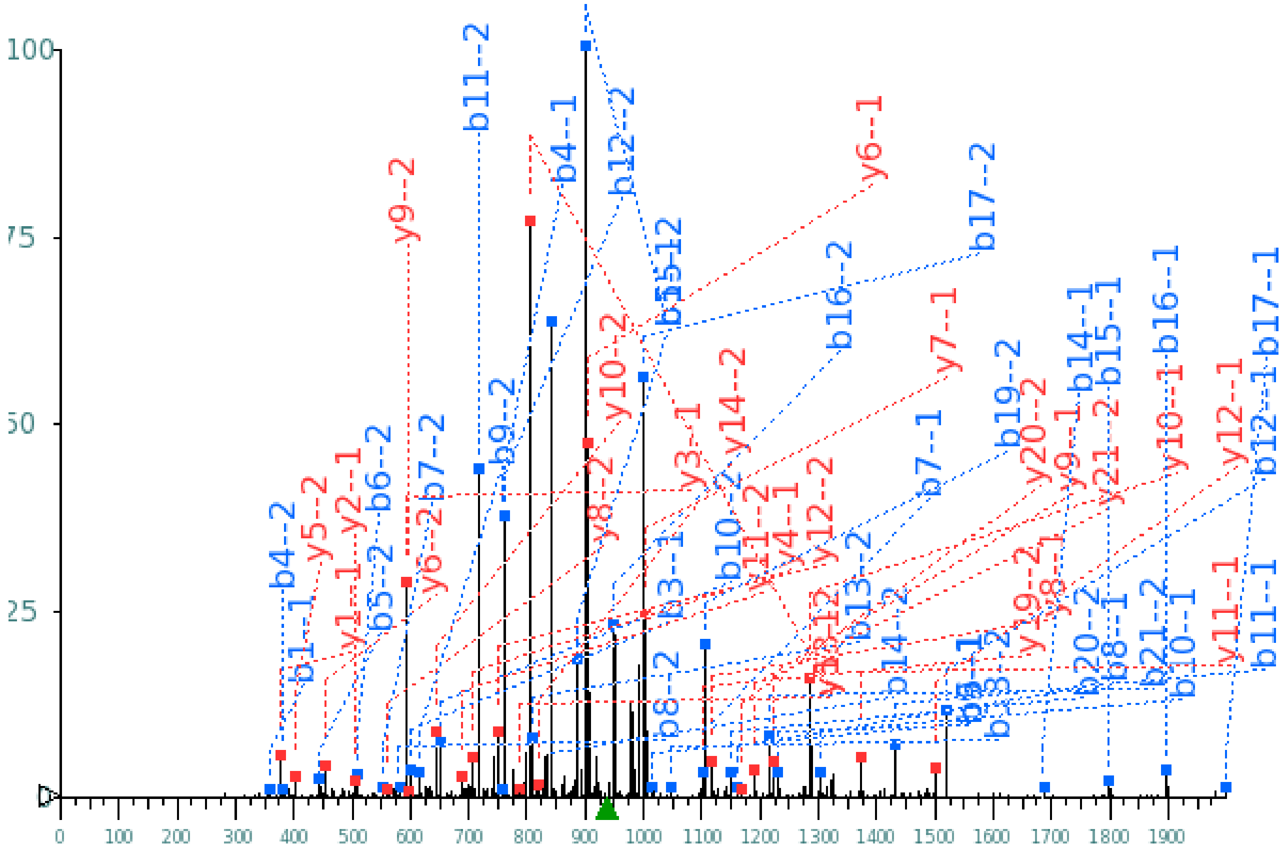

ELEREESGAAESPALVTPDSEK (+3)

Primary Reference: KTI12_HUMAN
Search ID: 41866
Search Name: 20130330_ananiav_TMT_GPP_10percent_fraction2_lysC_2MC_IAA
Scan#: 13484
Observed Mass: 934.819 (0.6 ppm)
PSM Score: 76.99

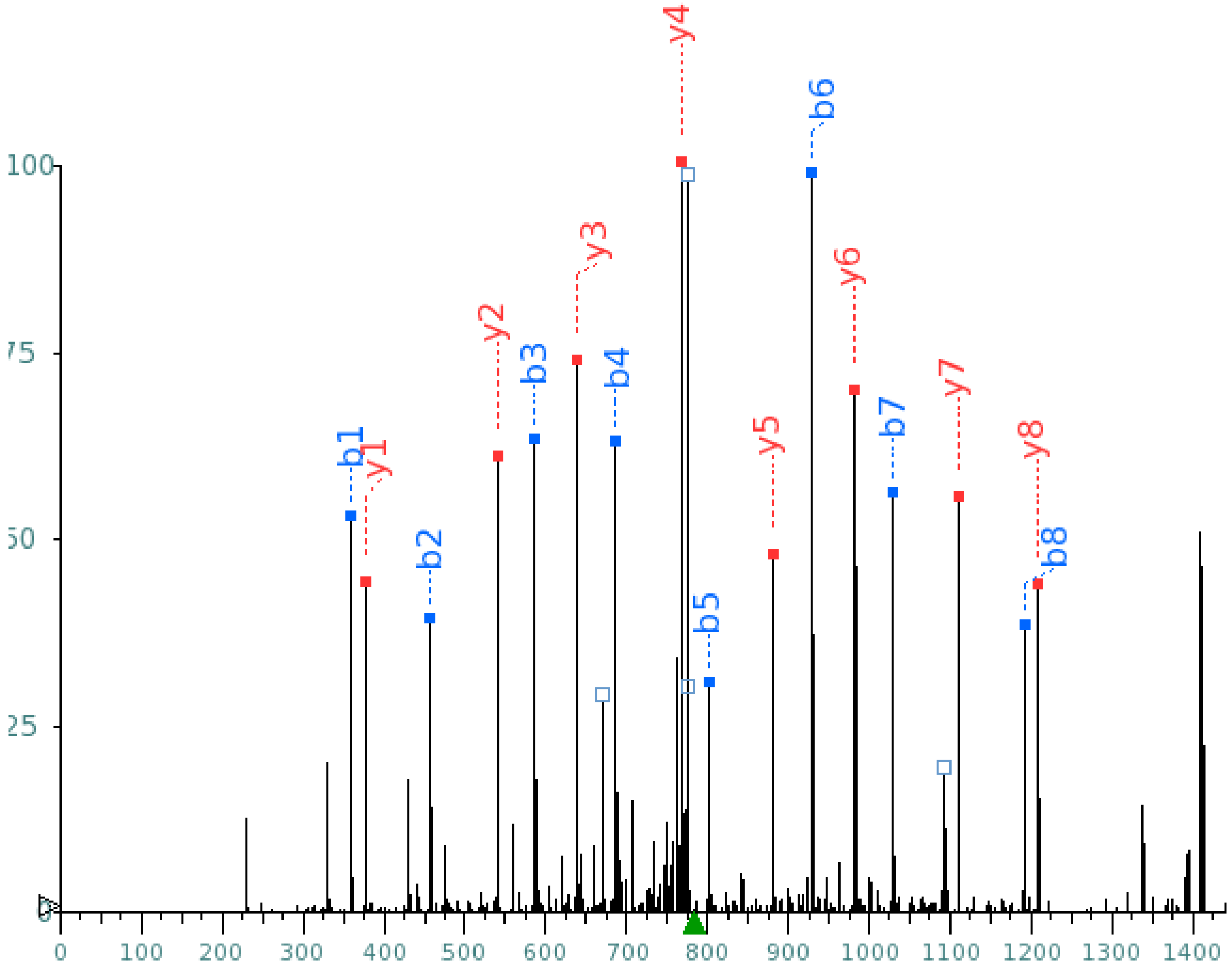

EVQTIEVYK (+2)

Primary Reference: THOC6_HUMAN
Search ID: 41866
Search Name: 20130330_ananiav_TMT_GPP_10percent_fraction2_lysC_2MC_IAA
Scan#: 14449
Observed Mass: 783.9611 (0.4 ppm)
PSM Score: 53.41

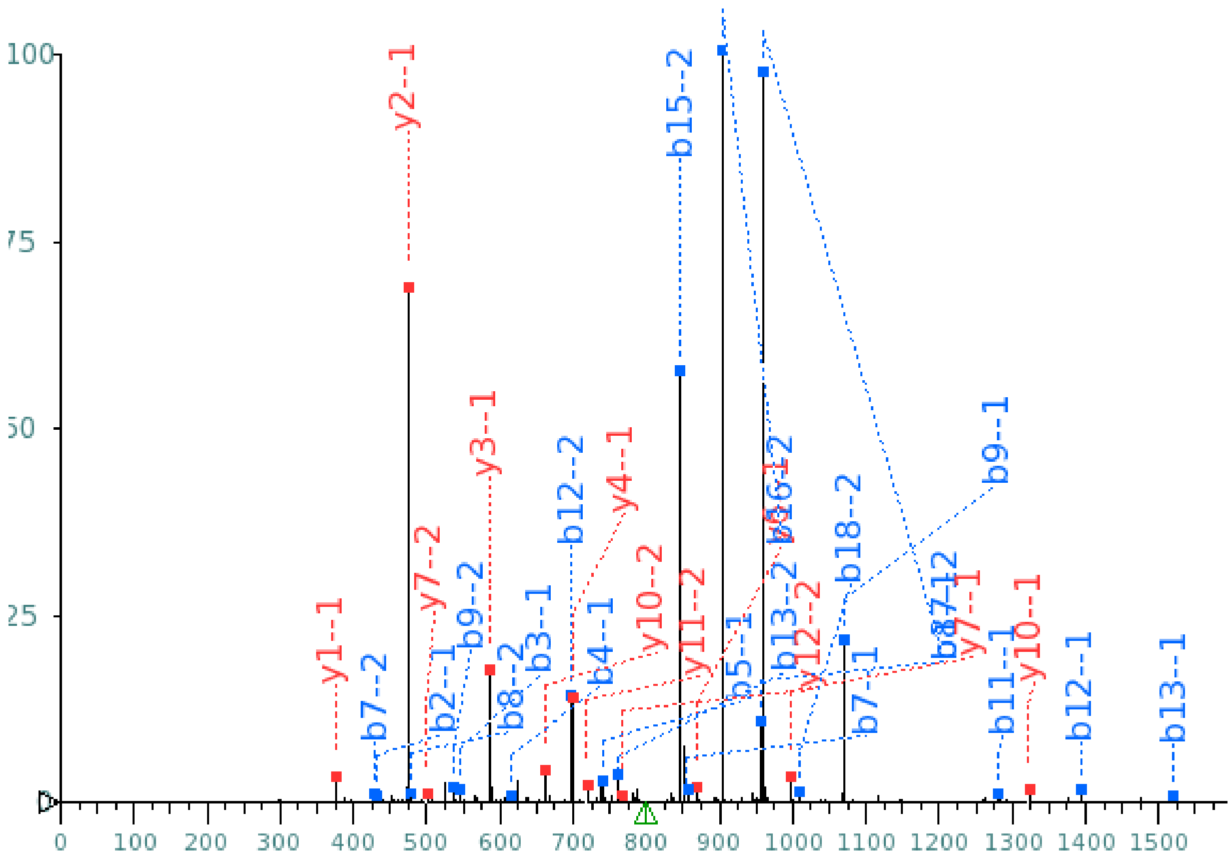

VTIAQGGVLPNIQAVLLPK (+3)

Primary Reference: H2A1D_HUMAN
Search ID: 41866
Search Name: 20130330_ananiav_TMT_GPP_10percent_fraction2_lysC_2MC_IAA
Scan#: 26600
Observed Mass: 797.1713 (1.9 ppm)
PSM Score: 41.32

File name: 2015-12-17 03.51.45PM.pdf

Creation time: 2015-12-17 03:53PM

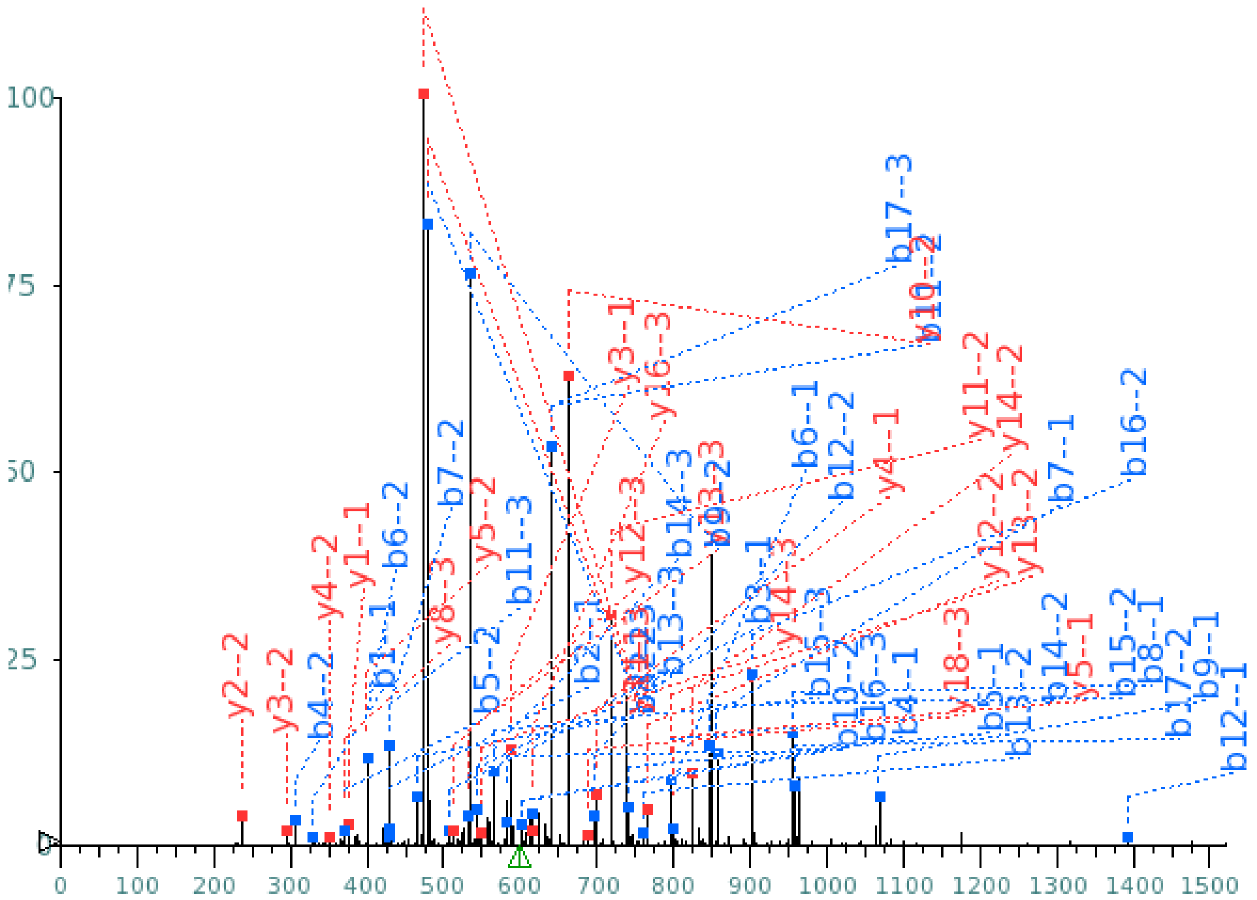

VTIAQGGVLPNIQAVLLPK (+4)

Primary Reference: H2A1D_HUMAN
Search ID: 41866
Search Name: 20130330_ananiav_TMT_GPP_10percent_fraction2_lysC_2MC_IAA
Scan#: 26604
Observed Mass: 598.1301 (1.7 ppm)
PSM Score: 57.65

File name: 2015-12-17 03.51.45PM.pdf

Creation time: 2015-12-17 03:53PM

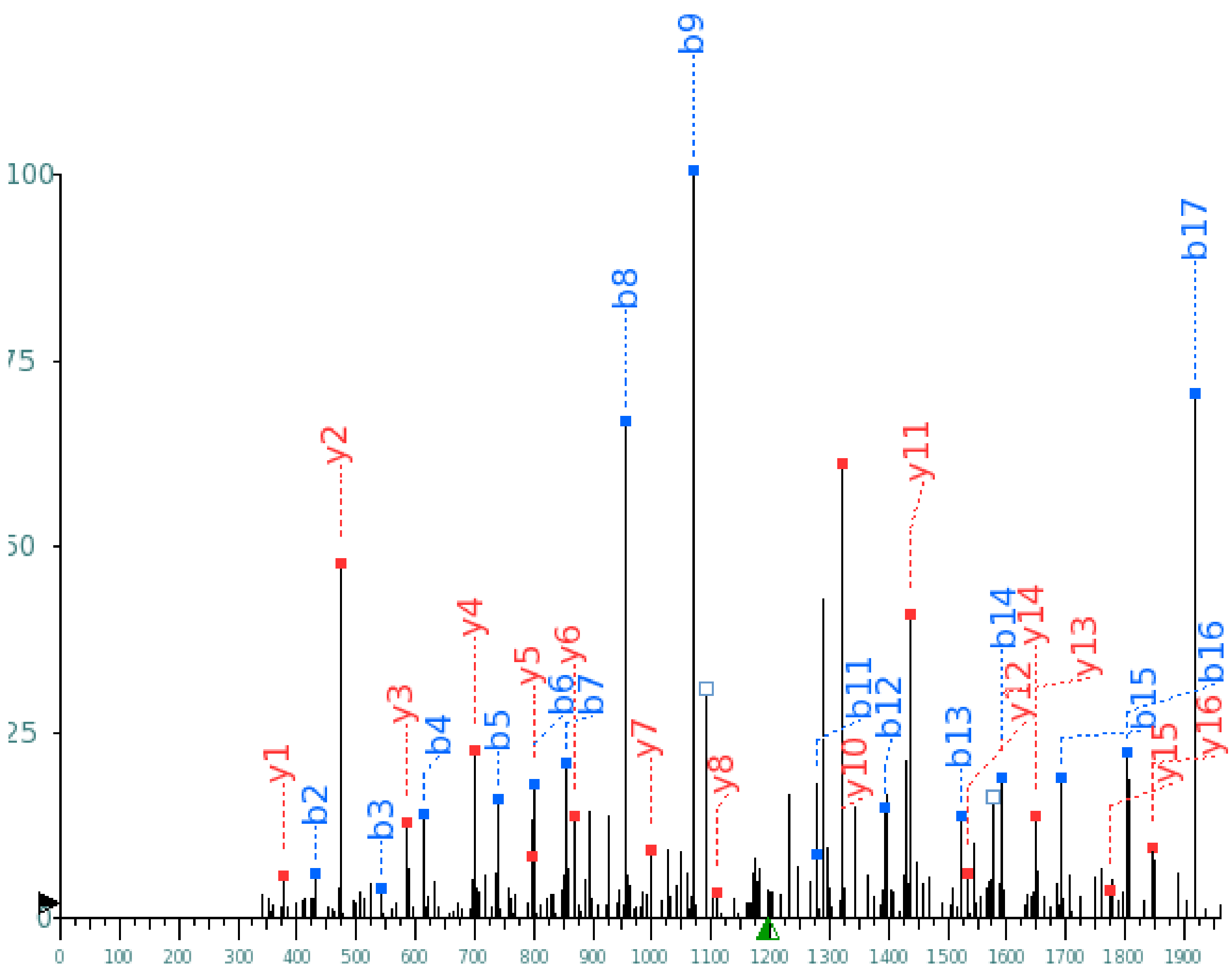

VTIAQGGVLPNIQAVLLPK (+2)

Primary Reference: H2A1D_HUMAN

Search ID: 41866

Search Name: 20130330_ananiav_TMT_GPP_10percent_fraction2_lysC_2MC_IAA

Scan#: 26634

Observed Mass: 1195.2511 (0.2 ppm)

PSM Score: 96.41

File name: 2015-12-17 03.51.45PM.pdf

Creation time: 2015-12-17 03:53PM

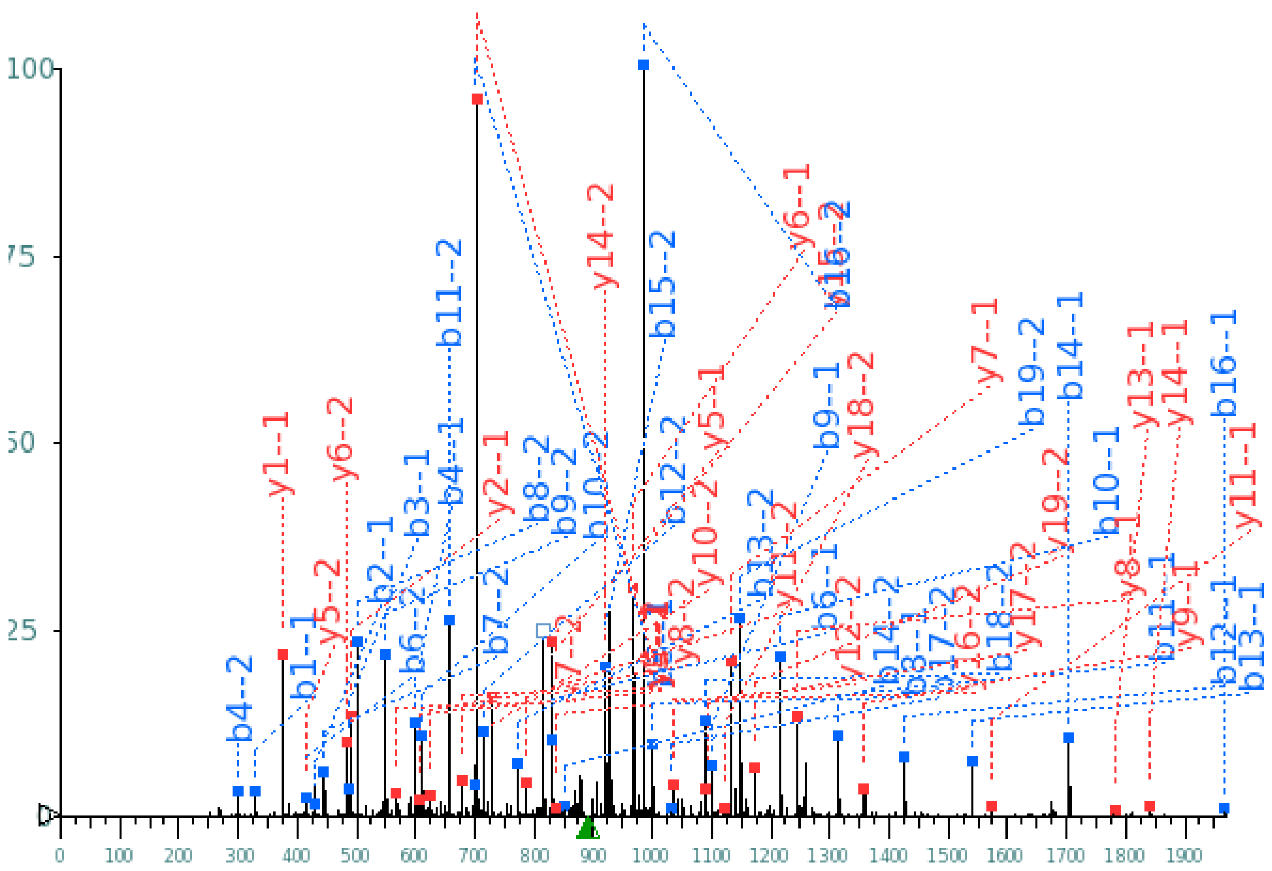

VTGLTEGLVDVILYHQPDDK (+3)

Primary Reference: Q6MZS5_HUMAN
Search ID: 41866
Search Name: 20130330_ananiav_TMT_GPP_10percent_fraction2_lysC_2MC_IAA
Scan#: 26735
Observed Mass: 890.8318 (2.1 ppm)
PSM Score: 70.82

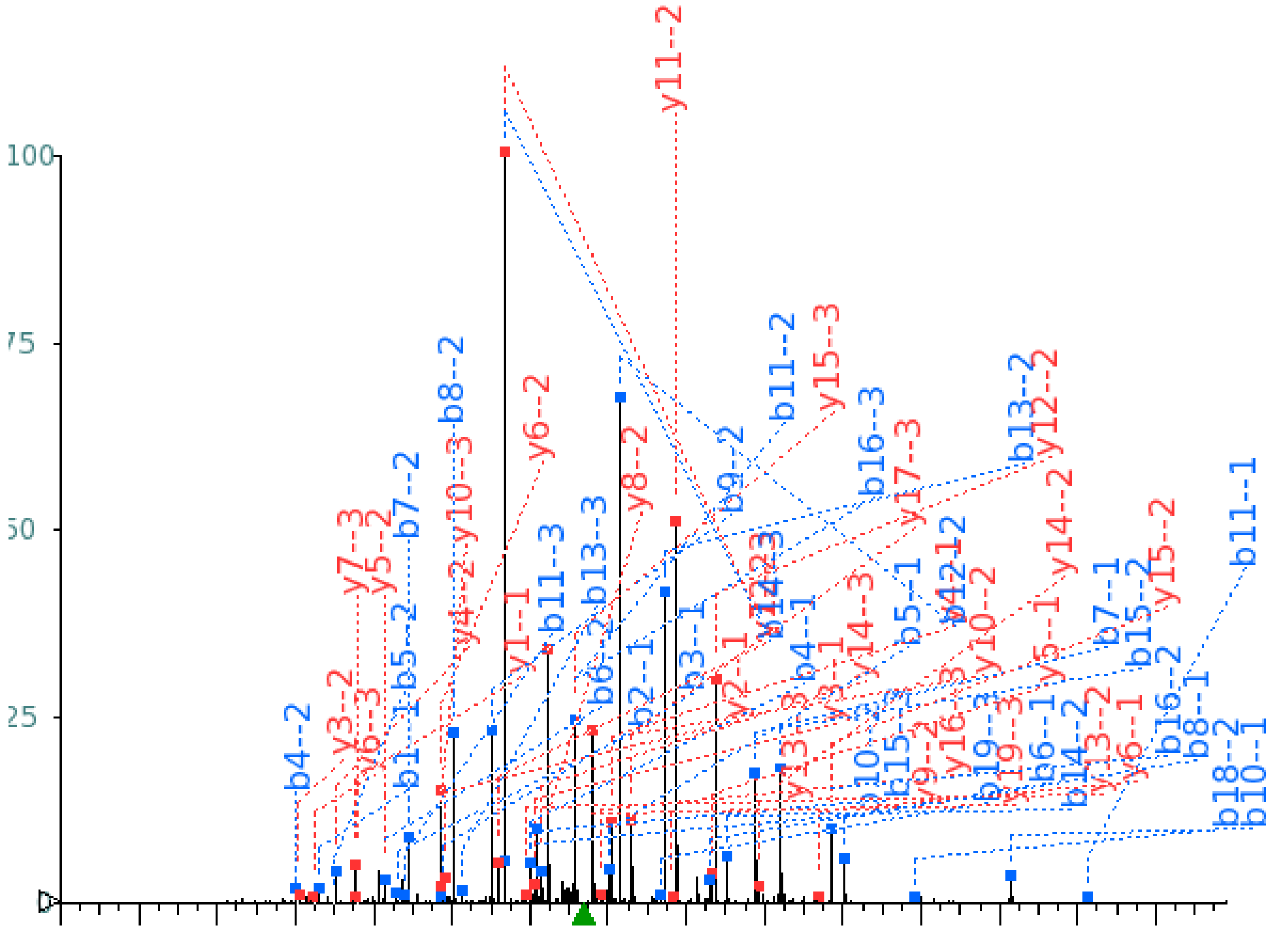

VTGLTEGLVDVILYHQPDDK (+4)

Primary Reference: Q6MZS5_HUMAN
Search ID: 41866
Search Name: 20130330_ananiav_TMT_GPP_10percent_fraction2_lysC_2MC_IAA
Scan#: 26739
Observed Mass: 668.3758 (2.1 ppm)
PSM Score: 77.87

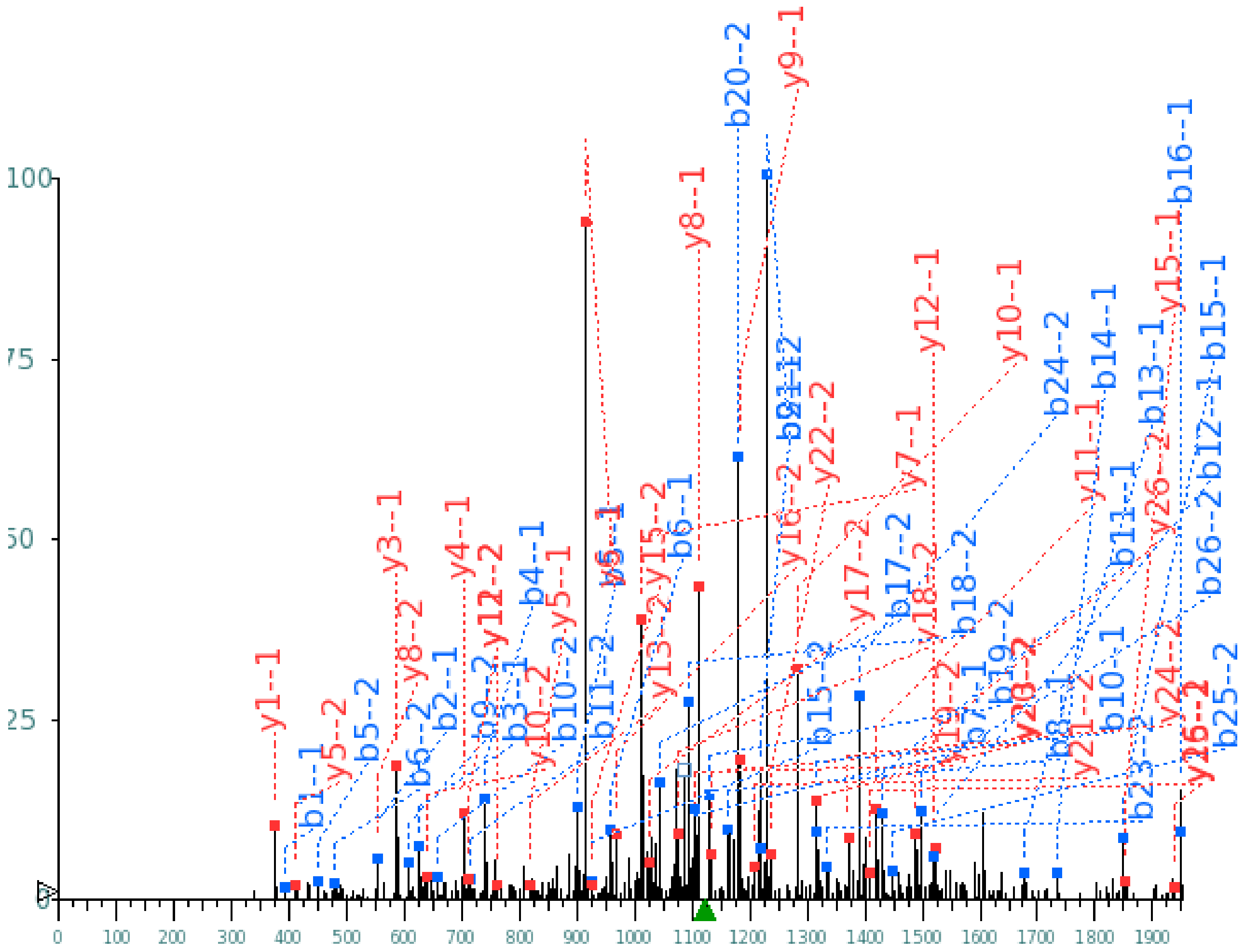

YYALC*GFGGVLSC*GLTHTAVVPLDLVK (+3)

Primary Reference: B2RE88_HUMAN

Search ID: 41866

Search Name: 20130330_ananiav_TMT_GPP_10percent_fraction2_lysC_2MC_IAA

Scan#: 26900

Observed Mass: 1123.6111 (1 ppm)

PSM Score: 72.48

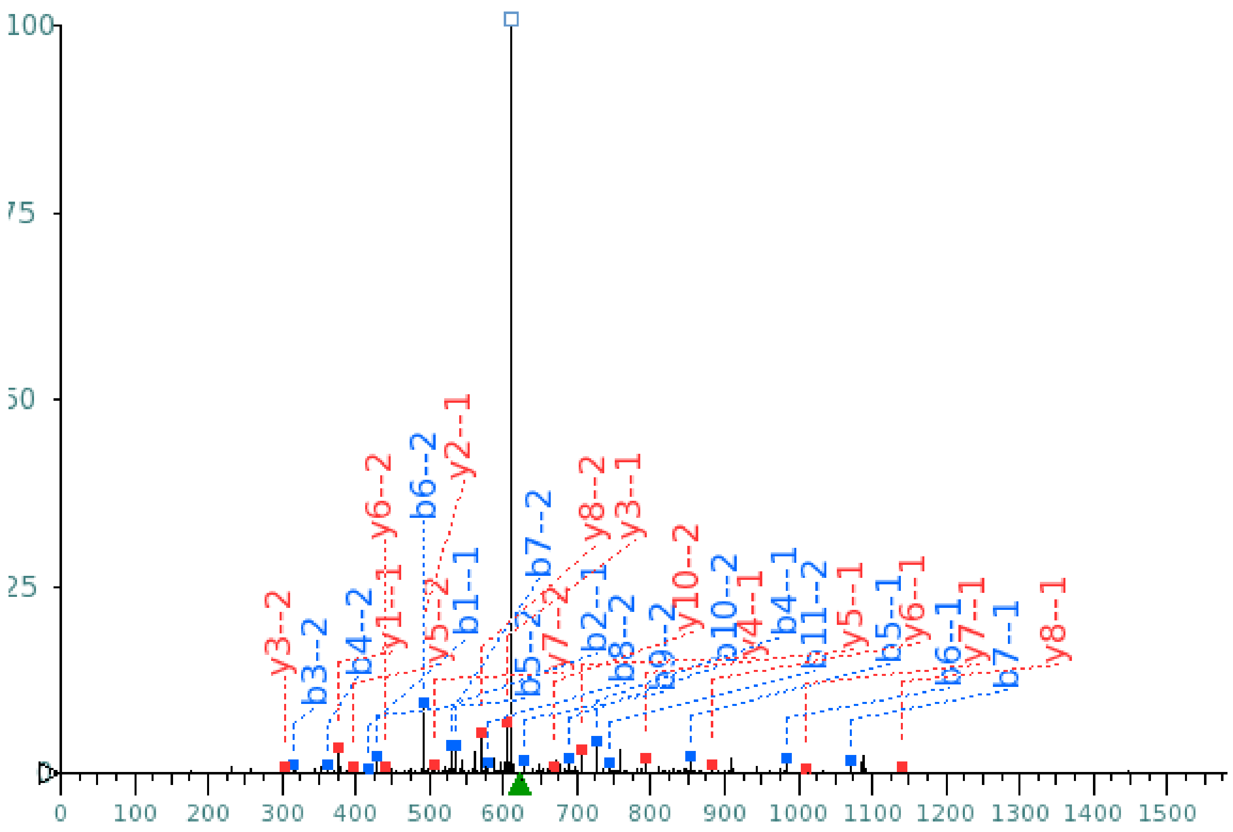

WLPVQESSTDDK (+3)

Primary Reference: SPCS1_HUMAN
Search ID: 41867
Search Name: 20130330_ananiav_TMT_GPP_10percent_fraction3_lysC_2MC_IAA
Scan#: 14999
Observed Mass: 621.6691 (1.4 ppm)
PSM Score: 32.6

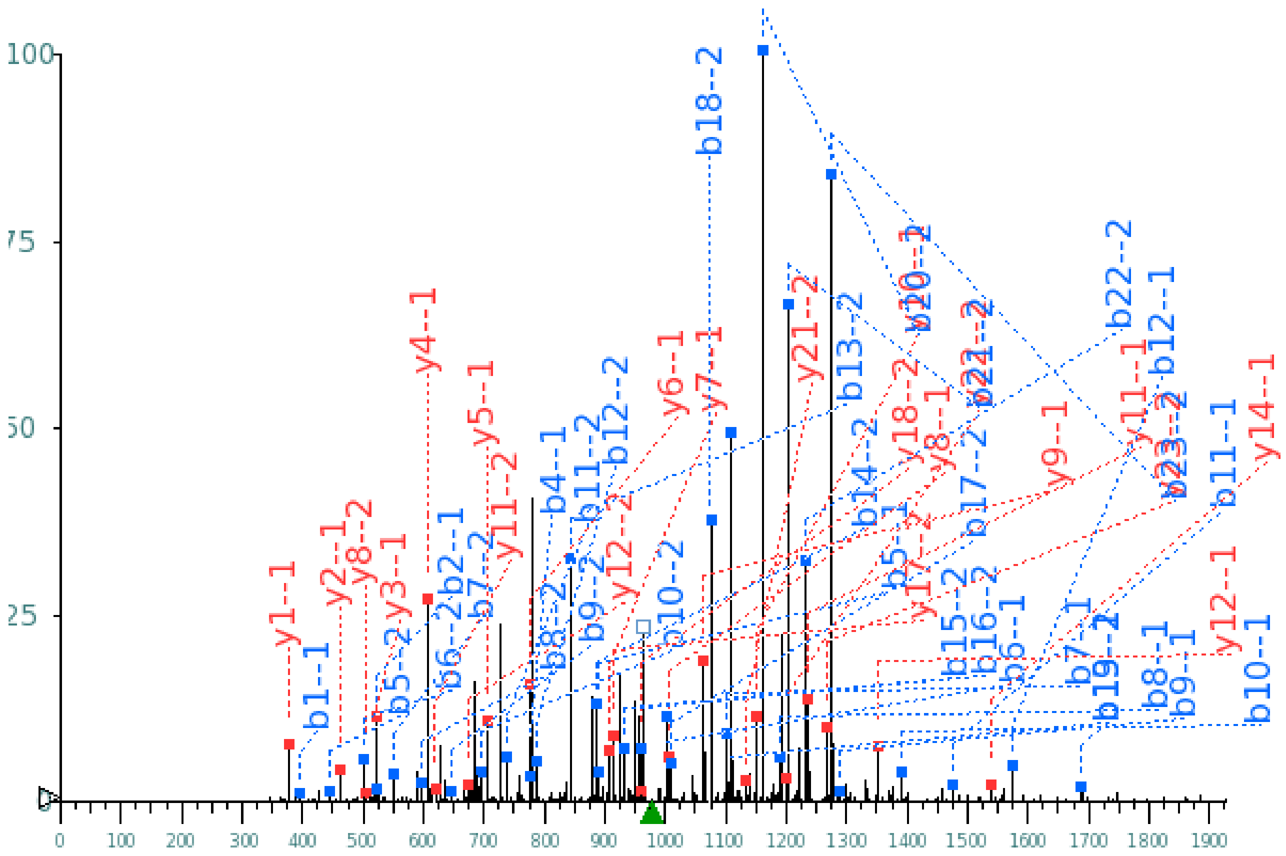

YEHDDIVSTVSVLSSGTQAVSGSK (+3)

Primary Reference: MEP50_HUMAN

Search ID: 41867

Search Name: 20130330_ananiav_TMT_GPP_10percent_fraction3_lysC_2MC_IAA

Scan#: 18452

Observed Mass: 975.5169 (3.7 ppm)

PSM Score: 104.96

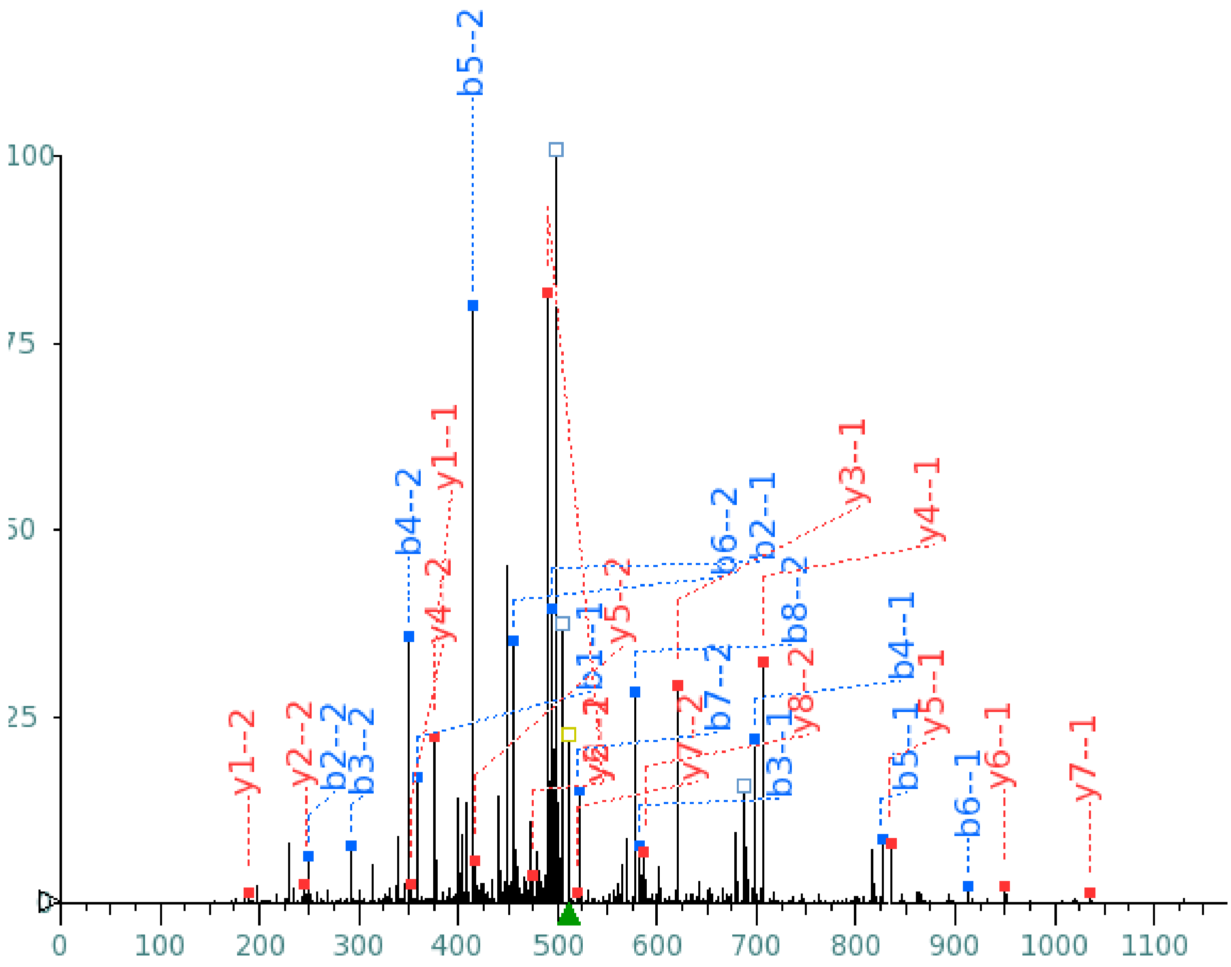

QHSDQSENK (+3)

Primary Reference: ZF64B_HUMAN
Search ID: 41868
Search Name: 20130330_ananiav_TMT_GPP_10percent_fraction4_lysC_2MC_IAA
Scan#: 2059
Observed Mass: 510.9362 (1.9 ppm)
PSM Score: 60.3

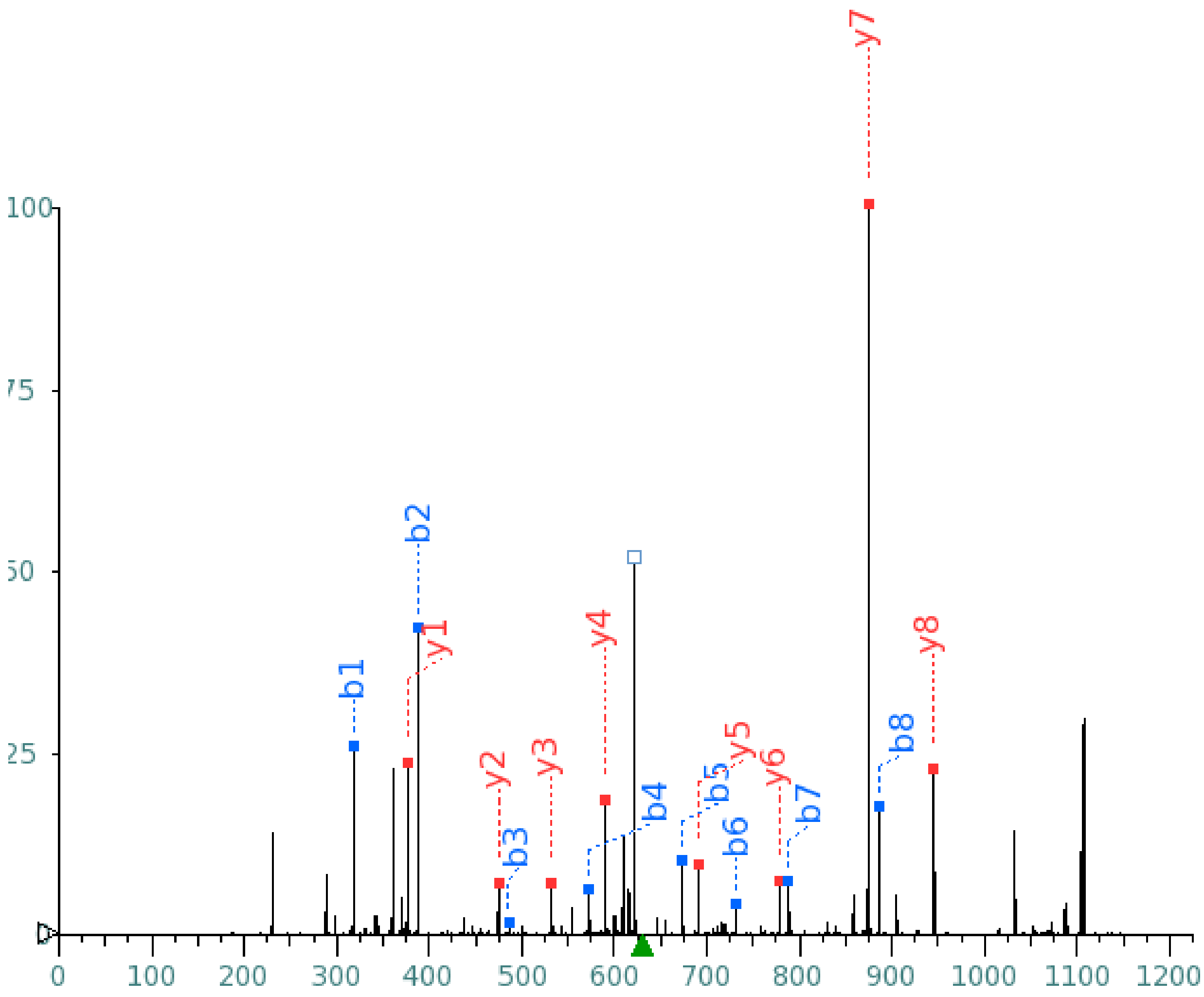

SAPSTGGVK (+2)

Primary Reference: H33_HUMAN
Search ID: 41868
Search Name: 20130330_ananiav_TMT_GPP_10percent_fraction4_lysC_2MC_IAA
Scan#: 6249
Observed Mass: 631.3806 (1.8 ppm)
PSM Score: 64.43

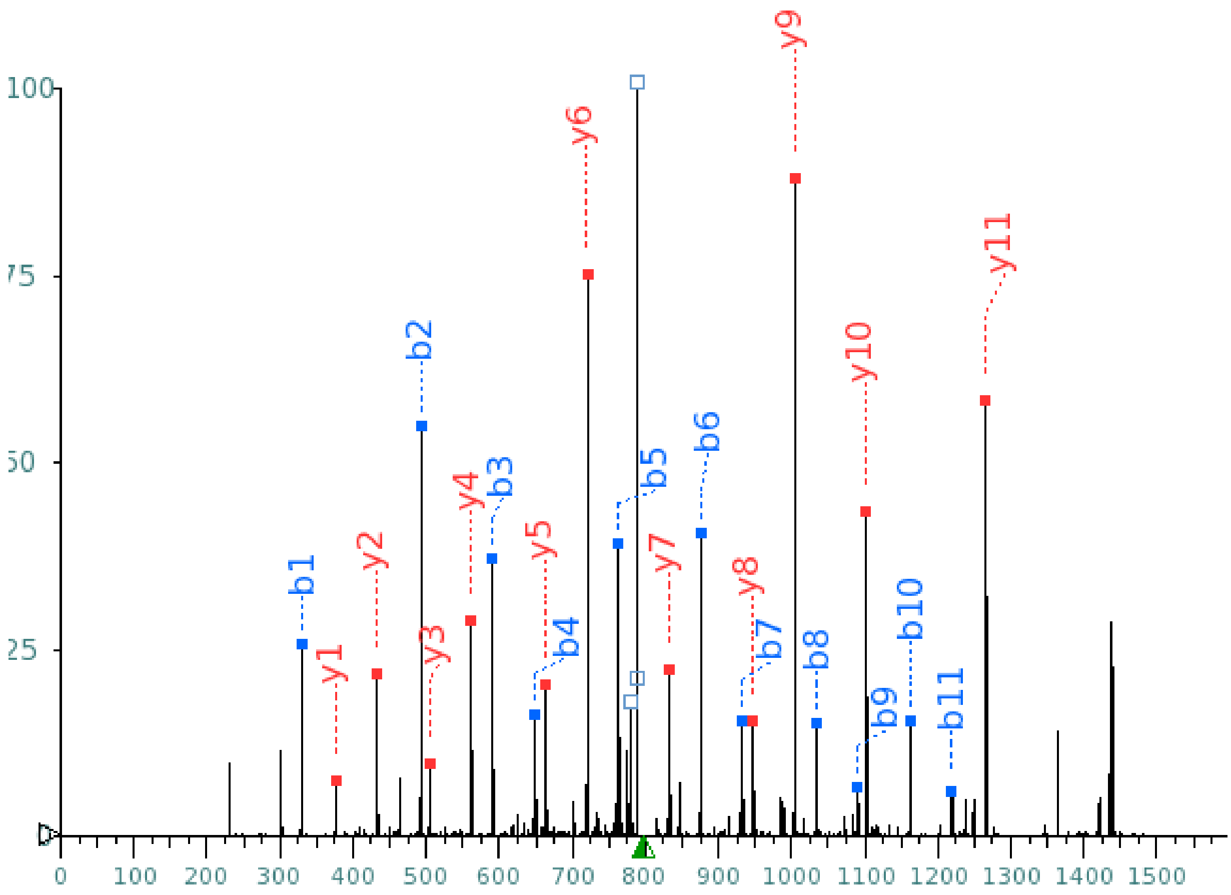

VYVGNLGTGAGK (+2)

Primary Reference: SRSF7_HUMAN
Search ID: 41868
Search Name: 20130330_ananiav_TMT_GPP_10percent_fraction4_lysC_2MC_IAA
Scan#: 13933
Observed Mass: 797.4731 (1.5 ppm)
PSM Score: 65.92

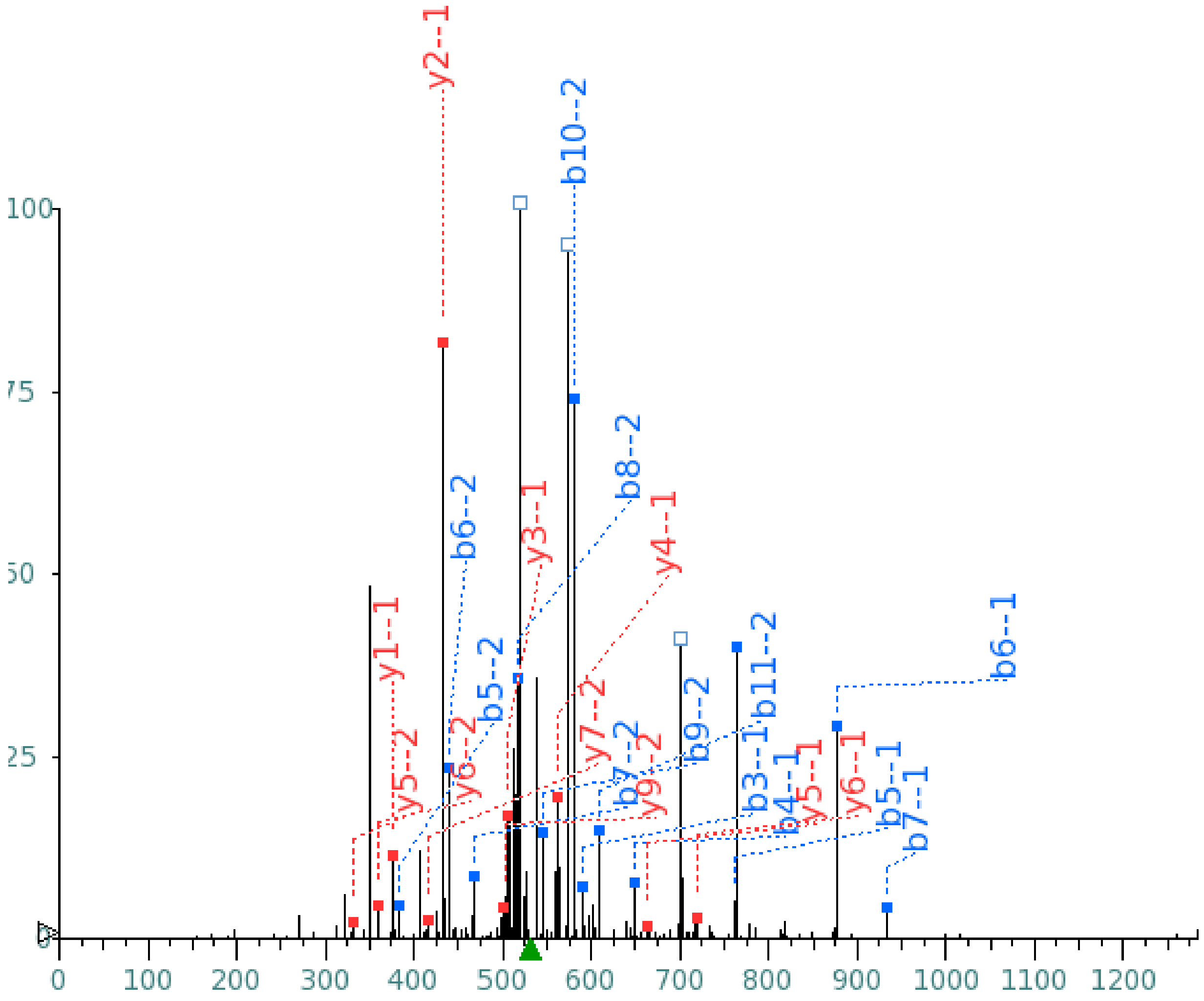

VYVGNLGTGAGK (+3)

Primary Reference: SRSF7_HUMAN
Search ID: 41868
Search Name: 20130330_ananiav_TMT_GPP_10percent_fraction4_lysC_2MC_IAA
Scan#: 13950
Observed Mass: 531.985 (2.5 ppm)
PSM Score: 30.96

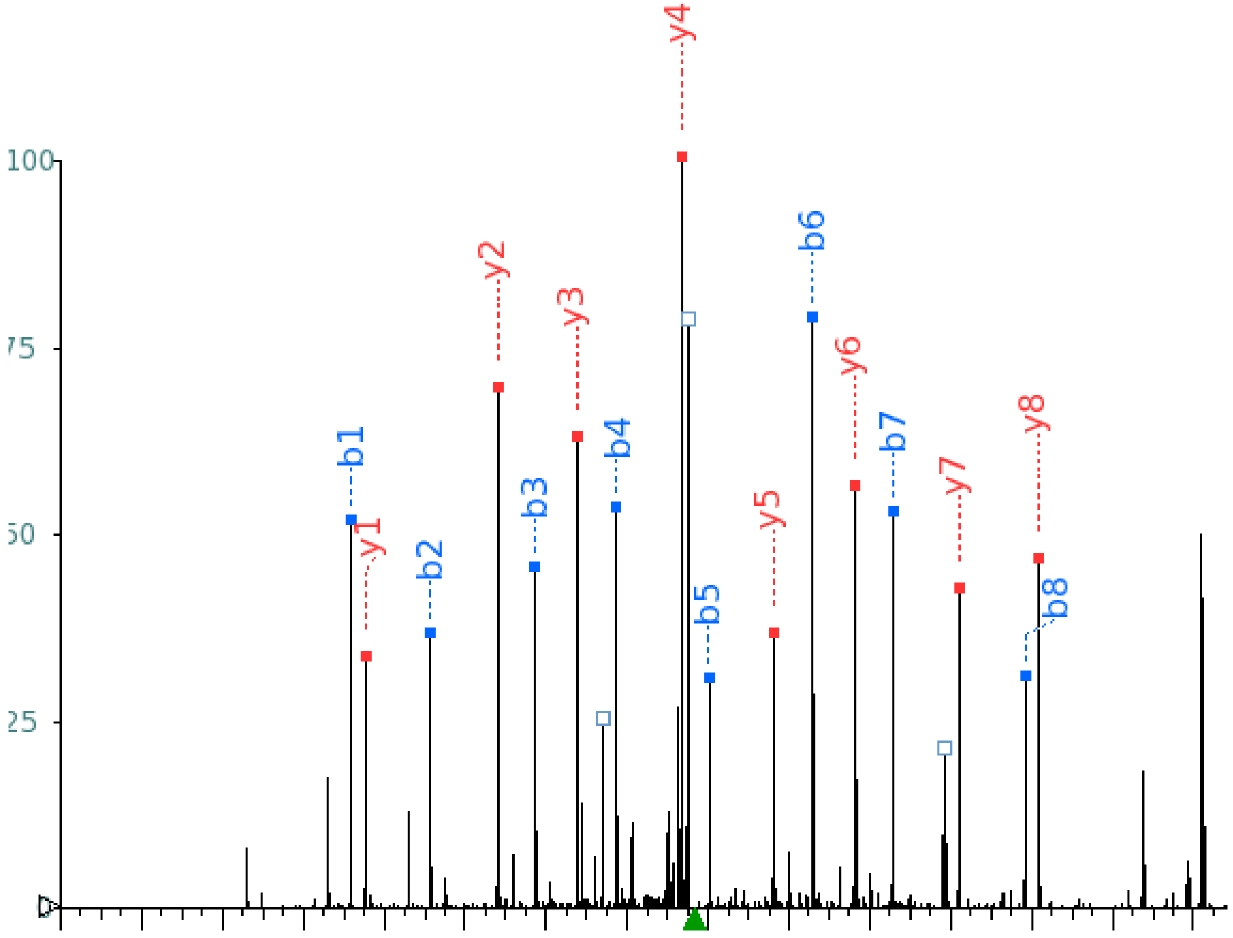

EVQTIEVYK (+2)

Primary Reference: THOC6_HUMAN
Search ID: 41868
Search Name: 20130330_ananiav_TMT_GPP_10percent_fraction4_lysC_2MC_IAA
Scan#: 14300
Observed Mass: 783.9628 (2.6 ppm)
PSM Score: 56.06

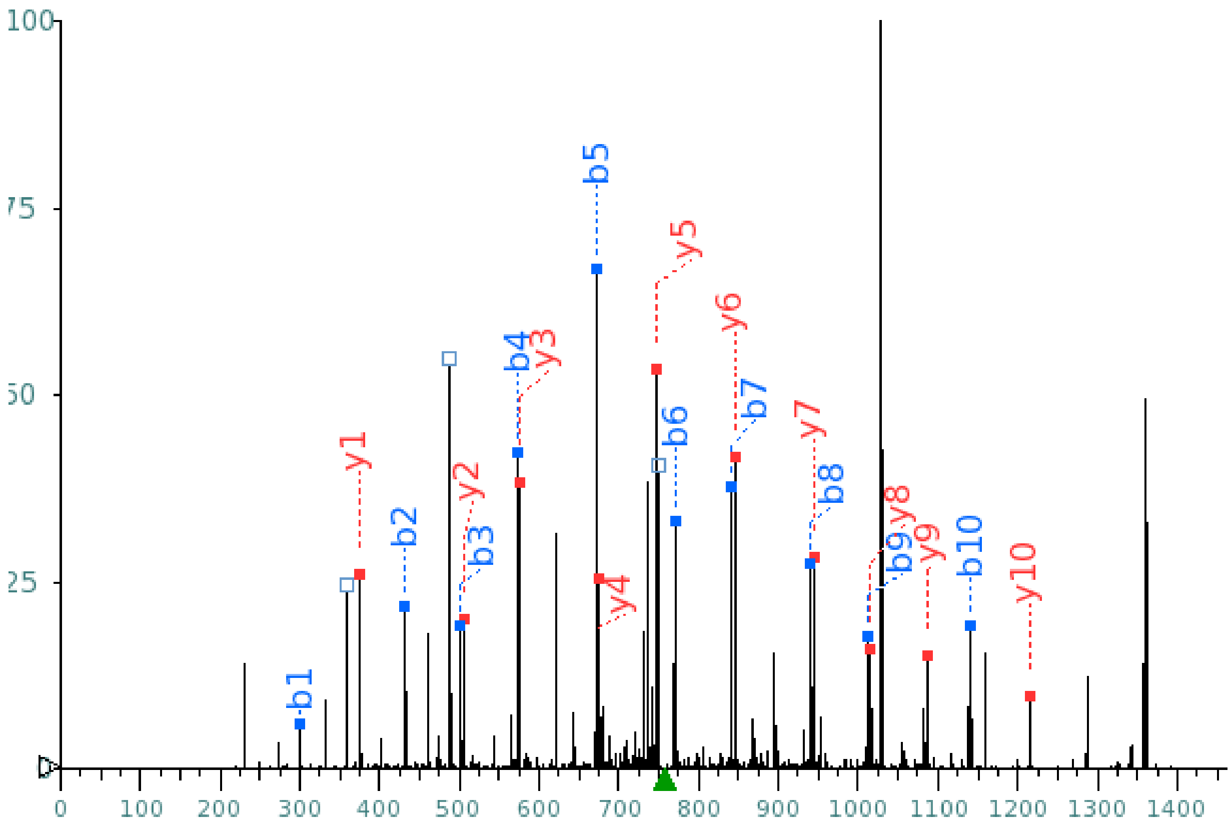

AEAAVVAVAEK (+2)

Primary Reference: SELH_HUMAN
Search ID: 41868
Search Name: 20130330_ananiav_TMT_GPP_10percent_fraction4_lysC_2MC_IAA
Scan#: 14755
Observed Mass: 758.4626 (2.2 ppm)
PSM Score: 56.83

File name: 2015-12-17 03.51.45PM.pdf

Creation time: 2015-12-17 03:53PM

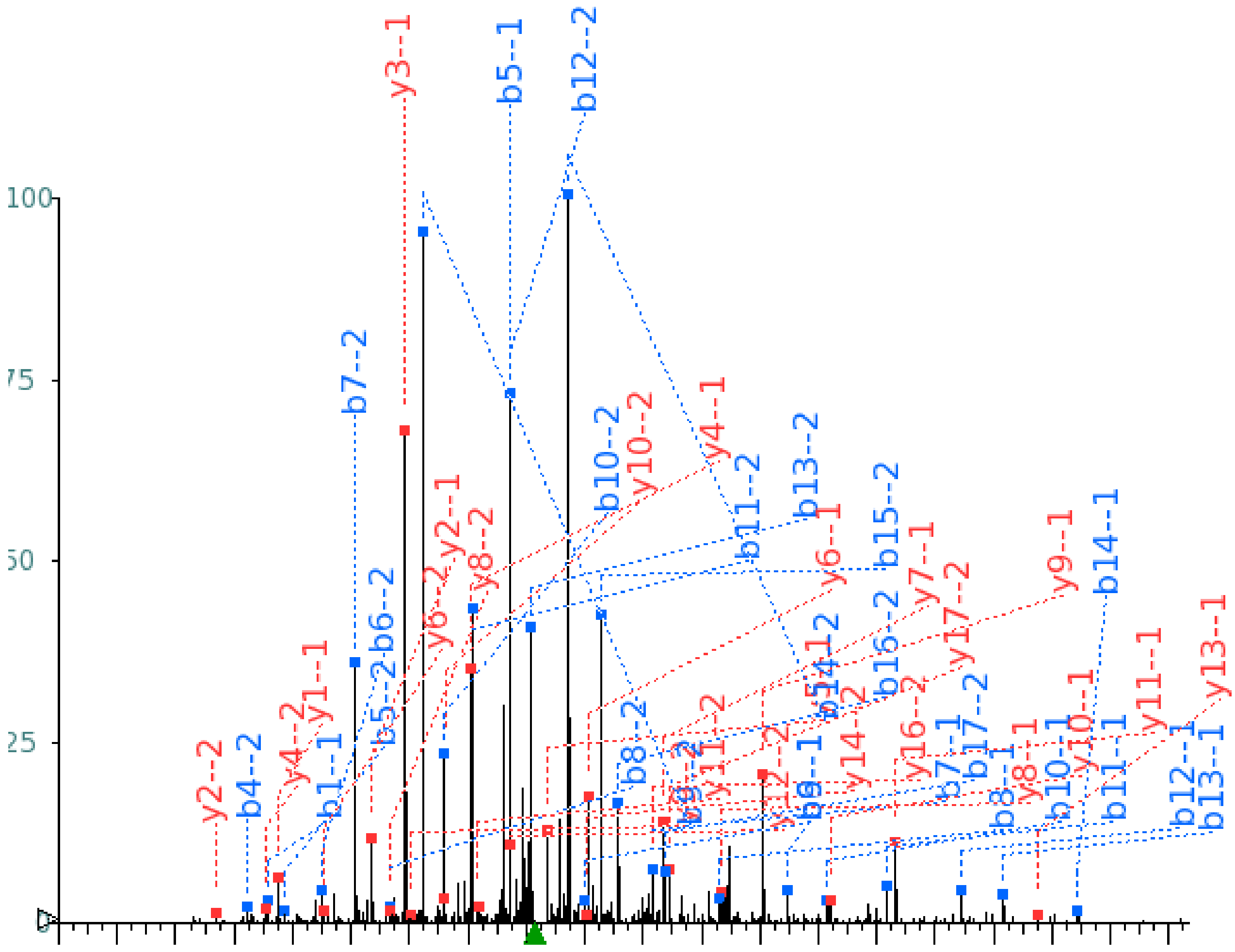

EGAREEDLDAVEAQIGC*K (+3)

Primary Reference: FBX3_HUMAN
Search ID: 41868
Search Name: 20130330_ananiav_TMT_GPP_10percent_fraction4_lysC_2MC_IAA
Scan#: 15727
Observed Mass: 816.7538 (1.2 ppm)
PSM Score: 75.95

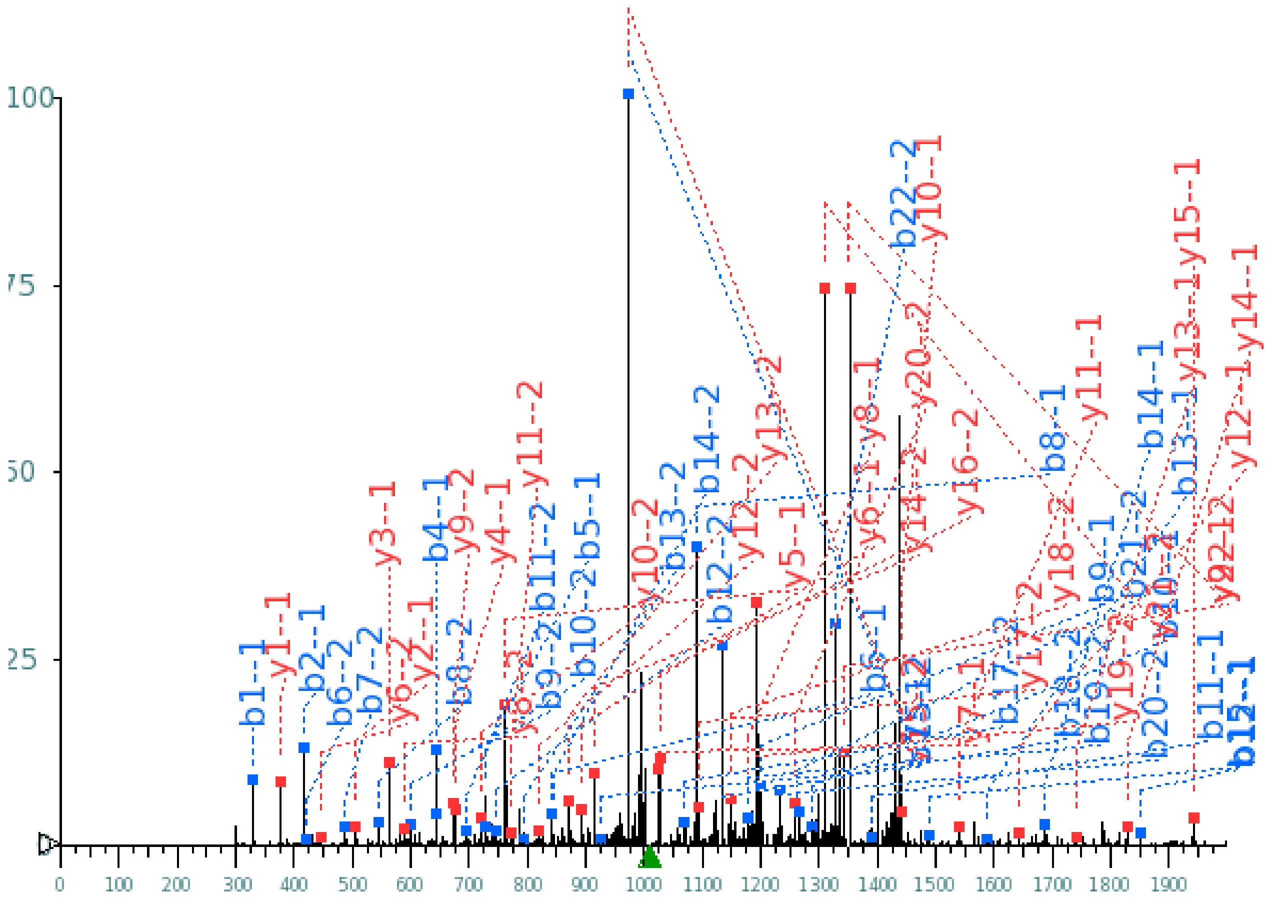

VSPESNEDISTTVVYRMESLGEK (+3)

Primary Reference: B8ZZA8_HUMAN

Search ID: 41868

Search Name: 20130330_ananiav_TMT_GPP_10percent_fraction4_lysC_2MC_IAA

Scan#: 21720

Observed Mass: 1010.1922 (2.4 ppm)

PSM Score: 70.16

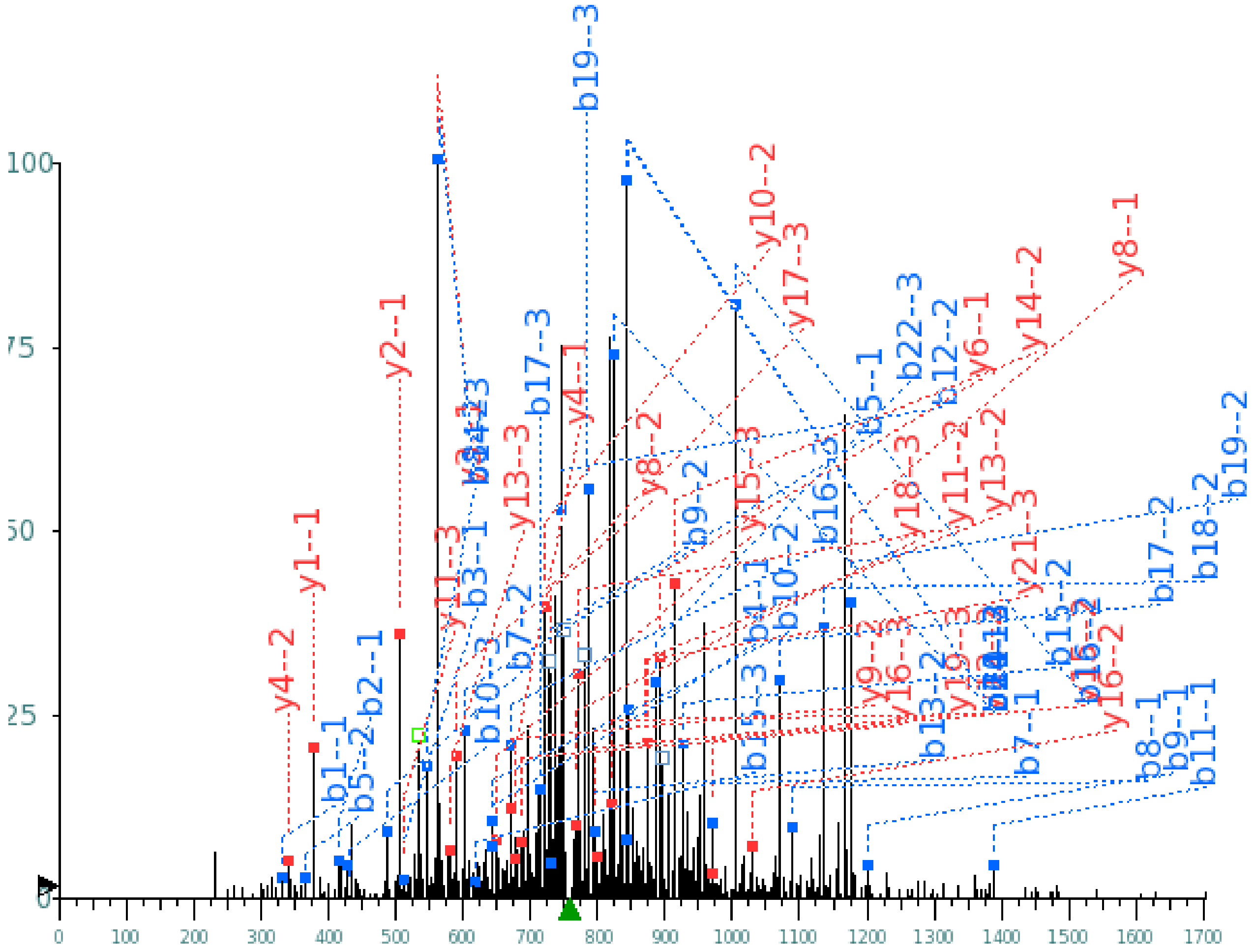

VSPESNEDISTTVVYRMESLGEK (+4)

Primary Reference: B8ZZA8_HUMAN
Search ID: 41868
Search Name: 20130330_ananiav_TMT_GPP_10percent_fraction4_lysC_2MC_IAA
Scan#: 21732
Observed Mass: 757.897 (3.7 ppm)
PSM Score: 25.01

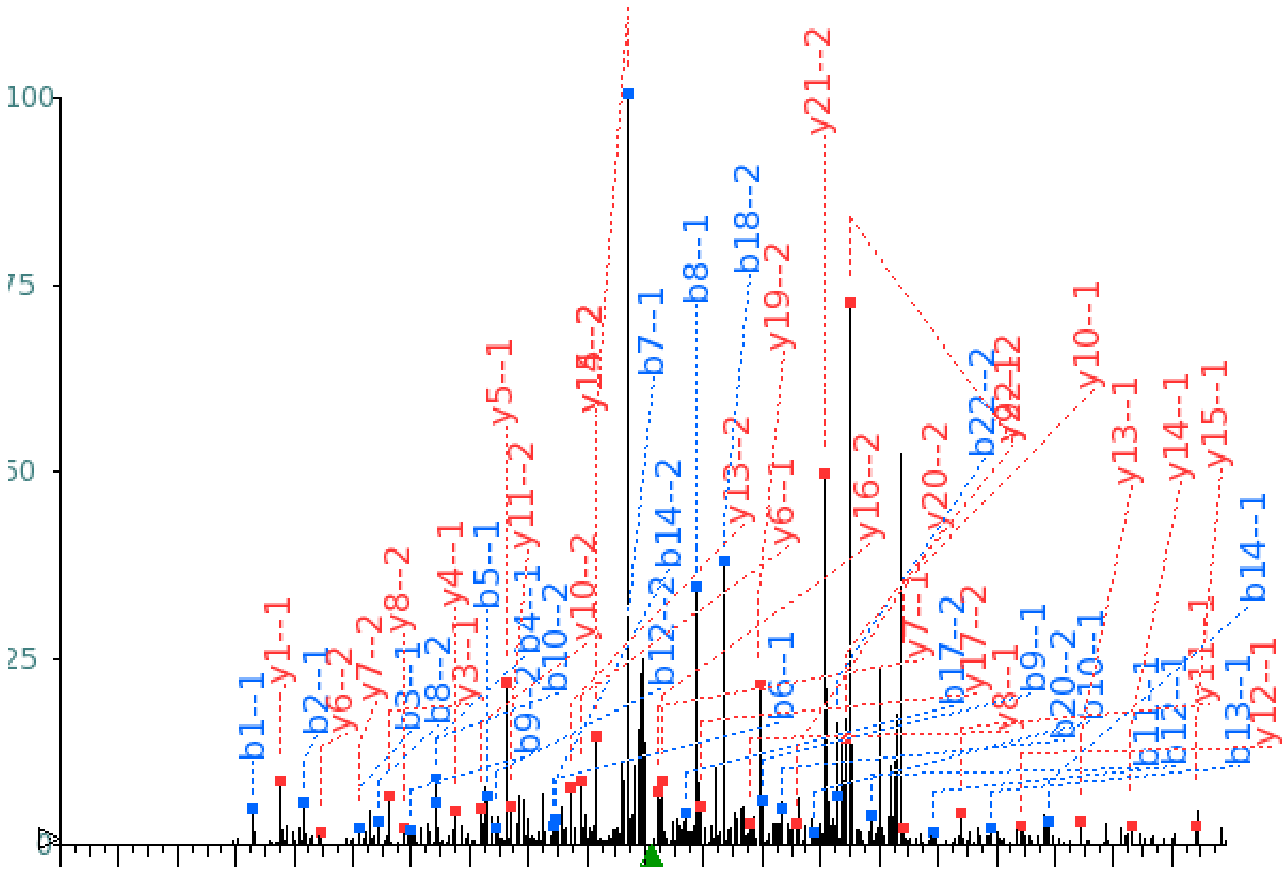

VSPESNEDISTTVVYRMESLGEK (+3)

Primary Reference: B8ZZA8_HUMAN
Search ID: 41868
Search Name: 20130330_ananiav_TMT_GPP_10percent_fraction4_lysC_2MC_IAA
Scan#: 21819
Observed Mass: 1010.1924 (2.6 ppm)
PSM Score: 40.73

File name: 2015-12-17 03.51.45PM.pdf

Creation time: 2015-12-17 03:53PM

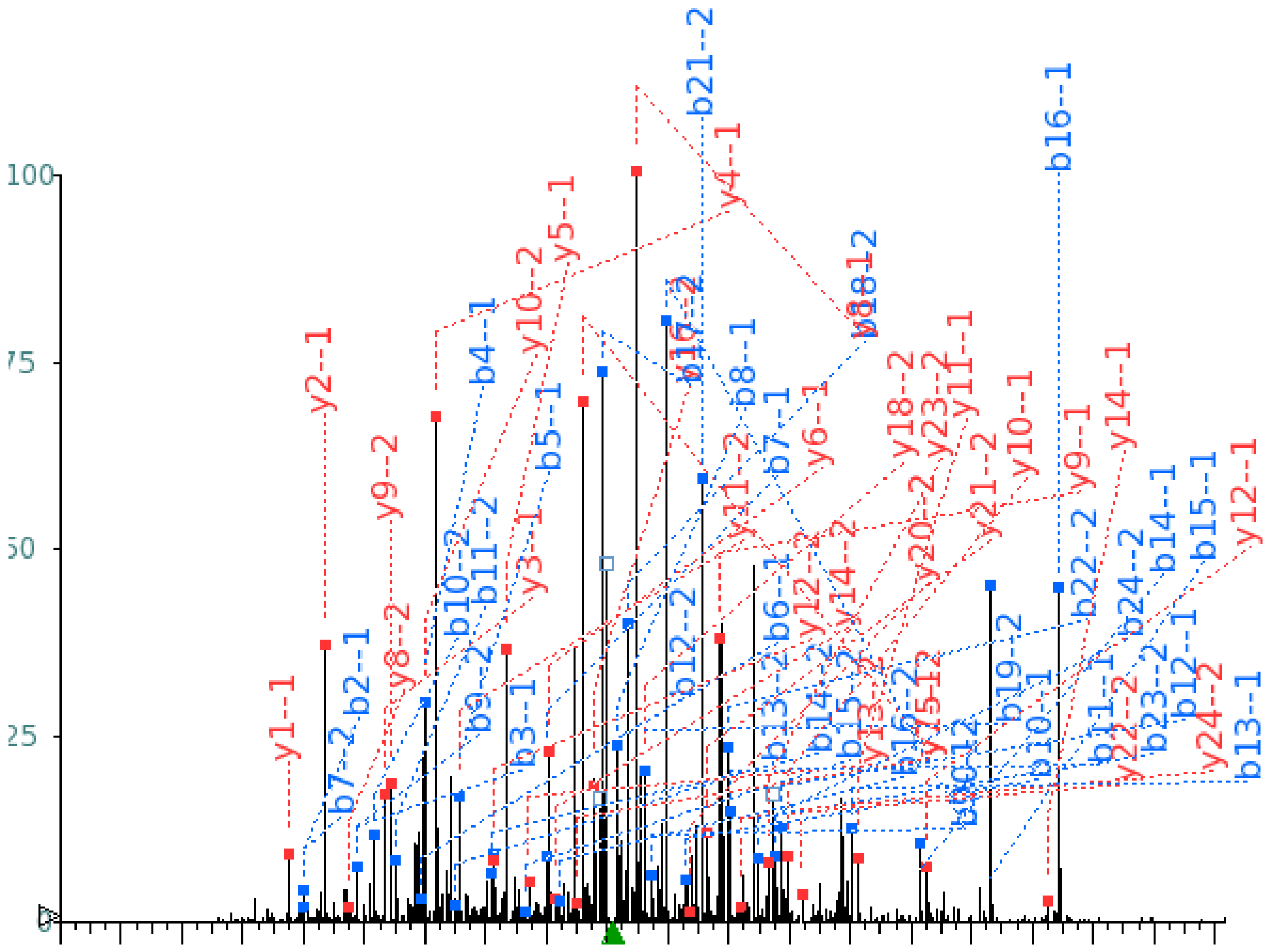

GLSLGGSDGGASILDLHSGALSVGK (+3)

Primary Reference: CQ101_HUMAN
Search ID: 41868
Search Name: 20130330_ananiav_TMT_GPP_10percent_fraction4_lysC_2MC_IAA
Scan#: 22337
Observed Mass: 909.5092 (1.6 ppm)
PSM Score: 79.87

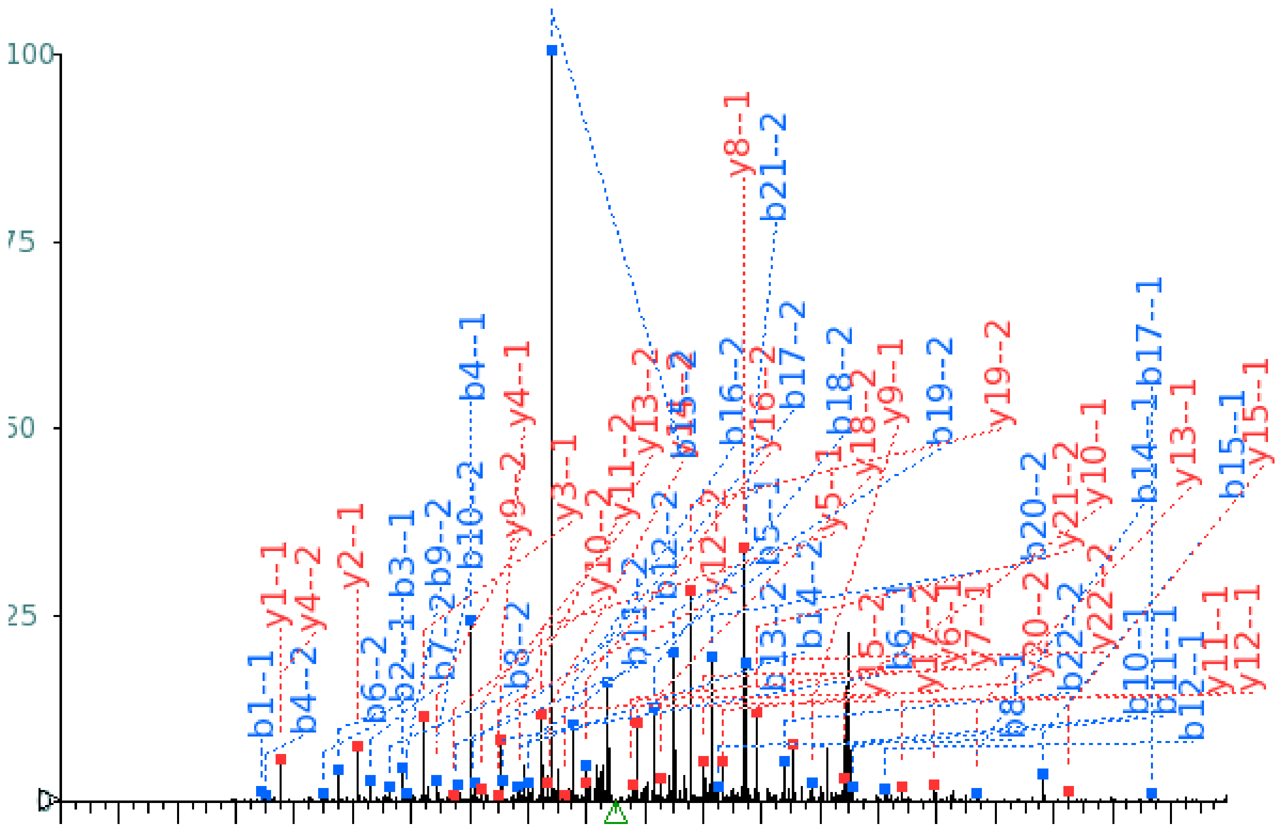

NEIDVVRAGTAGPGDALYAMLMK (+3)

Primary Reference: TR10A_HUMAN
Search ID: 41868
Search Name: 20130330_ananiav_TMT_GPP_10percent_fraction4_lysC_2MC_IAA
Scan#: 25984
Observed Mass: 950.8507 (4.2 ppm)
PSM Score: 69.08

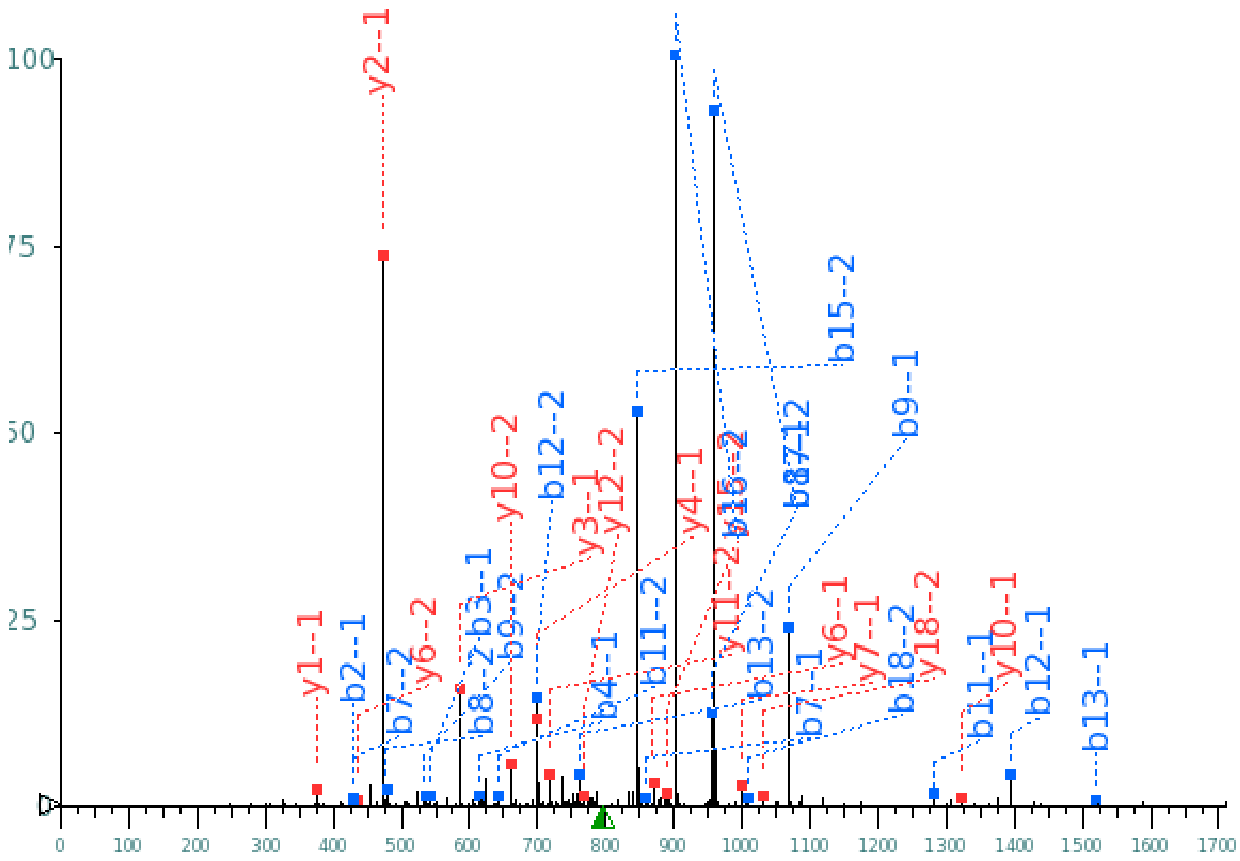

VTIAQGGVLPNIQAVLLPK (+3)

Primary Reference: H2A1D_HUMAN
Search ID: 41868
Search Name: 20130330_ananiav_TMT_GPP_10percent_fraction4_lysC_2MC_IAA
Scan#: 26466
Observed Mass: 797.1724 (3.4 ppm)
PSM Score: 39.02

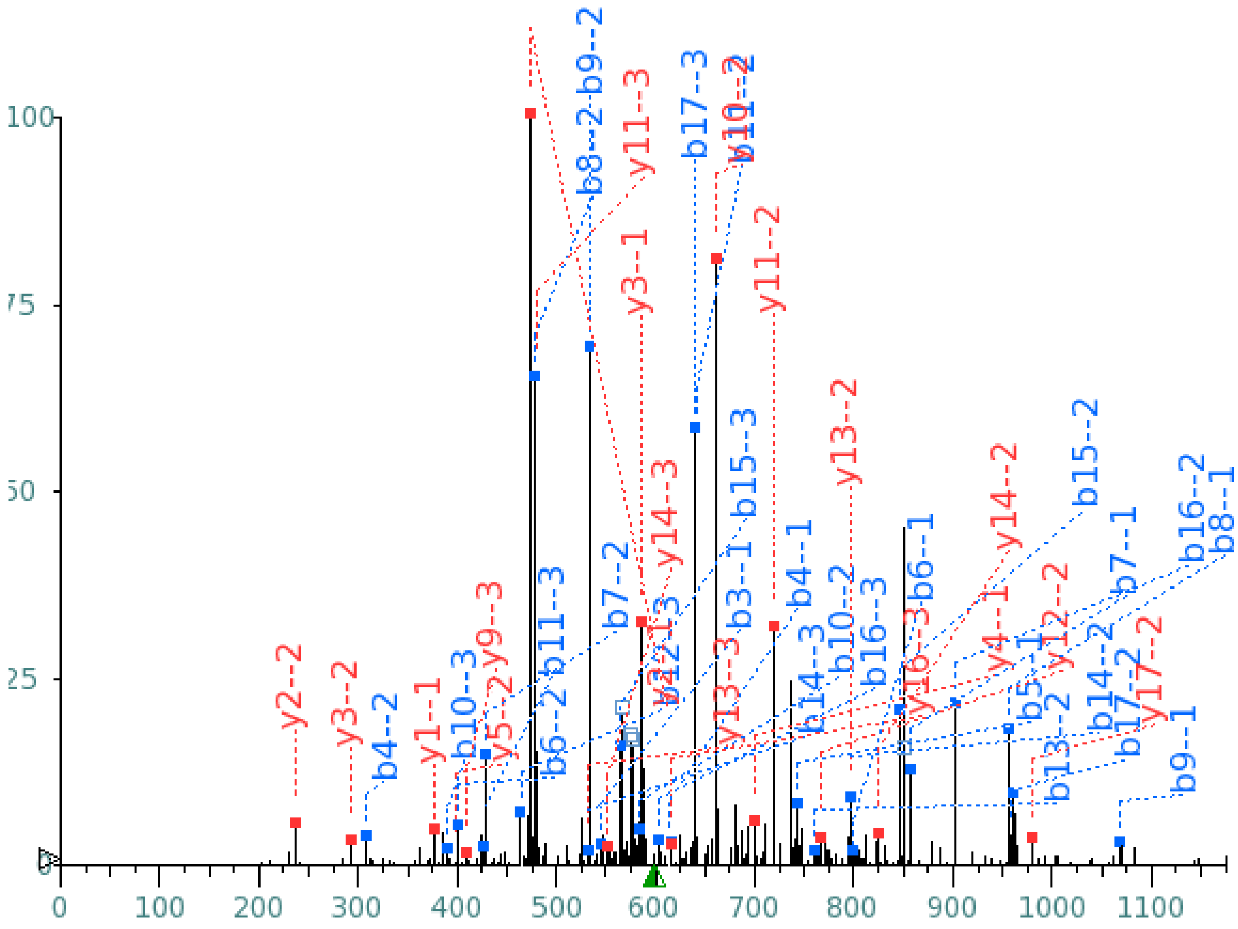

VTIAQGGVLPNIQAVLLPK (+4)

Primary Reference: H2A1D_HUMAN
Search ID: 41868
Search Name: 20130330_ananiav_TMT_GPP_10percent_fraction4_lysC_2MC_IAA
Scan#: 26470
Observed Mass: 598.1314 (3.8 ppm)
PSM Score: 51.64

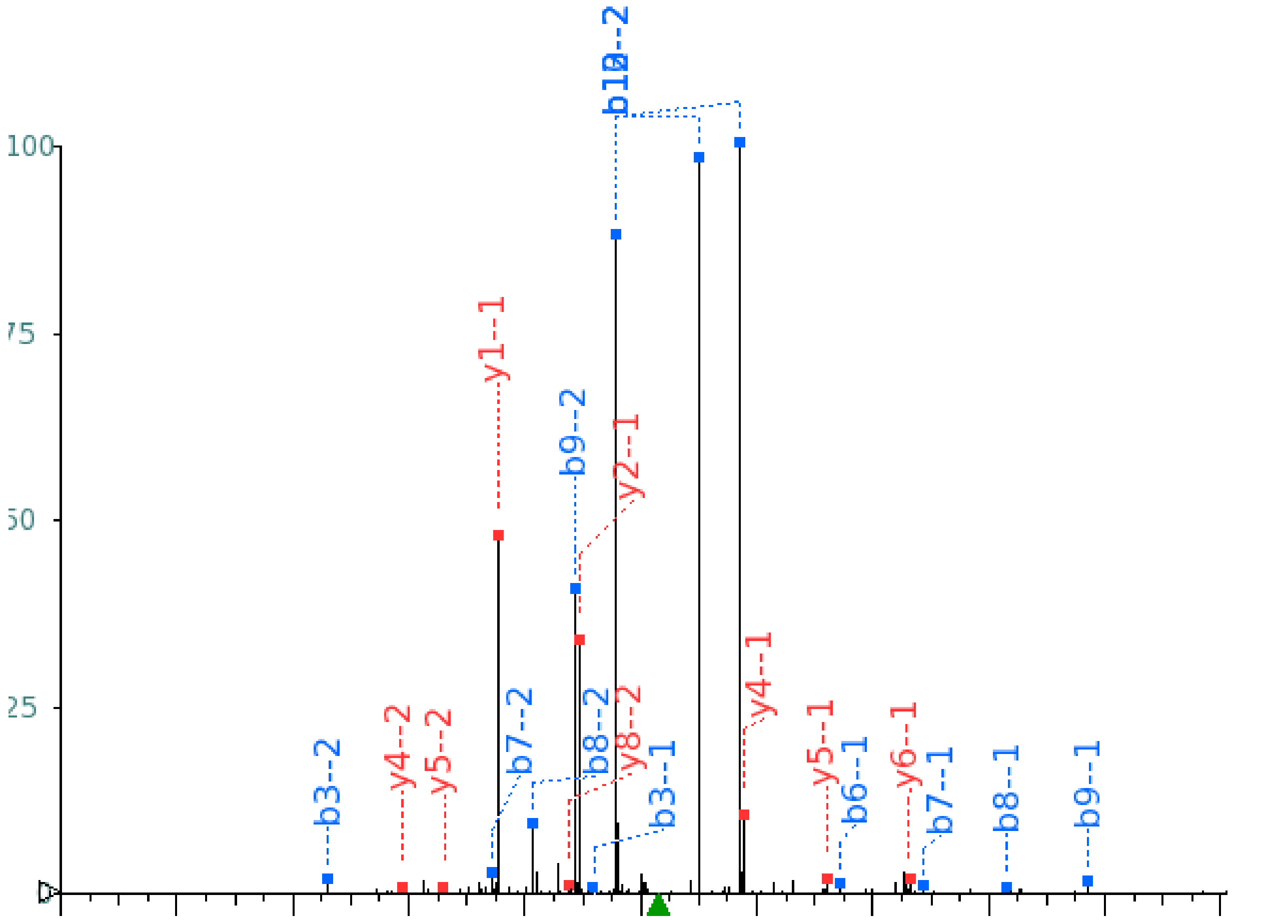

GTAAAAAAAAAAAK (+3)

Primary Reference: A8K3Q9_HUMAN
Search ID: 41869
Search Name: 20130330_ananiav_TMT_GPP_10percent_fraction5_lysC_2MC_IAA
Scan#: 12216
Observed Mass: 515.6449 (2.6 ppm)
PSM Score: 34.92

File name: 2015-12-17 03.51.45PM.pdf

Creation time: 2015-12-17 03:53PM

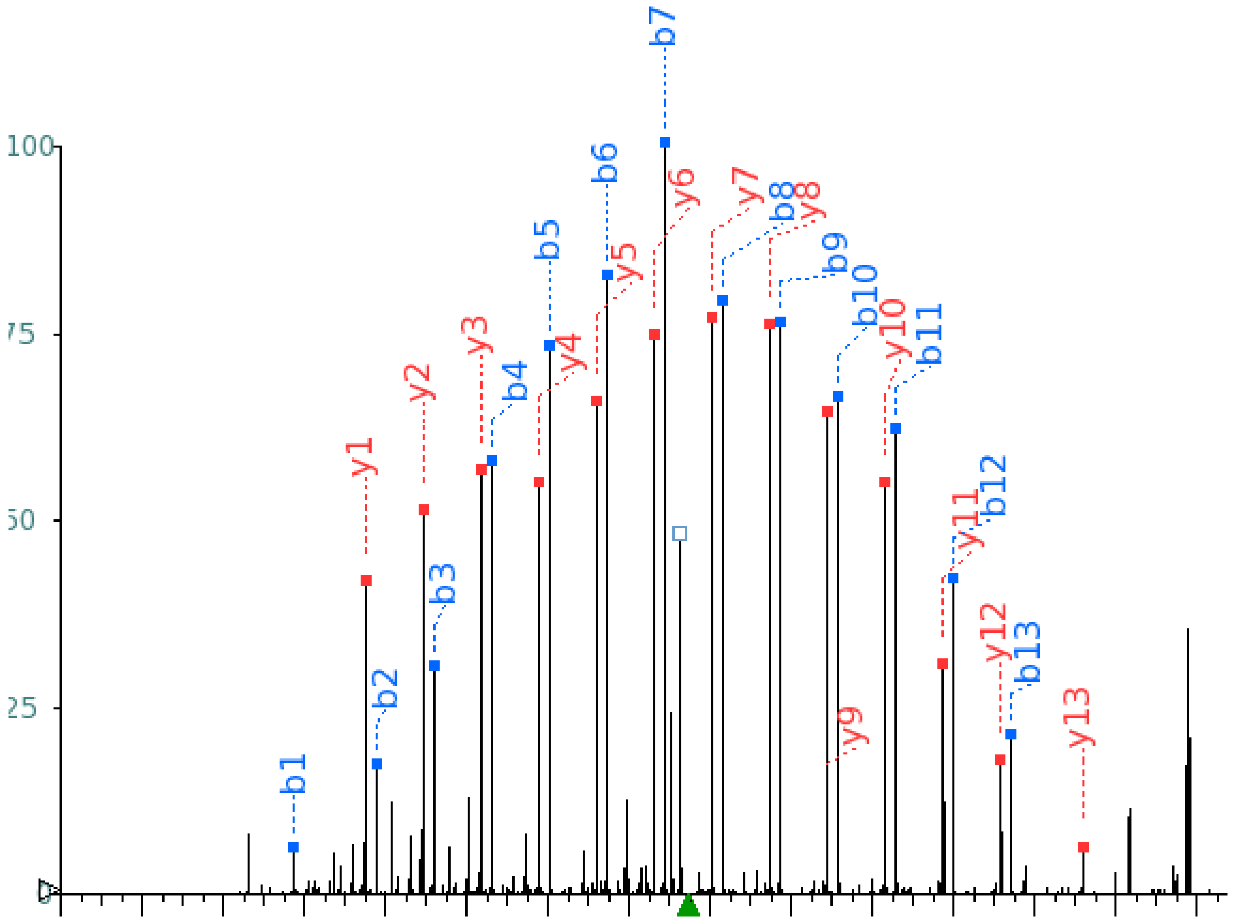

GTAAAAAAAAAAAK (+2)

Primary Reference: A8K3Q9_HUMAN
Search ID: 41869
Search Name: 20130330_ananiav_TMT_GPP_10percent_fraction5_lysC_2MC_IAA
Scan#: 12224
Observed Mass: 772.9629 (1.6 ppm)
PSM Score: 117.24

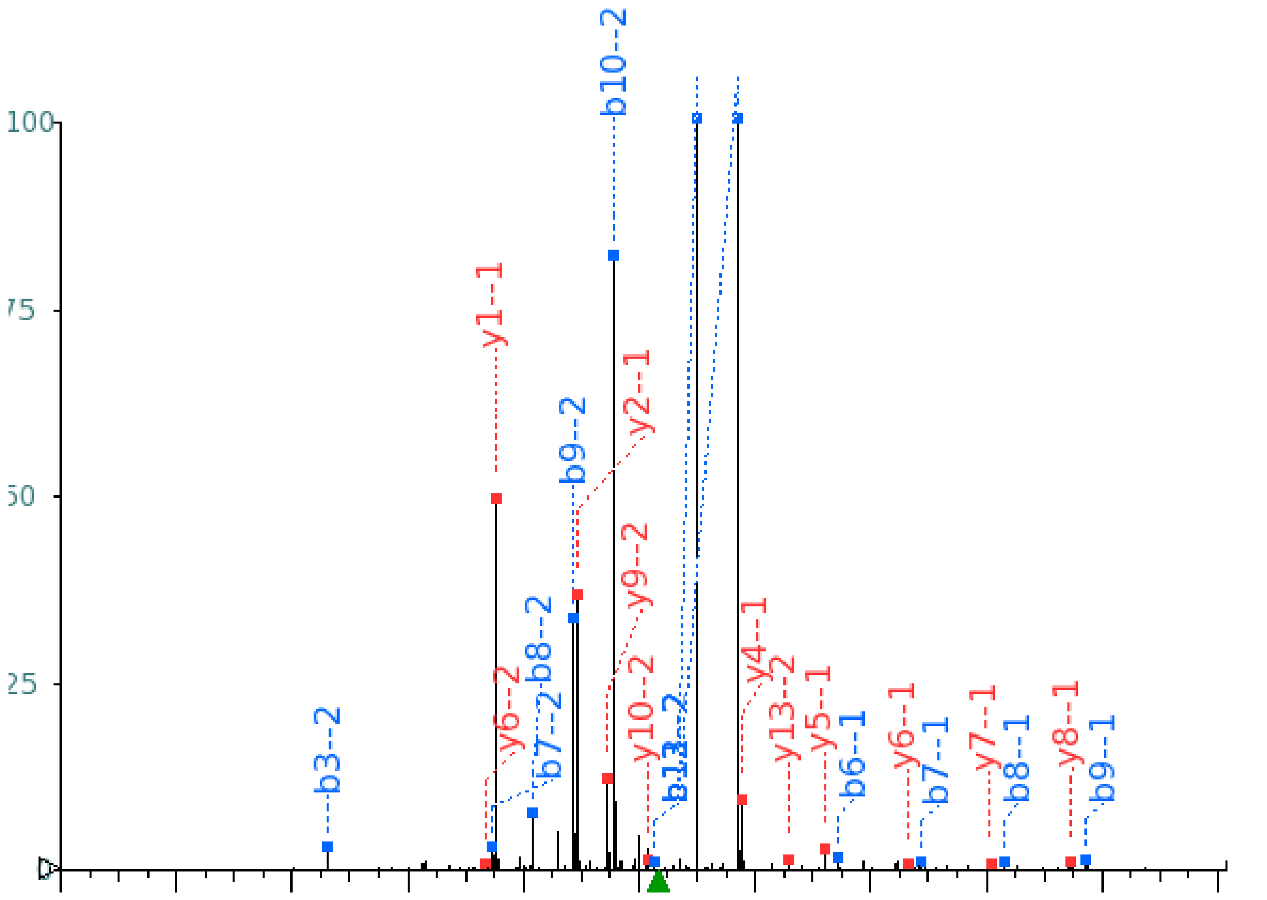

GTAAAAAAAAAAAK (+3)

Primary Reference: A8K3Q9_HUMAN
Search ID: 41869
Search Name: 20130330_ananiav_TMT_GPP_10percent_fraction5_lysC_2MC_IAA
Scan#: 12321
Observed Mass: 515.6449 (2.7 ppm)
PSM Score: 39.17

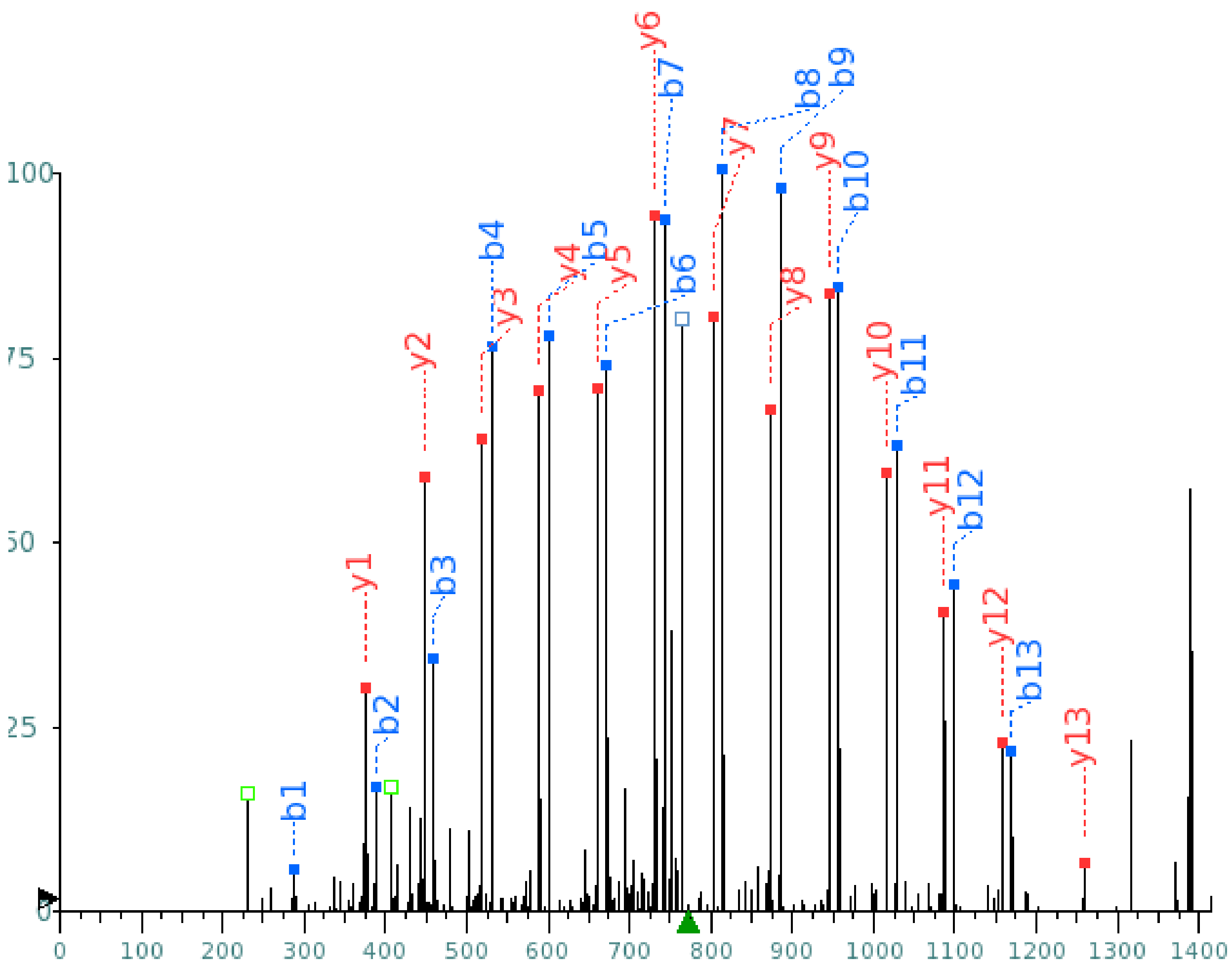

GTAAAAAAAAAAAK (+2)

Primary Reference: A8K3Q9_HUMAN
Search ID: 41869
Search Name: 20130330_ananiav_TMT_GPP_10percent_fraction5_lysC_2MC_IAA
Scan#: 12327
Observed Mass: 772.9629 (1.7 ppm)
PSM Score: 117.8

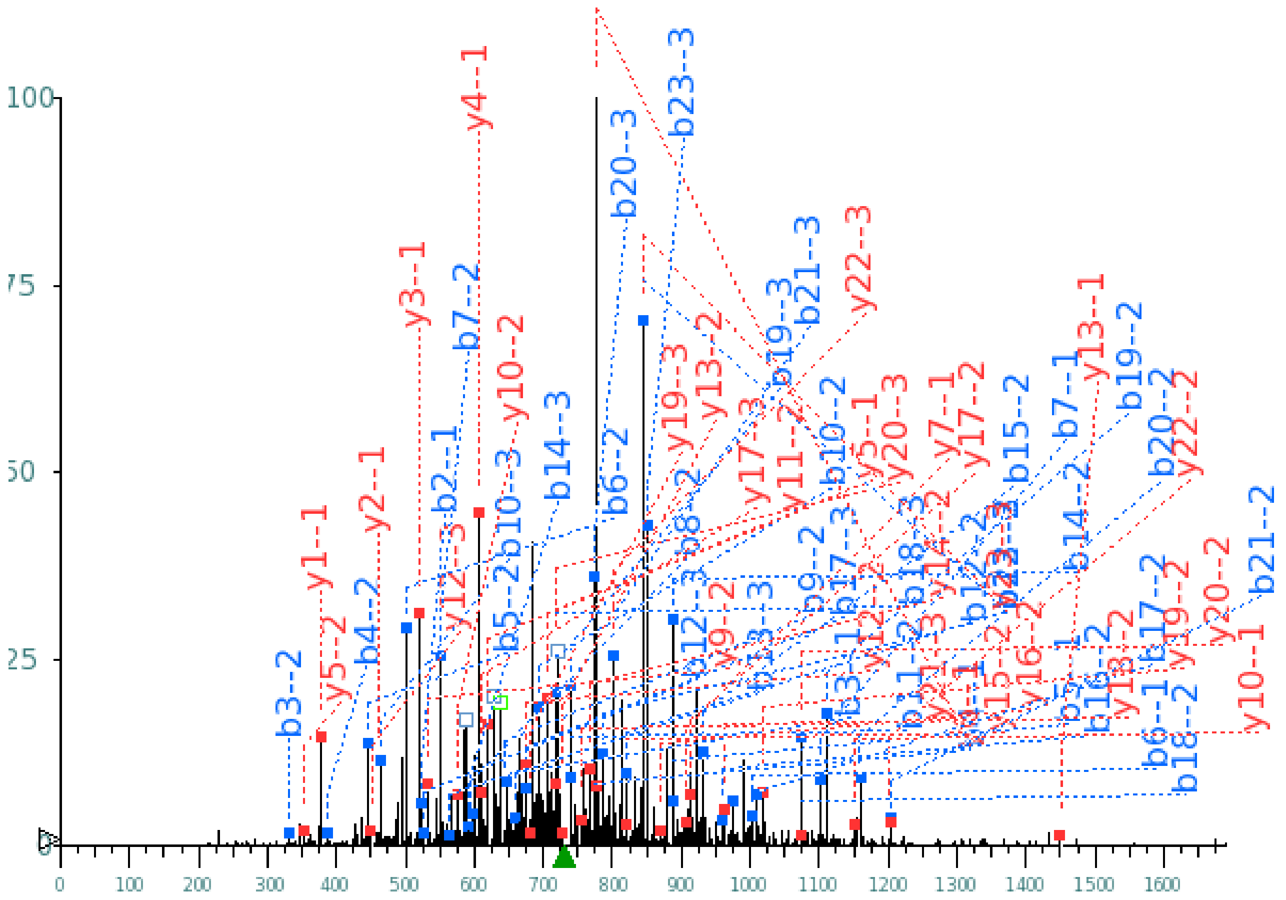

YEHDDIVSTVSVLSSGTQAVSGSK (+4)

Primary Reference: MEP50_HUMAN
Search ID: 41869
Search Name: 20130330_ananiav_TMT_GPP_10percent_fraction5_lysC_2MC_IAA
Scan#: 19381
Observed Mass: 731.8893 (3.5 ppm)
PSM Score: 69.66

File name: 2015-12-17 03.51.45PM.pdf

Creation time: 2015-12-17 03:53PM

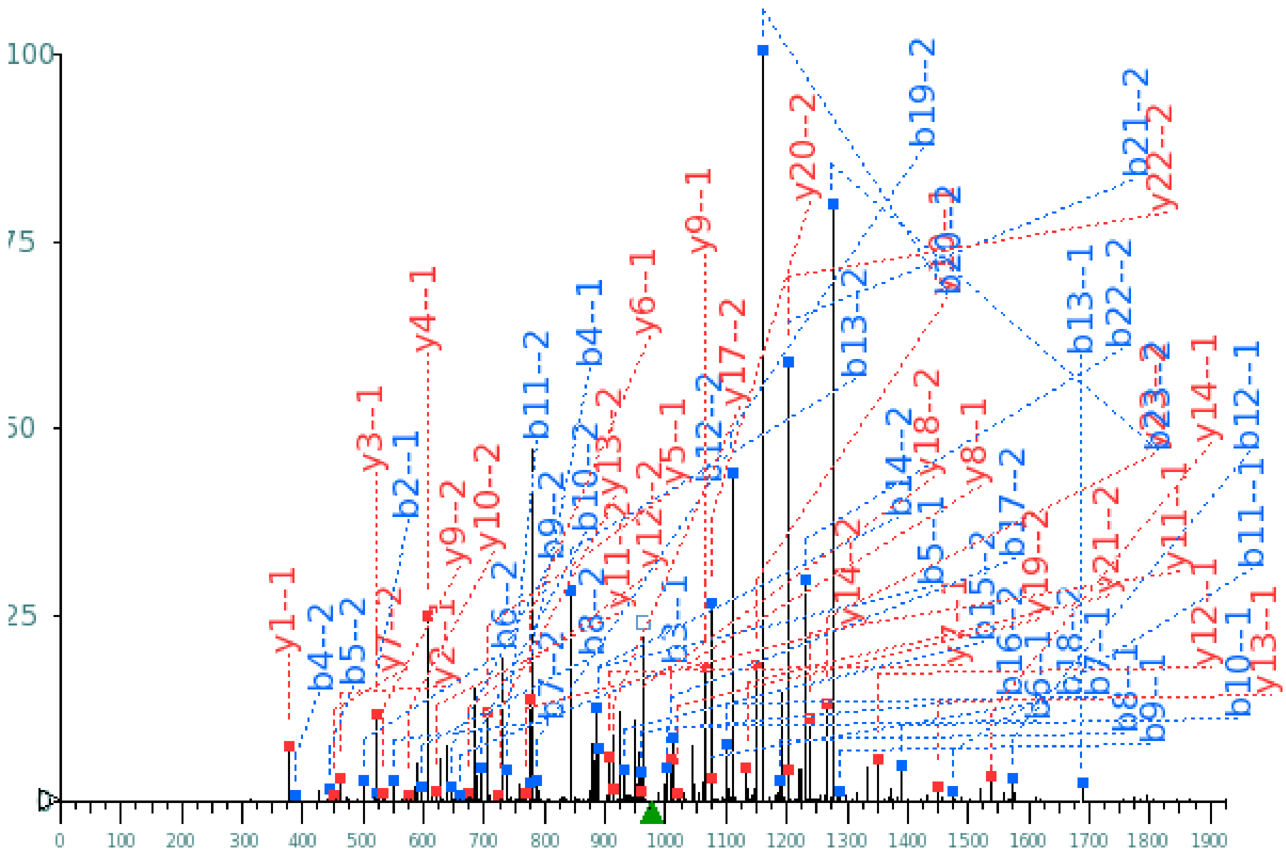

YEHDDIVSTVSVLSSGTQAVSGSK (+3)

Primary Reference: MEP50_HUMAN
Search ID: 41869
Search Name: 20130330_ananiav_TMT_GPP_10percent_fraction5_lysC_2MC_IAA
Scan#: 19383
Observed Mass: 975.5158 (2.5 ppm)
PSM Score: 109.83

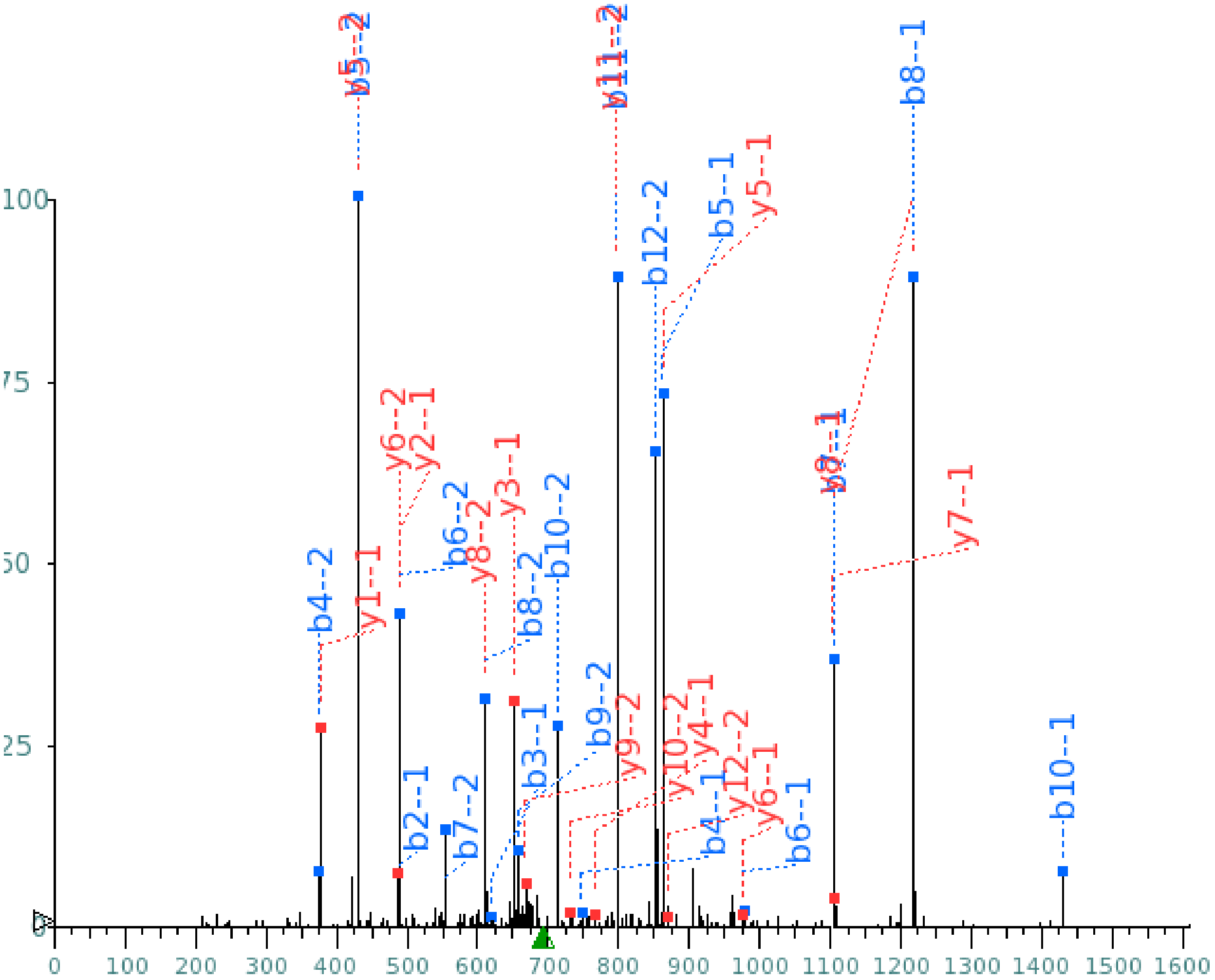

LFQEDDEIPLYLK (+3)

Primary Reference: CX7A2_HUMAN
Search ID: 41869
Search Name: 20130330_ananiav_TMT_GPP_10percent_fraction5_lysC_2MC_IAA
Scan#: 23384
Observed Mass: 694.3932 (3.8 ppm)
PSM Score: 60.68

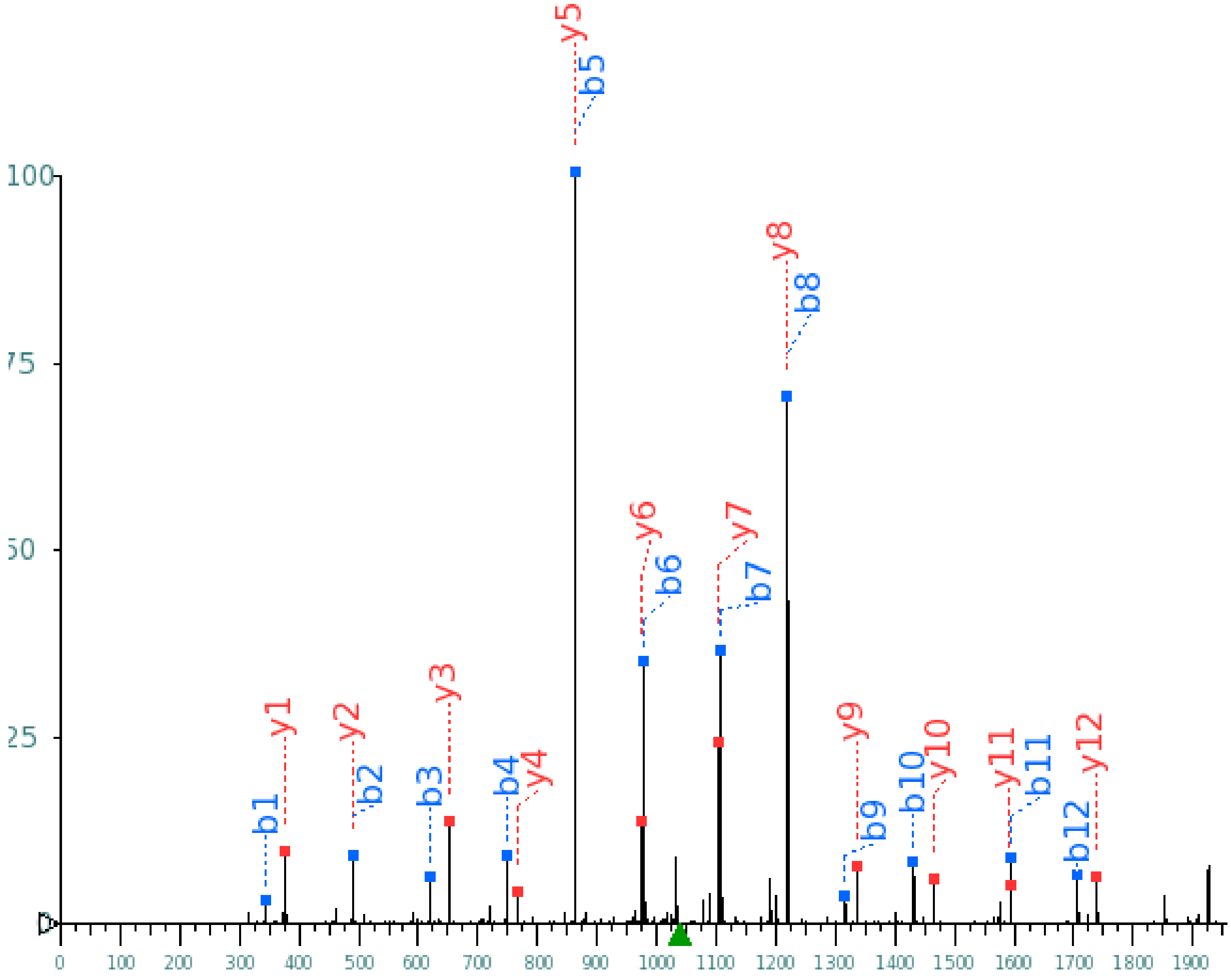

LFQEDDEIPLYLK (+2)

Primary Reference: CX7A2_HUMAN
Search ID: 41869
Search Name: 20130330_ananiav_TMT_GPP_10percent_fraction5_lysC_2MC_IAA
Scan#: 23411
Observed Mass: 1041.0849 (2.6 ppm)
PSM Score: 71

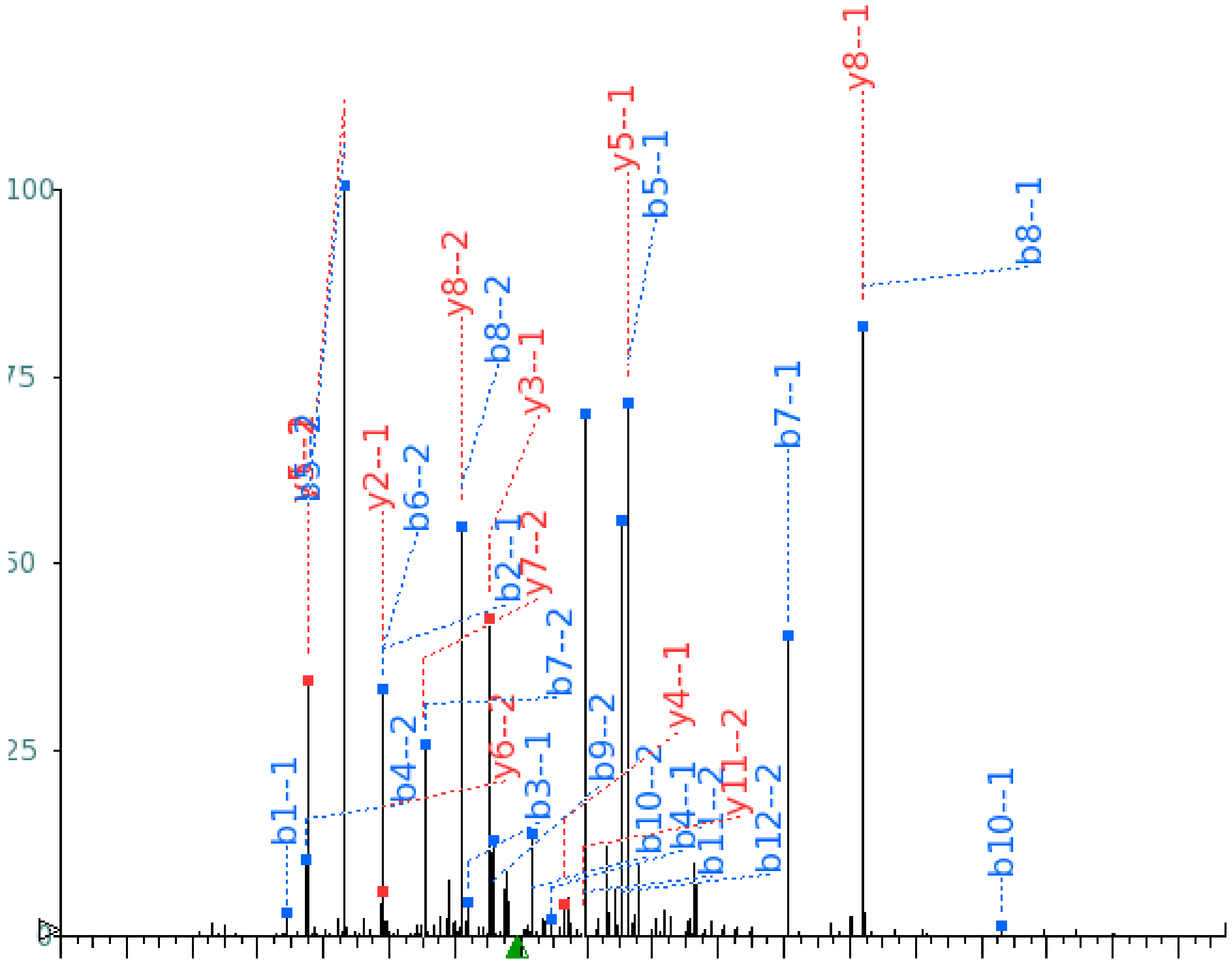

LFQEDDEIPLYLK (+3)

Primary Reference: CX7A2_HUMAN
Search ID: 41869
Search Name: 20130330_ananiav_TMT_GPP_10percent_fraction5_lysC_2MC_IAA
Scan#: 23467
Observed Mass: 694.3933 (4 ppm)
PSM Score: 56.83

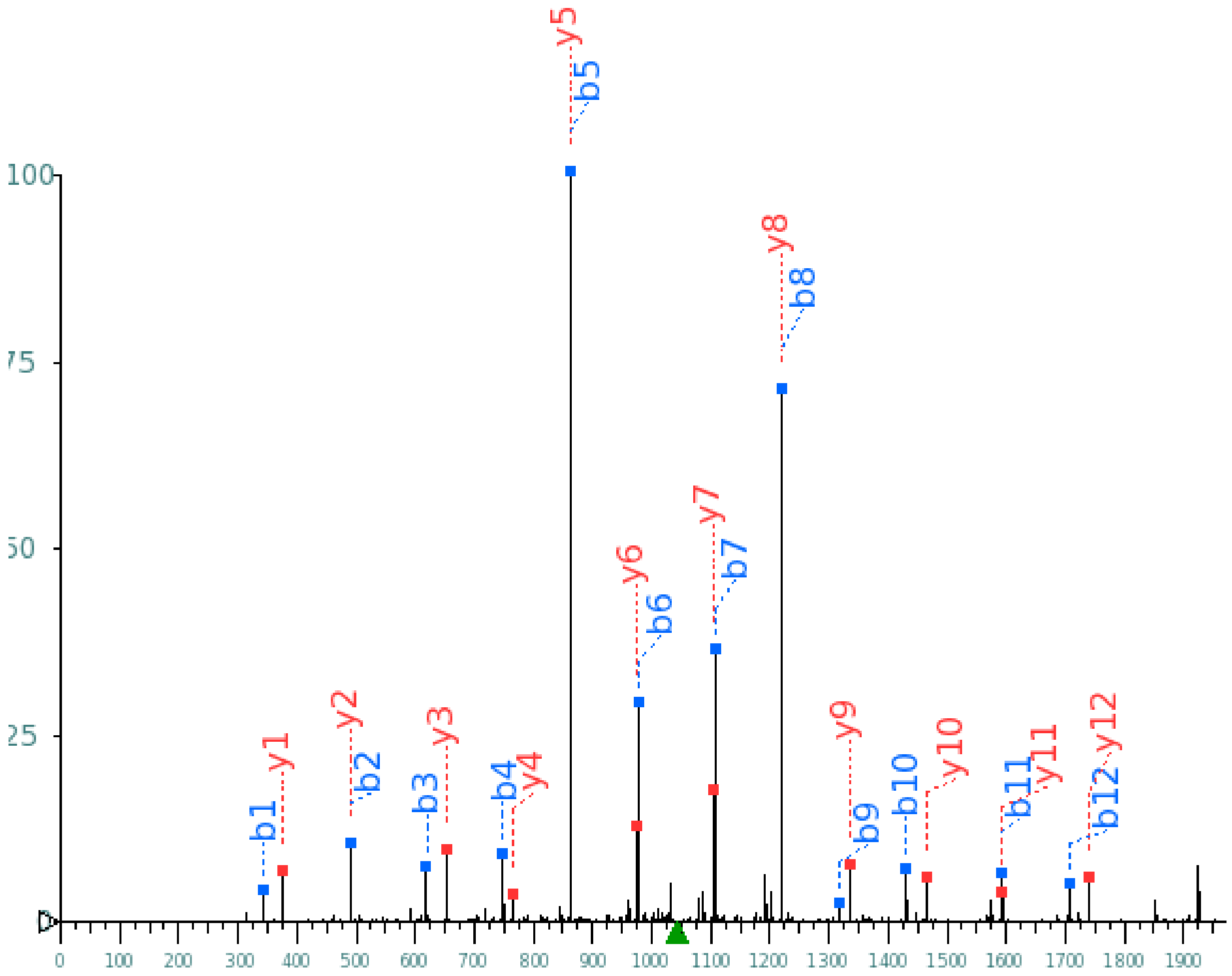

LFQEDDEIPLYLK (+2)

Primary Reference: CX7A2_HUMAN
Search ID: 41869
Search Name: 20130330_ananiav_TMT_GPP_10percent_fraction5_lysC_2MC_IAA
Scan#: 23504
Observed Mass: 1041.0851 (2.8 ppm)
PSM Score: 89.85

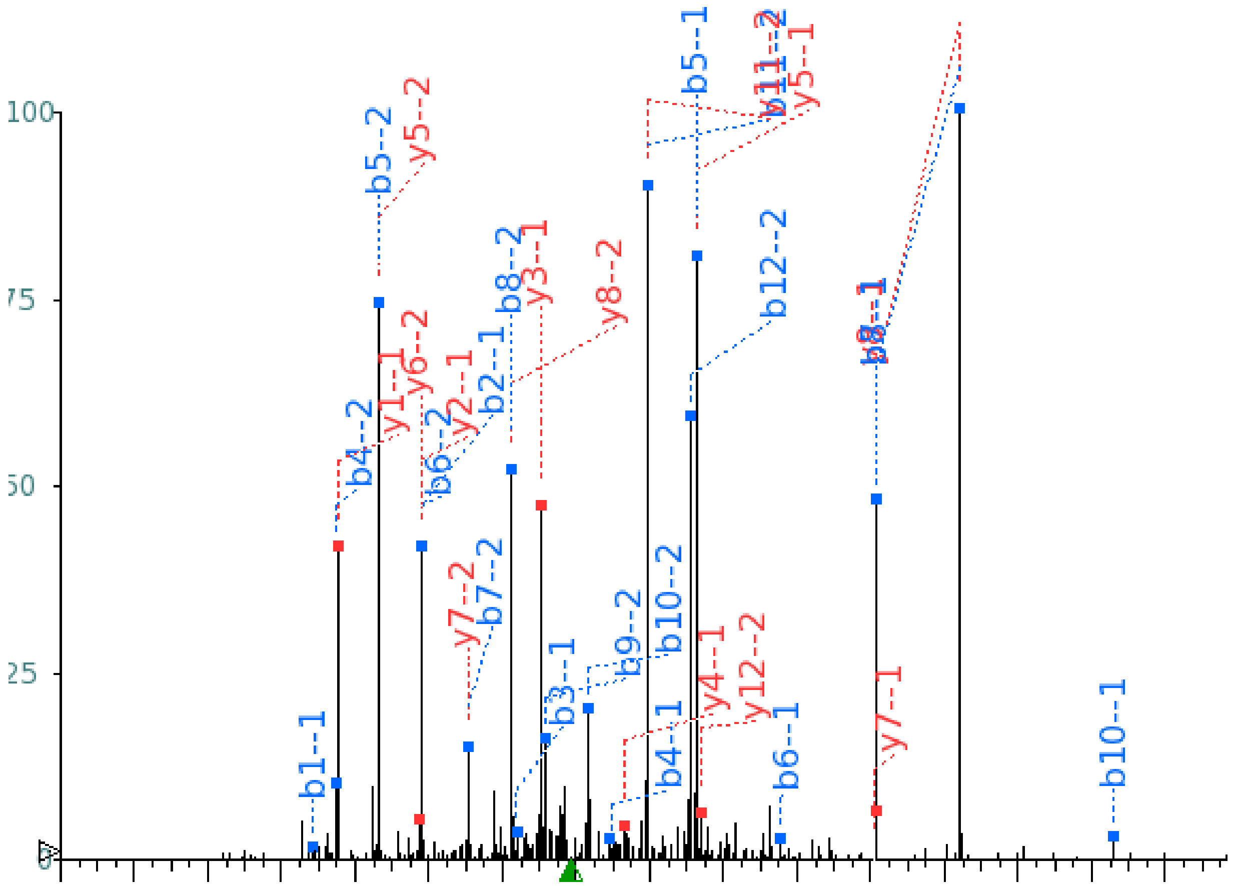

LFQEDDEIPLYLK (+3)

Primary Reference: CX7A2_HUMAN
Search ID: 41869
Search Name: 20130330_ananiav_TMT_GPP_10percent_fraction5_lysC_2MC_IAA
Scan#: 23549
Observed Mass: 694.3933 (3.9 ppm)
PSM Score: 44.94

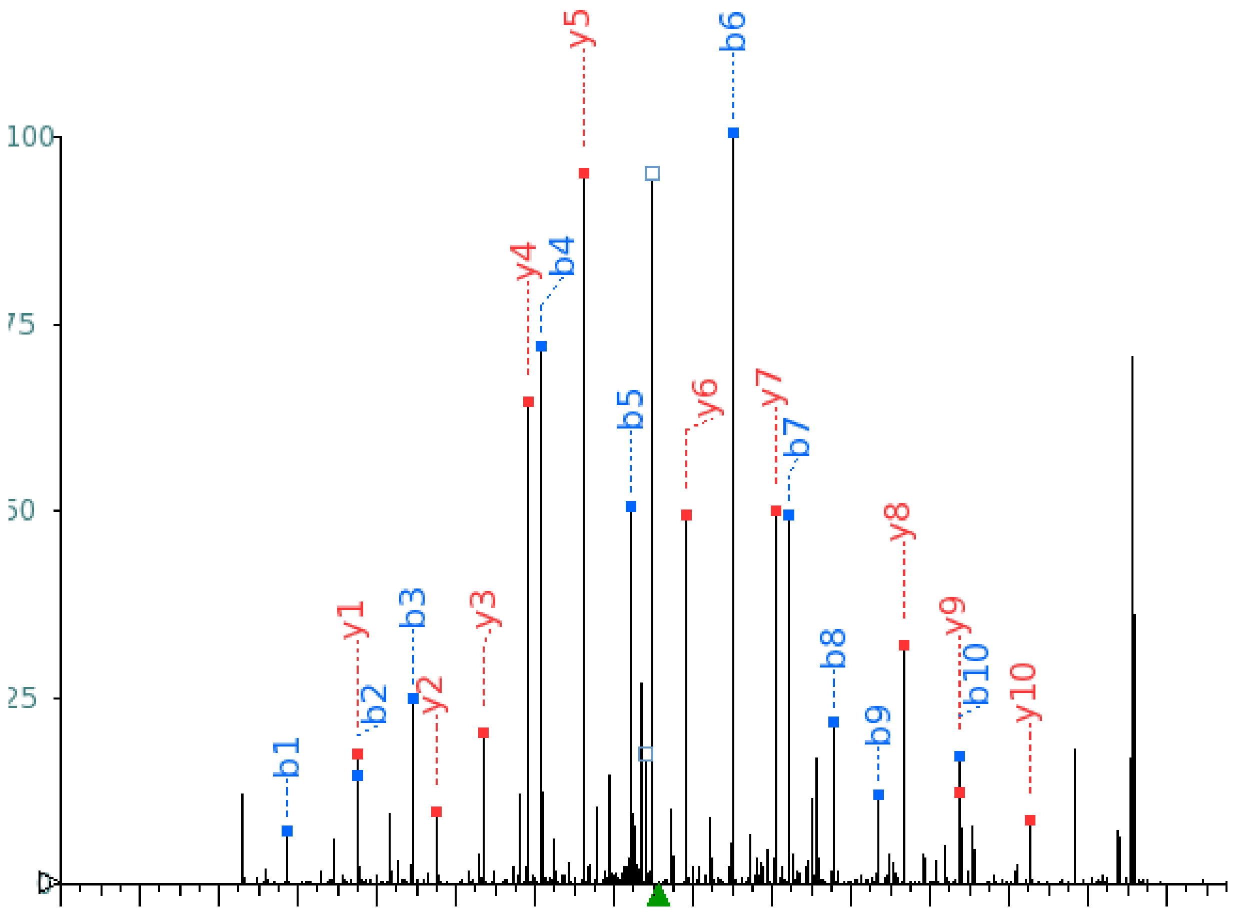

GSAYLEAGGTK (+2)

Primary Reference: EXOS6_HUMAN
Search ID: 41870
Search Name: 20130330_ananiav_TMT_GPP_10percent_fraction6_lysC_2MC_IAA
Scan#: 9982
Observed Mass: 756.4276 (0.7 ppm)
PSM Score: 73.38

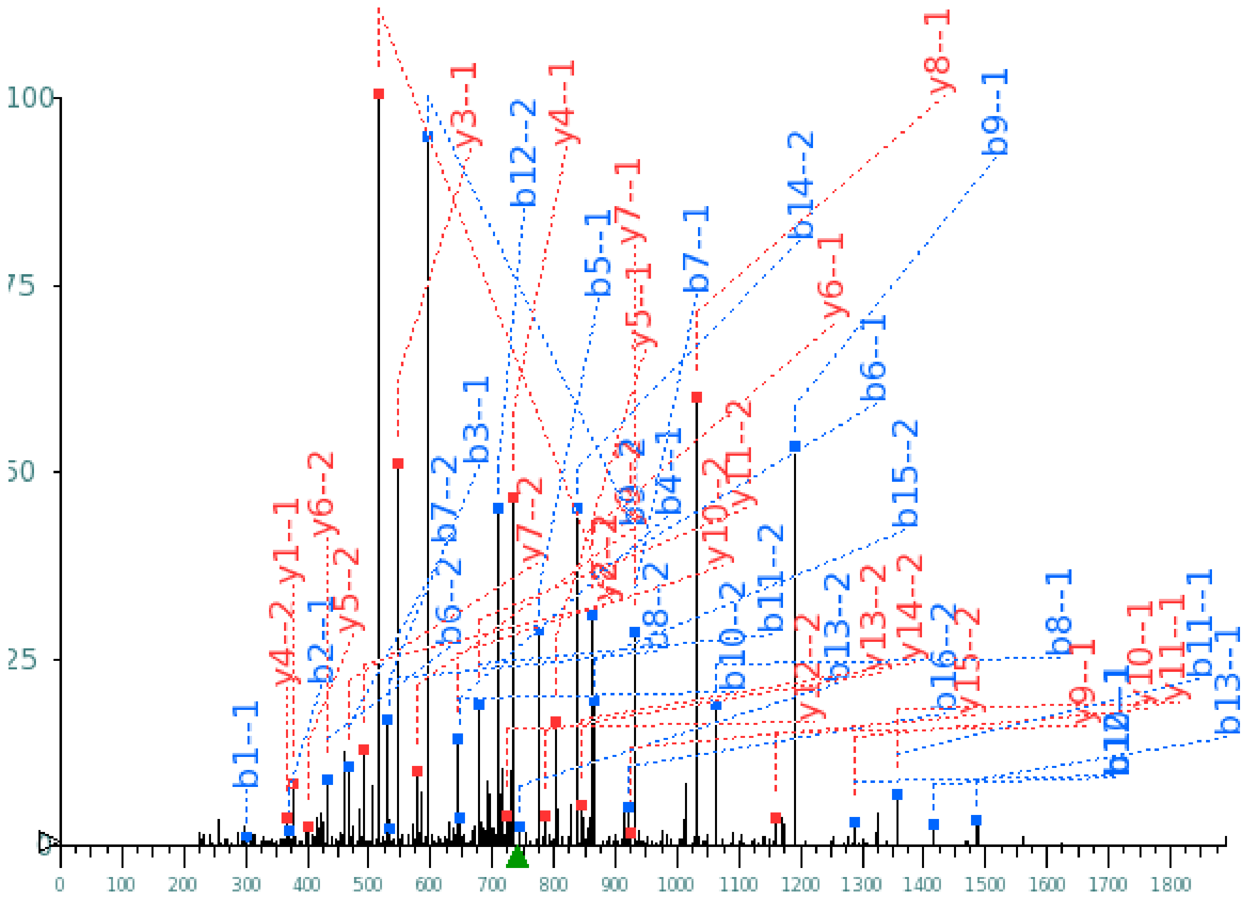

AAC*LESAQEPAGAWGNK (+3)

Primary Reference: B2R5V9_HUMAN
Search ID: 41870
Search Name: 20130330_ananiav_TMT_GPP_10percent_fraction6_lysC_2MC_IAA
Scan#: 14391
Observed Mass: 740.0499 (1.2 ppm)
PSM Score: 62.76

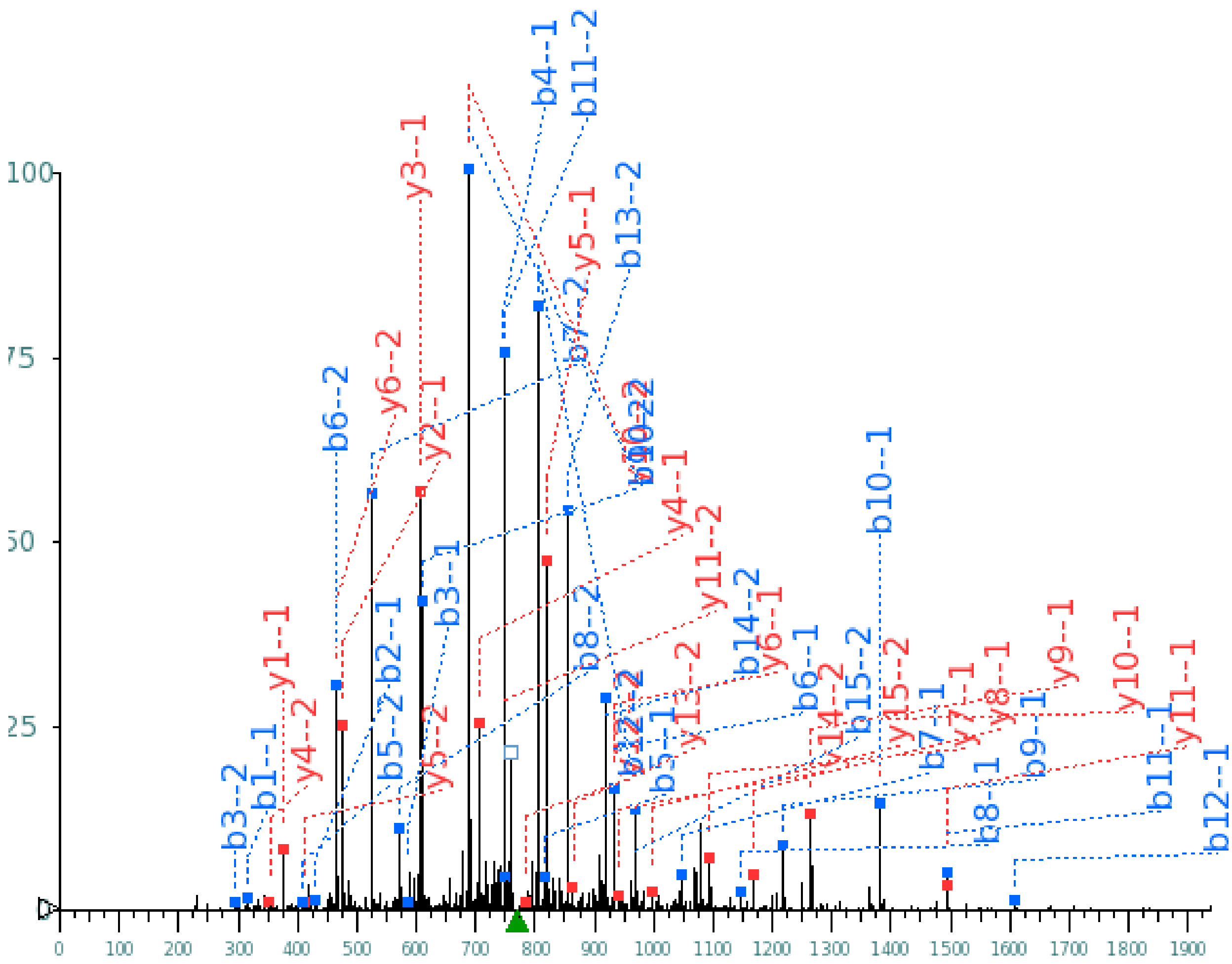

SLRC*ADDVAYINVETK (+3)

Primary Reference: GSKIP_HUMAN
Search ID: 41870
Search Name: 20130330_ananiav_TMT_GPP_10percent_fraction6_lysC_2MC_IAA
Scan#: 15733
Observed Mass: 771.416 (0.6 ppm)
PSM Score: 63.47

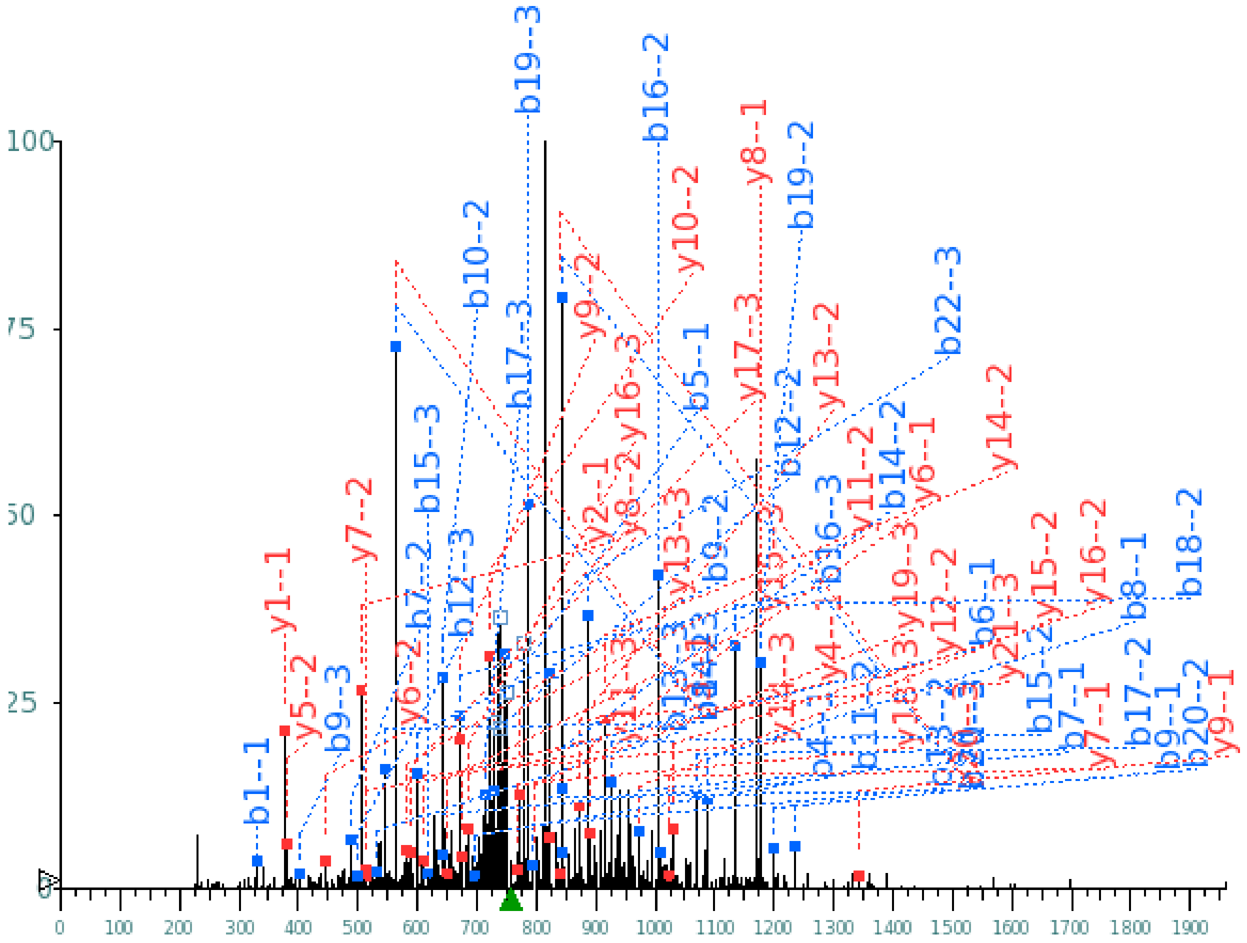

VSPESNEDISTTVVYRMESLGEK (+4)

Primary Reference: B8ZZA8_HUMAN
Search ID: 41870
Search Name: 20130330_ananiav_TMT_GPP_10percent_fraction6_lysC_2MC_IAA
Scan#: 21663
Observed Mass: 757.896 (2.4 ppm)
PSM Score: 38.34

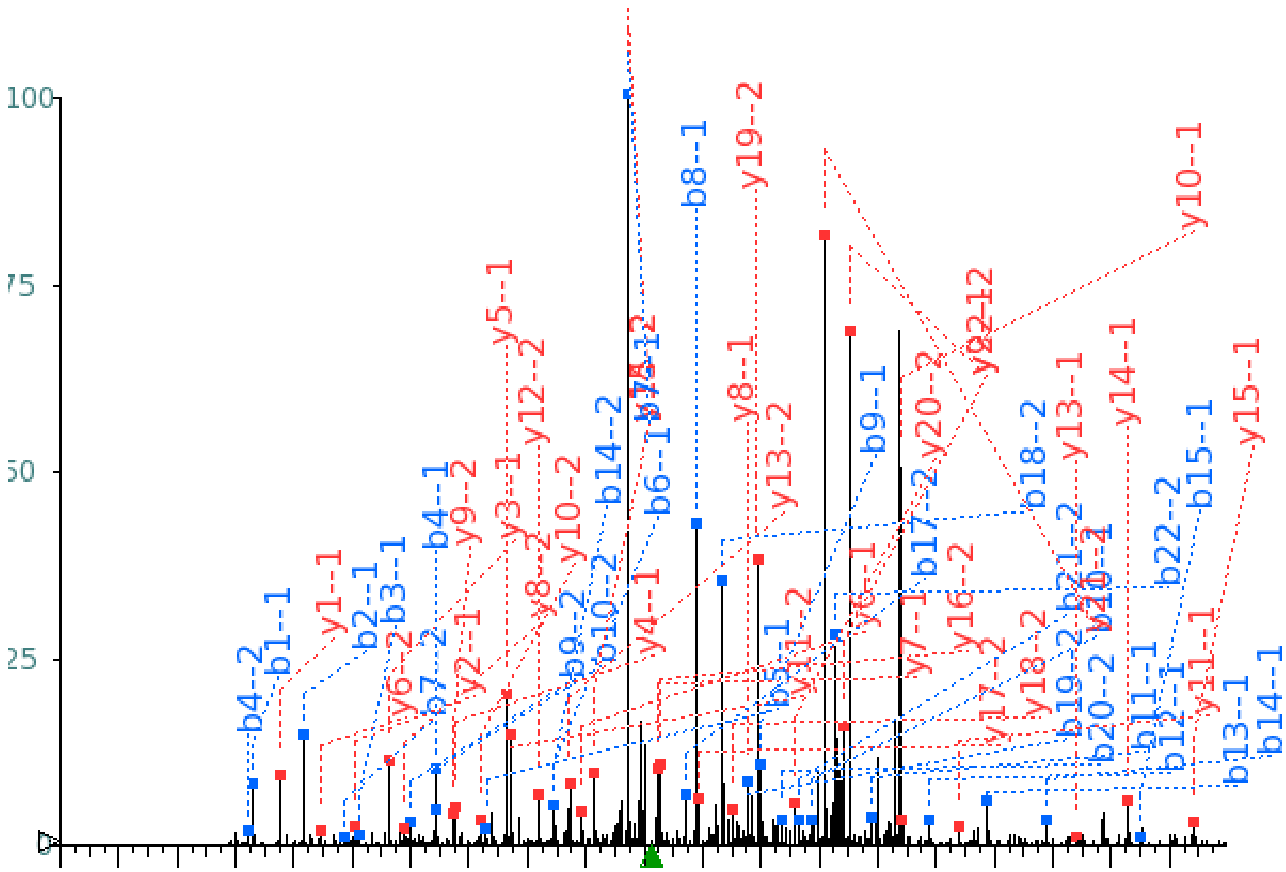

VSPESNEDISTTTVVYRMESLGEK (+3)

Primary Reference: B8ZZA8_HUMAN
Search ID: 41870
Search Name: 20130330_ananiav_TMT_GPP_10percent_fraction6_lysC_2MC_IAA
Scan#: 21677
Observed Mass: 1010.1913 (1.5 ppm)
PSM Score: 74.11

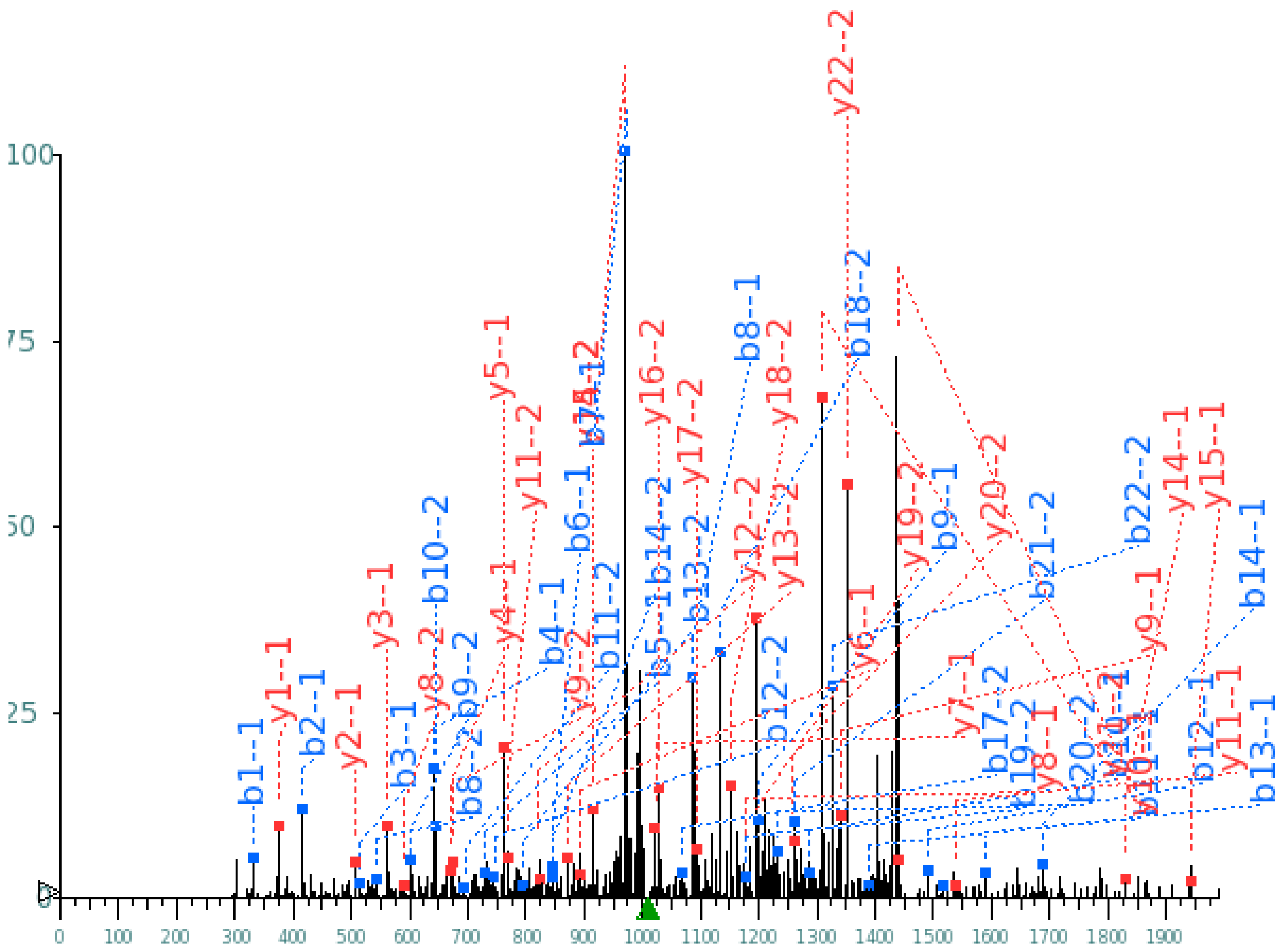

VSPESNEDISTTVVYRMESLGEK (+3)

Primary Reference: B8ZZA8_HUMAN
Search ID: 41870
Search Name: 20130330_ananiav_TMT_GPP_10percent_fraction6_lysC_2MC_IAA
Scan#: 21818
Observed Mass: 1010.1916 (1.8 ppm)
PSM Score: 32.86

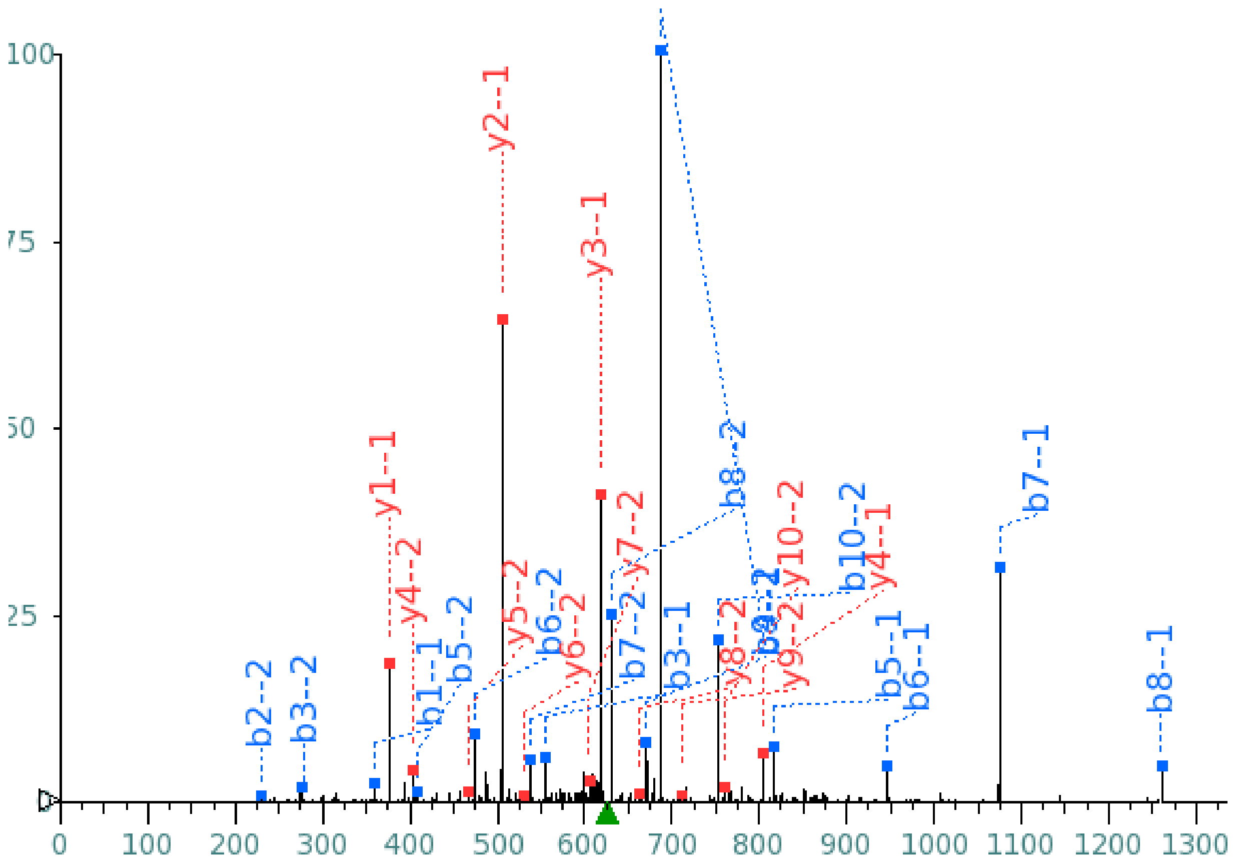

EPVDFEQWIEK (+3)

Primary Reference: HSB11_HUMAN
Search ID: 41870
Search Name: 20130330_ananiav_TMT_GPP_10percent_fraction6_lysC_2MC_IAA
Scan#: 22486
Observed Mass: 626.6747 (2.5 ppm)
PSM Score: 39.33

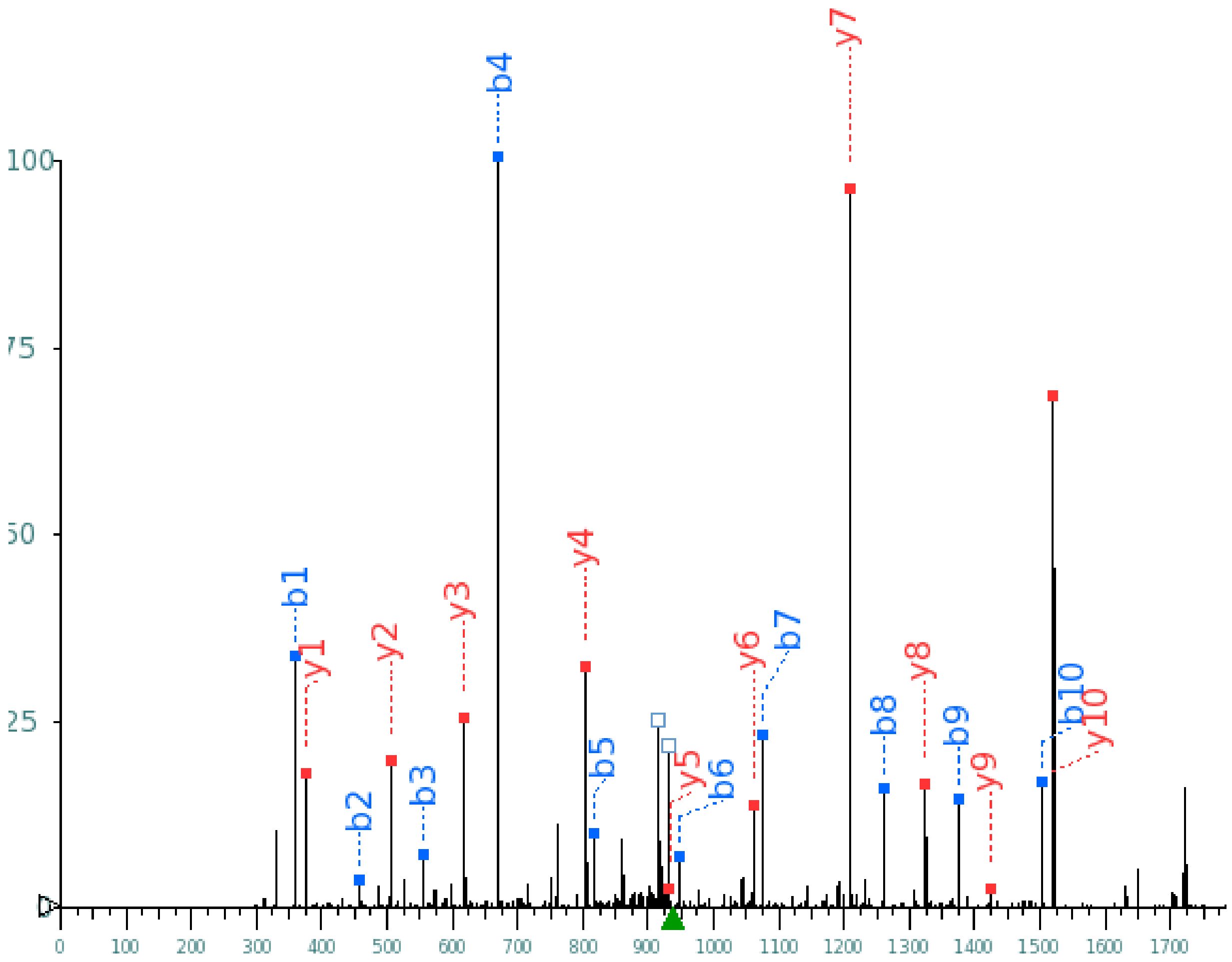

EPVDFEQWIEK (+2)

Primary Reference: HSB11_HUMAN
Search ID: 41870
Search Name: 20130330_ananiav_TMT_GPP_10percent_fraction6_lysC_2MC_IAA
Scan#: 22509
Observed Mass: 939.5078 (1.8 ppm)
PSM Score: 68.22

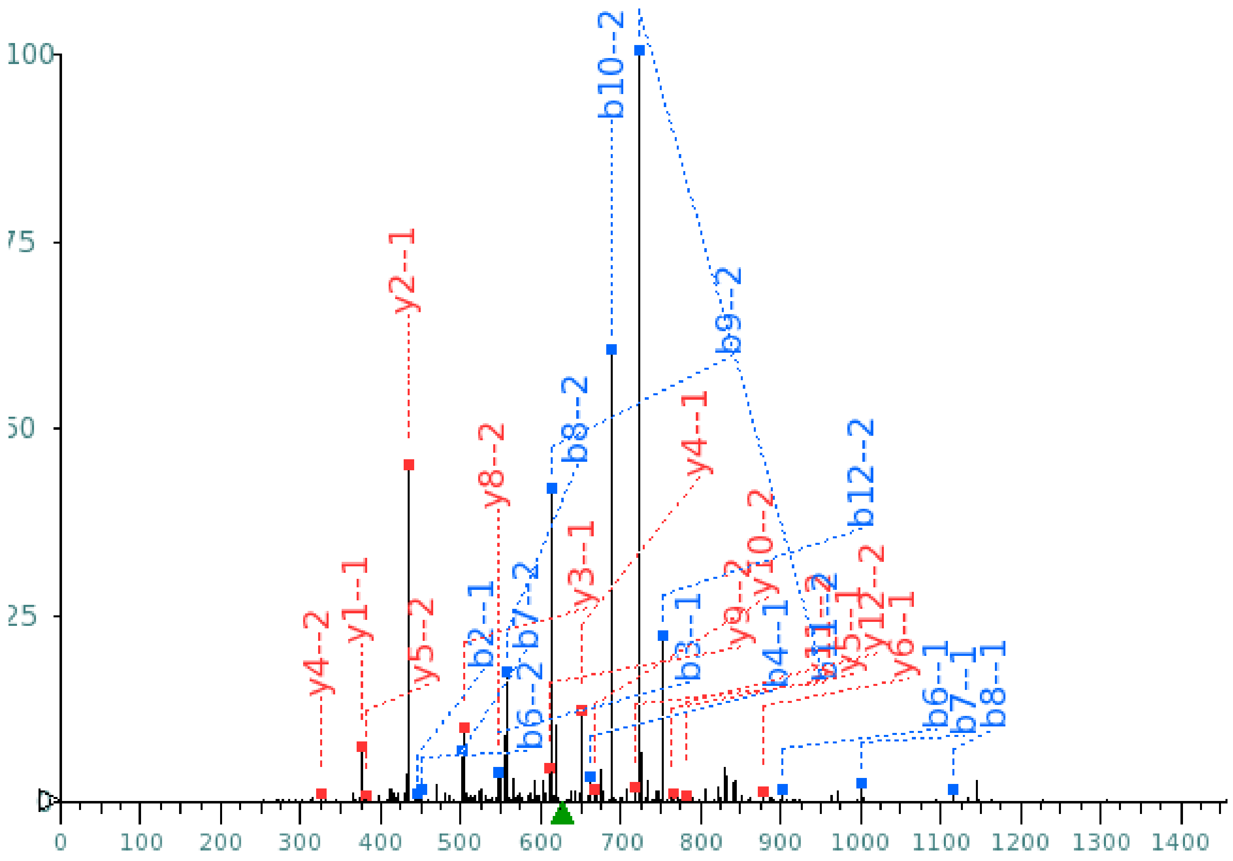

SQTDQLVLIFAGK (+3)

Primary Reference: UBQL2_HUMAN
Search ID: 41870
Search Name: 20130330_ananiav_TMT_GPP_10percent_fraction6_lysC_2MC_IAA
Scan#: 23357
Observed Mass: 626.71 (2.9 ppm)
PSM Score: 21.55

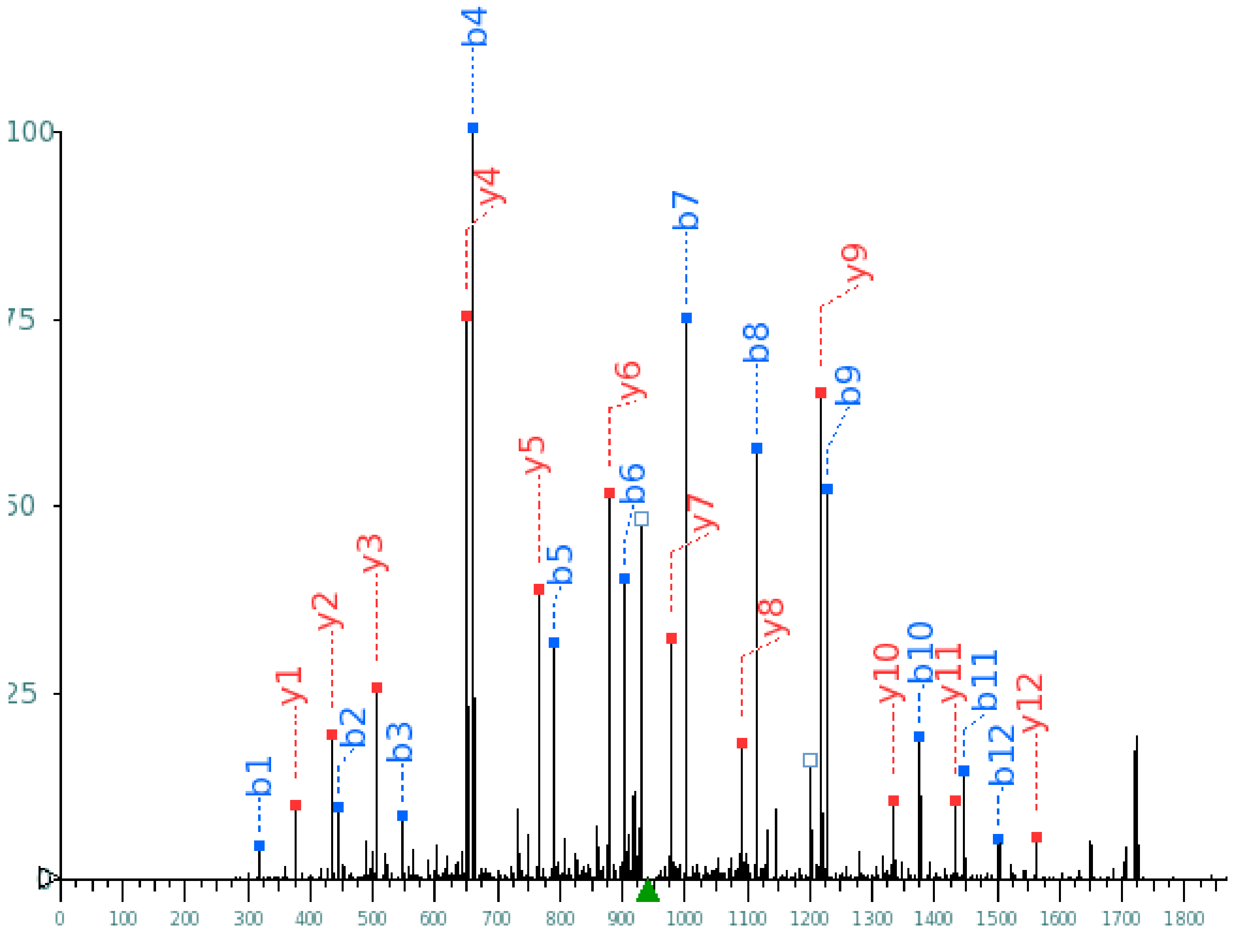

SQTDQLVLIFAGK (+2)

Primary Reference: UBQL2_HUMAN
Search ID: 41870
Search Name: 20130330_ananiav_TMT_GPP_10percent_fraction6_lysC_2MC_IAA
Scan#: 23367
Observed Mass: 939.5603 (1.7 ppm)
PSM Score: 95.21

File name: 2015-12-17 03.51.45PM.pdf

Creation time: 2015-12-17 03:53PM

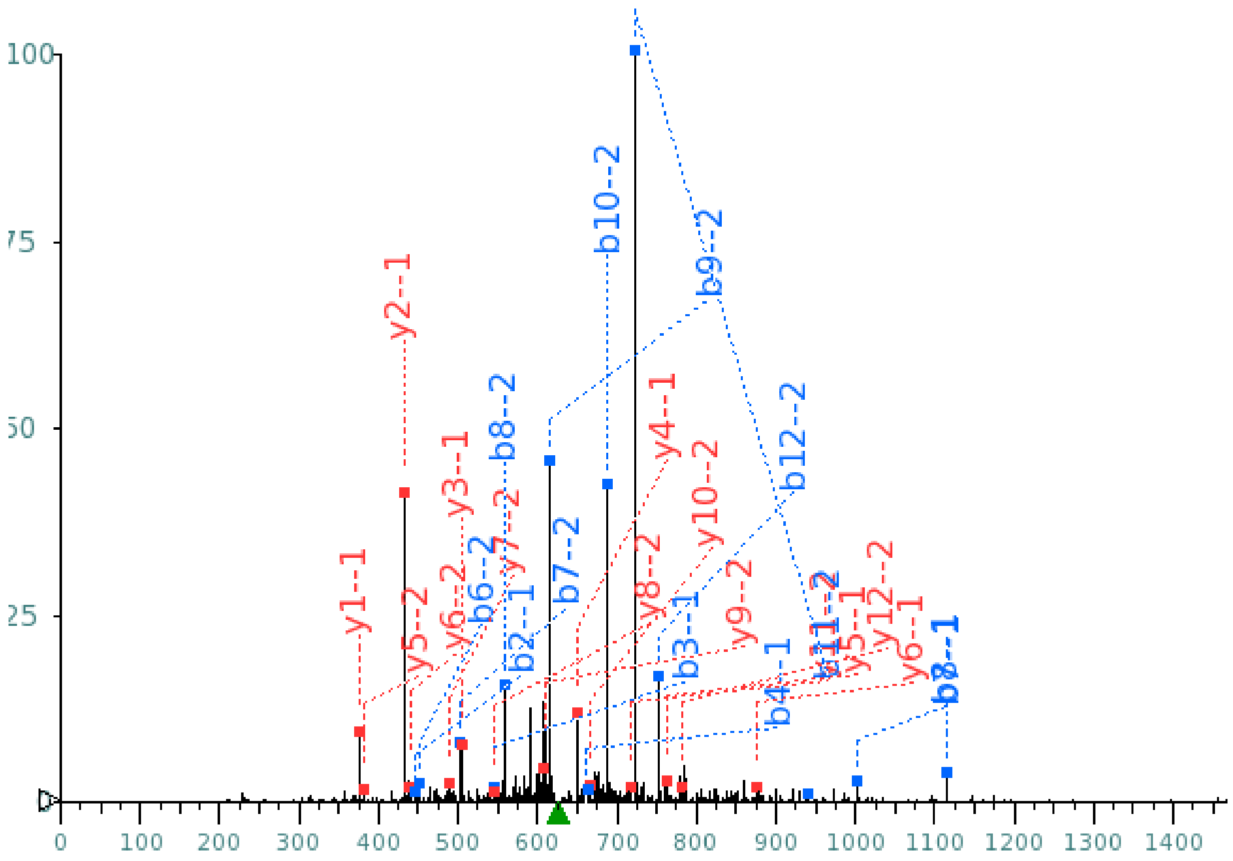

SQTDQLVLIFAGK (+3)

Primary Reference: UBQL2_HUMAN
Search ID: 41870
Search Name: 20130330_ananiav_TMT_GPP_10percent_fraction6_lysC_2MC_IAA
Scan#: 23468
Observed Mass: 626.7097 (2.3 ppm)
PSM Score: 25.25

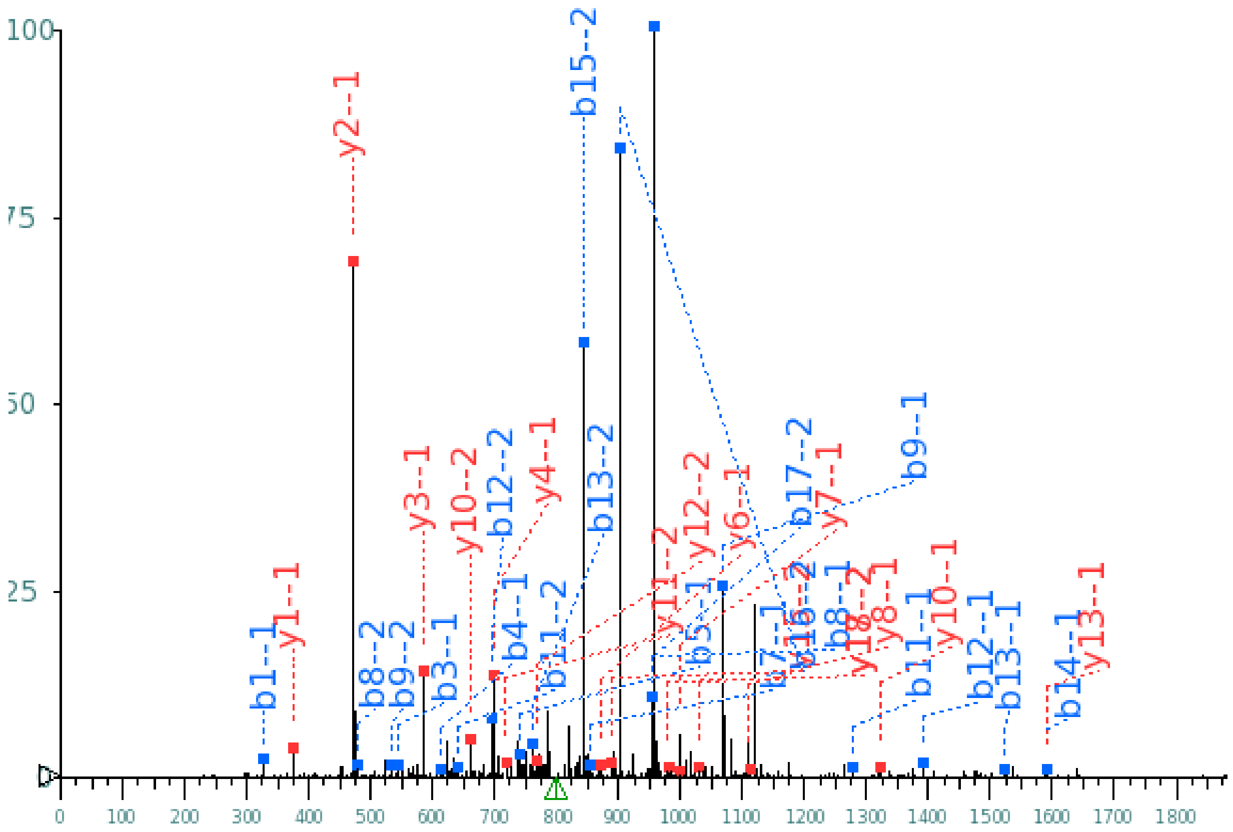

VTIAQGGVLPNIQAVLLPK (+3)

Primary Reference: H2A1D_HUMAN
Search ID: 41870
Search Name: 20130330_ananiav_TMT_GPP_10percent_fraction6_lysC_2MC_IAA
Scan#: 26179
Observed Mass: 797.1712 (1.8 ppm)
PSM Score: 29.84

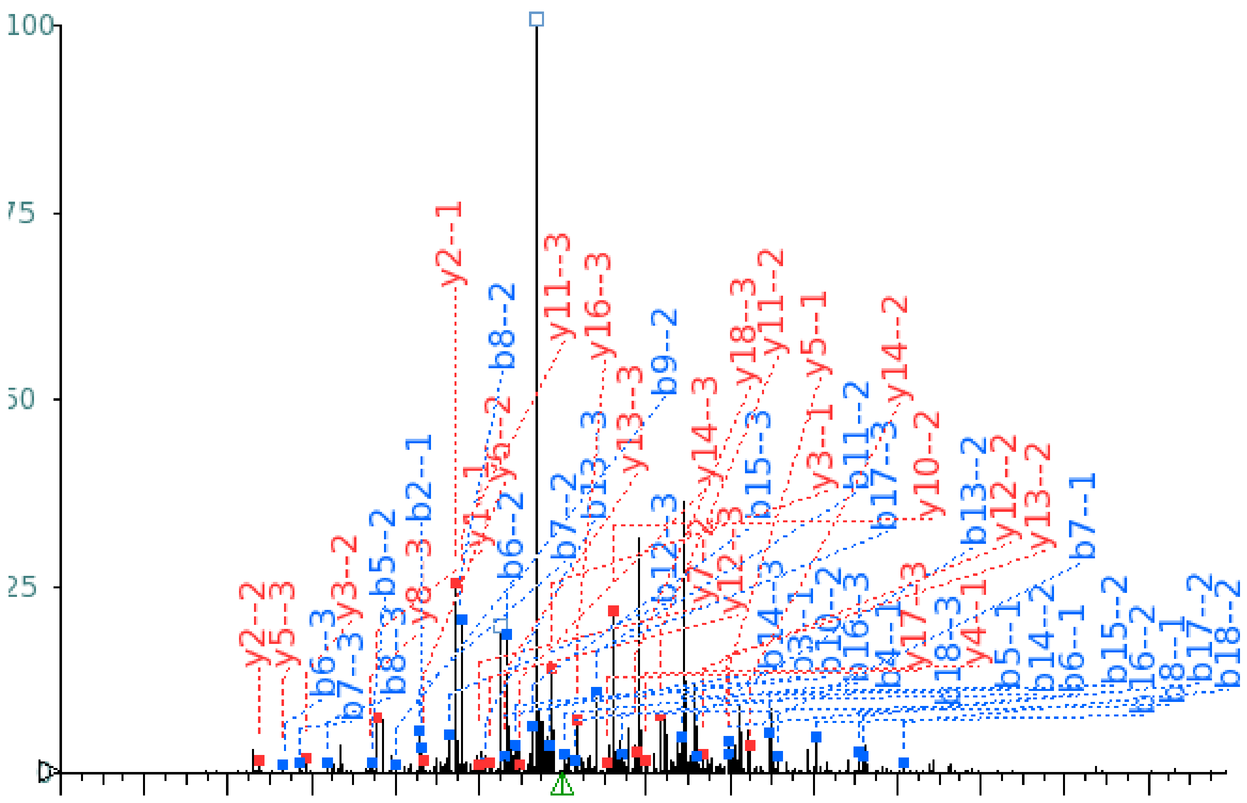

VTIAQGGVLPNIQAVLLPK (+4)

Primary Reference: H2A1D_HUMAN
Search ID: 41870
Search Name: 20130330_ananiav_TMT_GPP_10percent_fraction6_lysC_2MC_IAA
Scan#: 26188
Observed Mass: 598.1306 (2.4 ppm)
PSM Score: 16.24

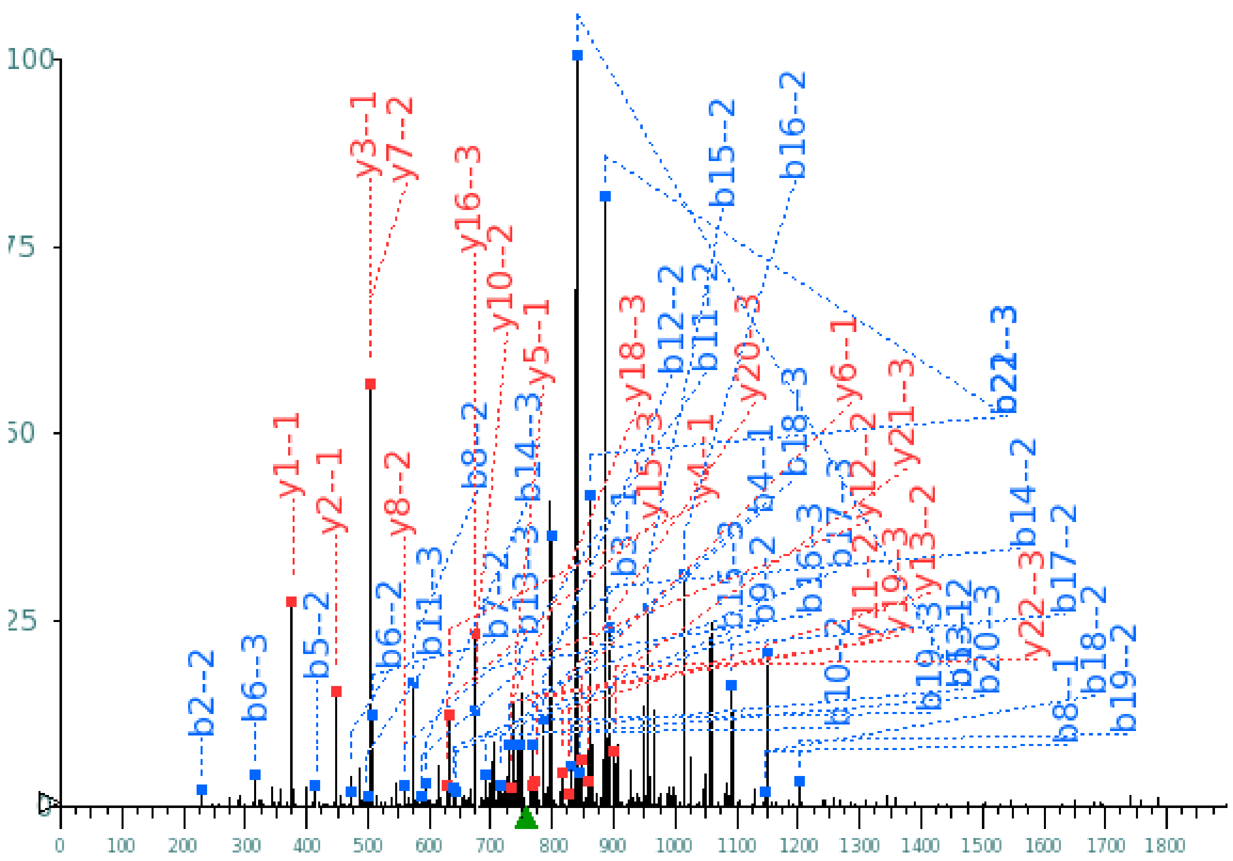

VEEEDDAEHVLALTMLC*LTEGAK (+4)

Primary Reference: NPM3_HUMAN
Search ID: 41870
Search Name: 20130330_ananiav_TMT_GPP_10percent_fraction6_lysC_2MC_IAA
Scan#: 26428
Observed Mass: 758.642 (3 ppm)
PSM Score: 21.27

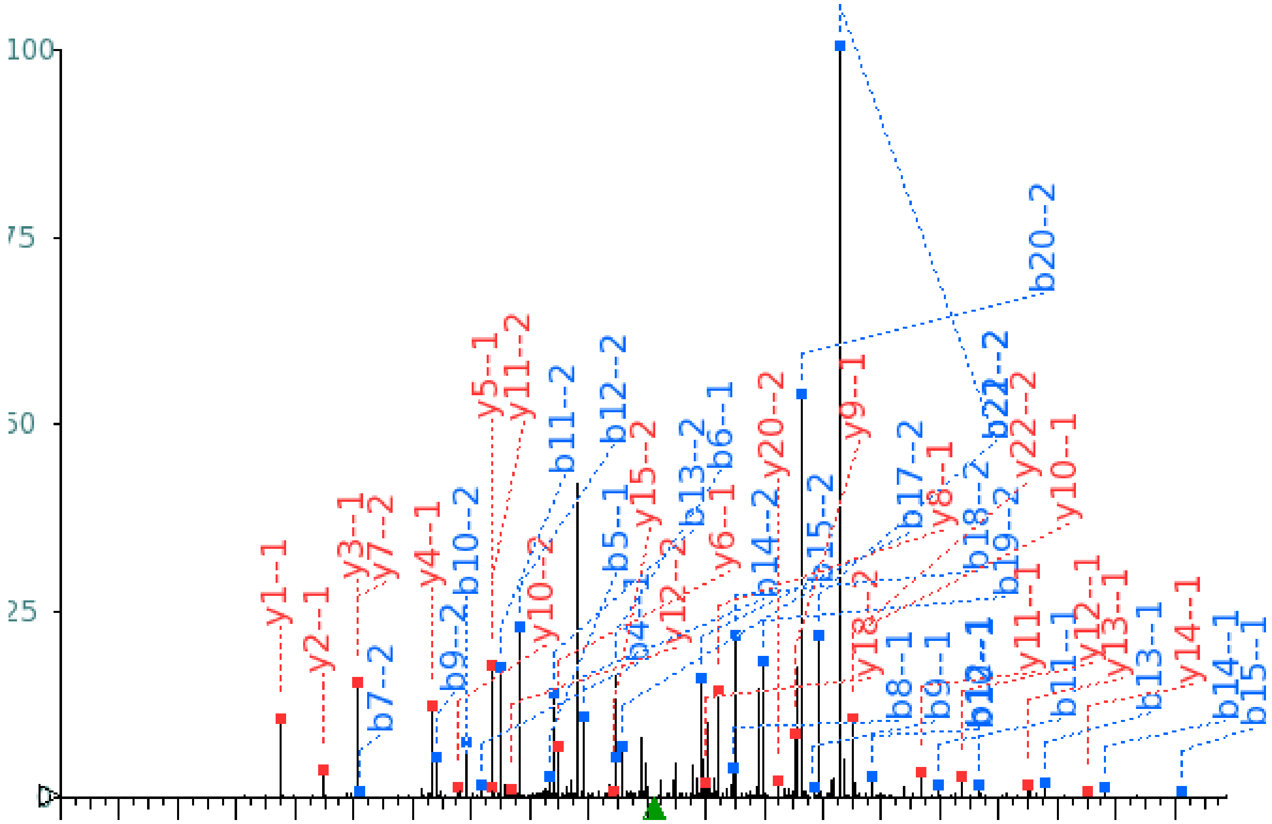

VEEEDDAEHVLALTMLC*LTEGAK (+3)

Primary Reference: NPM3_HUMAN
Search ID: 41870
Search Name: 20130330_ananiav_TMT_GPP_10percent_fraction6_lysC_2MC_IAA
Scan#: 26433
Observed Mass: 1011.1854 (1.6 ppm)
PSM Score: 74.03

File name: 2015-12-17 03.51.45PM.pdf

Creation time: 2015-12-17 03:53PM

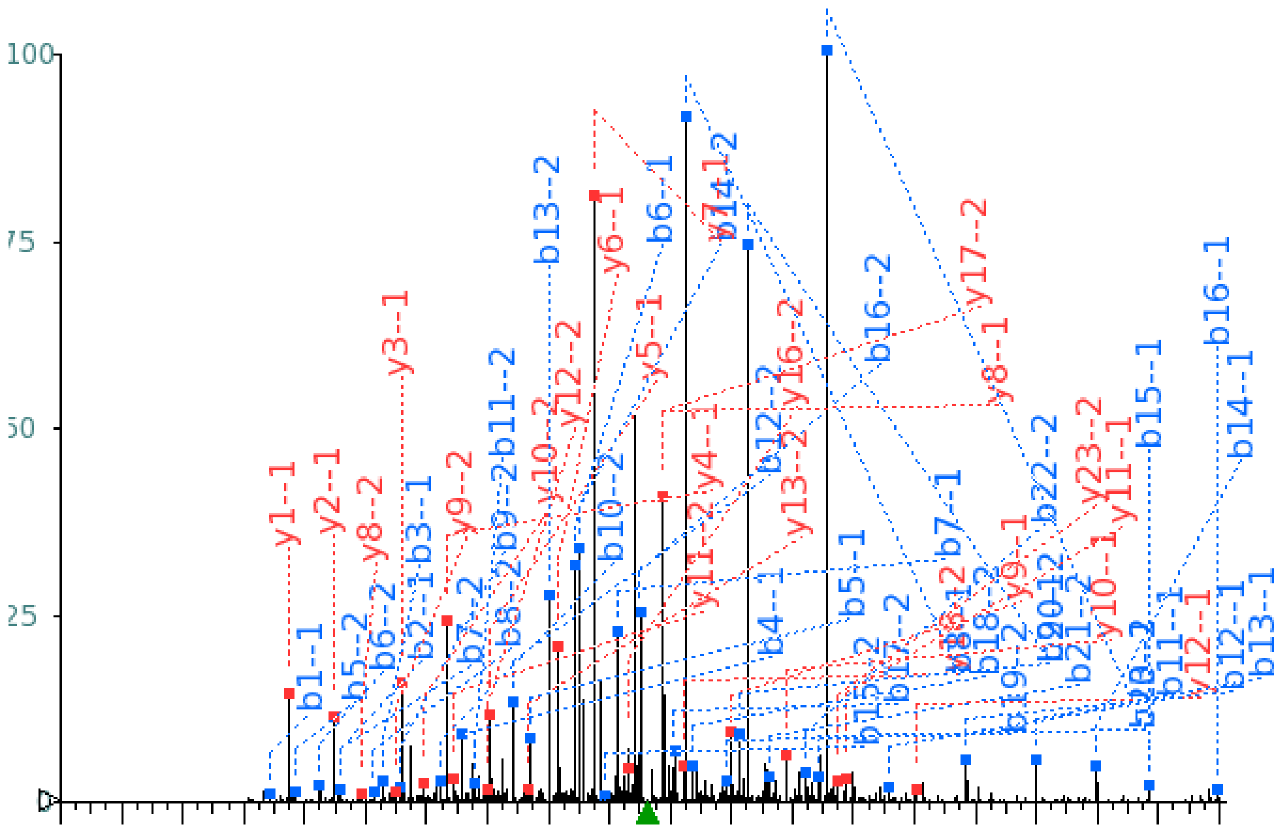

NIAELAALSQDELTSILGNAANAK (+3)

Primary Reference: XPF_HUMAN
Search ID: 41870
Search Name: 20130330_ananiav_TMT_GPP_10percent_fraction6_lysC_2MC_IAA
Scan#: 26787
Observed Mass: 962.5411 (3.6 ppm)
PSM Score: 89.9

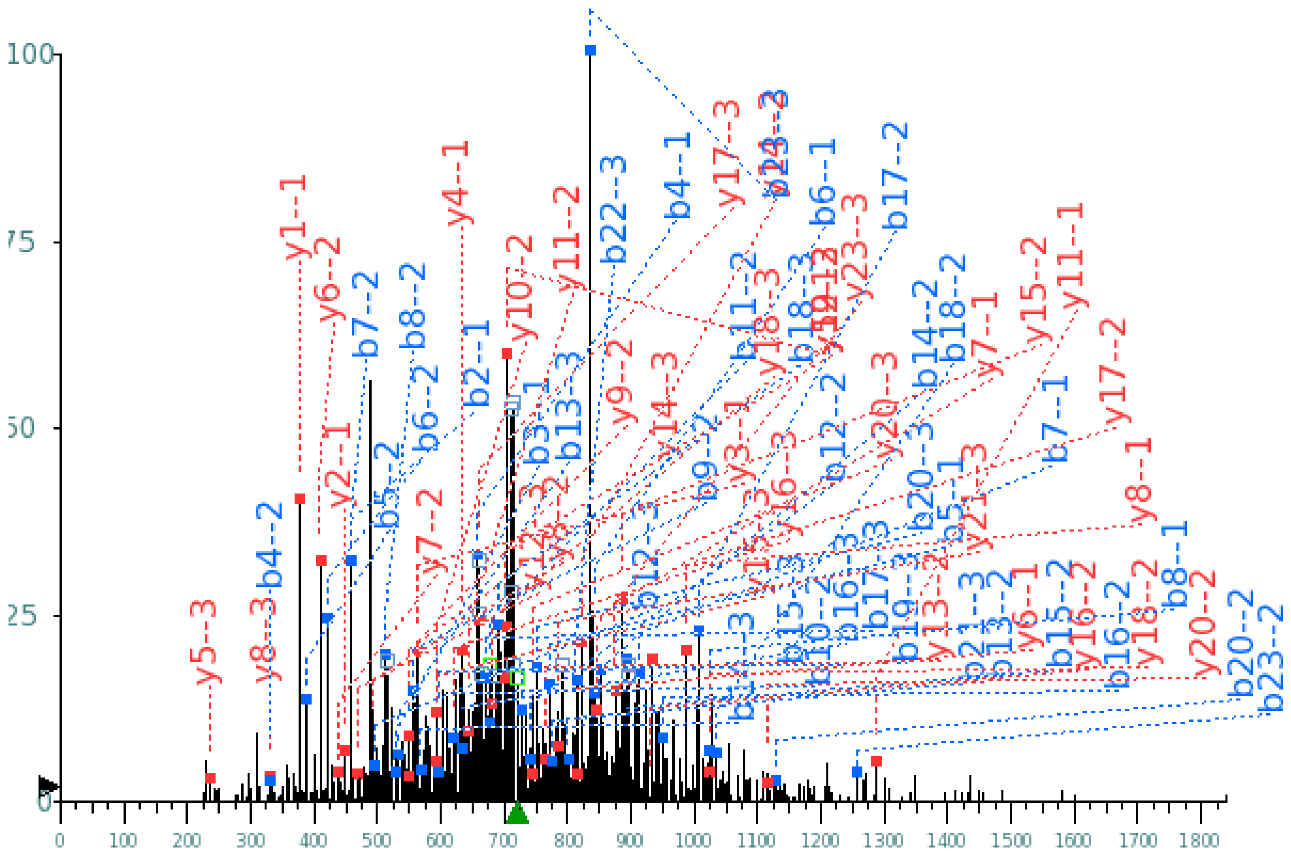

NIAELAALSQDELTSILGNAANAK (+4)

Primary Reference: XPF_HUMAN
Search ID: 41870
Search Name: 20130330_ananiav_TMT_GPP_10percent_fraction6_lysC_2MC_IAA
Scan#: 26812
Observed Mass: 722.158 (4.1 ppm)
PSM Score: 19.65

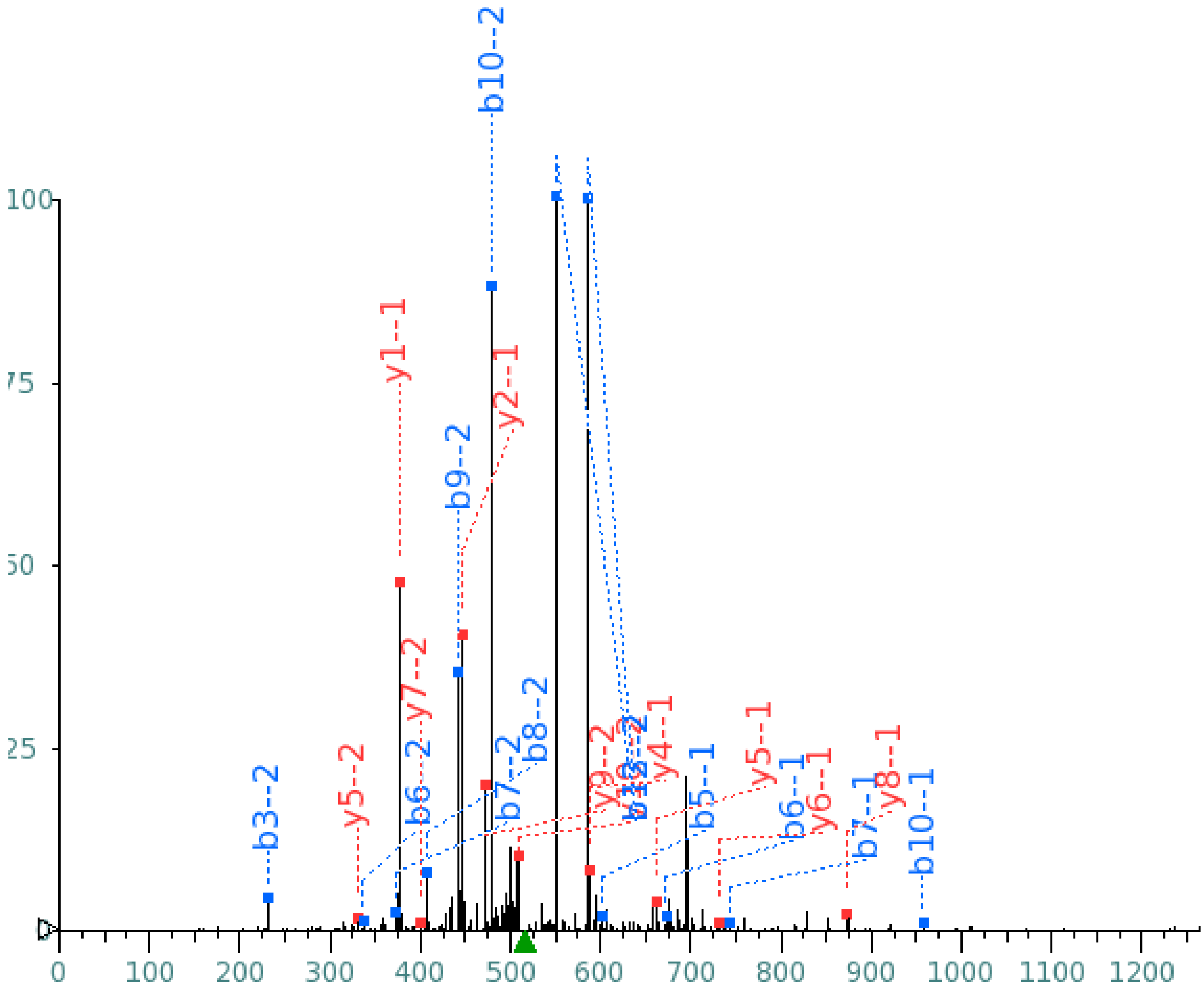

GTAAAAAAAAAAK (+3)

Primary Reference: A8K3Q9_HUMAN
Search ID: 41871
Search Name: 20130330_ananiav_TMT_GPP_10percent_fraction7_lysC_2MC_IAA
Scan#: 12803
Observed Mass: 515.6449 (2.8 ppm)
PSM Score: 21.86

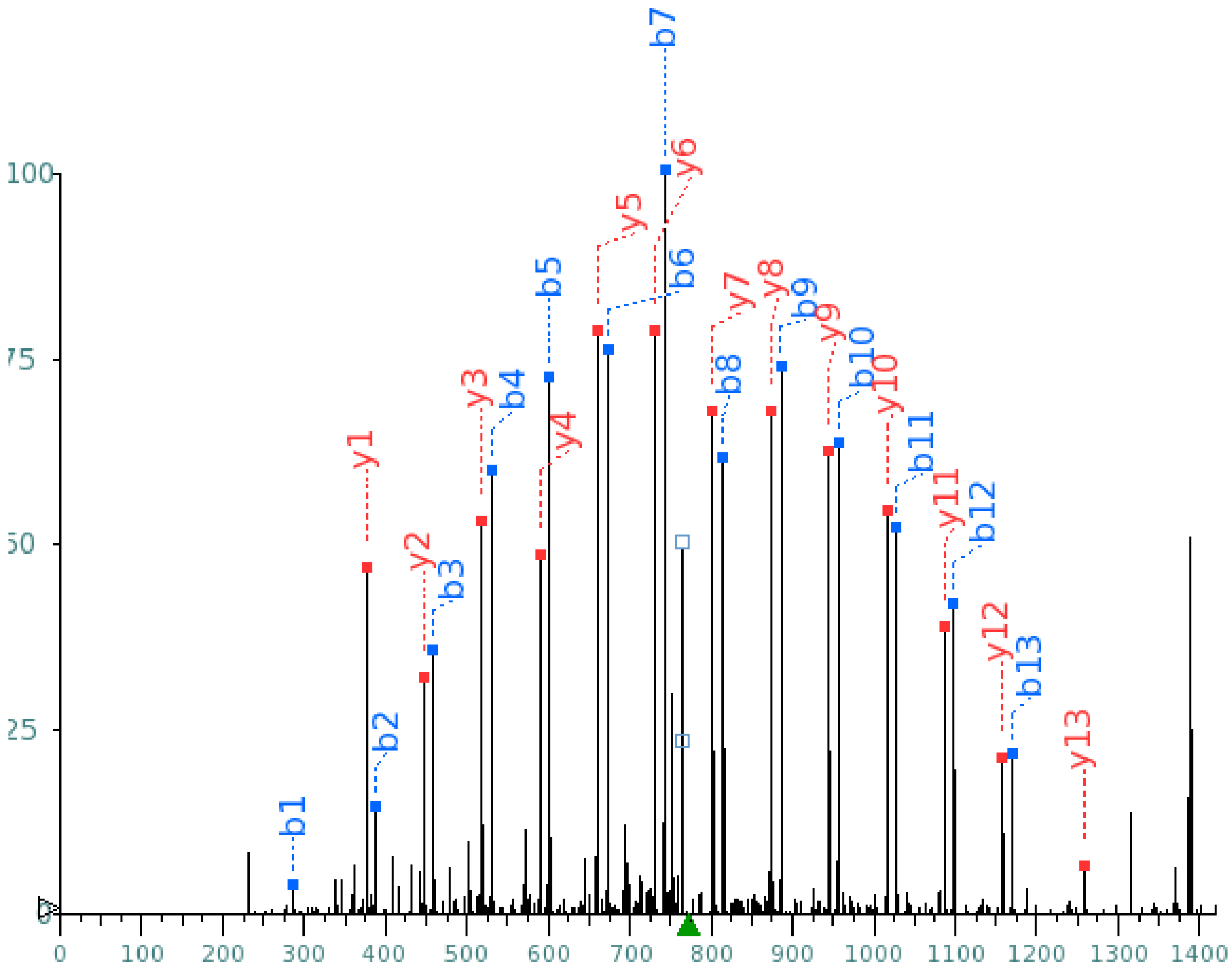

GTAAAAAAAAAAAK (+2)

Primary Reference: A8K3Q9_HUMAN
Search ID: 41871
Search Name: 20130330_ananiav_TMT_GPP_10percent_fraction7_lysC_2MC_IAA
Scan#: 12807
Observed Mass: 772.9624 (1 ppm)
PSM Score: 117.83

File name: 2015-12-17 03.51.45PM.pdf

Creation time: 2015-12-17 03:53PM

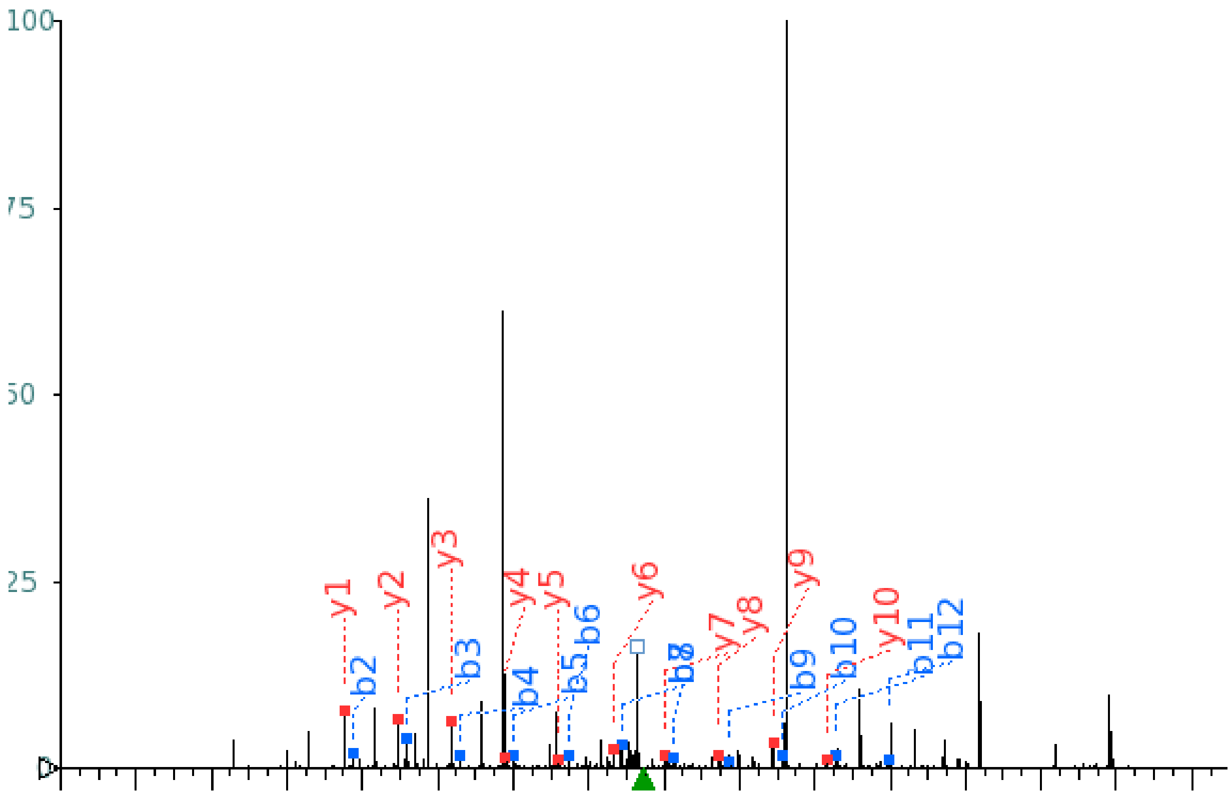

GTAAAAAAAAAAAK (+2)

Primary Reference: A8K3Q9_HUMAN
Search ID: 41871
Search Name: 20130330_ananiav_TMT_GPP_10percent_fraction7_lysC_2MC_IAA
Scan#: 12849
Observed Mass: 772.9624 (1 ppm)
PSM Score: 10.33

File name: 2015-12-17 03.51.45PM.pdf

Creation time: 2015-12-17 03:53PM

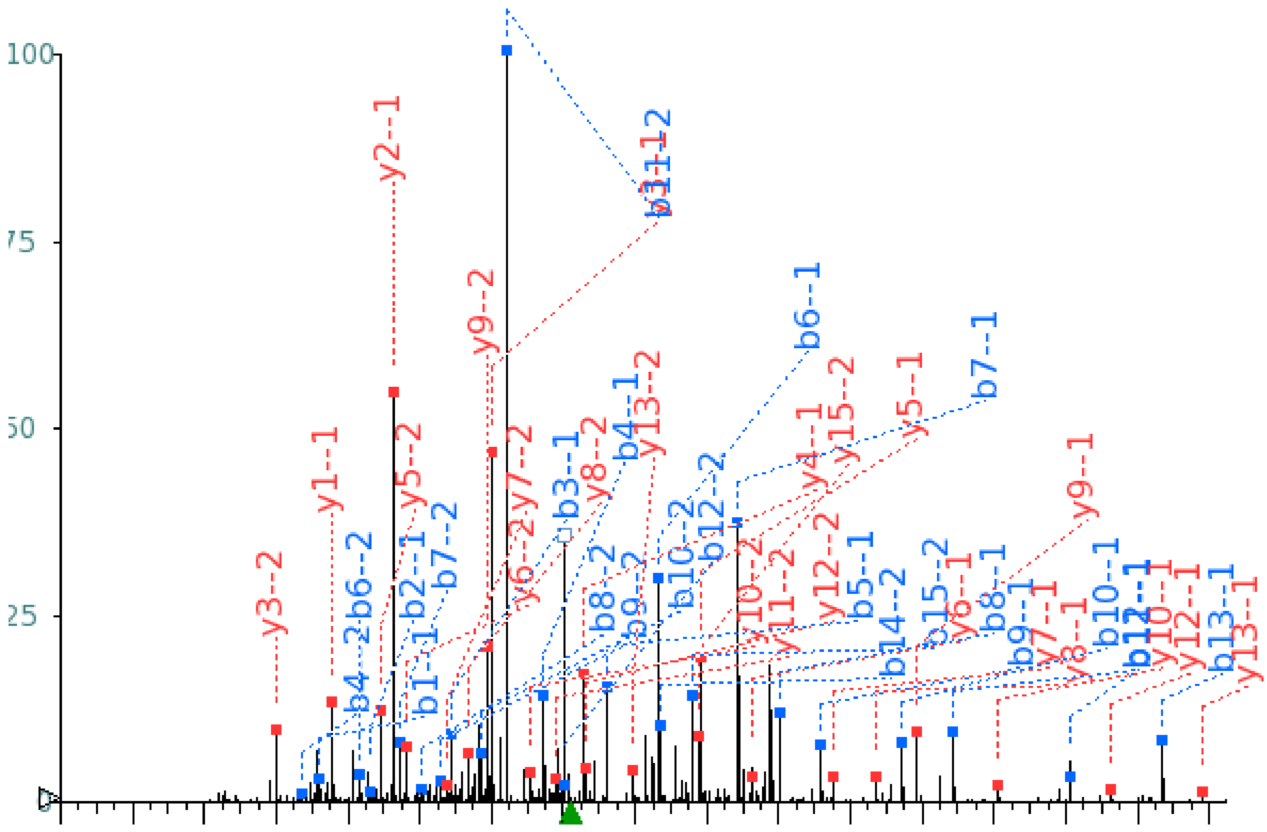

EAIESAIGGNAYQHSK (+3)

Primary Reference: DYLT1_HUMAN
Search ID: 41872
Search Name: 20130330_ananiav_TMT_GPP_10percent_fraction8_lysC_2MC_IAA
Scan#: 12560
Observed Mass: 711.7164 (0.3 ppm)
PSM Score: 74.03

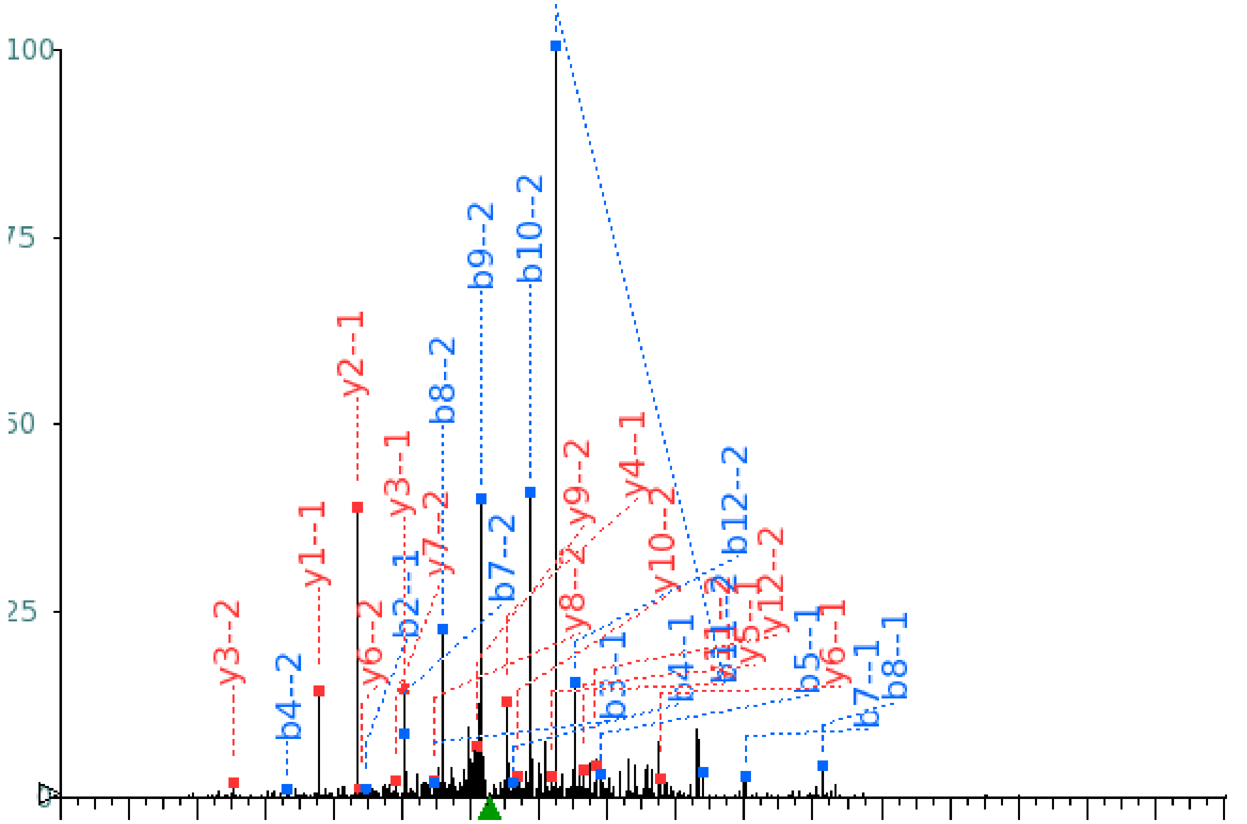

SQTDQLVLIFAGK (+3)

Primary Reference: UBQL2_HUMAN
Search ID: 41872
Search Name: 20130330_ananiav_TMT_GPP_10percent_fraction8_lysC_2MC_IAA
Scan#: 22809
Observed Mass: 626.709 (1.2 ppm)
PSM Score: 18.45

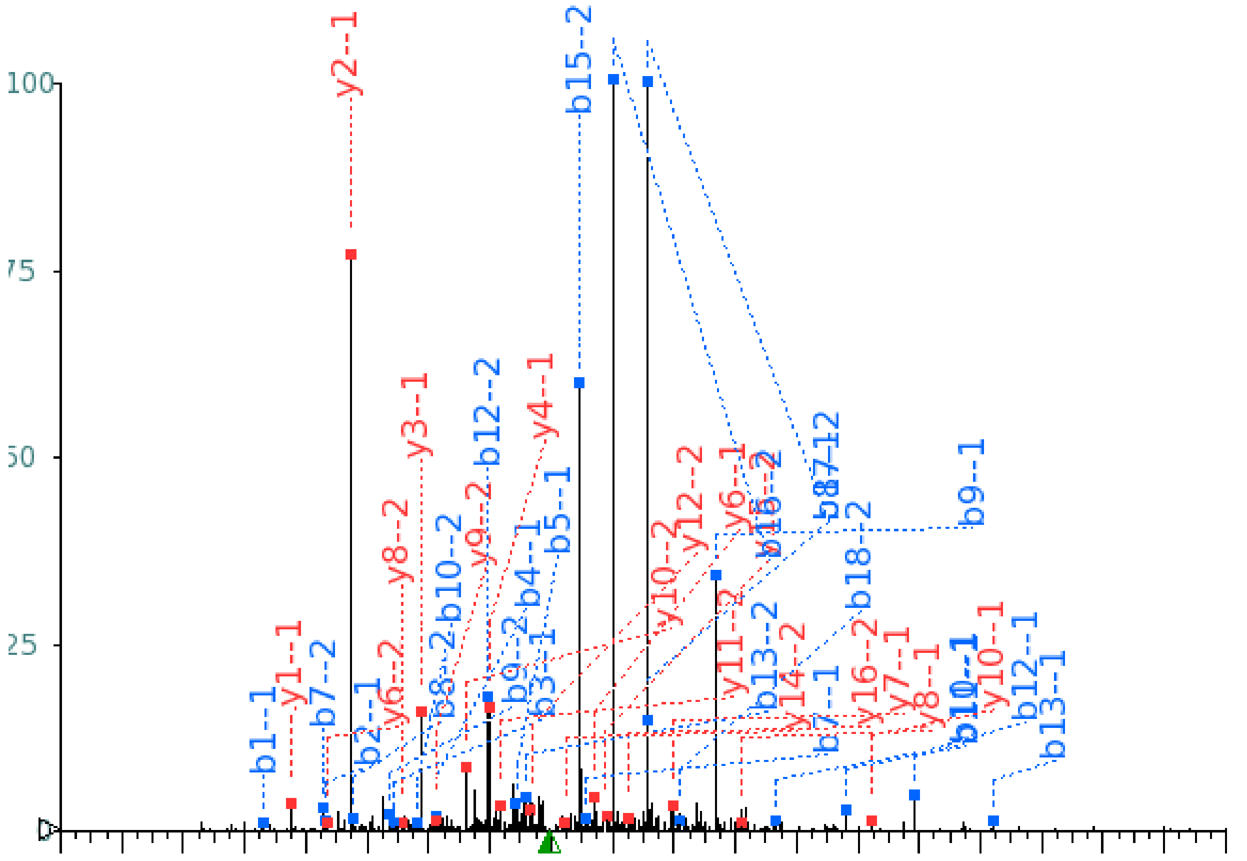

VTIAQGGVLPNIQAVLLPK (+3)

Primary Reference: H2A1D_HUMAN
Search ID: 41872
Search Name: 20130330_ananiav_TMT_GPP_10percent_fraction8_lysC_2MC_IAA
Scan#: 25434
Observed Mass: 797.1717 (2.5 ppm)
PSM Score: 25.12

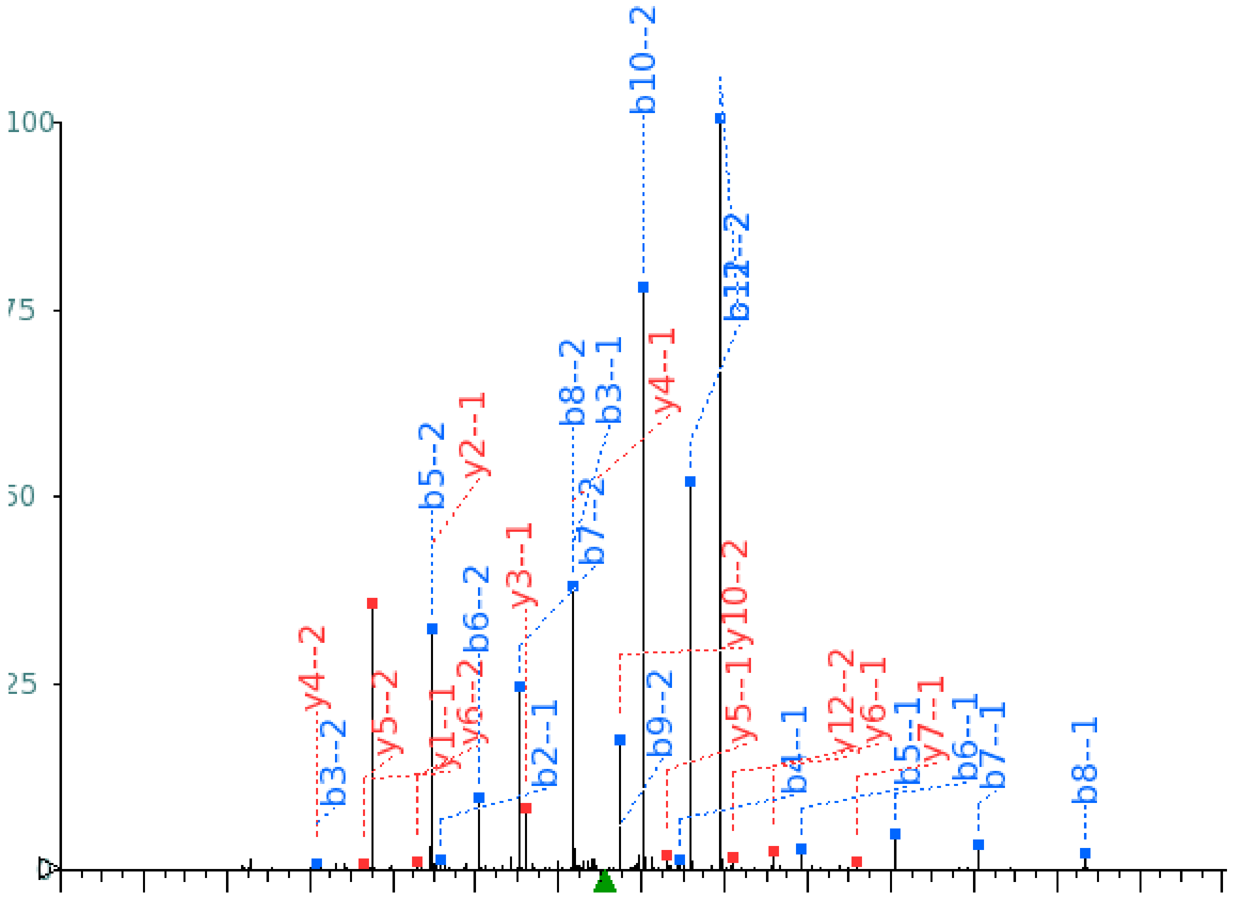

NIC*QFLVEIGLAK (+3)

Primary Reference: EIF1_HUMAN
Search ID: 41872
Search Name: 20130330_ananiav_TMT_GPP_10percent_fraction8_lysC_2MC_IAA
Scan#: 25821
Observed Mass: 655.0553 (3.1 ppm)
PSM Score: 51.27

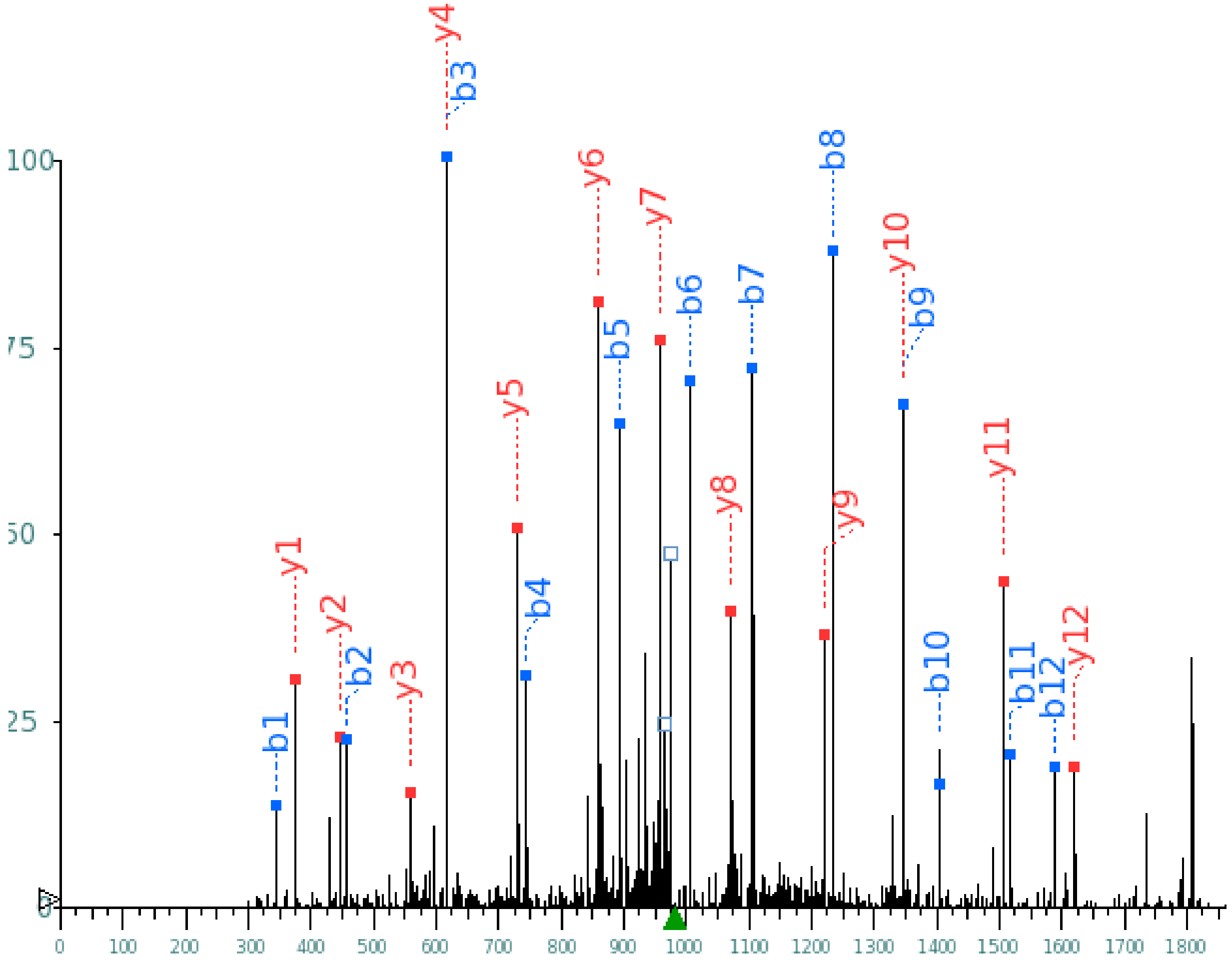

NIC*QFLVEIGLAK (+2)

Primary Reference: EIF1_HUMAN
Search ID: 41872
Search Name: 20130330_ananiav_TMT_GPP_10percent_fraction8_lysC_2MC_IAA
Scan#: 25831
Observed Mass: 982.0783 (2.2 ppm)
PSM Score: 89.95

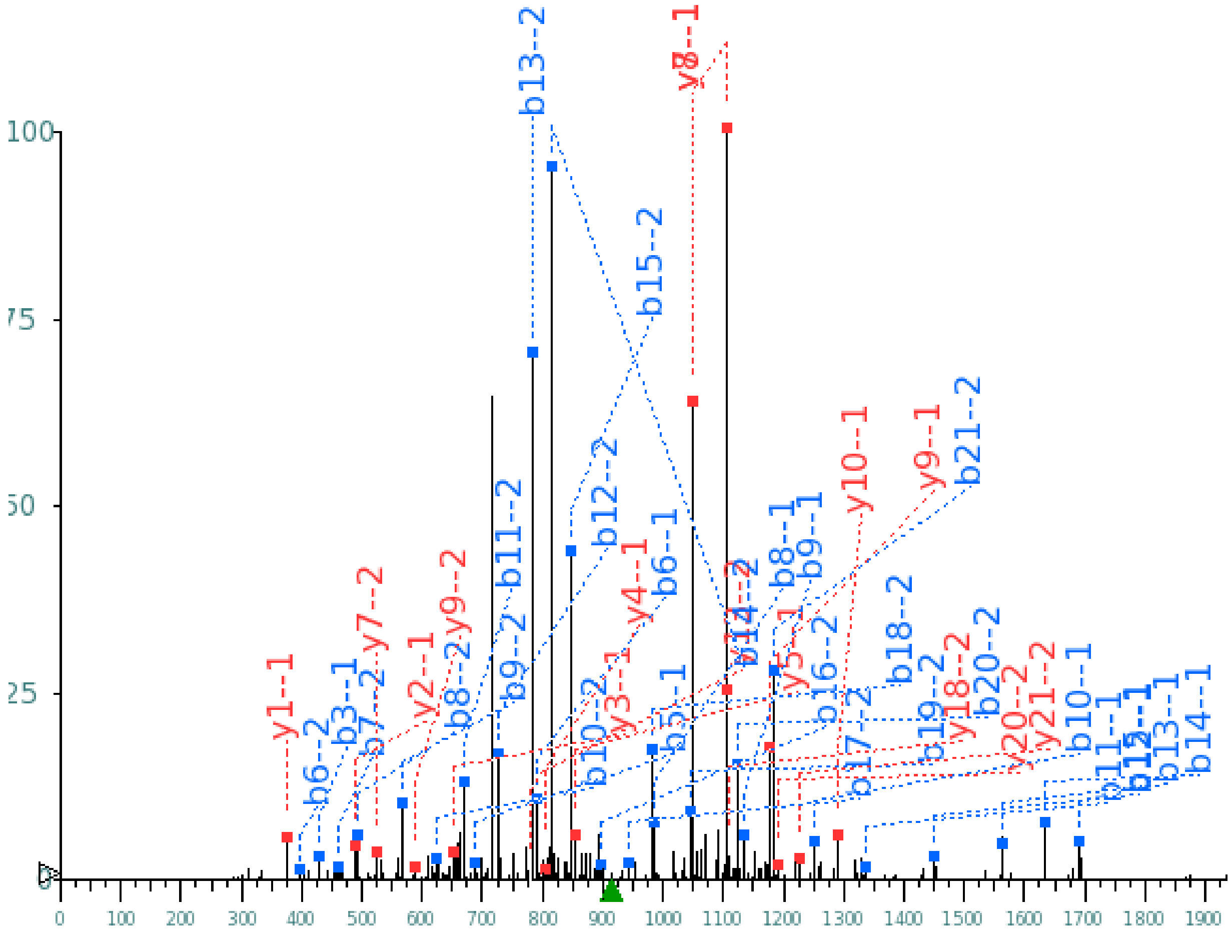

GAAPYVQAFDSLLAGPVAEYLK (+3)

Primary Reference: B4DNA3_HUMAN
Search ID: 41872
Search Name: 20130330_ananiav_TMT_GPP_10percent_fraction8_lysC_2MC_IAA
Scan#: 26021
Observed Mass: 913.5128 (2.6 ppm)
PSM Score: 71.83

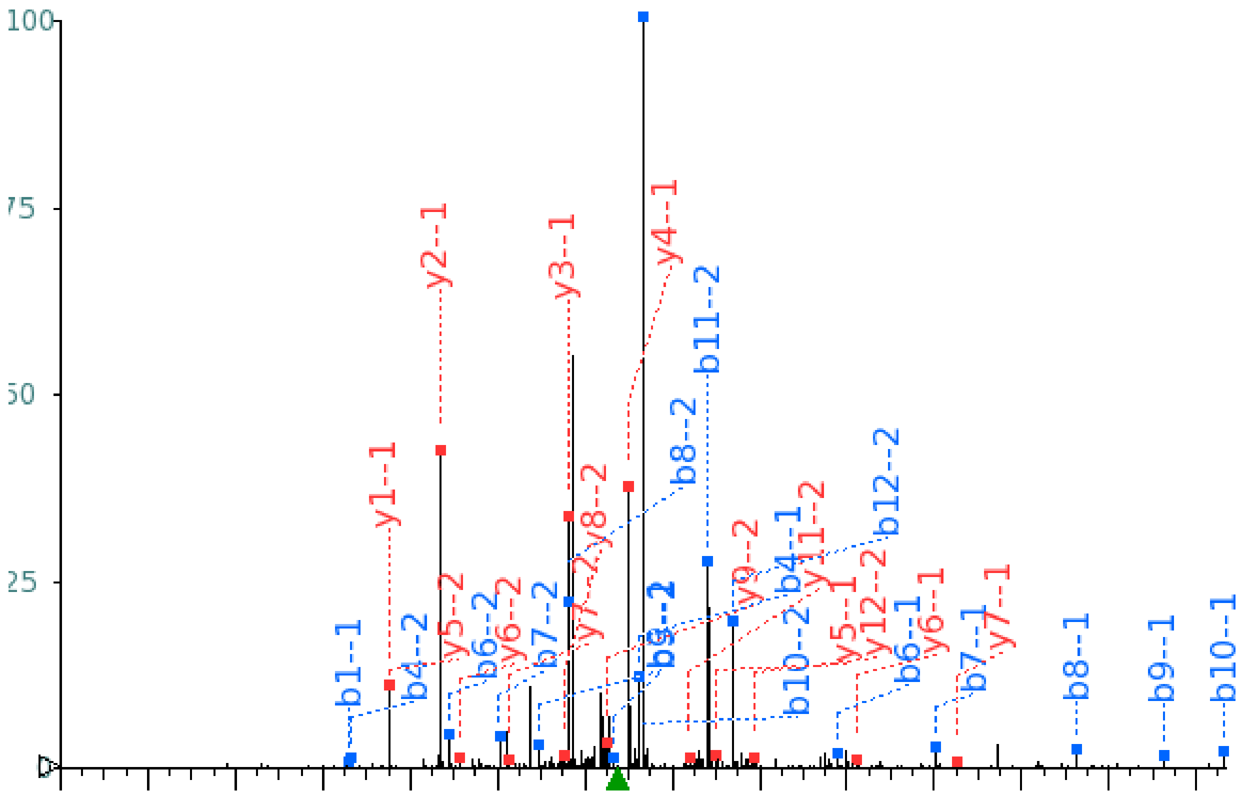

VFANPEDC*VAFGK (+3)

Primary Reference: MGST1_HUMAN
Search ID: 41873
Search Name: 20130330_ananiav_TMT_GPP_10percent_fraction9_lysC_2MC_IAA
Scan#: 18561
Observed Mass: 638.0075 (2.1 ppm)
PSM Score: 26.74

File name: 2015-12-17 03.51.45PM.pdf

Creation time: 2015-12-17 03:53PM

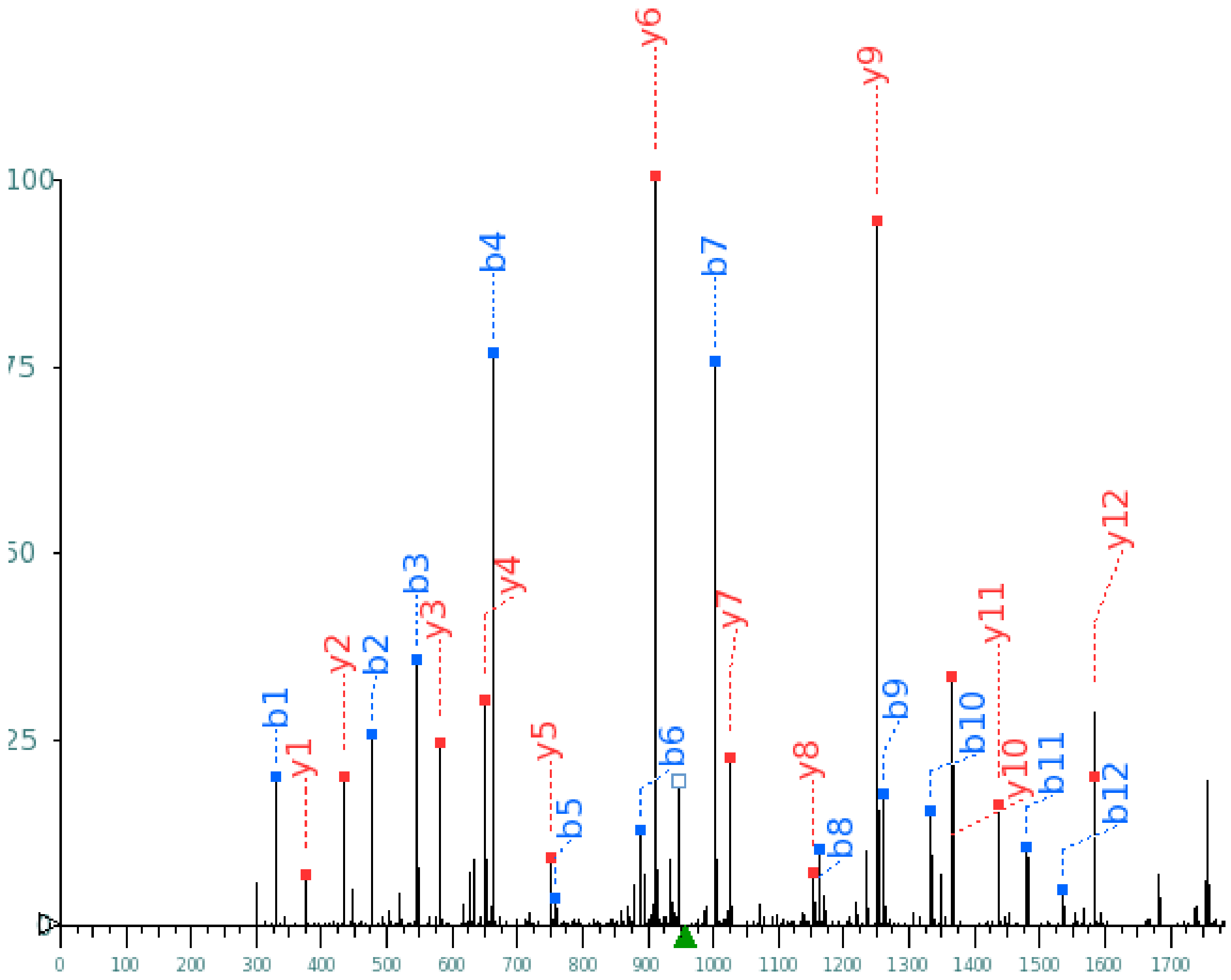

VFANPEDC*VAFGK (+2)

Primary Reference: MGST1_HUMAN
Search ID: 41873
Search Name: 20130330_ananiav_TMT_GPP_10percent_fraction9_lysC_2MC_IAA
Scan#: 18582
Observed Mass: 956.5069 (1.4 ppm)
PSM Score: 62.85

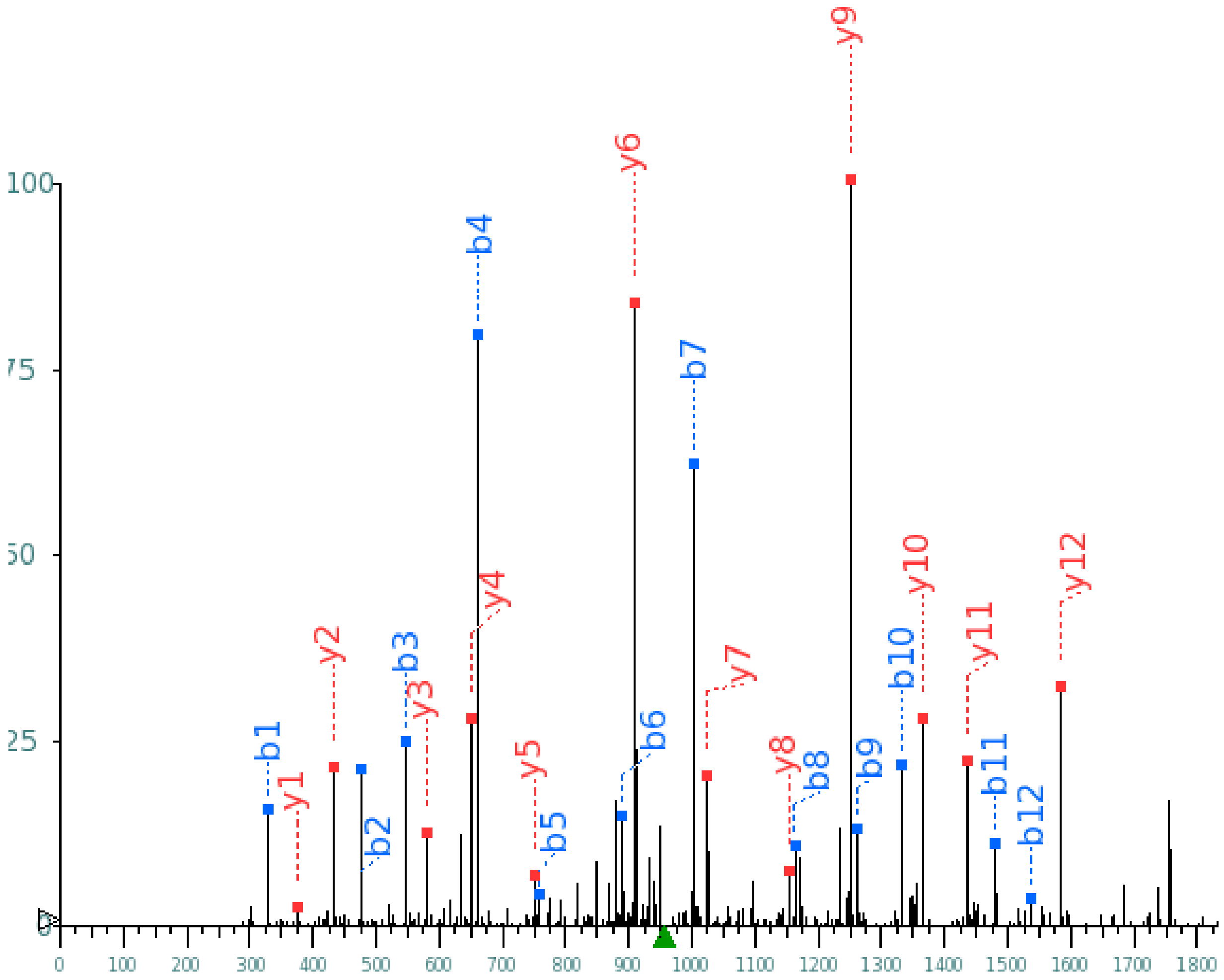

VFANPEDC*VAFGK (+2)

Primary Reference: MGST1_HUMAN
Search ID: 41873
Search Name: 20130330_ananiav_TMT_GPP_10percent_fraction9_lysC_2MC_IAA
Scan#: 18692
Observed Mass: 956.5067 (1.2 ppm)
PSM Score: 72.29

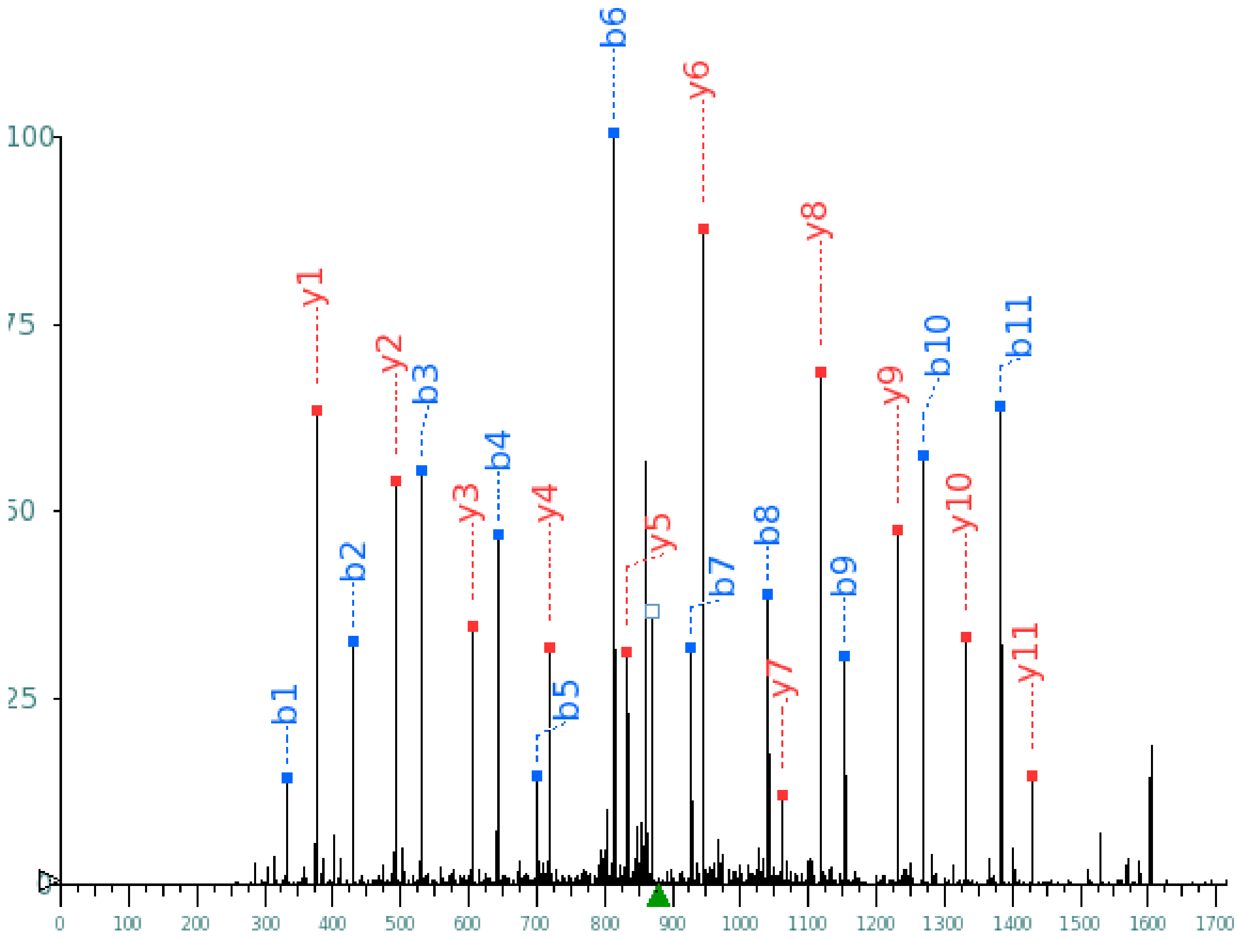

TVVLGDLLIDDK (+2)

Primary Reference: NT5C_HUMAN
Search ID: 41873
Search Name: 20130330_ananiav_TMT_GPP_10percent_fraction9_lysC_2MC_IAA
Scan#: 23274
Observed Mass: 880.0369 (2.7 ppm)
PSM Score: 75.55

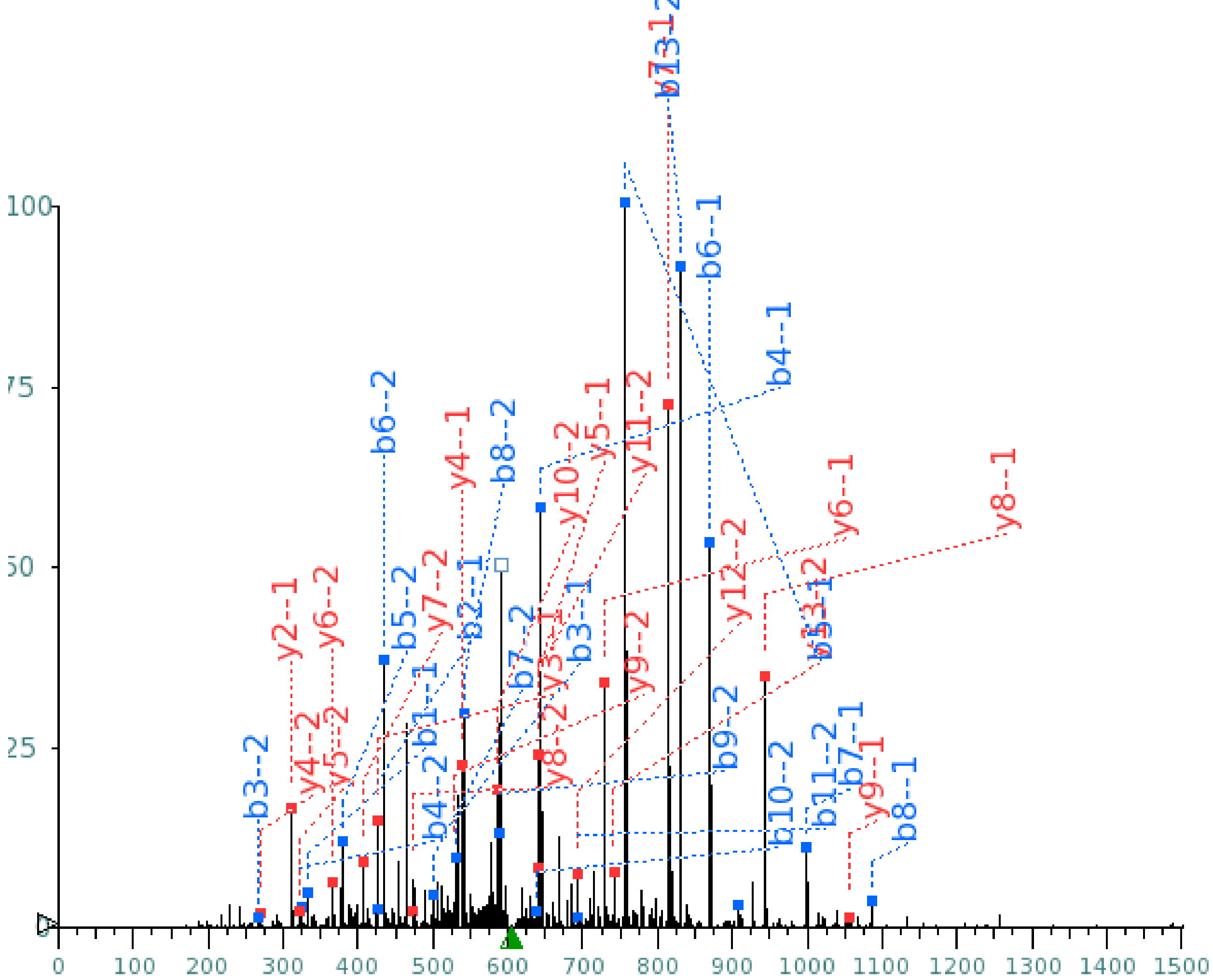

TPTDLLQSSTLDRH (+3)

Primary Reference: B7Z6I4_HUMAN
Search ID: 41874
Search Name: 20130330_ananiav_TMT_GPP_10percent_fraction10_lysC_2MC_IAA
Scan#: 14371
Observed Mass: 604.9944 (1.9 ppm)
PSM Score: 63.92

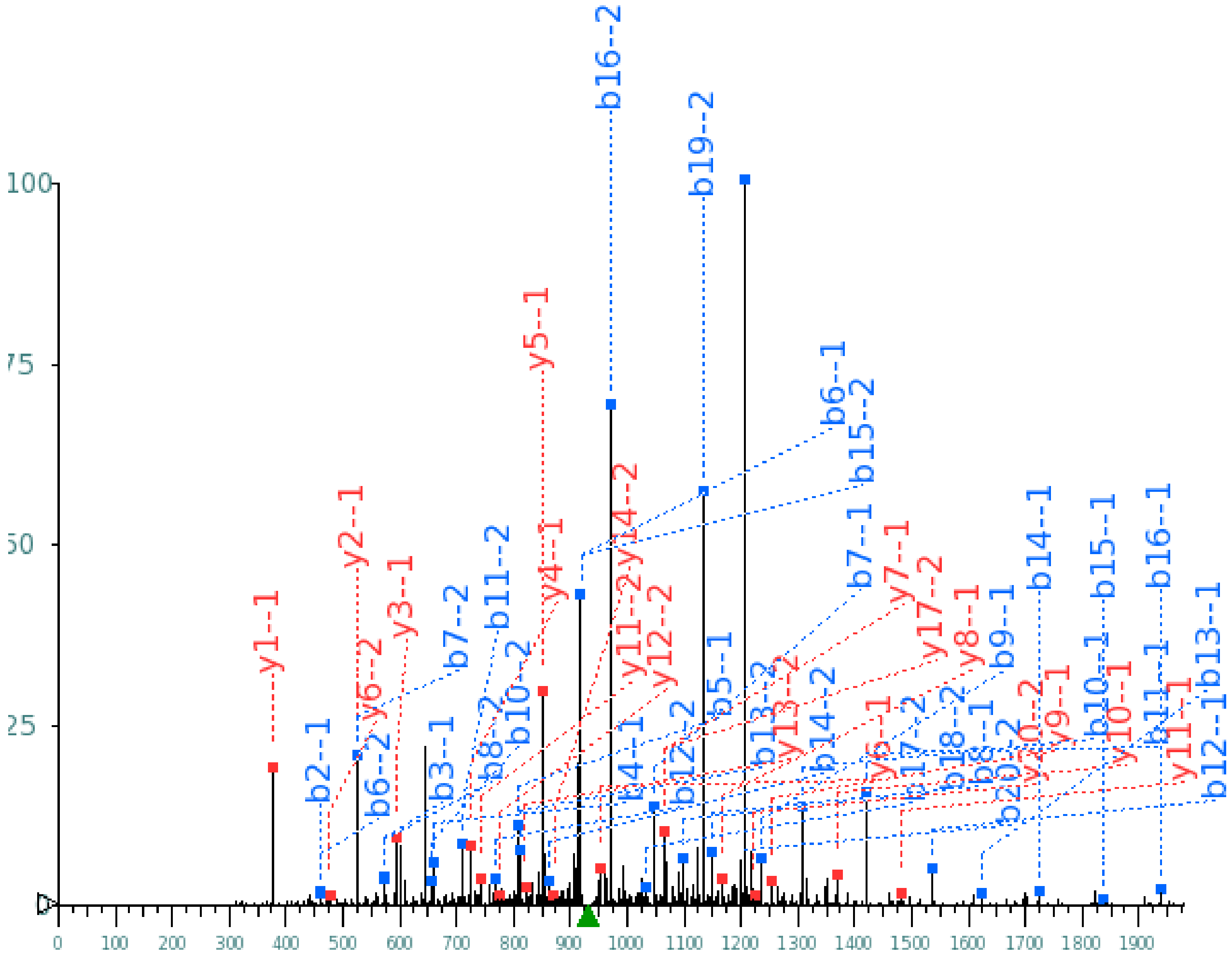

NNLSFIETSALDSTNVEEAFK (+3)

Primary Reference: RB11B_HUMAN
Search ID: 41874
Search Name: 20130330_ananiav_TMT_GPP_10percent_fraction10_lysC_2MC_IAA
Scan#: 24661
Observed Mass: 929.823 (3.3 ppm)
PSM Score: 92.6

File name: 2015-12-17 03.51.45PM.pdf

Creation time: 2015-12-17 03:53PM

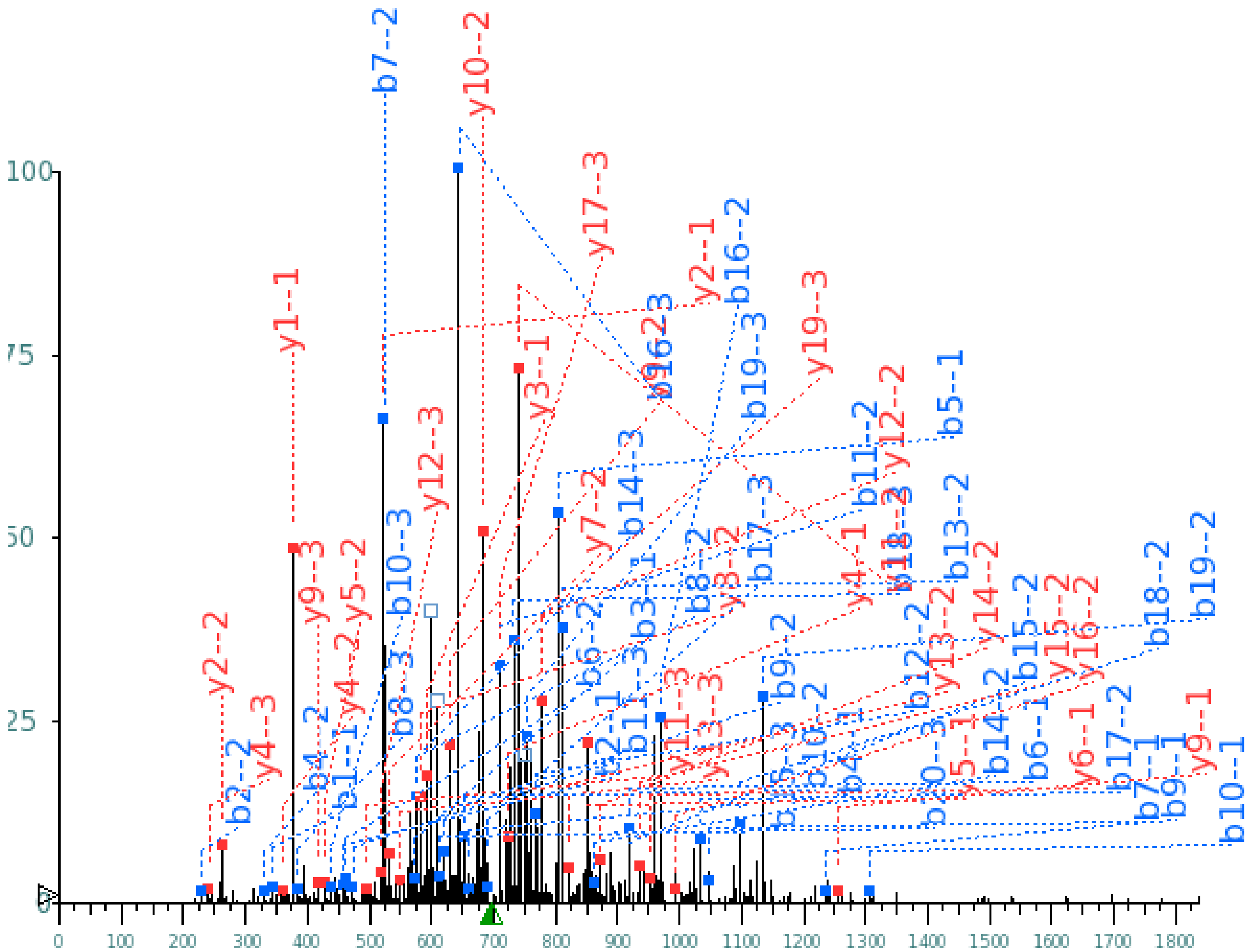

NNLSFIETSALDSTNVEEAFK (+4)

Primary Reference: RB11B_HUMAN
Search ID: 41874
Search Name: 20130330_ananiav_TMT_GPP_10percent_fraction10_lysC_2MC_IAA
Scan#: 24686
Observed Mass: 697.6191 (3.4 ppm)
PSM Score: 57.03

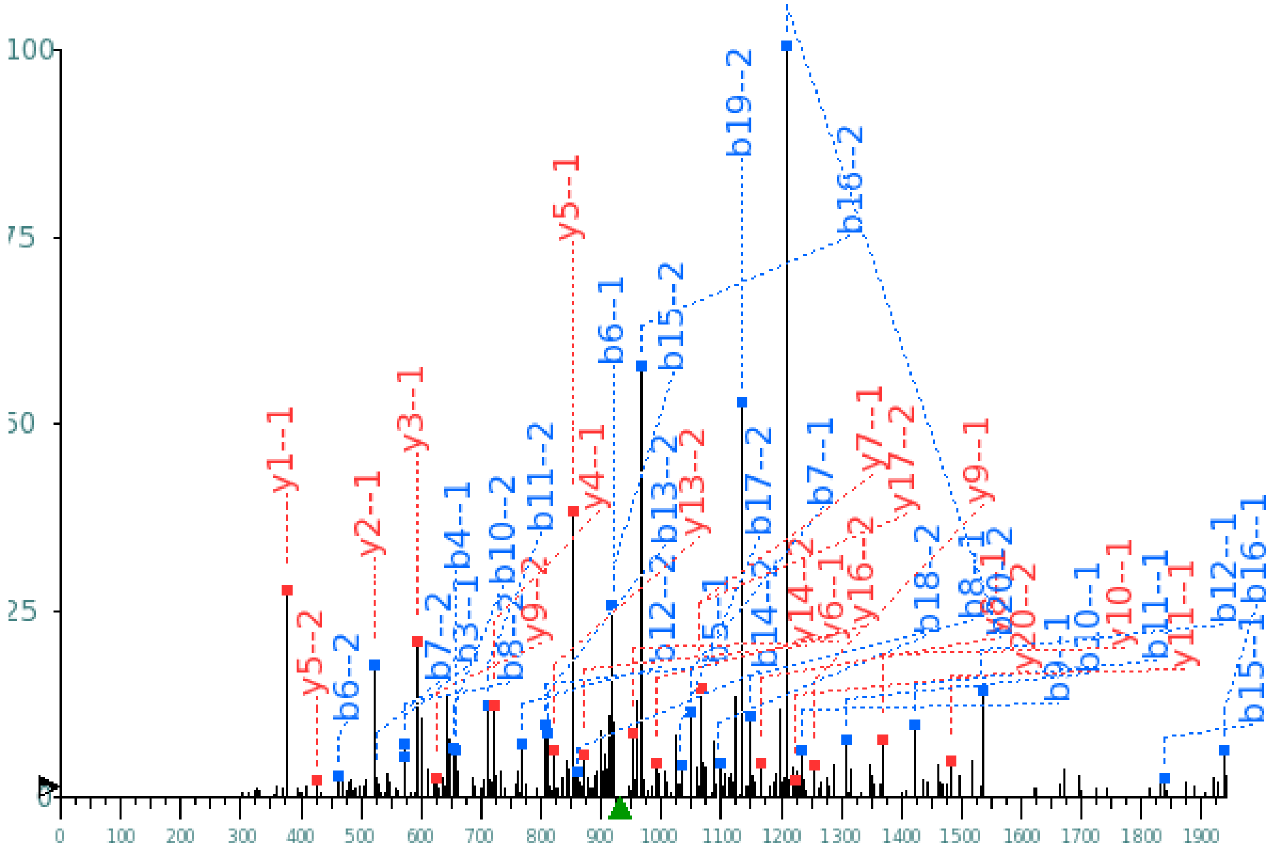

NNLSFIETSALDSTNVEEAFK (+3)

Primary Reference: RB11B_HUMAN
Search ID: 41874
Search Name: 20130330_ananiav_TMT_GPP_10percent_fraction10_lysC_2MC_IAA
Scan#: 24771
Observed Mass: 929.8231 (3.4 ppm)
PSM Score: 79.87

File name: 2015-12-17 03.51.45PM.pdf

Creation time: 2015-12-17 03:53PM

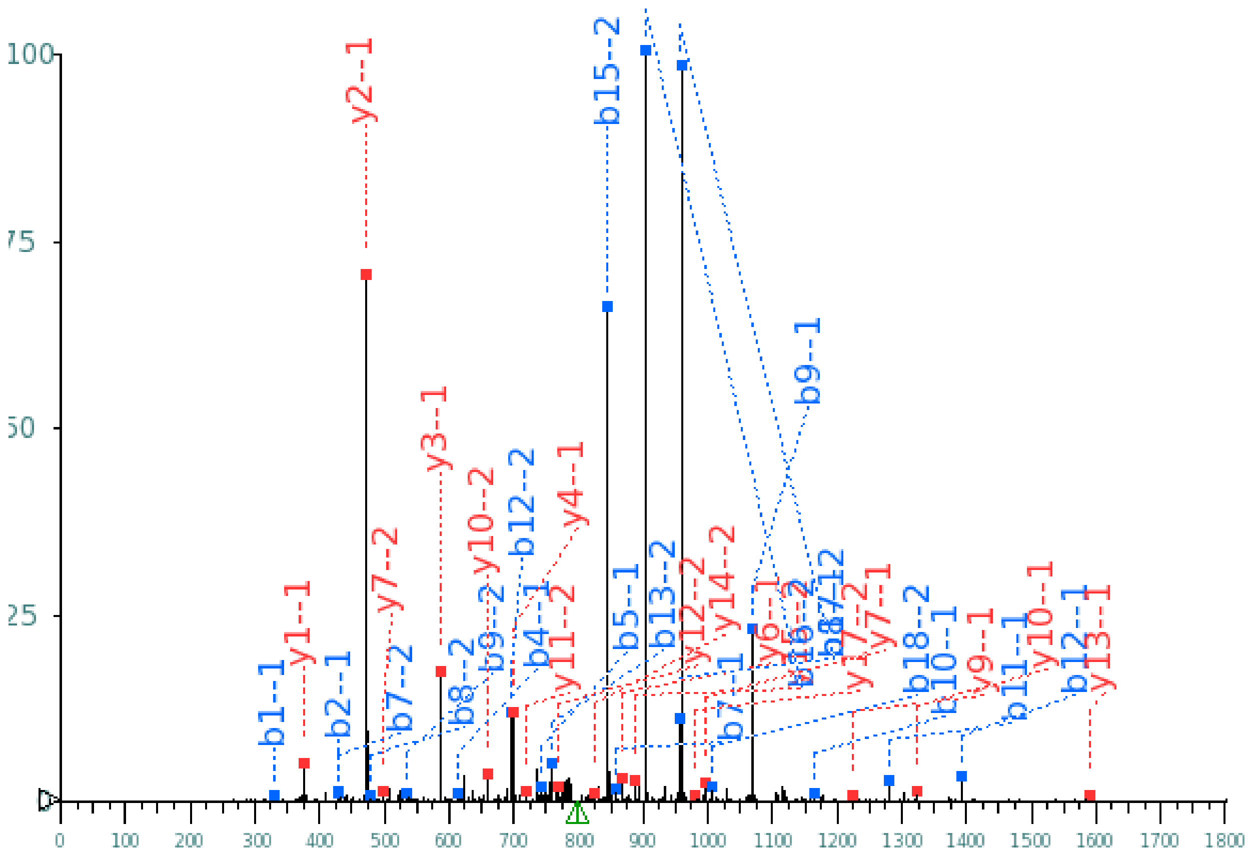

VTIAQGGVLPNIQAVLLPK (+3)

Primary Reference: H2A1D_HUMAN
Search ID: 41874
Search Name: 20130330_ananiav_TMT_GPP_10percent_fraction10_lysC_2MC_IAA
Scan#: 25907
Observed Mass: 797.1714 (2.1 ppm)
PSM Score: 31.33

File name: 2015-12-17 03.51.45PM.pdf

Creation time: 2015-12-17 03:53PM

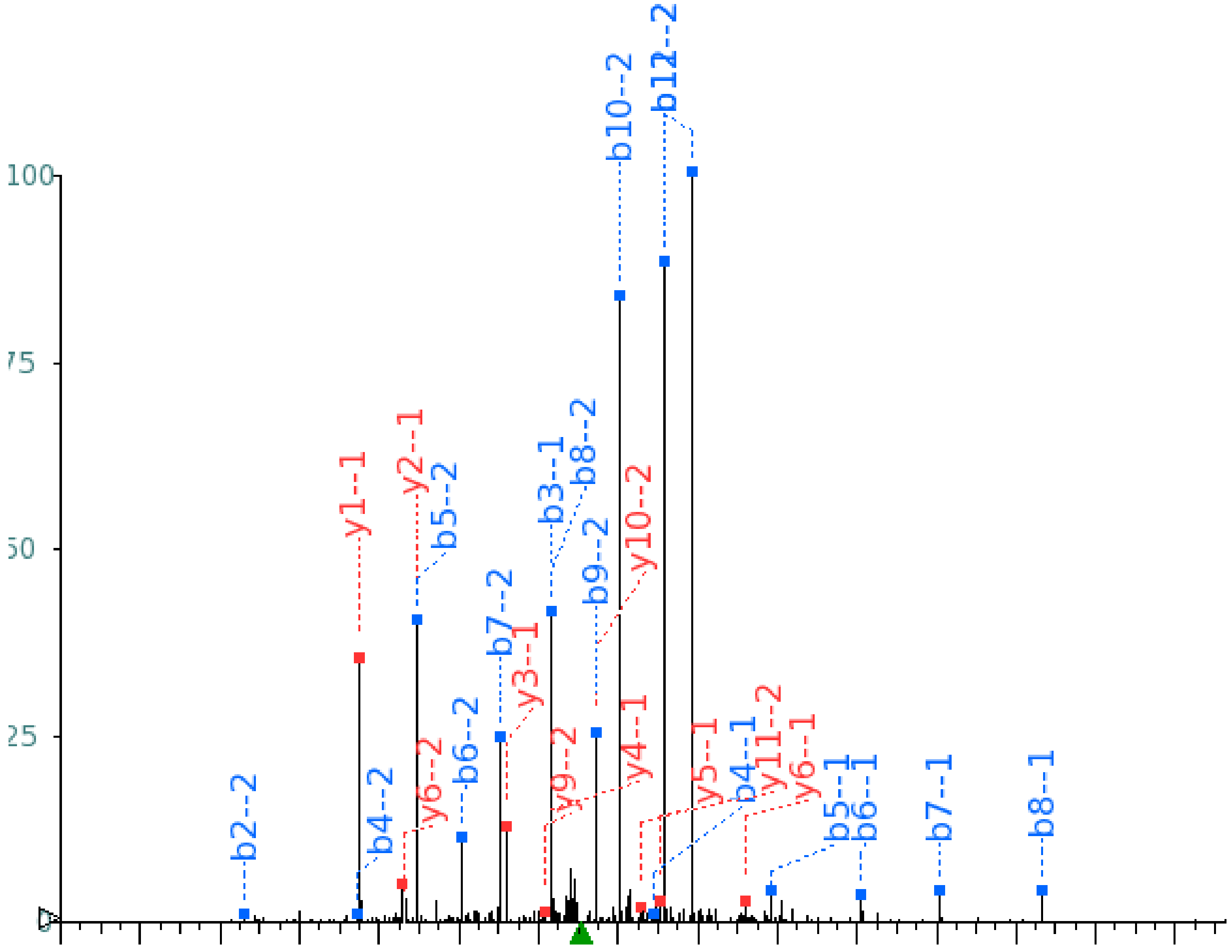

NIC*QFLVEIGLAK (+3)

Primary Reference: EIF1_HUMAN
Search ID: 41874
Search Name: 20130330_ananiav_TMT_GPP_10percent_fraction10_lysC_2MC_IAA
Scan#: 26321
Observed Mass: 655.0552 (3 ppm)
PSM Score: 43.5

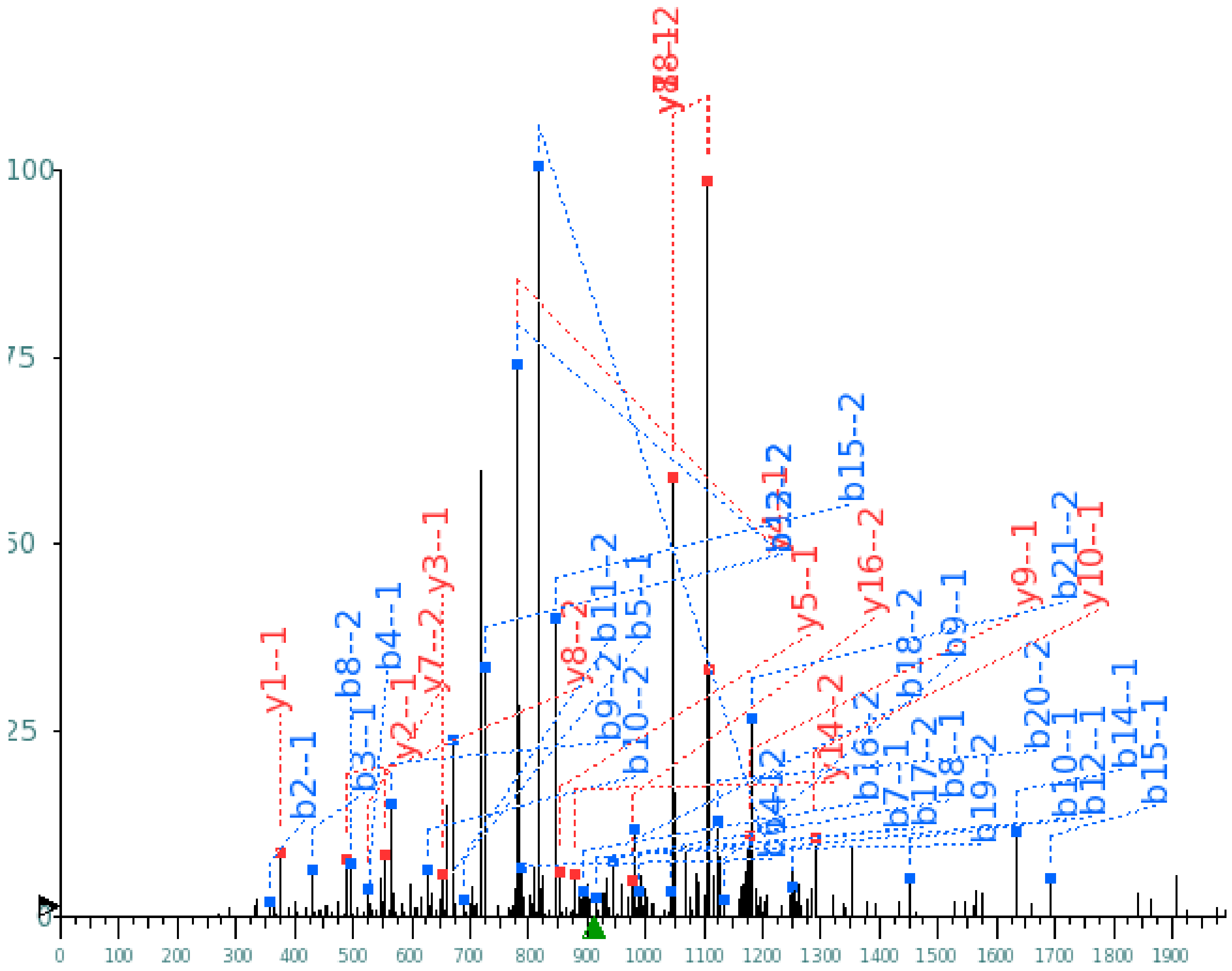

GAAPYVQAFDSLLAGPVAEYLK (+3)

Primary Reference: B4DNA3_HUMAN
Search ID: 41874
Search Name: 20130330_ananiav_TMT_GPP_10percent_fraction10_lysC_2MC_IAA
Scan#: 26524
Observed Mass: 913.5136 (3.4 ppm)
PSM Score: 60.81

File name: 2015-12-17 03.51.45PM.pdf

Creation time: 2015-12-17 03:53PM

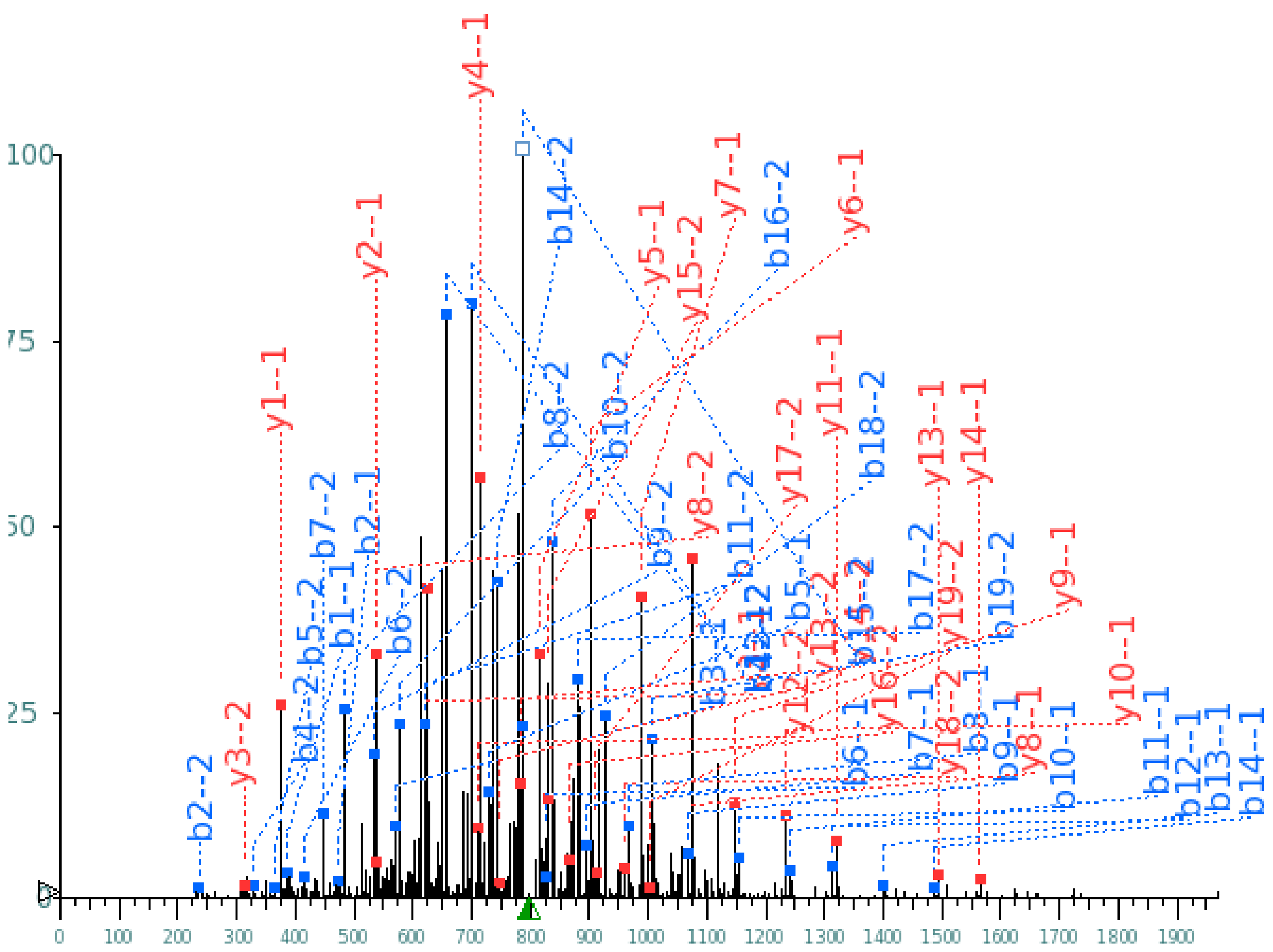

RSPSAPAATSSASSSTSSYK (+3)

Primary Reference: LRC41_HUMAN
Search ID: 41875
Search Name: 20130330_ananiav_TMT_GPP_10percent_fraction11_lysC_2MC_IAA
Scan#: 7500
Observed Mass: 796.7526 (1 ppm)
PSM Score: 82.13

File name: 2015-12-17 03.51.45PM.pdf

Creation time: 2015-12-17 03:53PM

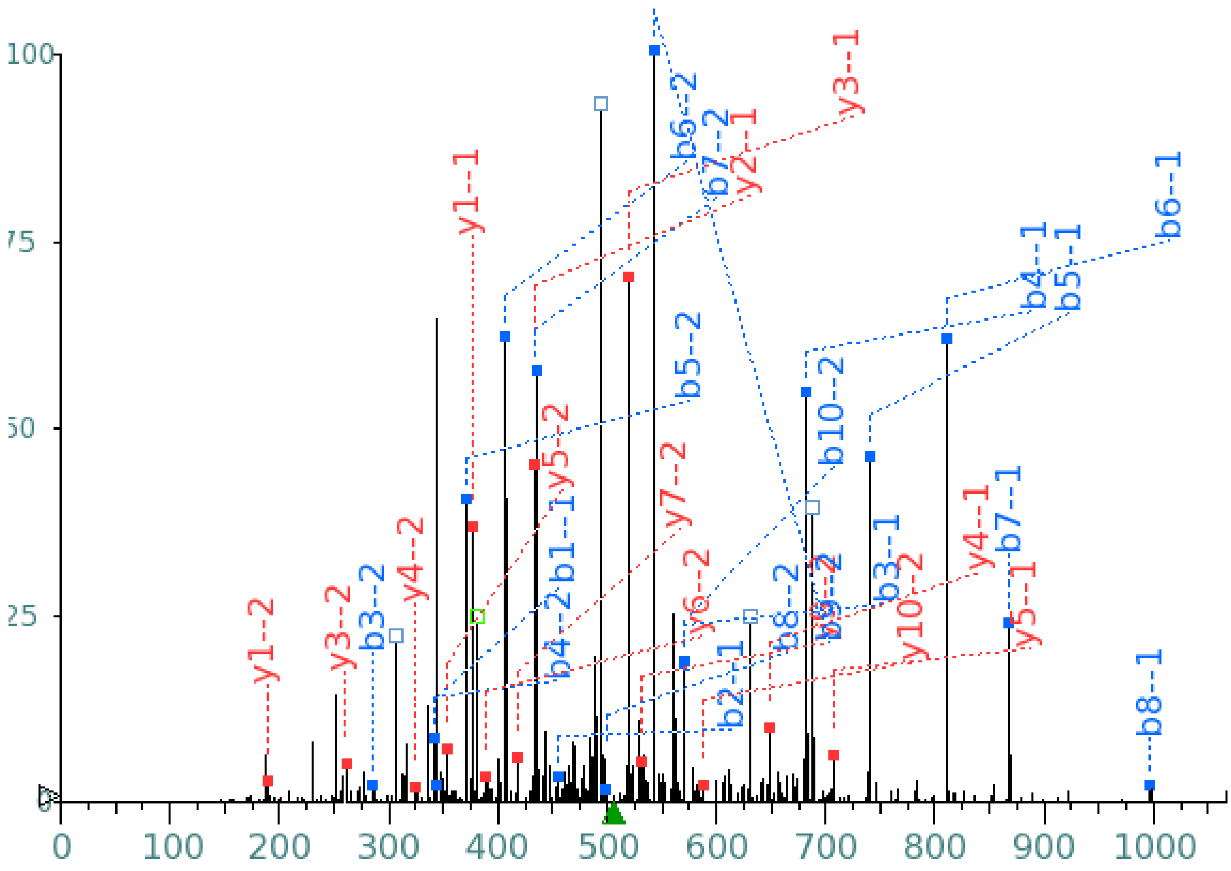

ILLLGAGESGK (+3)

Primary Reference: GNA12_HUMAN
Search ID: 41875
Search Name: 20130330_ananiav_TMT_GPP_10percent_fraction11_lysC_2MC_IAA
Scan#: 19117
Observed Mass: 505.9893 (1.6 ppm)
PSM Score: 31.14

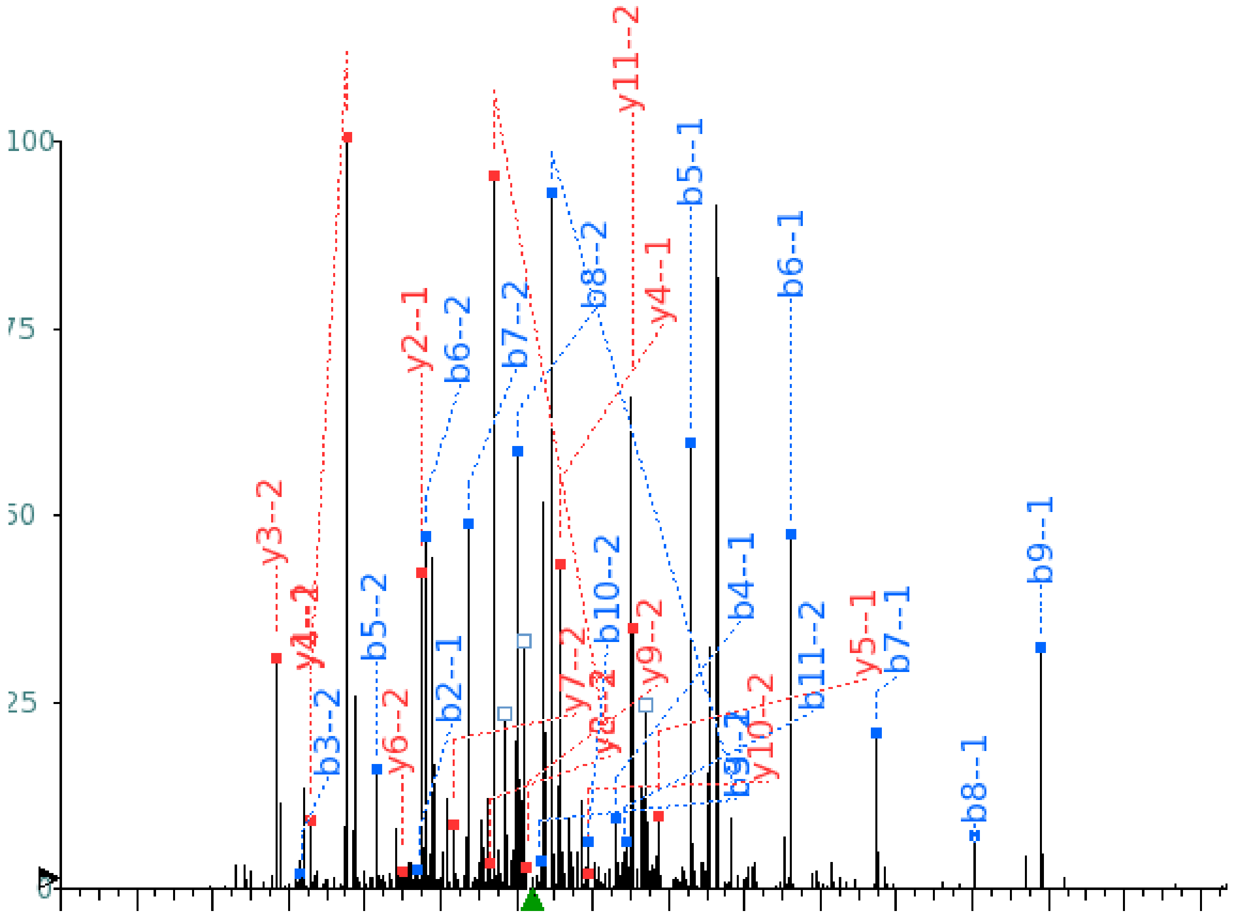

QIC*VVMLESPPK (+3)

Primary Reference: PCBP3_HUMAN
Search ID: 41875
Search Name: 20130330_ananiav_TMT_GPP_10percent_fraction11_lysC_2MC_IAA
Scan#: 19401
Observed Mass: 620.3576 (2.5 ppm)
PSM Score: 16.75

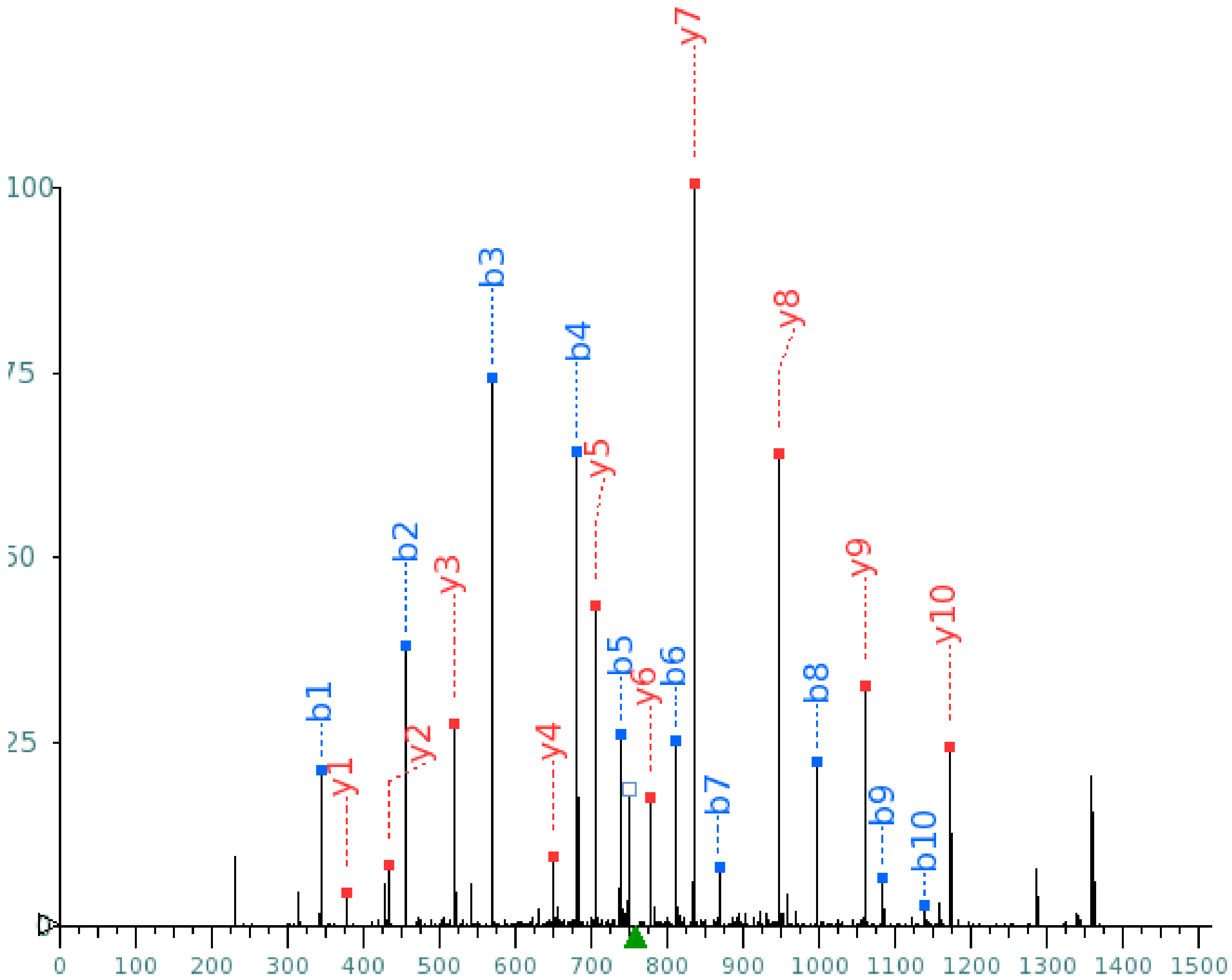

ILLLGAGESGK (+2)

Primary Reference: GNA12_HUMAN
Search ID: 41875
Search Name: 20130330_ananiav_TMT_GPP_10percent_fraction11_lysC_2MC_IAA
Scan#: 19660
Observed Mass: 758.4801 (1.3 ppm)
PSM Score: 55.34

File name: 2015-12-17 03.51.45PM.pdf

Creation time: 2015-12-17 03:53PM

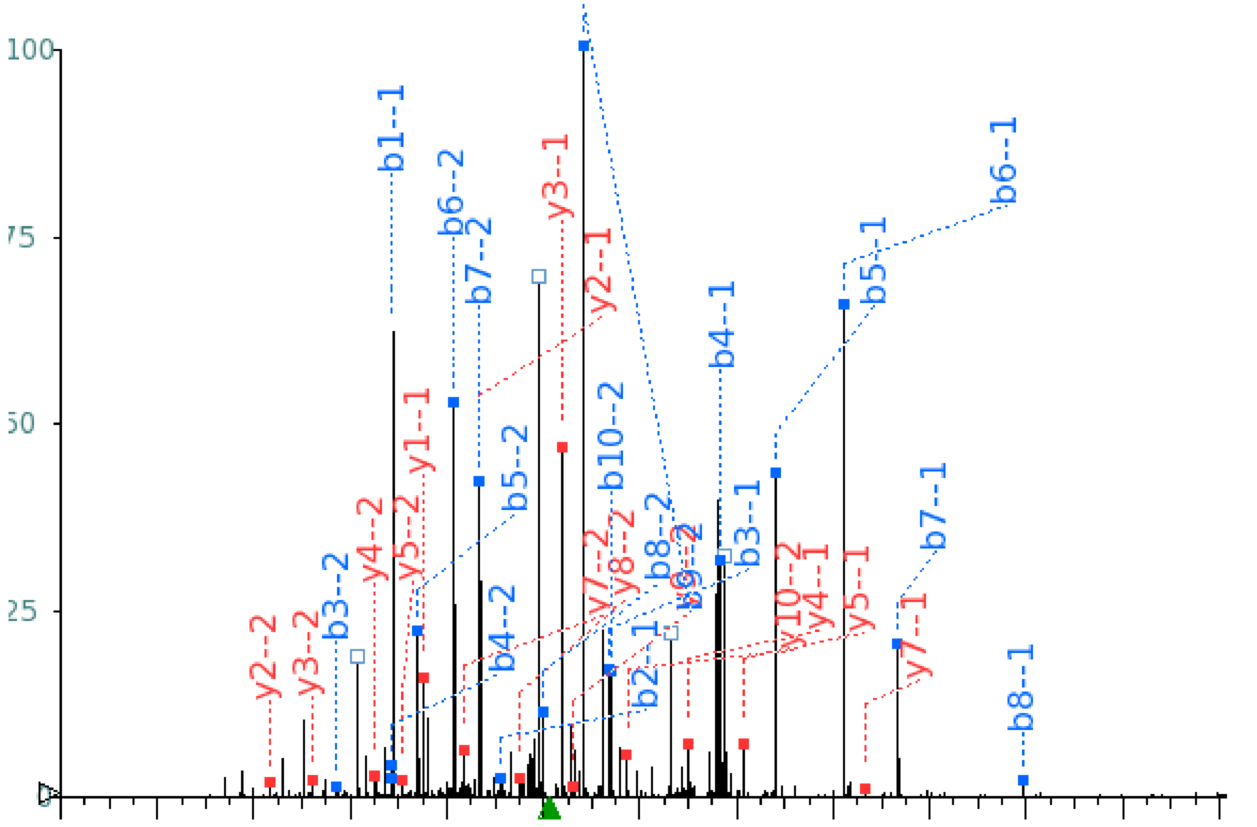

ILLLGAGESGK (+3)

Primary Reference: GNA12_HUMAN
Search ID: 41875
Search Name: 20130330_ananiav_TMT_GPP_10percent_fraction11_lysC_2MC_IAA
Scan#: 19666
Observed Mass: 505.9897 (2.3 ppm)
PSM Score: 30.88

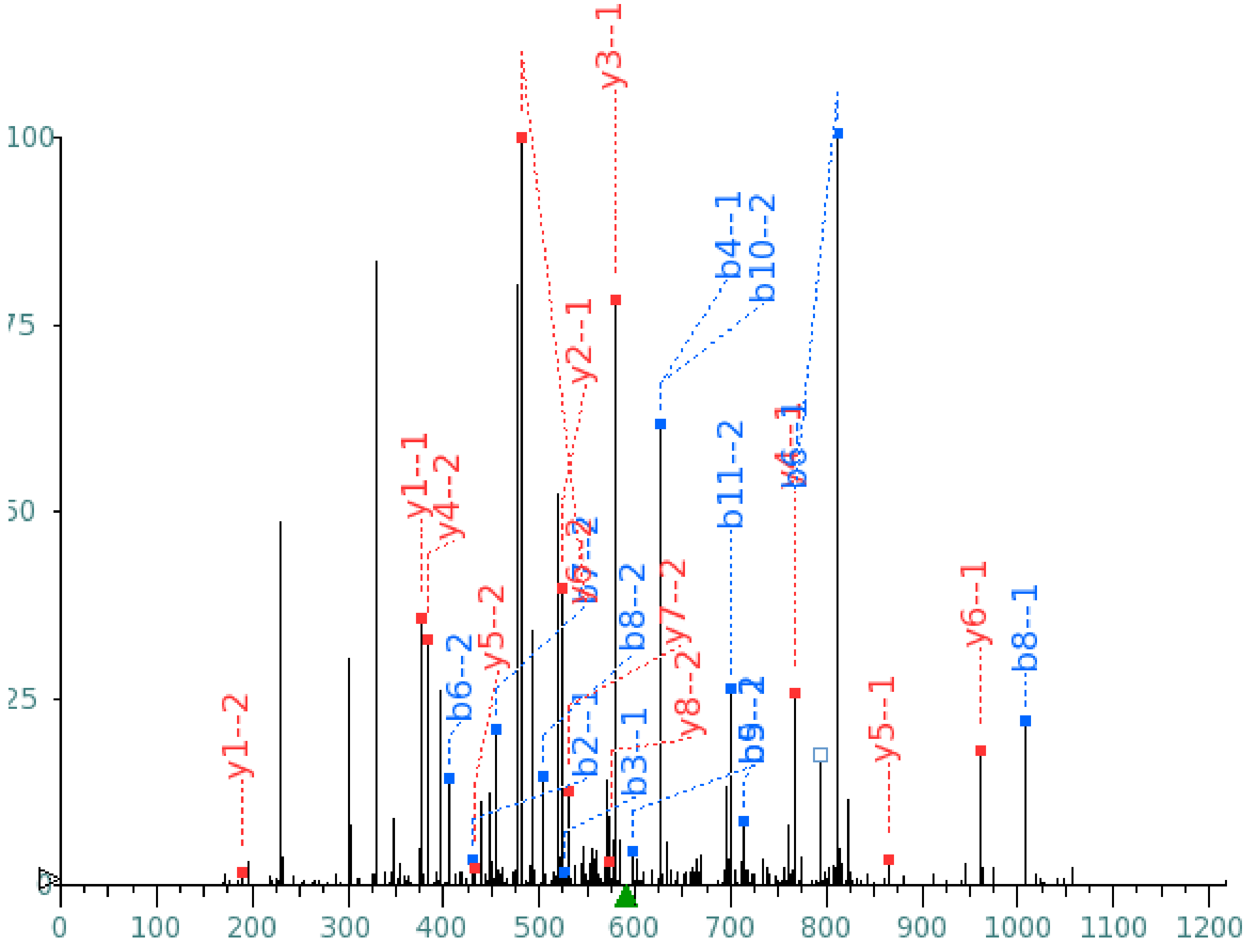

SLPVSVPVWGFK (+3)

Primary Reference: AKTS1_HUMAN
Search ID: 41875
Search Name: 20130330_ananiav_TMT_GPP_10percent_fraction11_lysC_2MC_IAA
Scan#: 24757
Observed Mass: 592.0288 (2.9 ppm)
PSM Score: 17.53

File name: 2015-12-17 03.51.45PM.pdf

Creation time: 2015-12-17 03:53PM

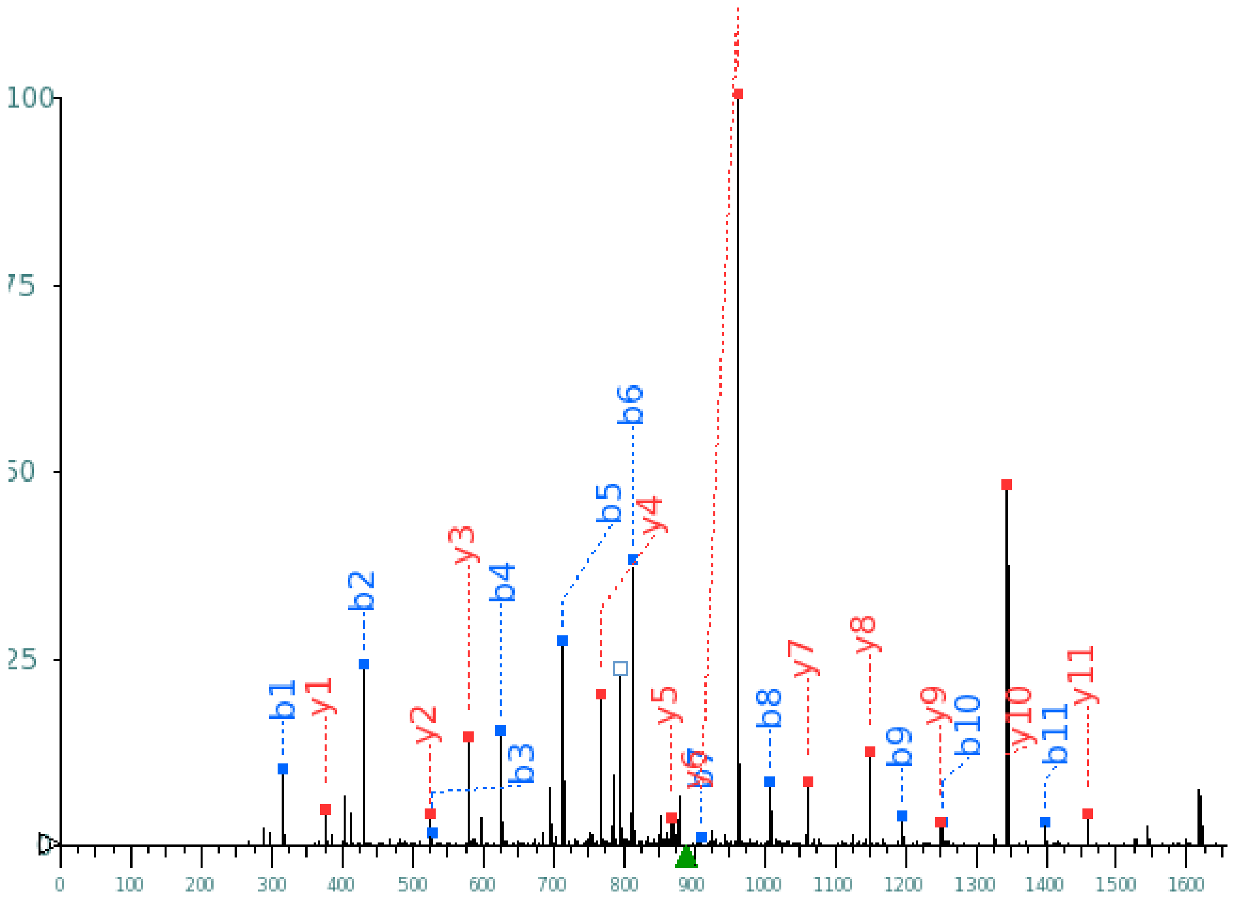

SLPVSVPVWGFK (+2)

Primary Reference: AKTS1_HUMAN
Search ID: 41875
Search Name: 20130330_ananiav_TMT_GPP_10percent_fraction11_lysC_2MC_IAA
Scan#: 24763
Observed Mass: 887.5377 (0.8 ppm)
PSM Score: 62.11

File name: 2015-12-17 03.51.45PM.pdf

Creation time: 2015-12-17 03:53PM

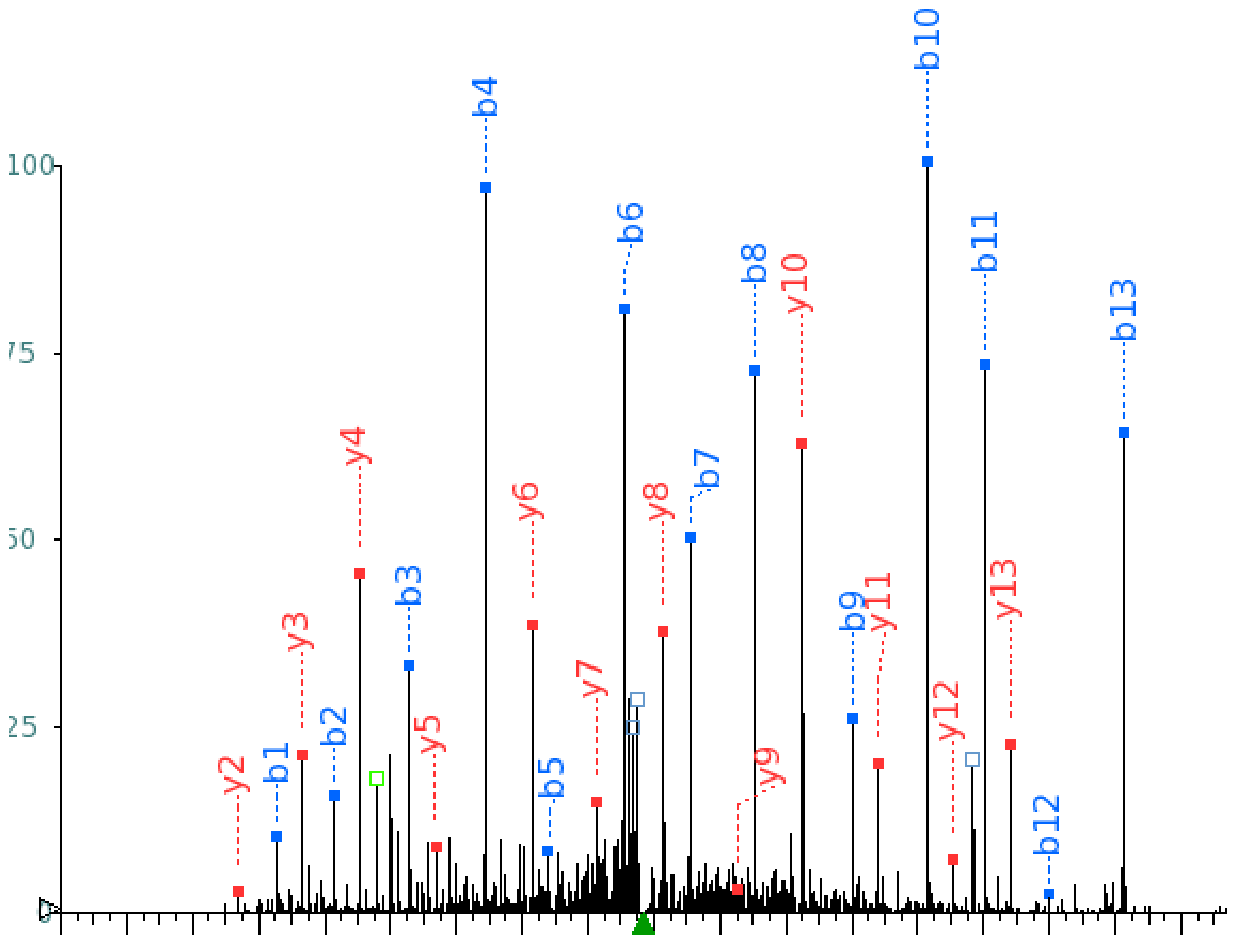

VSILPDVVFDSPLH (+2)

Primary Reference: SUFU_HUMAN
Search ID: 41875
Search Name: 20130330_ananiav_TMT_GPP_10percent_fraction11_lysC_2MC_IAA
Scan#: 26129
Observed Mass: 884.0007 (2.9 ppm)
PSM Score: 81.67

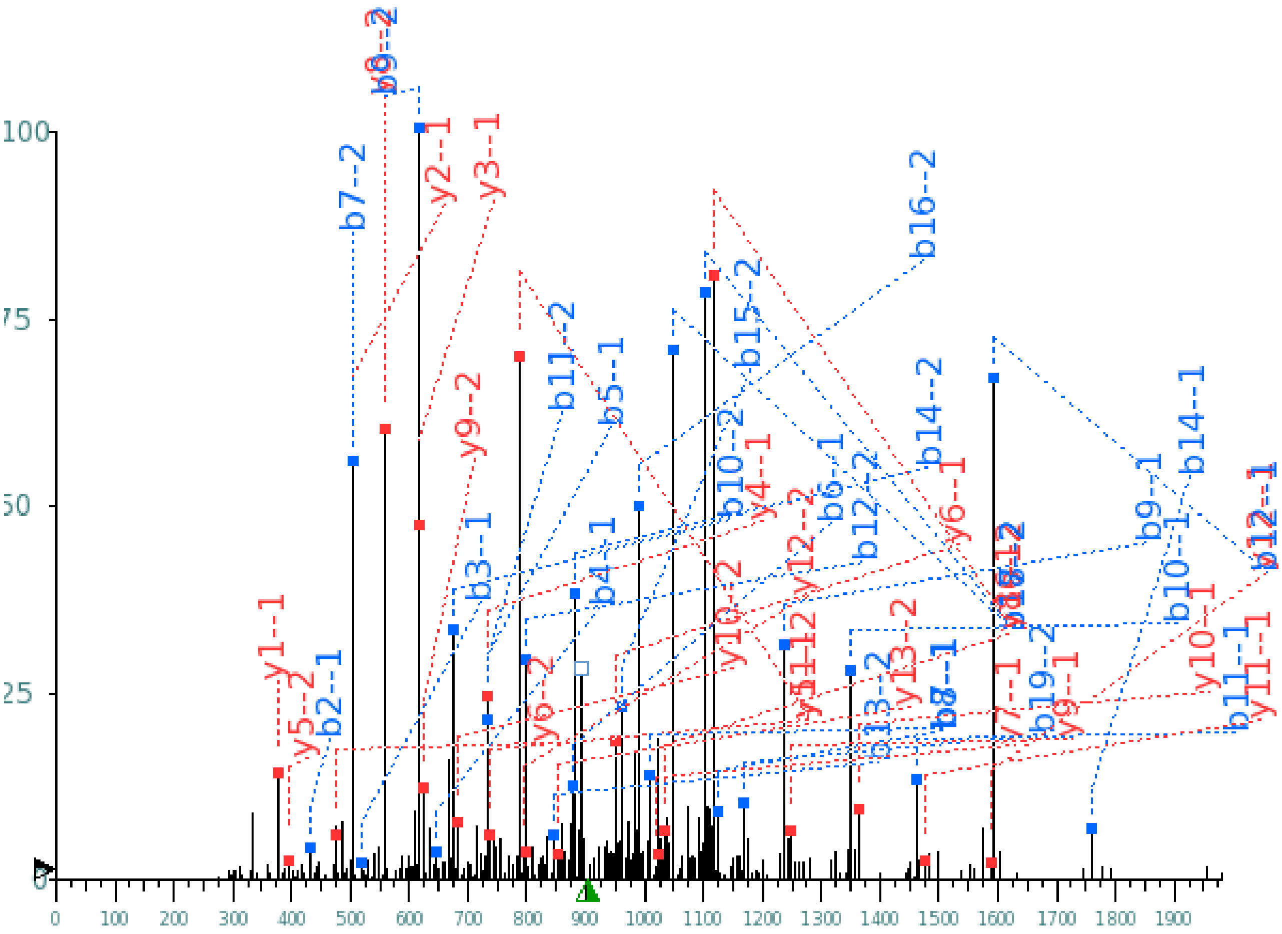

SLSQSFENLLDEPAYGLIQK (+3)

Primary Reference: B4DMT9_HUMAN
Search ID: 41875
Search Name: 20130330_ananiav_TMT_GPP_10percent_fraction11_lysC_2MC_IAA
Scan#: 26781
Observed Mass: 904.163 (1.5 ppm)
PSM Score: 56.91

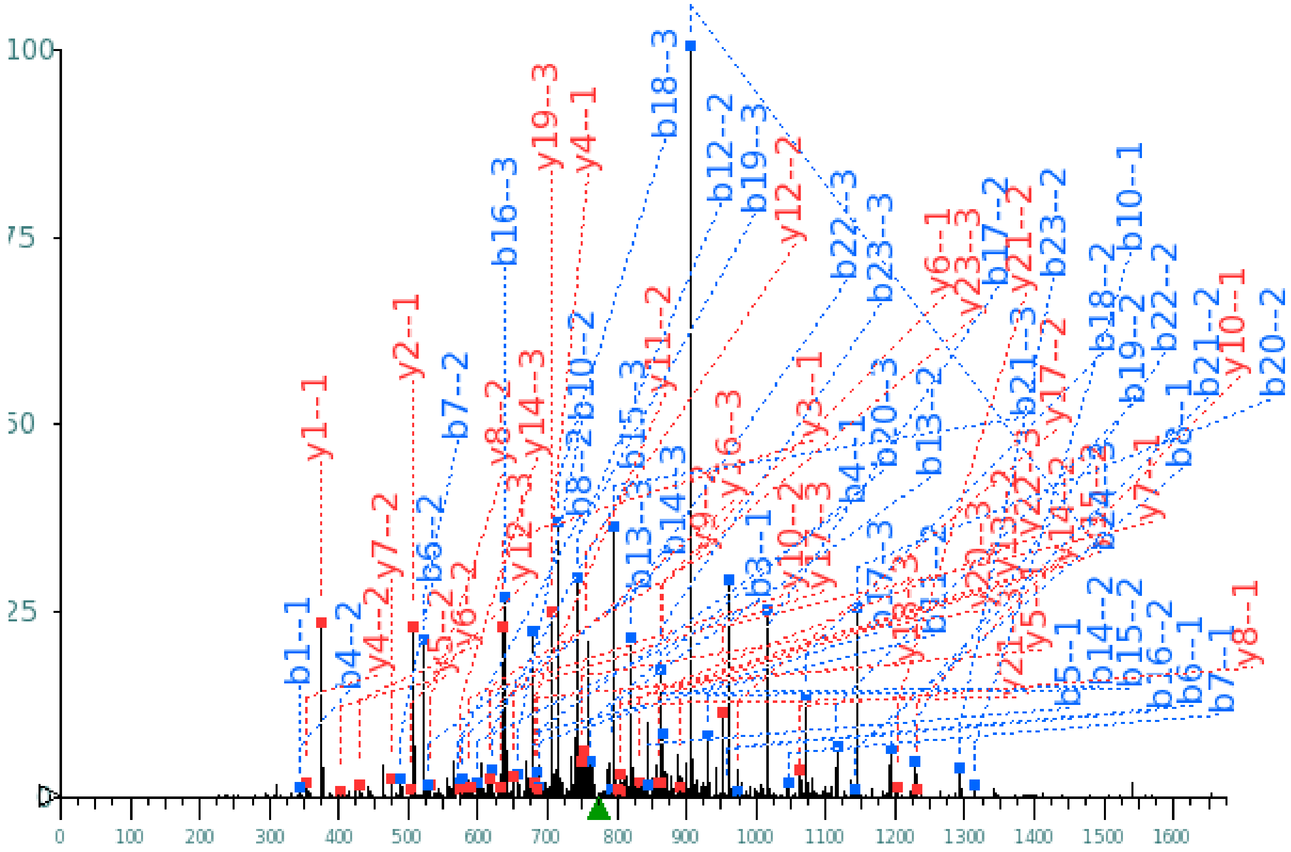

LRVSC*EAPGDGDPFQGLLSGVAQMK (+4)

Primary Reference: CN142_HUMAN
Search ID: 41876
Search Name: 20130330_ananiav_TMT_GPP_10percent_fraction12_lysC_2MC_IAA
Scan#: 25884
Observed Mass: 773.409 (0.8 ppm)
PSM Score: 43.9

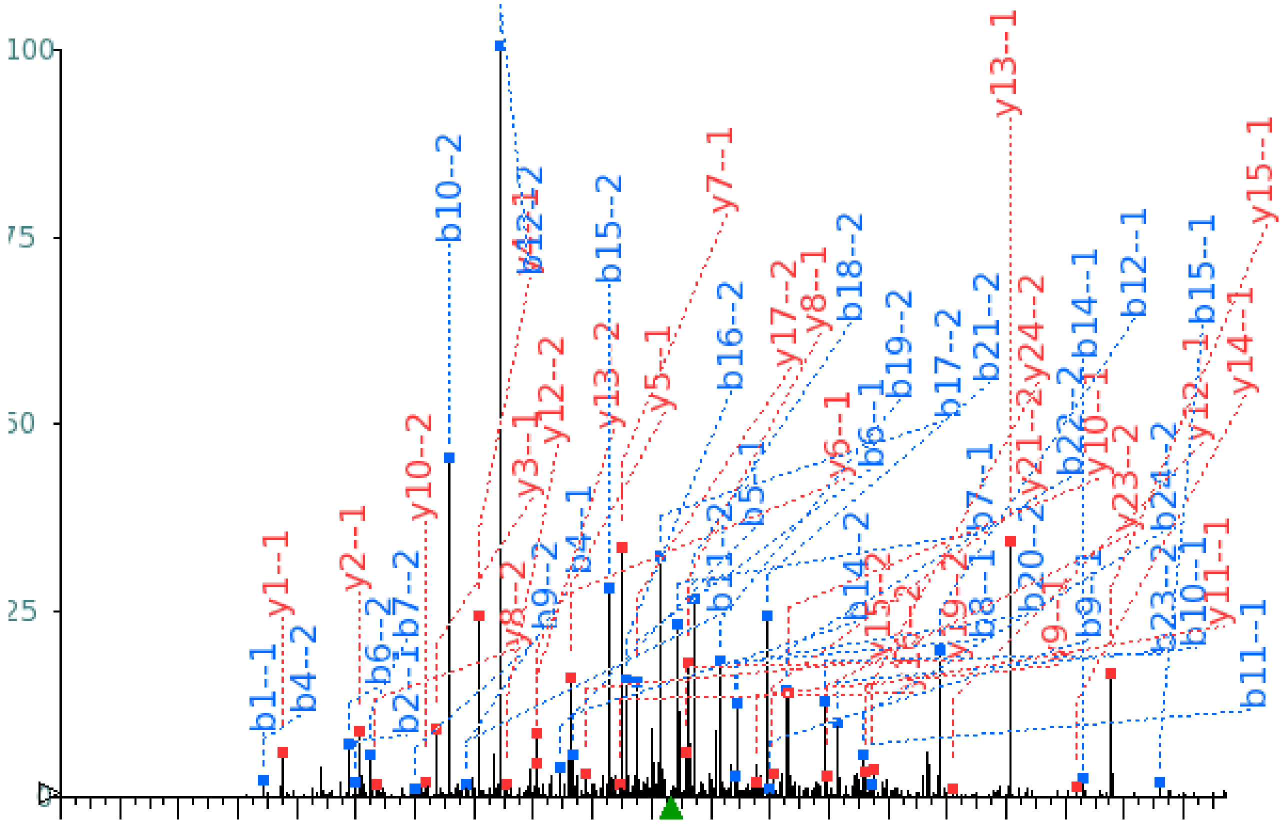

LRVSC*EAPGDGDPFQGLLSGVAQMK (+3)

Primary Reference: CN142_HUMAN
Search ID: 41876
Search Name: 20130330_ananiav_TMT_GPP_10percent_fraction12_lysC_2MC_IAA
Scan#: 25903
Observed Mass: 1030.8778 (2.4 ppm)
PSM Score: 101.93

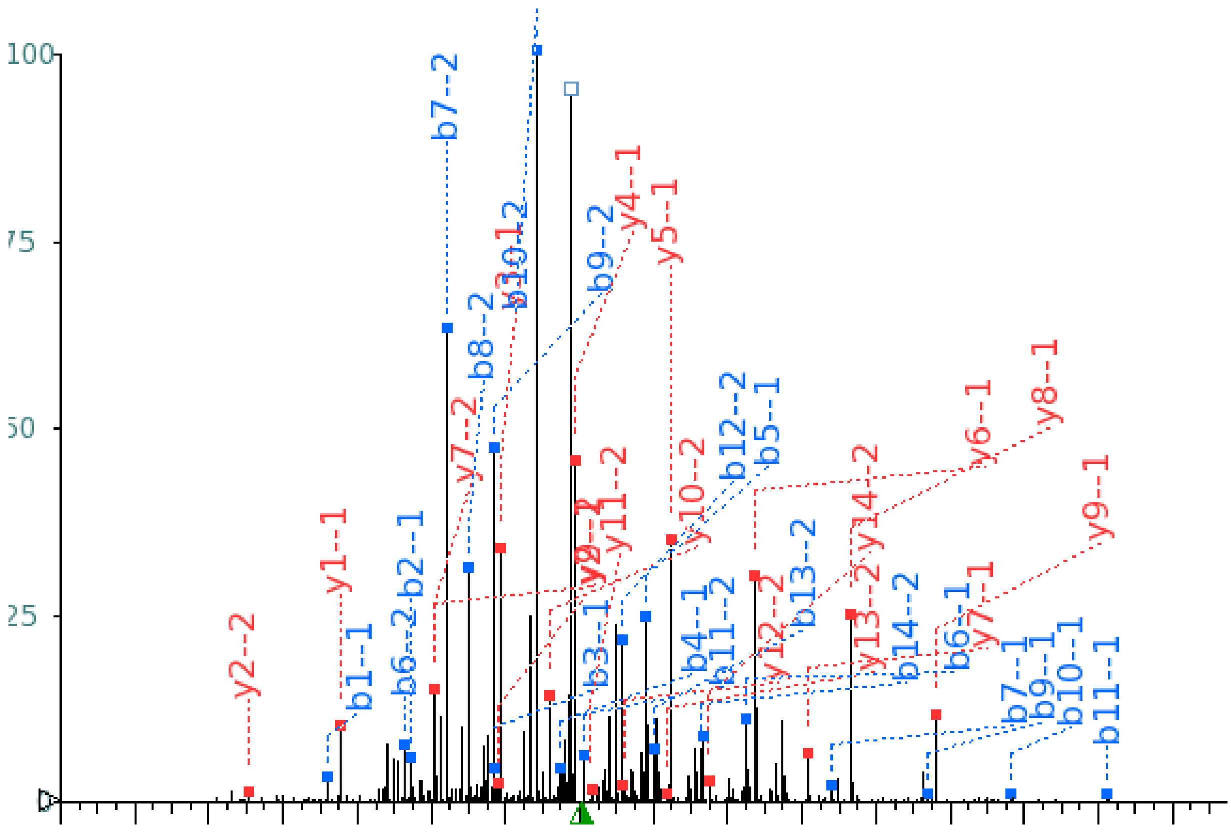

ELNSNHDGADETSEK (+3)

Primary Reference: B2RCX0_HUMAN
Search ID: 41884
Search Name: 20130330_ananiav_TMT_GPP_10percent_fraction13_lysC_2MC_IAA
Scan#: 6383
Observed Mass: 702.0121 (1 ppm)
PSM Score: 39.45

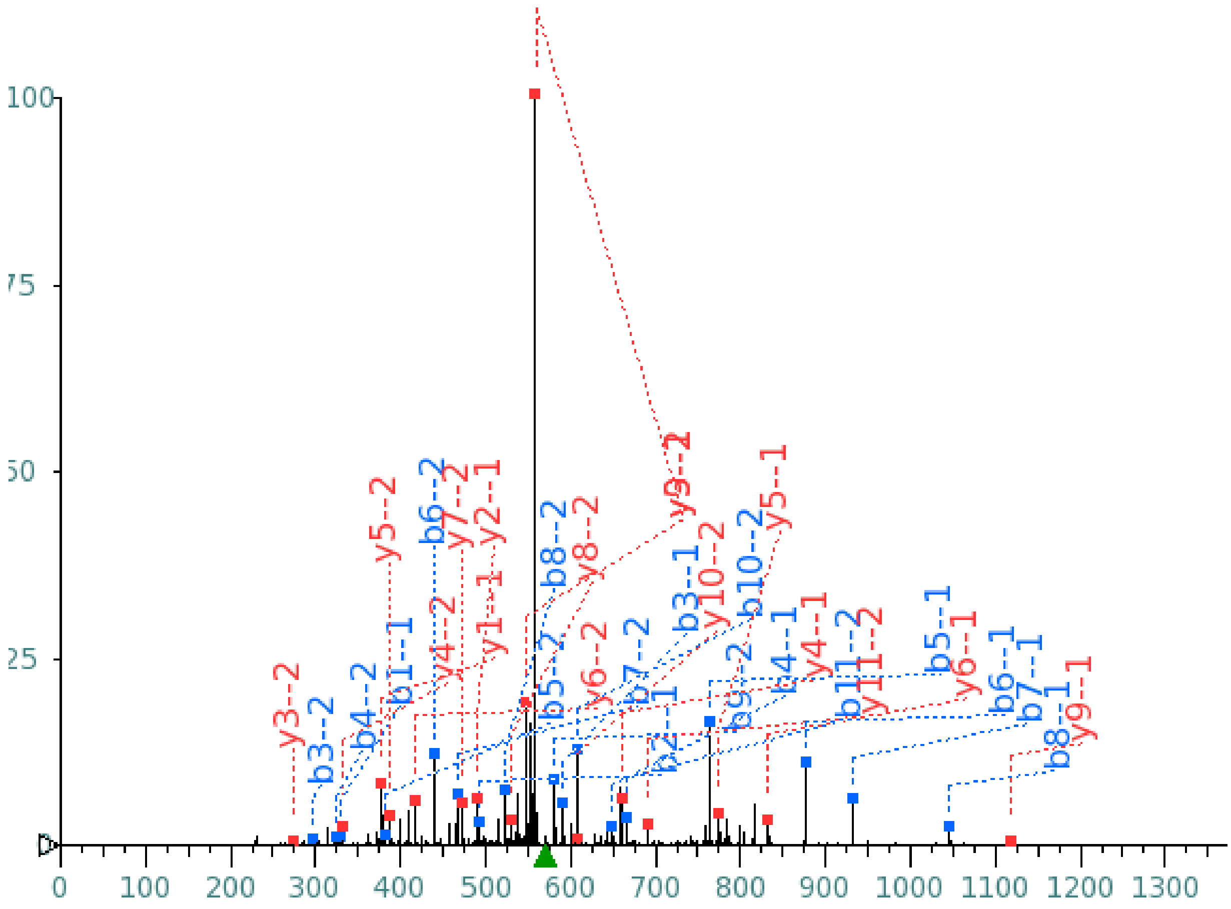

VYVGNLGNNGNK (+3)

Primary Reference: SRSF3_HUMAN
Search ID: 41884
Search Name: 20130330_ananiav_TMT_GPP_10percent_fraction13_lysC_2MC_IAA
Scan#: 12598
Observed Mass: 569.6588 (1.7 ppm)
PSM Score: 36.14

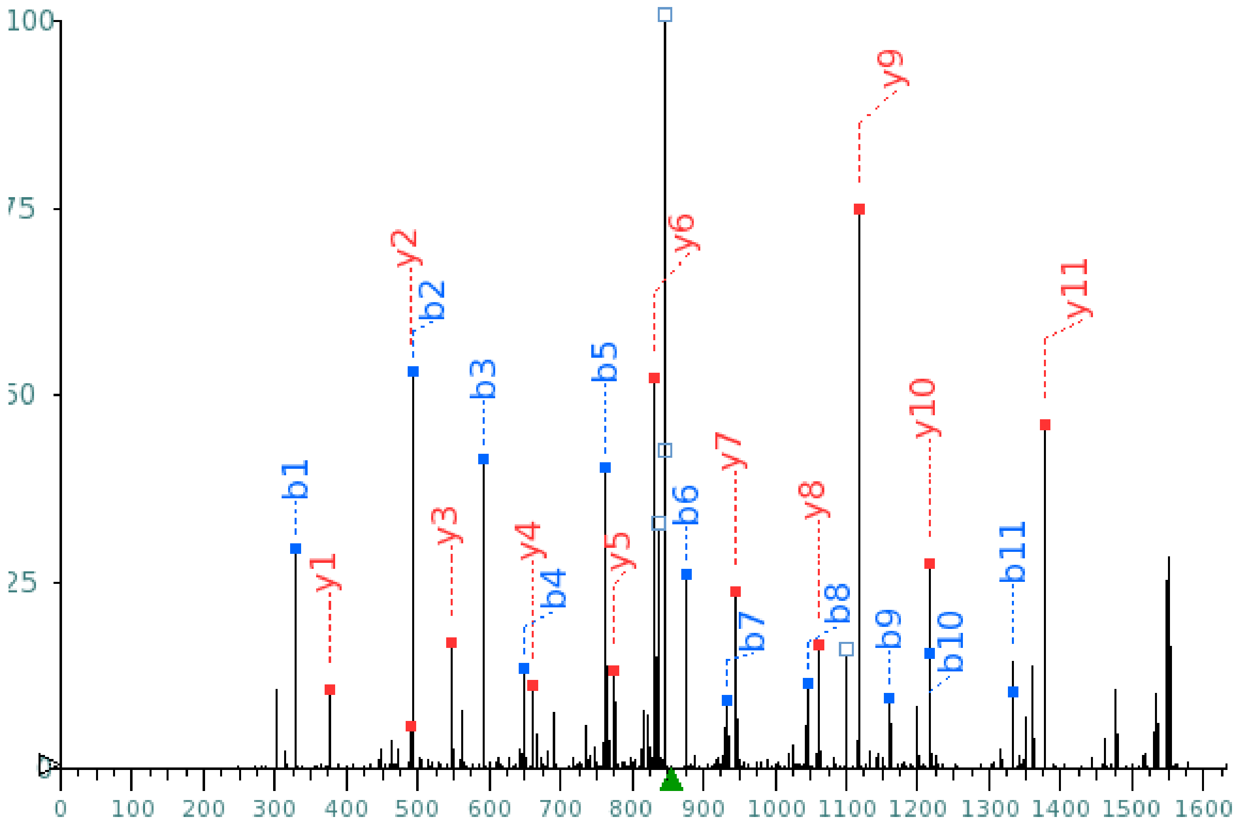

VYVGNLGNNGNK (+2)

Primary Reference: SRSF3_HUMAN
Search ID: 41884
Search Name: 20130330_ananiav_TMT_GPP_10percent_fraction13_lysC_2MC_IAA
Scan#: 12619
Observed Mass: 853.984 (1 ppm)
PSM Score: 51.43

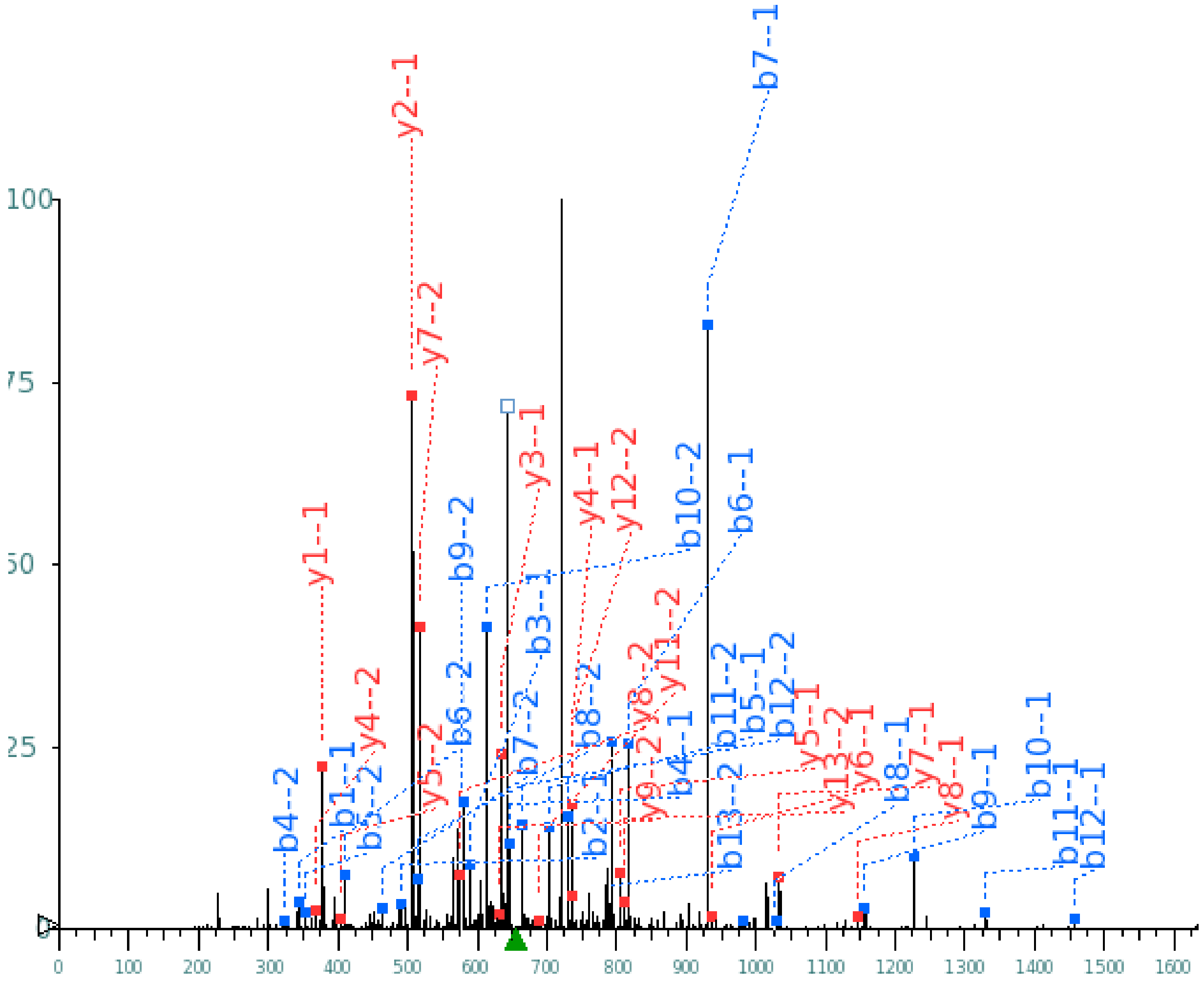

IFVGGLNPEATEEK (+3)

Primary Reference: B4DMY3_HUMAN
Search ID: 41884
Search Name: 20130330_ananiav_TMT_GPP_10percent_fraction13_lysC_2MC_IAA
Scan#: 19039
Observed Mass: 654.7052 (3.1 ppm)
PSM Score: 41.34

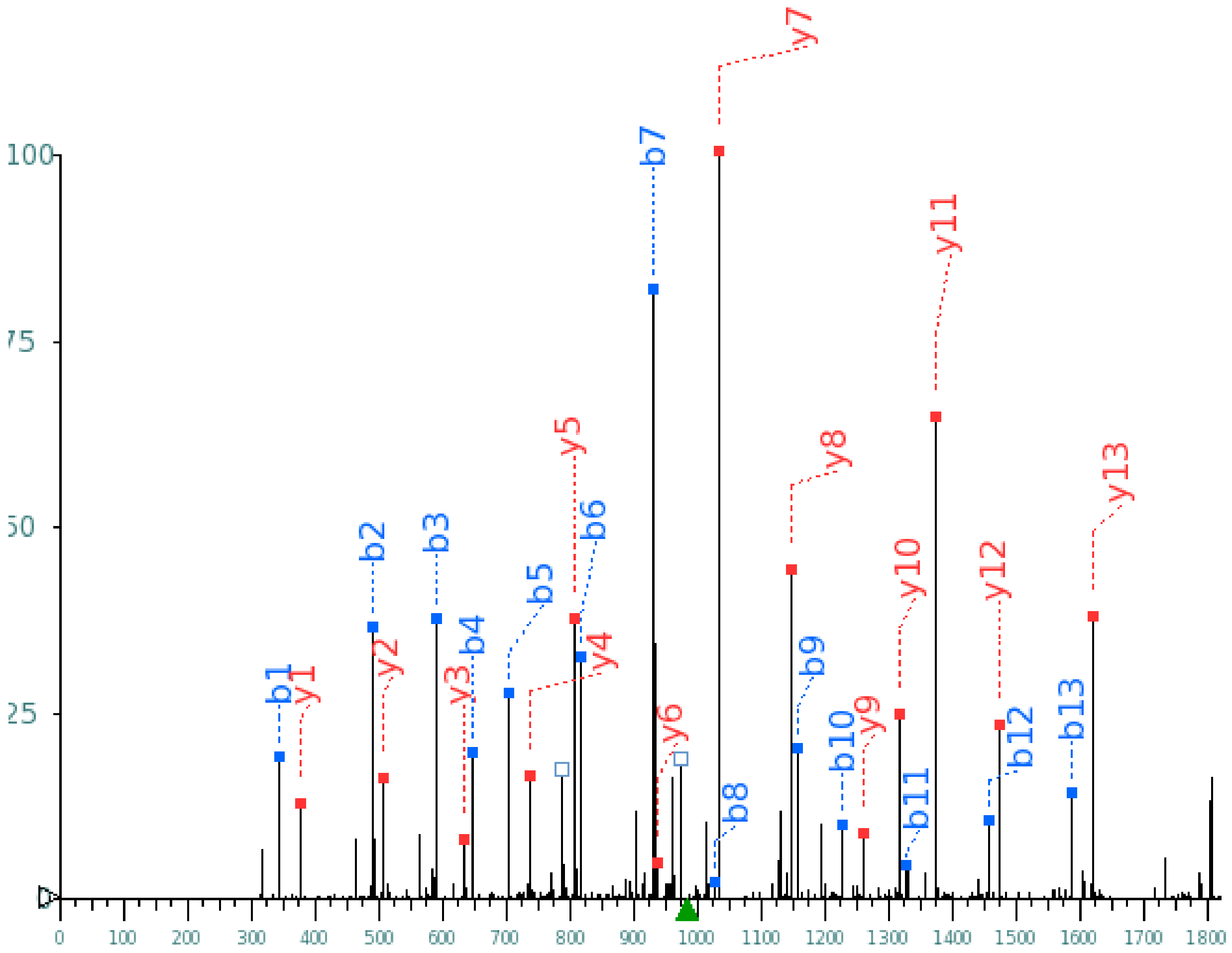

IFVGGLNPEATEEK (+2)

Primary Reference: B4DMY3_HUMAN
Search ID: 41884
Search Name: 20130330_ananiav_TMT_GPP_10percent_fraction13_lysC_2MC_IAA
Scan#: 19060
Observed Mass: 981.552 (0.9 ppm)
PSM Score: 77.84

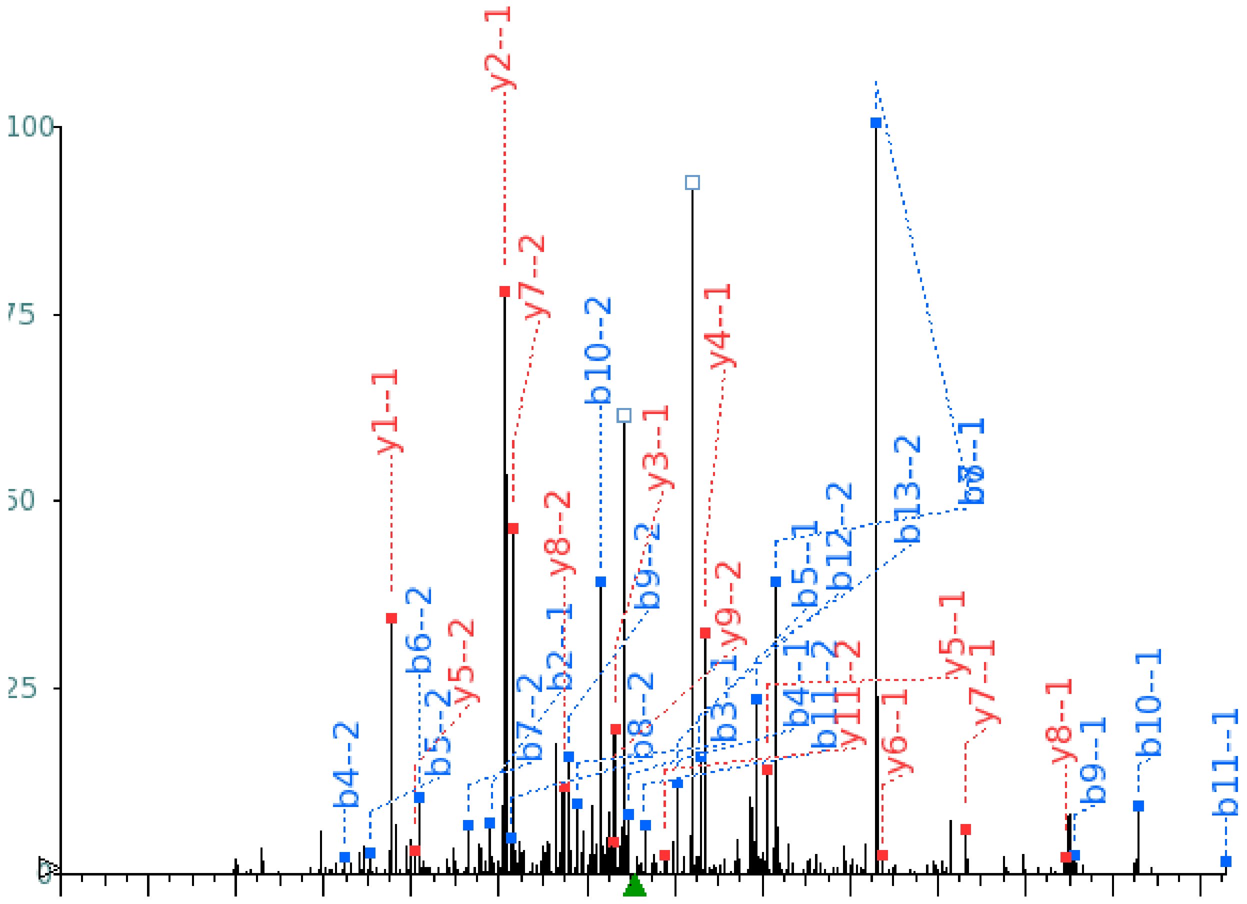

IFVGGLNPEATEEK (+3)

Primary Reference: B4DMY3_HUMAN
Search ID: 41884
Search Name: 20130330_ananiav_TMT_GPP_10percent_fraction13_lysC_2MC_IAA
Scan#: 19139
Observed Mass: 654.7051 (3 ppm)
PSM Score: 28.67

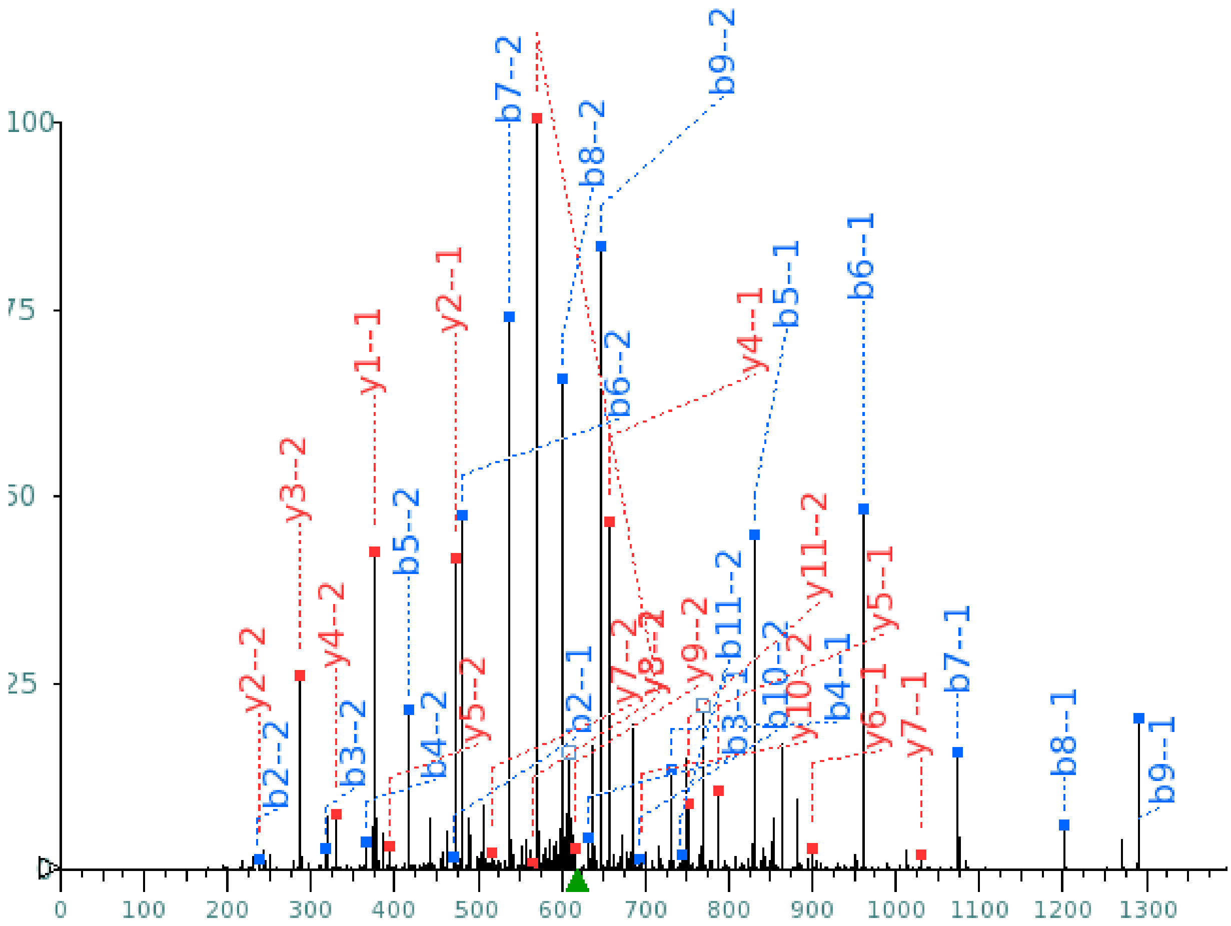

QIC*VVMLESPPK (+3)

Primary Reference: PCBP3_HUMAN
Search ID: 41884
Search Name: 20130330_ananiav_TMT_GPP_10percent_fraction13_lysC_2MC_IAA
Scan#: 19472
Observed Mass: 620.3577 (2.7 ppm)
PSM Score: 31.71

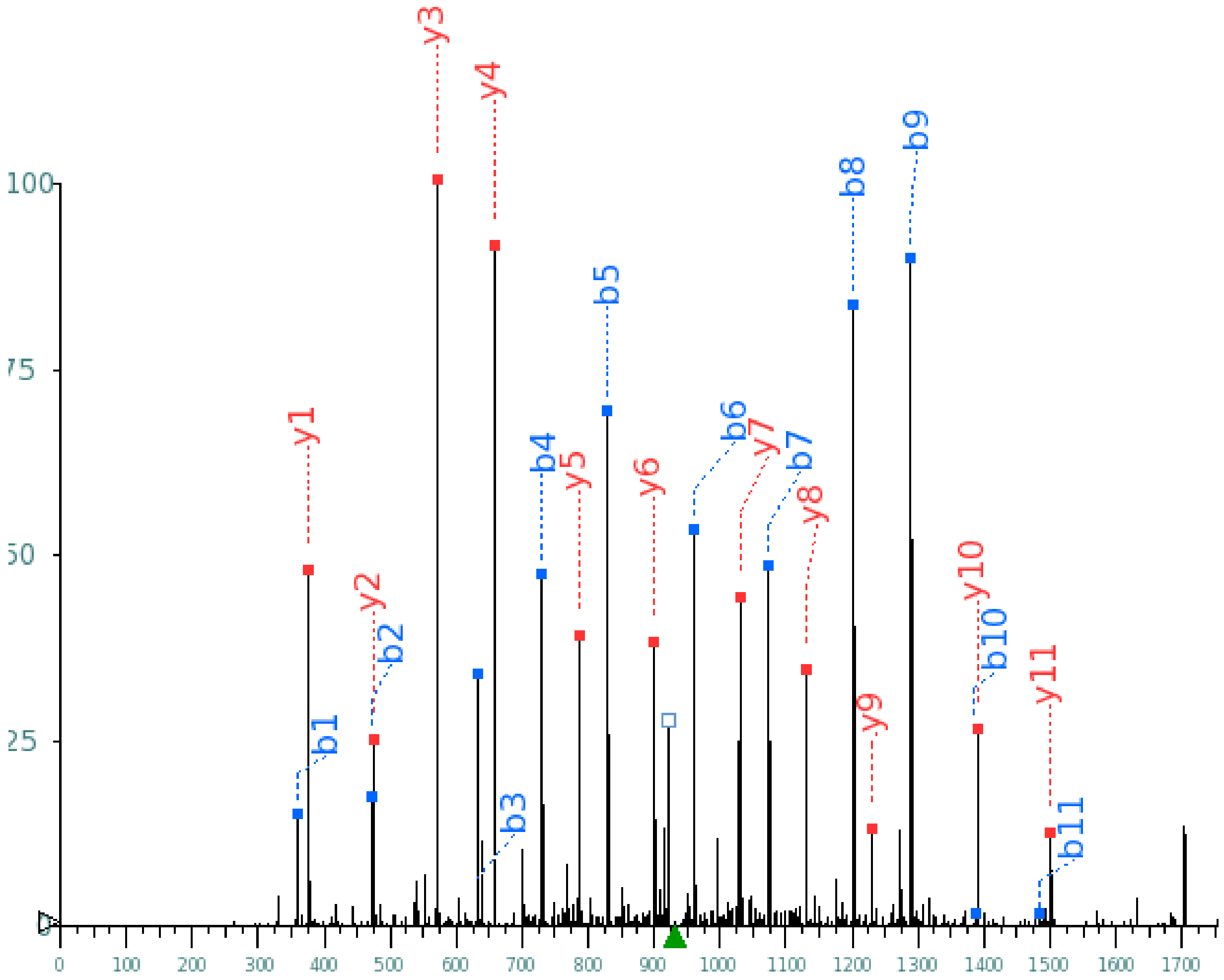

QIC*VVMLESPPK (+2)

Primary Reference: PCBP3_HUMAN
Search ID: 41884
Search Name: 20130330_ananiav_TMT_GPP_10percent_fraction13_lysC_2MC_IAA
Scan#: 19521
Observed Mass: 930.0312 (0.8 ppm)
PSM Score: 73.89

File name: 2015-12-17 03.51.45PM.pdf

Creation time: 2015-12-17 03:53PM

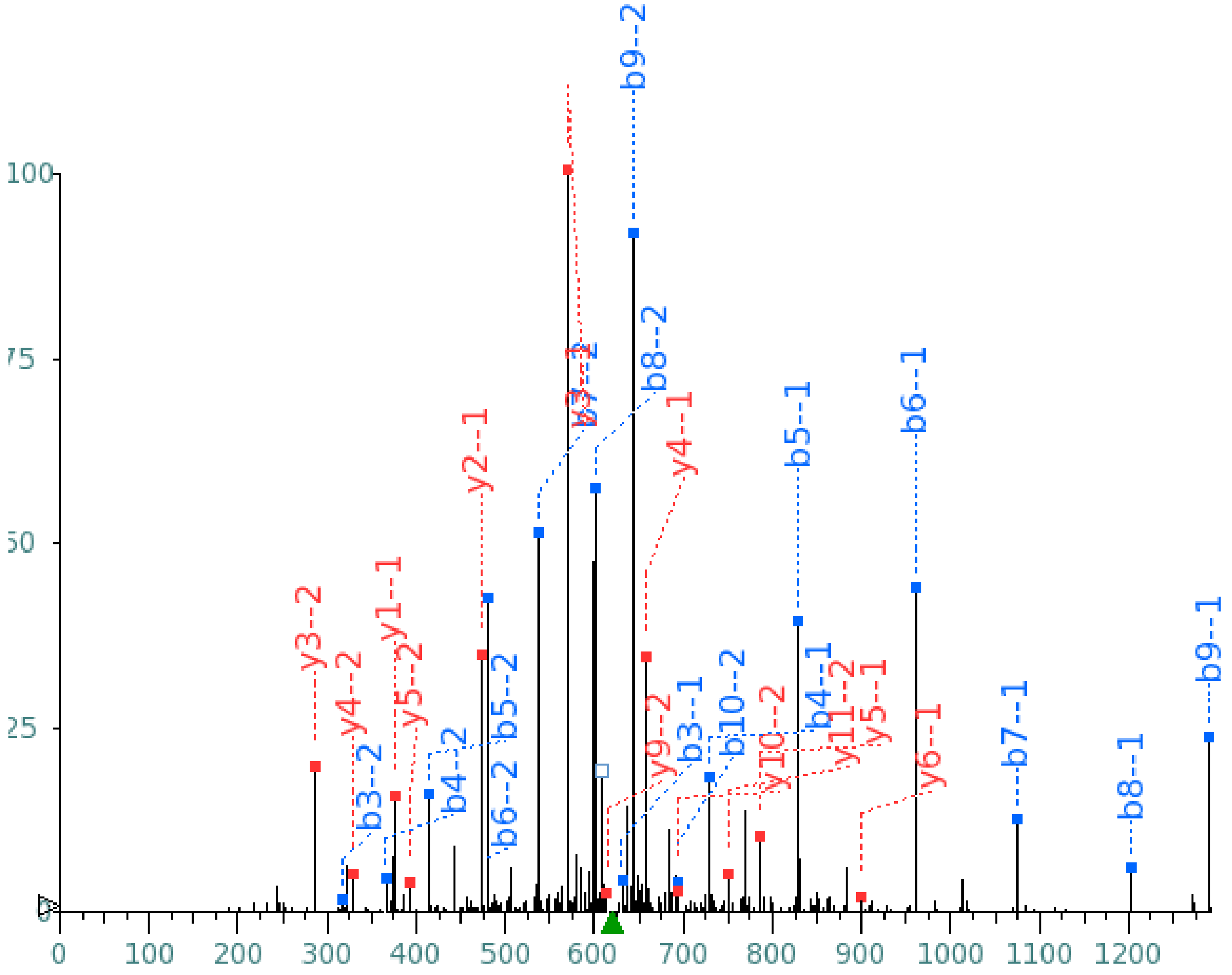

QIC*VVMLESPPK (+3)

Primary Reference: PCBP3_HUMAN
Search ID: 41884
Search Name: 20130330_ananiav_TMT_GPP_10percent_fraction13_lysC_2MC_IAA
Scan#: 19559
Observed Mass: 620.3575 (2.3 ppm)
PSM Score: 45.09

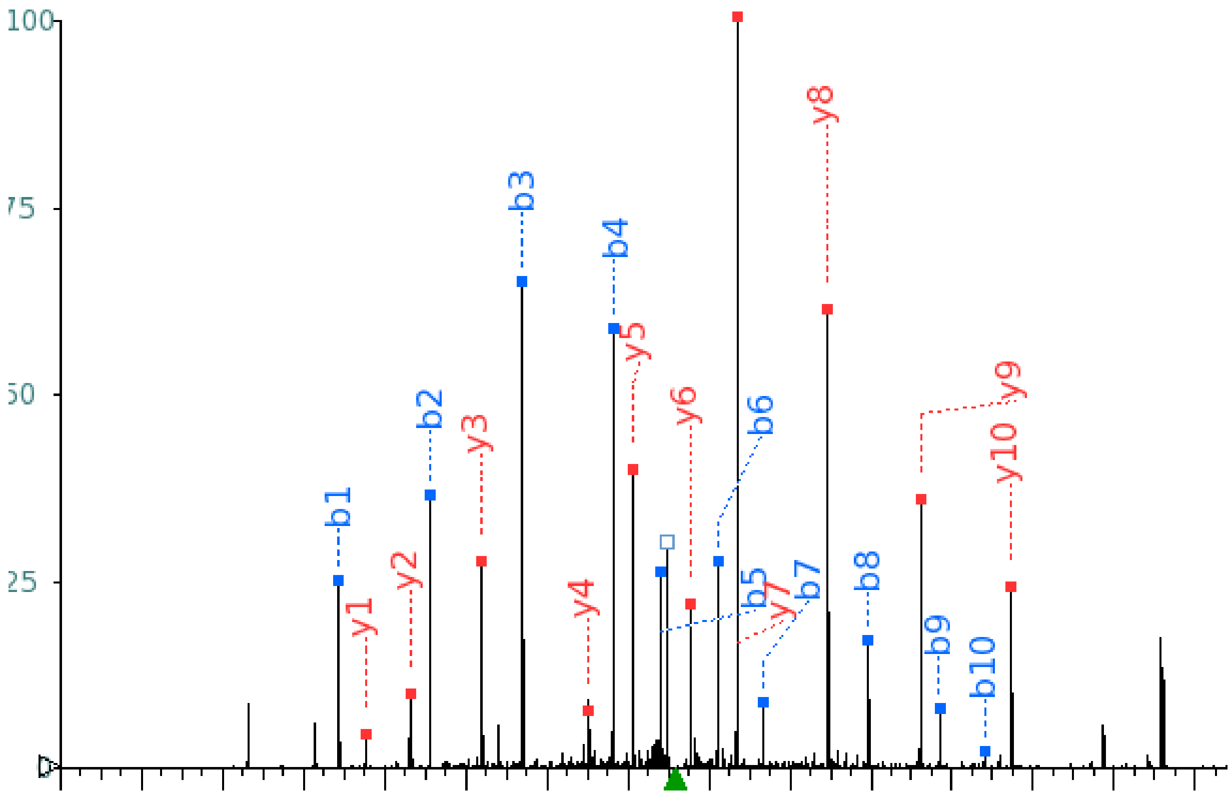

ILLLGAGESGK (+2)

Primary Reference: GNA12_HUMAN

Search ID: 41884

Search Name: 20130330_ananiav_TMT_GPP_10percent_fraction13_lysC_2MC_IAA

Scan#: 19771

Observed Mass: 758.4807 (2 ppm)

PSM Score: 54.5

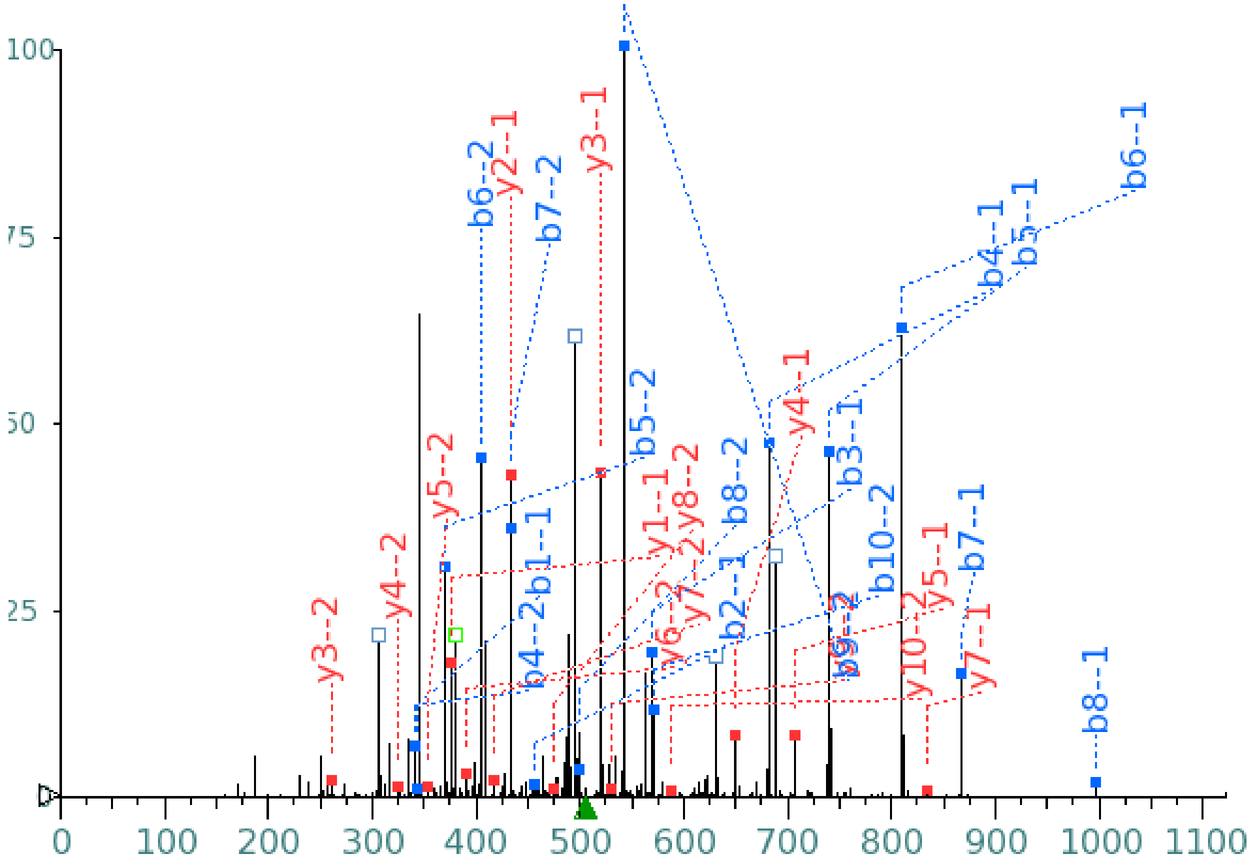

ILLLGAGESGK (+3)

Primary Reference: GNA12_HUMAN
Search ID: 41884
Search Name: 20130330_ananiav_TMT_GPP_10percent_fraction13_lysC_2MC_IAA
Scan#: 19775
Observed Mass: 505.9898 (2.6 ppm)
PSM Score: 37.67

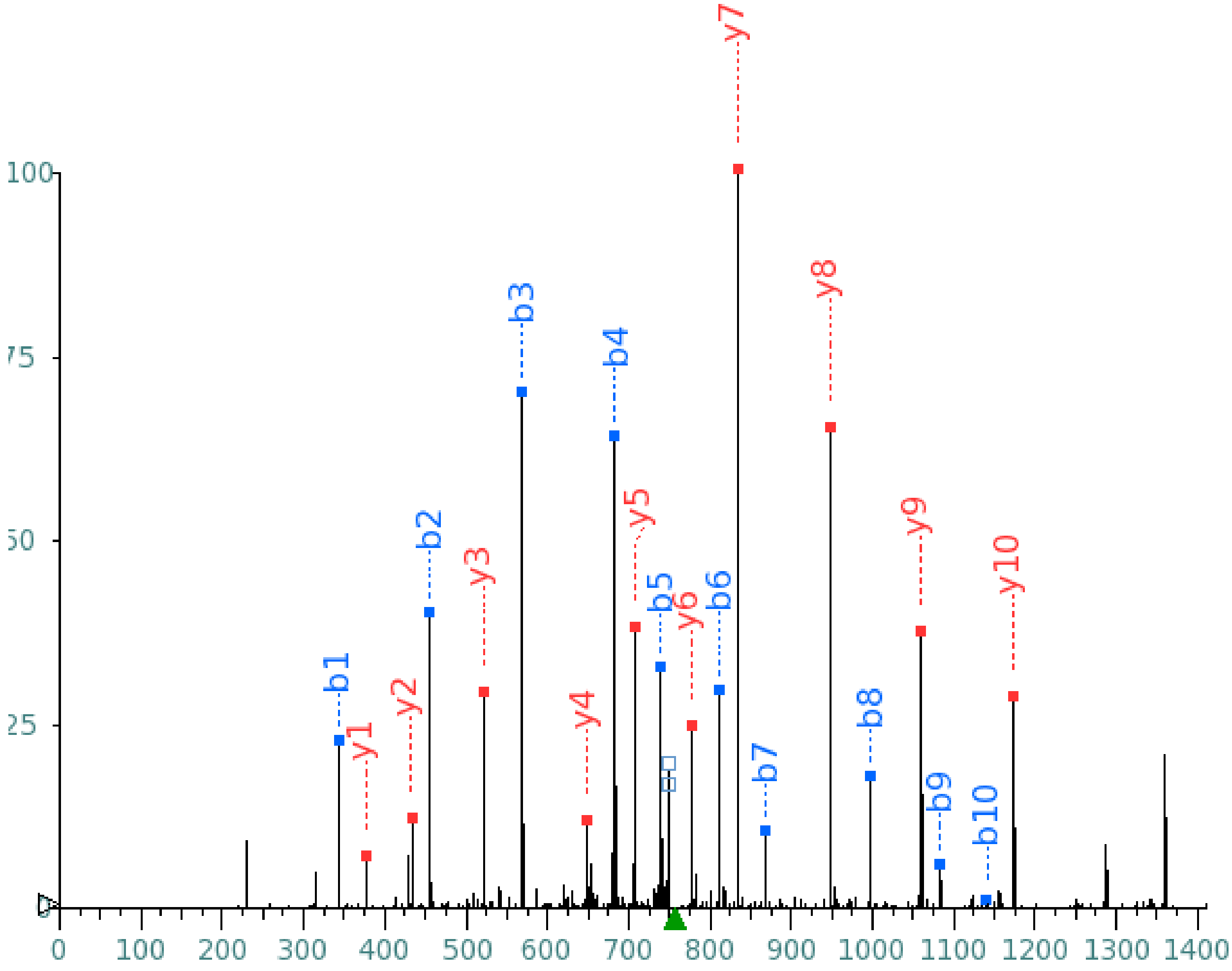

ILLLGAGESGK (+2)

Primary Reference: GNA12_HUMAN
Search ID: 41884
Search Name: 20130330_ananiav_TMT_GPP_10percent_fraction13_lysC_2MC_IAA
Scan#: 19874
Observed Mass: 758.4805 (1.8 ppm)
PSM Score: 61.86

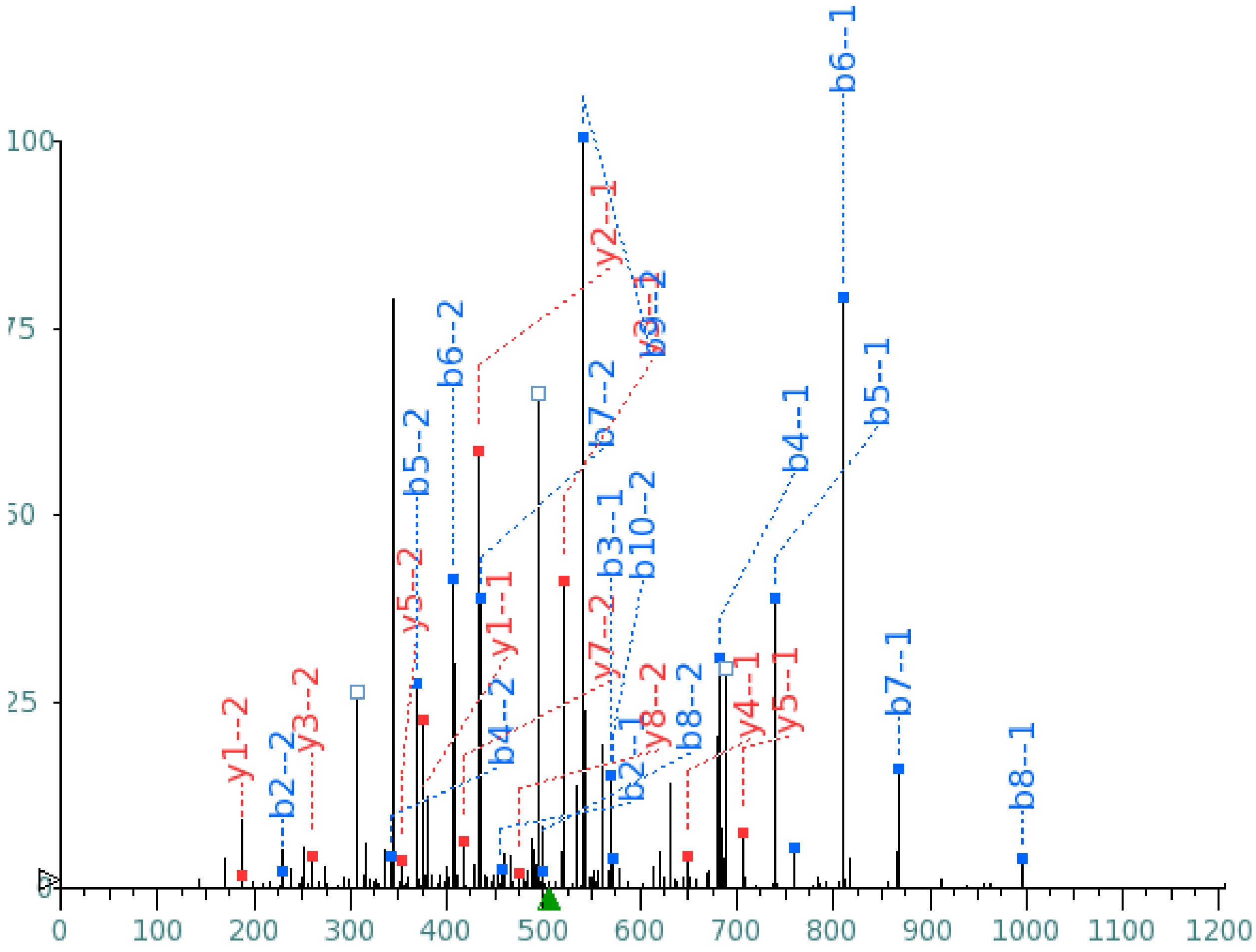

ILLLGAGESGK (+3)

Primary Reference: GNA12_HUMAN
Search ID: 41884
Search Name: 20130330_ananiav_TMT_GPP_10percent_fraction13_lysC_2MC_IAA
Scan#: 19877
Observed Mass: 505.9897 (2.4 ppm)
PSM Score: 35.51

File name: 2015-12-17 03.51.45PM.pdf

Creation time: 2015-12-17 03:53PM

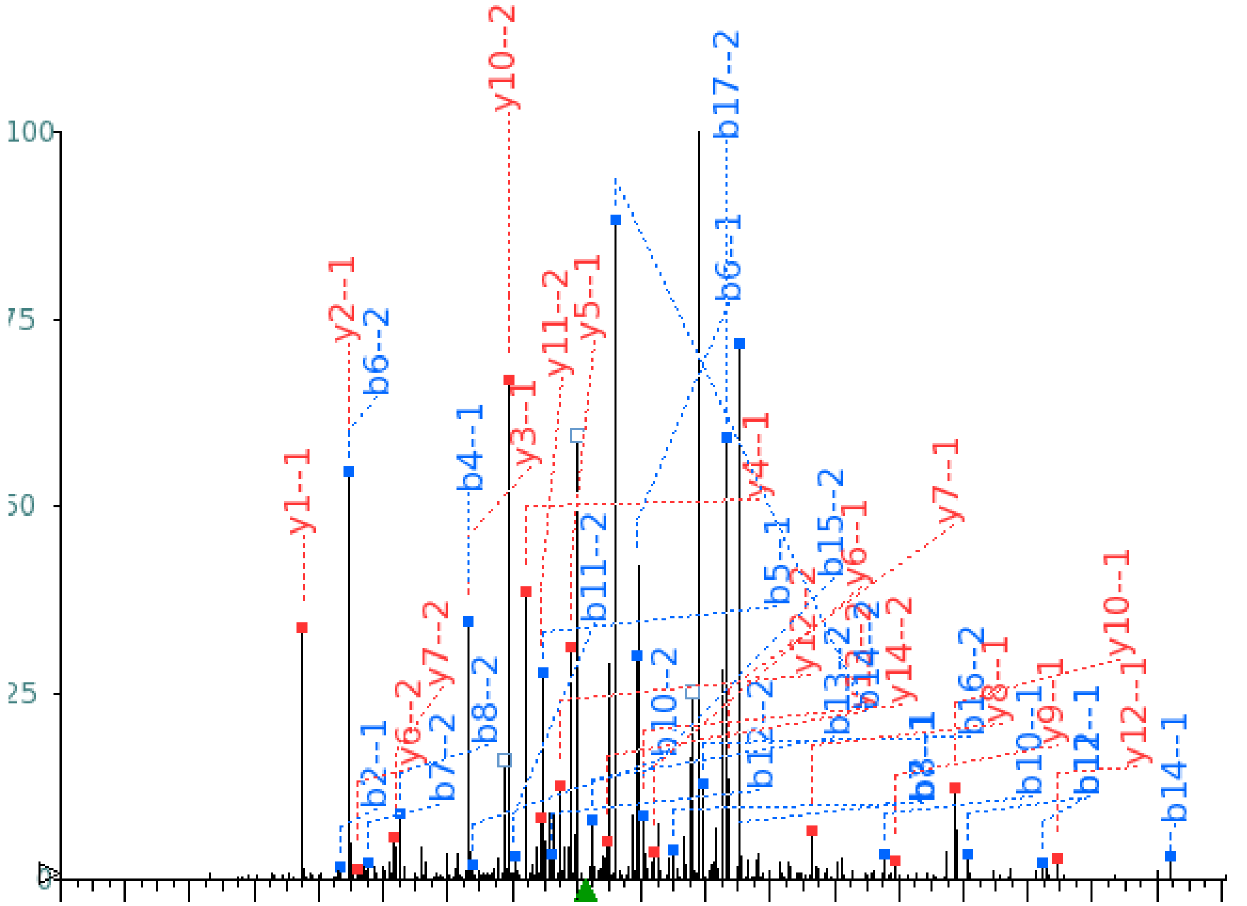

MASNIFGTPEENQASWAK (+3)

Primary Reference: HN1_HUMAN
Search ID: 41884
Search Name: 20130330_ananiav_TMT_GPP_10percent_fraction13_lysC_2MC_IAA
Scan#: 19995
Observed Mass: 813.7528 (2.5 ppm)
PSM Score: 61.78

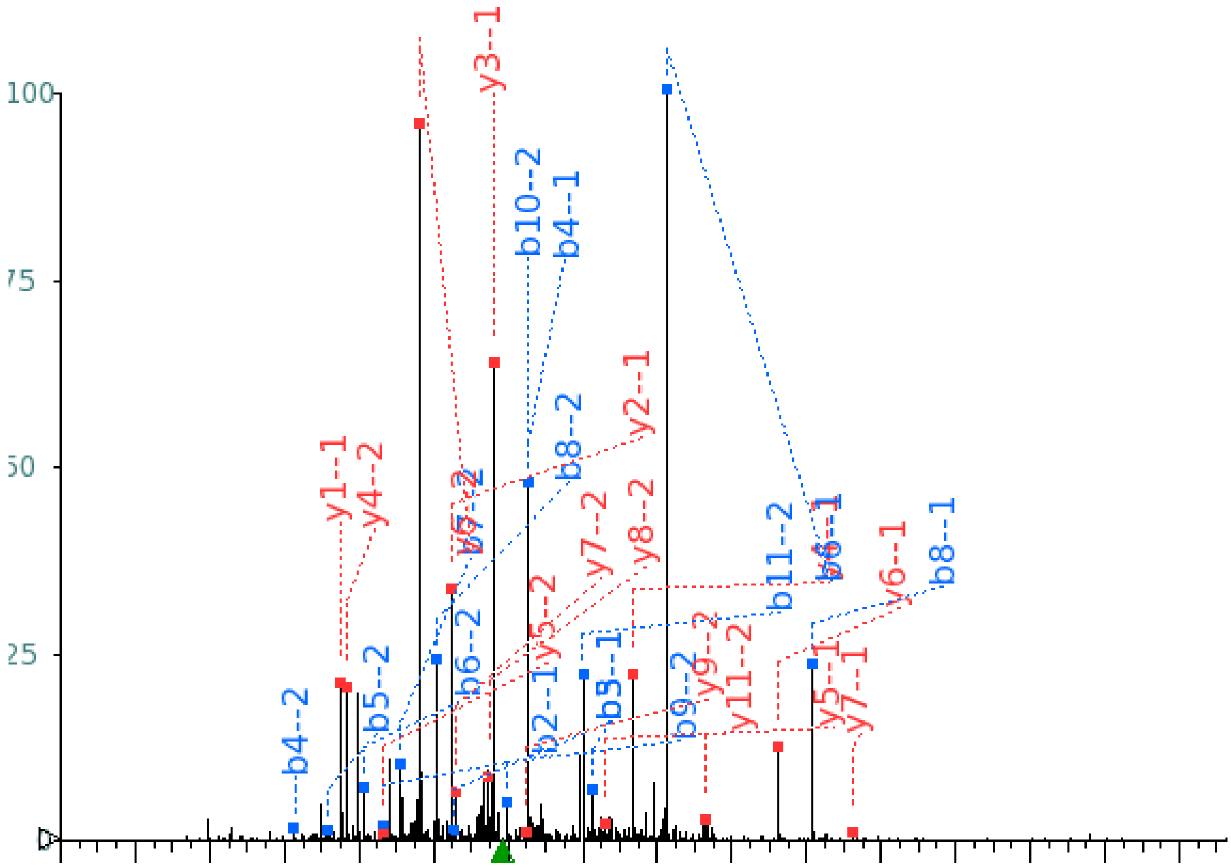

SLPVSVPVWGFK (+3)

Primary Reference: AKTS1_HUMAN
Search ID: 41884
Search Name: 20130330_ananiav_TMT_GPP_10percent_fraction13_lysC_2MC_IAA
Scan#: 24748
Observed Mass: 592.0293 (3.7 ppm)
PSM Score: 21.31

File name: 2015-12-17 03.51.45PM.pdf

Creation time: 2015-12-17 03:53PM

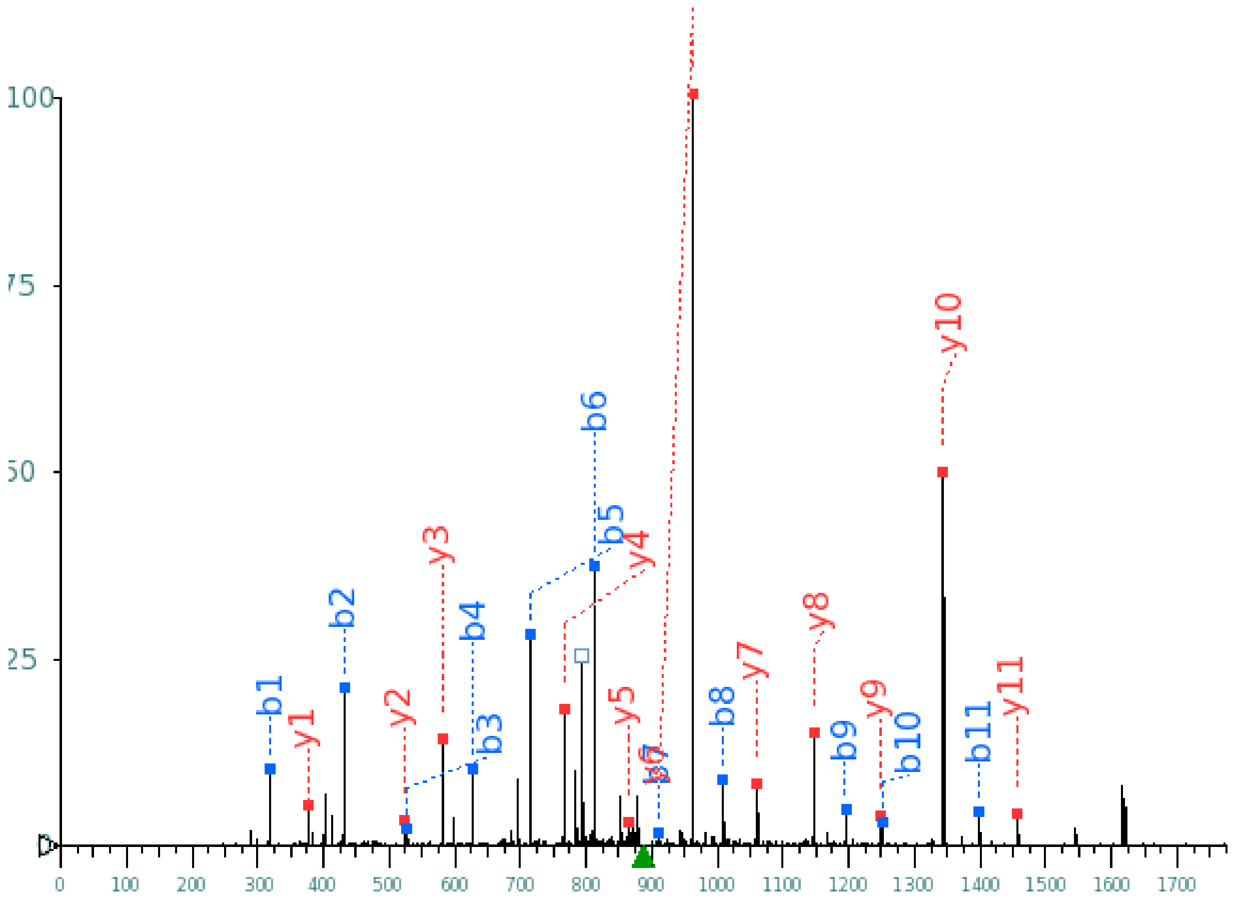

SLPVSVPVWGFK (+2)

Primary Reference: AKTS1_HUMAN
Search ID: 41884
Search Name: 20130330_ananiav_TMT_GPP_10percent_fraction13_lysC_2MC_IAA
Scan#: 24771
Observed Mass: 887.5392 (2.5 ppm)
PSM Score: 55.08

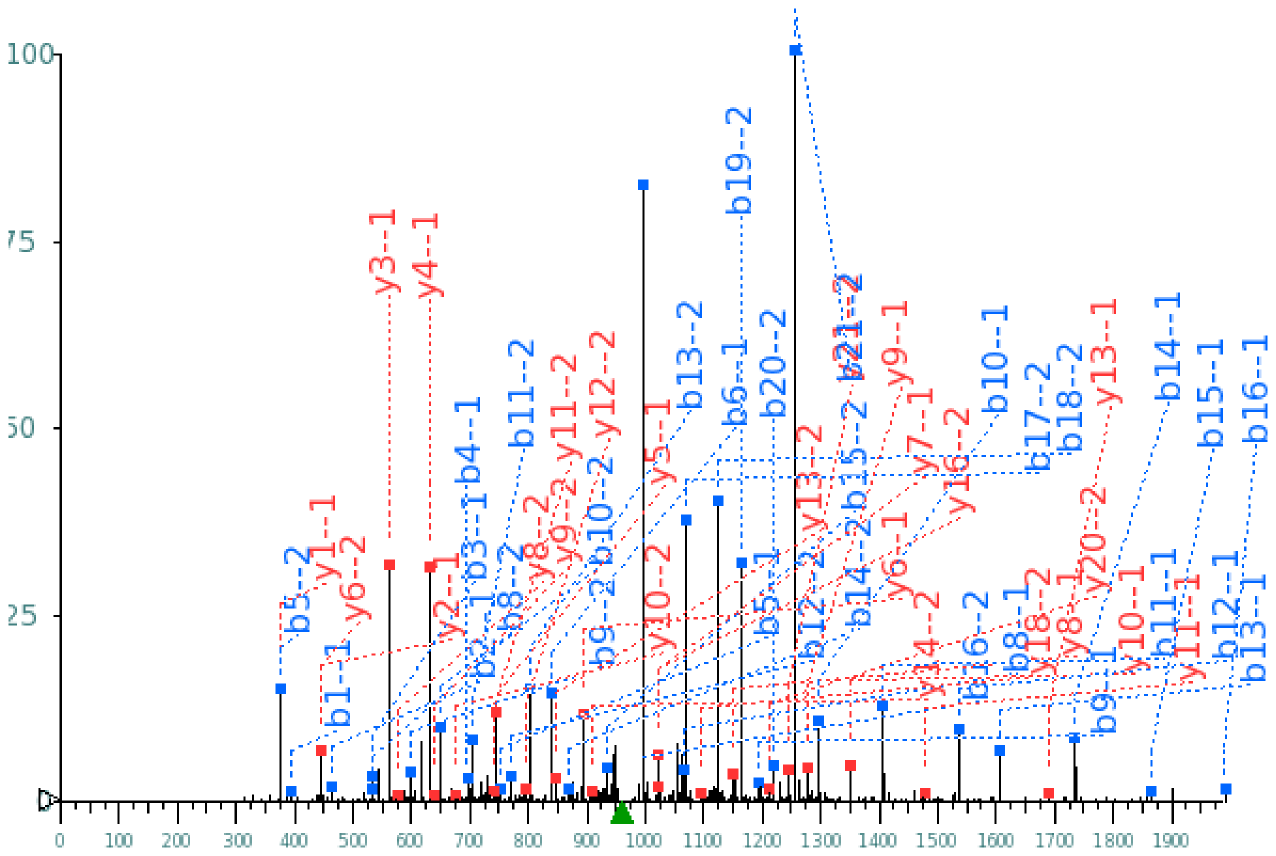

YAAC*GSPEEVLQAEQEFLANAK (+3)

Primary Reference: TSR3_HUMAN
Search ID: 41884
Search Name: 20130330_ananiav_TMT_GPP_10percent_fraction13_lysC_2MC_IAA
Scan#: 25501
Observed Mass: 961.8277 (3 ppm)
PSM Score: 86.75

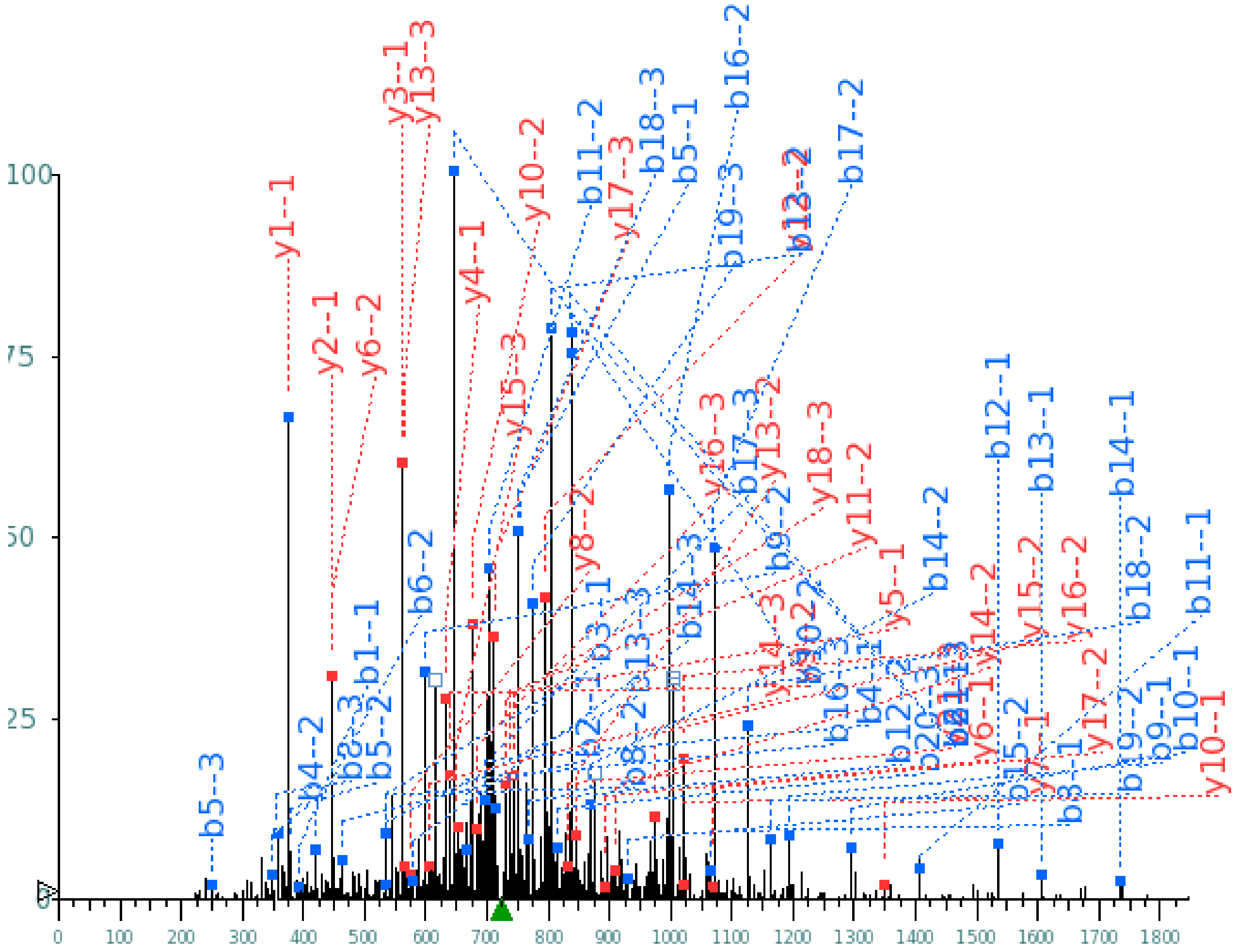

YAAC*GSPEEVLQAEQEFLANAK (+4)

Primary Reference: TSR3_HUMAN
Search ID: 41884
Search Name: 20130330_ananiav_TMT_GPP_10percent_fraction13_lysC_2MC_IAA
Scan#: 25525
Observed Mass: 721.6234 (4.1 ppm)
PSM Score: 46.61

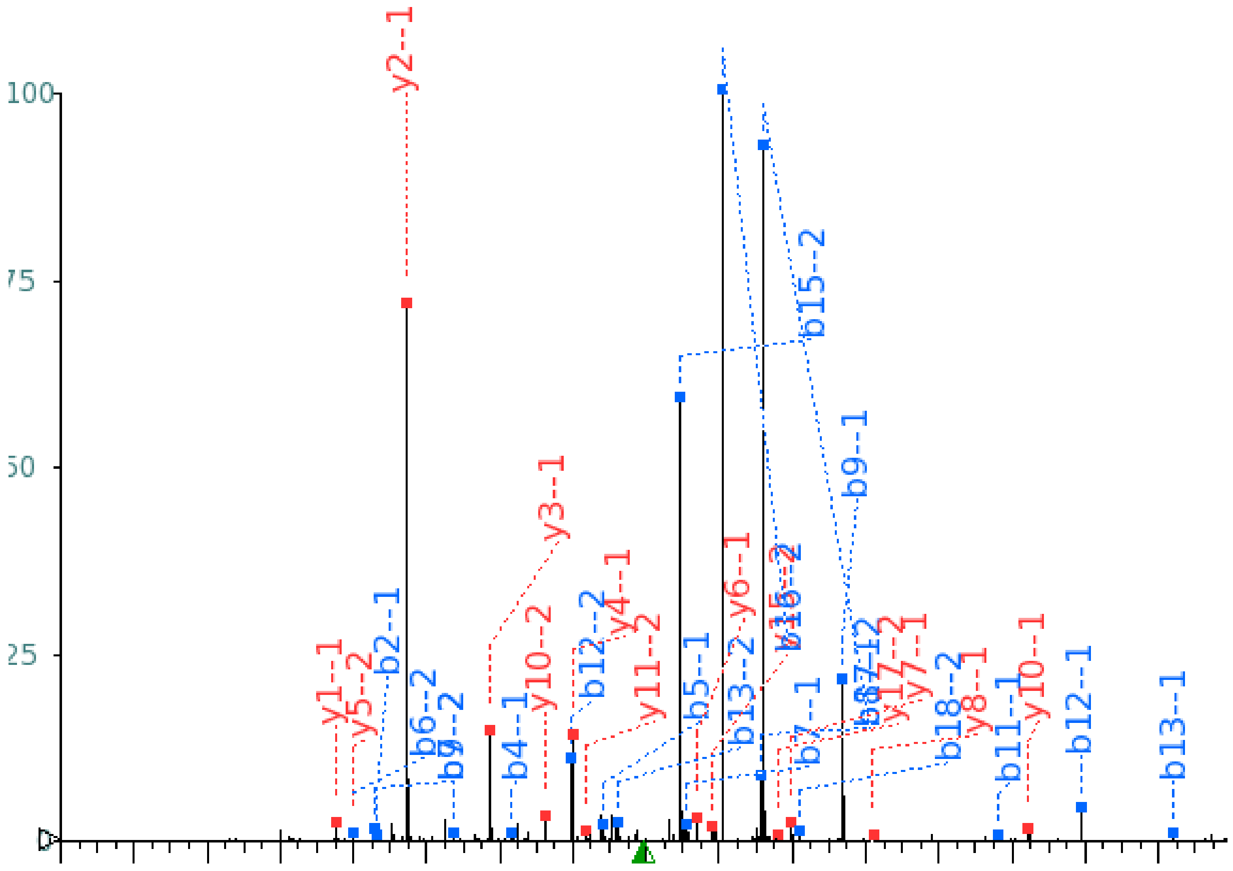

VTIAQGGVLPNIQAVLLPK (+3)

Primary Reference: H2A1D_HUMAN
Search ID: 41884
Search Name: 20130330_ananiav_TMT_GPP_10percent_fraction13_lysC_2MC_IAA
Scan#: 26397
Observed Mass: 797.1717 (2.5 ppm)
PSM Score: 33.48

File name: 2015-12-17 03.51.45PM.pdf

Creation time: 2015-12-17 03:53PM

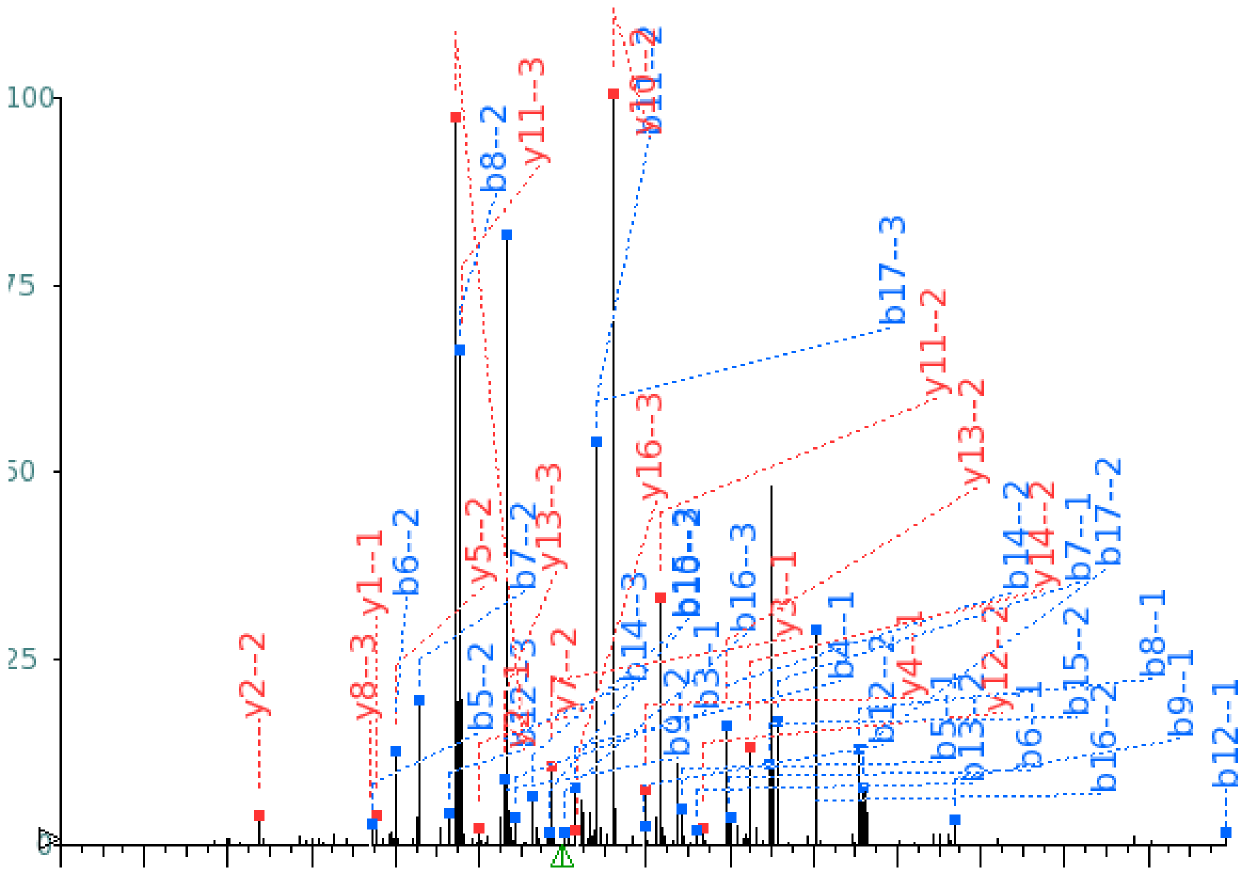

VTIAQGGVLPNIQAVLLPK (+4)

Primary Reference: H2A1D_HUMAN
Search ID: 41884
Search Name: 20130330_ananiav_TMT_GPP_10percent_fraction13_lysC_2MC_IAA
Scan#: 26415
Observed Mass: 598.1308 (2.7 ppm)
PSM Score: 53.59

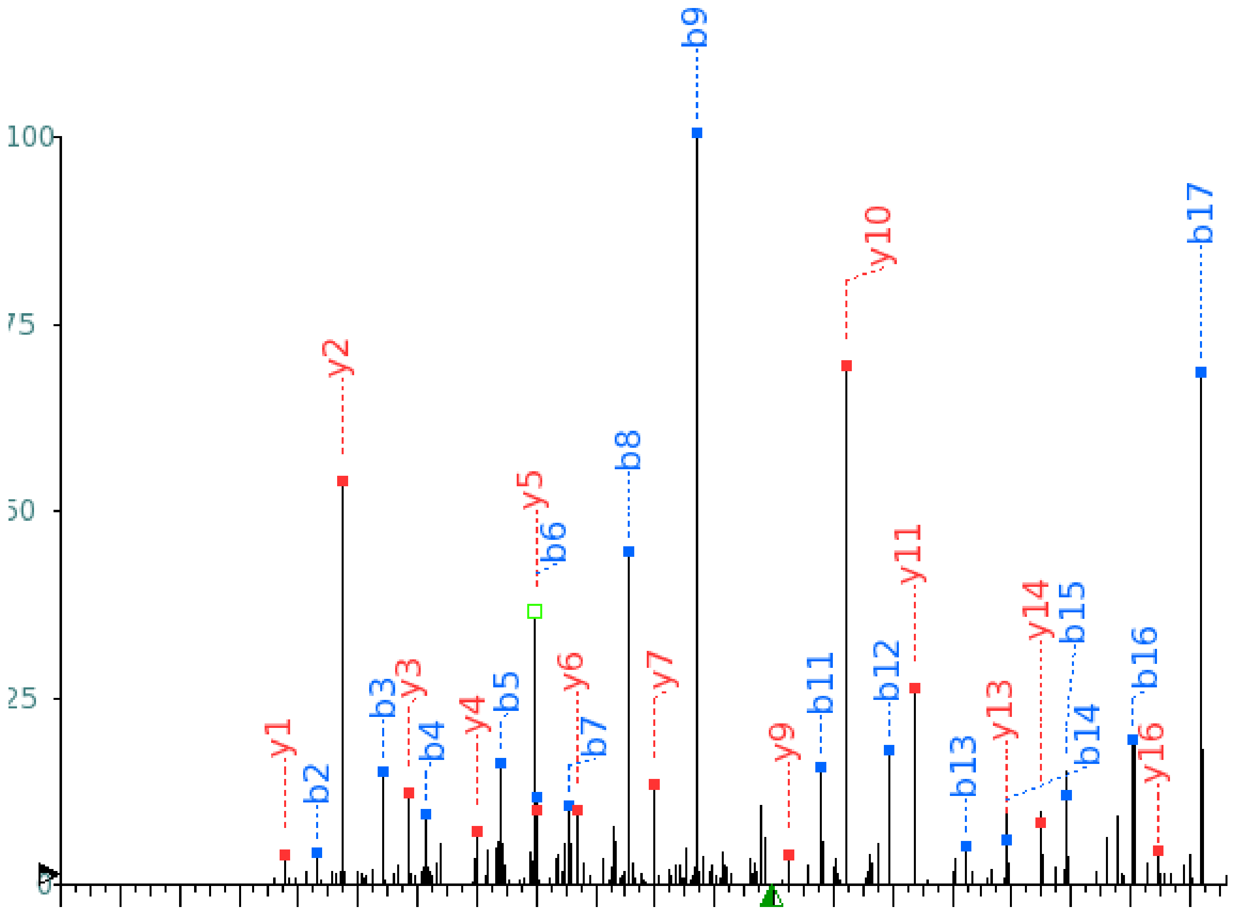

VTIAQGGVLPNIQAVLLPK (+2)

Primary Reference: H2A1D_HUMAN
Search ID: 41884
Search Name: 20130330_ananiav_TMT_GPP_10percent_fraction13_lysC_2MC_IAA
Scan#: 26430
Observed Mass: 1195.2523 (1.1 ppm)
PSM Score: 82

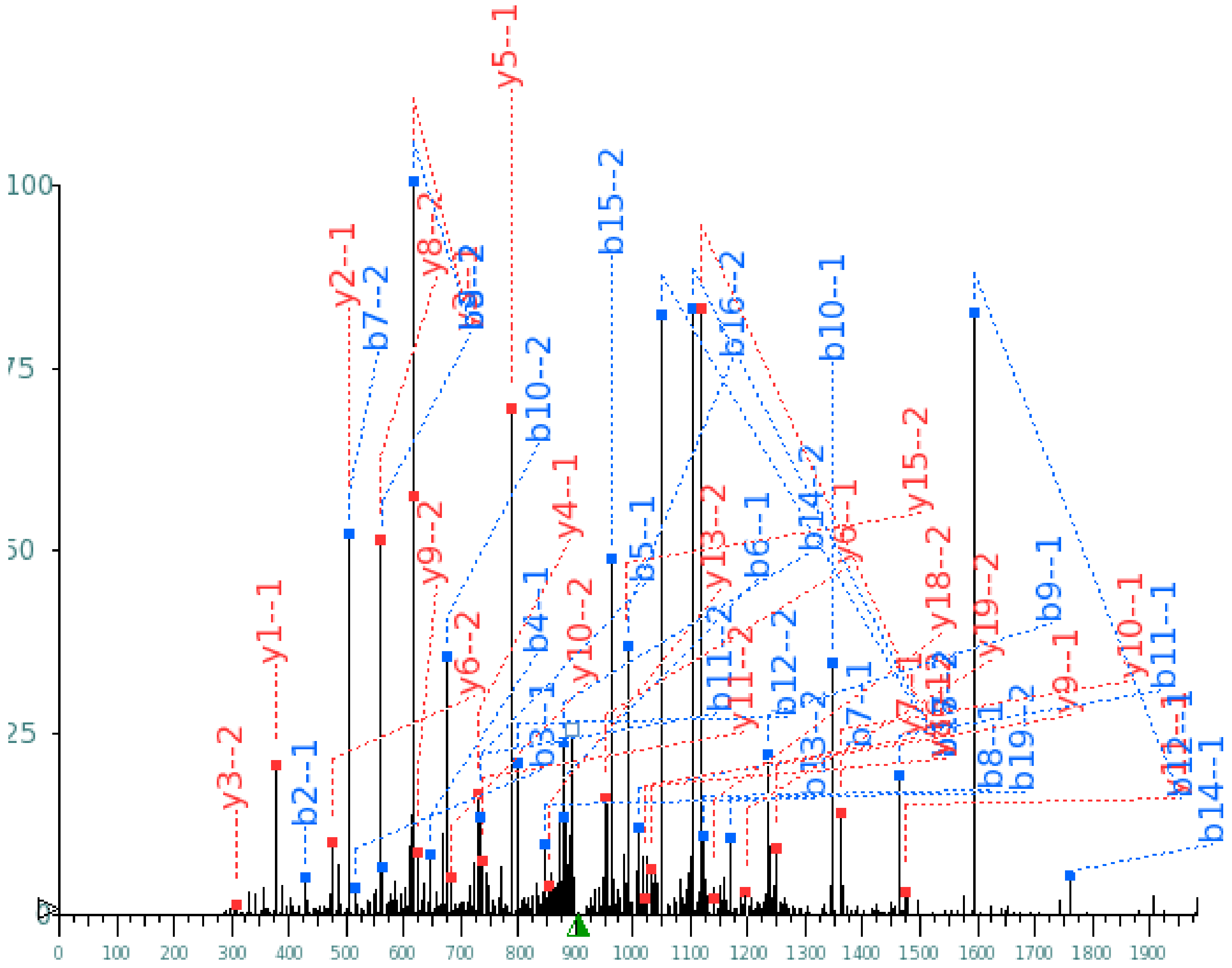

SLSQSFENLLDEPAYGLIQK (+3)

Primary Reference: B4DMT9_HUMAN
Search ID: 41884
Search Name: 20130330_ananiav_TMT_GPP_10percent_fraction13_lysC_2MC_IAA
Scan#: 26486
Observed Mass: 904.1638 (2.4 ppm)
PSM Score: 78.19

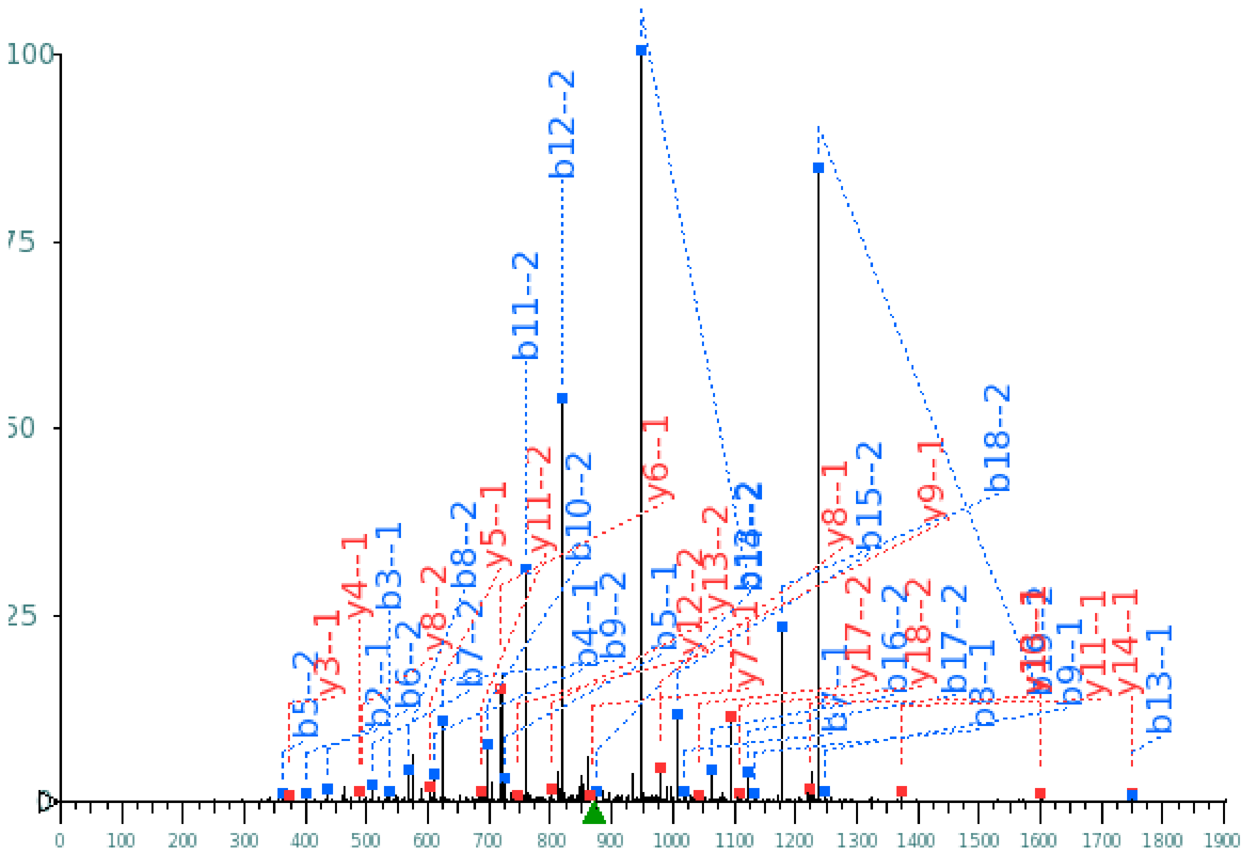

AVHADFFNDFEDLFDDDDIQ (+3)

Primary Reference: MYOV2_HUMAN
Search ID: 41884
Search Name: 20130330_ananiav_TMT_GPP_10percent_fraction13_lysC_2MC_IAA
Scan#: 26682
Observed Mass: 873.0596 (2.8 ppm)
PSM Score: 76.3

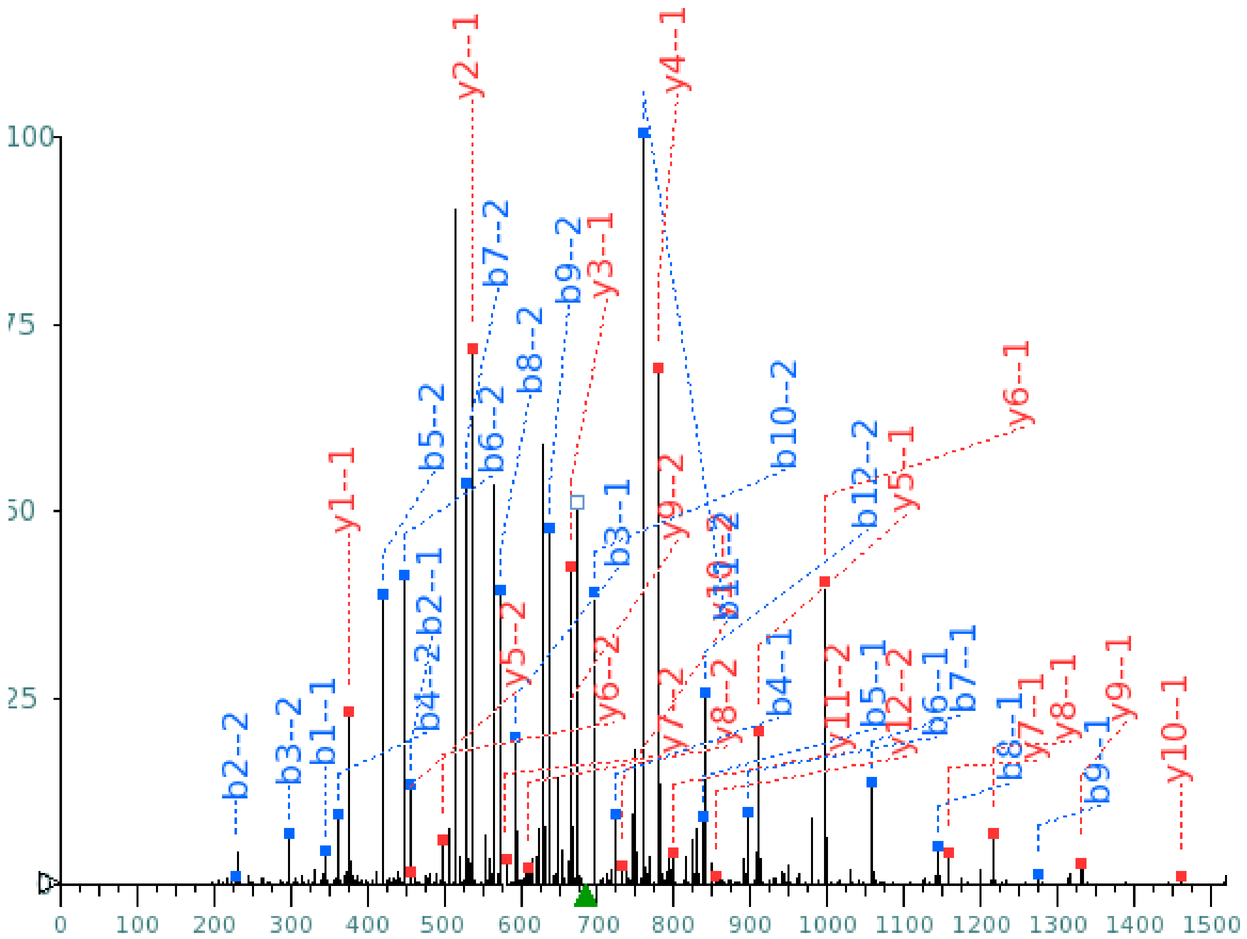

IIHEDGYSEDEC*K (+3)

Primary Reference: GNAI3_HUMAN
Search ID: 41885
Search Name: 20130330_ananiav_TMT_GPP_10percent_fraction14_lysC_2MC_IAA
Scan#: 10489
Observed Mass: 685.0041 (1.4 ppm)
PSM Score: 60.73

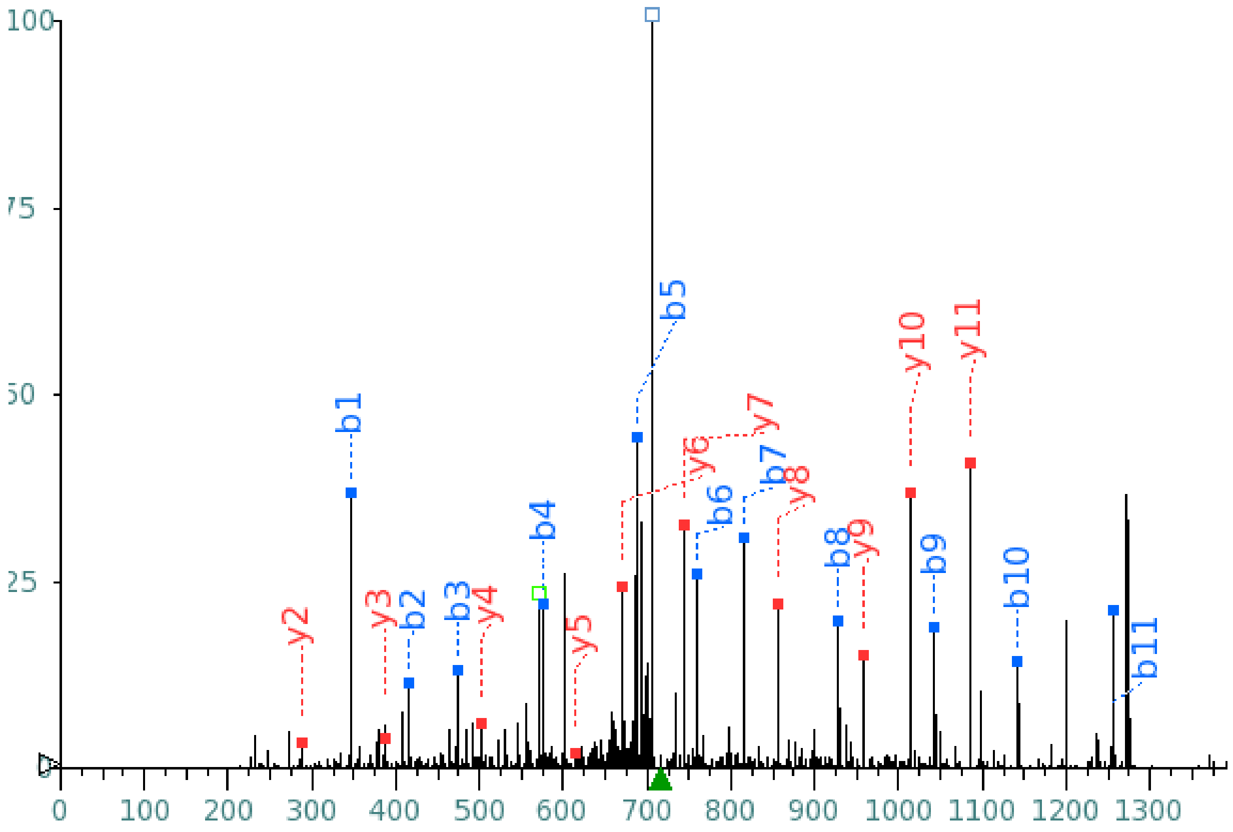

DAGTIAGLNVLR (+2)

Primary Reference: E9PQQ4_HUMAN
Search ID: 41885
Search Name: 20130330_ananiav_TMT_GPP_10percent_fraction14_lysC_2MC_IAA
Scan#: 17472
Observed Mass: 714.9245 (3.2 ppm)
PSM Score: 68.5

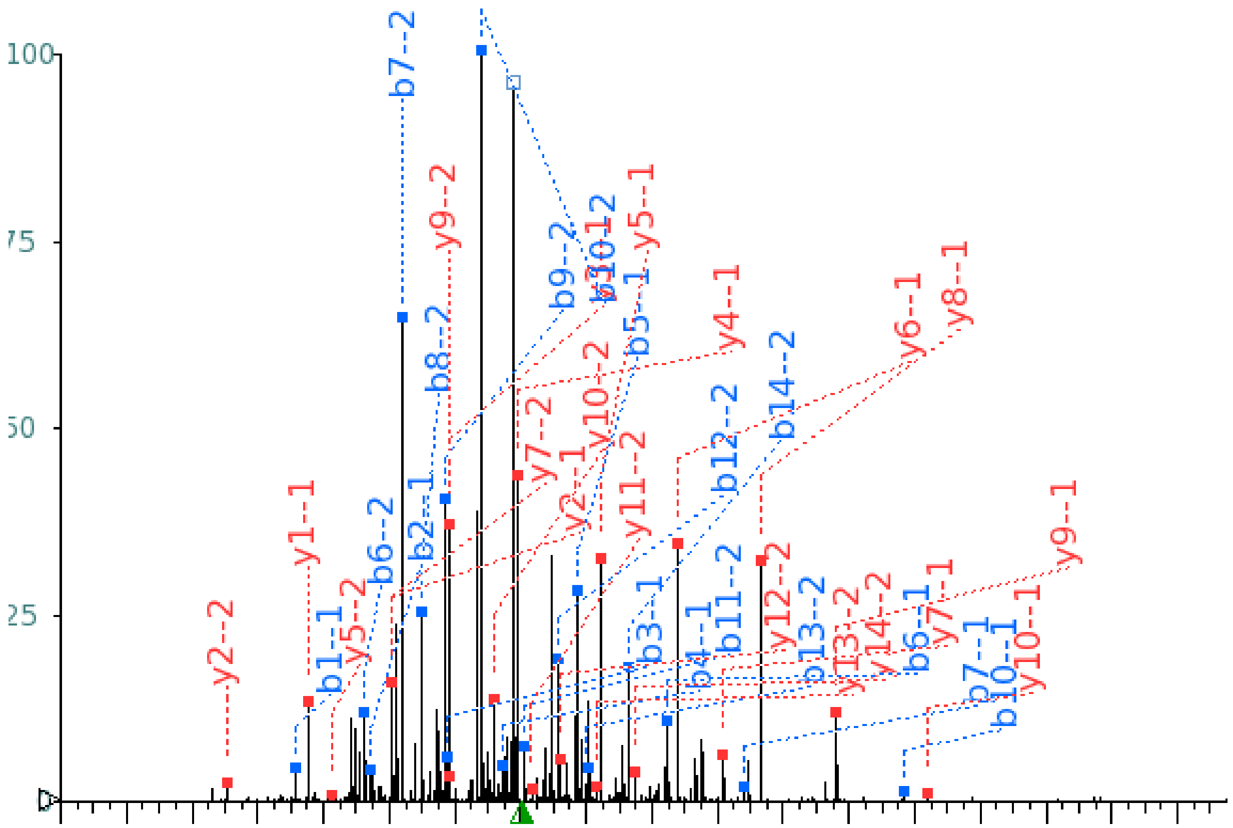

ELNSNHDGADETSEK (+3)

Primary Reference: B2RCX0_HUMAN
Search ID: 41886
Search Name: 20130330_ananiav_TMT_GPP_10percent_fraction15_lysC_2MC_IAA
Scan#: 6497
Observed Mass: 702.0119 (0.9 ppm)
PSM Score: 66.52

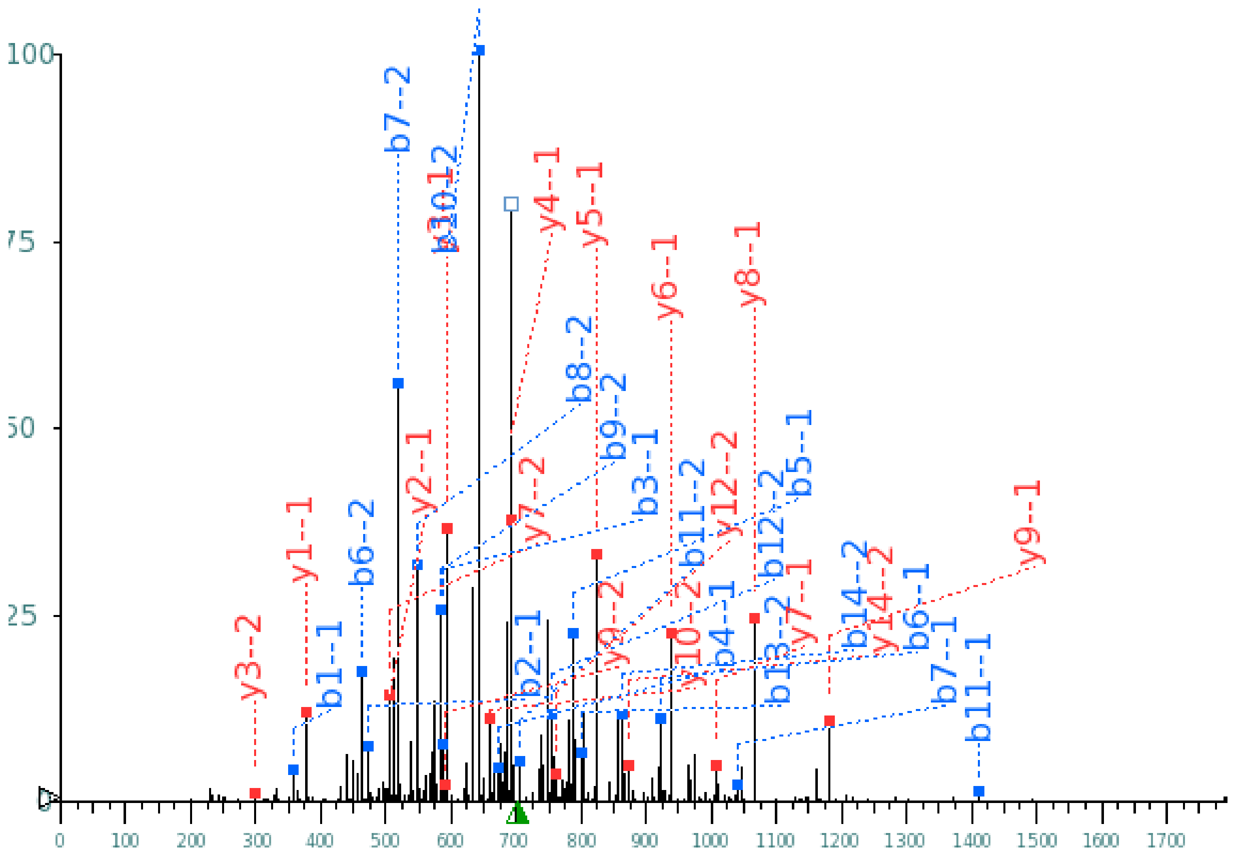

ELNSNHDGADETSEK (+3)

Primary Reference: B2RCX0_HUMAN
Search ID: 41886
Search Name: 20130330_ananiav_TMT_GPP_10percent_fraction15_lysC_2MC_IAA
Scan#: 6601
Observed Mass: 702.0119 (0.8 ppm)
PSM Score: 45.92

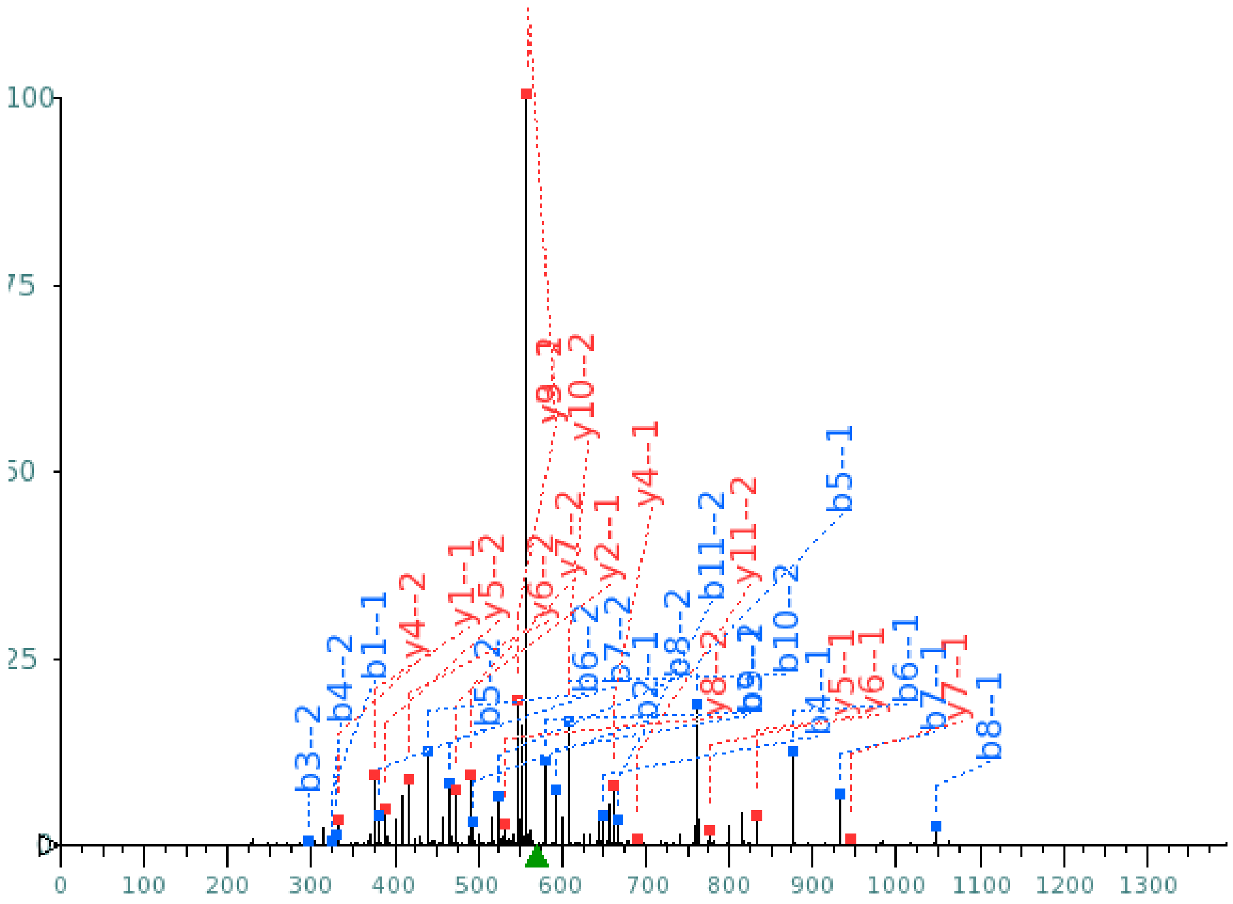

VYVGNLGNNGNK (+3)

Primary Reference: SRSF3_HUMAN
Search ID: 41886
Search Name: 20130330_ananiav_TMT_GPP_10percent_fraction15_lysC_2MC_IAA
Scan#: 12633
Observed Mass: 569.6589 (1.9 ppm)
PSM Score: 36.39

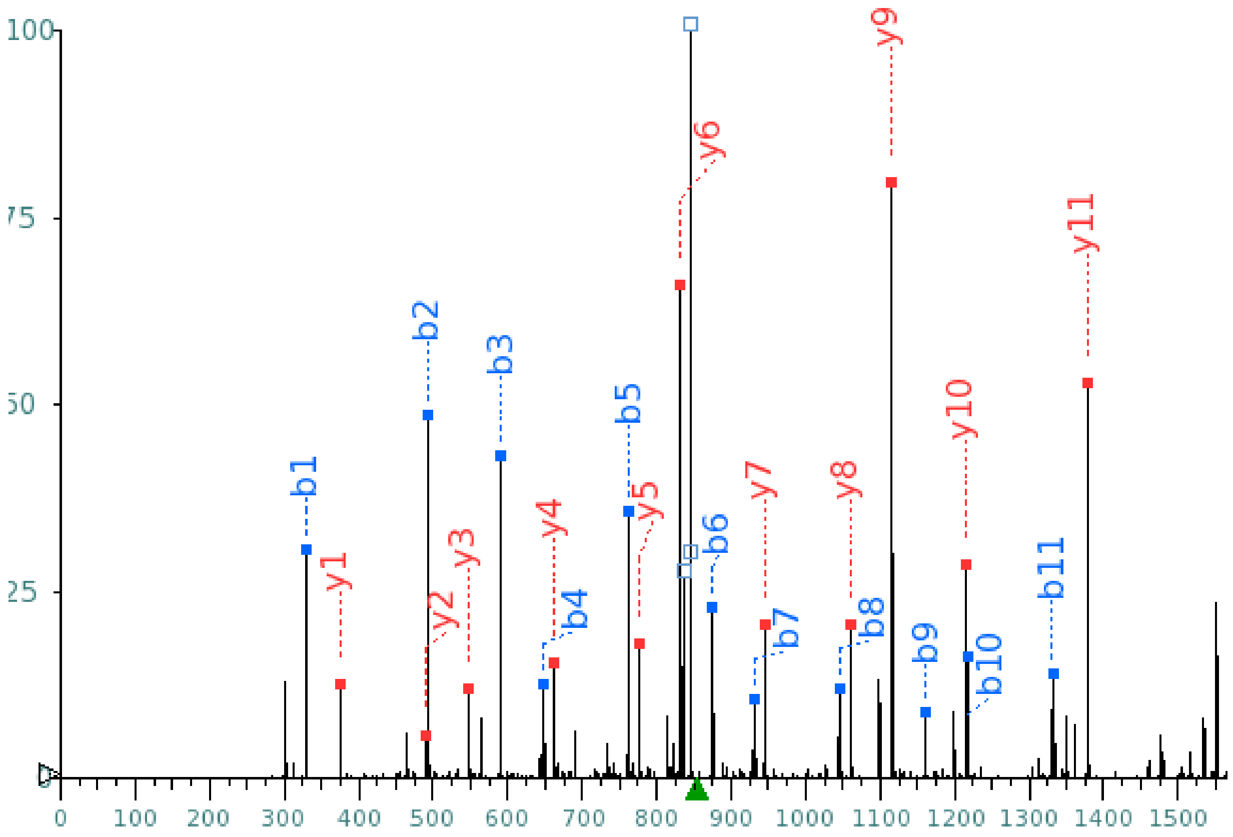

VYVGNLGNNGNK (+2)

Primary Reference: SRSF3_HUMAN
Search ID: 41886
Search Name: 20130330_ananiav_TMT_GPP_10percent_fraction15_lysC_2MC_IAA
Scan#: 12704
Observed Mass: 853.984 (1 ppm)
PSM Score: 64.93

File name: 2015-12-17 03.51.45PM.pdf

Creation time: 2015-12-17 03:53PM

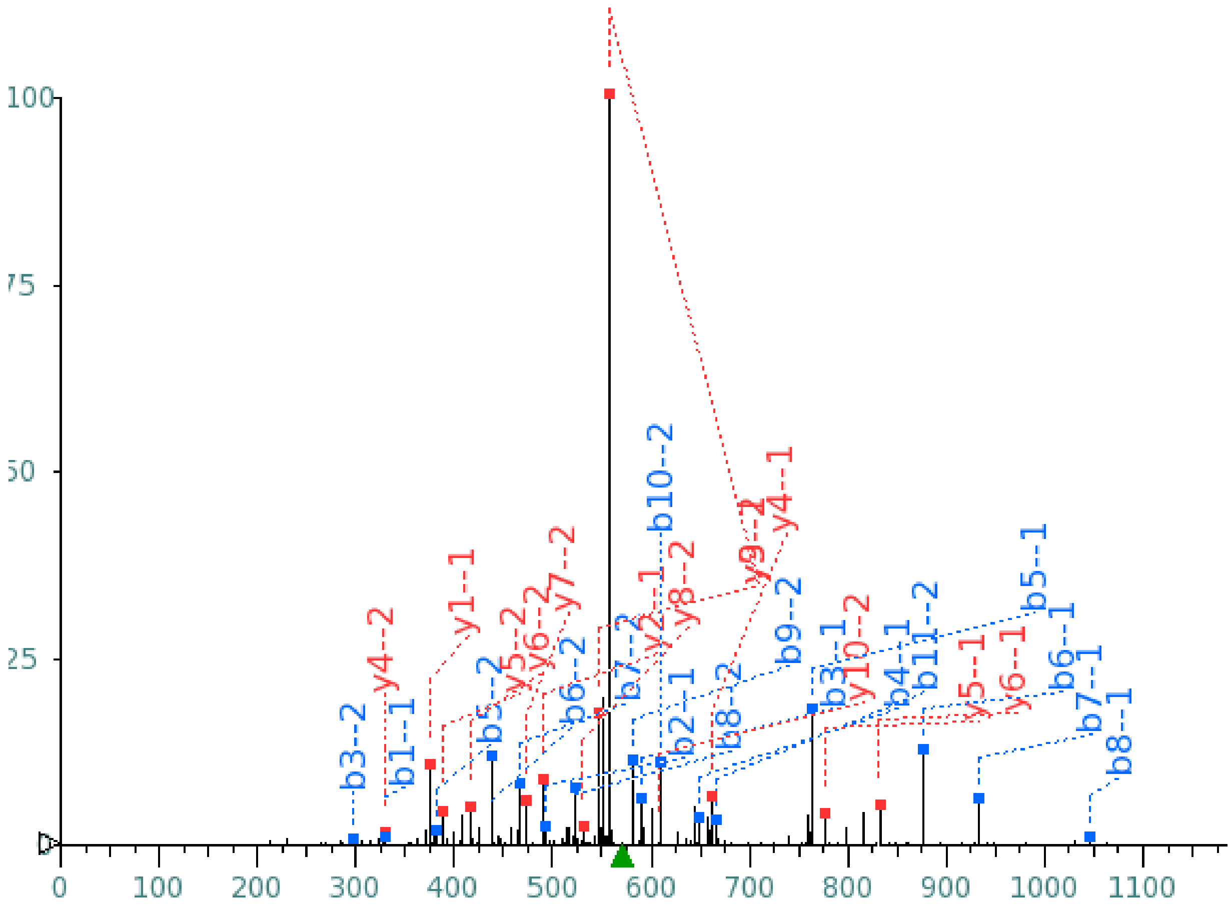

VYVGNLGNNGNK (+3)

Primary Reference: SRSF3_HUMAN
Search ID: 41886
Search Name: 20130330_ananiav_TMT_GPP_10percent_fraction15_lysC_2MC_IAA
Scan#: 12746
Observed Mass: 569.6587 (1.5 ppm)
PSM Score: 42.36

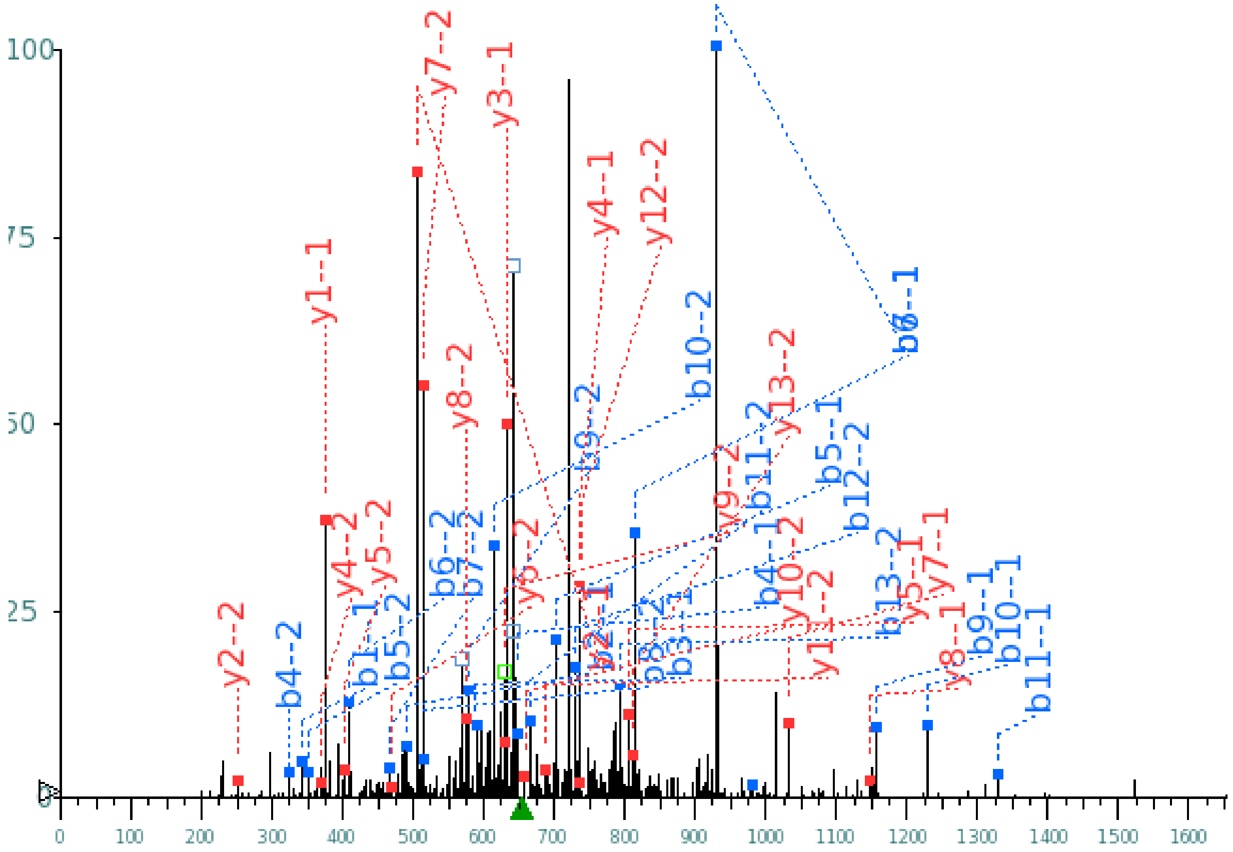

IFVGGLNPEATEEK (+3)

Primary Reference: B4DMY3_HUMAN
Search ID: 41886
Search Name: 20130330_ananiav_TMT_GPP_10percent_fraction15_lysC_2MC_IAA
Scan#: 19192
Observed Mass: 654.7053 (3.3 ppm)
PSM Score: 41.37

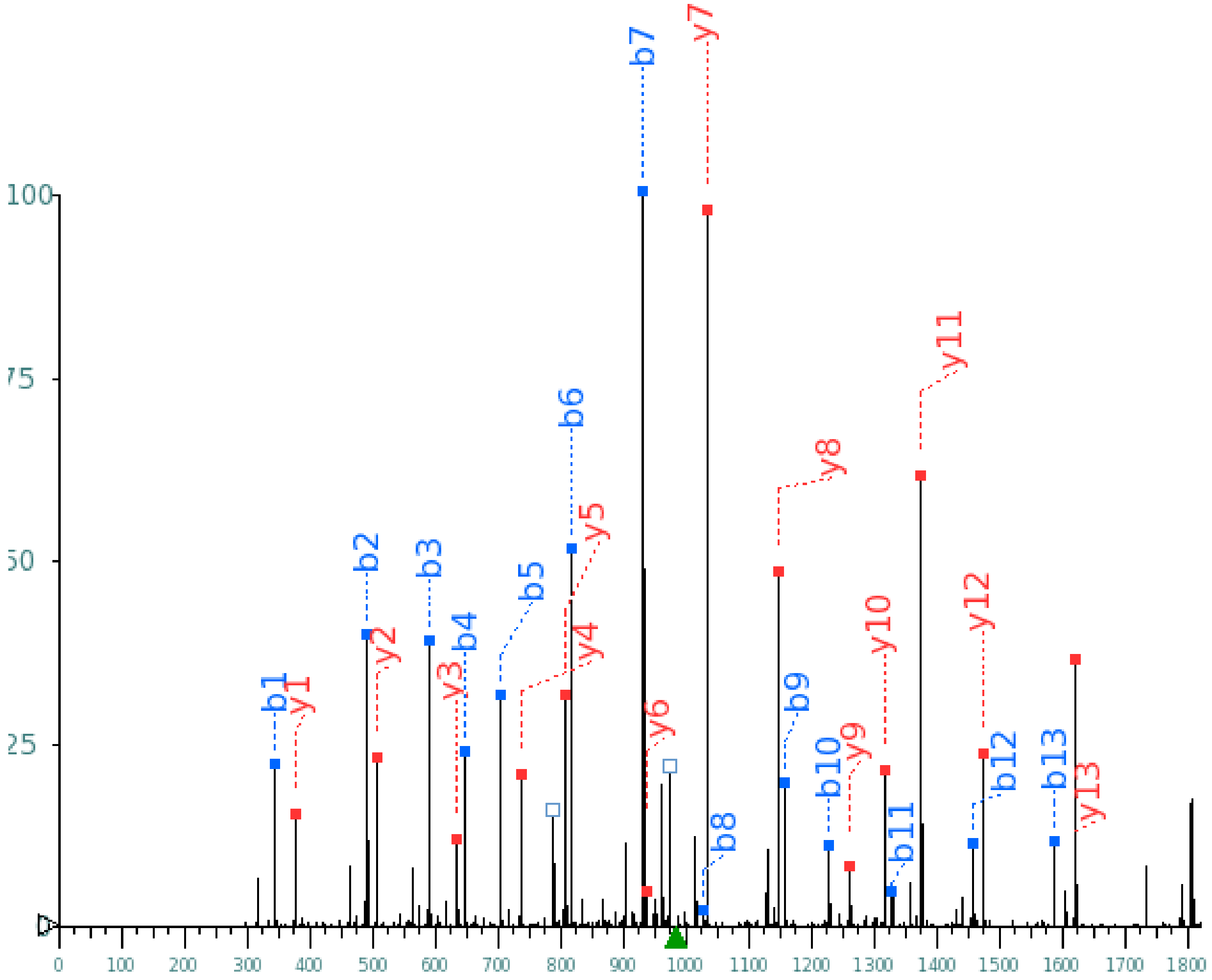

IFVGGLNPEATEEK (+2)

Primary Reference: B4DMY3_HUMAN
Search ID: 41886
Search Name: 20130330_ananiav_TMT_GPP_10percent_fraction15_lysC_2MC_IAA
Scan#: 19215
Observed Mass: 981.5528 (1.8 ppm)
PSM Score: 78.01

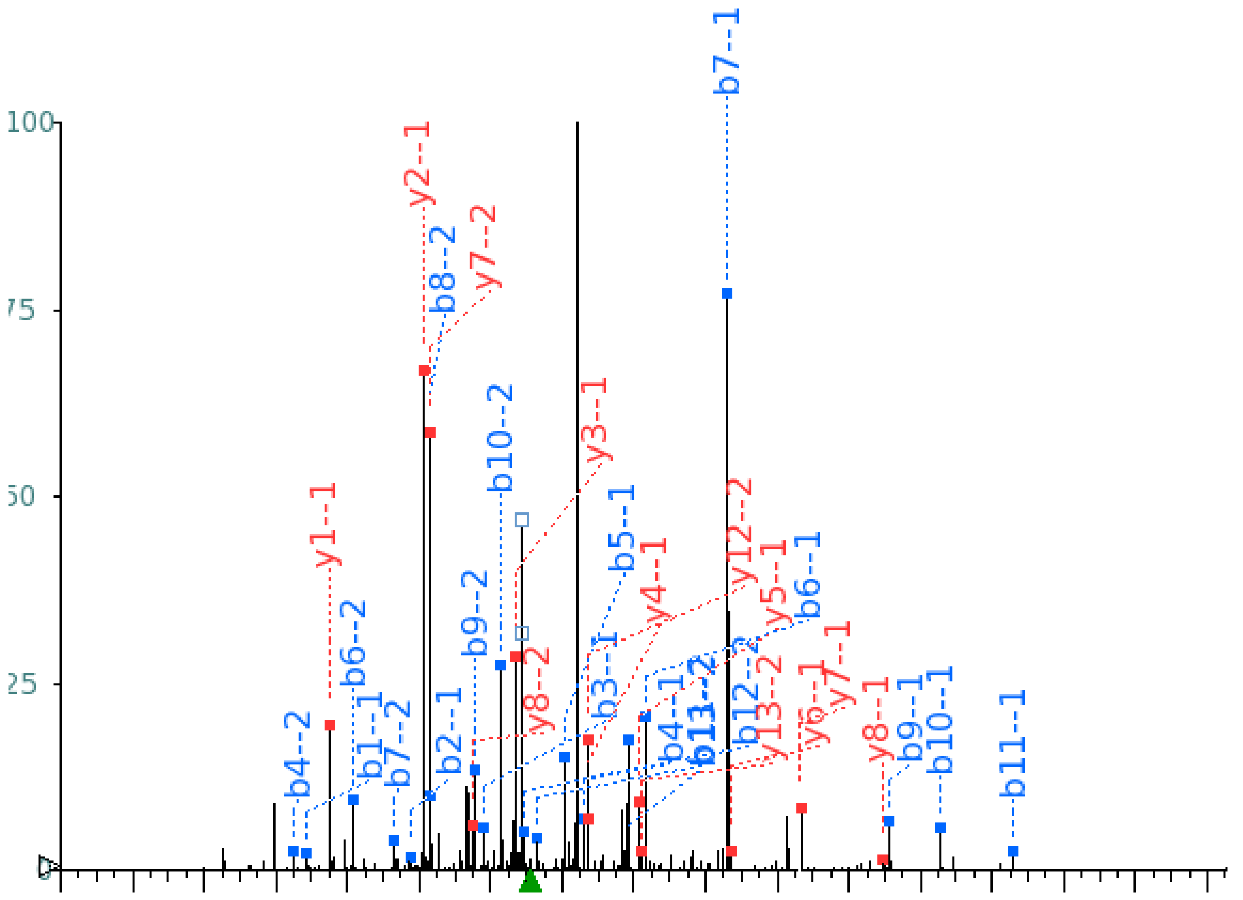

IFVGGLNPEATEEK (+3)

Primary Reference: B4DMY3_HUMAN
Search ID: 41886
Search Name: 20130330_ananiav_TMT_GPP_10percent_fraction15_lysC_2MC_IAA
Scan#: 19299
Observed Mass: 654.7053 (3.3 ppm)
PSM Score: 46.85

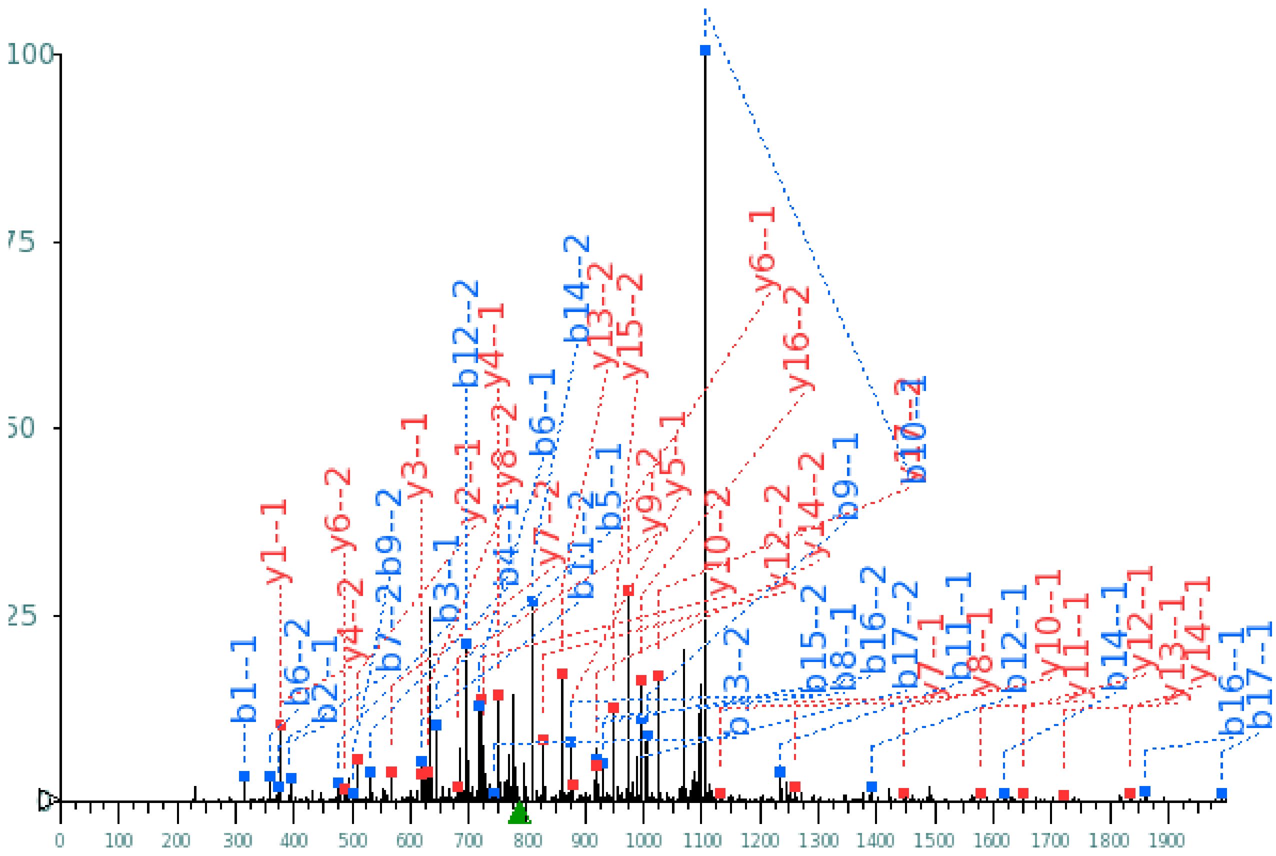

SGTGIAAMSVMRPEQIMK (+3)

Primary Reference: VATL_HUMAN
Search ID: 41886
Search Name: 20130330_ananiav_TMT_GPP_10percent_fraction15_lysC_2MC_IAA
Scan#: 19485
Observed Mass: 789.0997 (1.7 ppm)
PSM Score: 62.51

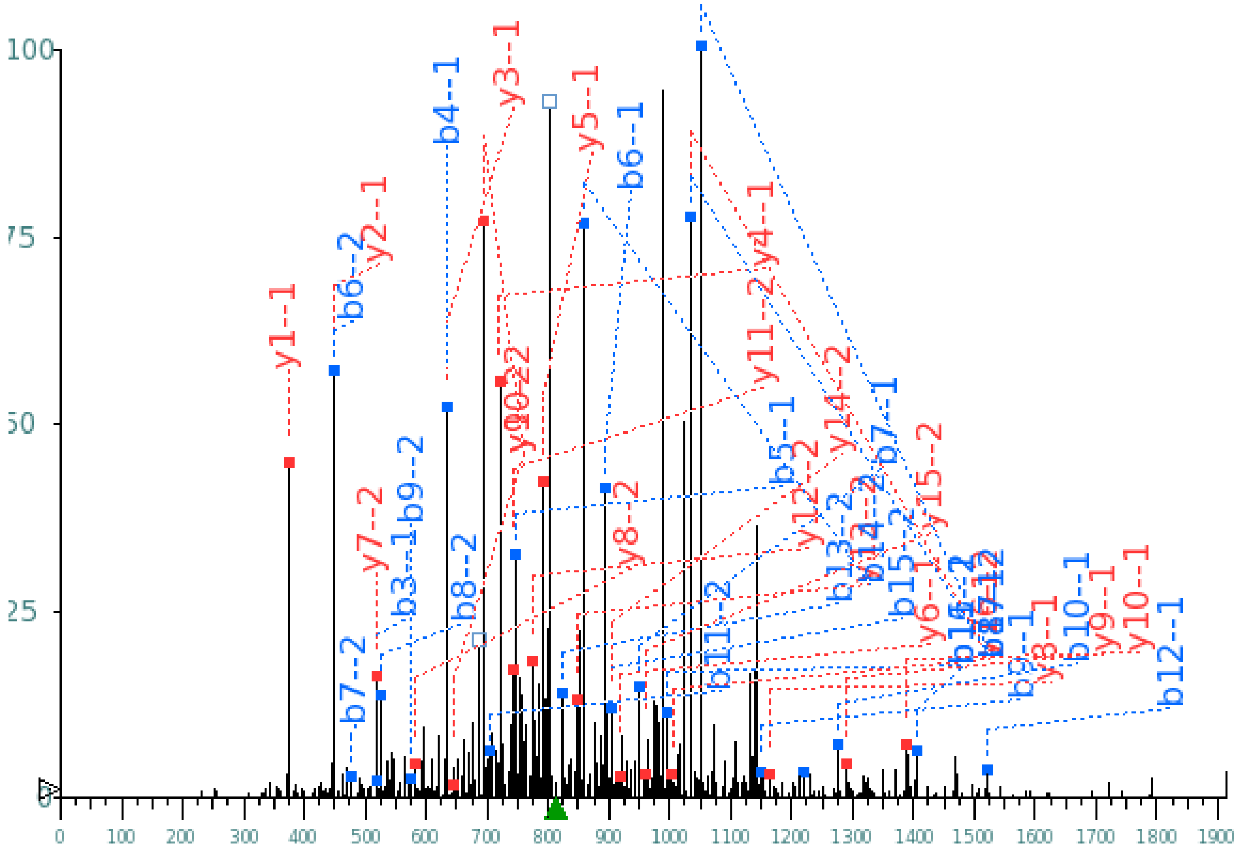

MASNIFGTPEENQASWAK (+3)

Primary Reference: HN1_HUMAN
Search ID: 41886
Search Name: 20130330_ananiav_TMT_GPP_10percent_fraction15_lysC_2MC_IAA
Scan#: 20146
Observed Mass: 813.7516 (1 ppm)
PSM Score: 39.7

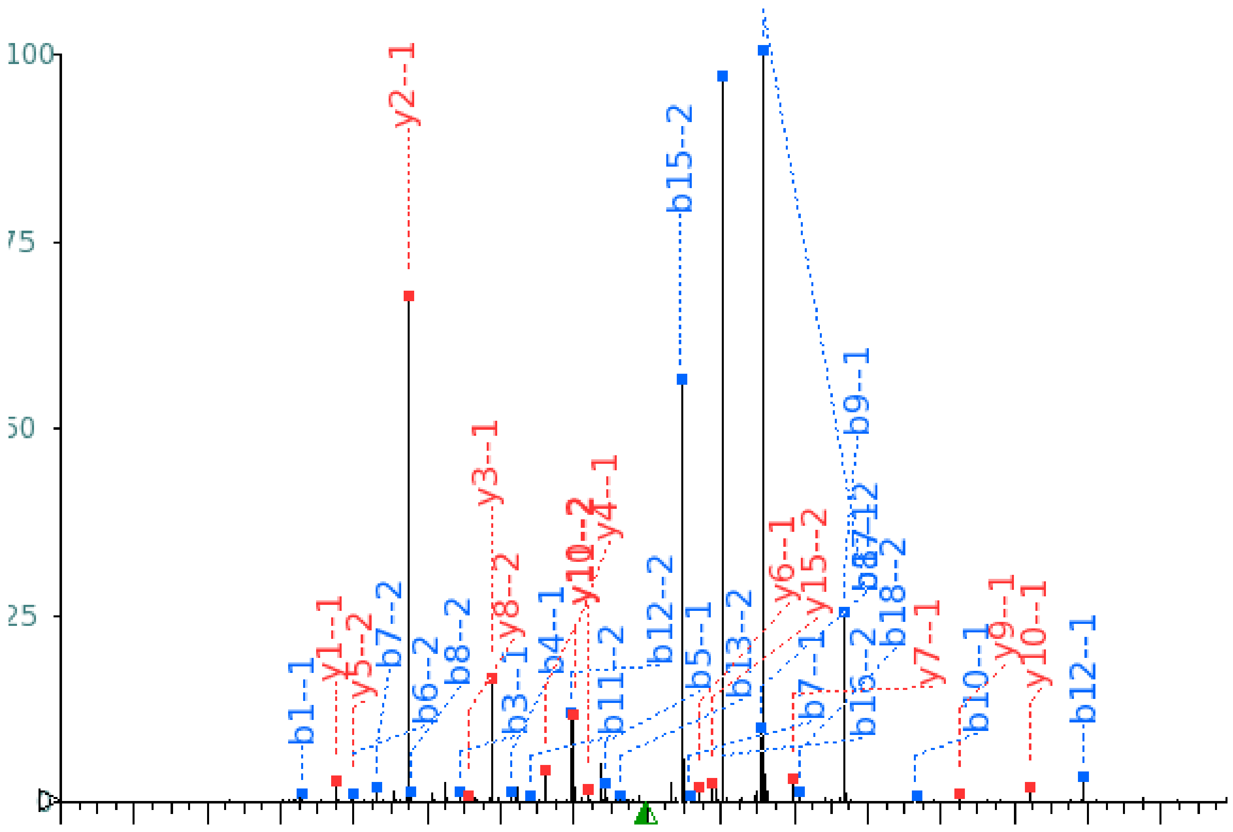

VTIAQGGVLPNIQAVLLPK (+3)

Primary Reference: H2A1D_HUMAN
Search ID: 41886
Search Name: 20130330_ananiav_TMT_GPP_10percent_fraction15_lysC_2MC_IAA
Scan#: 26273
Observed Mass: 797.173 (4.2 ppm)
PSM Score: 45.96

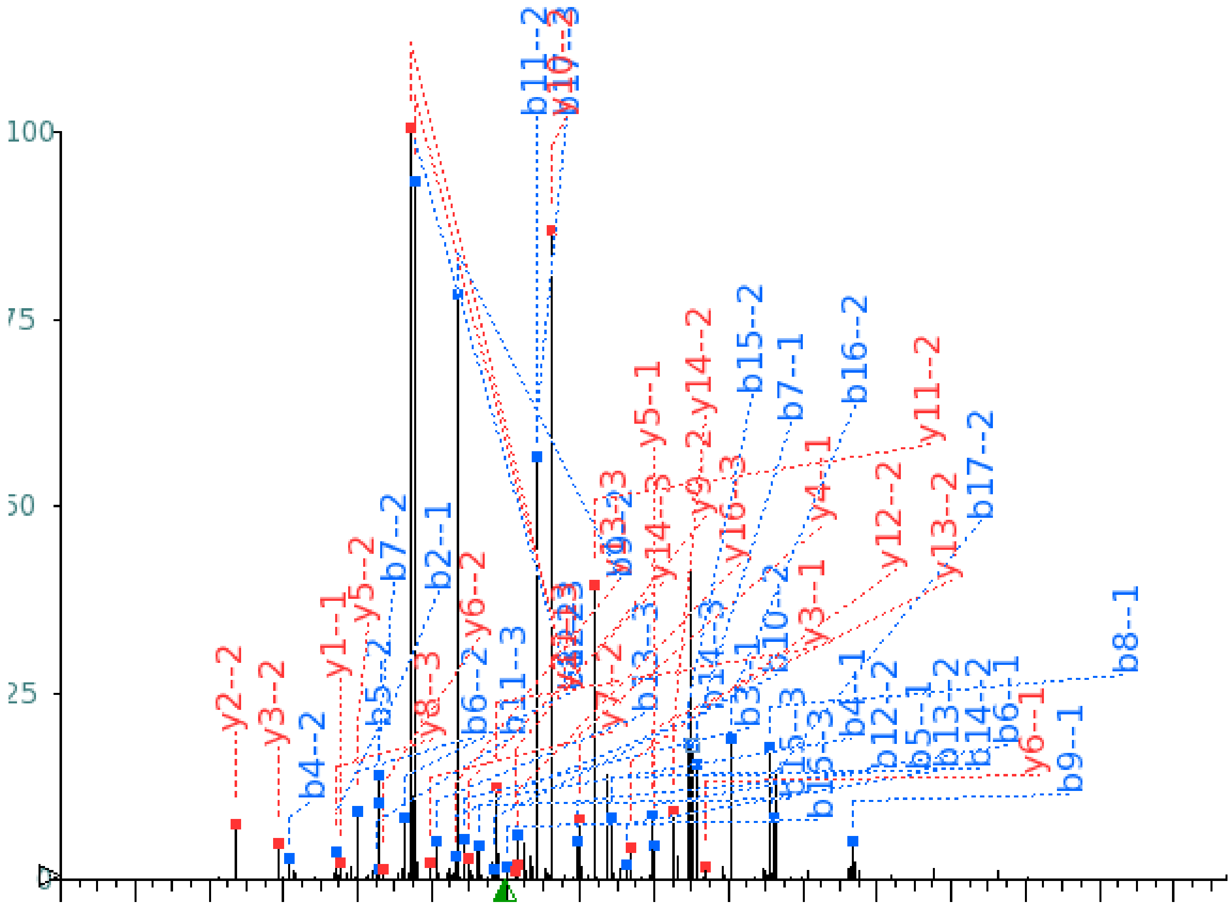

VTIAQGGVLPNIQAVLLPK (+4)

Primary Reference: H2A1D_HUMAN
Search ID: 41886
Search Name: 20130330_ananiav_TMT_GPP_10percent_fraction15_lysC_2MC_IAA
Scan#: 26275
Observed Mass: 598.1312 (3.5 ppm)
PSM Score: 76.96

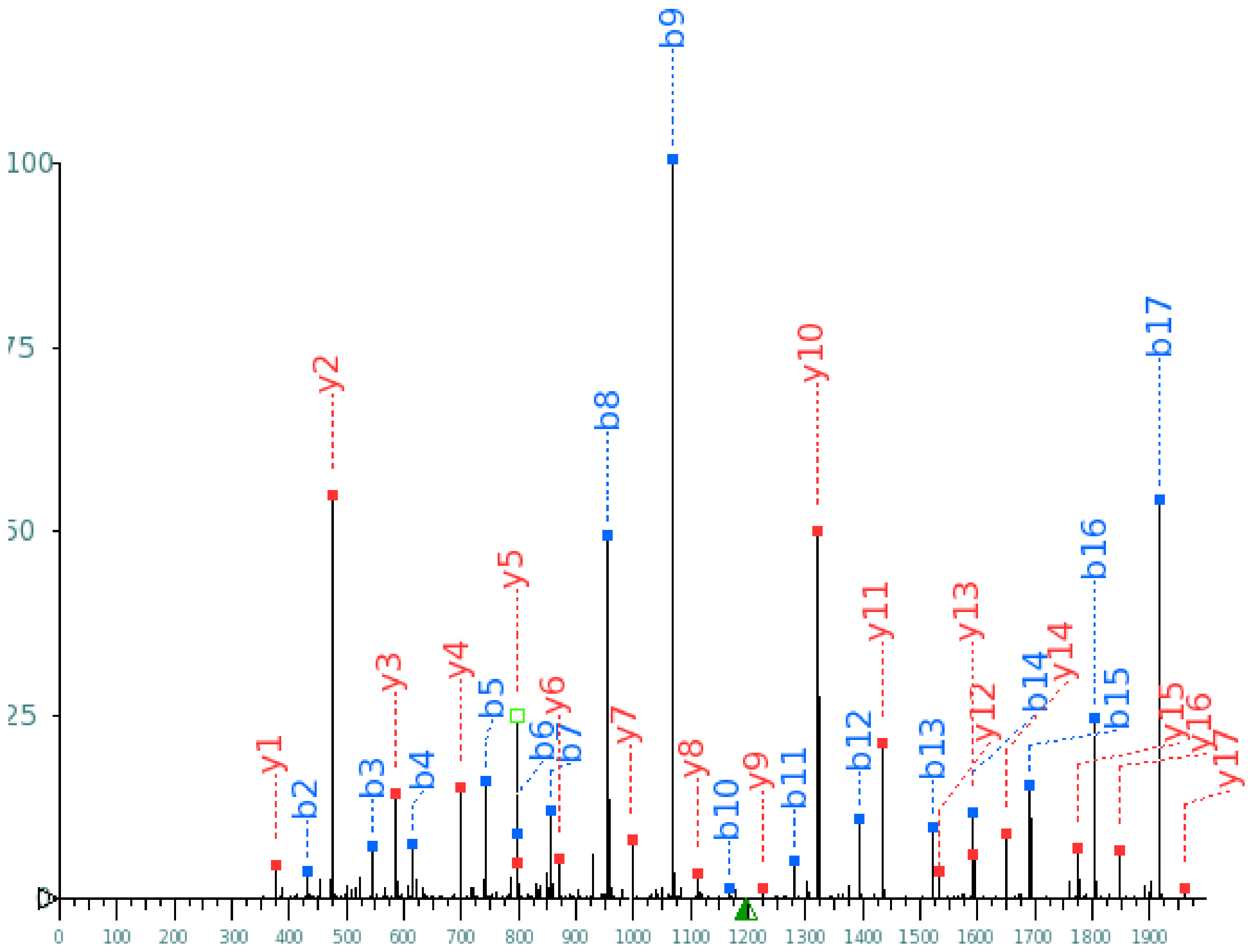

VTIAQGGVLPNIQAVLLPK (+2)

Primary Reference: H2A1D_HUMAN
Search ID: 41886
Search Name: 20130330_ananiav_TMT_GPP_10percent_fraction15_lysC_2MC_IAA
Scan#: 26279
Observed Mass: 1195.2529 (1.7 ppm)
PSM Score: 114.96

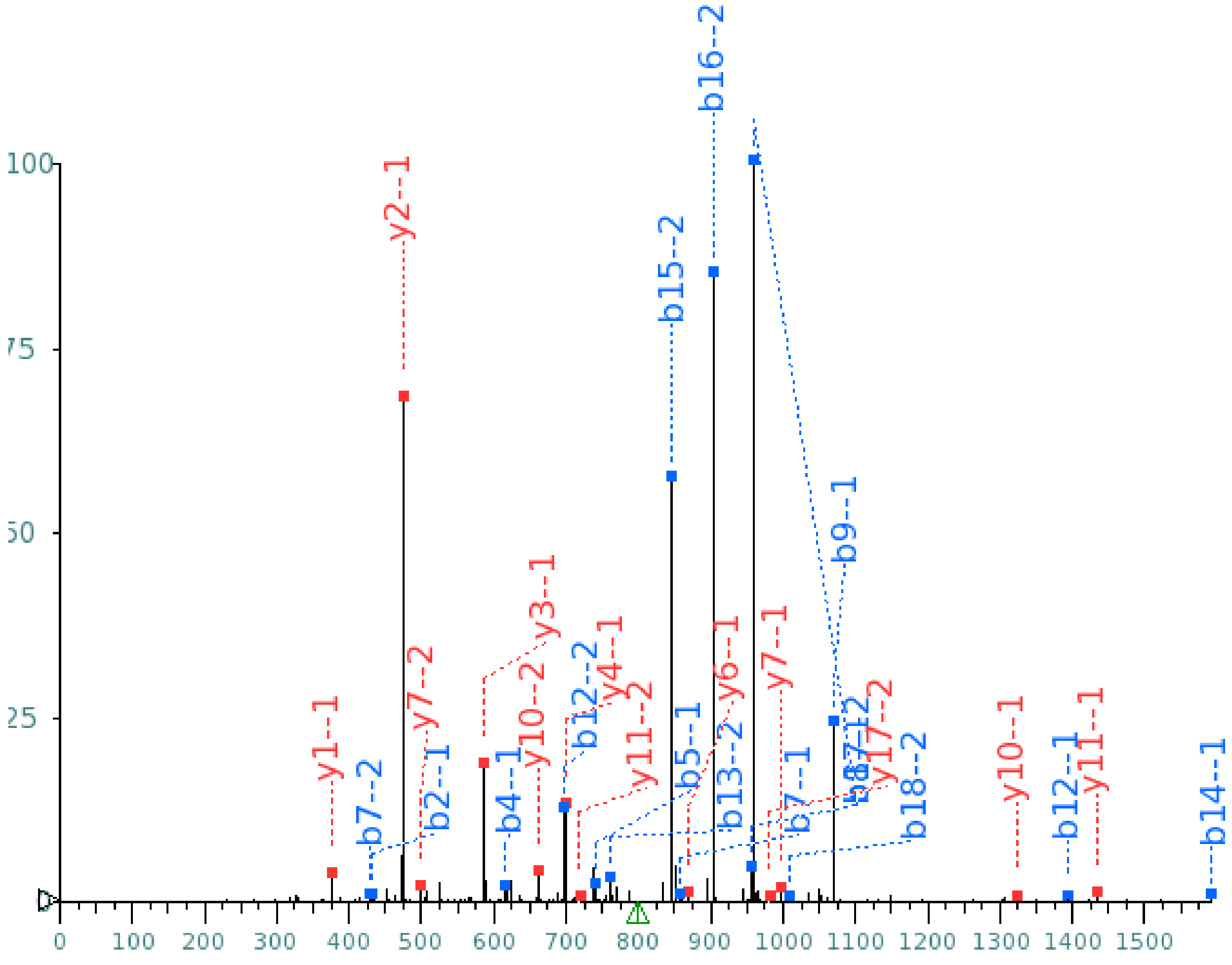

VTIAQGGVLPNIQAVLLPK (+3)

Primary Reference: H2A1D_HUMAN
Search ID: 41886
Search Name: 20130330_ananiav_TMT_GPP_10percent_fraction15_lysC_2MC_IAA
Scan#: 26383
Observed Mass: 797.1727 (3.8 ppm)
PSM Score: 32.57

File name: 2015-12-17 03.51.45PM.pdf

Creation time: 2015-12-17 03:53PM

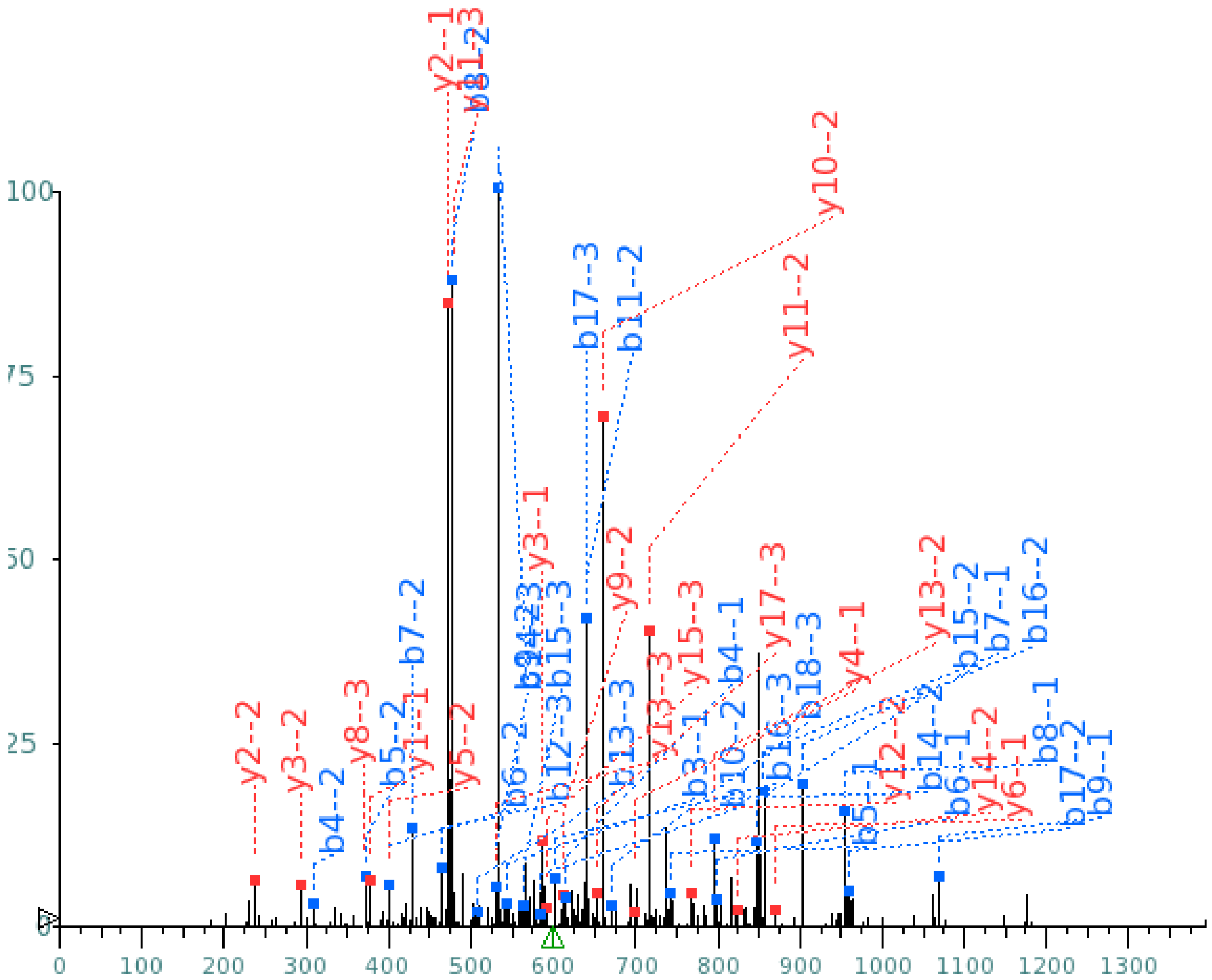

VTIAQGGVLPNIQAVLLPK (+4)

Primary Reference: H2A1D_HUMAN
Search ID: 41886
Search Name: 20130330_ananiav_TMT_GPP_10percent_fraction15_lysC_2MC_IAA
Scan#: 26448
Observed Mass: 598.131 (3.1 ppm)
PSM Score: 53.89

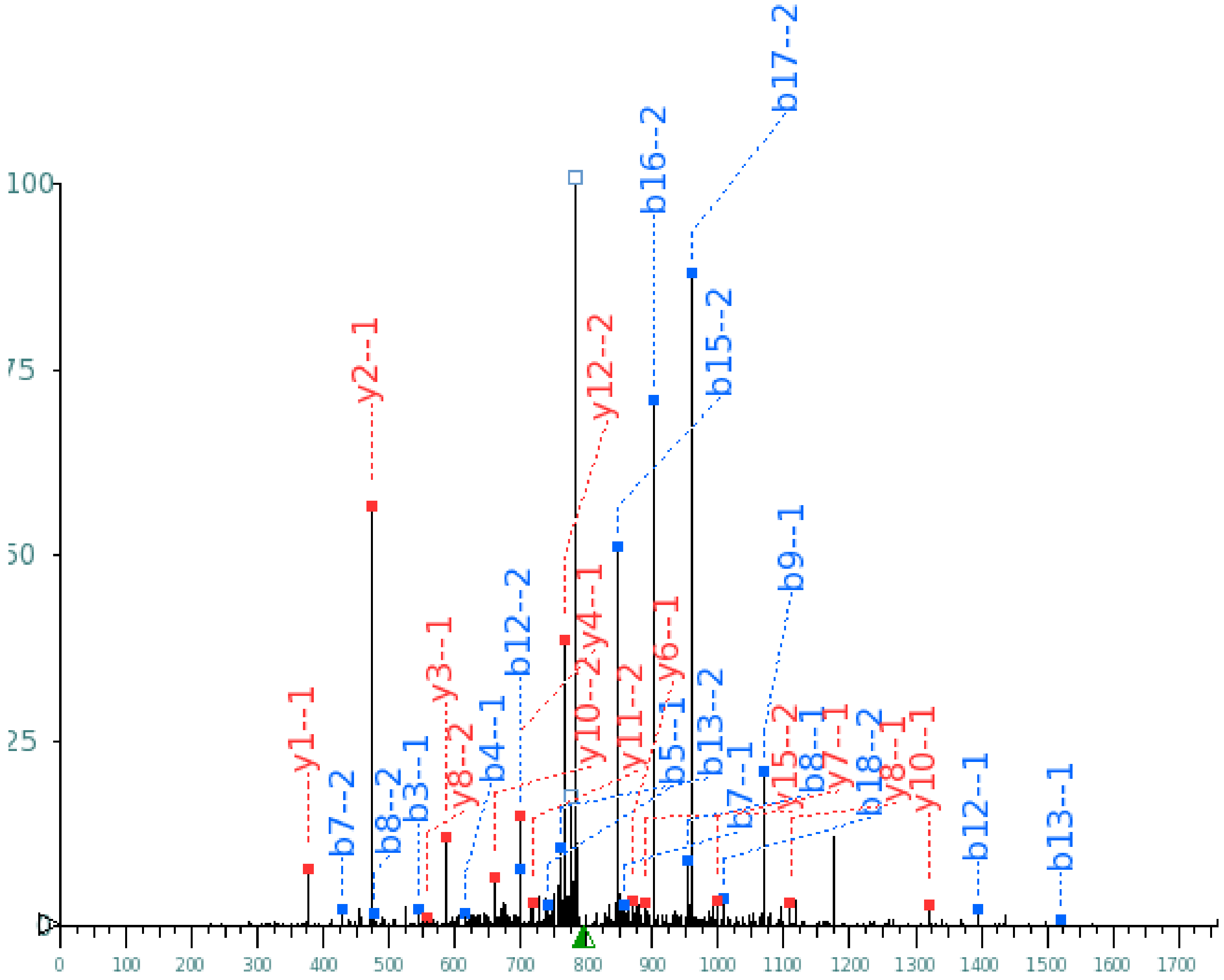

VTIAQGGVLPNIQAVLLPK (+3)

Primary Reference: H2A1D_HUMAN
Search ID: 41886
Search Name: 20130330_ananiav_TMT_GPP_10percent_fraction15_lysC_2MC_IAA
Scan#: 26511
Observed Mass: 797.1722 (3.1 ppm)
PSM Score: 30.6

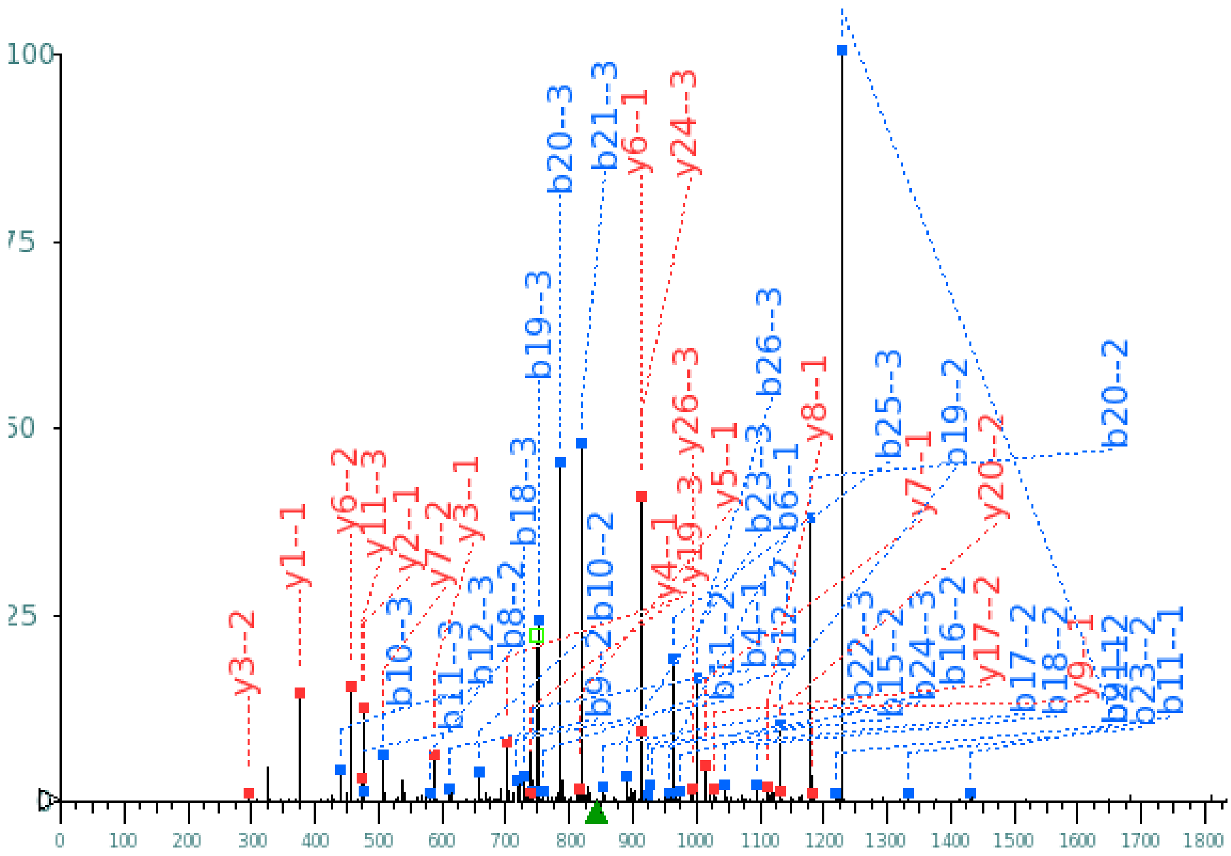

YYALC*GFGGVLSC*GLTHTAVVPLDLVK (+4)

Primary Reference: B2RE88_HUMAN
Search ID: 41886
Search Name: 20130330_ananiav_TMT_GPP_10percent_fraction15_lysC_2MC_IAA
Scan#: 26592
Observed Mass: 842.9617 (2.9 ppm)
PSM Score: 22.25

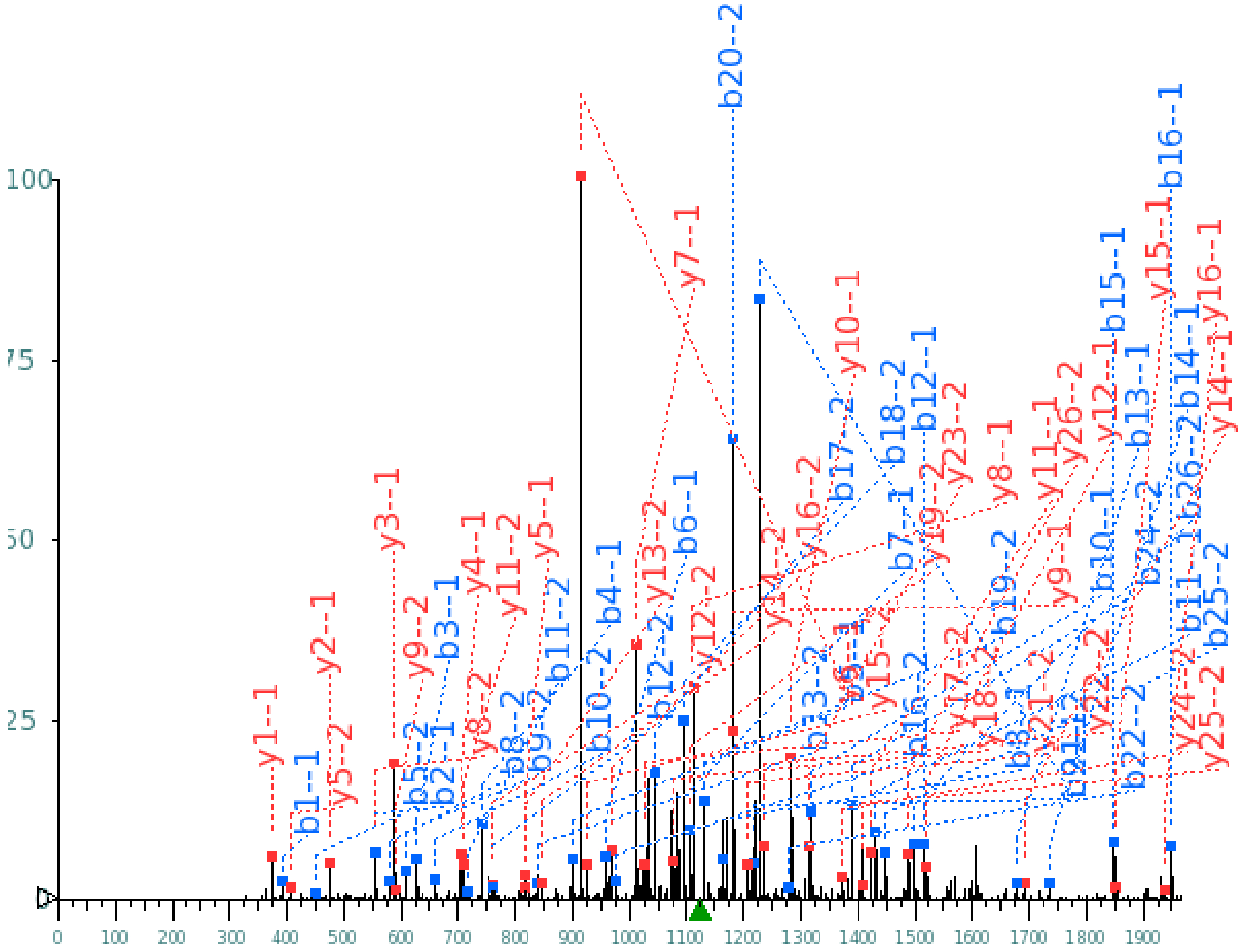

YYALC*GFGGVLSC*GLTHTAVVPLDLVK (+3)

Primary Reference: B2RE88_HUMAN
Search ID: 41886
Search Name: 20130330_ananiav_TMT_GPP_10percent_fraction15_lysC_2MC_IAA
Scan#: 26594
Observed Mass: 1123.6126 (2.4 ppm)
PSM Score: 102.7

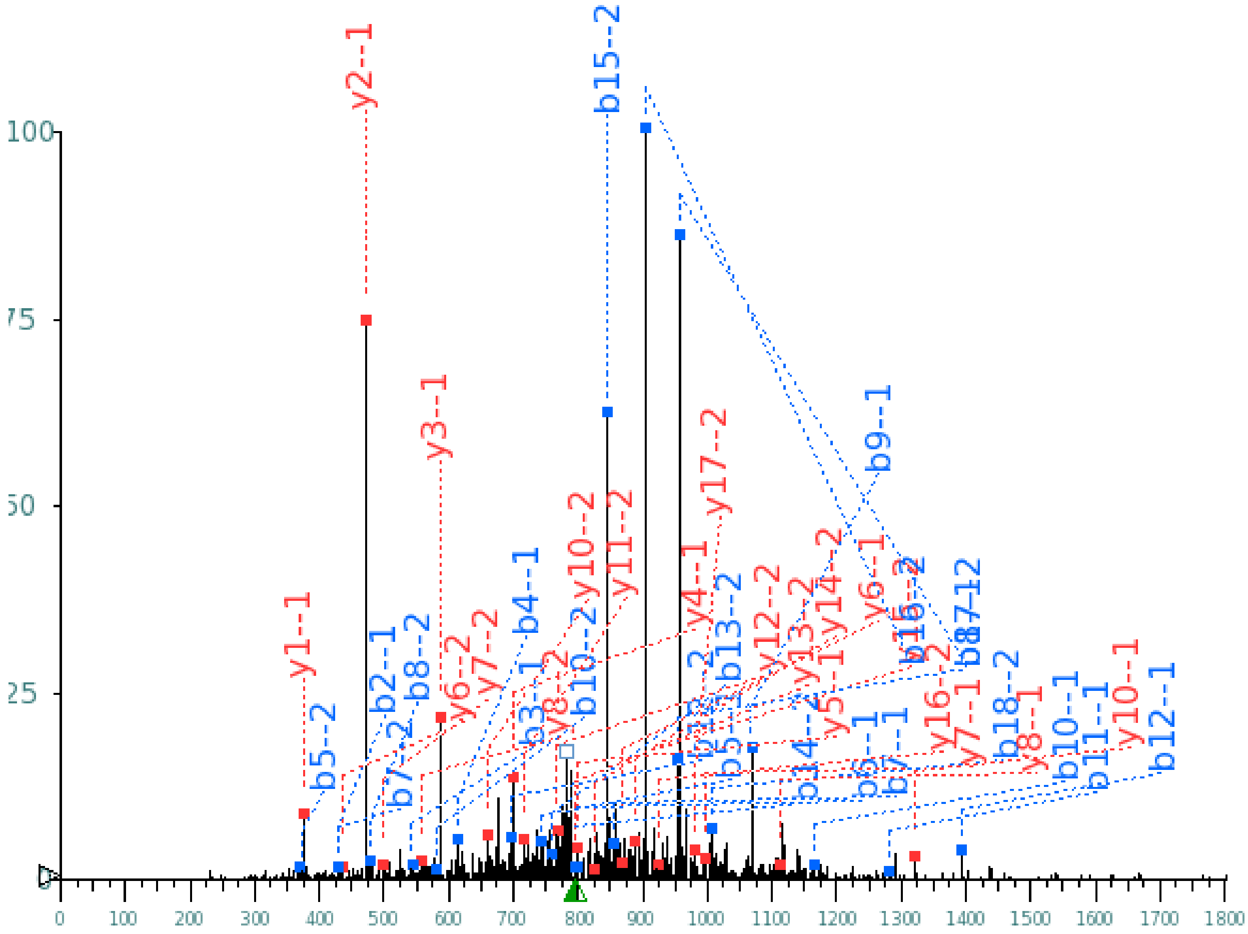

VTIAQGGVLPNIQAVLLPK (+3)

Primary Reference: H2A1D_HUMAN
Search ID: 41886
Search Name: 20130330_ananiav_TMT_GPP_10percent_fraction15_lysC_2MC_IAA
Scan#: 26697
Observed Mass: 797.1723 (3.2 ppm)
PSM Score: 19.47

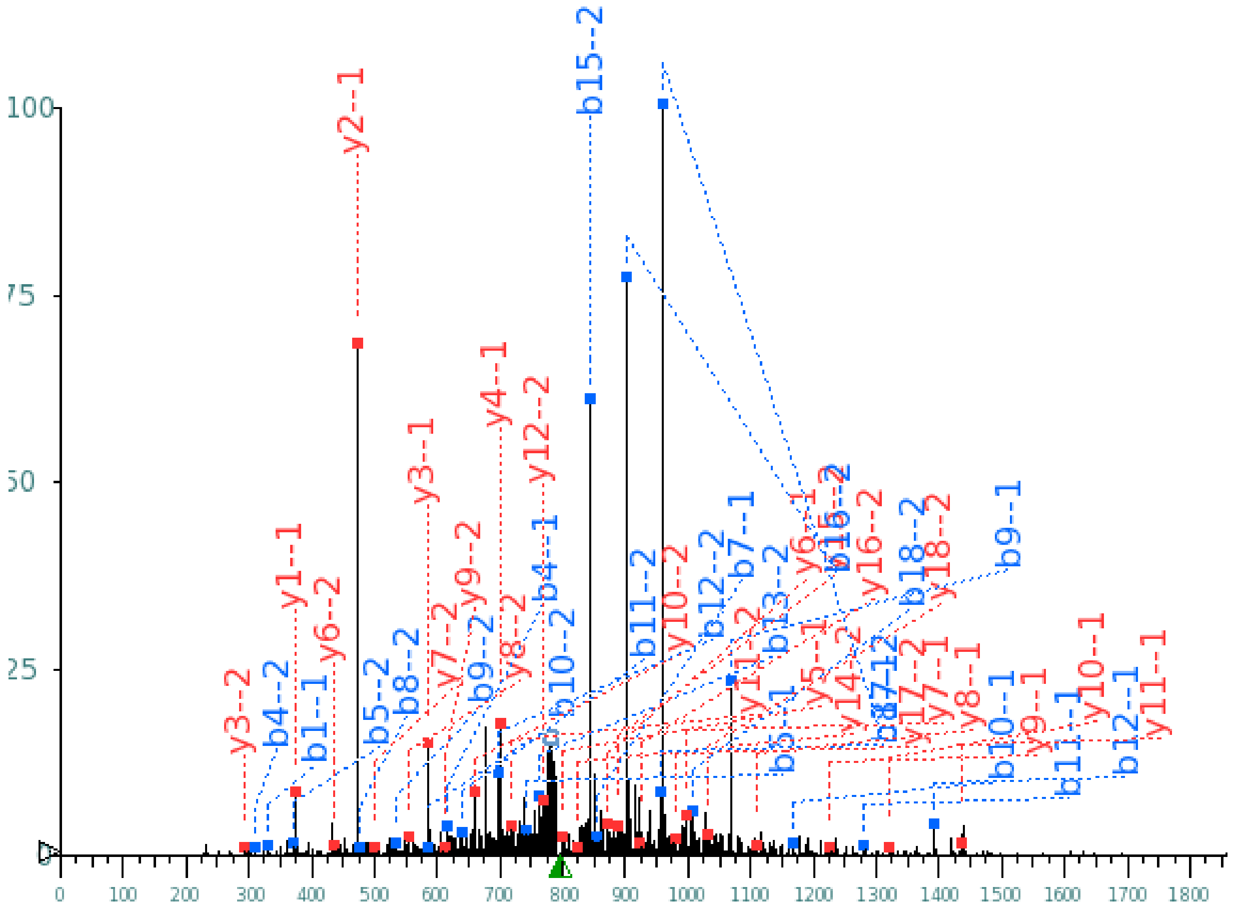

VTIAQGGVLPNIQAVLLPK (+3)

Primary Reference: H2A1D_HUMAN
Search ID: 41886
Search Name: 20130330_ananiav_TMT_GPP_10percent_fraction15_lysC_2MC_IAA
Scan#: 27019
Observed Mass: 797.1725 (3.5 ppm)
PSM Score: 18.48

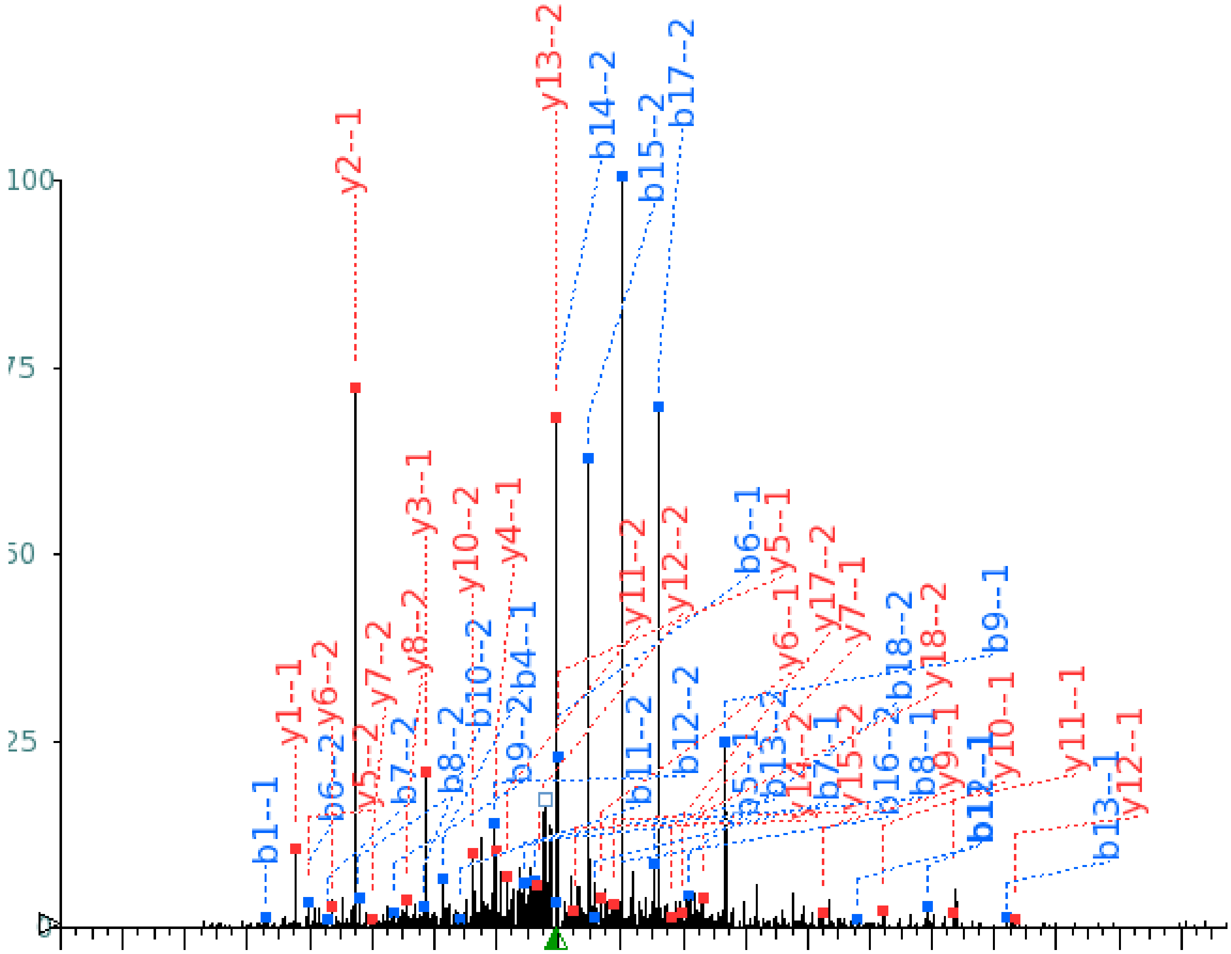

VTIAQGGVLPNIQAVLLPK (+3)

Primary Reference: H2A1D_HUMAN
Search ID: 41886
Search Name: 20130330_ananiav_TMT_GPP_10percent_fraction15_lysC_2MC_IAA
Scan#: 27261
Observed Mass: 797.1719 (2.7 ppm)
PSM Score: 24.07

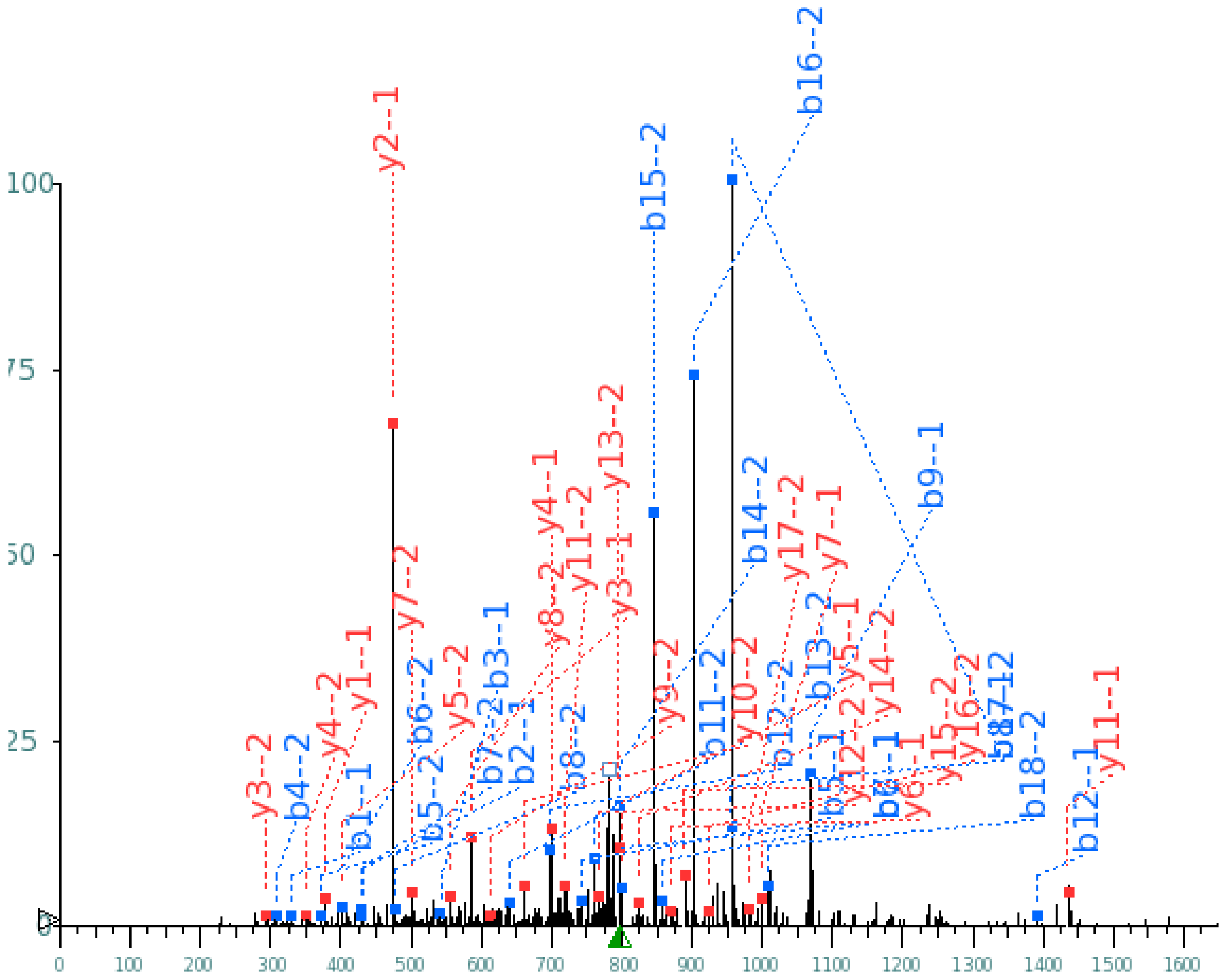

VTIAQGGVLPNIQAVLLPK (+3)

Primary Reference: H2A1D_HUMAN
Search ID: 41886
Search Name: 20130330_ananiav_TMT_GPP_10percent_fraction15_lysC_2MC_IAA
Scan#: 27399
Observed Mass: 797.1718 (2.6 ppm)
PSM Score: 22.45

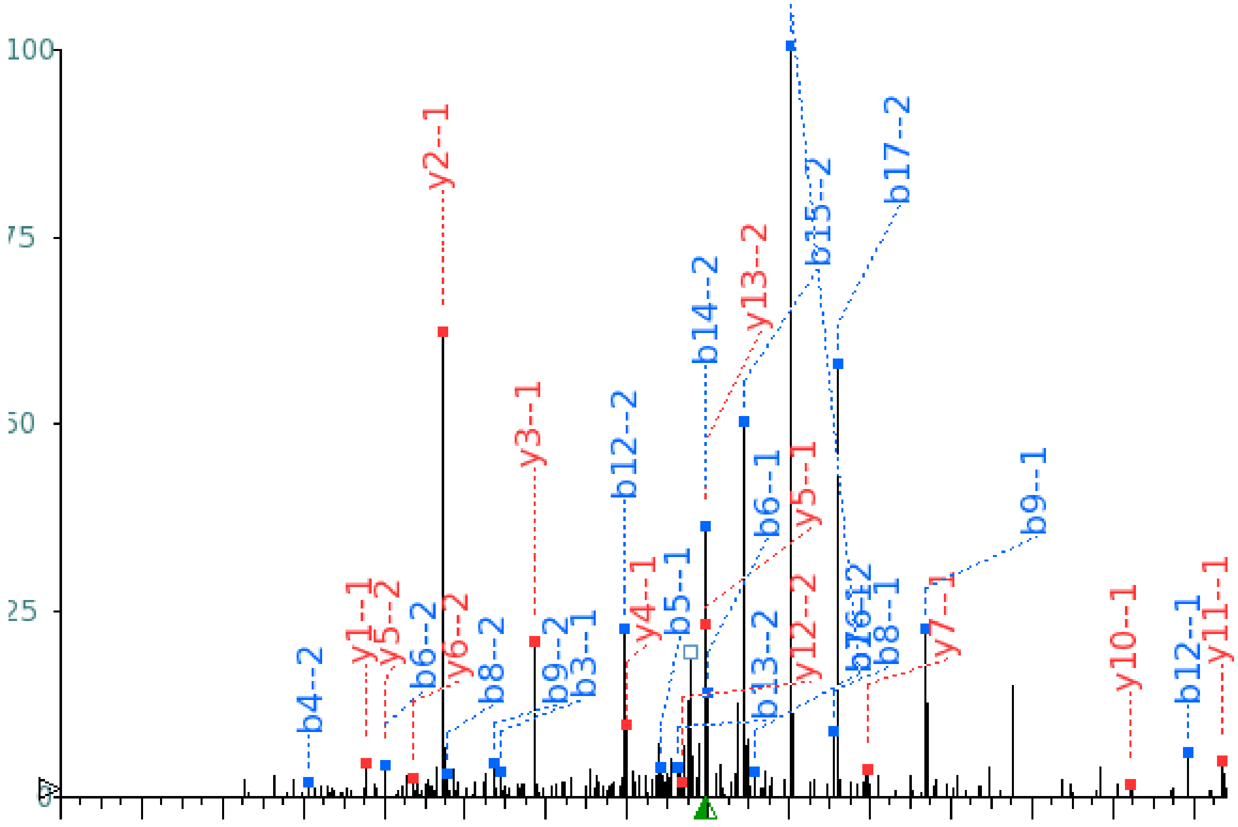

VTIAQGGVLPNIQAVLLPK (+3)

Primary Reference: H2A1D_HUMAN
Search ID: 41886
Search Name: 20130330_ananiav_TMT_GPP_10percent_fraction15_lysC_2MC_IAA
Scan#: 27464
Observed Mass: 797.1719 (2.7 ppm)
PSM Score: 26.84

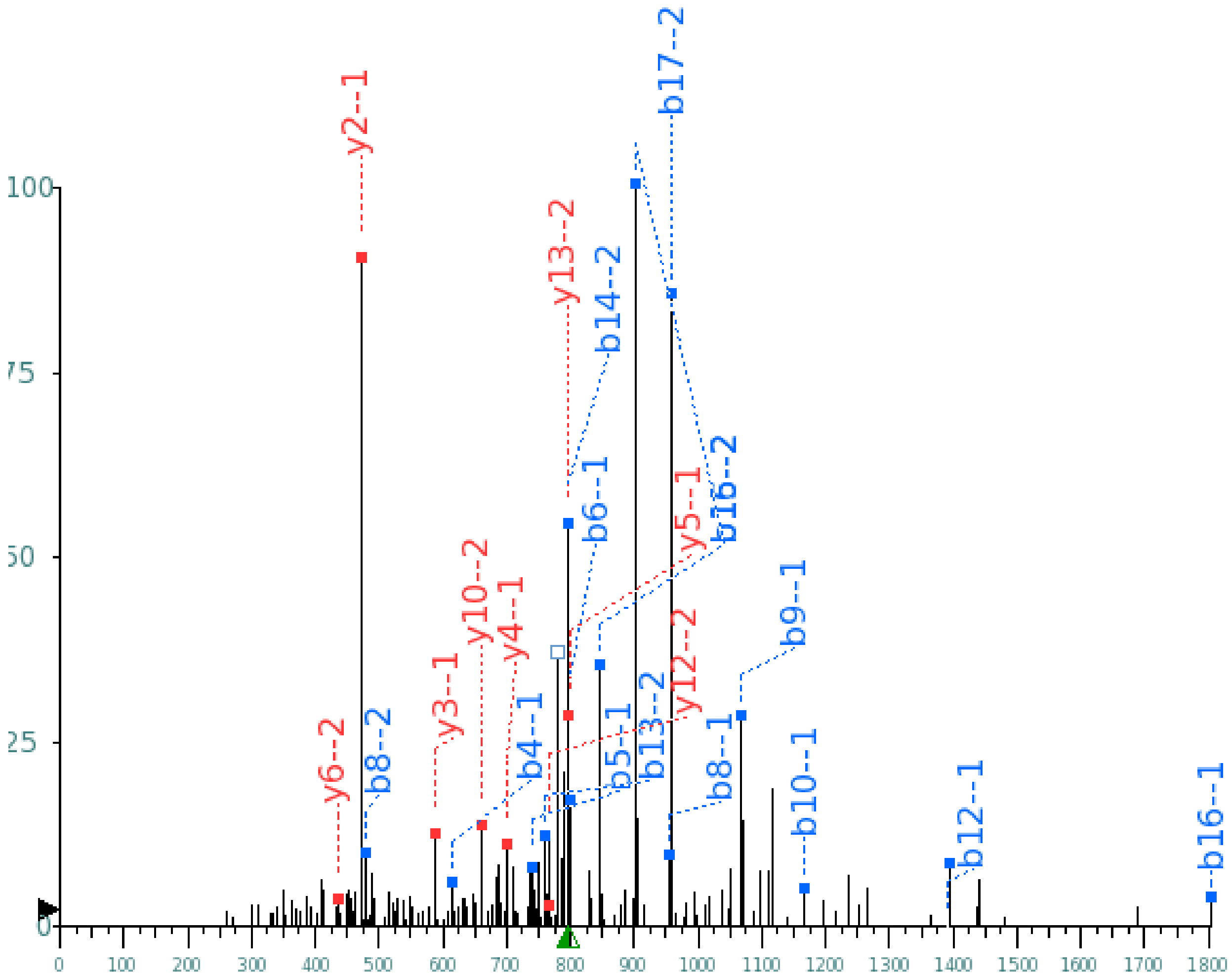

VTIAQGGVLPNIQAVLLPK (+3)

Primary Reference: H2A1D_HUMAN
Search ID: 41886
Search Name: 20130330_ananiav_TMT_GPP_10percent_fraction15_lysC_2MC_IAA
Scan#: 27530
Observed Mass: 797.1721 (3 ppm)
PSM Score: 19.72

File name: 2015-12-17 03.51.45PM.pdf

Creation time: 2015-12-17 03:53PM

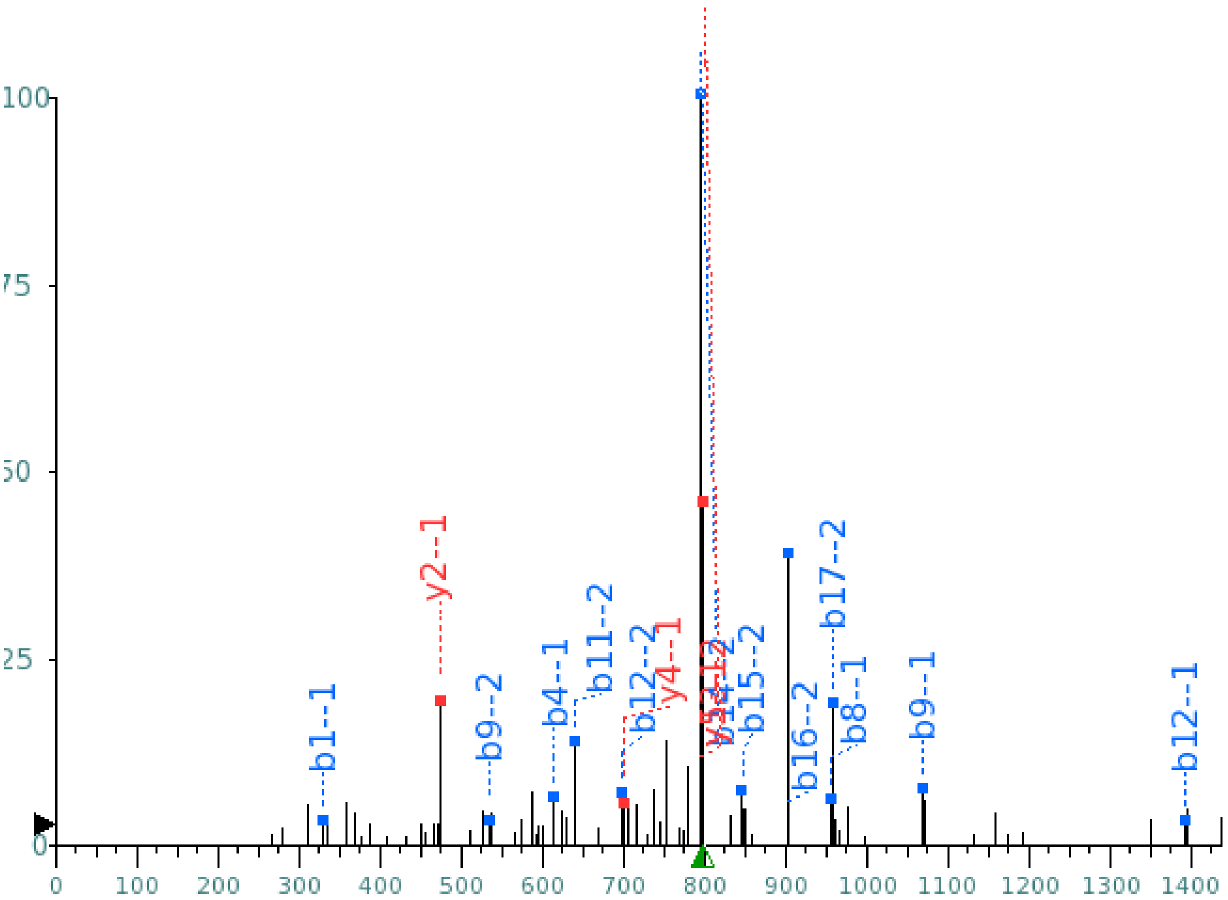

VTIAQGGVLPNIQAVLLPK (+3)

Primary Reference: H2A1D_HUMAN
Search ID: 41886
Search Name: 20130330_ananiav_TMT_GPP_10percent_fraction15_lysC_2MC_IAA
Scan#: 27801
Observed Mass: 797.1714 (2.1 ppm)
PSM Score: 18.52

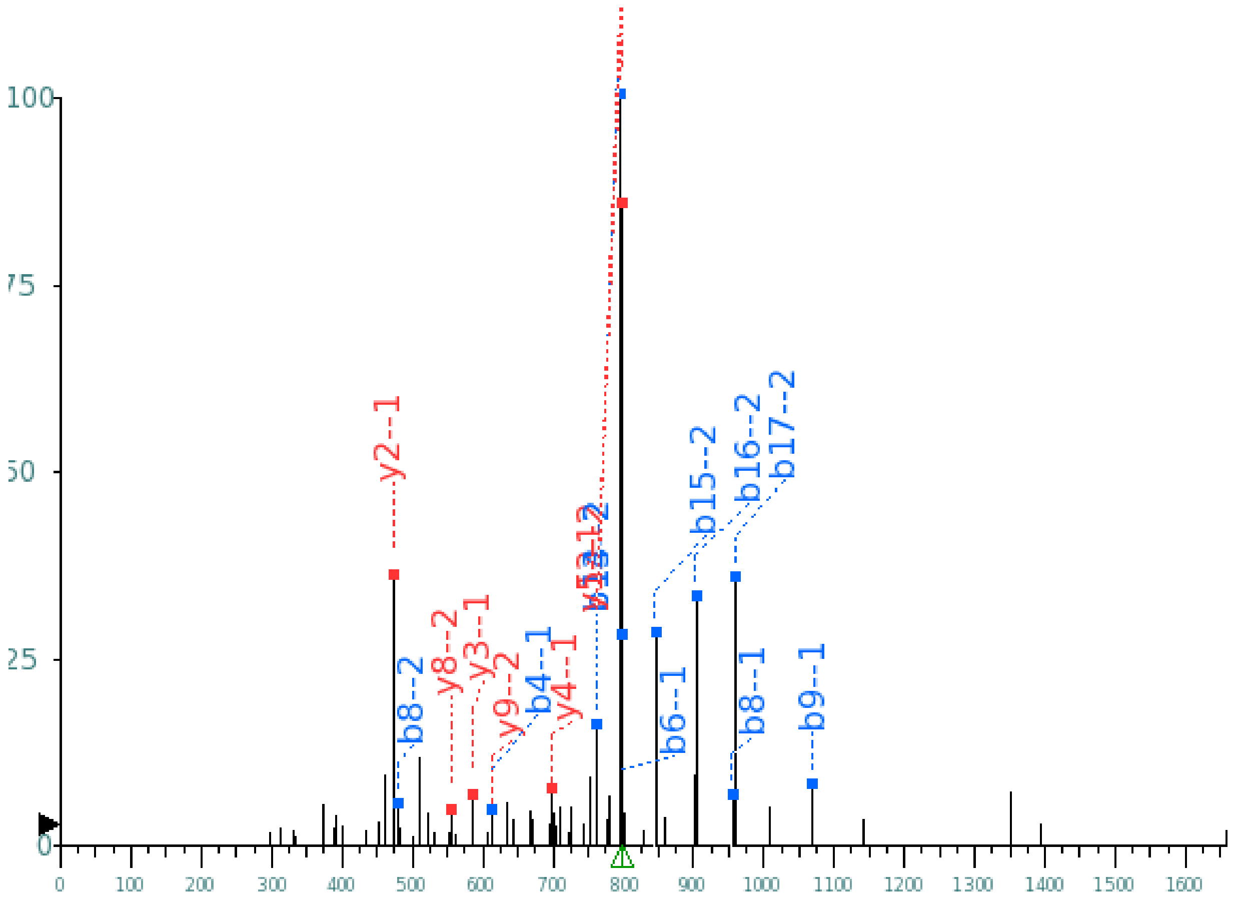

VTIAQGGVLPNIQAVLLPK (+3)

Primary Reference: H2A1D_HUMAN
Search ID: 41886
Search Name: 20130330_ananiav_TMT_GPP_10percent_fraction15_lysC_2MC_IAA
Scan#: 27818
Observed Mass: 797.172 (2.9 ppm)
PSM Score: 18.51

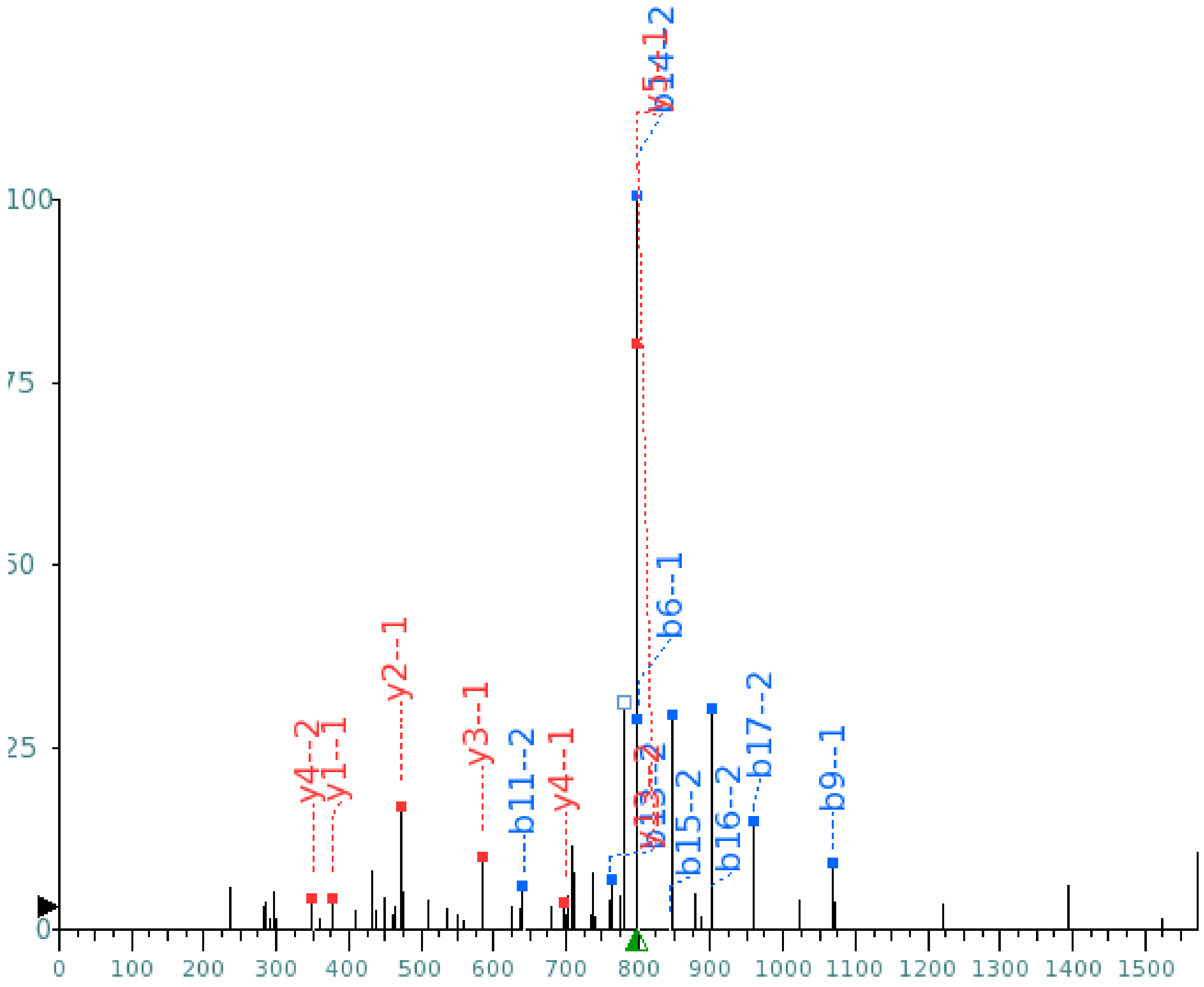

VTIAQGGVLPNIQAVLLPK (+3)

Primary Reference: H2A1D_HUMAN
Search ID: 41886
Search Name: 20130330_ananiav_TMT_GPP_10percent_fraction15_lysC_2MC_IAA
Scan#: 27859
Observed Mass: 797.1712 (1.9 ppm)
PSM Score: 12.15

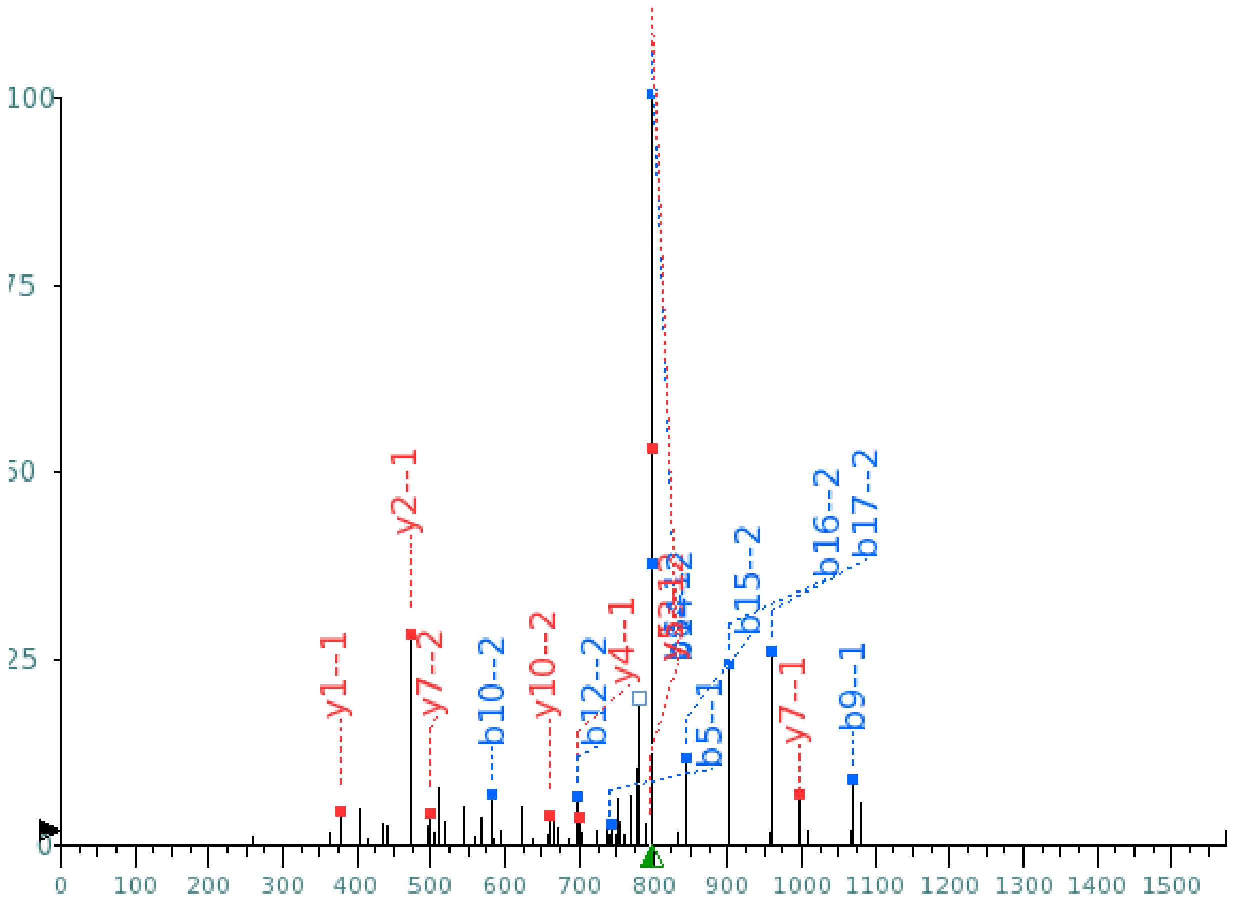

VTIAQGGVLPNIQAVLLPK (+3)

Primary Reference: H2A1D_HUMAN
Search ID: 41886
Search Name: 20130330_ananiav_TMT_GPP_10percent_fraction15_lysC_2MC_IAA
Scan#: 28304
Observed Mass: 797.1707 (1.2 ppm)
PSM Score: 24.72

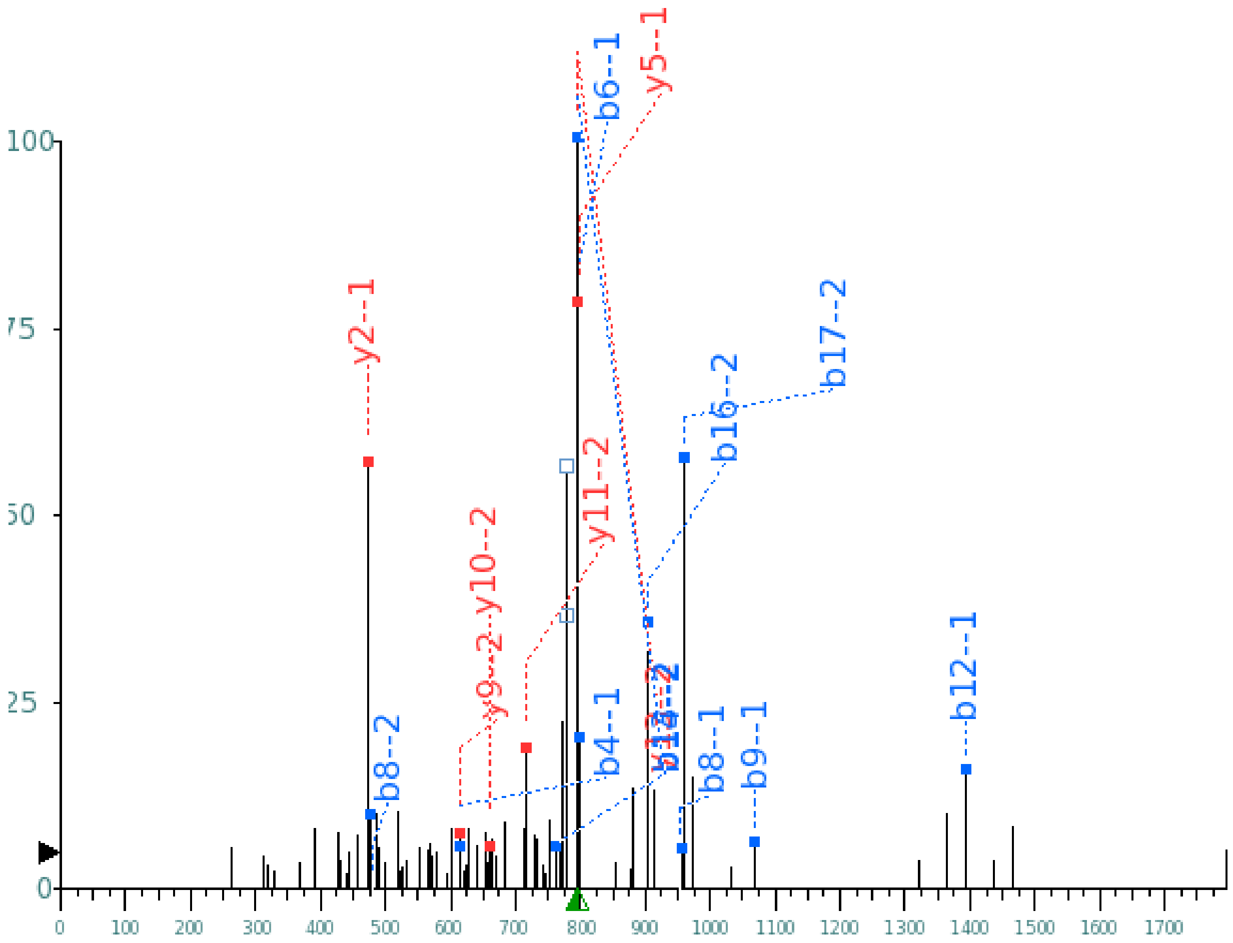

VTIAQGGVLPNIQAVLLPK (+3)

Primary Reference: H2A1D_HUMAN
Search ID: 41886
Search Name: 20130330_ananiav_TMT_GPP_10percent_fraction15_lysC_2MC_IAA
Scan#: 28417
Observed Mass: 797.1725 (3.5 ppm)
PSM Score: 15.75

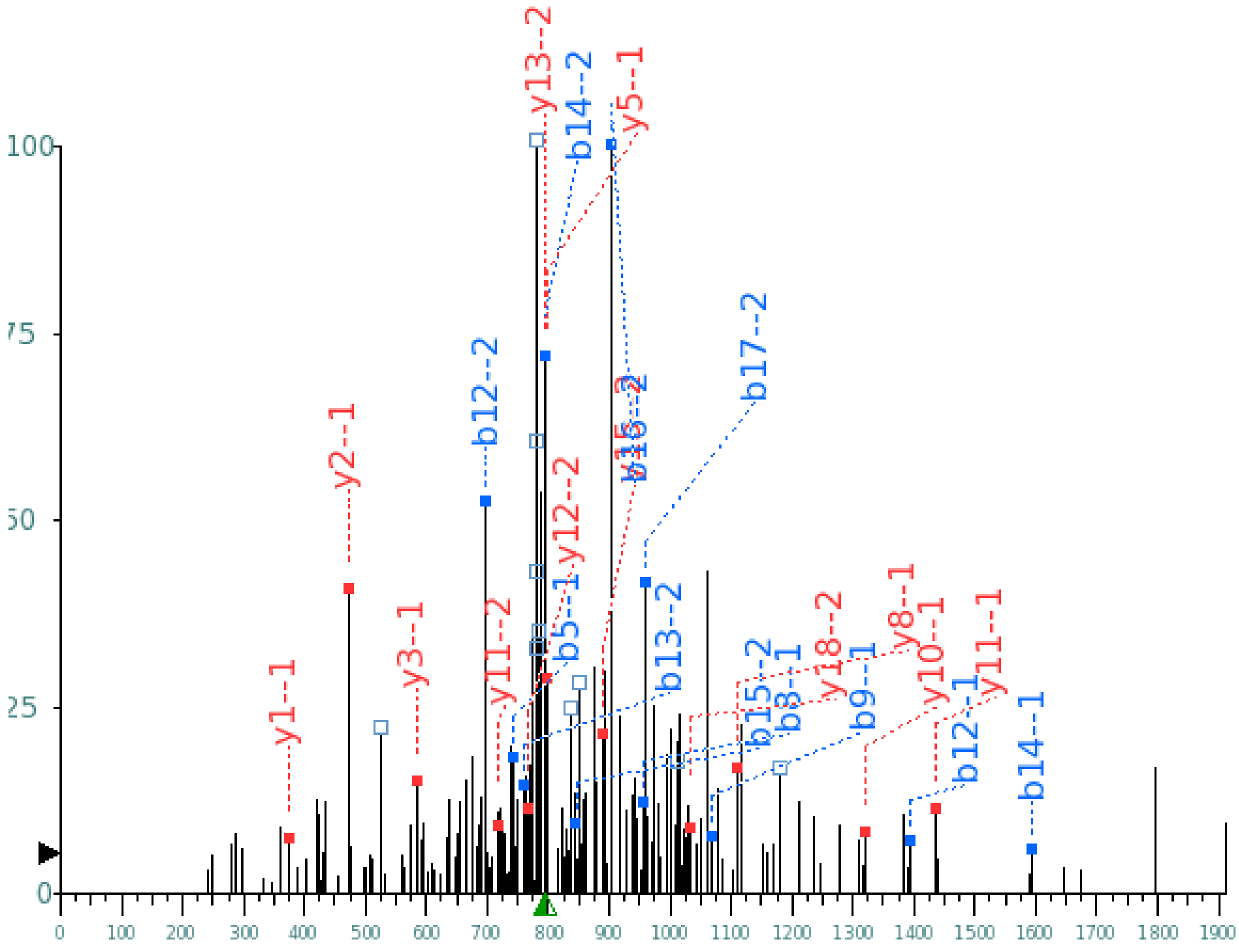

VTIAQGGVLPNIQAVLLPK (+3)

Primary Reference: H2A1D_HUMAN
Search ID: 41886
Search Name: 20130330_ananiav_TMT_GPP_10percent_fraction15_lysC_2MC_IAA
Scan#: 28774
Observed Mass: 797.1726 (3.5 ppm)
PSM Score: 7.6

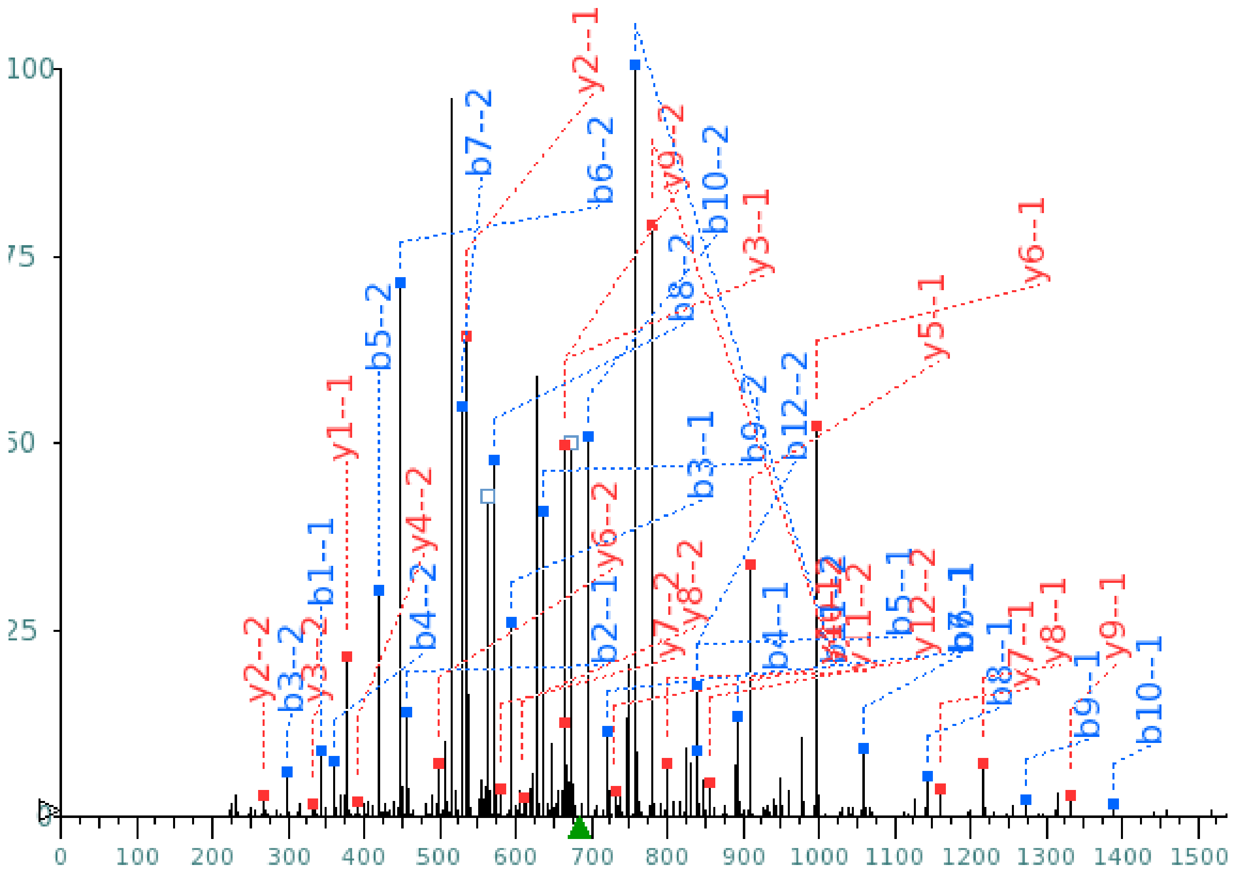

IIHEDGYSEDEC*K (+3)

Primary Reference: GNAI3_HUMAN
Search ID: 41887
Search Name: 20130330_ananiav_TMT_GPP_10percent_fraction16_lysC_2MC_IAA
Scan#: 10197
Observed Mass: 685.004 (1.2 ppm)
PSM Score: 64.05

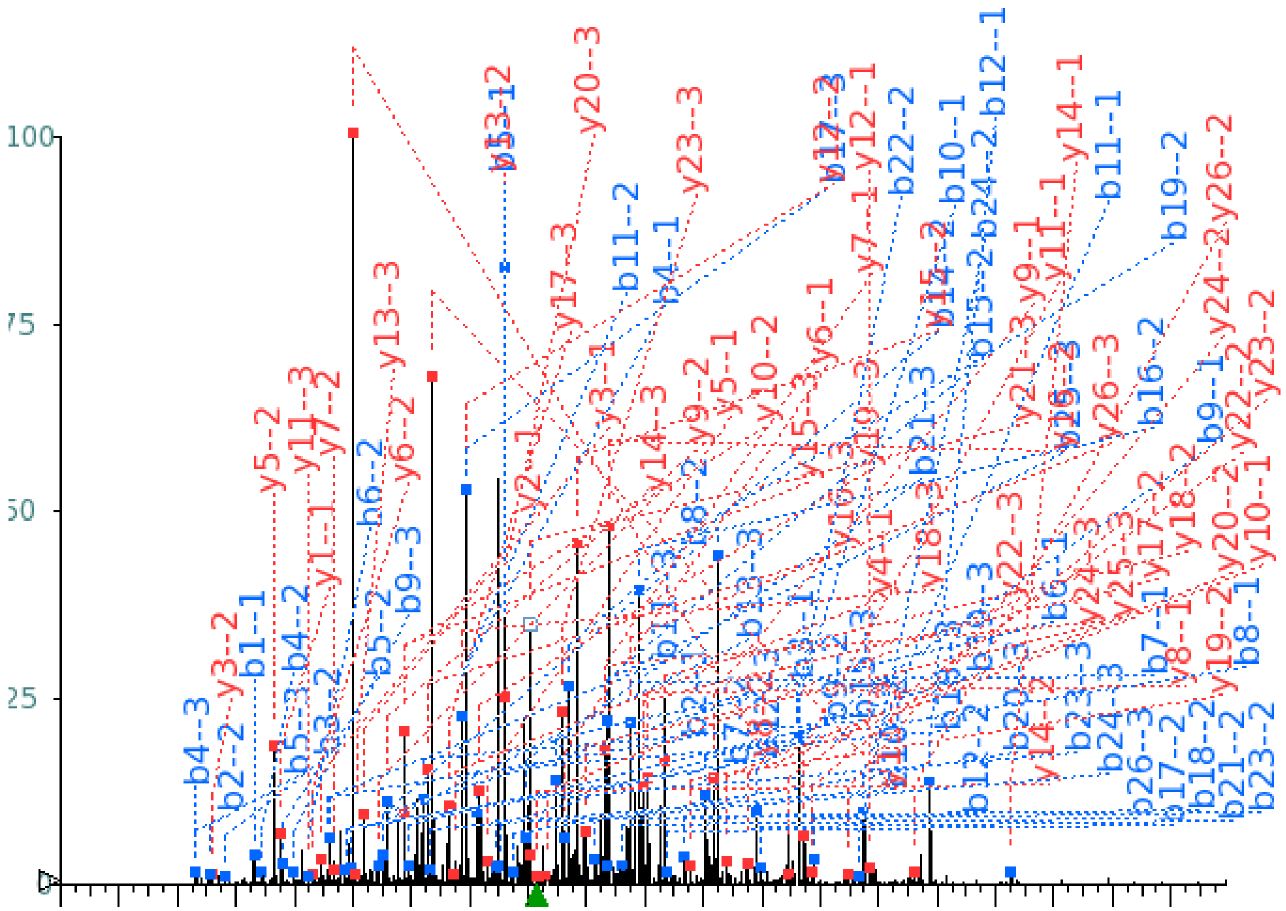

TQVEASEESALNHLQNPGDAAEGRAAK (+4)

Primary Reference: MOFA1_HUMAN
Search ID: 41887
Search Name: 20130330_ananiav_TMT_GPP_10percent_fraction16_lysC_2MC_IAA
Scan#: 14457
Observed Mass: 813.6732 (1.6 ppm)
PSM Score: 77.09

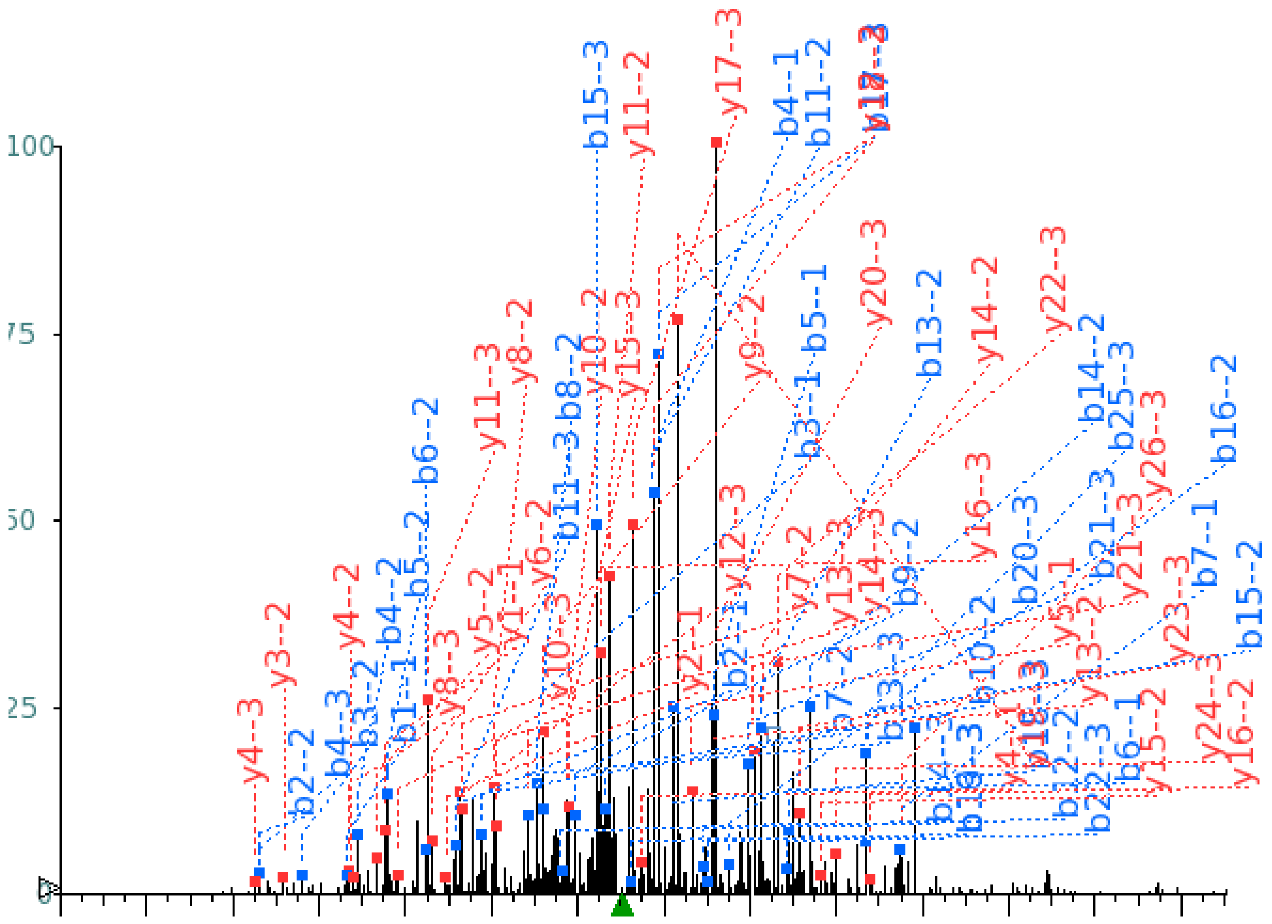

TQVEASEESALNHLQNPGDAAEGRAAK (+5)

Primary Reference: MOFA1_HUMAN
Search ID: 41887
Search Name: 20130330_ananiav_TMT_GPP_10percent_fraction16_lysC_2MC_IAA
Scan#: 14480
Observed Mass: 651.1404 (2.3 ppm)
PSM Score: 43.62

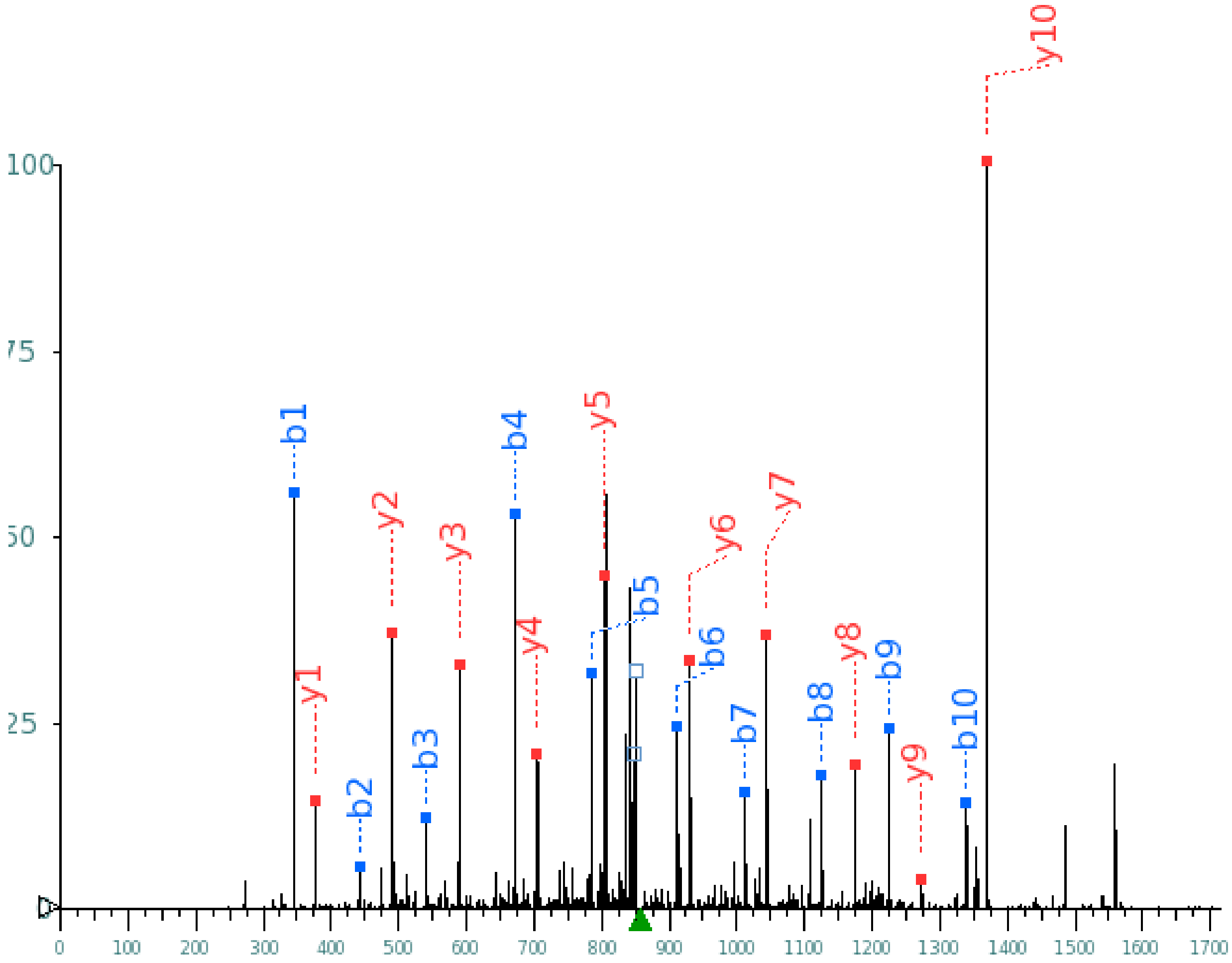

DPVEIQTLVNK (+2)

Primary Reference: LYRM4_HUMAN
Search ID: 41887
Search Name: 20130330_ananiav_TMT_GPP_10percent_fraction16_lysC_2MC_IAA
Scan#: 18129
Observed Mass: 857.5129 (2 ppm)
PSM Score: 72.07

File name: 2015-12-17 03.51.45PM.pdf

Creation time: 2015-12-17 03:53PM

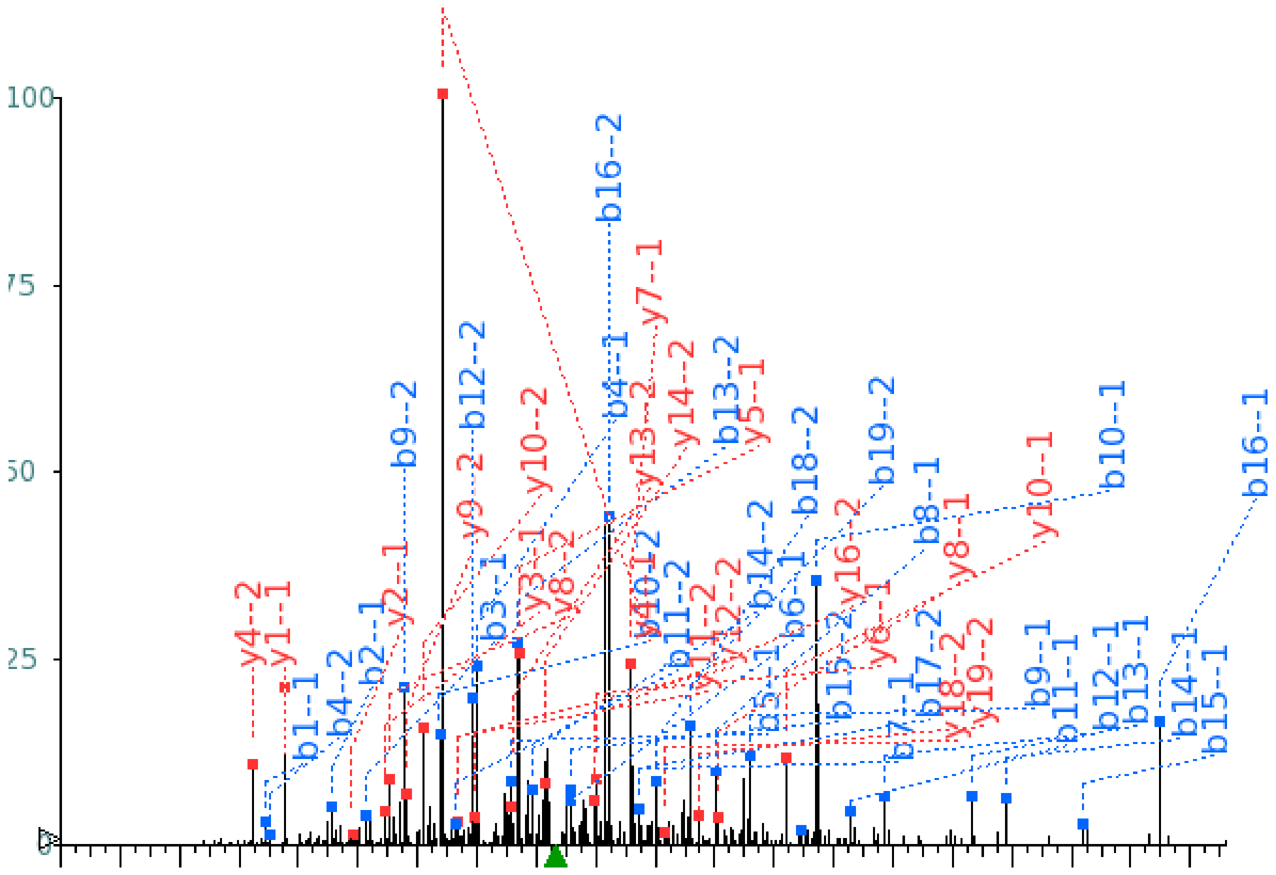

NIGWGTDQGIGGFGEEPGIK (+3)

Primary Reference: IR3IP_HUMAN
Search ID: 41887
Search Name: 20130330_ananiav_TMT_GPP_10percent_fraction16_lysC_2MC_IAA
Scan#: 20718
Observed Mass: 830.7738 (1.5 ppm)
PSM Score: 81.6
