# Supplementary material for: Uncovering a Dual Regulatory Role for Caspases During Endoplasmic Reticulum Stress-induced Cell Death
Source: Mol Cell Proteomics. 2016 Apr 28;15(7):2293–307. doi: 10.1074/mcp.M115.055376 (PMC4937505; doi:10.1074/mcp.M115.055376)
Supplement: Supplemental Data [file 10.1074_M115.055376_mcp.M115.055376-16.pdf]

Supplemental Figures and Tables

Supplemental Figure 1. Cellular response to treatment with tunicamycin. (A) Cells were treated for 0 to 72 hours with tunicamycin or a cross-linked version of the extrinsic apoptosis inducer Apo2L/TRAIL for 4 hours and activation of caspase-2, -3, -4, -7, -8, -9, and -10 was assessed by Western blot. As a marker of caspase substrate cleavage, cleaved PARP levels over the time course were also analyzed. To determine levels of ER stress induction during tunicamycin treatment, protein levels of BiP, phospho-IRE1, IRE1, PERK, eIF2a, JNK, phospho-JNK were all examined by Western blot analysis. GAPDH was included as a loading control. (B) Densitometry quantifying expression levels of BiP, phospho-IRE1, IRE1, PERK, eIF2a, JNK, and phospho-JNK. Quantified bands were normalized to the GAPDH loading control.

Supplemental Figure 2. MS/MS spectra for identified caspase substrates. MS/MS spectra for C-terminal Aspartic acid containing peptides identified using C-terminal Aspartic Acid peptide enrichment during treatment of HeLa cells with tunicamycin.

Supplemental Figure 3. Rb and YAP are cleaved and degraded in a caspase-dependent manner during ER stress. (A) The panel on the right shows TMT reporter ion quantification for caspase cleaved peptides of Rb at the peptide spectral match (PSM) level, peptide level, or protein level over time. The left panel is a Western blot against Rb using samples from cells that were treated with tunicamycin alone or tunicamycin + z-VAD-FMK. β-actin is provided as a loading control. (B) Same as in A but for YAP.

Supplemental Figure 4. Correlation between transcript and protein abundance of caspase substrates. This scatter plot shows the correlation between transcript level and protein abundance of caspase substrates during the tunicamycin time course. A linear regression was performed and using the Pearson method, the correlation between protein and transcript is 0.57 with a p-value<2.2e-16. The shaded area represents the 95% confidence region.

Supplemental Figure 5. Correlation plots for caspase substrates. Plots show the relative protein abundance (red), transcript abundance (green), and caspase cleavage product abundance (blue) plotted over time. GPP cluster #5 and #6 were overlaid on CSP clusters based on matched protein names. Transcripts that map to these proteins were also overlaid. Pearson correlations among the GPP, CSP and transcripts were calculated by an internal function cor.test() in R.

Supplemental Figure 6. Effect of caspase inhibition on gene expression levels. This volcano plot illustrates the log-scaled mean fold changes and adjusted p-values for all human genes resulting from the differential expression analysis between TM and TMZ at 72 hours. SRF, CREB1, and STAT6 are highlighted in blue.

Supplemental Figure 7. Single peptide identifications from global protein profiling. Annotated spectra from peptides identified from the global protein profiling experiment that were the only peptide identified from that protein (one hit wonders).

Supplemental Table 1. Caspase substrate cluster membership list. Sheet 1 shows the average expression levels of caspase-cleaved peptides clustered using fuzzy c-means clustering with mfuzz. The average relative abundance of each of the cleaved peptides for all identified proteins is listed along with the CSP cluster to which it was assigned. Sheet 2 shows the un-normalized peptide intensities for each caspase substrate identified.

Supplemental Table 2. Protein cluster membership list. Sheet 1 shows the average expression levels of proteins clustered using fuzzy c-means clustering with mfuzz. The average relative abundance of each of the proteins is listed along with the GPP cluster to which it was assigned. Sheet 2 shows the unnormalized protein intensities for each protein identified.

Supplemental Table 3. Transcript gene cluster membership list. The average expression levels of gene transcripts were clustered using fuzzy c-means clustering with mfuzz. The average relative abundance of each of the transcripts is listed along with the RNAseq cluster to which it was assigned.

Supplemental Table 4. Transcriptional changes after tunicamycin treatment. RPKM values, representing the transcriptional response to tunicamycin treatment, are listed for each replicate and time point.

Supplemental Table 5. Transcriptional changes after tunicamycin treatment alone or in combination with a pan caspase inhibitor. RPKM values, representing the transcriptional response to tunicamycin treatment with or without the pan caspase inhibitor z-VAD-FMK, are listed for each replicate and time point. TM is tunicamycin treatment alone and TMZ is tunicamycin plus z-VAD-FMK treatment.

Supplemental Table 6. Differential expression analysis: tunicamycin treatment alone versus combination with a pan caspase inhibitor. Genes, which were significantly differentially expressed after pan caspase inhibition (when compared to tunicamycin alone) after 24 (Sheet 1) or 72 hours (Sheet 2), are listed along with corresponding p-values.

Supplemental Table 7. Gene set enrichment analysis. MSigDB gene sets representing transcription factor target genes, which tended to show higher expression after pan caspase inhibition, are listed along with associated p-values and FDR.

Supplemental Table 8. TMT quantitative data for single peptide identifications from global protein profiling. Reporter ion intensities for the single peptide identifications from the global protein profiling are listed in this table.
