# Supplementary material for: Uncovering a Dual Regulatory Role for Caspases During Endoplasmic Reticulum Stress-induced Cell Death
Source: Mol Cell Proteomics. 2016 Apr 28;15(7):2293–307. doi: 10.1074/mcp.M115.055376 (PMC4937505; doi:10.1074/mcp.M115.055376)
Supplement: Supplemental Data [file 10.1074_M115.055376_mcp.M115.055376-2.pdf]

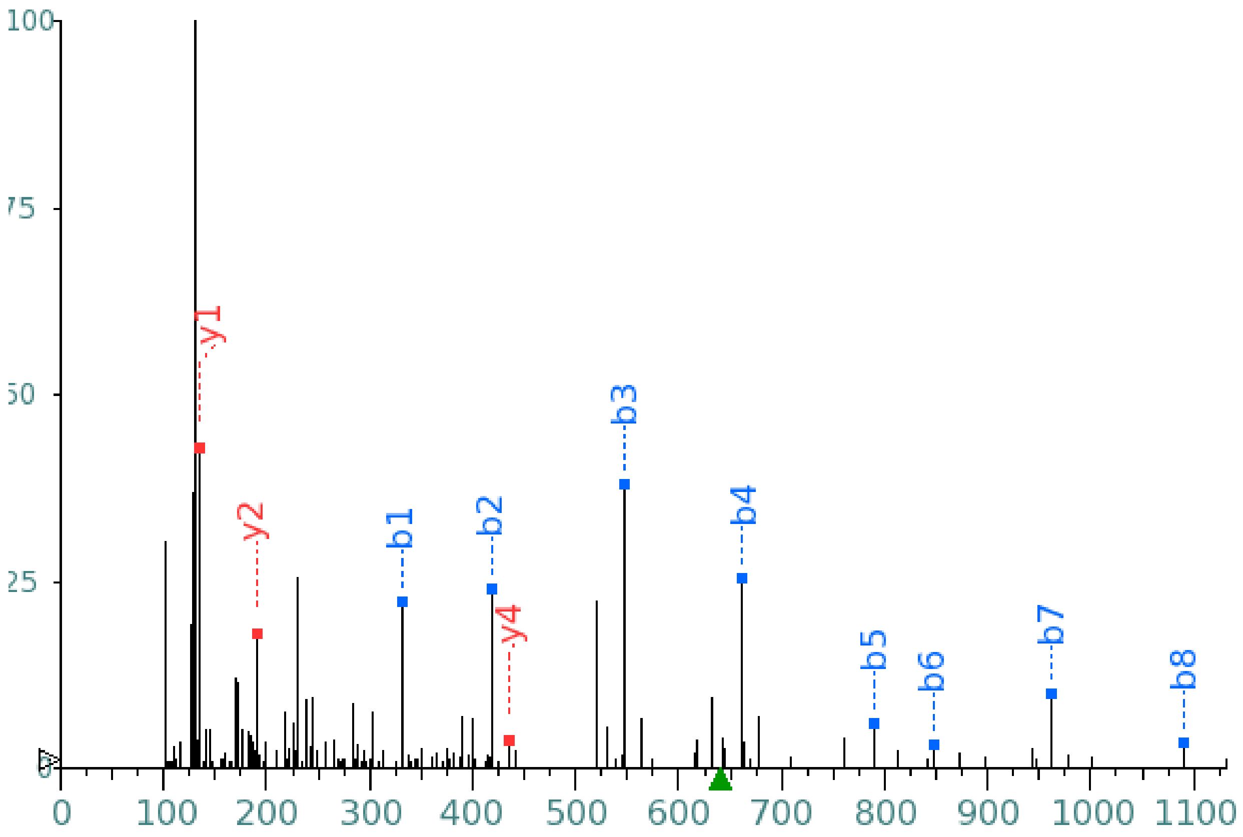

TSENQGDEGD (+2)

Primary Reference: 1433B_HUMAN
Search ID: 41758
Search Name: 20130328_ananiav_ERstress_TMT_TM_timecourse_chymo_inj1_4MC_IAA
Scan#: 2308
Observed Mass: 640.7761 (0.8 ppm)
PSM Score: 66.58

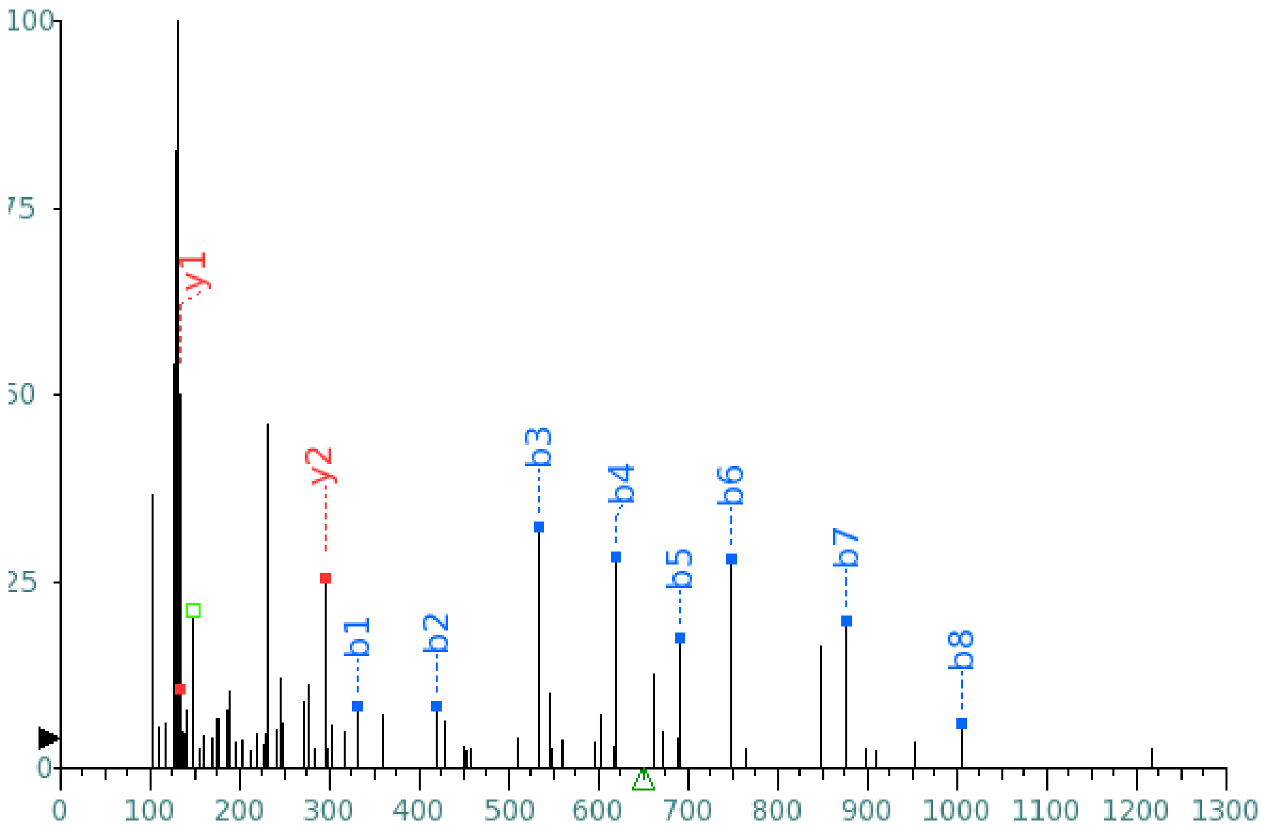

TSDSAGEEC*D (+2)

Primary Reference: 1433T_HUMAN
Search ID: 41758
Search Name: 20130328_ananiav_ERstress_TMT_TM_timecourse_chymo_inj1_4MC_IAA
Scan#: 2967
Observed Mass: 650.2639 (-0.2 ppm)
PSM Score: 48.58

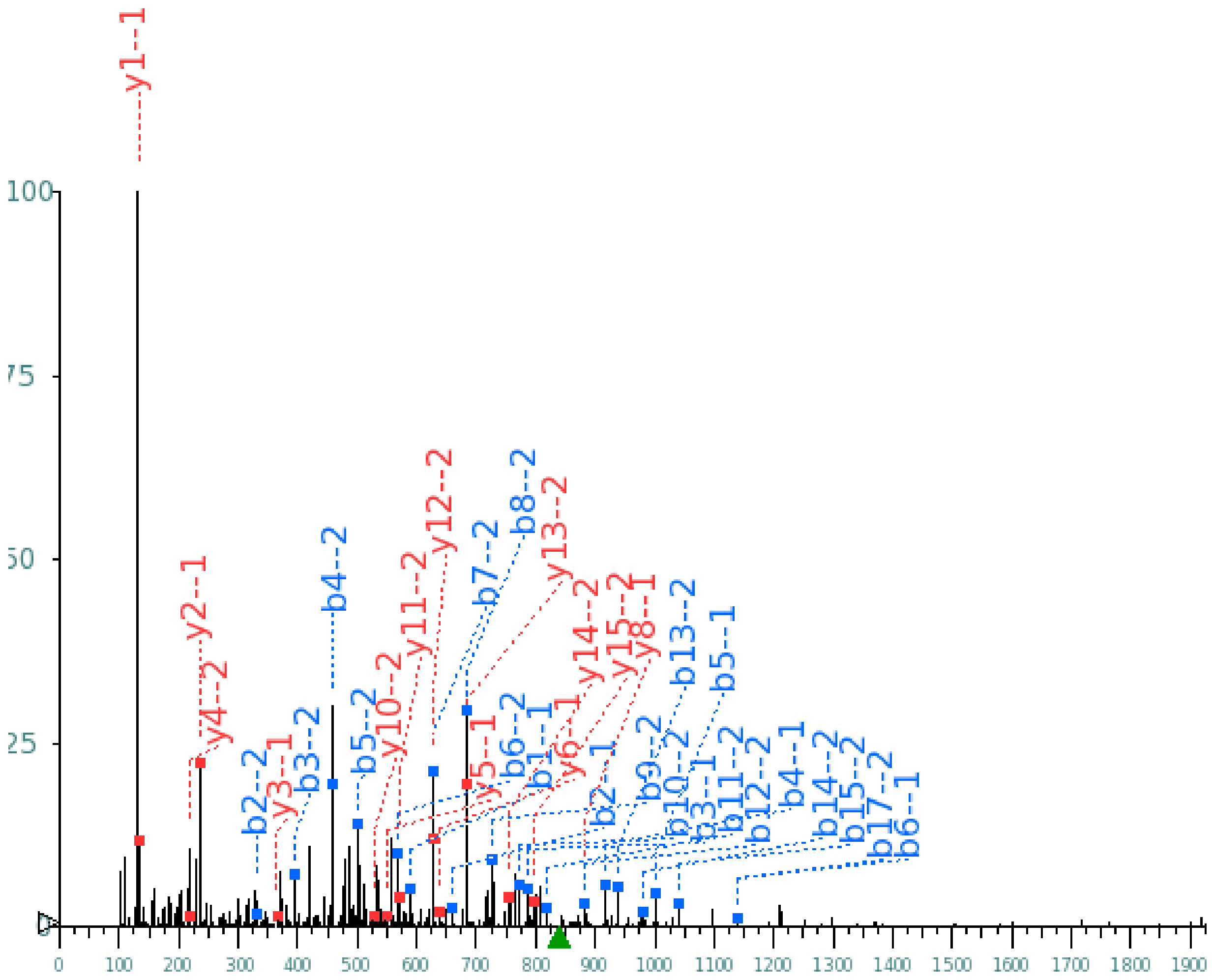

KAEQSHDDSSSEDSDAETD (+3)

Primary Reference: STK24_HUMAN
Search ID: 41758
Search Name: 20130328_ananiav_ERstress_TMT_TM_timecourse_chymo_inj1_4MC_IAA
Scan#: 4050
Observed Mass: 837.7048 (-0.3 ppm)
PSM Score: 53.77

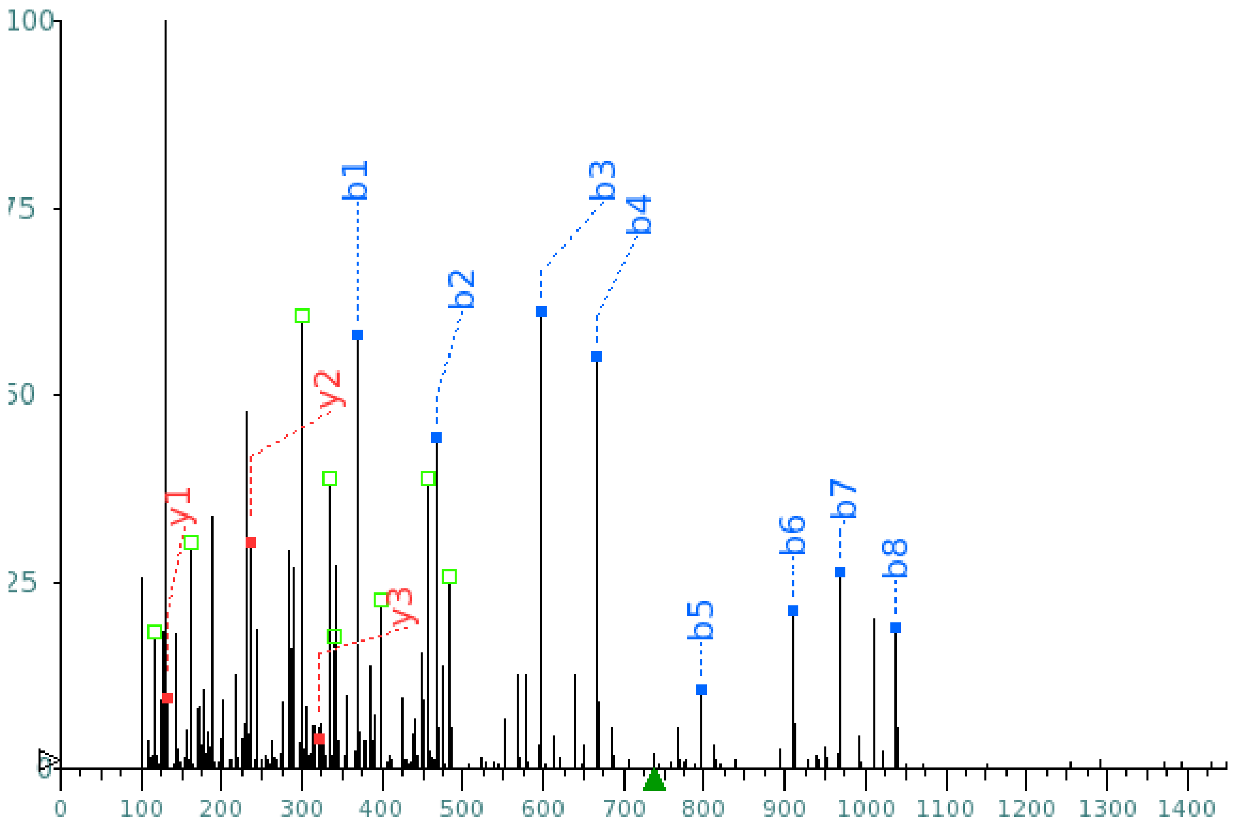

HTQAQDGAISTD (+2)

Primary Reference: TPP2_HUMAN
Search ID: 41758
Search Name: 20130328_ananiav_ERstress_TMT_TM_timecourse_chymo_inj1_4MC_IAA
Scan#: 4322
Observed Mass: 736.8627 (0.2 ppm)
PSM Score: 55.34

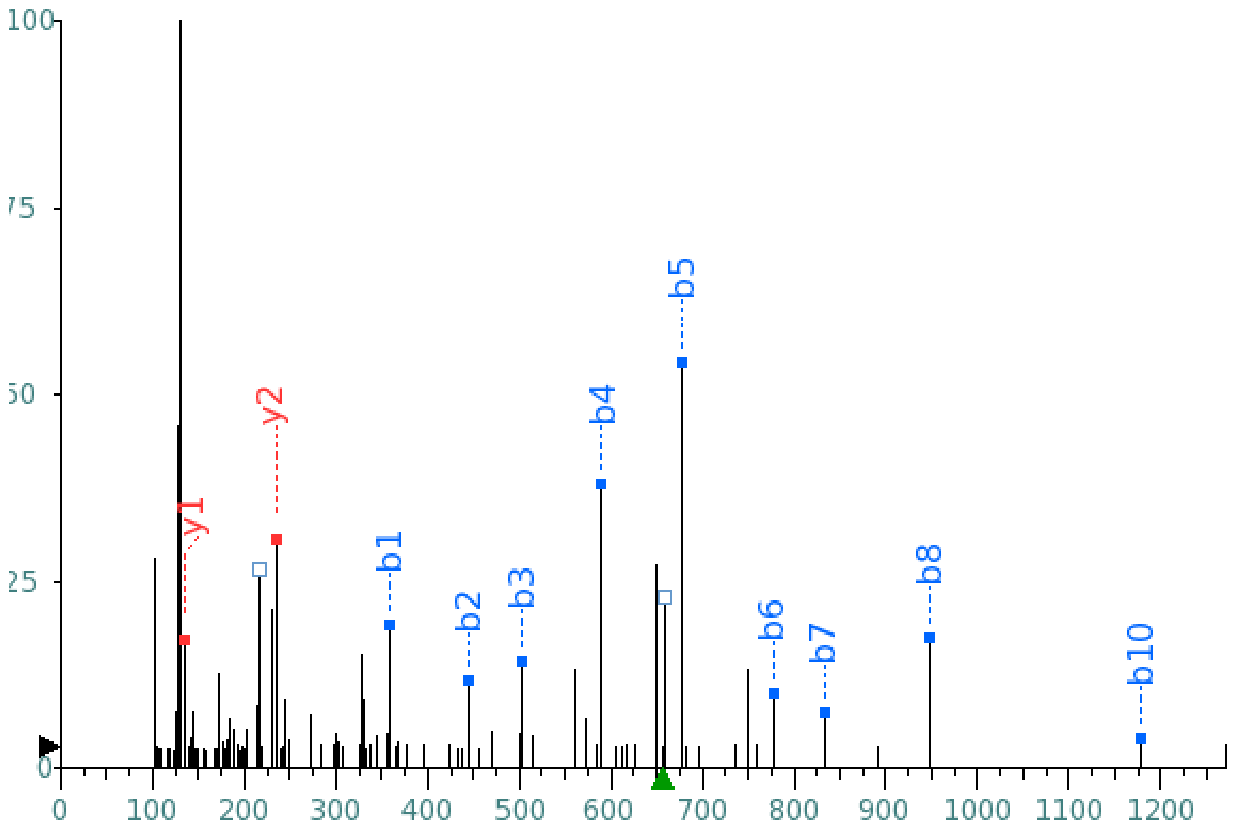

ESGSSVGDETD (+2)

Primary Reference: MATR3_HUMAN
Search ID: 41758
Search Name: 20130328_ananiav_ERstress_TMT_TM_timecourse_chymo_inj1_4MC_IAA
Scan#: 4338
Observed Mass: 656.2917 (0.9 ppm)
PSM Score: 60.92

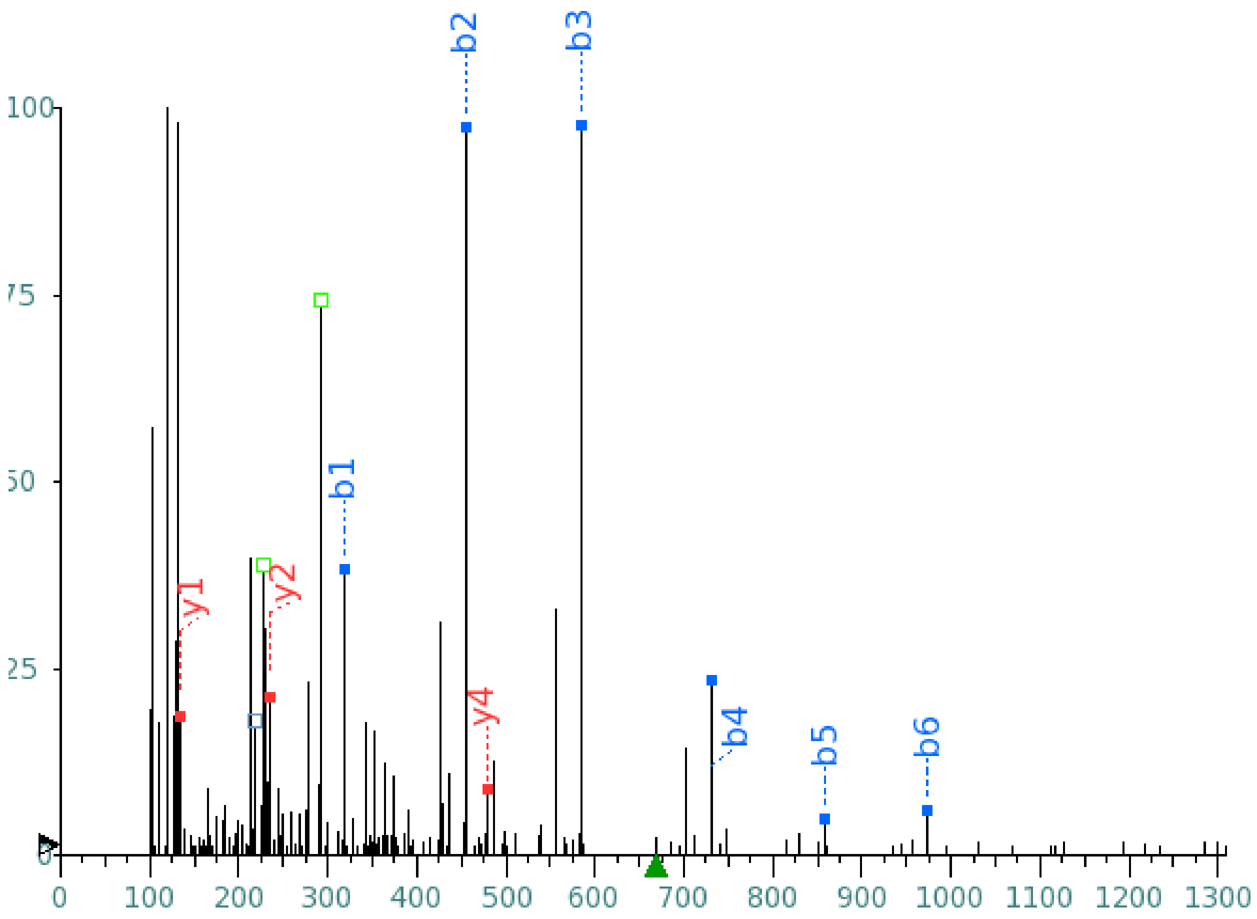

SHEFQDETD (+2)

Primary Reference: CBPD_HUMAN
Search ID: 41758
Search Name: 20130328_ananiav_ERstress_TMT_TM_timecourse_chymo_inj1_4MC_IAA
Scan#: 5165
Observed Mass: 668.7965 (0.2 ppm)
PSM Score: 31.45

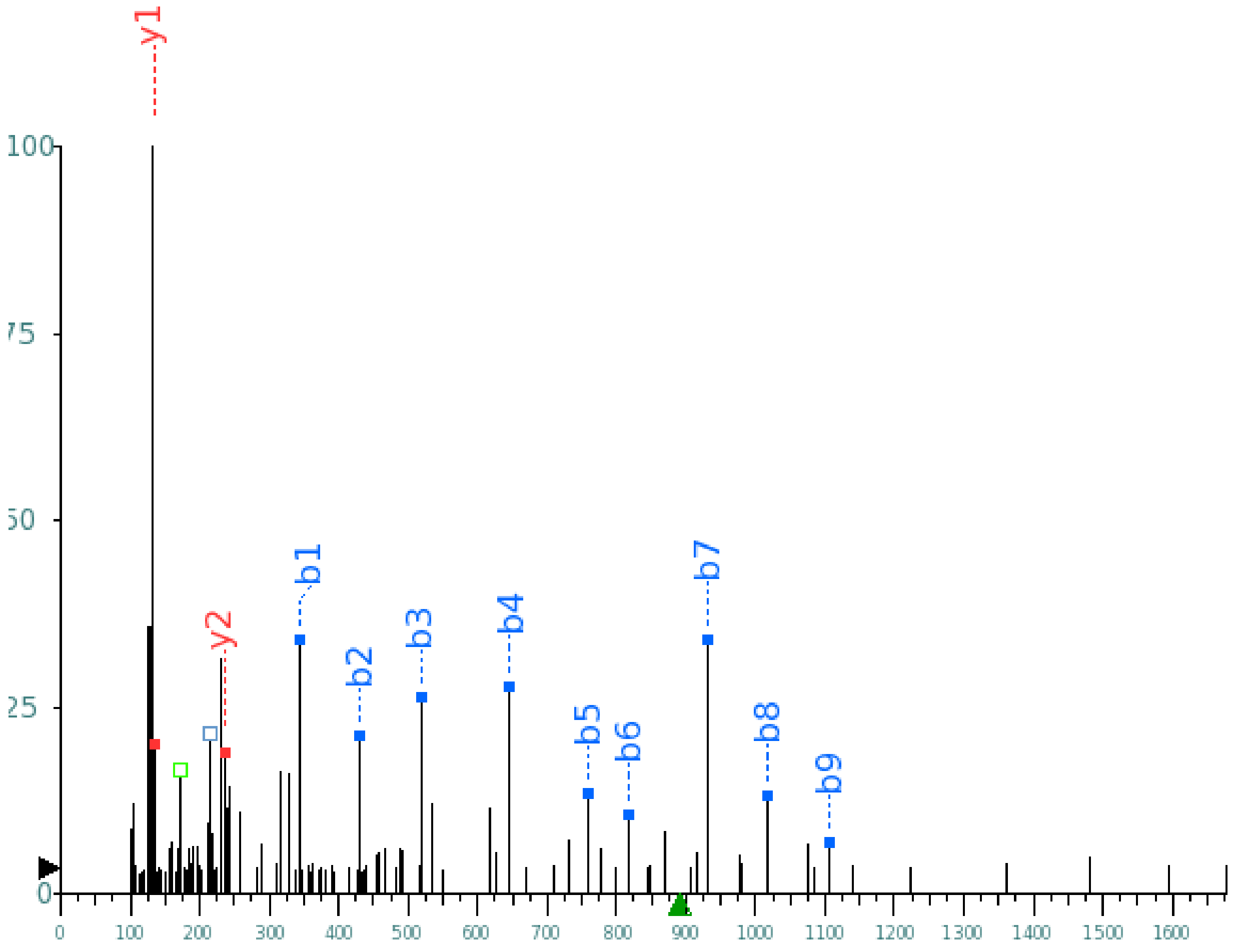

LSSQNGDSSTSQETD (+2)

Primary Reference: SRPK1_HUMAN
Search ID: 41758
Search Name: 20130328_ananiav_ERstress_TMT_TM_timecourse_chymo_inj1_4MC_IAA
Scan#: 5807
Observed Mass: 892.9037 (1 ppm)
PSM Score: 58.33

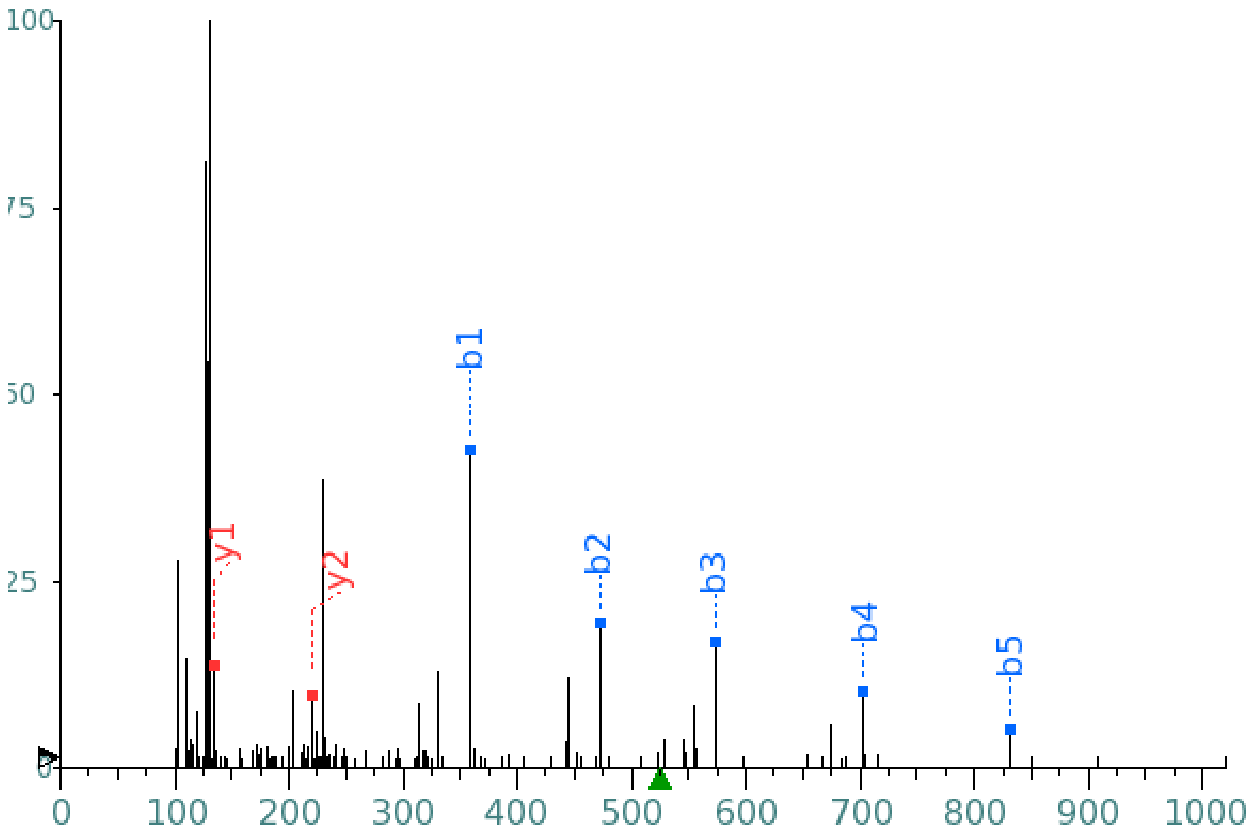

EITEESD (+2)

Primary Reference: PTRF_HUMAN
Search ID: 41758
Search Name: 20130328_ananiav_ERstress_TMT_TM_timecourse_chymo_inj1_4MC_IAA
Scan#: 6010
Observed Mass: 526.2535 (0.4 ppm)
PSM Score: 25.66

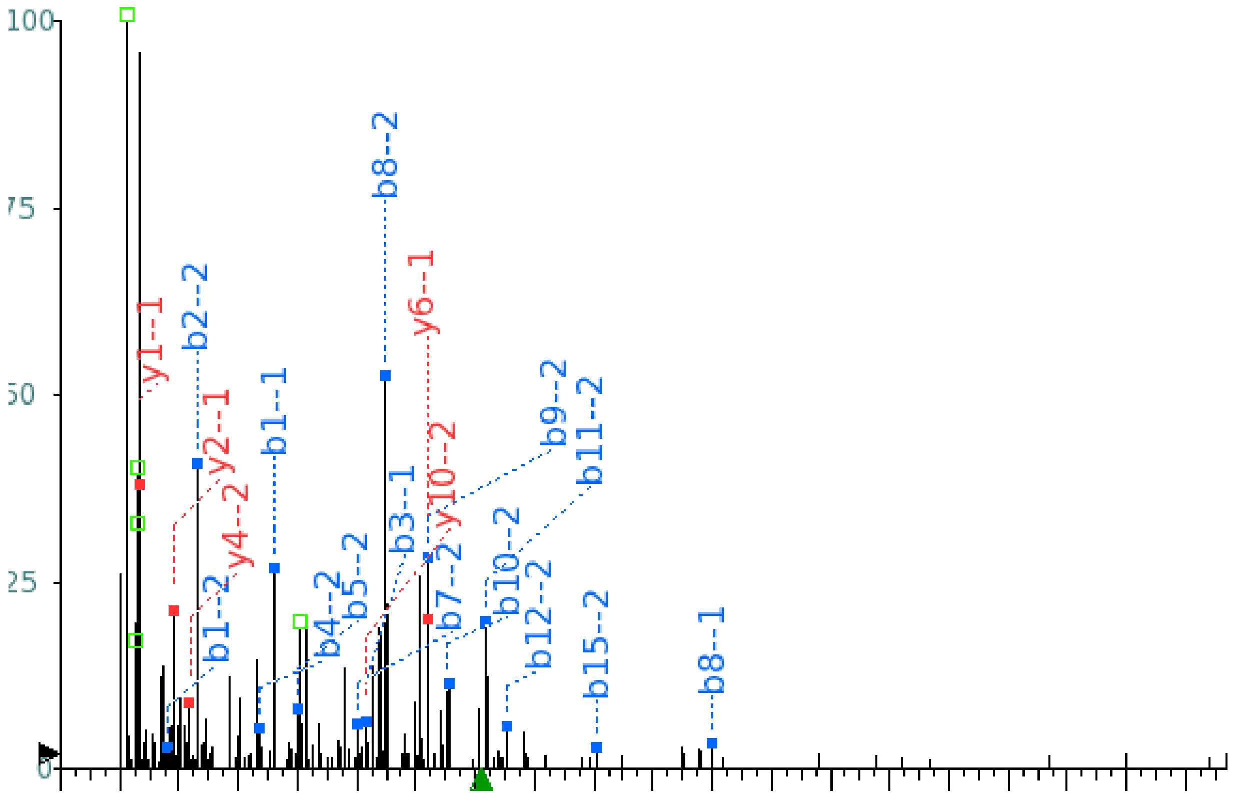

MPGREPTTHAEAQGDEGD (+3)

Primary Reference: AHNK2_HUMAN
Search ID: 41758
Search Name: 20130328_ananiav_ERstress_TMT_TM_timecourse_chymo_inj1_4MC_IAA
Scan#: 6442
Observed Mass: 709.6588 (0.4 ppm)
PSM Score: 24.63

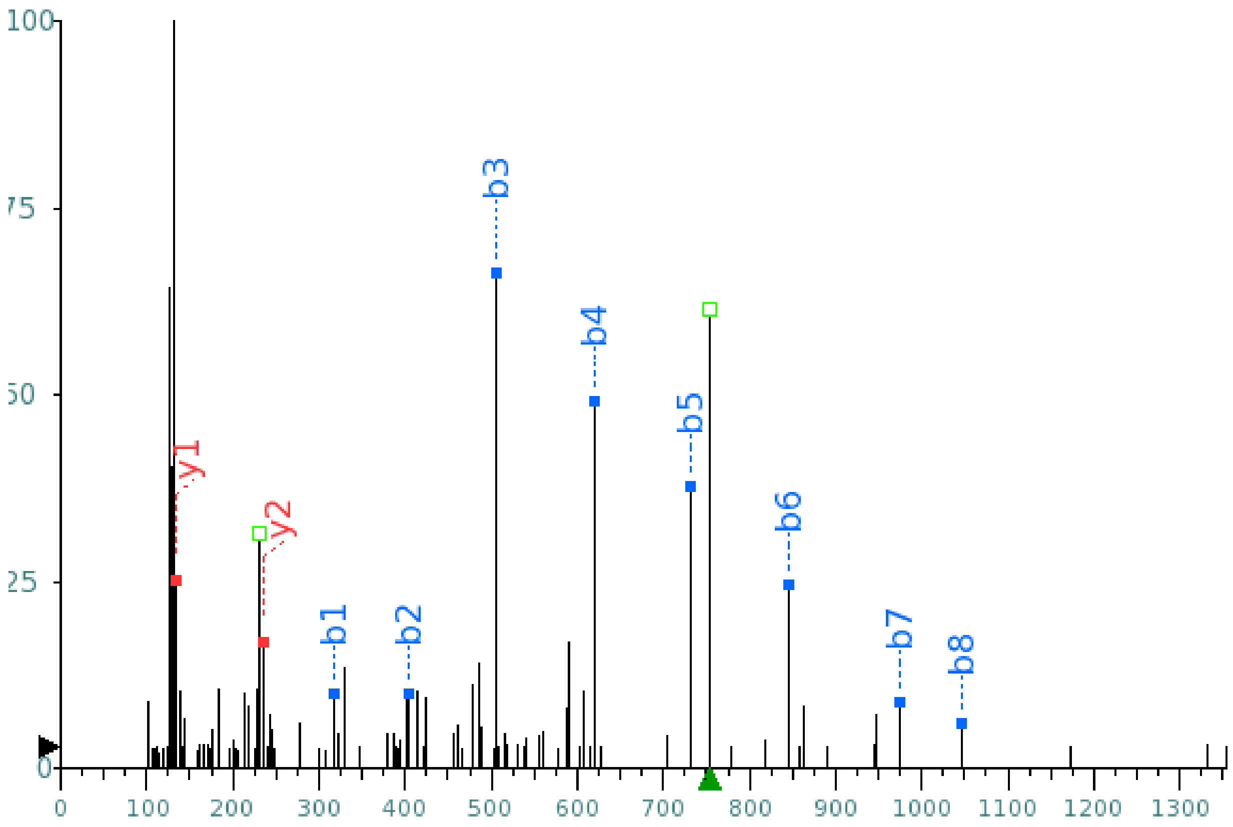

SSTINNQAVETD (+2)

Primary Reference: RHG12_HUMAN
Search ID: 41758
Search Name: 20130328_ananiav_ERstress_TMT_TM_timecourse_chymo_inj1_4MC_IAA
Scan#: 7606
Observed Mass: 754.376 (0.6 ppm)
PSM Score: 28.9

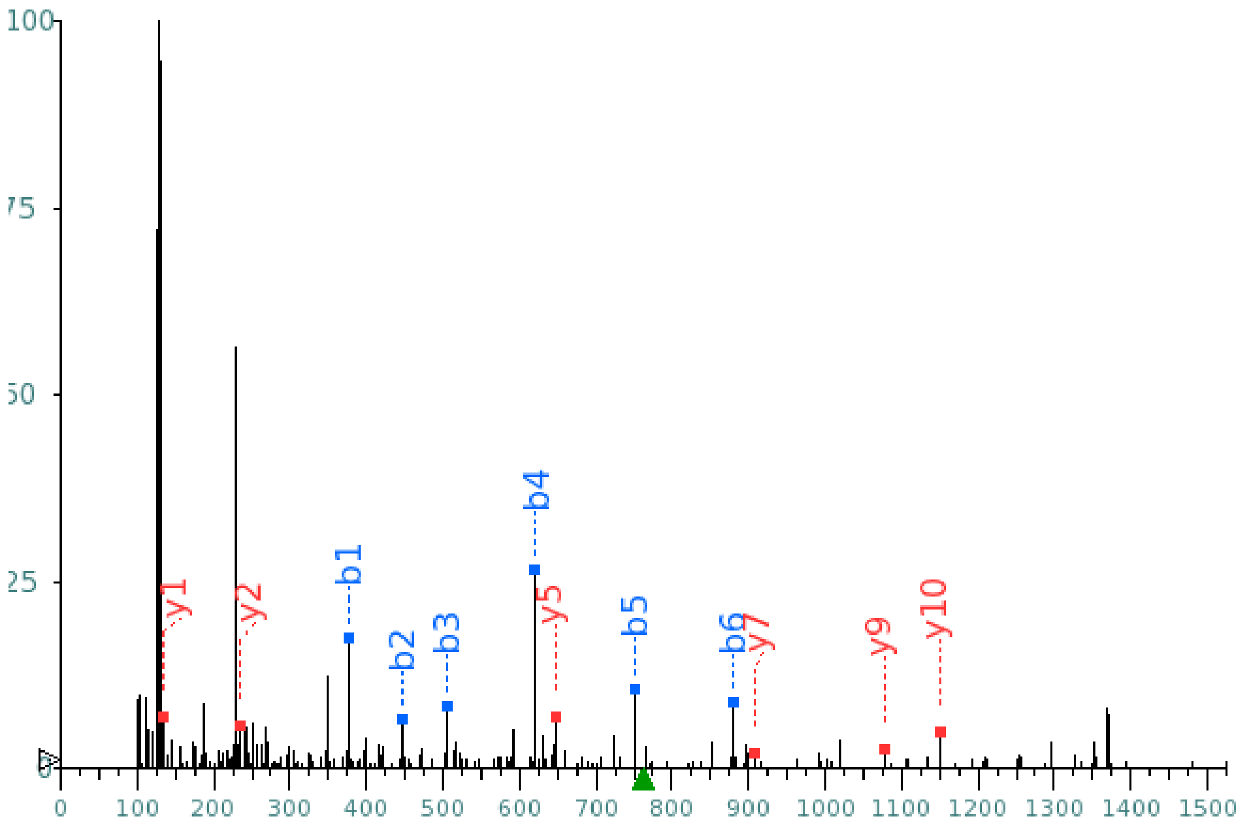

FAGNMERQQTD (+2)

Primary Reference: VAV2_HUMAN
Search ID: 41758
Search Name: 20130328_ananiav_ERstress_TMT_TM_timecourse_chymo_inj1_4MC_IAA
Scan#: 8050
Observed Mass: 763.3668 (-0.2 ppm)
PSM Score: 26.75

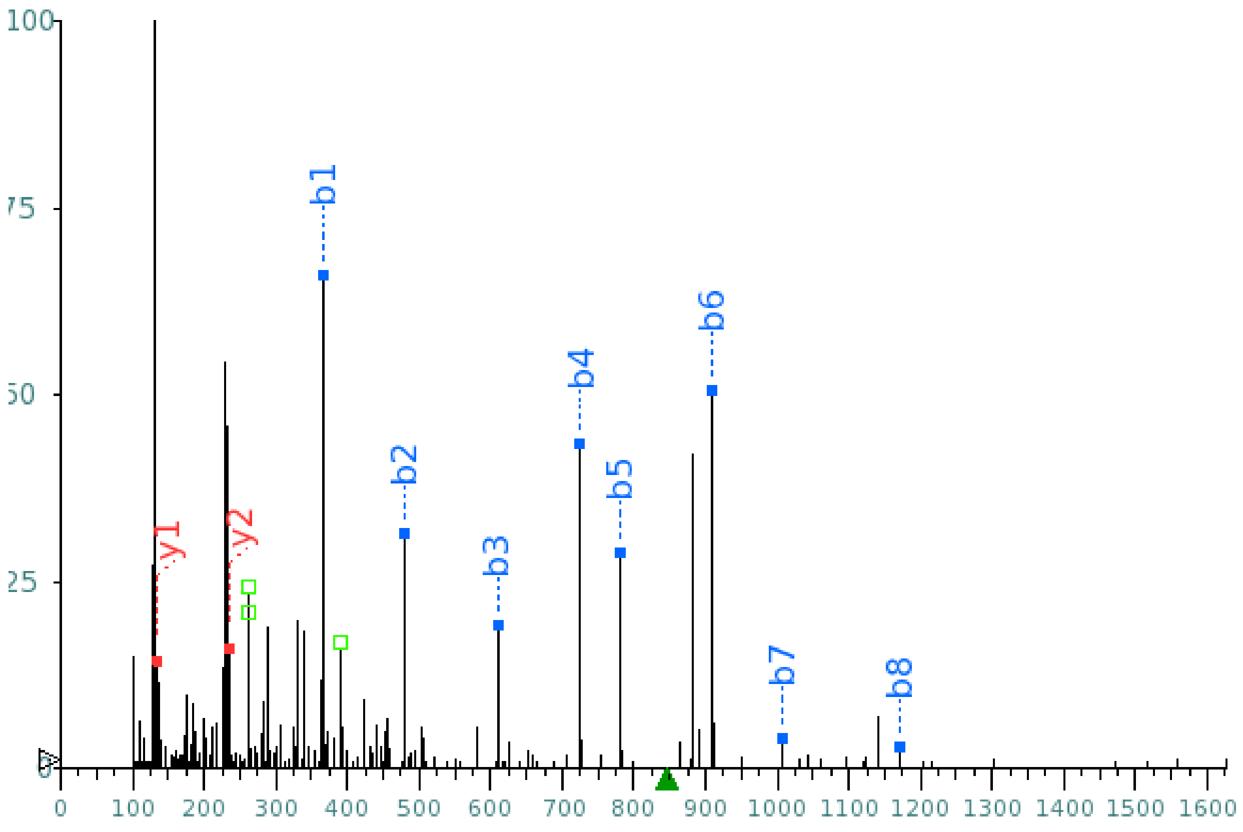

HLEDGEPYC*ETD (+2)

Primary Reference: PDLI5_HUMAN
Search ID: 41758
Search Name: 20130328_ananiav_ERstress_TMT_TM_timecourse_chymo_inj1_4MC_IAA
Scan#: 8688
Observed Mass: 847.3643 (-0 ppm)
PSM Score: 33.3

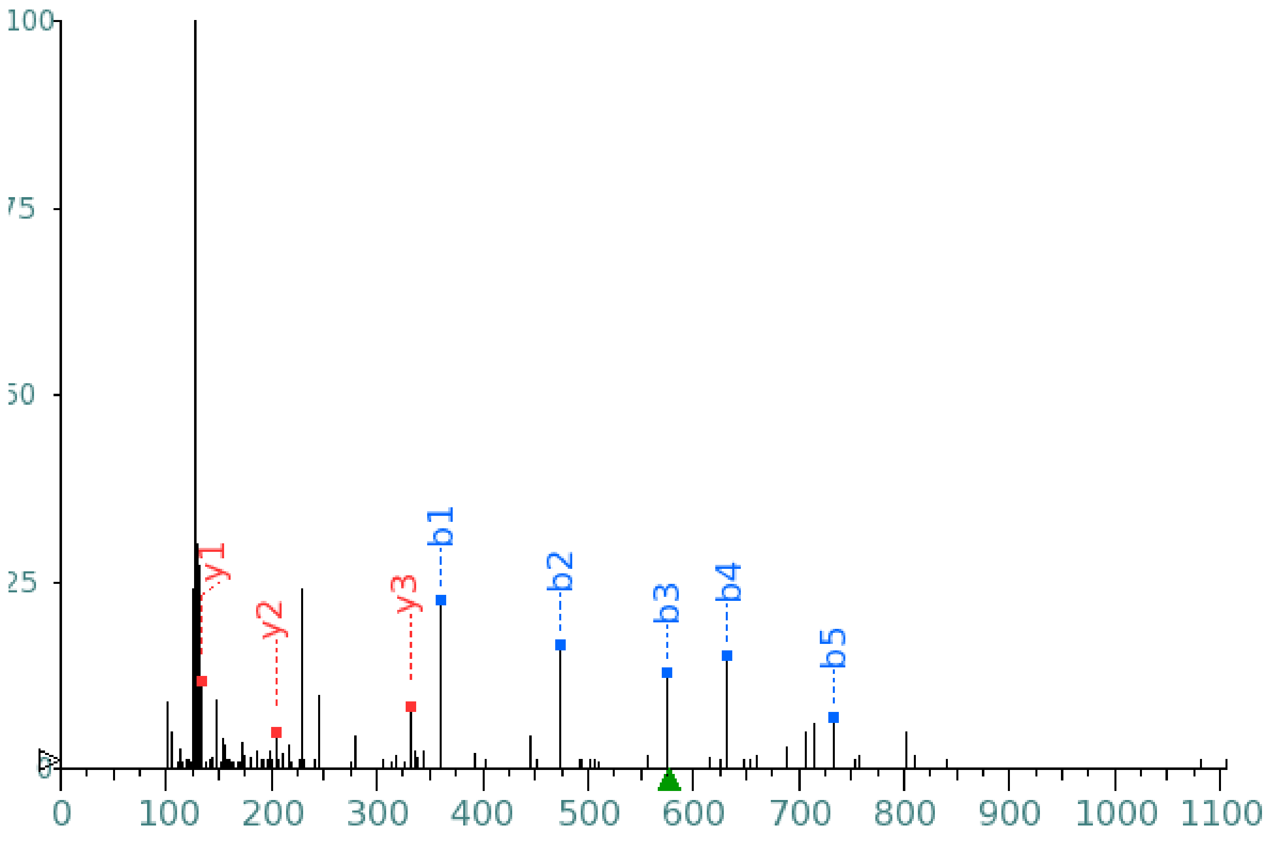

MITGTSQAD (+2)

Primary Reference: Q8IUB0_HUMAN
Search ID: 41758
Search Name: 20130328_ananiav_ERstress_TMT_TM_timecourse_chymo_inj1_4MC_IAA
Scan#: 8694
Observed Mass: 576.7923 (0.4 ppm)
PSM Score: 28.97

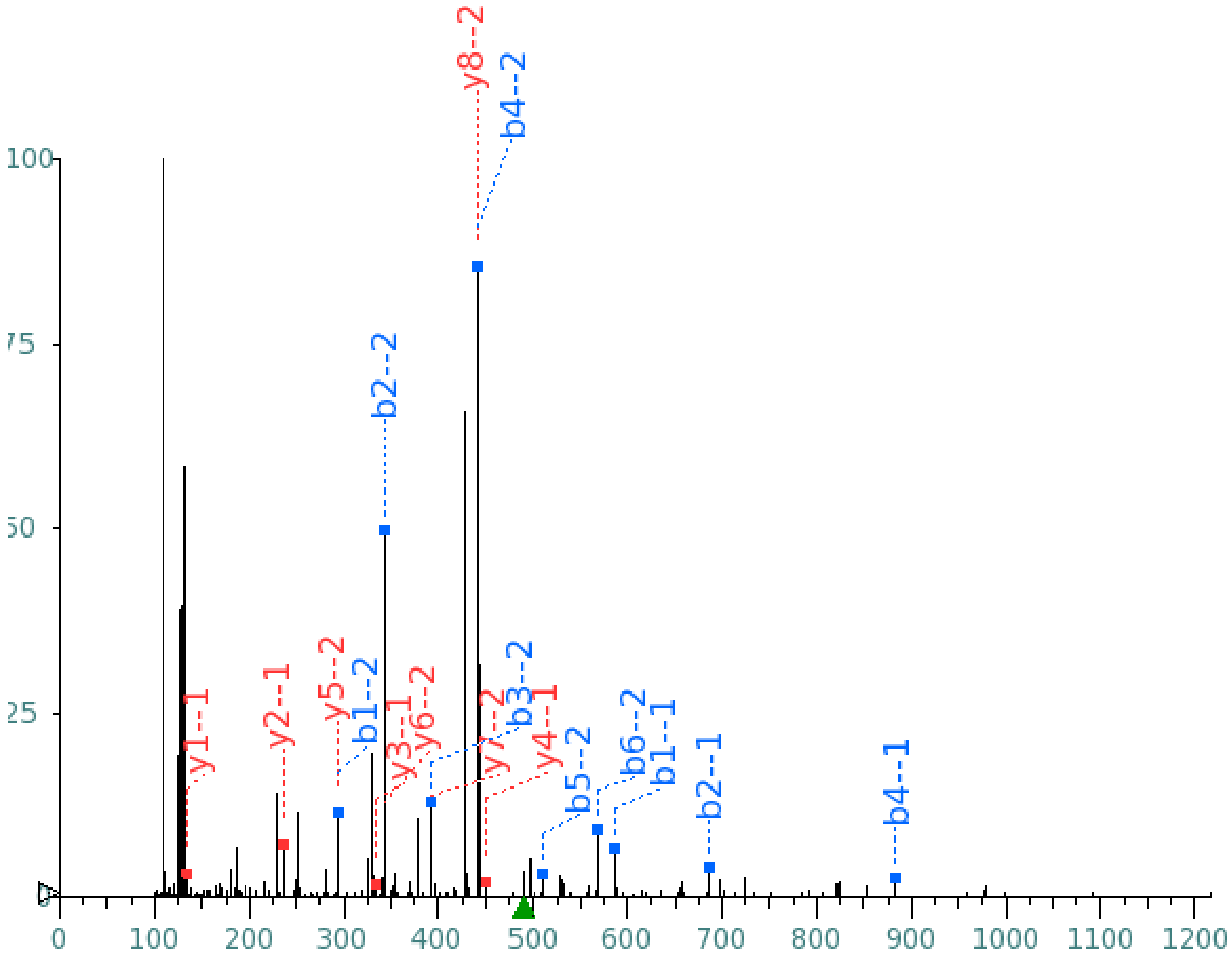

KVPVHDVTD (+3)

Primary Reference: FLNA_HUMAN
Search ID: 41758
Search Name: 20130328_ananiav_ERstress_TMT_TM_timecourse_chymo_inj1_4MC_IAA
Scan#: 8716
Observed Mass: 489.9578 (1.1 ppm)
PSM Score: 16.04

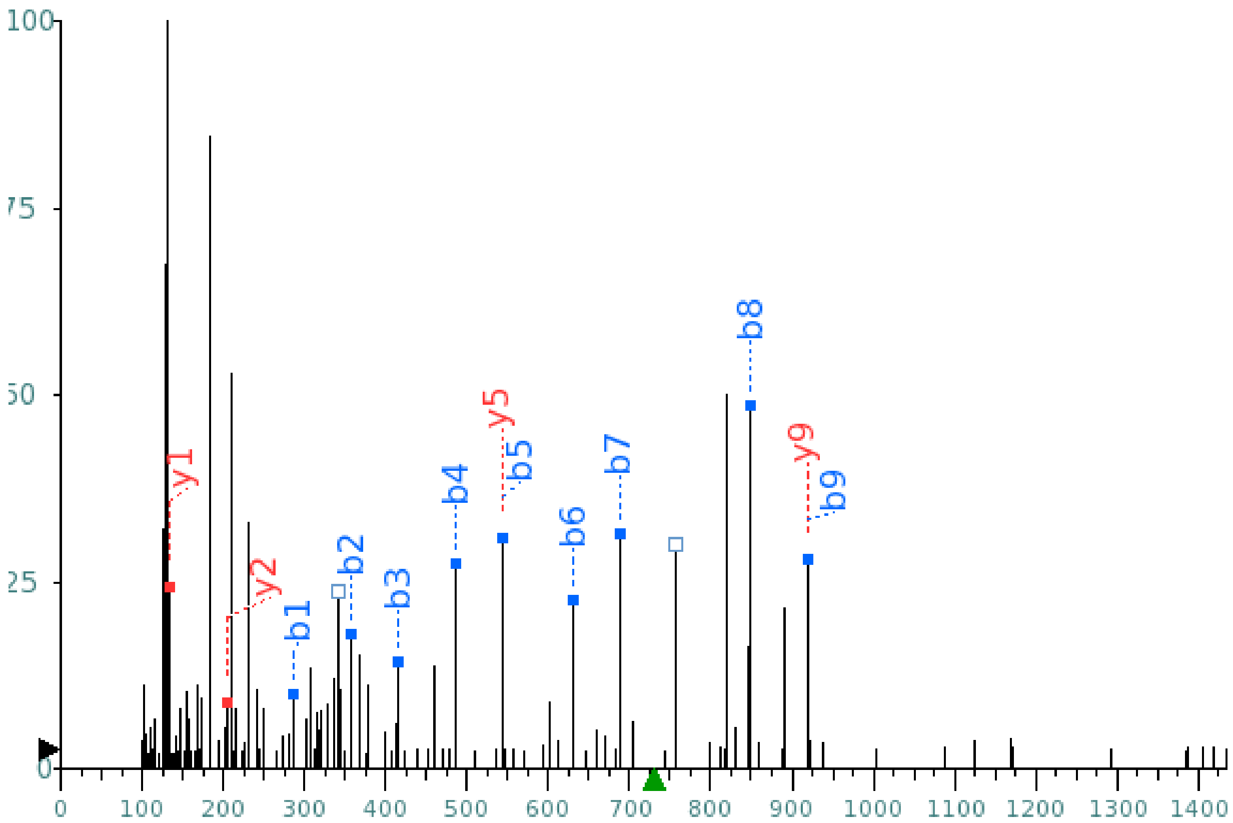

GAGAGSGC*APLEAD (+2)

Primary Reference: SC16A_HUMAN
Search ID: 41758
Search Name: 20130328_ananiav_ERstress_TMT_TM_timecourse_chymo_inj1_4MC_IAA
Scan#: 8718
Observed Mass: 731.3458 (0.2 ppm)
PSM Score: 27.11

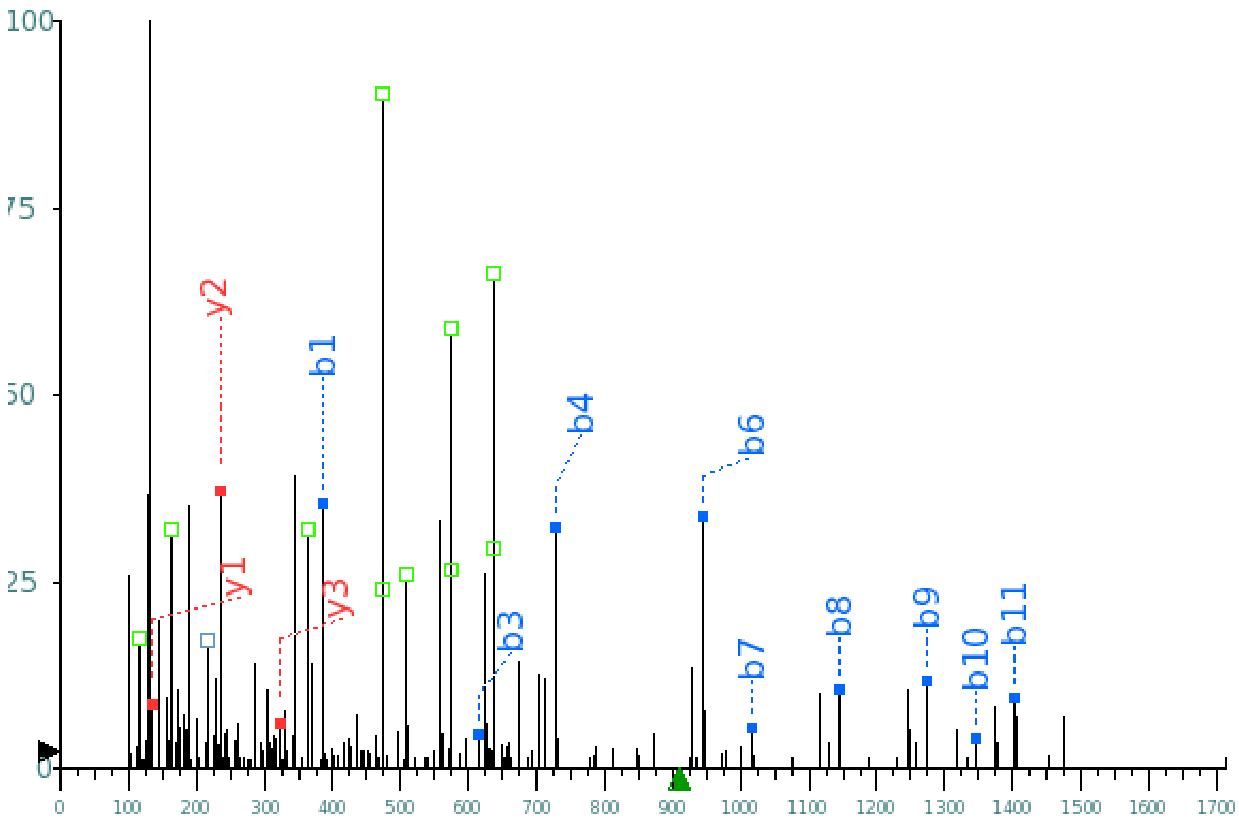

RPMDTDAEEAGVSTD (+2)

Primary Reference: DCBD1_HUMAN
Search ID: 41758
Search Name: 20130328_ananiav_ERstress_TMT_TM_timecourse_chymo_inj1_4MC_IAA
Scan#: 8725
Observed Mass: 911.9193 (-0.7 ppm)
PSM Score: 32.73

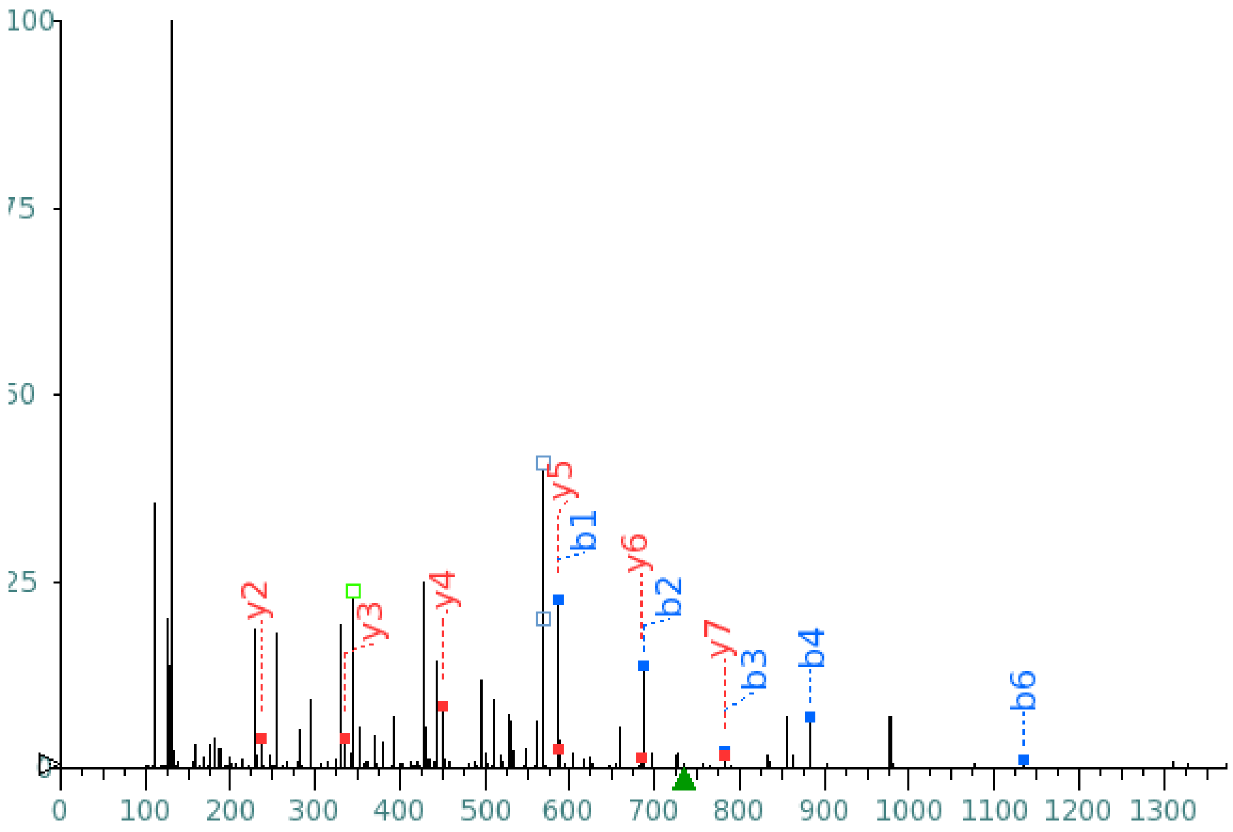

KVPVHDVTD (+2)

Primary Reference: FLNA_HUMAN
Search ID: 41758
Search Name: 20130328_ananiav_ERstress_TMT_TM_timecourse_chymo_inj1_4MC_IAA
Scan#: 8729
Observed Mass: 734.4327 (0.6 ppm)
PSM Score: 16.69

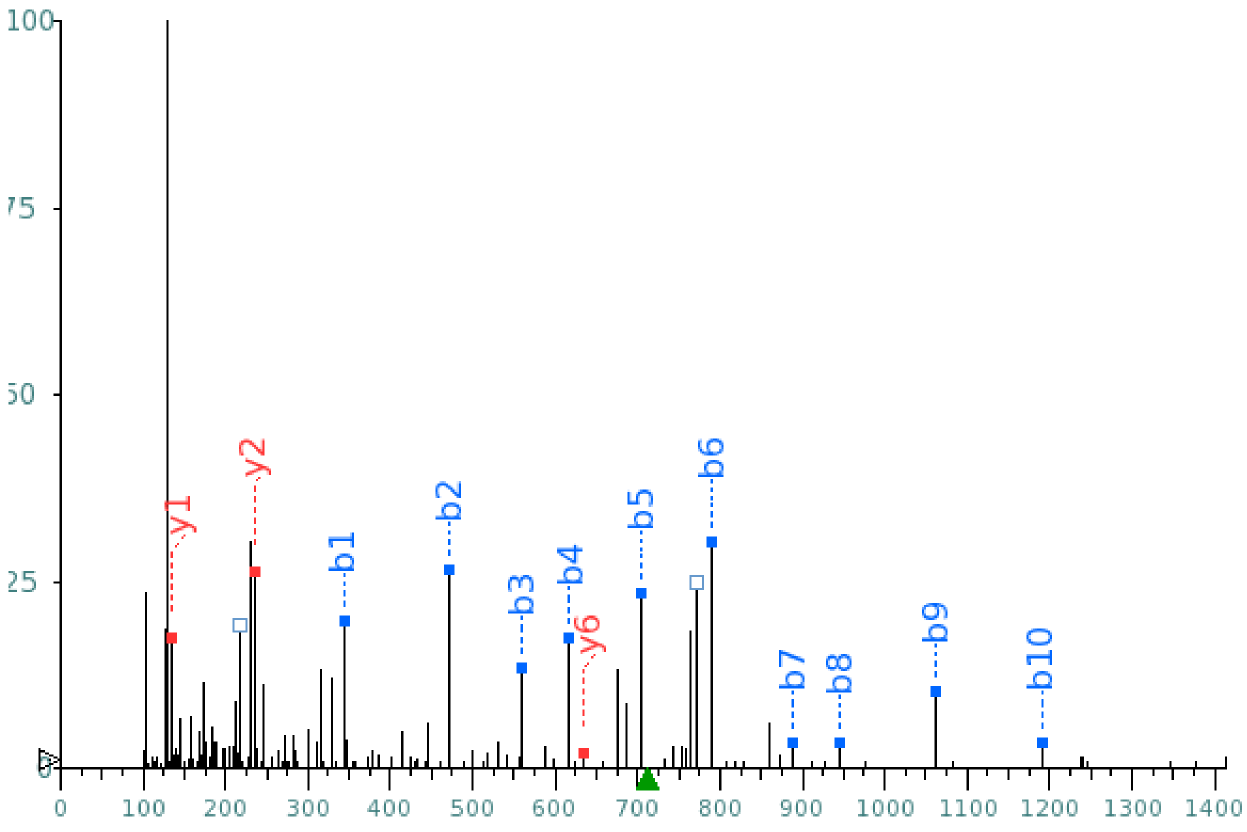

LESGGSSVGDETD (+2)

Primary Reference: MATR3_HUMAN
Search ID: 41758
Search Name: 20130328_ananiav_ERstress_TMT_TM_timecourse_chymo_inj1_4MC_IAA
Scan#: 8773
Observed Mass: 712.833 (-0.2 ppm)
PSM Score: 55.5

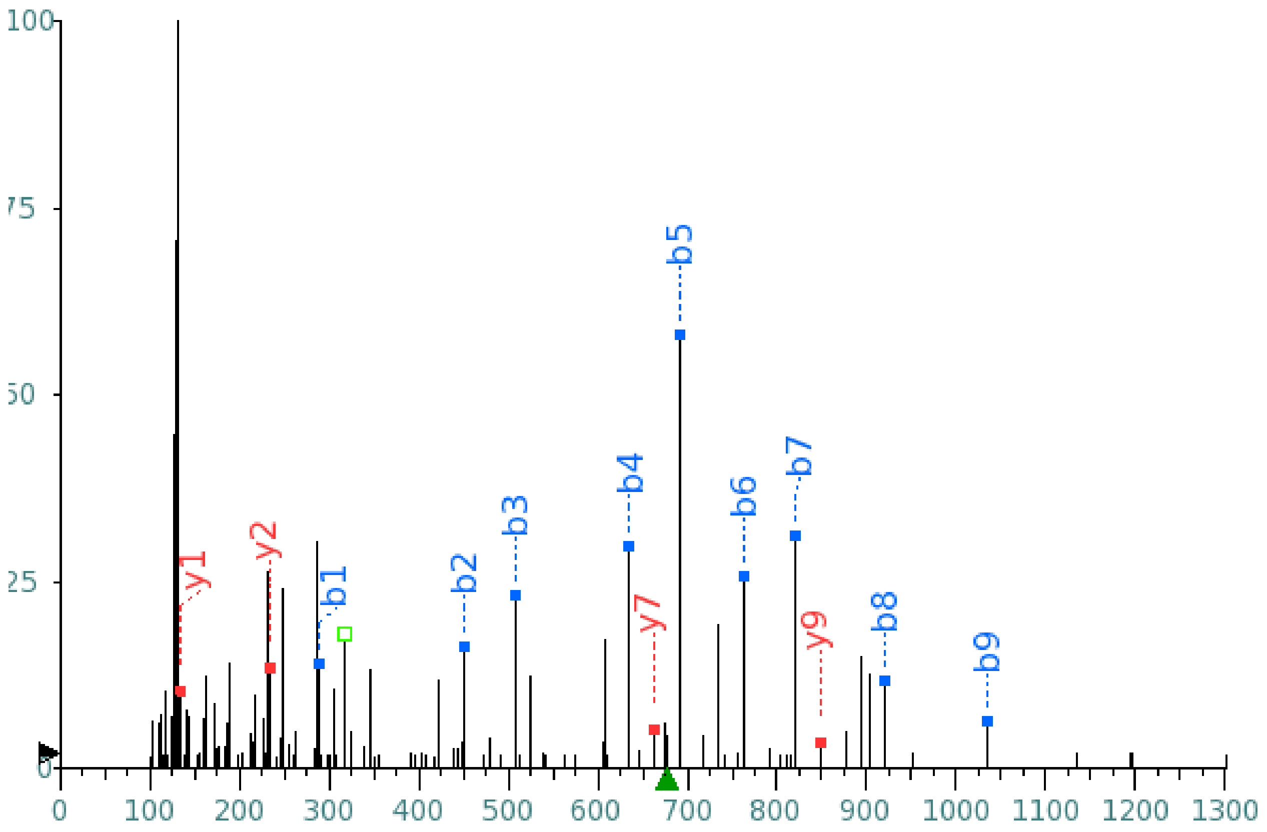

GYGQGAGTLSTD (+2)

Primary Reference: CSRP1_HUMAN
Search ID: 41758
Search Name: 20130328_ananiav_ERstress_TMT_TM_timecourse_chymo_inj1_4MC_IAA
Scan#: 8872
Observed Mass: 678.3357 (0 ppm)
PSM Score: 42.51

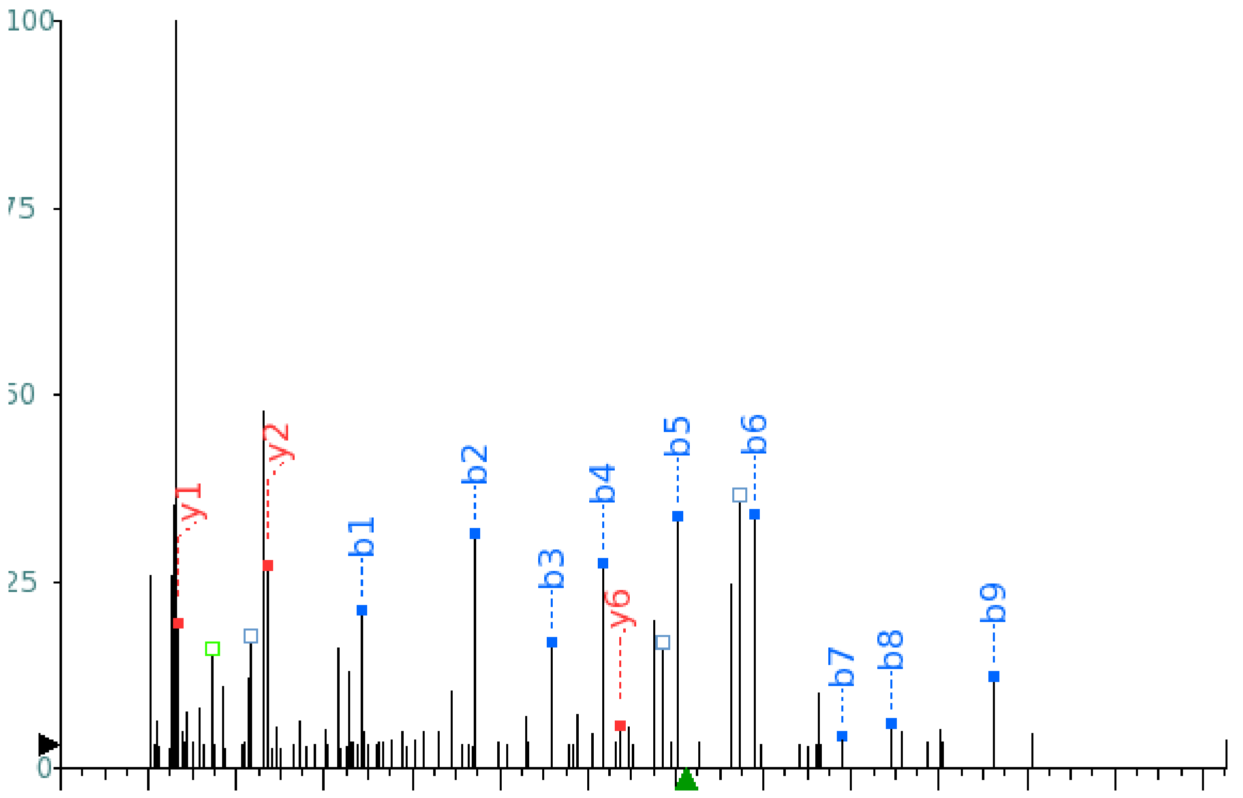

LESGSSVGDETD (+2)

Primary Reference: MATR3_HUMAN
Search ID: 41758
Search Name: 20130328_ananiav_ERstress_TMT_TM_timecourse_chymo_inj1_4MC_IAA
Scan#: 8881
Observed Mass: 712.8333 (0.2 ppm)
PSM Score: 44.5

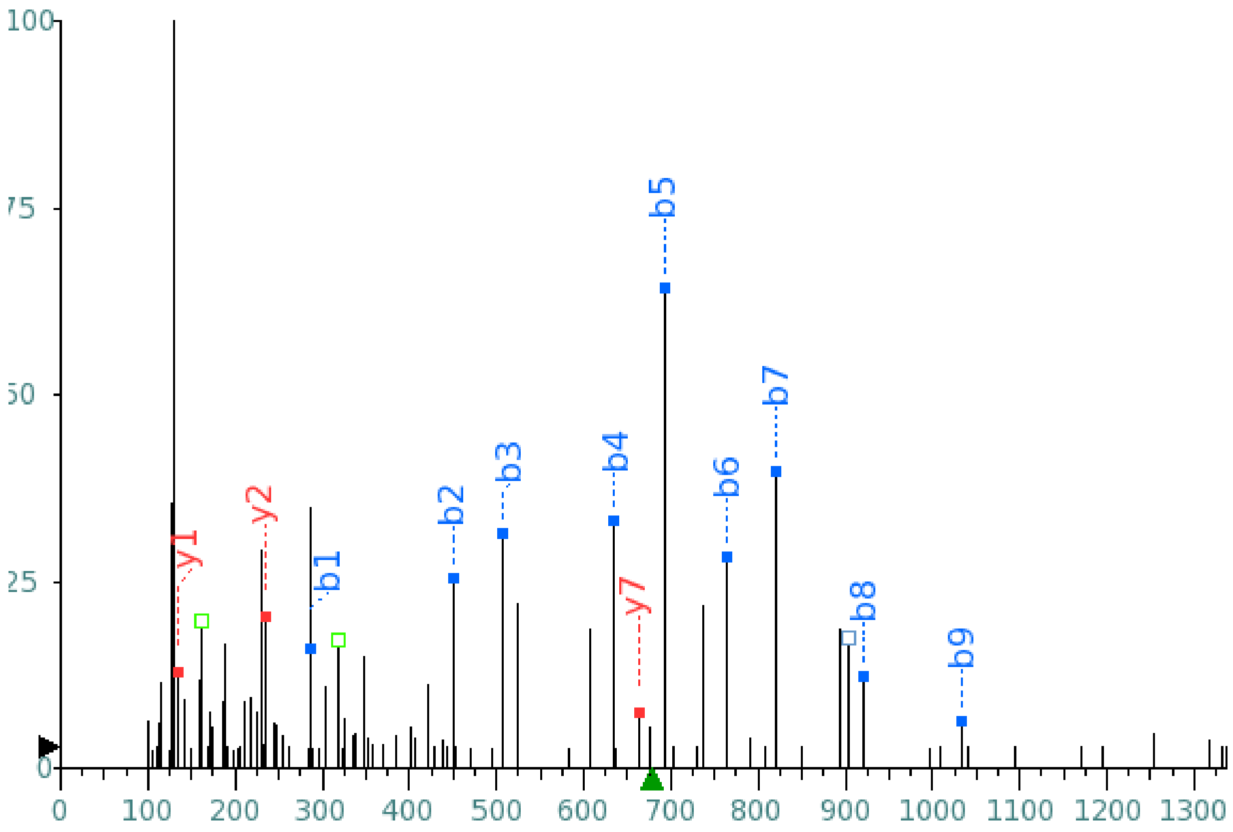

GYGQGAGTLSTD (+2)

Primary Reference: CSRP1_HUMAN
Search ID: 41758
Search Name: 20130328_ananiav_ERstress_TMT_TM_timecourse_chymo_inj1_4MC_IAA
Scan#: 8981
Observed Mass: 678.3357 (0 ppm)
PSM Score: 38.27

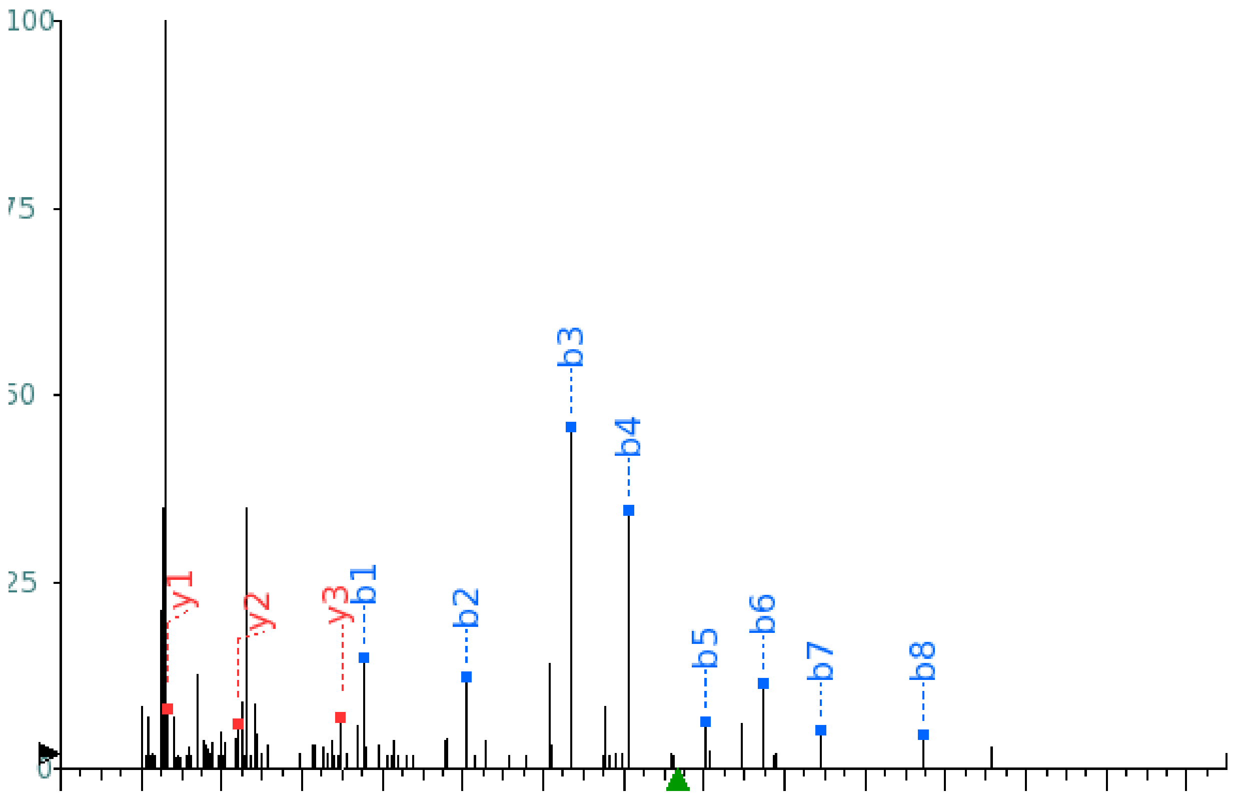

FQEAPAAQNESD (+2)

Primary Reference: OTUD4_HUMAN
Search ID: 41758
Search Name: 20130328_ananiav_ERstress_TMT_TM_timecourse_chymo_inj1_4MC_IAA
Scan#: 9054
Observed Mass: 768.3624 (0.1 ppm)
PSM Score: 44.62

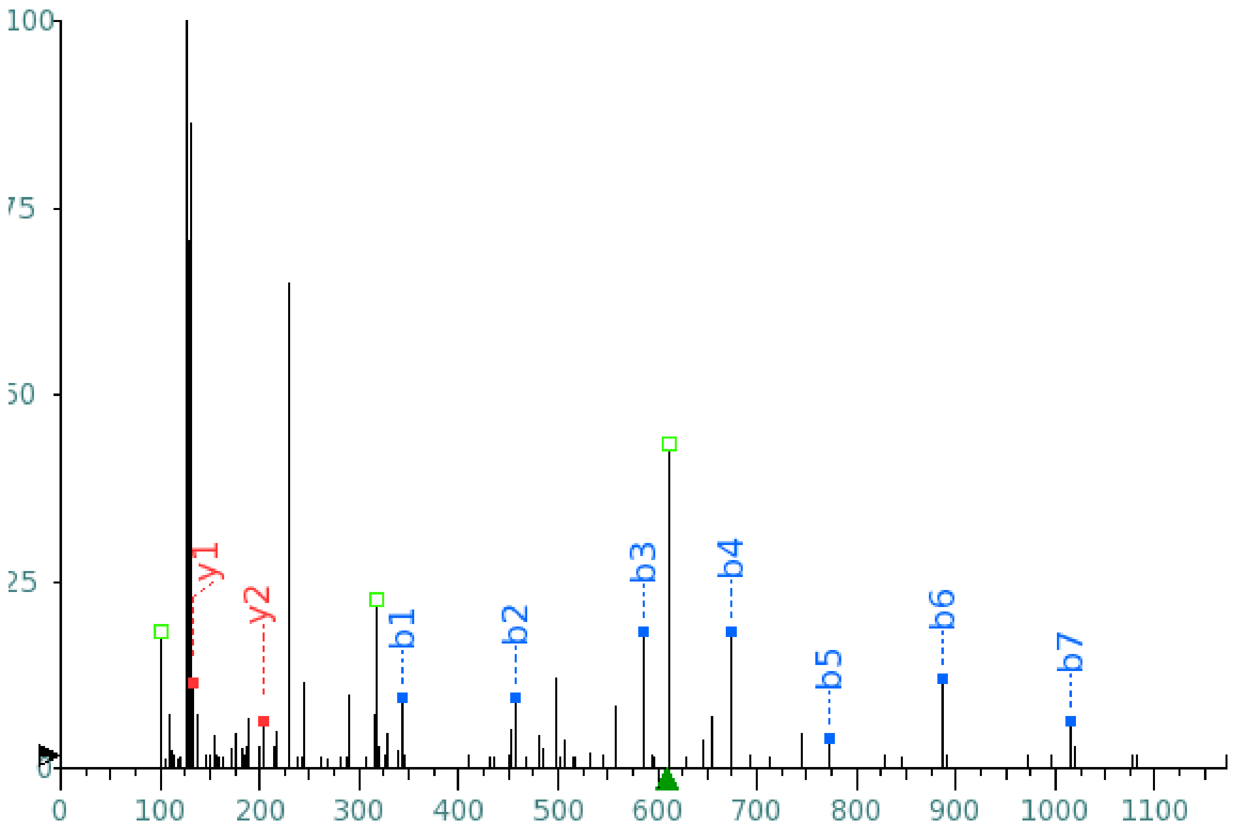

INESVDEAD (+2)

Primary Reference: RANB3_HUMAN
Search ID: 41758
Search Name: 20130328_ananiav_ERstress_TMT_TM_timecourse_chymo_inj1_4MC_IAA
Scan#: 9222
Observed Mass: 610.7962 (0.6 ppm)
PSM Score: 31.42

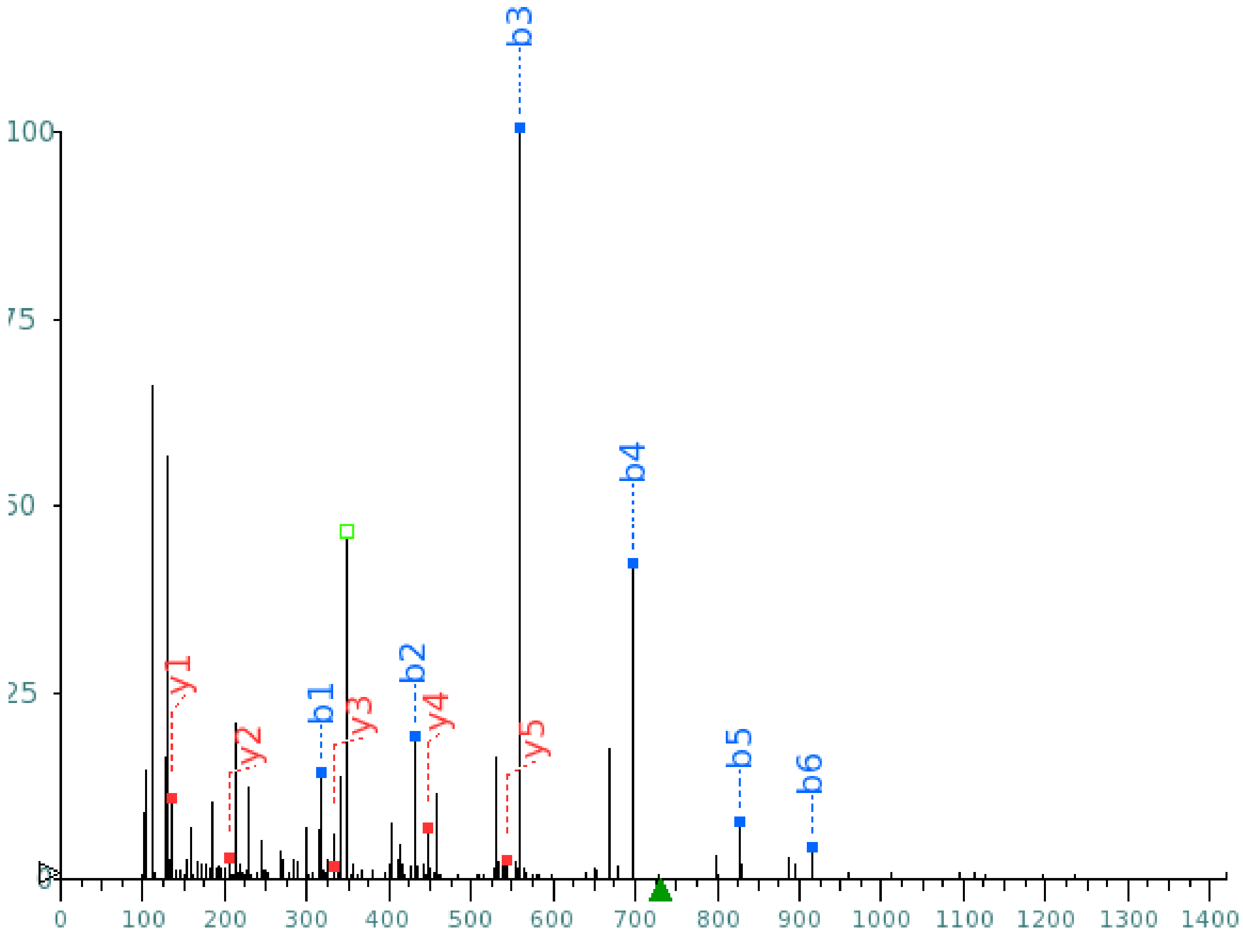

SLEHMSPDQAD (+2)

Primary Reference: SYNE2_HUMAN
Search ID: 41758
Search Name: 20130328_ananiav_ERstress_TMT_TM_timecourse_chymo_inj1_4MC_IAA
Scan#: 9235
Observed Mass: 729.8402 (-0.1 ppm)
PSM Score: 33.93

File name: 2015-02-04 04.01.53PM.pdf

Creation time: 2015-02-04 04:09PM

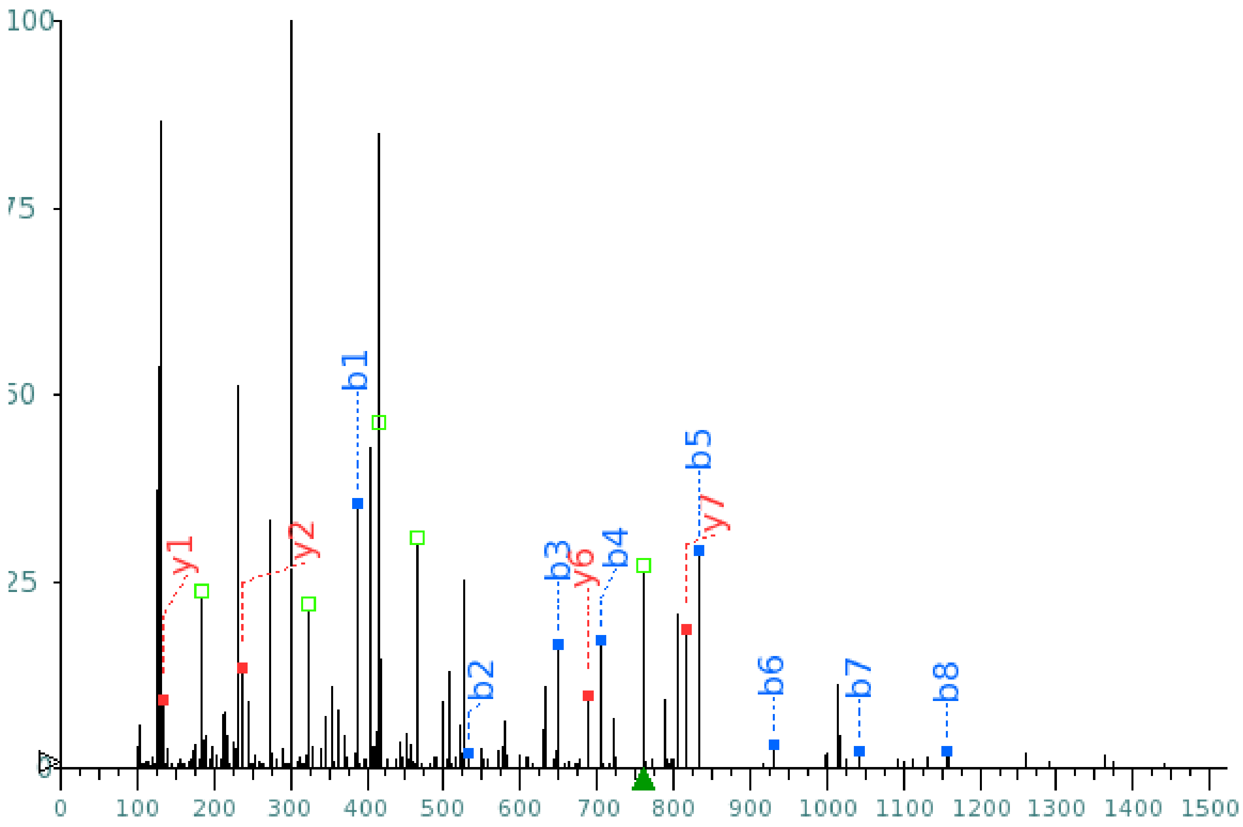

RFDGQPINETD (+2)

Primary Reference: SUMO2_HUMAN
Search ID: 41758
Search Name: 20130328_ananiav_ERstress_TMT_TM_timecourse_chymo_inj1_4MC_IAA
Scan#: 9478
Observed Mass: 760.8811 (0.5 ppm)
PSM Score: 25.35

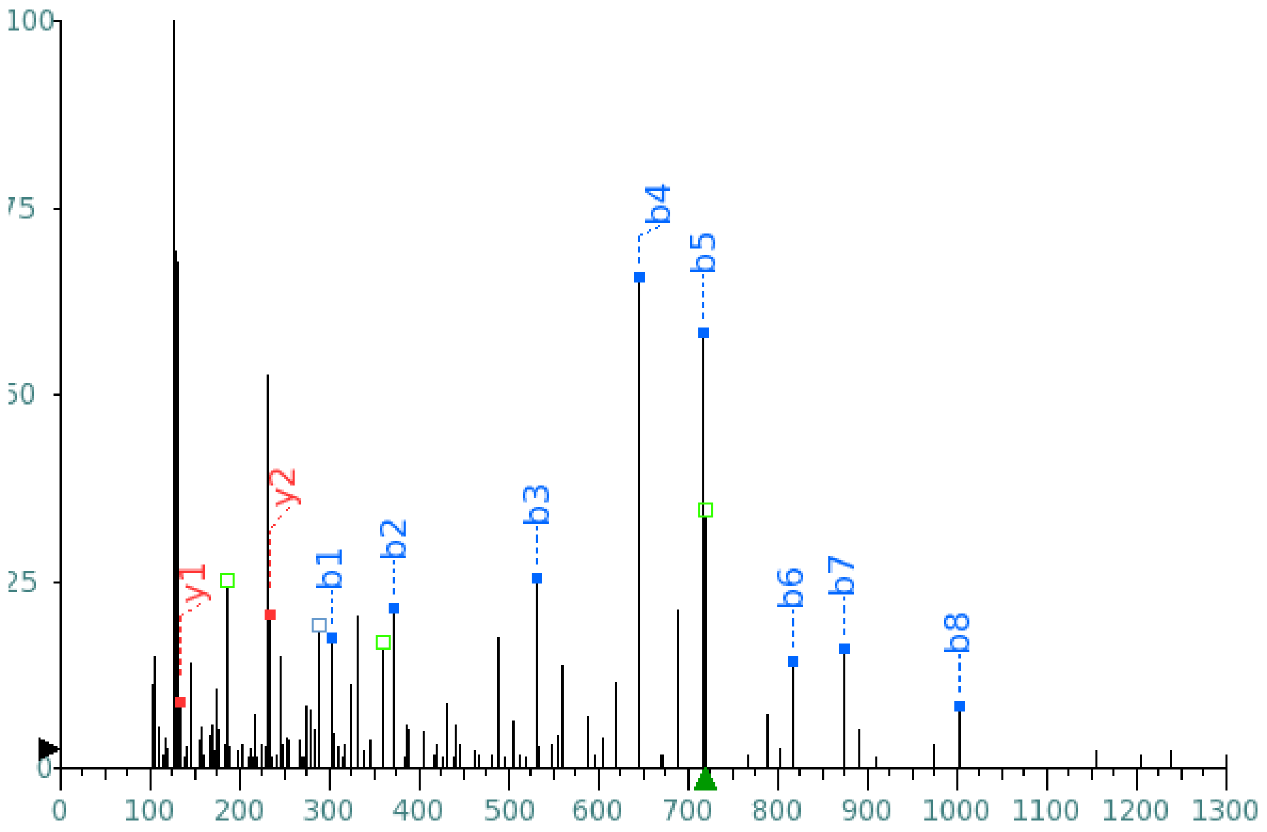

AAC*NAVGQMATD (+2)

Primary Reference: IPO5_HUMAN
Search ID: 41758
Search Name: 20130328_ananiav_ERstress_TMT_TM_timecourse_chymo_inj1_4MC_IAA
Scan#: 9655
Observed Mass: 719.338 (1.6 ppm)
PSM Score: 32.25

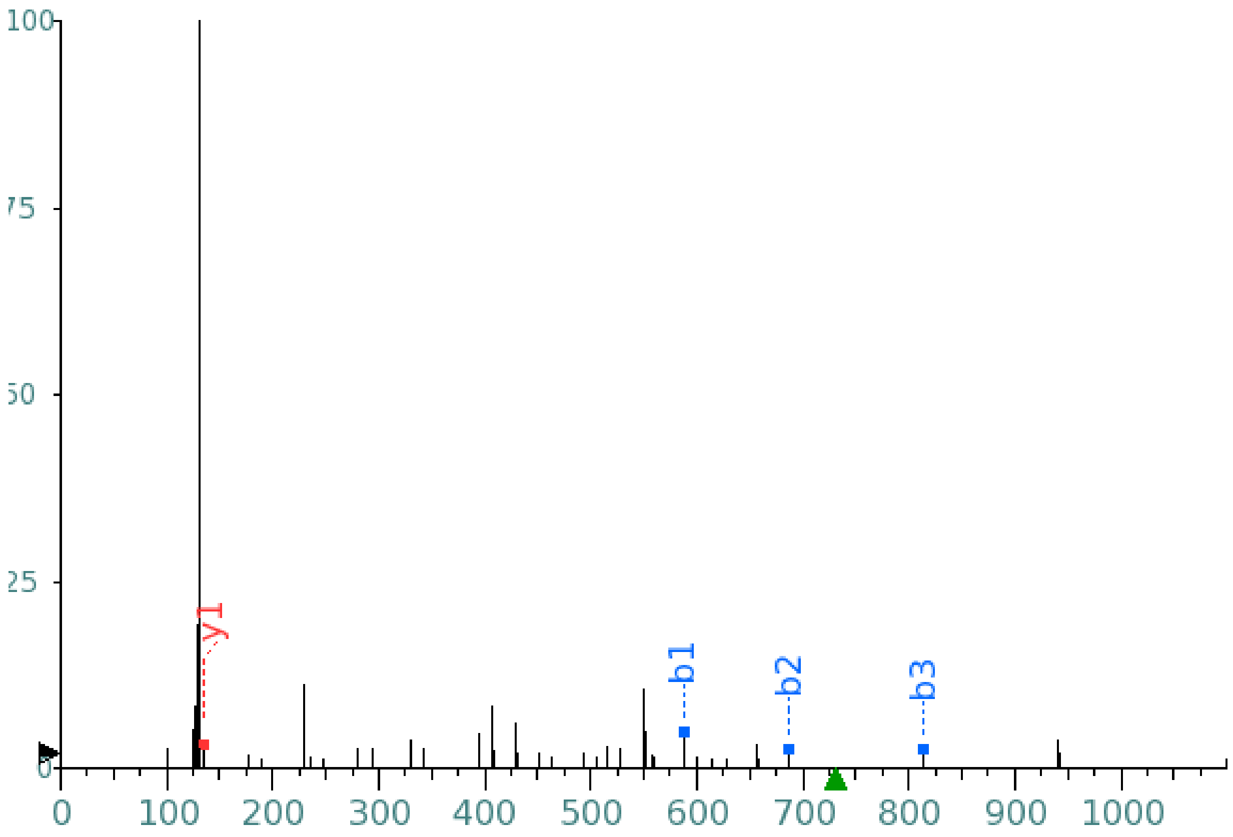

KVQLGDQTD (+2)

Primary Reference: MDN1_HUMAN
Search ID: 41758
Search Name: 20130328_ananiav_ERstress_TMT_TM_timecourse_chymo_inj1_4MC_IAA
Scan#: 9856
Observed Mass: 731.4199 (0.8 ppm)
PSM Score: 22.48

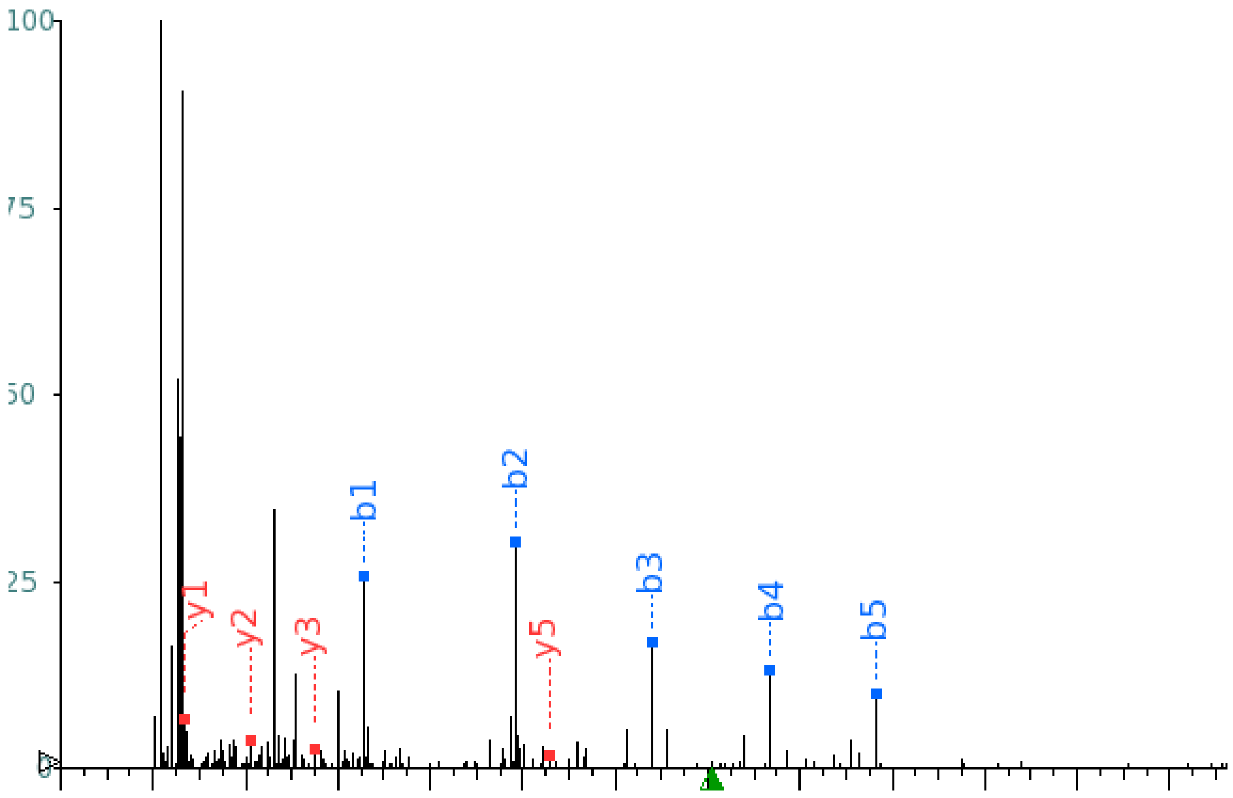

VYFQNHDAAD (+2)

Primary Reference: ROA0_HUMAN
Search ID: 41758
Search Name: 20130328_ananiav_ERstress_TMT_TM_timecourse_chymo_inj1_4MC_IAA
Scan#: 9913
Observed Mass: 704.8379 (-0.7 ppm)
PSM Score: 22.22

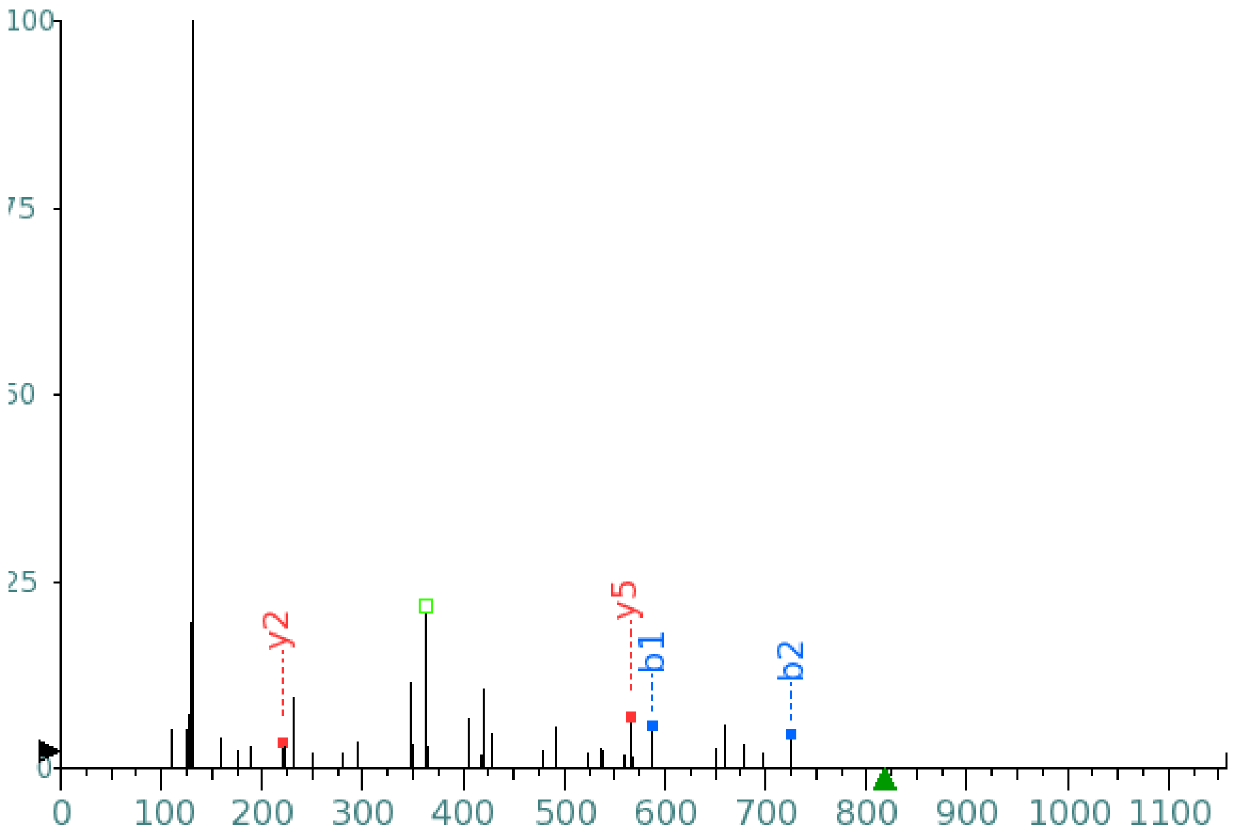

KHIFSEDTSD (+2)

Primary Reference: SPB5_HUMAN
Search ID: 41758
Search Name: 20130328_ananiav_ERstress_TMT_TM_timecourse_chymo_inj1_4MC_IAA
Scan#: 10120
Observed Mass: 818.9333 (0.6 ppm)
PSM Score: 28.83

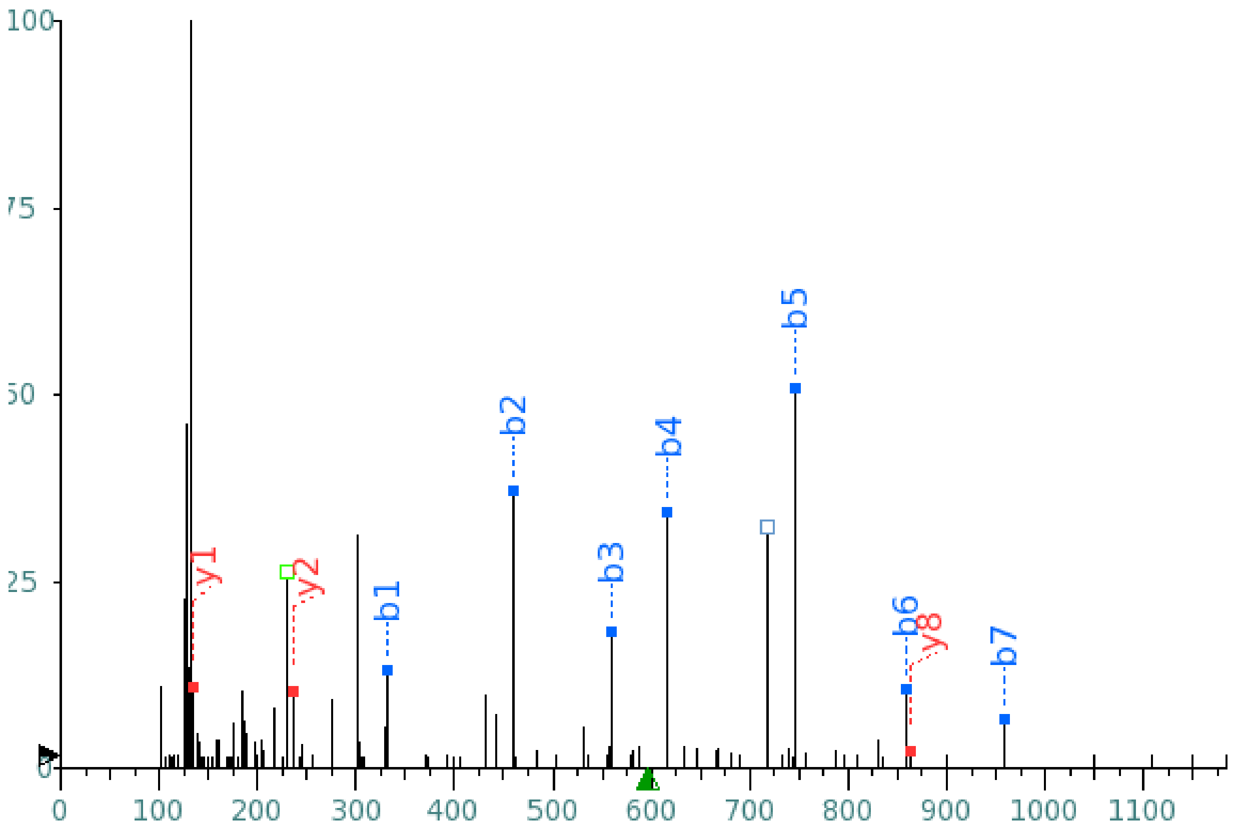

TEVGEITTD (+2)

Primary Reference: SP16H_HUMAN
Search ID: 41758
Search Name: 20130328_ananiav_ERstress_TMT_TM_timecourse_chymo_inj1_4MC_IAA
Scan#: 10322
Observed Mass: 597.3097 (1.9 ppm)
PSM Score: 57.79

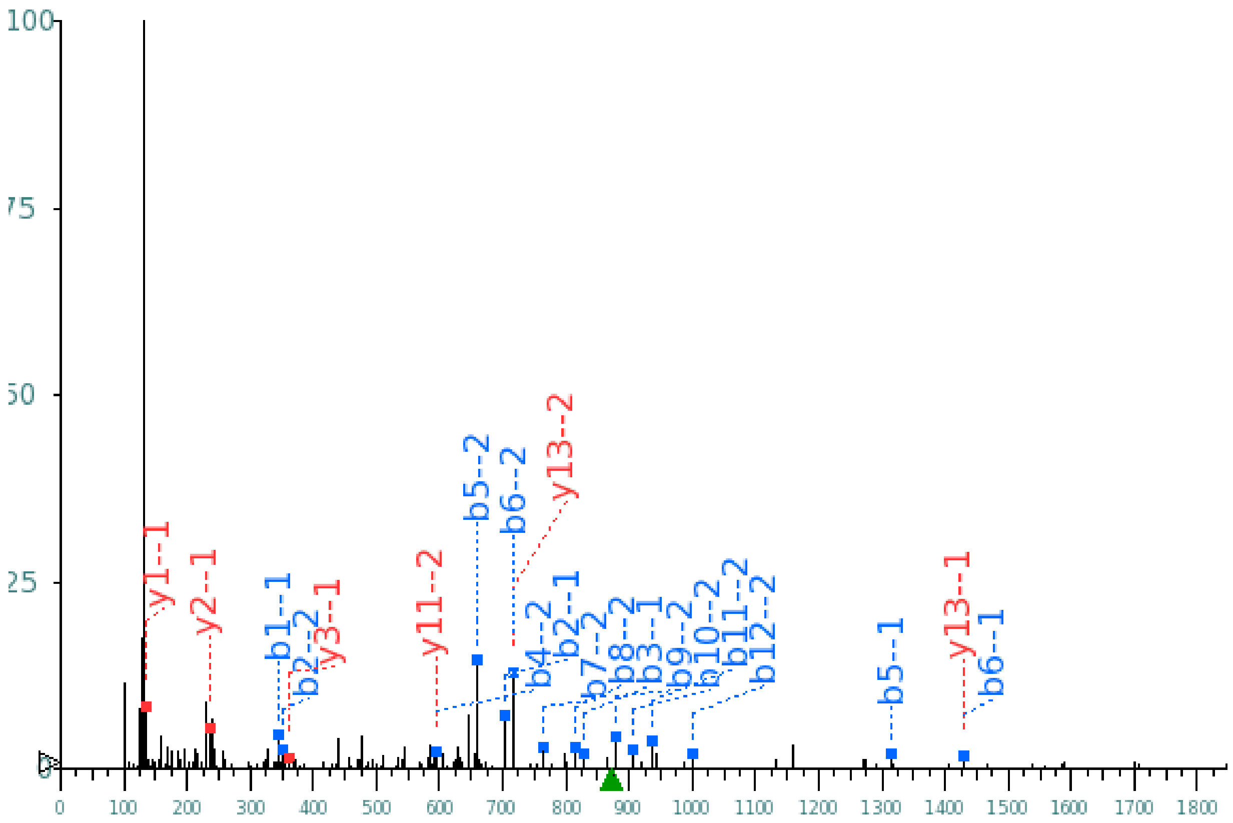

NKEKEIPVEGGQEQQTD (+3)

Primary Reference: B1AKC7_HUMAN
Search ID: 41758
Search Name: 20130328_ananiav_ERstress_TMT_TM_timecourse_chymo_inj1_4MC_IAA
Scan#: 10603
Observed Mass: 872.808 (0.4 ppm)
PSM Score: 28.78

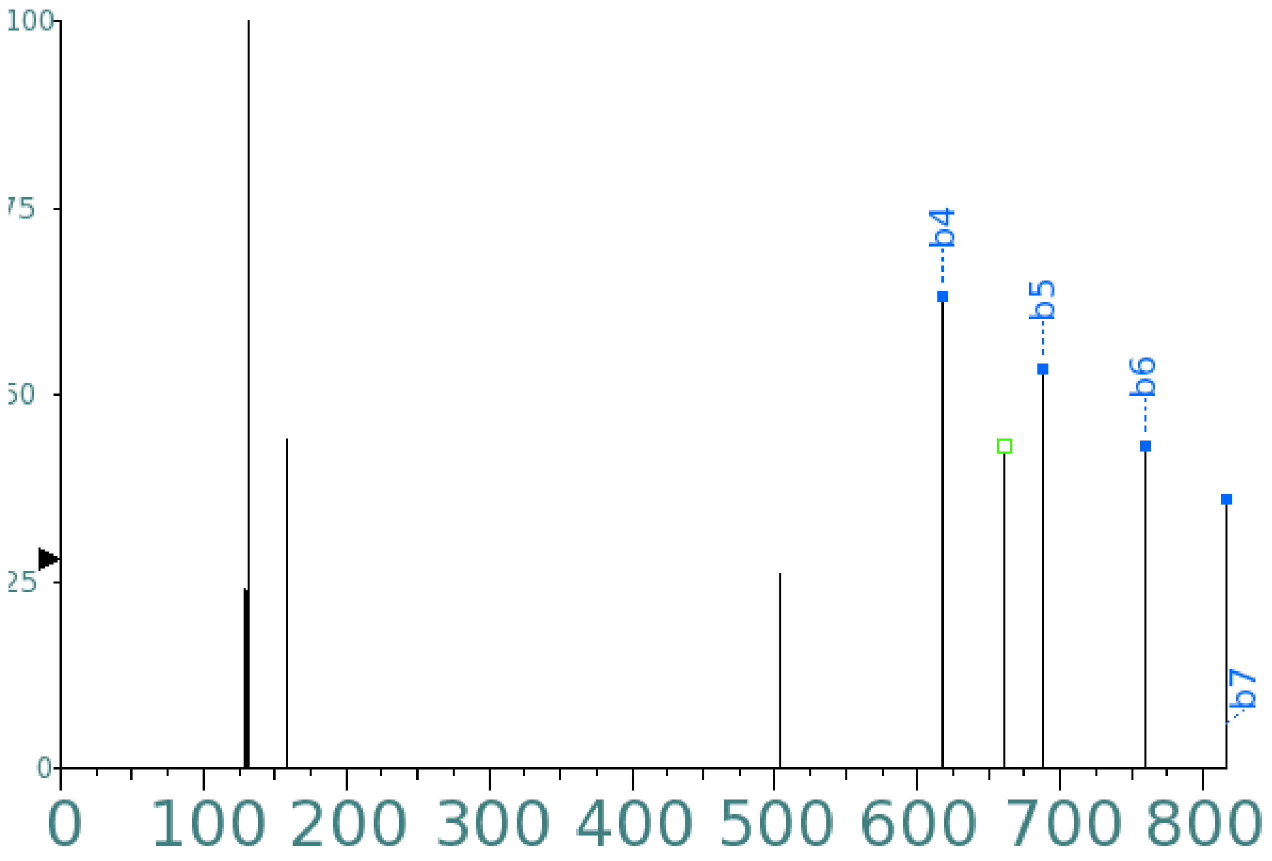

GSENAAGPC*NWDEAD (+2)

Primary Reference: MAGD2_HUMAN
Search ID: 41758
Search Name: 20130328_ananiav_ERstress_TMT_TM_timecourse_chymo_inj1_4MC_IAA
Scan#: 10791
Observed Mass: 911.3805 (-0.5 ppm)
PSM Score: 35.02

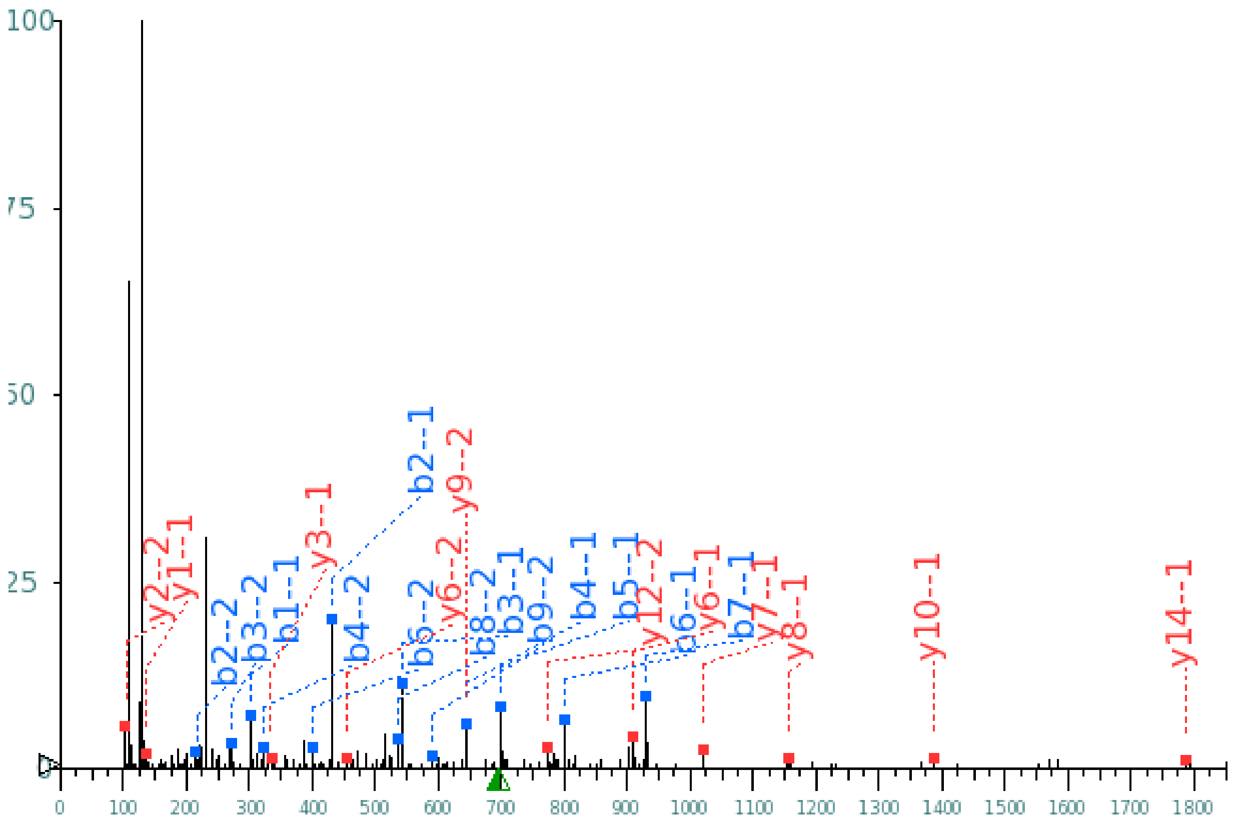

AQITGTEHLKESEAD (+3)

Primary Reference: PDLI5_HUMAN
Search ID: 41758
Search Name: 20130328_ananiav_ERstress_TMT_TM_timecourse_chymo_inj1_4MC_IAA
Scan#: 10938
Observed Mass: 696.3724 (0.3 ppm)
PSM Score: 24.48

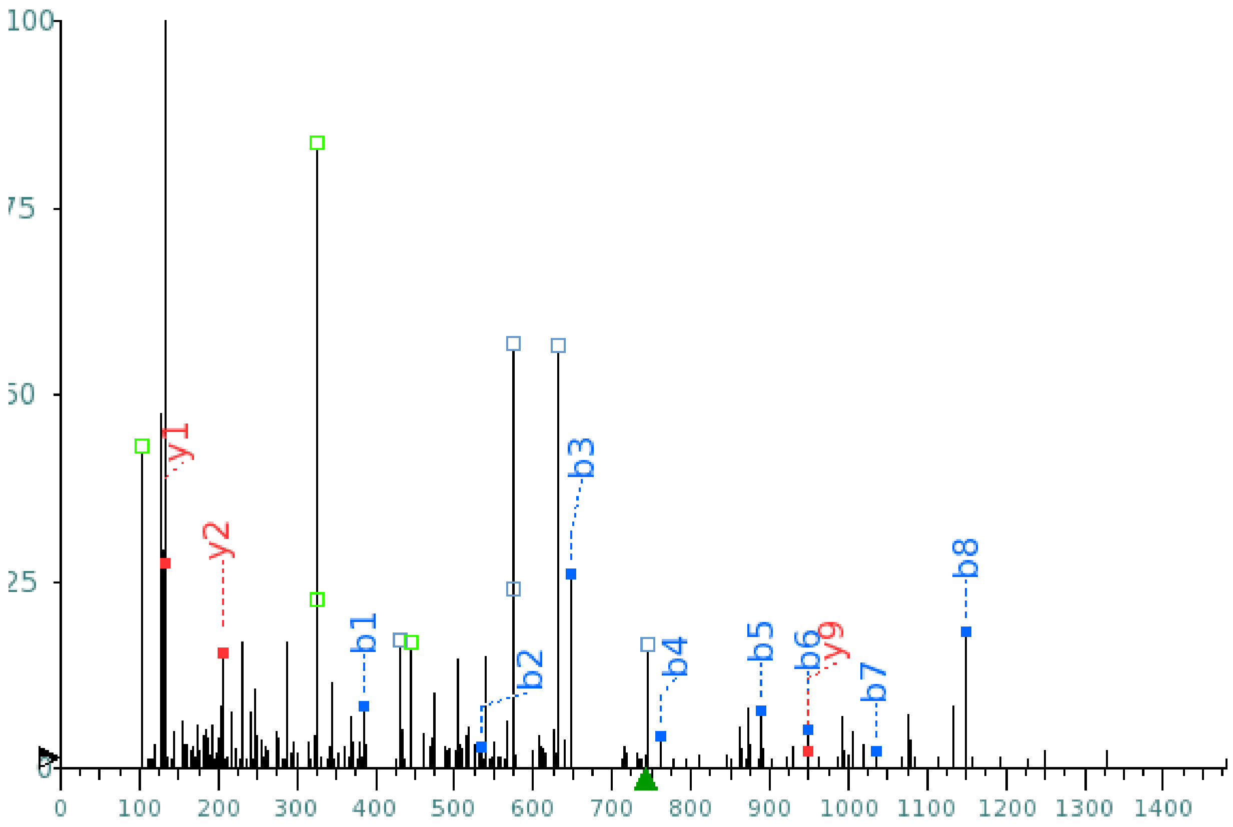

RFDIEGSDEAD (+2)

Primary Reference: RB_HUMAN
Search ID: 41758
Search Name: 20130328_ananiav_ERstress_TMT_TM_timecourse_chymo_inj1_4MC_IAA
Scan#: 10962
Observed Mass: 741.8496 (0.6 ppm)
PSM Score: 22.37

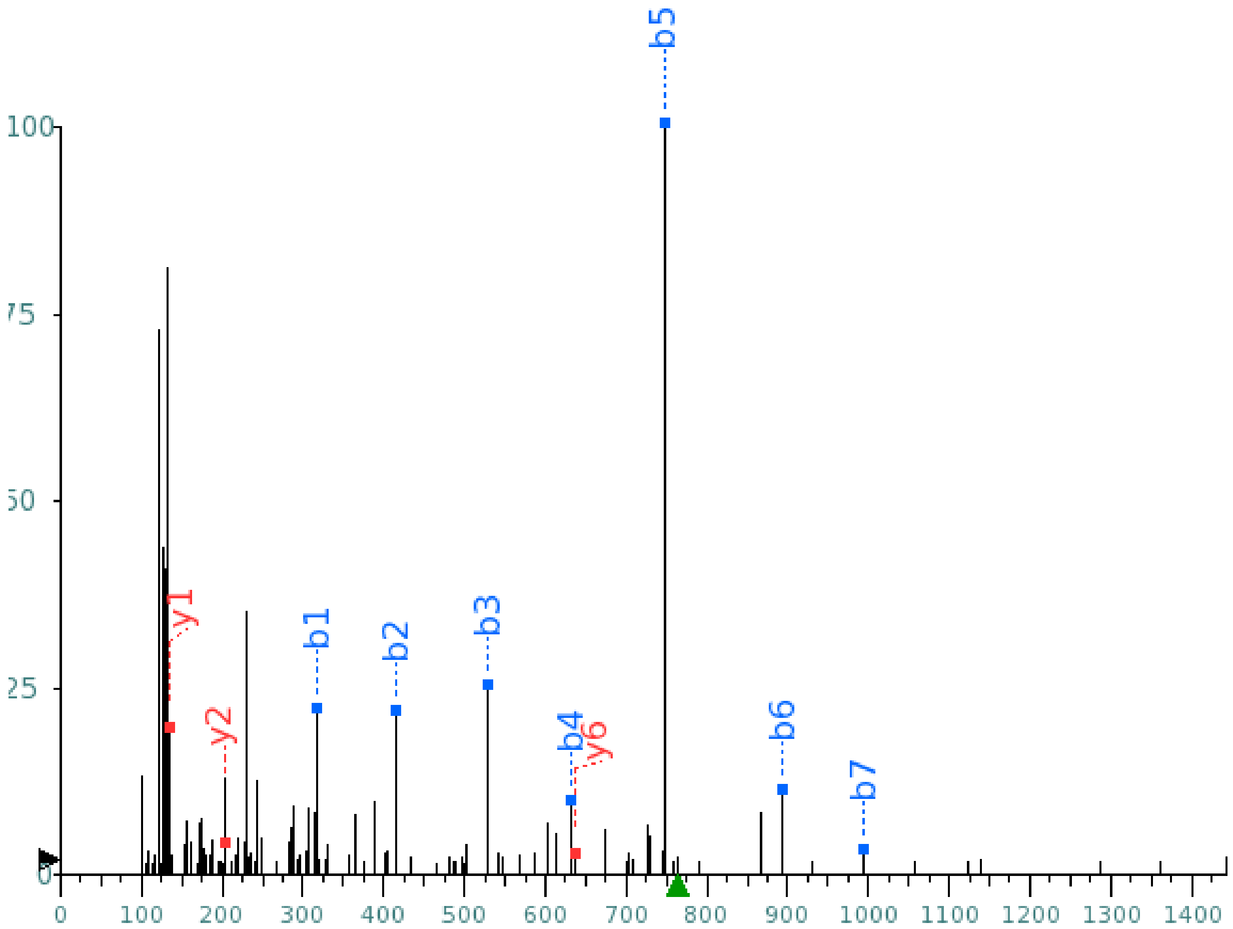

SVNTDFTSDQAD (+2)

Primary Reference: RED1_HUMAN
Search ID: 41758
Search Name: 20130328_ananiav_ERstress_TMT_TM_timecourse_chymo_inj1_4MC_IAA
Scan#: 11043
Observed Mass: 764.8521 (0.3 ppm)
PSM Score: 40.53

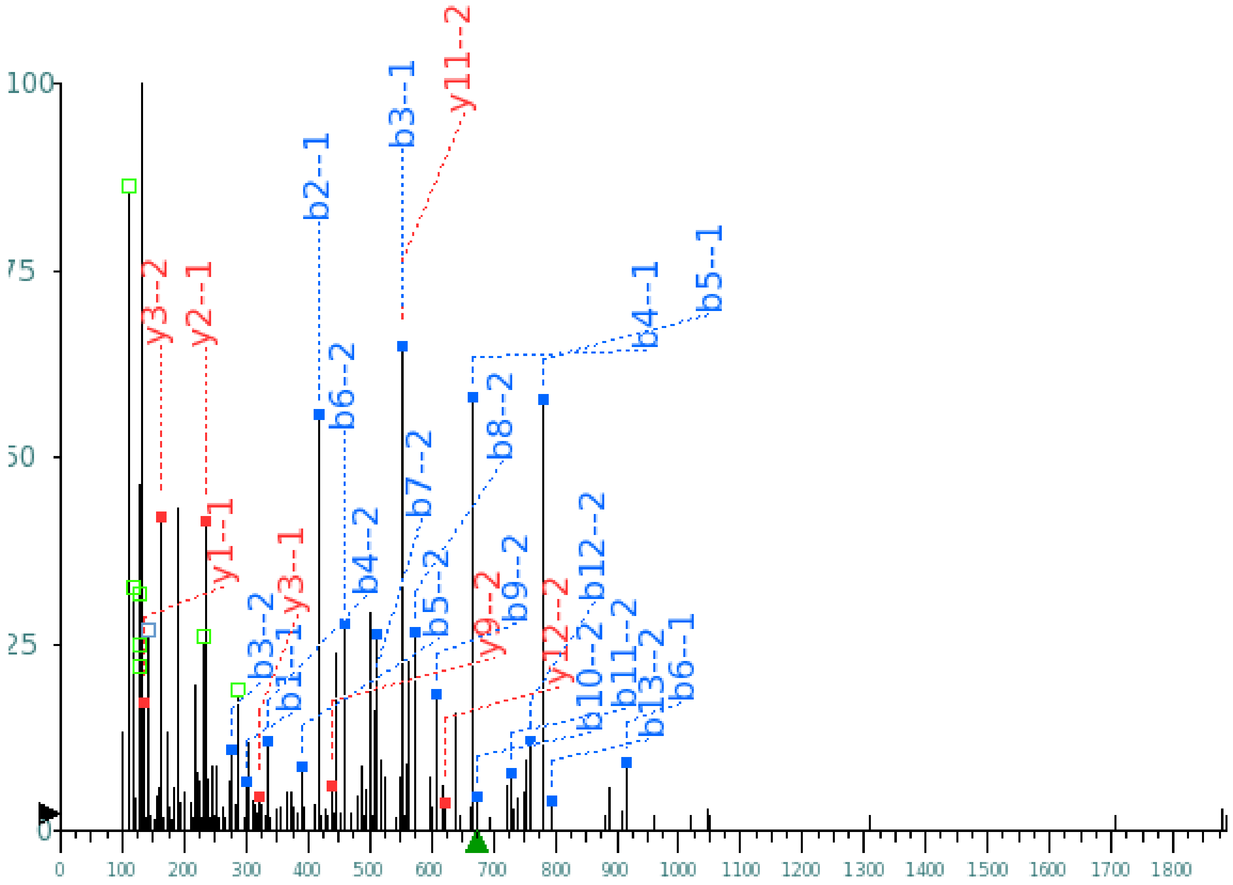

ADHLLHTQAQDGAISTD (+3)

Primary Reference: TPP2_HUMAN
Search ID: 41758
Search Name: 20130328_ananiav_ERstress_TMT_TM_timecourse_chymo_inj1_4MC_IAA
Scan#: 11338
Observed Mass: 674.6749 (0.5 ppm)
PSM Score: 38.24

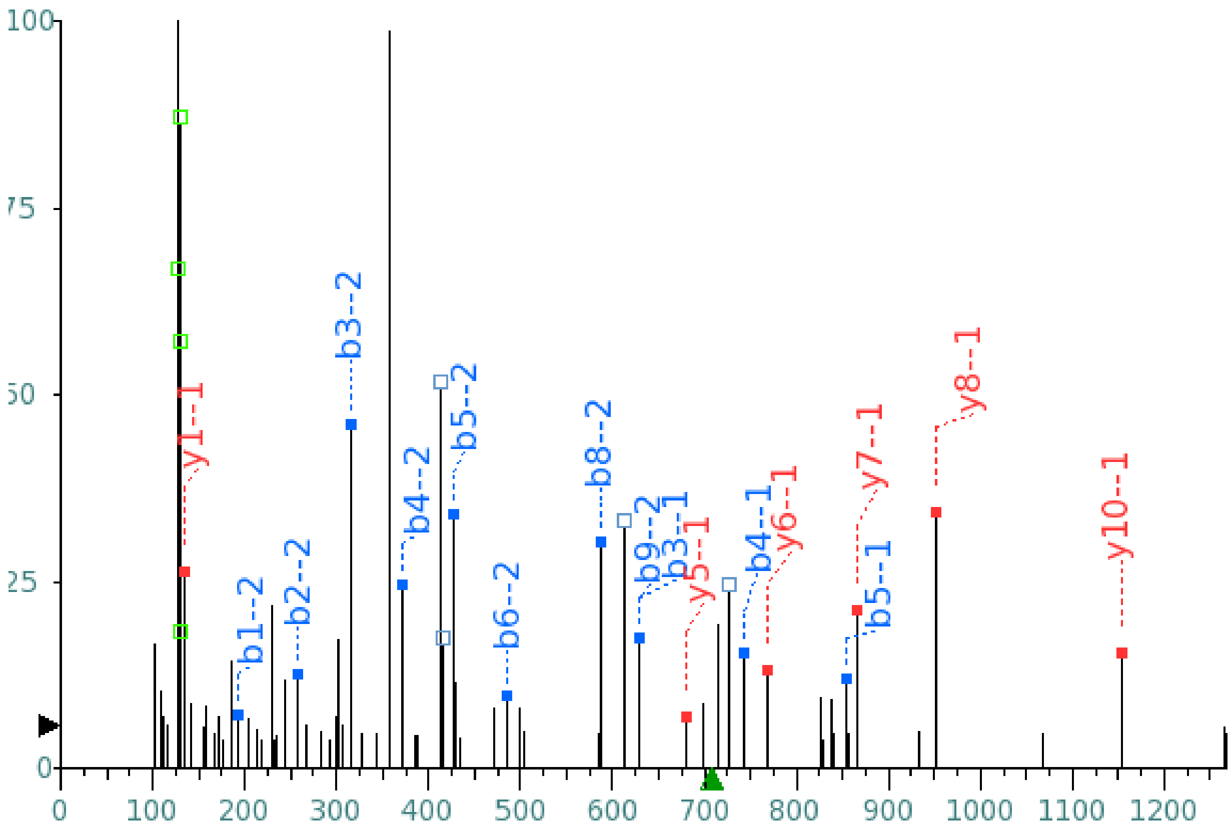

RENLLLSDSPSC*RETD (+3)

Primary Reference: ROCK2_HUMAN
Search ID: 41758
Search Name: 20130328_ananiav_ERstress_TMT_TM_timecourse_chymo_inj1_4MC_IAA
Scan#: 11353
Observed Mass: 707.6808 (-7.8 ppm)
PSM Score: 32.86

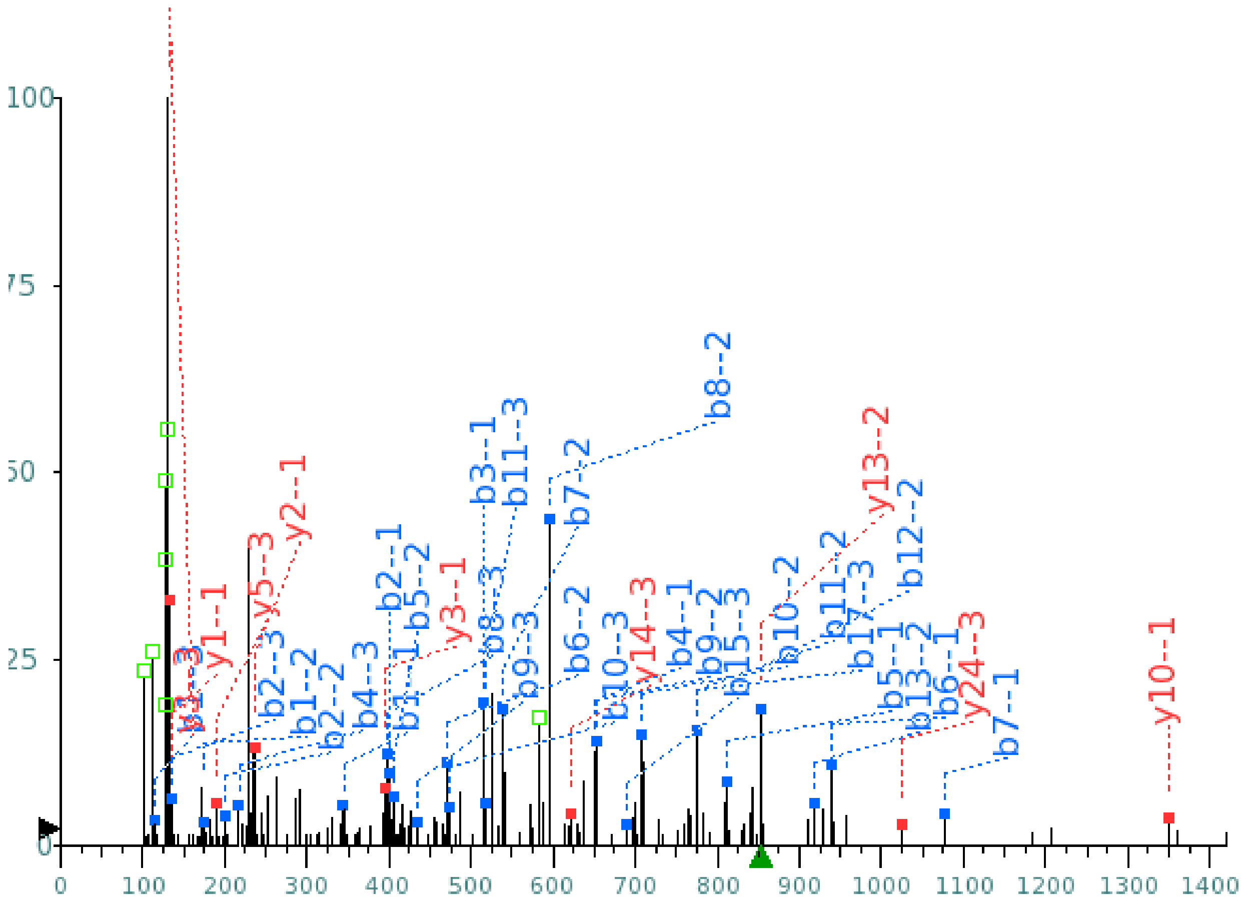

NGIHC*QHNINEC*EVEPC*KNGGIC*TD (+4)

Primary Reference: EDIL3_HUMAN
Search ID: 41758
Search Name: 20130328_ananiav_ERstress_TMT_TM_timecourse_chymo_inj1_4MC_IAA
Scan#: 11375
Observed Mass: 854.1431 (0.3 ppm)
PSM Score: 35.53

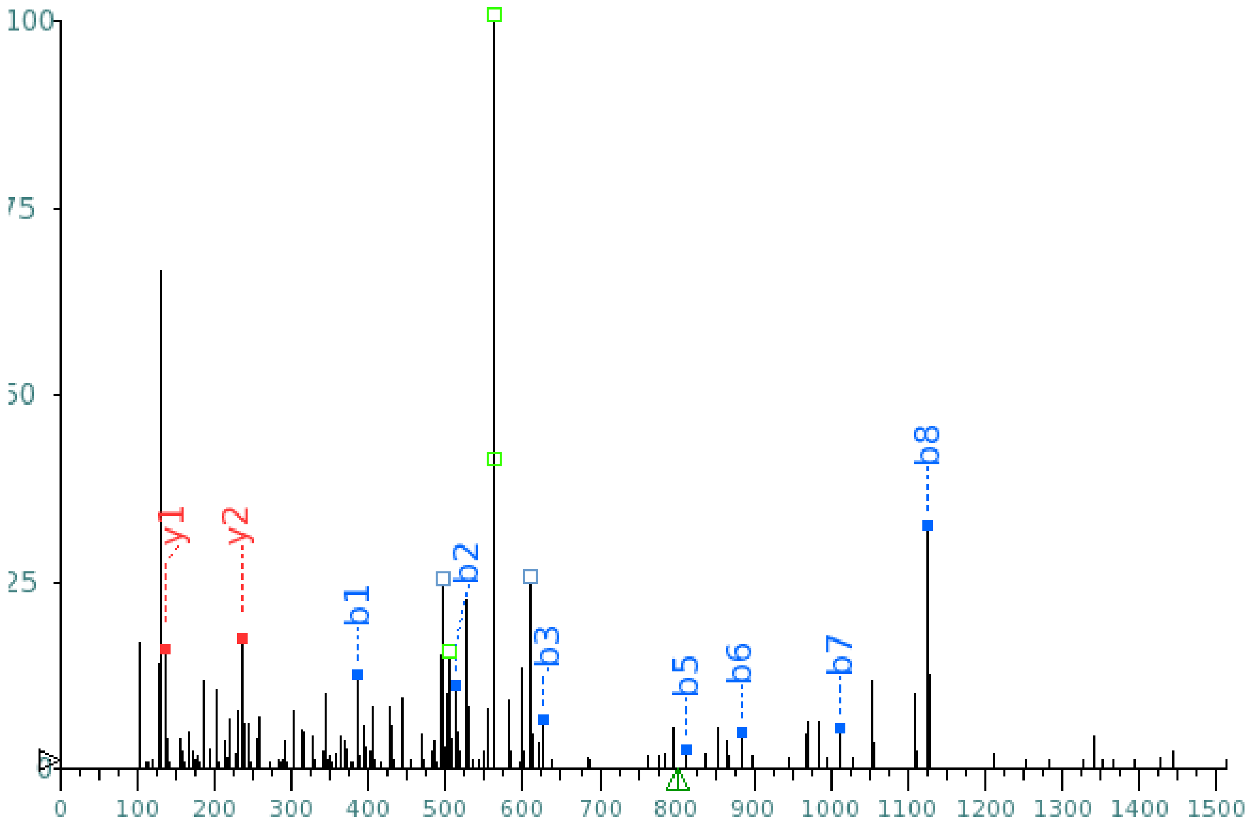

RQIPSAEDLETD (+2)

Primary Reference: HDAC7_HUMAN
Search ID: 41758
Search Name: 20130328_ananiav_ERstress_TMT_TM_timecourse_chymo_inj1_4MC_IAA
Scan#: 11467
Observed Mass: 801.9132 (1.2 ppm)
PSM Score: 25.14

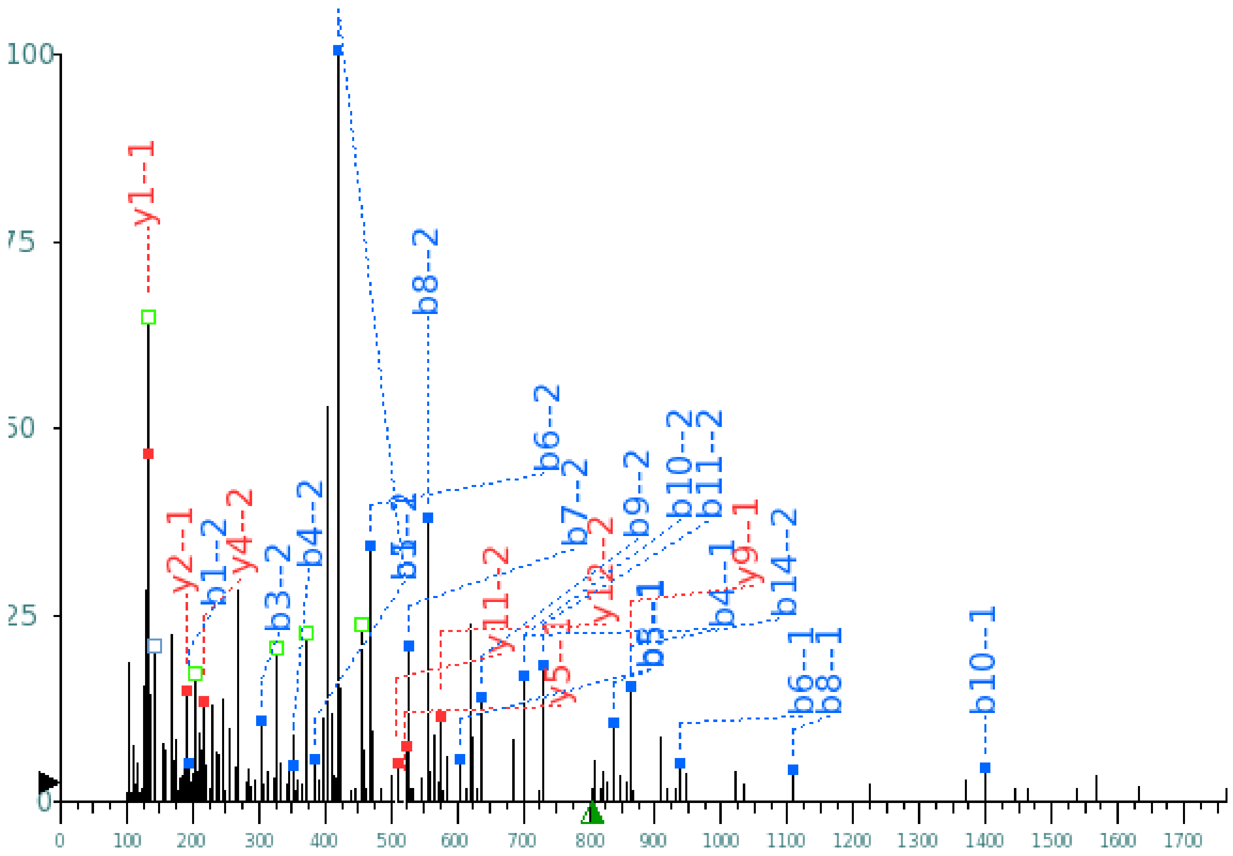

RGC*PHVLGYEGPGDPASDEGD (+3)

Primary Reference: RUND1_HUMAN
Search ID: 41758
Search Name: 20130328_ananiav_ERstress_TMT_TM_timecourse_chymo_inj1_4MC_IAA
Scan#: 11540
Observed Mass: 805.3672 (-0.4 ppm)
PSM Score: 34.18

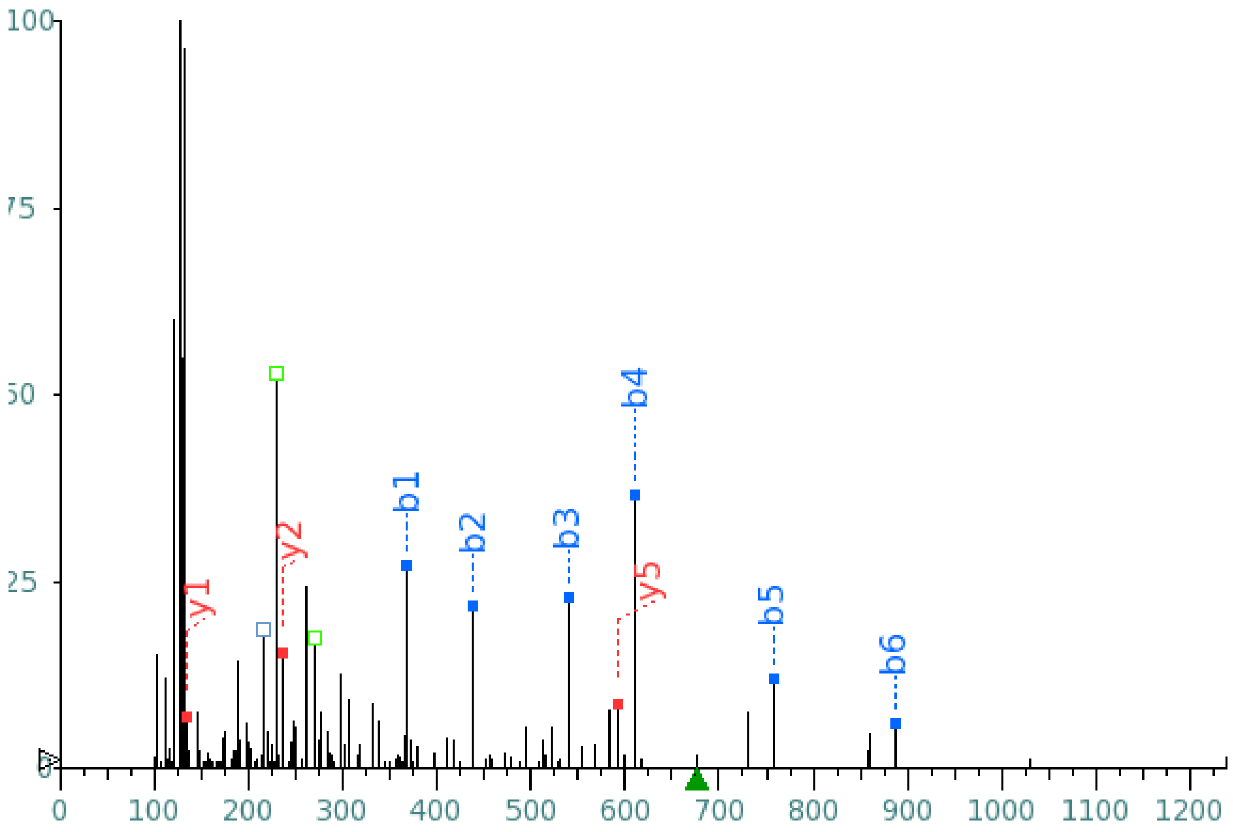

HATAFELDTD (+2)

Primary Reference: SERPH_HUMAN
Search ID: 41758
Search Name: 20130328_ananiav_ERstress_TMT_TM_timecourse_chymo_inj1_4MC_IAA
Scan#: 11852
Observed Mass: 674.8333 (0.9 ppm)
PSM Score: 36.1

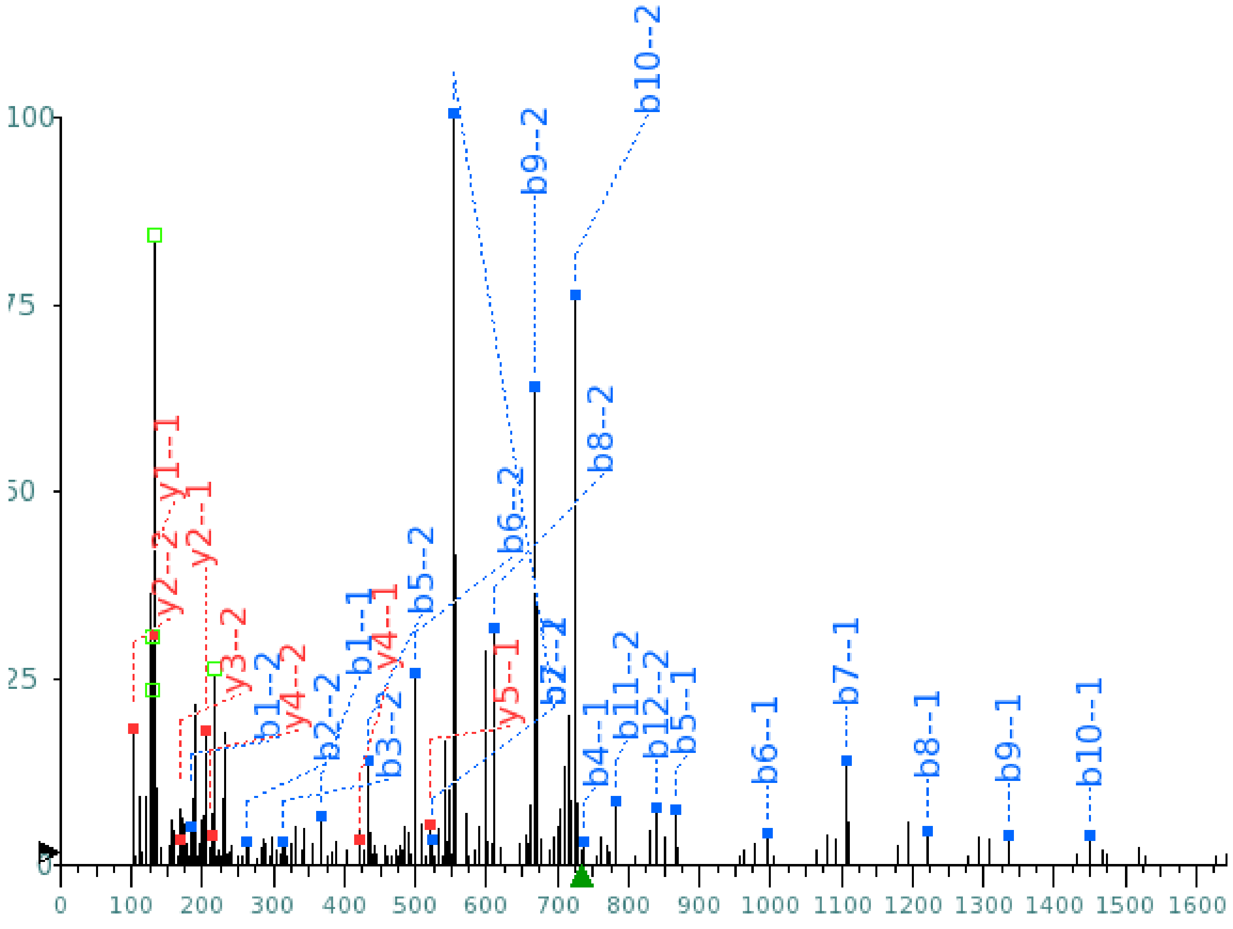

HRTIEQNINNLNVSEAD (+3)

Primary Reference: CND2_HUMAN
Search ID: 41758
Search Name: 20130328_ananiav_ERstress_TMT_TM_timecourse_chymo_inj1_4MC_IAA
Scan#: 12152
Observed Mass: 732.7124 (1 ppm)
PSM Score: 35.09

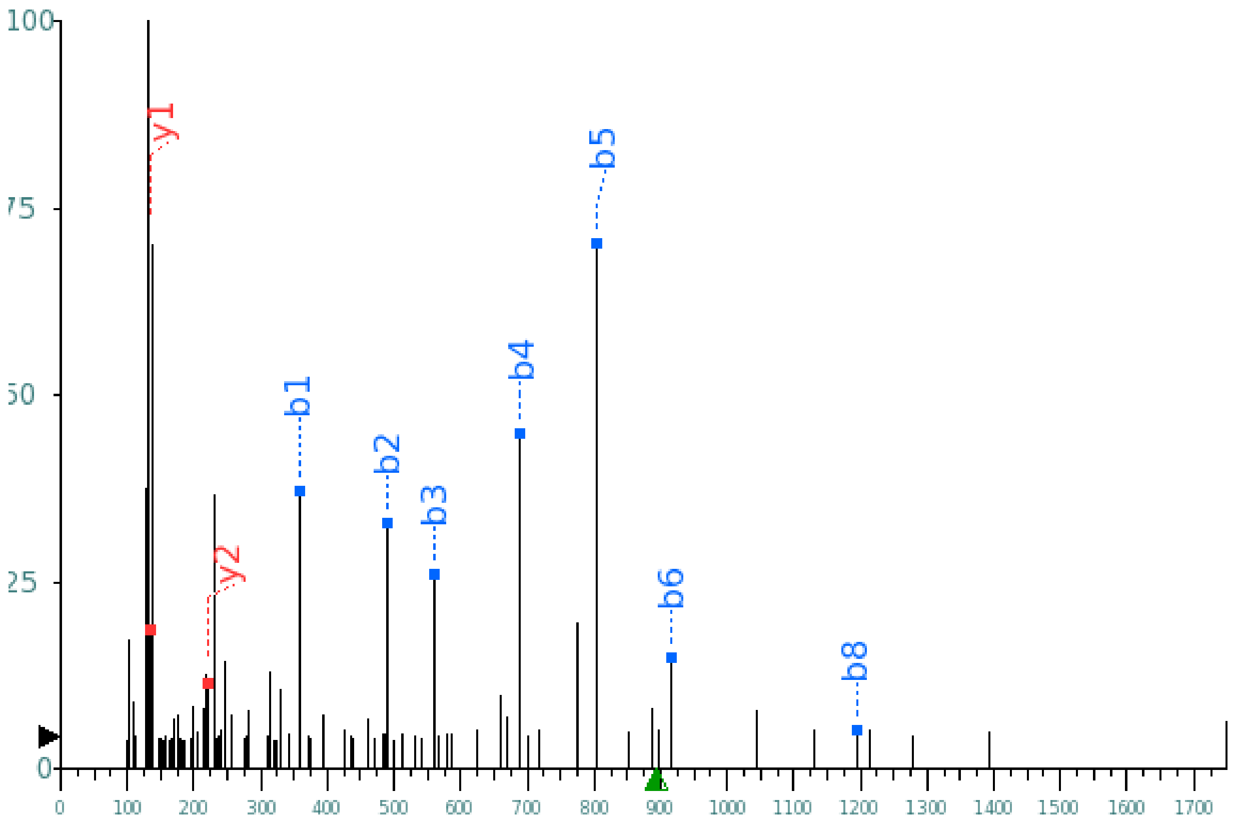

QMAENIYNDEESD (+2)

Primary Reference: RBBP7_HUMAN
Search ID: 41758
Search Name: 20130328_ananiav_ERstress_TMT_TM_timecourse_chymo_inj1_4MC_IAA
Scan#: 12200
Observed Mass: 893.8864 (0.9 ppm)
PSM Score: 29.17

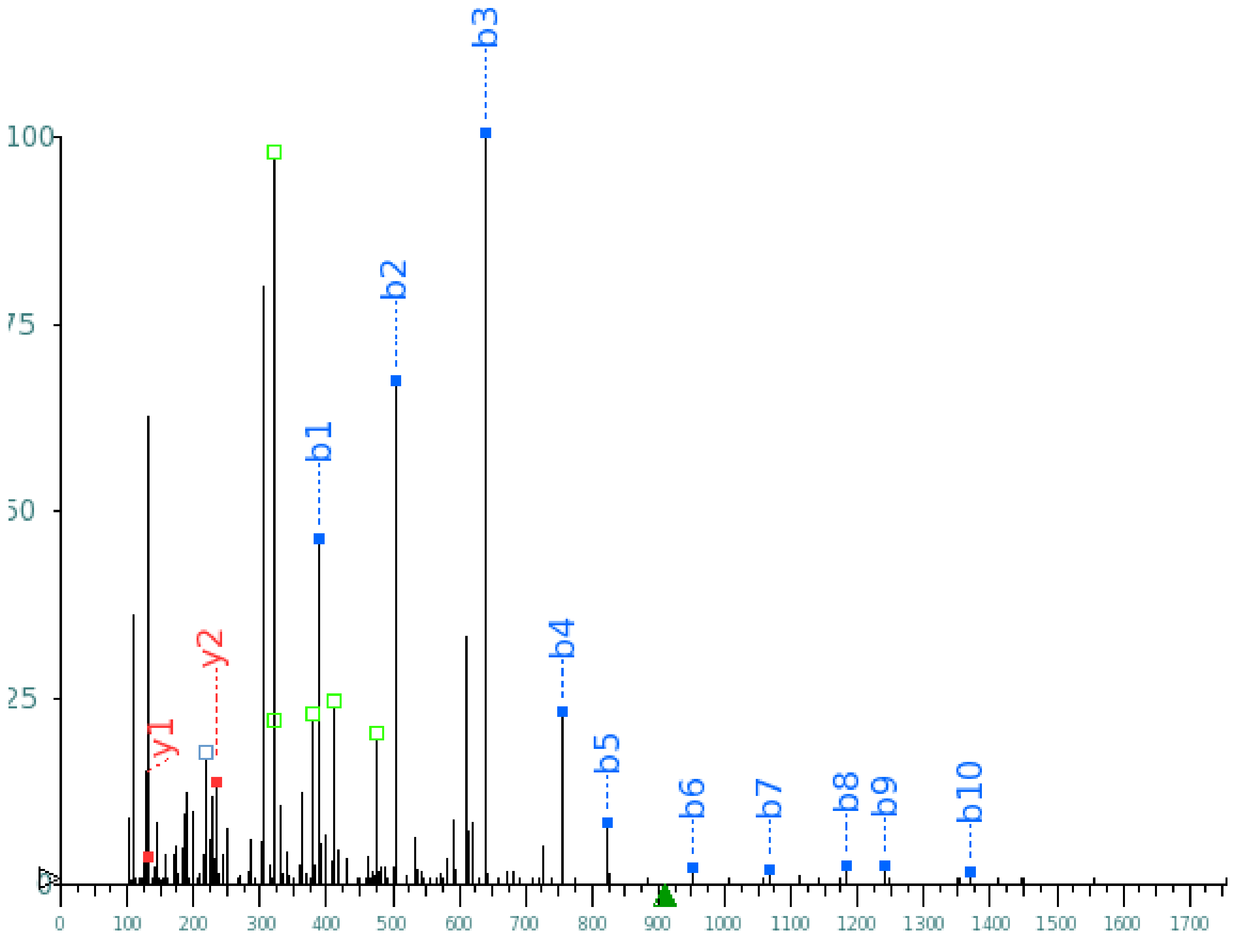

C*LHIAEDDGEVDTD (+2)

Primary Reference: SIN1_HUMAN
Search ID: 41758
Search Name: 20130328_ananiav_ERstress_TMT_TM_timecourse_chymo_inj1_4MC_IAA
Scan#: 12505
Observed Mass: 909.4071 (0.5 ppm)
PSM Score: 52.52

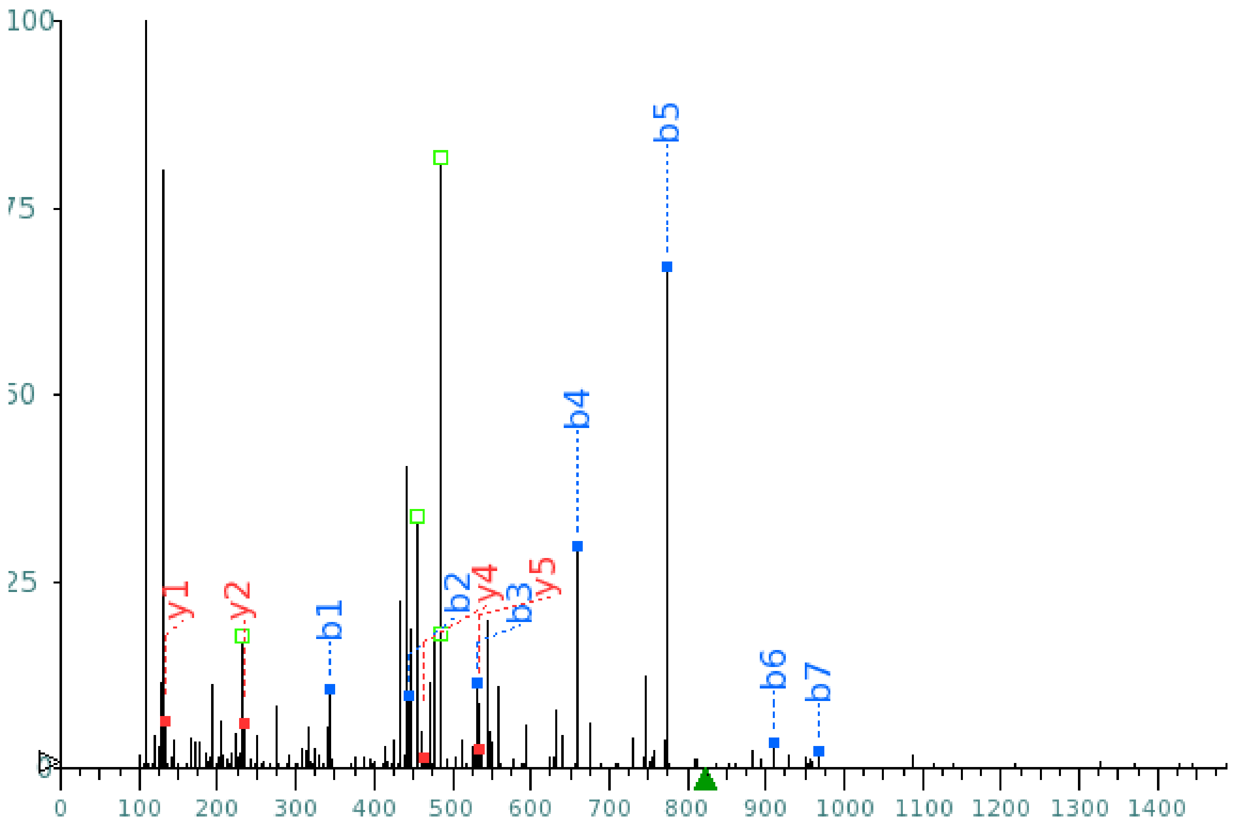

LTSQNHGFAVETD (+2)

Primary Reference: PYR1_HUMAN
Search ID: 41758
Search Name: 20130328_ananiav_ERstress_TMT_TM_timecourse_chymo_inj1_4MC_IAA
Scan#: 12545
Observed Mass: 824.4129 (0.6 ppm)
PSM Score: 21.43

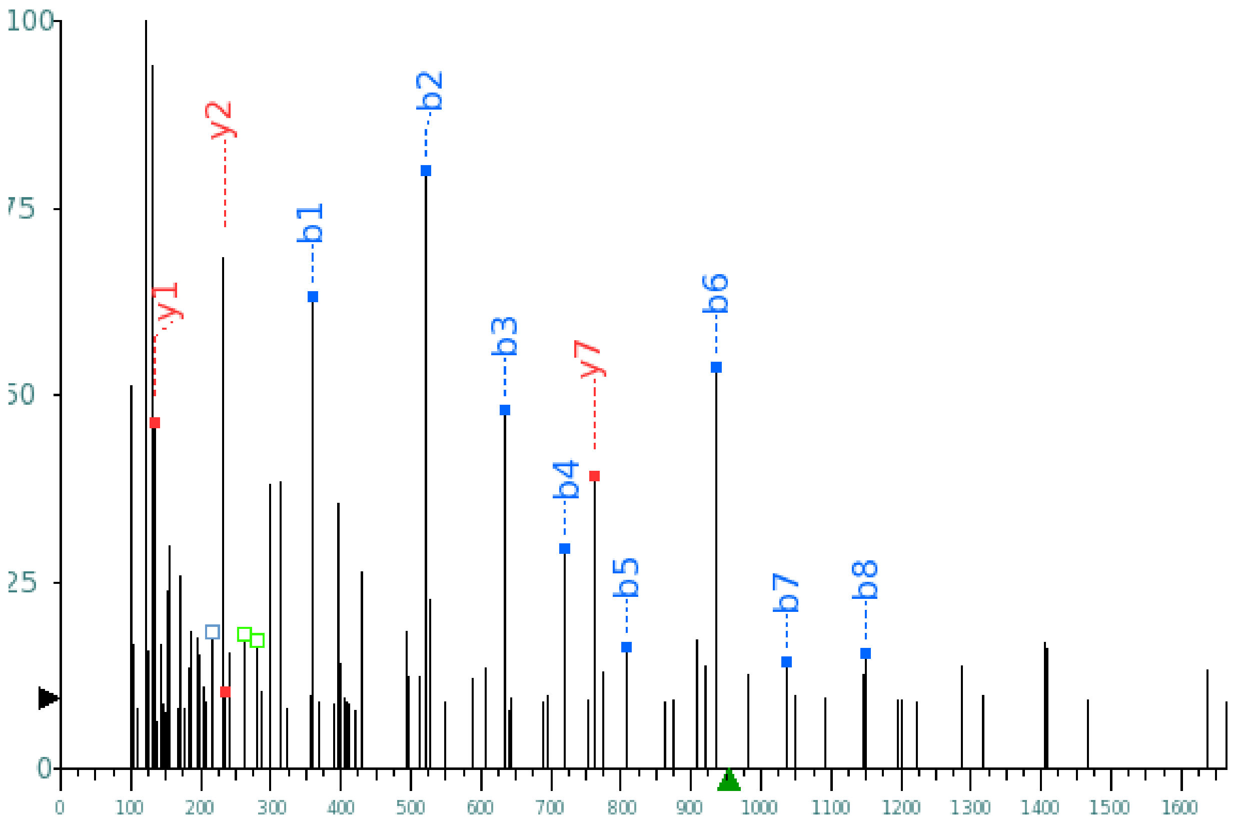

QYISSEVNPPSDETD (+2)

Primary Reference: IF4G2_HUMAN
Search ID: 41758
Search Name: 20130328_ananiav_ERstress_TMT_TM_timecourse_chymo_inj1_4MC_IAA
Scan#: 12622
Observed Mass: 955.4461 (-0.8 ppm)
PSM Score: 33.38

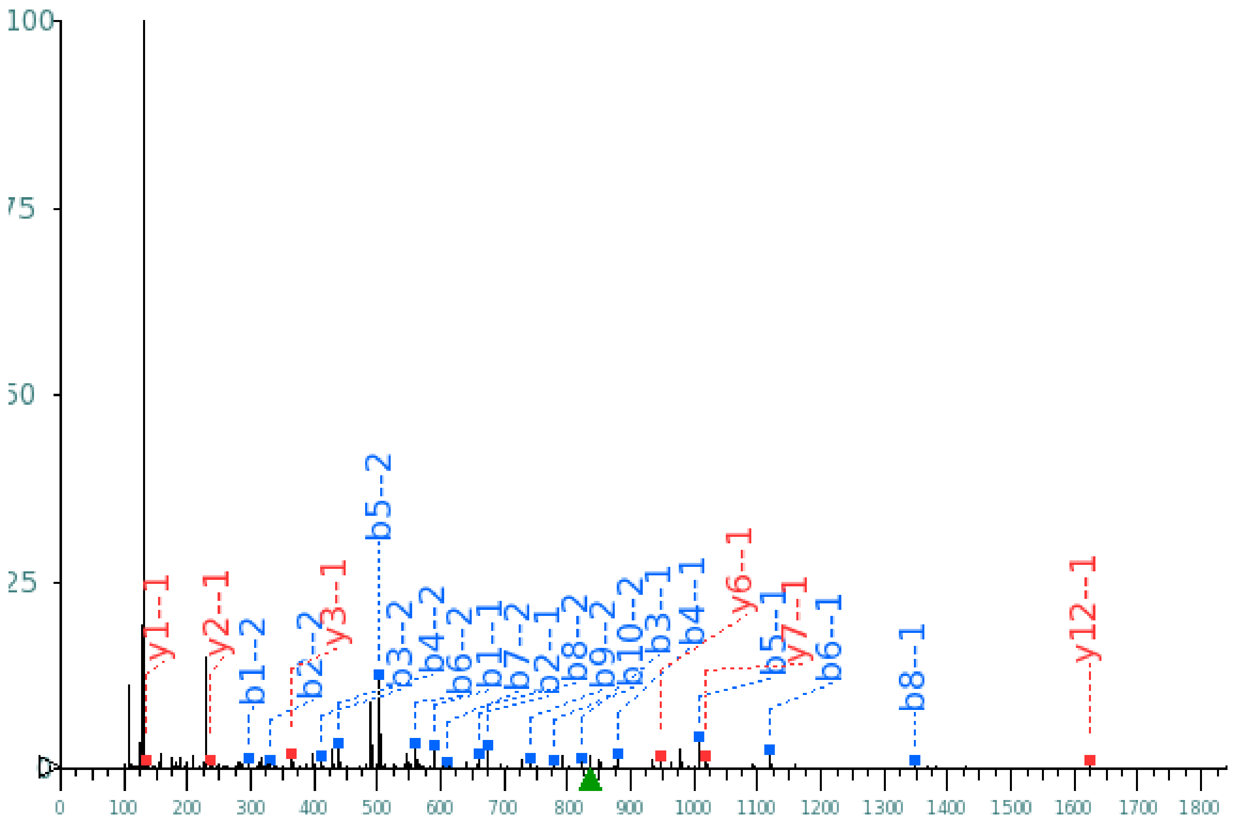

KAYGELPEHAKINETD (+3)

Primary Reference: IF1AX_HUMAN
Search ID: 41758
Search Name: 20130328_ananiav_ERstress_TMT_TM_timecourse_chymo_inj1_4MC_IAA
Scan#: 12666
Observed Mass: 834.799 (0.7 ppm)
PSM Score: 24.55

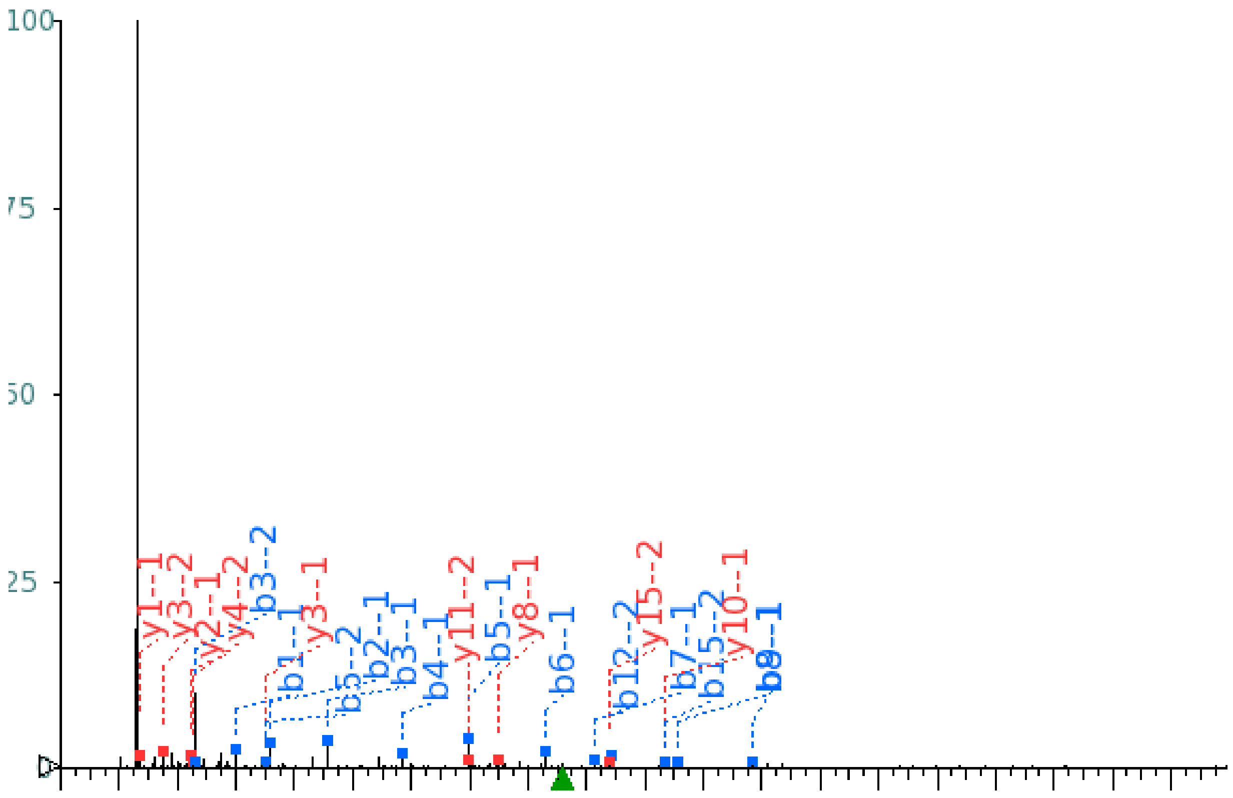

AGVQDQLIEKREPGSGTESD (+3)

Primary Reference: EAF6_HUMAN
Search ID: 41758
Search Name: 20130328_ananiav_ERstress_TMT_TM_timecourse_chymo_inj1_4MC_IAA
Scan#: 12707
Observed Mass: 858.7858 (0.7 ppm)
PSM Score: 32.39

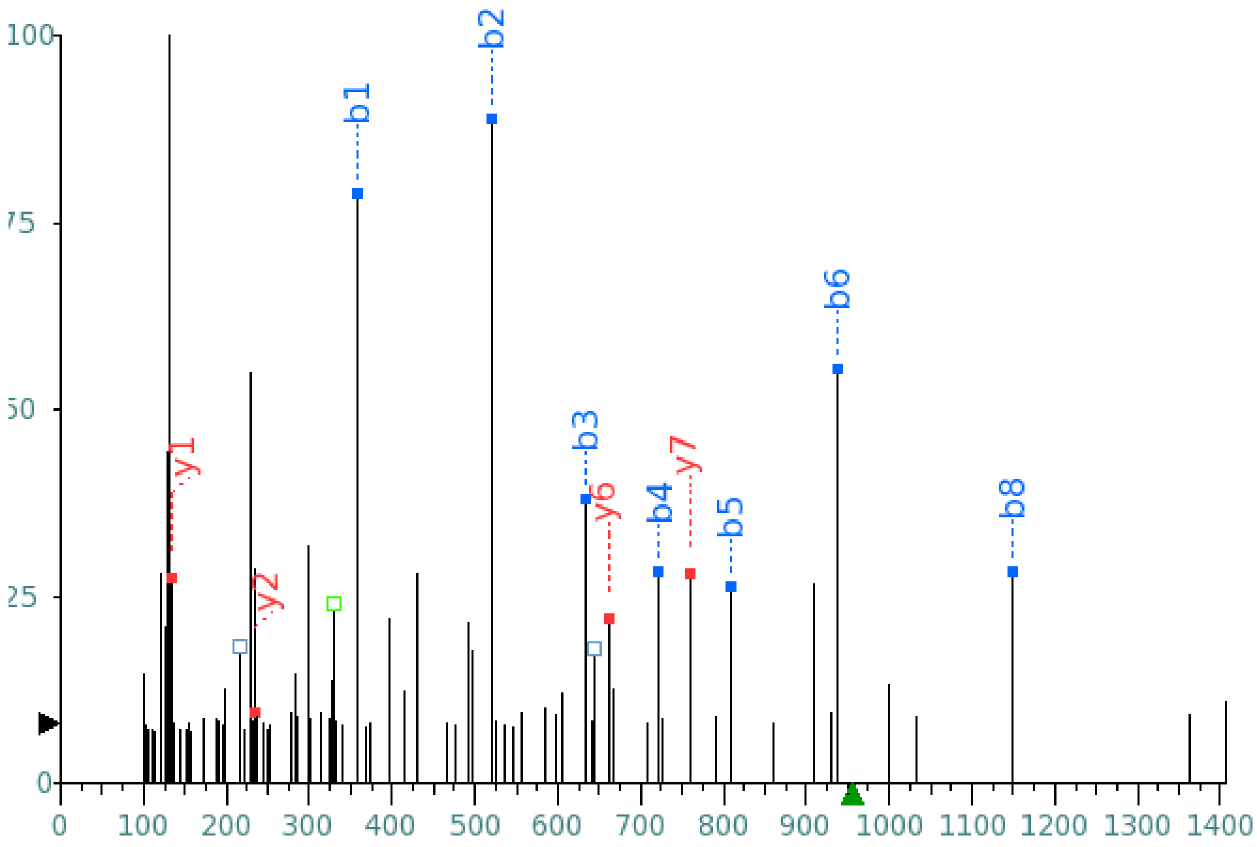

QYISSEVNPPSDETD (+2)

Primary Reference: IF4G2_HUMAN
Search ID: 41758
Search Name: 20130328_ananiav_ERstress_TMT_TM_timecourse_chymo_inj1_4MC_IAA
Scan#: 12720
Observed Mass: 955.447 (0.1 ppm)
PSM Score: 39.29

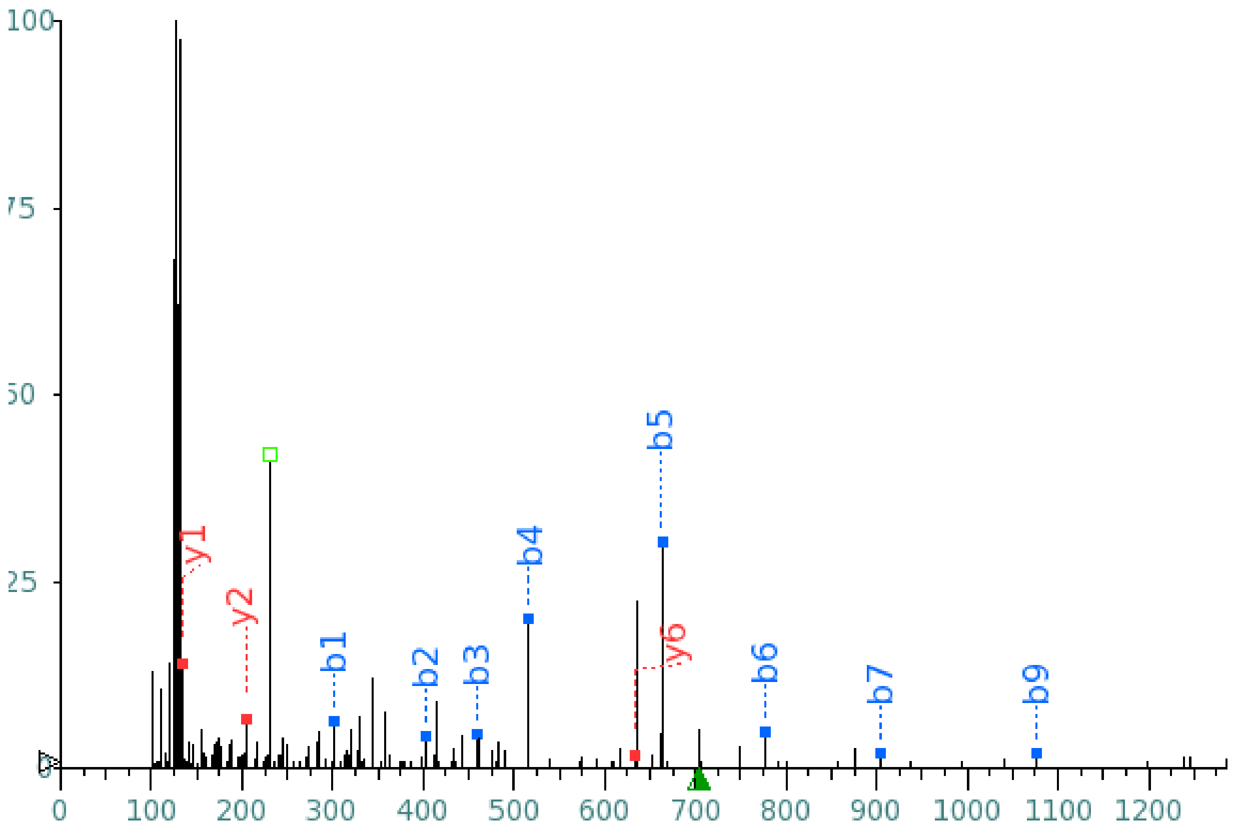

ATGGFLQGDEAD (+2)

Primary Reference: BRAT1_HUMAN
Search ID: 41758
Search Name: 20130328_ananiav_ERstress_TMT_TM_timecourse_chymo_inj1_4MC_IAA
Scan#: 12913
Observed Mass: 705.3409 (-0.1 ppm)
PSM Score: 24.53

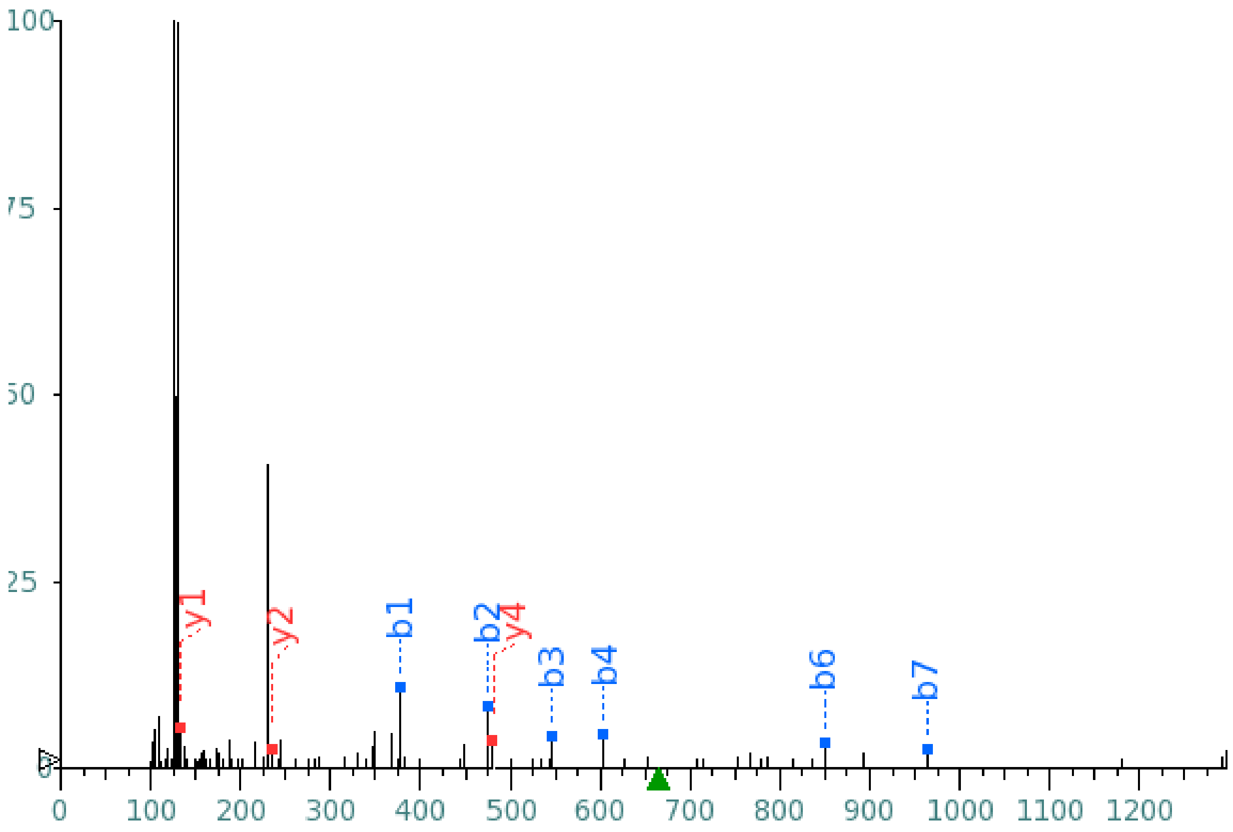

FVAGEDDMTD (+2)

Primary Reference: NXN_HUMAN
Search ID: 41758
Search Name: 20130328_ananiav_ERstress_TMT_TM_timecourse_chymo_inj1_4MC_IAA
Scan#: 13152
Observed Mass: 664.7981 (0.8 ppm)
PSM Score: 30.3

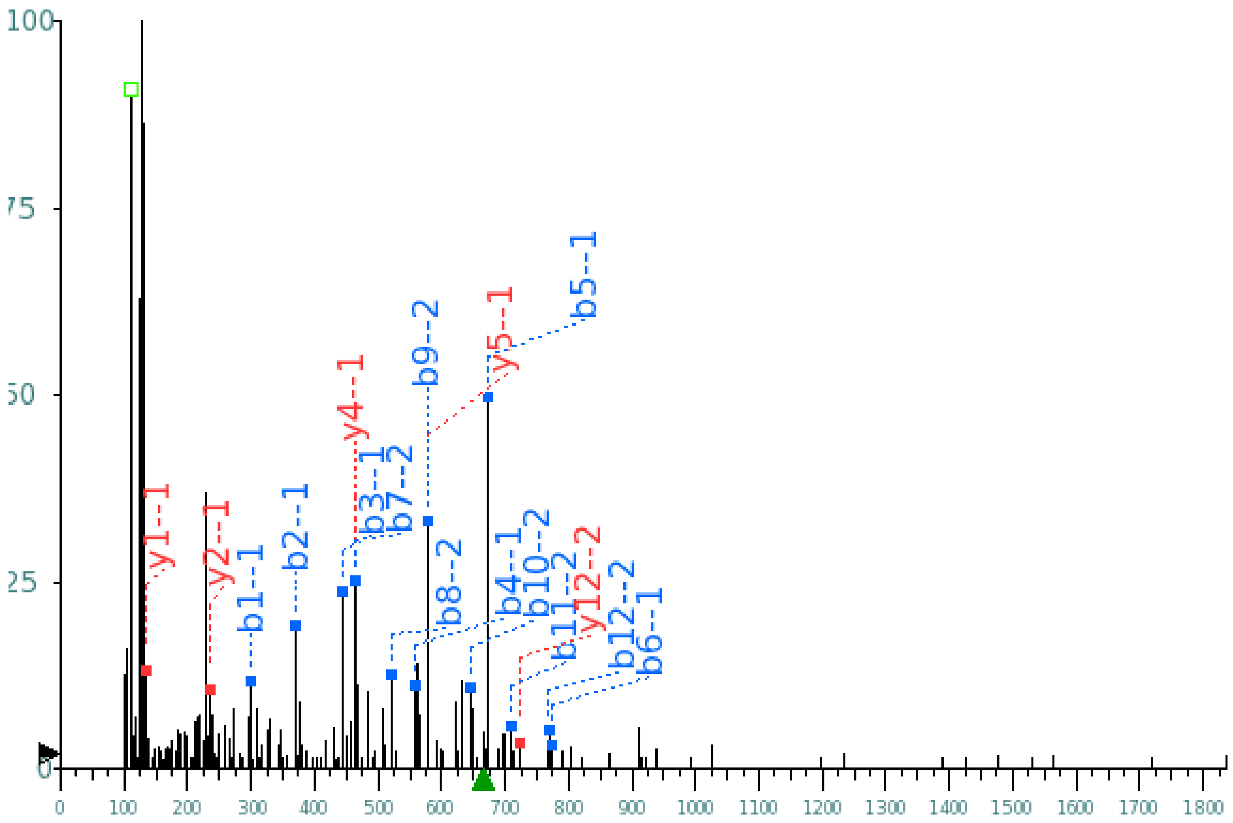

AAAIDTRILHENTQTD (+3)

Primary Reference: C1TM_HUMAN
Search ID: 41758
Search Name: 20130328_ananiav_ERstress_TMT_TM_timecourse_chymo_inj1_4MC_IAA
Scan#: 13158
Observed Mass: 666.6875 (1.3 ppm)
PSM Score: 28.77

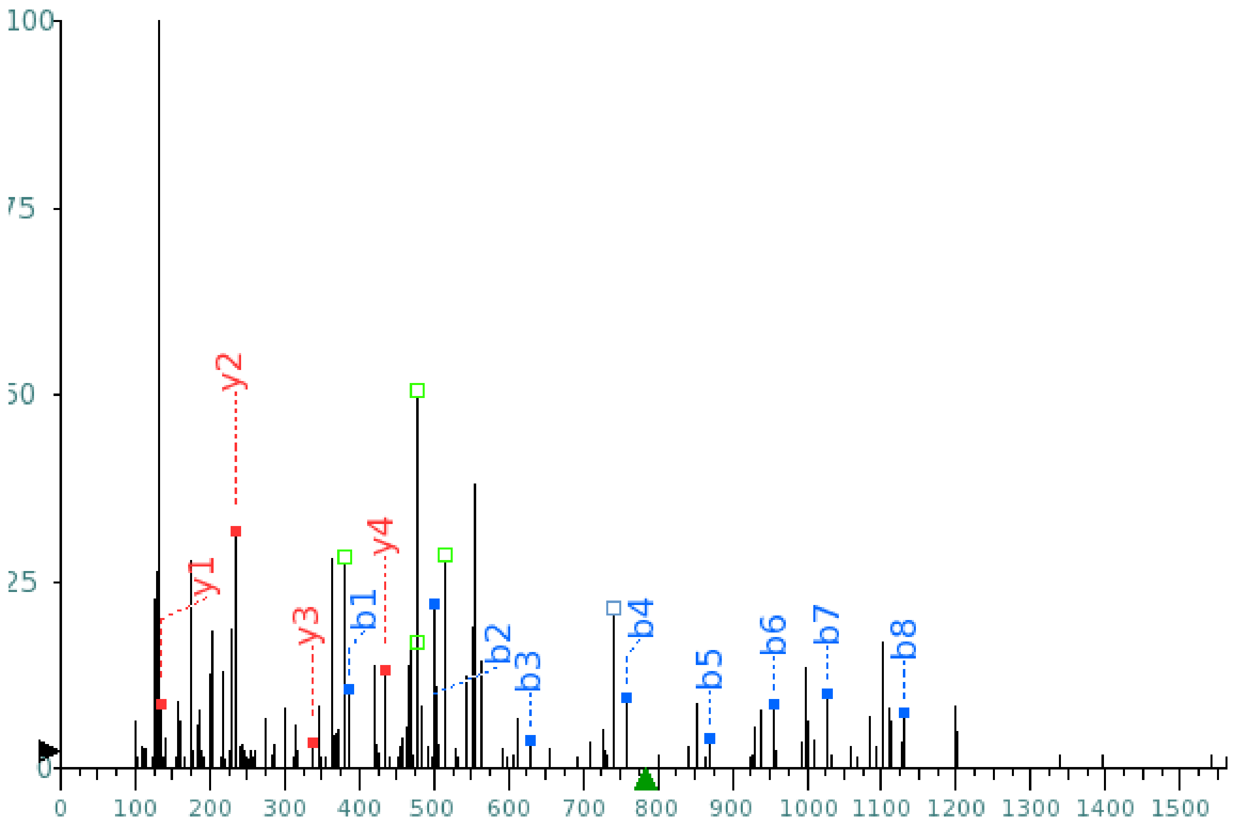

RIQELSATVTTD (+2)

Primary Reference: TRIP6_HUMAN
Search ID: 41758
Search Name: 20130328_ananiav_ERstress_TMT_TM_timecourse_chymo_inj1_4MC_IAA
Scan#: 13173
Observed Mass: 781.934 (1.3 ppm)
PSM Score: 29.04

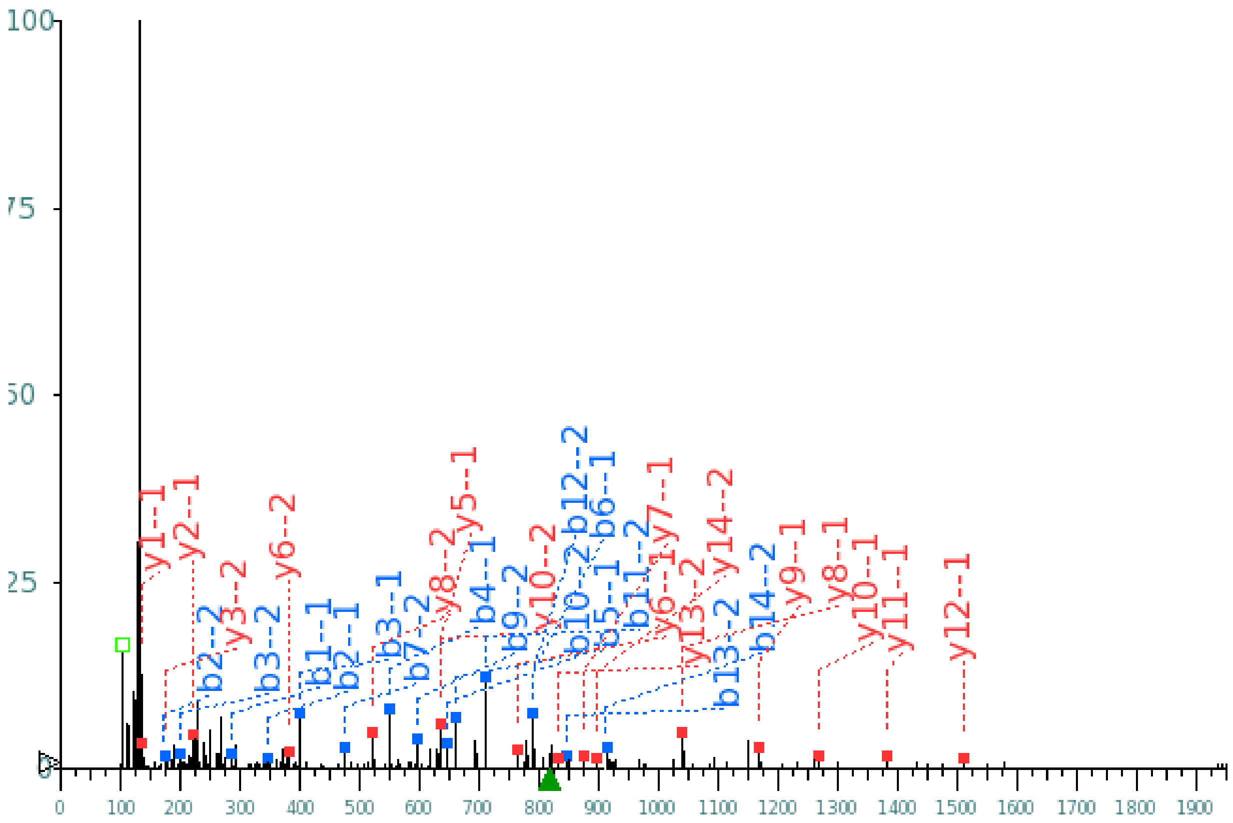

GGGFNERENVEYIEREESD (+3)

Primary Reference: ZRAB2_HUMAN
Search ID: 41758
Search Name: 20130328_ananiav_ERstress_TMT_TM_timecourse_chymo_inj1_4MC_IAA
Scan#: 13179
Observed Mass: 820.0488 (-0 ppm)
PSM Score: 29.23

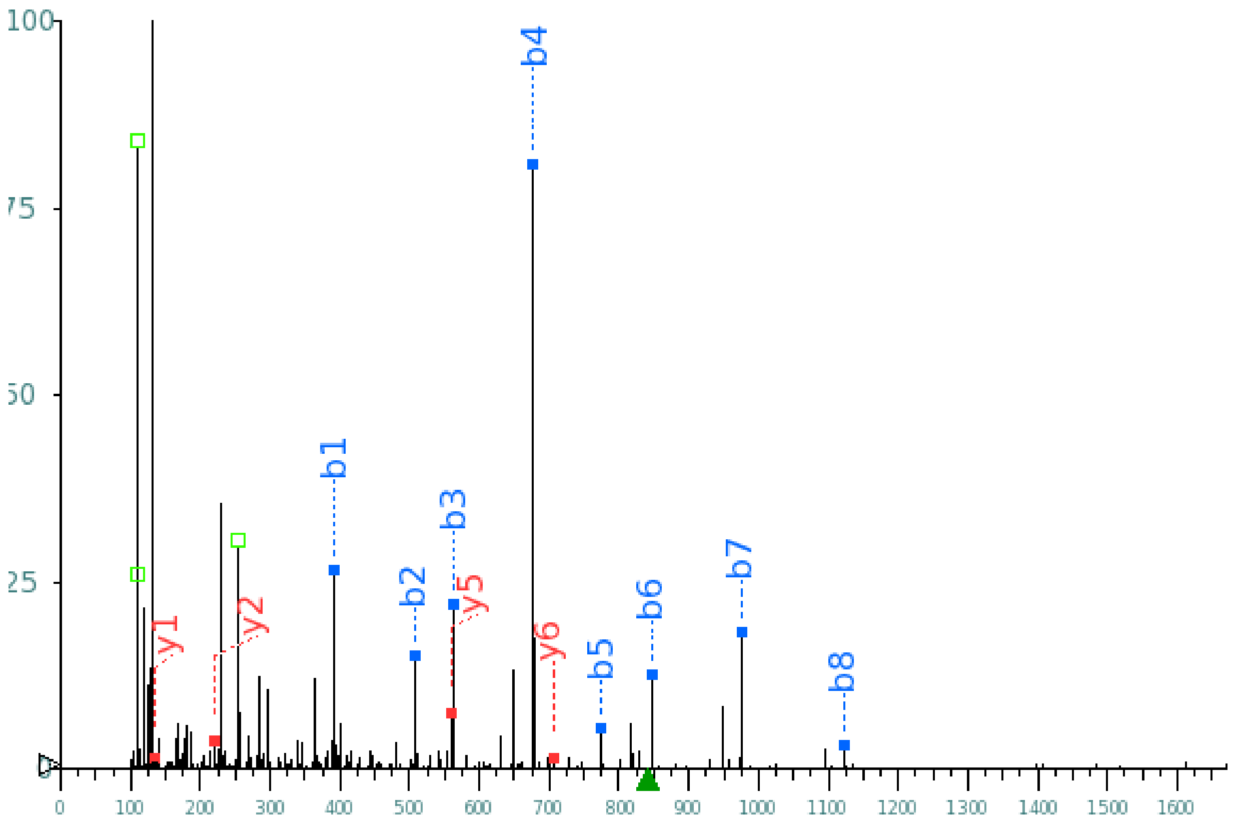

YLGNPAEFHDSSD (+2)

Primary Reference: RCN1_HUMAN
Search ID: 41758
Search Name: 20130328_ananiav_ERstress_TMT_TM_timecourse_chymo_inj1_4MC_IAA
Scan#: 13235
Observed Mass: 840.8889 (0.1 ppm)
PSM Score: 38.1

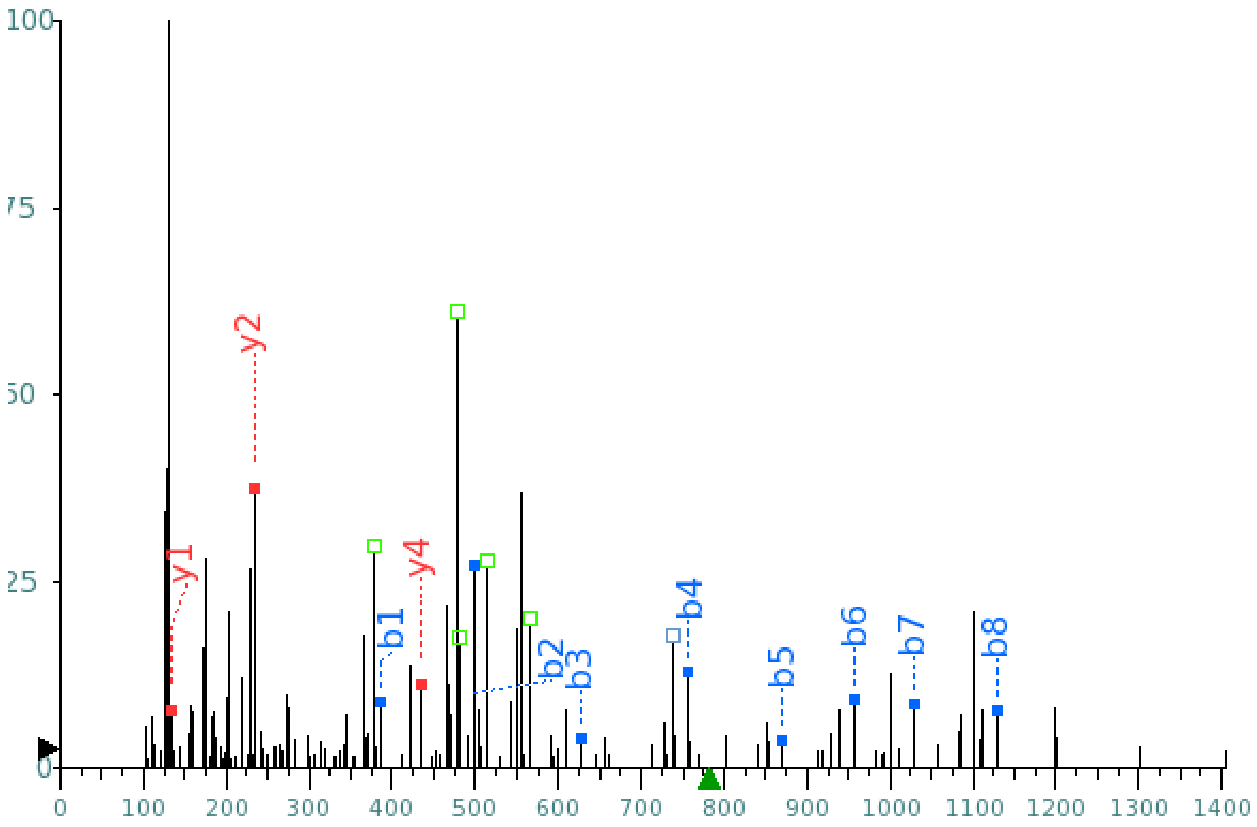

RIQELSATVTTD (+2)

Primary Reference: TRIP6_HUMAN
Search ID: 41758
Search Name: 20130328_ananiav_ERstress_TMT_TM_timecourse_chymo_inj1_4MC_IAA
Scan#: 13283
Observed Mass: 781.9354 (3 ppm)
PSM Score: 23.54

File name: 2015-02-04 04.01.53PM.pdf

Creation time: 2015-02-04 04:09PM

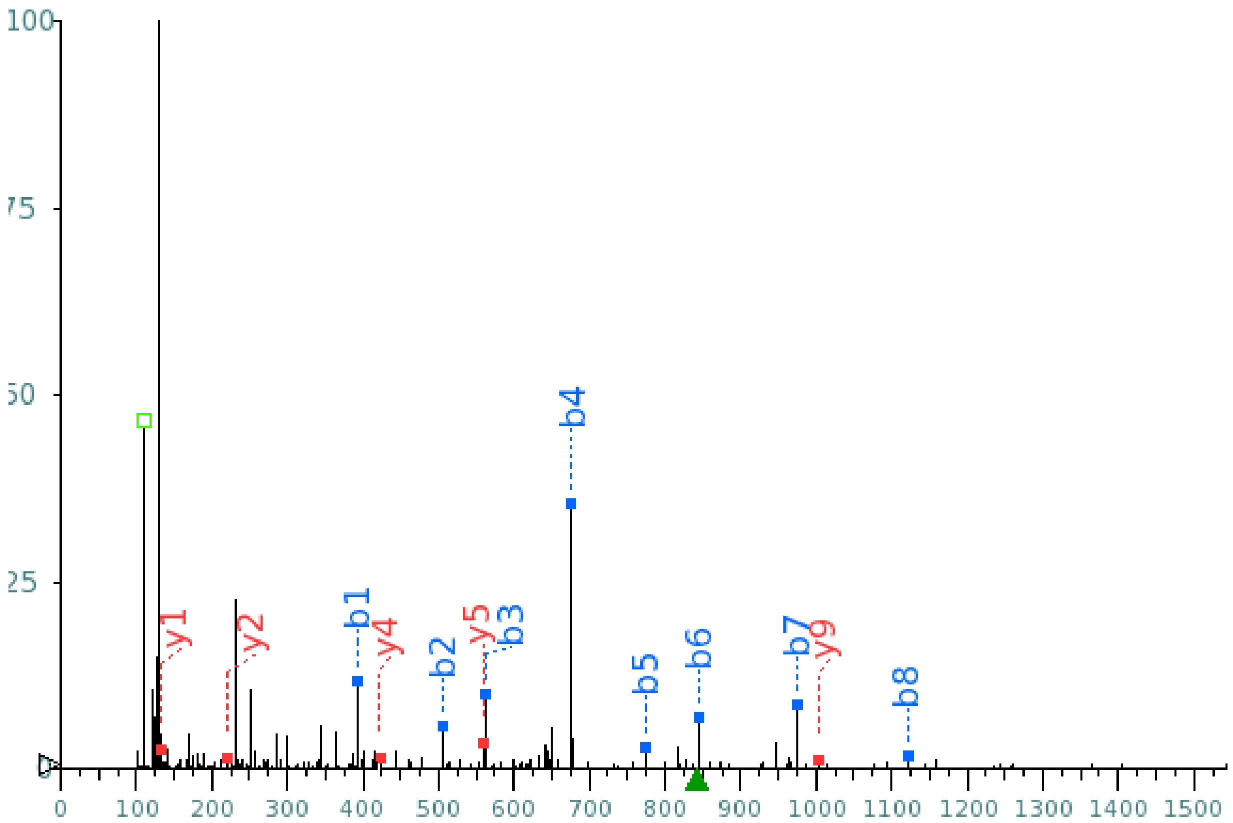

YLGNPAEFHDSSD (+2)

Primary Reference: RCN1_HUMAN
Search ID: 41758
Search Name: 20130328_ananiav_ERstress_TMT_TM_timecourse_chymo_inj1_4MC_IAA
Scan#: 13328
Observed Mass: 840.8888 (-0 ppm)
PSM Score: 37.05

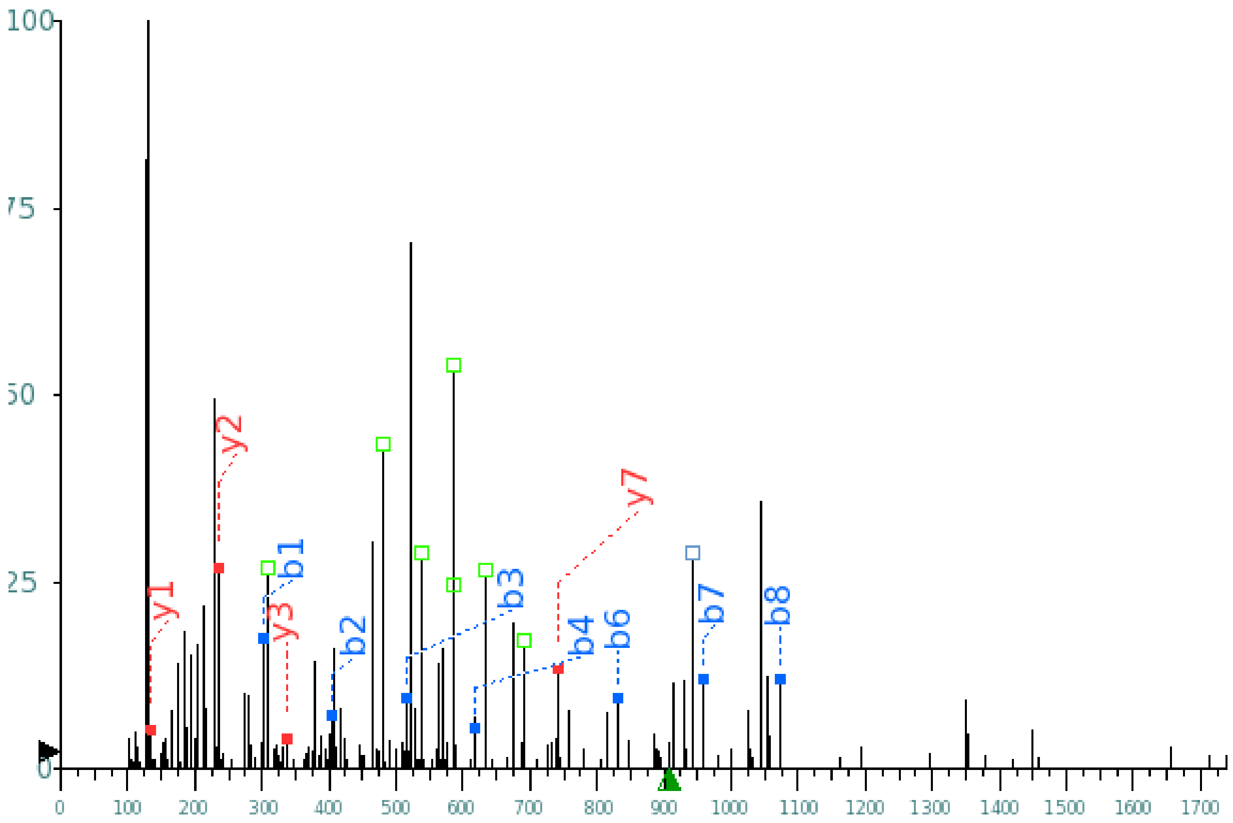

ATNTRGQIPPLVTTD (+2)

Primary Reference: SC24D_HUMAN
Search ID: 41758
Search Name: 20130328_ananiav_ERstress_TMT_TM_timecourse_chymo_inj1_4MC_IAA
Scan#: 13356
Observed Mass: 907.005 (0.6 ppm)
PSM Score: 29.22

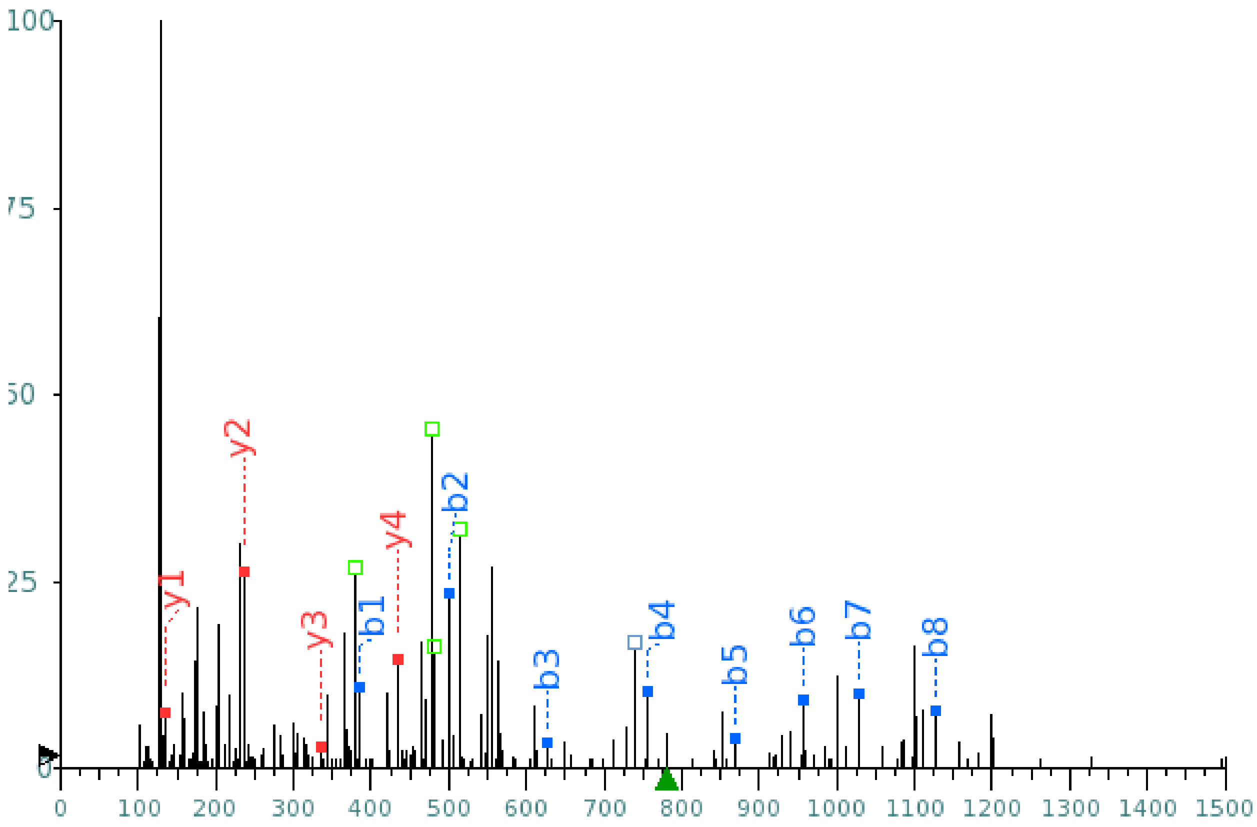

RIQELSATVTTD (+2)

Primary Reference: TRIP6_HUMAN
Search ID: 41758
Search Name: 20130328_ananiav_ERstress_TMT_TM_timecourse_chymo_inj1_4MC_IAA
Scan#: 13399
Observed Mass: 781.9339 (1.2 ppm)
PSM Score: 26.21

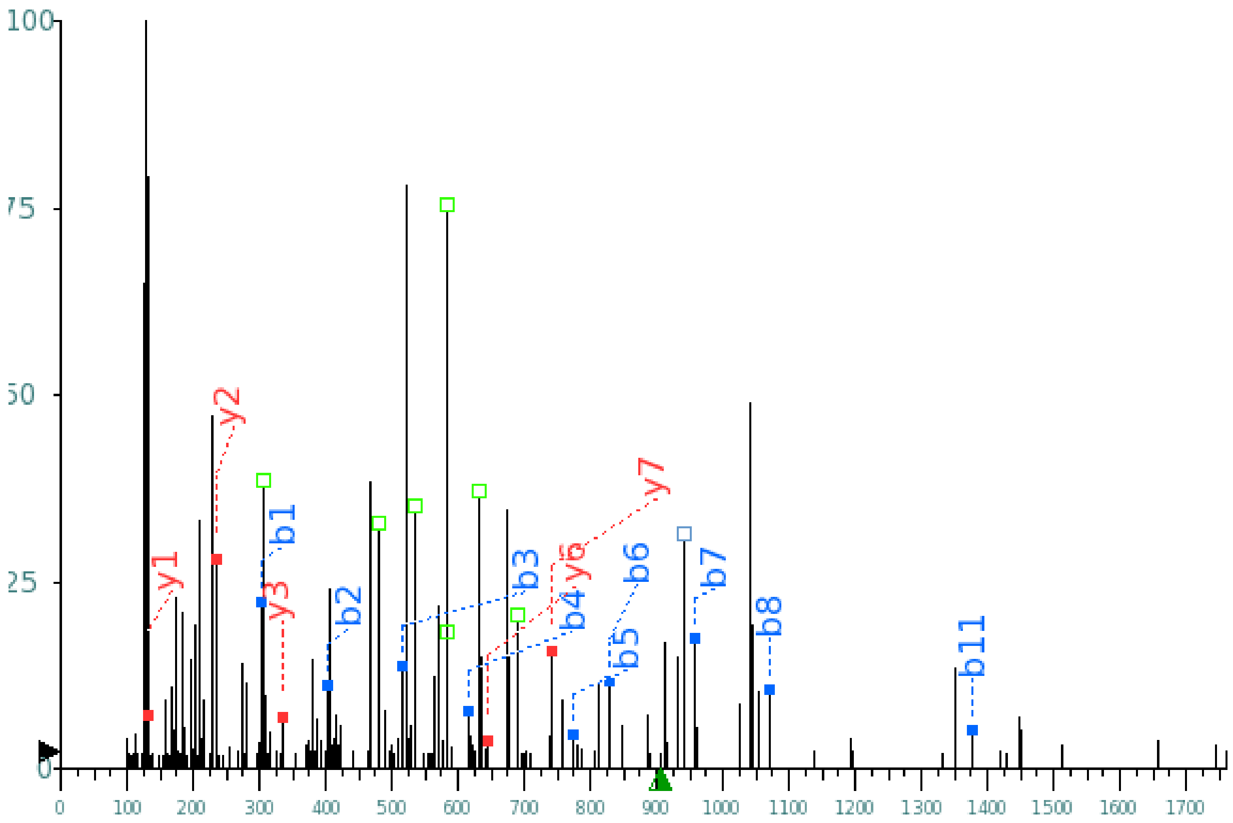

ATNTRGQIPPLVTTD (+2)

Primary Reference: SC24D_HUMAN
Search ID: 41758
Search Name: 20130328_ananiav_ERstress_TMT_TM_timecourse_chymo_inj1_4MC_IAA
Scan#: 13446
Observed Mass: 907.0053 (0.9 ppm)
PSM Score: 28.34

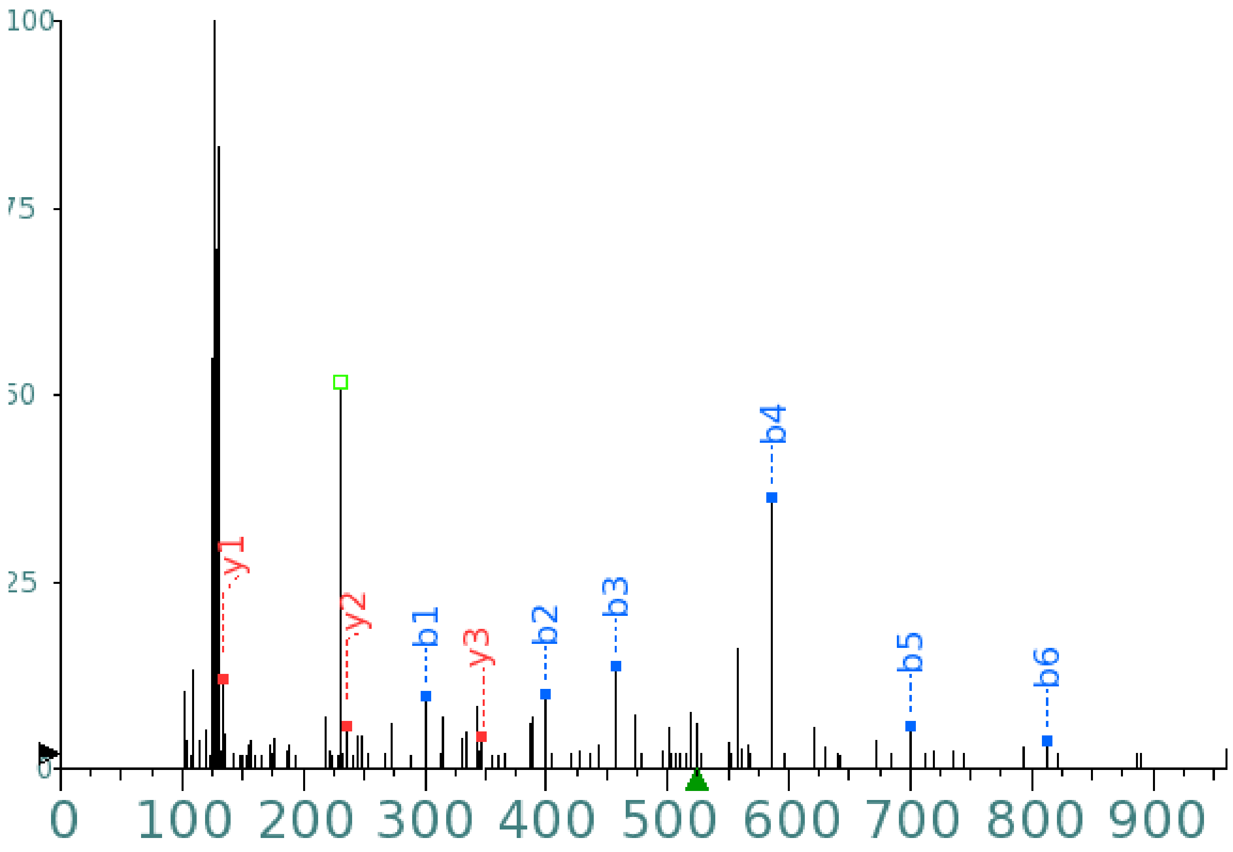

AVGEIITD (+2)

Primary Reference: VRK1_HUMAN
Search ID: 41758
Search Name: 20130328_ananiav_ERstress_TMT_TM_timecourse_chymo_inj1_4MC_IAA
Scan#: 13622
Observed Mass: 523.8013 (2.1 ppm)
PSM Score: 26.78

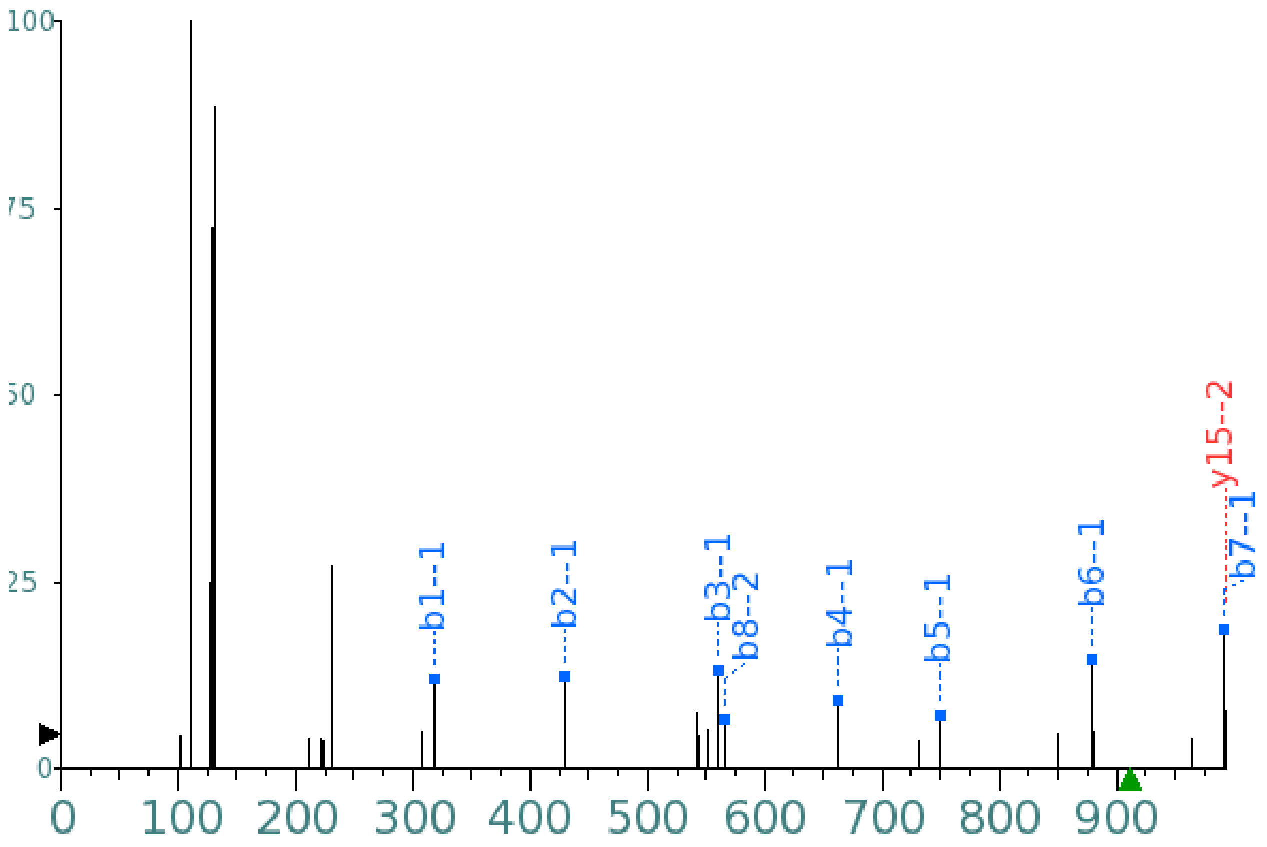

SIMTSENHLNNSDKEVDEVD (+3)

Primary Reference: FERM2_HUMAN
Search ID: 41758
Search Name: 20130328_ananiav_ERstress_TMT_TM_timecourse_chymo_inj1_4MC_IAA
Scan#: 13640
Observed Mass: 912.1147 (1.9 ppm)
PSM Score: 45.45

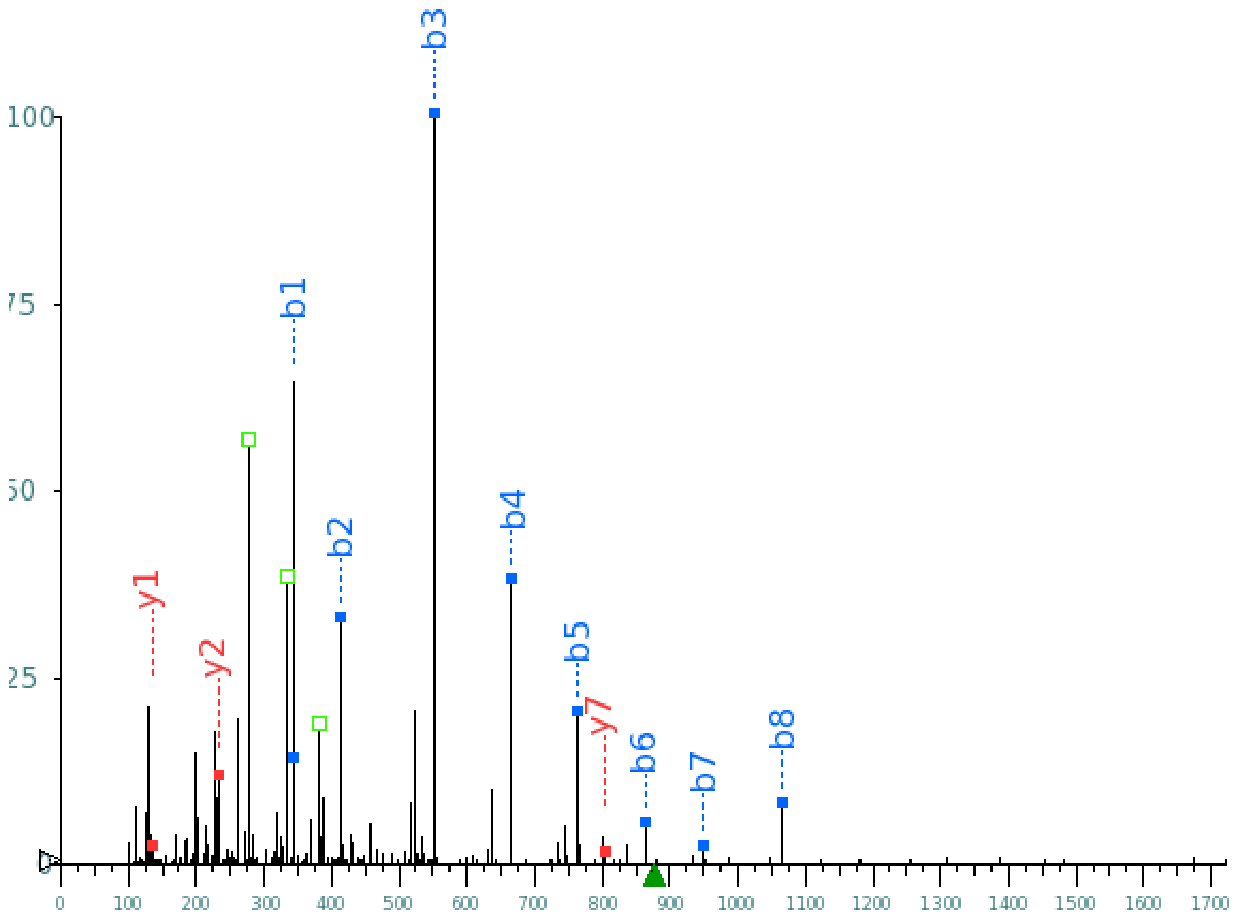

NAHIVVSDPEDITD (+2)

Primary Reference: PKN2_HUMAN
Search ID: 41758
Search Name: 20130328_ananiav_ERstress_TMT_TM_timecourse_chymo_inj1_4MC_IAA
Scan#: 13822
Observed Mass: 877.445 (1.2 ppm)
PSM Score: 35.42

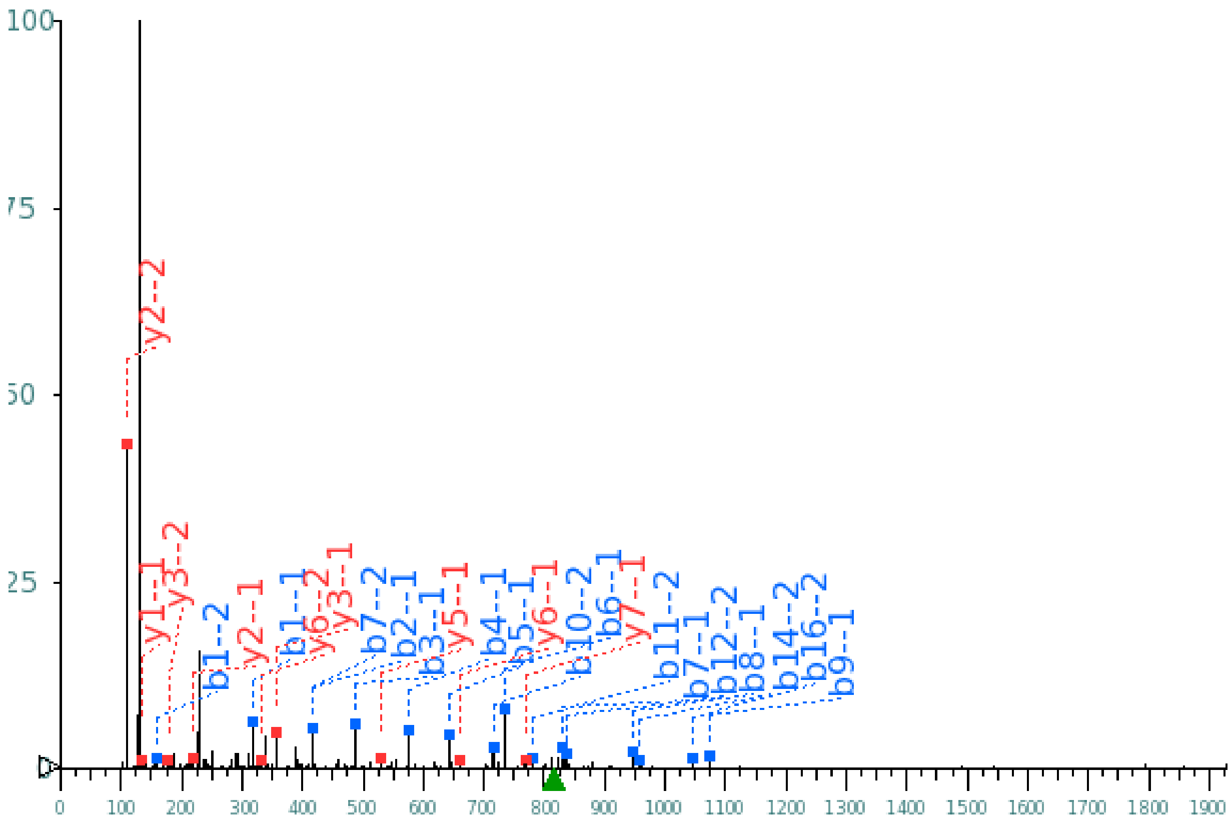

SVASASVIEKQNLEGDHSD (+3)

Primary Reference: HS74L_HUMAN
Search ID: 41758
Search Name: 20130328_ananiav_ERstress_TMT_TM_timecourse_chymo_inj1_4MC_IAA
Scan#: 13886
Observed Mass: 815.4279 (1 ppm)
PSM Score: 43.19

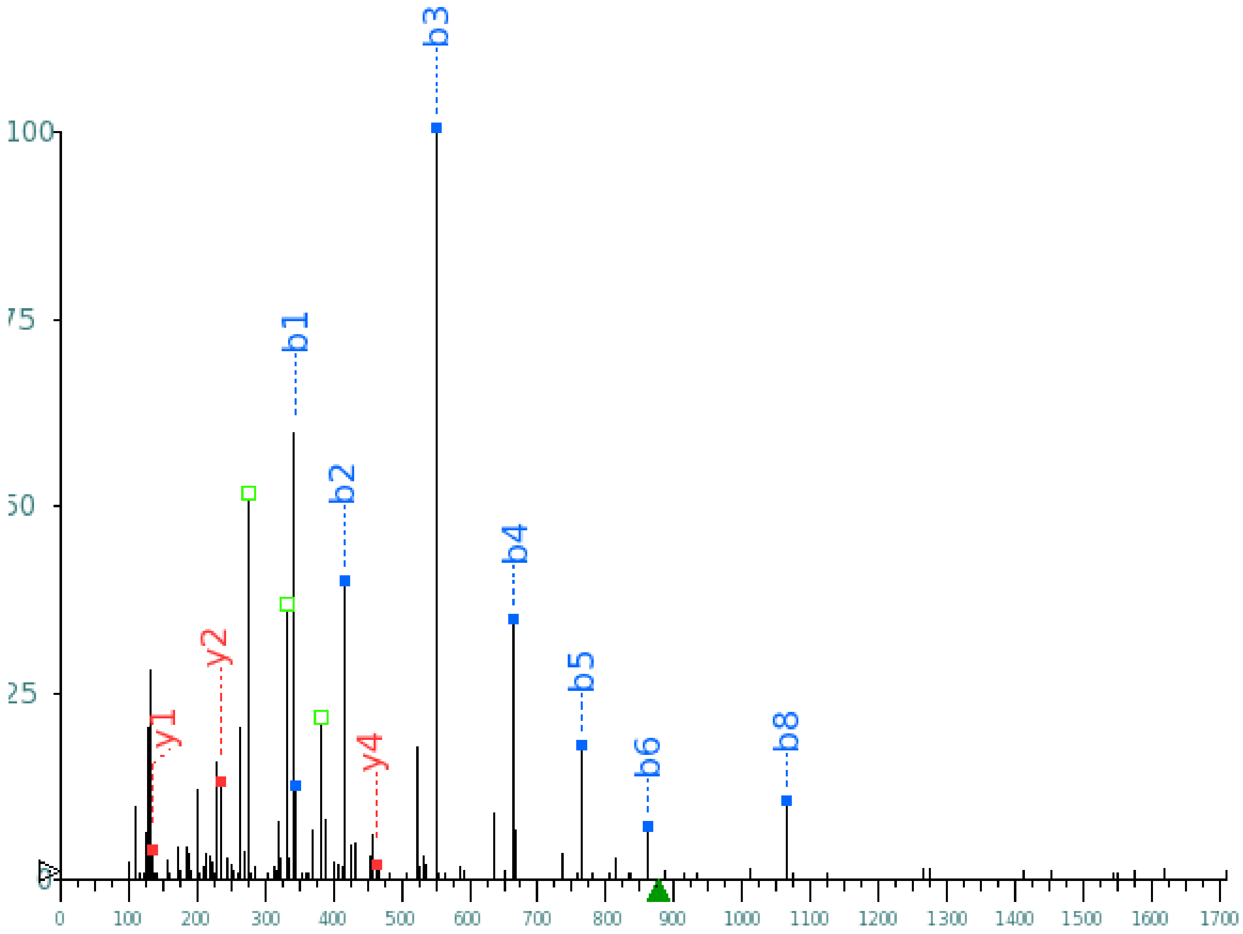

NAHIVVSDPEDITD (+2)

Primary Reference: PKN2_HUMAN
Search ID: 41758
Search Name: 20130328_ananiav_ERstress_TMT_TM_timecourse_chymo_inj1_4MC_IAA
Scan#: 13922
Observed Mass: 877.4445 (0.6 ppm)
PSM Score: 24.93

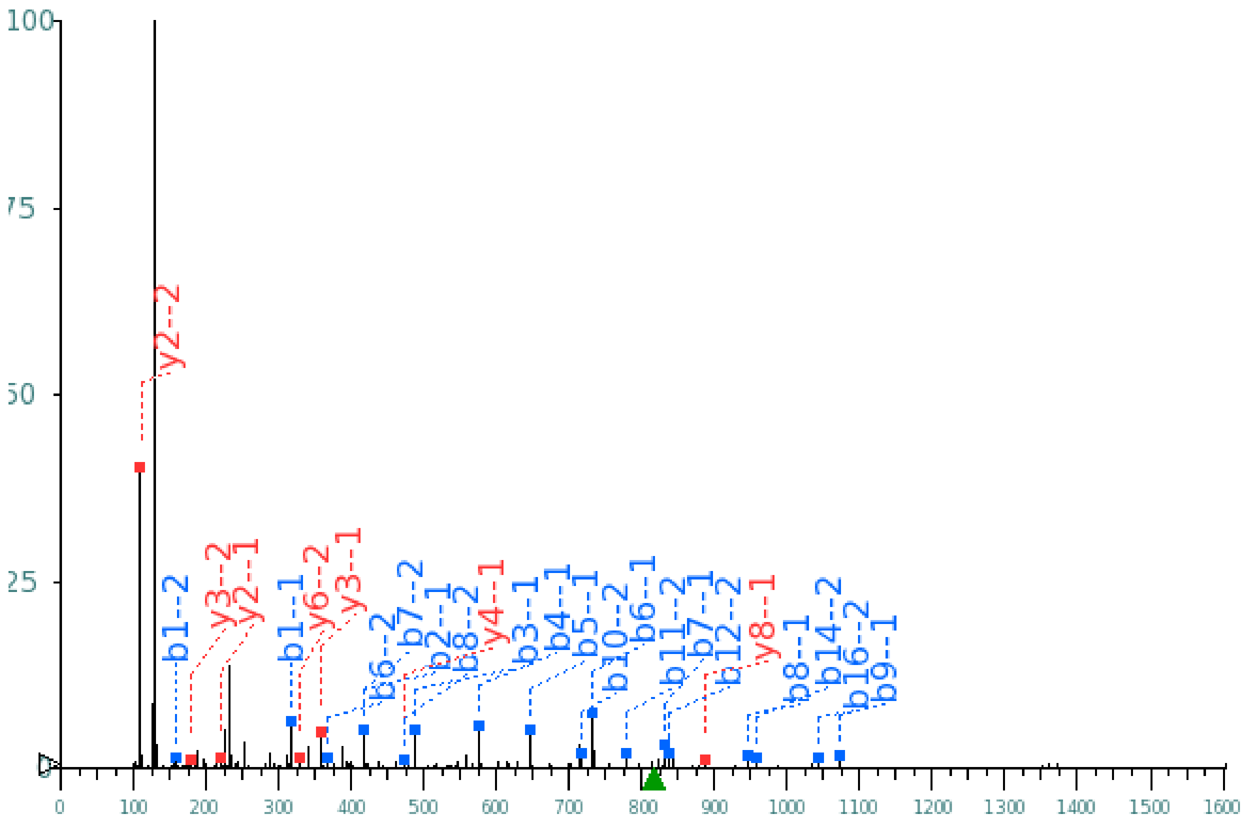

SVASASVIEKQNLEGDHSD (+3)

Primary Reference: HS74L_HUMAN
Search ID: 41758
Search Name: 20130328_ananiav_ERstress_TMT_TM_timecourse_chymo_inj1_4MC_IAA
Scan#: 14008
Observed Mass: 815.4274 (0.3 ppm)
PSM Score: 51.26

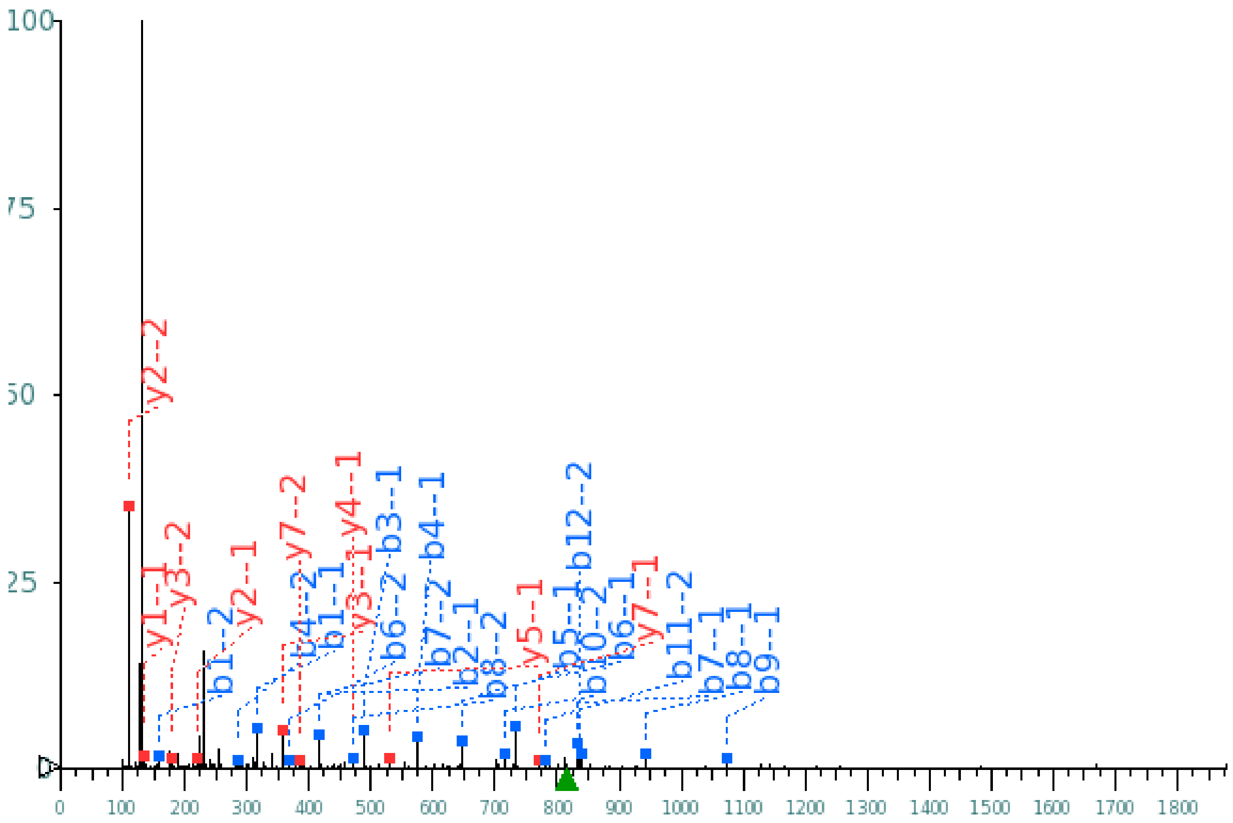

SVASASVIEKQNLEGDHSD (+3)

Primary Reference: HS74L_HUMAN
Search ID: 41758
Search Name: 20130328_ananiav_ERstress_TMT_TM_timecourse_chymo_inj1_4MC_IAA
Scan#: 14134
Observed Mass: 815.4278 (0.8 ppm)
PSM Score: 38.31

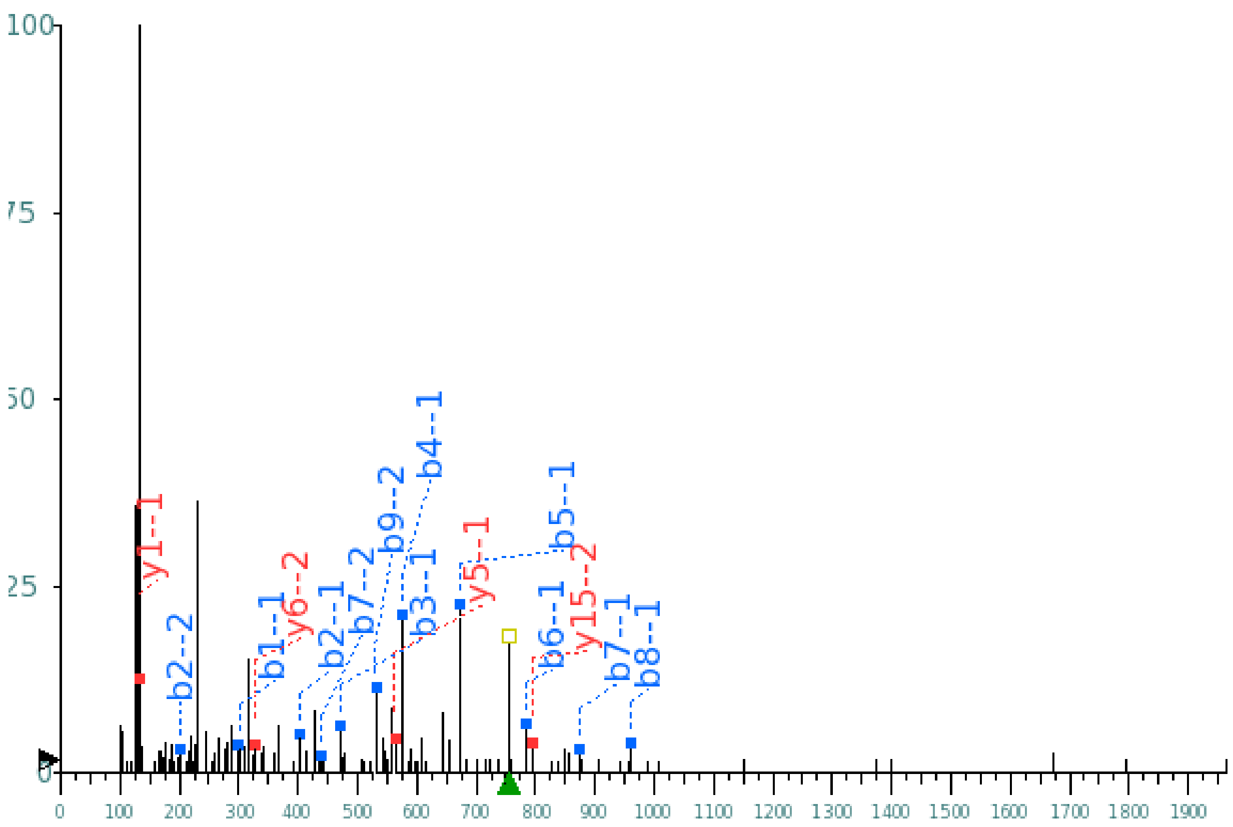

ATATVISSPSTEELSQDQGD (+3)

Primary Reference: RPGF2_HUMAN
Search ID: 41758
Search Name: 20130328_ananiav_ERstress_TMT_TM_timecourse_chymo_inj1_4MC_IAA
Scan#: 14491
Observed Mass: 755.7031 (0.7 ppm)
PSM Score: 27.66

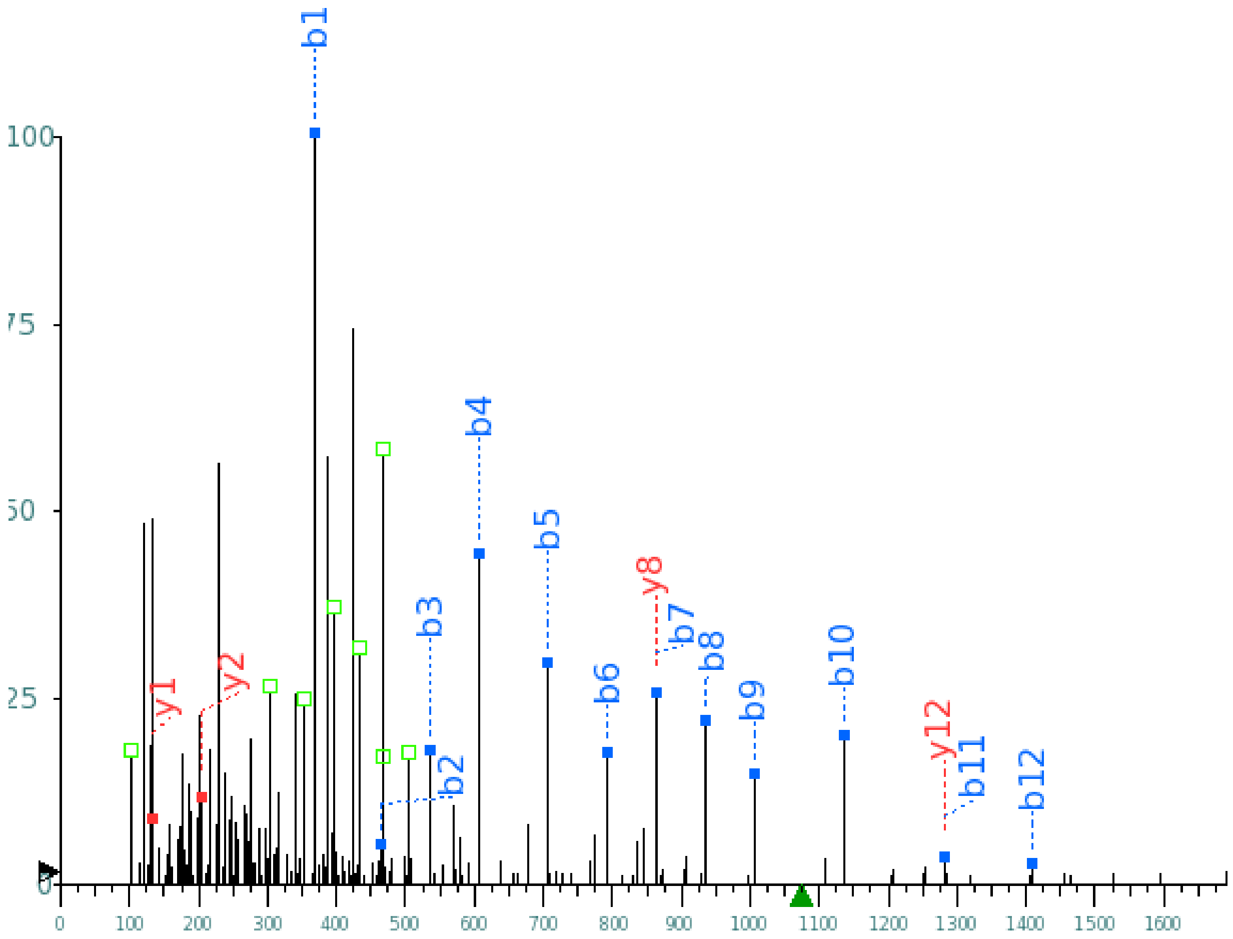

HPAAVSAAAEFENAESEAD (+2)

Primary Reference: C170L_HUMAN
Search ID: 41758
Search Name: 20130328_ananiav_ERstress_TMT_TM_timecourse_chymo_inj1_4MC_IAA
Scan#: 14505
Observed Mass: 1073.0005 (0.2 ppm)
PSM Score: 88.6

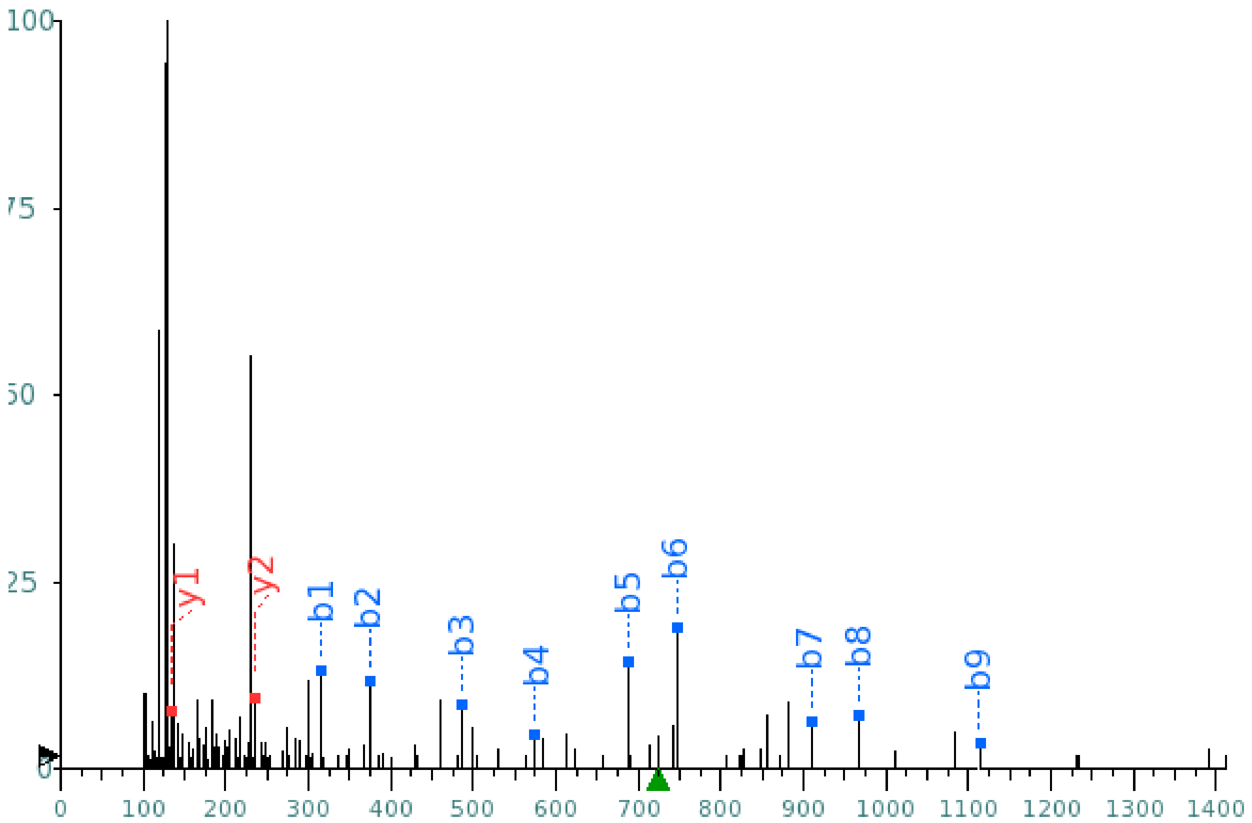

SGLSDGYGFTTD (+2)

Primary Reference: HNRPF_HUMAN
Search ID: 41758
Search Name: 20130328_ananiav_ERstress_TMT_TM_timecourse_chymo_inj1_4MC_IAA
Scan#: 14594
Observed Mass: 724.8405 (-0.4 ppm)
PSM Score: 44.12

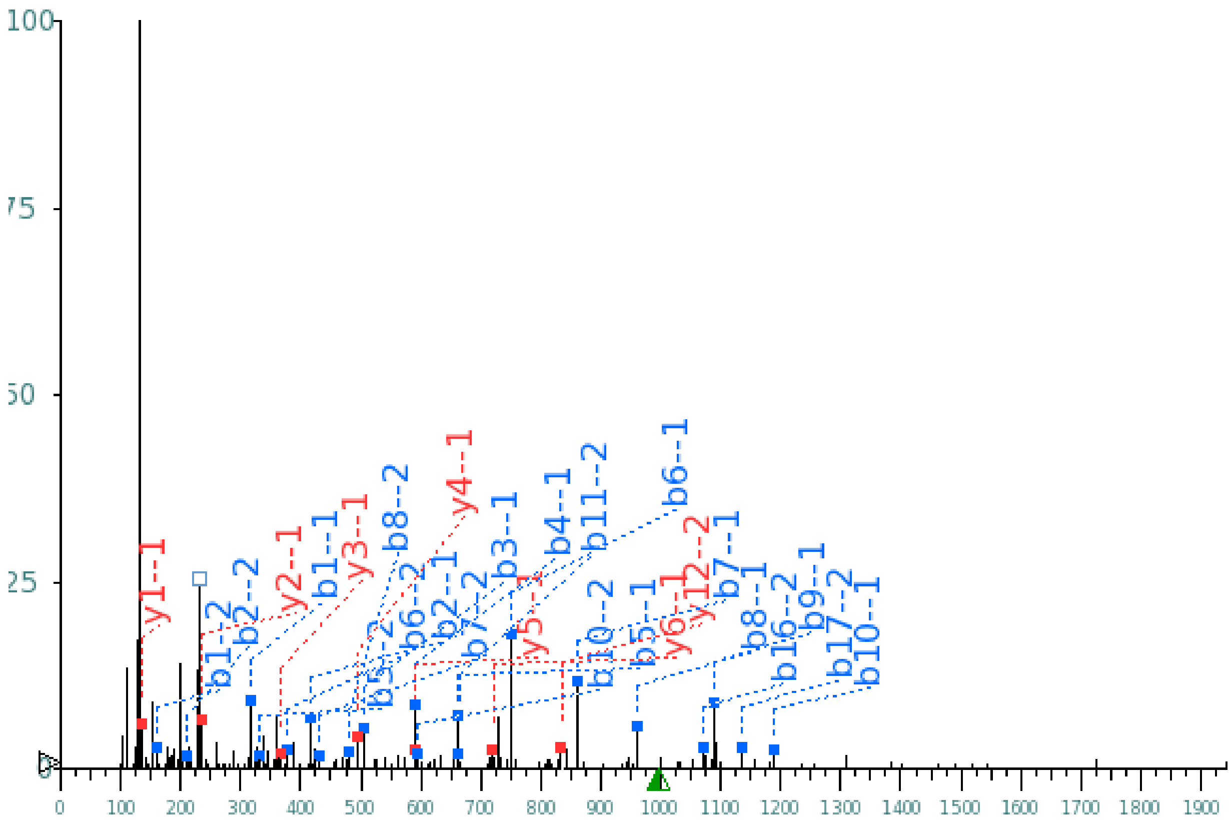

SVSSASLVEVHKSEENEEPMETD (+3)

Primary Reference: HSP74_HUMAN
Search ID: 41758
Search Name: 20130328_ananiav_ERstress_TMT_TM_timecourse_chymo_inj1_4MC_IAA
Scan#: 14627
Observed Mass: 997.8215 (-0.1 ppm)
PSM Score: 45.33

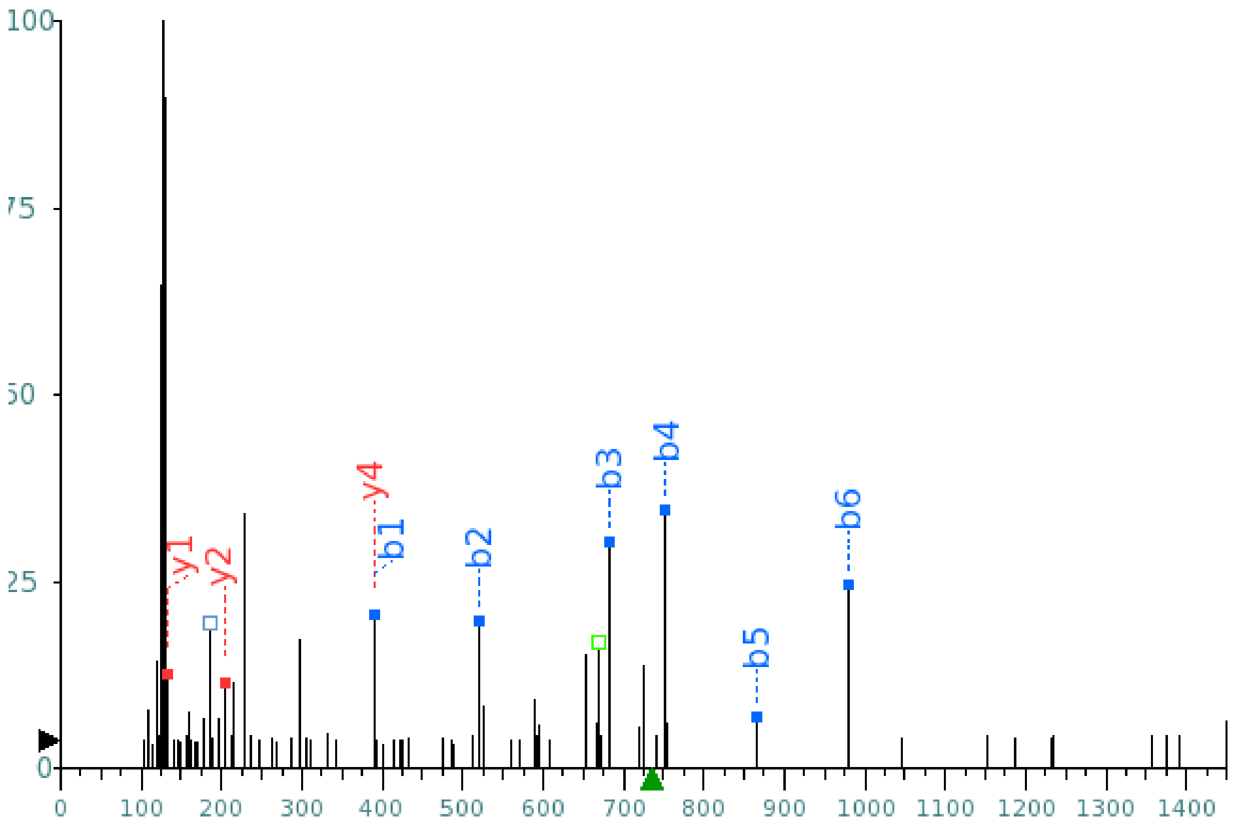

C*MC*ALDVDAAD (+2)

Primary Reference: CTU2_HUMAN
Search ID: 41758
Search Name: 20130328_ananiav_ERstress_TMT_TM_timecourse_chymo_inj1_4MC_IAA
Scan#: 14645
Observed Mass: 735.3175 (0.4 ppm)
PSM Score: 26.6

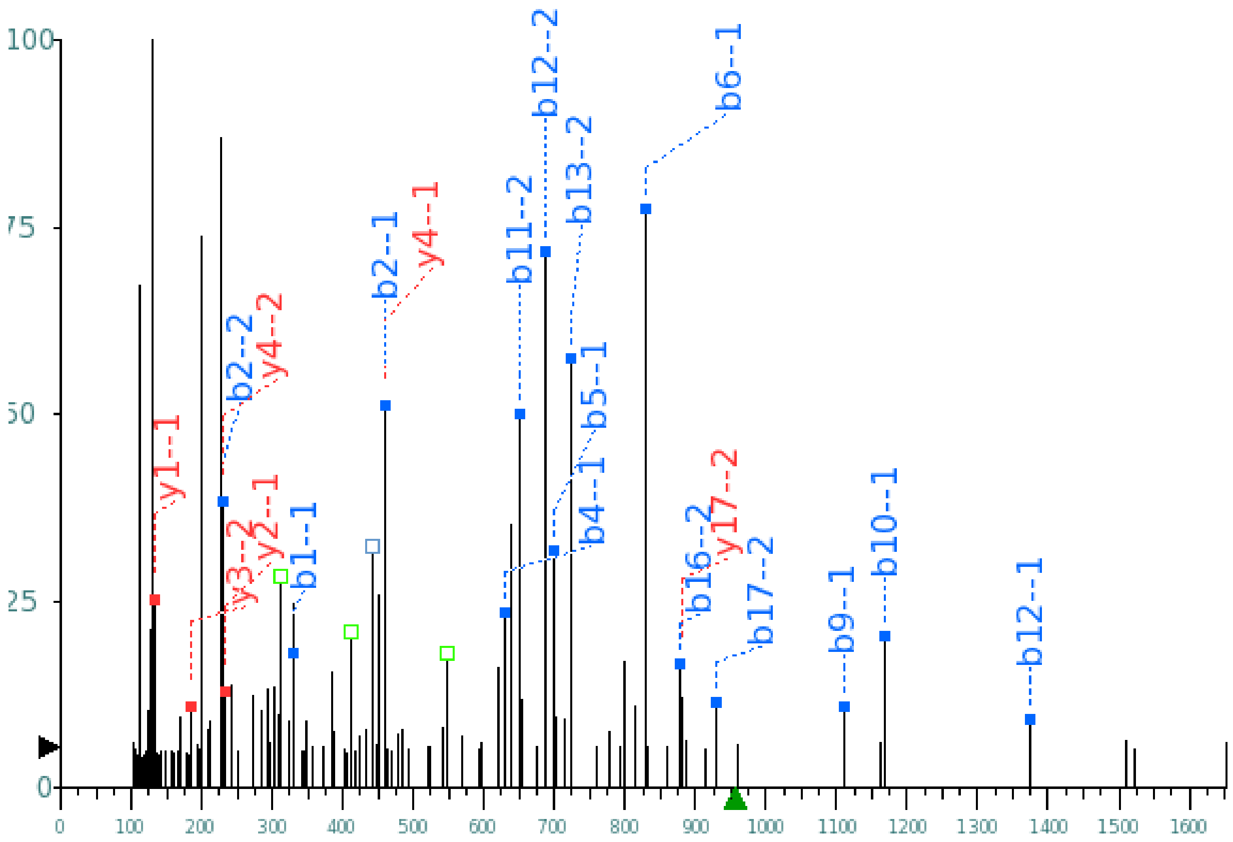

TEPAAQPGEGHAAPTNVVENPLPETD (+3)

Primary Reference: HGS_HUMAN
Search ID: 41758
Search Name: 20130328_ananiav_ERstress_TMT_TM_timecourse_chymo_inj1_4MC_IAA
Scan#: 14837
Observed Mass: 957.4722 (0.5 ppm)
PSM Score: 35.16

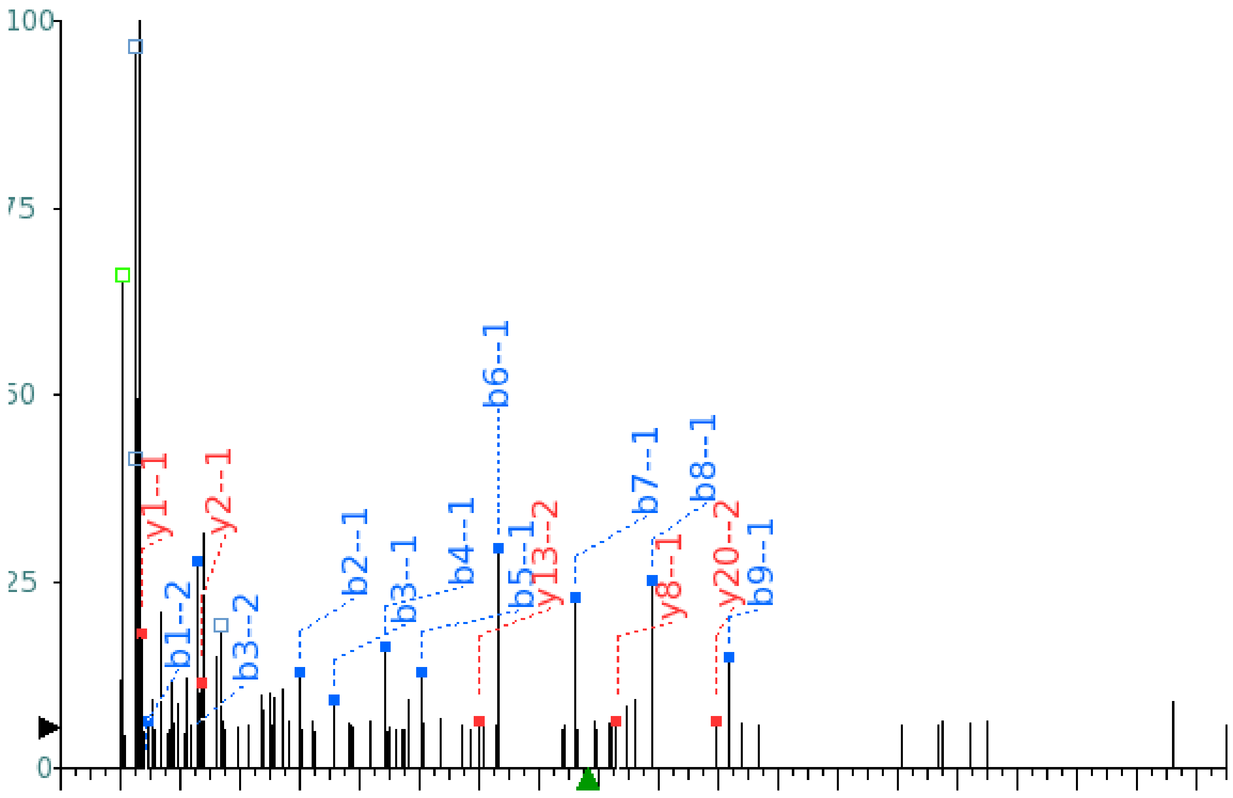

GNGSGEEEEEEDGPAVLVEQQTD (+3)

Primary Reference: GNL1_HUMAN
Search ID: 41758
Search Name: 20130328_ananiav_ERstress_TMT_TM_timecourse_chymo_inj1_4MC_IAA
Scan#: 14856
Observed Mass: 883.0623 (1.1 ppm)
PSM Score: 27.84

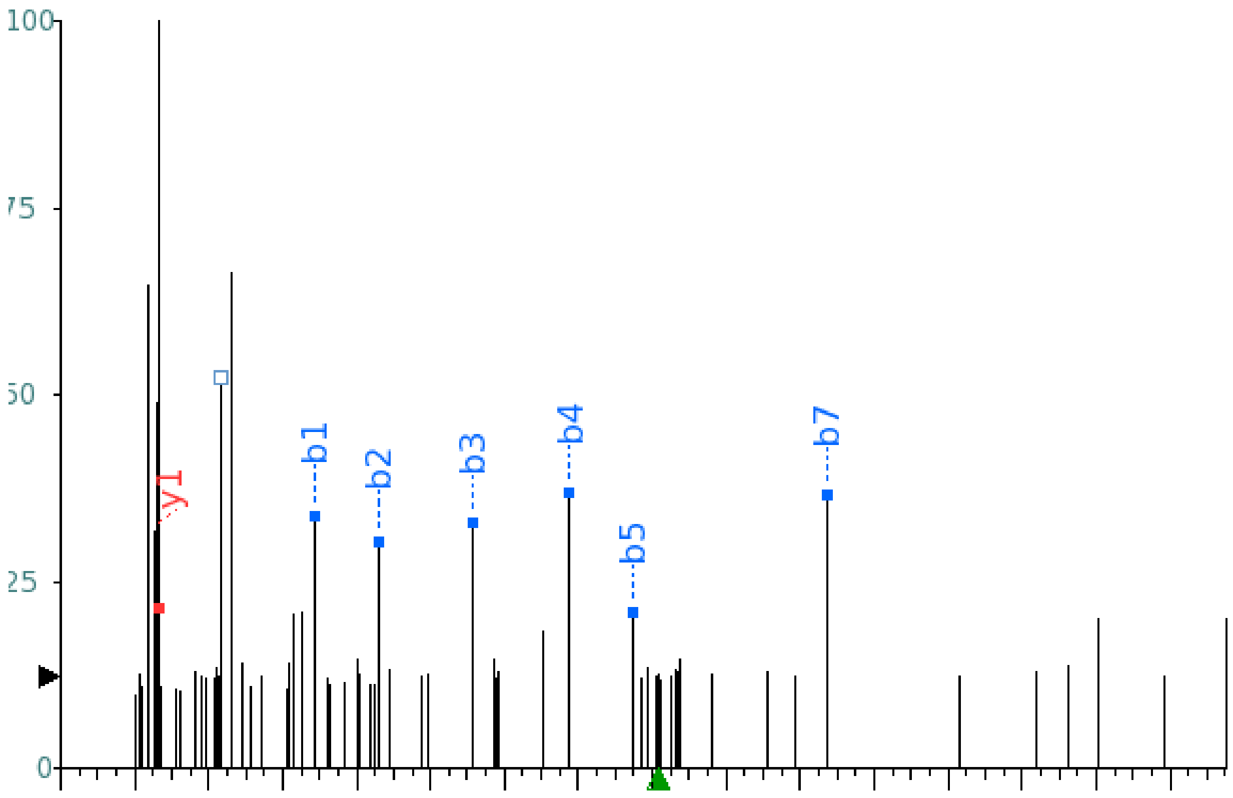

ISQESFDVDETD (+2)

Primary Reference: DFFA_HUMAN
Search ID: 41758
Search Name: 20130328_ananiav_ERstress_TMT_TM_timecourse_chymo_inj1_4MC_IAA
Scan#: 14898
Observed Mass: 807.3735 (1 ppm)
PSM Score: 28.41

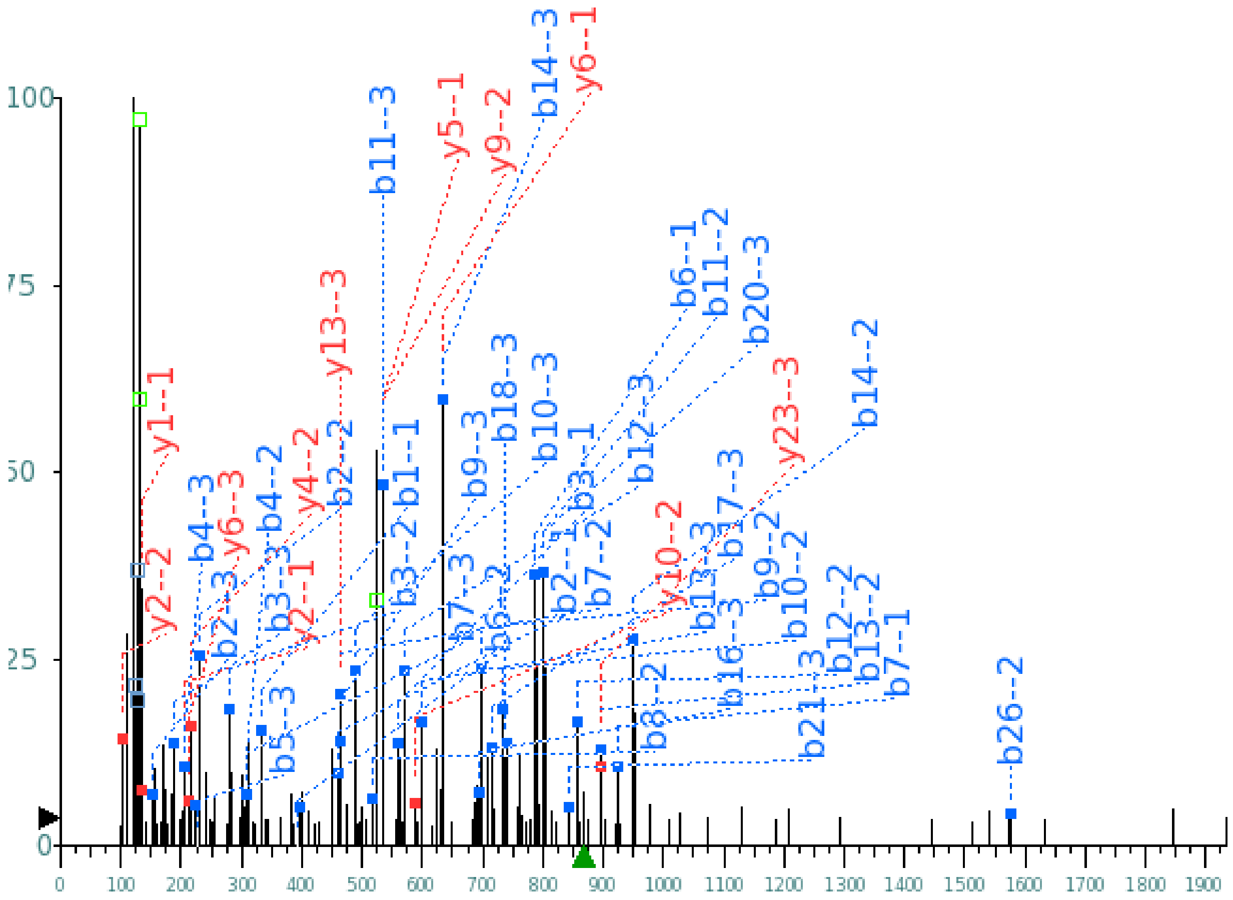

TQVGGDHIKAHIANPSGASTEC*FVTDNAD (+4)

Primary Reference: FLNB_HUMAN
Search ID: 41758
Search Name: 20130328_ananiav_ERstress_TMT_TM_timecourse_chymo_inj1_4MC_IAA
Scan#: 14958
Observed Mass: 868.4314 (0.8 ppm)
PSM Score: 47.29

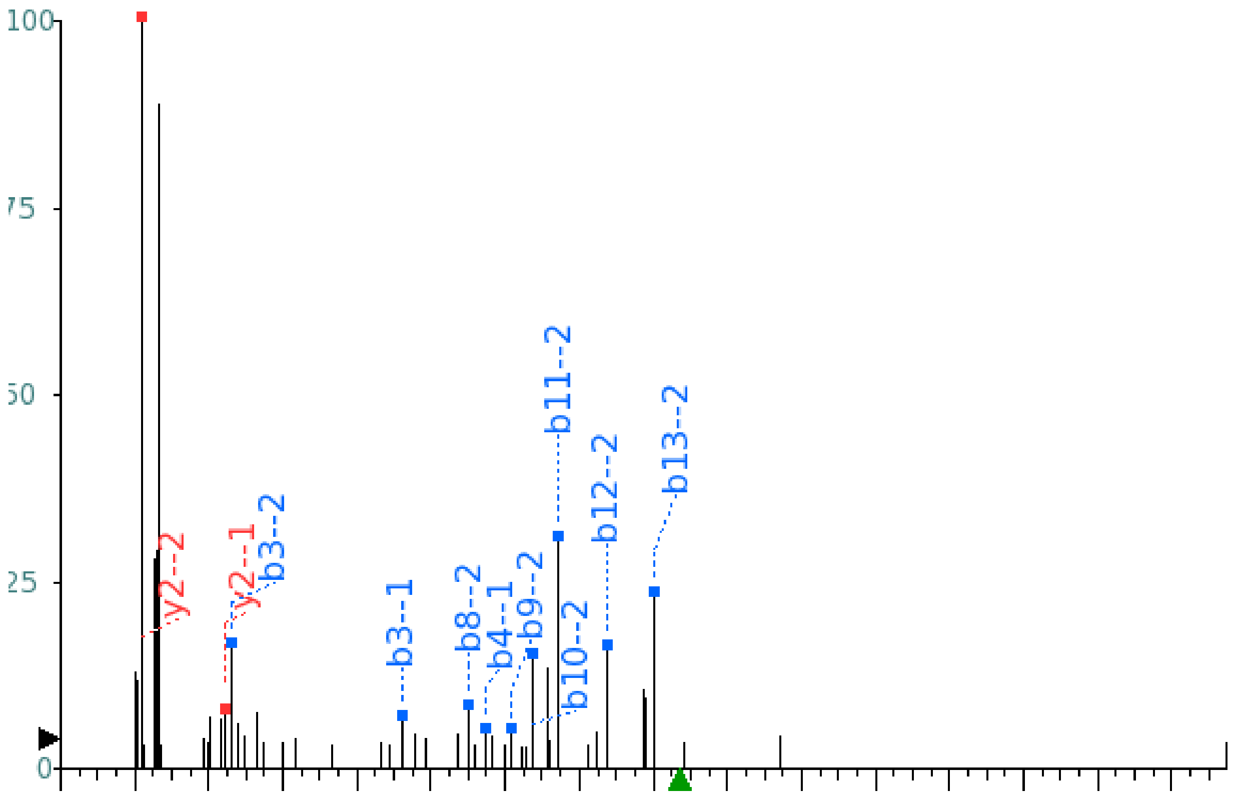

ASAIREELNGAMEHTNSNESD (+3)

Primary Reference: FOXP1_HUMAN
Search ID: 41758
Search Name: 20130328_ananiav_ERstress_TMT_TM_timecourse_chymo_inj1_4MC_IAA
Scan#: 15136
Observed Mass: 835.3886 (-0.3 ppm)
PSM Score: 42.55

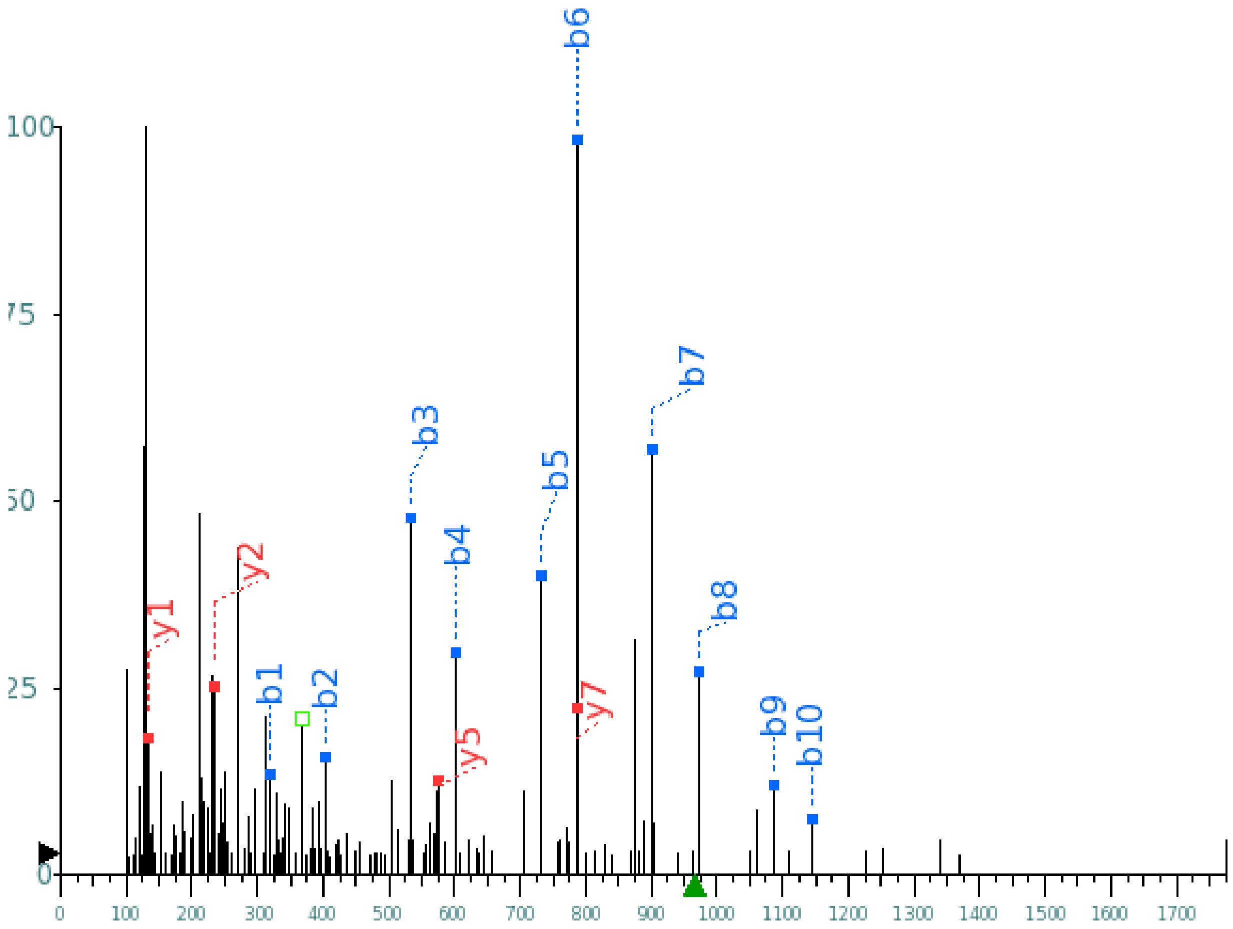

SSQAEGLANGPDVLETD (+2)

Primary Reference: EHMT1_HUMAN
Search ID: 41758
Search Name: 20130328_ananiav_ERstress_TMT_TM_timecourse_chymo_inj1_4MC_IAA
Scan#: 15287
Observed Mass: 966.4763 (3 ppm)
PSM Score: 42.34

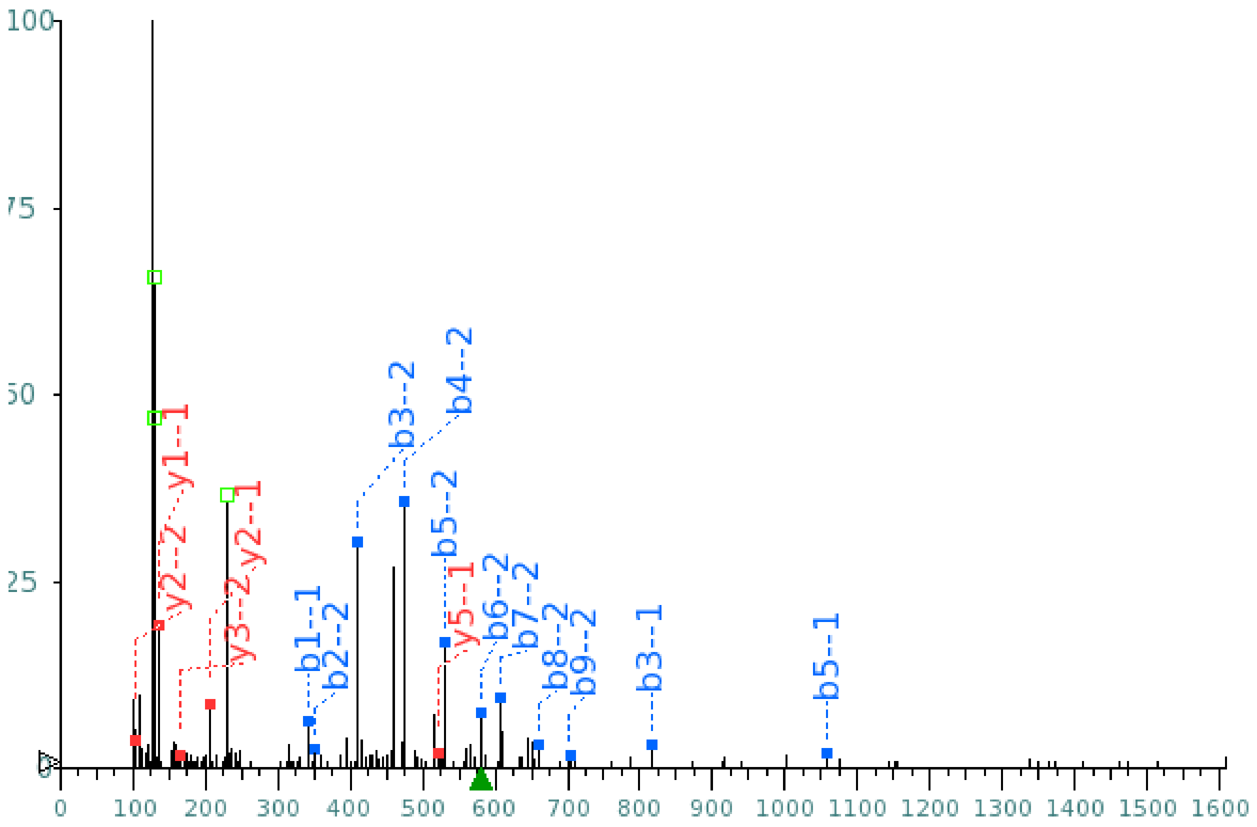

IKNMITGTSQAD (+3)

Primary Reference: EF1A1_HUMAN
Search ID: 41758
Search Name: 20130328_ananiav_ERstress_TMT_TM_timecourse_chymo_inj1_4MC_IAA
Scan#: 15394
Observed Mass: 579.6599 (2 ppm)
PSM Score: 13.28

File name: 2015-02-04 04.01.53PM.pdf

Creation time: 2015-02-04 04:09PM

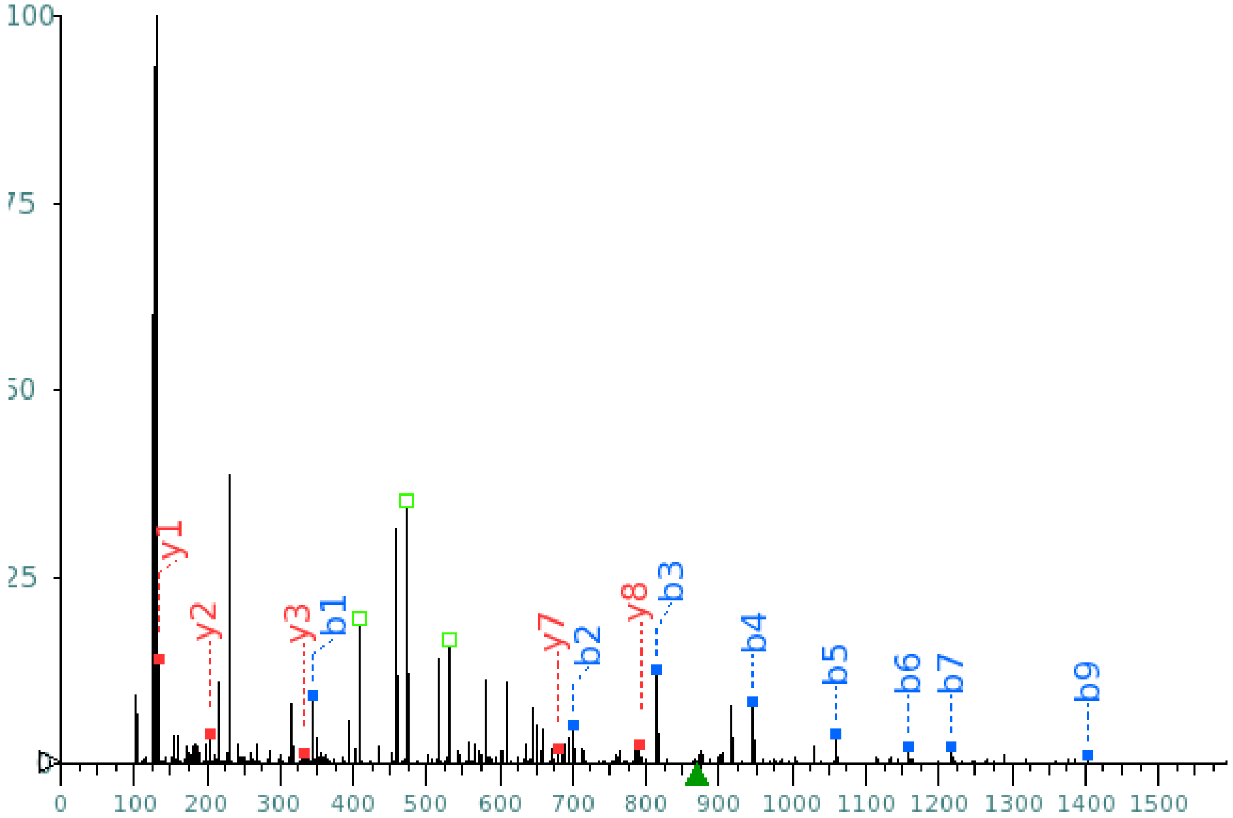

IKNMITGTSQAD (+2)

Primary Reference: EF1A1_HUMAN
Search ID: 41758
Search Name: 20130328_ananiav_ERstress_TMT_TM_timecourse_chymo_inj1_4MC_IAA
Scan#: 15399
Observed Mass: 868.9853 (0.9 ppm)
PSM Score: 44.32

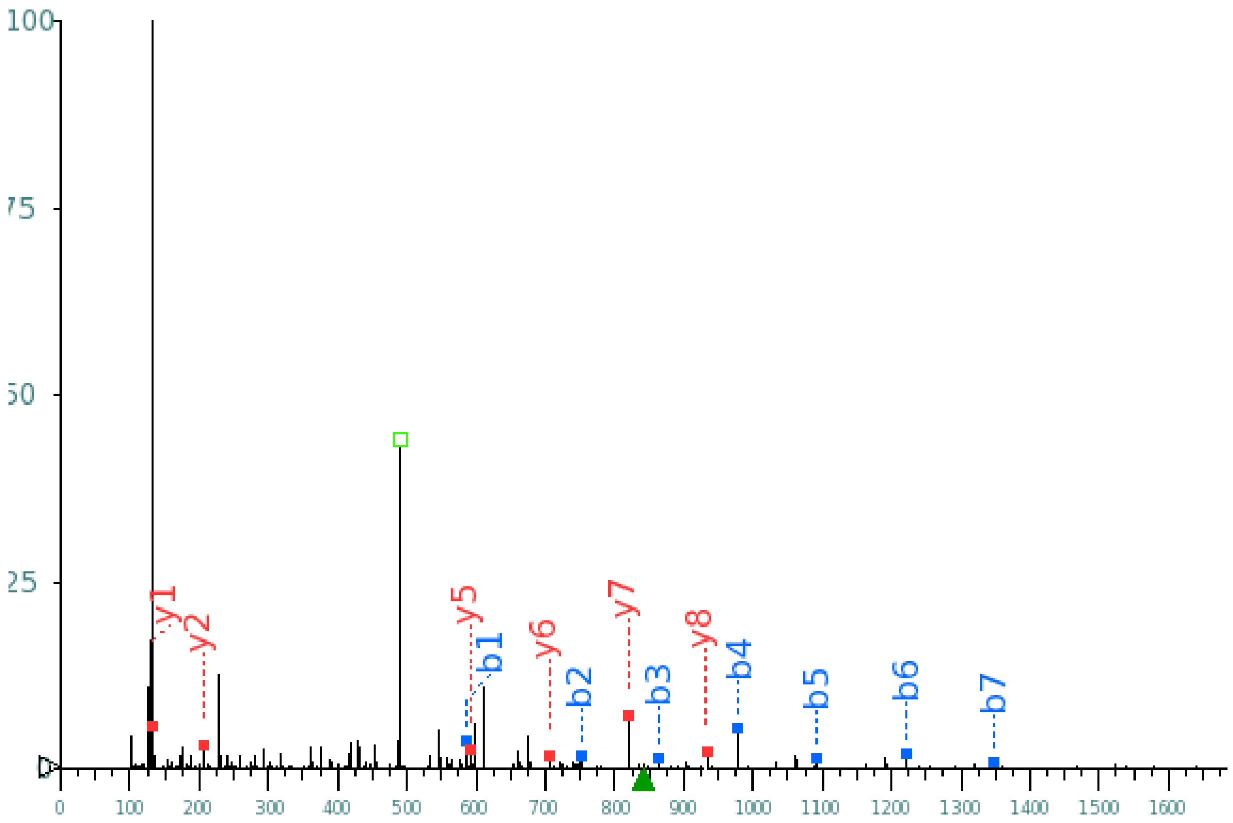

KYLDLEEEAD (+2)

Primary Reference: BAG5_HUMAN
Search ID: 41758
Search Name: 20130328_ananiav_ERstress_TMT_TM_timecourse_chymo_inj1_4MC_IAA
Scan#: 15443
Observed Mass: 841.9482 (0.2 ppm)
PSM Score: 25.26

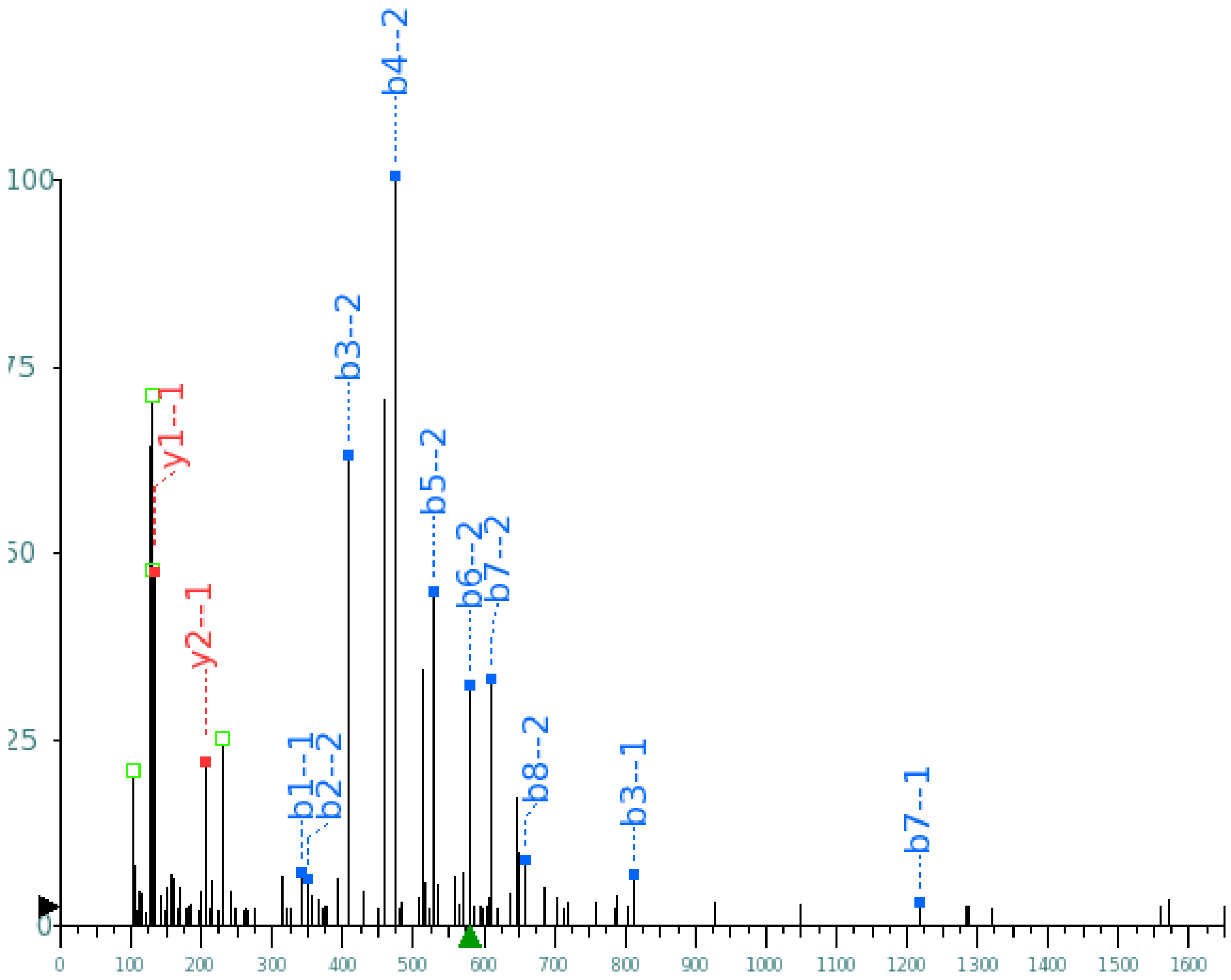

IKNMITGTSQAD (+3)

Primary Reference: EF1A1_HUMAN
Search ID: 41758
Search Name: 20130328_ananiav_ERstress_TMT_TM_timecourse_chymo_inj1_4MC_IAA
Scan#: 15492
Observed Mass: 579.6593 (1 ppm)
PSM Score: 16.82

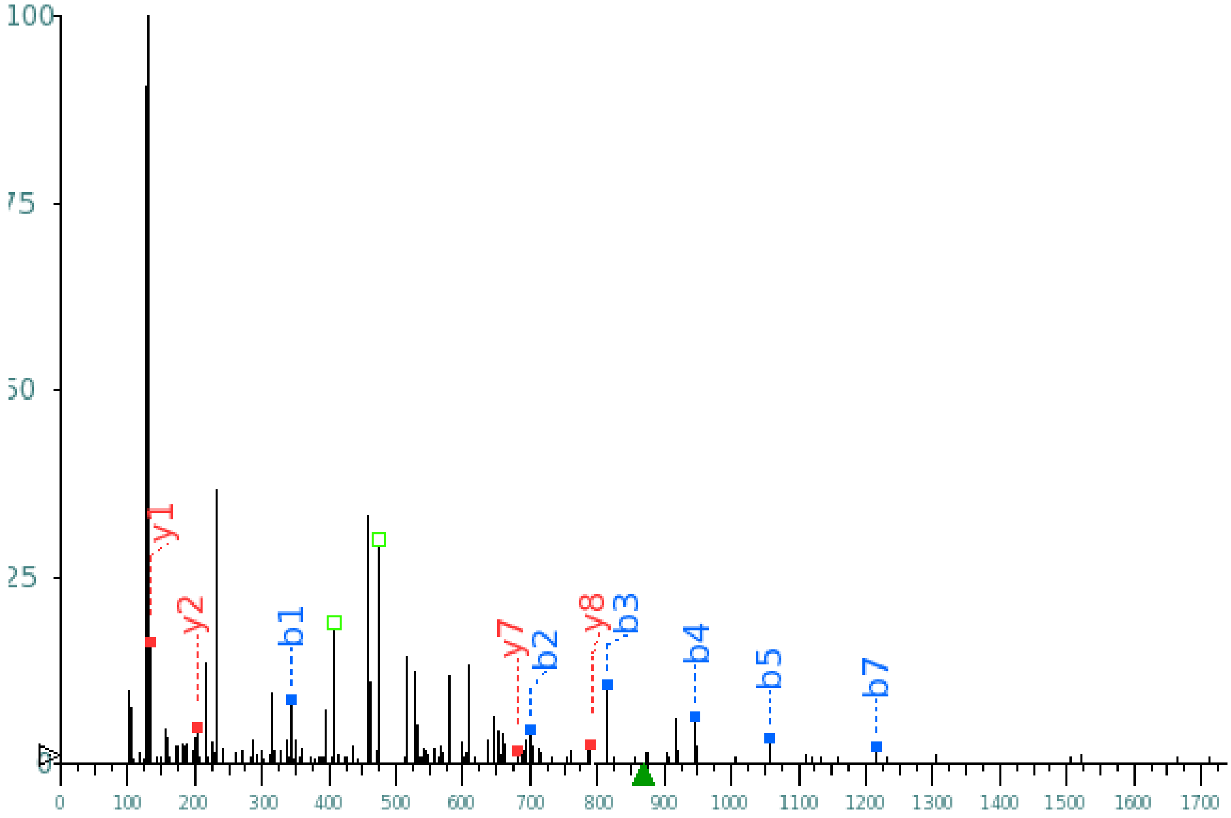

IKNMITGTSQAD (+2)

Primary Reference: EF1A1_HUMAN
Search ID: 41758
Search Name: 20130328_ananiav_ERstress_TMT_TM_timecourse_chymo_inj1_4MC_IAA
Scan#: 15493
Observed Mass: 868.9846 (0.1 ppm)
PSM Score: 18.06

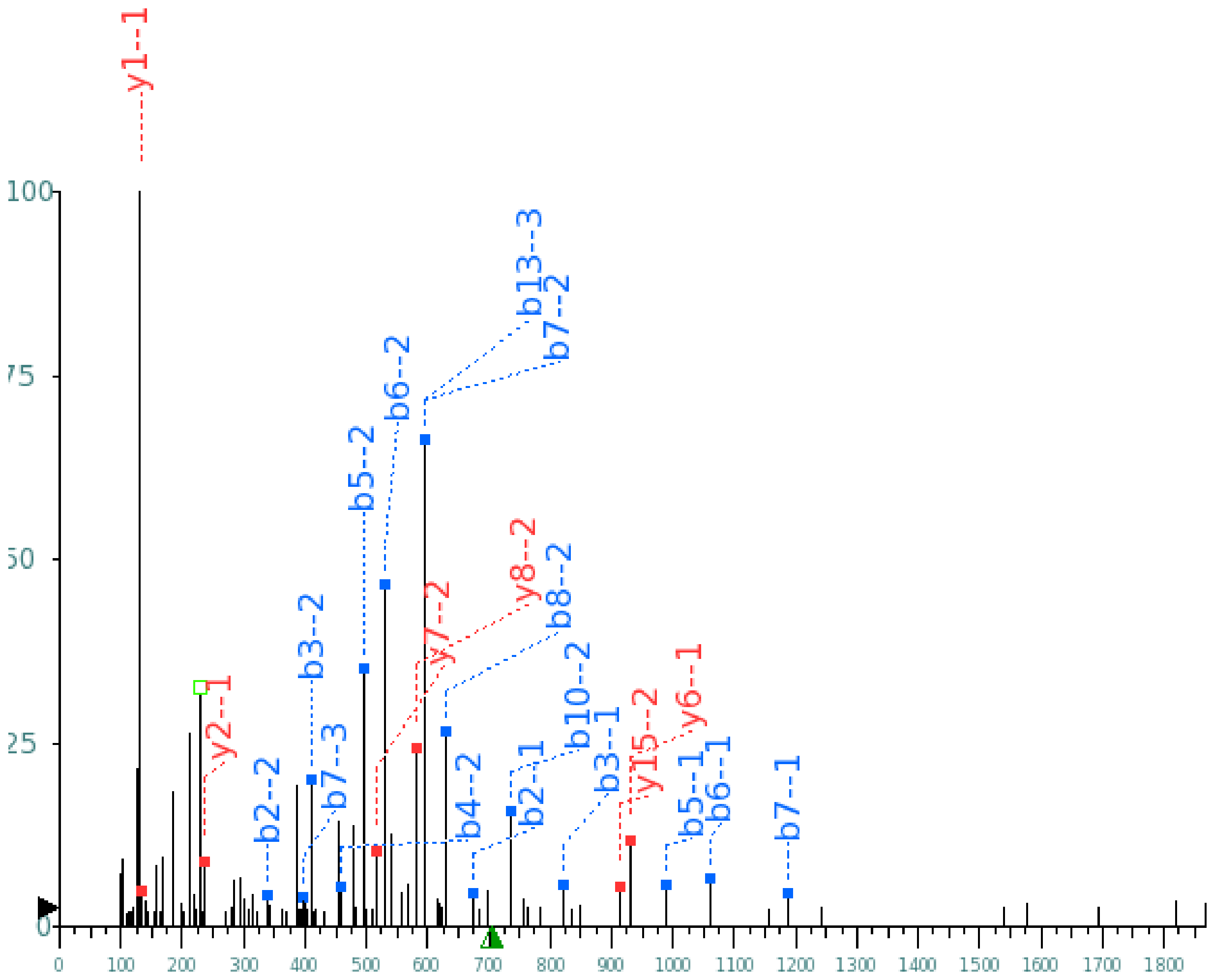

SKFPAAQAPDPSETPKIETD (+4)

Primary Reference: ABLM1_HUMAN
Search ID: 41758
Search Name: 20130328_ananiav_ERstress_TMT_TM_timecourse_chymo_inj1_4MC_IAA
Scan#: 15558
Observed Mass: 704.8884 (1.2 ppm)
PSM Score: 32.74

File name: 2015-02-04 04.01.53PM.pdf

Creation time: 2015-02-04 04:09PM

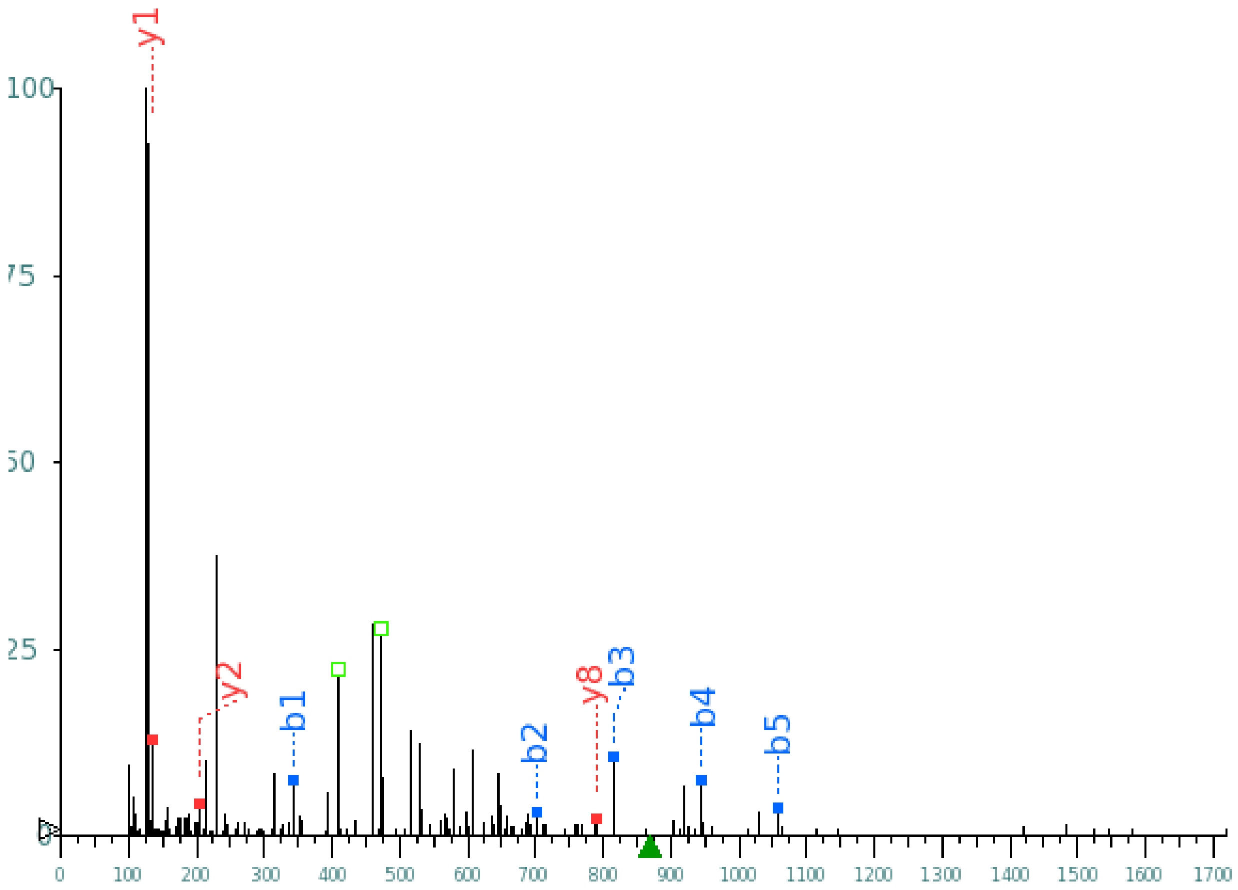

IKNMITGTSQAD (+2)

Primary Reference: EF1A1_HUMAN
Search ID: 41758
Search Name: 20130328_ananiav_ERstress_TMT_TM_timecourse_chymo_inj1_4MC_IAA
Scan#: 15595
Observed Mass: 868.9848 (0.3 ppm)
PSM Score: 11.59

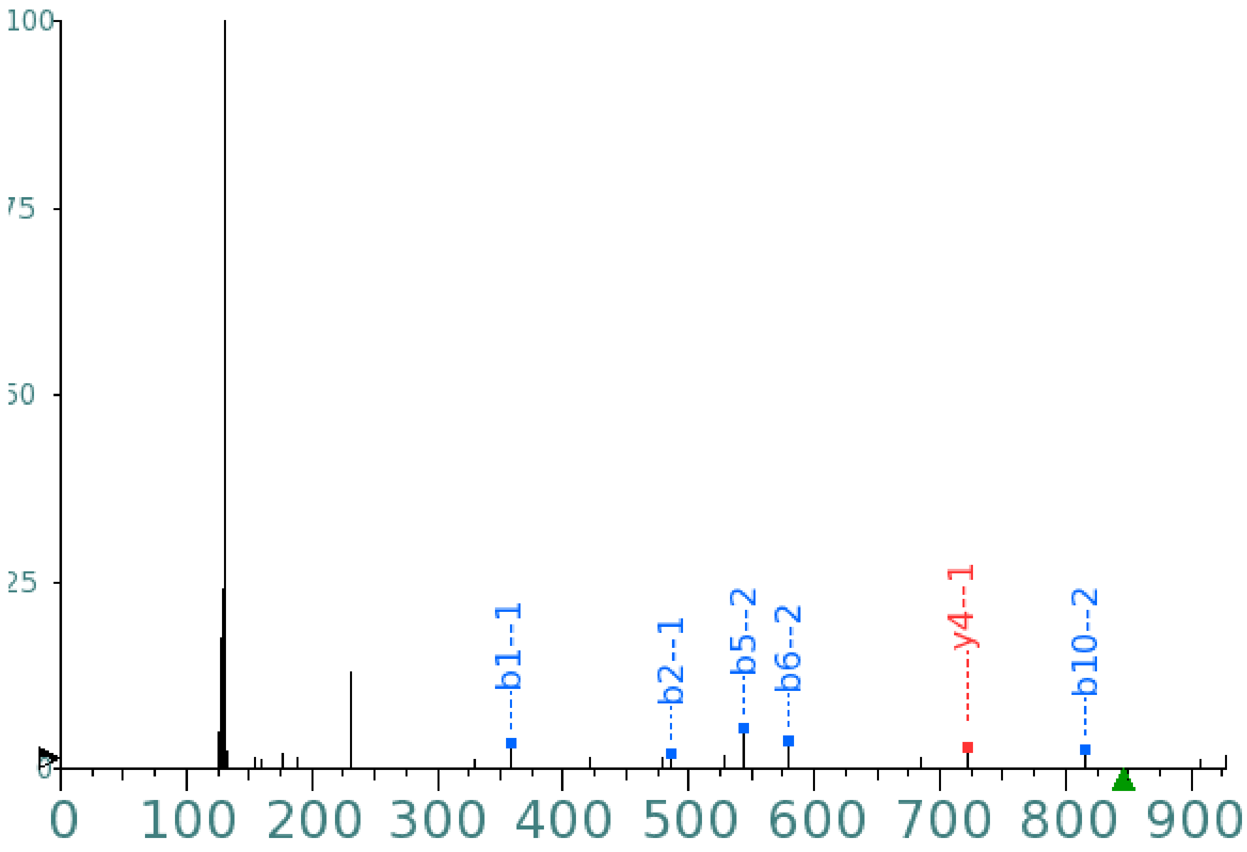

QQKLEAIEDDSVKETD (+3)

Primary Reference: UBP14_HUMAN
Search ID: 41758
Search Name: 20130328_ananiav_ERstress_TMT_TM_timecourse_chymo_inj1_4MC_IAA
Scan#: 15598
Observed Mass: 845.7977 (1.1 ppm)
PSM Score: 26.57

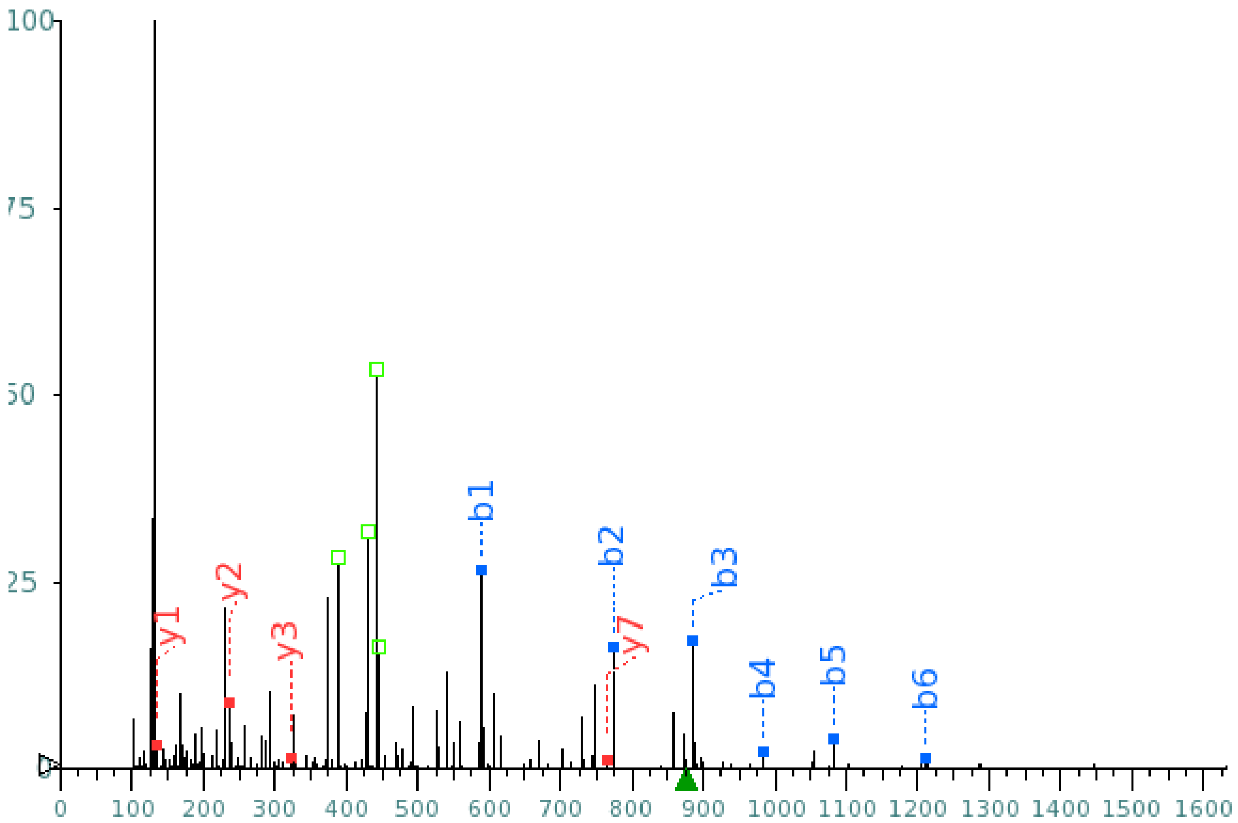

KWLPVQESSTD (+2)

Primary Reference: SPCS1_HUMAN
Search ID: 41758
Search Name: 20130328_ananiav_ERstress_TMT_TM_timecourse_chymo_inj1_4MC_IAA
Scan#: 15927
Observed Mass: 874.4861 (1.1 ppm)
PSM Score: 34.07

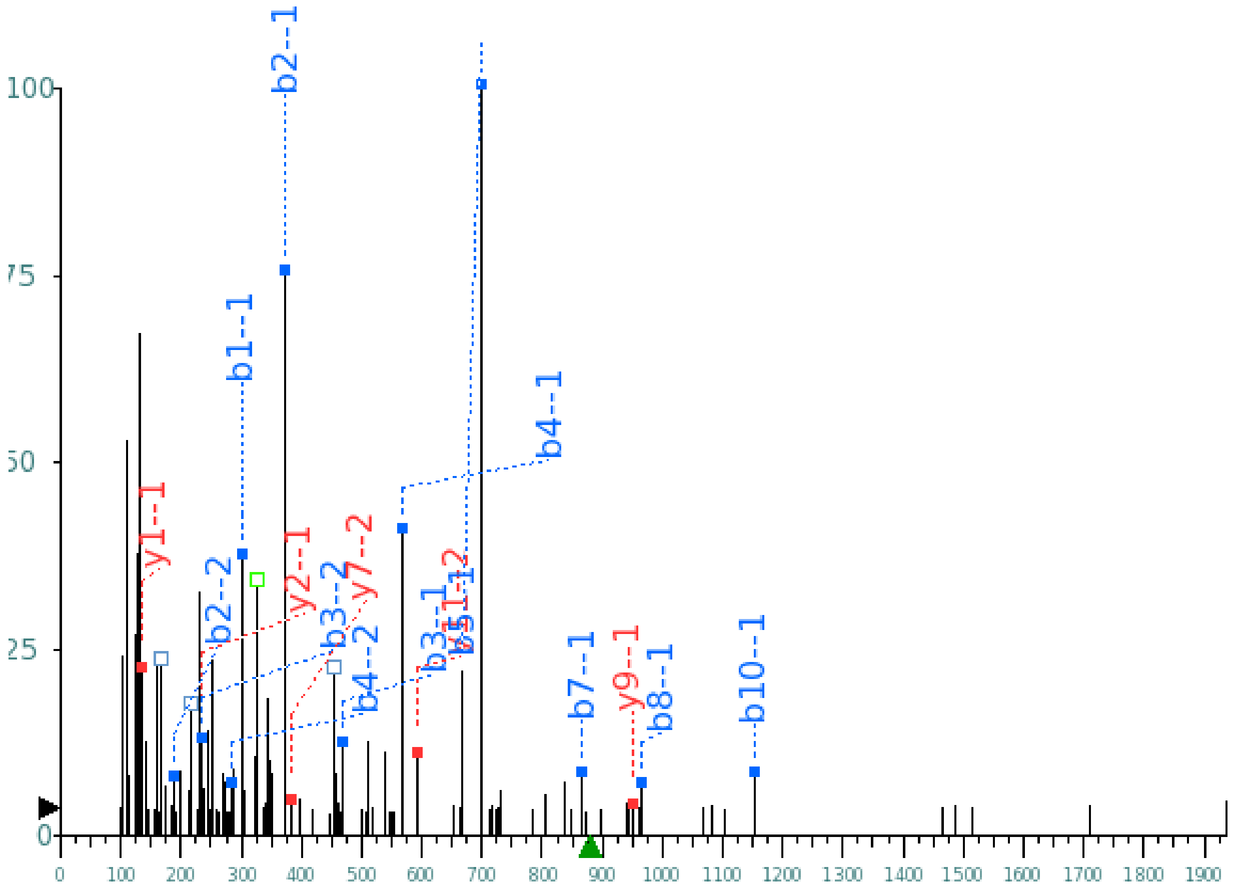

AAPVEPATADATEVHDAADTIETD (+3)

Primary Reference: B7Z2Z0_HUMAN
Search ID: 41758
Search Name: 20130328_ananiav_ERstress_TMT_TM_timecourse_chymo_inj1_4MC_IAA
Scan#: 15995
Observed Mass: 880.4221 (-0.1 ppm)
PSM Score: 25.56

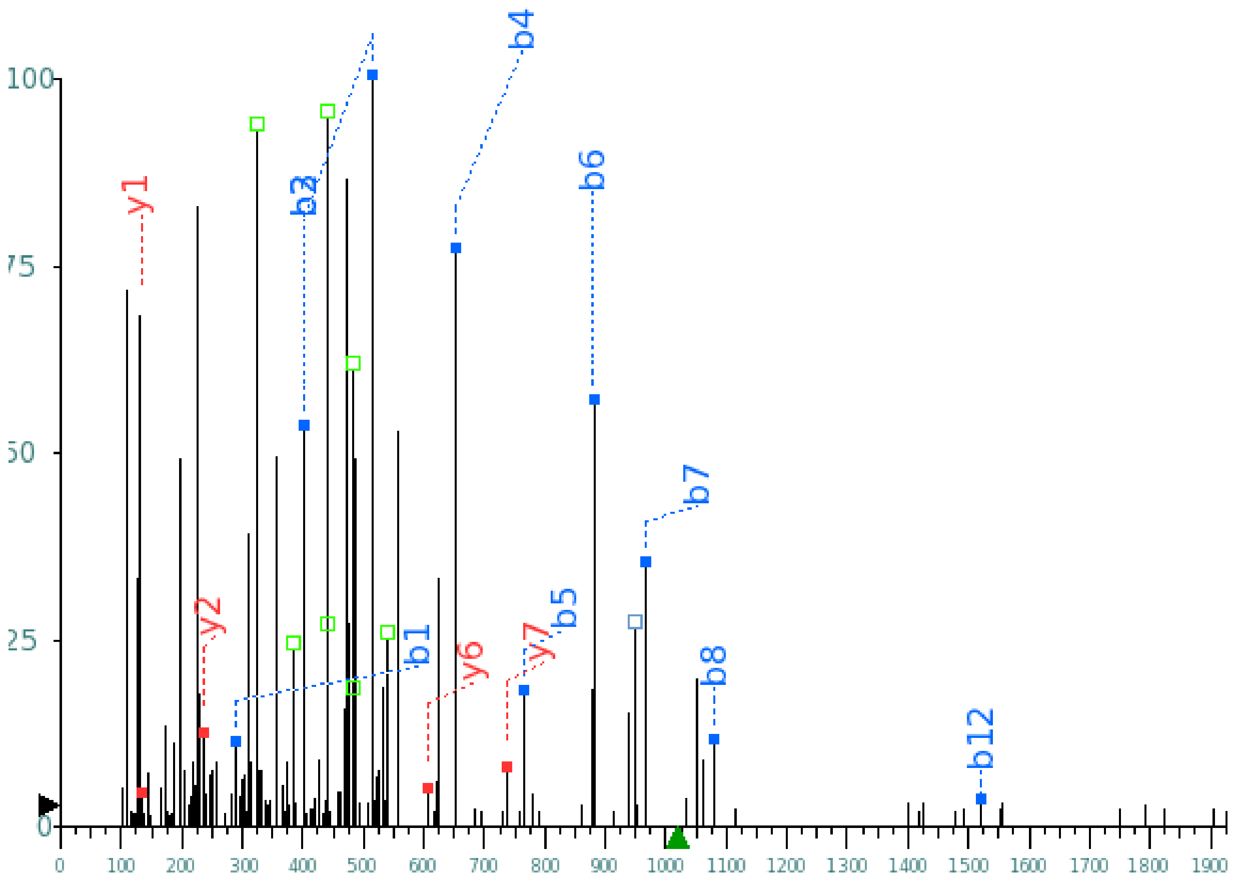

GNLHLDSLPEESDVATD (+2)

Primary Reference: SRR1L_HUMAN
Search ID: 41758
Search Name: 20130328_ananiav_ERstress_TMT_TM_timecourse_chymo_inj1_4MC_IAA
Scan#: 16111
Observed Mass: 1020.9994 (-0.4 ppm)
PSM Score: 26.58

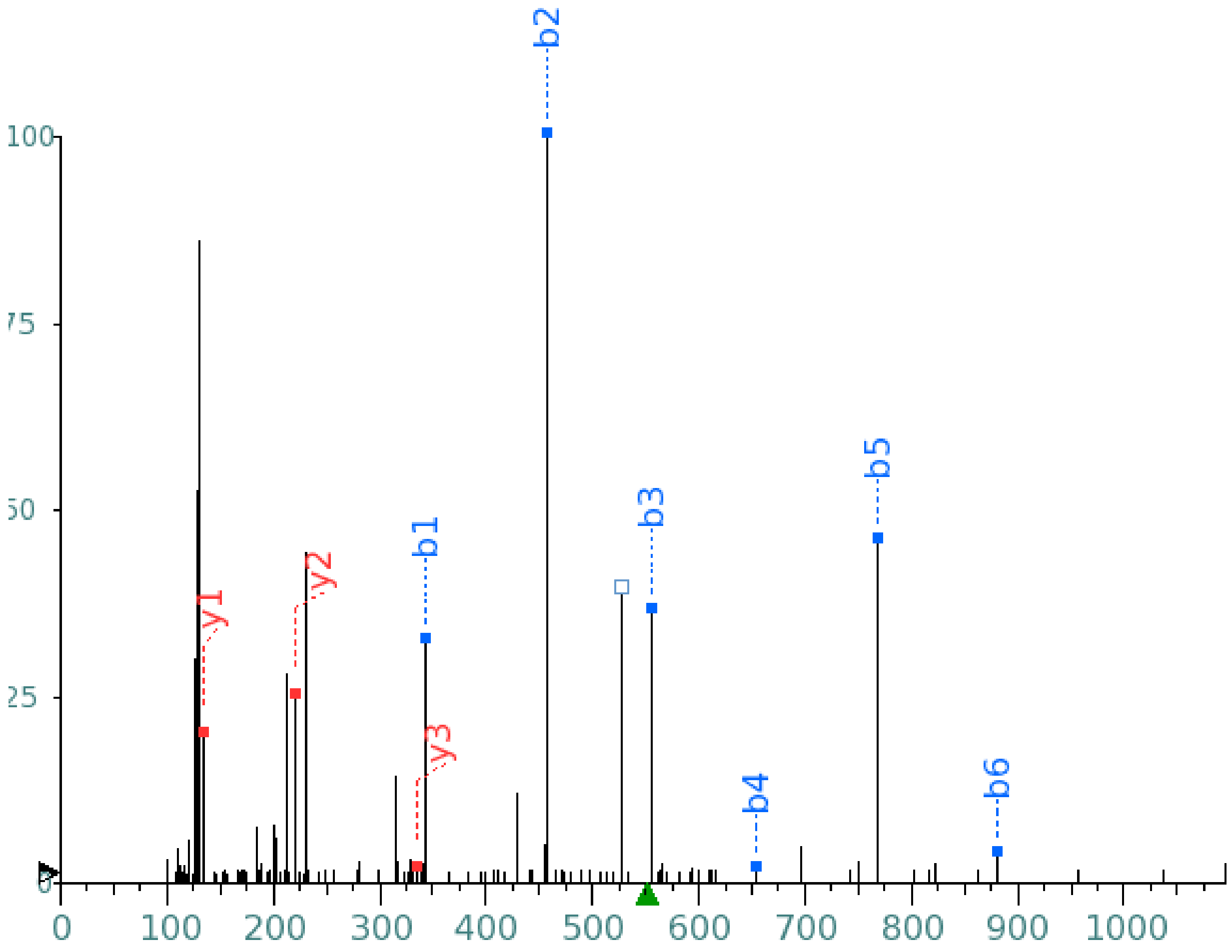

LNVPDLSD (+2)

Primary Reference: ZO1_HUMAN
Search ID: 41758
Search Name: 20130328_ananiav_ERstress_TMT_TM_timecourse_chymo_inj1_4MC_IAA
Scan#: 16801
Observed Mass: 551.3038 (1.3 ppm)
PSM Score: 22.95

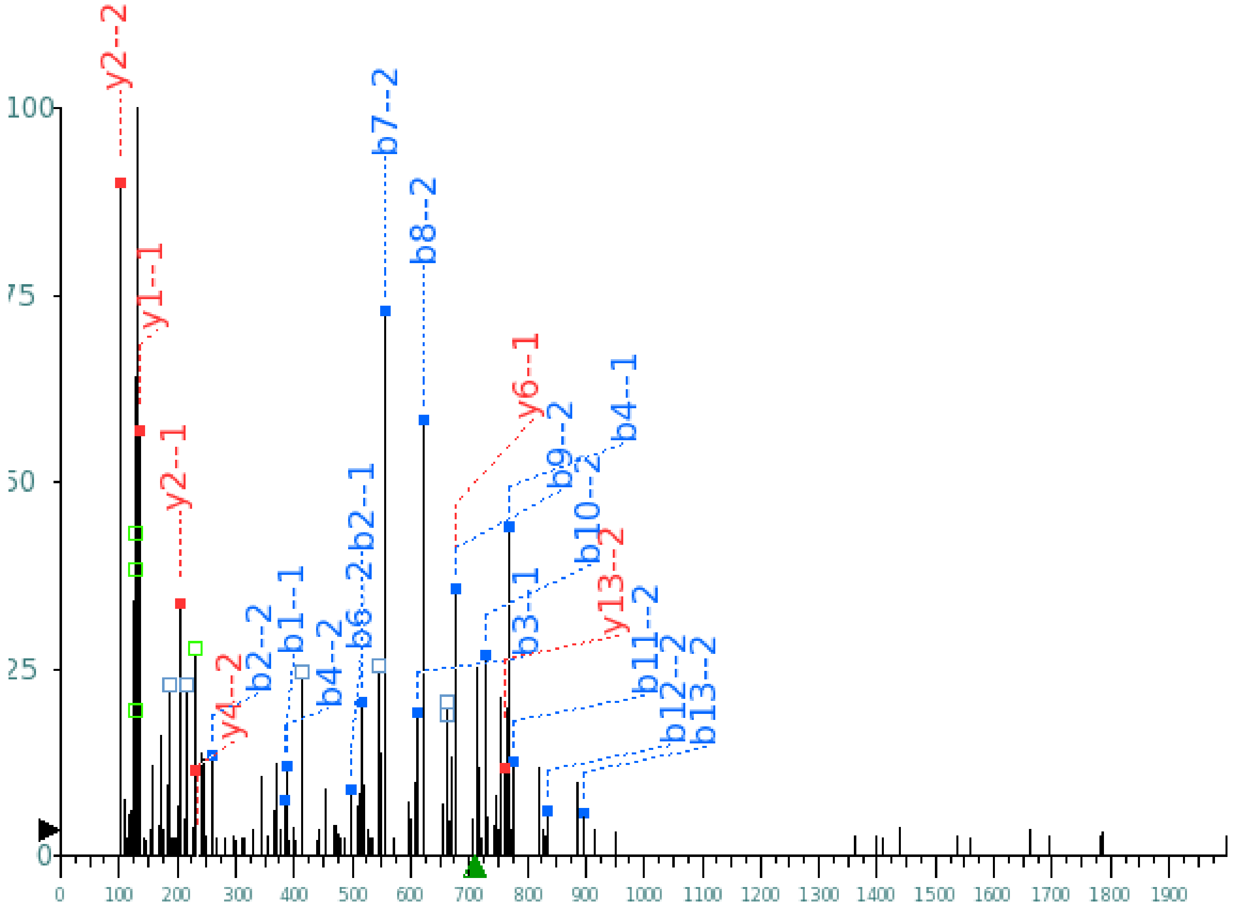

REPRPMDELVTLEEAD (+3)

Primary Reference: CACO1_HUMAN
Search ID: 41758
Search Name: 20130328_ananiav_ERstress_TMT_TM_timecourse_chymo_inj1_4MC_IAA
Scan#: 17573
Observed Mass: 710.3638 (1.1 ppm)
PSM Score: 25.17

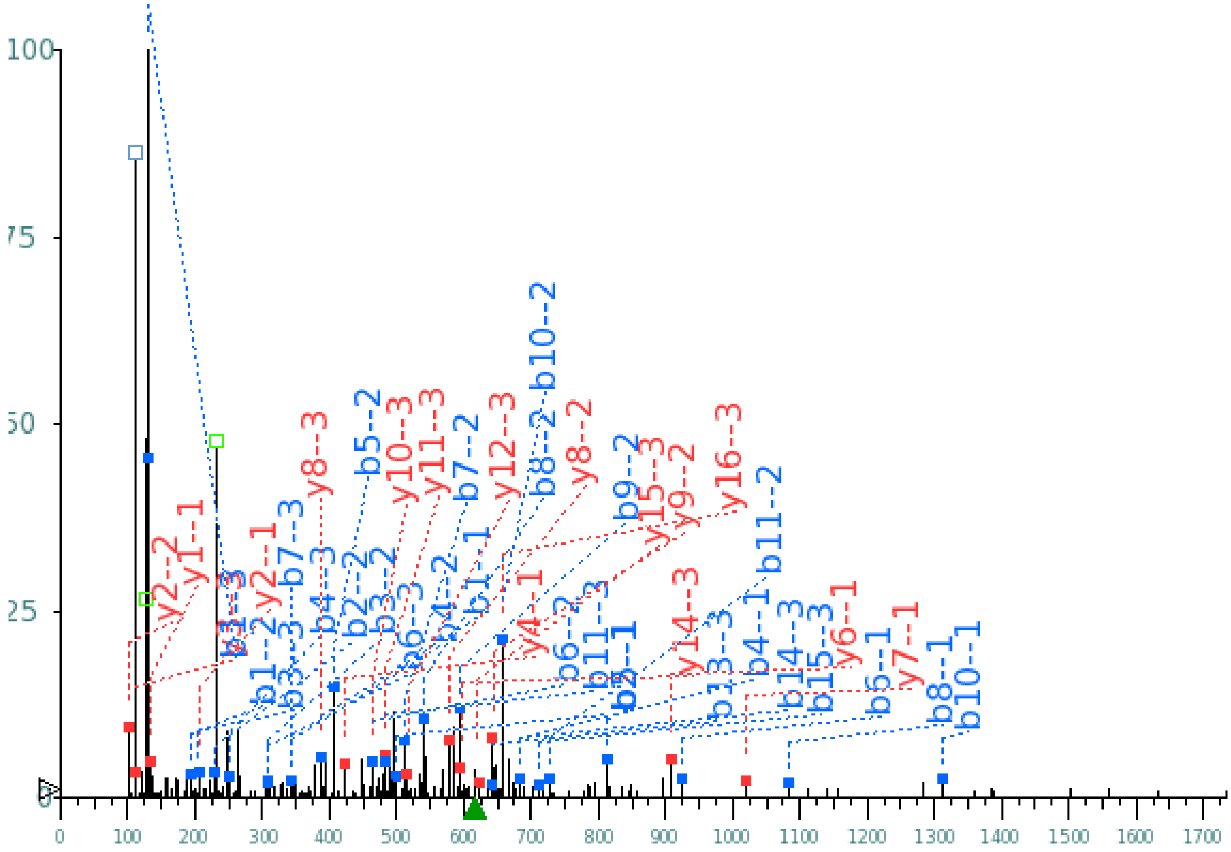

RILAQITGTEHLKESEAD (+4)

Primary Reference: PDLI5_HUMAN
Search ID: 41758
Search Name: 20130328_ananiav_ERstress_TMT_TM_timecourse_chymo_inj1_4MC_IAA
Scan#: 17631
Observed Mass: 618.0992 (1.5 ppm)
PSM Score: 26.48

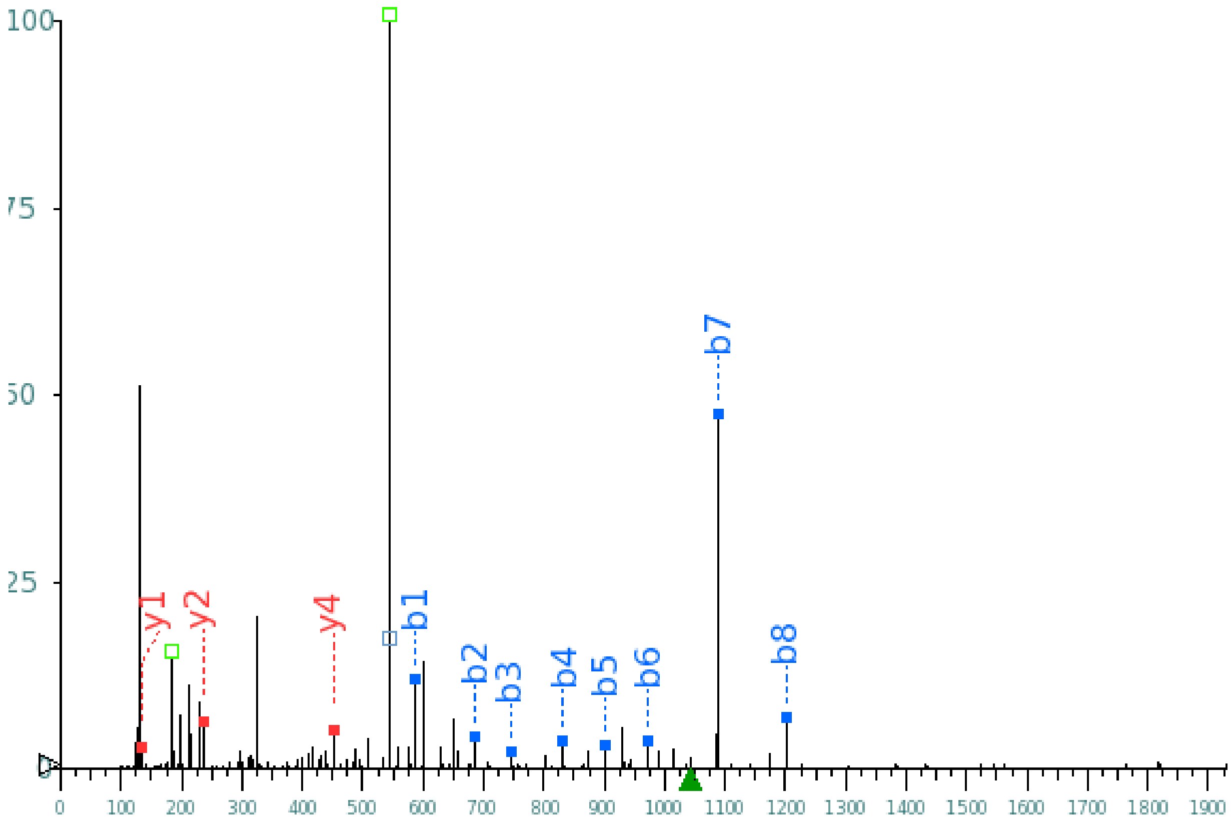

KVGSAADIPINISETD (+2)

Primary Reference: FLNA_HUMAN
Search ID: 41758
Search Name: 20130328_ananiav_ERstress_TMT_TM_timecourse_chymo_inj1_4MC_IAA
Scan#: 17639
Observed Mass: 1044.5842 (1.1 ppm)
PSM Score: 25.95

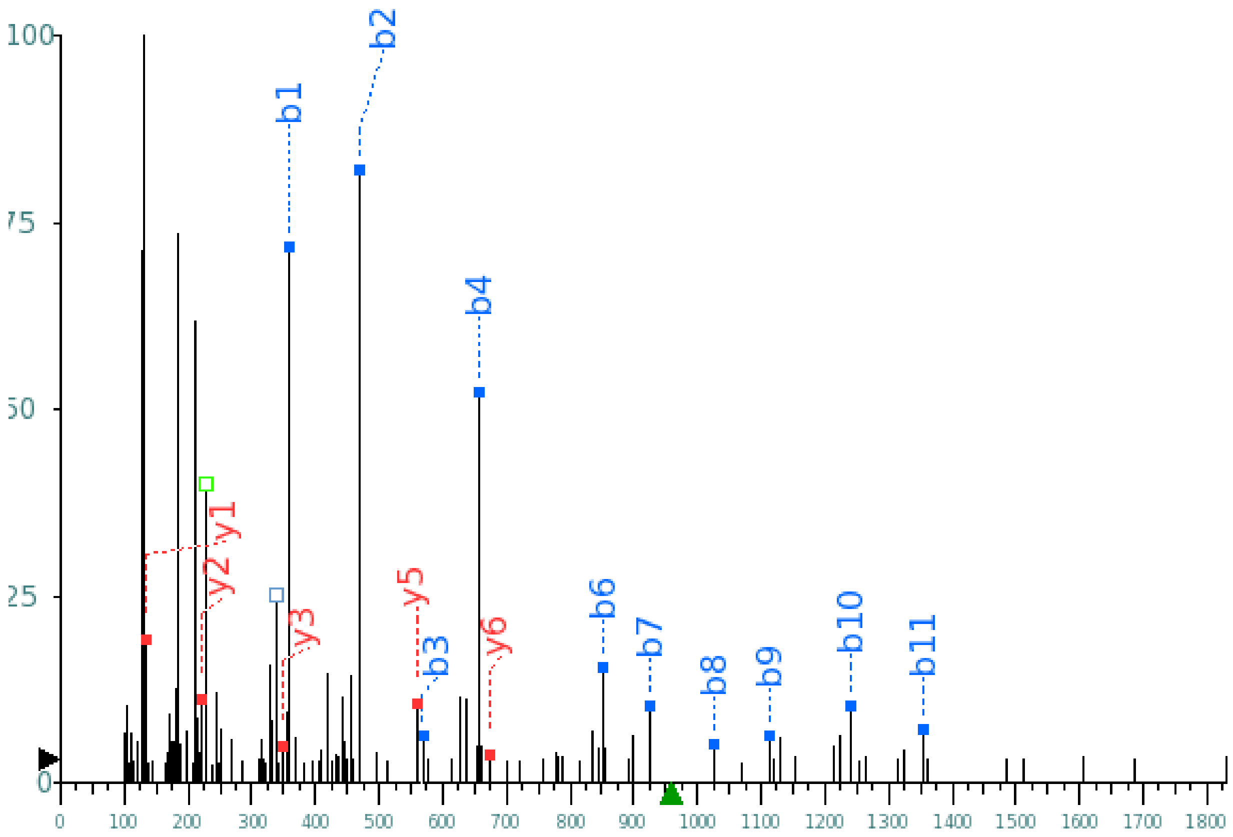

QLPSPTATSQLPLESD (+2)

Primary Reference: HNRPK_HUMAN
Search ID: 41758
Search Name: 20130328_ananiav_ERstress_TMT_TM_timecourse_chymo_inj1_4MC_IAA
Scan#: 17926
Observed Mass: 957.0077 (0.8 ppm)
PSM Score: 50.84

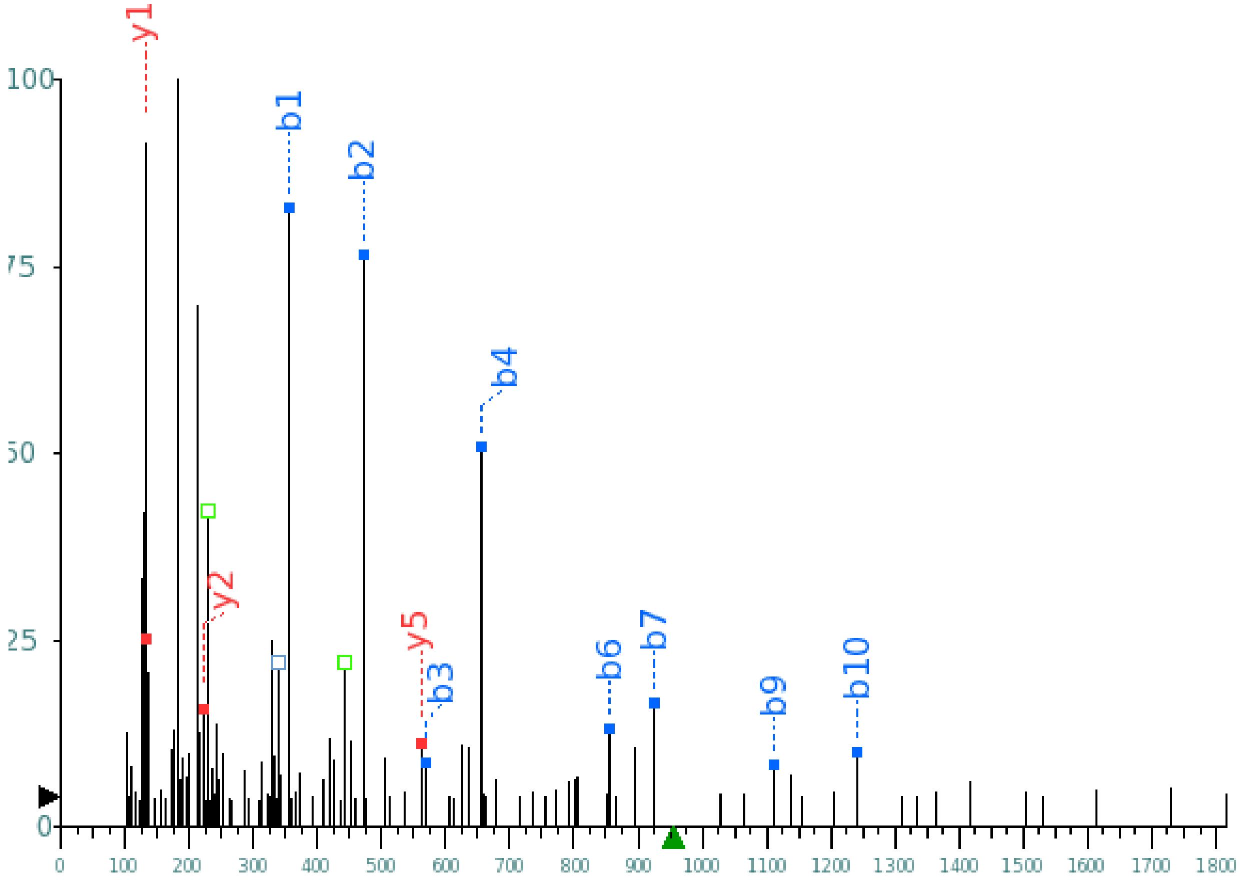

QLPSPTATSQLPLESD (+2)

Primary Reference: HNRPK_HUMAN
Search ID: 41758
Search Name: 20130328_ananiav_ERstress_TMT_TM_timecourse_chymo_inj1_4MC_IAA
Scan#: 18012
Observed Mass: 957.0074 (0.6 ppm)
PSM Score: 28.75

File name: 2015-02-04 04.01.53PM.pdf

Creation time: 2015-02-04 04:09PM

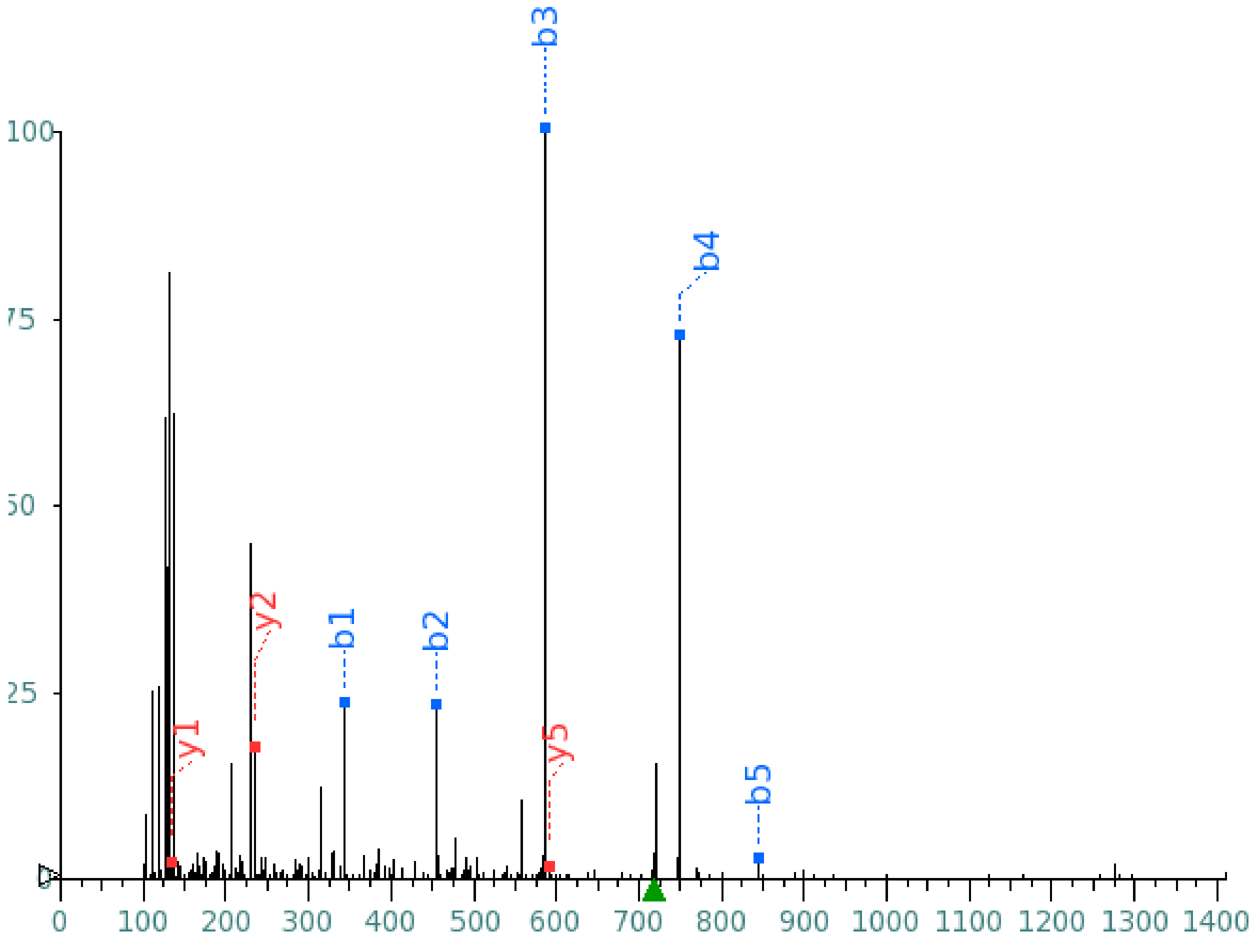

IIEYPHFATD (+2)

Primary Reference: NNTM_HUMAN
Search ID: 41758
Search Name: 20130328_ananiav_ERstress_TMT_TM_timecourse_chymo_inj1_4MC_IAA
Scan#: 18149
Observed Mass: 717.8767 (-0.3 ppm)
PSM Score: 21.58

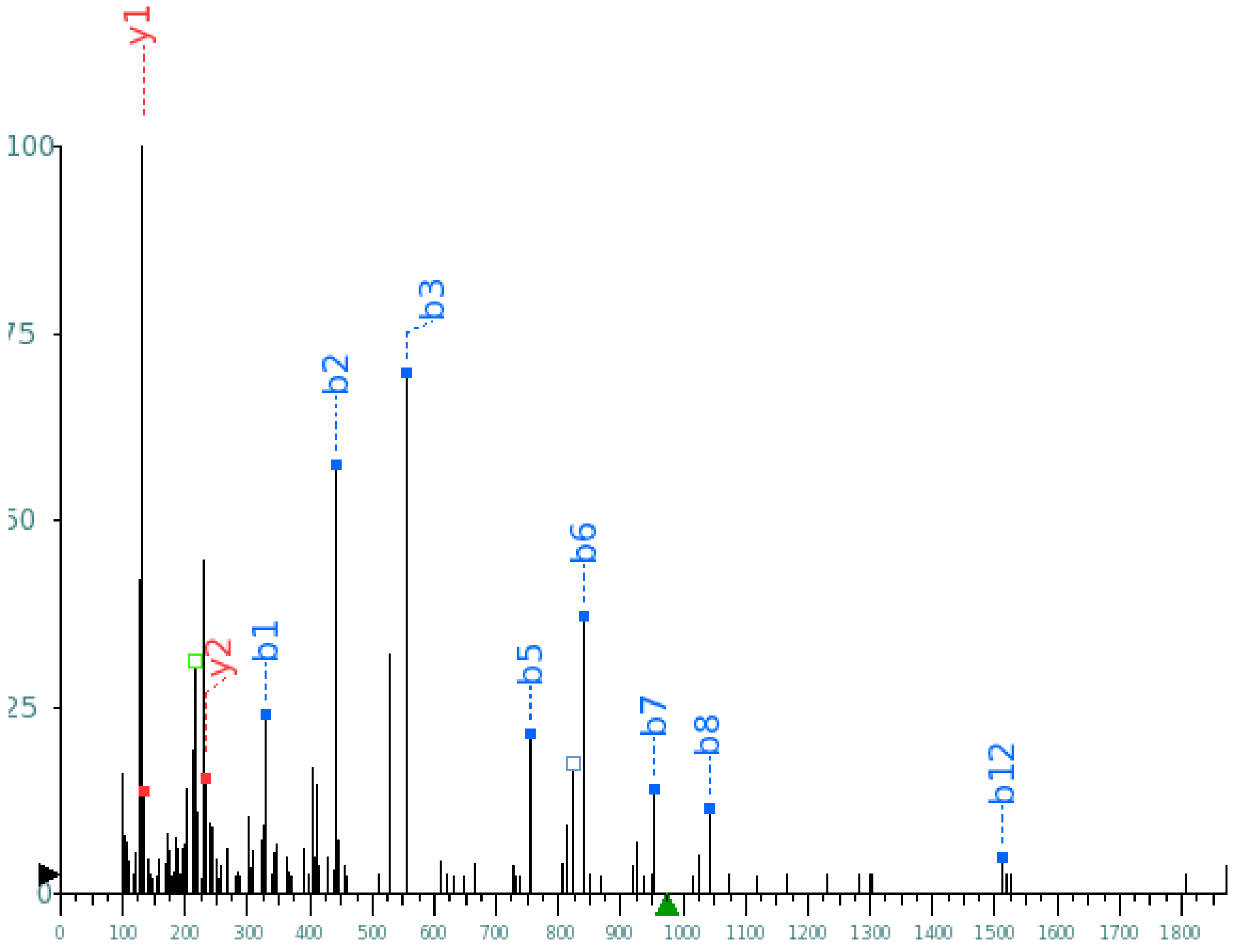

VNLPTSISQQTIAETD (+2)

Primary Reference: COPT2_HUMAN
Search ID: 41758
Search Name: 20130328_ananiav_ERstress_TMT_TM_timecourse_chymo_inj1_4MC_IAA
Scan#: 18401
Observed Mass: 973.5182 (0.6 ppm)
PSM Score: 46.57

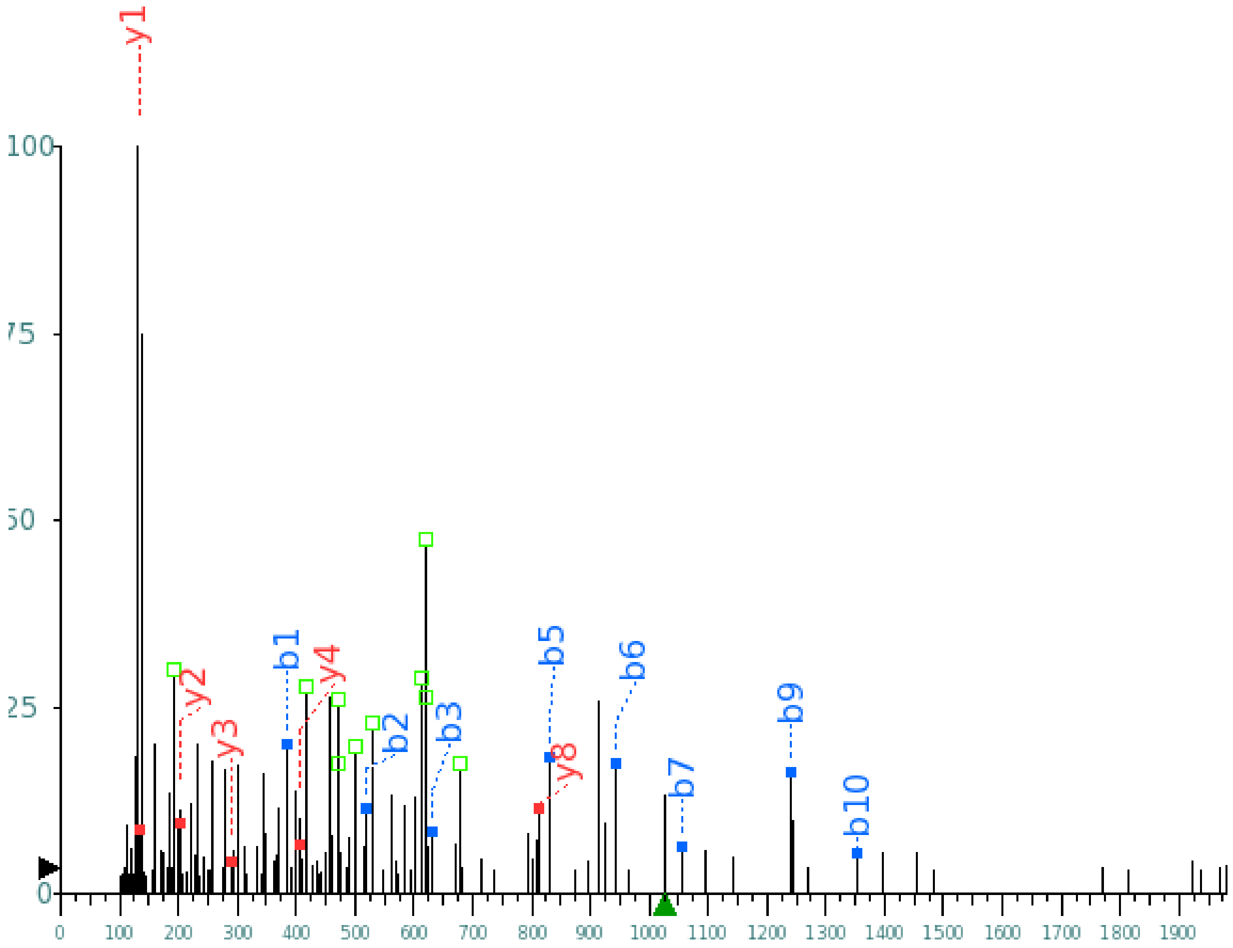

RMLPTIIADNAGYDSAD (+2)

Primary Reference: TCPB_HUMAN
Search ID: 41758
Search Name: 20130328_ananiav_ERstress_TMT_TM_timecourse_chymo_inj1_4MC_IAA
Scan#: 18613
Observed Mass: 1026.5172 (0.1 ppm)
PSM Score: 37.89

File name: 2015-02-04 04.01.53PM.pdf

Creation time: 2015-02-04 04:09PM

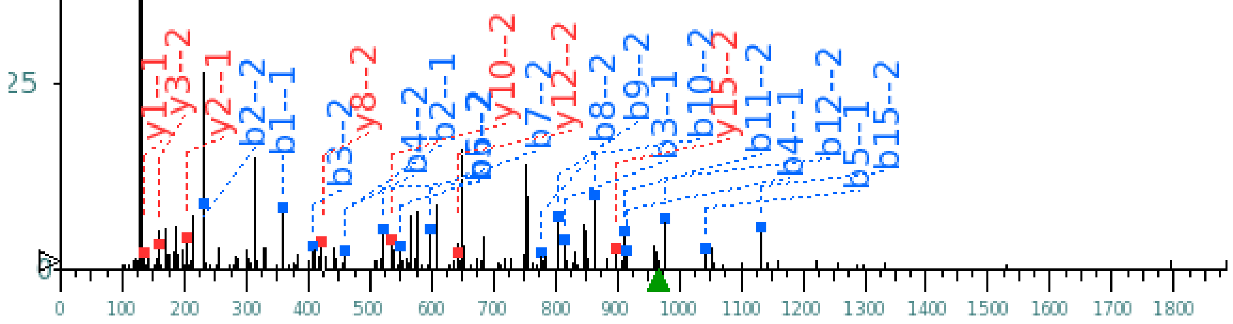

QVKVQGPKGLVEPVDVVDNAD (+3)

Primary Reference: FLNA_HUMAN
Search ID: 41758
Search Name: 20130328_ananiav_ERstress_TMT_TM_timecourse_chymo_inj1_4MC_IAA
Scan#: 18728
Observed Mass: 965.2257 (0.8 ppm)
PSM Score: 16.66

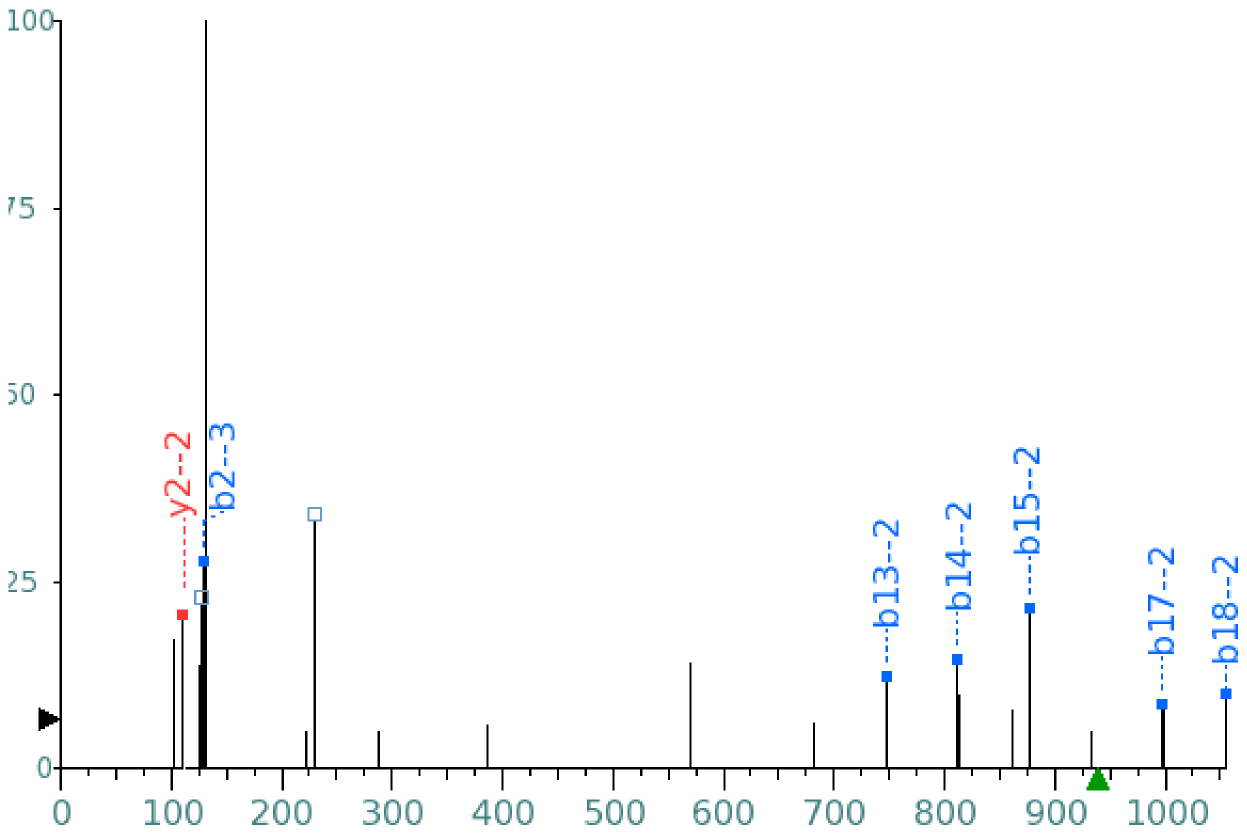

GVSGSQSRGSTMEEELENITTKHIVSNDSSD (+4)

Primary Reference: PA24A_HUMAN
Search ID: 41758
Search Name: 20130328_ananiav_ERstress_TMT_TM_timecourse_chymo_inj1_4MC_IAA
Scan#: 19305
Observed Mass: 938.959 (-3.8 ppm)
PSM Score: 34.64

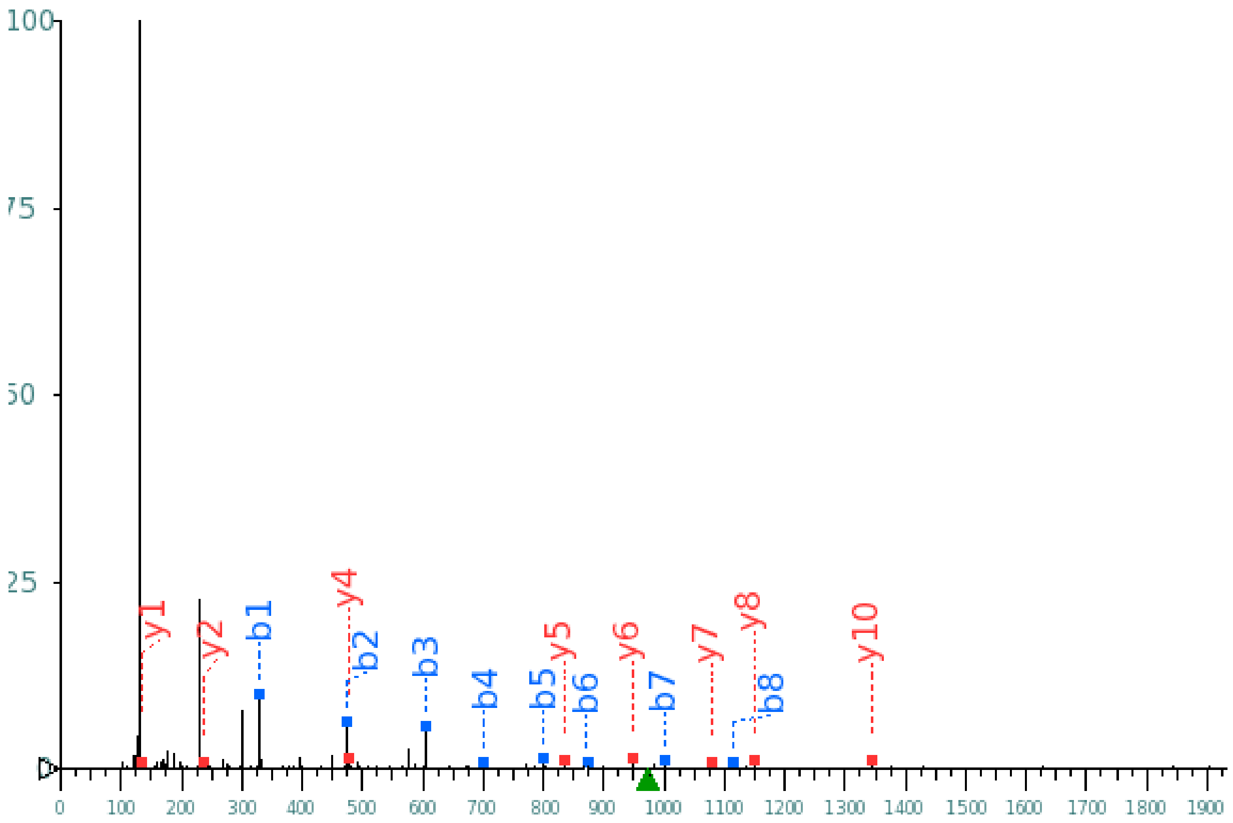

VFQPVAELKDQTD (+2)

Primary Reference: K6PF_HUMAN
Search ID: 41758
Search Name: 20130328_ananiav_ERstress_TMT_TM_timecourse_chymo_inj1_4MC_IAA
Scan#: 19378
Observed Mass: 974.5451 (1.9 ppm)
PSM Score: 24.11

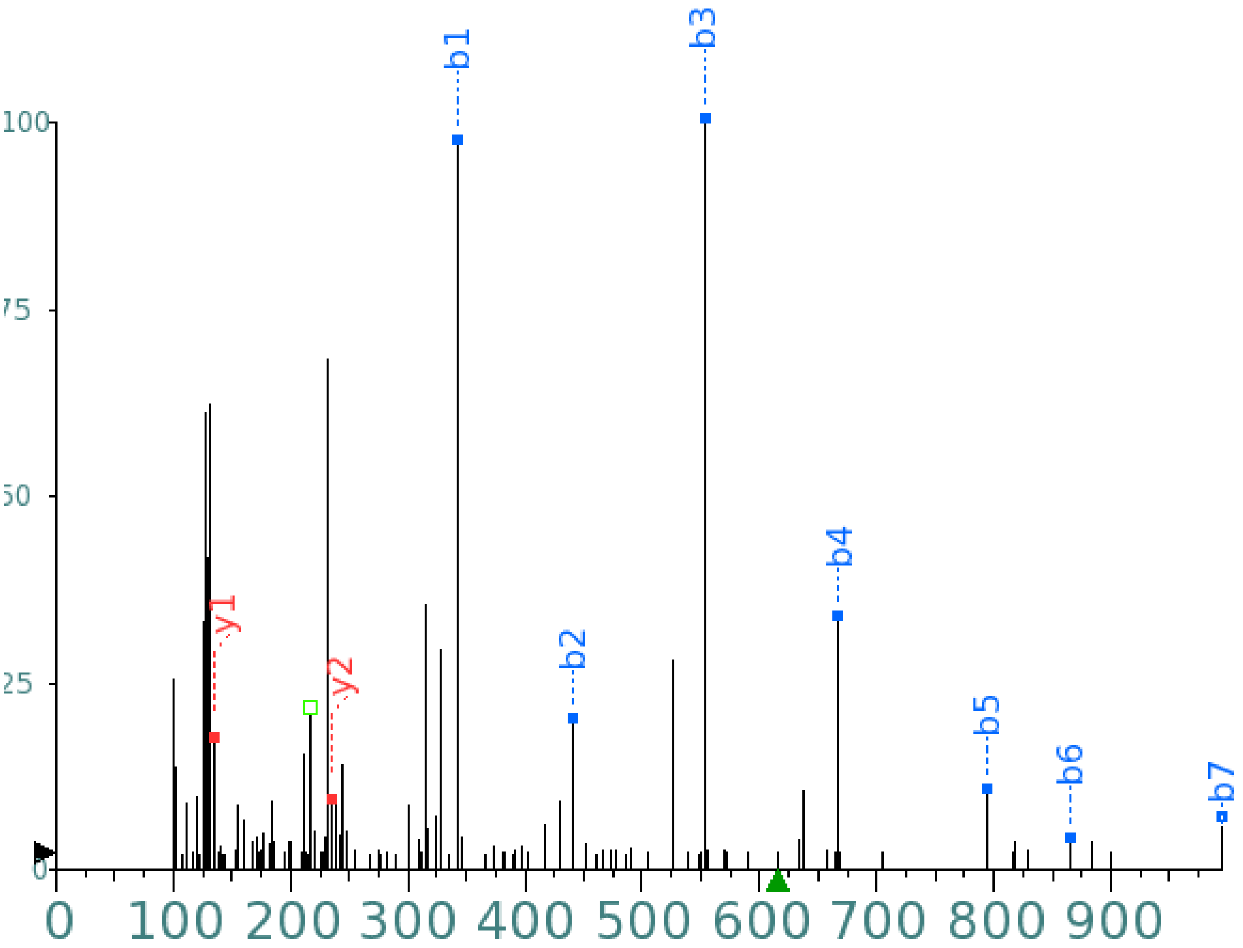

LPLLQAETD (+2)

Primary Reference: NDUAD_HUMAN
Search ID: 41758
Search Name: 20130328_ananiav_ERstress_TMT_TM_timecourse_chymo_inj1_4MC_IAA
Scan#: 19441
Observed Mass: 614.8542 (2 ppm)
PSM Score: 43.26

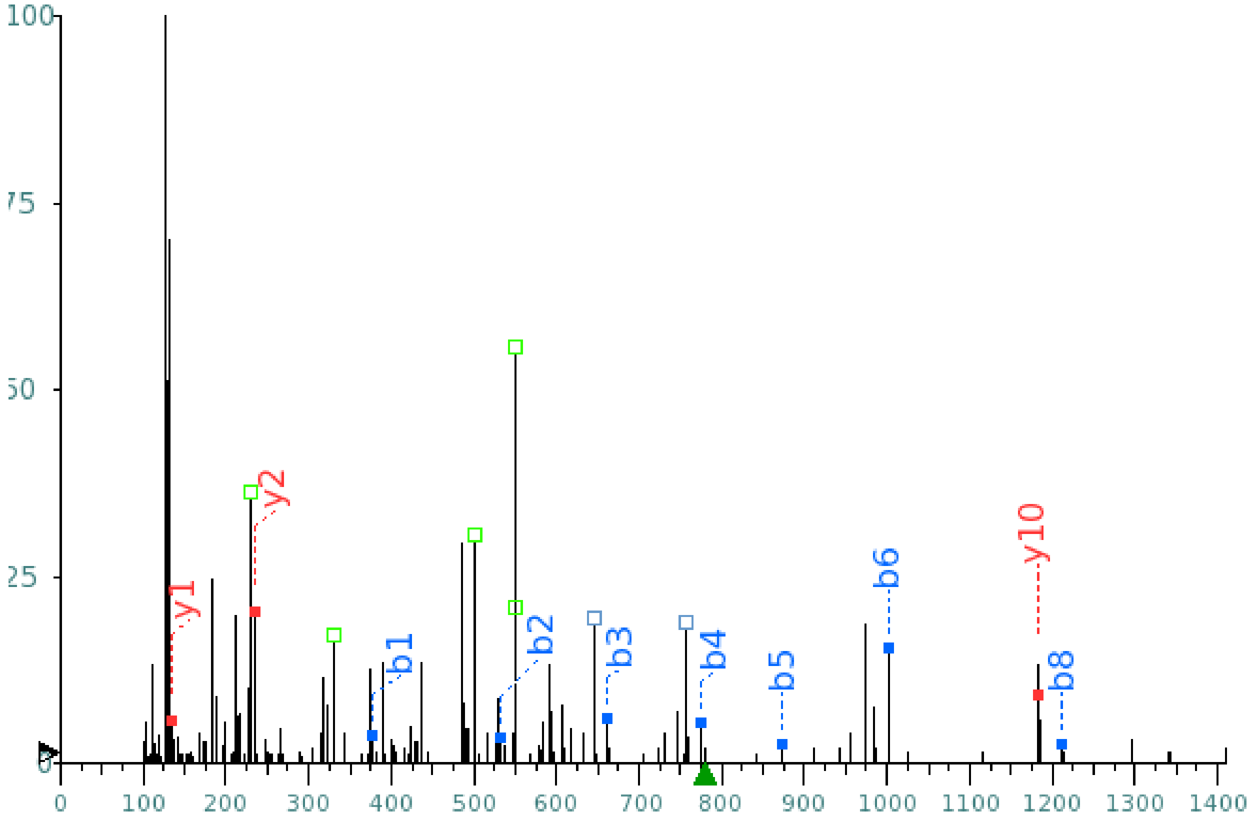

FRELPEPLLTD (+2)

Primary Reference: ABR_HUMAN
Search ID: 41758
Search Name: 20130328_ananiav_ERstress_TMT_TM_timecourse_chymo_inj1_4MC_IAA
Scan#: 19708
Observed Mass: 779.939 (1.8 ppm)
PSM Score: 25.83

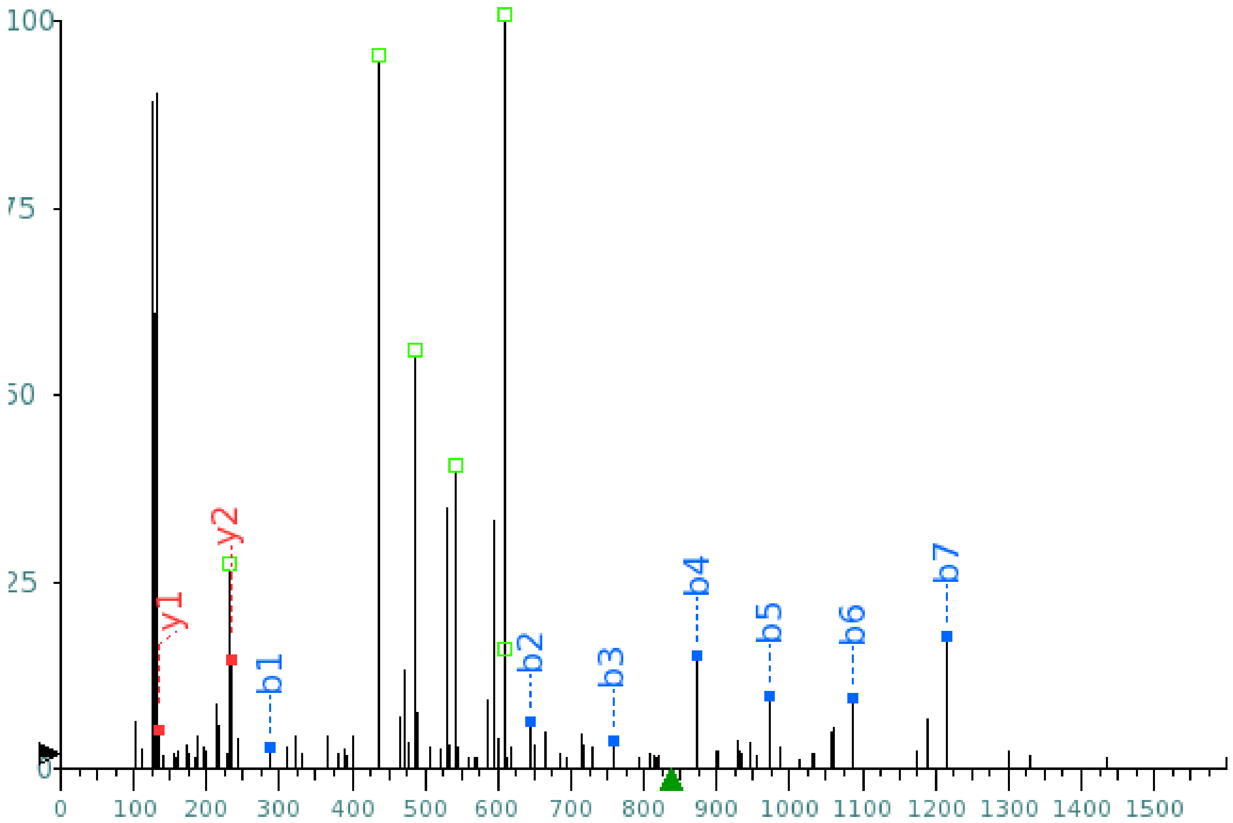

GKIDTIEIITD (+2)

Primary Reference: ROA2_HUMAN
Search ID: 41758
Search Name: 20130328_ananiav_ERstress_TMT_TM_timecourse_chymo_inj1_4MC_IAA
Scan#: 19735
Observed Mass: 838.5004 (3.2 ppm)
PSM Score: 21.89

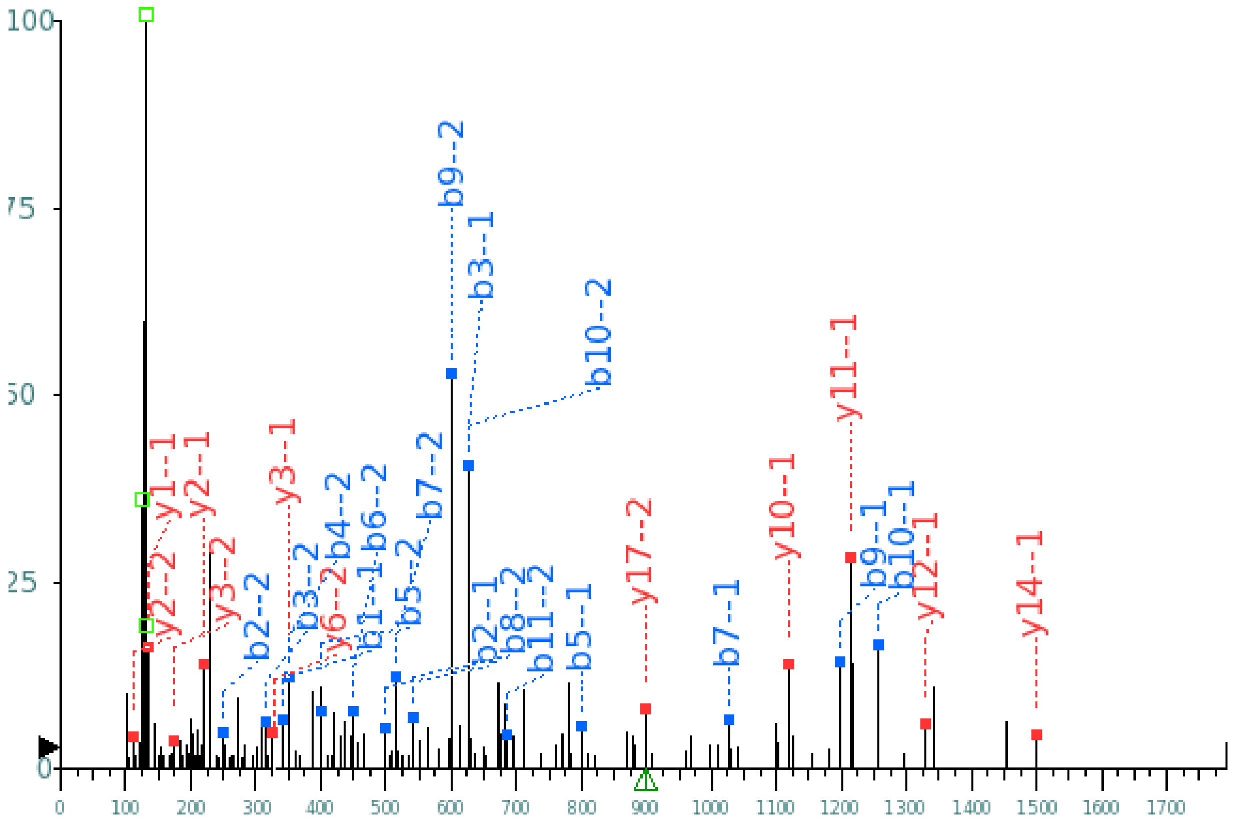

LREAVVEGDGILPPLRTEATESD (+3)

Primary Reference: ZGPAT_HUMAN
Search ID: 41758
Search Name: 20130328_ananiav_ERstress_TMT_TM_timecourse_chymo_inj1_4MC_IAA
Scan#: 19863
Observed Mass: 899.4845 (3.1 ppm)
PSM Score: 31.18

File name: 2015-02-04 04.01.53PM.pdf

Creation time: 2015-02-04 04:09PM

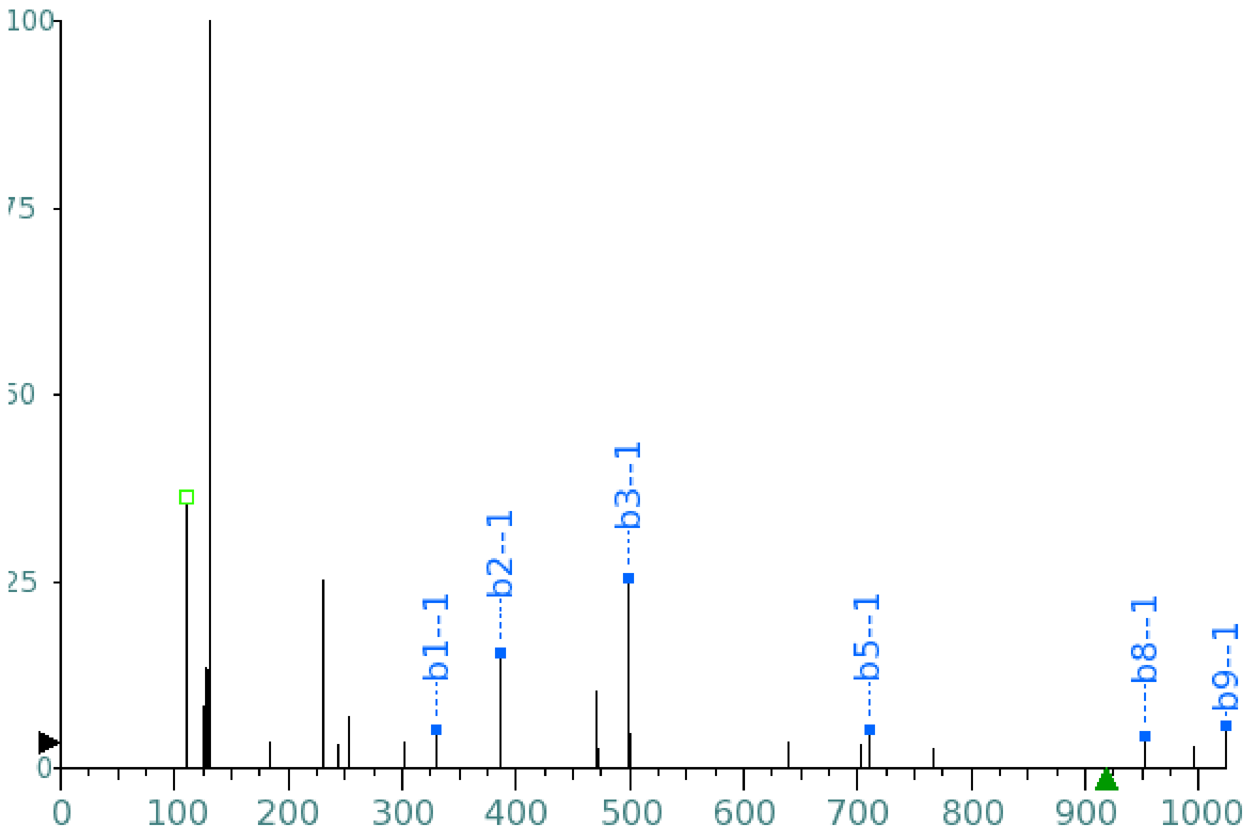

VGIPIGEGAHDEEVLKTIDEGD (+3)

Primary Reference: KDM3B_HUMAN
Search ID: 41758
Search Name: 20130328_ananiav_ERstress_TMT_TM_timecourse_chymo_inj1_4MC_IAA
Scan#: 20218
Observed Mass: 917.8228 (3.1 ppm)
PSM Score: 35.46

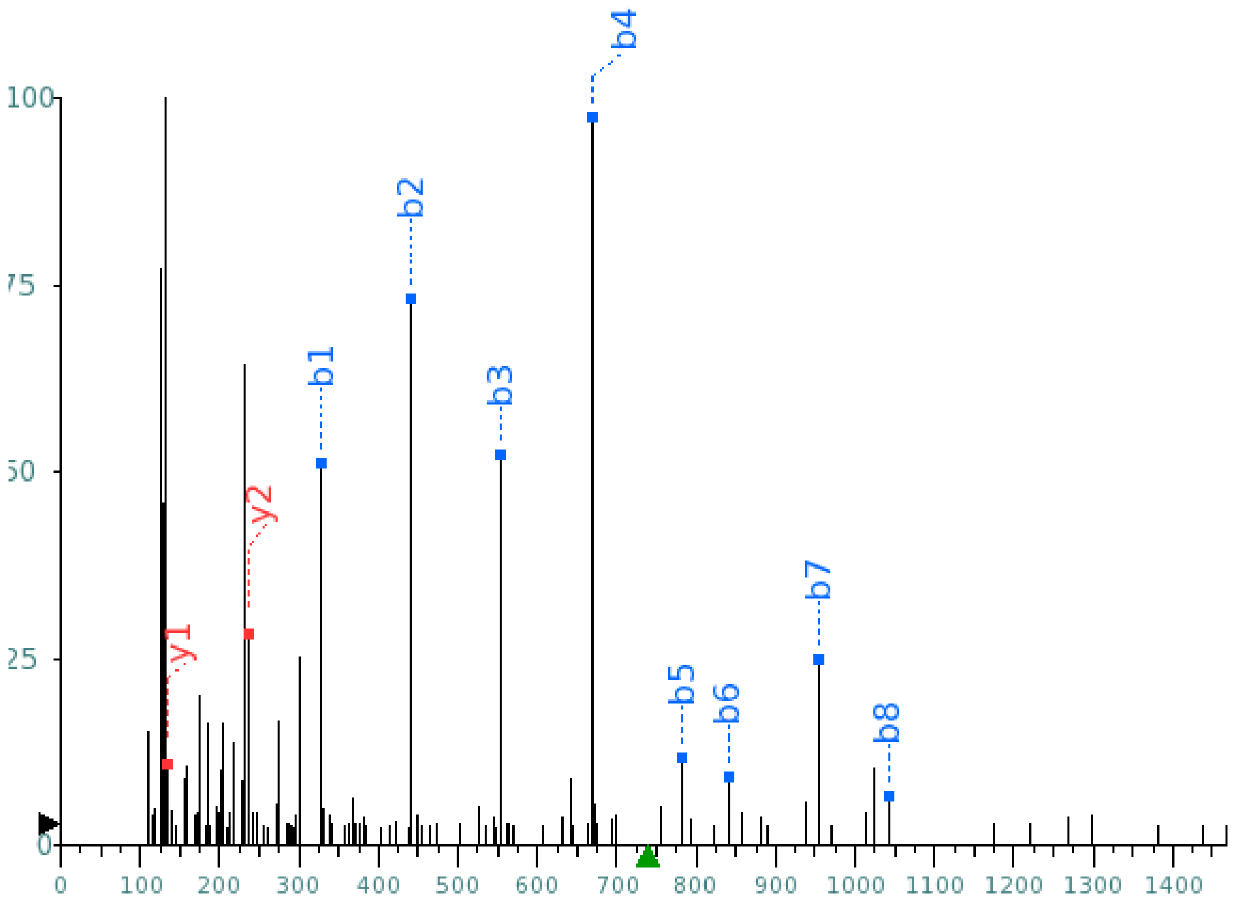

VLLNLGDSVTTD (+2)

Primary Reference: ACOC_HUMAN
Search ID: 41758
Search Name: 20130328_ananiav_ERstress_TMT_TM_timecourse_chymo_inj1_4MC_IAA
Scan#: 20376
Observed Mass: 738.4121 (1 ppm)
PSM Score: 44.58

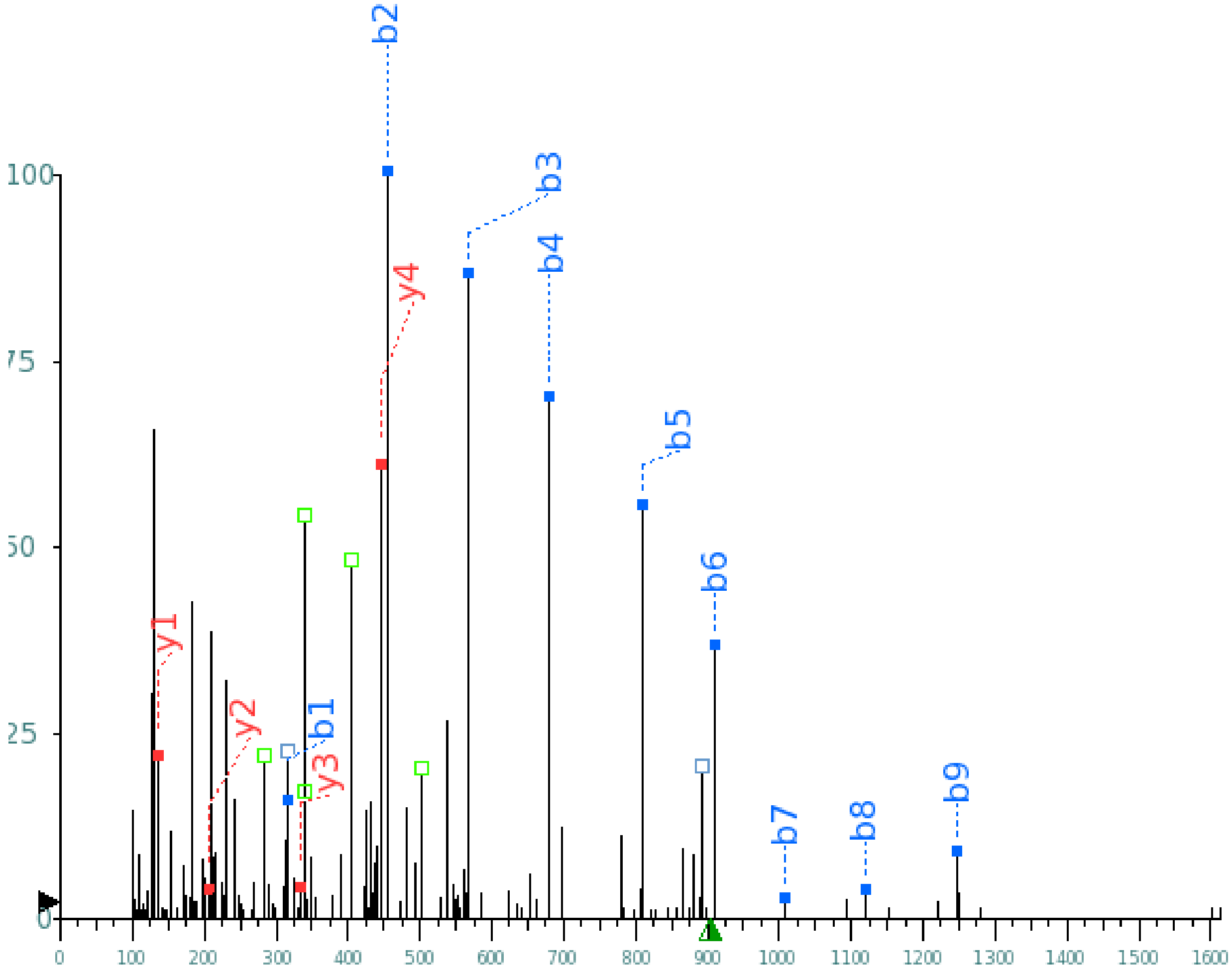

SHILQTPIEIIQAD (+2)

Primary Reference: OTU6B_HUMAN
Search ID: 41758
Search Name: 20130328_ananiav_ERstress_TMT_TM_timecourse_chymo_inj1_4MC_IAA
Scan#: 20767
Observed Mass: 904.0128 (1.2 ppm)
PSM Score: 53.71

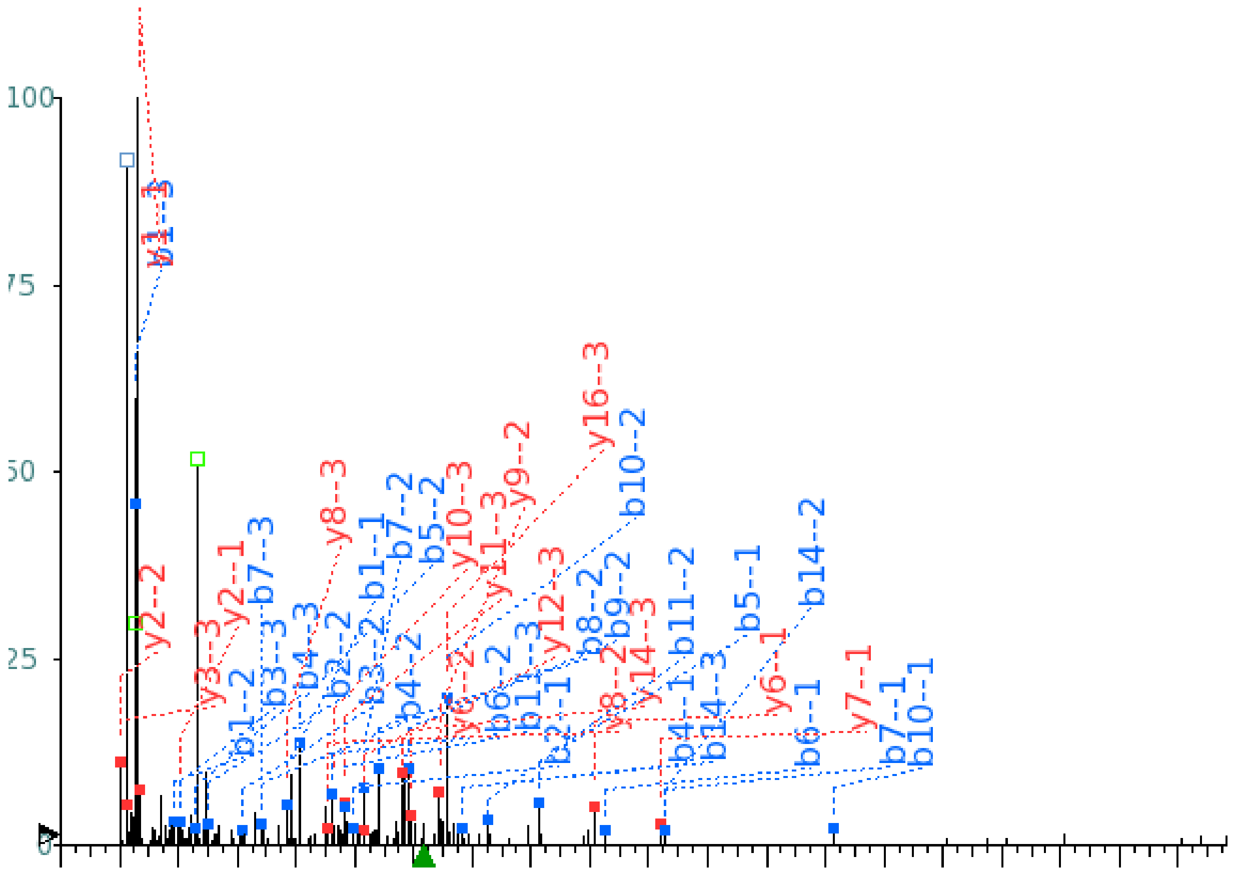

RILAQITGTEHLKESEAD (+4)

Primary Reference: PDLI5_HUMAN
Search ID: 41758
Search Name: 20130328_ananiav_ERstress_TMT_TM_timecourse_chymo_inj1_4MC_IAA
Scan#: 21012
Observed Mass: 618.0993 (1.7 ppm)
PSM Score: 24.5

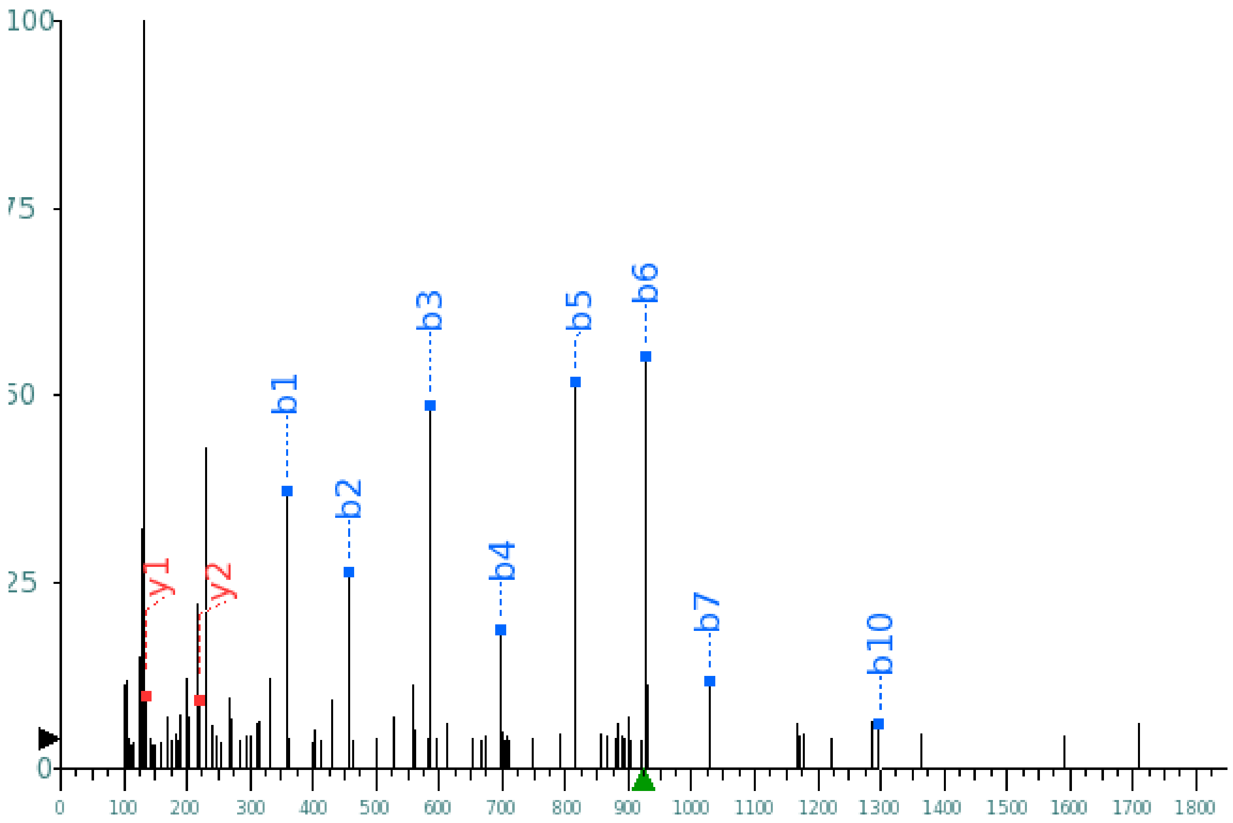

QVELDNVTGLLSQSD (+2)

Primary Reference: MYH9_HUMAN
Search ID: 41758
Search Name: 20130328_ananiav_ERstress_TMT_TM_timecourse_chymo_inj1_4MC_IAA
Scan#: 21058
Observed Mass: 923.9868 (3.7 ppm)
PSM Score: 39.07

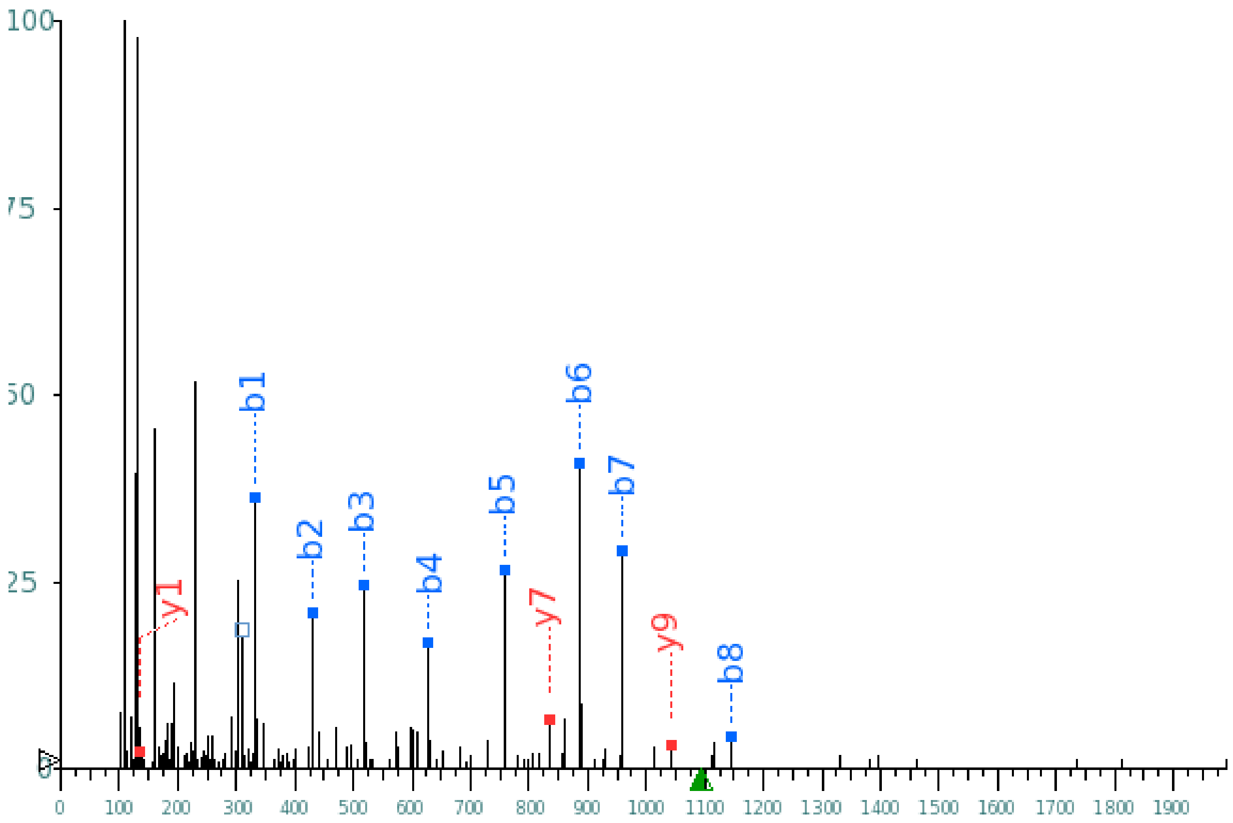

VVSLEEAWPLHGDC*ETD (+2)

Primary Reference: MDC1_HUMAN
Search ID: 41758
Search Name: 20130328_ananiav_ERstress_TMT_TM_timecourse_chymo_inj1_4MC_IAA
Scan#: 21149
Observed Mass: 1093.5169 (-0.4 ppm)
PSM Score: 31.27

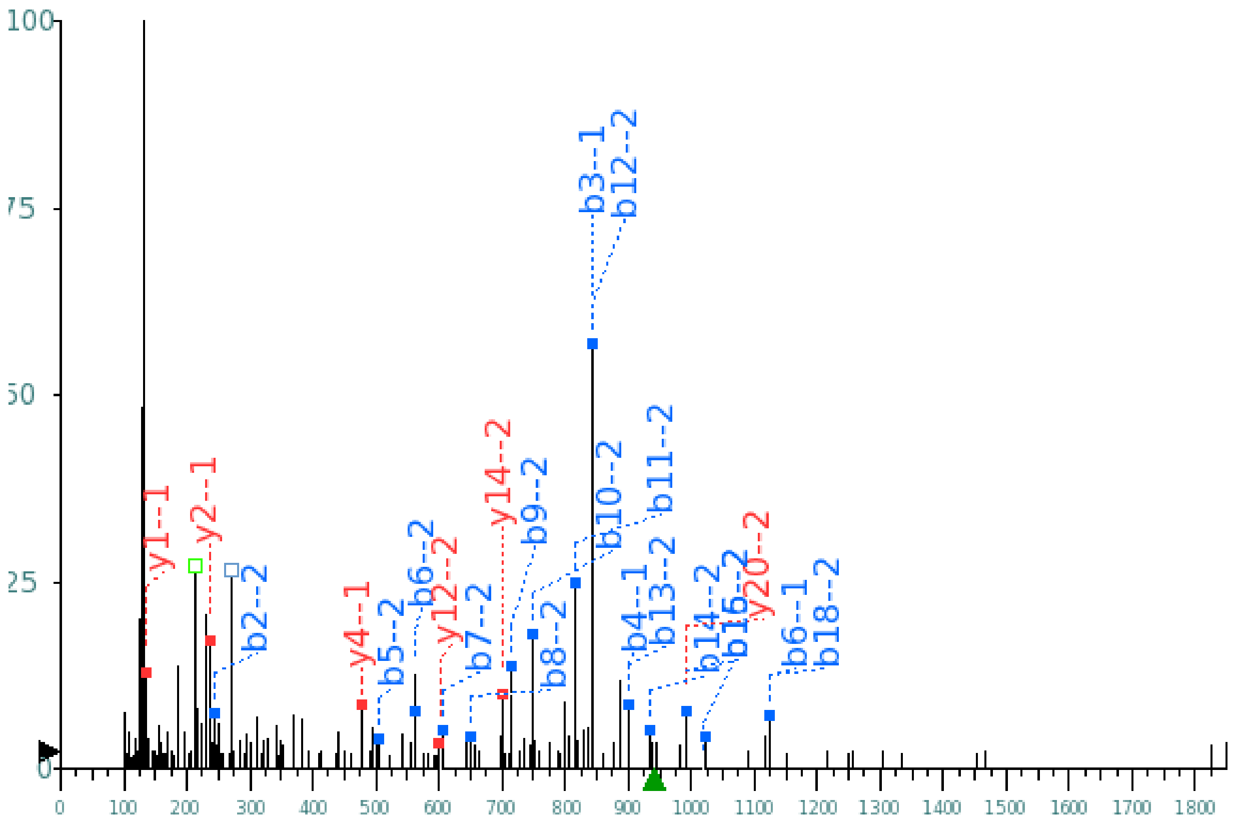

RVKGILSSQAEGLANGPDVLETD (+3)

Primary Reference: EHMT1_HUMAN
Search ID: 41758
Search Name: 20130328_ananiav_ERstress_TMT_TM_timecourse_chymo_inj1_4MC_IAA
Scan#: 21198
Observed Mass: 943.1896 (-0.8 ppm)
PSM Score: 50.19

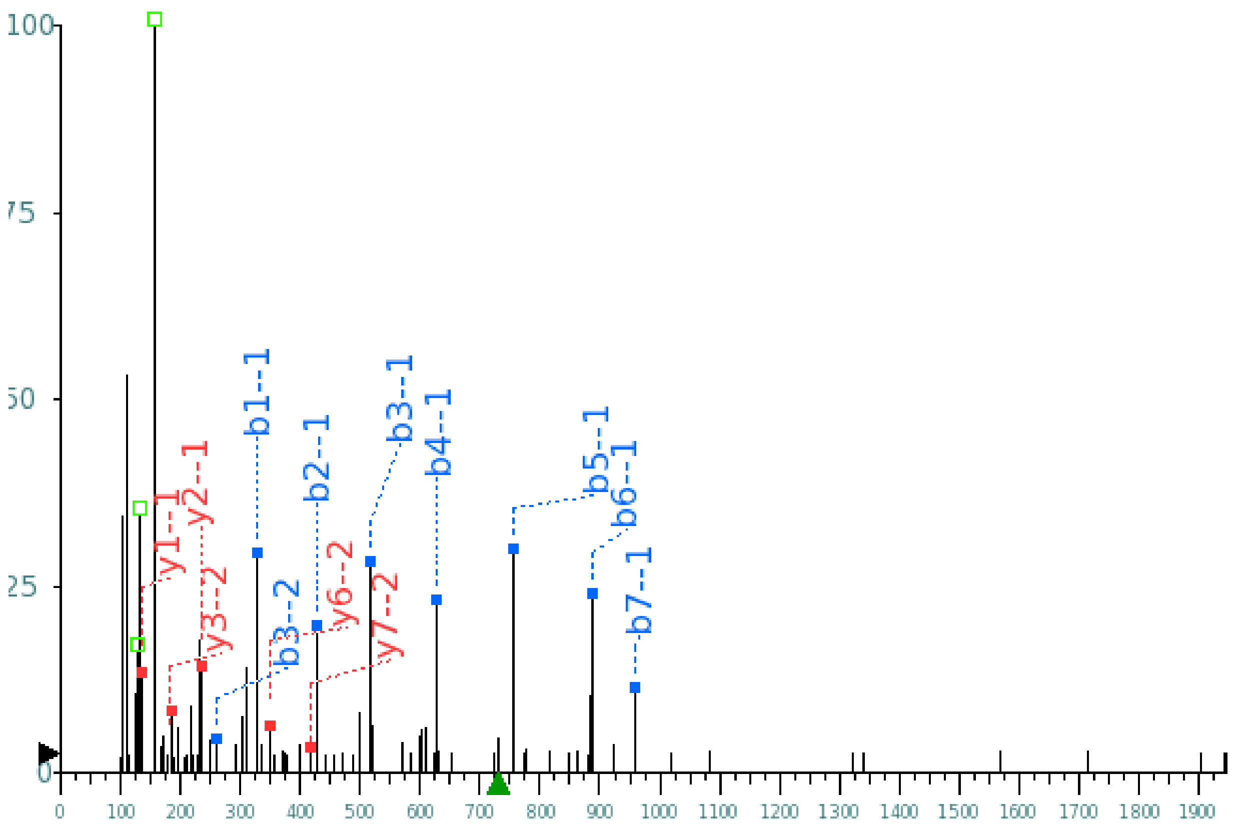

VVSLEEAWPLHGDC*ETD (+3)

Primary Reference: MDC1_HUMAN
Search ID: 41758
Search Name: 20130328_ananiav_ERstress_TMT_TM_timecourse_chymo_inj1_4MC_IAA
Scan#: 21212
Observed Mass: 729.3477 (0.6 ppm)
PSM Score: 35.38

File name: 2015-02-04 04.01.53PM.pdf

Creation time: 2015-02-04 04:09PM

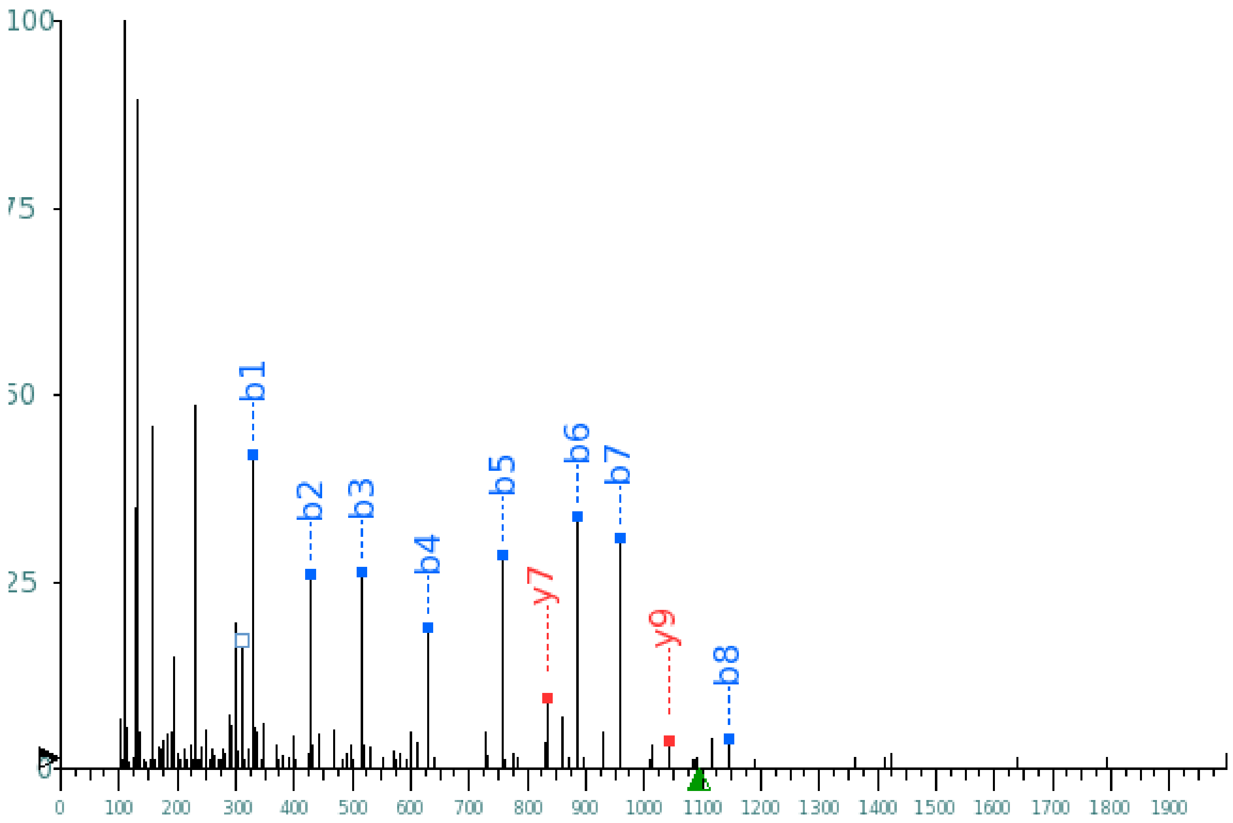

VVSLEEAWPLHGDC*ETD (+2)

Primary Reference: MDC1_HUMAN
Search ID: 41758
Search Name: 20130328_ananiav_ERstress_TMT_TM_timecourse_chymo_inj1_4MC_IAA
Scan#: 21223
Observed Mass: 1093.5173 (-0 ppm)
PSM Score: 31.4

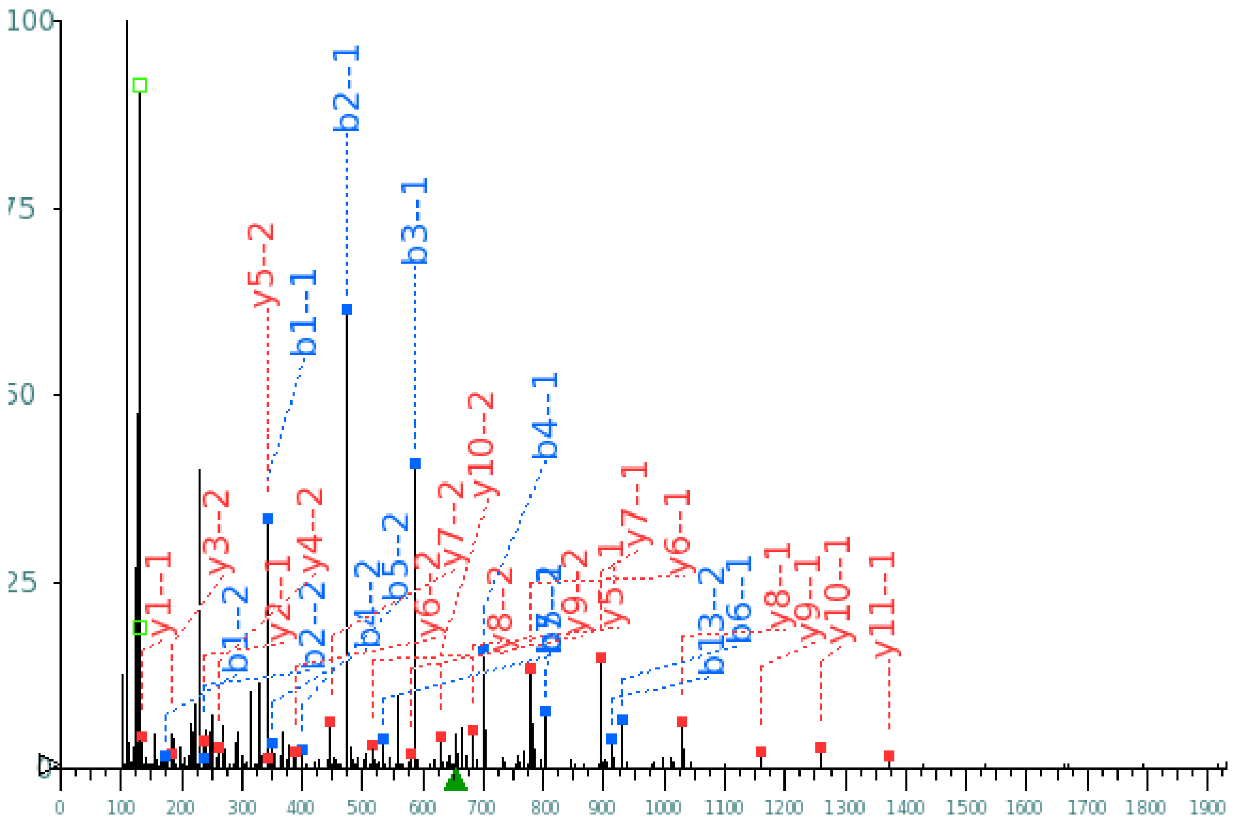

IMILTEHLVRC*ETD (+3)

Primary Reference: NCBP1_HUMAN
Search ID: 41758
Search Name: 20130328_ananiav_ERstress_TMT_TM_timecourse_chymo_inj1_4MC_IAA
Scan#: 21385
Observed Mass: 653.6816 (3.2 ppm)
PSM Score: 35.4

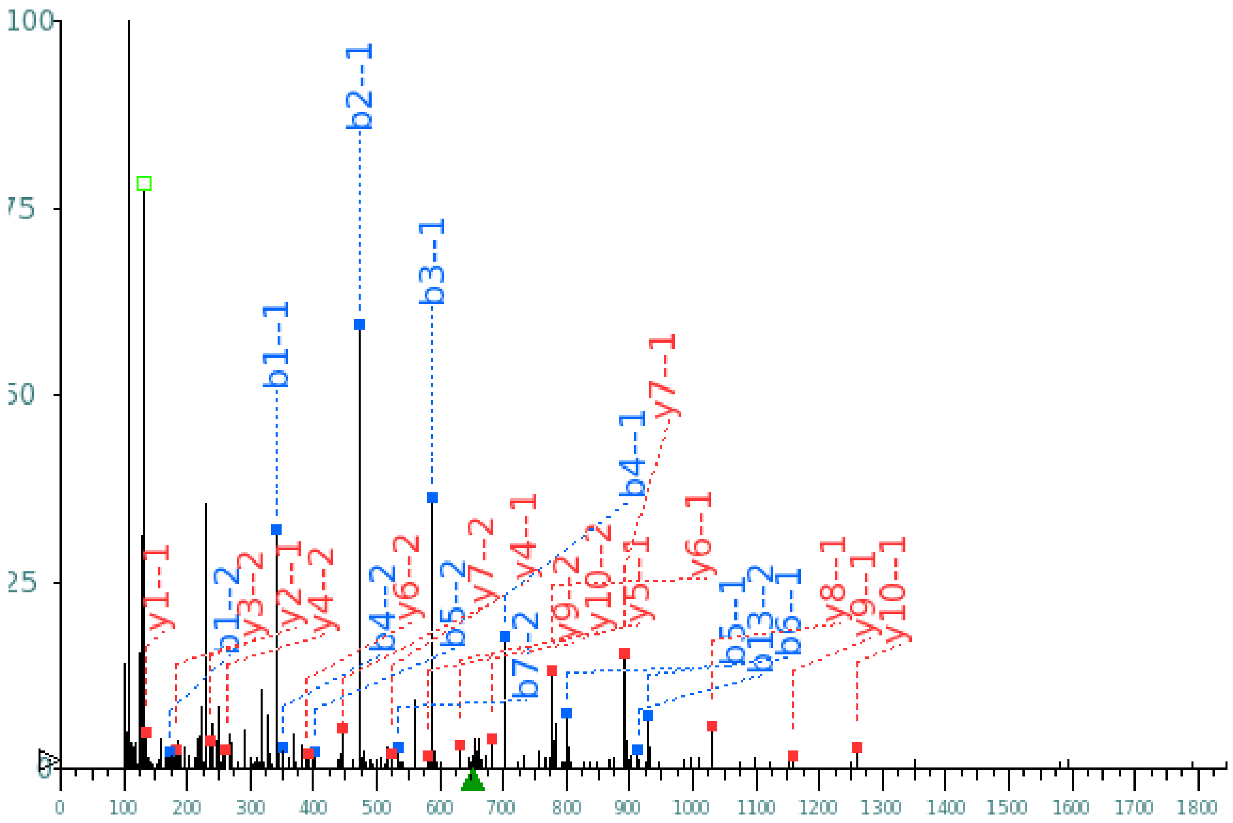

IMILTEHLVRC*ETD (+3)

Primary Reference: NCBP1_HUMAN
Search ID: 41758
Search Name: 20130328_ananiav_ERstress_TMT_TM_timecourse_chymo_inj1_4MC_IAA
Scan#: 21391
Observed Mass: 653.6816 (3.2 ppm)
PSM Score: 34.28

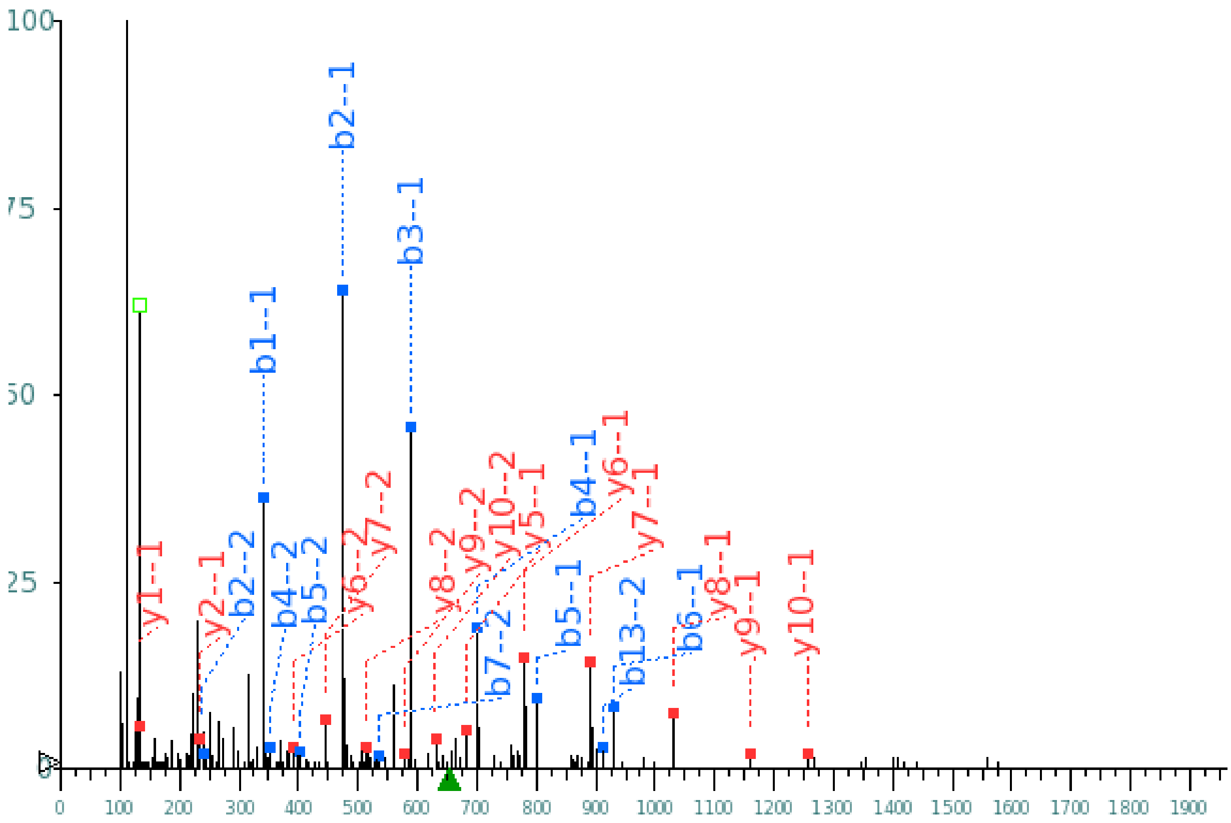

IMILTEHLVRC*ETD (+3)

Primary Reference: NCBP1_HUMAN
Search ID: 41758
Search Name: 20130328_ananiav_ERstress_TMT_TM_timecourse_chymo_inj1_4MC_IAA
Scan#: 21465
Observed Mass: 653.6811 (2.4 ppm)
PSM Score: 40.2

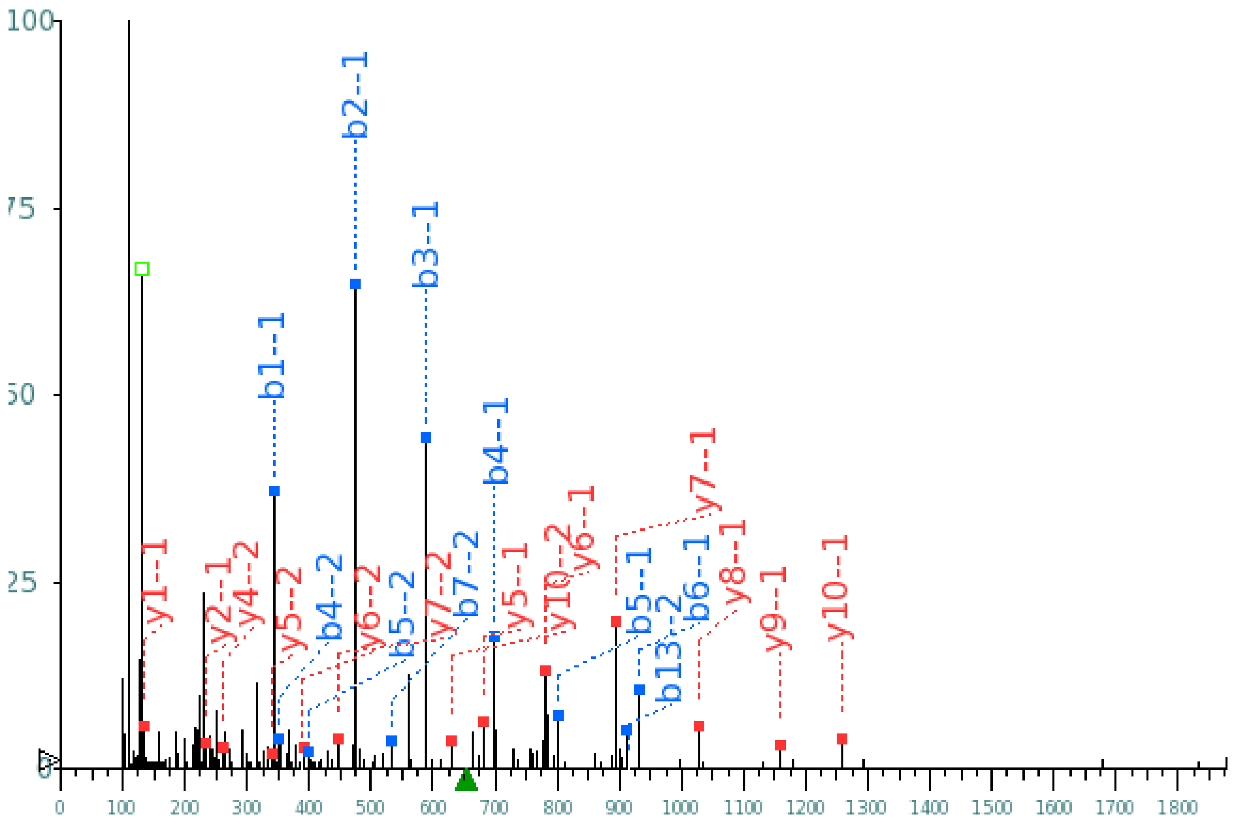

IMILTEHLVRC*ETD (+3)

Primary Reference: NCBP1_HUMAN
Search ID: 41758
Search Name: 20130328_ananiav_ERstress_TMT_TM_timecourse_chymo_inj1_4MC_IAA
Scan#: 21542
Observed Mass: 653.6813 (2.6 ppm)
PSM Score: 39.97

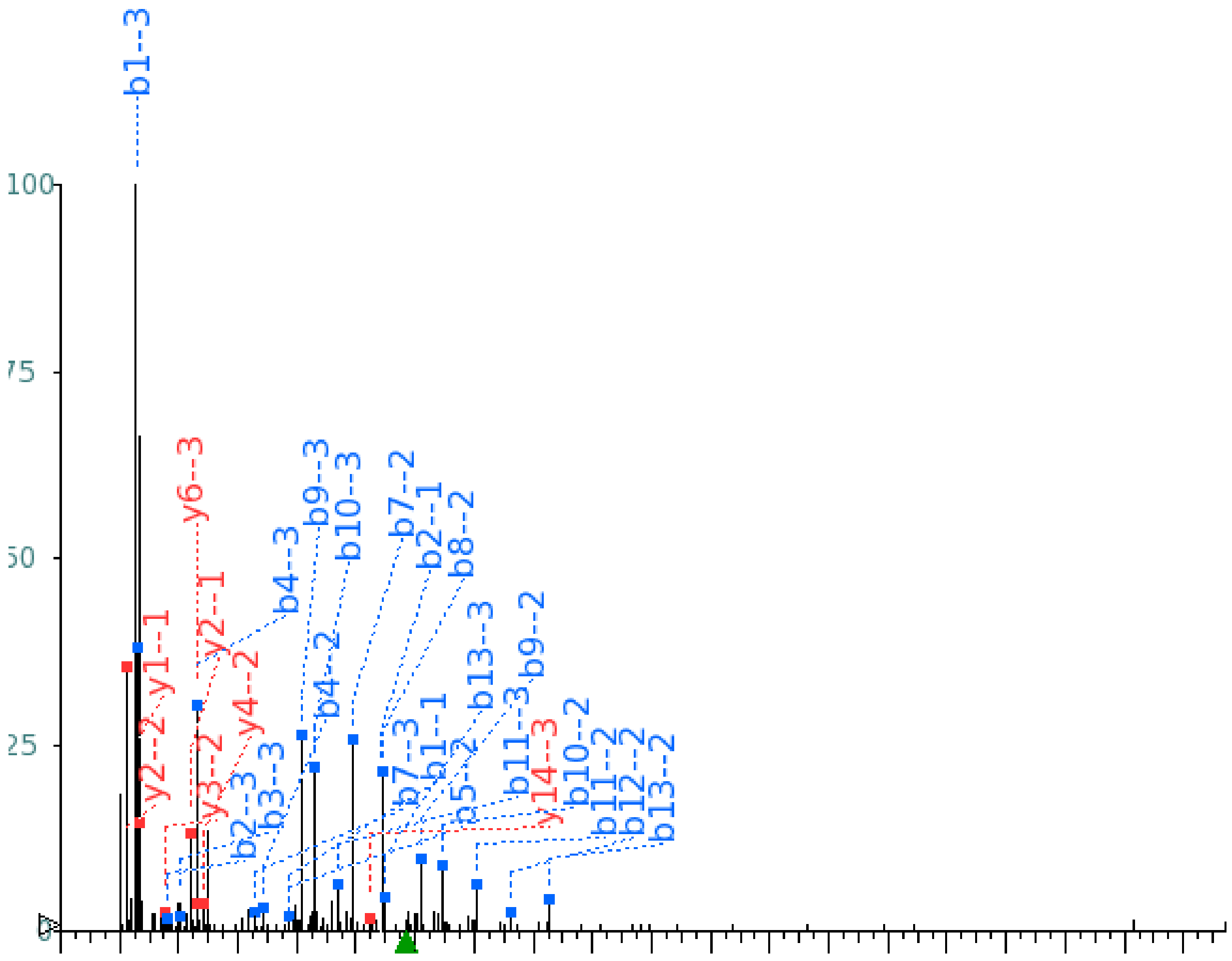

RRGSSPDVHALLEITEESD (+4)

Primary Reference: PTRF_HUMAN
Search ID: 41758
Search Name: 20130328_ananiav_ERstress_TMT_TM_timecourse_chymo_inj1_4MC_IAA
Scan#: 21768
Observed Mass: 585.8066 (2.2 ppm)
PSM Score: 28.15

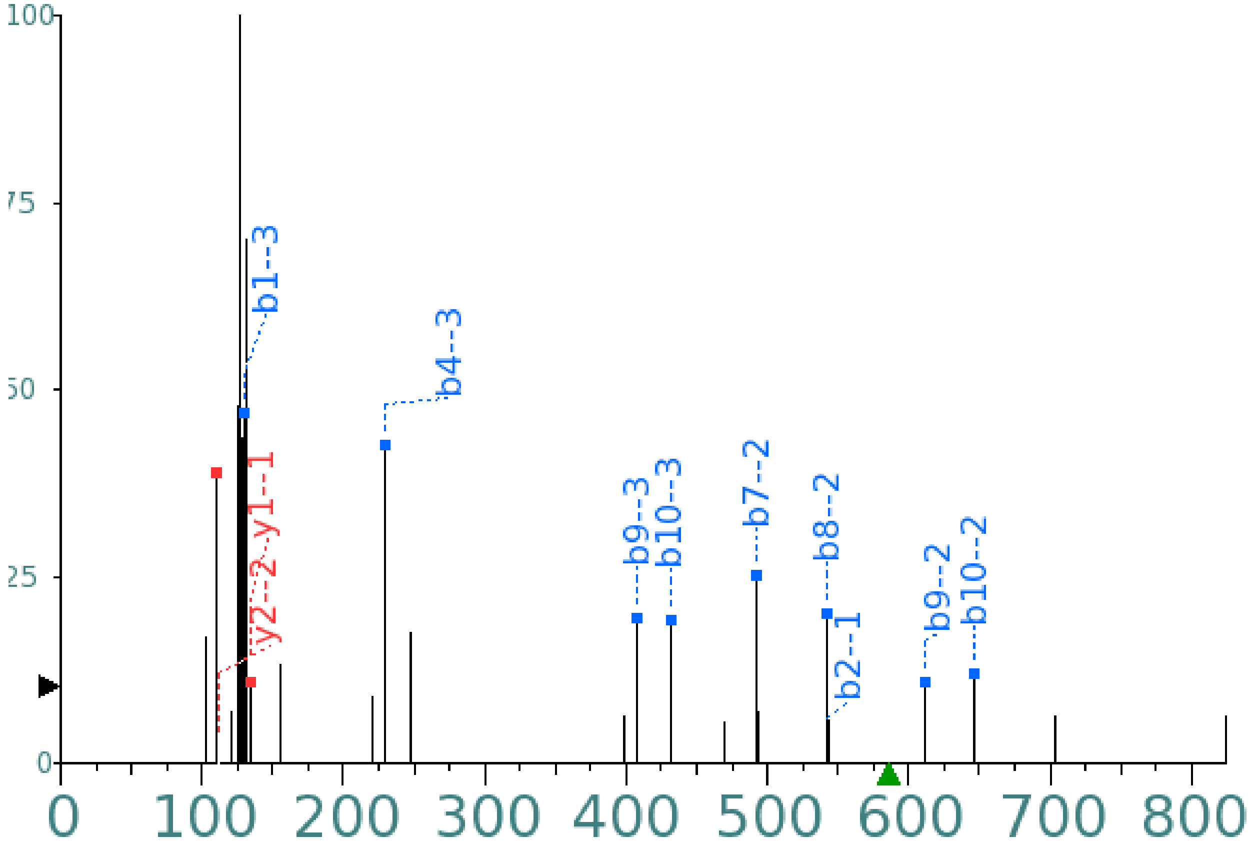

RRGSSPDVHALLEITEESD (+4)

Primary Reference: PTRF_HUMAN
Search ID: 41758
Search Name: 20130328_ananiav_ERstress_TMT_TM_timecourse_chymo_inj1_4MC_IAA
Scan#: 22528
Observed Mass: 585.8059 (1.1 ppm)
PSM Score: 25.23

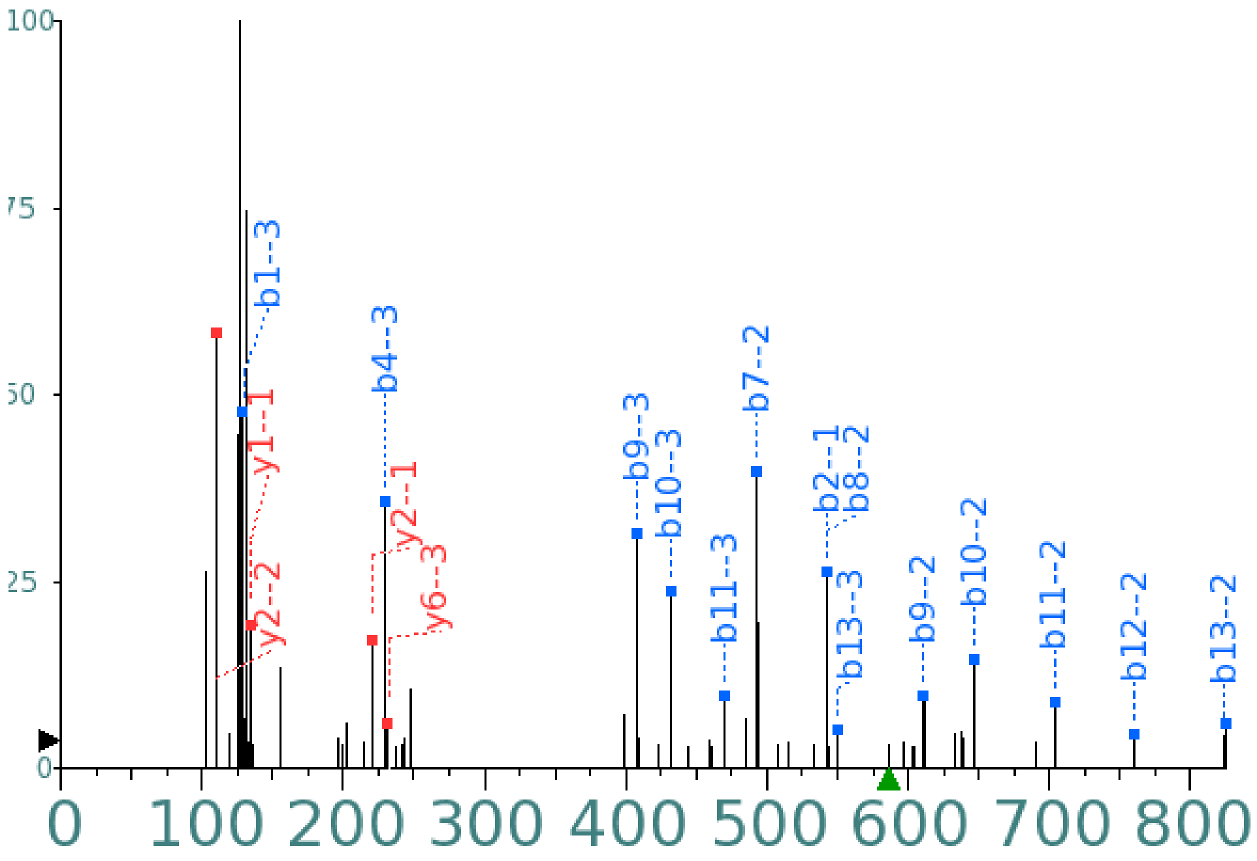

RRGSSPDVHALLEITEESD (+4)

Primary Reference: PTRF_HUMAN
Search ID: 41758
Search Name: 20130328_ananiav_ERstress_TMT_TM_timecourse_chymo_inj1_4MC_IAA
Scan#: 22551
Observed Mass: 585.8052 (-0.1 ppm)
PSM Score: 28.19

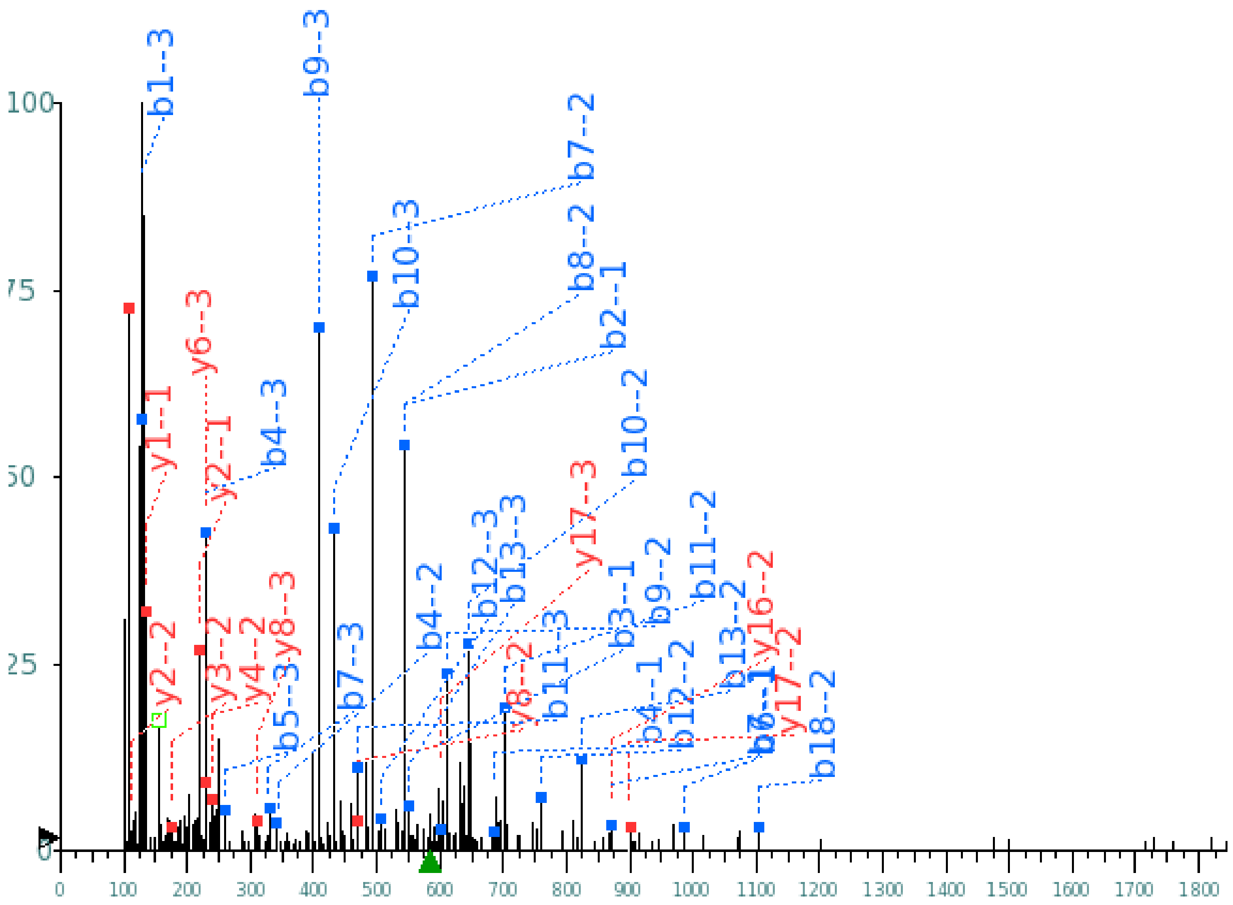

RRGSSPDVHALLEITEESD (+4)

Primary Reference: PTRF_HUMAN
Search ID: 41758
Search Name: 20130328_ananiav_ERstress_TMT_TM_timecourse_chymo_inj1_4MC_IAA
Scan#: 22640
Observed Mass: 585.8061 (1.4 ppm)
PSM Score: 26.04

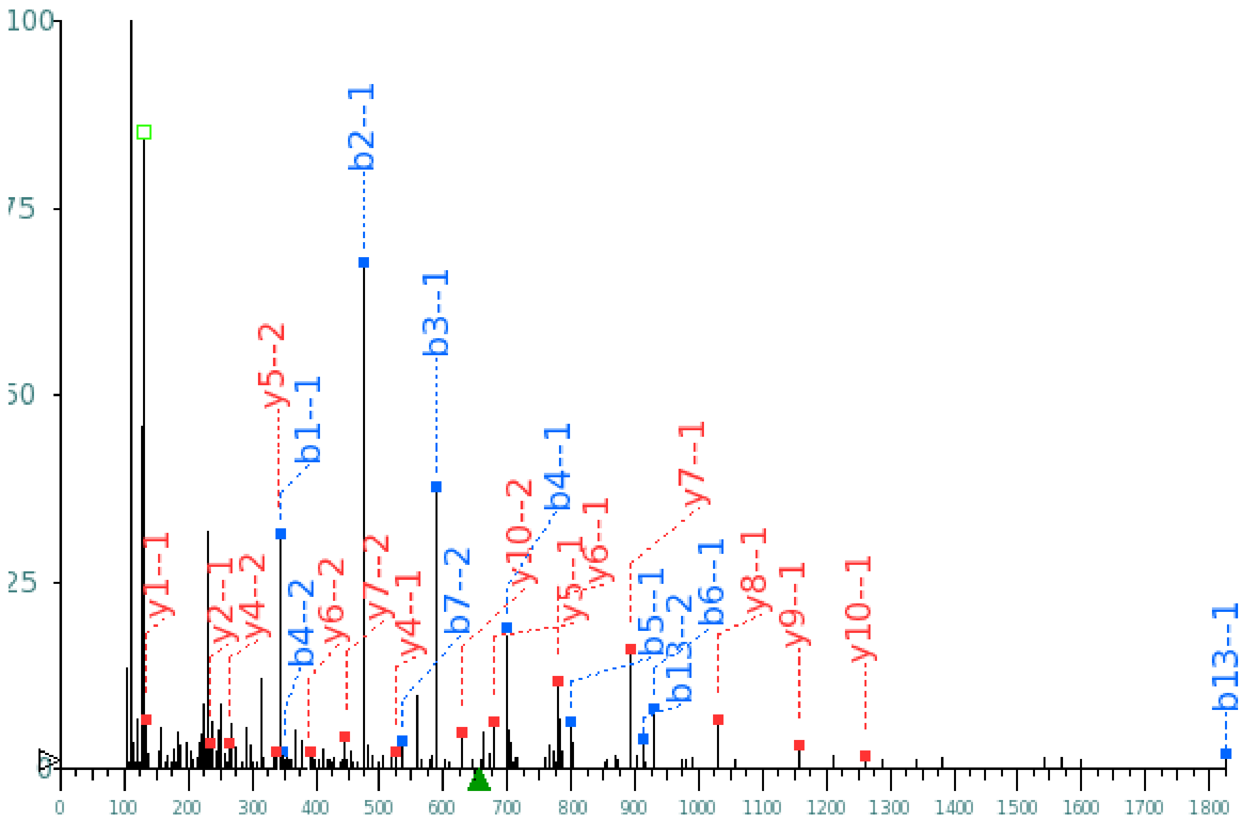

IMILTEHLVRC*ETD (+3)

Primary Reference: NCBP1_HUMAN
Search ID: 41758
Search Name: 20130328_ananiav_ERstress_TMT_TM_timecourse_chymo_inj1_4MC_IAA
Scan#: 22822
Observed Mass: 653.6813 (2.8 ppm)
PSM Score: 39.98

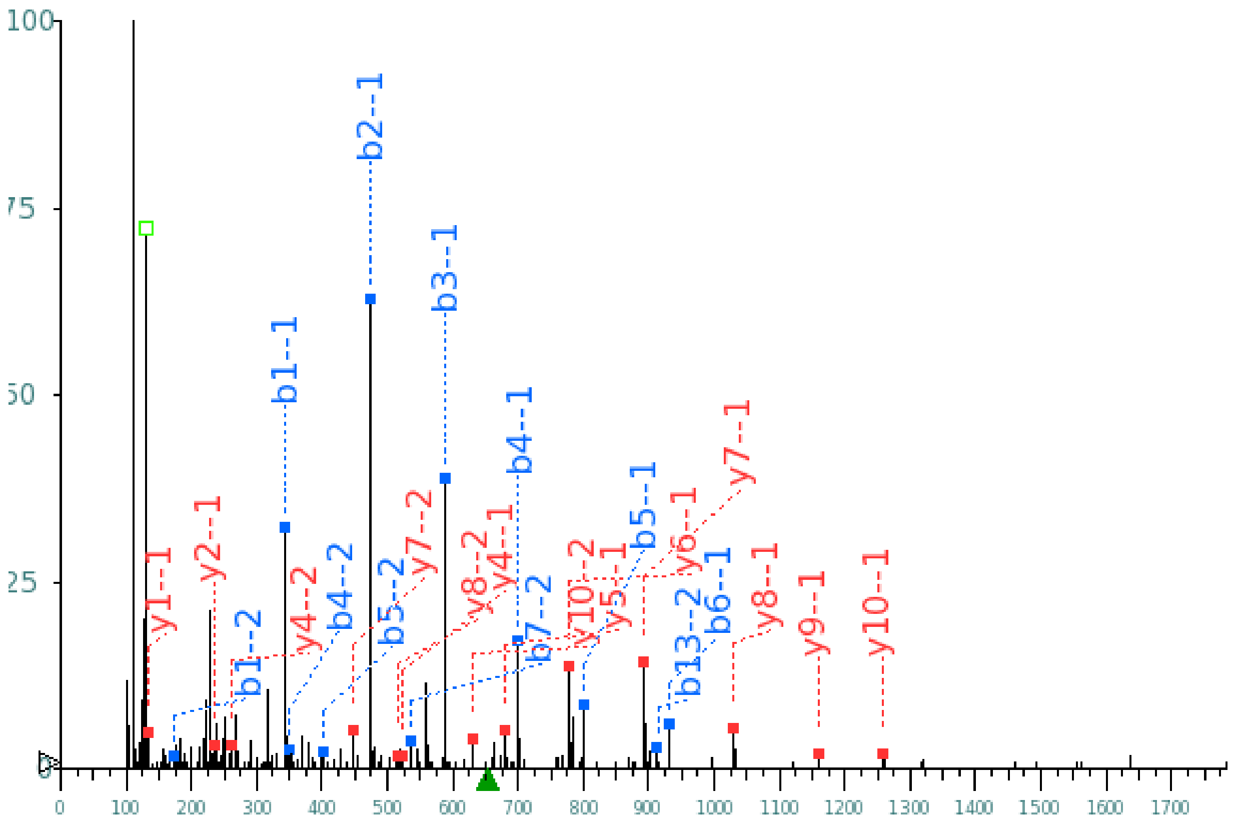

IMILTEHLVRC*ETD (+3)

Primary Reference: NCBP1_HUMAN
Search ID: 41758
Search Name: 20130328_ananiav_ERstress_TMT_TM_timecourse_chymo_inj1_4MC_IAA
Scan#: 22896
Observed Mass: 653.6811 (2.4 ppm)
PSM Score: 27.38

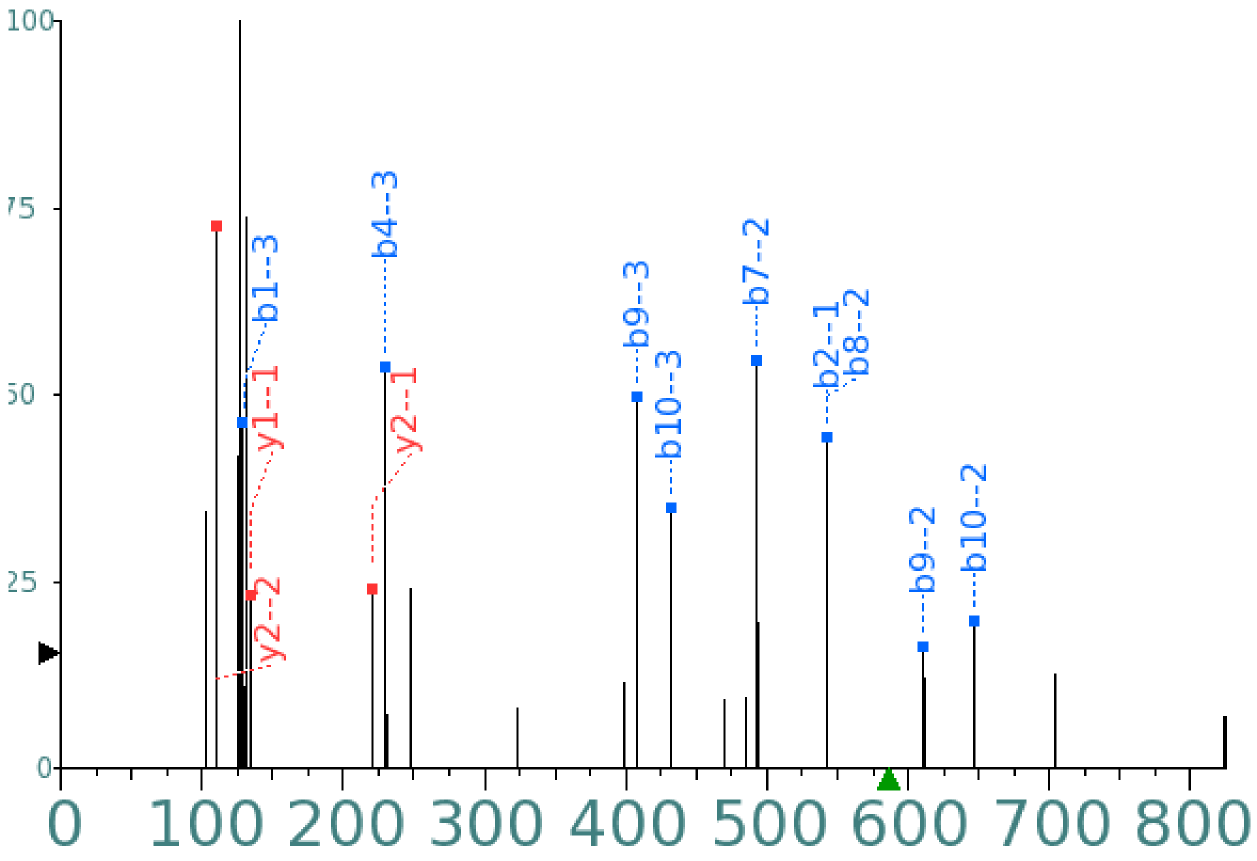

RRGSSPDVHALLEITEESD (+4)

Primary Reference: PTRF_HUMAN
Search ID: 41758
Search Name: 20130328_ananiav_ERstress_TMT_TM_timecourse_chymo_inj1_4MC_IAA
Scan#: 22968
Observed Mass: 585.8066 (2.3 ppm)
PSM Score: 25.45

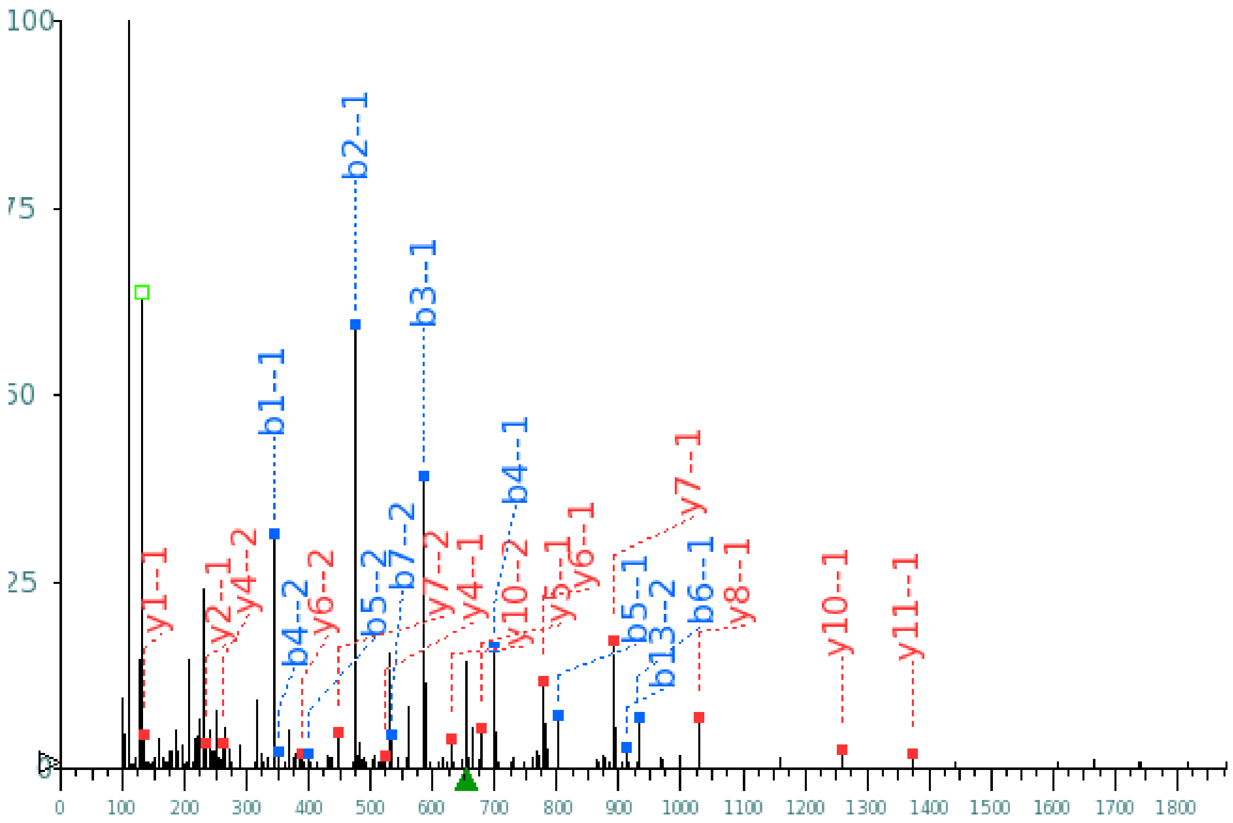

IMILTEHLVRC*ETD (+3)

Primary Reference: NCBP1_HUMAN
Search ID: 41758
Search Name: 20130328_ananiav_ERstress_TMT_TM_timecourse_chymo_inj1_4MC_IAA
Scan#: 22975
Observed Mass: 653.6815 (3 ppm)
PSM Score: 29.69

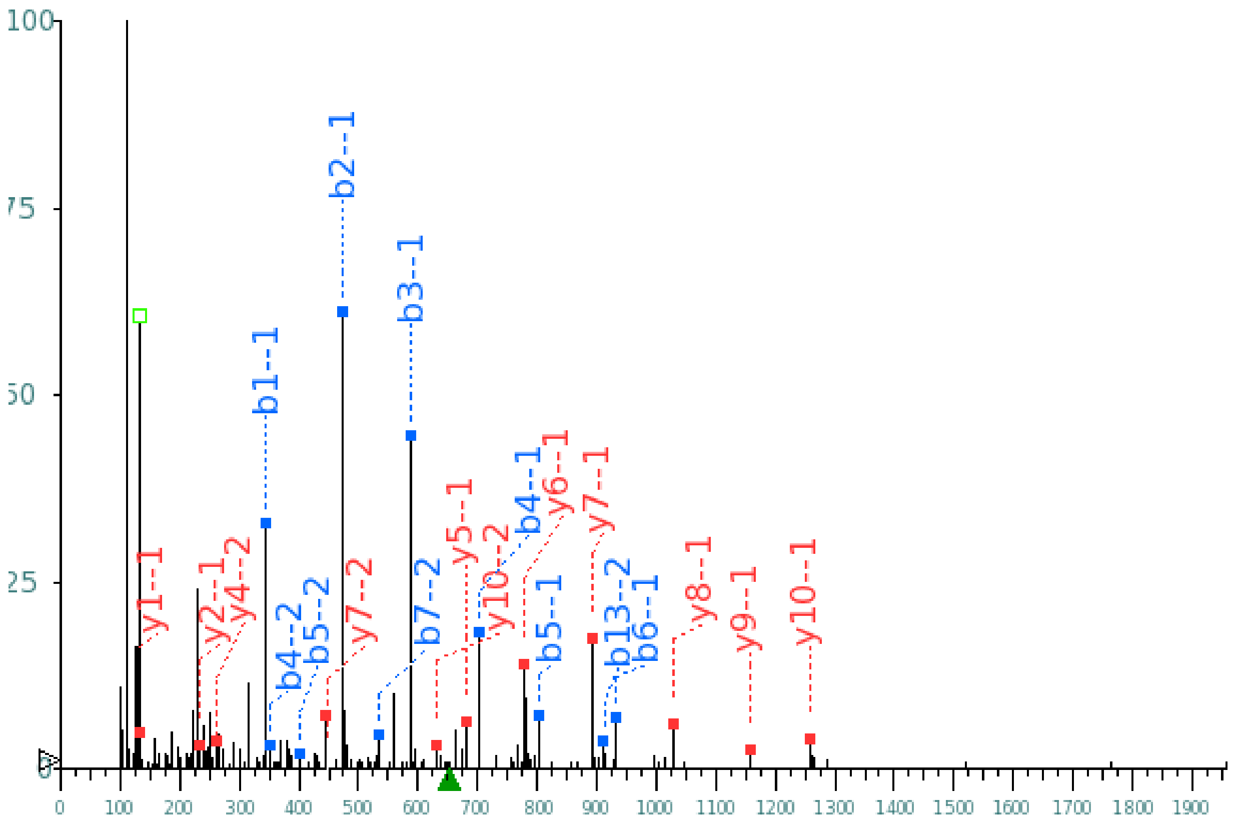

IMILTEHLVRC*ETD (+3)

Primary Reference: NCBP1_HUMAN
Search ID: 41758
Search Name: 20130328_ananiav_ERstress_TMT_TM_timecourse_chymo_inj1_4MC_IAA
Scan#: 23039
Observed Mass: 653.681 (2.2 ppm)
PSM Score: 37.23

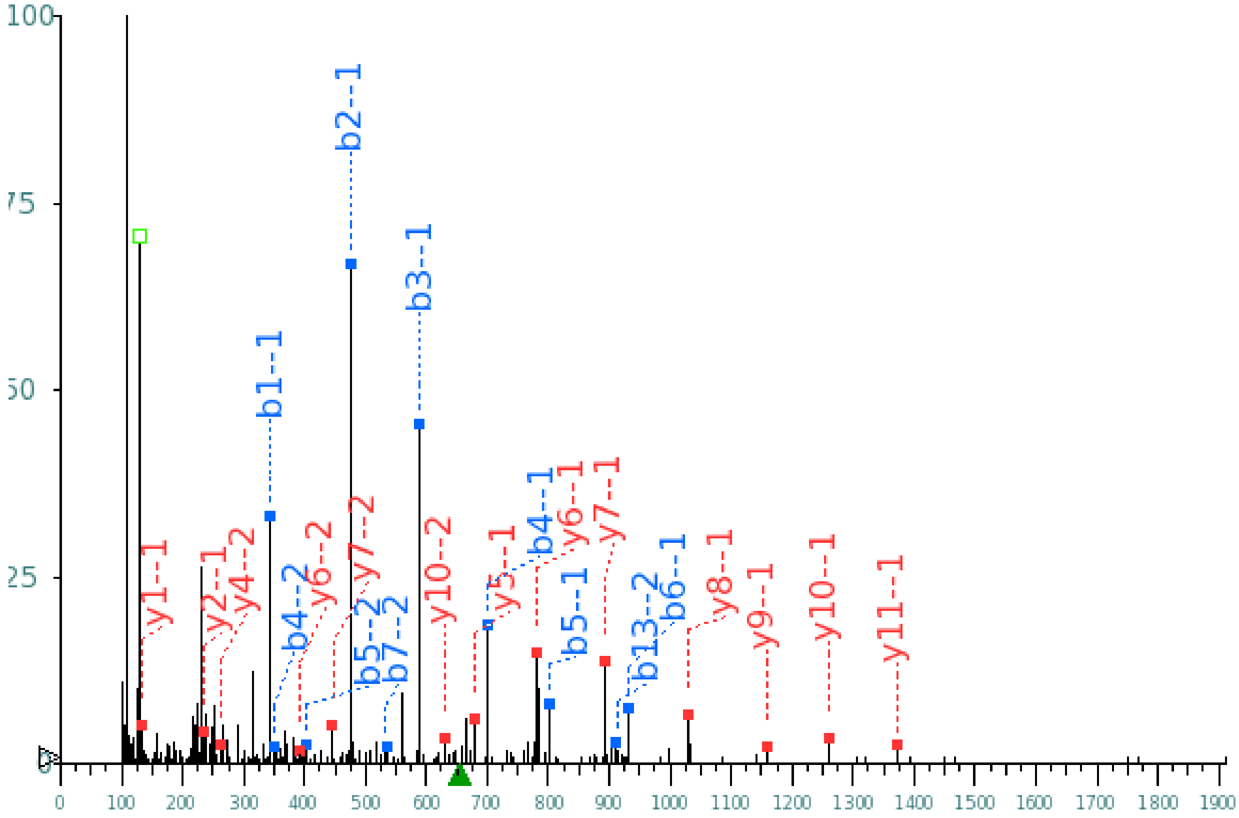

IMILTEHLVRC*ETD (+3)

Primary Reference: NCBP1_HUMAN
Search ID: 41758
Search Name: 20130328_ananiav_ERstress_TMT_TM_timecourse_chymo_inj1_4MC_IAA
Scan#: 23105
Observed Mass: 653.681 (2.3 ppm)
PSM Score: 40.86

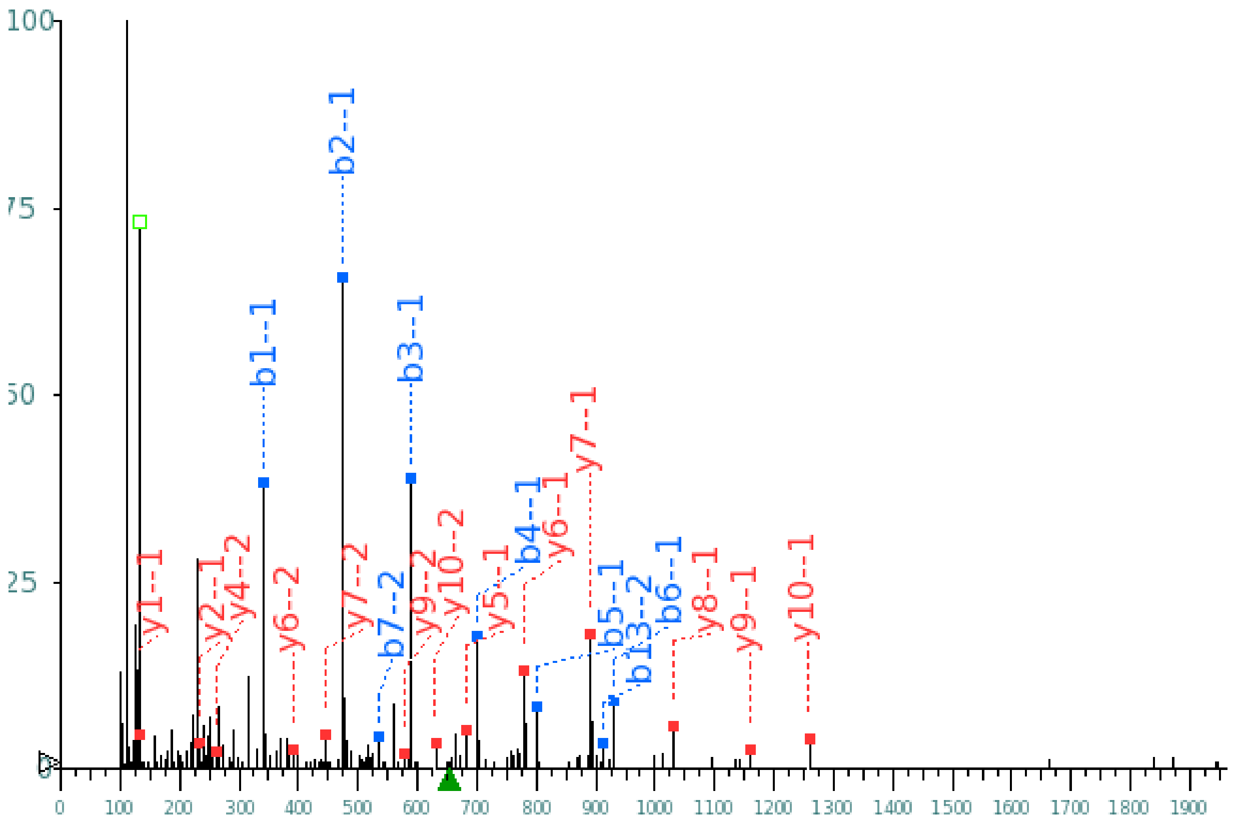

IMILTEHLVRC*ETD (+3)

Primary Reference: NCBP1_HUMAN
Search ID: 41758
Search Name: 20130328_ananiav_ERstress_TMT_TM_timecourse_chymo_inj1_4MC_IAA
Scan#: 23174
Observed Mass: 653.6815 (2.9 ppm)
PSM Score: 39.95

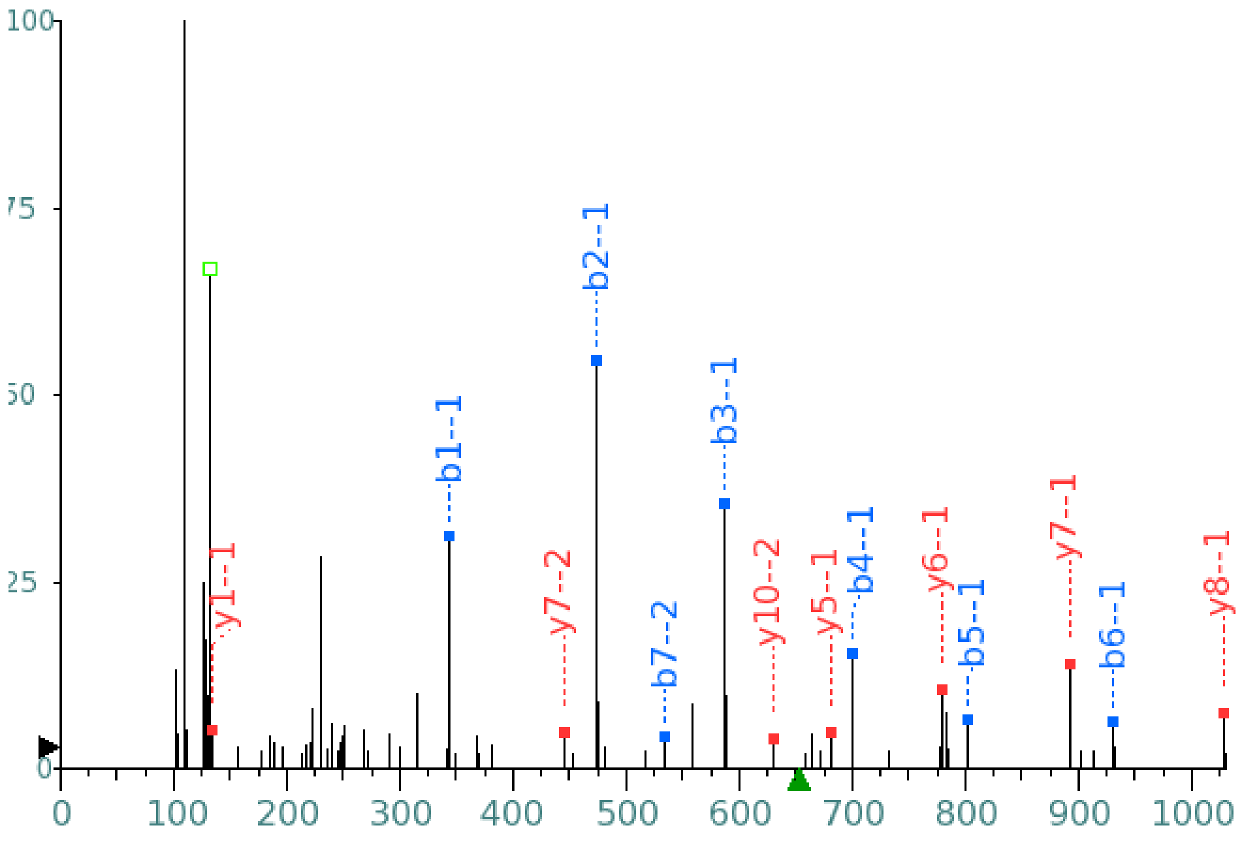

IMILTEHLVRC*ETD (+3)

Primary Reference: NCBP1_HUMAN
Search ID: 41758
Search Name: 20130328_ananiav_ERstress_TMT_TM_timecourse_chymo_inj1_4MC_IAA
Scan#: 23243
Observed Mass: 653.6813 (2.7 ppm)
PSM Score: 34.6

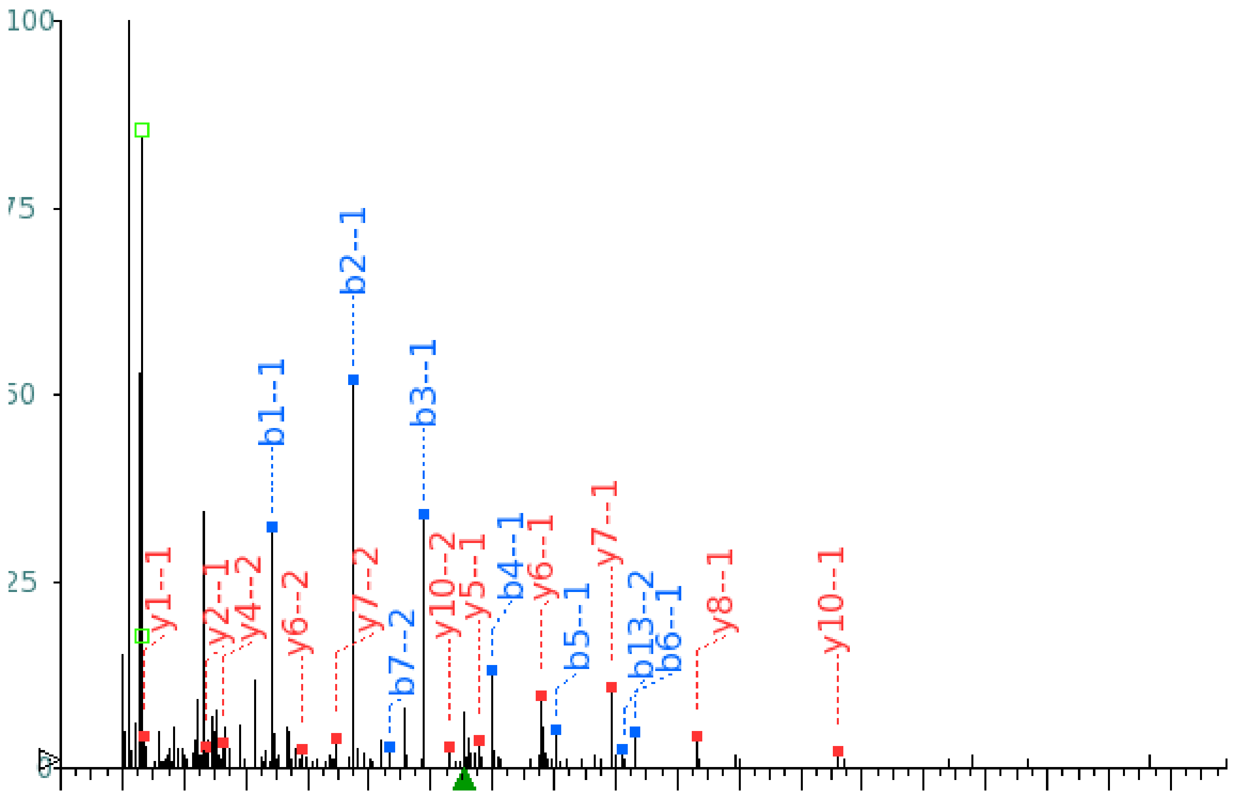

IMILTEHLVRC*ETD (+3)

Primary Reference: NCBP1_HUMAN
Search ID: 41758
Search Name: 20130328_ananiav_ERstress_TMT_TM_timecourse_chymo_inj1_4MC_IAA
Scan#: 23305
Observed Mass: 653.6807 (1.8 ppm)
PSM Score: 27.54

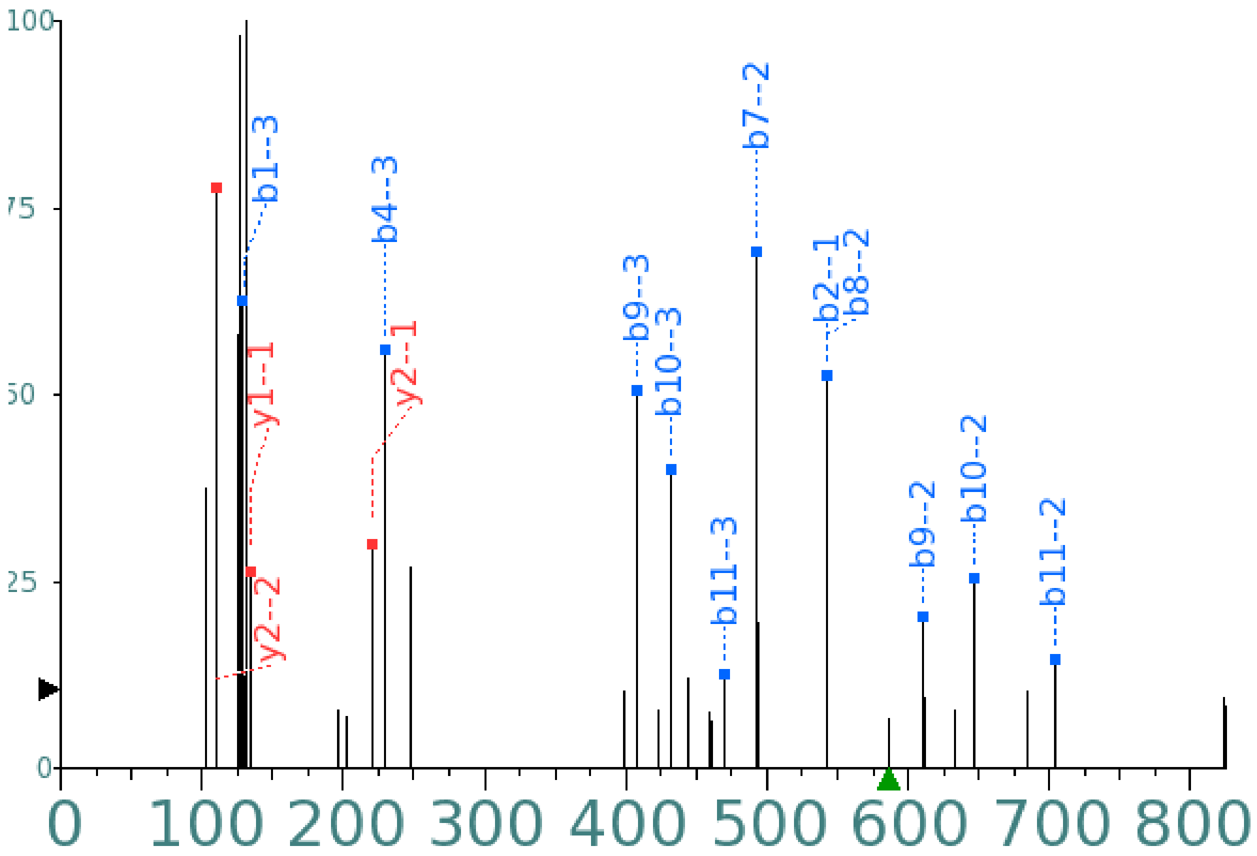

RRGSSPDVHALLEITEESD (+4)

Primary Reference: PTRF_HUMAN
Search ID: 41758
Search Name: 20130328_ananiav_ERstress_TMT_TM_timecourse_chymo_inj1_4MC_IAA
Scan#: 23320
Observed Mass: 585.8055 (0.4 ppm)
PSM Score: 27.86

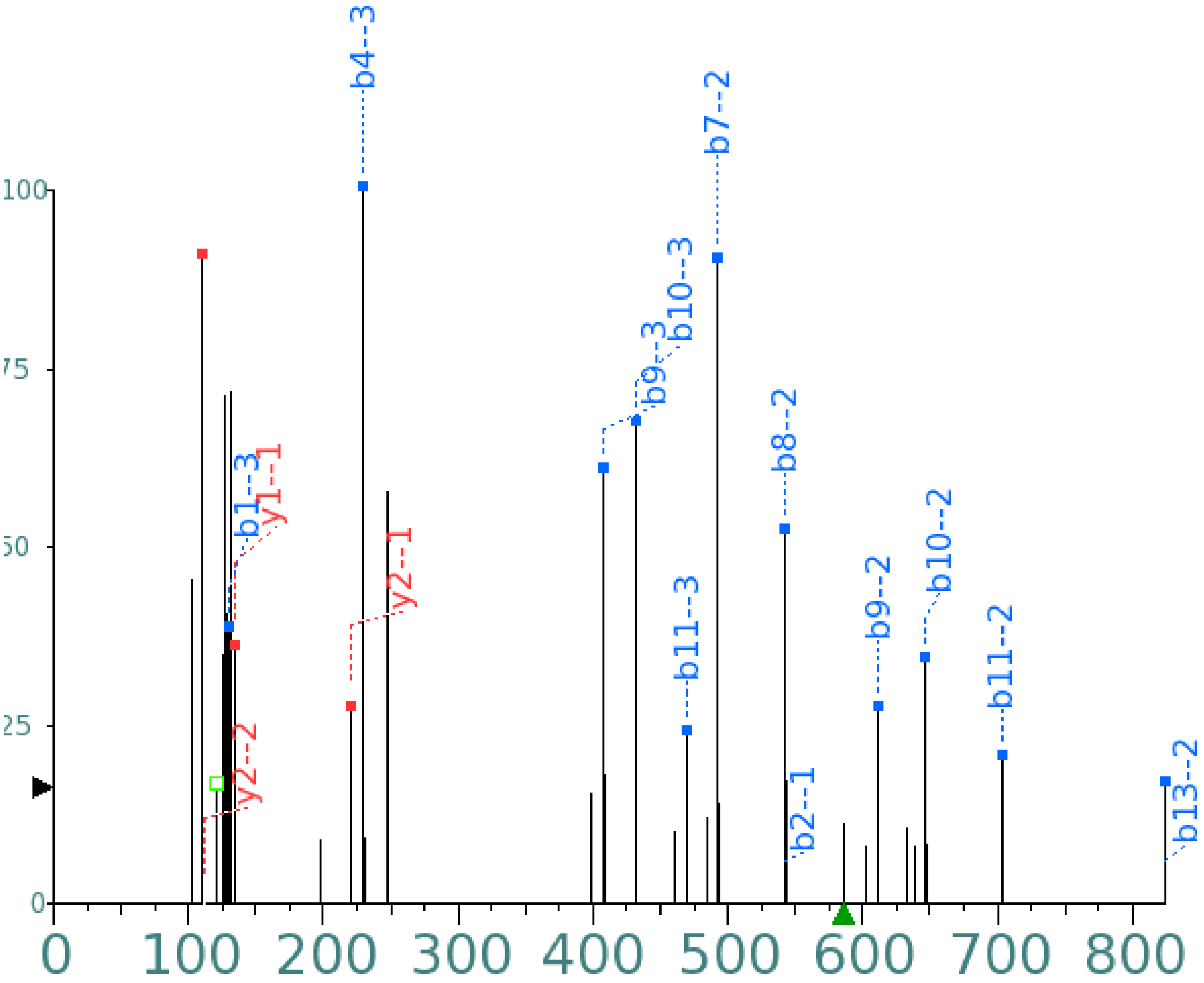

RRGSSPDVHALLEITEESD (+4)

Primary Reference: PTRF_HUMAN
Search ID: 41758
Search Name: 20130328_ananiav_ERstress_TMT_TM_timecourse_chymo_inj1_4MC_IAA
Scan#: 23639
Observed Mass: 585.8073 (3.5 ppm)
PSM Score: 27.93

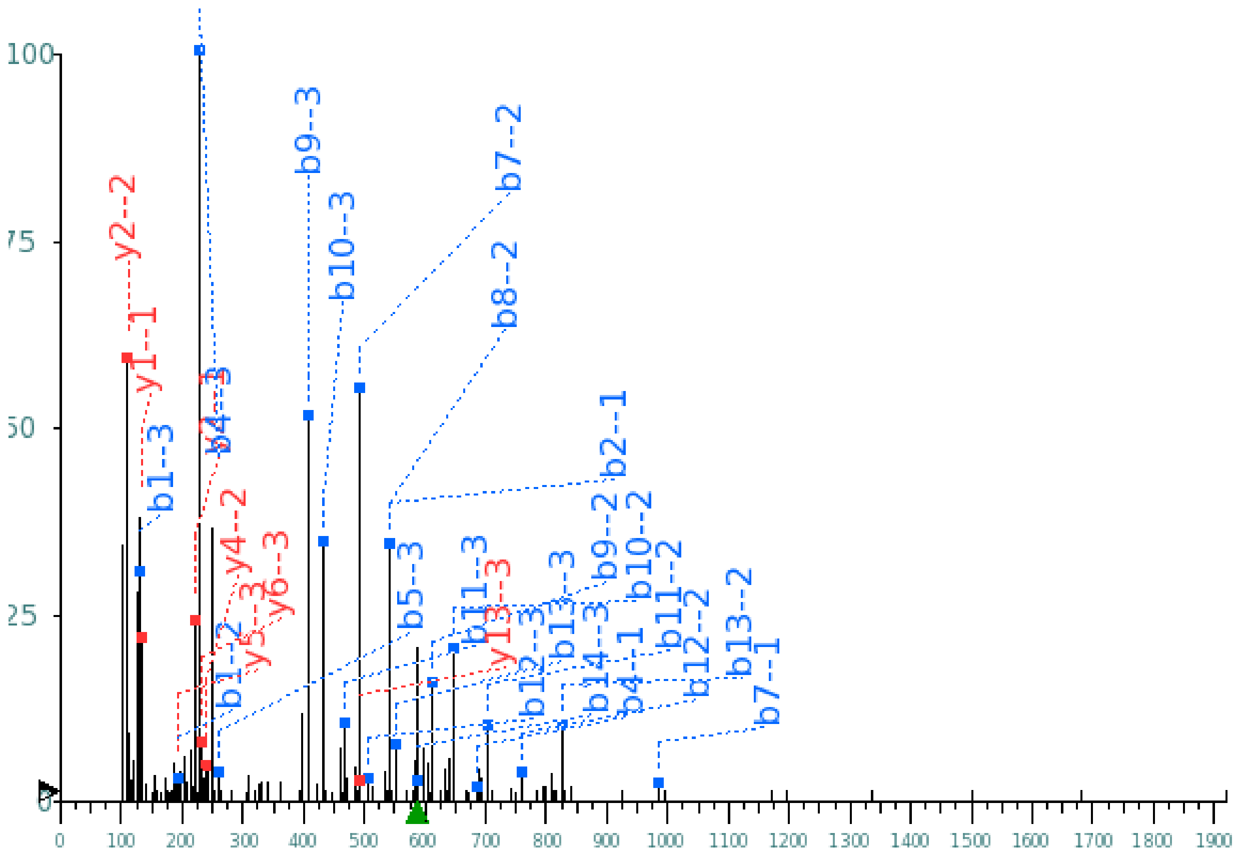

RRGSSPDVHALLEITEESD (+4)

Primary Reference: PTRF_HUMAN
Search ID: 41758
Search Name: 20130328_ananiav_ERstress_TMT_TM_timecourse_chymo_inj1_4MC_IAA
Scan#: 23779
Observed Mass: 585.8076 (3.9 ppm)
PSM Score: 27.46

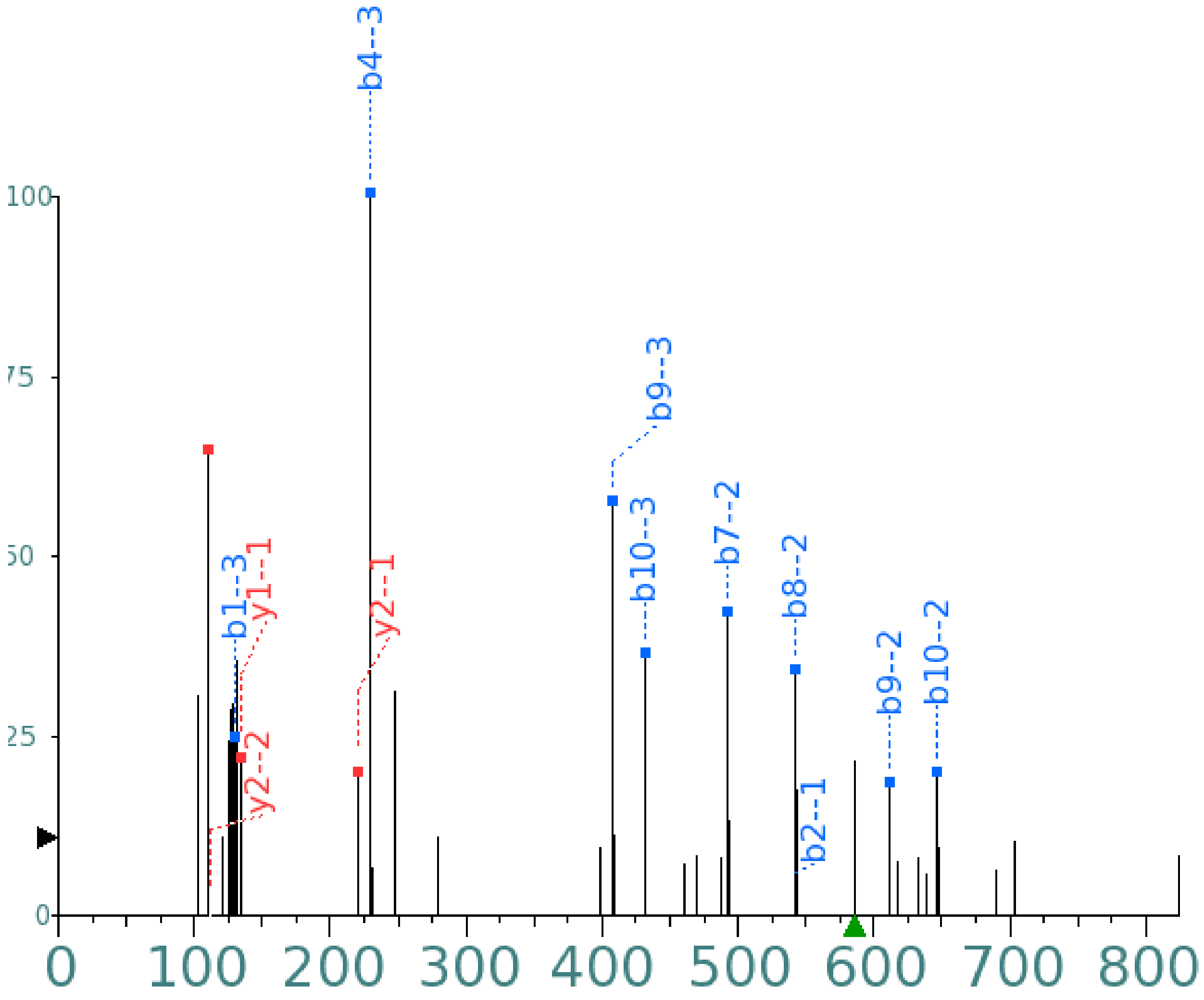

RRGSSPDVHALLEITEESD (+4)

Primary Reference: PTRF_HUMAN
Search ID: 41758
Search Name: 20130328_ananiav_ERstress_TMT_TM_timecourse_chymo_inj1_4MC_IAA
Scan#: 24068
Observed Mass: 585.8067 (2.4 ppm)
PSM Score: 26.53

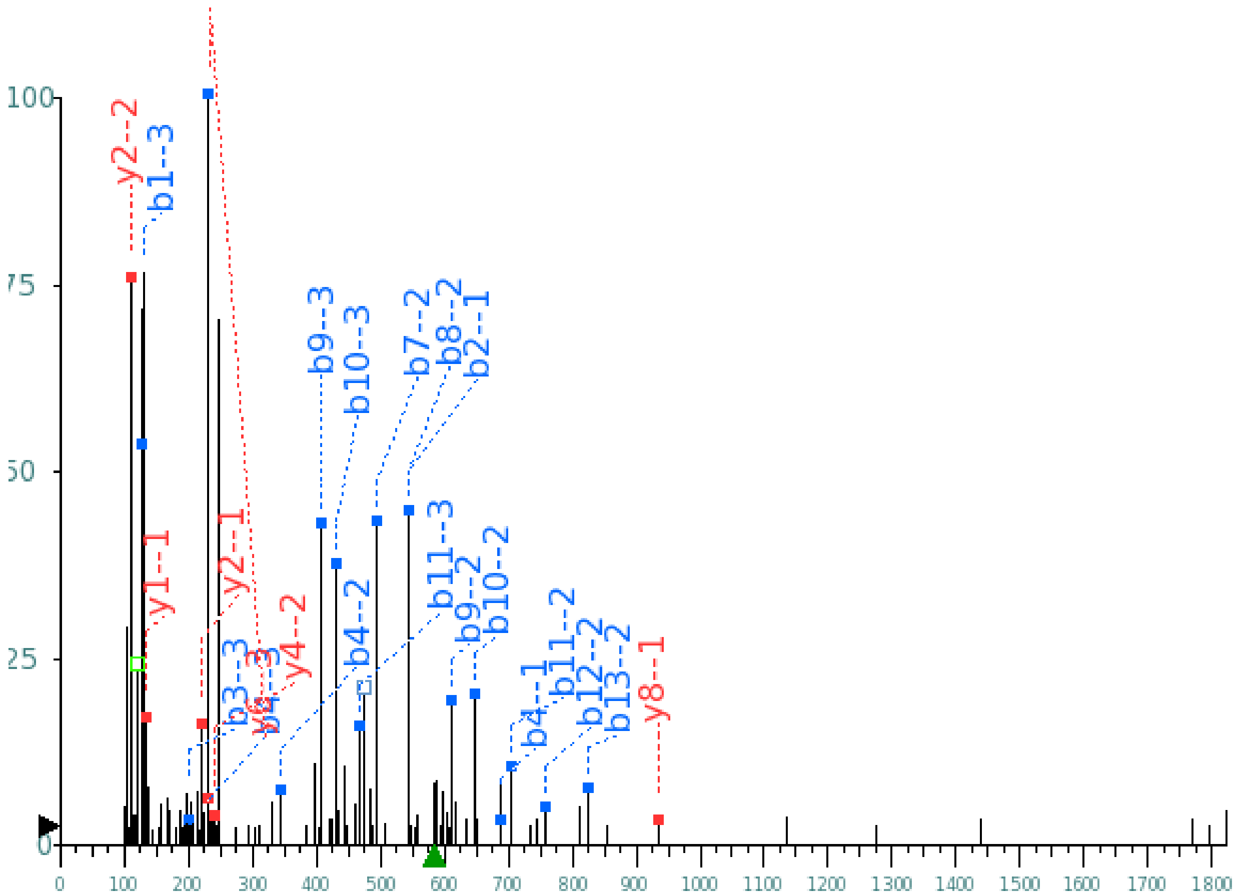

RRGSSPDVHALLEITEESD (+4)

Primary Reference: PTRF_HUMAN
Search ID: 41758
Search Name: 20130328_ananiav_ERstress_TMT_TM_timecourse_chymo_inj1_4MC_IAA
Scan#: 24235
Observed Mass: 585.8072 (3.3 ppm)
PSM Score: 26.55

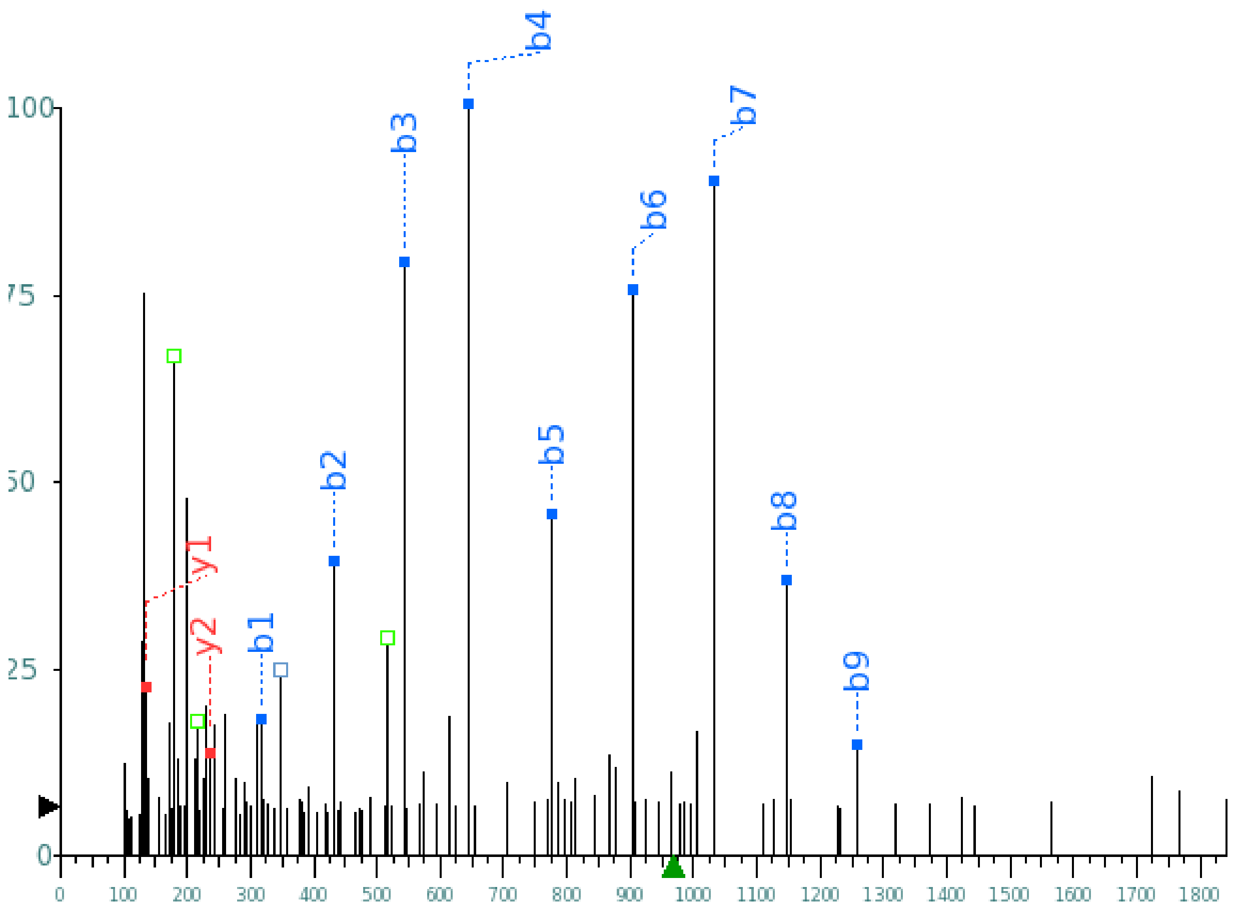

SNLVMEELLPTLQTD (+2)

Primary Reference: NIBAN_HUMAN
Search ID: 41758
Search Name: 20130328_ananiav_ERstress_TMT_TM_timecourse_chymo_inj1_4MC_IAA
Scan#: 24249
Observed Mass: 966.5162 (2.8 ppm)
PSM Score: 43.48

File name: 2015-02-04 04.01.53PM.pdf

Creation time: 2015-02-04 04:09PM

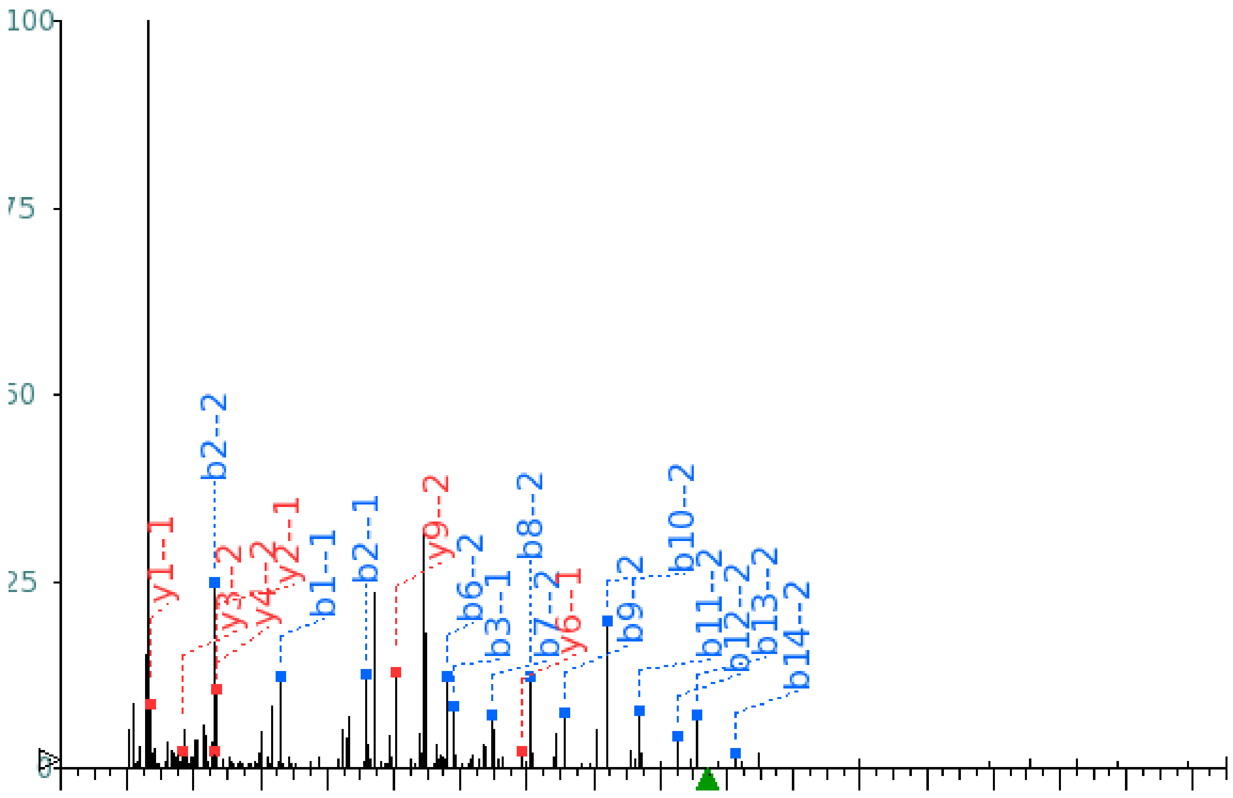

VMMPNKHNVEVLGILSDDVETD (+3)

Primary Reference: ERF3A_HUMAN
Search ID: 41758
Search Name: 20130328_ananiav_ERstress_TMT_TM_timecourse_chymo_inj1_4MC_IAA
Scan#: 24304
Observed Mass: 971.8443 (2.8 ppm)
PSM Score: 29.03

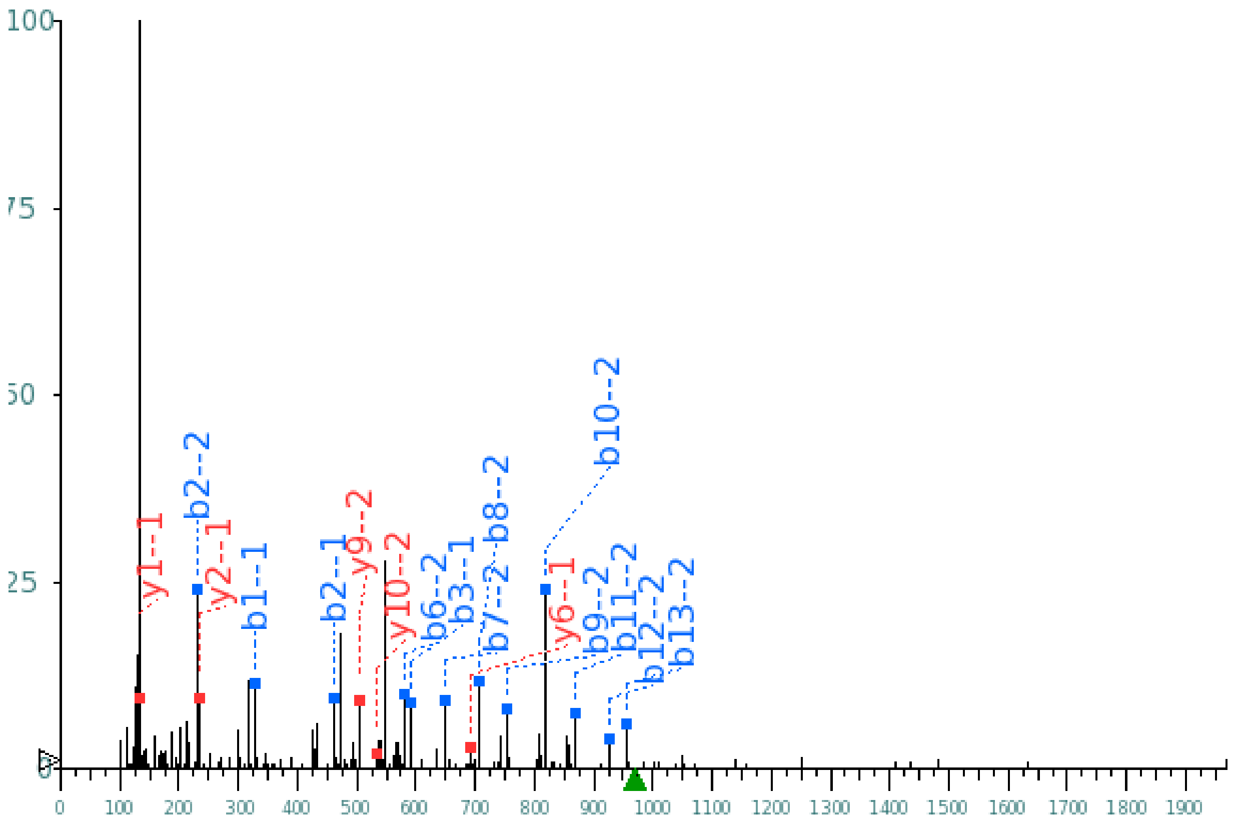

VMMPNKHNVEVLGILSDDVETD (+3)

Primary Reference: ERF3A_HUMAN
Search ID: 41758
Search Name: 20130328_ananiav_ERstress_TMT_TM_timecourse_chymo_inj1_4MC_IAA
Scan#: 24322
Observed Mass: 971.8438 (2.3 ppm)
PSM Score: 33.2

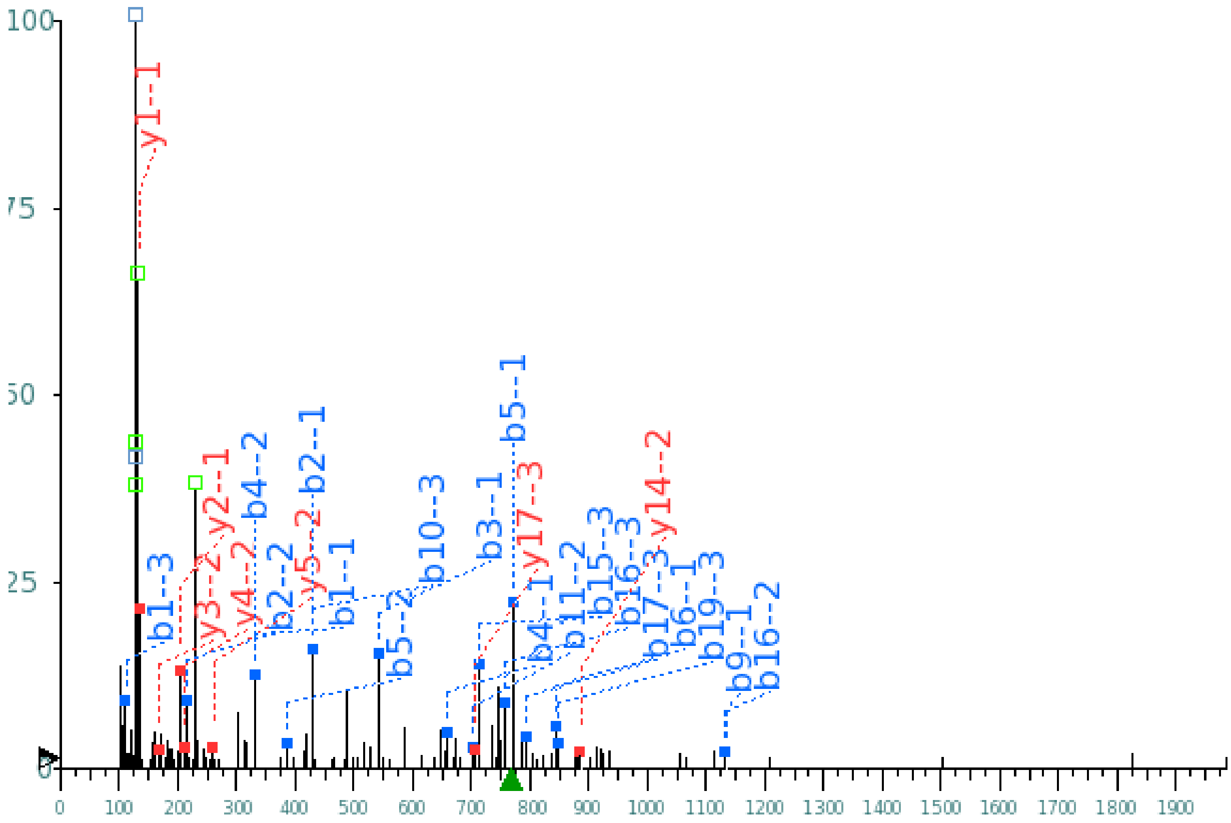

VTIIDAPGHRDFIKNMITGTSQAD (+4)

Primary Reference: EF1A1_HUMAN
Search ID: 41758
Search Name: 20130328_ananiav_ERstress_TMT_TM_timecourse_chymo_inj1_4MC_IAA
Scan#: 24425
Observed Mass: 765.4121 (-4.3 ppm)
PSM Score: 16.83

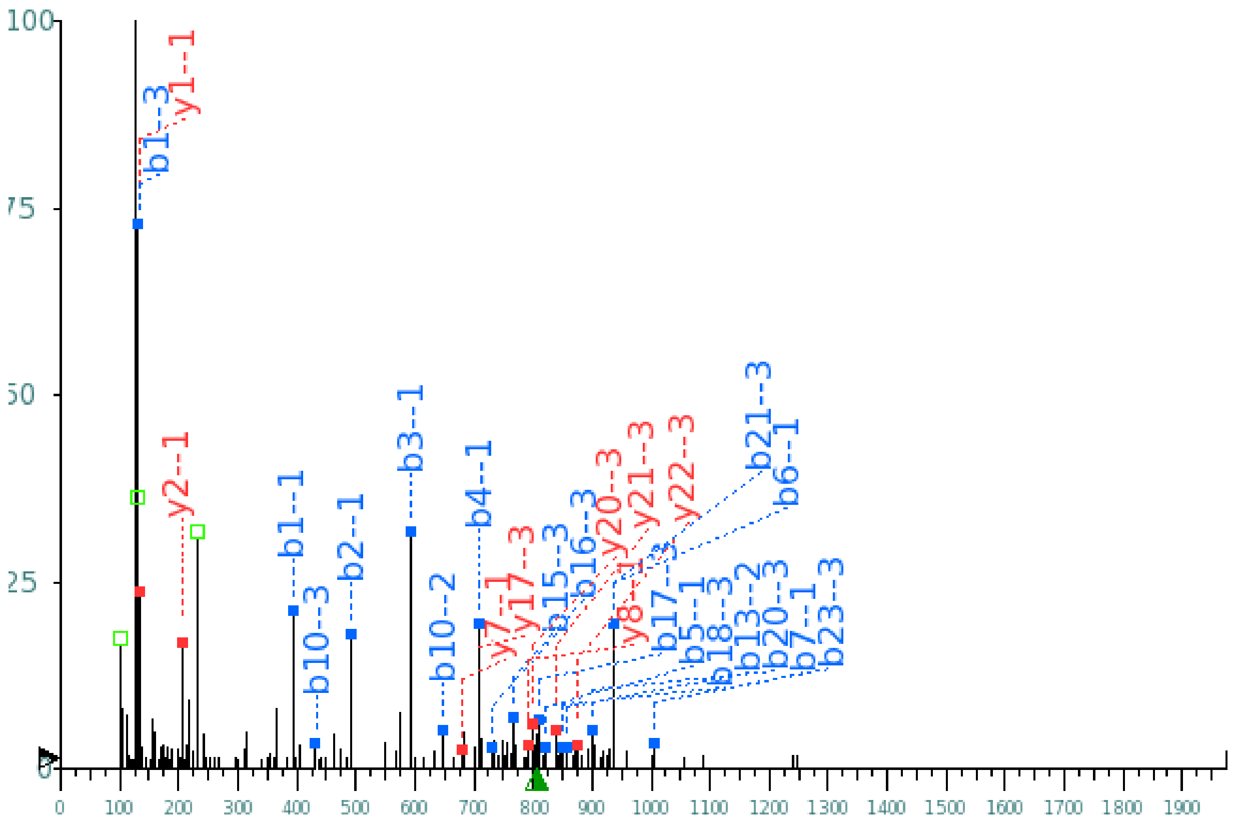

YVTIIDAPGHRDFIKNMITGTSQAD (+4)

Primary Reference: EF1A1_HUMAN
Search ID: 41758
Search Name: 20130328_ananiav_ERstress_TMT_TM_timecourse_chymo_inj1_4MC_IAA
Scan#: 24589
Observed Mass: 806.1827 (1.9 ppm)
PSM Score: 22.79

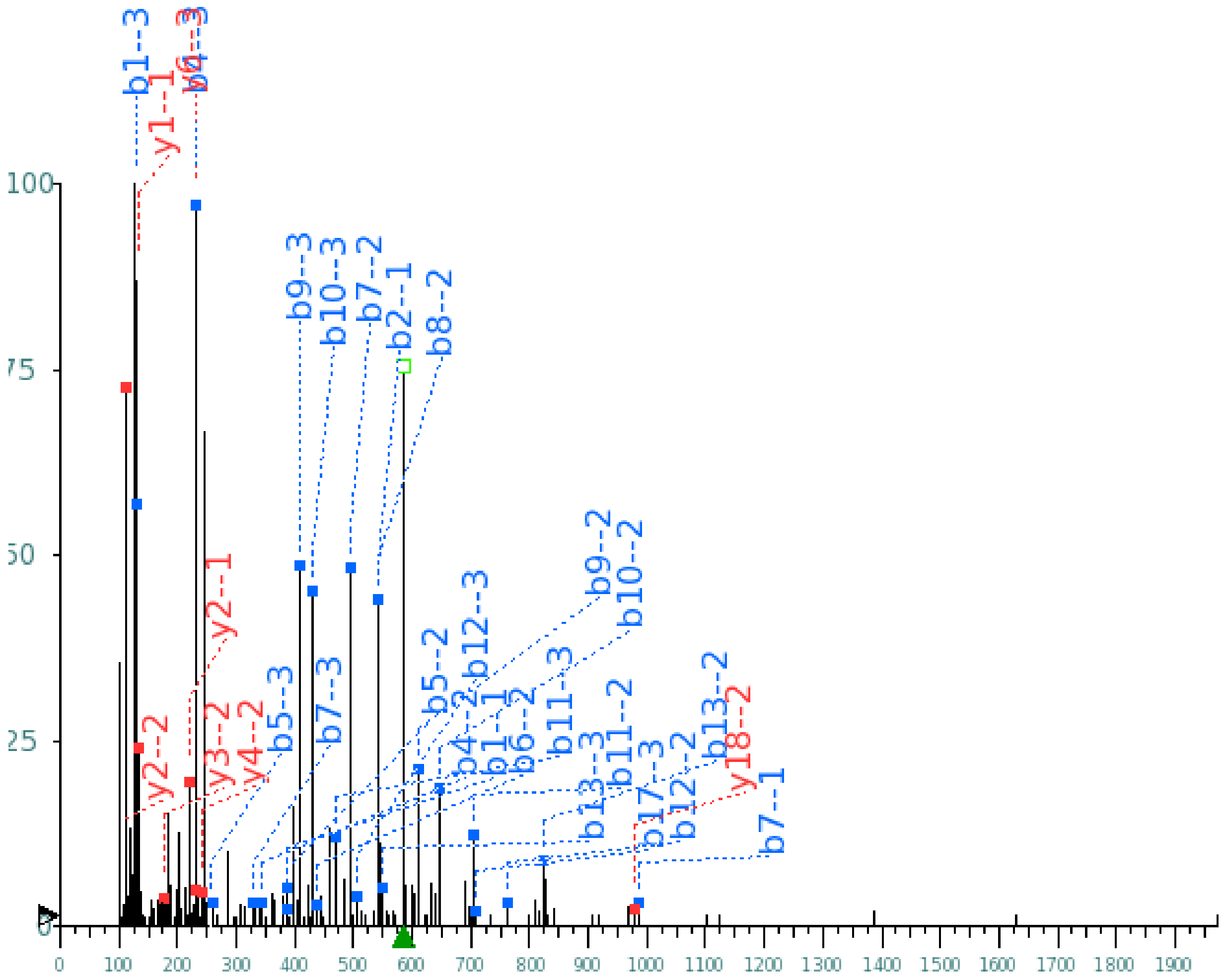

RRGSSPDVHALLEITEESD (+4)

Primary Reference: PTRF_HUMAN
Search ID: 41758
Search Name: 20130328_ananiav_ERstress_TMT_TM_timecourse_chymo_inj1_4MC_IAA
Scan#: 25178
Observed Mass: 585.8061 (1.5 ppm)
PSM Score: 31.66

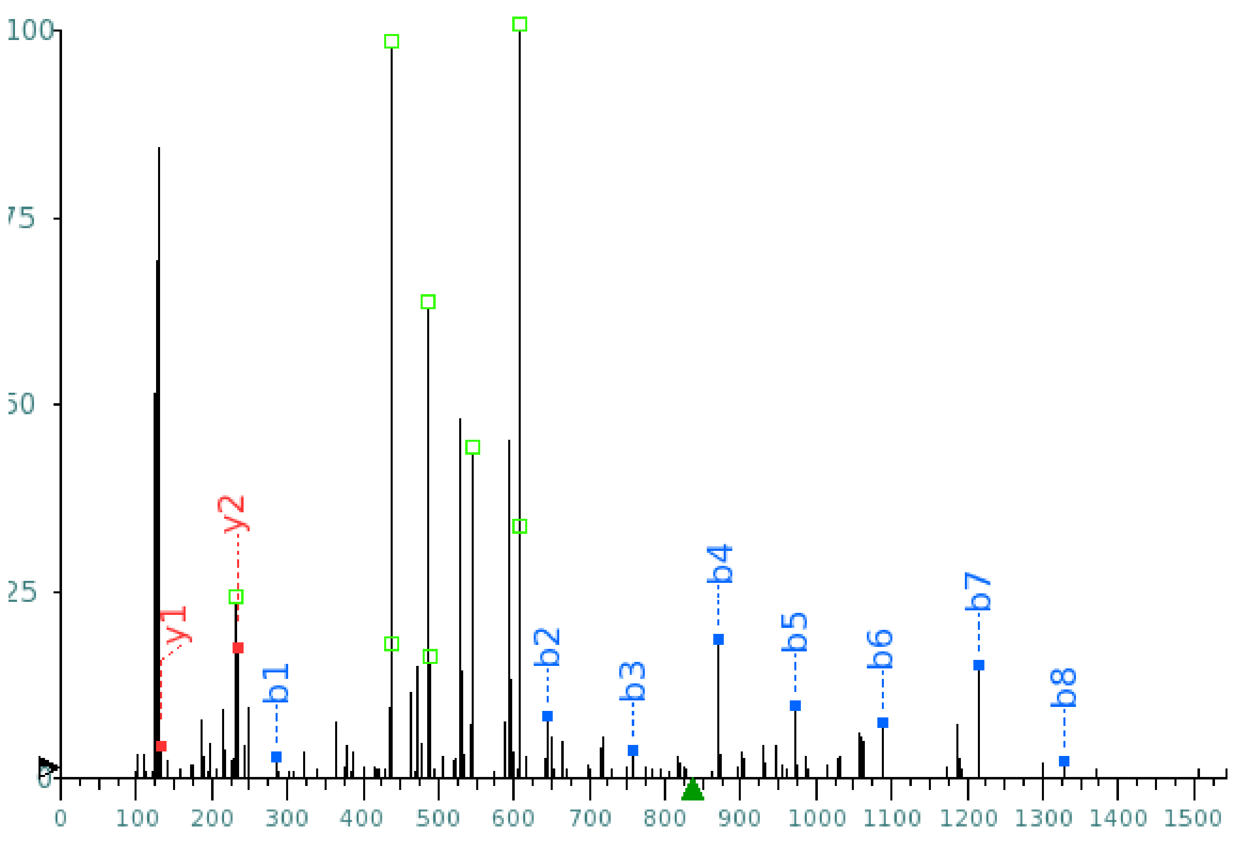

GKIDTIEIITD (+2)

Primary Reference: ROA2_HUMAN
Search ID: 41758
Search Name: 20130328_ananiav_ERstress_TMT_TM_timecourse_chymo_inj1_4MC_IAA
Scan#: 25417
Observed Mass: 838.4993 (1.8 ppm)
PSM Score: 25.87

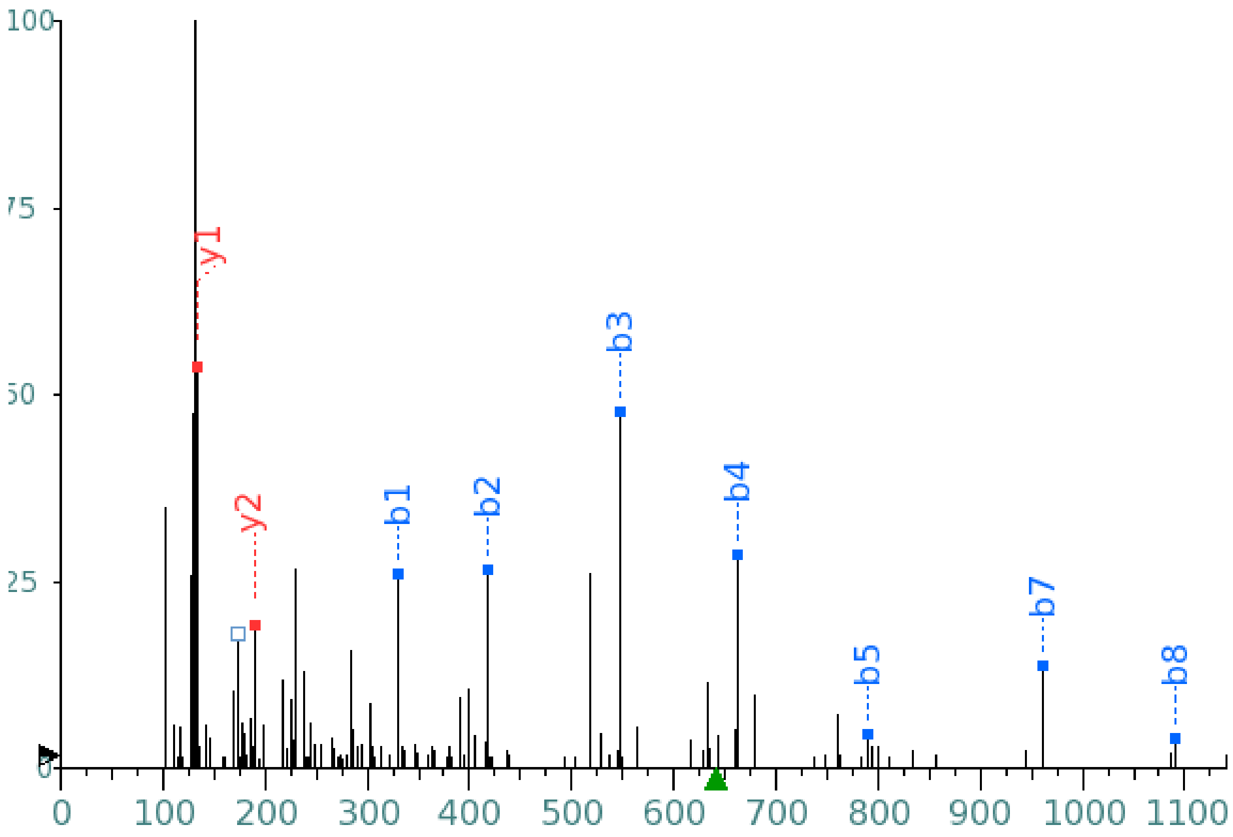

TSENQGDEGD (+2)

Primary Reference: 1433B_HUMAN
Search ID: 41759
Search Name: 20130328_ananiav_ERstress_TMT_TM_timecourse_chymo_inj2_4MC_IAA
Scan#: 2382
Observed Mass: 640.7763 (1.1 ppm)
PSM Score: 35.62

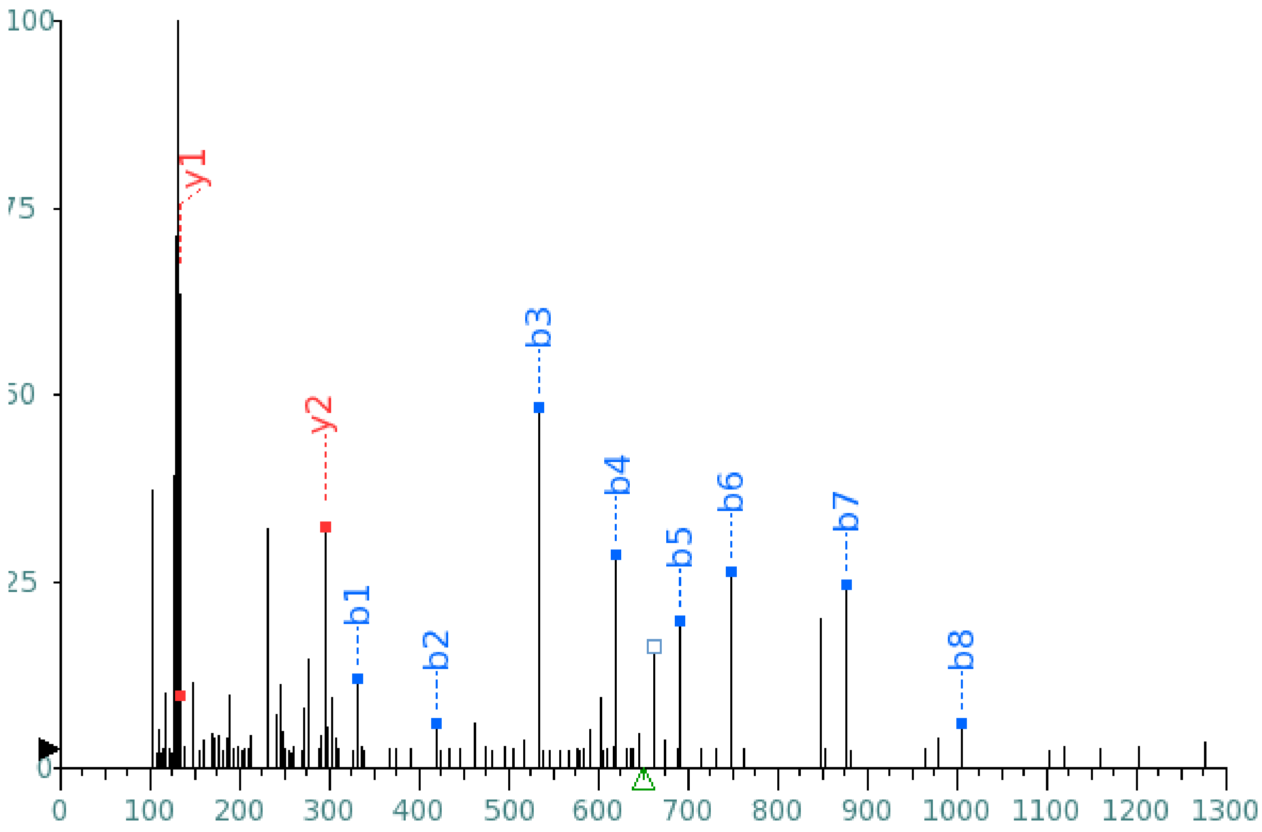

TSDSAGEEC*D (+2)

Primary Reference: 1433T_HUMAN
Search ID: 41759
Search Name: 20130328_ananiav_ERstress_TMT_TM_timecourse_chymo_inj2_4MC_IAA
Scan#: 3069
Observed Mass: 650.2634 (-1 ppm)
PSM Score: 38.78

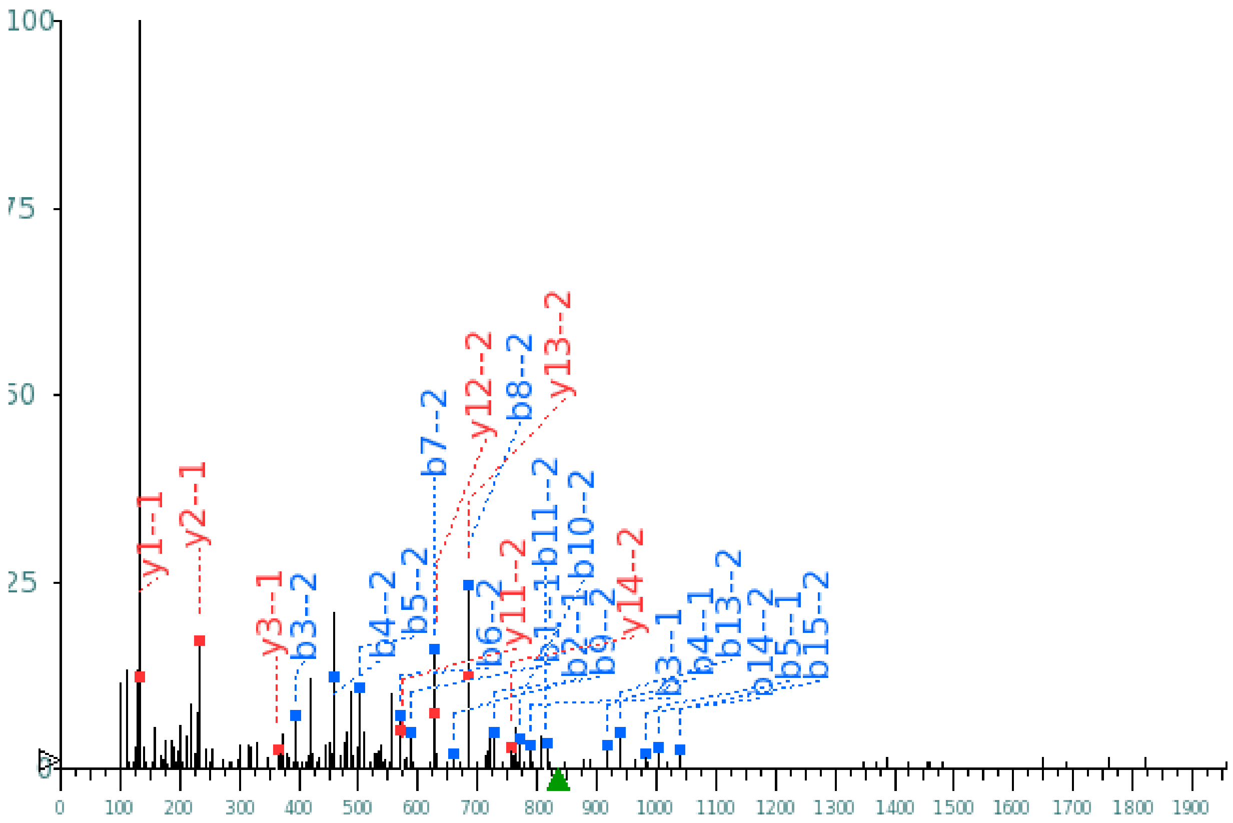

KAEQSHDDSSSEDSDAETD (+3)

Primary Reference: STK24_HUMAN
Search ID: 41759
Search Name: 20130328_ananiav_ERstress_TMT_TM_timecourse_chymo_inj2_4MC_IAA
Scan#: 4130
Observed Mass: 837.7052 (0.1 ppm)
PSM Score: 58.12

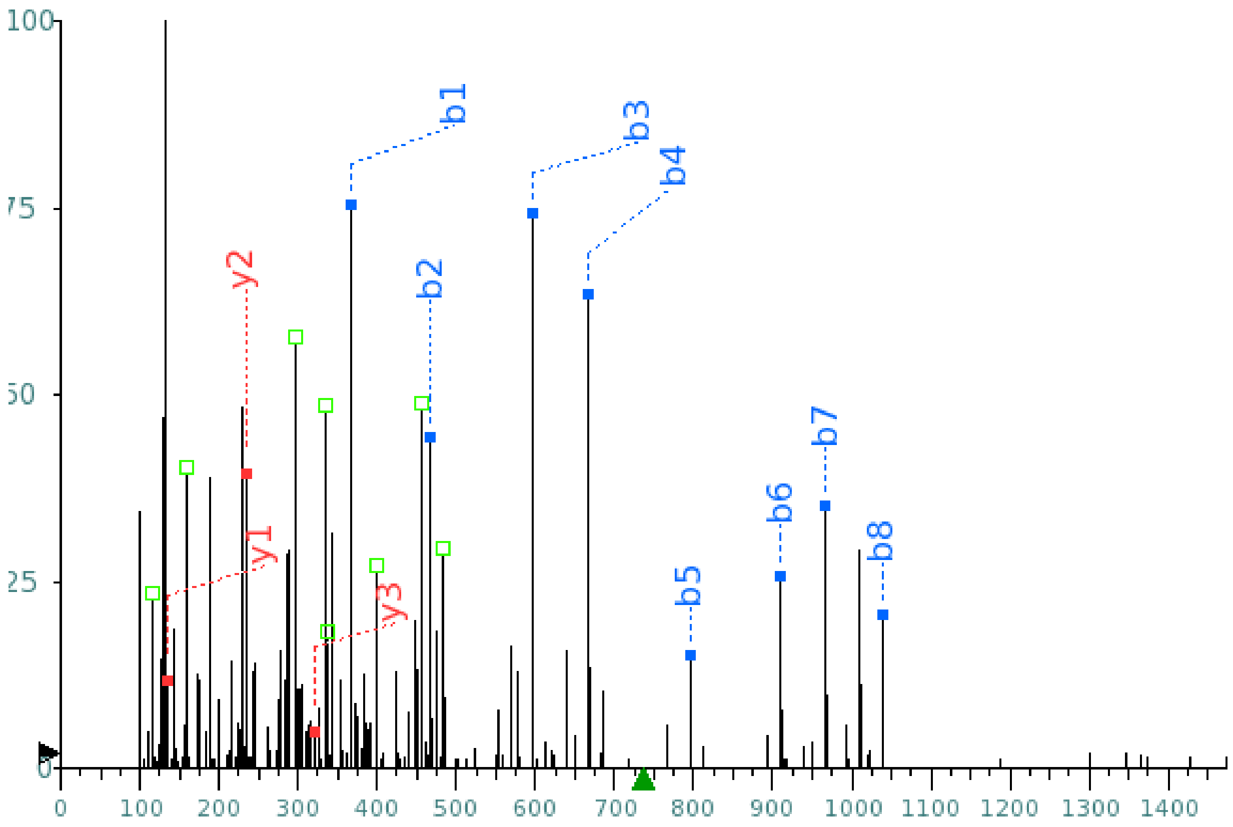

HTQAQDGAISTD (+2)

Primary Reference: TPP2_HUMAN
Search ID: 41759
Search Name: 20130328_ananiav_ERstress_TMT_TM_timecourse_chymo_inj2_4MC_IAA
Scan#: 4407
Observed Mass: 736.8632 (0.9 ppm)
PSM Score: 46.9

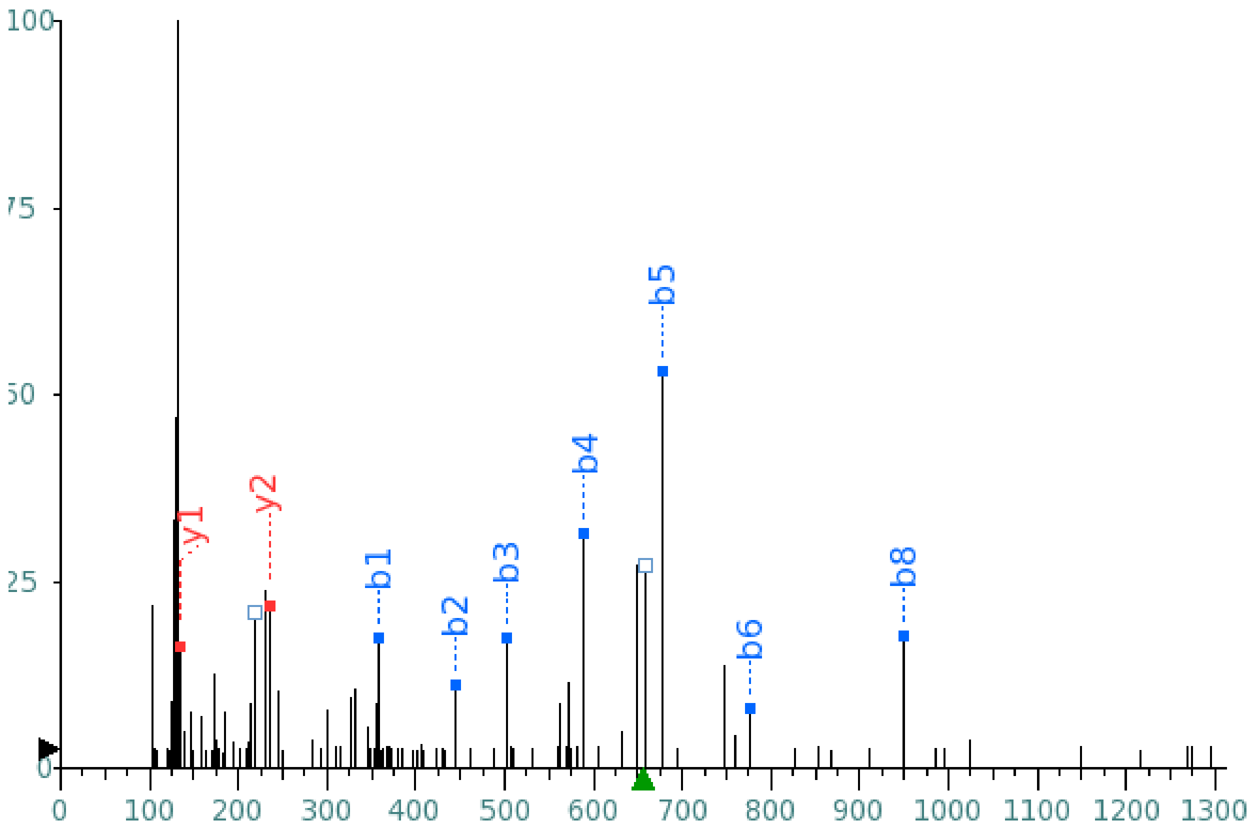

ESGSSVGDETD (+2)

Primary Reference: MATR3_HUMAN
Search ID: 41759
Search Name: 20130328_ananiav_ERstress_TMT_TM_timecourse_chymo_inj2_4MC_IAA
Scan#: 4437
Observed Mass: 656.2913 (0.3 ppm)
PSM Score: 34.83

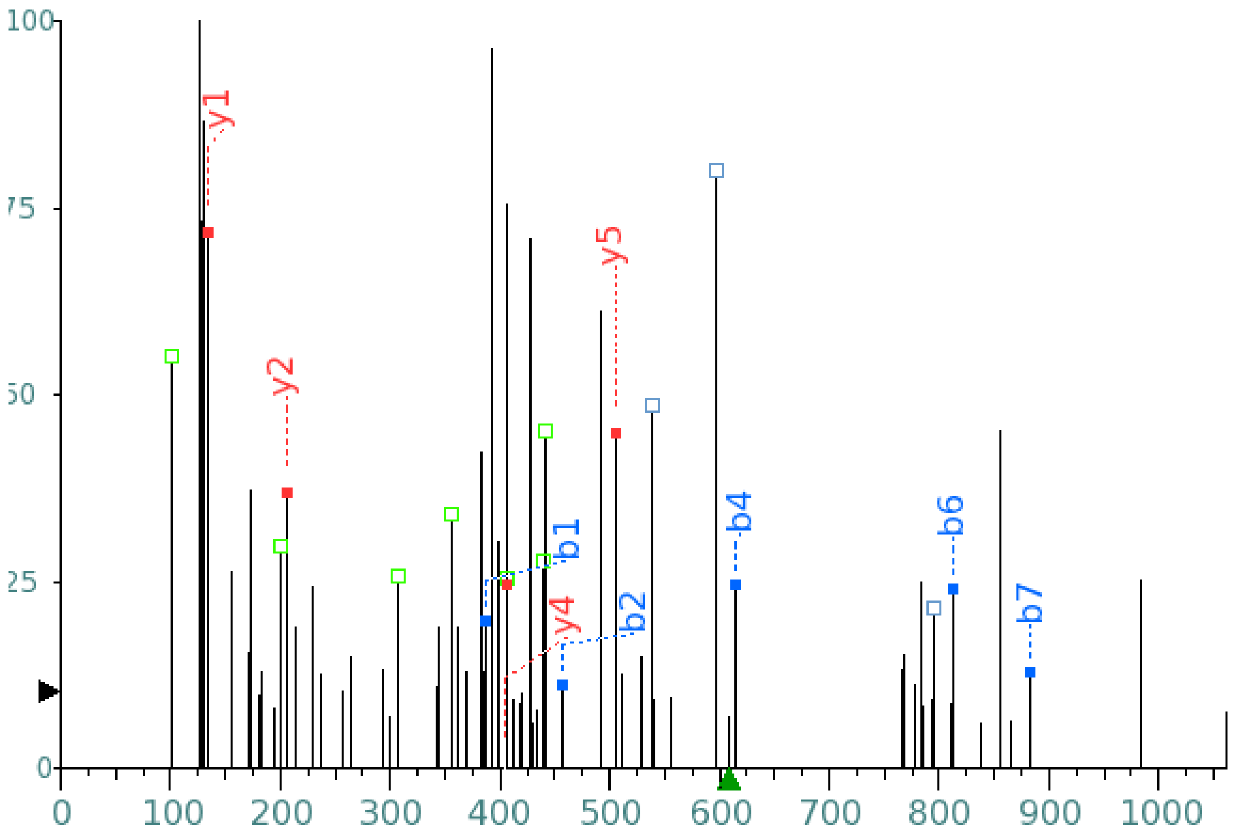

RAVGPTAEAD (+2)

Primary Reference: RED_HUMAN
Search ID: 41759
Search Name: 20130328_ananiav_ERstress_TMT_TM_timecourse_chymo_inj2_4MC_IAA
Scan#: 4796
Observed Mass: 608.3308 (1 ppm)
PSM Score: 23.65

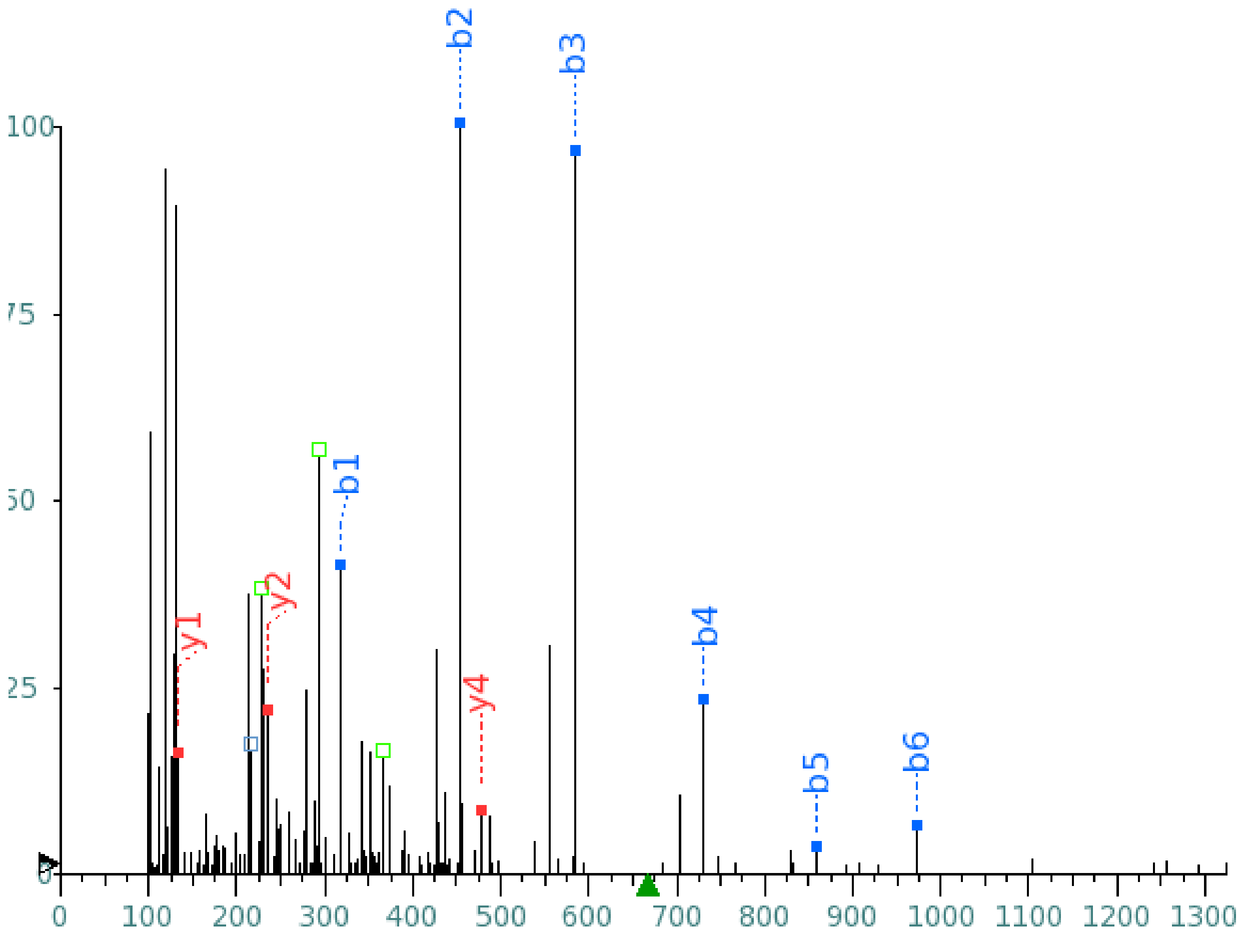

SHEFQDETD (+2)

Primary Reference: CBPD_HUMAN
Search ID: 41759
Search Name: 20130328_ananiav_ERstress_TMT_TM_timecourse_chymo_inj2_4MC_IAA
Scan#: 5251
Observed Mass: 668.7963 (-0.1 ppm)
PSM Score: 22.04

File name: 2015-02-04 04.01.53PM.pdf

Creation time: 2015-02-04 04:09PM

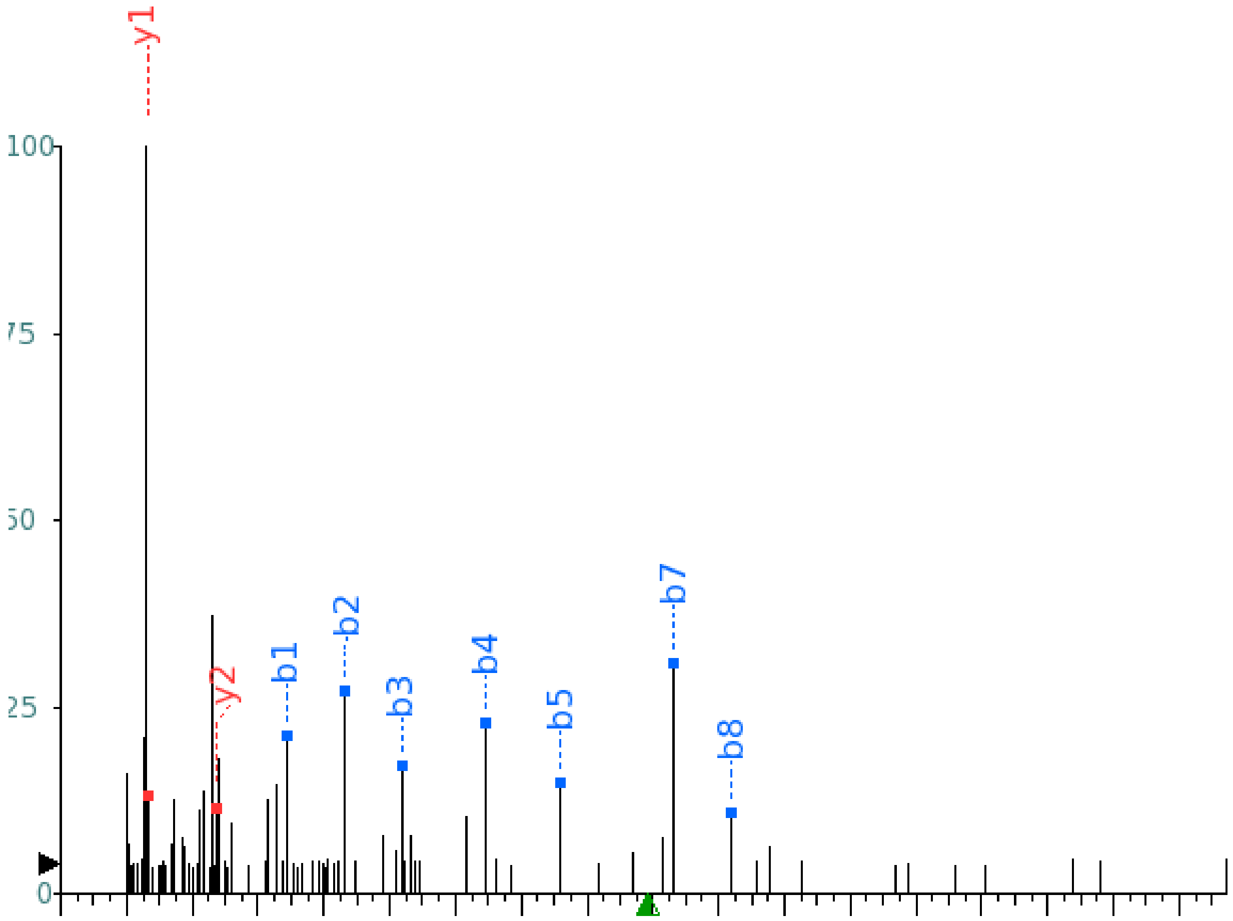

LSSQNGDSSTSQETD (+2)

Primary Reference: SRPK1_HUMAN
Search ID: 41759
Search Name: 20130328_ananiav_ERstress_TMT_TM_timecourse_chymo_inj2_4MC_IAA
Scan#: 5889
Observed Mass: 892.9034 (0.7 ppm)
PSM Score: 24.67

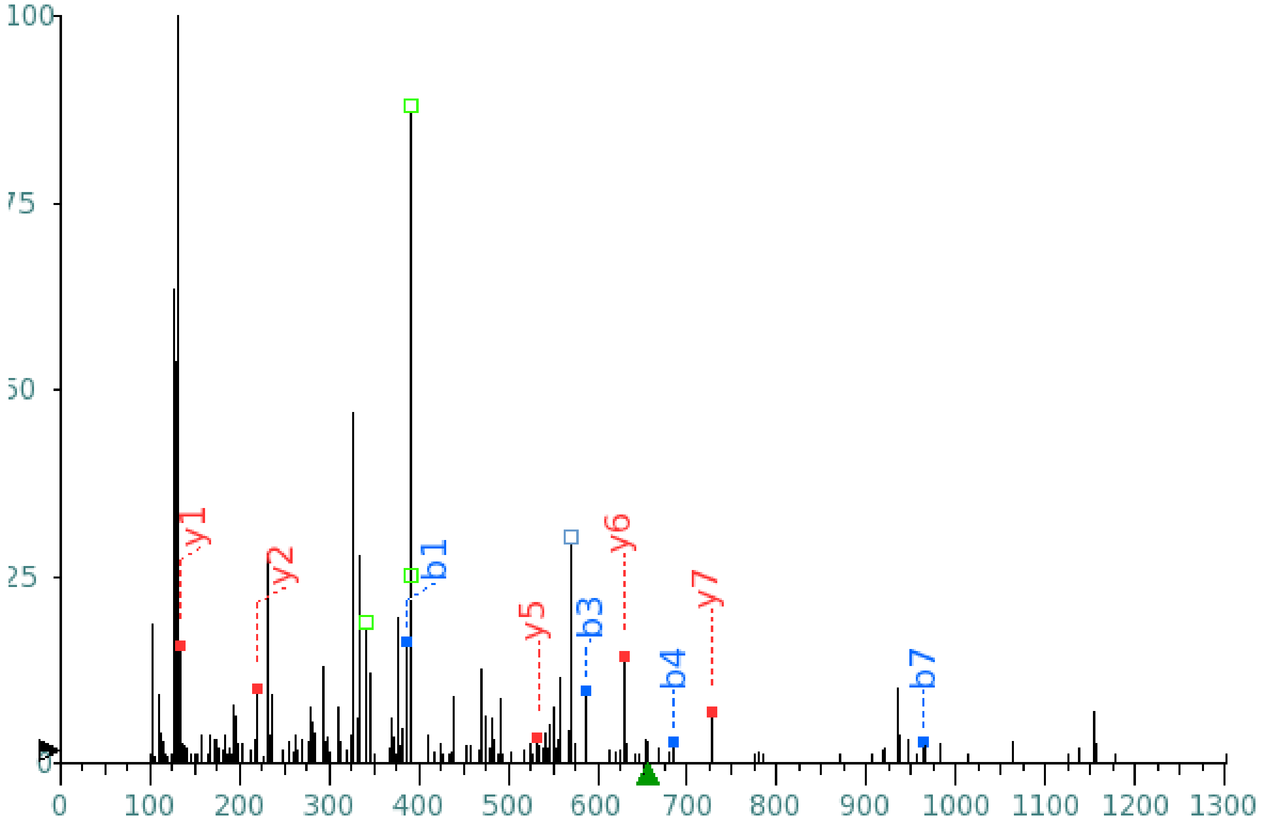

RLSPPPPSQSD (+2)

Primary Reference: GDF15_HUMAN
Search ID: 41759
Search Name: 20130328_ananiav_ERstress_TMT_TM_timecourse_chymo_inj2_4MC_IAA
Scan#: 6350
Observed Mass: 656.8572 (1 ppm)
PSM Score: 23.97

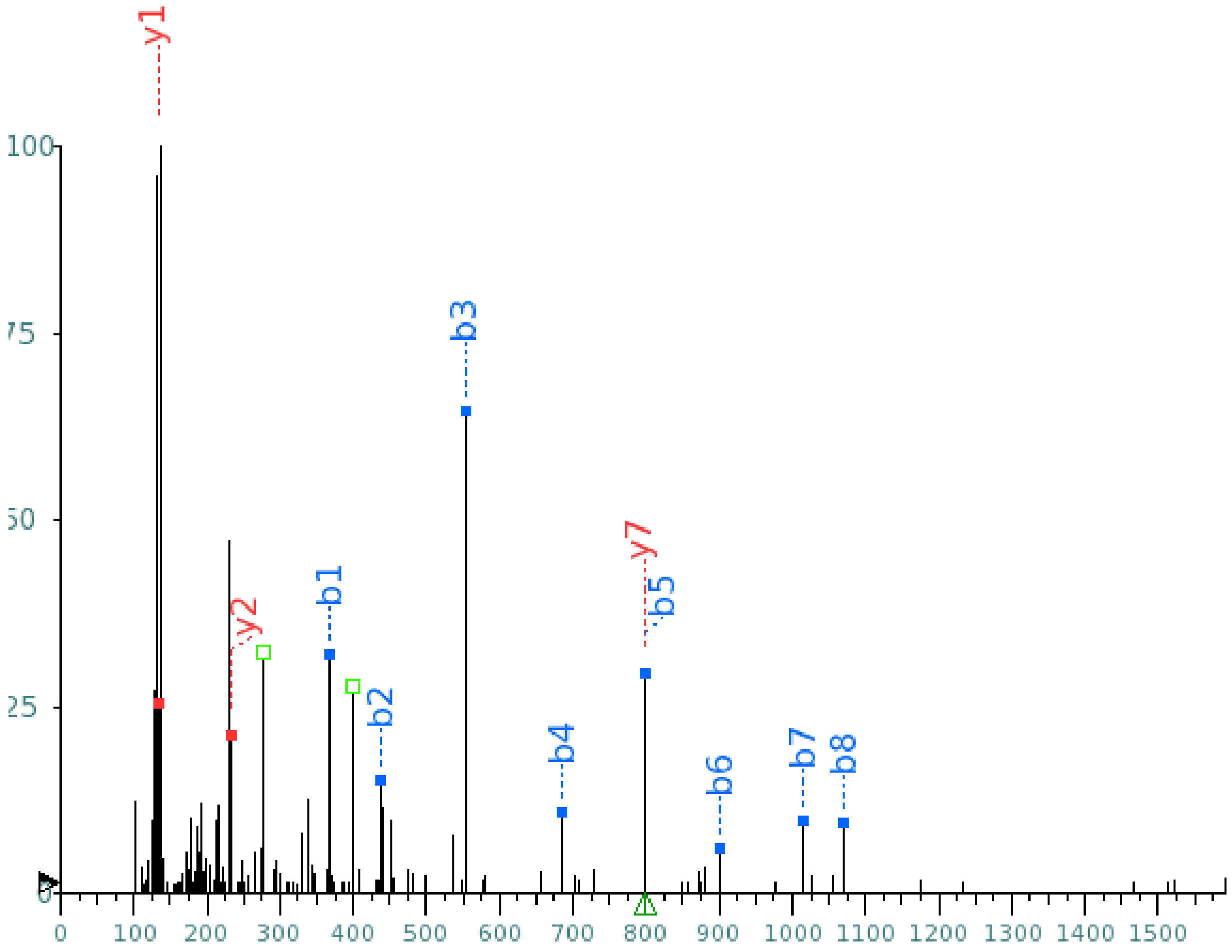

HADMDTNGYETD (+2)

Primary Reference: MIER1_HUMAN
Search ID: 41759
Search Name: 20130328_ananiav_ERstress_TMT_TM_timecourse_chymo_inj2_4MC_IAA
Scan#: 6817
Observed Mass: 799.3363 (1 ppm)
PSM Score: 34.74

File name: 2015-02-04 04.01.53PM.pdf

Creation time: 2015-02-04 04:09PM

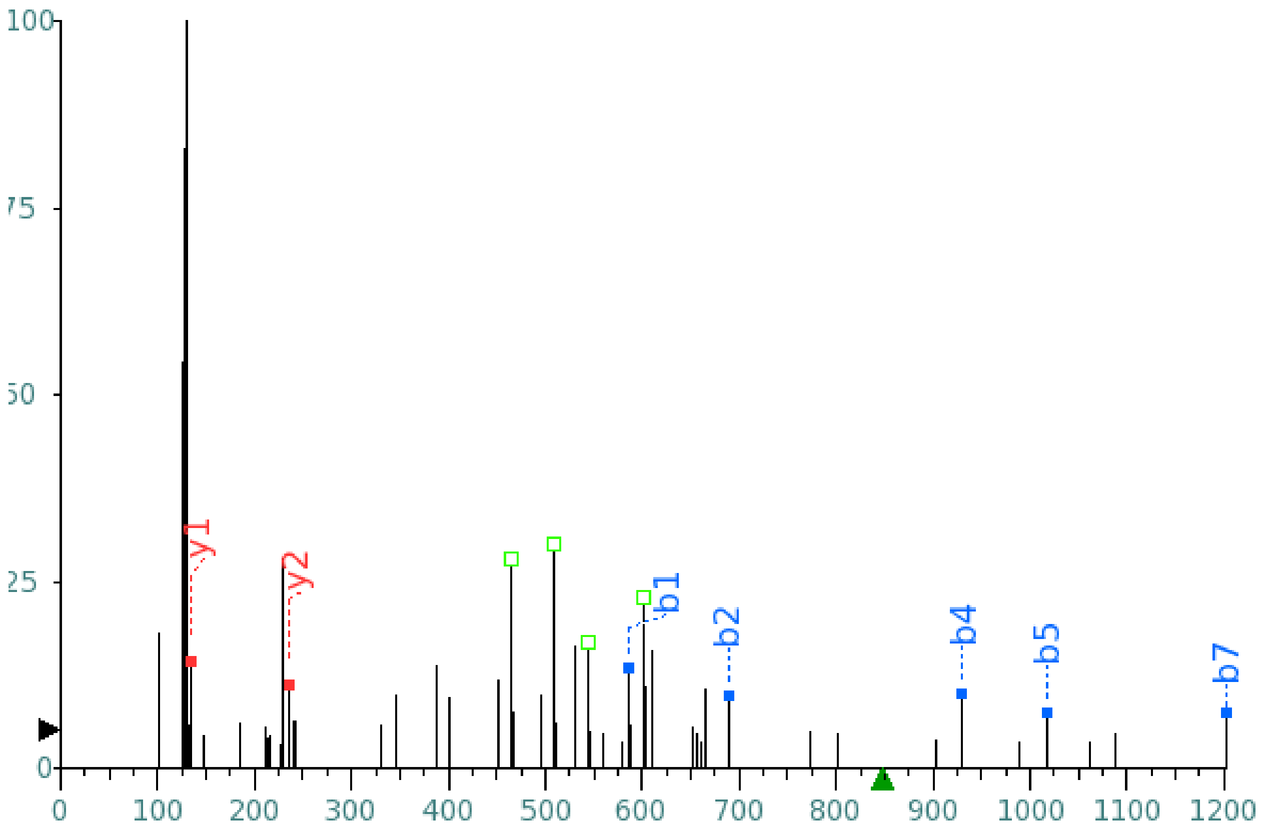

KTIESANQQTD (+2)

Primary Reference: SRP72_HUMAN
Search ID: 41759
Search Name: 20130328_ananiav_ERstress_TMT_TM_timecourse_chymo_inj2_4MC_IAA
Scan#: 7233
Observed Mass: 846.9625 (0.5 ppm)
PSM Score: 30.93

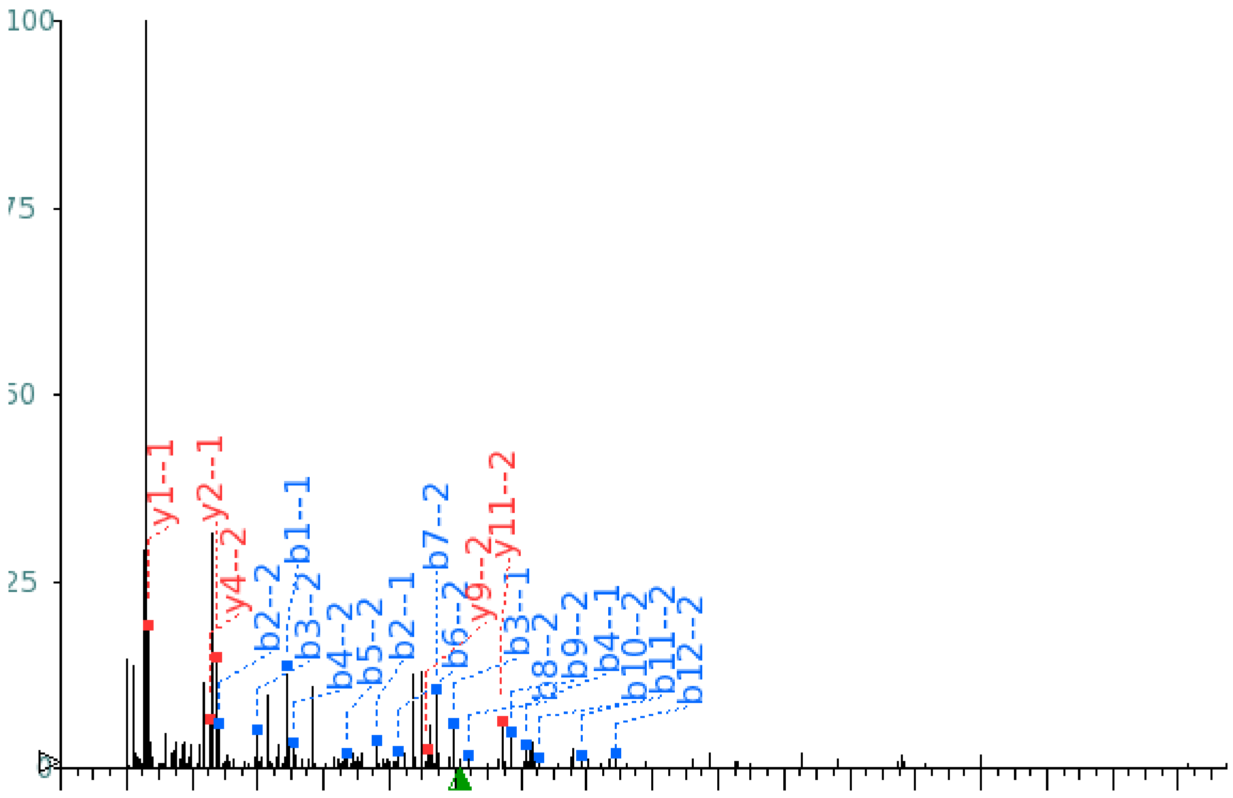

IHDNYRDPESETD (+3)

Primary Reference: PRKDC_HUMAN
Search ID: 41759
Search Name: 20130328_ananiav_ERstress_TMT_TM_timecourse_chymo_inj2_4MC_IAA
Scan#: 7350
Observed Mass: 607.282 (1.1 ppm)
PSM Score: 18.16

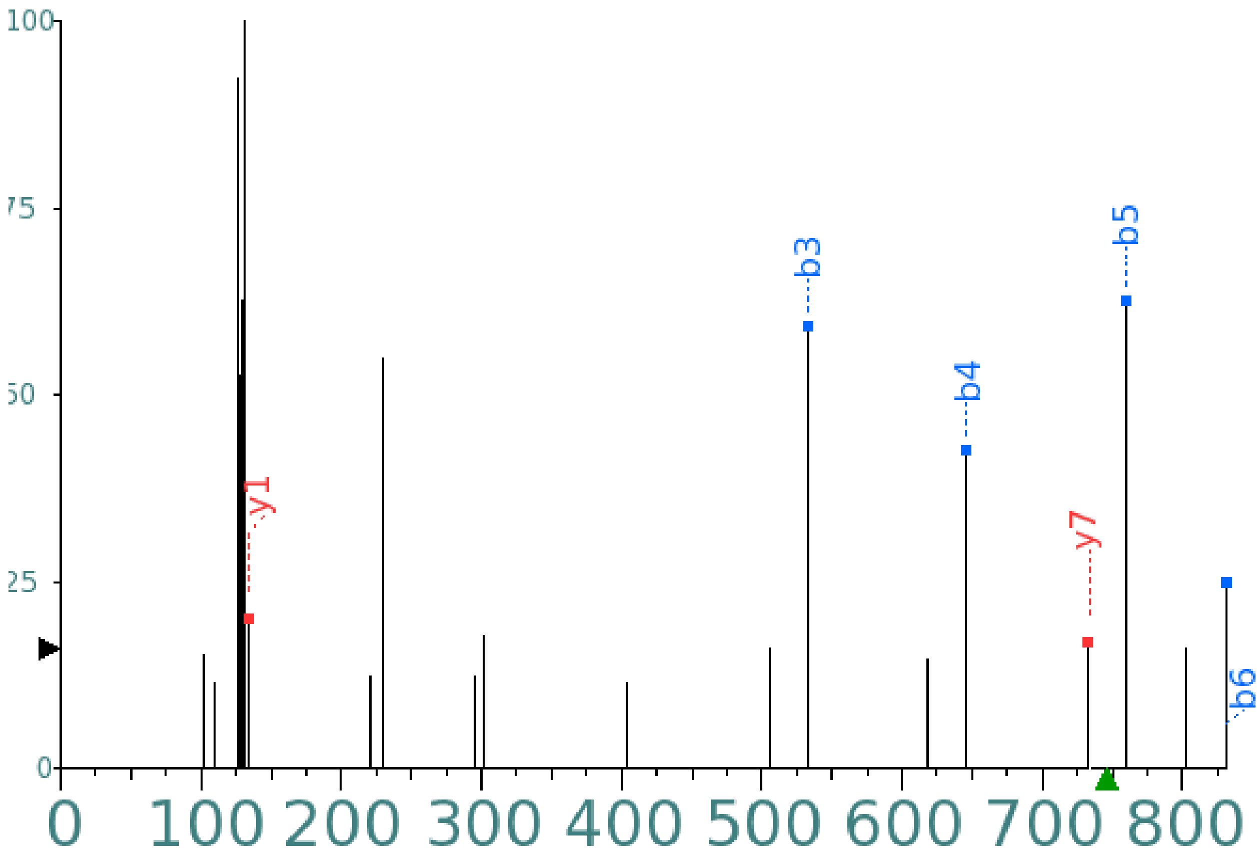

SSELNAPSQESD (+2)

Primary Reference: RBM25_HUMAN
Search ID: 41759
Search Name: 20130328_ananiav_ERstress_TMT_TM_timecourse_chymo_inj2_4MC_IAA
Scan#: 7508
Observed Mass: 746.8524 (0.7 ppm)
PSM Score: 30.99

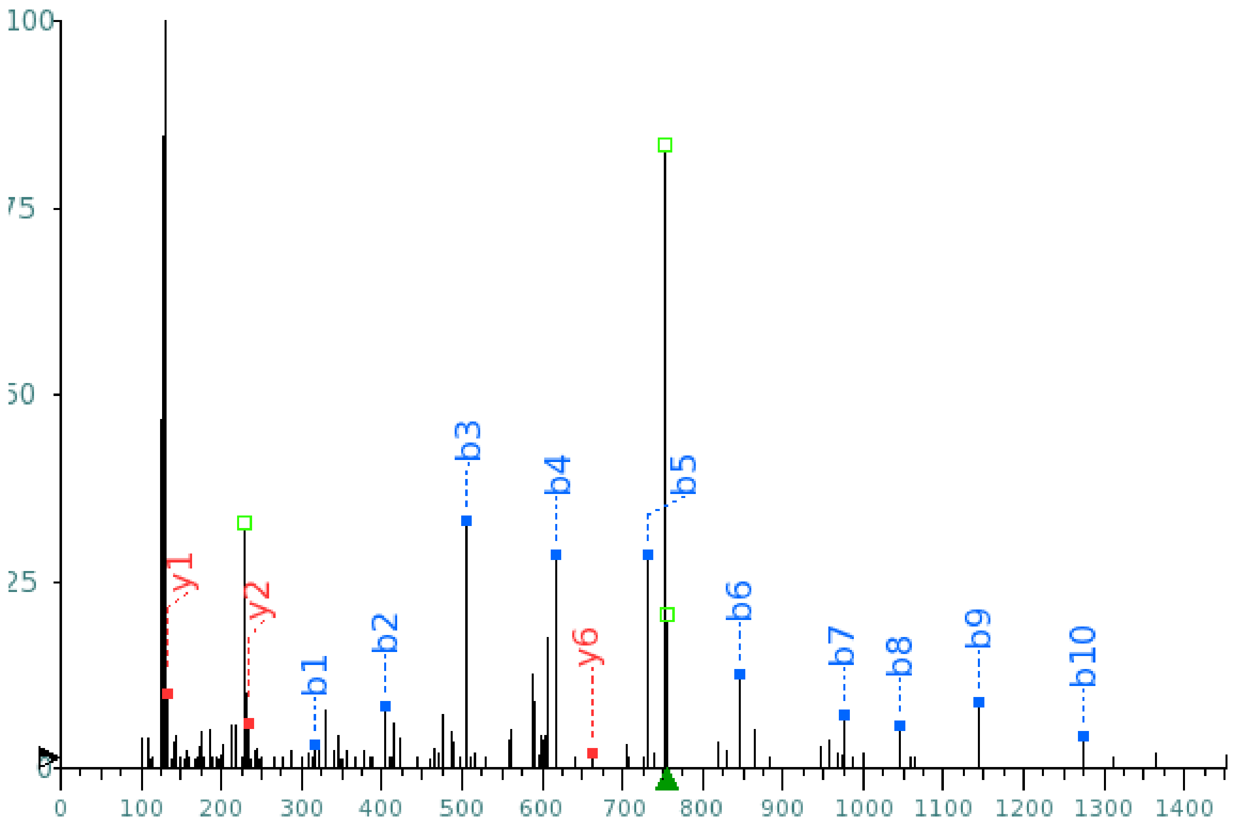

SSTINNQAVETD (+2)

Primary Reference: RHG12_HUMAN
Search ID: 41759
Search Name: 20130328_ananiav_ERstress_TMT_TM_timecourse_chymo_inj2_4MC_IAA
Scan#: 7671
Observed Mass: 754.3756 (0.1 ppm)
PSM Score: 50.86

File name: 2015-02-04 04.01.53PM.pdf

Creation time: 2015-02-04 04:09PM

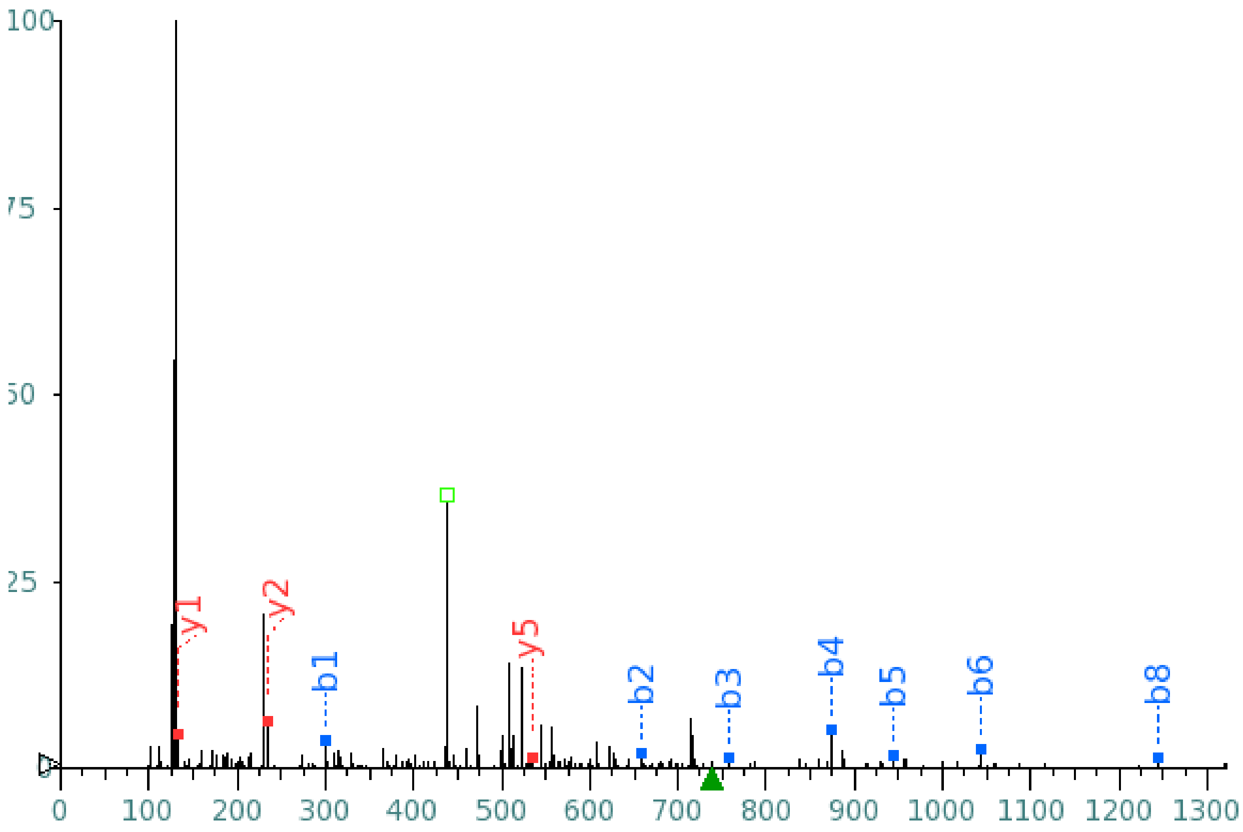

AKVDATAETD (+2)

Primary Reference: PDIA4_HUMAN
Search ID: 41759
Search Name: 20130328_ananiav_ERstress_TMT_TM_timecourse_chymo_inj2_4MC_IAA
Scan#: 7696
Observed Mass: 739.9109 (2.9 ppm)
PSM Score: 32.9

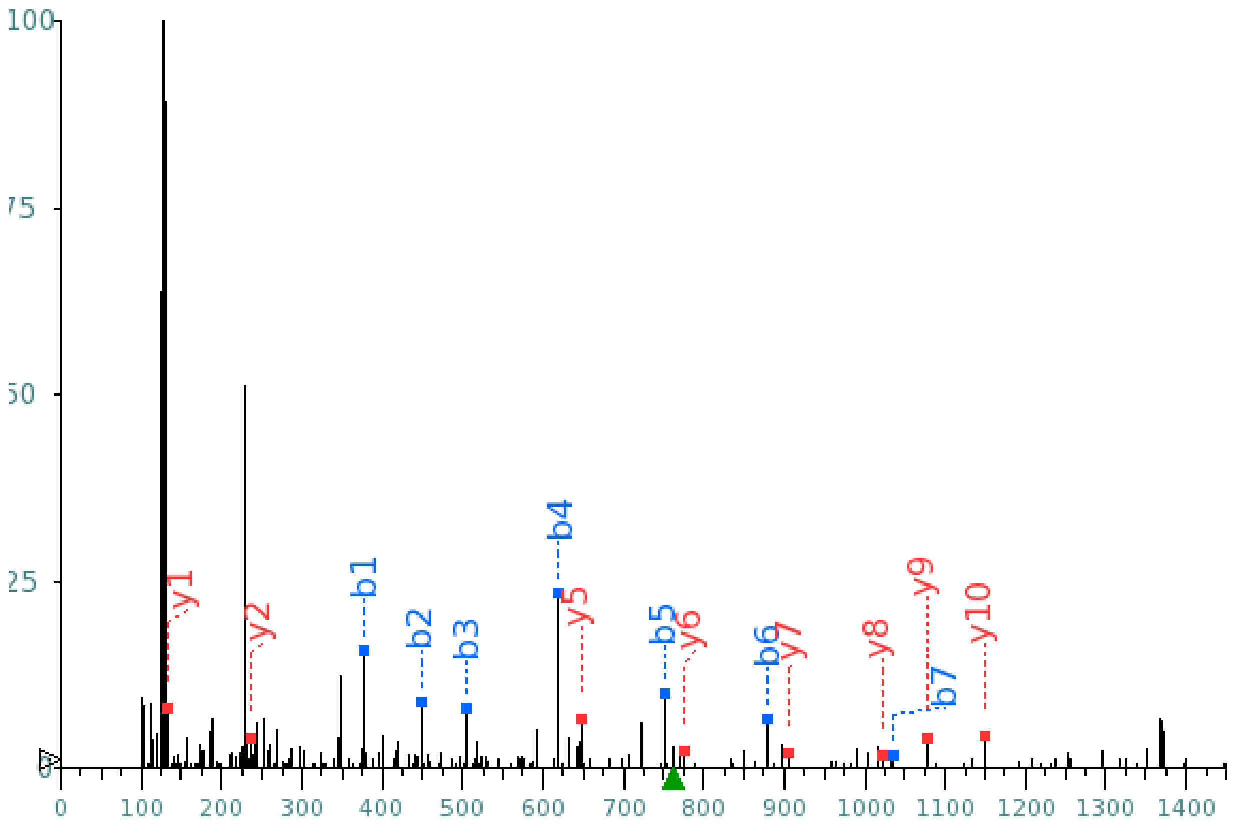

FAGNMERQQTD (+2)

Primary Reference: VAV2_HUMAN
Search ID: 41759
Search Name: 20130328_ananiav_ERstress_TMT_TM_timecourse_chymo_inj2_4MC_IAA
Scan#: 8122
Observed Mass: 763.367 (0.1 ppm)
PSM Score: 32.26

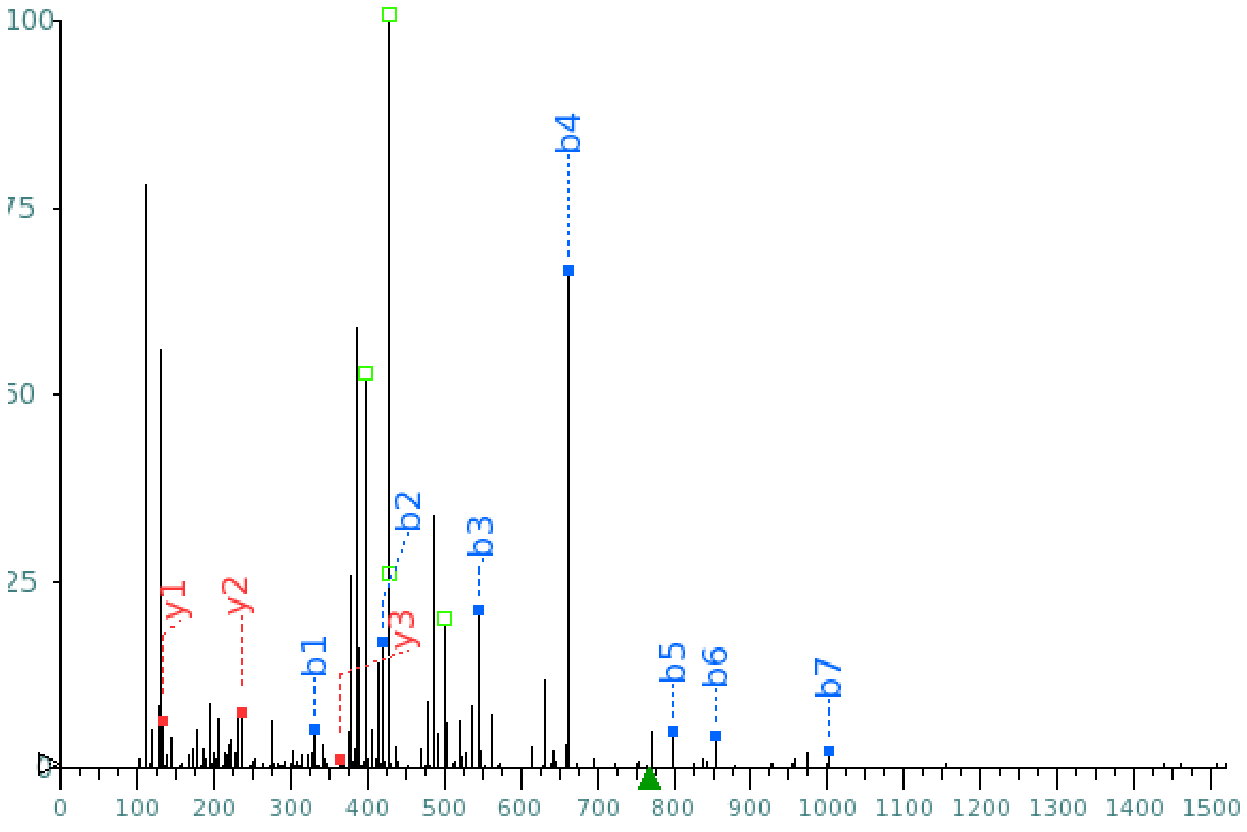

TSQNHGFAVETD (+2)

Primary Reference: PYR1_HUMAN
Search ID: 41759
Search Name: 20130328_ananiav_ERstress_TMT_TM_timecourse_chymo_inj2_4MC_IAA
Scan#: 8290
Observed Mass: 767.8708 (0.6 ppm)
PSM Score: 21.64

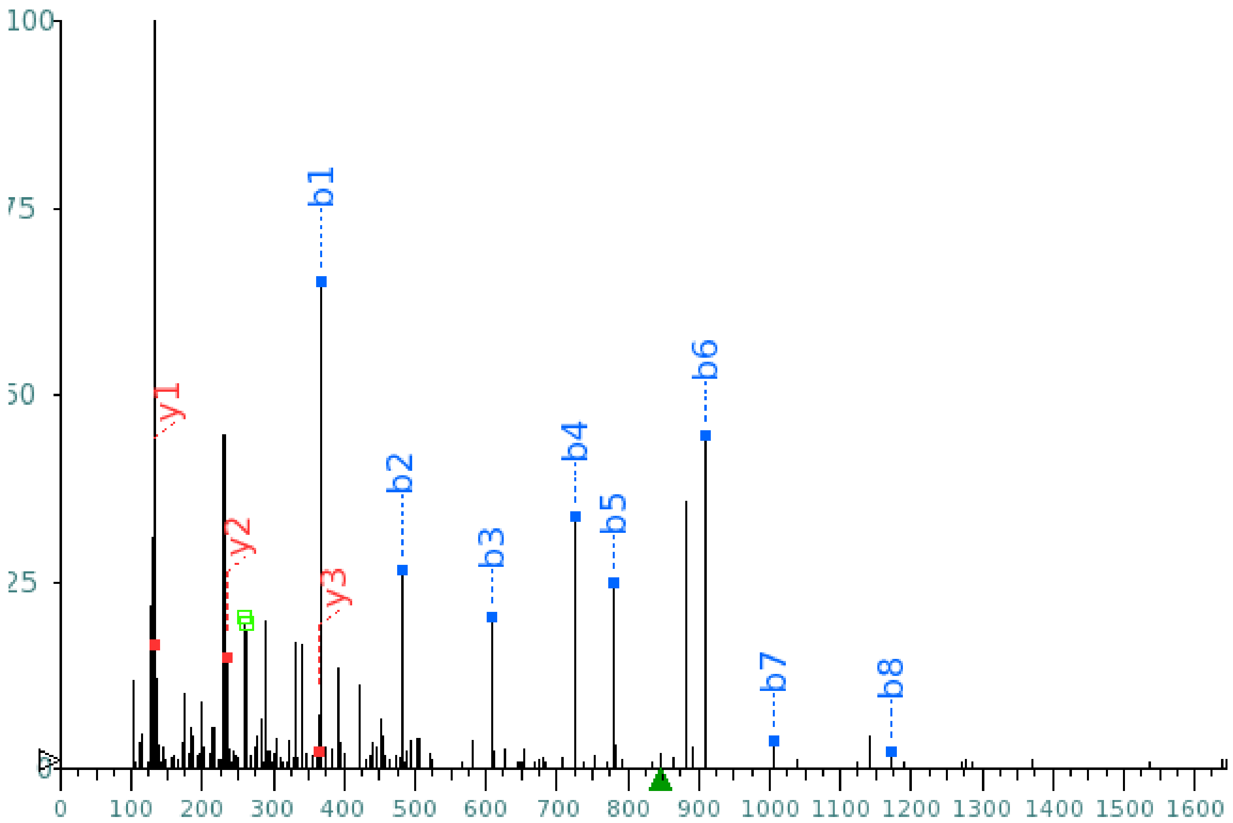

HLEDGEPYC*ETD (+2)

Primary Reference: PDLI5_HUMAN
Search ID: 41759
Search Name: 20130328_ananiav_ERstress_TMT_TM_timecourse_chymo_inj2_4MC_IAA
Scan#: 8760
Observed Mass: 847.3651 (0.9 ppm)
PSM Score: 32.17

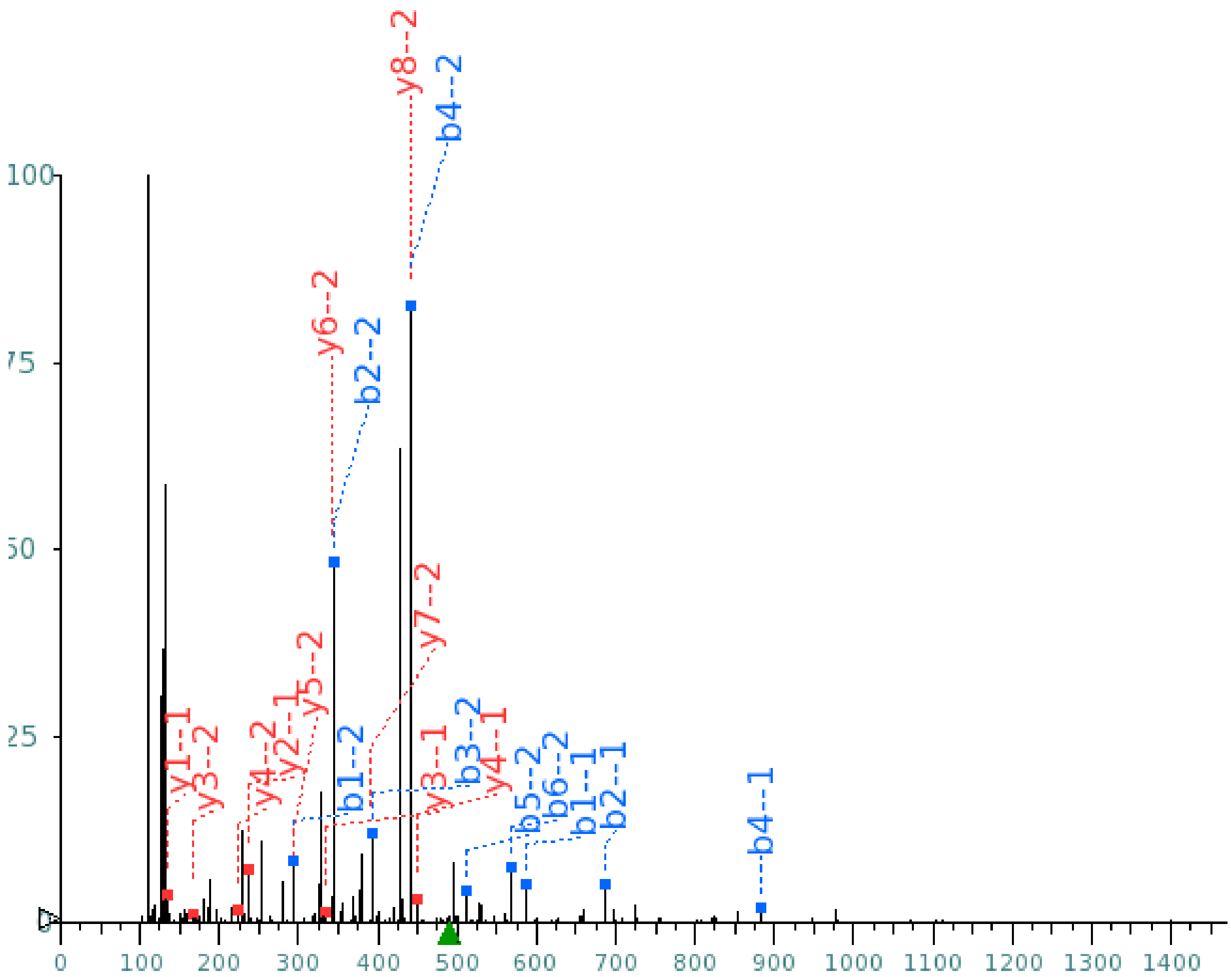

KVPVHDVTD (+3)

Primary Reference: FLNA_HUMAN
Search ID: 41759
Search Name: 20130328_ananiav_ERstress_TMT_TM_timecourse_chymo_inj2_4MC_IAA
Scan#: 8790
Observed Mass: 489.958 (1.6 ppm)
PSM Score: 16.74

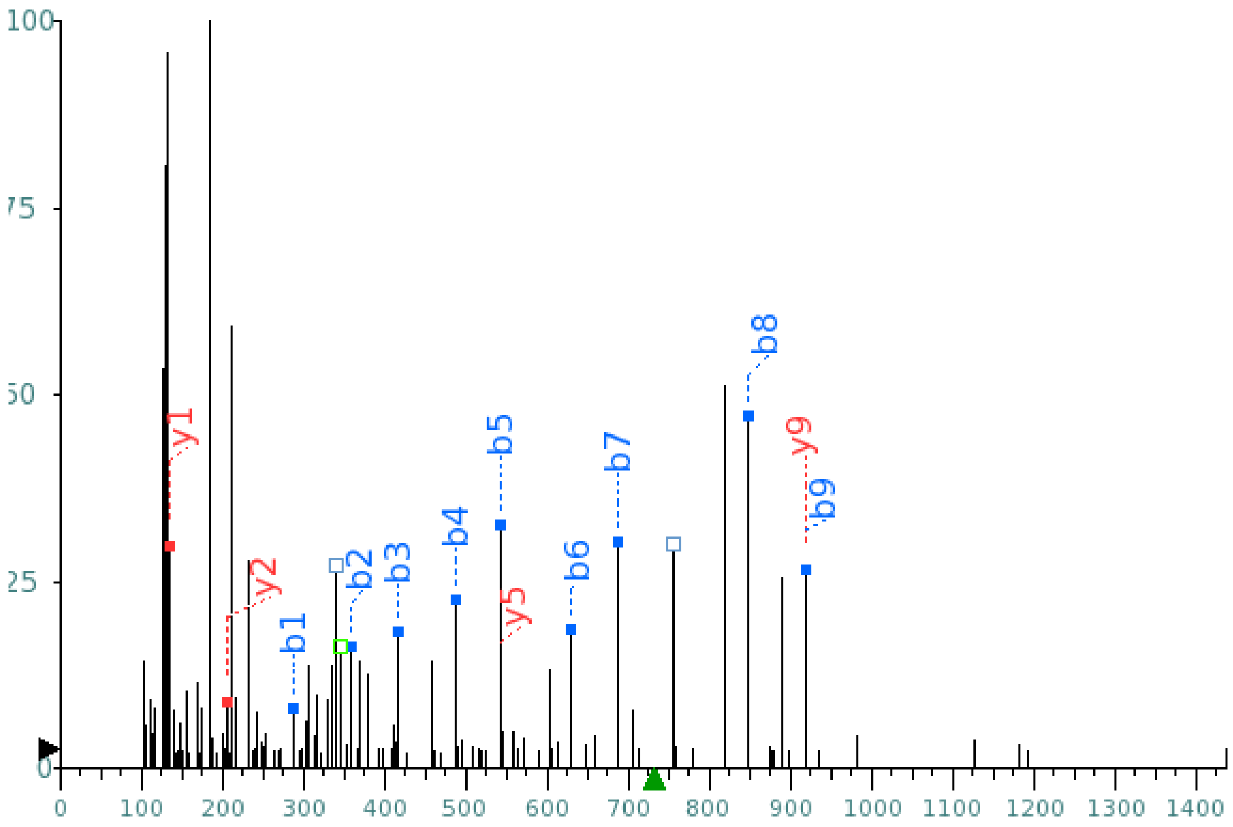

GAGAGSGC*APLEAD (+2)

Primary Reference: SC16A_HUMAN
Search ID: 41759
Search Name: 20130328_ananiav_ERstress_TMT_TM_timecourse_chymo_inj2_4MC_IAA
Scan#: 8800
Observed Mass: 731.3457 (0.1 ppm)
PSM Score: 38.14

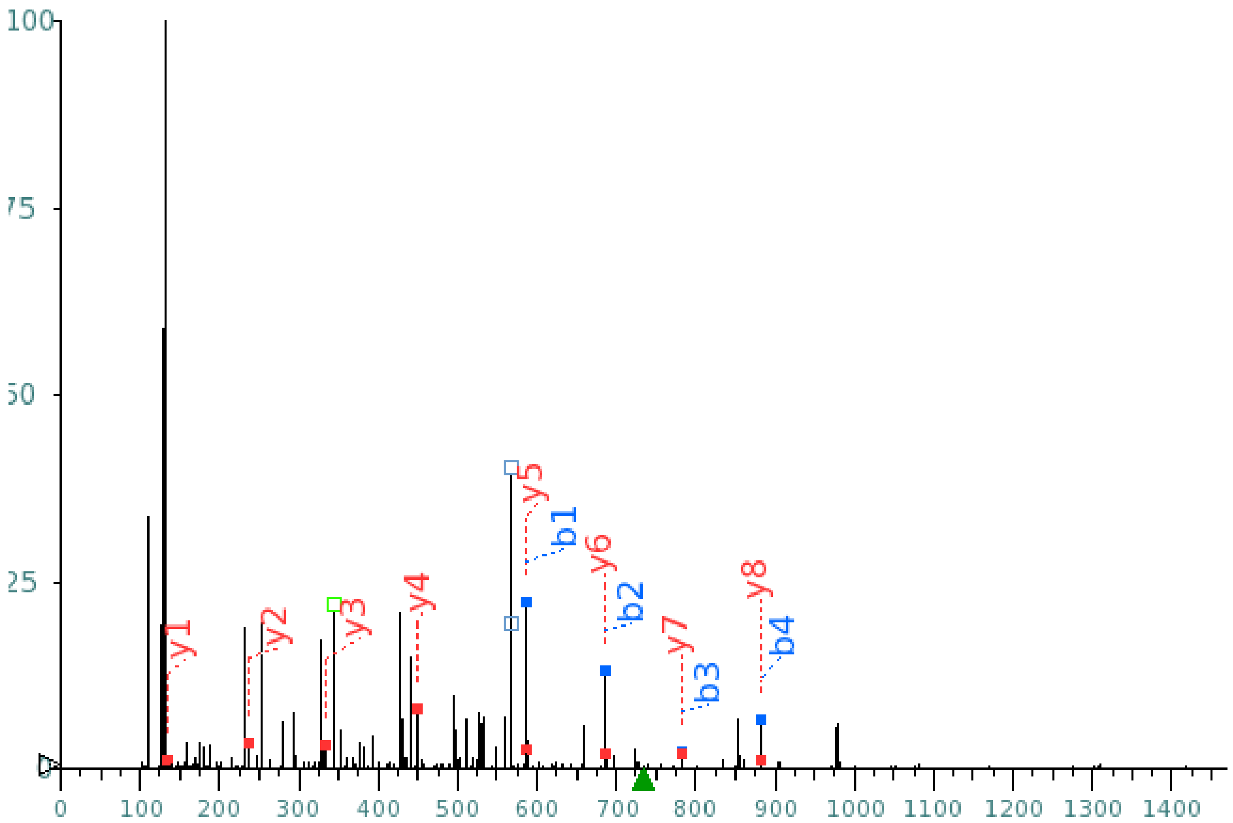

KVPVHDVTD (+2)

Primary Reference: FLNA_HUMAN
Search ID: 41759
Search Name: 20130328_ananiav_ERstress_TMT_TM_timecourse_chymo_inj2_4MC_IAA
Scan#: 8801
Observed Mass: 734.4322 (-0.1 ppm)
PSM Score: 14.34

File name: 2015-02-04 04.01.53PM.pdf

Creation time: 2015-02-04 04:09PM

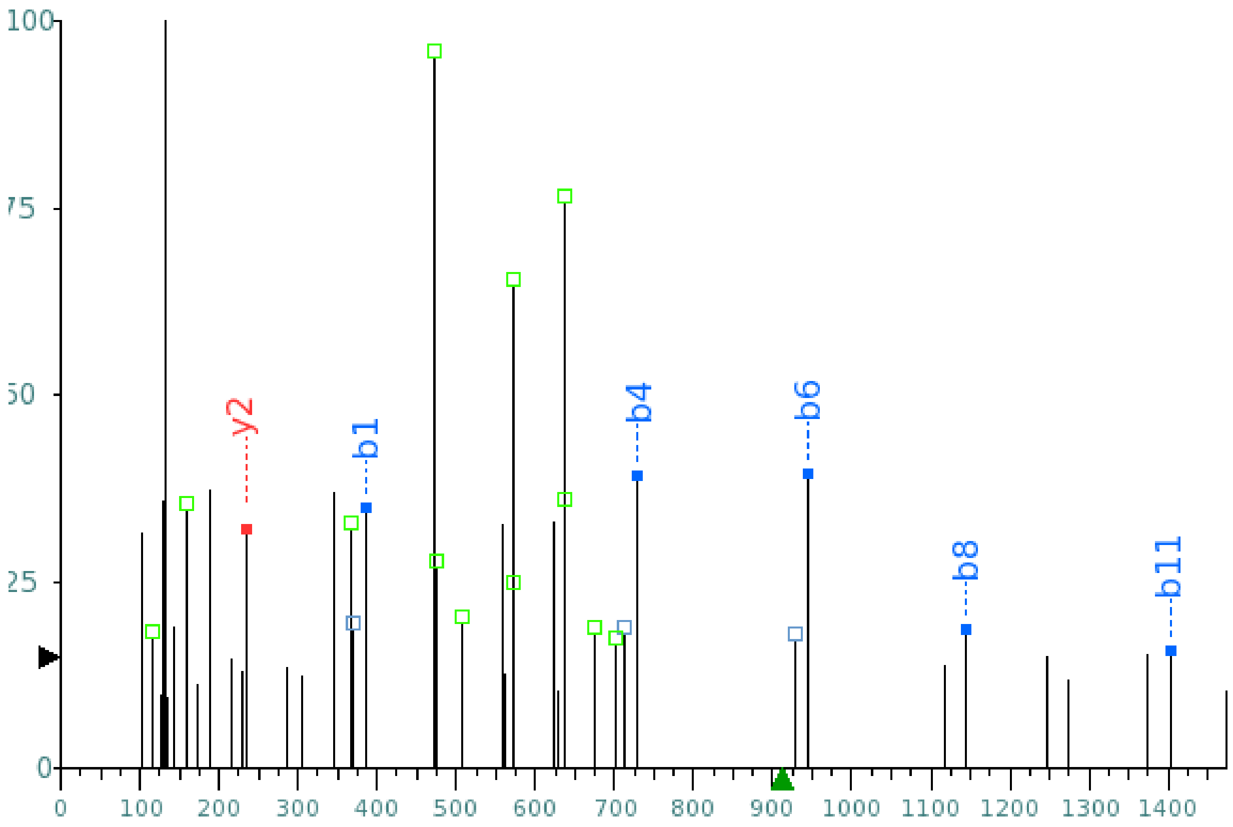

RPMDTDAEEAGVSTD (+2)

Primary Reference: DCBD1_HUMAN
Search ID: 41759
Search Name: 20130328_ananiav_ERstress_TMT_TM_timecourse_chymo_inj2_4MC_IAA
Scan#: 8806
Observed Mass: 911.9198 (-0.2 ppm)
PSM Score: 33.03

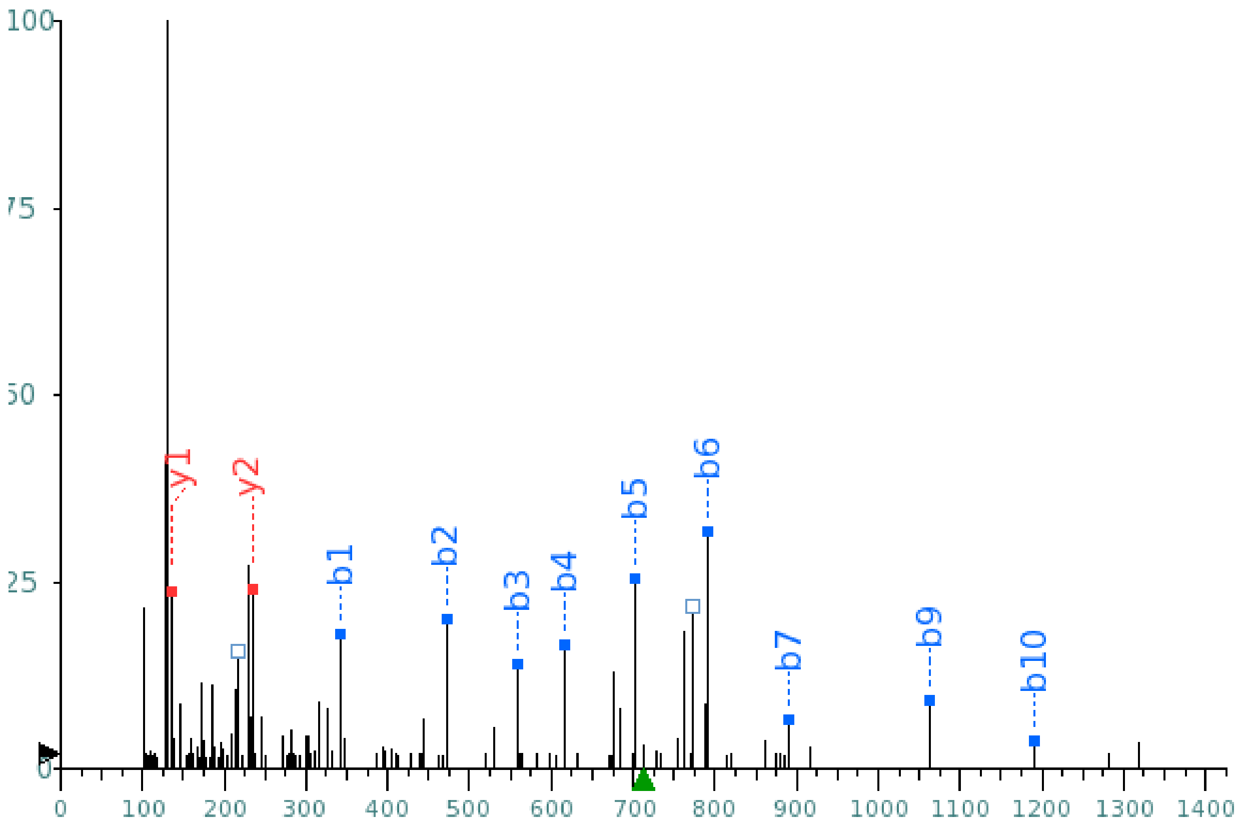

LESGSSVGDETD (+2)

Primary Reference: MATR3_HUMAN
Search ID: 41759
Search Name: 20130328_ananiav_ERstress_TMT_TM_timecourse_chymo_inj2_4MC_IAA
Scan#: 8854
Observed Mass: 712.8337 (0.8 ppm)
PSM Score: 56.94

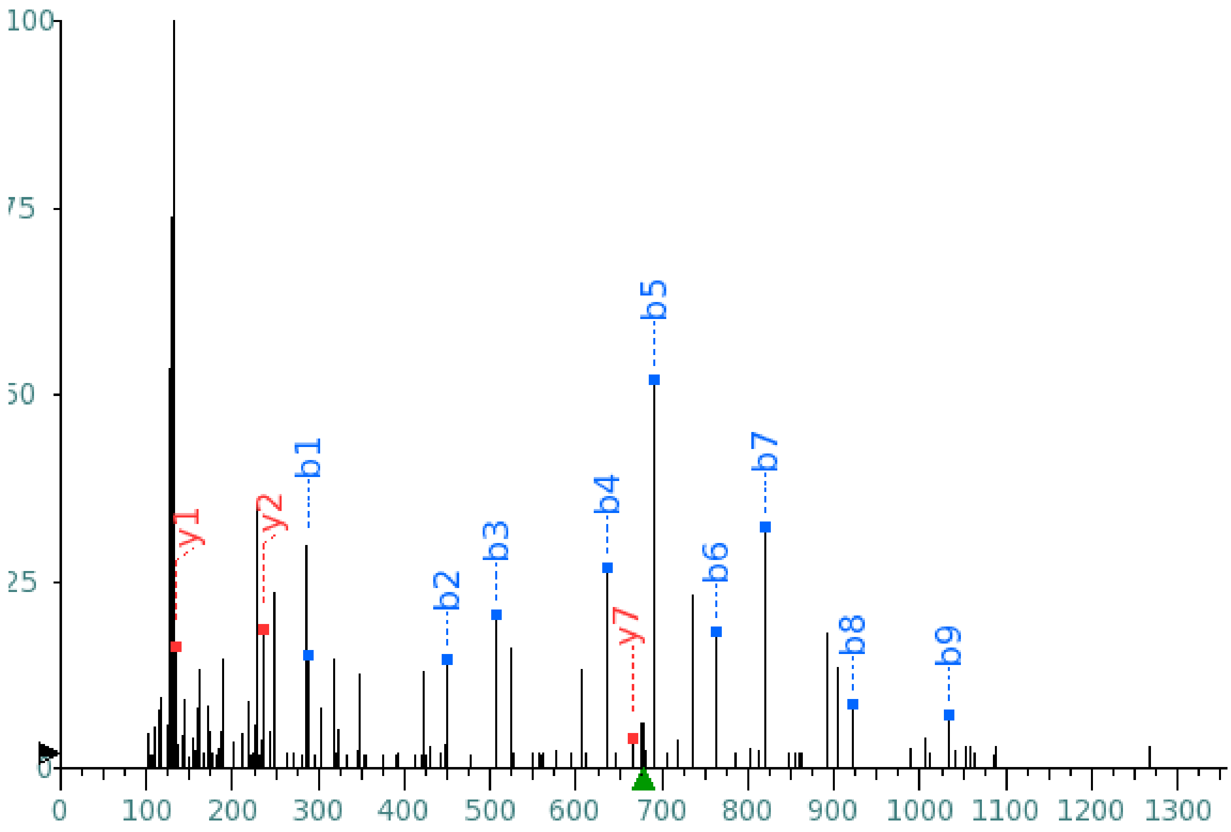

GYGQGAGTLSTD (+2)

Primary Reference: CSRP1_HUMAN
Search ID: 41759
Search Name: 20130328_ananiav_ERstress_TMT_TM_timecourse_chymo_inj2_4MC_IAA
Scan#: 8955
Observed Mass: 678.3363 (0.9 ppm)
PSM Score: 48.71

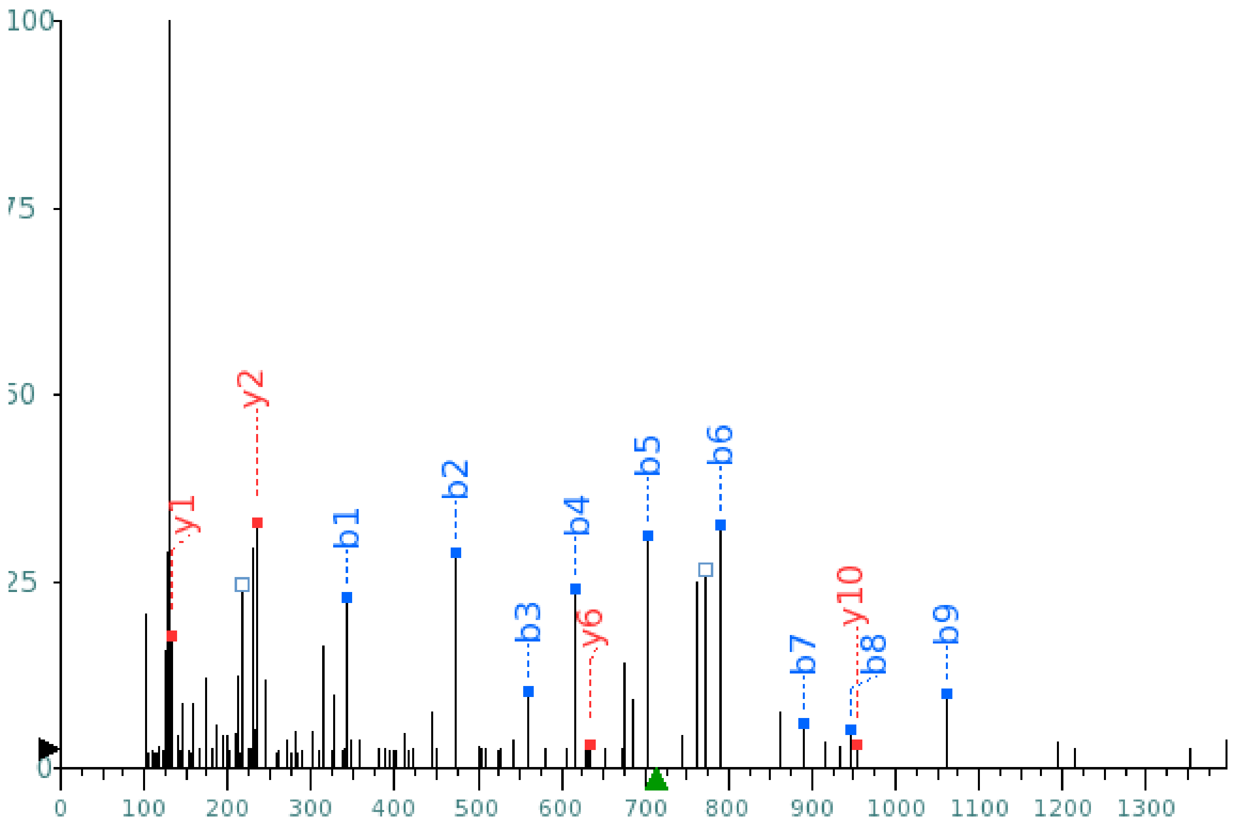

LESGSSVGDETD (+2)

Primary Reference: MATR3_HUMAN
Search ID: 41759
Search Name: 20130328_ananiav_ERstress_TMT_TM_timecourse_chymo_inj2_4MC_IAA
Scan#: 8964
Observed Mass: 712.8335 (0.5 ppm)
PSM Score: 53.37

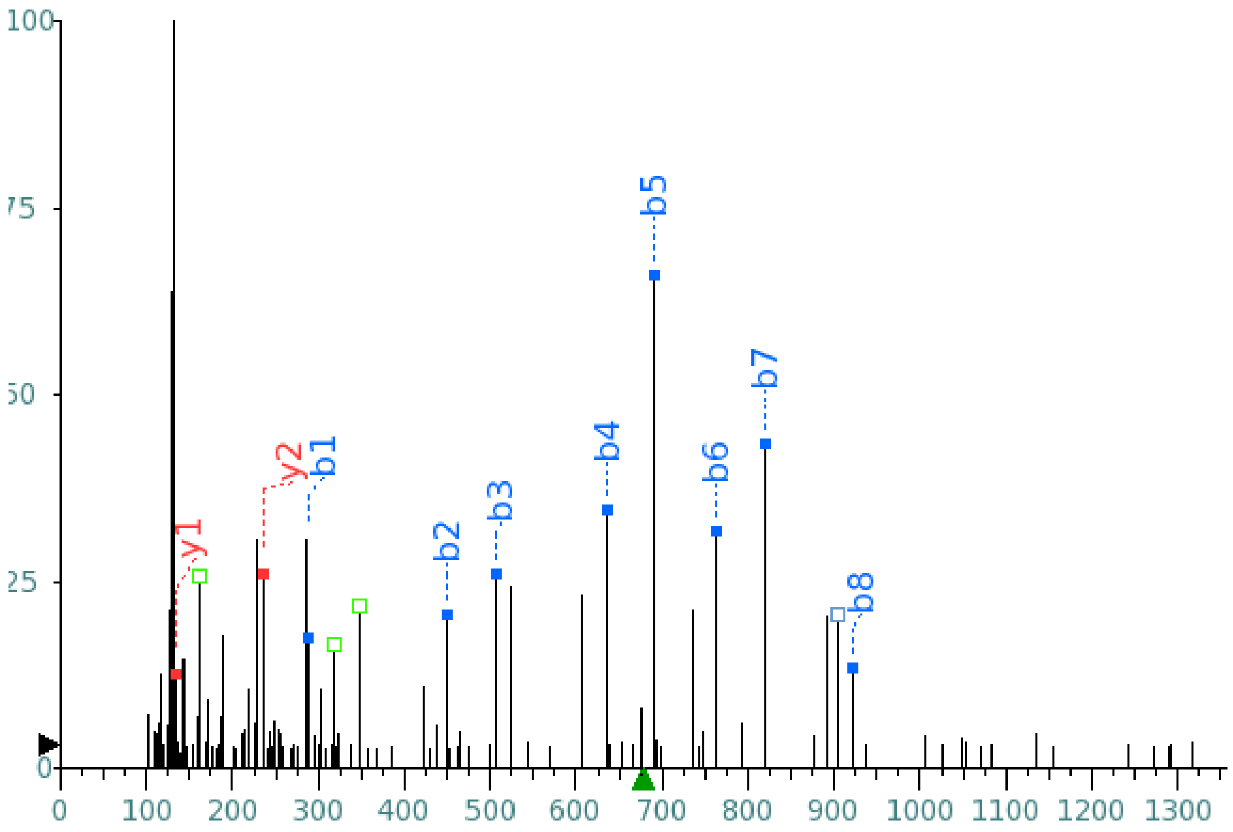

GYGQGAGTLSTD (+2)

Primary Reference: CSRP1_HUMAN
Search ID: 41759
Search Name: 20130328_ananiav_ERstress_TMT_TM_timecourse_chymo_inj2_4MC_IAA
Scan#: 9063
Observed Mass: 678.3365 (1.2 ppm)
PSM Score: 31.89

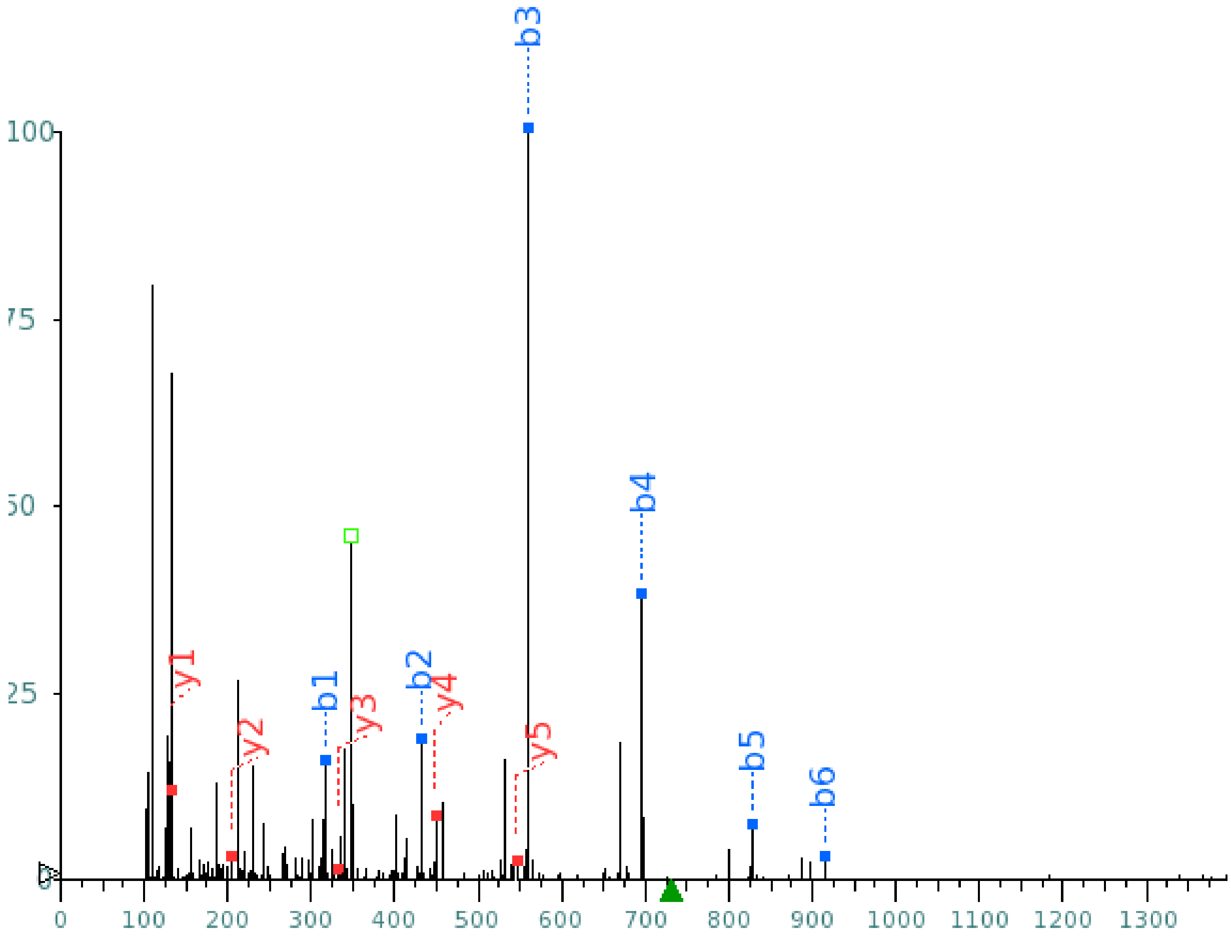

SLEHMSPDQAD (+2)

Primary Reference: SYNE2_HUMAN
Search ID: 41759
Search Name: 20130328_ananiav_ERstress_TMT_TM_timecourse_chymo_inj2_4MC_IAA
Scan#: 9303
Observed Mass: 729.8406 (0.4 ppm)
PSM Score: 24.01

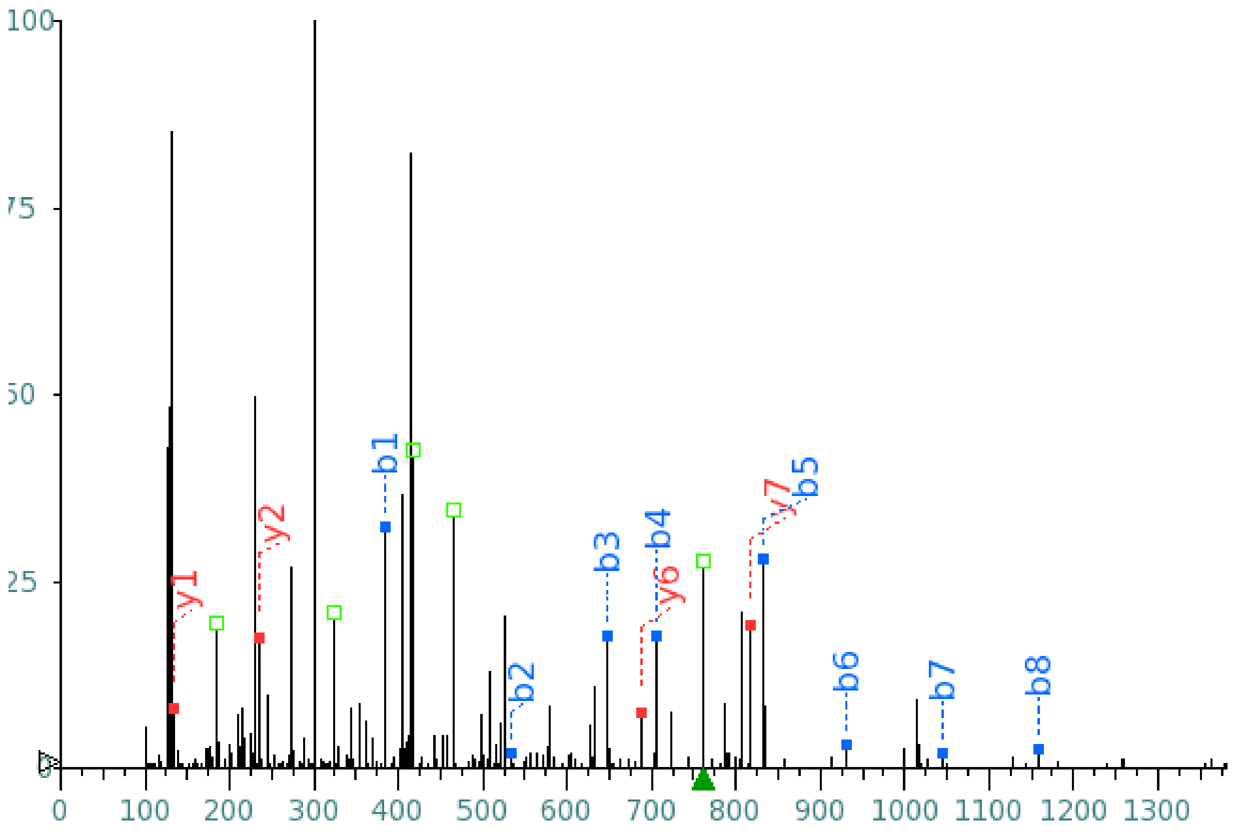

RFDGQPINETD (+2)

Primary Reference: SUMO2_HUMAN
Search ID: 41759
Search Name: 20130328_ananiav_ERstress_TMT_TM_timecourse_chymo_inj2_4MC_IAA
Scan#: 9548
Observed Mass: 760.8814 (0.8 ppm)
PSM Score: 25.49

File name: 2015-02-04 04.01.53PM.pdf

Creation time: 2015-02-04 04:09PM

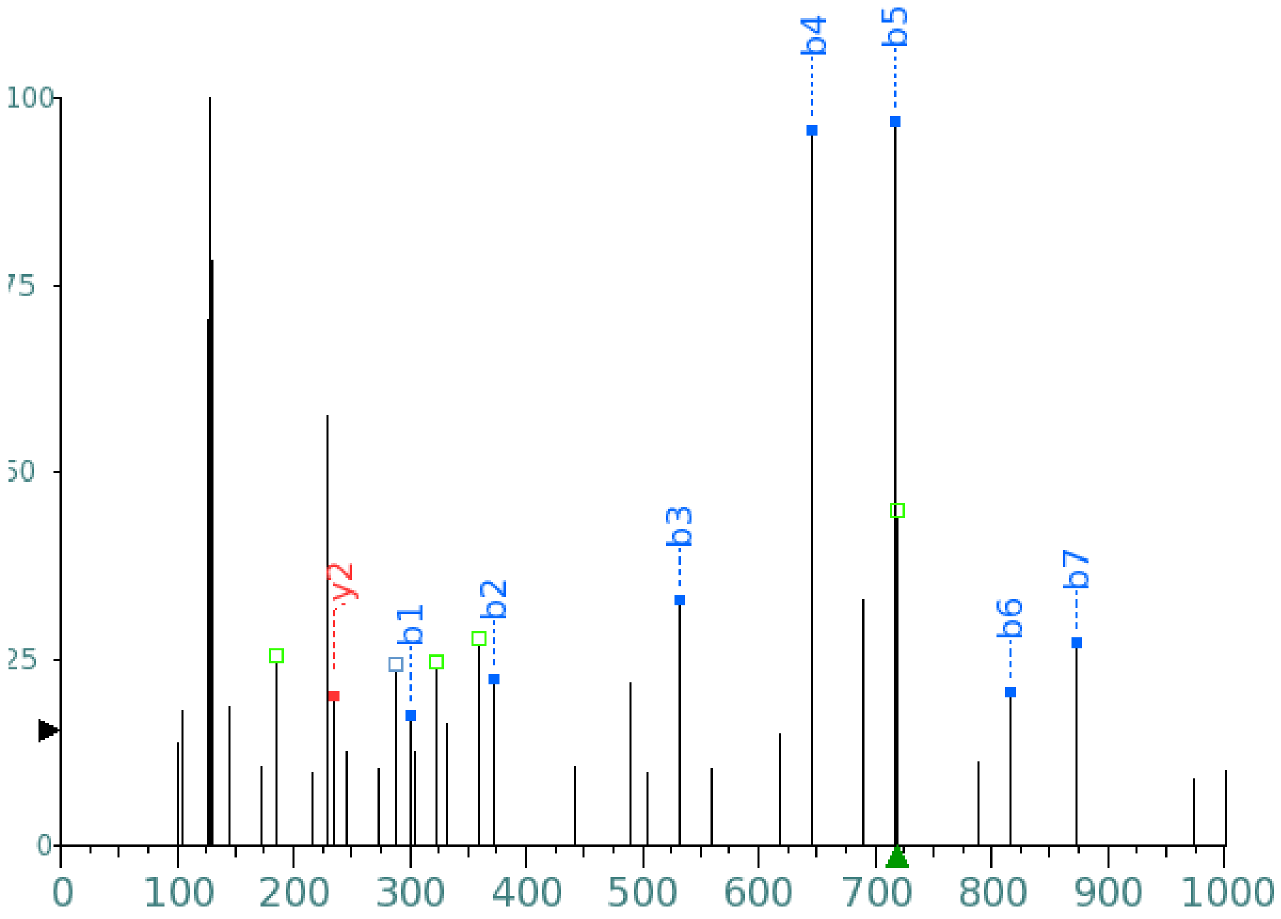

AAC*NAVGQMATD (+2)

Primary Reference: IPO5_HUMAN
Search ID: 41759
Search Name: 20130328_ananiav_ERstress_TMT_TM_timecourse_chymo_inj2_4MC_IAA
Scan#: 9730
Observed Mass: 719.3381 (1.8 ppm)
PSM Score: 39.9

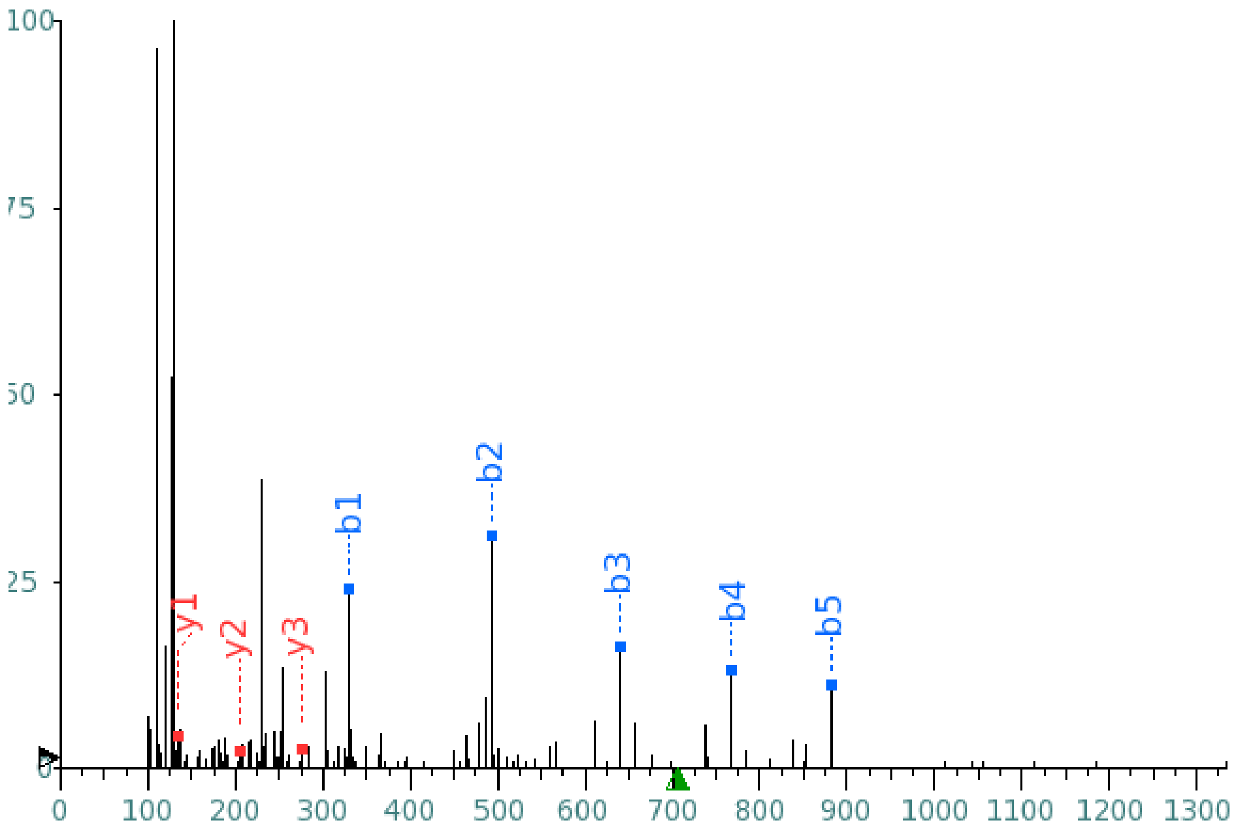

VYFQNHDAAD (+2)

Primary Reference: ROA0_HUMAN
Search ID: 41759
Search Name: 20130328_ananiav_ERstress_TMT_TM_timecourse_chymo_inj2_4MC_IAA
Scan#: 9995
Observed Mass: 704.8386 (0.3 ppm)
PSM Score: 23.98

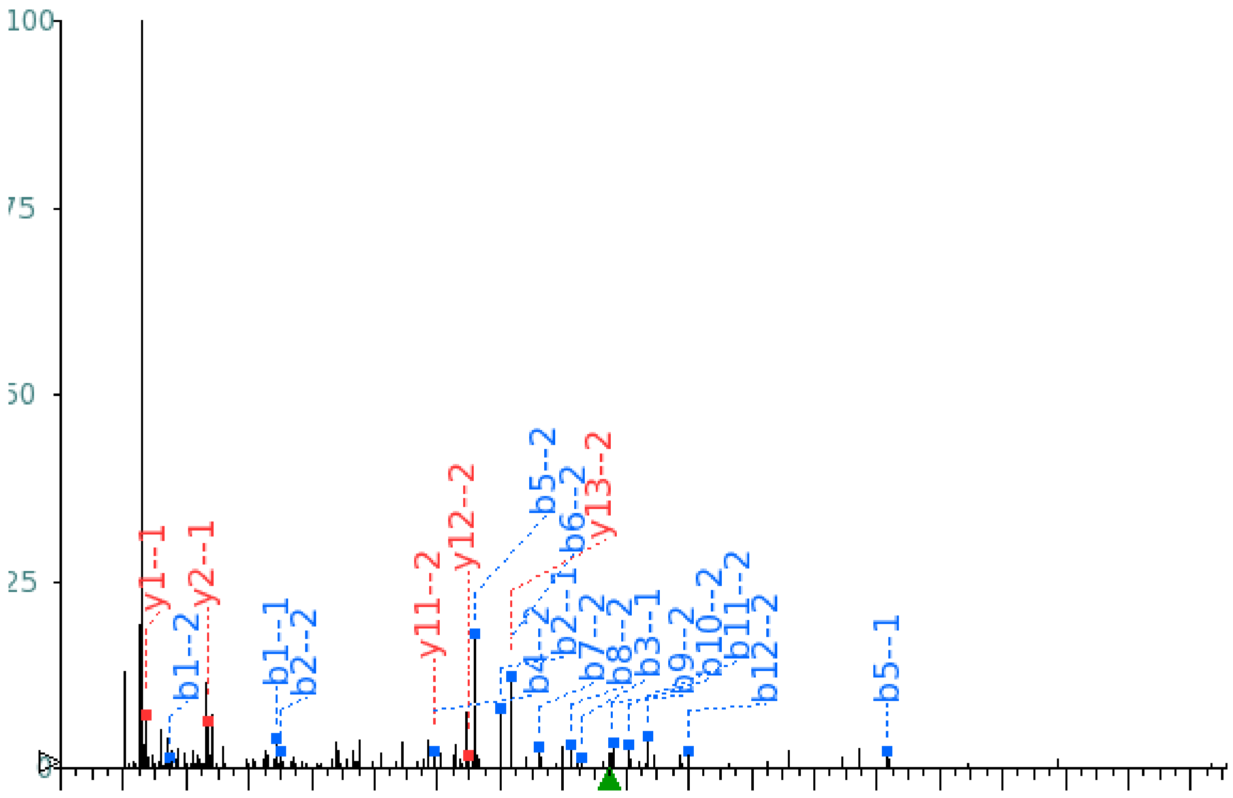

NKEKEIPVEGGGQEQQTD (+3)

Primary Reference: B1AKC7_HUMAN
Search ID: 41759
Search Name: 20130328_ananiav_ERstress_TMT_TM_timecourse_chymo_inj2_4MC_IAA
Scan#: 10673
Observed Mass: 872.8081 (0.5 ppm)
PSM Score: 30.91

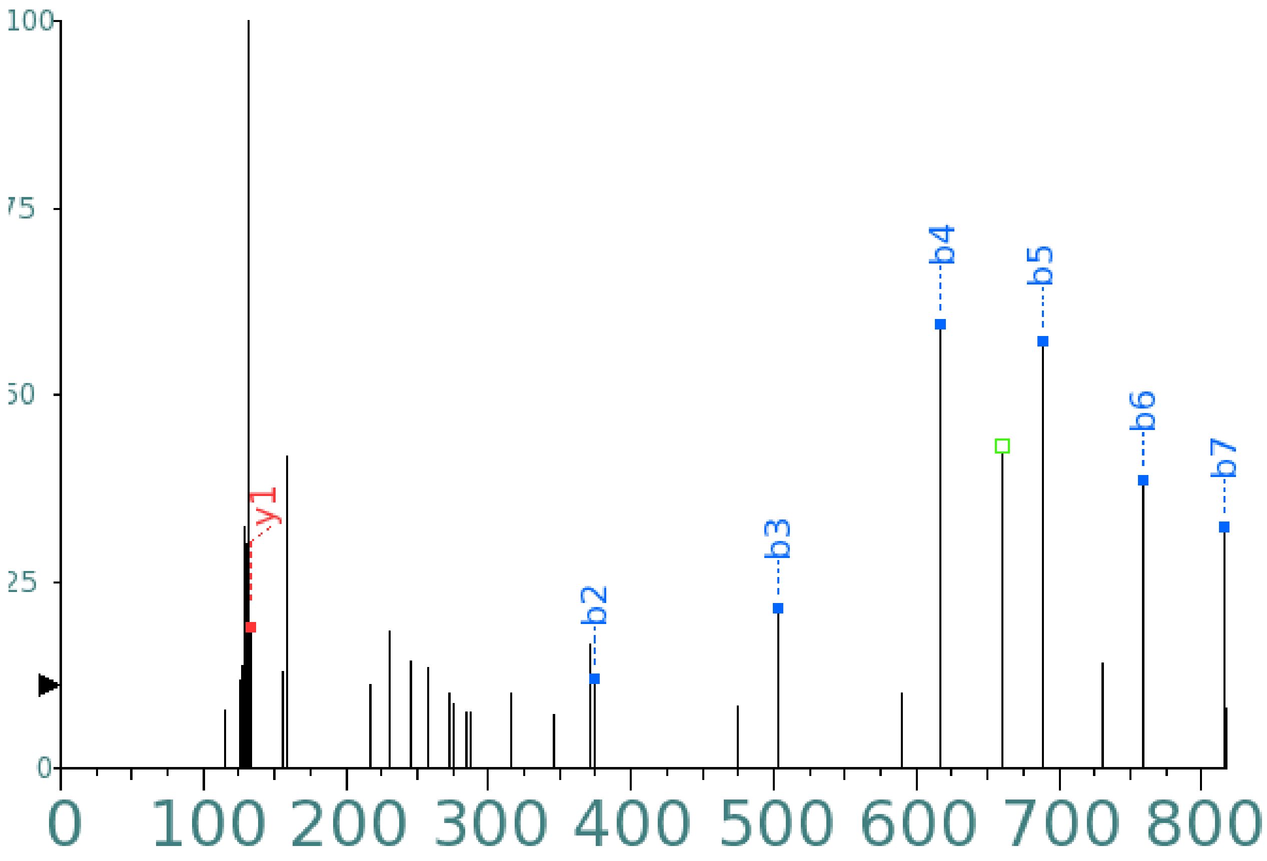

GSENAAGPC*NWDEAD (+2)

Primary Reference: MAGD2_HUMAN
Search ID: 41759
Search Name: 20130328_ananiav_ERstress_TMT_TM_timecourse_chymo_inj2_4MC_IAA
Scan#: 10864
Observed Mass: 911.3806 (-0.4 ppm)
PSM Score: 28.39

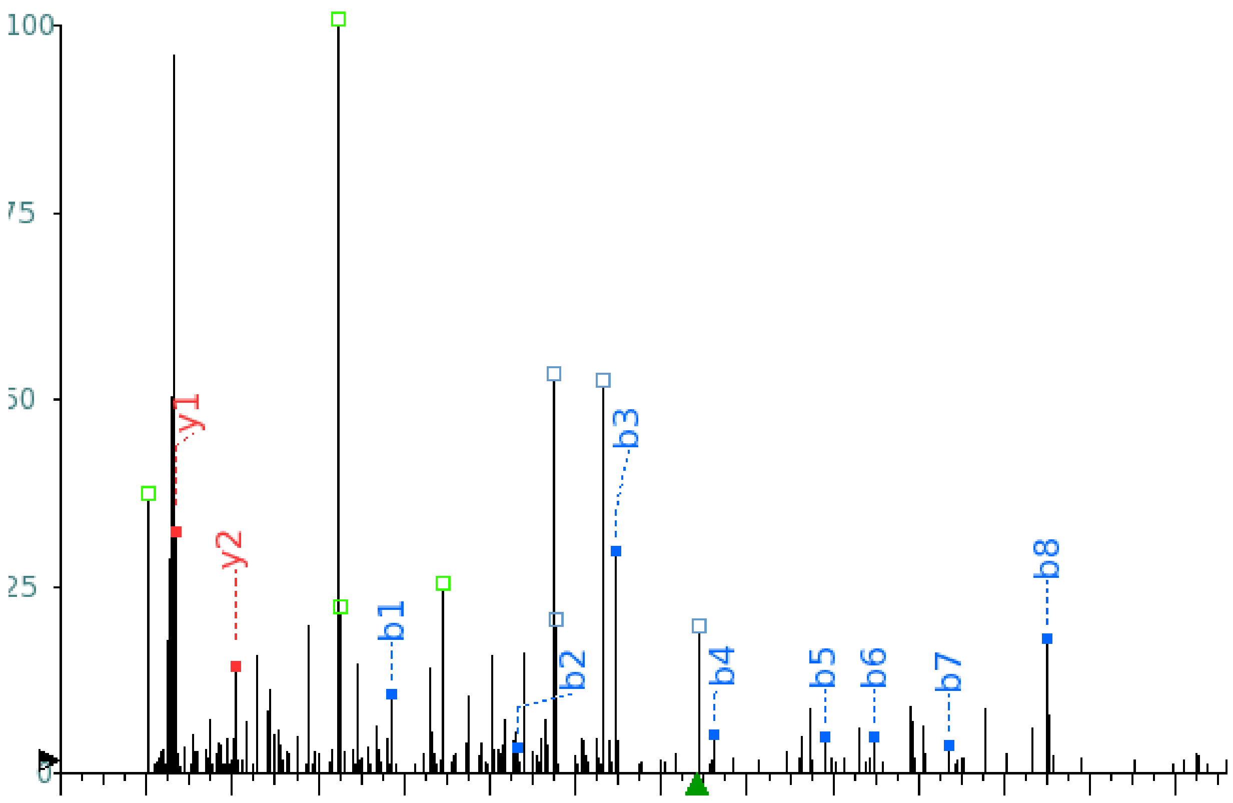

RFDIEGSDEAD (+2)

Primary Reference: RB_HUMAN
Search ID: 41759
Search Name: 20130328_ananiav_ERstress_TMT_TM_timecourse_chymo_inj2_4MC_IAA
Scan#: 11032
Observed Mass: 741.8494 (0.4 ppm)
PSM Score: 22.29

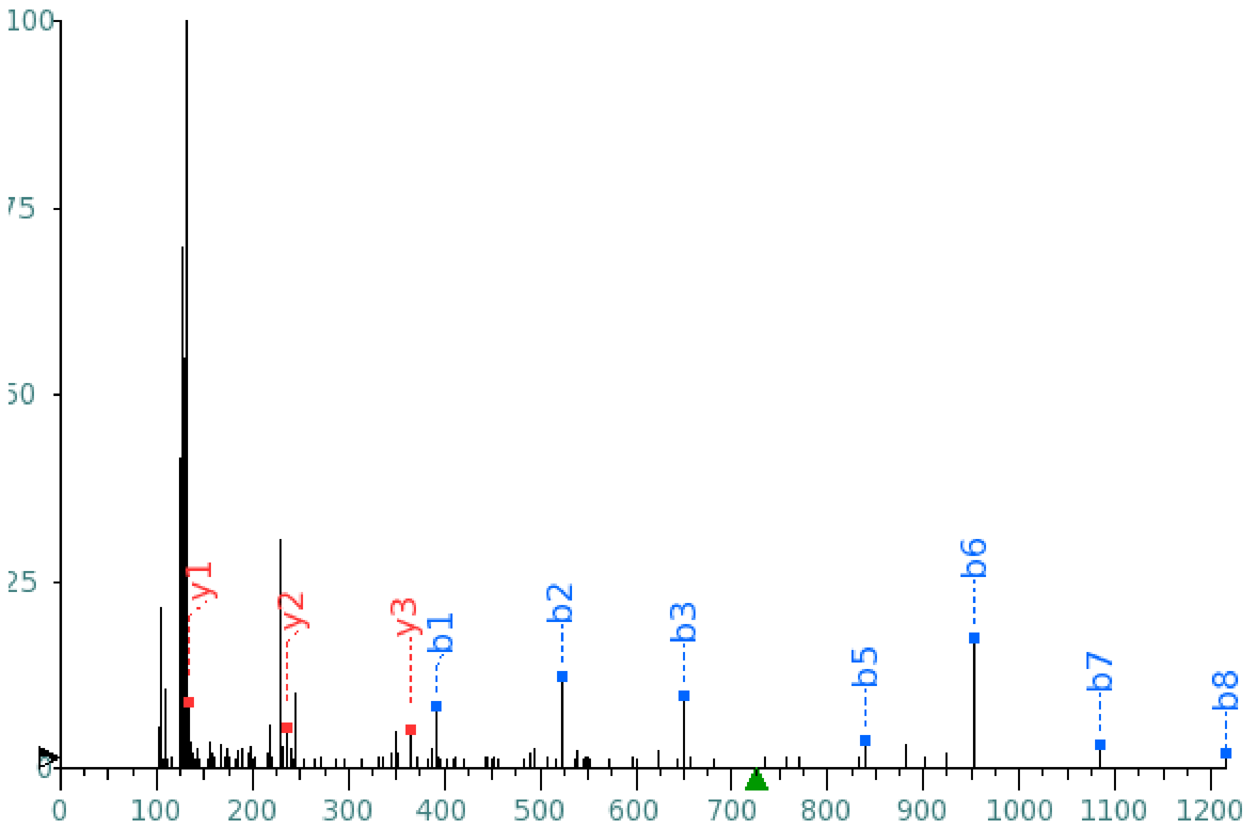

YEQTSDMMTD (+2)

Primary Reference: PCM1_HUMAN
Search ID: 41759
Search Name: 20130328_ananiav_ERstress_TMT_TM_timecourse_chymo_inj2_4MC_IAA
Scan#: 11087
Observed Mass: 725.308 (0.9 ppm)
PSM Score: 30.34

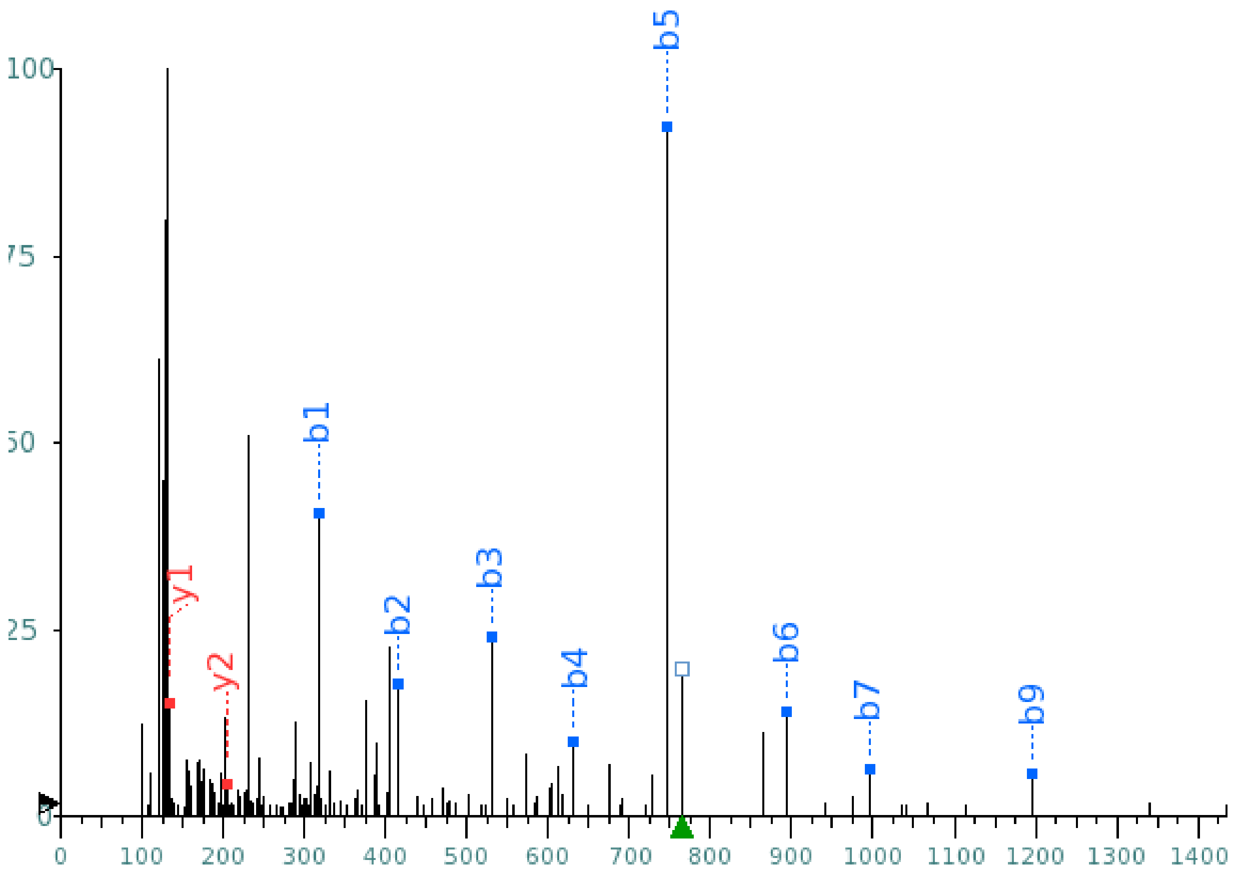

SVNTDFTSDQAD (+2)

Primary Reference: RED1_HUMAN
Search ID: 41759
Search Name: 20130328_ananiav_ERstress_TMT_TM_timecourse_chymo_inj2_4MC_IAA
Scan#: 11126
Observed Mass: 764.8521 (0.2 ppm)
PSM Score: 41.58

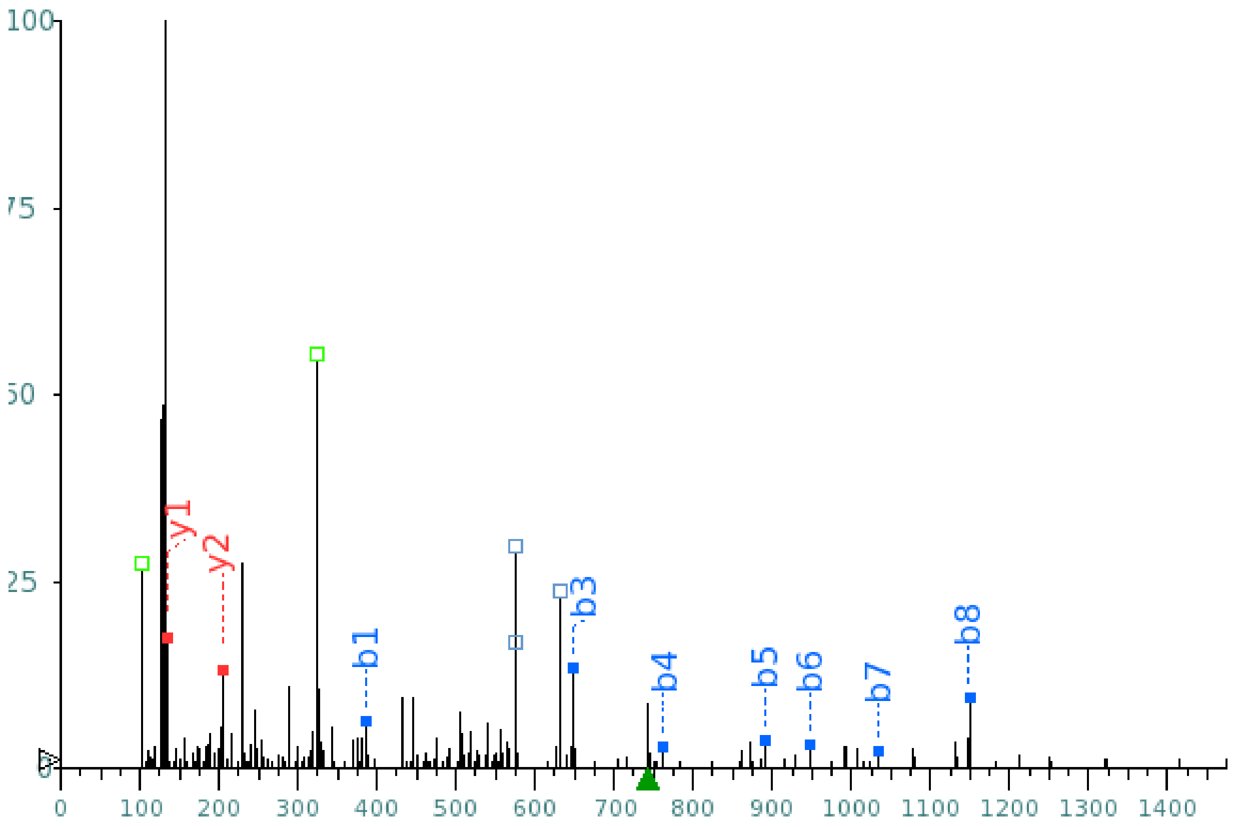

RFDIEGSDEAD (+2)

Primary Reference: RB_HUMAN
Search ID: 41759
Search Name: 20130328_ananiav_ERstress_TMT_TM_timecourse_chymo_inj2_4MC_IAA
Scan#: 11151
Observed Mass: 741.8499 (1 ppm)
PSM Score: 26.66

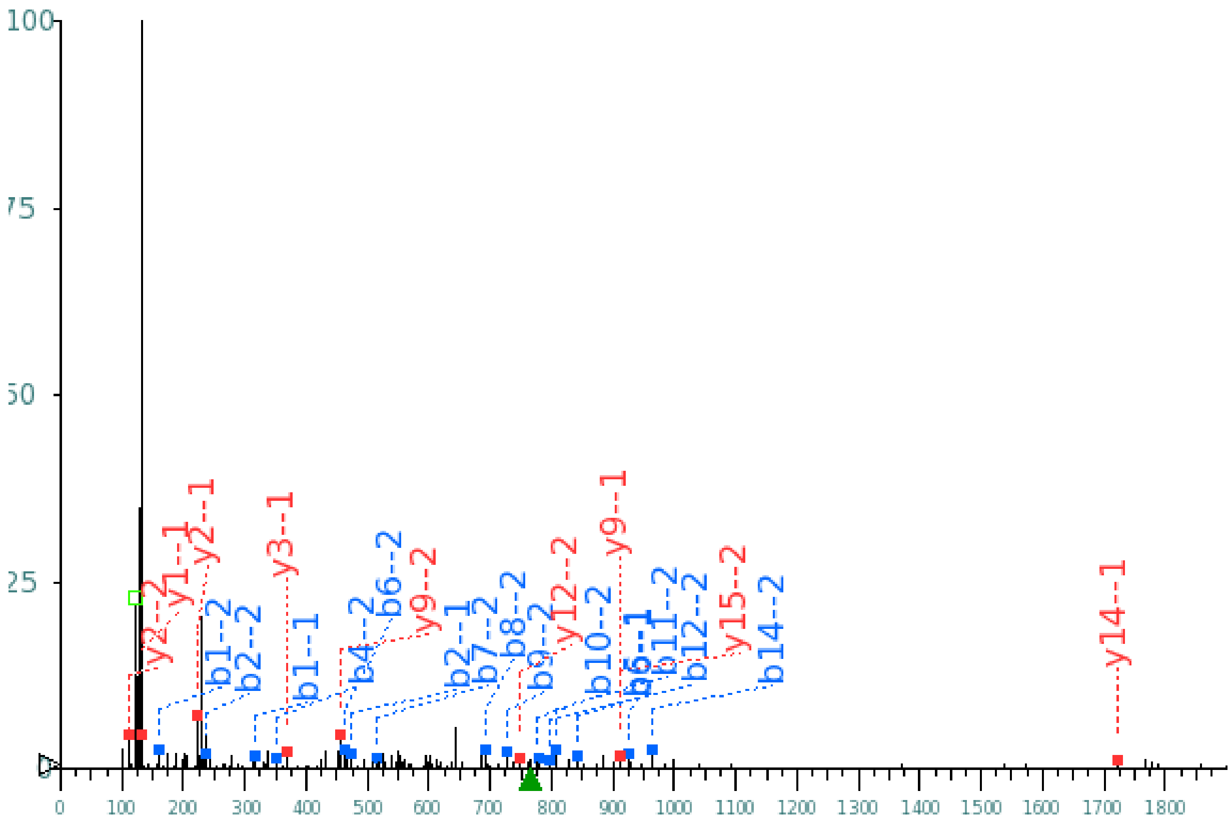

SRPMPEPKATGAQDFSD (+3)

Primary Reference: GT2D1_HUMAN
Search ID: 41759
Search Name: 20130328_ananiav_ERstress_TMT_TM_timecourse_chymo_inj2_4MC_IAA
Scan#: 11415
Observed Mass: 764.7283 (0.5 ppm)
PSM Score: 26.07

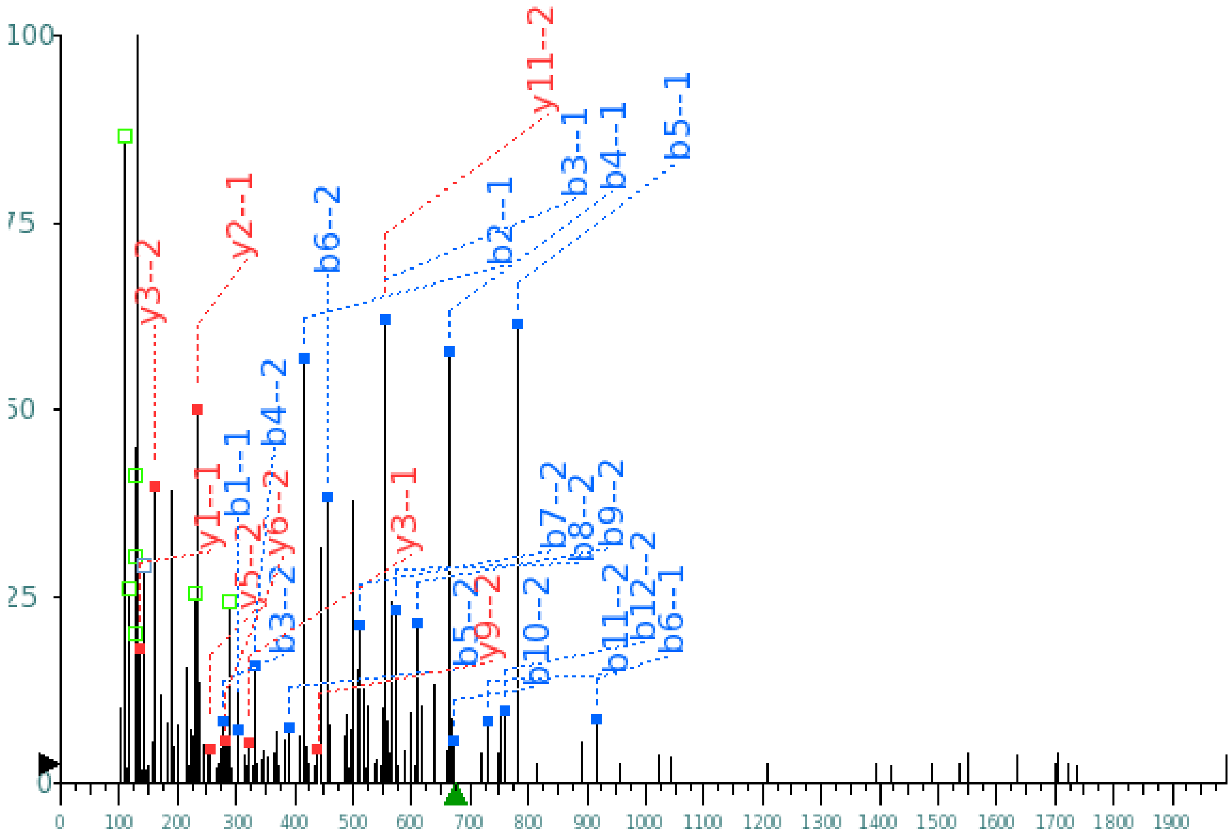

ADHLLHTQAQDGAISTD (+3)

Primary Reference: TPP2_HUMAN
Search ID: 41759
Search Name: 20130328_ananiav_ERstress_TMT_TM_timecourse_chymo_inj2_4MC_IAA
Scan#: 11429
Observed Mass: 674.6756 (1.6 ppm)
PSM Score: 34.88

File name: 2015-02-04 04.01.53PM.pdf

Creation time: 2015-02-04 04:09PM

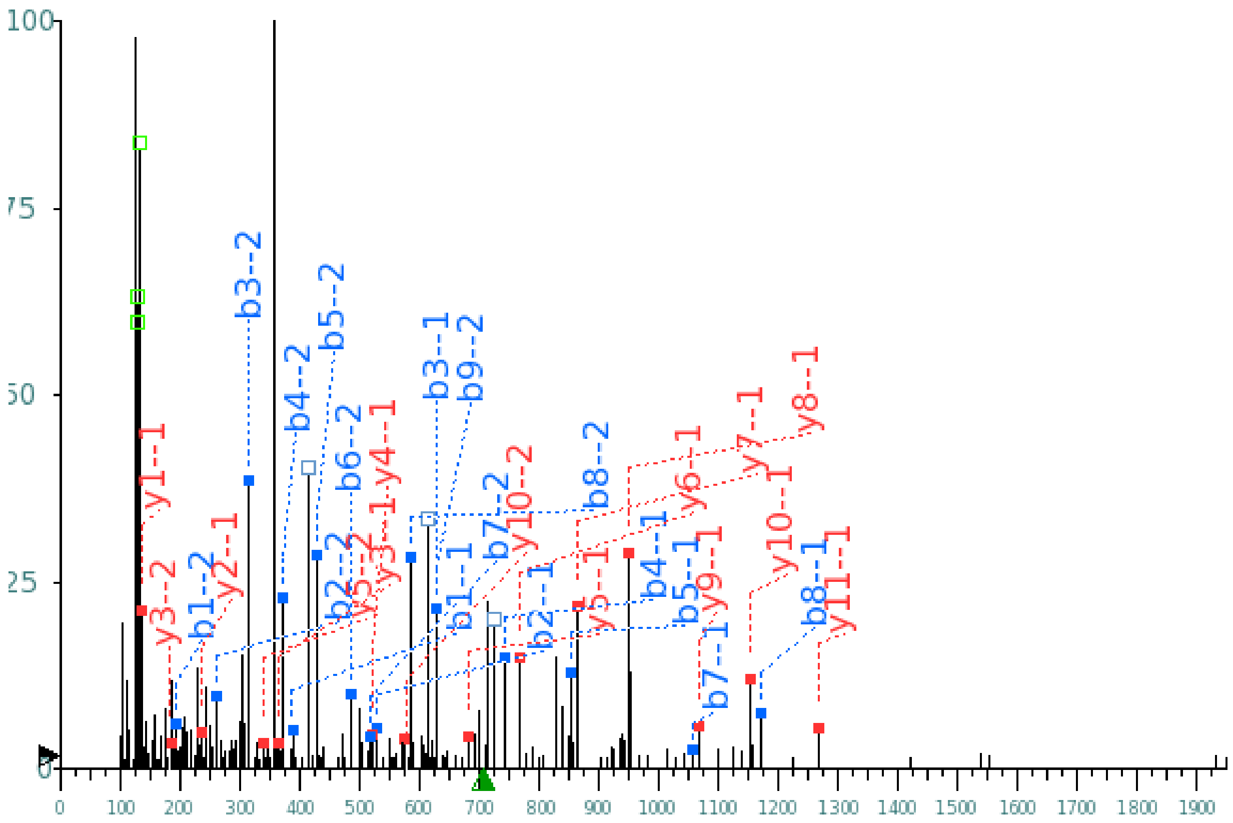

RENLLLSDSPSC*RETD (+3)

Primary Reference: ROCK2_HUMAN
Search ID: 41759
Search Name: 20130328_ananiav_ERstress_TMT_TM_timecourse_chymo_inj2_4MC_IAA
Scan#: 11433
Observed Mass: 707.6827 (-5.1 ppm)
PSM Score: 28.84

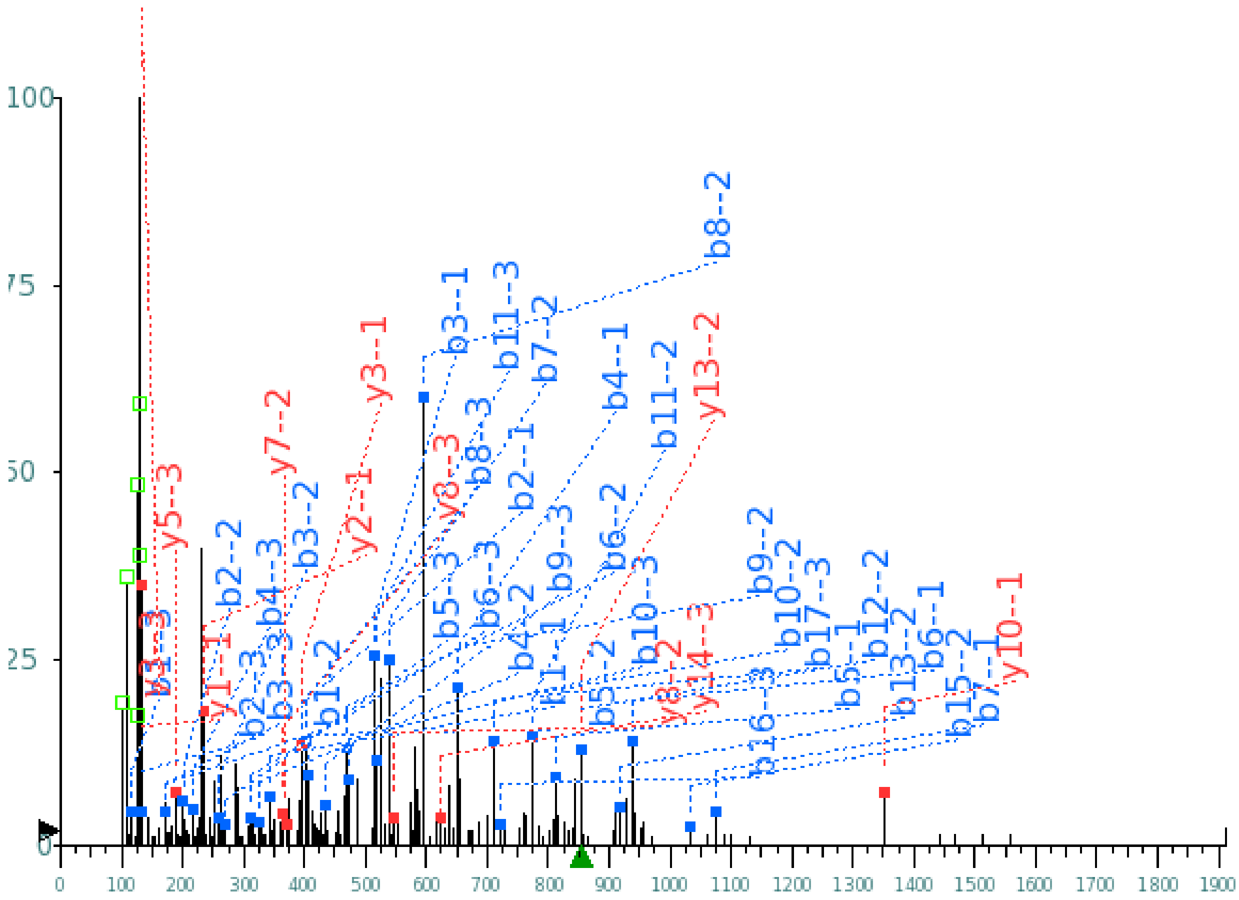

NGIHC*QHNINEC*EVEPC*KNGGIC*TD (+4)

Primary Reference: EDIL3_HUMAN
Search ID: 41759
Search Name: 20130328_ananiav_ERstress_TMT_TM_timecourse_chymo_inj2_4MC_IAA
Scan#: 11459
Observed Mass: 854.1435 (0.7 ppm)
PSM Score: 38.49

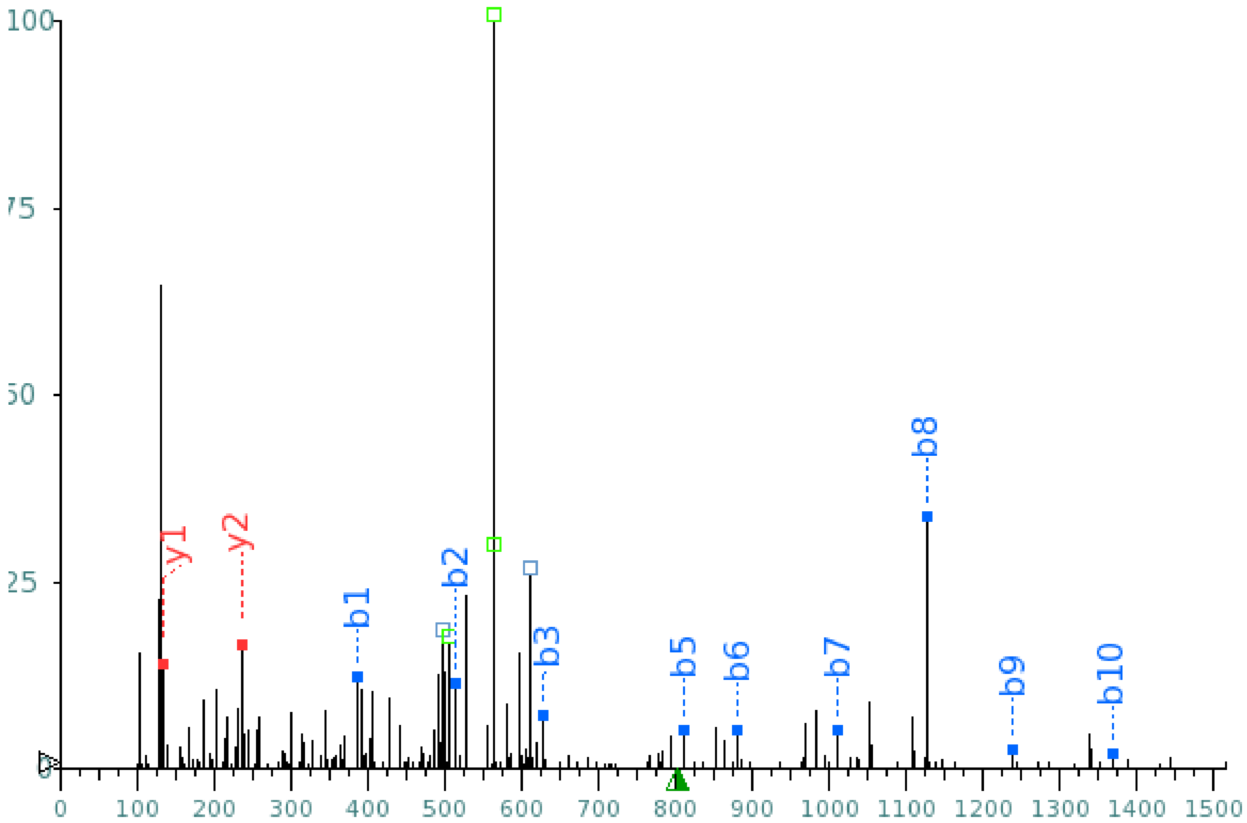

RQIPSAEDLETD (+2)

Primary Reference: HDAC7_HUMAN
Search ID: 41759
Search Name: 20130328_ananiav_ERstress_TMT_TM_timecourse_chymo_inj2_4MC_IAA
Scan#: 11563
Observed Mass: 801.9134 (1.4 ppm)
PSM Score: 30.56

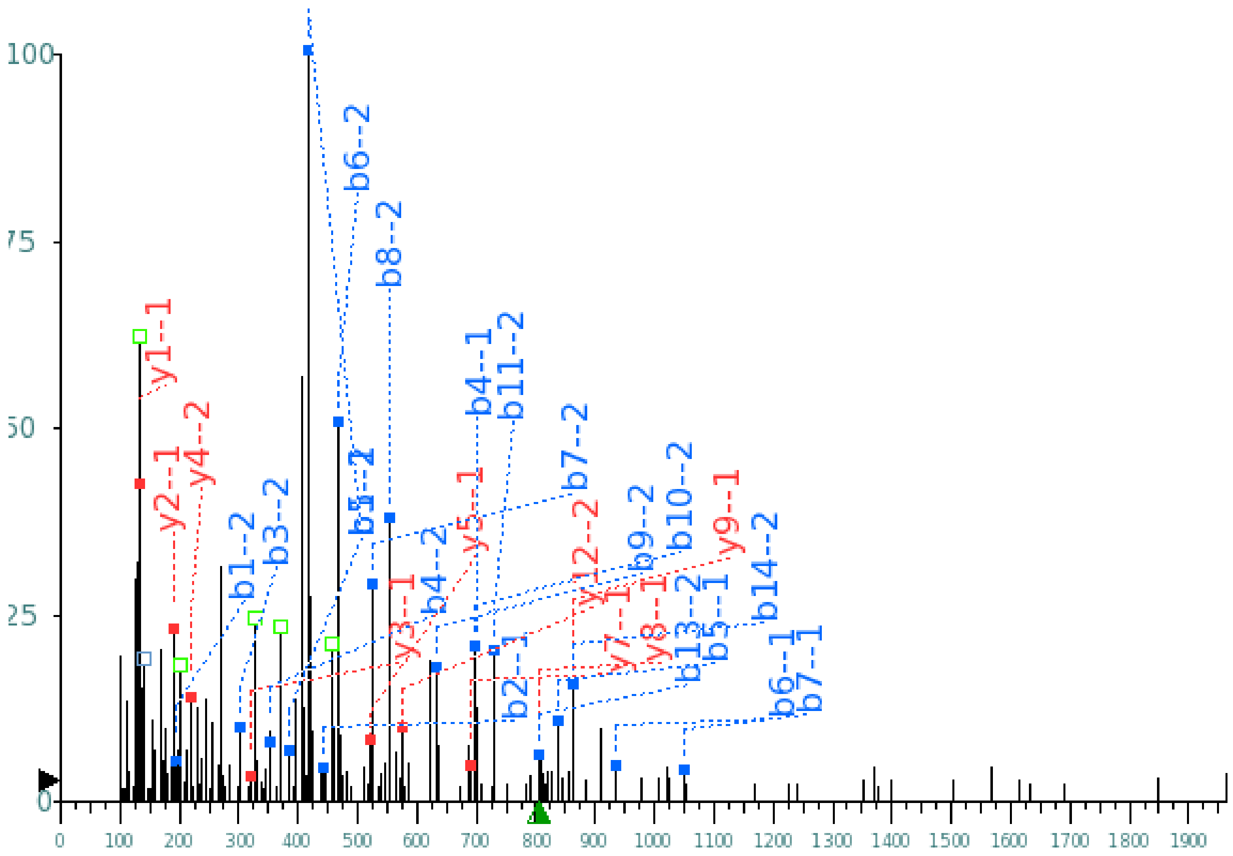

RGC*PHVLGYEGPGDPASDEGD (+3)

Primary Reference: RUND1_HUMAN
Search ID: 41759
Search Name: 20130328_ananiav_ERstress_TMT_TM_timecourse_chymo_inj2_4MC_IAA
Scan#: 11652
Observed Mass: 805.3687 (1.4 ppm)
PSM Score: 33.15

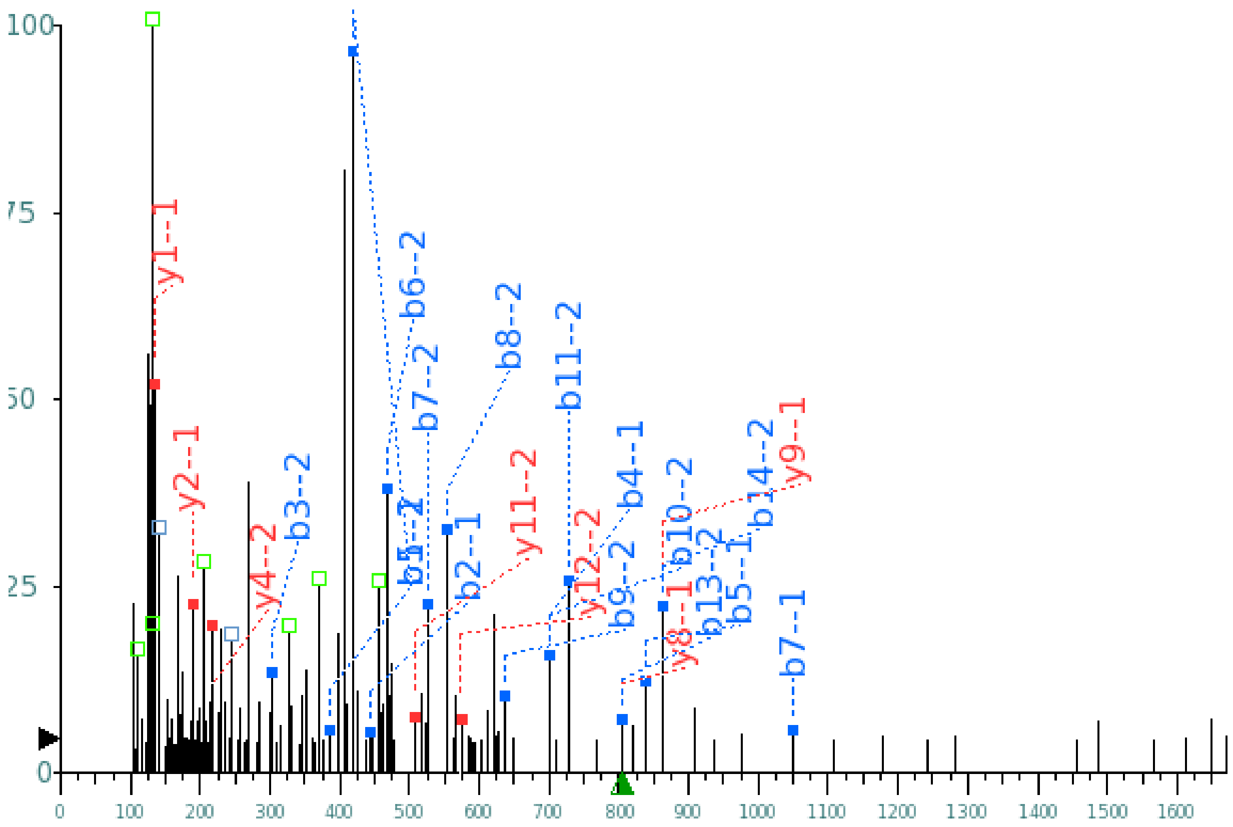

RGC*PHVLGYEGPGDPASDEGD (+3)

Primary Reference: RUND1_HUMAN
Search ID: 41759
Search Name: 20130328_ananiav_ERstress_TMT_TM_timecourse_chymo_inj2_4MC_IAA
Scan#: 11690
Observed Mass: 805.3689 (1.8 ppm)
PSM Score: 27.04

File name: 2015-02-04 04.01.53PM.pdf

Creation time: 2015-02-04 04:09PM

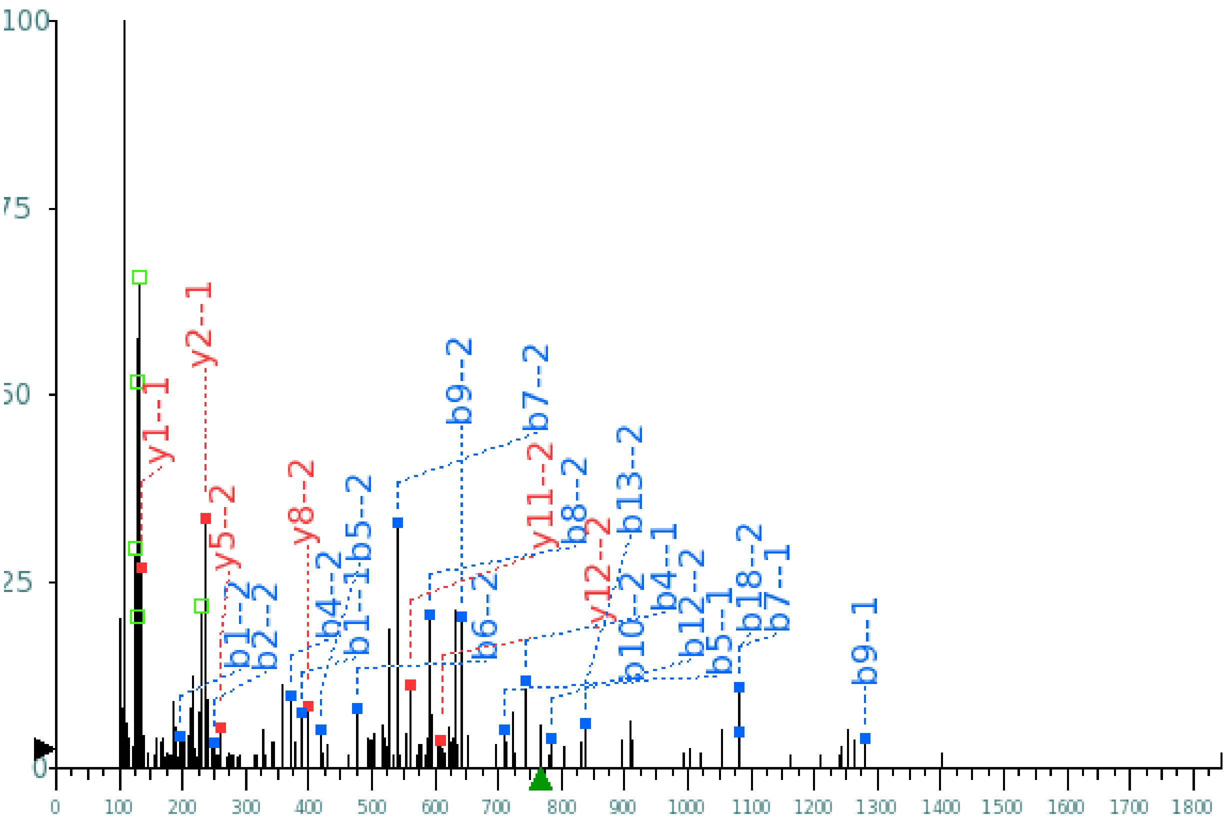

RIIEPNEVTHSGDTGVETD (+3)

Primary Reference: B4E2P4_HUMAN
Search ID: 41759
Search Name: 20130328_ananiav_ERstress_TMT_TM_timecourse_chymo_inj2_4MC_IAA
Scan#: 11839
Observed Mass: 766.7186 (0.1 ppm)
PSM Score: 37.29

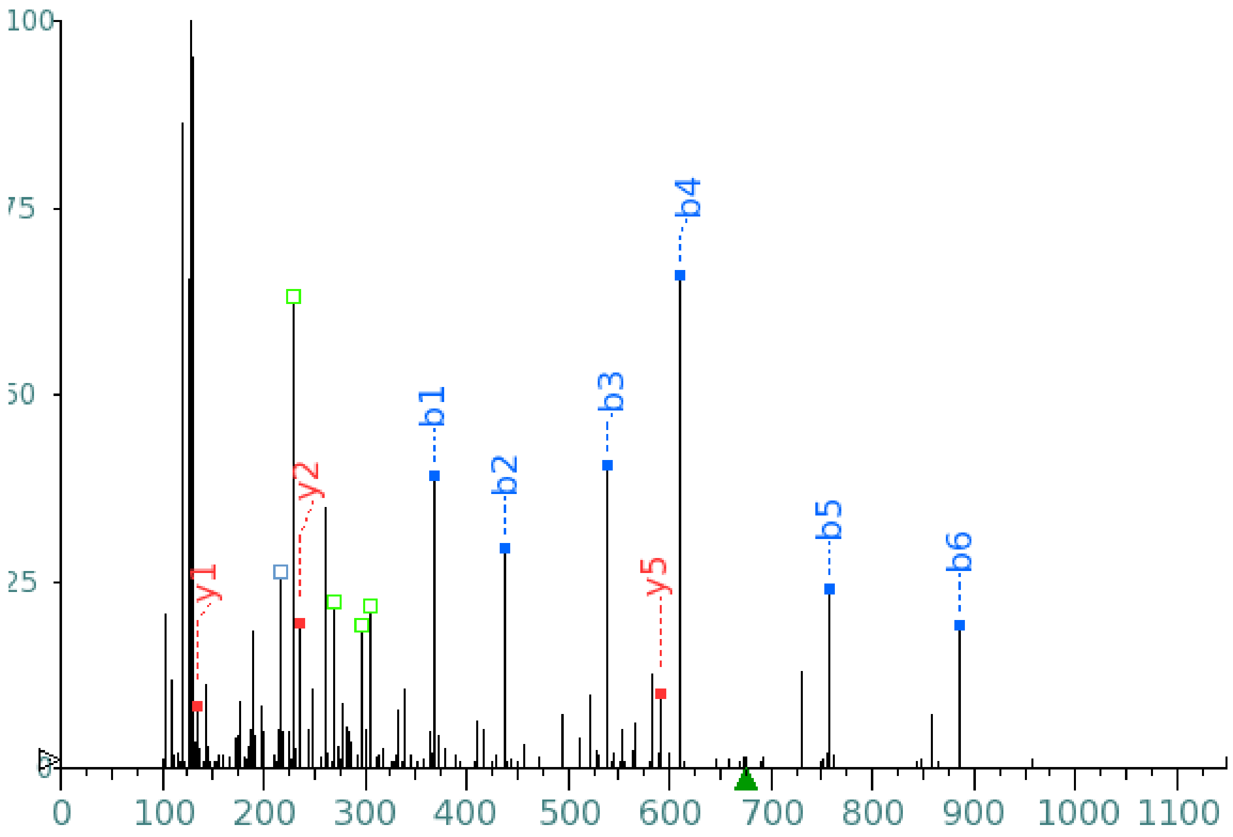

HATAFELDTD (+2)

Primary Reference: SERPH_HUMAN
Search ID: 41759
Search Name: 20130328_ananiav_ERstress_TMT_TM_timecourse_chymo_inj2_4MC_IAA
Scan#: 11935
Observed Mass: 674.8336 (1.3 ppm)
PSM Score: 36.97

File name: 2015-02-04 04.01.53PM.pdf

Creation time: 2015-02-04 04:09PM

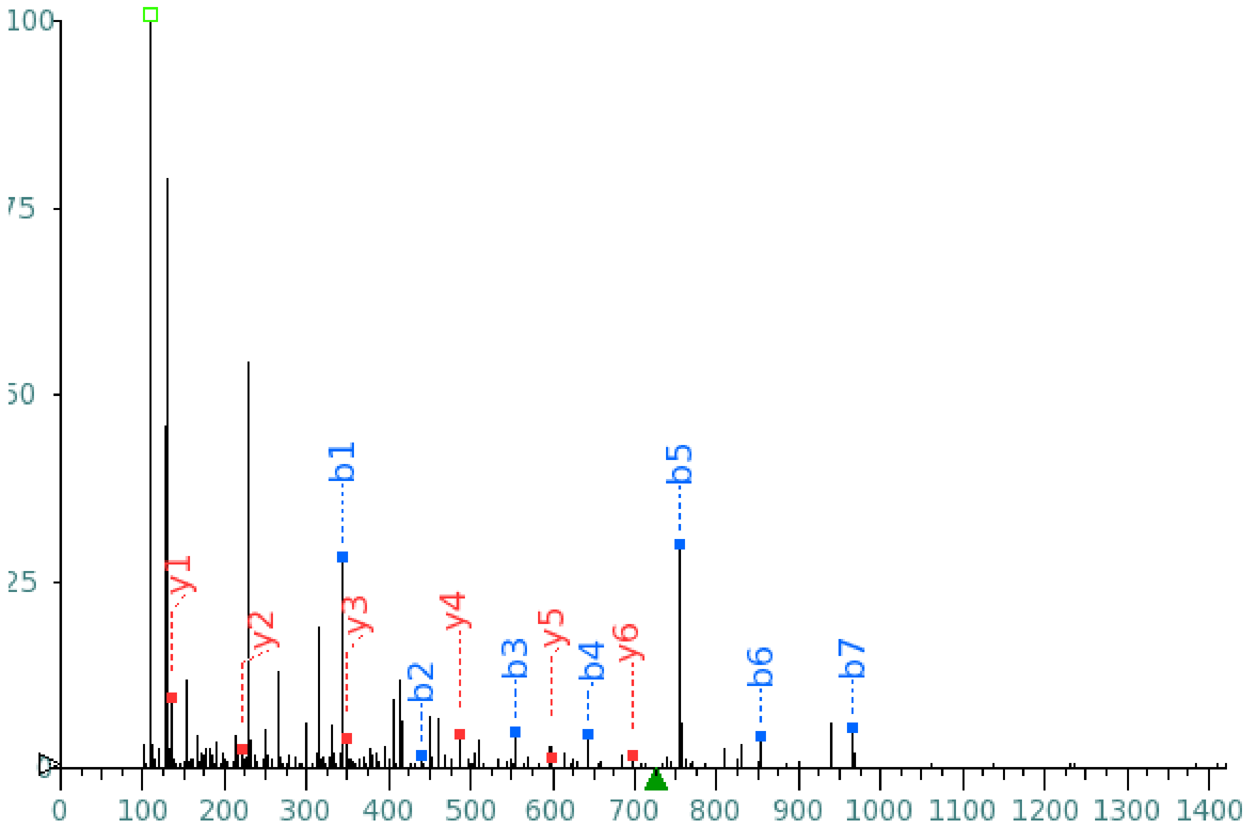

LPNSDPLHQSD (+2)

Primary Reference: ANR17_HUMAN
Search ID: 41759
Search Name: 20130328_ananiav_ERstress_TMT_TM_timecourse_chymo_inj2_4MC_IAA
Scan#: 12094
Observed Mass: 726.3722 (3 ppm)
PSM Score: 24.49

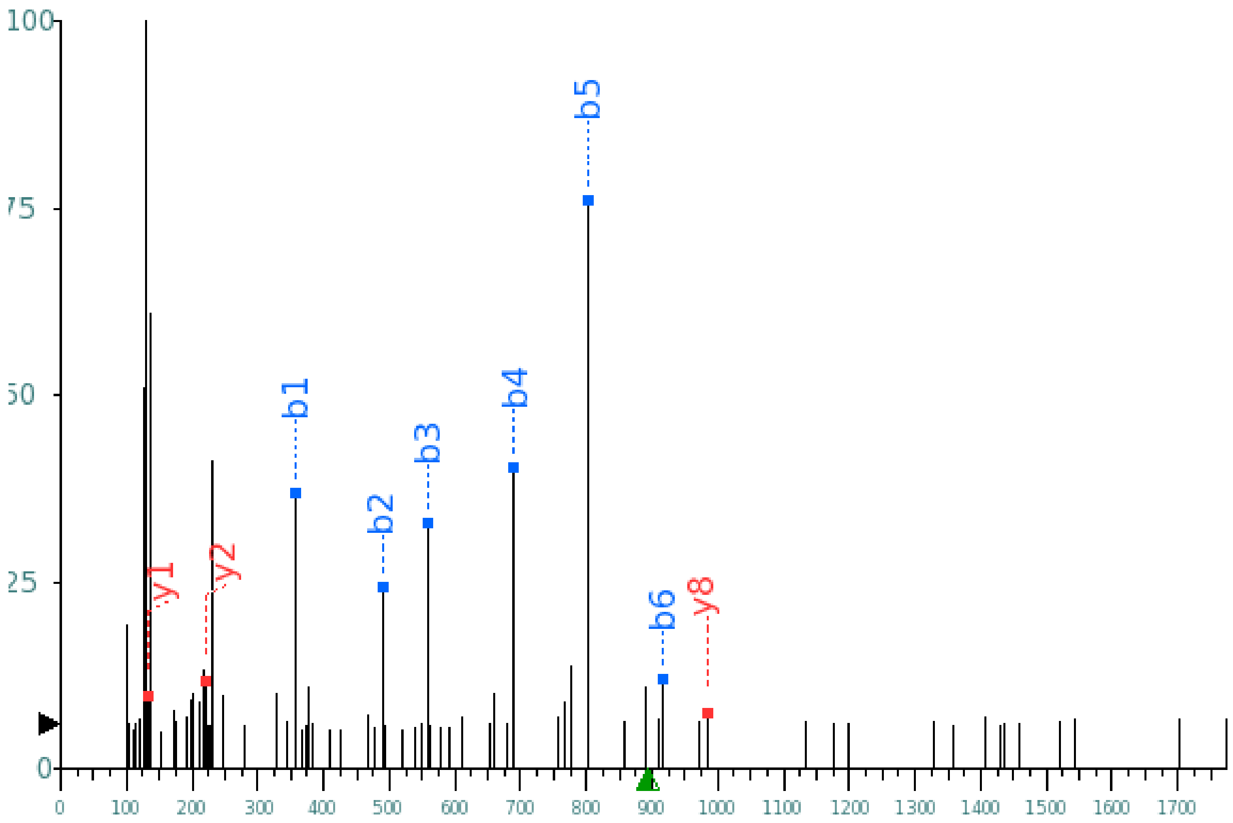

QMAENIYNDEESD (+2)

Primary Reference: RBBP7_HUMAN
Search ID: 41759
Search Name: 20130328_ananiav_ERstress_TMT_TM_timecourse_chymo_inj2_4MC_IAA
Scan#: 12306
Observed Mass: 893.886 (0.5 ppm)
PSM Score: 28.56

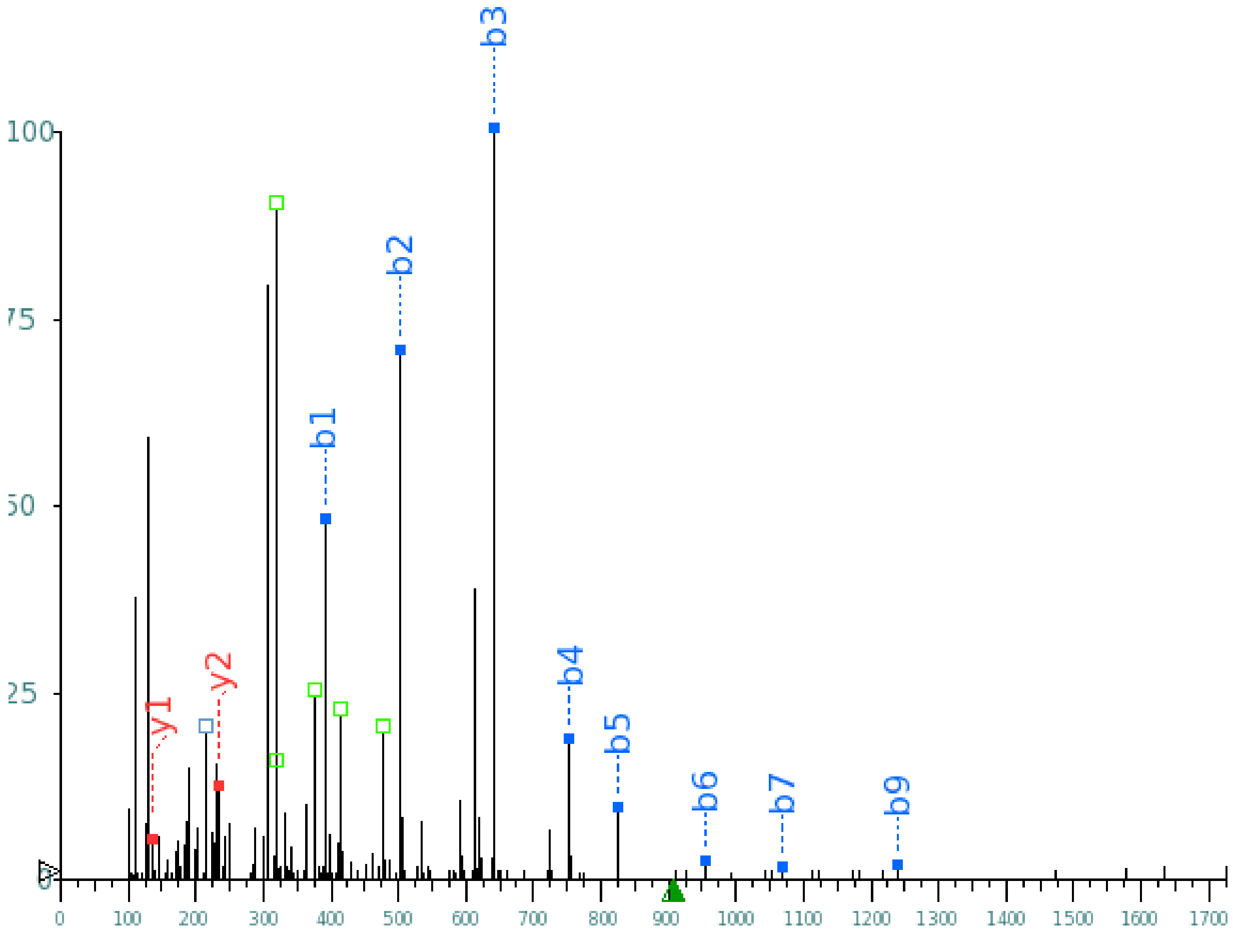

C*LHIAEDDGEVDTD (+2)

Primary Reference: SIN1_HUMAN
Search ID: 41759
Search Name: 20130328_ananiav_ERstress_TMT_TM_timecourse_chymo_inj2_4MC_IAA
Scan#: 12589
Observed Mass: 909.4077 (1.1 ppm)
PSM Score: 32.07

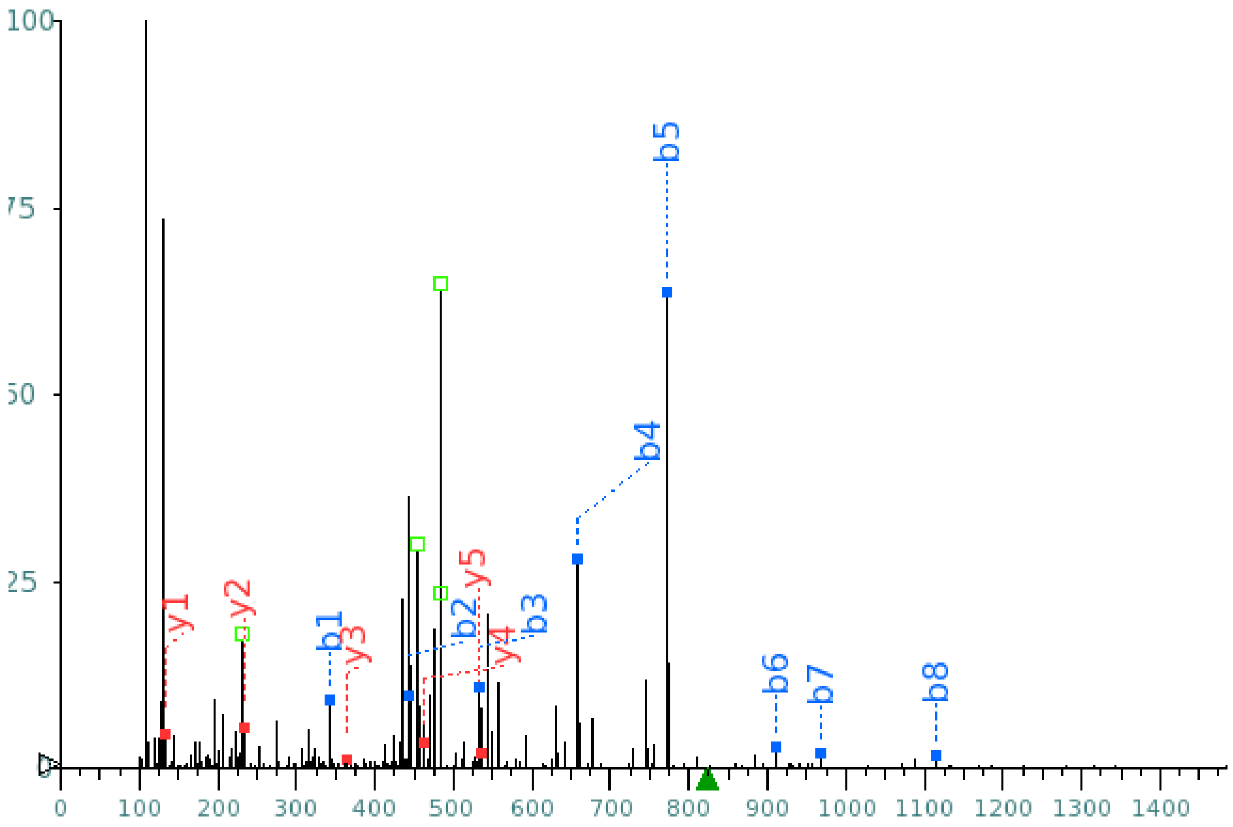

LTSQNHGFAVETD (+2)

Primary Reference: PYR1_HUMAN
Search ID: 41759
Search Name: 20130328_ananiav_ERstress_TMT_TM_timecourse_chymo_inj2_4MC_IAA
Scan#: 12622
Observed Mass: 824.413 (0.6 ppm)
PSM Score: 25.77

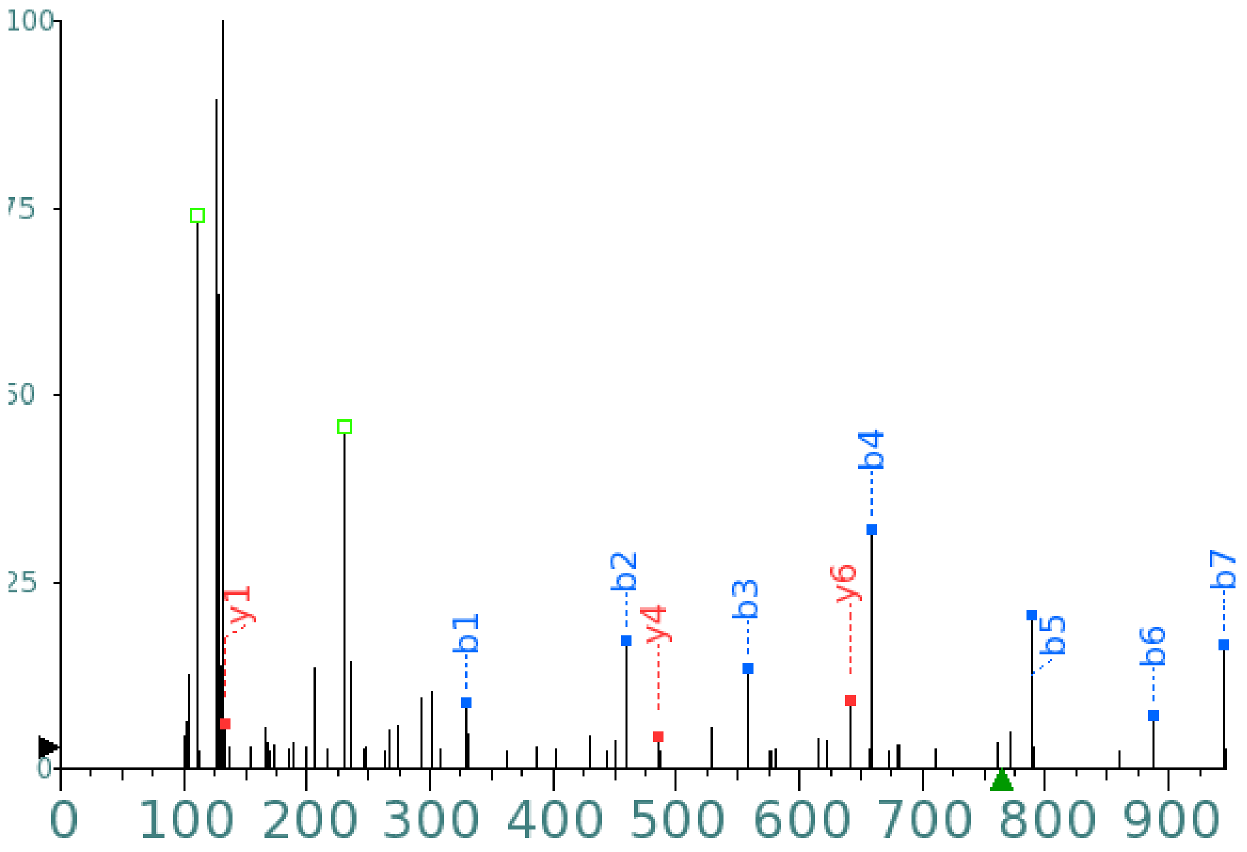

VEVTMVGPHQSD (+2)

Primary Reference: UN13B_HUMAN
Search ID: 41759
Search Name: 20130328_ananiav_ERstress_TMT_TM_timecourse_chymo_inj2_4MC_IAA
Scan#: 12657
Observed Mass: 764.3874 (0 ppm)
PSM Score: 45.78

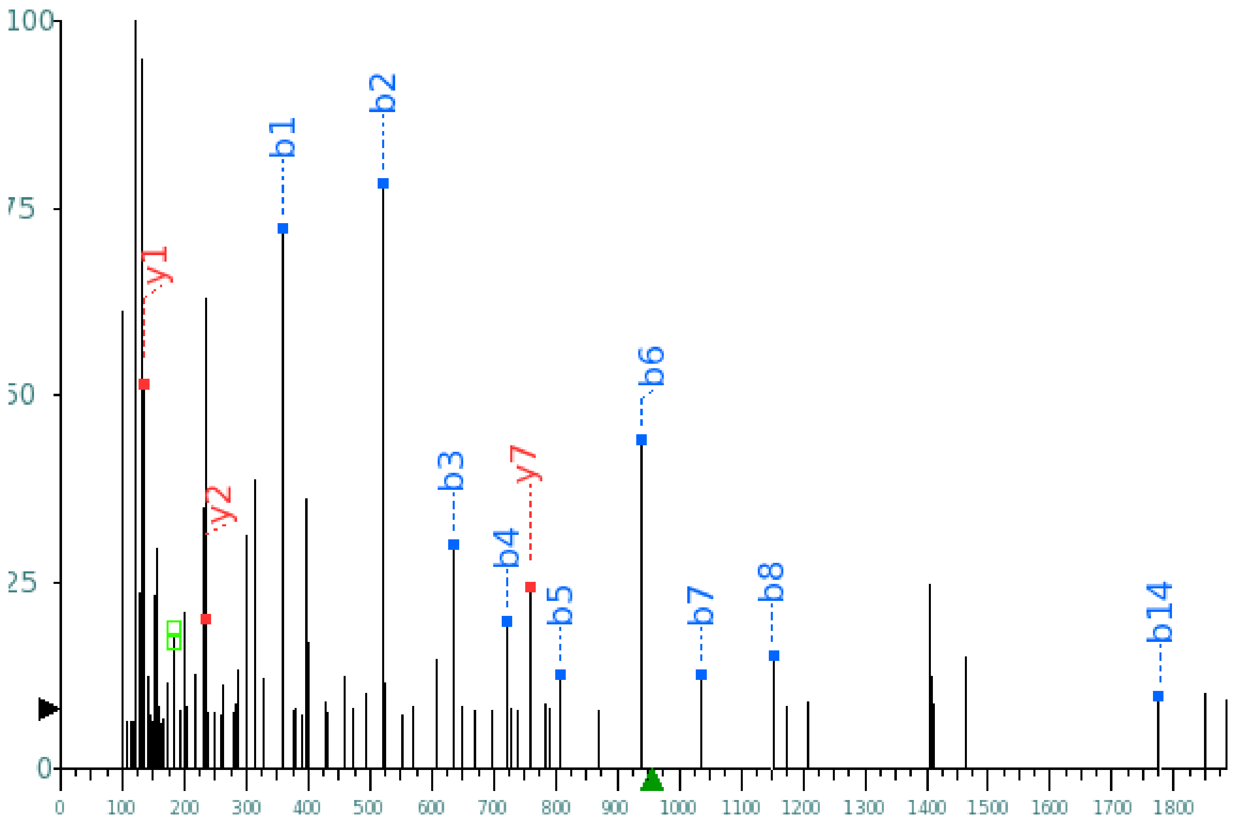

QYISSEVNPPSDETD (+2)

Primary Reference: IF4G2_HUMAN
Search ID: 41759
Search Name: 20130328_ananiav_ERstress_TMT_TM_timecourse_chymo_inj2_4MC_IAA
Scan#: 12705
Observed Mass: 955.4462 (-0.7 ppm)
PSM Score: 45.82

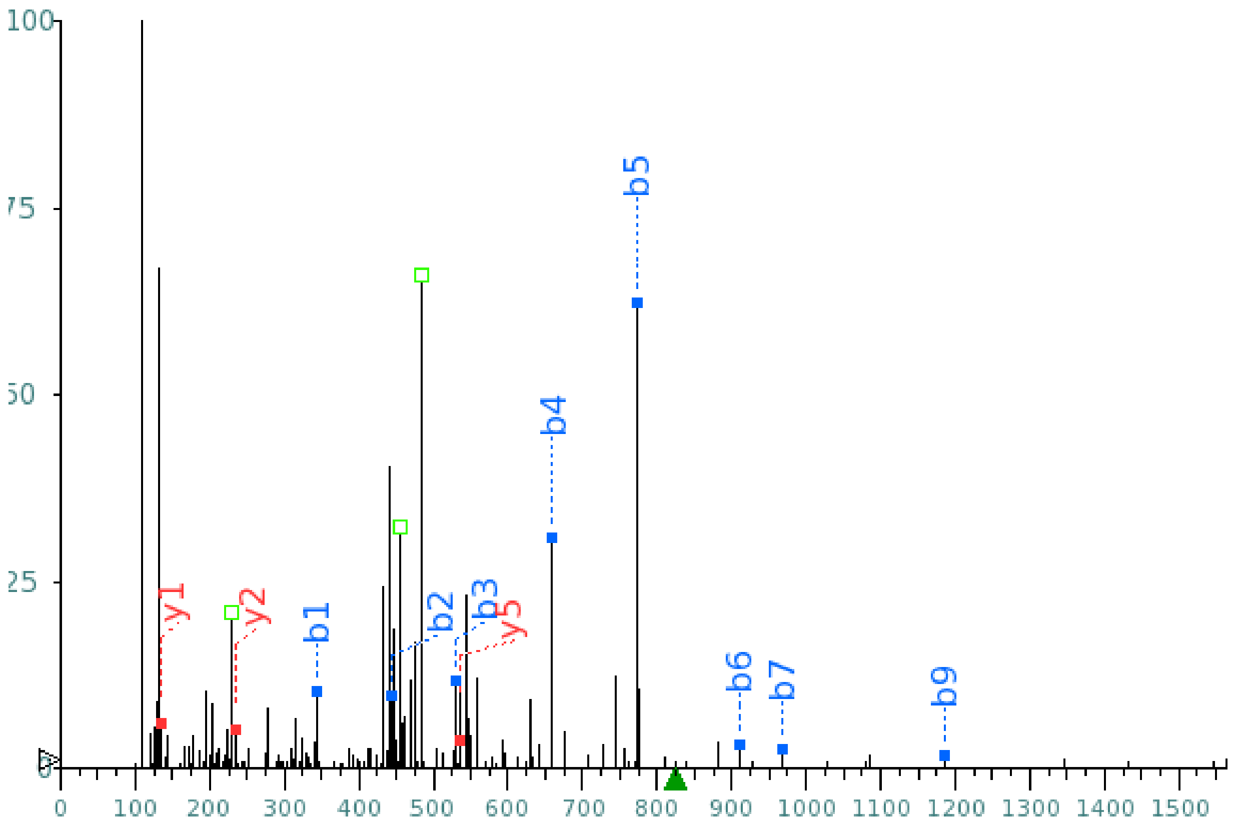

LTSQNHGFAVETD (+2)

Primary Reference: PYR1_HUMAN
Search ID: 41759
Search Name: 20130328_ananiav_ERstress_TMT_TM_timecourse_chymo_inj2_4MC_IAA
Scan#: 12715
Observed Mass: 824.4128 (0.4 ppm)
PSM Score: 22.37

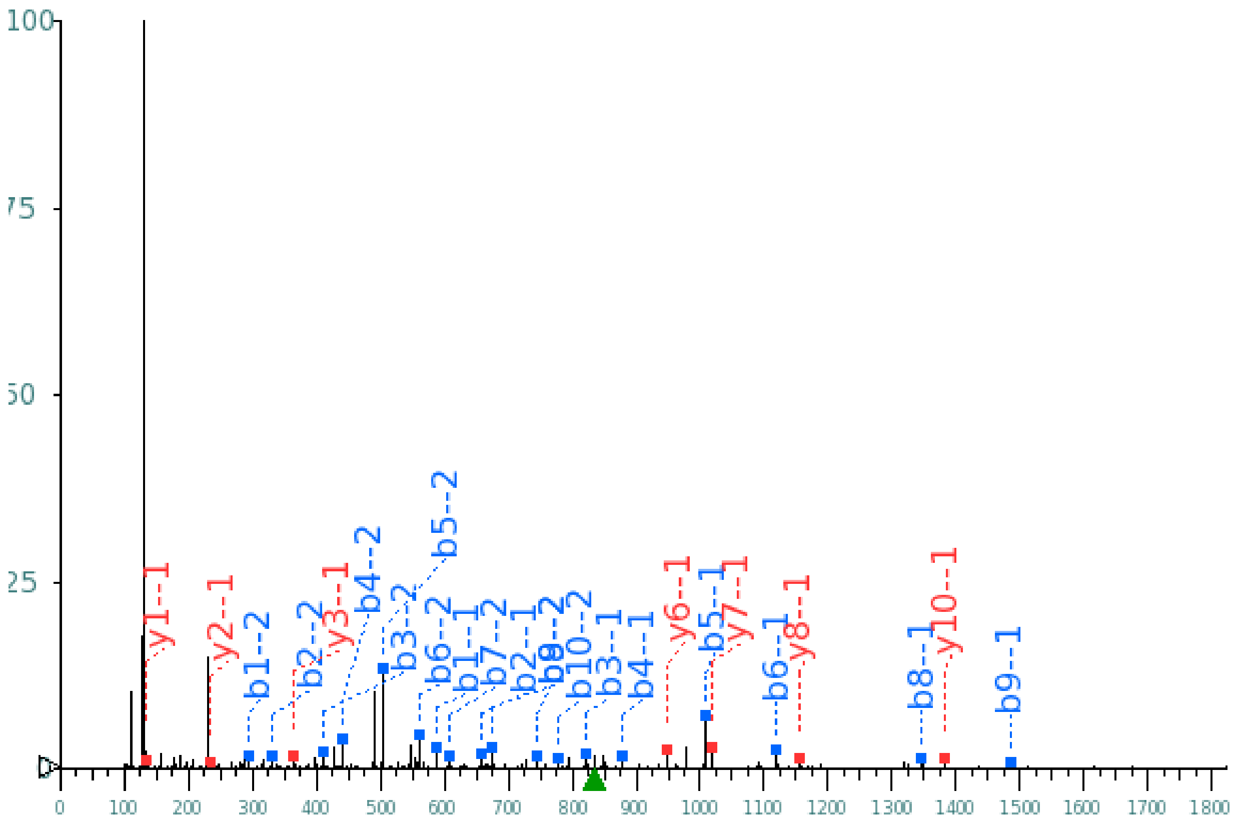

KAYGELPEHAKINETD (+3)

Primary Reference: IF1AX_HUMAN
Search ID: 41759
Search Name: 20130328_ananiav_ERstress_TMT_TM_timecourse_chymo_inj2_4MC_IAA
Scan#: 12721
Observed Mass: 834.7992 (0.9 ppm)
PSM Score: 33.51

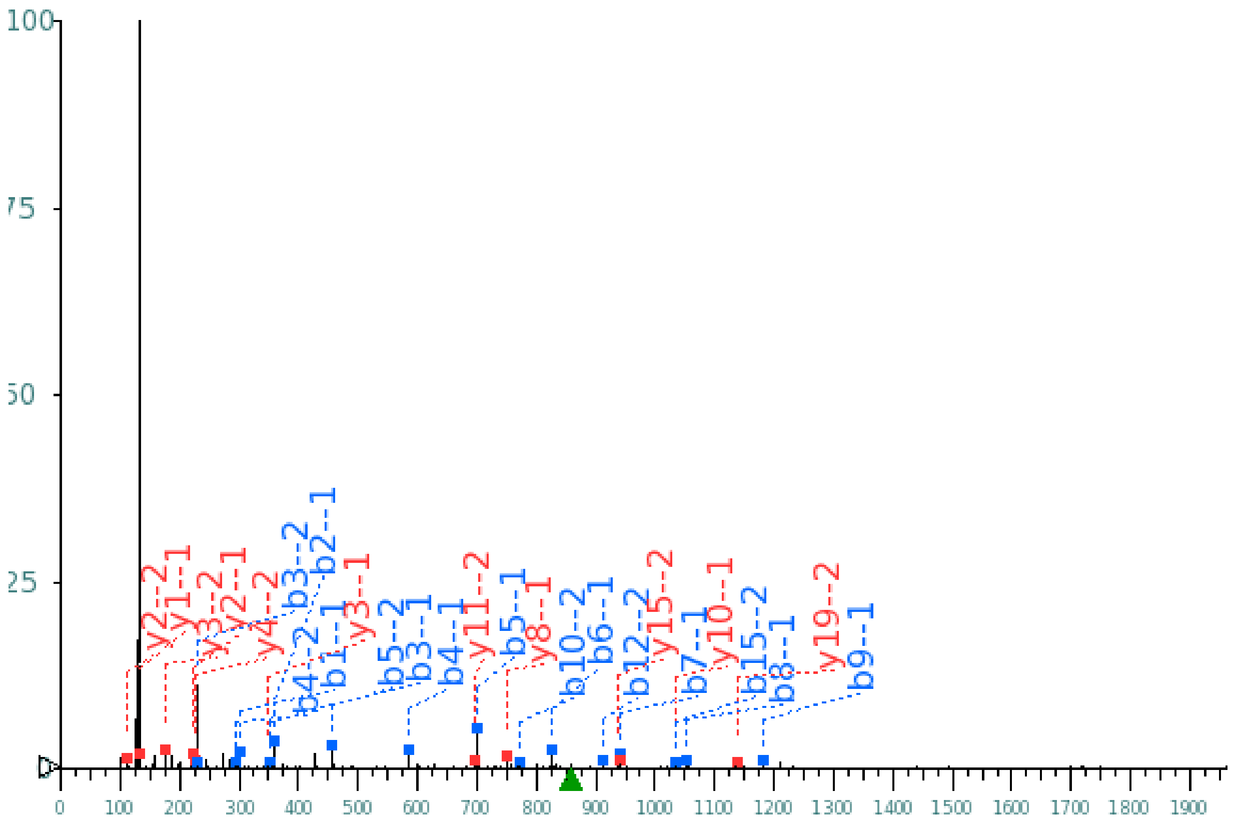

AGVQDQLIEKREPGSGTESD (+3)

Primary Reference: EAF6_HUMAN
Search ID: 41759
Search Name: 20130328_ananiav_ERstress_TMT_TM_timecourse_chymo_inj2_4MC_IAA
Scan#: 12763
Observed Mass: 858.7867 (1.7 ppm)
PSM Score: 34.5

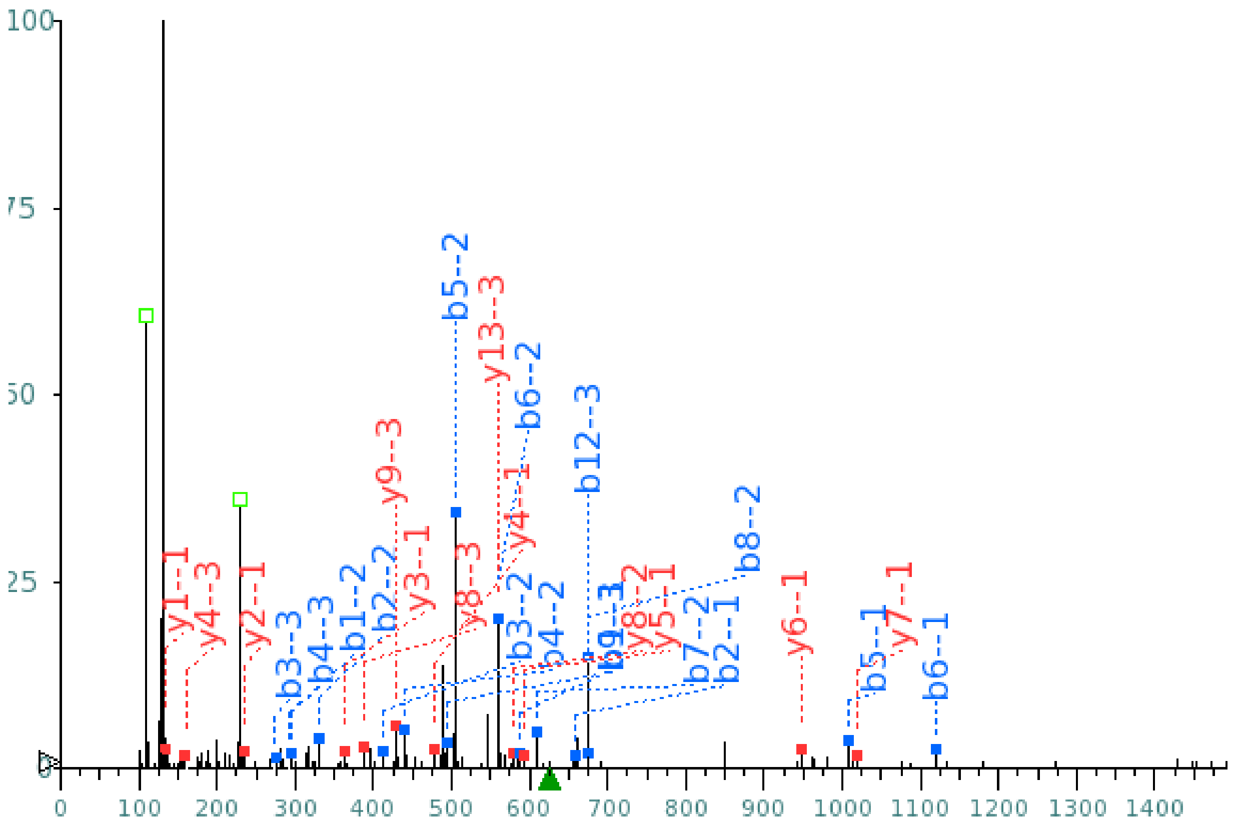

KAYGELPEHAKINETD (+4)

Primary Reference: IF1AX_HUMAN
Search ID: 41759
Search Name: 20130328_ananiav_ERstress_TMT_TM_timecourse_chymo_inj2_4MC_IAA
Scan#: 12795
Observed Mass: 626.3519 (2.1 ppm)
PSM Score: 24.06

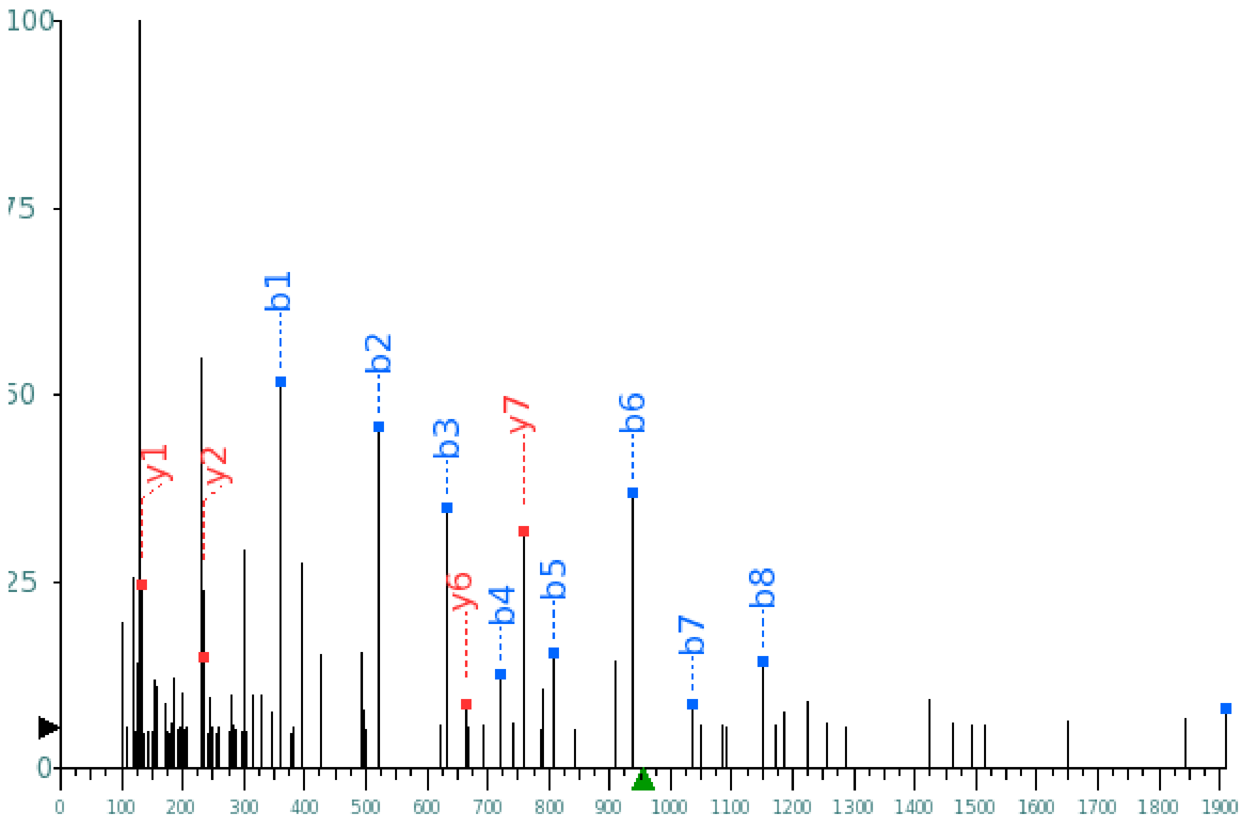

QYISSEVNPPSDETD (+2)

Primary Reference: IF4G2_HUMAN
Search ID: 41759
Search Name: 20130328_ananiav_ERstress_TMT_TM_timecourse_chymo_inj2_4MC_IAA
Scan#: 12805
Observed Mass: 955.4478 (1 ppm)
PSM Score: 32.43

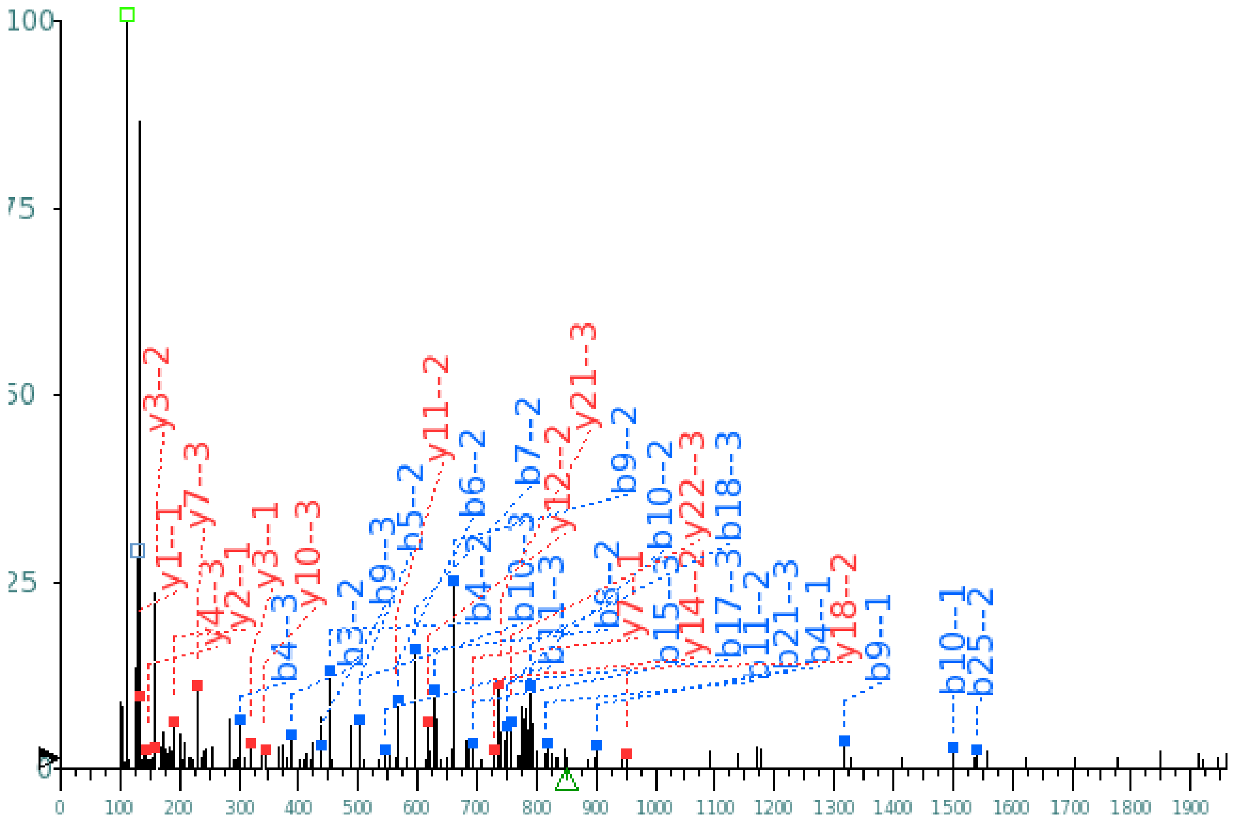

KGEEVEGAGWMPGREPTTHAEAQGDEGD (+4)

Primary Reference: AHNK2_HUMAN
Search ID: 41759
Search Name: 20130328_ananiav_ERstress_TMT_TM_timecourse_chymo_inj2_4MC_IAA
Scan#: 13007
Observed Mass: 850.4046 (0.2 ppm)
PSM Score: 23.17

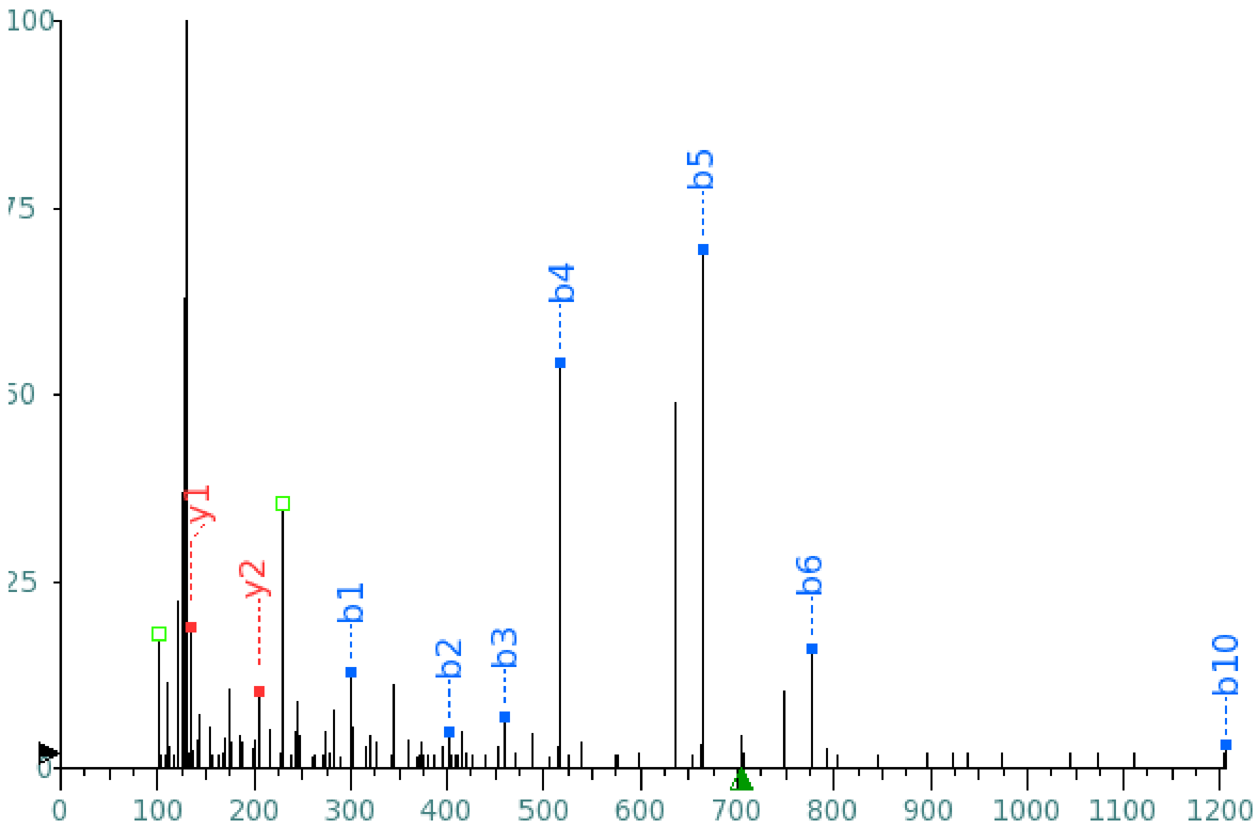

ATGGFLQGDEAD (+2)

Primary Reference: BRAT1_HUMAN
Search ID: 41759
Search Name: 20130328_ananiav_ERstress_TMT_TM_timecourse_chymo_inj2_4MC_IAA
Scan#: 13016
Observed Mass: 705.3411 (0.3 ppm)
PSM Score: 33.22

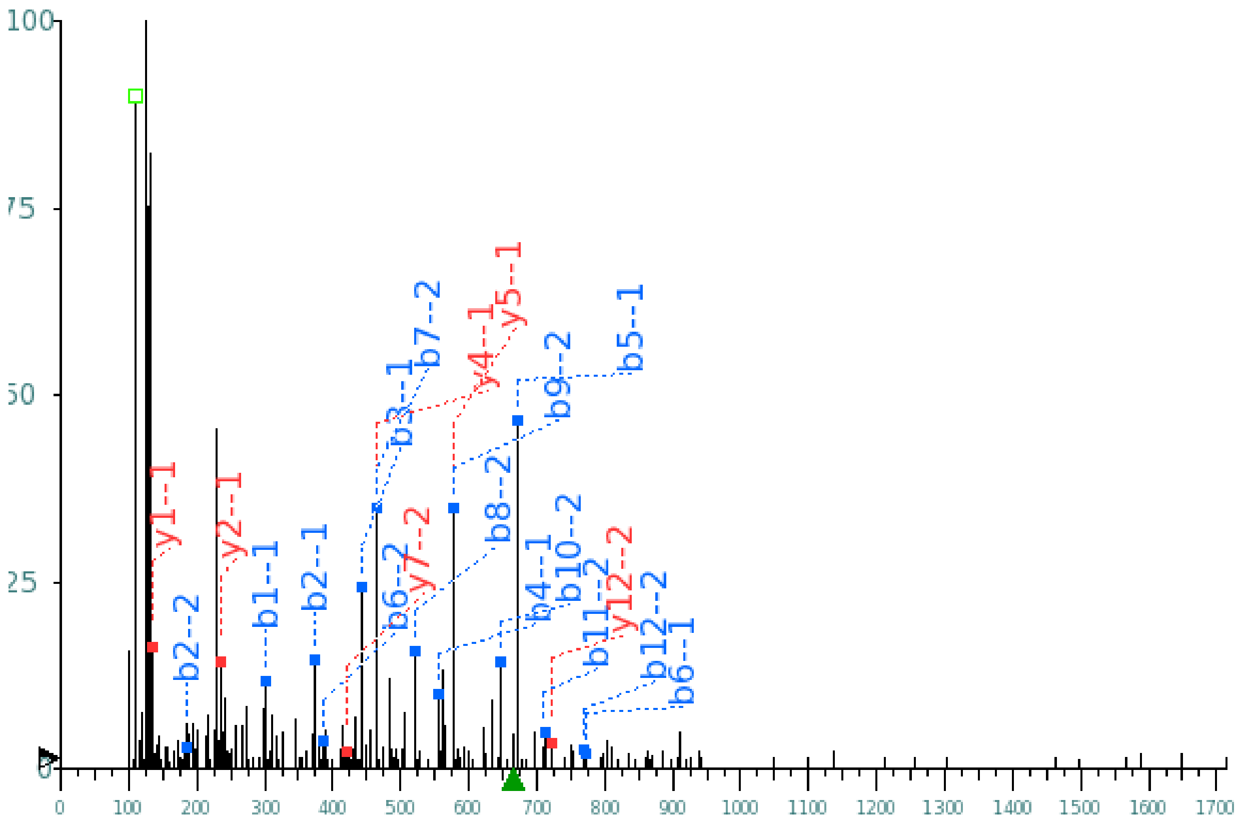

AAAIDTRILHENTQTD (+3)

Primary Reference: C1TM_HUMAN
Search ID: 41759
Search Name: 20130328_ananiav_ERstress_TMT_TM_timecourse_chymo_inj2_4MC_IAA
Scan#: 13226
Observed Mass: 666.6875 (1.4 ppm)
PSM Score: 25.44

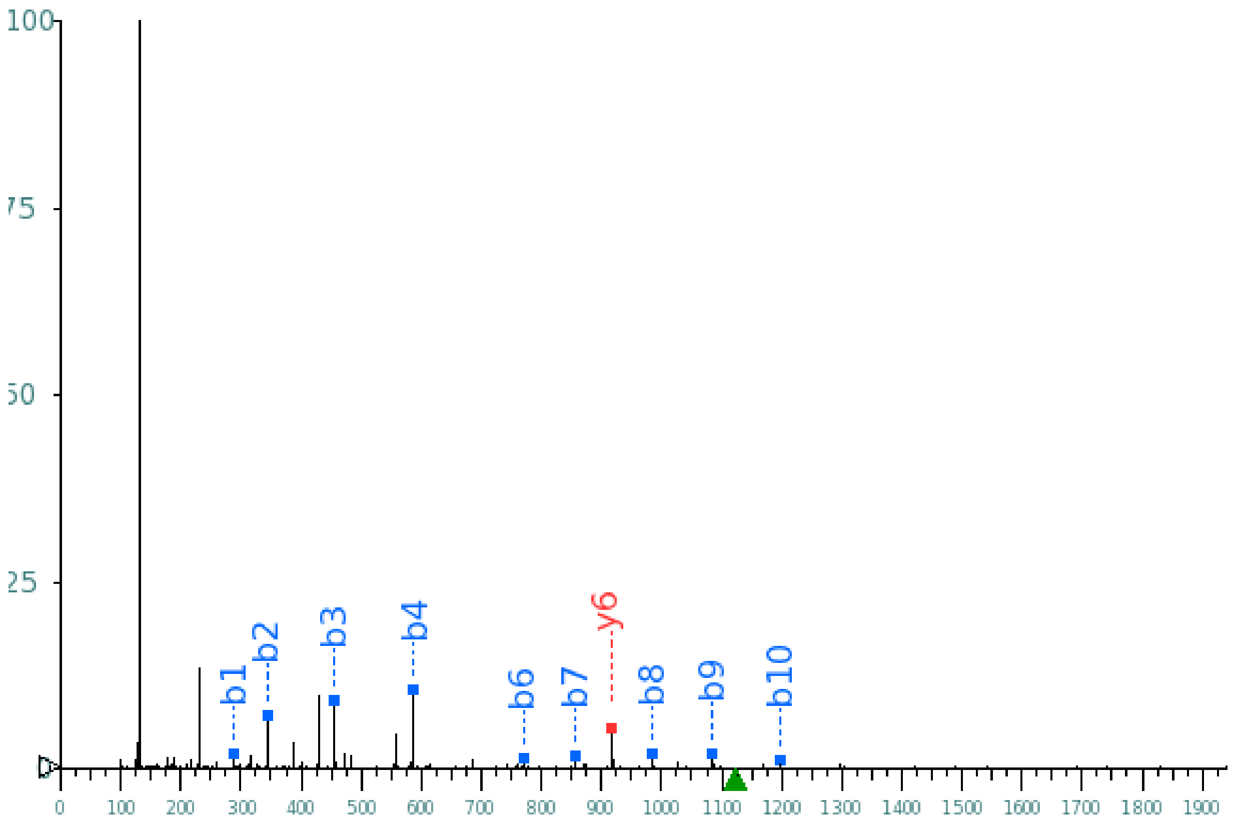

GGIQPSSQTIQPKVETD (+2)

Primary Reference: CDK13_HUMAN
Search ID: 41759
Search Name: 20130328_ananiav_ERstress_TMT_TM_timecourse_chymo_inj2_4MC_IAA
Scan#: 13252
Observed Mass: 1122.1183 (0.4 ppm)
PSM Score: 28.09

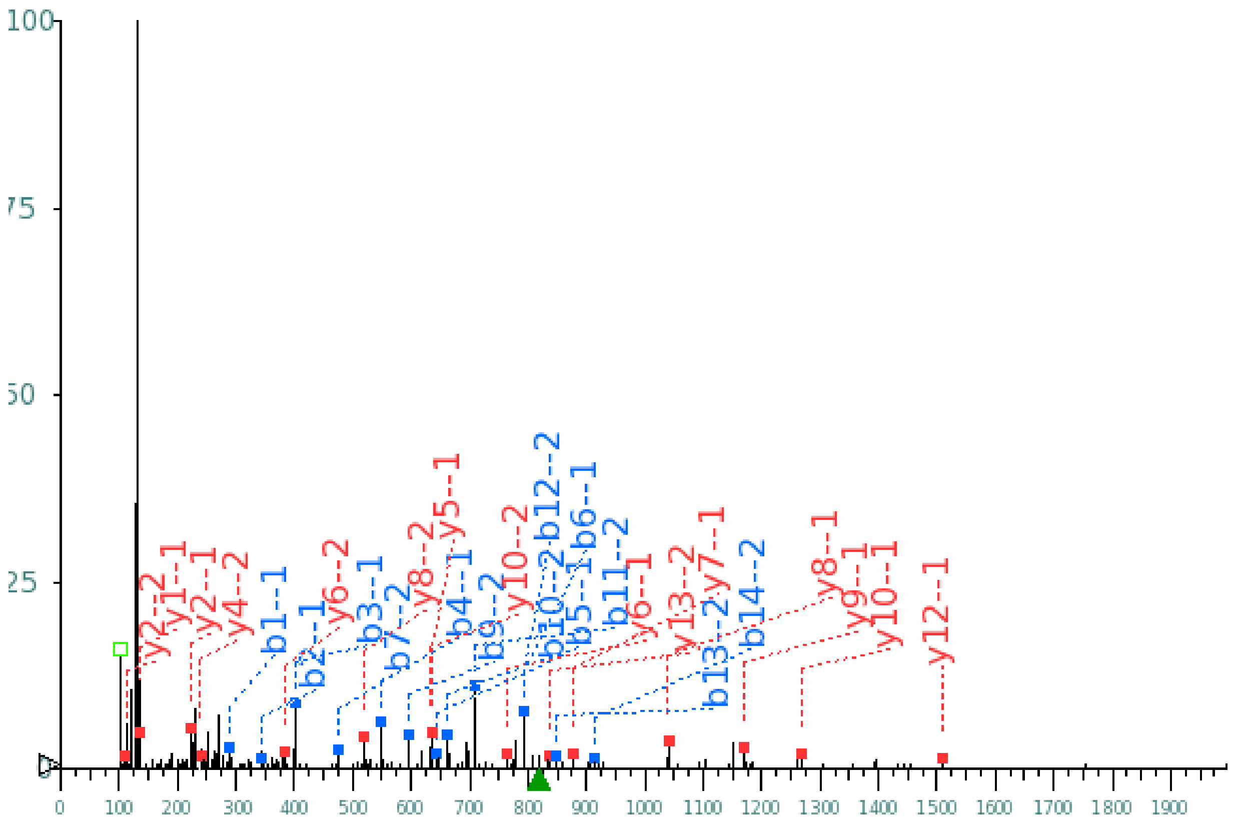

GGGFNERENVEYIEREESD (+3)

Primary Reference: ZRAB2_HUMAN
Search ID: 41759
Search Name: 20130328_ananiav_ERstress_TMT_TM_timecourse_chymo_inj2_4MC_IAA
Scan#: 13256
Observed Mass: 820.0491 (0.3 ppm)
PSM Score: 24.35

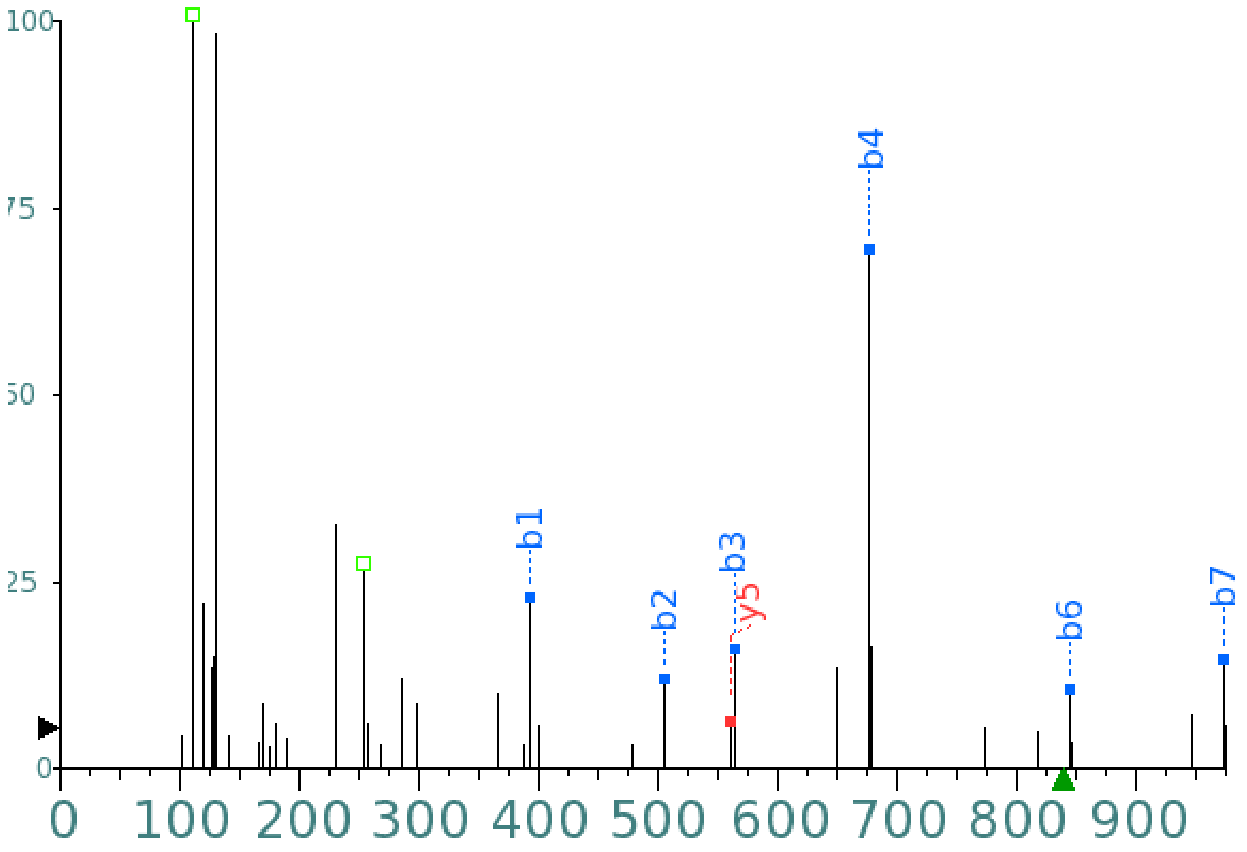

YLGNPAEFHDSSD (+2)

Primary Reference: RCN1_HUMAN
Search ID: 41759
Search Name: 20130328_ananiav_ERstress_TMT_TM_timecourse_chymo_inj2_4MC_IAA
Scan#: 13316
Observed Mass: 840.8894 (0.7 ppm)
PSM Score: 43.54

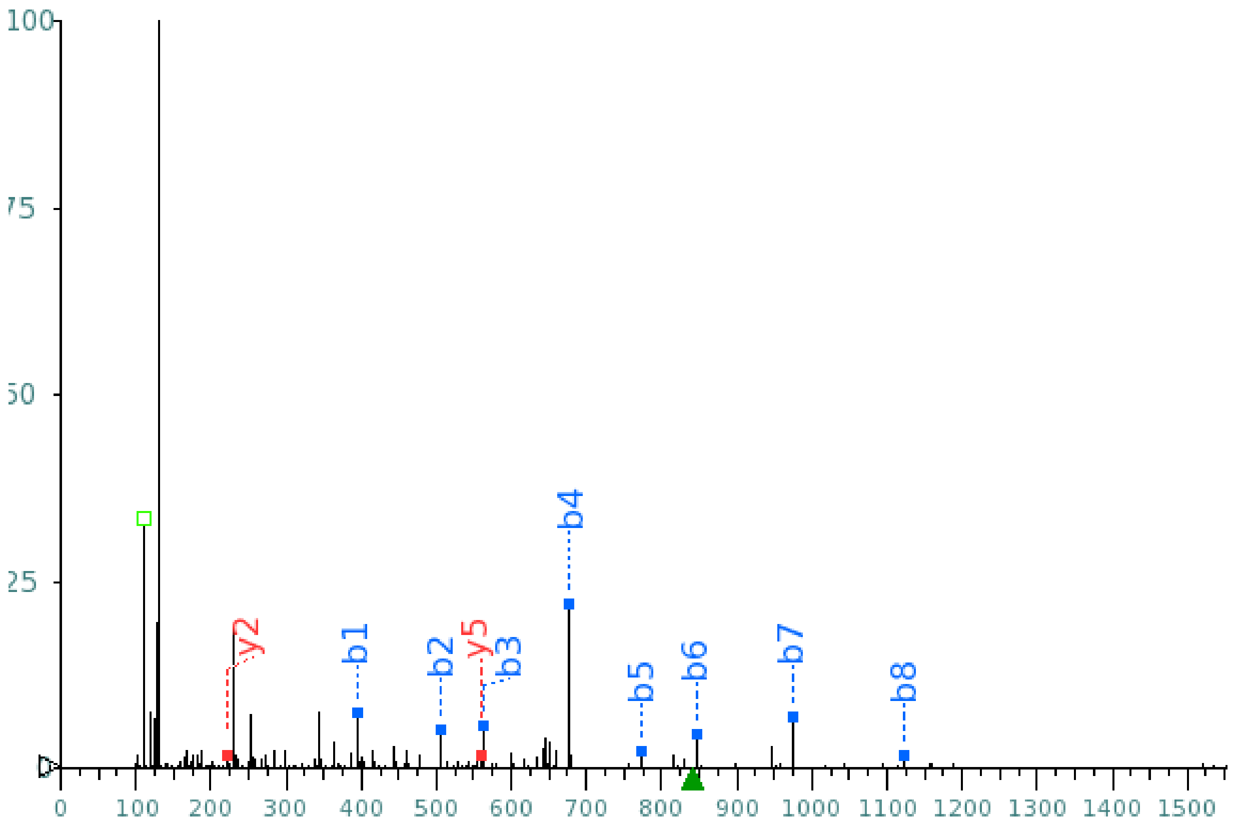

YLGNPAEFHDSSD (+2)

Primary Reference: RCN1_HUMAN
Search ID: 41759
Search Name: 20130328_ananiav_ERstress_TMT_TM_timecourse_chymo_inj2_4MC_IAA
Scan#: 13416
Observed Mass: 840.889 (0.3 ppm)
PSM Score: 35.24

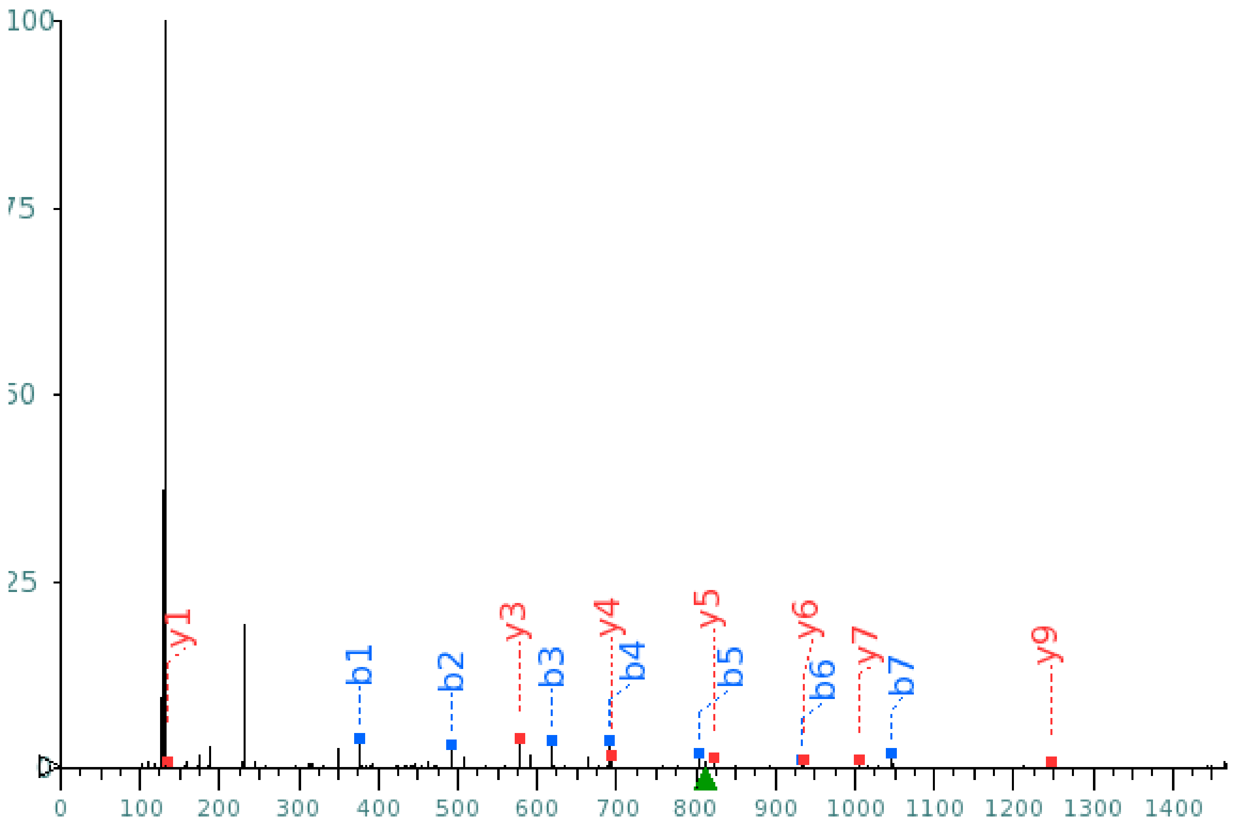

FNQALEDKSD (+2)

Primary Reference: P121A_HUMAN
Search ID: 41759
Search Name: 20130328_ananiav_ERstress_TMT_TM_timecourse_chymo_inj2_4MC_IAA
Scan#: 13442
Observed Mass: 812.9331 (0.4 ppm)
PSM Score: 31.91

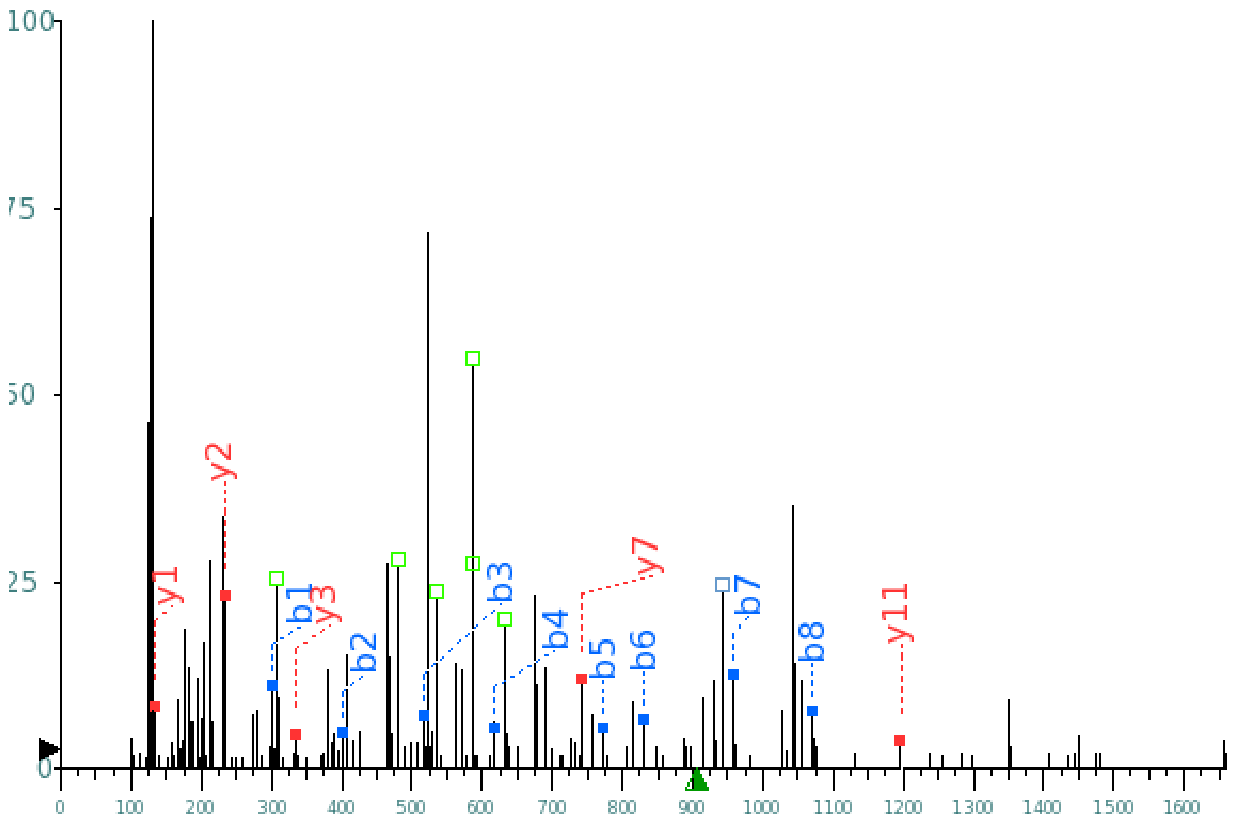

ATNTRGQIPPLVTTD (+2)

Primary Reference: SC24D_HUMAN
Search ID: 41759
Search Name: 20130328_ananiav_ERstress_TMT_TM_timecourse_chymo_inj2_4MC_IAA
Scan#: 13530
Observed Mass: 907.0052 (0.8 ppm)
PSM Score: 30.04

File name: 2015-02-04 04.01.53PM.pdf

Creation time: 2015-02-04 04:09PM

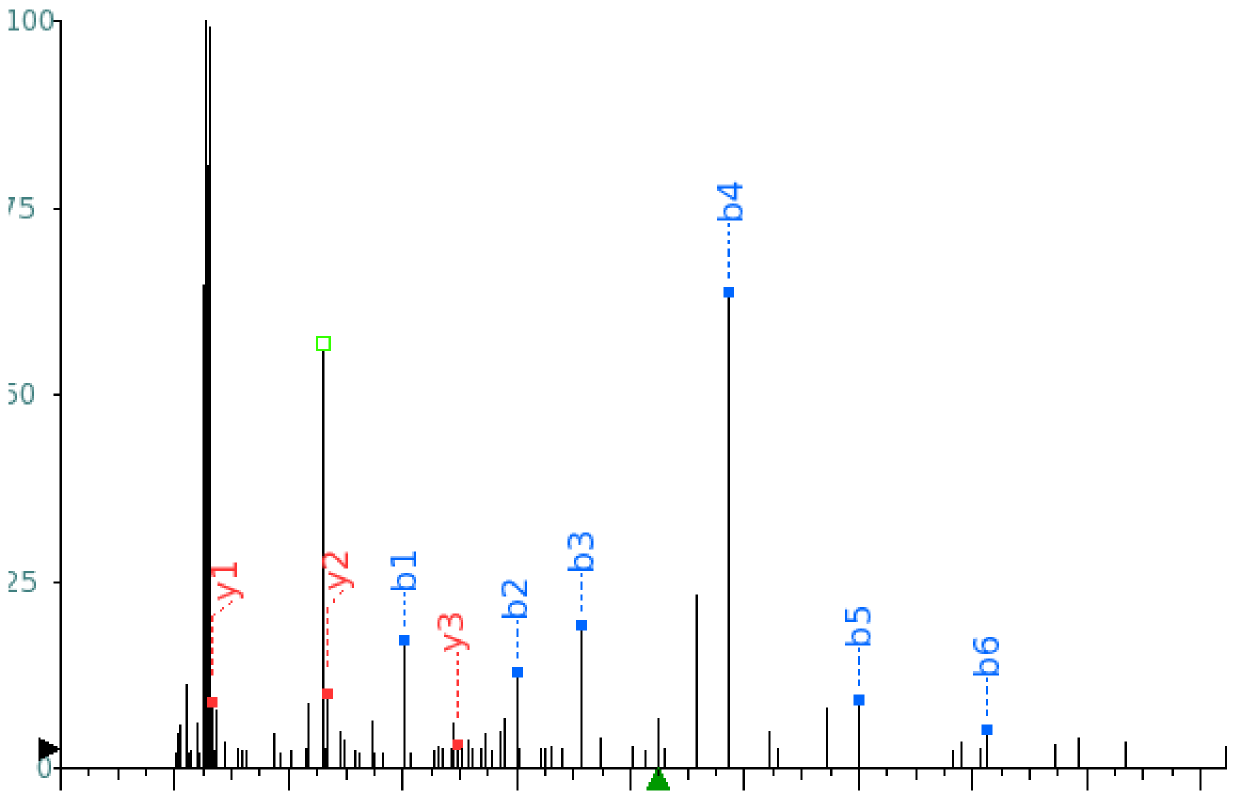

AVGEIITD (+2)

Primary Reference: VRK1_HUMAN
Search ID: 41759
Search Name: 20130328_ananiav_ERstress_TMT_TM_timecourse_chymo_inj2_4MC_IAA
Scan#: 13717
Observed Mass: 523.8013 (2.1 ppm)
PSM Score: 25.82

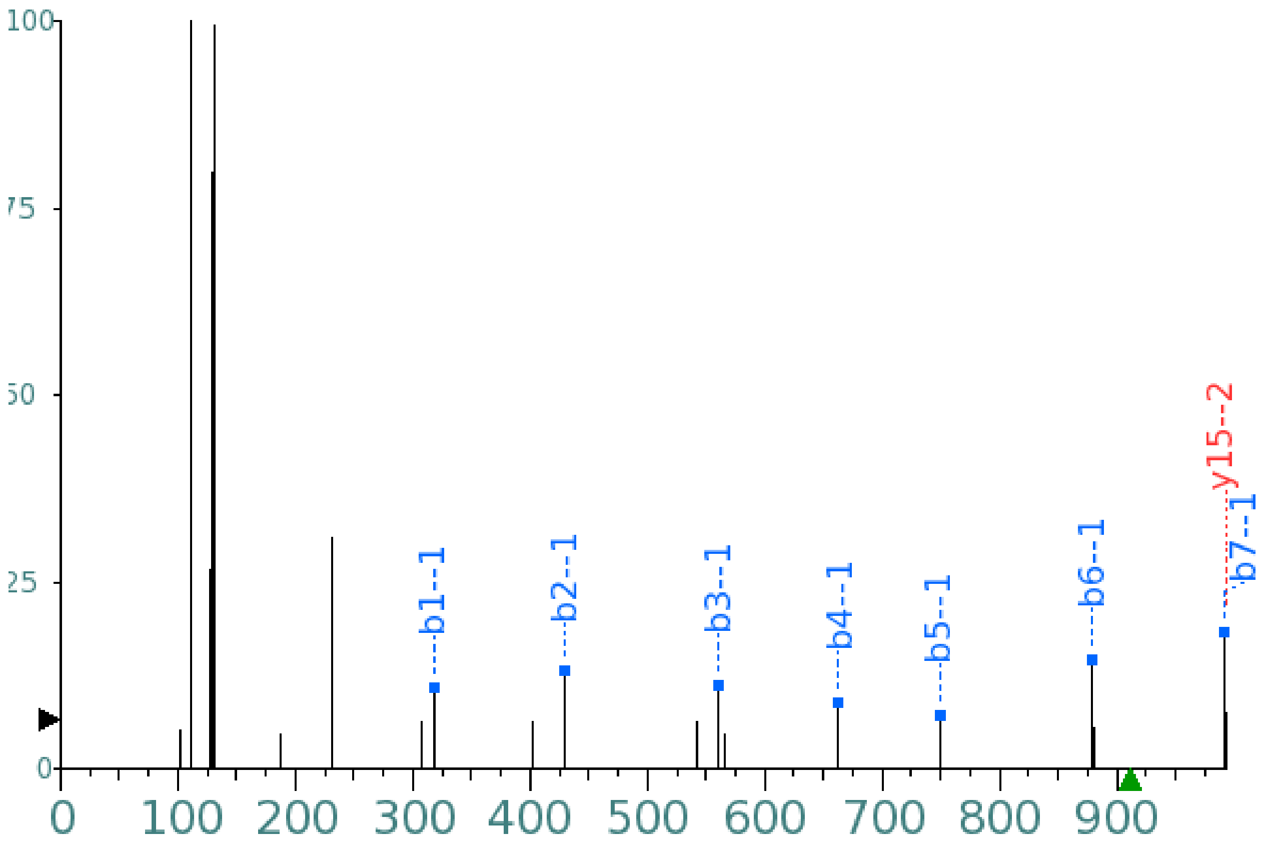

SIMTSENHLNNSDKEVDEVD (+3)

Primary Reference: FERM2_HUMAN
Search ID: 41759
Search Name: 20130328_ananiav_ERstress_TMT_TM_timecourse_chymo_inj2_4MC_IAA
Scan#: 13734
Observed Mass: 912.1132 (0.3 ppm)
PSM Score: 45.31

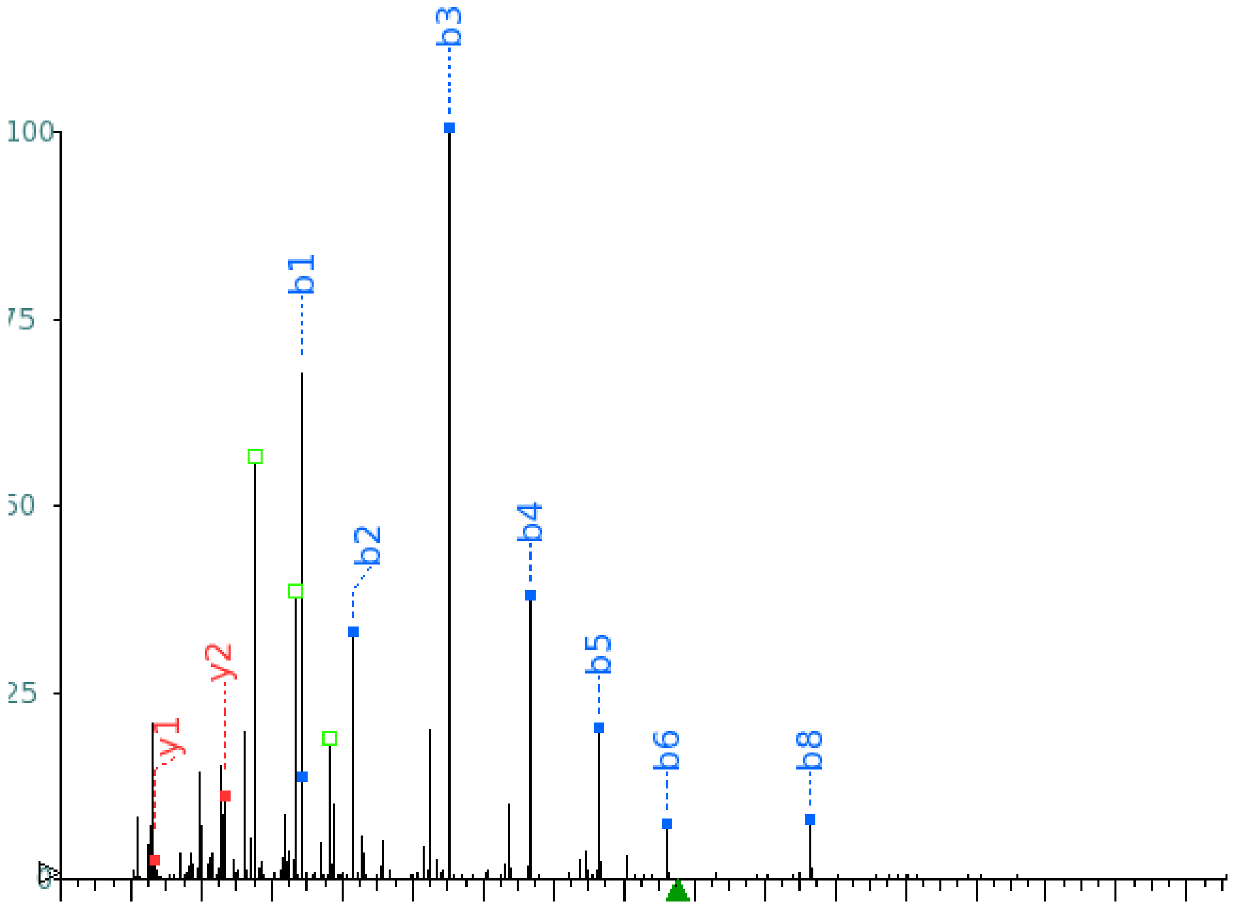

NAHIVVSDPEDITD (+2)

Primary Reference: PKN2_HUMAN
Search ID: 41759
Search Name: 20130328_ananiav_ERstress_TMT_TM_timecourse_chymo_inj2_4MC_IAA
Scan#: 13904
Observed Mass: 877.4453 (1.6 ppm)
PSM Score: 30.91

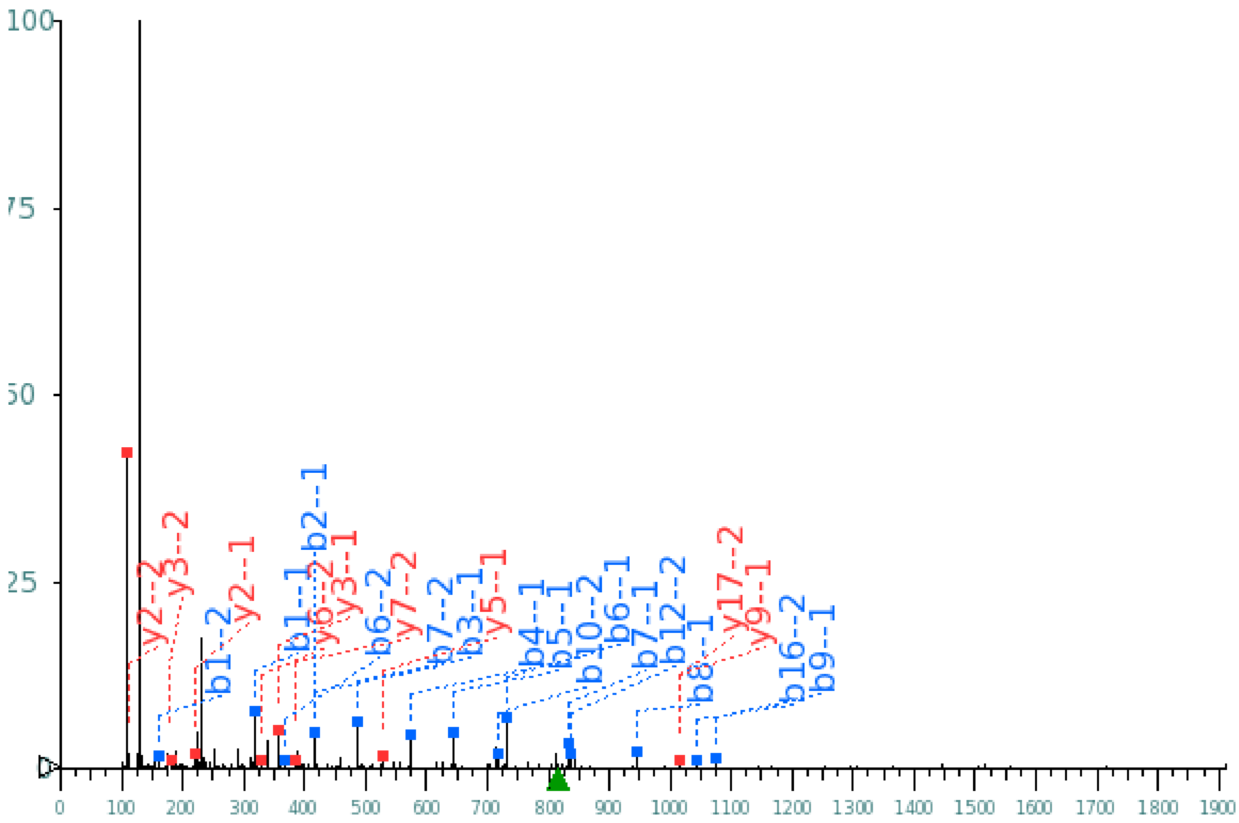

SVASASVIEKQNLEGDHSD (+3)

Primary Reference: HS74L_HUMAN
Search ID: 41759
Search Name: 20130328_ananiav_ERstress_TMT_TM_timecourse_chymo_inj2_4MC_IAA
Scan#: 13958
Observed Mass: 815.4281 (1.1 ppm)
PSM Score: 34.69

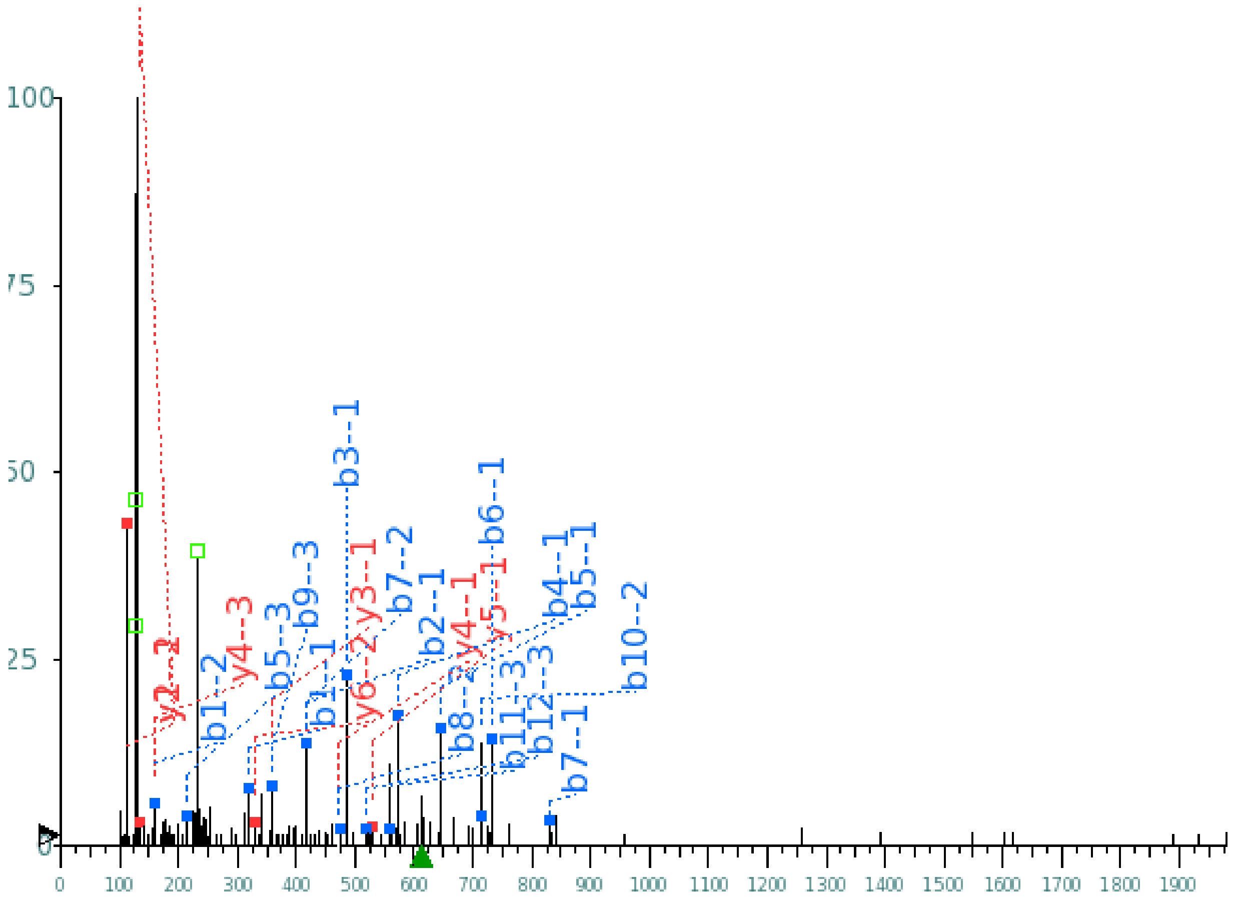

SVASASVIEKQNLEGDHSD (+4)

Primary Reference: HS74L_HUMAN
Search ID: 41759
Search Name: 20130328_ananiav_ERstress_TMT_TM_timecourse_chymo_inj2_4MC_IAA
Scan#: 13970
Observed Mass: 611.8232 (1.6 ppm)
PSM Score: 24.05

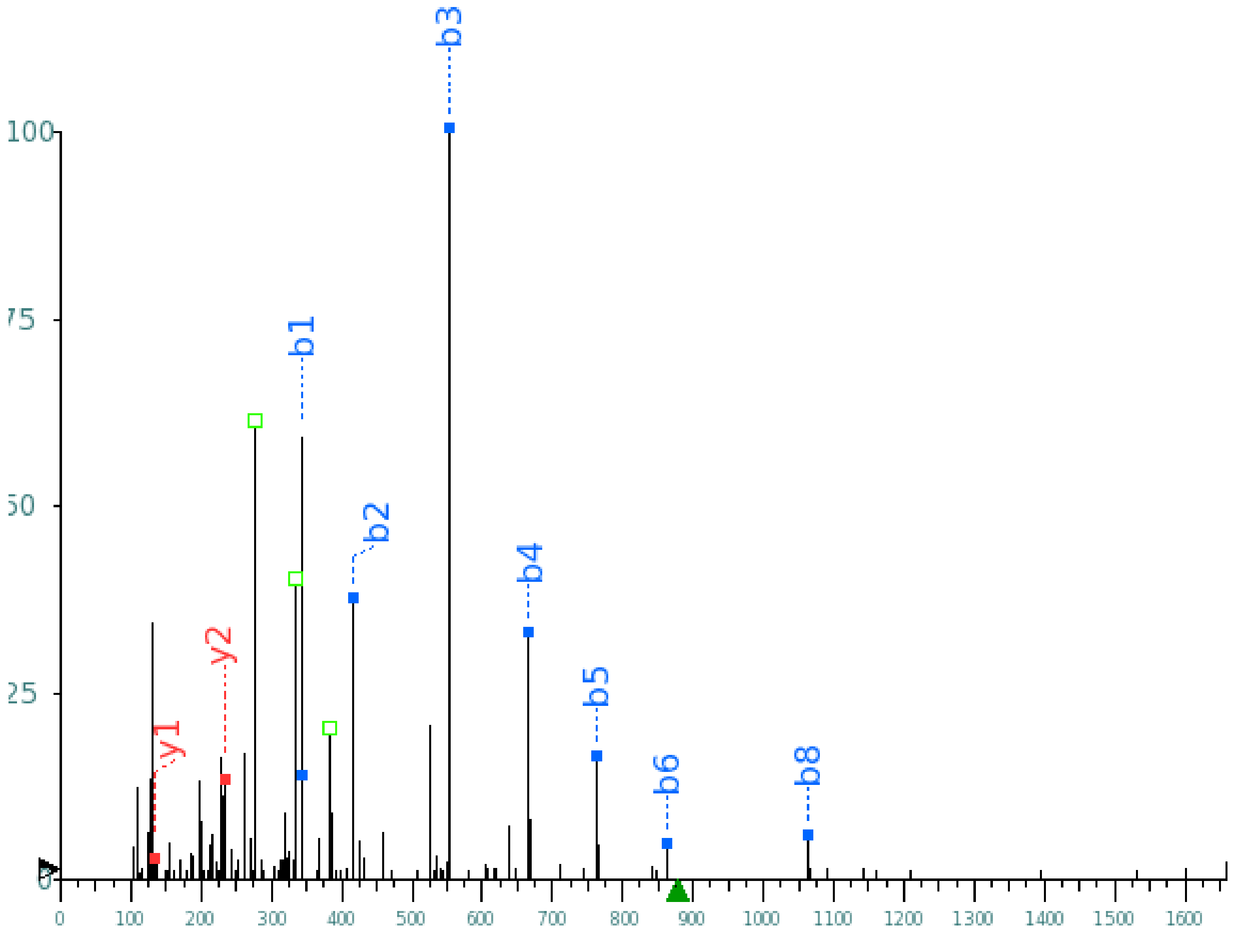

NAHIVVSDPEDITD (+2)

Primary Reference: PKN2_HUMAN
Search ID: 41759
Search Name: 20130328_ananiav_ERstress_TMT_TM_timecourse_chymo_inj2_4MC_IAA
Scan#: 14007
Observed Mass: 877.4448 (1 ppm)
PSM Score: 28.14

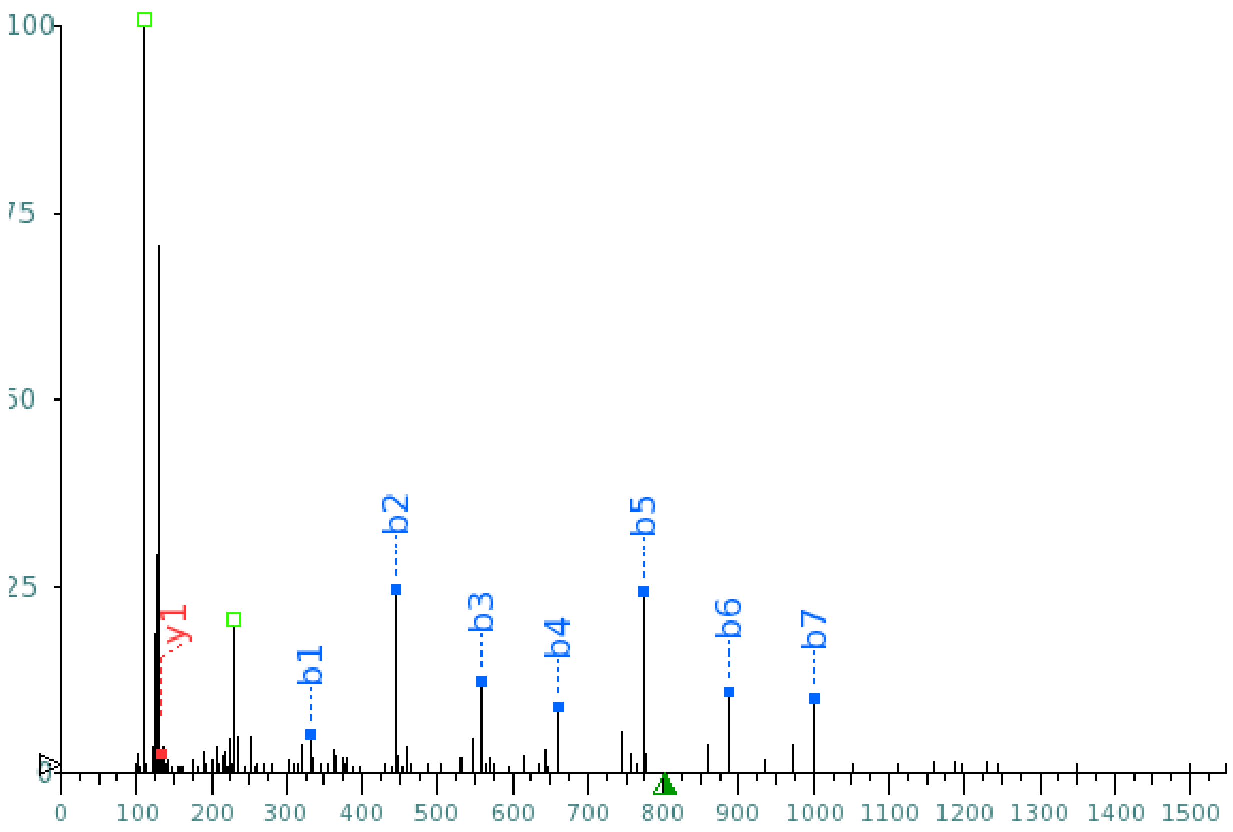

TDITNLNHLESD (+2)

Primary Reference: CTNL1_HUMAN
Search ID: 41759
Search Name: 20130328_ananiav_ERstress_TMT_TM_timecourse_chymo_inj2_4MC_IAA
Scan#: 14044
Observed Mass: 800.9069 (3 ppm)
PSM Score: 36.18

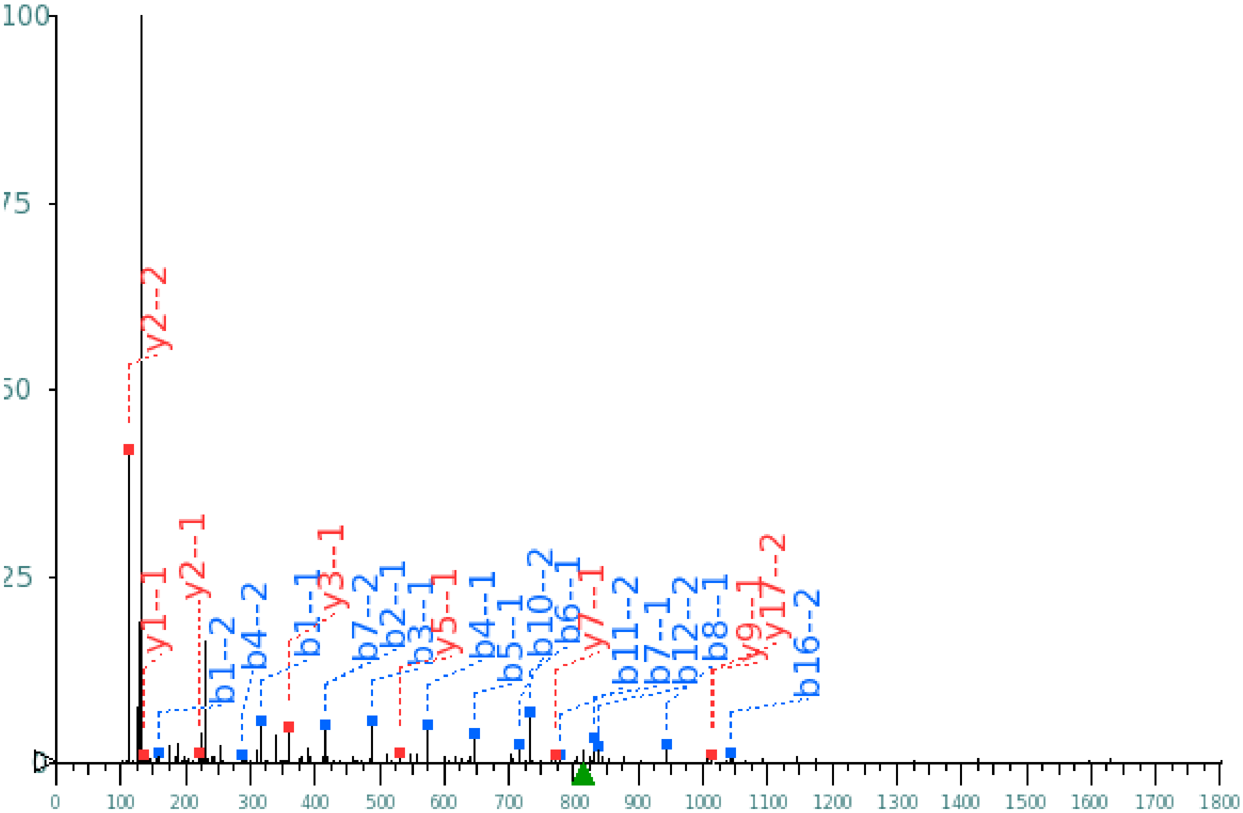

SVASASVIEKQNLEGDHSD (+3)

Primary Reference: HS74L_HUMAN
Search ID: 41759
Search Name: 20130328_ananiav_ERstress_TMT_TM_timecourse_chymo_inj2_4MC_IAA
Scan#: 14069
Observed Mass: 815.4284 (1.6 ppm)
PSM Score: 33.53

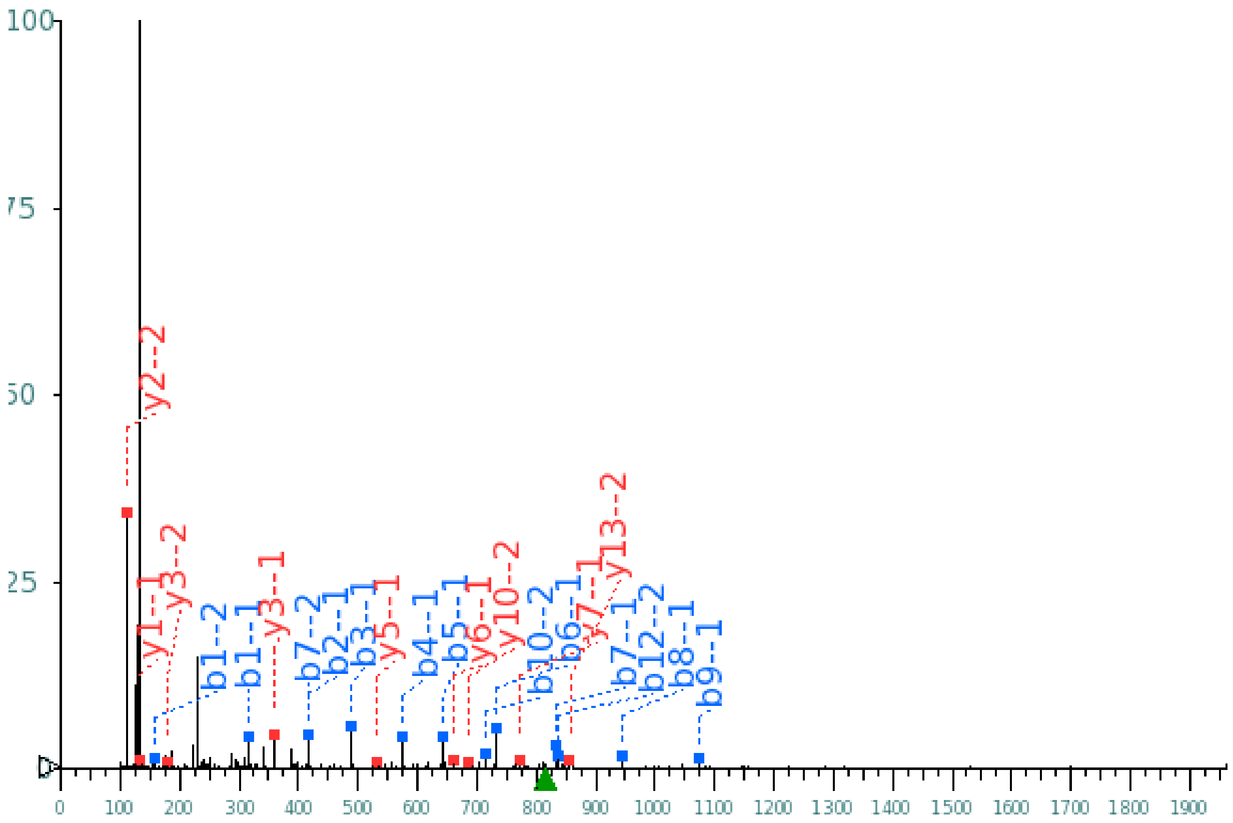

SVASASVIEKQNLEGDHSD (+3)

Primary Reference: HS74L_HUMAN
Search ID: 41759
Search Name: 20130328_ananiav_ERstress_TMT_TM_timecourse_chymo_inj2_4MC_IAA
Scan#: 14209
Observed Mass: 815.4283 (1.5 ppm)
PSM Score: 35.98

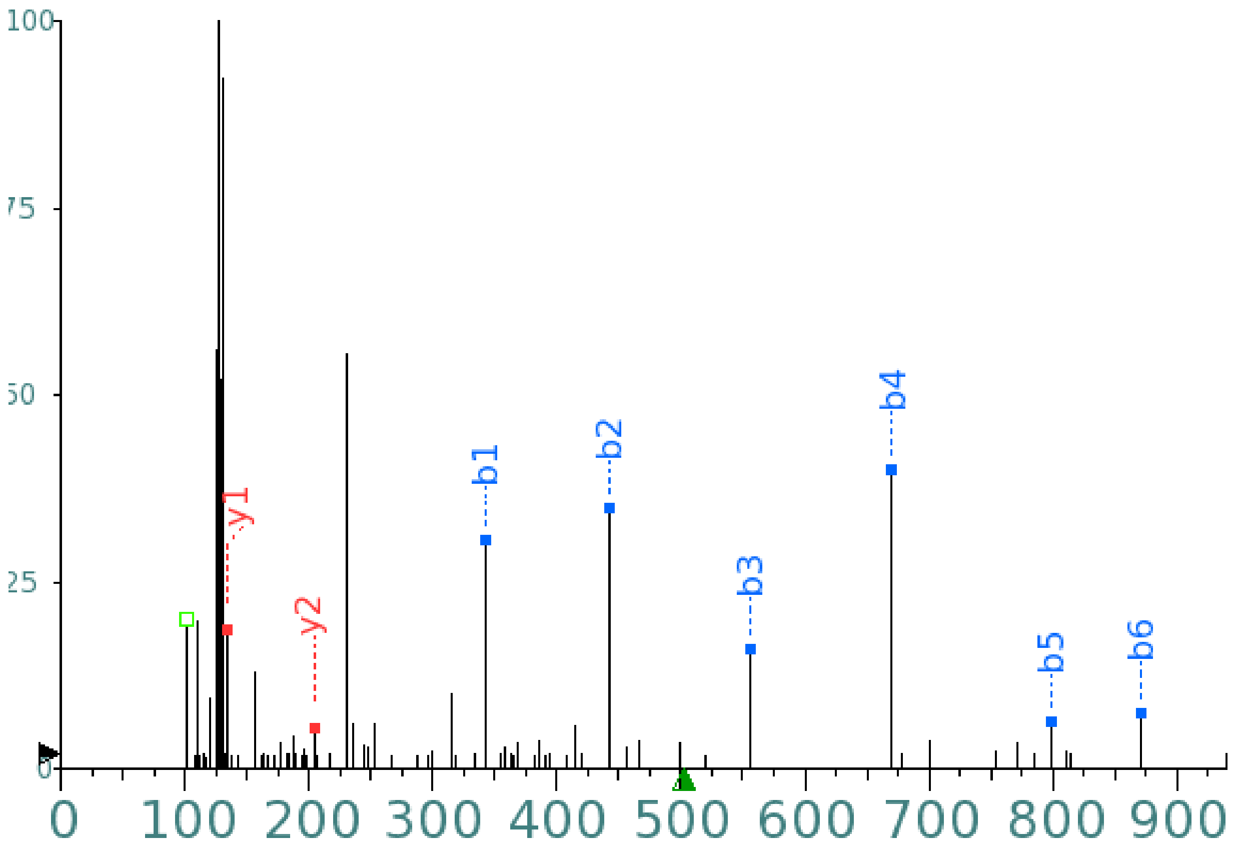

LVIDEAD (+2)

Primary Reference: DDX53_HUMAN
Search ID: 41759
Search Name: 20130328_ananiav_ERstress_TMT_TM_timecourse_chymo_inj2_4MC_IAA
Scan#: 14443
Observed Mass: 502.2802 (2.2 ppm)
PSM Score: 44.26

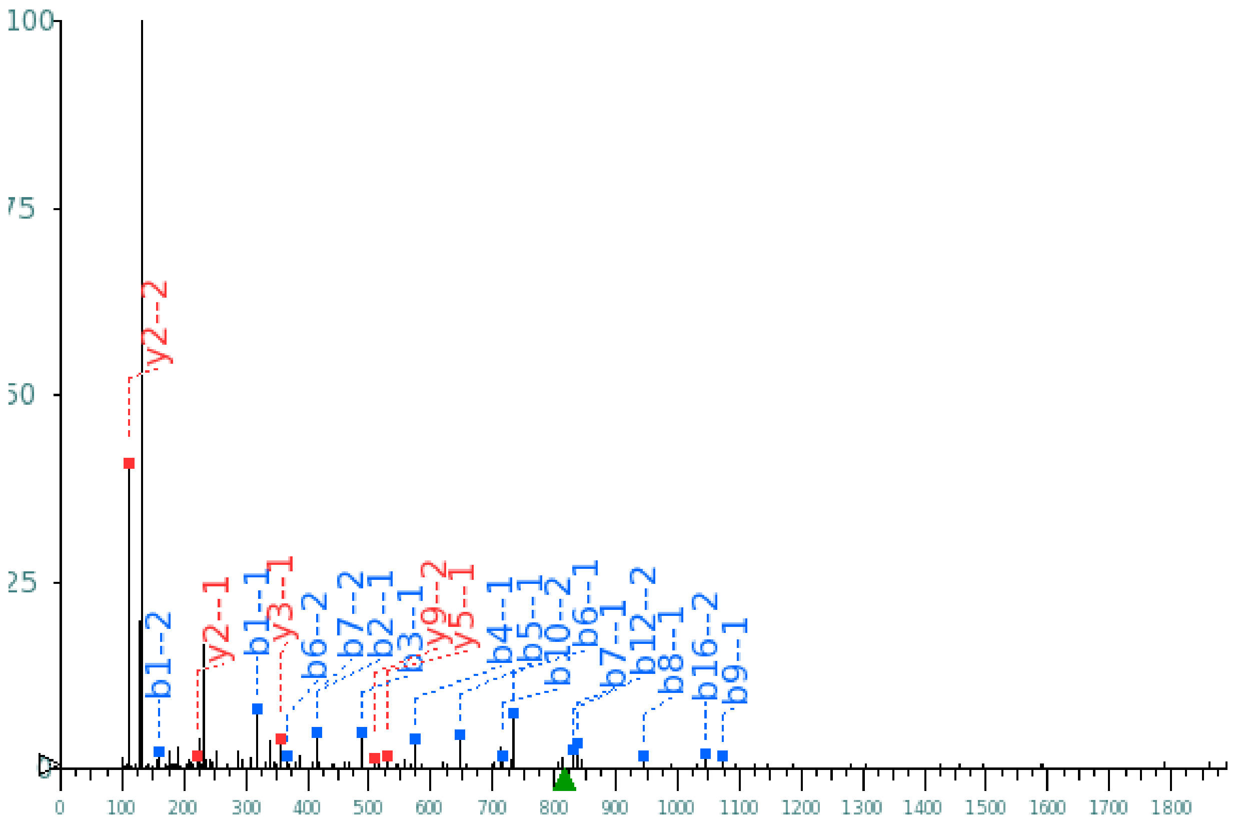

SVASASVIEKQNLEGDHSD (+3)

Primary Reference: HS74L_HUMAN
Search ID: 41759
Search Name: 20130328_ananiav_ERstress_TMT_TM_timecourse_chymo_inj2_4MC_IAA
Scan#: 14457
Observed Mass: 815.4246 (-3.2 ppm)
PSM Score: 35.1

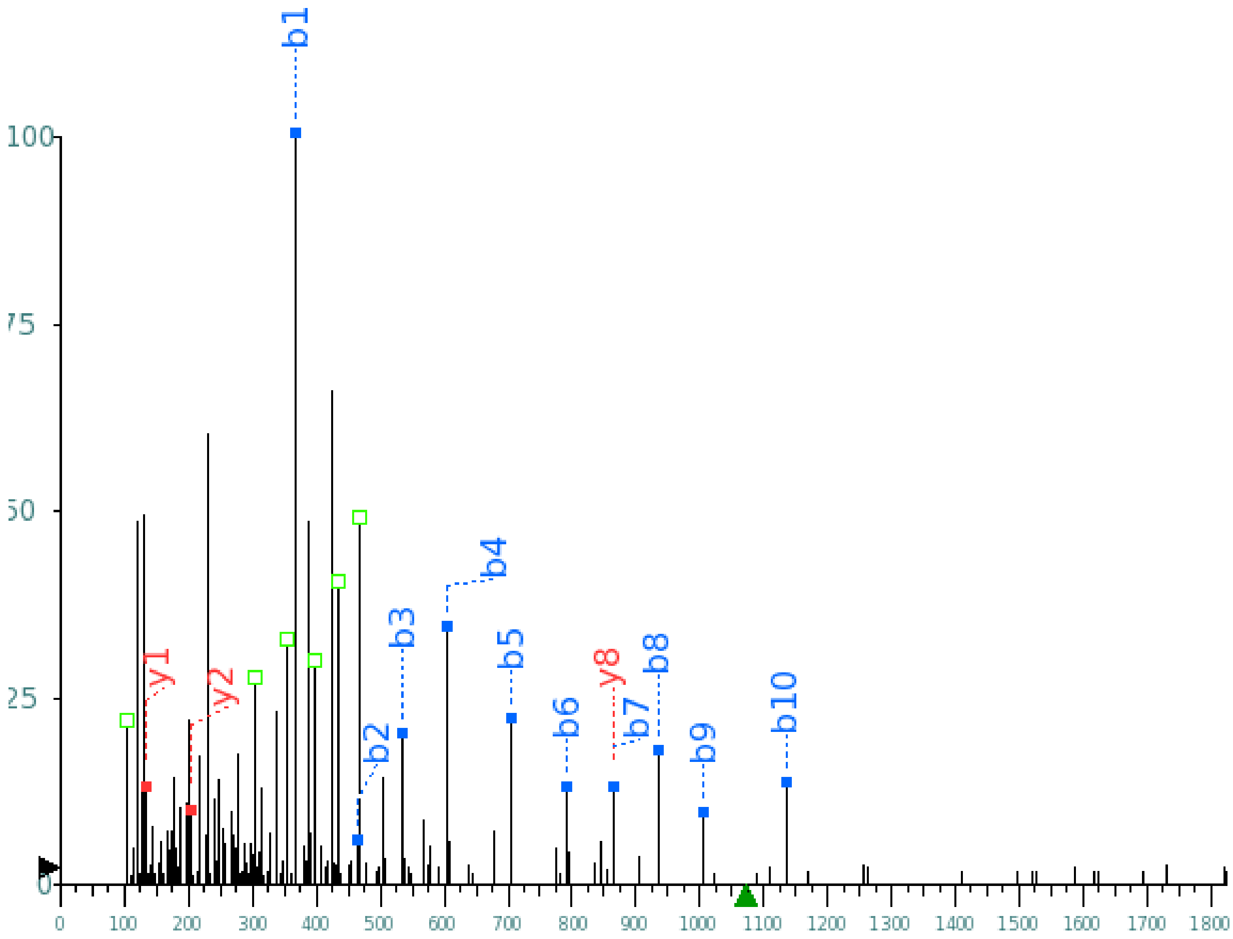

HPAAVSAAAEFENAESEAD (+2)

Primary Reference: C170L_HUMAN
Search ID: 41759
Search Name: 20130328_ananiav_ERstress_TMT_TM_timecourse_chymo_inj2_4MC_IAA
Scan#: 14609
Observed Mass: 1073.001 (0.6 ppm)
PSM Score: 51.18

File name: 2015-02-04 04.01.53PM.pdf

Creation time: 2015-02-04 04:09PM

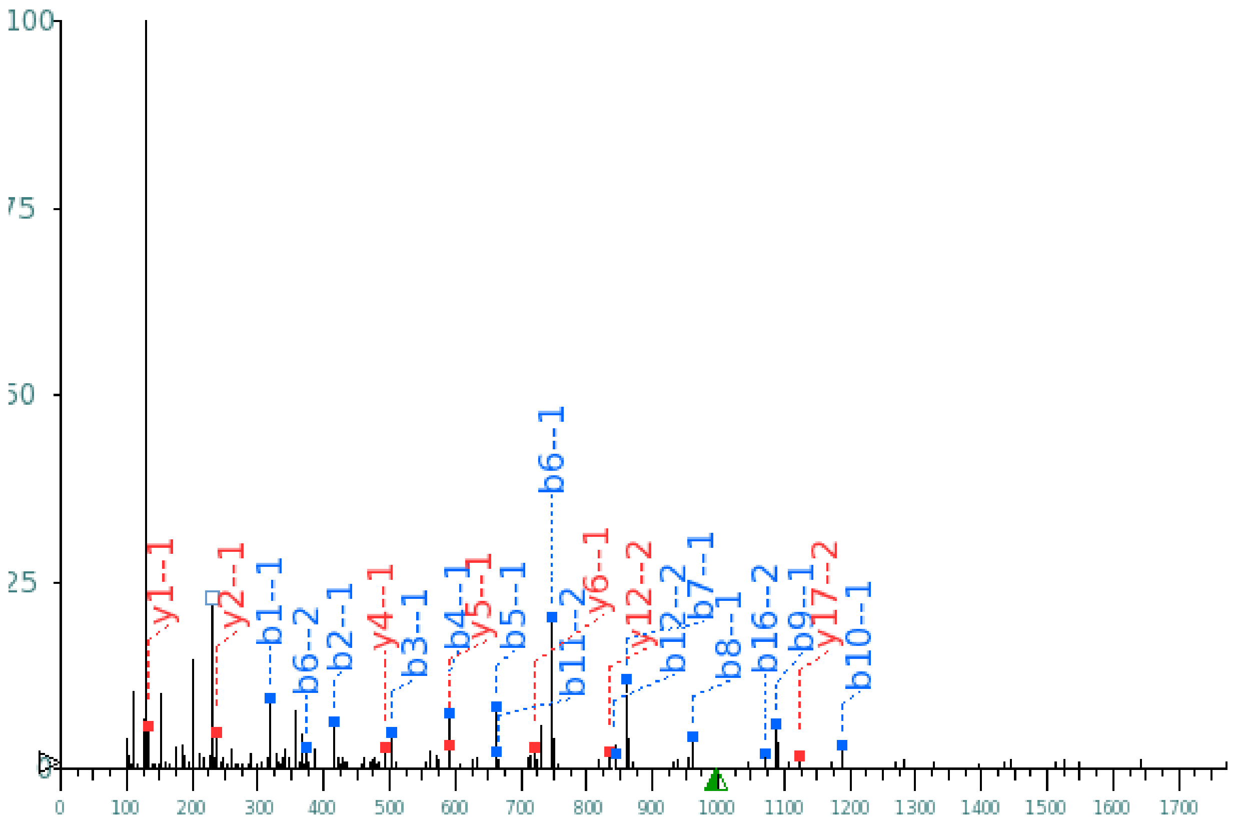

SVSSASLVEVHKSEENEEPMETD (+3)

Primary Reference: HSP74_HUMAN
Search ID: 41759
Search Name: 20130328_ananiav_ERstress_TMT_TM_timecourse_chymo_inj2_4MC_IAA
Scan#: 14717
Observed Mass: 997.8219 (0.2 ppm)
PSM Score: 57.87

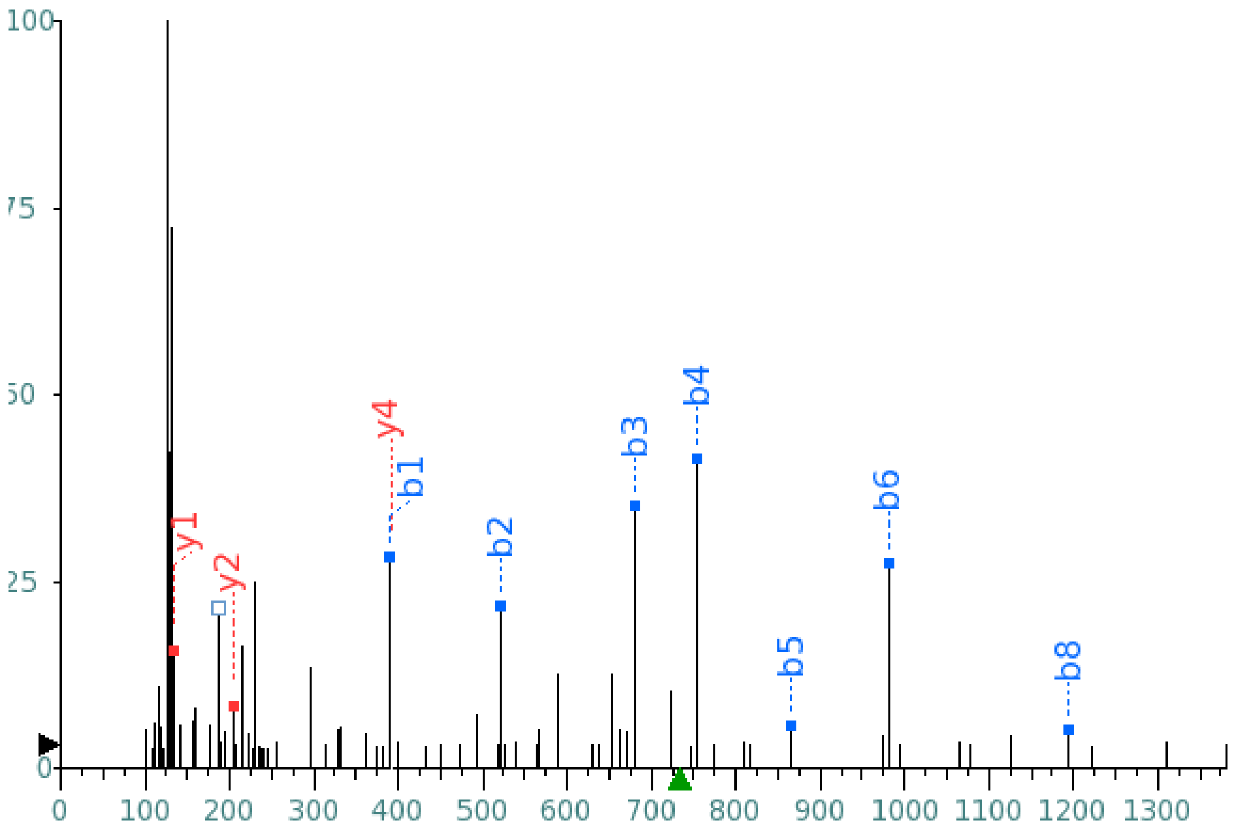

C*MC*ALDVDAAD (+2)

Primary Reference: CTU2_HUMAN
Search ID: 41759
Search Name: 20130328_ananiav_ERstress_TMT_TM_timecourse_chymo_inj2_4MC_IAA
Scan#: 14751
Observed Mass: 735.3184 (1.6 ppm)
PSM Score: 42.42

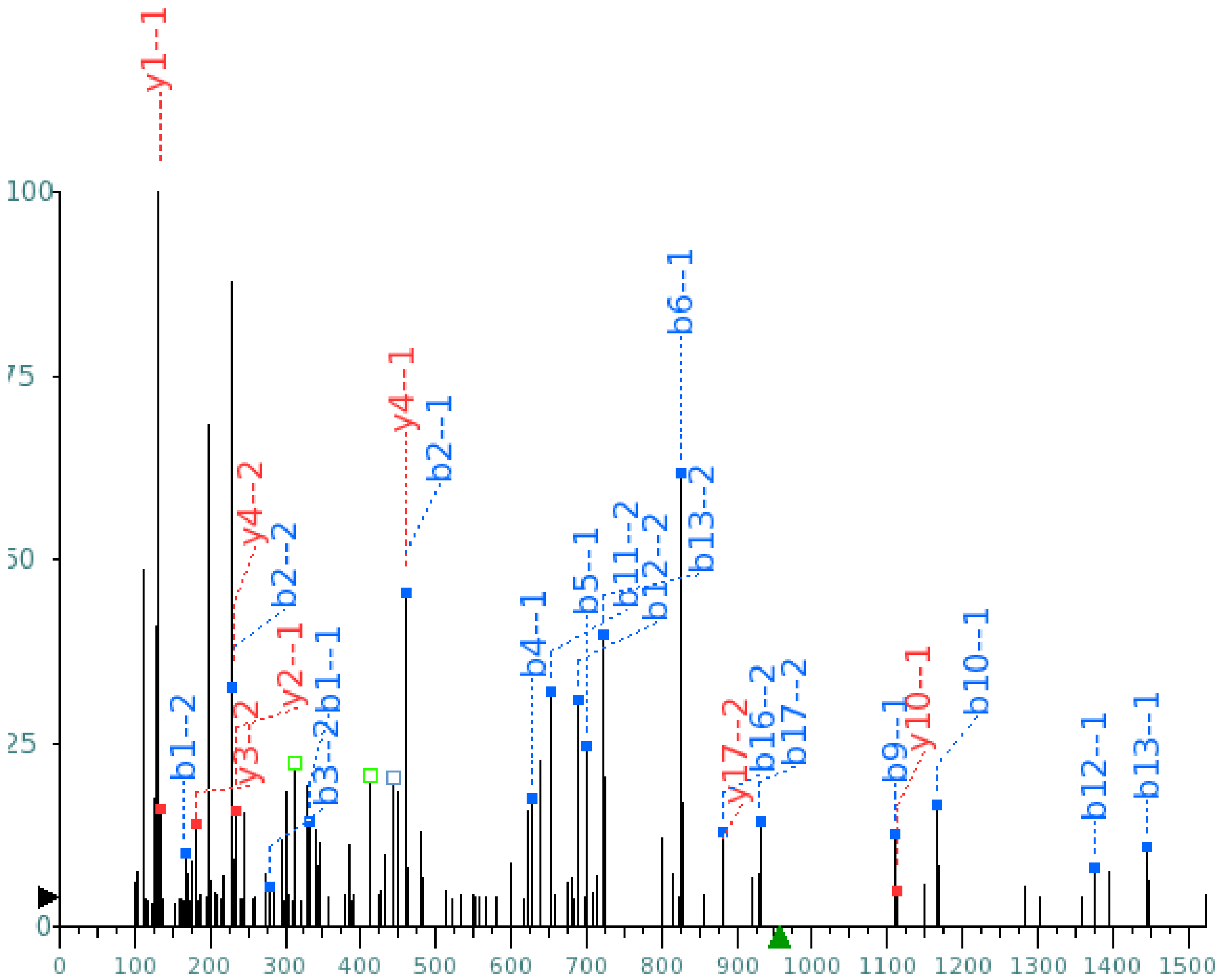

TEPAAQPGEGHAAPTNVVENPLPETD (+3)

Primary Reference: HGS_HUMAN
Search ID: 41759
Search Name: 20130328_ananiav_ERstress_TMT_TM_timecourse_chymo_inj2_4MC_IAA
Scan#: 14913
Observed Mass: 957.4725 (0.8 ppm)
PSM Score: 40.78

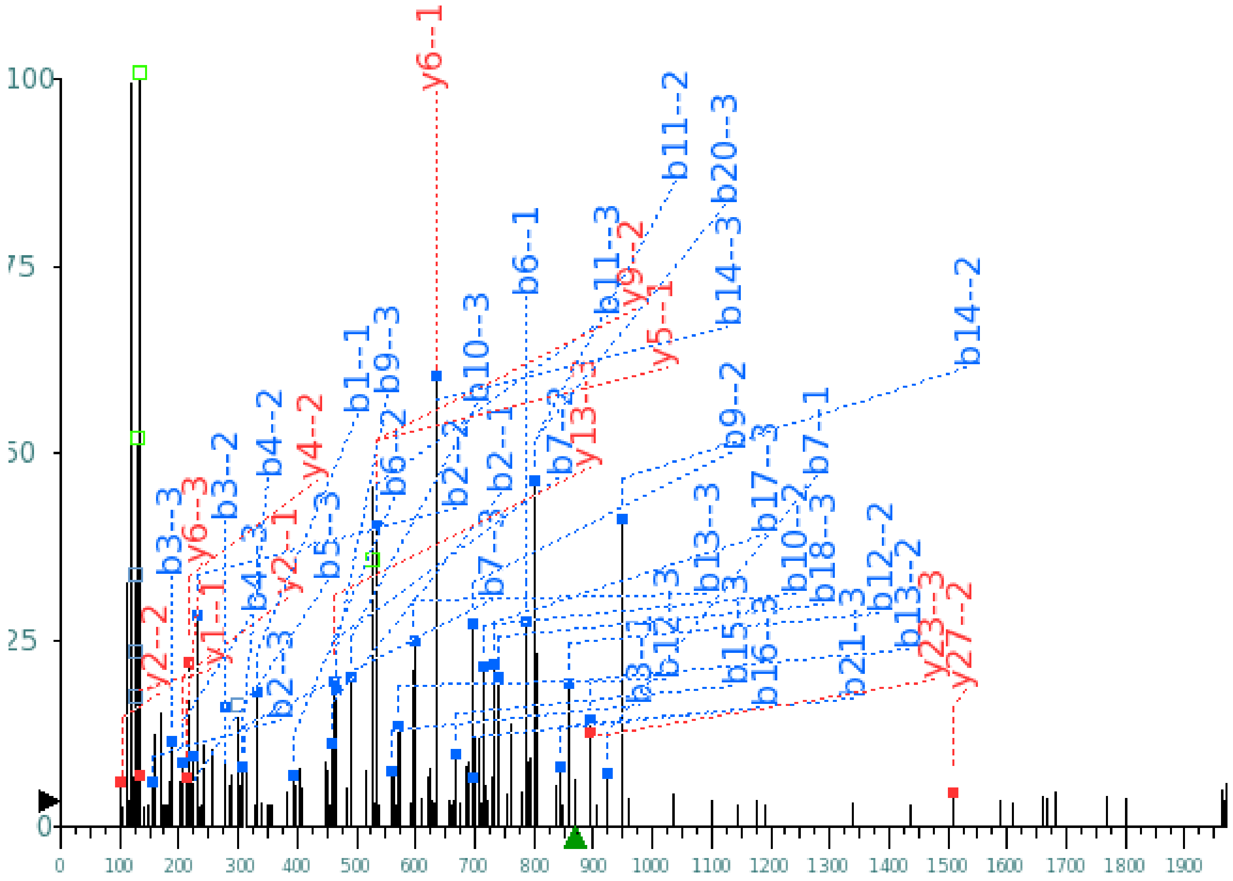

TQVGGDHIKAHIANPSGASTEC*FVTDNAD (+4)

Primary Reference: FLNB_HUMAN
Search ID: 41759
Search Name: 20130328_ananiav_ERstress_TMT_TM_timecourse_chymo_inj2_4MC_IAA
Scan#: 14986
Observed Mass: 868.4335 (3.2 ppm)
PSM Score: 41.1

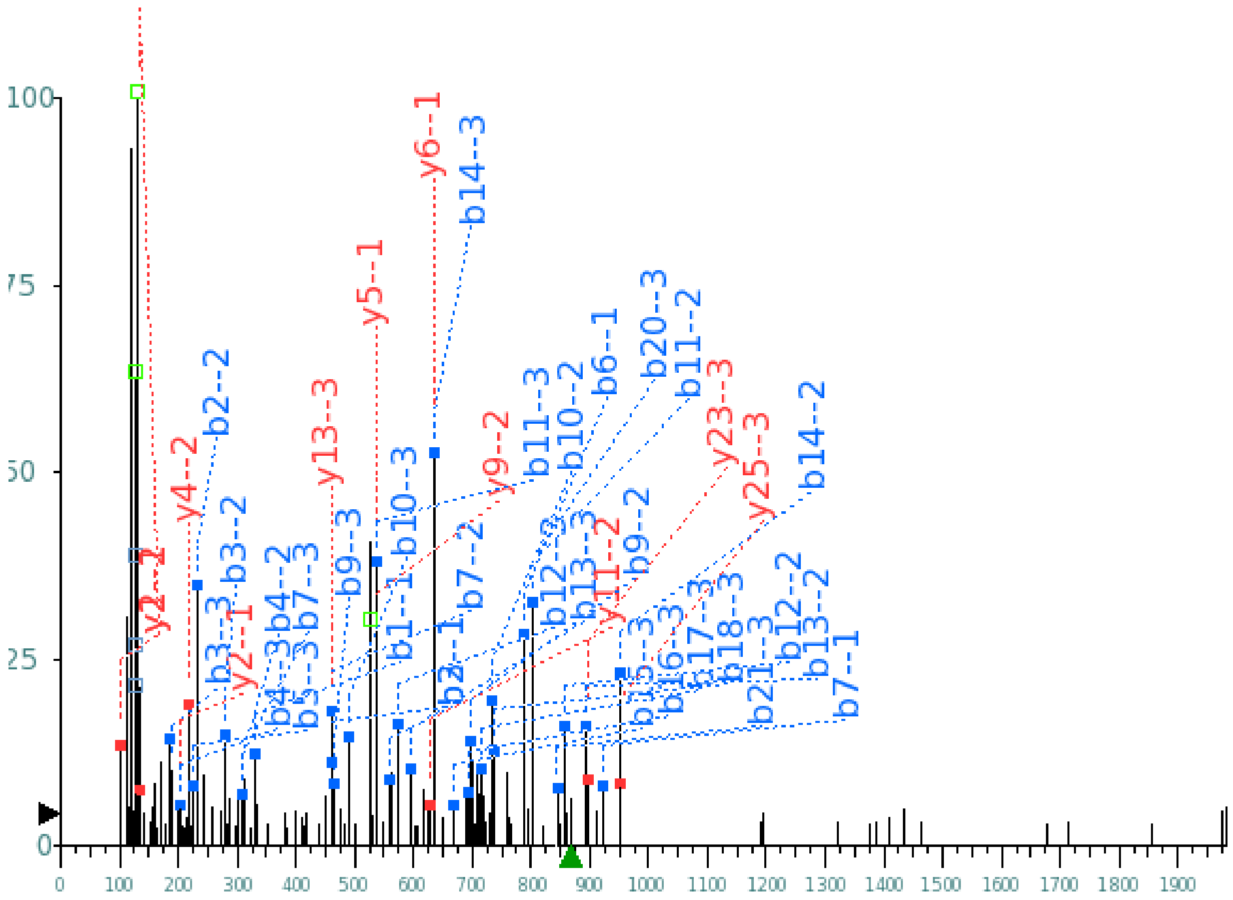

TQVGGDHIKAHIANPSGASTEC*FVTDNAD (+4)

Primary Reference: FLNB_HUMAN
Search ID: 41759
Search Name: 20130328_ananiav_ERstress_TMT_TM_timecourse_chymo_inj2_4MC_IAA
Scan#: 15030
Observed Mass: 868.4331 (2.8 ppm)
PSM Score: 31.7

File name: 2015-02-04 04.01.53PM.pdf

Creation time: 2015-02-04 04:09PM

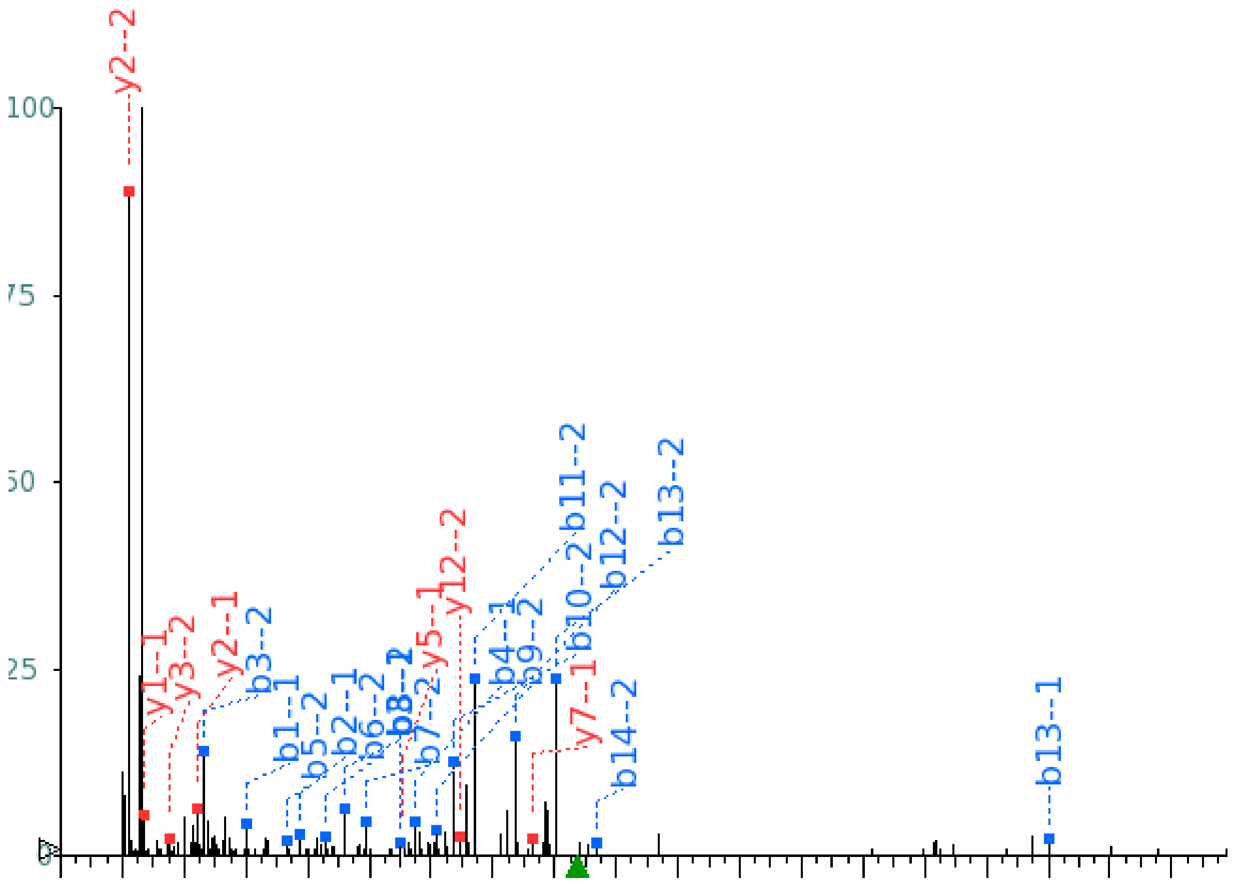

ASAIREELNGAMEHTNSNESD (+3)

Primary Reference: FOXP1_HUMAN
Search ID: 41759
Search Name: 20130328_ananiav_ERstress_TMT_TM_timecourse_chymo_inj2_4MC_IAA
Scan#: 15203
Observed Mass: 835.3903 (1.7 ppm)
PSM Score: 28.83

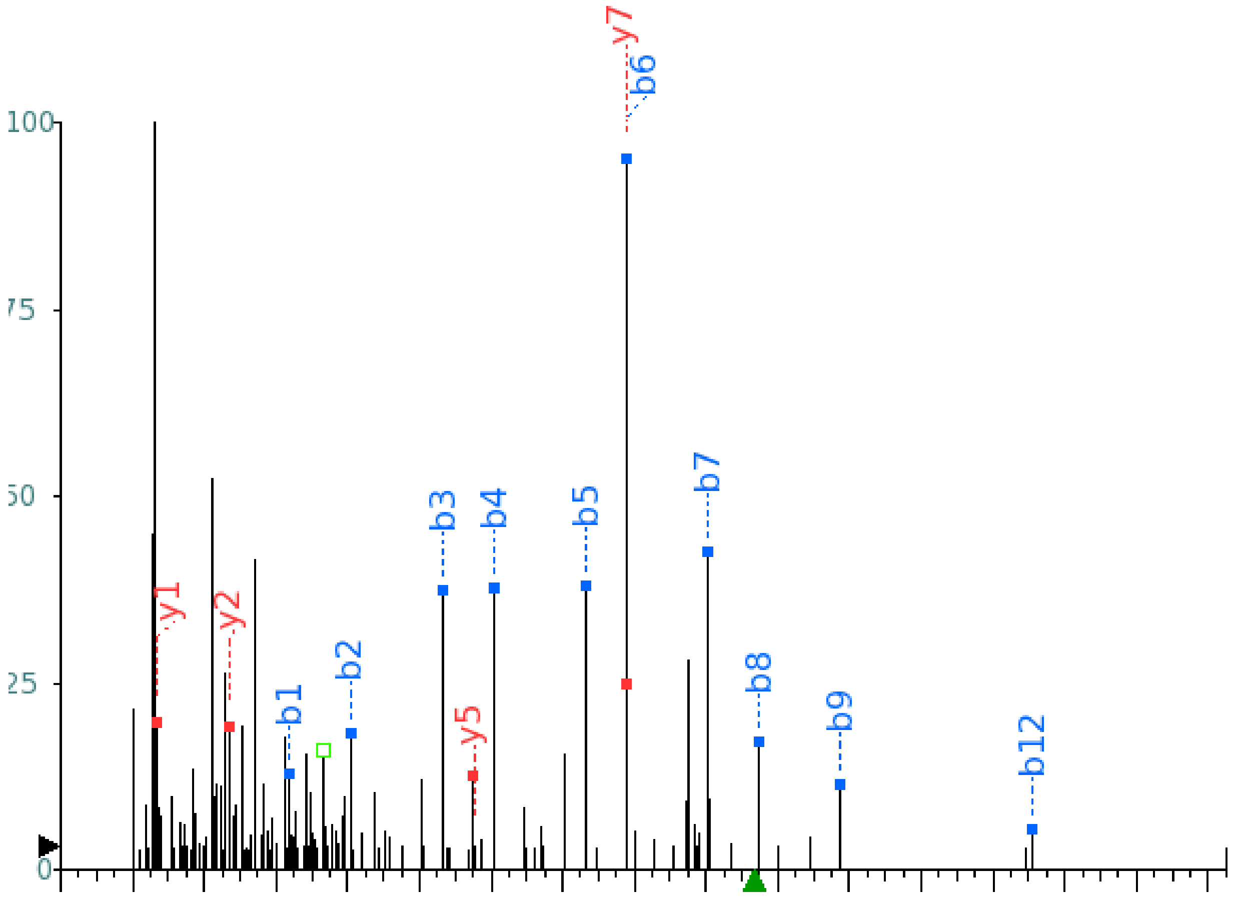

SSQAEGLANGPDVLETD (+2)

Primary Reference: EHMT1_HUMAN
Search ID: 41759
Search Name: 20130328_ananiav_ERstress_TMT_TM_timecourse_chymo_inj2_4MC_IAA
Scan#: 15379
Observed Mass: 966.4763 (3 ppm)
PSM Score: 45.82

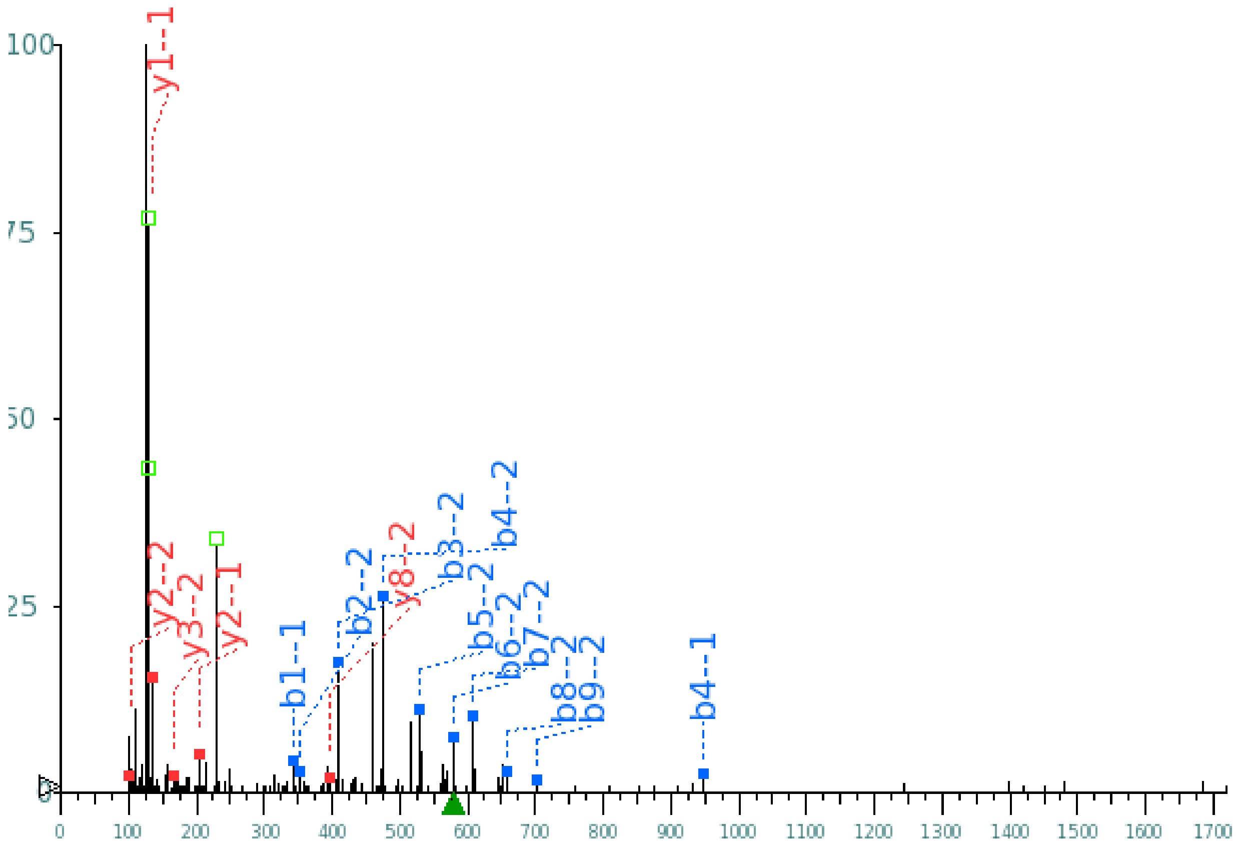

IKNMITGTSQAD (+3)

Primary Reference: EF1A1_HUMAN
Search ID: 41759
Search Name: 20130328_ananiav_ERstress_TMT_TM_timecourse_chymo_inj2_4MC_IAA
Scan#: 15447
Observed Mass: 579.6596 (1.4 ppm)
PSM Score: 14.2

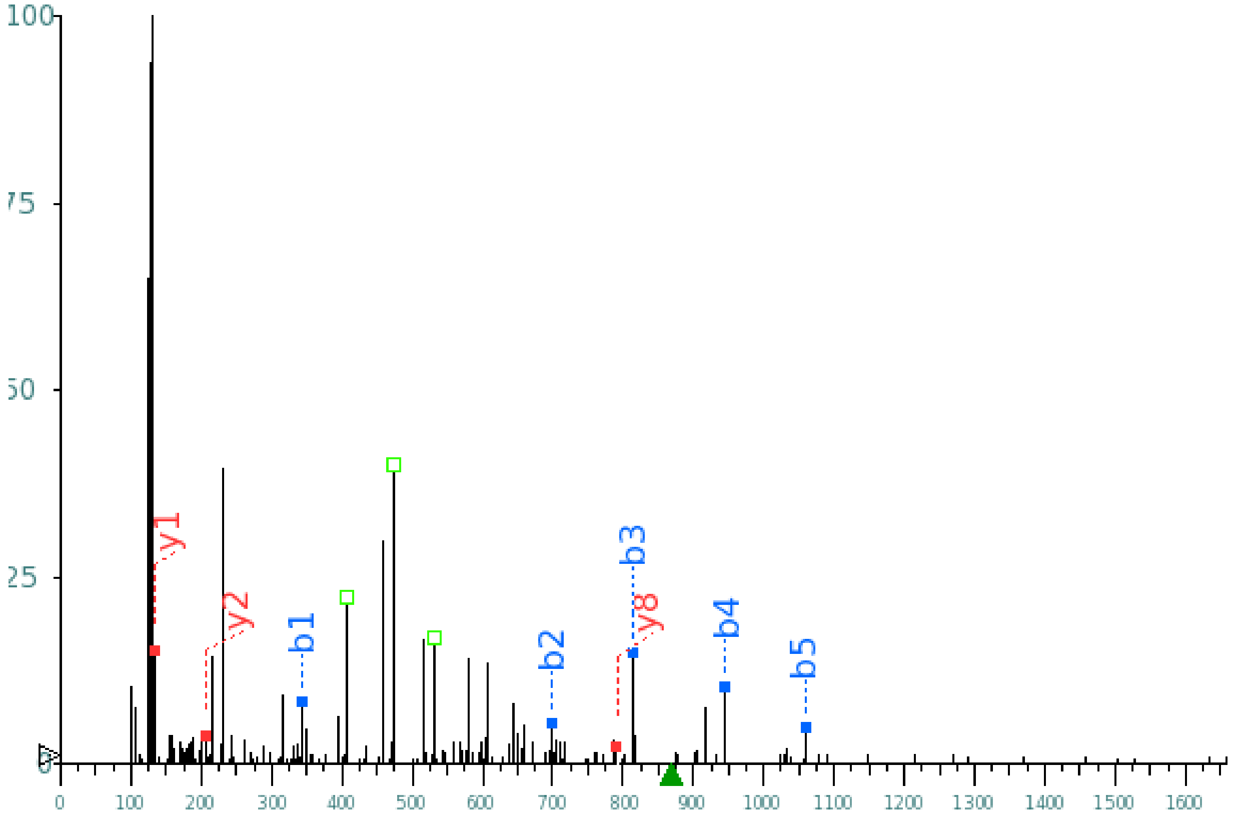

IKNMITGTSQAD (+2)

Primary Reference: EF1A1_HUMAN
Search ID: 41759
Search Name: 20130328_ananiav_ERstress_TMT_TM_timecourse_chymo_inj2_4MC_IAA
Scan#: 15454
Observed Mass: 868.9851 (0.7 ppm)
PSM Score: 21.01

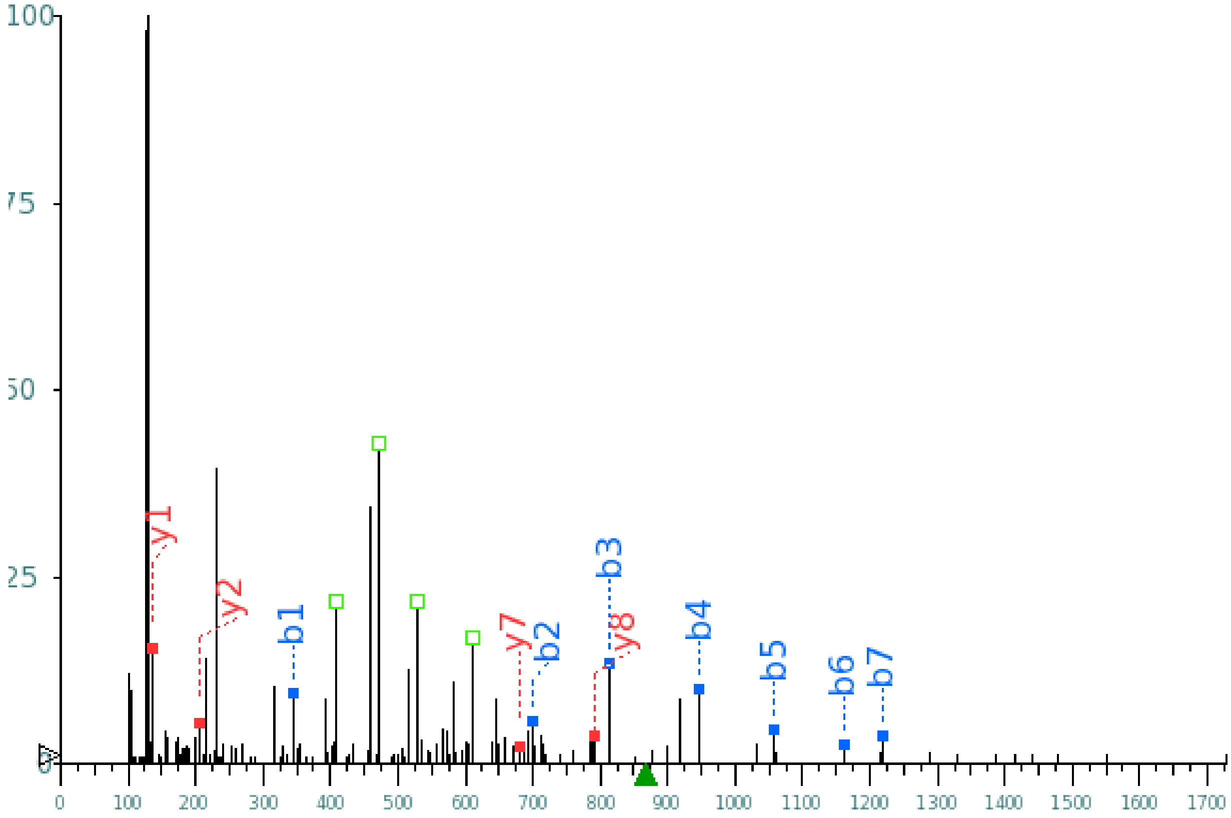

IKNMITGTSQAD (+2)

Primary Reference: EF1A1_HUMAN

Search ID: 41759

Search Name: 20130328_ananiav_ERstress_TMT_TM_timecourse_chymo_inj2_4MC_IAA

Scan#: 15553

Observed Mass: 868.9852 (0.9 ppm)

PSM Score: 19.63

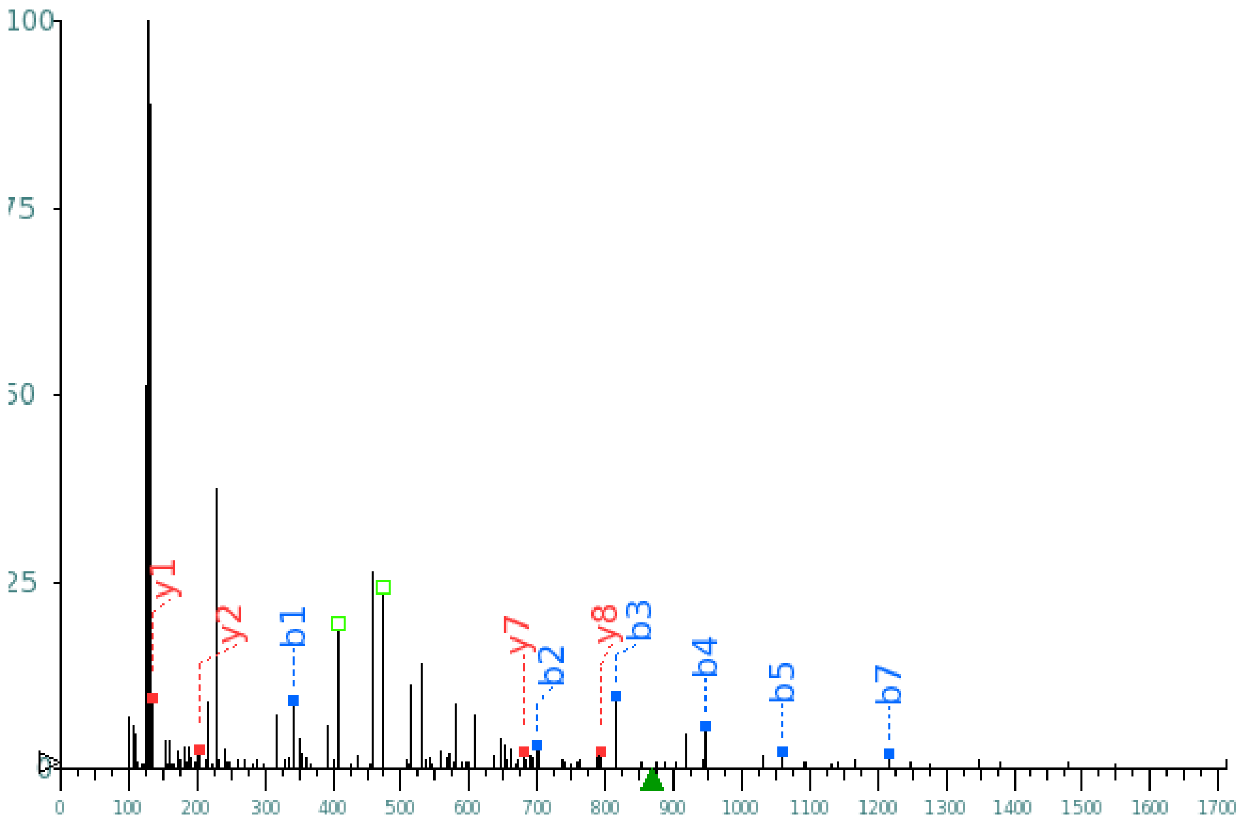

IKNMITGTSQAD (+2)

Primary Reference: EF1A1_HUMAN
Search ID: 41759
Search Name: 20130328_ananiav_ERstress_TMT_TM_timecourse_chymo_inj2_4MC_IAA
Scan#: 15655
Observed Mass: 868.9848 (0.3 ppm)
PSM Score: 20.22

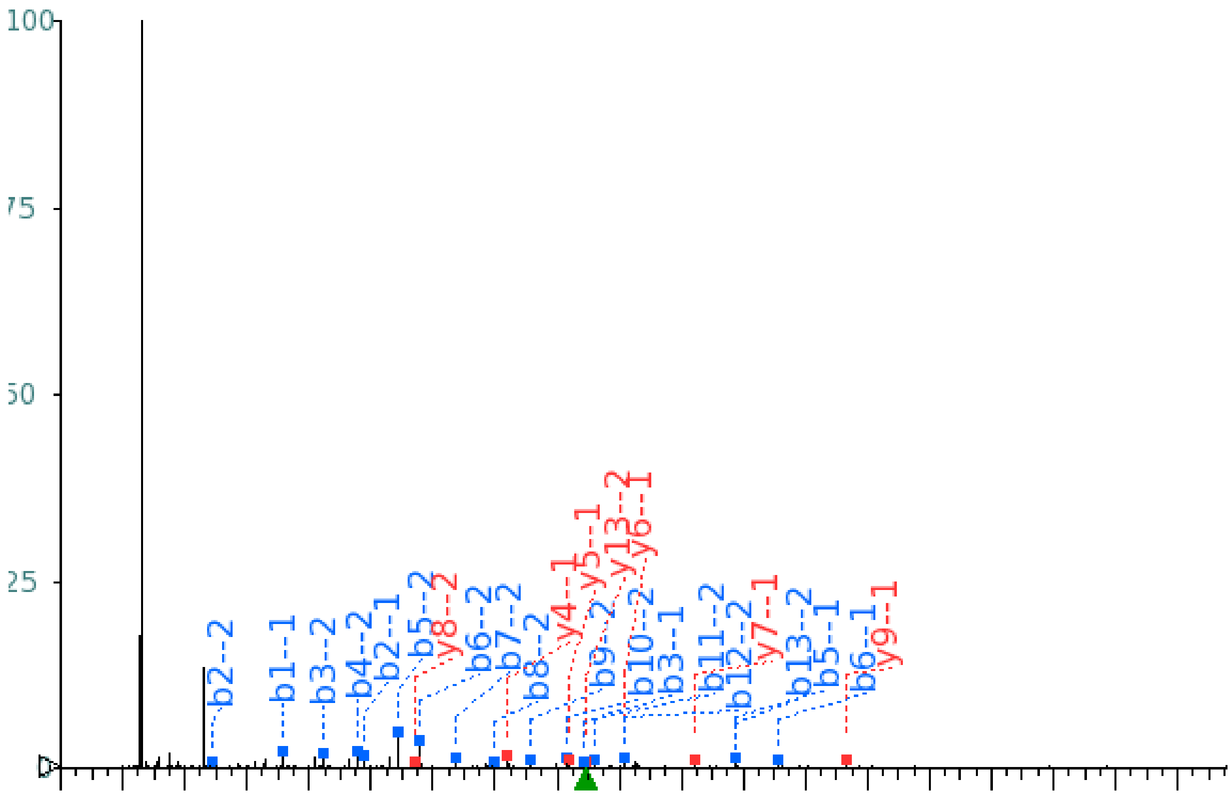

QQKLEAIEDDSVKETD (+3)

Primary Reference: UBP14_HUMAN
Search ID: 41759
Search Name: 20130328_ananiav_ERstress_TMT_TM_timecourse_chymo_inj2_4MC_IAA
Scan#: 15667
Observed Mass: 845.7976 (1 ppm)
PSM Score: 29.75

File name: 2015-02-04 04.01.53PM.pdf

Creation time: 2015-02-04 04:09PM

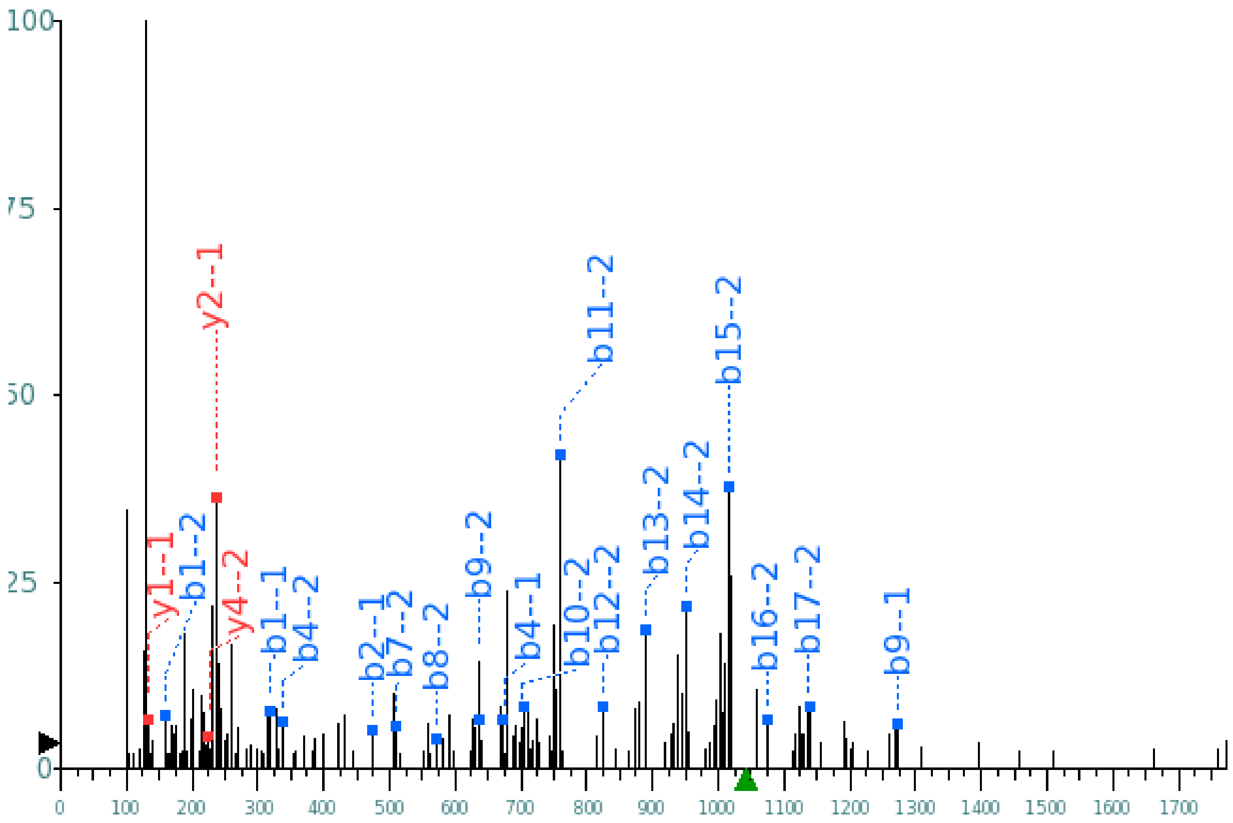

SRSLGREEEEEDEEEEELEEGTIDVTD (+3)

Primary Reference: DHX30_HUMAN
Search ID: 41759
Search Name: 20130328_ananiav_ERstress_TMT_TM_timecourse_chymo_inj2_4MC_IAA
Scan#: 15856
Observed Mass: 1042.1462 (3.7 ppm)
PSM Score: 44.13

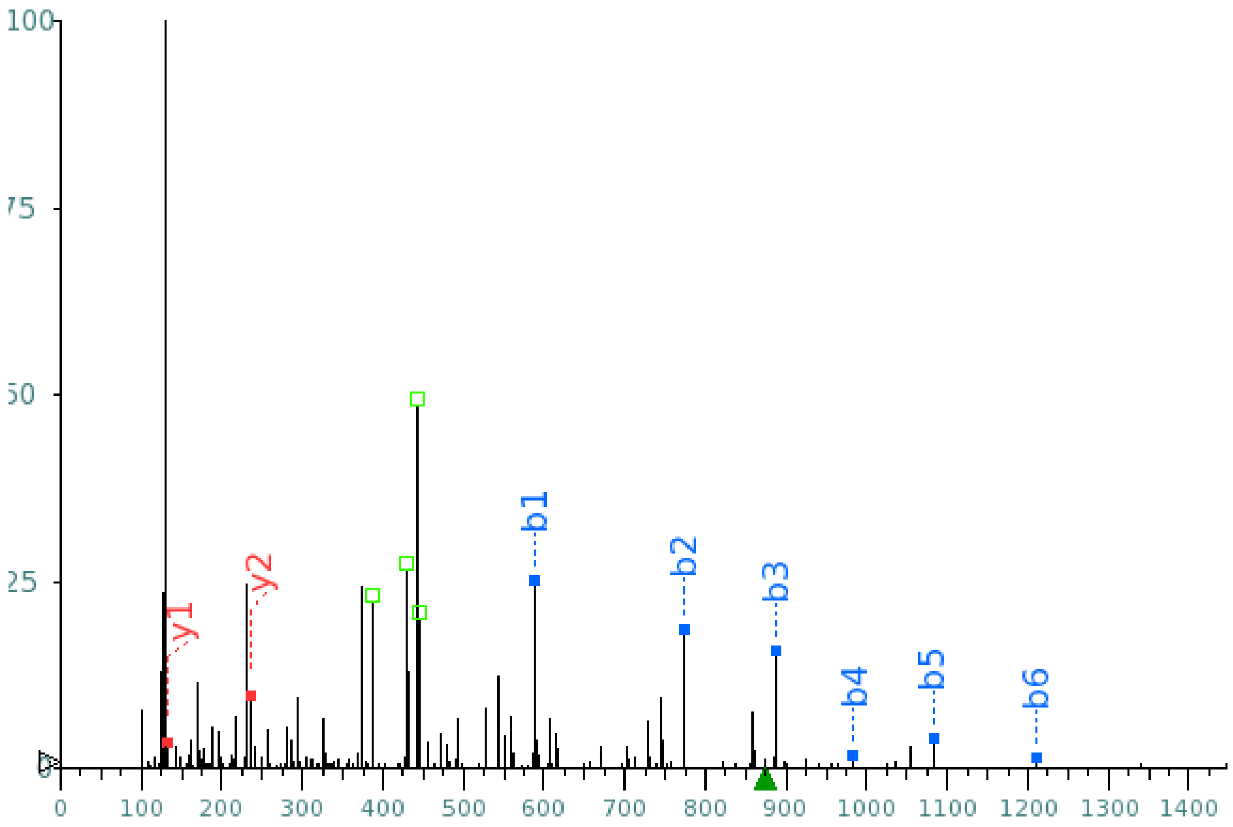

KWLPVQESSTD (+2)

Primary Reference: SPCS1_HUMAN
Search ID: 41759
Search Name: 20130328_ananiav_ERstress_TMT_TM_timecourse_chymo_inj2_4MC_IAA
Scan#: 15995
Observed Mass: 874.4875 (2.7 ppm)
PSM Score: 45.53

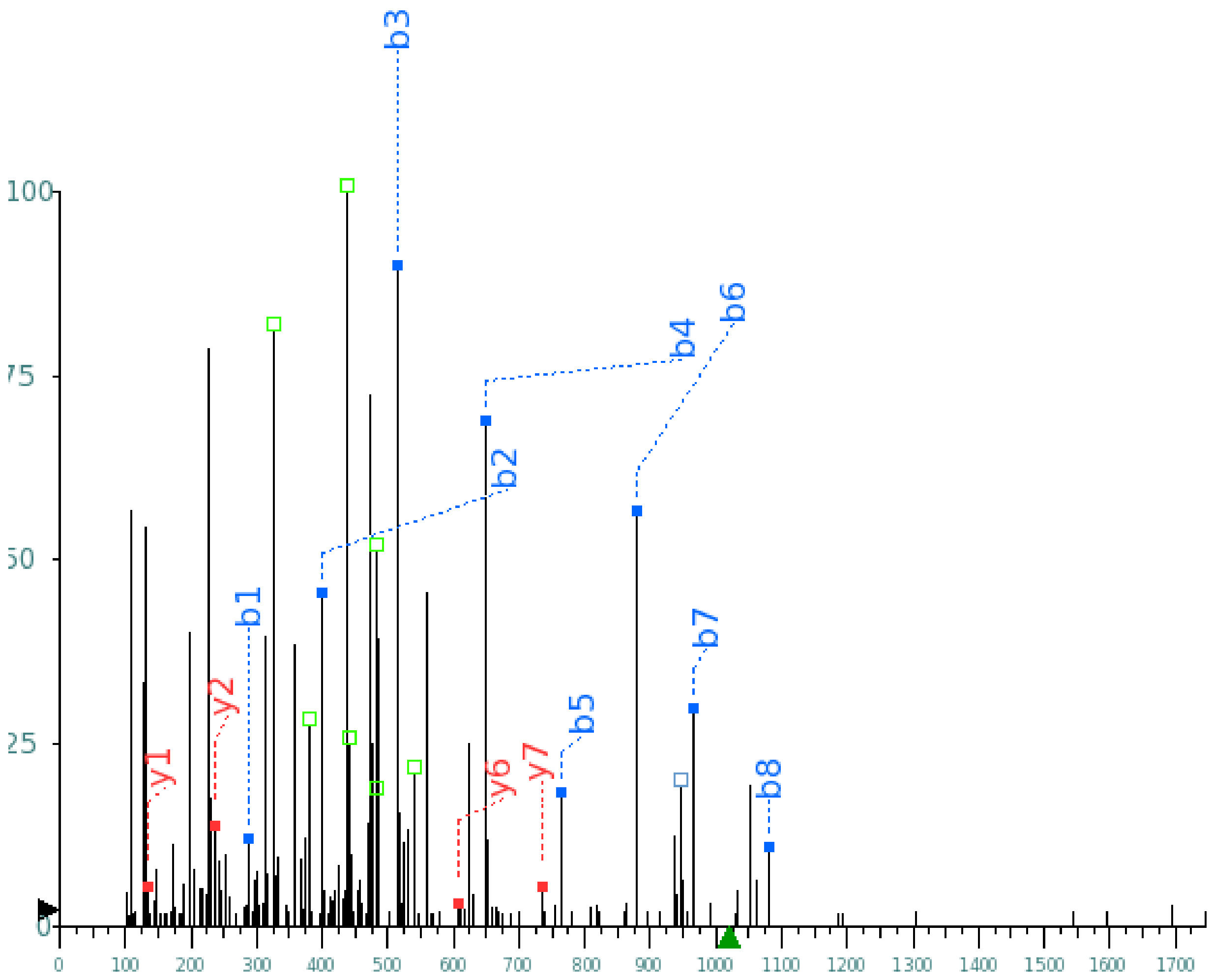

GNLHLDSLPEESDVATD (+2)

Primary Reference: SRR1L_HUMAN
Search ID: 41759
Search Name: 20130328_ananiav_ERstress_TMT_TM_timecourse_chymo_inj2_4MC_IAA
Scan#: 16193
Observed Mass: 1021.0008 (1 ppm)
PSM Score: 32.07

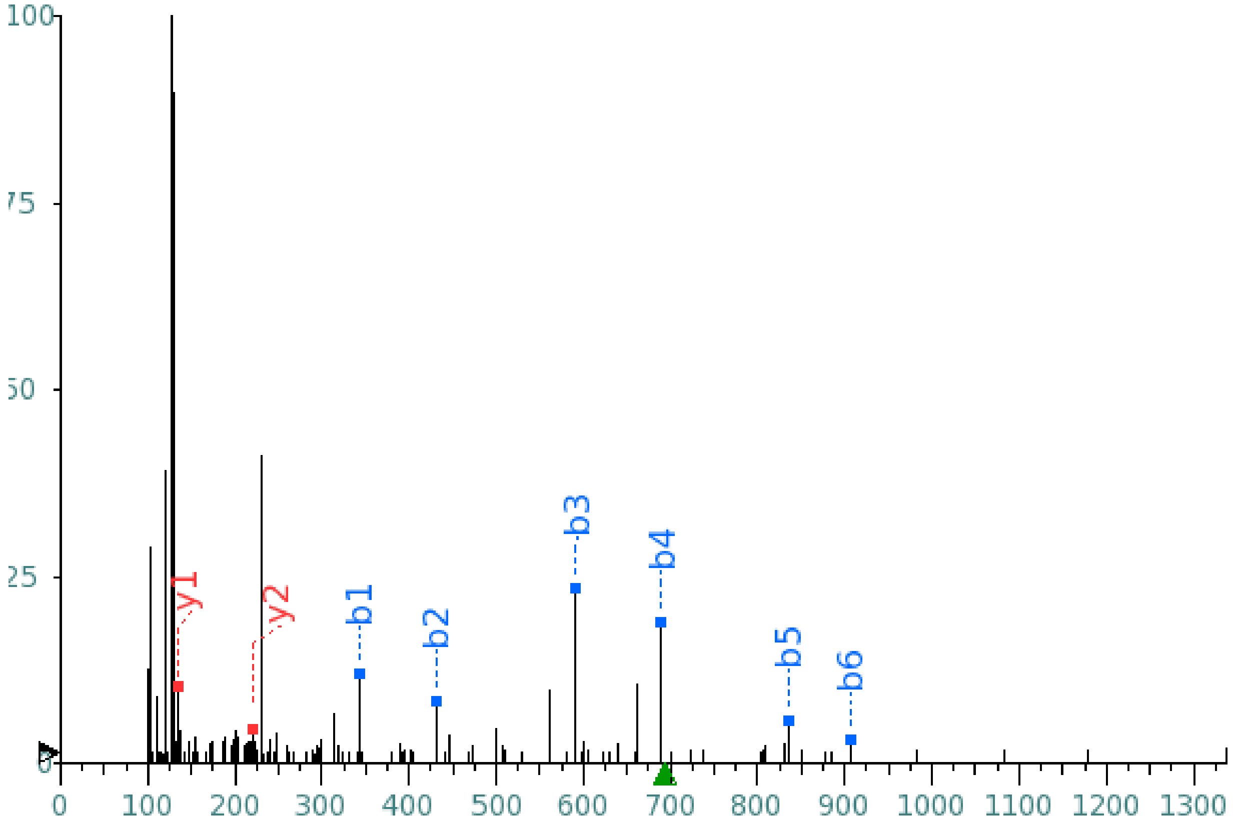

ISC*VFAEESD (+2)

Primary Reference: TF3C1_HUMAN
Search ID: 41759
Search Name: 20130328_ananiav_ERstress_TMT_TM_timecourse_chymo_inj2_4MC_IAA
Scan#: 16743
Observed Mass: 693.3278 (2 ppm)
PSM Score: 29.44

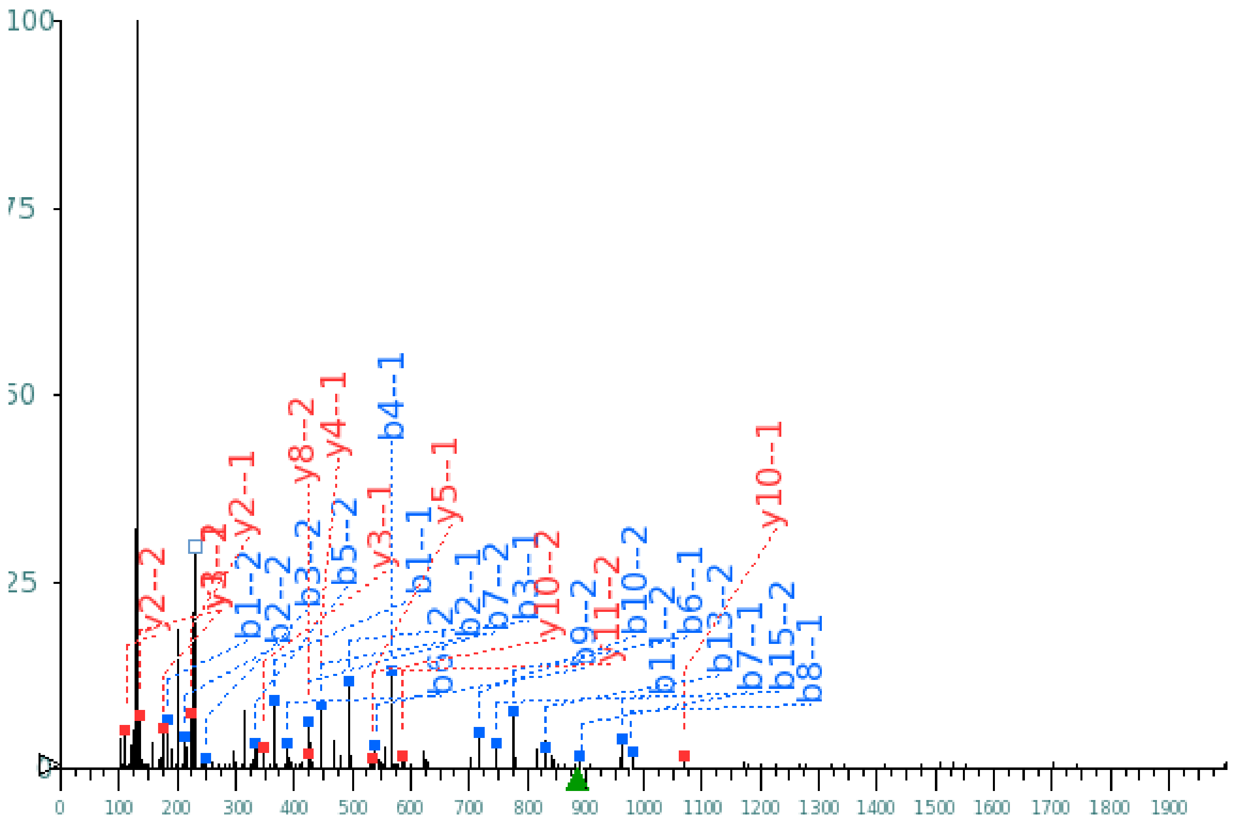

HGAAPDISVKGPAFNMASPESD (+3)

Primary Reference: AHNK_HUMAN
Search ID: 41759
Search Name: 20130328_ananiav_ERstress_TMT_TM_timecourse_chymo_inj2_4MC_IAA
Scan#: 17119
Observed Mass: 886.1214 (2.1 ppm)
PSM Score: 28.09

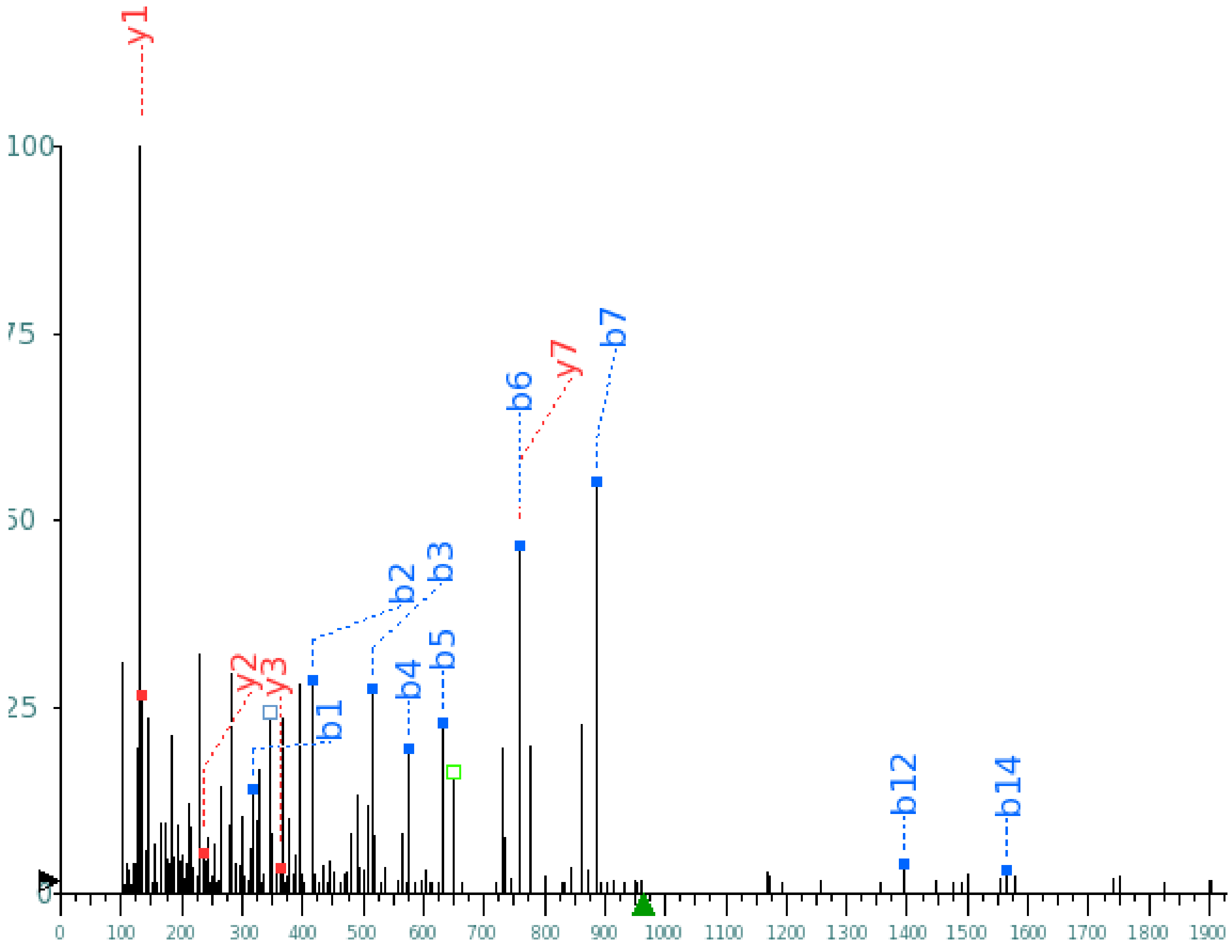

STVGGQQPPSLIGIQTD (+2)

Primary Reference: RCOR3_HUMAN
Search ID: 41759
Search Name: 20130328_ananiav_ERstress_TMT_TM_timecourse_chymo_inj2_4MC_IAA
Scan#: 17135
Observed Mass: 964.0224 (2.2 ppm)
PSM Score: 29.58

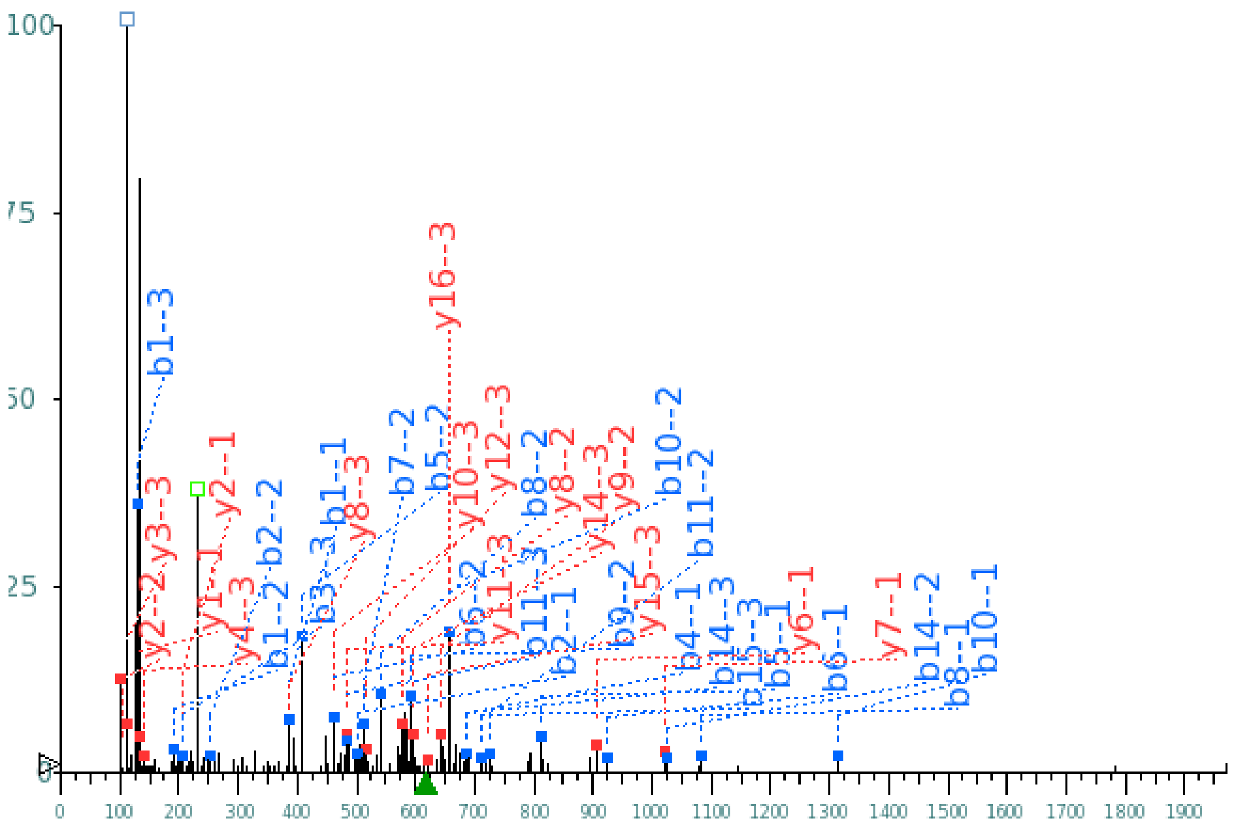

RILAQITGTEHLKESEAD (+4)

Primary Reference: PDLI5_HUMAN
Search ID: 41759
Search Name: 20130328_ananiav_ERstress_TMT_TM_timecourse_chymo_inj2_4MC_IAA
Scan#: 17228
Observed Mass: 618.0994 (1.8 ppm)
PSM Score: 26.25

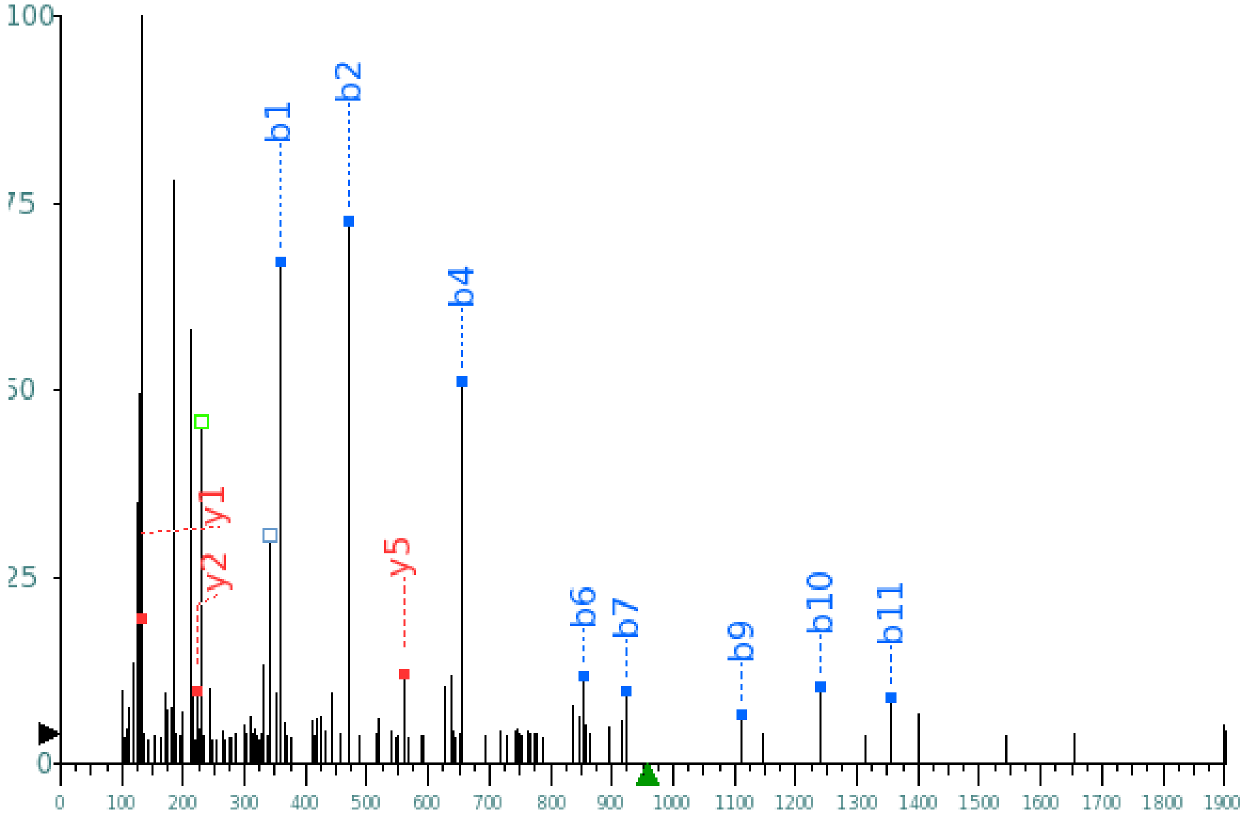

QLPSPTATSQLPLESD (+2)

Primary Reference: HNRPK_HUMAN
Search ID: 41759
Search Name: 20130328_ananiav_ERstress_TMT_TM_timecourse_chymo_inj2_4MC_IAA
Scan#: 18064
Observed Mass: 957.0077 (0.8 ppm)
PSM Score: 43.87

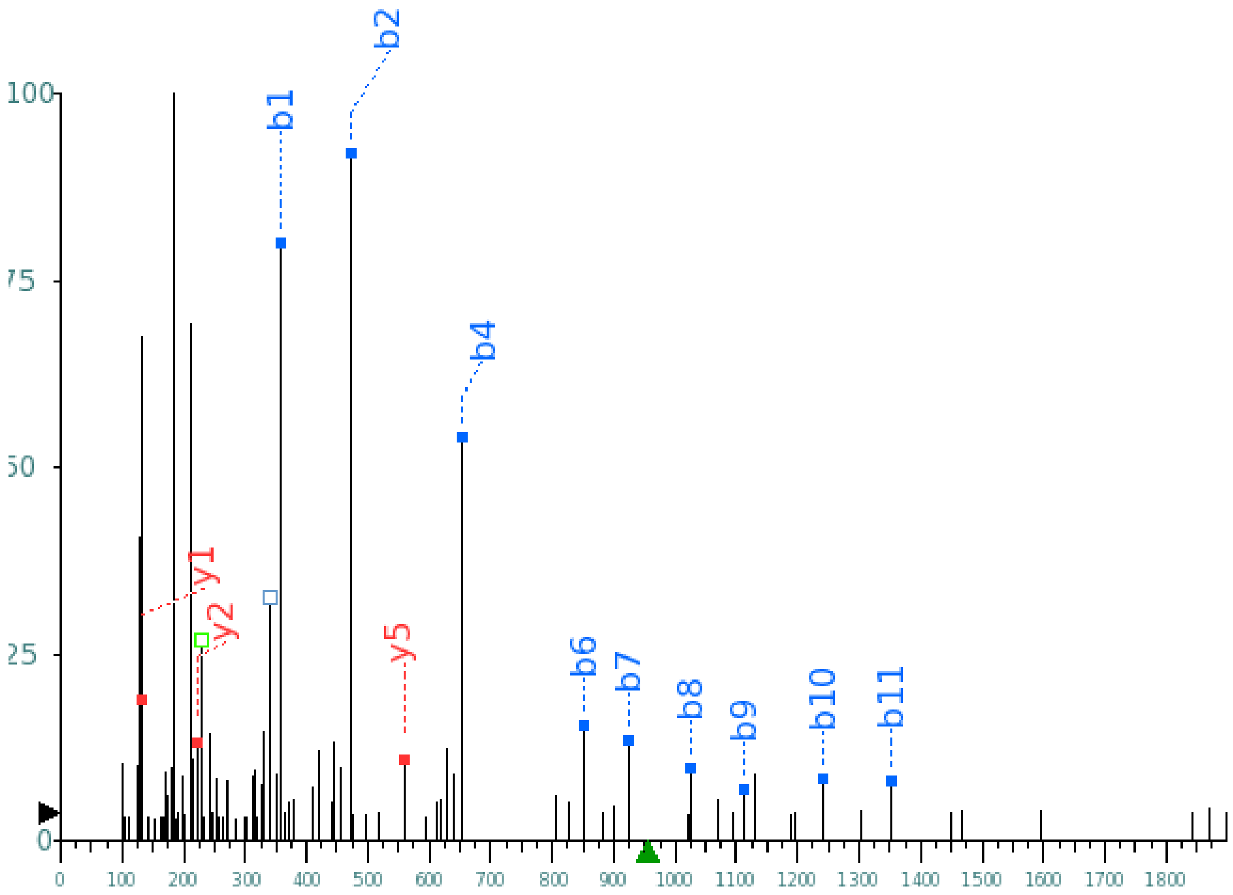

QLPSPTATSQLPLESD (+2)

Primary Reference: HNRPK_HUMAN
Search ID: 41759
Search Name: 20130328_ananiav_ERstress_TMT_TM_timecourse_chymo_inj2_4MC_IAA
Scan#: 18150
Observed Mass: 957.0084 (1.6 ppm)
PSM Score: 46.88

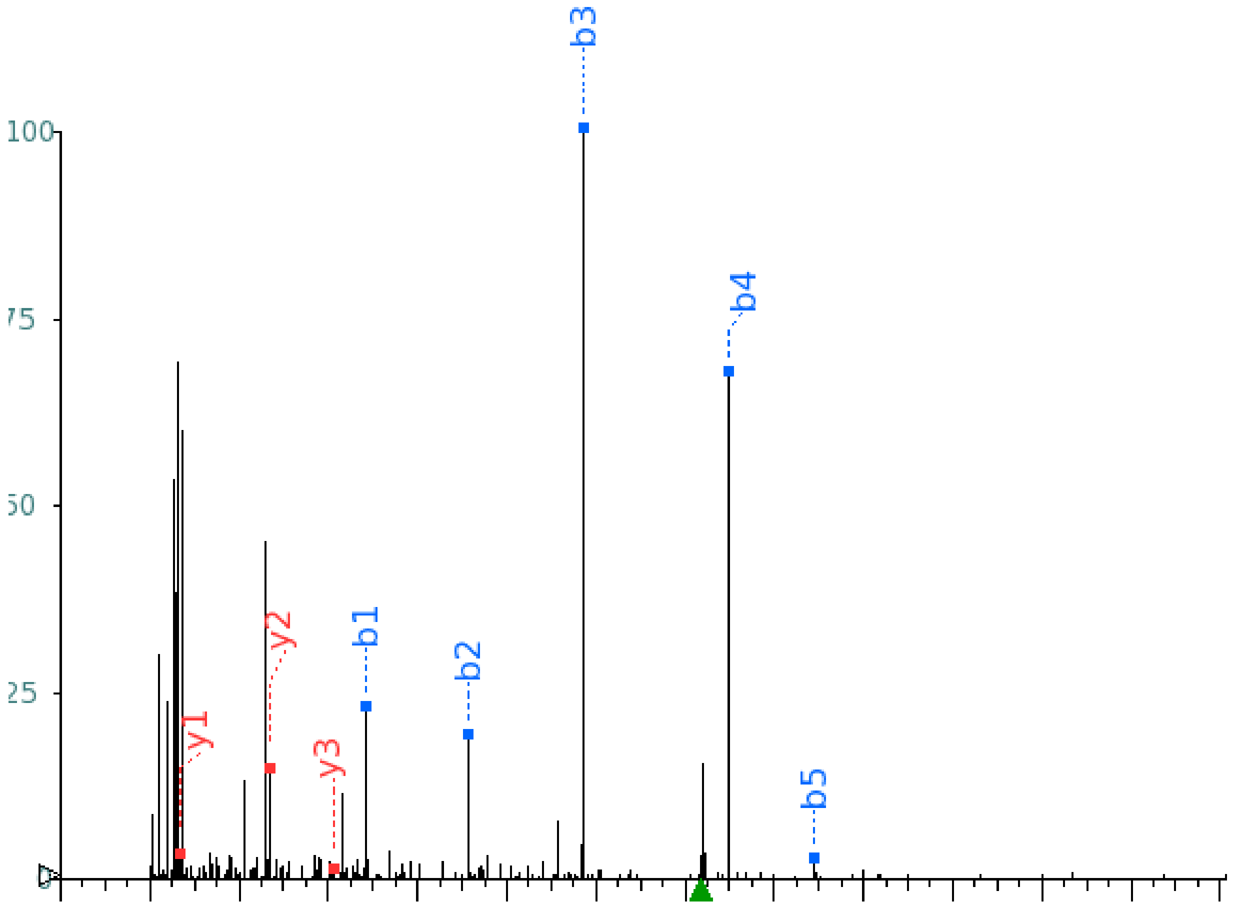

IIEYPHFATD (+2)

Primary Reference: NNTM_HUMAN
Search ID: 41759
Search Name: 20130328_ananiav_ERstress_TMT_TM_timecourse_chymo_inj2_4MC_IAA
Scan#: 18262
Observed Mass: 717.8779 (1.4 ppm)
PSM Score: 20

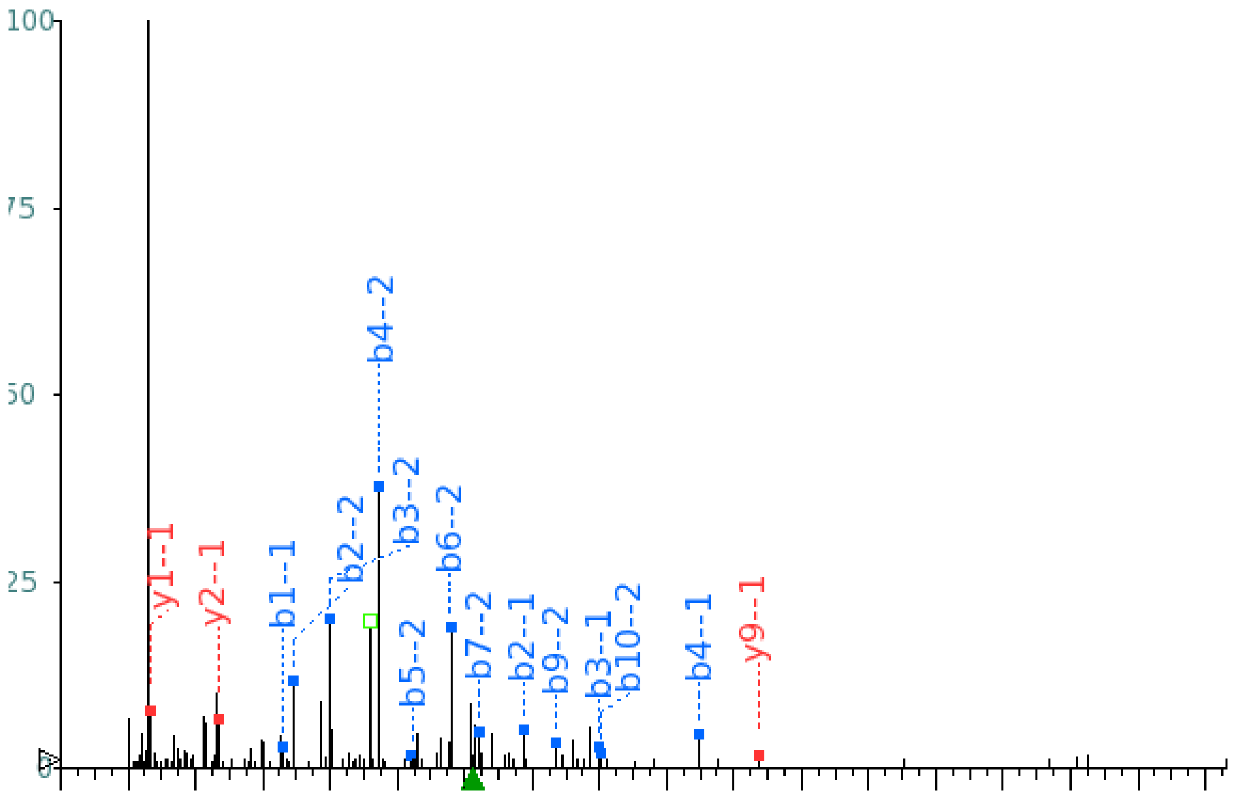

VKLFPNSLDQTD (+3)

Primary Reference: CALR_HUMAN
Search ID: 41759
Search Name: 20130328_ananiav_ERstress_TMT_TM_timecourse_chymo_inj2_4MC_IAA
Scan#: 18556
Observed Mass: 612.3496 (1.6 ppm)
PSM Score: 22.79

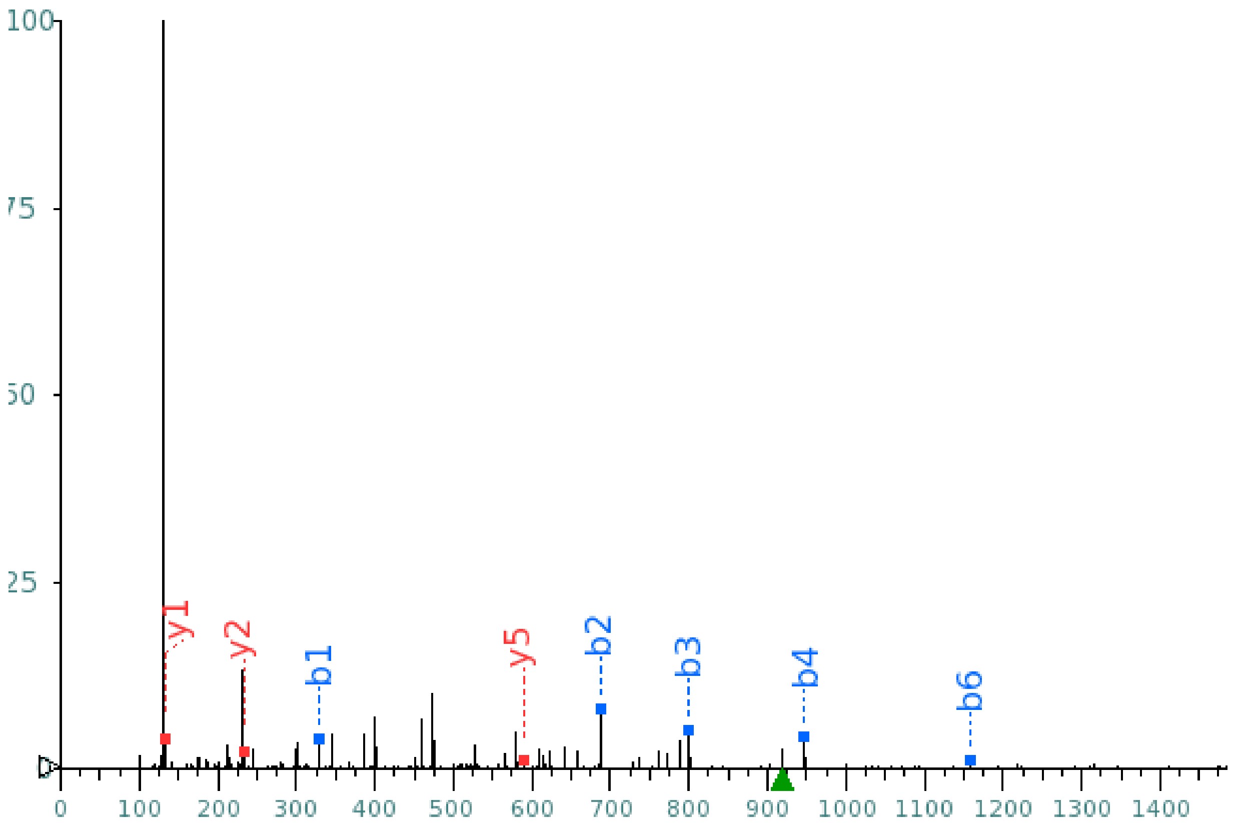

VKLFPNSLDQTD (+2)

Primary Reference: CALR_HUMAN
Search ID: 41759
Search Name: 20130328_ananiav_ERstress_TMT_TM_timecourse_chymo_inj2_4MC_IAA
Scan#: 18557
Observed Mass: 918.02 (0.6 ppm)
PSM Score: 20.32

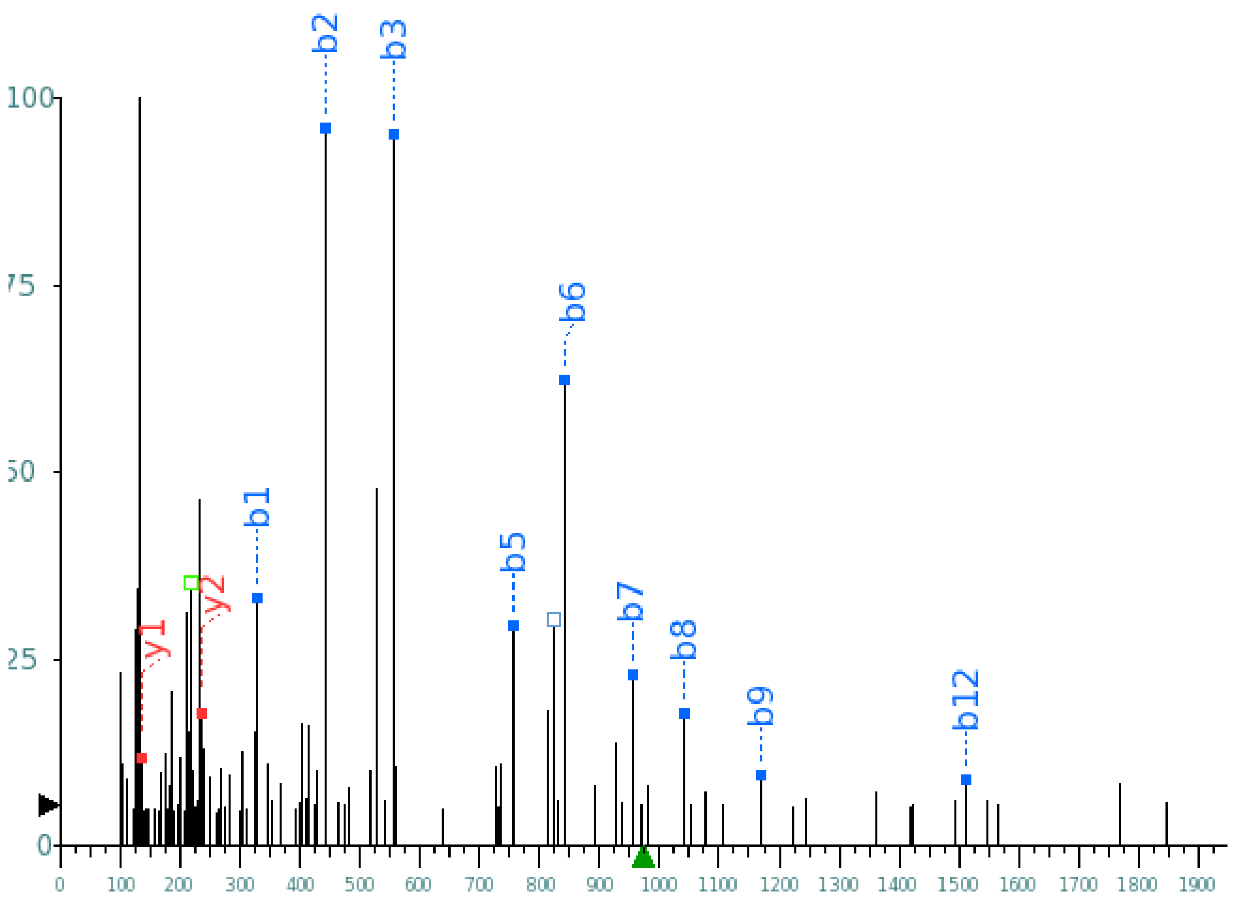

VNLPTSISQQTIAETD (+2)

Primary Reference: COPT2_HUMAN
Search ID: 41759
Search Name: 20130328_ananiav_ERstress_TMT_TM_timecourse_chymo_inj2_4MC_IAA
Scan#: 18561
Observed Mass: 973.5187 (1.1 ppm)
PSM Score: 55.1

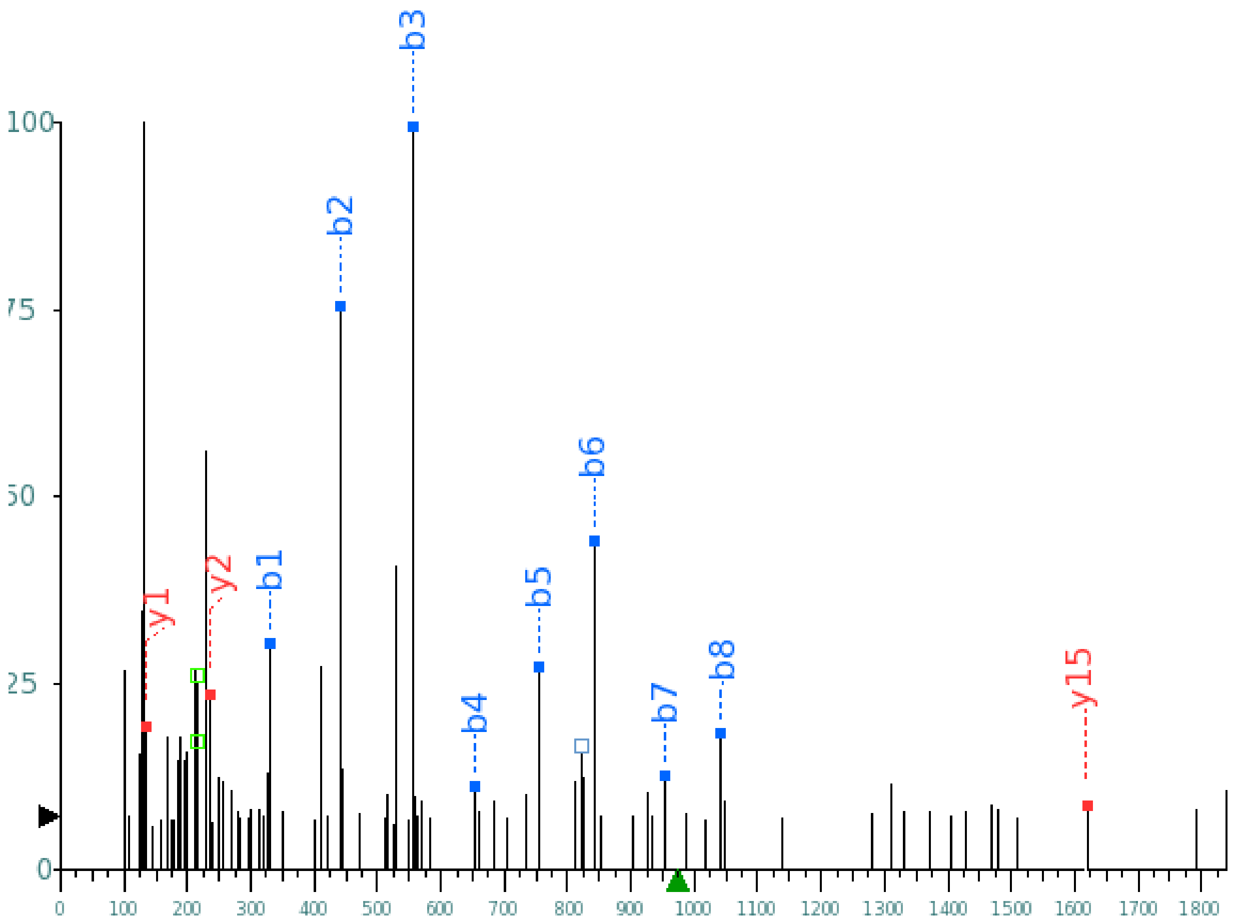

VNLPTSISQQTIAETD (+2)

Primary Reference: COPT2_HUMAN
Search ID: 41759
Search Name: 20130328_ananiav_ERstress_TMT_TM_timecourse_chymo_inj2_4MC_IAA
Scan#: 18660
Observed Mass: 973.5176 (-0.1 ppm)
PSM Score: 42.99

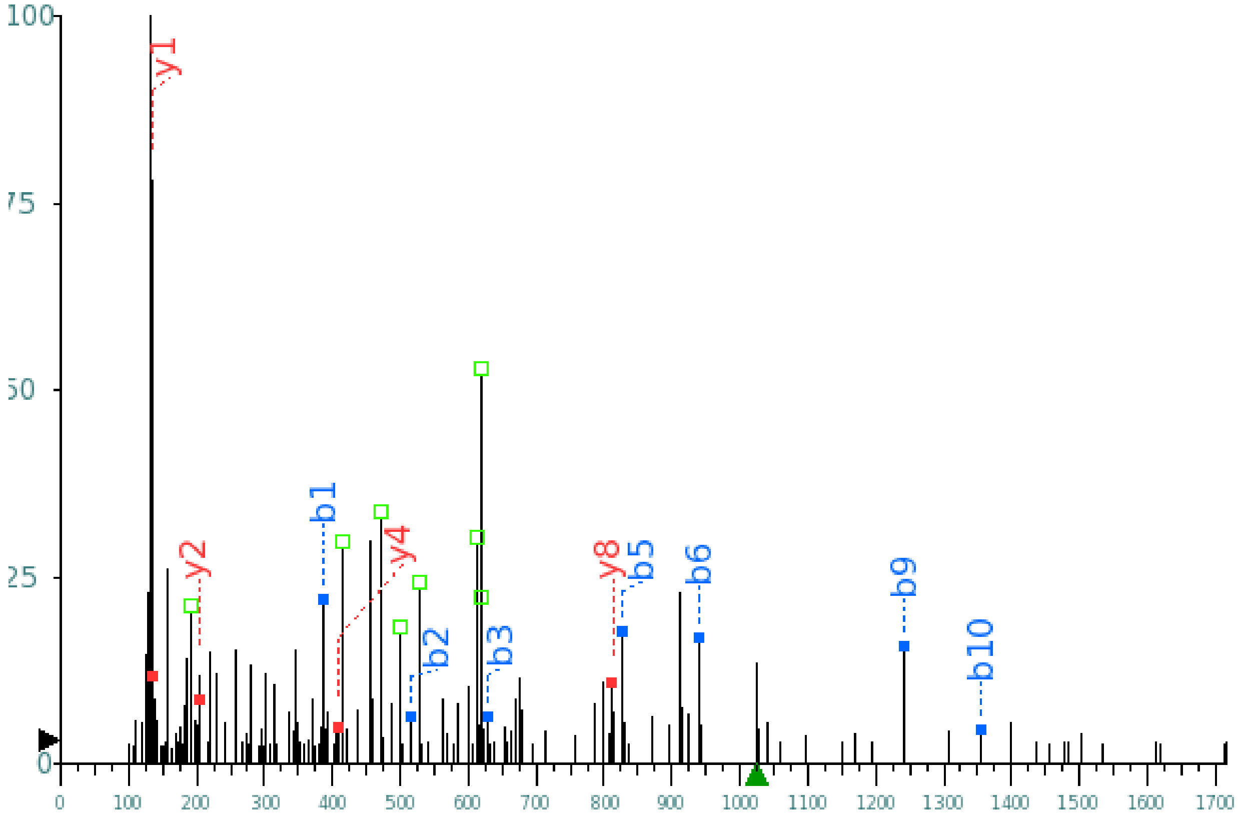

RMLPTIIADNAGYDSAD (+2)

Primary Reference: TCPB_HUMAN
Search ID: 41759
Search Name: 20130328_ananiav_ERstress_TMT_TM_timecourse_chymo_inj2_4MC_IAA
Scan#: 18715
Observed Mass: 1026.5165 (-0.6 ppm)
PSM Score: 27.26

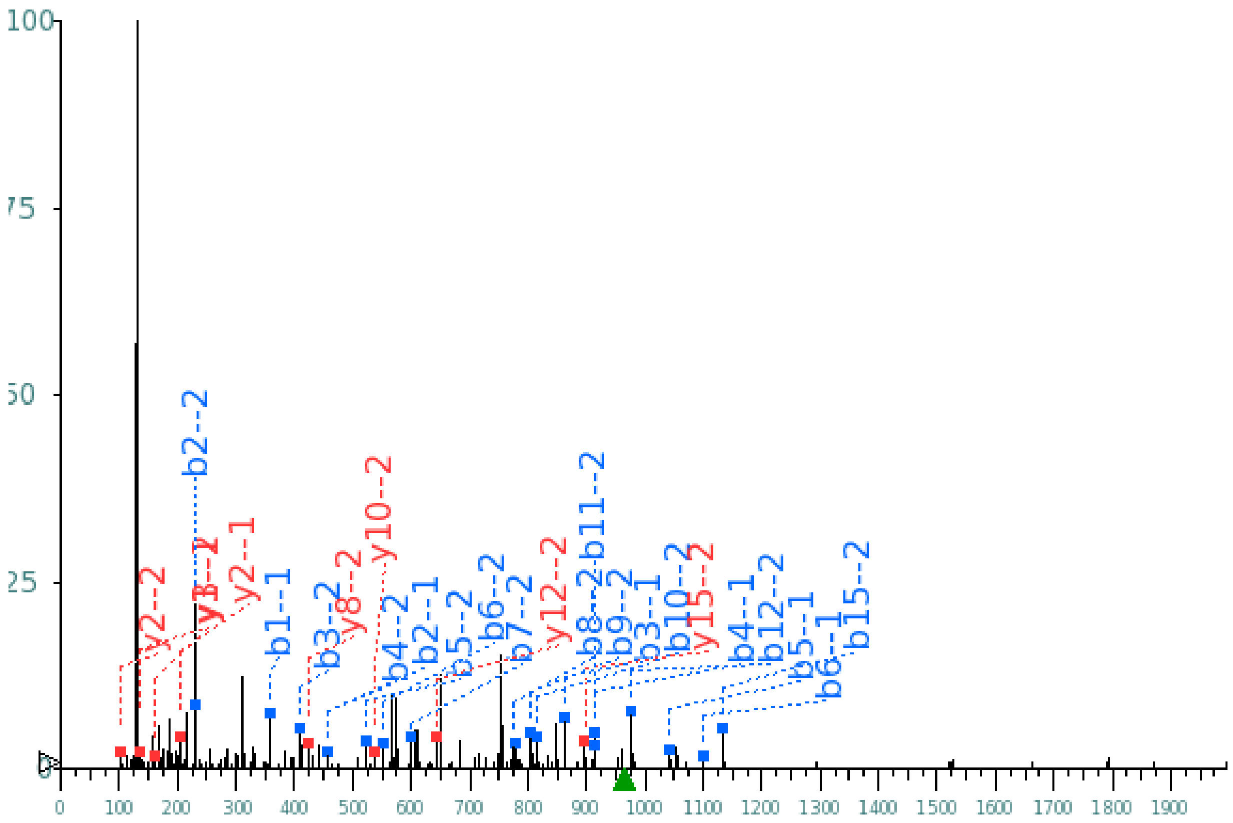

QVKVQGPKGLVEPVDVVDNAD (+3)

Primary Reference: FLNA_HUMAN
Search ID: 41759
Search Name: 20130328_ananiav_ERstress_TMT_TM_timecourse_chymo_inj2_4MC_IAA
Scan#: 18756
Observed Mass: 965.2261 (1.2 ppm)
PSM Score: 13.03

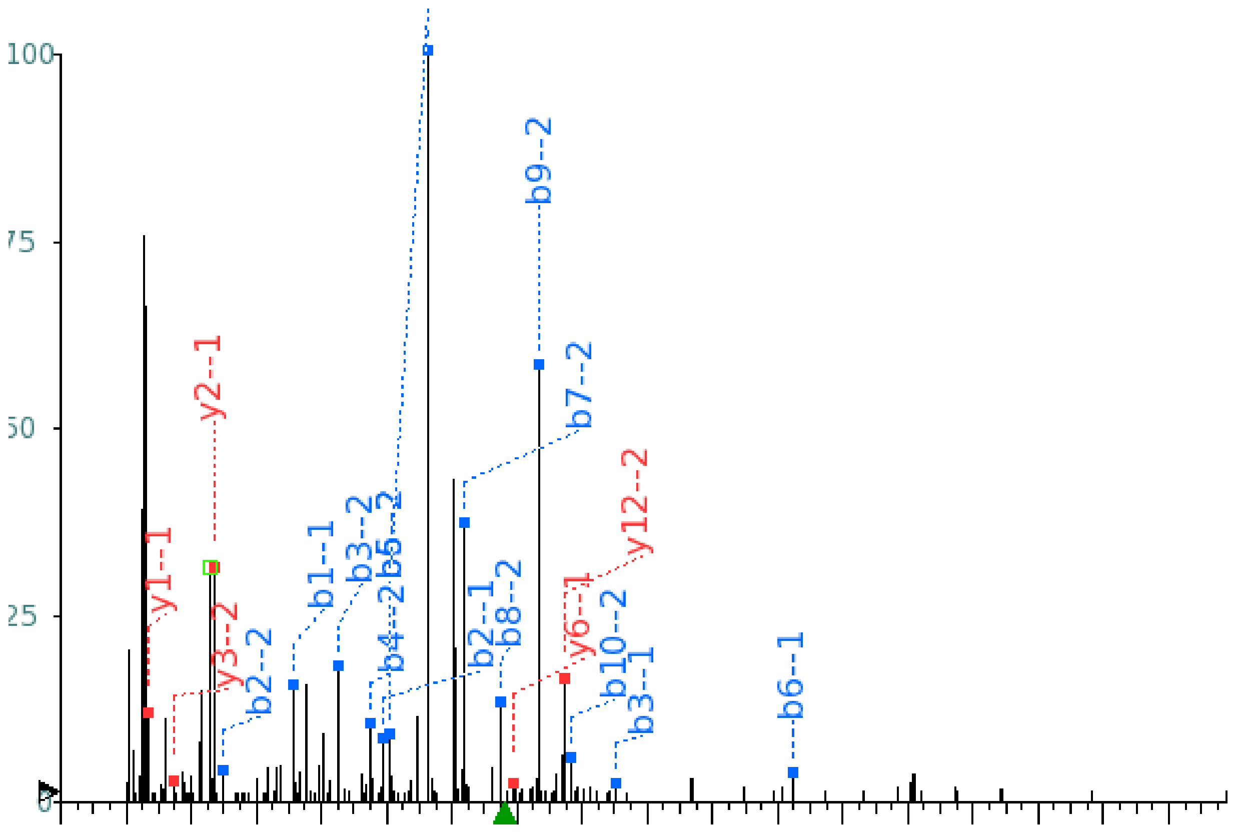

QHKVGNIIDTMITD (+3)

Primary Reference: SAMH1_HUMAN
Search ID: 41759
Search Name: 20130328_ananiav_ERstress_TMT_TM_timecourse_chymo_inj2_4MC_IAA
Scan#: 18952
Observed Mass: 681.7155 (0.5 ppm)
PSM Score: 23.98

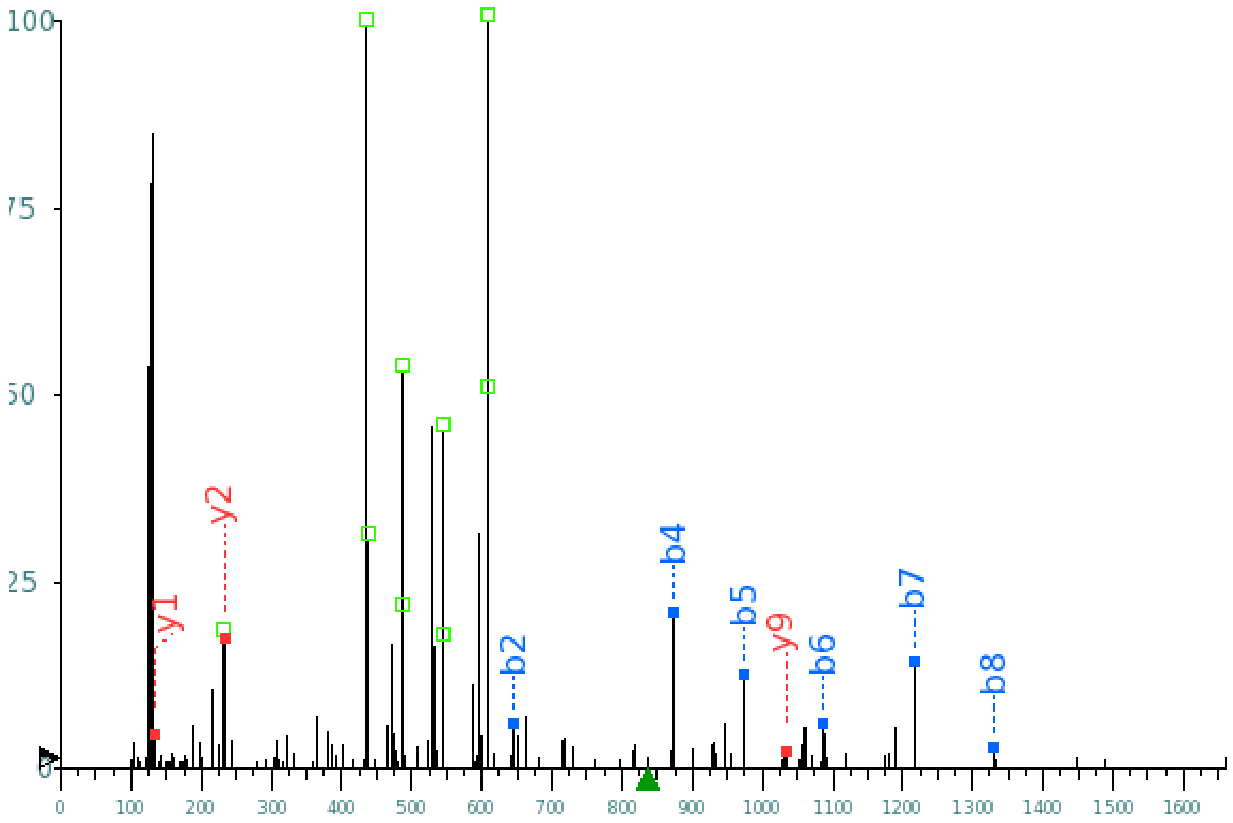

GKIDTIEIITD (+2)

Primary Reference: ROA2_HUMAN
Search ID: 41759
Search Name: 20130328_ananiav_ERstress_TMT_TM_timecourse_chymo_inj2_4MC_IAA
Scan#: 19272
Observed Mass: 838.4991 (1.6 ppm)
PSM Score: 22.27

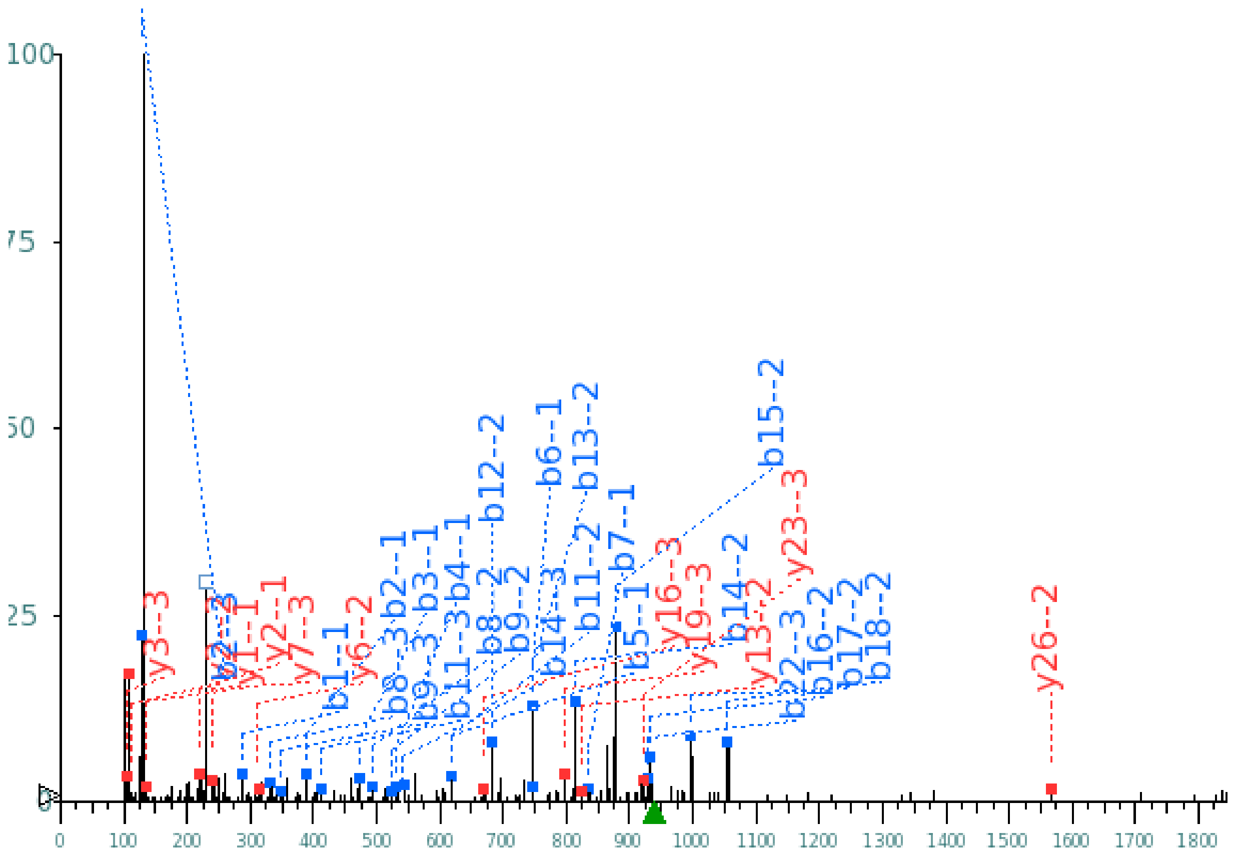

GVSGSQSRGSTMEEELENITTKHIVSNDSSD (+4)

Primary Reference: PA24A_HUMAN
Search ID: 41759
Search Name: 20130328_ananiav_ERstress_TMT_TM_timecourse_chymo_inj2_4MC_IAA
Scan#: 19376
Observed Mass: 938.9646 (2.1 ppm)
PSM Score: 27.34

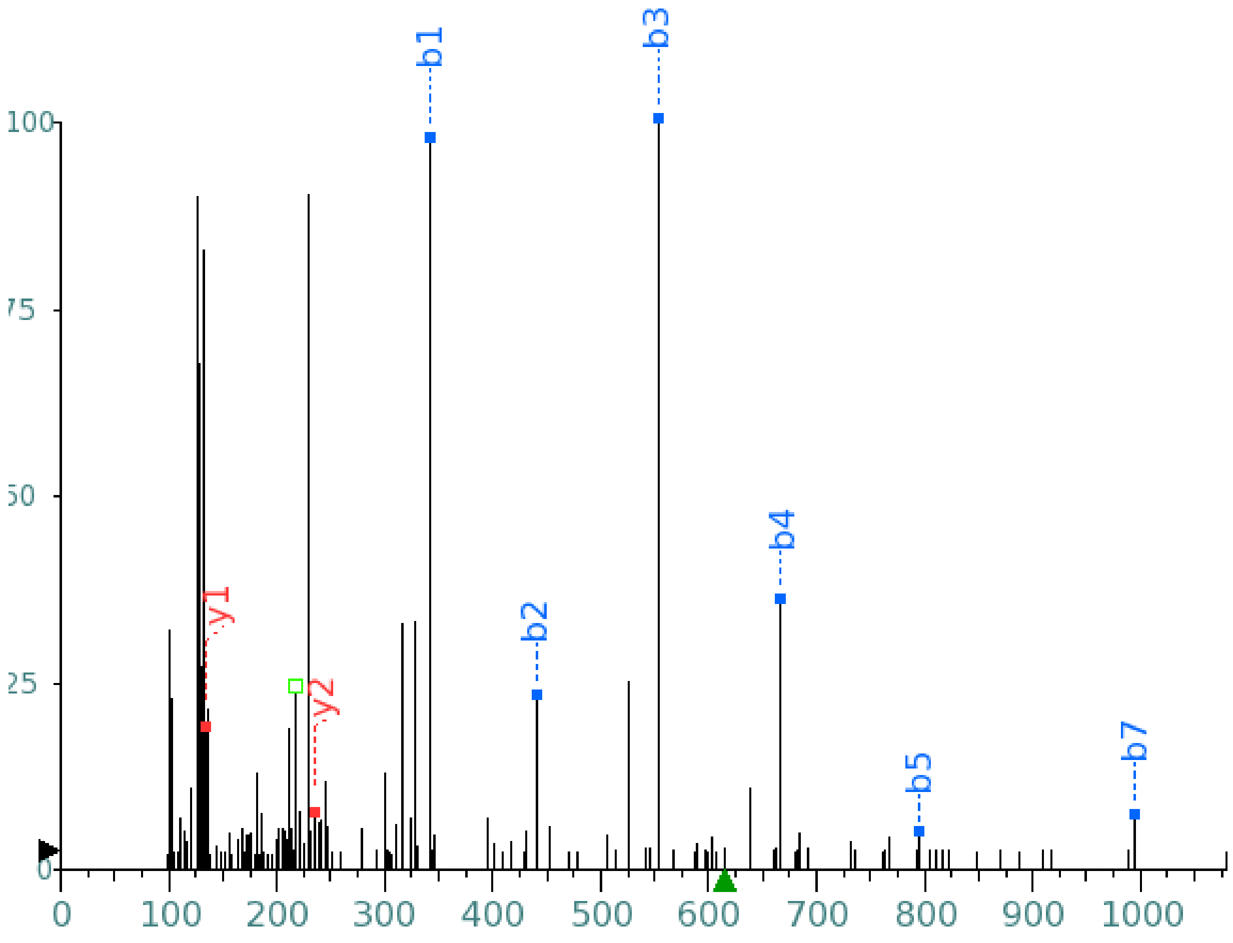

LPLLQAETD (+2)

Primary Reference: NDUAD_HUMAN
Search ID: 41759
Search Name: 20130328_ananiav_ERstress_TMT_TM_timecourse_chymo_inj2_4MC_IAA
Scan#: 19503
Observed Mass: 614.8546 (2.7 ppm)
PSM Score: 38.83

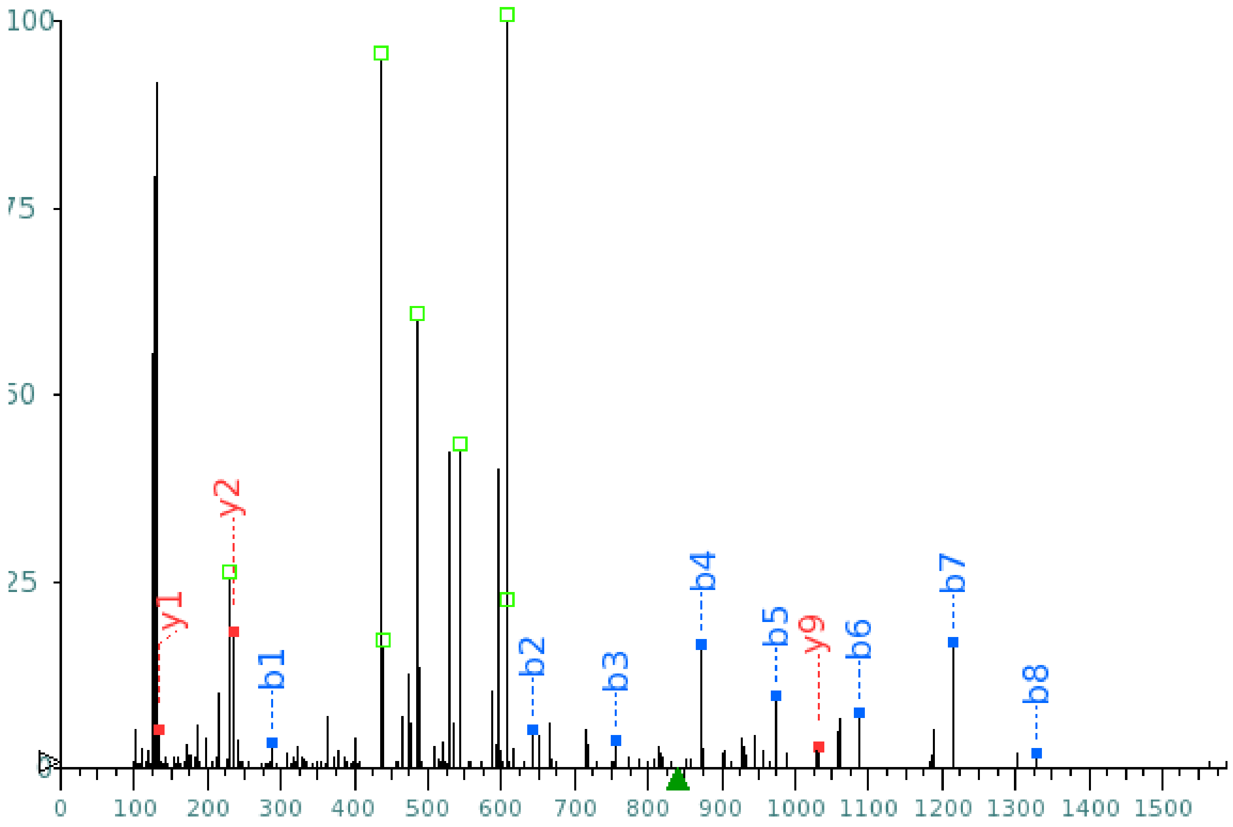

GKIDTIEIITD (+2)

Primary Reference: ROA2_HUMAN
Search ID: 41759
Search Name: 20130328_ananiav_ERstress_TMT_TM_timecourse_chymo_inj2_4MC_IAA
Scan#: 19593
Observed Mass: 838.5011 (3.9 ppm)
PSM Score: 23.55

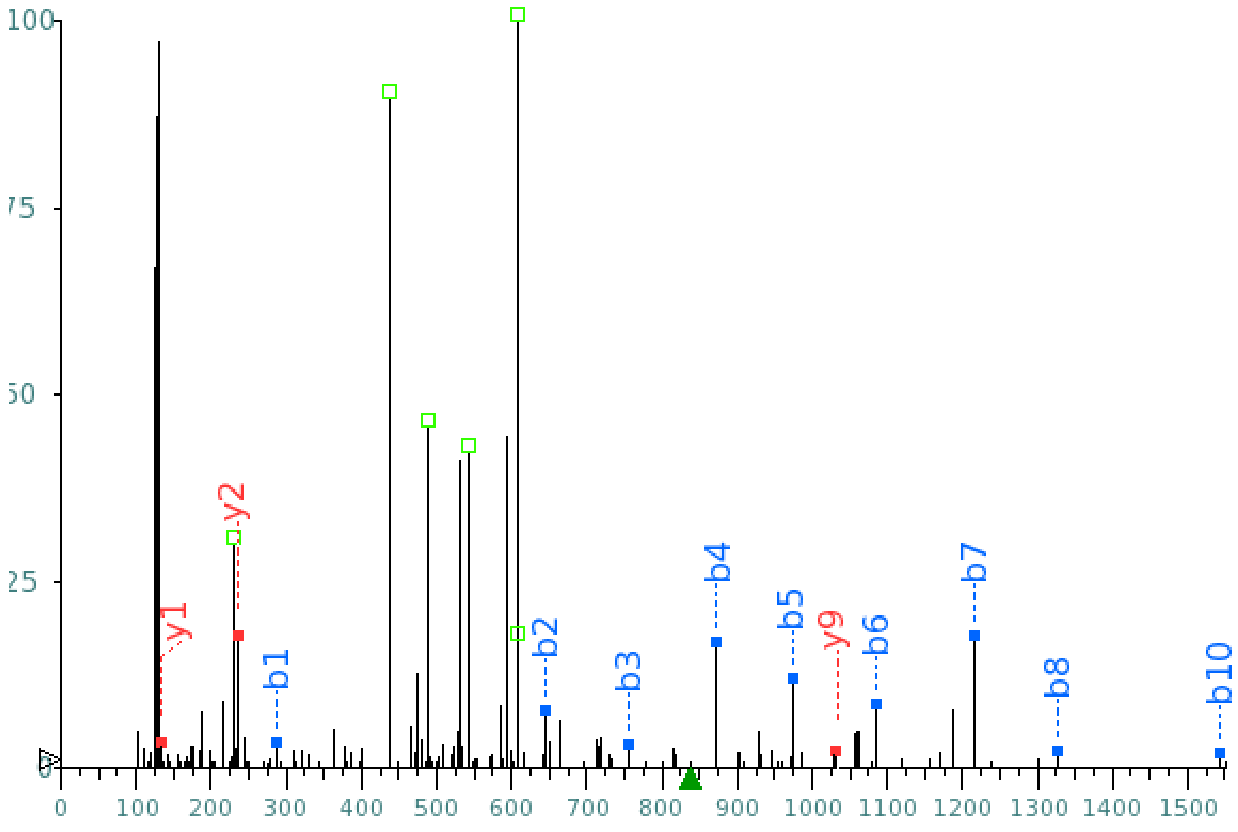

GKIDTIEIITD (+2)

Primary Reference: ROA2_HUMAN
Search ID: 41759
Search Name: 20130328_ananiav_ERstress_TMT_TM_timecourse_chymo_inj2_4MC_IAA
Scan#: 19771
Observed Mass: 838.5003 (3.1 ppm)
PSM Score: 31.31

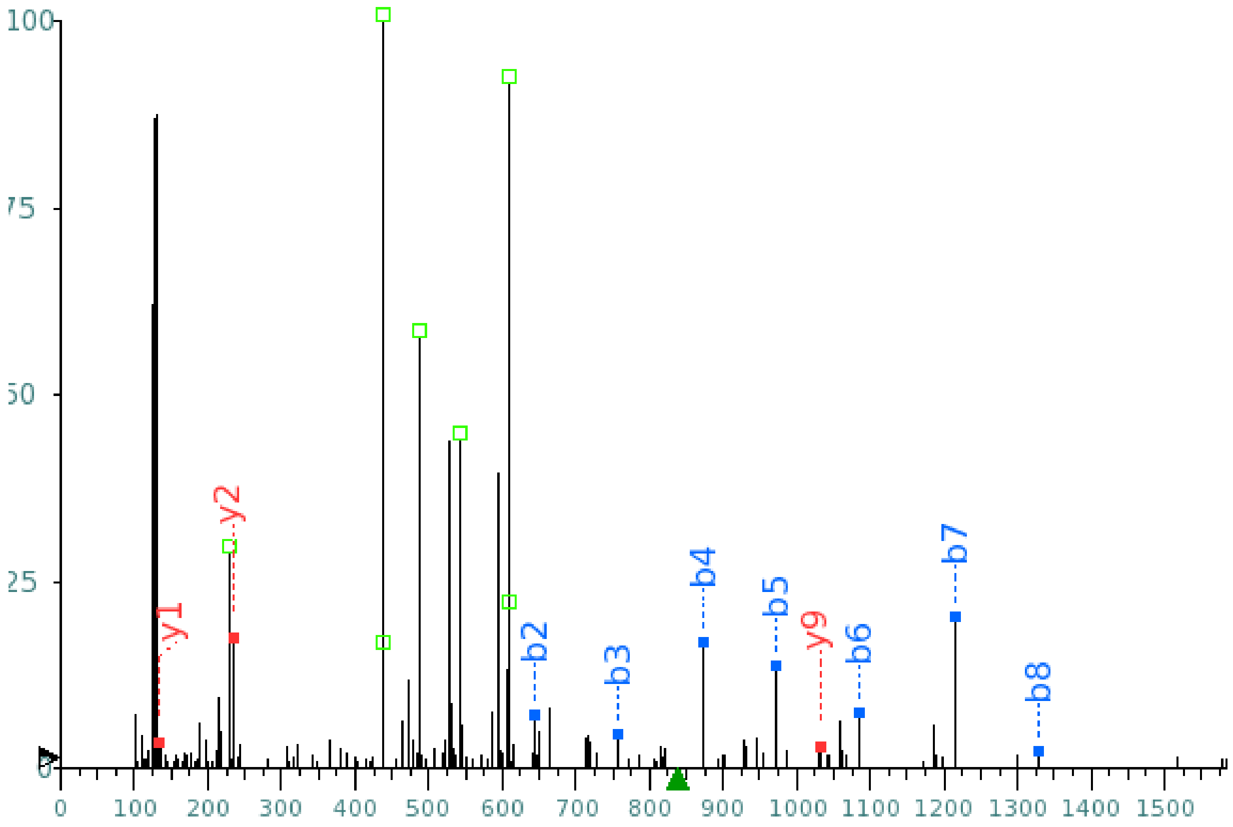

GKIDTIEIITD (+2)

Primary Reference: ROA2_HUMAN
Search ID: 41759
Search Name: 20130328_ananiav_ERstress_TMT_TM_timecourse_chymo_inj2_4MC_IAA
Scan#: 19868
Observed Mass: 838.4996 (2.2 ppm)
PSM Score: 23.35

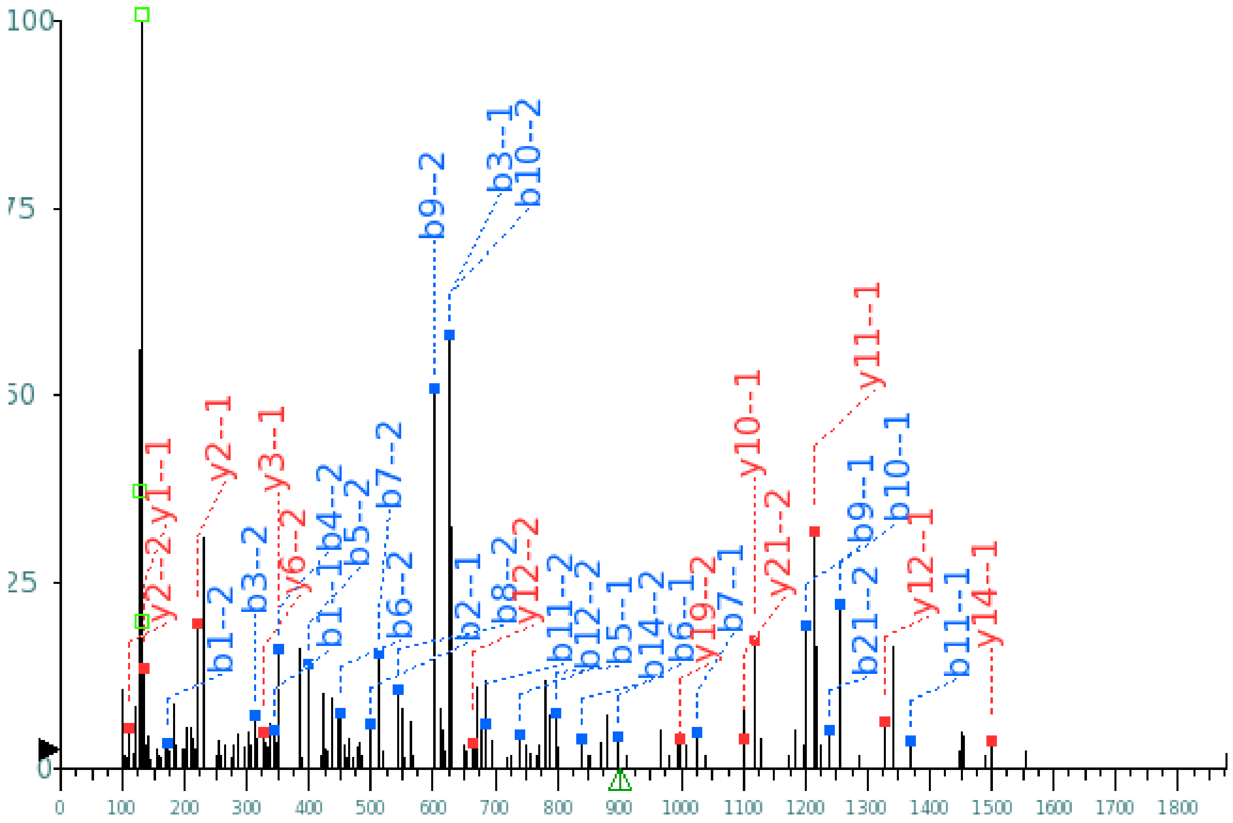

LREAVVEGDGILPPLRTEATESD (+3)

Primary Reference: ZGPAT_HUMAN
Search ID: 41759
Search Name: 20130328_ananiav_ERstress_TMT_TM_timecourse_chymo_inj2_4MC_IAA
Scan#: 19870
Observed Mass: 899.4843 (2.9 ppm)
PSM Score: 43.89

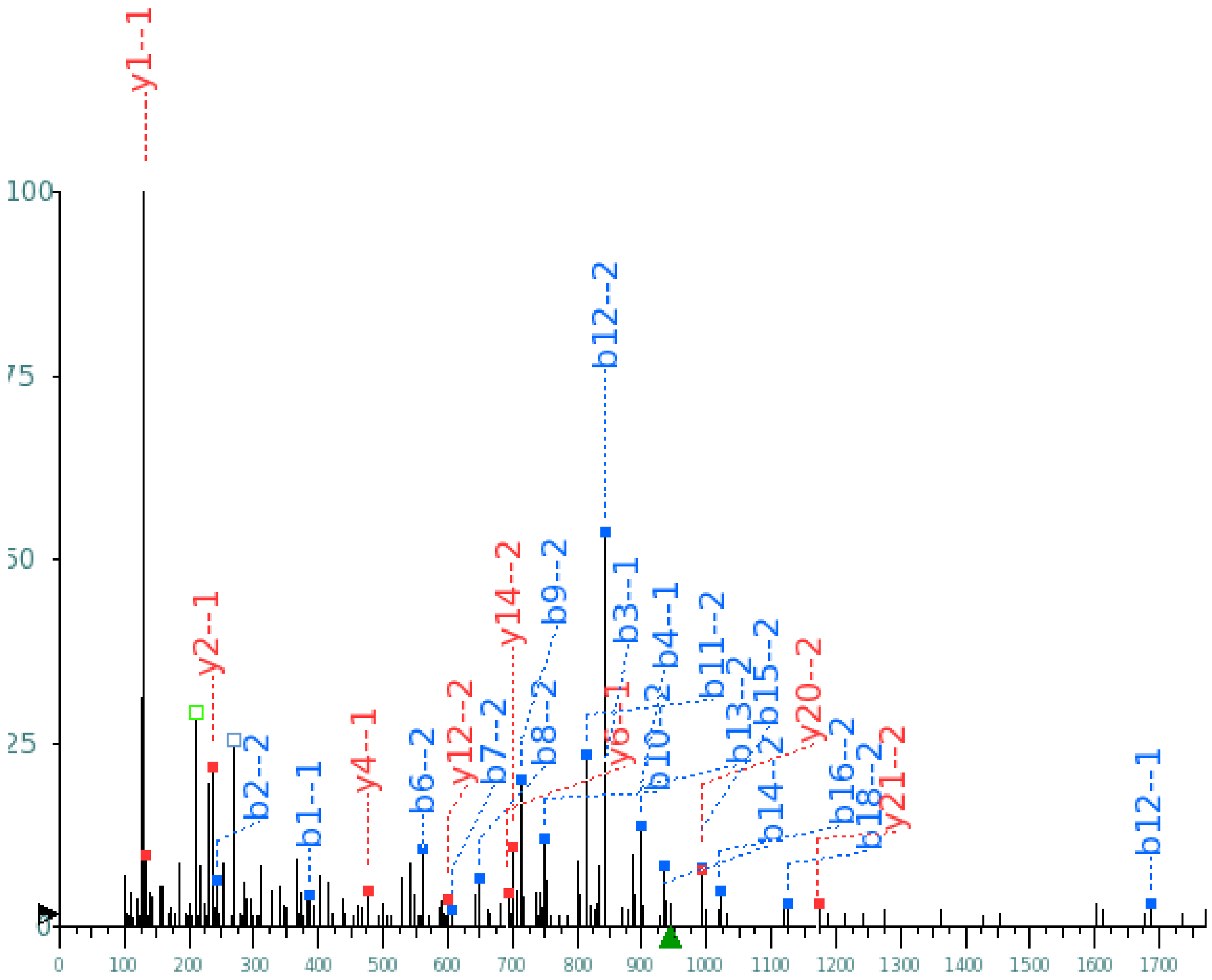

RVKGILSSQAEGLANGPDVLETD (+3)

Primary Reference: EHMT1_HUMAN
Search ID: 41759
Search Name: 20130328_ananiav_ERstress_TMT_TM_timecourse_chymo_inj2_4MC_IAA
Scan#: 19915
Observed Mass: 943.1925 (2.2 ppm)
PSM Score: 42.81

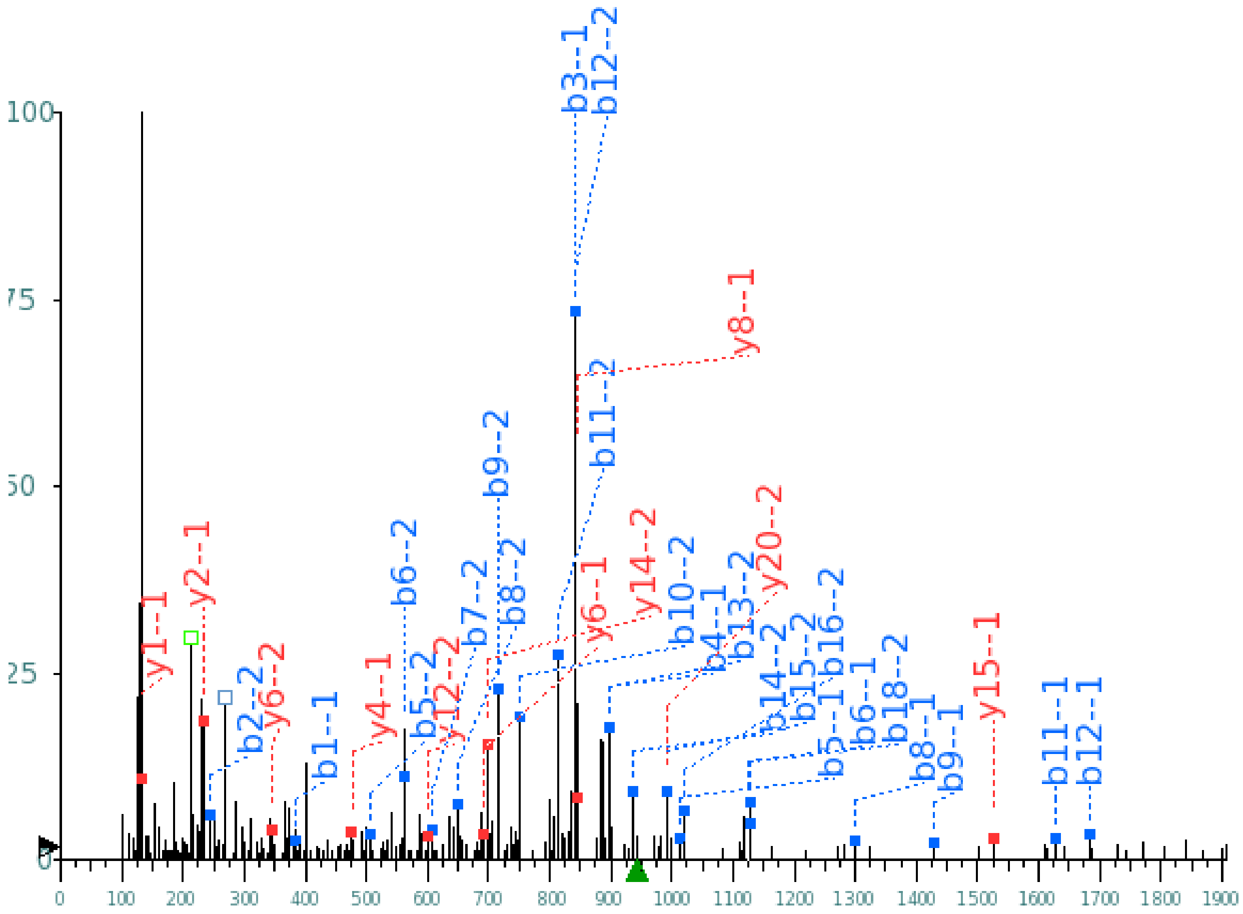

RVKGILSSQAEGLANGPDVLETD (+3)

Primary Reference: EHMT1_HUMAN
Search ID: 41759
Search Name: 20130328_ananiav_ERstress_TMT_TM_timecourse_chymo_inj2_4MC_IAA
Scan#: 19928
Observed Mass: 943.1925 (2.3 ppm)
PSM Score: 50.62

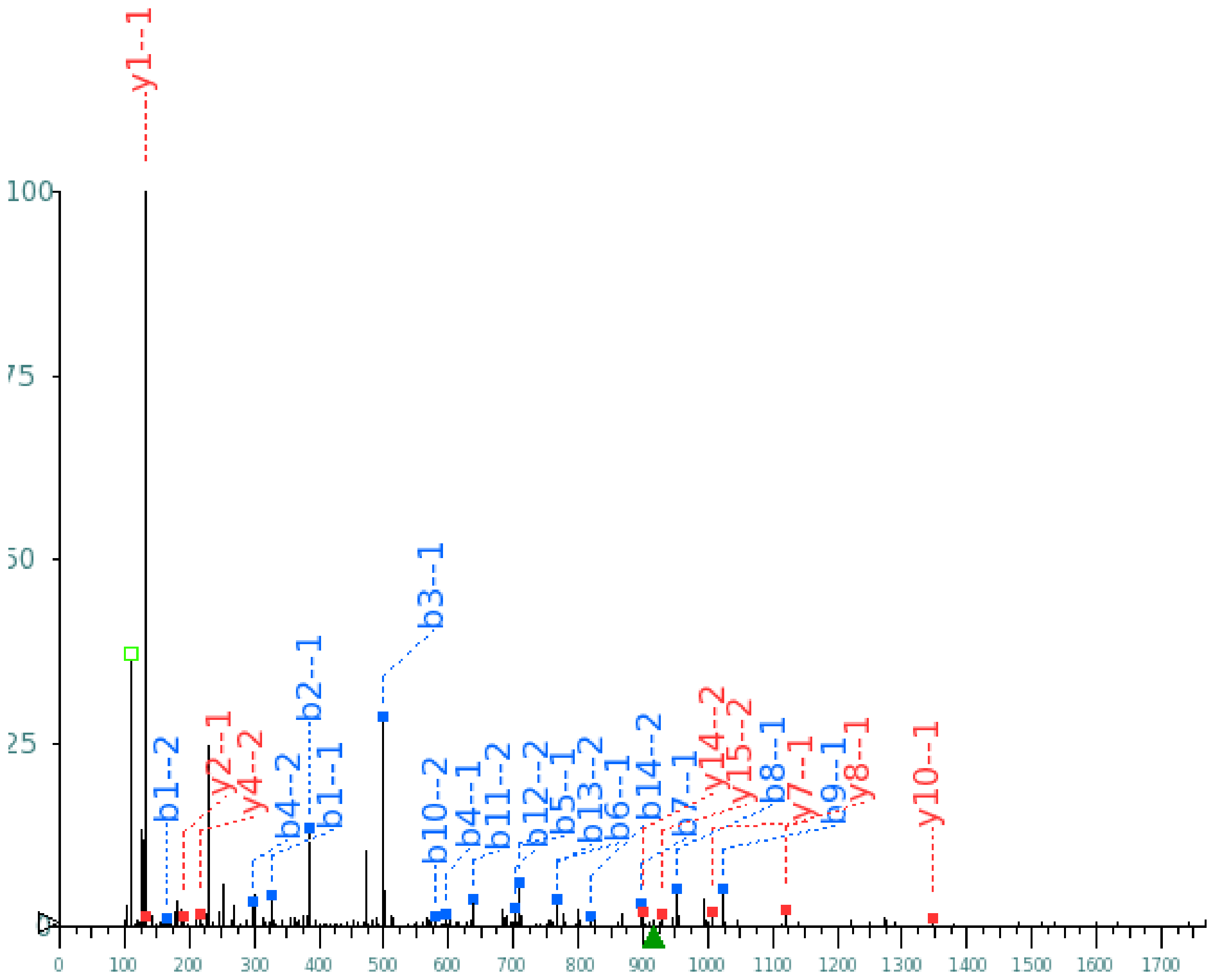

VGIPIGEGAHDEEVLKTIDEGD (+3)

Primary Reference: KDM3B_HUMAN
Search ID: 41759
Search Name: 20130328_ananiav_ERstress_TMT_TM_timecourse_chymo_inj2_4MC_IAA
Scan#: 20298
Observed Mass: 917.8216 (1.8 ppm)
PSM Score: 24.17

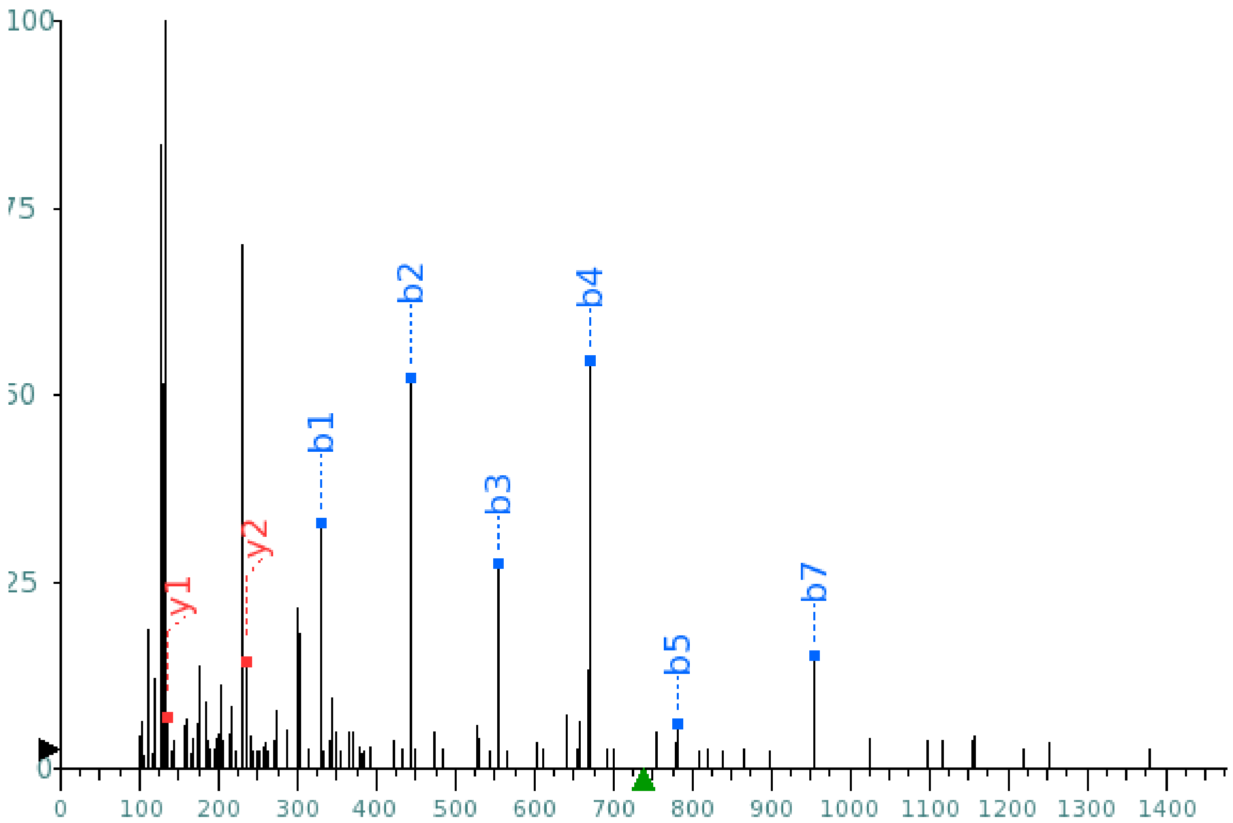

VLLNLGDSVTTD (+2)

Primary Reference: ACOC_HUMAN
Search ID: 41759
Search Name: 20130328_ananiav_ERstress_TMT_TM_timecourse_chymo_inj2_4MC_IAA
Scan#: 20503
Observed Mass: 738.4126 (1.7 ppm)
PSM Score: 32.29

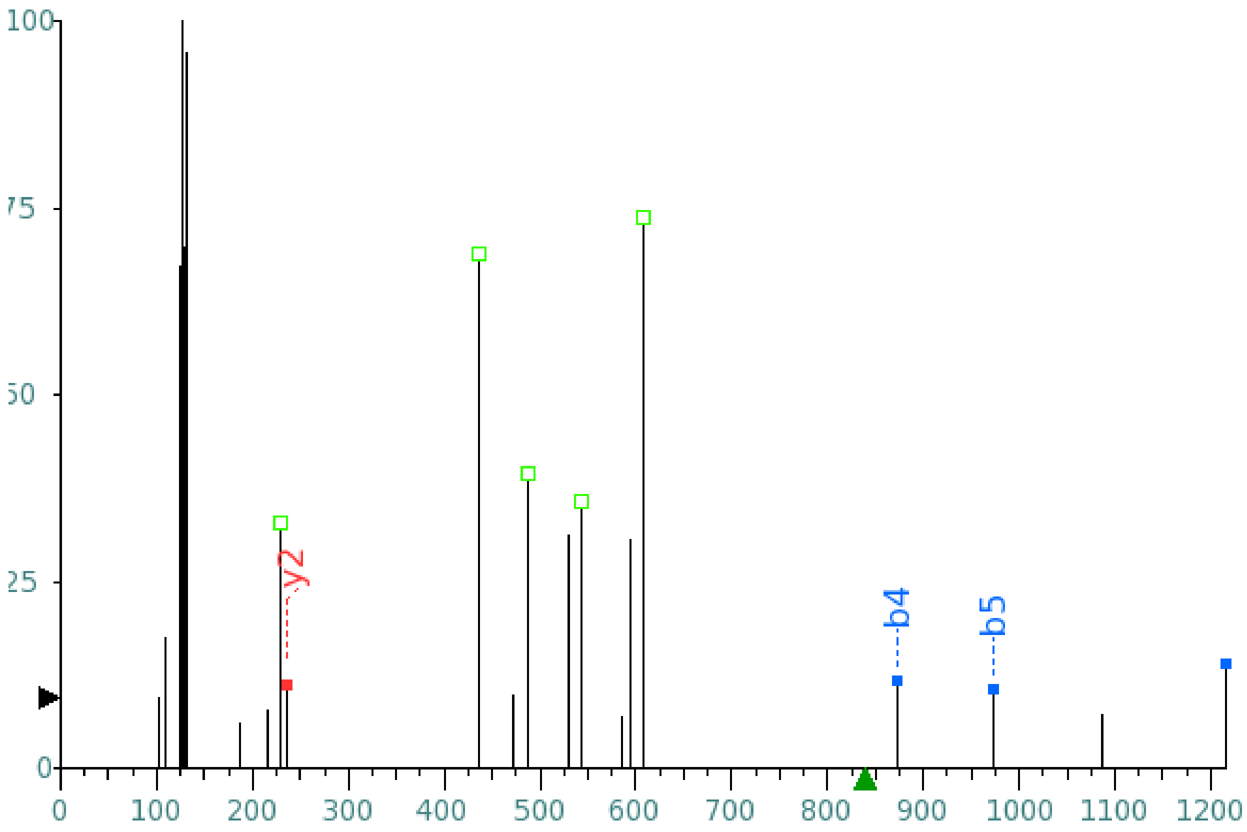

GKIDTIEIITD (+2)

Primary Reference: ROA2_HUMAN
Search ID: 41759
Search Name: 20130328_ananiav_ERstress_TMT_TM_timecourse_chymo_inj2_4MC_IAA
Scan#: 20554
Observed Mass: 838.5014 (4.4 ppm)
PSM Score: 23.19

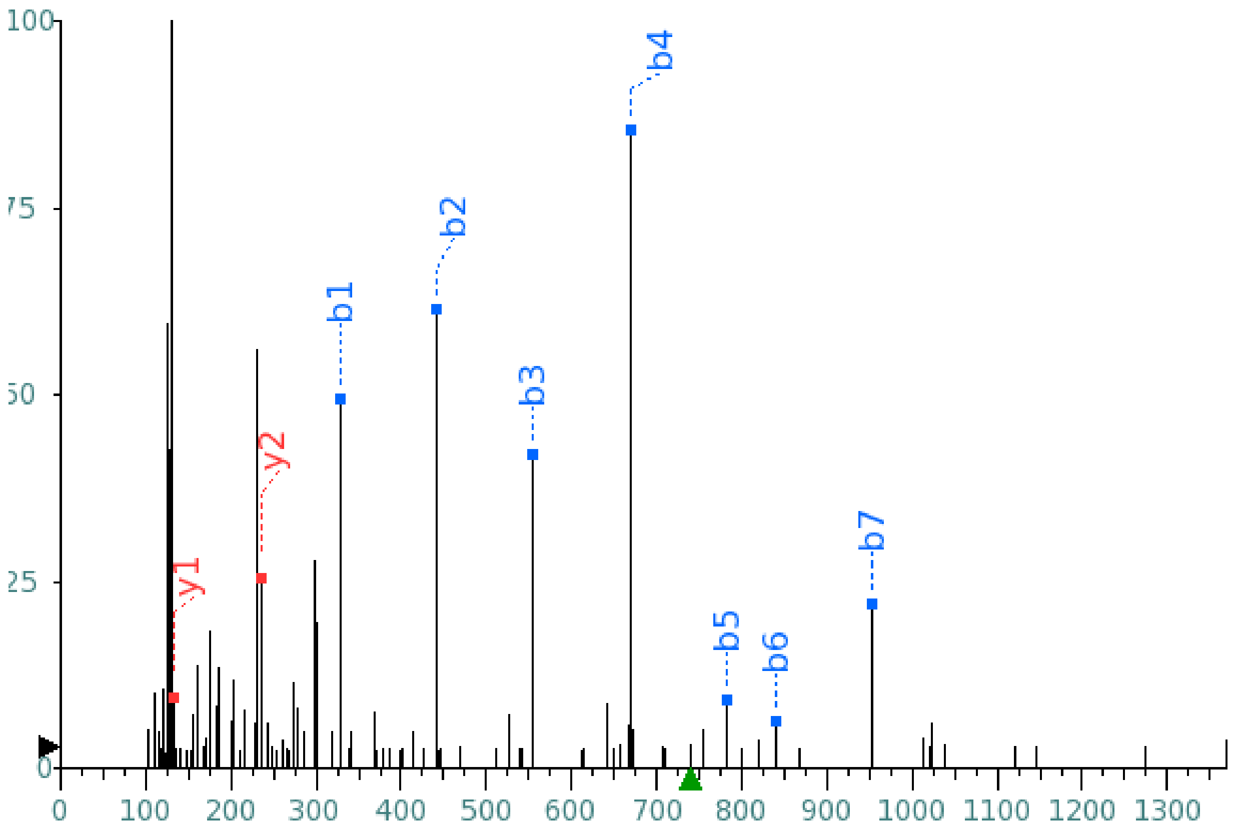

VLLNLGDSVTTD (+2)

Primary Reference: ACOC_HUMAN
Search ID: 41759
Search Name: 20130328_ananiav_ERstress_TMT_TM_timecourse_chymo_inj2_4MC_IAA
Scan#: 20580
Observed Mass: 738.4129 (2 ppm)
PSM Score: 46.93

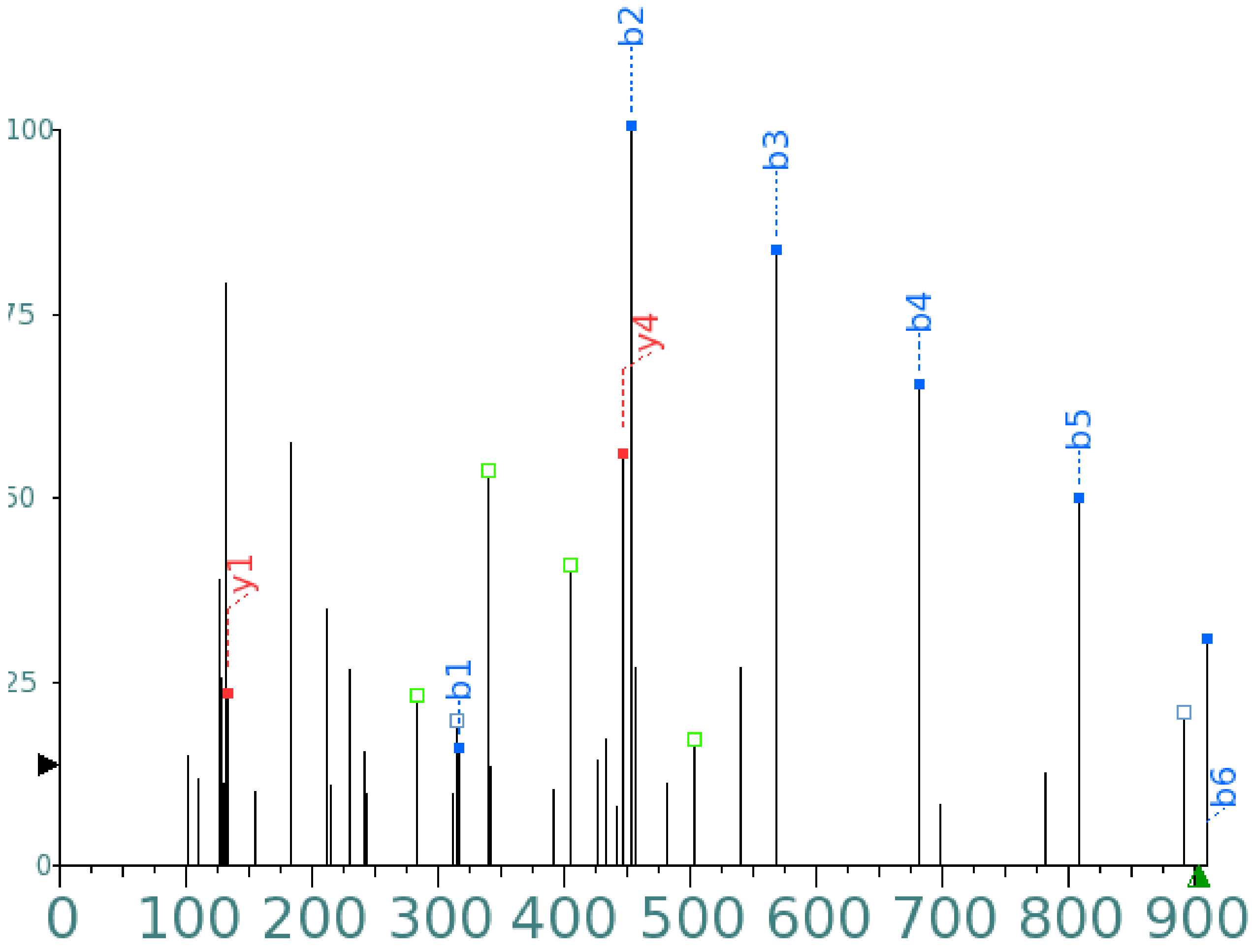

SHILQTPIEIIQAD (+2)

Primary Reference: OTU6B_HUMAN
Search ID: 41759
Search Name: 20130328_ananiav_ERstress_TMT_TM_timecourse_chymo_inj2_4MC_IAA
Scan#: 20862
Observed Mass: 904.0138 (2.2 ppm)
PSM Score: 31.27

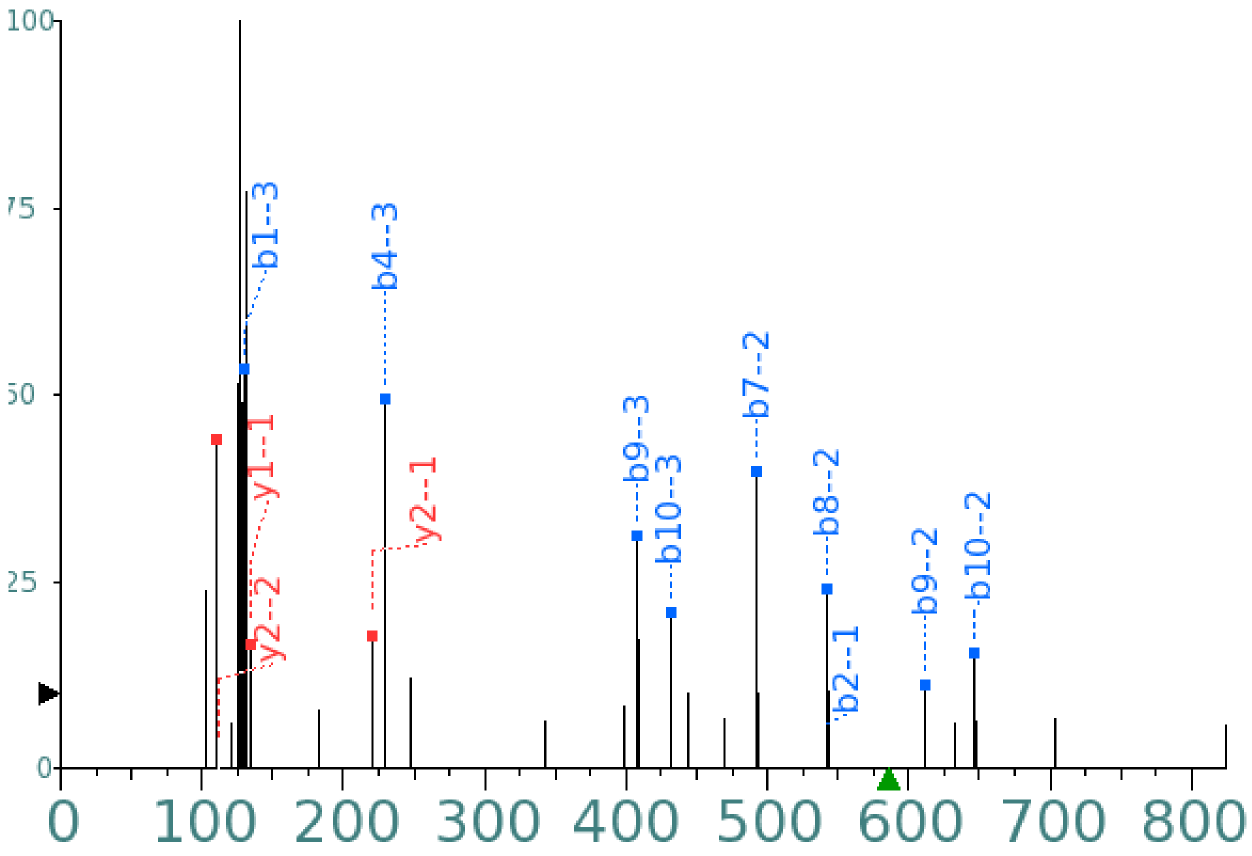

RRGSSPDVHALLEITEESD (+4)

Primary Reference: PTRF_HUMAN
Search ID: 41759
Search Name: 20130328_ananiav_ERstress_TMT_TM_timecourse_chymo_inj2_4MC_IAA
Scan#: 20961
Observed Mass: 585.8055 (0.4 ppm)
PSM Score: 35.22

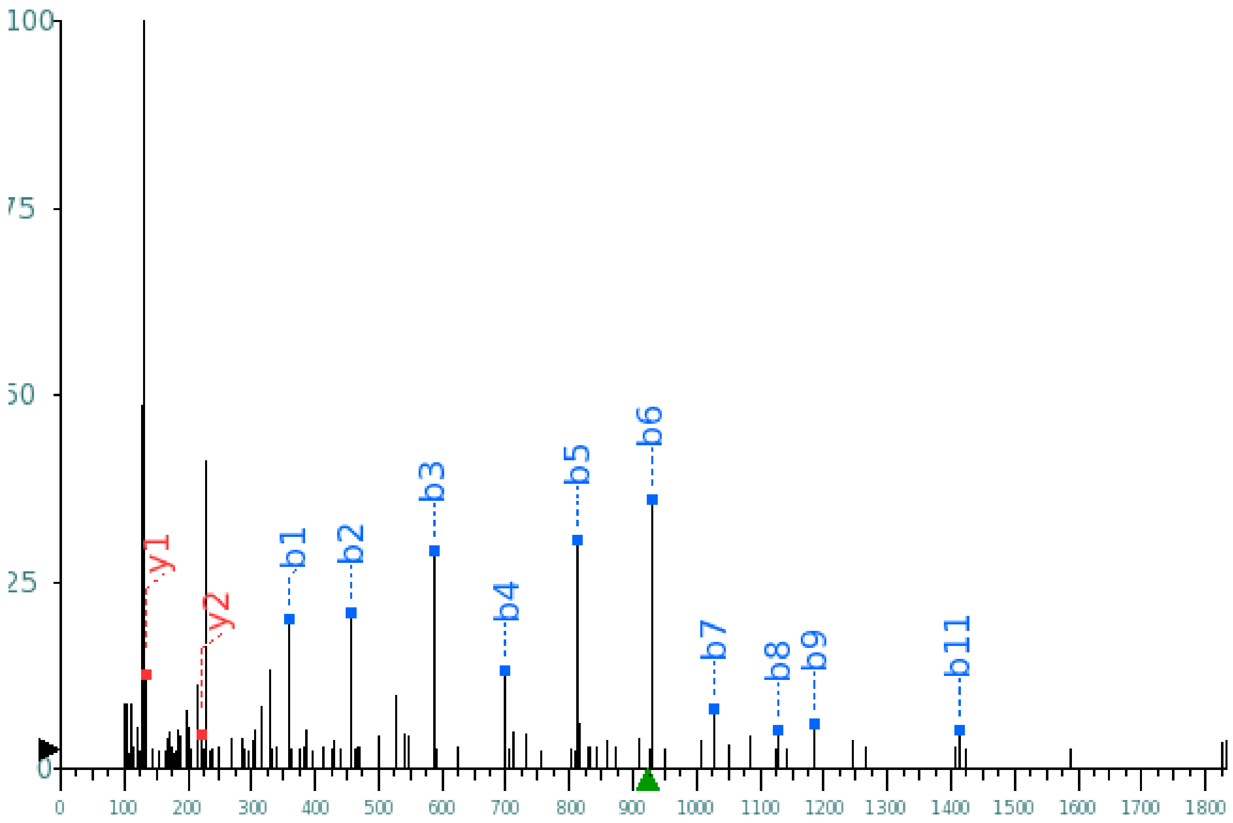

QVELDNVTGLLSQSD (+2)

Primary Reference: MYH9_HUMAN
Search ID: 41759
Search Name: 20130328_ananiav_ERstress_TMT_TM_timecourse_chymo_inj2_4MC_IAA
Scan#: 21198
Observed Mass: 923.9854 (2.1 ppm)
PSM Score: 47.35

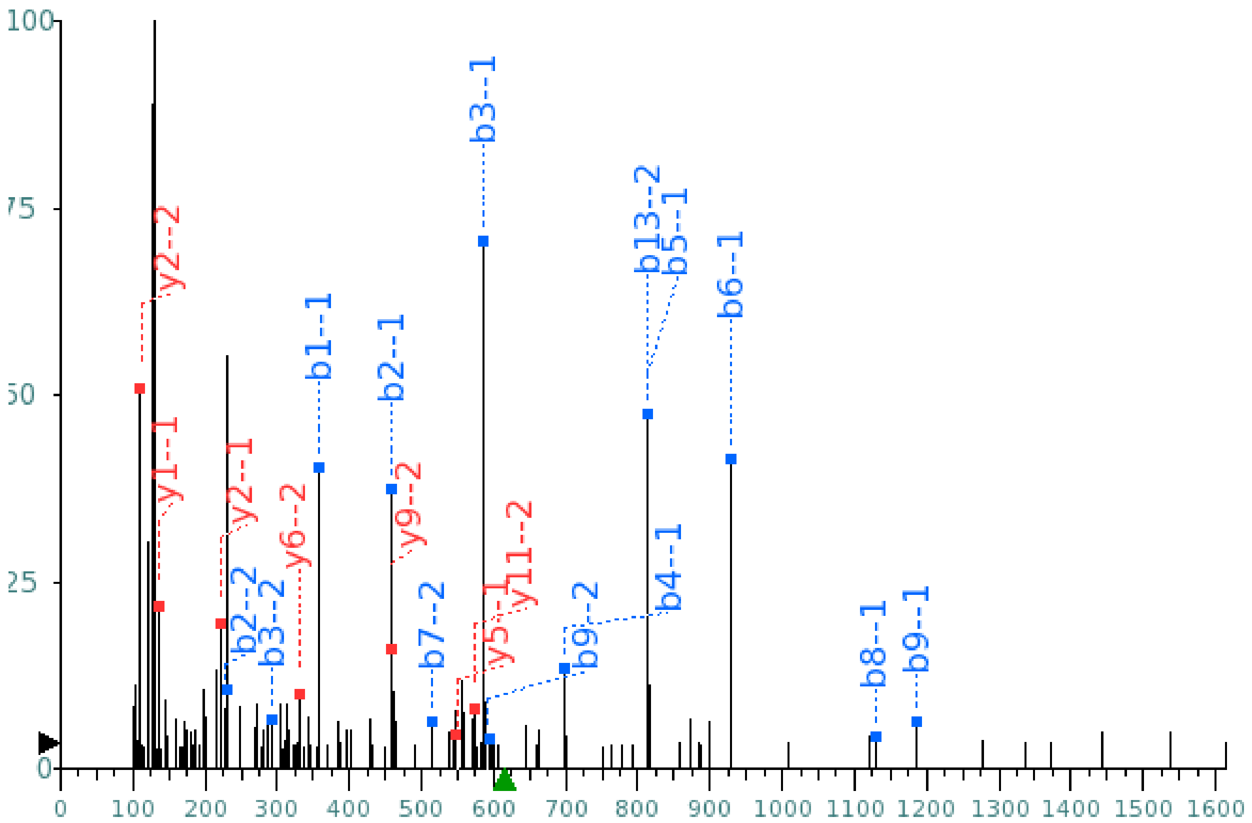

QVELDNVTGLLSQSD (+3)

Primary Reference: MYH9_HUMAN
Search ID: 41759
Search Name: 20130328_ananiav_ERstress_TMT_TM_timecourse_chymo_inj2_4MC_IAA
Scan#: 21207
Observed Mass: 616.3258 (1.8 ppm)
PSM Score: 32.76

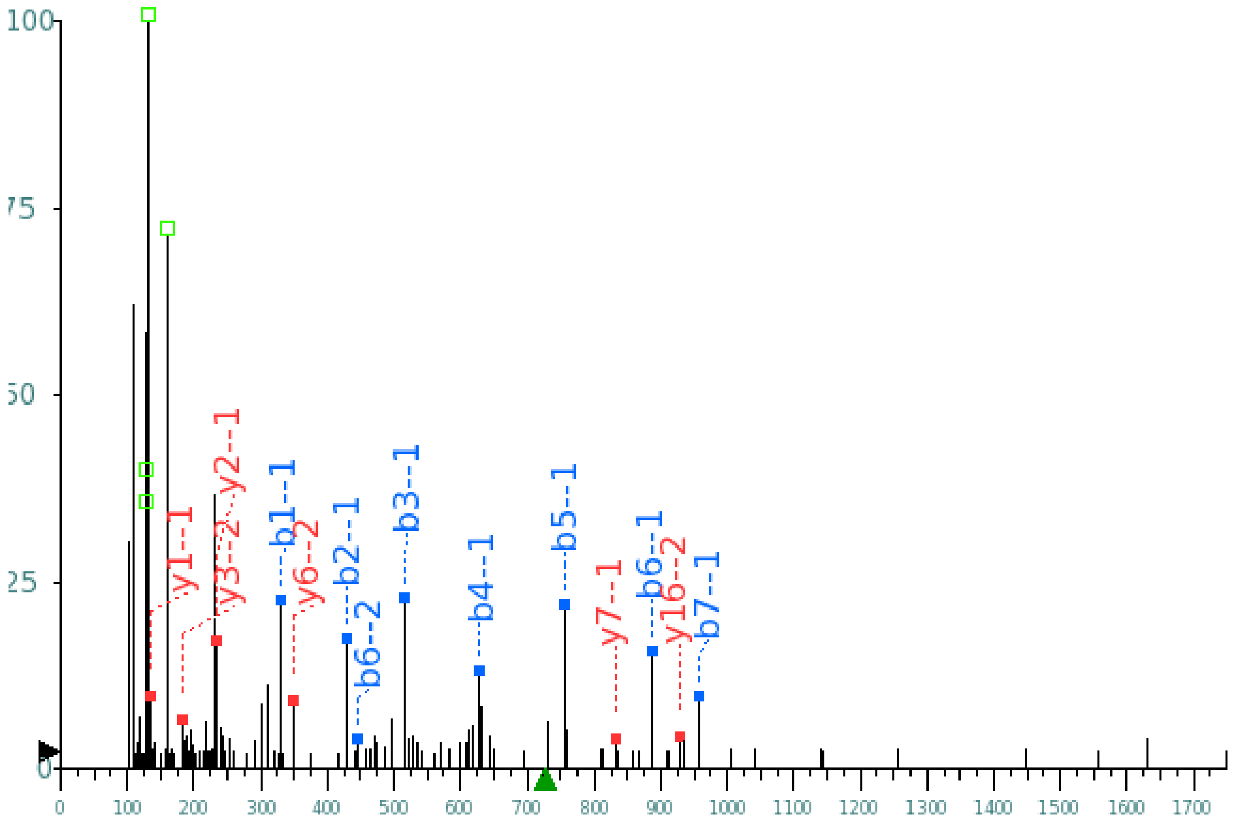

VVSLEEAWPLHGDC*ETD (+3)

Primary Reference: MDC1_HUMAN
Search ID: 41759
Search Name: 20130328_ananiav_ERstress_TMT_TM_timecourse_chymo_inj2_4MC_IAA
Scan#: 21253
Observed Mass: 729.3475 (0.2 ppm)
PSM Score: 28.58

File name: 2015-02-04 04.01.53PM.pdf

Creation time: 2015-02-04 04:09PM

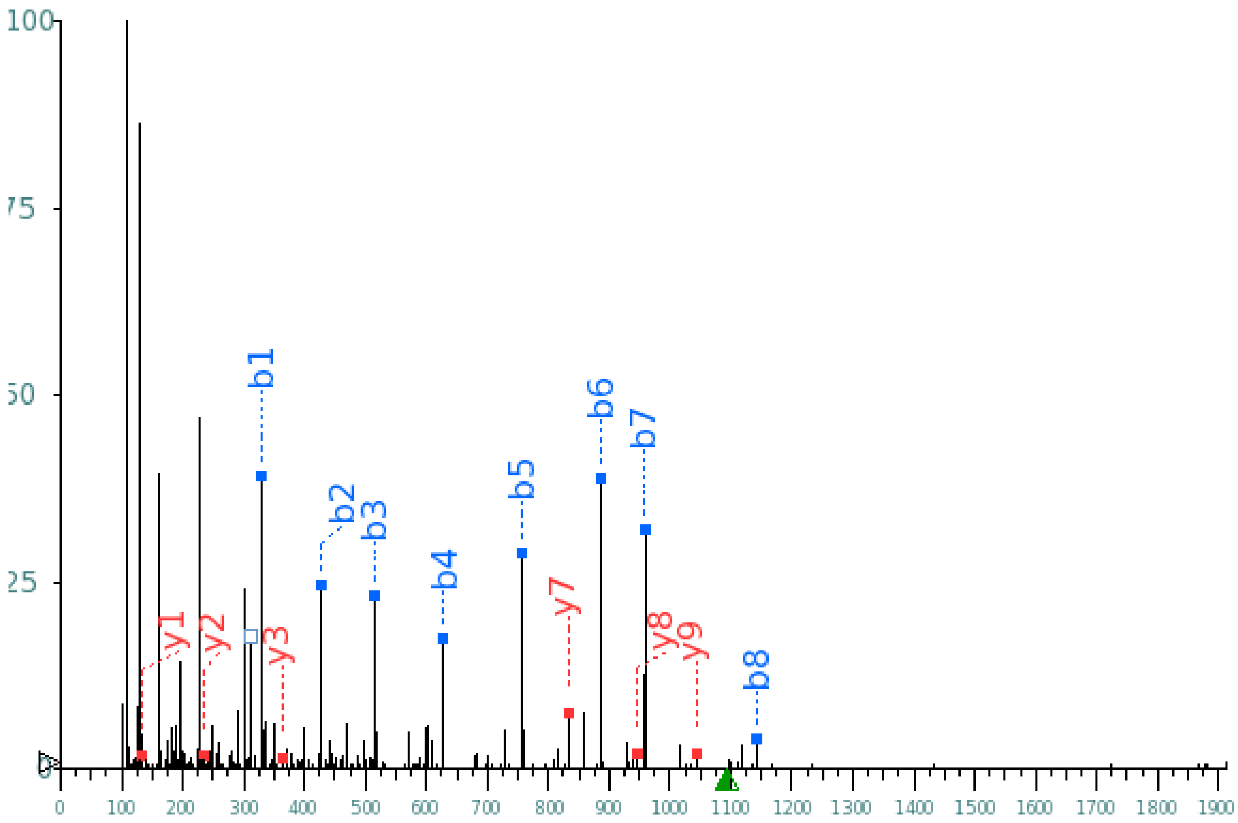

VVSLEEAWPLHGDC*ETD (+2)

Primary Reference: MDC1_HUMAN
Search ID: 41759
Search Name: 20130328_ananiav_ERstress_TMT_TM_timecourse_chymo_inj2_4MC_IAA
Scan#: 21271
Observed Mass: 1093.5161 (-1.1 ppm)
PSM Score: 42.67

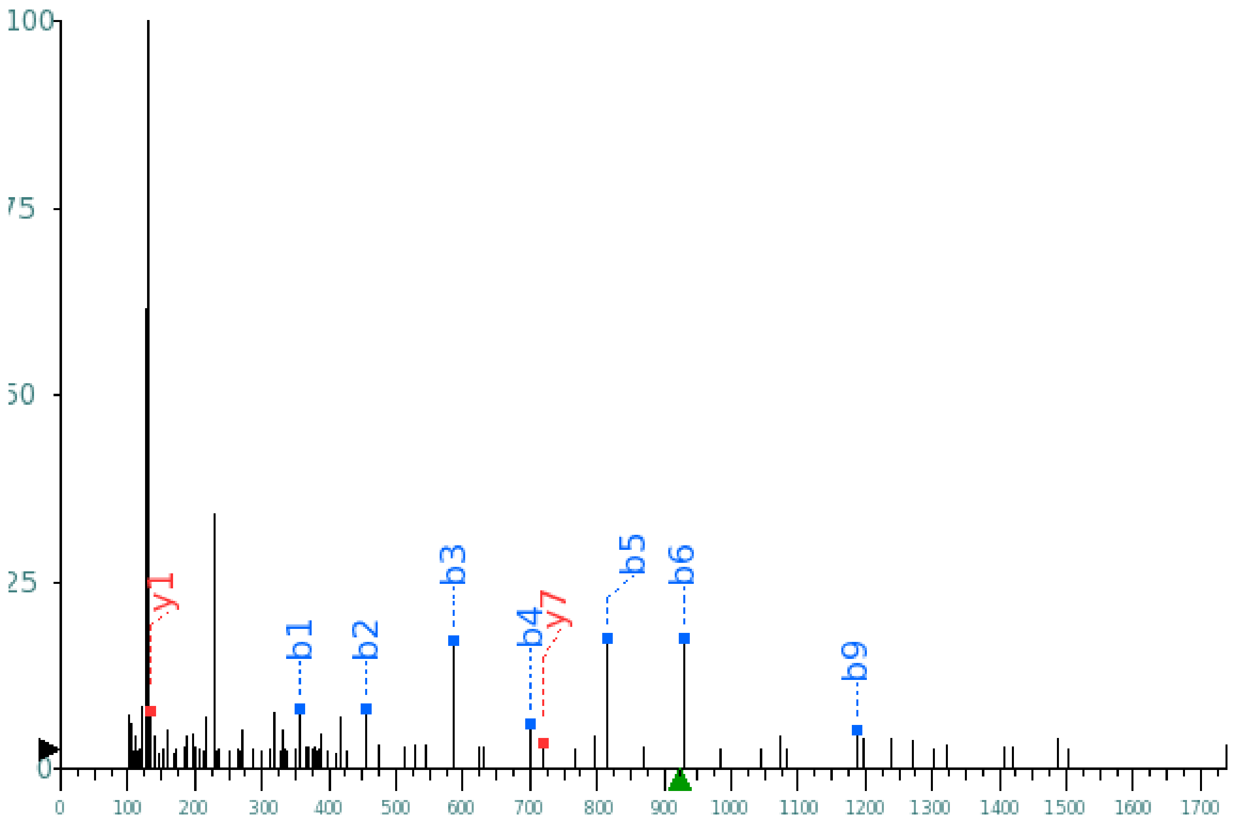

QVELDNVTGLLSQSD (+2)

Primary Reference: MYH9_HUMAN
Search ID: 41759
Search Name: 20130328_ananiav_ERstress_TMT_TM_timecourse_chymo_inj2_4MC_IAA
Scan#: 21274
Observed Mass: 923.9841 (0.8 ppm)
PSM Score: 33.93

File name: 2015-02-04 04.01.53PM.pdf

Creation time: 2015-02-04 04:09PM

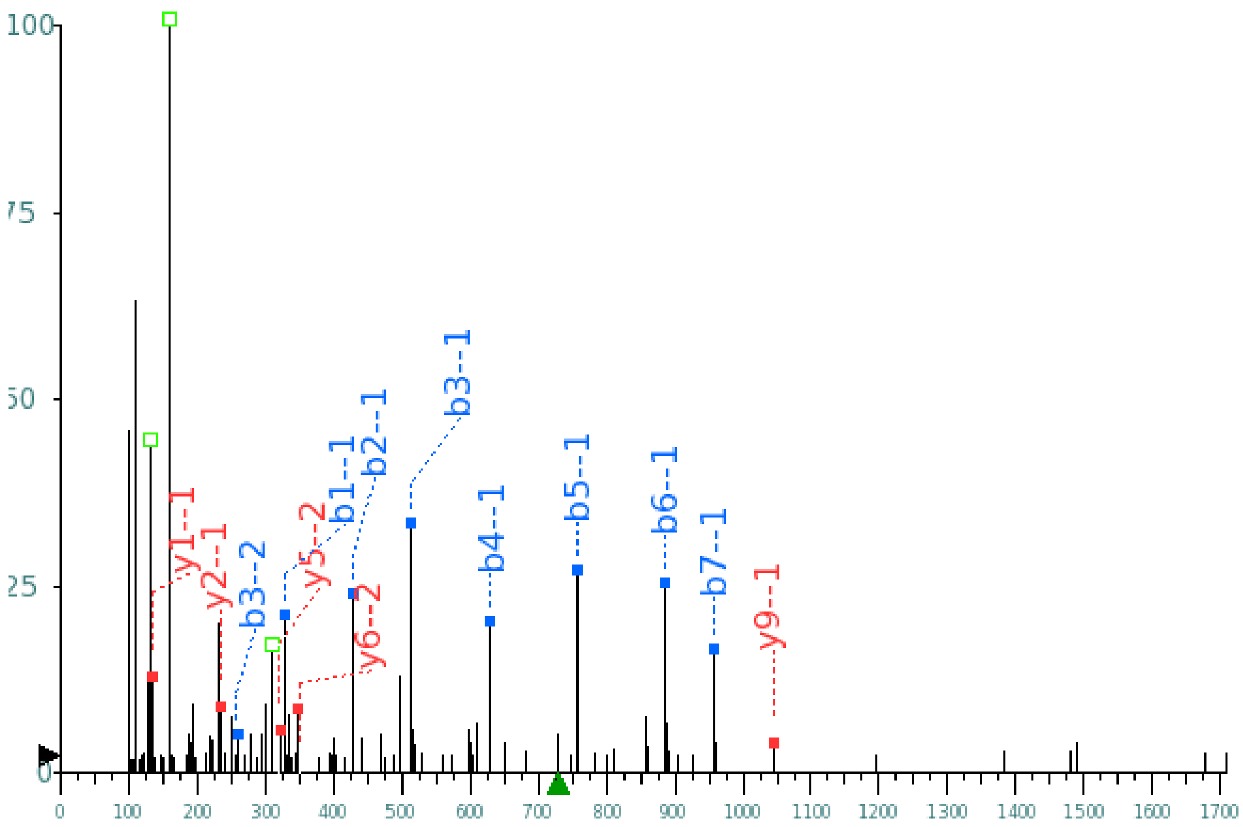

VVSLEEAWPLHGDC*ETD (+3)

Primary Reference: MDC1_HUMAN
Search ID: 41759
Search Name: 20130328_ananiav_ERstress_TMT_TM_timecourse_chymo_inj2_4MC_IAA
Scan#: 21339
Observed Mass: 729.3478 (0.8 ppm)
PSM Score: 32.78

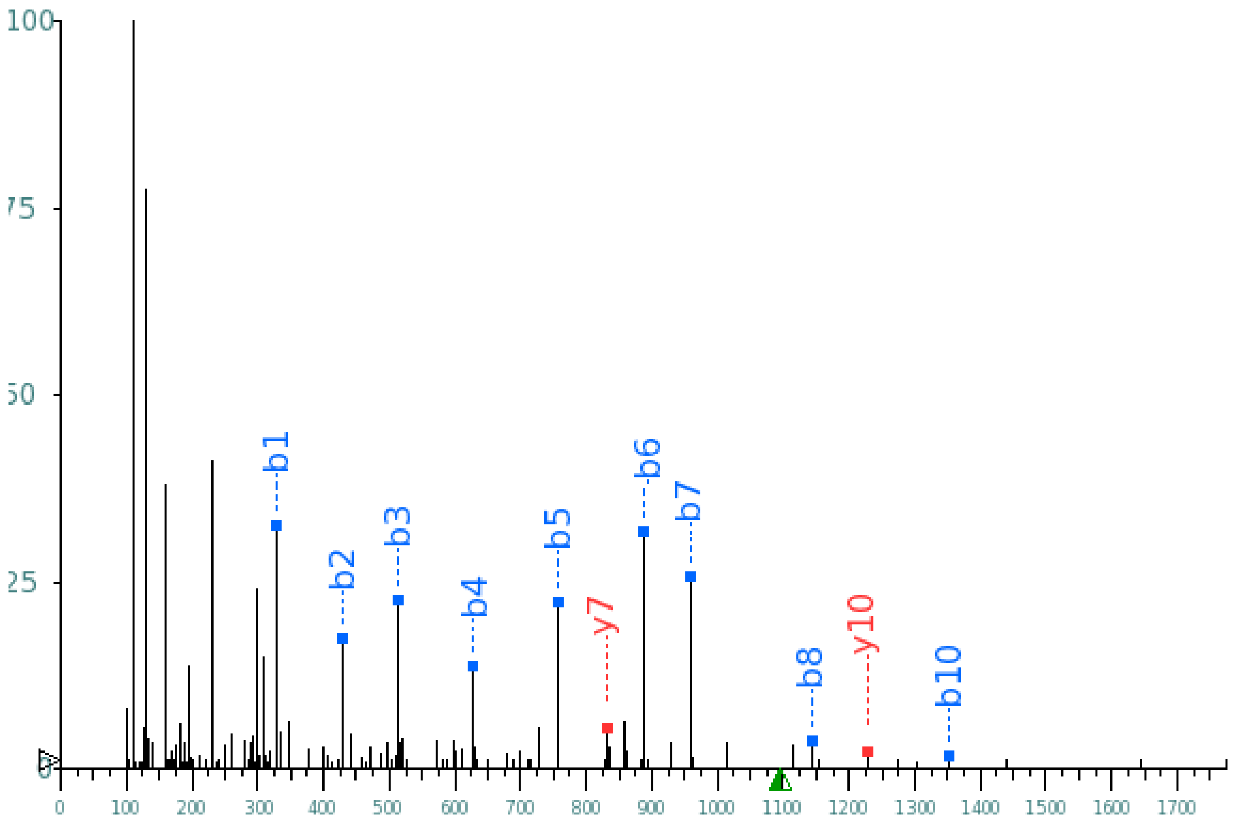

VVSLEEAWPLHGDC*ETD (+2)

Primary Reference: MDC1_HUMAN
Search ID: 41759
Search Name: 20130328_ananiav_ERstress_TMT_TM_timecourse_chymo_inj2_4MC_IAA
Scan#: 21341
Observed Mass: 1093.5172 (-0.1 ppm)
PSM Score: 45.47

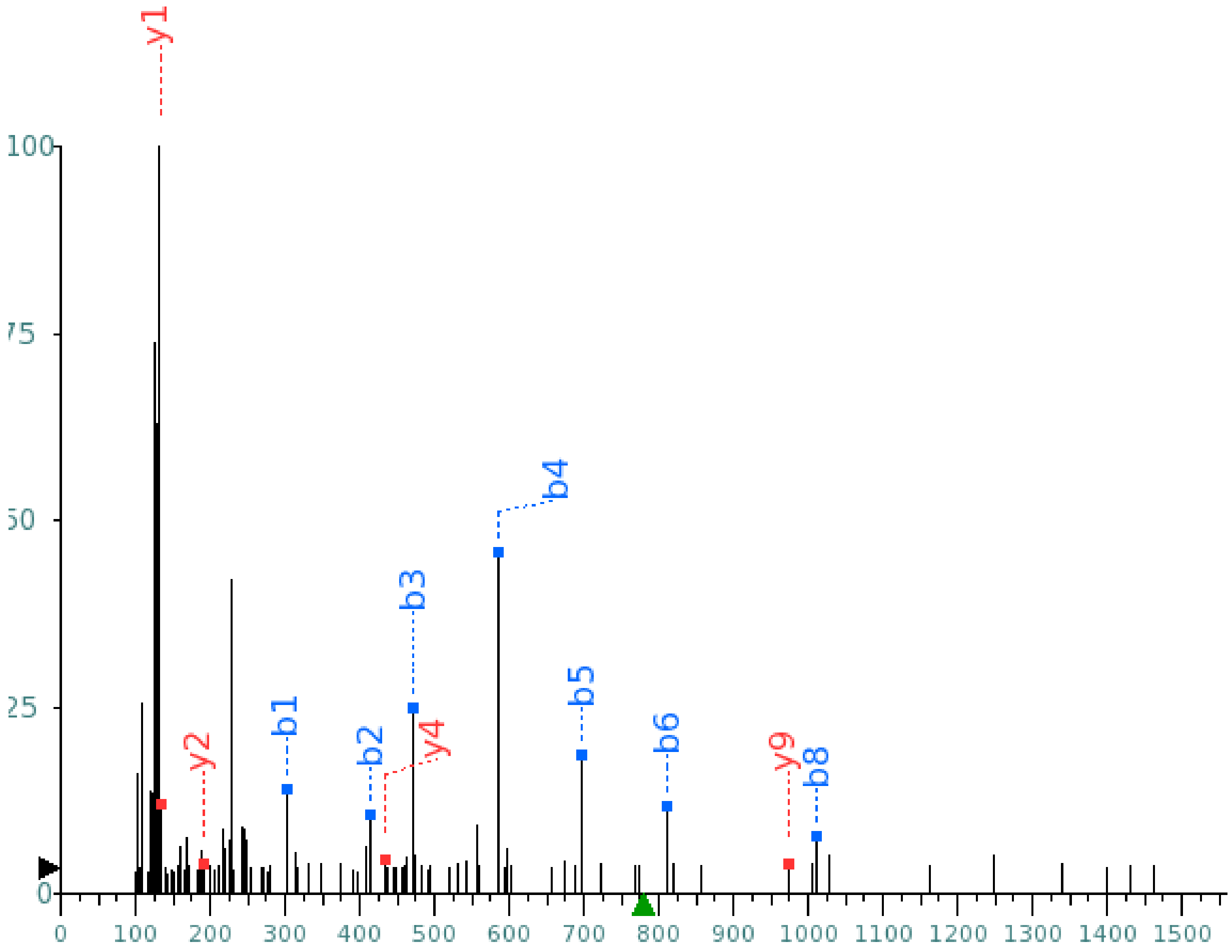

AIGLINEALDEGD (+2)

Primary Reference: IQGA1_HUMAN
Search ID: 41759
Search Name: 20130328_ananiav_ERstress_TMT_TM_timecourse_chymo_inj2_4MC_IAA
Scan#: 21605
Observed Mass: 779.9128 (1.4 ppm)
PSM Score: 24.89

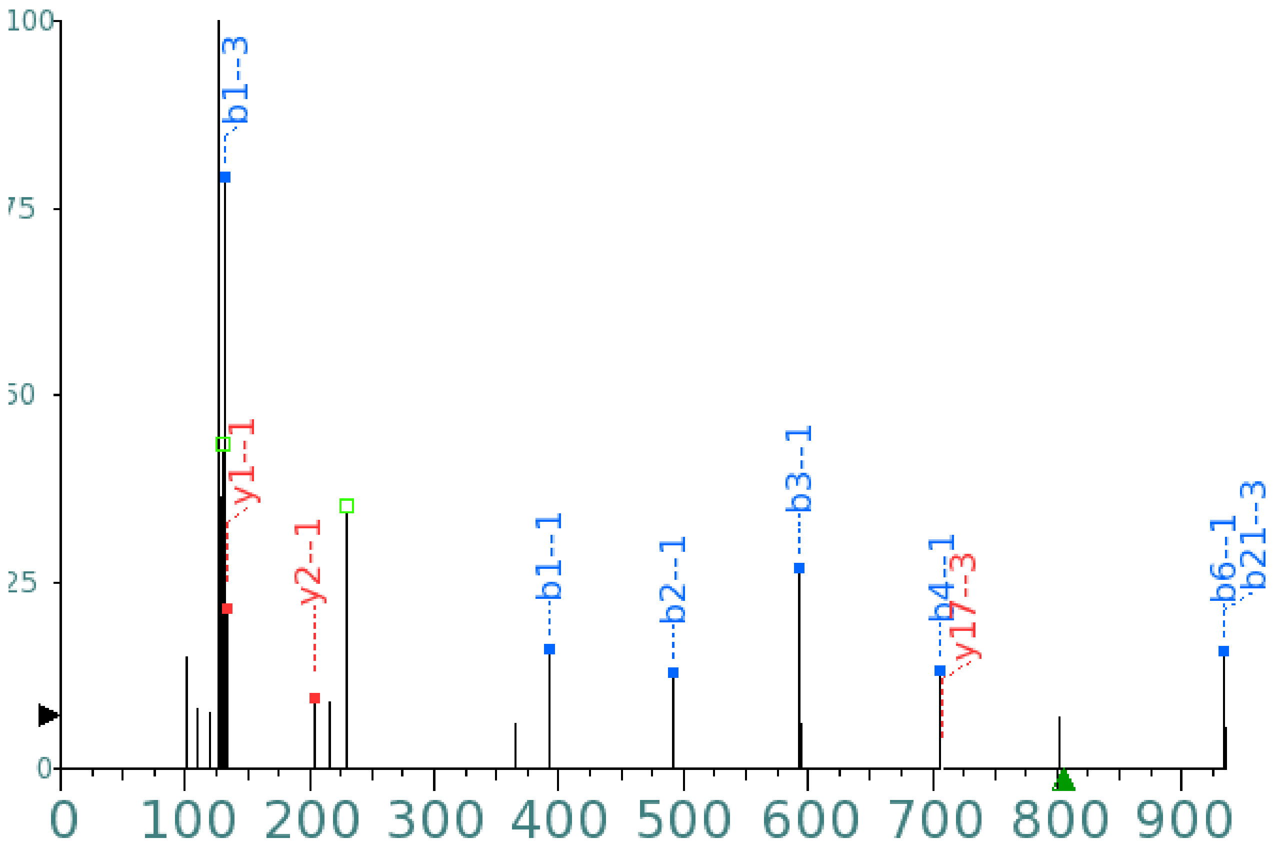

YVTIIDAPGHRDFIKNMITGTSQAD (+4)

Primary Reference: EF1A1_HUMAN
Search ID: 41759
Search Name: 20130328_ananiav_ERstress_TMT_TM_timecourse_chymo_inj2_4MC_IAA
Scan#: 22094
Observed Mass: 806.1836 (3 ppm)
PSM Score: 25.32

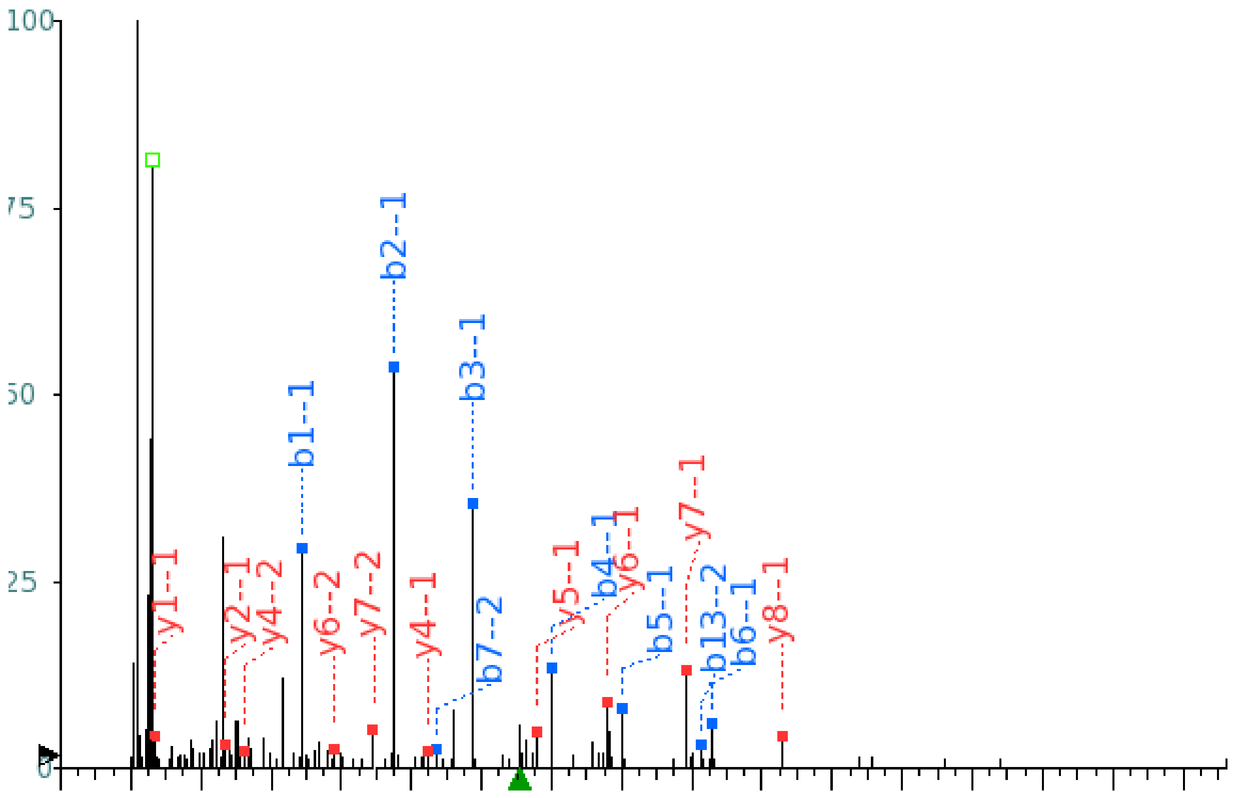

IMILTEHLVRC*ETD (+3)

Primary Reference: NCBP1_HUMAN
Search ID: 41759
Search Name: 20130328_ananiav_ERstress_TMT_TM_timecourse_chymo_inj2_4MC_IAA
Scan#: 22227
Observed Mass: 653.682 (3.7 ppm)
PSM Score: 38.72

File name: 2015-02-04 04.01.53PM.pdf

Creation time: 2015-02-04 04:09PM

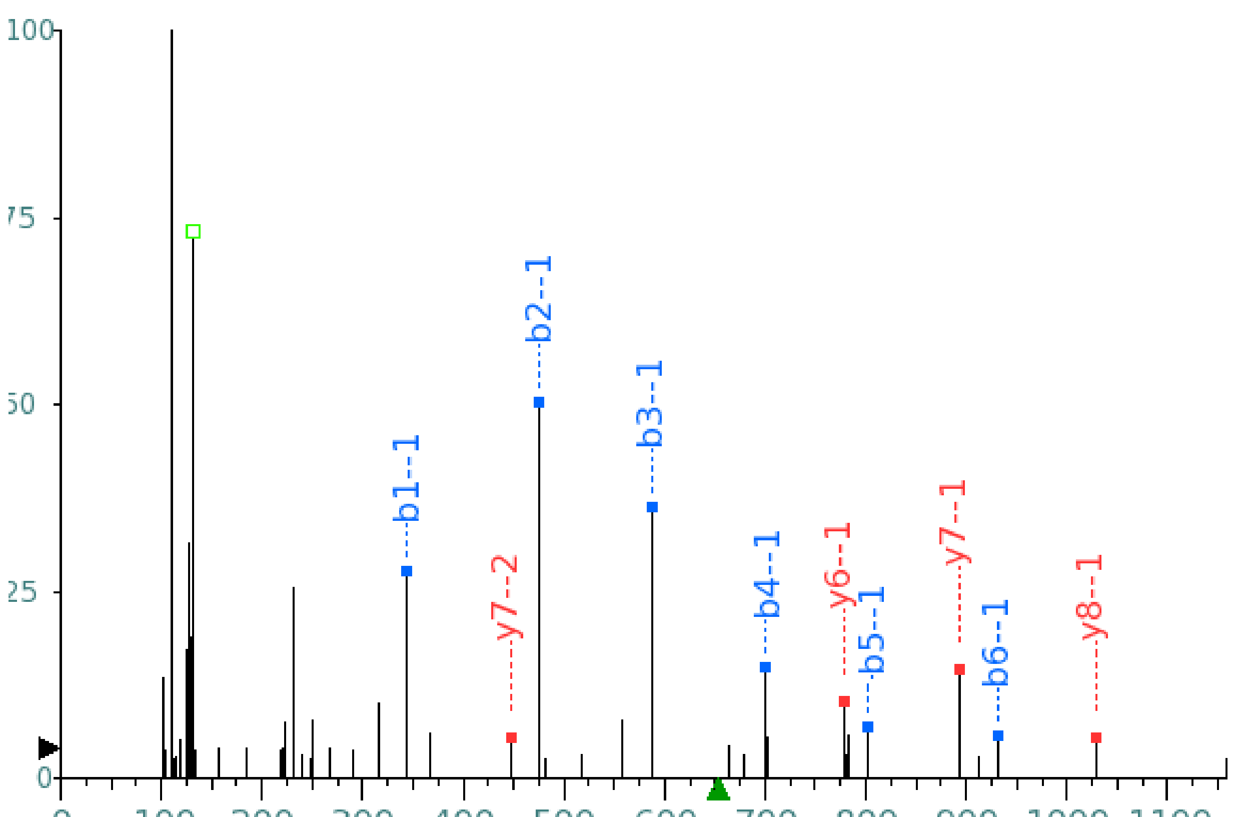

IMILTEHLVRC*ETD (+3)

Primary Reference: NCBP1_HUMAN
Search ID: 41759
Search Name: 20130328_ananiav_ERstress_TMT_TM_timecourse_chymo_inj2_4MC_IAA
Scan#: 22292
Observed Mass: 653.681 (2.2 ppm)
PSM Score: 42

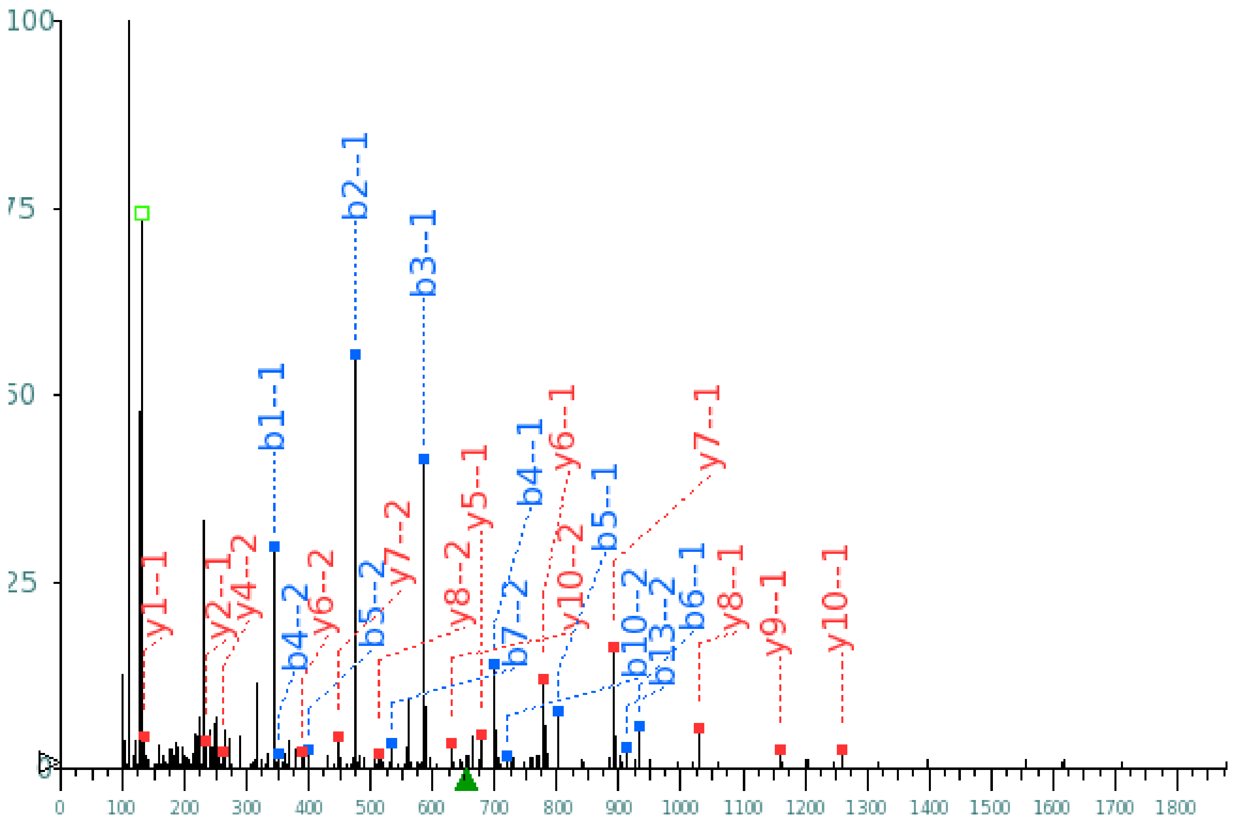

IMILTEHLVRC*ETD (+3)

Primary Reference: NCBP1_HUMAN
Search ID: 41759
Search Name: 20130328_ananiav_ERstress_TMT_TM_timecourse_chymo_inj2_4MC_IAA
Scan#: 22356
Observed Mass: 653.6814 (2.9 ppm)
PSM Score: 28.68

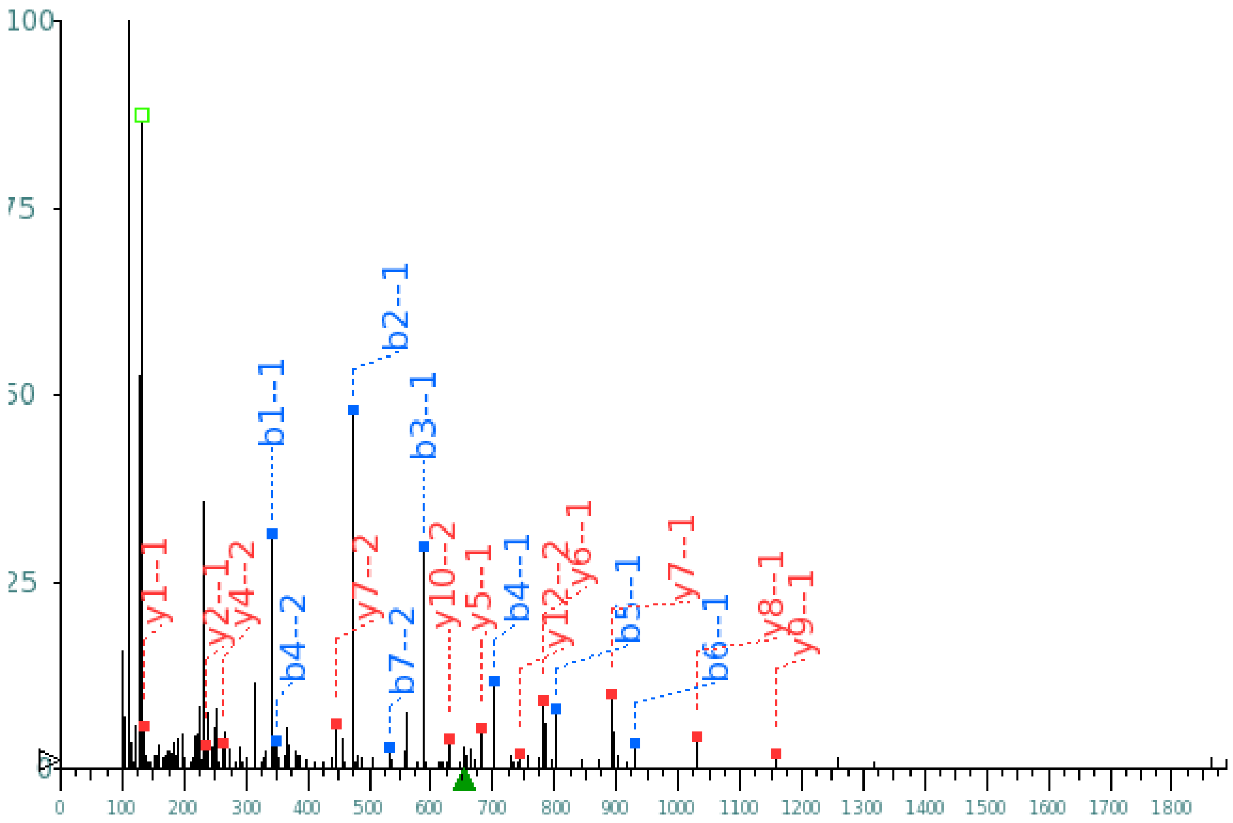

IMILTEHLVRC*ETD (+3)

Primary Reference: NCBP1_HUMAN
Search ID: 41759
Search Name: 20130328_ananiav_ERstress_TMT_TM_timecourse_chymo_inj2_4MC_IAA
Scan#: 22430
Observed Mass: 653.6814 (2.8 ppm)
PSM Score: 32.21

File name: 2015-02-04 04.01.53PM.pdf

Creation time: 2015-02-04 04:09PM

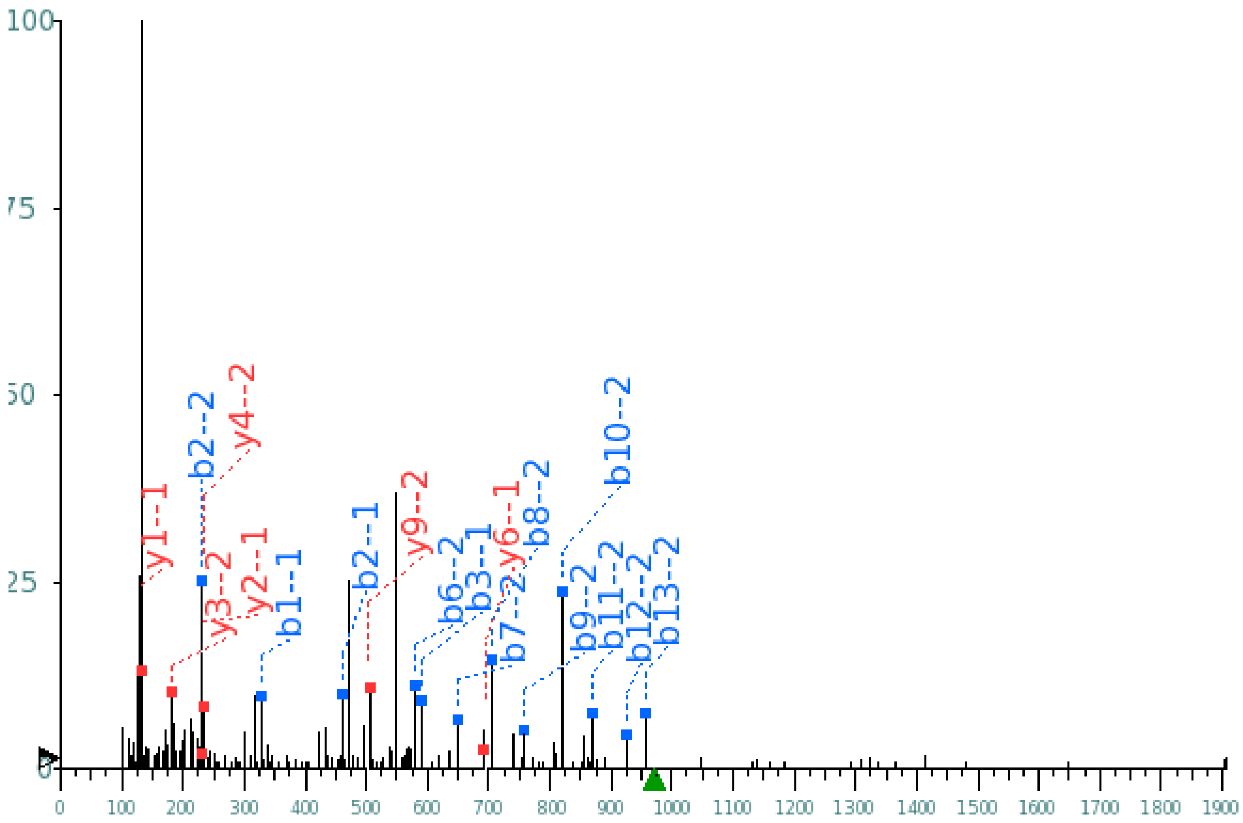

VMMPNKHNVEVLGILSDDVETD (+3)

Primary Reference: ERF3A_HUMAN
Search ID: 41759
Search Name: 20130328_ananiav_ERstress_TMT_TM_timecourse_chymo_inj2_4MC_IAA
Scan#: 22536
Observed Mass: 971.849 (7.7 ppm)
PSM Score: 35.02

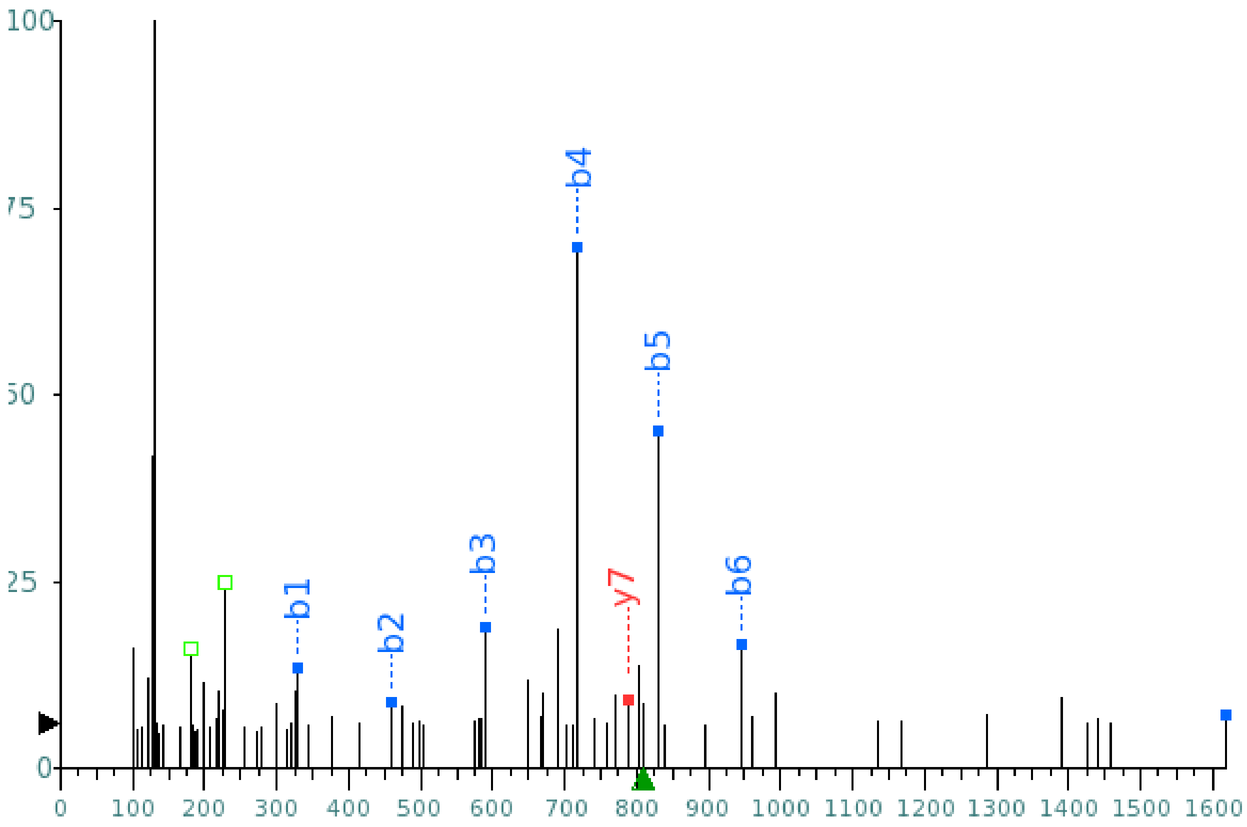

VMEELLPTLQTD (+2)

Primary Reference: NIBAN_HUMAN
Search ID: 41759
Search Name: 20130328_ananiav_ERstress_TMT_TM_timecourse_chymo_inj2_4MC_IAA
Scan#: 23033
Observed Mass: 809.4359 (2.4 ppm)
PSM Score: 29.25

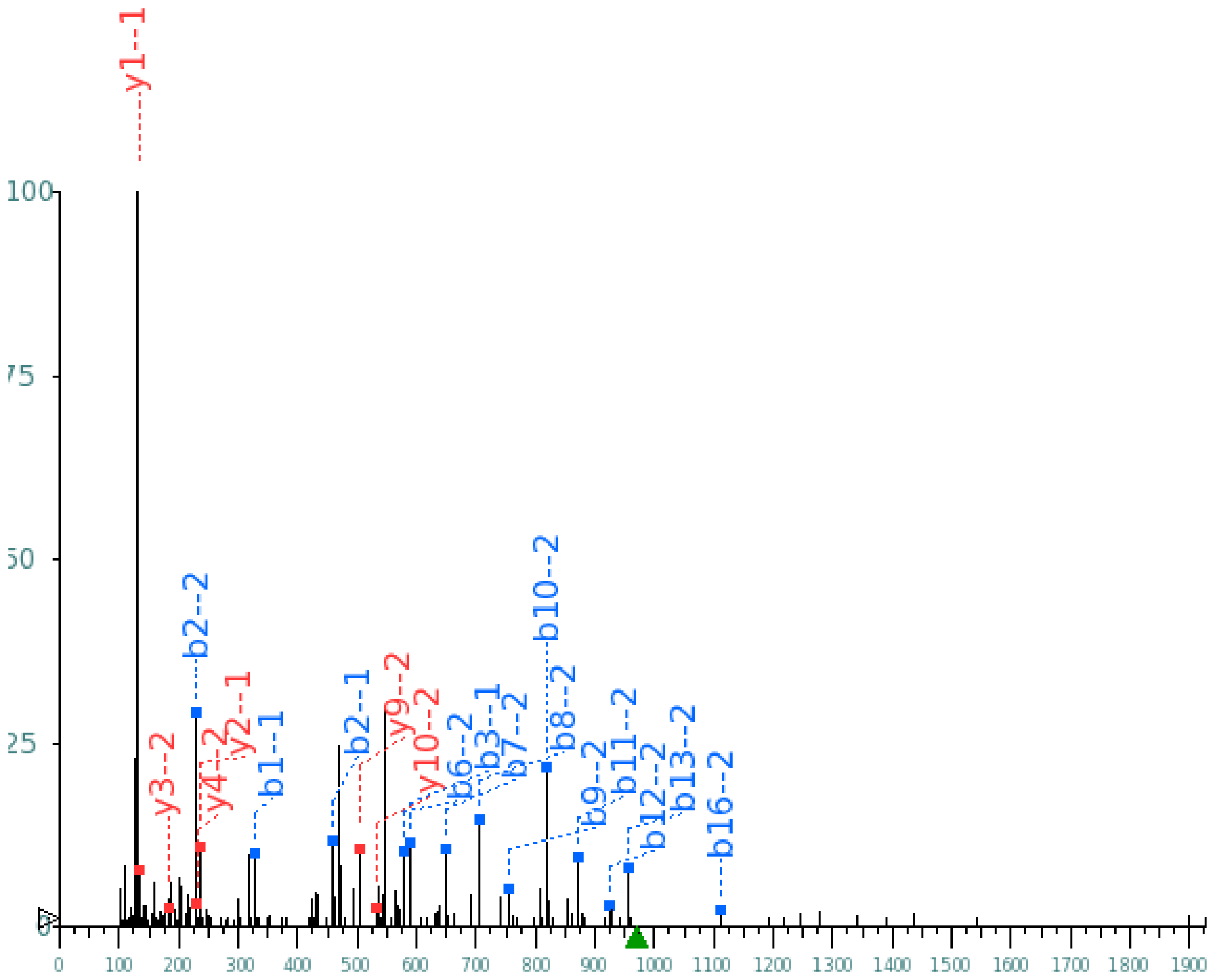

VMMPNKHNVEVLGILSDDVETD (+3)

Primary Reference: ERF3A_HUMAN
Search ID: 41759
Search Name: 20130328_ananiav_ERstress_TMT_TM_timecourse_chymo_inj2_4MC_IAA
Scan#: 23145
Observed Mass: 971.8416 (-0 ppm)
PSM Score: 43.03

File name: 2015-02-04 04.01.53PM.pdf

Creation time: 2015-02-04 04:09PM

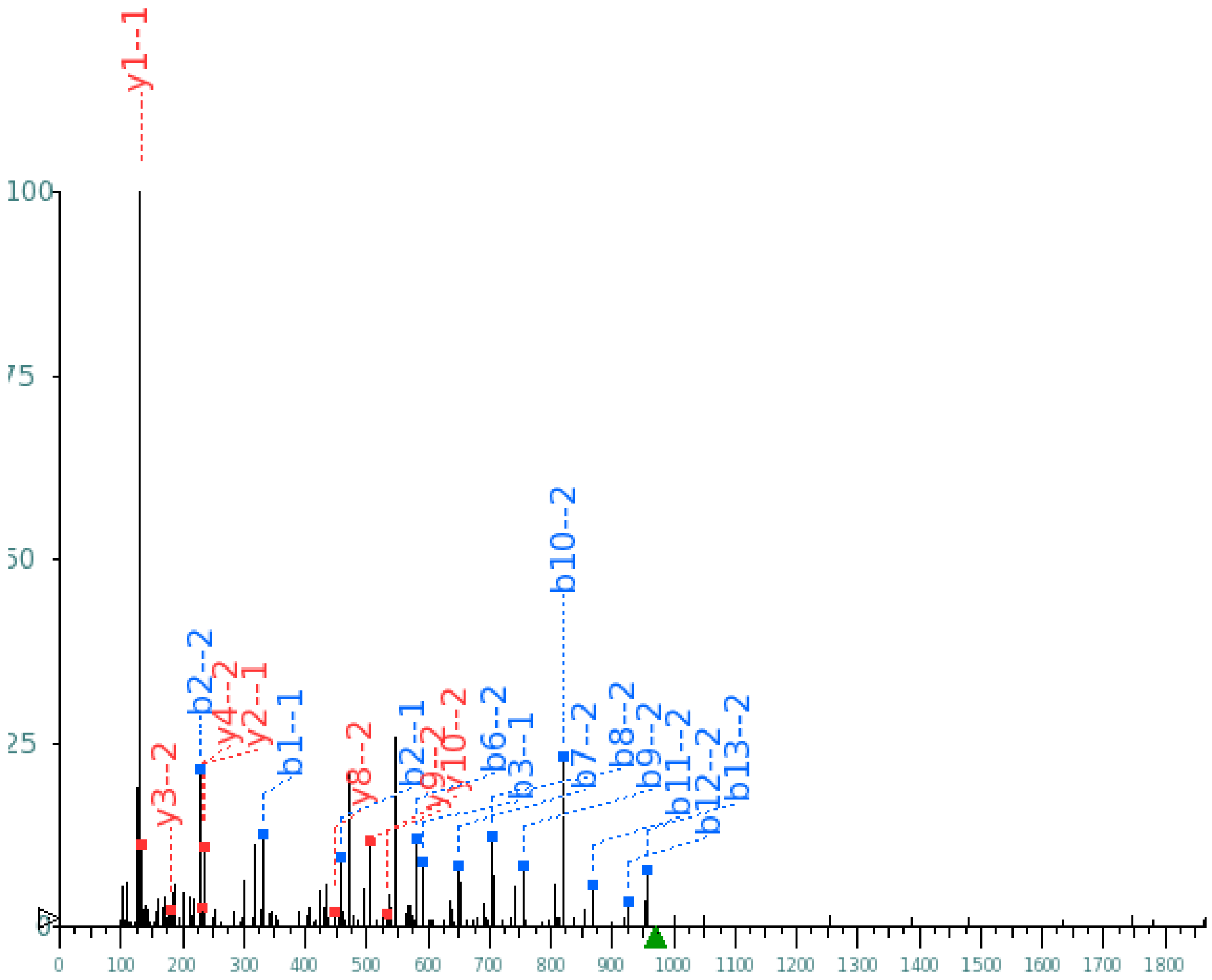

VMMPNKHNVEVLGILSDDVETD (+3)

Primary Reference: ERF3A_HUMAN
Search ID: 41759
Search Name: 20130328_ananiav_ERstress_TMT_TM_timecourse_chymo_inj2_4MC_IAA
Scan#: 23269
Observed Mass: 971.8415 (-0.1 ppm)
PSM Score: 23.78

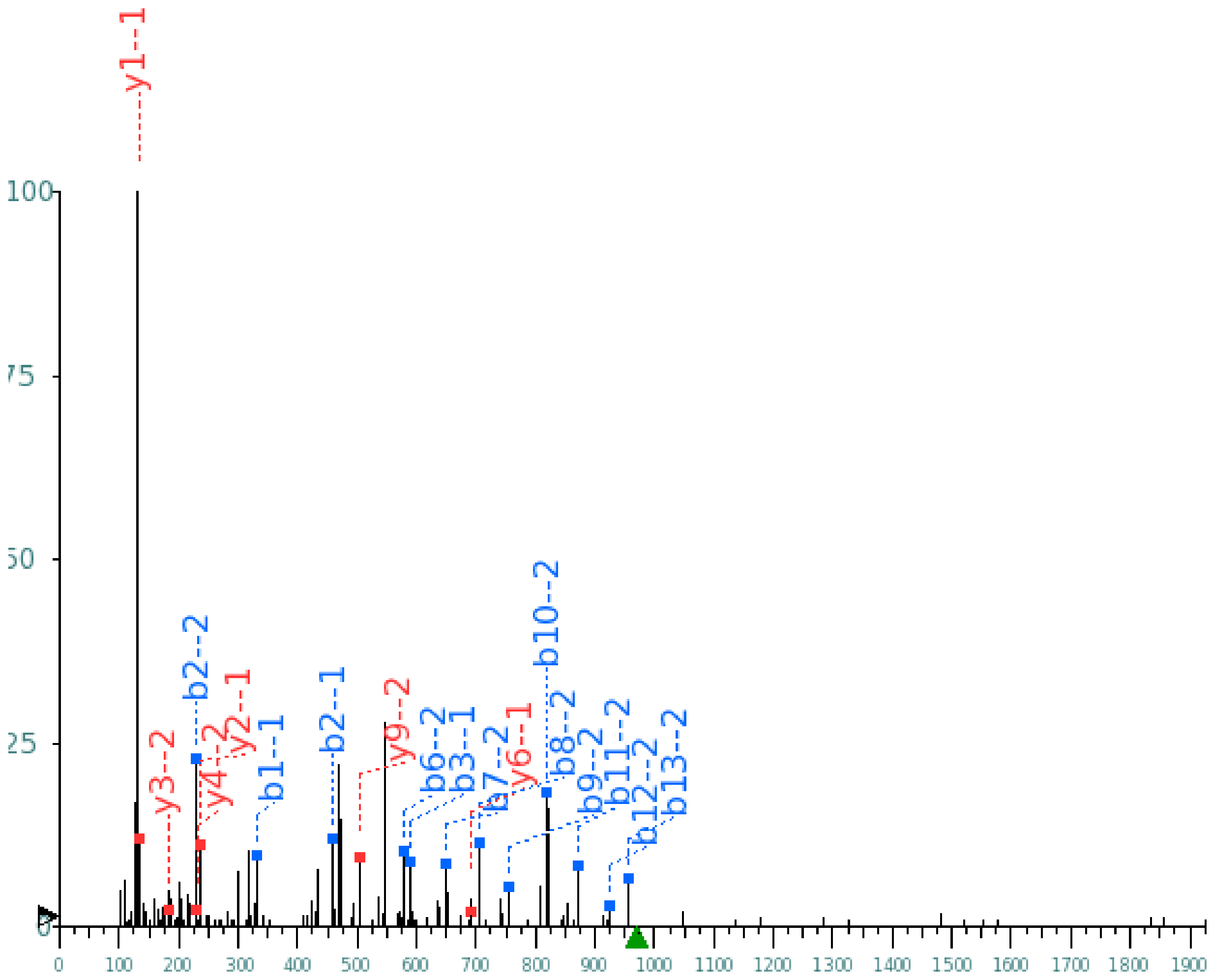

VMMPNKHNVEVLGILSDDVETD (+3)

Primary Reference: ERF3A_HUMAN
Search ID: 41759
Search Name: 20130328_ananiav_ERstress_TMT_TM_timecourse_chymo_inj2_4MC_IAA
Scan#: 23292
Observed Mass: 971.8413 (-0.3 ppm)
PSM Score: 25.37

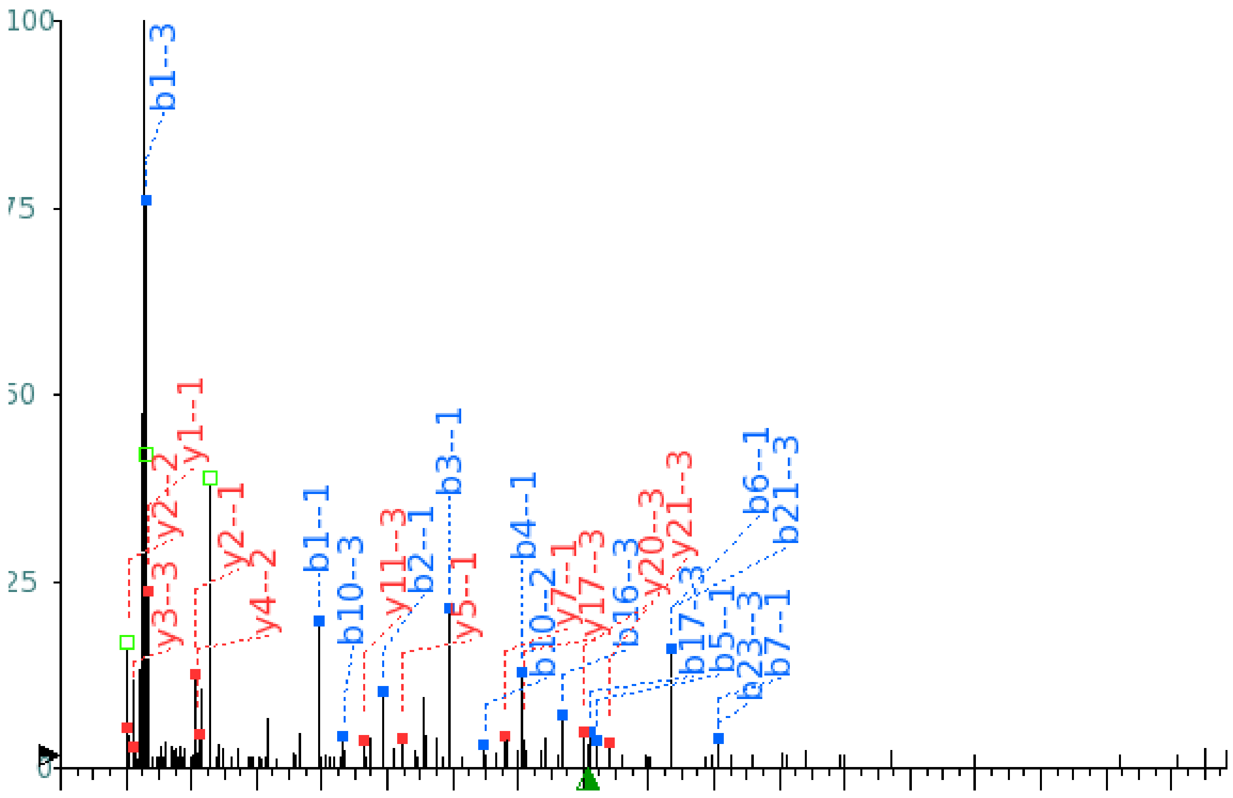

YVTIIDAPGHRDFIKNMITGTSQAD (+4)

Primary Reference: EF1A1_HUMAN

Search ID: 41759

Search Name: 20130328_ananiav_ERstress_TMT_TM_timecourse_chymo_inj2_4MC_IAA

Scan#: 23383

Observed Mass: 806.1808 (-0.5 ppm)

PSM Score: 16.69

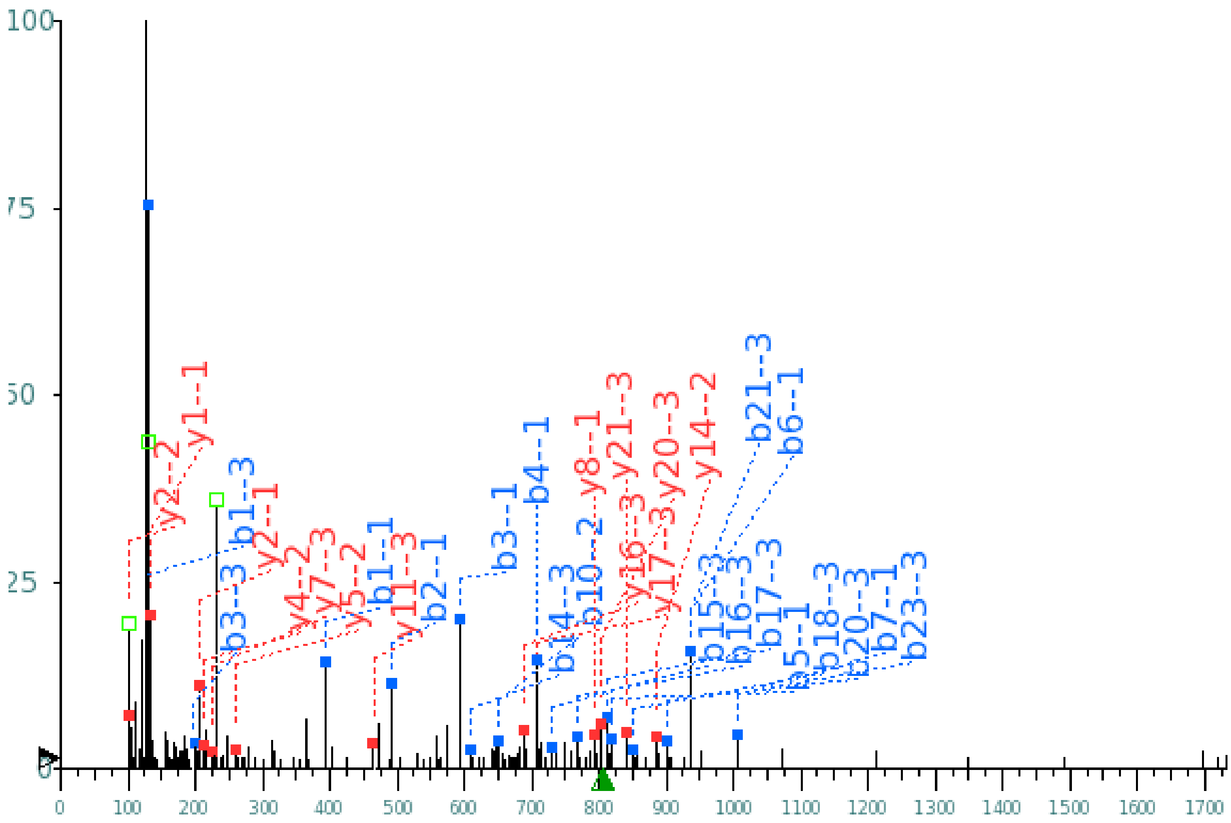

YVTIIDAPGHRDFIKNMITGTSQAD (+4)

Primary Reference: EF1A1_HUMAN
Search ID: 41759
Search Name: 20130328_ananiav_ERstress_TMT_TM_timecourse_chymo_inj2_4MC_IAA
Scan#: 23442
Observed Mass: 806.1845 (4.1 ppm)
PSM Score: 20.96

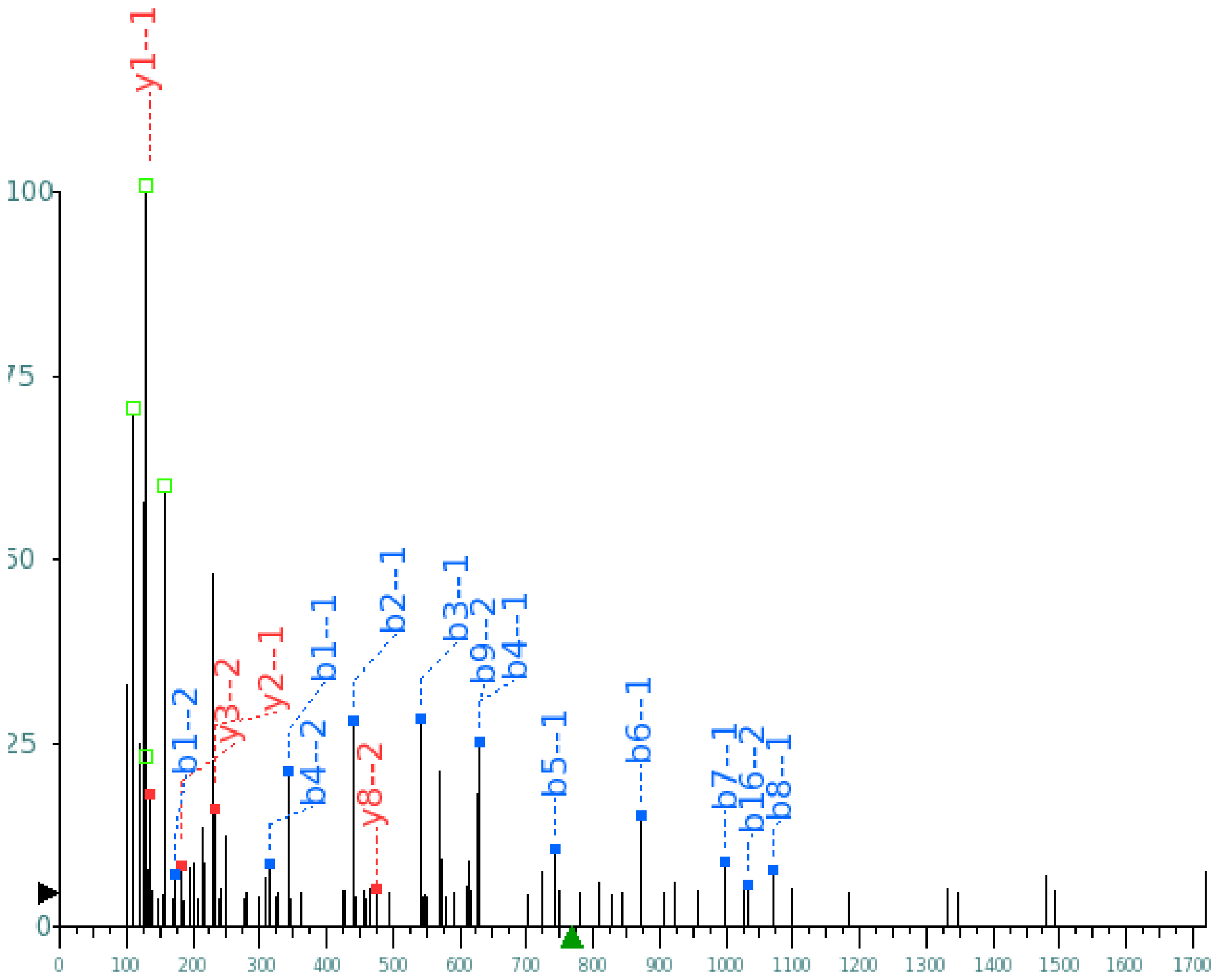

LVVSLEEAWPLHGDC*ETD (+3)

Primary Reference: MDC1_HUMAN
Search ID: 41759
Search Name: 20130328_ananiav_ERstress_TMT_TM_timecourse_chymo_inj2_4MC_IAA
Scan#: 24135
Observed Mass: 767.0439 (2.5 ppm)
PSM Score: 43.44

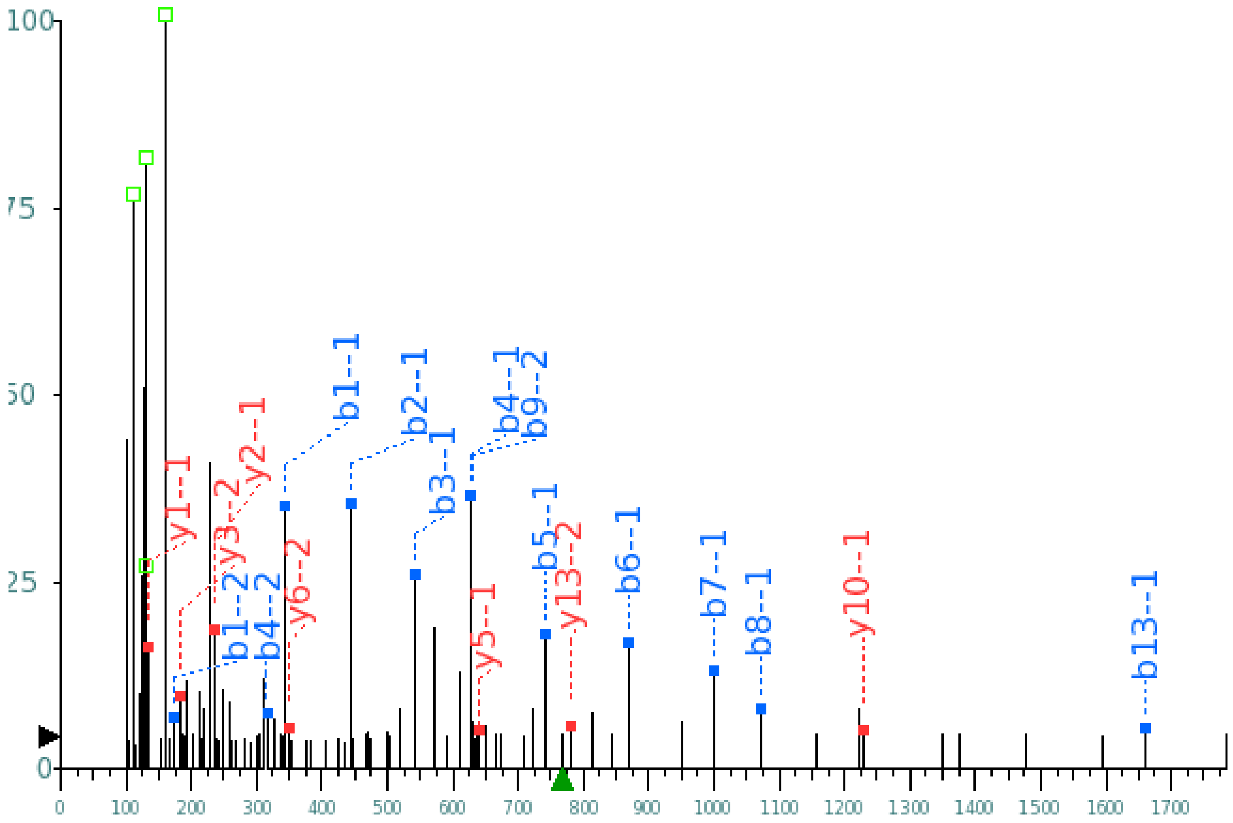

LVVSLEEAWPLHGDC*ETD (+3)

Primary Reference: MDC1_HUMAN
Search ID: 41759
Search Name: 20130328_ananiav_ERstress_TMT_TM_timecourse_chymo_inj2_4MC_IAA
Scan#: 24201
Observed Mass: 767.0435 (2 ppm)
PSM Score: 37.95

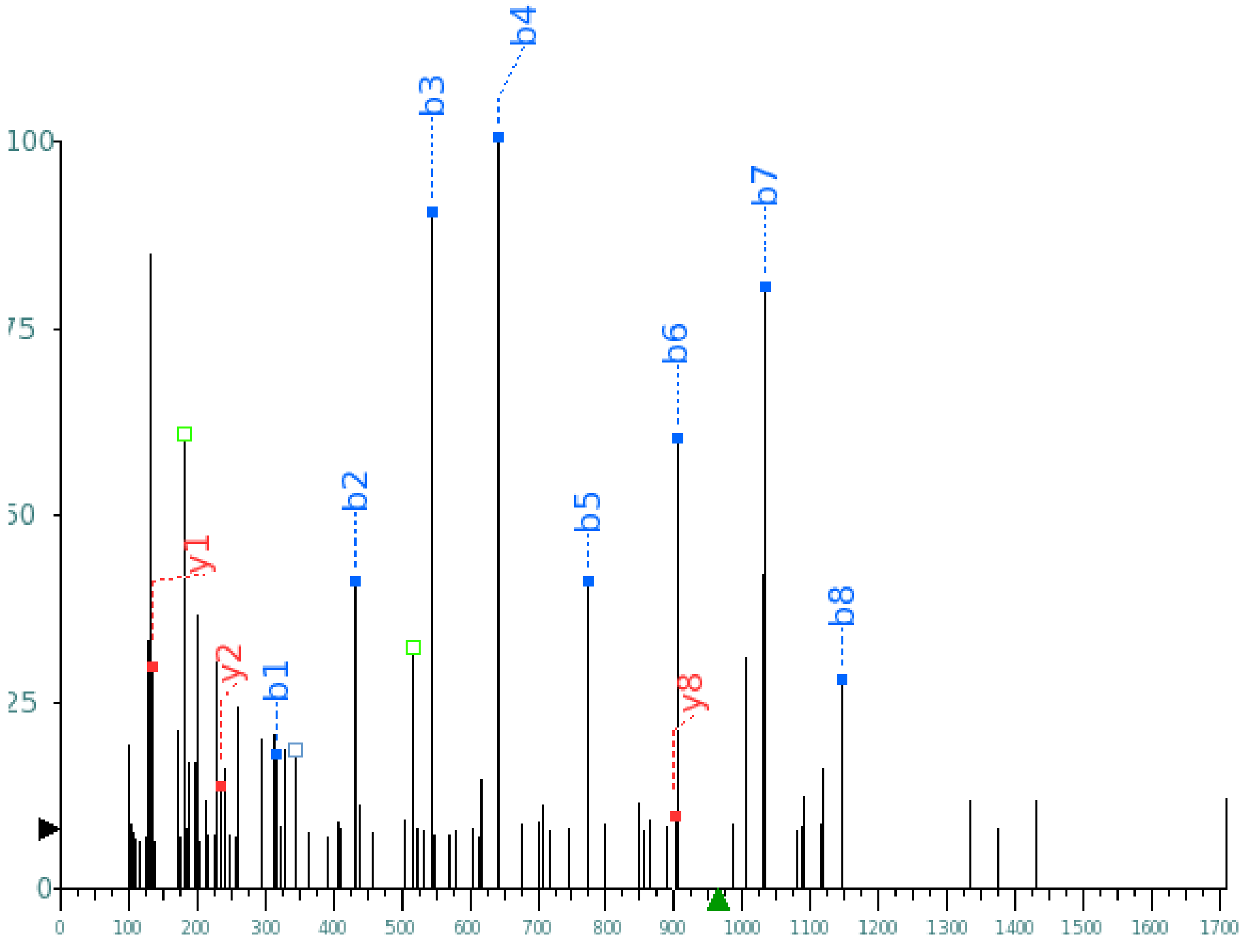

SNLVMEELLPTLQTD (+2)

Primary Reference: NIBAN_HUMAN
Search ID: 41759
Search Name: 20130328_ananiav_ERstress_TMT_TM_timecourse_chymo_inj2_4MC_IAA
Scan#: 24277
Observed Mass: 966.5161 (2.7 ppm)
PSM Score: 38.38

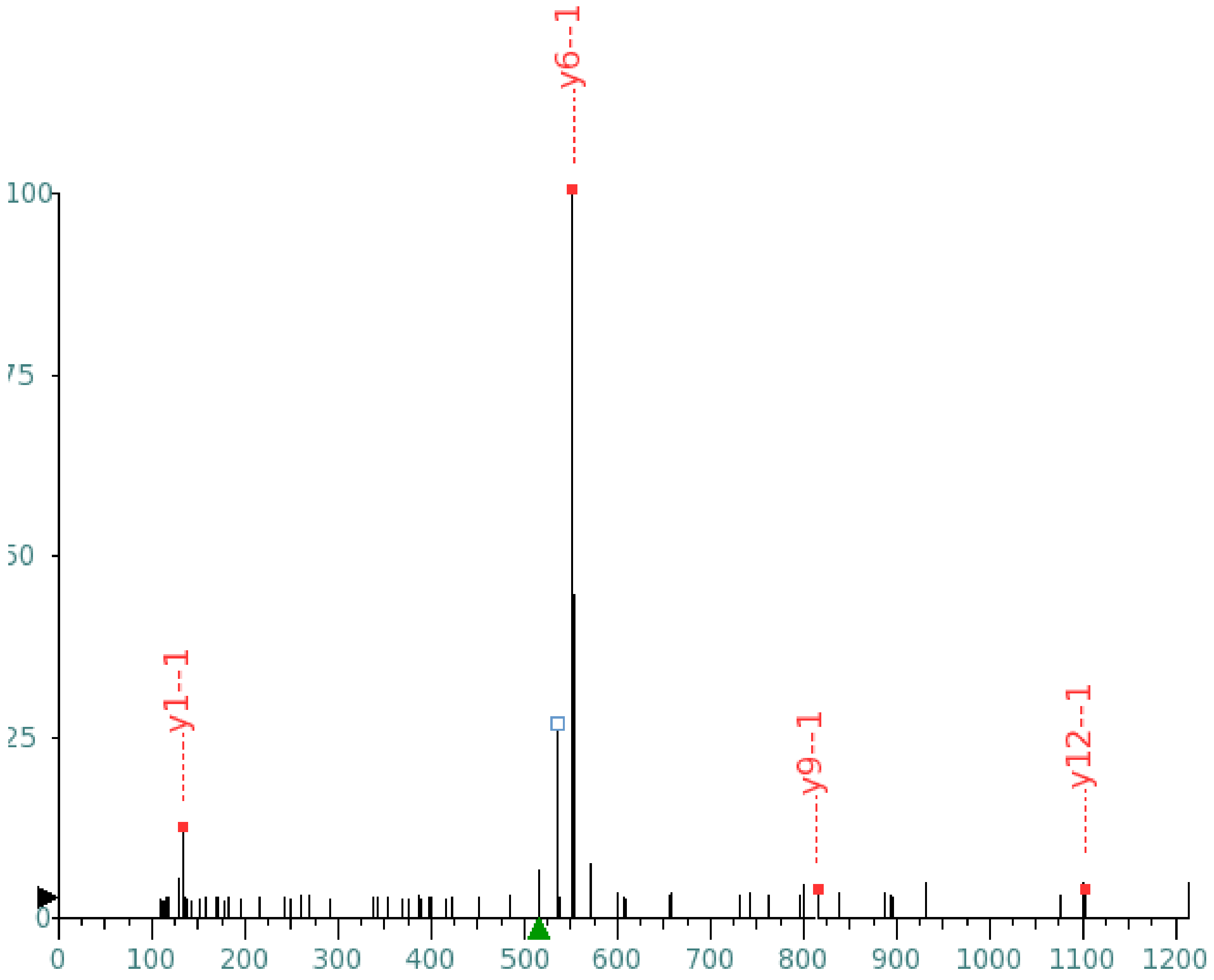

MSTEGGFGGTSSSD (+3)

Primary Reference: NFYC_HUMAN
Search ID: 41760
Search Name: 20130328_ananiav_ERstress_TMT_TM_timecourse_trypsin_inj1_4mc_iaa
Scan#: 358
Observed Mass: 516.8947 (0.6 ppm)
PSM Score: 10.98

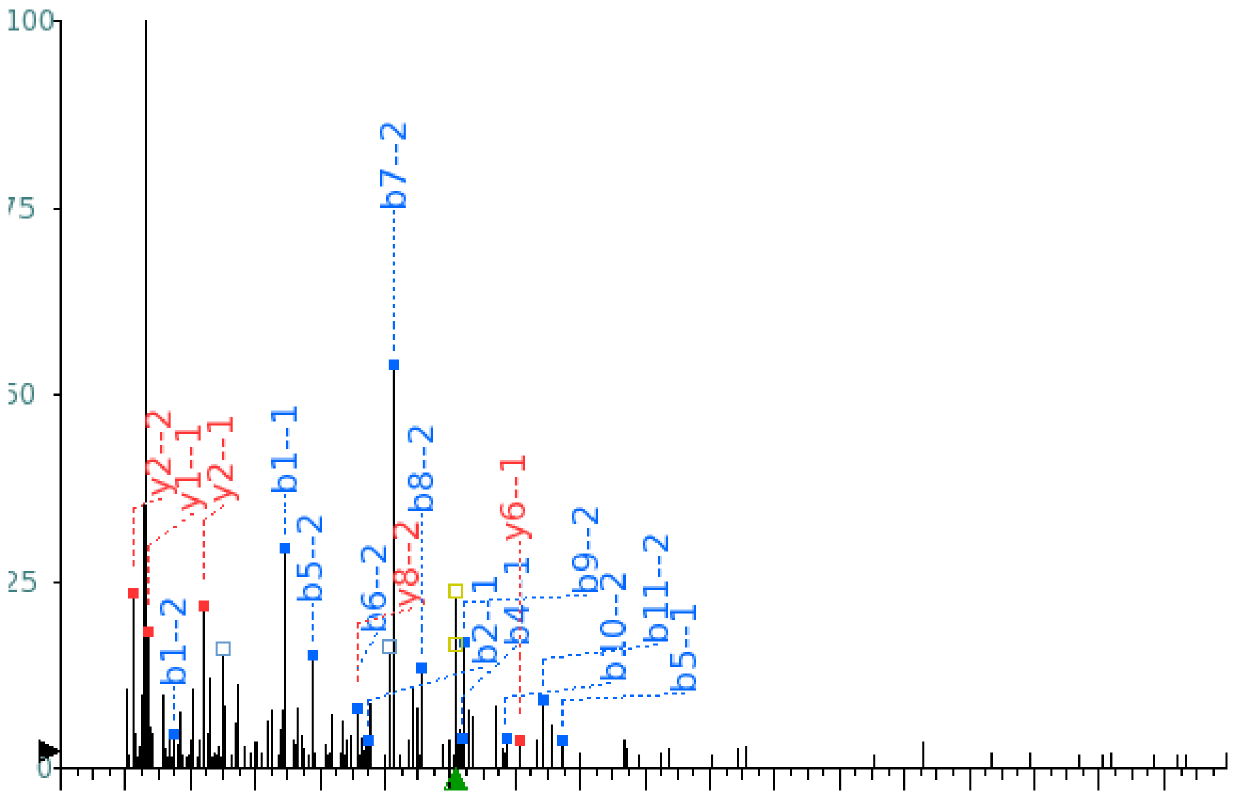

DQGSRHDSEQDNSD (+3)

Primary Reference: FUS_HUMAN
Search ID: 41760
Search Name: 20130328_ananiav_ERstress_TMT_TM_timecourse_trypsin_inj1_4mc_iaa
Scan#: 1679
Observed Mass: 606.9291 (2.2 ppm)
PSM Score: 9.1

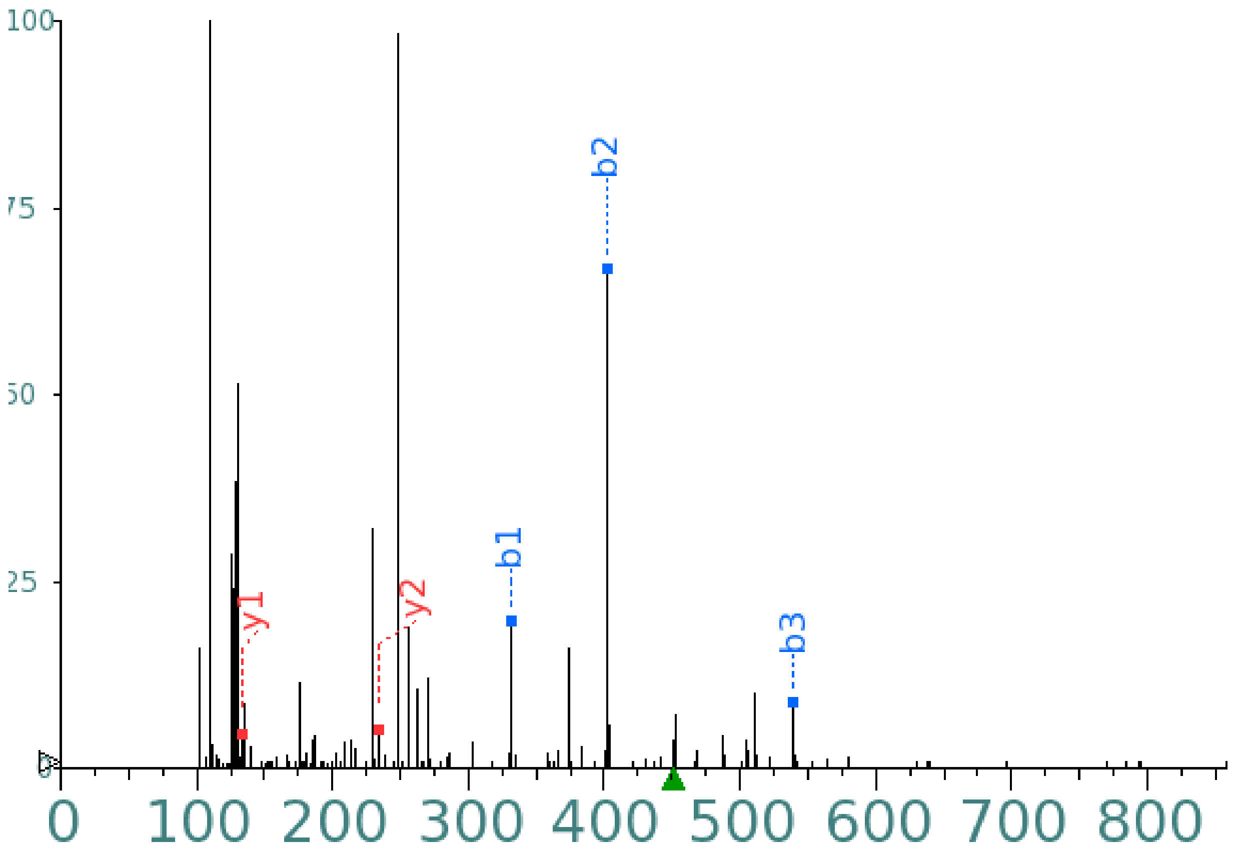

TAHETD (+2)

Primary Reference: CBX1_HUMAN
Search ID: 41760
Search Name: 20130328_ananiav_ERstress_TMT_TM_timecourse_trypsin_inj1_4mc_iaa
Scan#: 1714
Observed Mass: 451.7252 (1.6 ppm)
PSM Score: 8.29

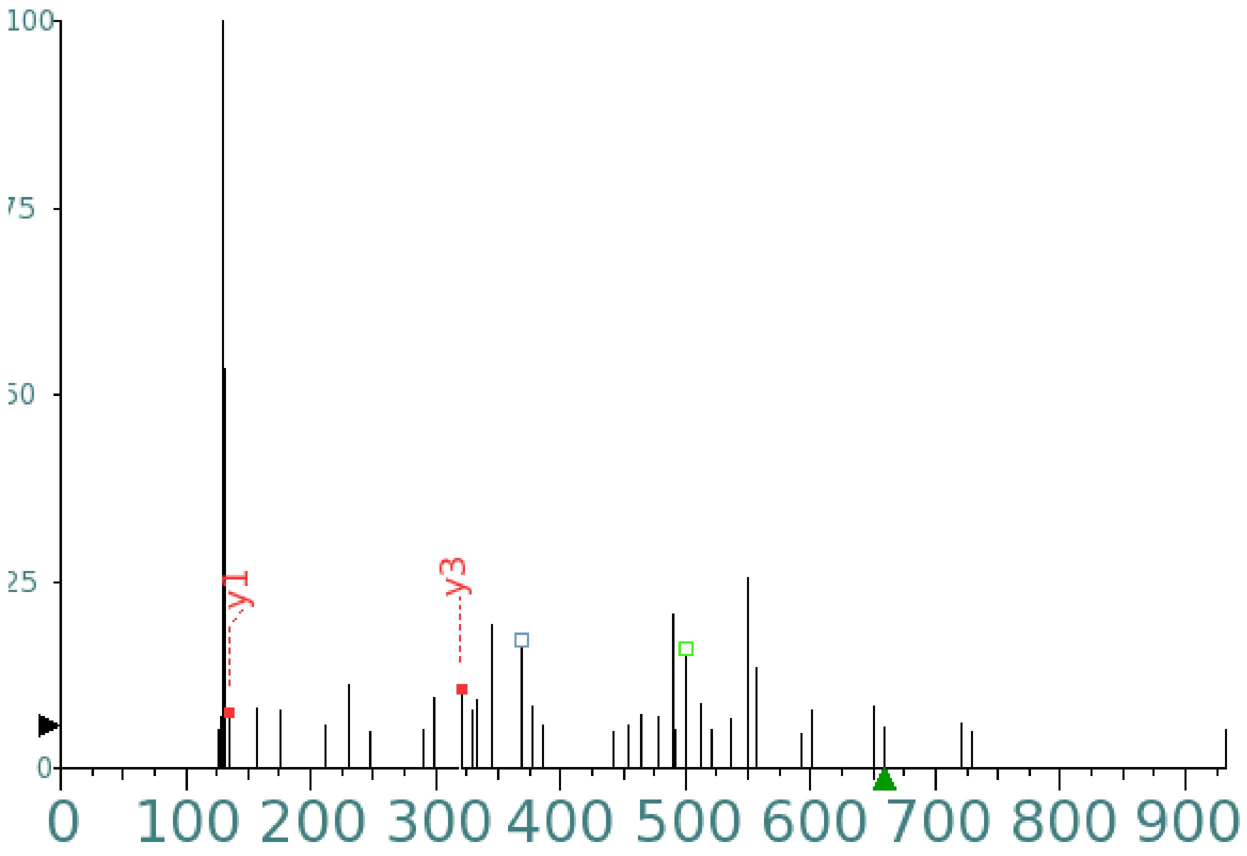

RPRDEDDAD (+2)

Primary Reference: TOP1_HUMAN
Search ID: 41760
Search Name: 20130328_ananiav_ERstress_TMT_TM_timecourse_trypsin_inj1_4mc_iaa
Scan#: 2009
Observed Mass: 659.3162 (1.4 ppm)
PSM Score: 13.93

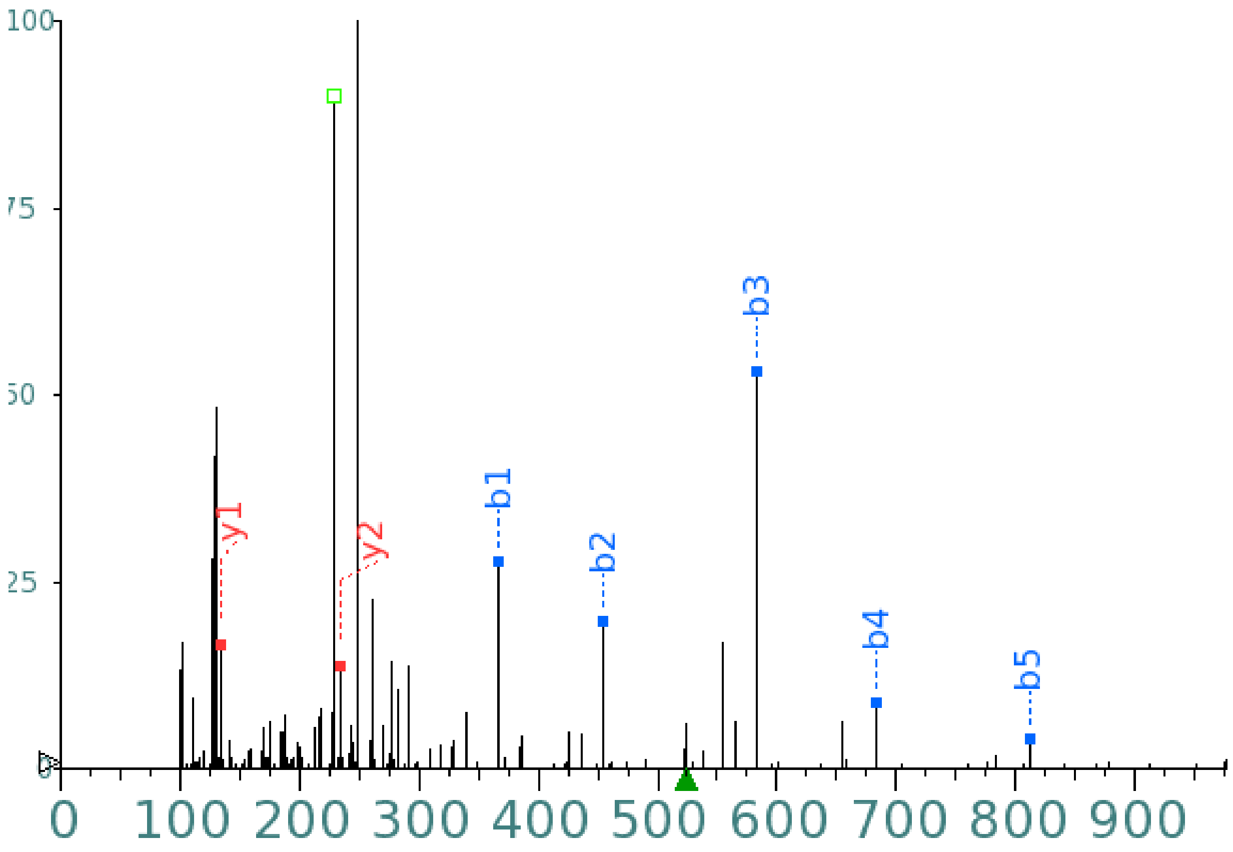

HSEVETD (+2)

Primary Reference: LYAR_HUMAN
Search ID: 41760
Search Name: 20130328_ananiav_ERstress_TMT_TM_timecourse_trypsin_inj1_4mc_iaa
Scan#: 2029
Observed Mass: 523.2543 (1.3 ppm)
PSM Score: 28.23

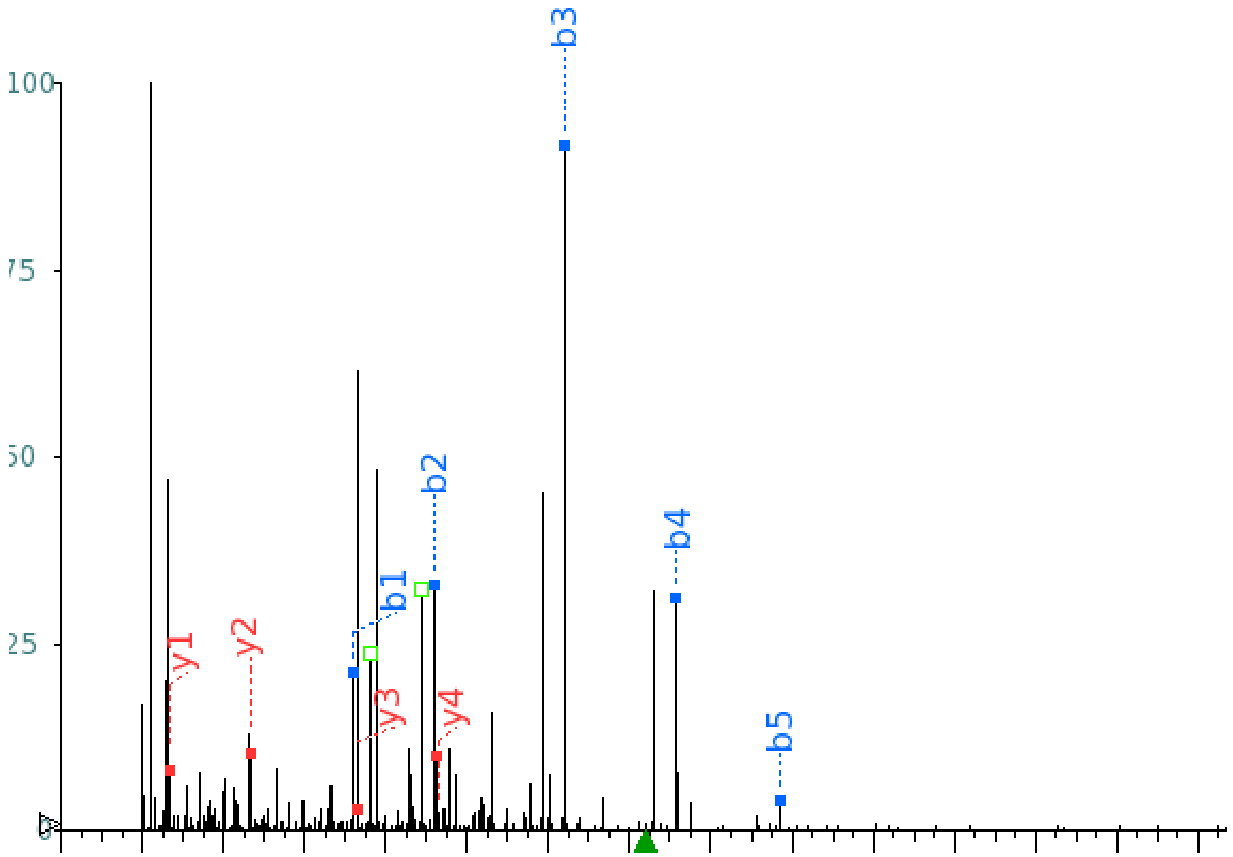

ETC*HQSTETD (+2)

Primary Reference: MORC3_HUMAN
Search ID: 41760
Search Name: 20130328_ananiav_ERstress_TMT_TM_timecourse_trypsin_inj1_4mc_iaa
Scan#: 2088
Observed Mass: 718.8135 (2.4 ppm)
PSM Score: 16.13

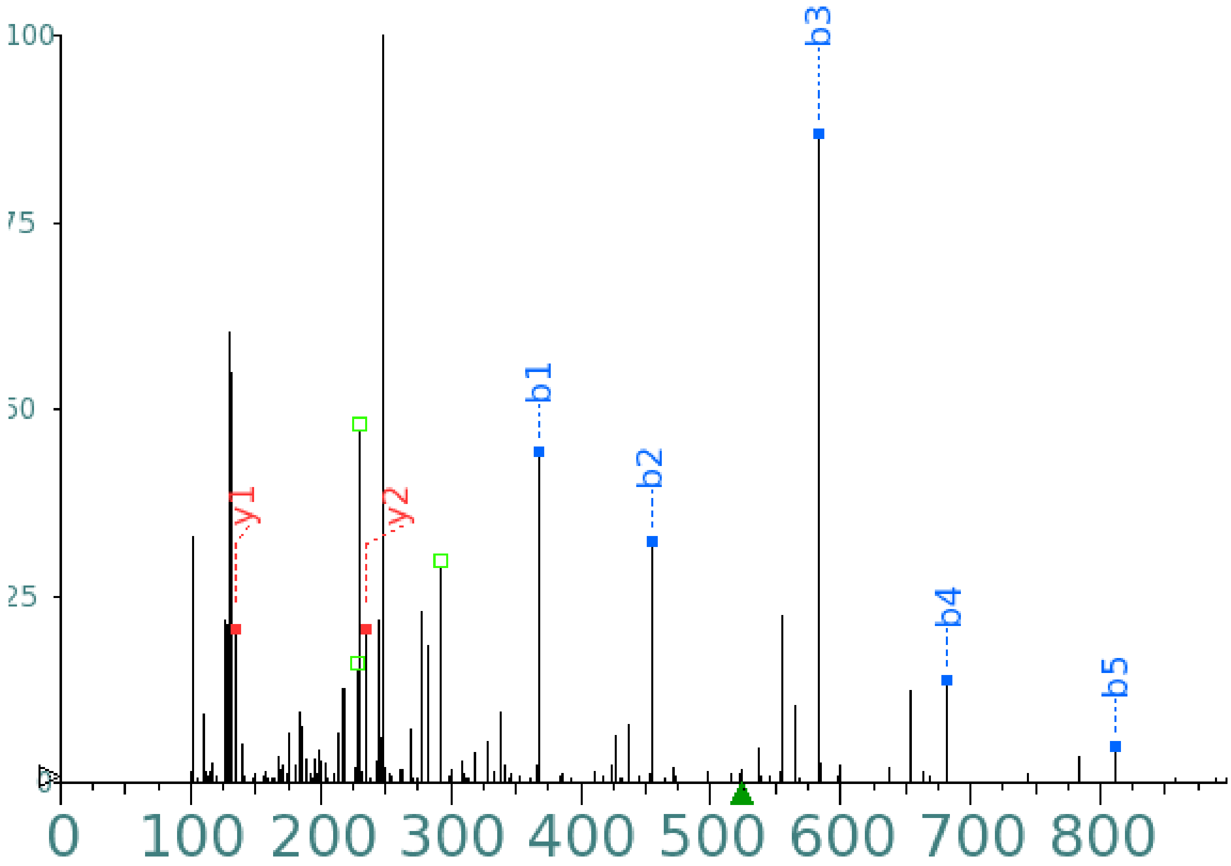

HSEVETD (+2)

Primary Reference: LYAR_HUMAN
Search ID: 41760
Search Name: 20130328_ananiav_ERstress_TMT_TM_timecourse_trypsin_inj1_4mc_iaa
Scan#: 2175
Observed Mass: 523.255 (2.7 ppm)
PSM Score: 32.46

File name: 2015-02-04 04.01.53PM.pdf

Creation time: 2015-02-04 04:09PM

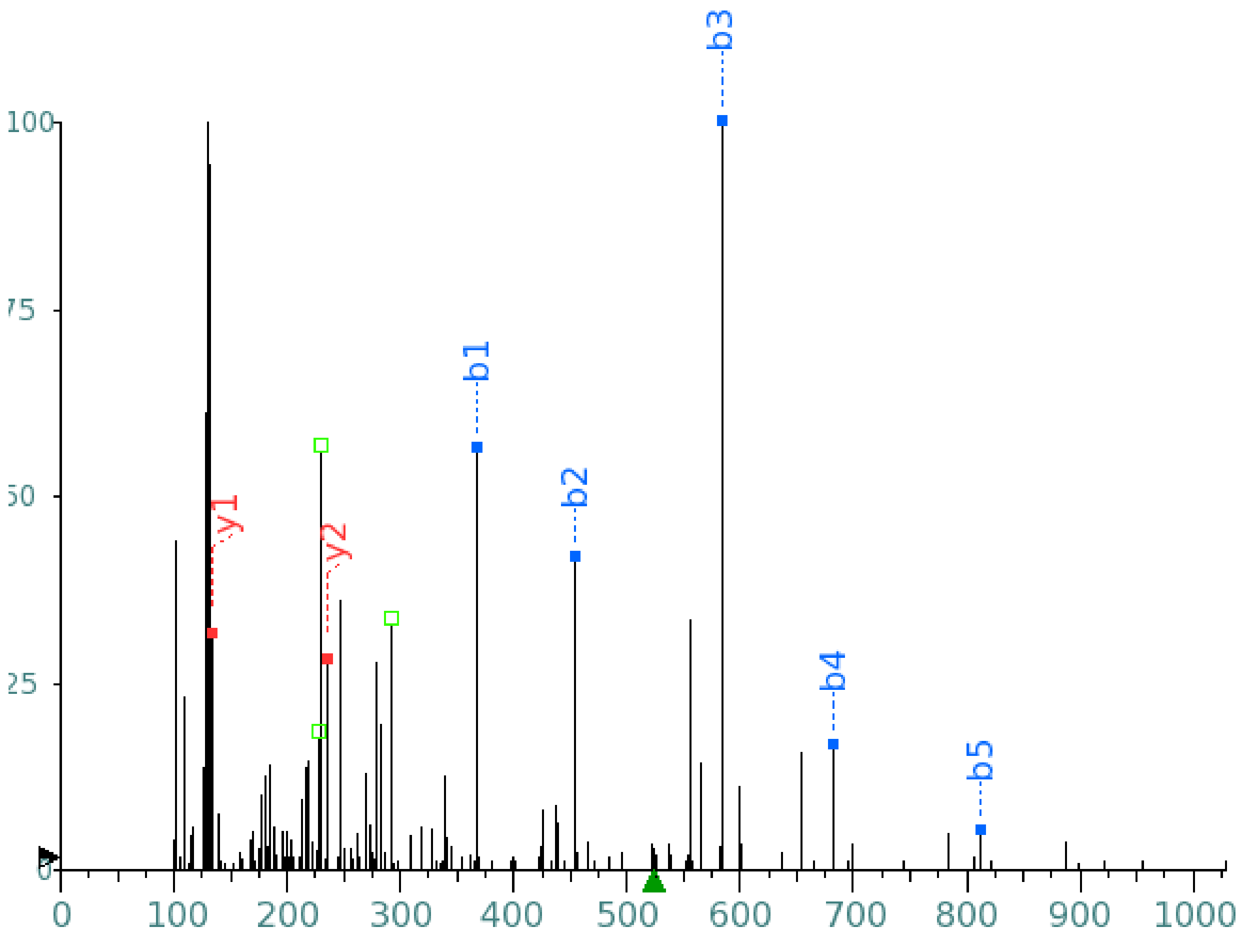

HSEVETD (+2)

Primary Reference: LYAR_HUMAN
Search ID: 41760
Search Name: 20130328_ananiav_ERstress_TMT_TM_timecourse_trypsin_inj1_4mc_iaa
Scan#: 2235
Observed Mass: 523.2558 (4.1 ppm)
PSM Score: 27.27

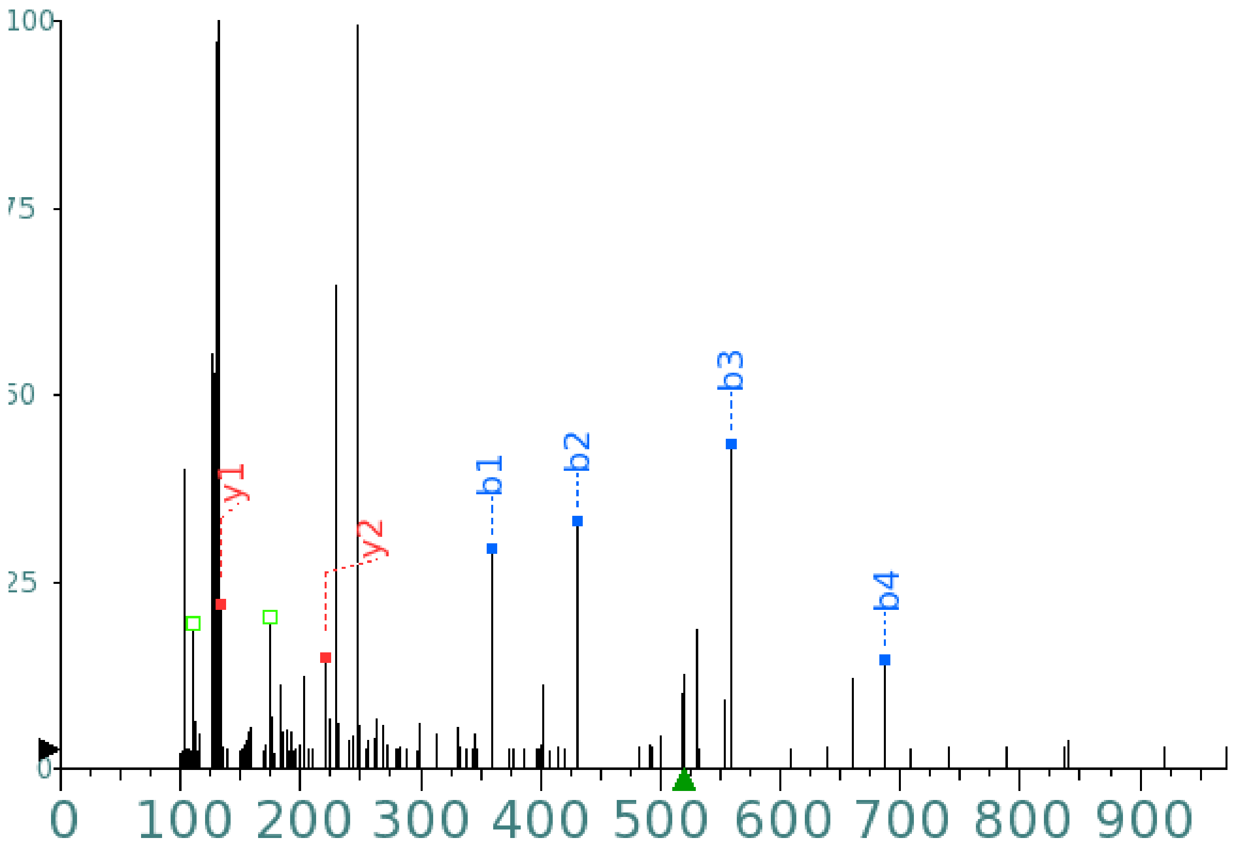

EAEEESD (+2)

Primary Reference: IF4G1_HUMAN
Search ID: 41760
Search Name: 20130328_ananiav_ERstress_TMT_TM_timecourse_trypsin_inj1_4mc_iaa
Scan#: 2261
Observed Mass: 519.2286 (2.6 ppm)
PSM Score: 15.03

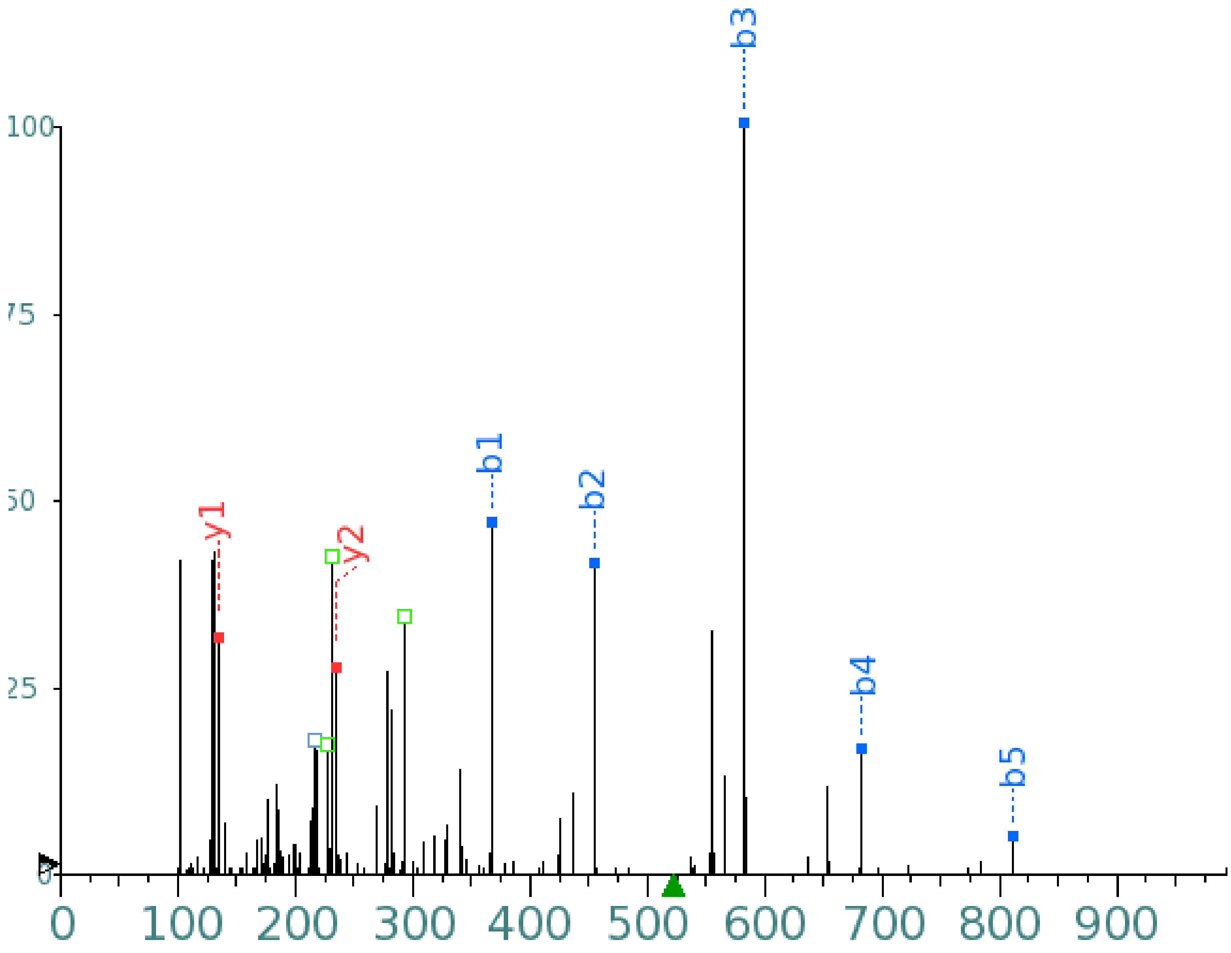

HSEVETD (+2)

Primary Reference: LYAR_HUMAN
Search ID: 41760
Search Name: 20130328_ananiav_ERstress_TMT_TM_timecourse_trypsin_inj1_4mc_iaa
Scan#: 2299
Observed Mass: 523.2552 (3.1 ppm)
PSM Score: 31.18

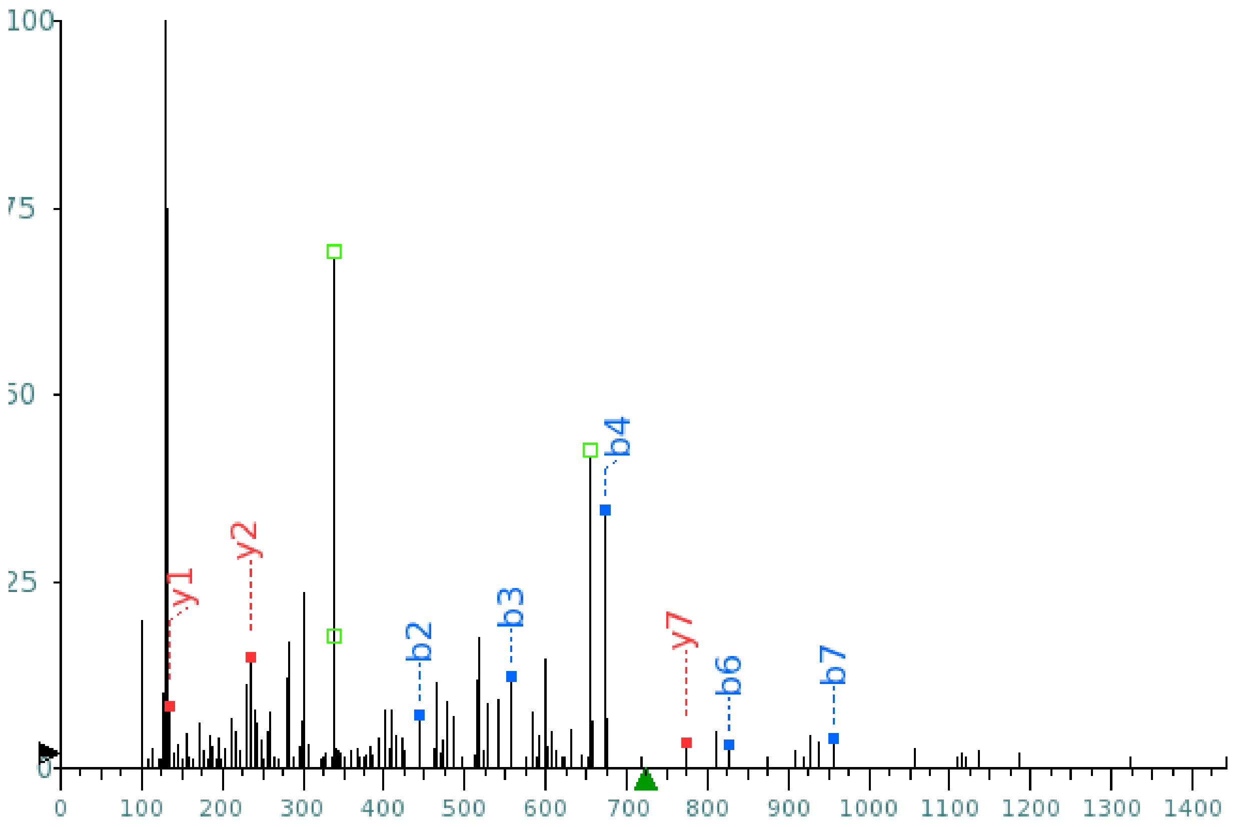

GRDDPGQQETD (+2)

Primary Reference: K1704_HUMAN
Search ID: 41760
Search Name: 20130328_ananiav_ERstress_TMT_TM_timecourse_trypsin_inj1_4mc_iaa
Scan#: 2327
Observed Mass: 723.8389 (3.3 ppm)
PSM Score: 14.11

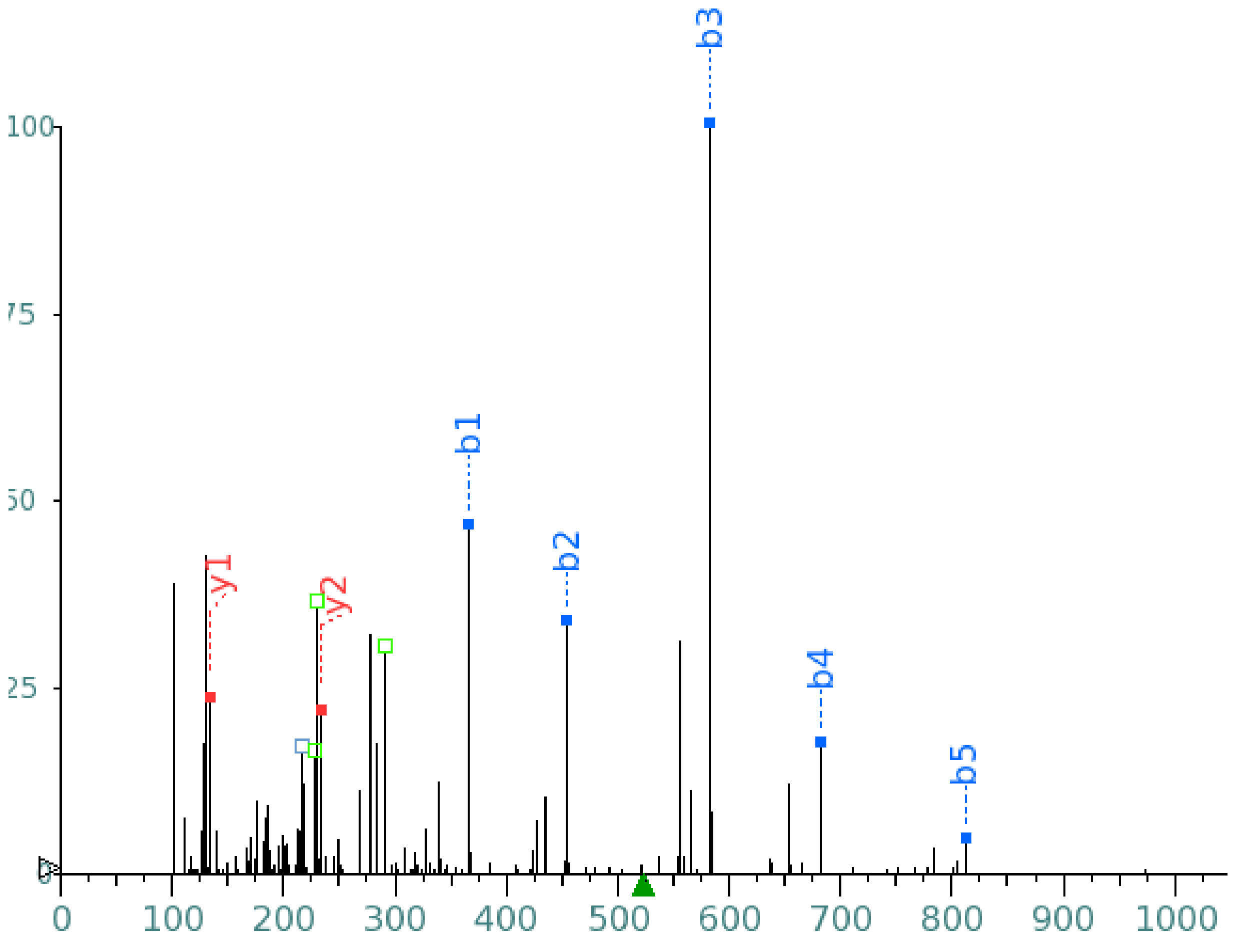

HSEVETD (+2)

Primary Reference: LYAR_HUMAN
Search ID: 41760
Search Name: 20130328_ananiav_ERstress_TMT_TM_timecourse_trypsin_inj1_4mc_iaa
Scan#: 2366
Observed Mass: 523.2552 (3 ppm)
PSM Score: 30.95

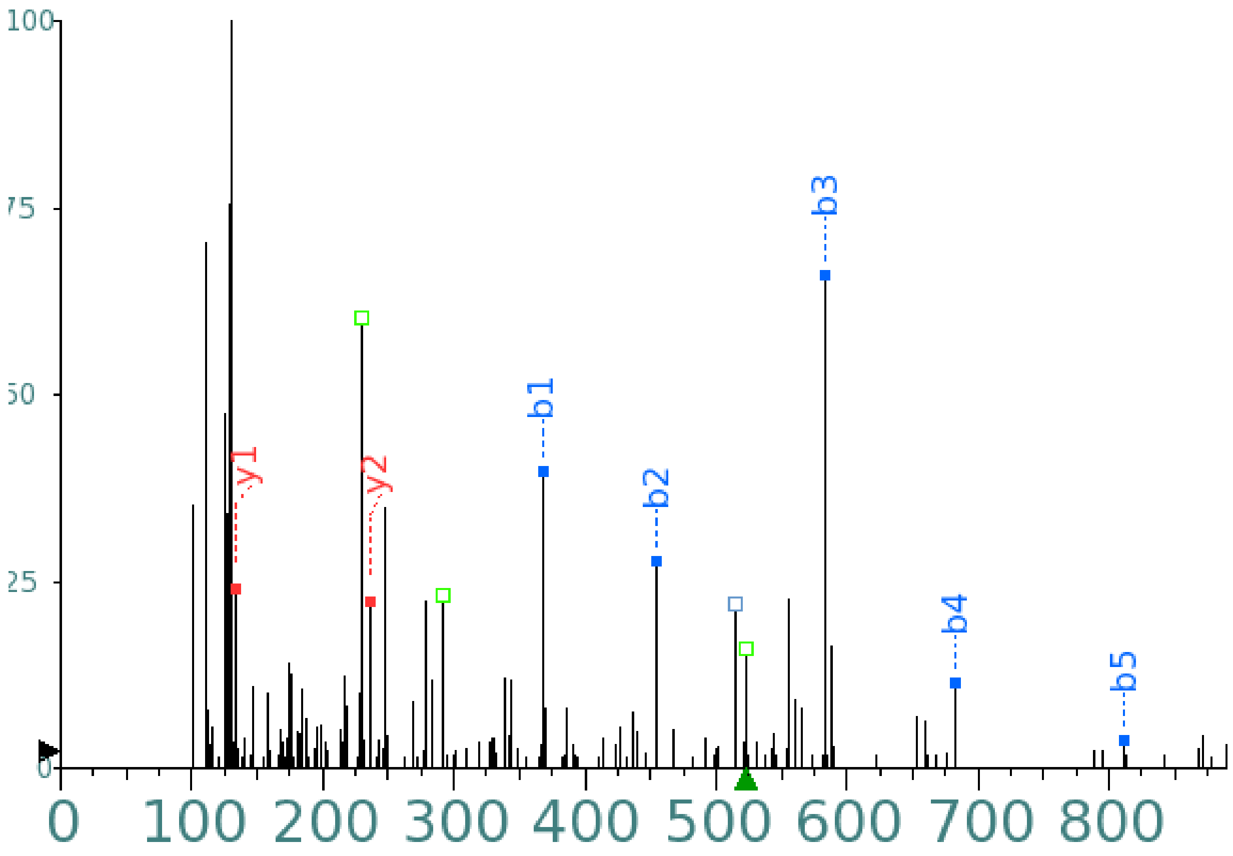

HSEVETD (+2)

Primary Reference: LYAR_HUMAN
Search ID: 41760
Search Name: 20130328_ananiav_ERstress_TMT_TM_timecourse_trypsin_inj1_4mc_iaa
Scan#: 2437
Observed Mass: 523.255 (2.6 ppm)
PSM Score: 17.34

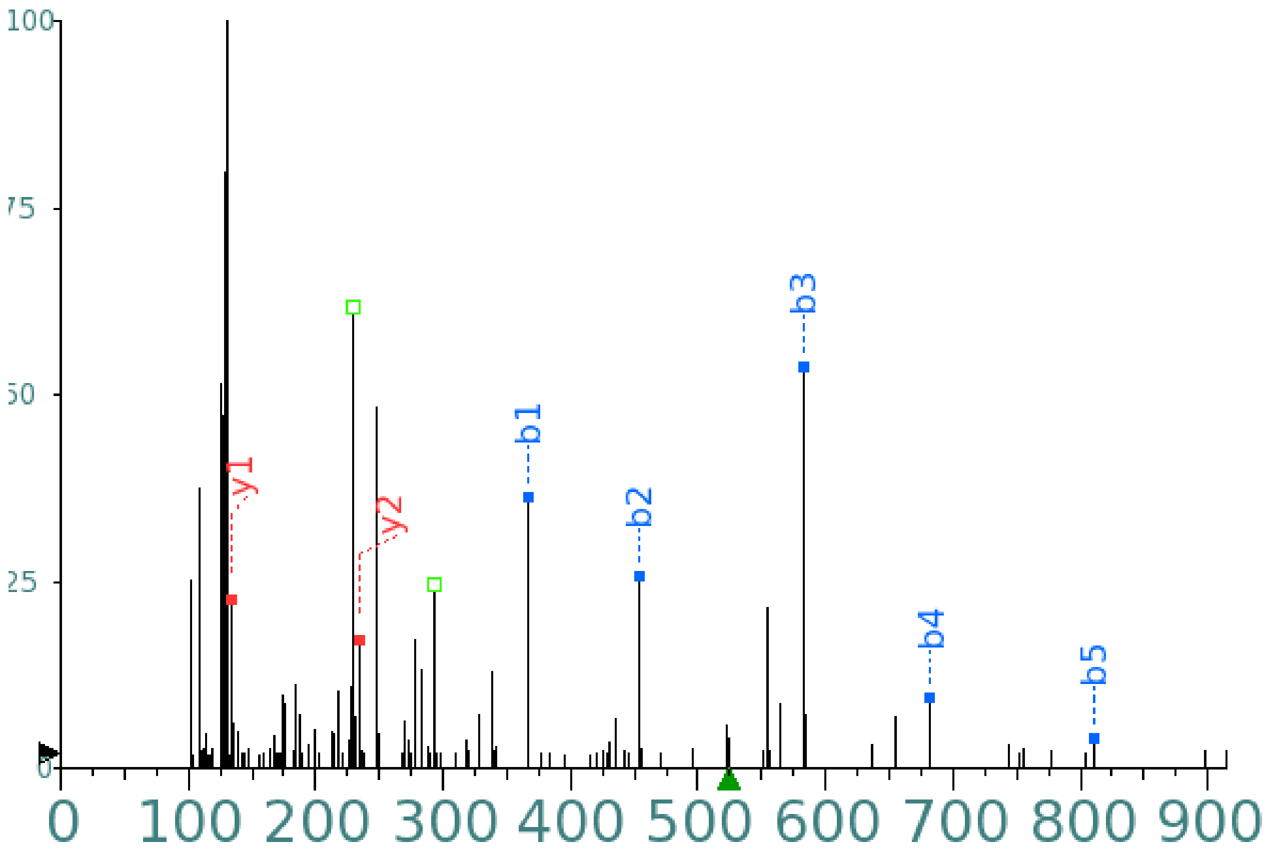

HSEVETD (+2)

Primary Reference: LYAR_HUMAN
Search ID: 41760
Search Name: 20130328_ananiav_ERstress_TMT_TM_timecourse_trypsin_inj1_4mc_iaa
Scan#: 2492
Observed Mass: 523.2552 (3 ppm)
PSM Score: 27.92

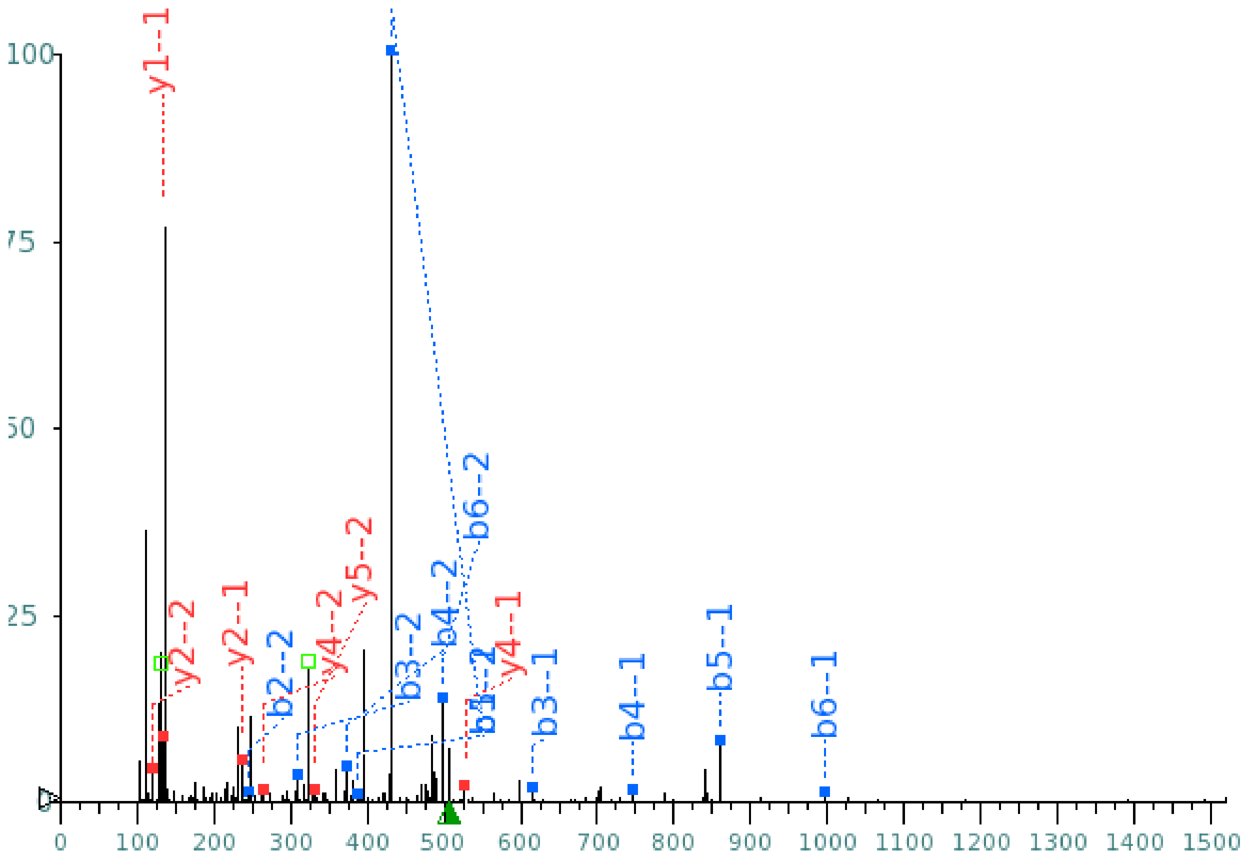

RTEQDHYETD (+3)

Primary Reference: OCLN_HUMAN
Search ID: 41760
Search Name: 20130328_ananiav_ERstress_TMT_TM_timecourse_trypsin_inj1_4mc_iaa
Scan#: 2509
Observed Mass: 508.239 (3.4 ppm)
PSM Score: 8.71

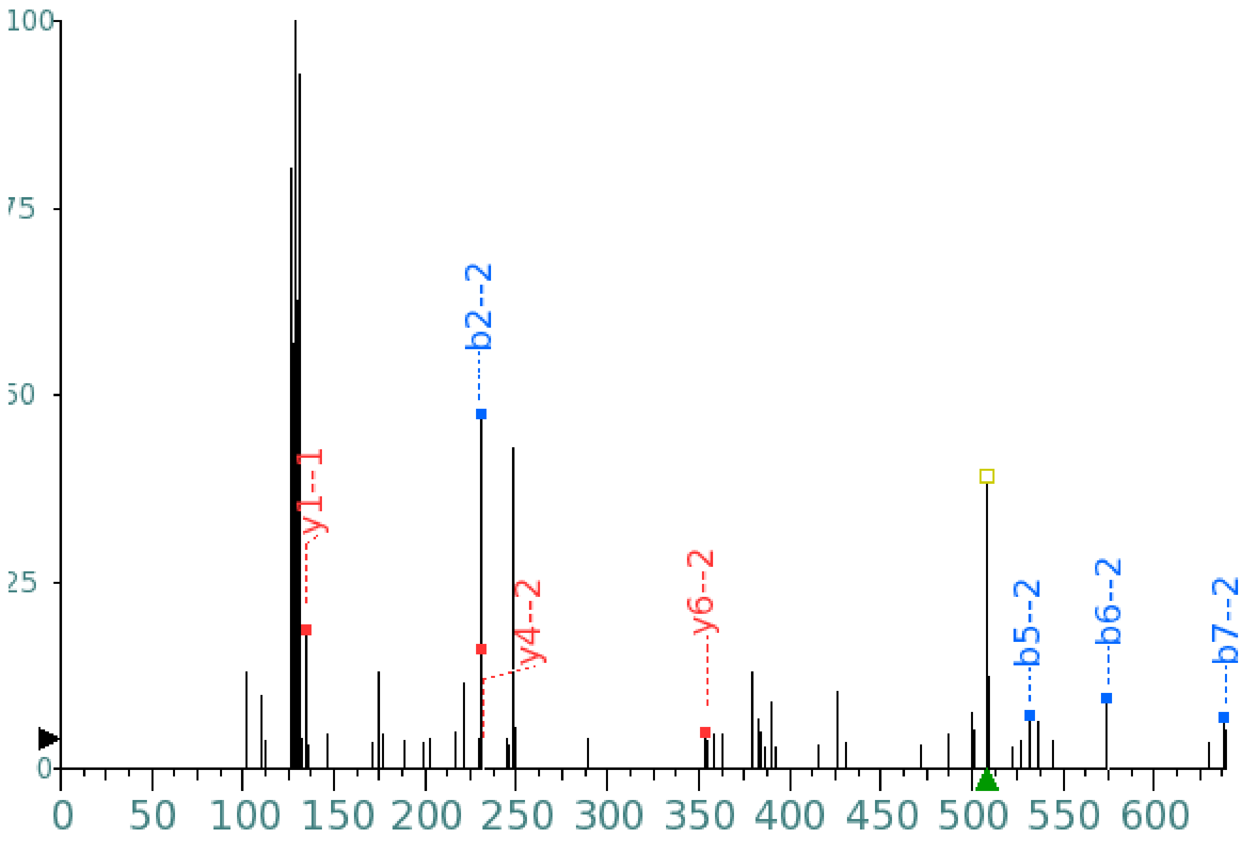

VEKDMSELD (+3)

Primary Reference: ##CTDP1_HUMAN
Search ID: 41760
Search Name: 20130328_ananiav_ERstress_TMT_TM_timecourse_trypsin_inj1_4mc_iaa
Scan#: 2557
Observed Mass: 508.6061 (0.8 ppm)
PSM Score: 9.34

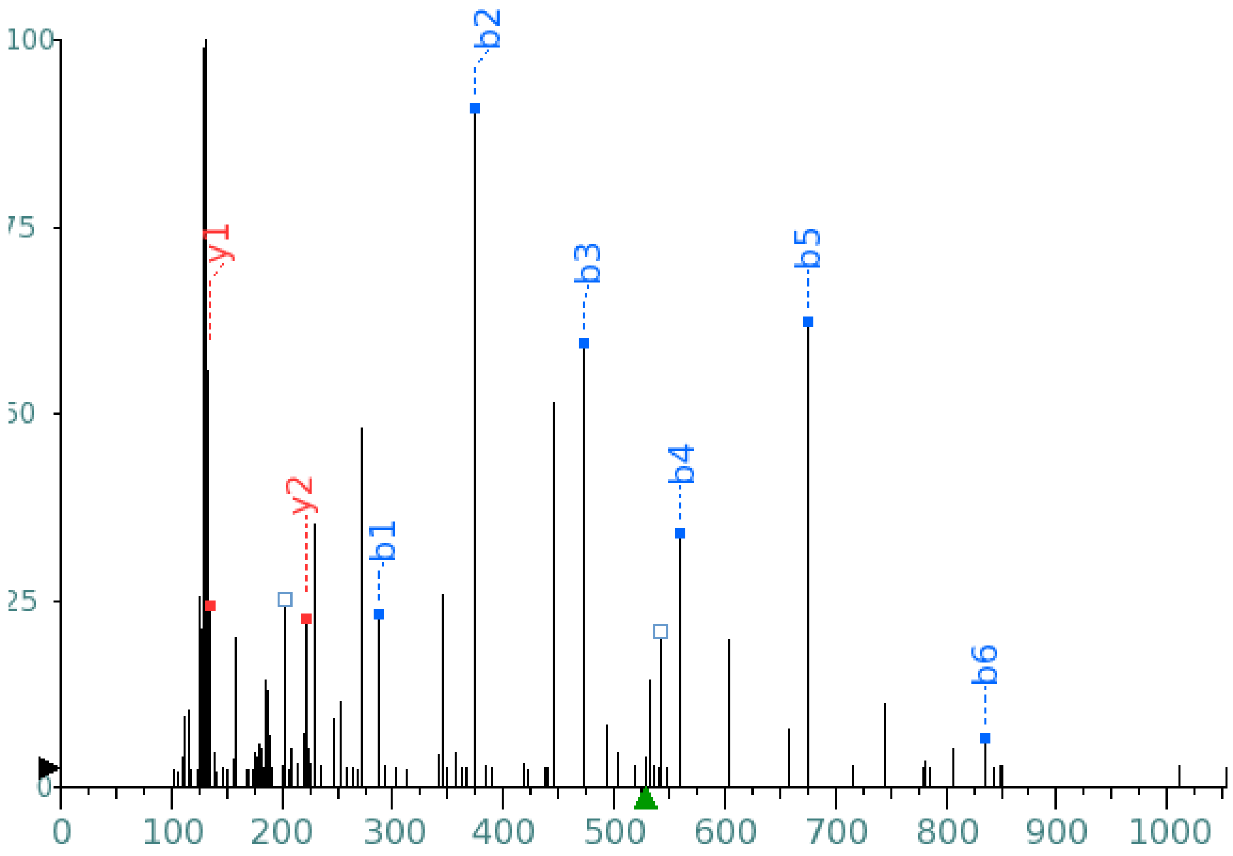

GSVSDC*SD (+2)

Primary Reference: ERF_HUMAN
Search ID: 41760
Search Name: 20130328_ananiav_ERstress_TMT_TM_timecourse_trypsin_inj1_4mc_iaa
Scan#: 2818
Observed Mass: 528.2307 (2.7 ppm)
PSM Score: 34.49

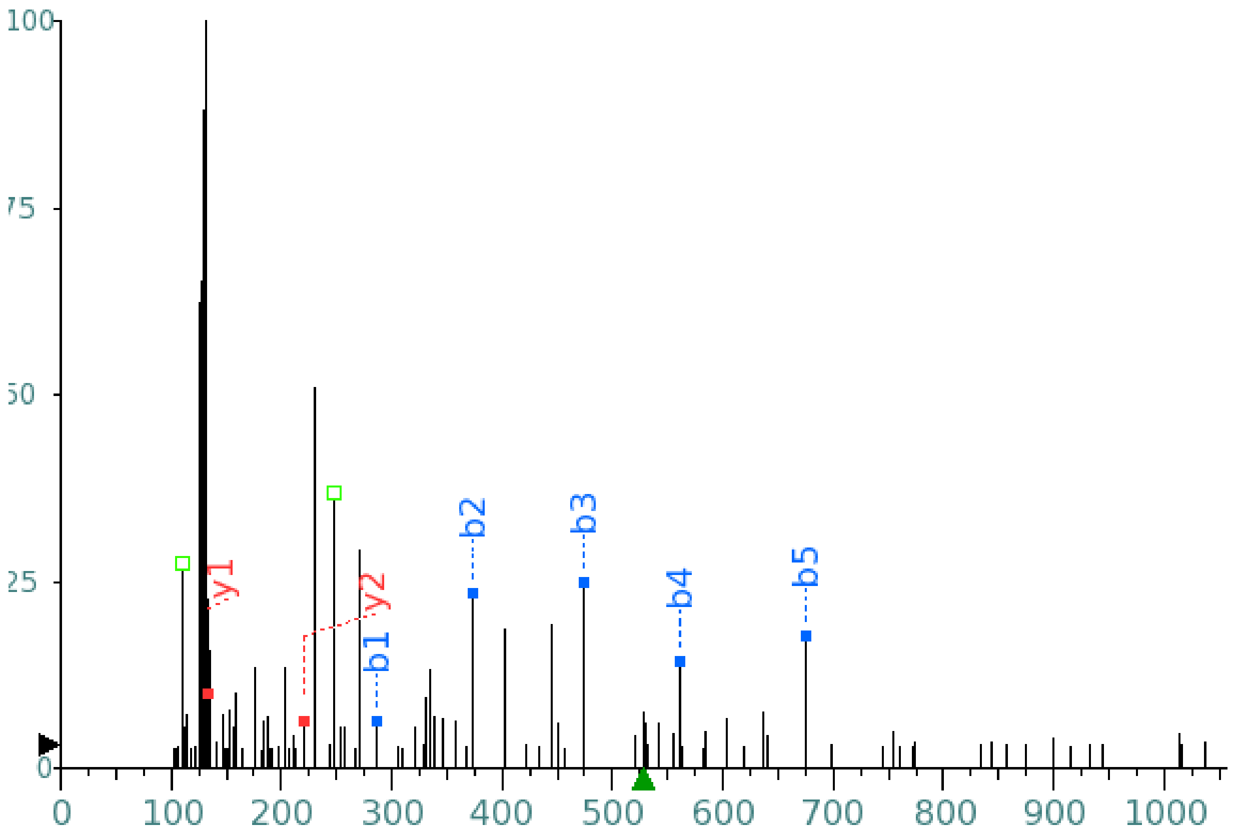

GSVSDC*SD (+2)

Primary Reference: ERF_HUMAN
Search ID: 41760
Search Name: 20130328_ananiav_ERstress_TMT_TM_timecourse_trypsin_inj1_4mc_iaa
Scan#: 2886
Observed Mass: 528.231 (3.2 ppm)
PSM Score: 11.61

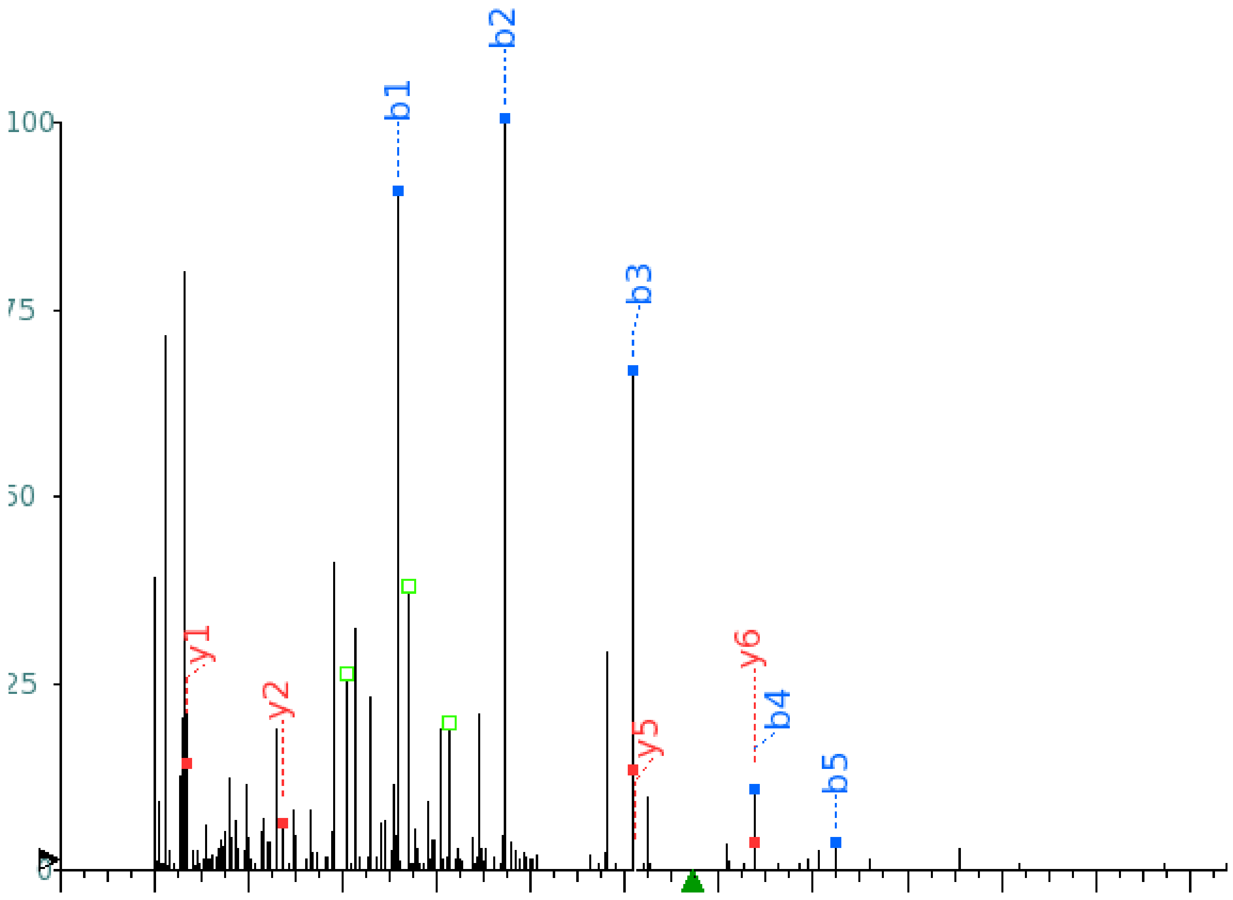

QLHQSC*QTD (+2)

Primary Reference: CSN2_HUMAN
Search ID: 41760
Search Name: 20130328_ananiav_ERstress_TMT_TM_timecourse_trypsin_inj1_4mc_iaa
Scan#: 2929
Observed Mass: 673.3241 (3.1 ppm)
PSM Score: 21.74

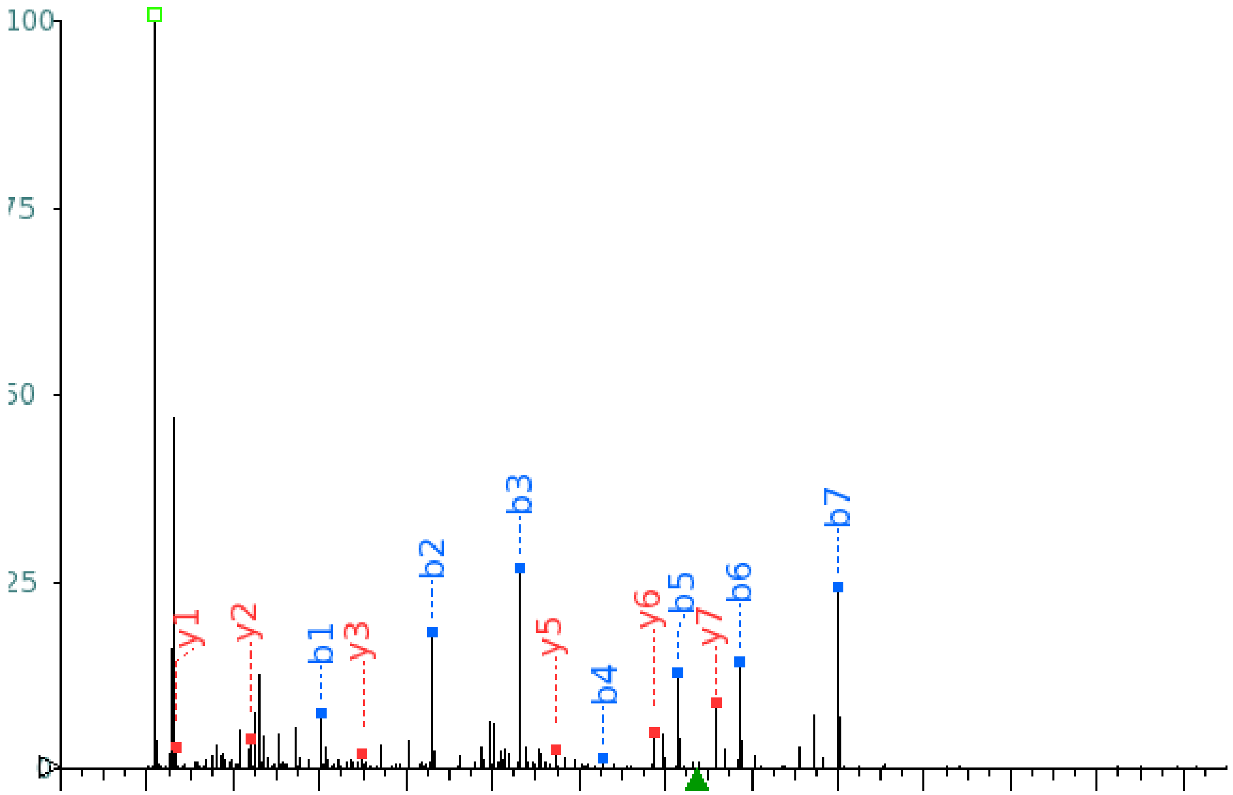

AETPSANHSESD (+2)

Primary Reference: YETS2_HUMAN
Search ID: 41760
Search Name: 20130328_ananiav_ERstress_TMT_TM_timecourse_trypsin_inj1_4mc_iaa
Scan#: 2954
Observed Mass: 737.3376 (1.6 ppm)
PSM Score: 22.31

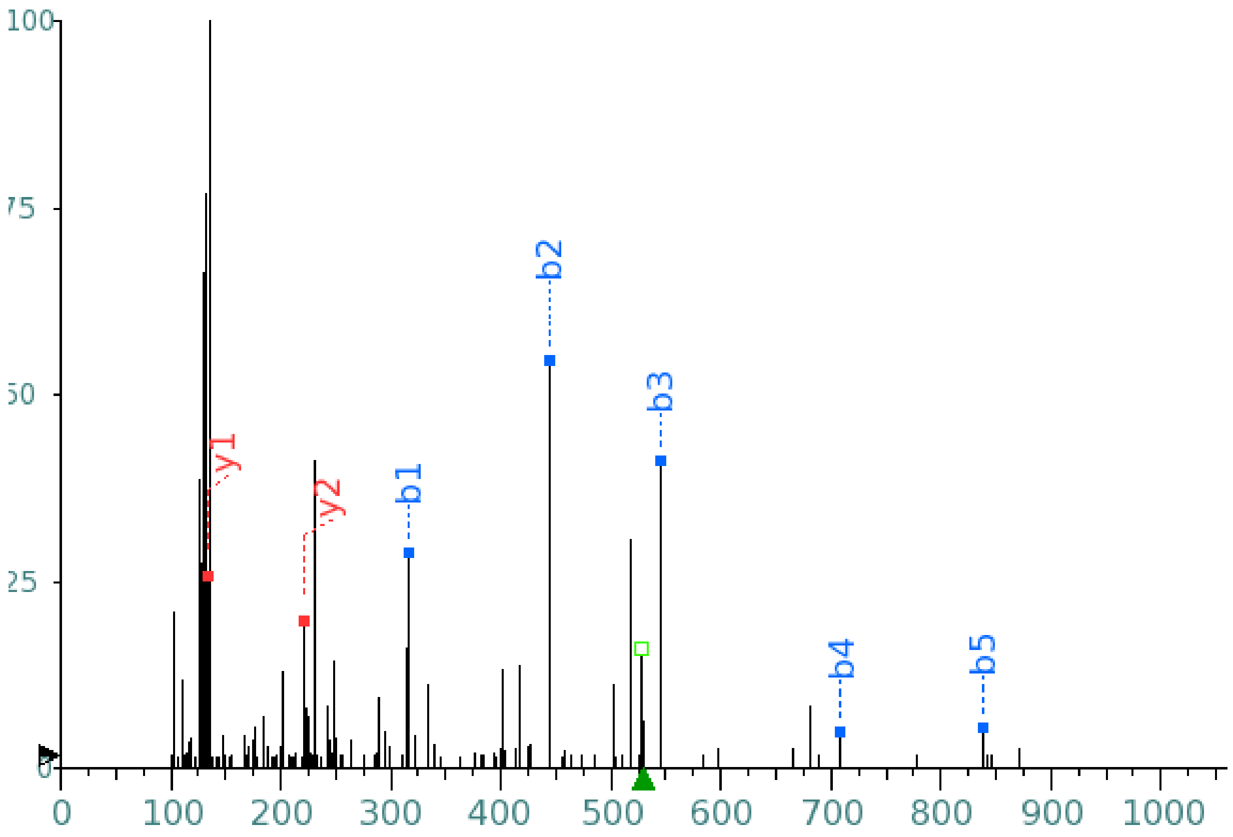

SQTYESD (+2)

Primary Reference: ADAM9_HUMAN
Search ID: 41760
Search Name: 20130328_ananiav_ERstress_TMT_TM_timecourse_trypsin_inj1_4mc_iaa
Scan#: 2971
Observed Mass: 529.747 (2.6 ppm)
PSM Score: 31.37

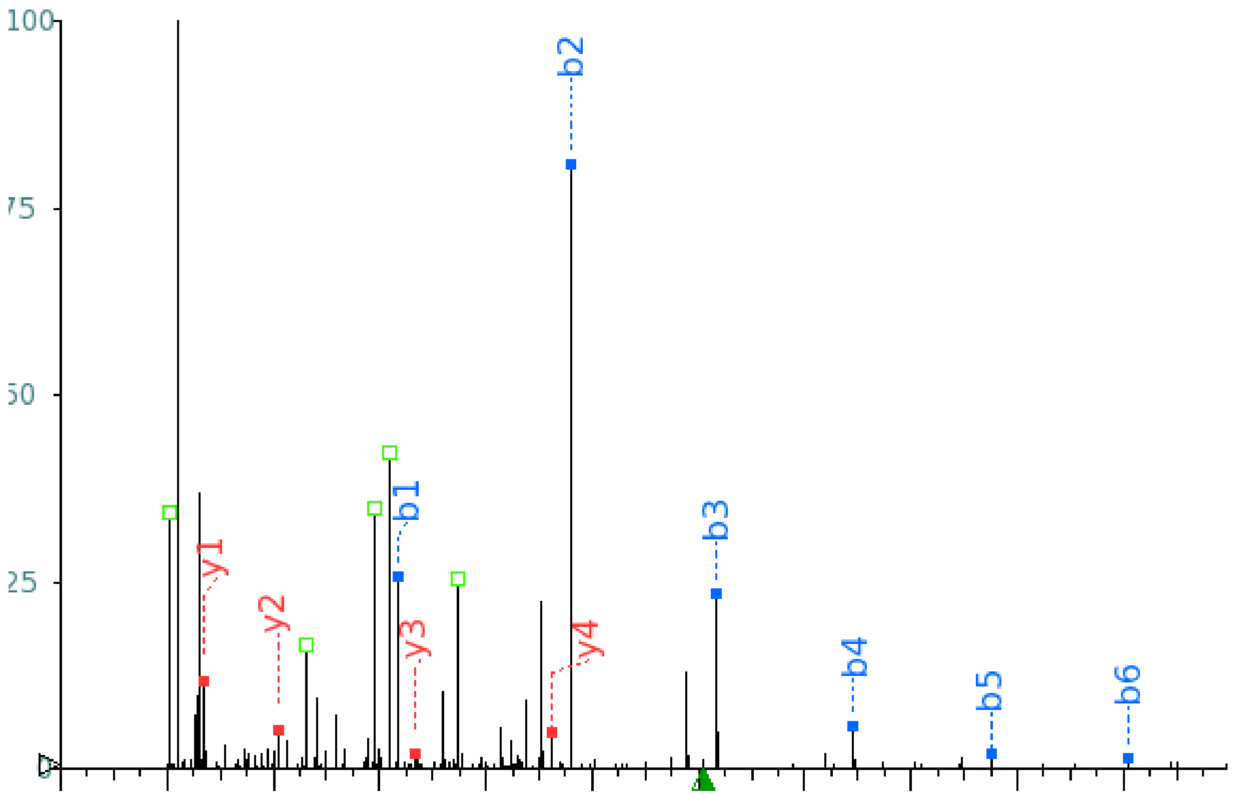

SYHEEEAD (+2)

Primary Reference: RAD9A_HUMAN
Search ID: 41760
Search Name: 20130328_ananiav_ERstress_TMT_TM_timecourse_trypsin_inj1_4mc_iaa
Scan#: 3019
Observed Mass: 604.7677 (1 ppm)
PSM Score: 20.79

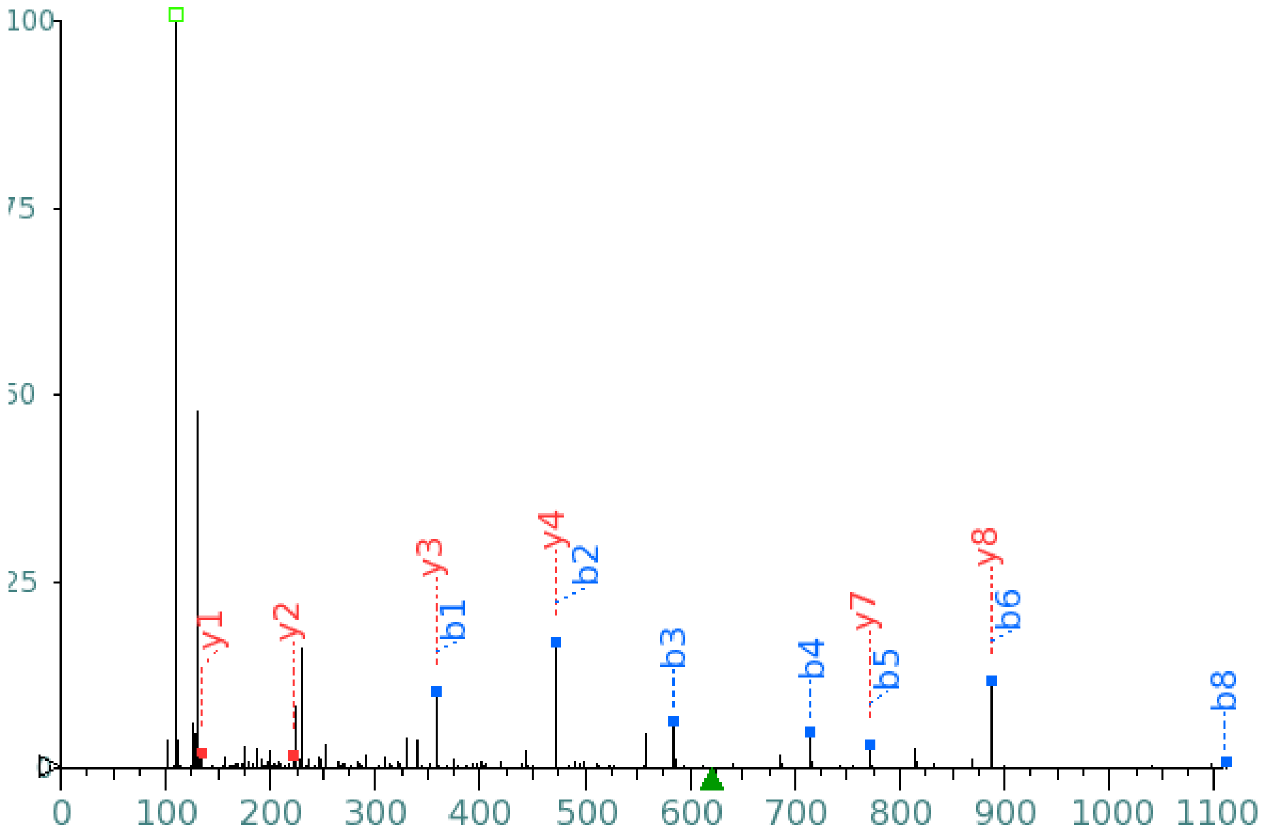

QNLEGDHSD (+2)

Primary Reference: HS74L_HUMAN
Search ID: 41760
Search Name: 20130328_ananiav_ERstress_TMT_TM_timecourse_trypsin_inj1_4mc_iaa
Scan#: 3107
Observed Mass: 622.2929 (2.6 ppm)
PSM Score: 25.92

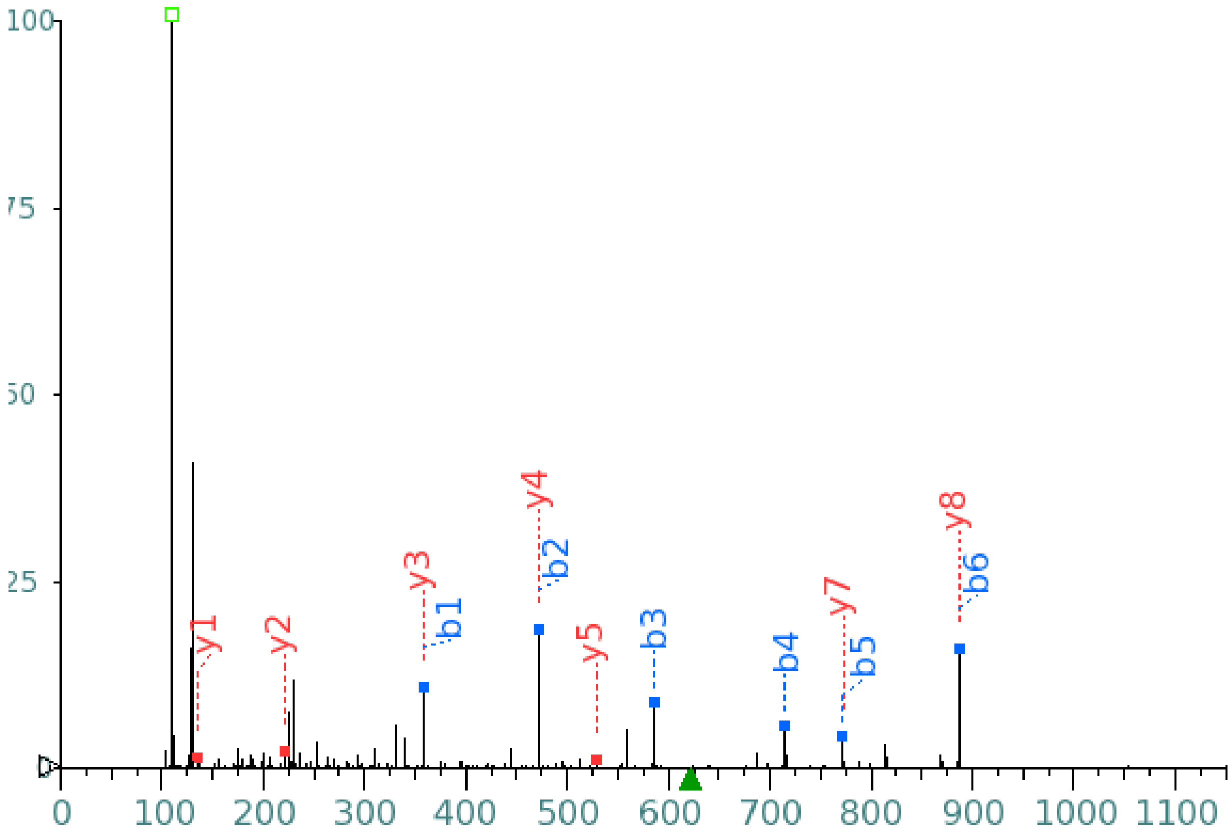

QNLEGDHSD (+2)

Primary Reference: HS74L_HUMAN
Search ID: 41760
Search Name: 20130328_ananiav_ERstress_TMT_TM_timecourse_trypsin_inj1_4mc_iaa
Scan#: 3168
Observed Mass: 622.2929 (2.6 ppm)
PSM Score: 29.98

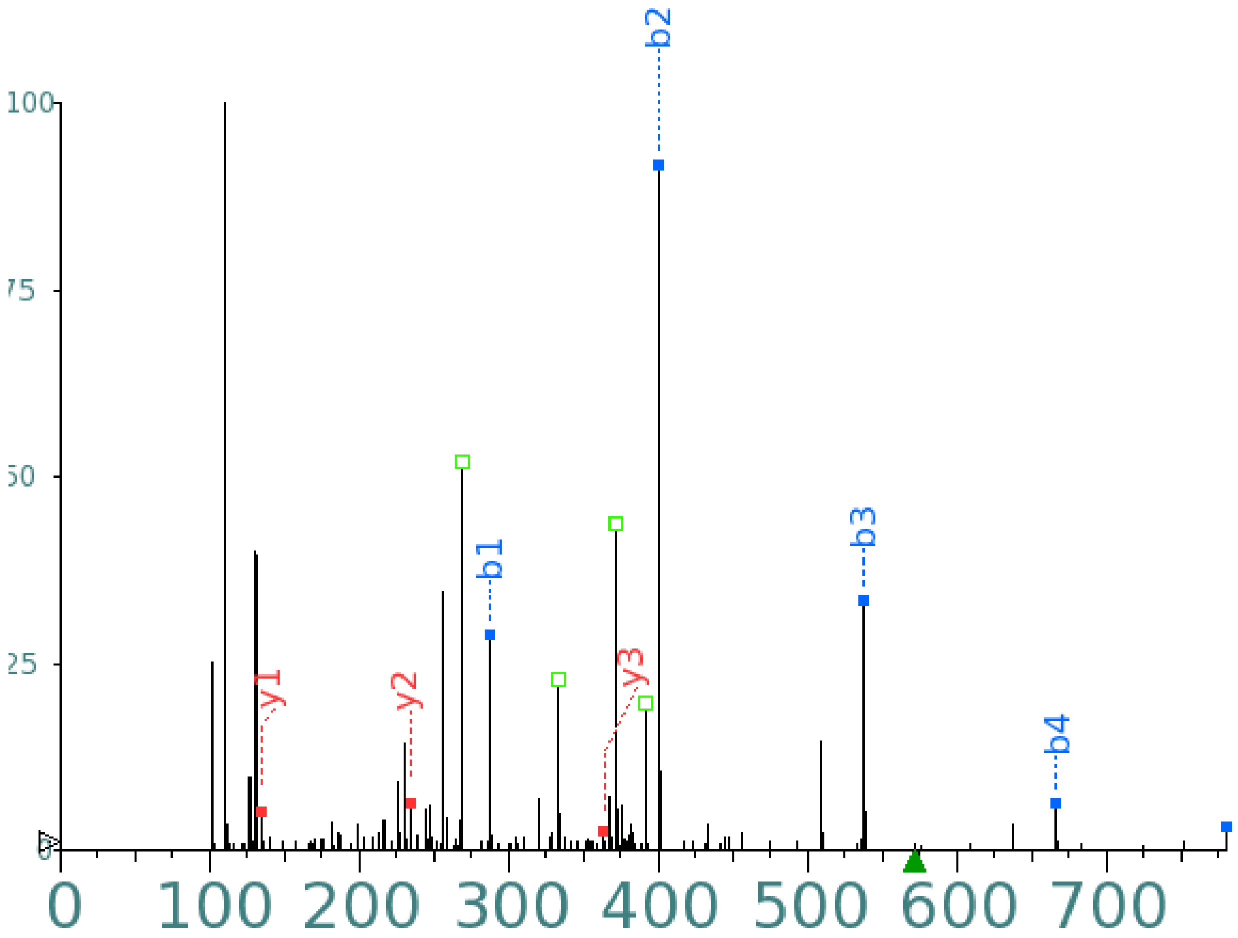

GIHENETD (+2)

Primary Reference: CC017_HUMAN
Search ID: 41760
Search Name: 20130328_ananiav_ERstress_TMT_TM_timecourse_trypsin_inj1_4mc_iaa
Scan#: 3176
Observed Mass: 572.2793 (3 ppm)
PSM Score: 21.84

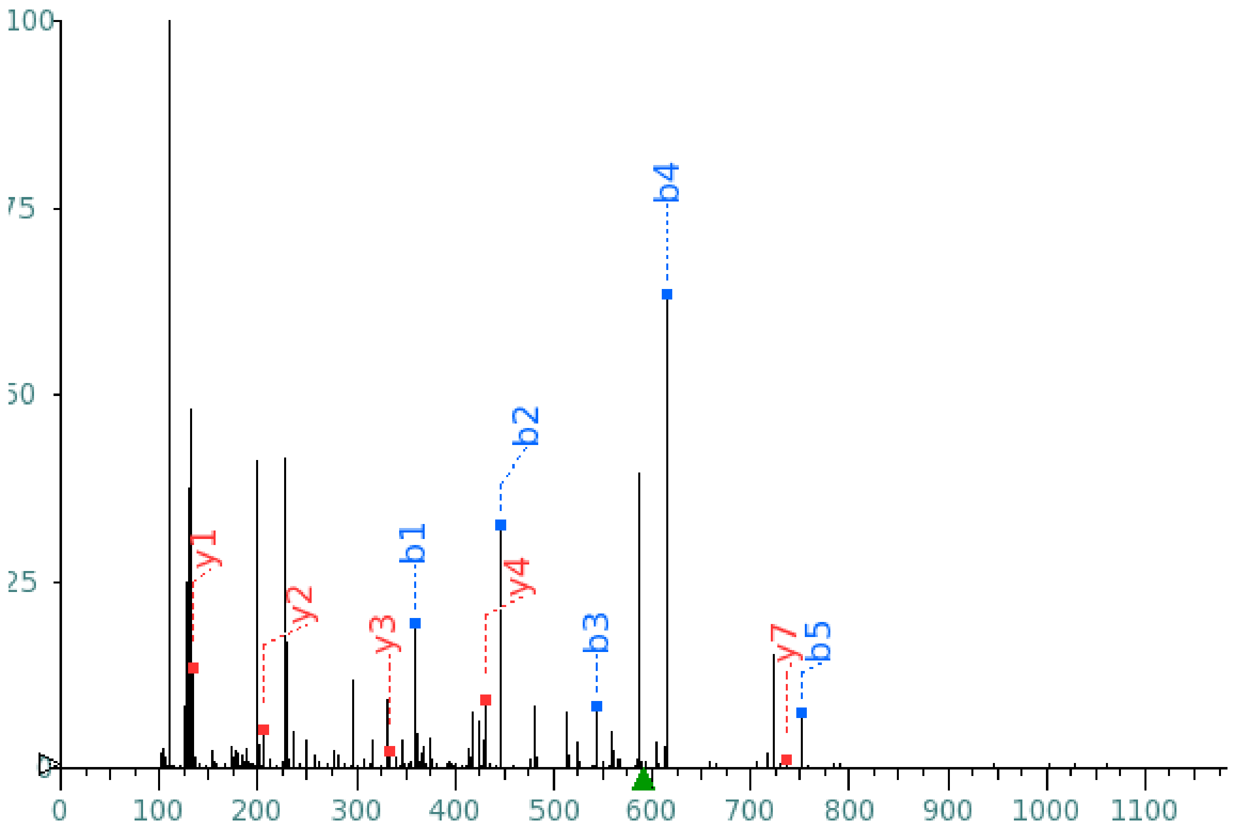

ESPAHPEAD (+2)

Primary Reference: AK17A_HUMAN
Search ID: 41760
Search Name: 20130328_ananiav_ERstress_TMT_TM_timecourse_trypsin_inj1_4mc_iaa
Scan#: 3224
Observed Mass: 591.2897 (7.3 ppm)
PSM Score: 13.78

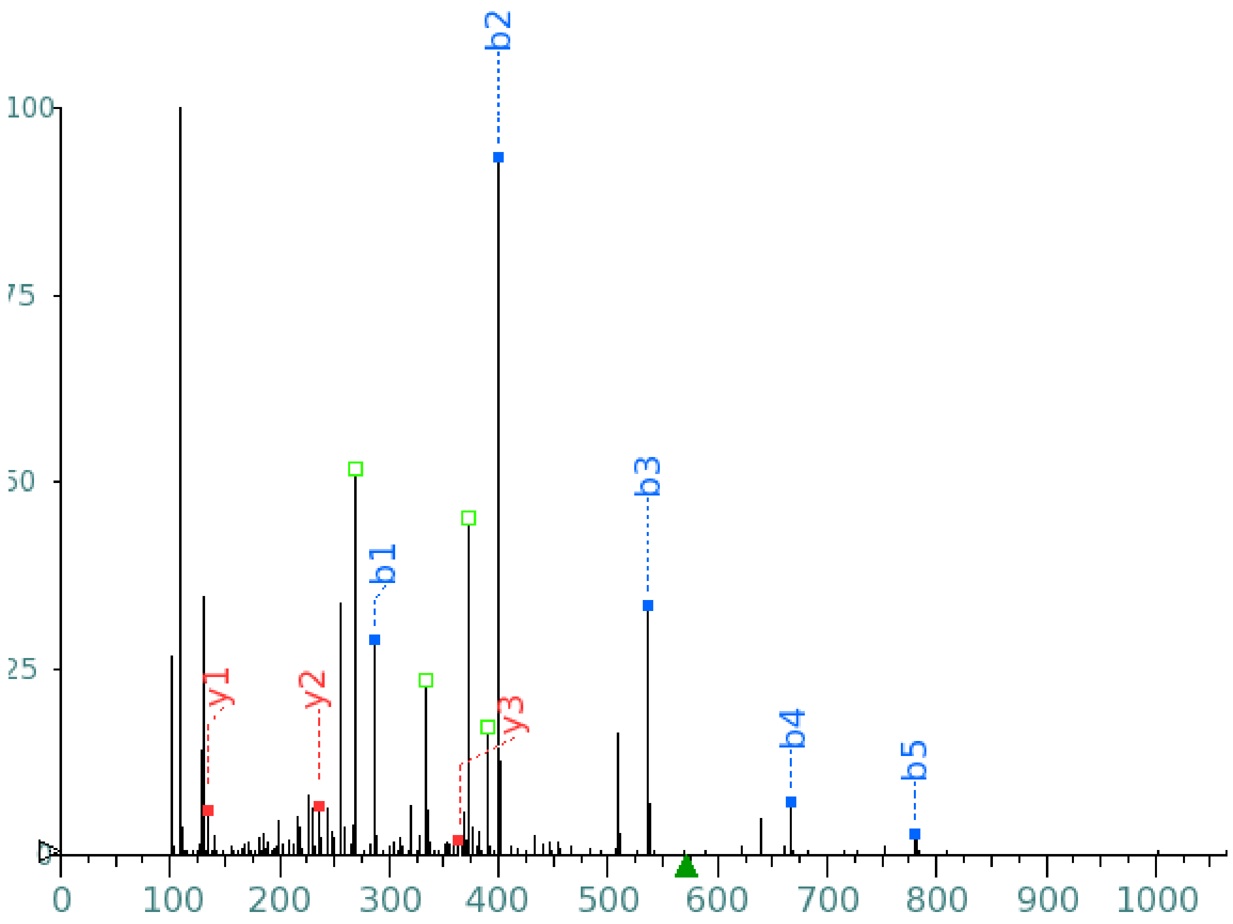

GIHENETD (+2)

Primary Reference: CC017_HUMAN
Search ID: 41760
Search Name: 20130328_ananiav_ERstress_TMT_TM_timecourse_trypsin_inj1_4mc_iaa
Scan#: 3234
Observed Mass: 572.2791 (2.6 ppm)
PSM Score: 17.8

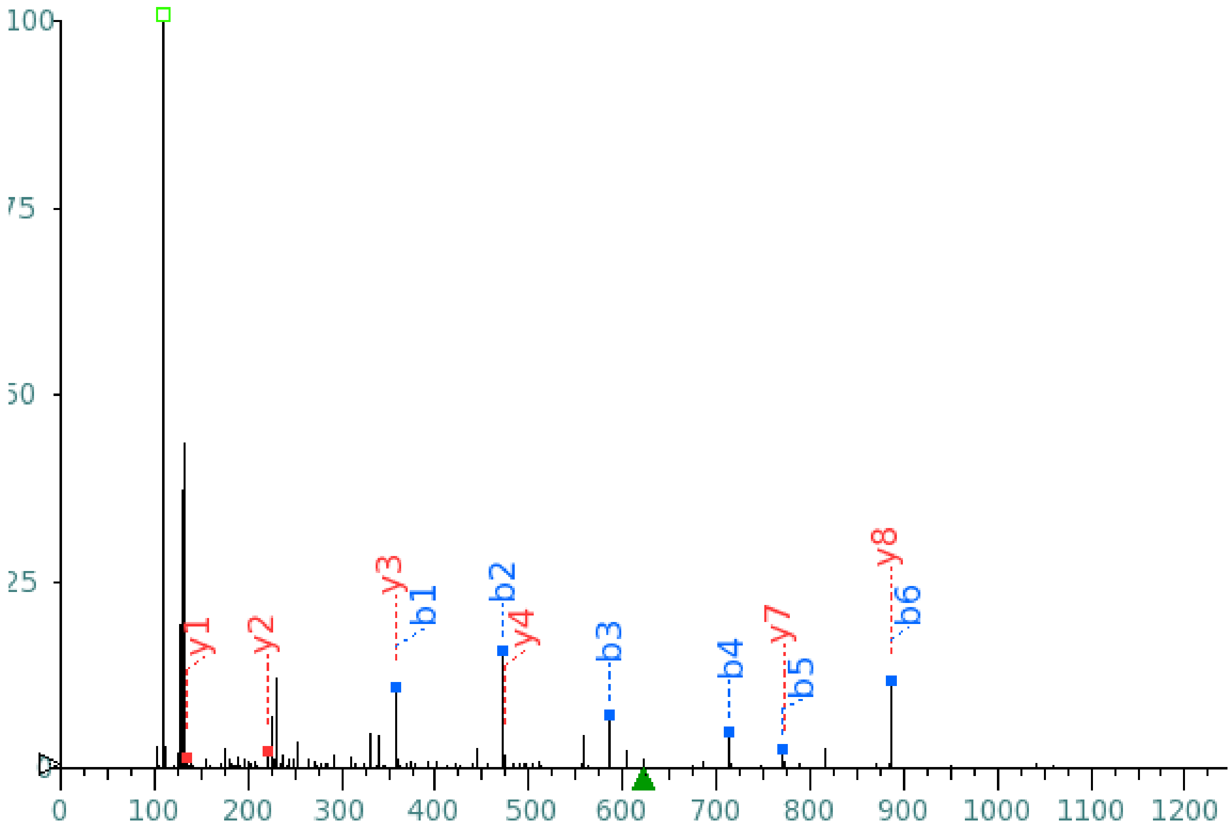

QNLEGDHSD (+2)

Primary Reference: HS74L_HUMAN
Search ID: 41760
Search Name: 20130328_ananiav_ERstress_TMT_TM_timecourse_trypsin_inj1_4mc_iaa
Scan#: 3238
Observed Mass: 622.2928 (2.5 ppm)
PSM Score: 28.47

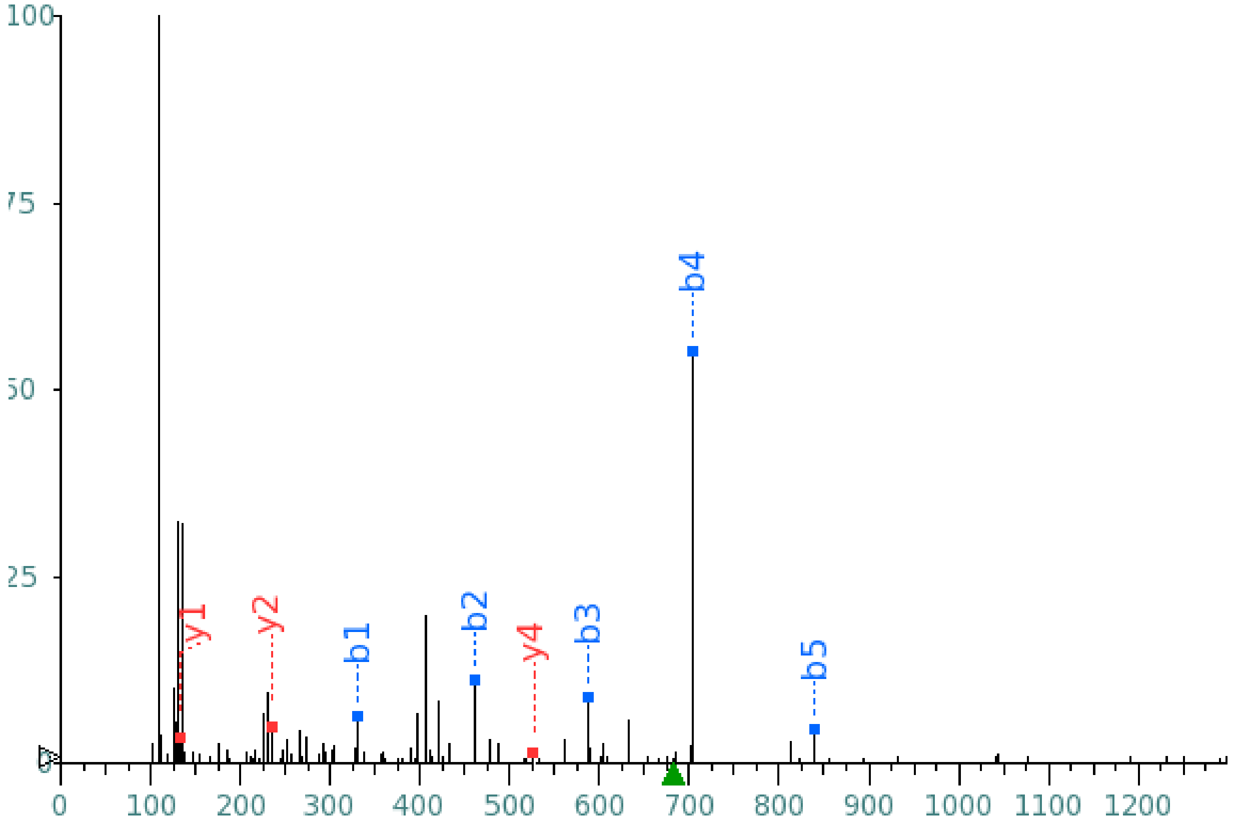

TEQDHYETD (+2)

Primary Reference: OCLN_HUMAN
Search ID: 41760
Search Name: 20130328_ananiav_ERstress_TMT_TM_timecourse_trypsin_inj1_4mc_iaa
Scan#: 3265
Observed Mass: 683.8029 (1.8 ppm)
PSM Score: 10.13

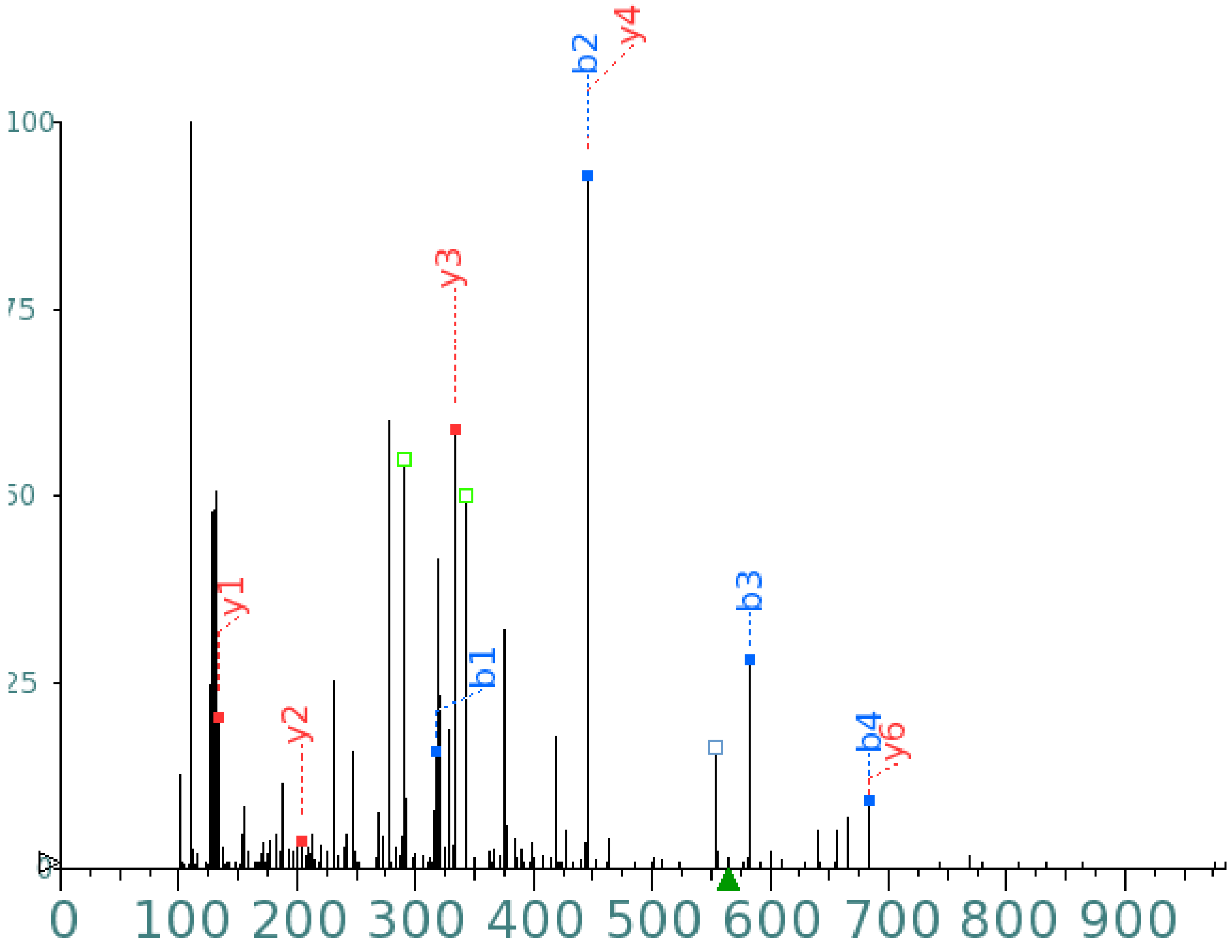

SQHTLQAD (+2)

Primary Reference: HD_HUMAN
Search ID: 41760
Search Name: 20130328_ananiav_ERstress_TMT_TM_timecourse_trypsin_inj1_4mc_iaa
Scan#: 3287
Observed Mass: 564.7968 (1.5 ppm)
PSM Score: 8.72

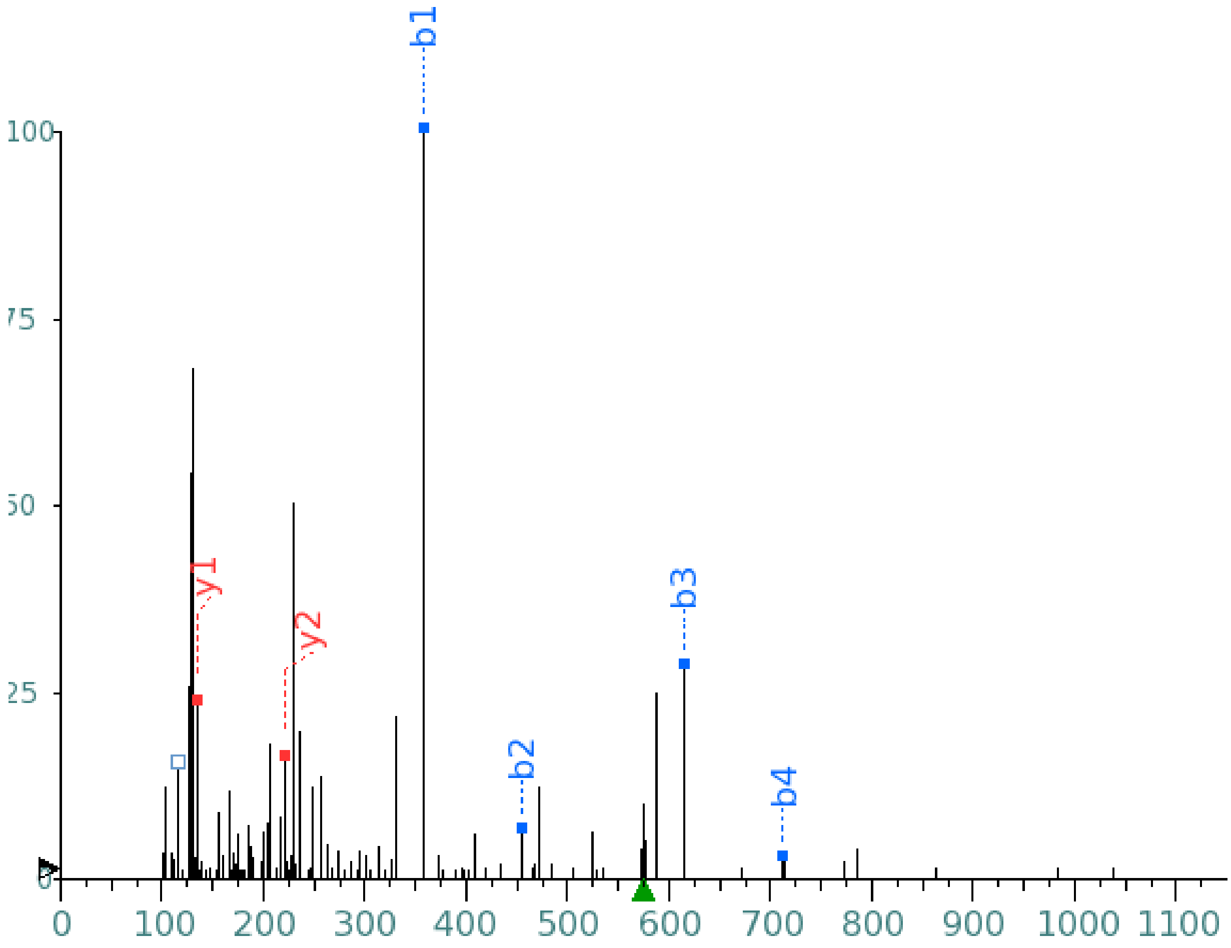

QPC*PSESD (+2)

Primary Reference: PSIP1_HUMAN
Search ID: 41760
Search Name: 20130328_ananiav_ERstress_TMT_TM_timecourse_trypsin_inj1_4mc_iaa
Scan#: 3292
Observed Mass: 574.7584 (0.4 ppm)
PSM Score: 7.31

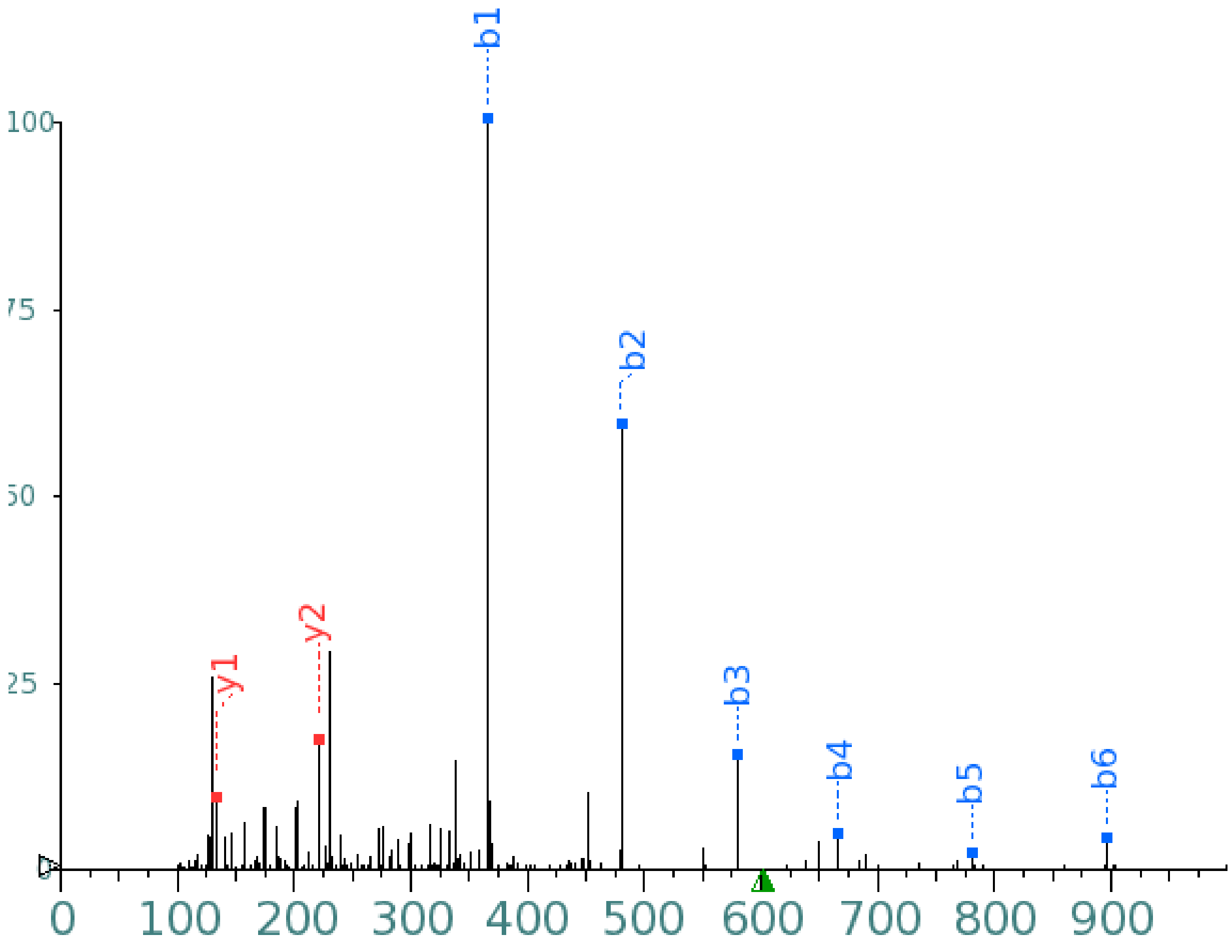

HIVSNDSSD (+2)

Primary Reference: PA24A_HUMAN
Search ID: 41760
Search Name: 20130328_ananiav_ERstress_TMT_TM_timecourse_trypsin_inj1_4mc_iaa
Scan#: 3444
Observed Mass: 601.7963 (0.3 ppm)
PSM Score: 45.49

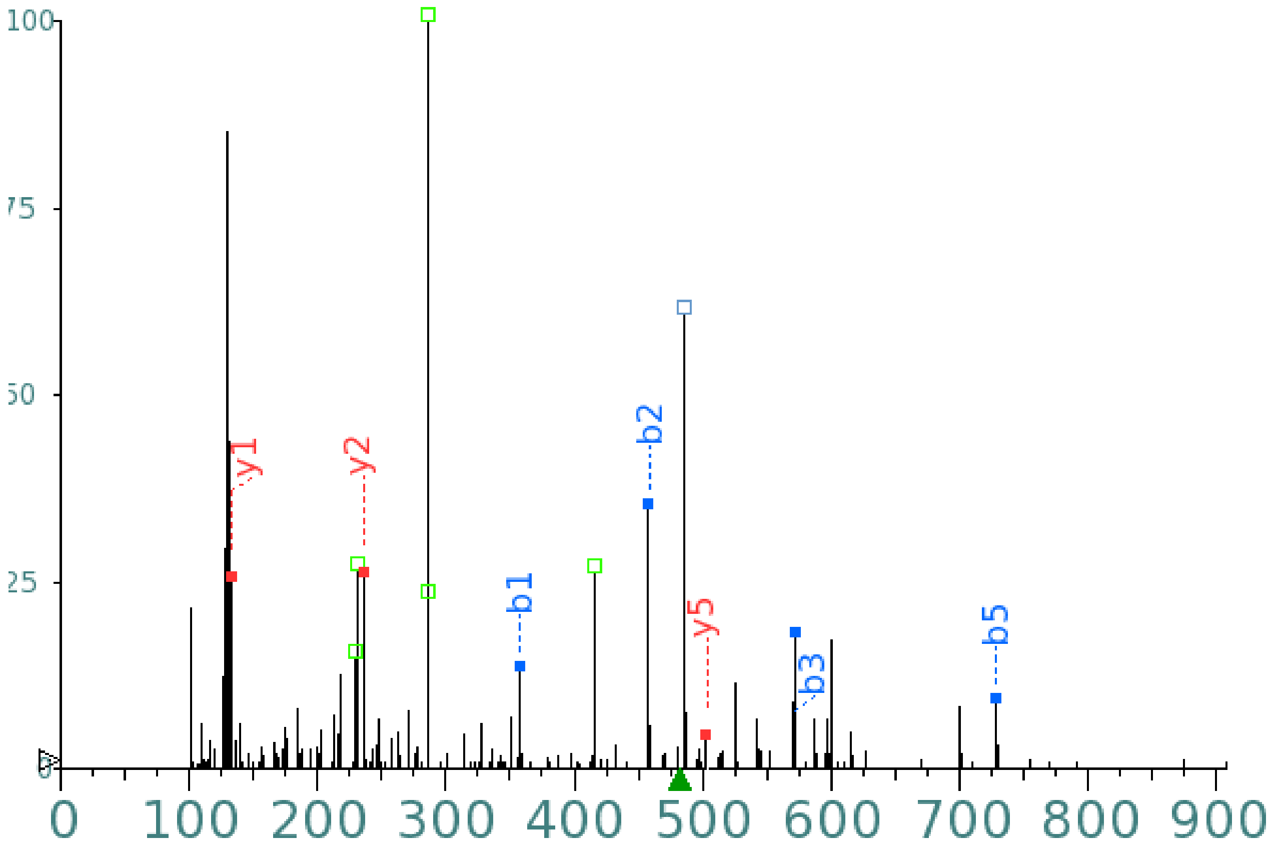

QTLVGTD (+2)

Primary Reference: F8WCT0_HUMAN
Search ID: 41760
Search Name: 20130328_ananiav_ERstress_TMT_TM_timecourse_trypsin_inj1_4mc_iaa
Scan#: 3476
Observed Mass: 481.7718 (0.7 ppm)
PSM Score: 12.83

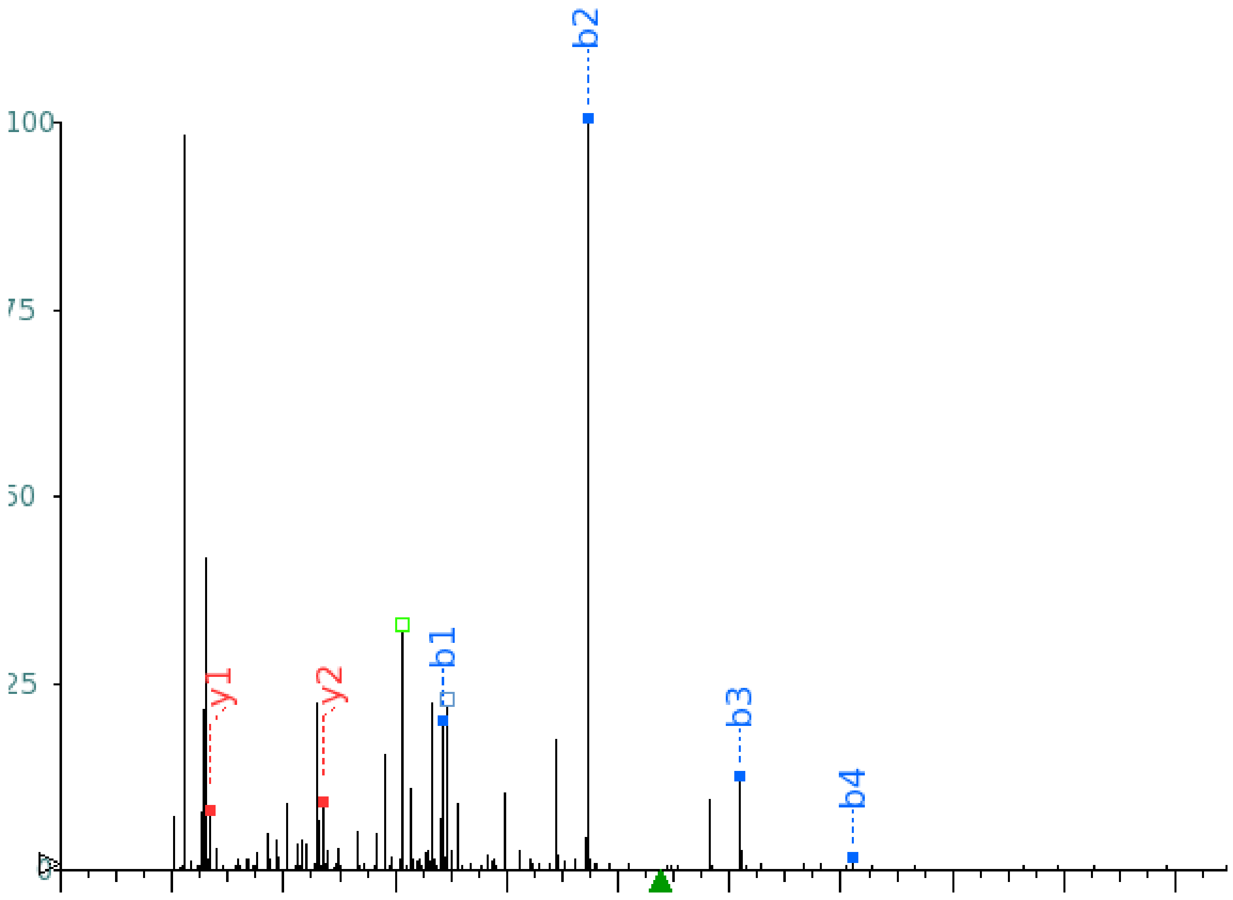

IEHTETD (+2)

Primary Reference: TM63B_HUMAN
Search ID: 41760
Search Name: 20130328_ananiav_ERstress_TMT_TM_timecourse_trypsin_inj1_4mc_iaa
Scan#: 3543
Observed Mass: 537.2702 (1.8 ppm)
PSM Score: 7.14

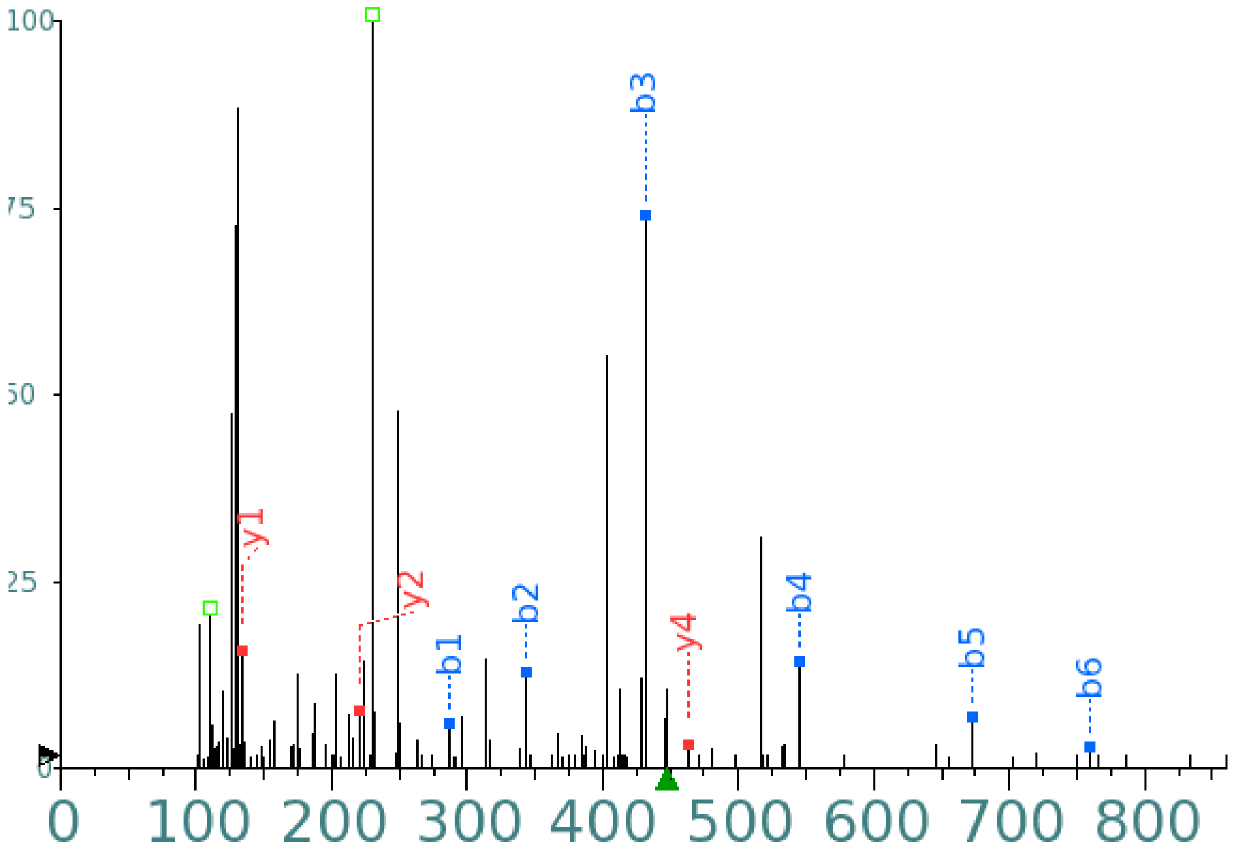

GGSLESD (+2)

Primary Reference: MECP2_HUMAN
Search ID: 41760
Search Name: 20130328_ananiav_ERstress_TMT_TM_timecourse_trypsin_inj1_4mc_iaa
Scan#: 3708
Observed Mass: 447.2256 (2.8 ppm)
PSM Score: 16.18

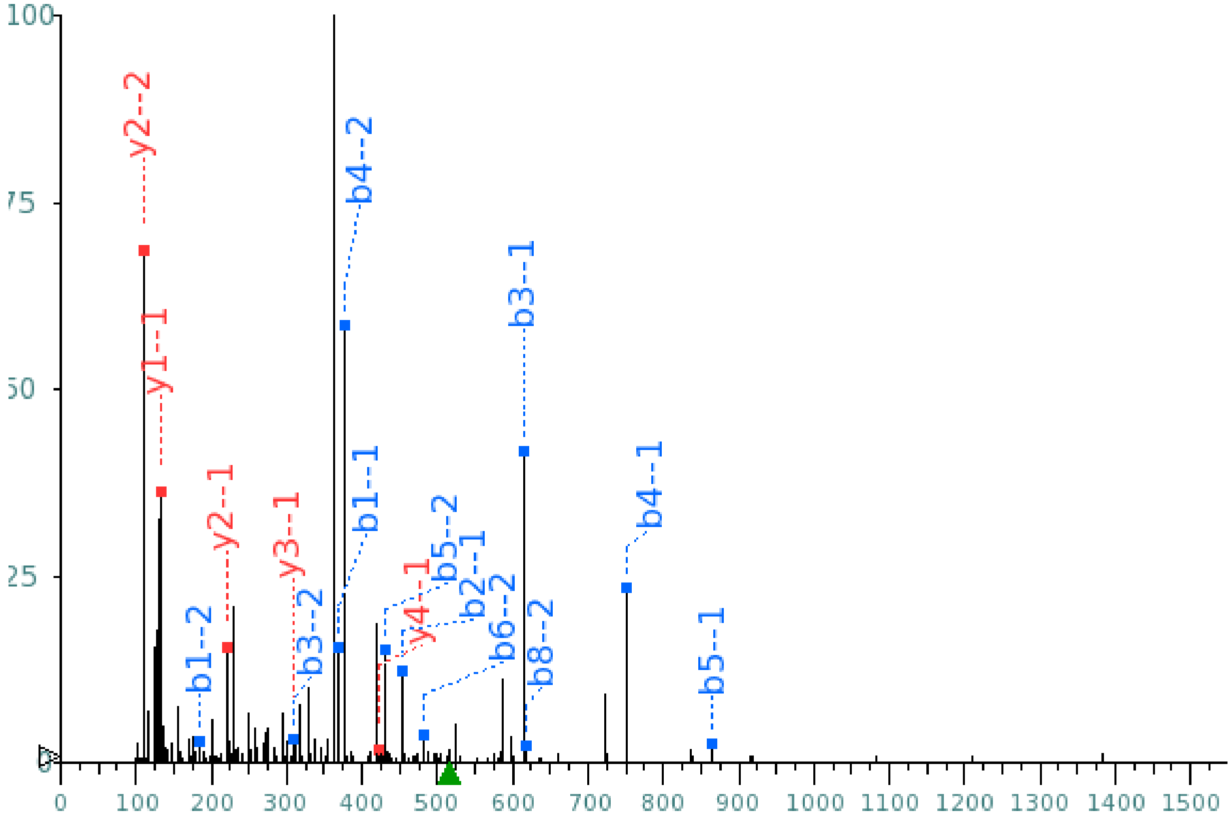

HSC*HLVC*DSSD (+3)

Primary Reference: RGS3_HUMAN
Search ID: 41760
Search Name: 20130328_ananiav_ERstress_TMT_TM_timecourse_trypsin_inj1_4mc_iaa
Scan#: 3757
Observed Mass: 515.8936 (2.4 ppm)
PSM Score: 10.4

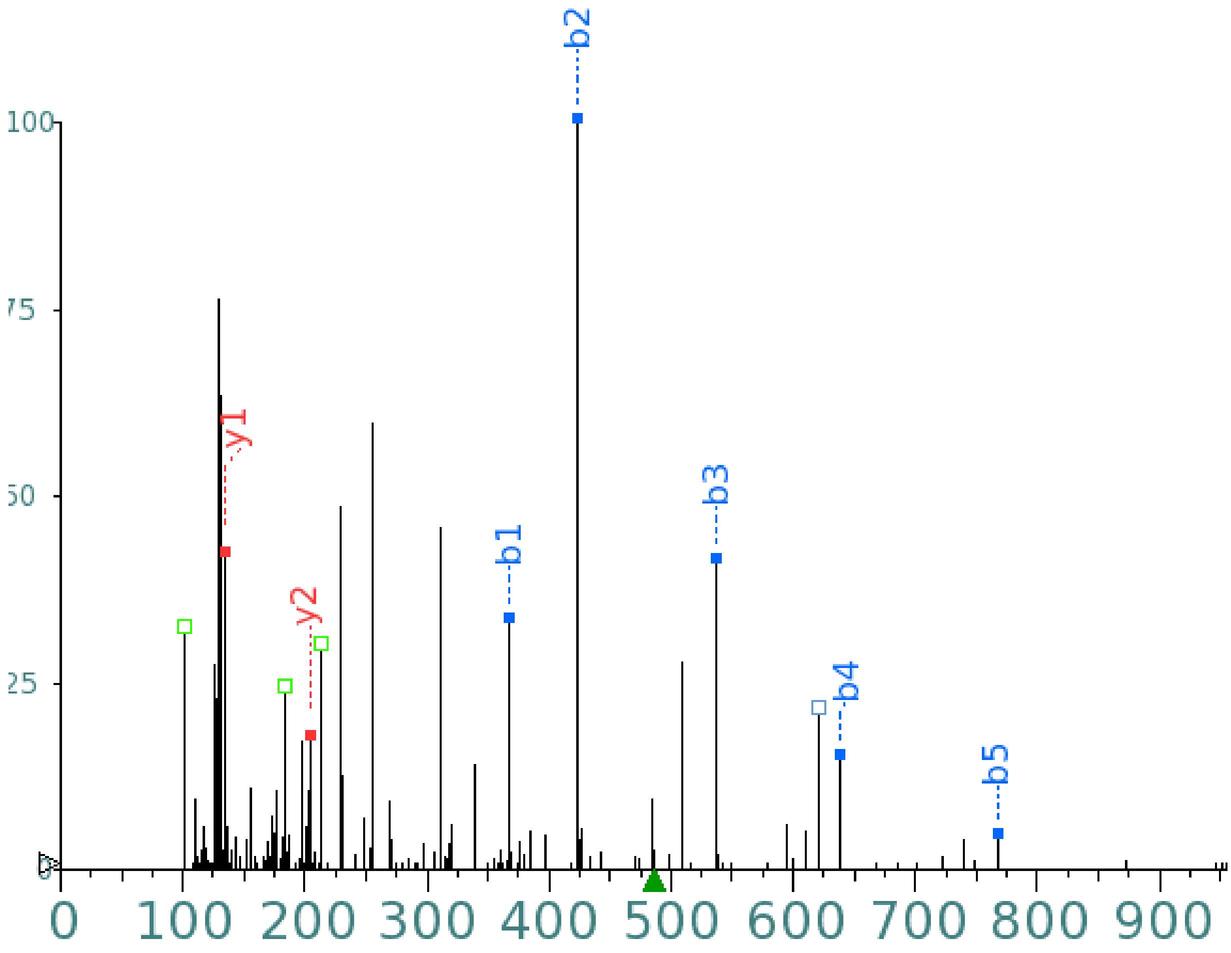

HGLTEAD (+2)

Primary Reference: PUS7_HUMAN
Search ID: 41760
Search Name: 20130328_ananiav_ERstress_TMT_TM_timecourse_trypsin_inj1_4mc_iaa
Scan#: 3787
Observed Mass: 486.2551 (3.4 ppm)
PSM Score: 22.99

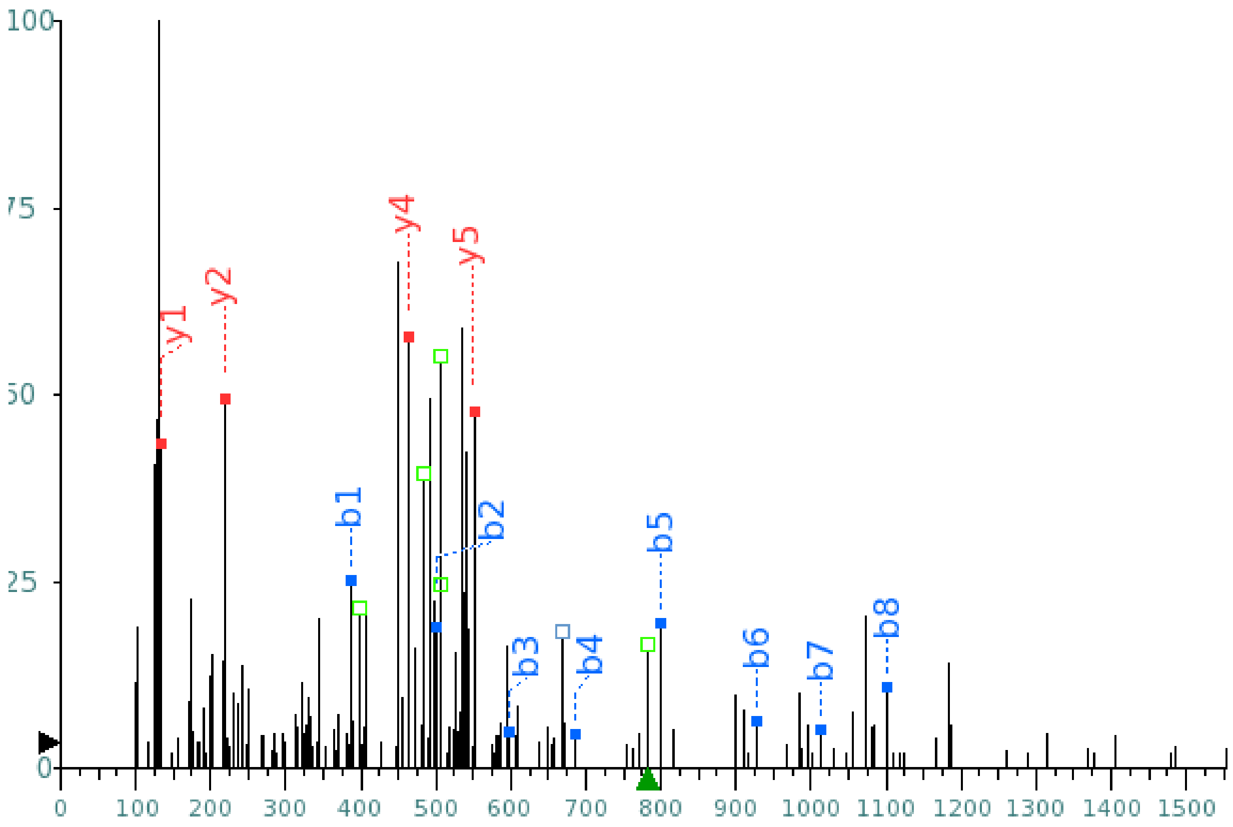

RNPSNQSSLESD (+2)

Primary Reference: MYO5B_HUMAN
Search ID: 41760
Search Name: 20130328_ananiav_ERstress_TMT_TM_timecourse_trypsin_inj1_4mc_iaa
Scan#: 3825
Observed Mass: 781.8852 (1.5 ppm)
PSM Score: 27.26

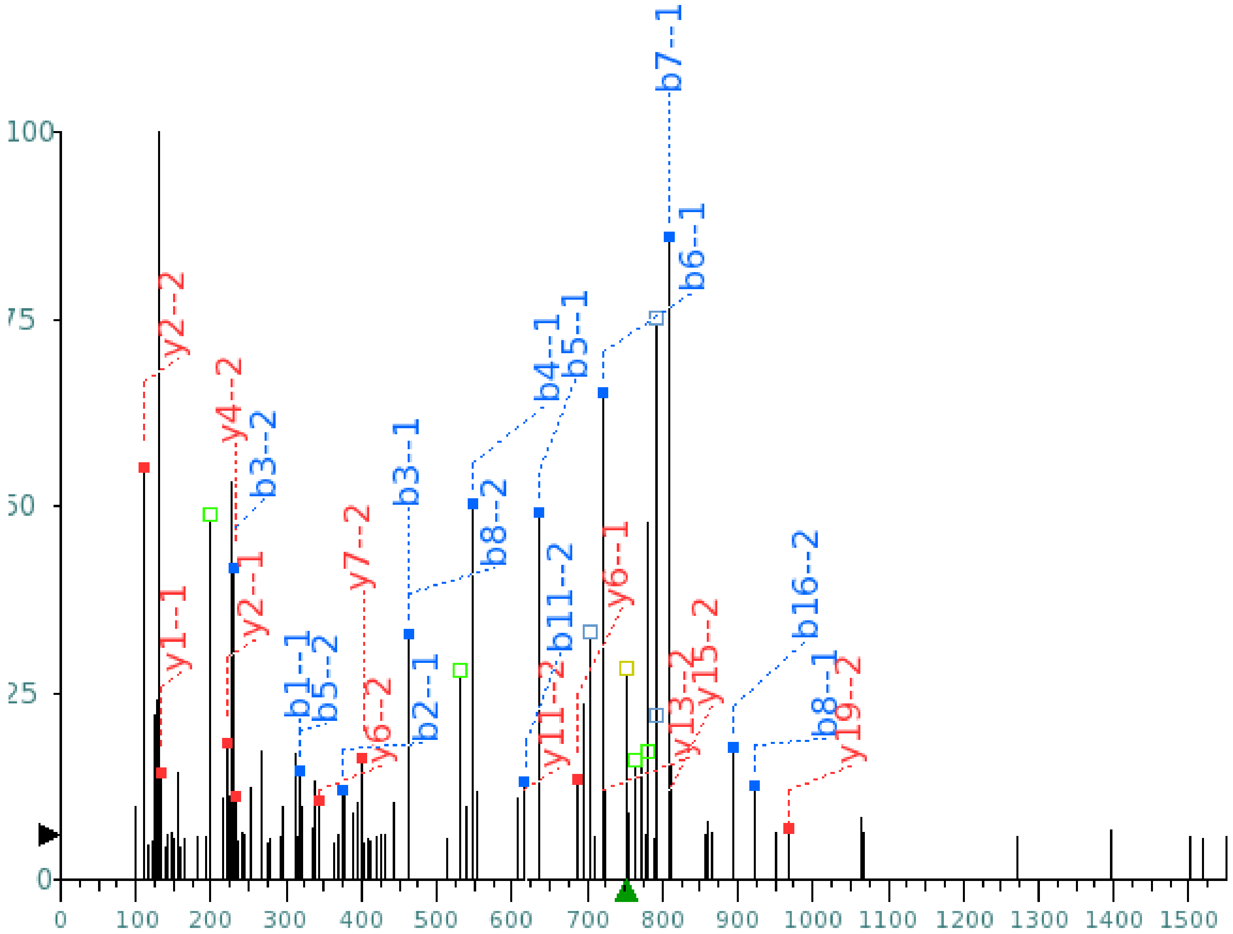

SGSSSSSSIPESQSNHSNQSD (+3)

Primary Reference: RAPH1_HUMAN
Search ID: 41760
Search Name: 20130328_ananiav_ERstress_TMT_TM_timecourse_trypsin_inj1_4mc_iaa
Scan#: 3842
Observed Mass: 751.0032 (1.9 ppm)
PSM Score: 18.71

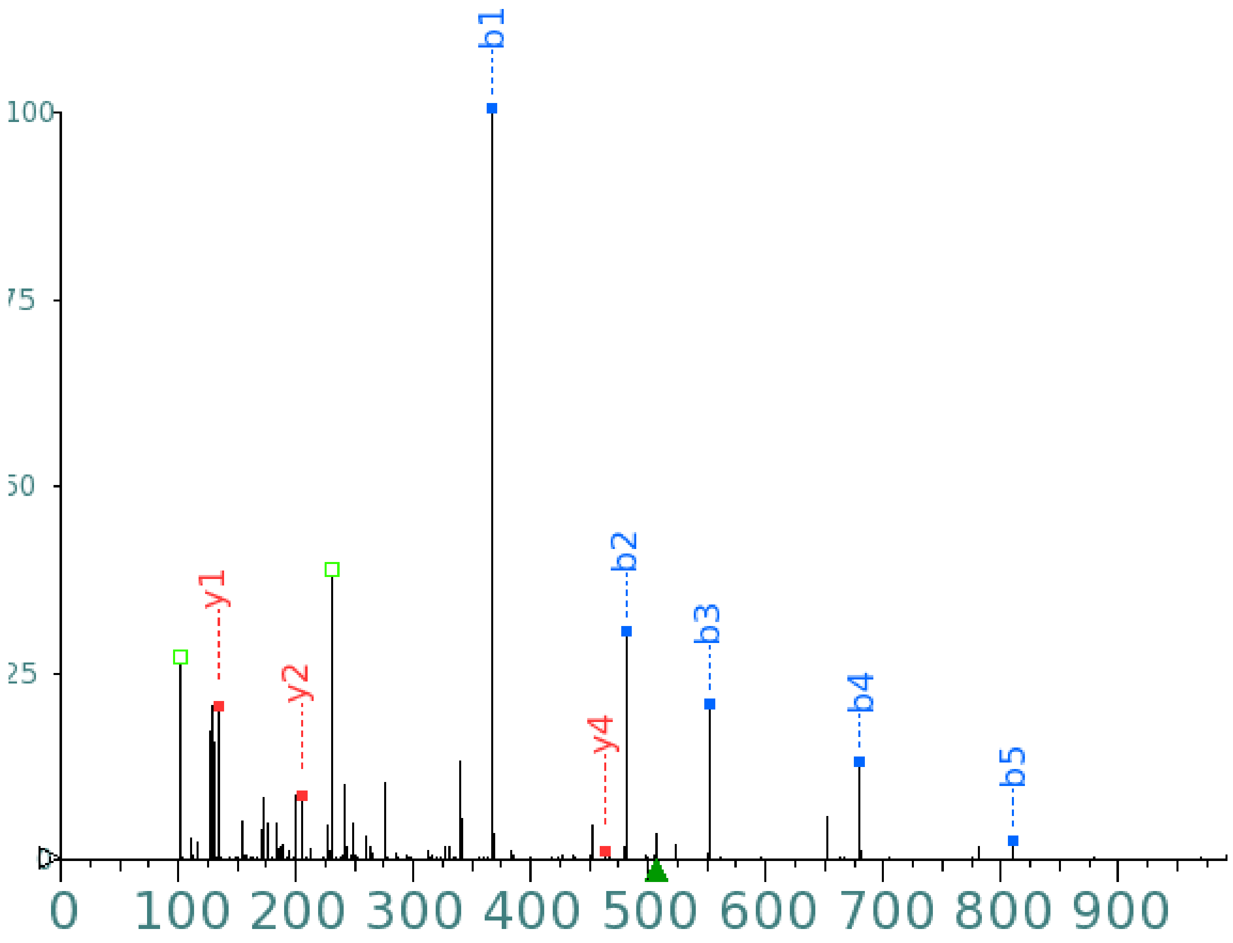

HIAEEAD (+2)

Primary Reference: TPM3L_HUMAN
Search ID: 41760
Search Name: 20130328_ananiav_ERstress_TMT_TM_timecourse_trypsin_inj1_4mc_iaa
Scan#: 3865
Observed Mass: 507.26 (2.5 ppm)
PSM Score: 32.11

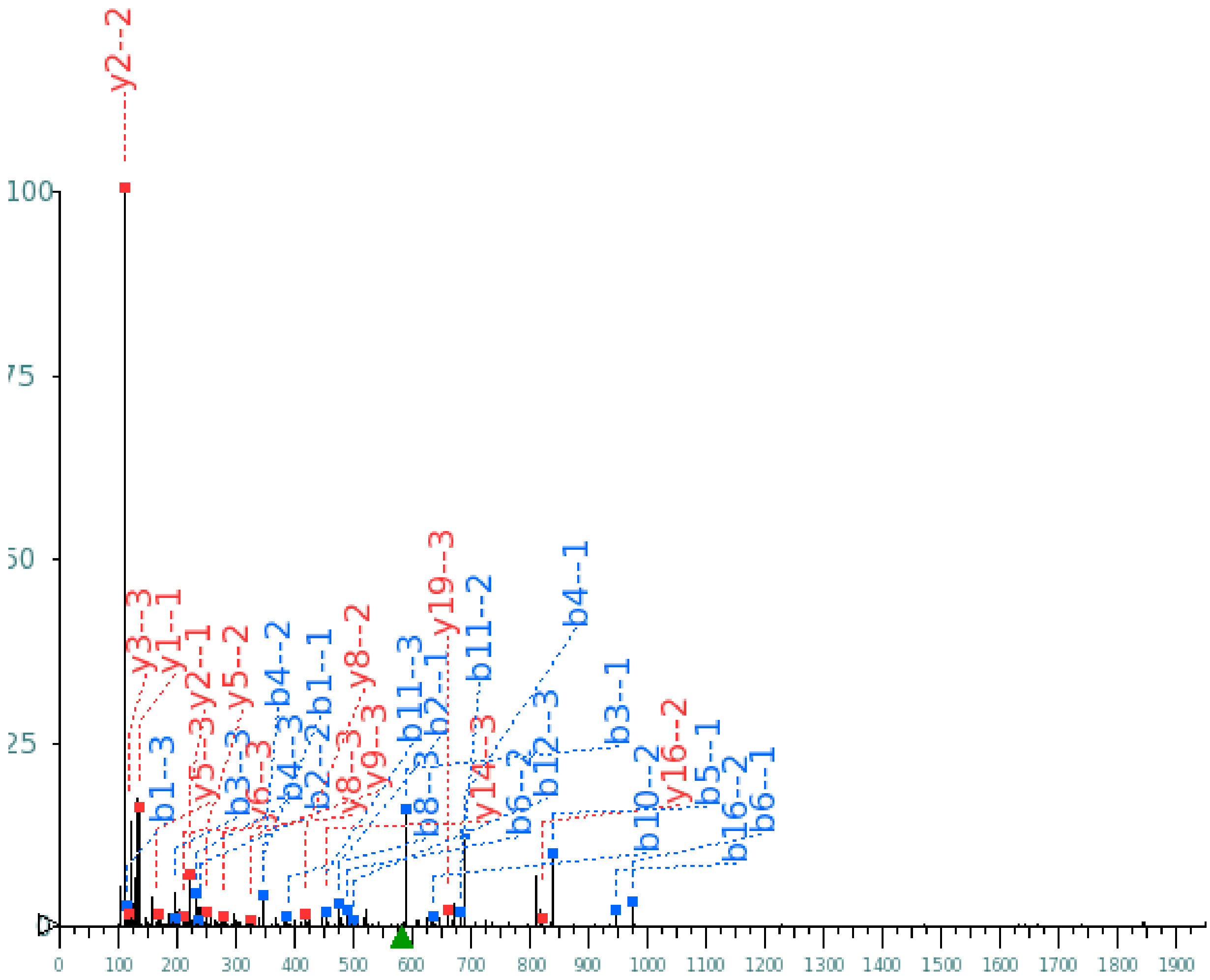

DQDTFHPSGGSHTTHGSESD (+4)

Primary Reference: A8K309_HUMAN
Search ID: 41760
Search Name: 20130328_ananiav_ERstress_TMT_TM_timecourse_trypsin_inj1_4mc_iaa
Scan#: 3909
Observed Mass: 582.7562 (2.8 ppm)
PSM Score: 12.69

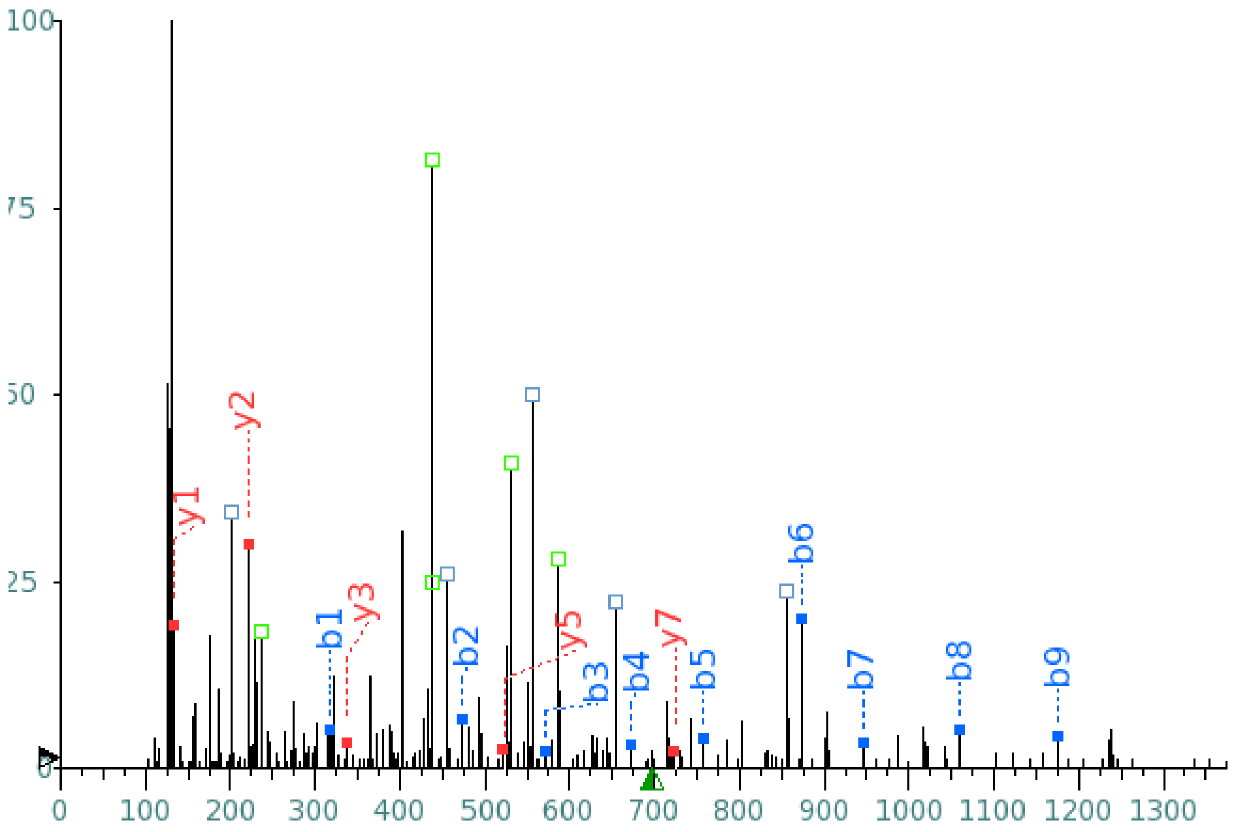

SRVVSDADDSD (+2)

Primary Reference: IWS1_HUMAN
Search ID: 41760
Search Name: 20130328_ananiav_ERstress_TMT_TM_timecourse_trypsin_inj1_4mc_iaa
Scan#: 3921
Observed Mass: 697.8362 (3.9 ppm)
PSM Score: 20.85

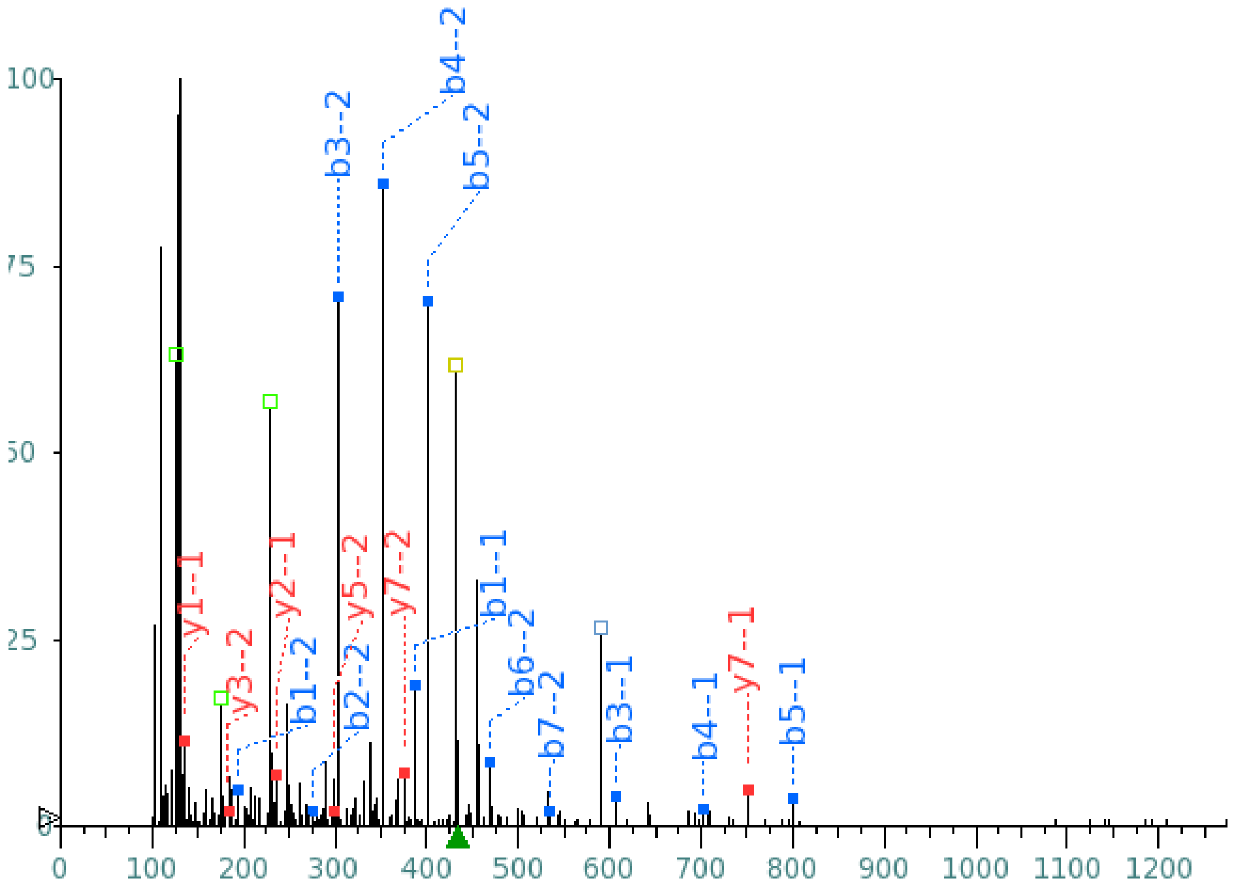

RYGPPHETD (+3)

Primary Reference: TCF20_HUMAN
Search ID: 41760
Search Name: 20130328_ananiav_ERstress_TMT_TM_timecourse_trypsin_inj1_4mc_iaa
Scan#: 3939
Observed Mass: 434.2219 (2.1 ppm)
PSM Score: 8.42

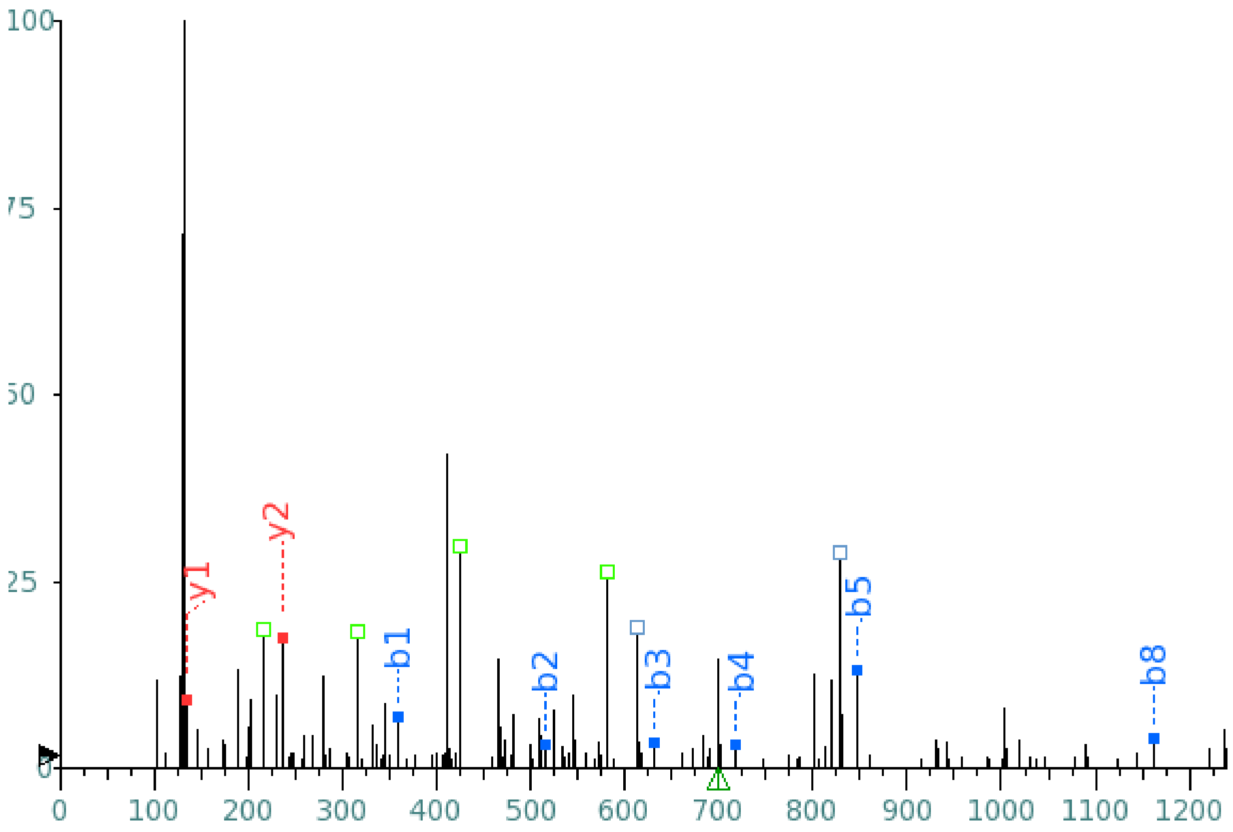

ERDSELSDTD (+2)

Primary Reference: UHRF1_HUMAN
Search ID: 41760
Search Name: 20130328_ananiav_ERstress_TMT_TM_timecourse_trypsin_inj1_4mc_iaa
Scan#: 4011
Observed Mass: 698.3281 (3.7 ppm)
PSM Score: 18.45

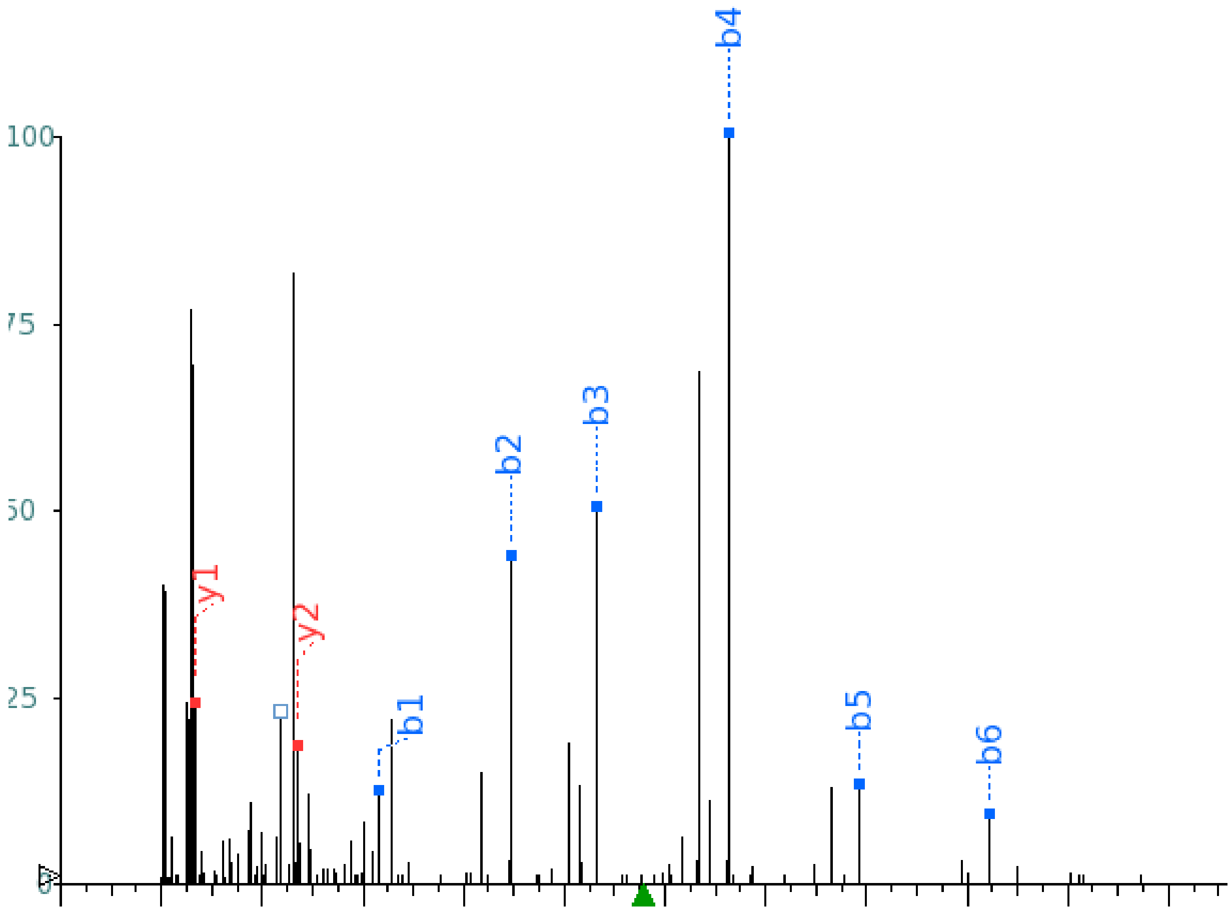

SESEMETD (+2)

Primary Reference: LARP7_HUMAN
Search ID: 41760
Search Name: 20130328_ananiav_ERstress_TMT_TM_timecourse_trypsin_inj1_4mc_iaa
Scan#: 4095
Observed Mass: 578.7489 (2.4 ppm)
PSM Score: 23.29

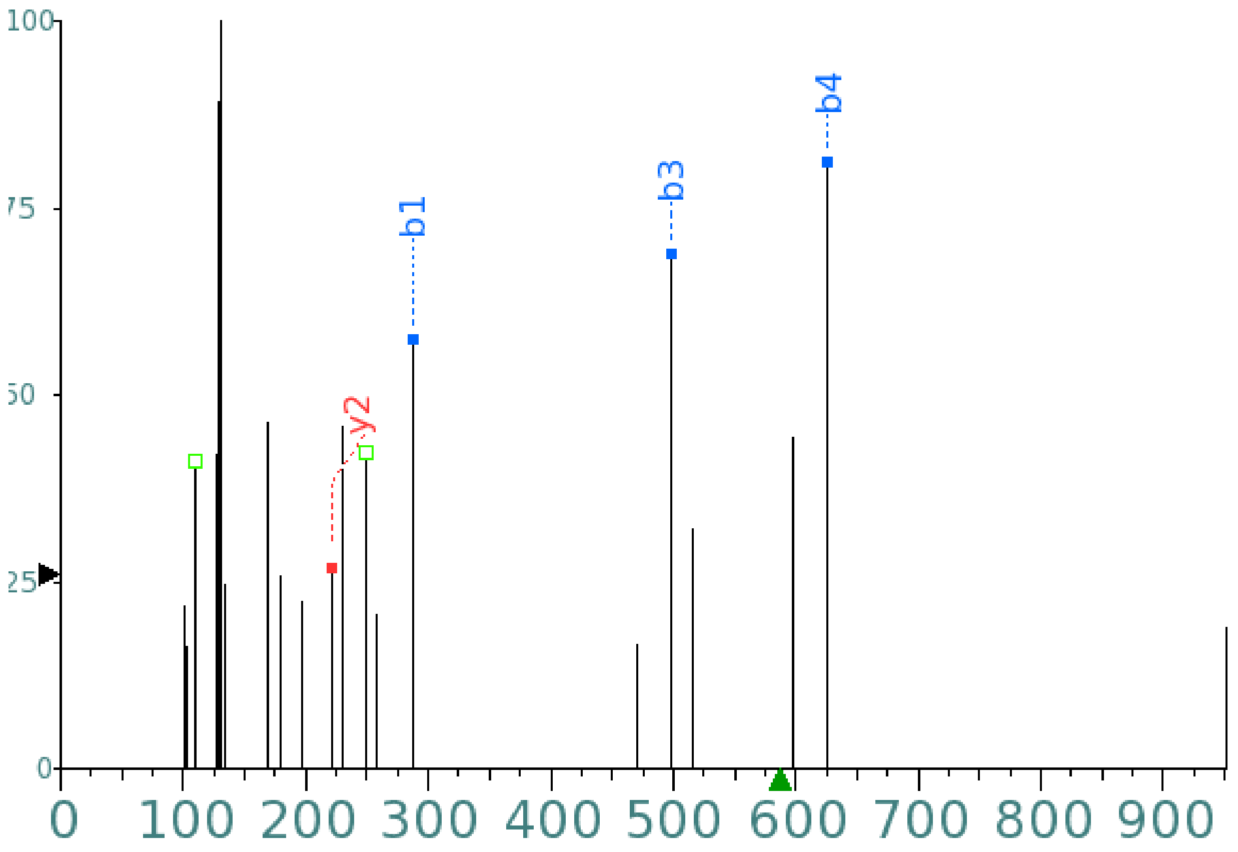

GPNQPVESD (+2)

Primary Reference: IRAK1_HUMAN
Search ID: 41760
Search Name: 20130328_ananiav_ERstress_TMT_TM_timecourse_trypsin_inj1_4mc_iaa
Scan#: 4143
Observed Mass: 586.2944 (2 ppm)
PSM Score: 24.83

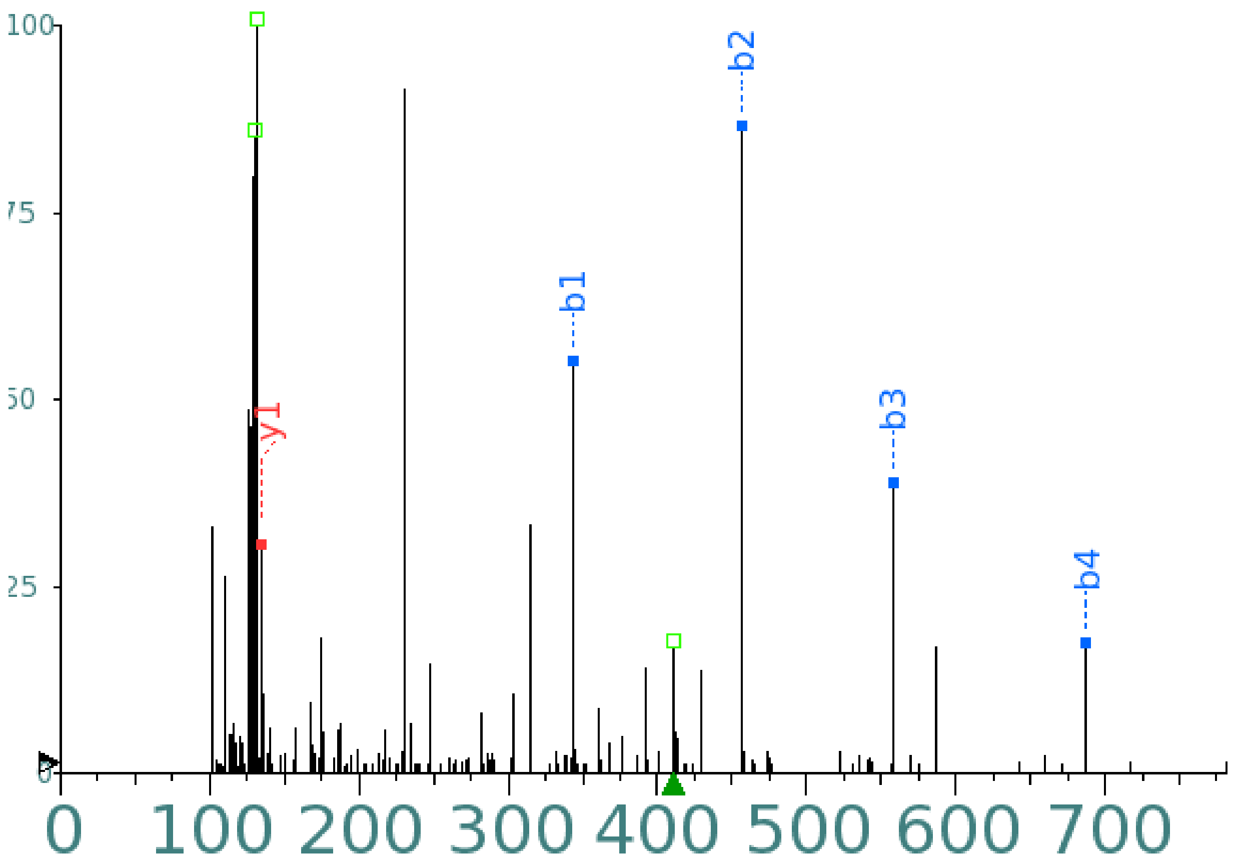

INTED (+2)

Primary Reference: CASQ2_HUMAN
Search ID: 41760
Search Name: 20130328_ananiav_ERstress_TMT_TM_timecourse_trypsin_inj1_4mc_iaa
Scan#: 4206
Observed Mass: 410.7163 (0.4 ppm)
PSM Score: 23.36

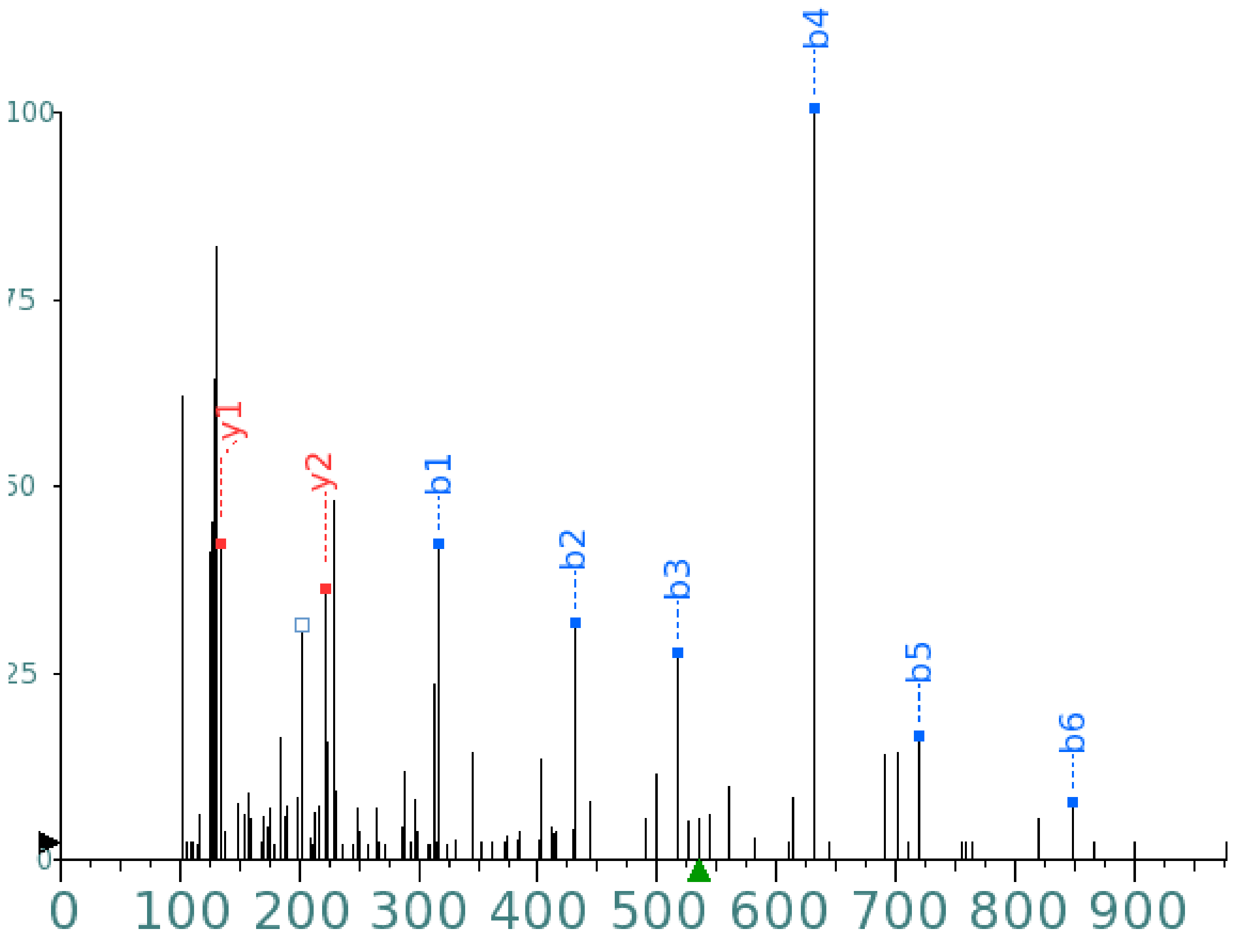

SLSDSESD (+2)

Primary Reference: CBX3_HUMAN
Search ID: 41760
Search Name: 20130328_ananiav_ERstress_TMT_TM_timecourse_trypsin_inj1_4mc_iaa
Scan#: 4292
Observed Mass: 534.7498 (2.8 ppm)
PSM Score: 44.99

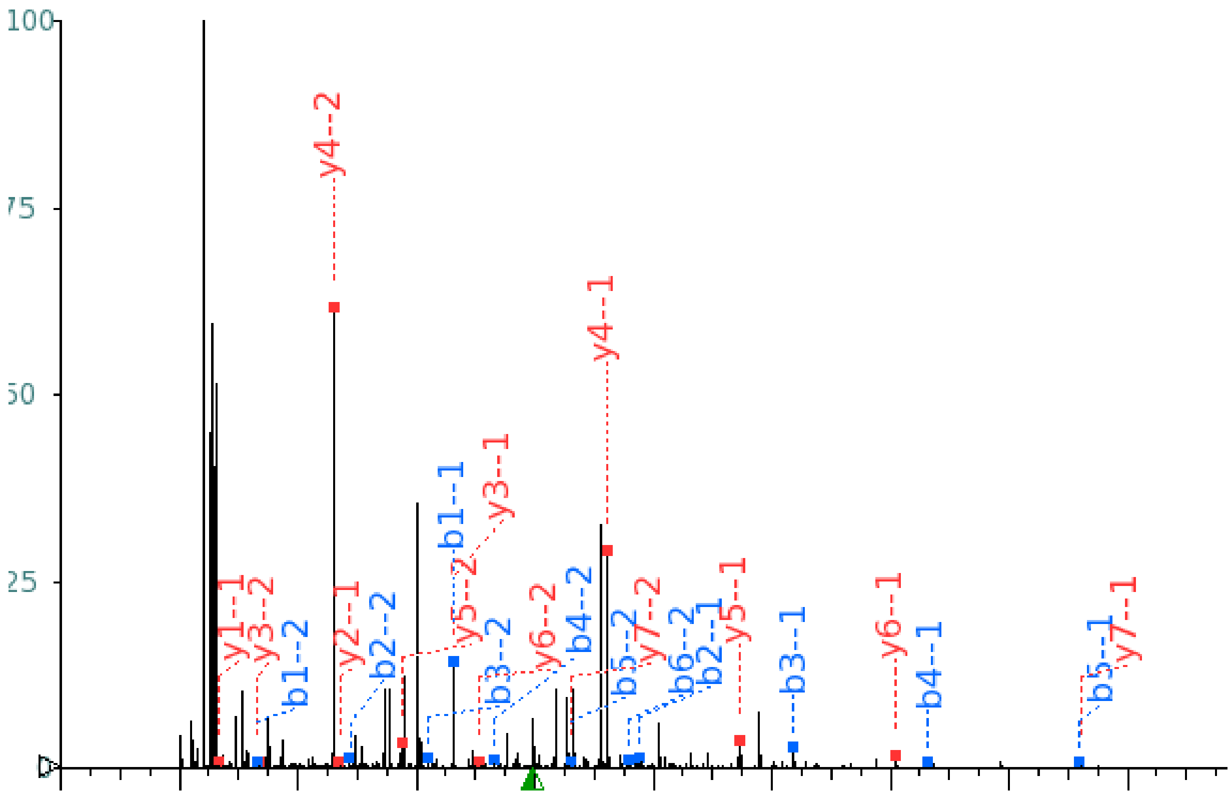

TRMLQPTD (+3)

Primary Reference: ##RASF2_HUMAN
Search ID: 41760
Search Name: 20130328_ananiav_ERstress_TMT_TM_timecourse_trypsin_inj1_4mc_iaa
Scan#: 4353
Observed Mass: 397.5546 (7.8 ppm)
PSM Score: 10.53

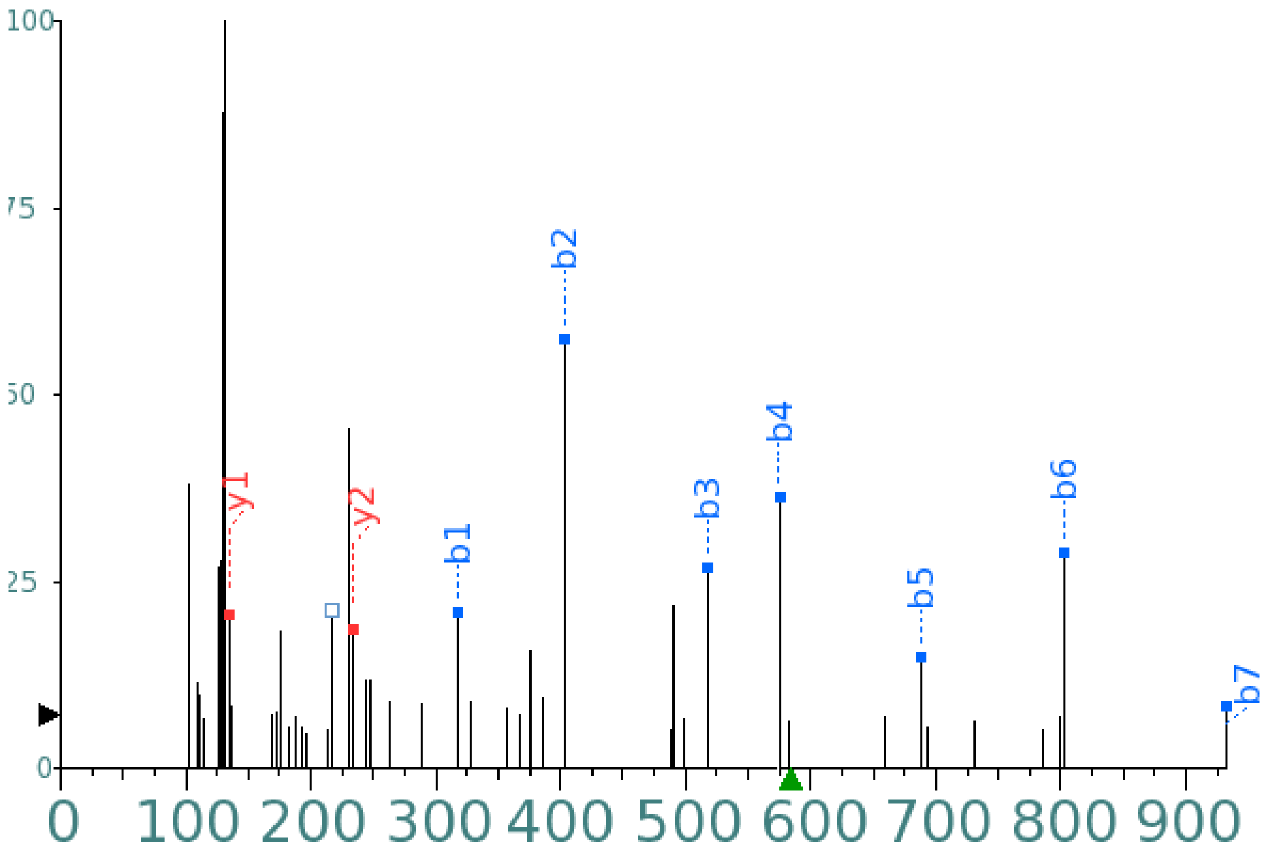

SSLGNDETD (+2)

Primary Reference: COBL_HUMAN
Search ID: 41760
Search Name: 20130328_ananiav_ERstress_TMT_TM_timecourse_trypsin_inj1_4mc_iaa
Scan#: 4363
Observed Mass: 583.7735 (2 ppm)
PSM Score: 43

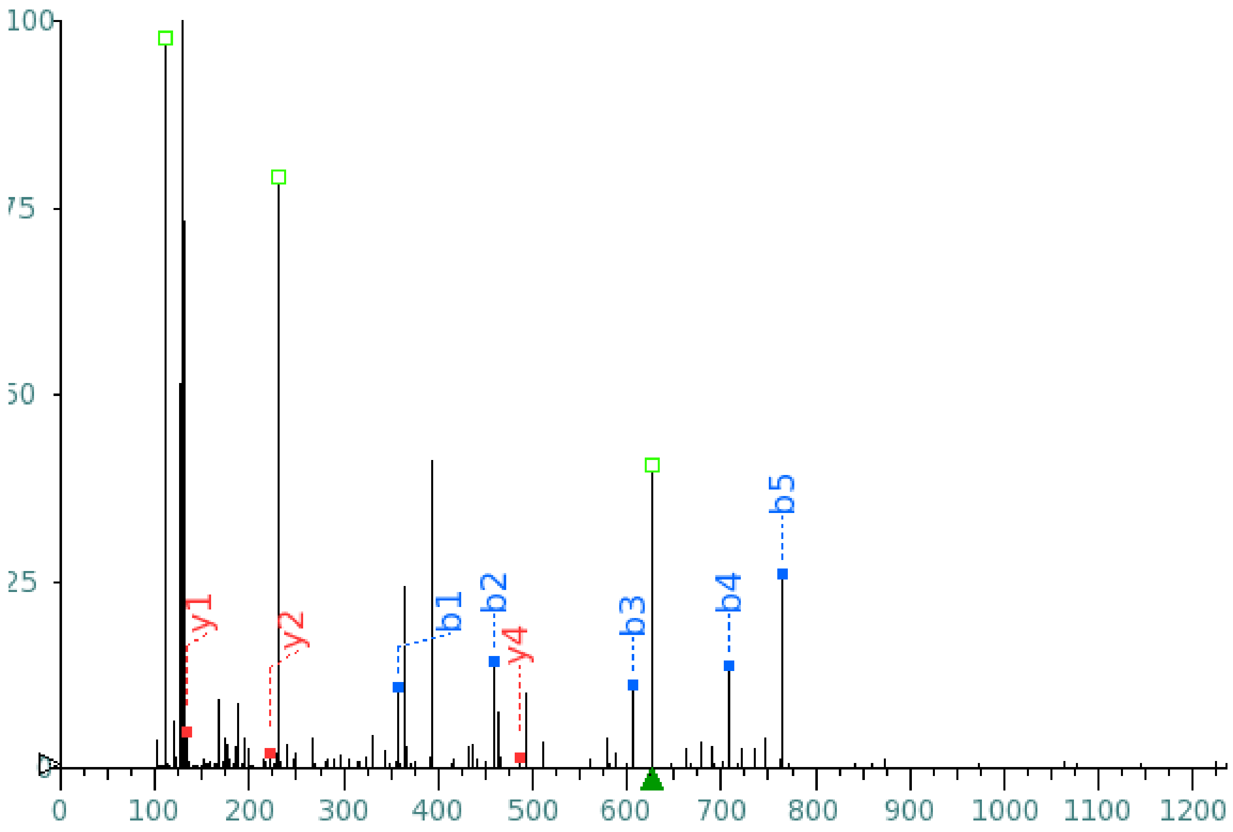

QTFTGHESD (+2)

Primary Reference: GBB1_HUMAN
Search ID: 41760
Search Name: 20130328_ananiav_ERstress_TMT_TM_timecourse_trypsin_inj1_4mc_iaa
Scan#: 4384
Observed Mass: 625.7976 (2.2 ppm)
PSM Score: 8.17

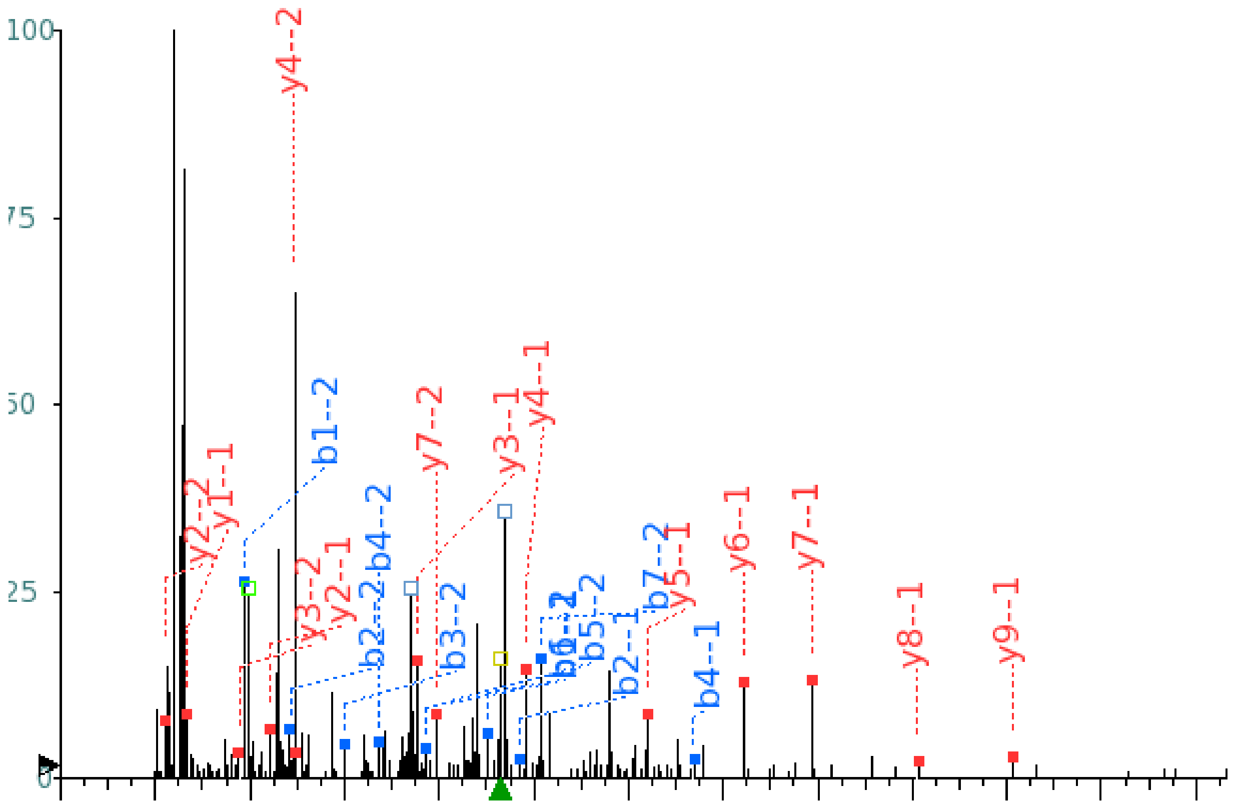

RVLATEDRSD (+3)

Primary Reference: RAB9A_HUMAN
Search ID: 41760
Search Name: 20130328_ananiav_ERstress_TMT_TM_timecourse_trypsin_inj1_4mc_iaa
Scan#: 4405
Observed Mass: 464.2546 (0.3 ppm)
PSM Score: 17.58

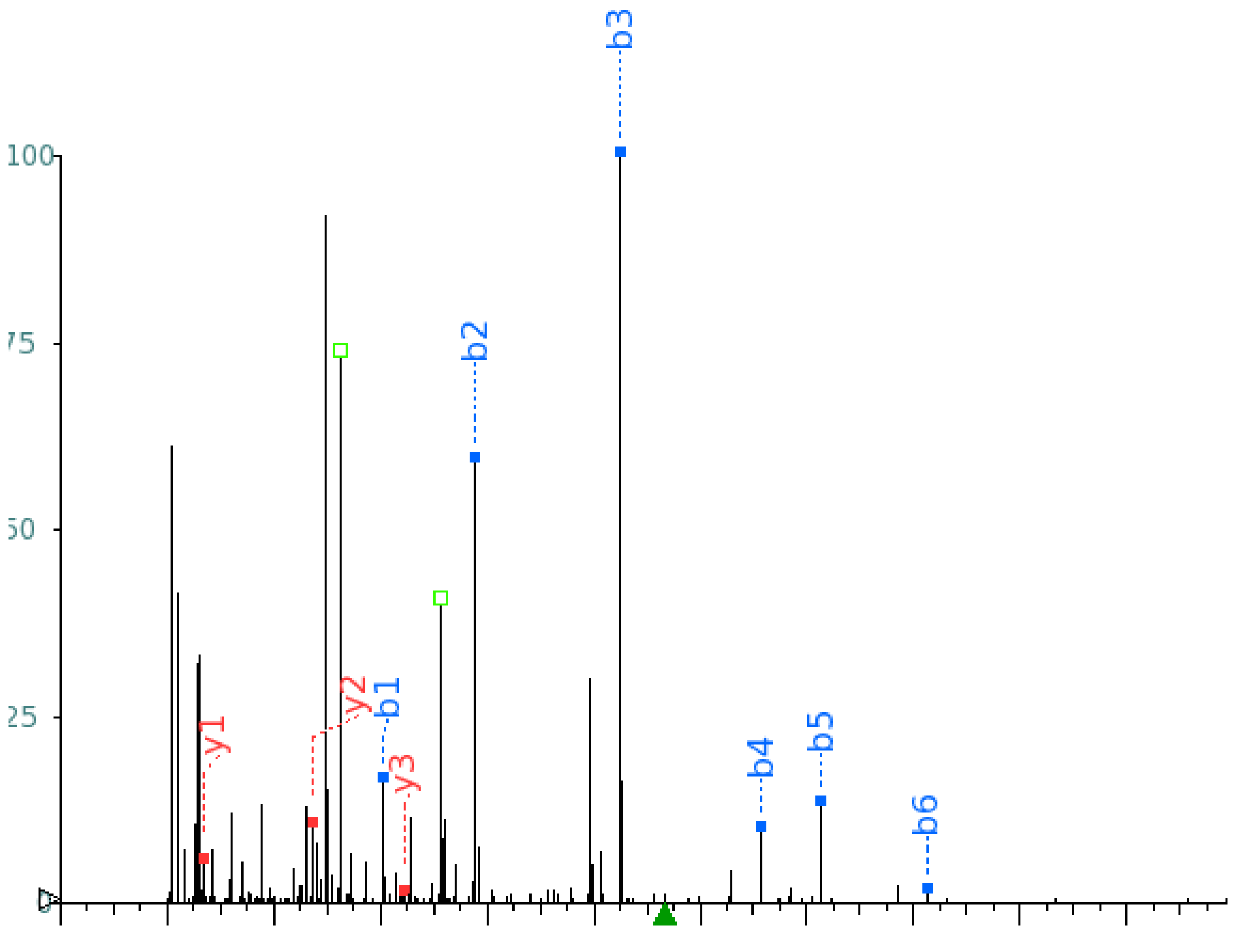

ASHMGVSTD (+2)

Primary Reference: LUZP1_HUMAN
Search ID: 41760
Search Name: 20130328_ananiav_ERstress_TMT_TM_timecourse_trypsin_inj1_4mc_iaa
Scan#: 4459
Observed Mass: 567.2769 (0.7 ppm)
PSM Score: 26.34

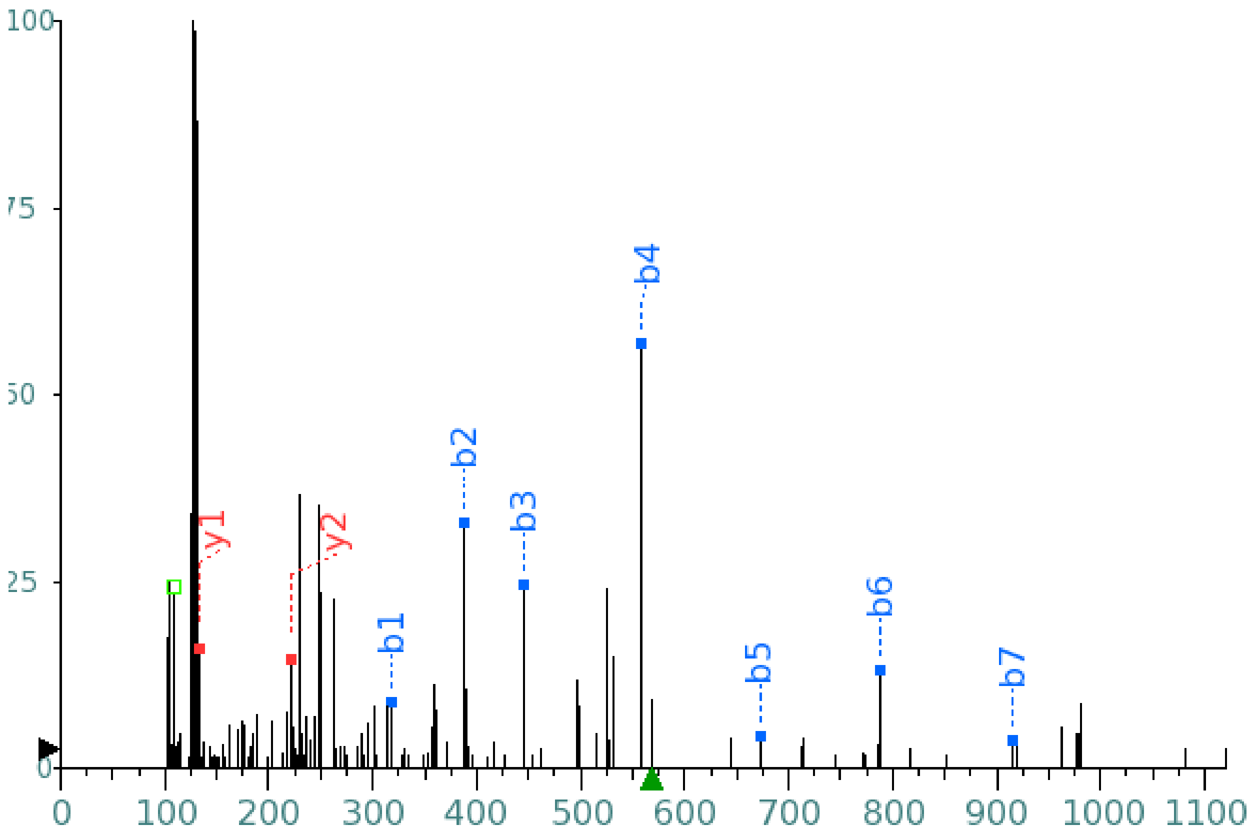

SAGNLDESD (+2)

Primary Reference: RABE1_HUMAN
Search ID: 41760
Search Name: 20130328_ananiav_ERstress_TMT_TM_timecourse_trypsin_inj1_4mc_iaa
Scan#: 4543
Observed Mass: 568.7681 (1.8 ppm)
PSM Score: 11.51

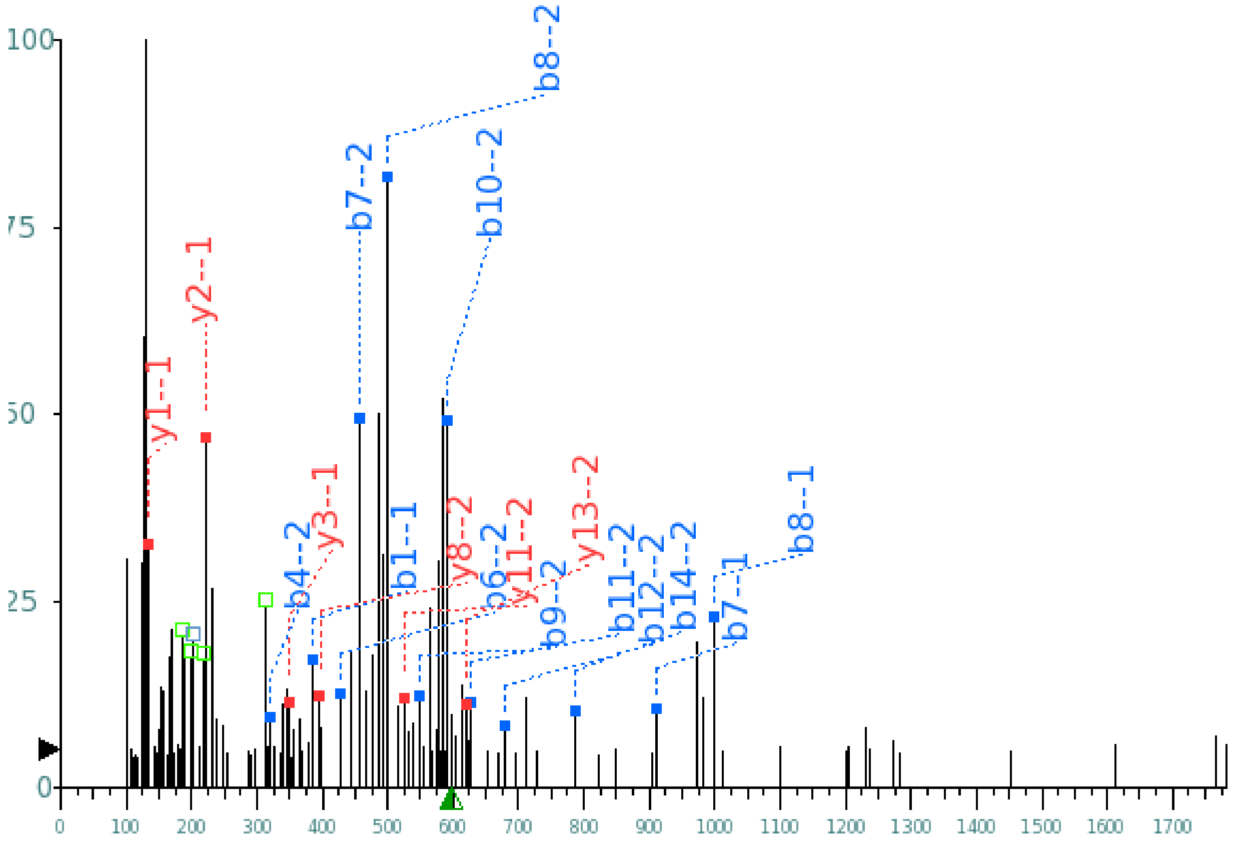

RPASVNGSPSATSESD (+3)

Primary Reference: ITCH_HUMAN
Search ID: 41760
Search Name: 20130328_ananiav_ERstress_TMT_TM_timecourse_trypsin_inj1_4mc_iaa
Scan#: 4561
Observed Mass: 597.6295 (1.2 ppm)
PSM Score: 14.35

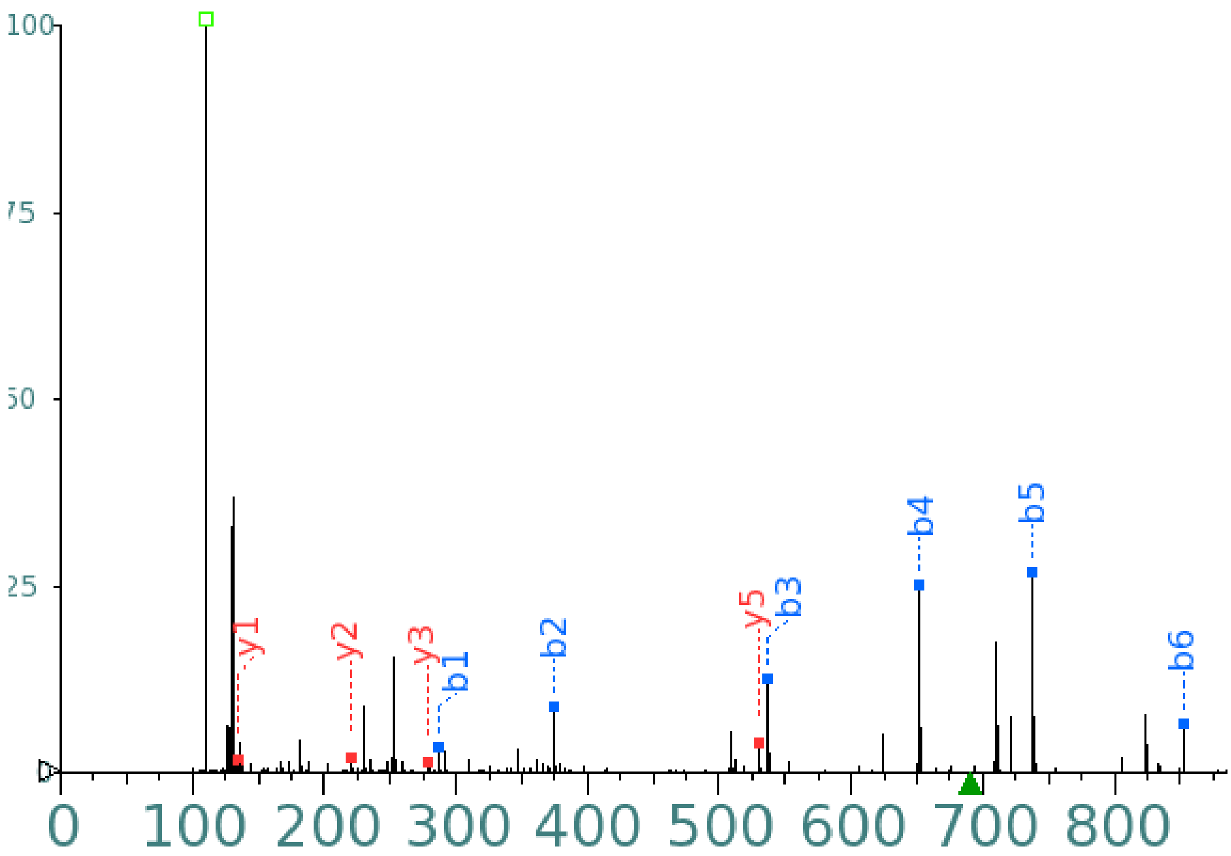

GSYNSLHDGSD (+2)

Primary Reference: CI150_HUMAN
Search ID: 41760
Search Name: 20130328_ananiav_ERstress_TMT_TM_timecourse_trypsin_inj1_4mc_iaa
Scan#: 4722
Observed Mass: 690.8157 (0.9 ppm)
PSM Score: 16.33

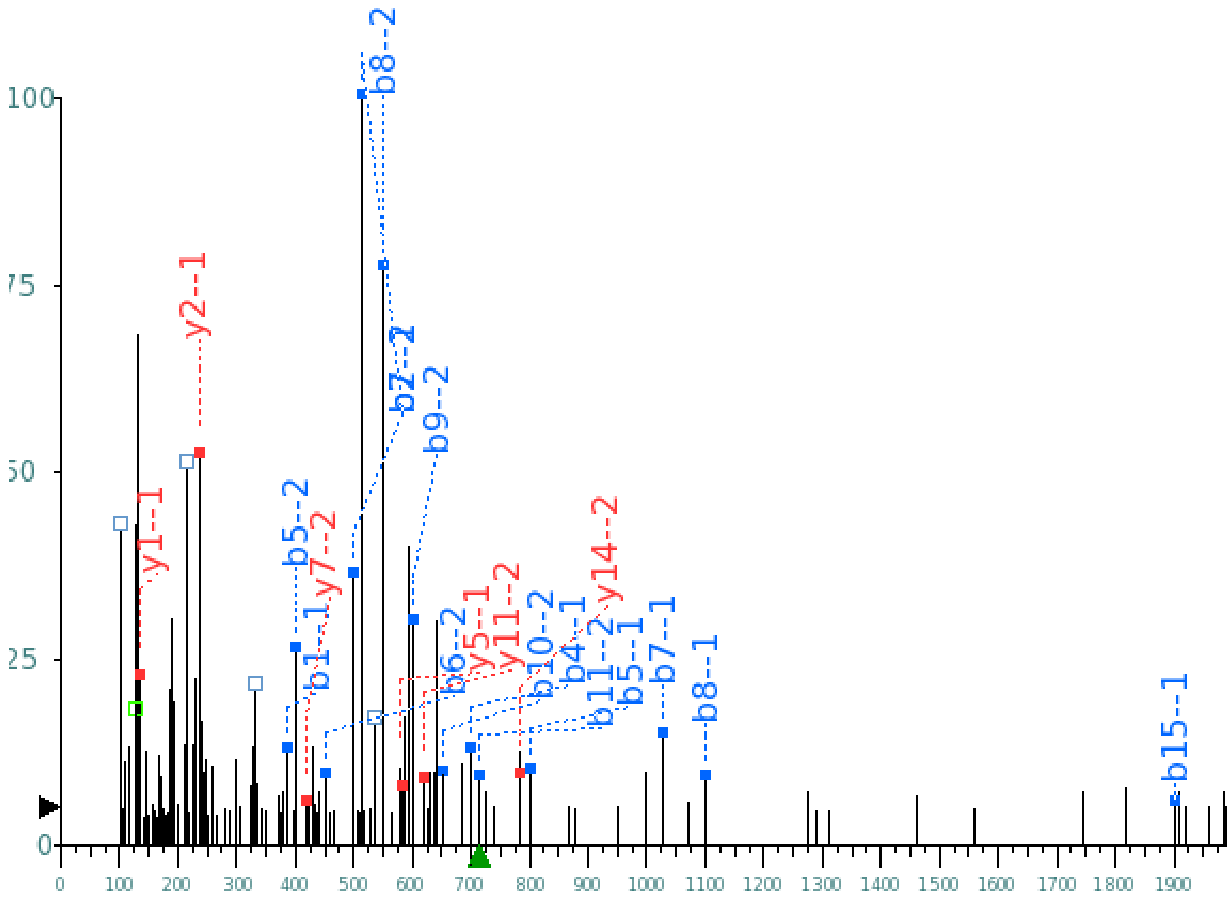

RNAETPEATTQQETDTD (+3)

Primary Reference: K1967_HUMAN
Search ID: 41760
Search Name: 20130328_ananiav_ERstress_TMT_TM_timecourse_trypsin_inj1_4mc_iaa
Scan#: 4729
Observed Mass: 712.6688 (1.3 ppm)
PSM Score: 24.05

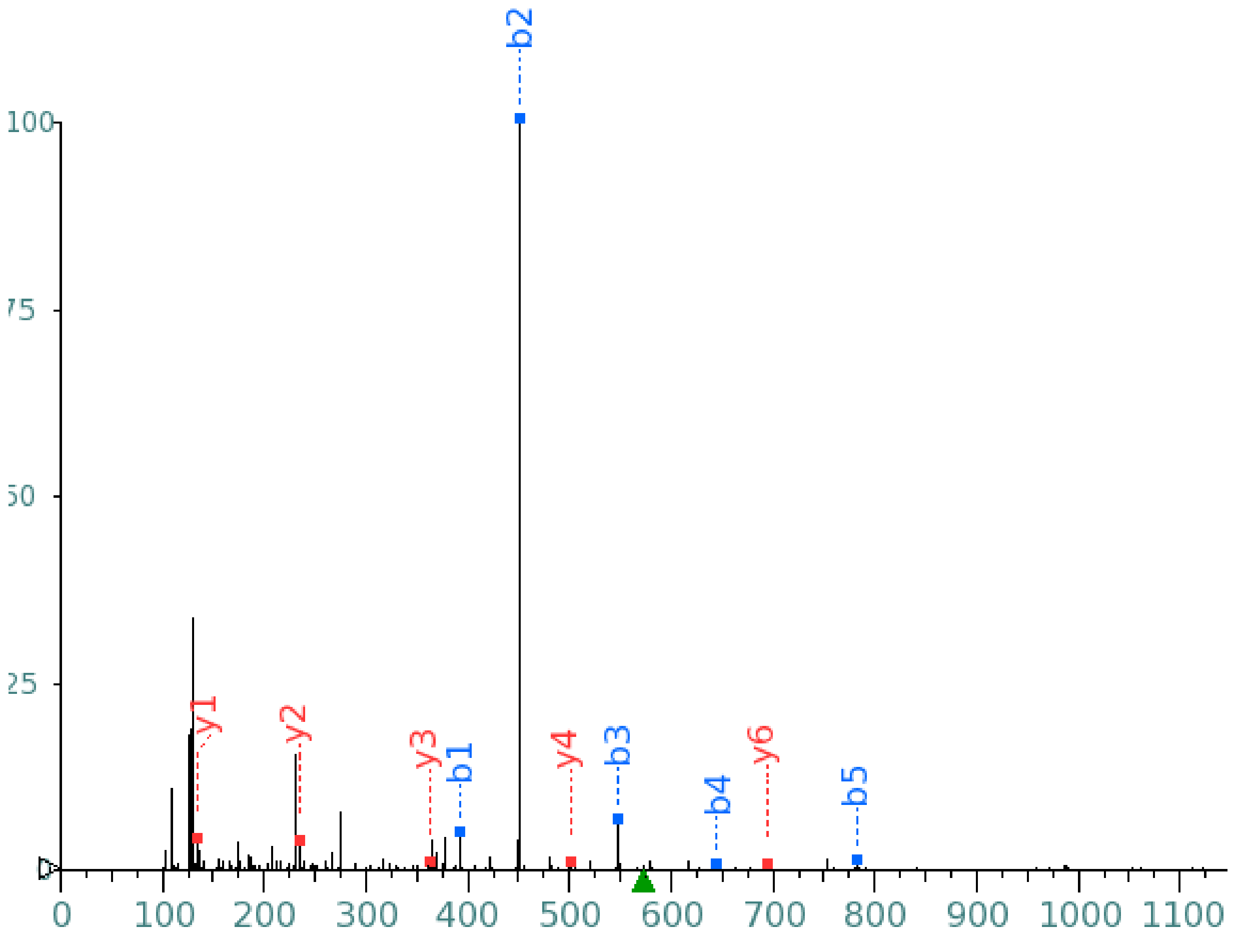

YGPPHETD (+2)

Primary Reference: TCF20_HUMAN
Search ID: 41760
Search Name: 20130328_ananiav_ERstress_TMT_TM_timecourse_trypsin_inj1_4mc_iaa
Scan#: 4734
Observed Mass: 572.7785 (2.1 ppm)
PSM Score: 14.16

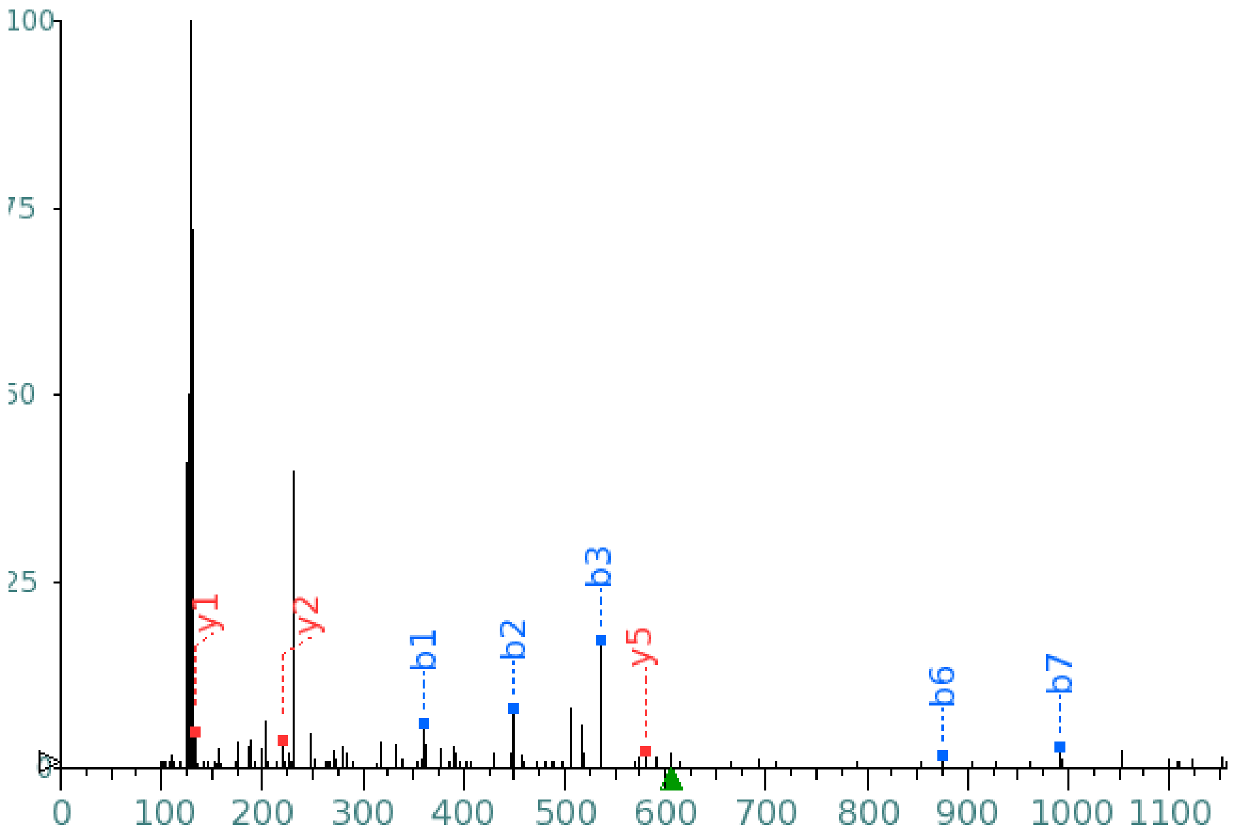

MSSPEDDSD (+2)

Primary Reference: SAFB1_HUMAN
Search ID: 41760
Search Name: 20130328_ananiav_ERstress_TMT_TM_timecourse_trypsin_inj1_4mc_iaa
Scan#: 4737
Observed Mass: 606.2515 (1.9 ppm)
PSM Score: 19.54

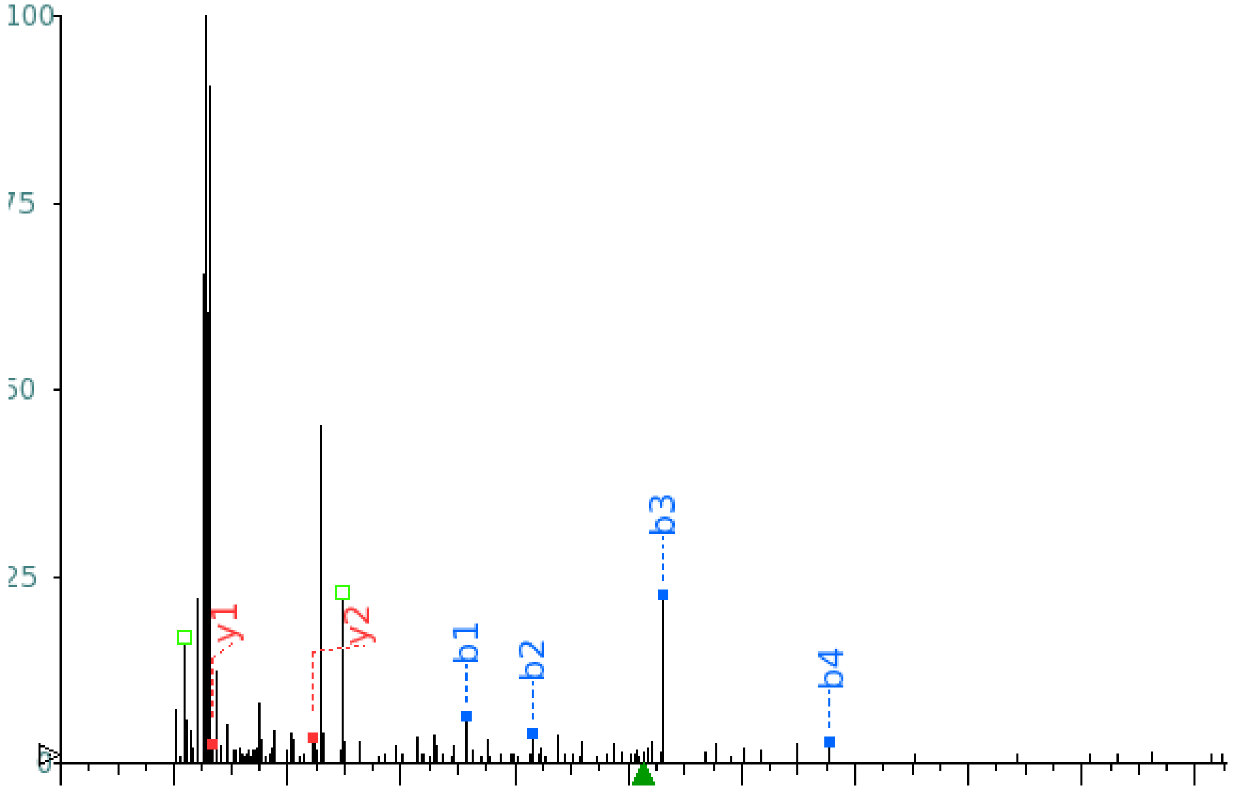

QGDFESD (+2)

Primary Reference: SMHD1_HUMAN
Search ID: 41760
Search Name: 20130328_ananiav_ERstress_TMT_TM_timecourse_trypsin_inj1_4mc_iaa
Scan#: 4789
Observed Mass: 513.7334 (1.8 ppm)
PSM Score: 5.16

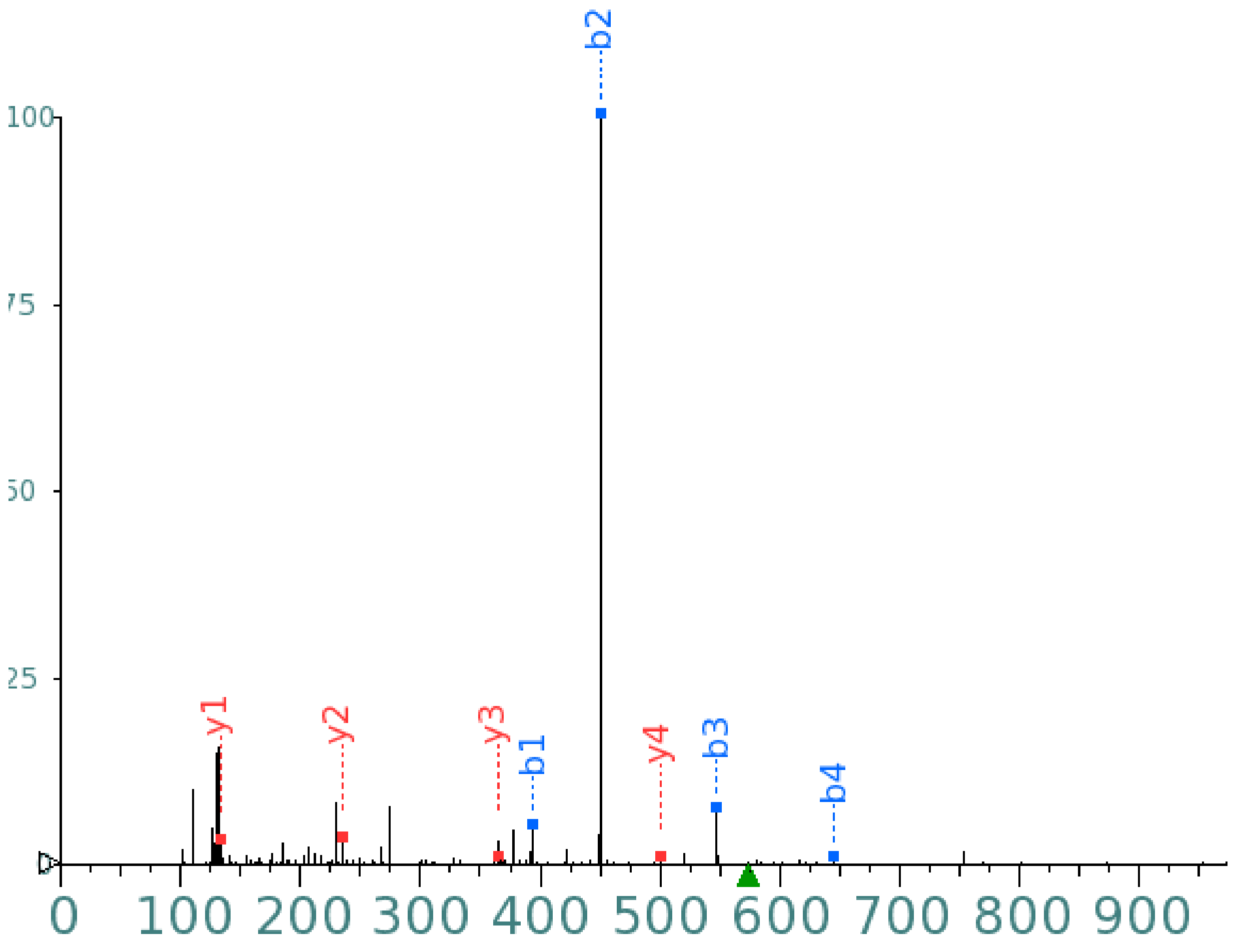

YGPPHETD (+2)

Primary Reference: TCF20_HUMAN
Search ID: 41760
Search Name: 20130328_ananiav_ERstress_TMT_TM_timecourse_trypsin_inj1_4mc_iaa
Scan#: 4813
Observed Mass: 572.7786 (2.4 ppm)
PSM Score: 11.84

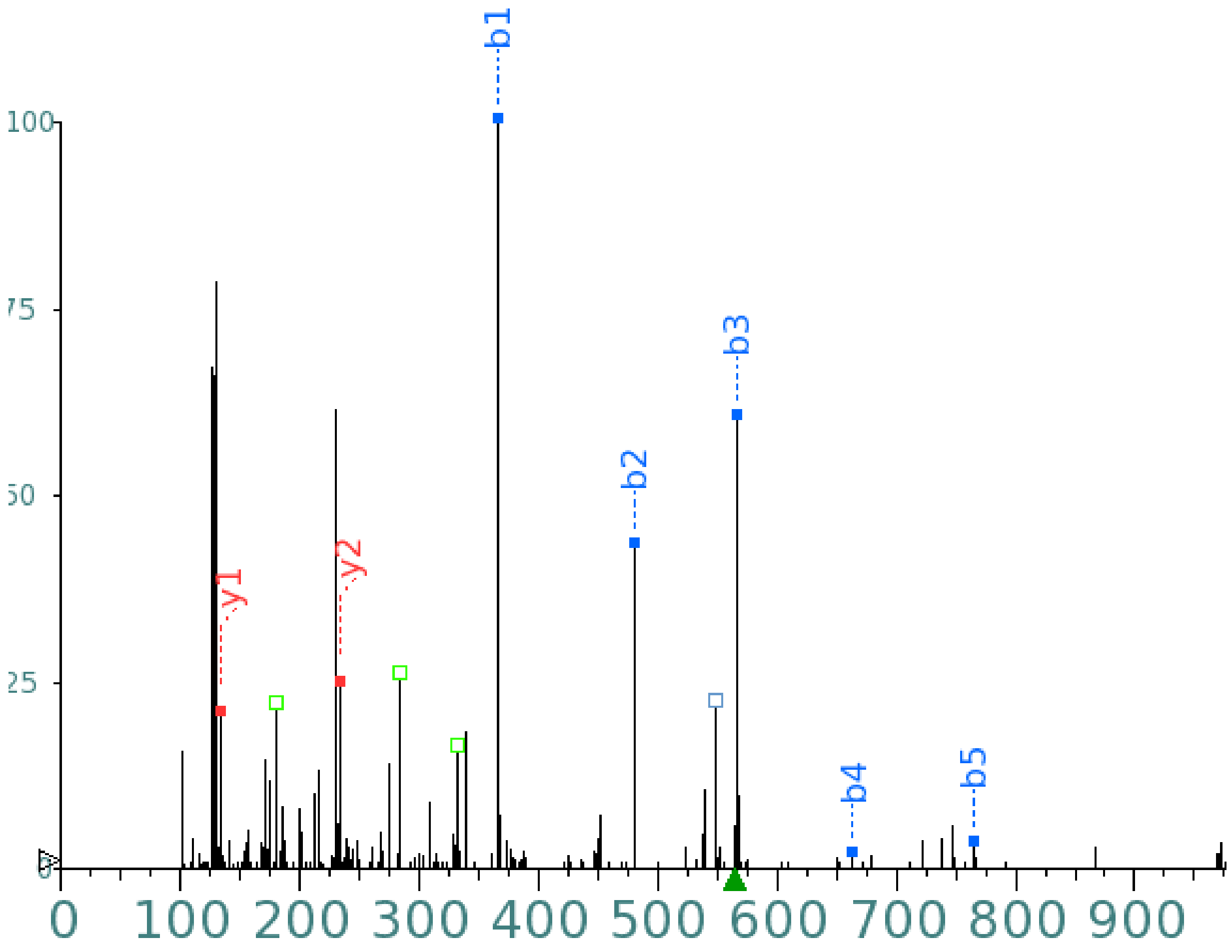

HLSPTETD (+2)

Primary Reference: KDIS_HUMAN
Search ID: 41760
Search Name: 20130328_ananiav_ERstress_TMT_TM_timecourse_trypsin_inj1_4mc_iaa
Scan#: 4824
Observed Mass: 564.7918 (2.6 ppm)
PSM Score: 10.61

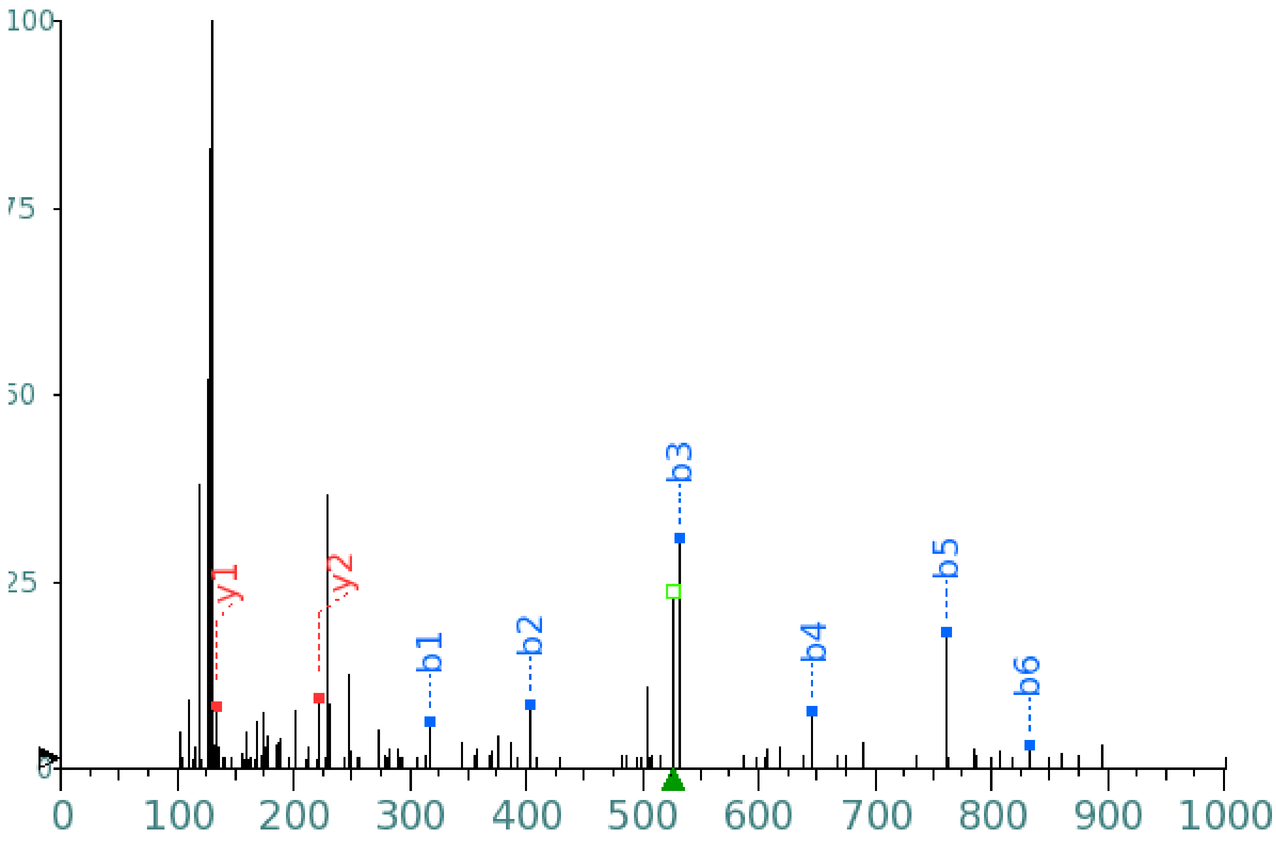

SSELDASD (+2)

Primary Reference: MARK3_HUMAN
Search ID: 41760
Search Name: 20130328_ananiav_ERstress_TMT_TM_timecourse_trypsin_inj1_4mc_iaa
Scan#: 4826
Observed Mass: 526.7521 (2.3 ppm)
PSM Score: 24.03

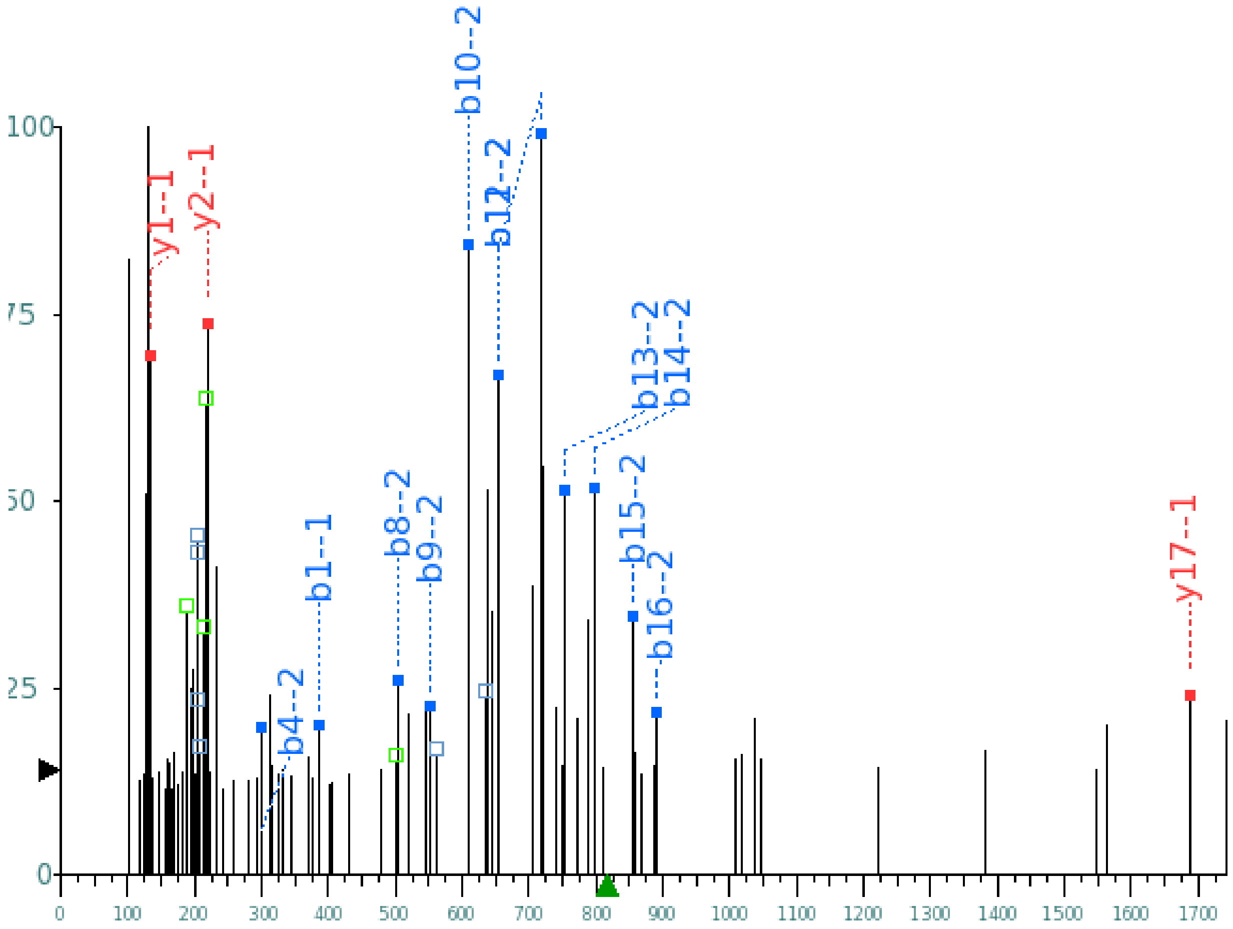

RGPGYTSGTNSEASNASETESD (+3)

Primary Reference: FMR1_HUMAN
Search ID: 41760
Search Name: 20130328_ananiav_ERstress_TMT_TM_timecourse_trypsin_inj1_4mc_iaa
Scan#: 4860
Observed Mass: 816.0335 (2.3 ppm)
PSM Score: 40.14

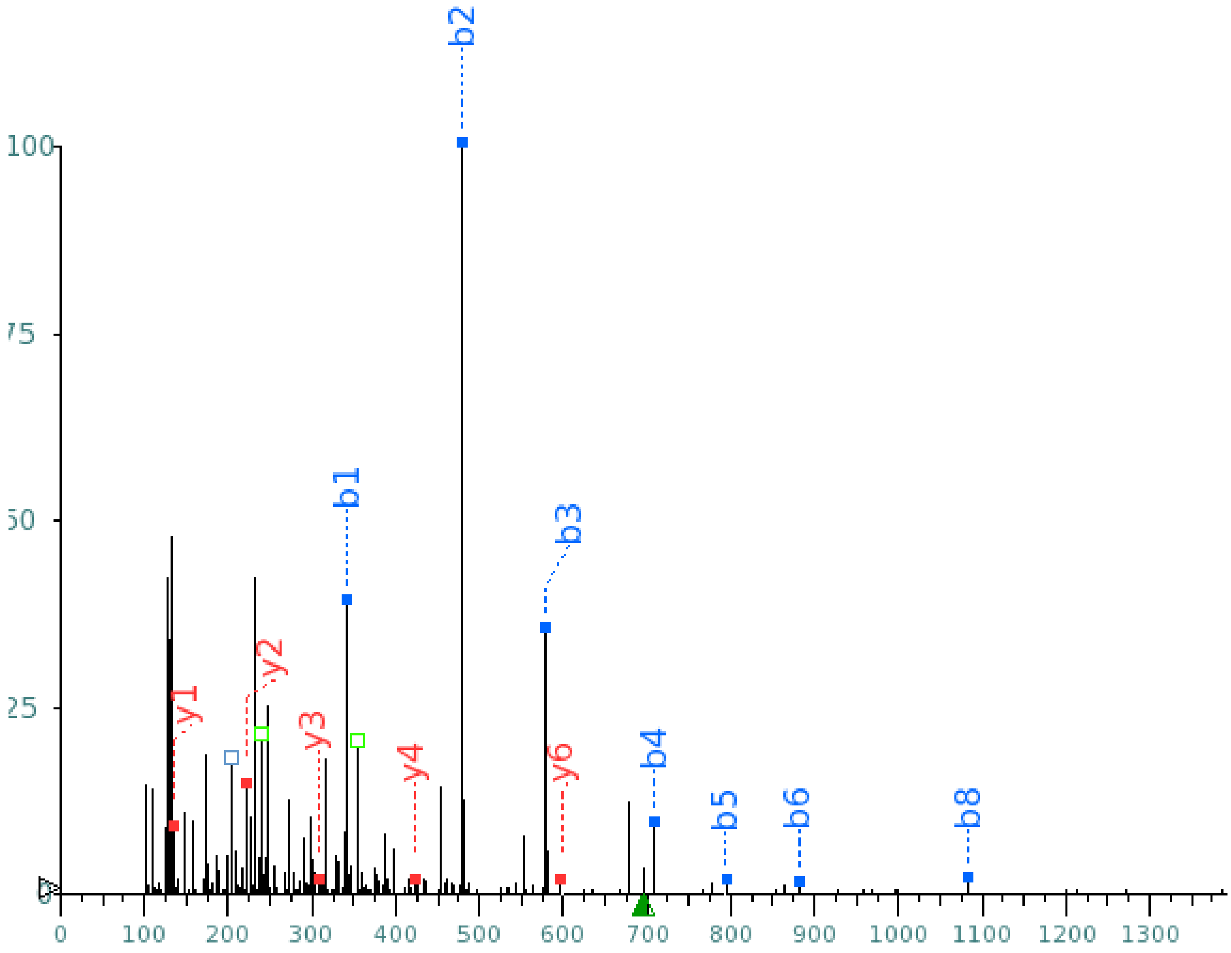

IHVQSSSDSSD (+2)

Primary Reference: F172A_HUMAN
Search ID: 41760
Search Name: 20130328_ananiav_ERstress_TMT_TM_timecourse_trypsin_inj1_4mc_iaa
Scan#: 4884
Observed Mass: 695.8385 (3.5 ppm)
PSM Score: 34.25

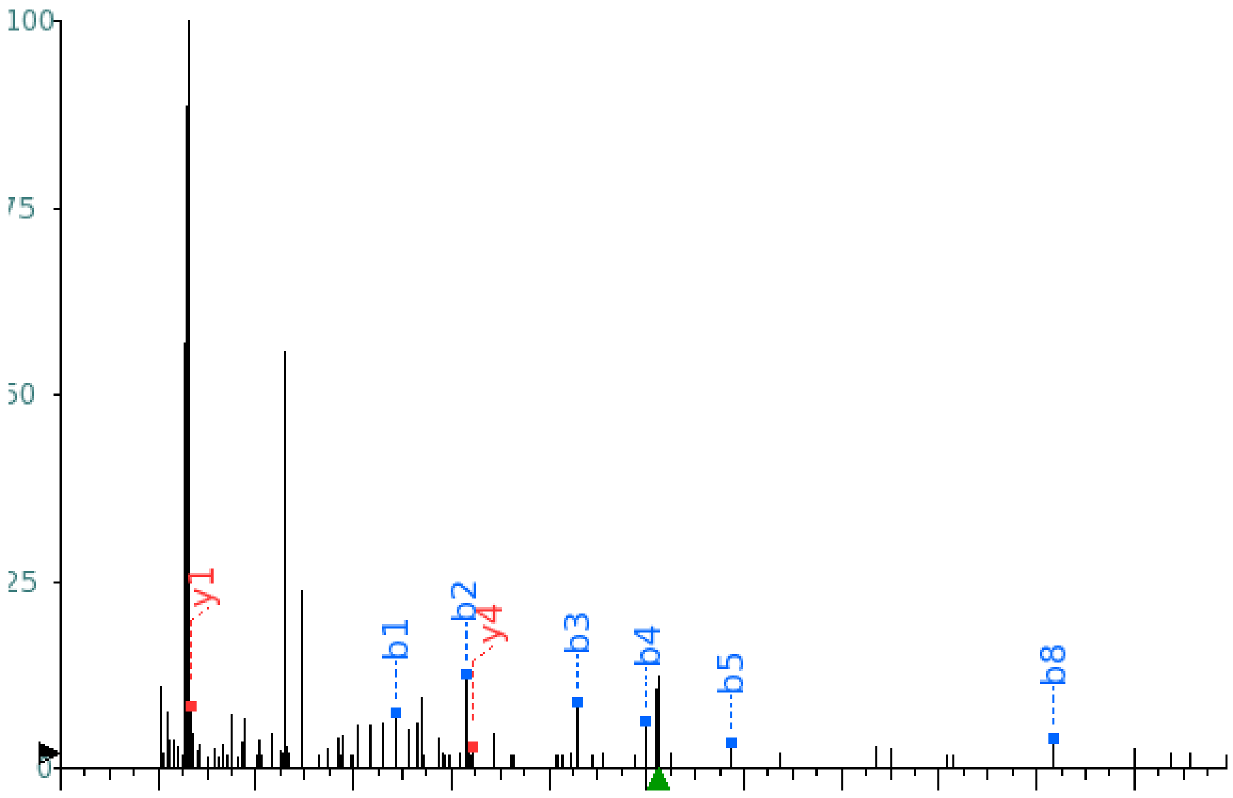

NAIASDSEAD (+2)

Primary Reference: LEO1_HUMAN
Search ID: 41760
Search Name: 20130328_ananiav_ERstress_TMT_TM_timecourse_trypsin_inj1_4mc_iaa
Scan#: 4887
Observed Mass: 611.2949 (2.4 ppm)
PSM Score: 16.45

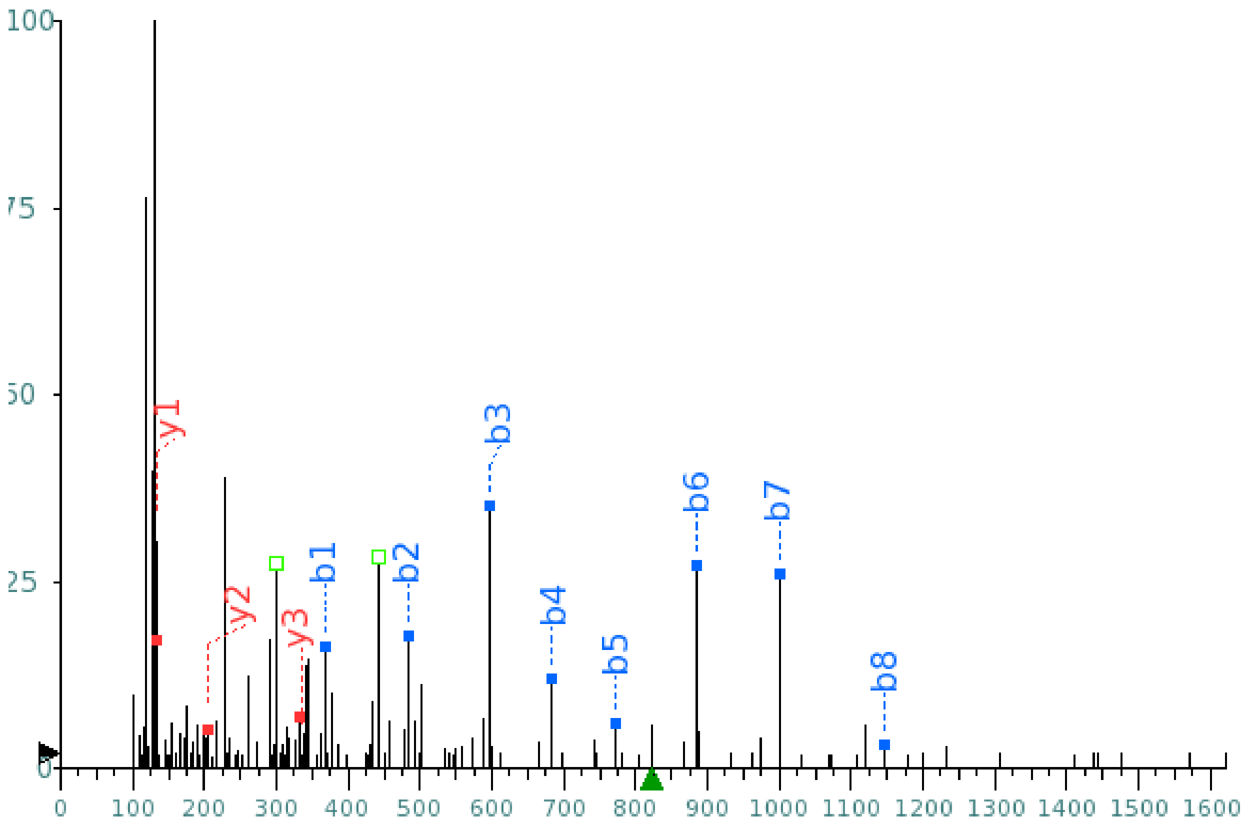

HDDSSDNFC*EAD (+2)

Primary Reference: ERO1A_HUMAN
Search ID: 41760
Search Name: 20130328_ananiav_ERstress_TMT_TM_timecourse_trypsin_inj1_4mc_iaa
Scan#: 4888
Observed Mass: 820.8216 (1.7 ppm)
PSM Score: 32.68

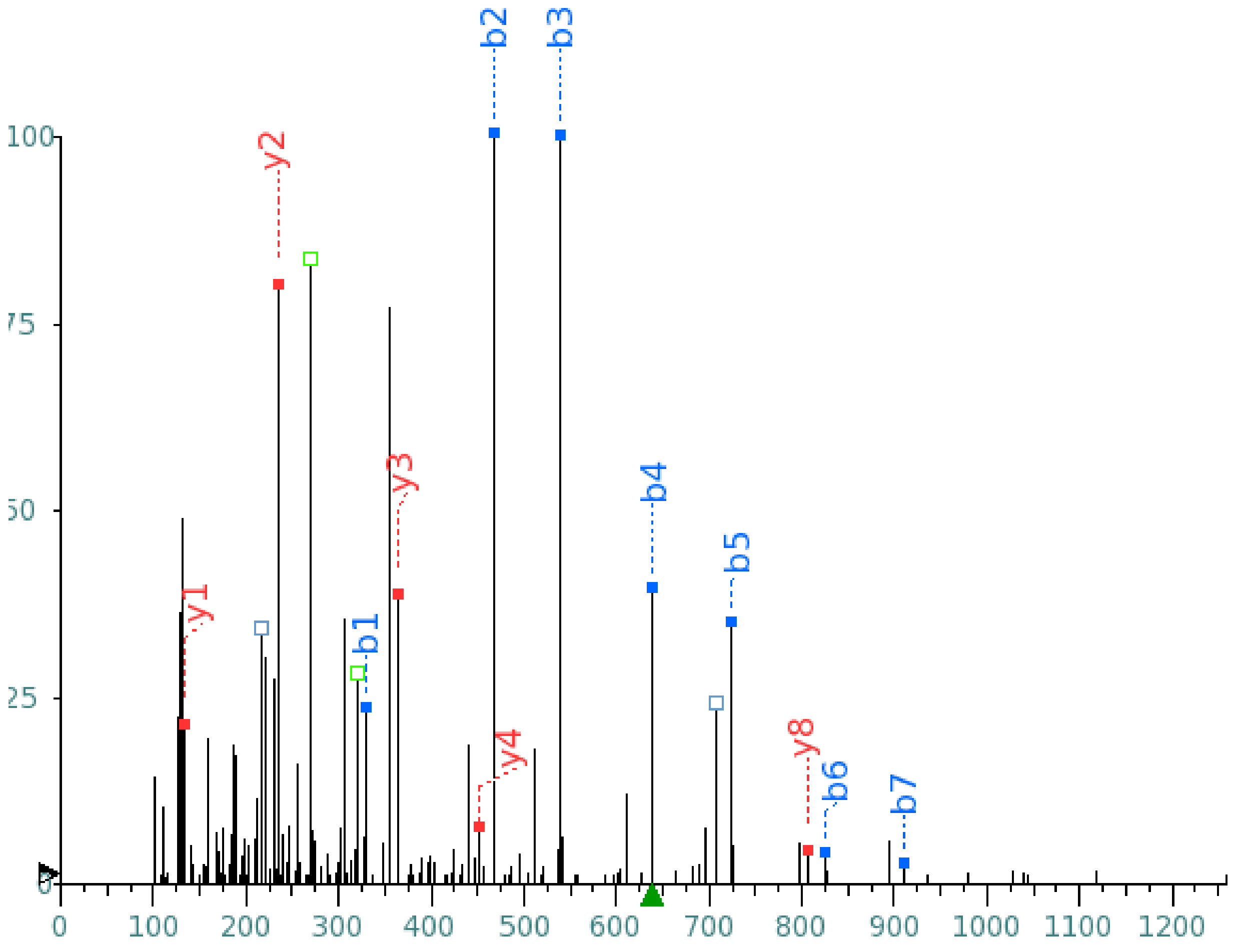

THAVSVSETD (+2)

Primary Reference: FAK1_HUMAN
Search ID: 41760
Search Name: 20130328_ananiav_ERstress_TMT_TM_timecourse_trypsin_inj1_4mc_iaa
Scan#: 4915
Observed Mass: 637.8263 (2.2 ppm)
PSM Score: 29.68

File name: 2015-02-04 04.01.53PM.pdf

Creation time: 2015-02-04 04:09PM

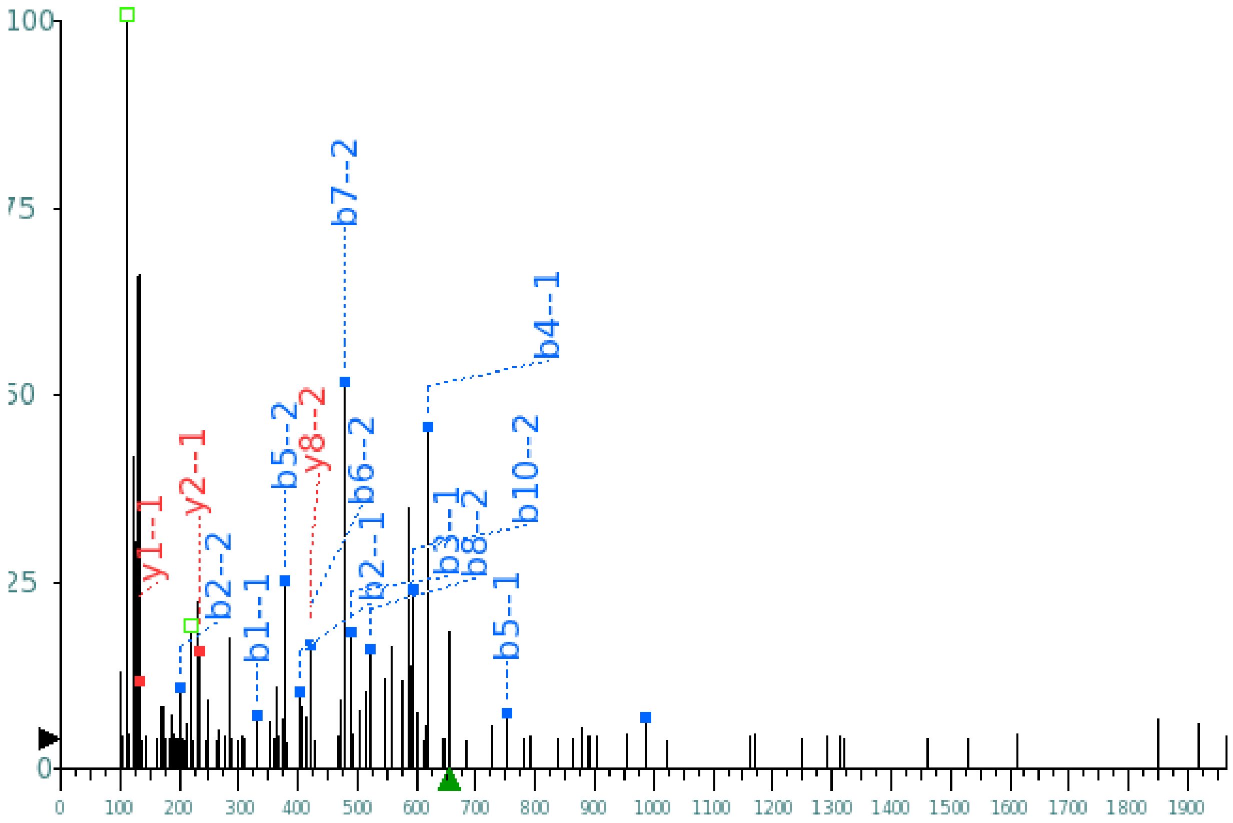

TASQHSDSSGFAEDSTD (+3)

Primary Reference: SSFA2_HUMAN
Search ID: 41760
Search Name: 20130328_ananiav_ERstress_TMT_TM_timecourse_trypsin_inj1_4mc_iaa
Scan#: 4951
Observed Mass: 657.6203 (2.6 ppm)
PSM Score: 9.72

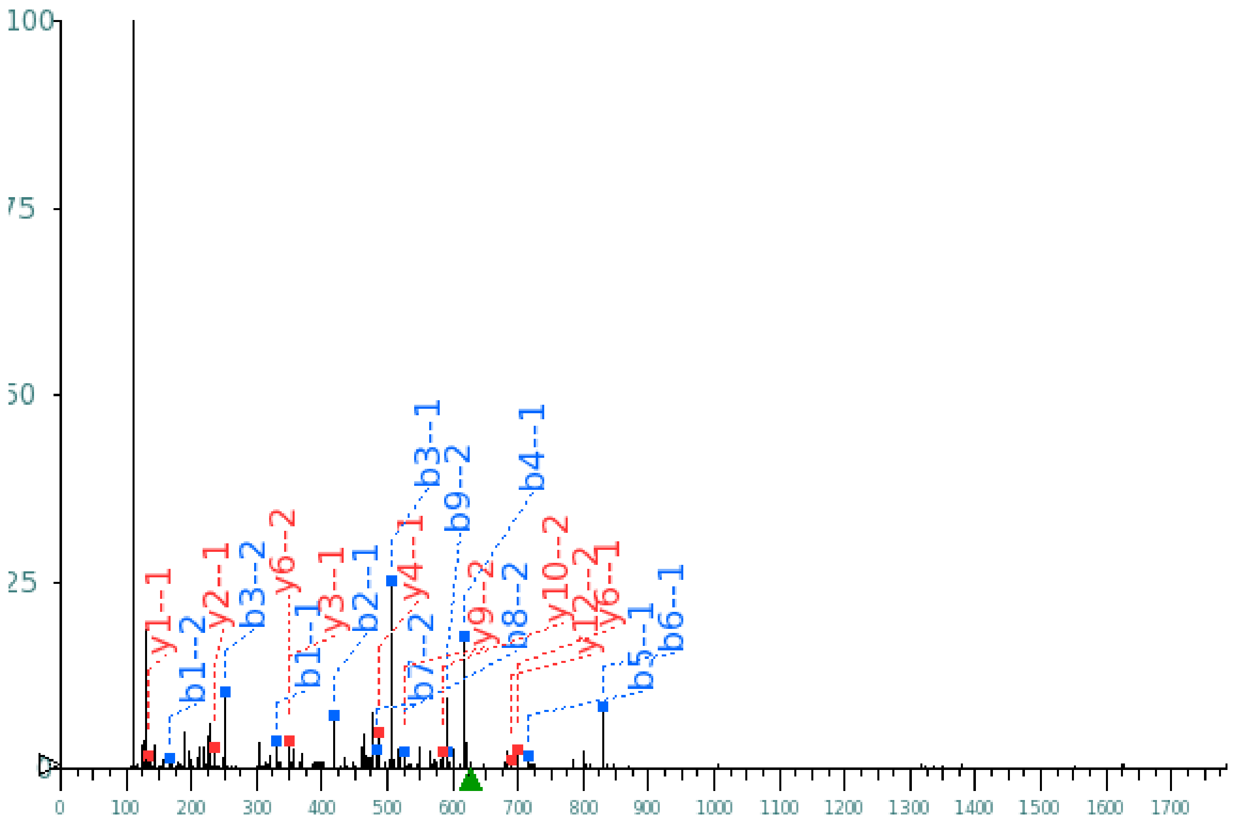

TSSLPNHSEPDHDTD (+3)

Primary Reference: VIR_HUMAN
Search ID: 41760
Search Name: 20130328_ananiav_ERstress_TMT_TM_timecourse_trypsin_inj1_4mc_iaa
Scan#: 4958
Observed Mass: 627.6215 (2.1 ppm)
PSM Score: 27.44

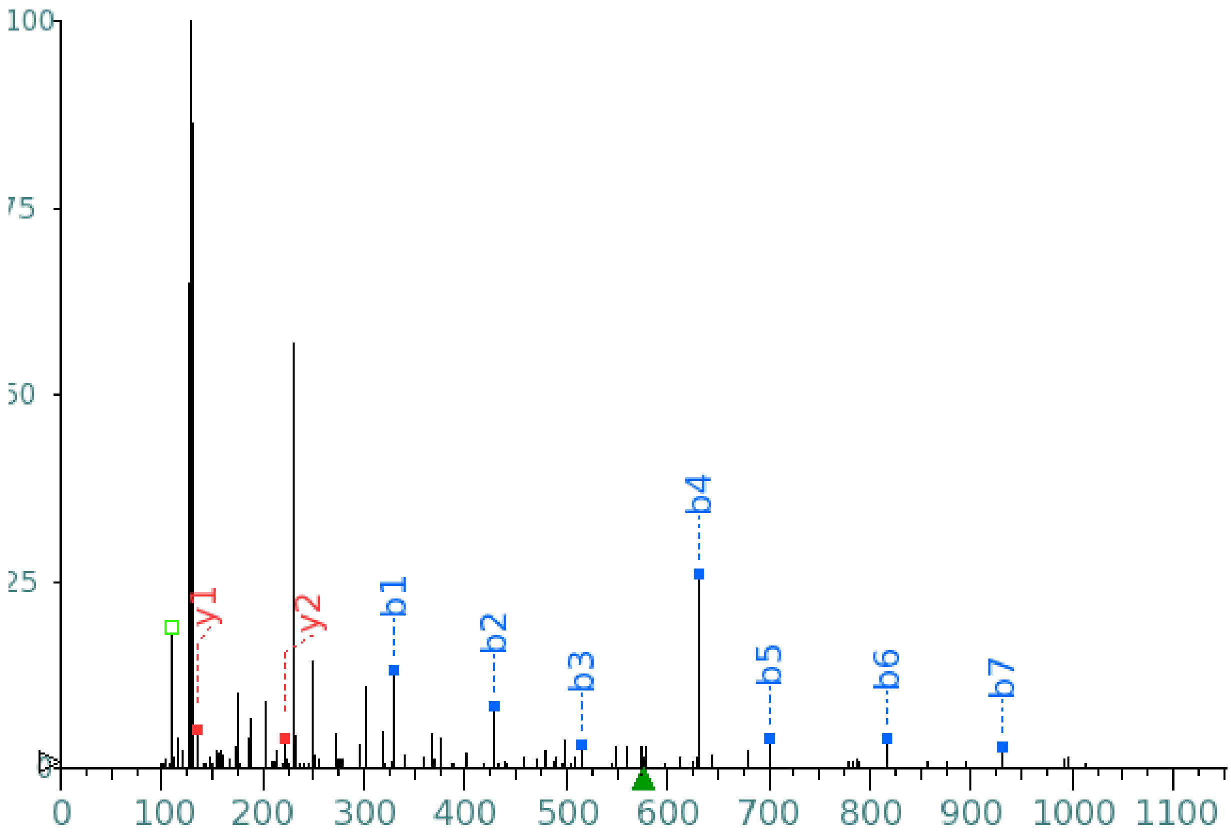

VVSDADDSD (+2)

Primary Reference: IWS1_HUMAN
Search ID: 41760
Search Name: 20130328_ananiav_ERstress_TMT_TM_timecourse_trypsin_inj1_4mc_iaa
Scan#: 4961
Observed Mass: 576.2683 (2.5 ppm)
PSM Score: 35.75

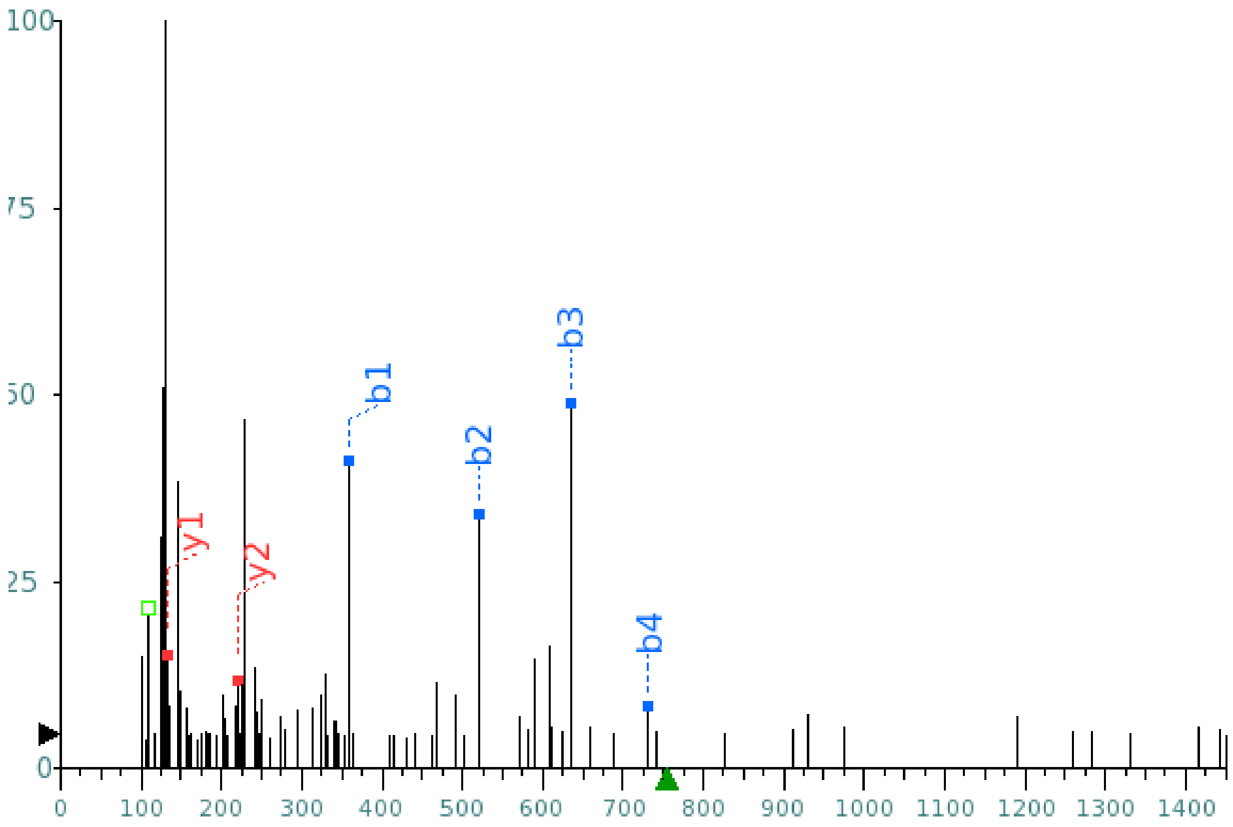

QYNPPQQDSSD (+2)

Primary Reference: GP125_HUMAN
Search ID: 41760
Search Name: 20130328_ananiav_ERstress_TMT_TM_timecourse_trypsin_inj1_4mc_iaa
Scan#: 4975
Observed Mass: 754.3486 (2.5 ppm)
PSM Score: 8.19

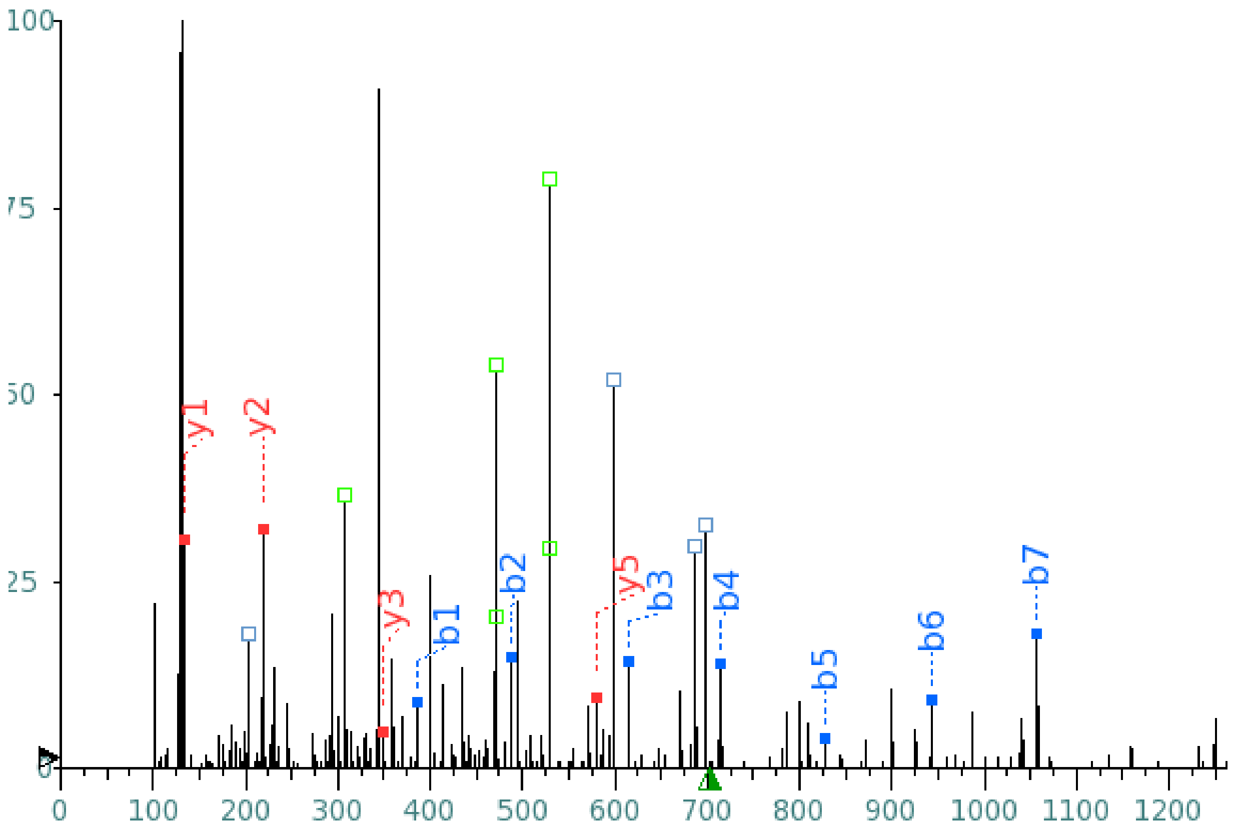

RTQVIDDESD (+2)

Primary Reference: TRIP4_HUMAN
Search ID: 41760
Search Name: 20130328_ananiav_ERstress_TMT_TM_timecourse_trypsin_inj1_4mc_iaa
Scan#: 5047
Observed Mass: 703.8532 (2.2 ppm)
PSM Score: 23.95

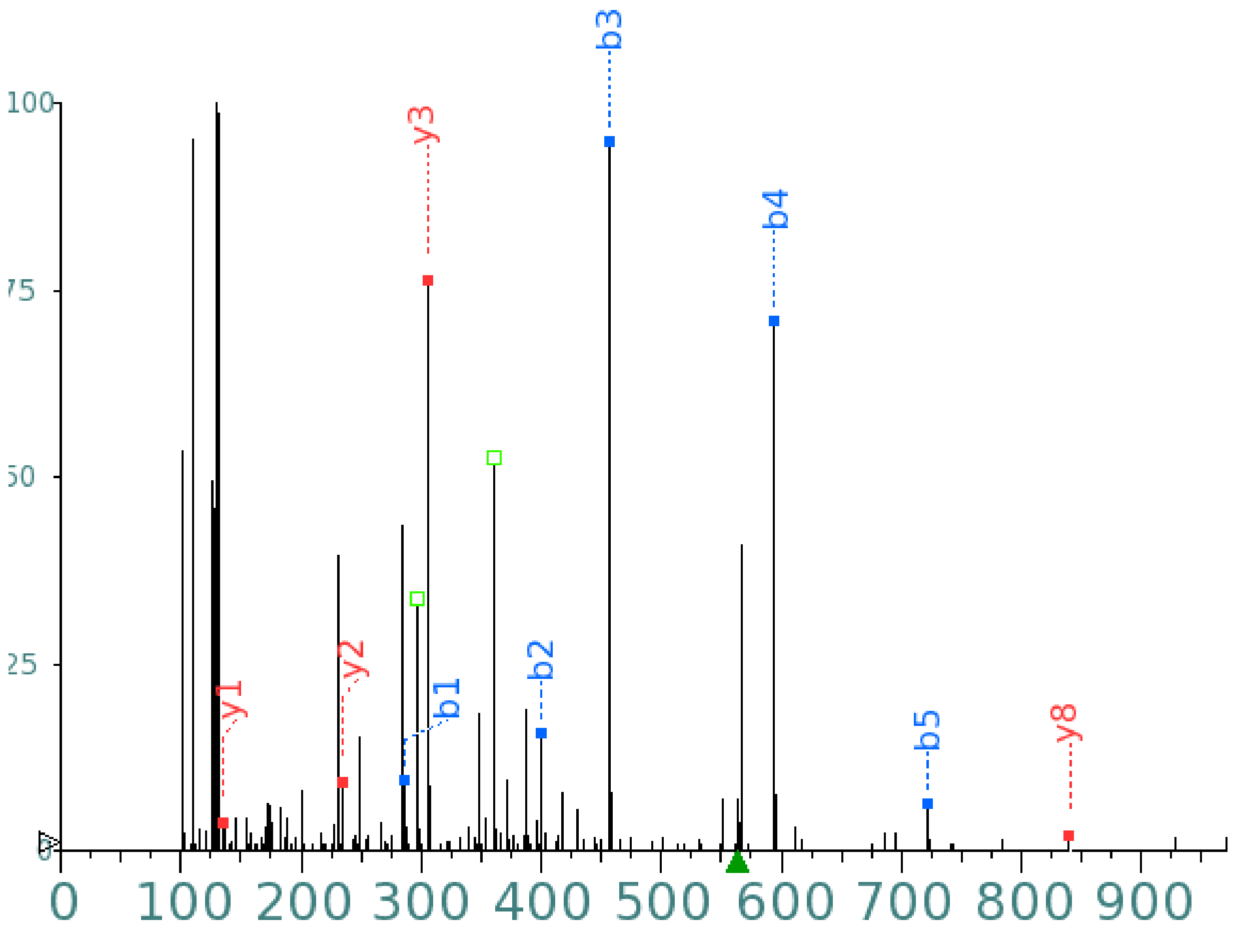

GLGHQVATD (+2)

Primary Reference: RTCB_HUMAN
Search ID: 41760
Search Name: 20130328_ananiav_ERstress_TMT_TM_timecourse_trypsin_inj1_4mc_iaa
Scan#: 5079
Observed Mass: 563.8072 (1.6 ppm)
PSM Score: 14.94

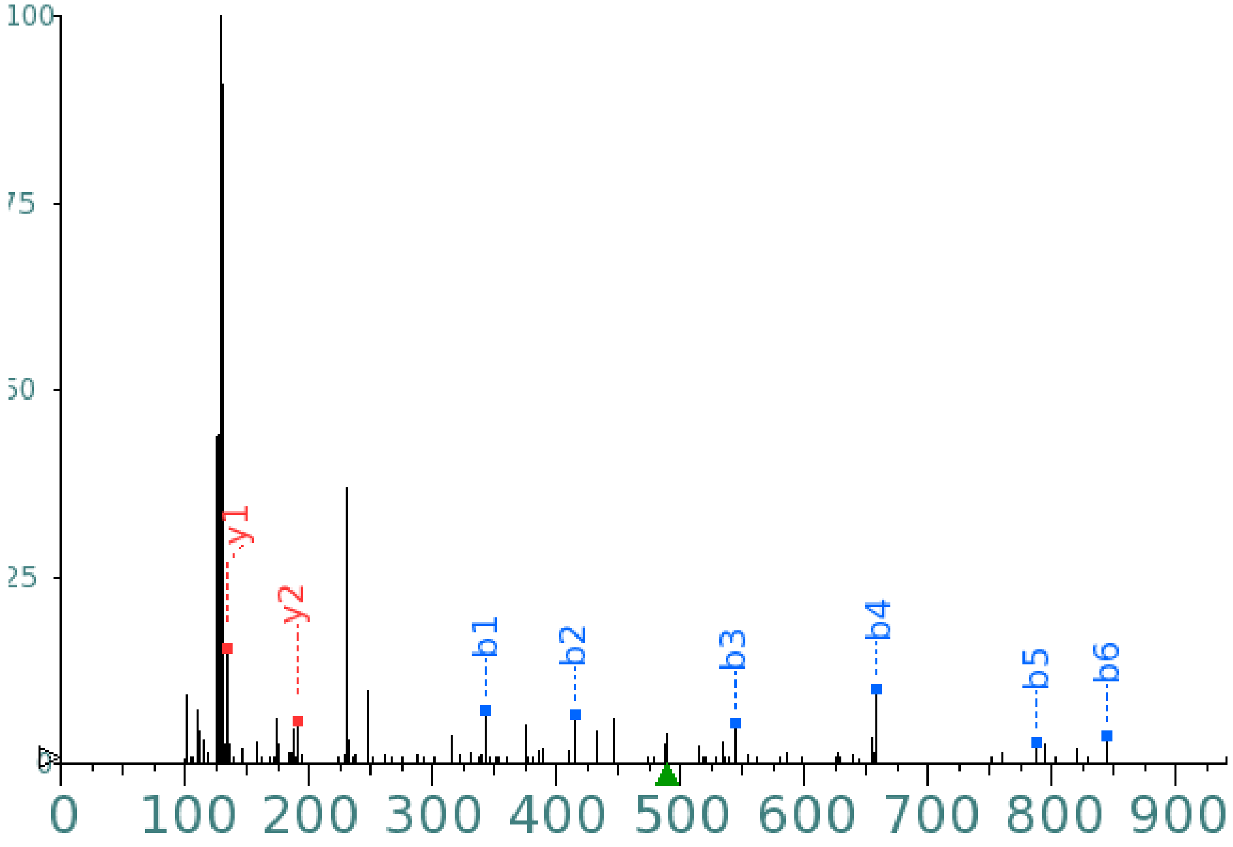

LAEDEGD (+2)

Primary Reference: BAZ1B_HUMAN
Search ID: 41760
Search Name: 20130328_ananiav_ERstress_TMT_TM_timecourse_trypsin_inj1_4mc_iaa
Scan#: 5110
Observed Mass: 489.2362 (2.6 ppm)
PSM Score: 29.09

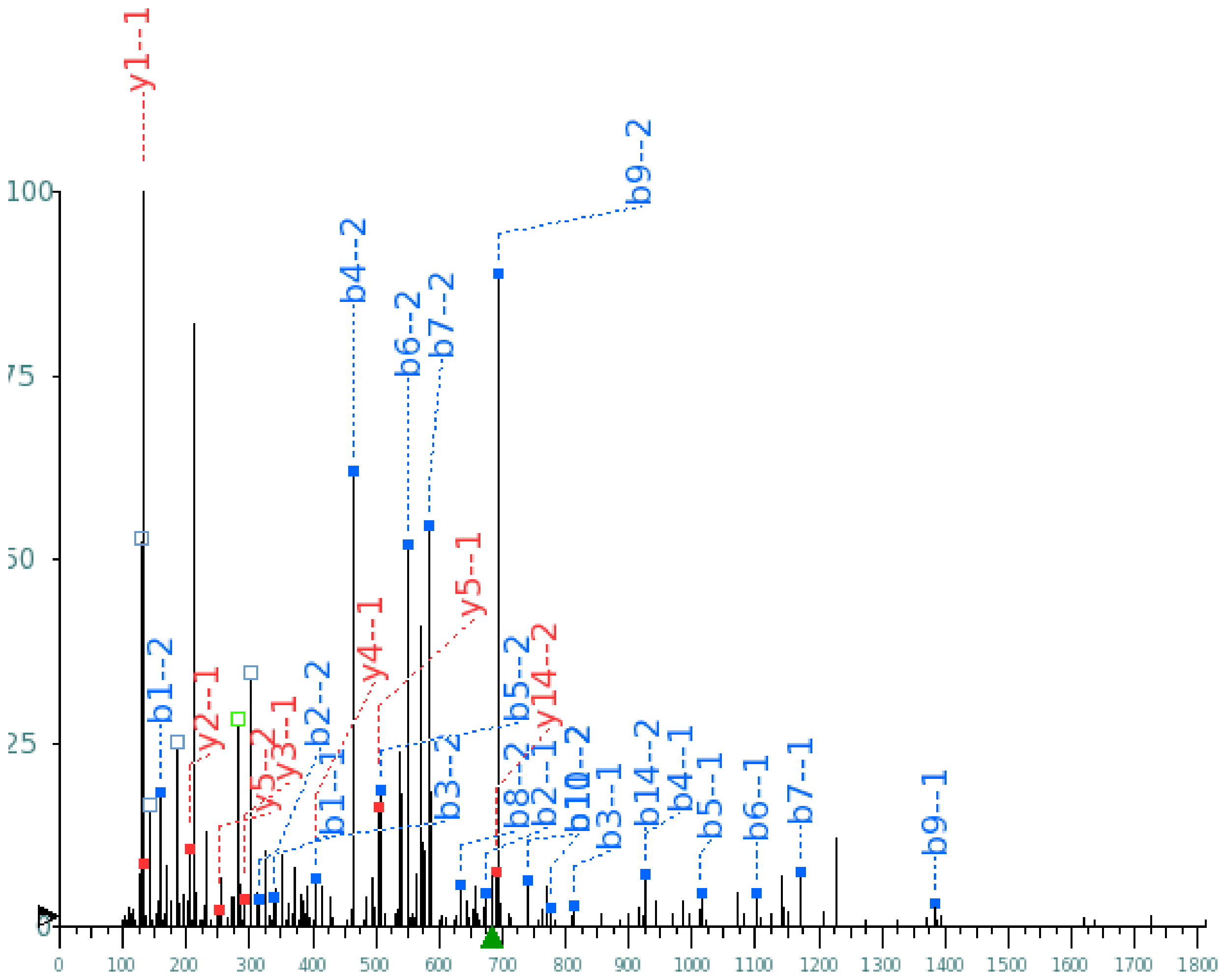

SKHDSSAPDPAPDSAD (+3)

Primary Reference: DIDO1_HUMAN
Search ID: 41760
Search Name: 20130328_ananiav_ERstress_TMT_TM_timecourse_trypsin_inj1_4mc_iaa
Scan#: 5114
Observed Mass: 685.674 (2.1 ppm)
PSM Score: 43.12

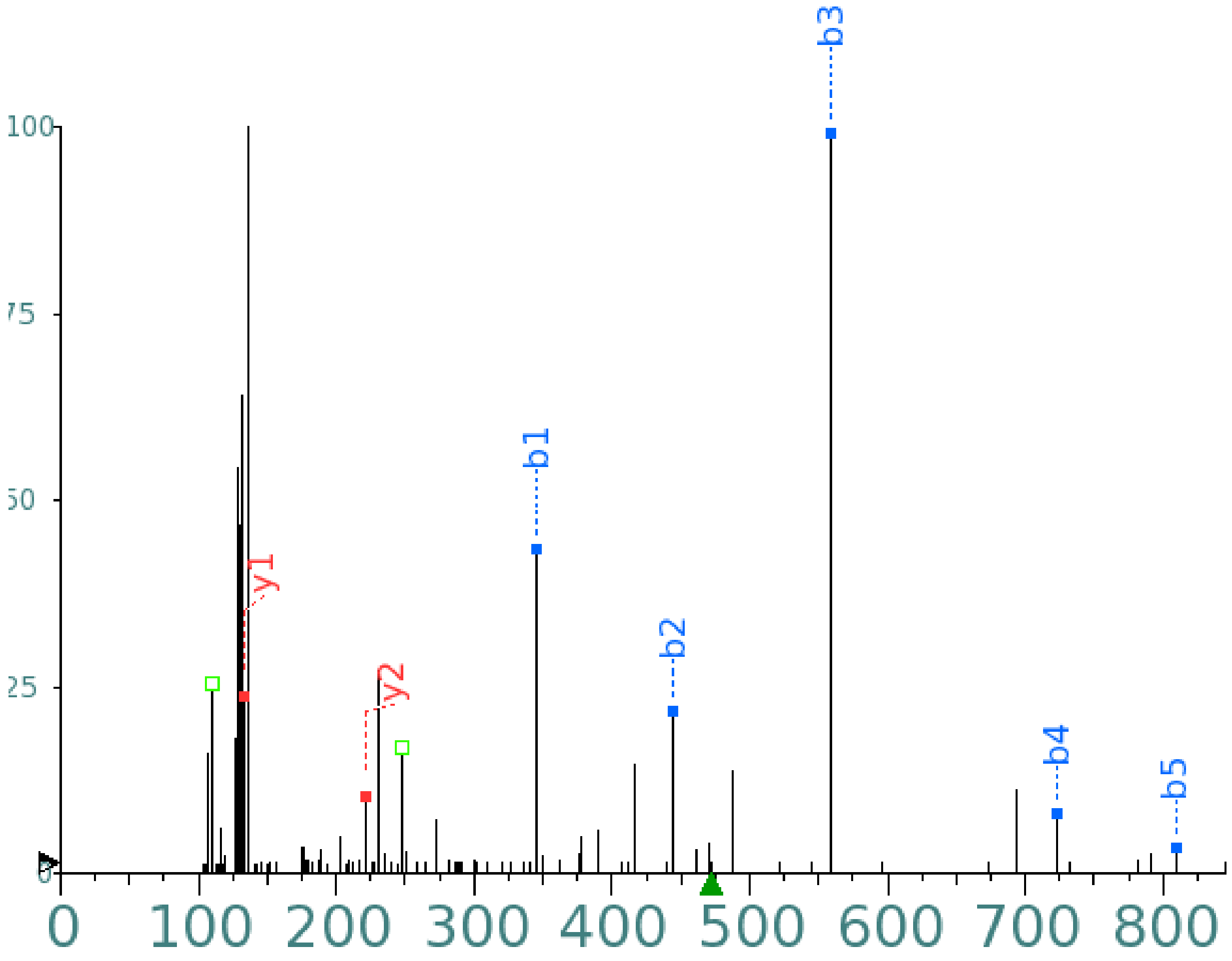

DVDYSD (+2)

Primary Reference: SMCA2_HUMAN
Search ID: 41760
Search Name: 20130328_ananiav_ERstress_TMT_TM_timecourse_trypsin_inj1_4mc_iaa
Scan#: 5121
Observed Mass: 471.7175 (2.4 ppm)
PSM Score: 34.72

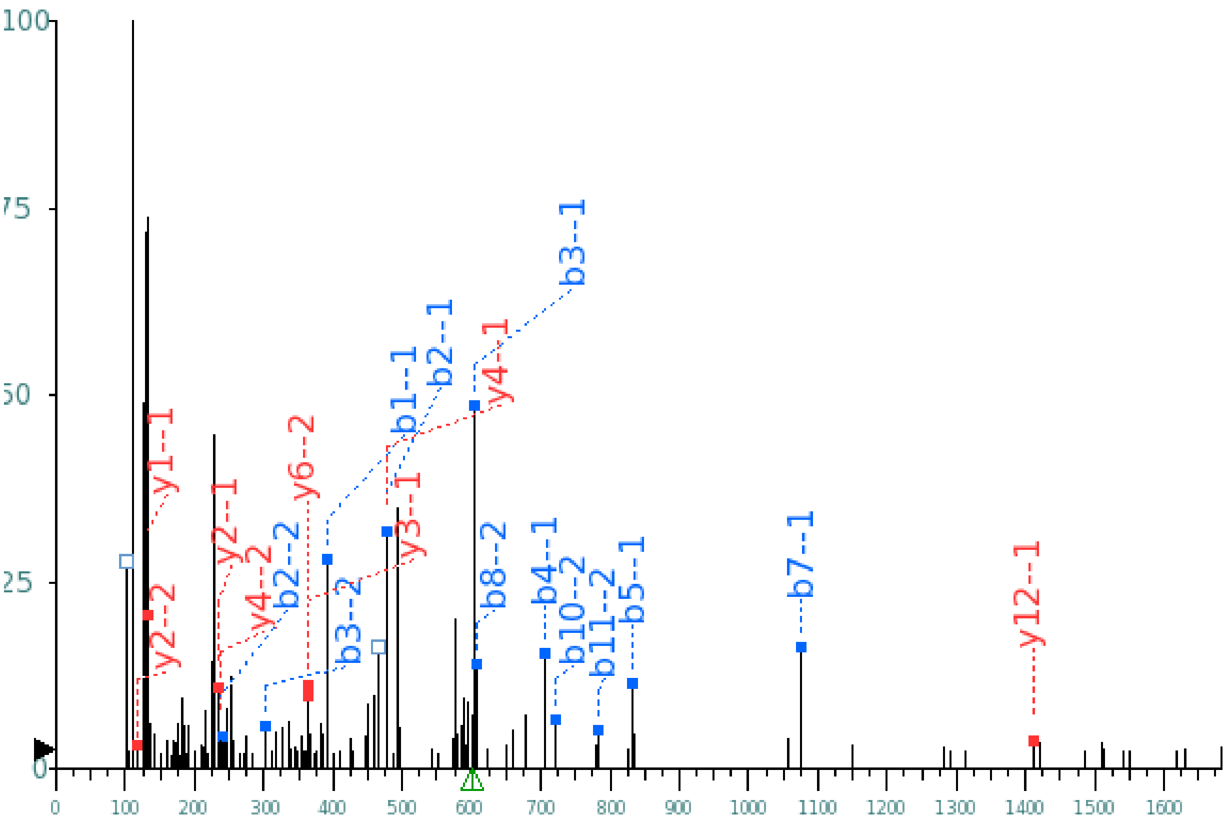

C*SQVQQDHLNQTD (+3)

Primary Reference: HOOK1_HUMAN
Search ID: 41760
Search Name: 20130328_ananiav_ERstress_TMT_TM_timecourse_trypsin_inj1_4mc_iaa
Scan#: 5169
Observed Mass: 601.2844 (2.8 ppm)
PSM Score: 17.34

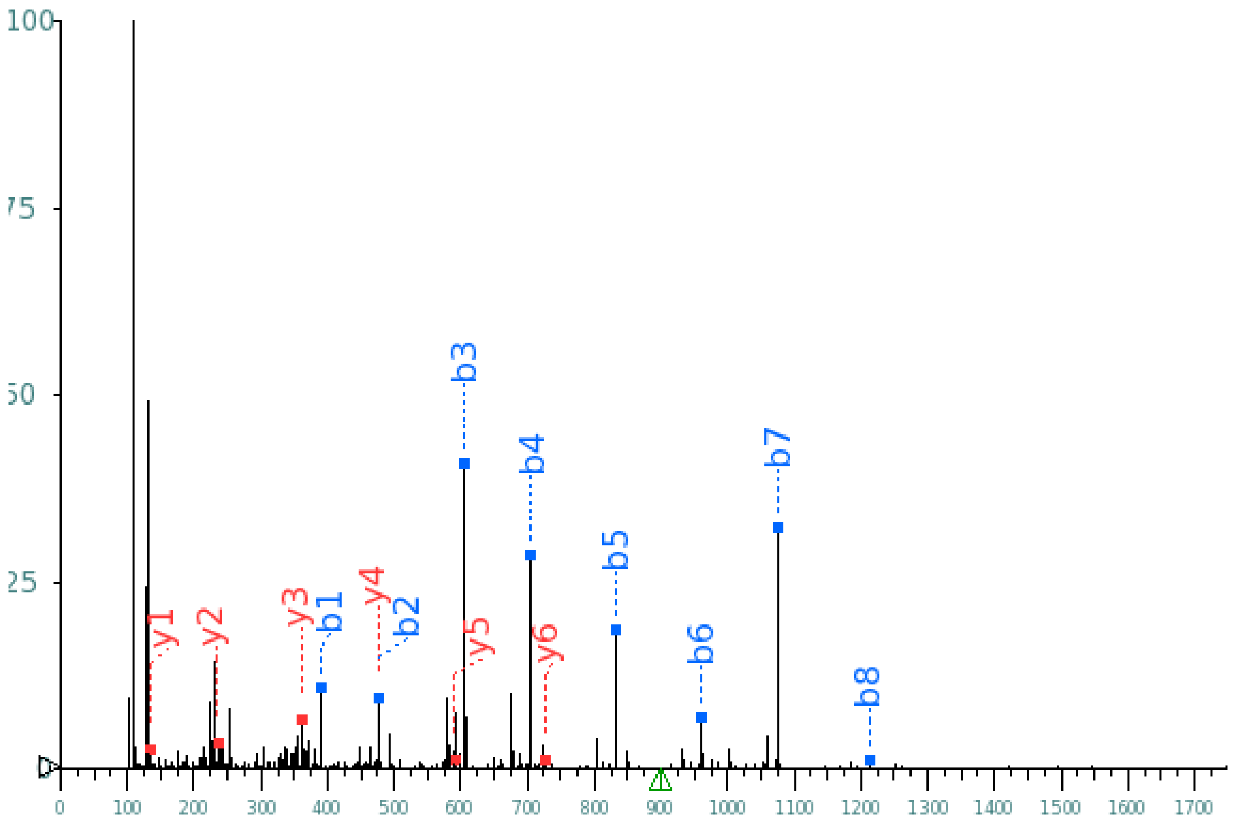

C*SQVQQDHLNQTD (+2)

Primary Reference: HOOK1_HUMAN
Search ID: 41760
Search Name: 20130328_ananiav_ERstress_TMT_TM_timecourse_trypsin_inj1_4mc_iaa
Scan#: 5173
Observed Mass: 901.4228 (2.6 ppm)
PSM Score: 41.1

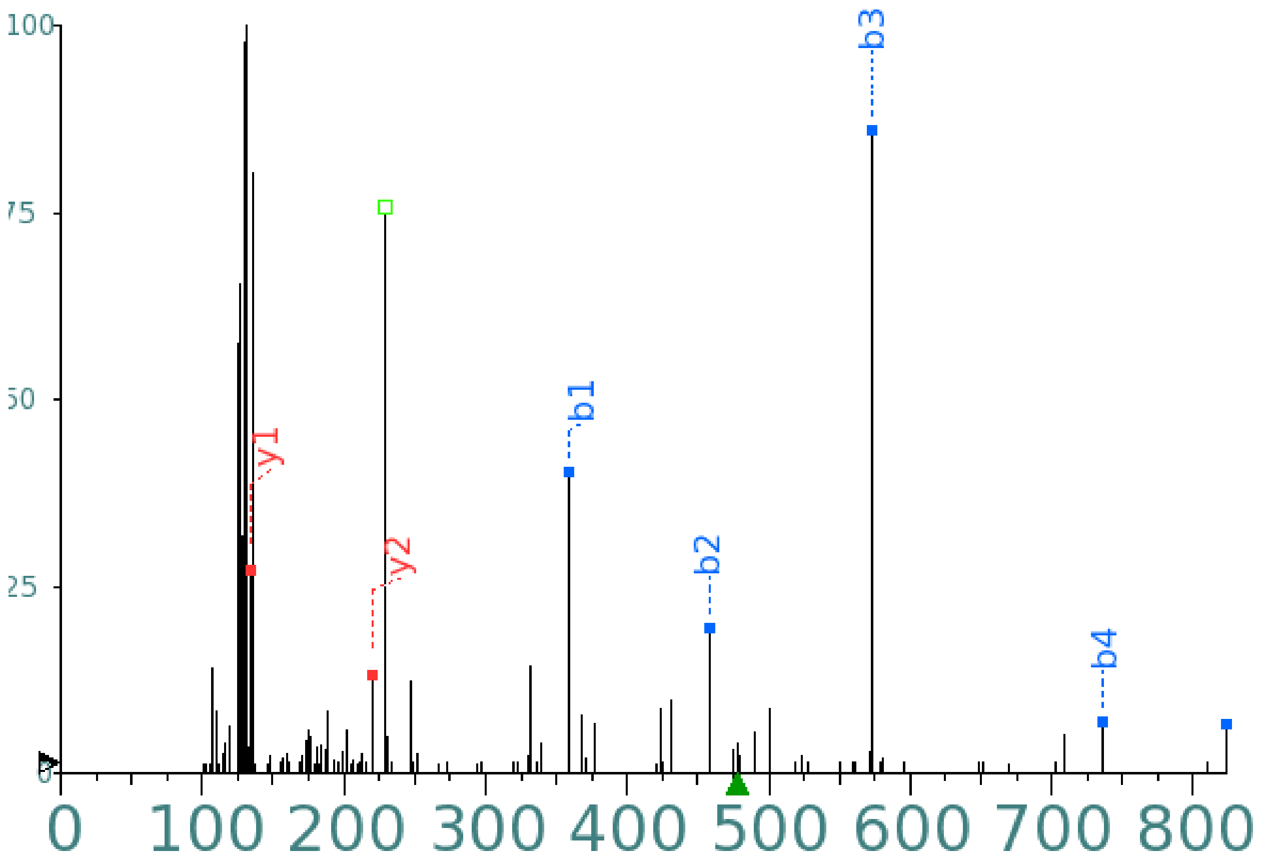

EVDYSD (+2)

Primary Reference: SMCA4_HUMAN
Search ID: 41760
Search Name: 20130328_ananiav_ERstress_TMT_TM_timecourse_trypsin_inj1_4mc_iaa
Scan#: 5243
Observed Mass: 478.7242 (0.1 ppm)
PSM Score: 34.66

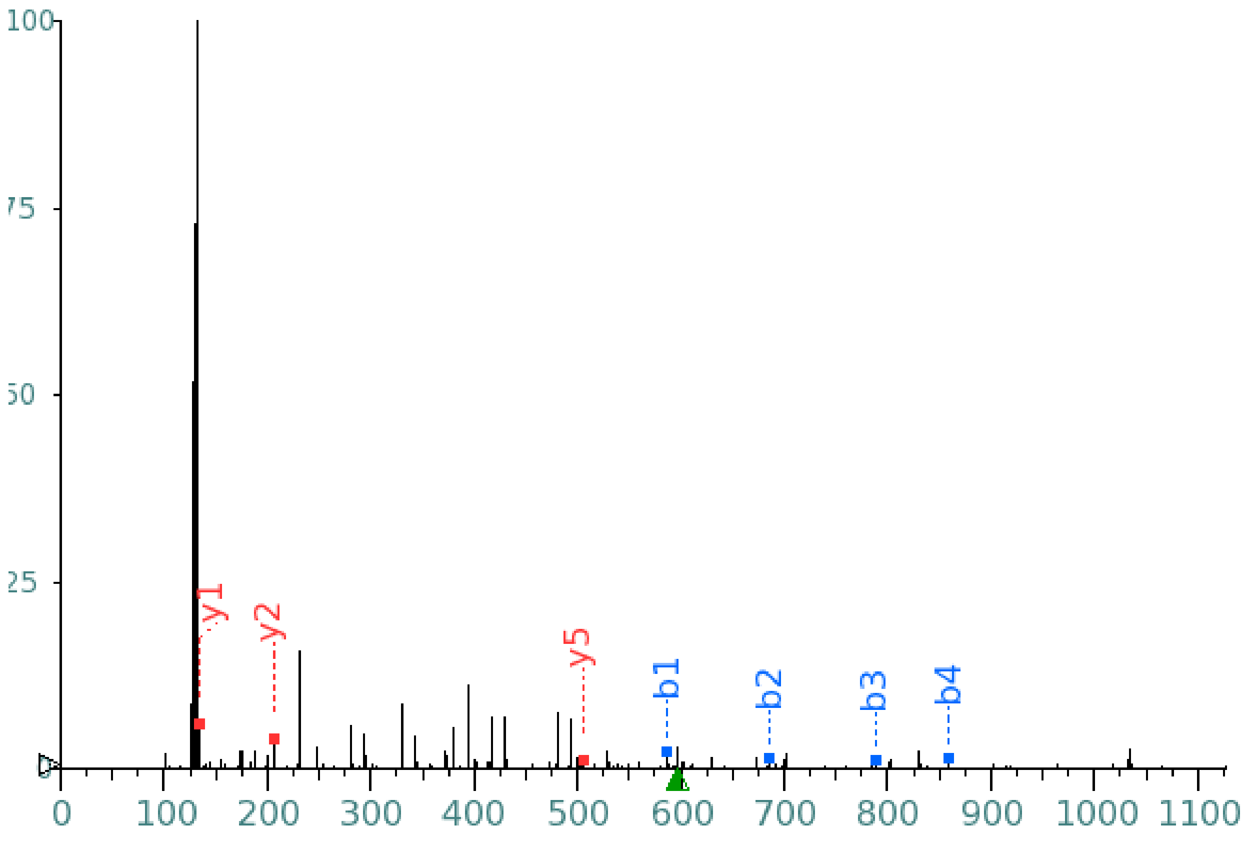

KVTAEAD (+2)

Primary Reference: SHOT1_HUMAN
Search ID: 41760
Search Name: 20130328_ananiav_ERstress_TMT_TM_timecourse_trypsin_inj1_4mc_iaa
Scan#: 5286
Observed Mass: 596.3537 (1.3 ppm)
PSM Score: 9.26

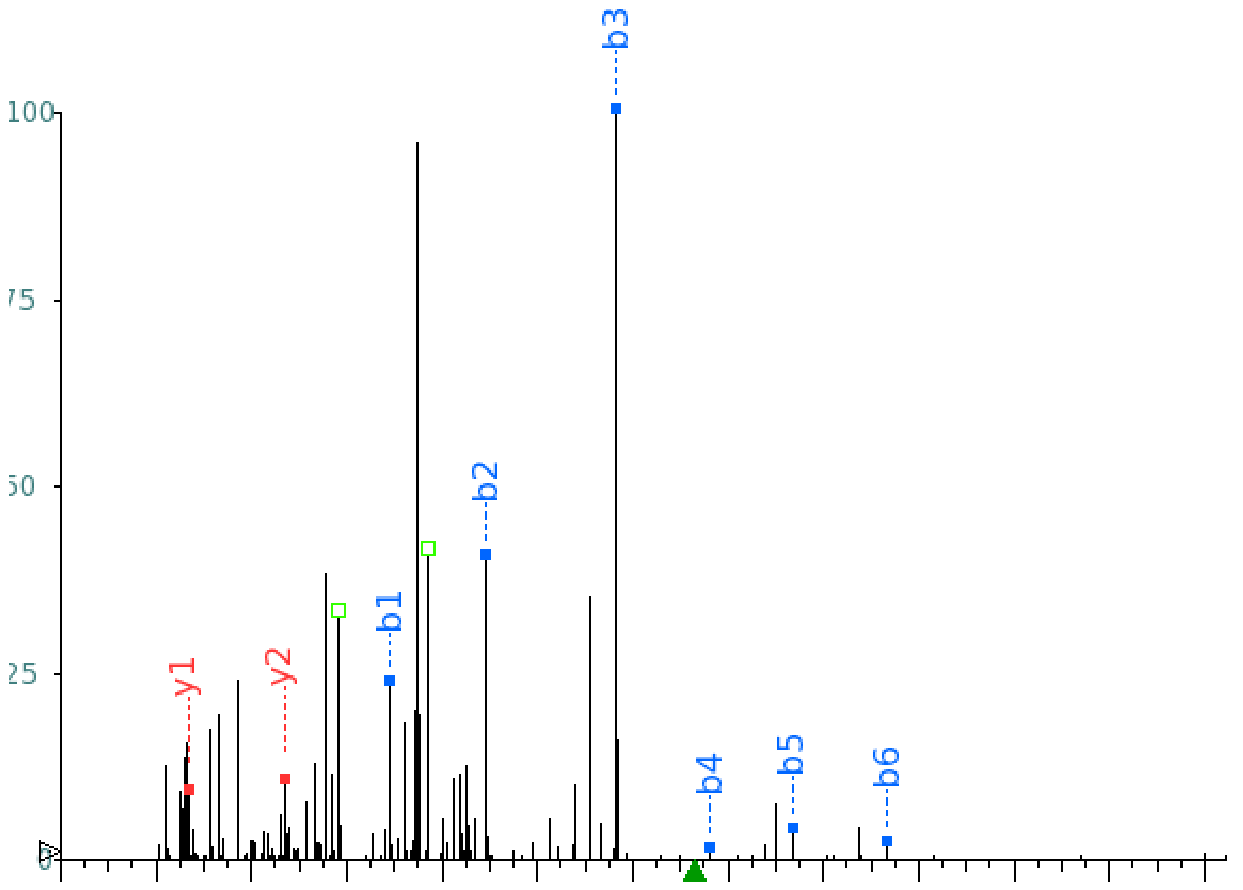

DTHPSVVETD (+2)

Primary Reference: ARHG5_HUMAN
Search ID: 41760
Search Name: 20130328_ananiav_ERstress_TMT_TM_timecourse_trypsin_inj1_4mc_iaa
Scan#: 5288
Observed Mass: 664.8309 (1 ppm)
PSM Score: 23.57

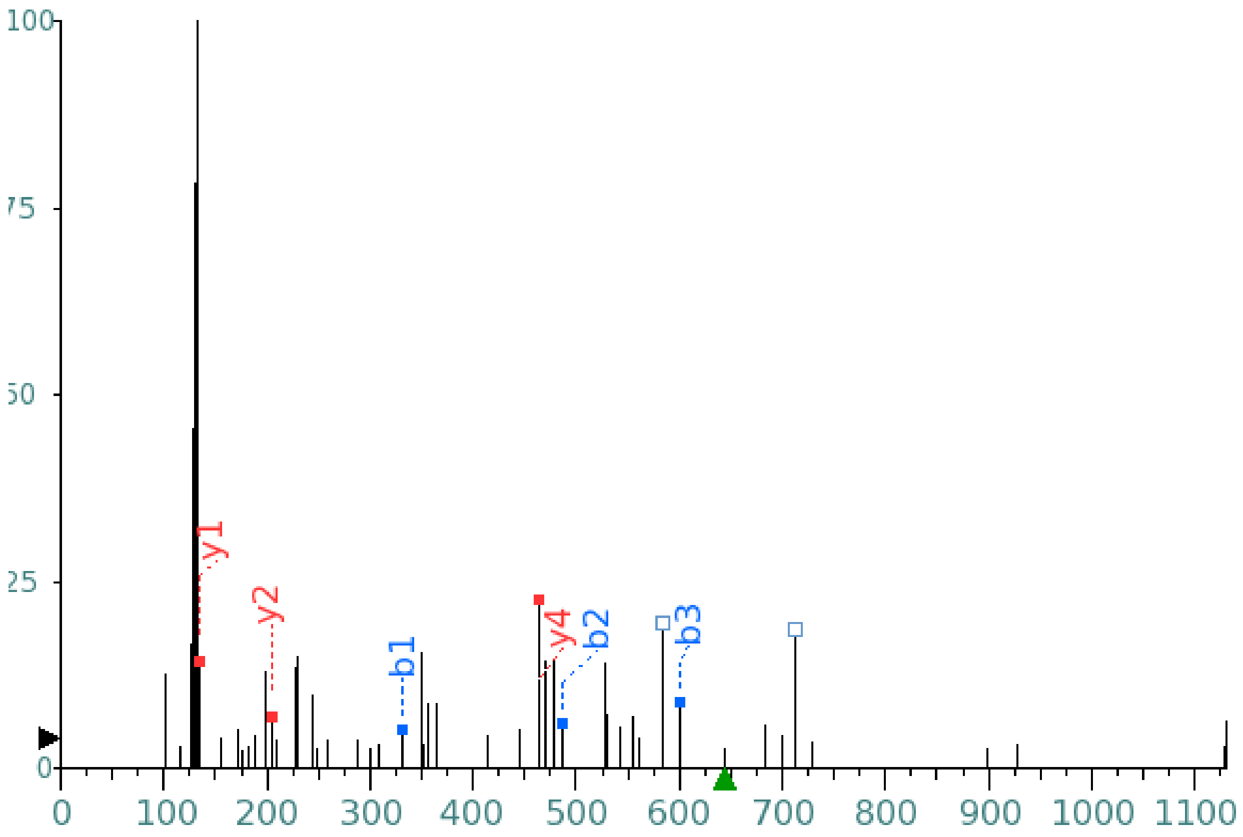

TRLQPEEAD (+2)

Primary Reference: DIDO1_HUMAN
Search ID: 41760
Search Name: 20130328_ananiav_ERstress_TMT_TM_timecourse_trypsin_inj1_4mc_iaa
Scan#: 5316
Observed Mass: 644.3442 (5.4 ppm)
PSM Score: 20.47

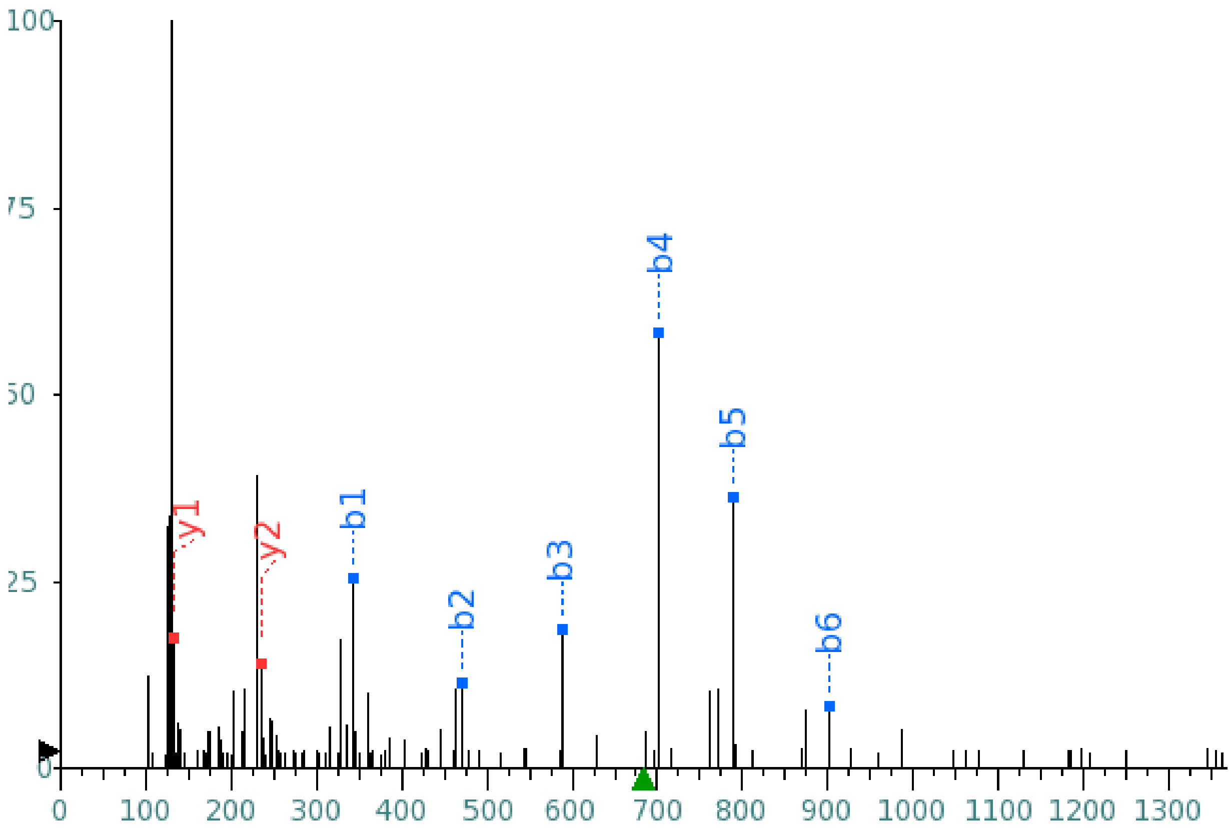

NQDDSLTETD (+2)

Primary Reference: PACS1_HUMAN
Search ID: 41760
Search Name: 20130328_ananiav_ERstress_TMT_TM_timecourse_trypsin_inj1_4mc_iaa
Scan#: 5352
Observed Mass: 683.8134 (1.7 ppm)
PSM Score: 17.95

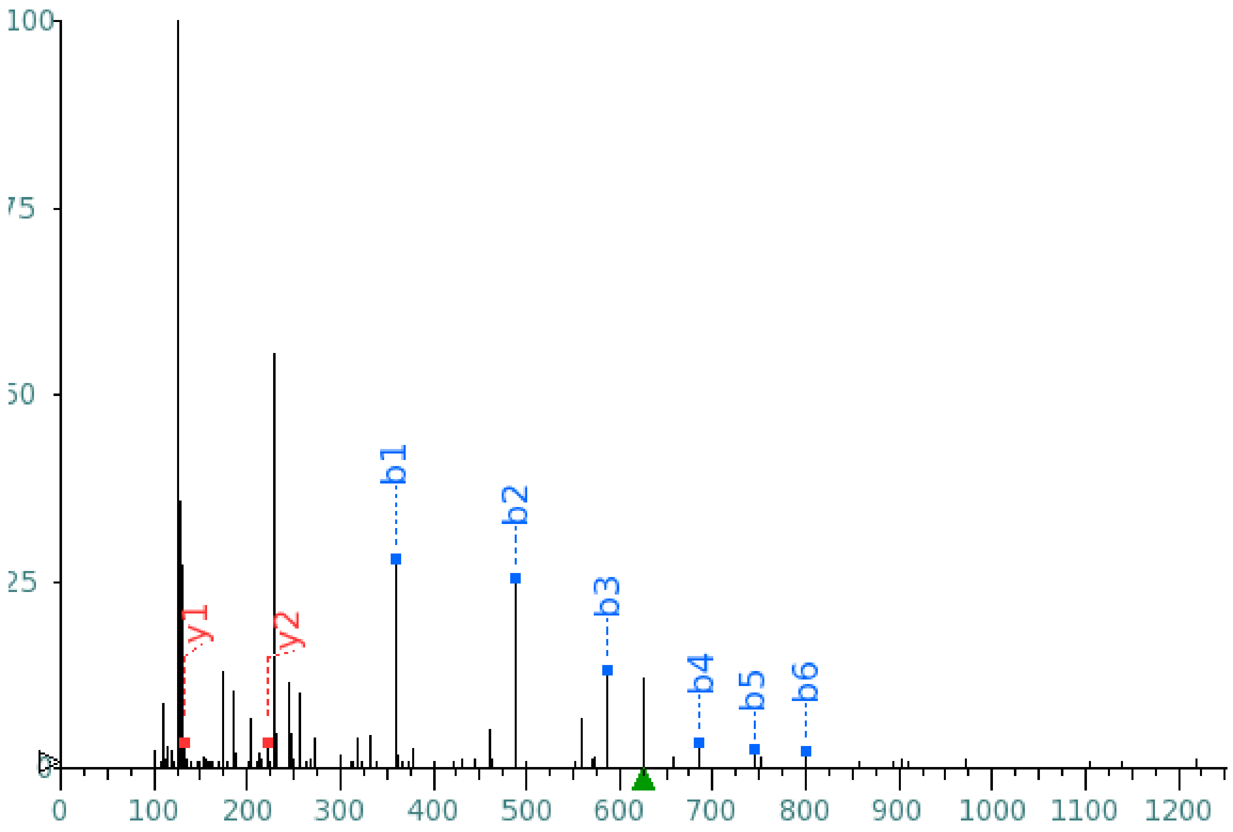

EEVVGGDDSD (+2)

Primary Reference: TYY1_HUMAN
Search ID: 41760
Search Name: 20130328_ananiav_ERstress_TMT_TM_timecourse_trypsin_inj1_4mc_iaa
Scan#: 5389
Observed Mass: 625.7846 (2.7 ppm)
PSM Score: 22.34

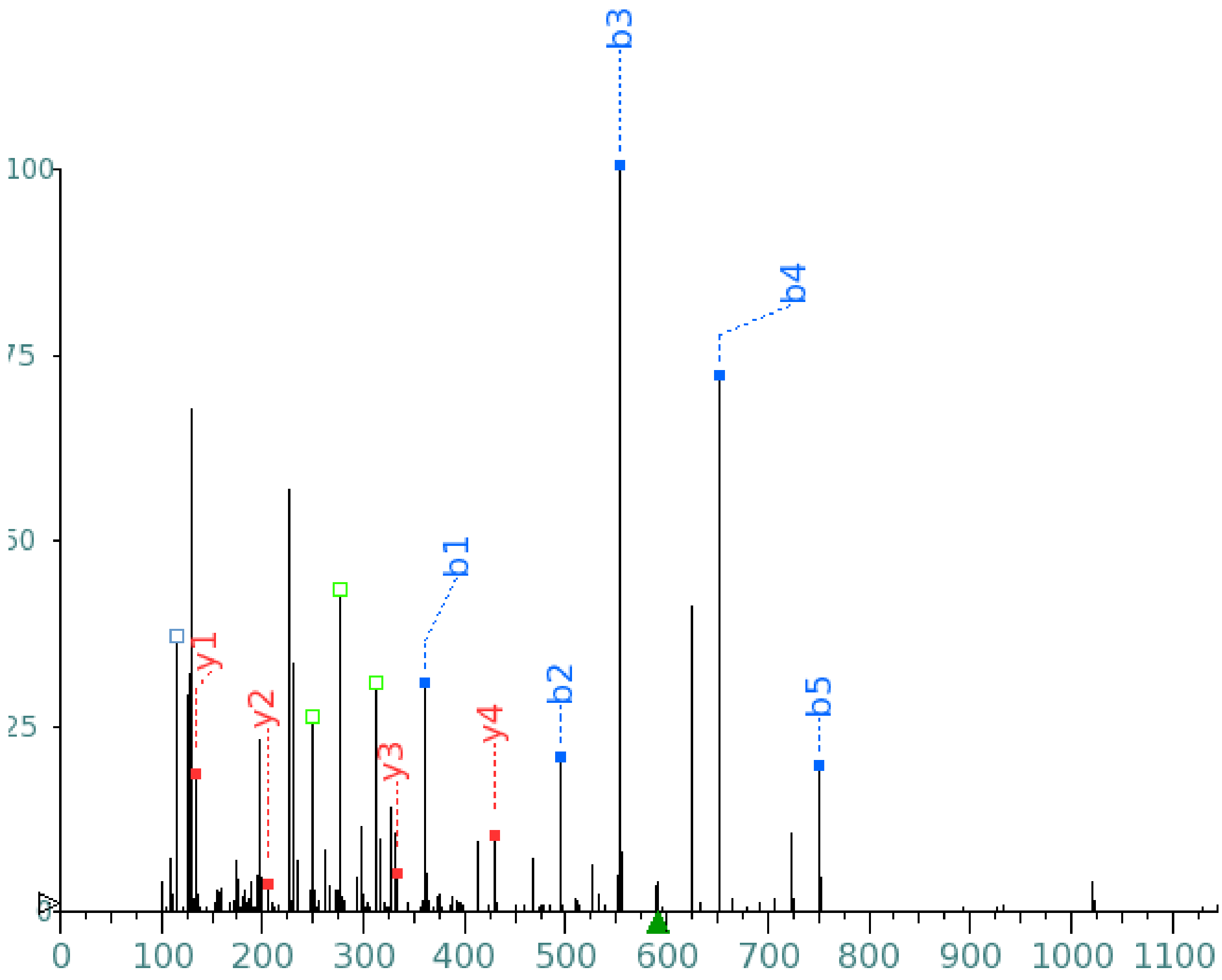

EHGVVPQAD (+2)

Primary Reference: DNJC2_HUMAN
Search ID: 41760
Search Name: 20130328_ananiav_ERstress_TMT_TM_timecourse_trypsin_inj1_4mc_iaa
Scan#: 5401
Observed Mass: 590.8133 (2.9 ppm)
PSM Score: 27.83

File name: 2015-02-04 04.01.53PM.pdf

Creation time: 2015-02-04 04:09PM

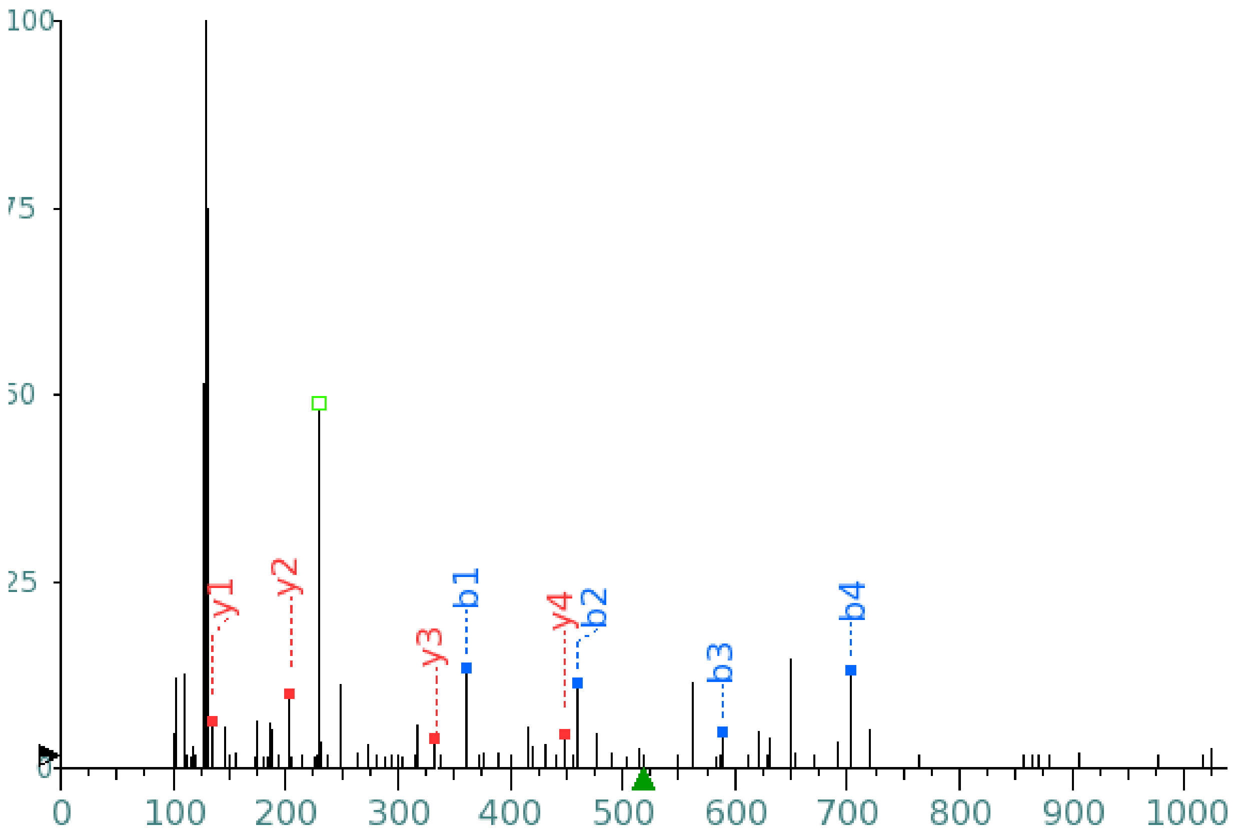

MVQDEAD (+2)

Primary Reference: SNX33_HUMAN
Search ID: 41760
Search Name: 20130328_ananiav_ERstress_TMT_TM_timecourse_trypsin_inj1_4mc_iaa
Scan#: 5418
Observed Mass: 518.7463 (3.3 ppm)
PSM Score: 7.22

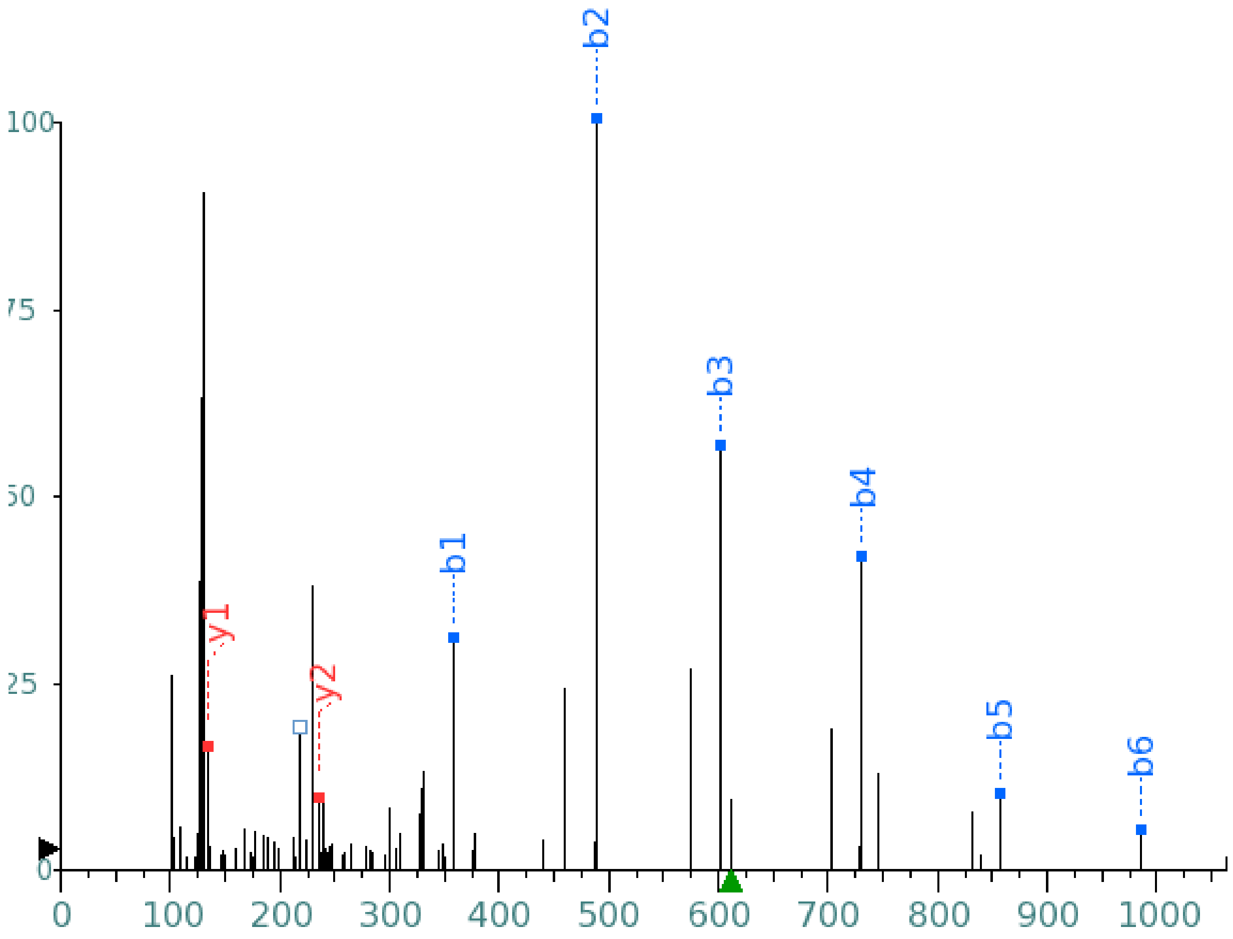

EELEQQTD (+2)

Primary Reference: SEC62_HUMAN
Search ID: 41760
Search Name: 20130328_ananiav_ERstress_TMT_TM_timecourse_trypsin_inj1_4mc_iaa
Scan#: 5425
Observed Mass: 610.7973 (2.5 ppm)
PSM Score: 41.07

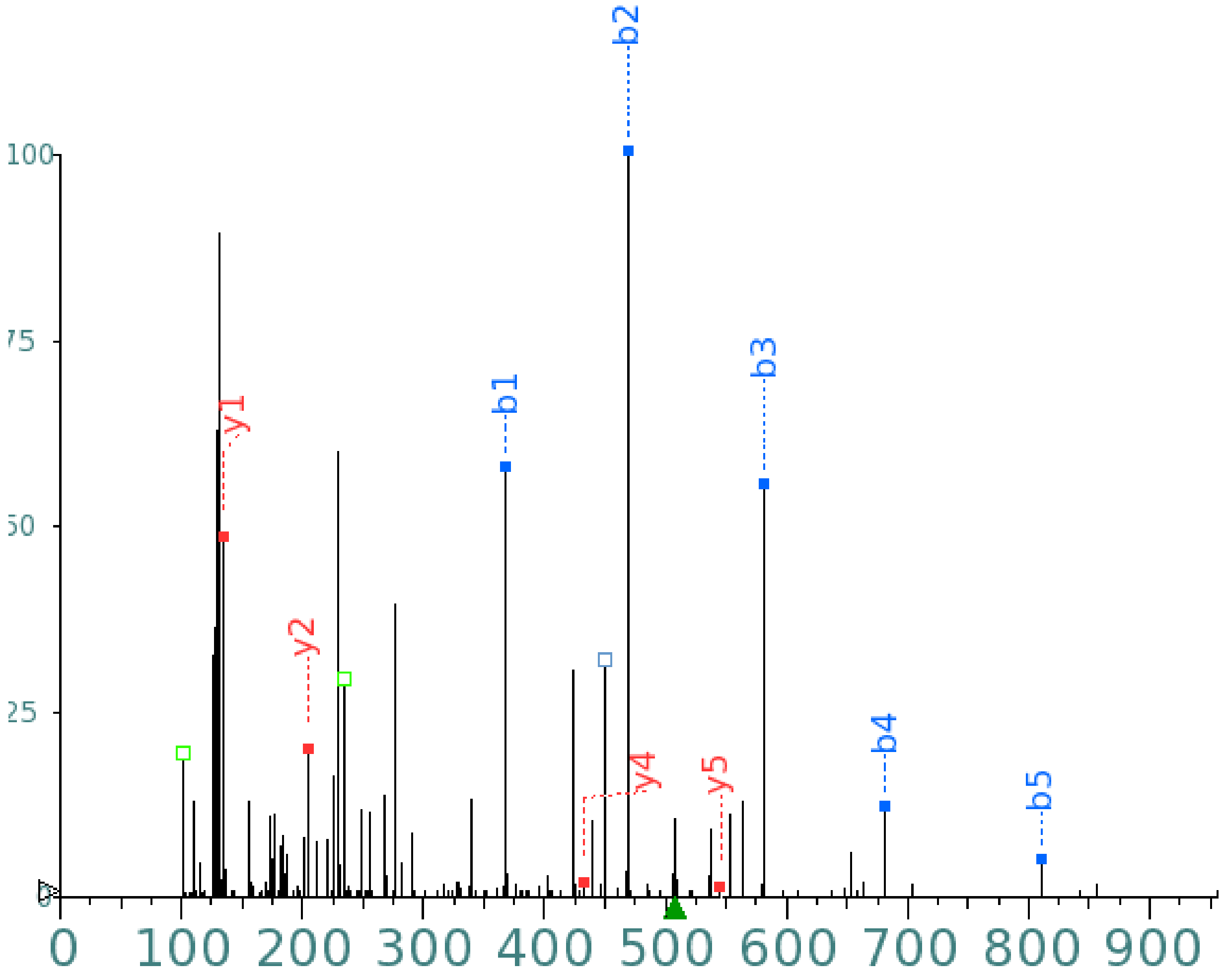

HTLVEAD (+2)

Primary Reference: SPTB2_HUMAN
Search ID: 41760
Search Name: 20130328_ananiav_ERstress_TMT_TM_timecourse_trypsin_inj1_4mc_iaa
Scan#: 5430
Observed Mass: 507.2782 (2.7 ppm)
PSM Score: 30.06

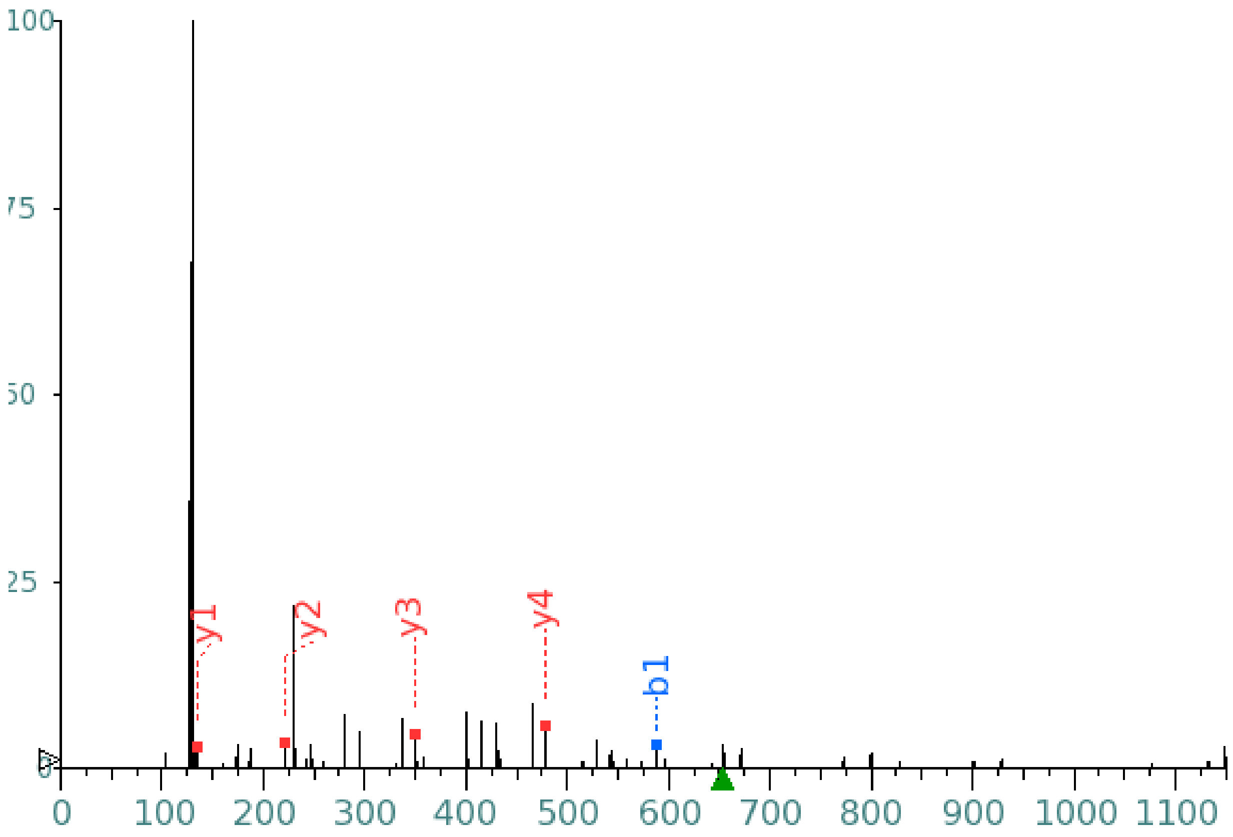

KLQEESD (+2)

Primary Reference: EIF3J_HUMAN
Search ID: 41760
Search Name: 20130328_ananiav_ERstress_TMT_TM_timecourse_trypsin_inj1_4mc_iaa
Scan#: 5447
Observed Mass: 653.8682 (2.8 ppm)
PSM Score: 8.05

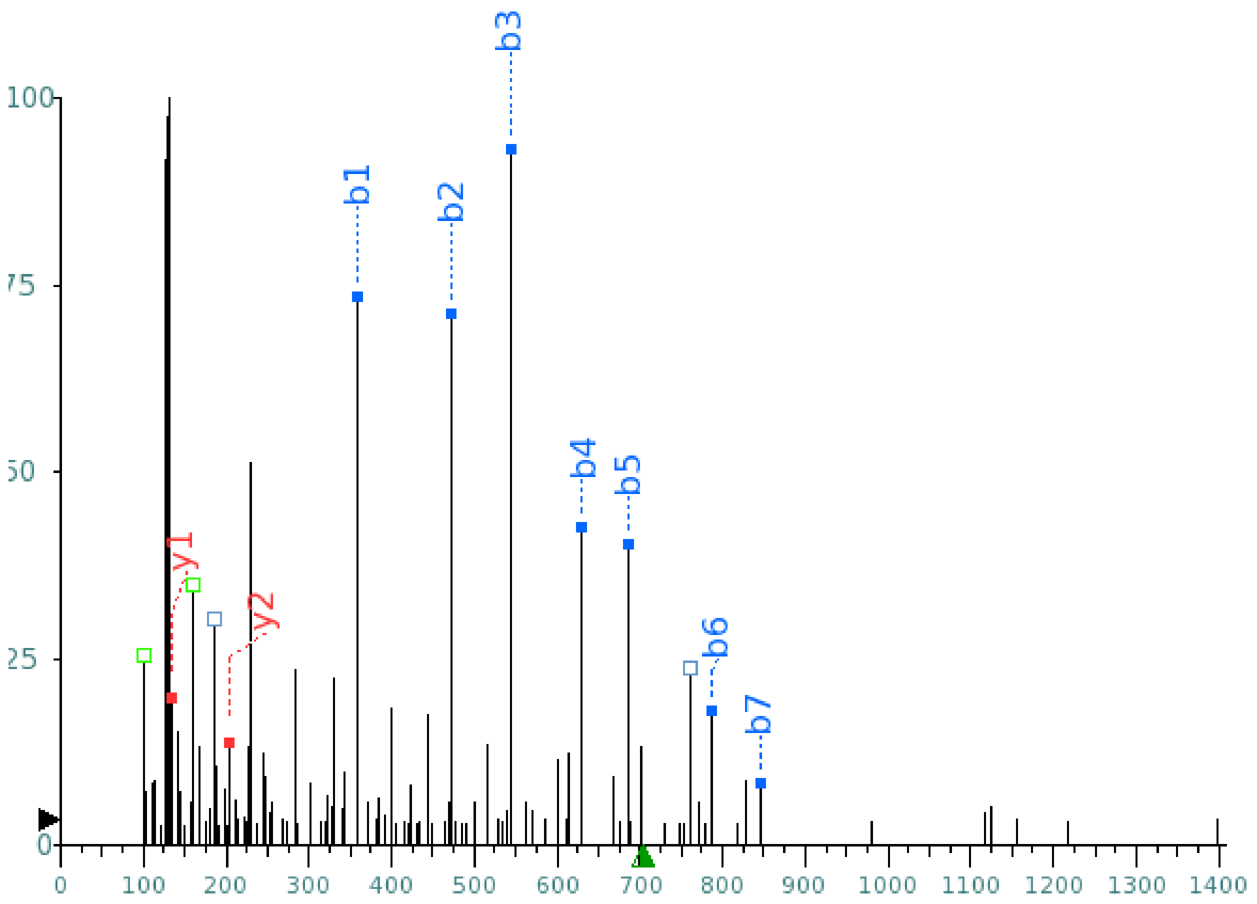

ELASGTGQDDAD (+2)

Primary Reference: E2QRJ1_HUMAN
Search ID: 41760
Search Name: 20130328_ananiav_ERstress_TMT_TM_timecourse_trypsin_inj1_4mc_iaa
Scan#: 5465
Observed Mass: 704.327 (2.2 ppm)
PSM Score: 32.15

File name: 2015-02-04 04.01.53PM.pdf

Creation time: 2015-02-04 04:09PM

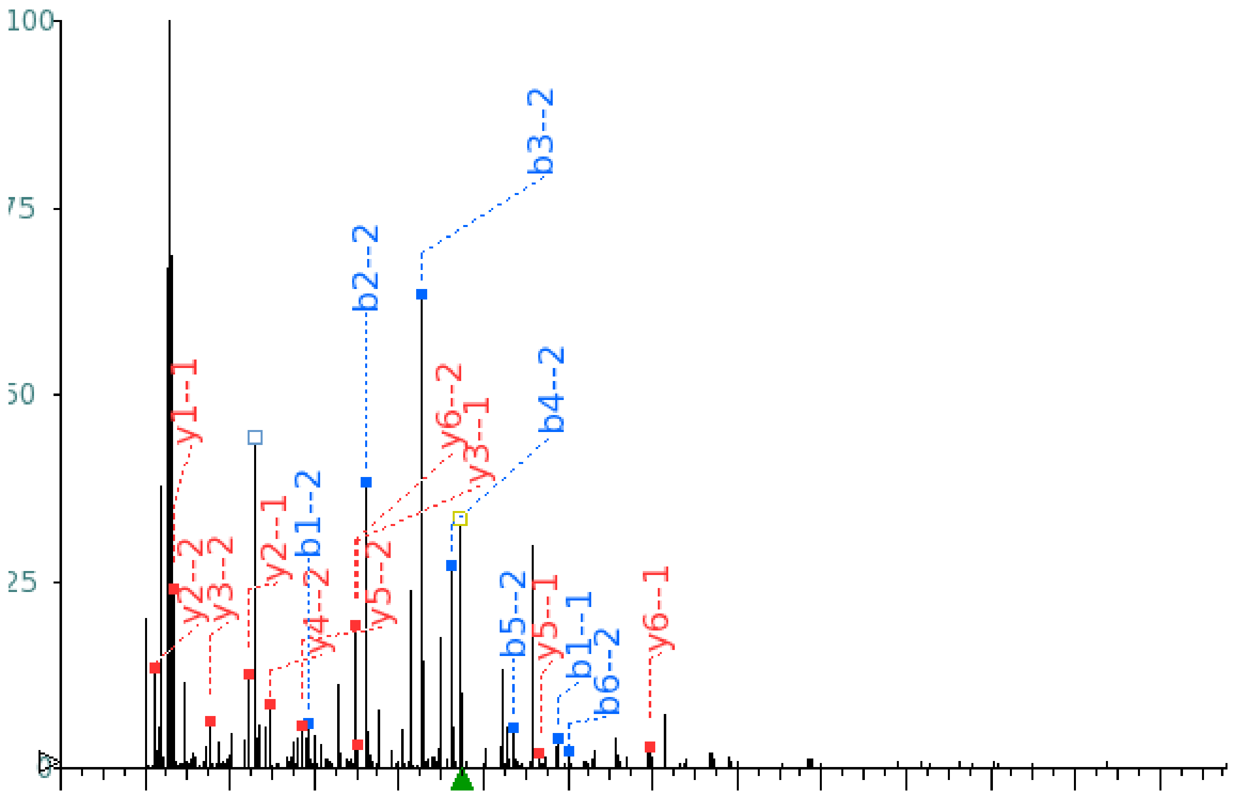

KHEAFESD (+3)

Primary Reference: ACTN1_HUMAN
Search ID: 41760
Search Name: 20130328_ananiav_ERstress_TMT_TM_timecourse_trypsin_inj1_4mc_iaa
Scan#: 5510
Observed Mass: 474.2546 (1.4 ppm)
PSM Score: 2.13

File name: 2015-02-04 04.01.53PM.pdf

Creation time: 2015-02-04 04:09PM

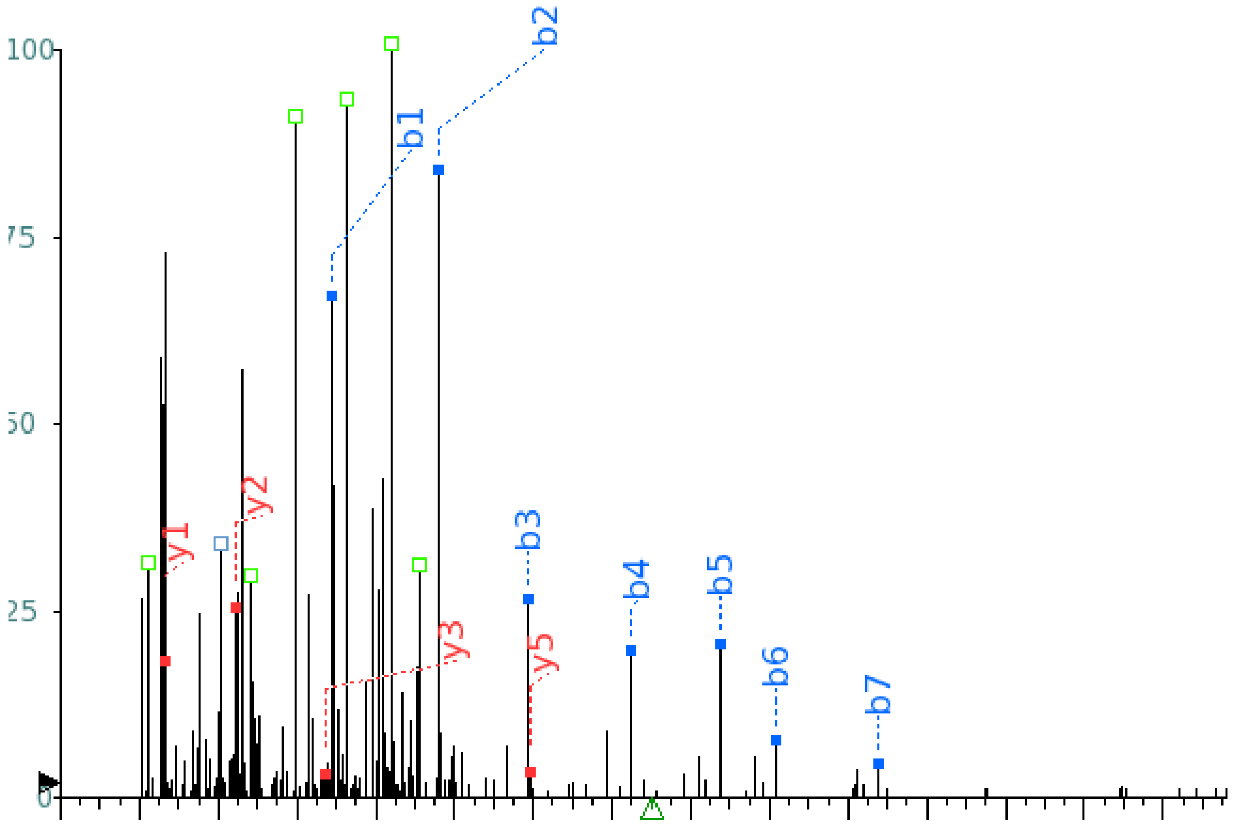

LHNENAEMDSD (+2)

Primary Reference: UBE2Z_HUMAN
Search ID: 41760
Search Name: 20130328_ananiav_ERstress_TMT_TM_timecourse_trypsin_inj1_4mc_iaa
Scan#: 5526
Observed Mass: 752.3342 (1.8 ppm)
PSM Score: 33.9

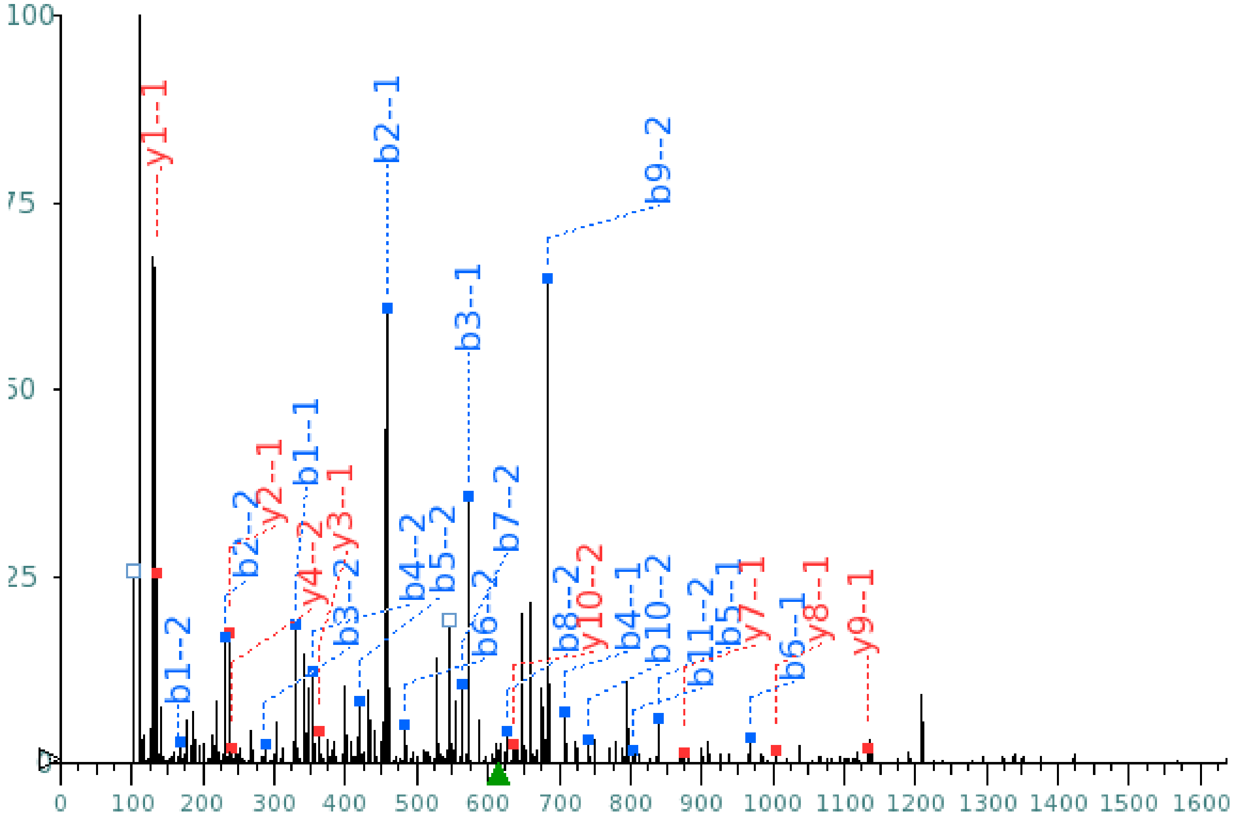

TQLHEERQDIQTD (+3)

Primary Reference: TRIPB_HUMAN
Search ID: 41760
Search Name: 20130328_ananiav_ERstress_TMT_TM_timecourse_trypsin_inj1_4mc_iaa
Scan#: 5536
Observed Mass: 614.646 (2.4 ppm)
PSM Score: 18.67

File name: 2015-02-04 04.01.53PM.pdf

Creation time: 2015-02-04 04:09PM

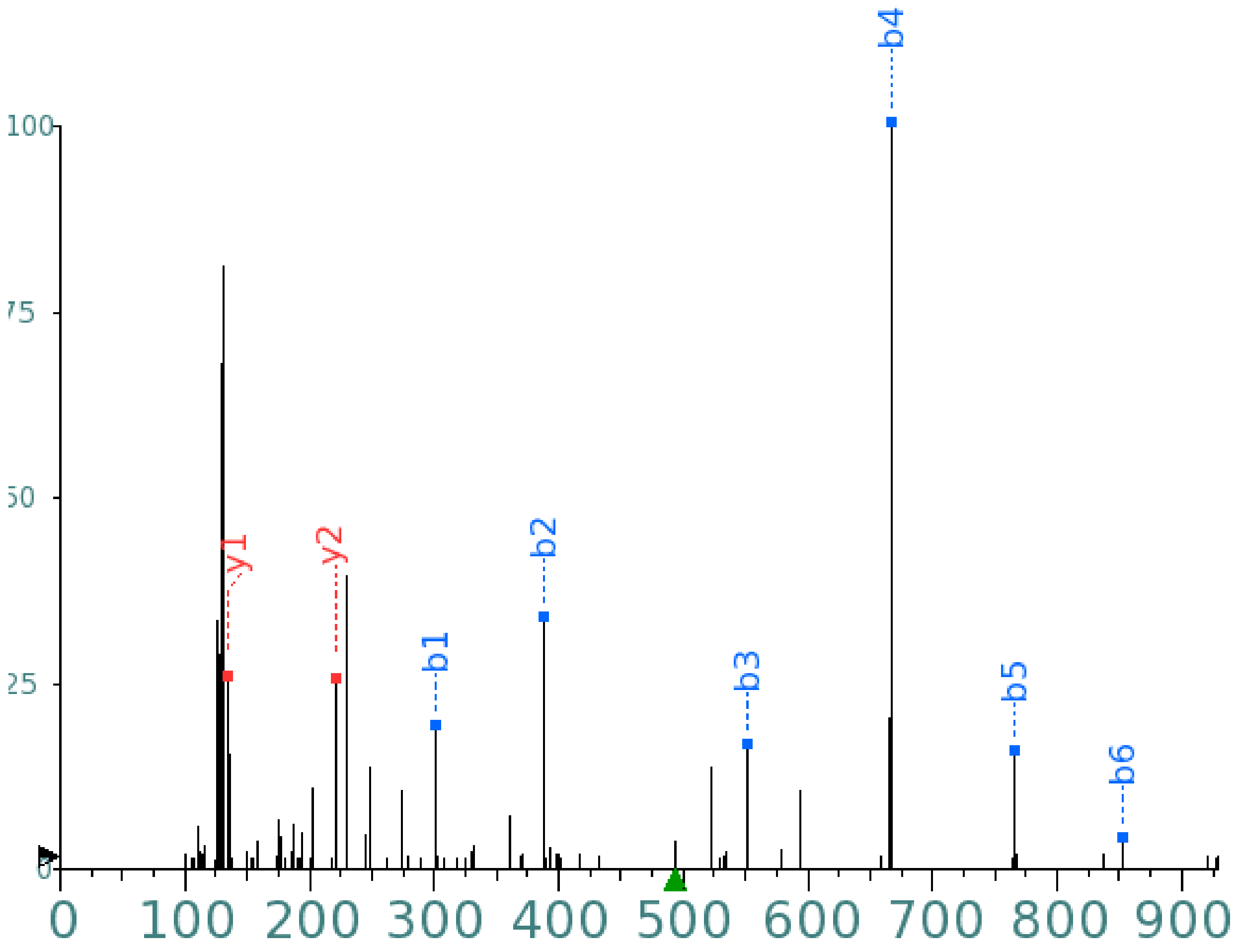

ASYDVSD (+2)

Primary Reference: K6PP_HUMAN
Search ID: 41760
Search Name: 20130328_ananiav_ERstress_TMT_TM_timecourse_trypsin_inj1_4mc_iaa
Scan#: 5584
Observed Mass: 493.2386 (2.4 ppm)
PSM Score: 32.21

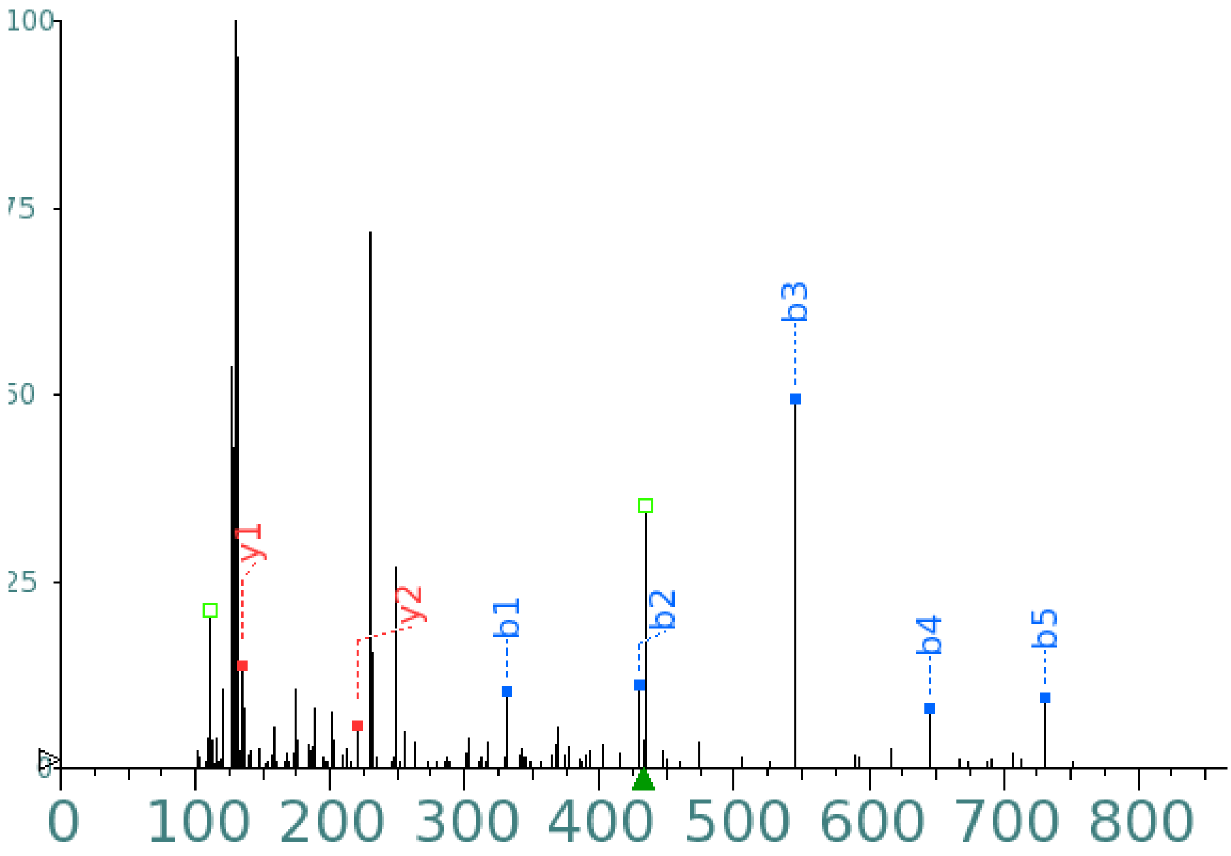

TVDVSD (+2)

Primary Reference: RPC4_HUMAN
Search ID: 41760
Search Name: 20130328_ananiav_ERstress_TMT_TM_timecourse_trypsin_inj1_4mc_iaa
Scan#: 5588
Observed Mass: 432.73 (1.7 ppm)
PSM Score: 36.14

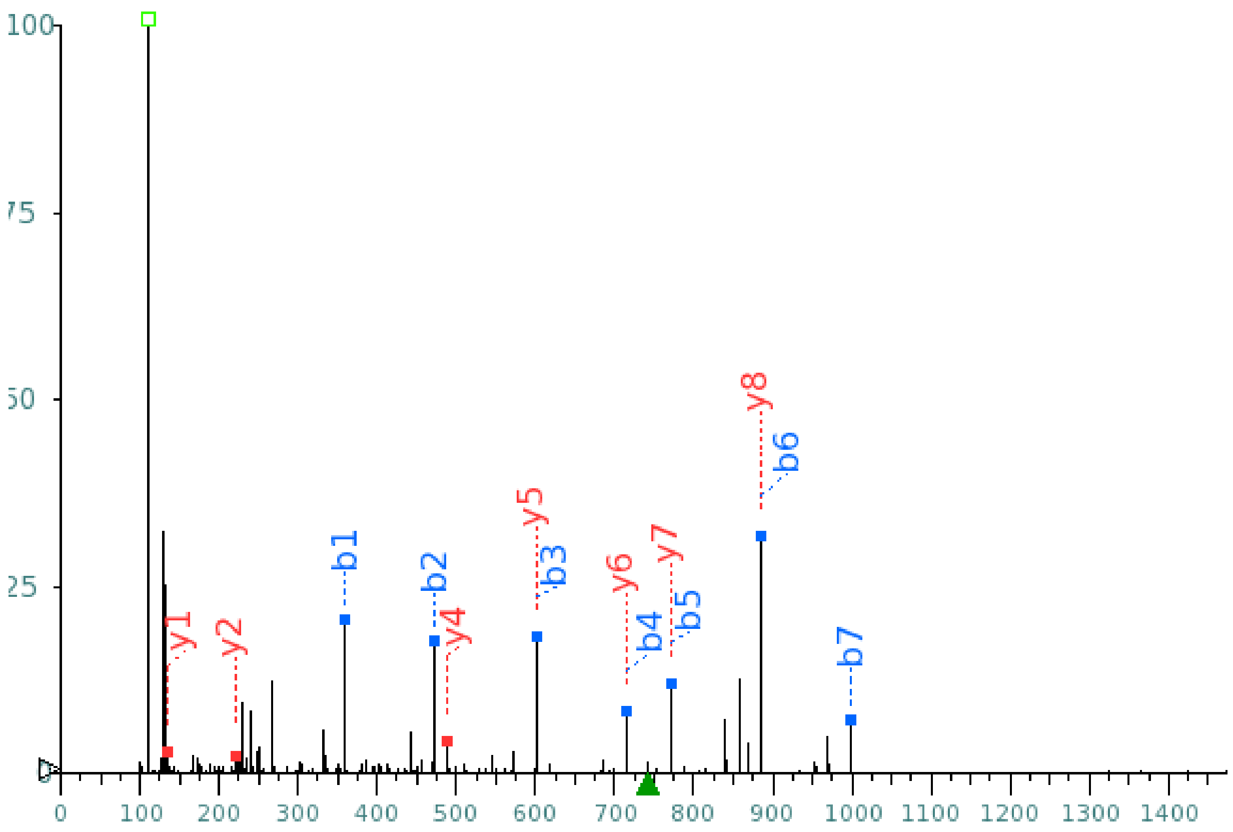

EIQNGNLHESD (+2)

Primary Reference: KTN1_HUMAN
Search ID: 41760
Search Name: 20130328_ananiav_ERstress_TMT_TM_timecourse_trypsin_inj1_4mc_iaa
Scan#: 5617
Observed Mass: 742.8637 (1.6 ppm)
PSM Score: 34.21

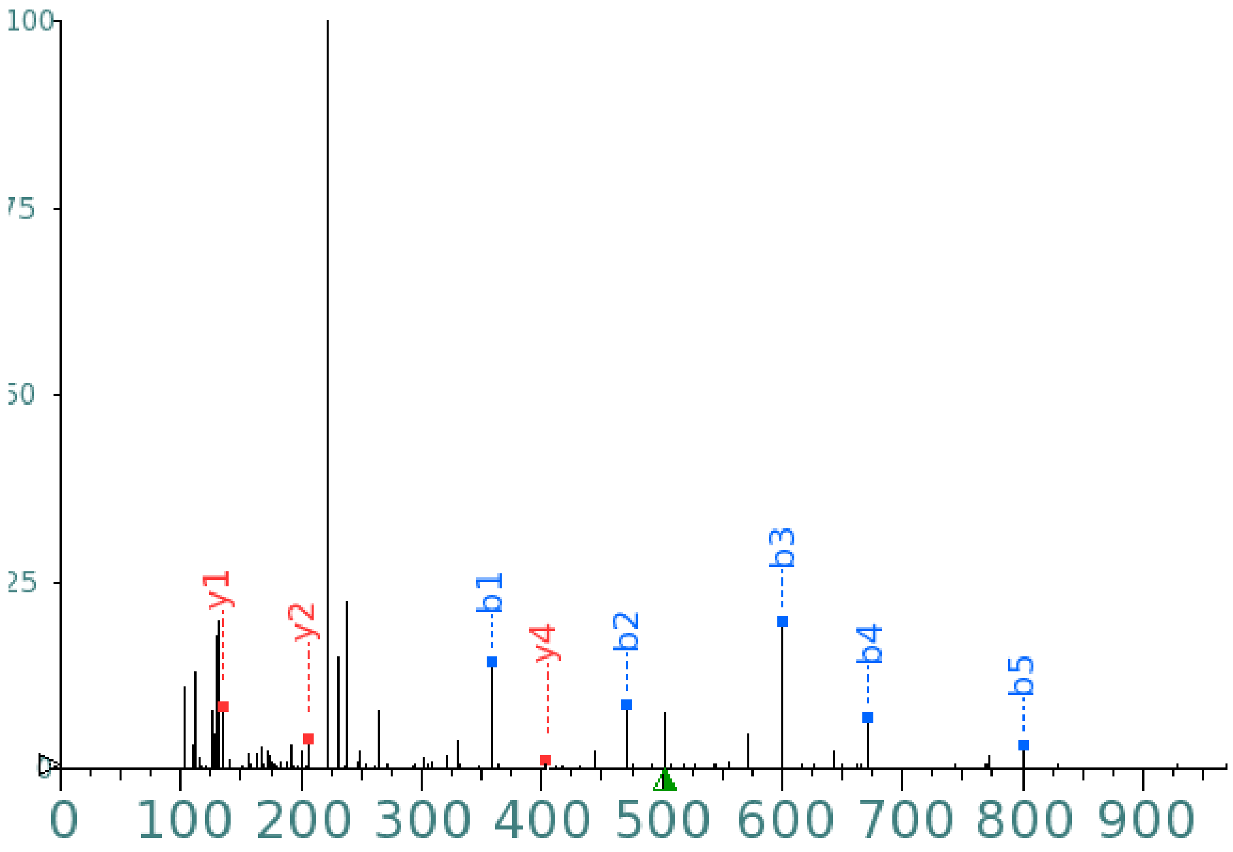

QLEAEAD (+2)

Primary Reference: HUWE1_HUMAN
Search ID: 41760
Search Name: 20130328_ananiav_ERstress_TMT_TM_timecourse_trypsin_inj1_4mc_iaa
Scan#: 5638
Observed Mass: 502.7594 (1.8 ppm)
PSM Score: 28.81

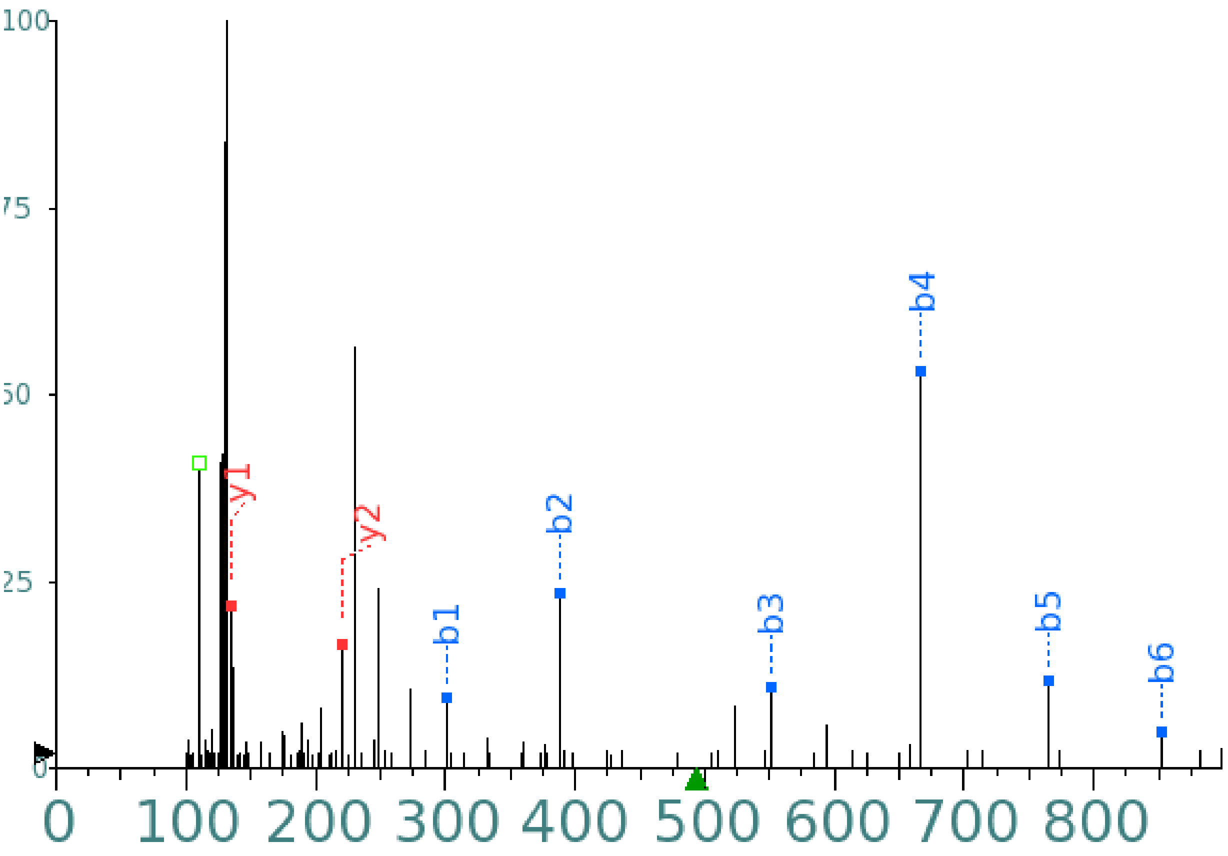

ASYDVSD (+2)

Primary Reference: K6PP_HUMAN
Search ID: 41760
Search Name: 20130328_ananiav_ERstress_TMT_TM_timecourse_trypsin_inj1_4mc_iaa
Scan#: 5671
Observed Mass: 493.2384 (2 ppm)
PSM Score: 32.25

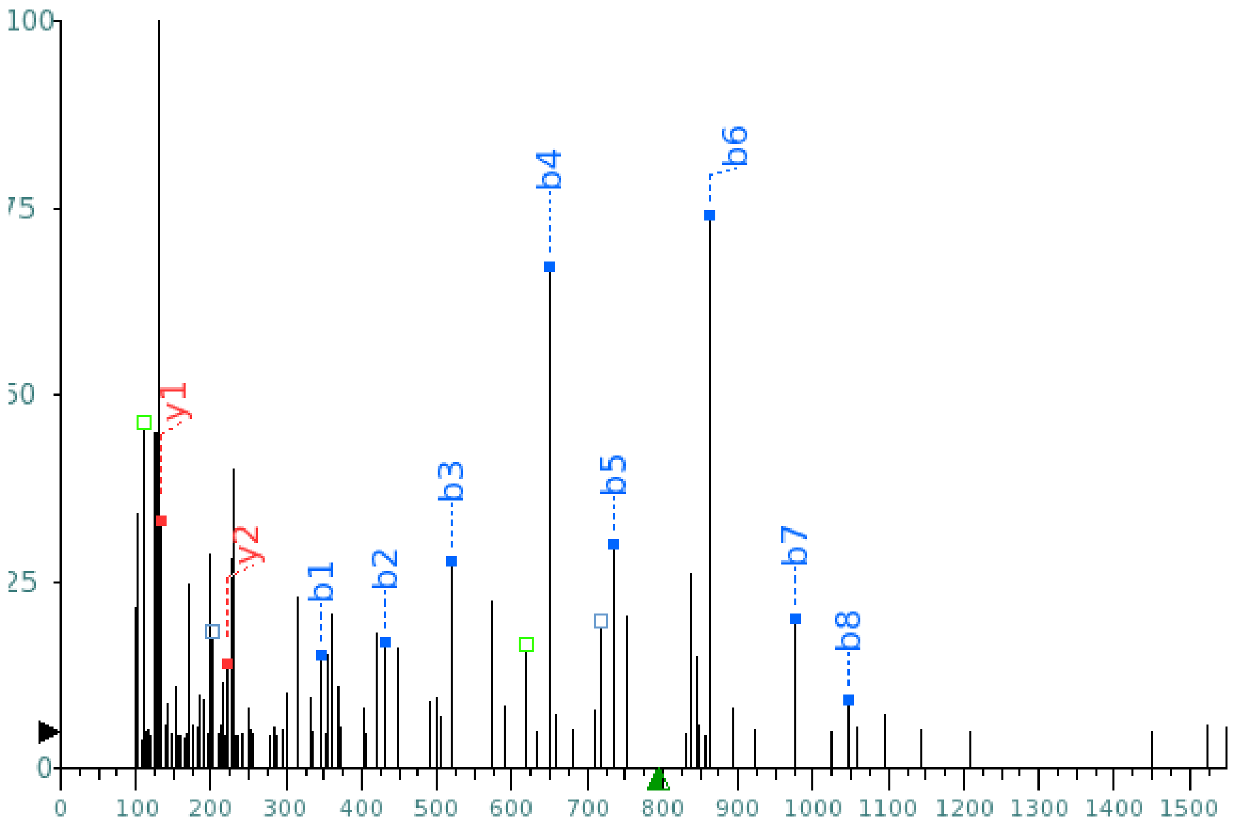

DSSESQLASTESD (+2)

Primary Reference: CR025_HUMAN
Search ID: 41760
Search Name: 20130328_ananiav_ERstress_TMT_TM_timecourse_trypsin_inj1_4mc_iaa
Scan#: 5677
Observed Mass: 792.8597 (2.9 ppm)
PSM Score: 29.29

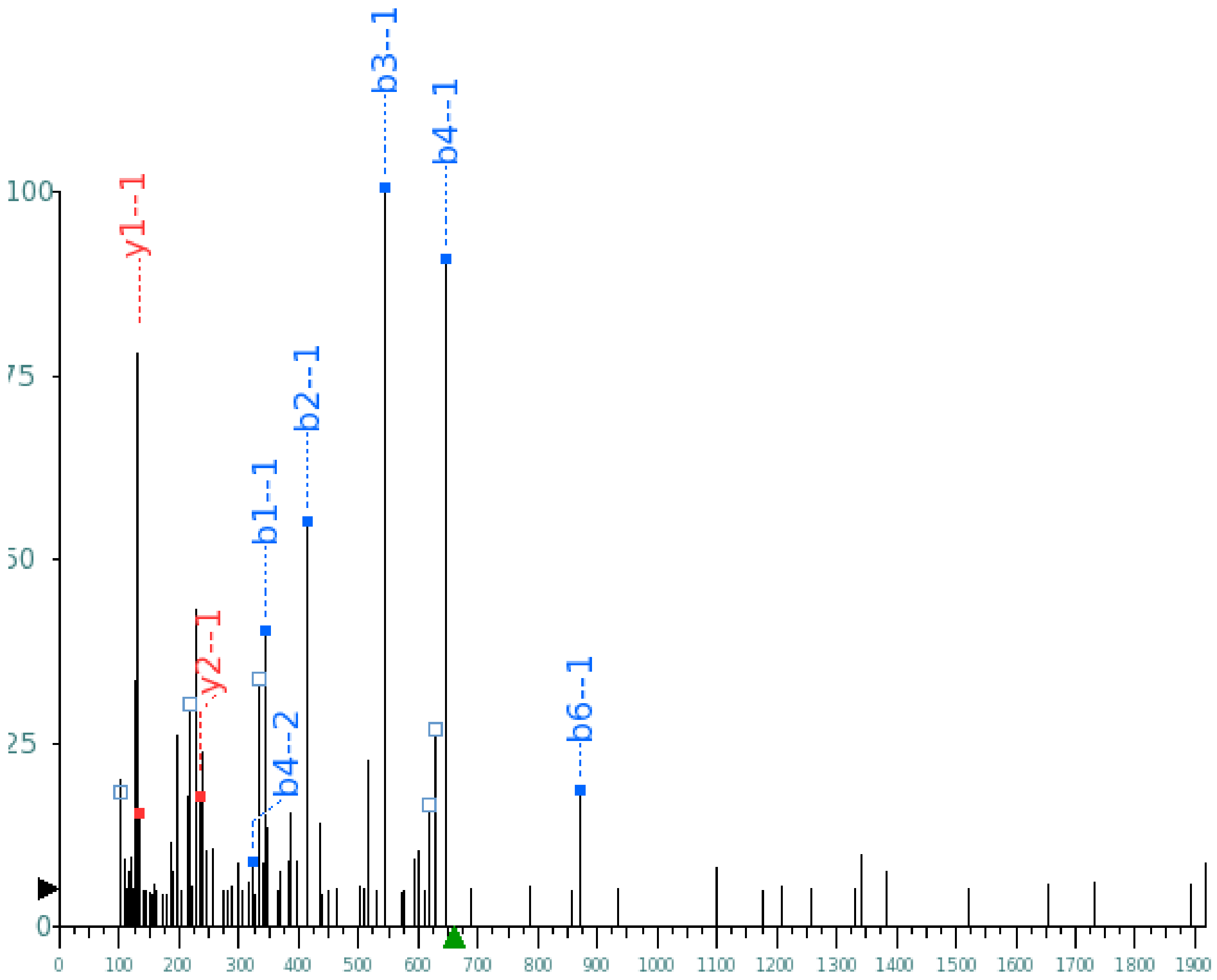

NAETPEATTQQETDTD (+3)

Primary Reference: K1967_HUMAN
Search ID: 41760
Search Name: 20130328_ananiav_ERstress_TMT_TM_timecourse_trypsin_inj1_4mc_iaa
Scan#: 5720
Observed Mass: 660.6353 (1.8 ppm)
PSM Score: 12.18

File name: 2015-02-04 04.01.53PM.pdf

Creation time: 2015-02-04 04:09PM

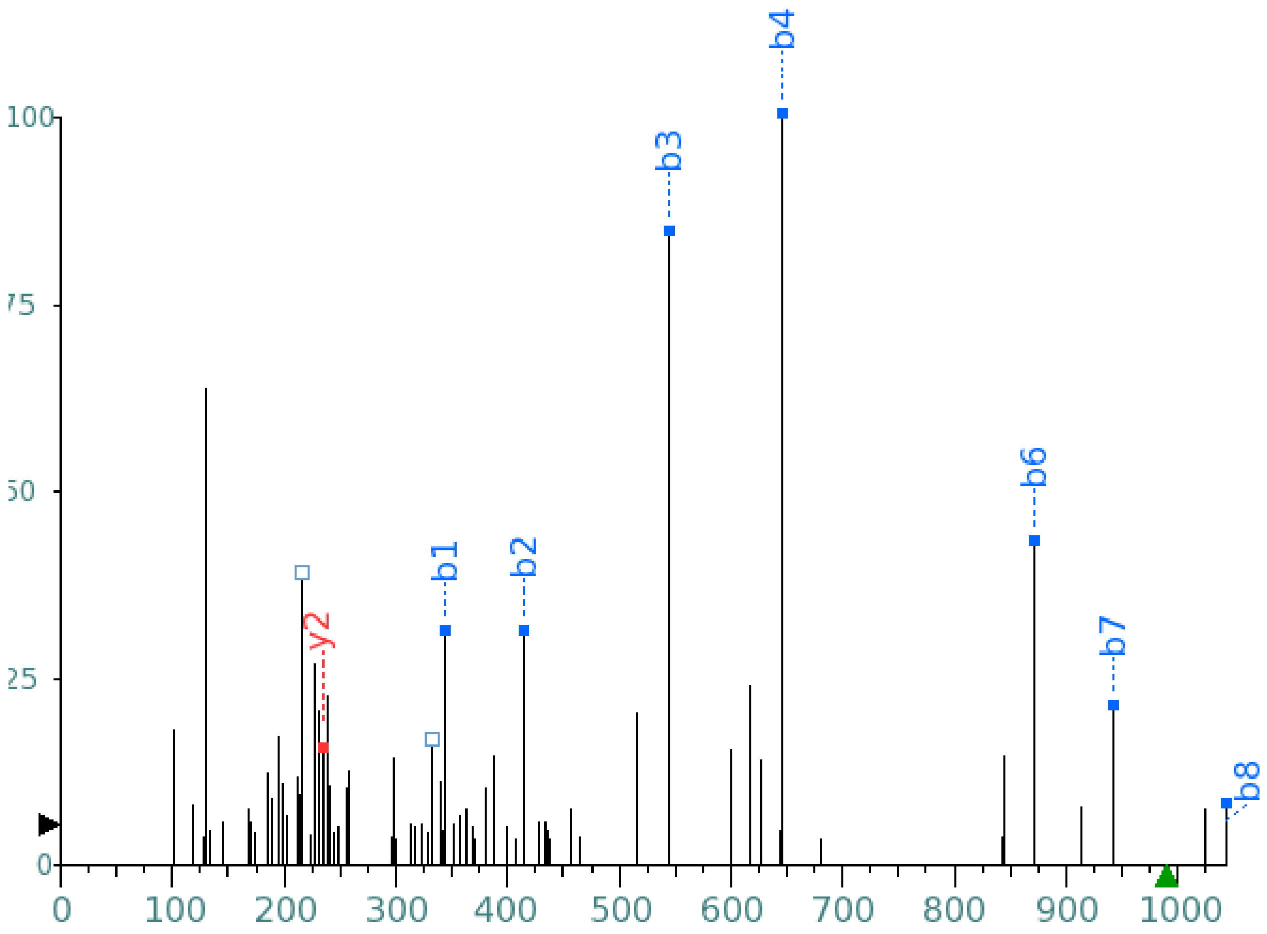

NAETPEATTQQETDTD (+2)

Primary Reference: K1967_HUMAN
Search ID: 41760
Search Name: 20130328_ananiav_ERstress_TMT_TM_timecourse_trypsin_inj1_4mc_iaa
Scan#: 5722
Observed Mass: 990.4488 (1.2 ppm)
PSM Score: 53.09

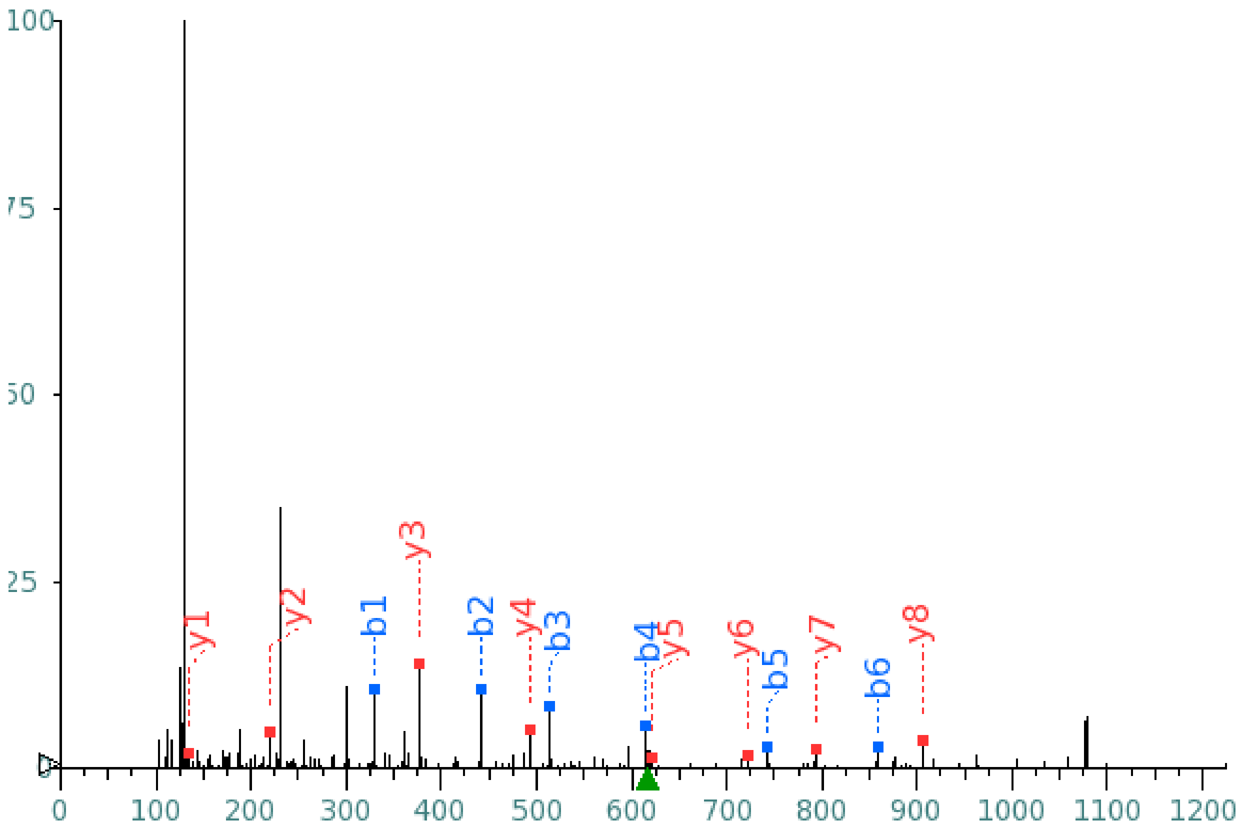

VLATEDRSD (+2)

Primary Reference: RAB9A_HUMAN
Search ID: 41760
Search Name: 20130328_ananiav_ERstress_TMT_TM_timecourse_trypsin_inj1_4mc_iaa
Scan#: 5805
Observed Mass: 617.8283 (1.4 ppm)
PSM Score: 28.14

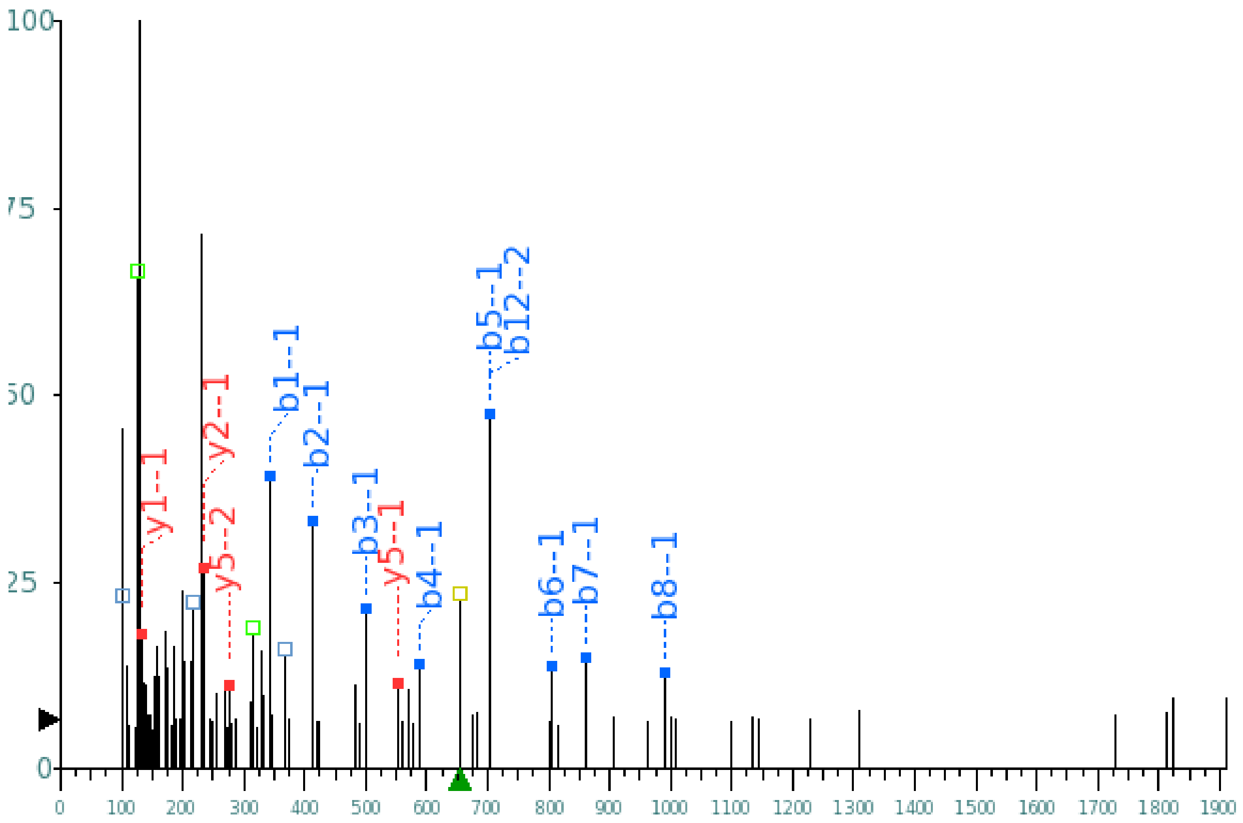

LASSDTGESDQSSTETD (+3)

Primary Reference: TTC28_HUMAN
Search ID: 41760
Search Name: 20130328_ananiav_ERstress_TMT_TM_timecourse_trypsin_inj1_4mc_iaa
Scan#: 5817
Observed Mass: 653.6227 (1.2 ppm)
PSM Score: 28.51

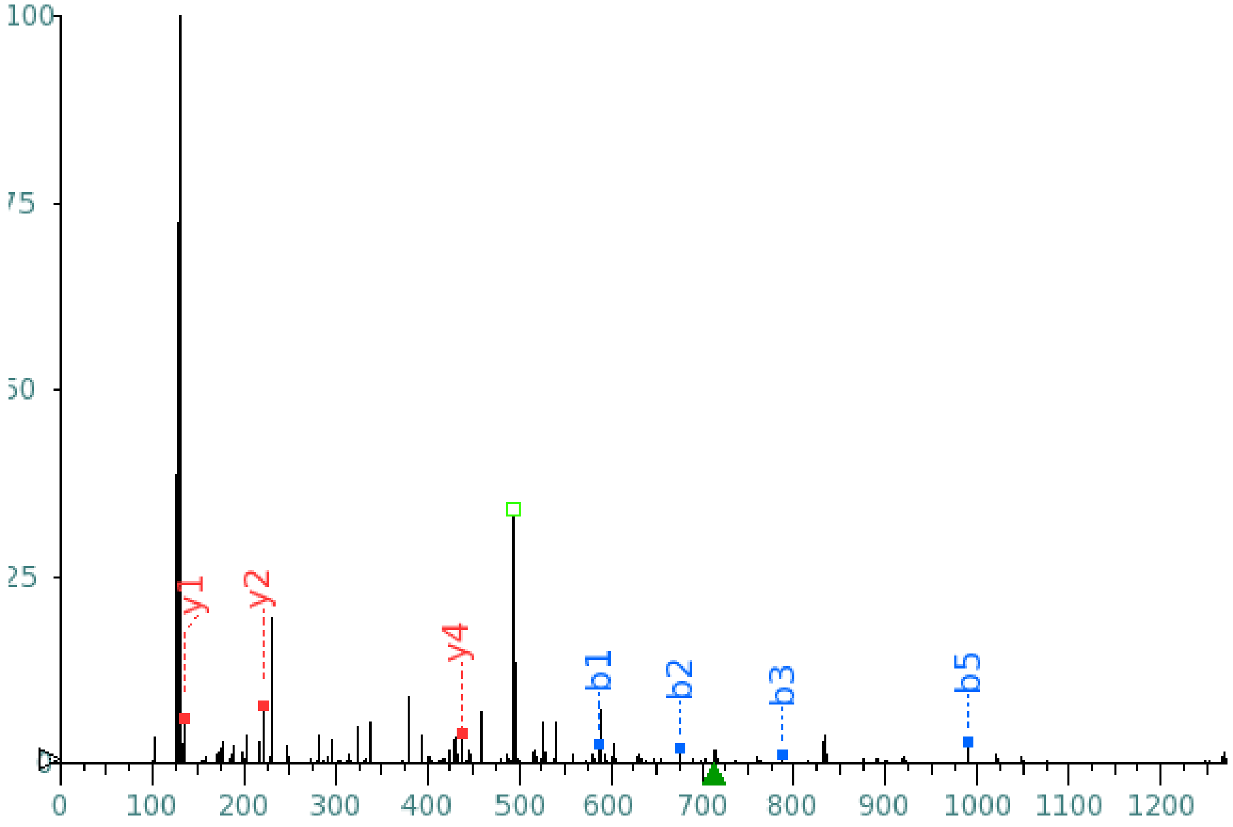

KSLSDSESD (+2)

Primary Reference: CBX3_HUMAN
Search ID: 41760
Search Name: 20130328_ananiav_ERstress_TMT_TM_timecourse_trypsin_inj1_4mc_iaa
Scan#: 5845
Observed Mass: 713.378 (1 ppm)
PSM Score: 10.84

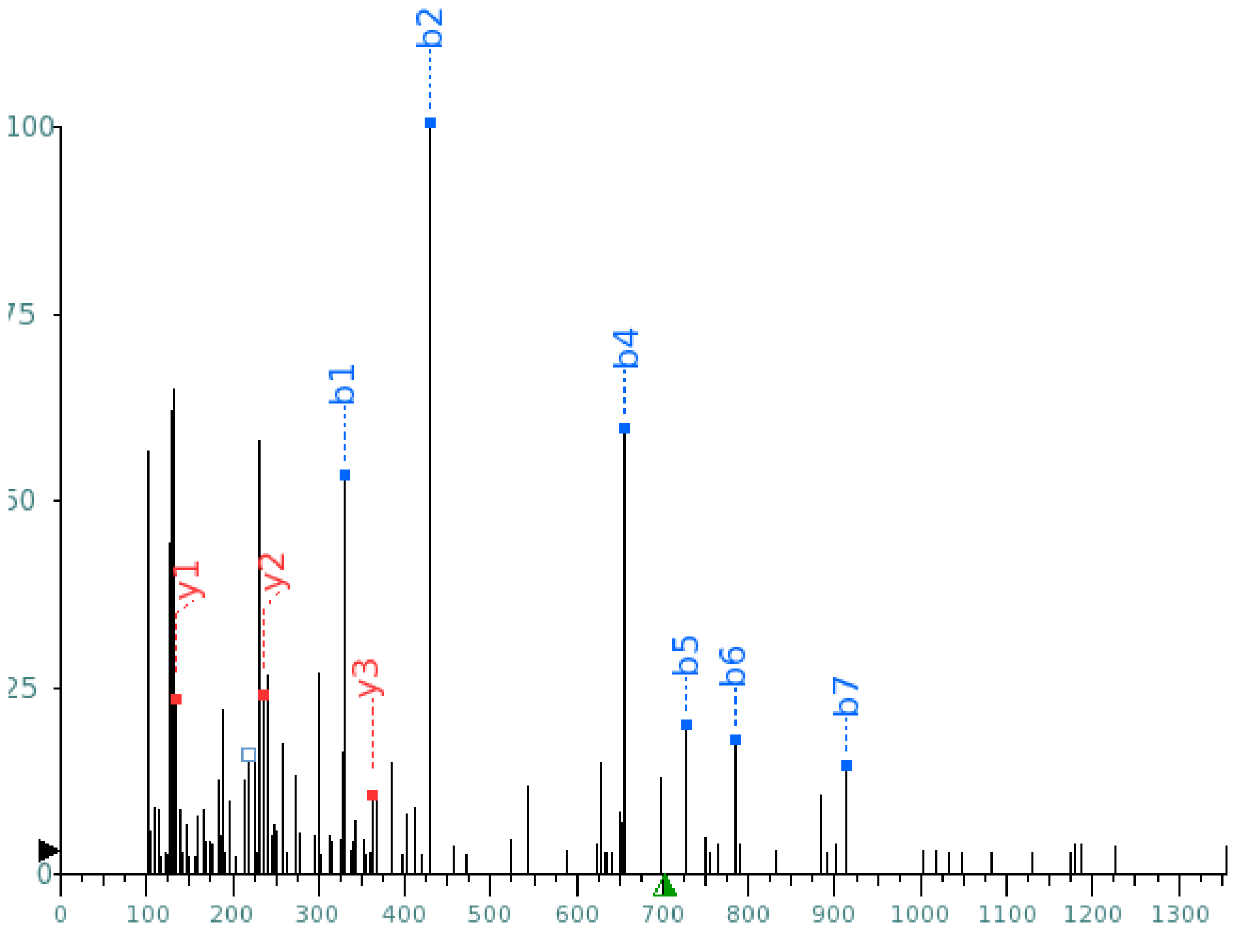

VTPQAGEEETD (+2)

Primary Reference: NACAD_HUMAN
Search ID: 41760
Search Name: 20130328_ananiav_ERstress_TMT_TM_timecourse_trypsin_inj1_4mc_iaa
Scan#: 5877
Observed Mass: 702.8389 (1 ppm)
PSM Score: 21.81

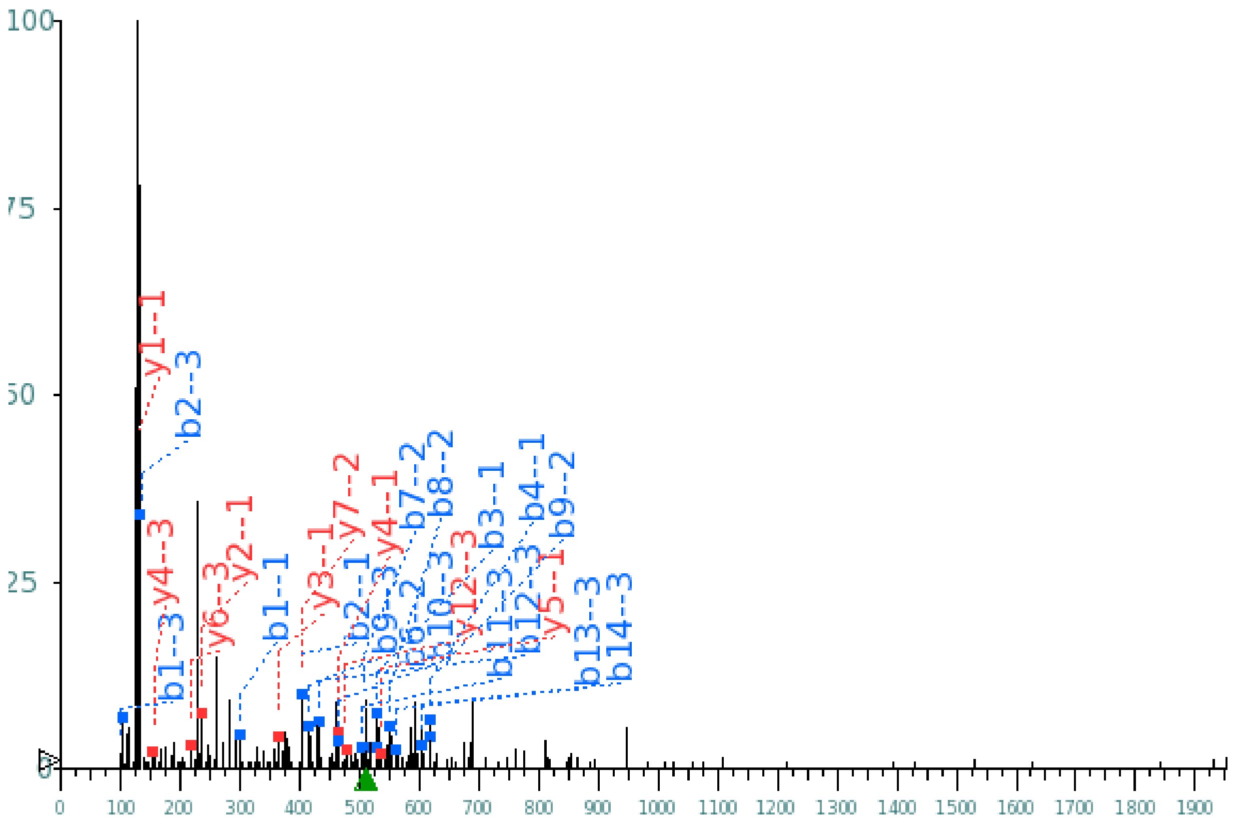

ATQSIERSHRIATETD (+4)

Primary Reference: VTI1B_HUMAN
Search ID: 41760
Search Name: 20130328_ananiav_ERstress_TMT_TM_timecourse_trypsin_inj1_4mc_iaa
Scan#: 5880
Observed Mass: 511.7726 (3.2 ppm)
PSM Score: 10.82

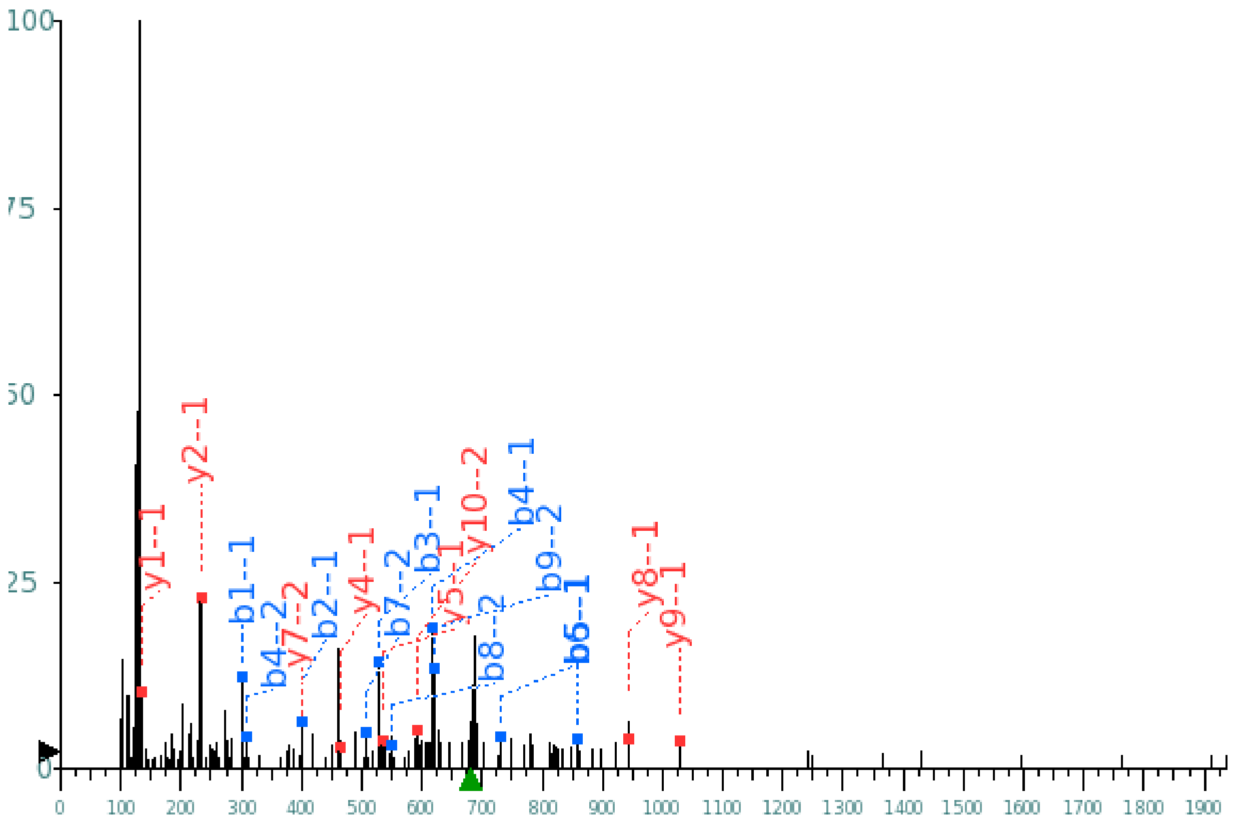

ATQSIERSHRIATETD (+3)

Primary Reference: VTI1B_HUMAN
Search ID: 41760
Search Name: 20130328_ananiav_ERstress_TMT_TM_timecourse_trypsin_inj1_4mc_iaa
Scan#: 5883
Observed Mass: 682.0262 (1 ppm)
PSM Score: 21.46

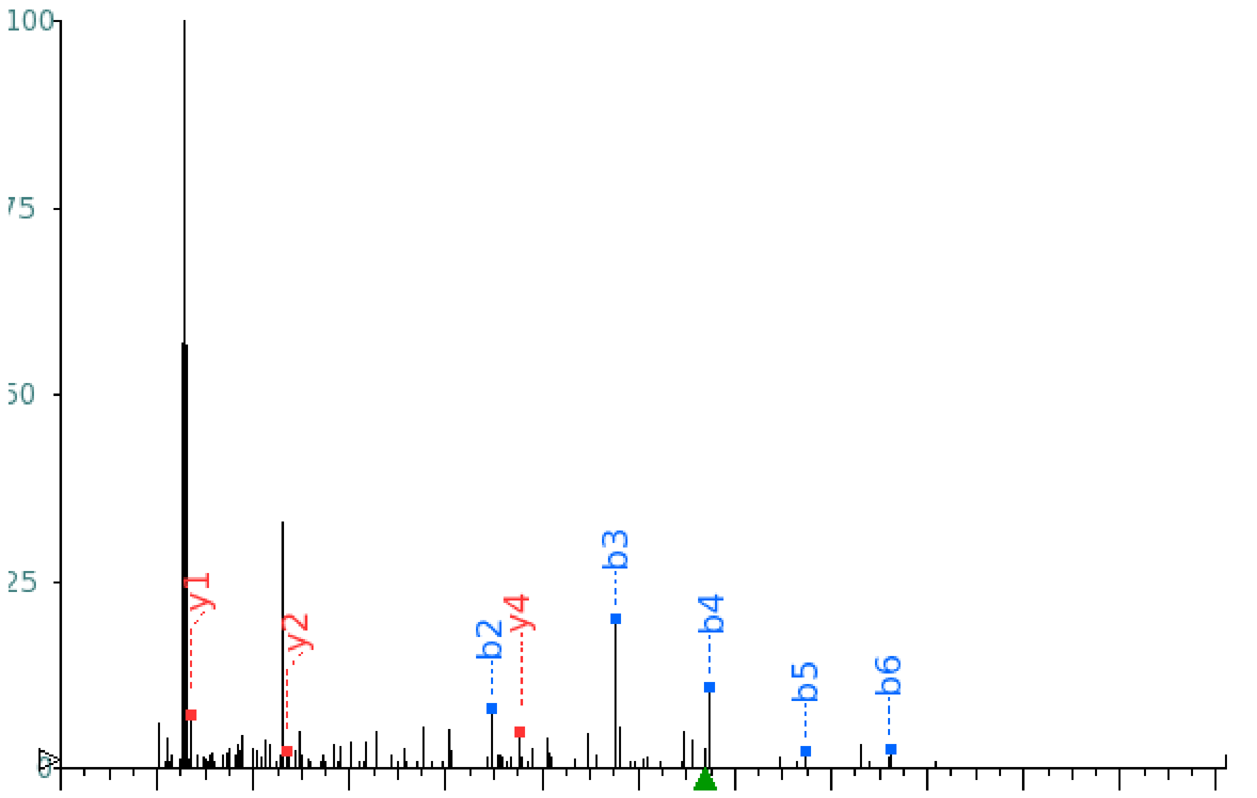

TDEVVSDQTD (+2)

Primary Reference: Z280D_HUMAN
Search ID: 41760
Search Name: 20130328_ananiav_ERstress_TMT_TM_timecourse_trypsin_inj1_4mc_iaa
Scan#: 5906
Observed Mass: 669.3178 (1 ppm)
PSM Score: 9.3

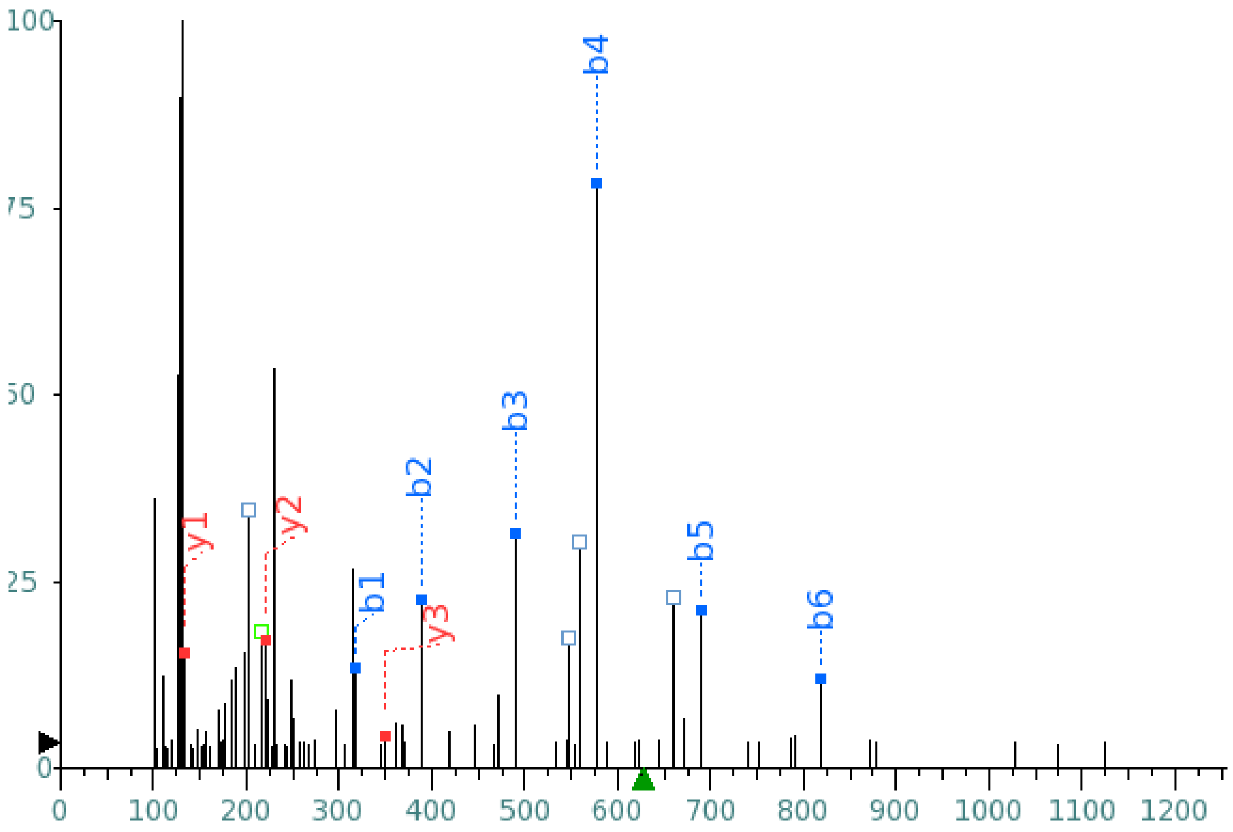

SATSLESESD (+2)

Primary Reference: RGNEF_HUMAN
Search ID: 41760
Search Name: 20130328_ananiav_ERstress_TMT_TM_timecourse_trypsin_inj1_4mc_iaa
Scan#: 5918
Observed Mass: 627.7994 (1.3 ppm)
PSM Score: 19.89

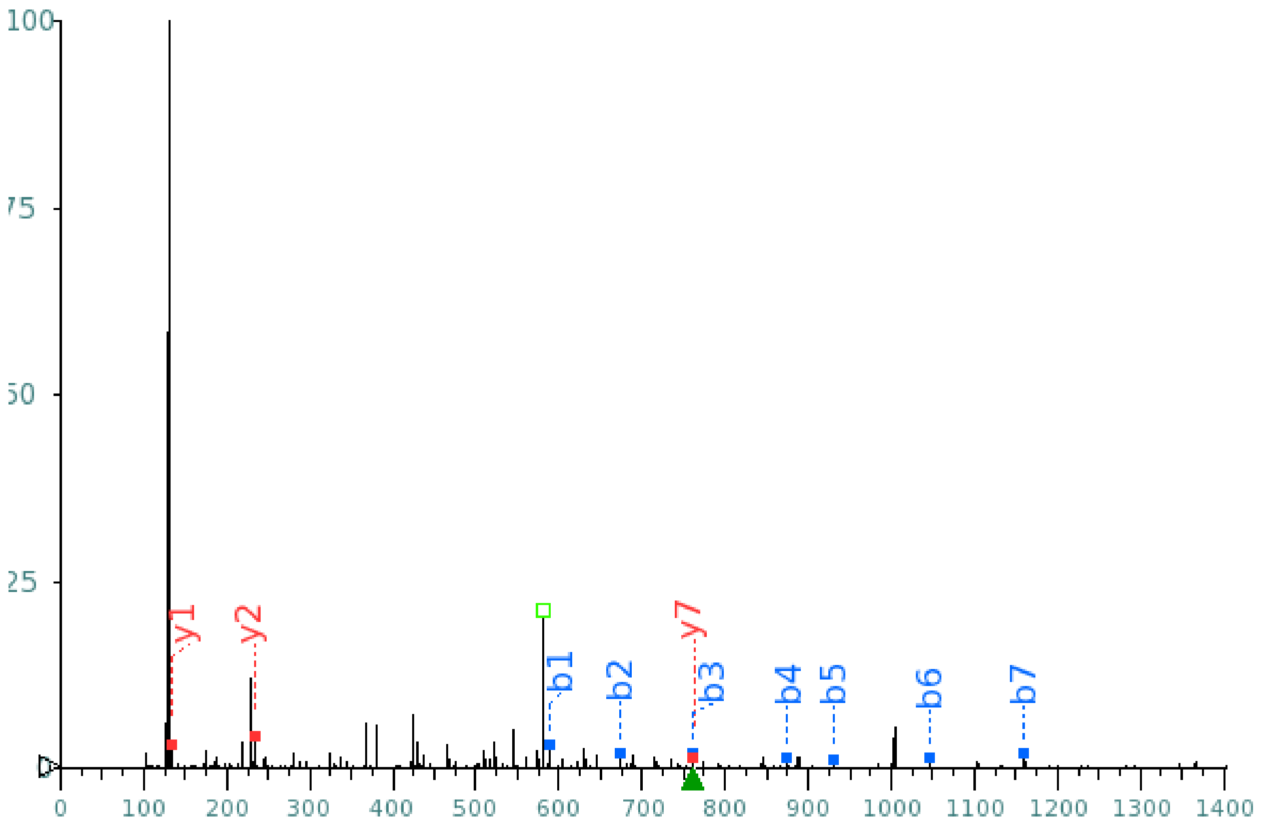

KSSLGNDETD (+2)

Primary Reference: COBL_HUMAN
Search ID: 41760
Search Name: 20130328_ananiav_ERstress_TMT_TM_timecourse_trypsin_inj1_4mc_iaa
Scan#: 5919
Observed Mass: 762.4021 (1.1 ppm)
PSM Score: 12.03

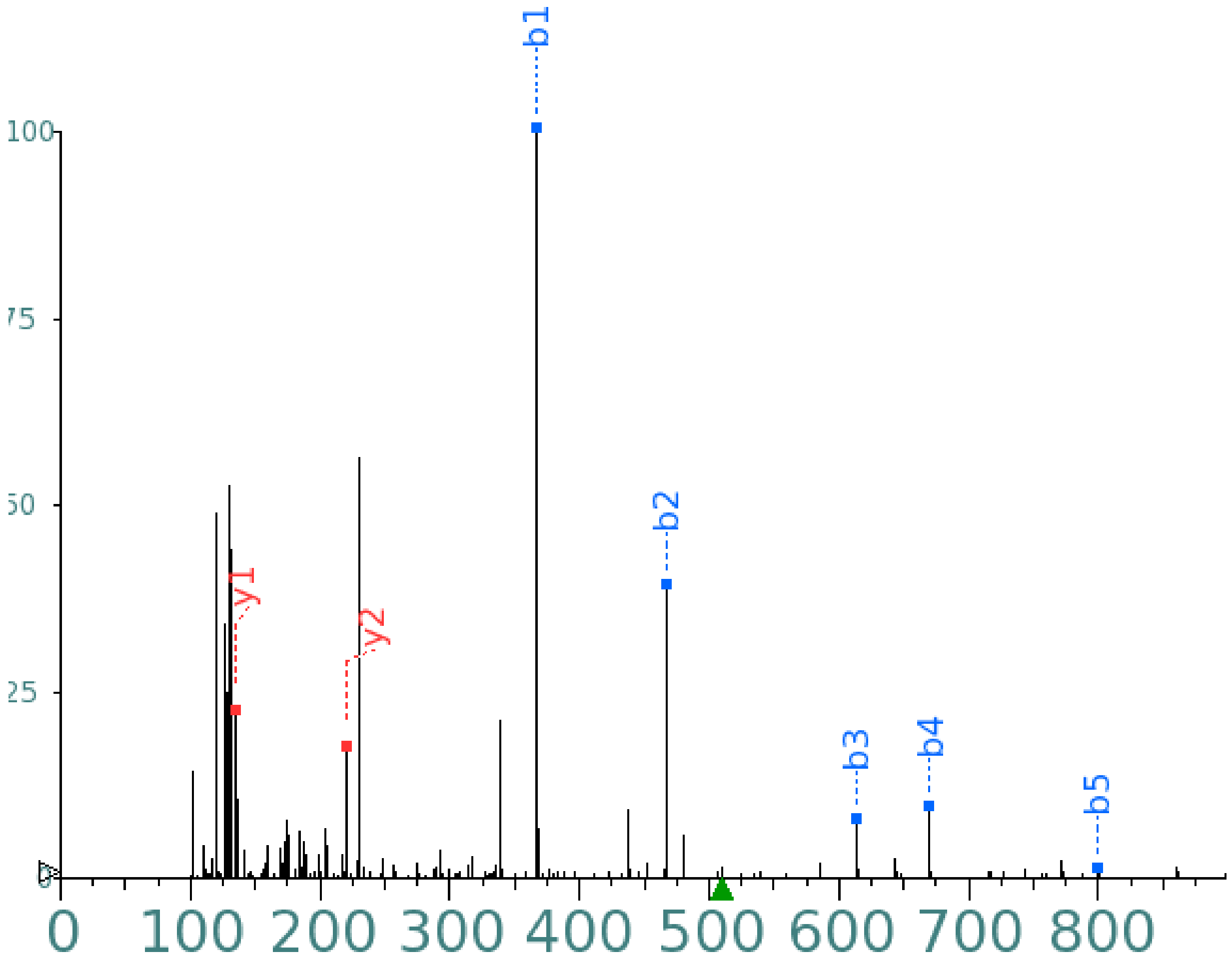

HVFGESD (+2)

Primary Reference: TPIS_HUMAN
Search ID: 41760
Search Name: 20130328_ananiav_ERstress_TMT_TM_timecourse_trypsin_inj1_4mc_iaa
Scan#: 5992
Observed Mass: 510.2546 (2.4 ppm)
PSM Score: 9.76

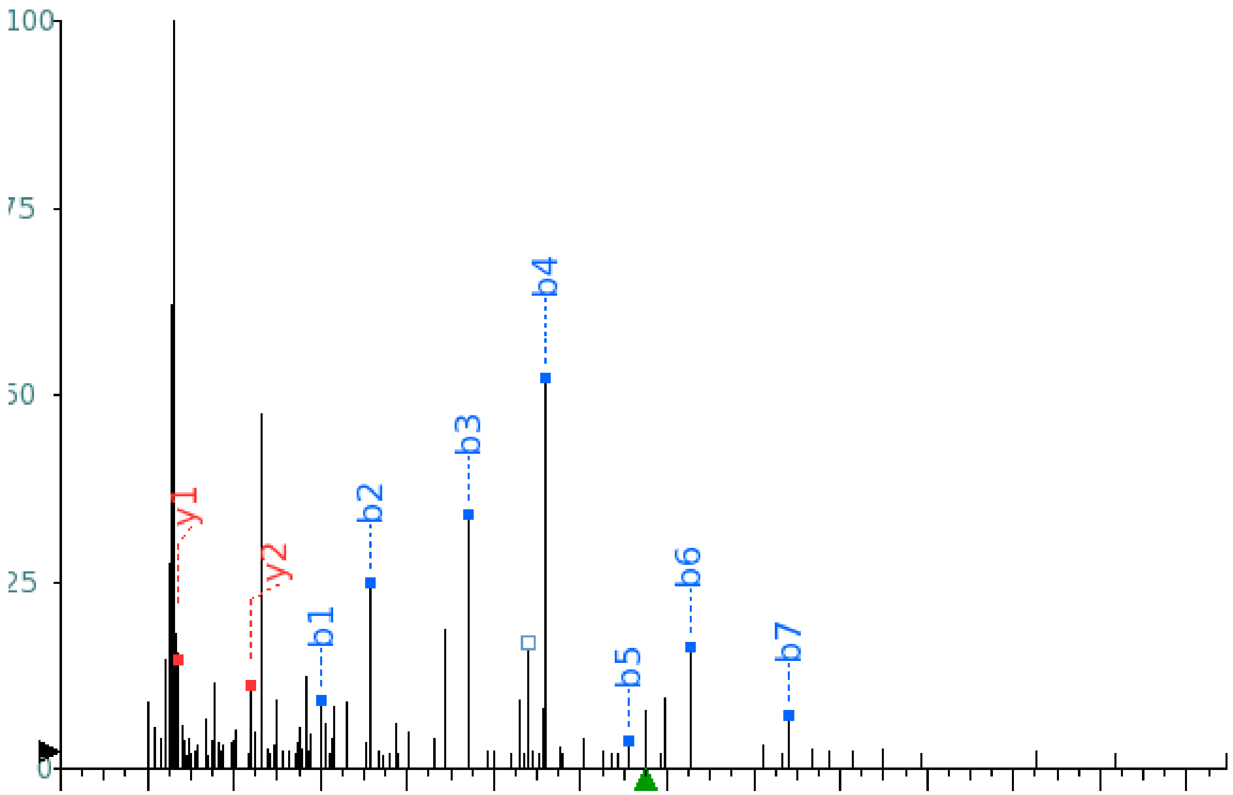

AGLSPANC*QSD (+2)

Primary Reference: BMS1_HUMAN
Search ID: 41760
Search Name: 20130328_ananiav_ERstress_TMT_TM_timecourse_trypsin_inj1_4mc_iaa
Scan#: 6042
Observed Mass: 674.8225 (1 ppm)
PSM Score: 23.25

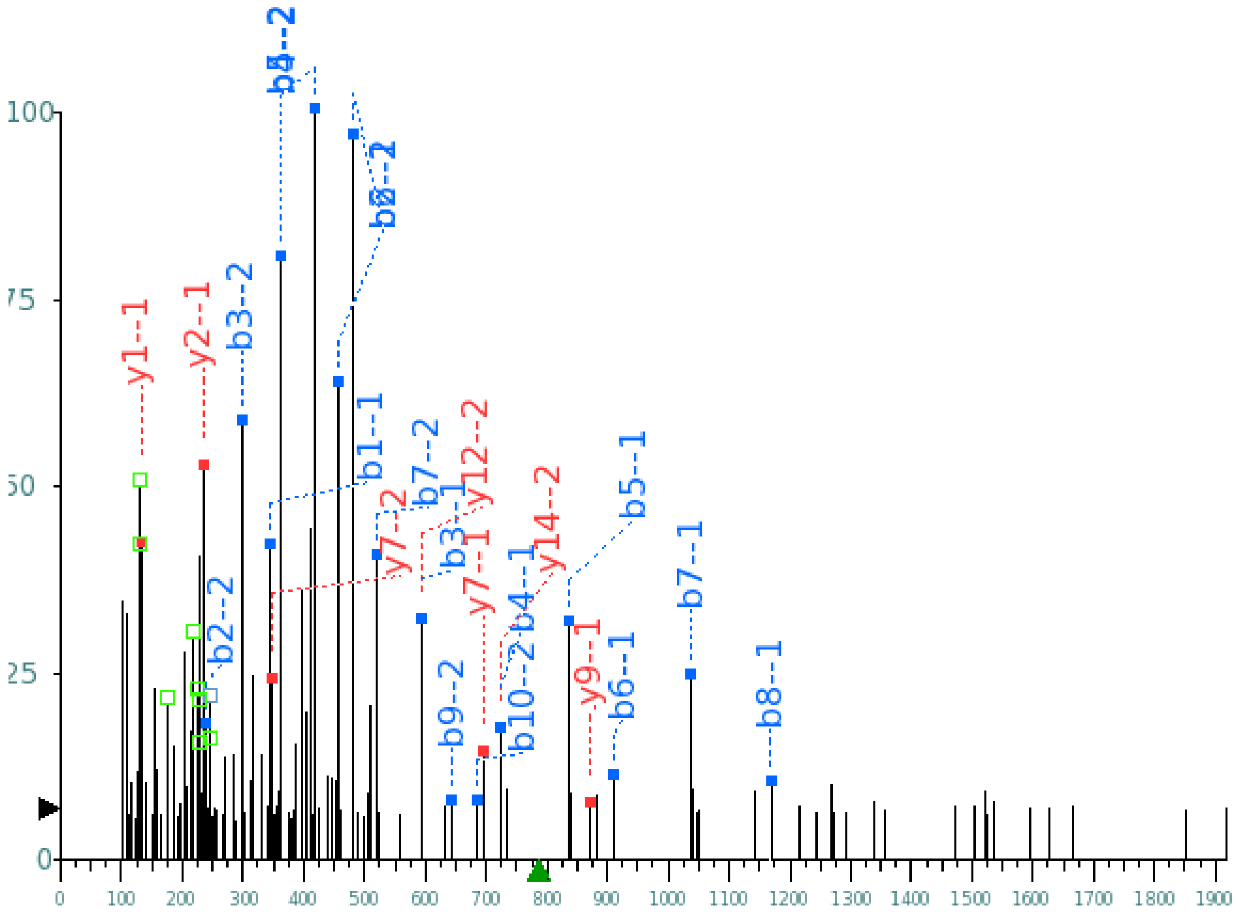

LHNENAEMDSDSSSSGTETD (+3)

Primary Reference: UBE2Z_HUMAN
Search ID: 41760
Search Name: 20130328_ananiav_ERstress_TMT_TM_timecourse_trypsin_inj1_4mc_iaa
Scan#: 6070
Observed Mass: 785.664 (2 ppm)
PSM Score: 33.01

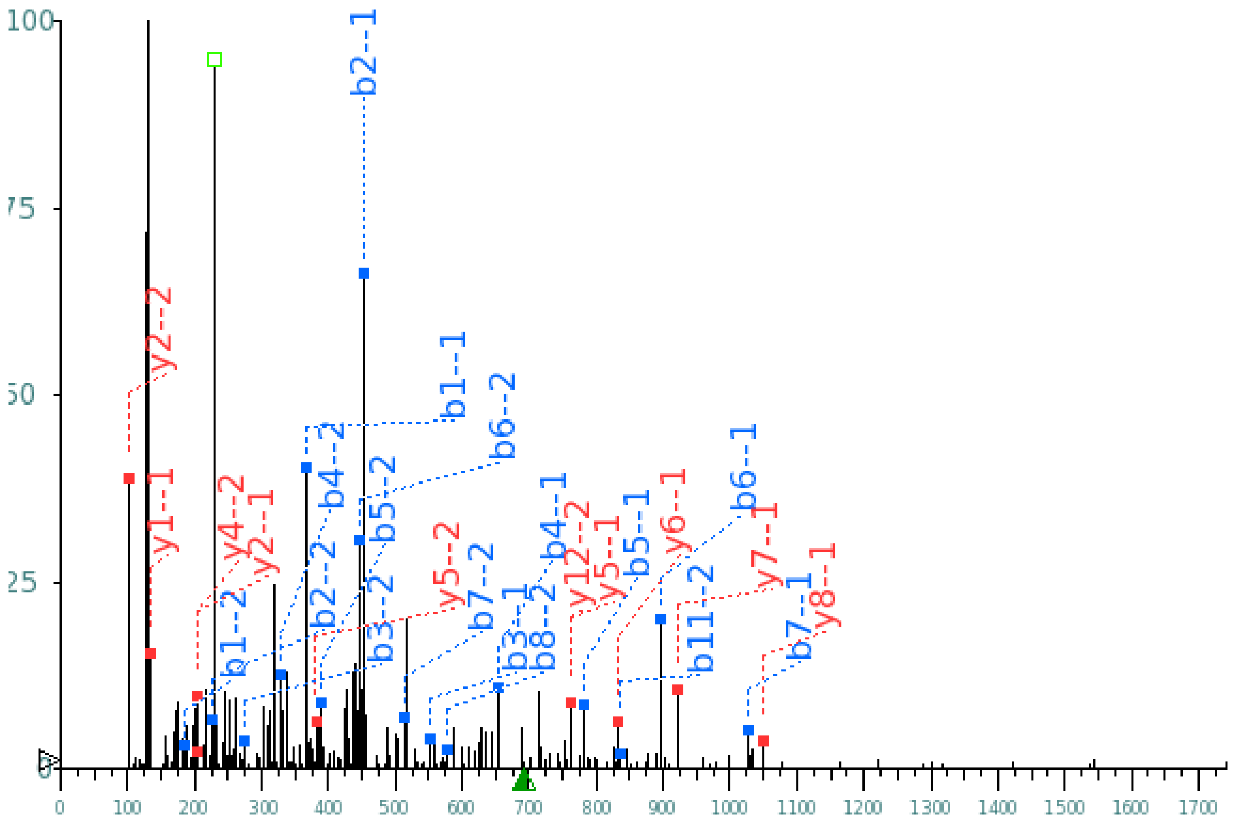

HSPTEDEESAKAEAD (+3)

Primary Reference: MAP1B_HUMAN
Search ID: 41760
Search Name: 20130328_ananiav_ERstress_TMT_TM_timecourse_trypsin_inj1_4mc_iaa
Scan#: 6075
Observed Mass: 692.0054 (2 ppm)
PSM Score: 27.64

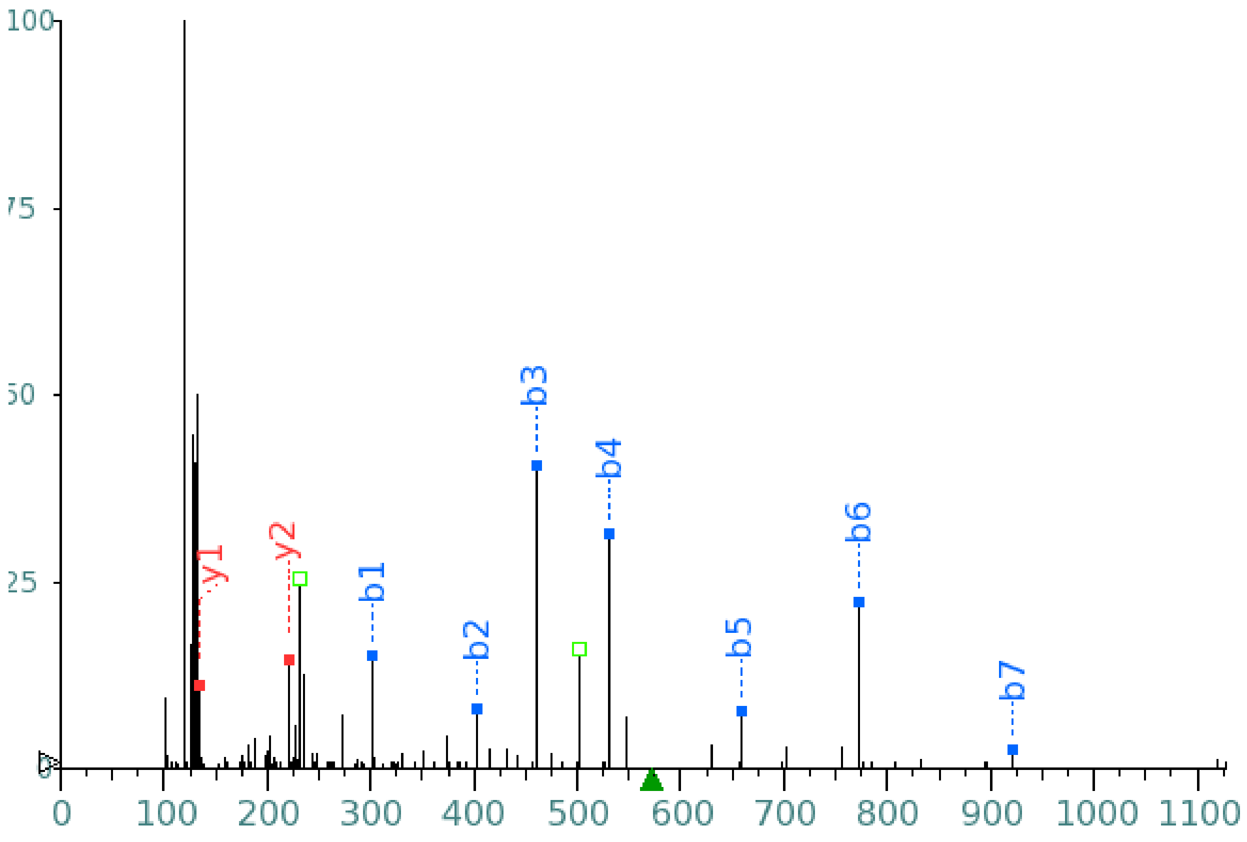

ATGAQDFSD (+2)

Primary Reference: GT2D1_HUMAN
Search ID: 41760
Search Name: 20130328_ananiav_ERstress_TMT_TM_timecourse_trypsin_inj1_4mc_iaa
Scan#: 6101
Observed Mass: 570.7733 (2 ppm)
PSM Score: 41.44

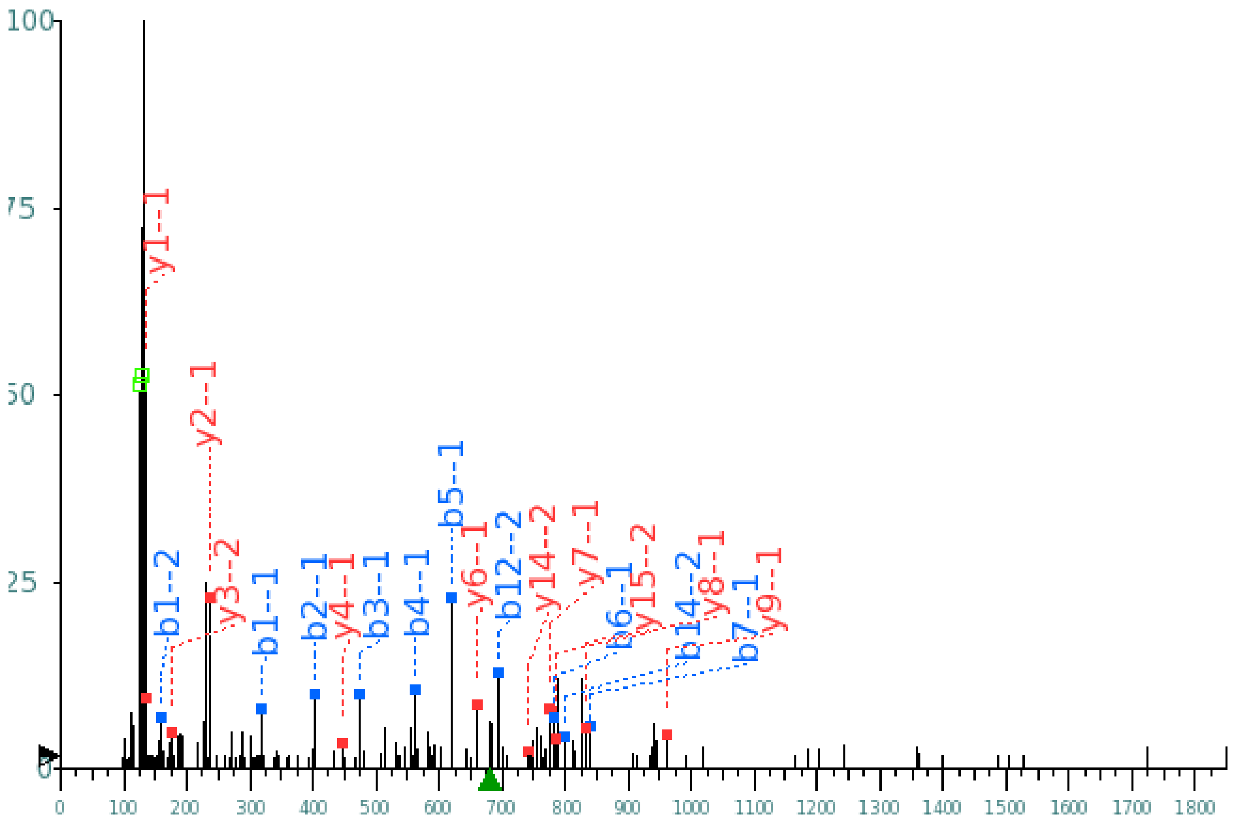

SSASGYGSREGDRGVITD (+3)

Primary Reference: SLTM_HUMAN
Search ID: 41760
Search Name: 20130328_ananiav_ERstress_TMT_TM_timecourse_trypsin_inj1_4mc_iaa
Scan#: 6136
Observed Mass: 681.6705 (1.5 ppm)
PSM Score: 31.14

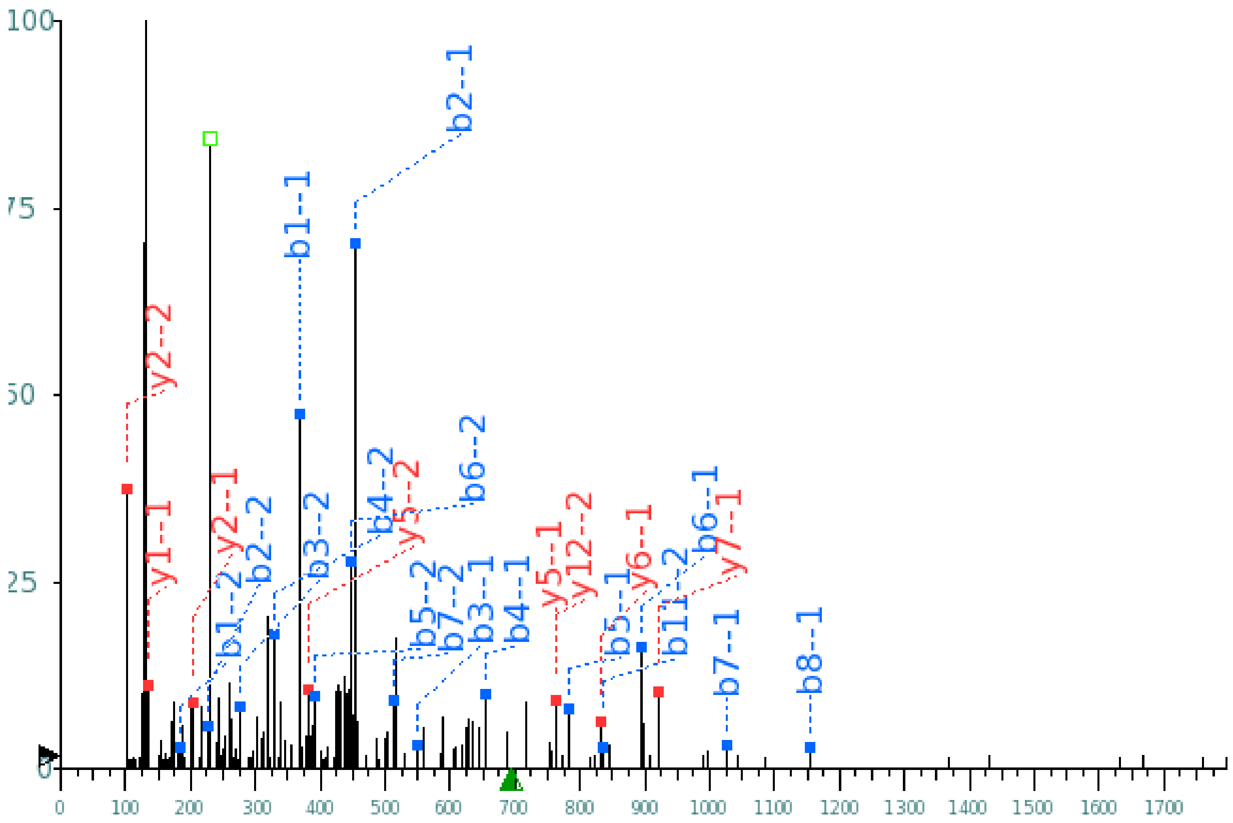

HSPTEDEESAKAEAD (+3)

Primary Reference: MAP1B_HUMAN
Search ID: 41760
Search Name: 20130328_ananiav_ERstress_TMT_TM_timecourse_trypsin_inj1_4mc_iaa
Scan#: 6155
Observed Mass: 692.005 (1.4 ppm)
PSM Score: 24.84

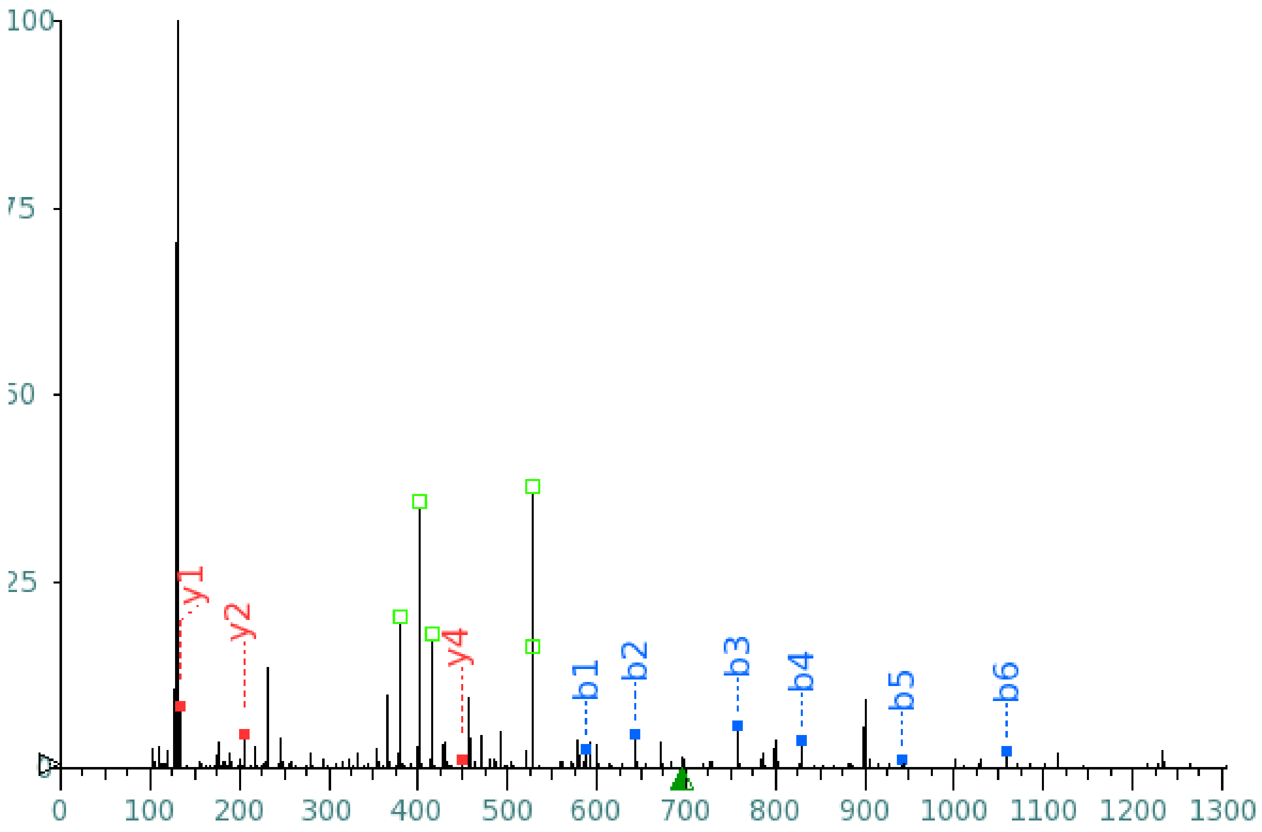

KGNAIDEAD (+2)

Primary Reference: C2D1A_HUMAN
Search ID: 41760
Search Name: 20130328_ananiav_ERstress_TMT_TM_timecourse_trypsin_inj1_4mc_iaa
Scan#: 6158
Observed Mass: 695.8838 (1.8 ppm)
PSM Score: 7.48

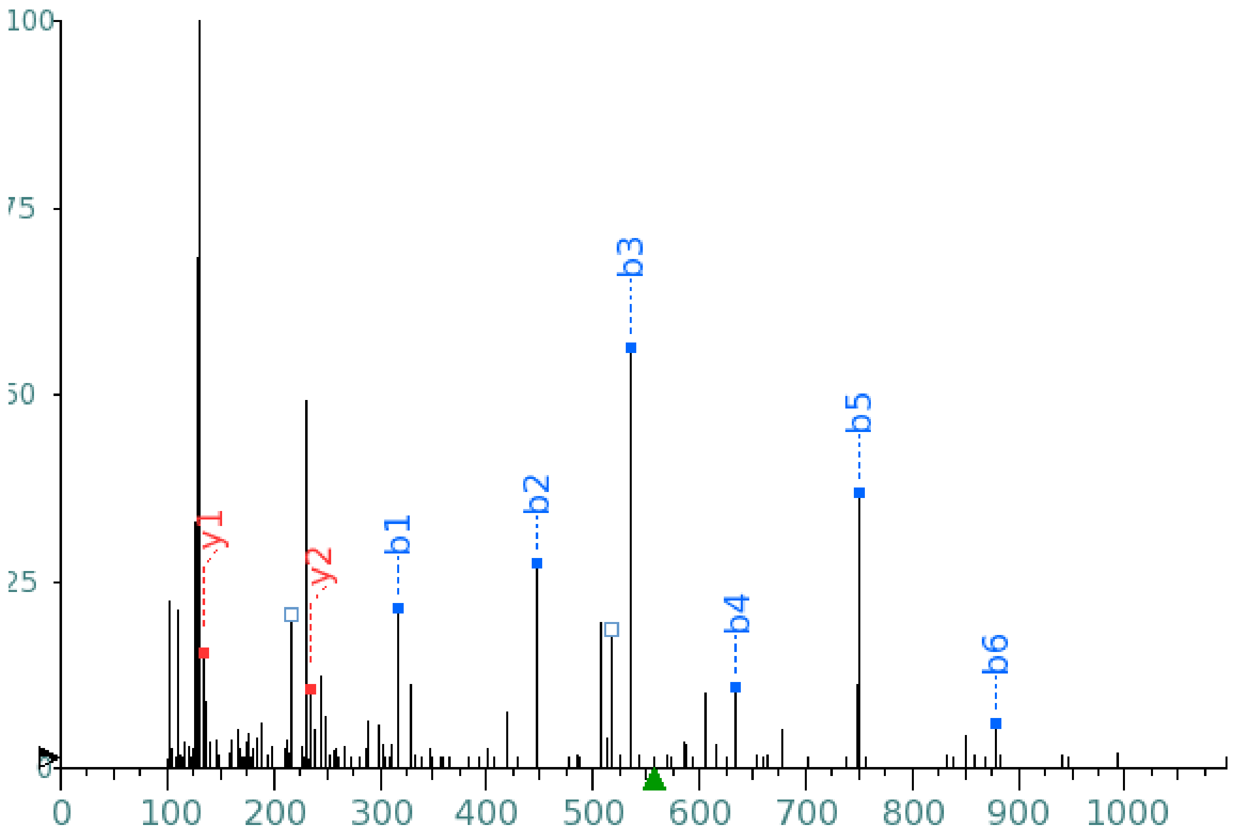

SMSVDETD (+2)

Primary Reference: COBL1_HUMAN
Search ID: 41760
Search Name: 20130328_ananiav_ERstress_TMT_TM_timecourse_trypsin_inj1_4mc_iaa
Scan#: 6168
Observed Mass: 556.7541 (2.6 ppm)
PSM Score: 39.84

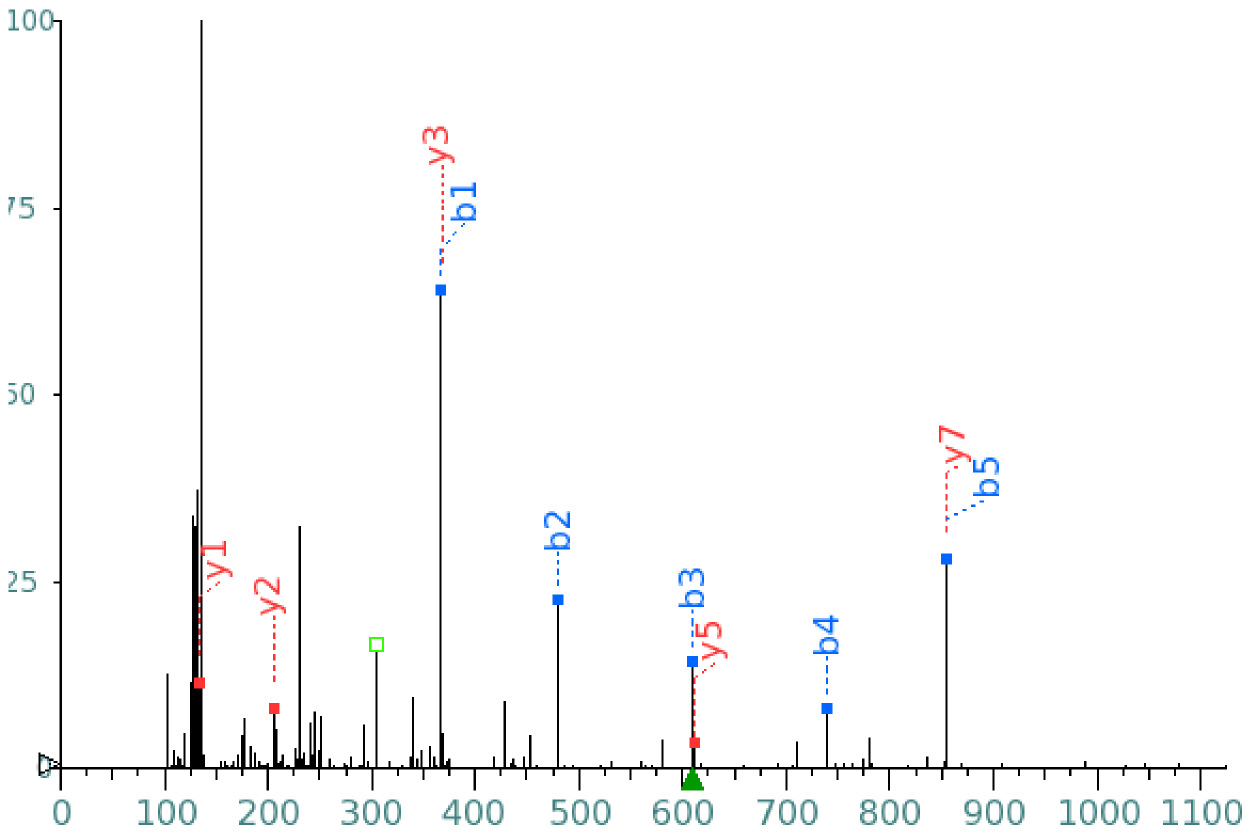

HLEEDYAD (+2)

Primary Reference: CACB3_HUMAN
Search ID: 41760
Search Name: 20130328_ananiav_ERstress_TMT_TM_timecourse_trypsin_inj1_4mc_iaa
Scan#: 6182
Observed Mass: 610.7865 (1.9 ppm)
PSM Score: 24.73

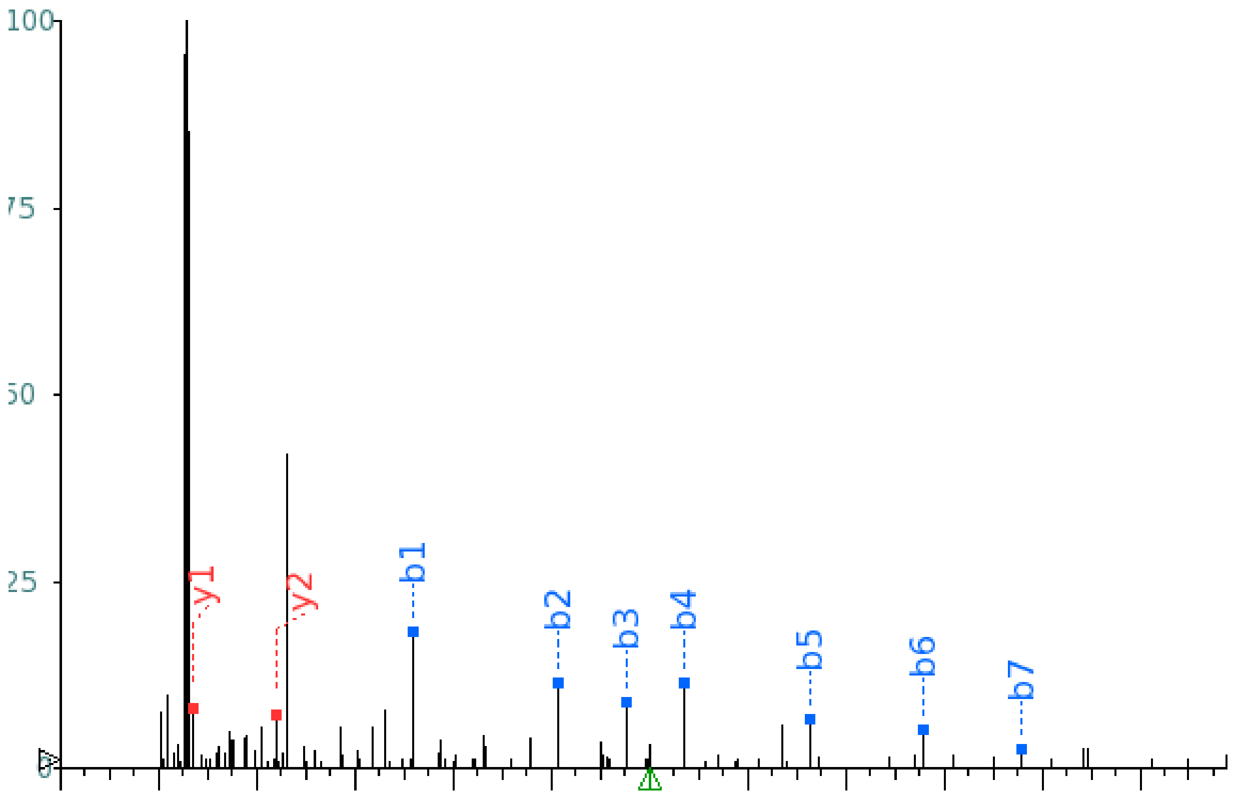

EFAGEDTSD (+2)

Primary Reference: XPO1_HUMAN
Search ID: 41760
Search Name: 20130328_ananiav_ERstress_TMT_TM_timecourse_trypsin_inj1_4mc_iaa
Scan#: 6244
Observed Mass: 600.2681 (2 ppm)
PSM Score: 36.69

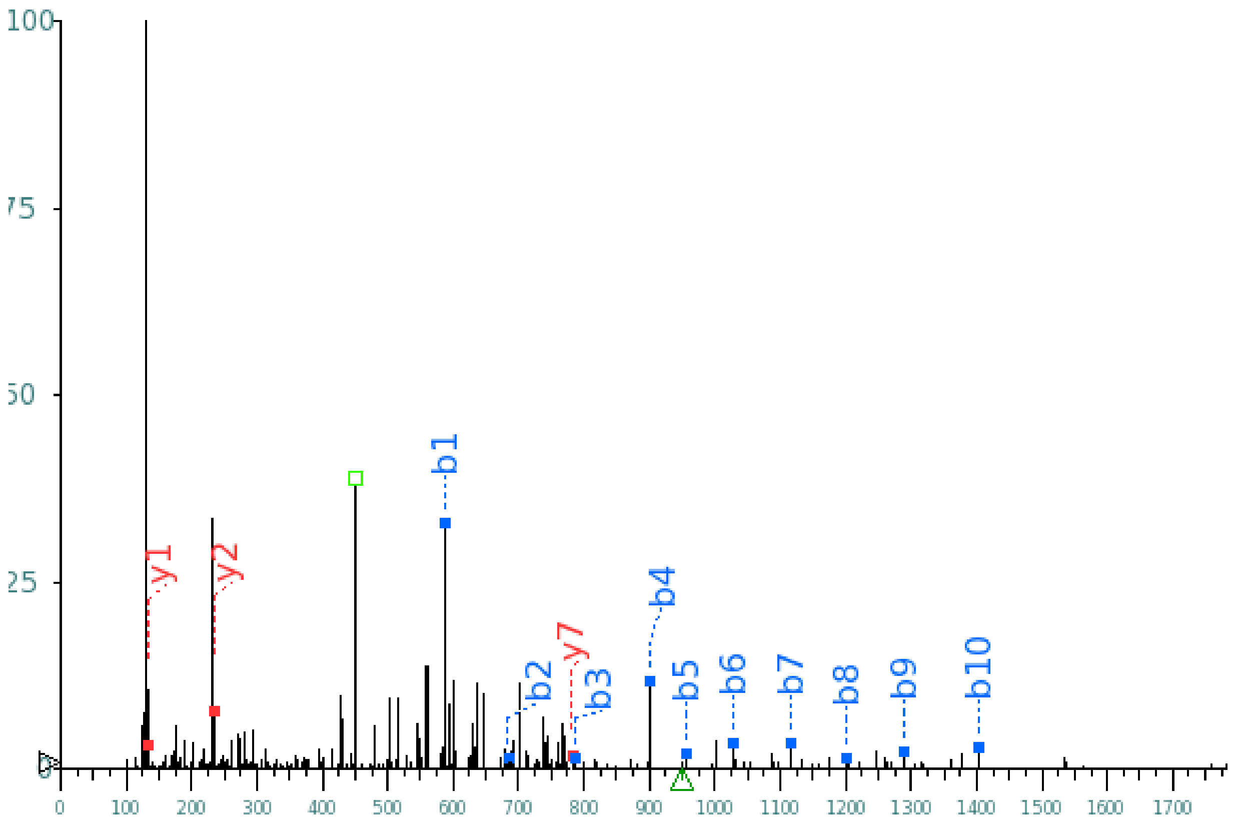

KPTDGASSSNC*VTD (+2)

Primary Reference: NASP_HUMAN
Search ID: 41760
Search Name: 20130328_ananiav_ERstress_TMT_TM_timecourse_trypsin_inj1_4mc_iaa
Scan#: 6251
Observed Mass: 948.9727 (0.4 ppm)
PSM Score: 26.97

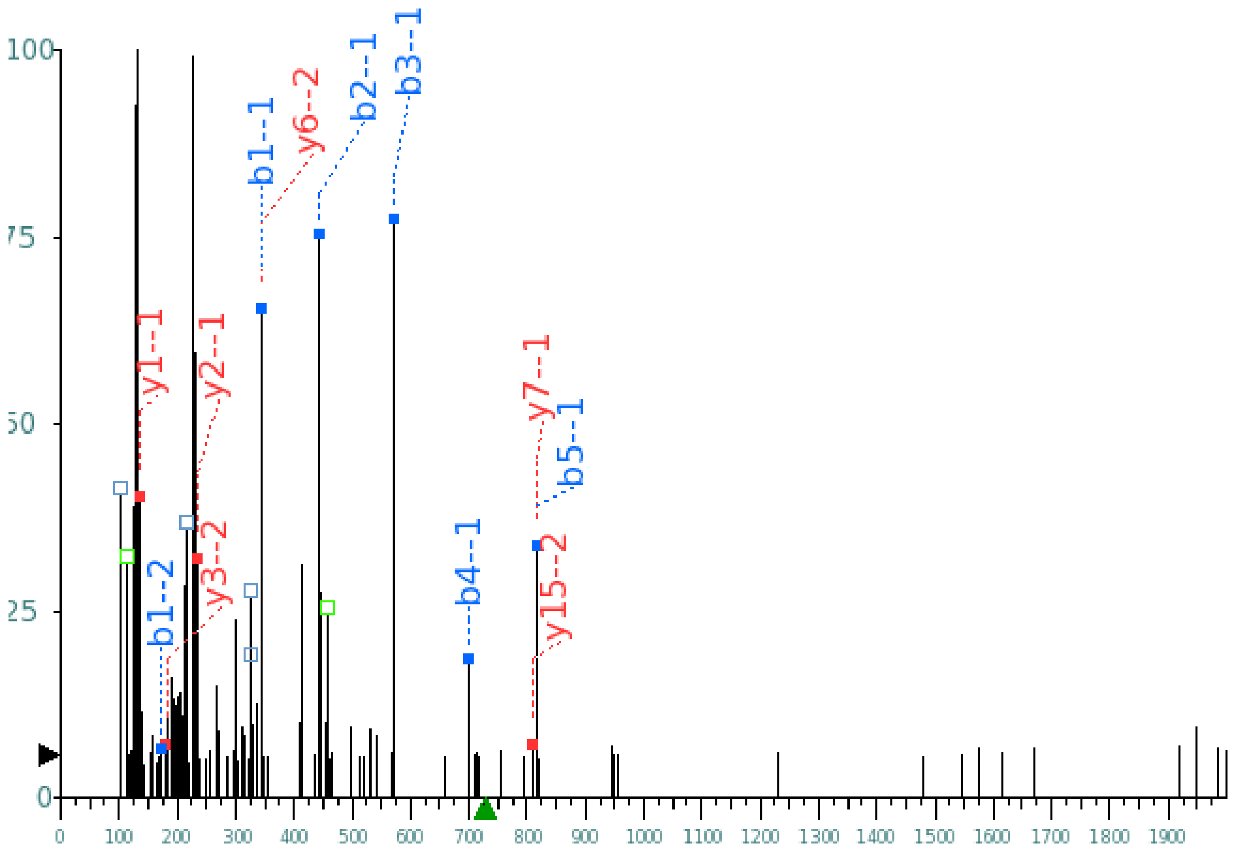

NVQQDNSEAGTQPQVQTD (+3)

Primary Reference: HS105_HUMAN
Search ID: 41760
Search Name: 20130328_ananiav_ERstress_TMT_TM_timecourse_trypsin_inj1_4mc_iaa
Scan#: 6280
Observed Mass: 730.017 (2.2 ppm)
PSM Score: 14.3

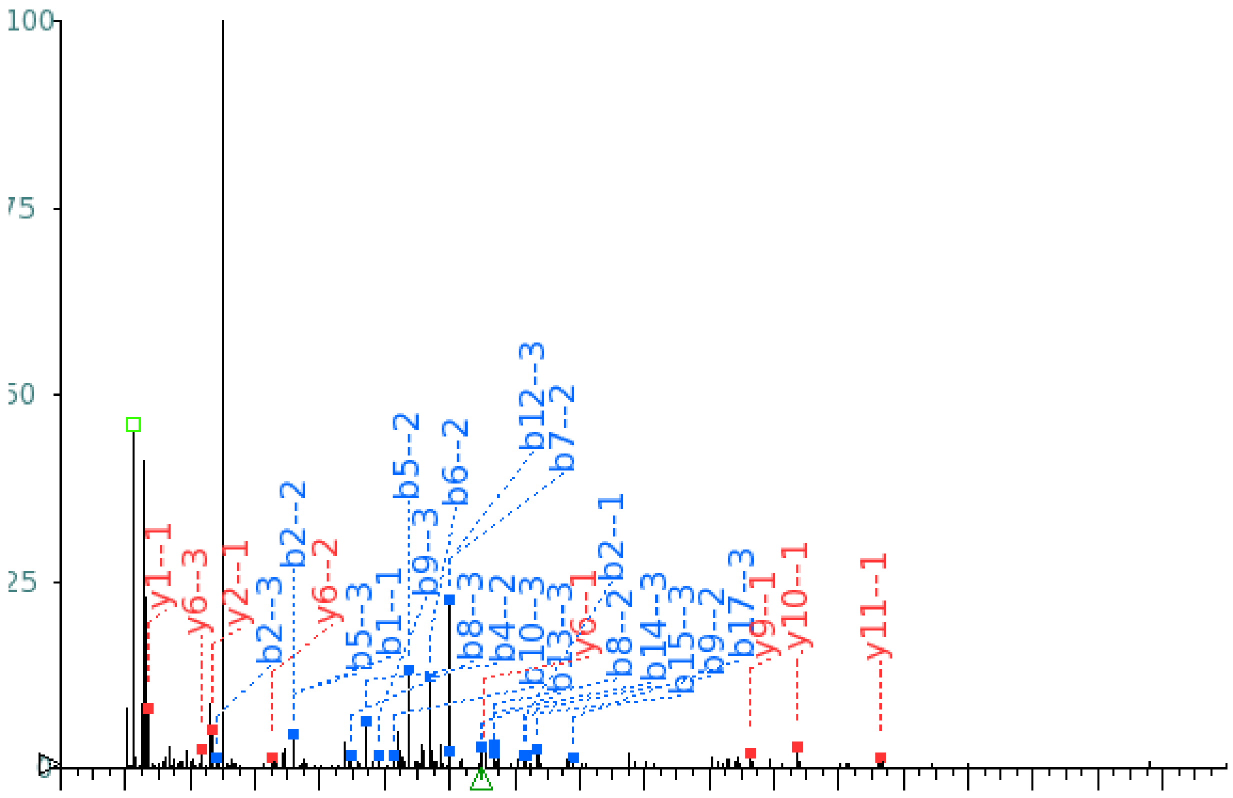

EKPEEAGHEAEERGETETD (+4)

Primary Reference: MDN1_HUMAN
Search ID: 41760
Search Name: 20130328_ananiav_ERstress_TMT_TM_timecourse_trypsin_inj1_4mc_iaa
Scan#: 6281
Observed Mass: 651.0647 (2.1 ppm)
PSM Score: 12.27

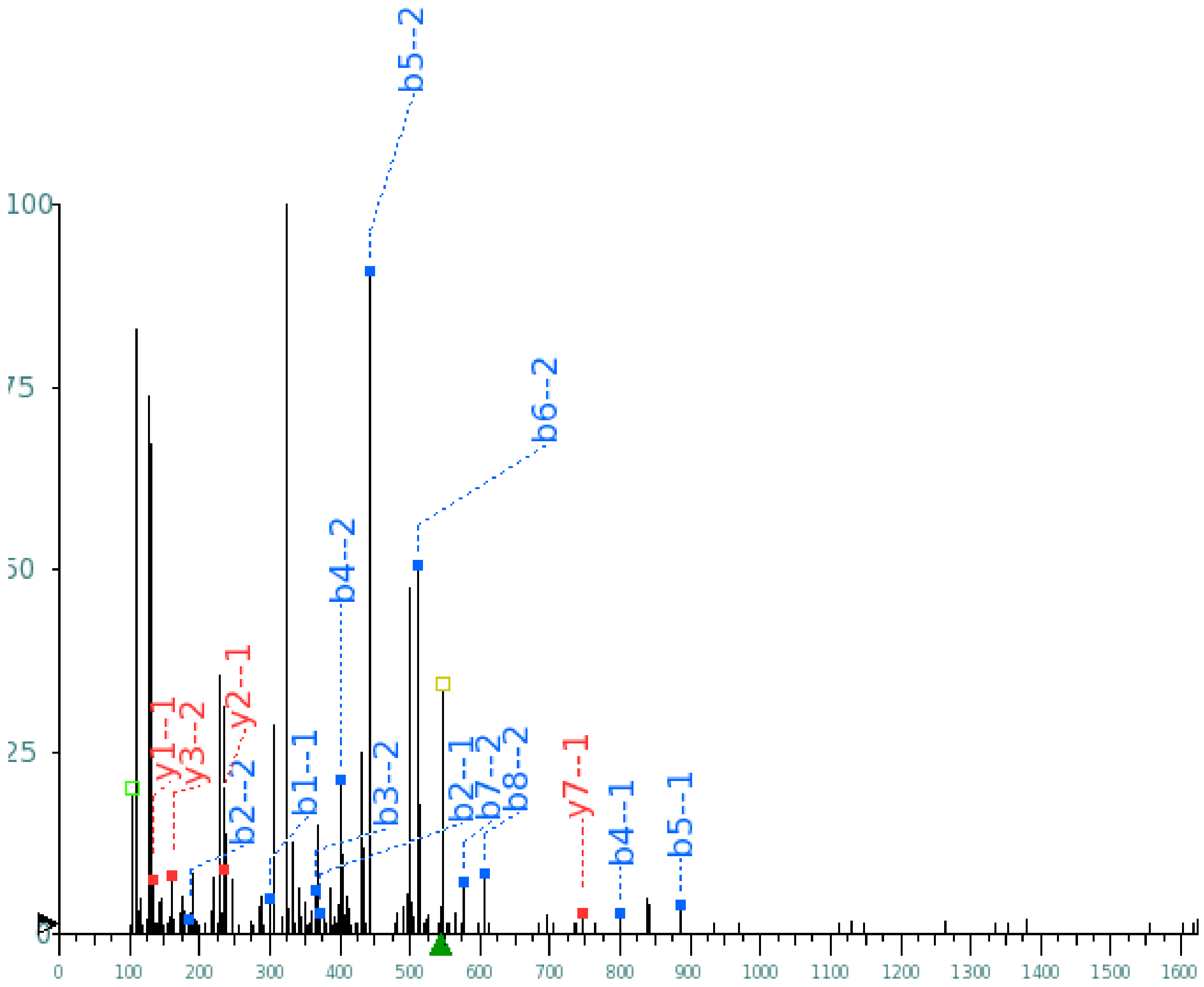

AAKASHMGVSTD (+3)

Primary Reference: LUZP1_HUMAN
Search ID: 41760
Search Name: 20130328_ananiav_ERstress_TMT_TM_timecourse_trypsin_inj1_4mc_iaa
Scan#: 6378
Observed Mass: 544.9648 (1.1 ppm)
PSM Score: 3.97

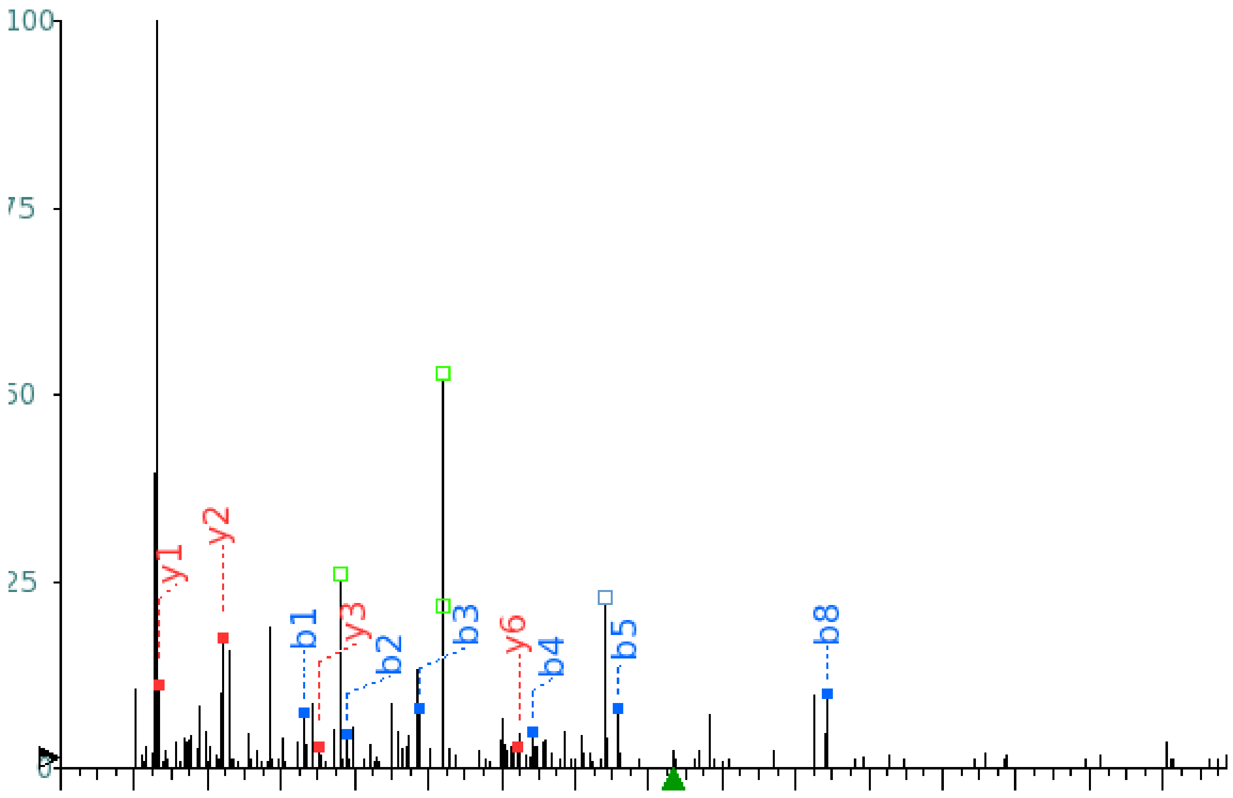

TGVRDPADVSSESD (+2)

Primary Reference: BAKOR_HUMAN
Search ID: 41760
Search Name: 20130328_ananiav_ERstress_TMT_TM_timecourse_trypsin_inj1_4mc_iaa
Scan#: 6455
Observed Mass: 832.403 (0.9 ppm)
PSM Score: 22.3

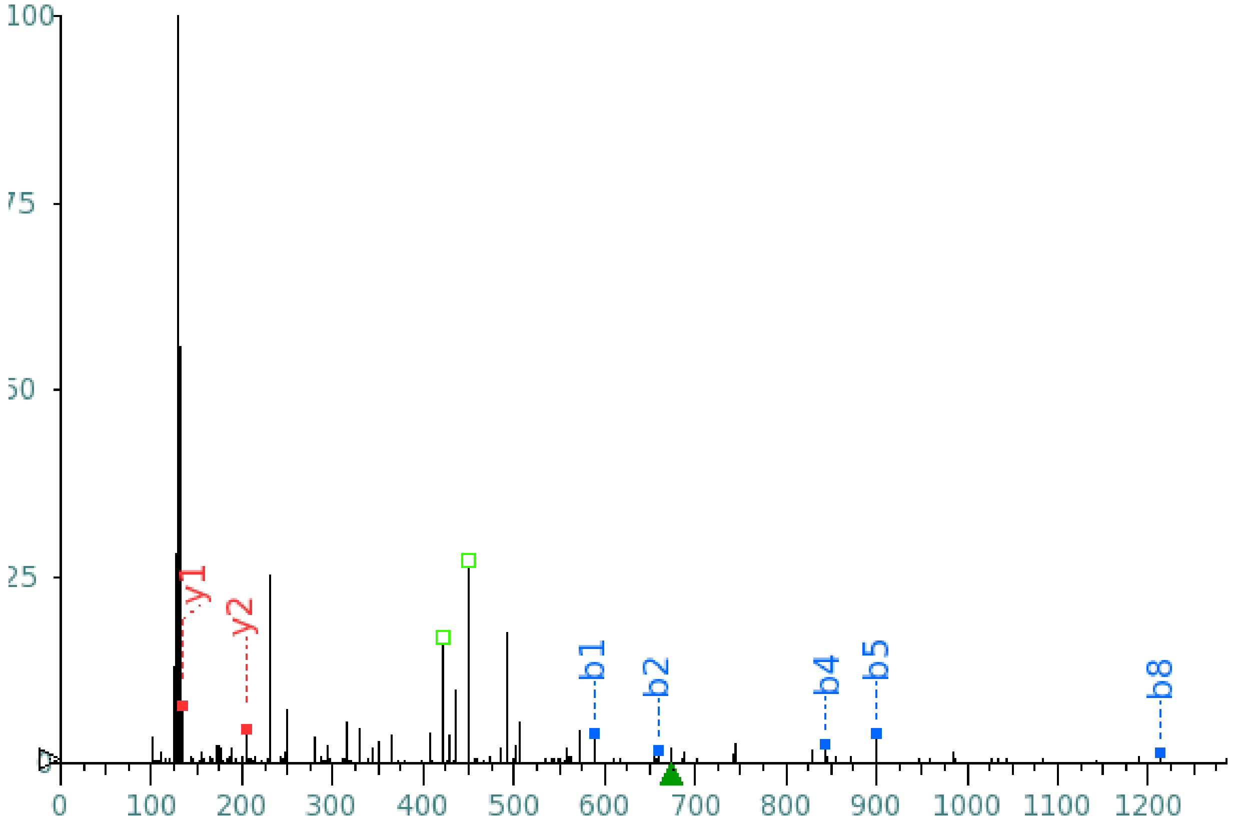

KAANGLEAD (+2)

Primary Reference: LUZP1_HUMAN
Search ID: 41760
Search Name: 20130328_ananiav_ERstress_TMT_TM_timecourse_trypsin_inj1_4mc_iaa
Scan#: 6479
Observed Mass: 673.8891 (2.3 ppm)
PSM Score: 23.04

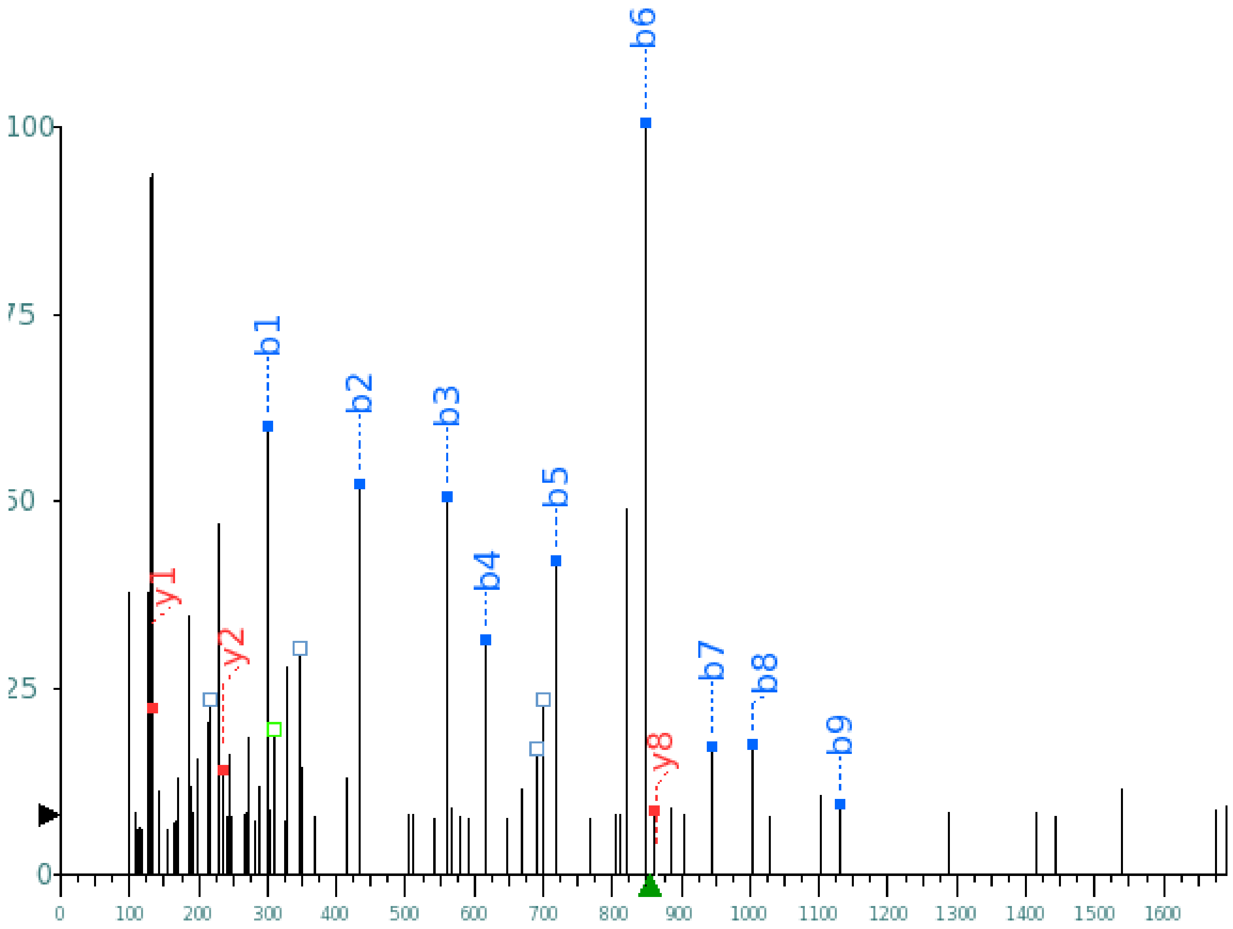

AMQGTEVGQTDQTD (+2)

Primary Reference: MIA3_HUMAN
Search ID: 41760
Search Name: 20130328_ananiav_ERstress_TMT_TM_timecourse_trypsin_inj1_4mc_iaa
Scan#: 6664
Observed Mass: 855.3957 (-0.5 ppm)
PSM Score: 52.6

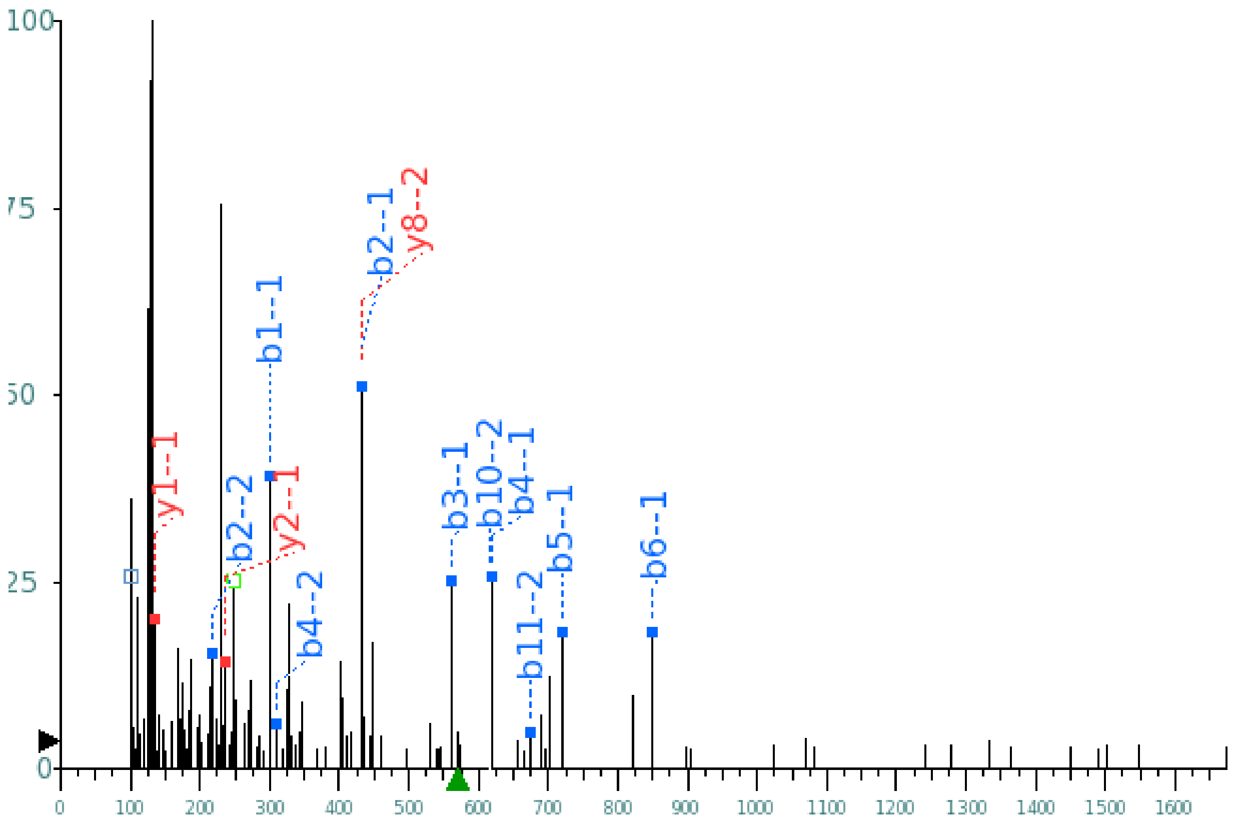

AMQGTEVGQTDQTD (+3)

Primary Reference: MIA3_HUMAN
Search ID: 41760
Search Name: 20130328_ananiav_ERstress_TMT_TM_timecourse_trypsin_inj1_4mc_iaa
Scan#: 6681
Observed Mass: 570.5994 (-0.9 ppm)
PSM Score: 25.94

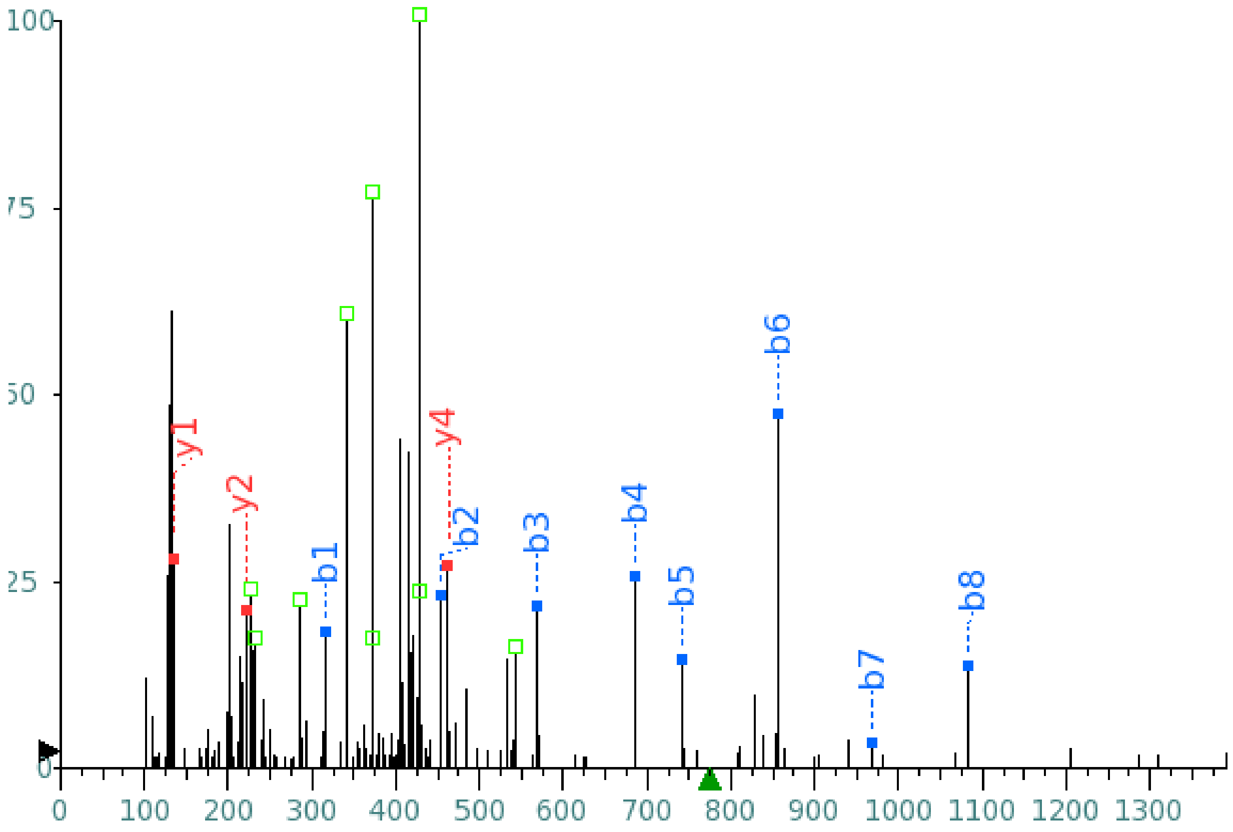

SHDDGNIDLESD (+2)

Primary Reference: SON_HUMAN
Search ID: 41760
Search Name: 20130328_ananiav_ERstress_TMT_TM_timecourse_trypsin_inj1_4mc_iaa
Scan#: 6710
Observed Mass: 773.3477 (0.9 ppm)
PSM Score: 36.48

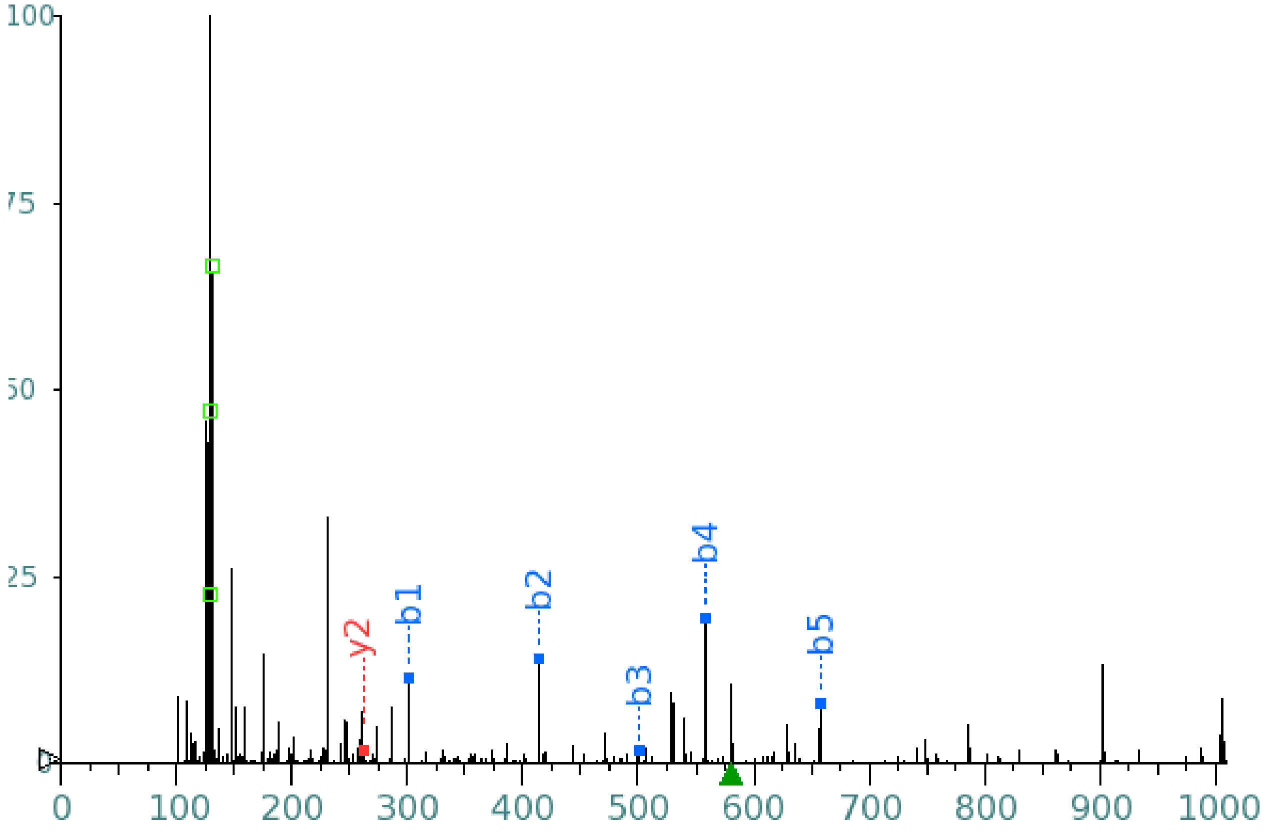

ALSGVLEQD (+2)

Primary Reference: HBS1L_HUMAN
Search ID: 41760
Search Name: 20130328_ananiav_ERstress_TMT_TM_timecourse_trypsin_inj1_4mc_iaa
Scan#: 6732
Observed Mass: 580.8228 (1.9 ppm)
PSM Score: 10.23

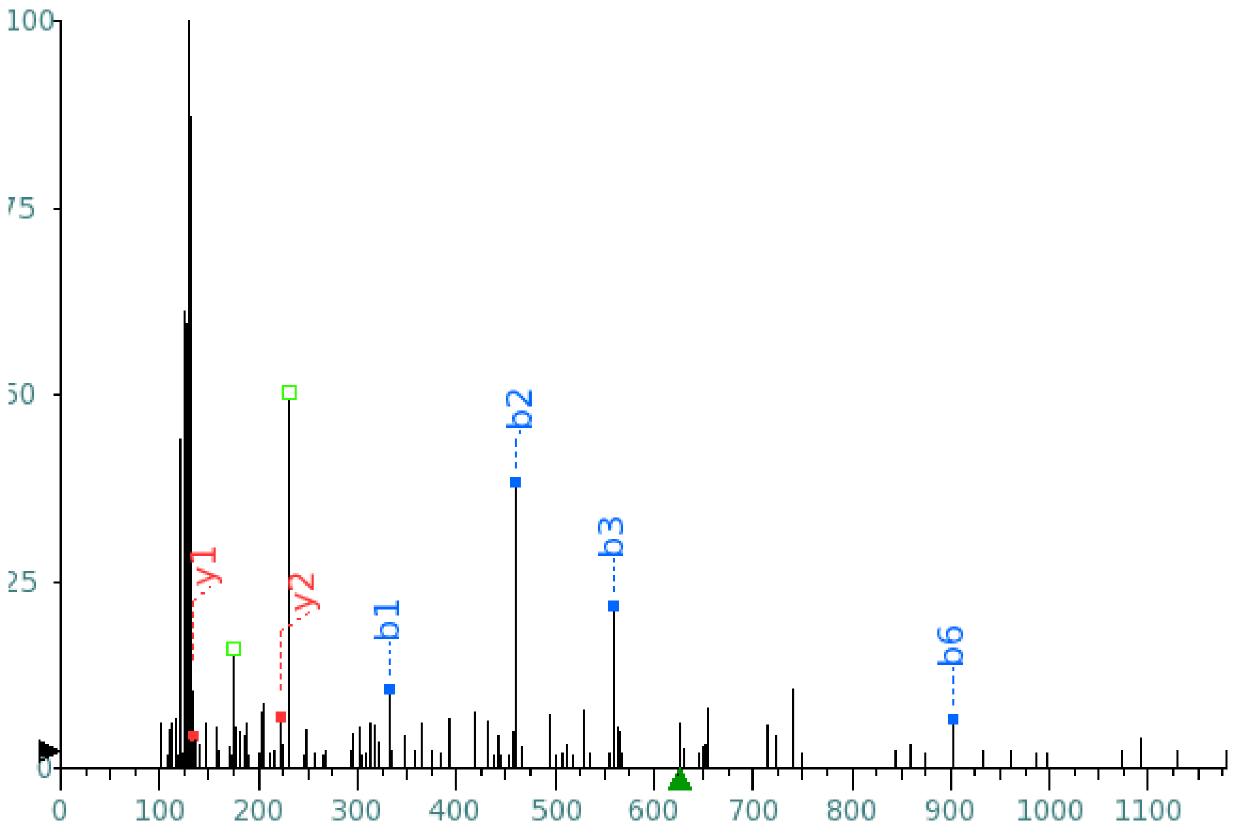

TQVIDDESD (+2)

Primary Reference: TRIP4_HUMAN
Search ID: 41760
Search Name: 20130328_ananiav_ERstress_TMT_TM_timecourse_trypsin_inj1_4mc_iaa
Scan#: 6797
Observed Mass: 625.8026 (2.4 ppm)
PSM Score: 13.87

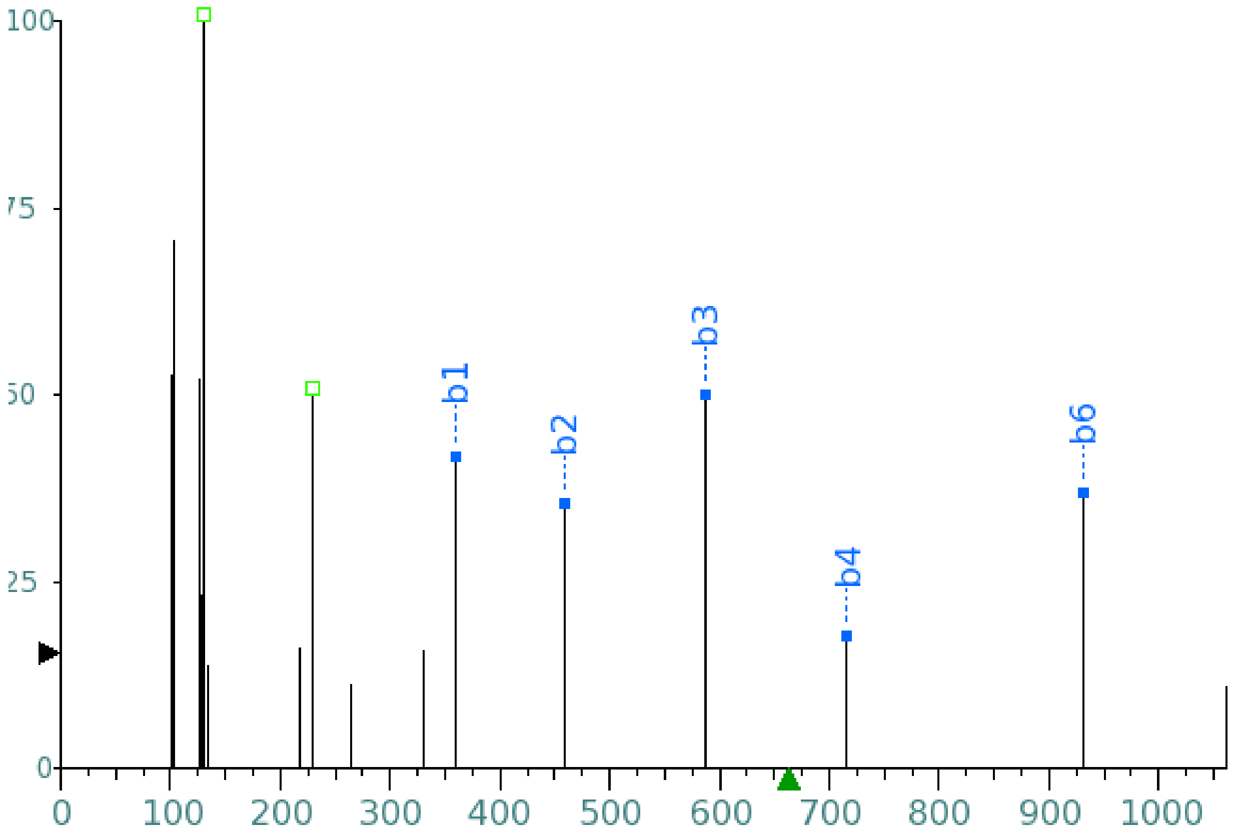

EVEETDEMD (+2)

Primary Reference: DNJA1_HUMAN
Search ID: 41760
Search Name: 20130328_ananiav_ERstress_TMT_TM_timecourse_trypsin_inj1_4mc_iaa
Scan#: 6800
Observed Mass: 663.2856 (1.8 ppm)
PSM Score: 52.06

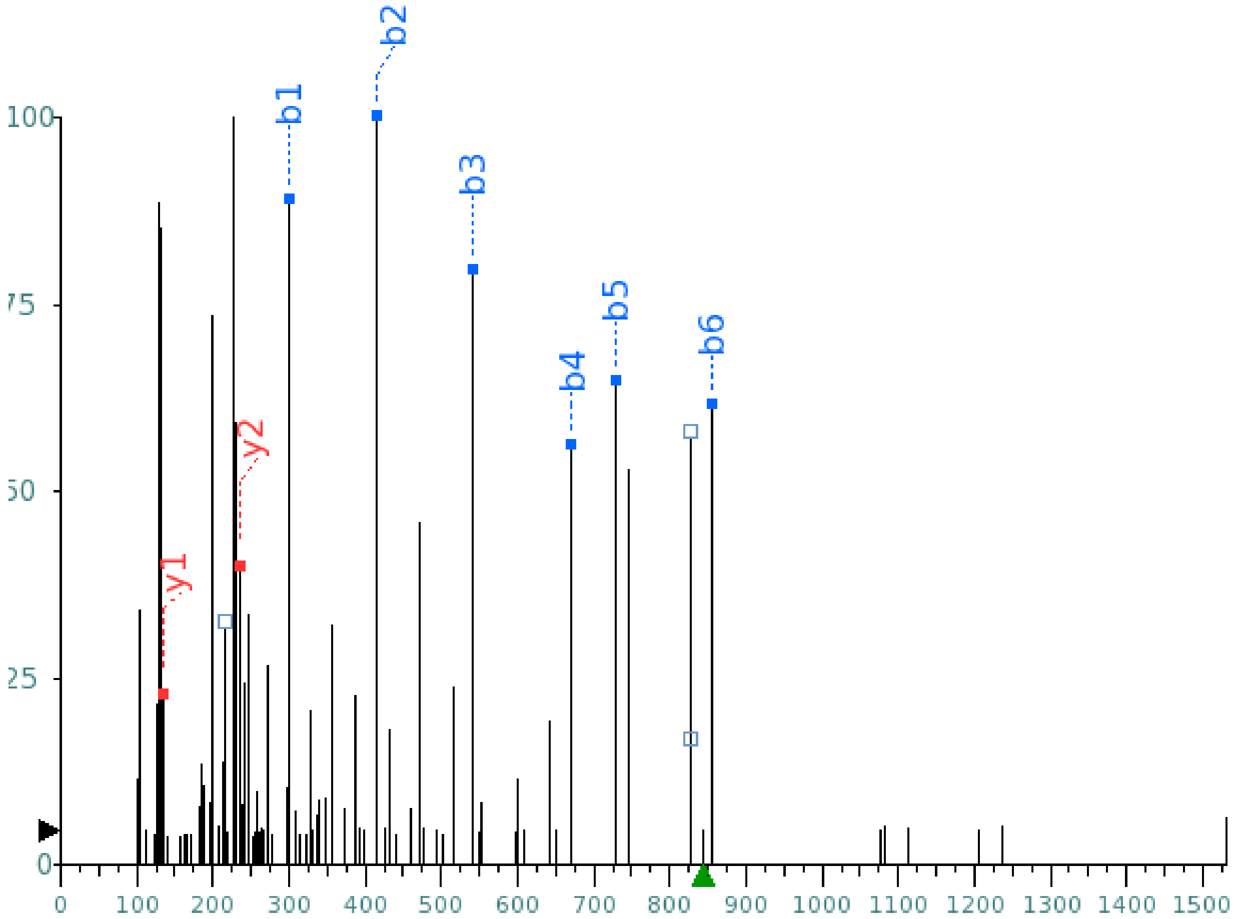

ALQEGQPEEDETD (+2)

Primary Reference: LENG1_HUMAN
Search ID: 41760
Search Name: 20130328_ananiav_ERstress_TMT_TM_timecourse_trypsin_inj1_4mc_iaa
Scan#: 6842
Observed Mass: 845.3879 (2 ppm)
PSM Score: 30.82

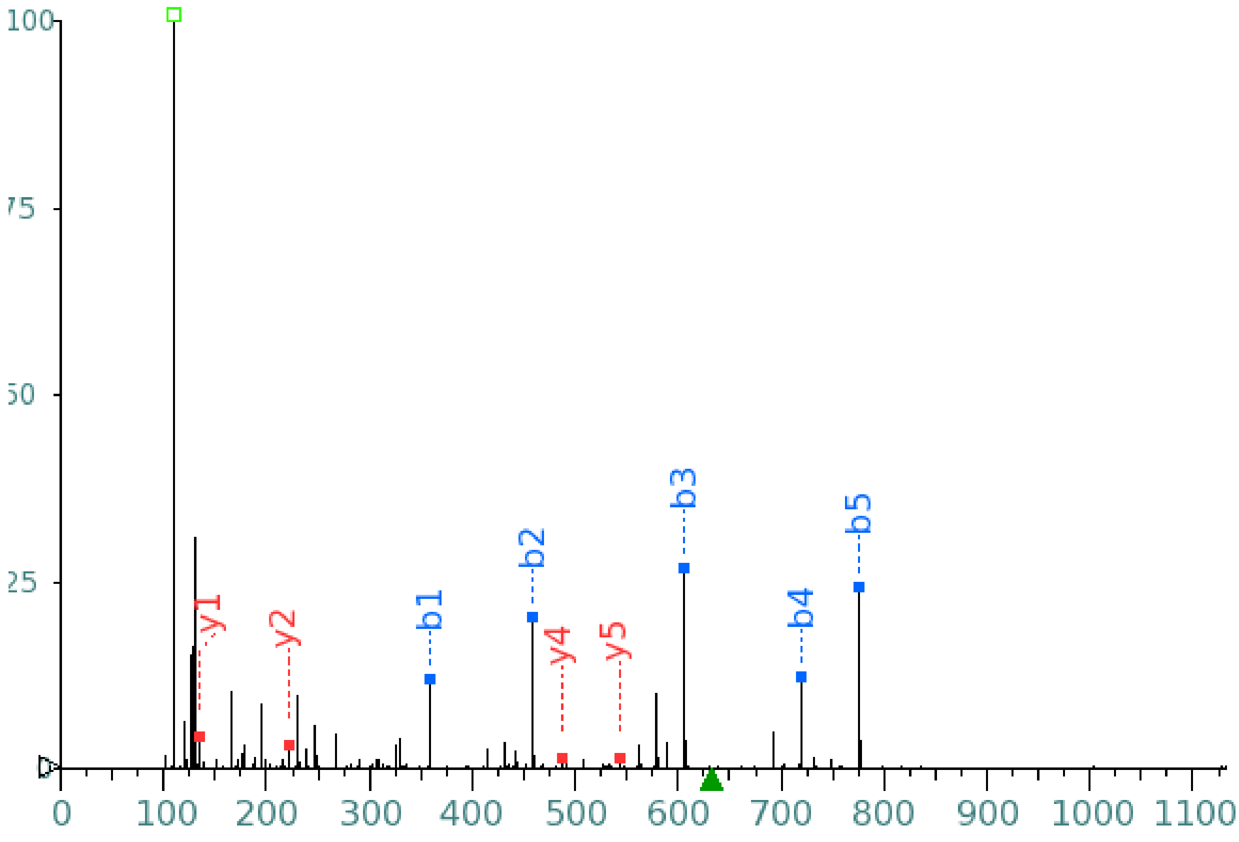

QTFIGHESD (+2)

Primary Reference: GBB2_HUMAN
Search ID: 41760
Search Name: 20130328_ananiav_ERstress_TMT_TM_timecourse_trypsin_inj1_4mc_iaa
Scan#: 6863
Observed Mass: 631.8156 (1.9 ppm)
PSM Score: 15.61

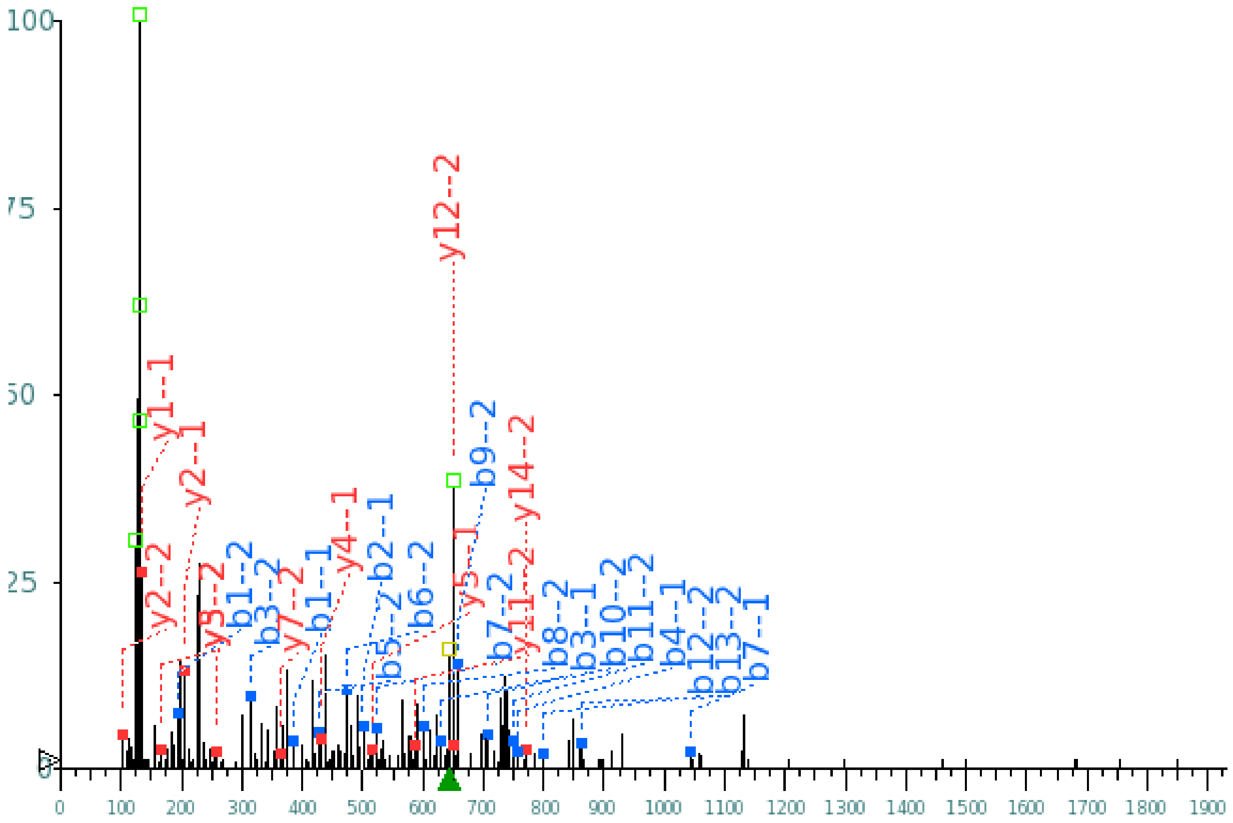

RLEQTSVRDPSPEAD (+3)

Primary Reference: CASC3_HUMAN
Search ID: 41760
Search Name: 20130328_ananiav_ERstress_TMT_TM_timecourse_trypsin_inj1_4mc_iaa
Scan#: 6907
Observed Mass: 643.669 (2.6 ppm)
PSM Score: 18.46

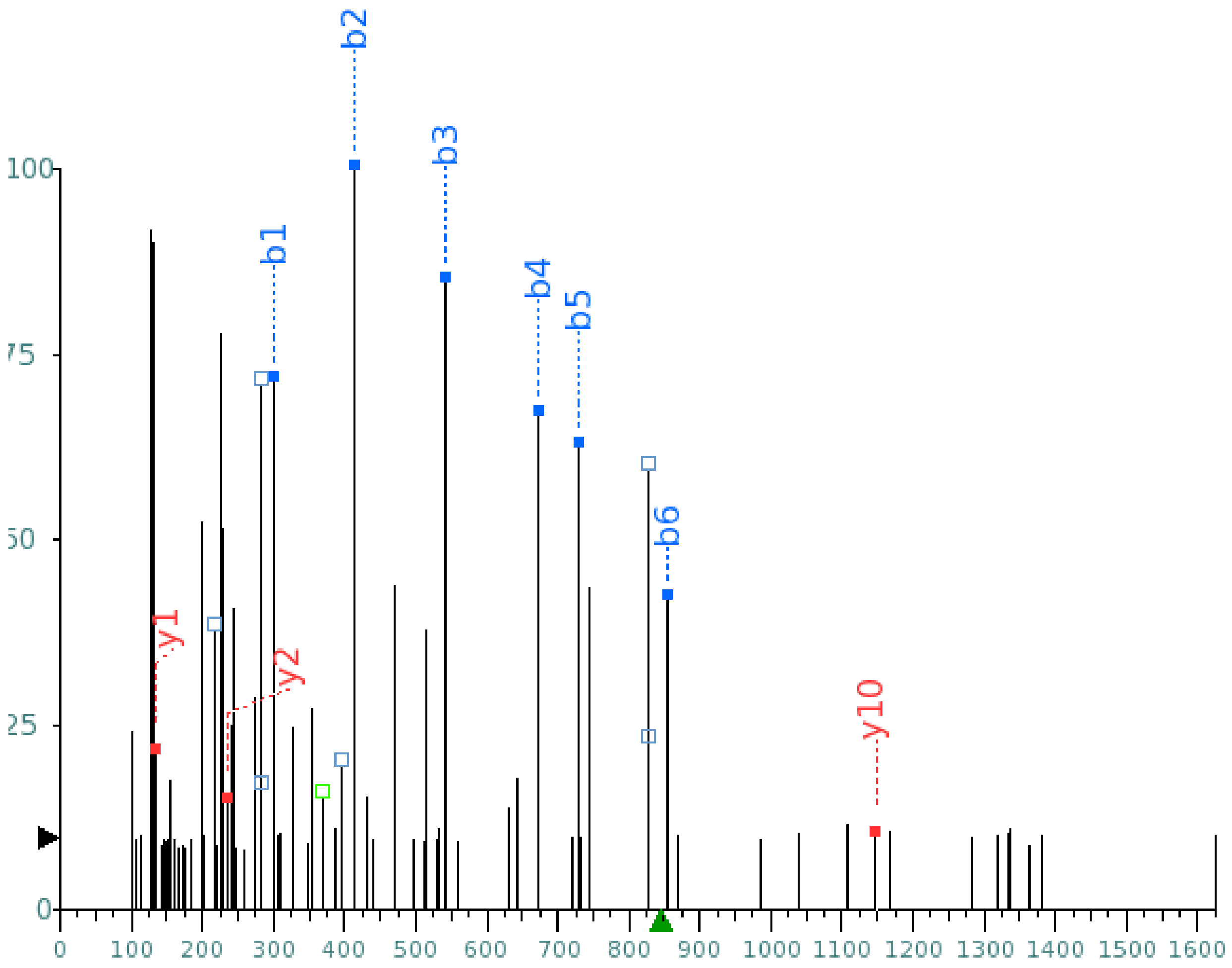

ALQEGQPEEDETD (+2)

Primary Reference: LENG1_HUMAN
Search ID: 41760
Search Name: 20130328_ananiav_ERstress_TMT_TM_timecourse_trypsin_inj1_4mc_iaa
Scan#: 6928
Observed Mass: 845.3877 (1.7 ppm)
PSM Score: 20.81

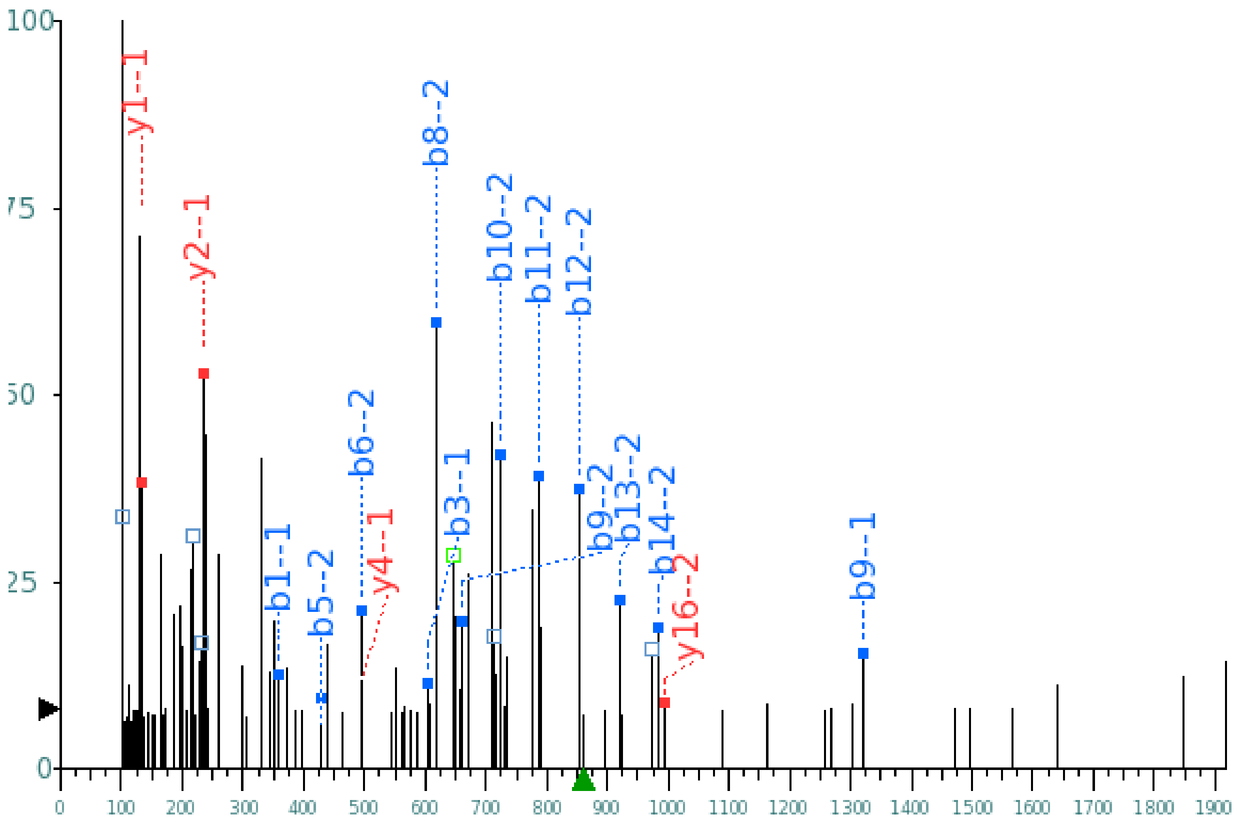

ESRQEEMNSQQEEEEEMETD (+3)

Primary Reference: SF3B2_HUMAN
Search ID: 41760
Search Name: 20130328_ananiav_ERstress_TMT_TM_timecourse_trypsin_inj1_4mc_iaa
Scan#: 6938
Observed Mass: 863.0272 (2.4 ppm)
PSM Score: 16.73

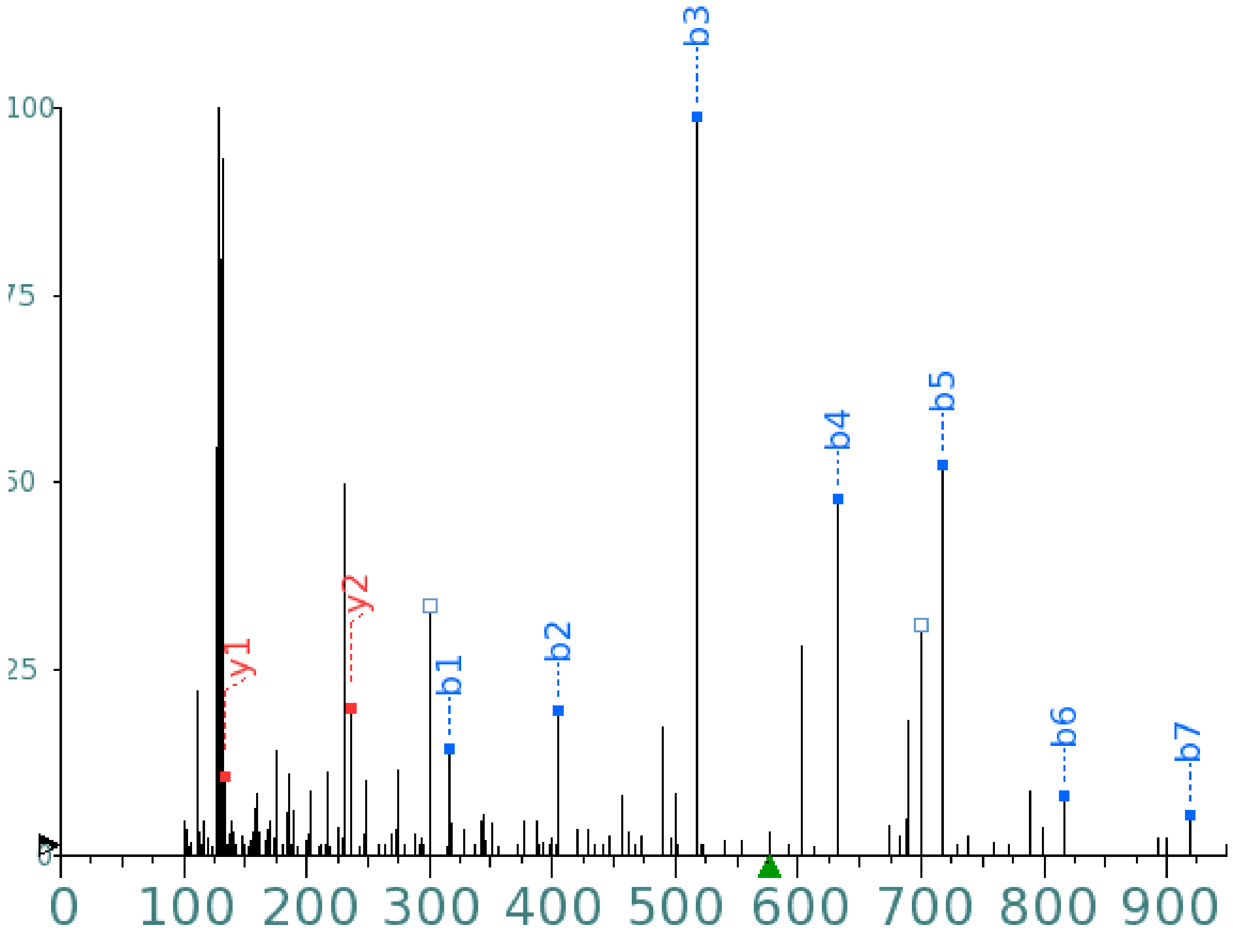

SSNLSVTTD (+2)

Primary Reference: LMO7_HUMAN
Search ID: 41760
Search Name: 20130328_ananiav_ERstress_TMT_TM_timecourse_trypsin_inj1_4mc_iaa
Scan#: 6996
Observed Mass: 576.8026 (2.9 ppm)
PSM Score: 59.77

File name: 2015-02-04 04.01.53PM.pdf

Creation time: 2015-02-04 04:09PM

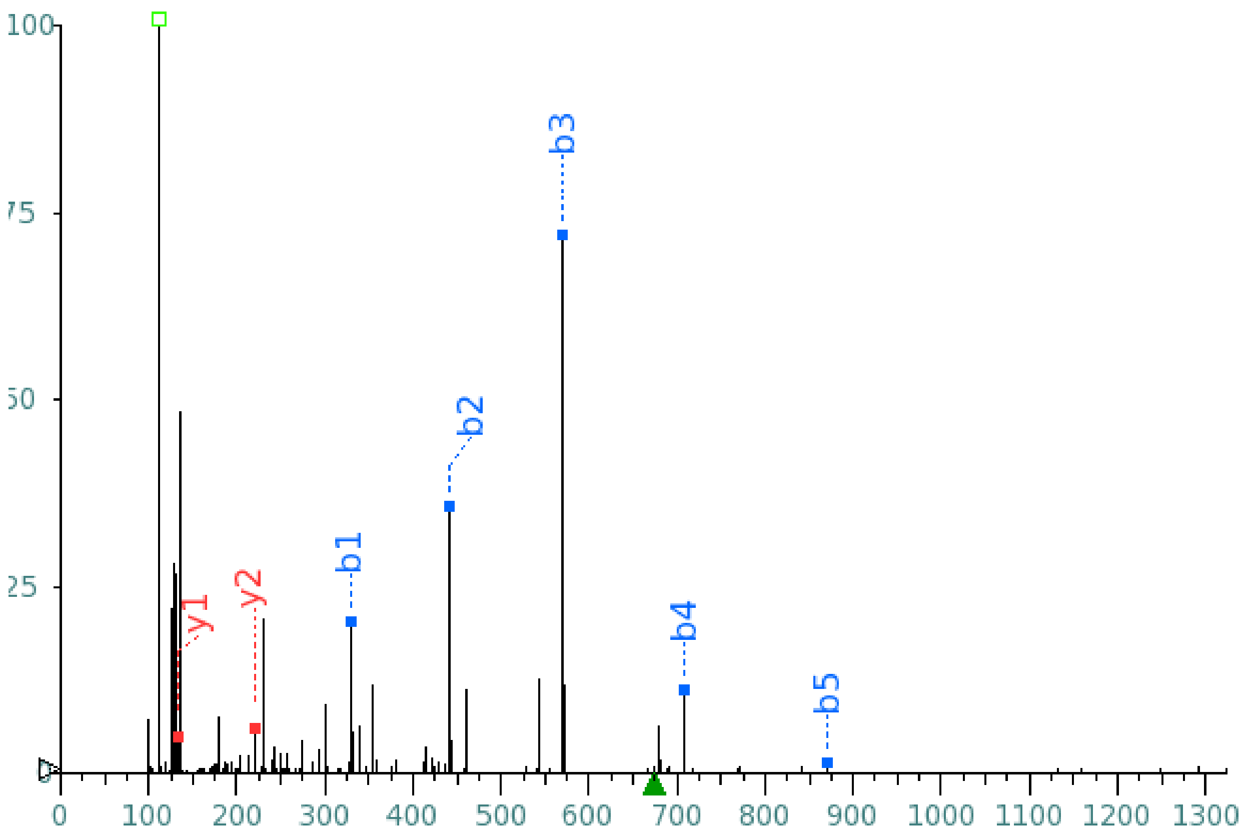

VLQHYQESD (+2)

Primary Reference: MTCH2_HUMAN
Search ID: 41760
Search Name: 20130328_ananiav_ERstress_TMT_TM_timecourse_trypsin_inj1_4mc_iaa
Scan#: 7017
Observed Mass: 674.3411 (0.6 ppm)
PSM Score: 23.02

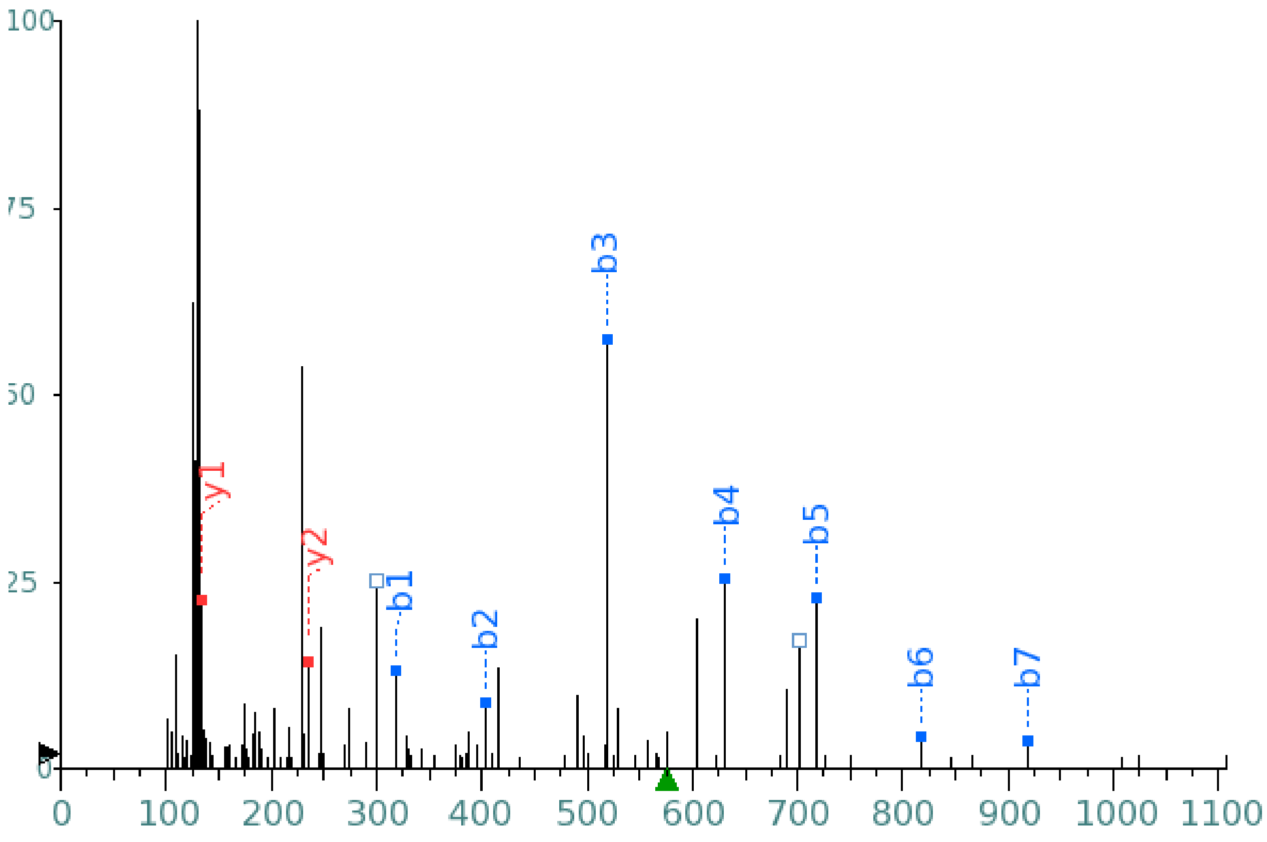

SSNLSVTTD (+2)

Primary Reference: LMO7_HUMAN
Search ID: 41760
Search Name: 20130328_ananiav_ERstress_TMT_TM_timecourse_trypsin_inj1_4mc_iaa
Scan#: 7081
Observed Mass: 576.8023 (2.4 ppm)
PSM Score: 35.68

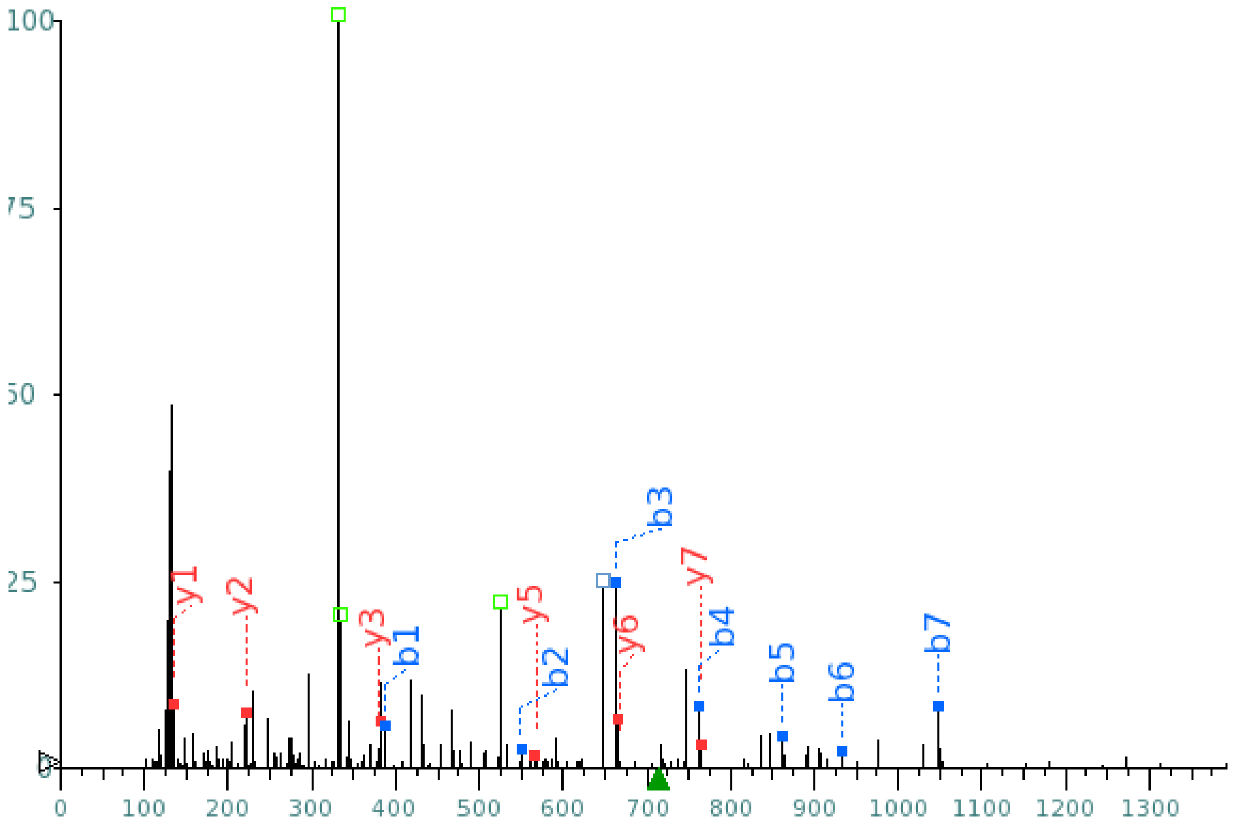

RYDVVADC*SD (+2)

Primary Reference: MOCS3_HUMAN
Search ID: 41760
Search Name: 20130328_ananiav_ERstress_TMT_TM_timecourse_trypsin_inj1_4mc_iaa
Scan#: 7085
Observed Mass: 714.8362 (1.8 ppm)
PSM Score: 21.35

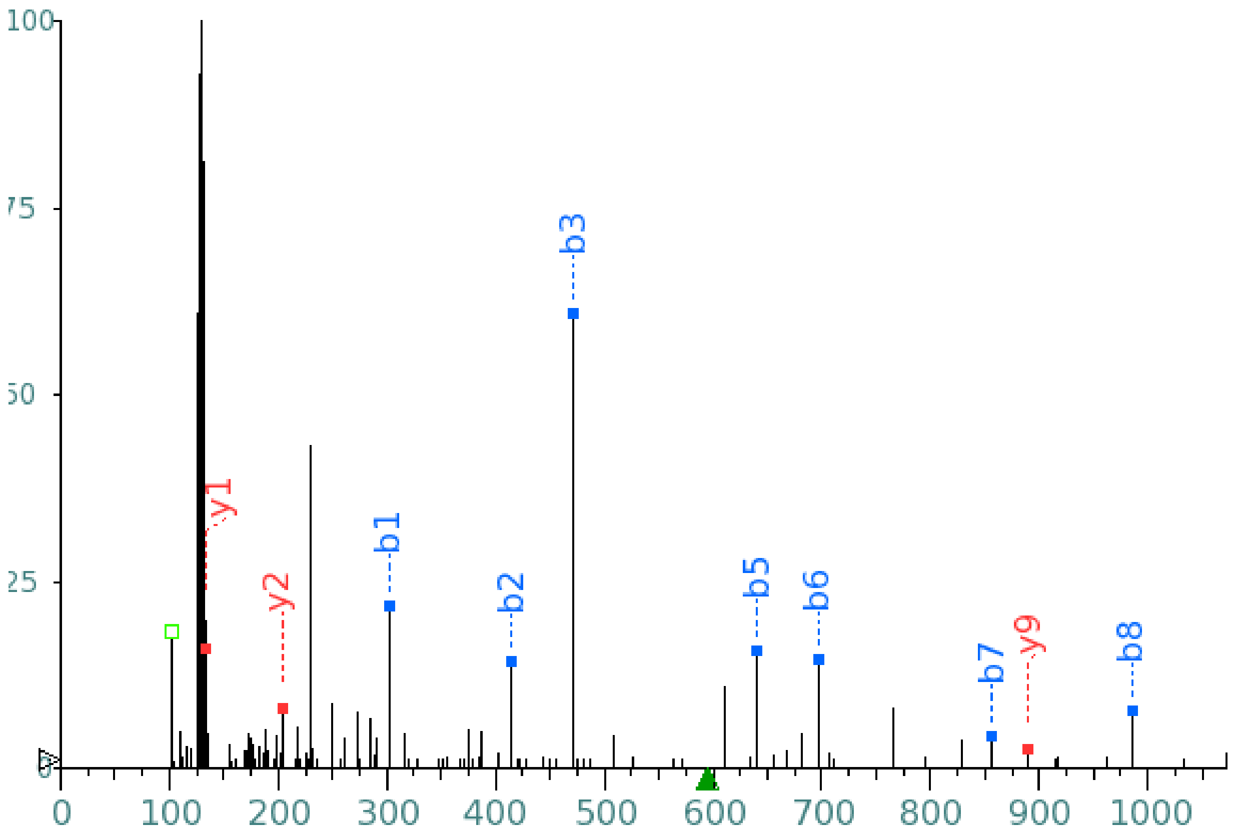

ALGPAGC*EAD (+2)

Primary Reference: AGRIN_HUMAN
Search ID: 41760
Search Name: 20130328_ananiav_ERstress_TMT_TM_timecourse_trypsin_inj1_4mc_iaa
Scan#: 7104
Observed Mass: 595.2907 (1.7 ppm)
PSM Score: 32.1

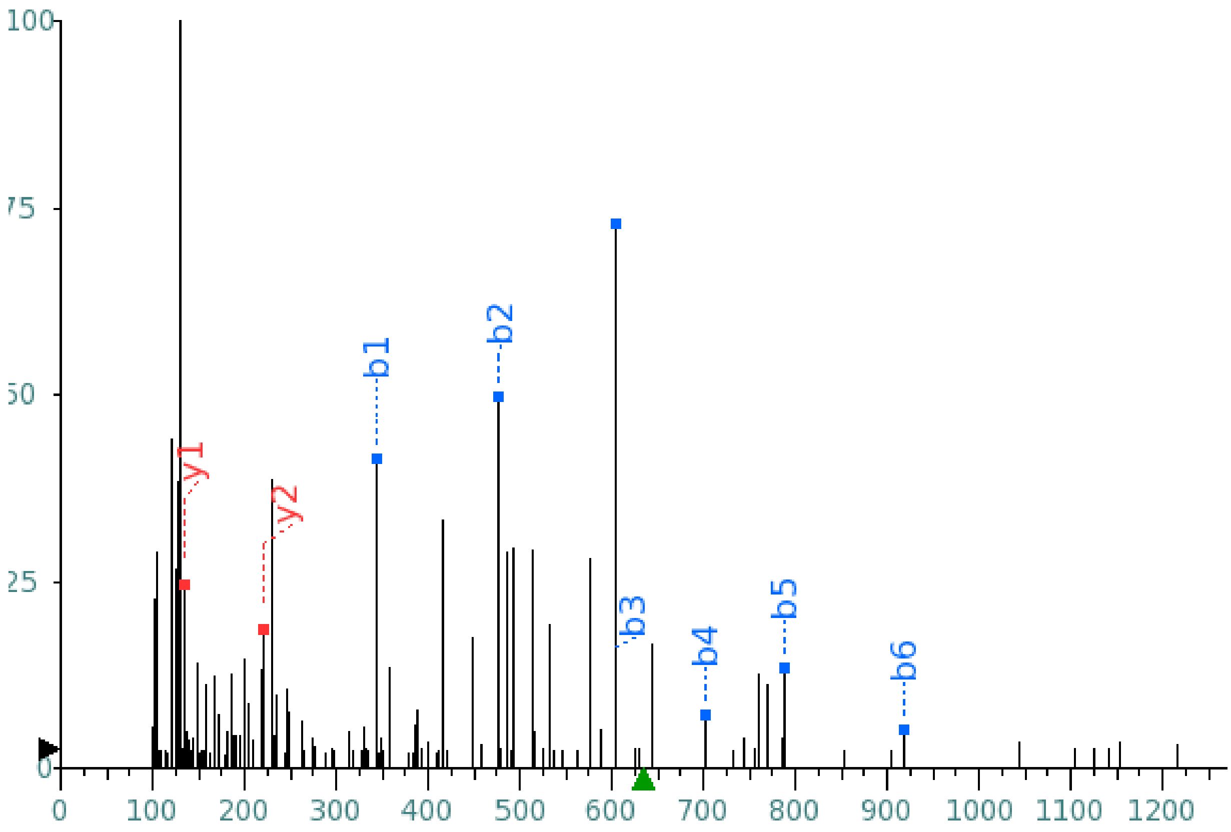

DMQPSMESD (+2)

Primary Reference: MAP4_HUMAN
Search ID: 41760
Search Name: 20130328_ananiav_ERstress_TMT_TM_timecourse_trypsin_inj1_4mc_iaa
Scan#: 7169
Observed Mass: 634.7717 (2 ppm)
PSM Score: 27.26

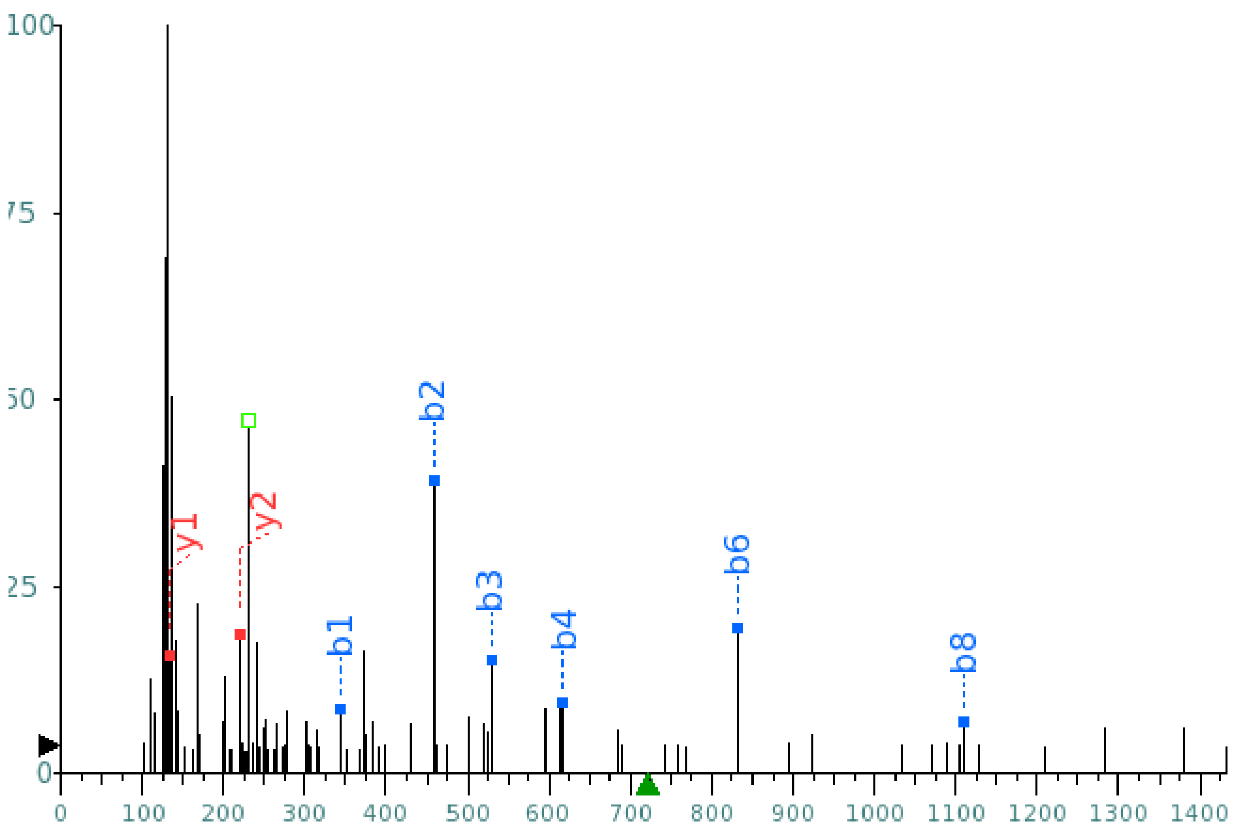

NNASTDYDLSD (+2)

Primary Reference: RL3_HUMAN
Search ID: 41760
Search Name: 20130328_ananiav_ERstress_TMT_TM_timecourse_trypsin_inj1_4mc_iaa
Scan#: 7315
Observed Mass: 722.3273 (2.6 ppm)
PSM Score: 18.3

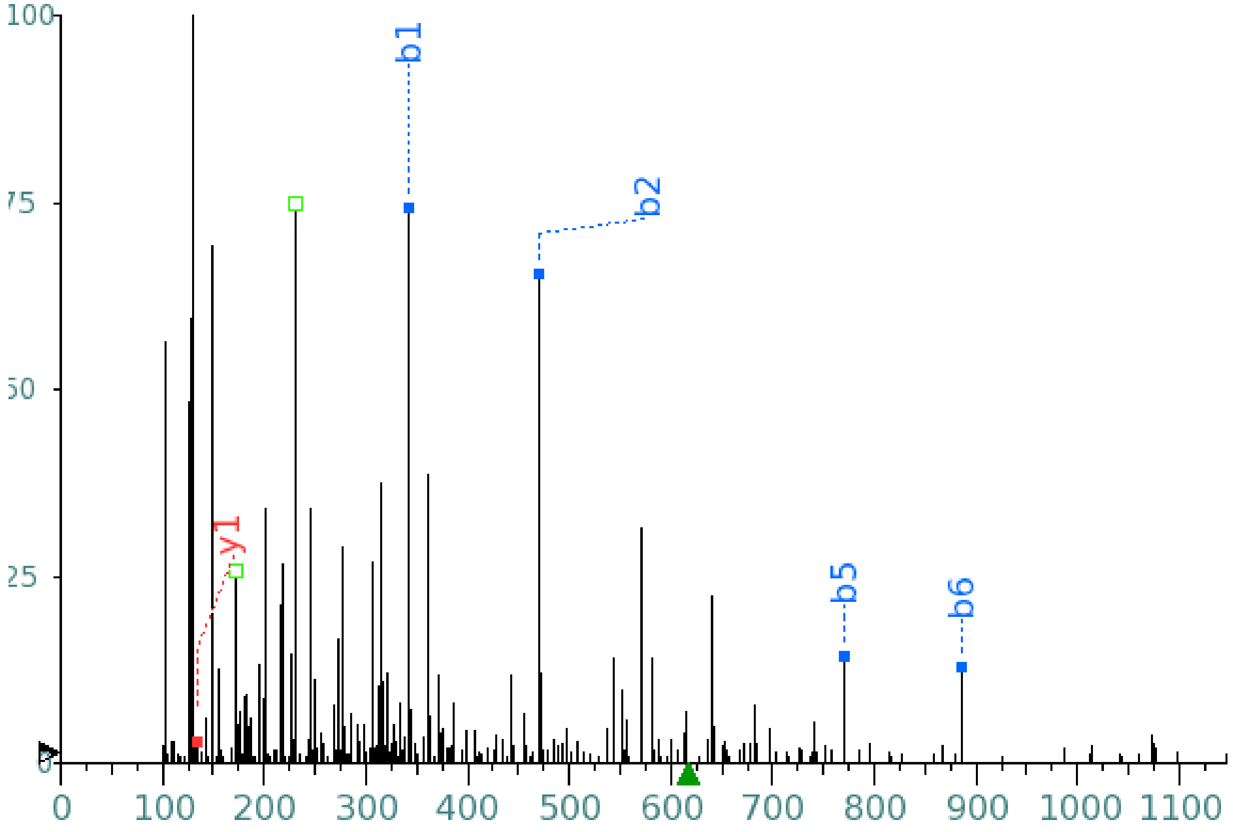

LQSVLDDVD (+2)

Primary Reference: ##TPR_HUMAN
Search ID: 41760
Search Name: 20130328_ananiav_ERstress_TMT_TM_timecourse_trypsin_inj1_4mc_iaa
Scan#: 7356
Observed Mass: 616.8333 (1.7 ppm)
PSM Score: 10.29

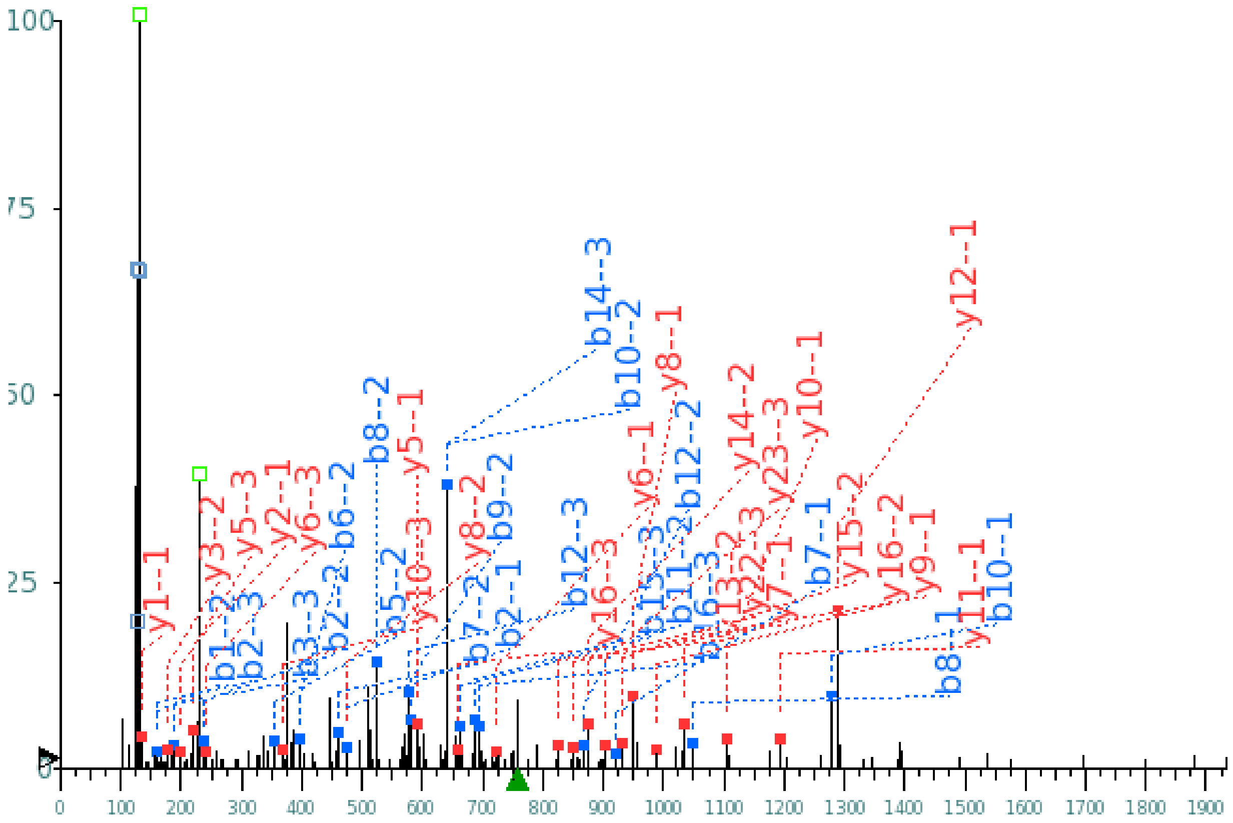

SRSGSSQELDVKPSASPQERSESD (+4)

Primary Reference: SRRM2_HUMAN
Search ID: 41760
Search Name: 20130328_ananiav_ERstress_TMT_TM_timecourse_trypsin_inj1_4mc_iaa
Scan#: 7360
Observed Mass: 756.1351 (2.1 ppm)
PSM Score: 26.63

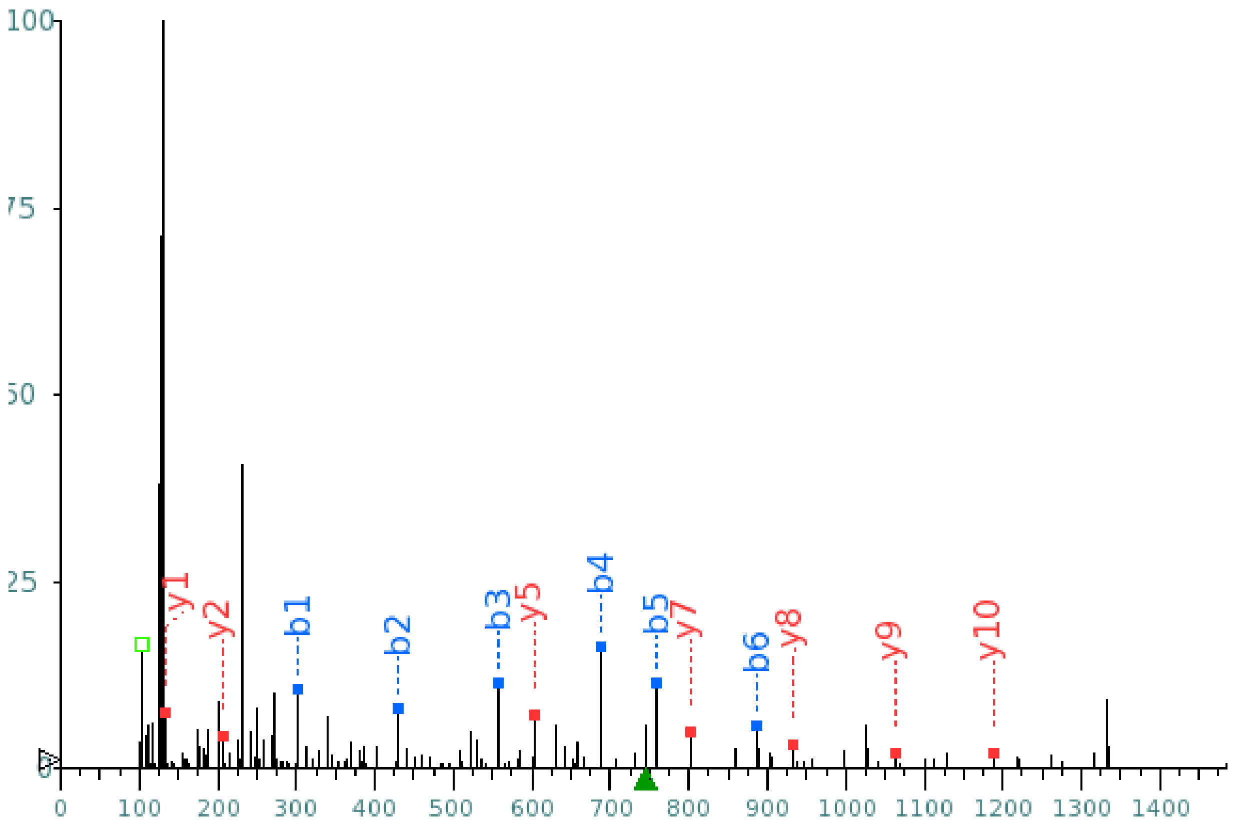

AQEEAERLEAD (+2)

Primary Reference: EZRI_HUMAN
Search ID: 41760
Search Name: 20130328_ananiav_ERstress_TMT_TM_timecourse_trypsin_inj1_4mc_iaa
Scan#: 7470
Observed Mass: 745.3704 (0.3 ppm)
PSM Score: 35.93

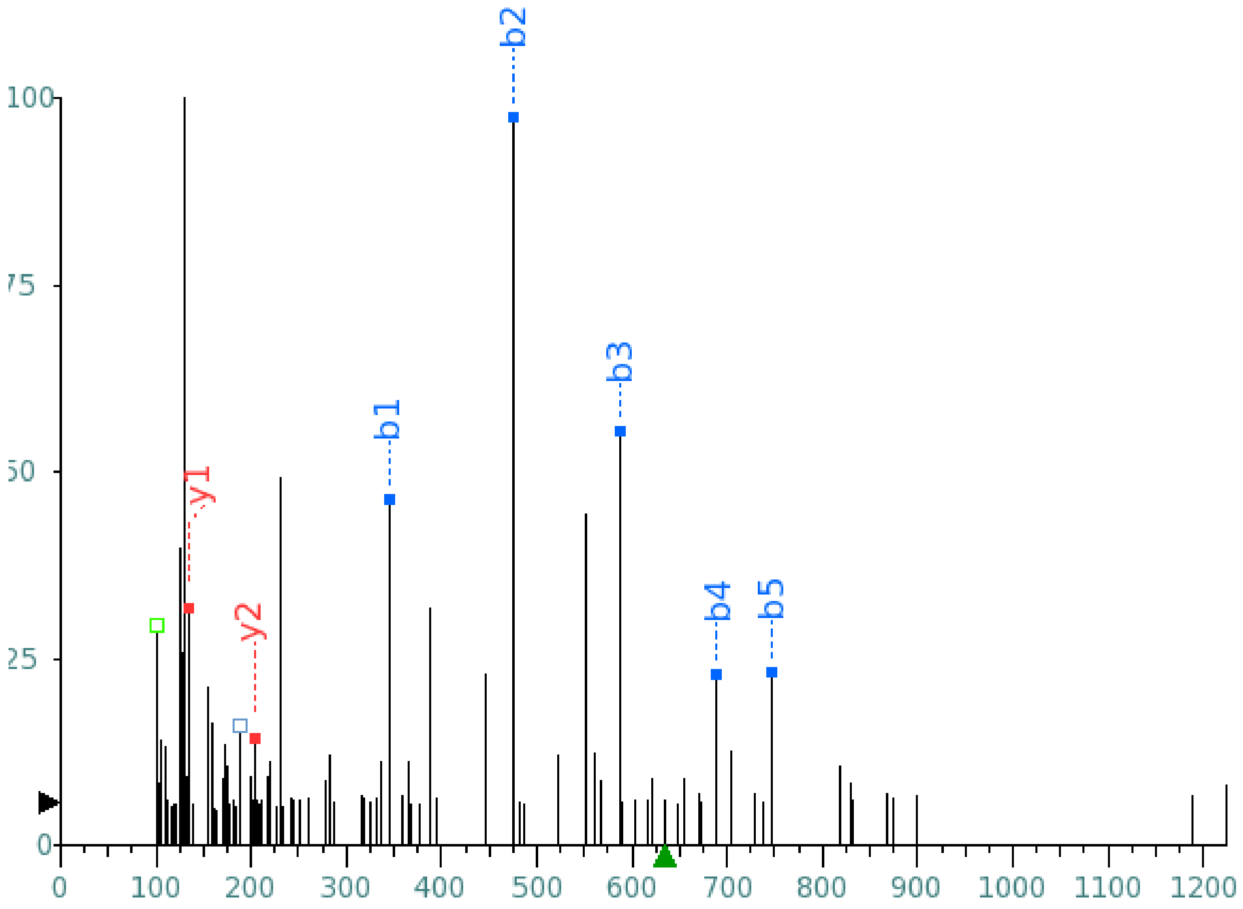

NMITGTSQAD (+2)

Primary Reference: EF1A1_HUMAN
Search ID: 41760
Search Name: 20130328_ananiav_ERstress_TMT_TM_timecourse_trypsin_inj1_4mc_iaa
Scan#: 7475
Observed Mass: 633.8161 (4.1 ppm)
PSM Score: 29.28

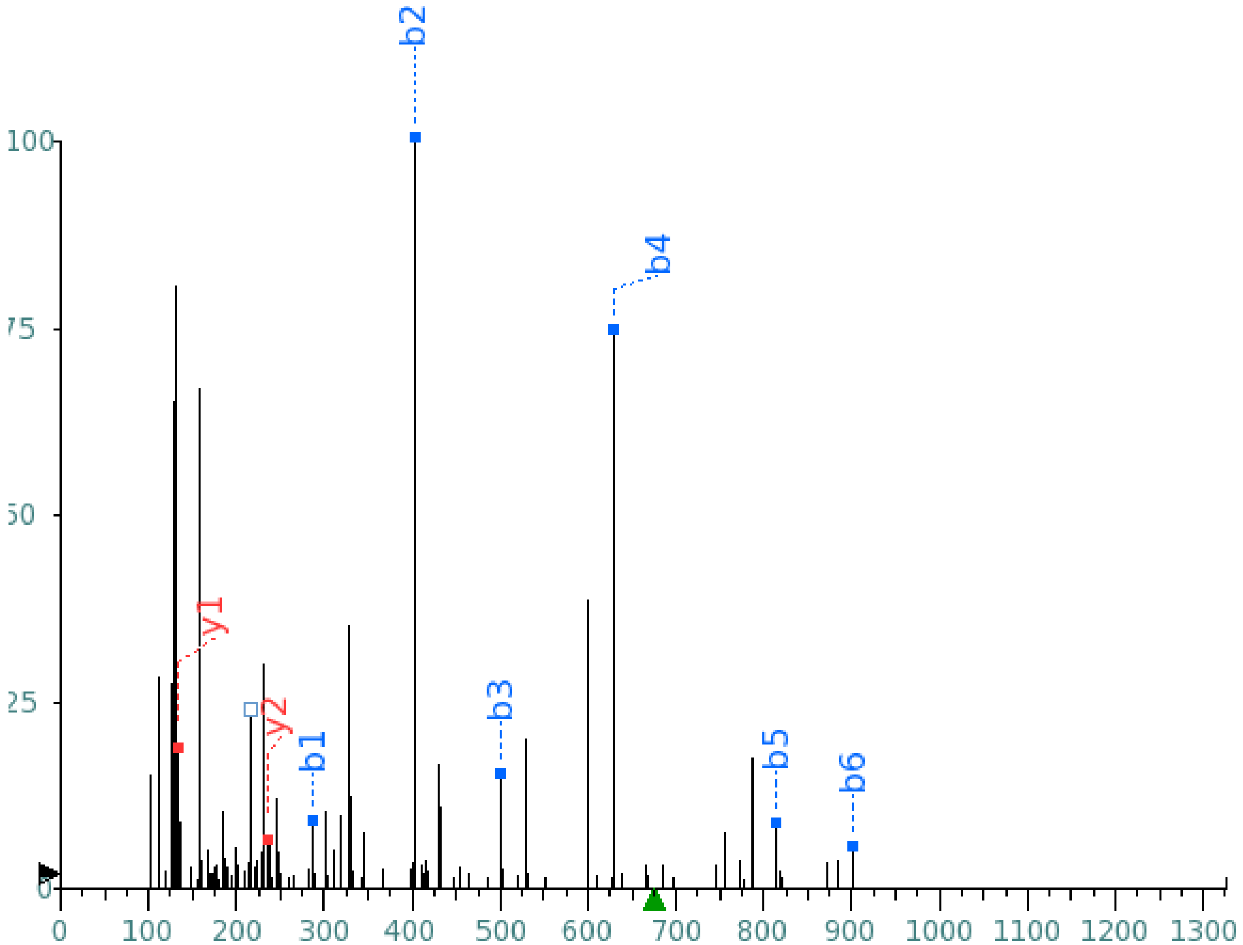

GDPEWSSETD (+2)

Primary Reference: T179B_HUMAN
Search ID: 41760
Search Name: 20130328_ananiav_ERstress_TMT_TM_timecourse_trypsin_inj1_4mc_iaa
Scan#: 7560
Observed Mass: 676.2973 (1.6 ppm)
PSM Score: 11.54

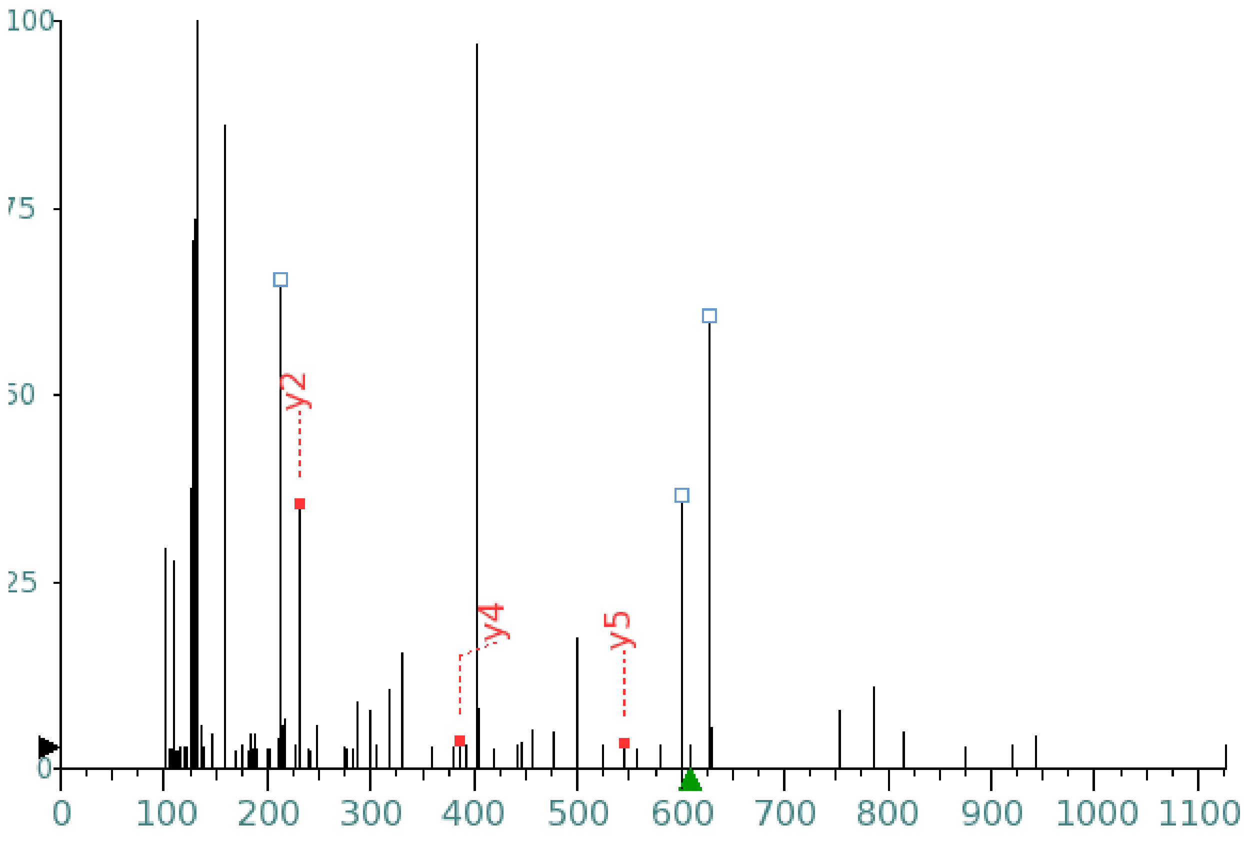

DIDTC*GPPD (+2)

Primary Reference: ##RRP44_HUMAN
Search ID: 41760
Search Name: 20130328_ananiav_ERstress_TMT_TM_timecourse_trypsin_inj1_4mc_iaa
Scan#: 7598
Observed Mass: 609.7784 (-1.2 ppm)
PSM Score: 10.18

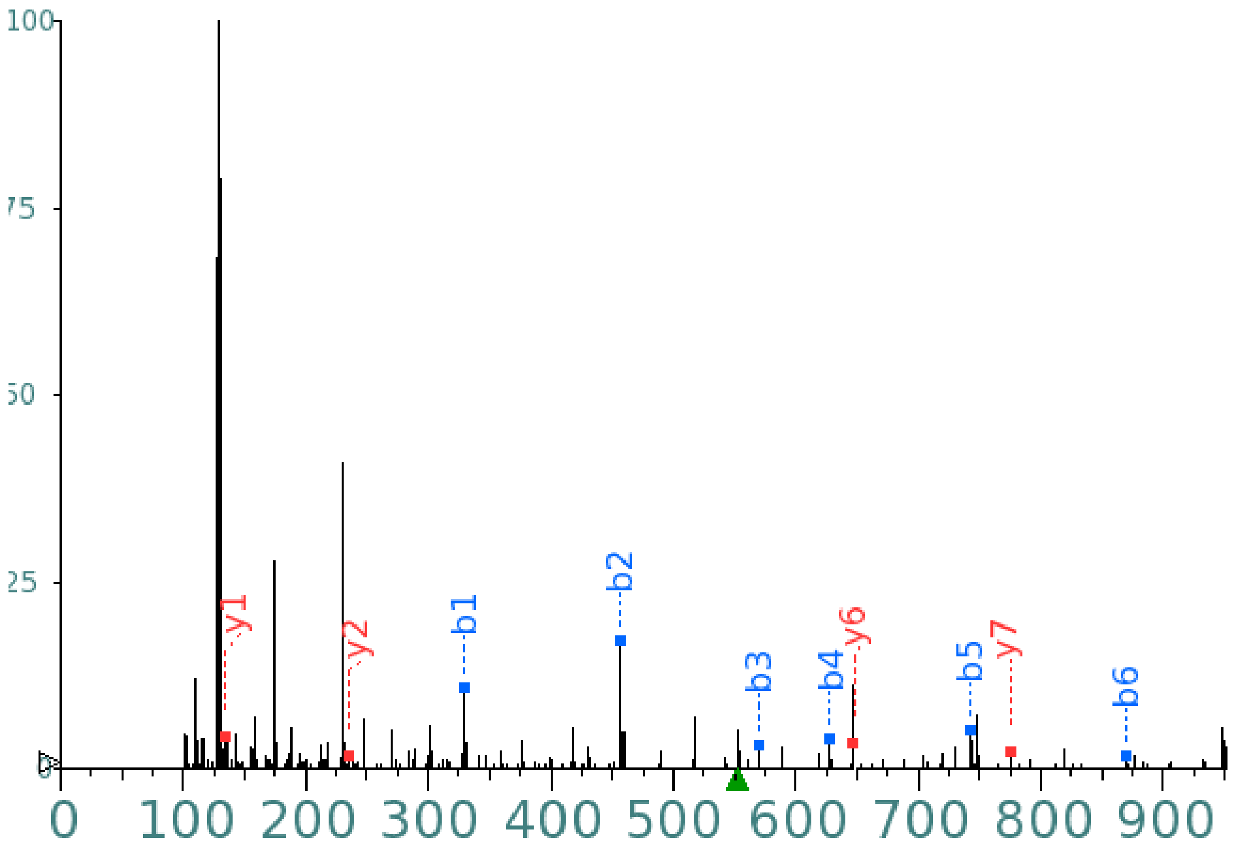

VQLGDQTD (+2)

Primary Reference: MDN1_HUMAN
Search ID: 41760
Search Name: 20130328_ananiav_ERstress_TMT_TM_timecourse_trypsin_inj1_4mc_iaa
Scan#: 7656
Observed Mass: 552.7915 (2.1 ppm)
PSM Score: 10.45

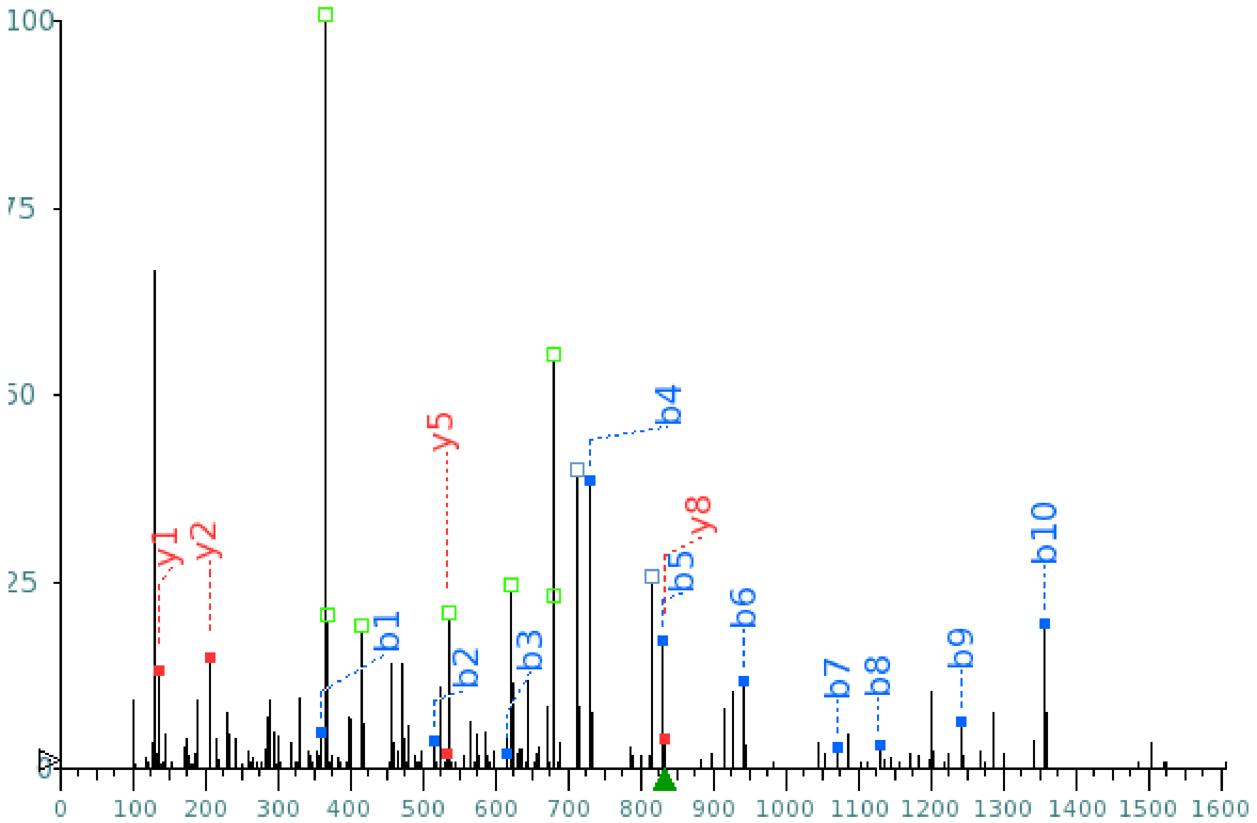

ERTDVIQGDDVAD (+2)

Primary Reference: RBP2_HUMAN
Search ID: 41760
Search Name: 20130328_ananiav_ERstress_TMT_TM_timecourse_trypsin_inj1_4mc_iaa
Scan#: 7695
Observed Mass: 831.4139 (1.5 ppm)
PSM Score: 22.54

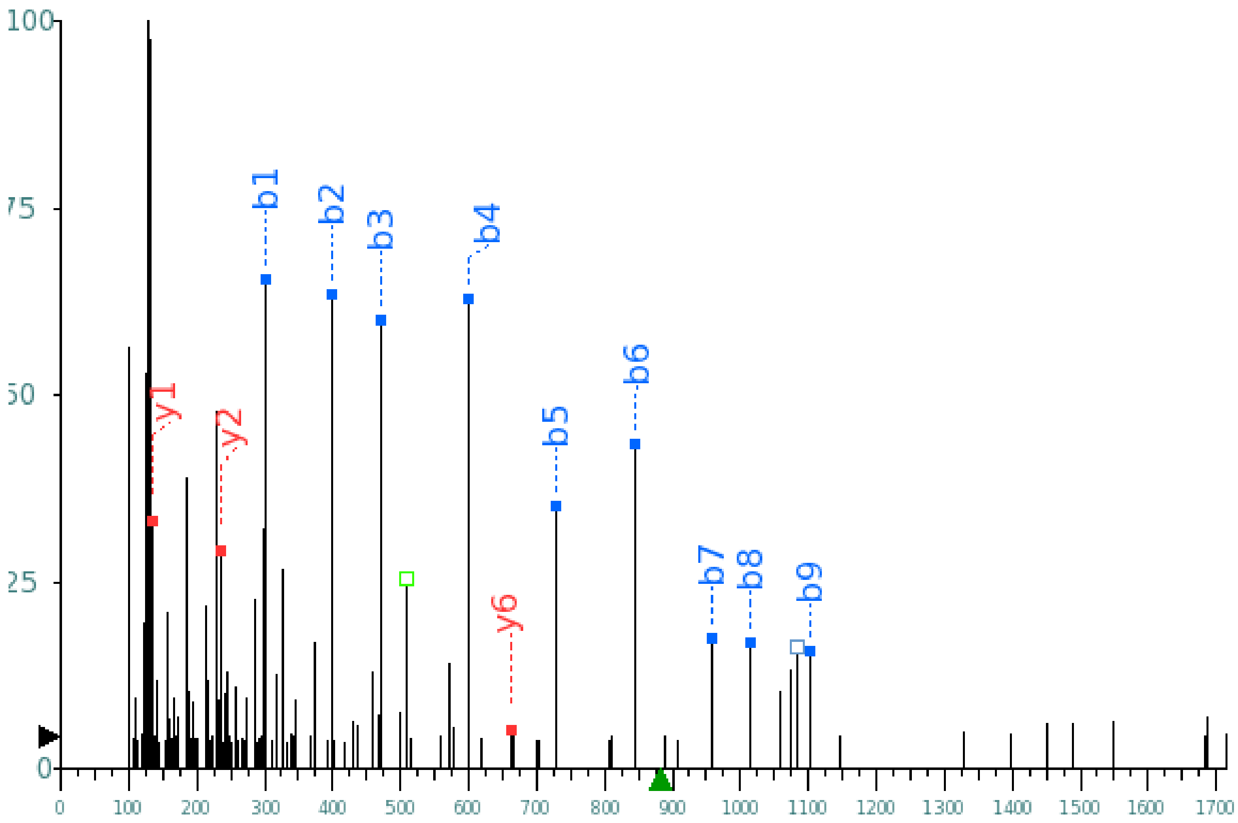

AVAEEDNGSIGEETD (+2)

Primary Reference: STIM1_HUMAN
Search ID: 41760
Search Name: 20130328_ananiav_ERstress_TMT_TM_timecourse_trypsin_inj1_4mc_iaa
Scan#: 7711
Observed Mass: 882.9038 (1.7 ppm)
PSM Score: 46.64

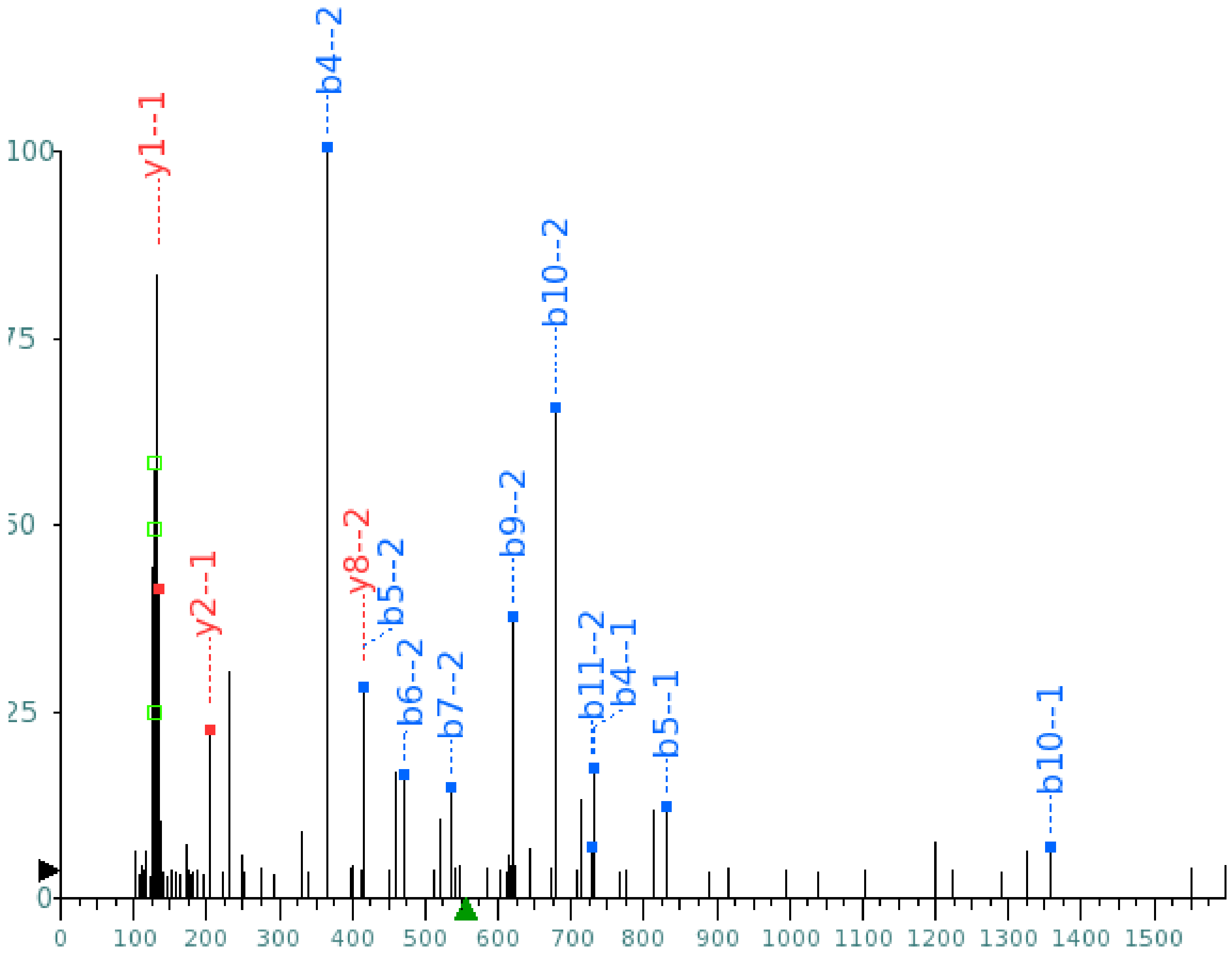

ERTDVIQGDDVAD (+3)

Primary Reference: RBP2_HUMAN
Search ID: 41760
Search Name: 20130328_ananiav_ERstress_TMT_TM_timecourse_trypsin_inj1_4mc_iaa
Scan#: 7719
Observed Mass: 554.6118 (1.6 ppm)
PSM Score: 24.39

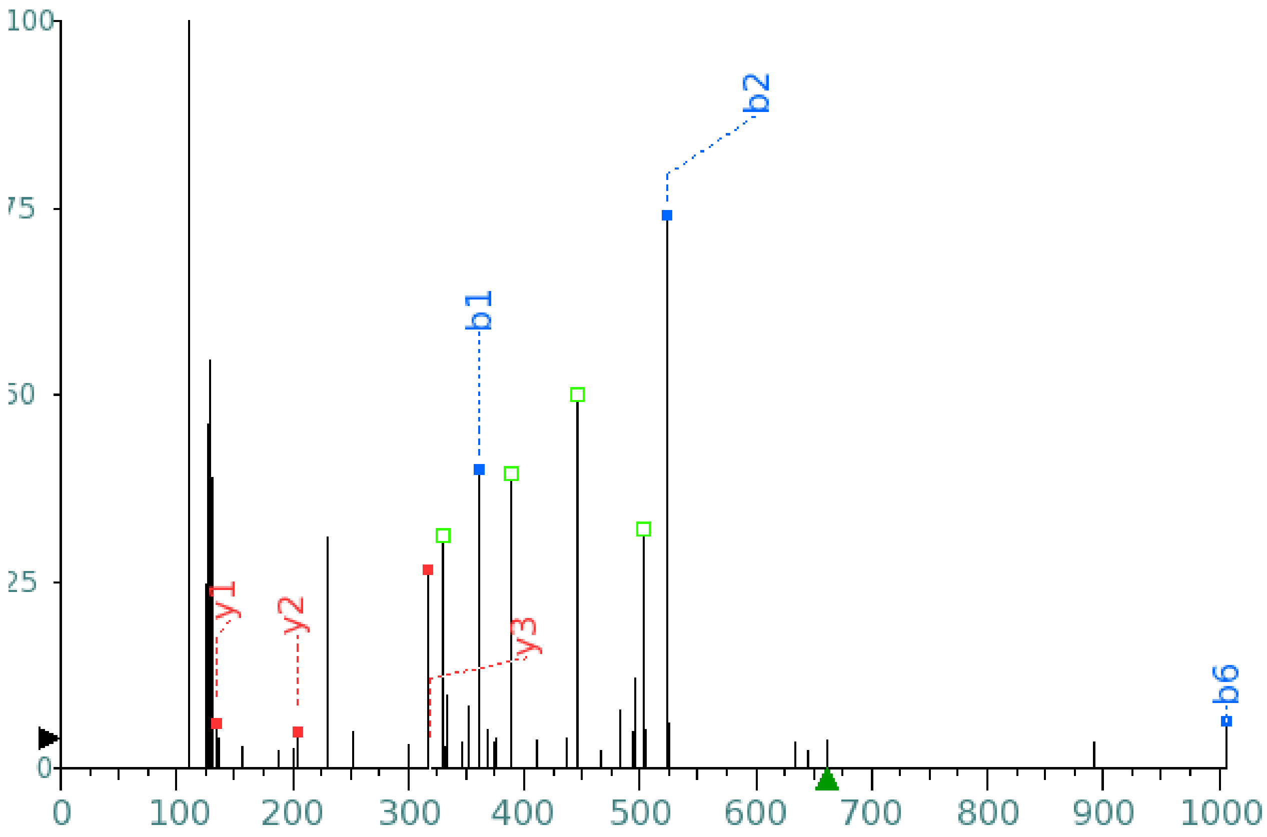

MYHDDDLAD (+2)

Primary Reference: MAP4_HUMAN
Search ID: 41760
Search Name: 20130328_ananiav_ERstress_TMT_TM_timecourse_trypsin_inj1_4mc_iaa
Scan#: 7746
Observed Mass: 662.2915 (2.5 ppm)
PSM Score: 31.03

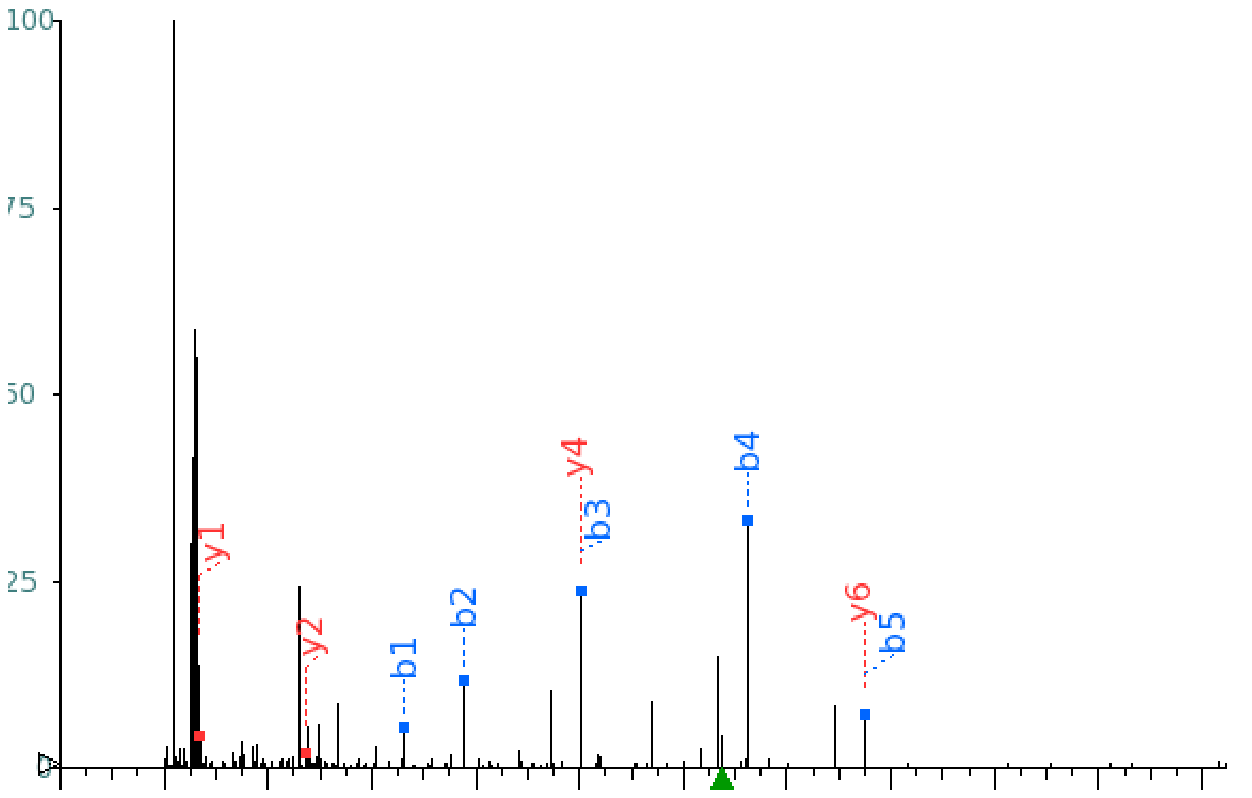

TGIC*IHETD (+2)

Primary Reference: UNK_HUMAN
Search ID: 41760
Search Name: 20130328_ananiav_ERstress_TMT_TM_timecourse_trypsin_inj1_4mc_iaa
Scan#: 7759
Observed Mass: 637.8169 (1.3 ppm)
PSM Score: 12.32

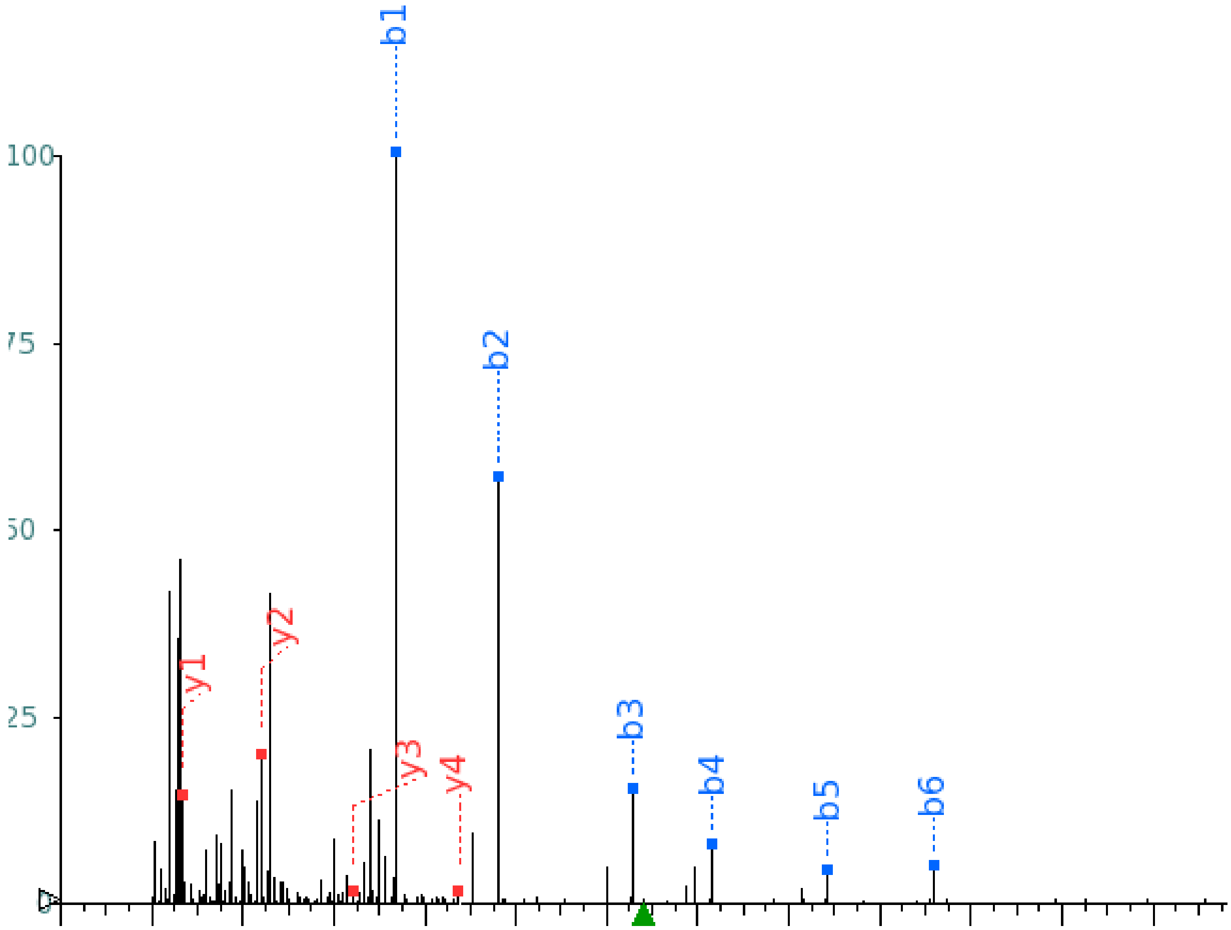

HIFSEDTSD (+2)

Primary Reference: SPB5_HUMAN
Search ID: 41760
Search Name: 20130328_ananiav_ERstress_TMT_TM_timecourse_trypsin_inj1_4mc_iaa
Scan#: 7764
Observed Mass: 640.3052 (2.2 ppm)
PSM Score: 35.08

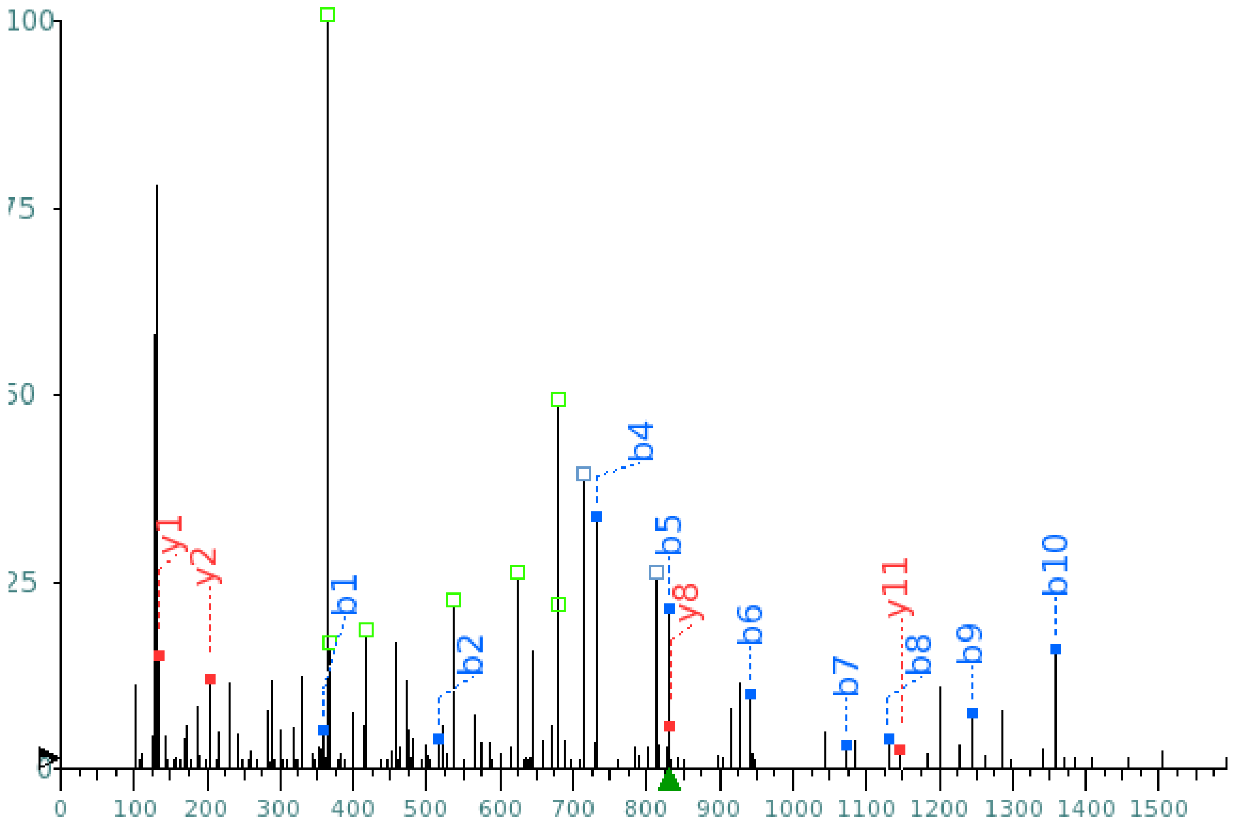

ERTDVIQGDDVAD (+2)

Primary Reference: RBP2_HUMAN
Search ID: 41760
Search Name: 20130328_ananiav_ERstress_TMT_TM_timecourse_trypsin_inj1_4mc_iaa
Scan#: 7785
Observed Mass: 831.414 (1.6 ppm)
PSM Score: 24.98

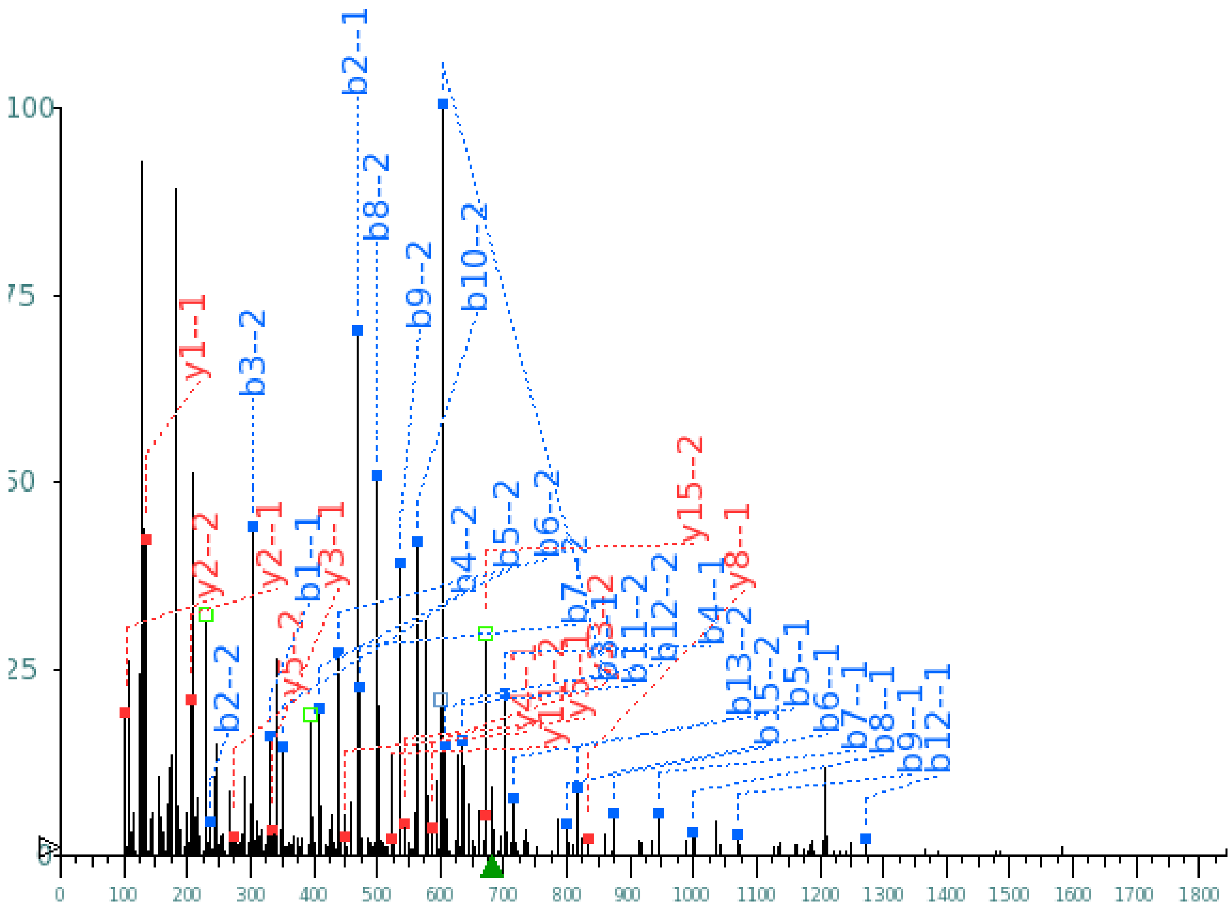

THHPLGAGAGSGC*APLEAD (+3)

Primary Reference: SC16A_HUMAN
Search ID: 41760
Search Name: 20130328_ananiav_ERstress_TMT_TM_timecourse_trypsin_inj1_4mc_iaa
Scan#: 7789
Observed Mass: 683.0018 (2.1 ppm)
PSM Score: 52.33

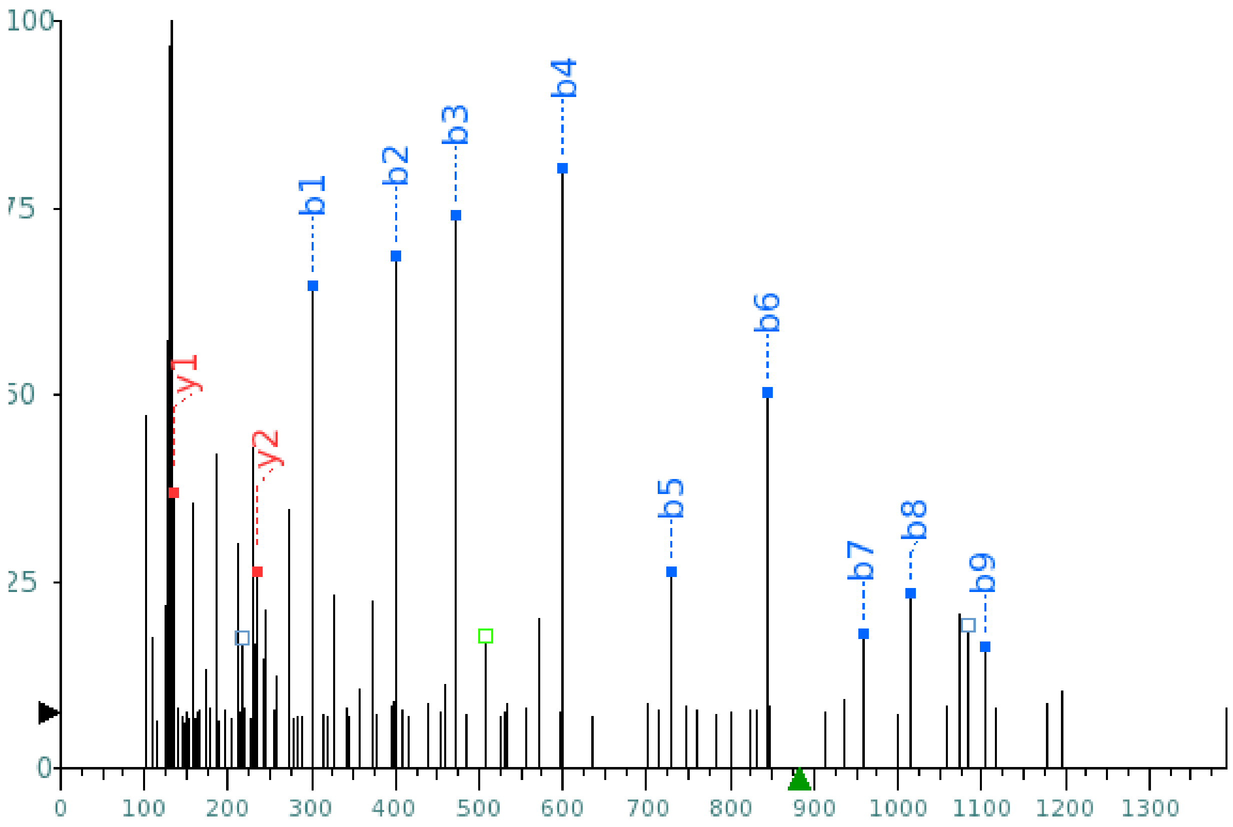

AVAEEDNGSIGEETD (+2)

Primary Reference: STIM1_HUMAN
Search ID: 41760
Search Name: 20130328_ananiav_ERstress_TMT_TM_timecourse_trypsin_inj1_4mc_iaa
Scan#: 7796
Observed Mass: 882.9039 (1.8 ppm)
PSM Score: 73.87

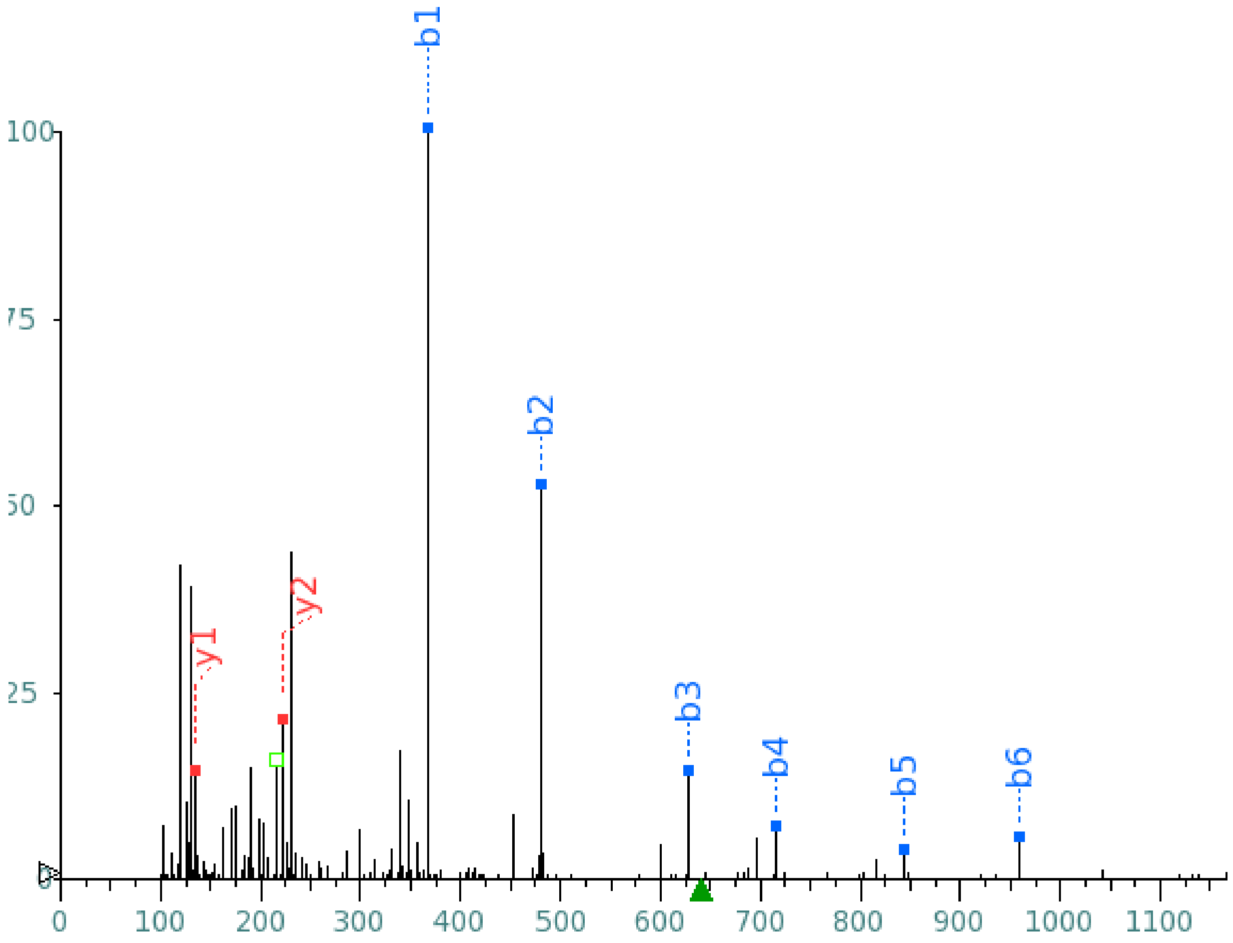

HIFSEDTSD (+2)

Primary Reference: SPB5_HUMAN
Search ID: 41760
Search Name: 20130328_ananiav_ERstress_TMT_TM_timecourse_trypsin_inj1_4mc_iaa
Scan#: 7850
Observed Mass: 640.3051 (2.1 ppm)
PSM Score: 40.95

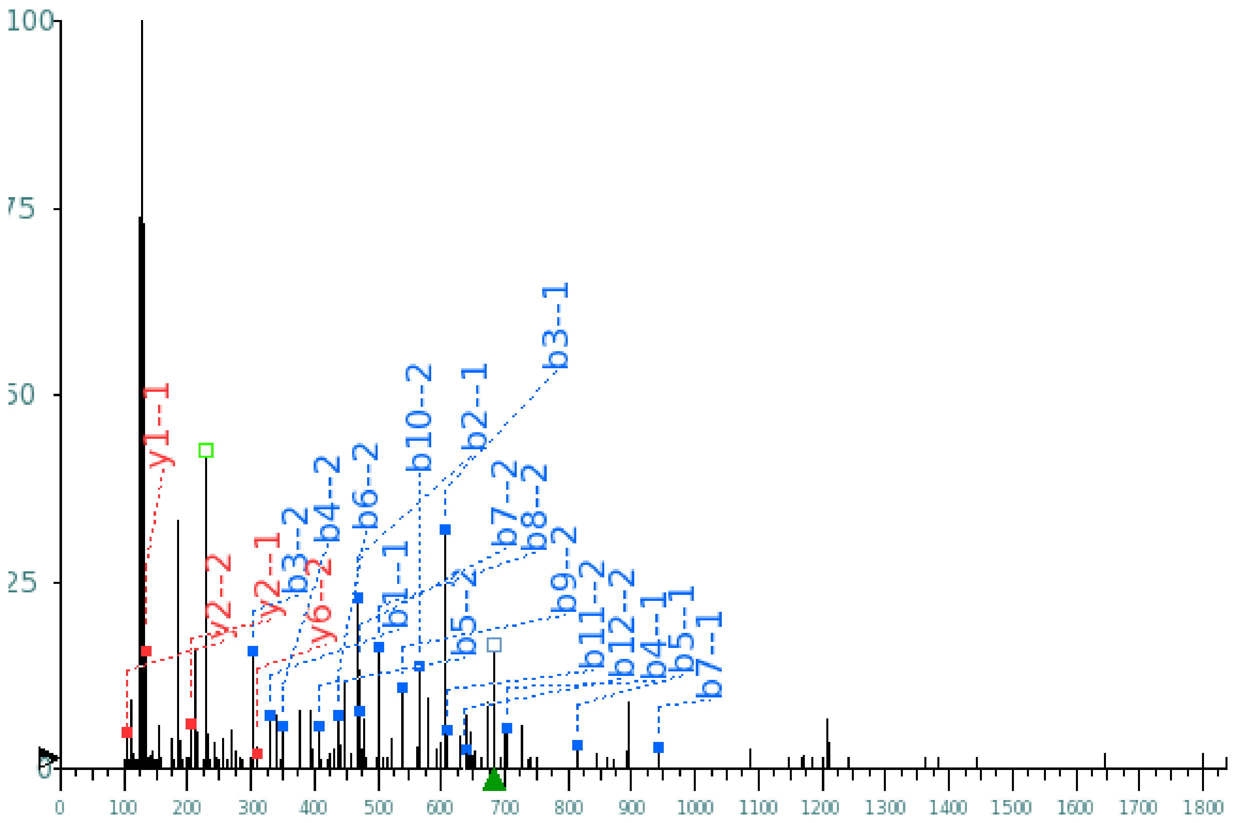

THHPLGAGAGSGC*APLEAD (+3)

Primary Reference: SC16A_HUMAN
Search ID: 41760
Search Name: 20130328_ananiav_ERstress_TMT_TM_timecourse_trypsin_inj1_4mc_iaa
Scan#: 7876
Observed Mass: 683.0017 (1.9 ppm)
PSM Score: 17.98

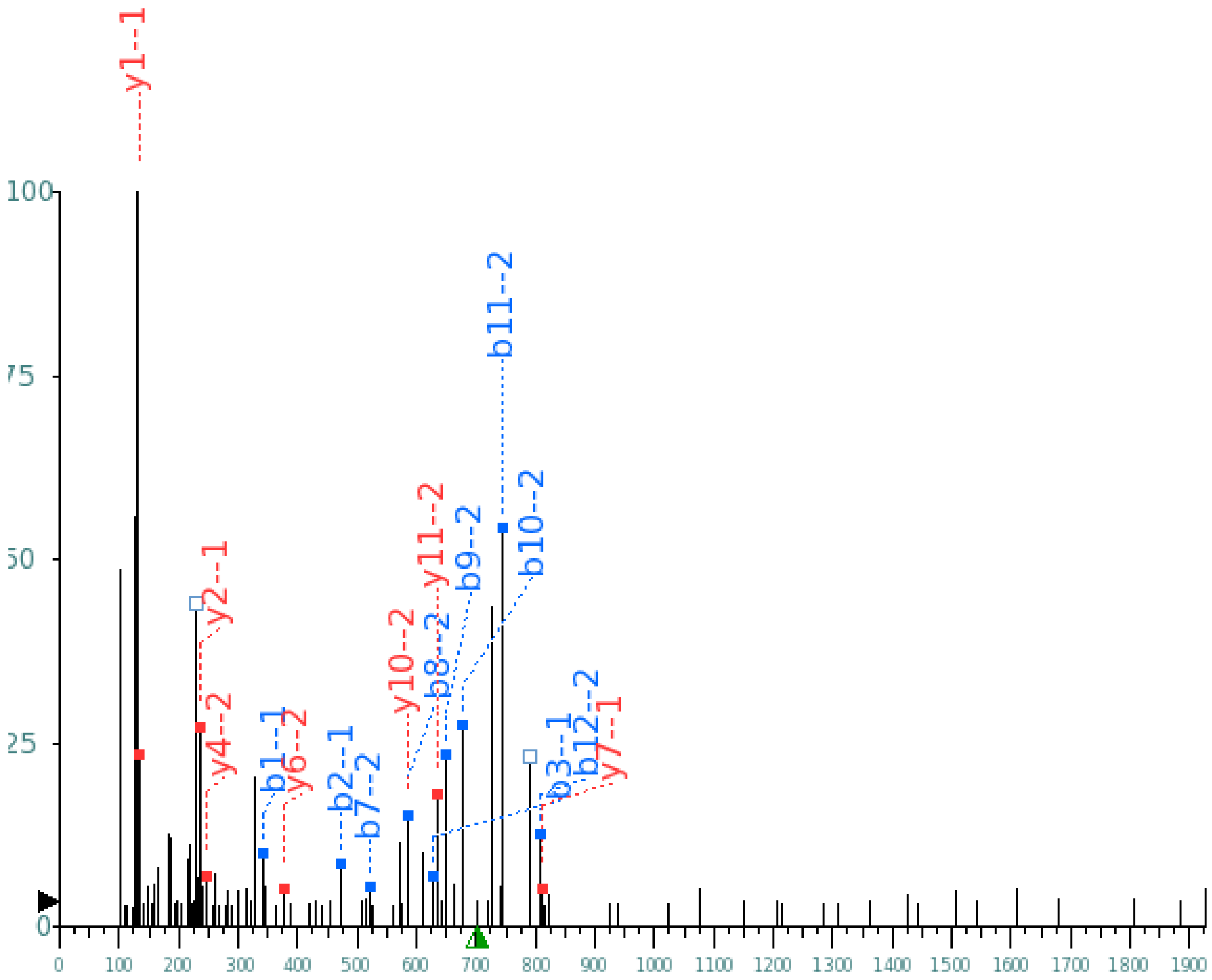

IQRPNTTQEGEEMETD (+3)

Primary Reference: HUWE1_HUMAN
Search ID: 41760
Search Name: 20130328_ananiav_ERstress_TMT_TM_timecourse_trypsin_inj1_4mc_iaa
Scan#: 7929
Observed Mass: 702.9997 (1.6 ppm)
PSM Score: 11.33

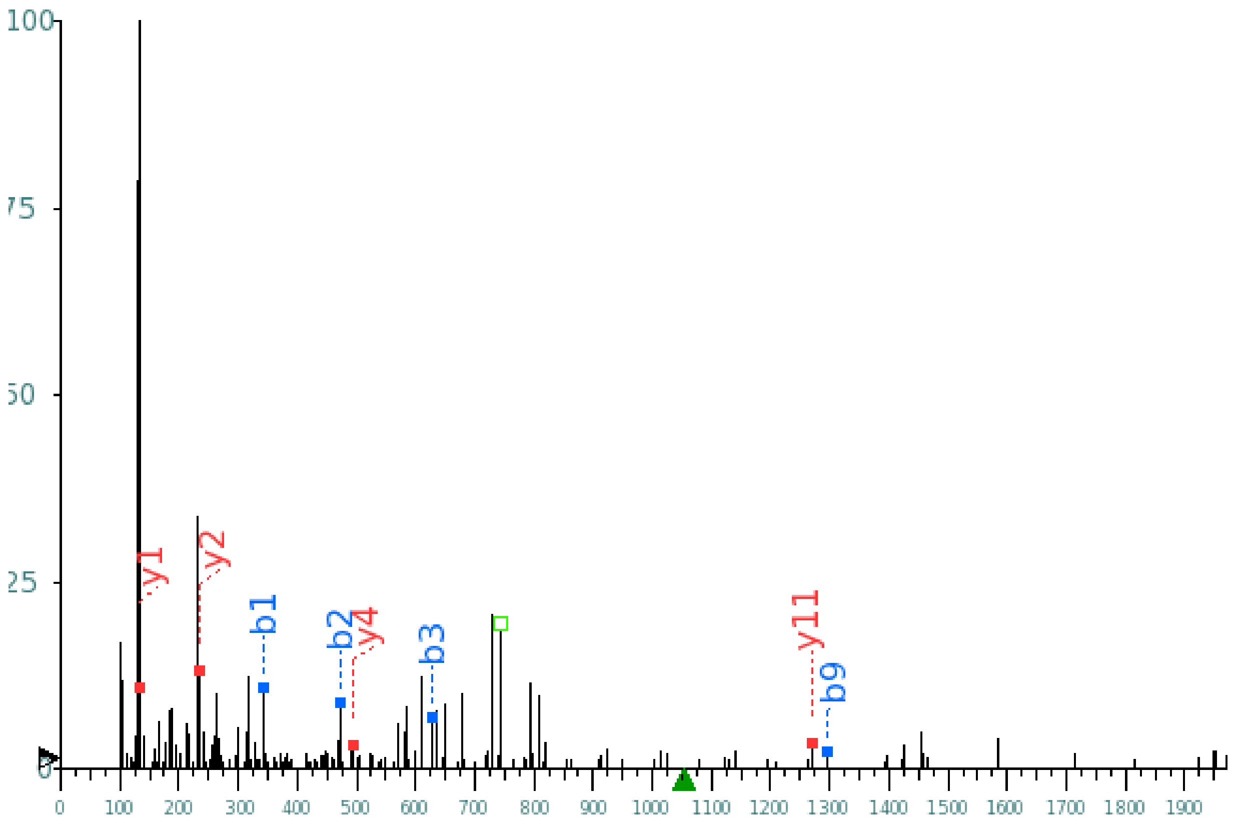

IQRPNTTQEGEEMETD (+2)

Primary Reference: HUWE1_HUMAN
Search ID: 41760
Search Name: 20130328_ananiav_ERstress_TMT_TM_timecourse_trypsin_inj1_4mc_iaa
Scan#: 7950
Observed Mass: 1053.9946 (0.4 ppm)
PSM Score: 9

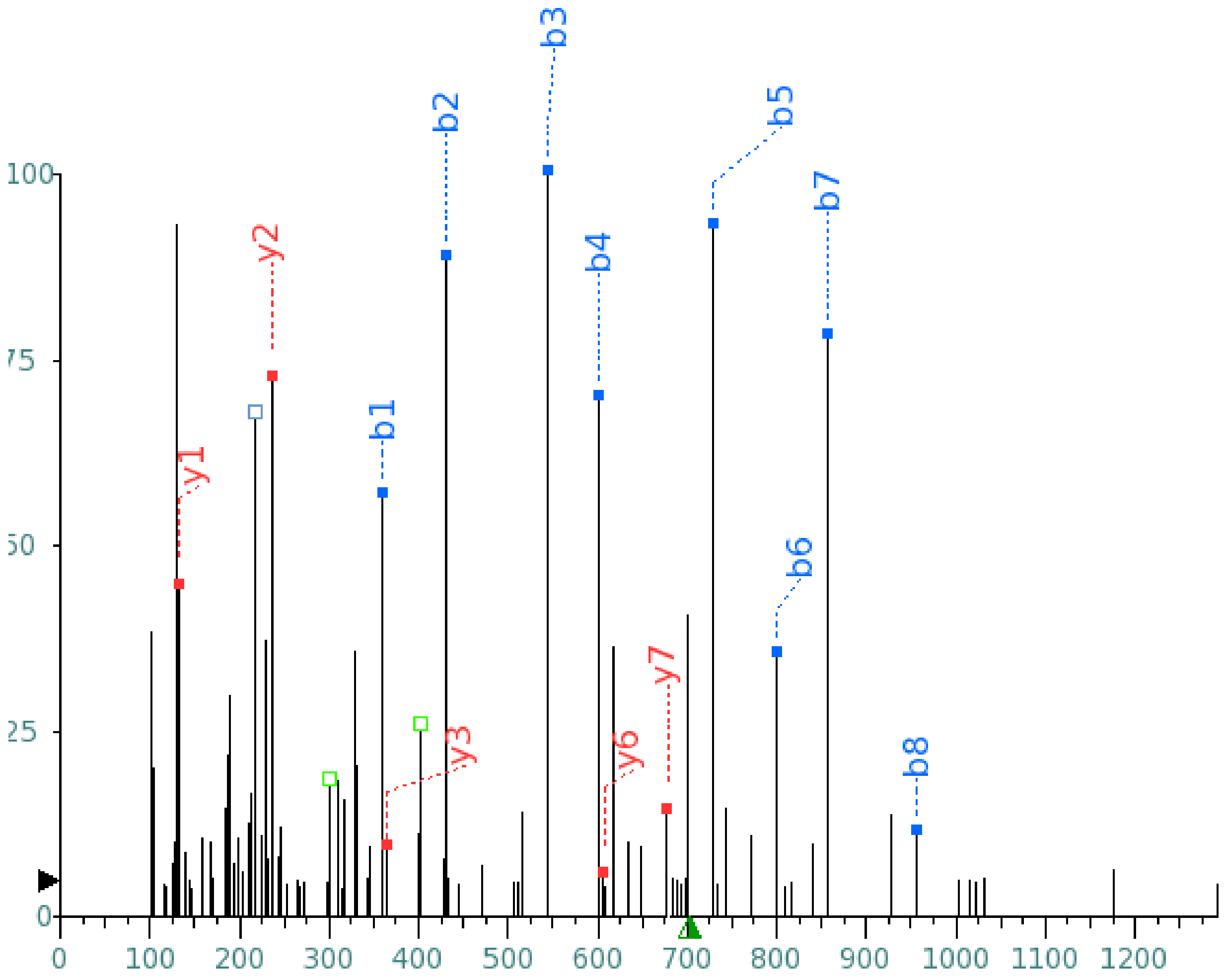

EALGQAGVSETD (+2)

Primary Reference: LIPA1_HUMAN
Search ID: 41760
Search Name: 20130328_ananiav_ERstress_TMT_TM_timecourse_trypsin_inj1_4mc_iaa
Scan#: 7992
Observed Mass: 703.3551 (1.4 ppm)
PSM Score: 44.3

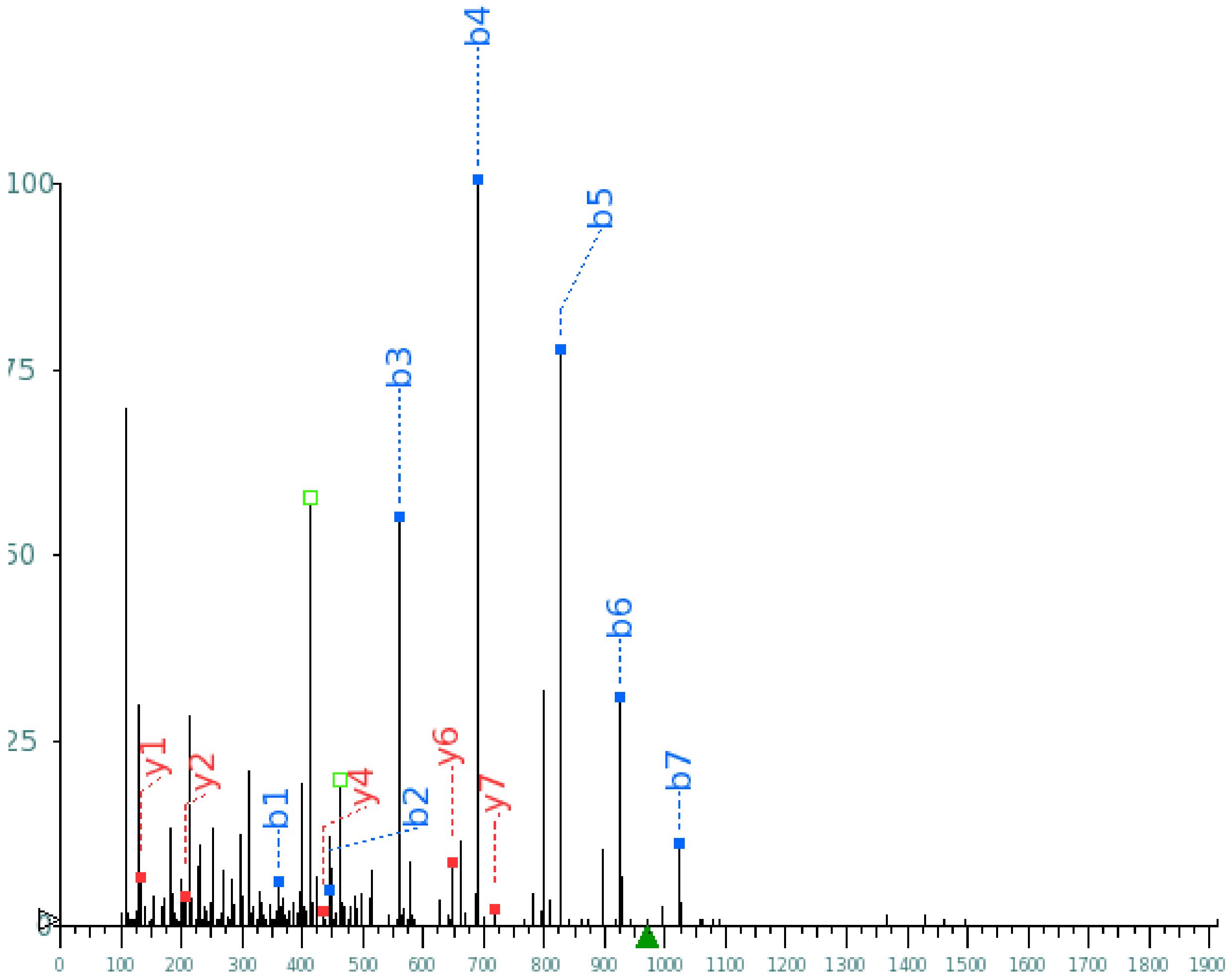

ESDQHVVPTAPDTEAD (+2)

Primary Reference: PIEZ1_HUMAN
Search ID: 41760
Search Name: 20130328_ananiav_ERstress_TMT_TM_timecourse_trypsin_inj1_4mc_iaa
Scan#: 8064
Observed Mass: 970.4614 (3.8 ppm)
PSM Score: 21.08

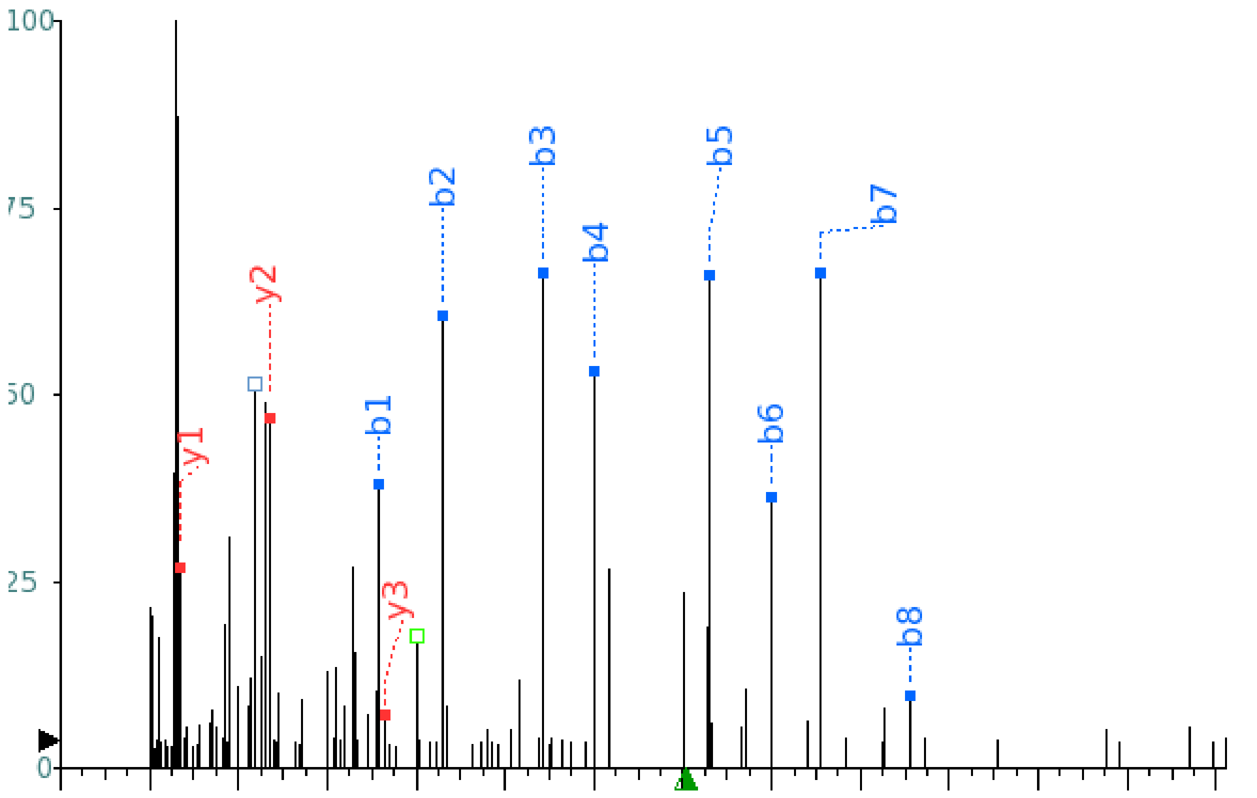

EALGQAGVSETD (+2)

Primary Reference: LIPA1_HUMAN
Search ID: 41760
Search Name: 20130328_ananiav_ERstress_TMT_TM_timecourse_trypsin_inj1_4mc_iaa
Scan#: 8082
Observed Mass: 703.3559 (2.6 ppm)
PSM Score: 44.51

File name: 2015-02-04 04.01.53PM.pdf

Creation time: 2015-02-04 04:09PM

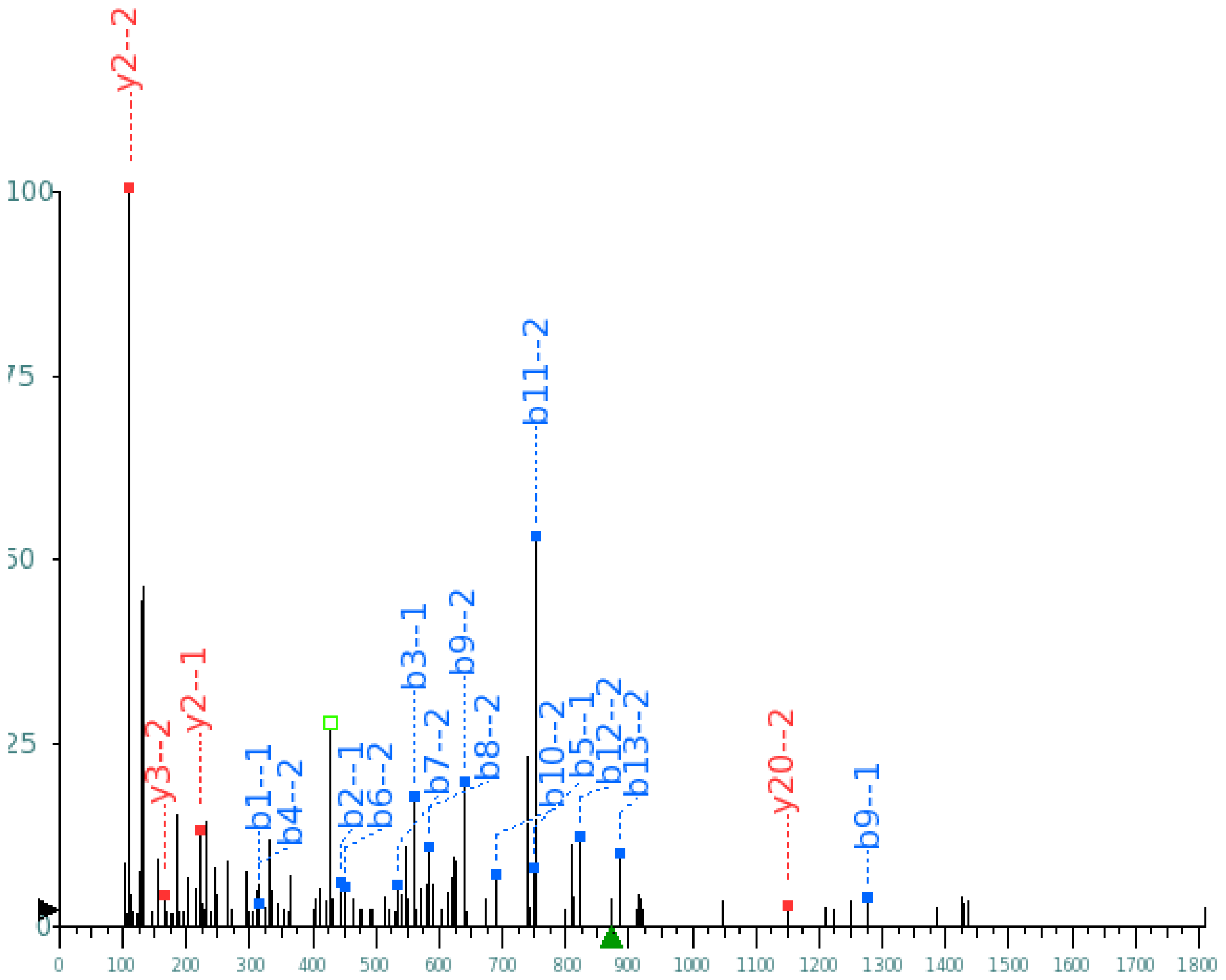

SEDADRC*TLPEHESPSQDISD (+3)

Primary Reference: LRRF1_HUMAN
Search ID: 41760
Search Name: 20130328_ananiav_ERstress_TMT_TM_timecourse_trypsin_inj1_4mc_iaa
Scan#: 8100
Observed Mass: 873.058 (2.8 ppm)
PSM Score: 20.28

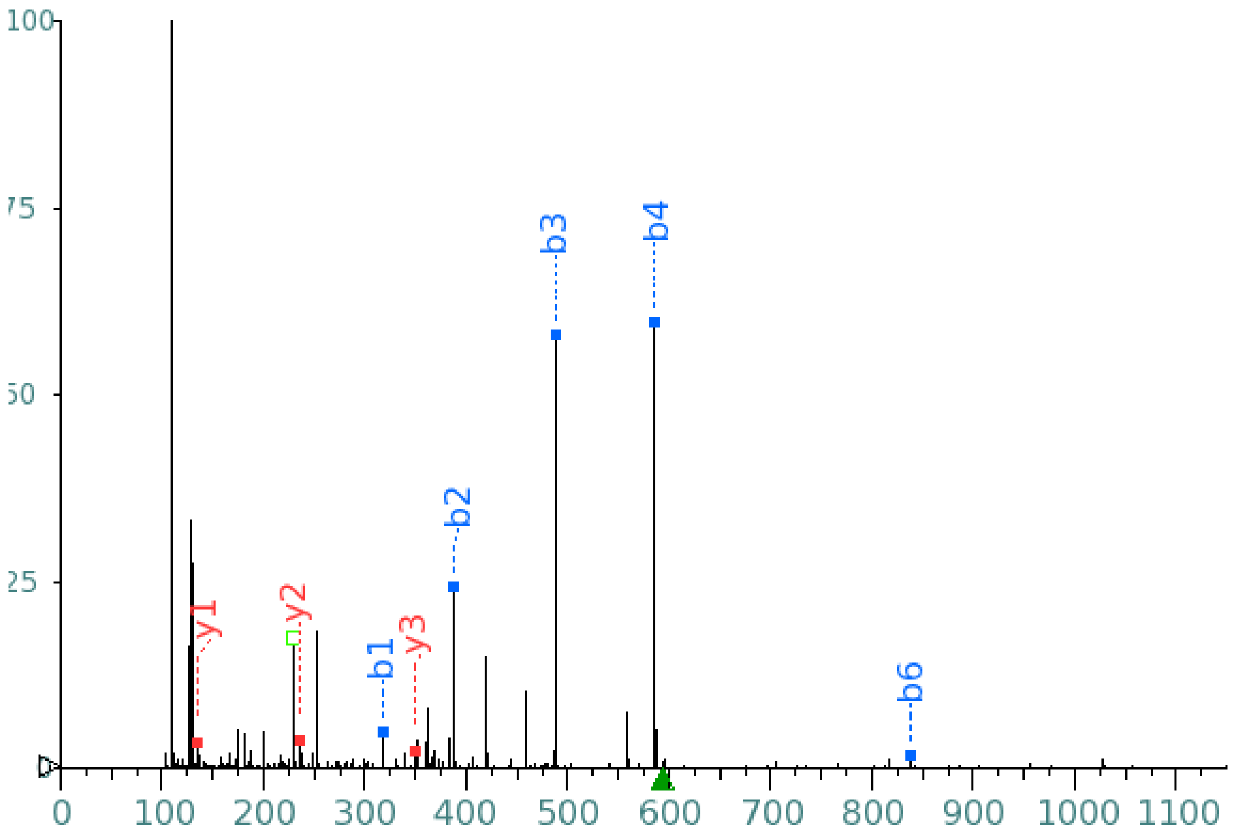

SAVVHDITD (+2)

Primary Reference: AKA12_HUMAN
Search ID: 41760
Search Name: 20130328_ananiav_ERstress_TMT_TM_timecourse_trypsin_inj1_4mc_iaa
Scan#: 8117
Observed Mass: 593.3205 (2.1 ppm)
PSM Score: 16.96

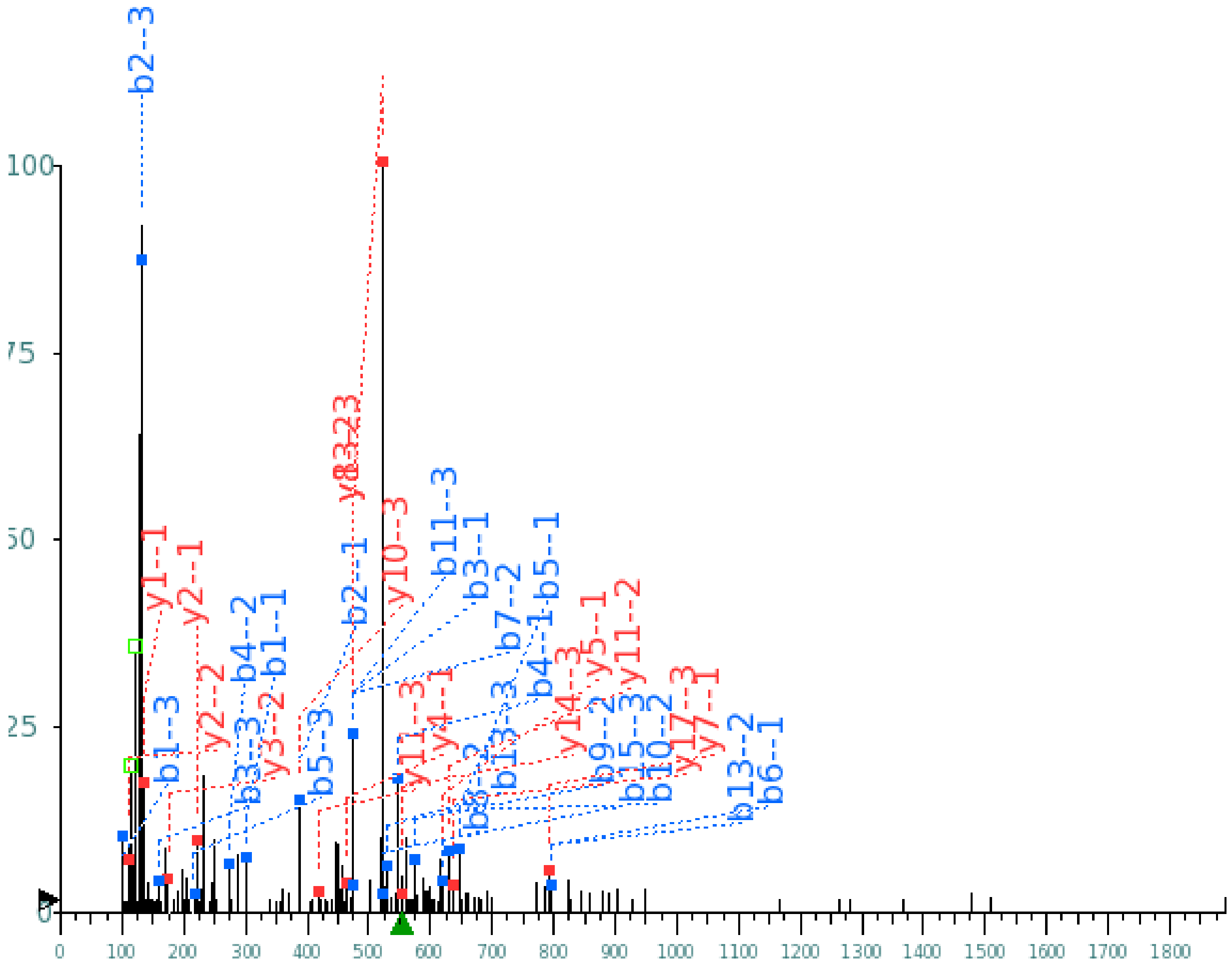

ASSATFRPVIRGDRDESD (+4)

Primary Reference: K0284_HUMAN
Search ID: 41760
Search Name: 20130328_ananiav_ERstress_TMT_TM_timecourse_trypsin_inj1_4mc_iaa
Scan#: 8147
Observed Mass: 552.7869 (2.4 ppm)
PSM Score: 26.25

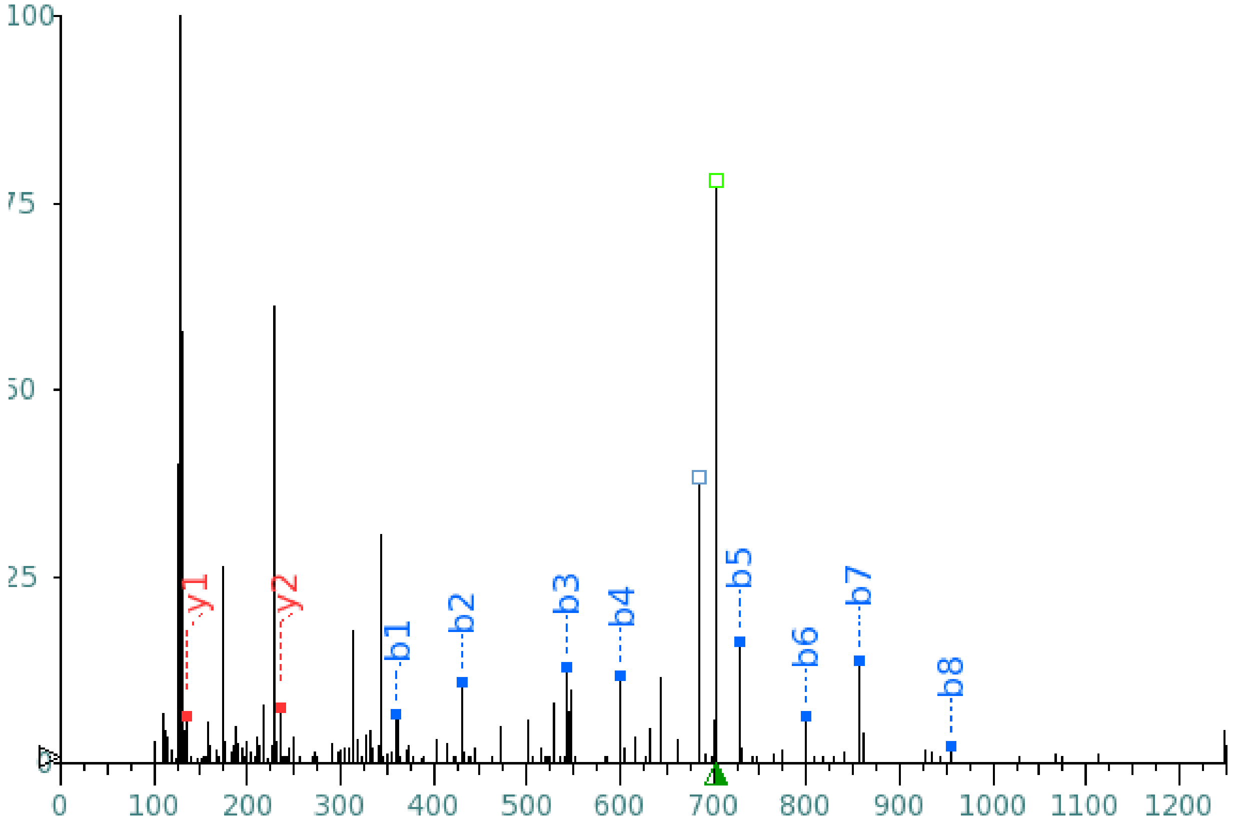

EALGQAGVSETD (+2)

Primary Reference: LIPA1_HUMAN
Search ID: 41760
Search Name: 20130328_ananiav_ERstress_TMT_TM_timecourse_trypsin_inj1_4mc_iaa
Scan#: 8173
Observed Mass: 703.3546 (0.7 ppm)
PSM Score: 20.83

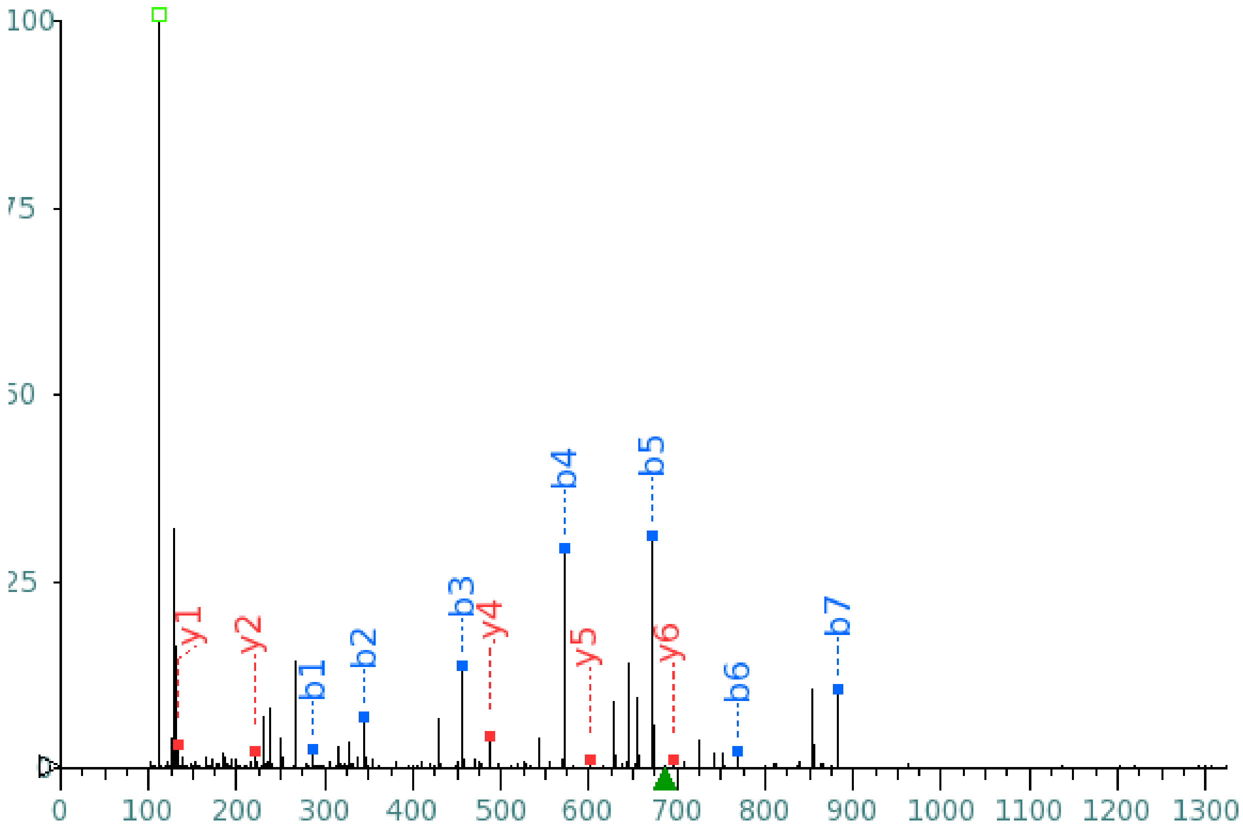

GGLNTPLHESD (+2)

Primary Reference: CDC5L_HUMAN
Search ID: 41760
Search Name: 20130328_ananiav_ERstress_TMT_TM_timecourse_trypsin_inj1_4mc_iaa
Scan#: 8181
Observed Mass: 684.8527 (1.7 ppm)
PSM Score: 12.47

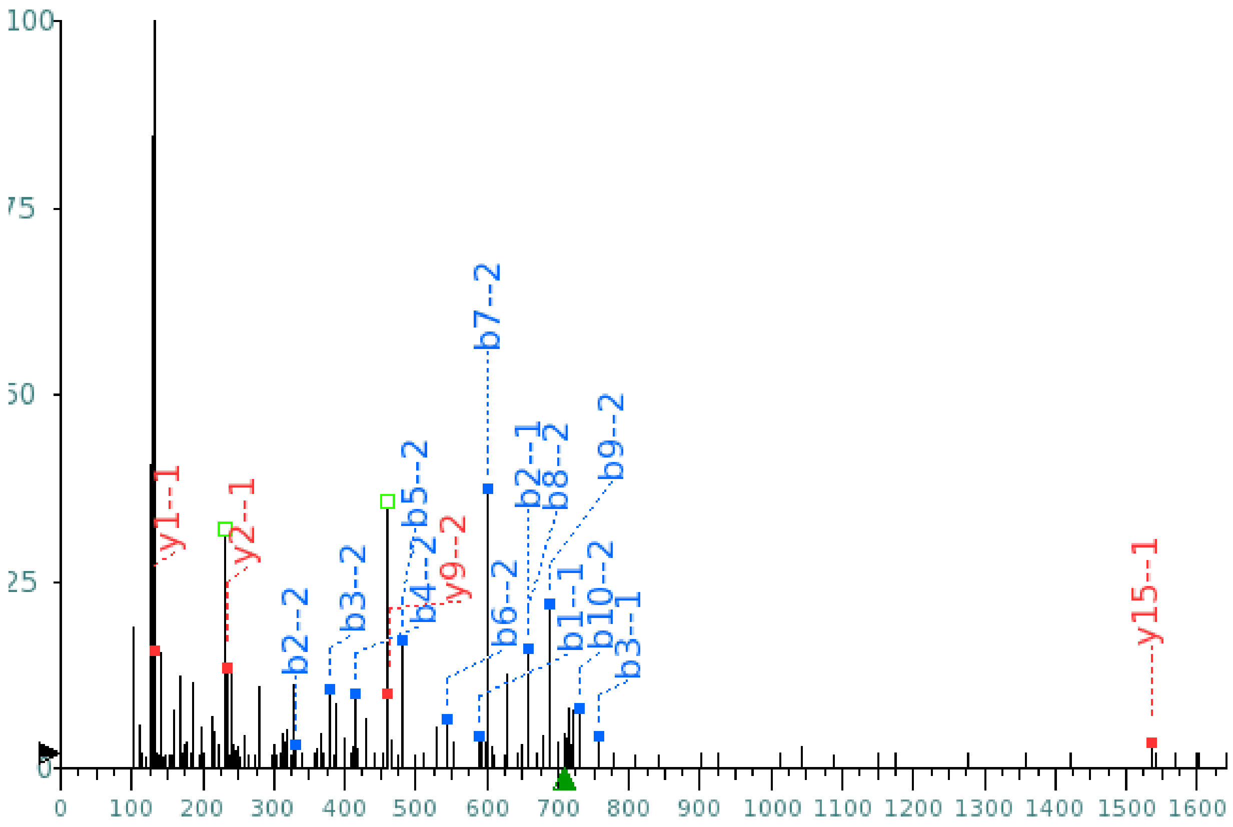

KAVAEEDNGSIGEETD (+3)

Primary Reference: STIM1_HUMAN
Search ID: 41760
Search Name: 20130328_ananiav_ERstress_TMT_TM_timecourse_trypsin_inj1_4mc_iaa
Scan#: 8225
Observed Mass: 708.0242 (1.4 ppm)
PSM Score: 20.4

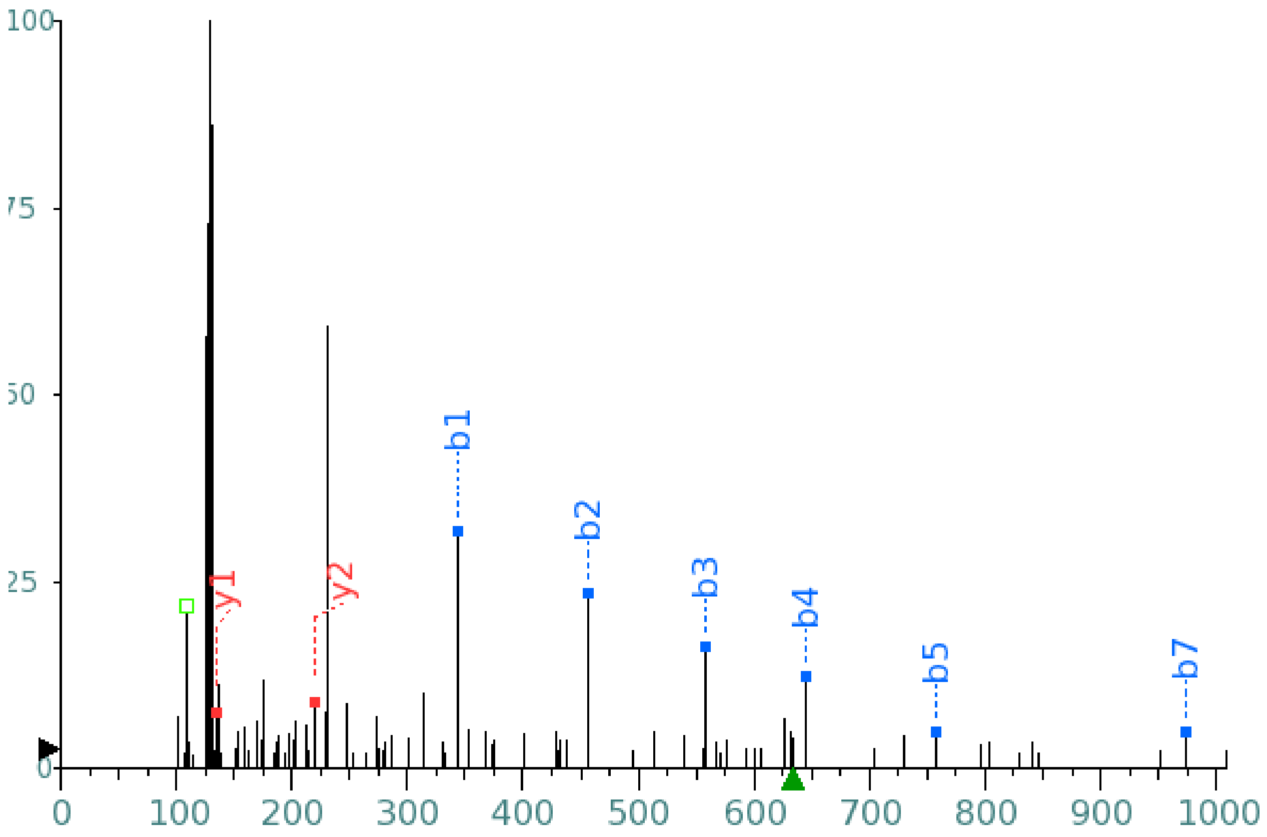

LITSNTDASD (+2)

Primary Reference: GOGB1_HUMAN
Search ID: 41760
Search Name: 20130328_ananiav_ERstress_TMT_TM_timecourse_trypsin_inj1_4mc_iaa
Scan#: 8247
Observed Mass: 633.3265 (2.8 ppm)
PSM Score: 39.63

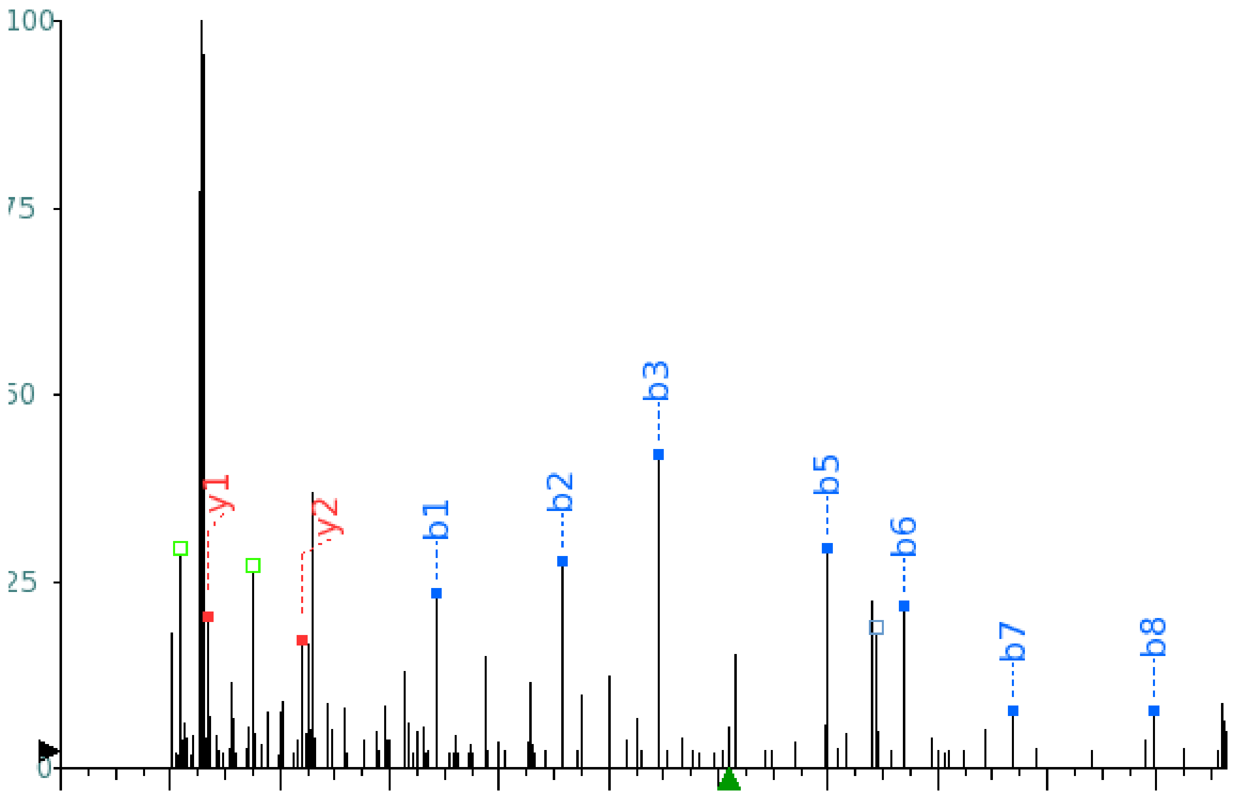

NLSPGAVESD (+2)

Primary Reference: COPA_HUMAN
Search ID: 41760
Search Name: 20130328_ananiav_ERstress_TMT_TM_timecourse_trypsin_inj1_4mc_iaa
Scan#: 8302
Observed Mass: 609.3148 (0.9 ppm)
PSM Score: 37.72

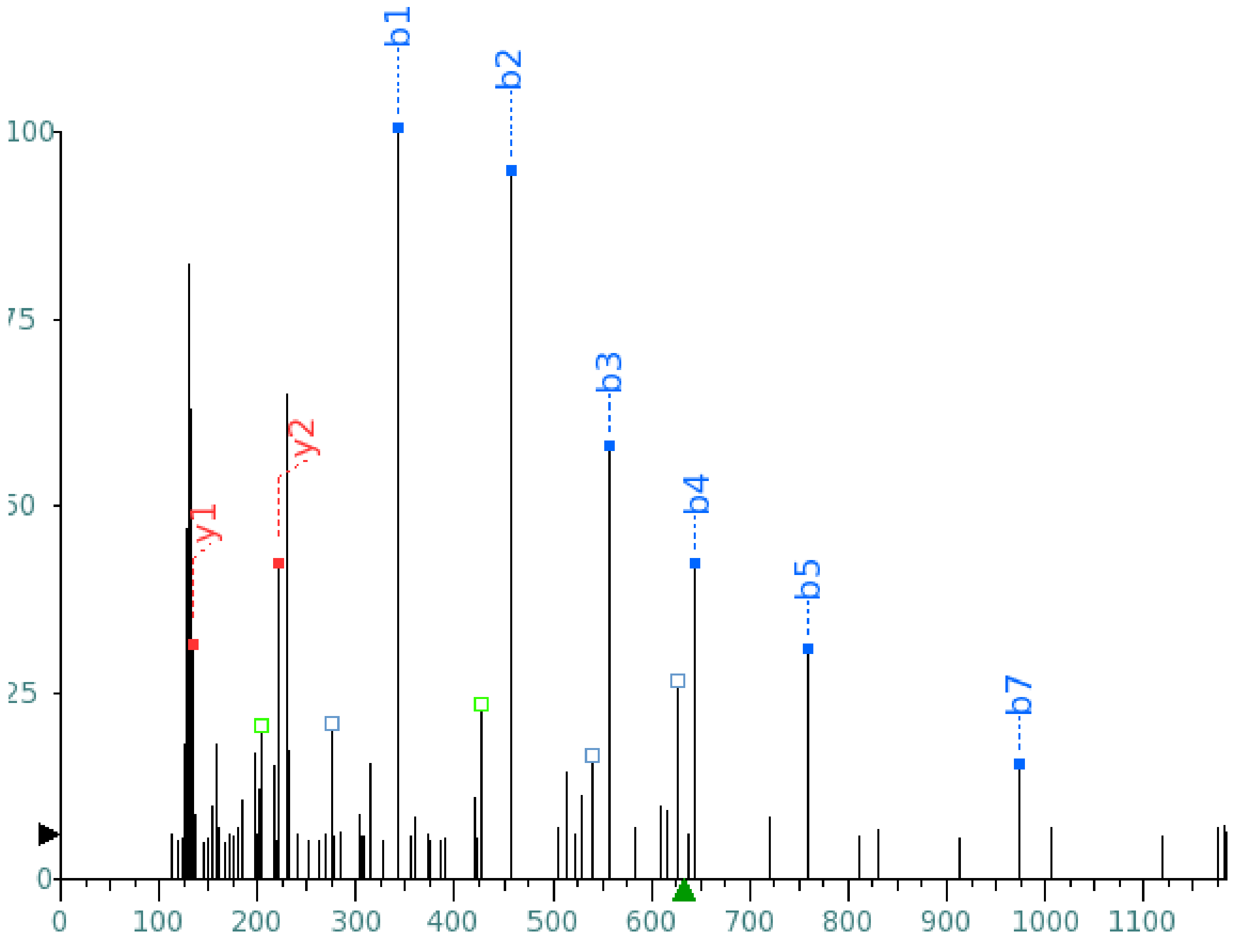

LITSNTDASD (+2)

Primary Reference: GOGB1_HUMAN
Search ID: 41760
Search Name: 20130328_ananiav_ERstress_TMT_TM_timecourse_trypsin_inj1_4mc_iaa
Scan#: 8322
Observed Mass: 633.3264 (2.5 ppm)
PSM Score: 40.29

File name: 2015-02-04 04.01.53PM.pdf

Creation time: 2015-02-04 04:09PM

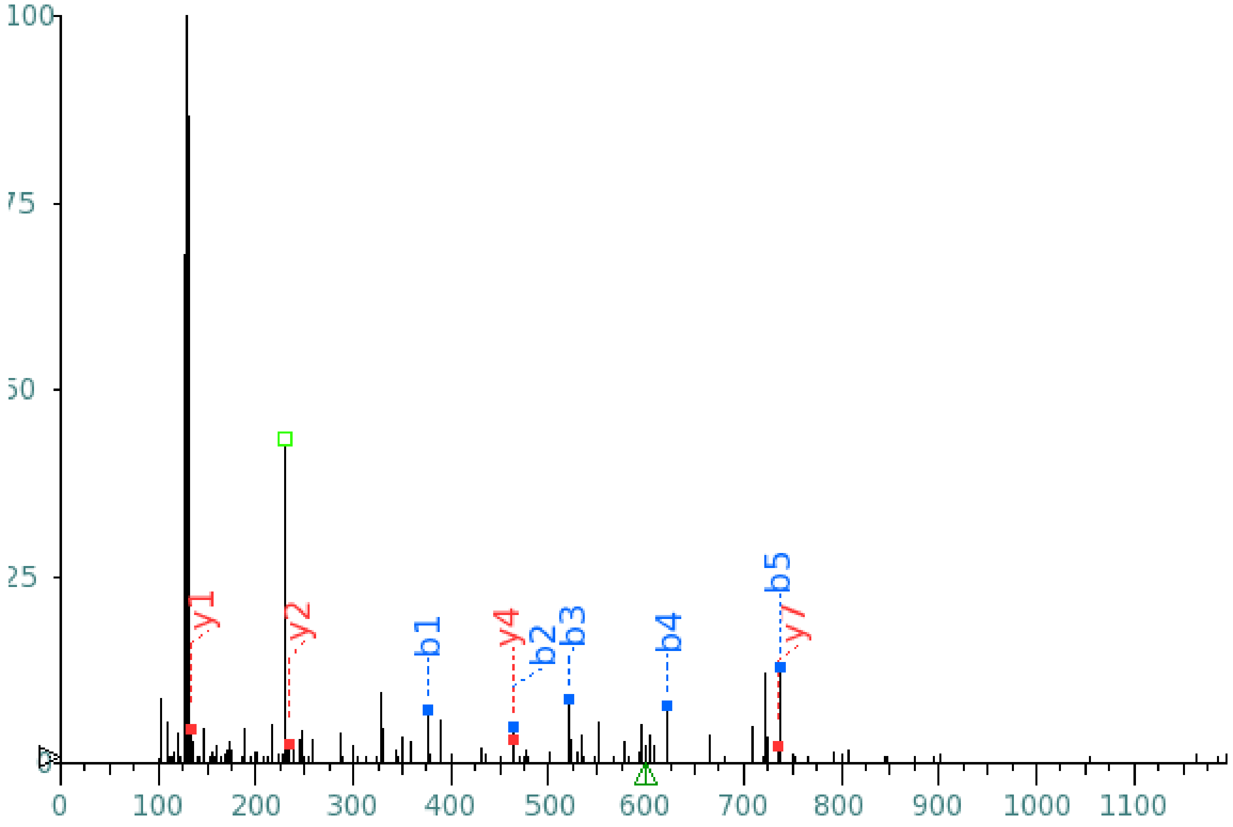

FSGTNVETD (+2)

Primary Reference: NEUL_HUMAN
Search ID: 41760
Search Name: 20130328_ananiav_ERstress_TMT_TM_timecourse_trypsin_inj1_4mc_iaa
Scan#: 8330
Observed Mass: 599.7939 (1.3 ppm)
PSM Score: 12.09

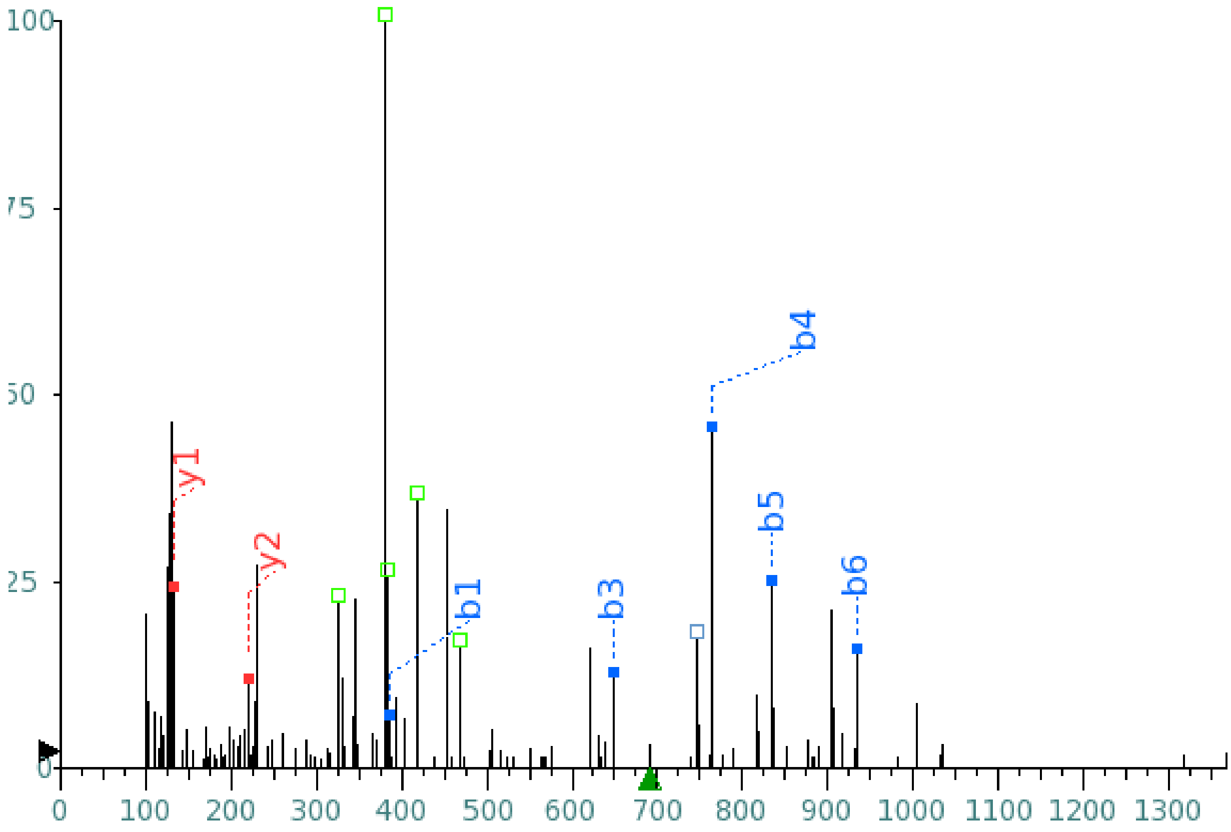

RFDDAVVQSD (+2)

Primary Reference: HSP7C_HUMAN
Search ID: 41760
Search Name: 20130328_ananiav_ERstress_TMT_TM_timecourse_trypsin_inj1_4mc_iaa
Scan#: 8364
Observed Mass: 690.8534 (2.8 ppm)
PSM Score: 11.6

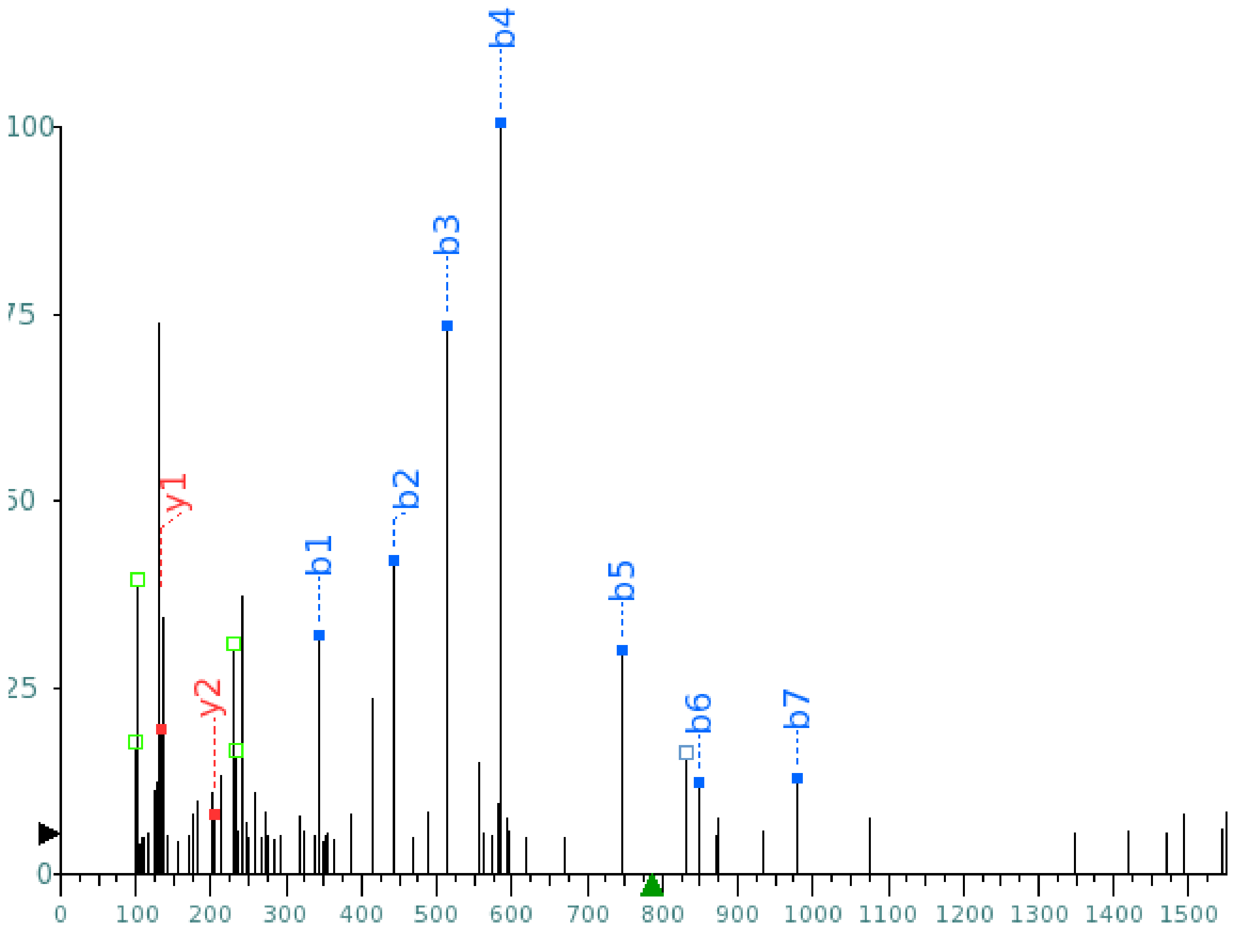

NVAAYTEQEEAD (+2)

Primary Reference: GOGA4_HUMAN
Search ID: 41760
Search Name: 20130328_ananiav_ERstress_TMT_TM_timecourse_trypsin_inj1_4mc_iaa
Scan#: 8377
Observed Mass: 784.8676 (0.1 ppm)
PSM Score: 30.71

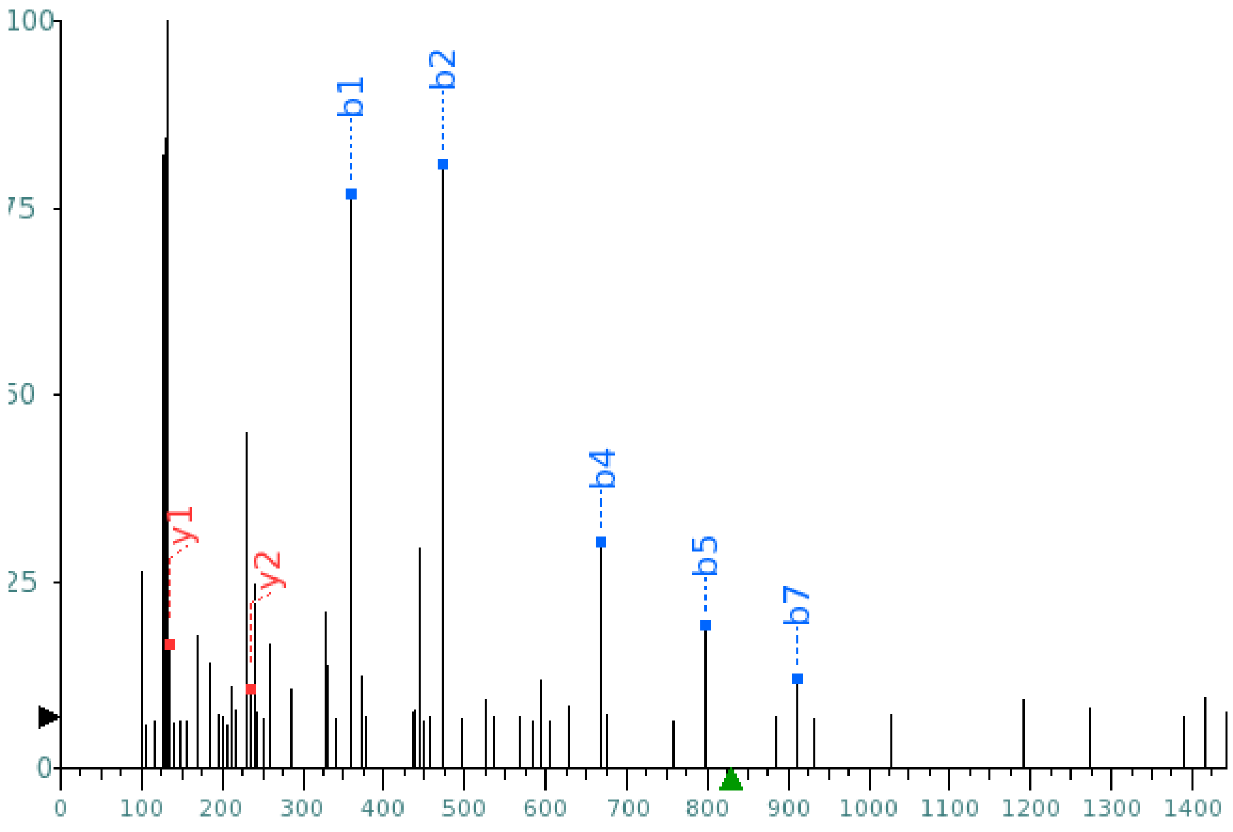

EIPVEGGQEQQTD (+2)

Primary Reference: B1AKC7_HUMAN
Search ID: 41760
Search Name: 20130328_ananiav_ERstress_TMT_TM_timecourse_trypsin_inj1_4mc_iaa
Scan#: 8396
Observed Mass: 829.9073 (0.1 ppm)
PSM Score: 16.54

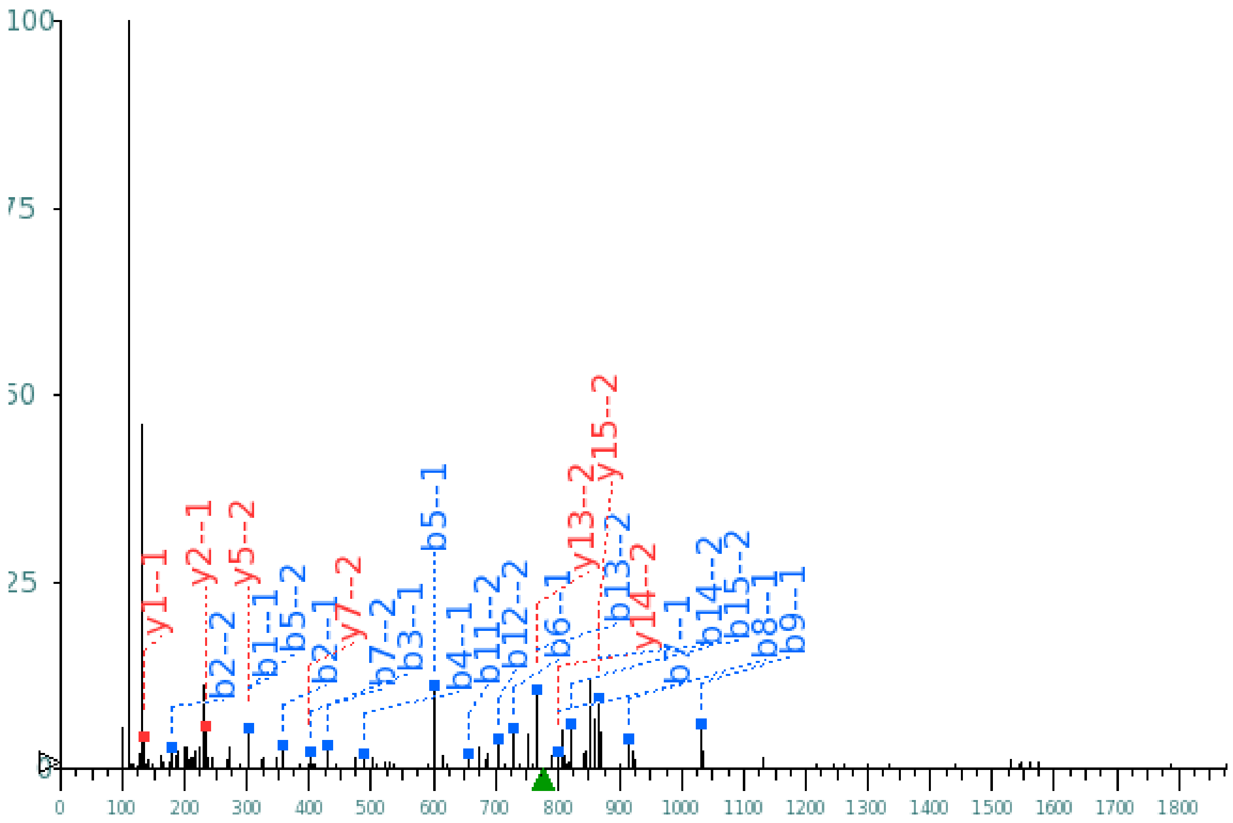

AGAGDEADDERSELSHVETD (+3)

Primary Reference: RHG23_HUMAN

Search ID: 41760

Search Name: 20130328_ananiav_ERstress_TMT_TM_timecourse_trypsin_inj1_4mc_iaa

Scan#: 8445

Observed Mass: 778.0181 (1 ppm)

PSM Score: 22.64

File name: 2015-02-04 04.01.53PM.pdf

Creation time: 2015-02-04 04:09PM

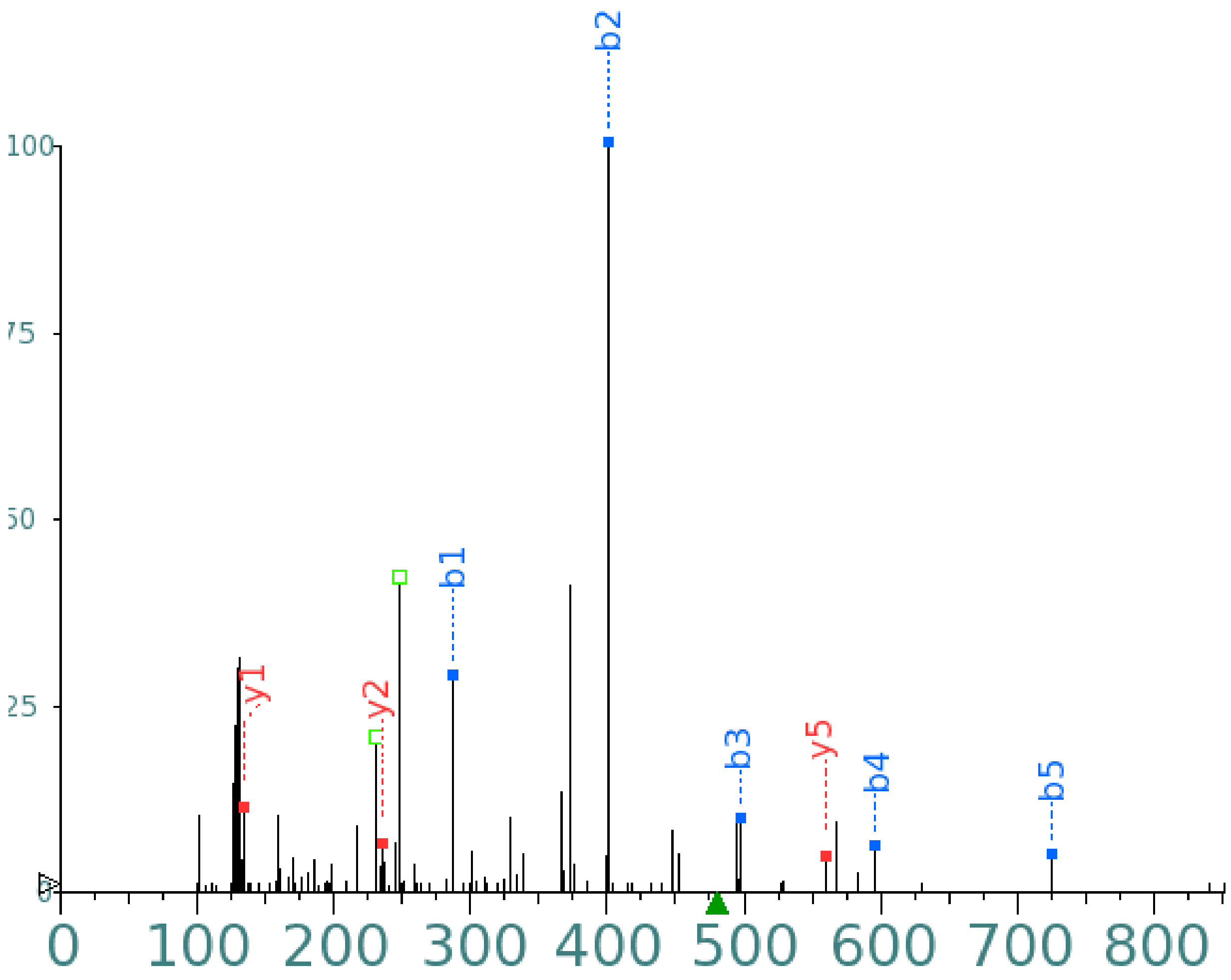

GIPVETD (+2)

Primary Reference: CASP8_HUMAN
Search ID: 41760
Search Name: 20130328_ananiav_ERstress_TMT_TM_timecourse_trypsin_inj1_4mc_iaa
Scan#: 8465
Observed Mass: 480.2667 (1.5 ppm)
PSM Score: 22.51

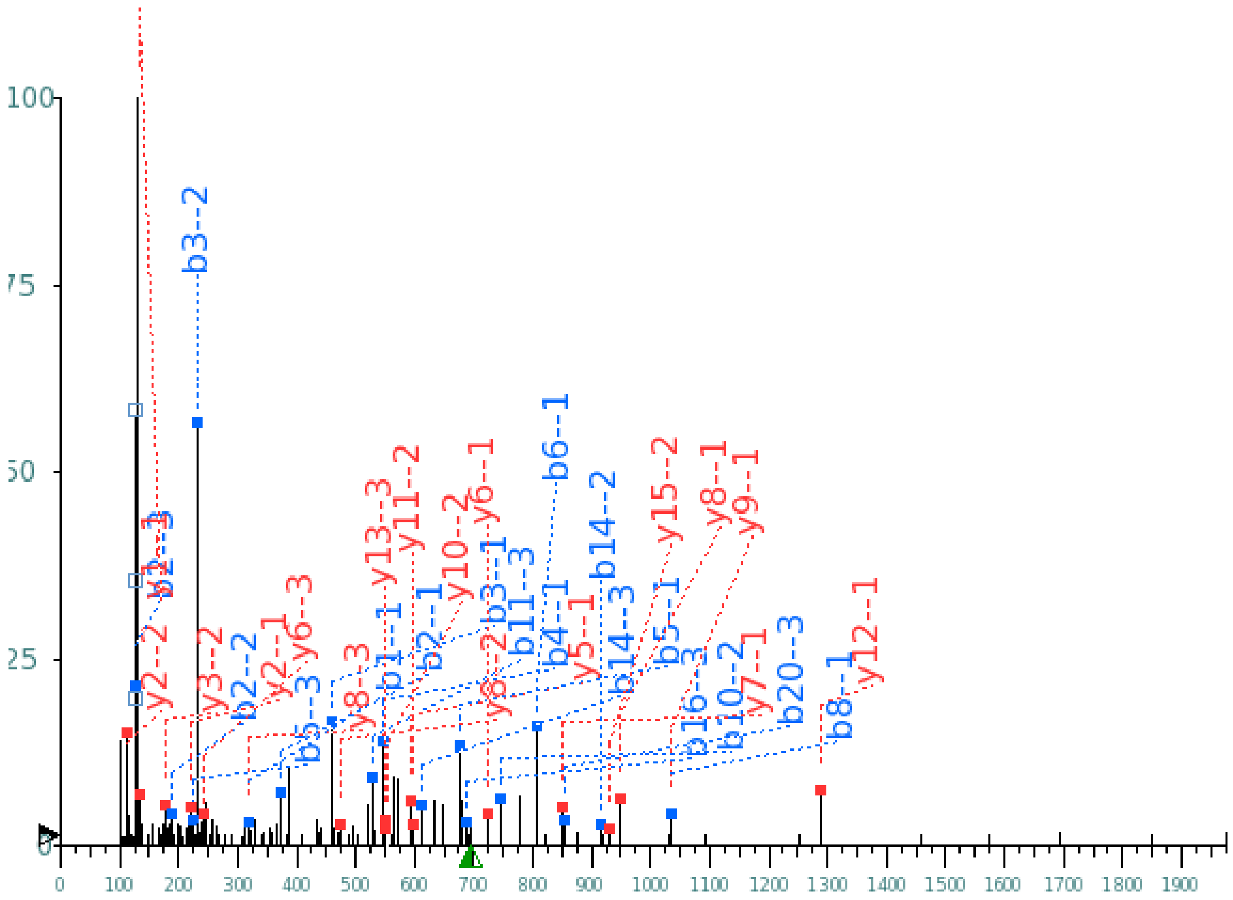

SGSSQELDVKPSASPQERSESD (+4)

Primary Reference: SRRM2_HUMAN
Search ID: 41760
Search Name: 20130328_ananiav_ERstress_TMT_TM_timecourse_trypsin_inj1_4mc_iaa
Scan#: 8485
Observed Mass: 695.3517 (2 ppm)
PSM Score: 21.39

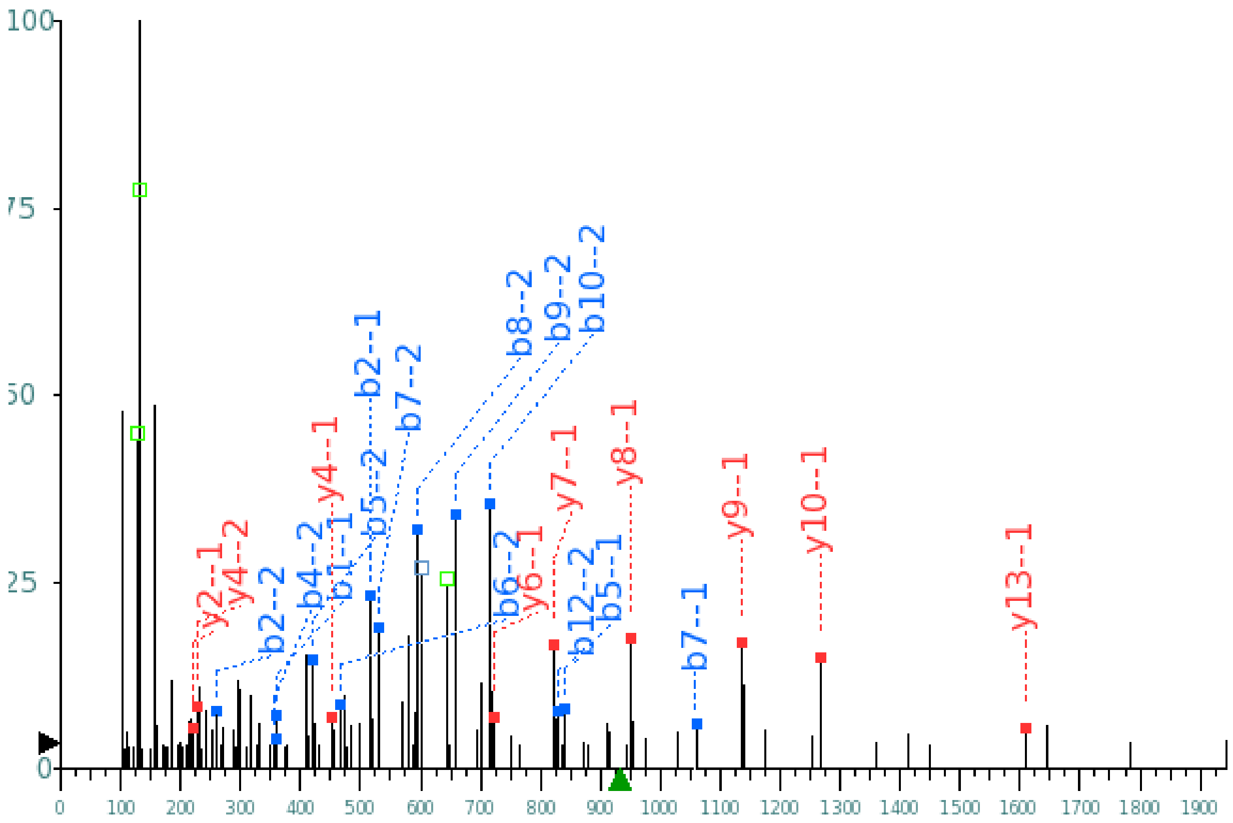

ERTPESEEEENVEWETNRDDSD (+3)

Primary Reference: ZC3HD_HUMAN
Search ID: 41760
Search Name: 20130328_ananiav_ERstress_TMT_TM_timecourse_trypsin_inj1_4mc_iaa
Scan#: 8502
Observed Mass: 932.4093 (2 ppm)
PSM Score: 35.03

File name: 2015-02-04 04.01.53PM.pdf

Creation time: 2015-02-04 04:09PM

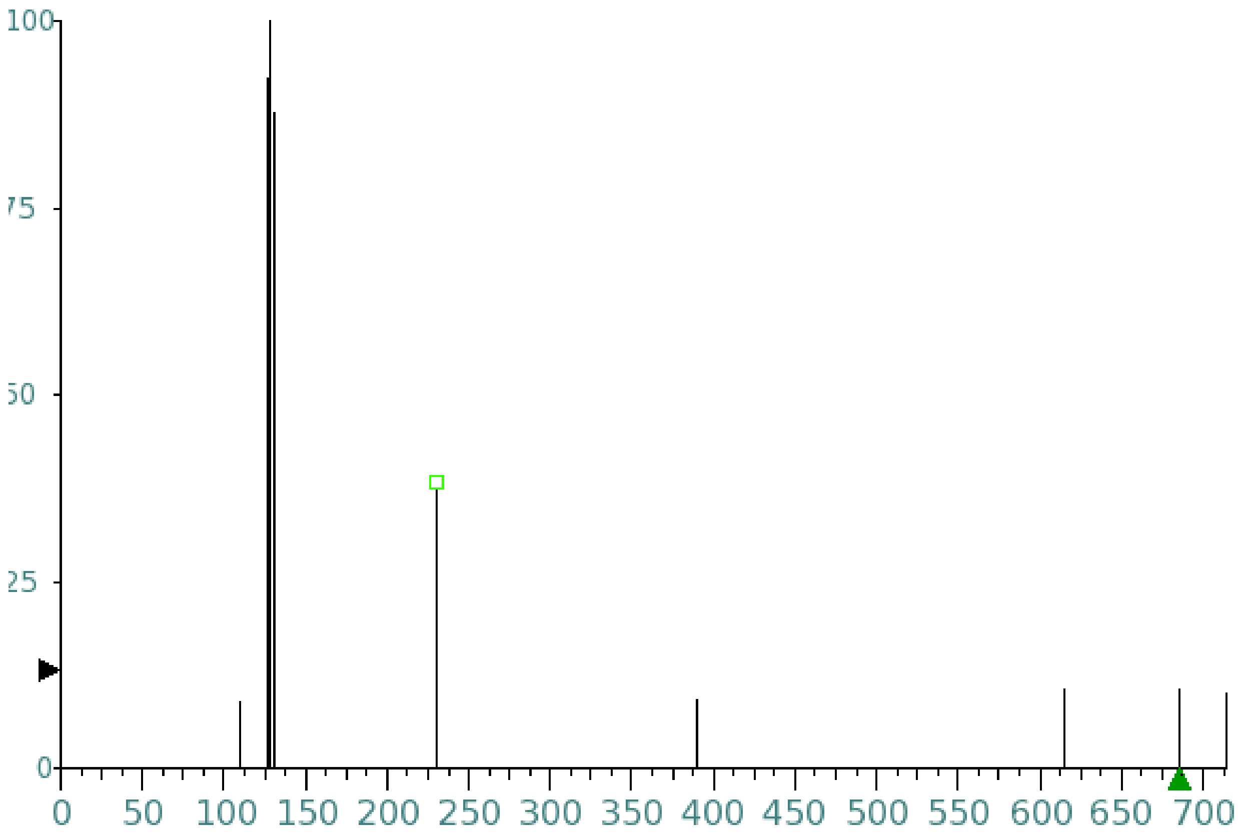

C*APGVVGPAEAD (+2)

Primary Reference: FLNA_HUMAN
Search ID: 41760
Search Name: 20130328_ananiav_ERstress_TMT_TM_timecourse_trypsin_inj1_4mc_iaa
Scan#: 8505
Observed Mass: 686.3436 (1.6 ppm)
PSM Score: 7.47

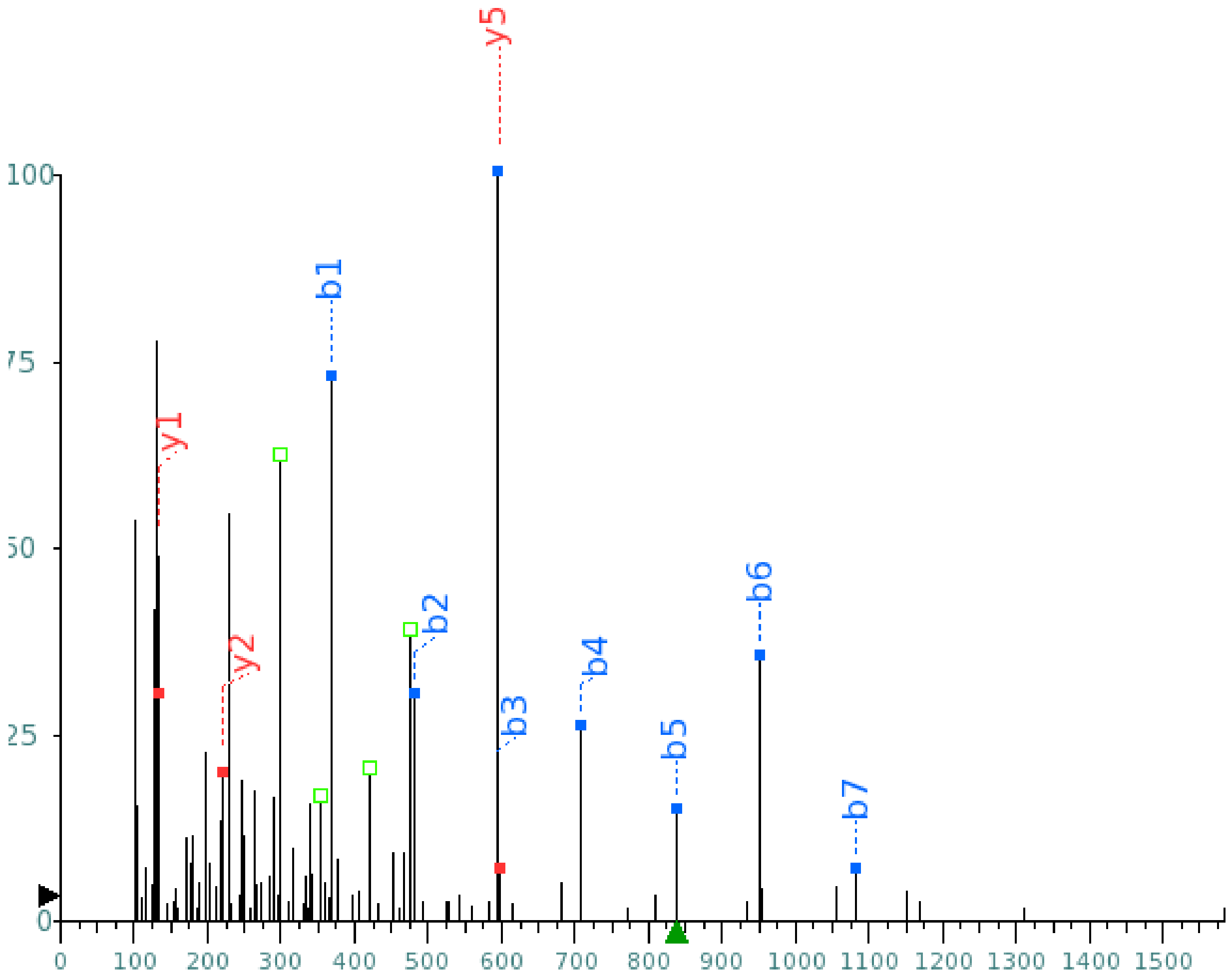

HIDLEDESC*ESD (+2)

Primary Reference: ZDBF2_HUMAN
Search ID: 41760
Search Name: 20130328_ananiav_ERstress_TMT_TM_timecourse_trypsin_inj1_4mc_iaa
Scan#: 8517
Observed Mass: 839.3599 (0.9 ppm)
PSM Score: 46.11

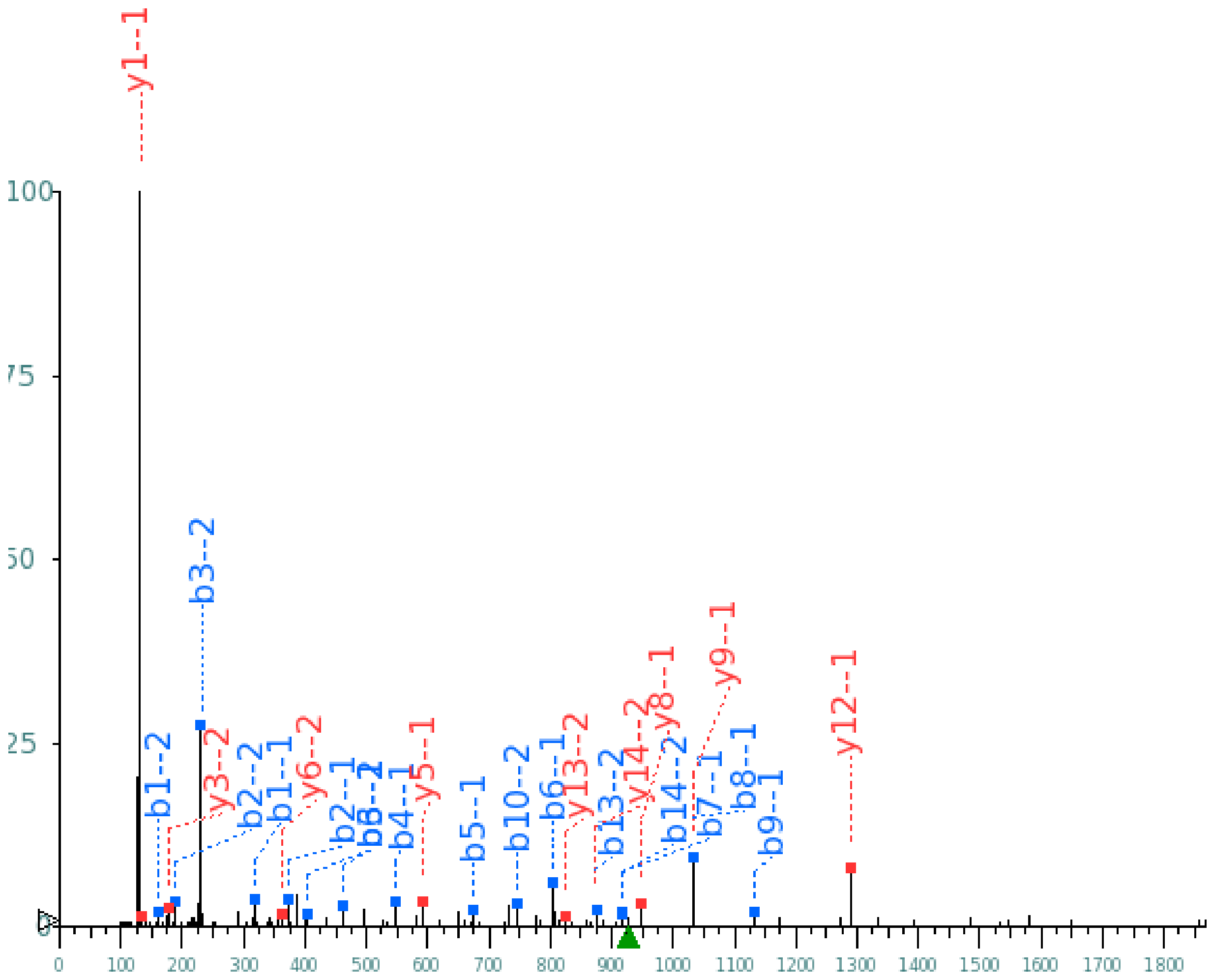

SGSSQELDVKPSASPQERSESD (+3)

Primary Reference: SRRM2_HUMAN
Search ID: 41760
Search Name: 20130328_ananiav_ERstress_TMT_TM_timecourse_trypsin_inj1_4mc_iaa
Scan#: 8523
Observed Mass: 926.7988 (0.9 ppm)
PSM Score: 32.8

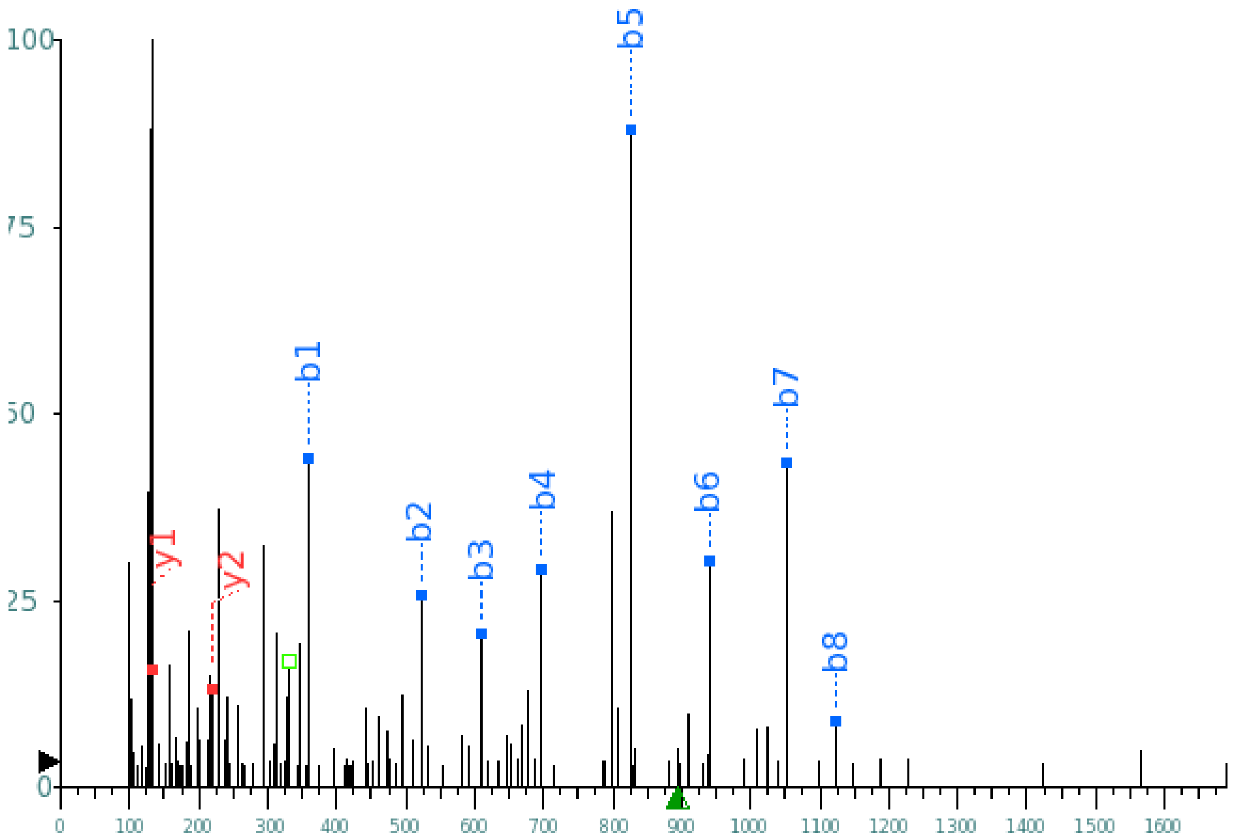

EYSSELNAPSQESD (+2)

Primary Reference: RBM25_HUMAN
Search ID: 41760
Search Name: 20130328_ananiav_ERstress_TMT_TM_timecourse_trypsin_inj1_4mc_iaa
Scan#: 8550
Observed Mass: 892.9059 (1.2 ppm)
PSM Score: 35.1

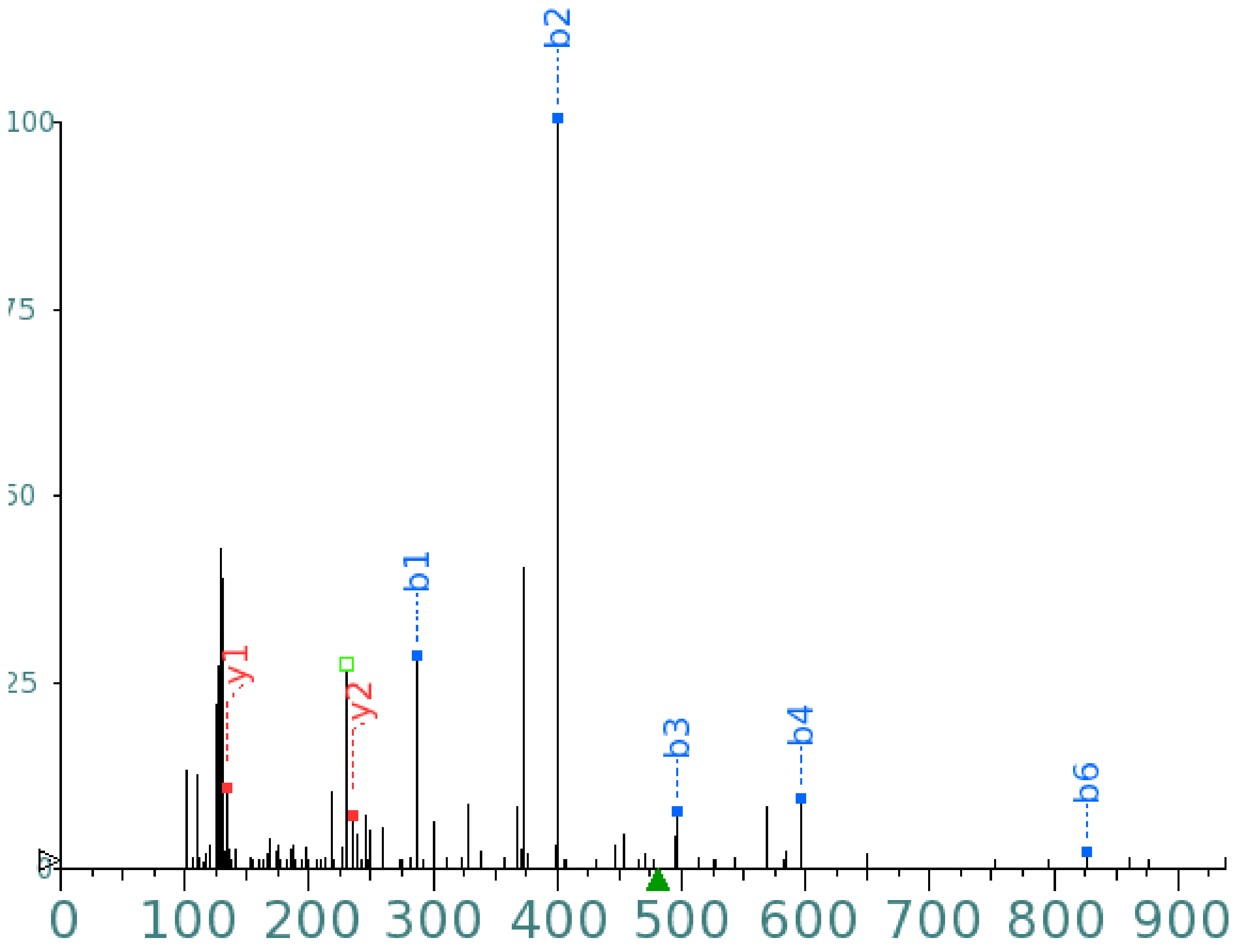

GIPVETD (+2)

Primary Reference: CASP8_HUMAN
Search ID: 41760
Search Name: 20130328_ananiav_ERstress_TMT_TM_timecourse_trypsin_inj1_4mc_iaa
Scan#: 8553
Observed Mass: 480.2672 (2.5 ppm)
PSM Score: 32.43

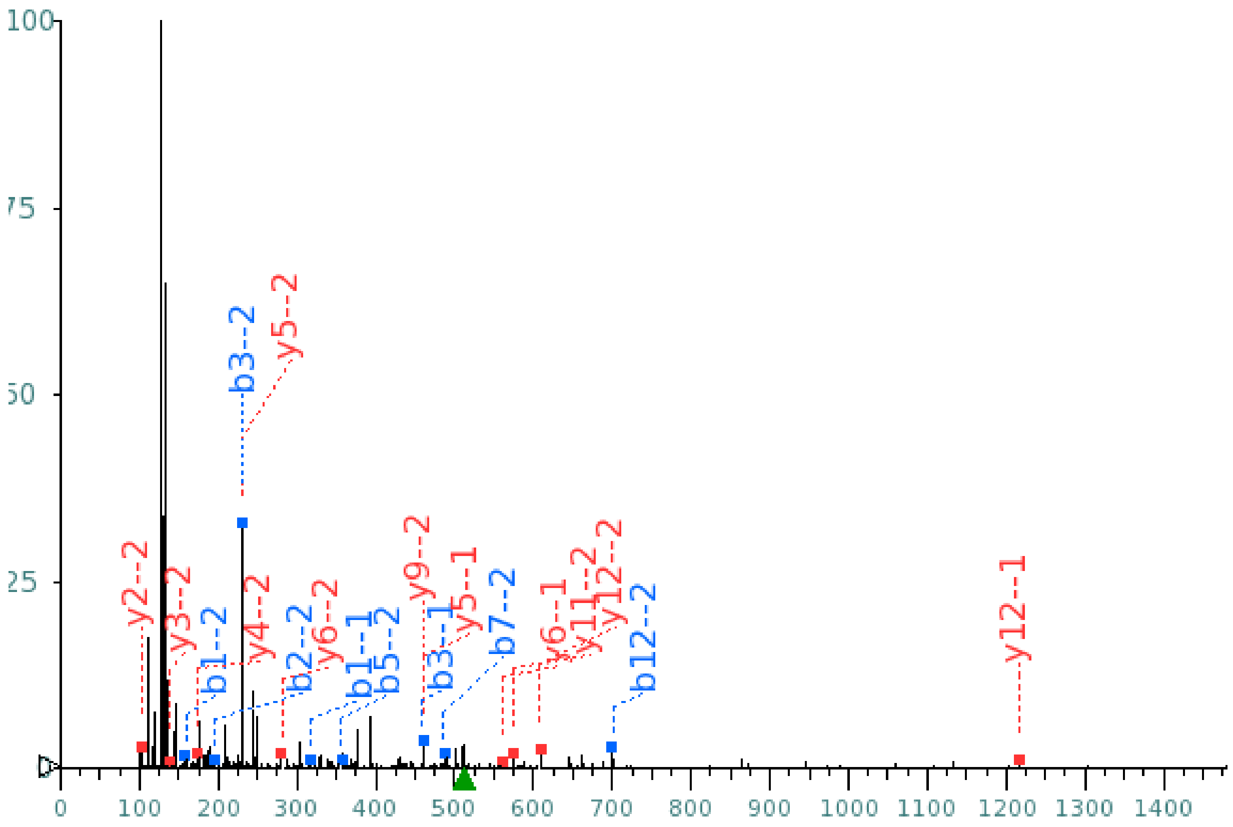

SAARPPYTLAAAD (+3)

Primary Reference: BEST3_HUMAN
Search ID: 41760
Search Name: 20130328_ananiav_ERstress_TMT_TM_timecourse_trypsin_inj1_4mc_iaa
Scan#: 8628
Observed Mass: 511.6143 (0.9 ppm)
PSM Score: 11.68

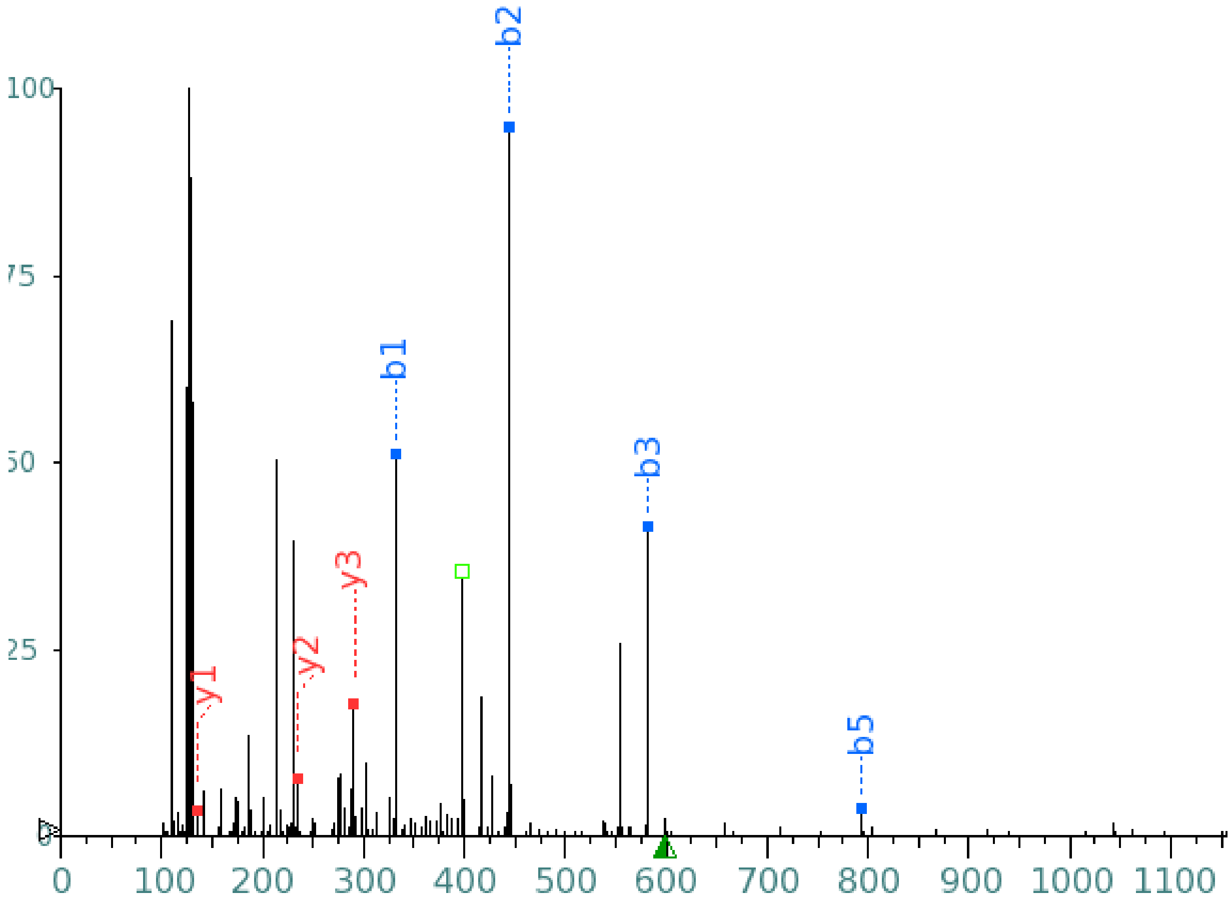

TLHPDLGTD (+2)

Primary Reference: LDHA_HUMAN
Search ID: 41760
Search Name: 20130328_ananiav_ERstress_TMT_TM_timecourse_trypsin_inj1_4mc_iaa
Scan#: 8665
Observed Mass: 599.3209 (2.7 ppm)
PSM Score: 16.51

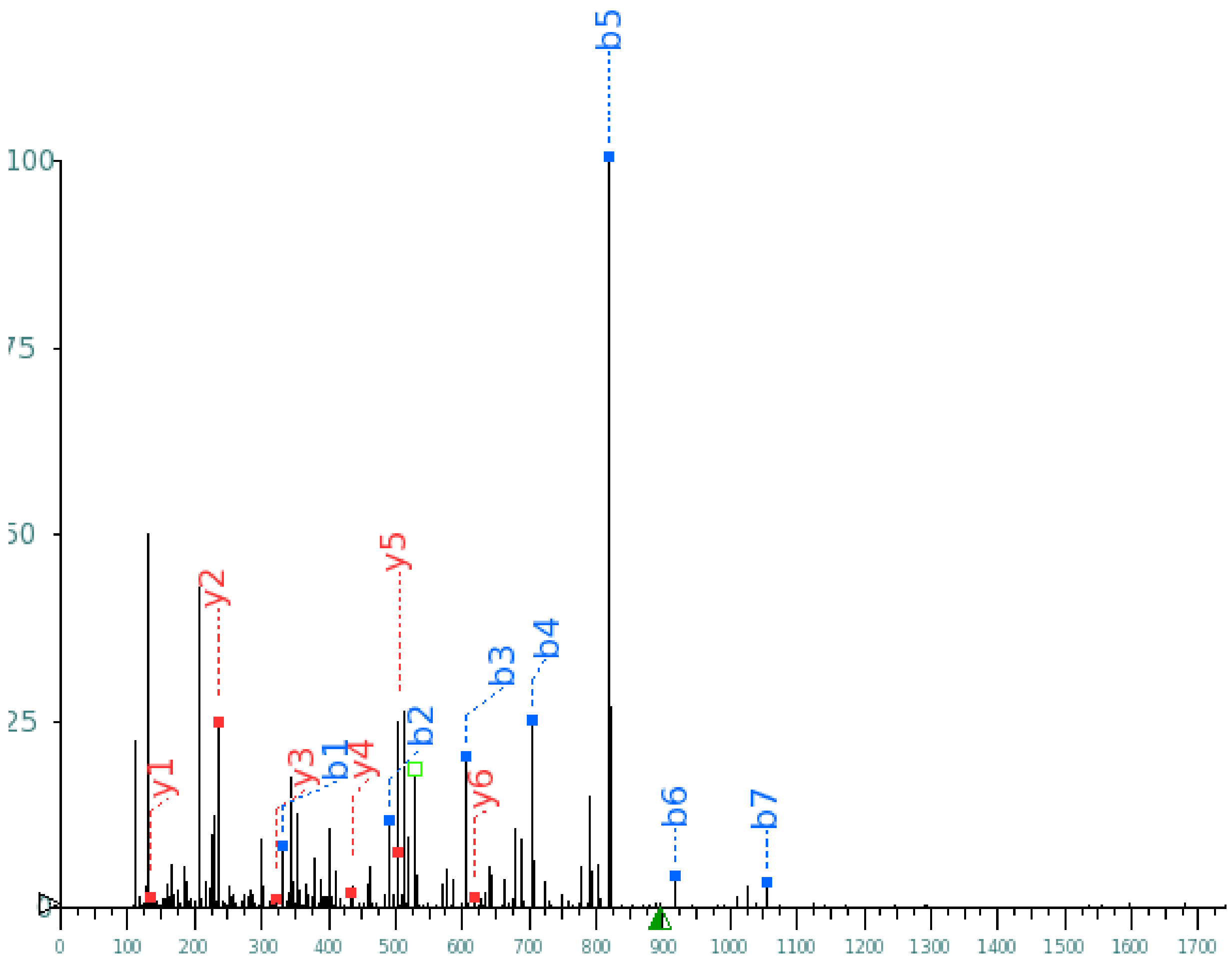

TC*NTNPHLNALSTD (+2)

Primary Reference: F120A_HUMAN
Search ID: 41760
Search Name: 20130328_ananiav_ERstress_TMT_TM_timecourse_trypsin_inj1_4mc_iaa
Scan#: 8679
Observed Mass: 893.9348 (1.8 ppm)
PSM Score: 21.67

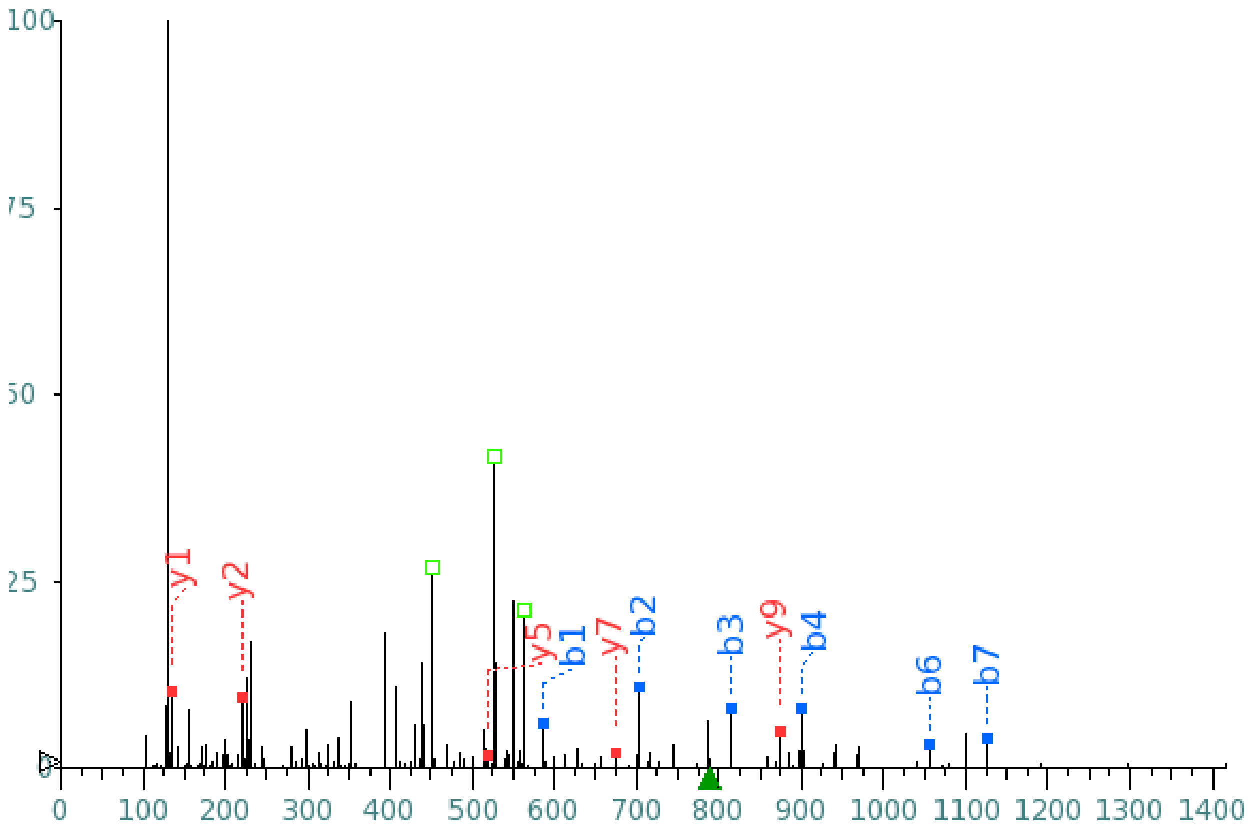

KNLSPGAVESD (+2)

Primary Reference: COPA_HUMAN
Search ID: 41760
Search Name: 20130328_ananiav_ERstress_TMT_TM_timecourse_trypsin_inj1_4mc_iaa
Scan#: 8724
Observed Mass: 787.9446 (1.9 ppm)
PSM Score: 12.54

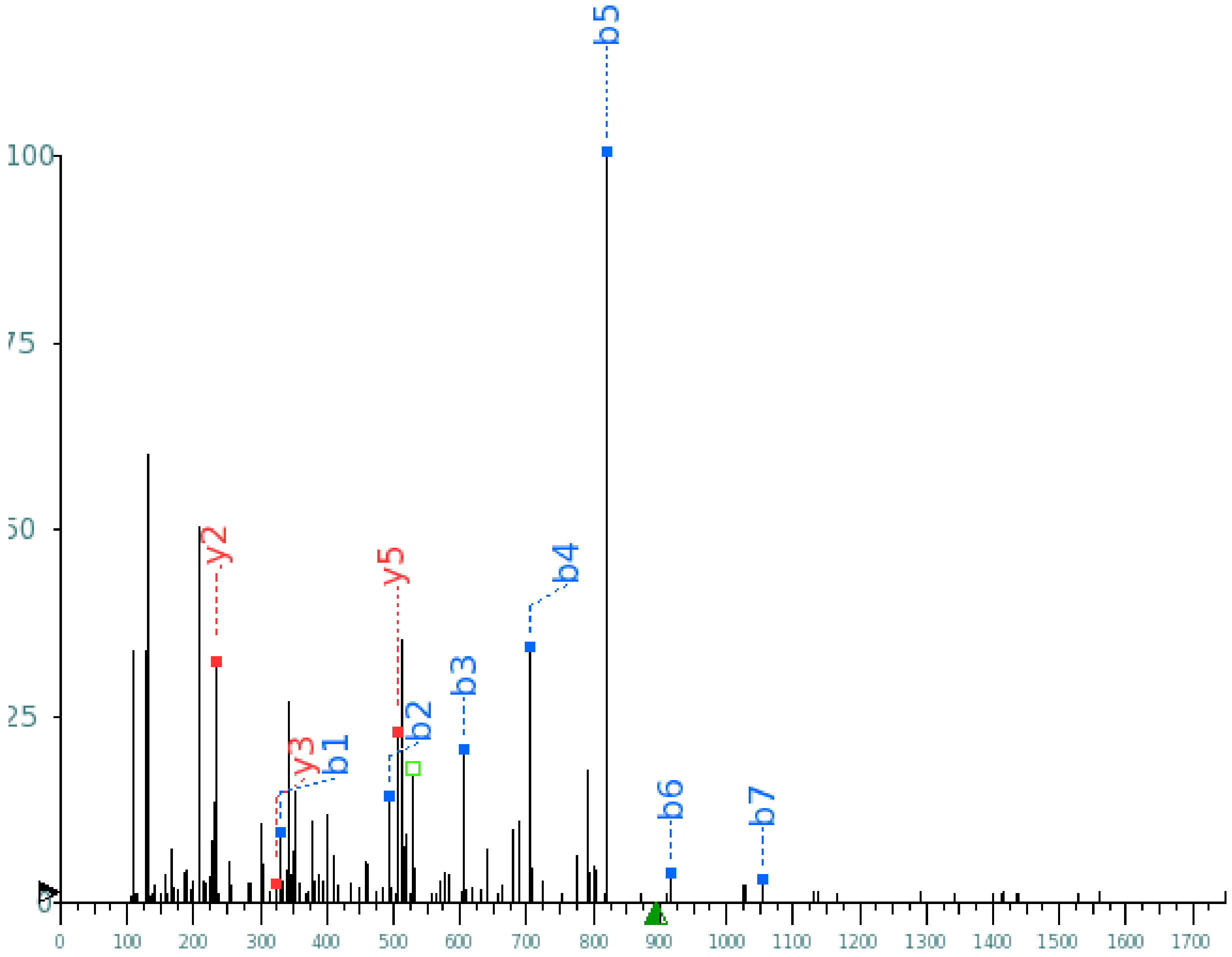

TC*NTNPHLNALSTD (+2)

Primary Reference: F120A_HUMAN
Search ID: 41760
Search Name: 20130328_ananiav_ERstress_TMT_TM_timecourse_trypsin_inj1_4mc_iaa
Scan#: 8763
Observed Mass: 893.9347 (1.6 ppm)
PSM Score: 25.93

File name: 2015-02-04 04.01.53PM.pdf

Creation time: 2015-02-04 04:09PM

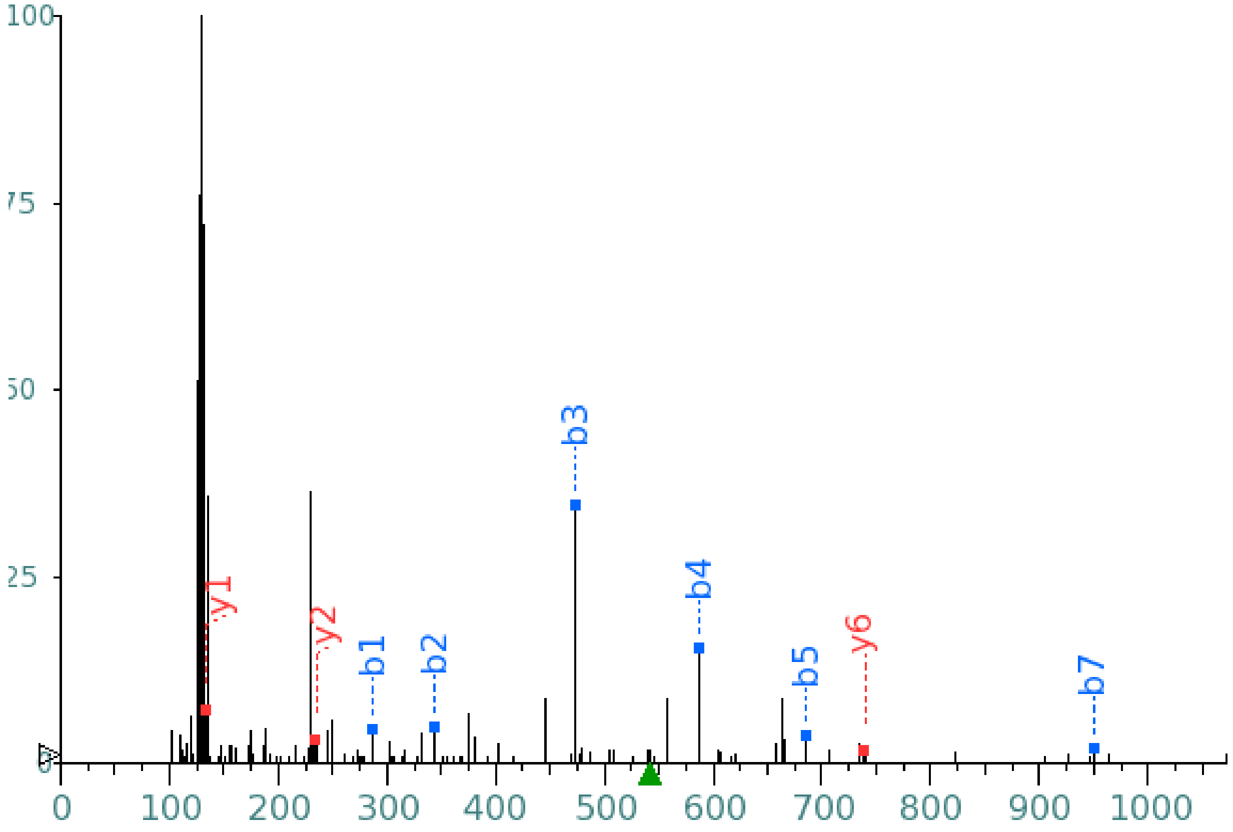

GGELVYTD (+2)

Primary Reference: PLEC_HUMAN
Search ID: 41760
Search Name: 20130328_ananiav_ERstress_TMT_TM_timecourse_trypsin_inj1_4mc_iaa
Scan#: 8873
Observed Mass: 541.7834 (2.6 ppm)
PSM Score: 8.56

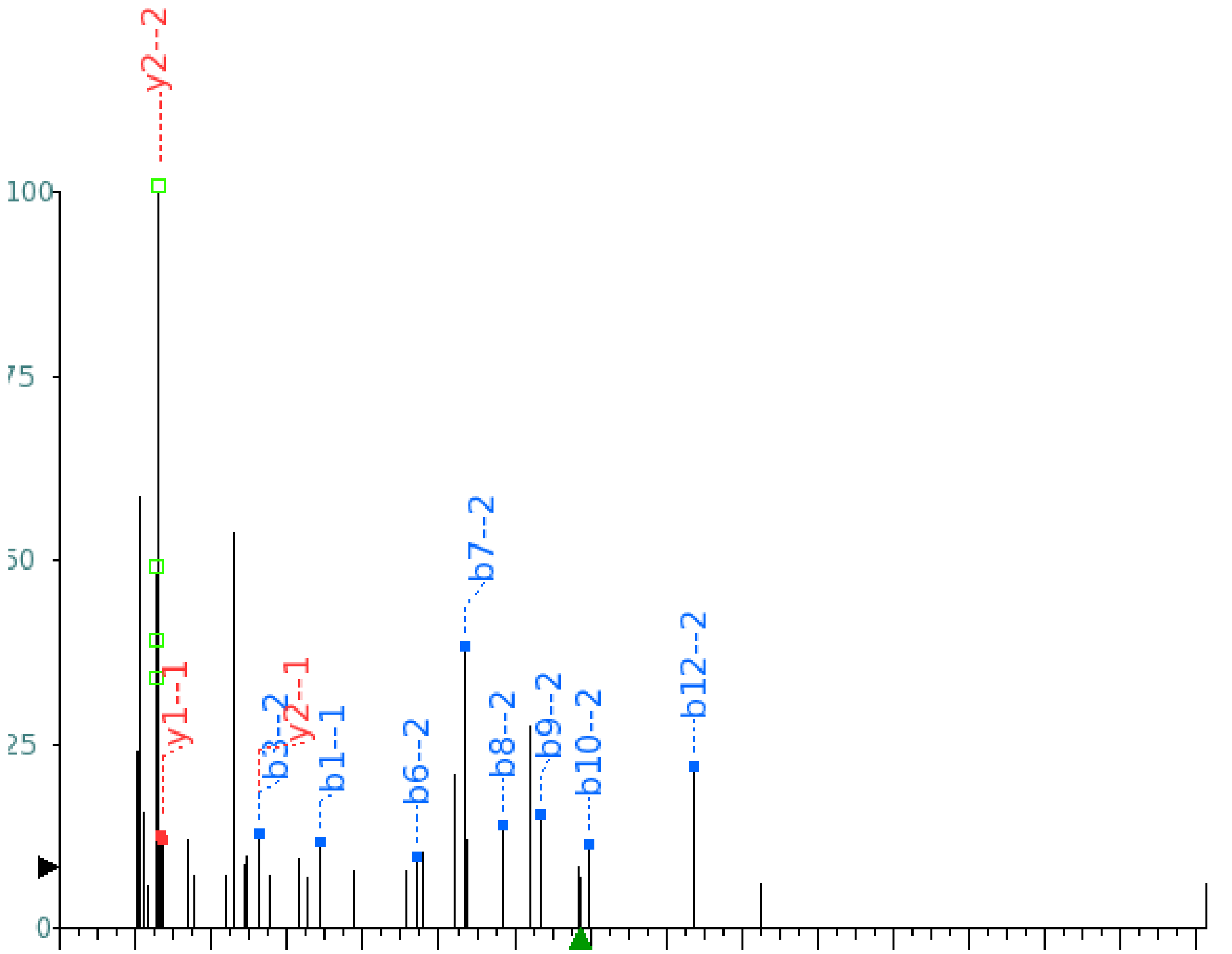

INAEREQPVQC*DEMD (+3)

Primary Reference: TUT4_HUMAN
Search ID: 41760
Search Name: 20130328_ananiav_ERstress_TMT_TM_timecourse_trypsin_inj1_4mc_iaa
Scan#: 8890
Observed Mass: 688.3177 (0.7 ppm)
PSM Score: 29.95

File name: 2015-02-04 04.01.53PM.pdf

Creation time: 2015-02-04 04:09PM

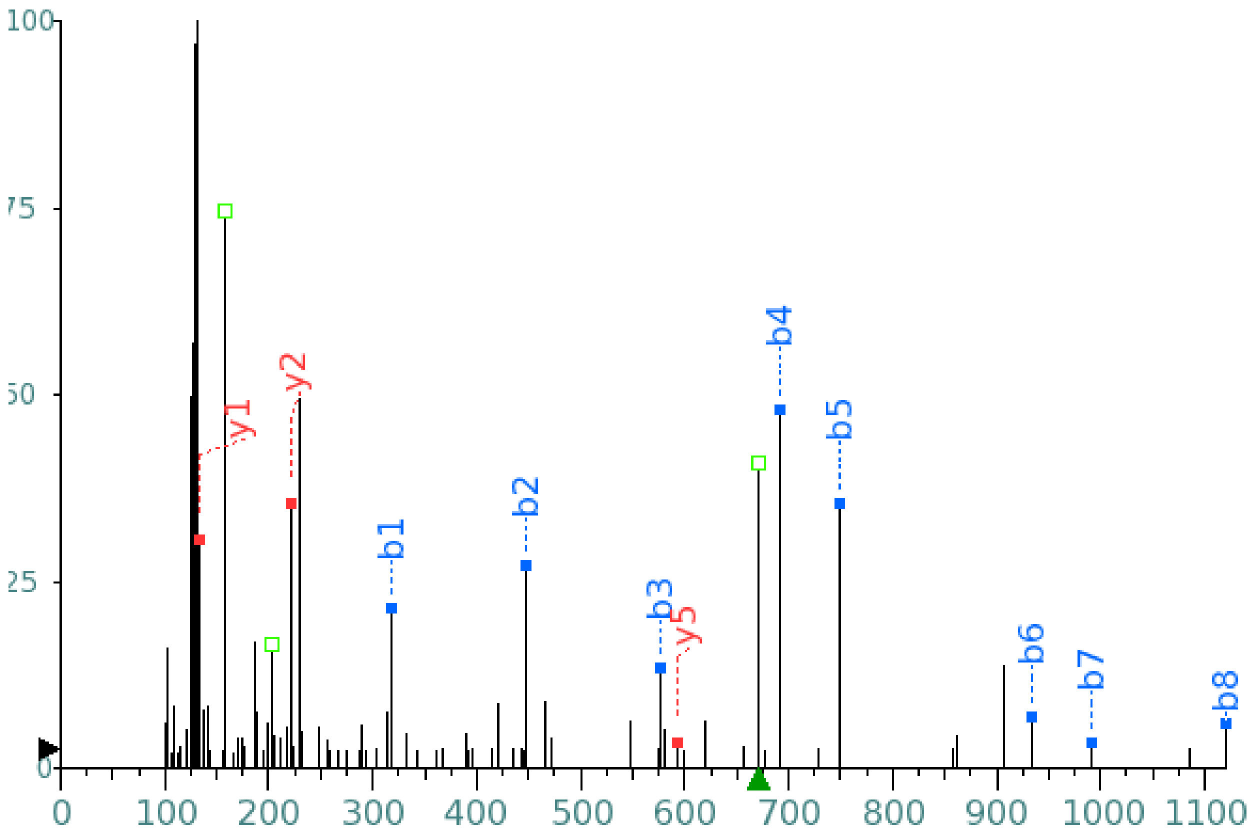

SMQDGWGESD (+2)

Primary Reference: TNR6B_HUMAN
Search ID: 41760
Search Name: 20130328_ananiav_ERstress_TMT_TM_timecourse_trypsin_inj1_4mc_iaa
Scan#: 8898
Observed Mass: 670.7866 (2.5 ppm)
PSM Score: 33.46

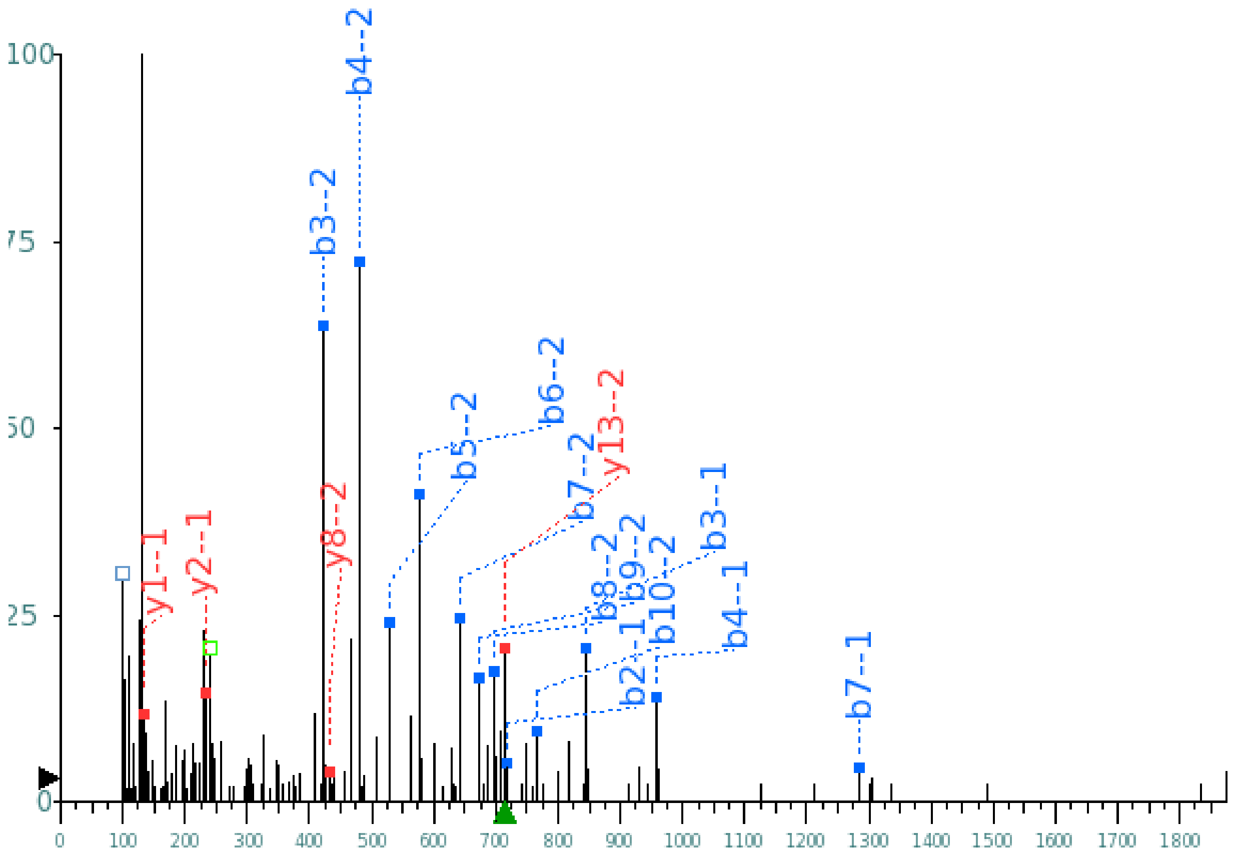

EKEIPVEGGGQEQQTD (+3)

Primary Reference: B1AKC7_HUMAN
Search ID: 41760
Search Name: 20130328_ananiav_ERstress_TMT_TM_timecourse_trypsin_inj1_4mc_iaa
Scan#: 8921
Observed Mass: 715.7091 (2.5 ppm)
PSM Score: 25.93

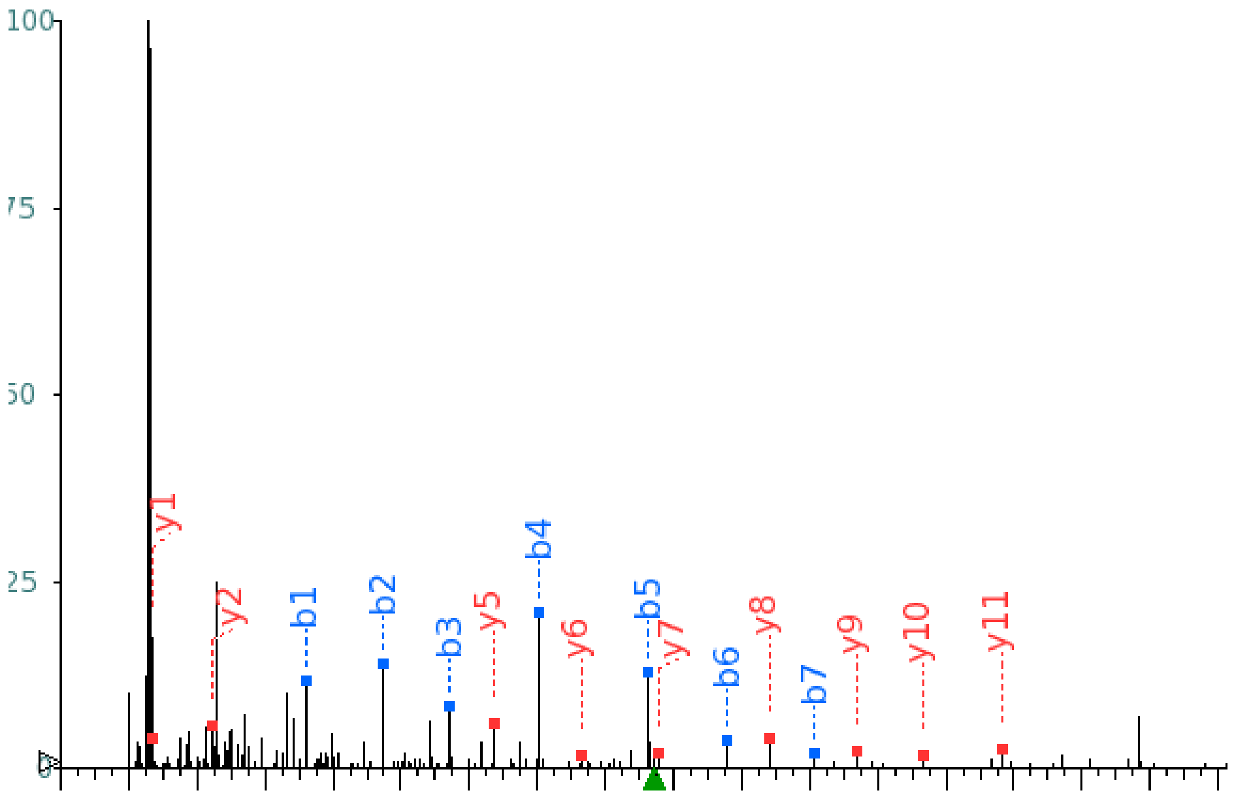

ENVEYIEREESD (+2)

Primary Reference: ZRAB2_HUMAN
Search ID: 41760
Search Name: 20130328_ananiav_ERstress_TMT_TM_timecourse_trypsin_inj1_4mc_iaa
Scan#: 8966
Observed Mass: 870.9113 (1.7 ppm)
PSM Score: 31.22

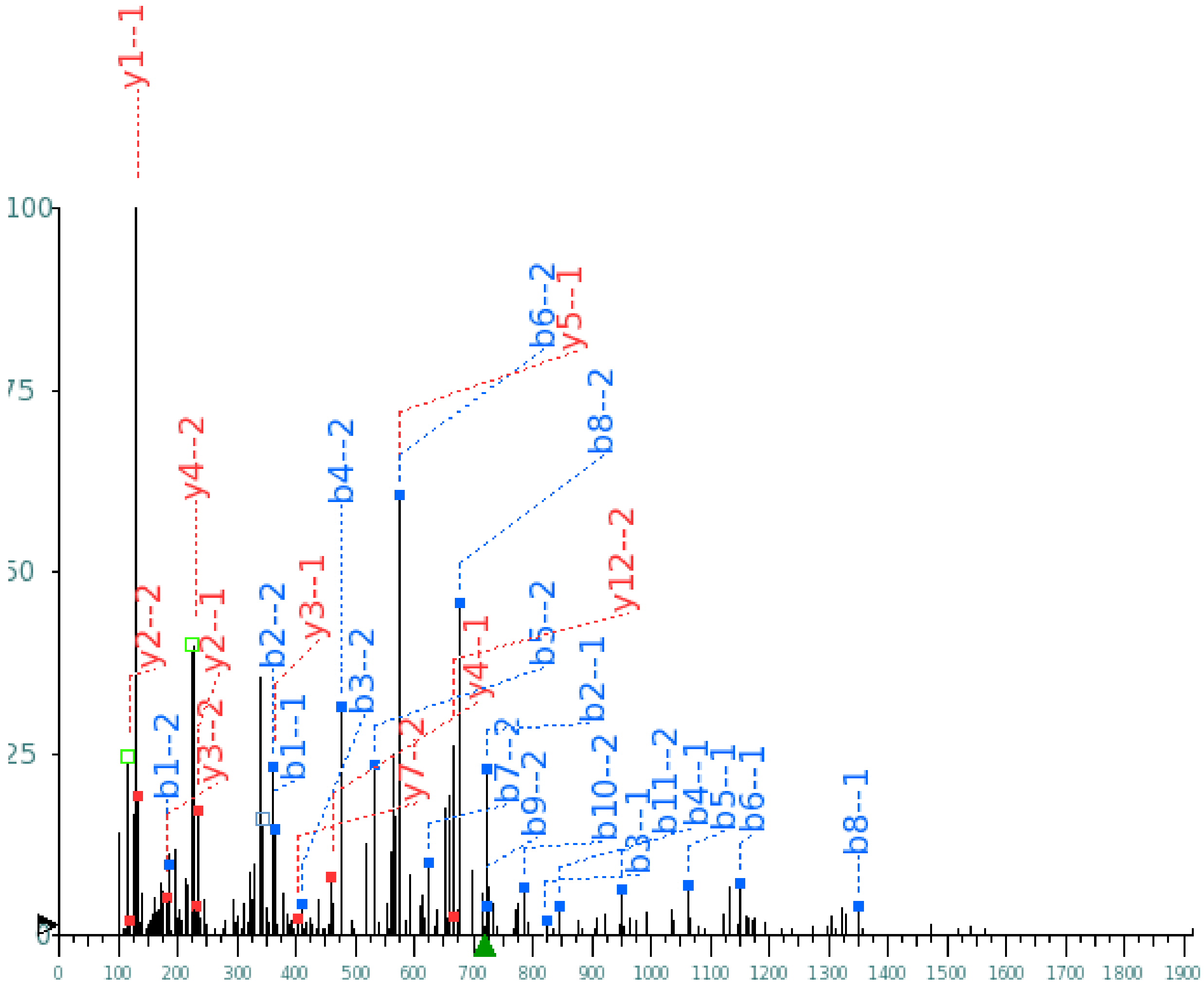

HKPELSPTPQNVQTD (+3)

Primary Reference: HAUS6_HUMAN
Search ID: 41760
Search Name: 20130328_ananiav_ERstress_TMT_TM_timecourse_trypsin_inj1_4mc_iaa
Scan#: 9023
Observed Mass: 717.0611 (1.6 ppm)
PSM Score: 31.25

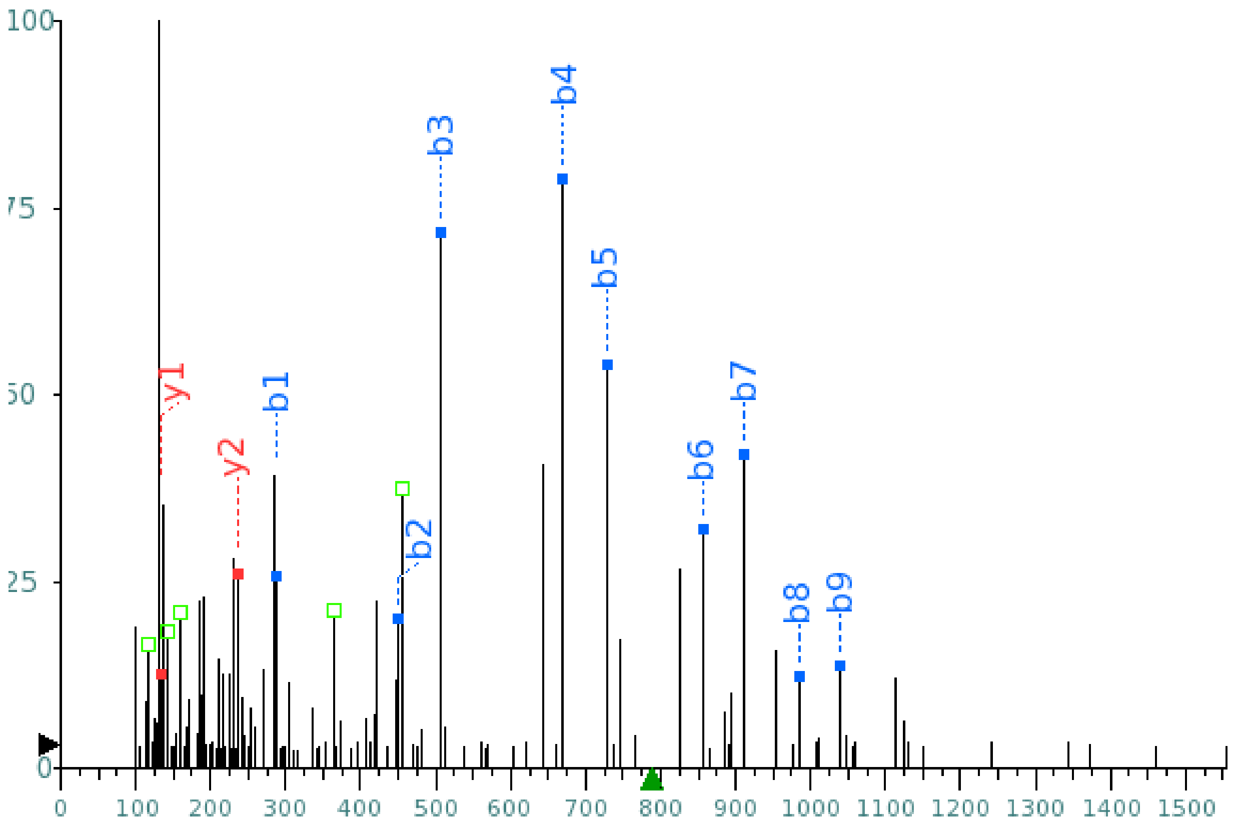

GYGYGQGAGTLSTD (+2)

Primary Reference: CSRP1_HUMAN
Search ID: 41760
Search Name: 20130328_ananiav_ERstress_TMT_TM_timecourse_trypsin_inj1_4mc_iaa
Scan#: 9202
Observed Mass: 788.38 (2.5 ppm)
PSM Score: 35.85

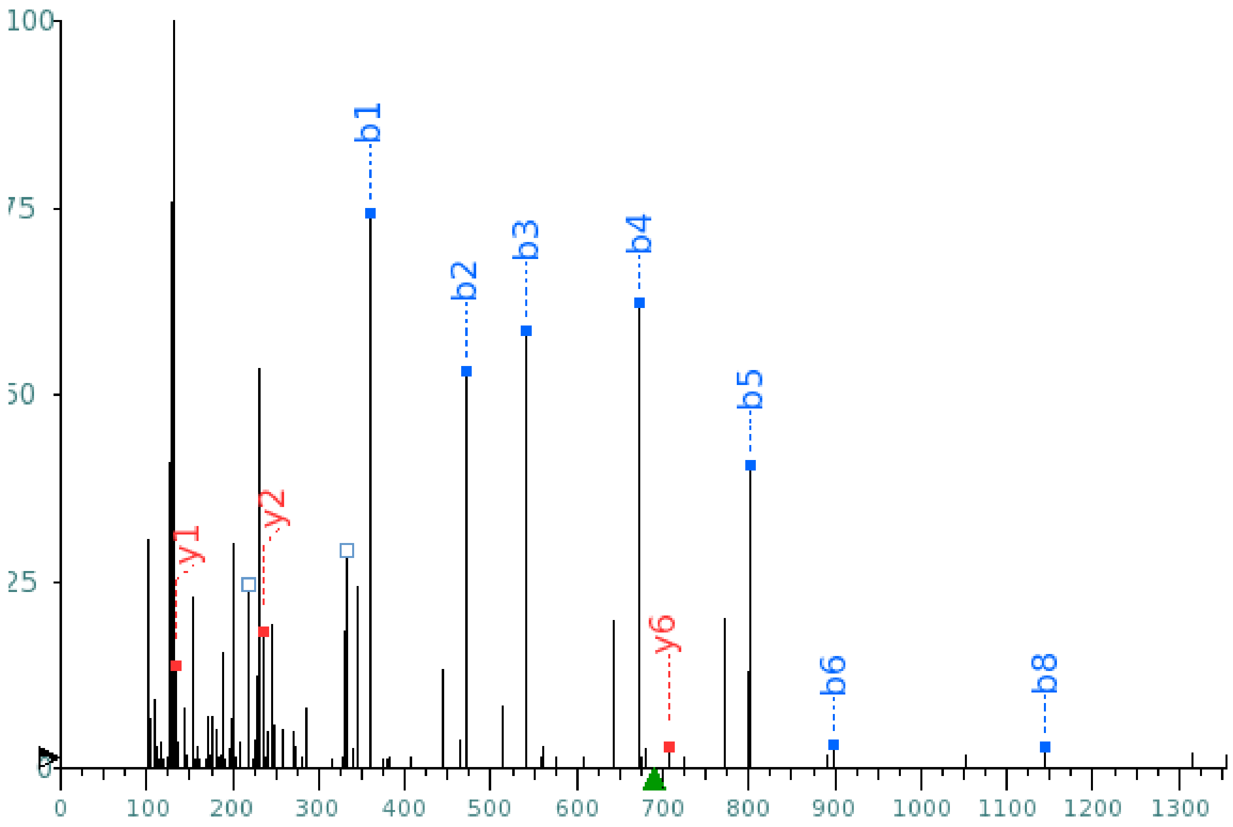

QLAEEPMDTD (+2)

Primary Reference: B4DKB3_HUMAN
Search ID: 41760
Search Name: 20130328_ananiav_ERstress_TMT_TM_timecourse_trypsin_inj1_4mc_iaa
Scan#: 9210
Observed Mass: 689.3254 (2.1 ppm)
PSM Score: 50.41

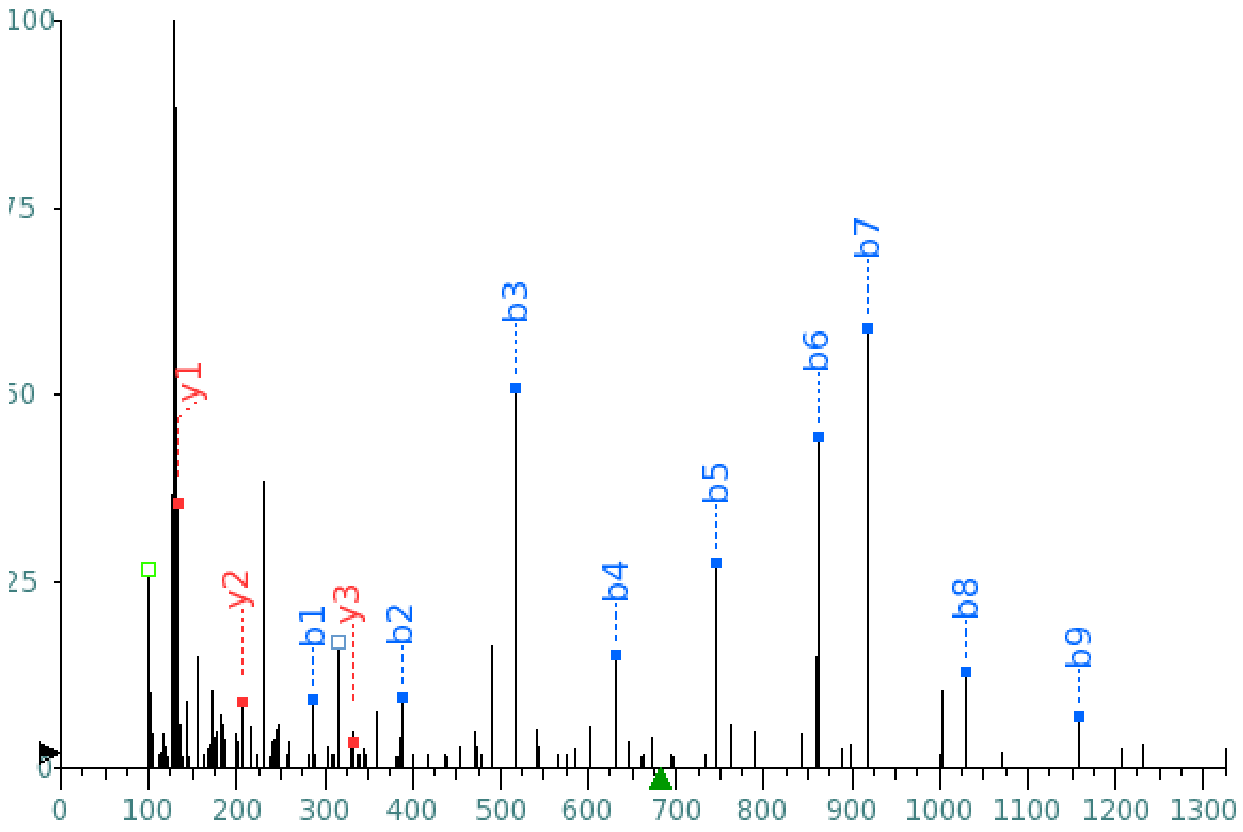

GTELDDGIQAD (+2)

Primary Reference: CASP7_HUMAN
Search ID: 41760
Search Name: 20130328_ananiav_ERstress_TMT_TM_timecourse_trypsin_inj1_4mc_iaa
Scan#: 9266
Observed Mass: 681.8348 (2.7 ppm)
PSM Score: 48.09

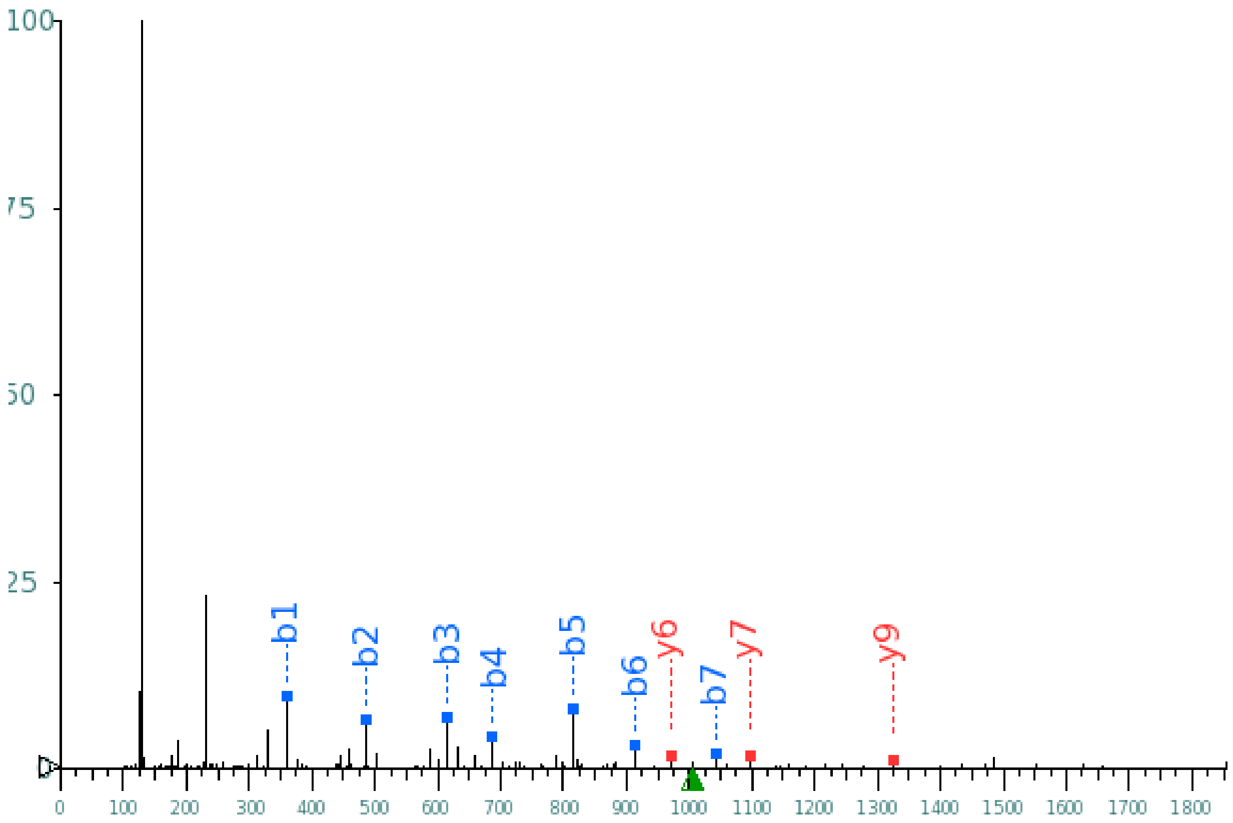

EQQAQVEKEDFSD (+2)

Primary Reference: SF3B2_HUMAN
Search ID: 41760
Search Name: 20130328_ananiav_ERstress_TMT_TM_timecourse_trypsin_inj1_4mc_iaa
Scan#: 9281
Observed Mass: 1006.0018 (-2.9 ppm)
PSM Score: 18.19

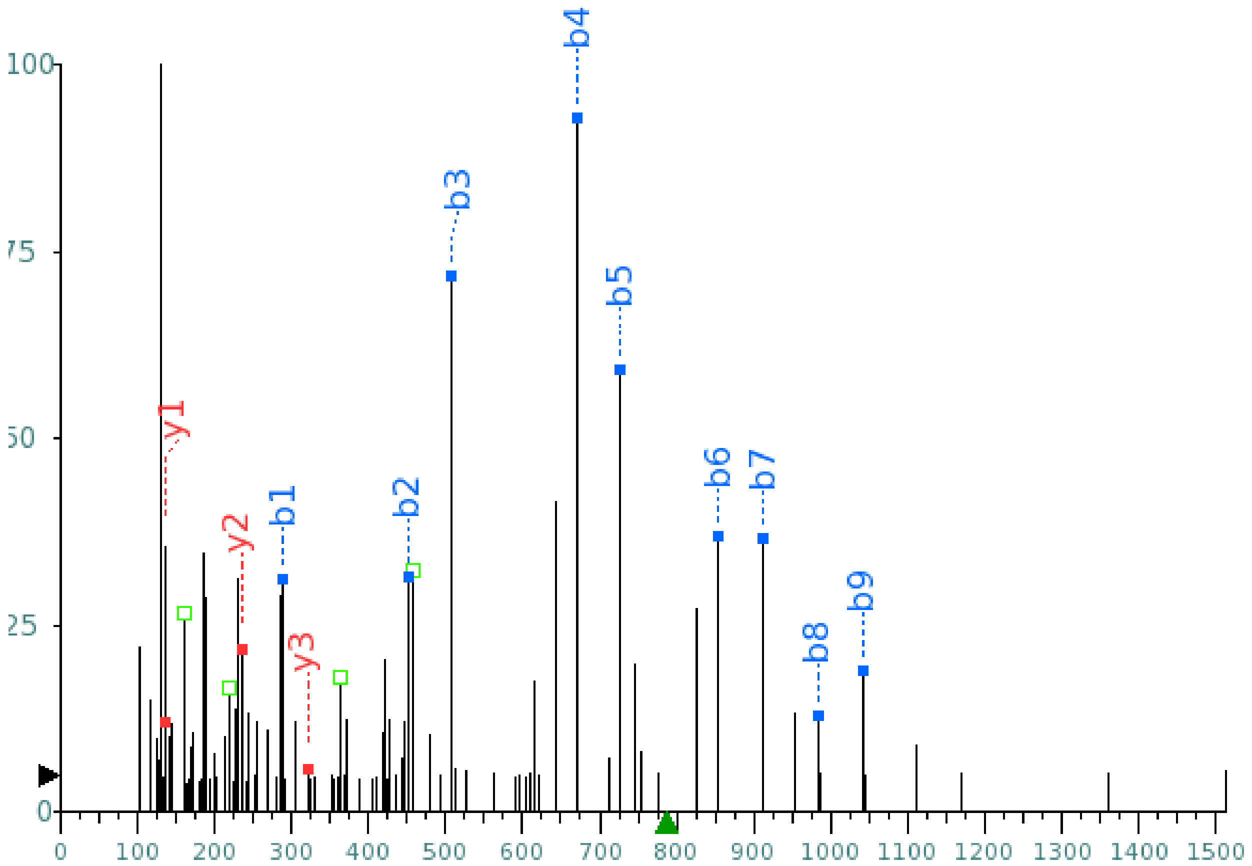

GYGYGGQGAGTLSTD (+2)

Primary Reference: CSRP1_HUMAN
Search ID: 41760
Search Name: 20130328_ananiav_ERstress_TMT_TM_timecourse_trypsin_inj1_4mc_iaa
Scan#: 9301
Observed Mass: 788.3798 (2.3 ppm)
PSM Score: 41.75

File name: 2015-02-04 04.01.53PM.pdf

Creation time: 2015-02-04 04:09PM

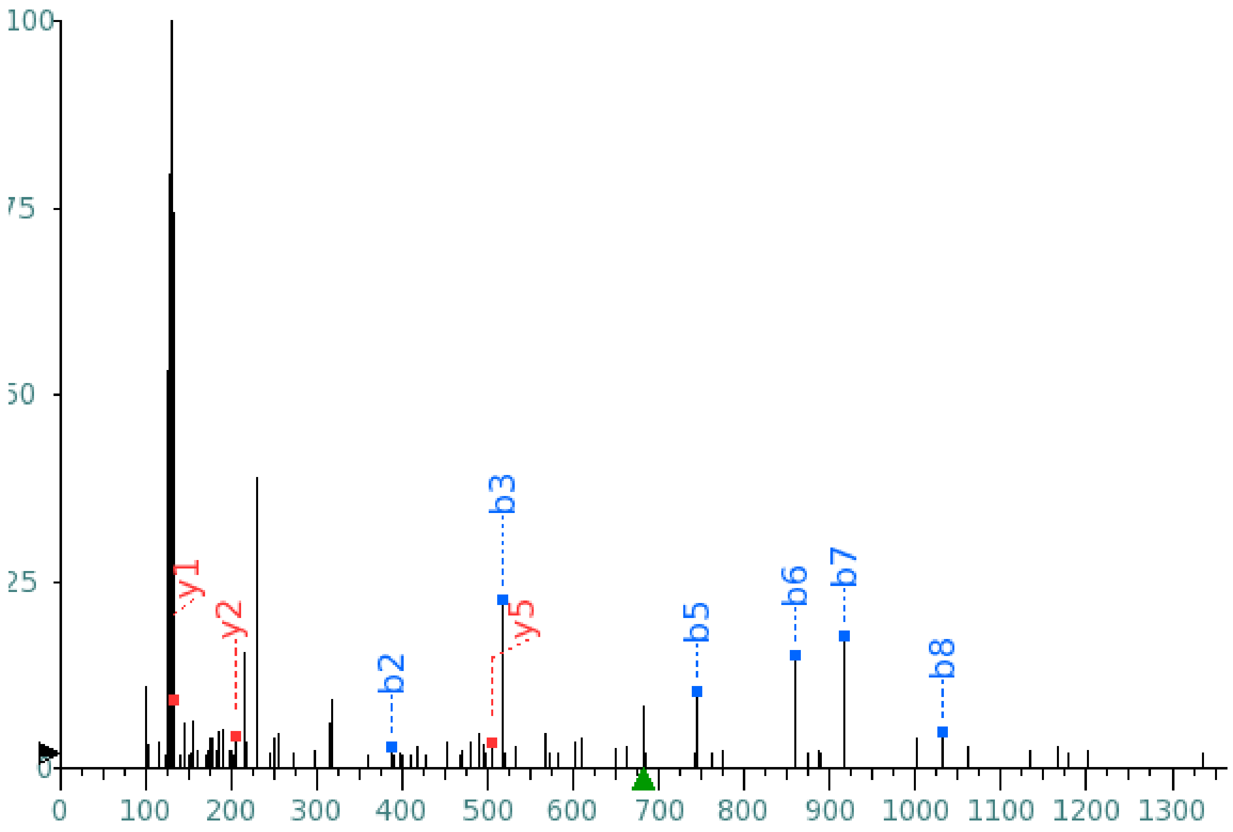

GTELDDGIQAD (+2)

Primary Reference: CASP7_HUMAN
Search ID: 41760
Search Name: 20130328_ananiav_ERstress_TMT_TM_timecourse_trypsin_inj1_4mc_iaa
Scan#: 9359
Observed Mass: 681.8343 (2 ppm)
PSM Score: 17.91

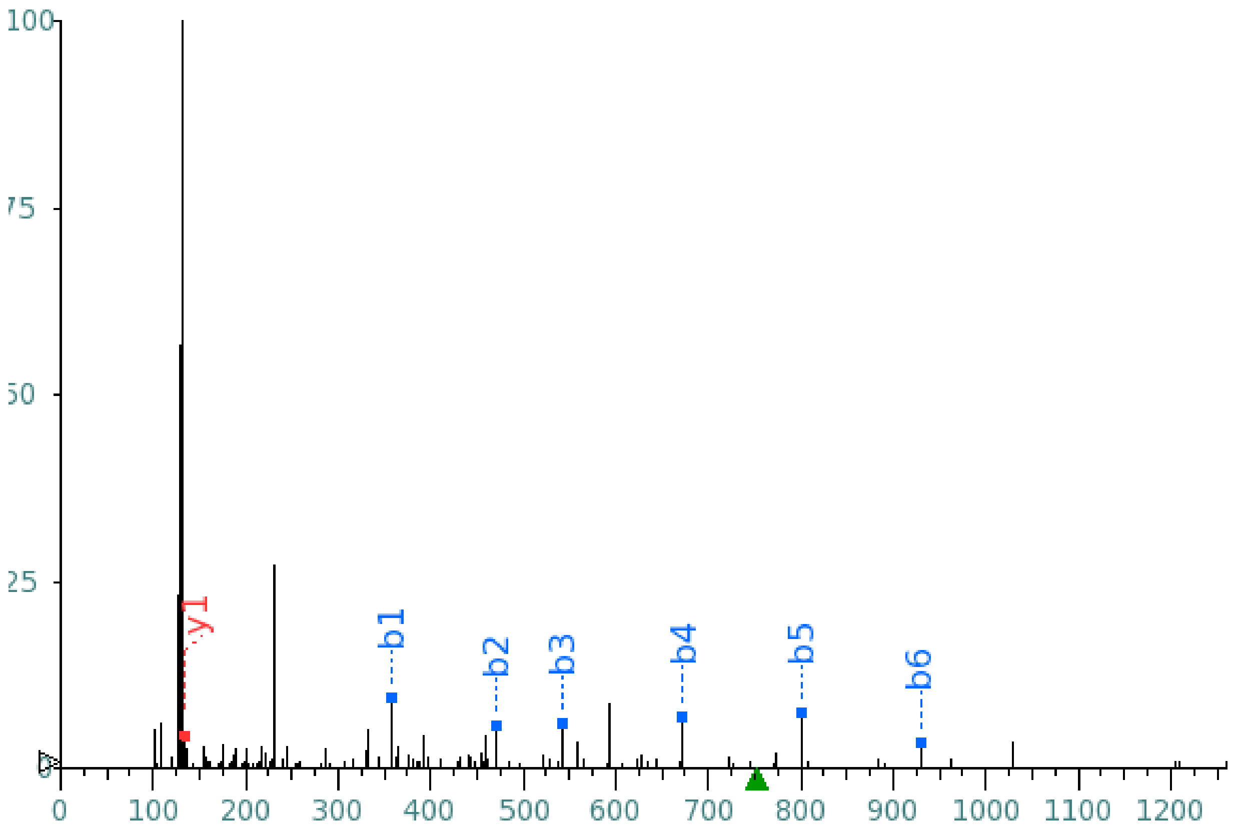

QLAEEEEPMDTD (+2)

Primary Reference: UCHL5_HUMAN
Search ID: 41760
Search Name: 20130328_ananiav_ERstress_TMT_TM_timecourse_trypsin_inj1_4mc_iaa
Scan#: 9381
Observed Mass: 753.8468 (2.1 ppm)
PSM Score: 17.14

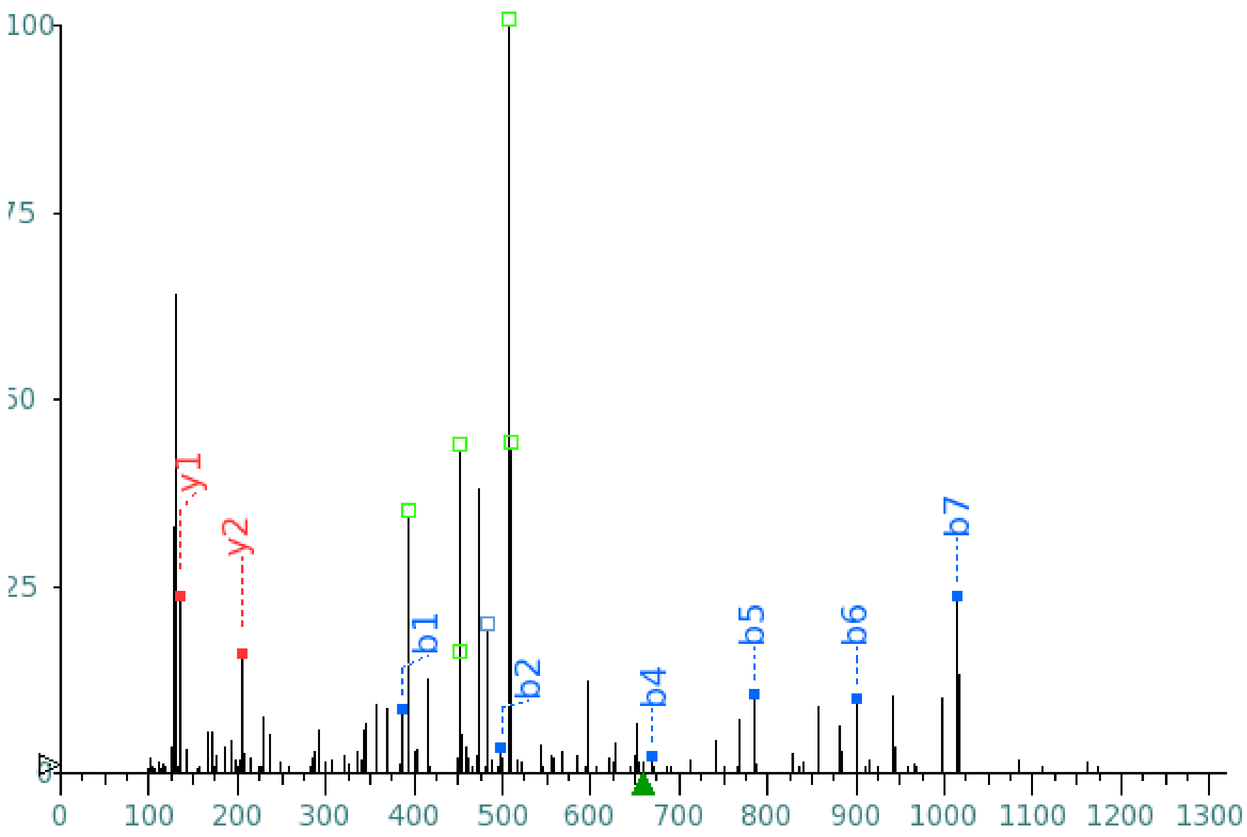

RLLGDDDVAD (+2)

Primary Reference: WDR62_HUMAN
Search ID: 41760
Search Name: 20130328_ananiav_ERstress_TMT_TM_timecourse_trypsin_inj1_4mc_iaa
Scan#: 9389
Observed Mass: 659.3475 (2.2 ppm)
PSM Score: 14.62

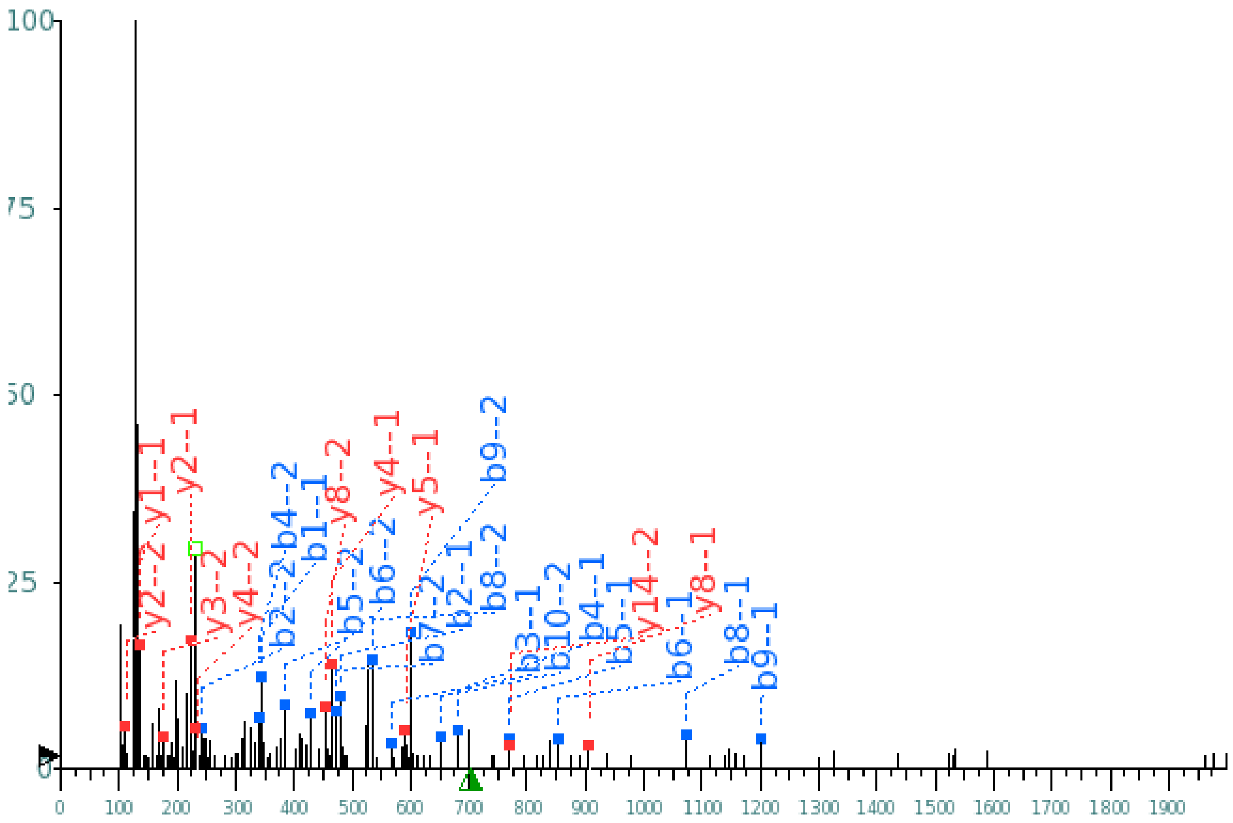

IHSNSSSEEVSQELESD (+3)

Primary Reference: PCLI1_HUMAN
Search ID: 41760
Search Name: 20130328_ananiav_ERstress_TMT_TM_timecourse_trypsin_inj1_4mc_iaa
Scan#: 9392
Observed Mass: 702.6621 (2.1 ppm)
PSM Score: 29.52

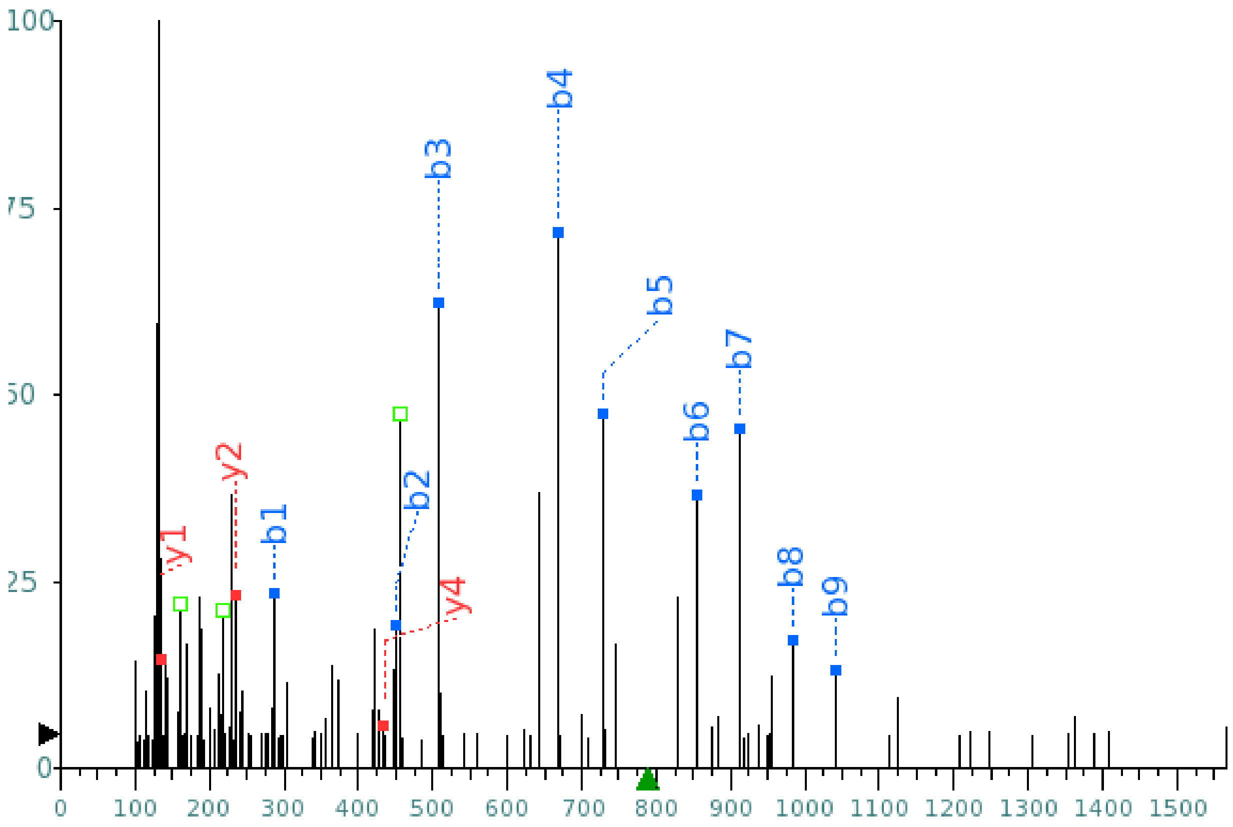

GYGYGQGAGTLSTD (+2)

Primary Reference: CSRP1_HUMAN
Search ID: 41760
Search Name: 20130328_ananiav_ERstress_TMT_TM_timecourse_trypsin_inj1_4mc_iaa
Scan#: 9401
Observed Mass: 788.3801 (2.6 ppm)
PSM Score: 33.67

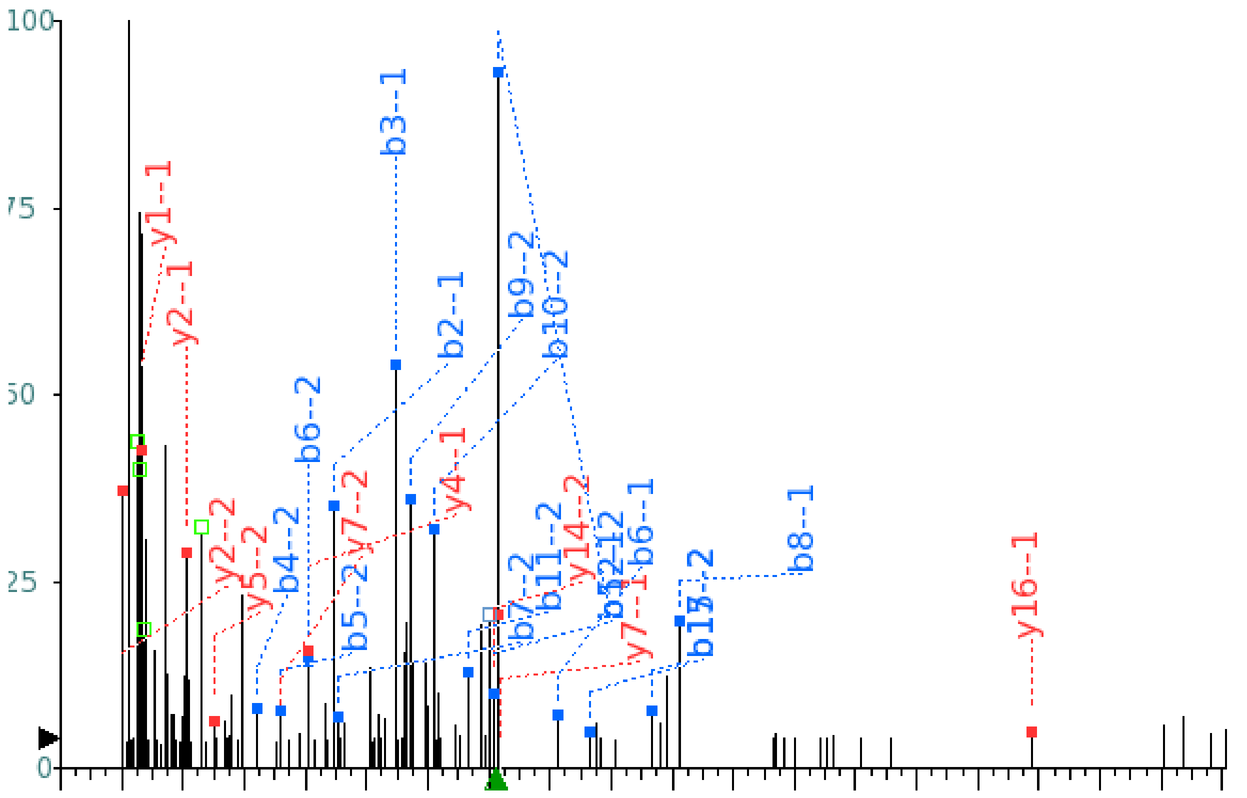

TDTPAPPTHALSQSPAEAD (+3)

Primary Reference: SNPC4_HUMAN
Search ID: 41760
Search Name: 20130328_ananiav_ERstress_TMT_TM_timecourse_trypsin_inj1_4mc_iaa
Scan#: 9412
Observed Mass: 712.3555 (3 ppm)
PSM Score: 38.58

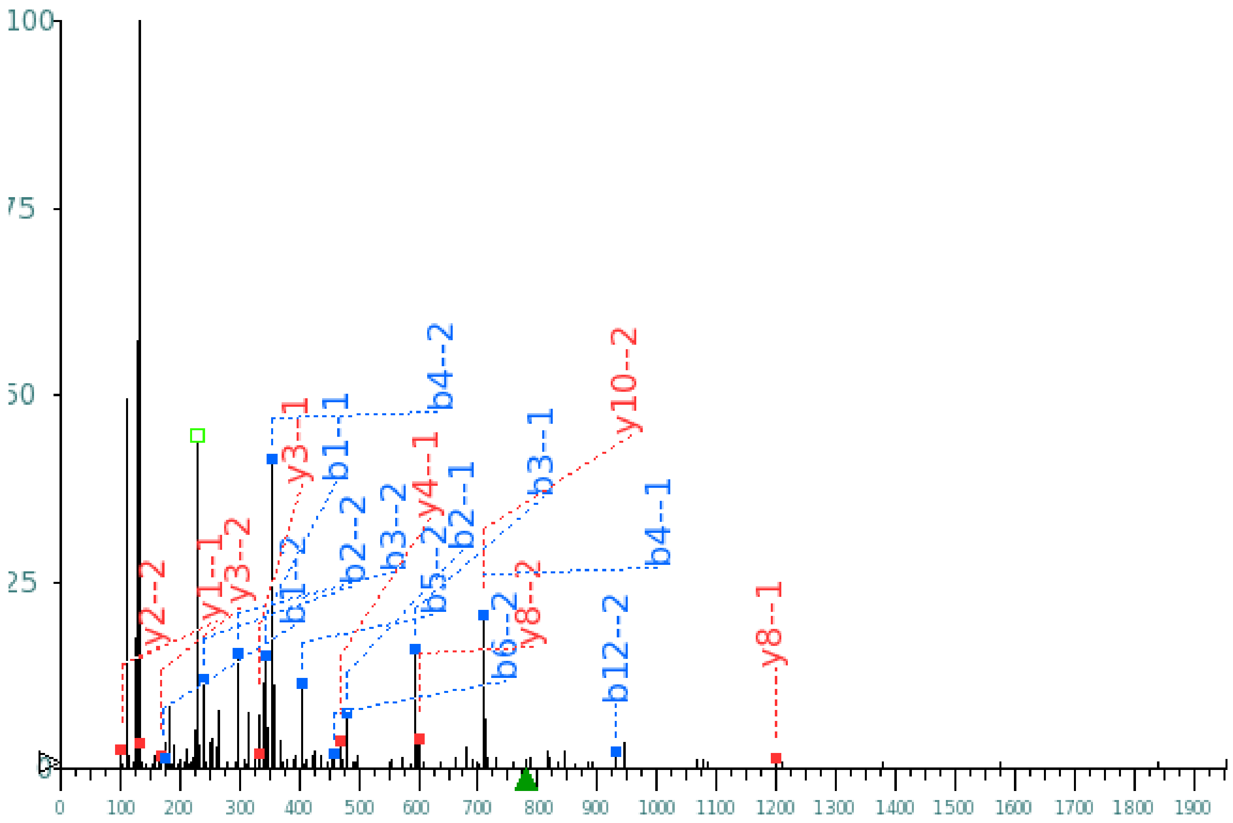

LHDNPLTDENKEHEAD (+3)

Primary Reference: NAA15_HUMAN
Search ID: 41760
Search Name: 20130328_ananiav_ERstress_TMT_TM_timecourse_trypsin_inj1_4mc_iaa
Scan#: 9414
Observed Mass: 779.0587 (2.1 ppm)
PSM Score: 14.99

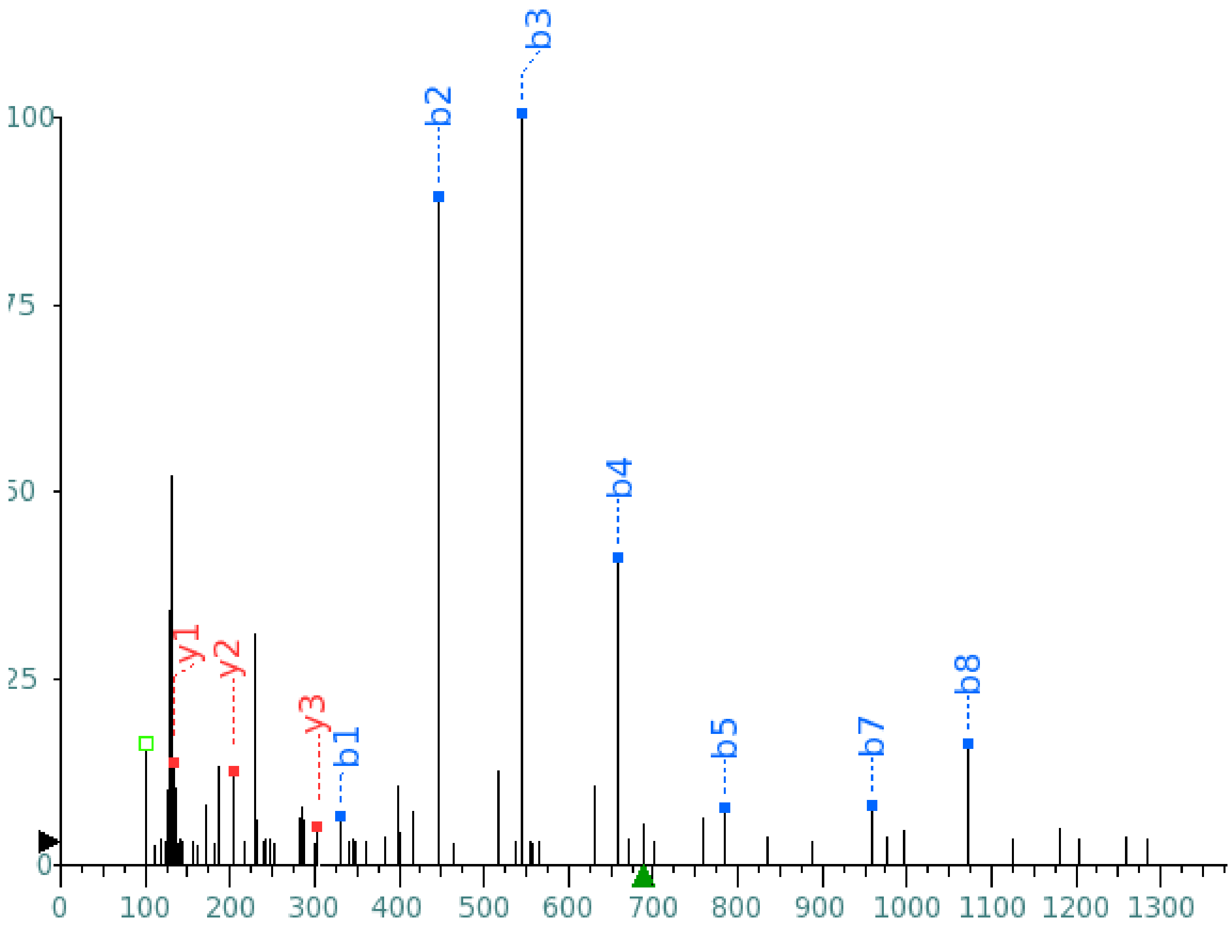

TDVIQGDDVAD (+2)

Primary Reference: RBP2_HUMAN
Search ID: 41760
Search Name: 20130328_ananiav_ERstress_TMT_TM_timecourse_trypsin_inj1_4mc_iaa
Scan#: 9422
Observed Mass: 688.8414 (1 ppm)
PSM Score: 35.34

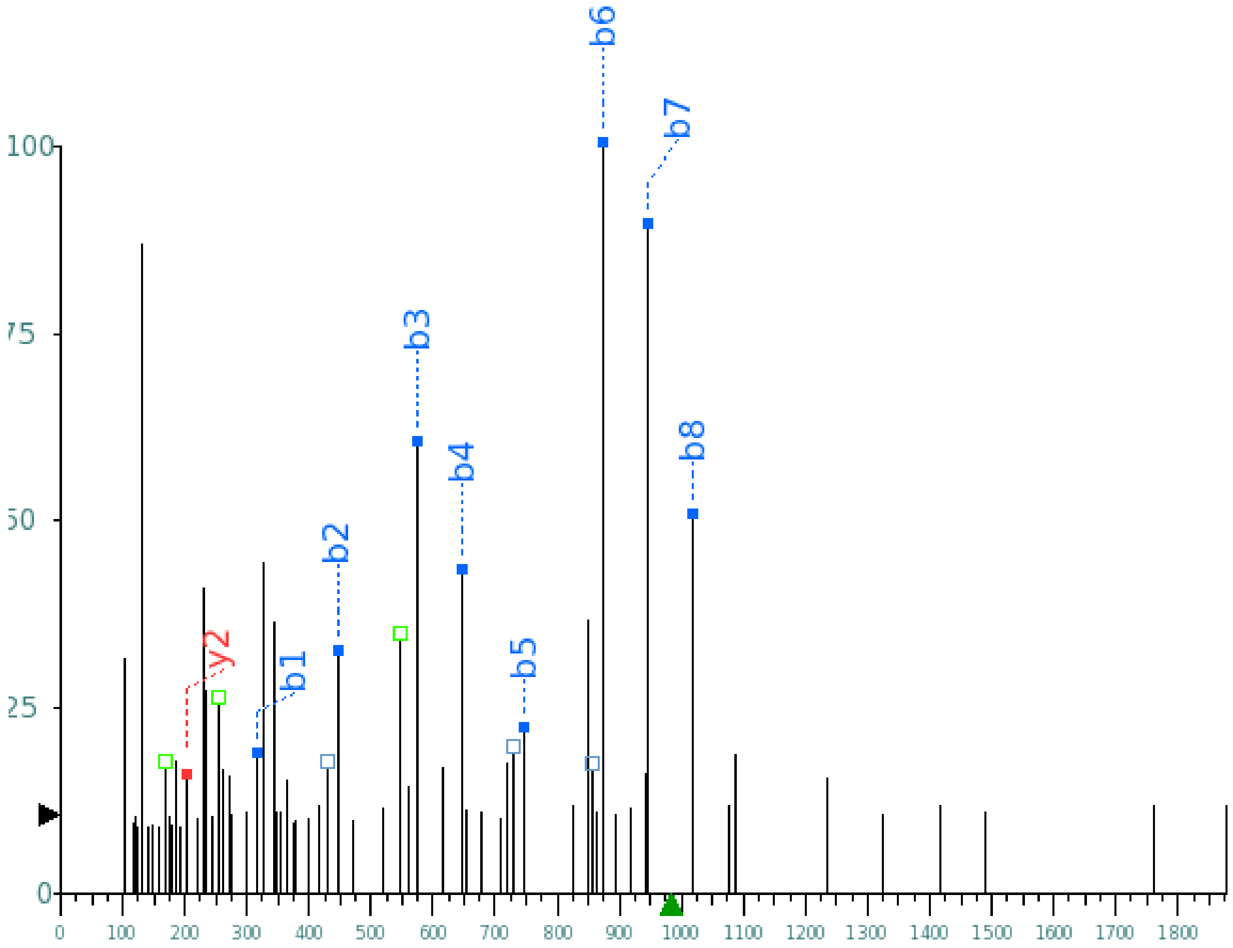

SQEATEAAPSC*VGDMAD (+2)

Primary Reference: SEPT9_HUMAN
Search ID: 41760
Search Name: 20130328_ananiav_ERstress_TMT_TM_timecourse_trypsin_inj1_4mc_iaa
Scan#: 9467
Observed Mass: 984.4312 (1.4 ppm)
PSM Score: 35.21

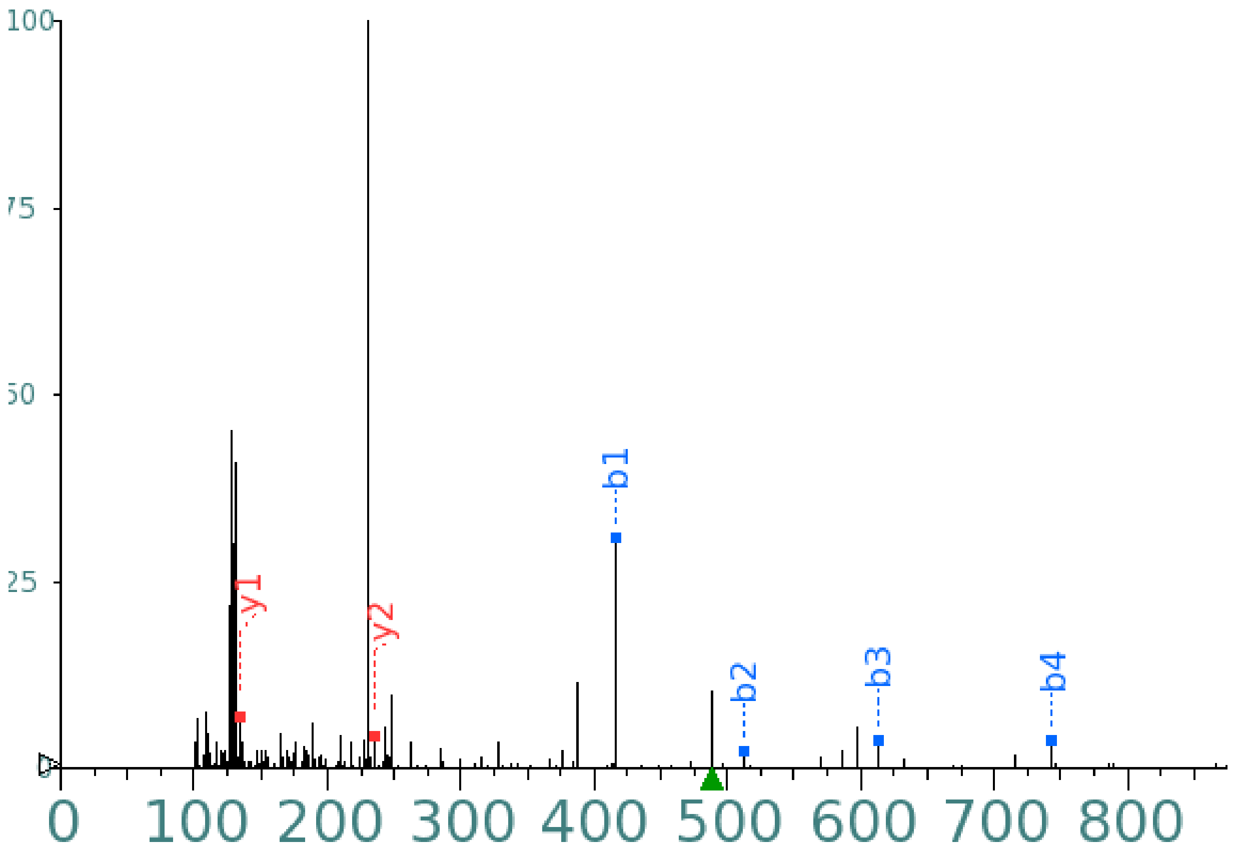

WPTETD (+2)

Primary Reference: MAP4_HUMAN
Search ID: 41760
Search Name: 20130328_ananiav_ERstress_TMT_TM_timecourse_trypsin_inj1_4mc_iaa
Scan#: 9481
Observed Mass: 489.2436 (2.2 ppm)
PSM Score: 10.21

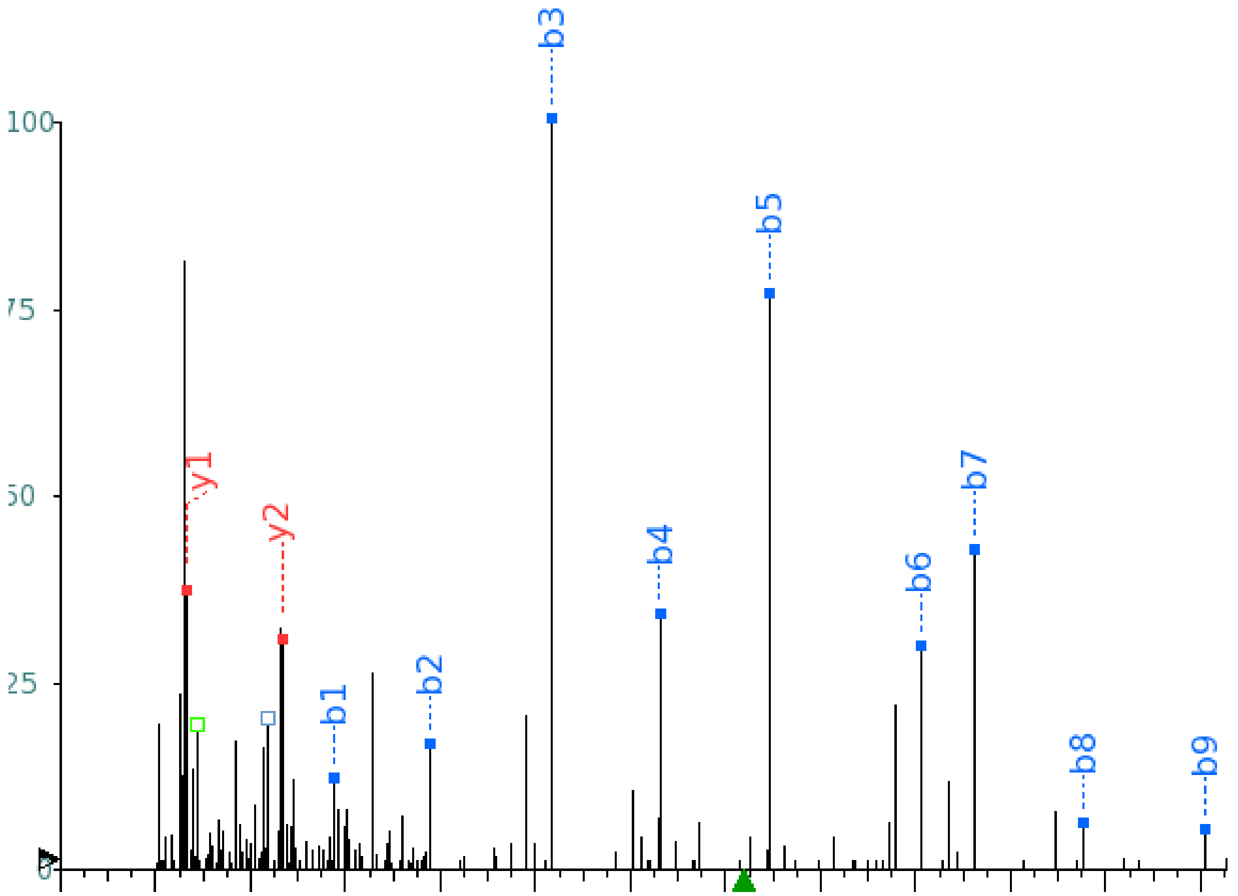

GTELDC*GIETD (+2)

Primary Reference: CASP3_HUMAN
Search ID: 41760
Search Name: 20130328_ananiav_ERstress_TMT_TM_timecourse_trypsin_inj1_4mc_iaa
Scan#: 9486
Observed Mass: 719.8333 (1.7 ppm)
PSM Score: 43.12

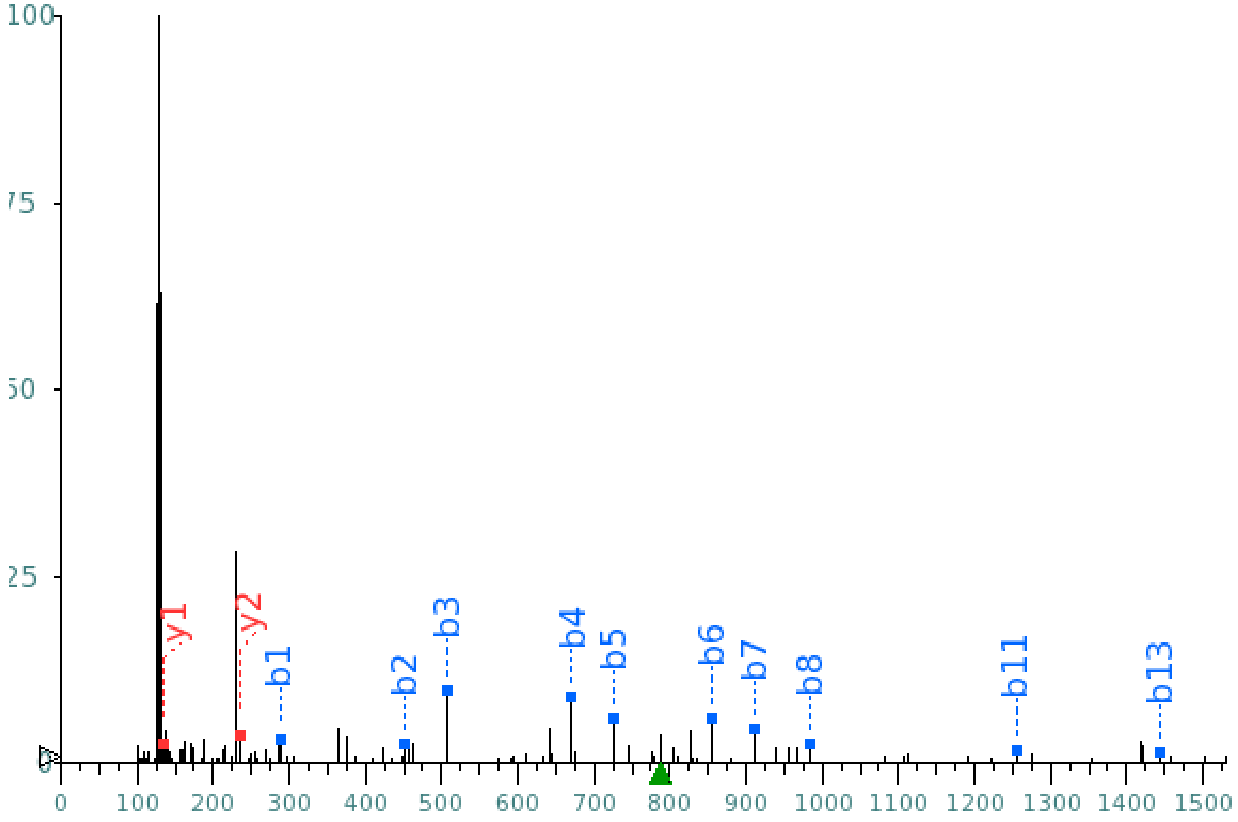

GYGYGQGAGTLSTD (+2)

Primary Reference: CSRP1_HUMAN
Search ID: 41760
Search Name: 20130328_ananiav_ERstress_TMT_TM_timecourse_trypsin_inj1_4mc_iaa
Scan#: 9496
Observed Mass: 788.3792 (1.4 ppm)
PSM Score: 25.85

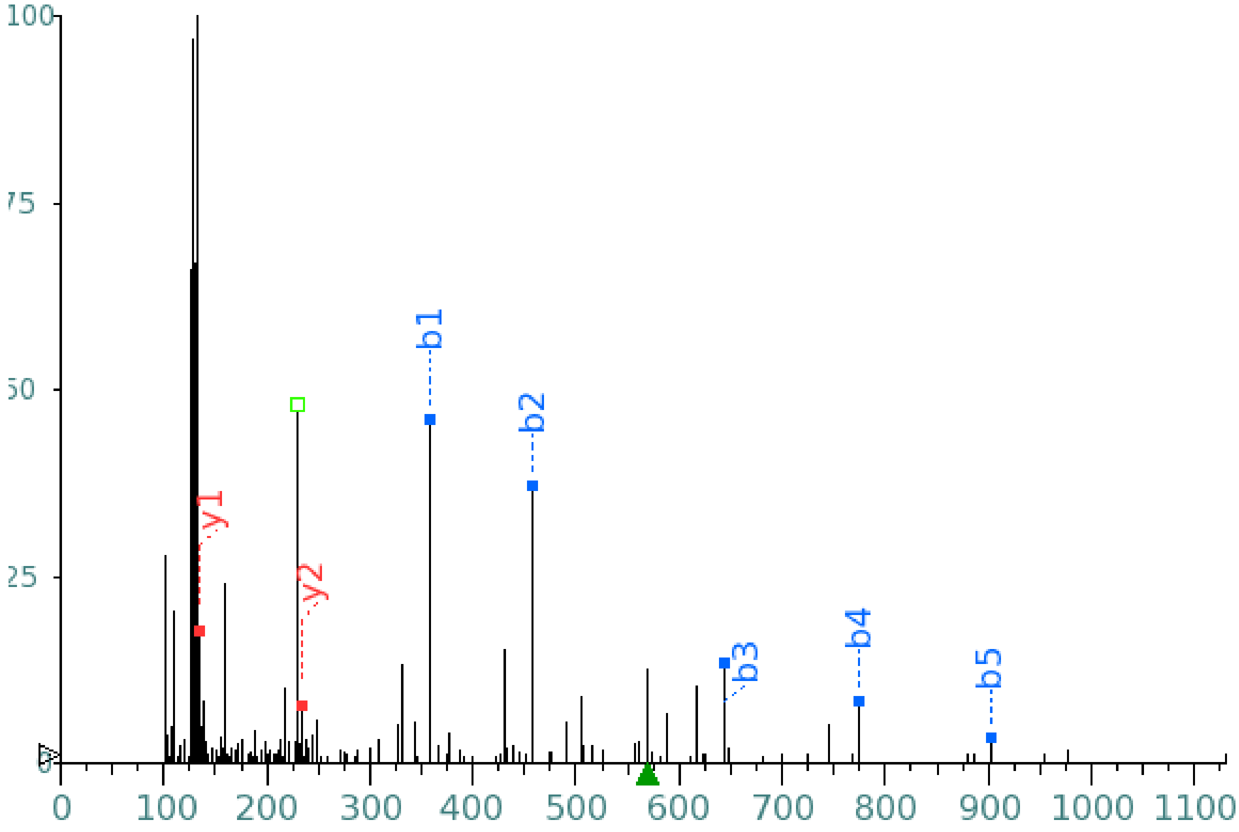

EVWEETD (+2)

Primary Reference: NUCB2_HUMAN
Search ID: 41760
Search Name: 20130328_ananiav_ERstress_TMT_TM_timecourse_trypsin_inj1_4mc_iaa
Scan#: 9515
Observed Mass: 568.7703 (2.1 ppm)
PSM Score: 25.87

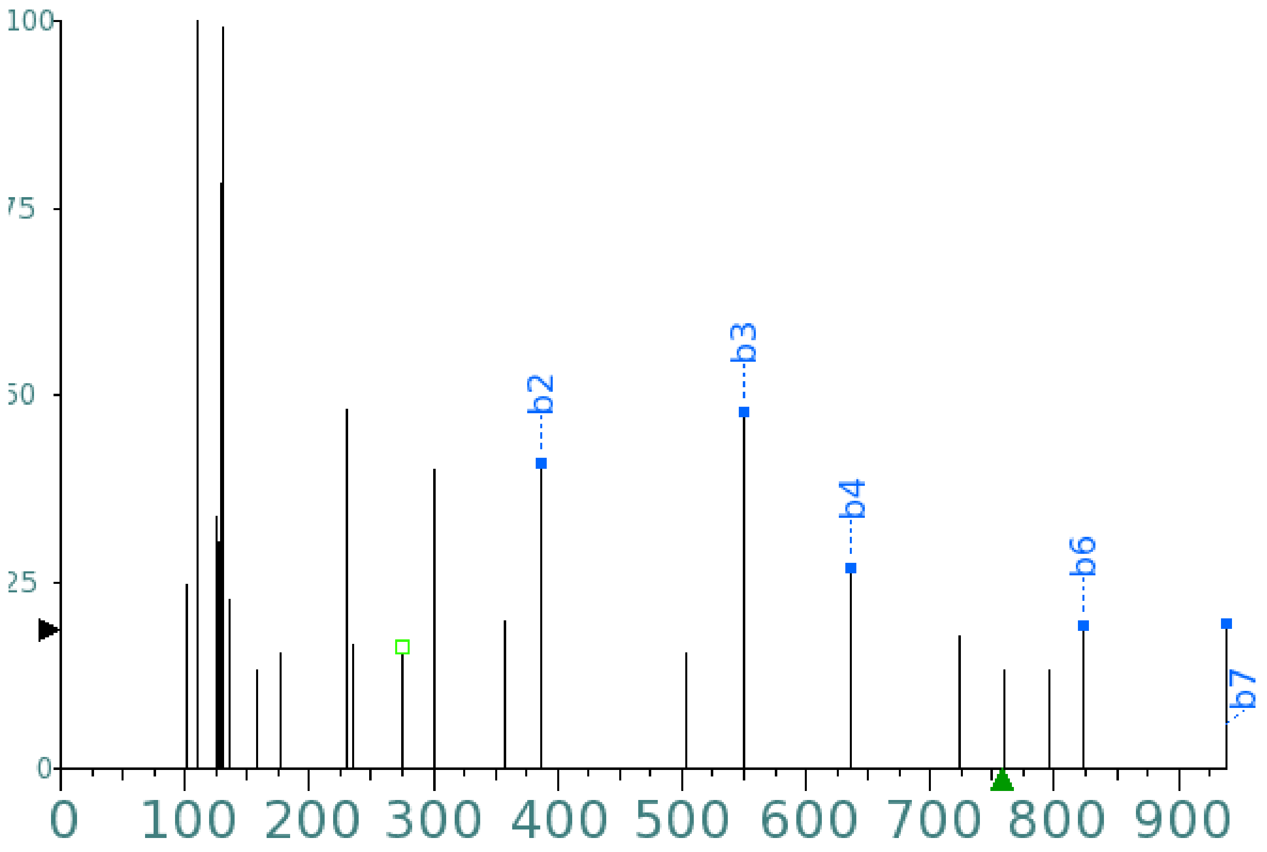

GVYSSTNELTTD (+2)

Primary Reference: N4BP1_HUMAN
Search ID: 41760
Search Name: 20130328_ananiav_ERstress_TMT_TM_timecourse_trypsin_inj1_4mc_iaa
Scan#: 9517
Observed Mass: 758.3731 (0.9 ppm)
PSM Score: 43.14

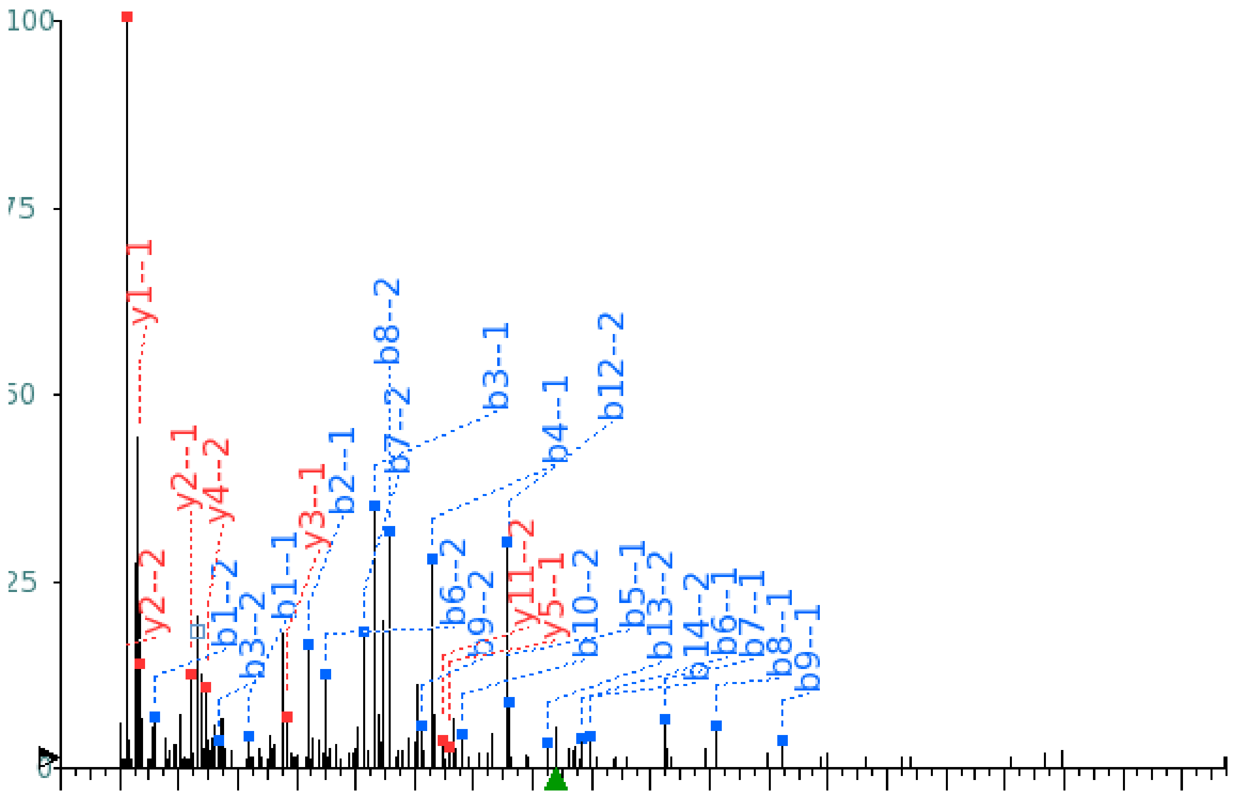

STLVQHESIHTGENPYDC*SD (+3)

Primary Reference: ZN134_HUMAN
Search ID: 41760
Search Name: 20130328_ananiav_ERstress_TMT_TM_timecourse_trypsin_inj1_4mc_iaa
Scan#: 9525
Observed Mass: 840.0513 (1.5 ppm)
PSM Score: 38.55

File name: 2015-02-04 04.01.53PM.pdf

Creation time: 2015-02-04 04:09PM

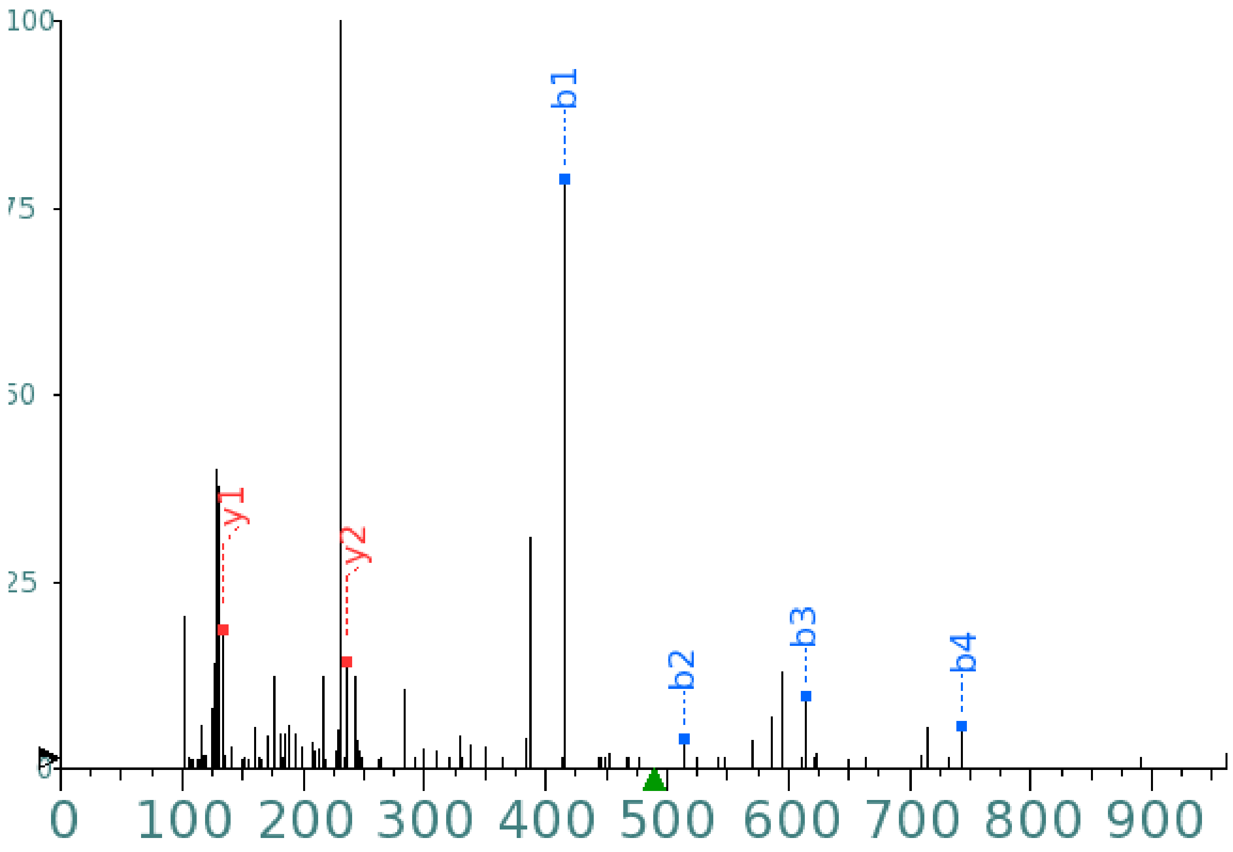

WPTETD (+2)

Primary Reference: MAP4_HUMAN
Search ID: 41760
Search Name: 20130328_ananiav_ERstress_TMT_TM_timecourse_trypsin_inj1_4mc_iaa
Scan#: 9576
Observed Mass: 489.2437 (2.4 ppm)
PSM Score: 4.18

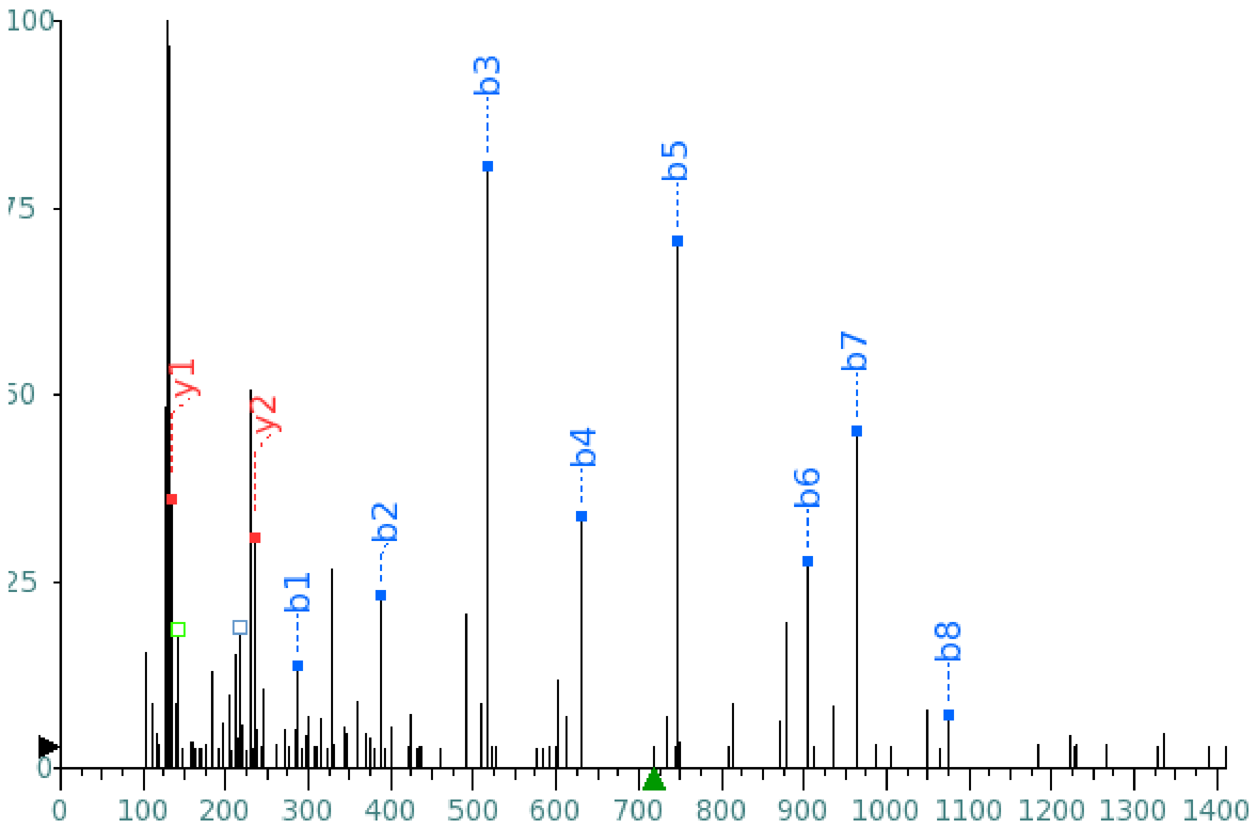

GTELDC*GIETD (+2)

Primary Reference: CASP3_HUMAN
Search ID: 41760
Search Name: 20130328_ananiav_ERstress_TMT_TM_timecourse_trypsin_inj1_4mc_iaa
Scan#: 9579
Observed Mass: 719.8335 (2 ppm)
PSM Score: 24.84

File name: 2015-02-04 04.01.53PM.pdf

Creation time: 2015-02-04 04:09PM

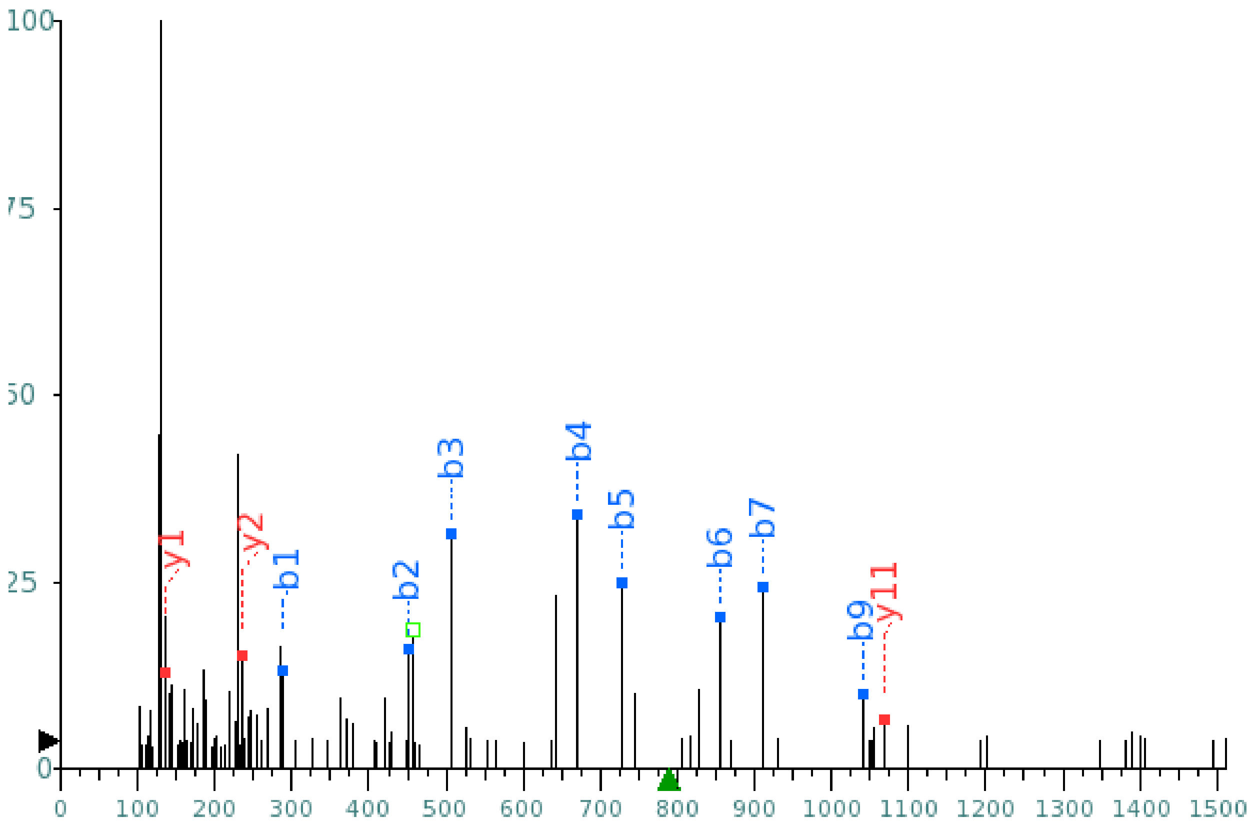

GYGYGQGAGTLSTD (+2)

Primary Reference: CSRP1_HUMAN
Search ID: 41760
Search Name: 20130328_ananiav_ERstress_TMT_TM_timecourse_trypsin_inj1_4mc_iaa
Scan#: 9585
Observed Mass: 788.3789 (1.1 ppm)
PSM Score: 23.93

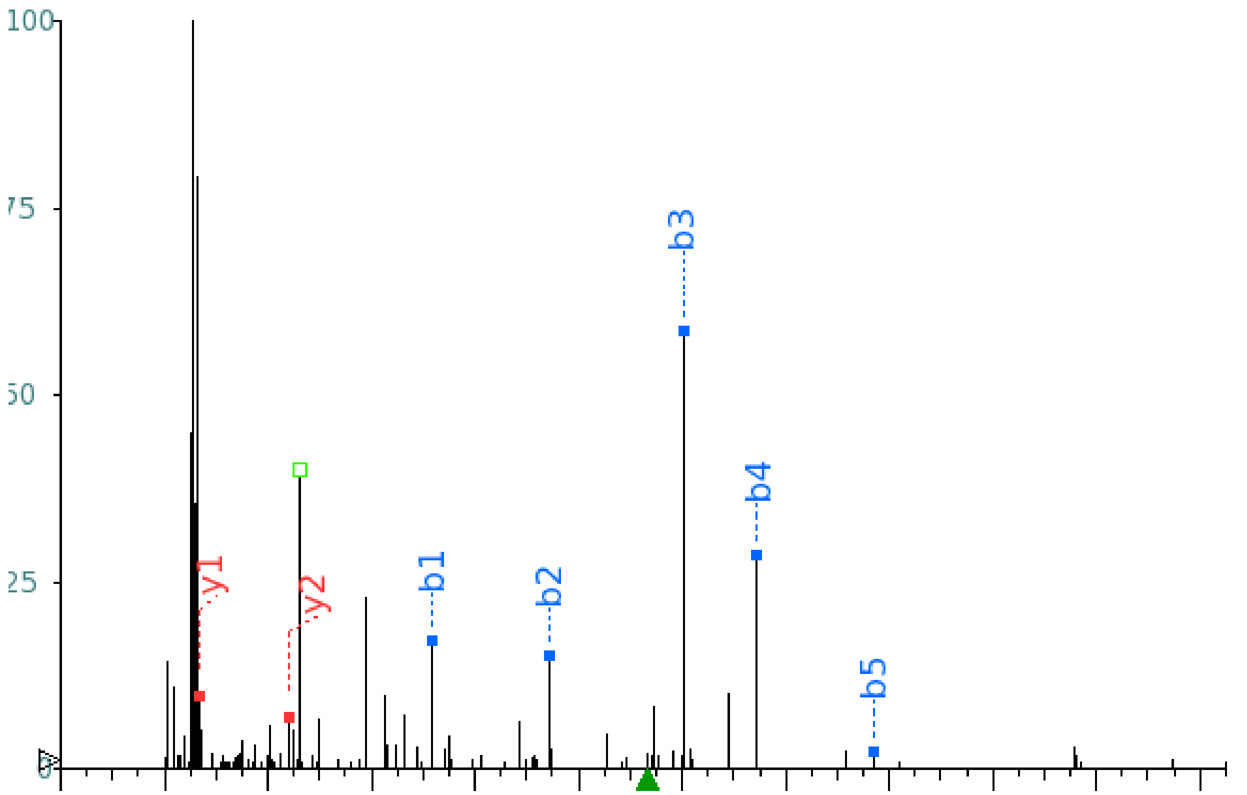

EIEAIESD (+2)

Primary Reference: RSRC1_HUMAN
Search ID: 41760
Search Name: 20130328_ananiav_ERstress_TMT_TM_timecourse_trypsin_inj1_4mc_iaa
Scan#: 9623
Observed Mass: 567.7912 (2.1 ppm)
PSM Score: 12.52

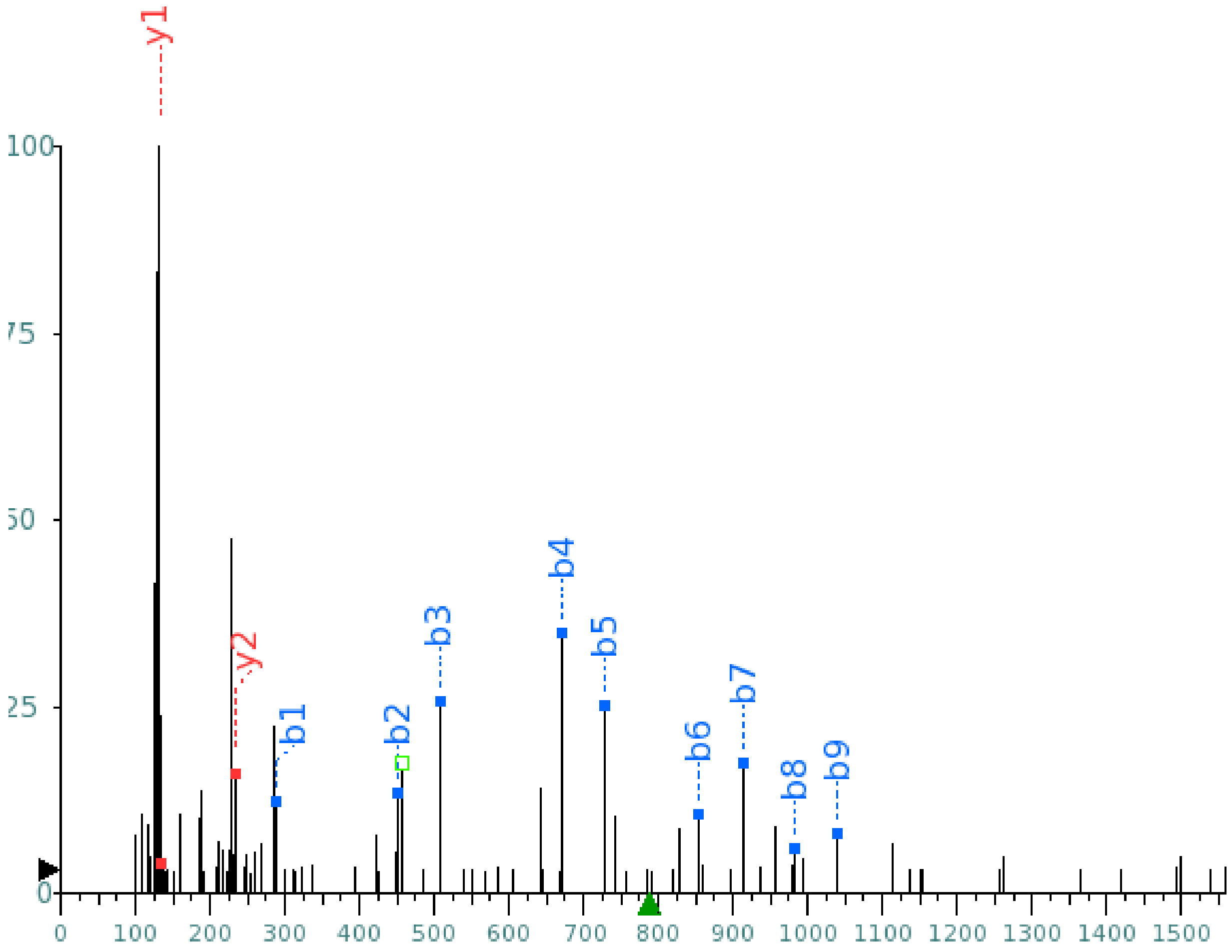

GYGYGQGAGTLSTD (+2)

Primary Reference: CSRP1_HUMAN
Search ID: 41760
Search Name: 20130328_ananiav_ERstress_TMT_TM_timecourse_trypsin_inj1_4mc_iaa
Scan#: 9669
Observed Mass: 788.3793 (1.6 ppm)
PSM Score: 29.4

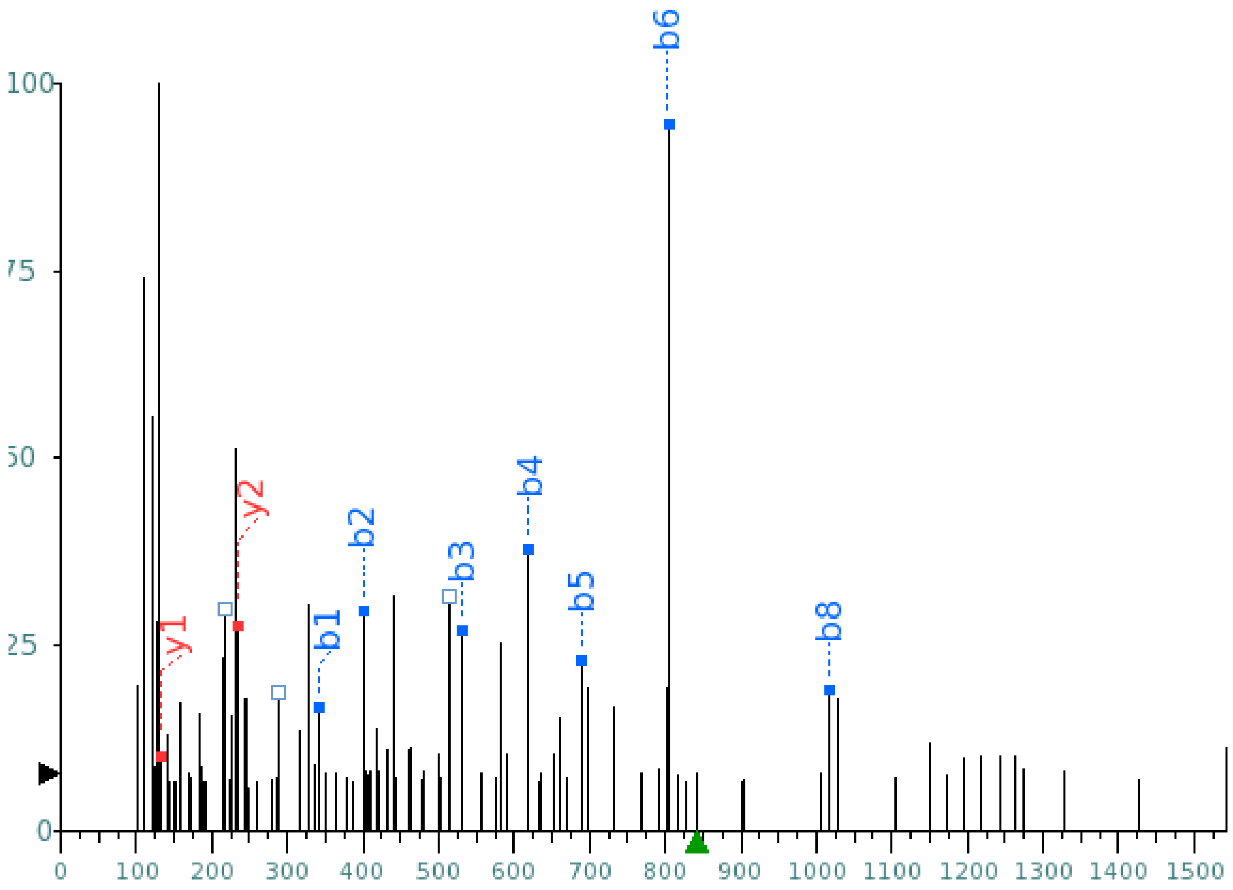

LGMSADPDNEDATD (+2)

Primary Reference: ZC11A_HUMAN
Search ID: 41760
Search Name: 20130328_ananiav_ERstress_TMT_TM_timecourse_trypsin_inj1_4mc_iaa
Scan#: 9708
Observed Mass: 840.3691 (2.5 ppm)
PSM Score: 15.11

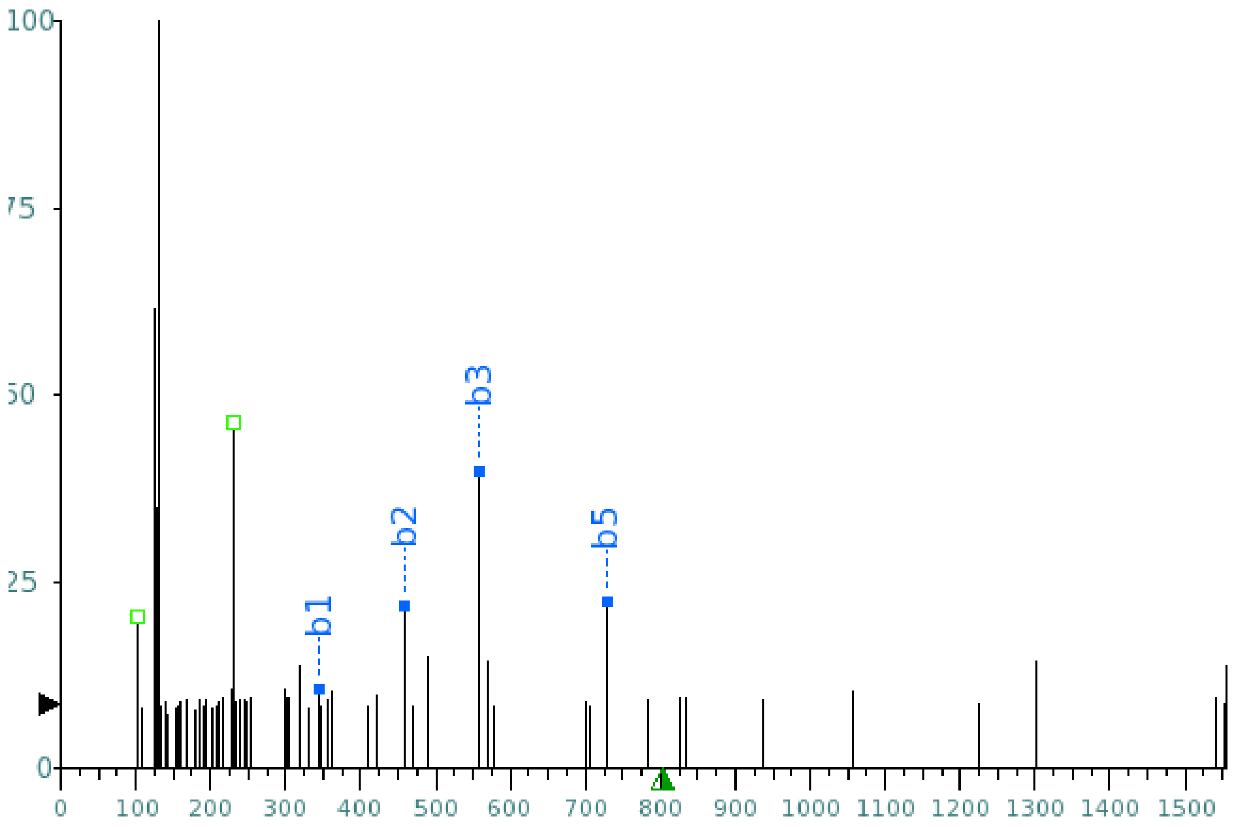

DLTPAVTDNDEAD (+2)

Primary Reference: SELB_HUMAN
Search ID: 41760
Search Name: 20130328_ananiav_ERstress_TMT_TM_timecourse_trypsin_inj1_4mc_iaa
Scan#: 9724
Observed Mass: 802.8787 (0.8 ppm)
PSM Score: 12.34

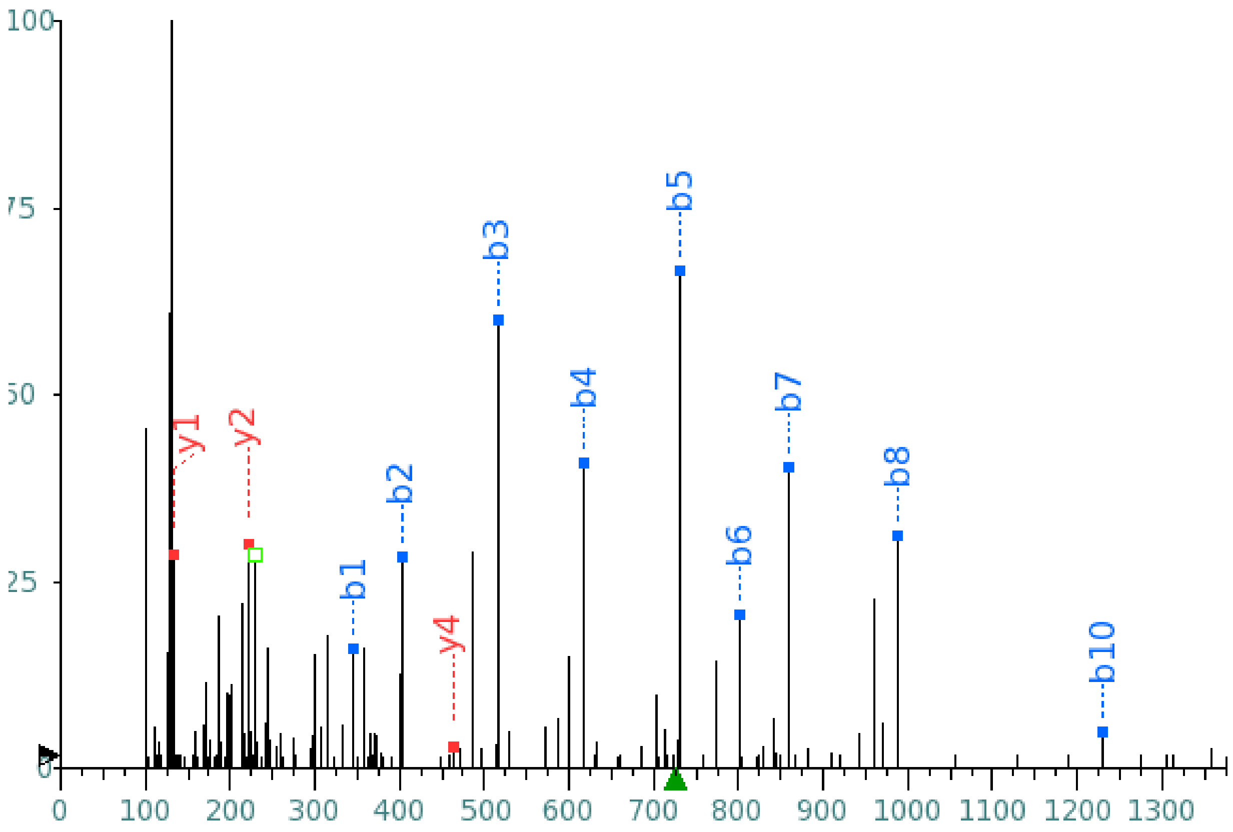

DGLTNAGELESD (+2)

Primary Reference: BMI1_HUMAN
Search ID: 41760
Search Name: 20130328_ananiav_ERstress_TMT_TM_timecourse_trypsin_inj1_4mc_iaa
Scan#: 9822
Observed Mass: 725.3497 (1.1 ppm)
PSM Score: 54.38

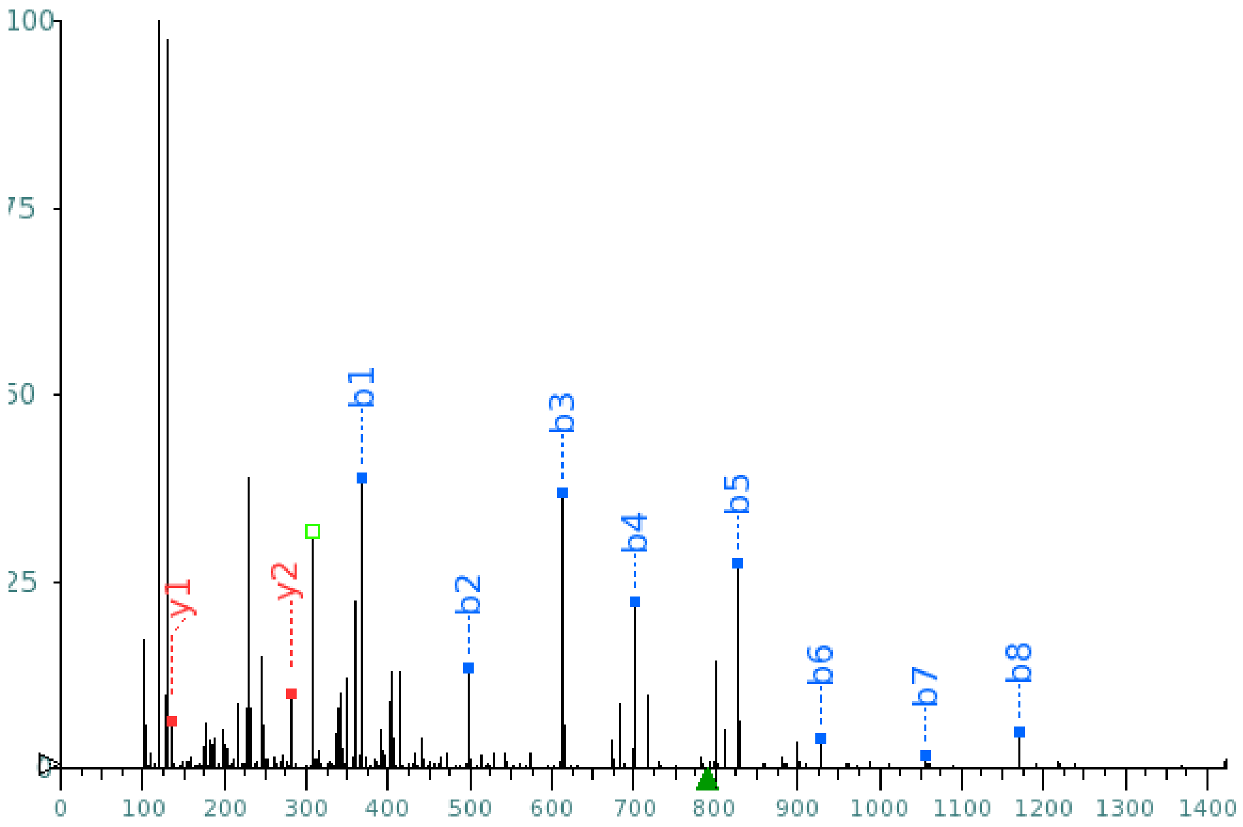

HMDSQVEDEFD (+2)

Primary Reference: SLBP_HUMAN
Search ID: 41760
Search Name: 20130328_ananiav_ERstress_TMT_TM_timecourse_trypsin_inj1_4mc_iaa
Scan#: 9826
Observed Mass: 790.8413 (1 ppm)
PSM Score: 30.76

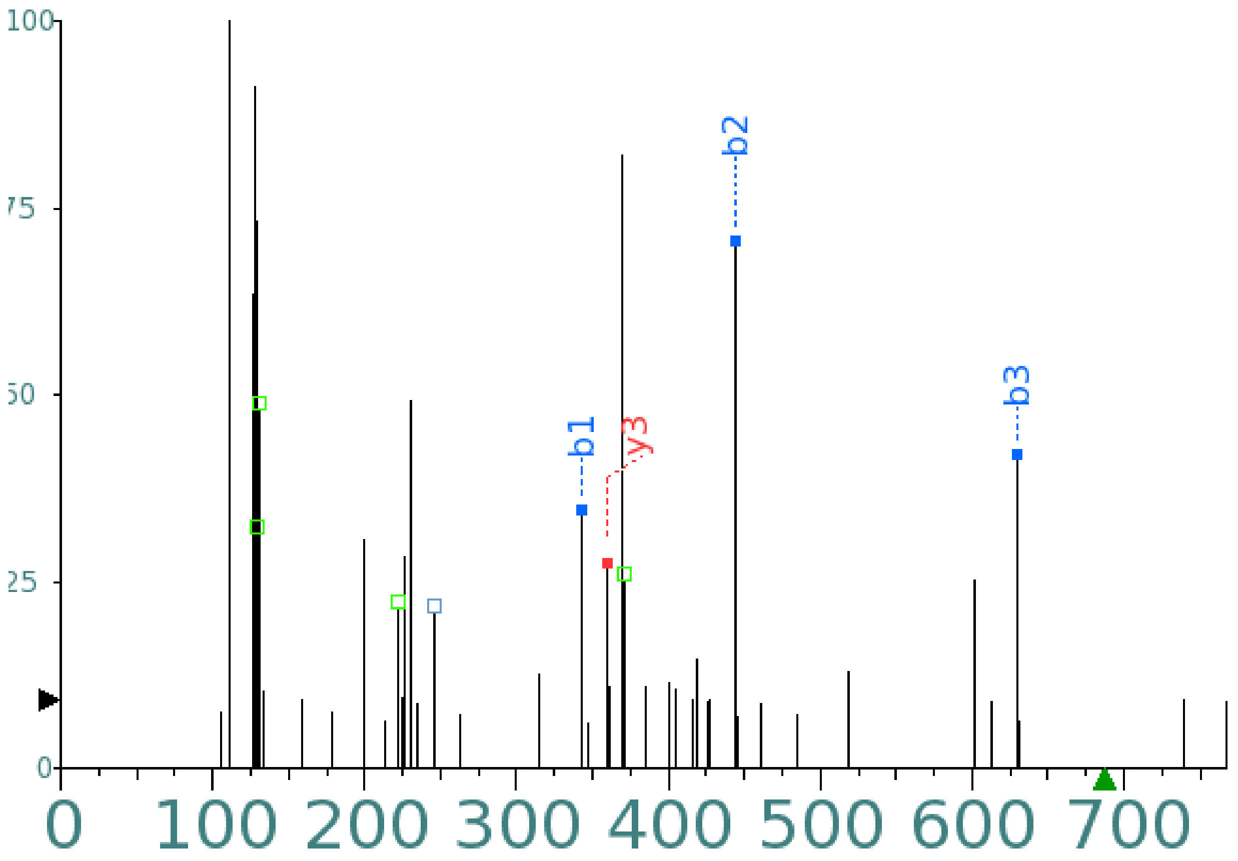

LTWHSC*PED (+2)

Primary Reference: CBX3_HUMAN
Search ID: 41760
Search Name: 20130328_ananiav_ERstress_TMT_TM_timecourse_trypsin_inj1_4mc_iaa
Scan#: 9847
Observed Mass: 687.3214 (-0.1 ppm)
PSM Score: 11.61

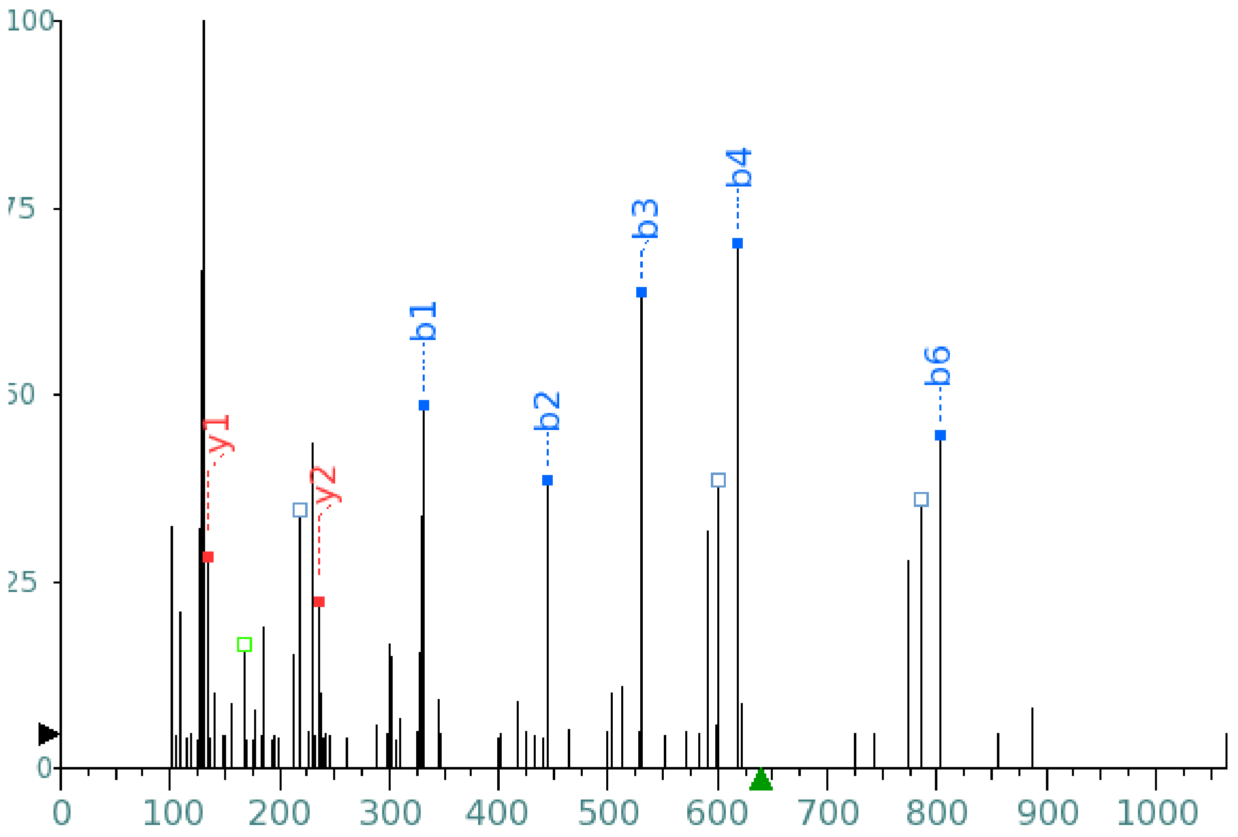

TLSSPSLQTD (+2)

Primary Reference: DOCK5_HUMAN
Search ID: 41760
Search Name: 20130328_ananiav_ERstress_TMT_TM_timecourse_trypsin_inj1_4mc_iaa
Scan#: 9906
Observed Mass: 639.3438 (1.3 ppm)
PSM Score: 32.14

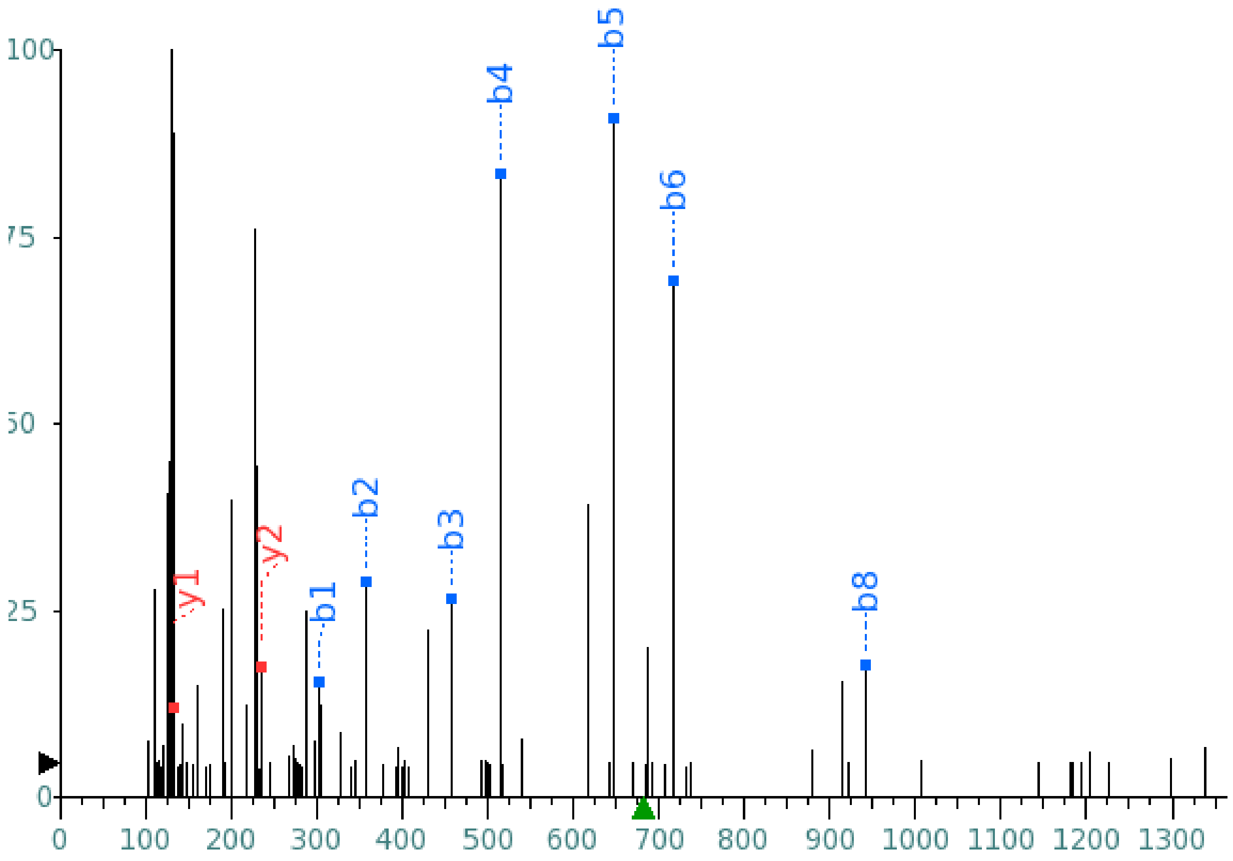

AGVGMAPEVSTD (+2)

Primary Reference: MA7D3_HUMAN
Search ID: 41760
Search Name: 20130328_ananiav_ERstress_TMT_TM_timecourse_trypsin_inj1_4mc_iaa
Scan#: 9931
Observed Mass: 681.8429 (1 ppm)
PSM Score: 31.51

File name: 2015-02-04 04.01.53PM.pdf

Creation time: 2015-02-04 04:09PM

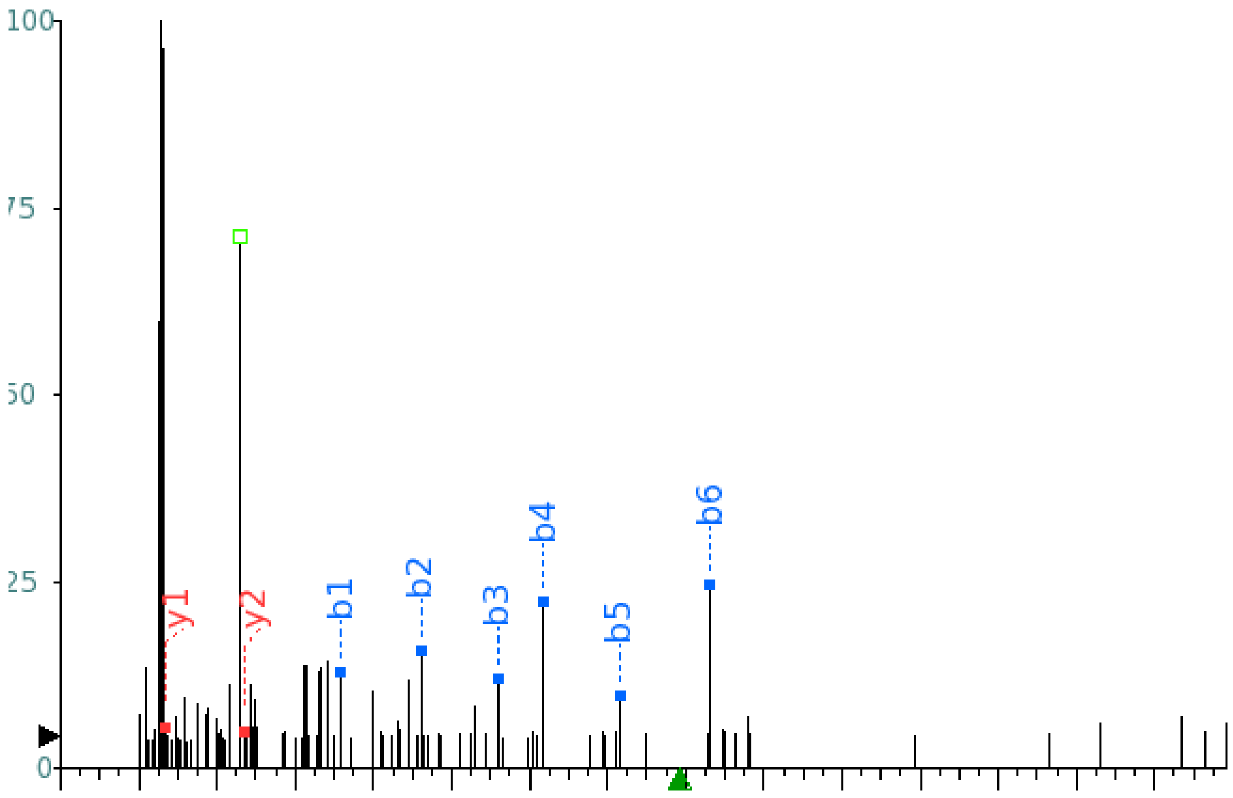

ETVGVDATTMDTD (+2)

Primary Reference: RICTR_HUMAN
Search ID: 41760
Search Name: 20130328_ananiav_ERstress_TMT_TM_timecourse_trypsin_inj1_4mc_iaa
Scan#: 9988
Observed Mass: 792.3688 (-0.3 ppm)
PSM Score: 18.5

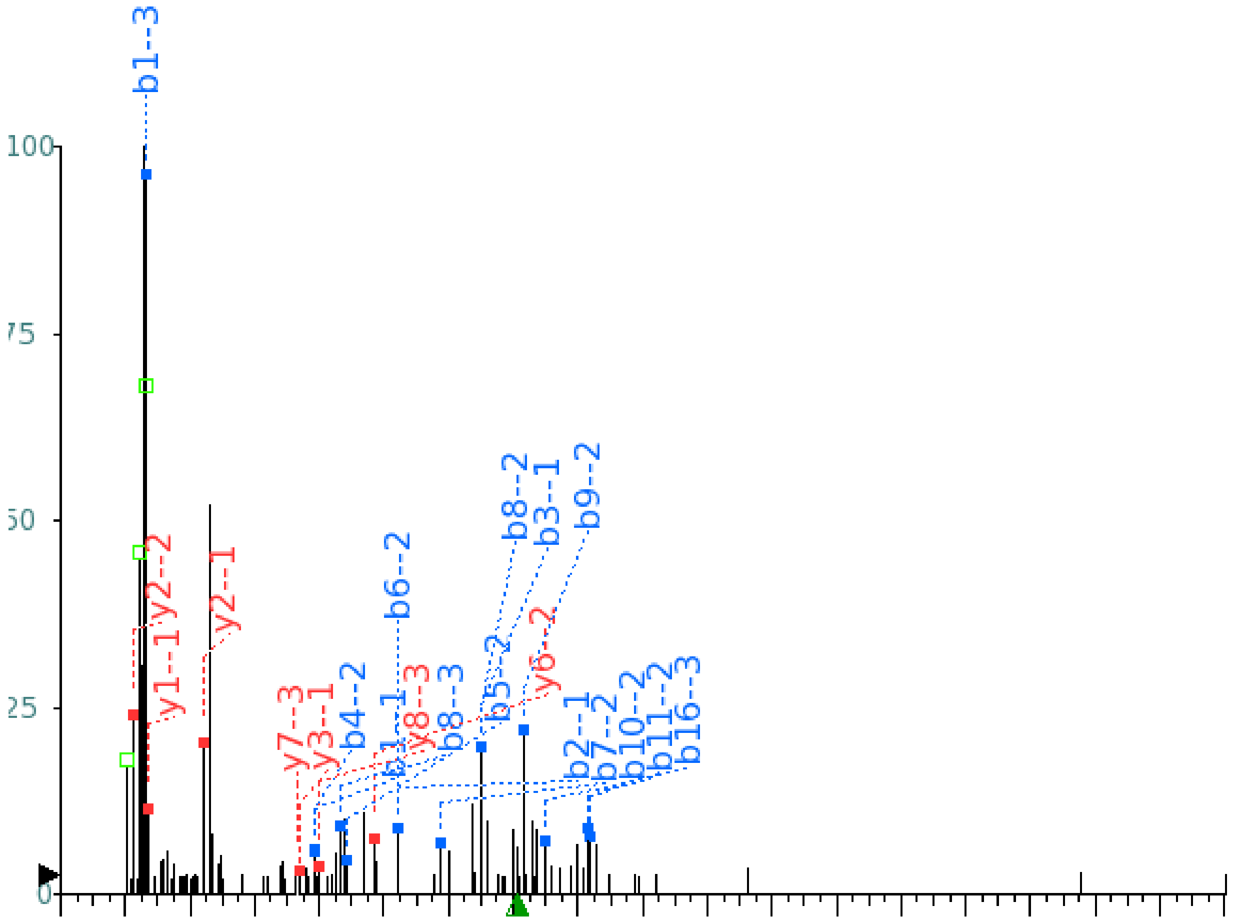

YEEHVREQQAQVEKEDFSD (+4)

Primary Reference: SF3B2_HUMAN
Search ID: 41760
Search Name: 20130328_ananiav_ERstress_TMT_TM_timecourse_trypsin_inj1_4mc_iaa
Scan#: 9992
Observed Mass: 706.849 (-1.8 ppm)
PSM Score: 15.57

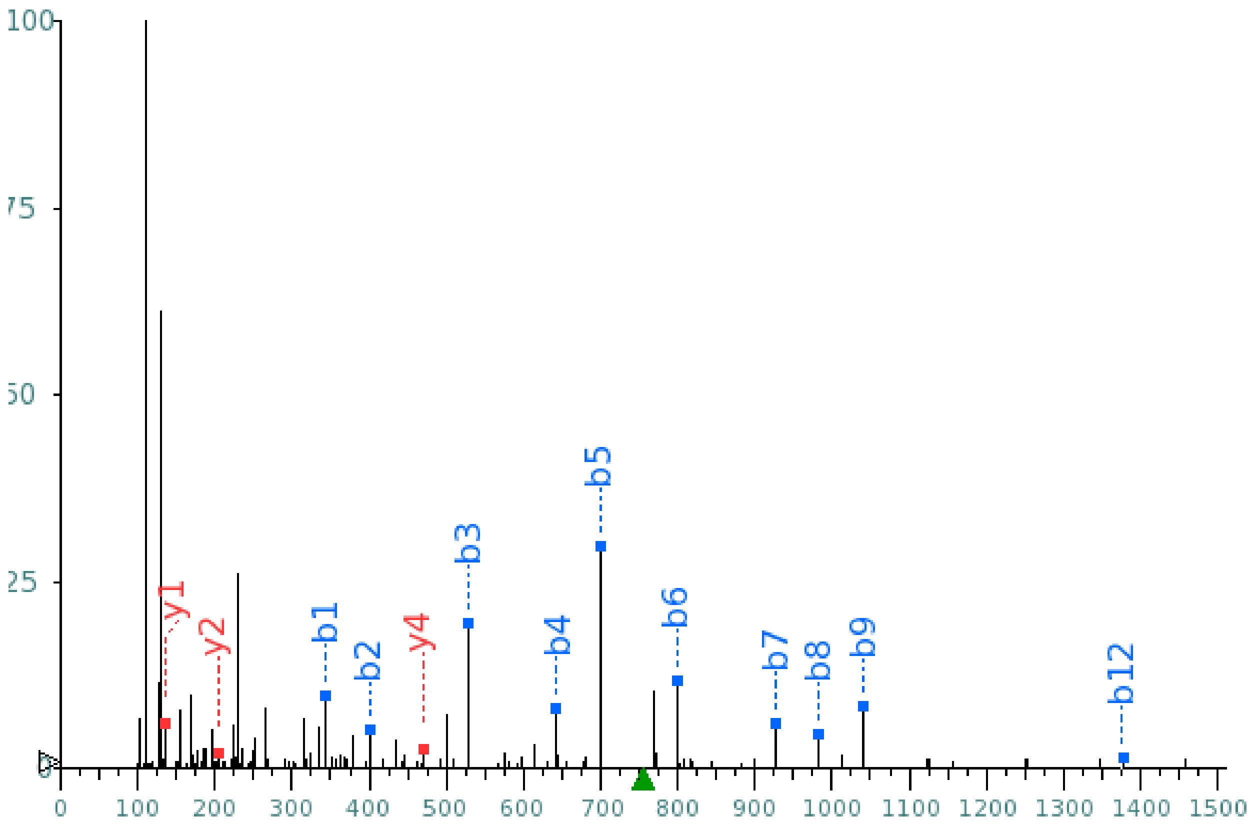

LGELGVQGGHQAD (+2)

Primary Reference: CC165_HUMAN
Search ID: 41760
Search Name: 20130328_ananiav_ERstress_TMT_TM_timecourse_trypsin_inj1_4mc_iaa
Scan#: 10030
Observed Mass: 755.3984 (2.4 ppm)
PSM Score: 41.31

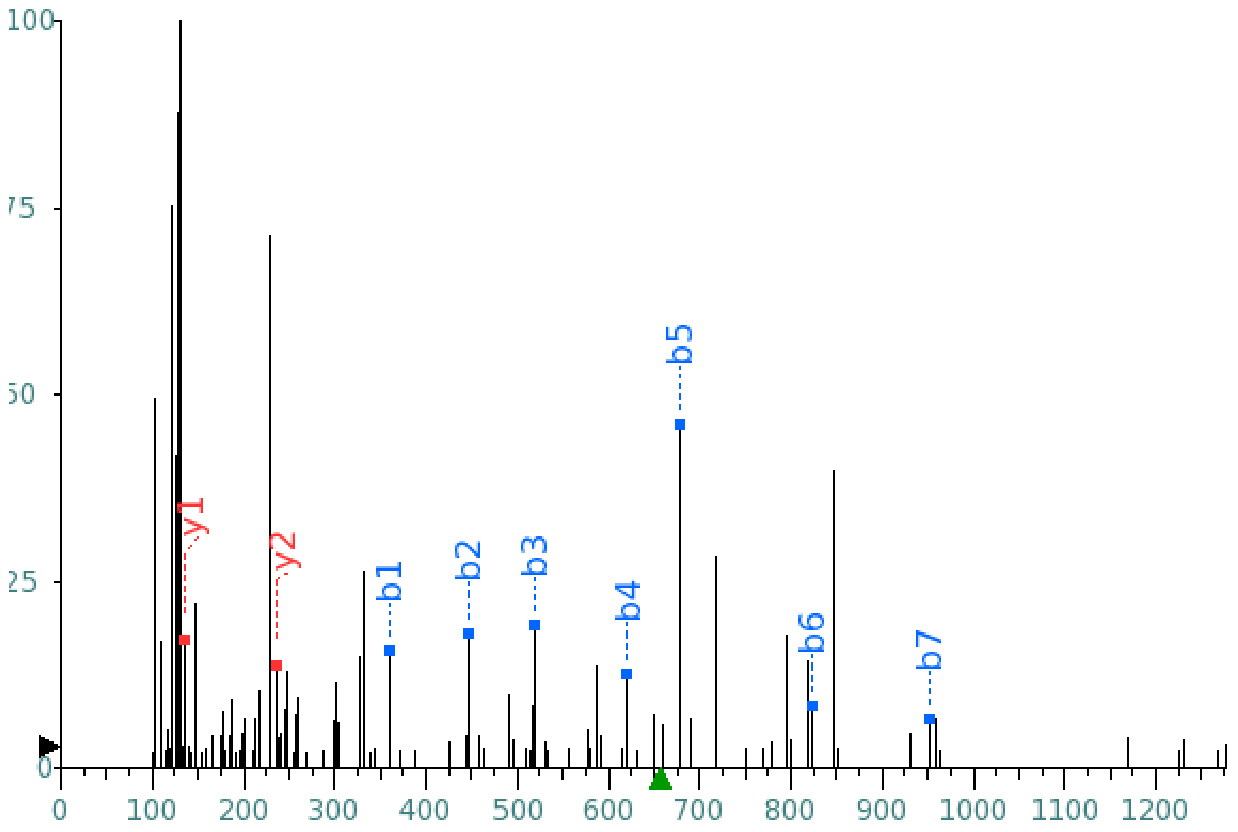

MSATGFEETD (+2)

Primary Reference: SRF_HUMAN
Search ID: 41760
Search Name: 20130328_ananiav_ERstress_TMT_TM_timecourse_trypsin_inj1_4mc_iaa
Scan#: 10072
Observed Mass: 658.7979 (0.6 ppm)
PSM Score: 17.29

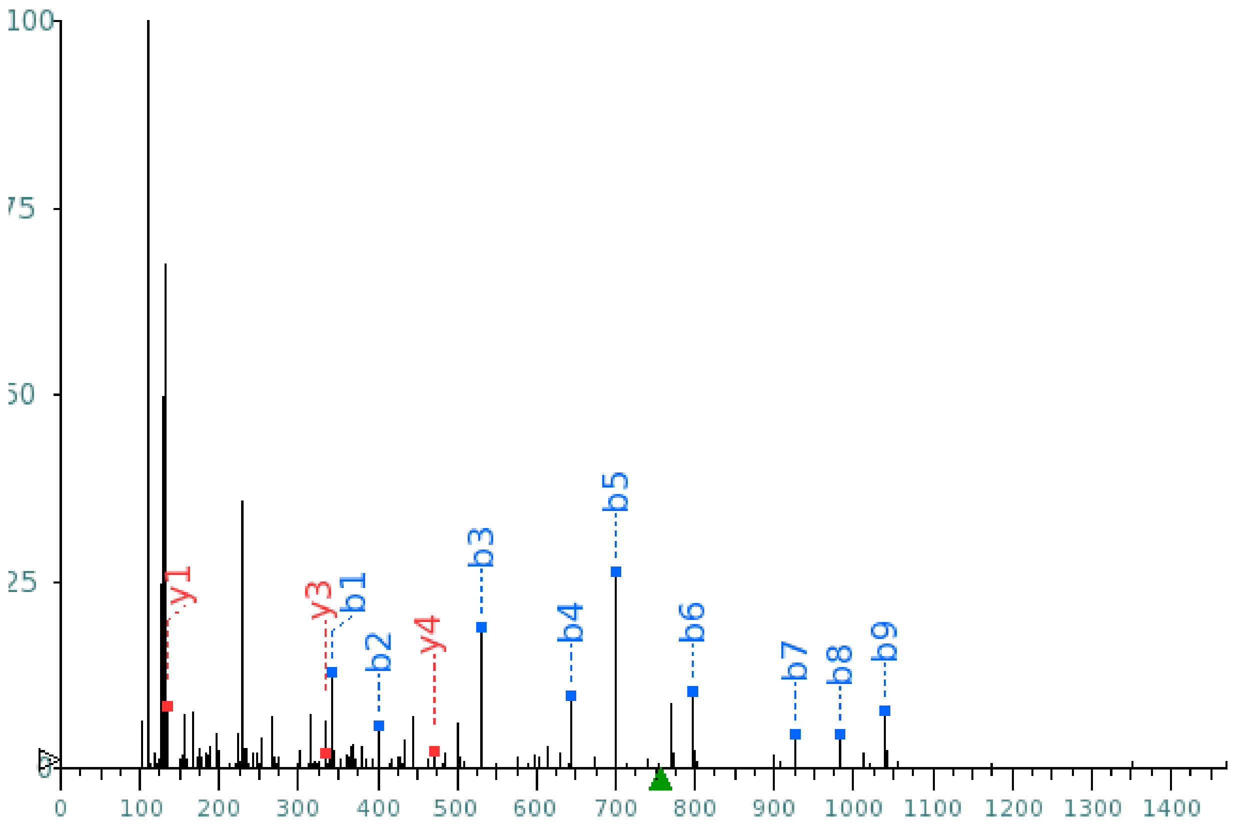

LGELGVQGGHQAD (+2)

Primary Reference: CC165_HUMAN
Search ID: 41760
Search Name: 20130328_ananiav_ERstress_TMT_TM_timecourse_trypsin_inj1_4mc_iaa
Scan#: 10088
Observed Mass: 755.3974 (1.1 ppm)
PSM Score: 34.8

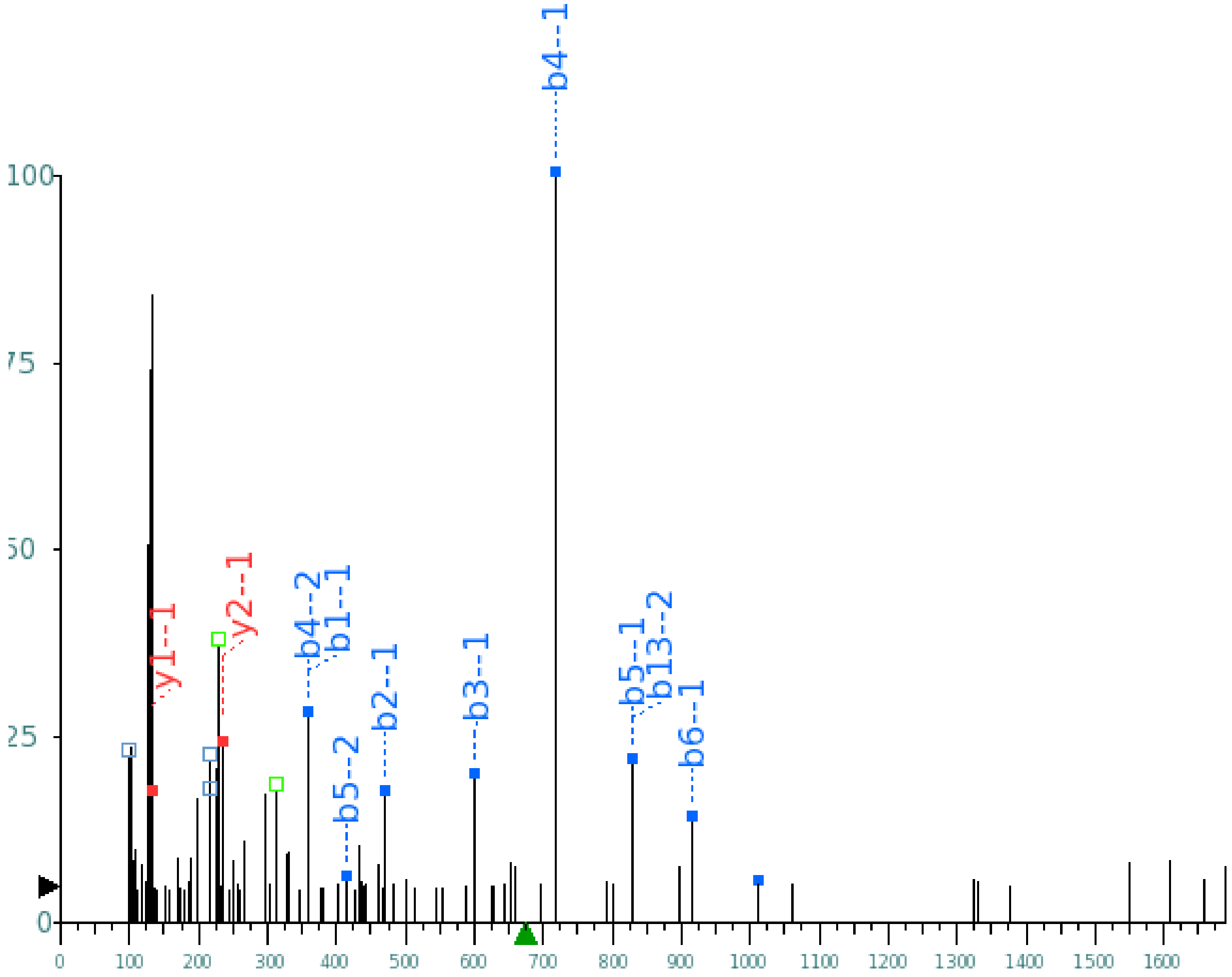

EIEDLSQSQSPESETD (+3)

Primary Reference: NBEA_HUMAN
Search ID: 41760
Search Name: 20130328_ananiav_ERstress_TMT_TM_timecourse_trypsin_inj1_4mc_iaa
Scan#: 10238
Observed Mass: 674.9784 (0.8 ppm)
PSM Score: 26.26

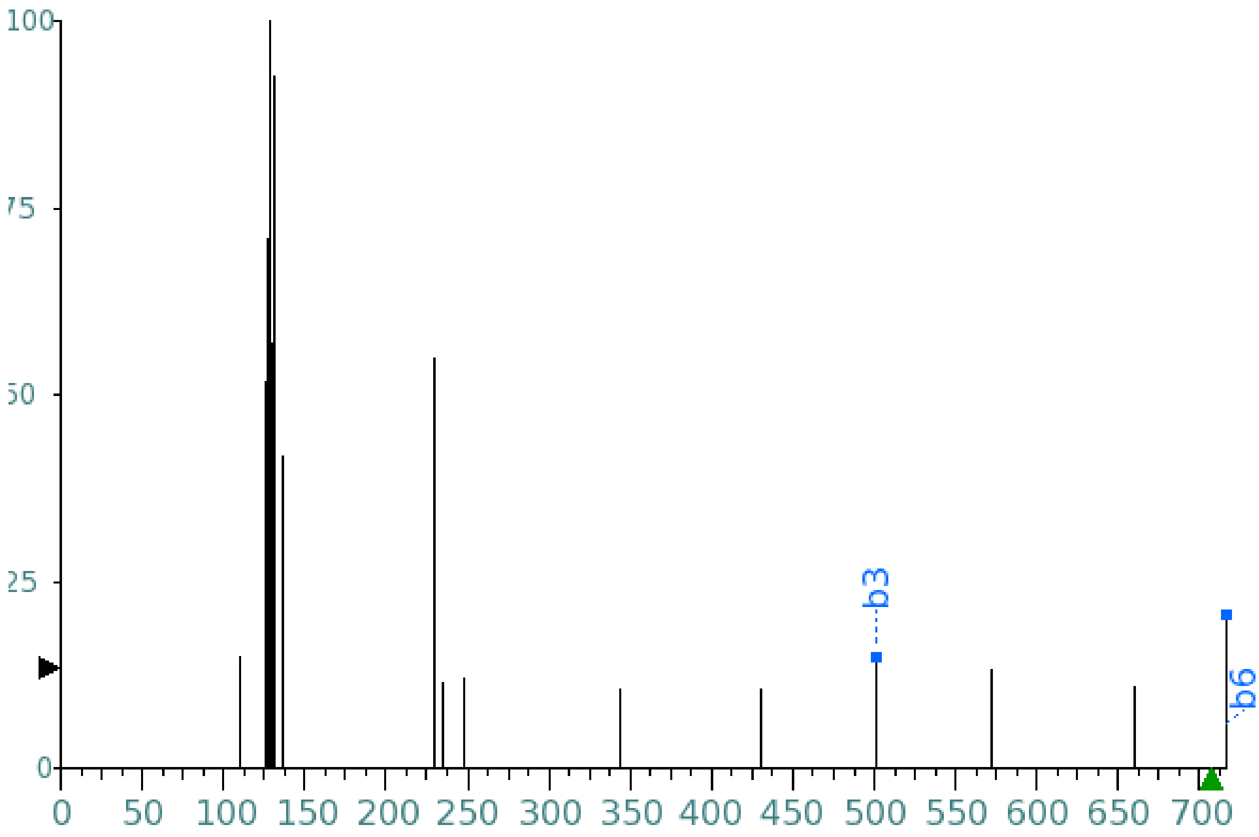

LSAASGYSDVTD (+2)

Primary Reference: PM2PB_HUMAN
Search ID: 41760
Search Name: 20130328_ananiav_ERstress_TMT_TM_timecourse_trypsin_inj1_4mc_iaa
Scan#: 10496
Observed Mass: 707.8499 (1.9 ppm)
PSM Score: 35.55

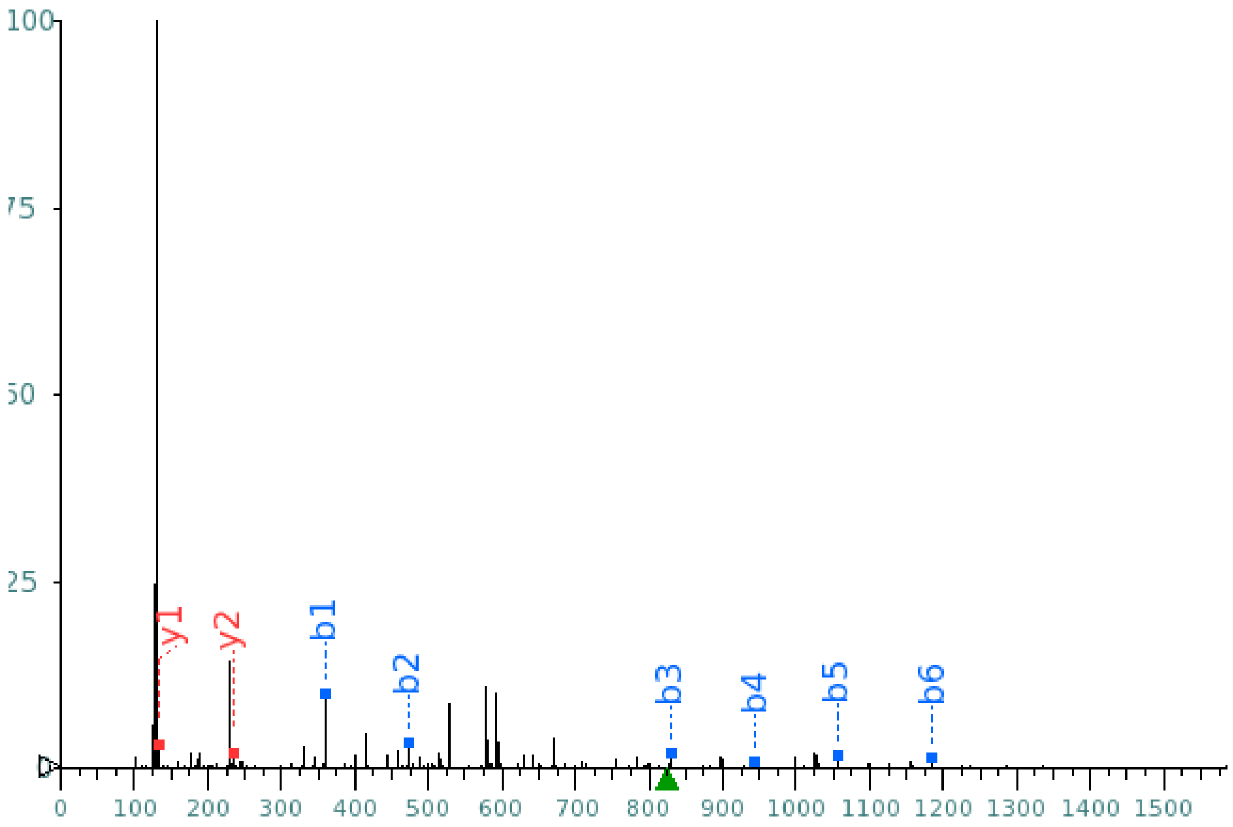

QIKINEVQTD (+2)

Primary Reference: TWF1_HUMAN
Search ID: 41760
Search Name: 20130328_ananiav_ERstress_TMT_TM_timecourse_trypsin_inj1_4mc_iaa
Scan#: 10513
Observed Mass: 823.4814 (1.8 ppm)
PSM Score: 15.88

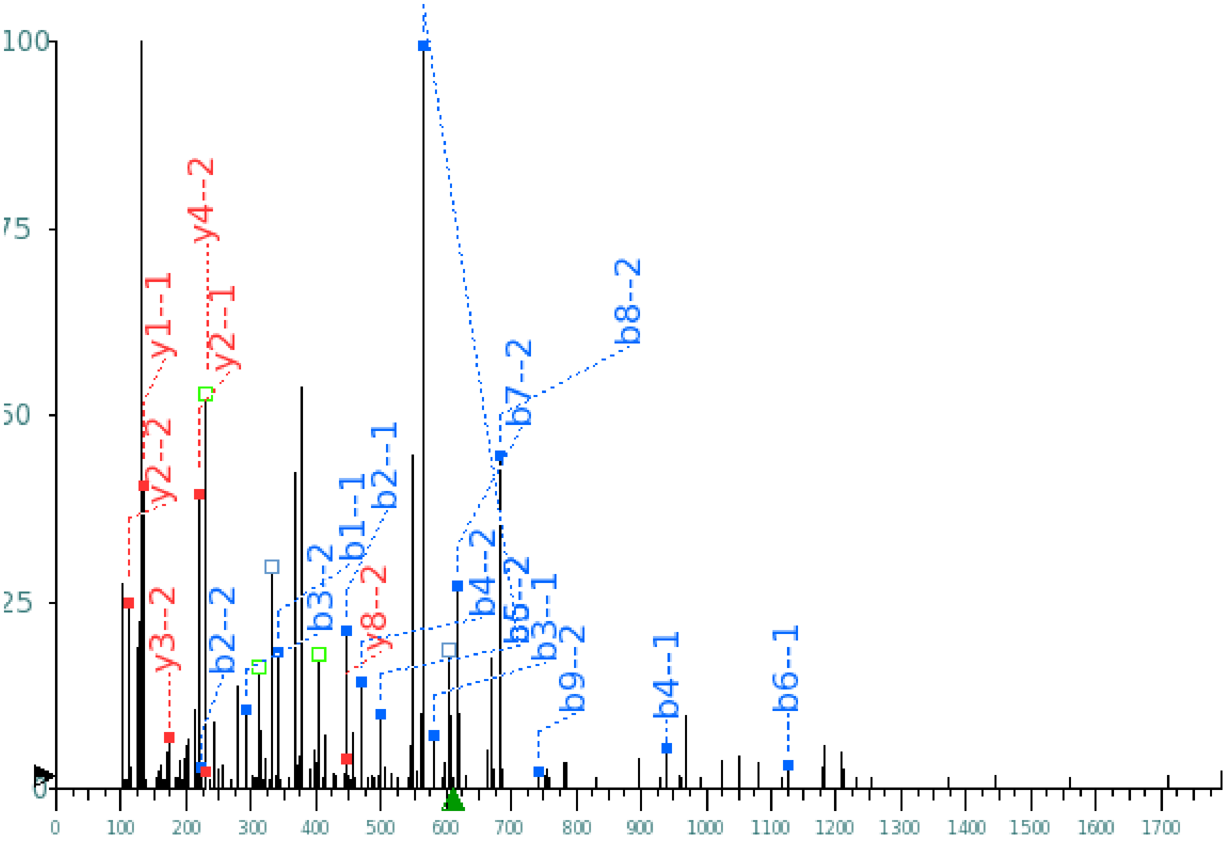

ITHKGEIEIESD (+3)

Primary Reference: PHF3_HUMAN
Search ID: 41760
Search Name: 20130328_ananiav_ERstress_TMT_TM_timecourse_trypsin_inj1_4mc_iaa
Scan#: 10699
Observed Mass: 610.3414 (2.2 ppm)
PSM Score: 11.41

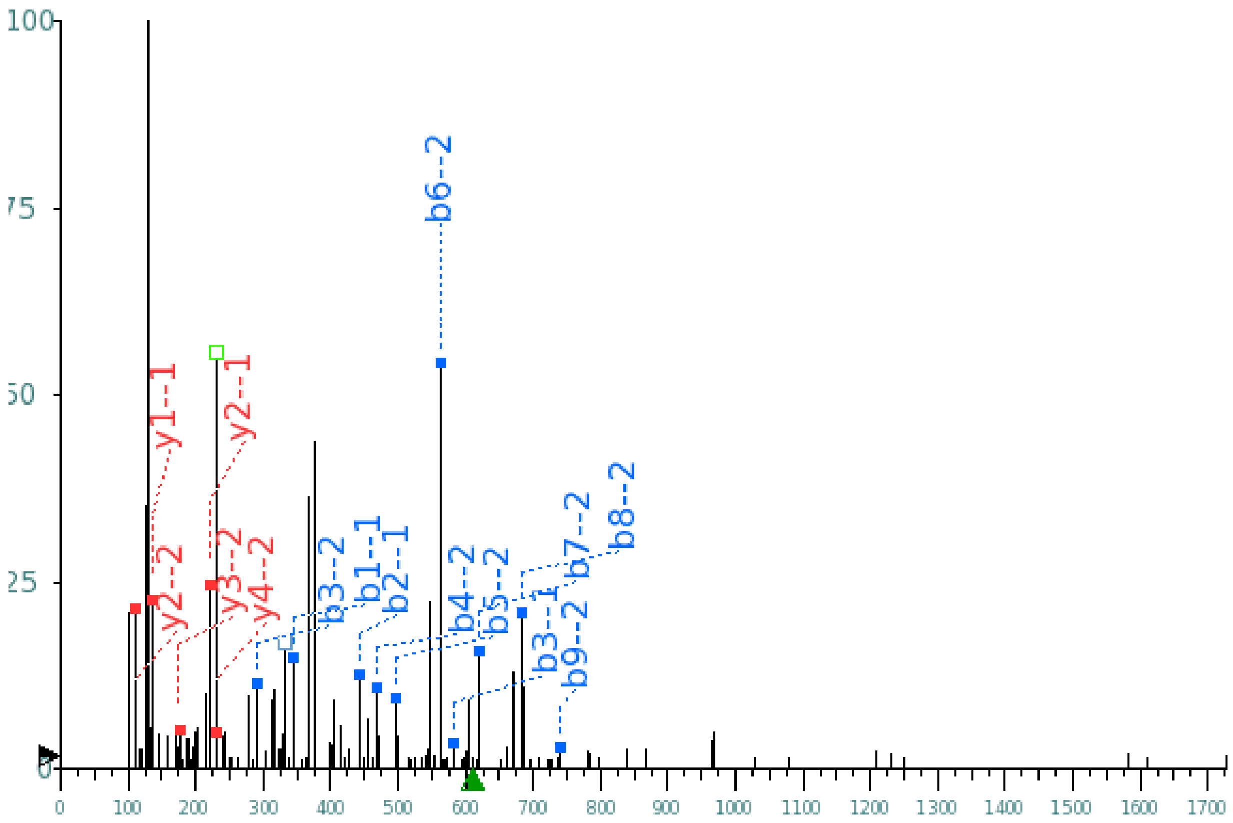

ITHKGEIEIESD (+3)

Primary Reference: PHF3_HUMAN
Search ID: 41760
Search Name: 20130328_ananiav_ERstress_TMT_TM_timecourse_trypsin_inj1_4mc_iaa
Scan#: 10809
Observed Mass: 610.341 (1.6 ppm)
PSM Score: 17.3

File name: 2015-02-04 04.01.53PM.pdf

Creation time: 2015-02-04 04:09PM

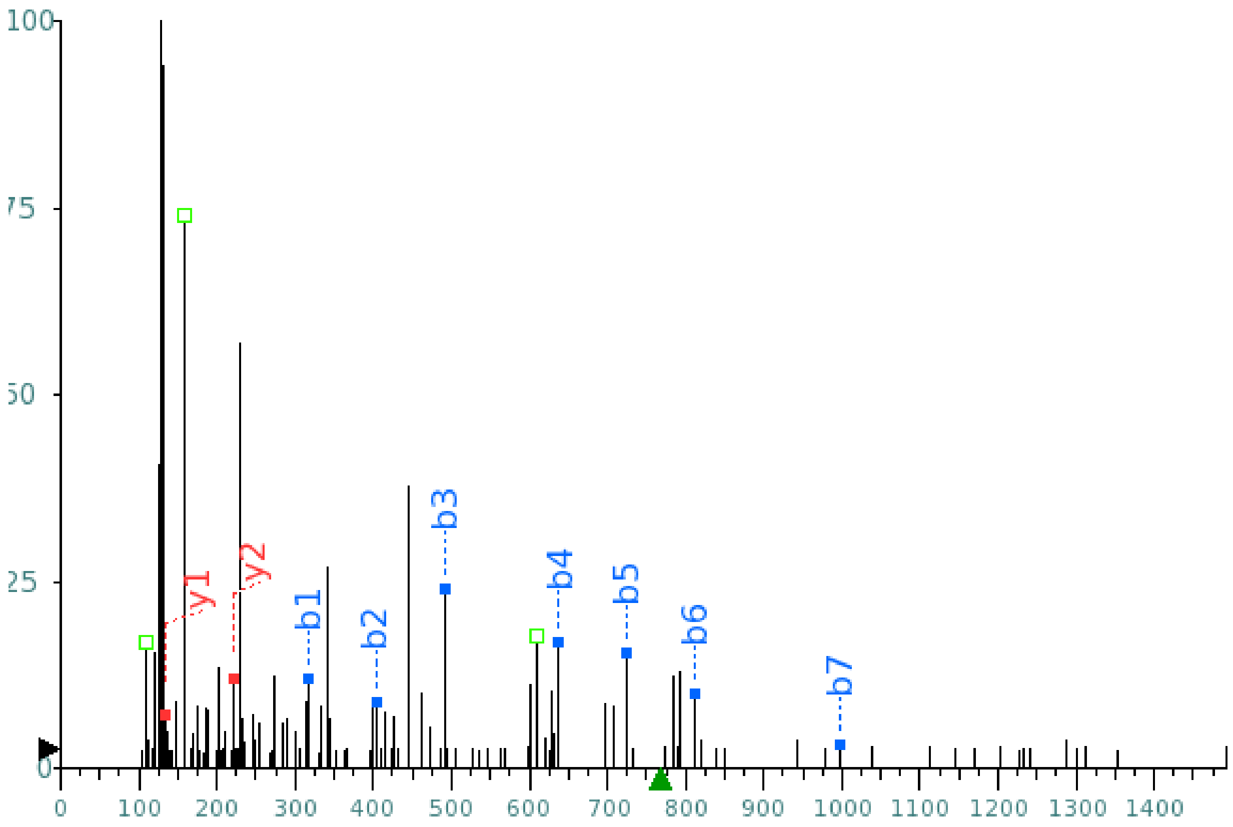

SSSFSSWDDSSD (+2)

Primary Reference: ARFG3_HUMAN
Search ID: 41760
Search Name: 20130328_ananiav_ERstress_TMT_TM_timecourse_trypsin_inj1_4mc_iaa
Scan#: 10825
Observed Mass: 768.3221 (2.2 ppm)
PSM Score: 16.19

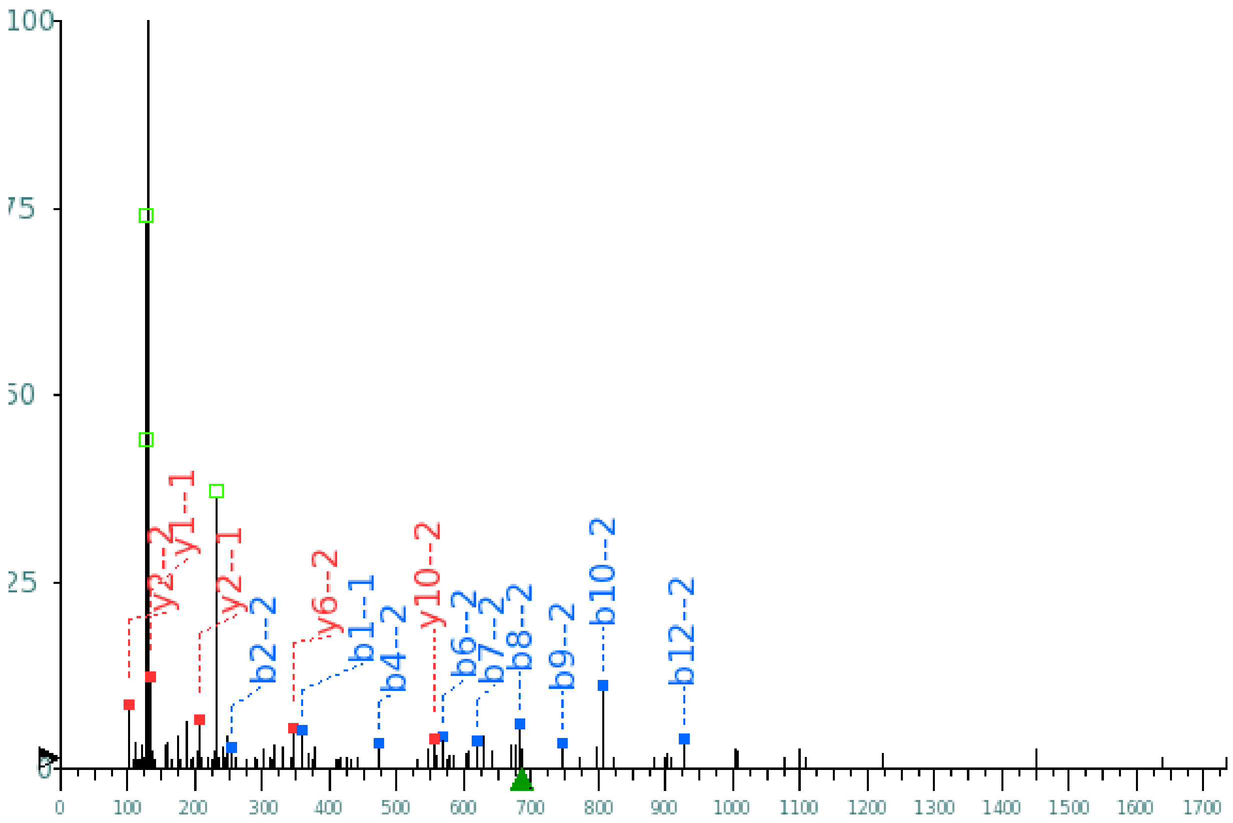

QFSKGETEQDLEAD (+3)

Primary Reference: ZMYM4_HUMAN
Search ID: 41760
Search Name: 20130328_ananiav_ERstress_TMT_TM_timecourse_trypsin_inj1_4mc_iaa
Scan#: 10846
Observed Mass: 685.6824 (2.2 ppm)
PSM Score: 11.64

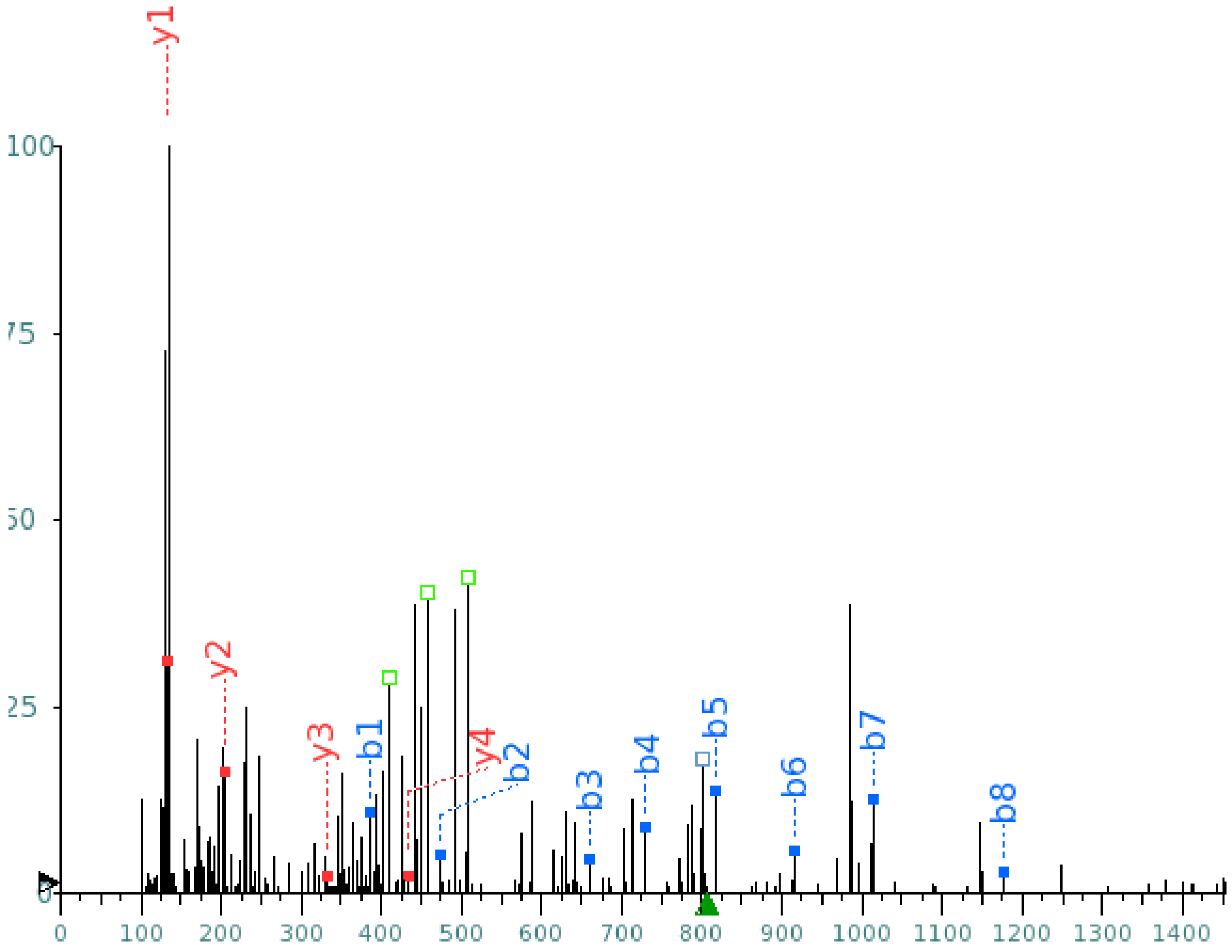

RSWASPVYTEAD (+2)

Primary Reference: LMO7_HUMAN
Search ID: 41760
Search Name: 20130328_ananiav_ERstress_TMT_TM_timecourse_trypsin_inj1_4mc_iaa
Scan#: 10861
Observed Mass: 805.9063 (2.6 ppm)
PSM Score: 14.64

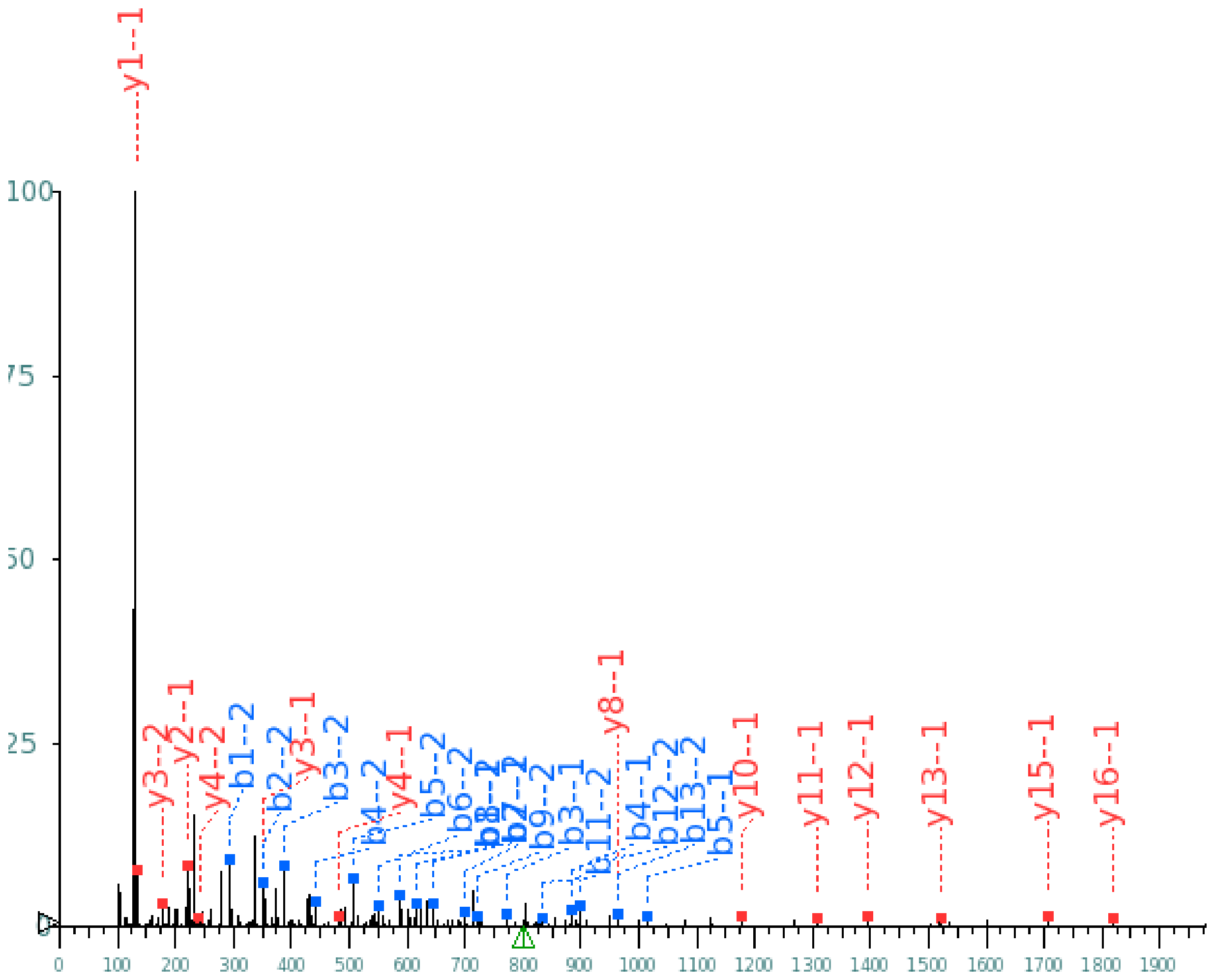

KIALESEGRPEEQMESD (+3)

Primary Reference: SQSTM_HUMAN
Search ID: 41760
Search Name: 20130328_ananiav_ERstress_TMT_TM_timecourse_trypsin_inj1_4mc_iaa
Scan#: 10877
Observed Mass: 802.7476 (2.5 ppm)
PSM Score: 44.15

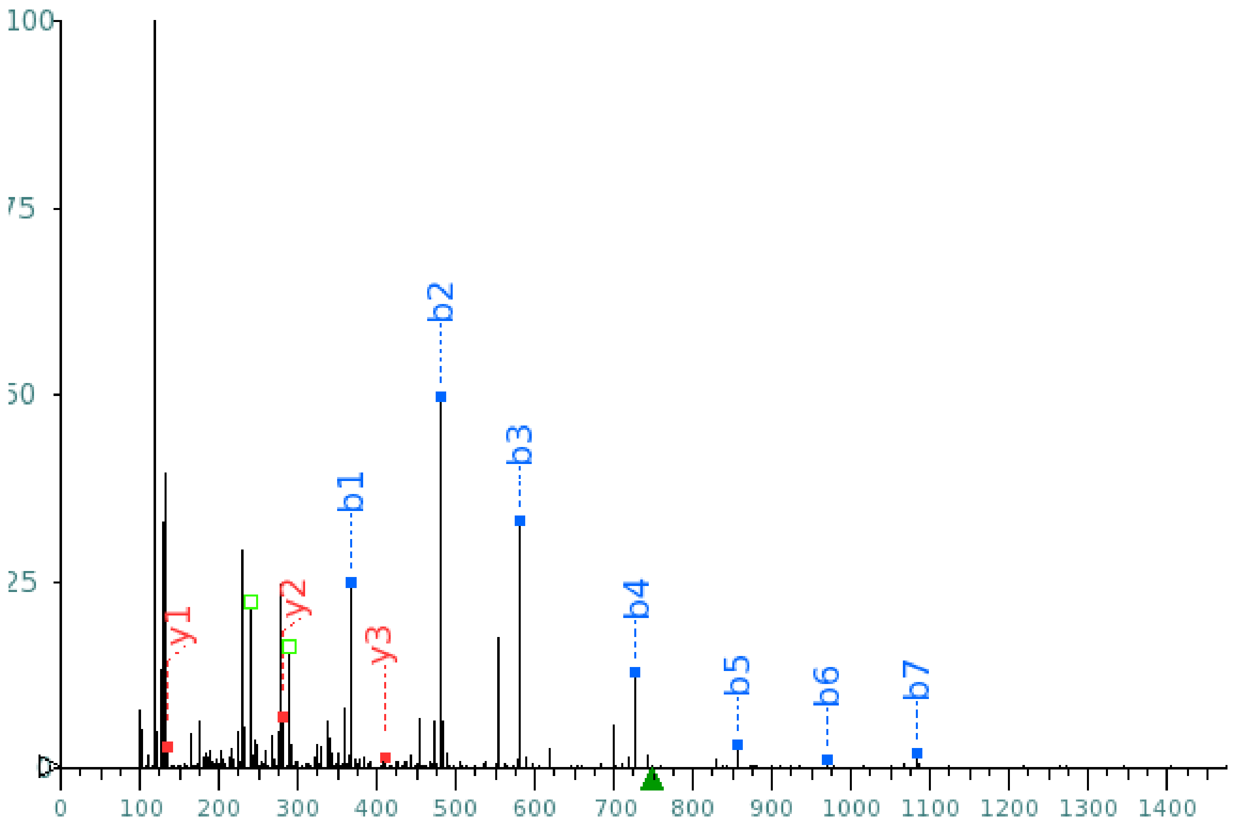

HNVFQNDEFD (+2)

Primary Reference: ASCC2_HUMAN
Search ID: 41760
Search Name: 20130328_ananiav_ERstress_TMT_TM_timecourse_trypsin_inj1_4mc_iaa
Scan#: 10882
Observed Mass: 747.3483 (2.4 ppm)
PSM Score: 42.48

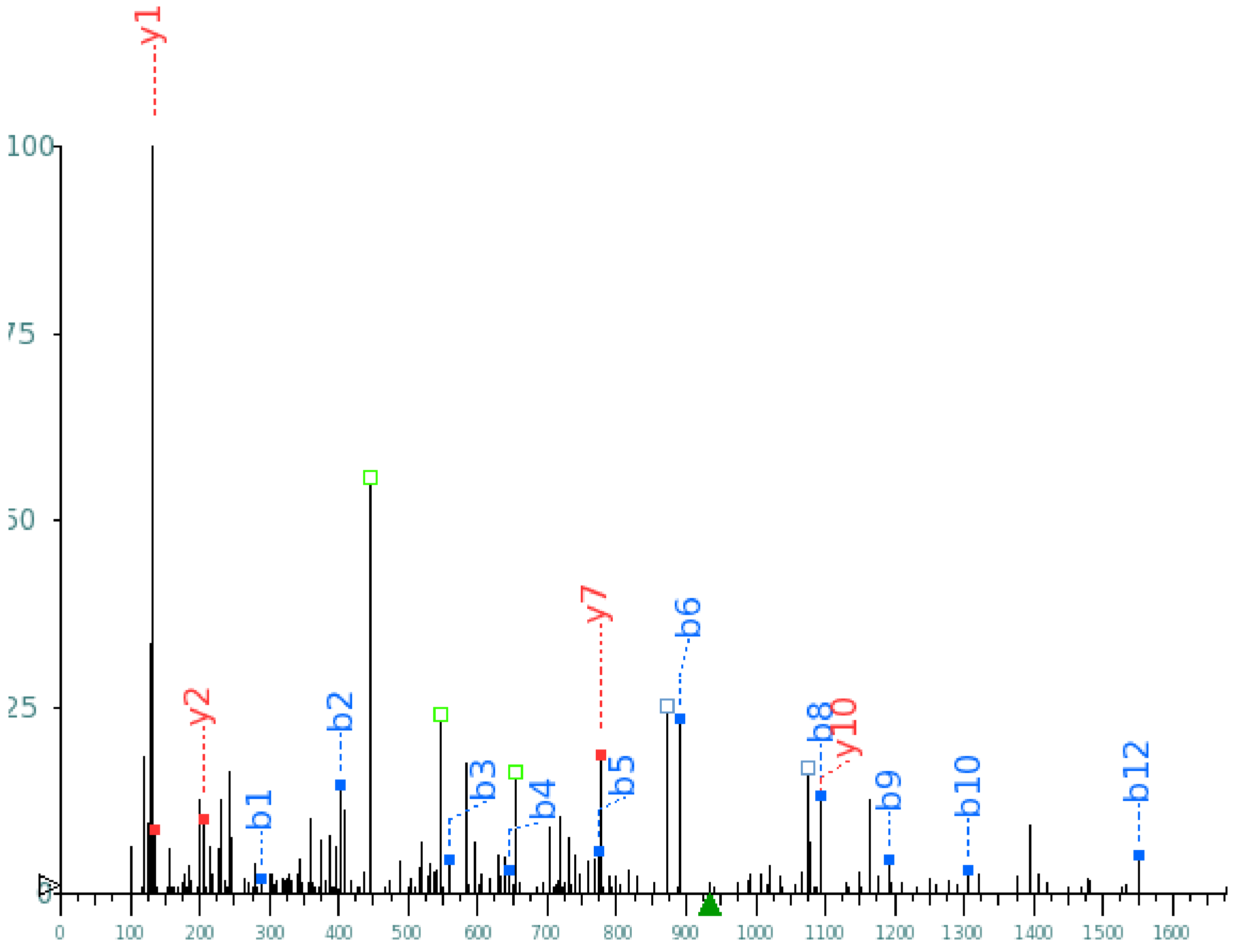

GDRSEDFGVNEDLAD (+2)

Primary Reference: ANXA1_HUMAN
Search ID: 41760
Search Name: 20130328_ananiav_ERstress_TMT_TM_timecourse_trypsin_inj1_4mc_iaa
Scan#: 10896
Observed Mass: 934.4303 (1.4 ppm)
PSM Score: 28.63

File name: 2015-02-04 04.01.53PM.pdf

Creation time: 2015-02-04 04:09PM

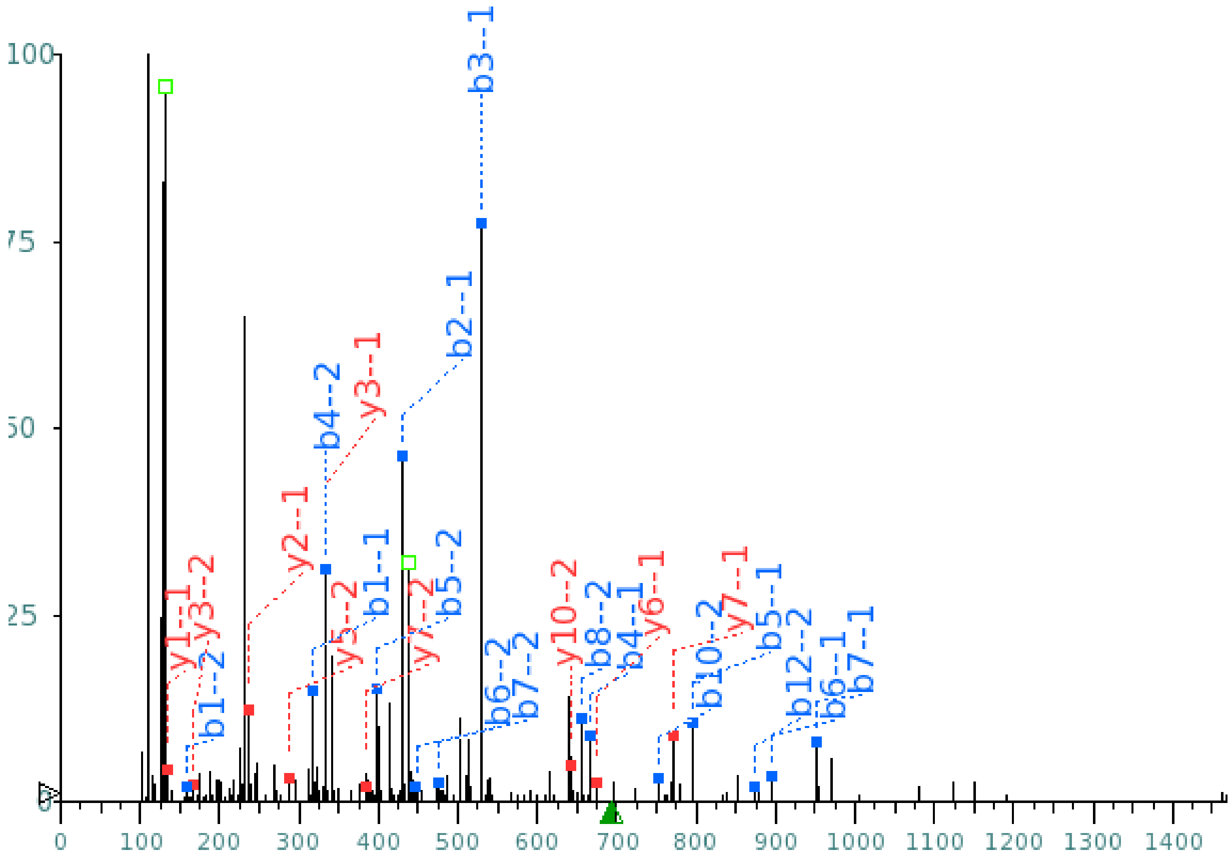

SLVHEVGKPPQDVTD (+3)

Primary Reference: MINT_HUMAN
Search ID: 41760
Search Name: 20130328_ananiav_ERstress_TMT_TM_timecourse_trypsin_inj1_4mc_iaa
Scan#: 10913
Observed Mass: 693.7227 (2.3 ppm)
PSM Score: 18.47

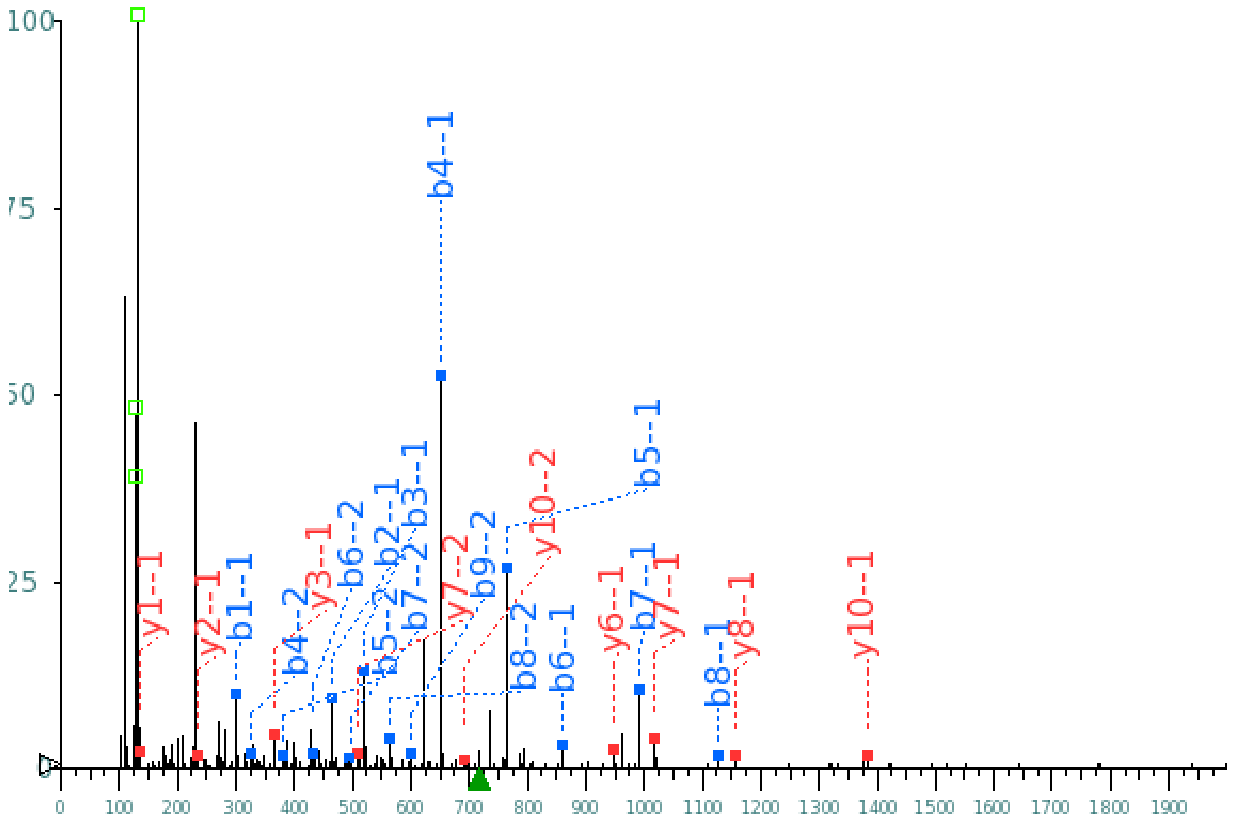

AYGELPEHAKINETD (+3)

Primary Reference: IF1AX_HUMAN
Search ID: 41760
Search Name: 20130328_ananiav_ERstress_TMT_TM_timecourse_trypsin_inj1_4mc_iaa
Scan#: 10919
Observed Mass: 715.7141 (2.2 ppm)
PSM Score: 37.75

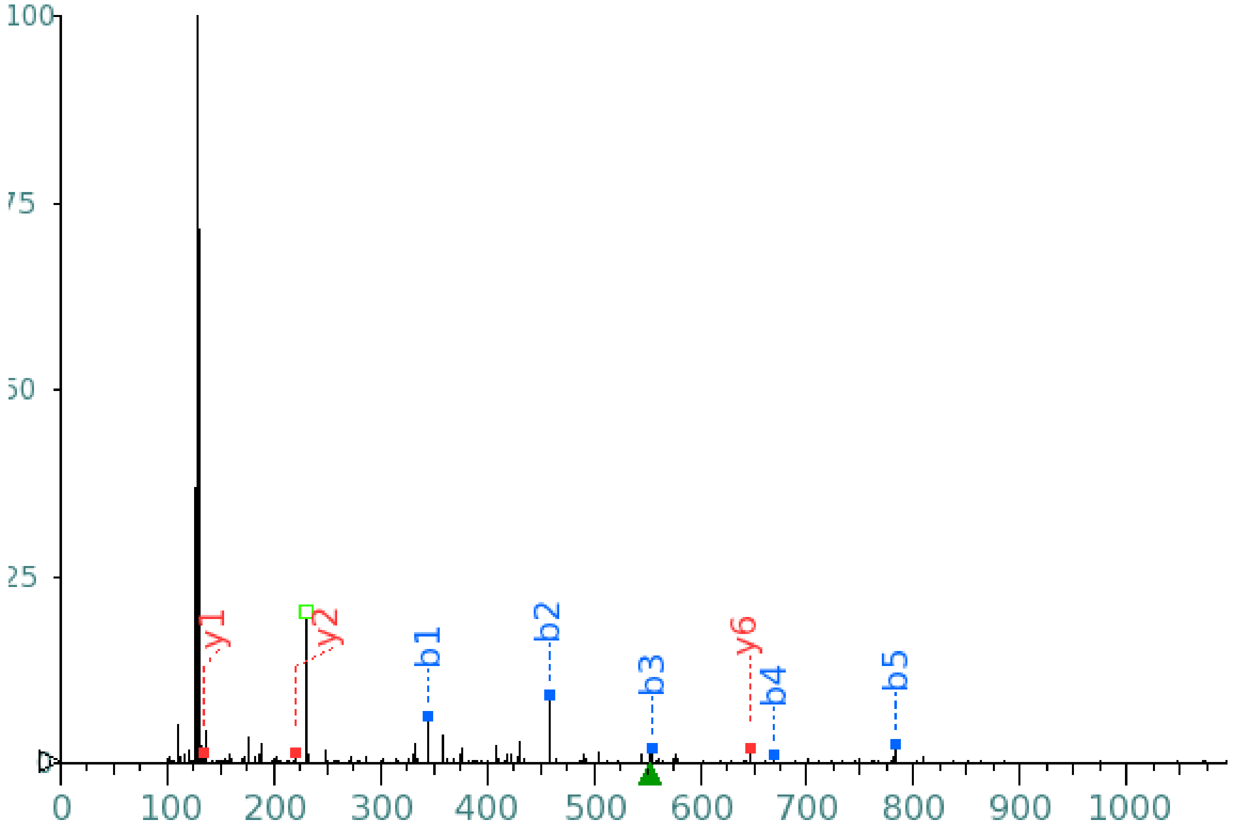

DLPLDTSD (+2)

Primary Reference: 3BP2_HUMAN
Search ID: 41760
Search Name: 20130328_ananiav_ERstress_TMT_TM_timecourse_trypsin_inj1_4mc_iaa
Scan#: 10944
Observed Mass: 552.7857 (1.7 ppm)
PSM Score: 12.17

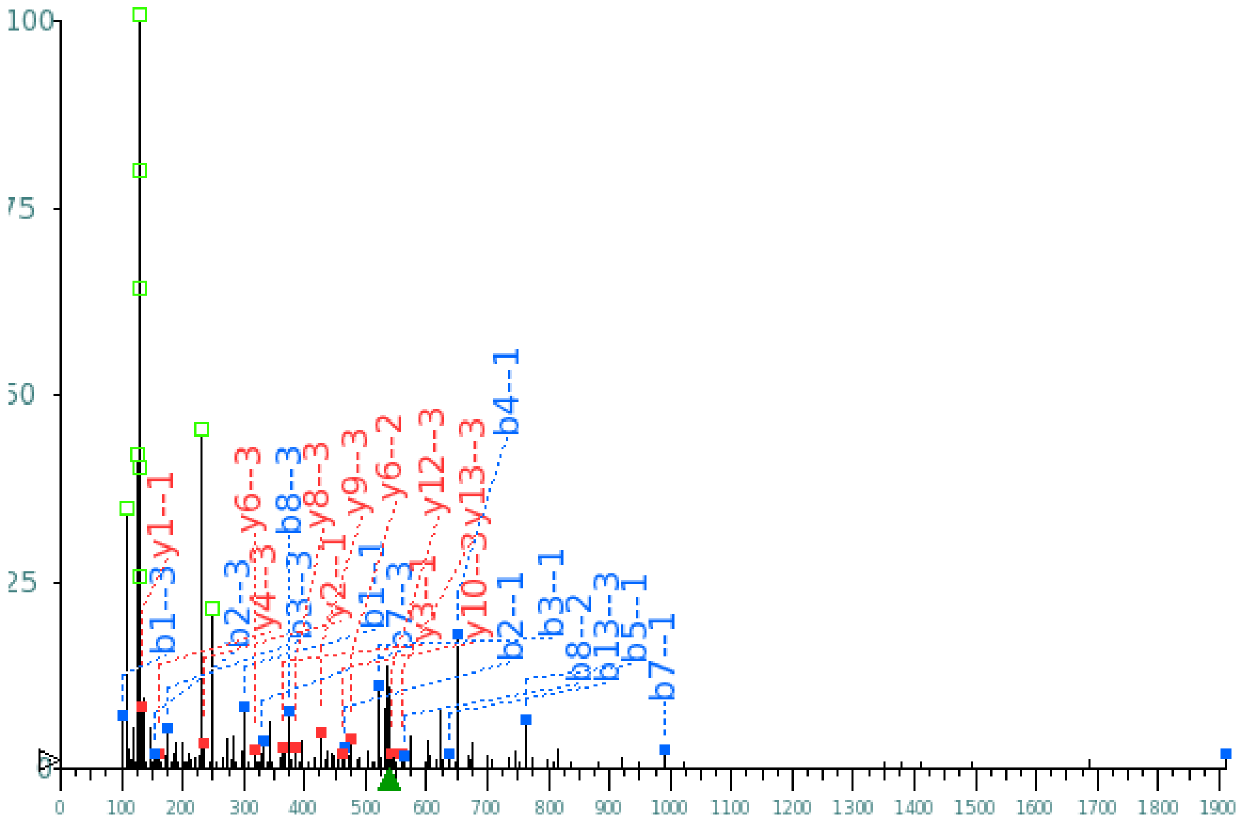

AYGELPEHAKINETD (+4)

Primary Reference: IF1AX_HUMAN
Search ID: 41760
Search Name: 20130328_ananiav_ERstress_TMT_TM_timecourse_trypsin_inj1_4mc_iaa
Scan#: 10948
Observed Mass: 537.0368 (1.2 ppm)
PSM Score: 18.98

File name: 2015-02-04 04.01.53PM.pdf

Creation time: 2015-02-04 04:09PM

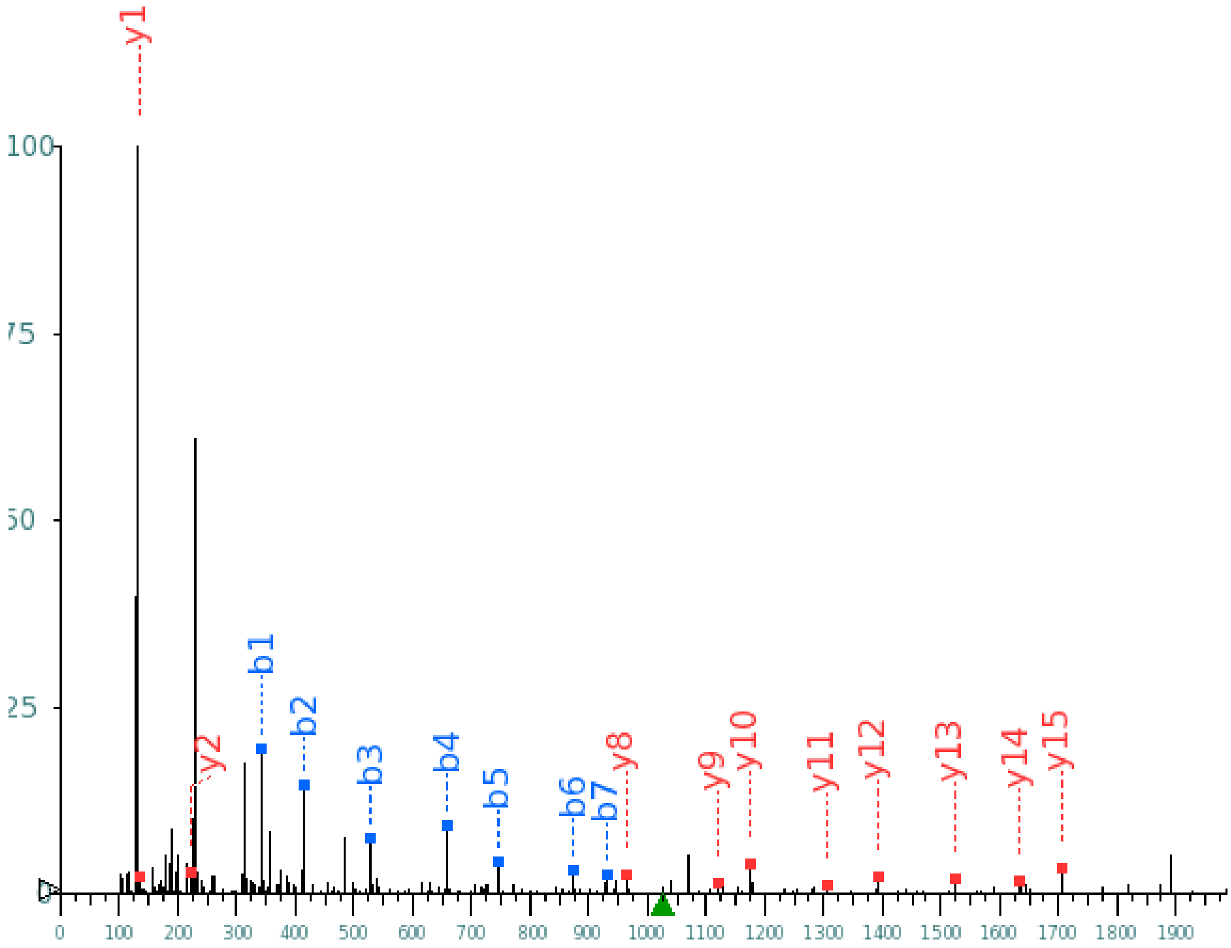

IALESEGRPEEQMESD (+2)

Primary Reference: SQSTM_HUMAN
Search ID: 41760
Search Name: 20130328_ananiav_ERstress_TMT_TM_timecourse_trypsin_inj1_4mc_iaa
Scan#: 10977
Observed Mass: 1024.9877 (1.9 ppm)
PSM Score: 35.84

File name: 2015-02-04 04.01.53PM.pdf

Creation time: 2015-02-04 04:09PM

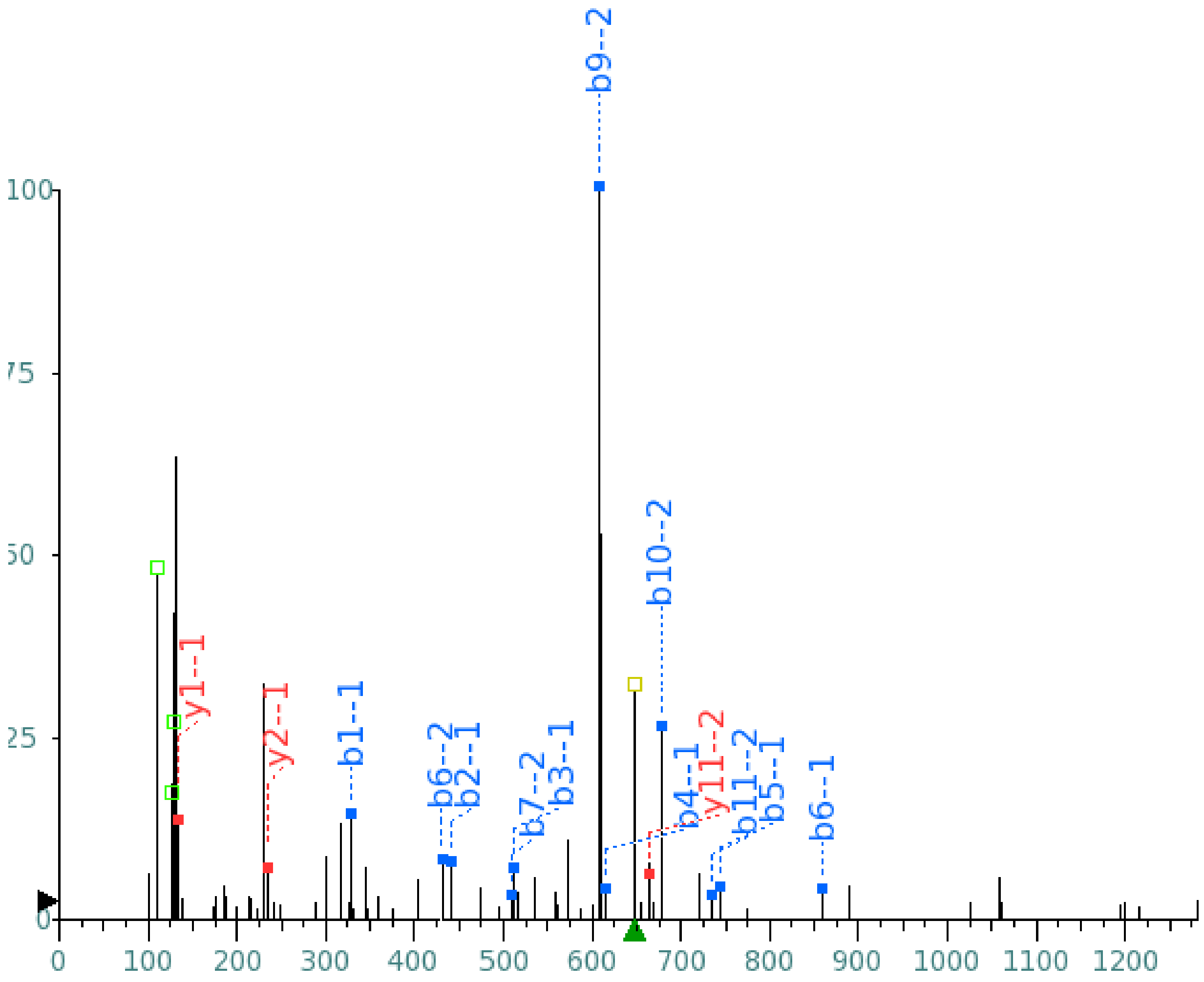

VLATEDRSDHLIQTD (+3)

Primary Reference: RAB9A_HUMAN
Search ID: 41760
Search Name: 20130328_ananiav_ERstress_TMT_TM_timecourse_trypsin_inj1_4mc_iaa
Scan#: 10991
Observed Mass: 648.0093 (2.8 ppm)
PSM Score: 21.17

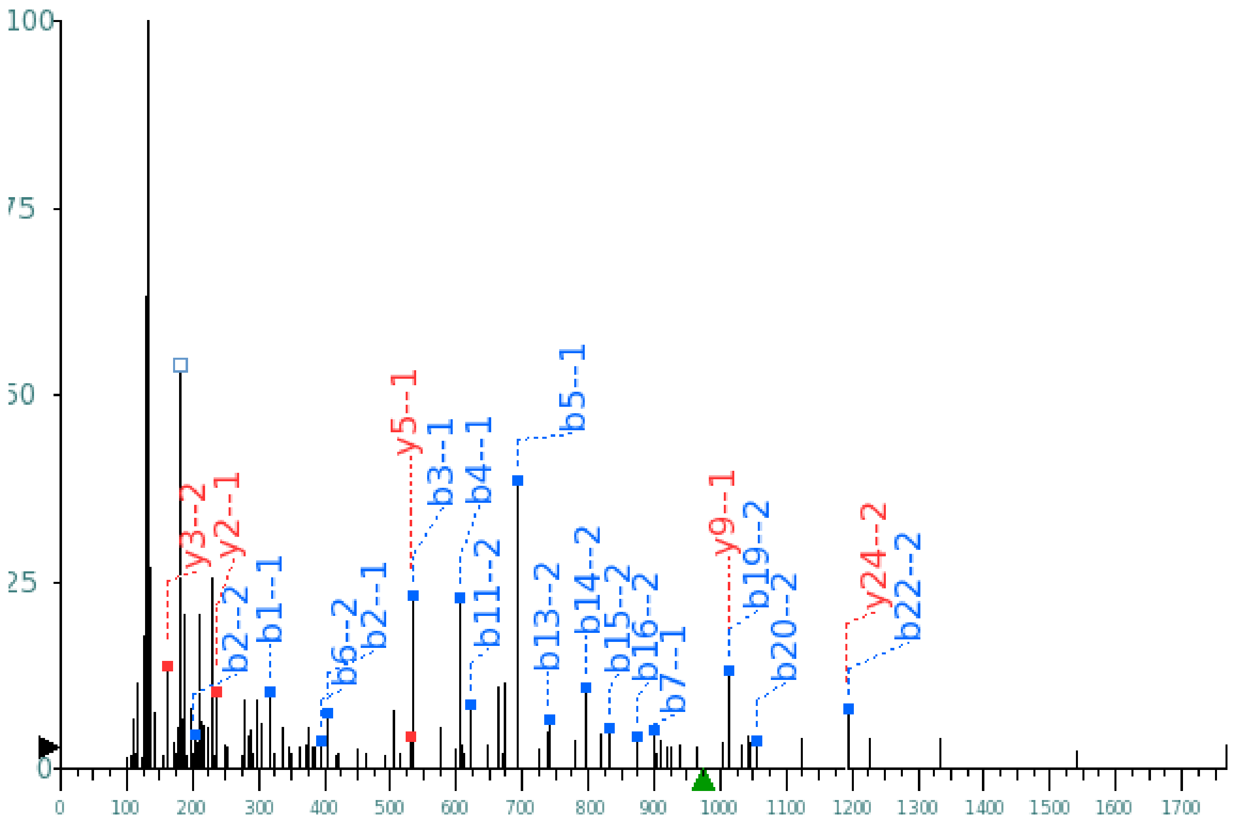

SSEASPLGAGRPHNASSADSYDPISTD (+3)

Primary Reference: GLI2_HUMAN
Search ID: 41760
Search Name: 20130328_ananiav_ERstress_TMT_TM_timecourse_trypsin_inj1_4mc_iaa
Scan#: 11010
Observed Mass: 973.4596 (1.4 ppm)
PSM Score: 40.27

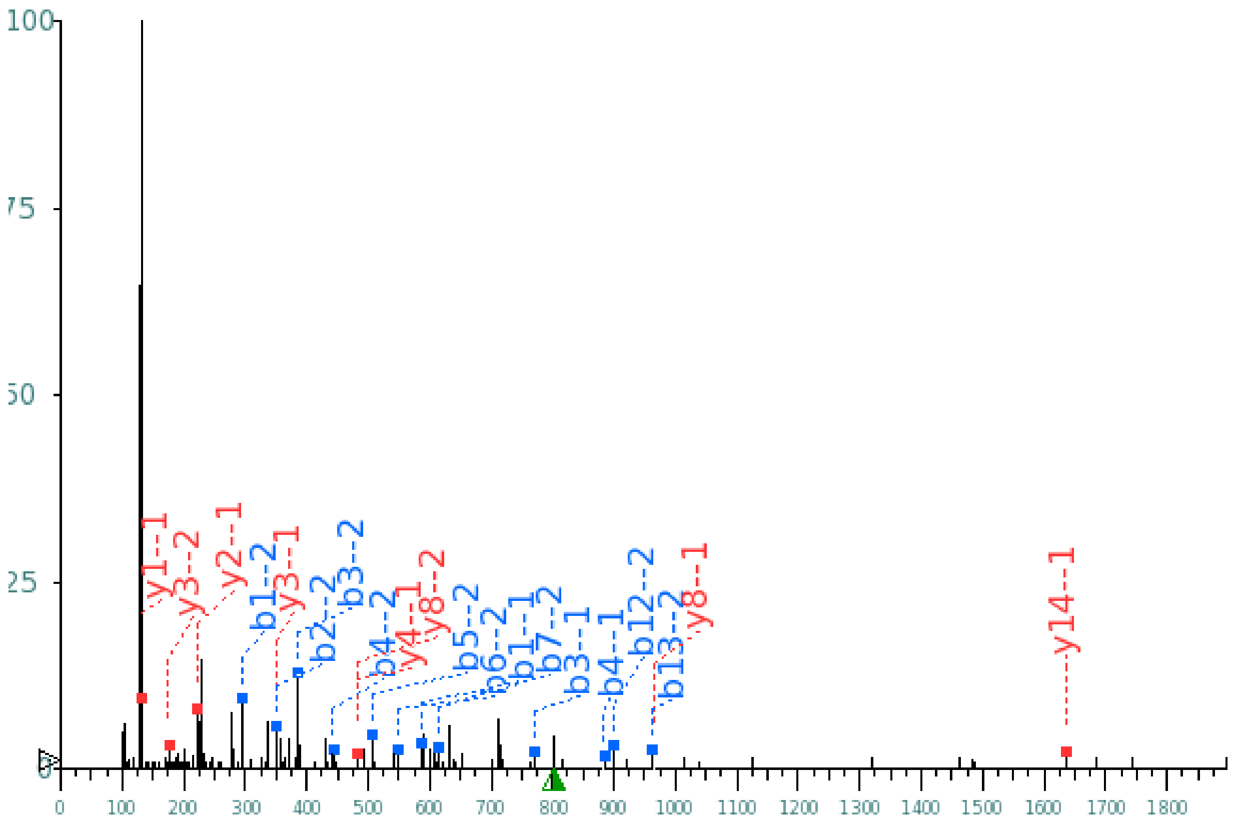

KIALESEGRPEEQMESD (+3)

Primary Reference: SQSTM_HUMAN
Search ID: 41760
Search Name: 20130328_ananiav_ERstress_TMT_TM_timecourse_trypsin_inj1_4mc_iaa
Scan#: 11021
Observed Mass: 802.7473 (2.1 ppm)
PSM Score: 19.55

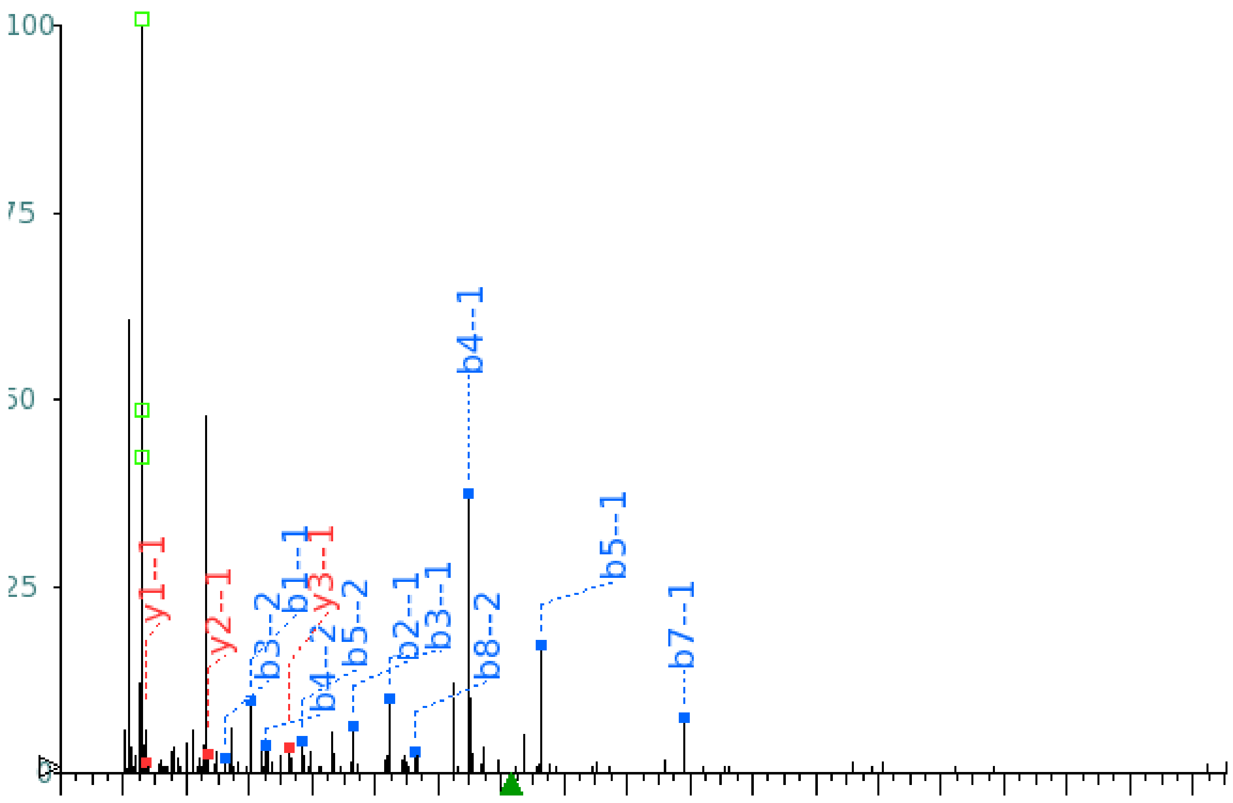

AYGELPEHAKINETD (+3)

Primary Reference: IF1AX_HUMAN
Search ID: 41760
Search Name: 20130328_ananiav_ERstress_TMT_TM_timecourse_trypsin_inj1_4mc_iaa
Scan#: 11023
Observed Mass: 715.7141 (2.3 ppm)
PSM Score: 22.73

File name: 2015-02-04 04.01.53PM.pdf

Creation time: 2015-02-04 04:09PM

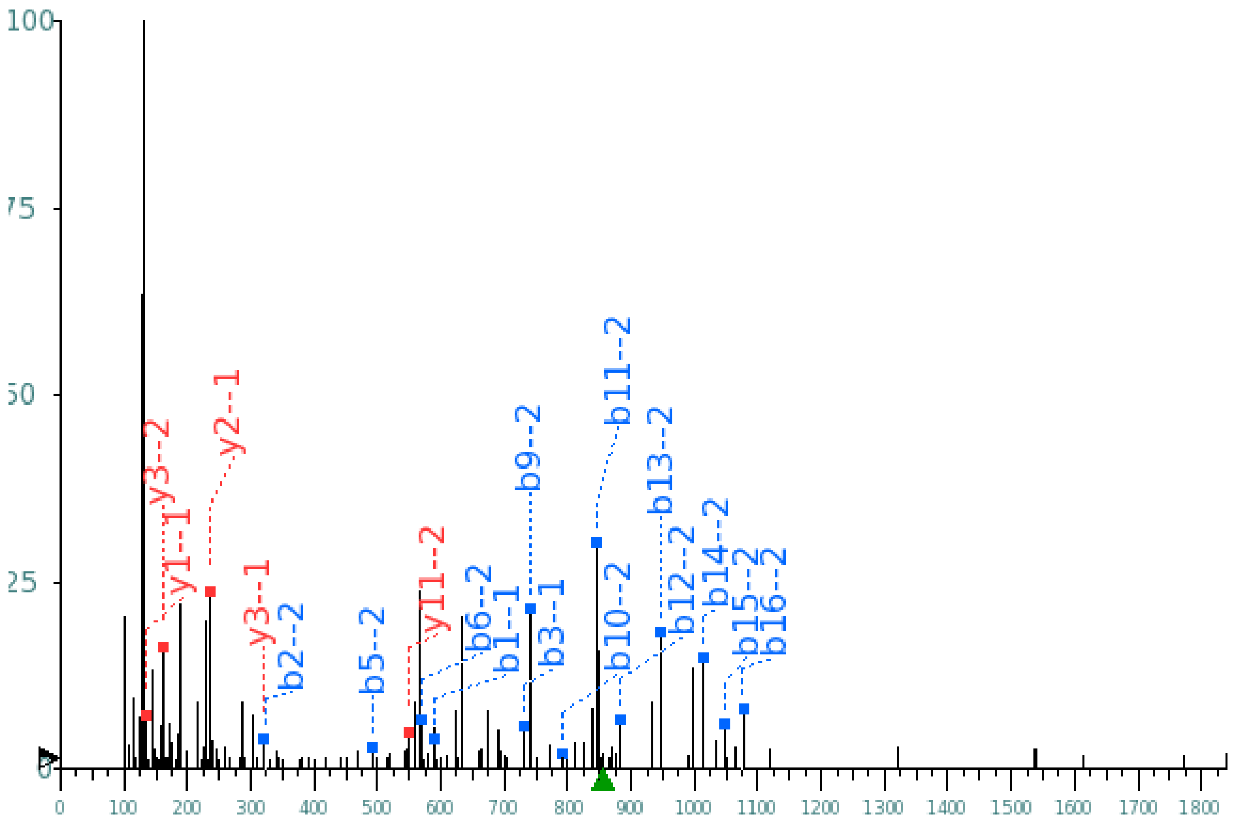

KGSTFRPMDTDAEEAGVSTD (+3)

Primary Reference: DCBD1_HUMAN
Search ID: 41760
Search Name: 20130328_ananiav_ERstress_TMT_TM_timecourse_trypsin_inj1_4mc_iaa
Scan#: 11032
Observed Mass: 858.0926 (1.2 ppm)
PSM Score: 29.75

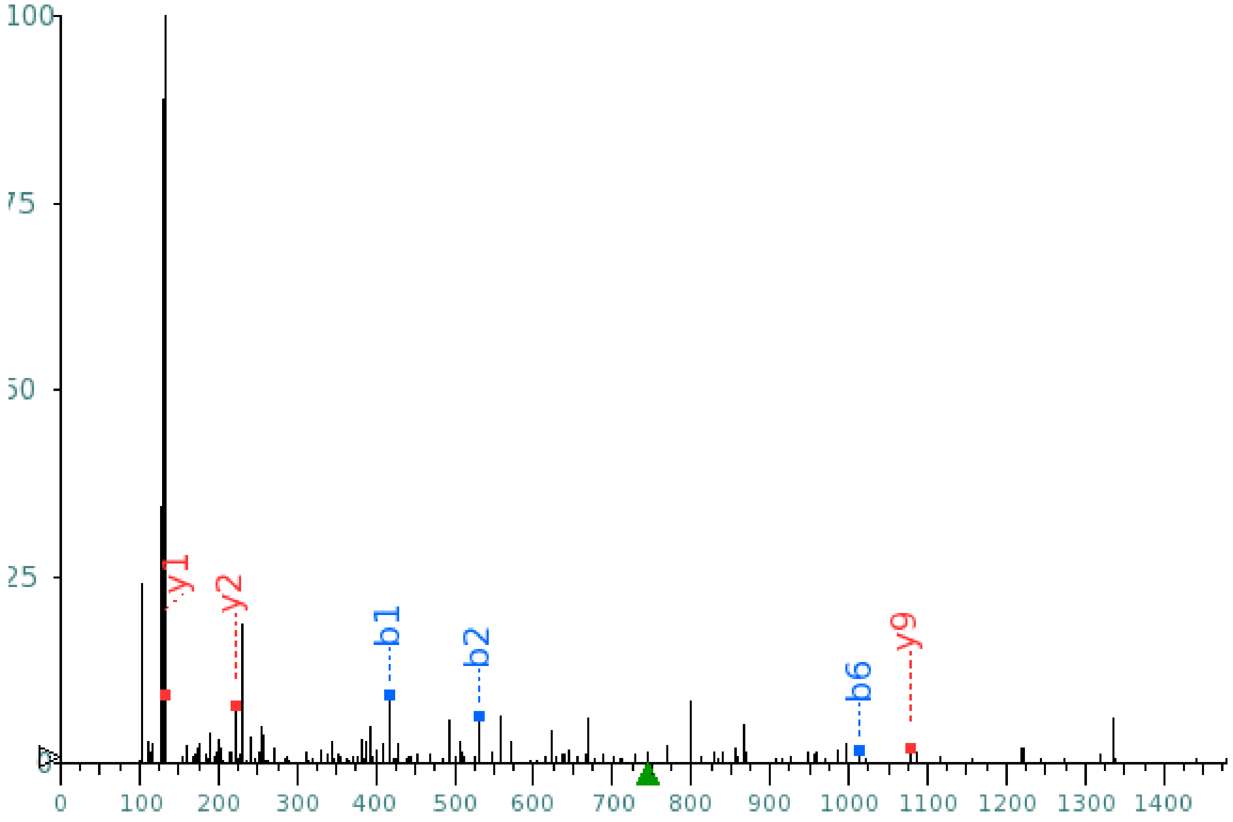

WLREAEEESD (+2)

Primary Reference: IF4G1_HUMAN
Search ID: 41760
Search Name: 20130328_ananiav_ERstress_TMT_TM_timecourse_trypsin_inj1_4mc_iaa
Scan#: 11050
Observed Mass: 746.8612 (2.3 ppm)
PSM Score: 7.84

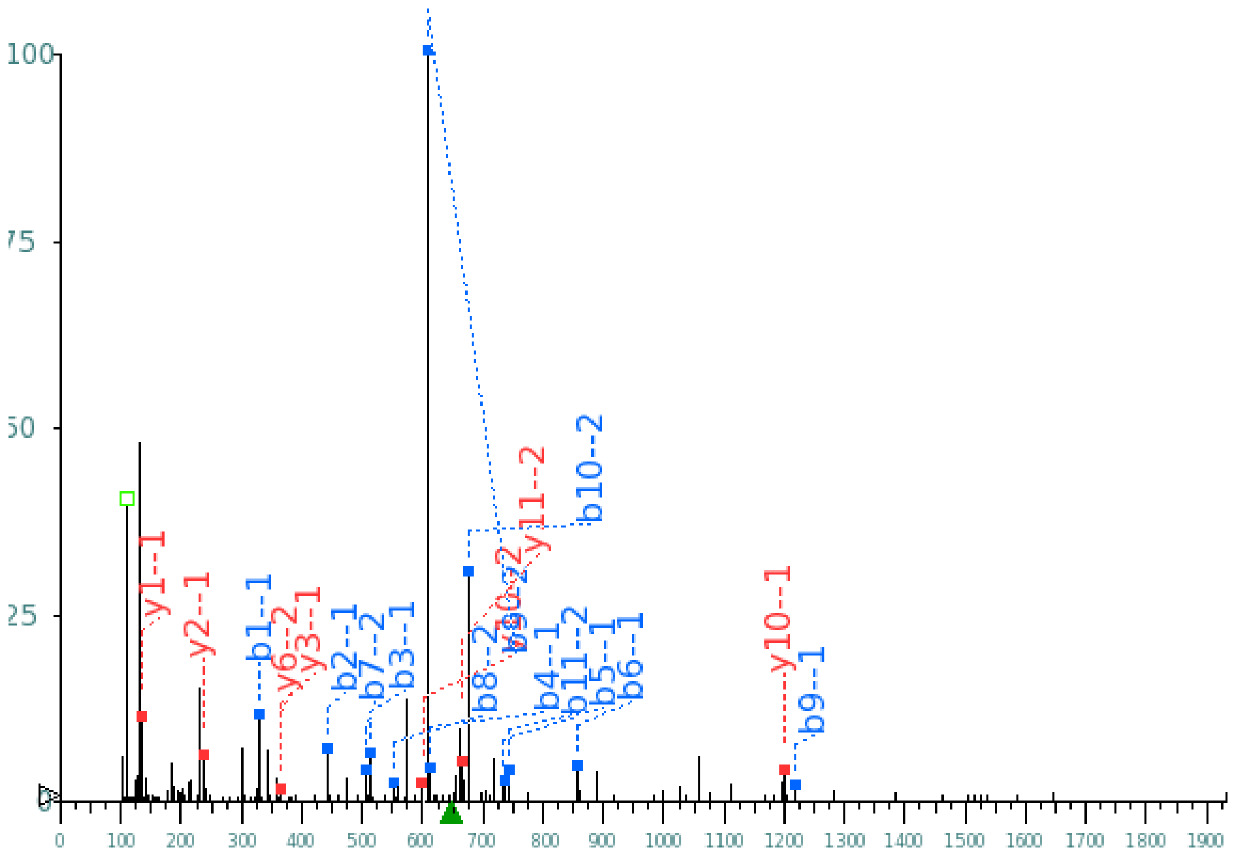

VLATEDRSDHLIQTD (+3)

Primary Reference: RAB9A_HUMAN
Search ID: 41760
Search Name: 20130328_ananiav_ERstress_TMT_TM_timecourse_trypsin_inj1_4mc_iaa
Scan#: 11072
Observed Mass: 648.0093 (2.8 ppm)
PSM Score: 20.86

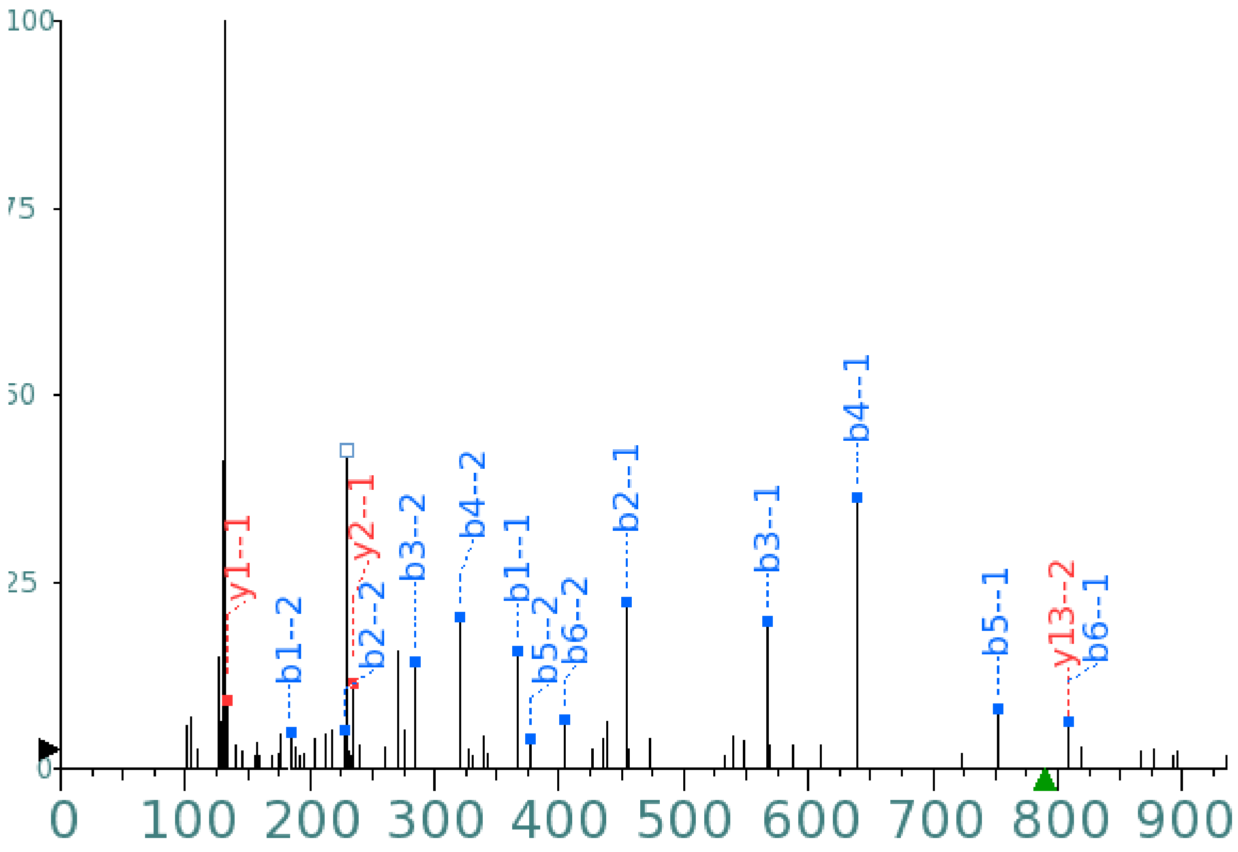

HSLALGSATEDKDSMETD (+3)

Primary Reference: NU188_HUMAN
Search ID: 41760
Search Name: 20130328_ananiav_ERstress_TMT_TM_timecourse_trypsin_inj1_4mc_iaa
Scan#: 11112
Observed Mass: 789.0596 (2.1 ppm)
PSM Score: 28.9

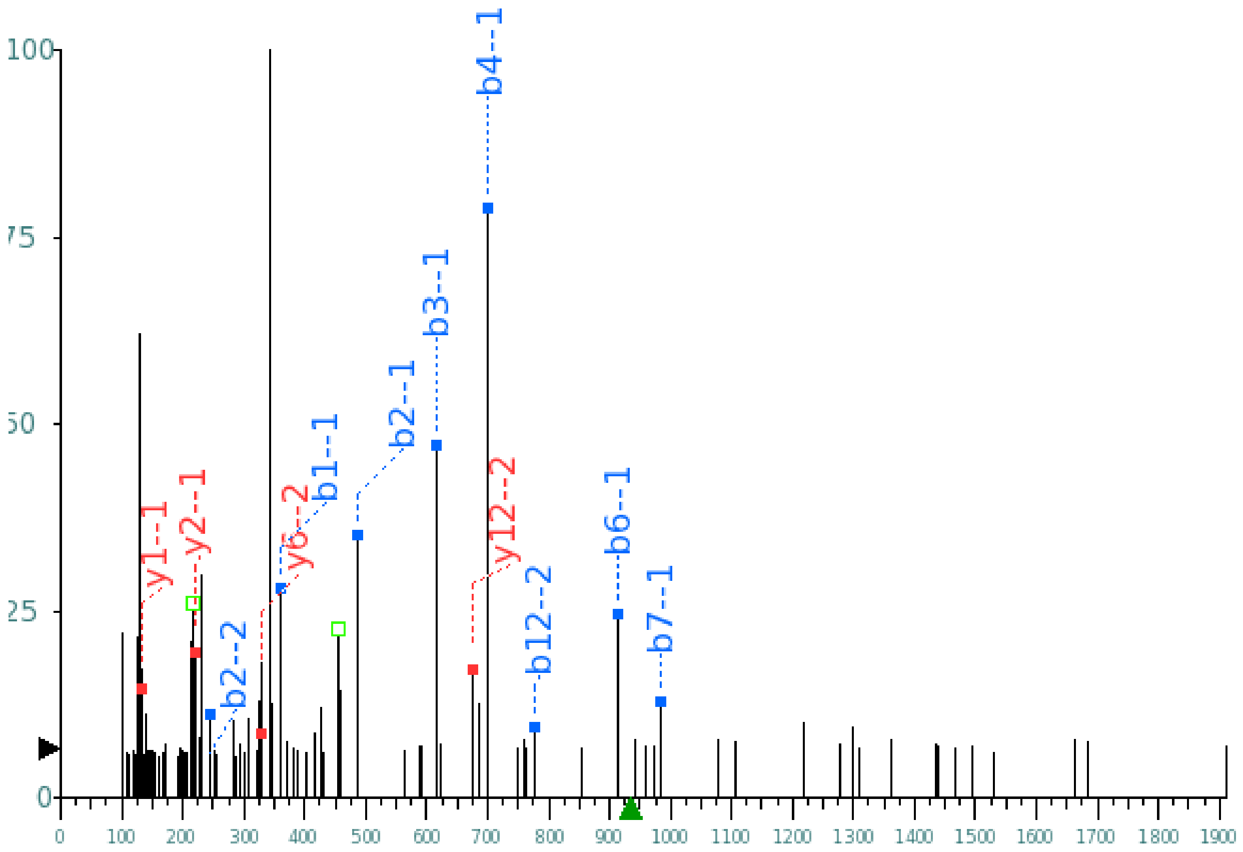

QEQSPNANNDQPEDLIQGC*SESD (+3)

Primary Reference: BPTF_HUMAN
Search ID: 41760
Search Name: 20130328_ananiav_ERstress_TMT_TM_timecourse_trypsin_inj1_4mc_iaa
Scan#: 11122
Observed Mass: 935.4104 (1.8 ppm)
PSM Score: 16.61

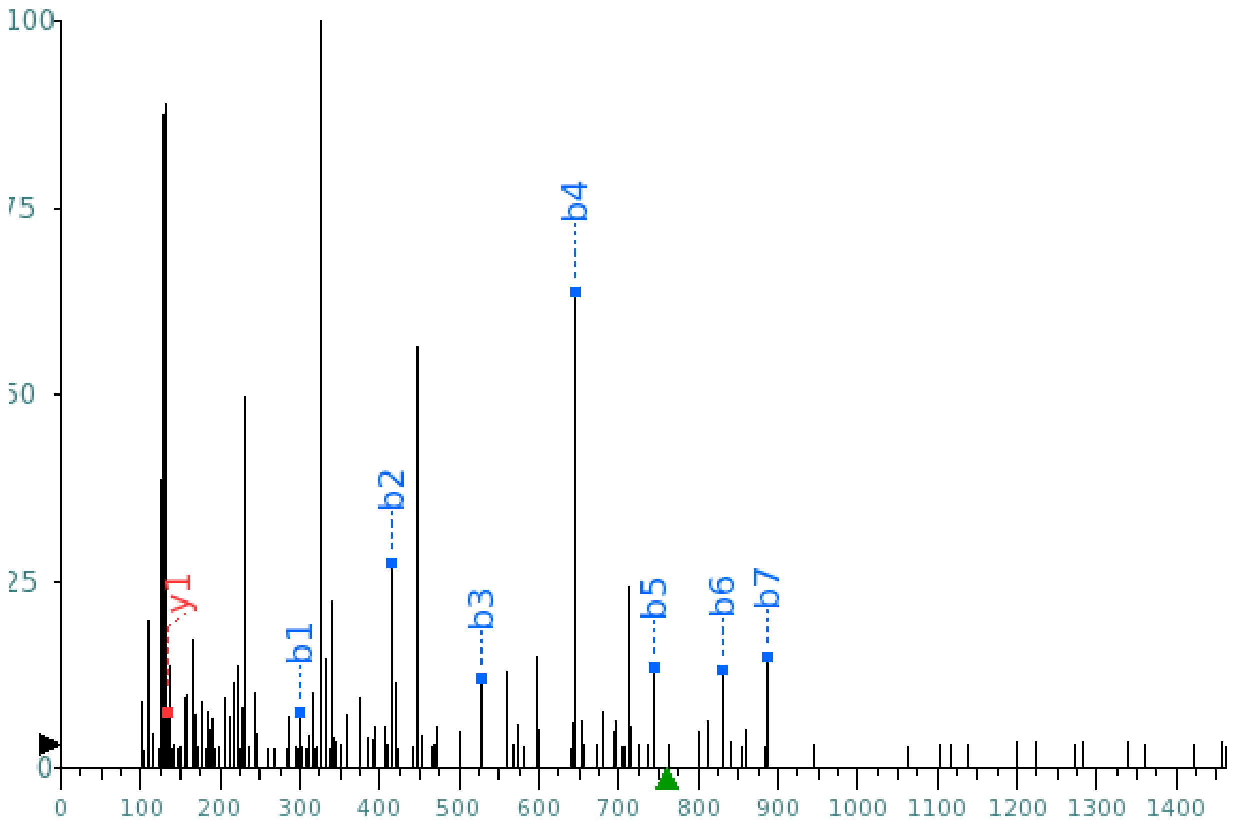

ADIDVSGPSVDTD (+2)

Primary Reference: AHNK_HUMAN
Search ID: 41760
Search Name: 20130328_ananiav_ERstress_TMT_TM_timecourse_trypsin_inj1_4mc_iaa
Scan#: 11129
Observed Mass: 760.3711 (1.5 ppm)
PSM Score: 5.17

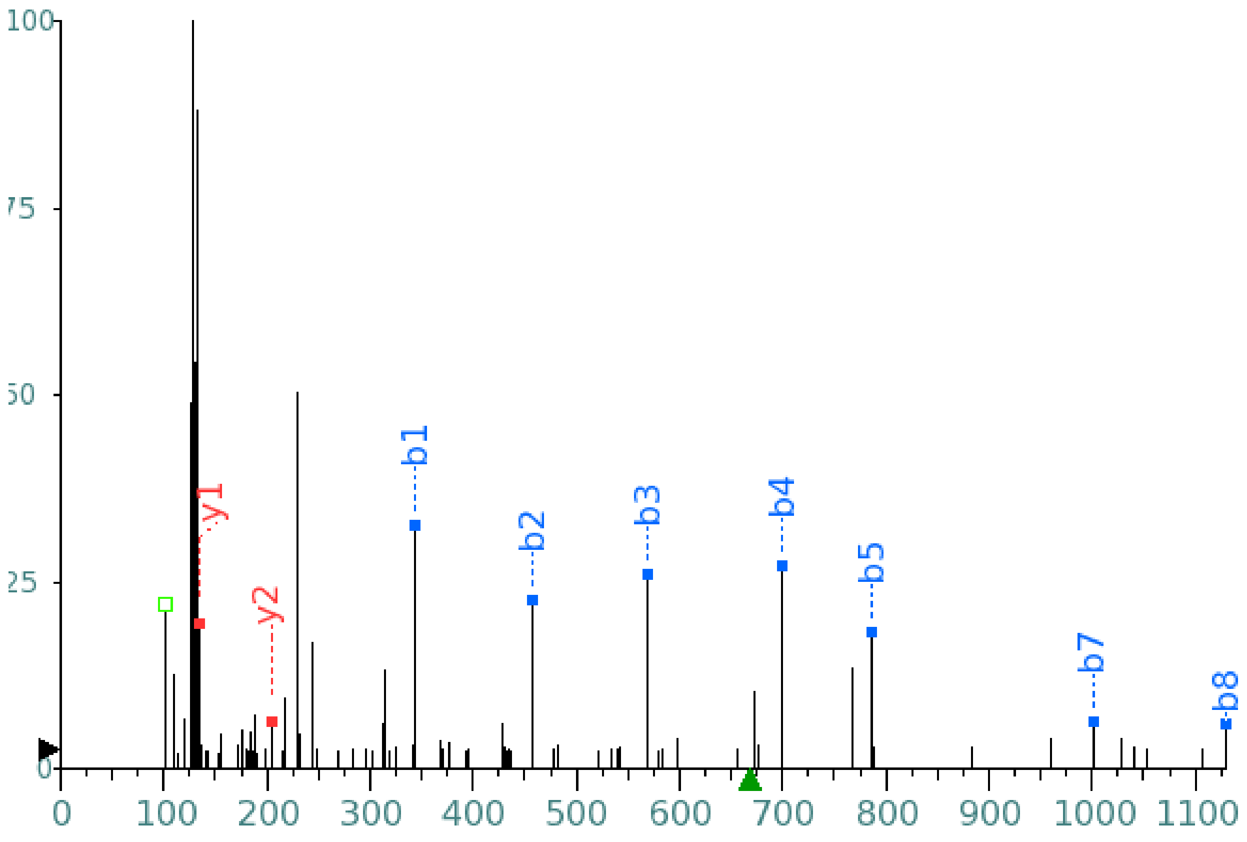

LINESVDEAD (+2)

Primary Reference: RANB3_HUMAN
Search ID: 41760
Search Name: 20130328_ananiav_ERstress_TMT_TM_timecourse_trypsin_inj1_4mc_iaa
Scan#: 11138
Observed Mass: 667.339 (1.6 ppm)
PSM Score: 48.25

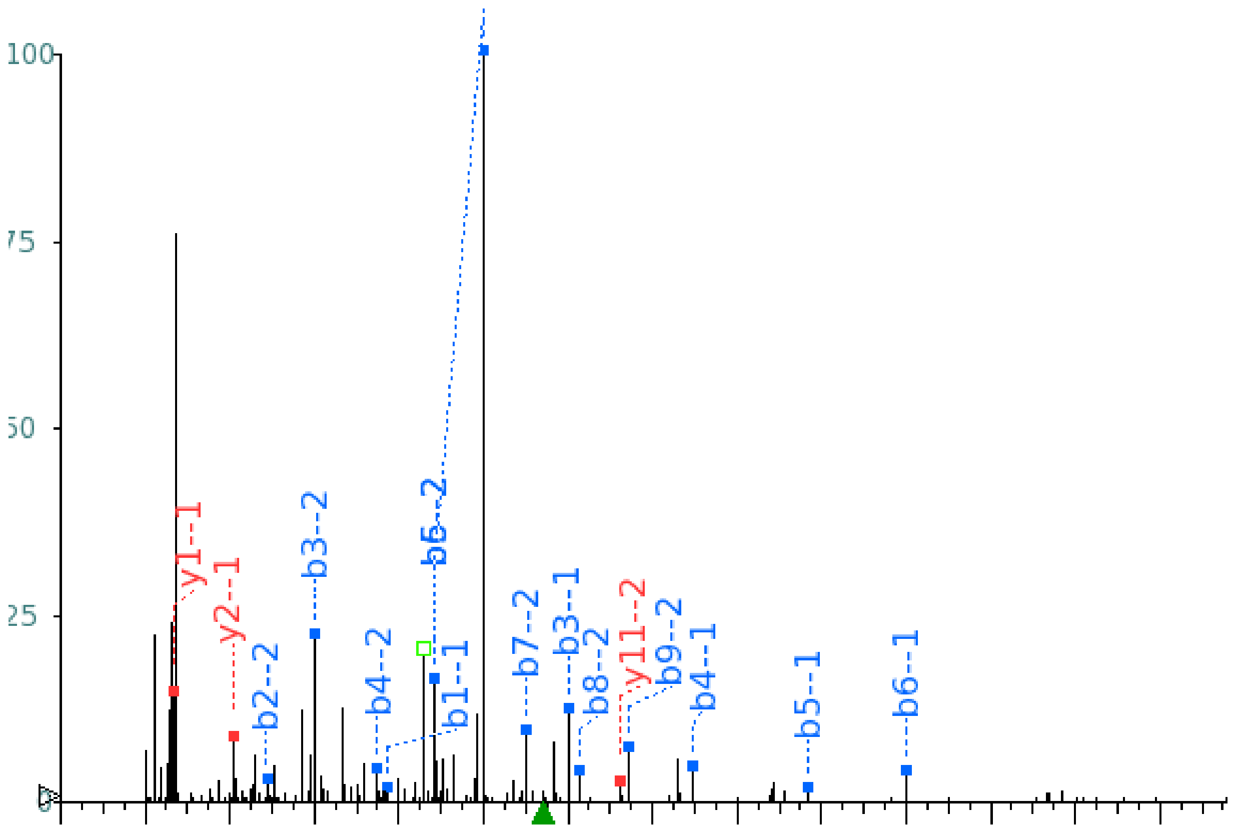

RVDFHDVQDYAD (+3)

Primary Reference: RAB24_HUMAN
Search ID: 41760
Search Name: 20130328_ananiav_ERstress_TMT_TM_timecourse_trypsin_inj1_4mc_iaa
Scan#: 11161
Observed Mass: 570.2769 (1.9 ppm)
PSM Score: 26.06

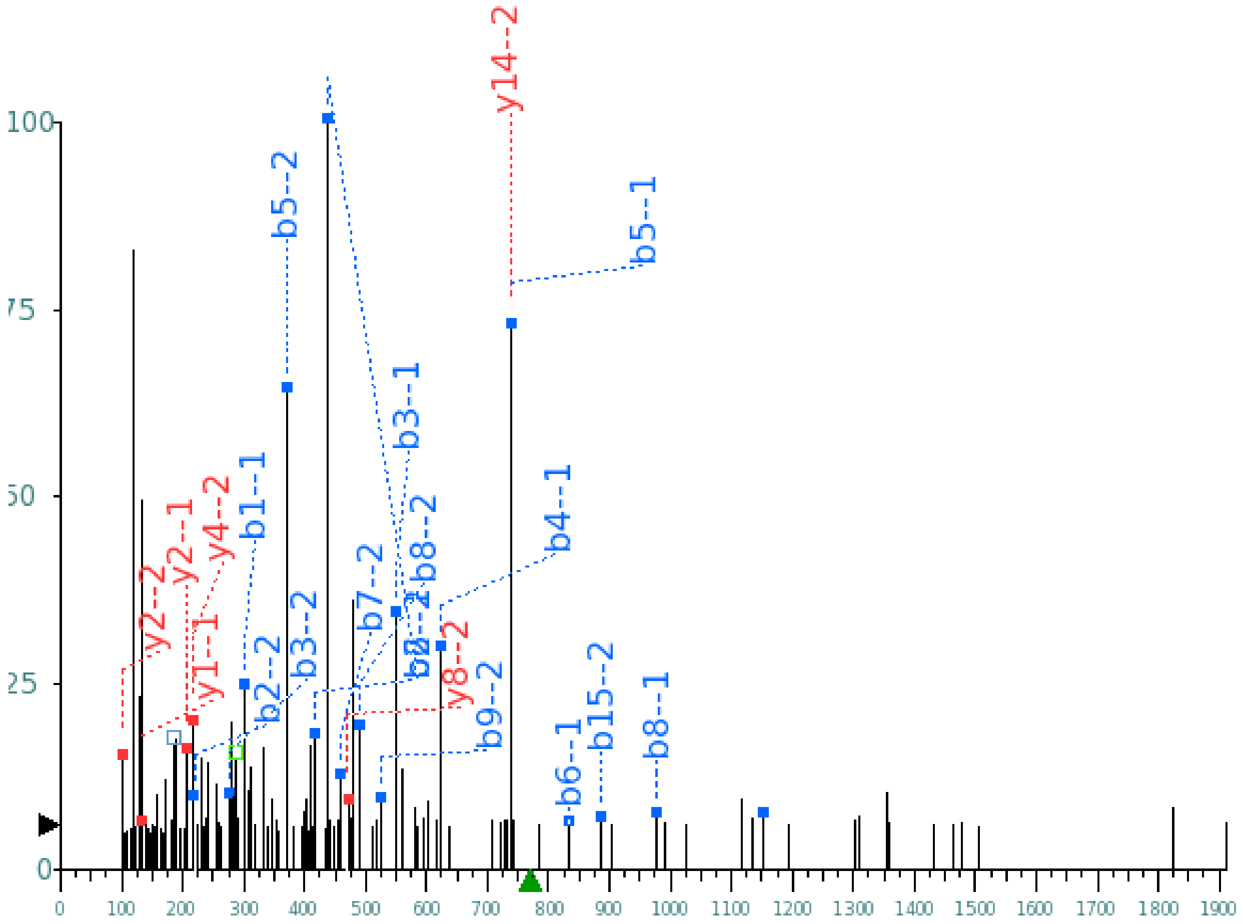

AHIANPSGASTEC*FVTDNAD (+3)

Primary Reference: FLNB_HUMAN
Search ID: 41760
Search Name: 20130328_ananiav_ERstress_TMT_TM_timecourse_trypsin_inj1_4mc_iaa
Scan#: 11182
Observed Mass: 769.3553 (-1.9 ppm)
PSM Score: 25.91

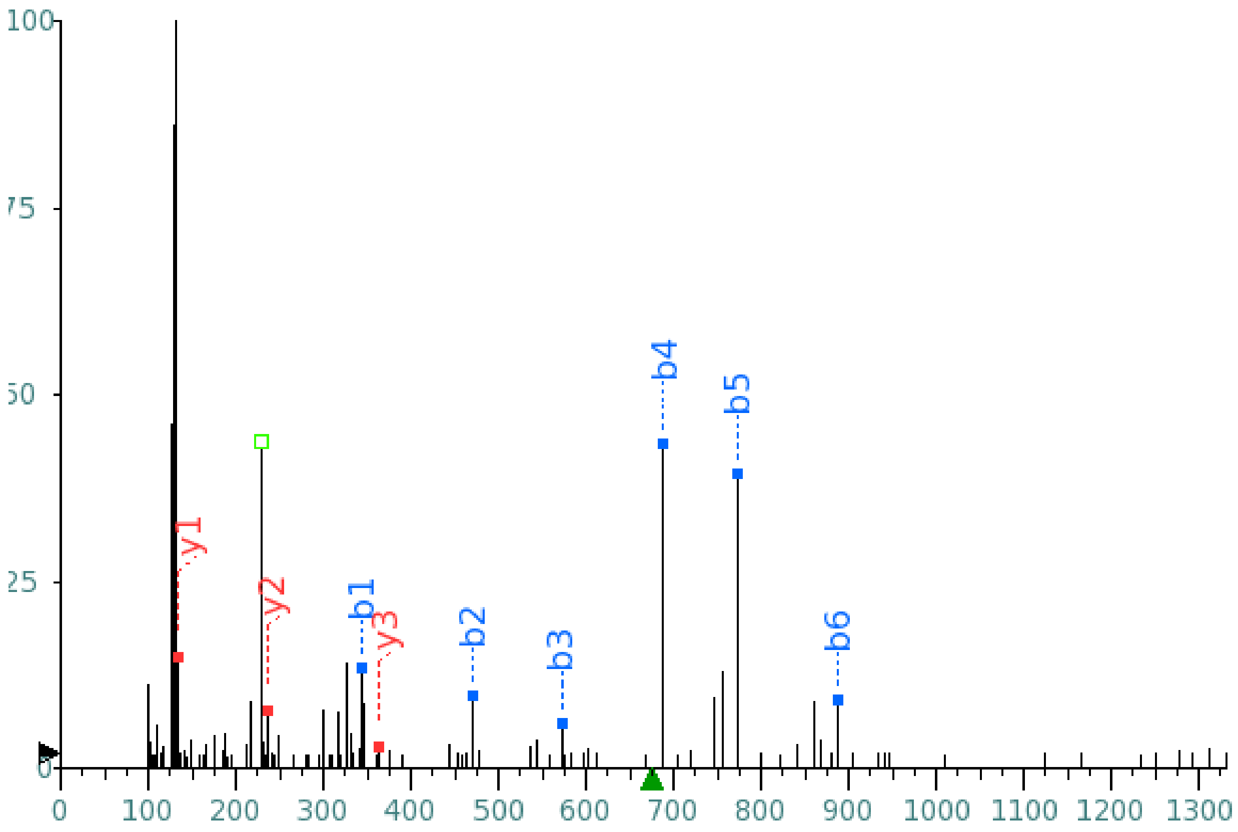

LQTDSIVQTD (+2)

Primary Reference: F1772_HUMAN
Search ID: 41760
Search Name: 20130328_ananiav_ERstress_TMT_TM_timecourse_trypsin_inj1_4mc_iaa
Scan#: 11198
Observed Mass: 674.8626 (1.6 ppm)
PSM Score: 20.46

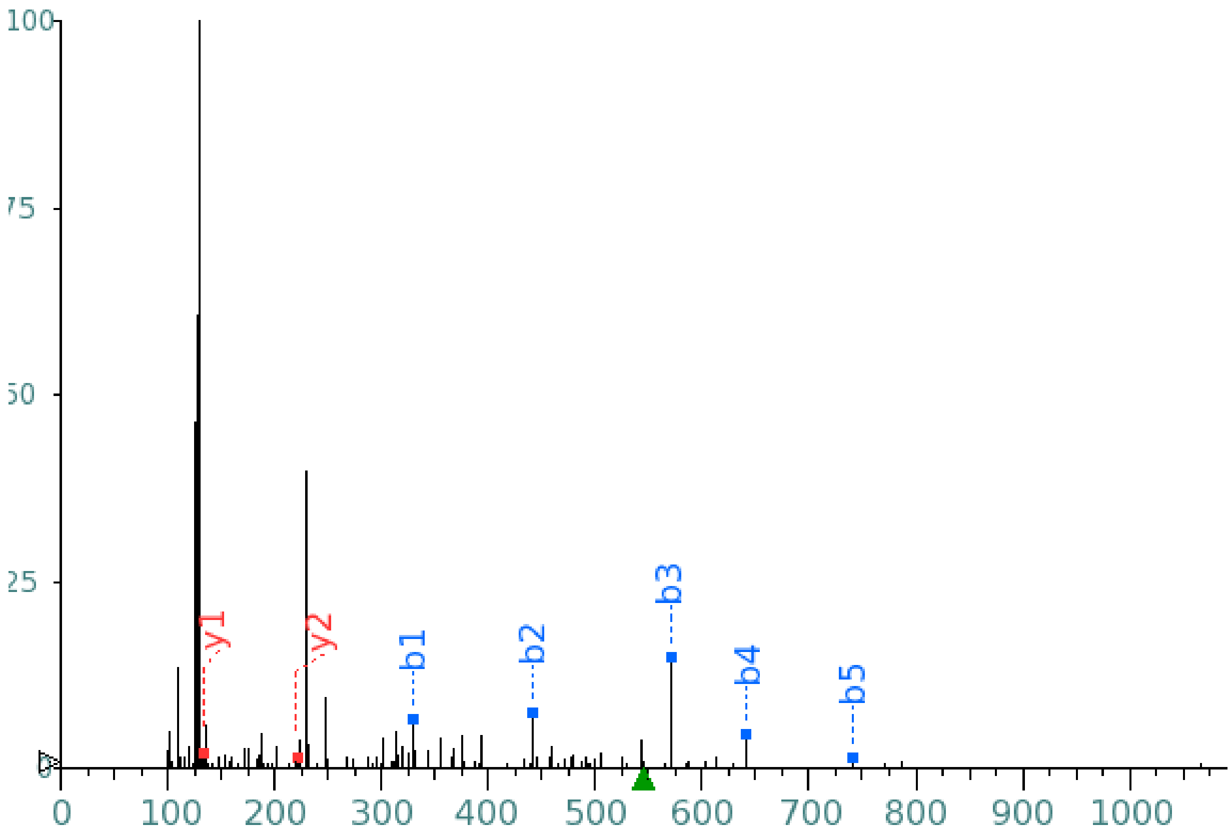

VLQAVESD (+2)

Primary Reference: K0556_HUMAN
Search ID: 41760
Search Name: 20130328_ananiav_ERstress_TMT_TM_timecourse_trypsin_inj1_4mc_iaa
Scan#: 11232
Observed Mass: 545.3048 (3 ppm)
PSM Score: 27.24

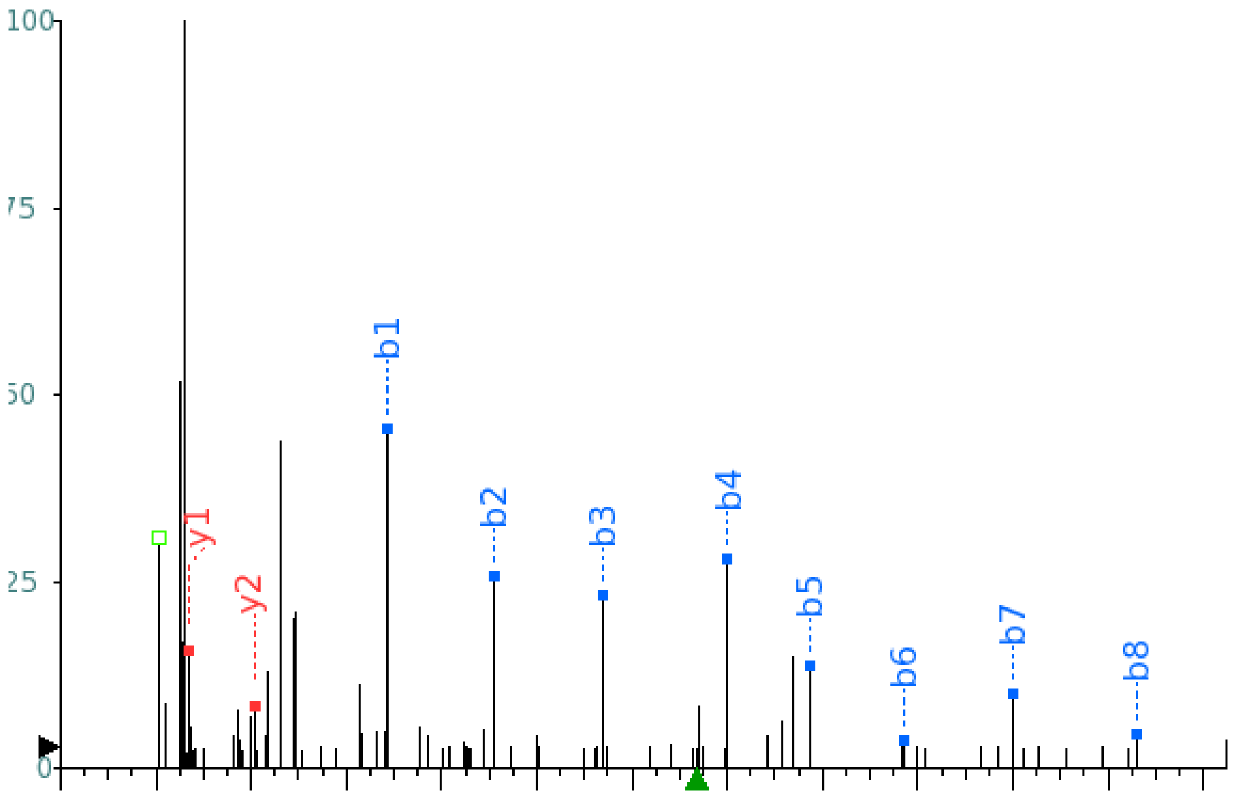

LINESVDEAD (+2)

Primary Reference: RANB3_HUMAN
Search ID: 41760
Search Name: 20130328_ananiav_ERstress_TMT_TM_timecourse_trypsin_inj1_4mc_iaa
Scan#: 11237
Observed Mass: 667.3391 (1.9 ppm)
PSM Score: 49.03

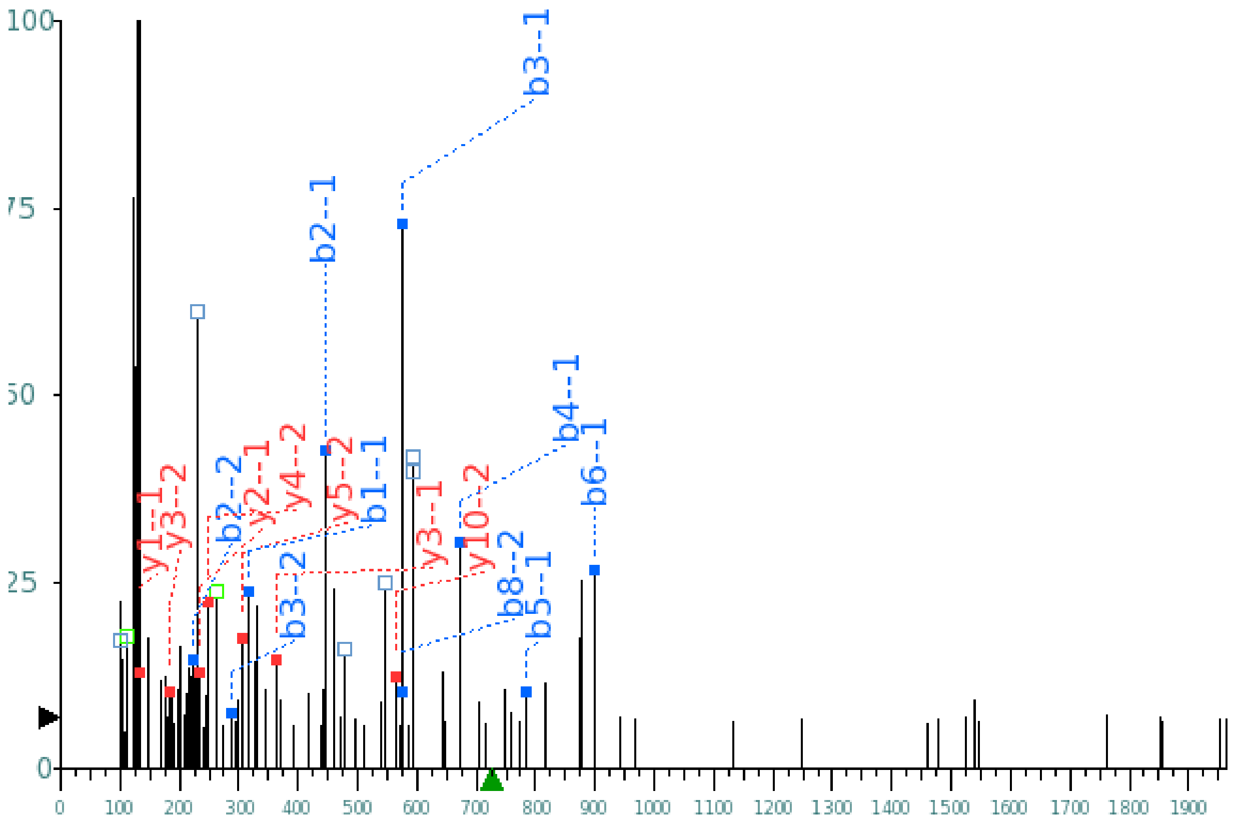

SQQVNNFTSNDVDMETD (+3)

Primary Reference: RANB9_HUMAN
Search ID: 41760
Search Name: 20130328_ananiav_ERstress_TMT_TM_timecourse_trypsin_inj1_4mc_iaa
Scan#: 11305
Observed Mass: 724.9918 (2.6 ppm)
PSM Score: 12.57

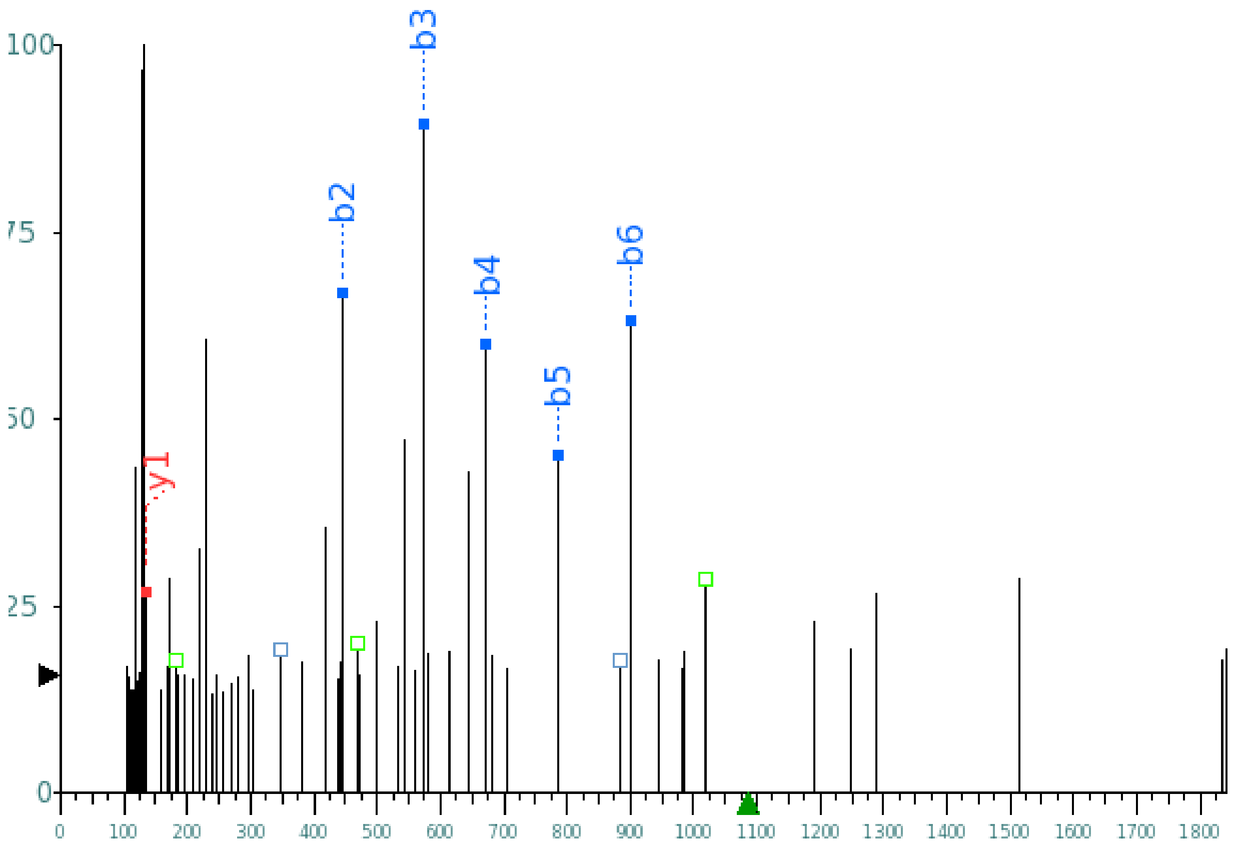

SQQVNNFTSNDVDMETD (+2)

Primary Reference: RANB9_HUMAN
Search ID: 41760
Search Name: 20130328_ananiav_ERstress_TMT_TM_timecourse_trypsin_inj1_4mc_iaa
Scan#: 11315
Observed Mass: 1086.9837 (2.2 ppm)
PSM Score: 14.9

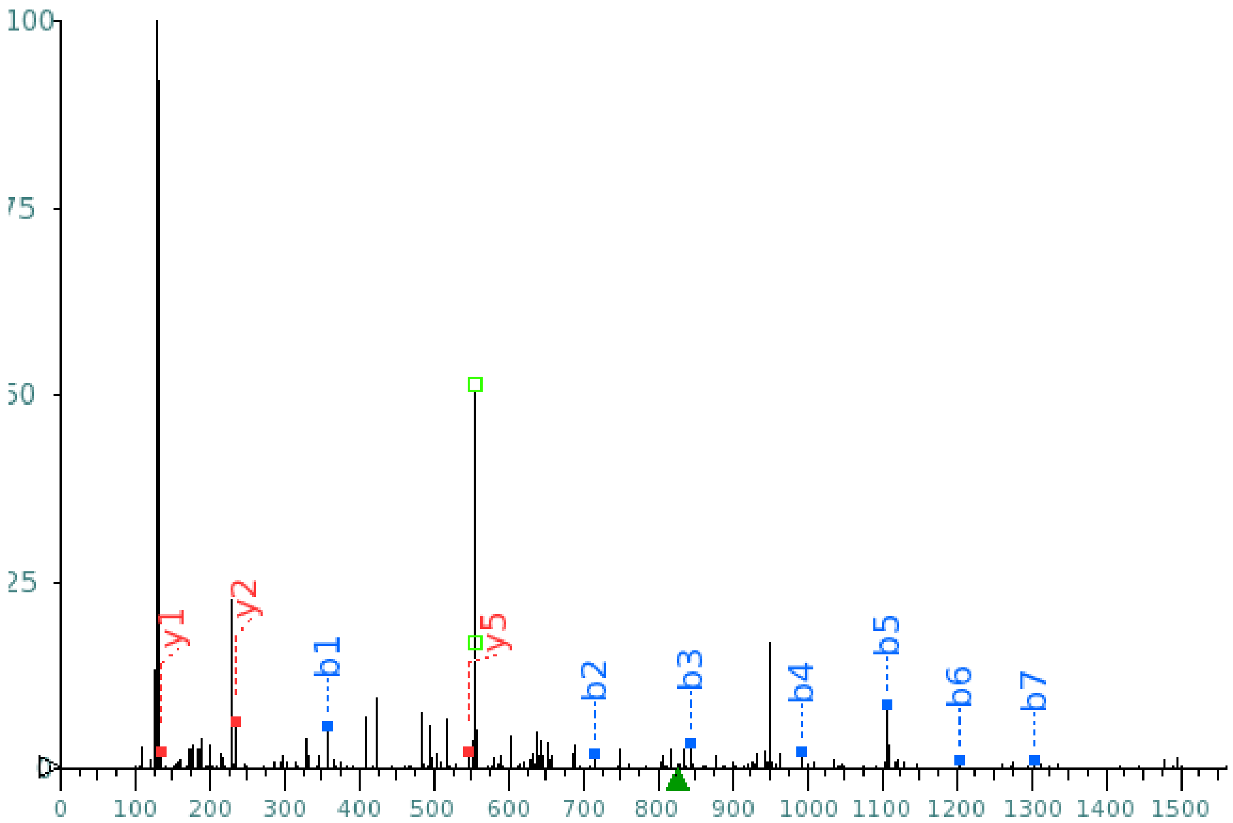

QKEFDPTITD (+2)

Primary Reference: CALD1_HUMAN
Search ID: 41760
Search Name: 20130328_ananiav_ERstress_TMT_TM_timecourse_trypsin_inj1_4mc_iaa
Scan#: 11325
Observed Mass: 826.4527 (2.3 ppm)
PSM Score: 11.3

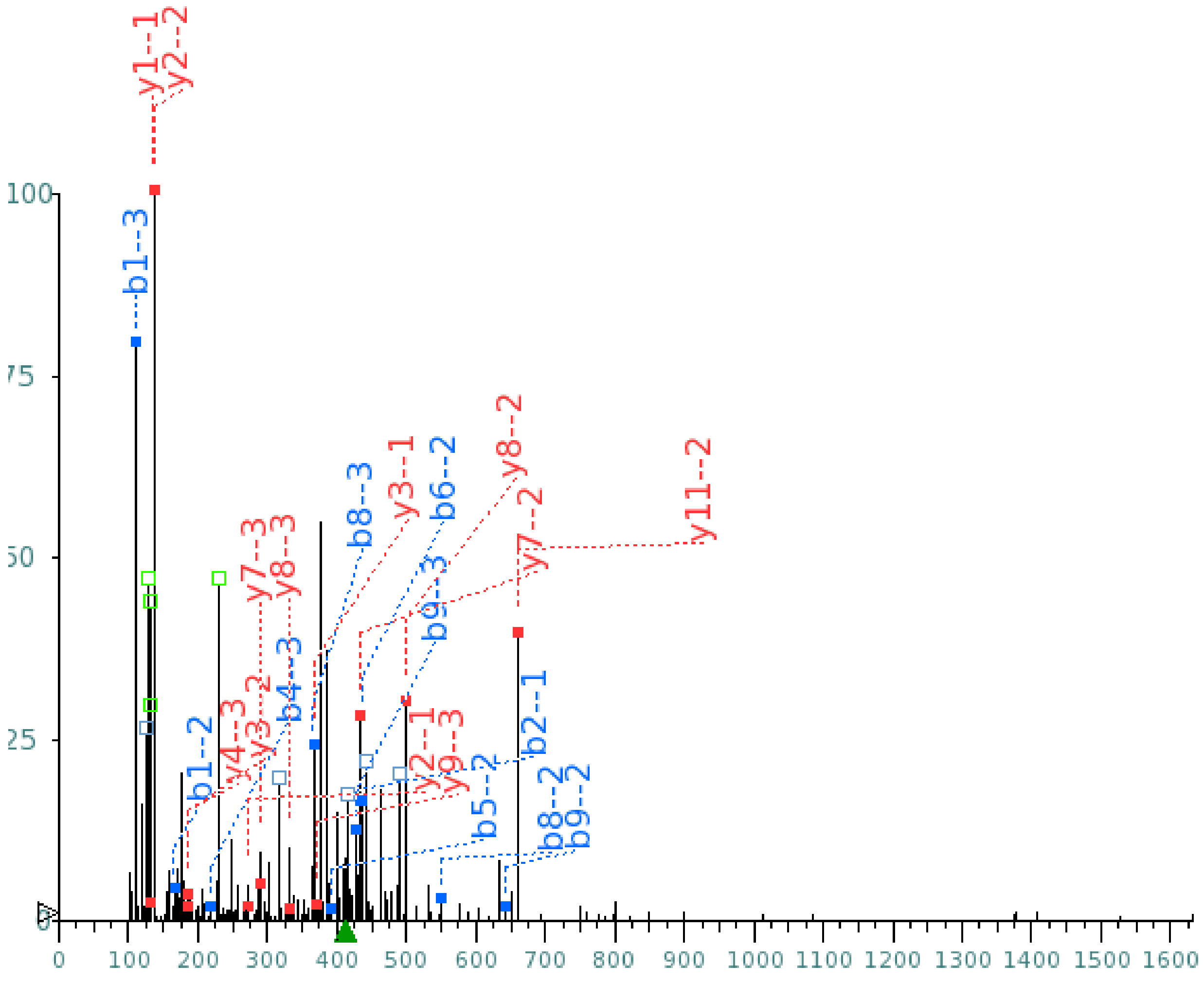

VVLNQSLLWPHD (+4)

Primary Reference: ##ACLY_HUMAN
Search ID: 41760
Search Name: 20130328_ananiav_ERstress_TMT_TM_timecourse_trypsin_inj1_4mc_iaa
Scan#: 11380
Observed Mass: 413.2371 (3.1 ppm)
PSM Score: 6.24

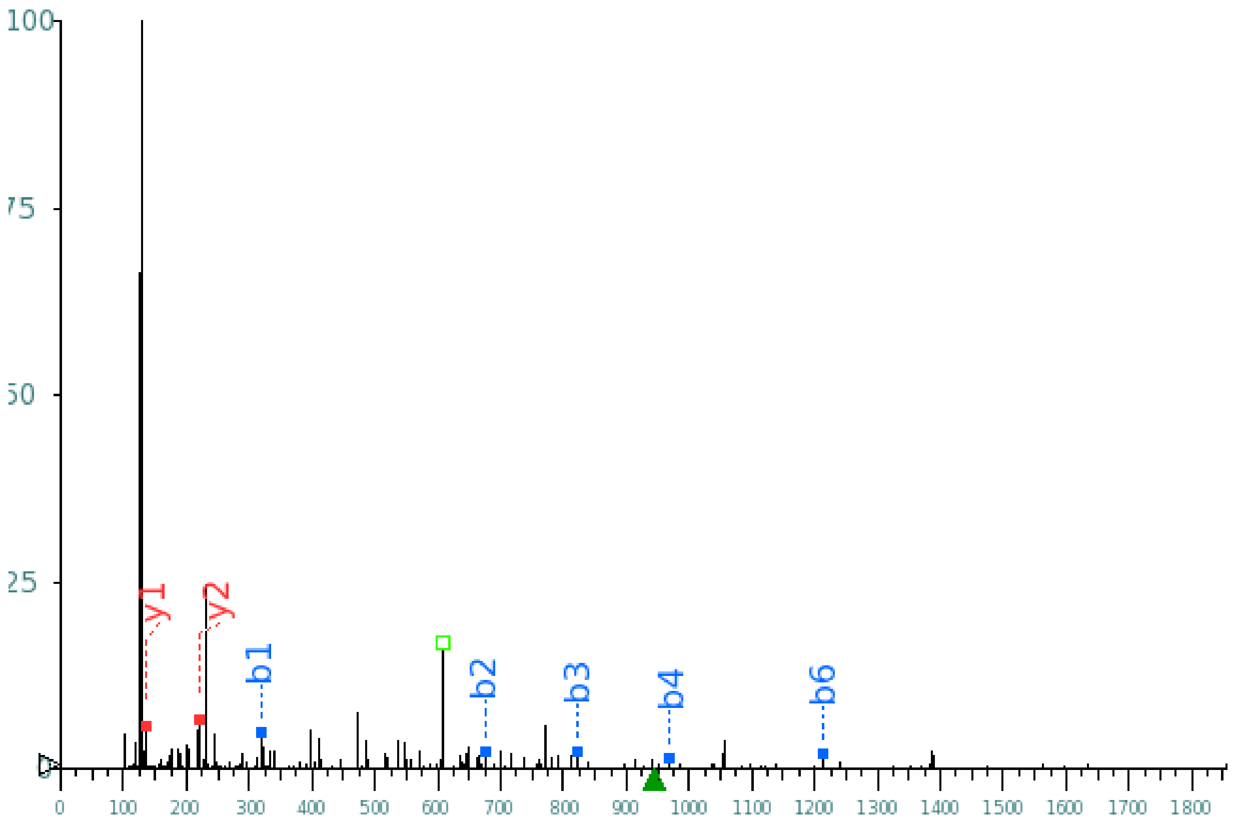

SKFFEDSEDESD (+2)

Primary Reference: PPM1G_HUMAN
Search ID: 41760
Search Name: 20130328_ananiav_ERstress_TMT_TM_timecourse_trypsin_inj1_4mc_iaa
Scan#: 11396
Observed Mass: 946.9456 (2 ppm)
PSM Score: 12.93

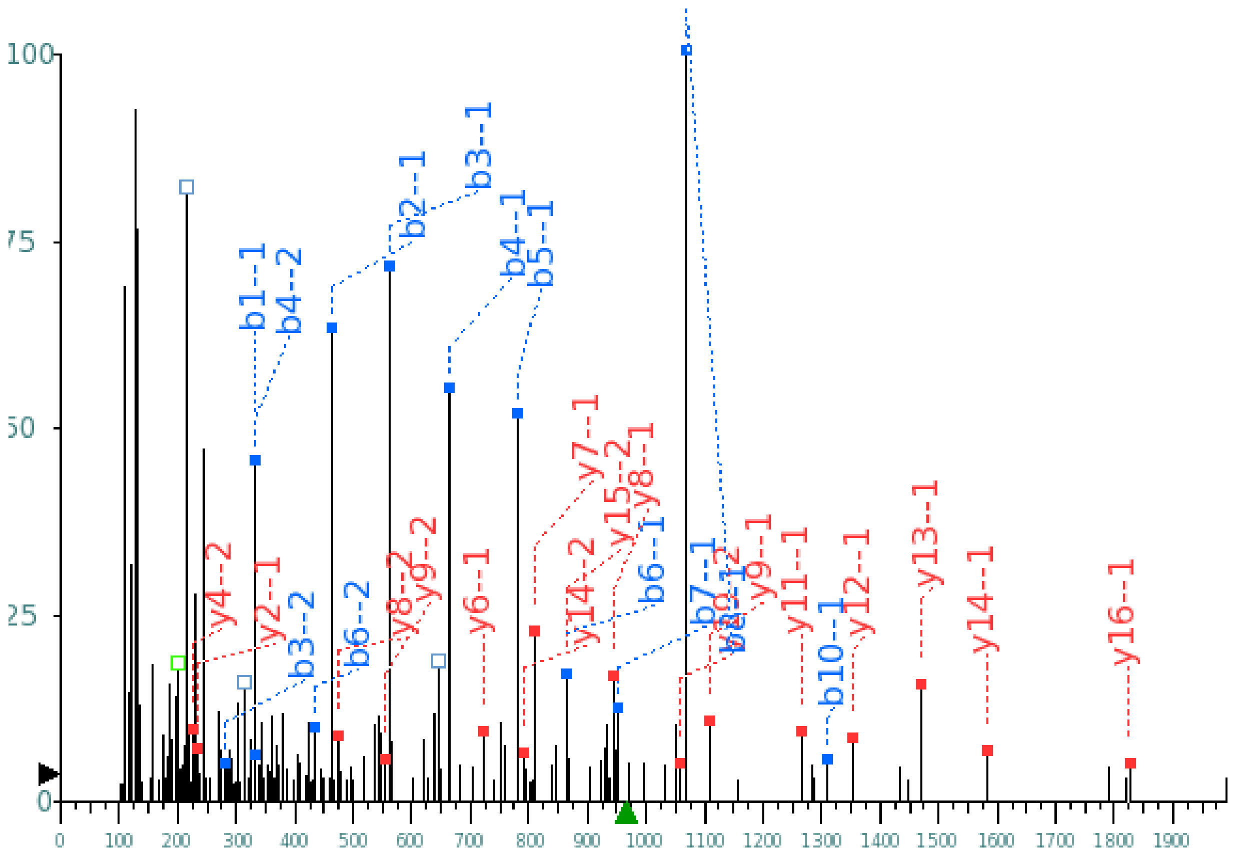

TMTTNSSDPFLNSGTYHSRDESTD (+3)

Primary Reference: YAP1_HUMAN
Search ID: 41760
Search Name: 20130328_ananiav_ERstress_TMT_TM_timecourse_trypsin_inj1_4mc_iaa
Scan#: 11417
Observed Mass: 964.7665 (2 ppm)
PSM Score: 63.42

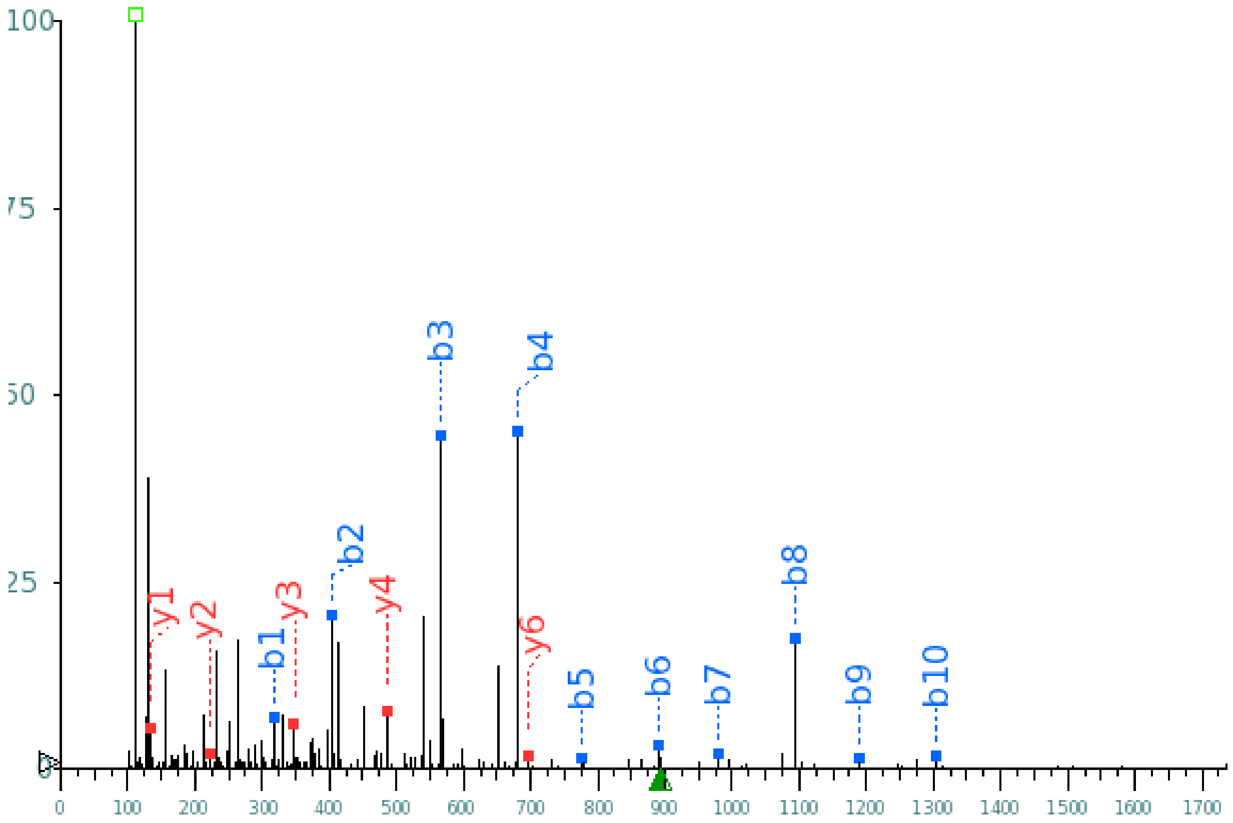

SSYLPNSDPLHQSD (+2)

Primary Reference: ANR17_HUMAN
Search ID: 41760
Search Name: 20130328_ananiav_ERstress_TMT_TM_timecourse_trypsin_inj1_4mc_iaa
Scan#: 11463
Observed Mass: 894.9346 (1 ppm)
PSM Score: 52.65

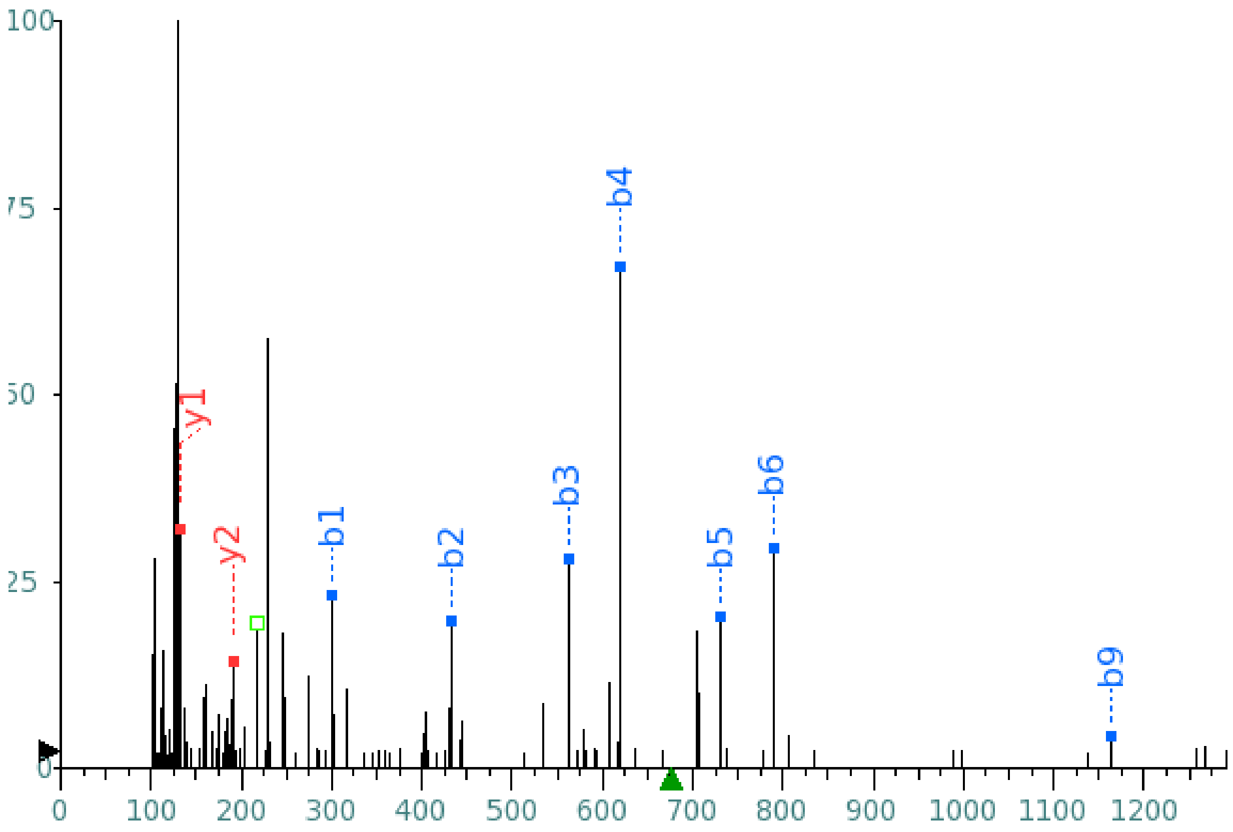

AMEGLGMDEGD (+2)

Primary Reference: PEX19_HUMAN
Search ID: 41760
Search Name: 20130328_ananiav_ERstress_TMT_TM_timecourse_trypsin_inj1_4mc_iaa
Scan#: 11491
Observed Mass: 677.2985 (2.4 ppm)
PSM Score: 34.43

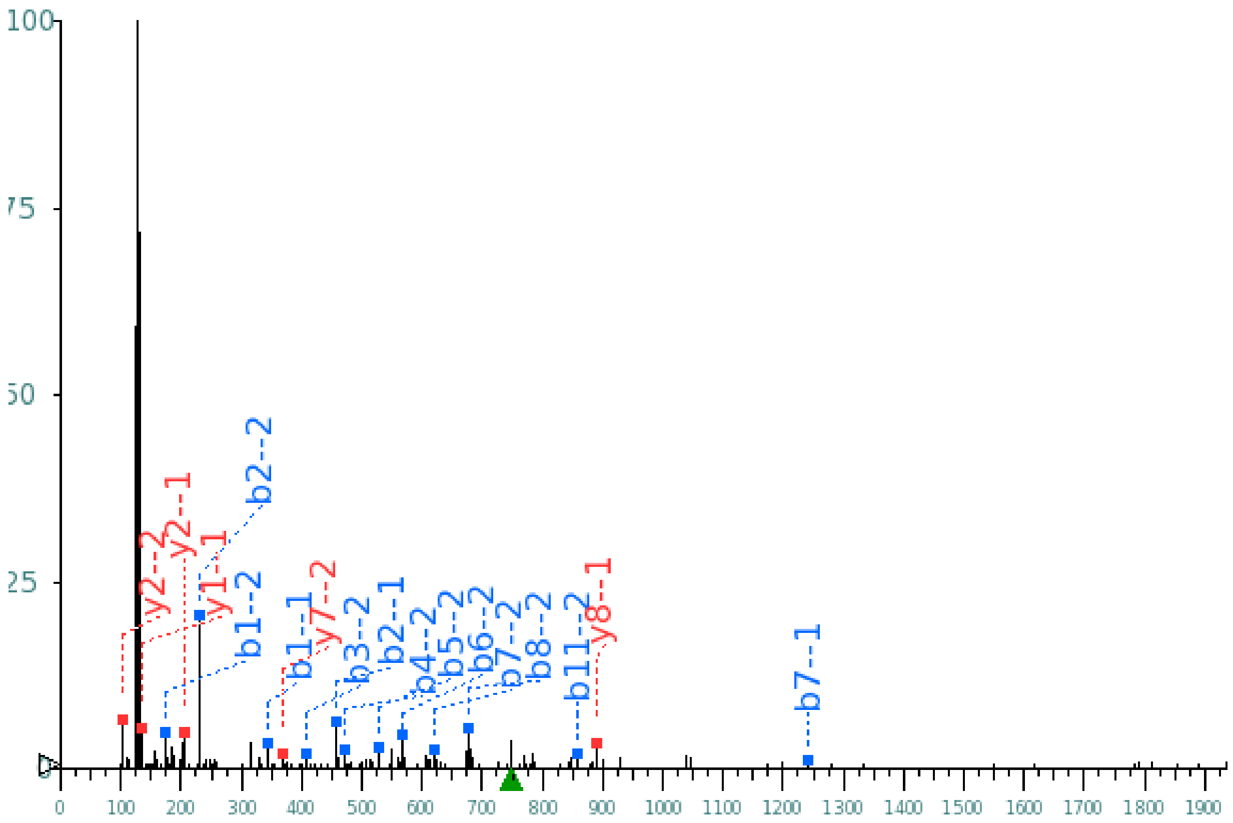

LDKENAIDRAEQAEAD (+3)

Primary Reference: TPM2_HUMAN
Search ID: 41760
Search Name: 20130328_ananiav_ERstress_TMT_TM_timecourse_trypsin_inj1_4mc_iaa
Scan#: 11590
Observed Mass: 749.3949 (1.6 ppm)
PSM Score: 16.64

File name: 2015-02-04 04.01.53PM.pdf

Creation time: 2015-02-04 04:09PM

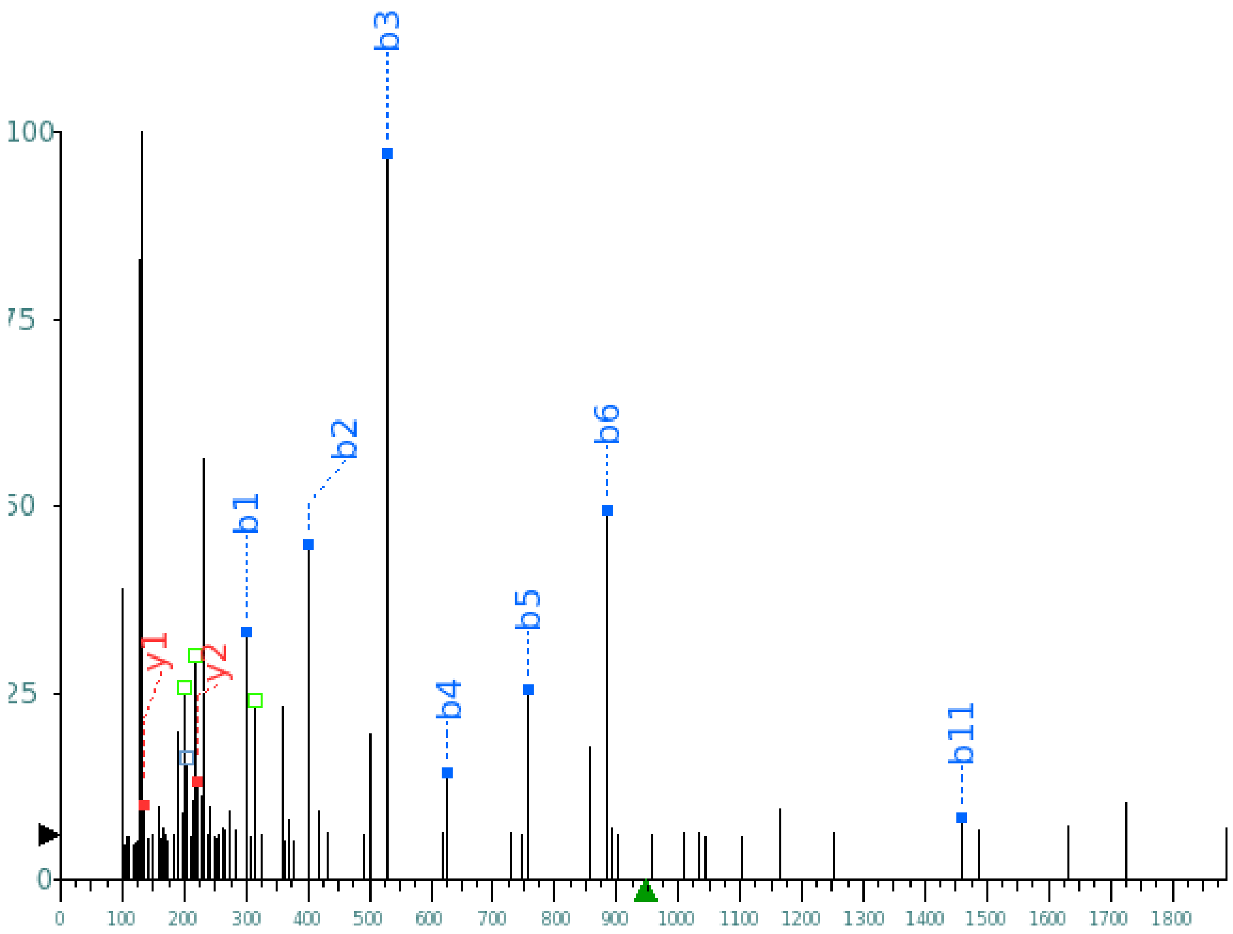

AVEPMEIDSEESESD (+2)

Primary Reference: ERCC5_HUMAN
Search ID: 41760
Search Name: 20130328_ananiav_ERstress_TMT_TM_timecourse_trypsin_inj1_4mc_iaa
Scan#: 11628
Observed Mass: 948.4196 (2.8 ppm)
PSM Score: 21.79

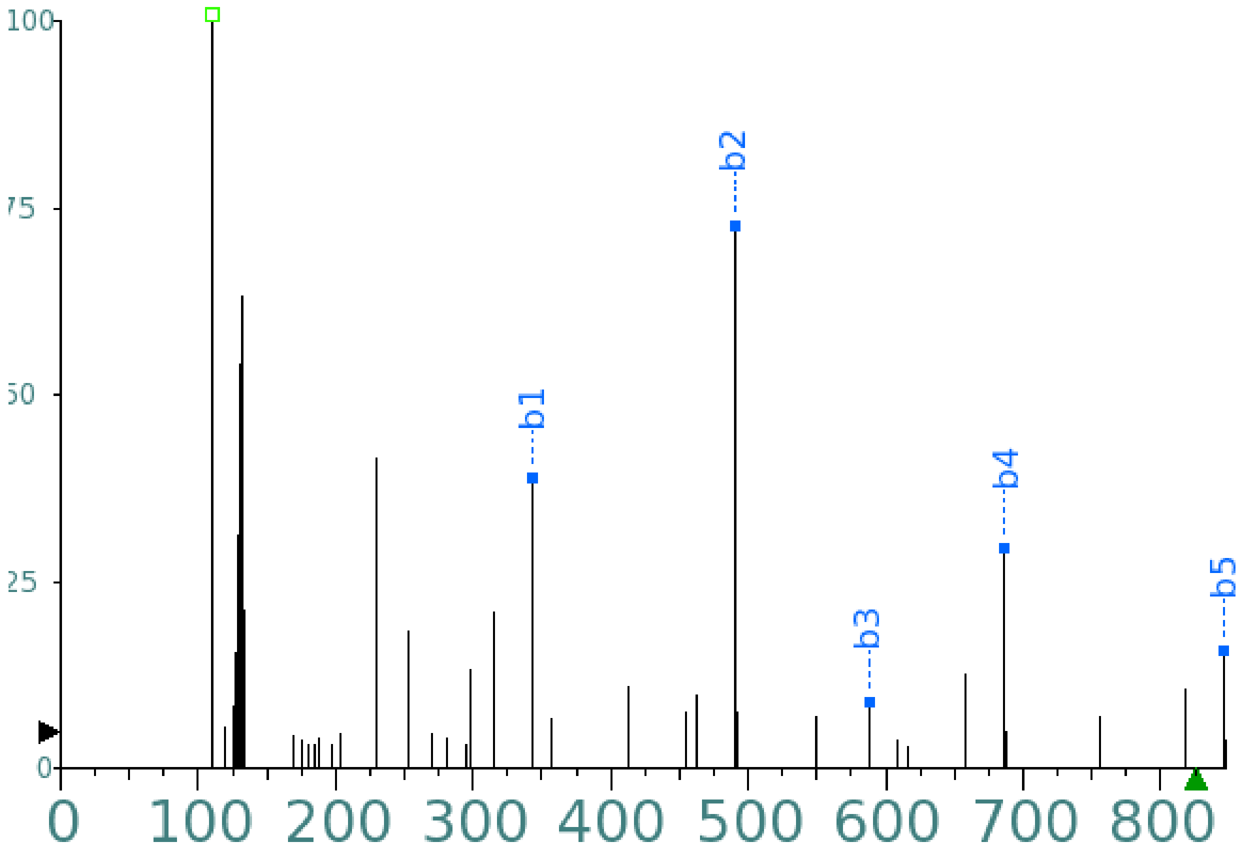

LFPVC*HDSDESD (+2)

Primary Reference: DNM3A_HUMAN
Search ID: 41760
Search Name: 20130328_ananiav_ERstress_TMT_TM_timecourse_trypsin_inj1_4mc_iaa
Scan#: 11673
Observed Mass: 825.3694 (0 ppm)
PSM Score: 29.02

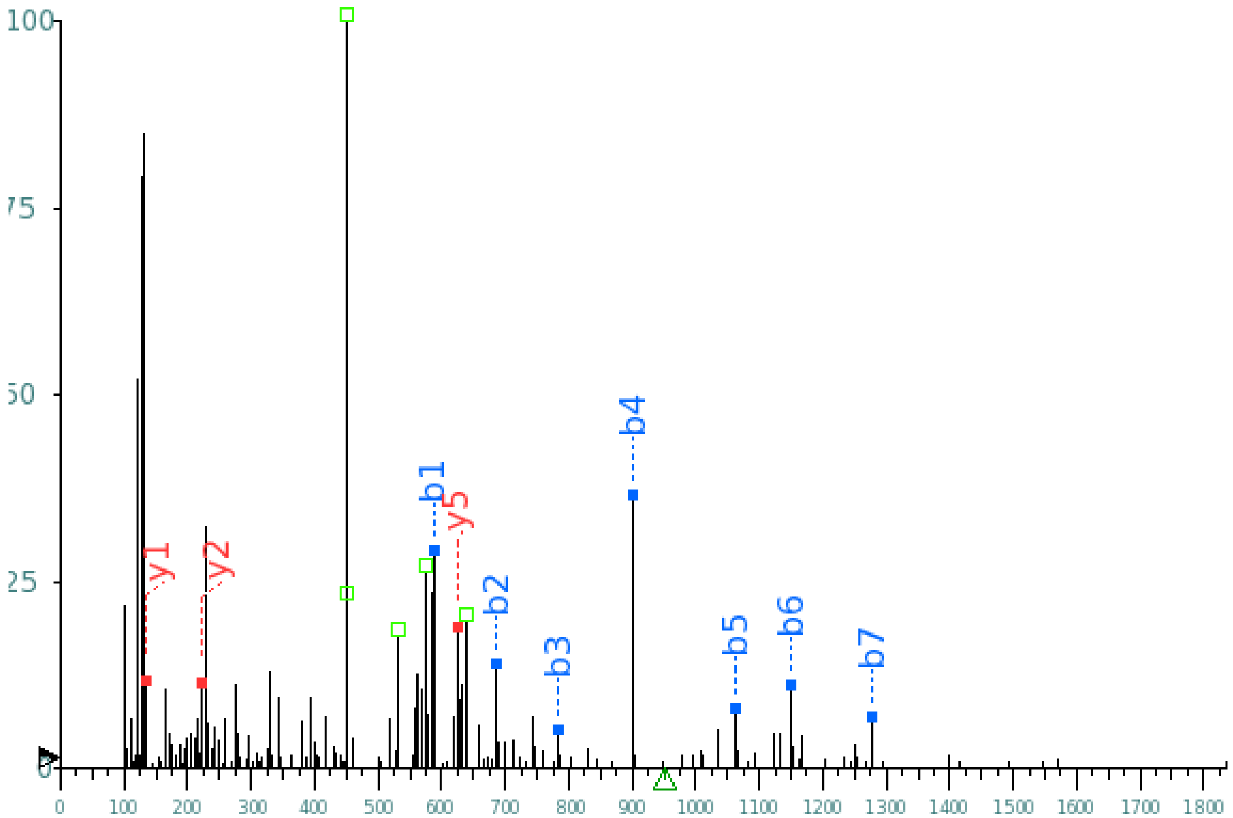

KVVDYSQFQESD (+2)

Primary Reference: NUCKS_HUMAN
Search ID: 41760
Search Name: 20130328_ananiav_ERstress_TMT_TM_timecourse_trypsin_inj1_4mc_iaa
Scan#: 11729
Observed Mass: 951.9976 (1.5 ppm)
PSM Score: 19.4

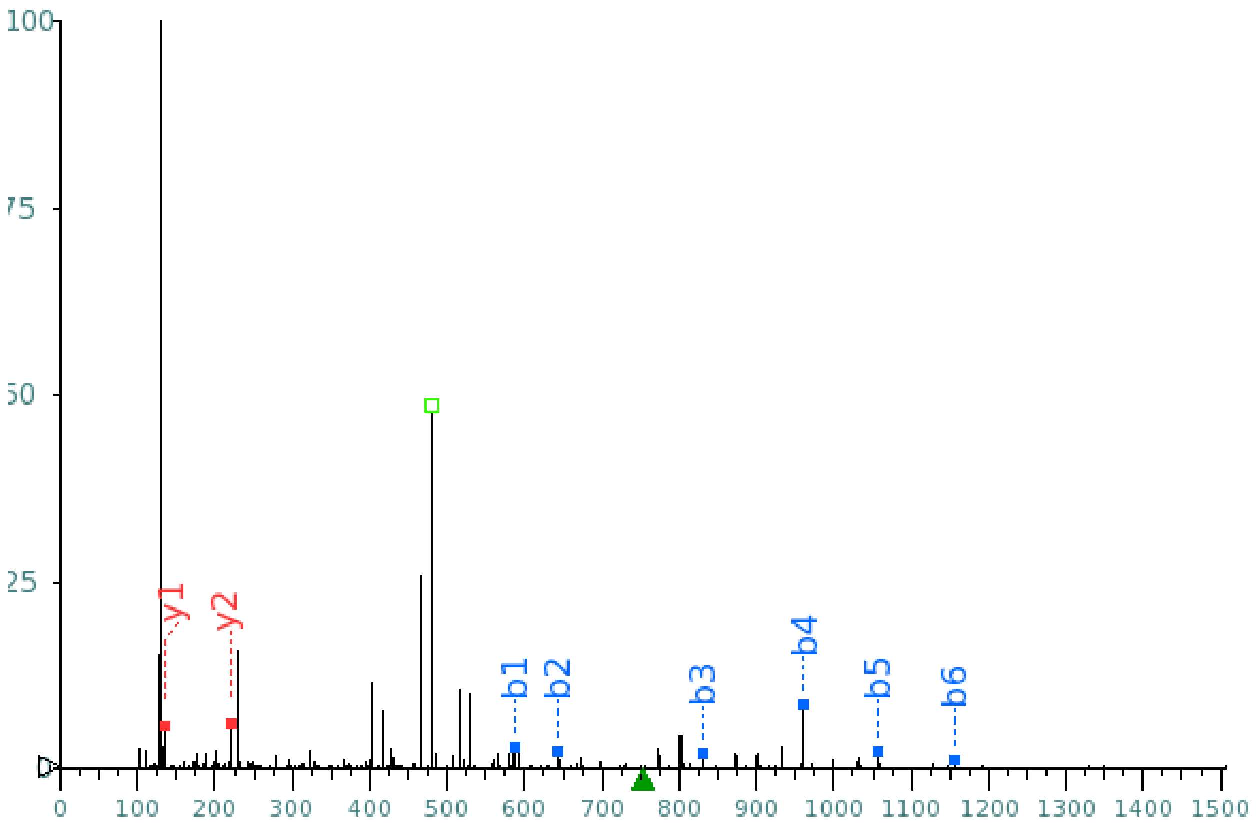

KGWEVVESD (+2)

Primary Reference: NQO1_HUMAN
Search ID: 41760
Search Name: 20130328_ananiav_ERstress_TMT_TM_timecourse_trypsin_inj1_4mc_iaa
Scan#: 11779
Observed Mass: 753.9144 (0.8 ppm)
PSM Score: 8.95

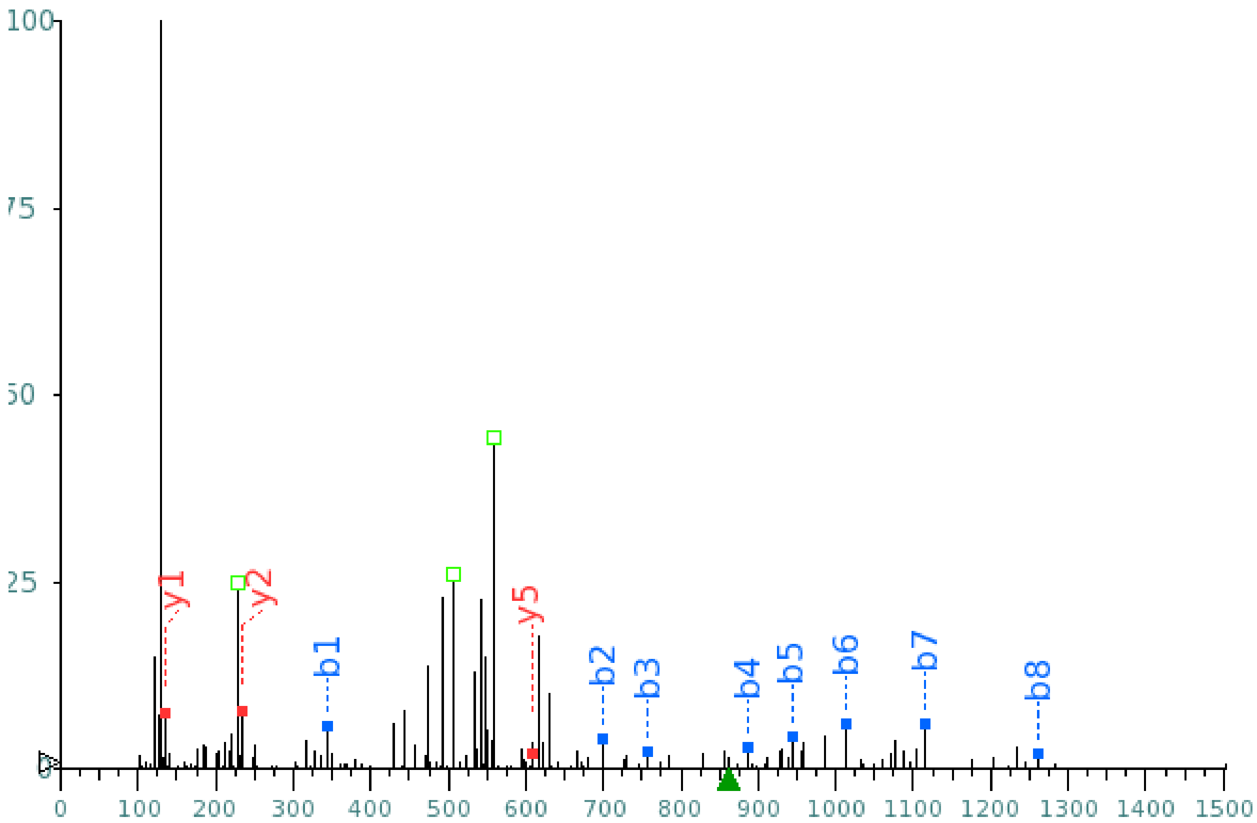

LKGQGATFVETD (+2)

Primary Reference: ZN407_HUMAN
Search ID: 41760
Search Name: 20130328_ananiav_ERstress_TMT_TM_timecourse_trypsin_inj1_4mc_iaa
Scan#: 11804
Observed Mass: 862.4873 (2.4 ppm)
PSM Score: 18.53

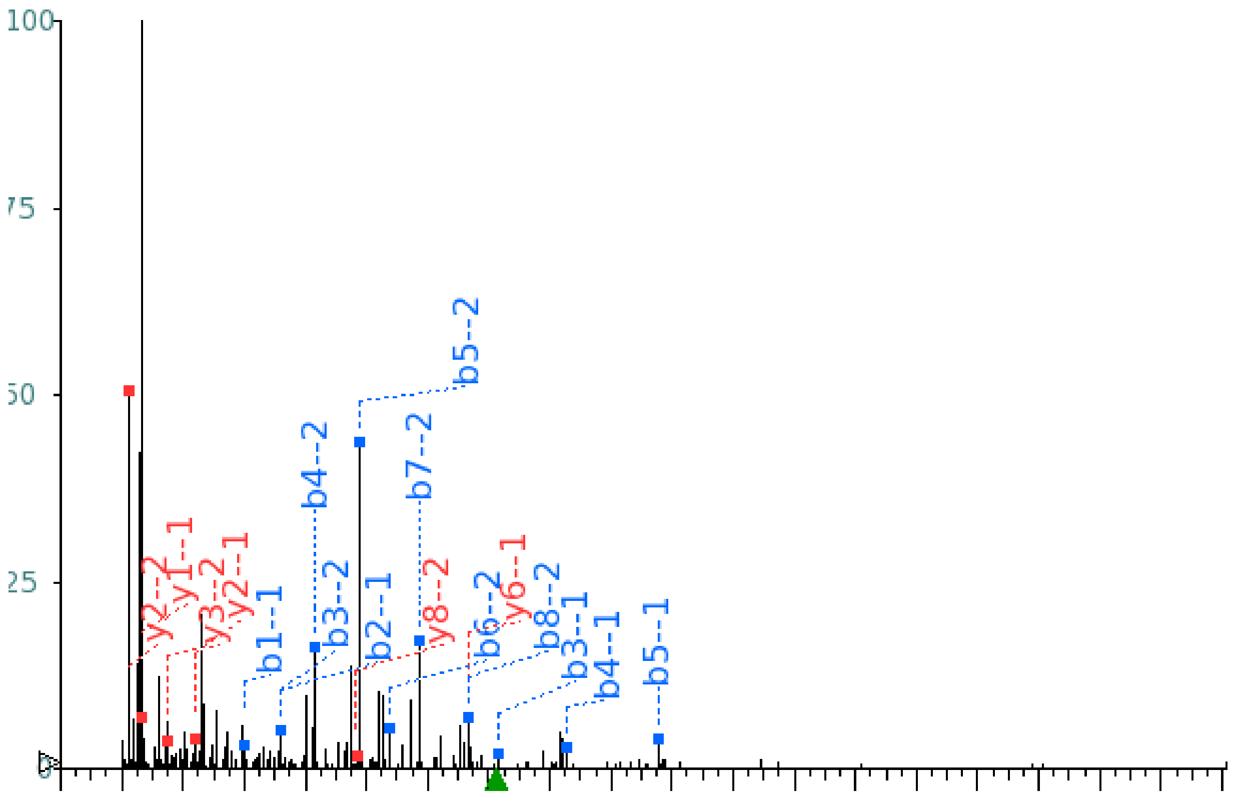

AGKLFPVC*HDSDESD (+3)

Primary Reference: DNM3A_HUMAN
Search ID: 41760
Search Name: 20130328_ananiav_ERstress_TMT_TM_timecourse_trypsin_inj1_4mc_iaa
Scan#: 11847
Observed Mass: 712.3539 (-0.4 ppm)
PSM Score: 10.99

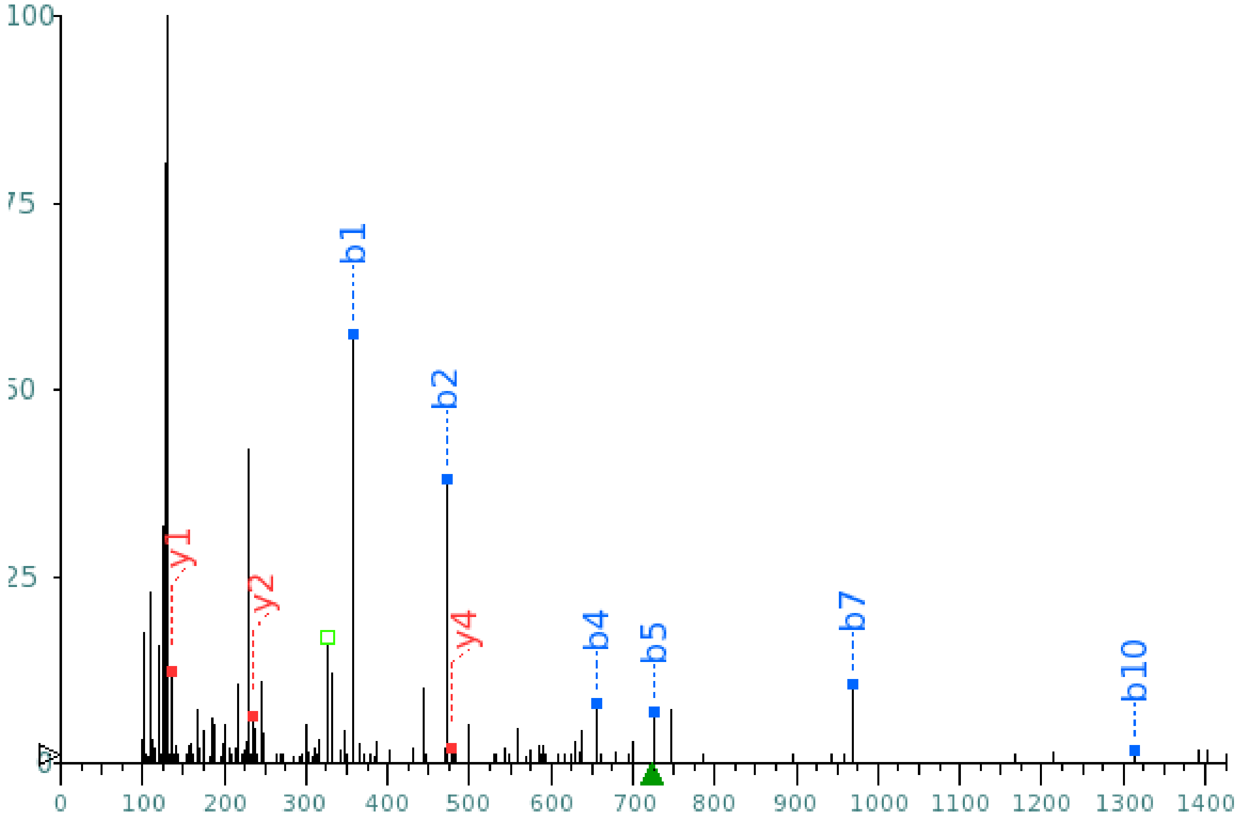

QIPSAEDLETD (+2)

Primary Reference: HDAC7_HUMAN
Search ID: 41760
Search Name: 20130328_ananiav_ERstress_TMT_TM_timecourse_trypsin_inj1_4mc_iaa
Scan#: 11858
Observed Mass: 723.8637 (2.8 ppm)
PSM Score: 17.64

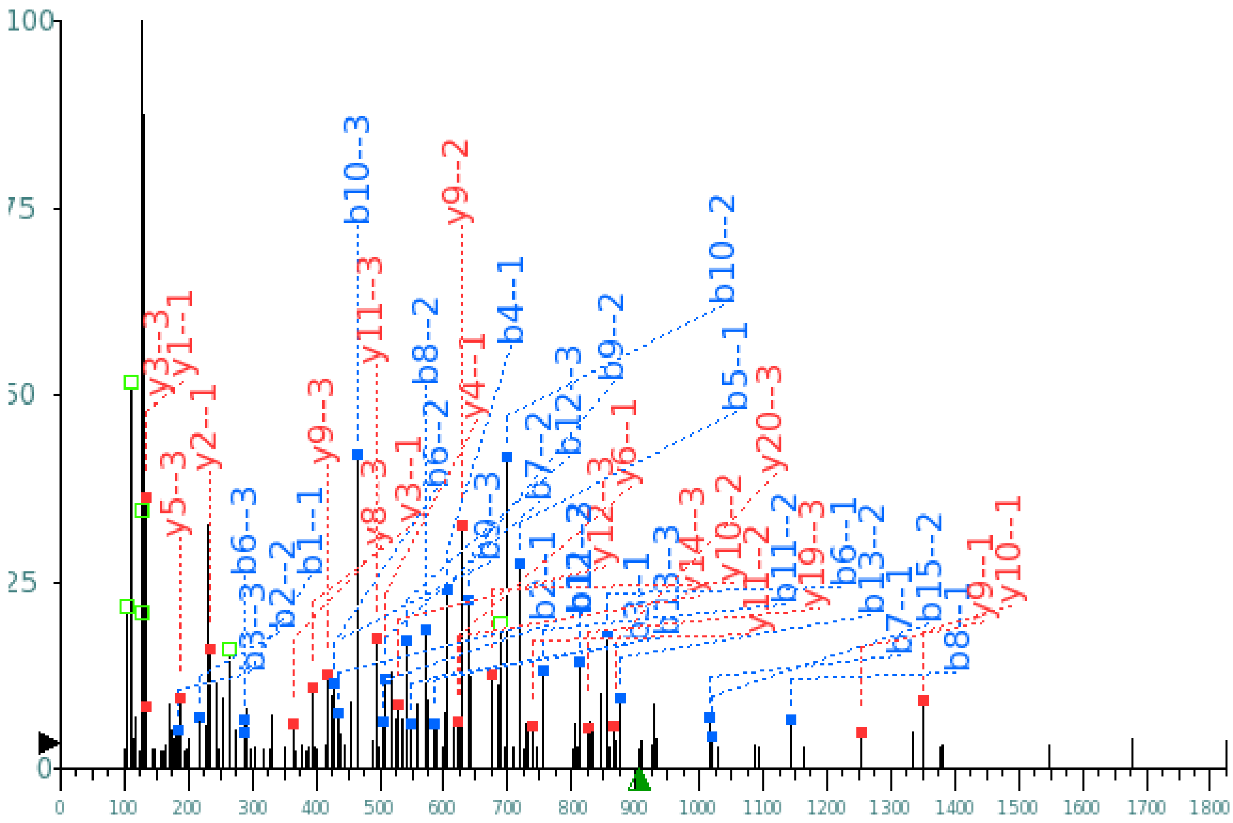

GFNGIHC*QHNINEC*EVEPC*KNGGIC*TD (+4)

Primary Reference: EDIL3_HUMAN
Search ID: 41760
Search Name: 20130328_ananiav_ERstress_TMT_TM_timecourse_trypsin_inj1_4mc_iaa
Scan#: 11864
Observed Mass: 905.1677 (2.6 ppm)
PSM Score: 28.94

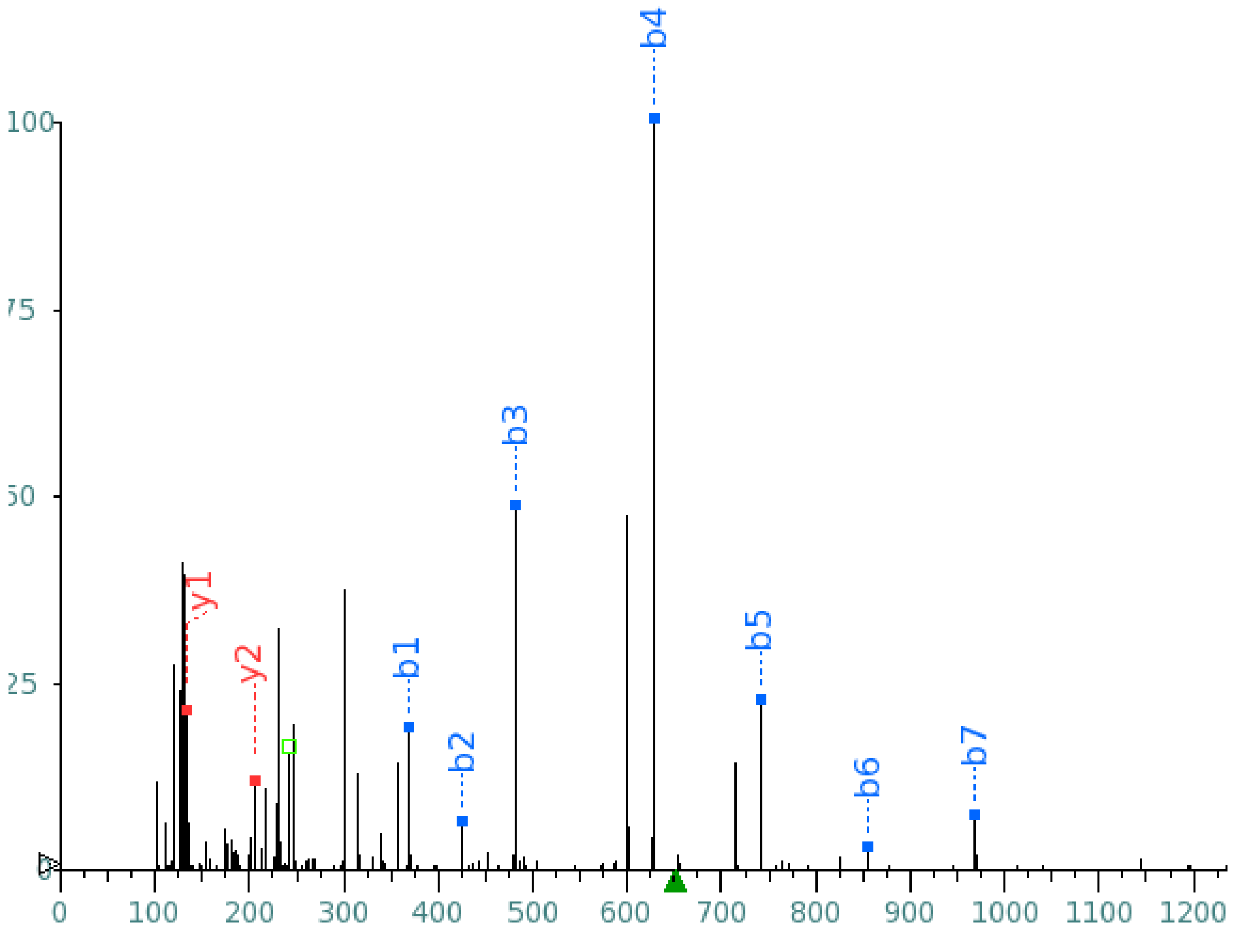

HGGFILDEAD (+2)

Primary Reference: SPT5H_HUMAN
Search ID: 41760
Search Name: 20130328_ananiav_ERstress_TMT_TM_timecourse_trypsin_inj1_4mc_iaa
Scan#: 11889
Observed Mass: 651.8312 (1.8 ppm)
PSM Score: 34.56

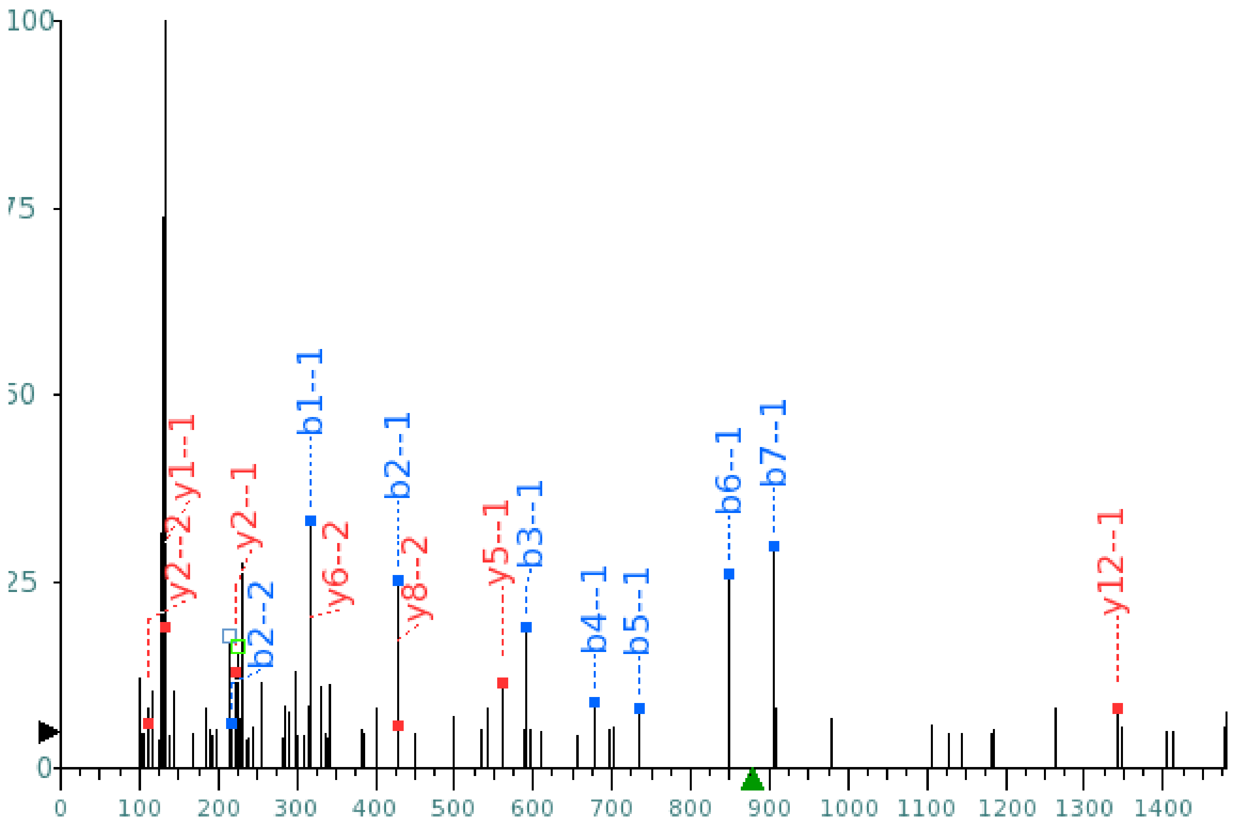

SLC*SGDGLGDTEISRPQAPDQSD (+3)

Primary Reference: HAUS5_HUMAN
Search ID: 41760
Search Name: 20130328_ananiav_ERstress_TMT_TM_timecourse_trypsin_inj1_4mc_iaa
Scan#: 11911
Observed Mass: 878.7454 (2.5 ppm)
PSM Score: 34

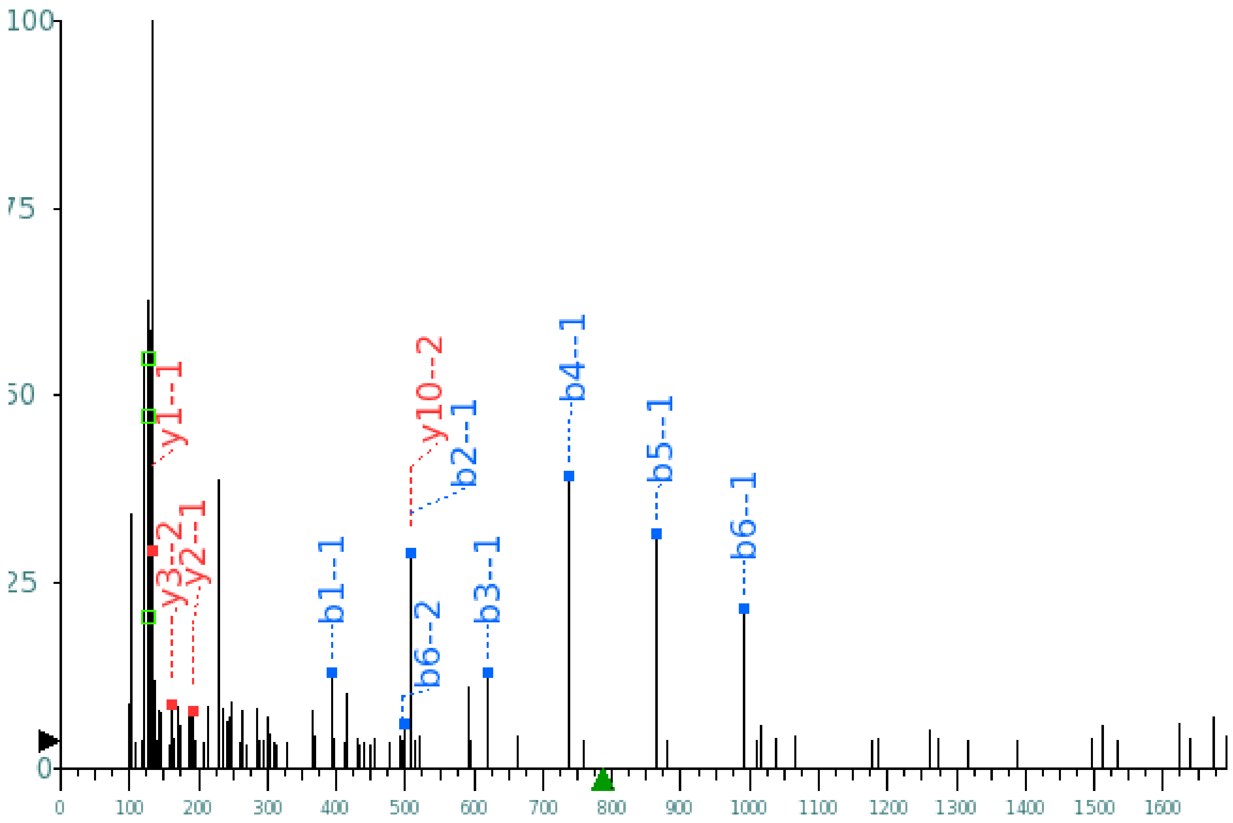

YNLDEETTSAGSGDDFDQGD (+3)

Primary Reference: TM9S1_HUMAN
Search ID: 41760
Search Name: 20130328_ananiav_ERstress_TMT_TM_timecourse_trypsin_inj1_4mc_iaa
Scan#: 11925
Observed Mass: 789.0005 (3.4 ppm)
PSM Score: 21.33

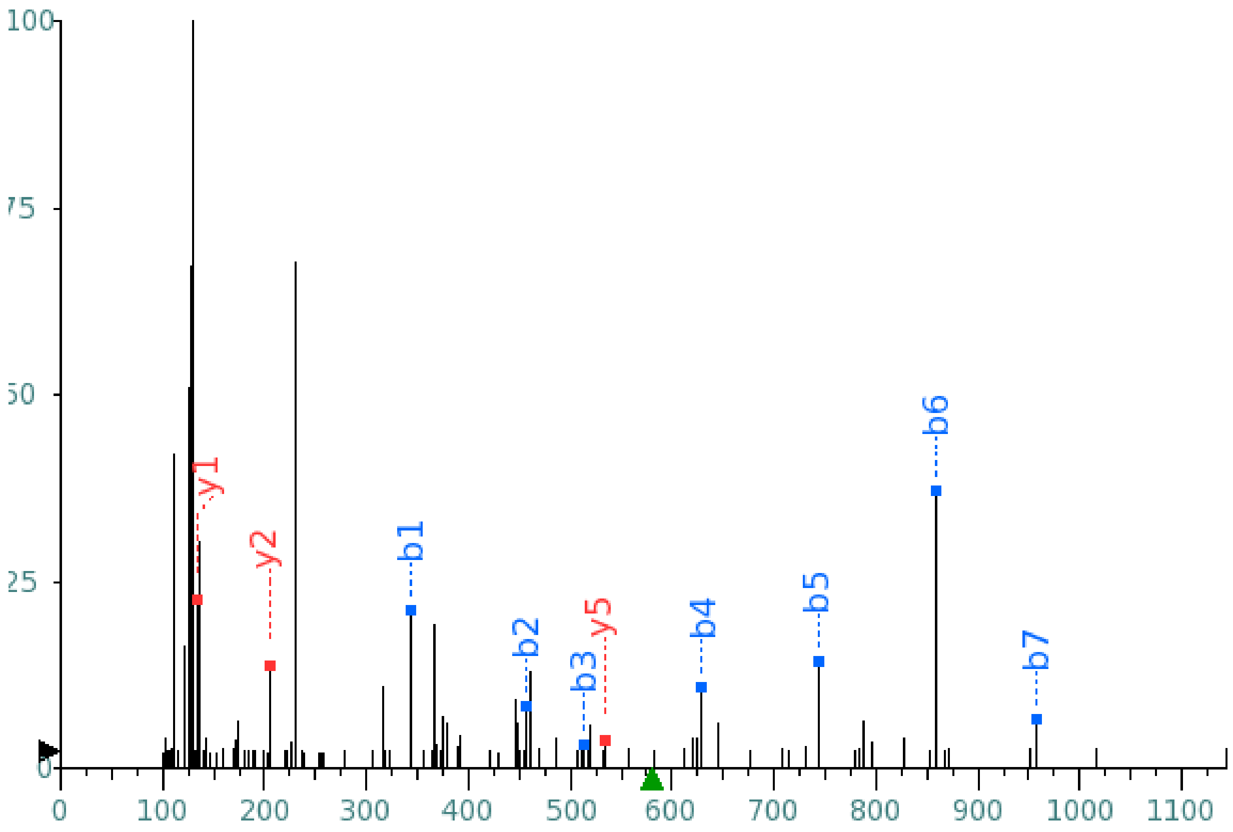

LLGDDDVAD (+2)

Primary Reference: WDR62_HUMAN
Search ID: 41760
Search Name: 20130328_ananiav_ERstress_TMT_TM_timecourse_trypsin_inj1_4mc_iaa
Scan#: 11930
Observed Mass: 581.2969 (2.5 ppm)
PSM Score: 24.64

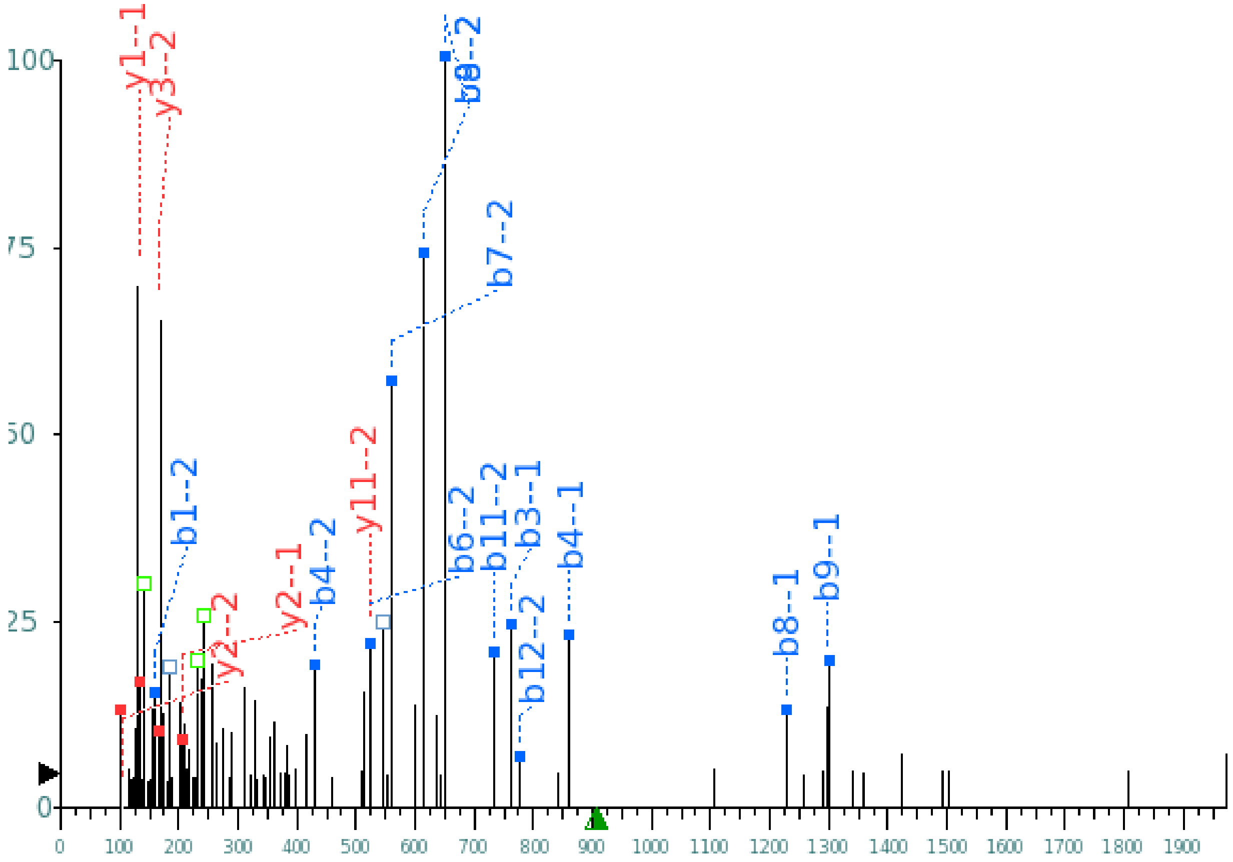

SSKVPSALAPASQEPSPAASAEAD (+3)

Primary Reference: DNM1L_HUMAN
Search ID: 41760
Search Name: 20130328_ananiav_ERstress_TMT_TM_timecourse_trypsin_inj1_4mc_iaa
Scan#: 11931
Observed Mass: 909.482 (2.4 ppm)
PSM Score: 24.9

File name: 2015-02-04 04.01.53PM.pdf

Creation time: 2015-02-04 04:09PM

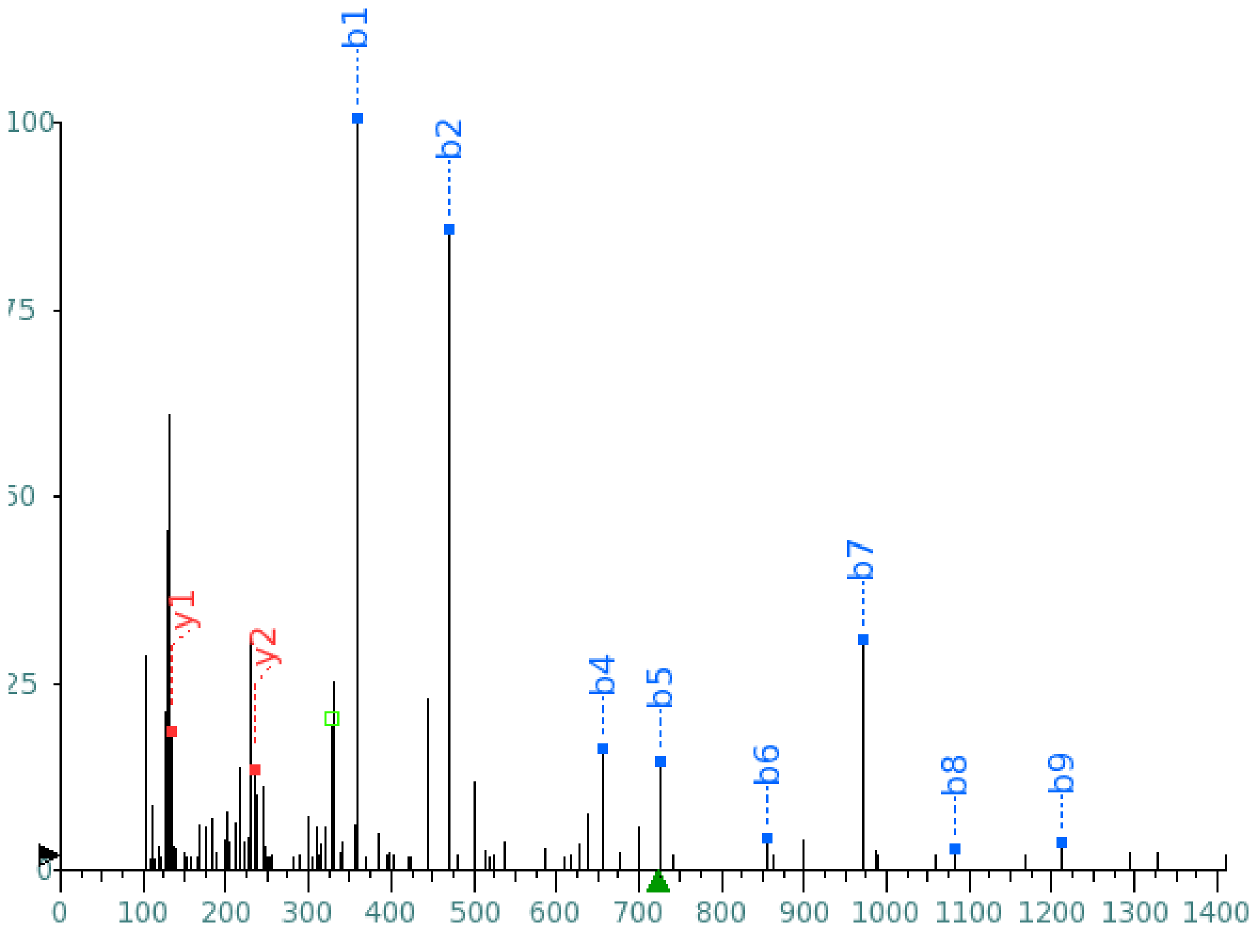

QIPSAEDLETD (+2)

Primary Reference: HDAC7_HUMAN
Search ID: 41760
Search Name: 20130328_ananiav_ERstress_TMT_TM_timecourse_trypsin_inj1_4mc_iaa
Scan#: 11942
Observed Mass: 723.8639 (3 ppm)
PSM Score: 44.75

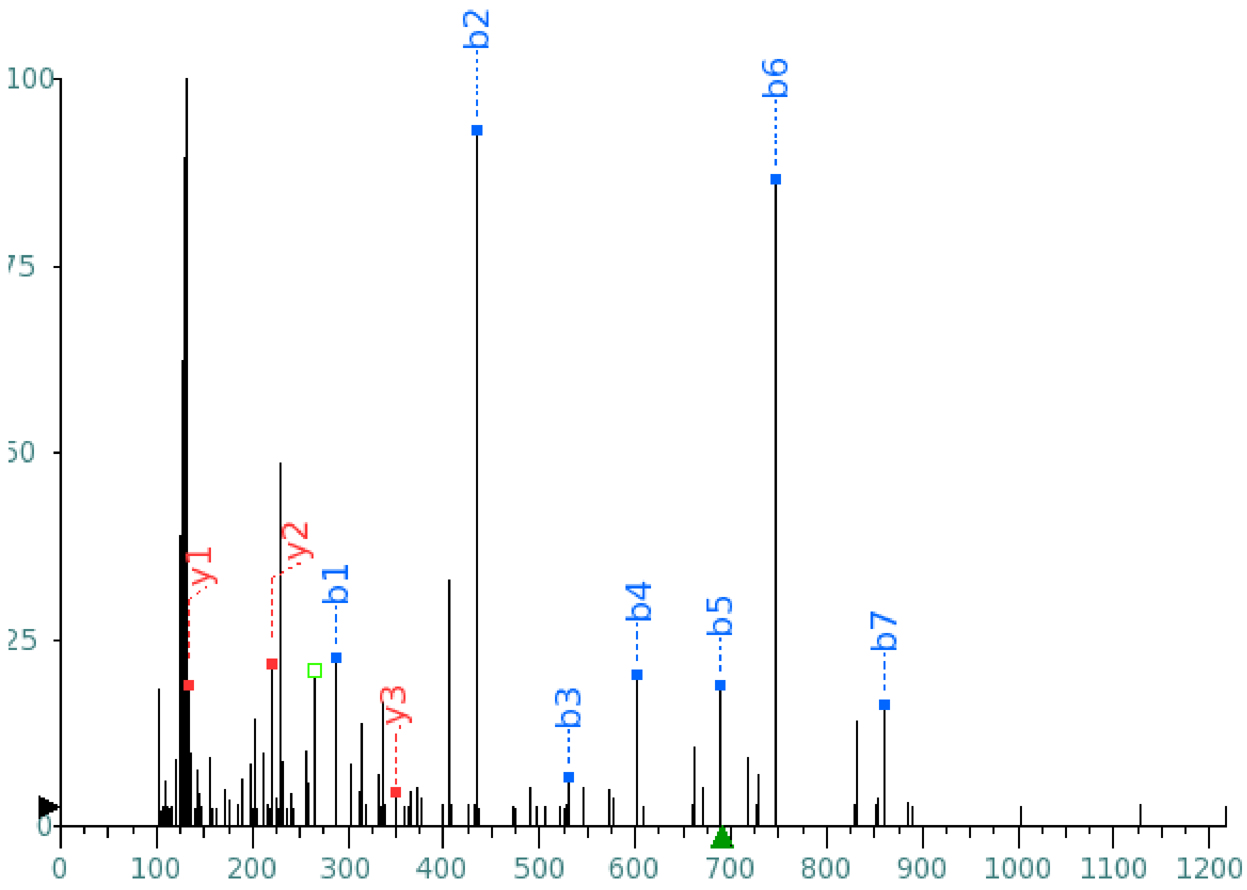

GFPASGLATESD (+2)

Primary Reference: AMRA1_HUMAN
Search ID: 41760
Search Name: 20130328_ananiav_ERstress_TMT_TM_timecourse_trypsin_inj1_4mc_iaa
Scan#: 11963
Observed Mass: 690.8474 (2.3 ppm)
PSM Score: 27.68

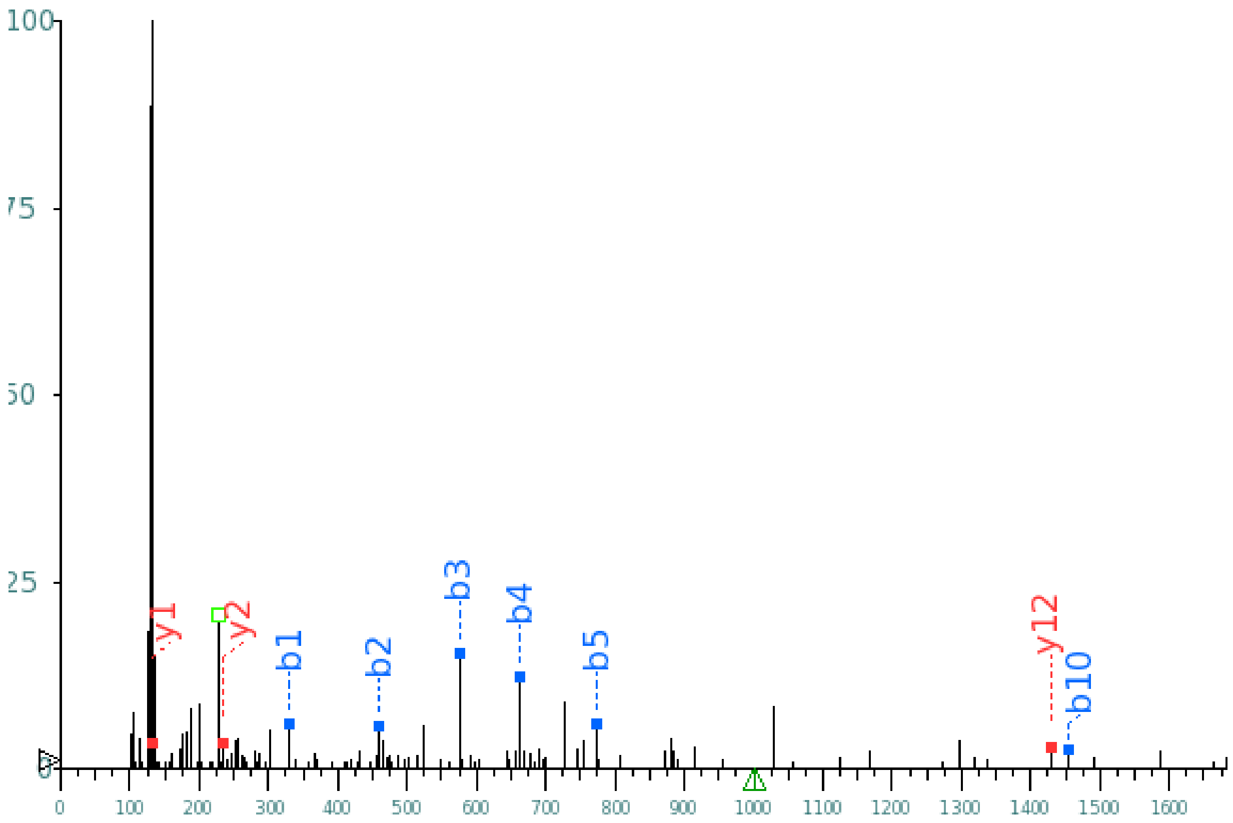

TEDSIRDYEDGMEVD (+2)

Primary Reference: ICLN_HUMAN
Search ID: 41760
Search Name: 20130328_ananiav_ERstress_TMT_TM_timecourse_trypsin_inj1_4mc_iaa
Scan#: 11988
Observed Mass: 1001.9426 (1.5 ppm)
PSM Score: 26.94

File name: 2015-02-04 04.01.53PM.pdf

Creation time: 2015-02-04 04:09PM

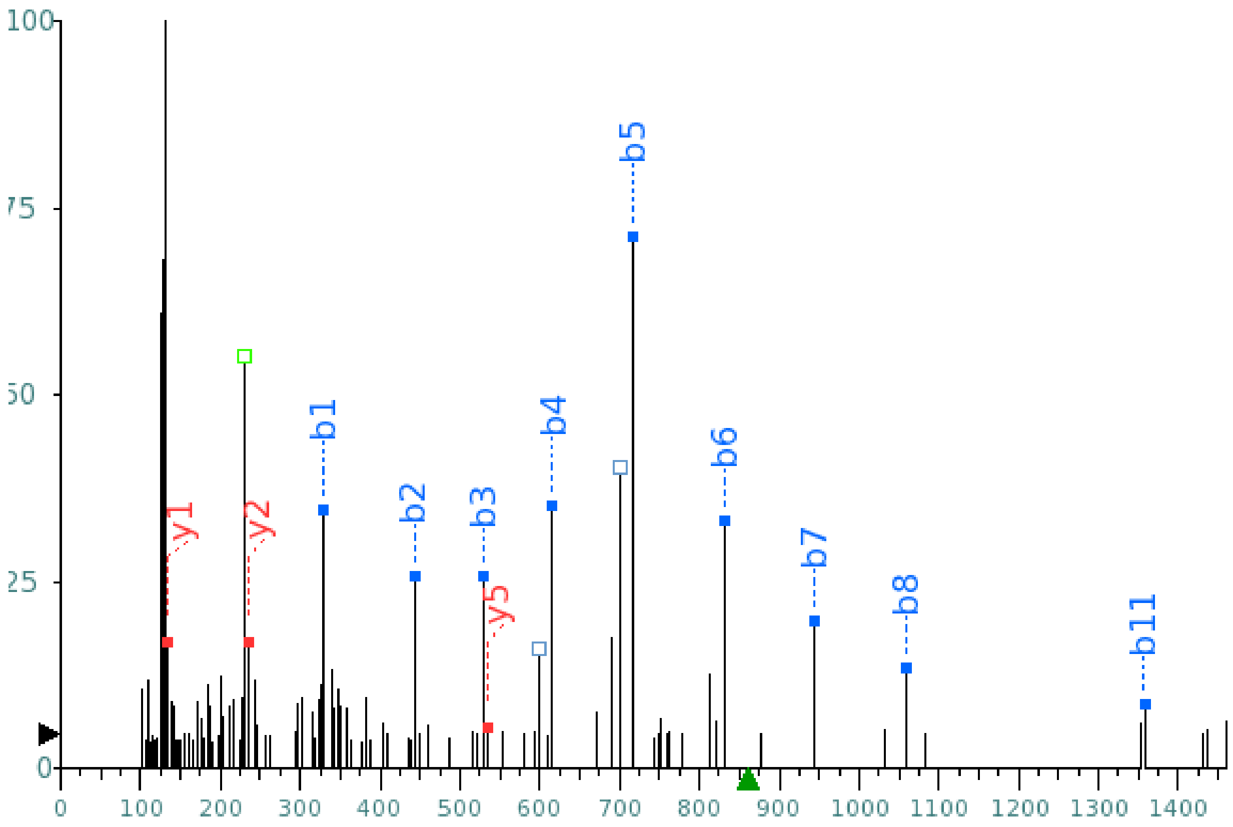

VLSSTINNQAVETD (+2)

Primary Reference: RHG12_HUMAN
Search ID: 41760
Search Name: 20130328_ananiav_ERstress_TMT_TM_timecourse_trypsin_inj1_4mc_iaa
Scan#: 12032
Observed Mass: 860.4536 (2.1 ppm)
PSM Score: 61.4

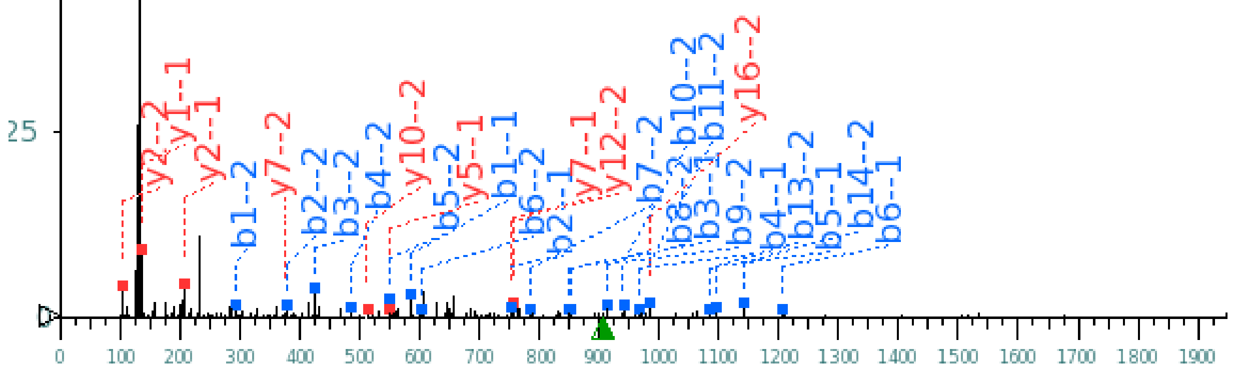

KYTLENKEEGSLSDTEAD (+3)

Primary Reference: LRC47_HUMAN
Search ID: 41760
Search Name: 20130328_ananiav_ERstress_TMT_TM_timecourse_trypsin_inj1_4mc_iaa
Scan#: 12122
Observed Mass: 906.1469 (4.8 ppm)
PSM Score: 25.09

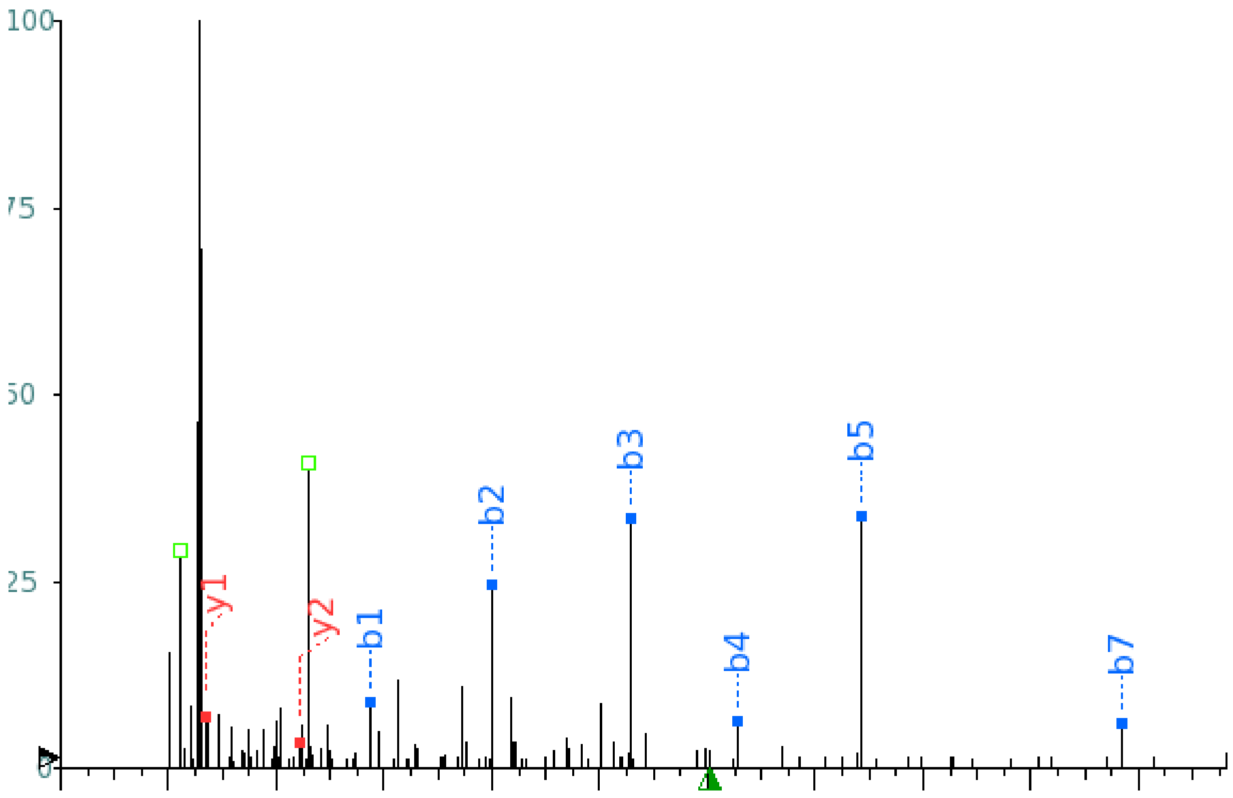

GLQVDLQSD (+2)

Primary Reference: MCM4_HUMAN
Search ID: 41760
Search Name: 20130328_ananiav_ERstress_TMT_TM_timecourse_trypsin_inj1_4mc_iaa
Scan#: 12128
Observed Mass: 602.3258 (2 ppm)
PSM Score: 25.15

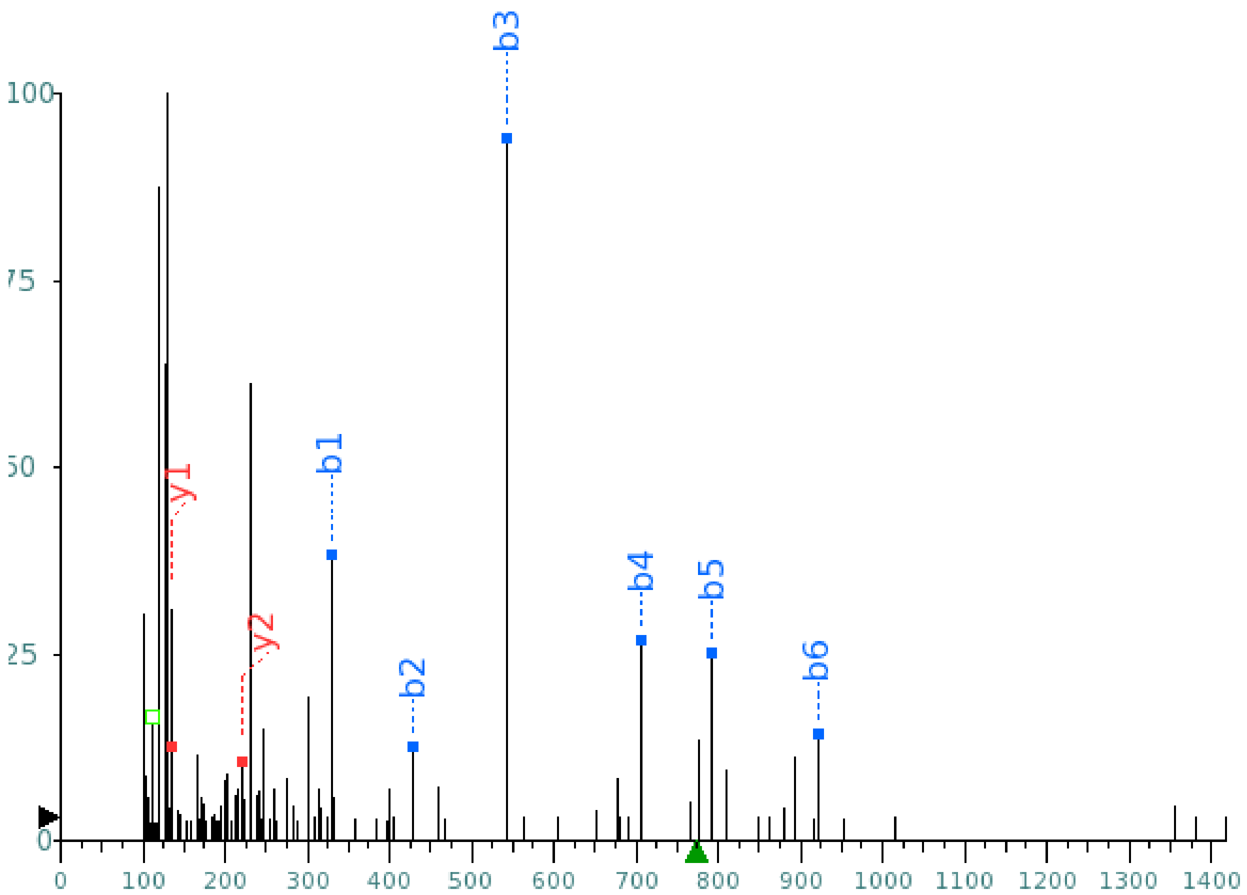

VVDYSQFQESD (+2)

Primary Reference: NUCKS_HUMAN
Search ID: 41760
Search Name: 20130328_ananiav_ERstress_TMT_TM_timecourse_trypsin_inj1_4mc_iaa
Scan#: 12154
Observed Mass: 773.369 (2.3 ppm)
PSM Score: 19.51

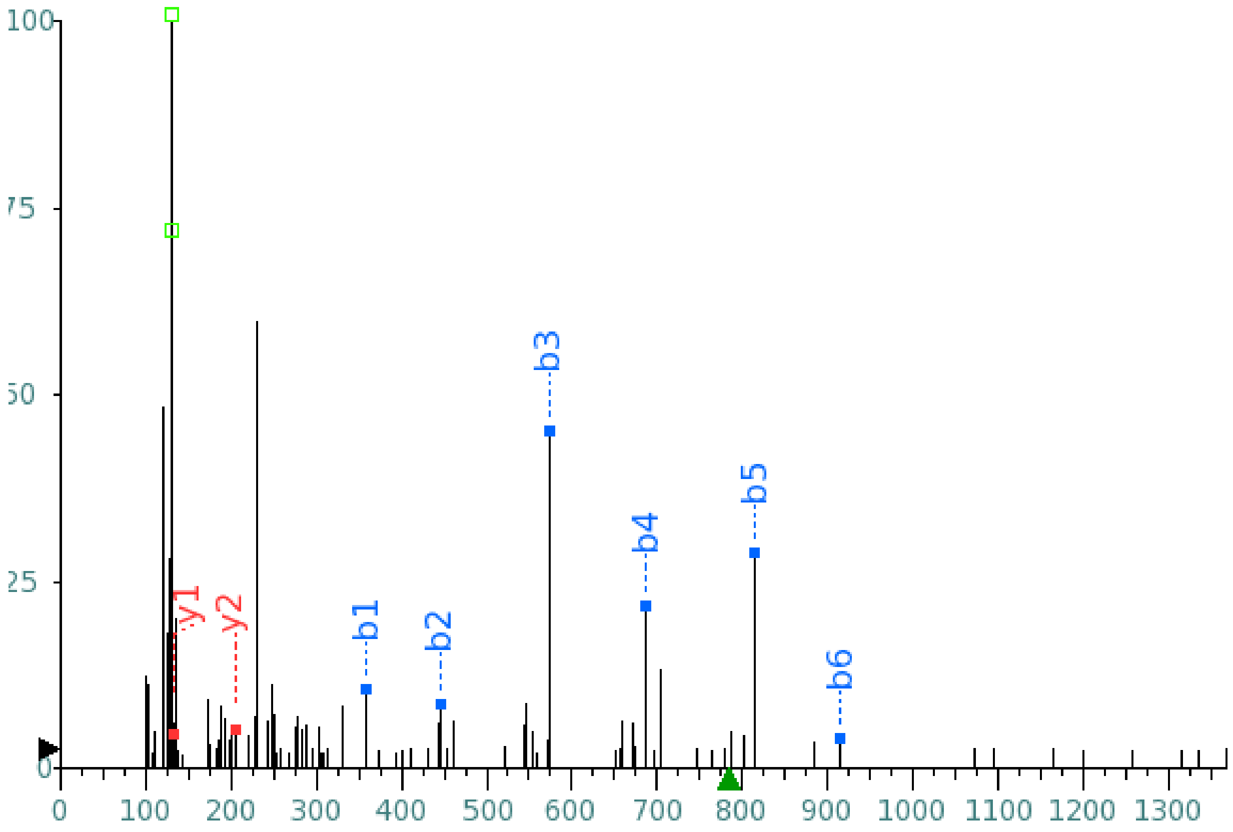

QSQIQVFEDGAD (+2)

Primary Reference: DCTN1_HUMAN
Search ID: 41760
Search Name: 20130328_ananiav_ERstress_TMT_TM_timecourse_trypsin_inj1_4mc_iaa
Scan#: 12195
Observed Mass: 783.3874 (1.9 ppm)
PSM Score: 29.93

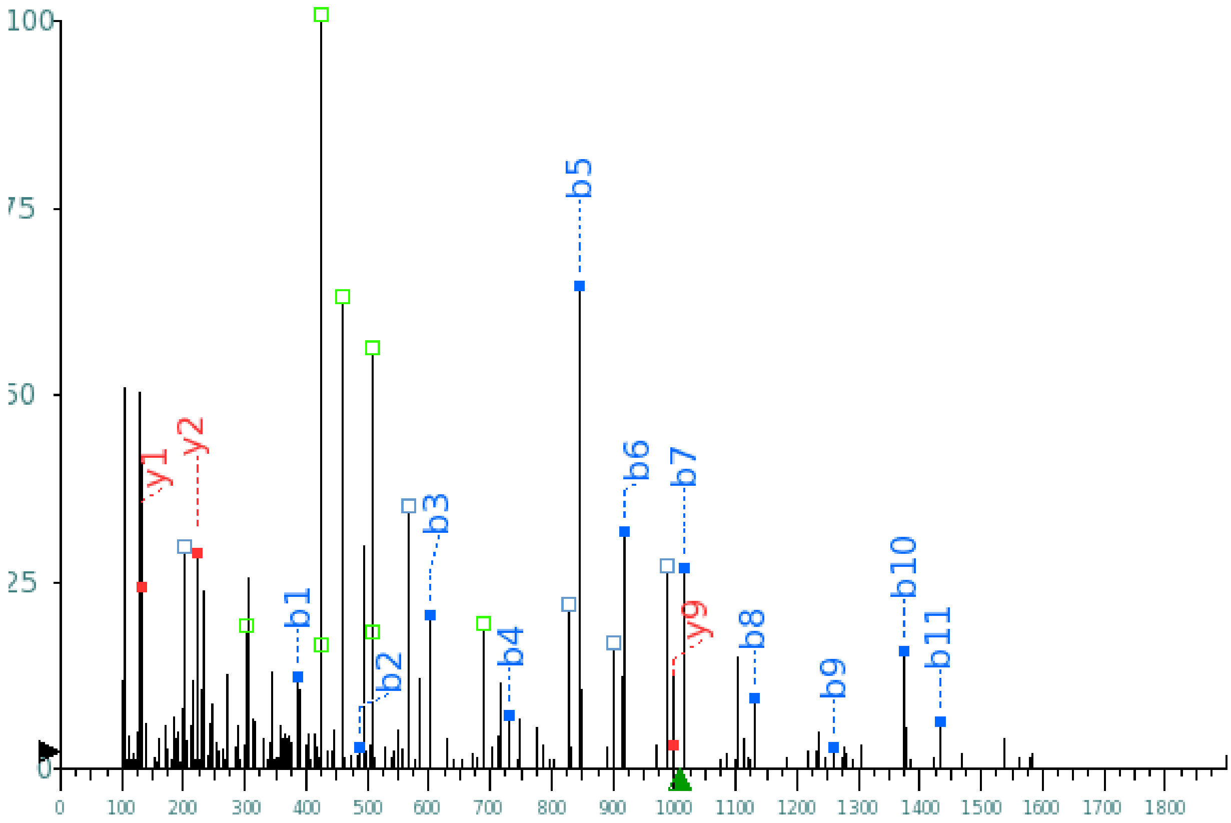

RTDEDAVLMDGMTESD (+2)

Primary Reference: FXR1_HUMAN
Search ID: 41760
Search Name: 20130328_ananiav_ERstress_TMT_TM_timecourse_trypsin_inj1_4mc_iaa
Scan#: 12233
Observed Mass: 1007.4526 (1.8 ppm)
PSM Score: 52.8

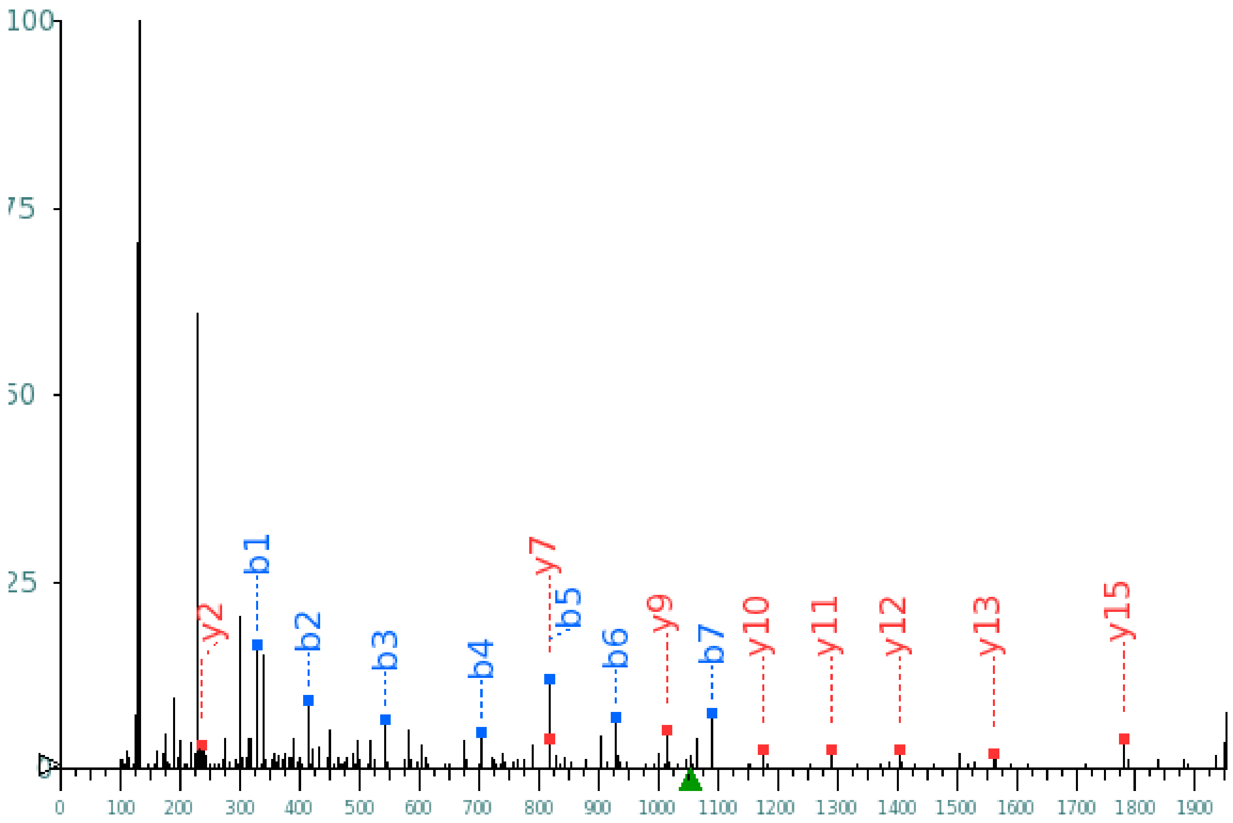

VSQC*NLC*PTRIEVSTD (+2)

Primary Reference: SPS2L_HUMAN
Search ID: 41760
Search Name: 20130328_ananiav_ERstress_TMT_TM_timecourse_trypsin_inj1_4mc_iaa
Scan#: 12250
Observed Mass: 1054.5209 (1.5 ppm)
PSM Score: 27.03

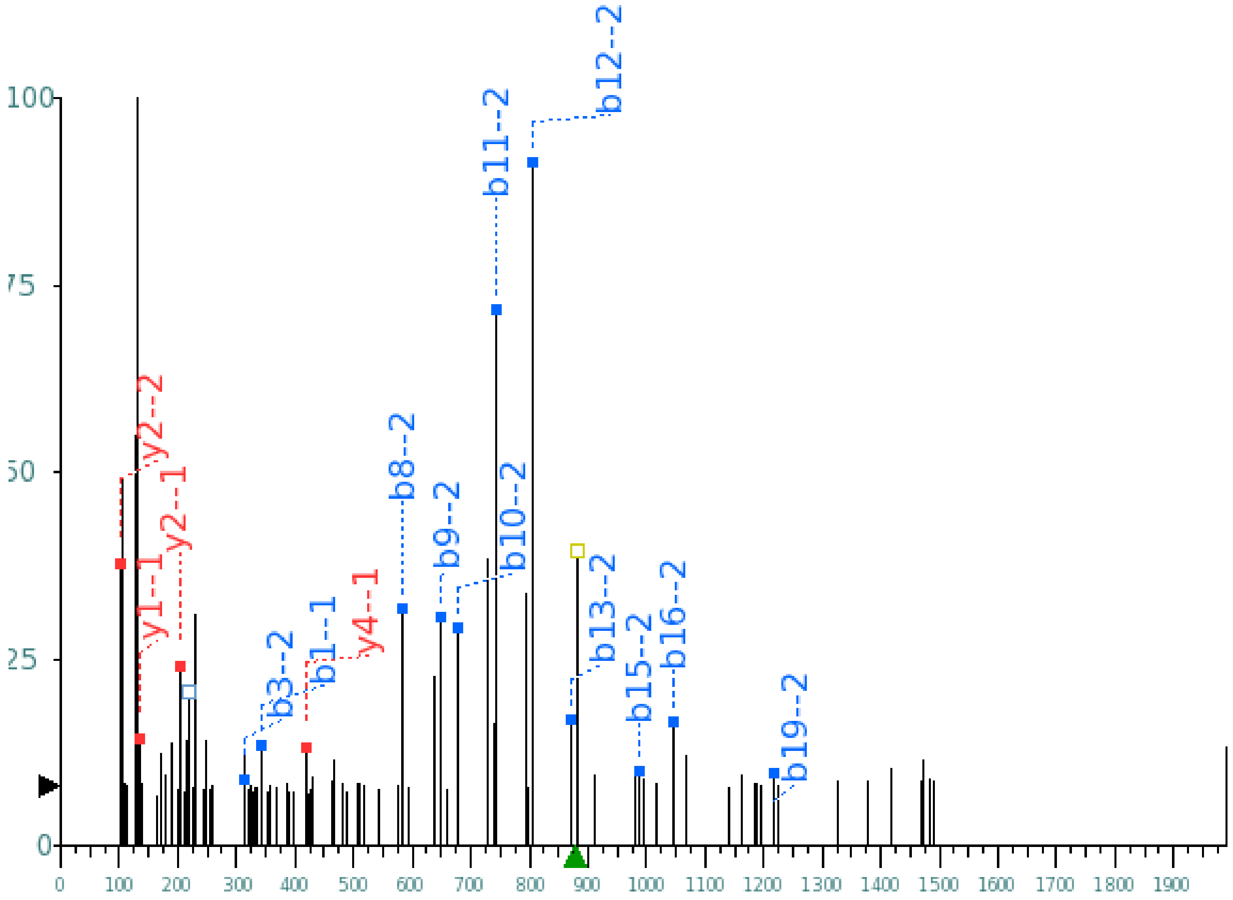

IQRPNTTQEGEEMETDMDVAD (+3)

Primary Reference: E9PEH3_HUMAN
Search ID: 41760
Search Name: 20130328_ananiav_ERstress_TMT_TM_timecourse_trypsin_inj1_4mc_iaa
Scan#: 12297
Observed Mass: 880.0675 (2.6 ppm)
PSM Score: 23.16

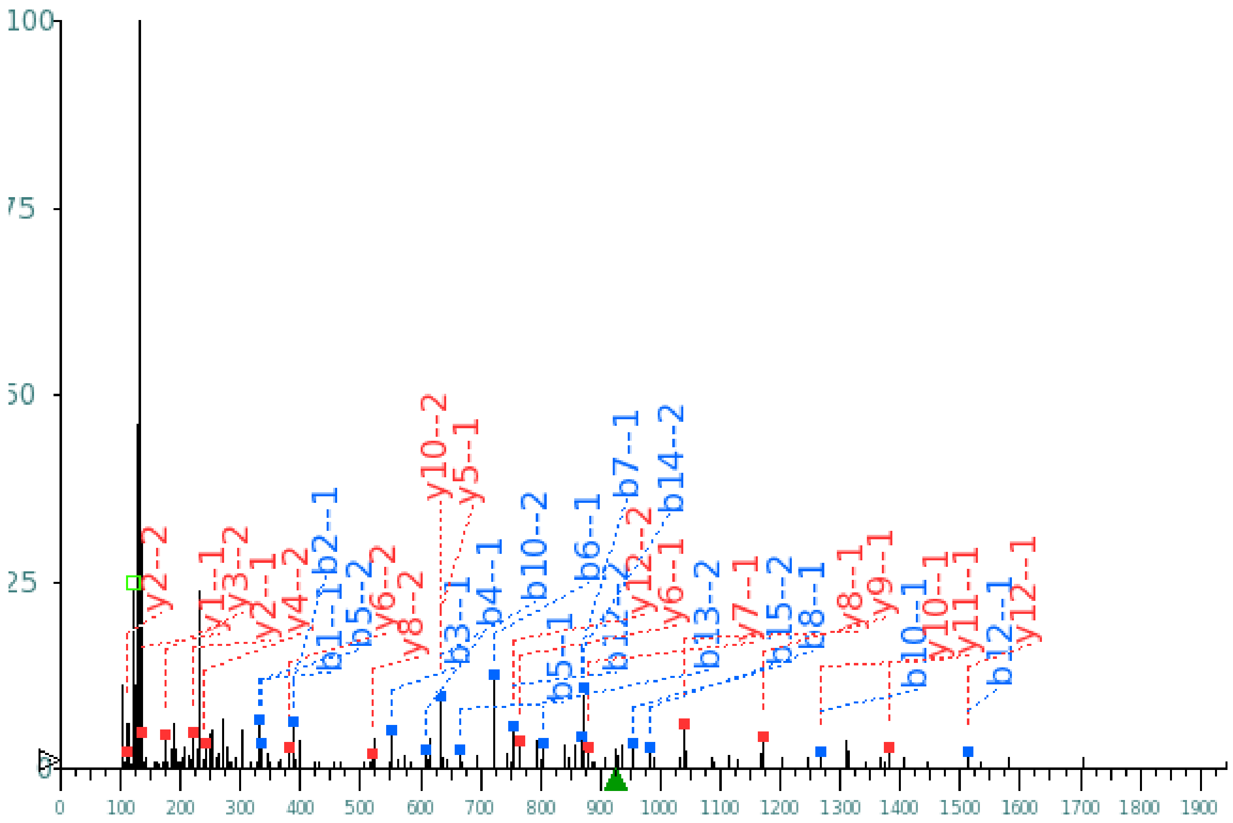

TGYGGGFNERENVEYIEREESD (+3)

Primary Reference: ZRAB2_HUMAN
Search ID: 41760
Search Name: 20130328_ananiav_ERstress_TMT_TM_timecourse_trypsin_inj1_4mc_iaa
Scan#: 12363
Observed Mass: 927.0931 (0.1 ppm)
PSM Score: 28.68

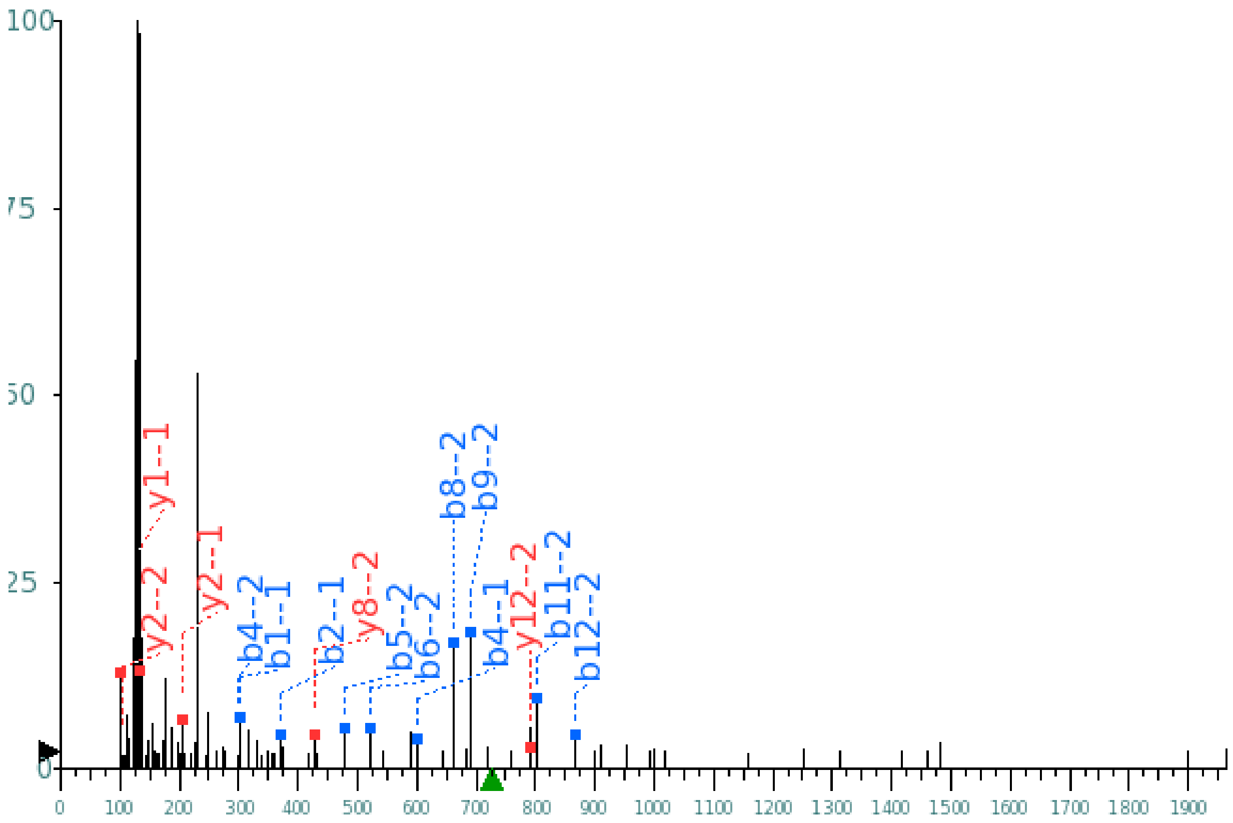

AAIDKSYDGPEEIEAD (+3)

Primary Reference: PAR3L_HUMAN
Search ID: 41760
Search Name: 20130328_ananiav_ERstress_TMT_TM_timecourse_trypsin_inj1_4mc_iaa
Scan#: 12375
Observed Mass: 727.7048 (1.6 ppm)
PSM Score: 15.59

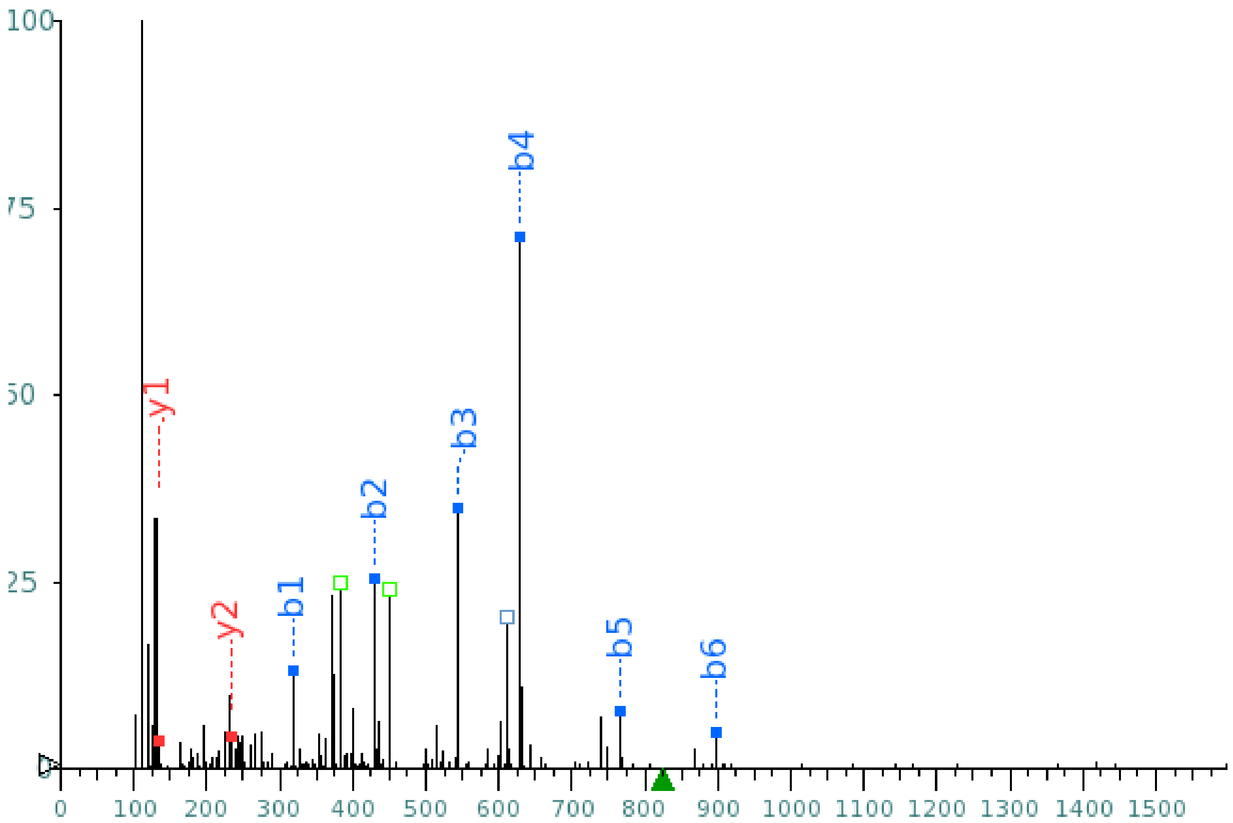

SLLSHEFQDETD (+2)

Primary Reference: CBPD_HUMAN
Search ID: 41760
Search Name: 20130328_ananiav_ERstress_TMT_TM_timecourse_trypsin_inj1_4mc_iaa
Scan#: 12384
Observed Mass: 825.398 (1.9 ppm)
PSM Score: 16.36

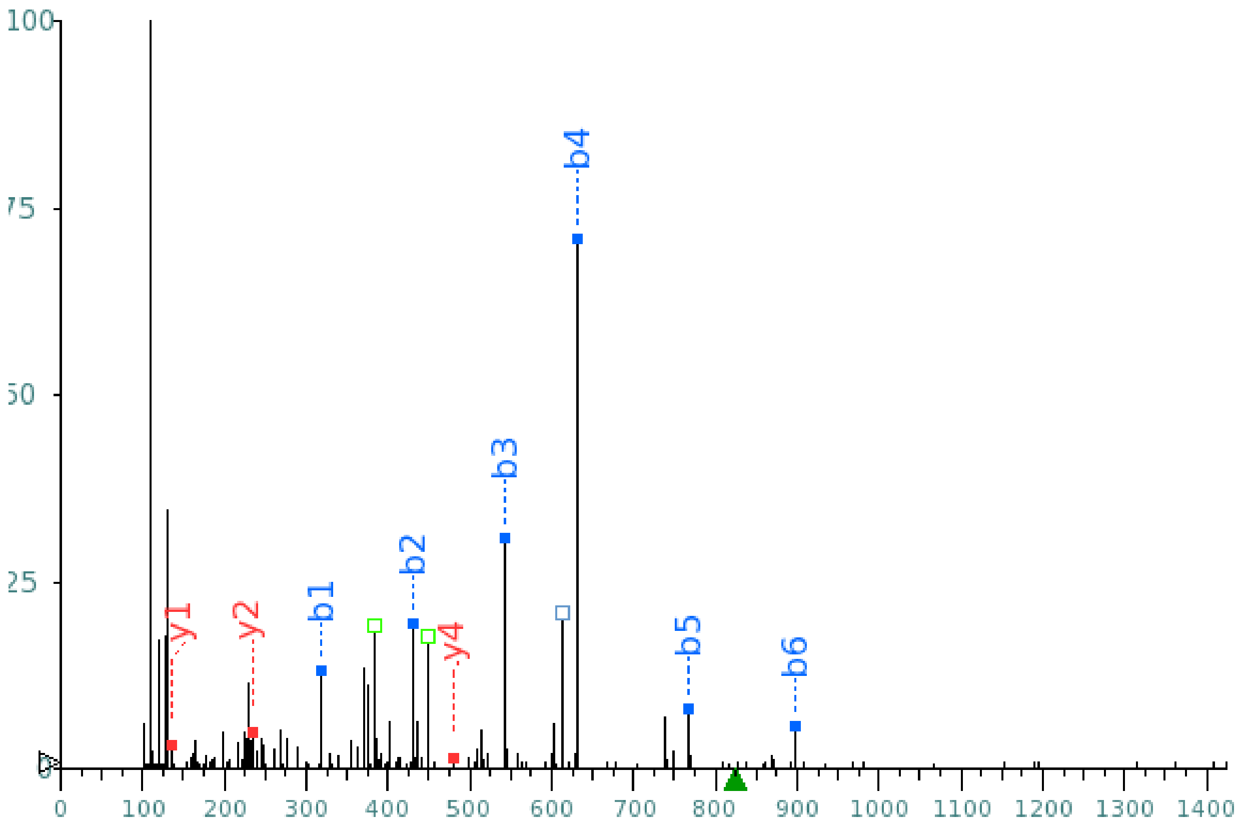

SLLSHEFQDETD (+2)

Primary Reference: CBPD_HUMAN
Search ID: 41760
Search Name: 20130328_ananiav_ERstress_TMT_TM_timecourse_trypsin_inj1_4mc_iaa
Scan#: 12471
Observed Mass: 825.3988 (2.9 ppm)
PSM Score: 12.88

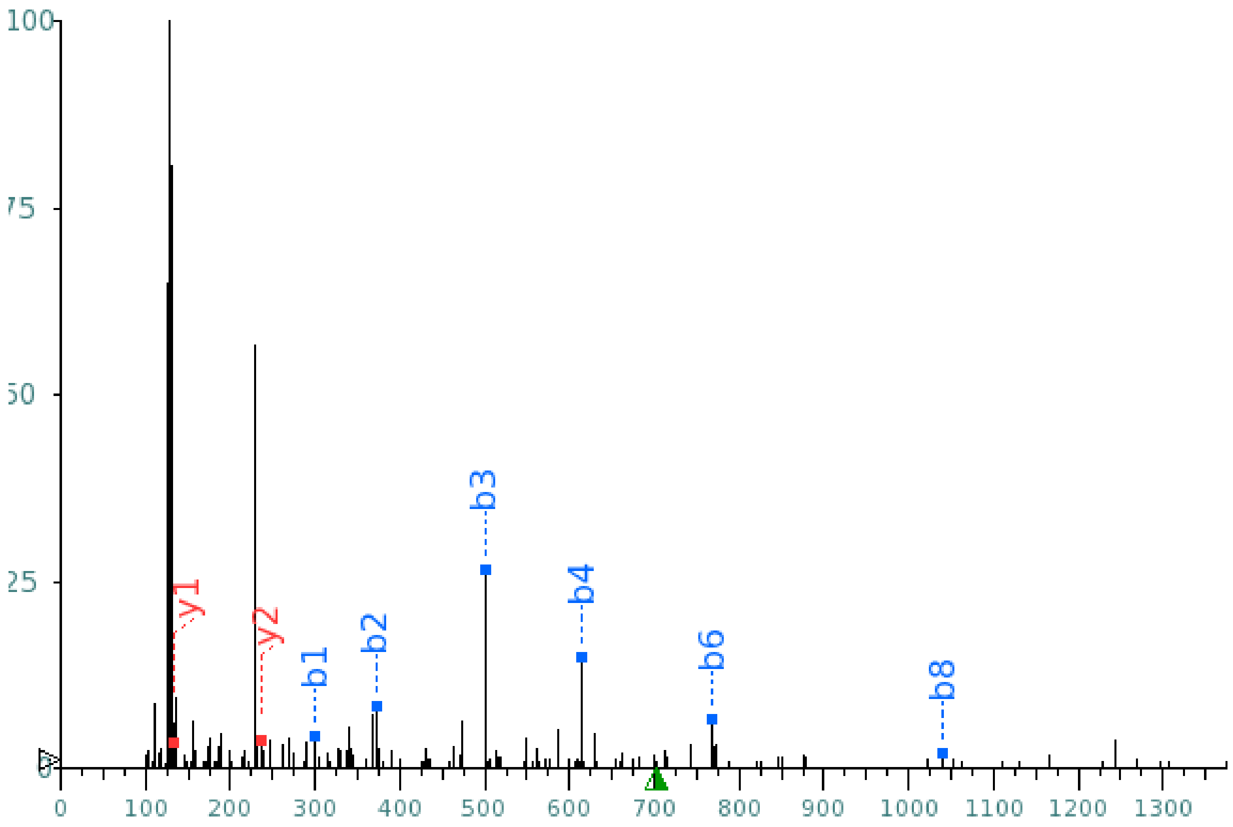

AAQLPGLC*ETD (+2)

Primary Reference: NCOA1_HUMAN
Search ID: 41760
Search Name: 20130328_ananiav_ERstress_TMT_TM_timecourse_trypsin_inj1_4mc_iaa
Scan#: 12519
Observed Mass: 702.3567 (1.7 ppm)
PSM Score: 17.25

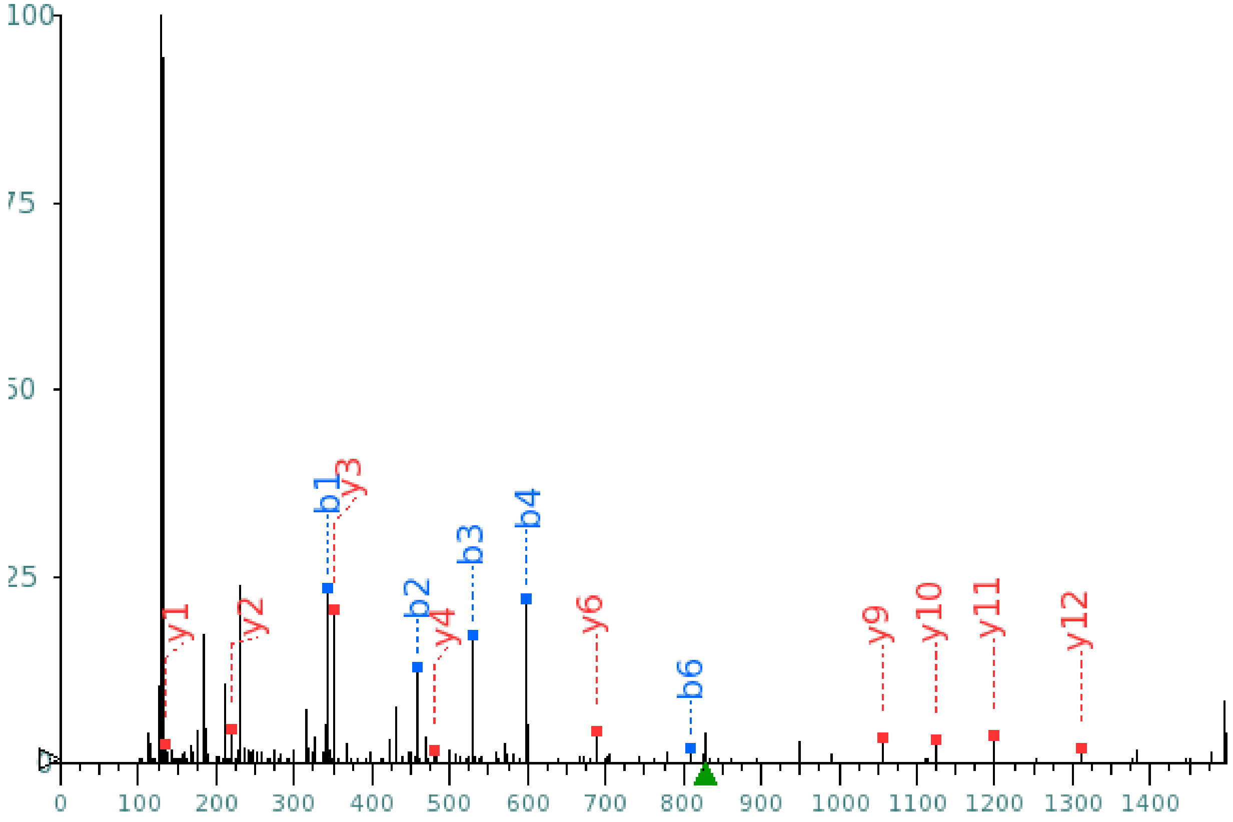

NLAAPIRPIEESD (+2)

Primary Reference: DCTN4_HUMAN
Search ID: 41760
Search Name: 20130328_ananiav_ERstress_TMT_TM_timecourse_trypsin_inj1_4mc_iaa
Scan#: 12537
Observed Mass: 827.4538 (-0.4 ppm)
PSM Score: 23.18

File name: 2015-02-04 04.01.53PM.pdf

Creation time: 2015-02-04 04:09PM

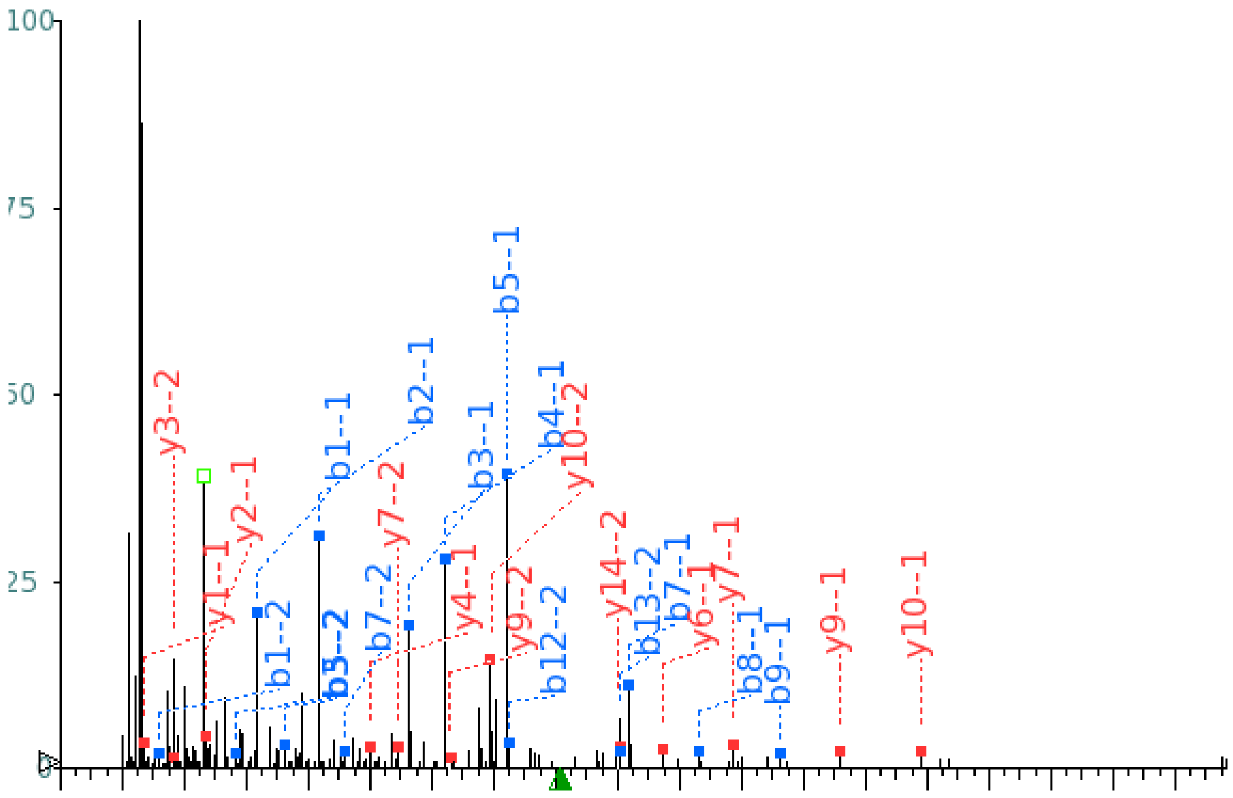

SVFGTPTLETANKNHETD (+3)

Primary Reference: RBP2_HUMAN
Search ID: 41760
Search Name: 20130328_ananiav_ERstress_TMT_TM_timecourse_trypsin_inj1_4mc_iaa
Scan#: 12538
Observed Mass: 807.0897 (1.6 ppm)
PSM Score: 30.97

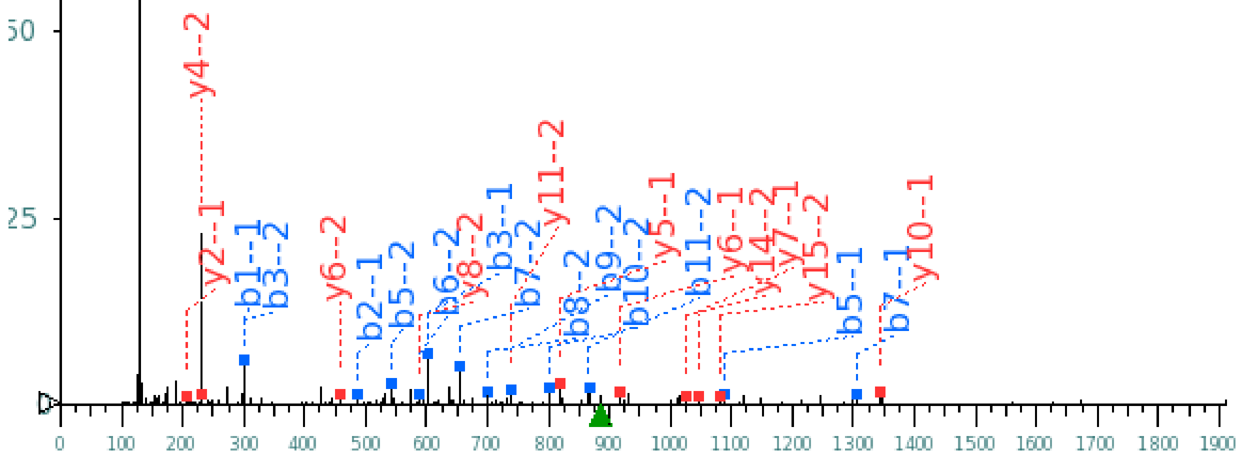

AWDKESEPAEEVKQEAD (+3)

Primary Reference: DPOLA_HUMAN
Search ID: 41760
Search Name: 20130328_ananiav_ERstress_TMT_TM_timecourse_trypsin_inj1_4mc_iaa
Scan#: 12540
Observed Mass: 883.4628 (3.1 ppm)
PSM Score: 19.57

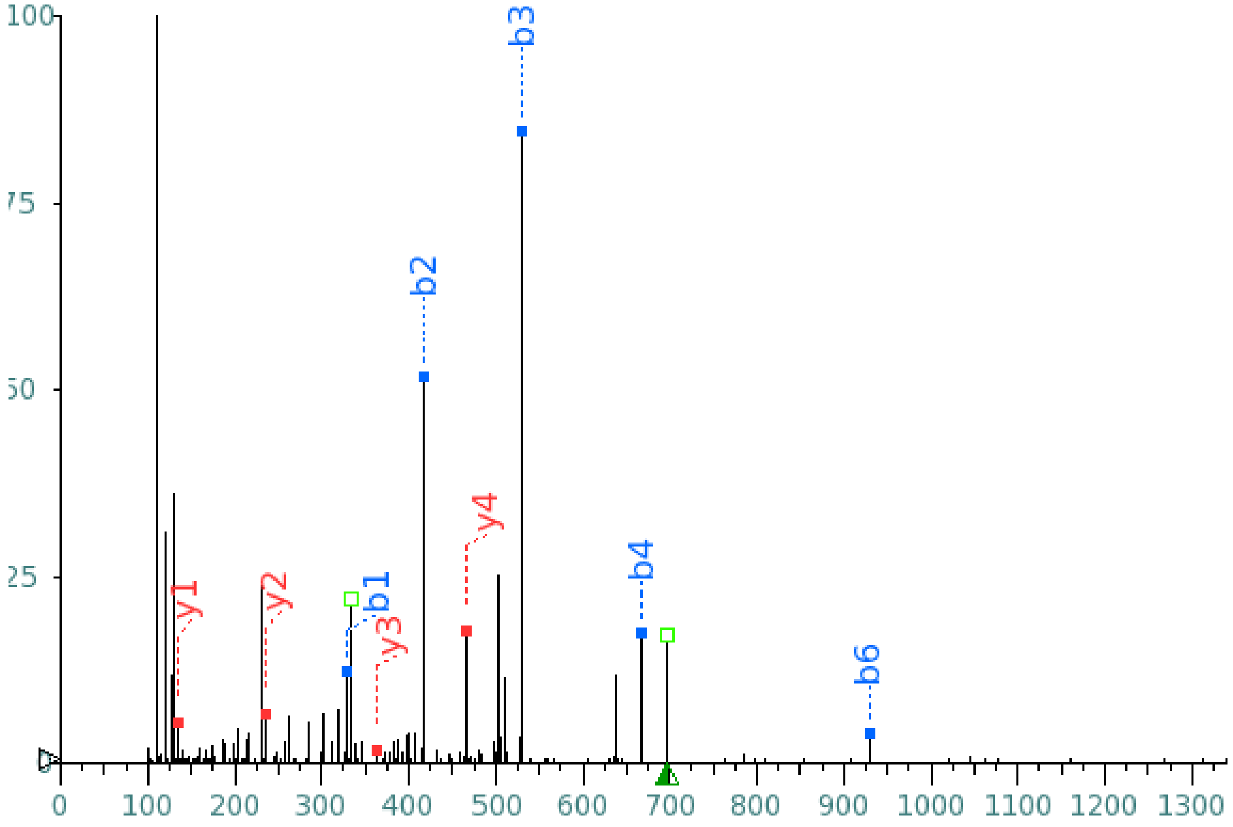

VSLHFDTETD (+2)

Primary Reference: JADE2_HUMAN
Search ID: 41760
Search Name: 20130328_ananiav_ERstress_TMT_TM_timecourse_trypsin_inj1_4mc_iaa
Scan#: 12610
Observed Mass: 696.8462 (0.5 ppm)
PSM Score: 15.67

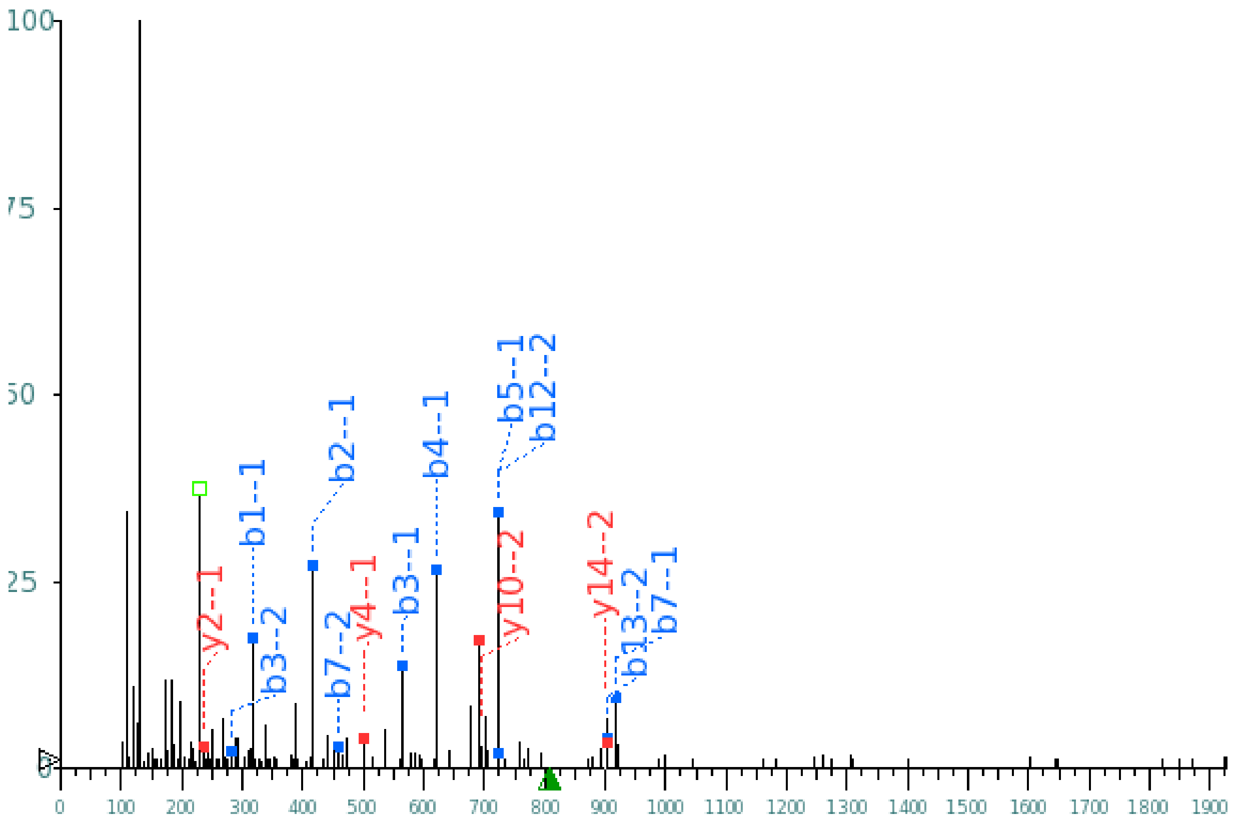

SVFGTPTLETANKNHETD (+3)

Primary Reference: RBP2_HUMAN
Search ID: 41760
Search Name: 20130328_ananiav_ERstress_TMT_TM_timecourse_trypsin_inj1_4mc_iaa
Scan#: 12623
Observed Mass: 807.0892 (1.1 ppm)
PSM Score: 21.85

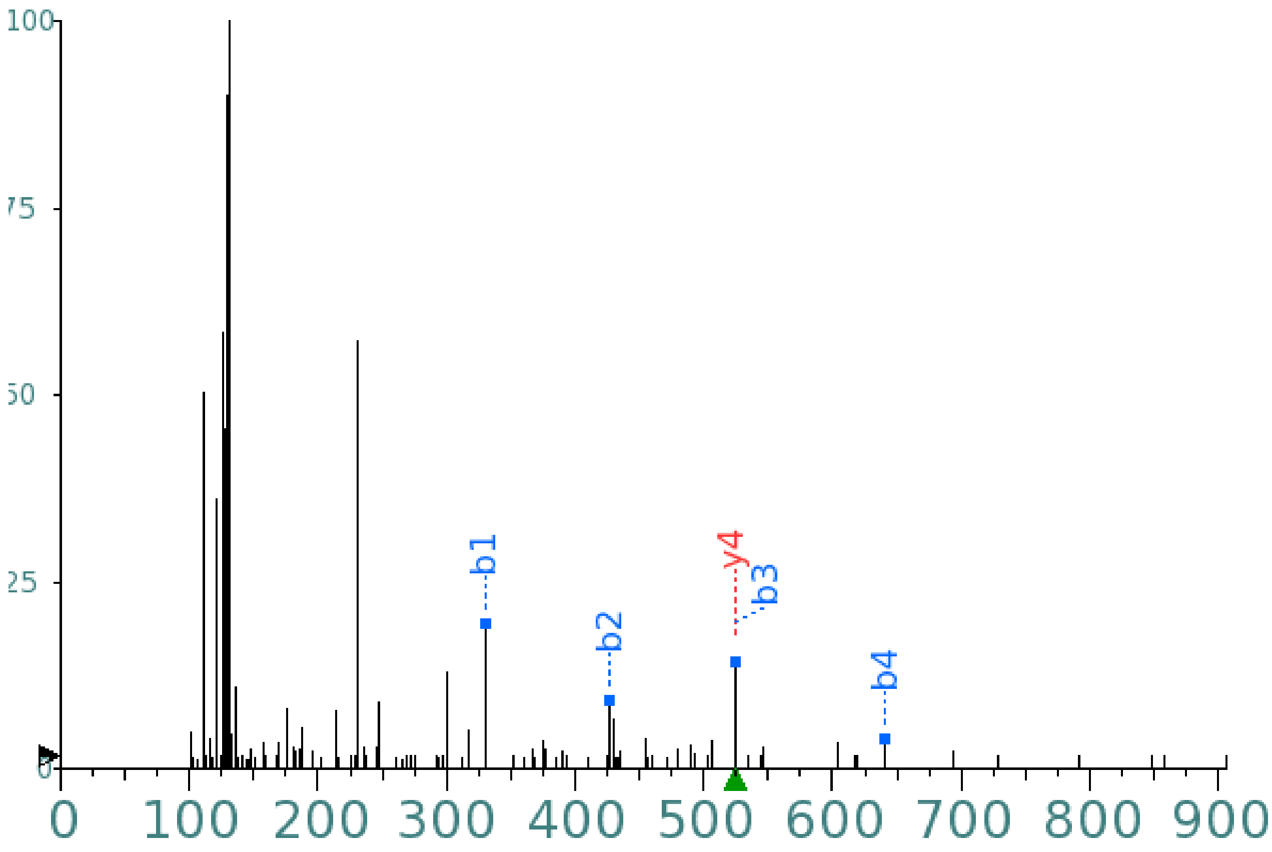

VPVDEFD (+2)

Primary Reference: DDX1_HUMAN
Search ID: 41760
Search Name: 20130328_ananiav_ERstress_TMT_TM_timecourse_trypsin_inj1_4mc_iaa
Scan#: 12626
Observed Mass: 525.2716 (0.6 ppm)
PSM Score: 3.79

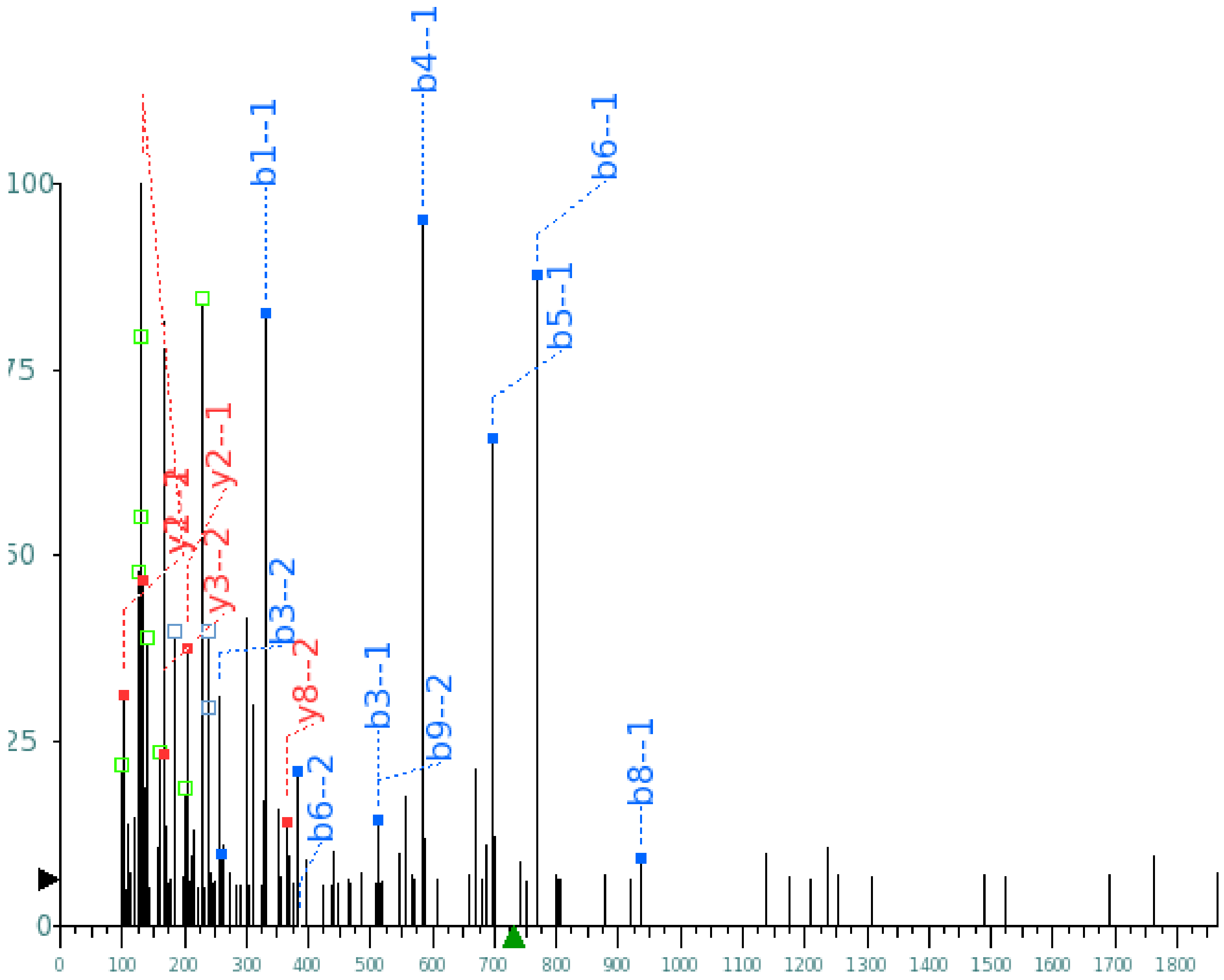

VPSALAPASQEPSPAASAEAD (+3)

Primary Reference: DNM1L_HUMAN
Search ID: 41760
Search Name: 20130328_ananiav_ERstress_TMT_TM_timecourse_trypsin_inj1_4mc_iaa
Scan#: 12682
Observed Mass: 732.3732 (1 ppm)
PSM Score: 11.75

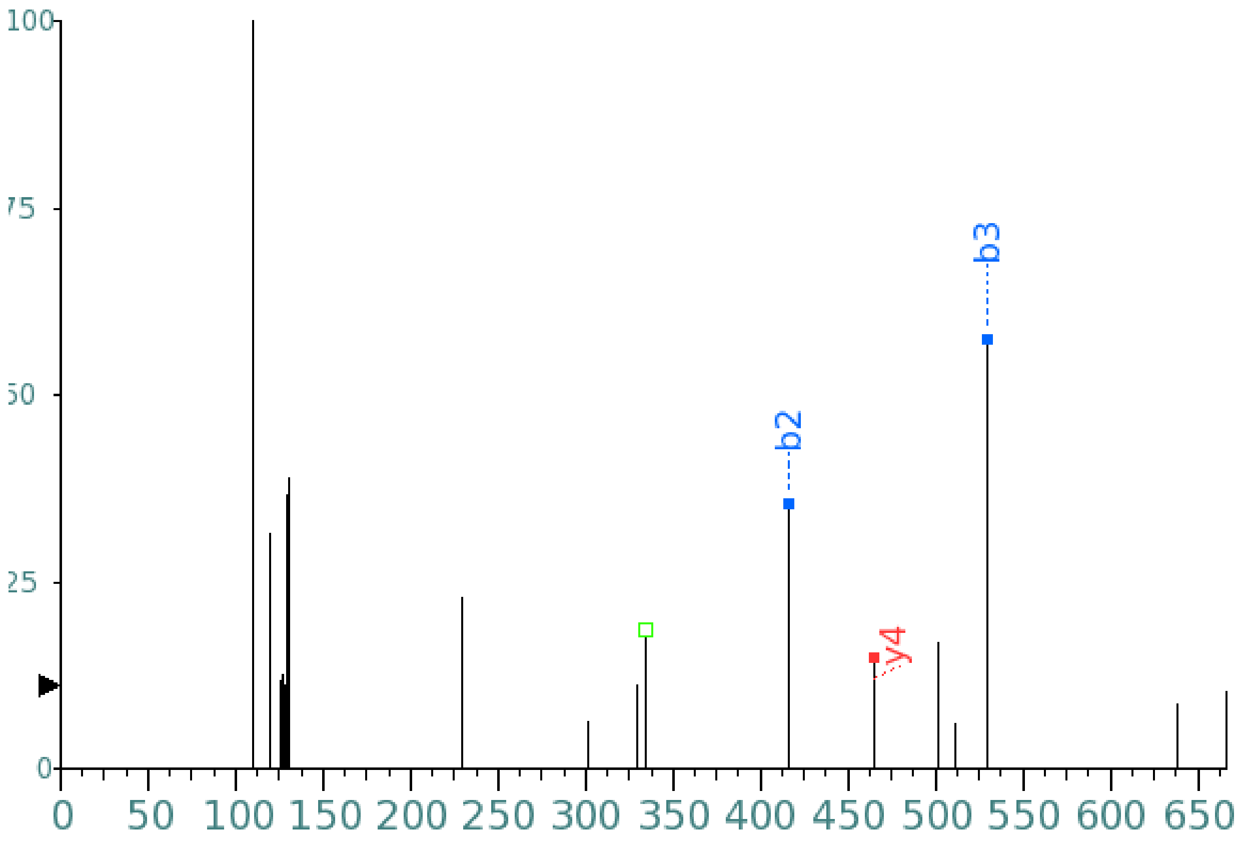

VSLHFDTETD (+2)

Primary Reference: JADE2_HUMAN
Search ID: 41760
Search Name: 20130328_ananiav_ERstress_TMT_TM_timecourse_trypsin_inj1_4mc_iaa
Scan#: 12696
Observed Mass: 696.8464 (0.7 ppm)
PSM Score: 15.09

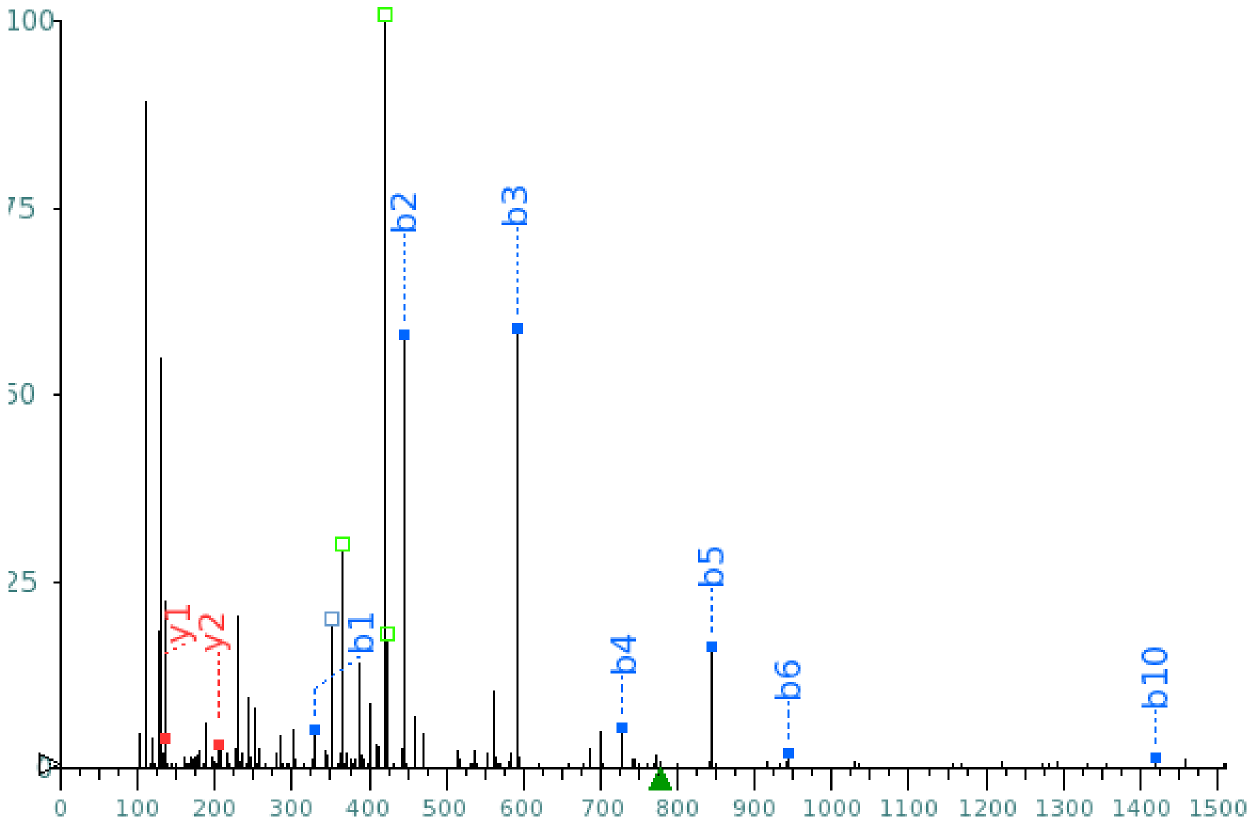

VDFHDVQDYAD (+2)

Primary Reference: RAB24_HUMAN
Search ID: 41760
Search Name: 20130328_ananiav_ERstress_TMT_TM_timecourse_trypsin_inj1_4mc_iaa
Scan#: 12737
Observed Mass: 776.859 (-0.7 ppm)
PSM Score: 16.46

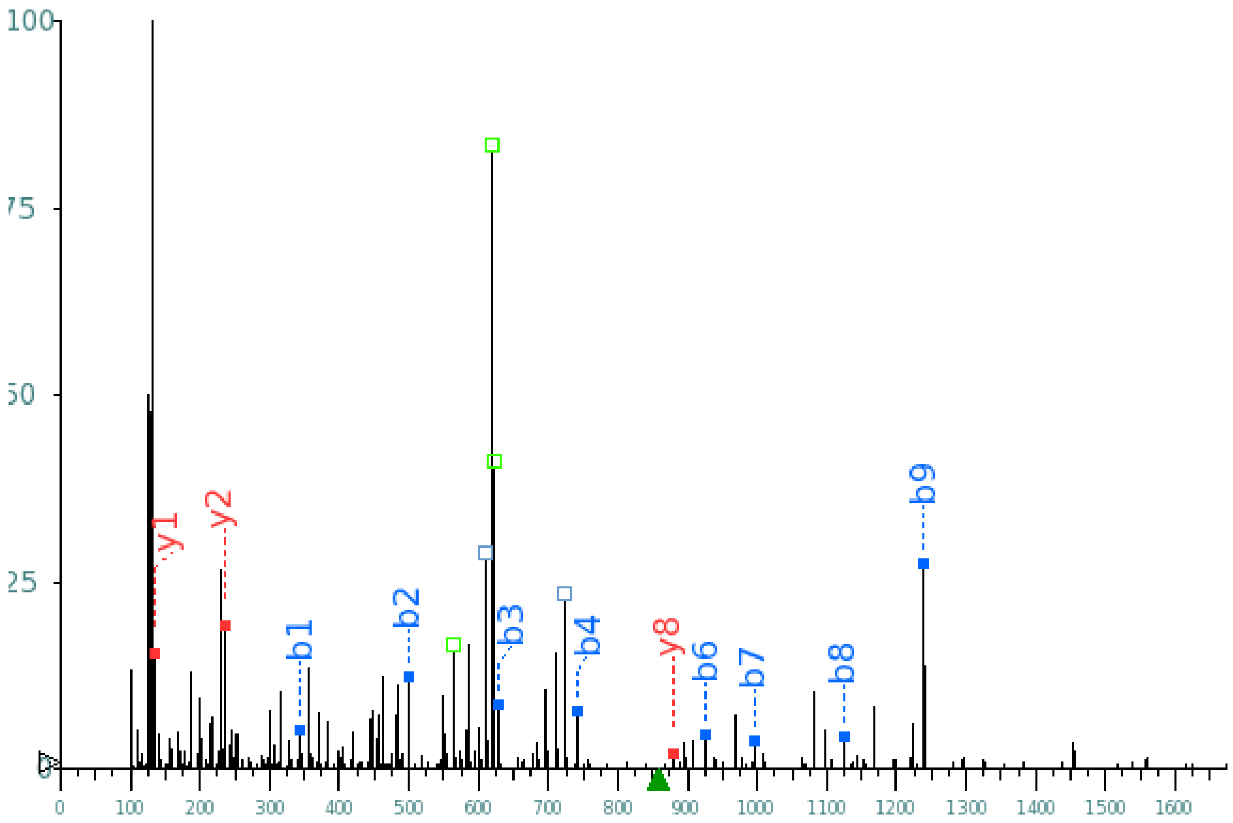

LRQIPSAEDLETD (+2)

Primary Reference: HDAC7_HUMAN
Search ID: 41760
Search Name: 20130328_ananiav_ERstress_TMT_TM_timecourse_trypsin_inj1_4mc_iaa
Scan#: 12752
Observed Mass: 858.4561 (2.2 ppm)
PSM Score: 16.32

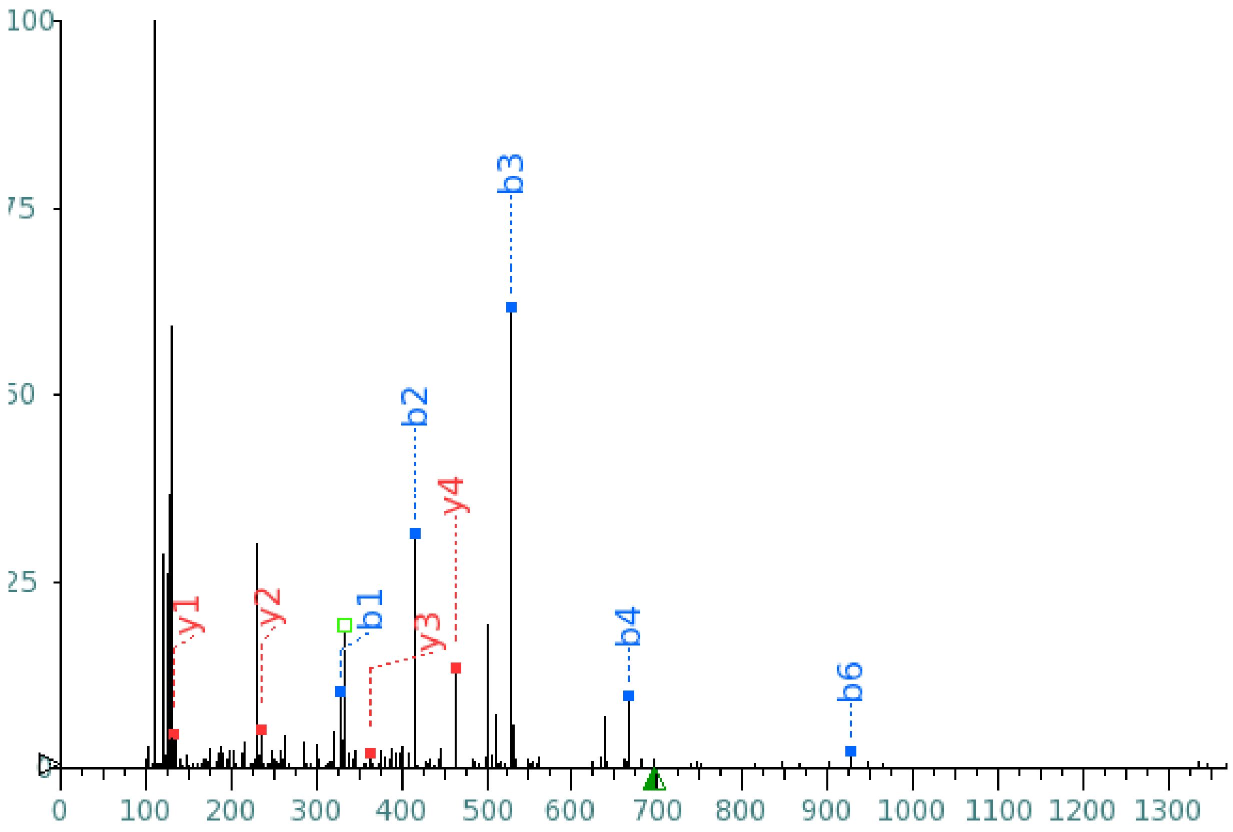

VSLHFDTETD (+2)

Primary Reference: JADE2_HUMAN
Search ID: 41760
Search Name: 20130328_ananiav_ERstress_TMT_TM_timecourse_trypsin_inj1_4mc_iaa
Scan#: 12780
Observed Mass: 696.8467 (1.1 ppm)
PSM Score: 18.47

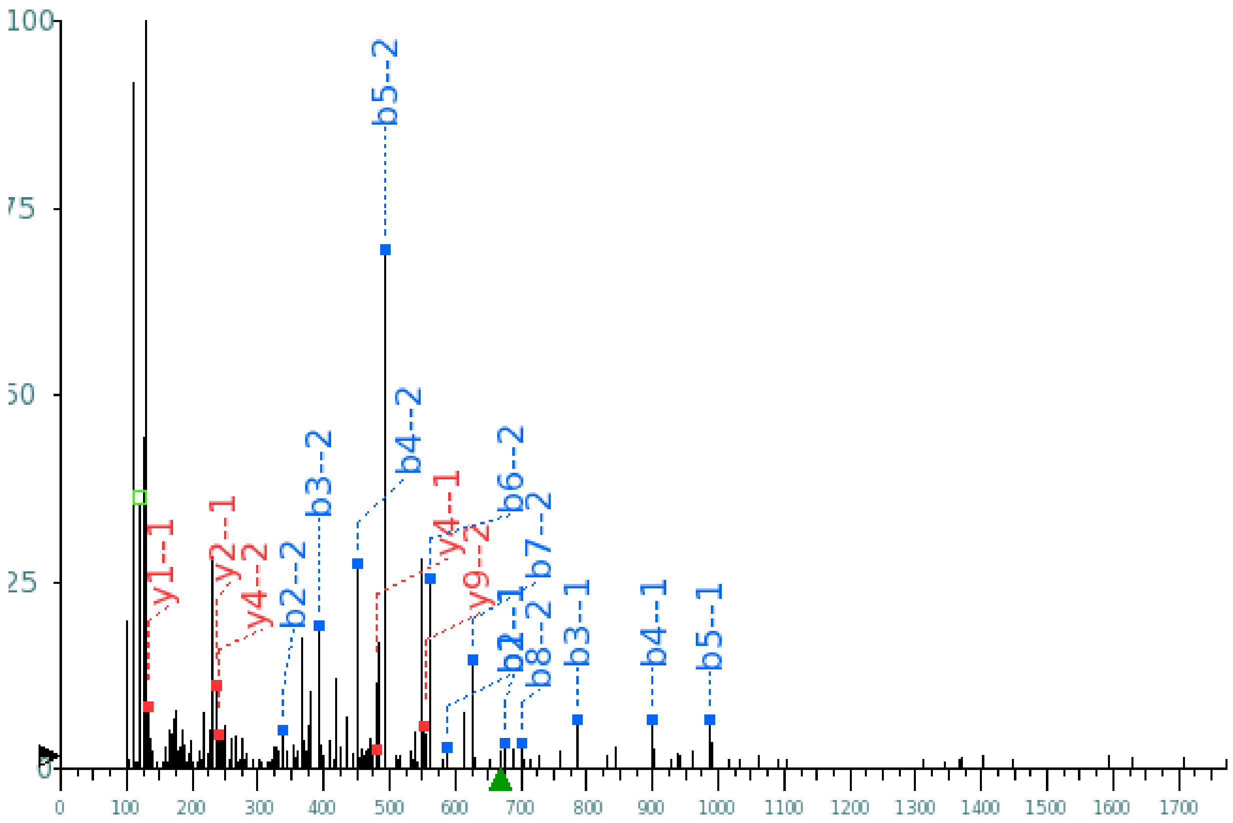

KSLLLSHEFQDETD (+3)

Primary Reference: CBPD_HUMAN
Search ID: 41760
Search Name: 20130328_ananiav_ERstress_TMT_TM_timecourse_trypsin_inj1_4mc_iaa
Scan#: 12852
Observed Mass: 669.6869 (1.3 ppm)
PSM Score: 10.39

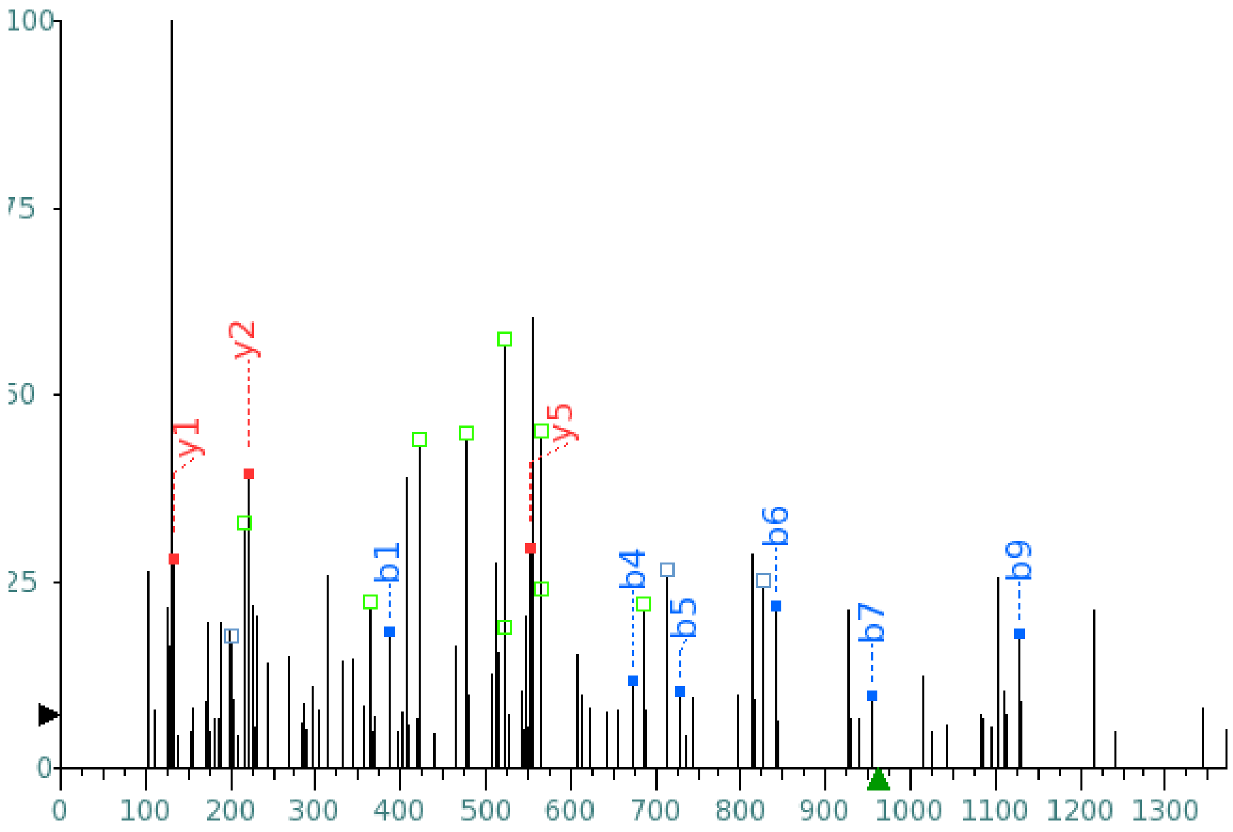

RASQGLLSSIENSESD (+2)

Primary Reference: RIF1_HUMAN
Search ID: 41760
Search Name: 20130328_ananiav_ERstress_TMT_TM_timecourse_trypsin_inj1_4mc_iaa
Scan#: 12885
Observed Mass: 961.4897 (3 ppm)
PSM Score: 40.59

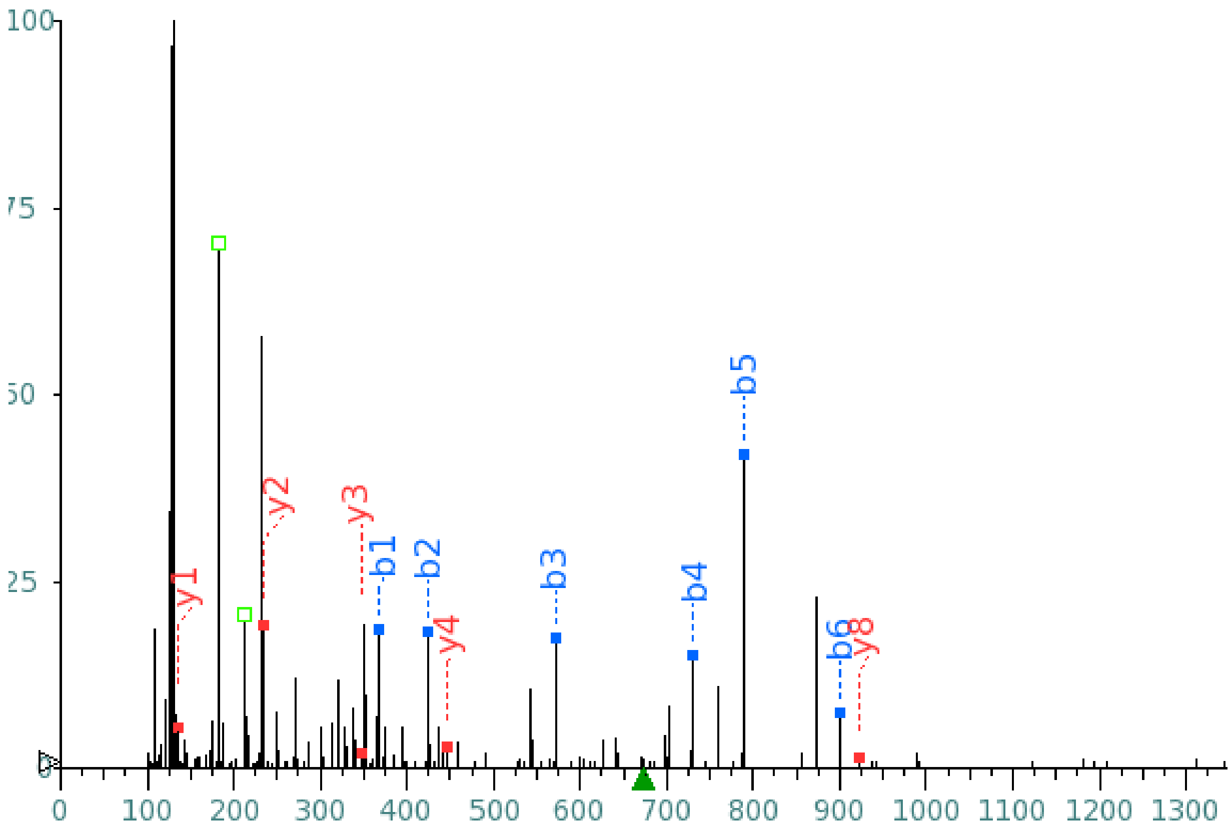

HGFC*GIPITD (+2)

Primary Reference: IMDH2_HUMAN
Search ID: 41760
Search Name: 20130328_ananiav_ERstress_TMT_TM_timecourse_trypsin_inj1_4mc_iaa
Scan#: 12901
Observed Mass: 673.3448 (3.8 ppm)
PSM Score: 14.7

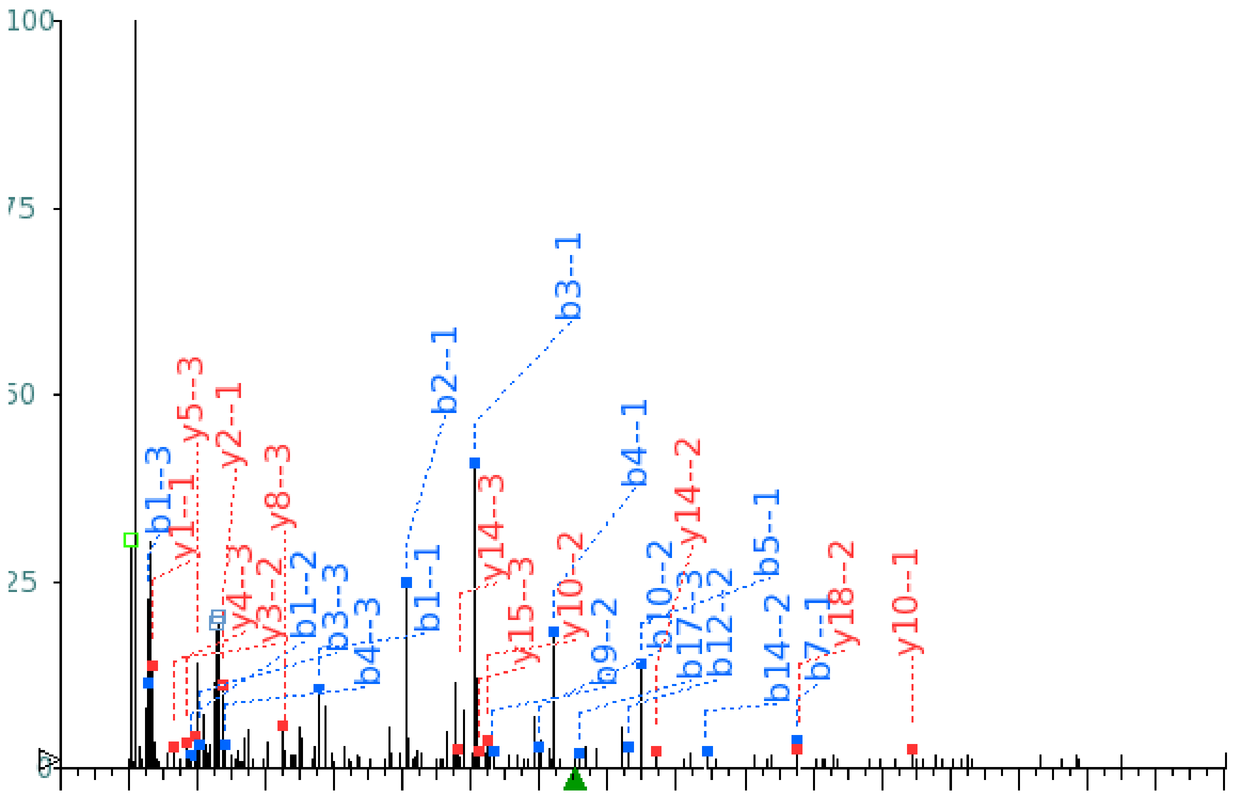

FETLEPEVSHEETEHSYHVEETD (+4)

Primary Reference: B4DQ07_HUMAN
Search ID: 41760
Search Name: 20130328_ananiav_ERstress_TMT_TM_timecourse_trypsin_inj1_4mc_iaa
Scan#: 12912
Observed Mass: 751.3407 (1.1 ppm)
PSM Score: 19.47

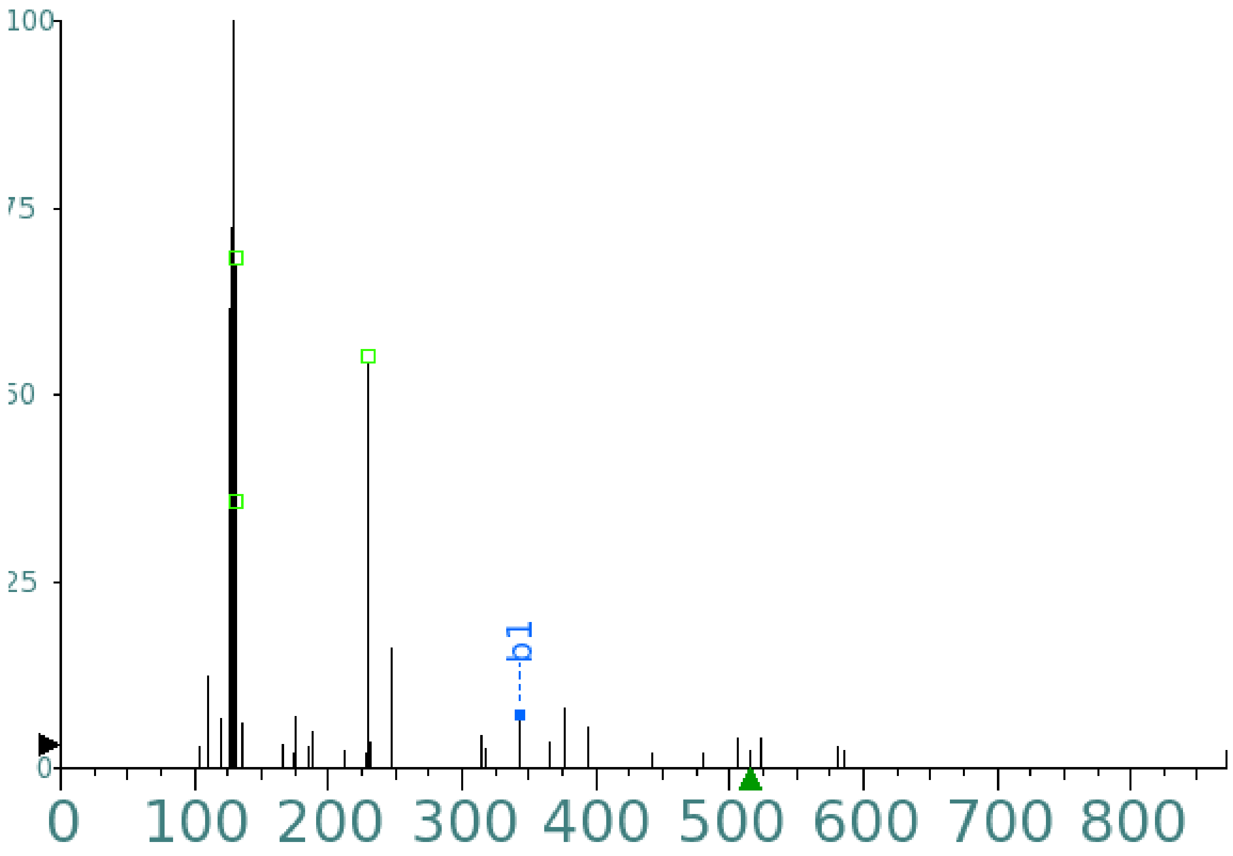

LILVVQD (+2)

Primary Reference: ##PKHD1_HUMAN
Search ID: 41760
Search Name: 20130328_ananiav_ERstress_TMT_TM_timecourse_trypsin_inj1_4mc_iaa
Scan#: 12927
Observed Mass: 514.8325 (2.4 ppm)
PSM Score: 5.28

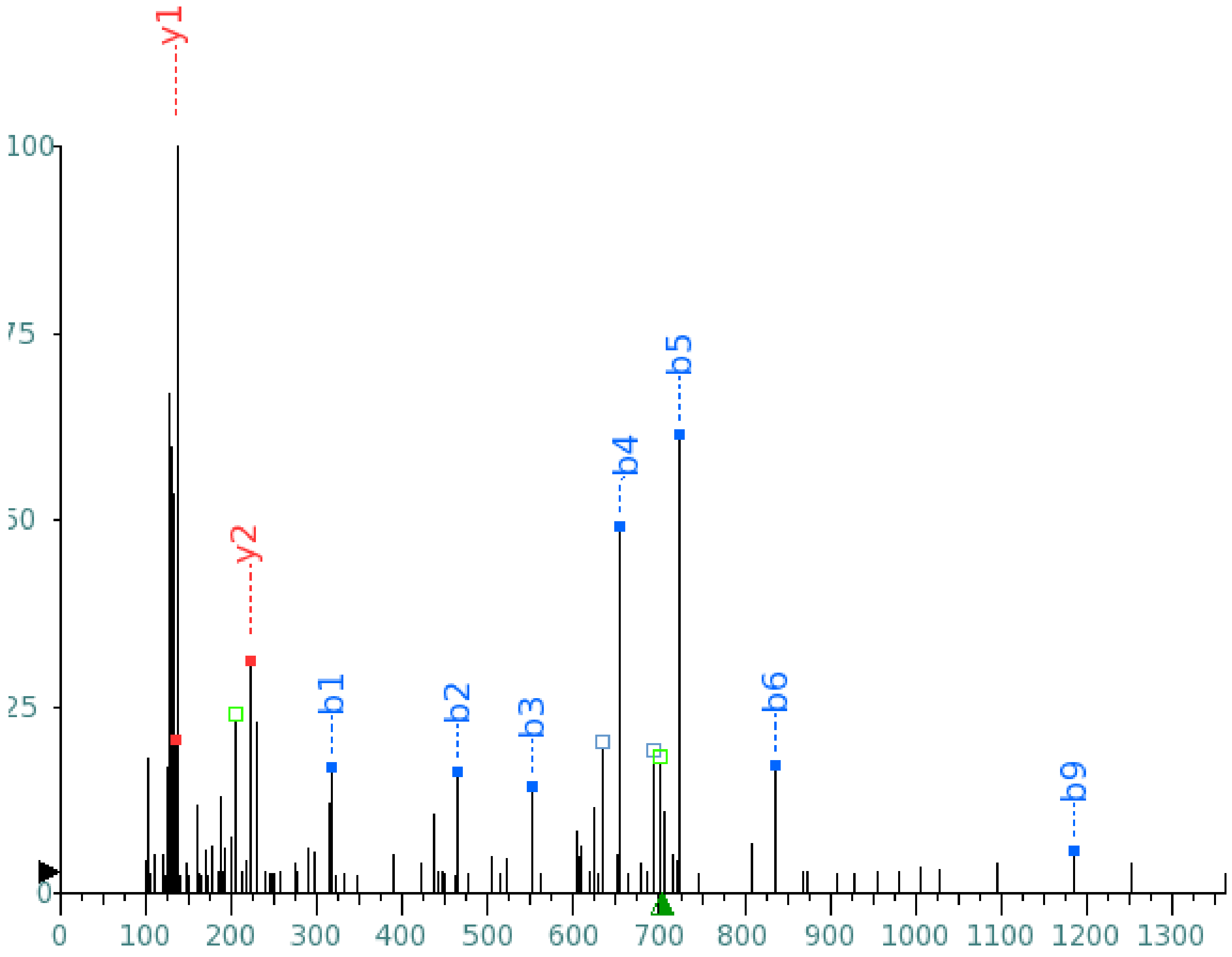

SFSTALYGESD (+2)

Primary Reference: ACTN4_HUMAN
Search ID: 41760
Search Name: 20130328_ananiav_ERstress_TMT_TM_timecourse_trypsin_inj1_4mc_iaa
Scan#: 12942
Observed Mass: 703.3391 (1.8 ppm)
PSM Score: 43.92

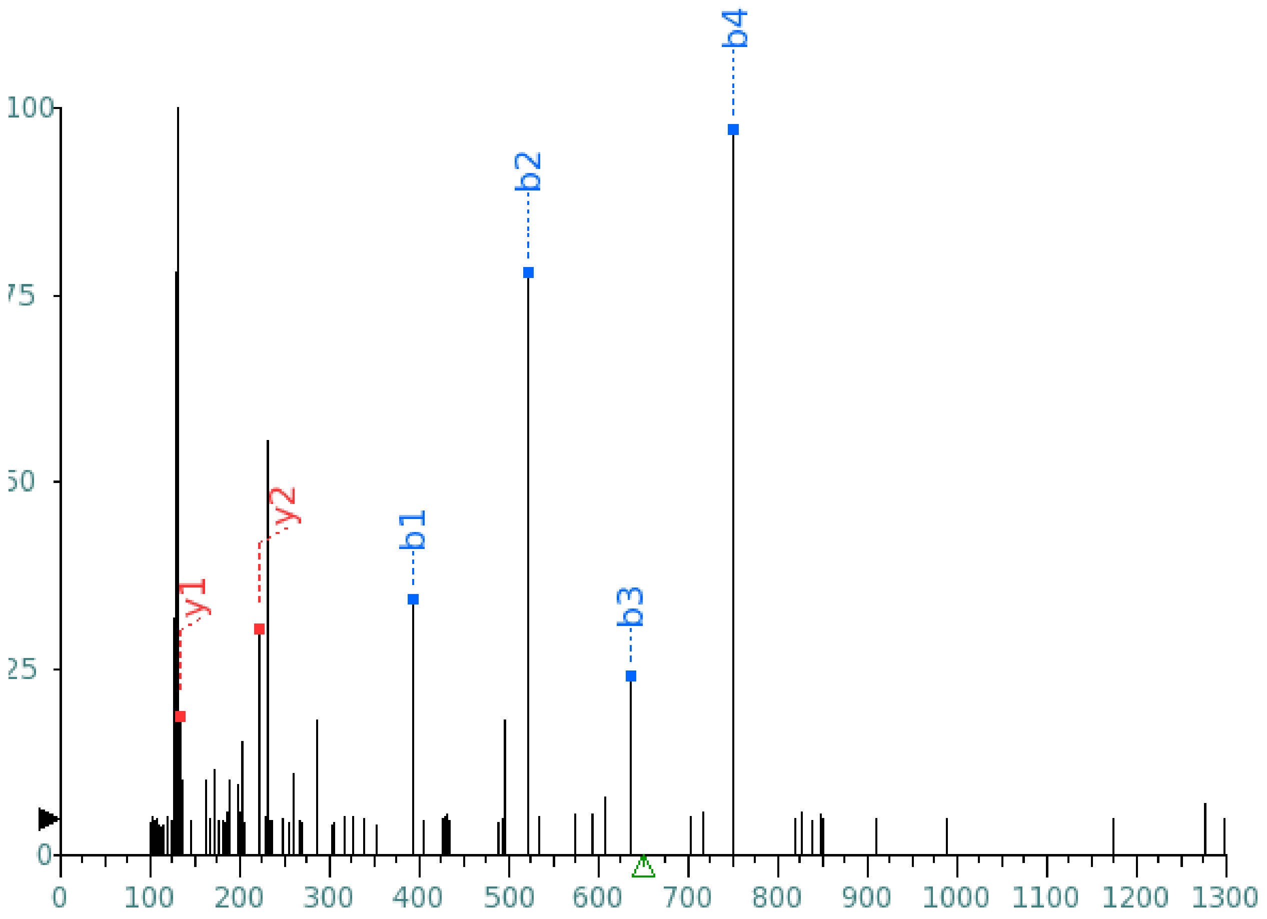

YEIDLDTSD (+2)

Primary Reference: K6PF_HUMAN
Search ID: 41760
Search Name: 20130328_ananiav_ERstress_TMT_TM_timecourse_trypsin_inj1_4mc_iaa
Scan#: 12943
Observed Mass: 650.3128 (2.3 ppm)
PSM Score: 14.01

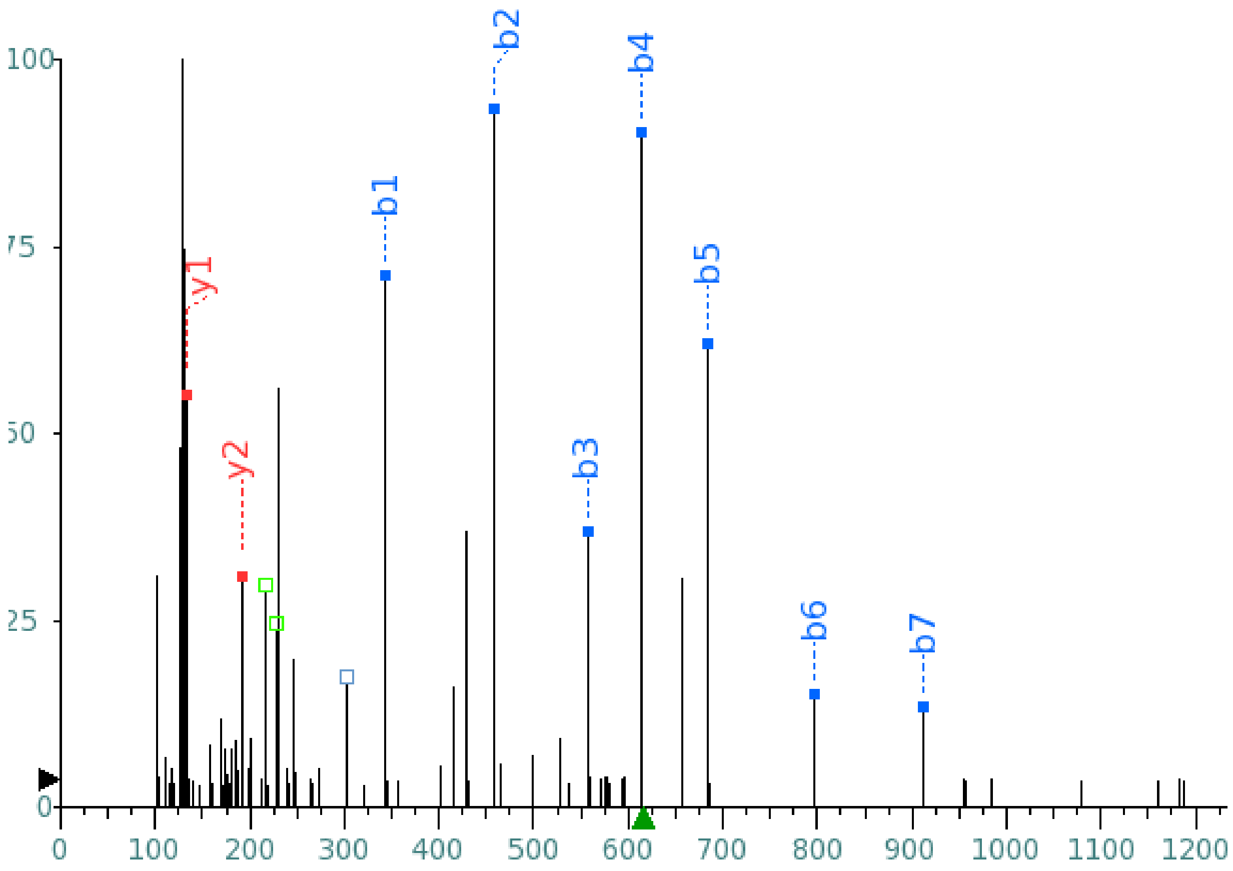

NLVGALDEGD (+2)

Primary Reference: ANR57_HUMAN
Search ID: 41760
Search Name: 20130328_ananiav_ERstress_TMT_TM_timecourse_trypsin_inj1_4mc_iaa
Scan#: 12964
Observed Mass: 616.3237 (2.7 ppm)
PSM Score: 43.68

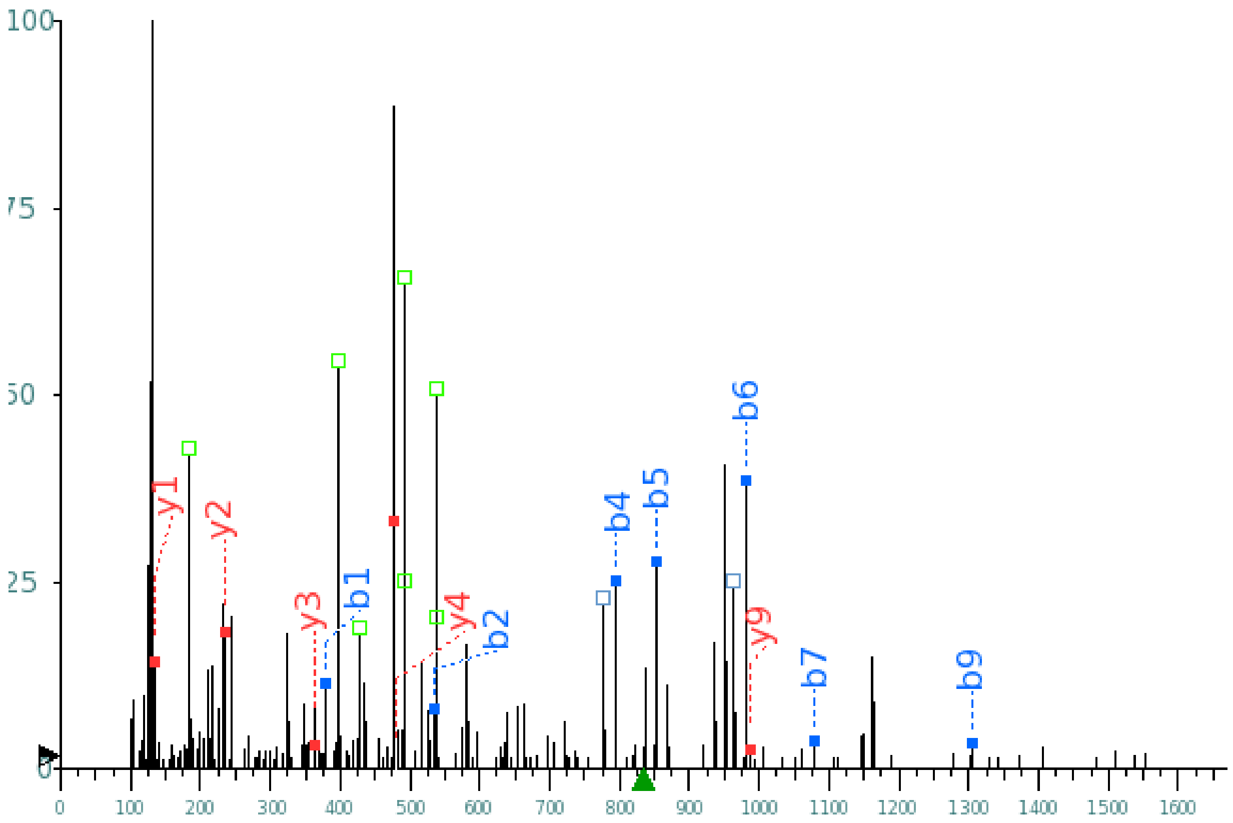

FRFDGQPINETD (+2)

Primary Reference: SUMO2_HUMAN
Search ID: 41760
Search Name: 20130328_ananiav_ERstress_TMT_TM_timecourse_trypsin_inj1_4mc_iaa
Scan#: 12975
Observed Mass: 834.4165 (1.8 ppm)
PSM Score: 19.24

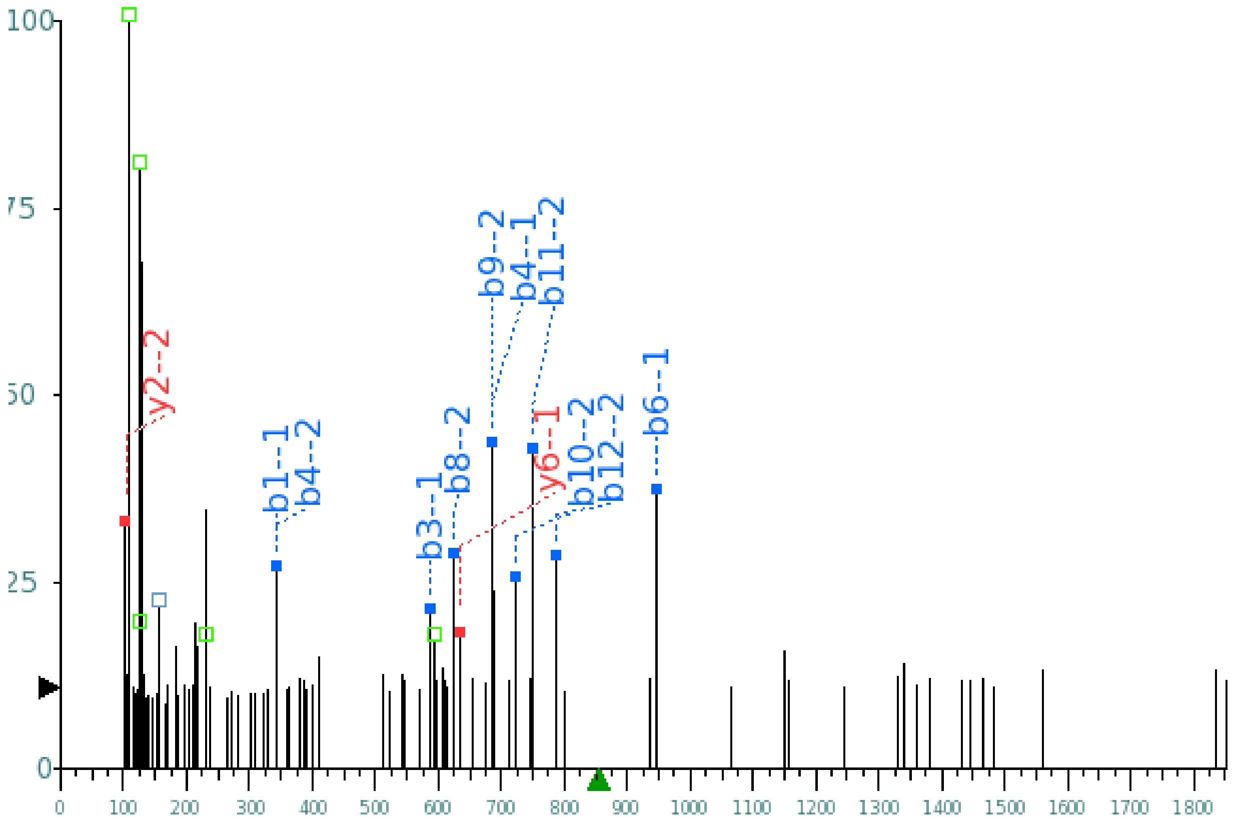

LLETPYHC*EAGAATDAEATEAD (+3)

Primary Reference: SELO_HUMAN
Search ID: 41760
Search Name: 20130328_ananiav_ERstress_TMT_TM_timecourse_trypsin_inj1_4mc_iaa
Scan#: 12986
Observed Mass: 855.3947 (1.4 ppm)
PSM Score: 15.66

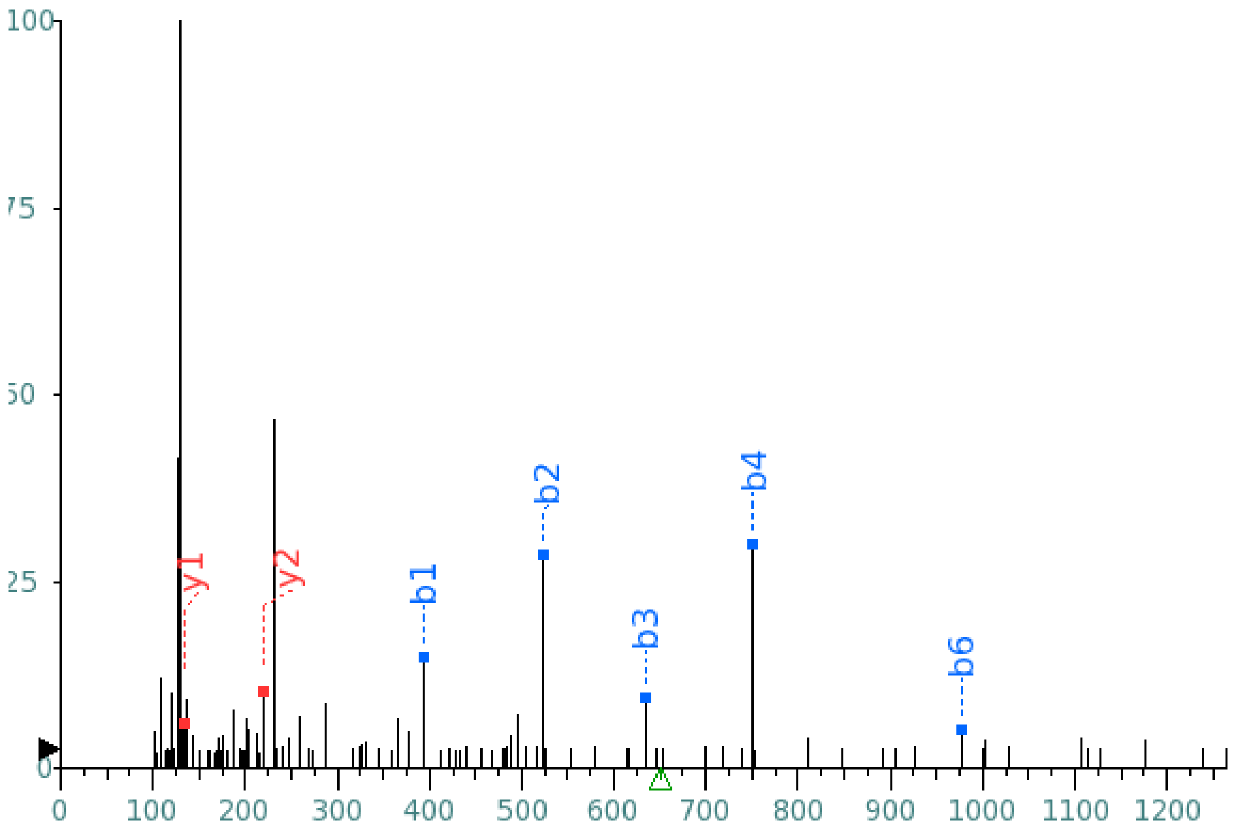

YEIDLDTSD (+2)

Primary Reference: K6PF_HUMAN
Search ID: 41760
Search Name: 20130328_ananiav_ERstress_TMT_TM_timecourse_trypsin_inj1_4mc_iaa
Scan#: 13030
Observed Mass: 650.3124 (1.7 ppm)
PSM Score: 19.34

File name: 2015-02-04 04.01.53PM.pdf

Creation time: 2015-02-04 04:09PM

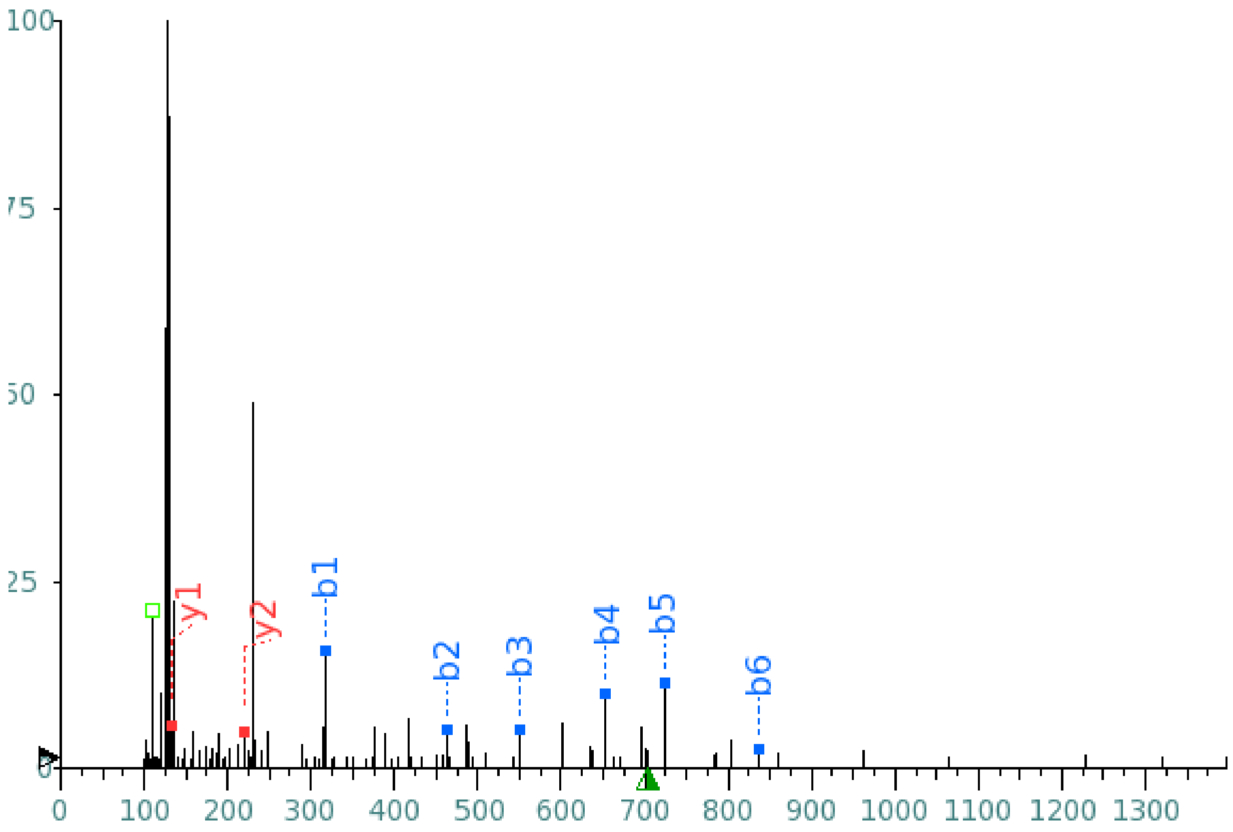

SFSTALYGESD (+2)

Primary Reference: ACTN4_HUMAN
Search ID: 41760
Search Name: 20130328_ananiav_ERstress_TMT_TM_timecourse_trypsin_inj1_4mc_iaa
Scan#: 13031
Observed Mass: 703.3395 (2.3 ppm)
PSM Score: 15.69

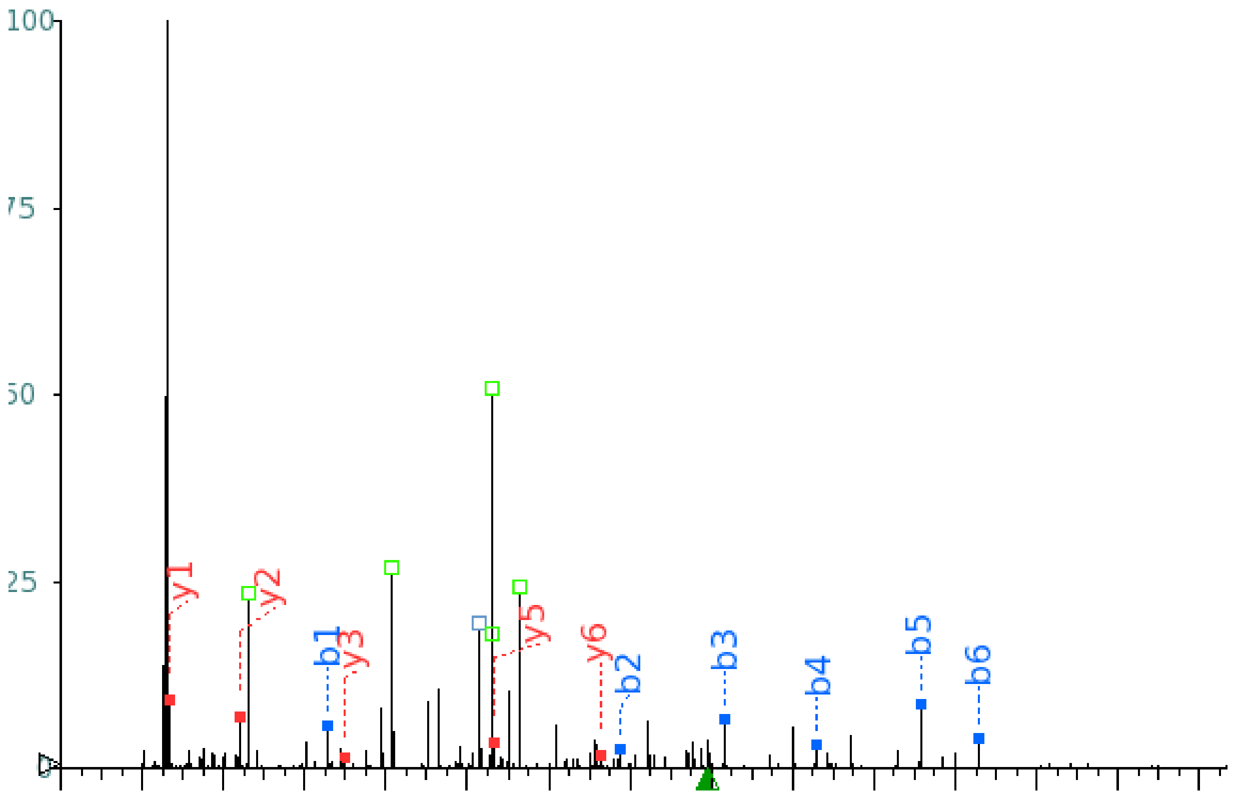

VKEIEAIESD (+2)

Primary Reference: RSRC1_HUMAN
Search ID: 41760
Search Name: 20130328_ananiav_ERstress_TMT_TM_timecourse_trypsin_inj1_4mc_iaa
Scan#: 13035
Observed Mass: 795.9542 (1.3 ppm)
PSM Score: 18.75

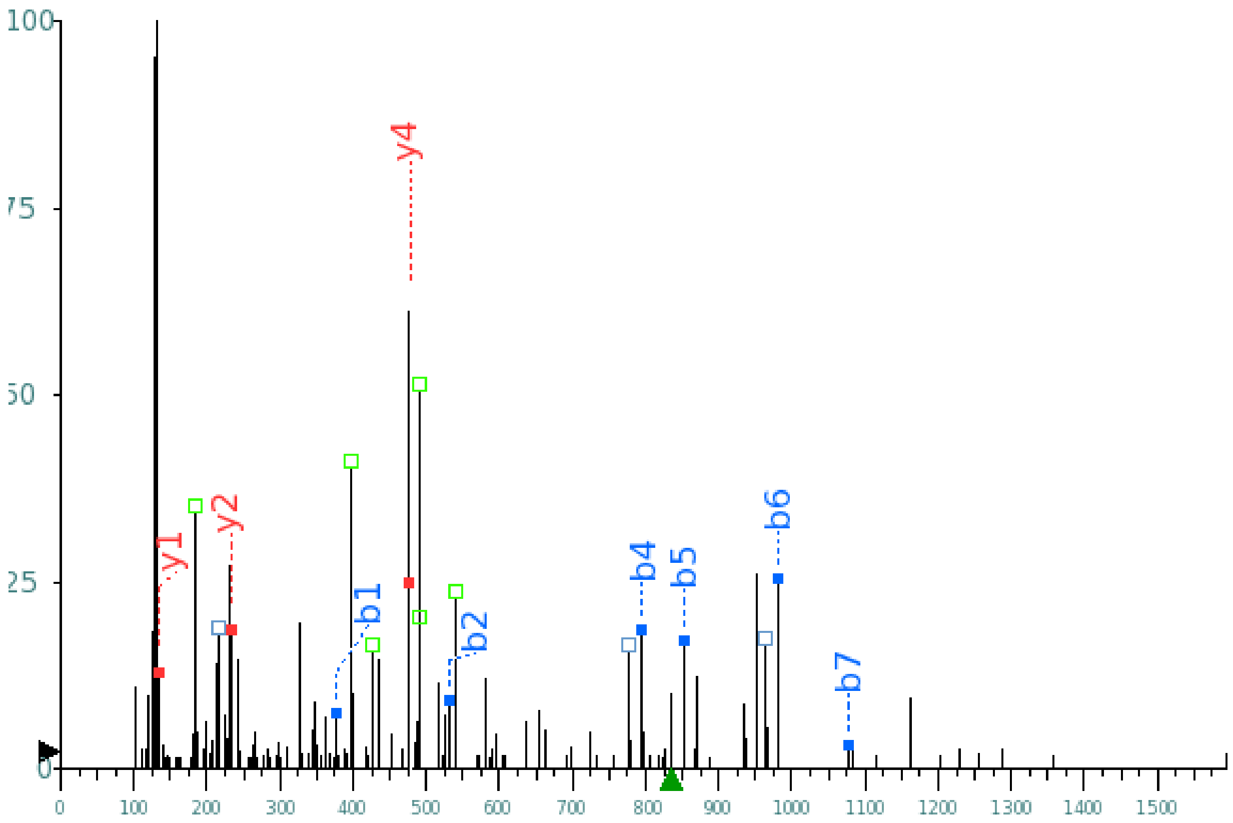

FRFDGQPINETD (+2)

Primary Reference: SUMO2_HUMAN
Search ID: 41760
Search Name: 20130328_ananiav_ERstress_TMT_TM_timecourse_trypsin_inj1_4mc_iaa
Scan#: 13065
Observed Mass: 834.4162 (1.5 ppm)
PSM Score: 13.73

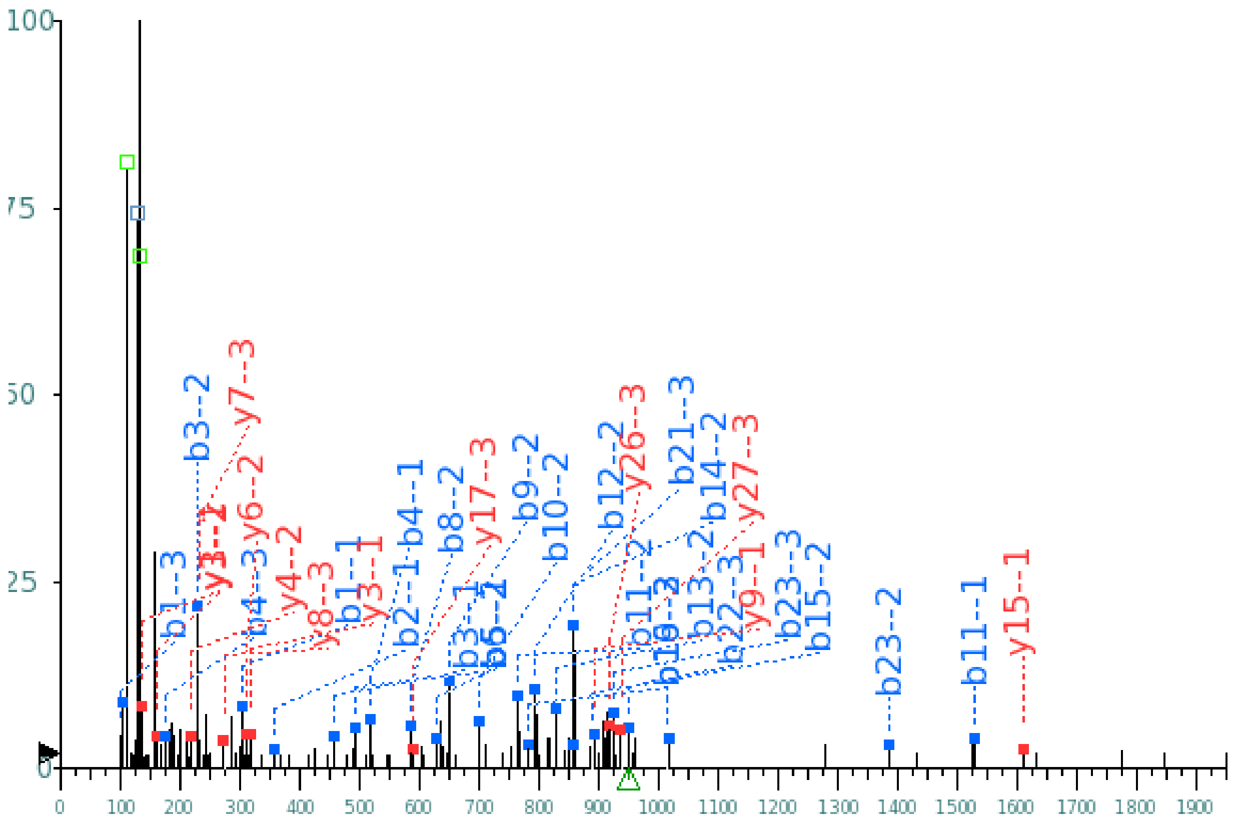

AGTGLKGEEVEGAGWMPGREPTTHAEAQGDEGD (+4)

Primary Reference: AHNK2_HUMAN
Search ID: 41760
Search Name: 20130328_ananiav_ERstress_TMT_TM_timecourse_trypsin_inj1_4mc_iaa
Scan#: 13218
Observed Mass: 950.2091 (1.7 ppm)
PSM Score: 40.13

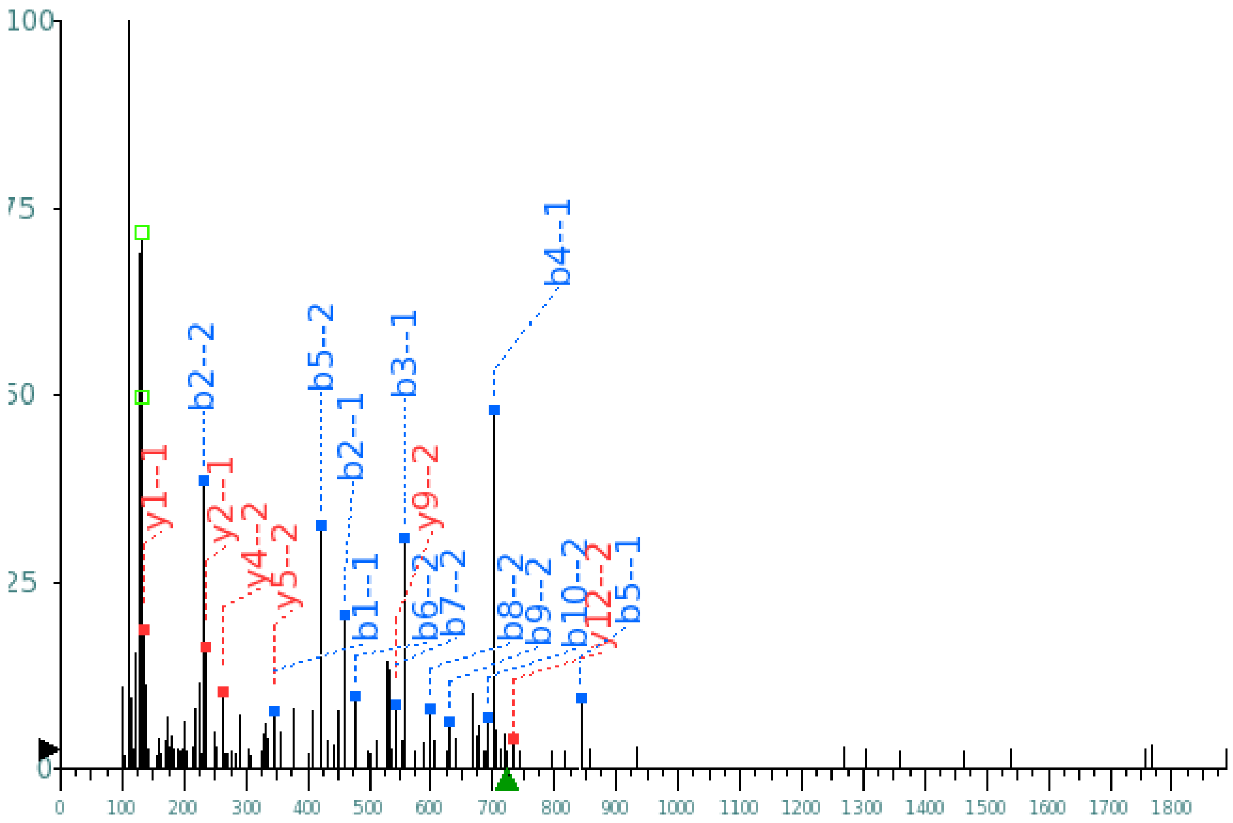

NNVFHLEDGEPYC*ETD (+3)

Primary Reference: PDLI5_HUMAN
Search ID: 41760
Search Name: 20130328_ananiav_ERstress_TMT_TM_timecourse_trypsin_inj1_4mc_iaa
Scan#: 13319
Observed Mass: 723.3208 (1.8 ppm)
PSM Score: 15.11

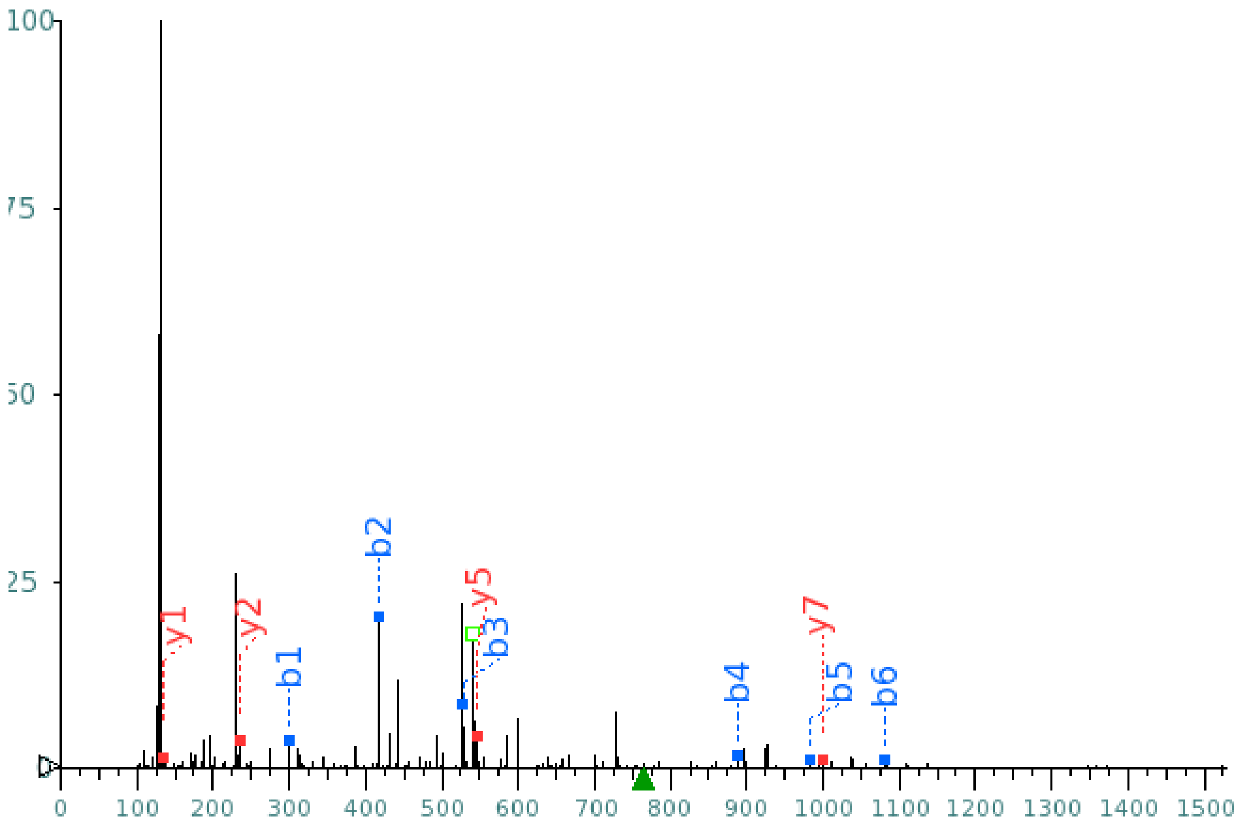

ADIKPVIVTD (+2)

Primary Reference: PHF12_HUMAN
Search ID: 41760
Search Name: 20130328_ananiav_ERstress_TMT_TM_timecourse_trypsin_inj1_4mc_iaa
Scan#: 13324
Observed Mass: 764.9719 (1 ppm)
PSM Score: 8.95

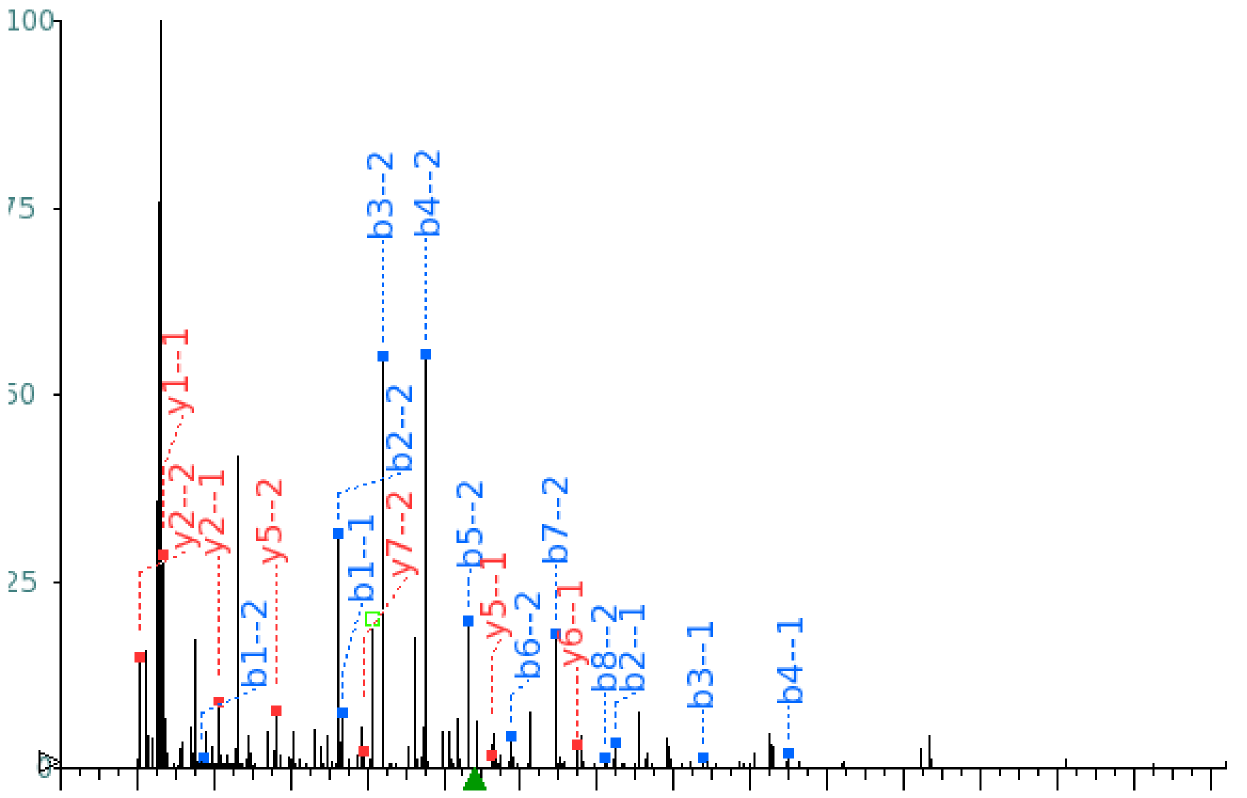

HKIIILDEAD (+3)

Primary Reference: RFC2_HUMAN
Search ID: 41760
Search Name: 20130328_ananiav_ERstress_TMT_TM_timecourse_trypsin_inj1_4mc_iaa
Scan#: 13327
Observed Mass: 542.3275 (0.4 ppm)
PSM Score: 10.74

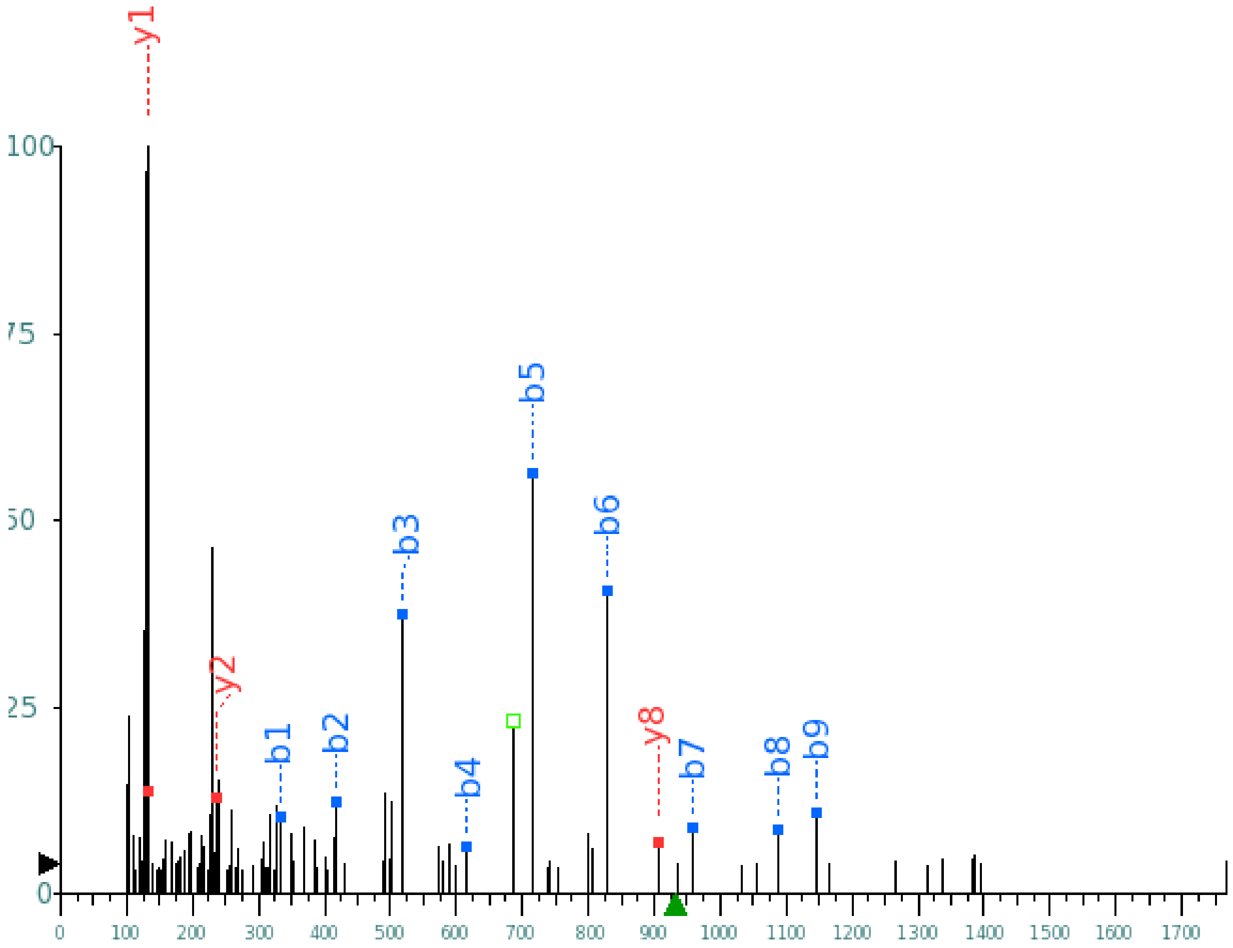

TSTPVIMEGVQEETD (+2)

Primary Reference: BOD1L_HUMAN
Search ID: 41760
Search Name: 20130328_ananiav_ERstress_TMT_TM_timecourse_trypsin_inj1_4mc_iaa
Scan#: 13333
Observed Mass: 932.9564 (0.4 ppm)
PSM Score: 30.88

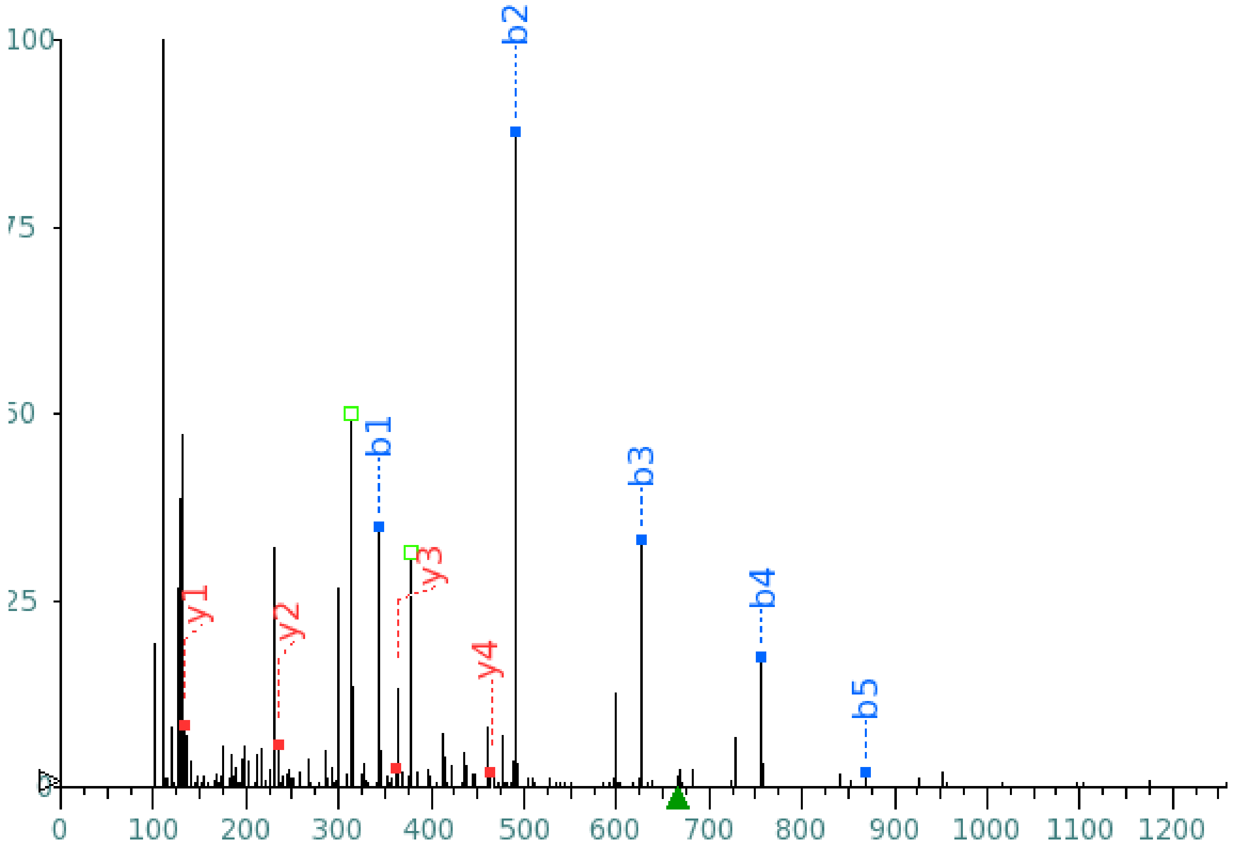

IFHELTQTD (+2)

Primary Reference: C1TC_HUMAN
Search ID: 41760
Search Name: 20130328_ananiav_ERstress_TMT_TM_timecourse_trypsin_inj1_4mc_iaa
Scan#: 13341
Observed Mass: 666.8533 (-0.3 ppm)
PSM Score: 12.54

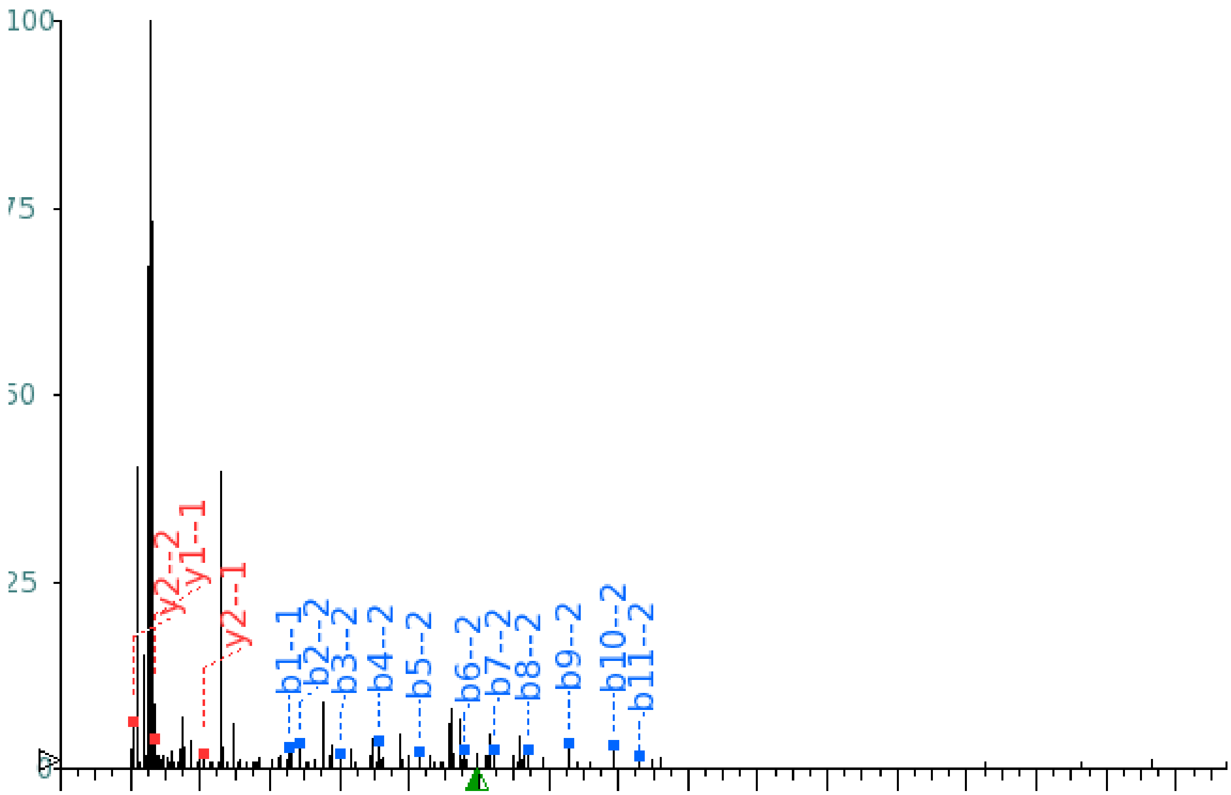

VKLINESVDEAD (+3)

Primary Reference: RANB3_HUMAN
Search ID: 41760
Search Name: 20130328_ananiav_ERstress_TMT_TM_timecourse_trypsin_inj1_4mc_iaa
Scan#: 13342
Observed Mass: 597.3367 (0.5 ppm)
PSM Score: 11.89

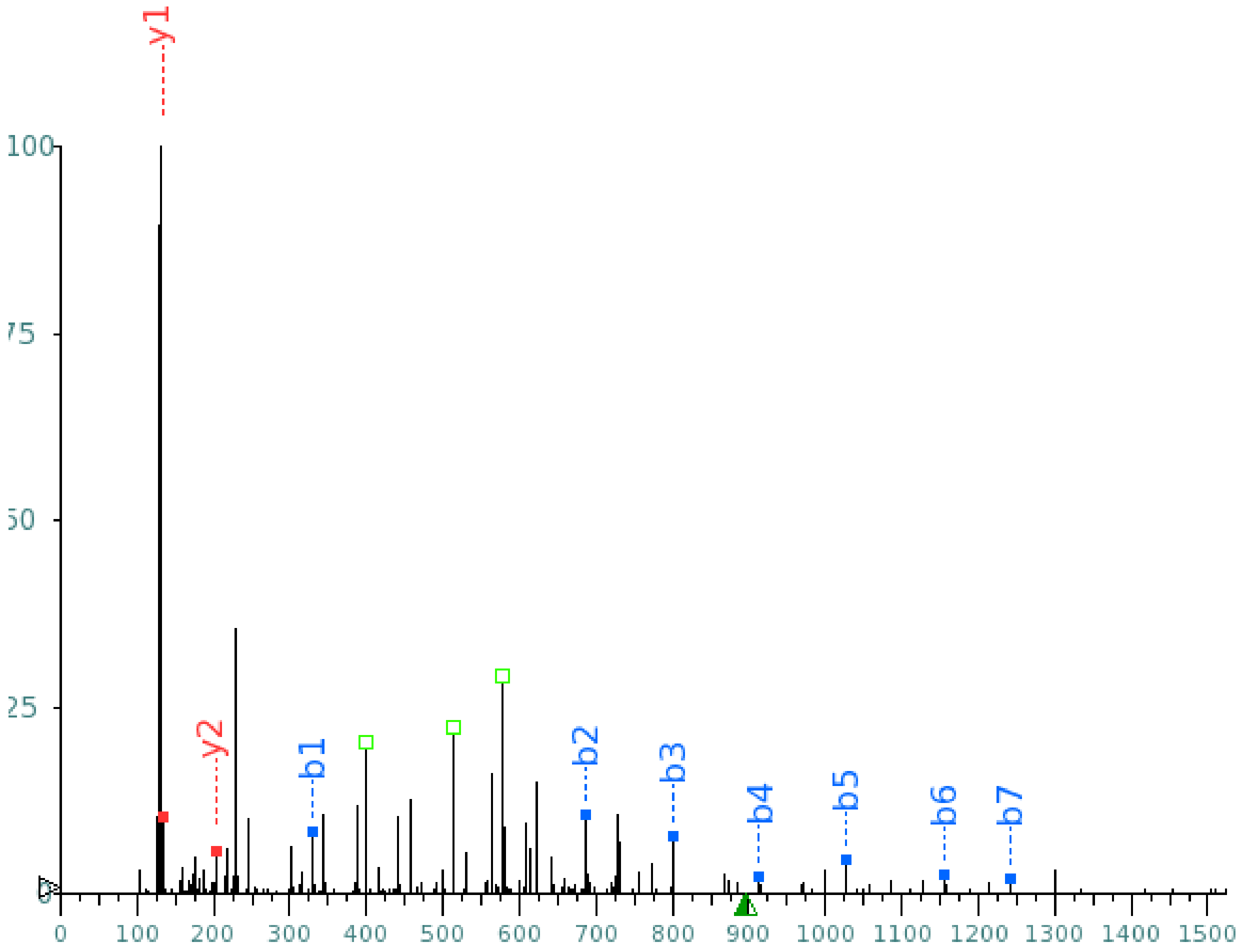

VKLINESVDEAD (+2)

Primary Reference: RANB3_HUMAN
Search ID: 41760
Search Name: 20130328_ananiav_ERstress_TMT_TM_timecourse_trypsin_inj1_4mc_iaa
Scan#: 13352
Observed Mass: 895.5027 (1.9 ppm)
PSM Score: 17.19

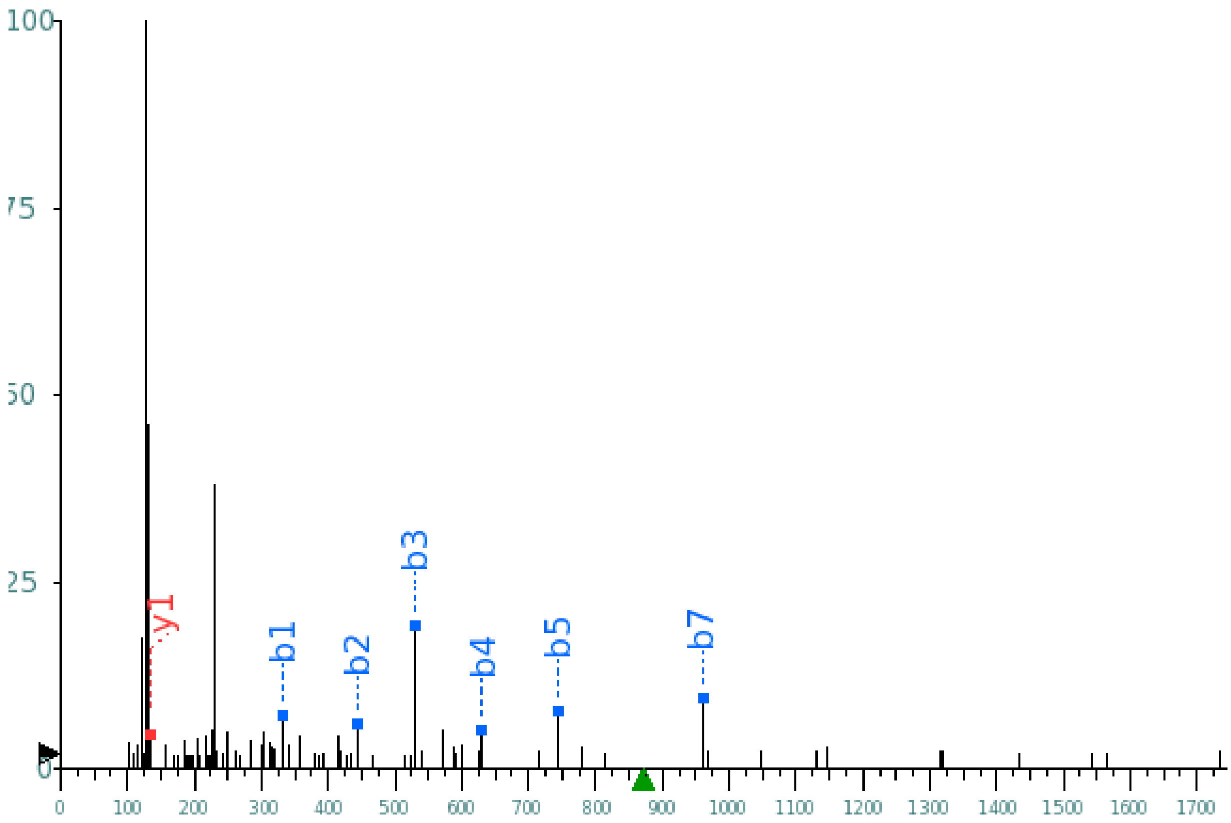

TLSVNTDFTSDQAD (+2)

Primary Reference: RED1_HUMAN
Search ID: 41760
Search Name: 20130328_ananiav_ERstress_TMT_TM_timecourse_trypsin_inj1_4mc_iaa
Scan#: 13407
Observed Mass: 871.9198 (2.4 ppm)
PSM Score: 21.35

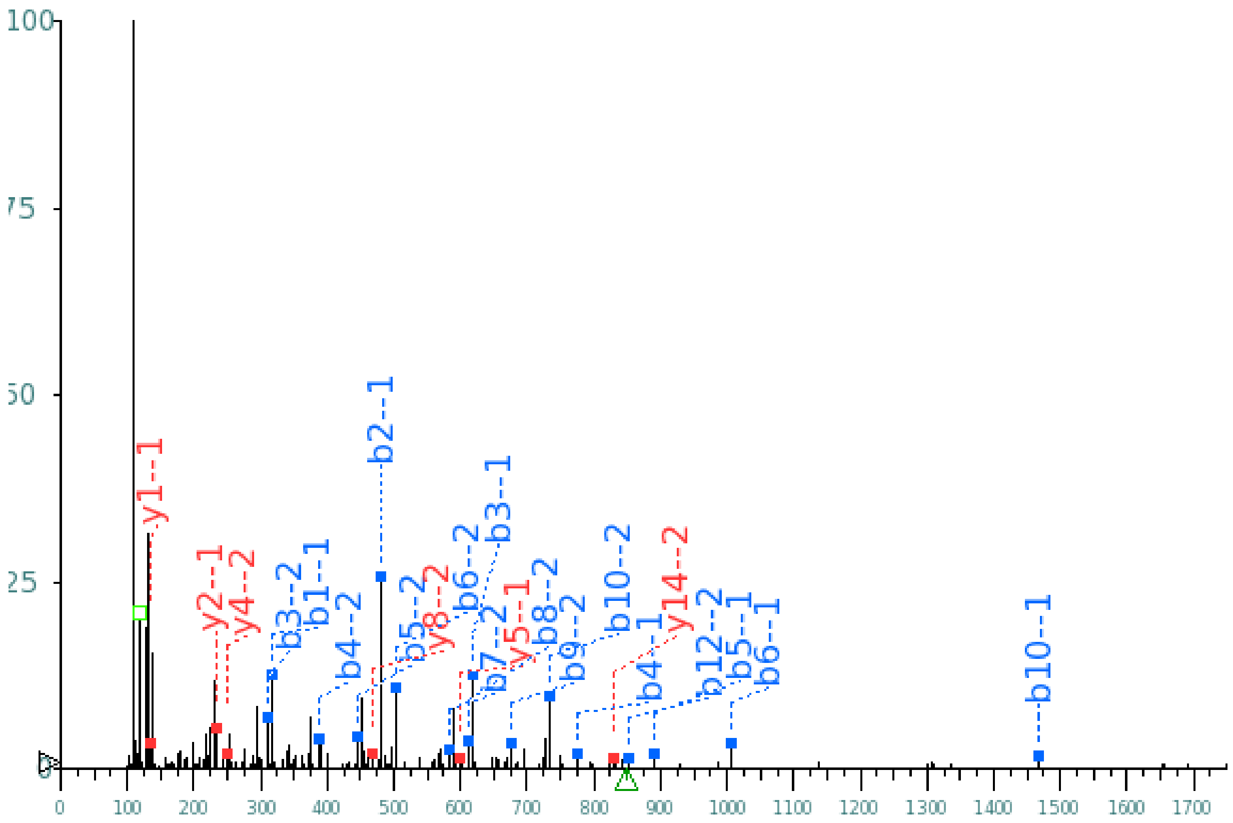

SYHC*NDC*GEDFSHITDFTD (+3)

Primary Reference: ZFP90_HUMAN
Search ID: 41760
Search Name: 20130328_ananiav_ERstress_TMT_TM_timecourse_trypsin_inj1_4mc_iaa
Scan#: 13413
Observed Mass: 850.346 (2 ppm)
PSM Score: 27.32

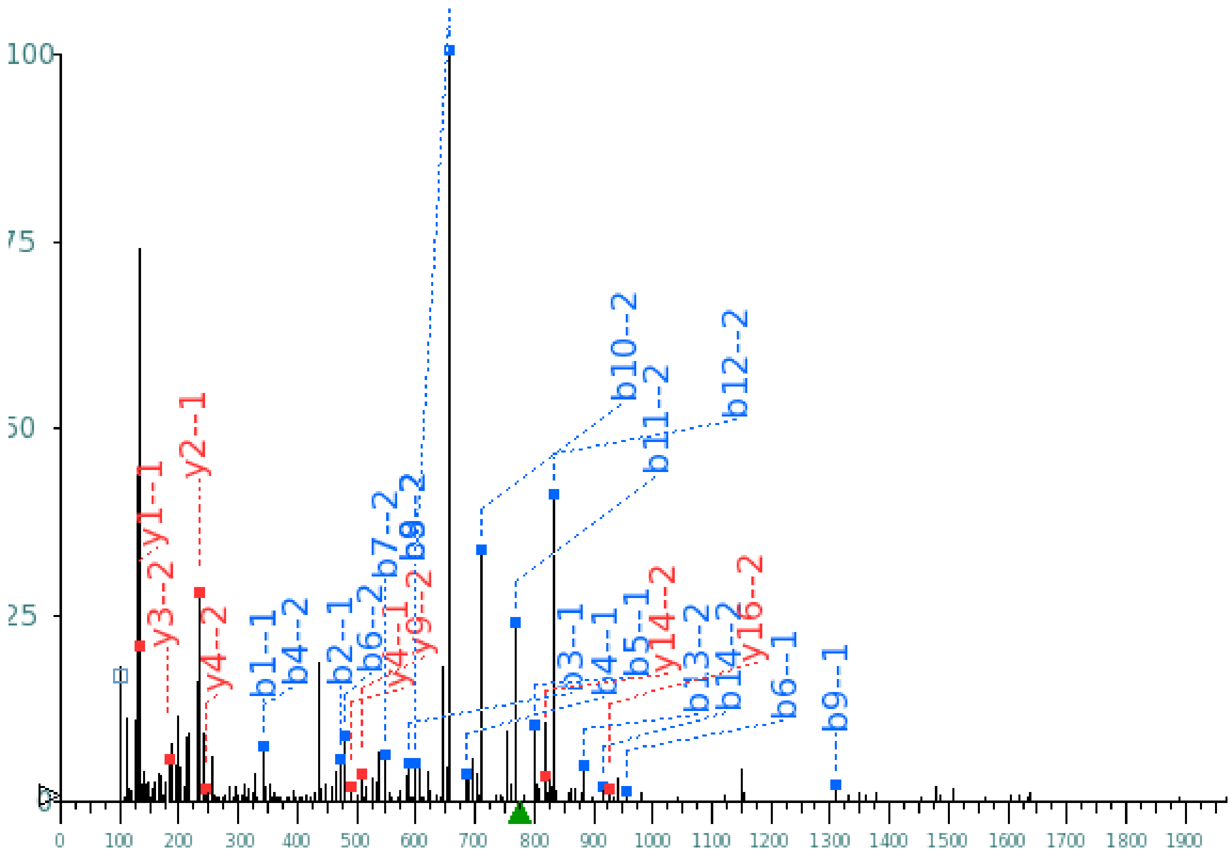

NENVDRHVDLLEVAQETD (+3)

Primary Reference: ATAD1_HUMAN
Search ID: 41760
Search Name: 20130328_ananiav_ERstress_TMT_TM_timecourse_trypsin_inj1_4mc_iaa
Scan#: 13457
Observed Mass: 775.7234 (1.5 ppm)
PSM Score: 26.46

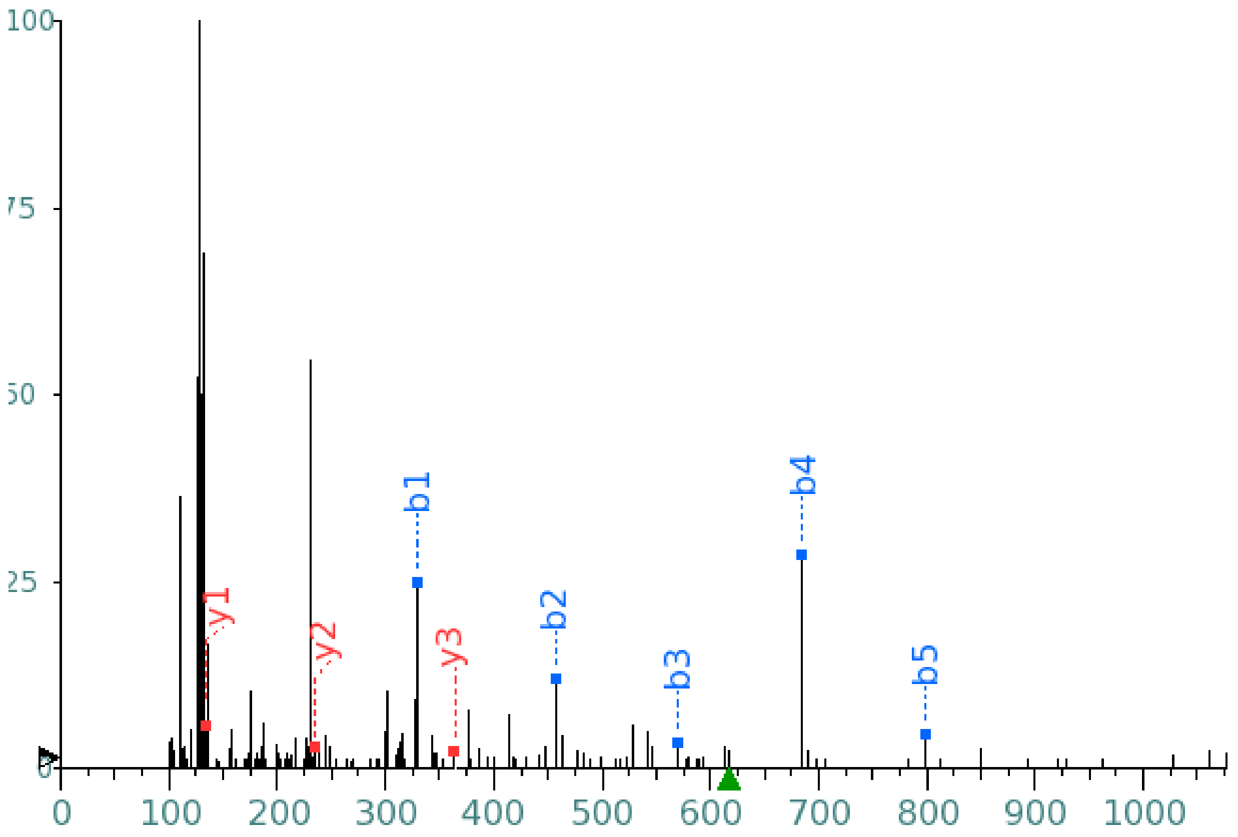

VQLDLAETD (+2)

Primary Reference: ESYT1_HUMAN
Search ID: 41760
Search Name: 20130328_ananiav_ERstress_TMT_TM_timecourse_trypsin_inj1_4mc_iaa
Scan#: 13482
Observed Mass: 616.8321 (-0.2 ppm)
PSM Score: 11.27

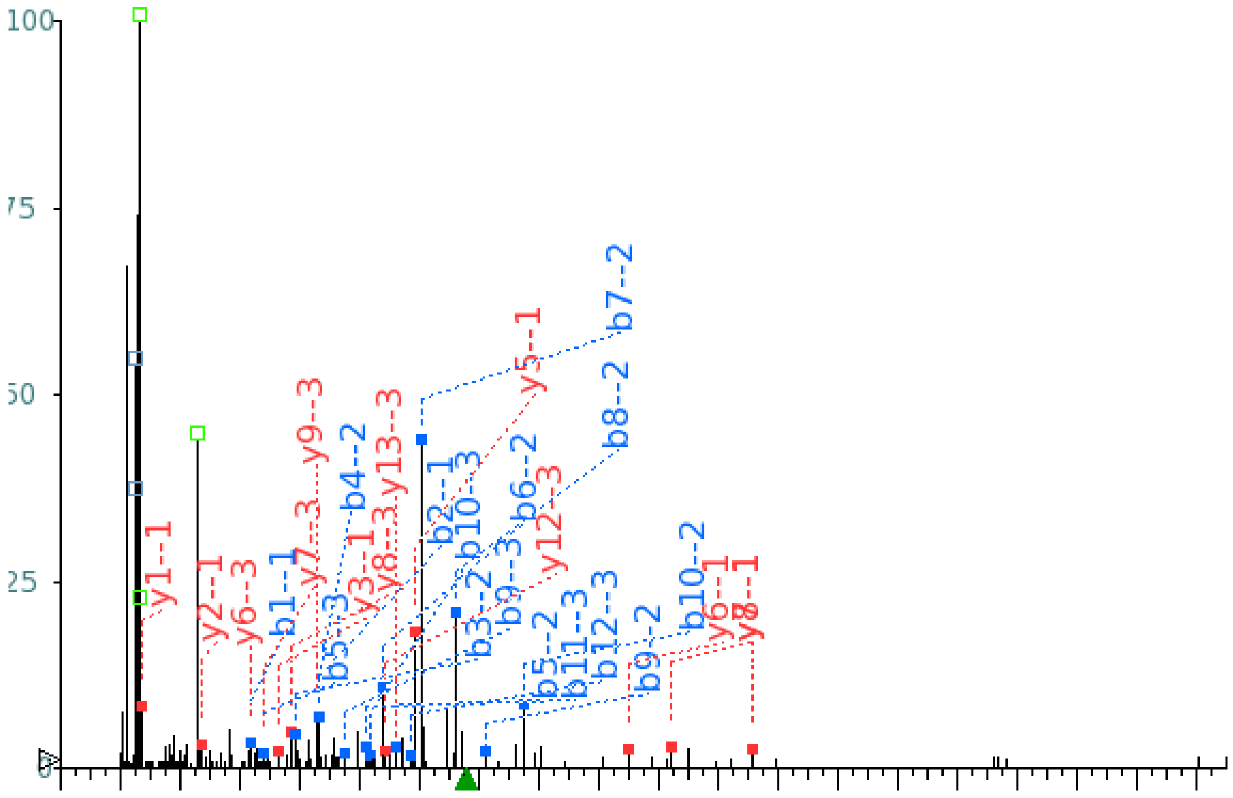

SLKAYGELPEHAKINETD (+4)

Primary Reference: IF1AX_HUMAN
Search ID: 41760
Search Name: 20130328_ananiav_ERstress_TMT_TM_timecourse_trypsin_inj1_4mc_iaa
Scan#: 13506
Observed Mass: 676.3777 (-3 ppm)
PSM Score: 16.4

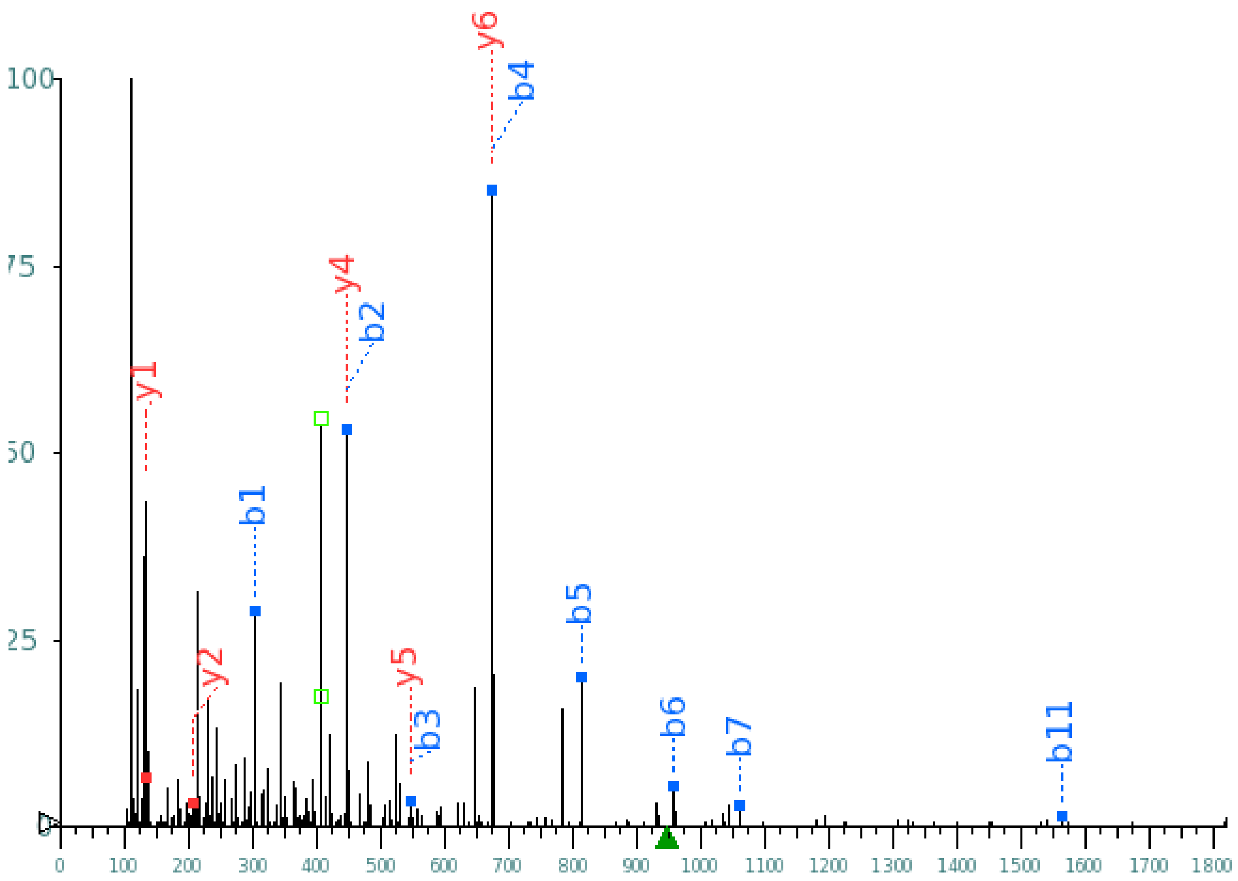

AFPEHFTYEPNEAD (+2)

Primary Reference: GYS1_HUMAN

Search ID: 41760

Search Name: 20130328_ananiav_ERstress_TMT_TM_timecourse_trypsin_inj1_4mc_iaa

Scan#: 13527

Observed Mass: 948.4374 (1.4 ppm)

PSM Score: 22.5

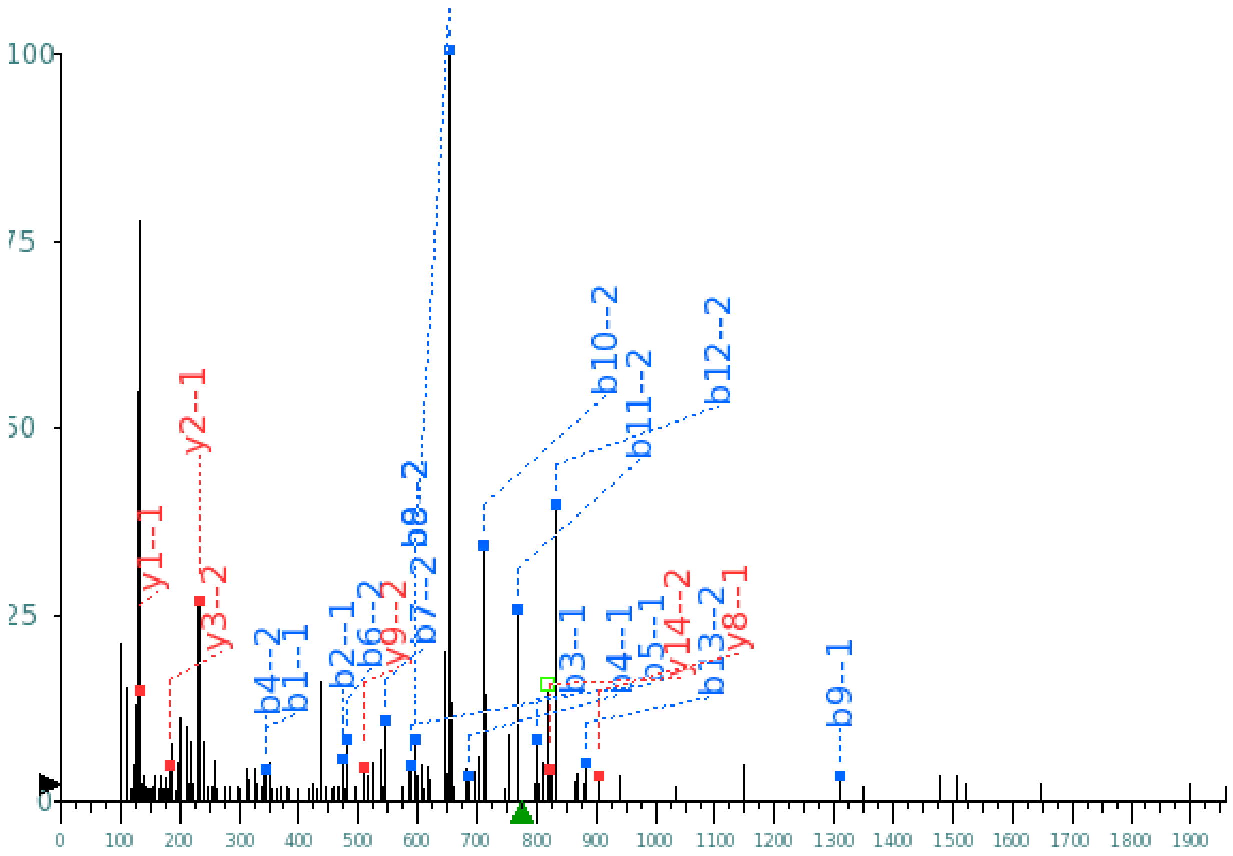

NENVDRHVDLLEVAQETD (+3)

Primary Reference: ATAD1_HUMAN
Search ID: 41760
Search Name: 20130328_ananiav_ERstress_TMT_TM_timecourse_trypsin_inj1_4mc_iaa
Scan#: 13542
Observed Mass: 775.723 (1.1 ppm)
PSM Score: 23.29

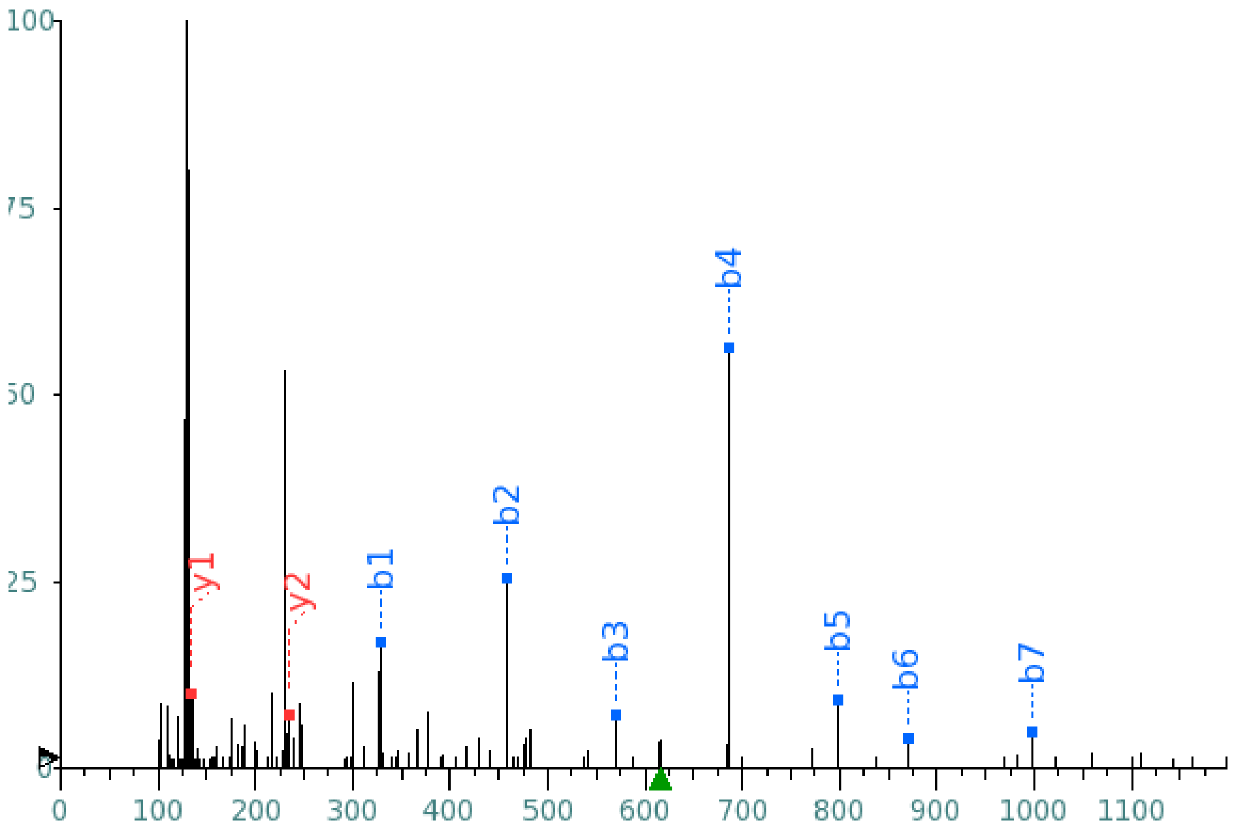

VQLDLAETD (+2)

Primary Reference: ESYT1_HUMAN
Search ID: 41760
Search Name: 20130328_ananiav_ERstress_TMT_TM_timecourse_trypsin_inj1_4mc_iaa
Scan#: 13570
Observed Mass: 616.8331 (1.4 ppm)
PSM Score: 49.88

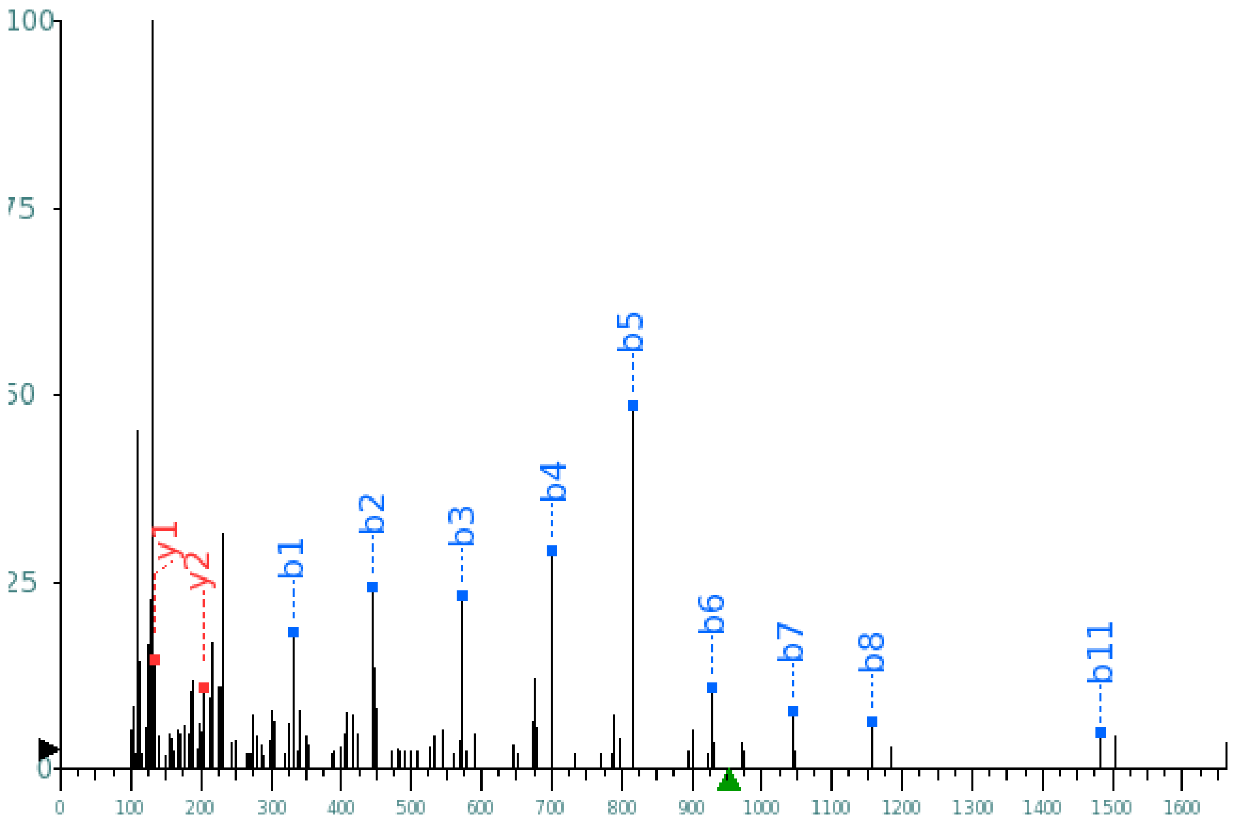

TIEQNINNLNVSEAD (+2)

Primary Reference: CND2_HUMAN
Search ID: 41760
Search Name: 20130328_ananiav_ERstress_TMT_TM_timecourse_trypsin_inj1_4mc_iaa
Scan#: 13578
Observed Mass: 951.9859 (2.1 ppm)
PSM Score: 62.28

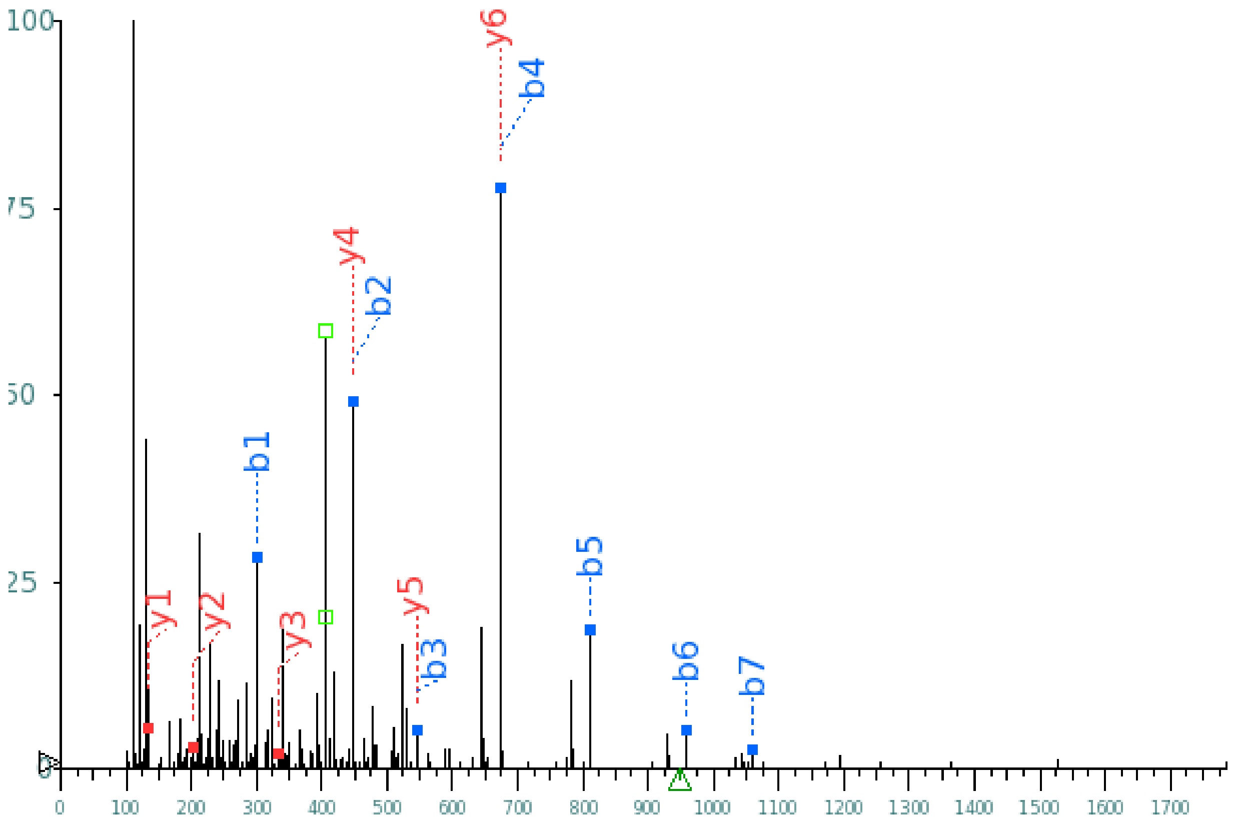

AFPEHFTYEPNEAD (+2)

Primary Reference: GYS1_HUMAN
Search ID: 41760
Search Name: 20130328_ananiav_ERstress_TMT_TM_timecourse_trypsin_inj1_4mc_iaa
Scan#: 13615
Observed Mass: 948.438 (2 ppm)
PSM Score: 20.77

File name: 2015-02-04 04.01.53PM.pdf

Creation time: 2015-02-04 04:09PM

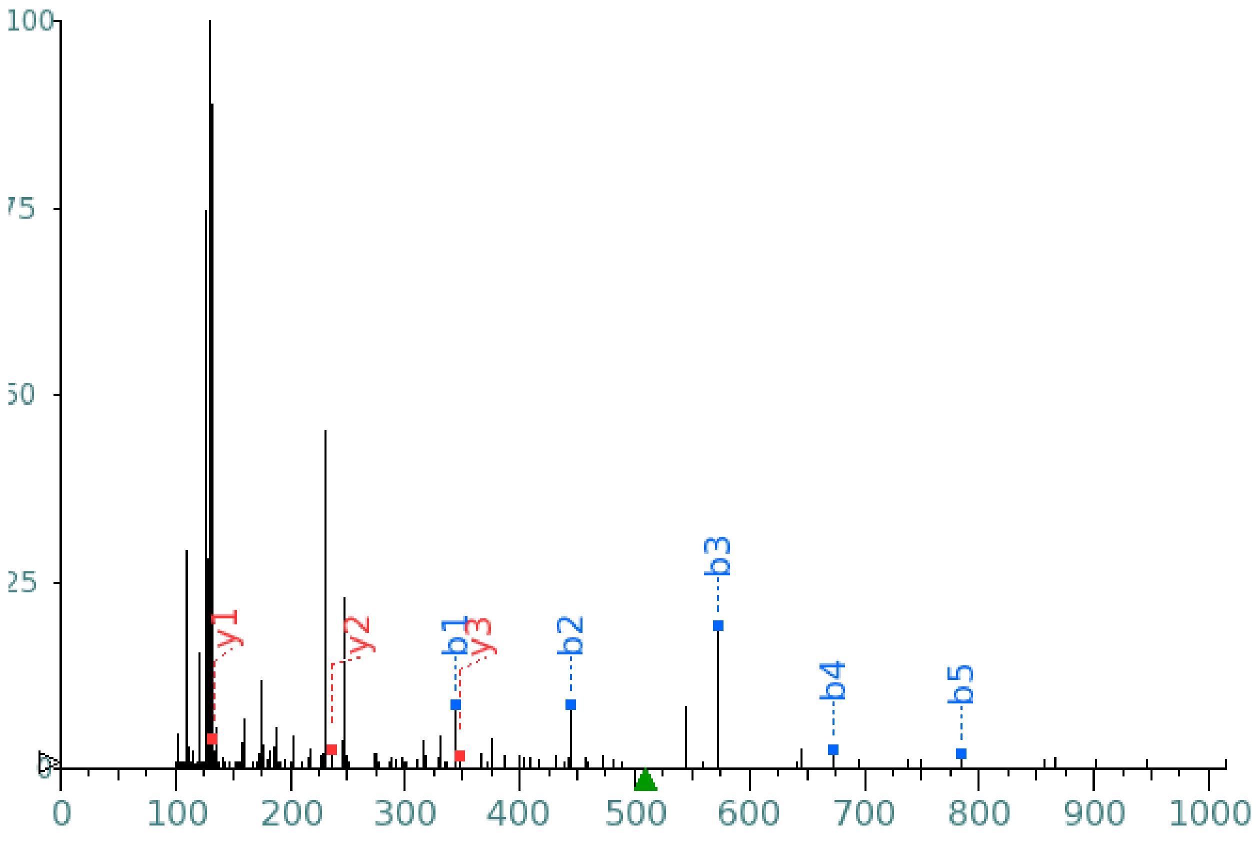

LTEVLTD (+2)

Primary Reference: GCN1L_HUMAN
Search ID: 41760
Search Name: 20130328_ananiav_ERstress_TMT_TM_timecourse_trypsin_inj1_4mc_iaa
Scan#: 13696
Observed Mass: 510.2957 (1.8 ppm)
PSM Score: 10.25

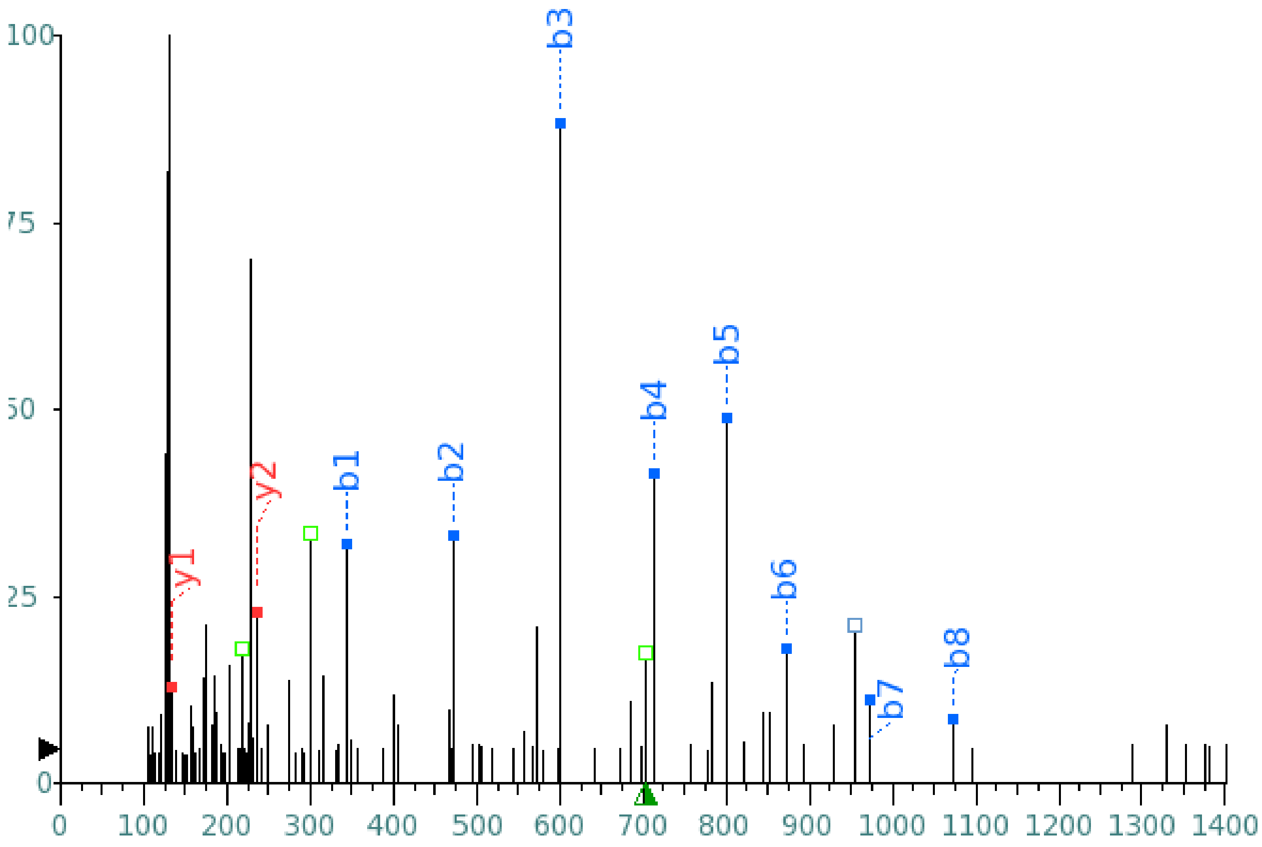

IQELSATVTTD (+2)

Primary Reference: TRIP6_HUMAN
Search ID: 41760
Search Name: 20130328_ananiav_ERstress_TMT_TM_timecourse_trypsin_inj1_4mc_iaa
Scan#: 13723
Observed Mass: 703.884 (2.2 ppm)
PSM Score: 46.6

File name: 2015-02-04 04.01.53PM.pdf

Creation time: 2015-02-04 04:09PM

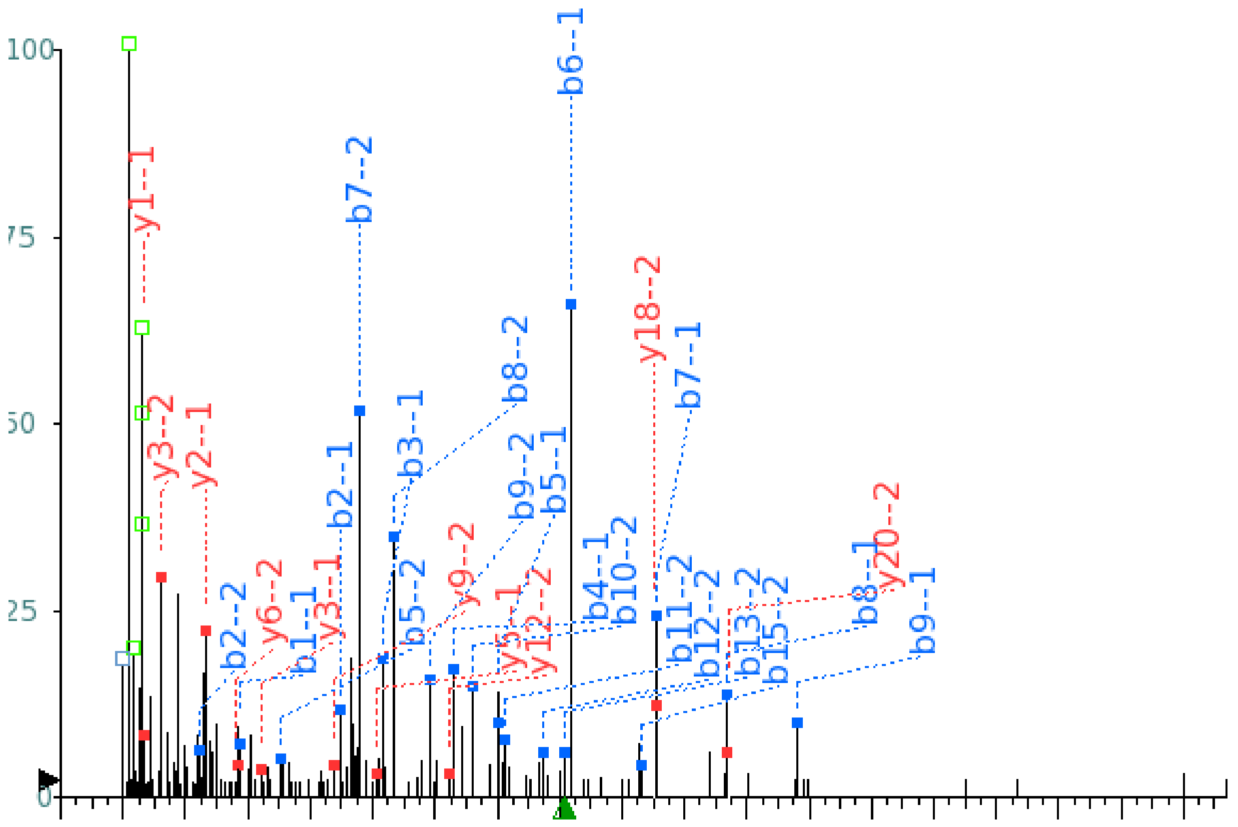

GC*ALADHLLHTQAQDGAISTD (+3)

Primary Reference: TPP2_HUMAN
Search ID: 41760
Search Name: 20130328_ananiav_ERstress_TMT_TM_timecourse_trypsin_inj1_4mc_iaa
Scan#: 13789
Observed Mass: 808.3999 (1.2 ppm)
PSM Score: 33.93

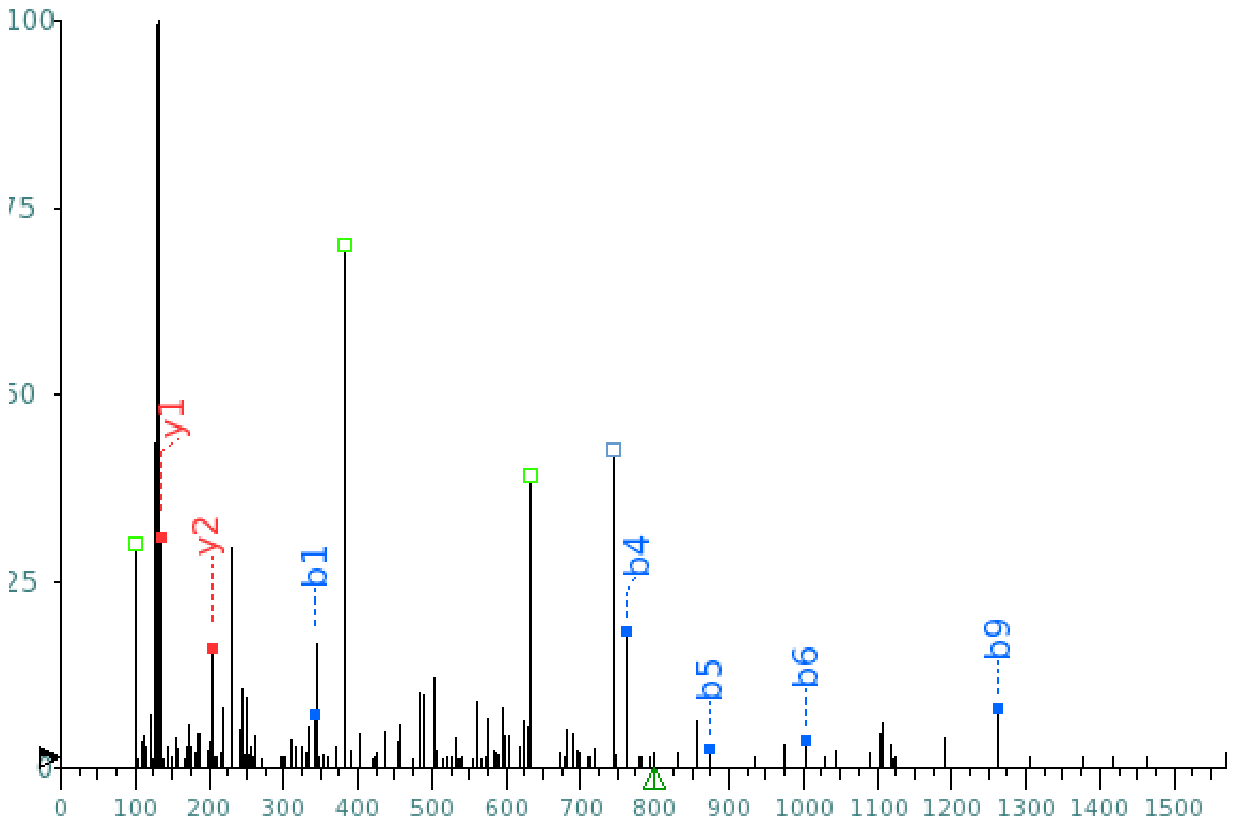

LRFDIEGSDEAD (+2)

Primary Reference: RB_HUMAN
Search ID: 41760
Search Name: 20130328_ananiav_ERstress_TMT_TM_timecourse_trypsin_inj1_4mc_iaa
Scan#: 13805
Observed Mass: 798.3914 (0.4 ppm)
PSM Score: 19.02

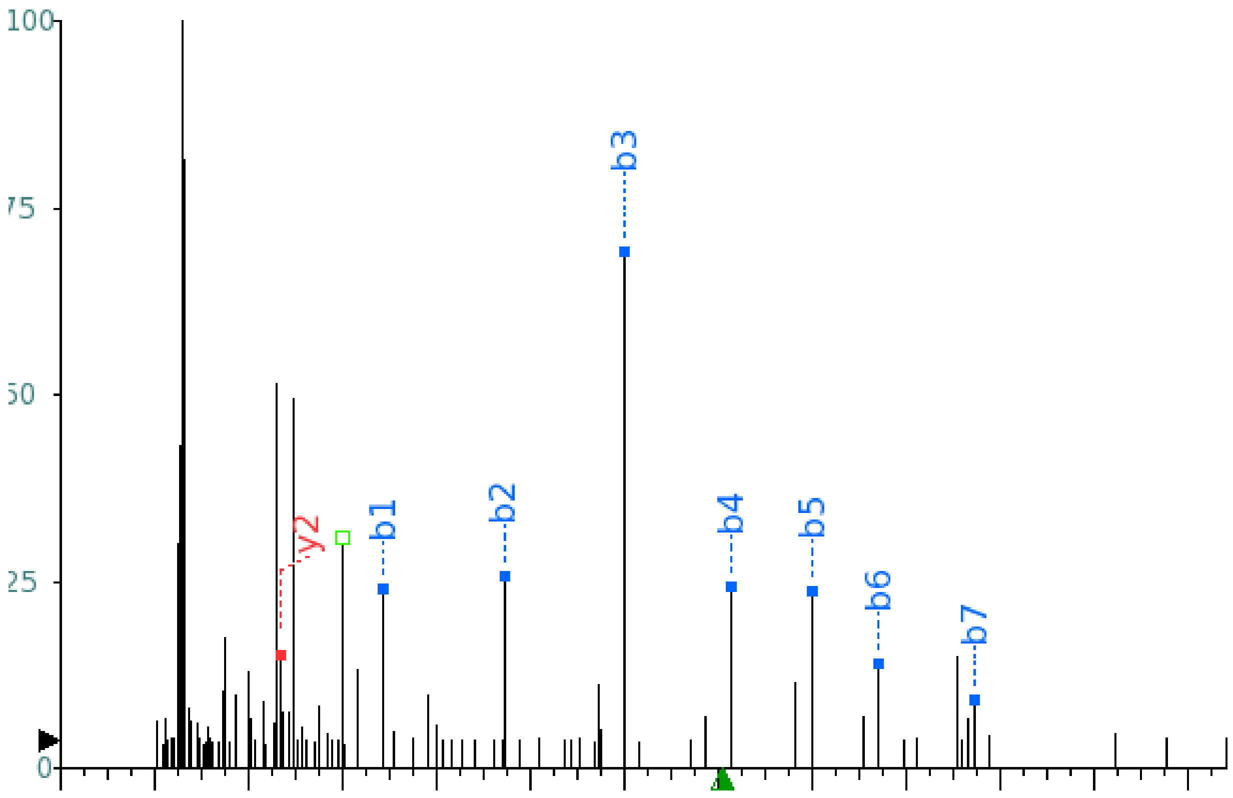

IQELSATVTTD (+2)

Primary Reference: TRIP6_HUMAN
Search ID: 41760
Search Name: 20130328_ananiav_ERstress_TMT_TM_timecourse_trypsin_inj1_4mc_iaa
Scan#: 13811
Observed Mass: 703.884 (2.2 ppm)
PSM Score: 36.48

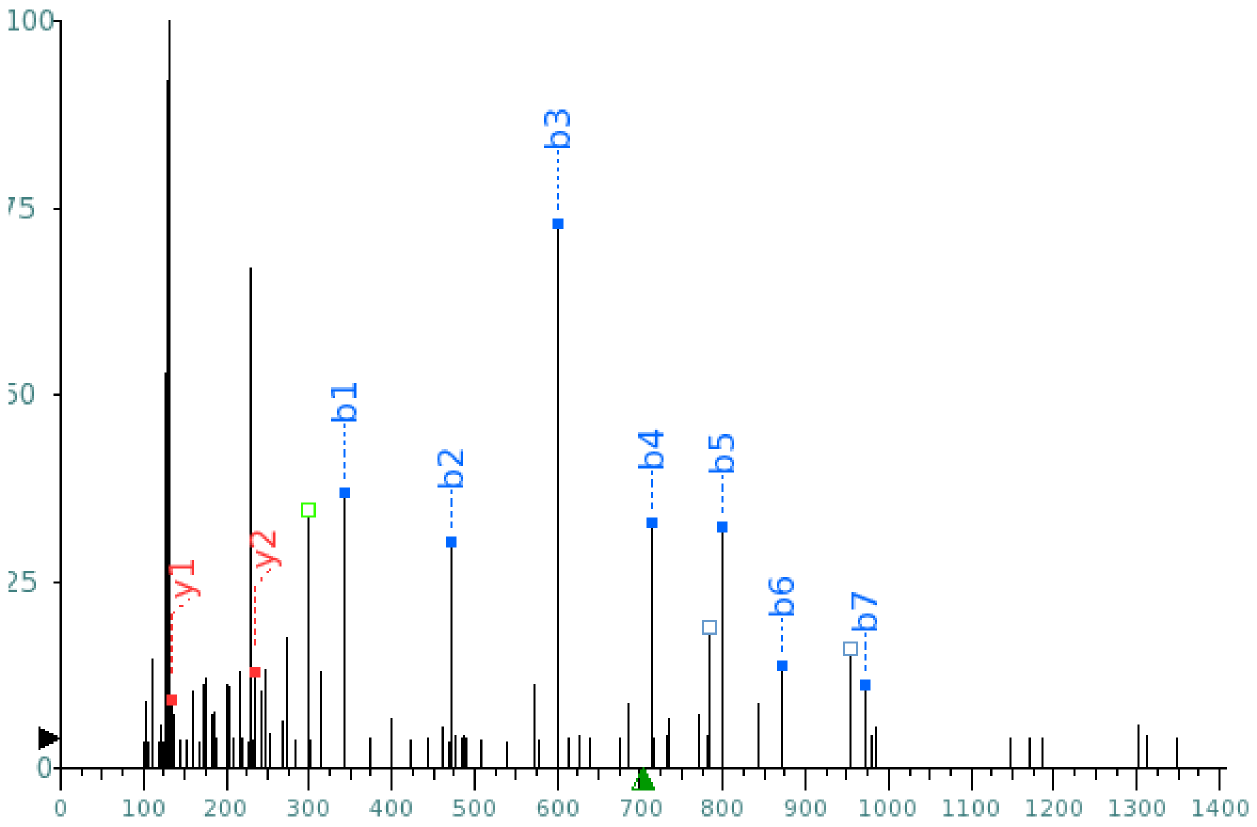

IQELSATVTTD (+2)

Primary Reference: TRIP6_HUMAN
Search ID: 41760
Search Name: 20130328_ananiav_ERstress_TMT_TM_timecourse_trypsin_inj1_4mc_iaa
Scan#: 13899
Observed Mass: 703.8842 (2.5 ppm)
PSM Score: 37.82

File name: 2015-02-04 04.01.53PM.pdf

Creation time: 2015-02-04 04:09PM

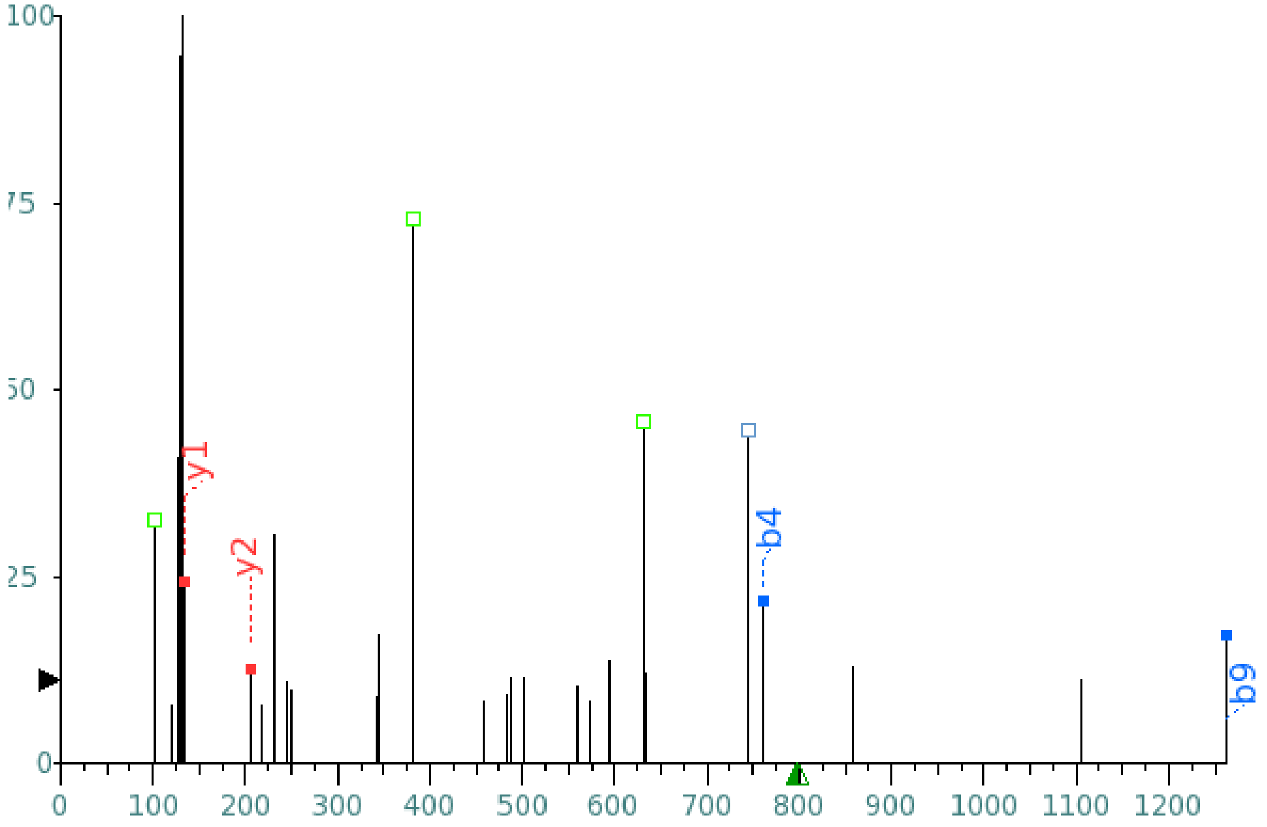

LRFDIEGSDEAD (+2)

Primary Reference: RB_HUMAN
Search ID: 41760
Search Name: 20130328_ananiav_ERstress_TMT_TM_timecourse_trypsin_inj1_4mc_iaa
Scan#: 13917
Observed Mass: 798.3923 (1.5 ppm)
PSM Score: 18.78

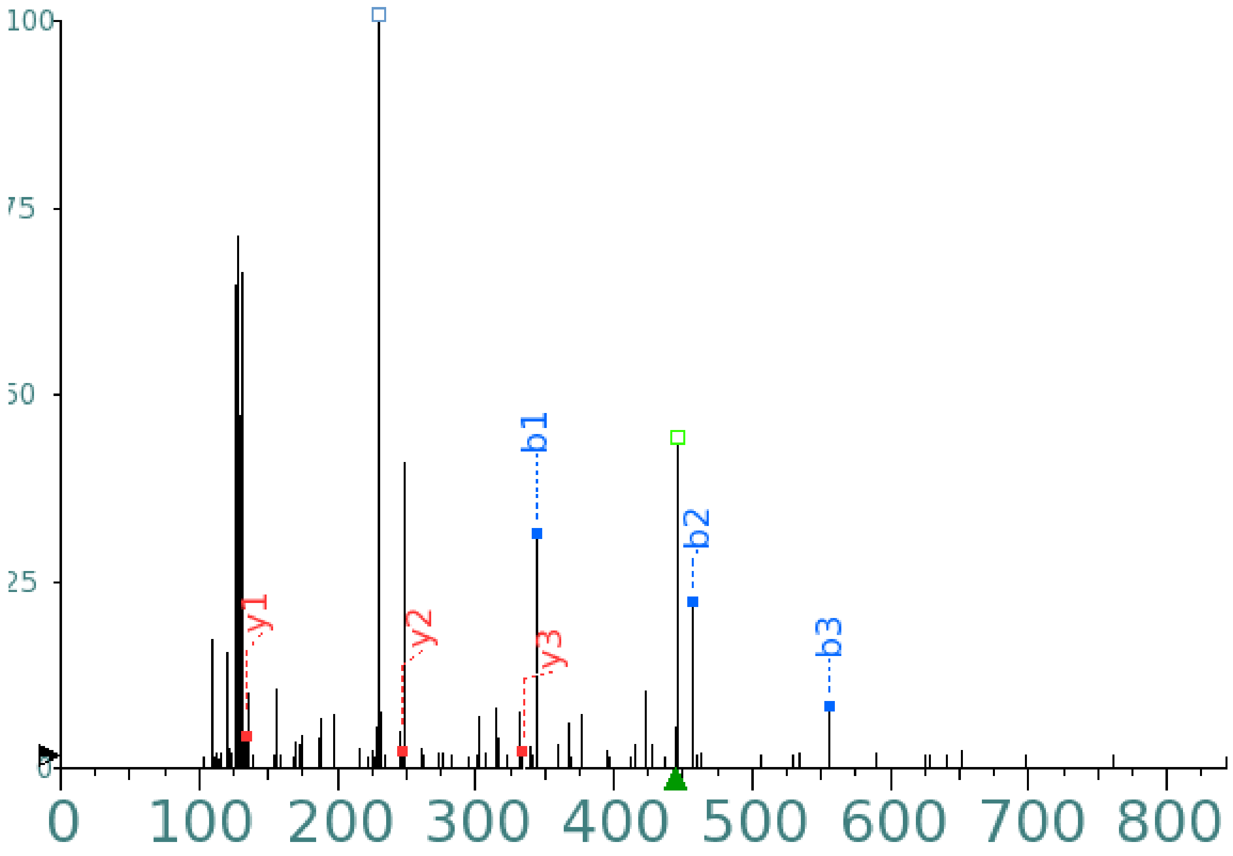

ILVSLD (+2)

Primary Reference: ##C9J6I7_HUMAN
Search ID: 41760
Search Name: 20130328_ananiav_ERstress_TMT_TM_timecourse_trypsin_inj1_4mc_iaa
Scan#: 13933
Observed Mass: 444.7851 (2.9 ppm)
PSM Score: 8.61

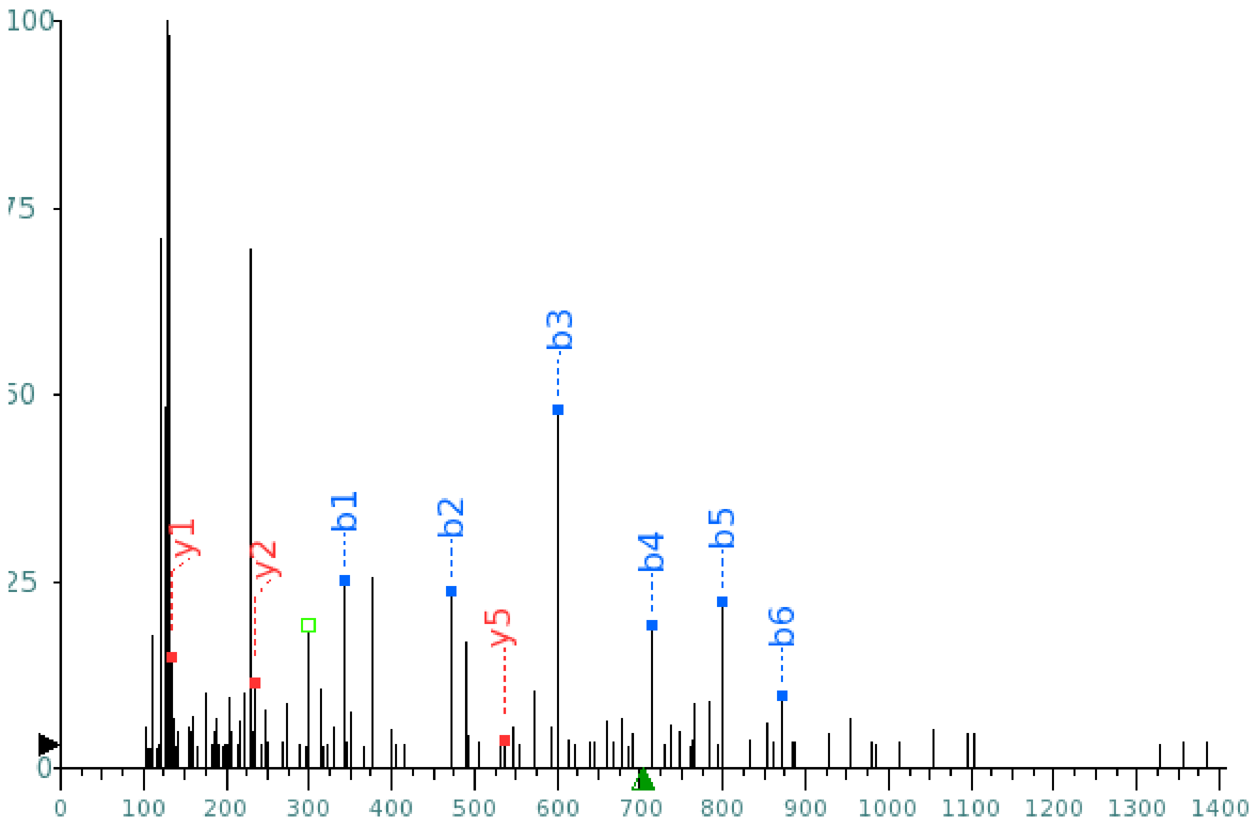

IQELSATVTTD (+2)

Primary Reference: TRIP6_HUMAN
Search ID: 41760
Search Name: 20130328_ananiav_ERstress_TMT_TM_timecourse_trypsin_inj1_4mc_iaa
Scan#: 13977
Observed Mass: 703.8853 (4.1 ppm)
PSM Score: 21.55

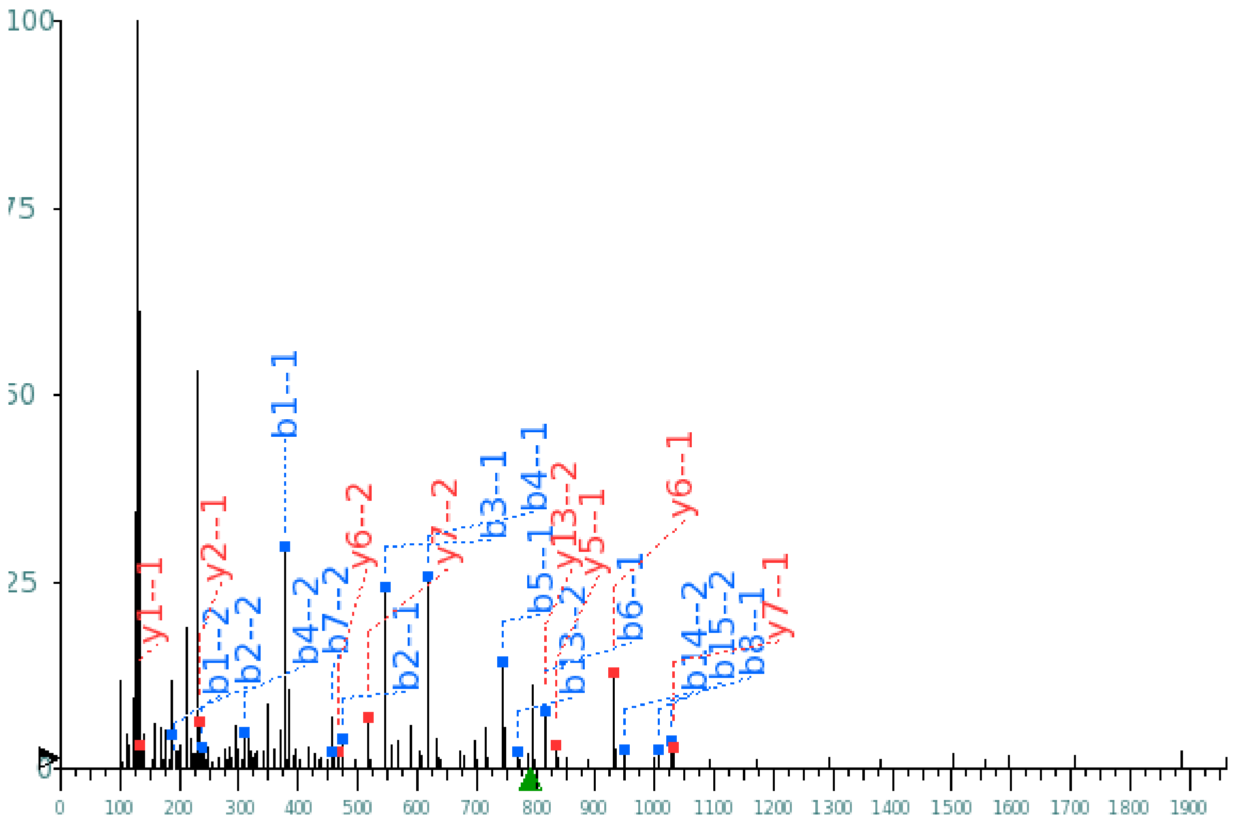

FPAAQAPDPSETPKIETD (+3)

Primary Reference: ABLM1_HUMAN
Search ID: 41760
Search Name: 20130328_ananiav_ERstress_TMT_TM_timecourse_trypsin_inj1_4mc_iaa
Scan#: 13991
Observed Mass: 791.4208 (3.9 ppm)
PSM Score: 22.56

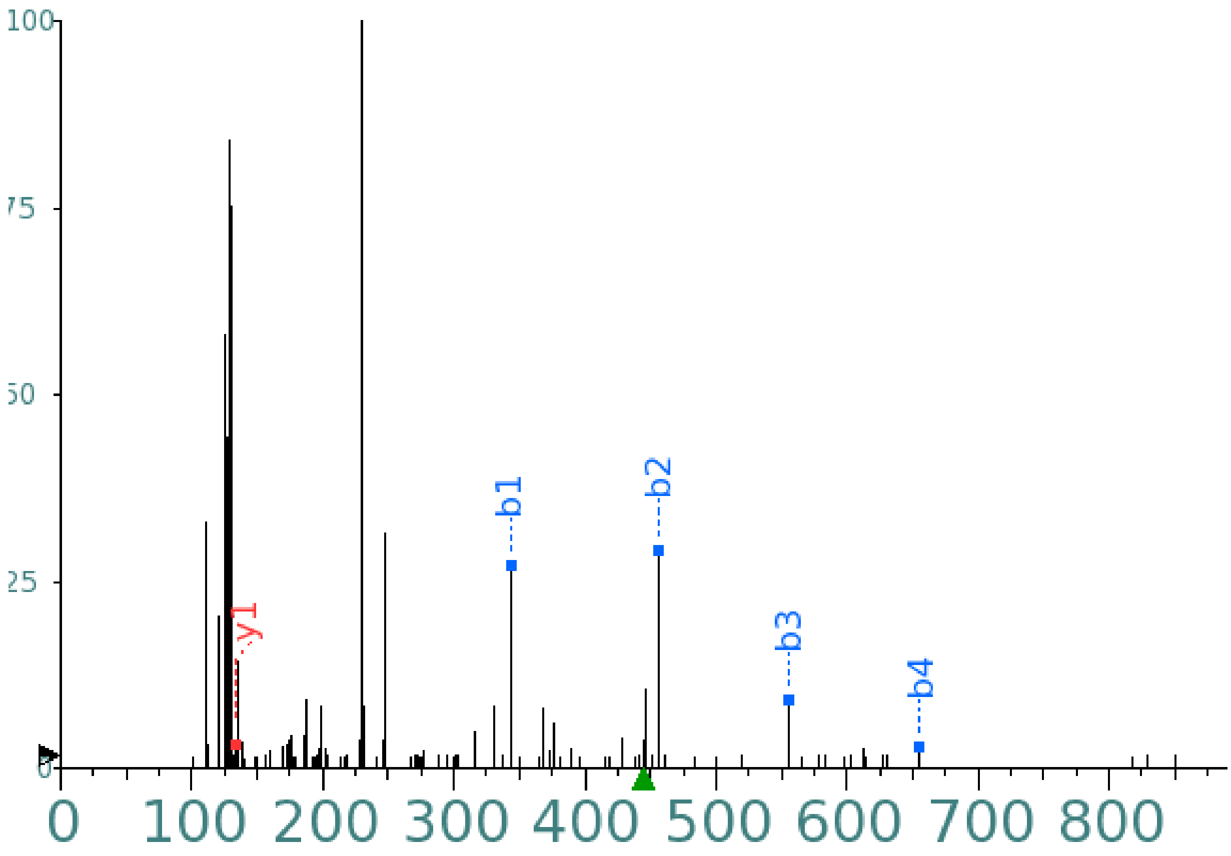

LIVVTD (+2)

Primary Reference: ZN187_HUMAN
Search ID: 41760
Search Name: 20130328_ananiav_ERstress_TMT_TM_timecourse_trypsin_inj1_4mc_iaa
Scan#: 14013
Observed Mass: 444.7851 (2.9 ppm)
PSM Score: 8.47

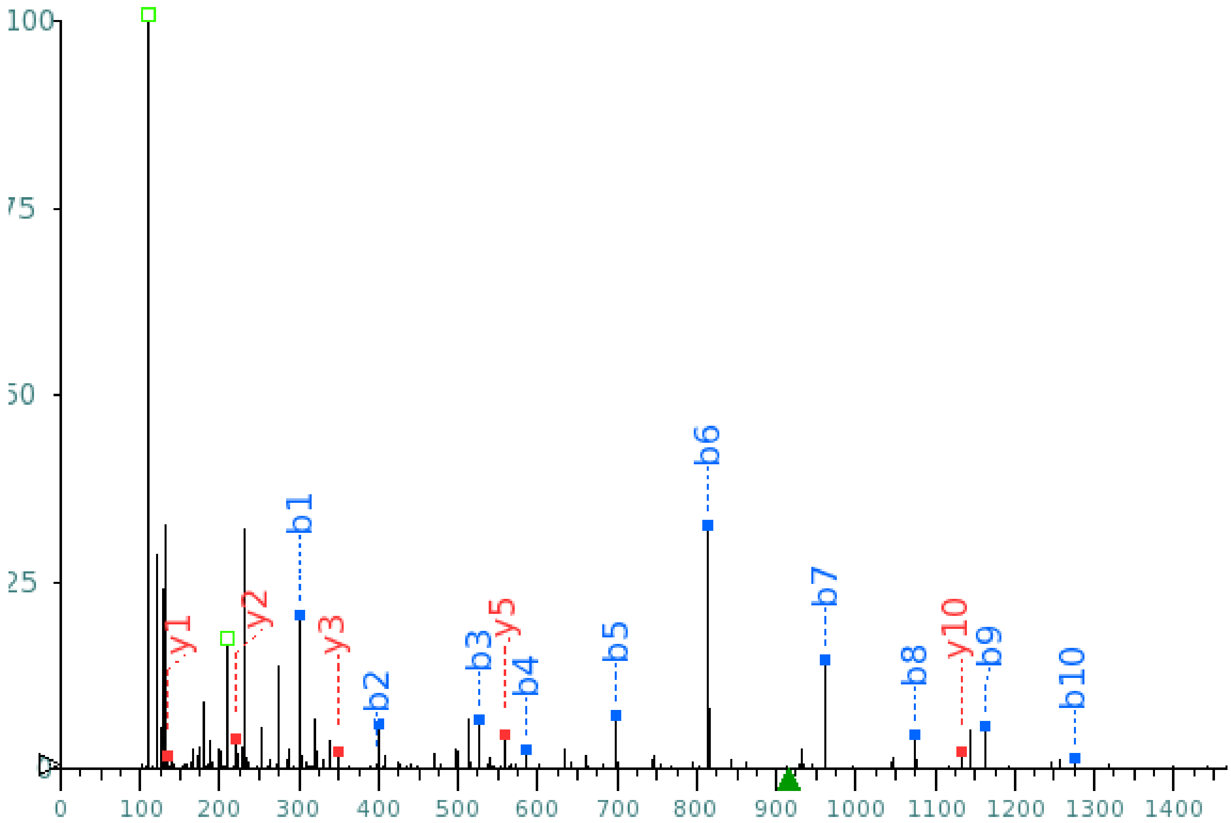

APEGDDFLSLHAESD (+2)

Primary Reference: SFR19_HUMAN
Search ID: 41760
Search Name: 20130328_ananiav_ERstress_TMT_TM_timecourse_trypsin_inj1_4mc_iaa
Scan#: 14029
Observed Mass: 916.4337 (3 ppm)
PSM Score: 22.26

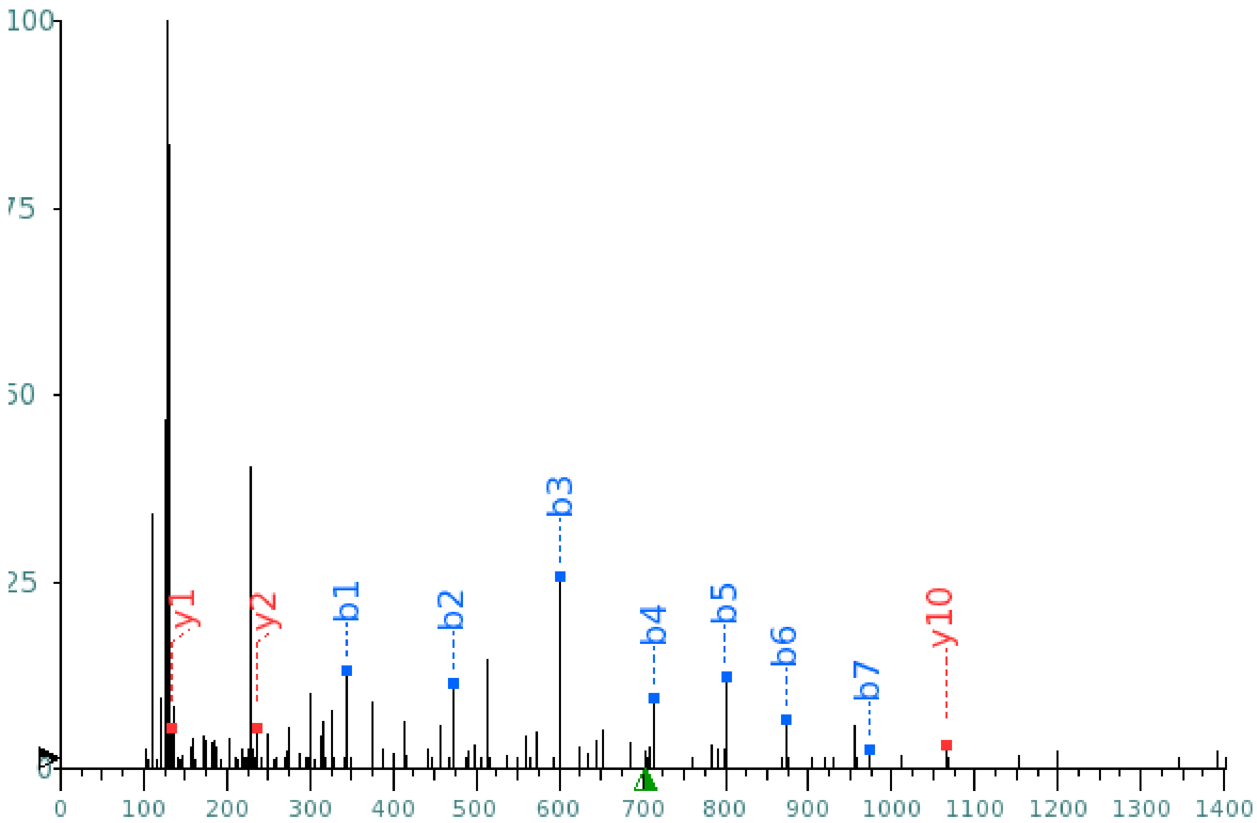

IQELSATVTTD (+2)

Primary Reference: TRIP6_HUMAN
Search ID: 41760
Search Name: 20130328_ananiav_ERstress_TMT_TM_timecourse_trypsin_inj1_4mc_iaa
Scan#: 14054
Observed Mass: 703.8851 (3.8 ppm)
PSM Score: 26.52

File name: 2015-02-04 04.01.53PM.pdf

Creation time: 2015-02-04 04:09PM

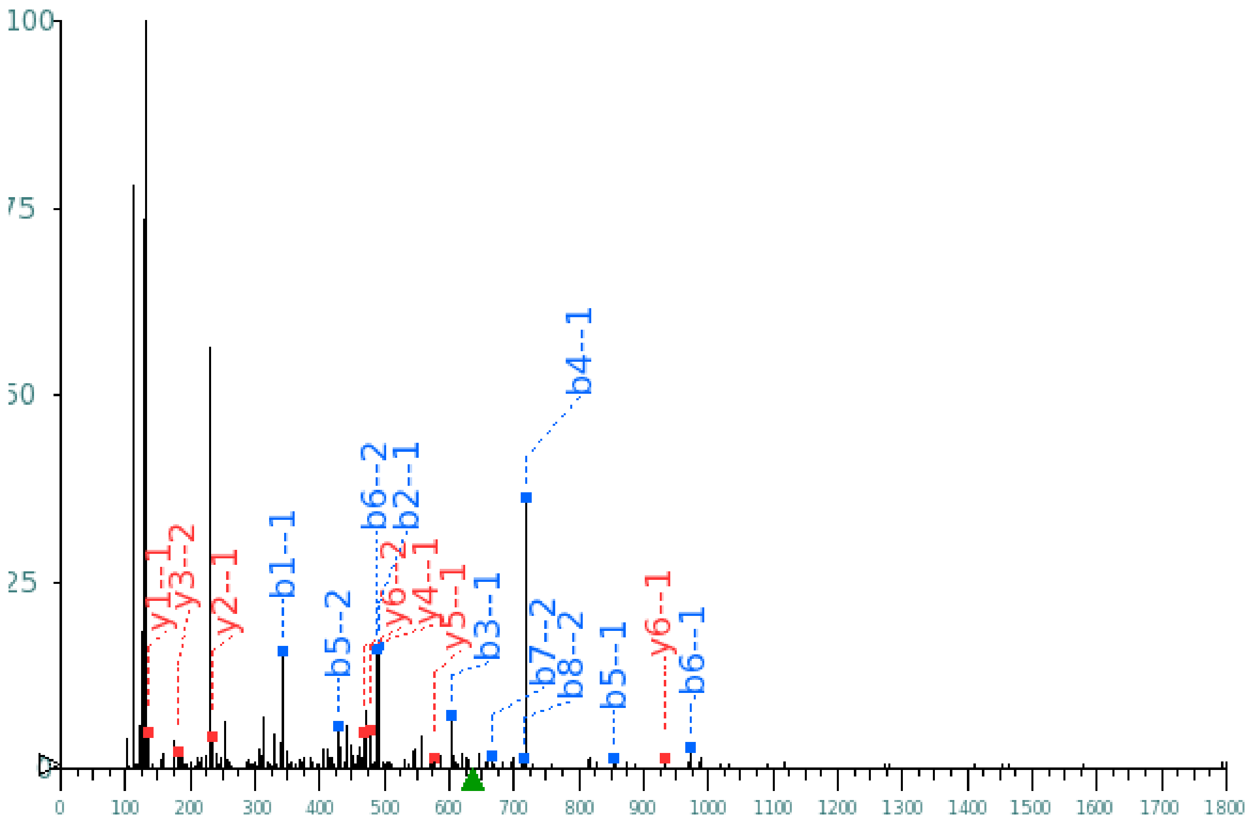

LFLDHDKTLQTD (+3)

Primary Reference: RB_HUMAN
Search ID: 41760
Search Name: 20130328_ananiav_ERstress_TMT_TM_timecourse_trypsin_inj1_4mc_iaa
Scan#: 14076
Observed Mass: 635.3595 (5.7 ppm)
PSM Score: 13.94

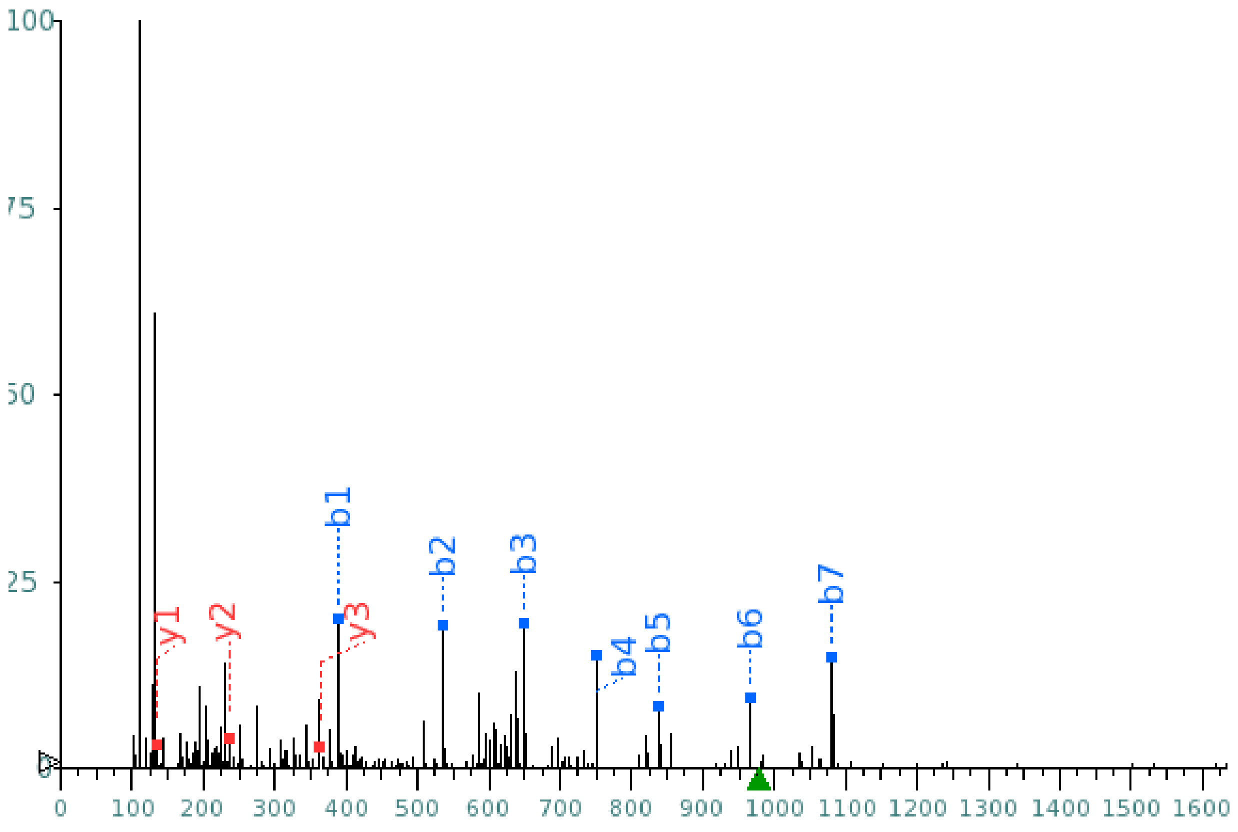

C*FLTSQNHGFAVETD (+2)

Primary Reference: PYR1_HUMAN
Search ID: 41760
Search Name: 20130328_ananiav_ERstress_TMT_TM_timecourse_trypsin_inj1_4mc_iaa
Scan#: 14099
Observed Mass: 977.9641 (2.1 ppm)
PSM Score: 31.14

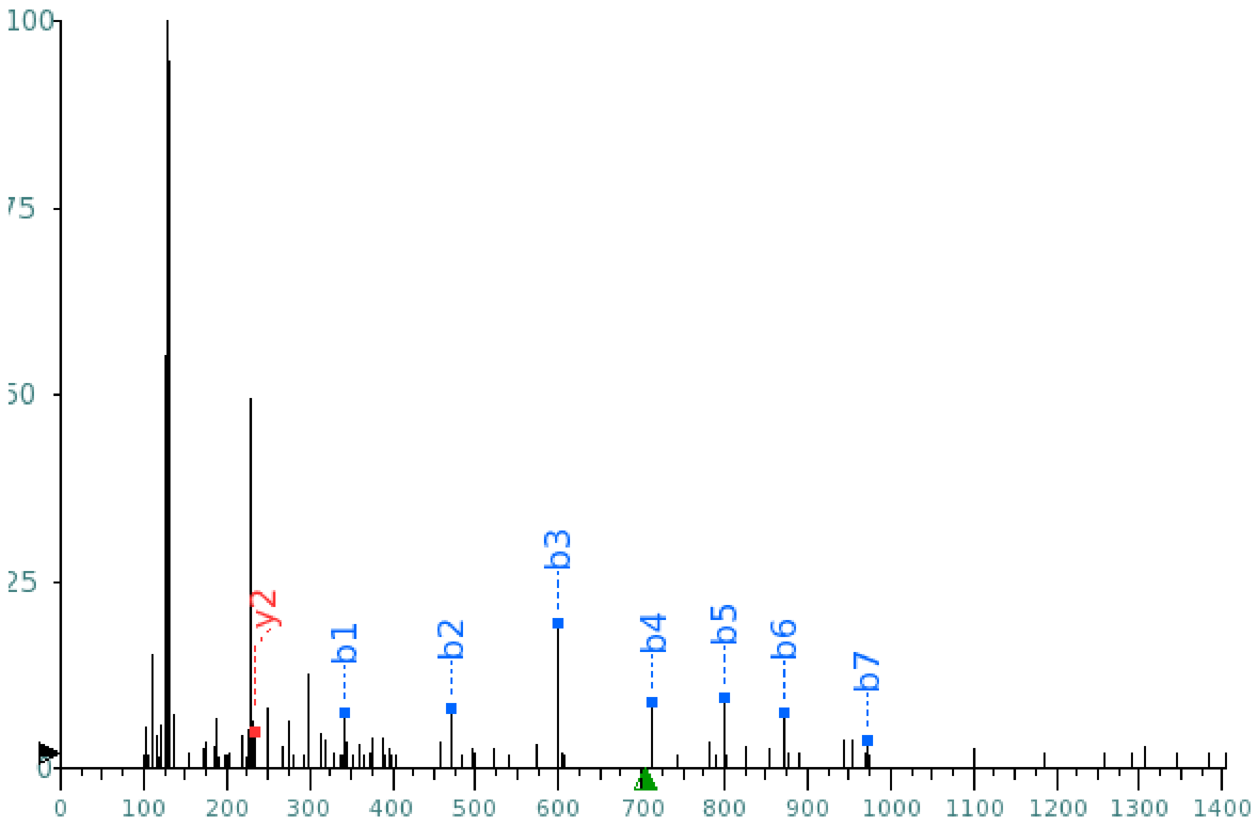

IQELSATVTTD (+2)

Primary Reference: TRIP6_HUMAN
Search ID: 41760
Search Name: 20130328_ananiav_ERstress_TMT_TM_timecourse_trypsin_inj1_4mc_iaa
Scan#: 14130
Observed Mass: 703.8847 (3.3 ppm)
PSM Score: 18.44

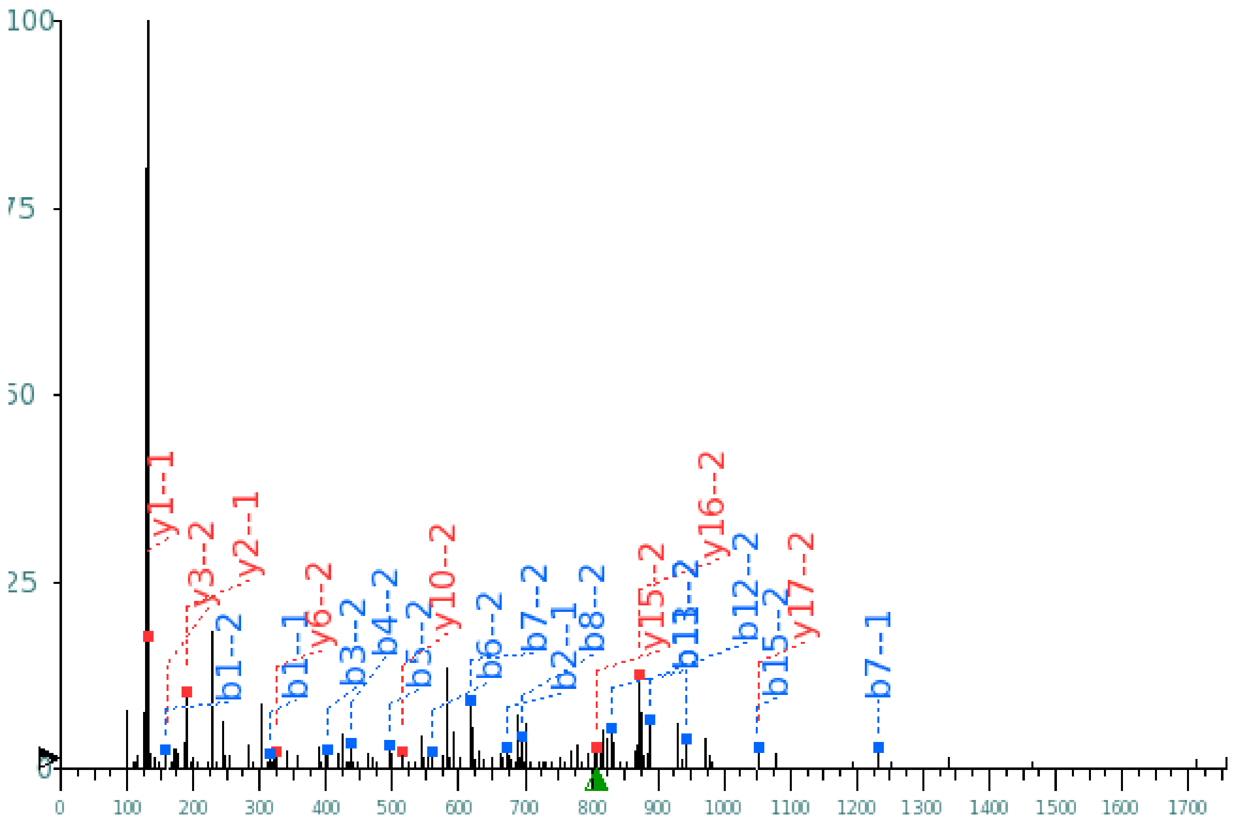

SKMALEDRPSSLLVDQGD (+3)

Primary Reference: TRIO_HUMAN
Search ID: 41760
Search Name: 20130328_ananiav_ERstress_TMT_TM_timecourse_trypsin_inj1_4mc_iaa
Scan#: 14131
Observed Mass: 807.104 (3 ppm)
PSM Score: 15.22

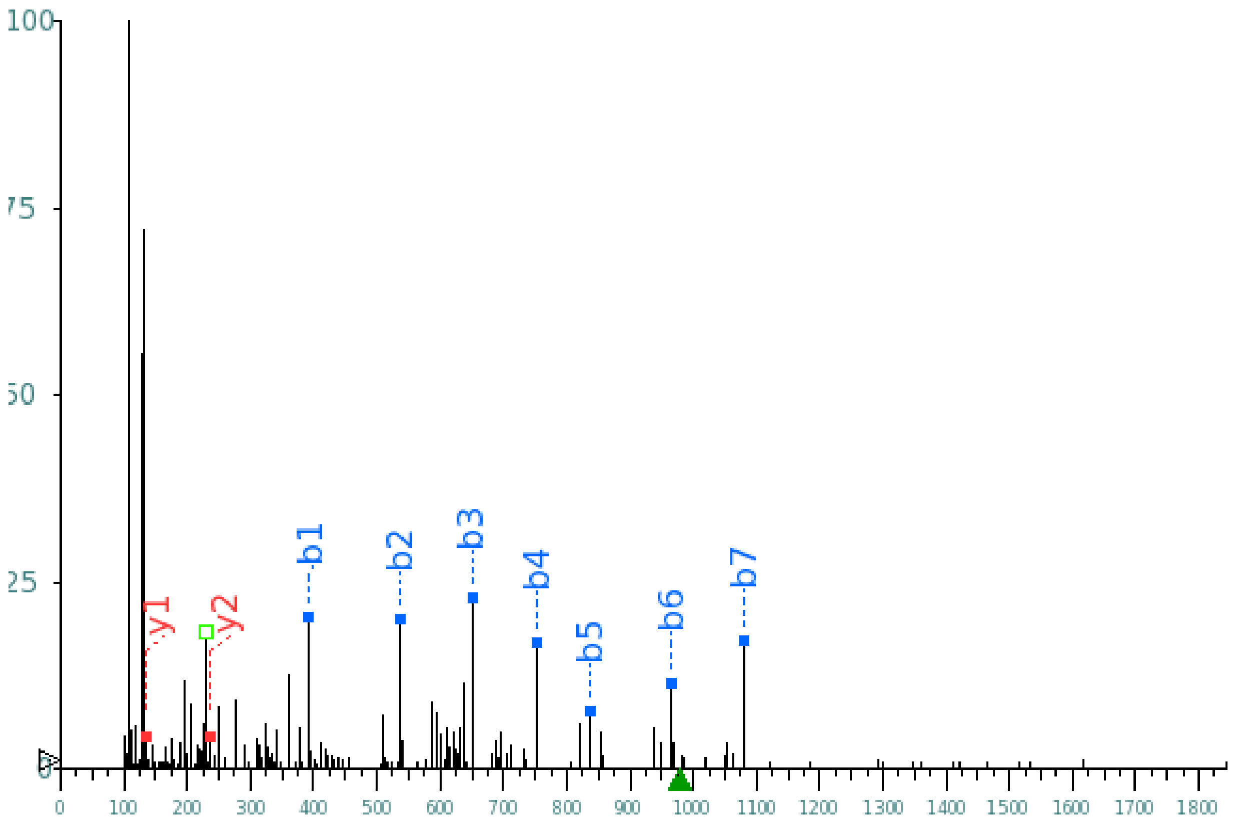

C*FLTSQNHGFAVETD (+2)

Primary Reference: PYR1_HUMAN
Search ID: 41760
Search Name: 20130328_ananiav_ERstress_TMT_TM_timecourse_trypsin_inj1_4mc_iaa
Scan#: 14175
Observed Mass: 977.9647 (2.8 ppm)
PSM Score: 29.4

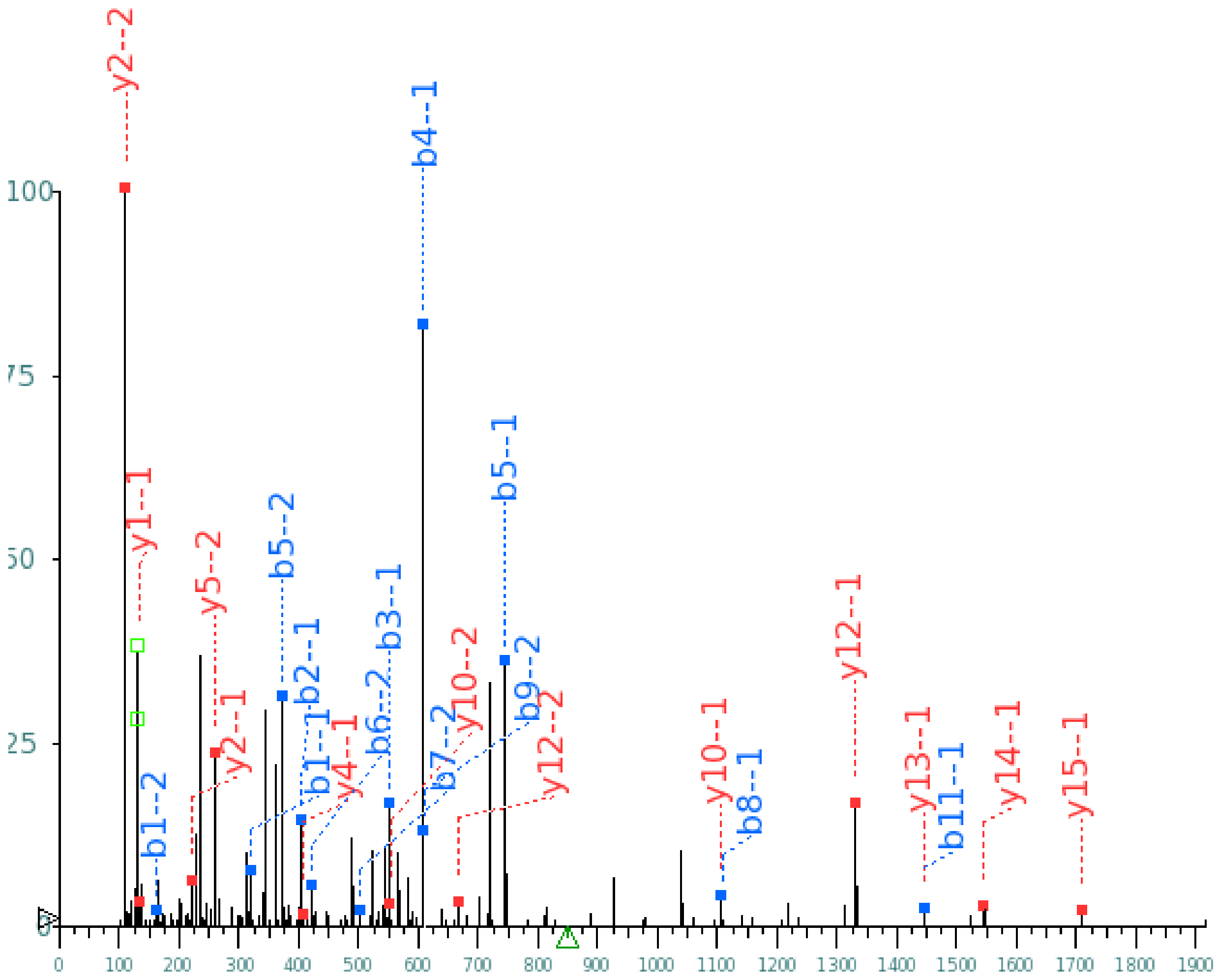

SSFGHPYTIPESLRVQDDASD (+3)

Primary Reference: TTC28_HUMAN

Search ID: 41760

Search Name: 20130328_ananiav_ERstress_TMT_TM_timecourse_trypsin_inj1_4mc_iaa

Scan#: 14183

Observed Mass: 850.7522 (4.3 ppm)

PSM Score: 28.79

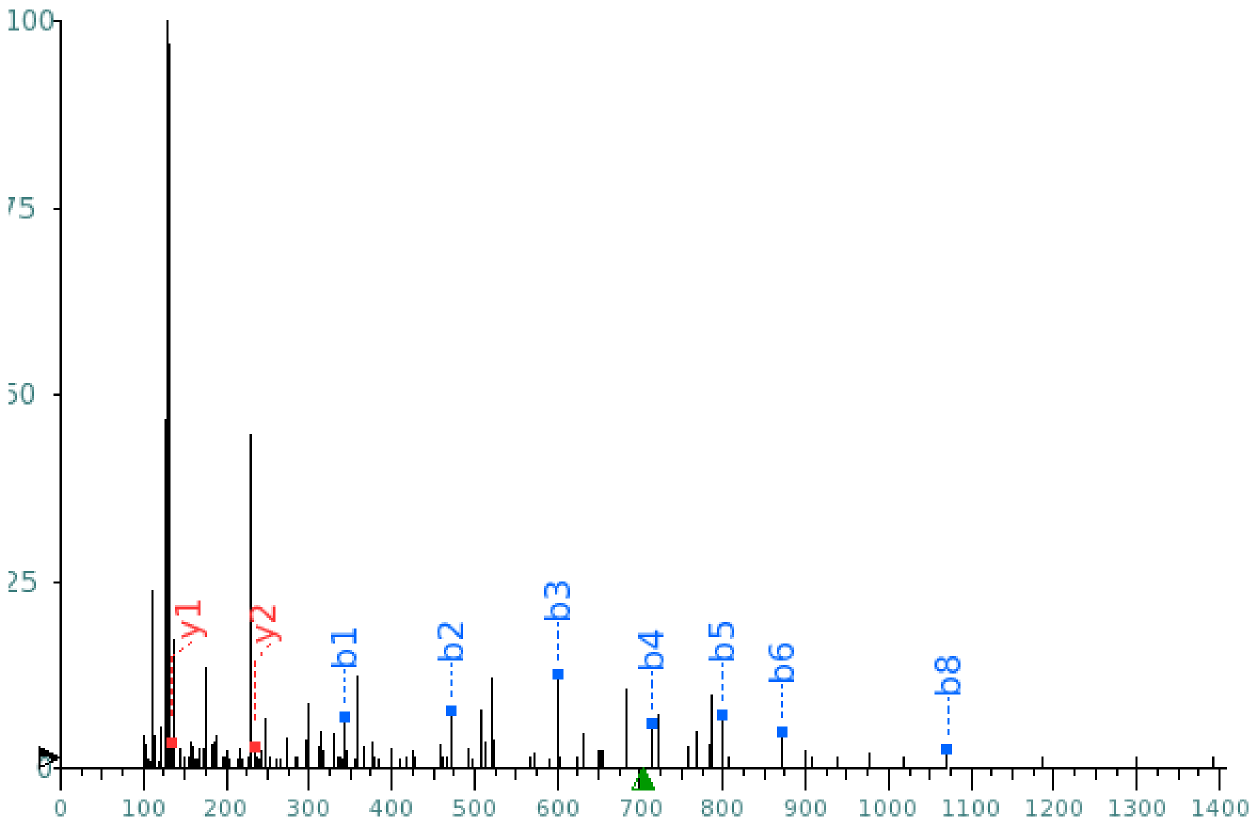

IQELSATVTTD (+2)

Primary Reference: TRIP6_HUMAN
Search ID: 41760
Search Name: 20130328_ananiav_ERstress_TMT_TM_timecourse_trypsin_inj1_4mc_iaa
Scan#: 14210
Observed Mass: 703.8842 (2.5 ppm)
PSM Score: 11.83

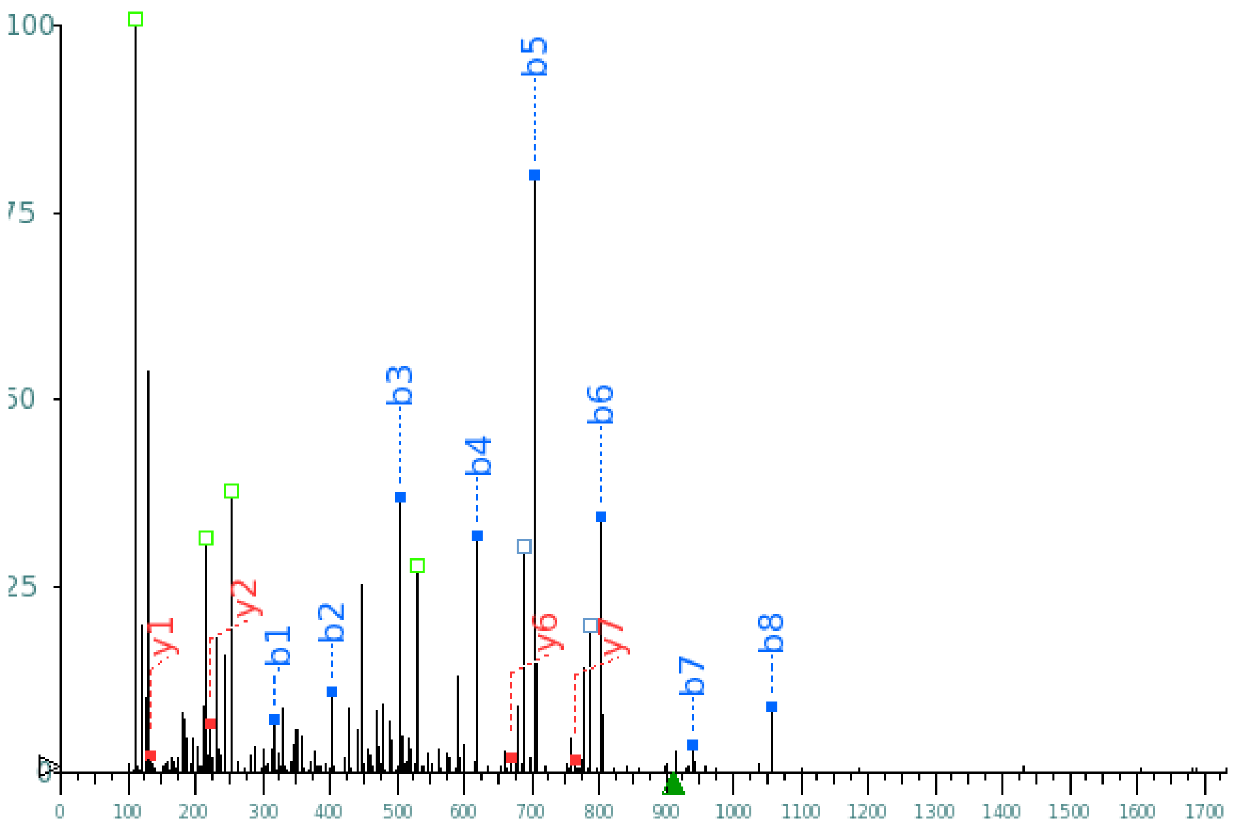

SSTISVHDPFSDVSD (+2)

Primary Reference: ARI1B_HUMAN
Search ID: 41760
Search Name: 20130328_ananiav_ERstress_TMT_TM_timecourse_trypsin_inj1_4mc_iaa
Scan#: 14211
Observed Mass: 911.4409 (2.2 ppm)
PSM Score: 40.36

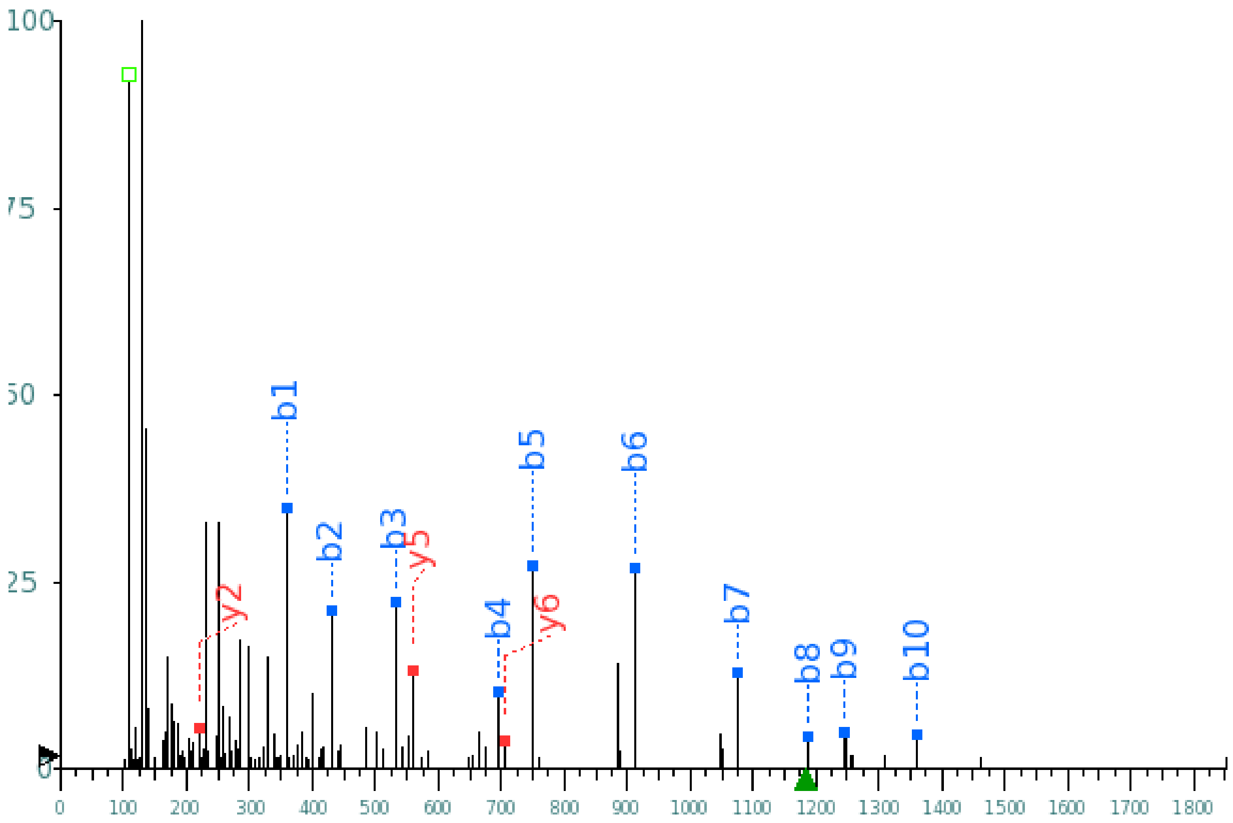

QATYGYYLGNPAEFHDSSD (+2)

Primary Reference: RCN1_HUMAN
Search ID: 41760
Search Name: 20130328_ananiav_ERstress_TMT_TM_timecourse_trypsin_inj1_4mc_iaa
Scan#: 14267
Observed Mass: 1182.5374 (2.4 ppm)
PSM Score: 69.26

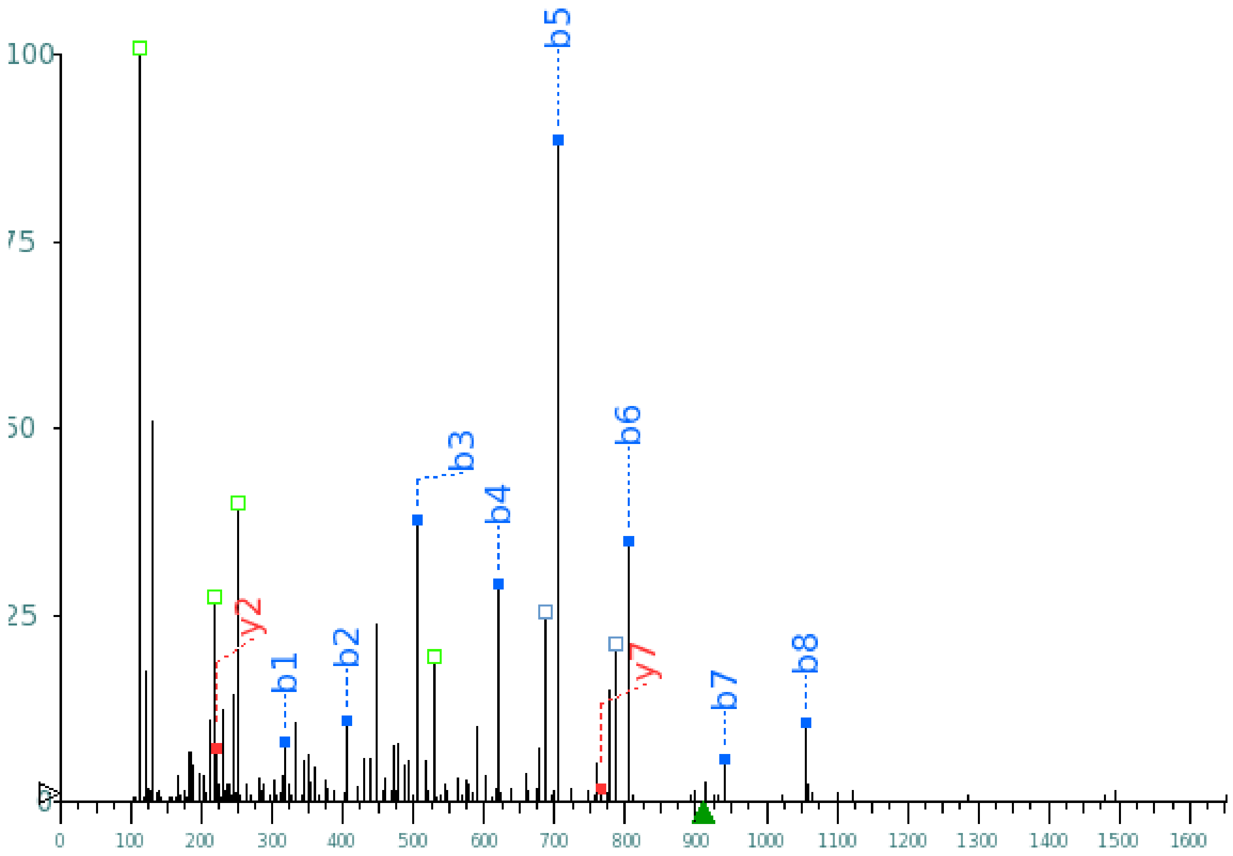

SSTISVHDPFSDVSD (+2)

Primary Reference: ARI1B_HUMAN
Search ID: 41760
Search Name: 20130328_ananiav_ERstress_TMT_TM_timecourse_trypsin_inj1_4mc_iaa
Scan#: 14285
Observed Mass: 911.4409 (2.3 ppm)
PSM Score: 40.62

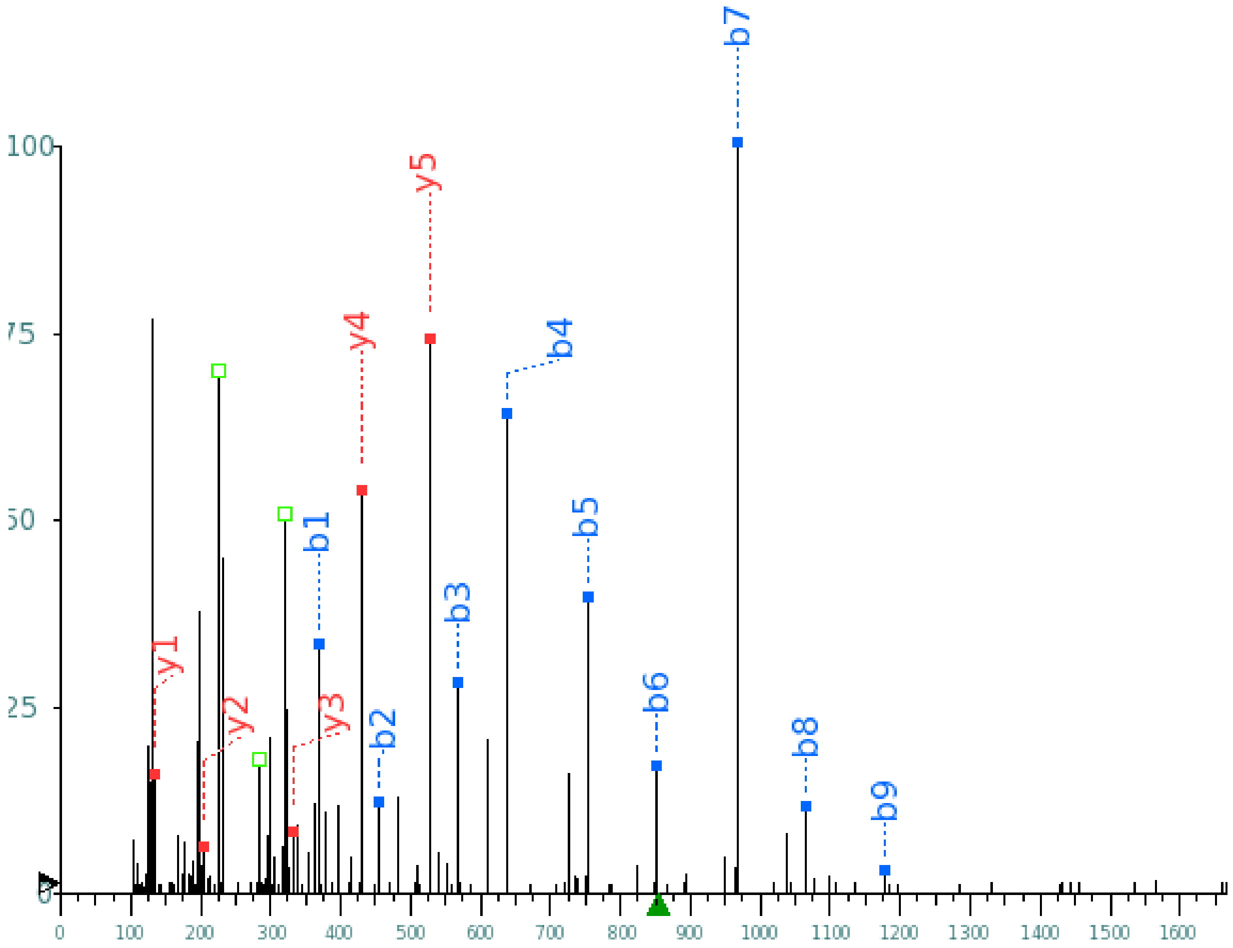

HSNALVDVLPPEAD (+2)

Primary Reference: PRS6B_HUMAN
Search ID: 41760
Search Name: 20130328_ananiav_ERstress_TMT_TM_timecourse_trypsin_inj1_4mc_iaa
Scan#: 14300
Observed Mass: 853.4553 (4.3 ppm)
PSM Score: 39.08

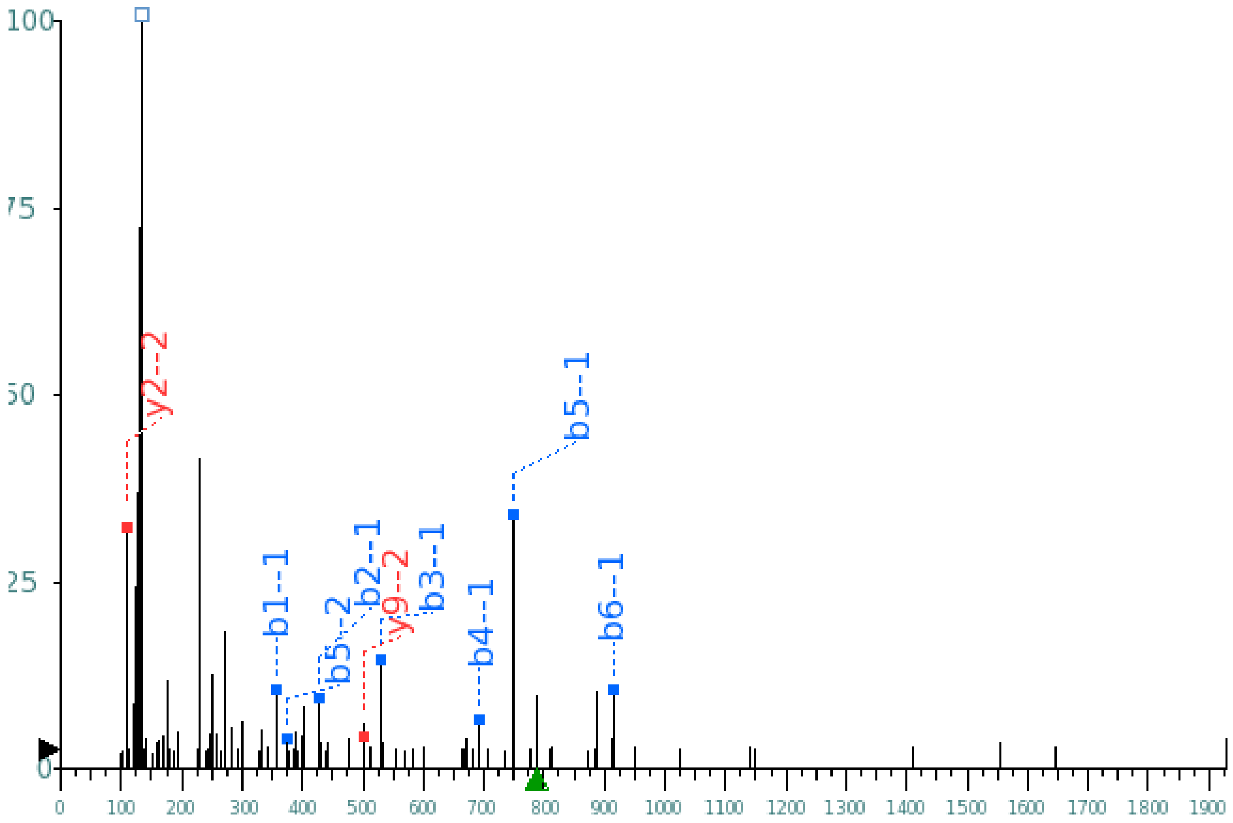

QATYGYYLGNPAEFHDSSD (+3)

Primary Reference: RCN1_HUMAN
Search ID: 41760
Search Name: 20130328_ananiav_ERstress_TMT_TM_timecourse_trypsin_inj1_4mc_iaa
Scan#: 14310
Observed Mass: 788.6933 (1.6 ppm)
PSM Score: 18.74

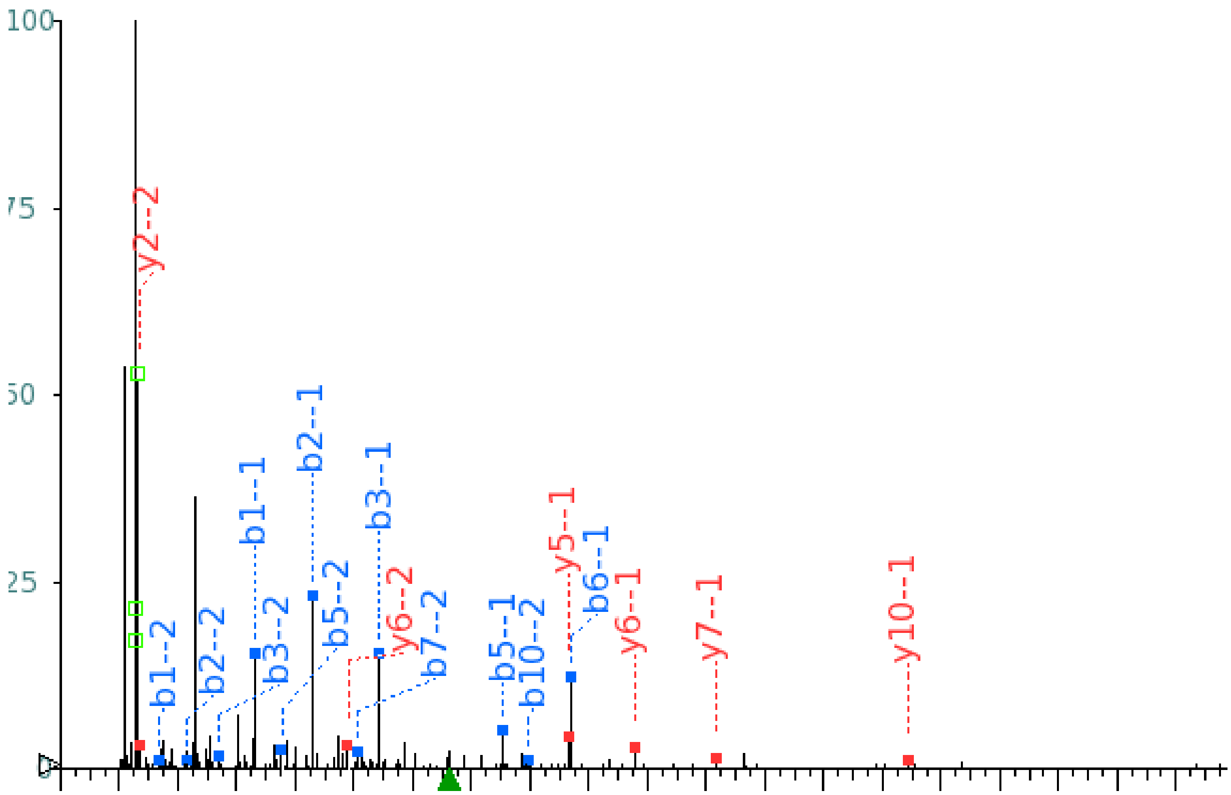

VVLPDDHLKDEMD (+3)

Primary Reference: RTN3_HUMAN
Search ID: 41760
Search Name: 20130328_ananiav_ERstress_TMT_TM_timecourse_trypsin_inj1_4mc_iaa
Scan#: 14365
Observed Mass: 662.0216 (2.1 ppm)
PSM Score: 12.39

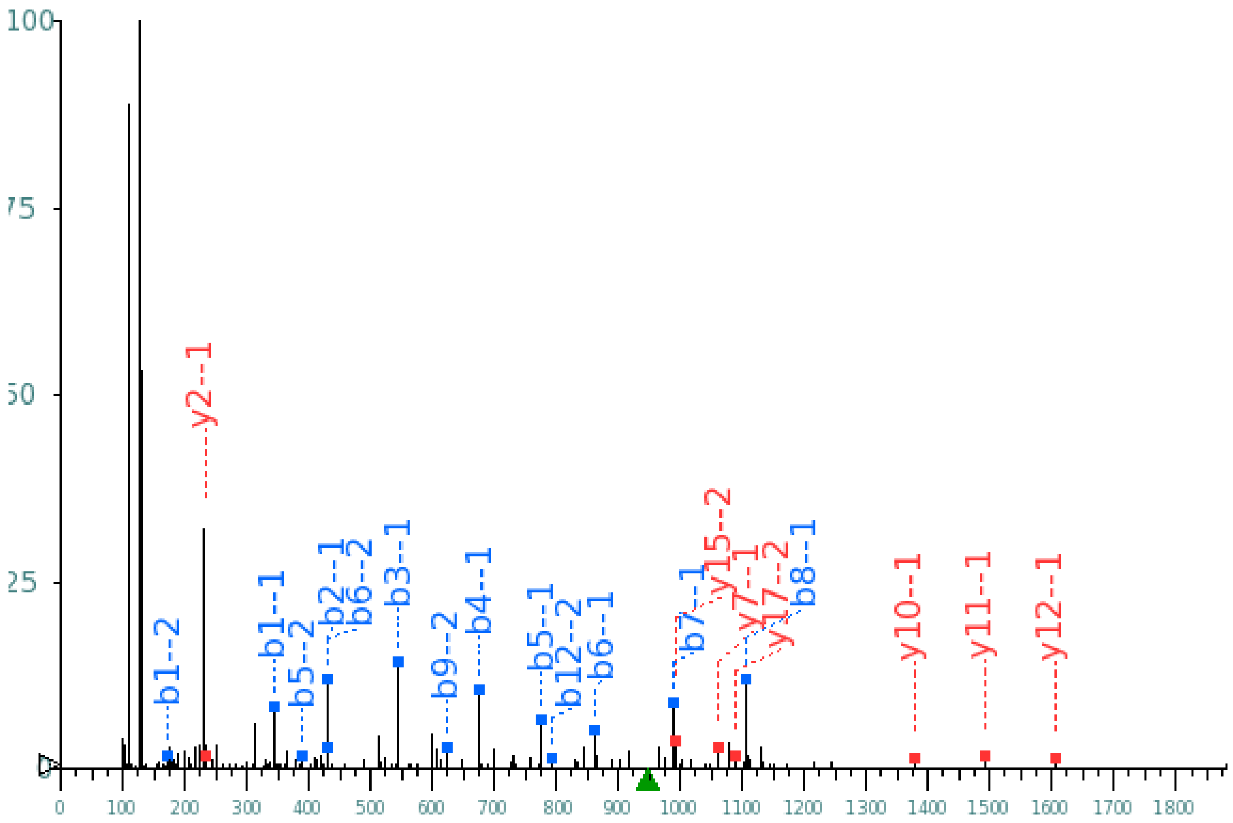

LSIMTSENHLNNSDKEVDEVD (+3)

Primary Reference: FERM2_HUMAN
Search ID: 41760
Search Name: 20130328_ananiav_ERstress_TMT_TM_timecourse_trypsin_inj1_4mc_iaa
Scan#: 14386
Observed Mass: 949.8095 (2 ppm)
PSM Score: 49.66

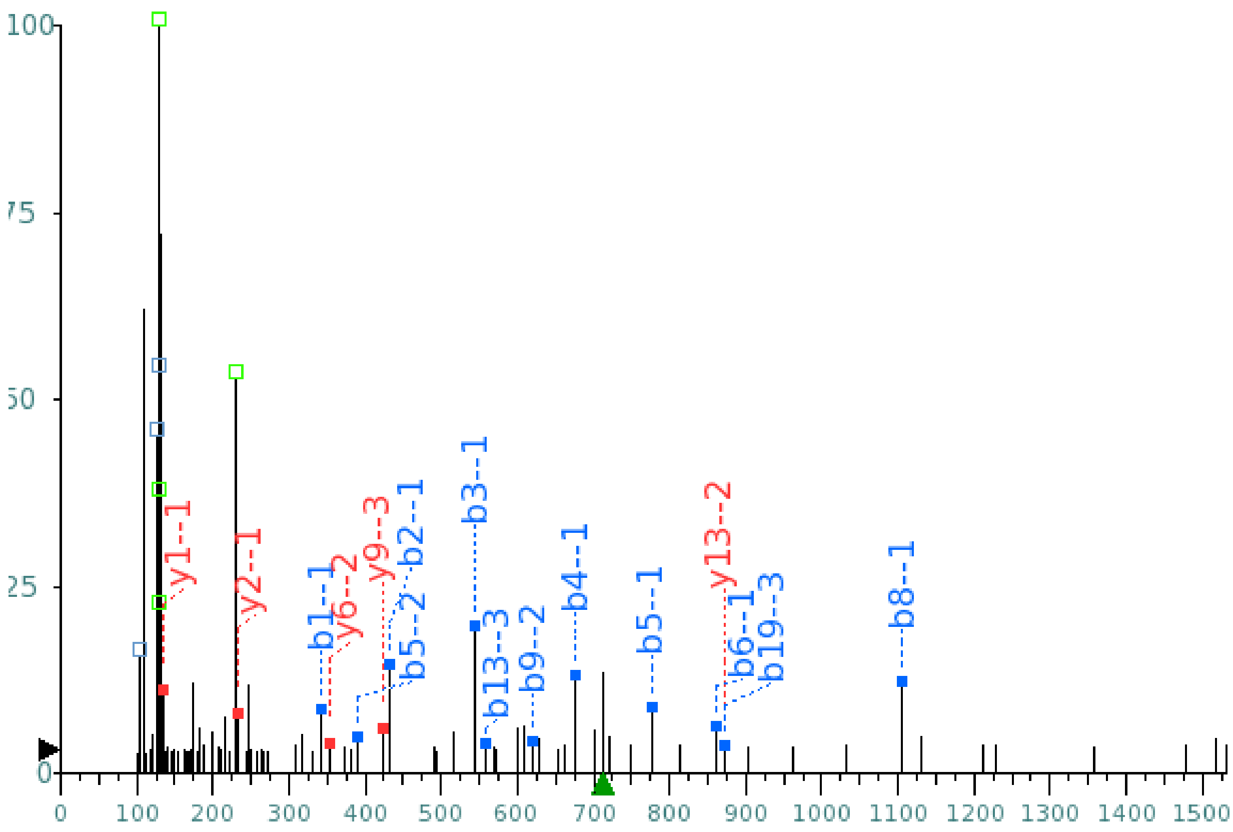

LSIMTSENHLNNSDKEVDEVD (+4)

Primary Reference: FERM2_HUMAN
Search ID: 41760
Search Name: 20130328_ananiav_ERstress_TMT_TM_timecourse_trypsin_inj1_4mc_iaa
Scan#: 14407
Observed Mass: 712.6091 (2.2 ppm)
PSM Score: 25.9

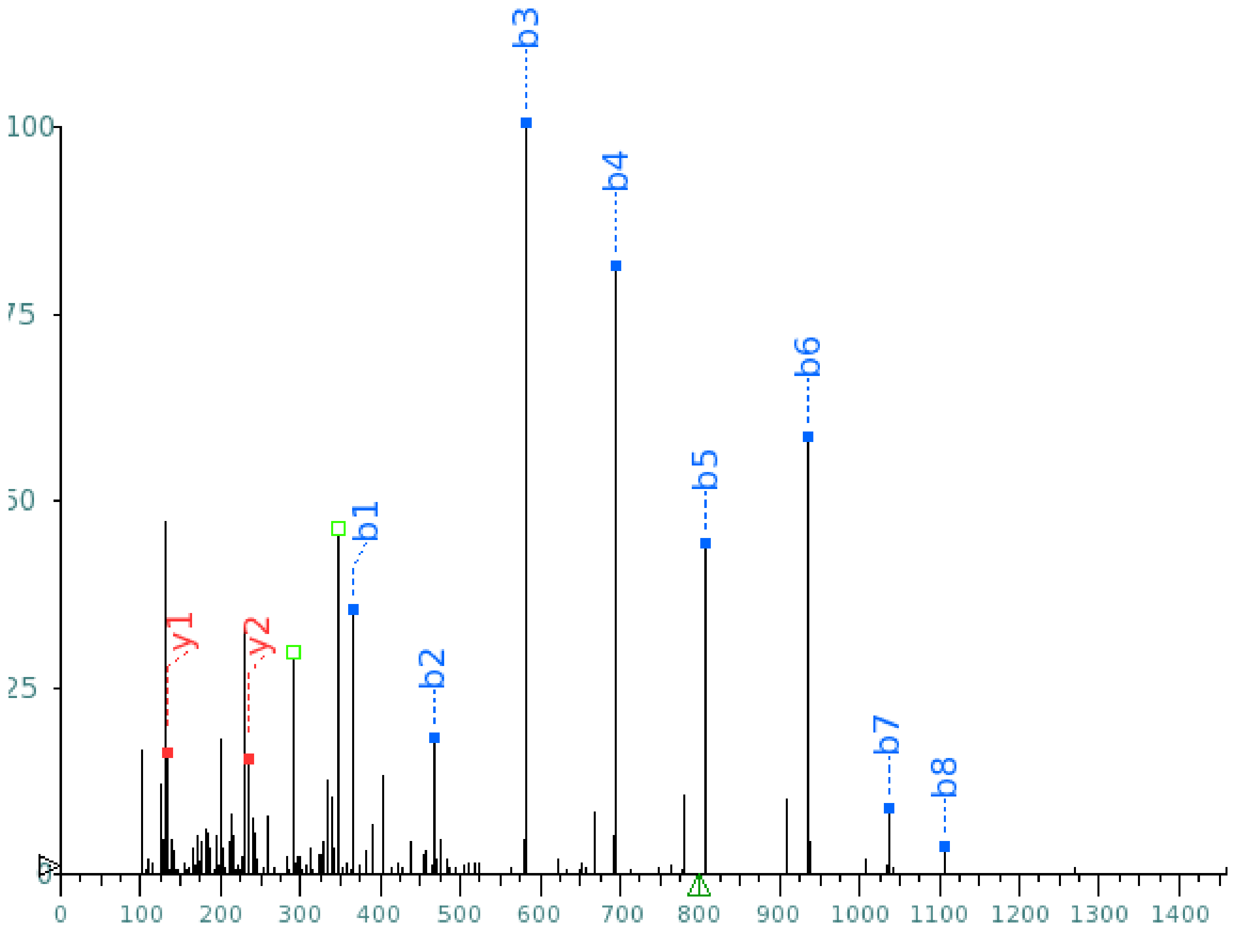

HVDLLEVAQETD (+2)

Primary Reference: ATAD1_HUMAN
Search ID: 41760
Search Name: 20130328_ananiav_ERstress_TMT_TM_timecourse_trypsin_inj1_4mc_iaa
Scan#: 14417
Observed Mass: 799.4182 (1.3 ppm)
PSM Score: 45.17

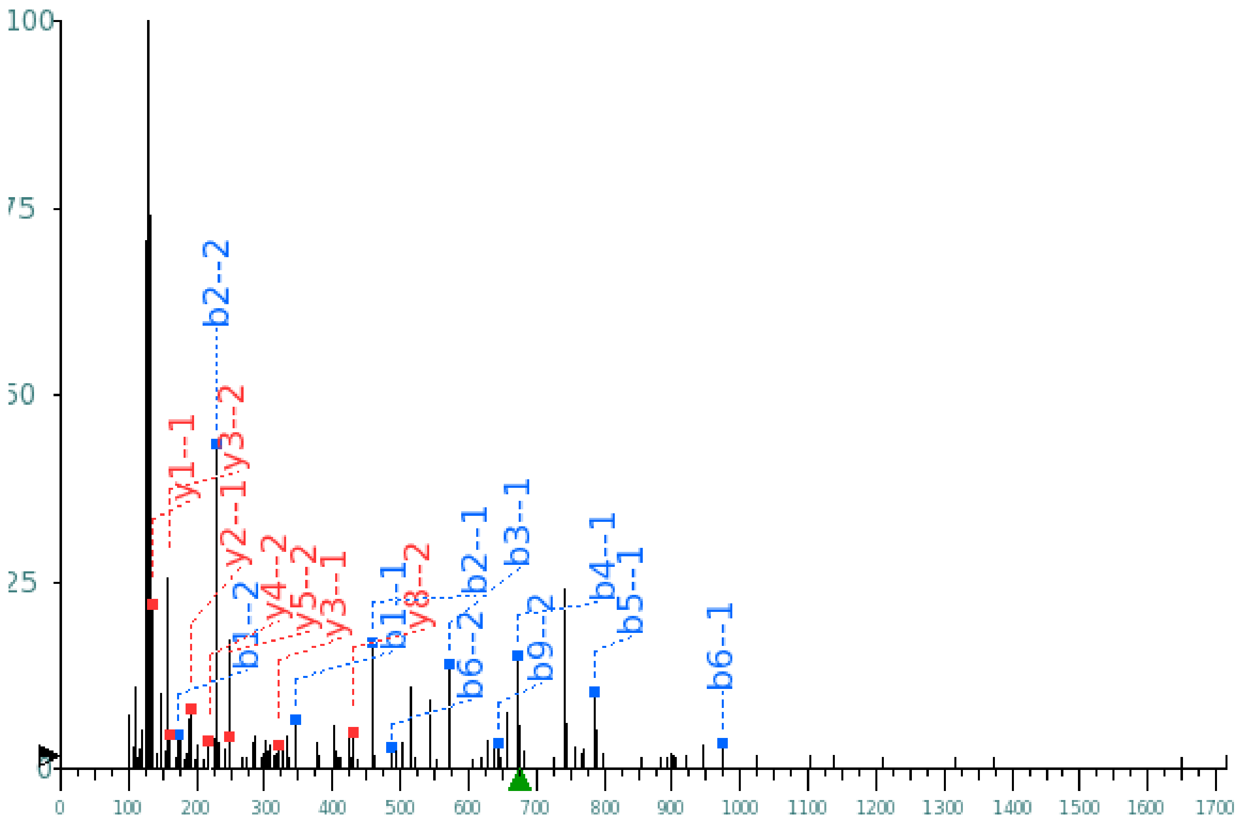

DNLTLWTSENQGDEGD (+3)

Primary Reference: 1433B_HUMAN
Search ID: 41760
Search Name: 20130328_ananiav_ERstress_TMT_TM_timecourse_trypsin_inj1_4mc_iaa
Scan#: 14420
Observed Mass: 674.9762 (2.6 ppm)
PSM Score: 11.11

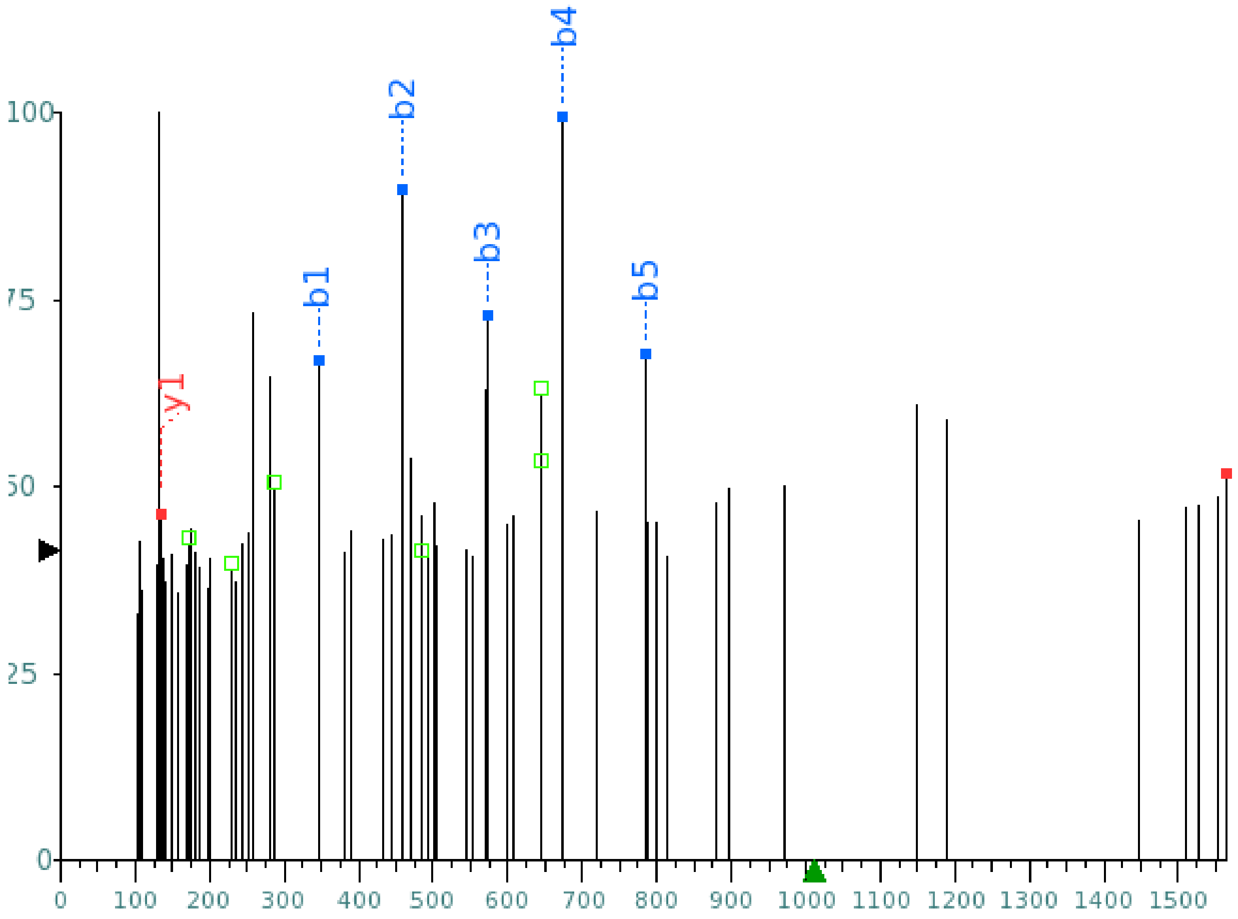

DNLTLWTSENQGDEGD (+2)

Primary Reference: 1433B_HUMAN
Search ID: 41760
Search Name: 20130328_ananiav_ERstress_TMT_TM_timecourse_trypsin_inj1_4mc_iaa
Scan#: 14427
Observed Mass: 1011.9598 (1.6 ppm)
PSM Score: 8.71

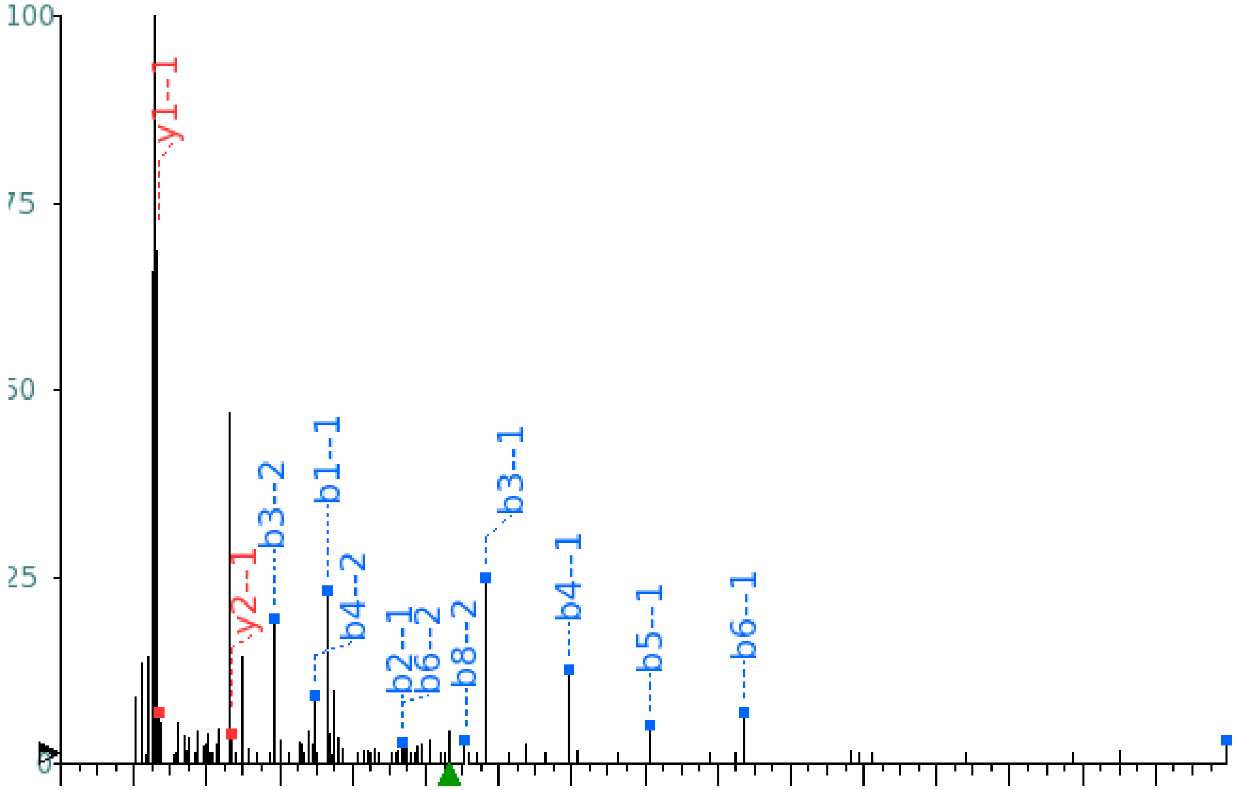

HVDLLEVAQETD (+3)

Primary Reference: ATAD1_HUMAN
Search ID: 41760
Search Name: 20130328_ananiav_ERstress_TMT_TM_timecourse_trypsin_inj1_4mc_iaa
Scan#: 14433
Observed Mass: 533.2813 (1.4 ppm)
PSM Score: 13.97

File name: 2015-02-04 04.01.53PM.pdf

Creation time: 2015-02-04 04:09PM

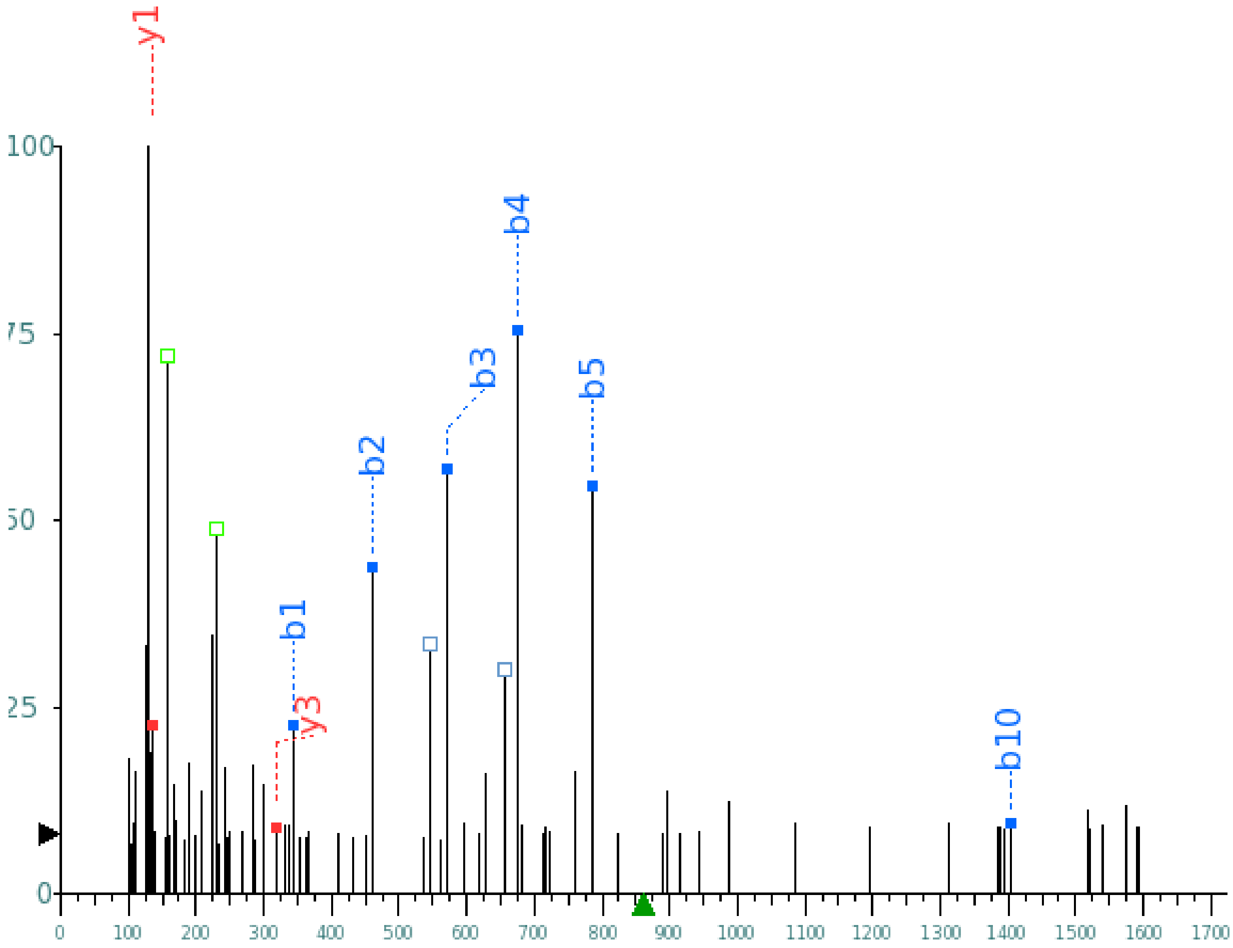

DNLTLWTSENQGD (+2)

Primary Reference: 1433B_HUMAN
Search ID: 41760
Search Name: 20130328_ananiav_ERstress_TMT_TM_timecourse_trypsin_inj1_4mc_iaa
Scan#: 14482
Observed Mass: 861.4141 (1.7 ppm)
PSM Score: 16.64

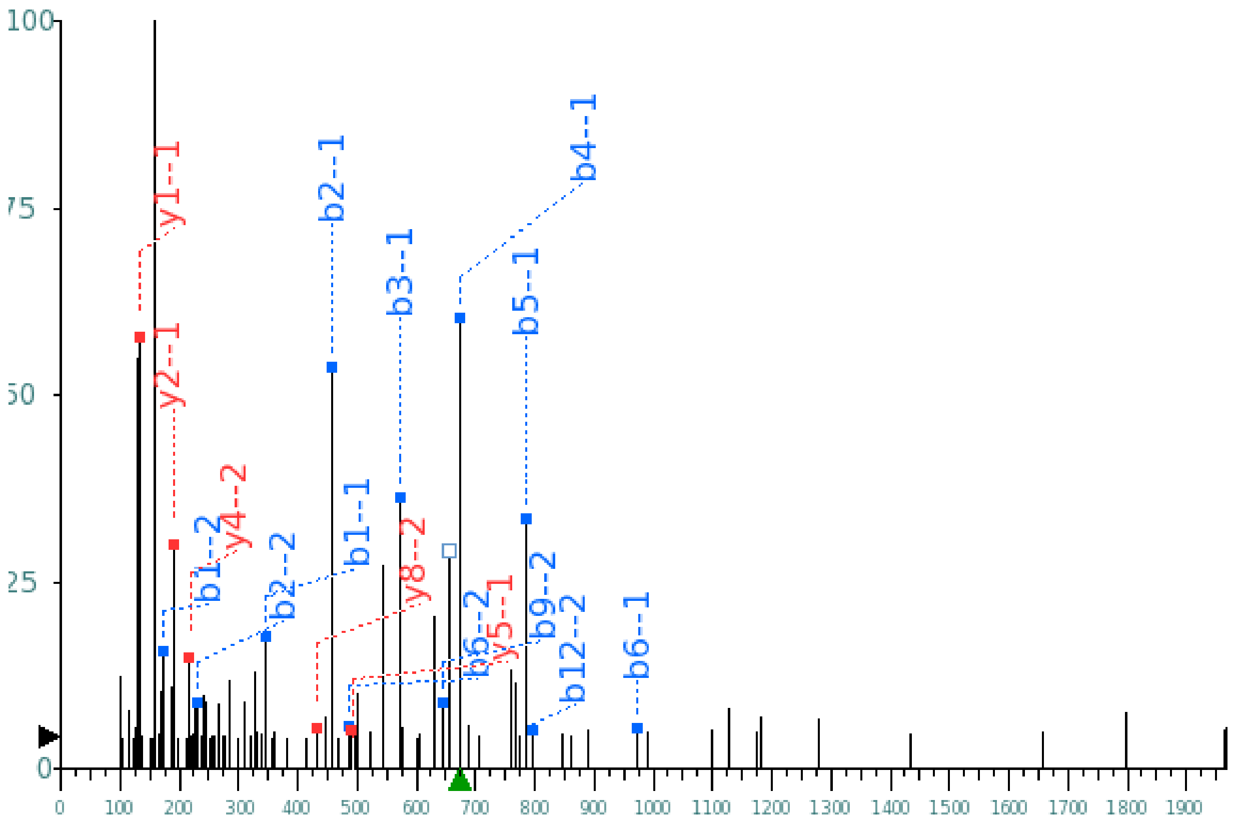

DNLTLWTSENQGDEGD (+3)

Primary Reference: 1433B_HUMAN
Search ID: 41760
Search Name: 20130328_ananiav_ERstress_TMT_TM_timecourse_trypsin_inj1_4mc_iaa
Scan#: 14504
Observed Mass: 674.976 (2.2 ppm)
PSM Score: 21.53

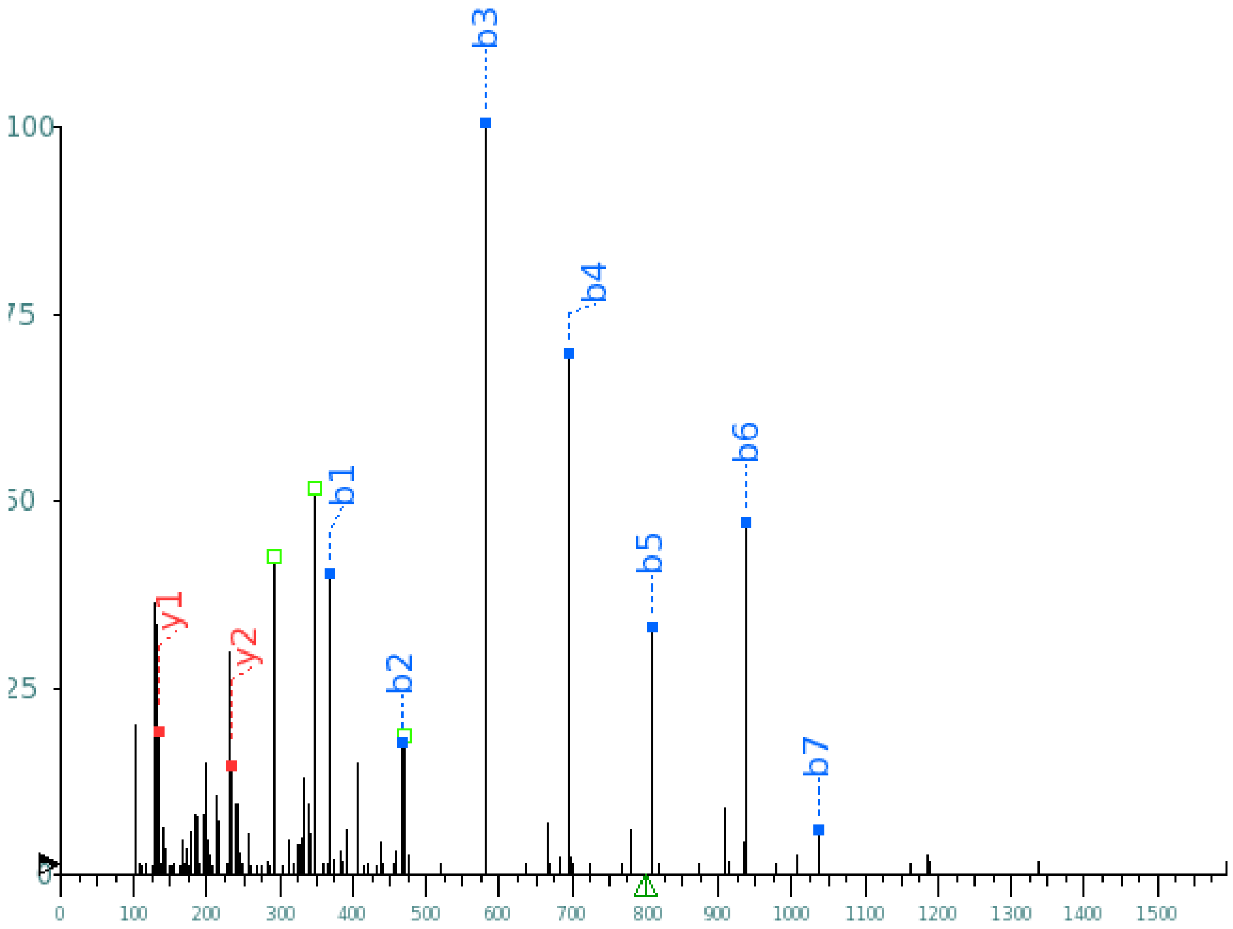

HVDLLEVAQETD (+2)

Primary Reference: ATAD1_HUMAN
Search ID: 41760
Search Name: 20130328_ananiav_ERstress_TMT_TM_timecourse_trypsin_inj1_4mc_iaa
Scan#: 14505
Observed Mass: 799.4181 (1.2 ppm)
PSM Score: 33.92

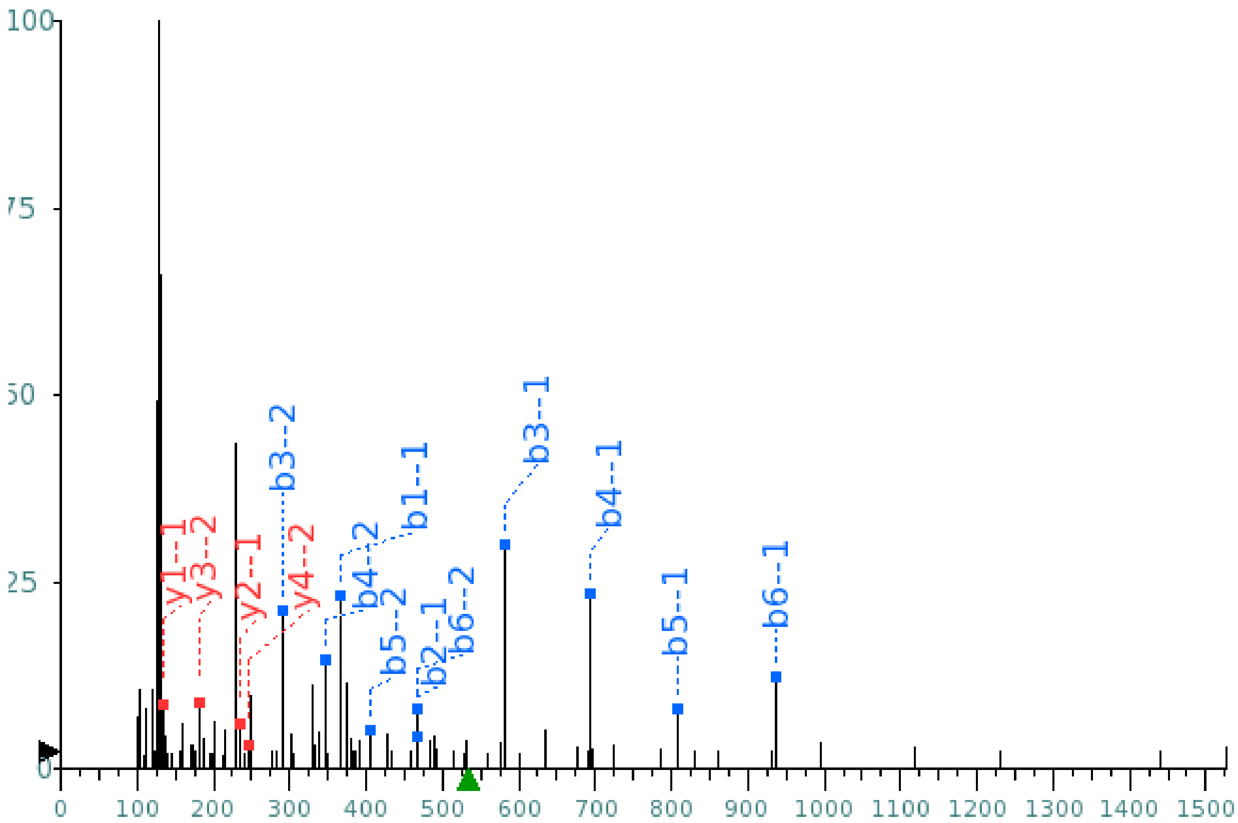

HVDLLEVAQETD (+3)

Primary Reference: ATAD1_HUMAN
Search ID: 41760
Search Name: 20130328_ananiav_ERstress_TMT_TM_timecourse_trypsin_inj1_4mc_iaa
Scan#: 14518
Observed Mass: 533.2812 (1.3 ppm)
PSM Score: 16.49

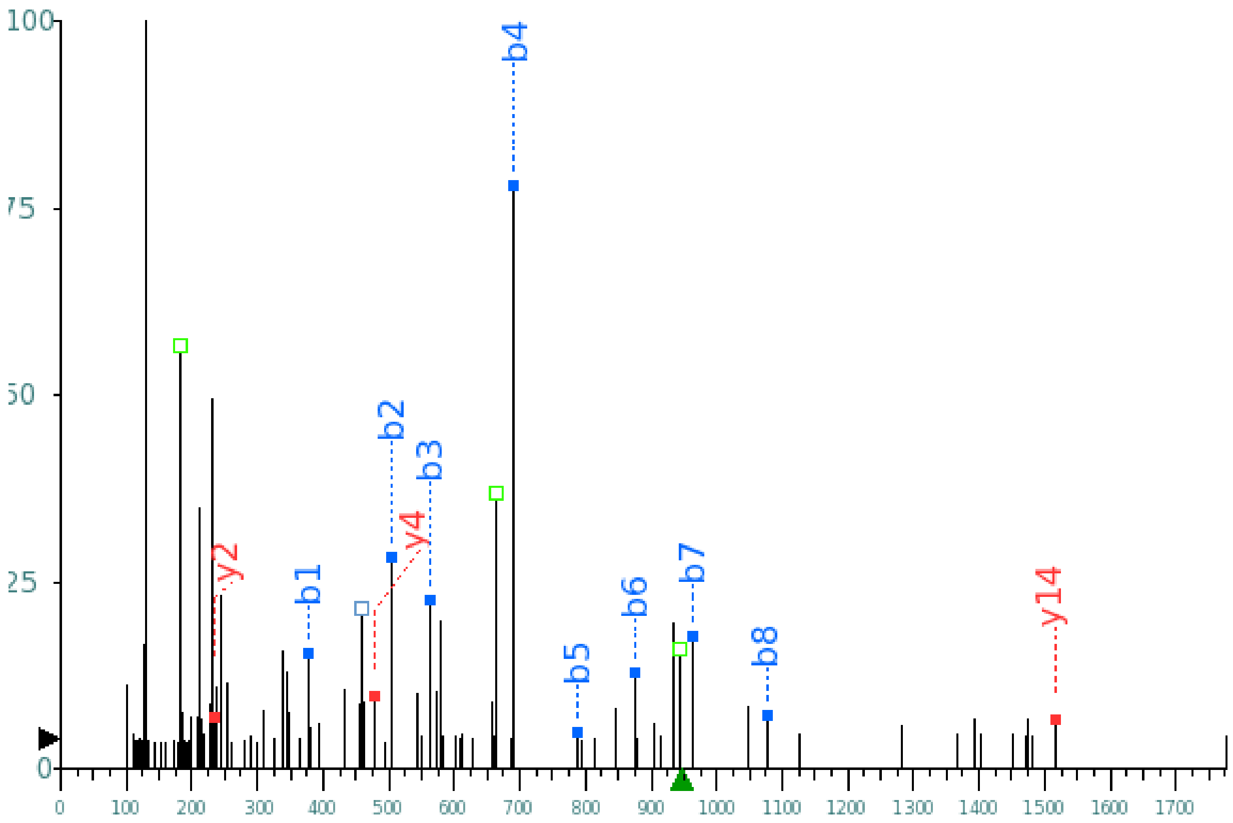

FEGQPSSLPLENETD (+2)

Primary Reference: ZN462_HUMAN
Search ID: 41760
Search Name: 20130328_ananiav_ERstress_TMT_TM_timecourse_trypsin_inj1_4mc_iaa
Scan#: 14532
Observed Mass: 946.4595 (-0.3 ppm)
PSM Score: 20.47

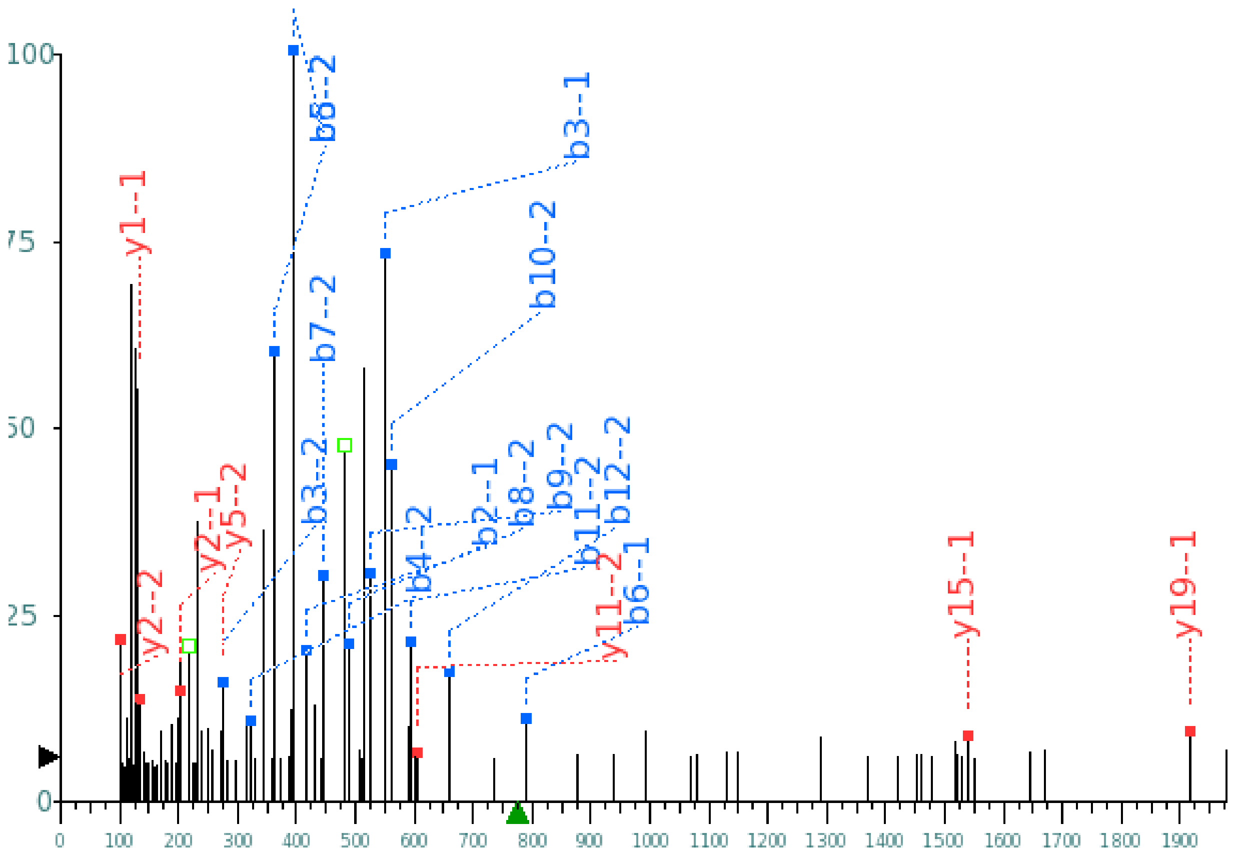

ALHPAAVSAAAEFENAESEAD (+3)

Primary Reference: C170L_HUMAN
Search ID: 41760
Search Name: 20130328_ananiav_ERstress_TMT_TM_timecourse_trypsin_inj1_4mc_iaa
Scan#: 14548
Observed Mass: 777.0447 (2.1 ppm)
PSM Score: 39.9

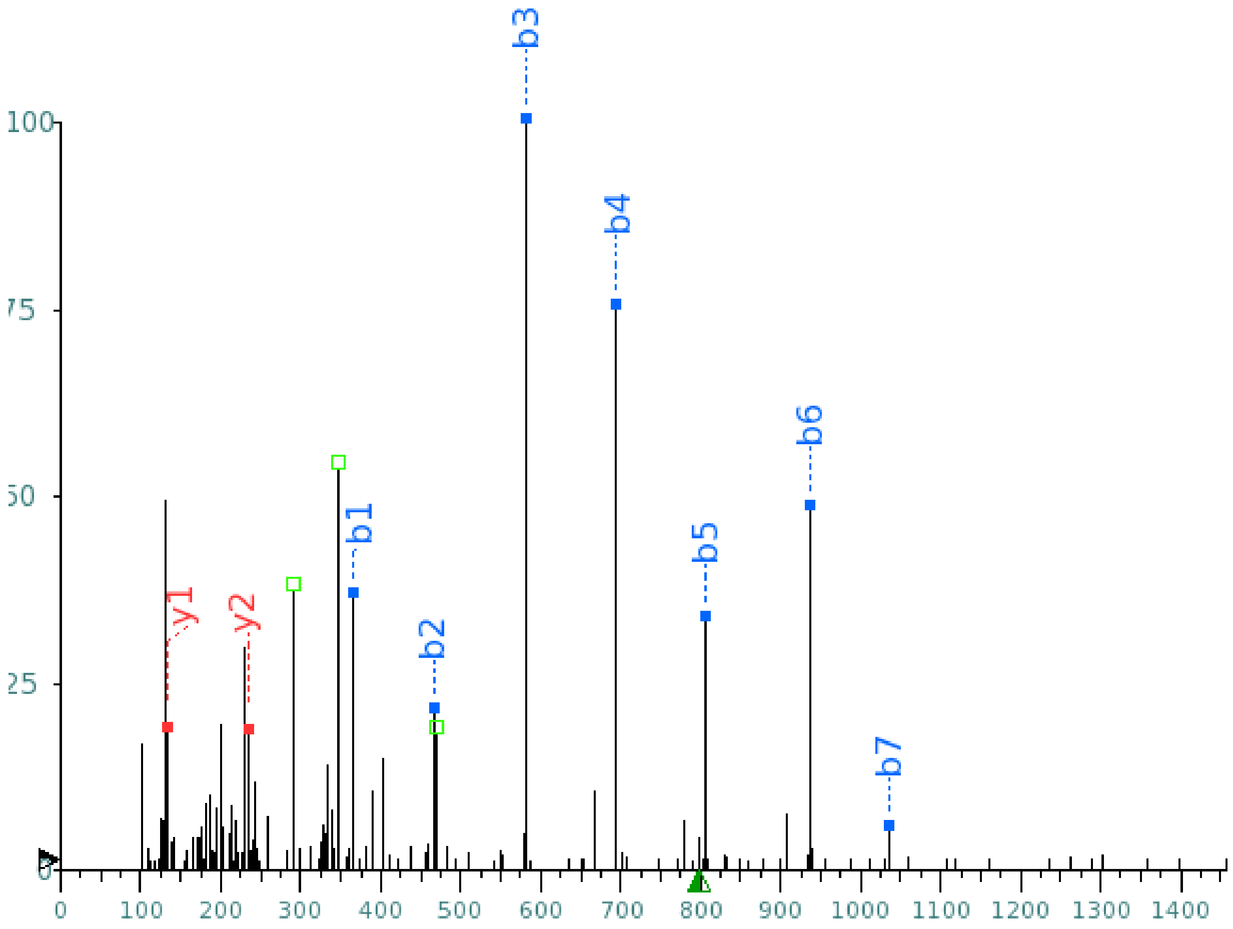

HVDLLEVAQETD (+2)

Primary Reference: ATAD1_HUMAN
Search ID: 41760
Search Name: 20130328_ananiav_ERstress_TMT_TM_timecourse_trypsin_inj1_4mc_iaa
Scan#: 14593
Observed Mass: 799.4189 (2.2 ppm)
PSM Score: 29.58

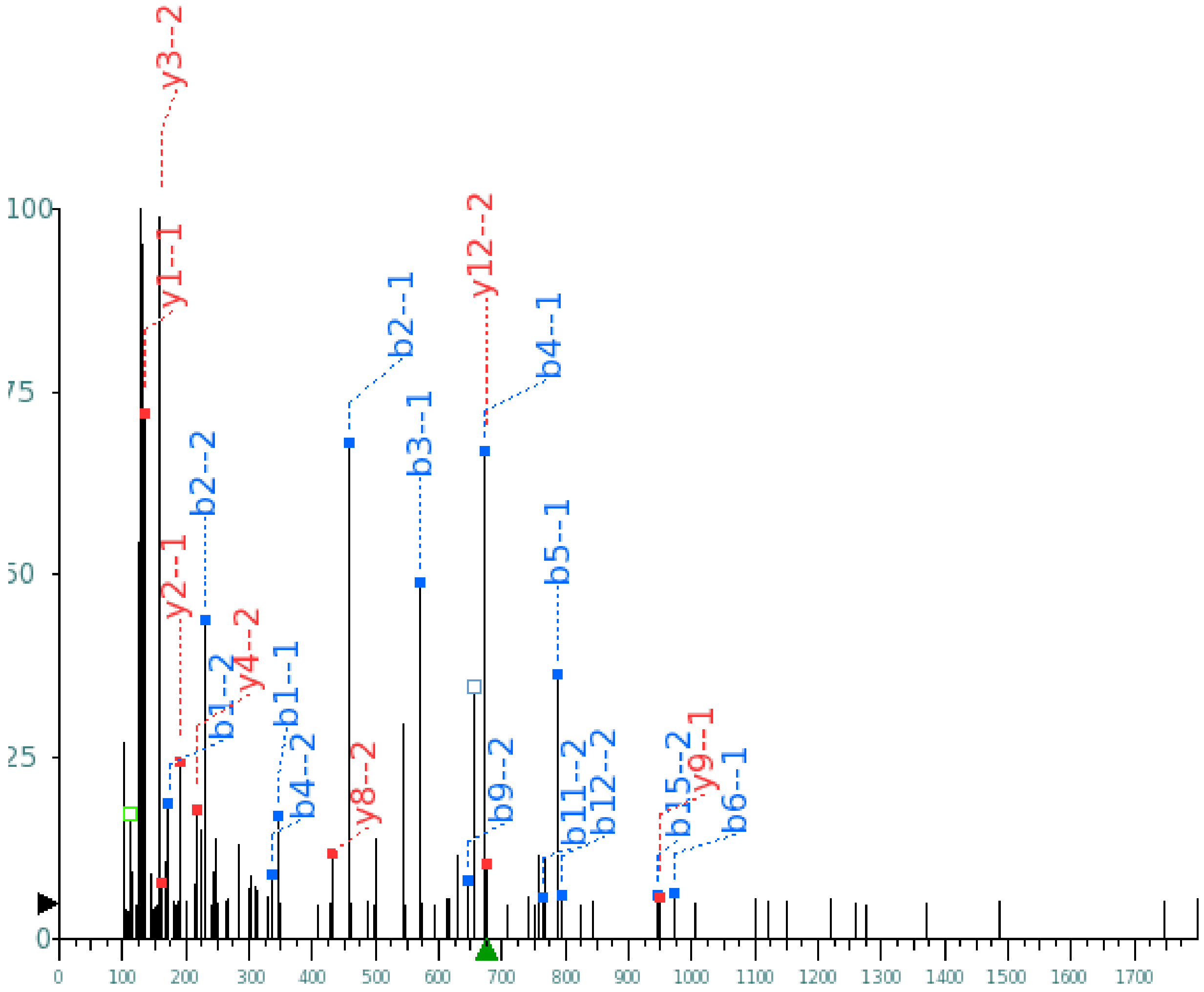

DNLTLWTSENQGDEGD (+3)

Primary Reference: 1433B_HUMAN
Search ID: 41760
Search Name: 20130328_ananiav_ERstress_TMT_TM_timecourse_trypsin_inj1_4mc_iaa
Scan#: 14594
Observed Mass: 674.9762 (2.5 ppm)
PSM Score: 25.24

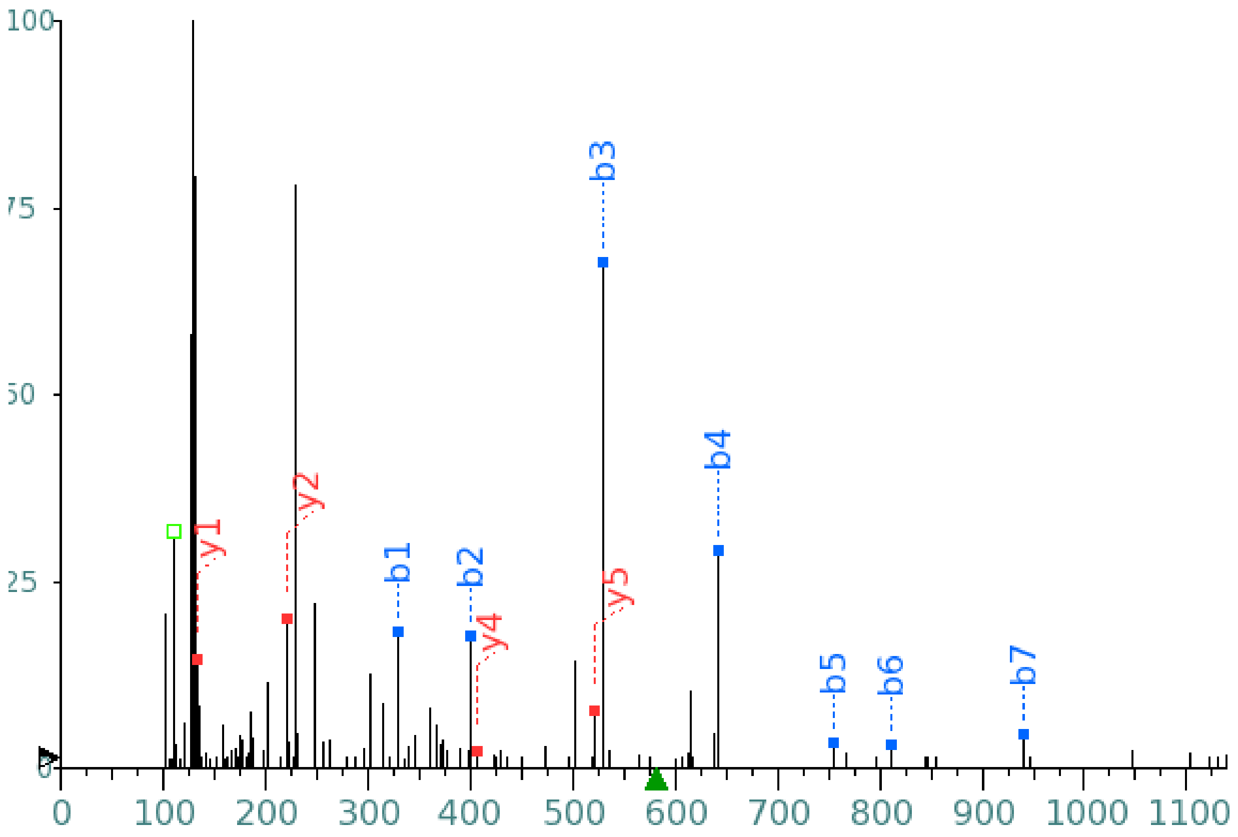

VAELLGESD (+2)

Primary Reference: KLC4_HUMAN
Search ID: 41760
Search Name: 20130328_ananiav_ERstress_TMT_TM_timecourse_trypsin_inj1_4mc_iaa
Scan#: 14614
Observed Mass: 581.314 (0.5 ppm)
PSM Score: 35.84

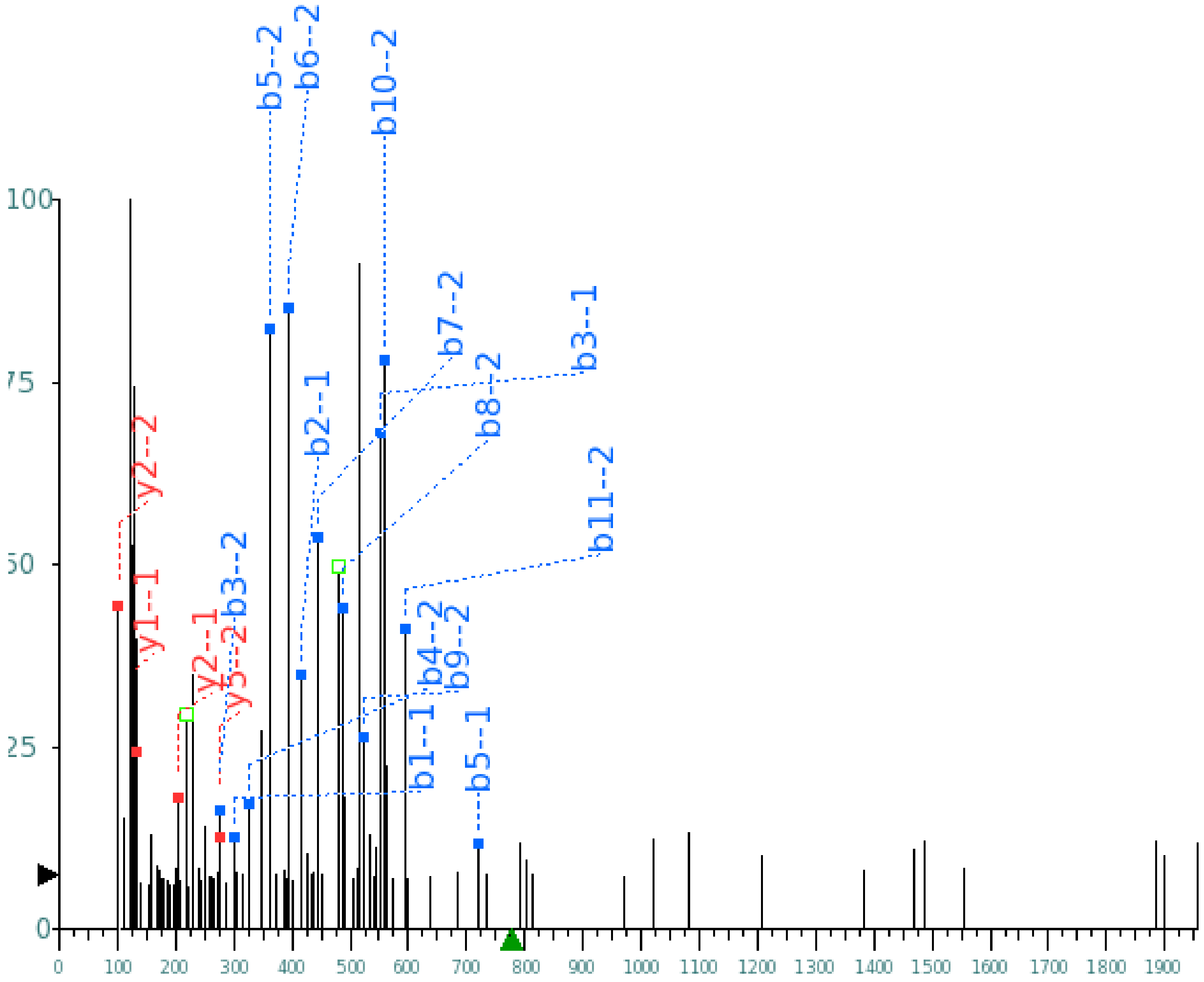

ALHPAAVSAAAEFENAESEAD (+3)

Primary Reference: C170L_HUMAN
Search ID: 41760
Search Name: 20130328_ananiav_ERstress_TMT_TM_timecourse_trypsin_inj1_4mc_iaa
Scan#: 14625
Observed Mass: 777.0463 (4.3 ppm)
PSM Score: 32.9

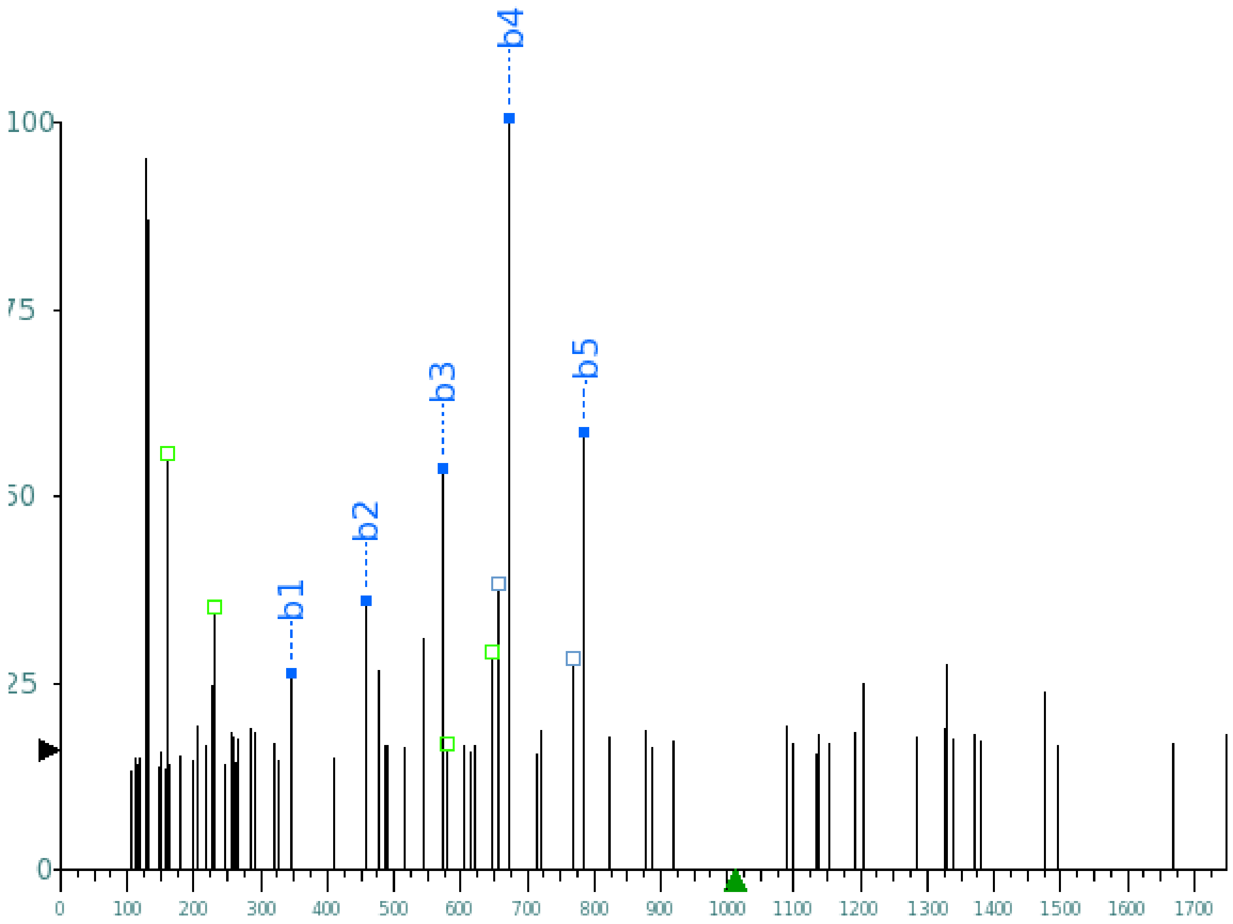

DNLTLWTSENQGDEGD (+2)

Primary Reference: 1433B_HUMAN
Search ID: 41760
Search Name: 20130328_ananiav_ERstress_TMT_TM_timecourse_trypsin_inj1_4mc_iaa
Scan#: 14684
Observed Mass: 1011.9608 (2.6 ppm)
PSM Score: 12.01

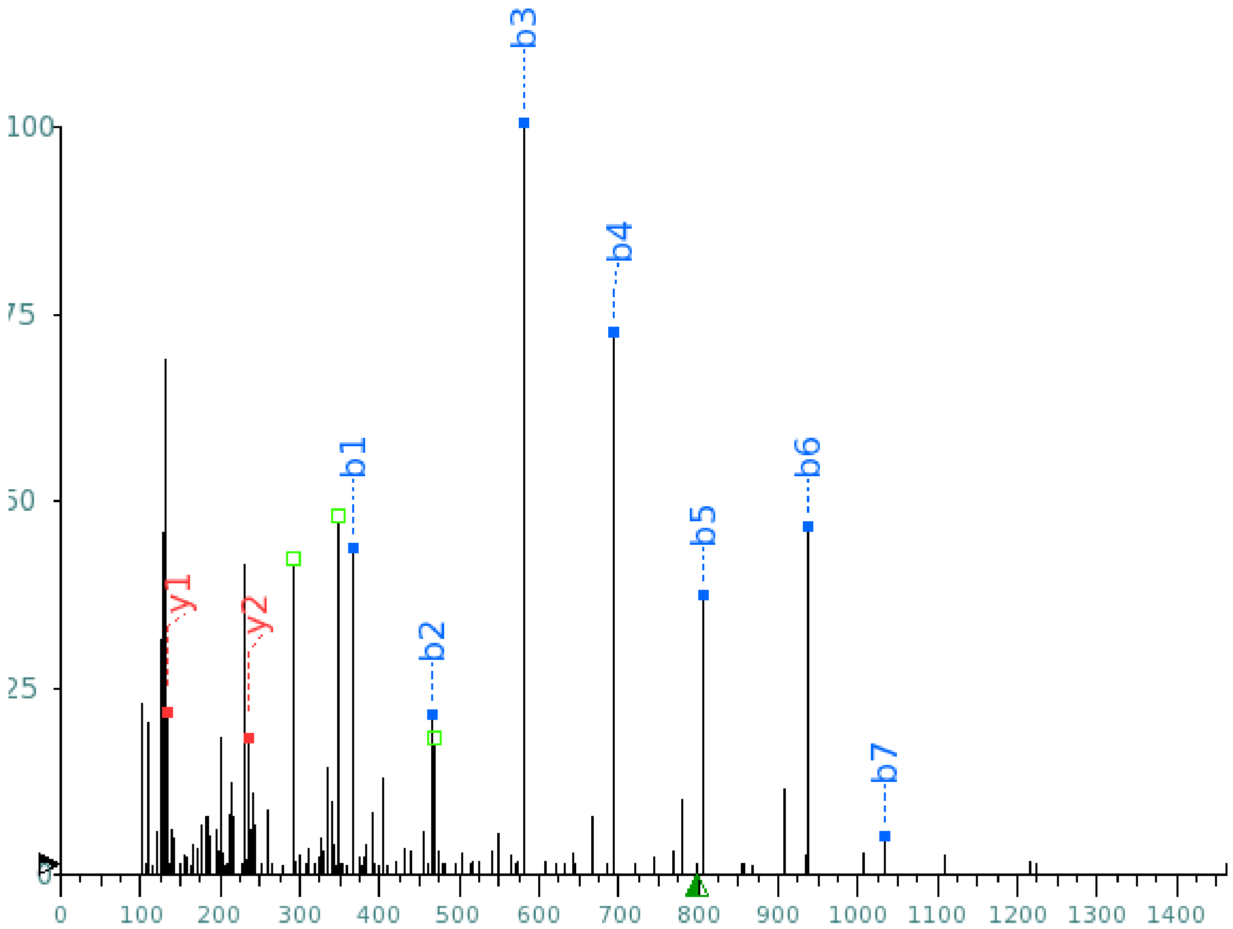

HVDLLEVAQETD (+2)

Primary Reference: ATAD1_HUMAN
Search ID: 41760
Search Name: 20130328_ananiav_ERstress_TMT_TM_timecourse_trypsin_inj1_4mc_iaa
Scan#: 14725
Observed Mass: 799.4193 (2.7 ppm)
PSM Score: 32.49

File name: 2015-02-04 04.01.53PM.pdf

Creation time: 2015-02-04 04:09PM

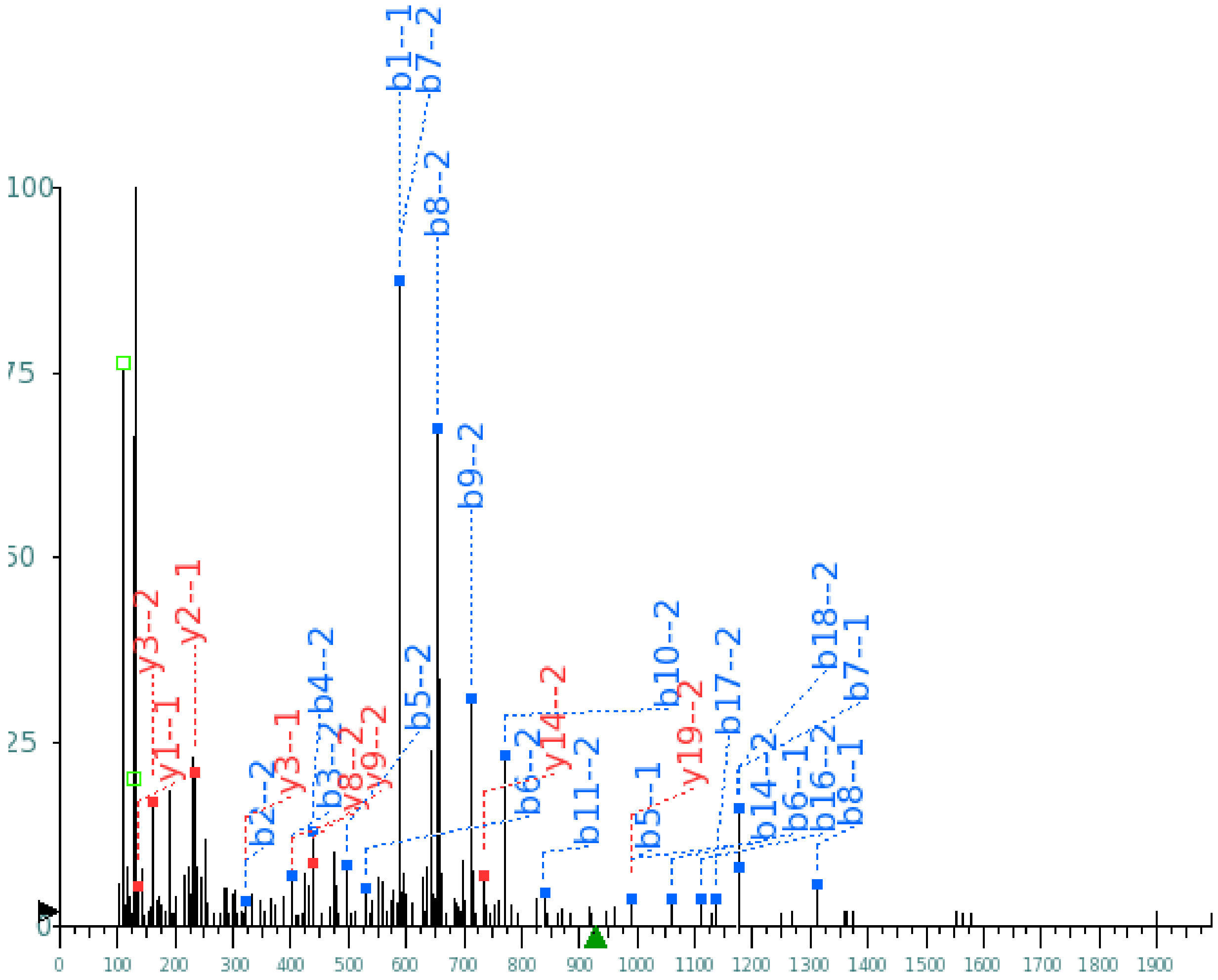

KGC*ALADHLLHTQAQDGAISTD (+3)

Primary Reference: TPP2_HUMAN
Search ID: 41760
Search Name: 20130328_ananiav_ERstress_TMT_TM_timecourse_trypsin_inj1_4mc_iaa
Scan#: 14752
Observed Mass: 927.4893 (4.8 ppm)
PSM Score: 32.77

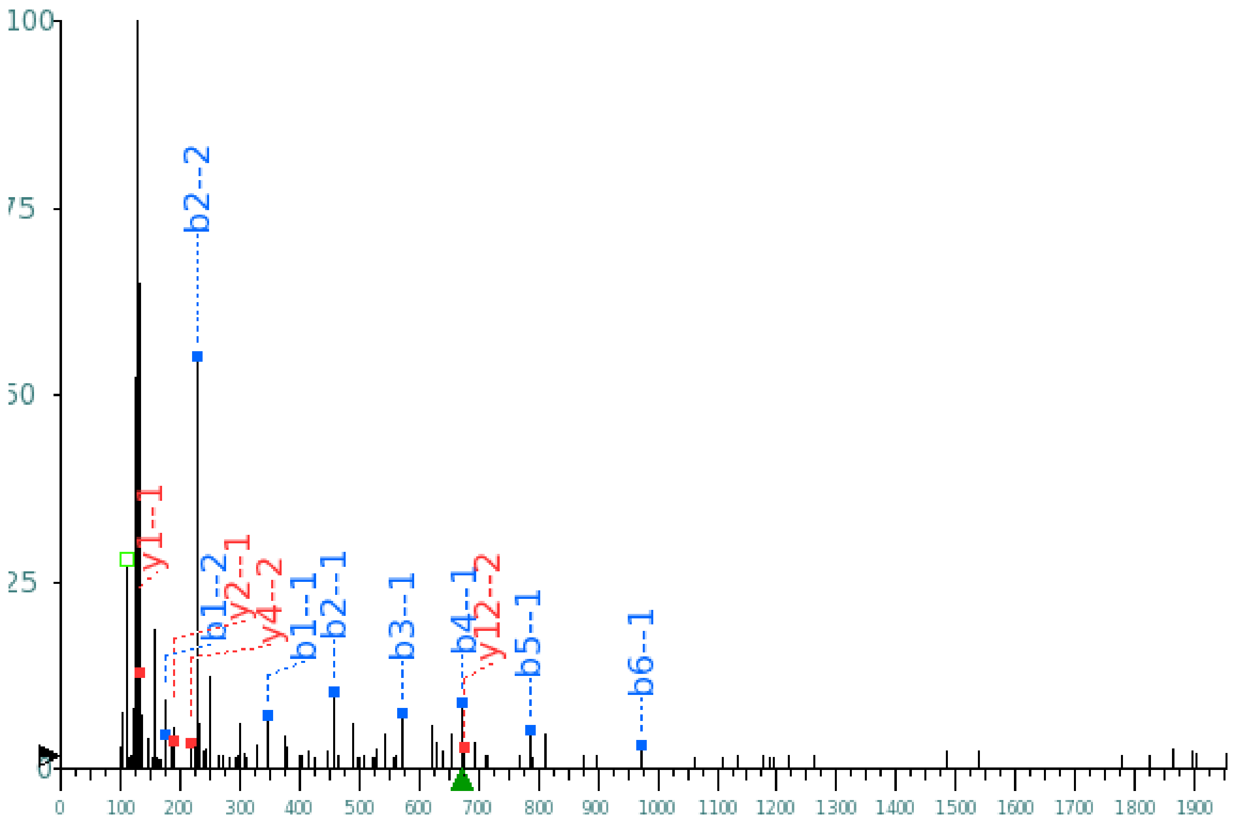

DNLTLWTSENQGDEGD (+3)

Primary Reference: 1433B_HUMAN
Search ID: 41760
Search Name: 20130328_ananiav_ERstress_TMT_TM_timecourse_trypsin_inj1_4mc_iaa
Scan#: 14763
Observed Mass: 674.976 (2.2 ppm)
PSM Score: 17.86

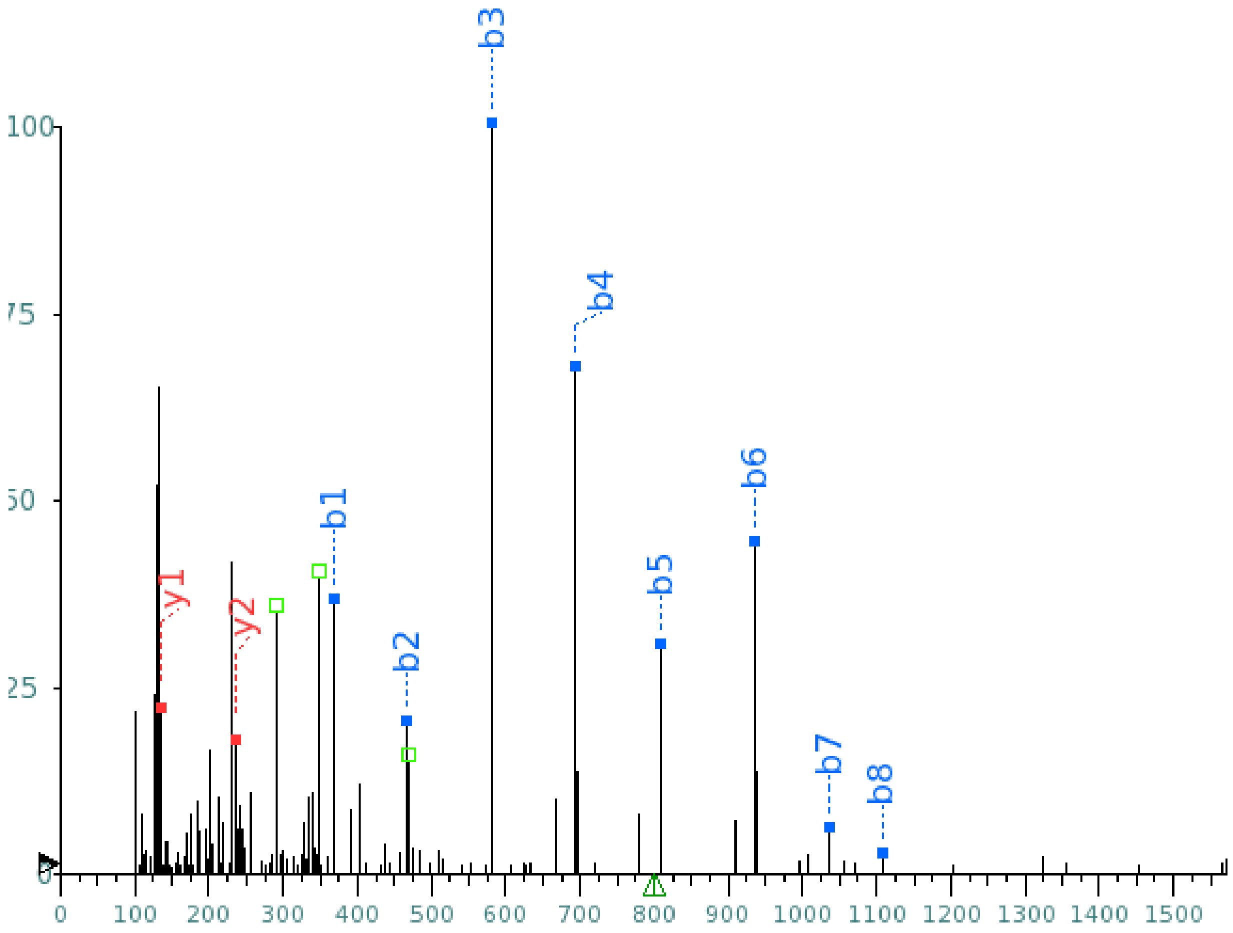

HVDLLEVAQETD (+2)

Primary Reference: ATAD1_HUMAN
Search ID: 41760
Search Name: 20130328_ananiav_ERstress_TMT_TM_timecourse_trypsin_inj1_4mc_iaa
Scan#: 14821
Observed Mass: 799.4201 (3.7 ppm)
PSM Score: 38.75

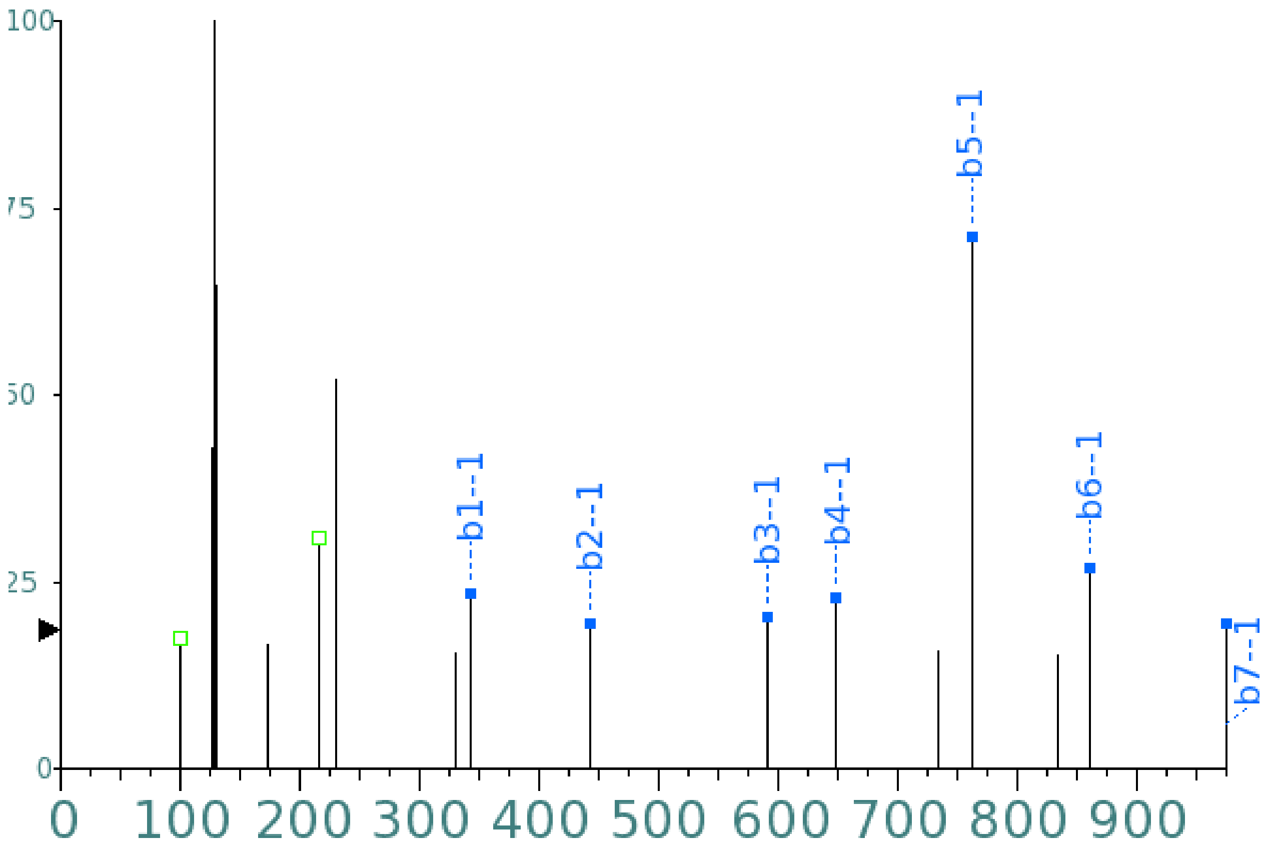

ITFGNVNGC*STAEESVSQNVEGTQAD (+3)

Primary Reference: AN13A_HUMAN
Search ID: 41760
Search Name: 20130328_ananiav_ERstress_TMT_TM_timecourse_trypsin_inj1_4mc_iaa
Scan#: 14830
Observed Mass: 981.7911 (3.8 ppm)
PSM Score: 46.9

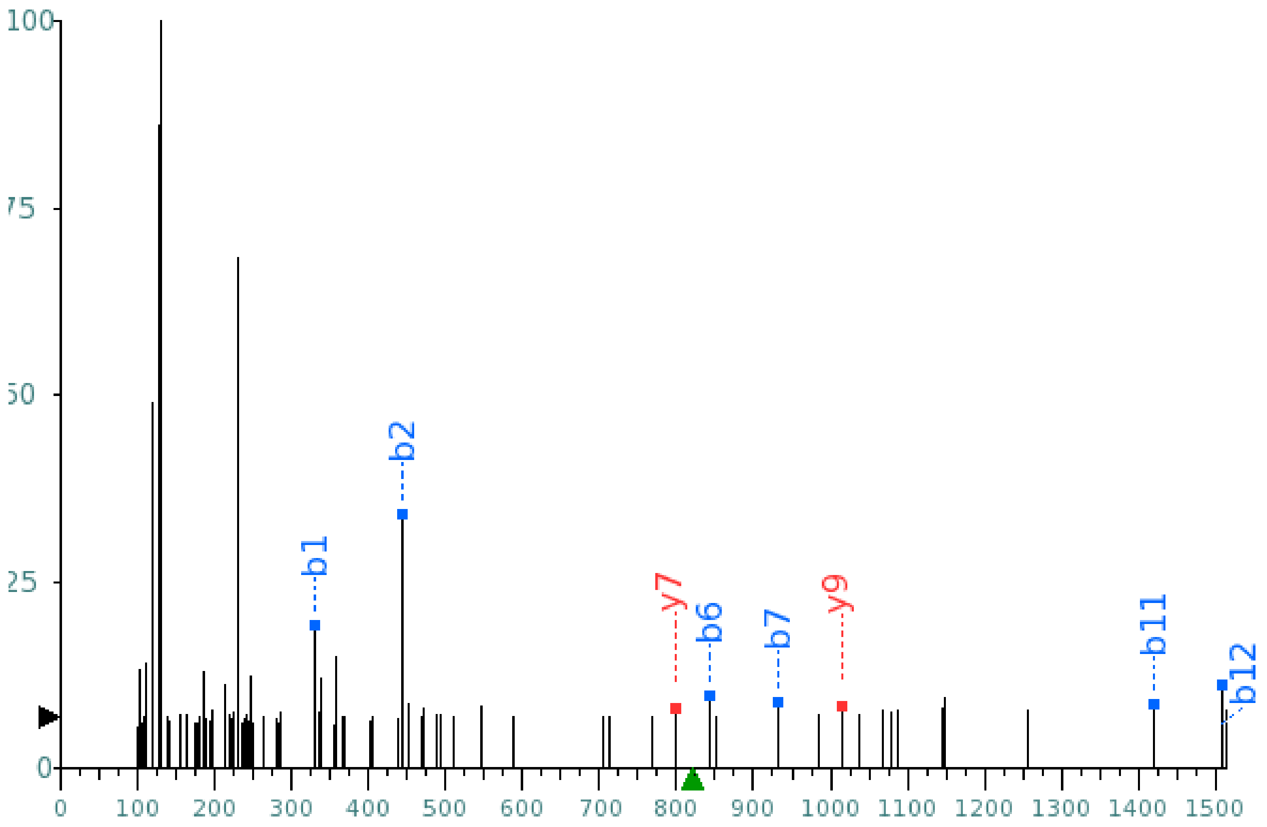

TLPSTDSLDDFSD (+2)

Primary Reference: Z3H7B_HUMAN
Search ID: 41760
Search Name: 20130328_ananiav_ERstress_TMT_TM_timecourse_trypsin_inj1_4mc_iaa
Scan#: 14867
Observed Mass: 821.39 (2.1 ppm)
PSM Score: 34.55

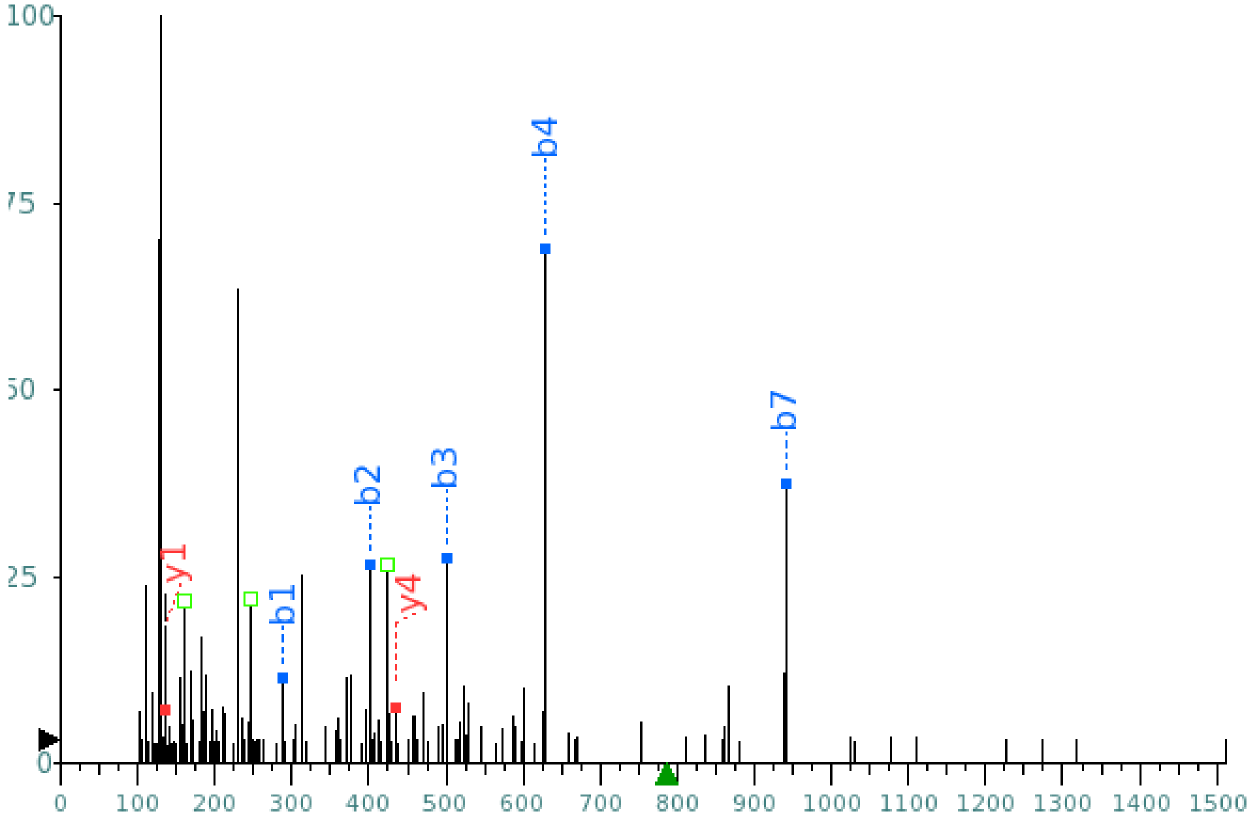

GLVEPVDVVDNAD (+2)

Primary Reference: FLNA_HUMAN
Search ID: 41760
Search Name: 20130328_ananiav_ERstress_TMT_TM_timecourse_trypsin_inj1_4mc_iaa
Scan#: 14869
Observed Mass: 785.9135 (2.3 ppm)
PSM Score: 7.5

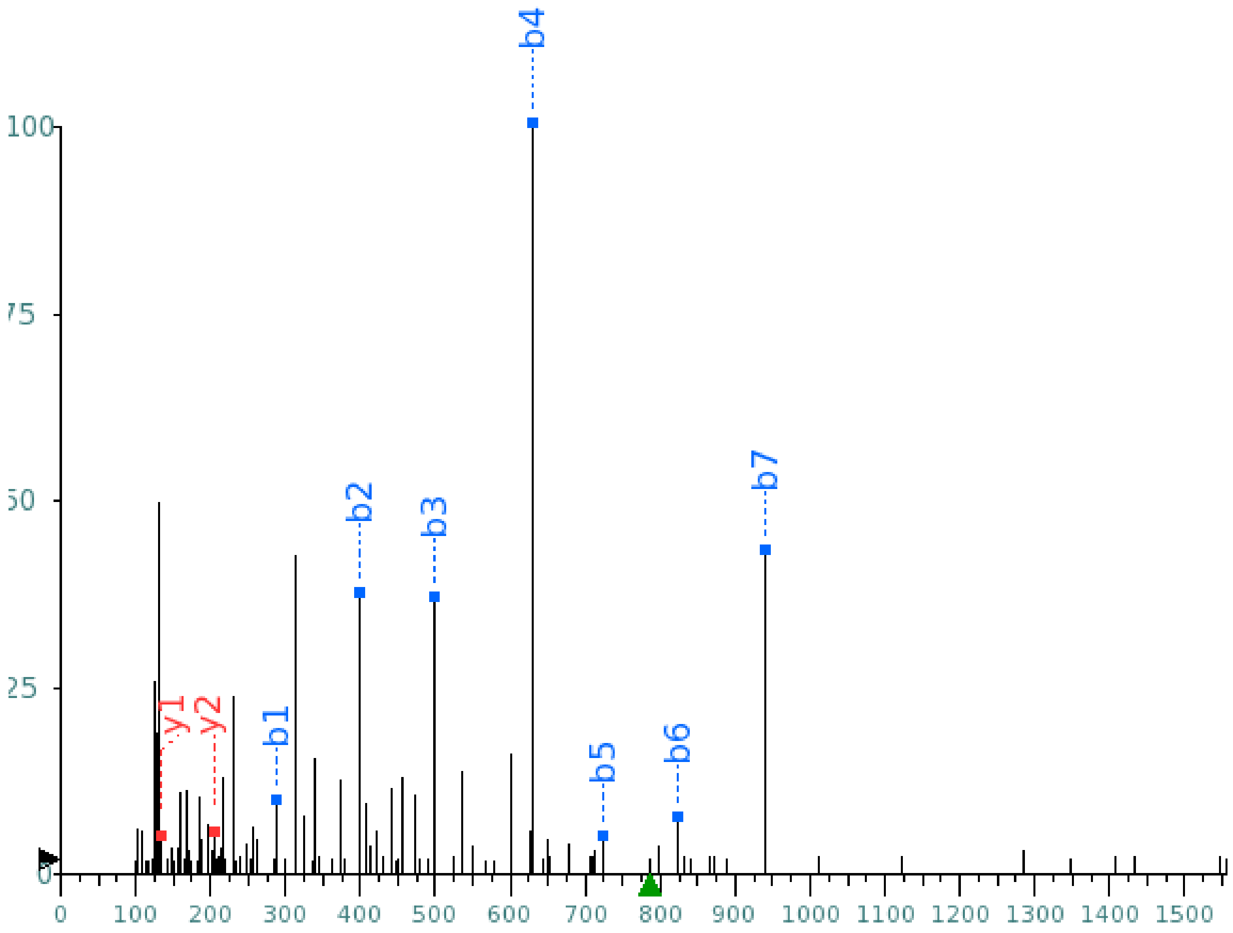

GLVEPVDVVDNAD (+2)

Primary Reference: FLNA_HUMAN
Search ID: 41760
Search Name: 20130328_ananiav_ERstress_TMT_TM_timecourse_trypsin_inj1_4mc_iaa
Scan#: 14955
Observed Mass: 785.9138 (2.6 ppm)
PSM Score: 18.85

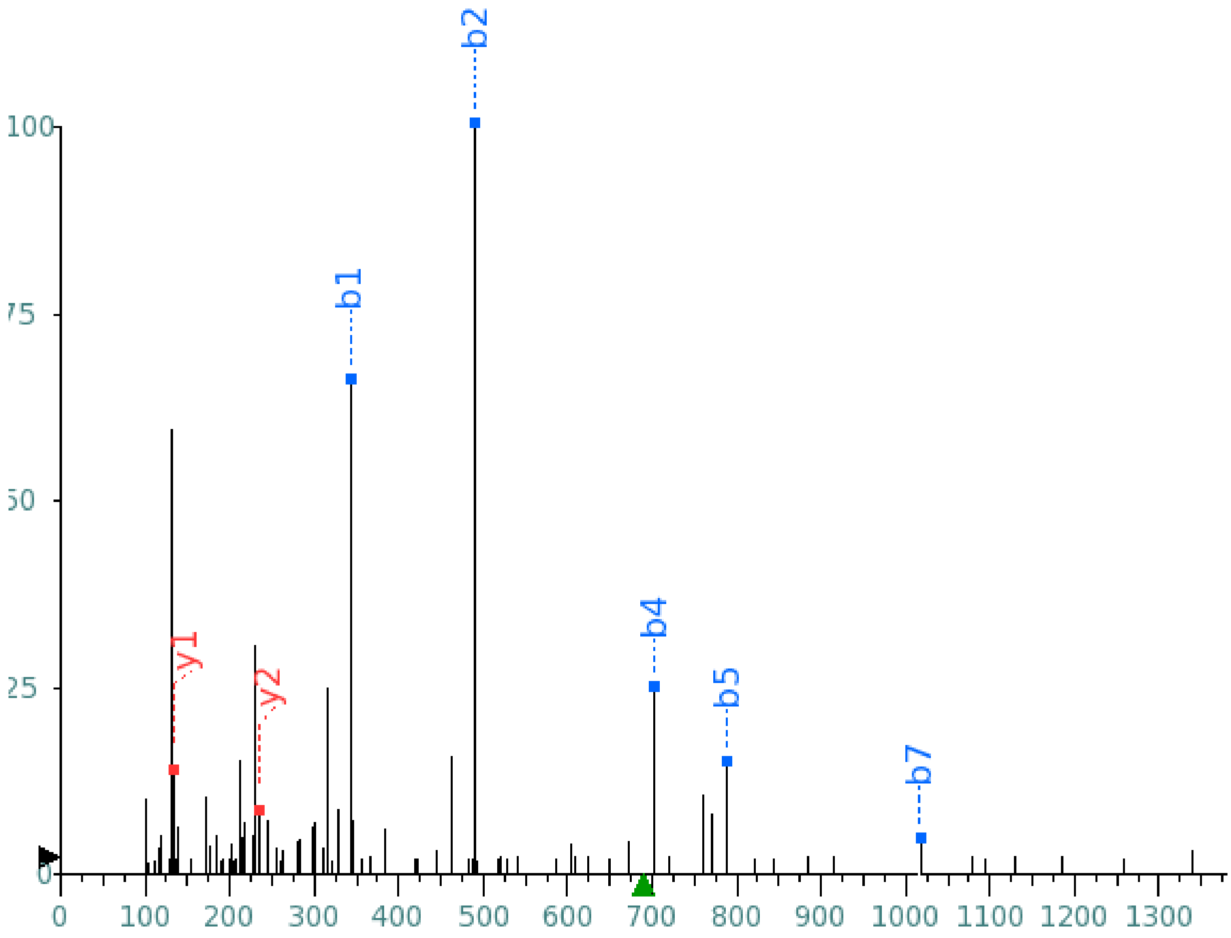

LFPNSLDQTD (+2)

Primary Reference: CALR_HUMAN
Search ID: 41760
Search Name: 20130328_ananiav_ERstress_TMT_TM_timecourse_trypsin_inj1_4mc_iaa
Scan#: 15065
Observed Mass: 689.8573 (1.6 ppm)
PSM Score: 14.18

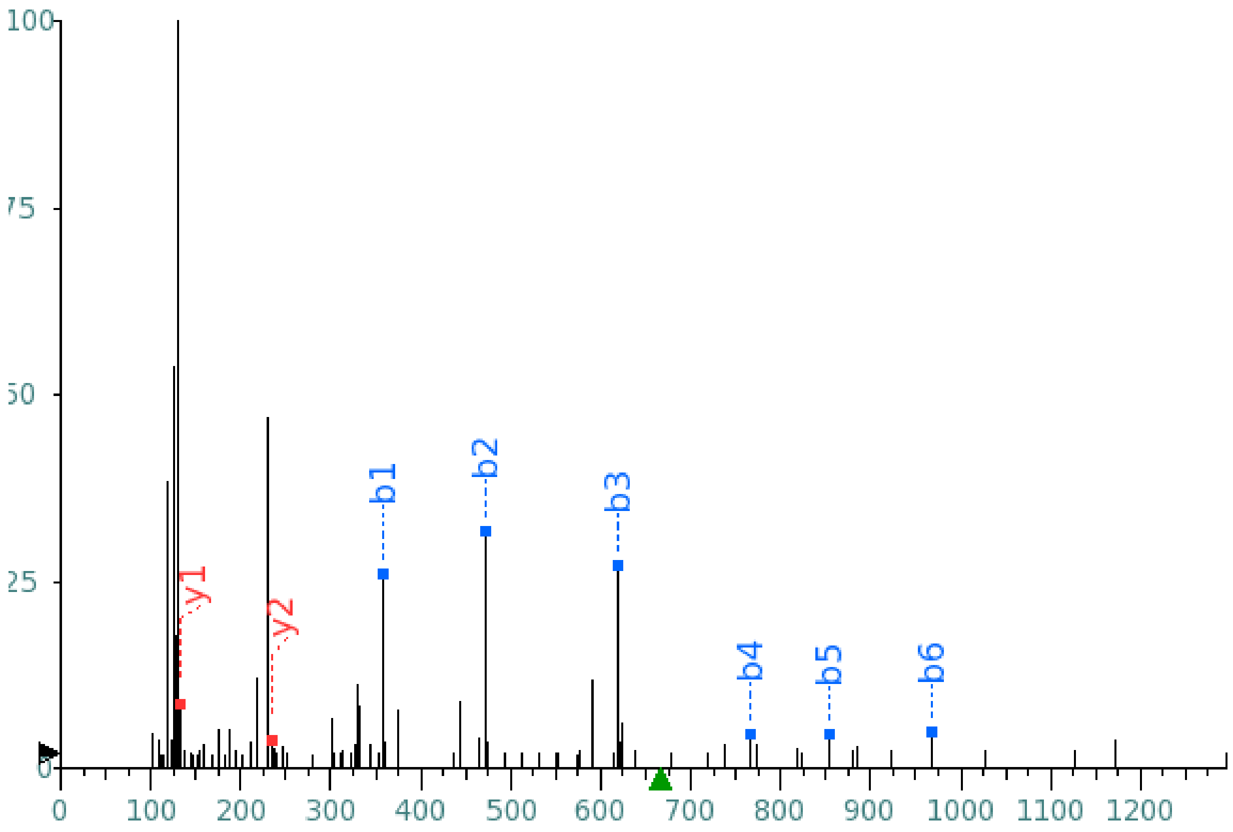

ELFFSDQTD (+2)

Primary Reference: PASK_HUMAN
Search ID: 41760
Search Name: 20130328_ananiav_ERstress_TMT_TM_timecourse_trypsin_inj1_4mc_iaa
Scan#: 15068
Observed Mass: 665.8232 (2 ppm)
PSM Score: 38.23

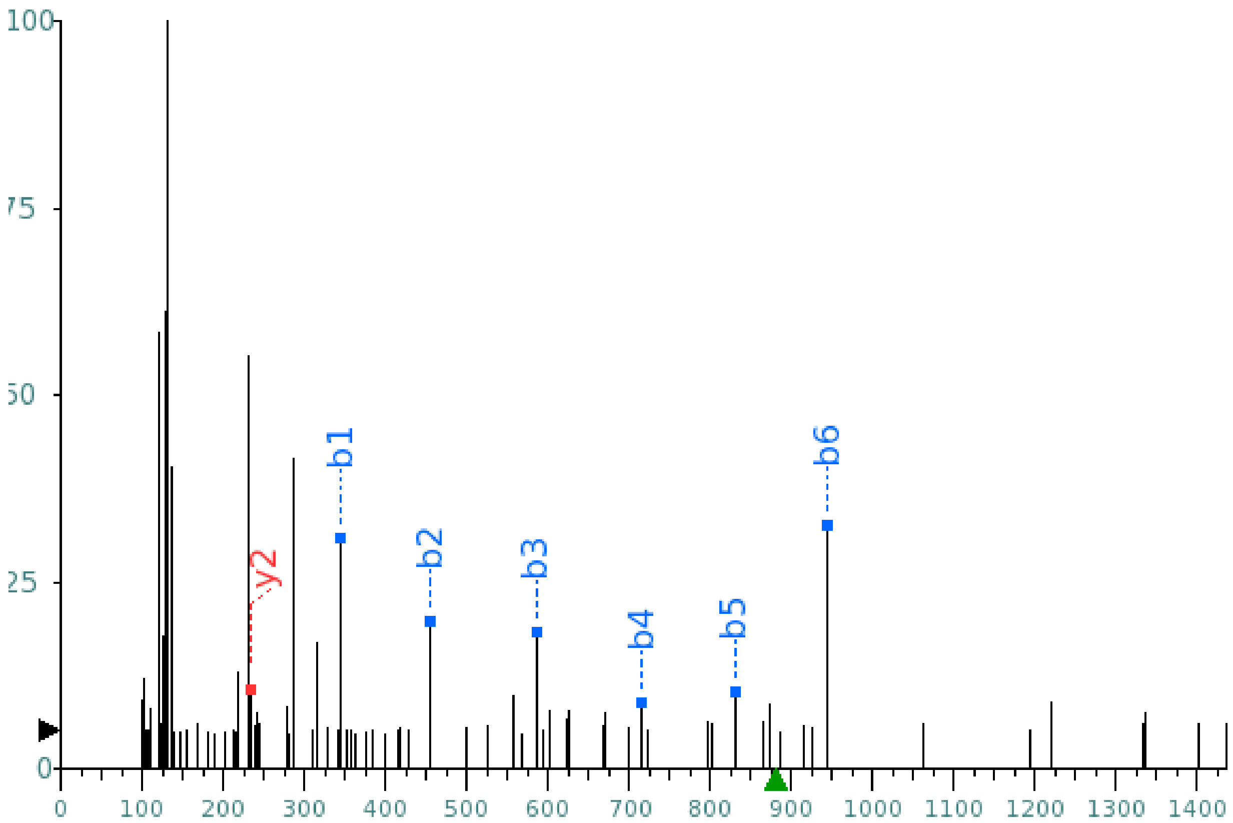

IIEEDDAYDFSTD (+2)

Primary Reference: GNL3_HUMAN
Search ID: 41760
Search Name: 20130328_ananiav_ERstress_TMT_TM_timecourse_trypsin_inj1_4mc_iaa
Scan#: 15135
Observed Mass: 881.4006 (2 ppm)
PSM Score: 23.96

File name: 2015-02-04 04.01.53PM.pdf

Creation time: 2015-02-04 04:09PM

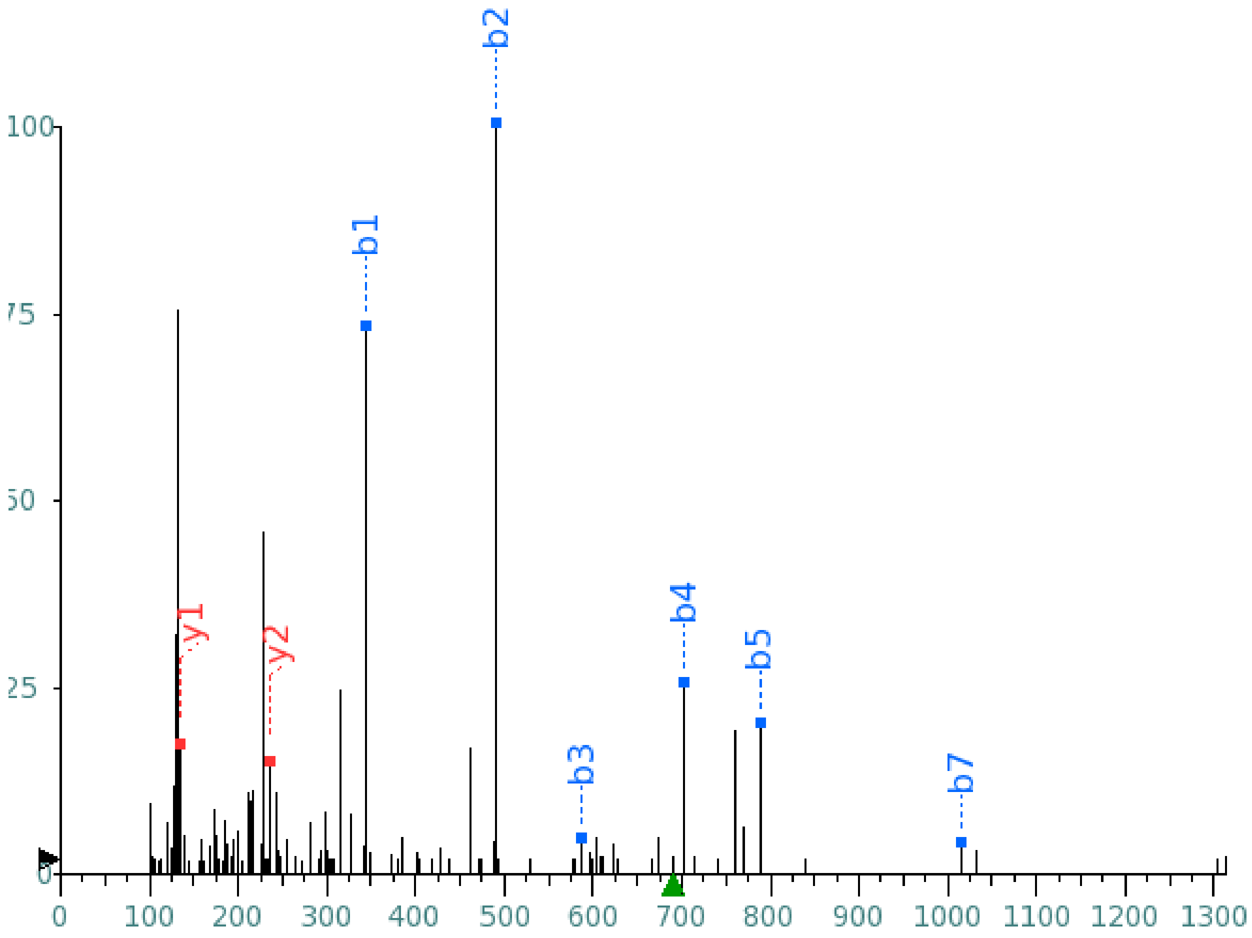

LFPNSLDQTD (+2)

Primary Reference: CALR_HUMAN
Search ID: 41760
Search Name: 20130328_ananiav_ERstress_TMT_TM_timecourse_trypsin_inj1_4mc_iaa
Scan#: 15153
Observed Mass: 689.8576 (1.9 ppm)
PSM Score: 27.09

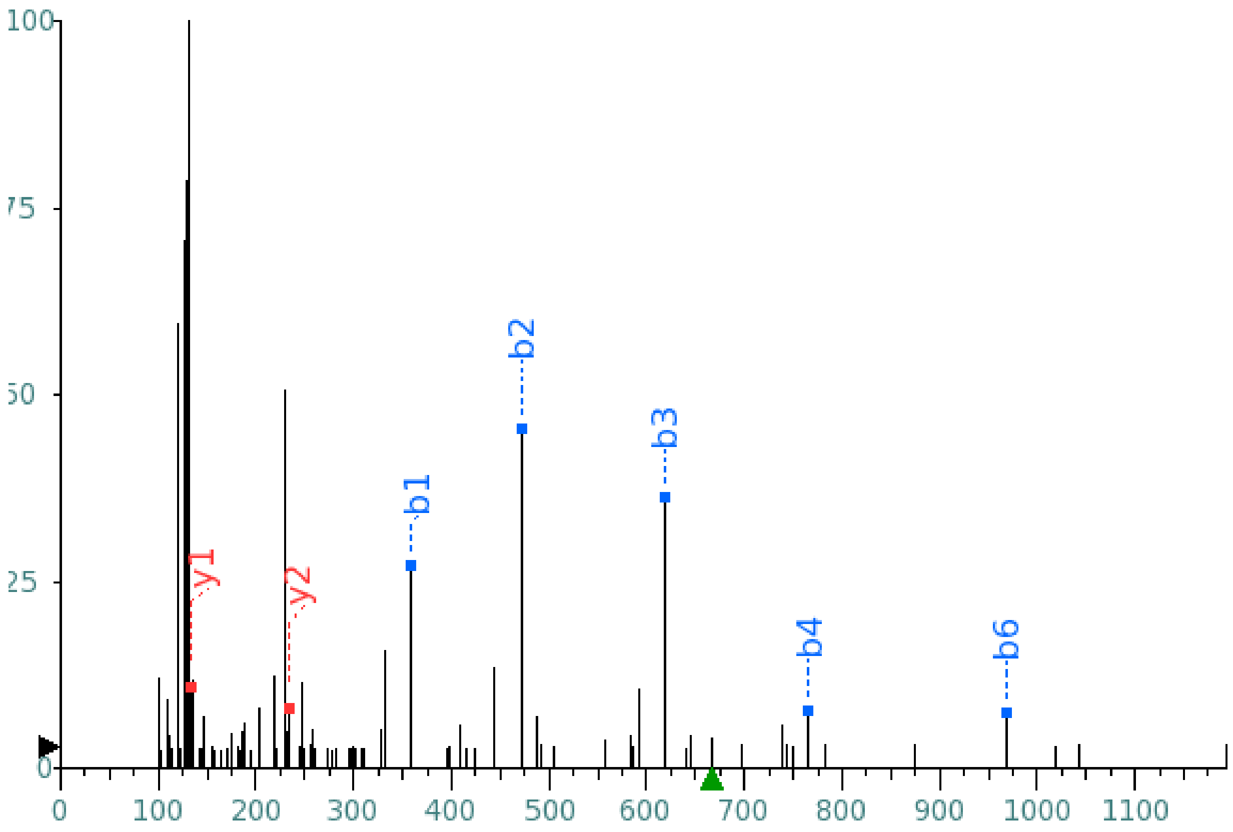

ELFFSDQTD (+2)

Primary Reference: PASK_HUMAN
Search ID: 41760
Search Name: 20130328_ananiav_ERstress_TMT_TM_timecourse_trypsin_inj1_4mc_iaa
Scan#: 15155
Observed Mass: 665.8234 (2.3 ppm)
PSM Score: 25.04

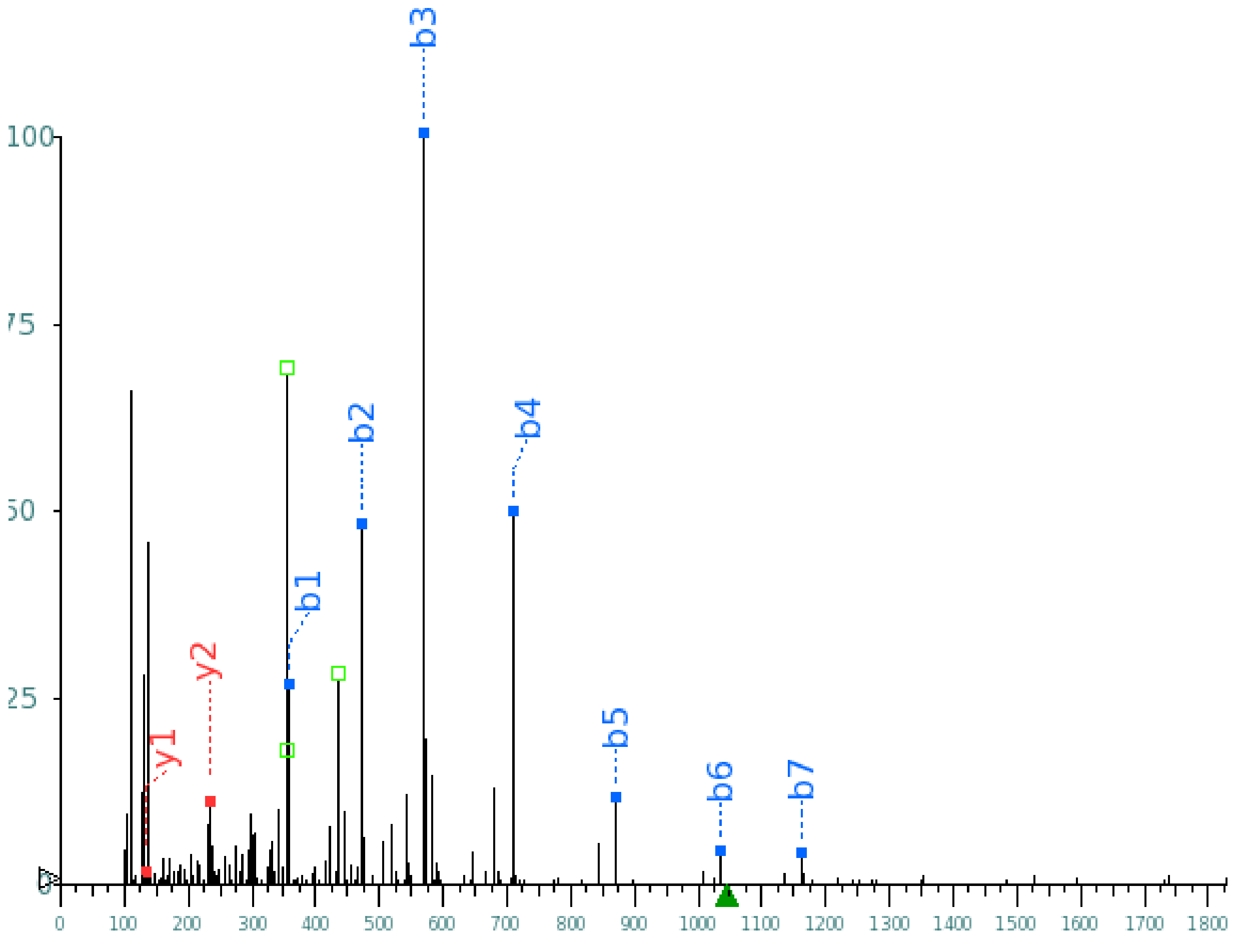

ELVHYYEQTSDMMTD (+2)

Primary Reference: PCM1_HUMAN
Search ID: 41760
Search Name: 20130328_ananiav_ERstress_TMT_TM_timecourse_trypsin_inj1_4mc_iaa
Scan#: 15183
Observed Mass: 1045.9646 (-1.4 ppm)
PSM Score: 31.13

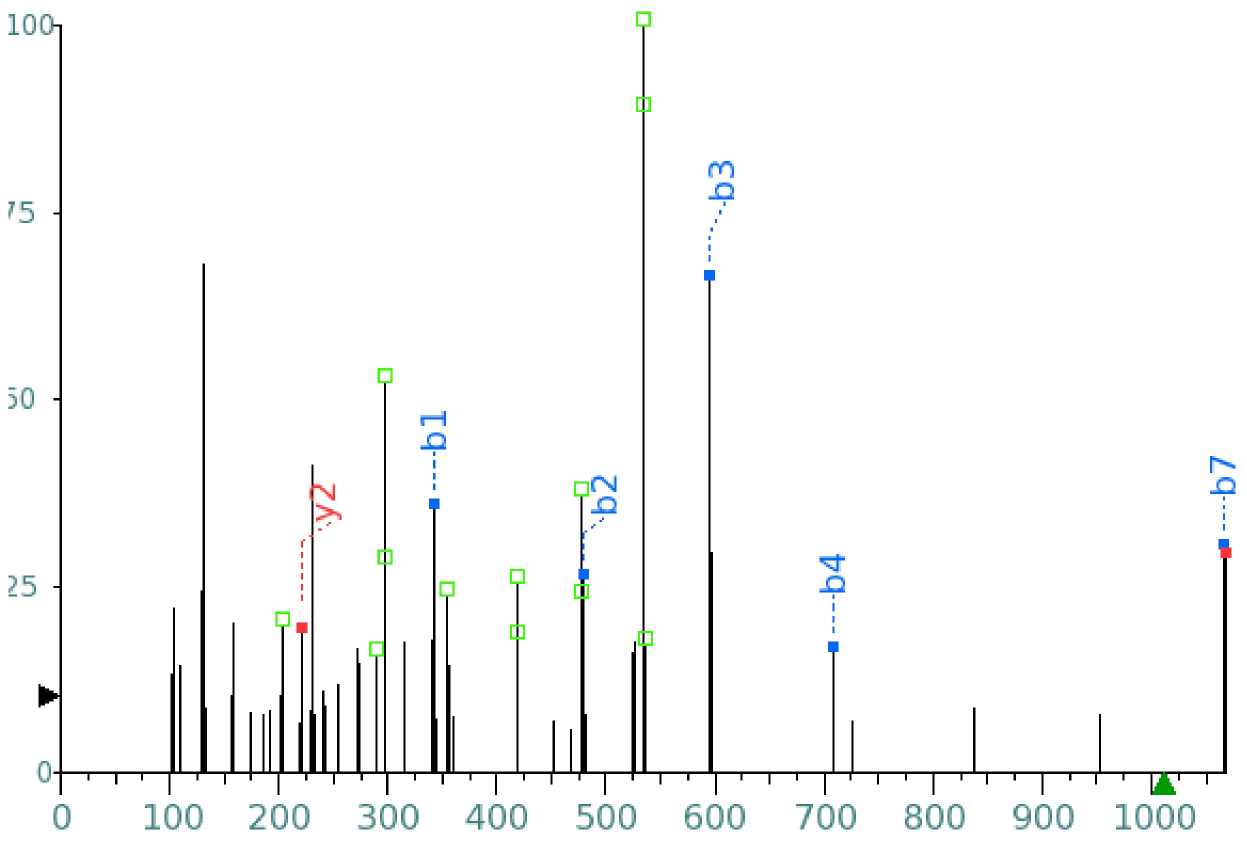

IHDLEDDLEMSSDASD (+2)

Primary Reference: T2FA_HUMAN
Search ID: 41760
Search Name: 20130328_ananiav_ERstress_TMT_TM_timecourse_trypsin_inj1_4mc_iaa
Scan#: 15193
Observed Mass: 1010.9487 (2.3 ppm)
PSM Score: 38.54

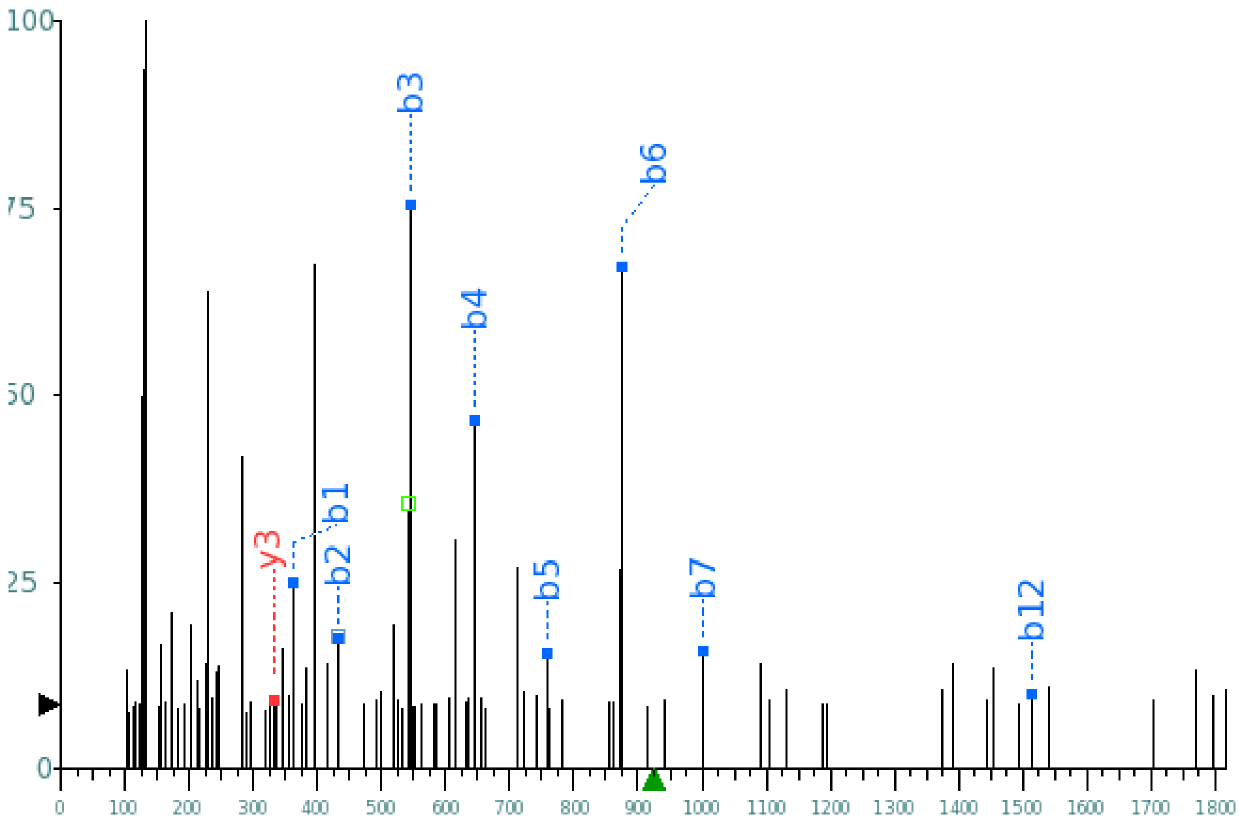

MANVLDEPGGQDLEAD (+2)

Primary Reference: ZMYM3_HUMAN
Search ID: 41760
Search Name: 20130328_ananiav_ERstress_TMT_TM_timecourse_trypsin_inj1_4mc_iaa
Scan#: 15200
Observed Mass: 923.4434 (3.1 ppm)
PSM Score: 16.1

File name: 2015-02-04 04.01.53PM.pdf

Creation time: 2015-02-04 04:09PM

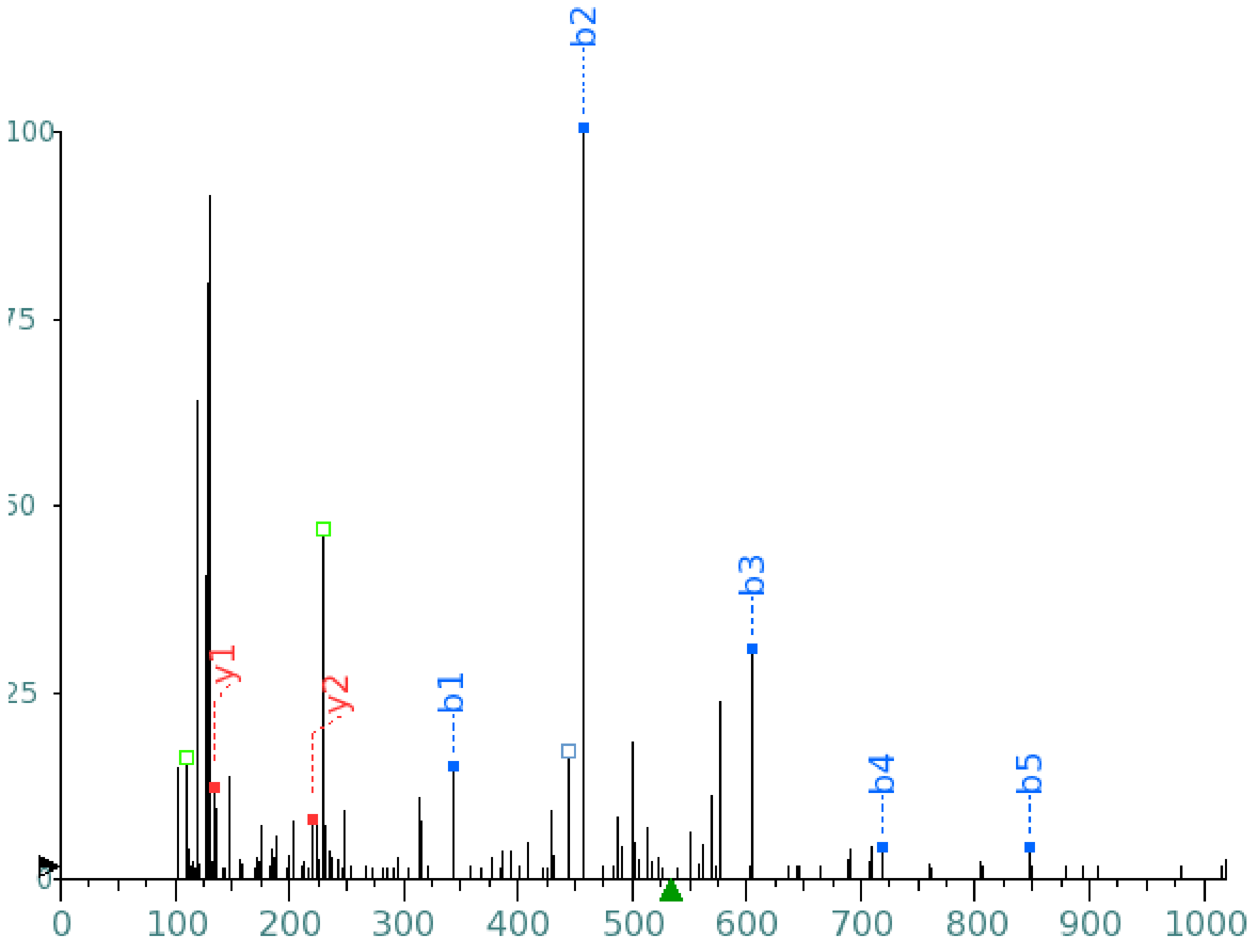

LDFIESD (+2)

Primary Reference: IF4G3_HUMAN
Search ID: 41760
Search Name: 20130328_ananiav_ERstress_TMT_TM_timecourse_trypsin_inj1_4mc_iaa
Scan#: 15219
Observed Mass: 534.2782 (3.1 ppm)
PSM Score: 13.74

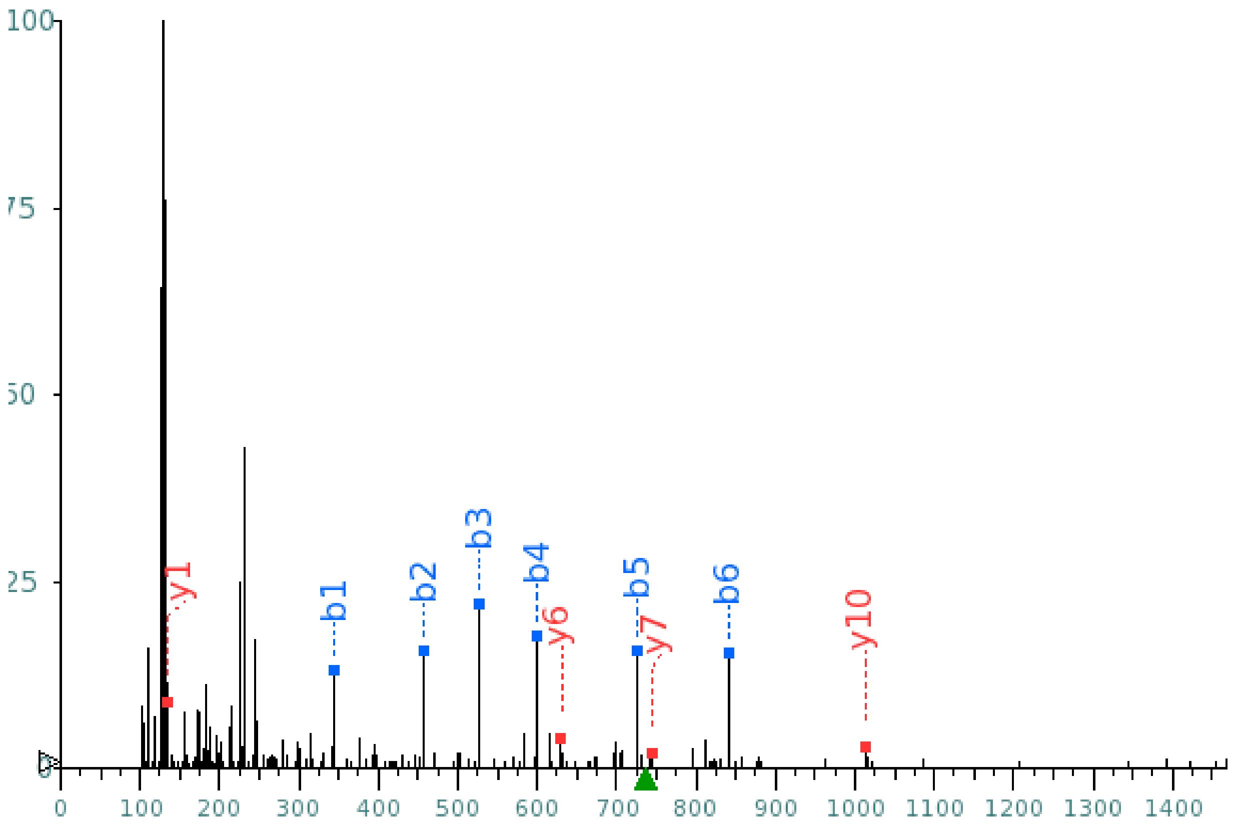

LLAAQNPLSQAD (+2)

Primary Reference: SF3B4_HUMAN
Search ID: 41760
Search Name: 20130328_ananiav_ERstress_TMT_TM_timecourse_trypsin_inj1_4mc_iaa
Scan#: 15222
Observed Mass: 735.4135 (2.4 ppm)
PSM Score: 19.47

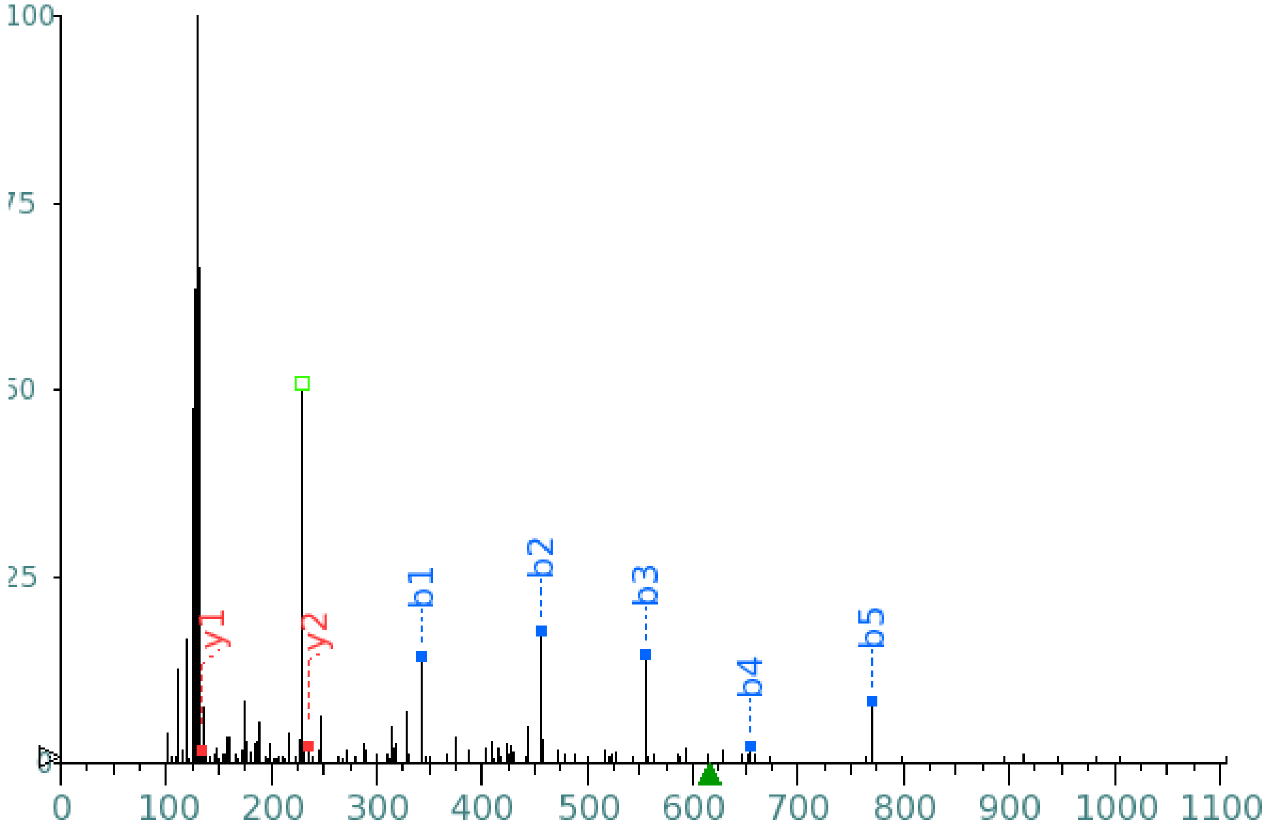

LLVVDPETD (+2)

Primary Reference: NHRF1_HUMAN
Search ID: 41760
Search Name: 20130328_ananiav_ERstress_TMT_TM_timecourse_trypsin_inj1_4mc_iaa
Scan#: 15231
Observed Mass: 615.3471 (3.5 ppm)
PSM Score: 9.73

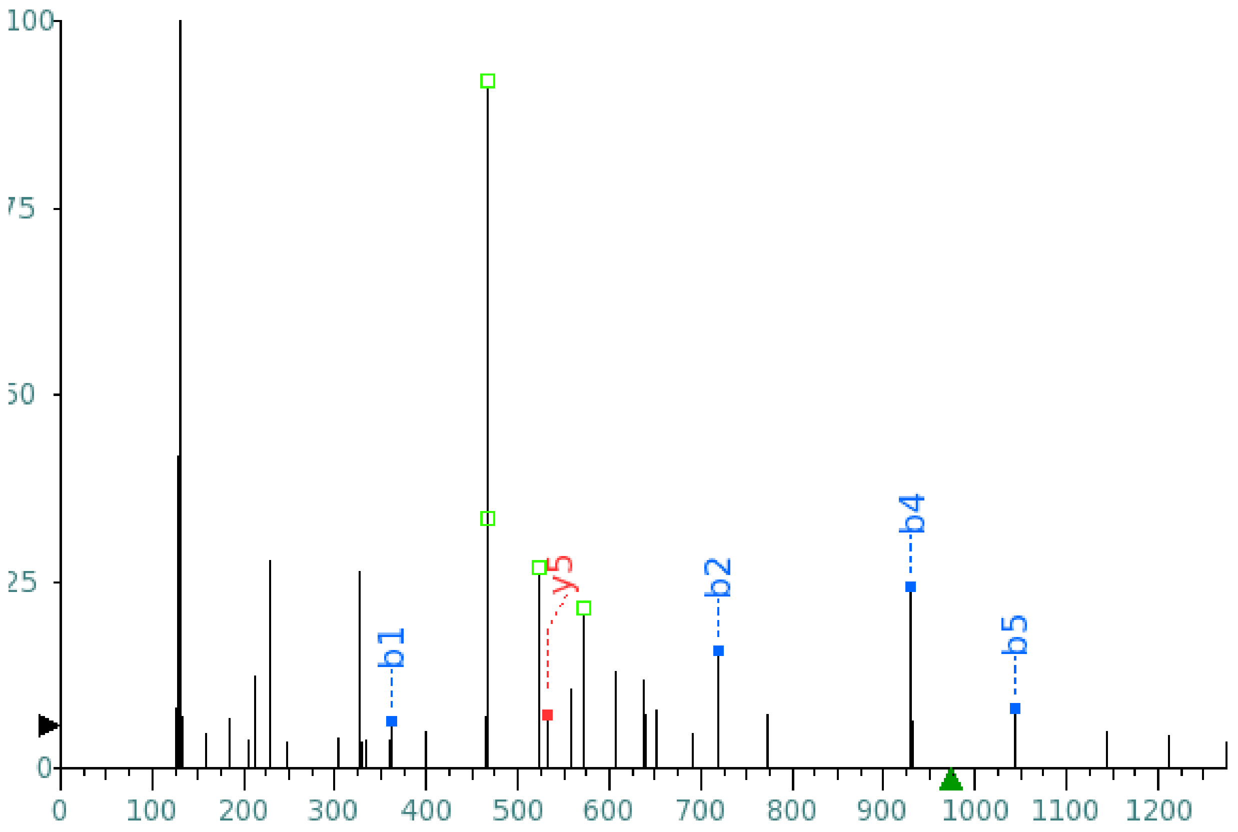

MKPDIVC*IPDDAD (+2)

Primary Reference: EI2BG_HUMAN
Search ID: 41760
Search Name: 20130328_ananiav_ERstress_TMT_TM_timecourse_trypsin_inj1_4mc_iaa
Scan#: 15237
Observed Mass: 974.003 (1 ppm)
PSM Score: 18.41

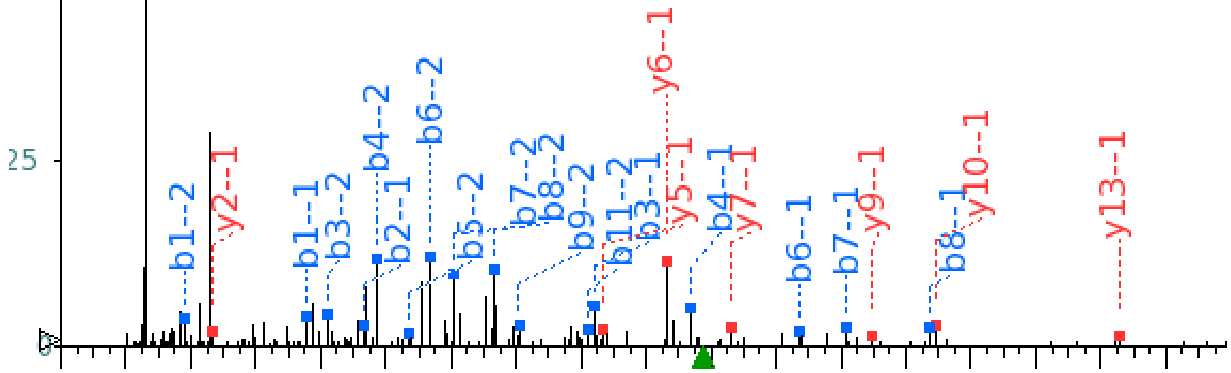

FSKFPAAQAPDPSETPKIETD (+3)

Primary Reference: ABLM1_HUMAN
Search ID: 41760
Search Name: 20130328_ananiav_ERstress_TMT_TM_timecourse_trypsin_inj1_4mc_iaa
Scan#: 15351
Observed Mass: 988.5395 (2.4 ppm)
PSM Score: 22.99

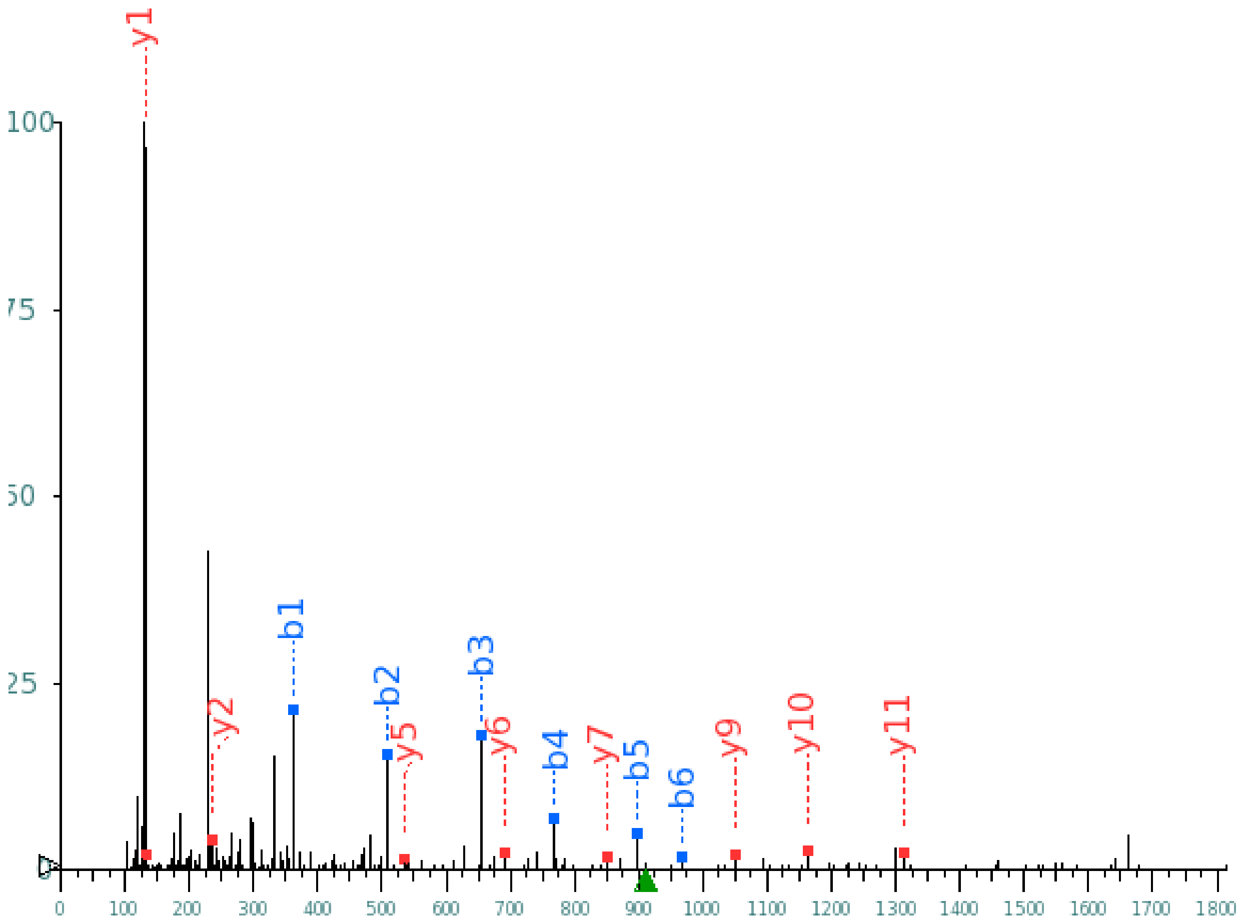

MFFIQAC*RGDETD (+2)

Primary Reference: CASP2_HUMAN
Search ID: 41760
Search Name: 20130328_ananiav_ERstress_TMT_TM_timecourse_trypsin_inj1_4mc_iaa
Scan#: 15386
Observed Mass: 909.9236 (2.5 ppm)
PSM Score: 22.81

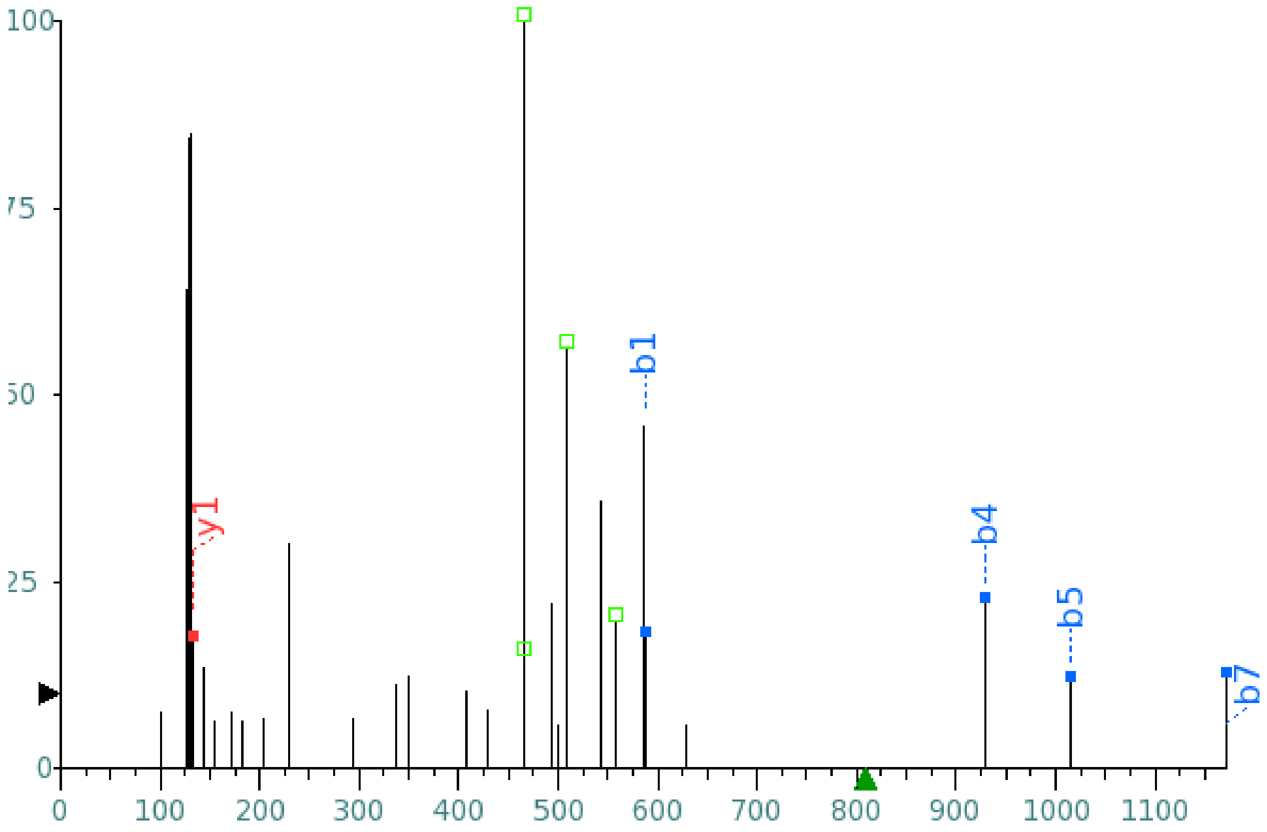

KILDSVGIEAD (+2)

Primary Reference: RLA2_HUMAN
Search ID: 41760
Search Name: 20130328_ananiav_ERstress_TMT_TM_timecourse_trypsin_inj1_4mc_iaa
Scan#: 15413
Observed Mass: 809.4807 (4.8 ppm)
PSM Score: 38.05

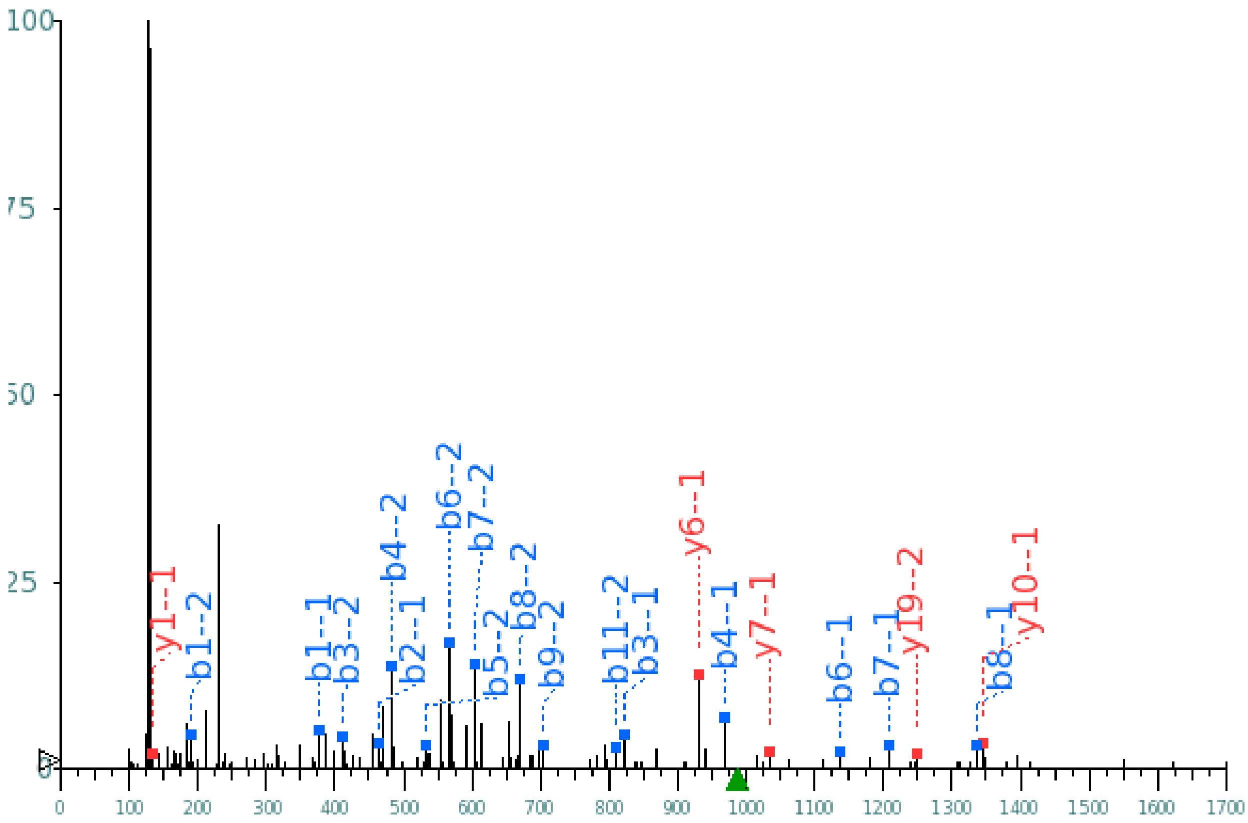

FSKFPAAQAPDPSETPKIETD (+3)

Primary Reference: ABLM1_HUMAN
Search ID: 41760
Search Name: 20130328_ananiav_ERstress_TMT_TM_timecourse_trypsin_inj1_4mc_iaa
Scan#: 15429
Observed Mass: 988.5394 (2.2 ppm)
PSM Score: 21.78

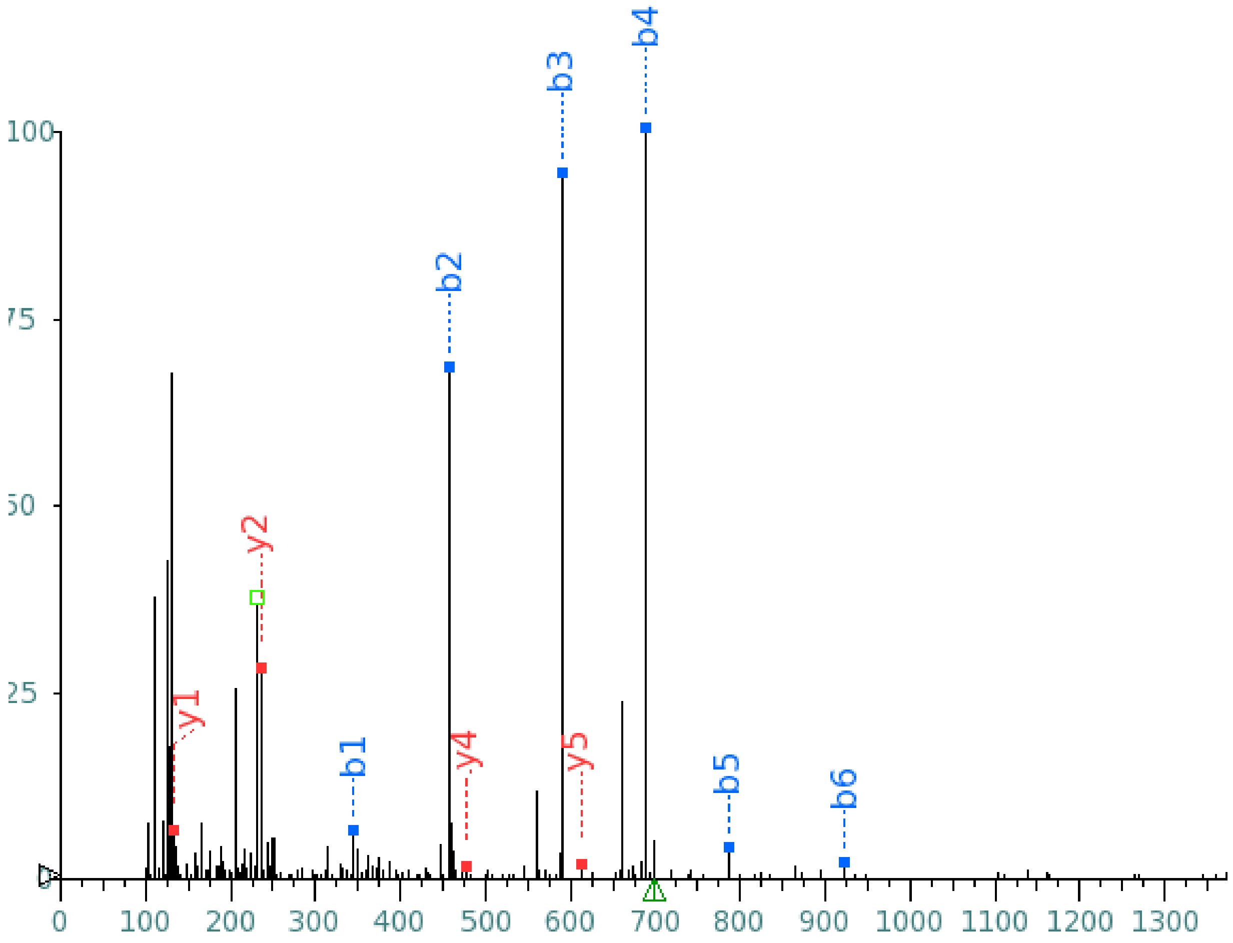

LDMVPHLETD (+2)

Primary Reference: AKP8L_HUMAN
Search ID: 41760
Search Name: 20130328_ananiav_ERstress_TMT_TM_timecourse_trypsin_inj1_4mc_iaa
Scan#: 15430
Observed Mass: 699.8625 (2.9 ppm)
PSM Score: 34.6

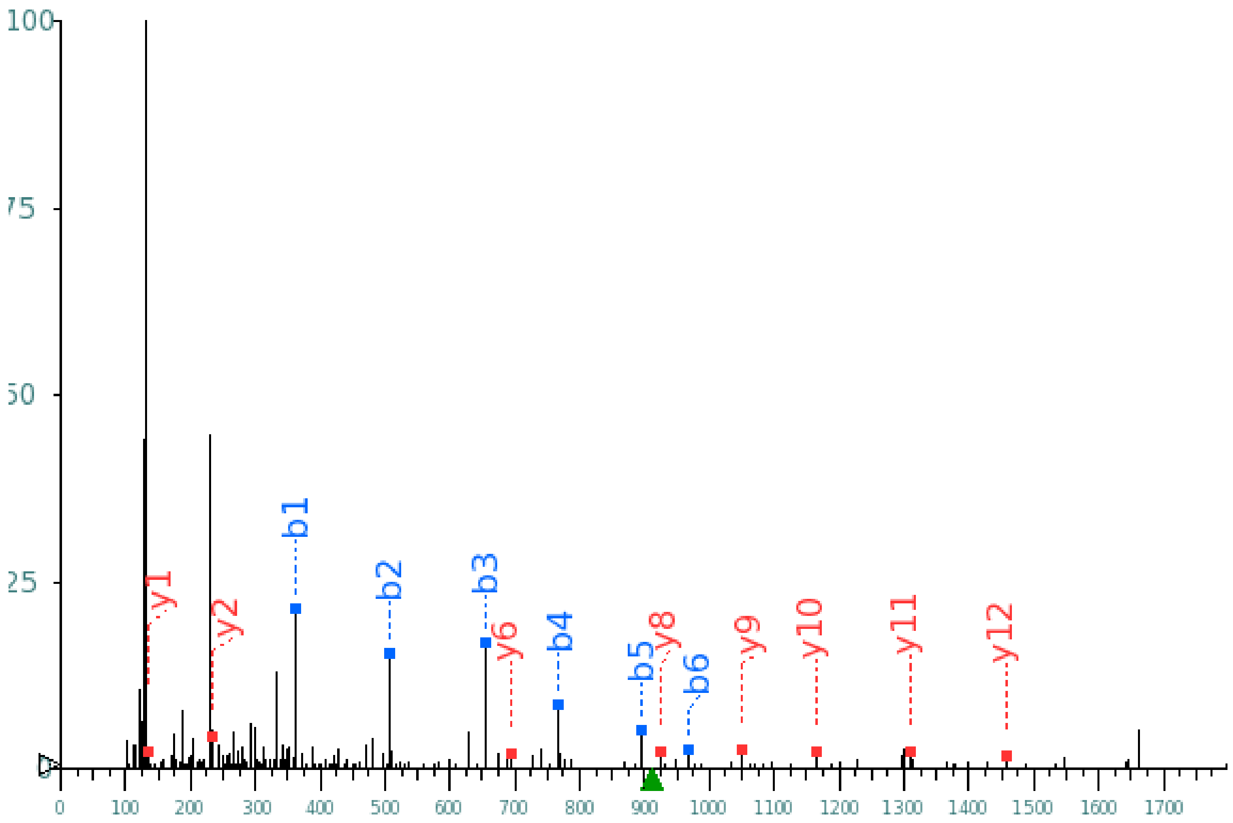

MFFIQAC*RGDETD (+2)

Primary Reference: CASP2_HUMAN
Search ID: 41760
Search Name: 20130328_ananiav_ERstress_TMT_TM_timecourse_trypsin_inj1_4mc_iaa
Scan#: 15461
Observed Mass: 909.9239 (2.9 ppm)
PSM Score: 25.09

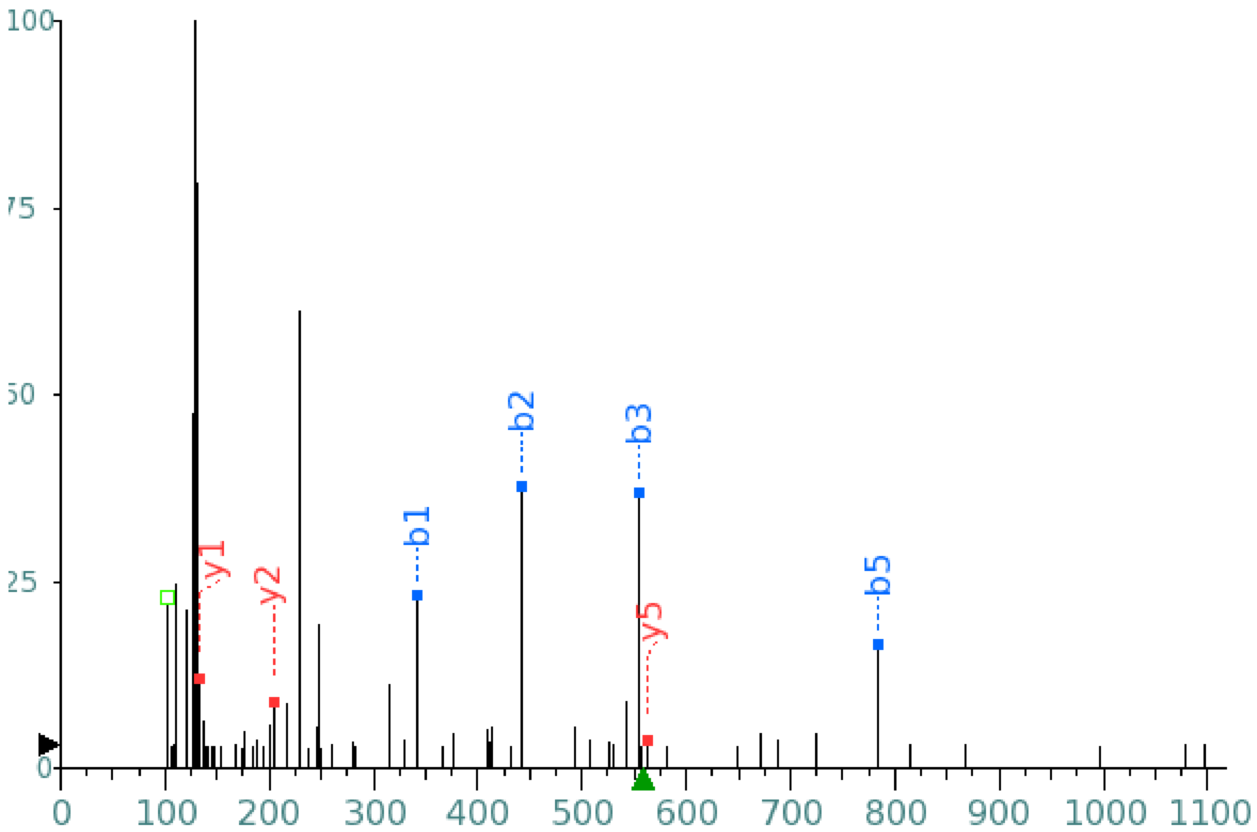

IVILDEAD (+2)

Primary Reference: RFC4_HUMAN
Search ID: 41760
Search Name: 20130328_ananiav_ERstress_TMT_TM_timecourse_trypsin_inj1_4mc_iaa
Scan#: 15571
Observed Mass: 558.8232 (3.7 ppm)
PSM Score: 13.72

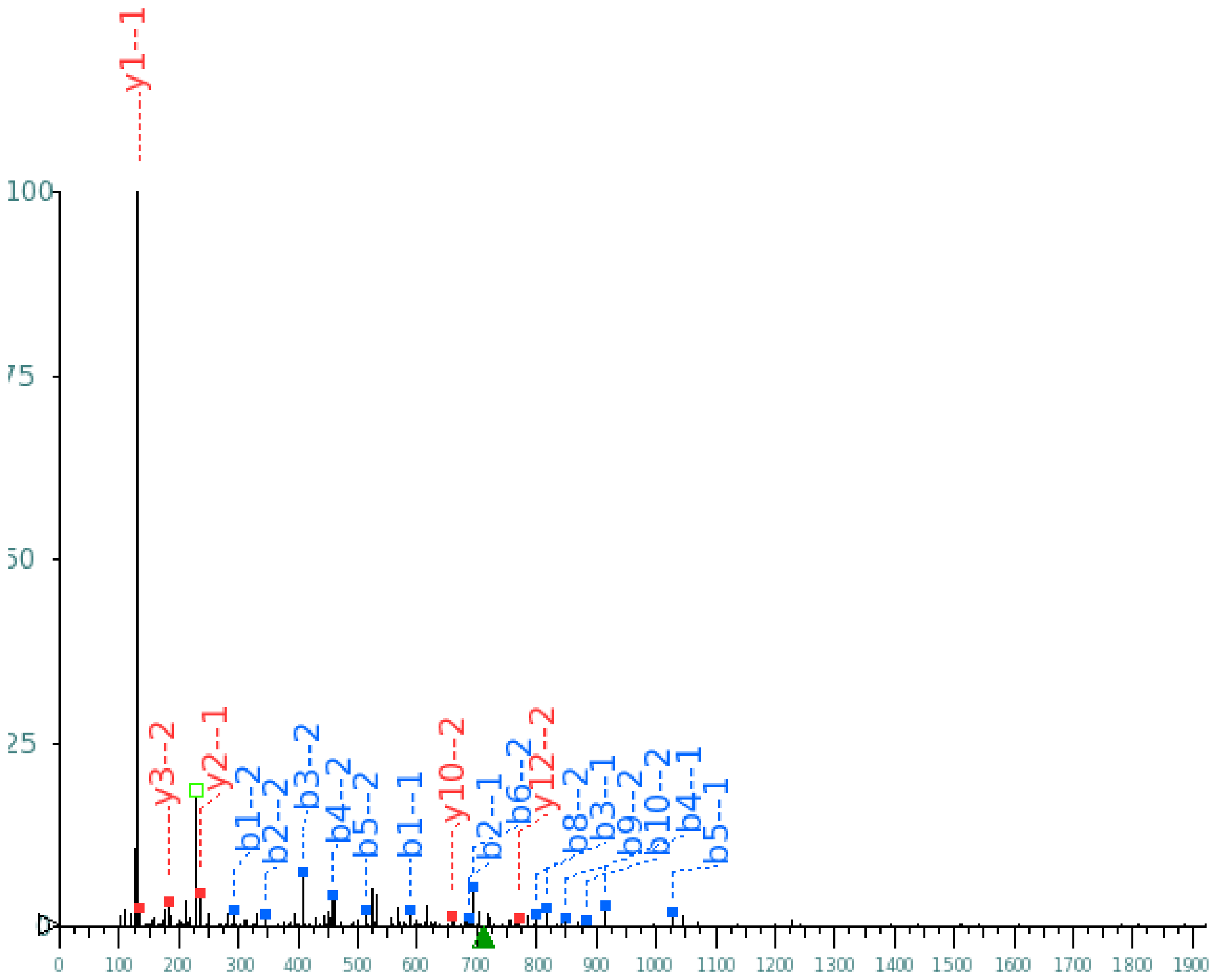

KVEVIKPLVAETD (+3)

Primary Reference: HMDH_HUMAN
Search ID: 41760
Search Name: 20130328_ananiav_ERstress_TMT_TM_timecourse_trypsin_inj1_4mc_iaa
Scan#: 15595
Observed Mass: 710.1136 (3.2 ppm)
PSM Score: 12.84

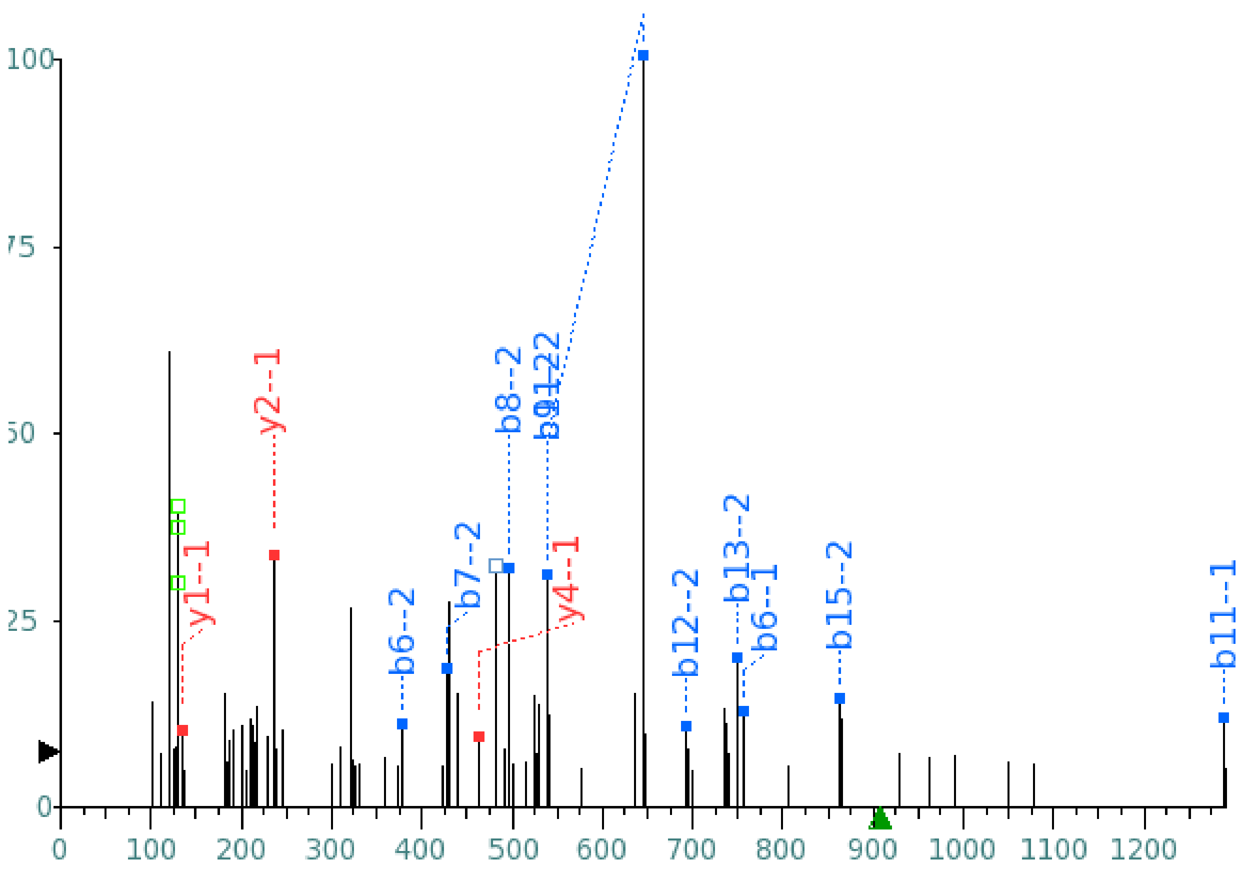

RNGAAGPHSPDPLLDEQAFGDLTD (+3)

Primary Reference: BORG4_HUMAN
Search ID: 41760
Search Name: 20130328_ananiav_ERstress_TMT_TM_timecourse_trypsin_inj1_4mc_iaa
Scan#: 15974
Observed Mass: 908.1147 (0.9 ppm)
PSM Score: 46.71

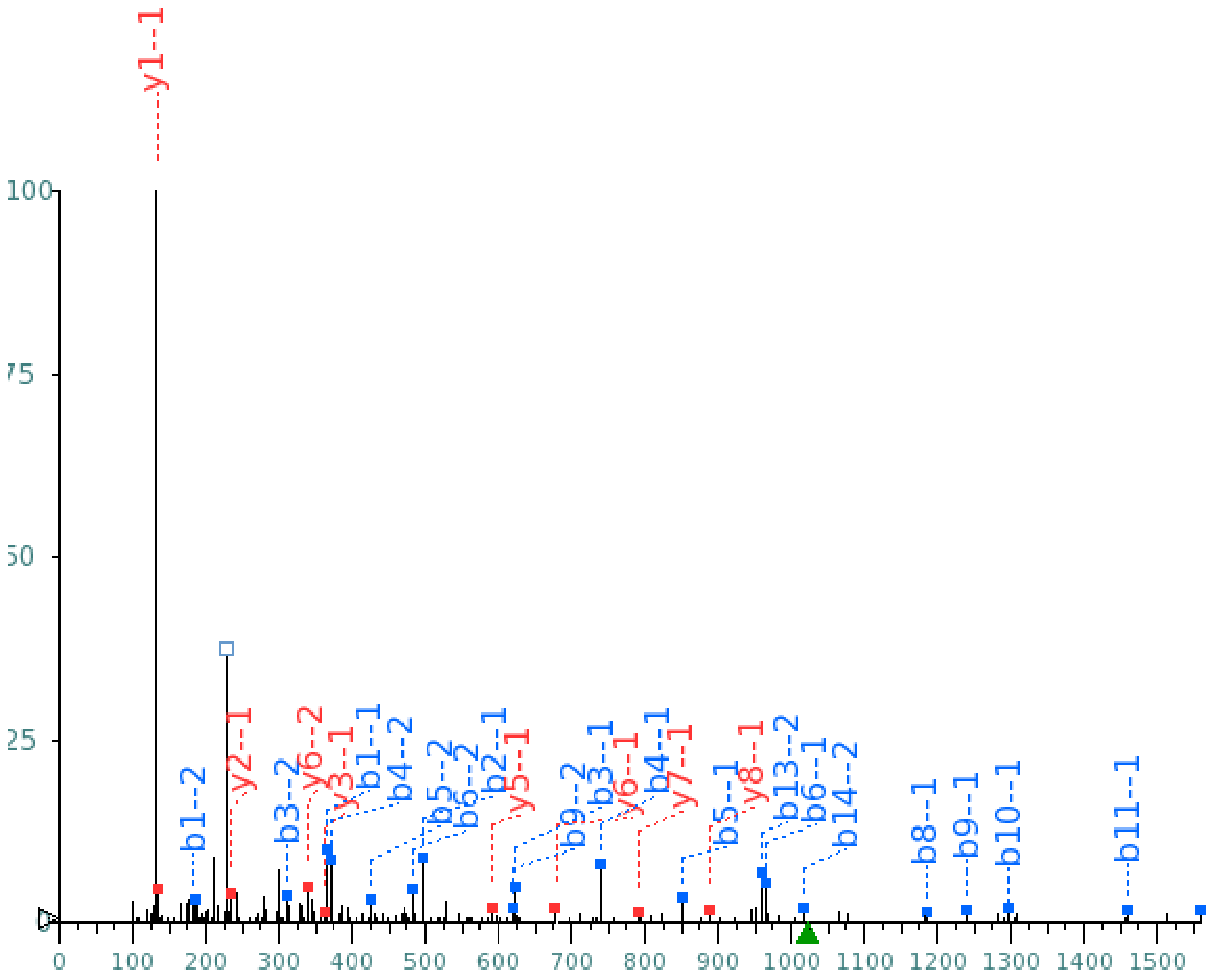

HEQNIDC*GGGYVKLFPNSLDQTD (+3)

Primary Reference: CALR_HUMAN
Search ID: 41760
Search Name: 20130328_ananiav_ERstress_TMT_TM_timecourse_trypsin_inj1_4mc_iaa
Scan#: 15978
Observed Mass: 1022.5103 (4.1 ppm)
PSM Score: 58.2

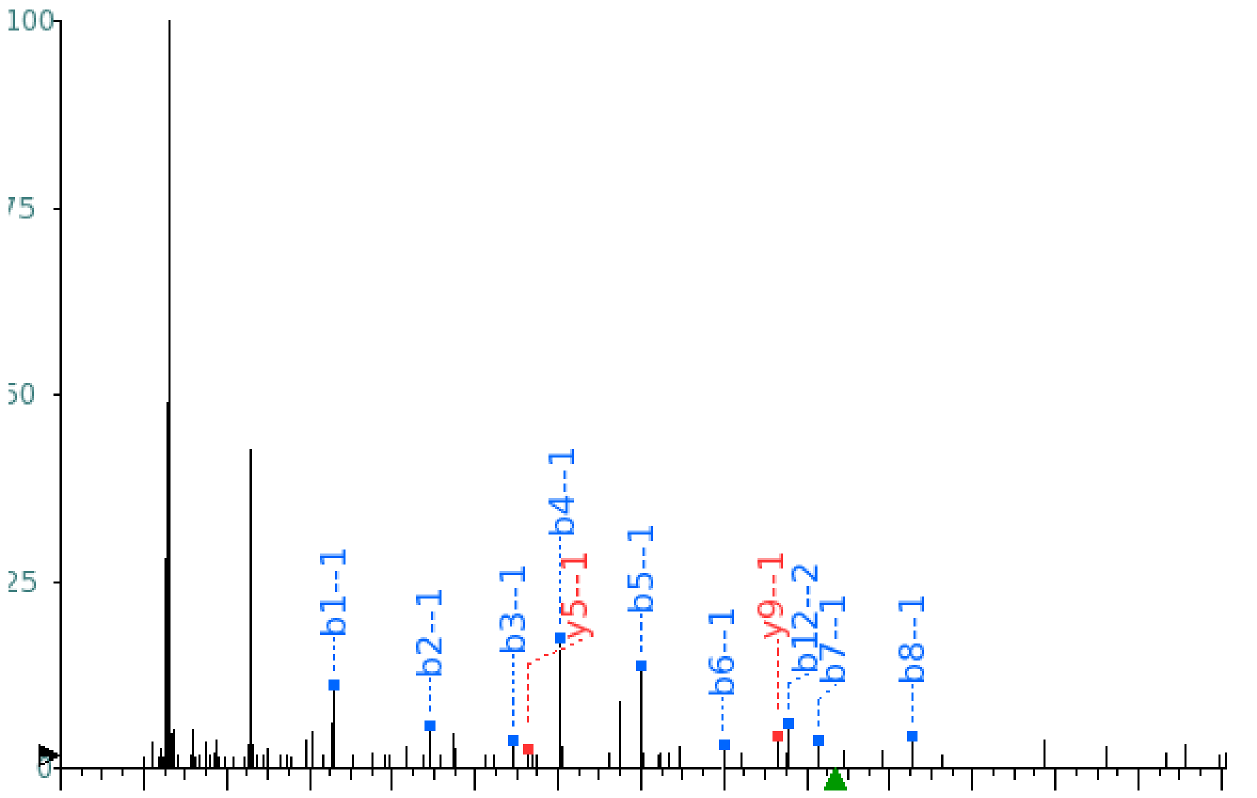

TITGVPDNIQKELASGTGQDDAD (+3)

Primary Reference: E2QRJ1_HUMAN
Search ID: 41760
Search Name: 20130328_ananiav_ERstress_TMT_TM_timecourse_trypsin_inj1_4mc_iaa
Scan#: 16016
Observed Mass: 935.1516 (1.4 ppm)
PSM Score: 28.65

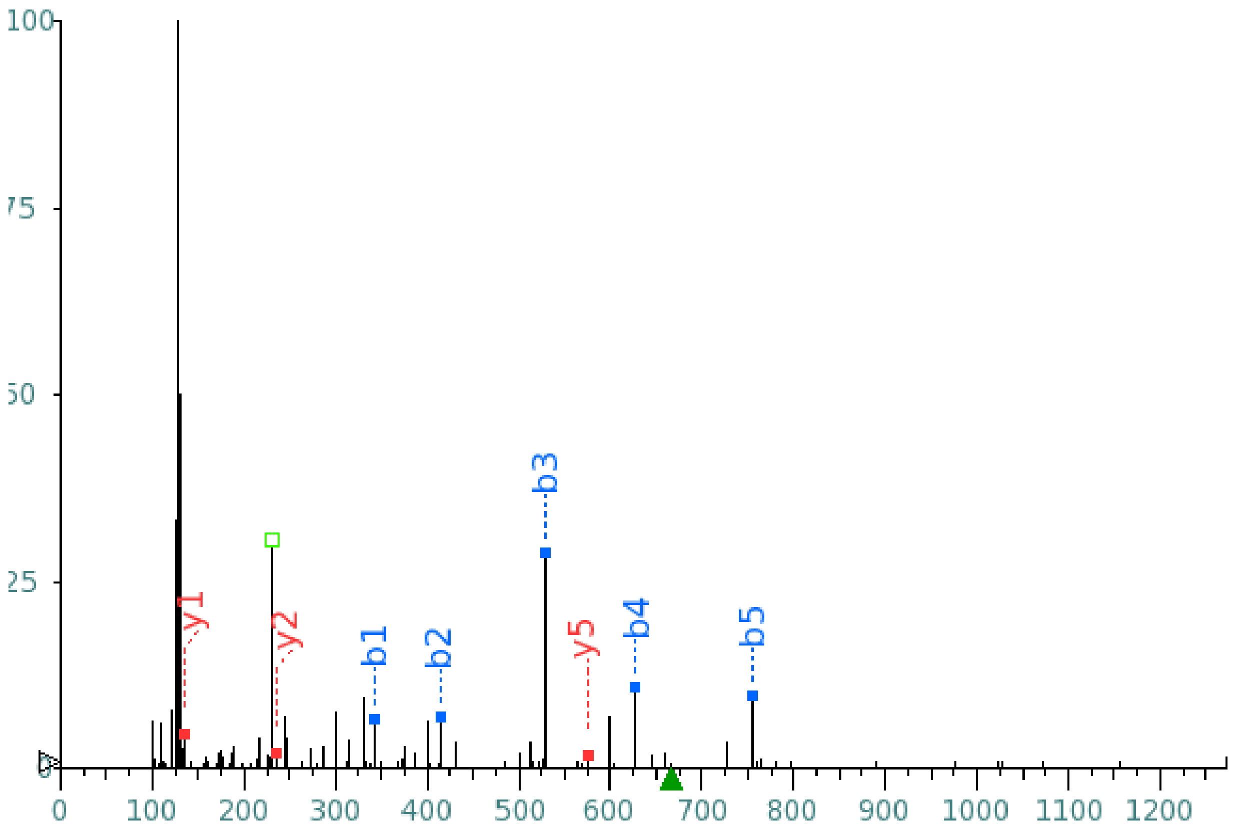

LANVQLLDTD (+2)

Primary Reference: PA1B2_HUMAN
Search ID: 41760
Search Name: 20130328_ananiav_ERstress_TMT_TM_timecourse_trypsin_inj1_4mc_iaa
Scan#: 16068
Observed Mass: 665.8761 (2.5 ppm)
PSM Score: 9.62

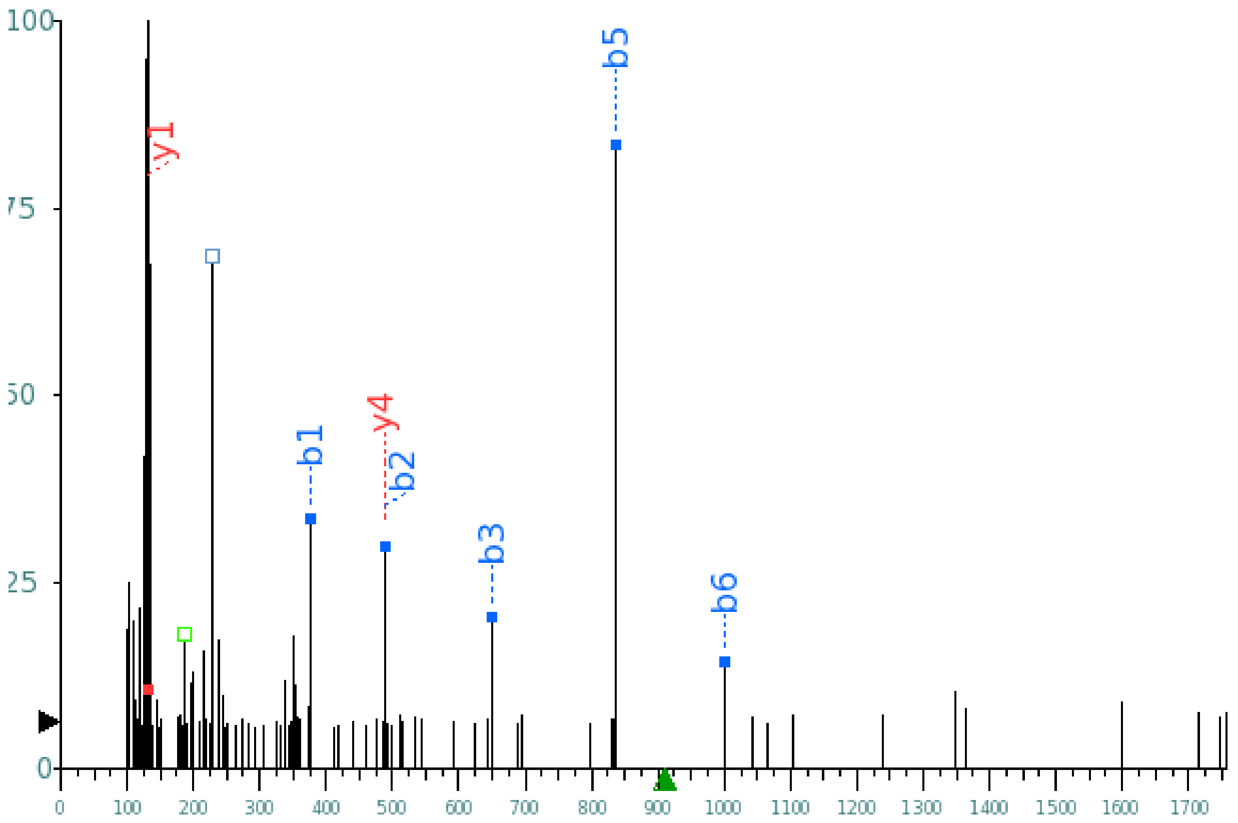

FLC*ADYAEQDELD (+2)

Primary Reference: ACINU_HUMAN
Search ID: 41760
Search Name: 20130328_ananiav_ERstress_TMT_TM_timecourse_trypsin_inj1_4mc_iaa
Scan#: 16090
Observed Mass: 909.4099 (1.4 ppm)
PSM Score: 13.94

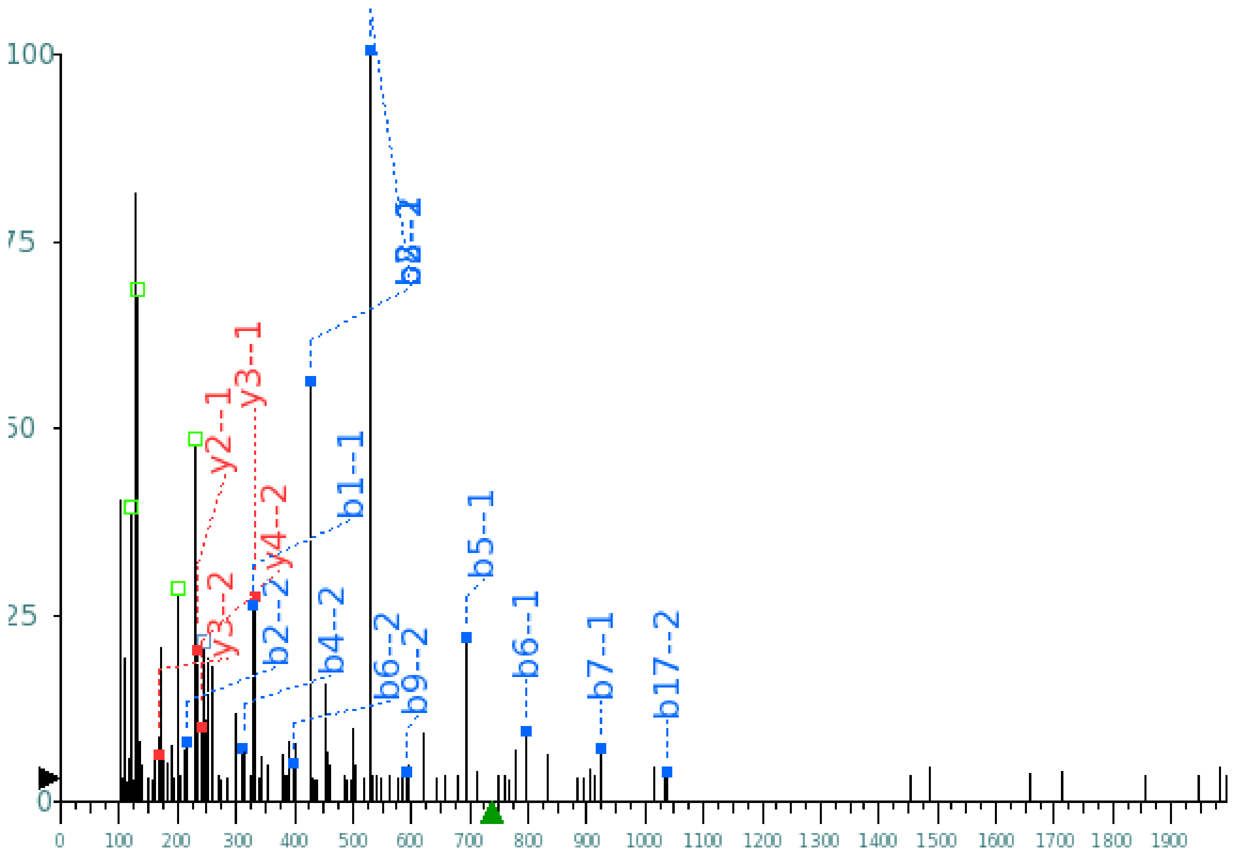

VVVPATEEEEAEVDEFPTD (+3)

Primary Reference: PESC_HUMAN
Search ID: 41760
Search Name: 20130328_ananiav_ERstress_TMT_TM_timecourse_trypsin_inj1_4mc_iaa
Scan#: 16111
Observed Mass: 735.6958 (3.7 ppm)
PSM Score: 16.93

File name: 2015-02-04 04.01.53PM.pdf

Creation time: 2015-02-04 04:09PM

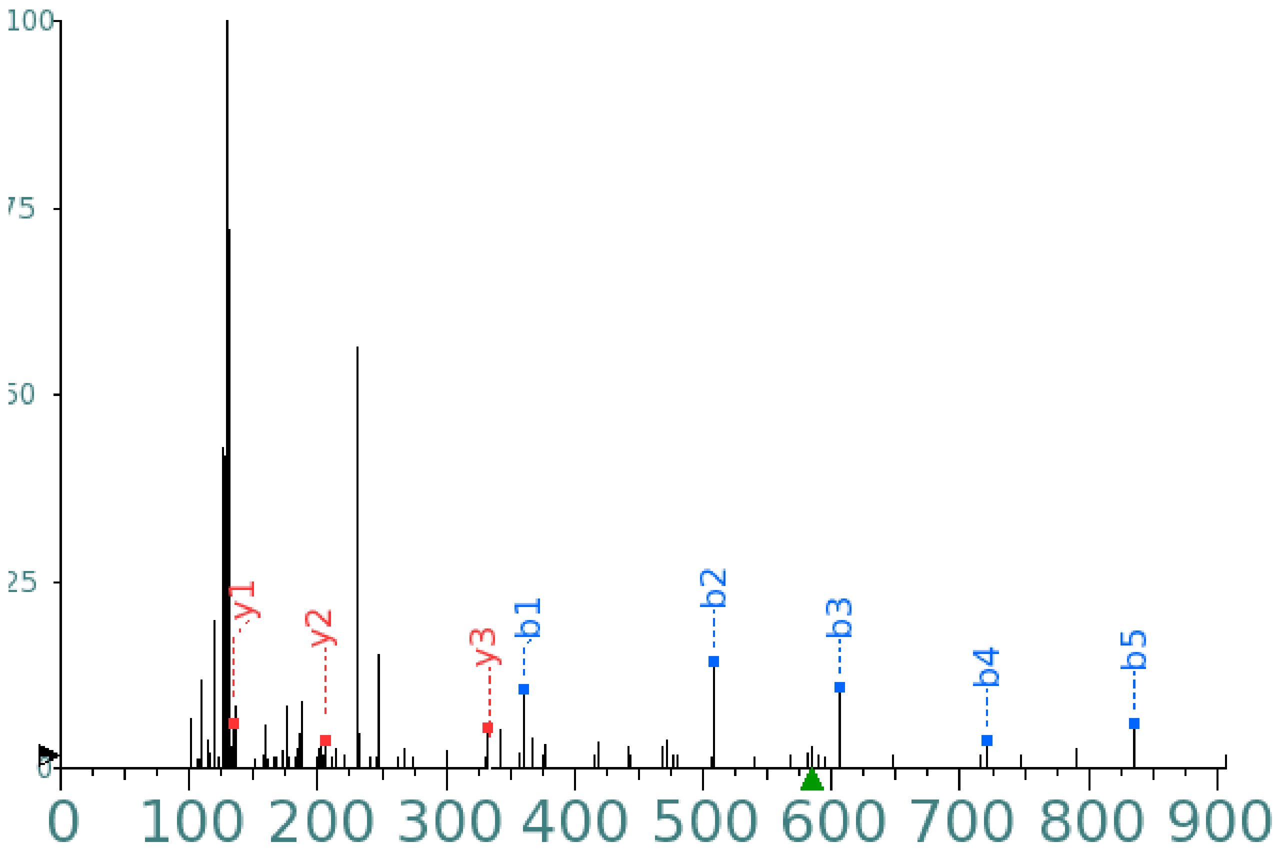

MFVLDEAD (+2)

Primary Reference: IF4A1_HUMAN
Search ID: 41760
Search Name: 20130328_ananiav_ERstress_TMT_TM_timecourse_trypsin_inj1_4mc_iaa
Scan#: 16133
Observed Mass: 584.7928 (2.3 ppm)
PSM Score: 24.28

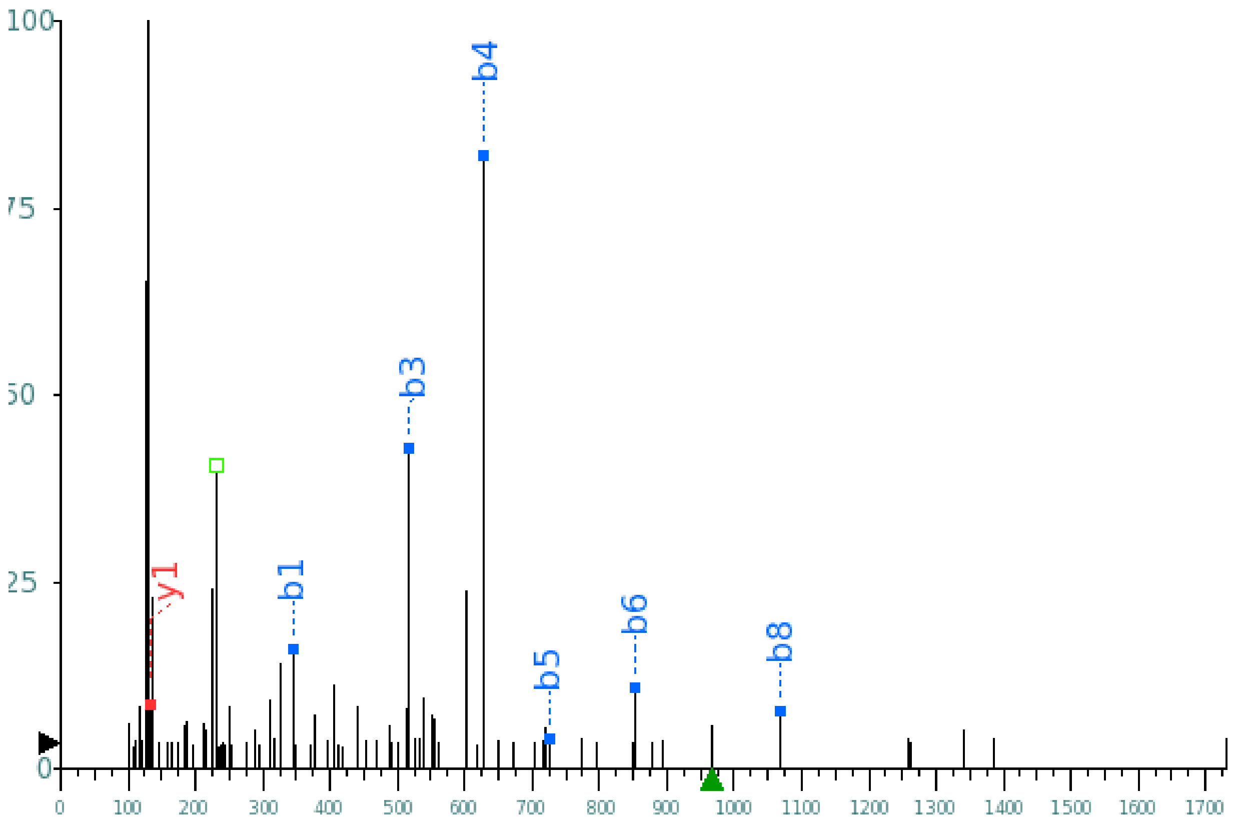

DLGLPQTNVEYIQTD (+2)

Primary Reference: HTRA2_HUMAN
Search ID: 41760
Search Name: 20130328_ananiav_ERstress_TMT_TM_timecourse_trypsin_inj1_4mc_iaa
Scan#: 16136
Observed Mass: 967.9996 (0.6 ppm)
PSM Score: 13.89

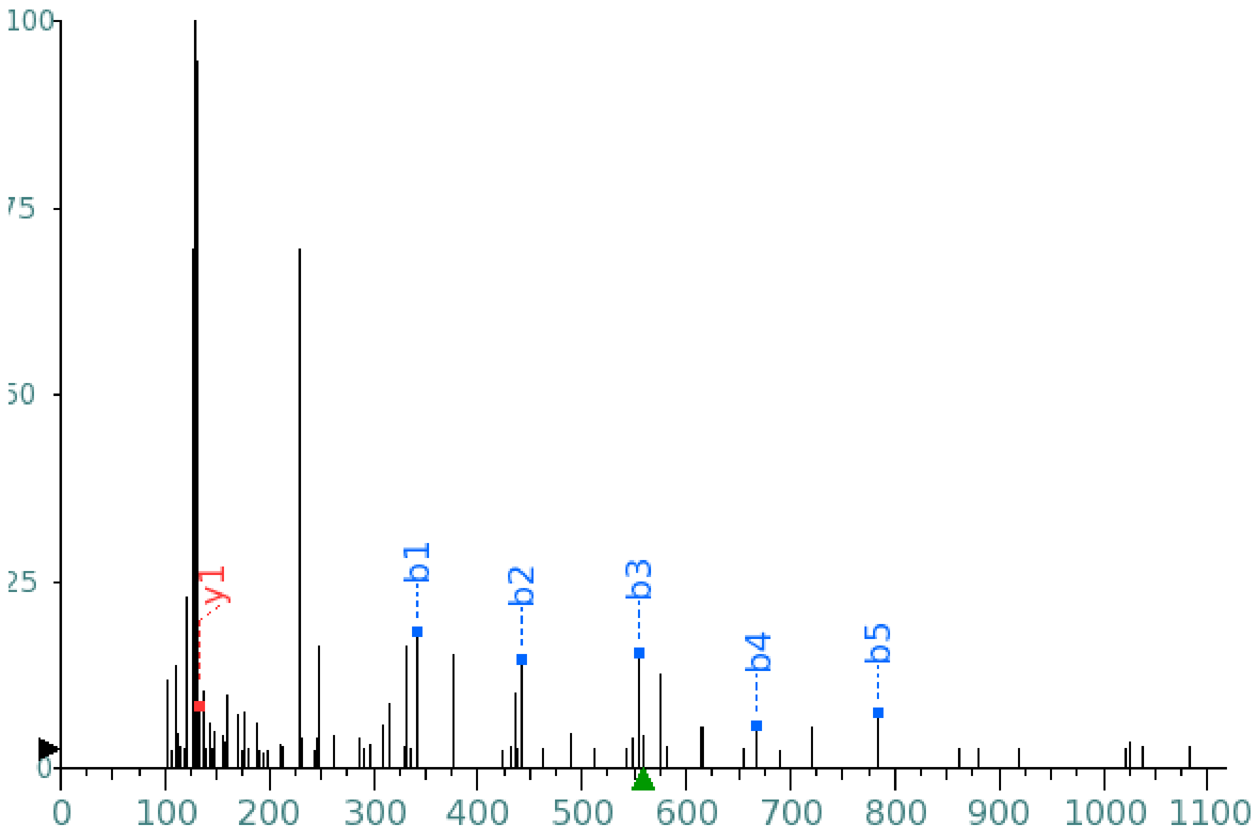

IVILDEAD (+2)

Primary Reference: RFC4_HUMAN
Search ID: 41760
Search Name: 20130328_ananiav_ERstress_TMT_TM_timecourse_trypsin_inj1_4mc_iaa
Scan#: 16154
Observed Mass: 558.8222 (1.9 ppm)
PSM Score: 10.17

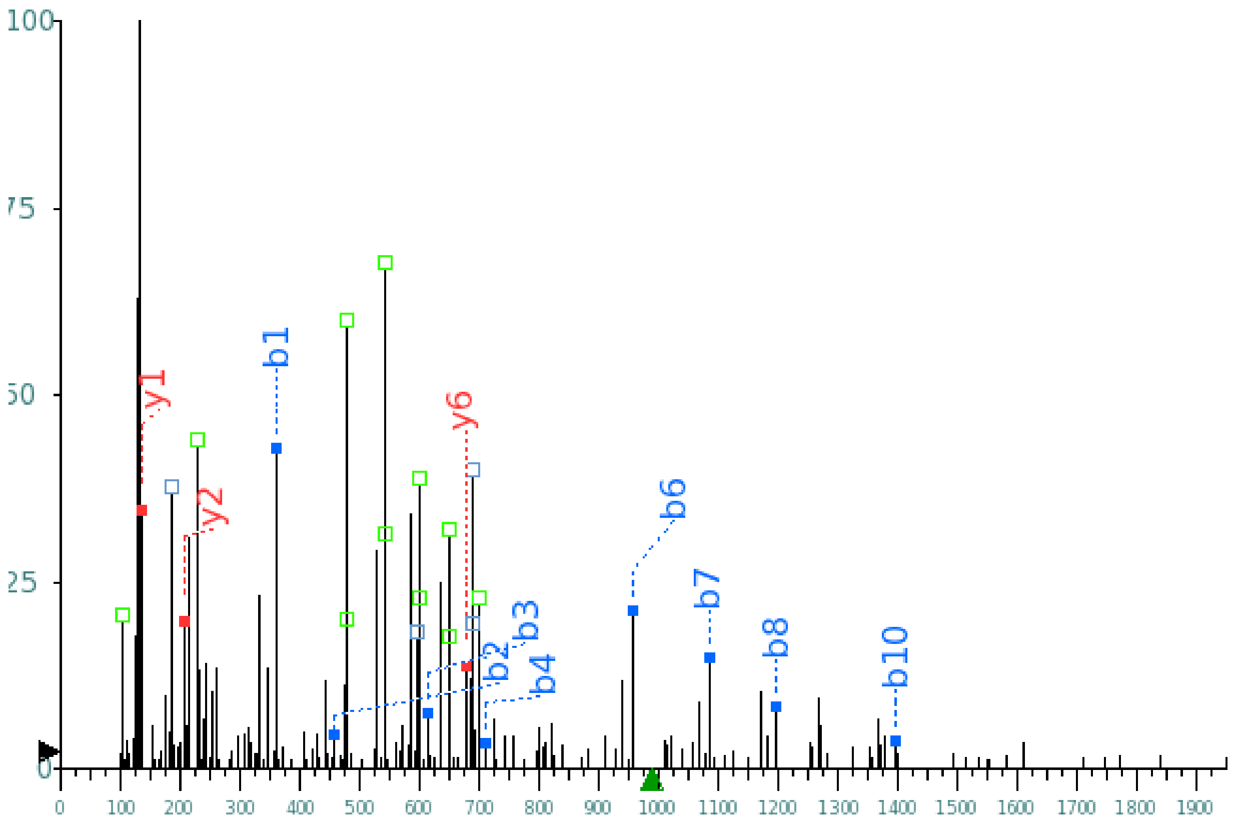

EPRPMDELVTLEEAD (+2)

Primary Reference: CACO1_HUMAN
Search ID: 41760
Search Name: 20130328_ananiav_ERstress_TMT_TM_timecourse_trypsin_inj1_4mc_iaa
Scan#: 16229
Observed Mass: 986.9917 (1.4 ppm)
PSM Score: 19.78

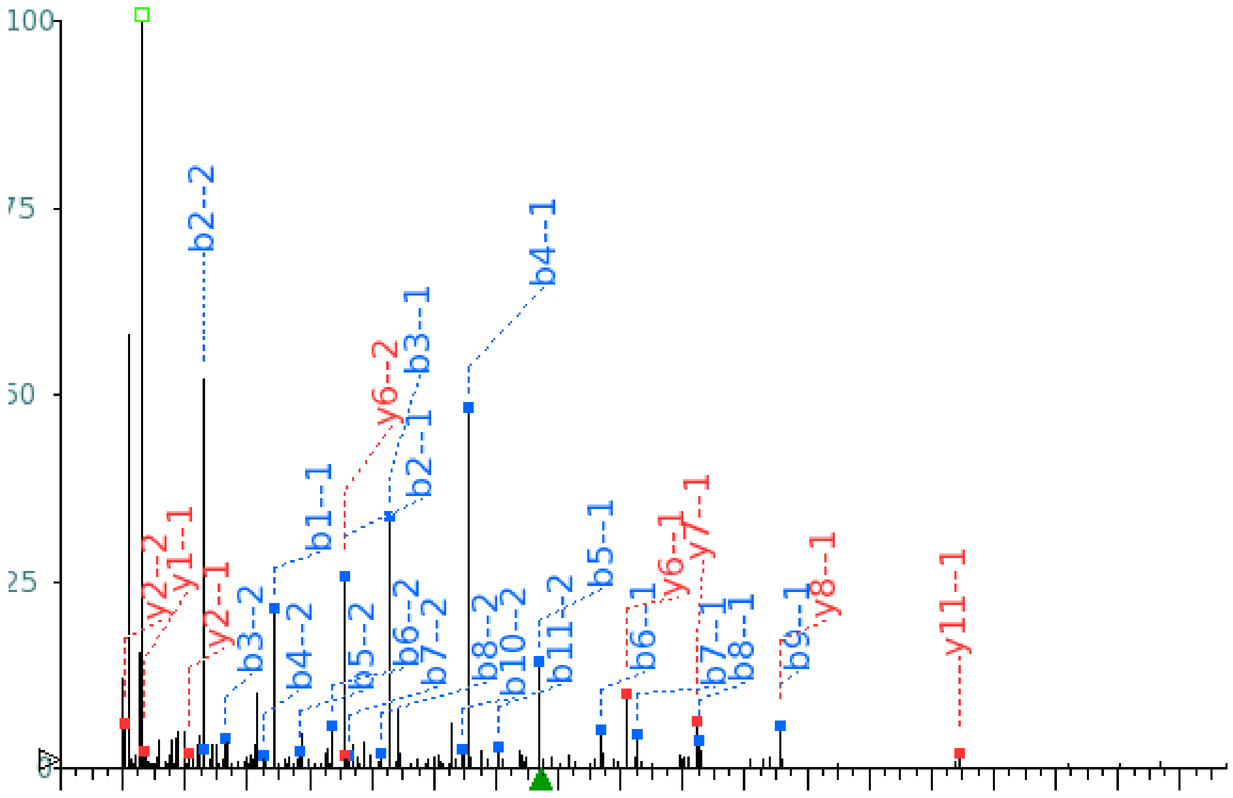

ILAQITGTEHLKESEAD (+3)

Primary Reference: PDLI5_HUMAN
Search ID: 41760
Search Name: 20130328_ananiav_ERstress_TMT_TM_timecourse_trypsin_inj1_4mc_iaa
Scan#: 16245
Observed Mass: 771.7634 (2.3 ppm)
PSM Score: 37.66

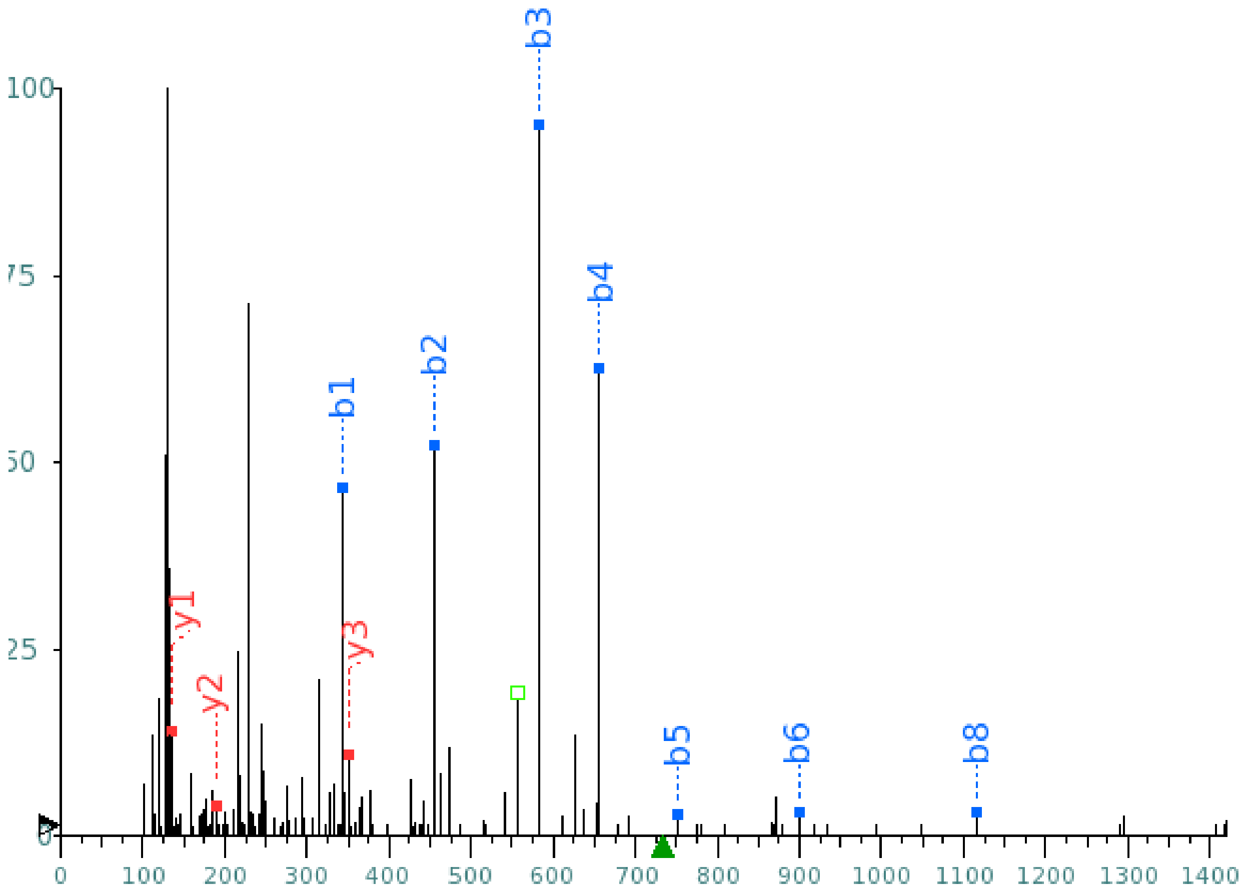

LLQAPFTDC*GD (+2)

Primary Reference: ITPR1_HUMAN
Search ID: 41760
Search Name: 20130328_ananiav_ERstress_TMT_TM_timecourse_trypsin_inj1_4mc_iaa
Scan#: 16266
Observed Mass: 733.3644 (1.3 ppm)
PSM Score: 29.93

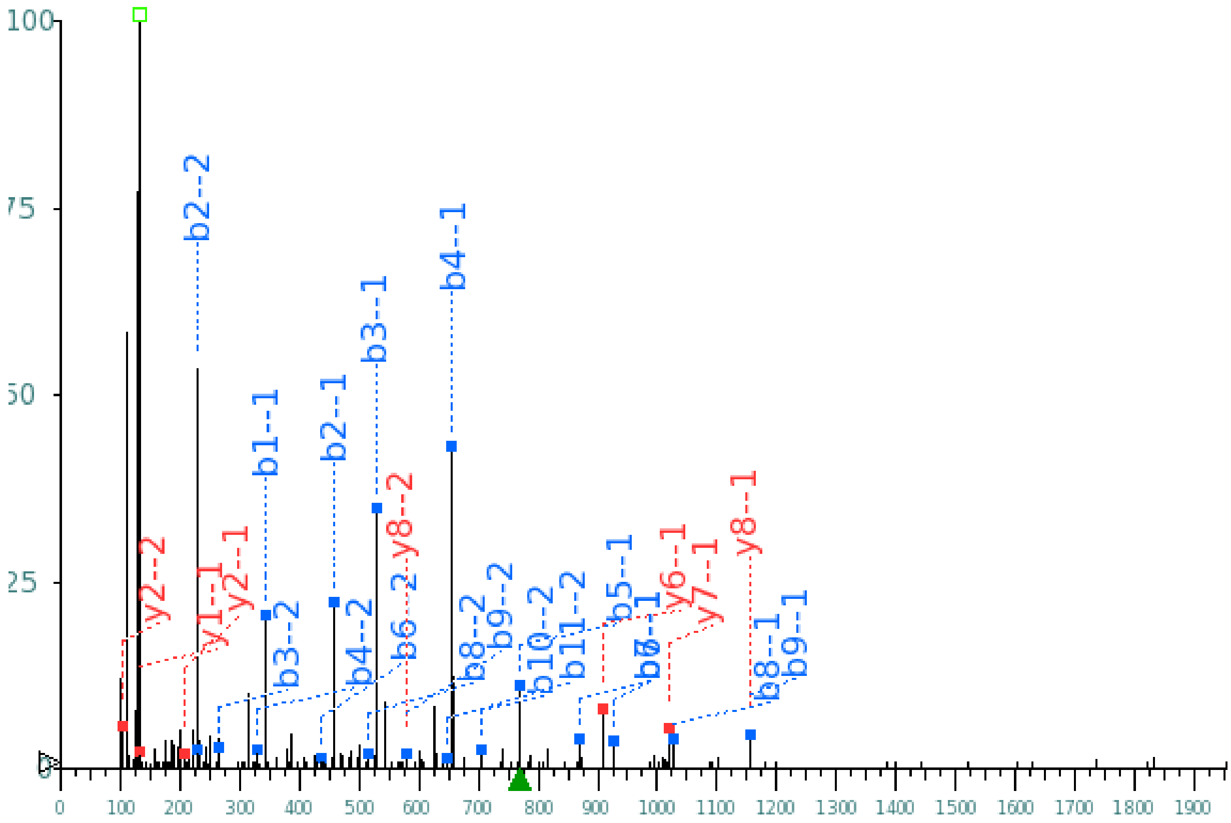

ILAQITGTEHLKESEAD (+3)

Primary Reference: PDLI5_HUMAN
Search ID: 41760
Search Name: 20130328_ananiav_ERstress_TMT_TM_timecourse_trypsin_inj1_4mc_iaa
Scan#: 16319
Observed Mass: 771.7633 (2.3 ppm)
PSM Score: 35.5

File name: 2015-02-04 04.01.53PM.pdf

Creation time: 2015-02-04 04:09PM

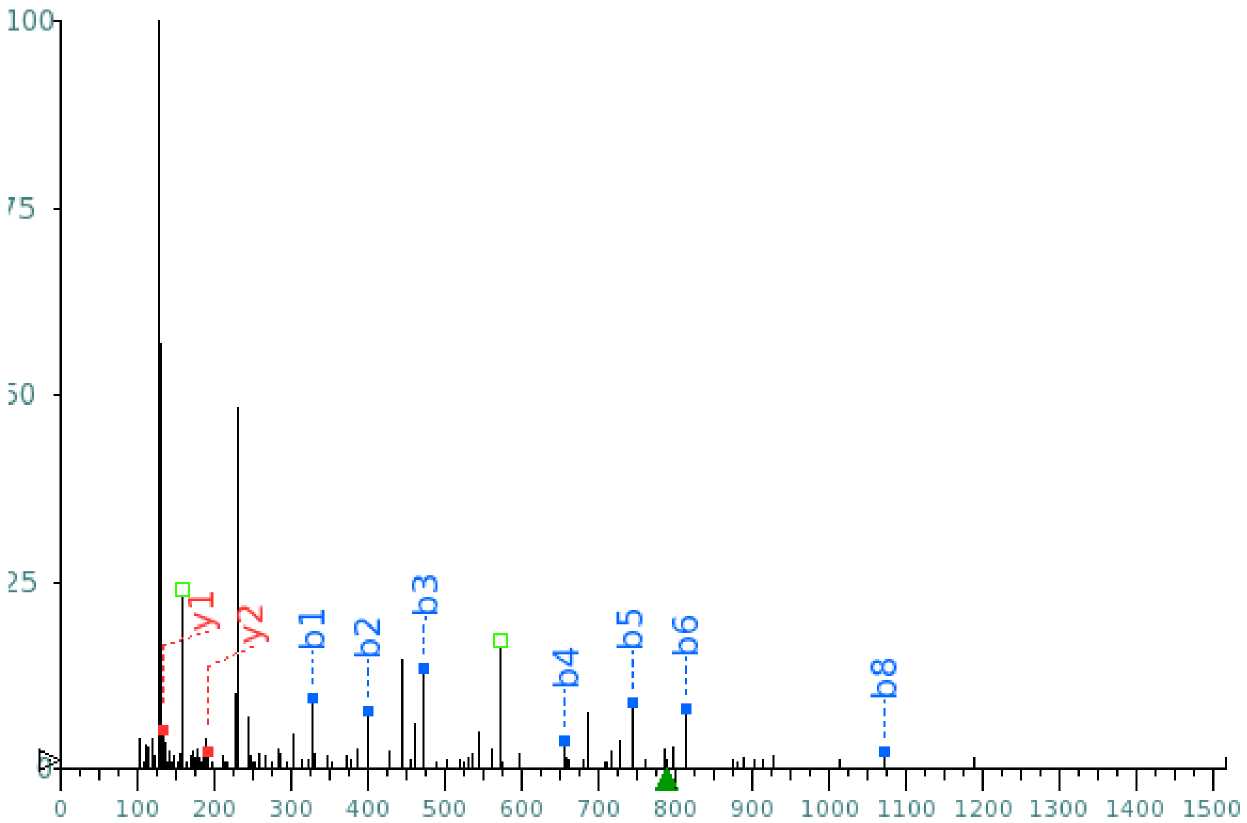

VAAWSAWAADQGD (+2)

Primary Reference: EPS8_HUMAN
Search ID: 41760
Search Name: 20130328_ananiav_ERstress_TMT_TM_timecourse_trypsin_inj1_4mc_iaa
Scan#: 16343
Observed Mass: 788.8853 (2.5 ppm)
PSM Score: 18.92

File name: 2015-02-04 04.01.53PM.pdf

Creation time: 2015-02-04 04:09PM

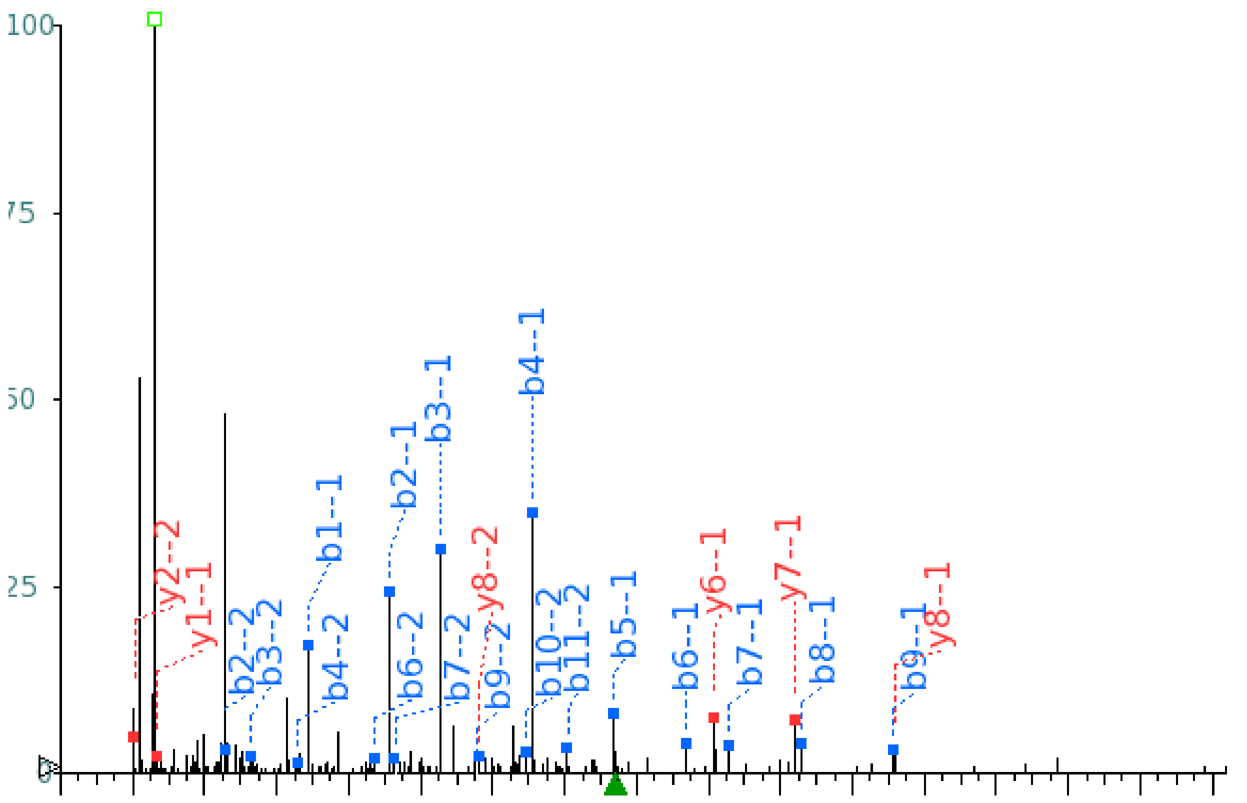

ILAQITGTEHLKESEAD (+3)

Primary Reference: PDLI5_HUMAN
Search ID: 41760
Search Name: 20130328_ananiav_ERstress_TMT_TM_timecourse_trypsin_inj1_4mc_iaa
Scan#: 16407
Observed Mass: 771.7636 (2.6 ppm)
PSM Score: 36.82

File name: 2015-02-04 04.01.53PM.pdf

Creation time: 2015-02-04 04:09PM

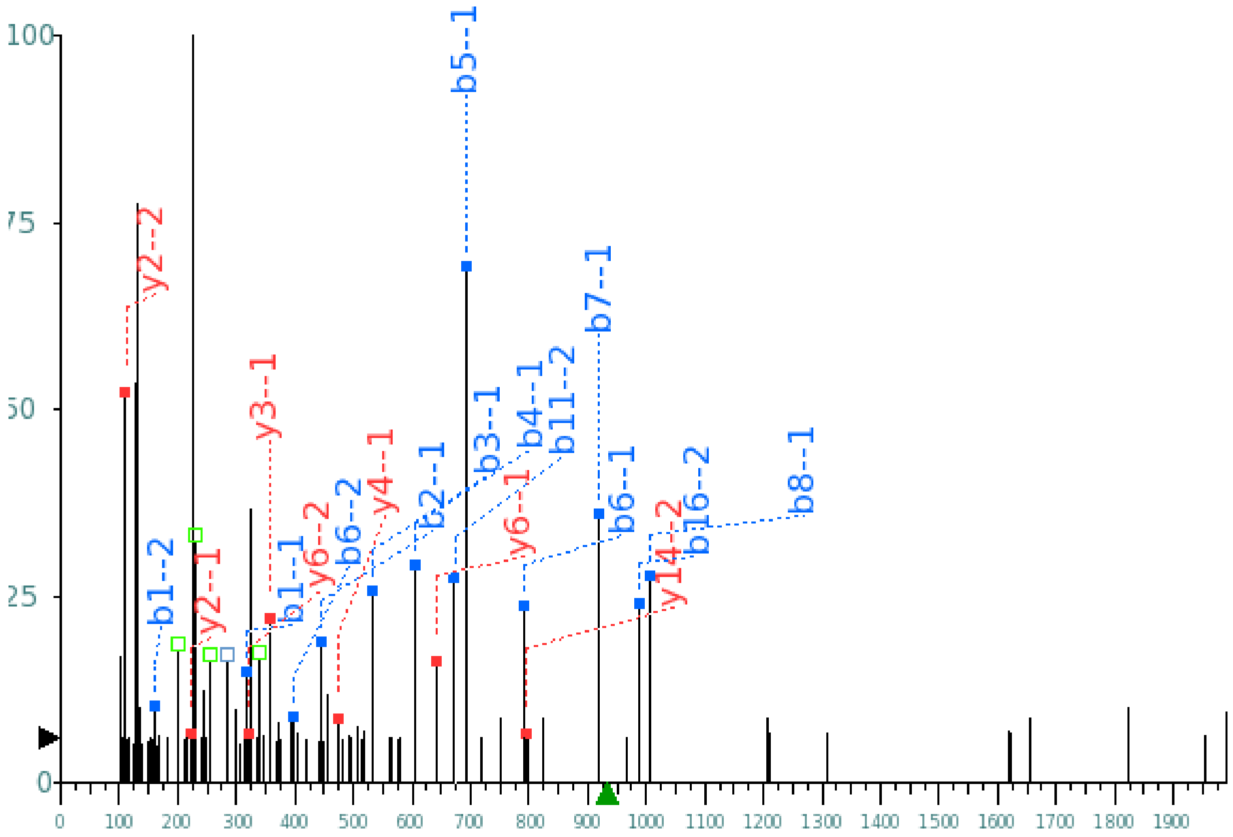

SQSASVESIPEVLEEC*TSPADHSD (+3)

Primary Reference: GBF1_HUMAN
Search ID: 41760
Search Name: 20130328_ananiav_ERstress_TMT_TM_timecourse_trypsin_inj1_4mc_iaa
Scan#: 16408
Observed Mass: 935.0998 (2.5 ppm)
PSM Score: 21.49

File name: 2015-02-04 04.01.53PM.pdf

Creation time: 2015-02-04 04:09PM

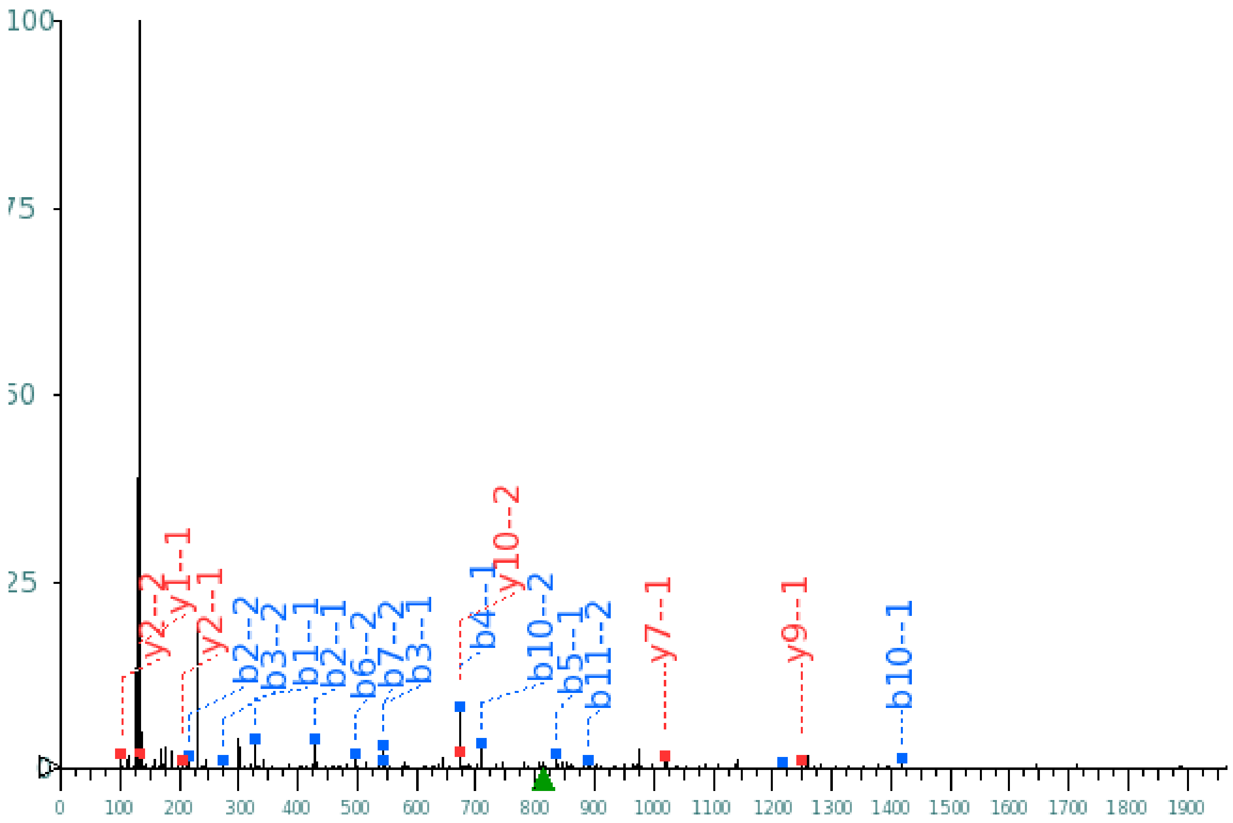

VTLEYRPVIDKTLNEAD (+3)

Primary Reference: DHSA_HUMAN
Search ID: 41760
Search Name: 20130328_ananiav_ERstress_TMT_TM_timecourse_trypsin_inj1_4mc_iaa
Scan#: 16423
Observed Mass: 812.1267 (2.5 ppm)
PSM Score: 15.11

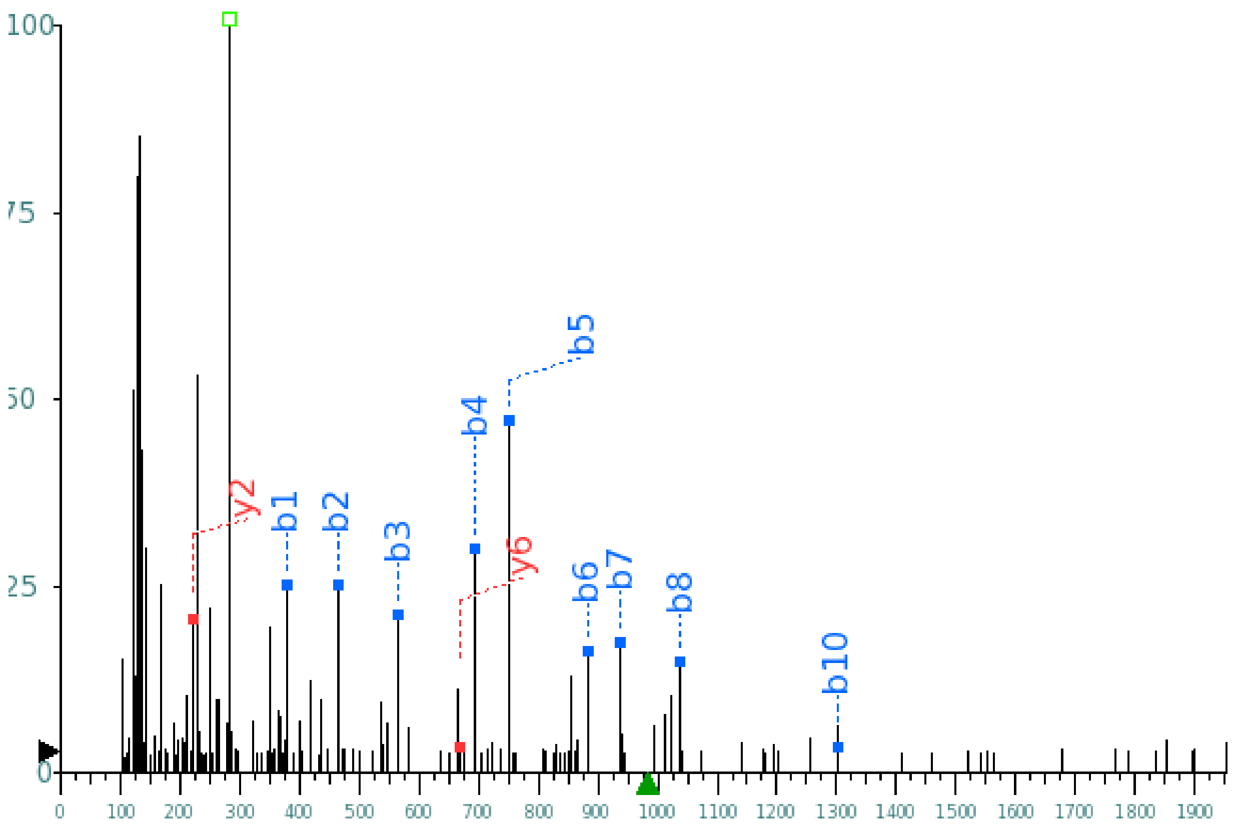

FSTQGMGTFNPADYSD (+2)

Primary Reference: UBAP2_HUMAN
Search ID: 41760
Search Name: 20130328_ananiav_ERstress_TMT_TM_timecourse_trypsin_inj1_4mc_iaa
Scan#: 16426
Observed Mass: 983.9404 (2.3 ppm)
PSM Score: 38.39

File name: 2015-02-04 04.01.53PM.pdf

Creation time: 2015-02-04 04:09PM

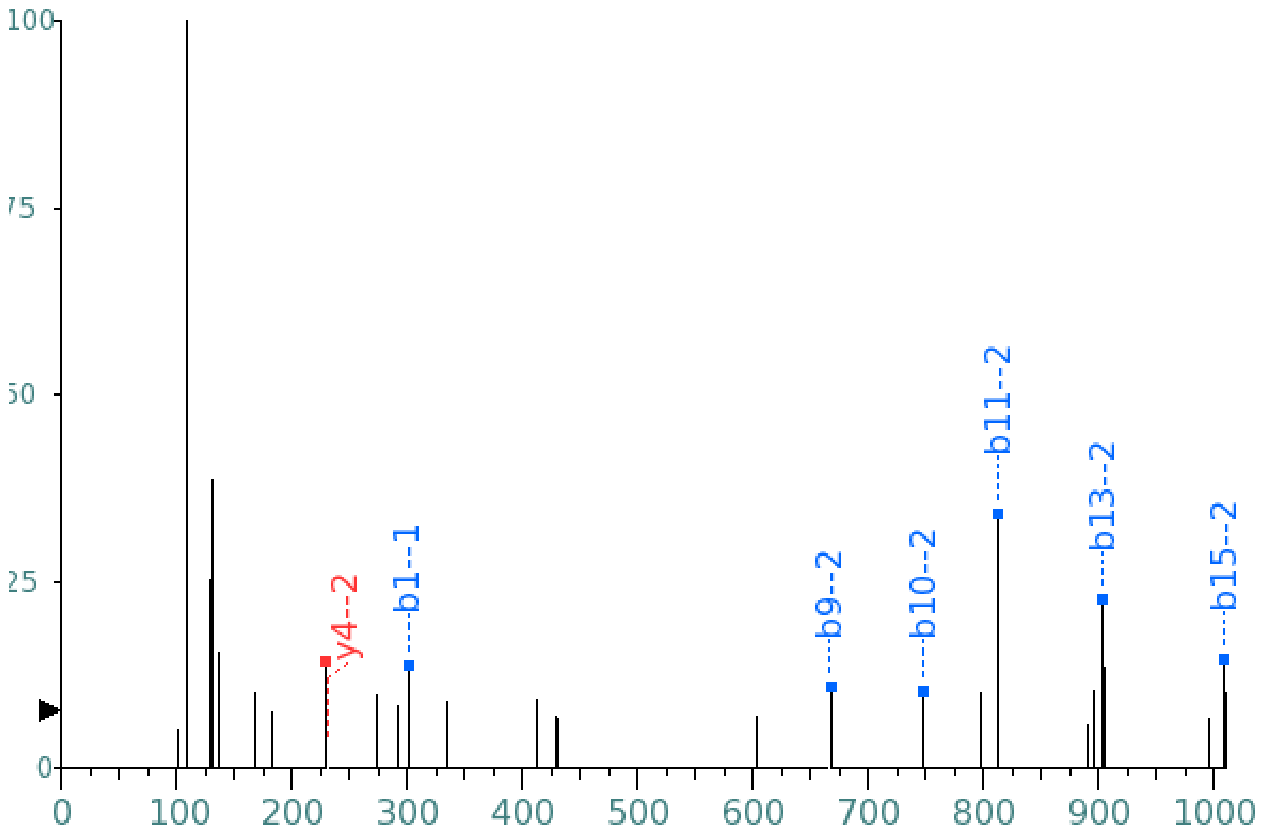

AEETRYPQQYQPSPLPVHYELLTD (+3)

Primary Reference: RB15B_HUMAN
Search ID: 41760
Search Name: 20130328_ananiav_ERstress_TMT_TM_timecourse_trypsin_inj1_4mc_iaa
Scan#: 16458
Observed Mass: 1035.1931 (2.4 ppm)
PSM Score: 25.05

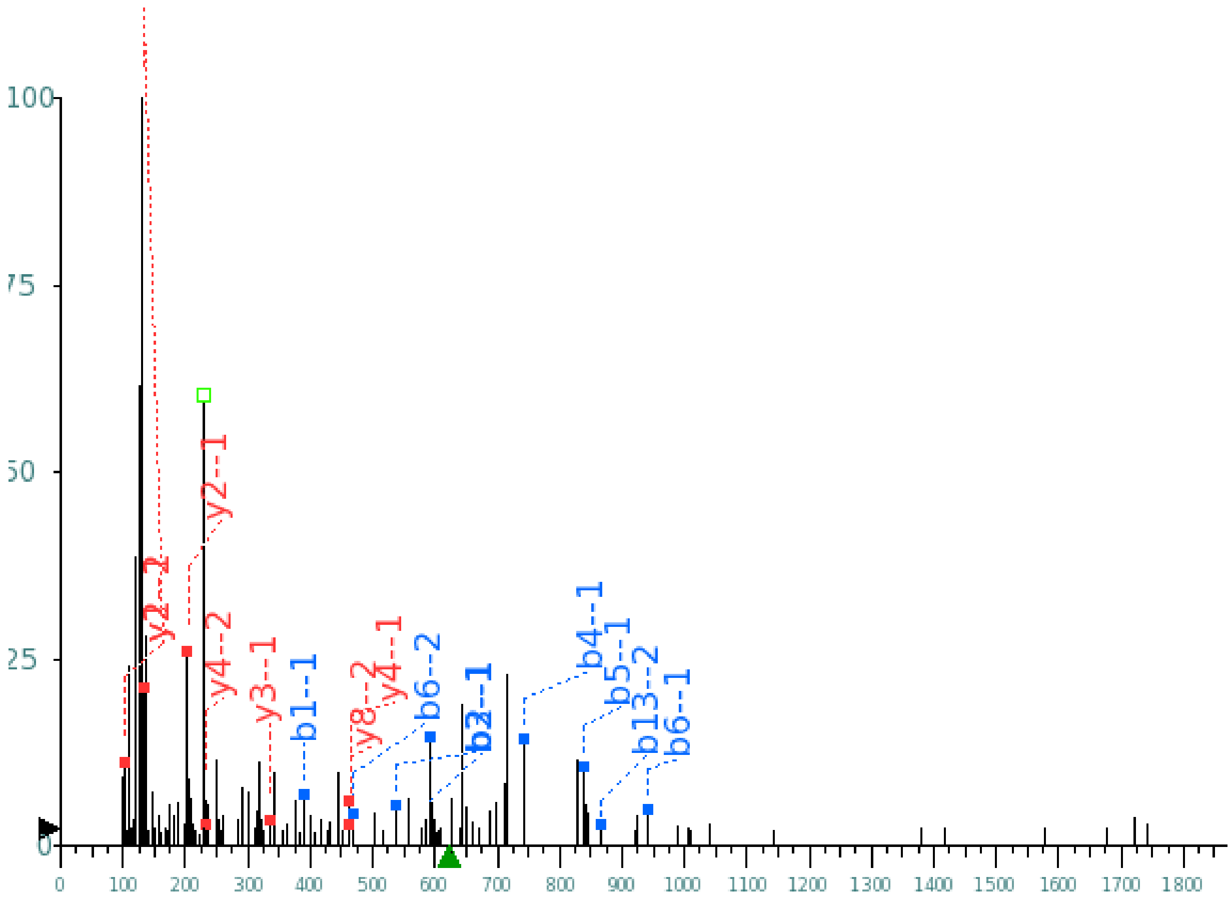

C*FGFVTYSNVEEAD (+3)

Primary Reference: ROA0_HUMAN
Search ID: 41760
Search Name: 20130328_ananiav_ERstress_TMT_TM_timecourse_trypsin_inj1_4mc_iaa
Scan#: 16467
Observed Mass: 622.9541 (3.2 ppm)
PSM Score: 2.91

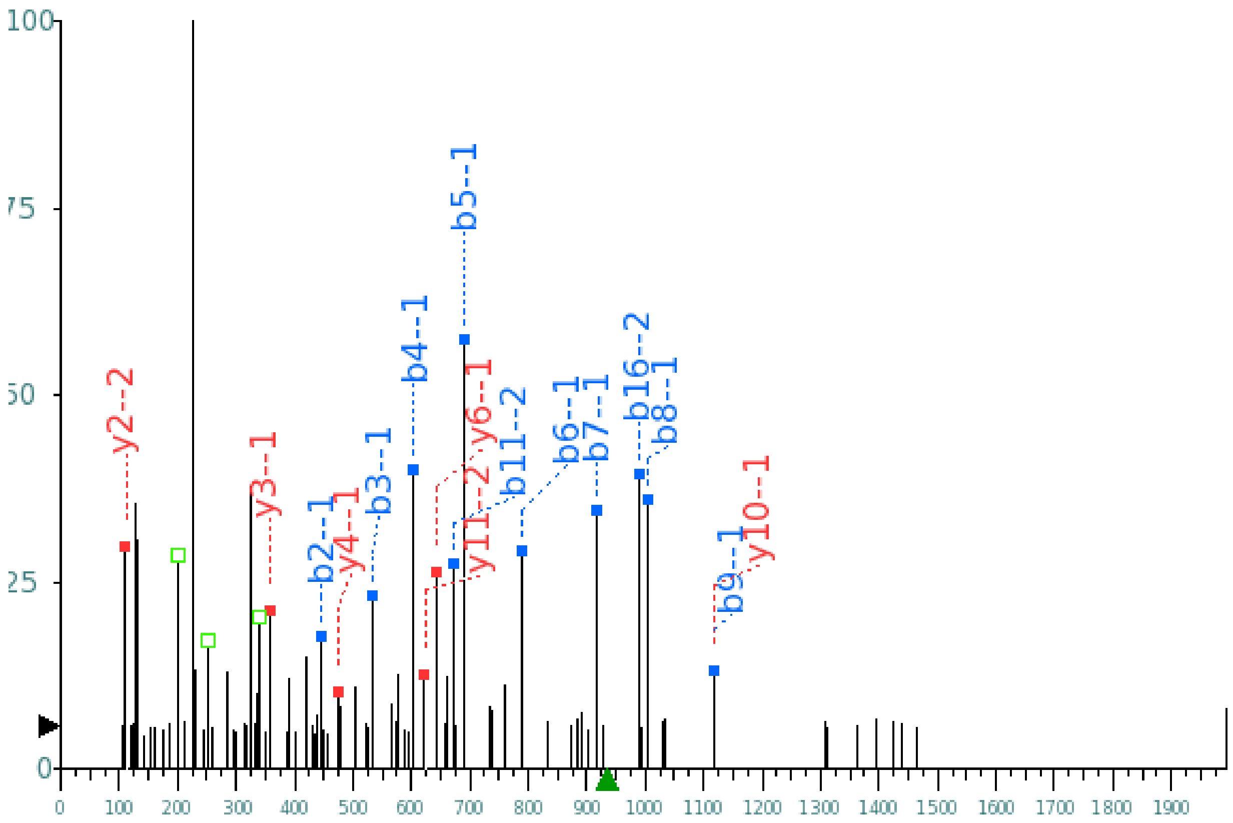

SQSASVESIPEVLEEC*TSPADHSD (+3)

Primary Reference: GBF1_HUMAN
Search ID: 41760
Search Name: 20130328_ananiav_ERstress_TMT_TM_timecourse_trypsin_inj1_4mc_iaa
Scan#: 16484
Observed Mass: 935.0999 (2.7 ppm)
PSM Score: 31.96

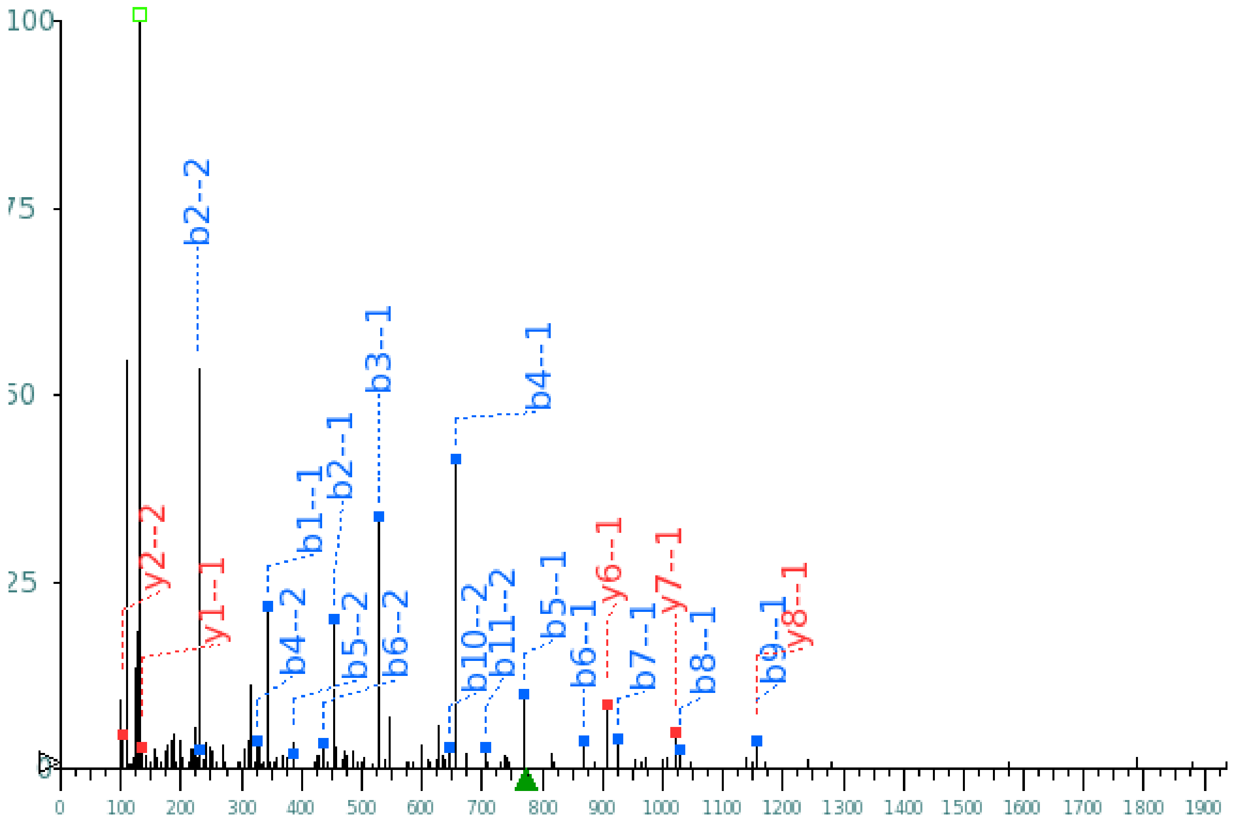

ILAQITGTEHLKESEAD (+3)

Primary Reference: PDLI5_HUMAN
Search ID: 41760
Search Name: 20130328_ananiav_ERstress_TMT_TM_timecourse_trypsin_inj1_4mc_iaa
Scan#: 16487
Observed Mass: 771.7634 (2.4 ppm)
PSM Score: 38.41

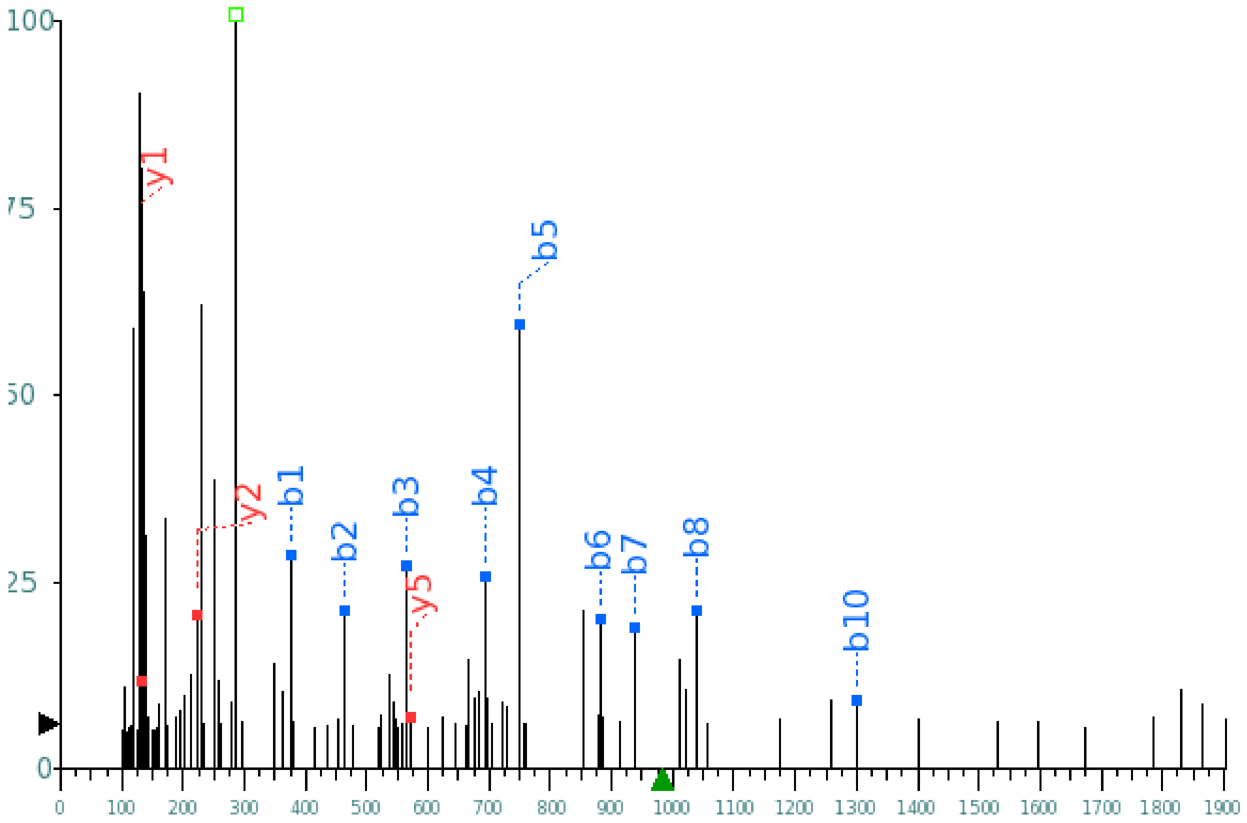

FSTQGMGTFNPADYSD (+2)

Primary Reference: UBAP2_HUMAN
Search ID: 41760
Search Name: 20130328_ananiav_ERstress_TMT_TM_timecourse_trypsin_inj1_4mc_iaa
Scan#: 16496
Observed Mass: 983.9403 (2.2 ppm)
PSM Score: 30.25

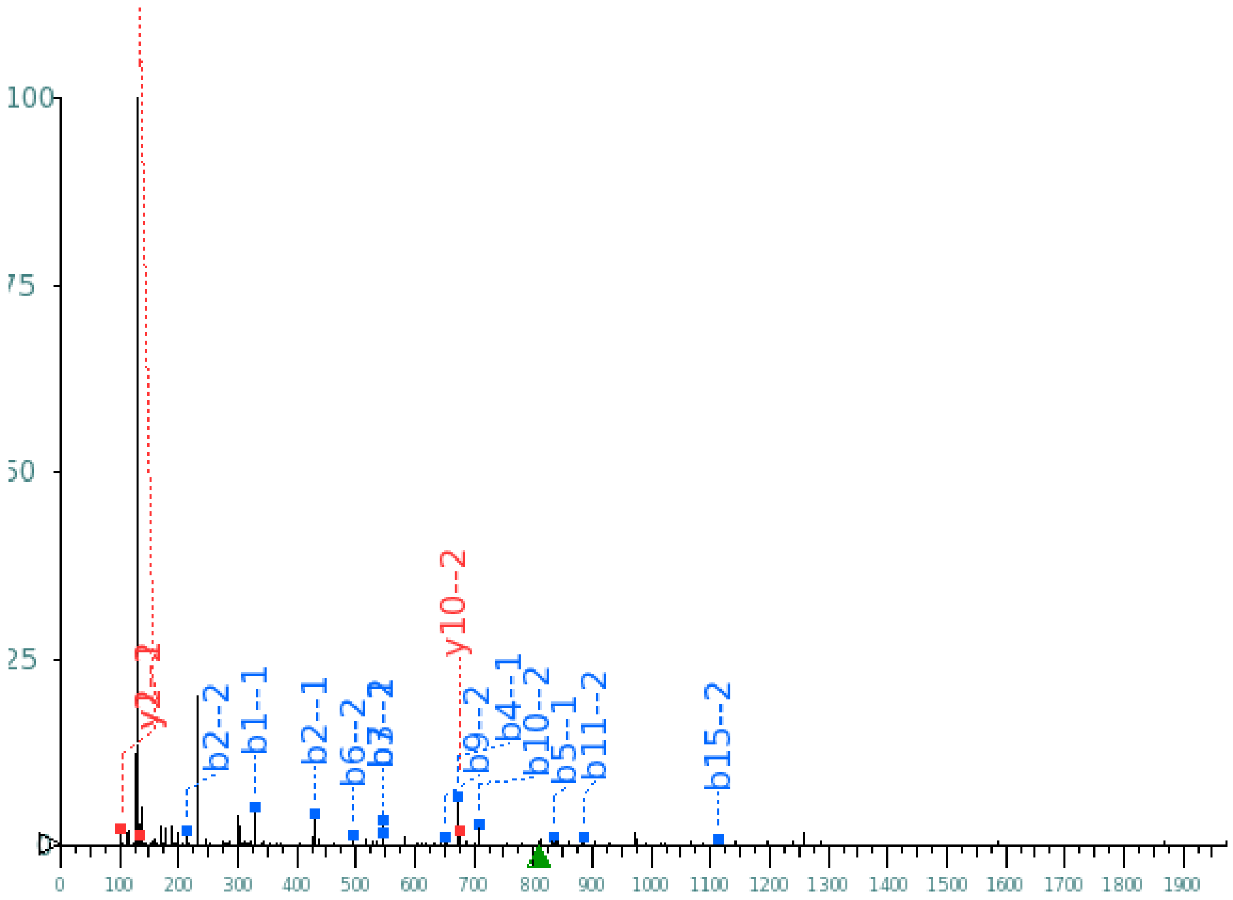

VTLEYRPVIDKTLNEAD (+3)

Primary Reference: DHSA_HUMAN
Search ID: 41760
Search Name: 20130328_ananiav_ERstress_TMT_TM_timecourse_trypsin_inj1_4mc_iaa
Scan#: 16498
Observed Mass: 812.1266 (2.4 ppm)
PSM Score: 11.15

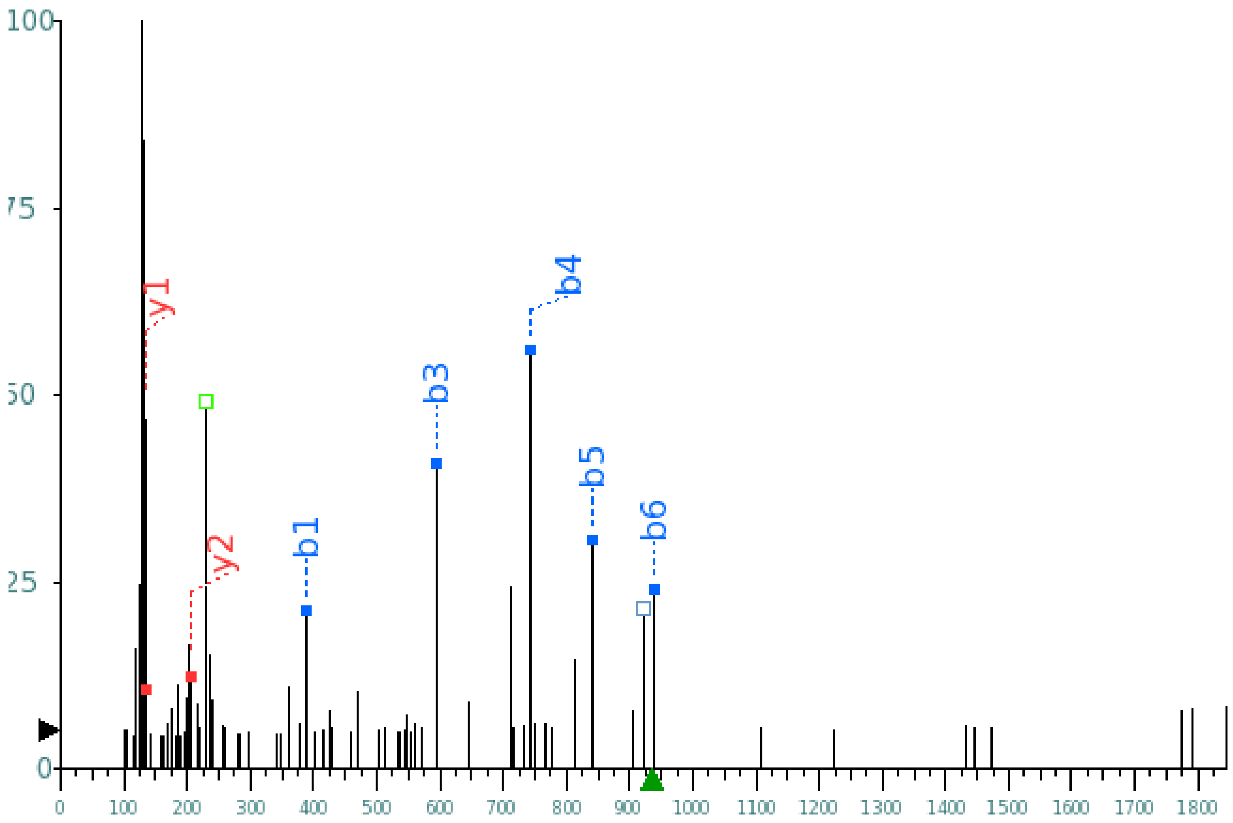

C*FGFVTYSNVEEAD (+2)

Primary Reference: ROA0_HUMAN
Search ID: 41760
Search Name: 20130328_ananiav_ERstress_TMT_TM_timecourse_trypsin_inj1_4mc_iaa
Scan#: 16539
Observed Mass: 933.9264 (2 ppm)
PSM Score: 15.41

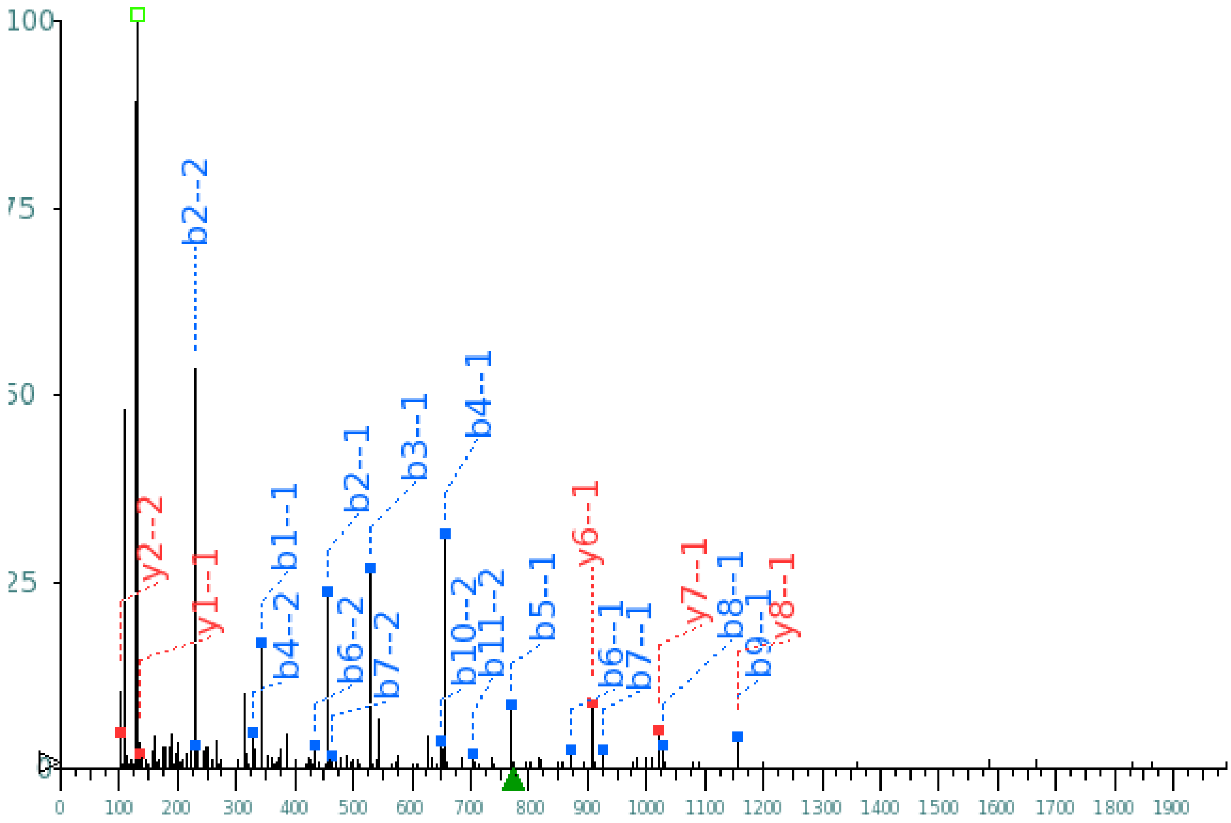

ILAQITGTEHLKESEAD (+3)

Primary Reference: PDLI5_HUMAN
Search ID: 41760
Search Name: 20130328_ananiav_ERstress_TMT_TM_timecourse_trypsin_inj1_4mc_iaa
Scan#: 16563
Observed Mass: 771.7638 (2.9 ppm)
PSM Score: 34.25

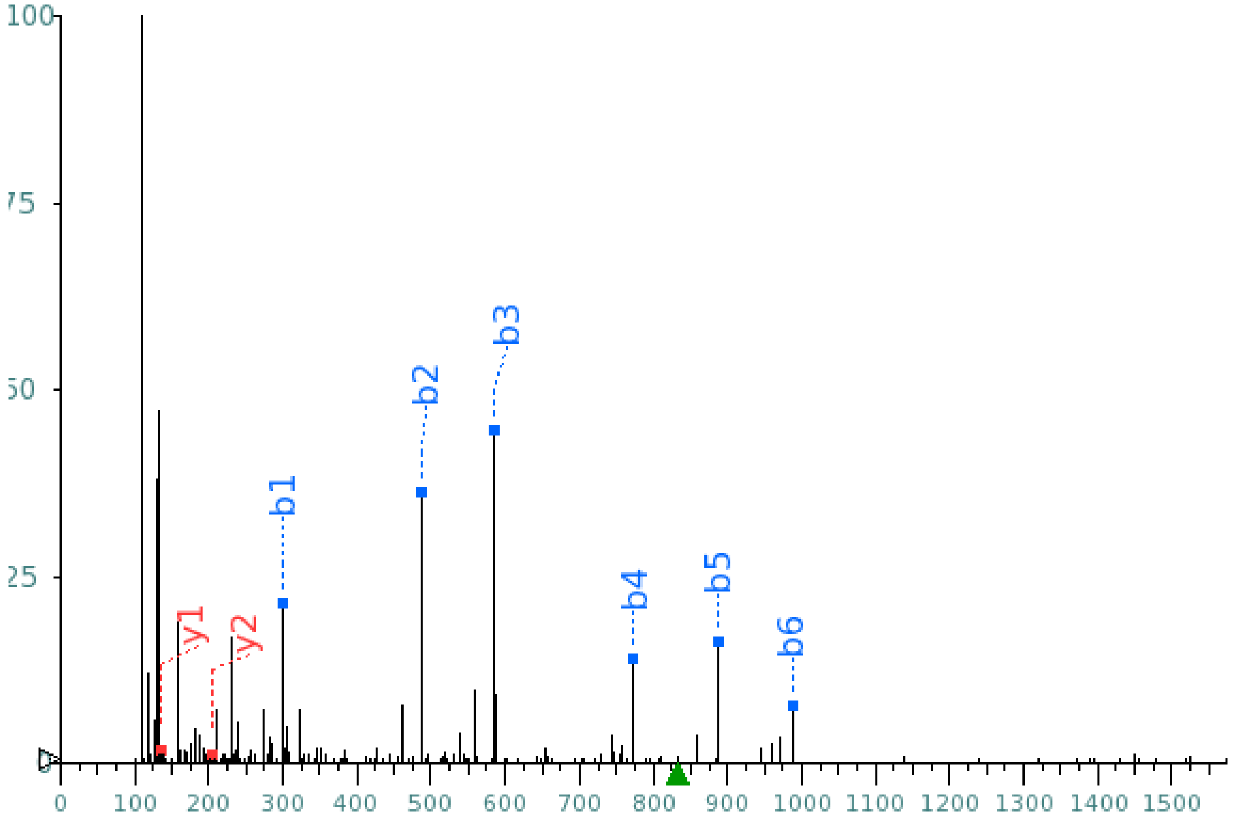

AWVWNTHADFAD (+2)

Primary Reference: RANG_HUMAN
Search ID: 41760
Search Name: 20130328_ananiav_ERstress_TMT_TM_timecourse_trypsin_inj1_4mc_iaa
Scan#: 16594
Observed Mass: 831.4016 (3 ppm)
PSM Score: 27.22

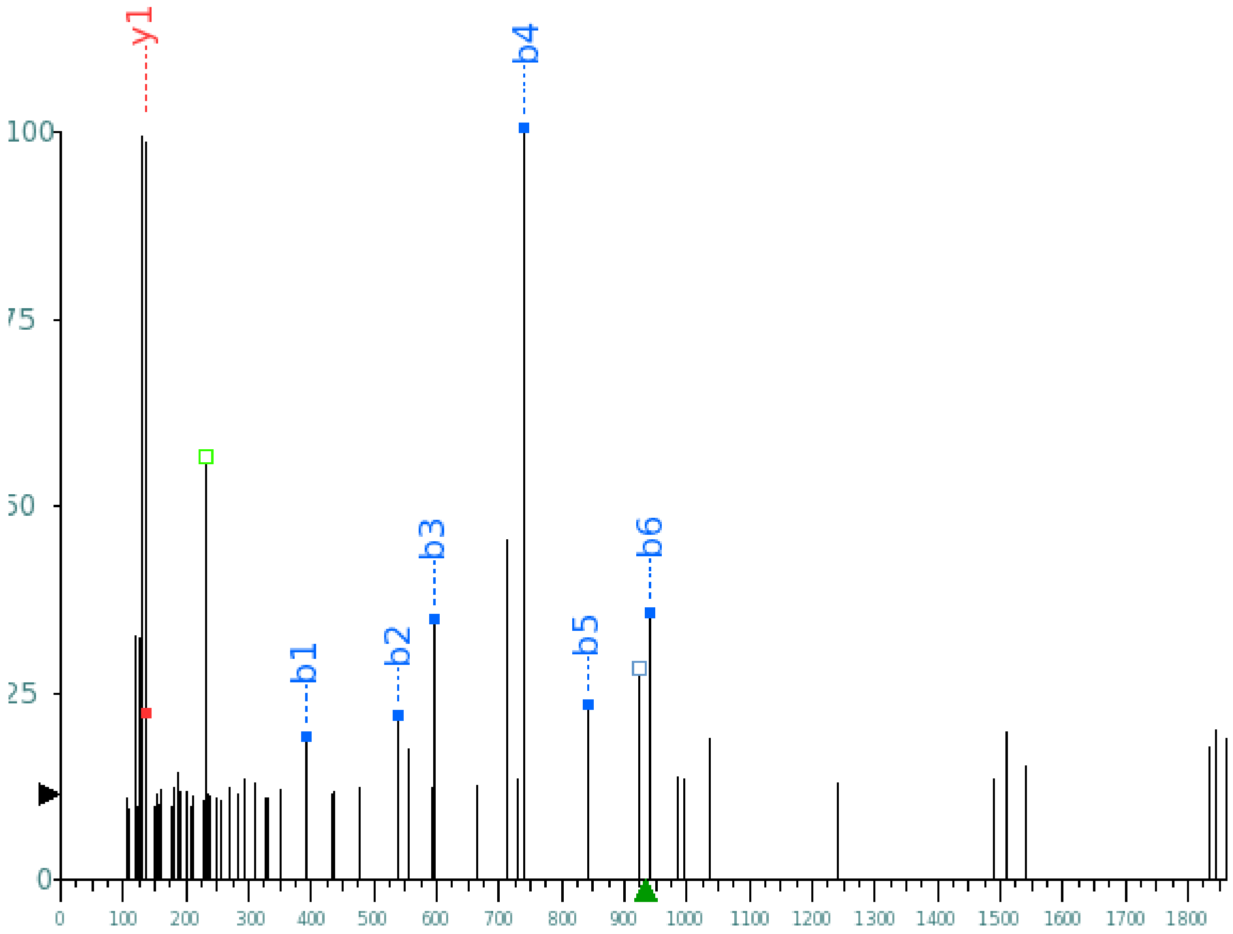

C*FGFVTYSNVEEAD (+2)

Primary Reference: ROA0_HUMAN
Search ID: 41760
Search Name: 20130328_ananiav_ERstress_TMT_TM_timecourse_trypsin_inj1_4mc_iaa
Scan#: 16616
Observed Mass: 933.9268 (2.4 ppm)
PSM Score: 16.41

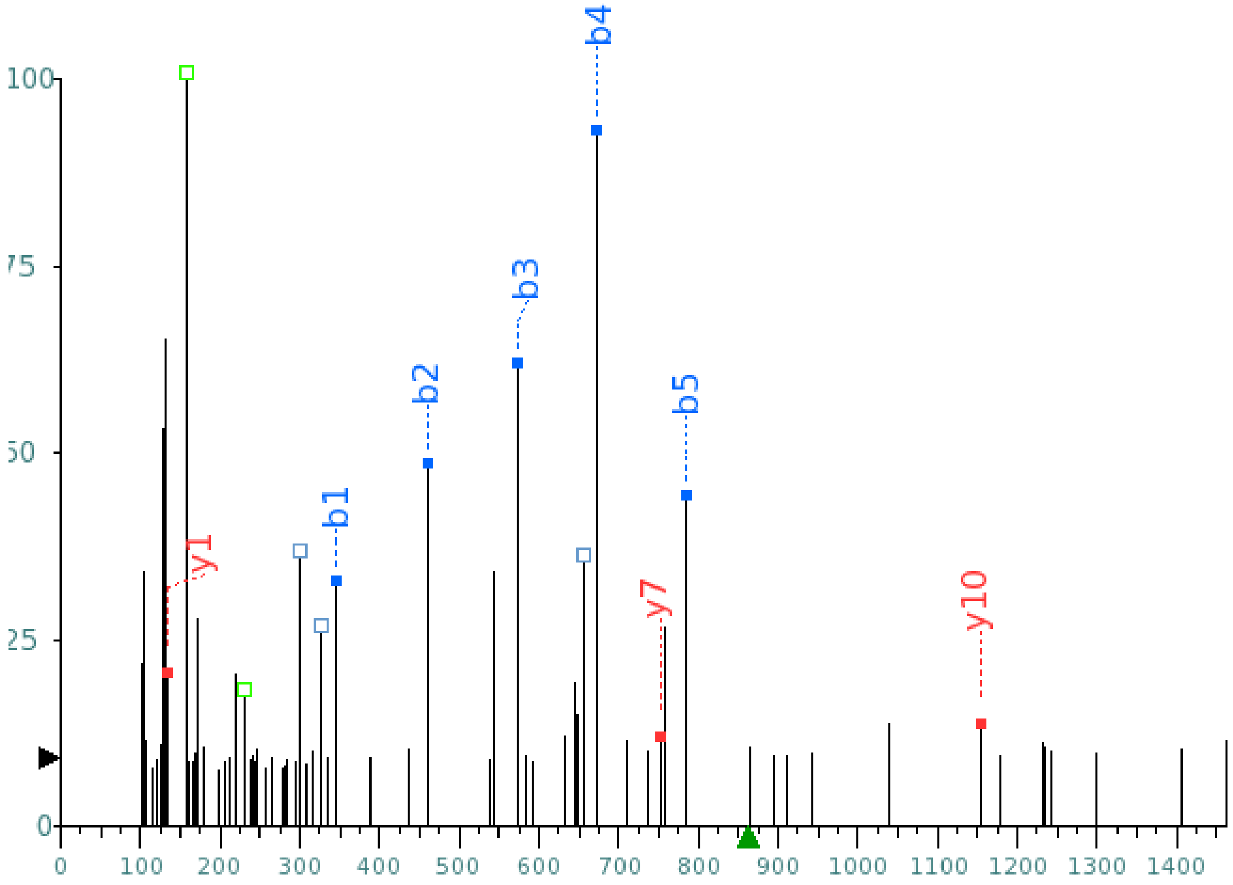

DNLTLWTSDMQGD (+2)

Primary Reference: 1433E_HUMAN
Search ID: 41760
Search Name: 20130328_ananiav_ERstress_TMT_TM_timecourse_trypsin_inj1_4mc_iaa
Scan#: 16638
Observed Mass: 862.9063 (3.1 ppm)
PSM Score: 11.69

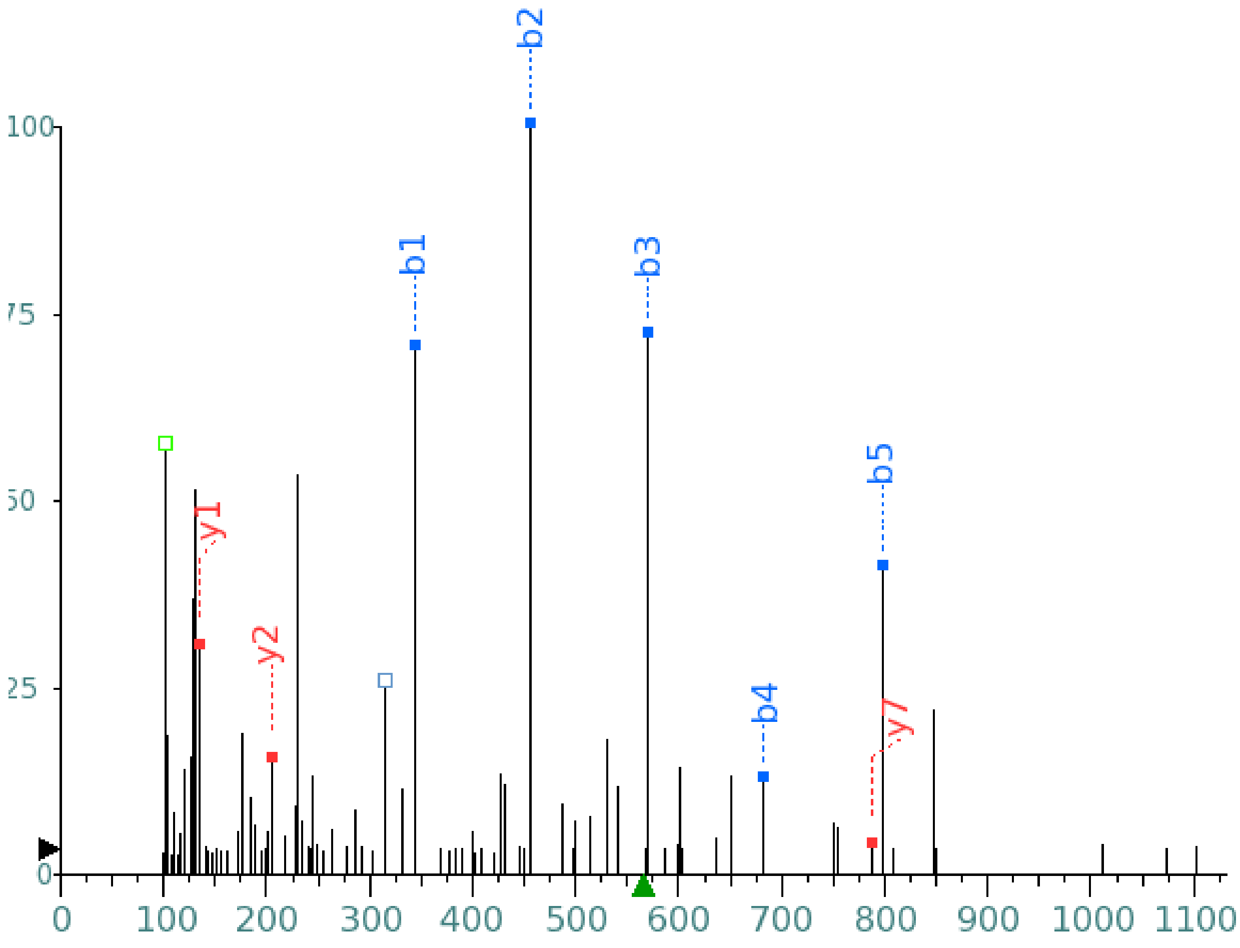

IIILDEAD (+2)

Primary Reference: RFC2_HUMAN
Search ID: 41760
Search Name: 20130328_ananiav_ERstress_TMT_TM_timecourse_trypsin_inj1_4mc_iaa
Scan#: 16639
Observed Mass: 565.8306 (2.9 ppm)
PSM Score: 16.8

File name: 2015-02-04 04.01.53PM.pdf

Creation time: 2015-02-04 04:09PM

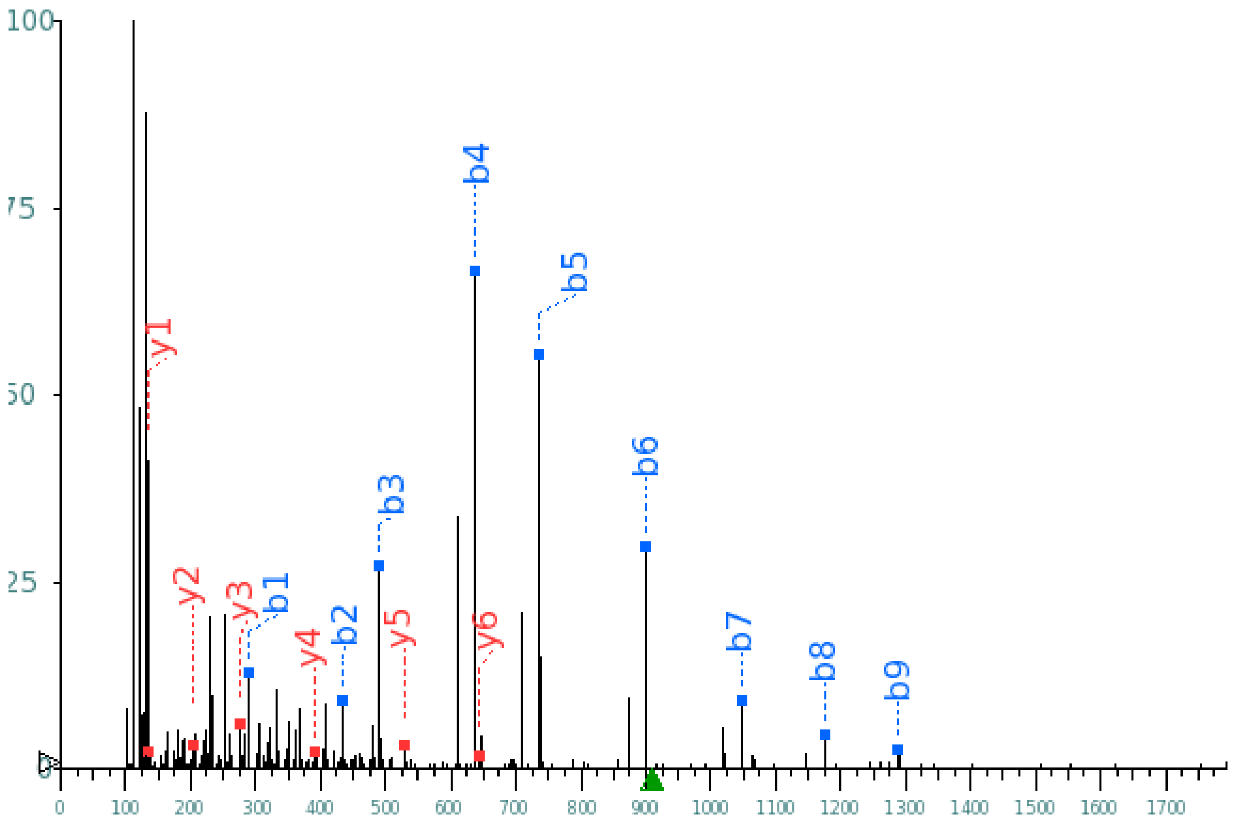

GFGFVYFQNHDAAD (+2)

Primary Reference: ROA0_HUMAN
Search ID: 41760
Search Name: 20130328_ananiav_ERstress_TMT_TM_timecourse_trypsin_inj1_4mc_iaa
Scan#: 16654
Observed Mass: 908.9308 (2.8 ppm)
PSM Score: 20.85

File name: 2015-02-04 04.01.53PM.pdf

Creation time: 2015-02-04 04:09PM

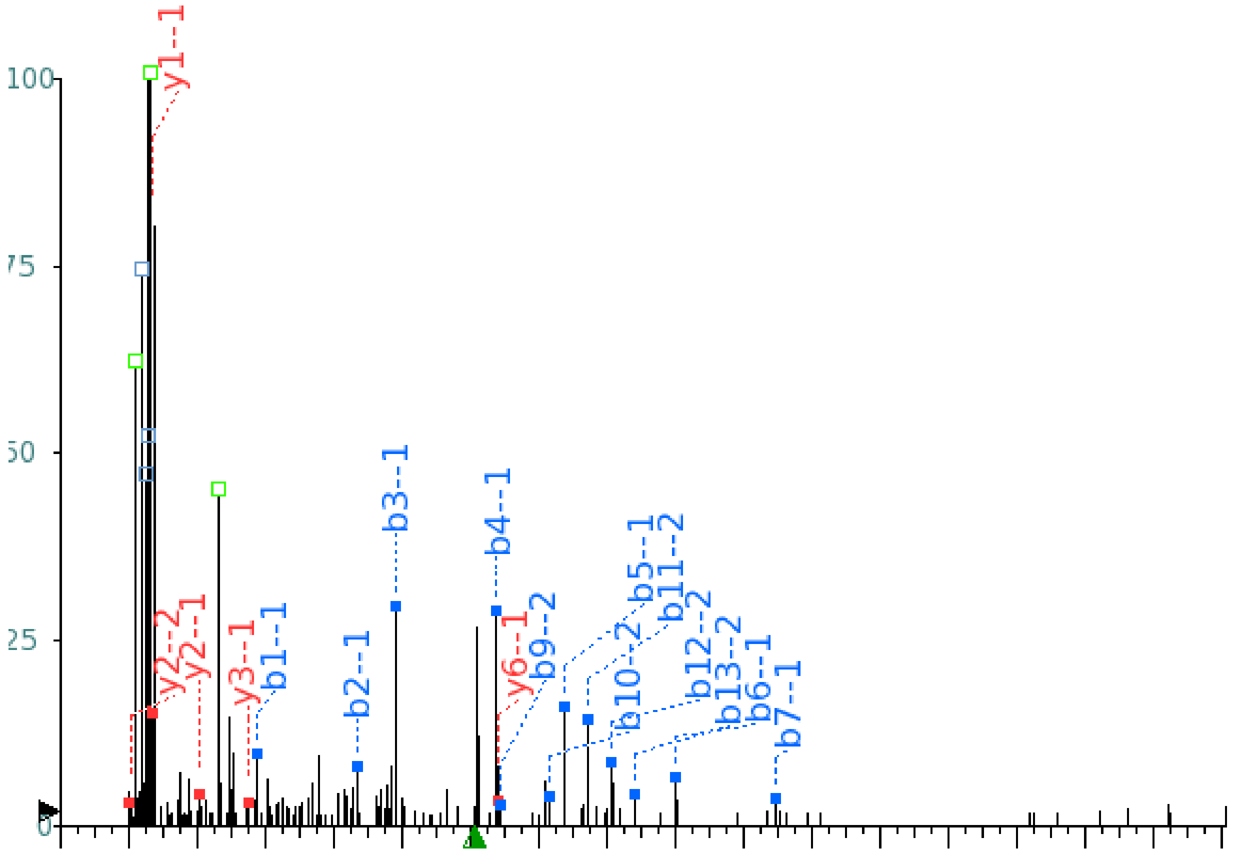

GFGFVYFQNHDAAD (+3)

Primary Reference: ROA0_HUMAN
Search ID: 41760
Search Name: 20130328_ananiav_ERstress_TMT_TM_timecourse_trypsin_inj1_4mc_iaa
Scan#: 16658
Observed Mass: 606.2897 (2.9 ppm)
PSM Score: 12.45

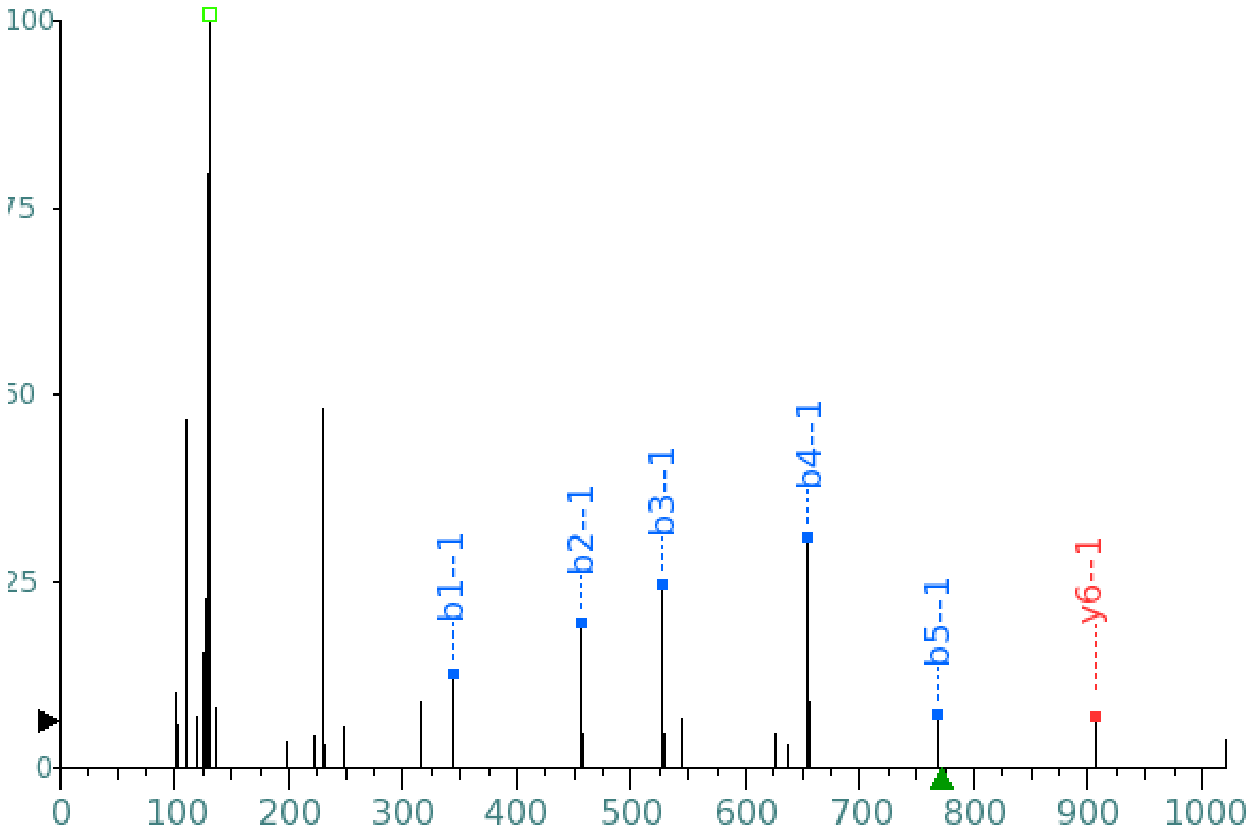

ILAQITGTEHLKESEAD (+3)

Primary Reference: PDLI5_HUMAN
Search ID: 41760
Search Name: 20130328_ananiav_ERstress_TMT_TM_timecourse_trypsin_inj1_4mc_iaa
Scan#: 16667
Observed Mass: 771.7648 (4.2 ppm)
PSM Score: 28.48

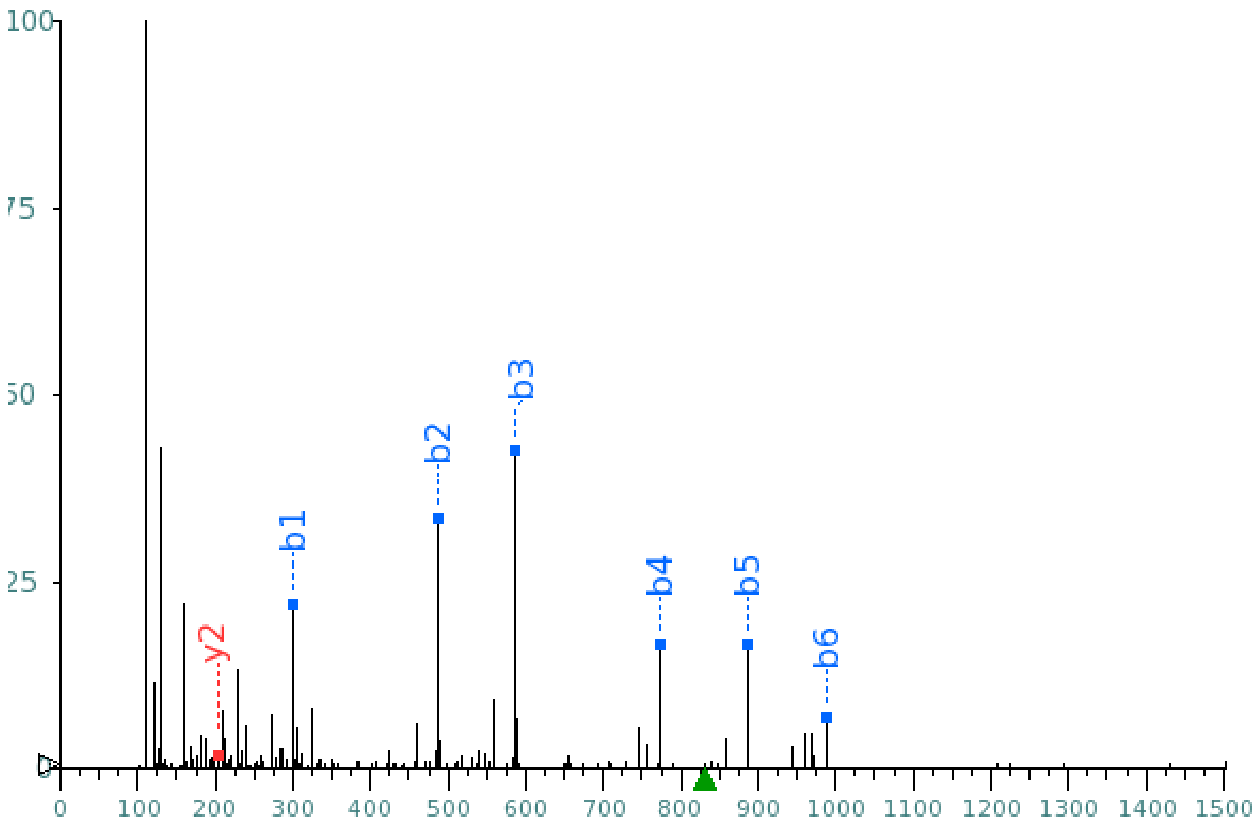

AWVWNTHADFAD (+2)

Primary Reference: RANG_HUMAN
Search ID: 41760
Search Name: 20130328_ananiav_ERstress_TMT_TM_timecourse_trypsin_inj1_4mc_iaa
Scan#: 16671
Observed Mass: 831.4012 (2.5 ppm)
PSM Score: 34.51

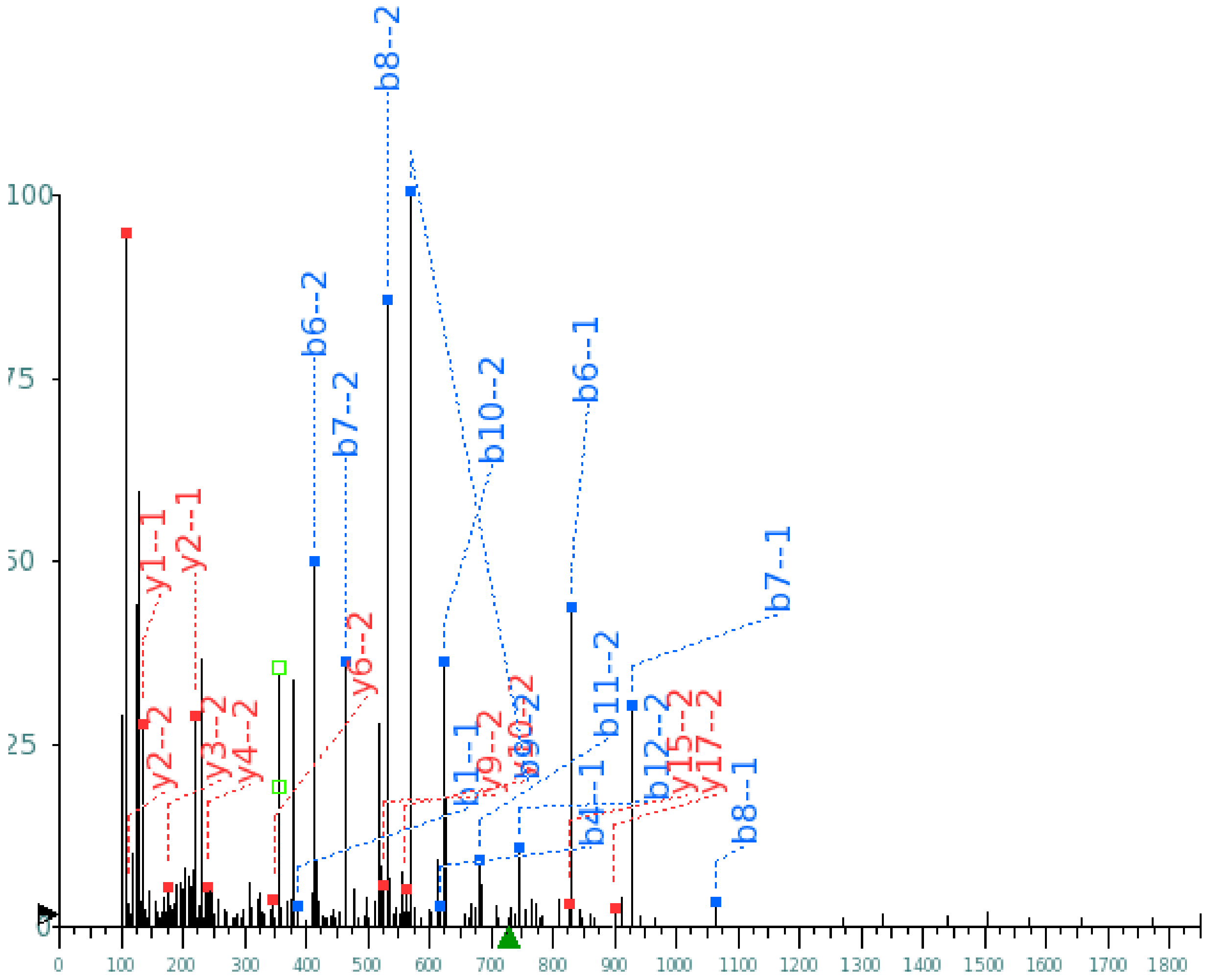

RGSSPDVHALLEITEESD (+3)

Primary Reference: PTRF_HUMAN
Search ID: 41760
Search Name: 20130328_ananiav_ERstress_TMT_TM_timecourse_trypsin_inj1_4mc_iaa
Scan#: 16677
Observed Mass: 728.7041 (-0.2 ppm)
PSM Score: 20.86

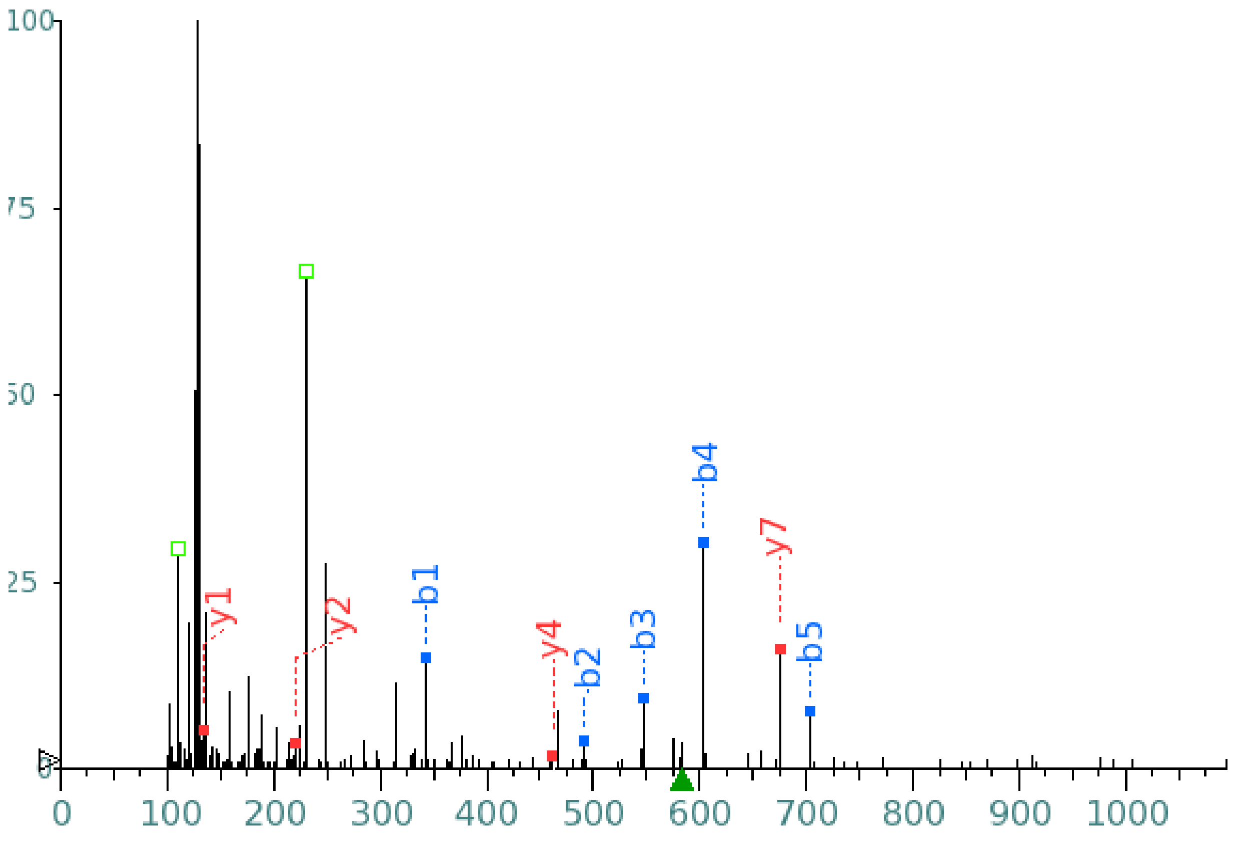

IFGGVLESD (+2)

Primary Reference: HAX1_HUMAN
Search ID: 41760
Search Name: 20130328_ananiav_ERstress_TMT_TM_timecourse_trypsin_inj1_4mc_iaa
Scan#: 16695
Observed Mass: 583.3203 (2.7 ppm)
PSM Score: 11.28

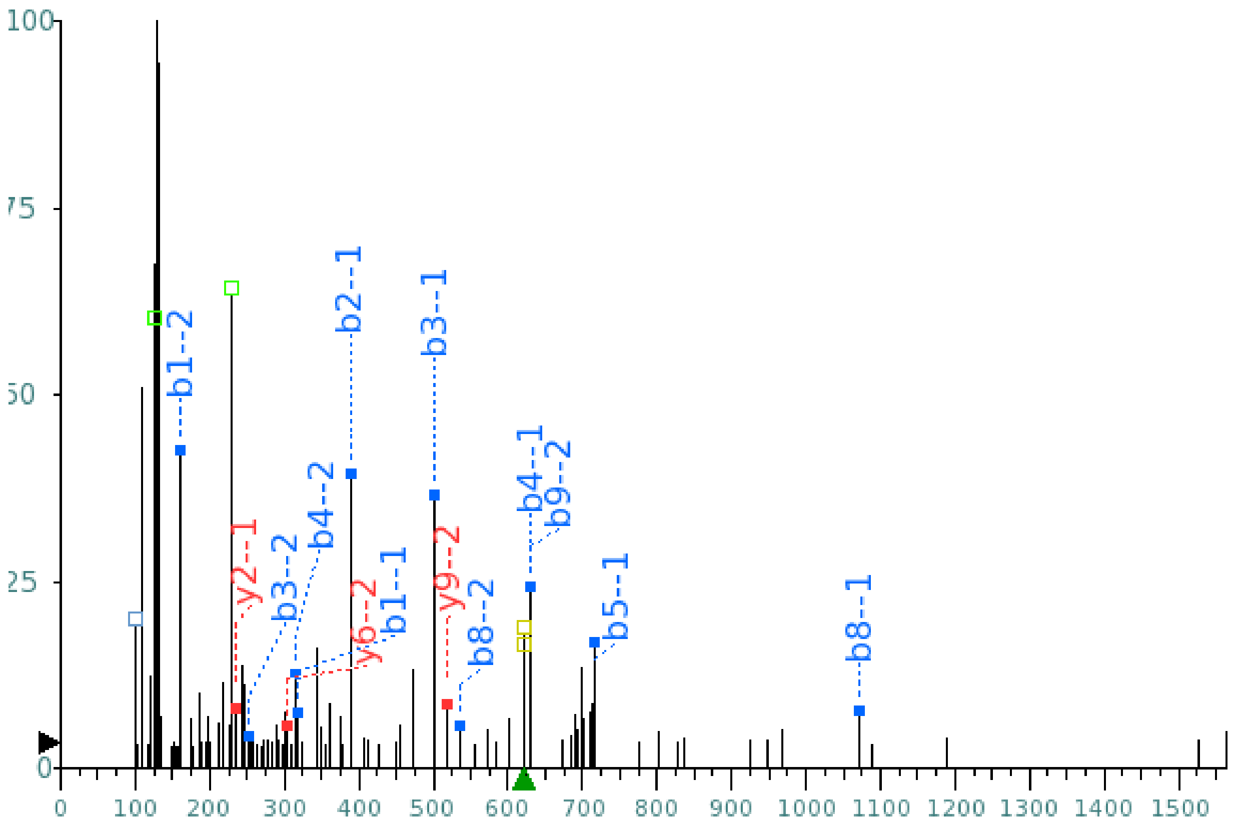

SALQSINEWAAQTTD (+3)

Primary Reference: SERPH_HUMAN
Search ID: 41760
Search Name: 20130328_ananiav_ERstress_TMT_TM_timecourse_trypsin_inj1_4mc_iaa
Scan#: 16715
Observed Mass: 621.983 (3.2 ppm)
PSM Score: 16.12

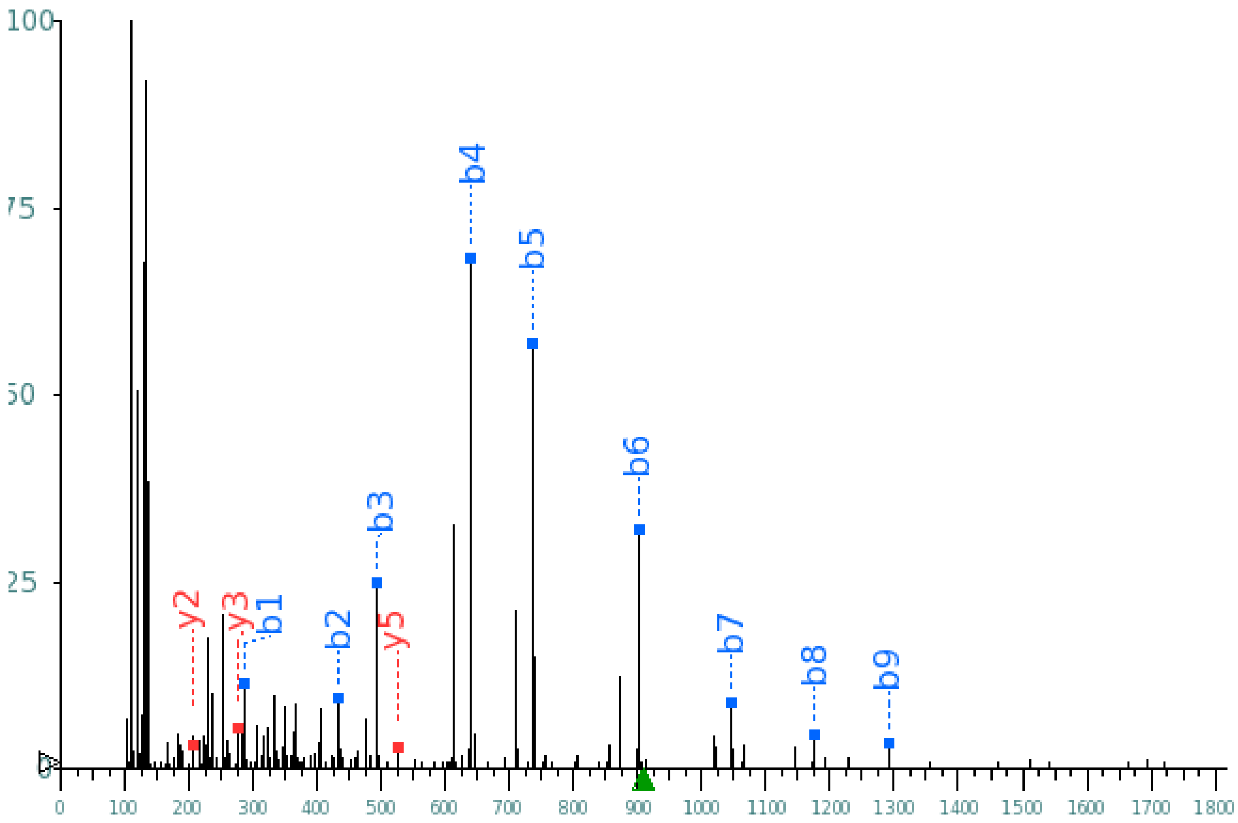

GFGFVYFQNHDAAD (+2)

Primary Reference: ROA0_HUMAN
Search ID: 41760
Search Name: 20130328_ananiav_ERstress_TMT_TM_timecourse_trypsin_inj1_4mc_iaa
Scan#: 16717
Observed Mass: 908.9308 (2.8 ppm)
PSM Score: 30.51

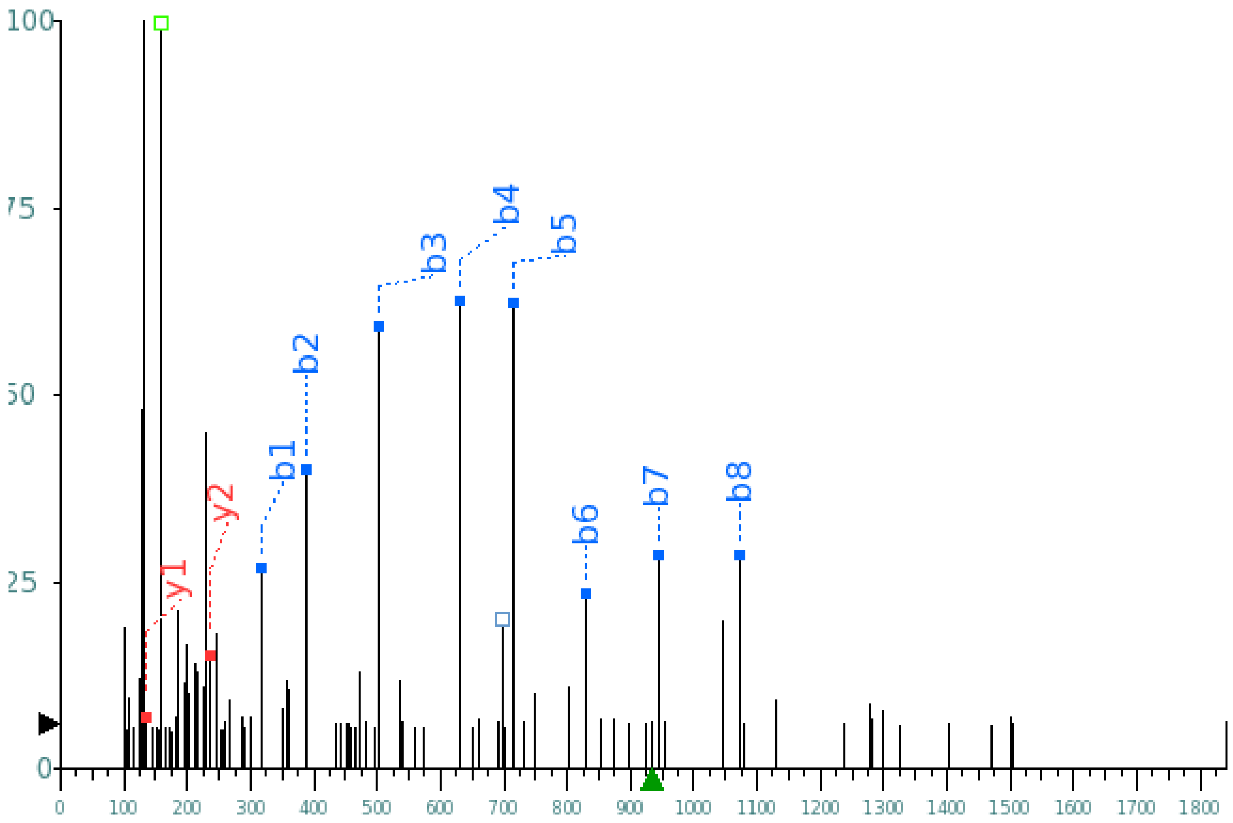

SALQSINEWAAQTTD (+2)

Primary Reference: SERPH_HUMAN
Search ID: 41760
Search Name: 20130328_ananiav_ERstress_TMT_TM_timecourse_trypsin_inj1_4mc_iaa
Scan#: 16726
Observed Mass: 932.4732 (5.7 ppm)
PSM Score: 37.02

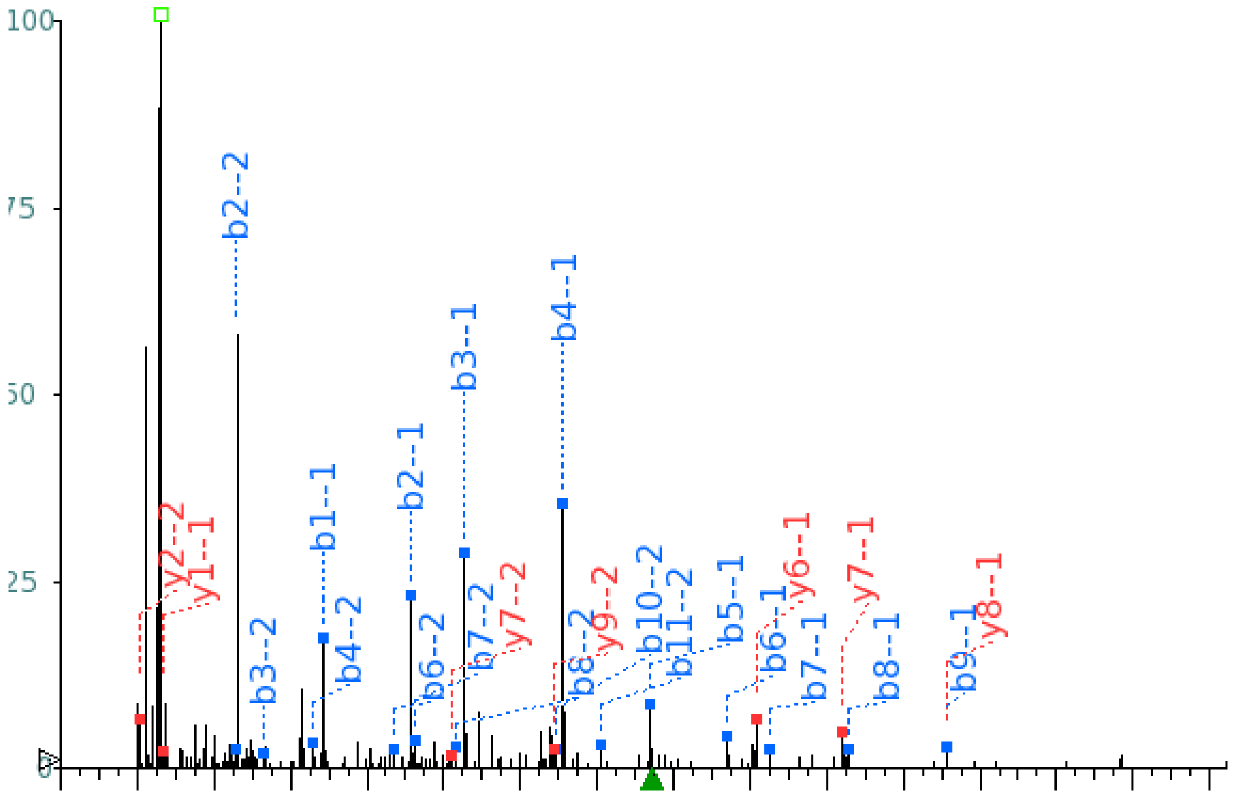

ILAQITGTEHLKESEAD (+3)

Primary Reference: PDLI5_HUMAN
Search ID: 41760
Search Name: 20130328_ananiav_ERstress_TMT_TM_timecourse_trypsin_inj1_4mc_iaa
Scan#: 16745
Observed Mass: 771.7647 (4.1 ppm)
PSM Score: 38.51

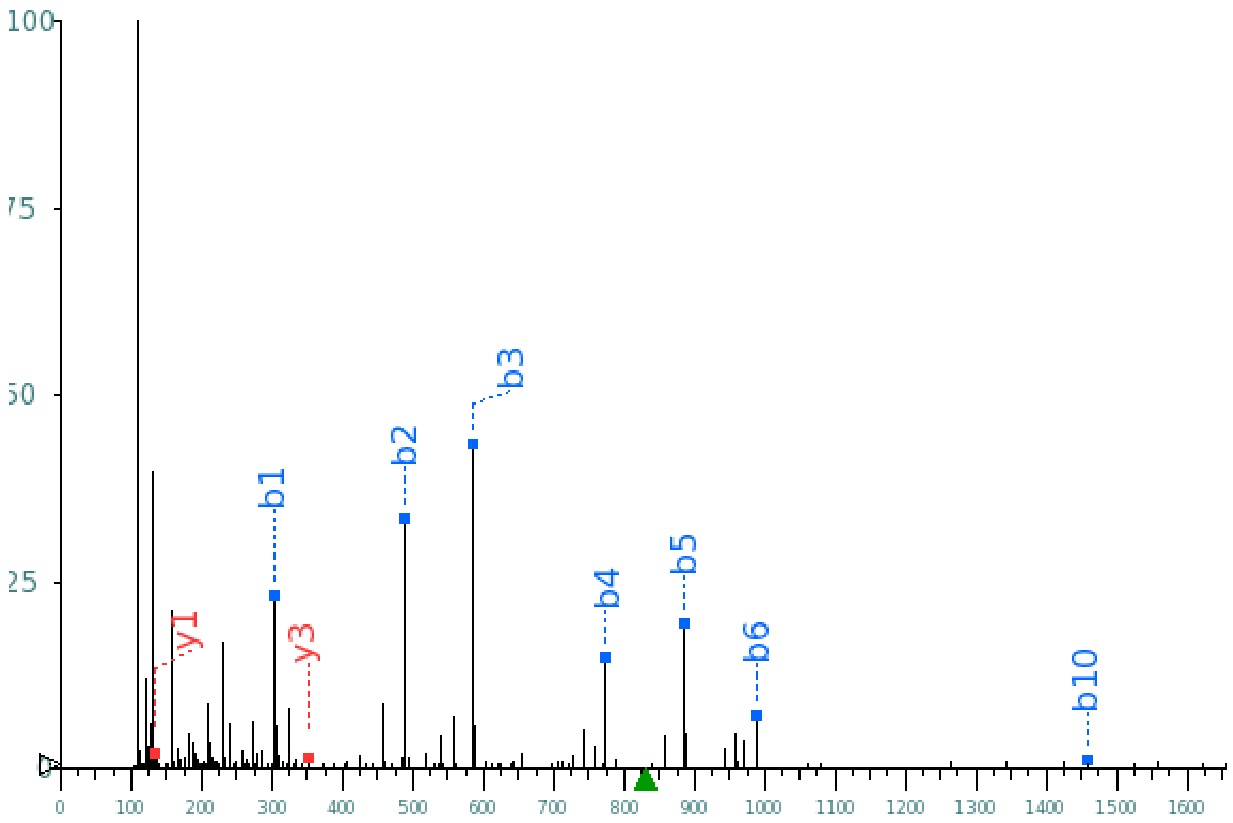

AWVWNTHADFAD (+2)

Primary Reference: RANG_HUMAN
Search ID: 41760
Search Name: 20130328_ananiav_ERstress_TMT_TM_timecourse_trypsin_inj1_4mc_iaa
Scan#: 16749
Observed Mass: 831.4012 (2.5 ppm)
PSM Score: 25.82

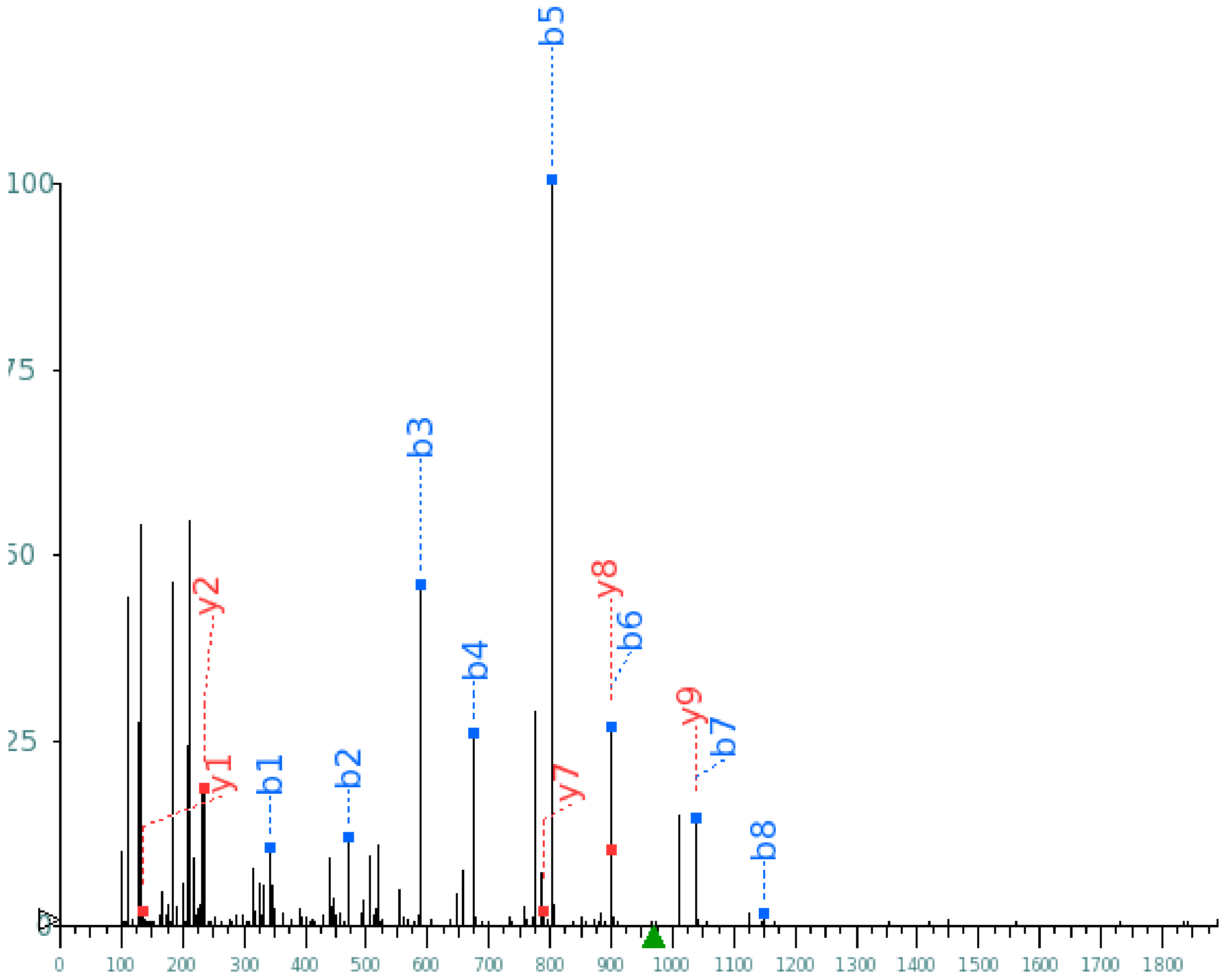

IEDSEPHIPLIDDTD (+2)

Primary Reference: AT2B1_HUMAN
Search ID: 41760
Search Name: 20130328_ananiav_ERstress_TMT_TM_timecourse_trypsin_inj1_4mc_iaa
Scan#: 16767
Observed Mass: 969.4832 (2.5 ppm)
PSM Score: 25.75

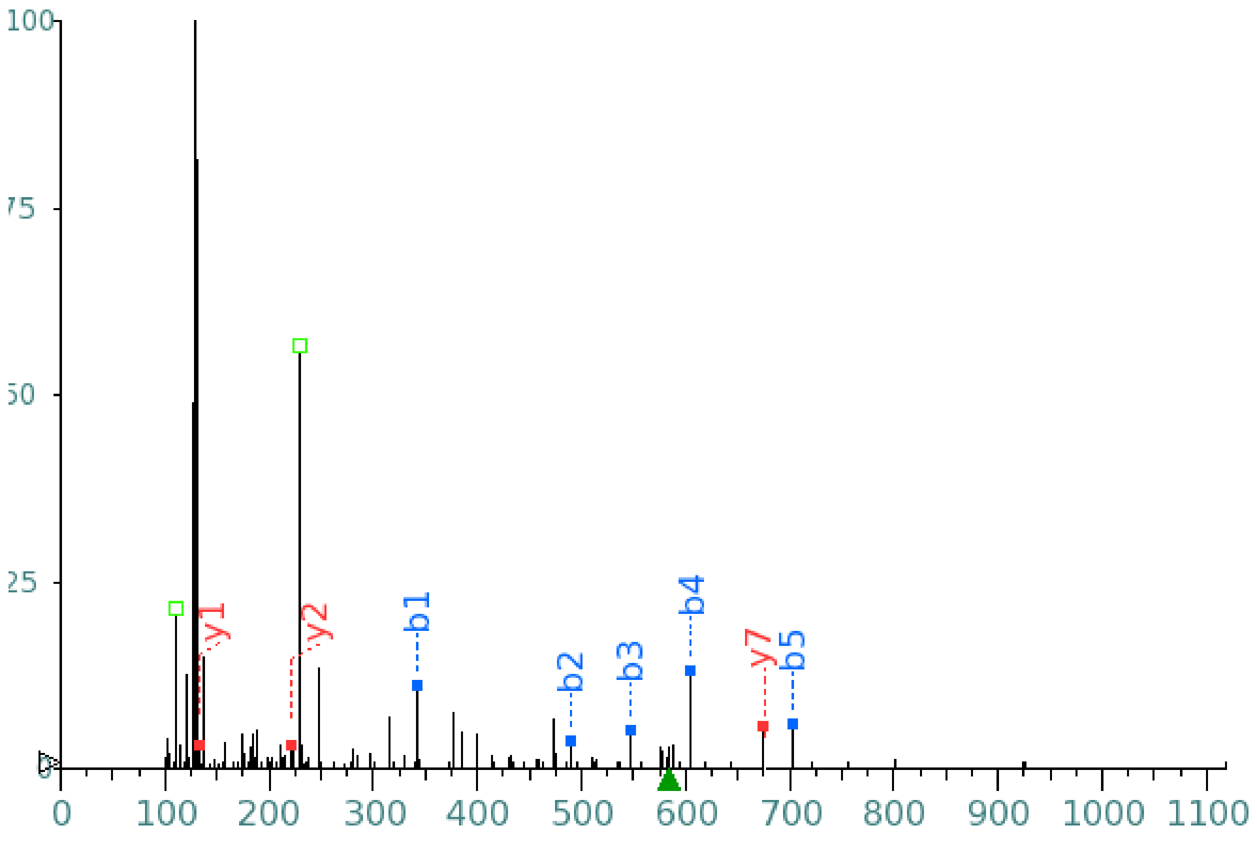

IFGGVLESD (+2)

Primary Reference: HAX1_HUMAN
Search ID: 41760
Search Name: 20130328_ananiav_ERstress_TMT_TM_timecourse_trypsin_inj1_4mc_iaa
Scan#: 16773
Observed Mass: 583.3204 (2.8 ppm)
PSM Score: 10.92

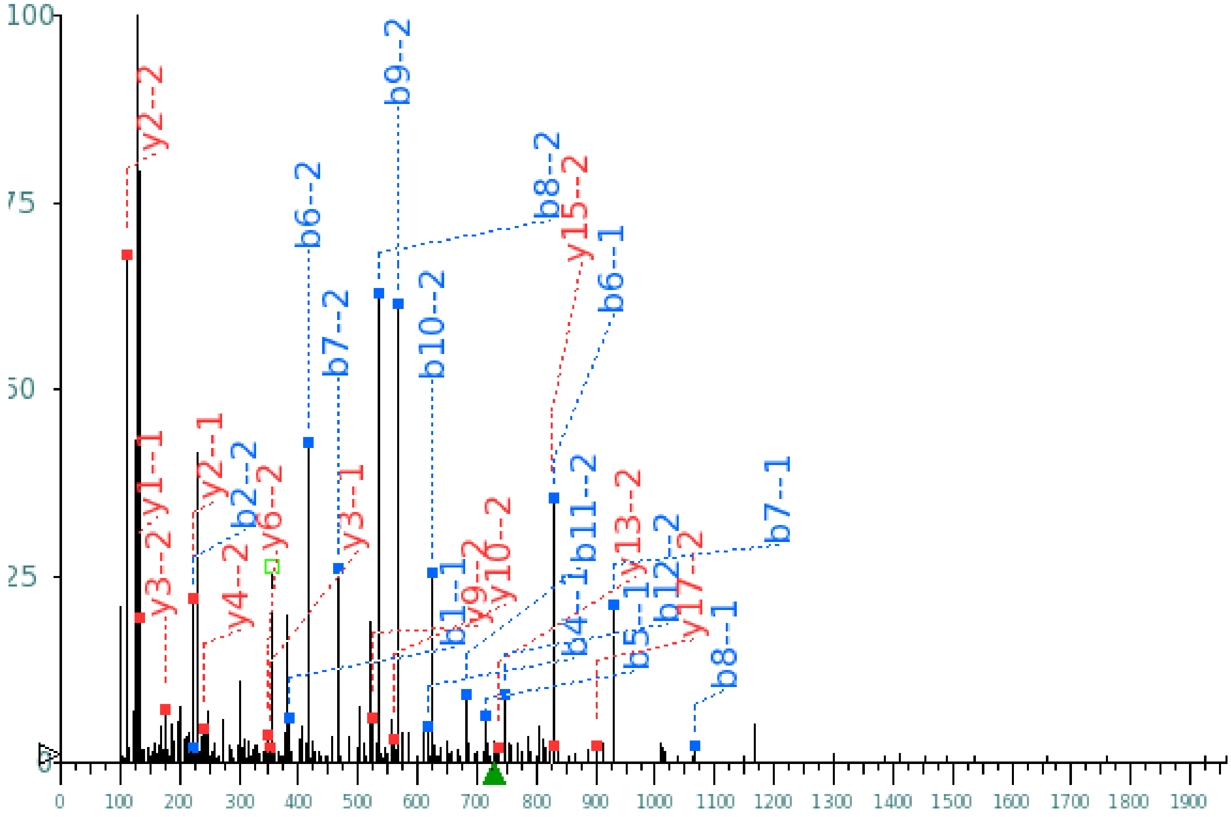

RGSSPDVHALLEITEESD (+3)

Primary Reference: PTRF_HUMAN
Search ID: 41760
Search Name: 20130328_ananiav_ERstress_TMT_TM_timecourse_trypsin_inj1_4mc_iaa
Scan#: 16787
Observed Mass: 728.7058 (2.2 ppm)
PSM Score: 17.79

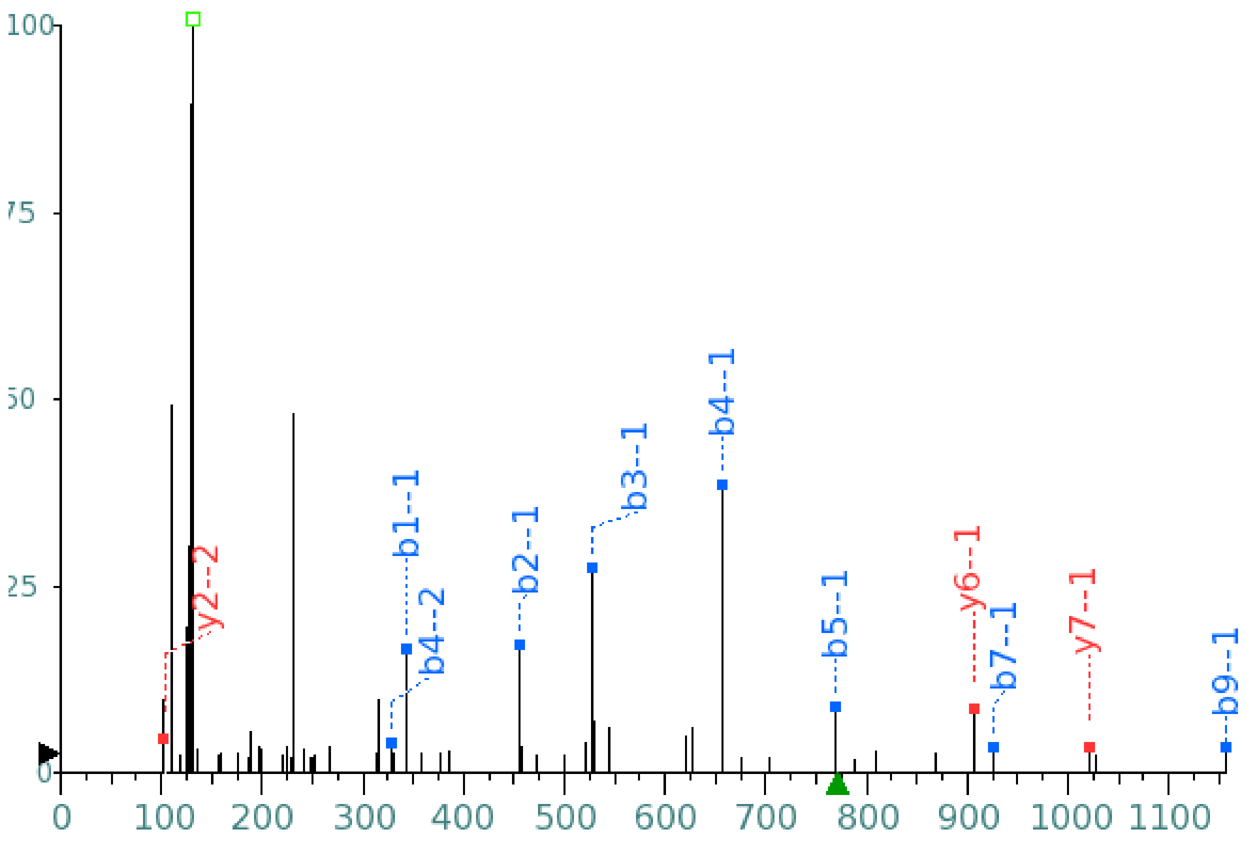

ILAQITGTEHLKESEAD (+3)

Primary Reference: PDLI5_HUMAN
Search ID: 41760
Search Name: 20130328_ananiav_ERstress_TMT_TM_timecourse_trypsin_inj1_4mc_iaa
Scan#: 16810
Observed Mass: 771.7638 (2.9 ppm)
PSM Score: 47.5

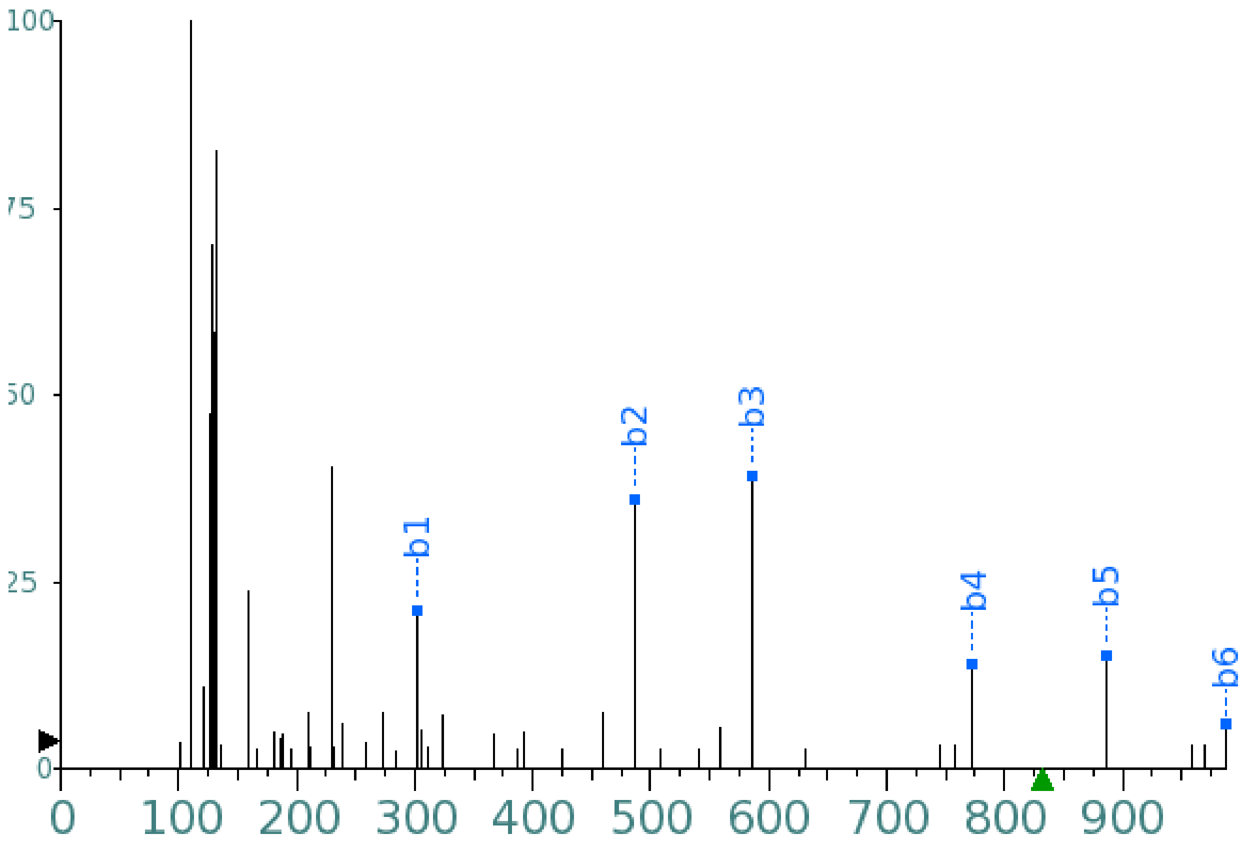

AWVWNTHADFAD (+2)

Primary Reference: RANG_HUMAN
Search ID: 41760
Search Name: 20130328_ananiav_ERstress_TMT_TM_timecourse_trypsin_inj1_4mc_iaa
Scan#: 16832
Observed Mass: 831.3995 (0.5 ppm)
PSM Score: 25.97

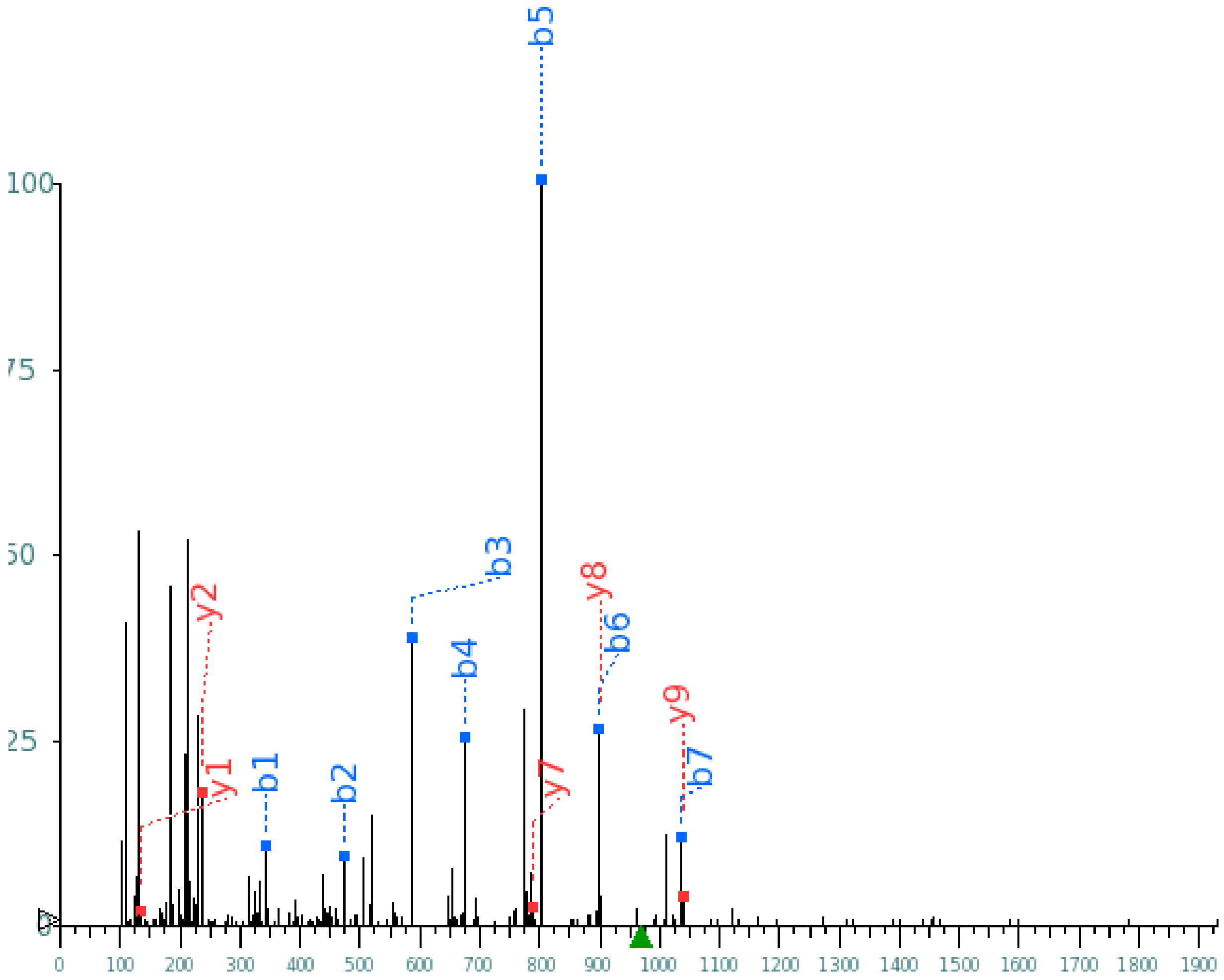

IEDSEPHIPLIDDTD (+2)

Primary Reference: AT2B1_HUMAN
Search ID: 41760
Search Name: 20130328_ananiav_ERstress_TMT_TM_timecourse_trypsin_inj1_4mc_iaa
Scan#: 16833
Observed Mass: 969.4839 (3.3 ppm)
PSM Score: 21.02

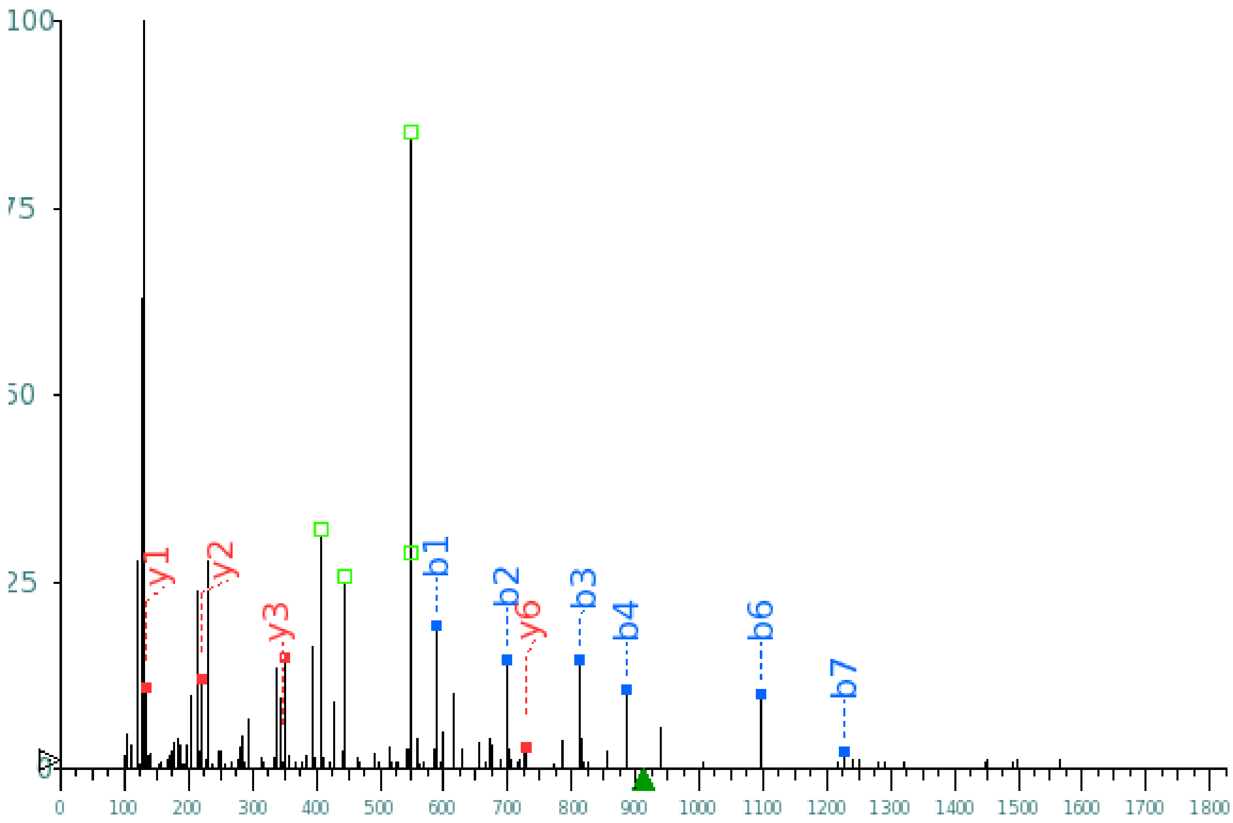

KLLAPDMFTESD (+2)

Primary Reference: PRP4B_HUMAN
Search ID: 41760
Search Name: 20130328_ananiav_ERstress_TMT_TM_timecourse_trypsin_inj1_4mc_iaa
Scan#: 16856
Observed Mass: 912.9983 (4.2 ppm)
PSM Score: 16.53

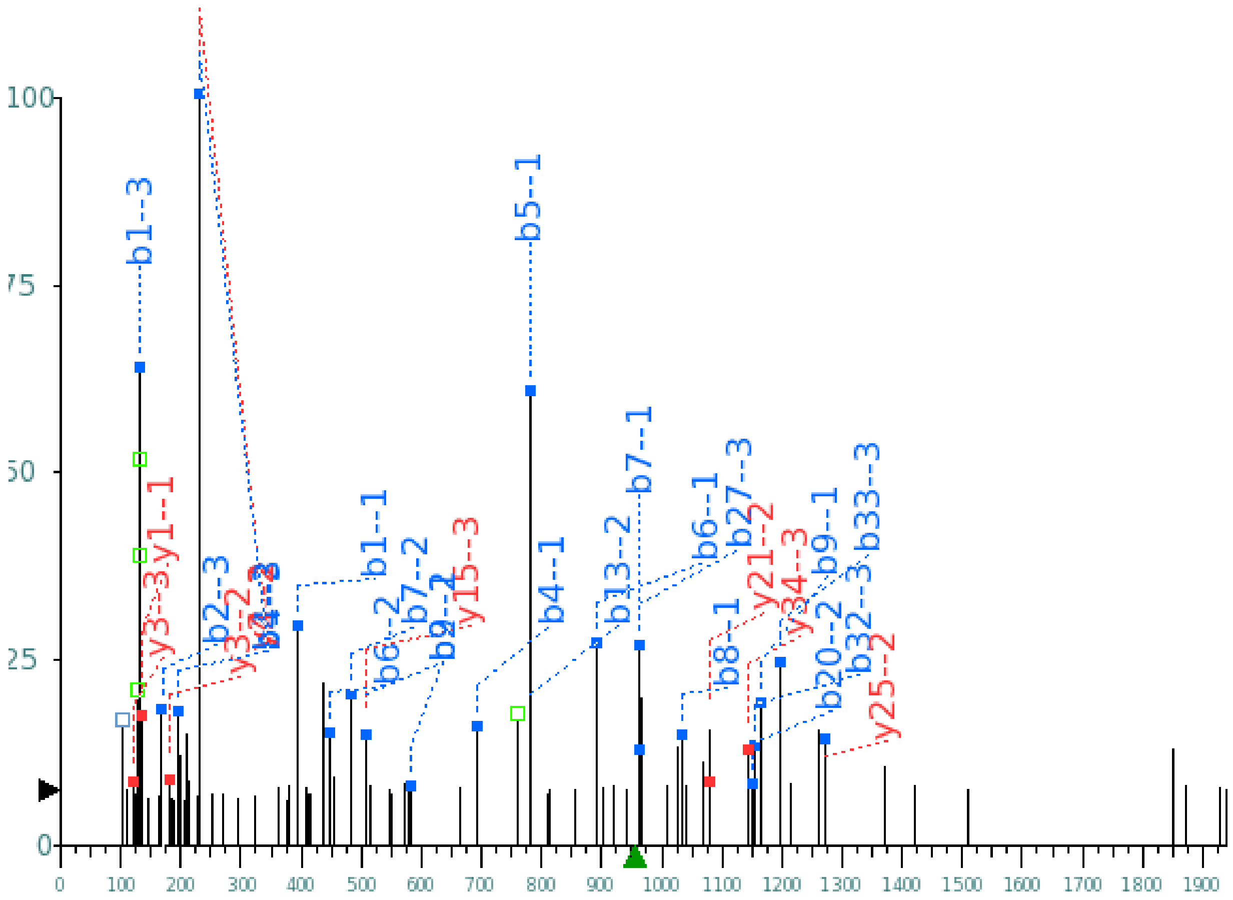

YIGESLAAAELPVSTQRPGSDTVGGGRGEEC*GVEPD (+4)

Primary Reference: MCM3A_HUMAN
Search ID: 41760
Search Name: 20130328_ananiav_ERstress_TMT_TM_timecourse_trypsin_inj1_4mc_iaa
Scan#: 16858
Observed Mass: 955.4669 (3.1 ppm)
PSM Score: 25.32

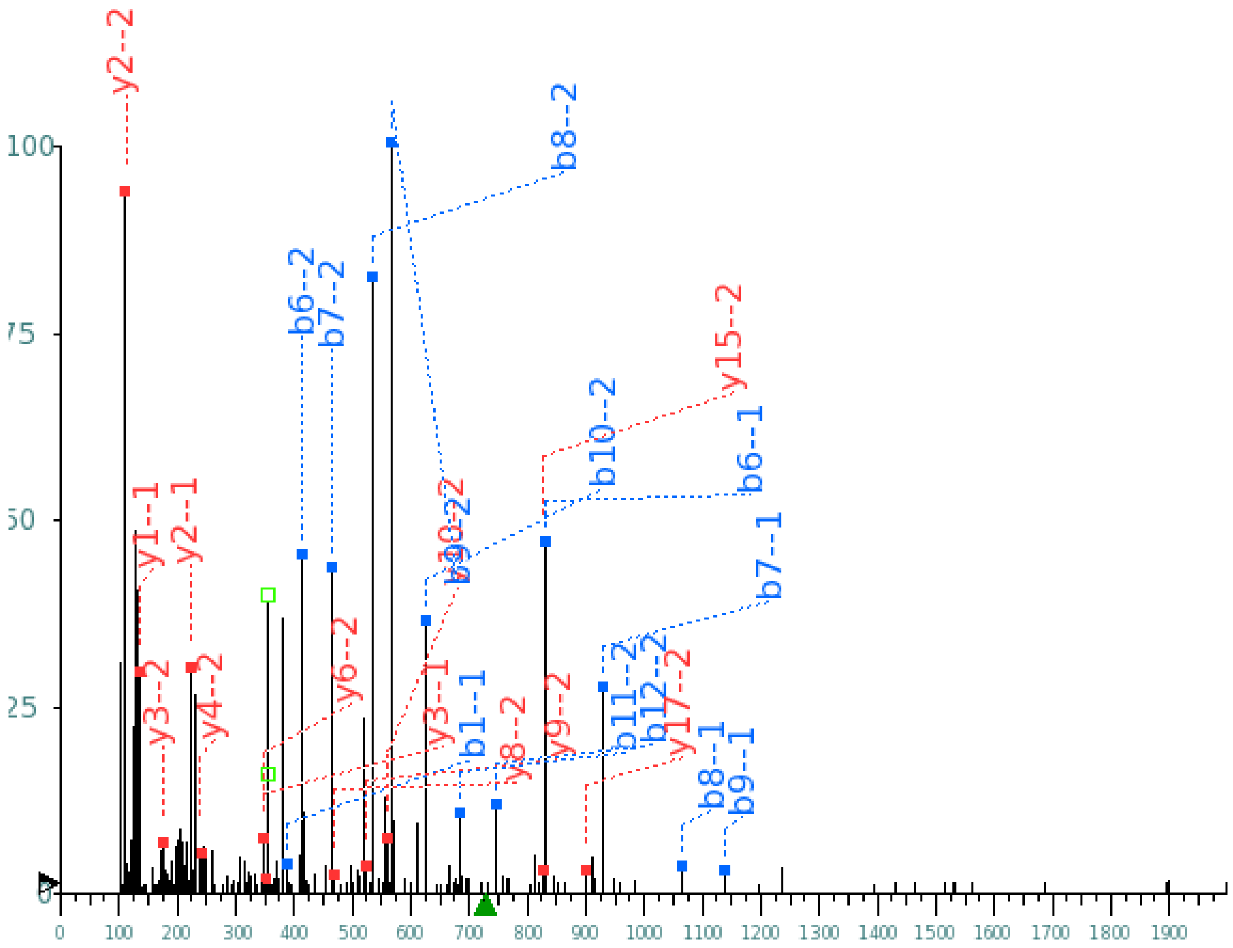

RGSSPDVHALLEITEESD (+3)

Primary Reference: PTRF_HUMAN
Search ID: 41760
Search Name: 20130328_ananiav_ERstress_TMT_TM_timecourse_trypsin_inj1_4mc_iaa
Scan#: 16862
Observed Mass: 728.7064 (3 ppm)
PSM Score: 22.68

File name: 2015-02-04 04.01.53PM.pdf

Creation time: 2015-02-04 04:09PM

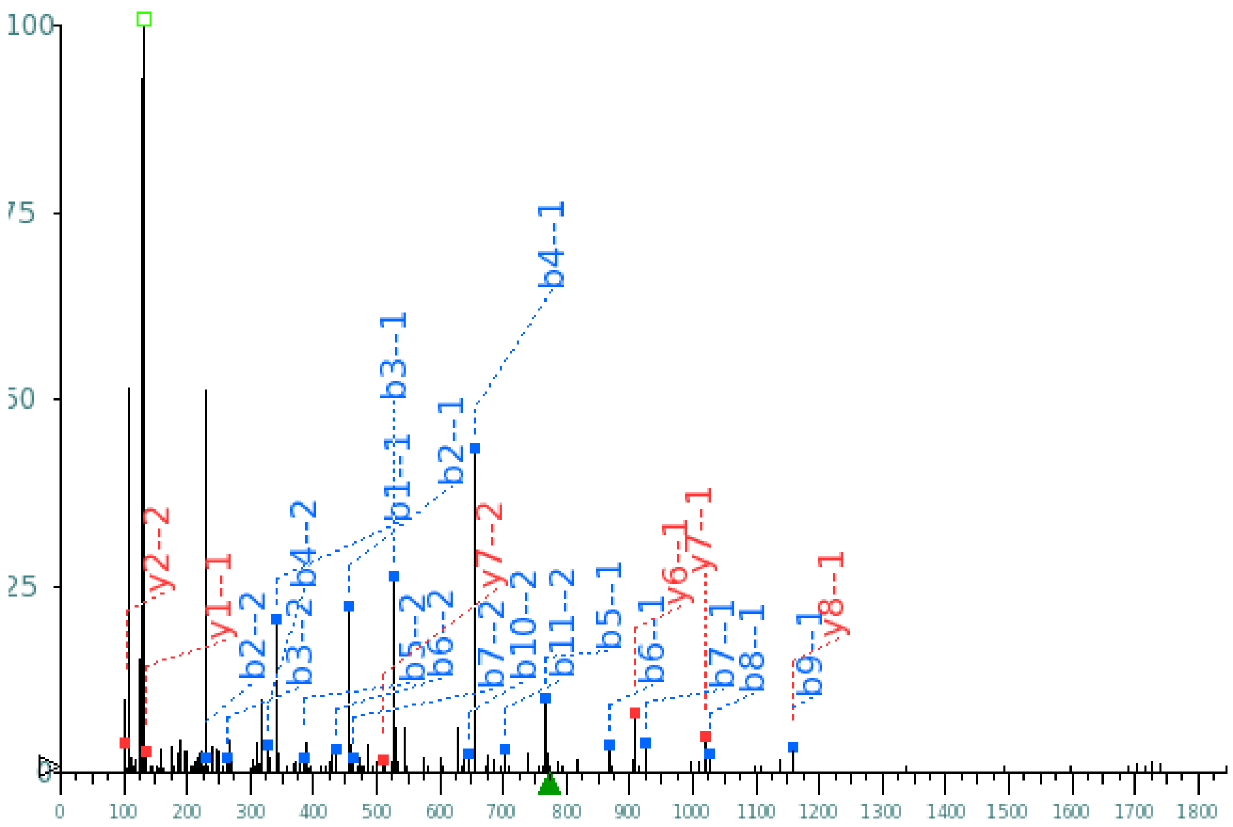

ILAQITGTEHLKESEAD (+3)

Primary Reference: PDLI5_HUMAN
Search ID: 41760
Search Name: 20130328_ananiav_ERstress_TMT_TM_timecourse_trypsin_inj1_4mc_iaa
Scan#: 16880
Observed Mass: 771.7637 (2.8 ppm)
PSM Score: 32.59

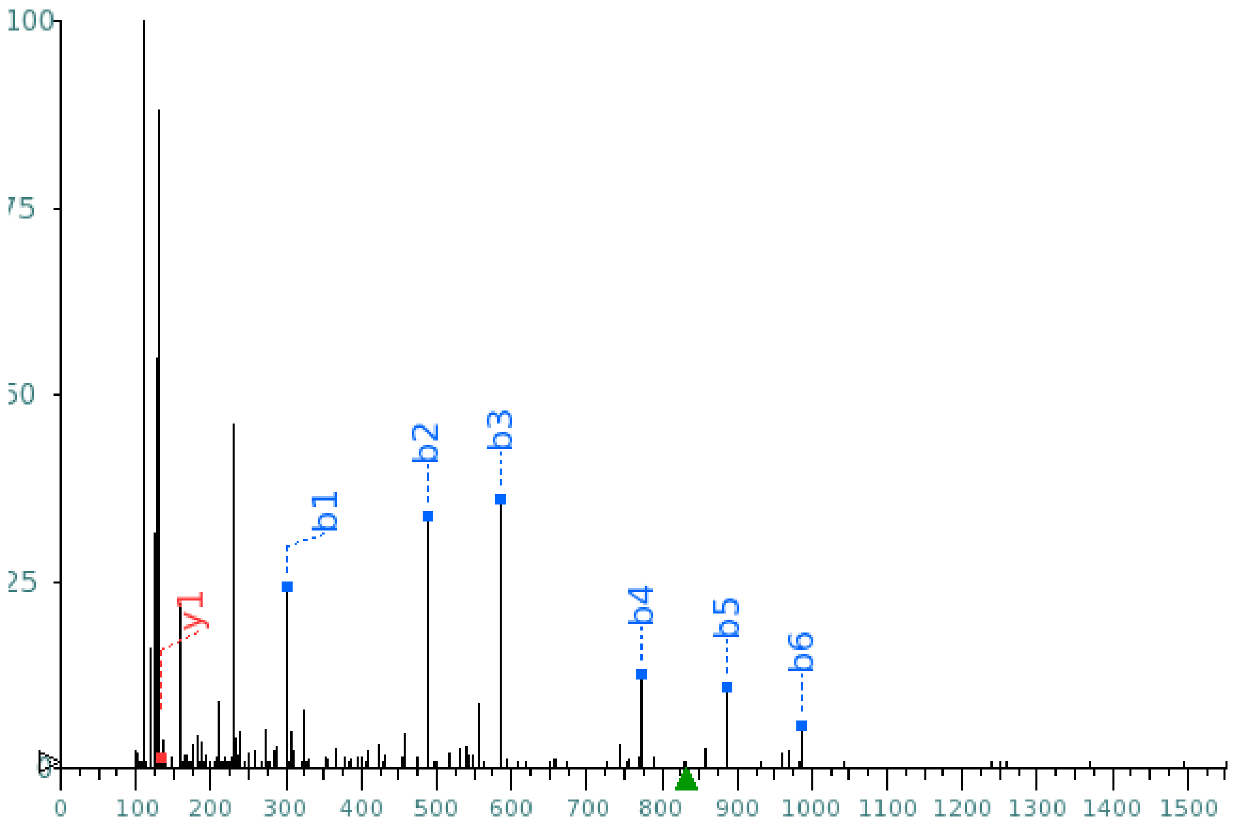

AWVWNTHADFAD (+2)

Primary Reference: RANG_HUMAN
Search ID: 41760
Search Name: 20130328_ananiav_ERstress_TMT_TM_timecourse_trypsin_inj1_4mc_iaa
Scan#: 16906
Observed Mass: 831.3997 (0.6 ppm)
PSM Score: 25.98

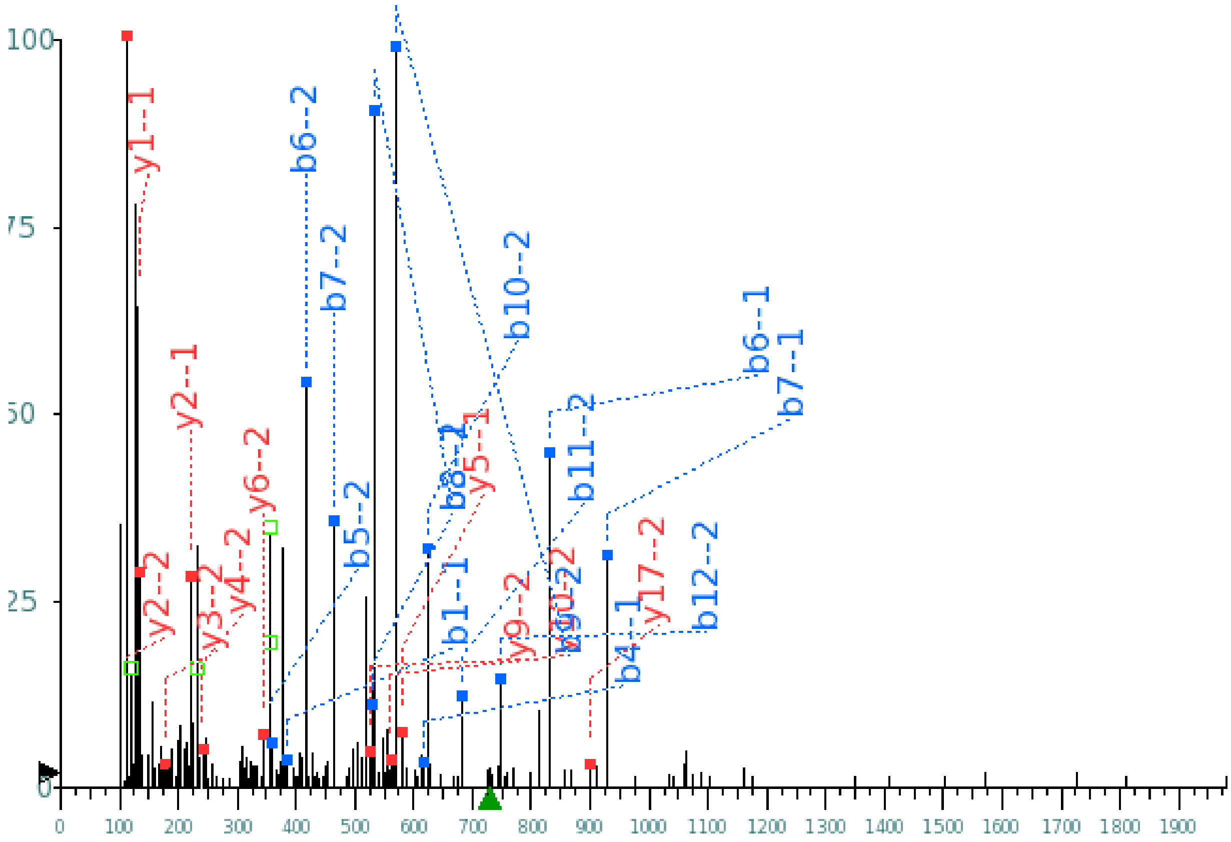

RGSSPDVHALLEITEESD (+3)

Primary Reference: PTRF_HUMAN
Search ID: 41760
Search Name: 20130328_ananiav_ERstress_TMT_TM_timecourse_trypsin_inj1_4mc_iaa
Scan#: 16930
Observed Mass: 728.7056 (1.9 ppm)
PSM Score: 21.5

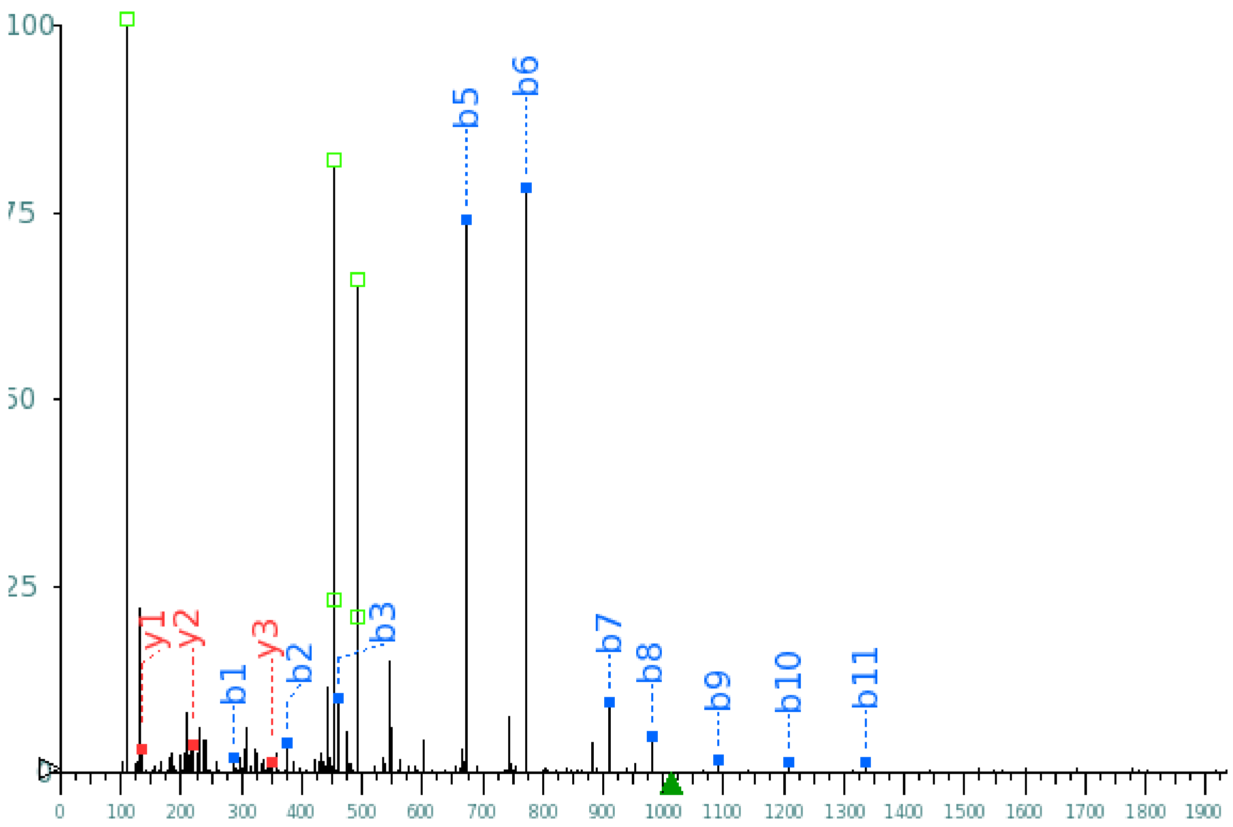

GSSPDVHALLEITEESD (+2)

Primary Reference: PTRF_HUMAN
Search ID: 41760
Search Name: 20130328_ananiav_ERstress_TMT_TM_timecourse_trypsin_inj1_4mc_iaa
Scan#: 16939
Observed Mass: 1014.5046 (2.4 ppm)
PSM Score: 29.33

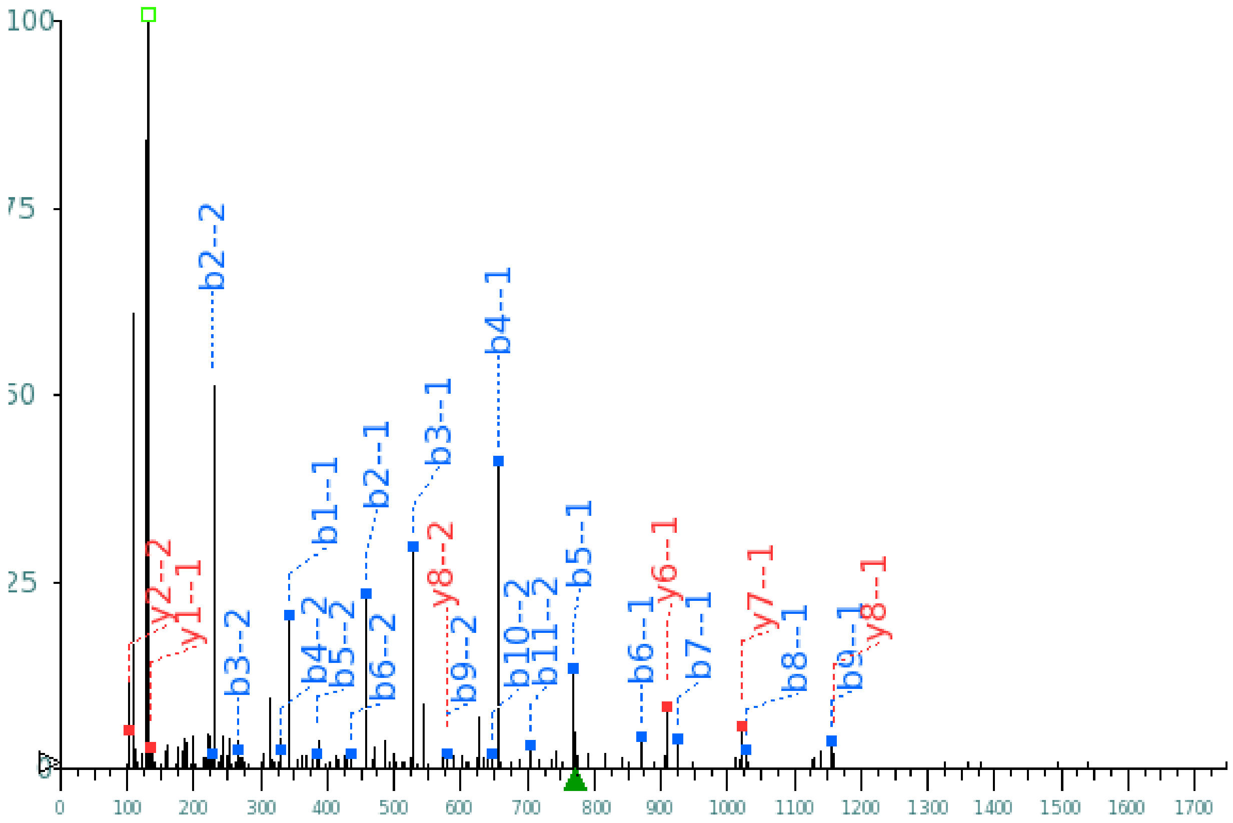

ILAQITGTEHLKESEAD (+3)

Primary Reference: PDLI5_HUMAN
Search ID: 41760
Search Name: 20130328_ananiav_ERstress_TMT_TM_timecourse_trypsin_inj1_4mc_iaa
Scan#: 16947
Observed Mass: 771.7638 (2.9 ppm)
PSM Score: 33.87

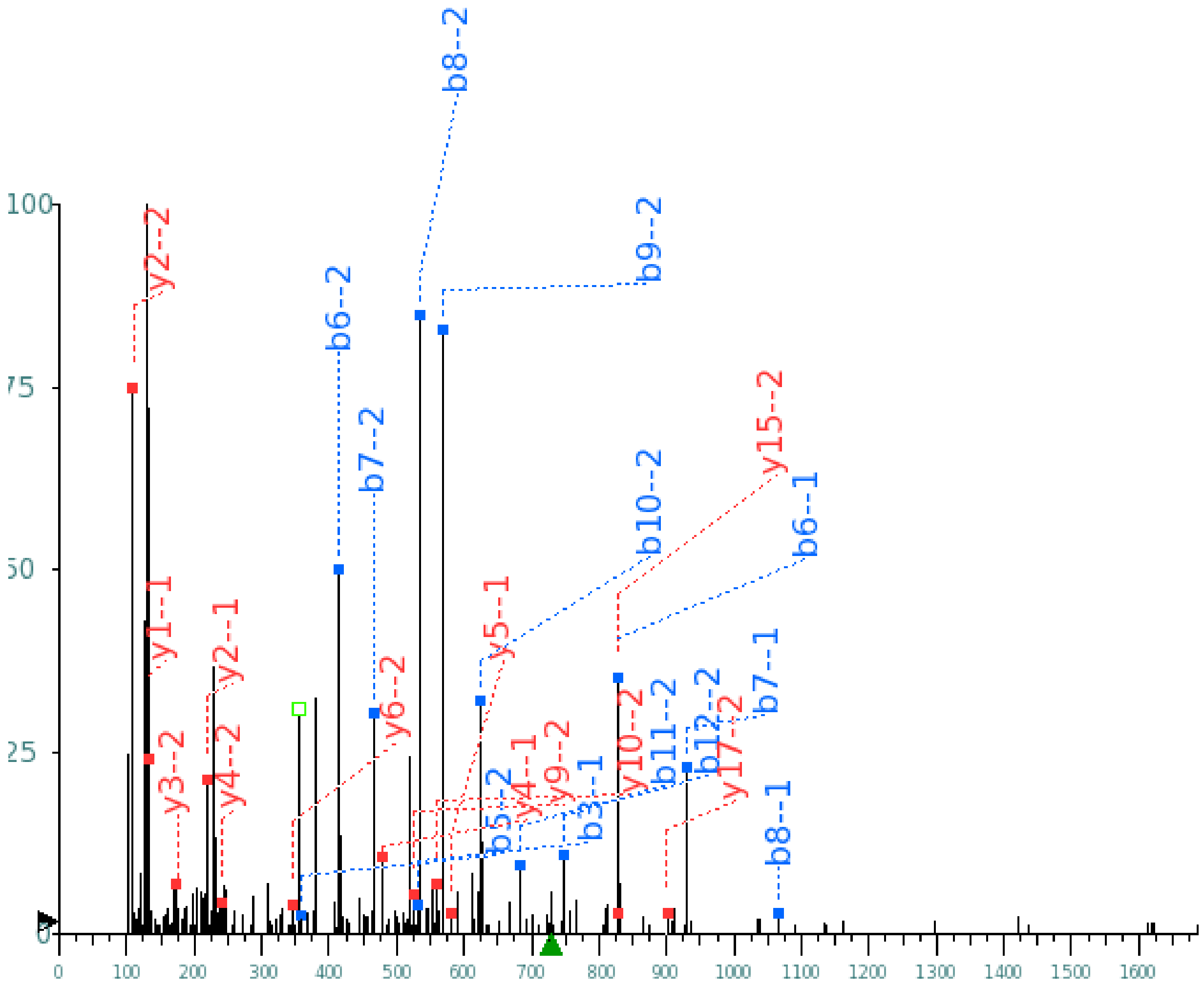

RGSSPDVHALLEITEESD (+3)

Primary Reference: PTRF_HUMAN
Search ID: 41760
Search Name: 20130328_ananiav_ERstress_TMT_TM_timecourse_trypsin_inj1_4mc_iaa
Scan#: 17004
Observed Mass: 728.7046 (0.5 ppm)
PSM Score: 20.72

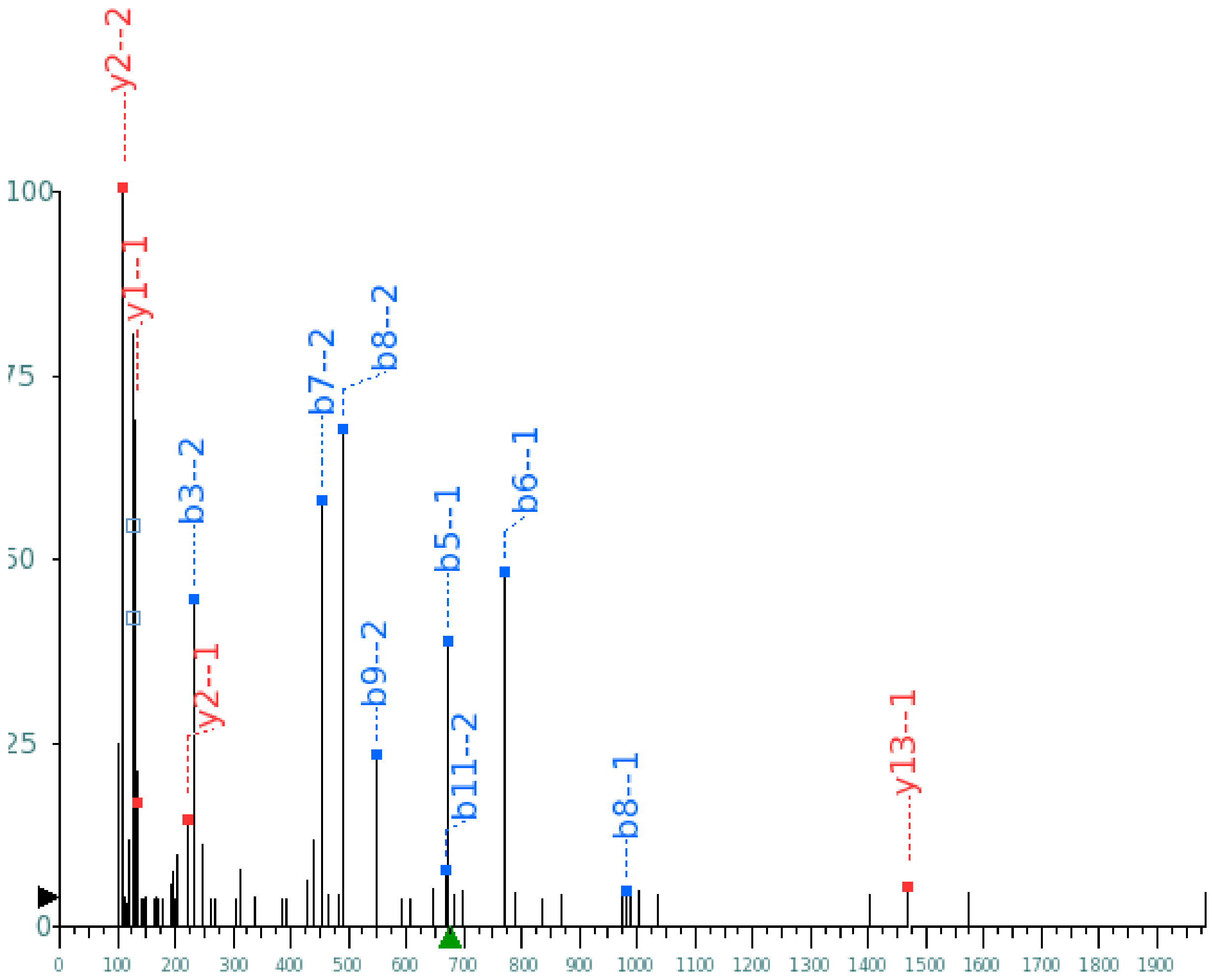

GSSPDVHALLEITEESD (+3)

Primary Reference: PTRF_HUMAN
Search ID: 41760
Search Name: 20130328_ananiav_ERstress_TMT_TM_timecourse_trypsin_inj1_4mc_iaa
Scan#: 17012
Observed Mass: 676.6722 (2.4 ppm)
PSM Score: 11.7

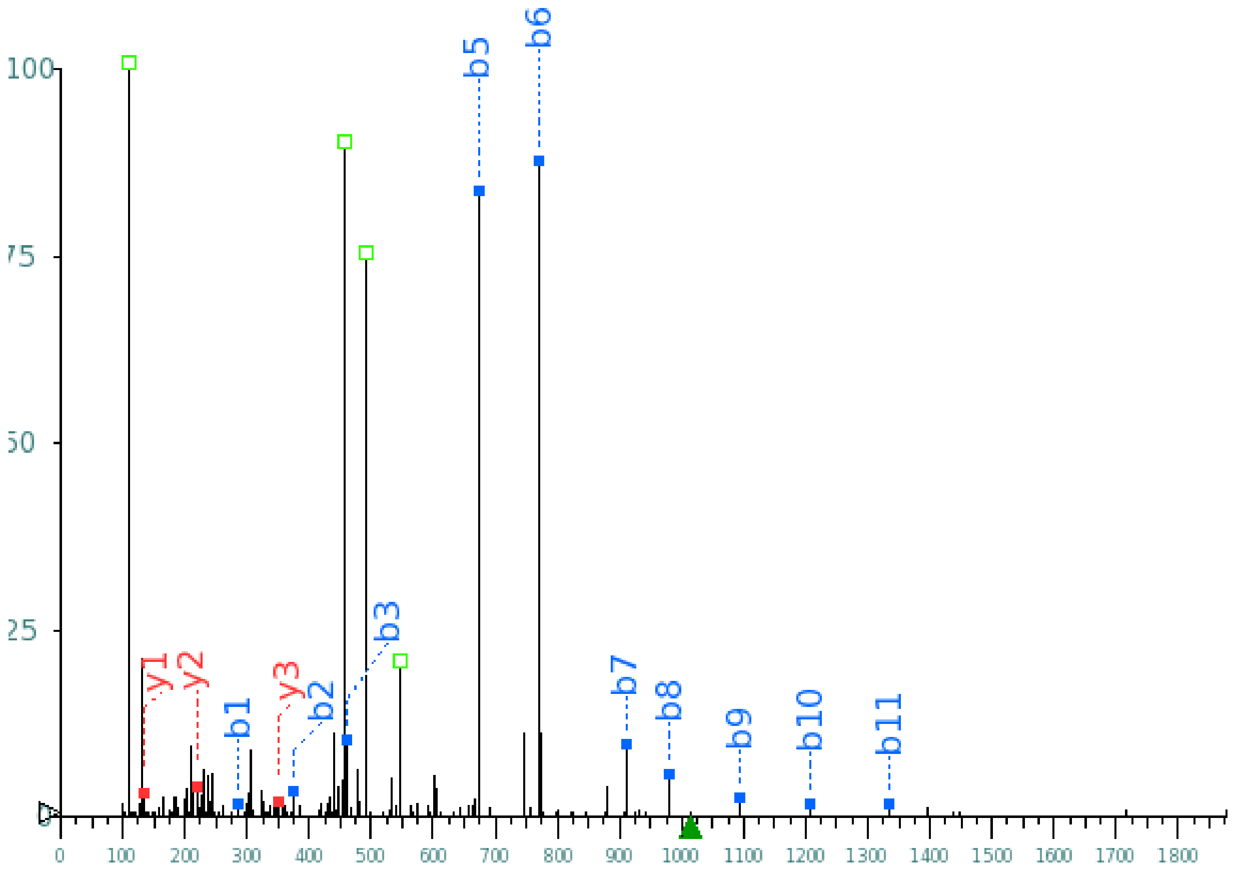

GSSPDVHALLEITEESD (+2)

Primary Reference: PTRF_HUMAN
Search ID: 41760
Search Name: 20130328_ananiav_ERstress_TMT_TM_timecourse_trypsin_inj1_4mc_iaa
Scan#: 17013
Observed Mass: 1014.5048 (2.6 ppm)
PSM Score: 37.31

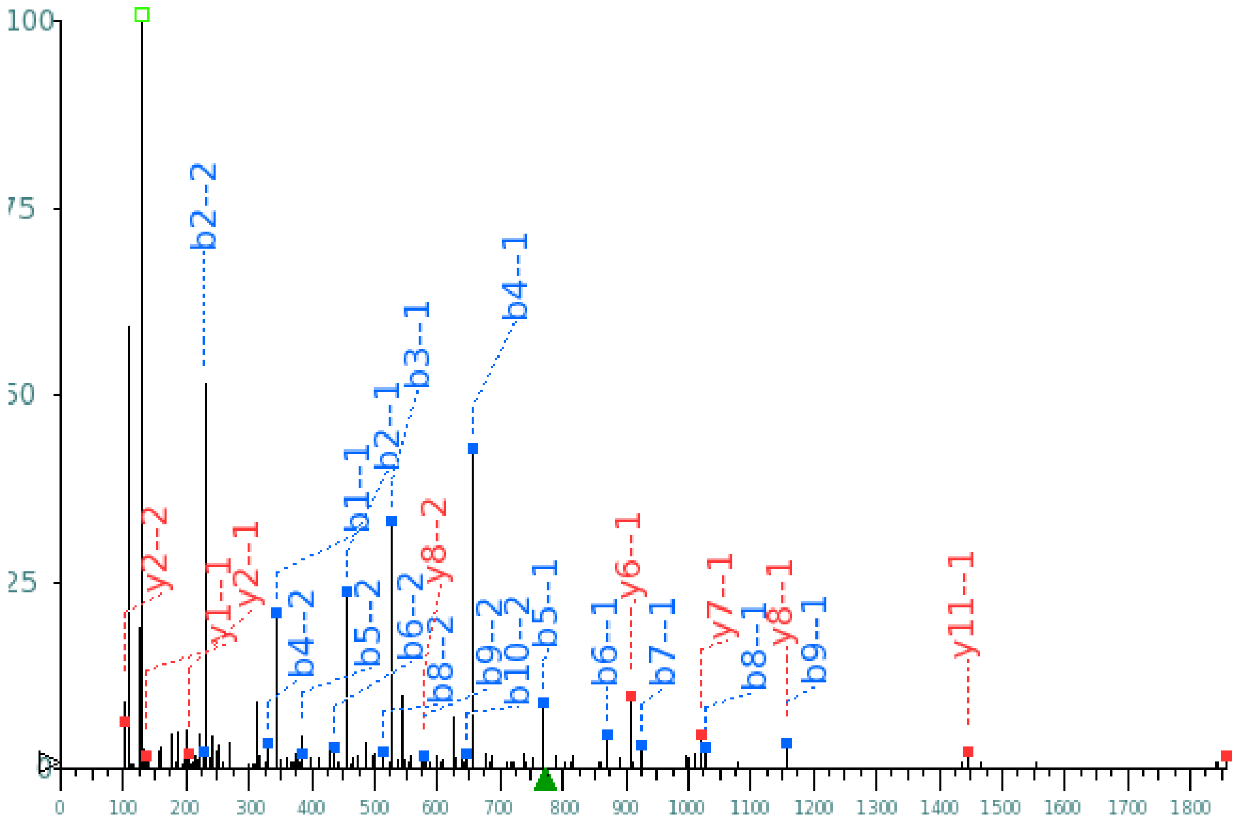

ILAQITGTEHLKESEAD (+3)

Primary Reference: PDLI5_HUMAN
Search ID: 41760
Search Name: 20130328_ananiav_ERstress_TMT_TM_timecourse_trypsin_inj1_4mc_iaa
Scan#: 17023
Observed Mass: 771.7639 (3 ppm)
PSM Score: 46.87

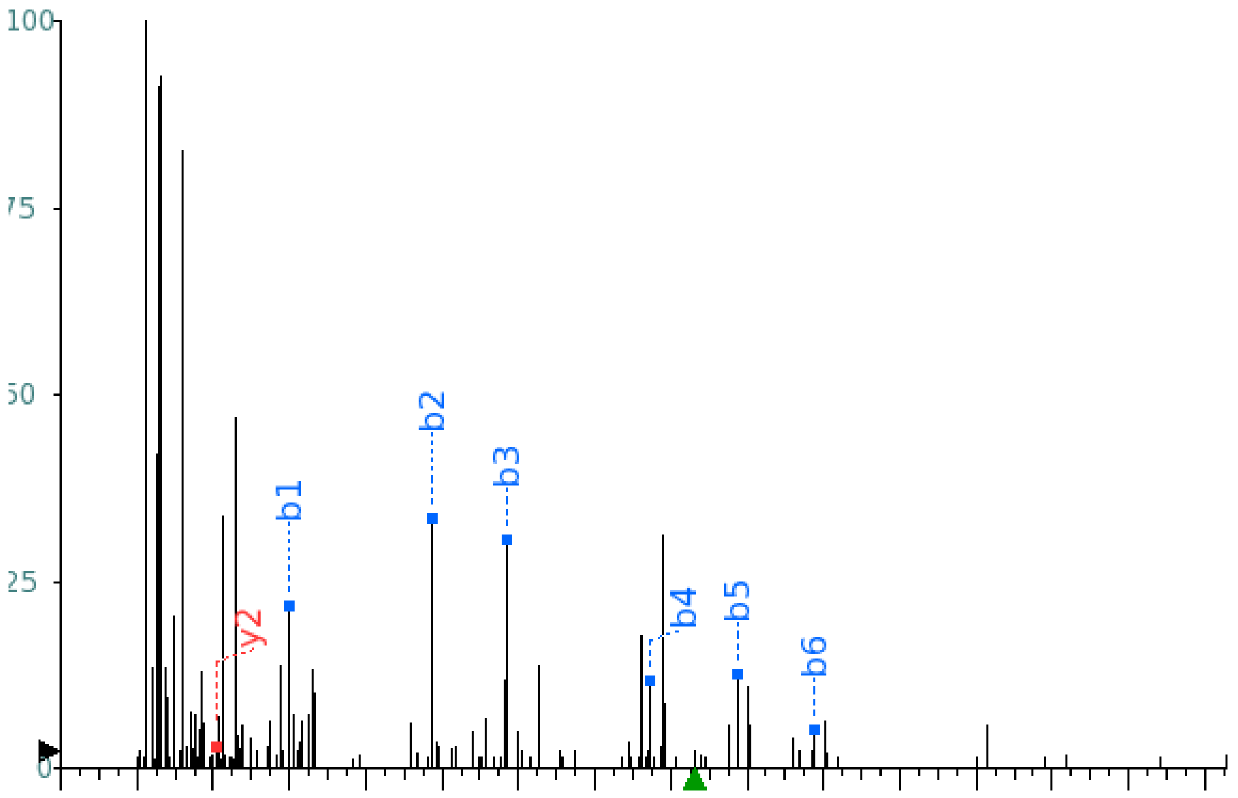

AWVWNTHADFAD (+2)

Primary Reference: RANG_HUMAN
Search ID: 41760
Search Name: 20130328_ananiav_ERstress_TMT_TM_timecourse_trypsin_inj1_4mc_iaa
Scan#: 17065
Observed Mass: 831.4054 (7.6 ppm)
PSM Score: 13.19

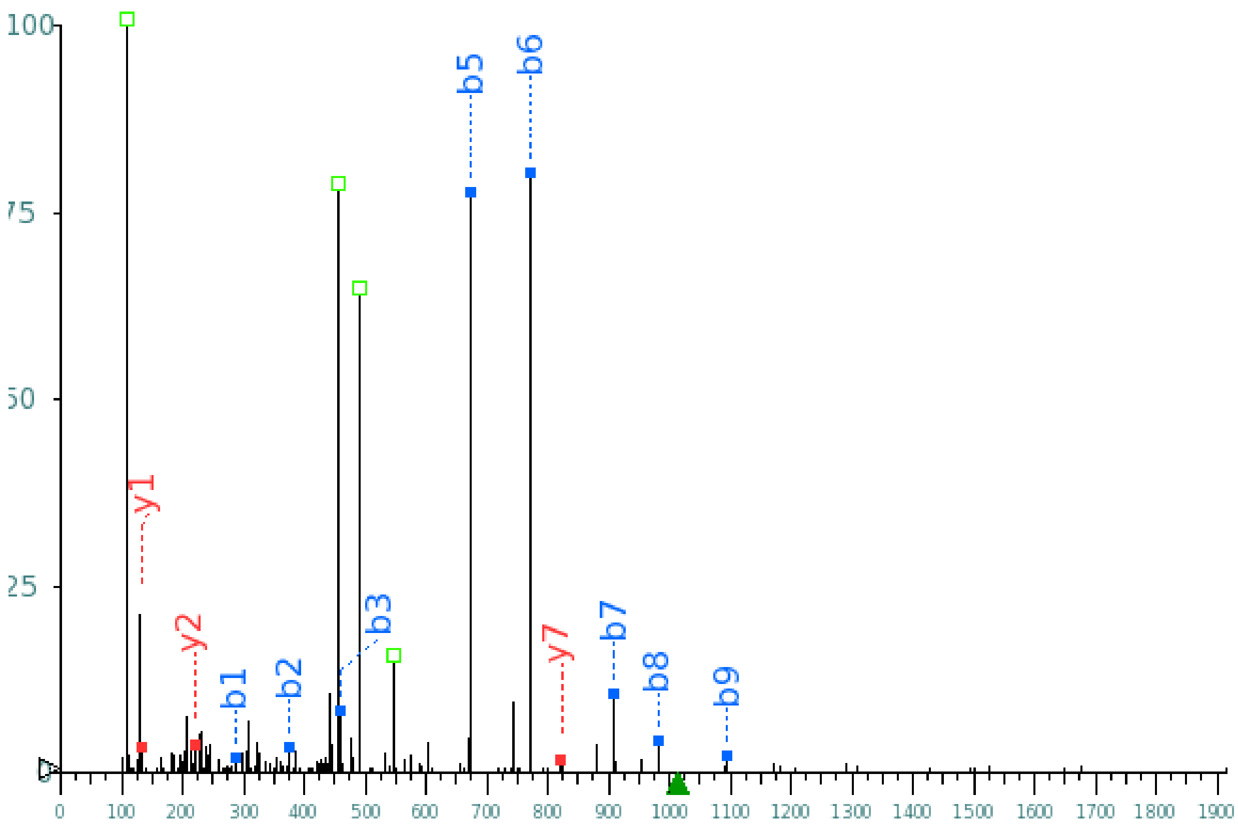

GSSPDVHALLEITEESD (+2)

Primary Reference: PTRF_HUMAN
Search ID: 41760
Search Name: 20130328_ananiav_ERstress_TMT_TM_timecourse_trypsin_inj1_4mc_iaa
Scan#: 17083
Observed Mass: 1014.5044 (2.2 ppm)
PSM Score: 19.8

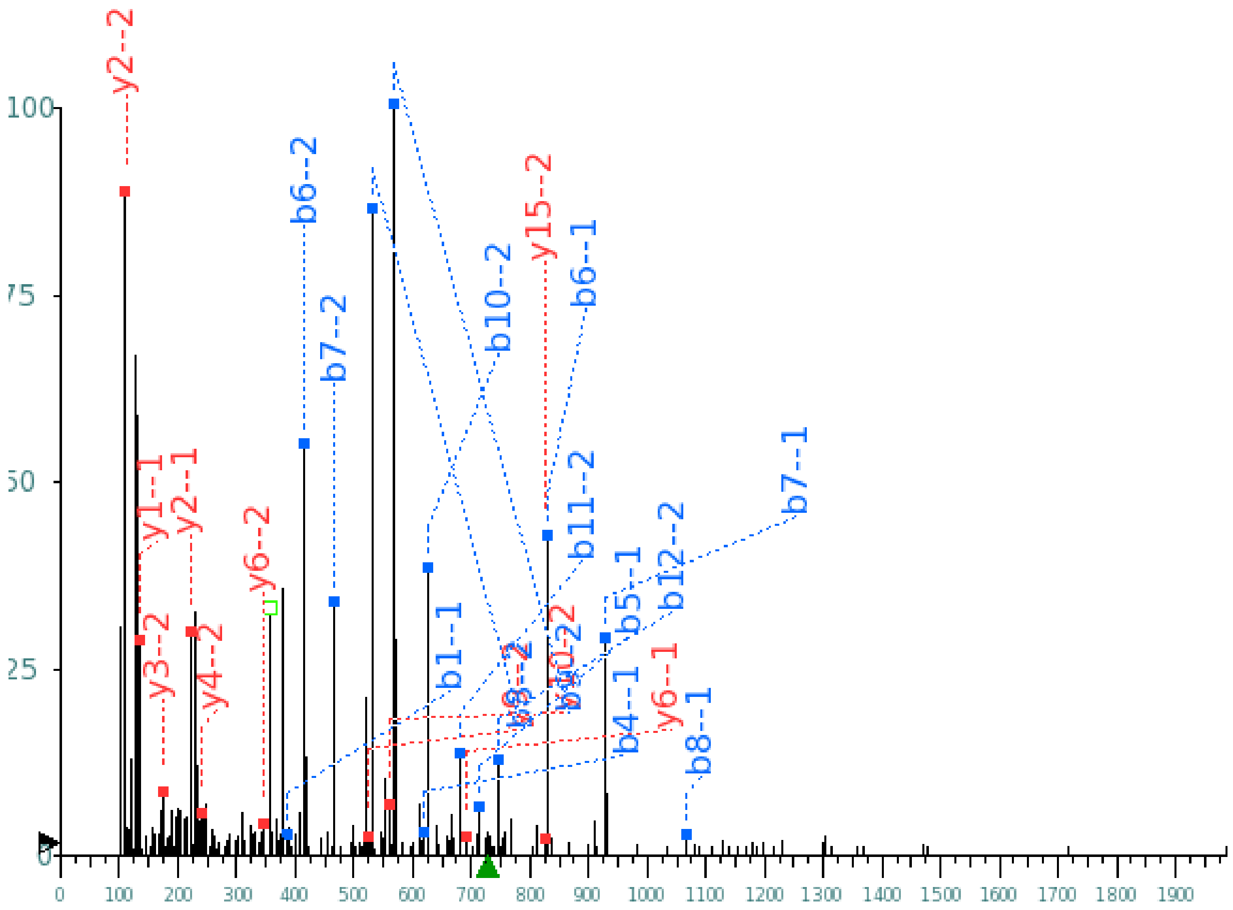

RGSSPDVHALLEITEESD (+3)

Primary Reference: PTRF_HUMAN
Search ID: 41760
Search Name: 20130328_ananiav_ERstress_TMT_TM_timecourse_trypsin_inj1_4mc_iaa
Scan#: 17094
Observed Mass: 728.7034 (-1.1 ppm)
PSM Score: 18.58

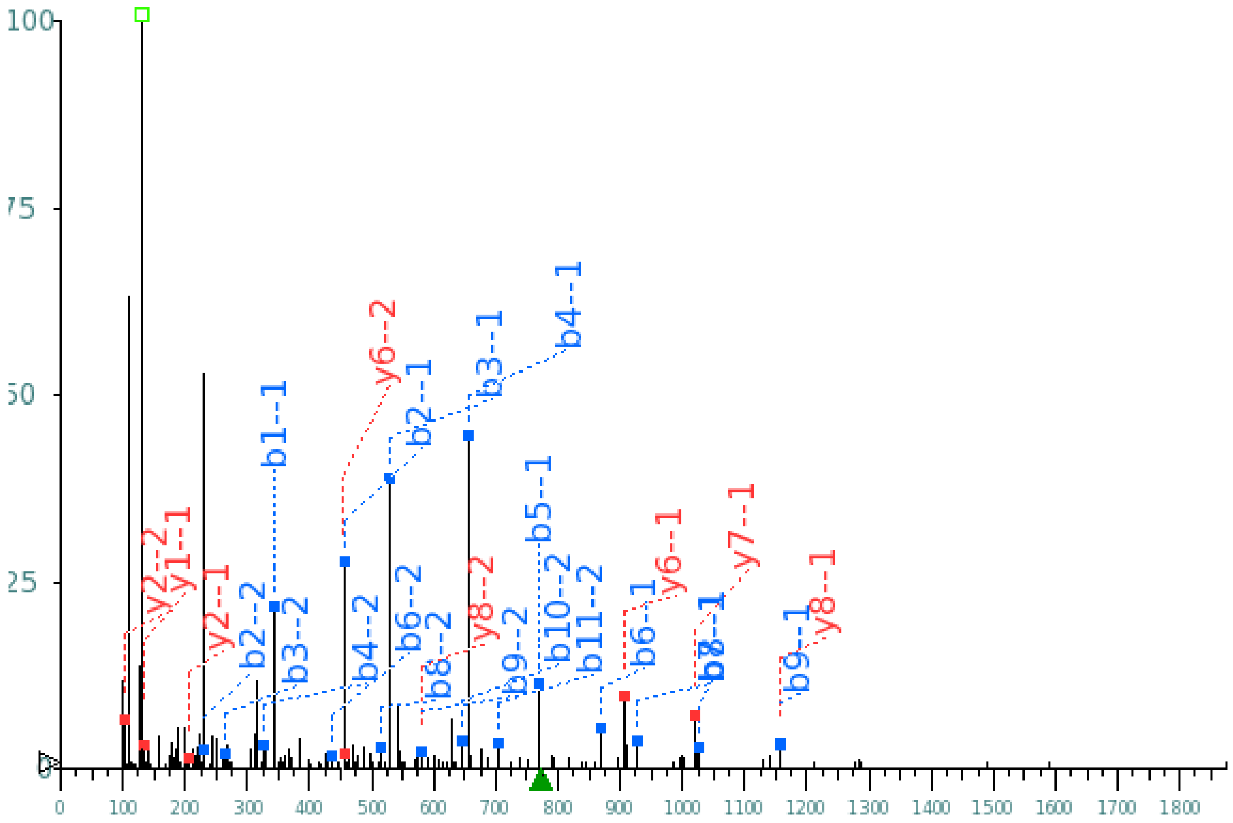

ILAQITGTEHLKESEAD (+3)

Primary Reference: PDLI5_HUMAN
Search ID: 41760
Search Name: 20130328_ananiav_ERstress_TMT_TM_timecourse_trypsin_inj1_4mc_iaa
Scan#: 17100
Observed Mass: 771.7634 (2.4 ppm)
PSM Score: 34.17

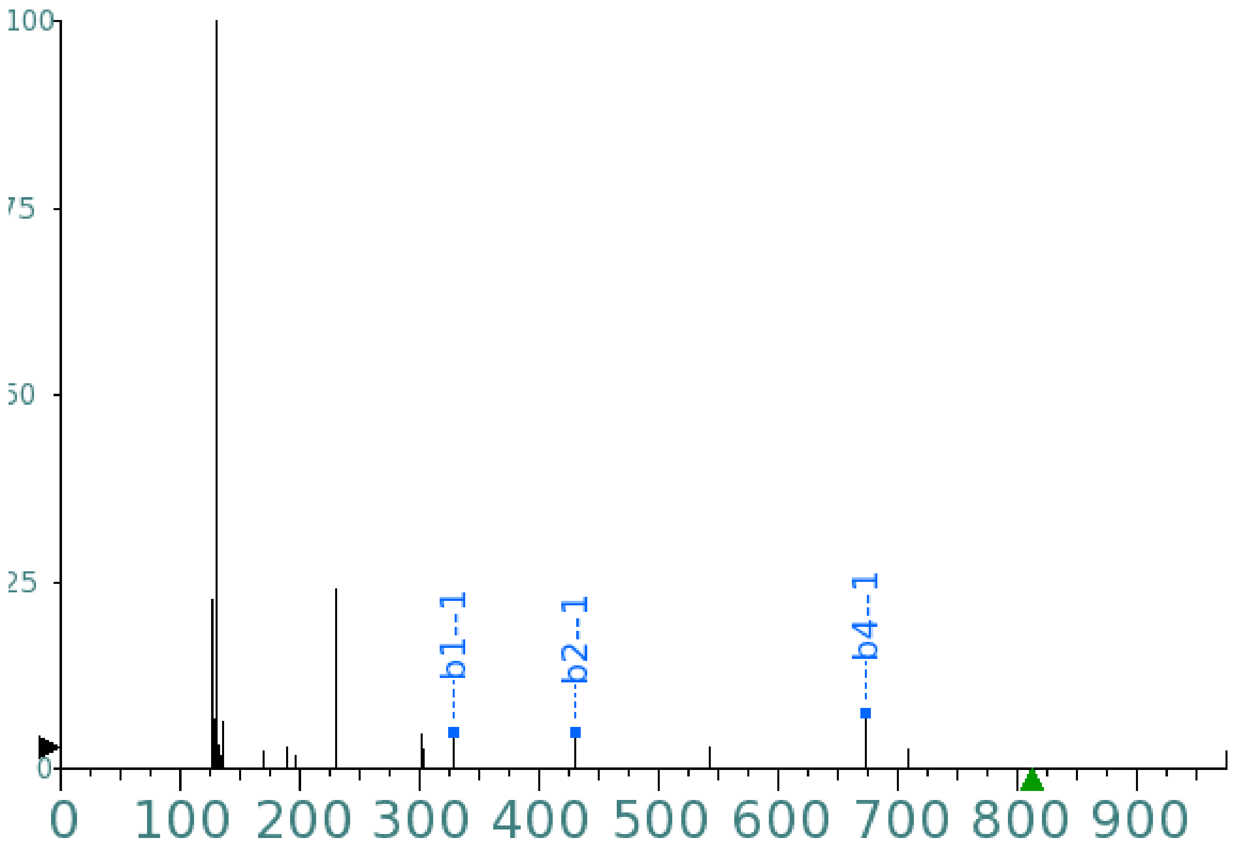

VTLEYRPVIDKTLNEAD (+3)

Primary Reference: DHSA_HUMAN
Search ID: 41760
Search Name: 20130328_ananiav_ERstress_TMT_TM_timecourse_trypsin_inj1_4mc_iaa
Scan#: 17129
Observed Mass: 812.1247 (0.1 ppm)
PSM Score: 14.7

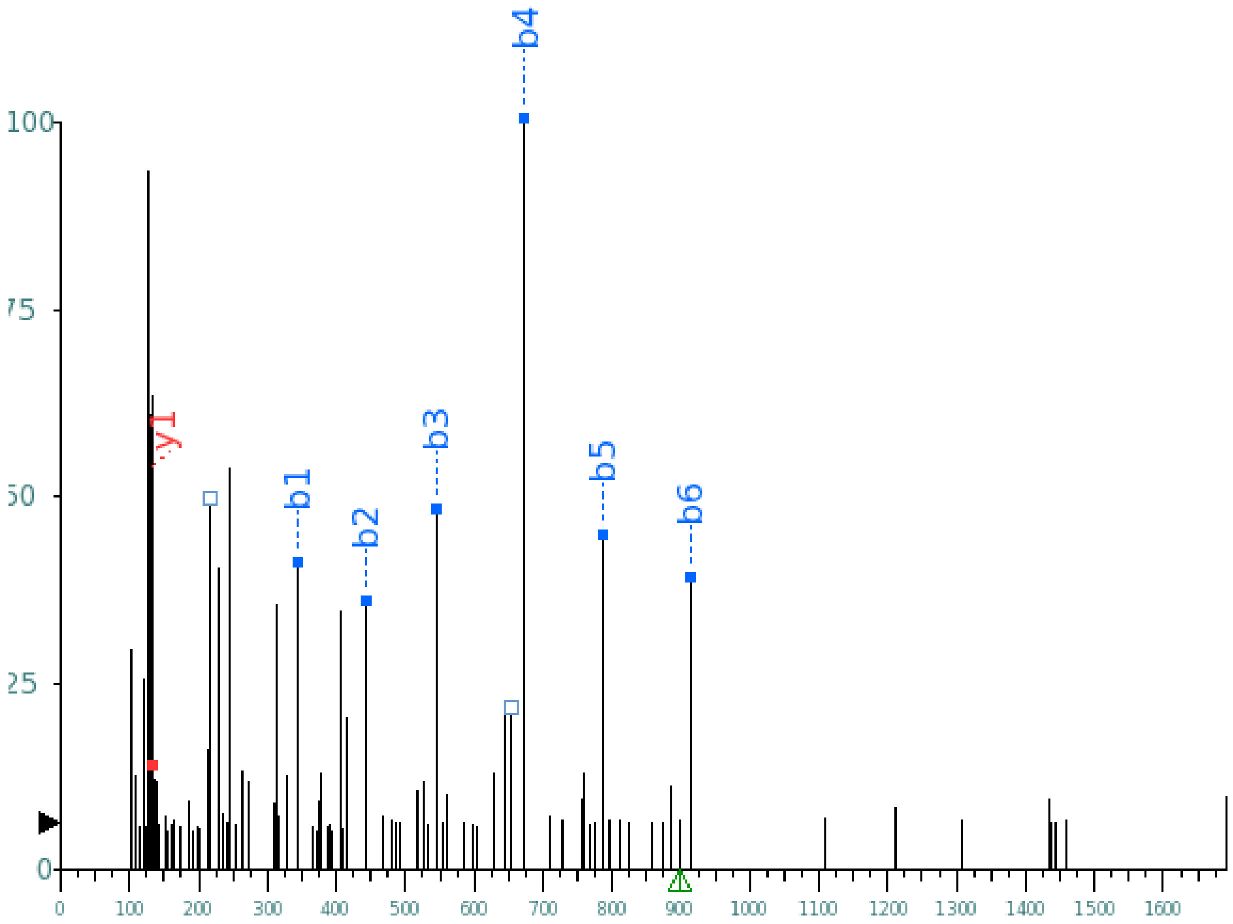

NVTQIEPFC*LETD (+2)

Primary Reference: TPX2_HUMAN
Search ID: 41760
Search Name: 20130328_ananiav_ERstress_TMT_TM_timecourse_trypsin_inj1_4mc_iaa
Scan#: 17133
Observed Mass: 897.9472 (5 ppm)
PSM Score: 25.59

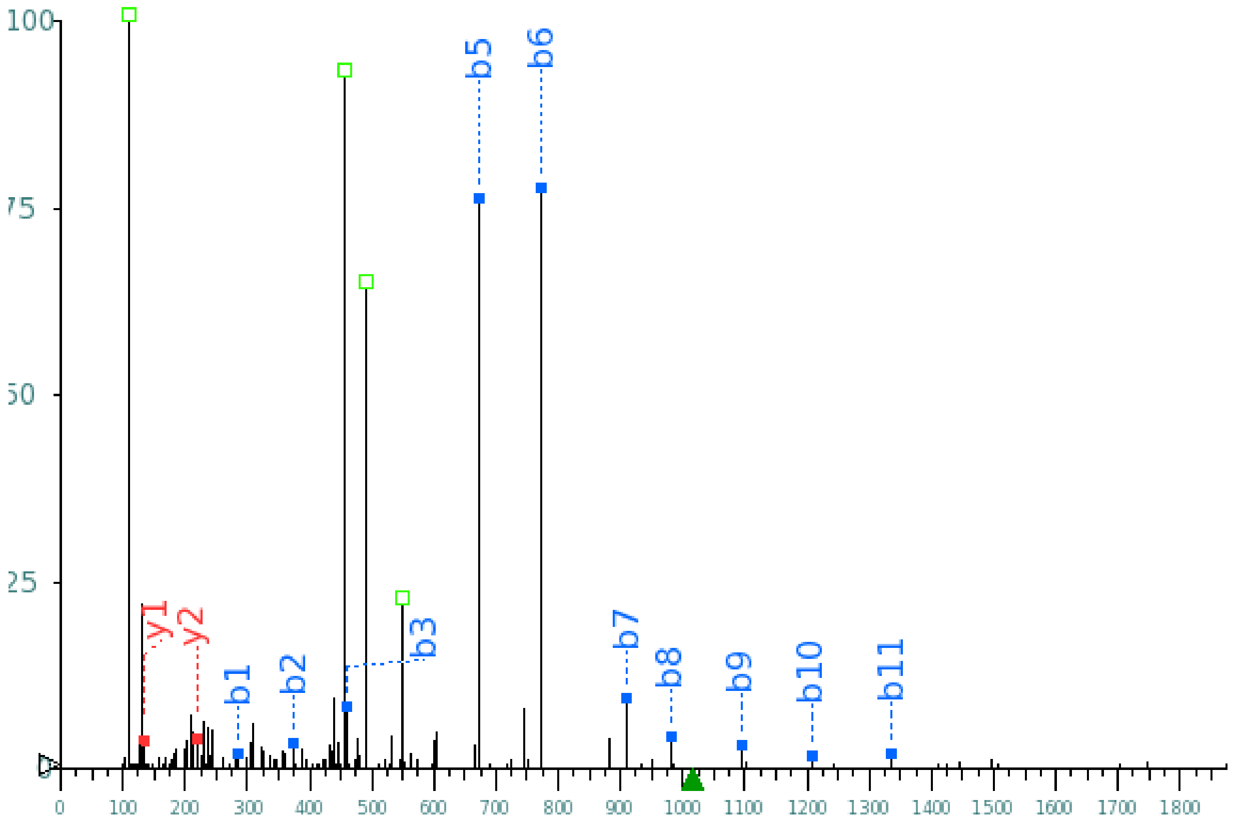

GSSPDVHALLEITEESD (+2)

Primary Reference: PTRF_HUMAN
Search ID: 41760
Search Name: 20130328_ananiav_ERstress_TMT_TM_timecourse_trypsin_inj1_4mc_iaa
Scan#: 17158
Observed Mass: 1014.5045 (2.3 ppm)
PSM Score: 31.75

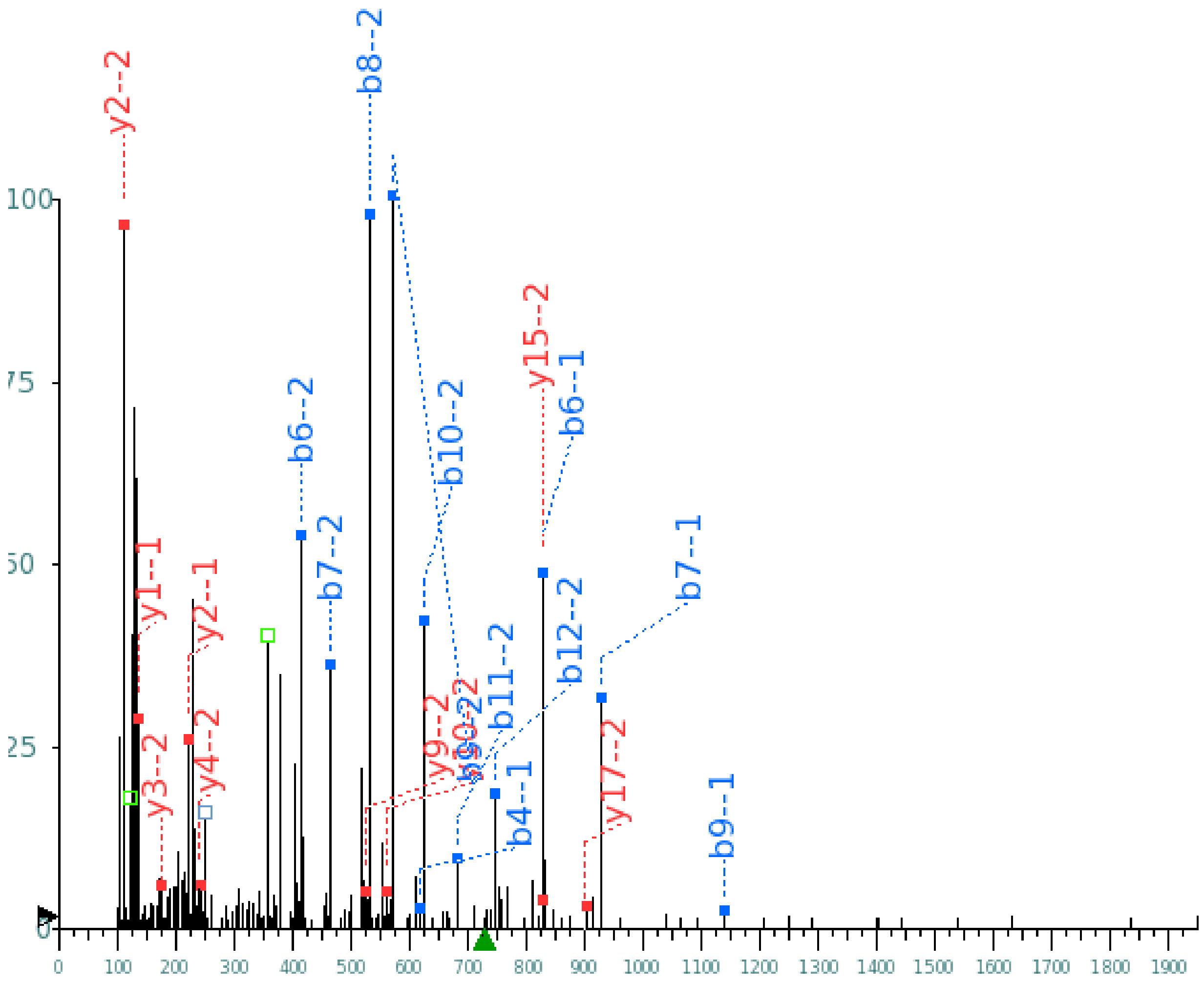

RGSSPDVHALLEITEESD (+3)

Primary Reference: PTRF_HUMAN
Search ID: 41760
Search Name: 20130328_ananiav_ERstress_TMT_TM_timecourse_trypsin_inj1_4mc_iaa
Scan#: 17163
Observed Mass: 728.705 (1 ppm)
PSM Score: 21.2

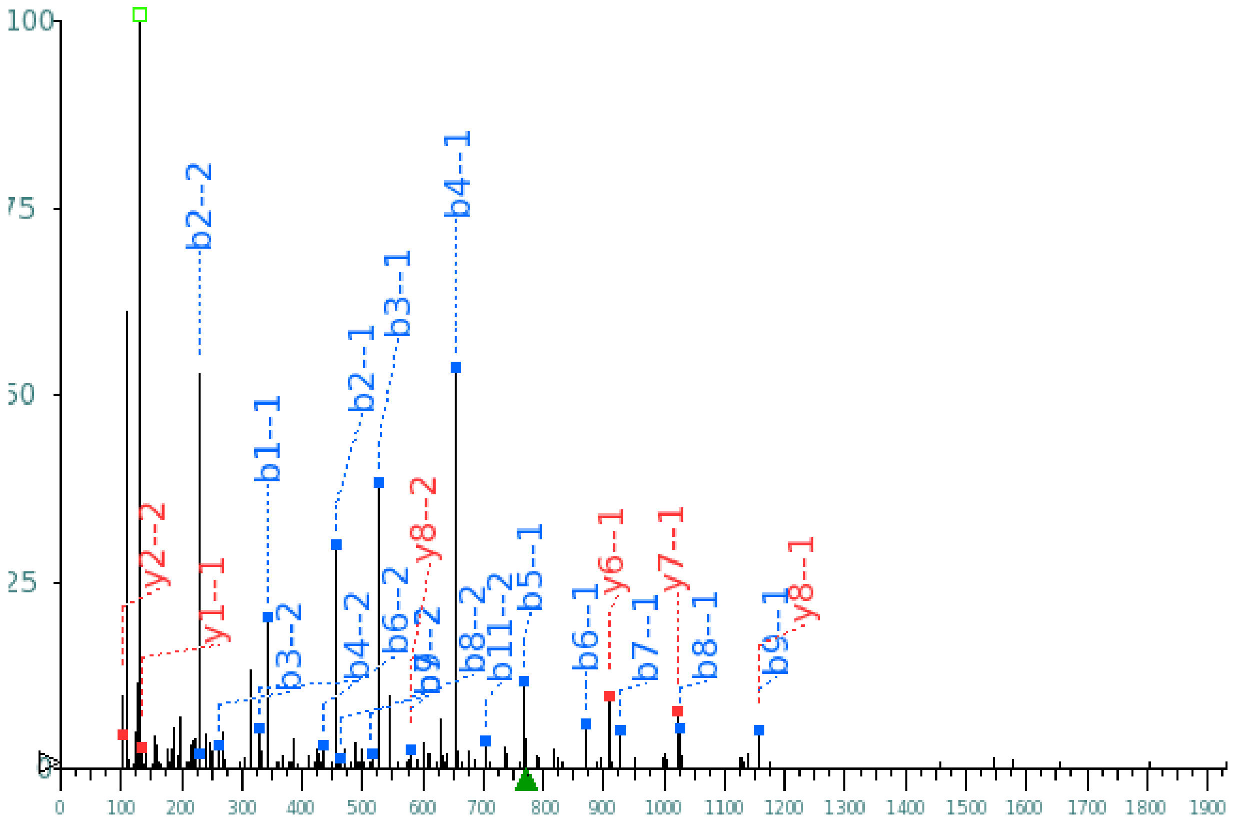

ILAQITGTEHLKESEAD (+3)

Primary Reference: PDLI5_HUMAN
Search ID: 41760
Search Name: 20130328_ananiav_ERstress_TMT_TM_timecourse_trypsin_inj1_4mc_iaa
Scan#: 17166
Observed Mass: 771.7633 (2.3 ppm)
PSM Score: 35.11

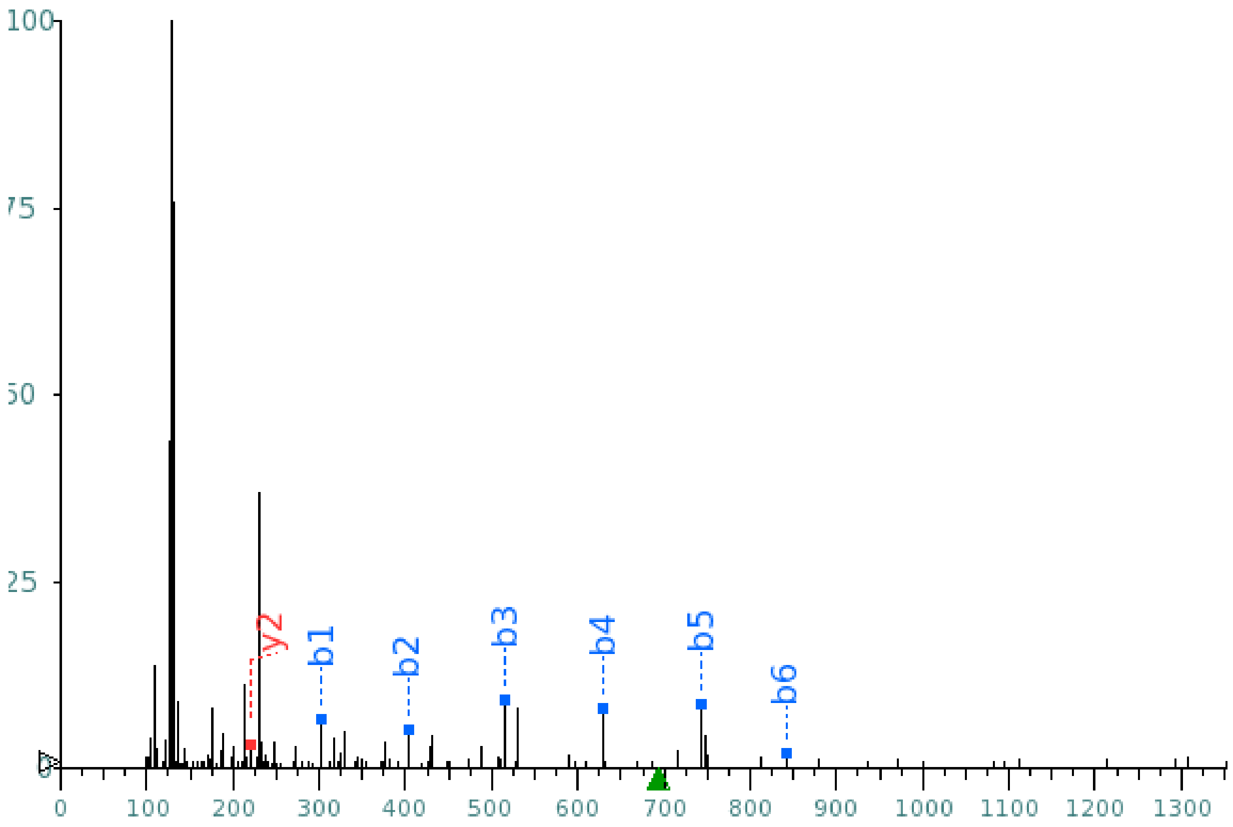

ATLLNVPDLSD (+2)

Primary Reference: ZO1_HUMAN
Search ID: 41760
Search Name: 20130328_ananiav_ERstress_TMT_TM_timecourse_trypsin_inj1_4mc_iaa
Scan#: 17167
Observed Mass: 693.8892 (2.4 ppm)
PSM Score: 14.73

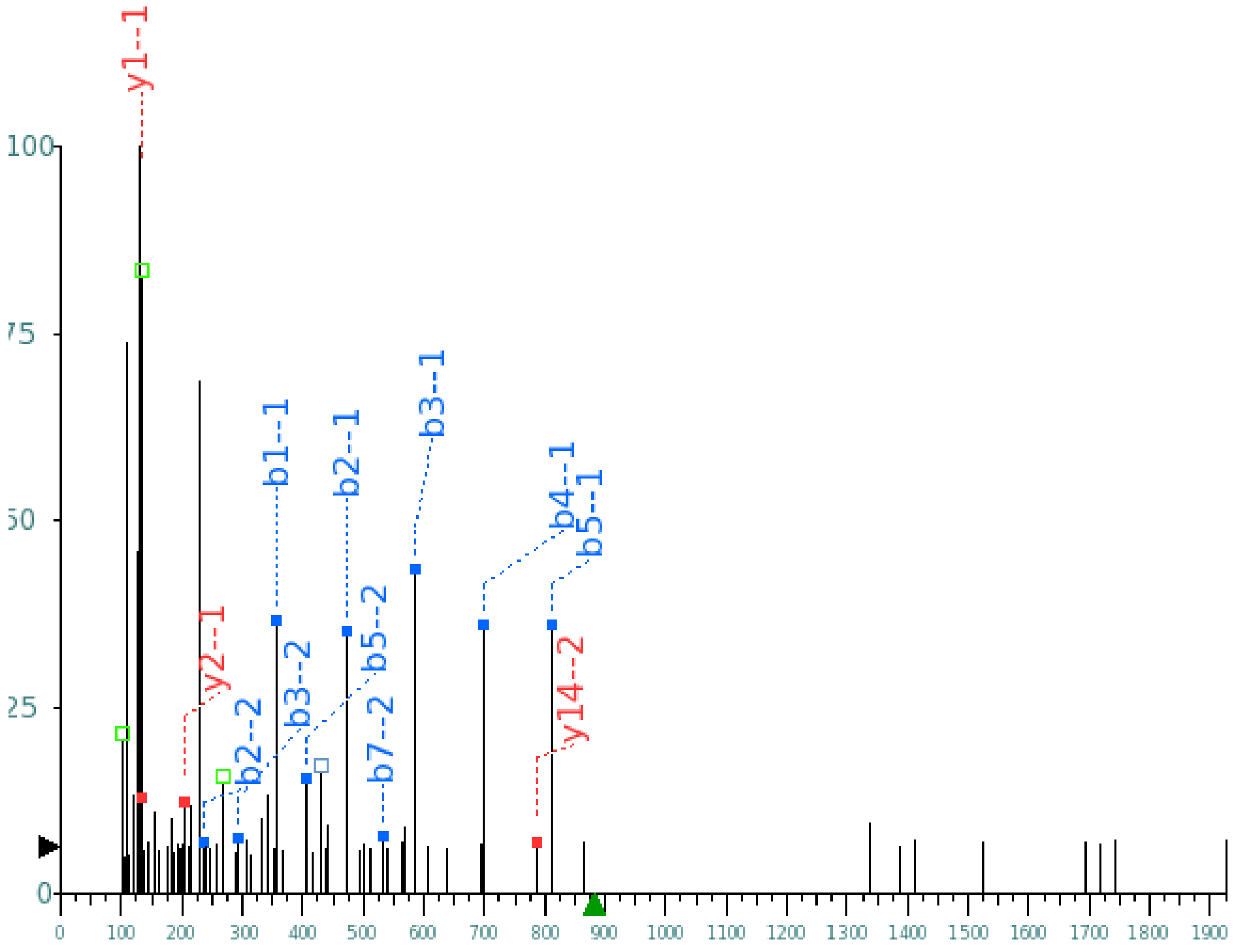

QILNNYSAQFSLEHMSPDQAD (+3)

Primary Reference: SYNE2_HUMAN
Search ID: 41760
Search Name: 20130328_ananiav_ERstress_TMT_TM_timecourse_trypsin_inj1_4mc_iaa
Scan#: 17179
Observed Mass: 879.7552 (2.2 ppm)
PSM Score: 12.82

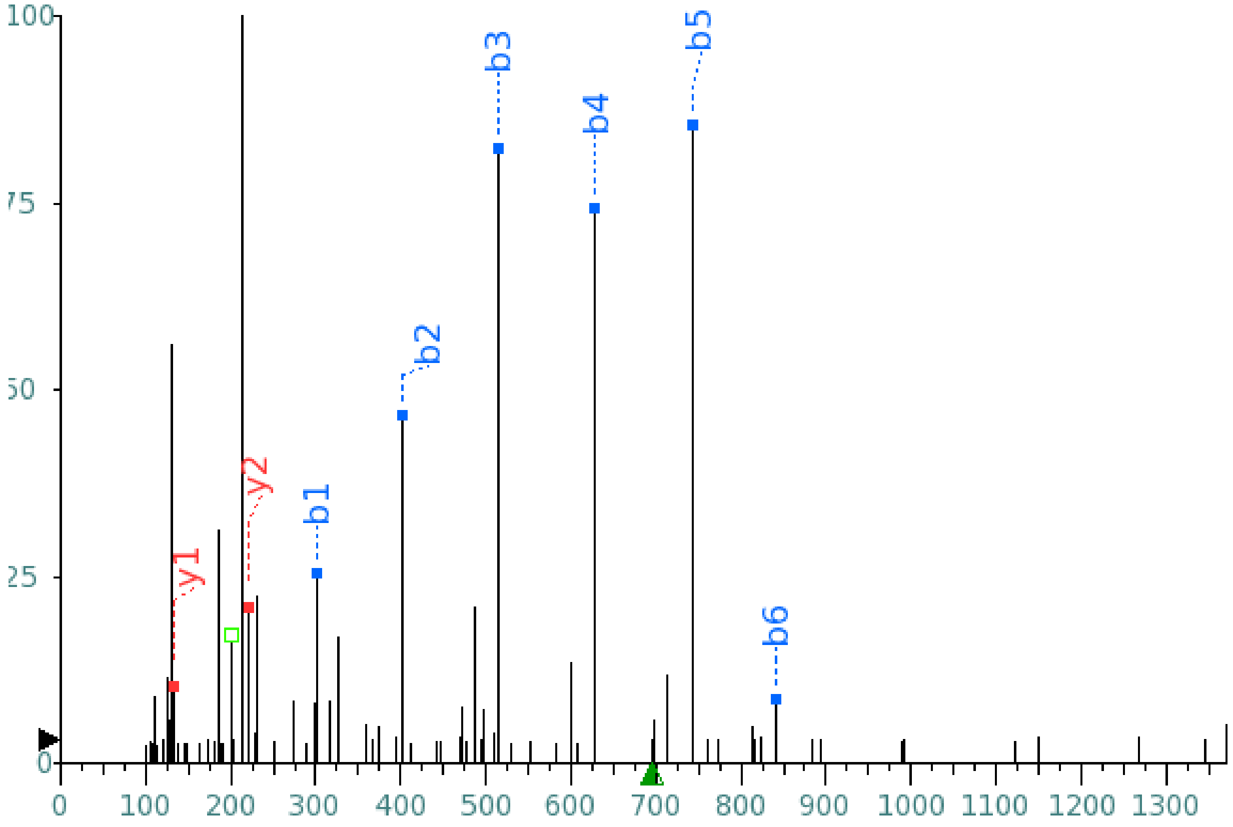

ATLLNVPDLSD (+2)

Primary Reference: ZO1_HUMAN
Search ID: 41760
Search Name: 20130328_ananiav_ERstress_TMT_TM_timecourse_trypsin_inj1_4mc_iaa
Scan#: 17243
Observed Mass: 693.8893 (2.6 ppm)
PSM Score: 35.05

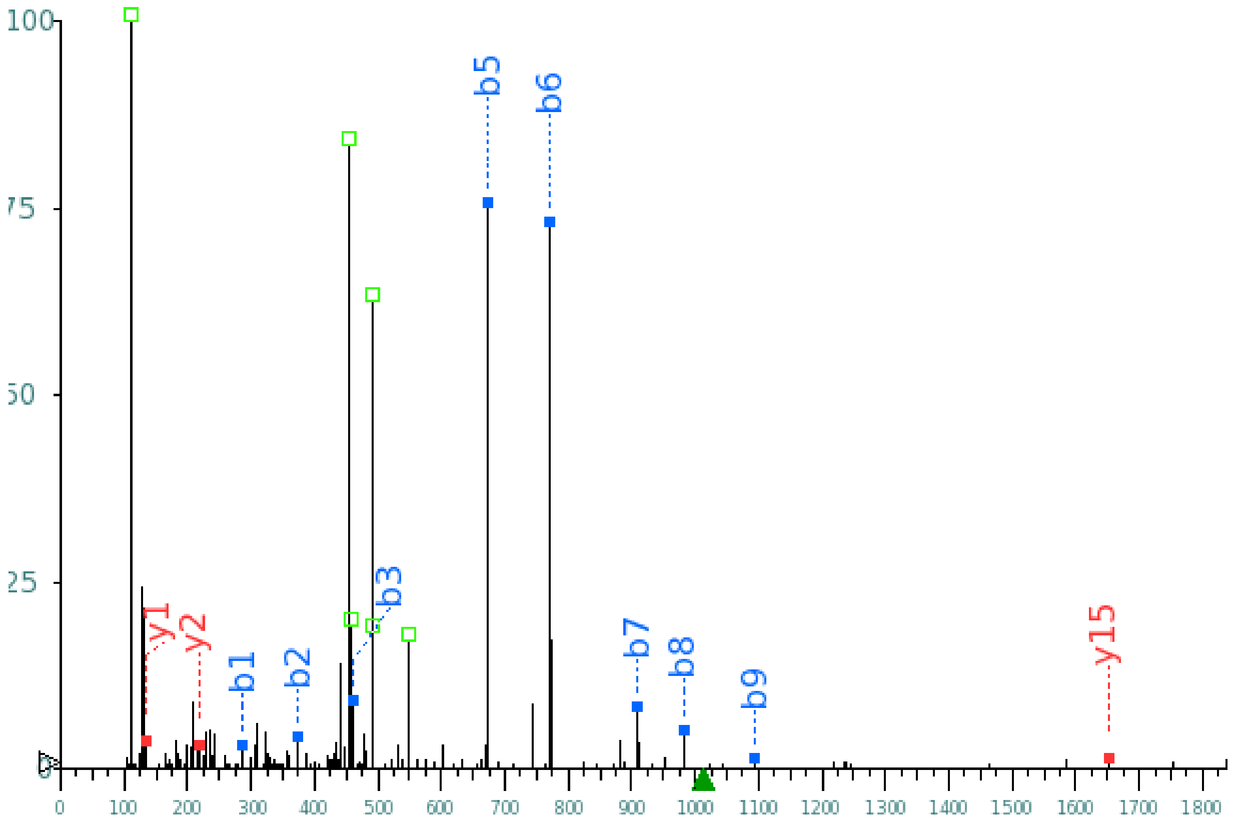

GSSPDVHALLEITEESD (+2)

Primary Reference: PTRF_HUMAN
Search ID: 41760
Search Name: 20130328_ananiav_ERstress_TMT_TM_timecourse_trypsin_inj1_4mc_iaa
Scan#: 17247
Observed Mass: 1014.5047 (2.5 ppm)
PSM Score: 19.52

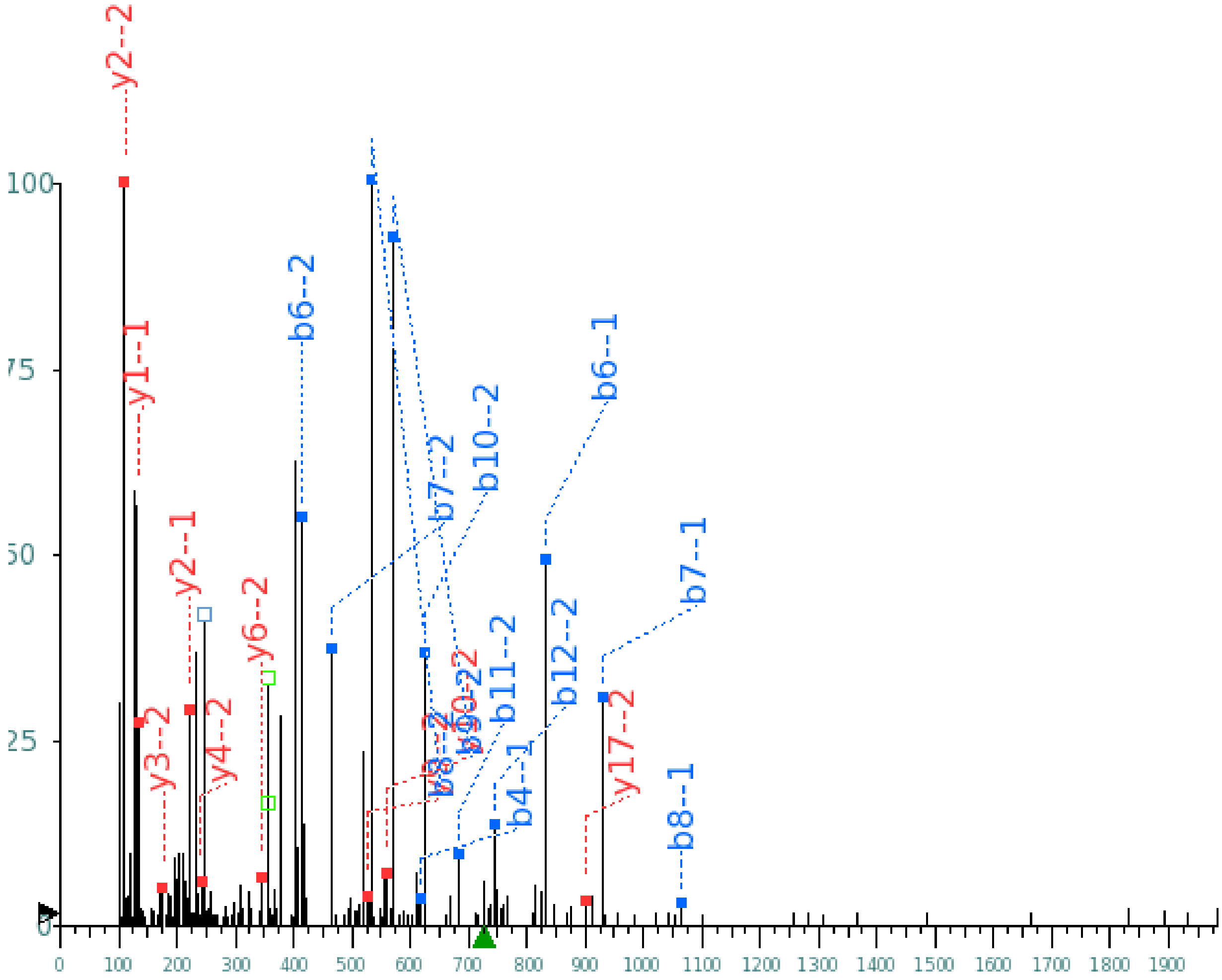

RGSSPDVHALLEITEESD (+3)

Primary Reference: PTRF_HUMAN

Search ID: 41760

Search Name: 20130328_ananiav_ERstress_TMT_TM_timecourse_trypsin_inj1_4mc_iaa

Scan#: 17249

Observed Mass: 728.7052 (1.4 ppm)

PSM Score: 20.23

File name: 2015-02-04 04.01.53PM.pdf

Creation time: 2015-02-04 04:09PM

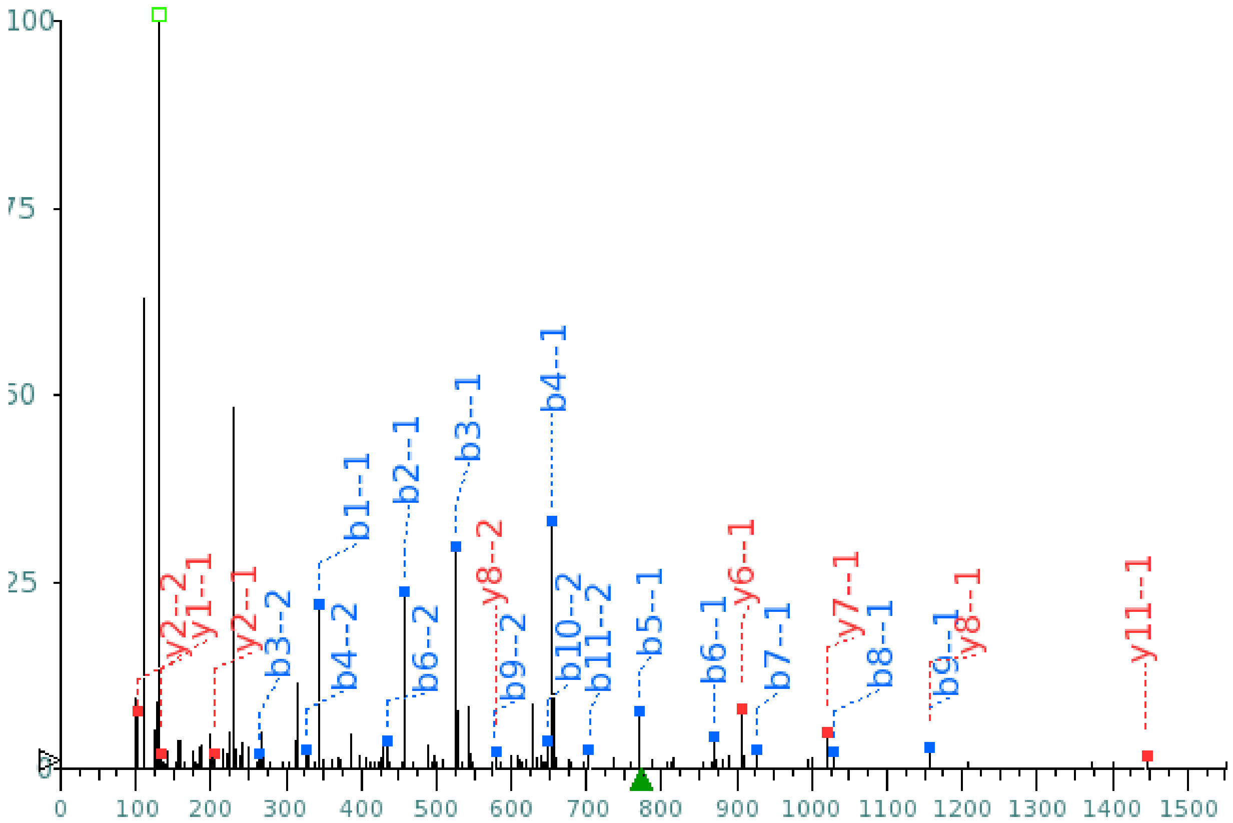

ILAQITGTEHLKESEAD (+3)

Primary Reference: PDLI5_HUMAN
Search ID: 41760
Search Name: 20130328_ananiav_ERstress_TMT_TM_timecourse_trypsin_inj1_4mc_iaa
Scan#: 17254
Observed Mass: 771.7633 (2.2 ppm)
PSM Score: 34.33

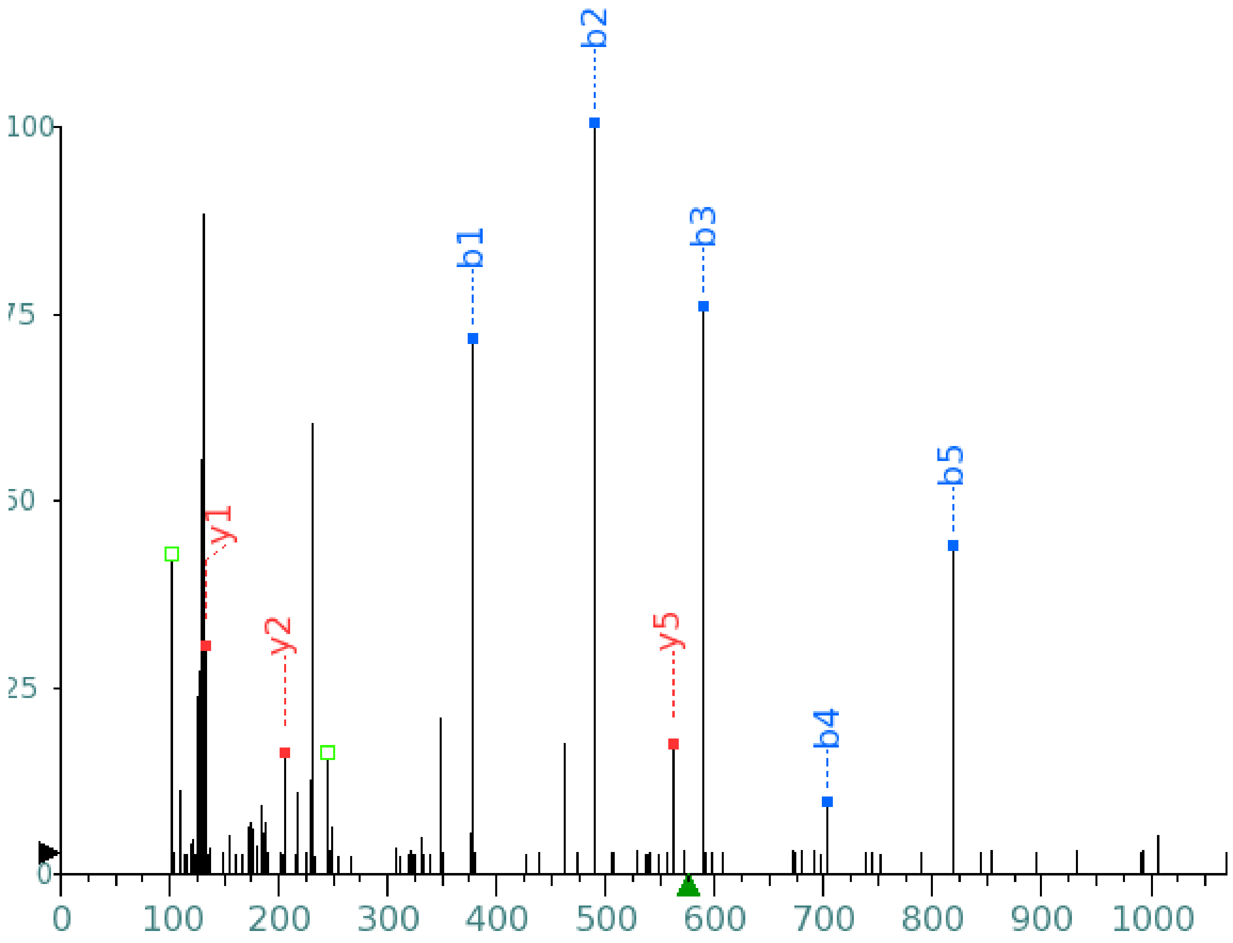

FLVLDEAD (+2)

Primary Reference: DDX1_HUMAN
Search ID: 41760
Search Name: 20130328_ananiav_ERstress_TMT_TM_timecourse_trypsin_inj1_4mc_iaa
Scan#: 17276
Observed Mass: 575.8148 (2.6 ppm)
PSM Score: 28.43

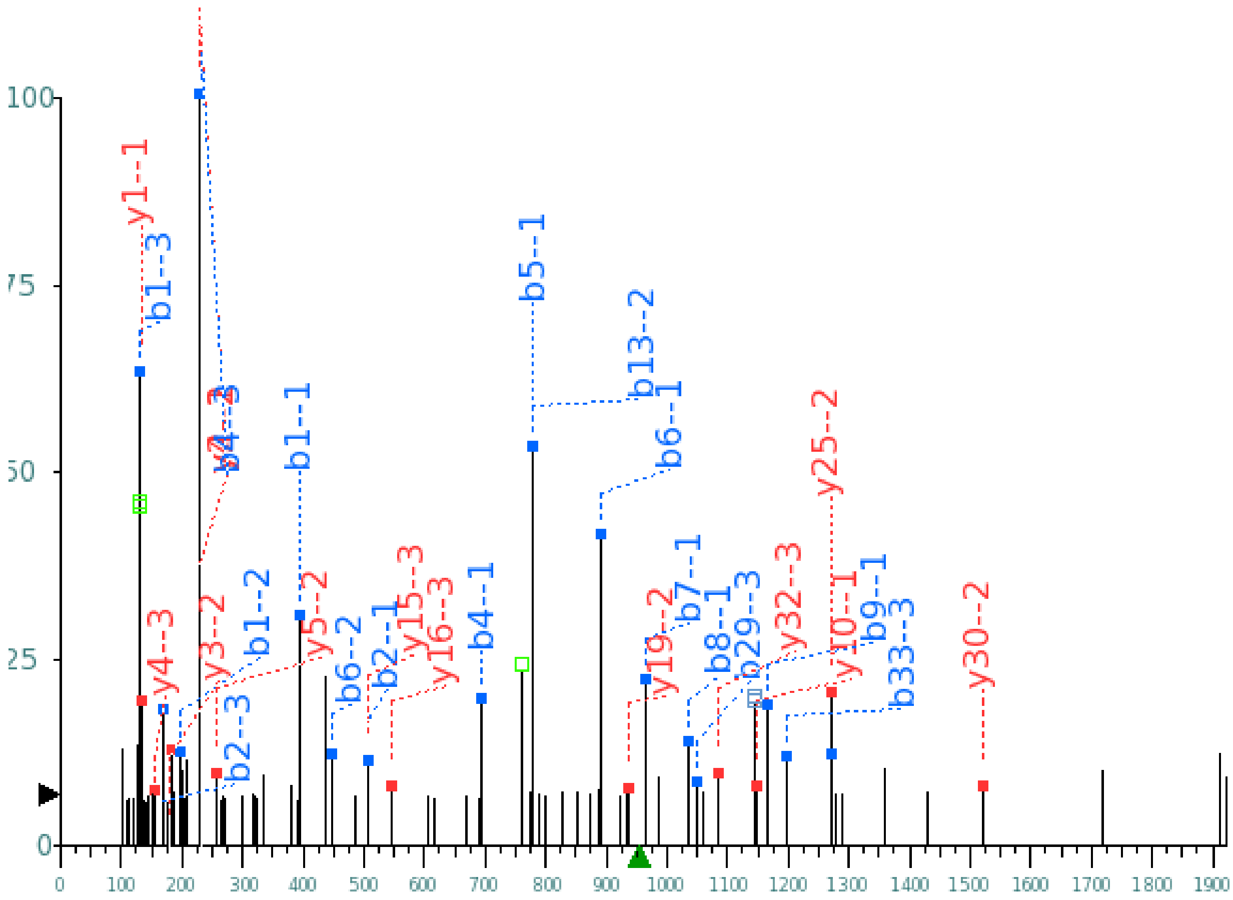

YIGESLAAELPVSTQRPGSDTVGGGRGEEC*GVEPD (+4)

Primary Reference: MCM3A_HUMAN
Search ID: 41760
Search Name: 20130328_ananiav_ERstress_TMT_TM_timecourse_trypsin_inj1_4mc_iaa
Scan#: 17300
Observed Mass: 955.4666 (2.7 ppm)
PSM Score: 34.15

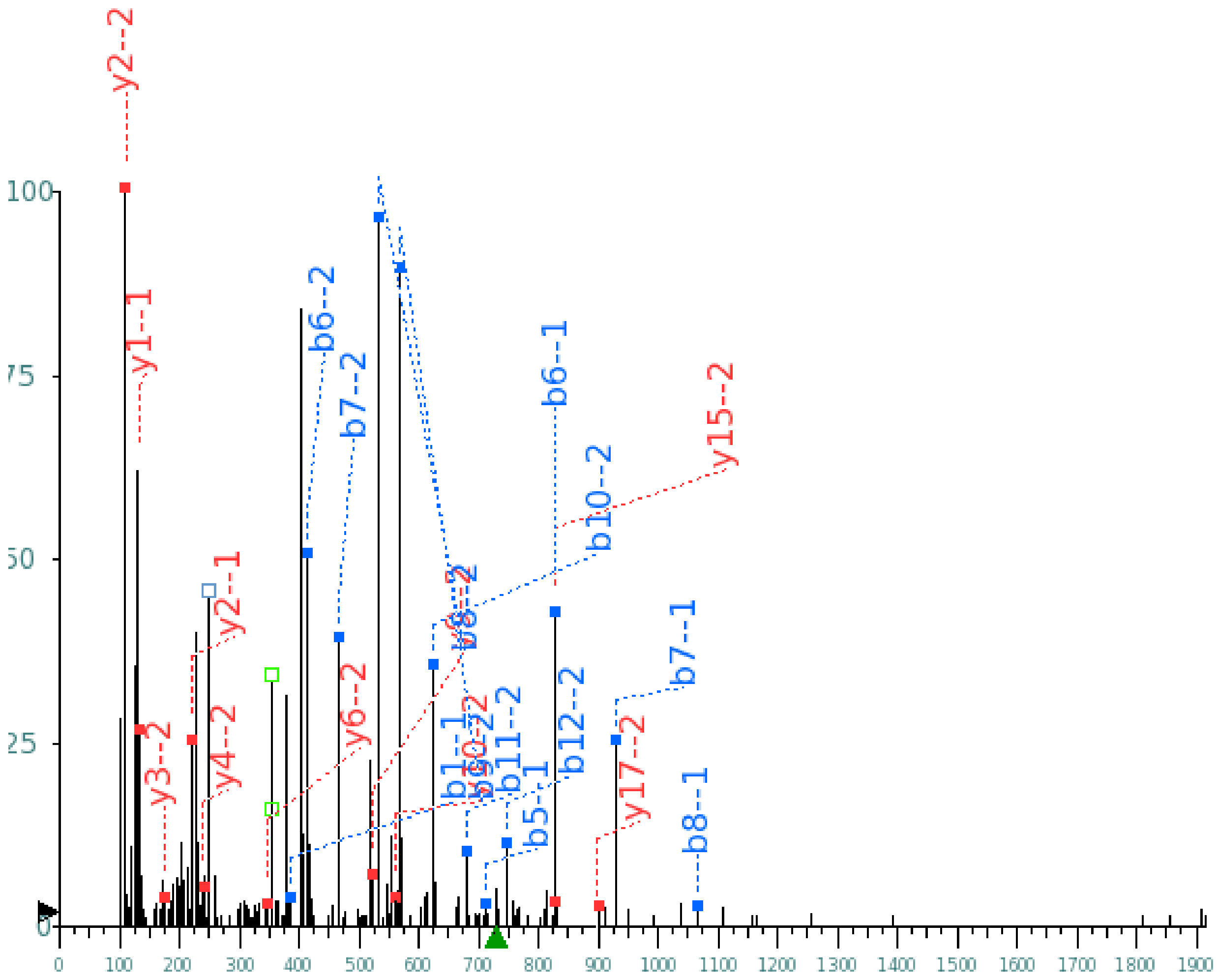

RGSSPDVHALLEITEESD (+3)

Primary Reference: PTRF_HUMAN
Search ID: 41760
Search Name: 20130328_ananiav_ERstress_TMT_TM_timecourse_trypsin_inj1_4mc_iaa
Scan#: 17326
Observed Mass: 728.7061 (2.6 ppm)
PSM Score: 20.62

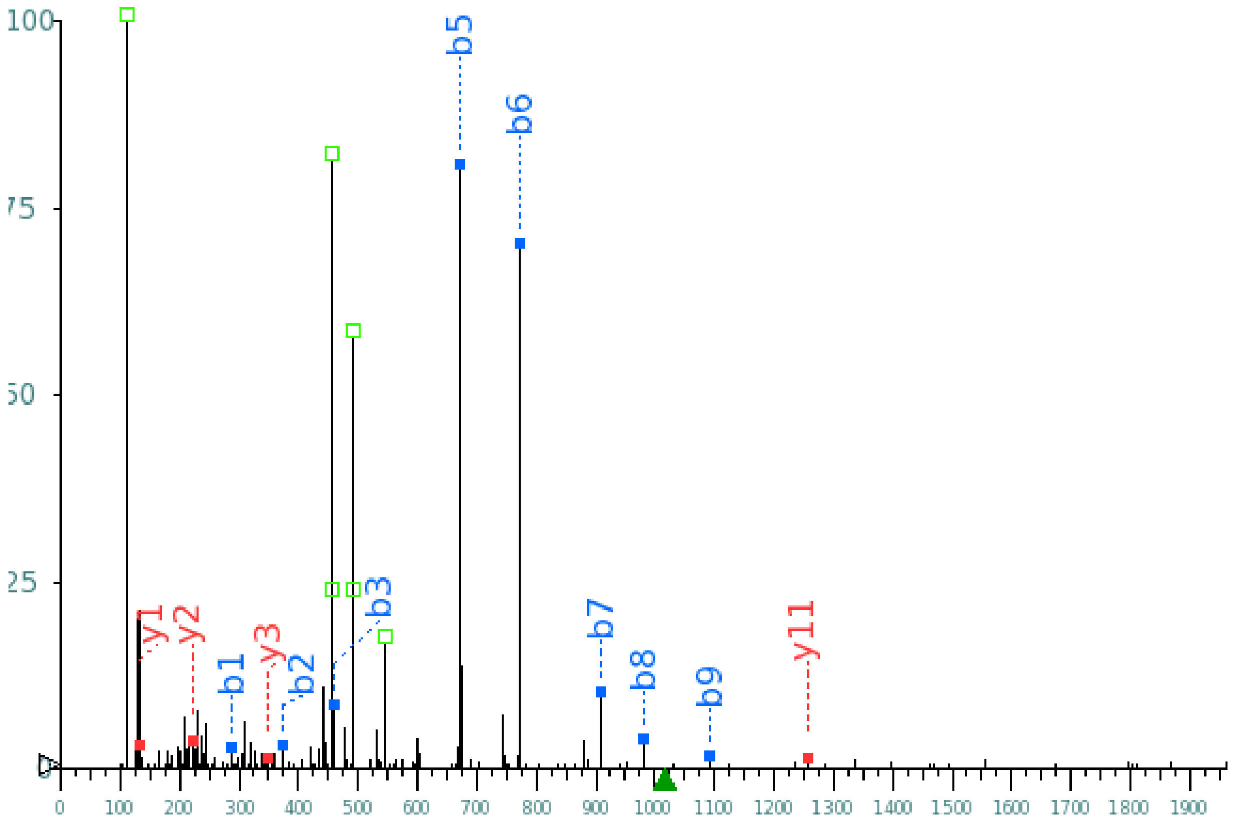

GSSPDVHALLEITEESD (+2)

Primary Reference: PTRF_HUMAN
Search ID: 41760
Search Name: 20130328_ananiav_ERstress_TMT_TM_timecourse_trypsin_inj1_4mc_iaa
Scan#: 17328
Observed Mass: 1014.5052 (3 ppm)
PSM Score: 19.12

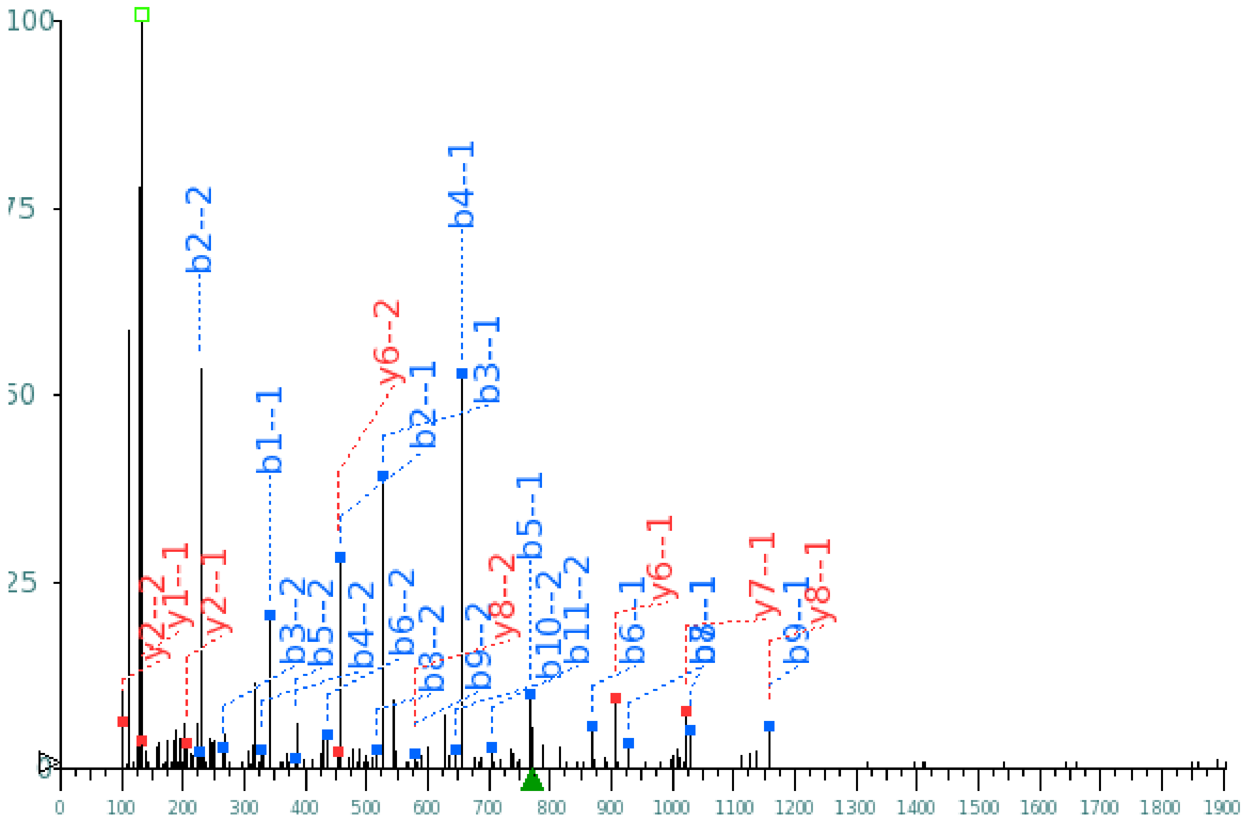

ILAQITGTEHLKESEAD (+3)

Primary Reference: PDLI5_HUMAN
Search ID: 41760
Search Name: 20130328_ananiav_ERstress_TMT_TM_timecourse_trypsin_inj1_4mc_iaa
Scan#: 17331
Observed Mass: 771.7639 (3.1 ppm)
PSM Score: 33.08

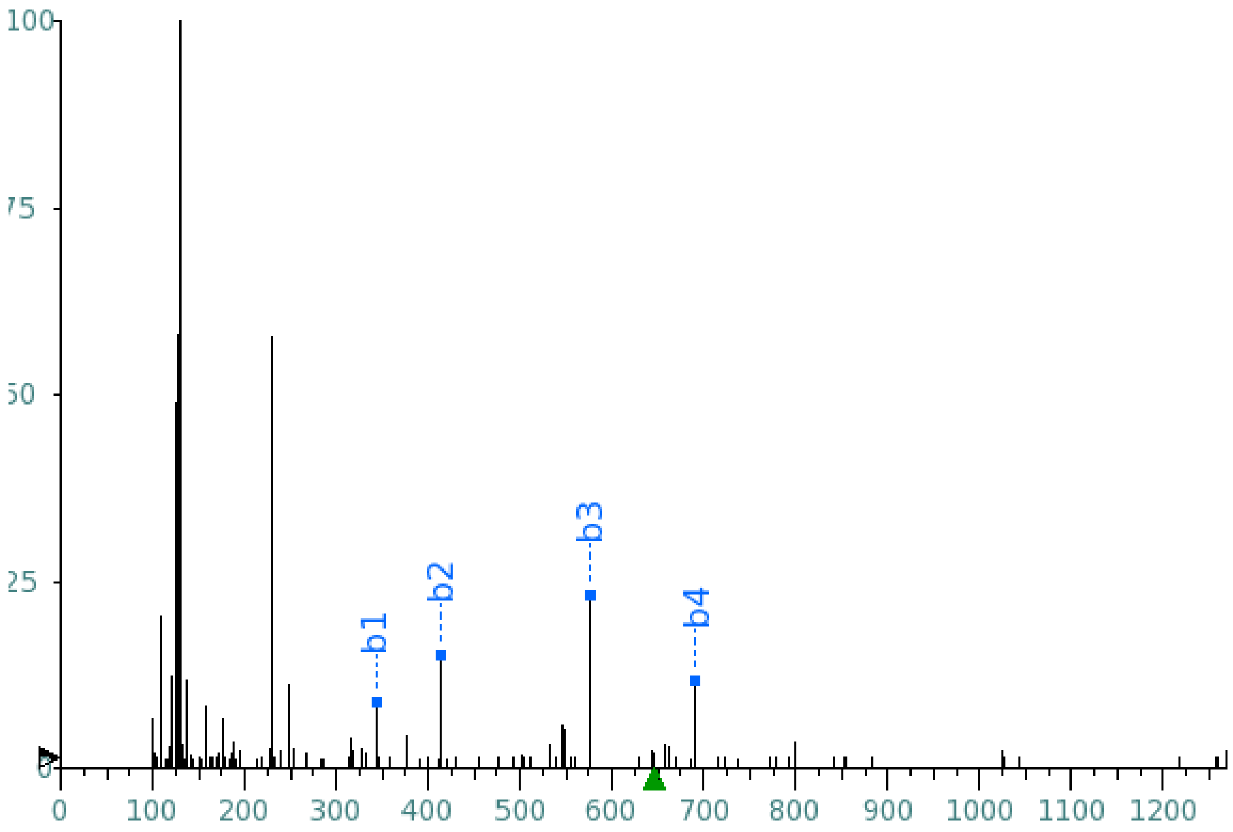

LAYLLQQTD (+2)

Primary Reference: SMCA2_HUMAN
Search ID: 41760
Search Name: 20130328_ananiav_ERstress_TMT_TM_timecourse_trypsin_inj1_4mc_iaa
Scan#: 17343
Observed Mass: 647.3691 (4.5 ppm)
PSM Score: 10.8

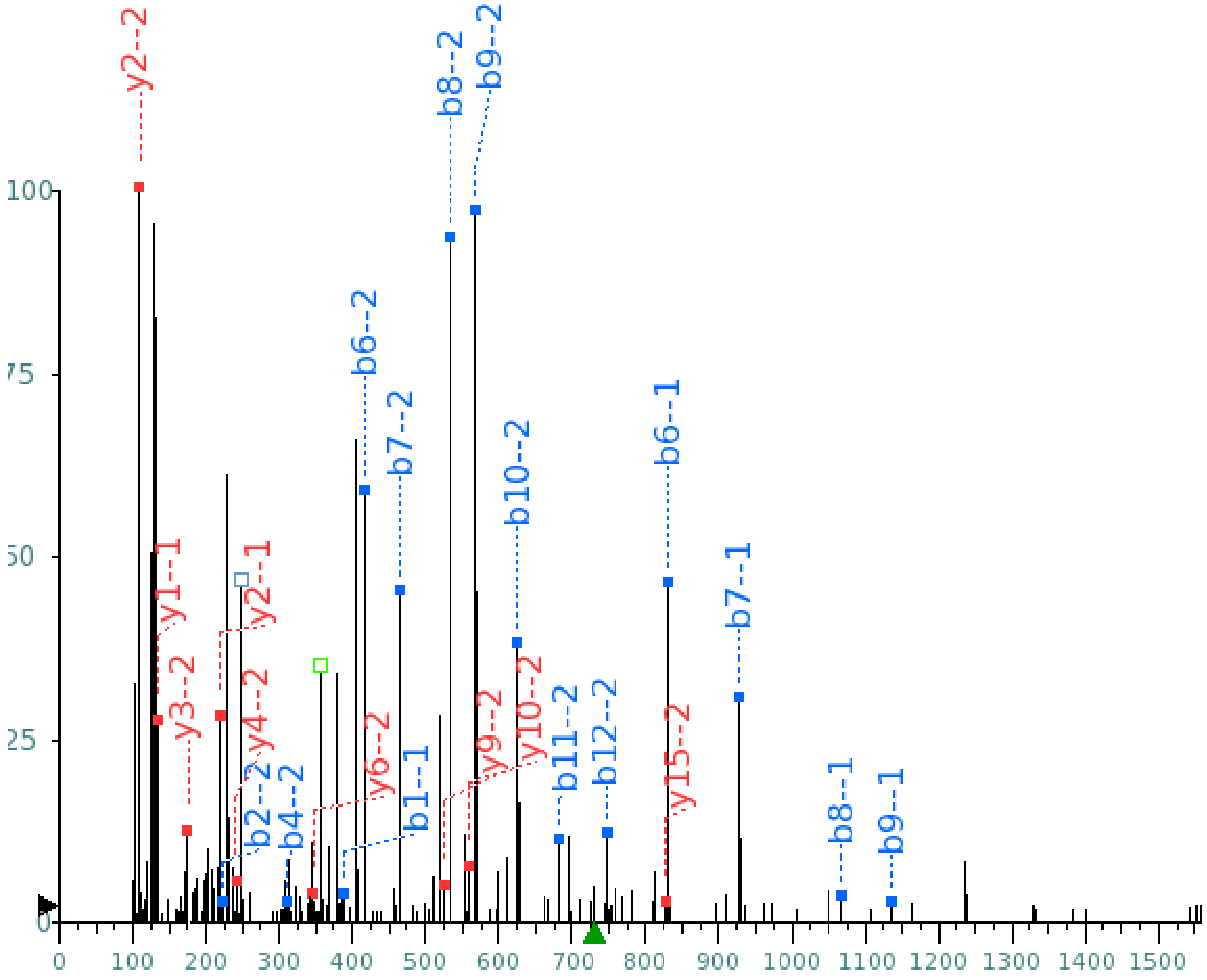

RGSSPDVHALLEITEESD (+3)

Primary Reference: PTRF_HUMAN
Search ID: 41760
Search Name: 20130328_ananiav_ERstress_TMT_TM_timecourse_trypsin_inj1_4mc_iaa
Scan#: 17391
Observed Mass: 728.7055 (1.7 ppm)
PSM Score: 17.48

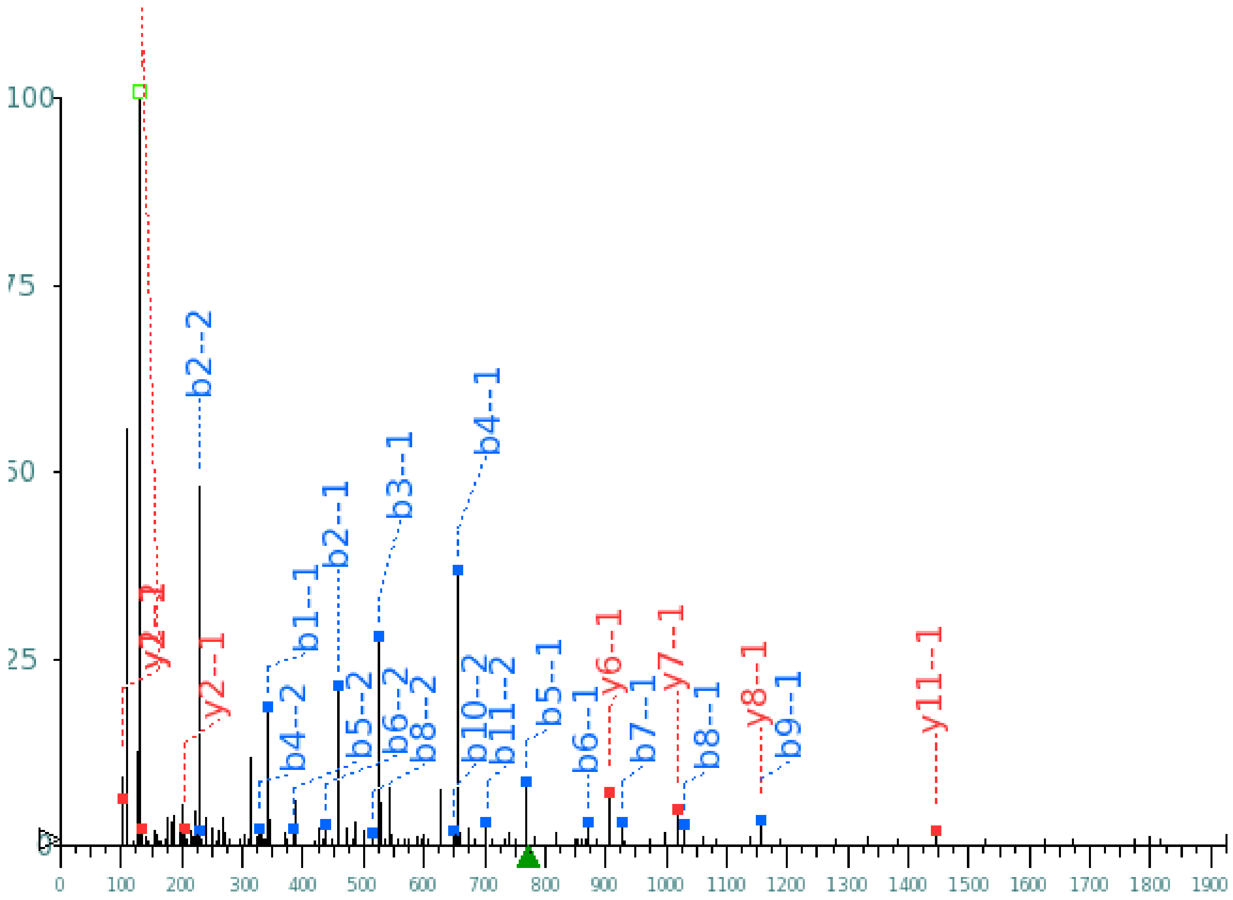

ILAQITGTEHLKESEAD (+3)

Primary Reference: PDLI5_HUMAN
Search ID: 41760
Search Name: 20130328_ananiav_ERstress_TMT_TM_timecourse_trypsin_inj1_4mc_iaa
Scan#: 17408
Observed Mass: 771.7639 (3 ppm)
PSM Score: 31.86

File name: 2015-02-04 04.01.53PM.pdf

Creation time: 2015-02-04 04:09PM

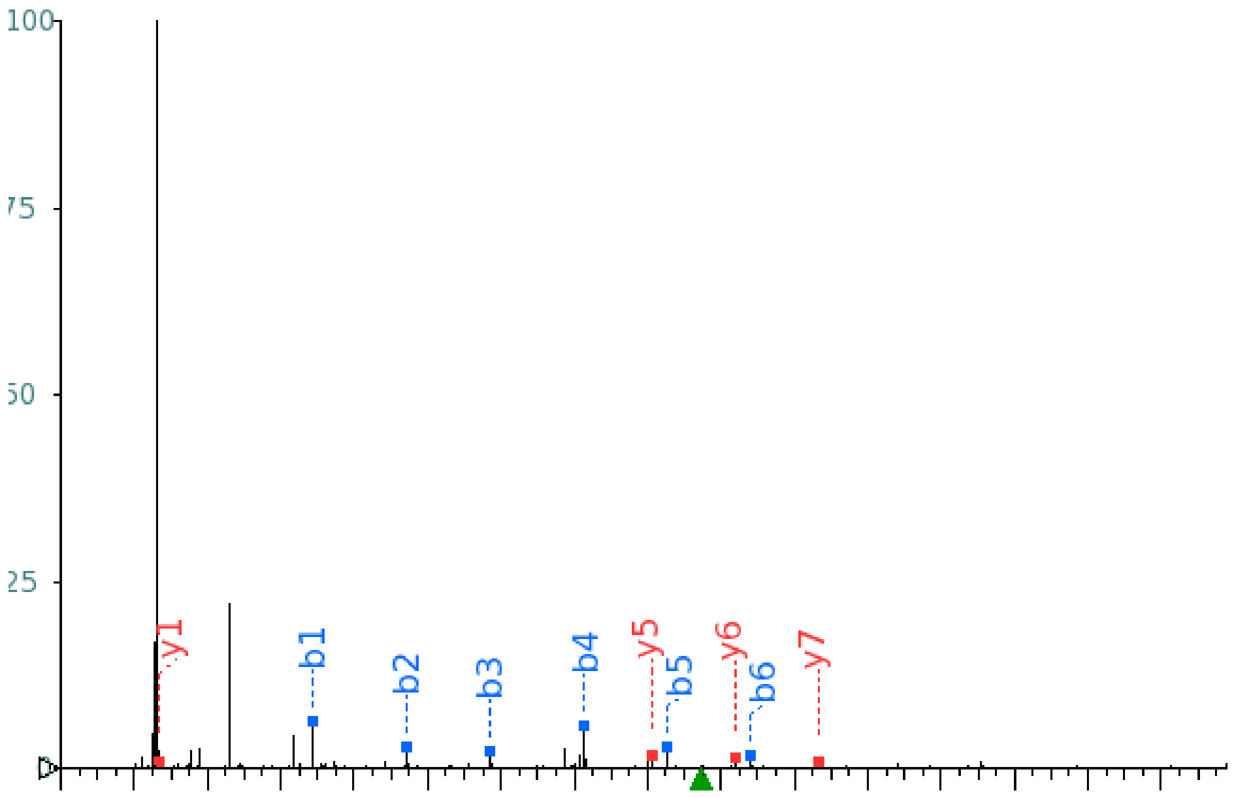

LQNEILKDLSD (+2)

Primary Reference: CKAP4_HUMAN
Search ID: 41760
Search Name: 20130328_ananiav_ERstress_TMT_TM_timecourse_trypsin_inj1_4mc_iaa
Scan#: 17442
Observed Mass: 873.511 (5.6 ppm)
PSM Score: 19.98

File name: 2015-02-04 04.01.53PM.pdf

Creation time: 2015-02-04 04:09PM

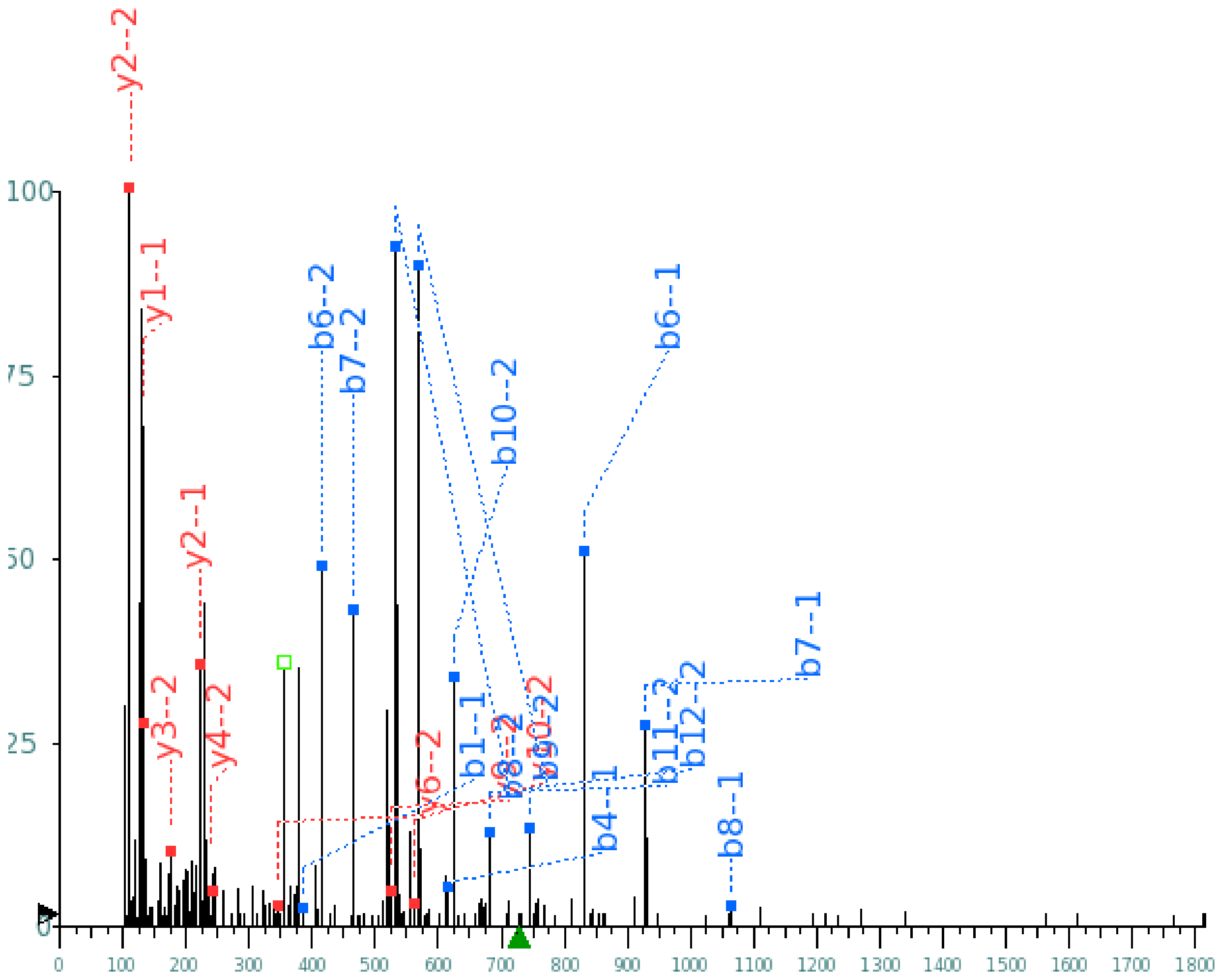

RGSSPDVHALLEITEESD (+3)

Primary Reference: PTRF_HUMAN
Search ID: 41760
Search Name: 20130328_ananiav_ERstress_TMT_TM_timecourse_trypsin_inj1_4mc_iaa
Scan#: 17471
Observed Mass: 728.7045 (0.3 ppm)
PSM Score: 20.66

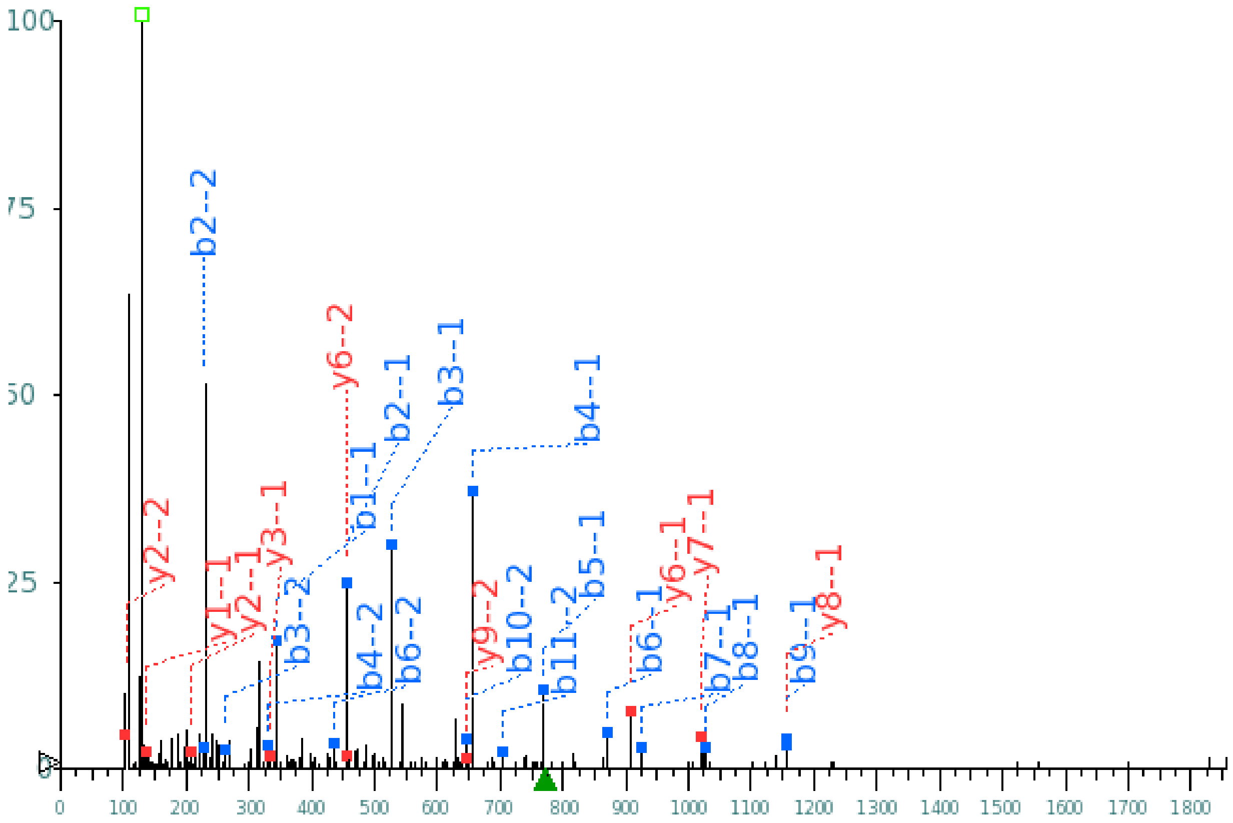

ILAQITGTEHLKESEAD (+3)

Primary Reference: PDLI5_HUMAN
Search ID: 41760
Search Name: 20130328_ananiav_ERstress_TMT_TM_timecourse_trypsin_inj1_4mc_iaa
Scan#: 17486
Observed Mass: 771.7645 (3.8 ppm)
PSM Score: 31.27

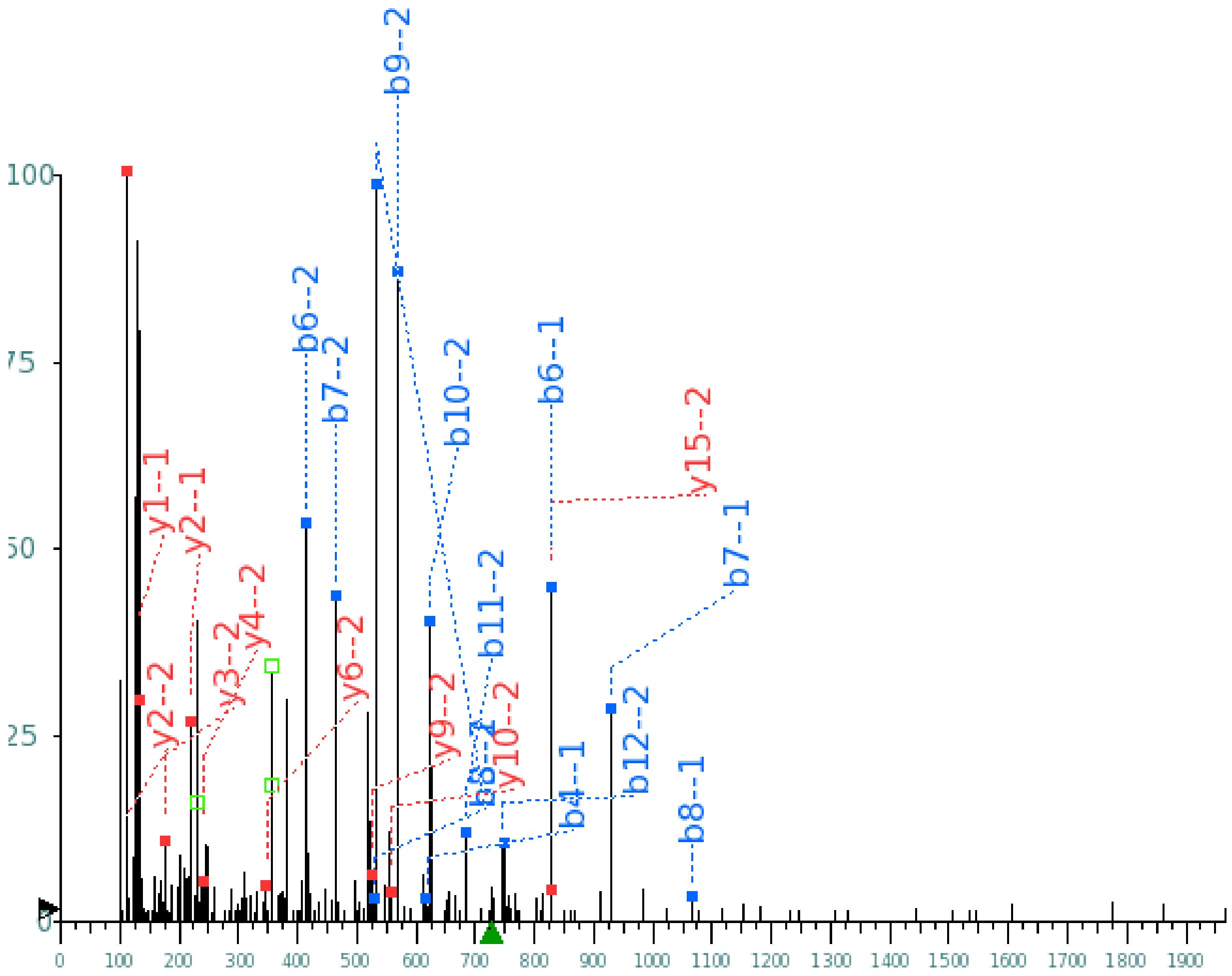

RGSSPDVHALLEITEESD (+3)

Primary Reference: PTRF_HUMAN
Search ID: 41760
Search Name: 20130328_ananiav_ERstress_TMT_TM_timecourse_trypsin_inj1_4mc_iaa
Scan#: 17534
Observed Mass: 728.7023 (-2.7 ppm)
PSM Score: 18.14

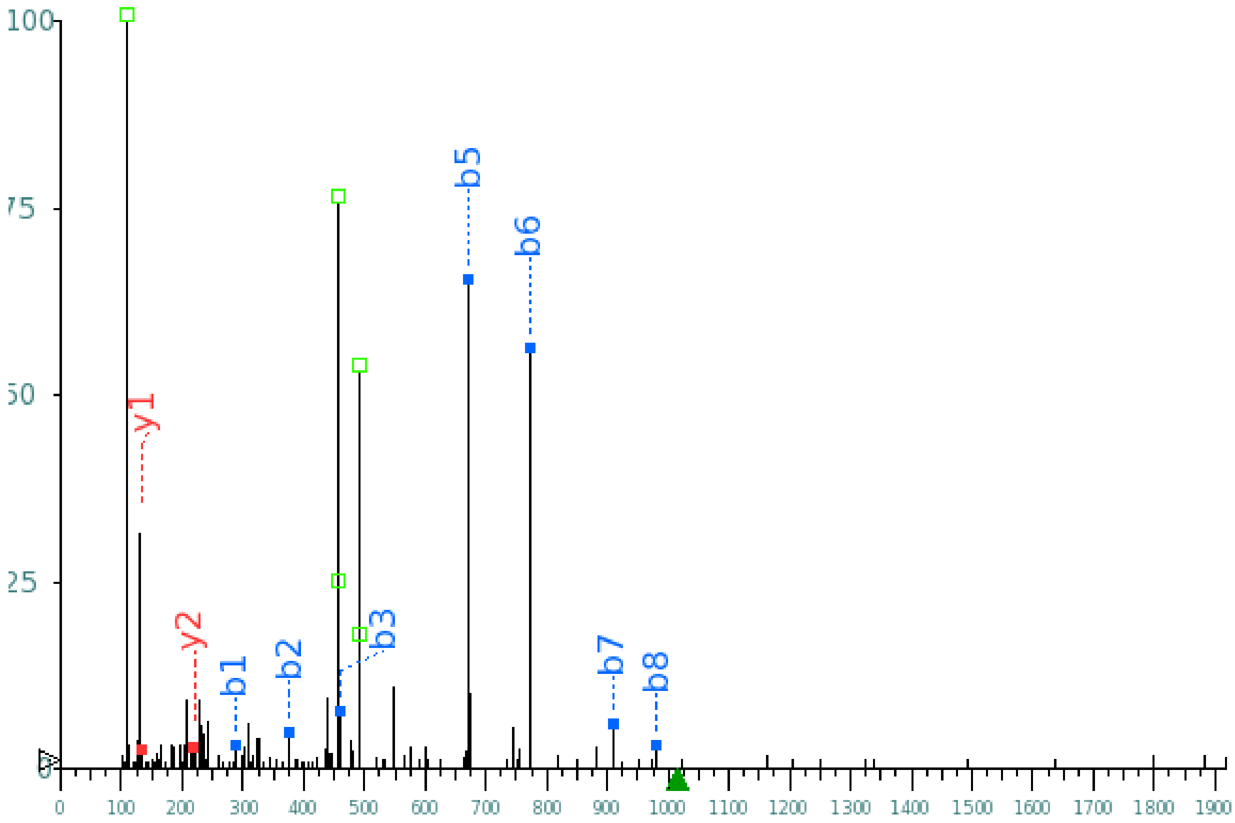

GSSPDVHALLEITEESD (+2)

Primary Reference: PTRF_HUMAN
Search ID: 41760
Search Name: 20130328_ananiav_ERstress_TMT_TM_timecourse_trypsin_inj1_4mc_iaa
Scan#: 17549
Observed Mass: 1014.506 (3.8 ppm)
PSM Score: 16.08

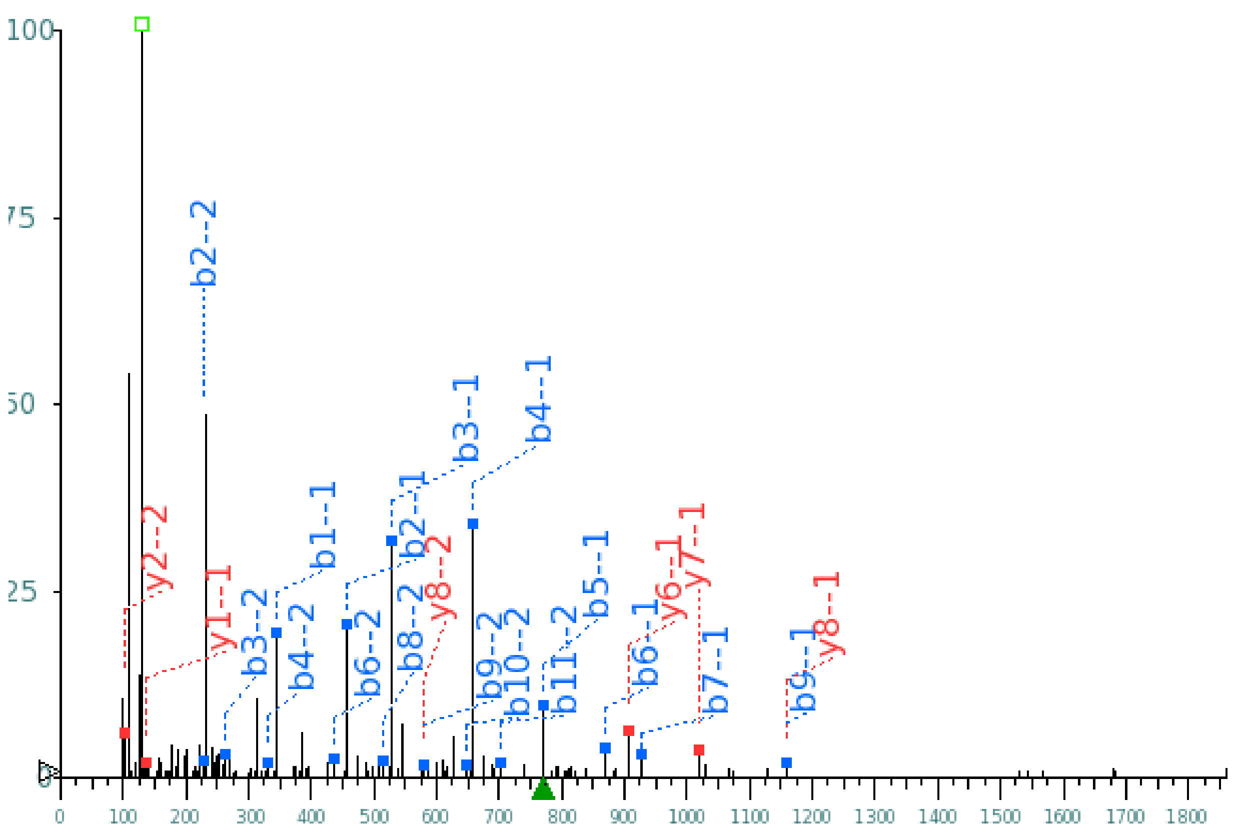

ILAQITGTEHLKESEAD (+3)

Primary Reference: PDLI5_HUMAN
Search ID: 41760
Search Name: 20130328_ananiav_ERstress_TMT_TM_timecourse_trypsin_inj1_4mc_iaa
Scan#: 17553
Observed Mass: 771.765 (4.4 ppm)
PSM Score: 39.41

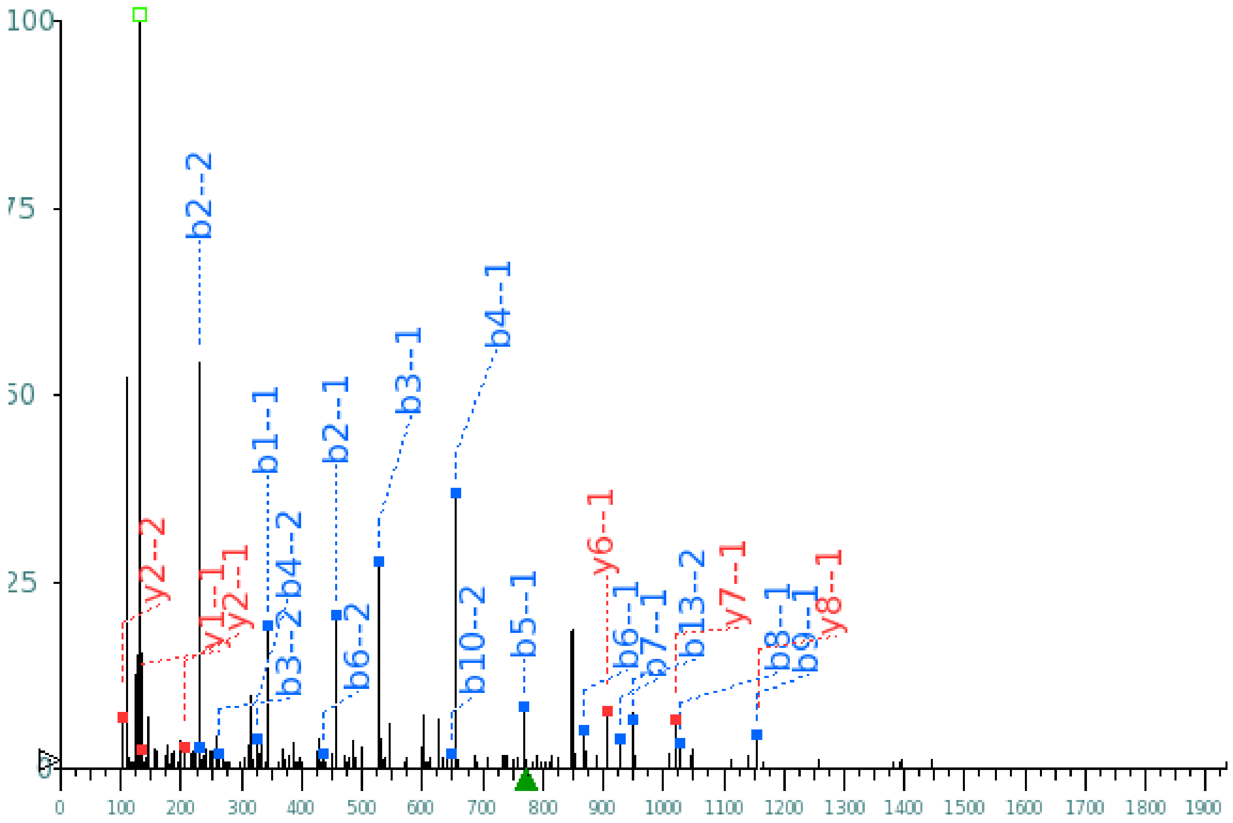

ILAQITGTEHLKESEAD (+3)

Primary Reference: PDLI5_HUMAN
Search ID: 41760
Search Name: 20130328_ananiav_ERstress_TMT_TM_timecourse_trypsin_inj1_4mc_iaa
Scan#: 17632
Observed Mass: 771.7627 (1.5 ppm)
PSM Score: 20.32

File name: 2015-02-04 04.01.53PM.pdf

Creation time: 2015-02-04 04:09PM

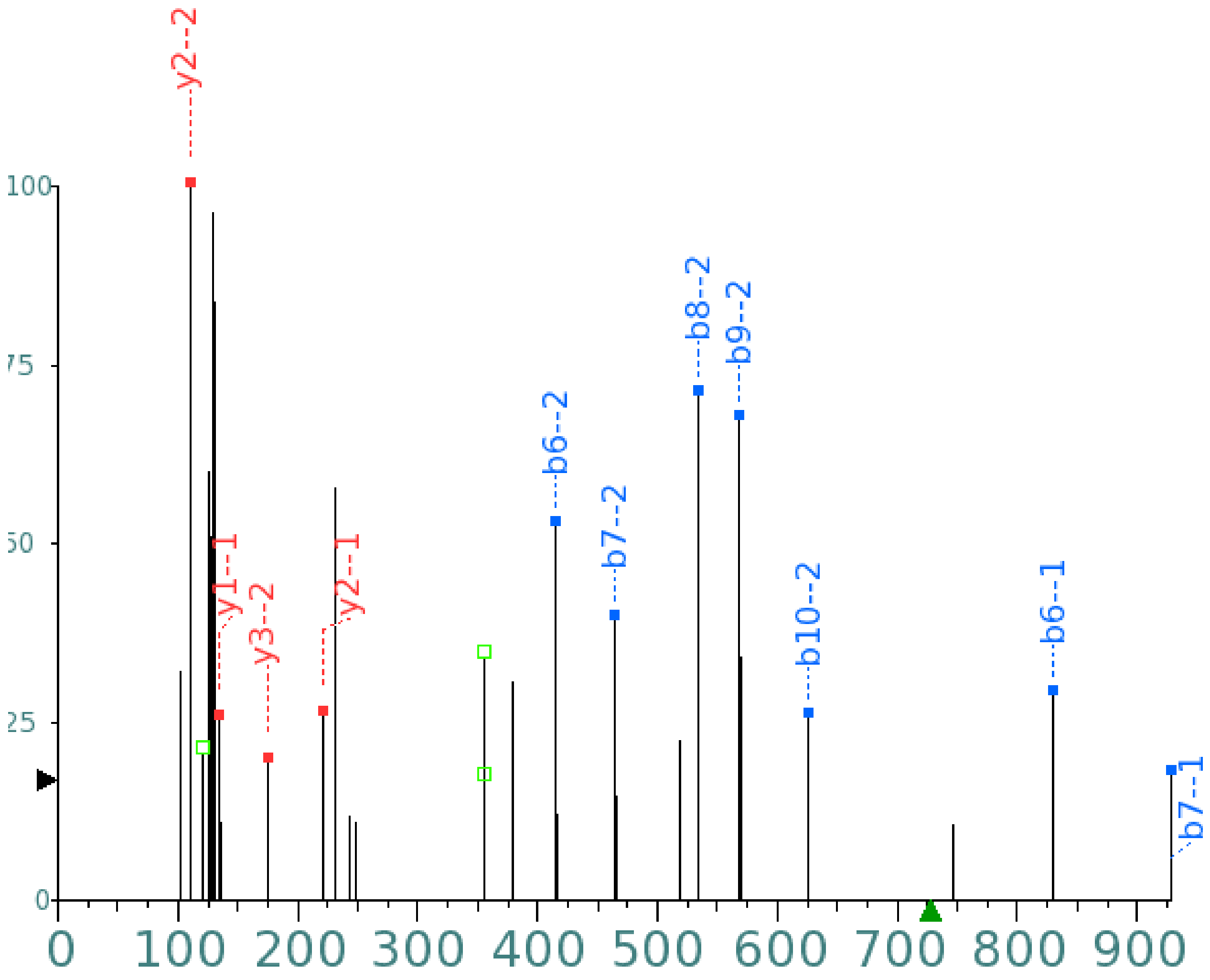

RGSSPDVHALLEITEESD (+3)

Primary Reference: PTRF_HUMAN
Search ID: 41760
Search Name: 20130328_ananiav_ERstress_TMT_TM_timecourse_trypsin_inj1_4mc_iaa
Scan#: 17636
Observed Mass: 728.7023 (-2.6 ppm)
PSM Score: 28.96

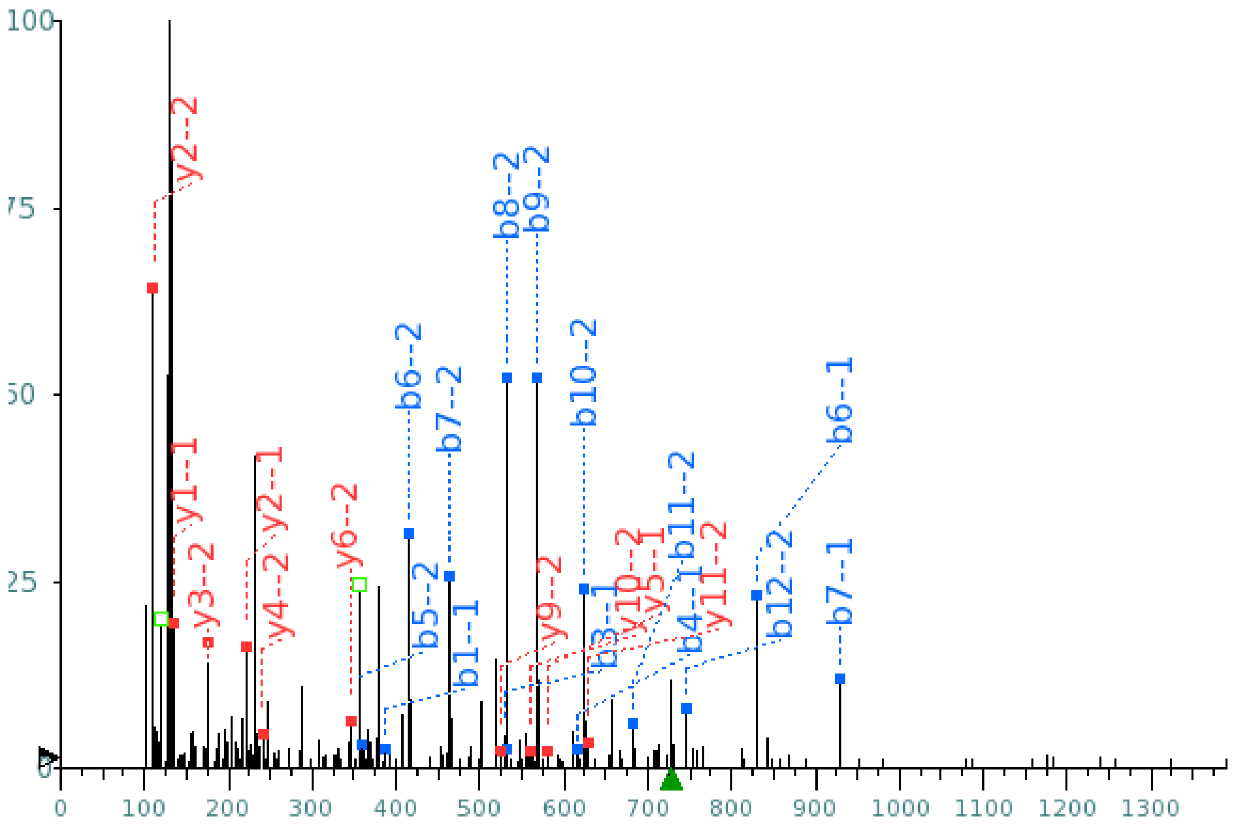

RGSSPDVHALLEITEESD (+3)

Primary Reference: PTRF_HUMAN
Search ID: 41760
Search Name: 20130328_ananiav_ERstress_TMT_TM_timecourse_trypsin_inj1_4mc_iaa
Scan#: 17722
Observed Mass: 728.7017 (-3.5 ppm)
PSM Score: 15.74

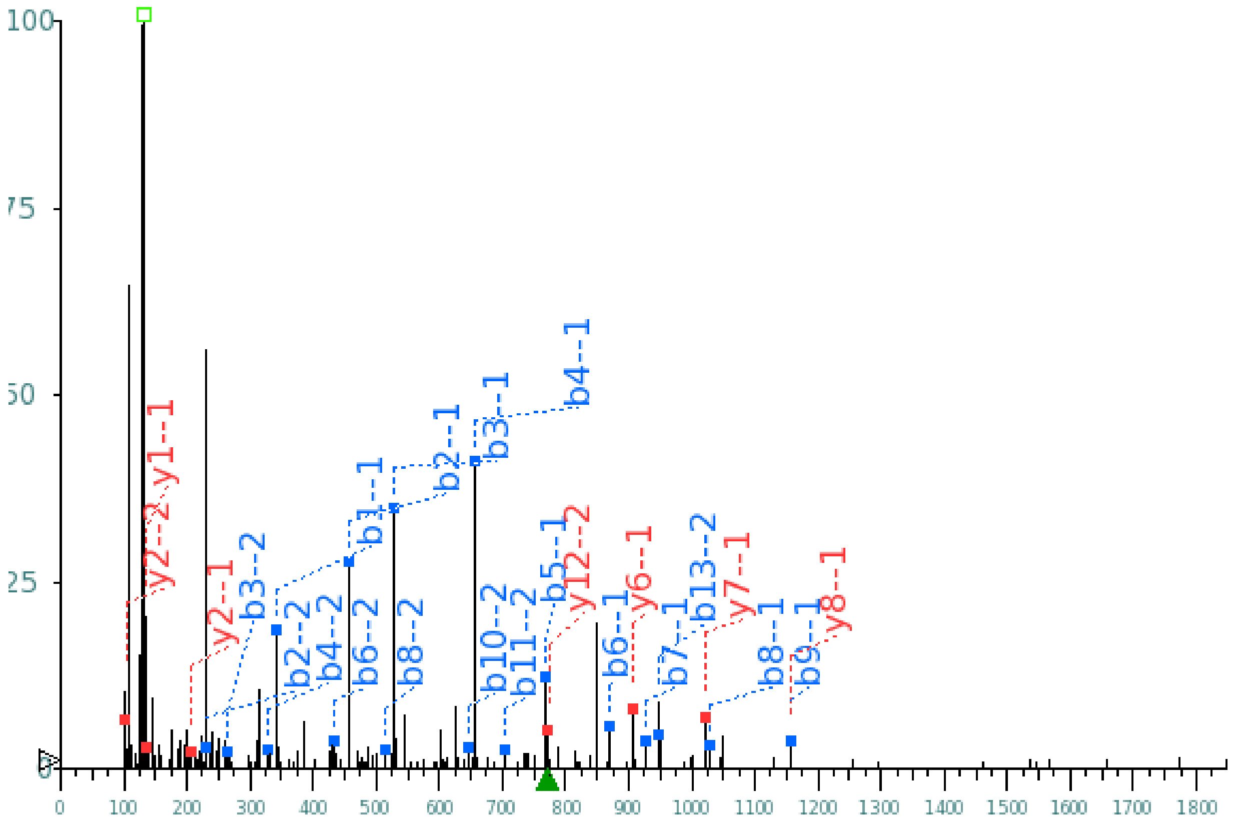

ILAQITGTEHLKESEAD (+3)

Primary Reference: PDLI5_HUMAN
Search ID: 41760
Search Name: 20130328_ananiav_ERstress_TMT_TM_timecourse_trypsin_inj1_4mc_iaa
Scan#: 17740
Observed Mass: 771.7634 (2.4 ppm)
PSM Score: 17.5

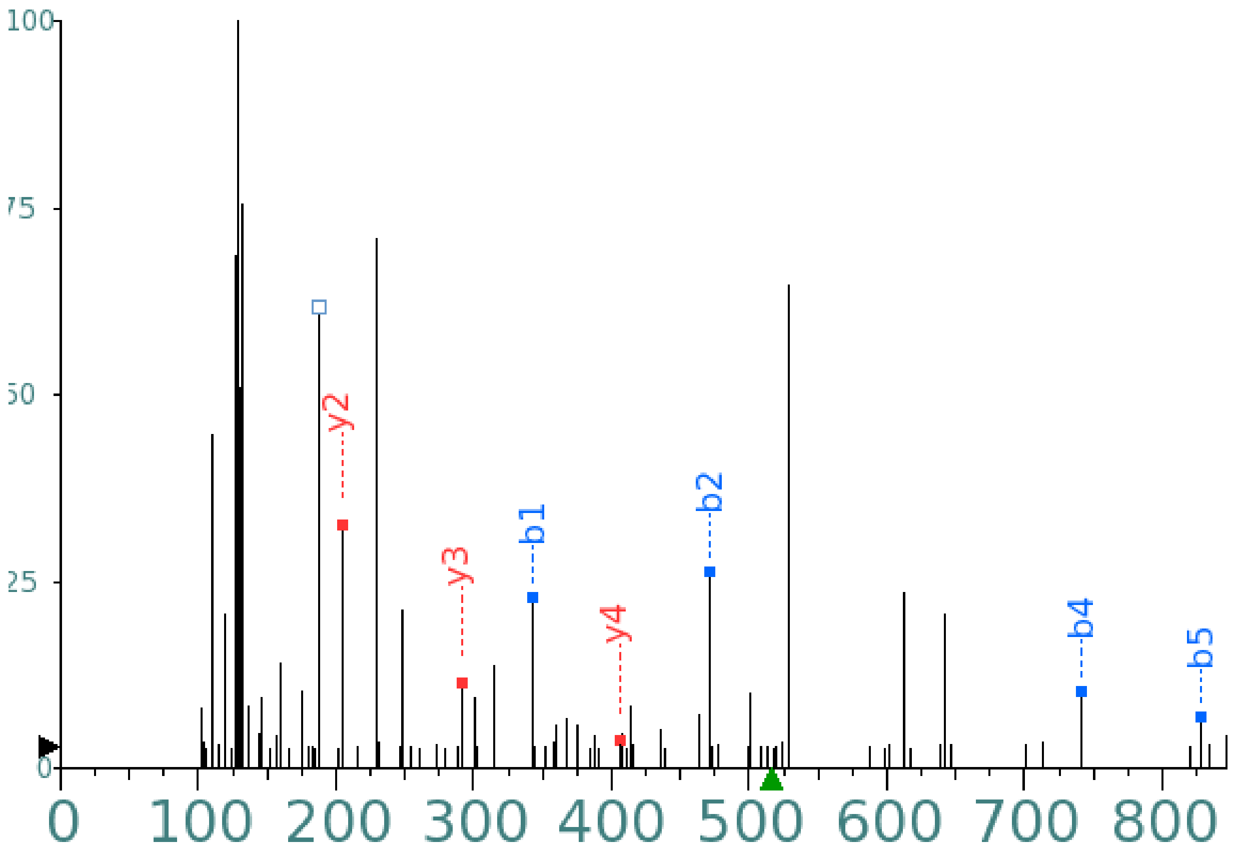

LQRLSAD (+2)

Primary Reference: ##RAB4B_HUMAN
Search ID: 41760
Search Name: 20130328_ananiav_ERstress_TMT_TM_timecourse_trypsin_inj1_4mc_iaa
Scan#: 17750
Observed Mass: 516.3094 (6.7 ppm)
PSM Score: 14.06

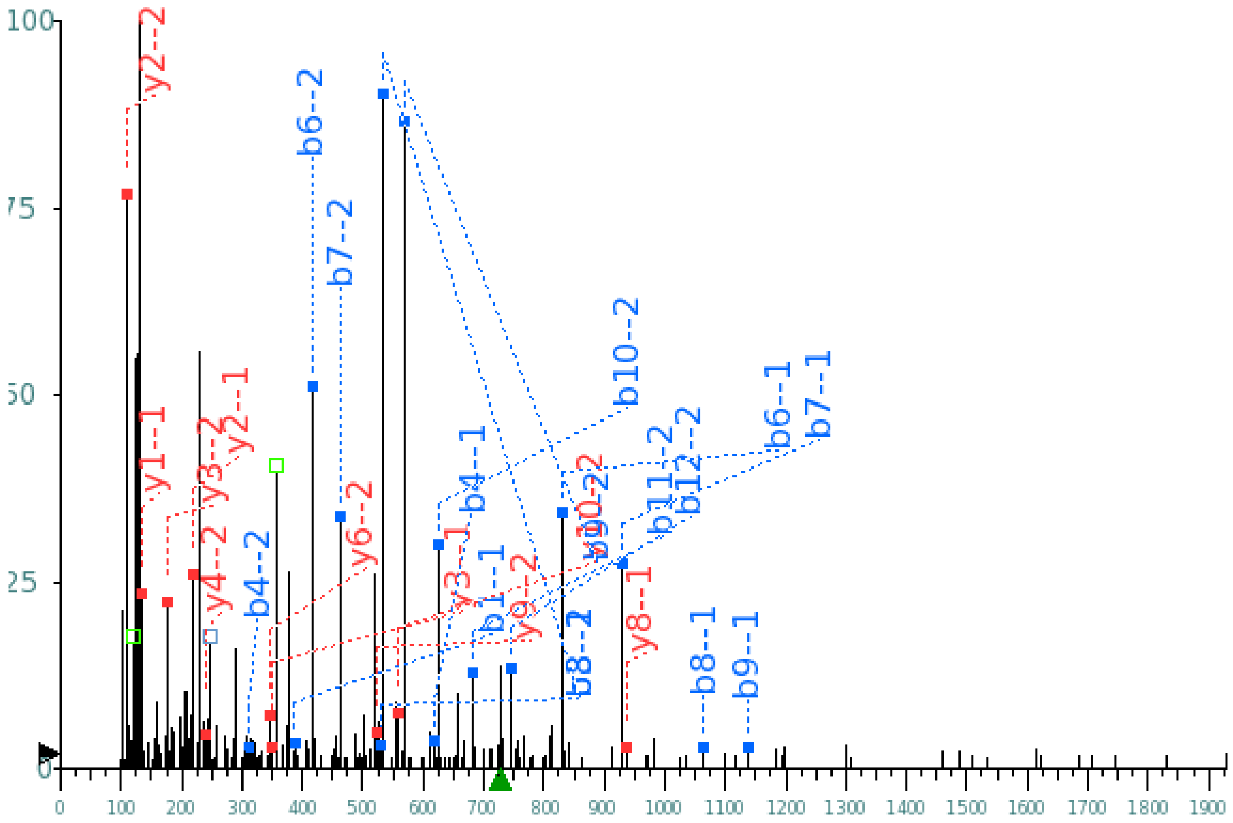

RGSSPDVHALLEITEESD (+3)

Primary Reference: PTRF_HUMAN
Search ID: 41760
Search Name: 20130328_ananiav_ERstress_TMT_TM_timecourse_trypsin_inj1_4mc_iaa
Scan#: 17791
Observed Mass: 728.7063 (2.9 ppm)
PSM Score: 16.91

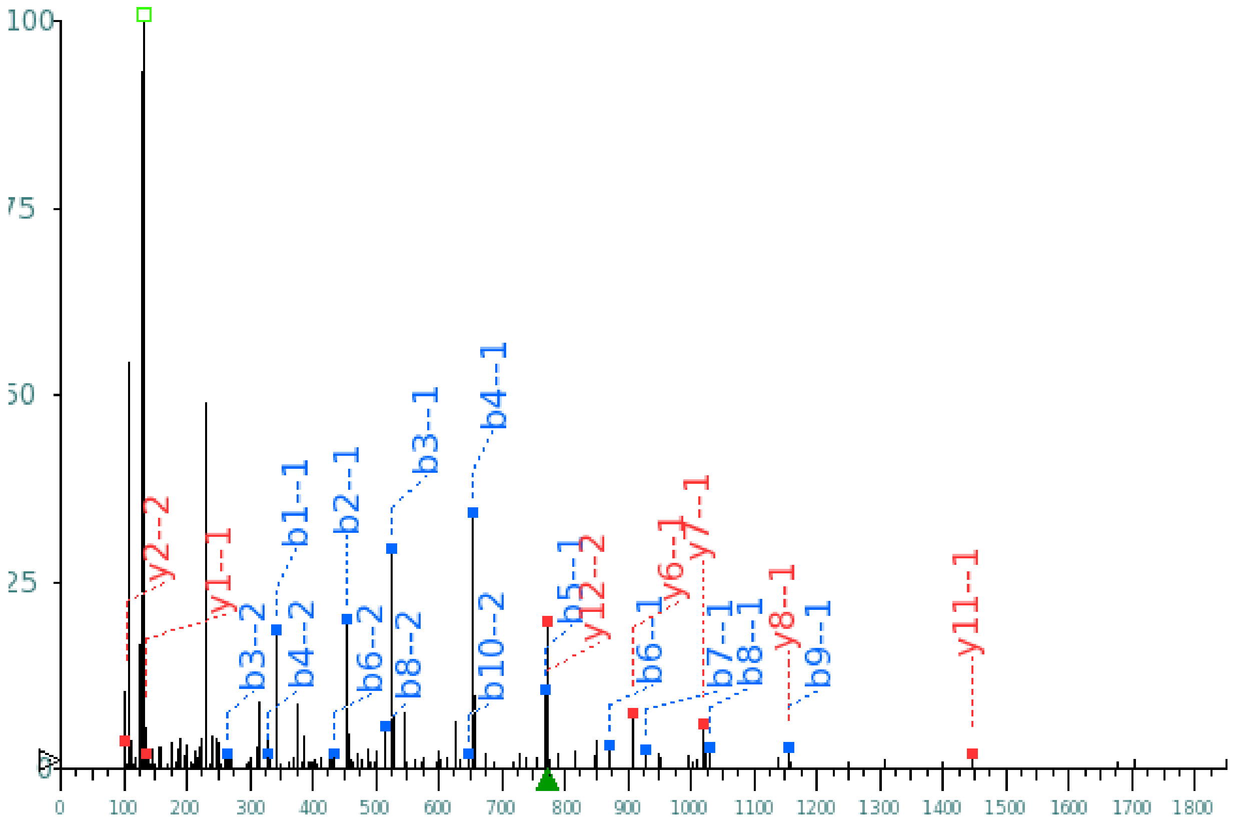

ILAQITGTEHLKESEAD (+3)

Primary Reference: PDLI5_HUMAN
Search ID: 41760
Search Name: 20130328_ananiav_ERstress_TMT_TM_timecourse_trypsin_inj1_4mc_iaa
Scan#: 17805
Observed Mass: 771.7639 (3.1 ppm)
PSM Score: 30.73

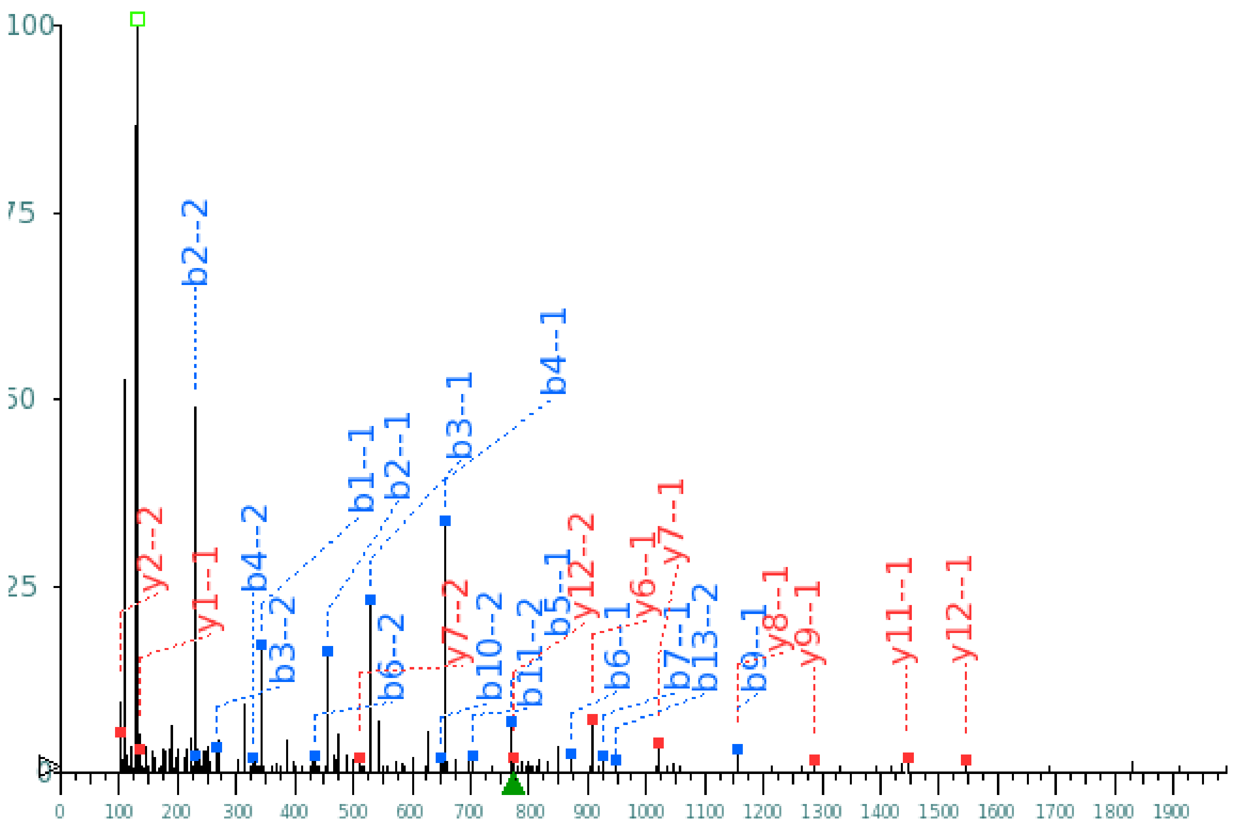

ILAQITGTEHLKESEAD (+3)

Primary Reference: PDLI5_HUMAN
Search ID: 41760
Search Name: 20130328_ananiav_ERstress_TMT_TM_timecourse_trypsin_inj1_4mc_iaa
Scan#: 17872
Observed Mass: 771.7631 (2 ppm)
PSM Score: 38.72

File name: 2015-02-04 04.01.53PM.pdf

Creation time: 2015-02-04 04:09PM

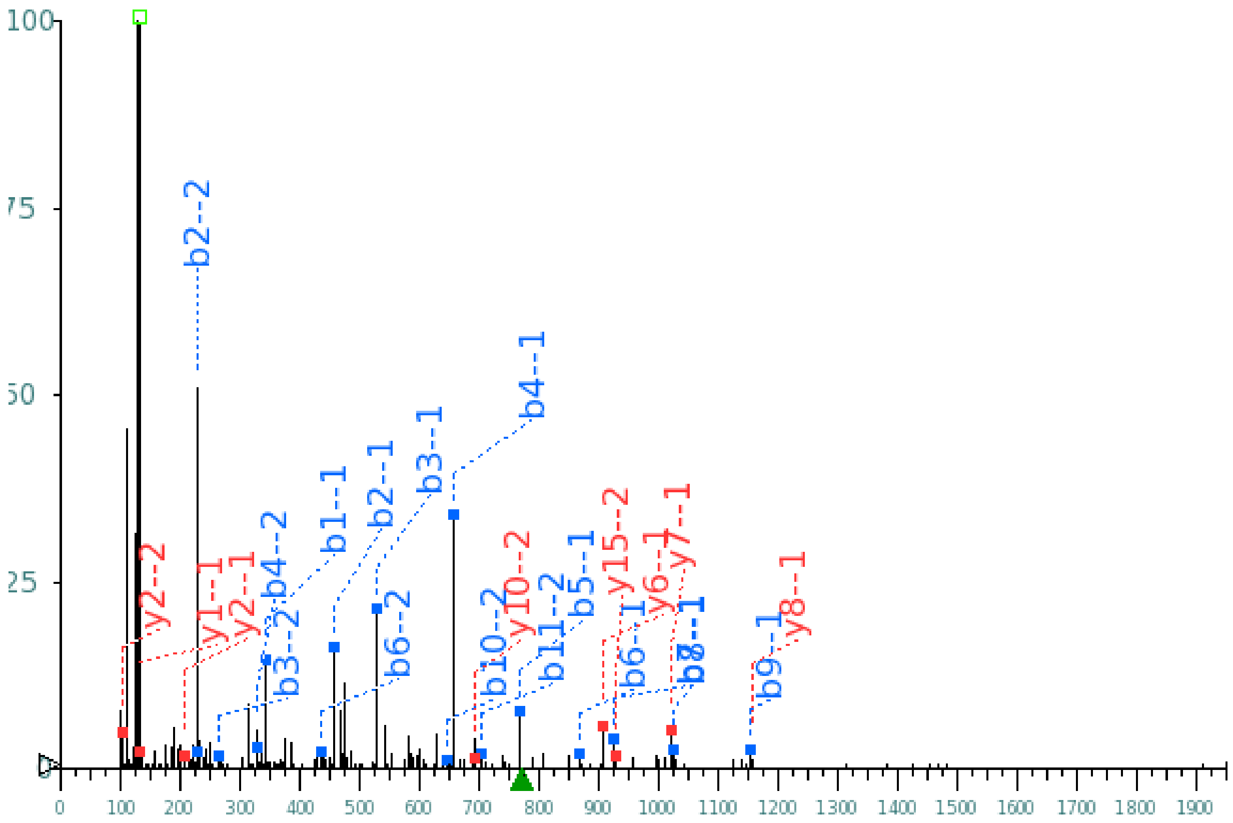

ILAQITGTEHLKESEAD (+3)

Primary Reference: PDLI5_HUMAN
Search ID: 41760
Search Name: 20130328_ananiav_ERstress_TMT_TM_timecourse_trypsin_inj1_4mc_iaa
Scan#: 17949
Observed Mass: 771.764 (3.2 ppm)
PSM Score: 27.56

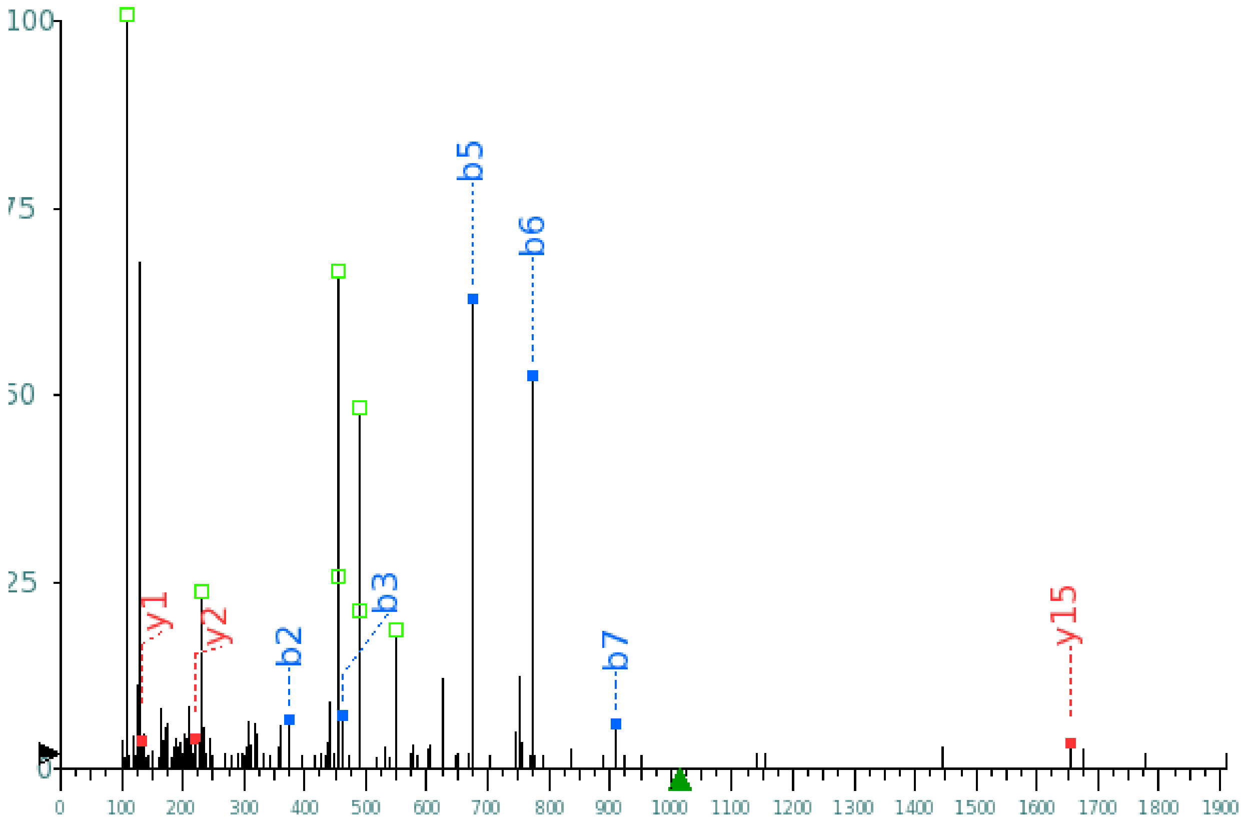

GSSPDVHALLEITEESD (+2)

Primary Reference: PTRF_HUMAN
Search ID: 41760
Search Name: 20130328_ananiav_ERstress_TMT_TM_timecourse_trypsin_inj1_4mc_iaa
Scan#: 17964
Observed Mass: 1014.5081 (5.8 ppm)
PSM Score: 14.65

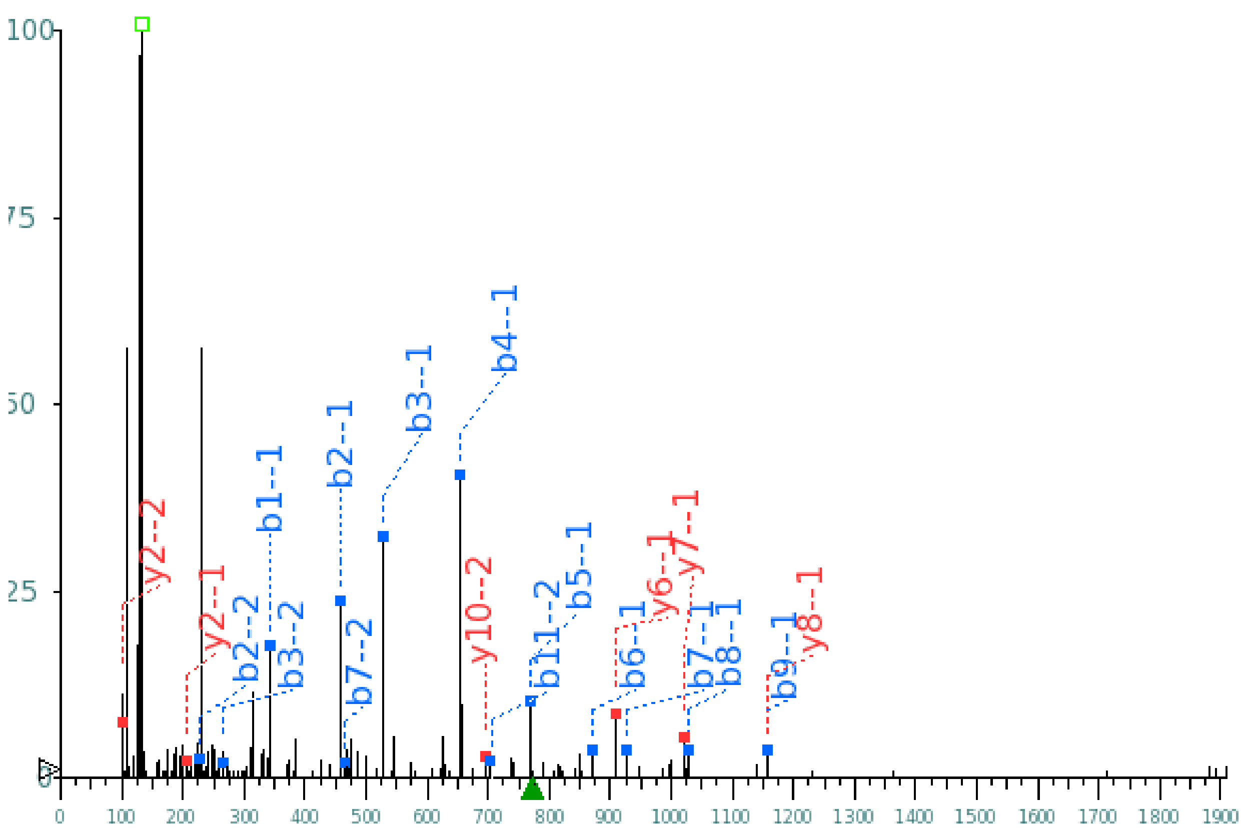

ILAQITGTEHLKESEAD (+3)

Primary Reference: PDLI5_HUMAN
Search ID: 41760
Search Name: 20130328_ananiav_ERstress_TMT_TM_timecourse_trypsin_inj1_4mc_iaa
Scan#: 18015
Observed Mass: 771.7648 (4.2 ppm)
PSM Score: 33.63

File name: 2015-02-04 04.01.53PM.pdf

Creation time: 2015-02-04 04:09PM

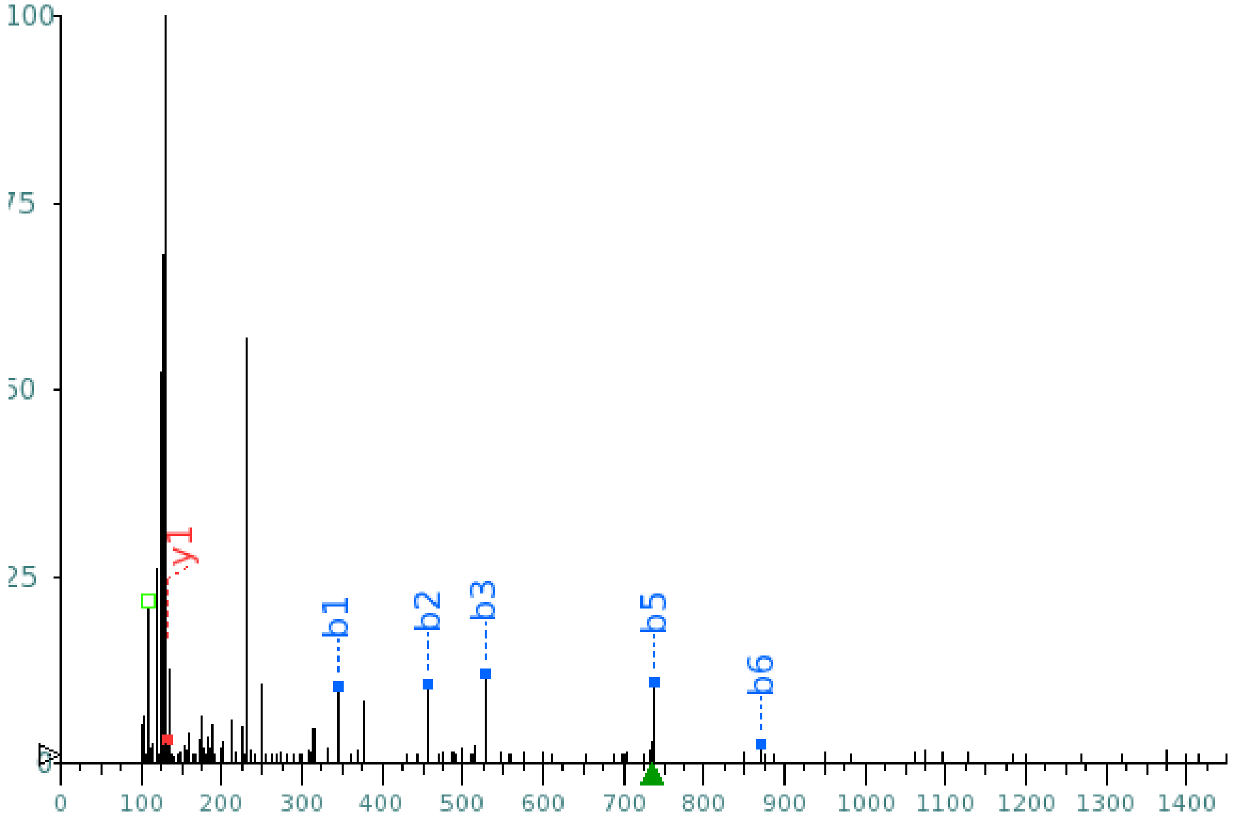

LLAPDMFTESD (+2)

Primary Reference: PRP4B_HUMAN
Search ID: 41760
Search Name: 20130328_ananiav_ERstress_TMT_TM_timecourse_trypsin_inj1_4mc_iaa
Scan#: 18049
Observed Mass: 734.3679 (3.2 ppm)
PSM Score: 14.42

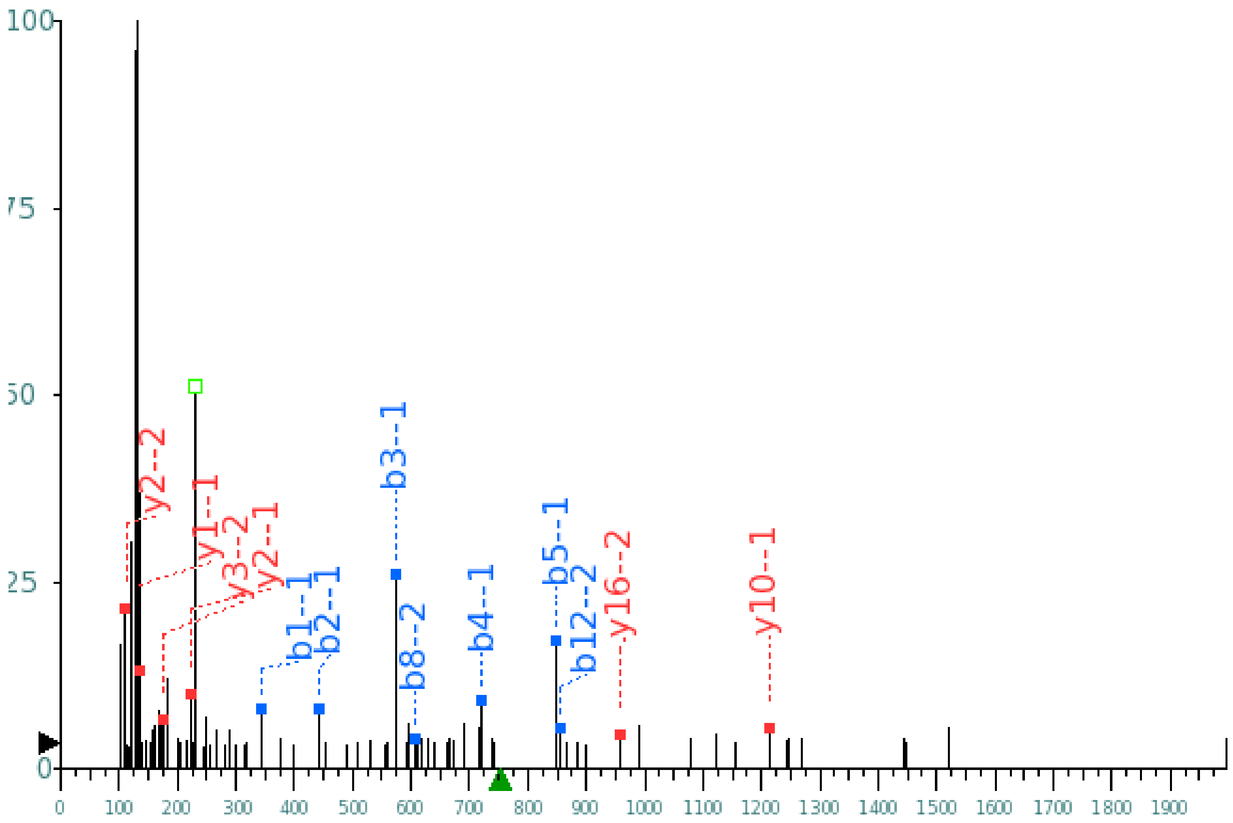

NVEFEPTYEEFPSLESD (+3)

Primary Reference: VPP2_HUMAN
Search ID: 41760
Search Name: 20130328_ananiav_ERstress_TMT_TM_timecourse_trypsin_inj1_4mc_iaa
Scan#: 18059
Observed Mass: 754.3514 (2.7 ppm)
PSM Score: 10.29

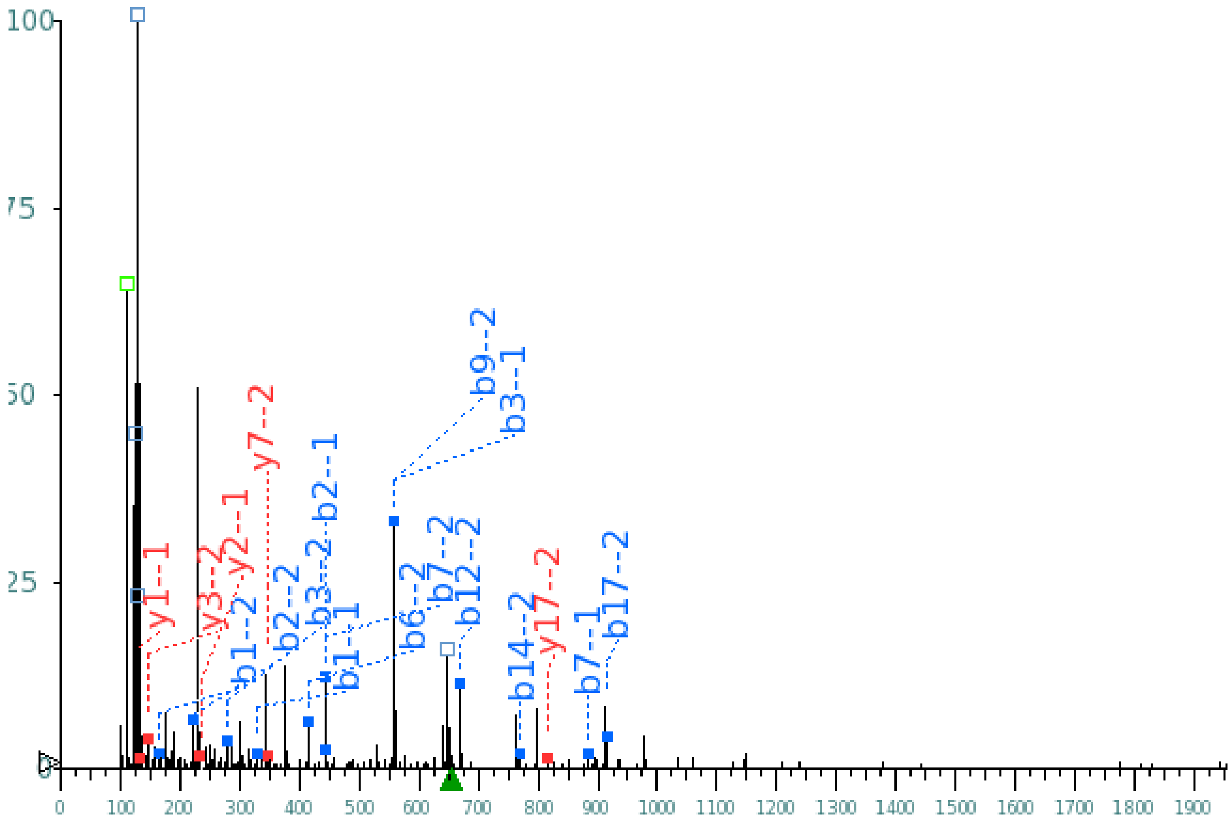

TLLSPSGTEPGAQAEGTD (+3)

Primary Reference: ADCY8_HUMAN
Search ID: 41760
Search Name: 20130328_ananiav_ERstress_TMT_TM_timecourse_trypsin_inj1_4mc_iaa
Scan#: 18074
Observed Mass: 653.9981 (4.6 ppm)
PSM Score: 12.67

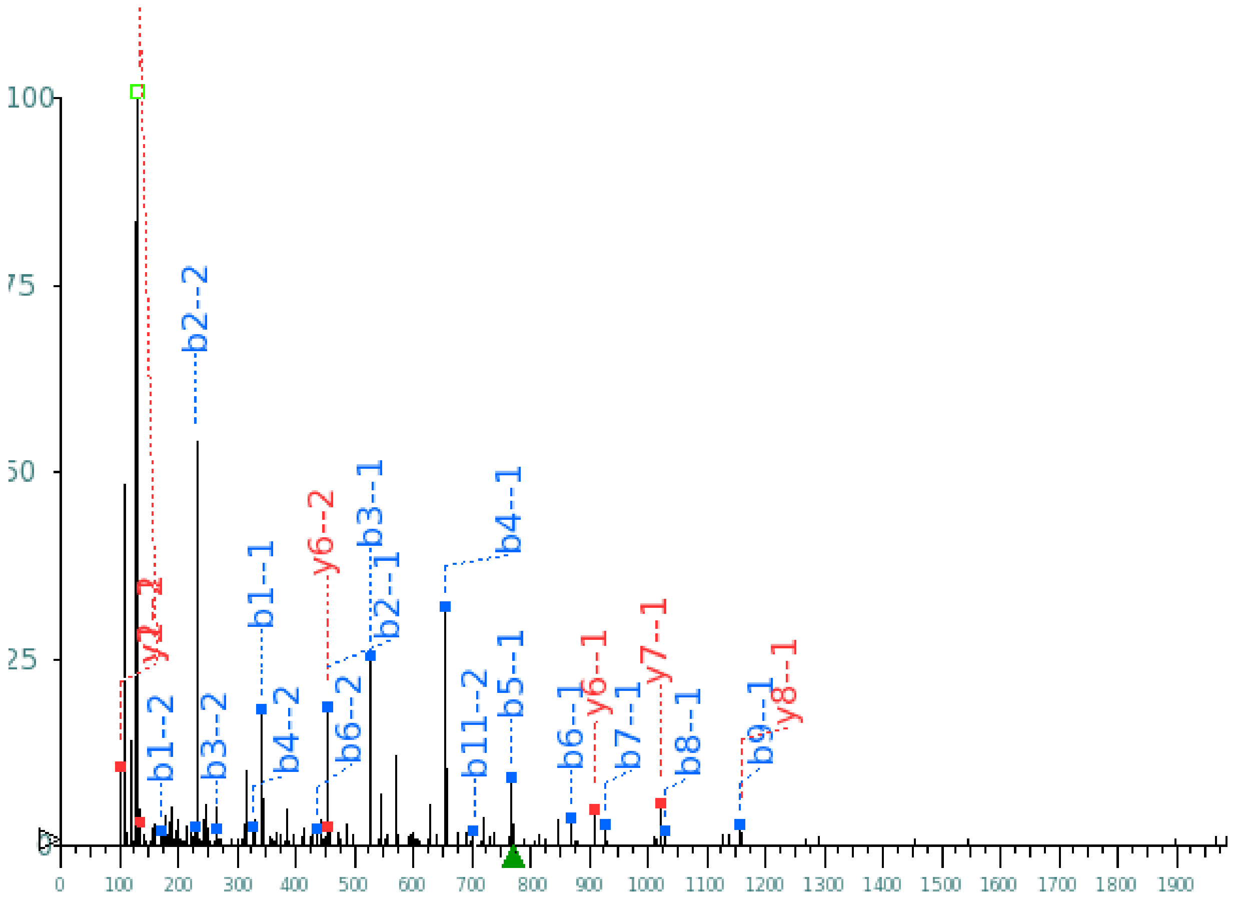

ILAQITGTEHLKESEAD (+3)

Primary Reference: PDLI5_HUMAN
Search ID: 41760
Search Name: 20130328_ananiav_ERstress_TMT_TM_timecourse_trypsin_inj1_4mc_iaa
Scan#: 18082
Observed Mass: 771.7643 (3.6 ppm)
PSM Score: 31.71

File name: 2015-02-04 04.01.53PM.pdf

Creation time: 2015-02-04 04:09PM

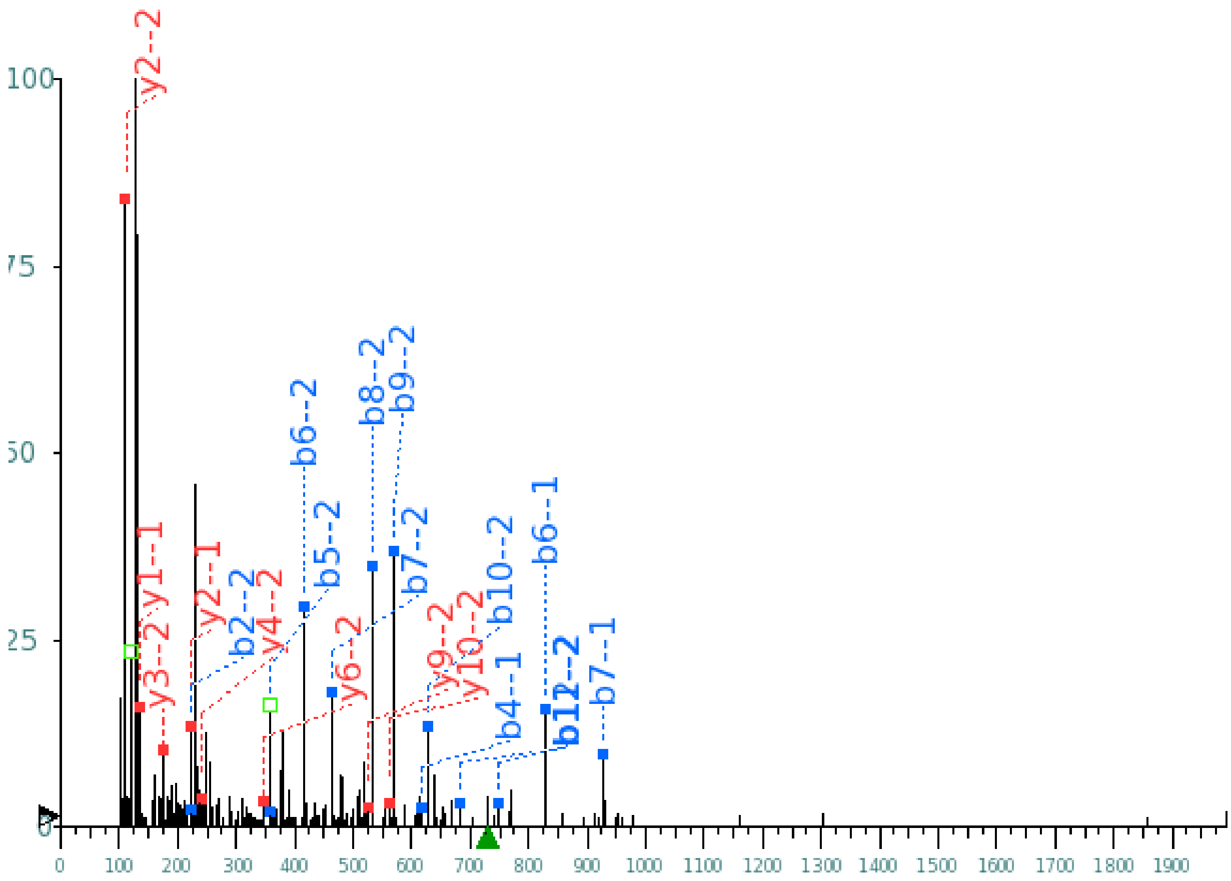

RGSSPDVHALLEITEESD (+3)

Primary Reference: PTRF_HUMAN
Search ID: 41760
Search Name: 20130328_ananiav_ERstress_TMT_TM_timecourse_trypsin_inj1_4mc_iaa
Scan#: 18118
Observed Mass: 728.7067 (3.4 ppm)
PSM Score: 18.52

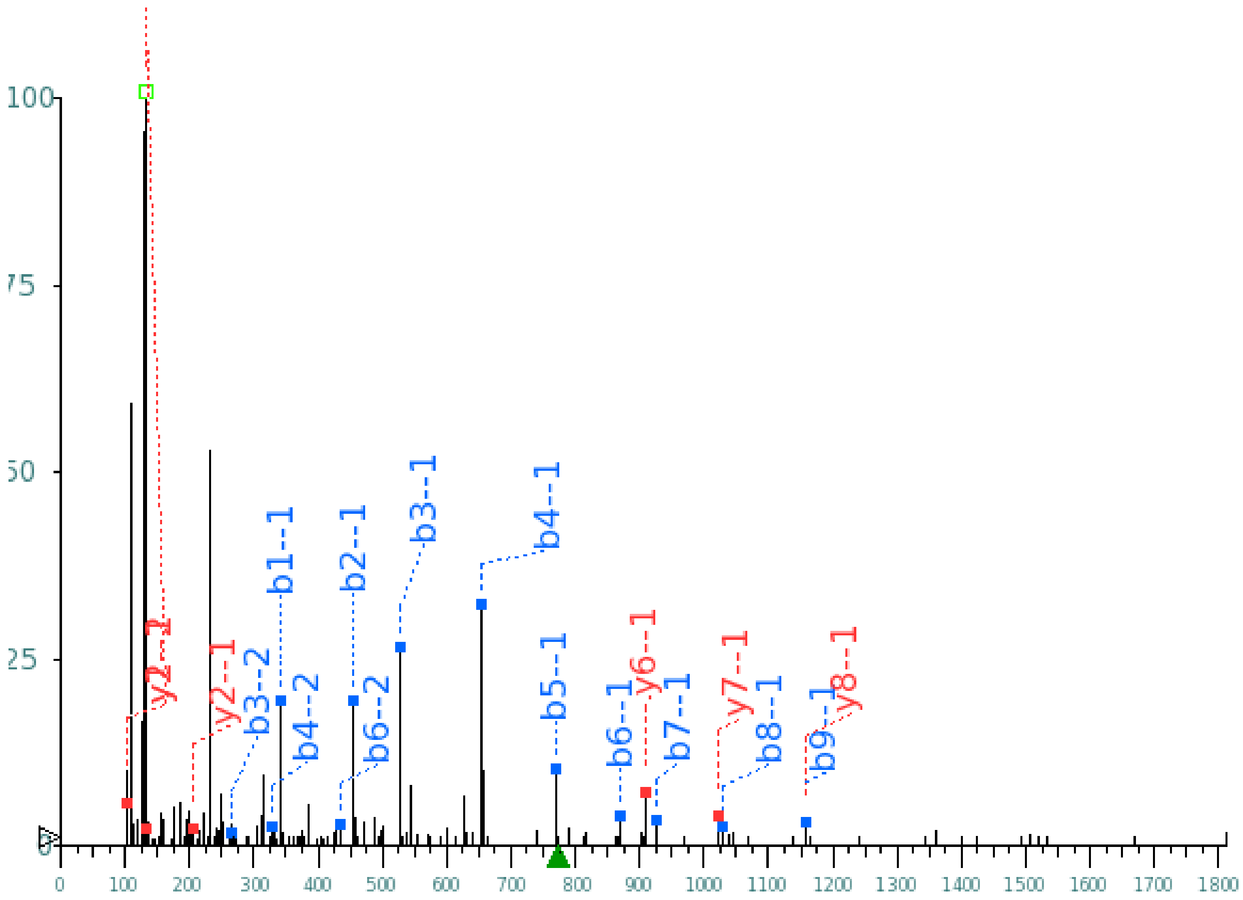

ILAQITGTEHLKESEAD (+3)

Primary Reference: PDLI5_HUMAN
Search ID: 41760
Search Name: 20130328_ananiav_ERstress_TMT_TM_timecourse_trypsin_inj1_4mc_iaa
Scan#: 18148
Observed Mass: 771.7635 (2.5 ppm)
PSM Score: 32.01

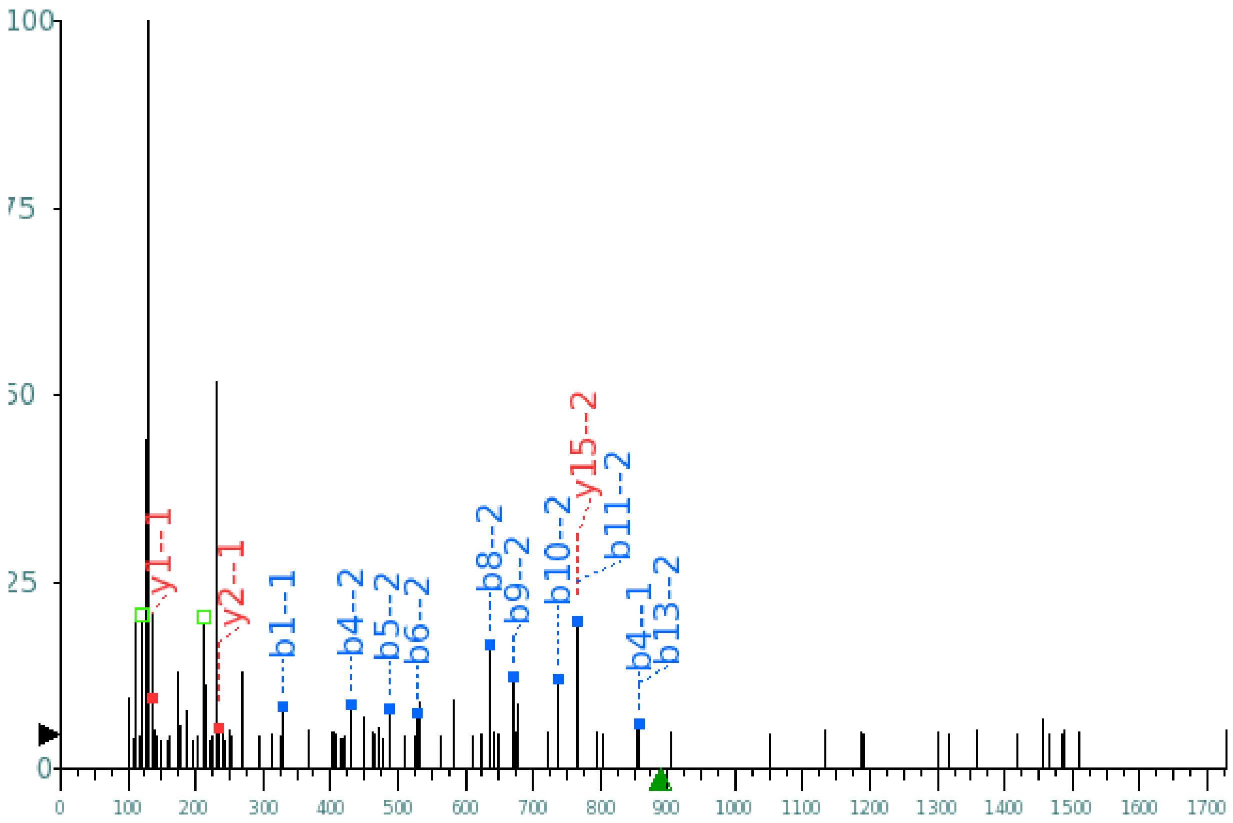

VKGILSSQAEGLANGPDVLETD (+3)

Primary Reference: EHMT1_HUMAN
Search ID: 41760
Search Name: 20130328_ananiav_ERstress_TMT_TM_timecourse_trypsin_inj1_4mc_iaa
Scan#: 18205
Observed Mass: 891.1578 (1.2 ppm)
PSM Score: 19.44

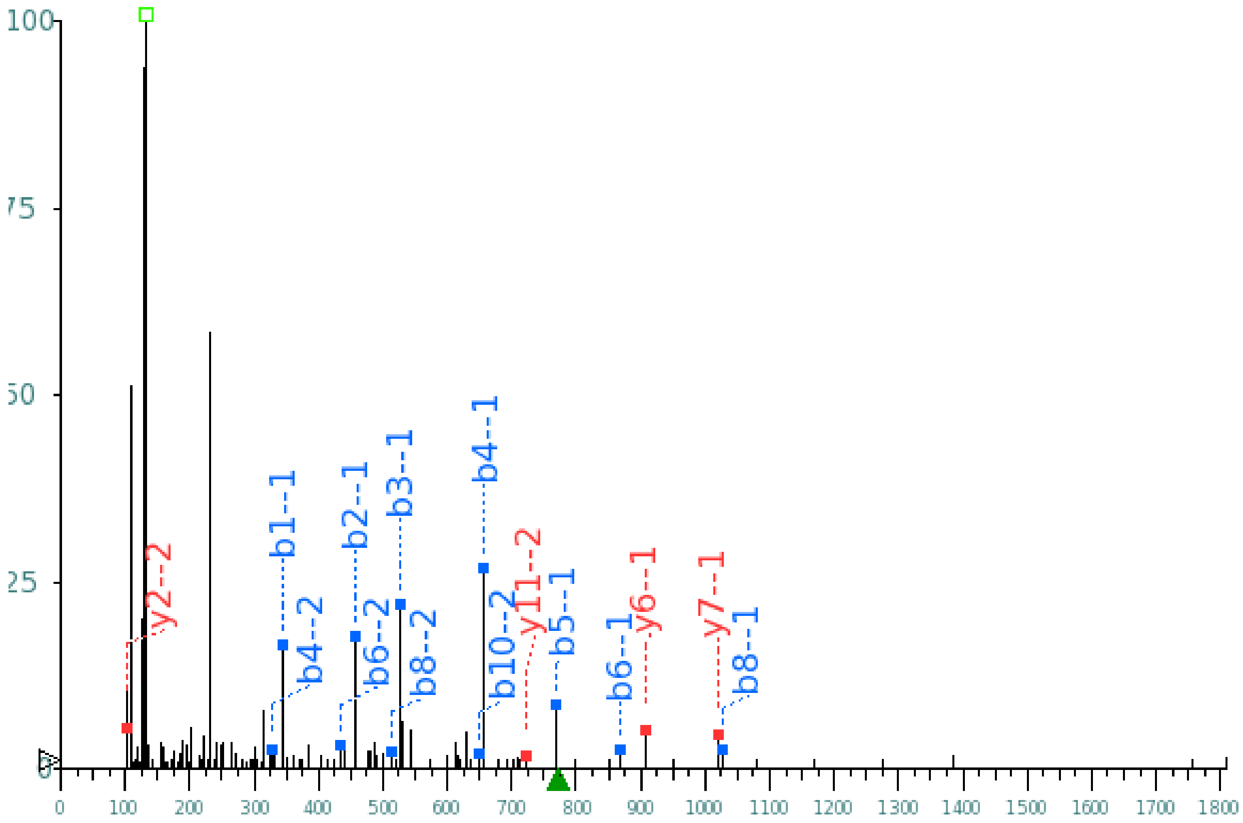

ILAQITGTEHLKESEAD (+3)

Primary Reference: PDLI5_HUMAN
Search ID: 41760
Search Name: 20130328_ananiav_ERstress_TMT_TM_timecourse_trypsin_inj1_4mc_iaa
Scan#: 18214
Observed Mass: 771.7634 (2.3 ppm)
PSM Score: 25.08

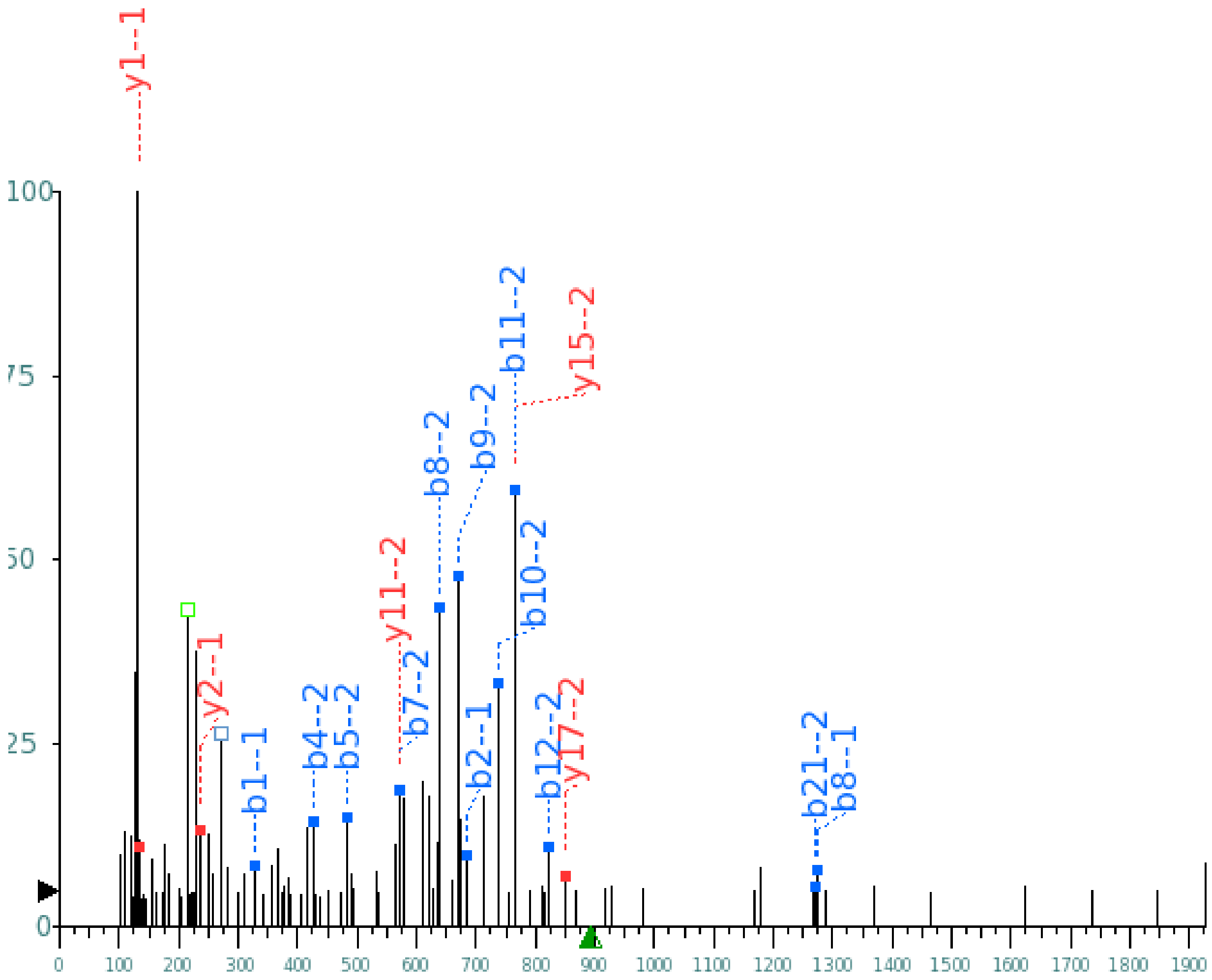

VKGILSSQAEGLANGPDVLETD (+3)

Primary Reference: EHMT1_HUMAN
Search ID: 41760
Search Name: 20130328_ananiav_ERstress_TMT_TM_timecourse_trypsin_inj1_4mc_iaa
Scan#: 18277
Observed Mass: 891.1587 (2.3 ppm)
PSM Score: 23.3

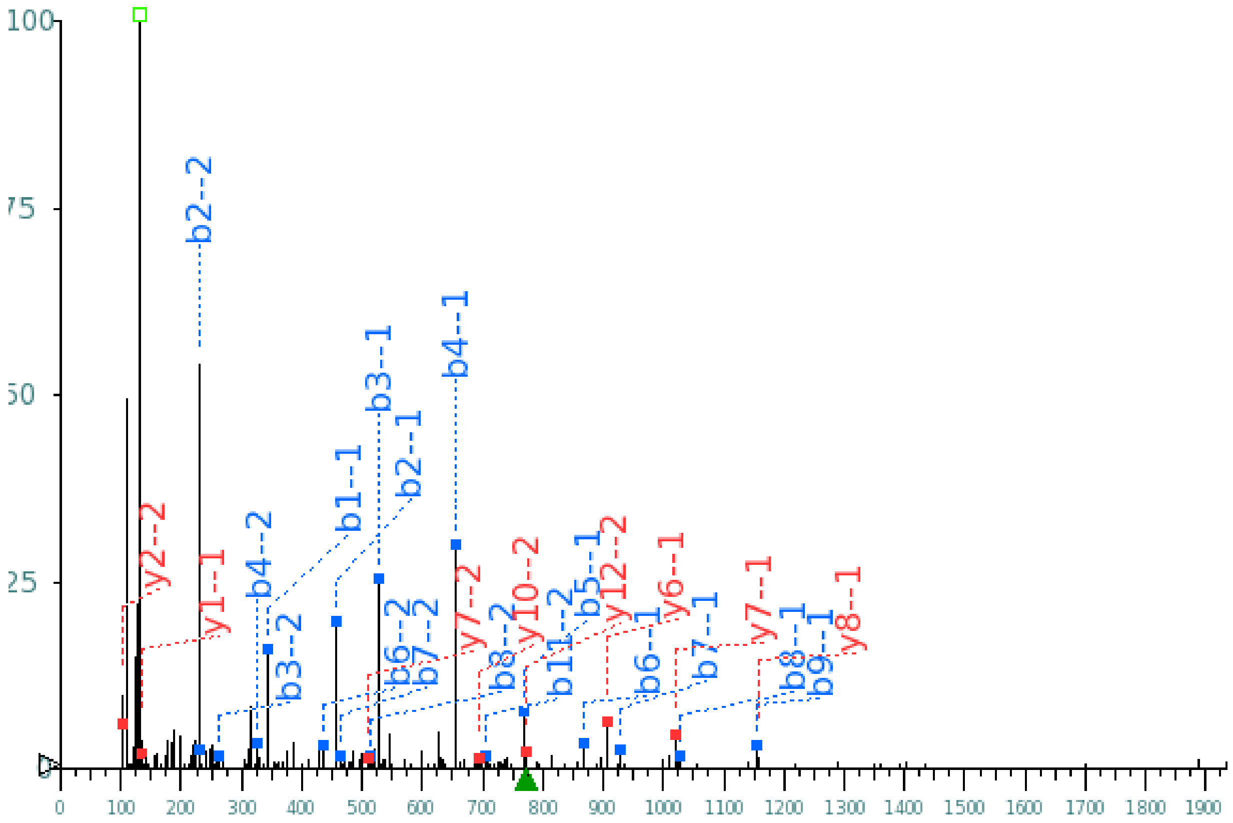

ILAQITGTEHLKESEAD (+3)

Primary Reference: PDLI5_HUMAN
Search ID: 41760
Search Name: 20130328_ananiav_ERstress_TMT_TM_timecourse_trypsin_inj1_4mc_iaa
Scan#: 18290
Observed Mass: 771.7626 (1.3 ppm)
PSM Score: 32.04

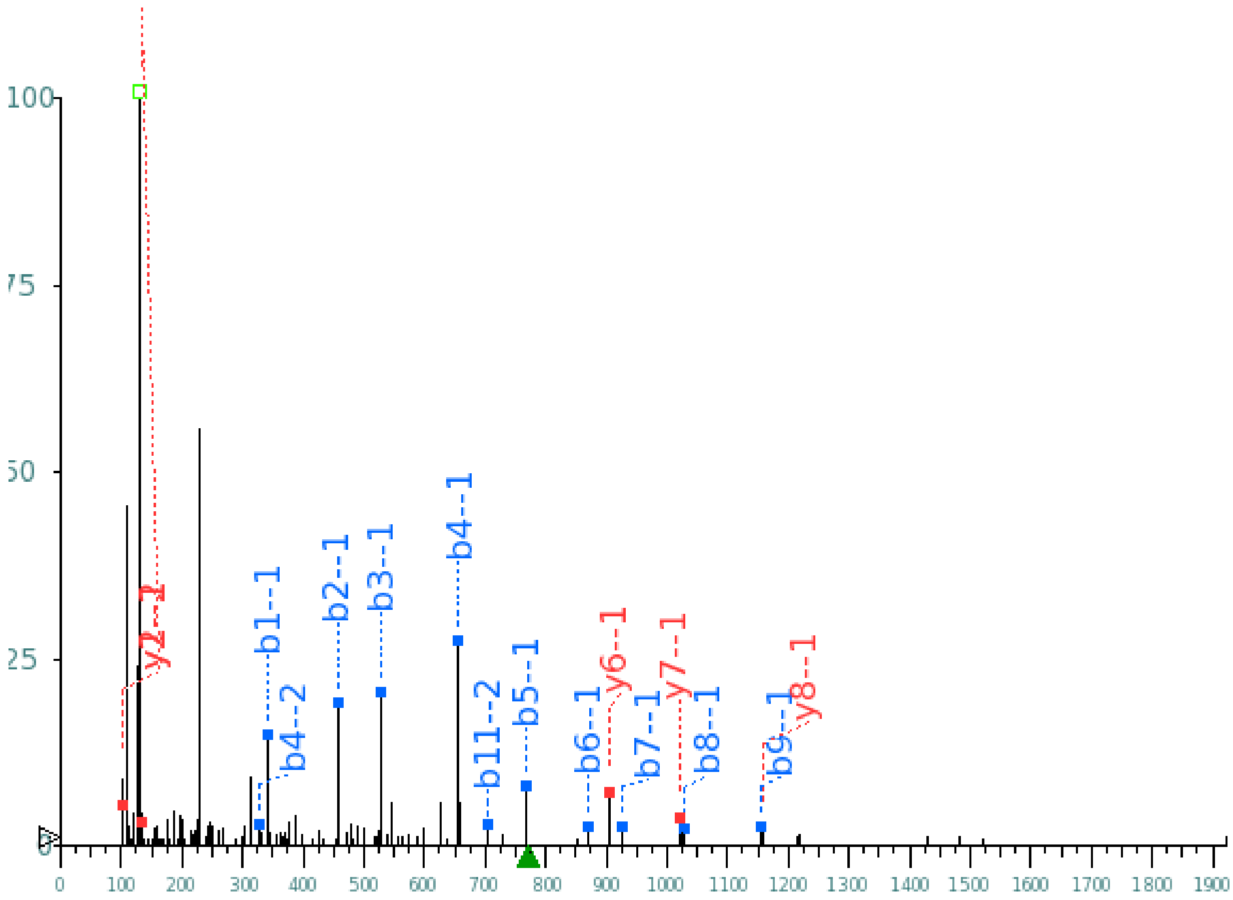

ILAQITGTEHLKESEAD (+3)

Primary Reference: PDLI5_HUMAN
Search ID: 41760
Search Name: 20130328_ananiav_ERstress_TMT_TM_timecourse_trypsin_inj1_4mc_iaa
Scan#: 18370
Observed Mass: 771.7645 (3.7 ppm)
PSM Score: 42.13

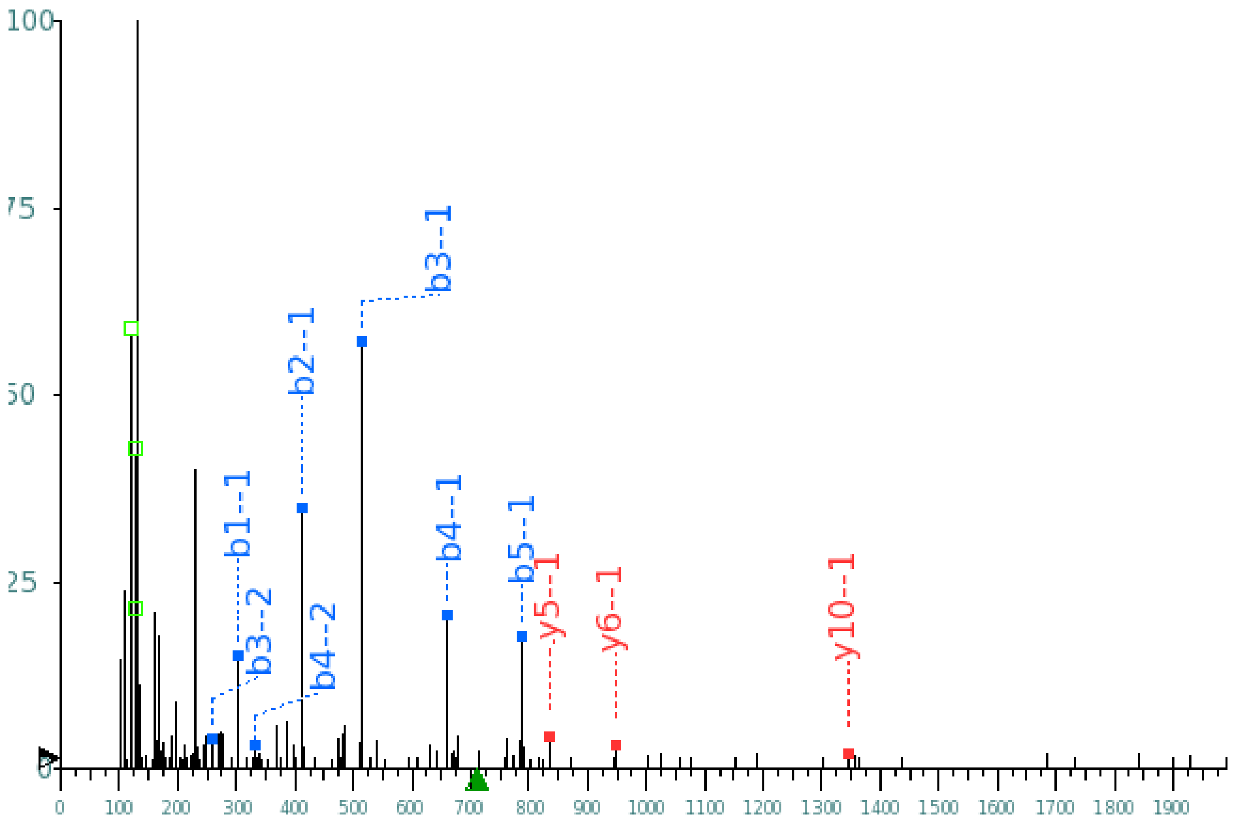

ALVFQPVAELKDQTD (+3)

Primary Reference: K6PF_HUMAN
Search ID: 41760
Search Name: 20130328_ananiav_ERstress_TMT_TM_timecourse_trypsin_inj1_4mc_iaa
Scan#: 18409
Observed Mass: 711.4069 (2.8 ppm)
PSM Score: 16.39

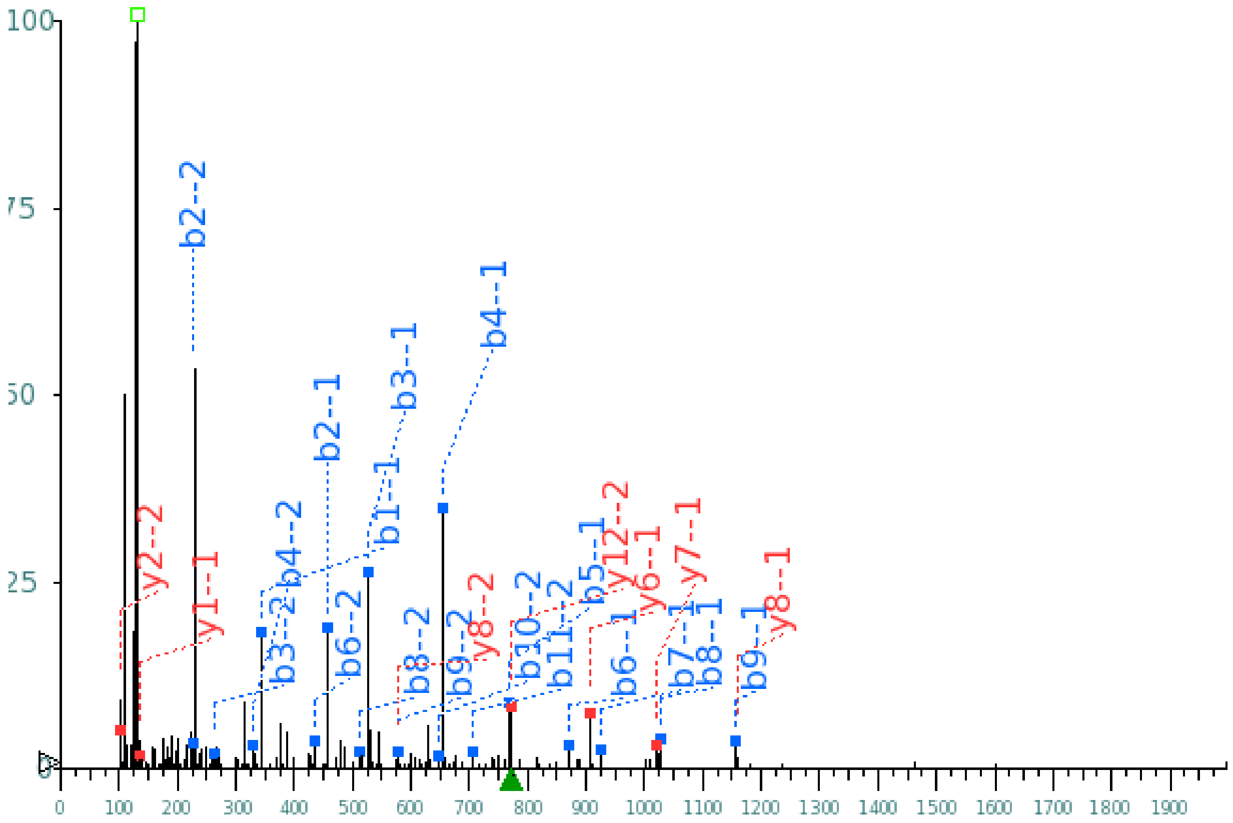

ILAQITGTEHLKESEAD (+3)

Primary Reference: PDLI5_HUMAN
Search ID: 41760
Search Name: 20130328_ananiav_ERstress_TMT_TM_timecourse_trypsin_inj1_4mc_iaa
Scan#: 18439
Observed Mass: 771.7646 (3.9 ppm)
PSM Score: 42.15

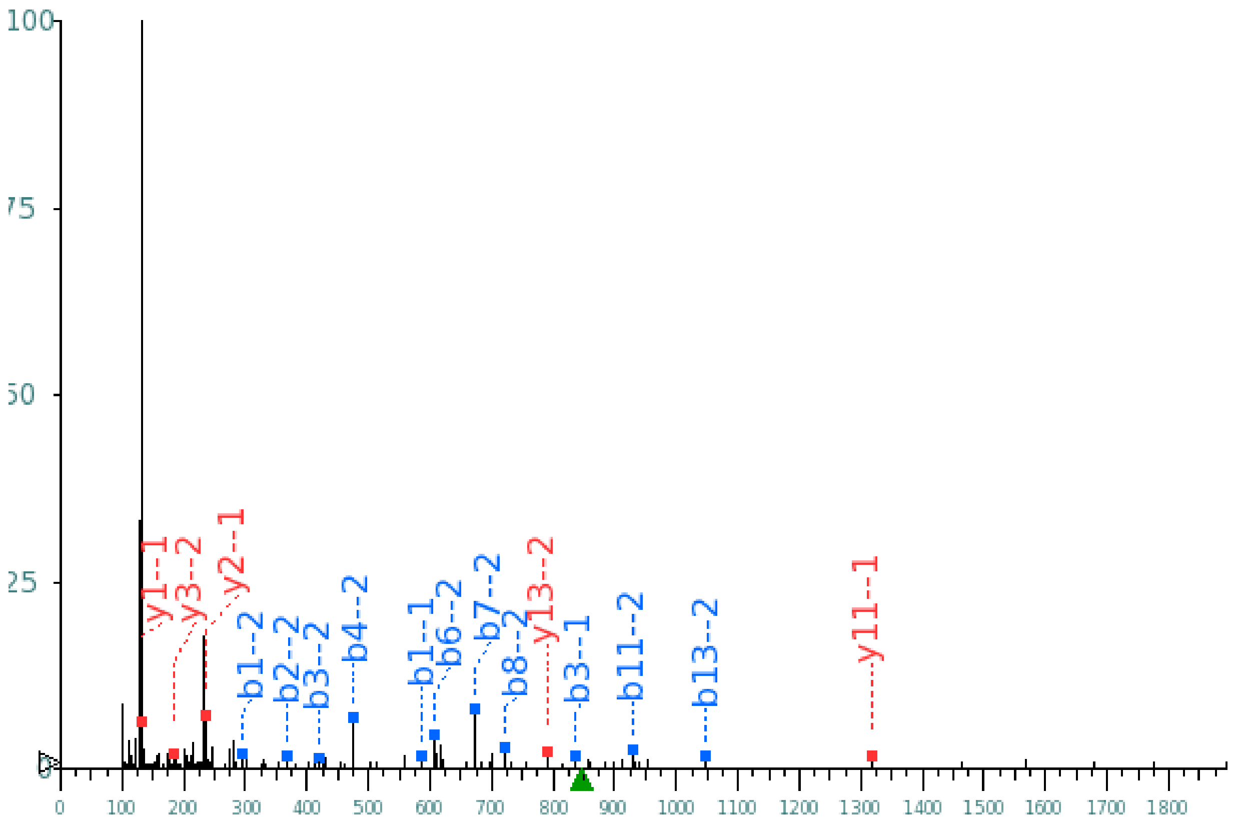

KFTDFDEVRQEIEAETD (+3)

Primary Reference: DYN2_HUMAN
Search ID: 41760
Search Name: 20130328_ananiav_ERstress_TMT_TM_timecourse_trypsin_inj1_4mc_iaa
Scan#: 18450
Observed Mass: 844.1017 (7.6 ppm)
PSM Score: 18.16

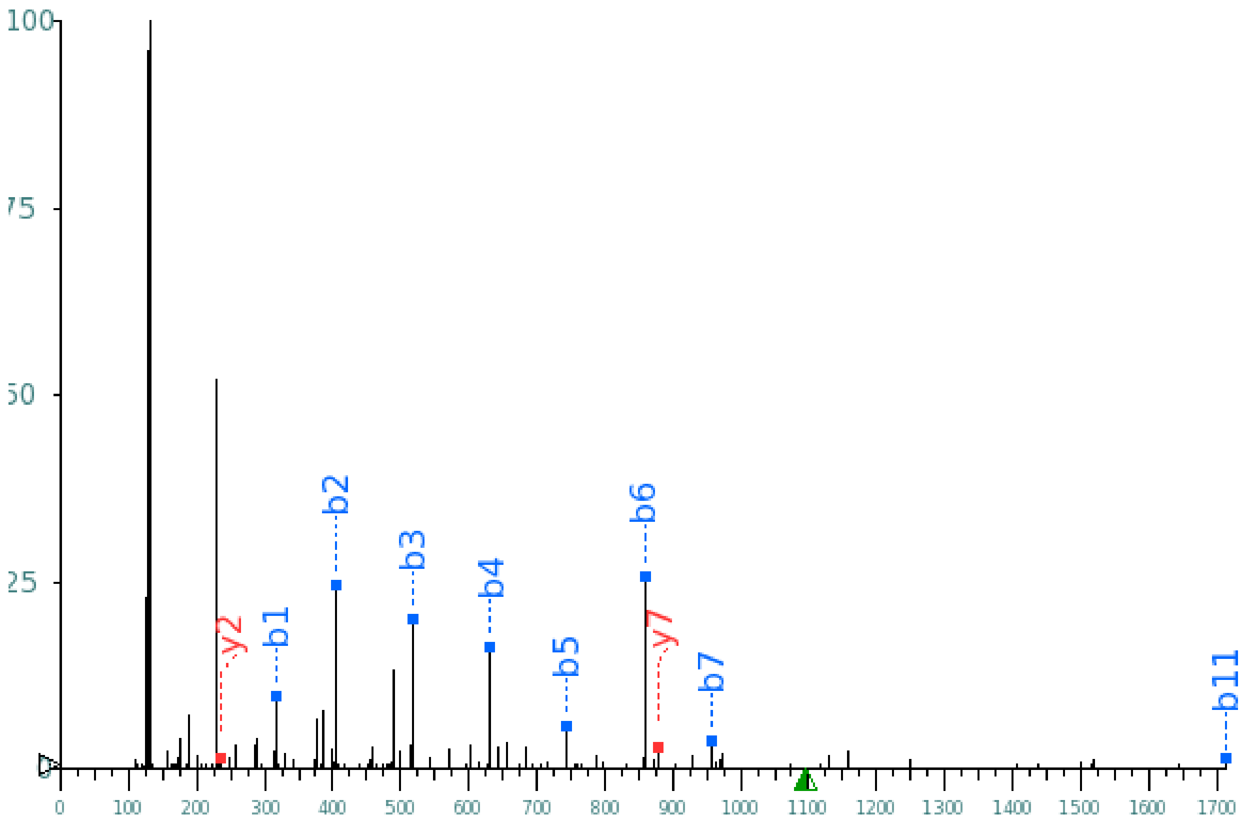

SSILLDVKPWDDETD (+2)

Primary Reference: EF1B_HUMAN
Search ID: 41760
Search Name: 20130328_ananiav_ERstress_TMT_TM_timecourse_trypsin_inj1_4mc_iaa
Scan#: 18476
Observed Mass: 1096.0824 (1.8 ppm)
PSM Score: 17.94

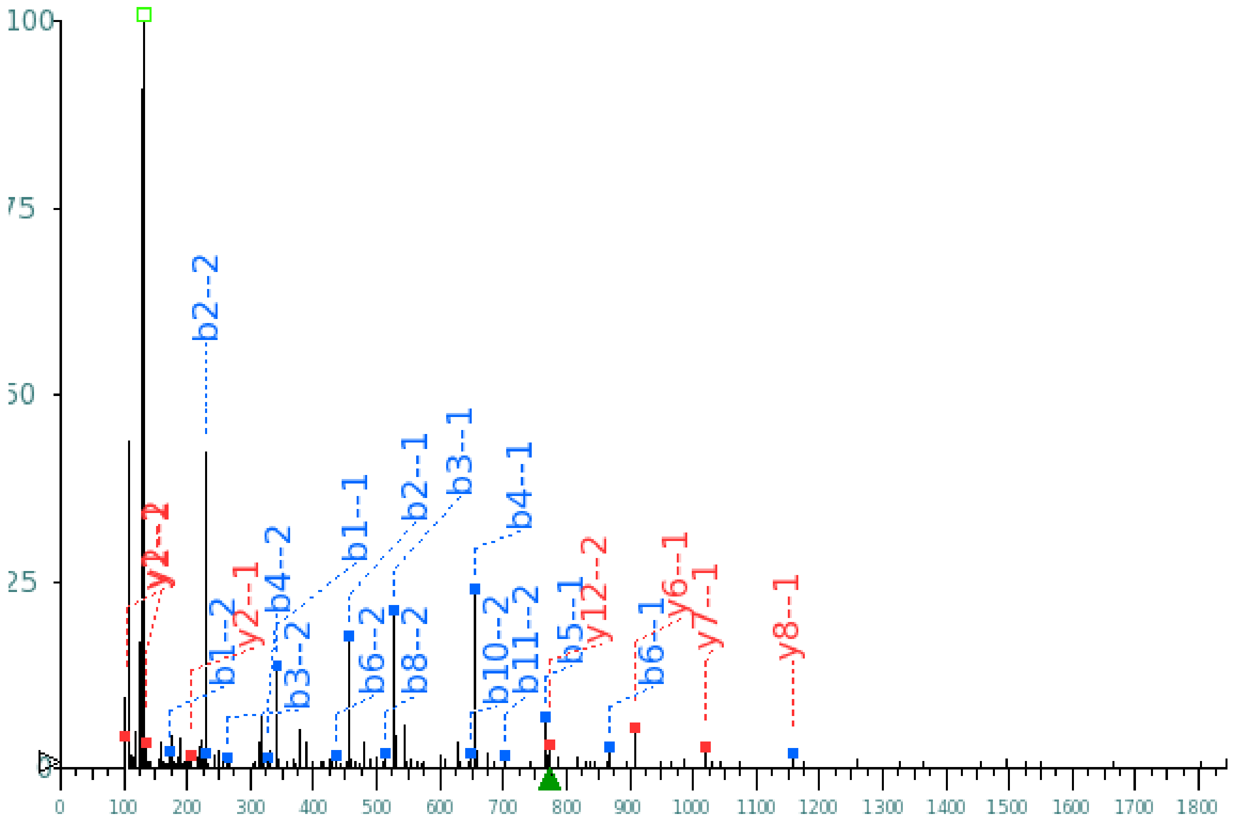

ILAQITGTEHLKESEAD (+3)

Primary Reference: PDLI5_HUMAN
Search ID: 41760
Search Name: 20130328_ananiav_ERstress_TMT_TM_timecourse_trypsin_inj1_4mc_iaa
Scan#: 18513
Observed Mass: 771.7638 (2.9 ppm)
PSM Score: 26.68

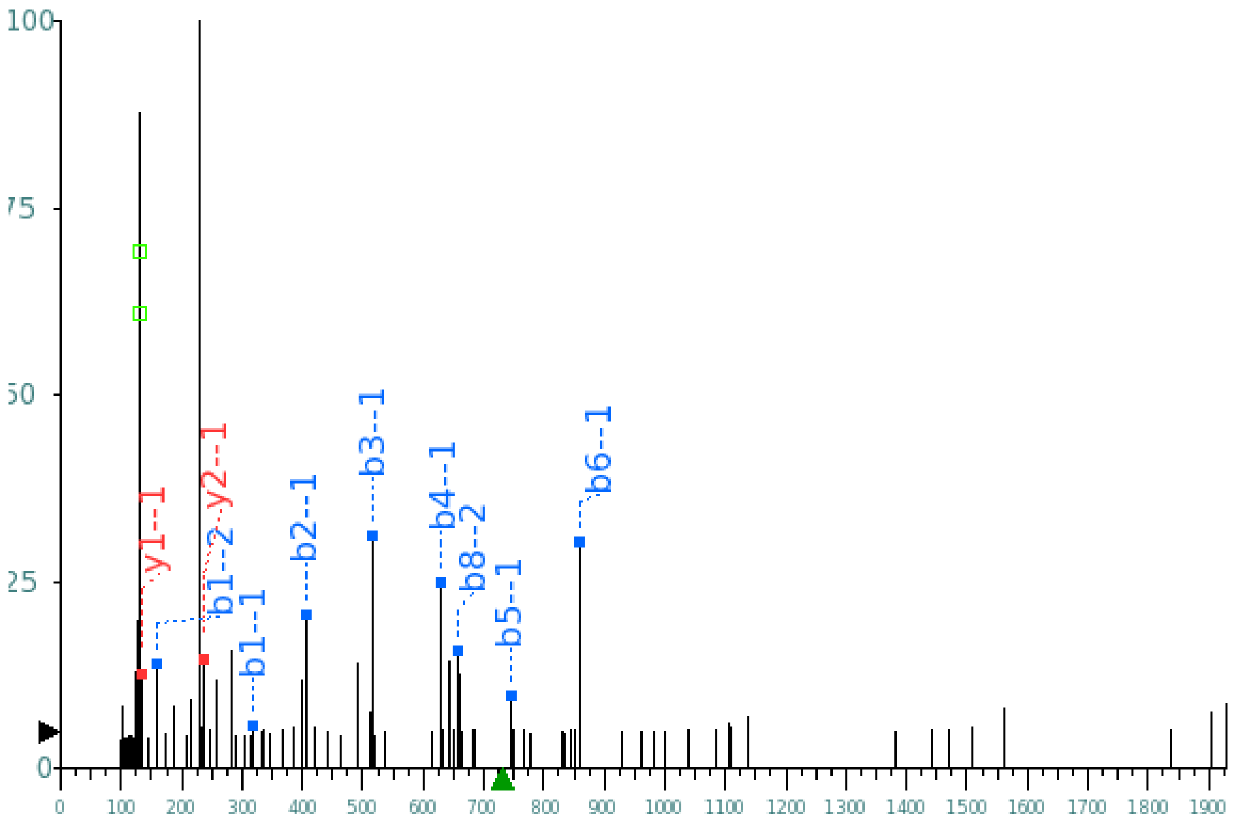

SSILLDVKPWDDETD (+3)

Primary Reference: EF1B_HUMAN
Search ID: 41760
Search Name: 20130328_ananiav_ERstress_TMT_TM_timecourse_trypsin_inj1_4mc_iaa
Scan#: 18530
Observed Mass: 731.0588 (3.8 ppm)
PSM Score: 10.65

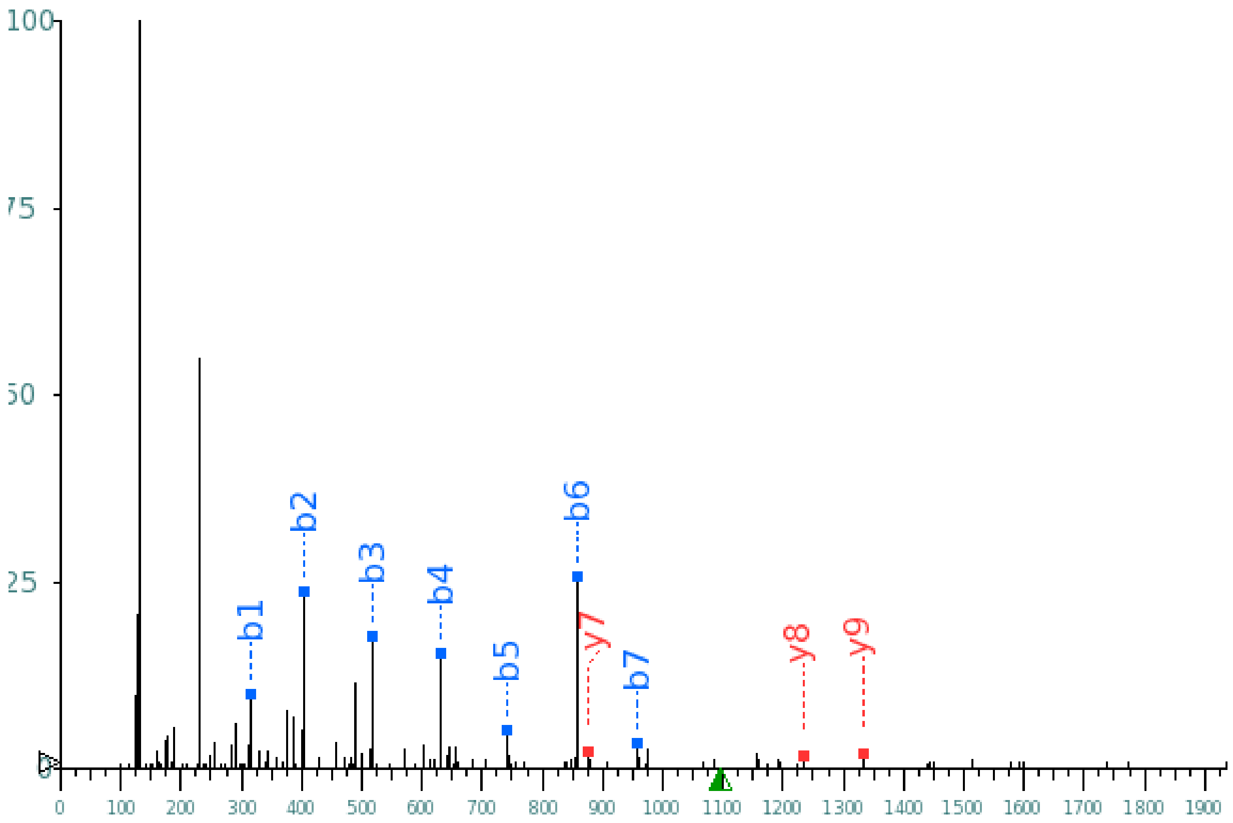

SSILLDVKPWDDETD (+2)

Primary Reference: EF1B_HUMAN
Search ID: 41760
Search Name: 20130328_ananiav_ERstress_TMT_TM_timecourse_trypsin_inj1_4mc_iaa
Scan#: 18552
Observed Mass: 1096.0826 (2 ppm)
PSM Score: 24.48

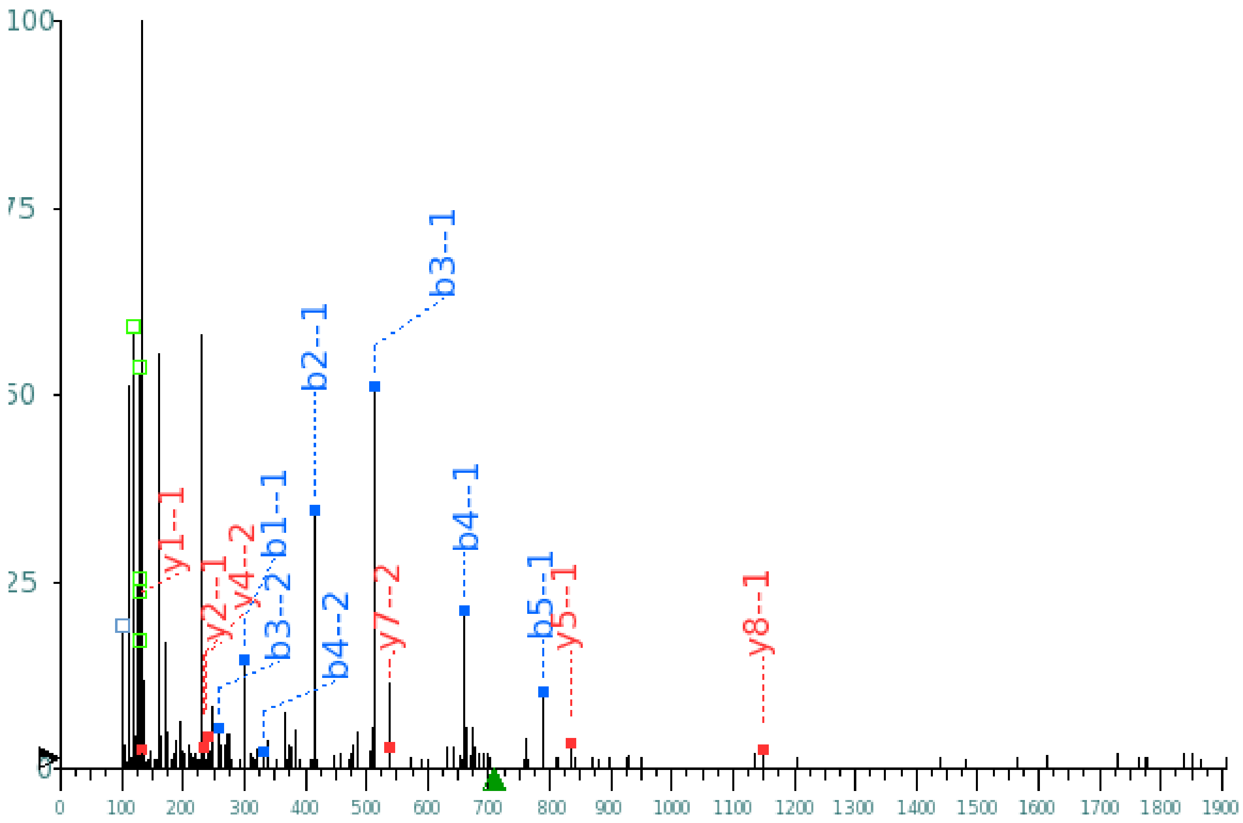

ALVFQPVAELKDQTD (+3)

Primary Reference: K6PF_HUMAN
Search ID: 41760
Search Name: 20130328_ananiav_ERstress_TMT_TM_timecourse_trypsin_inj1_4mc_iaa
Scan#: 18587
Observed Mass: 711.4057 (1 ppm)
PSM Score: 16.8

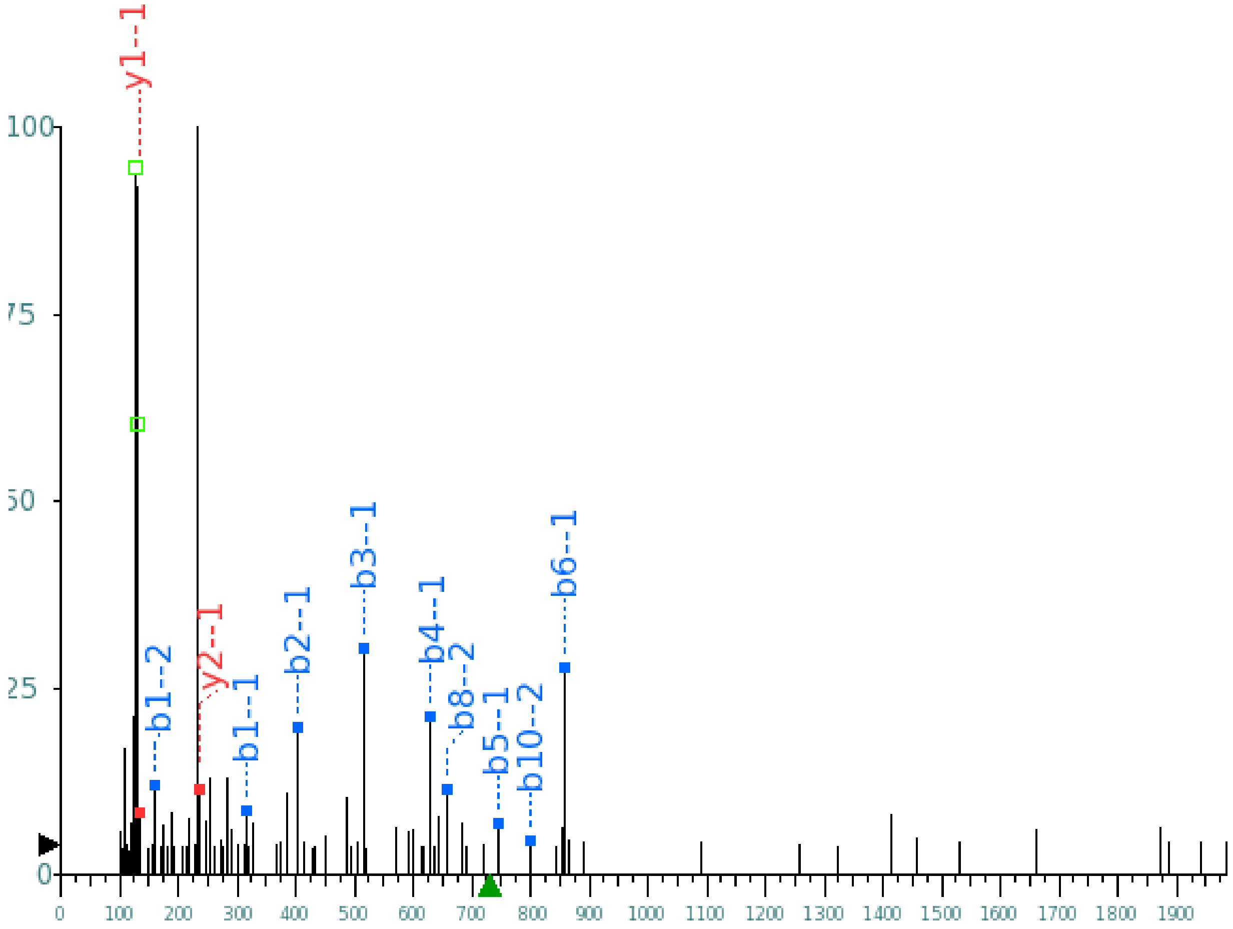

SSILLDVKPWDDETD (+3)

Primary Reference: EF1B_HUMAN
Search ID: 41760
Search Name: 20130328_ananiav_ERstress_TMT_TM_timecourse_trypsin_inj1_4mc_iaa
Scan#: 18596
Observed Mass: 731.0585 (3.5 ppm)
PSM Score: 12.2

File name: 2015-02-04 04.01.53PM.pdf

Creation time: 2015-02-04 04:09PM

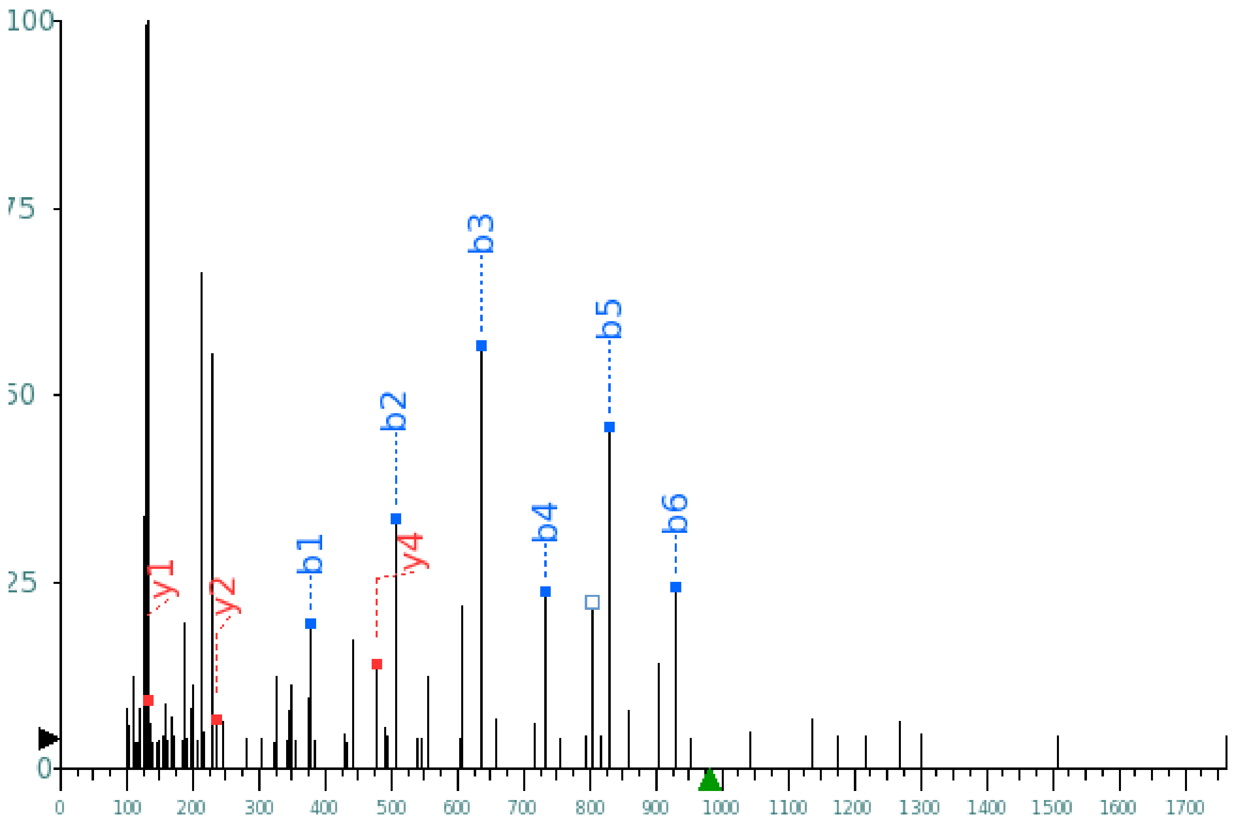

FEEPVVLPDLDDQTD (+2)

Primary Reference: NOTC1_HUMAN
Search ID: 41760
Search Name: 20130328_ananiav_ERstress_TMT_TM_timecourse_trypsin_inj1_4mc_iaa
Scan#: 18640
Observed Mass: 980.985 (1.9 ppm)
PSM Score: 23.18

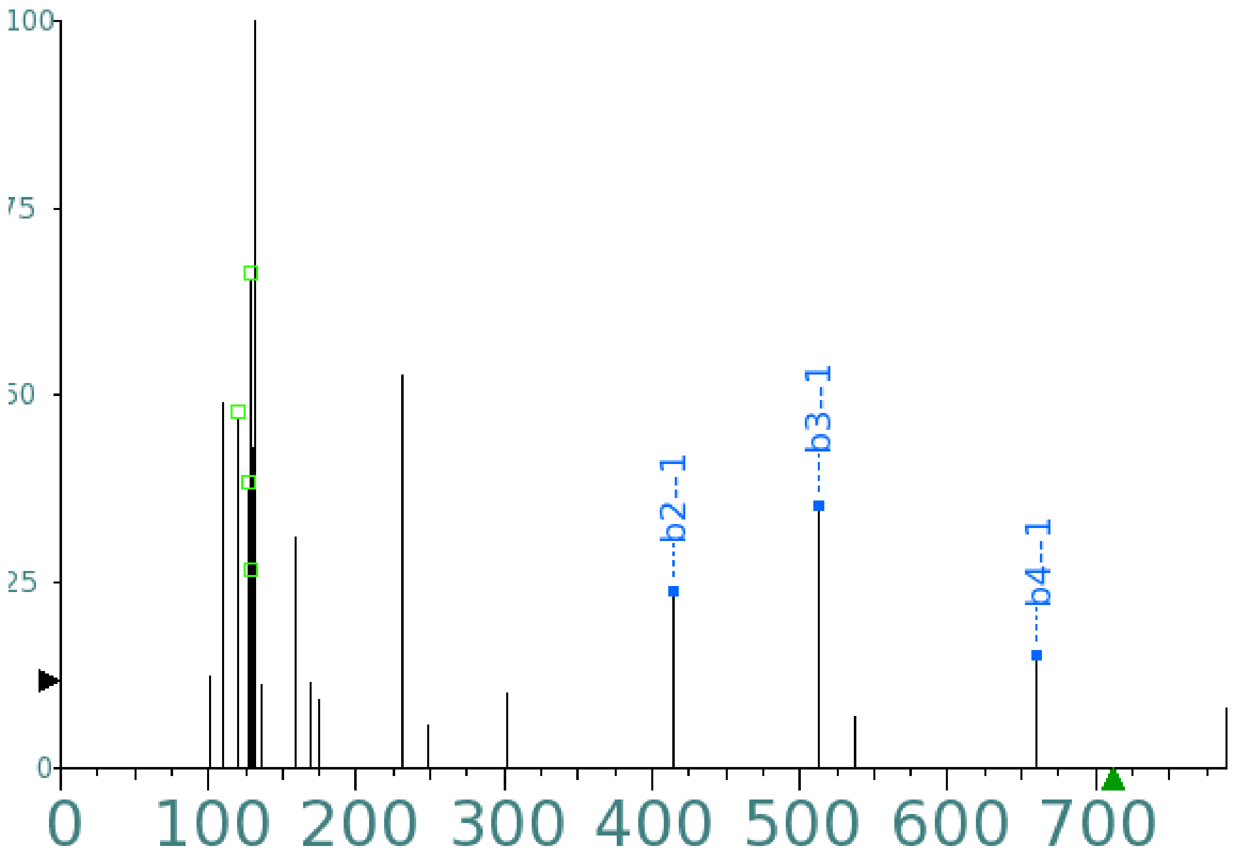

ALVFQPVAELKDQTD (+3)

Primary Reference: K6PF_HUMAN
Search ID: 41760
Search Name: 20130328_ananiav_ERstress_TMT_TM_timecourse_trypsin_inj1_4mc_iaa
Scan#: 18669
Observed Mass: 711.4075 (3.6 ppm)
PSM Score: 27.31

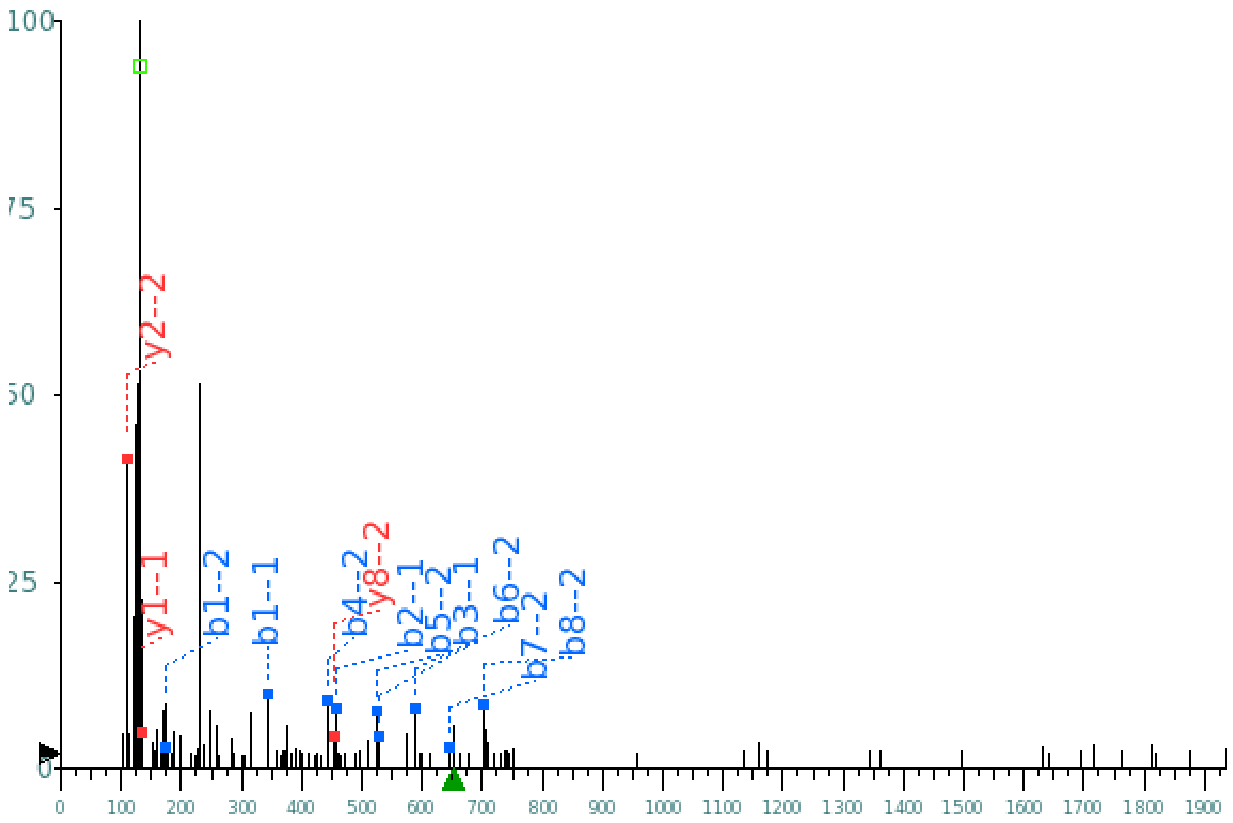

ILAKYEIDLDTSD (+3)

Primary Reference: K6PF_HUMAN
Search ID: 41760
Search Name: 20130328_ananiav_ERstress_TMT_TM_timecourse_trypsin_inj1_4mc_iaa
Scan#: 18731
Observed Mass: 652.0342 (4.8 ppm)
PSM Score: 6.85

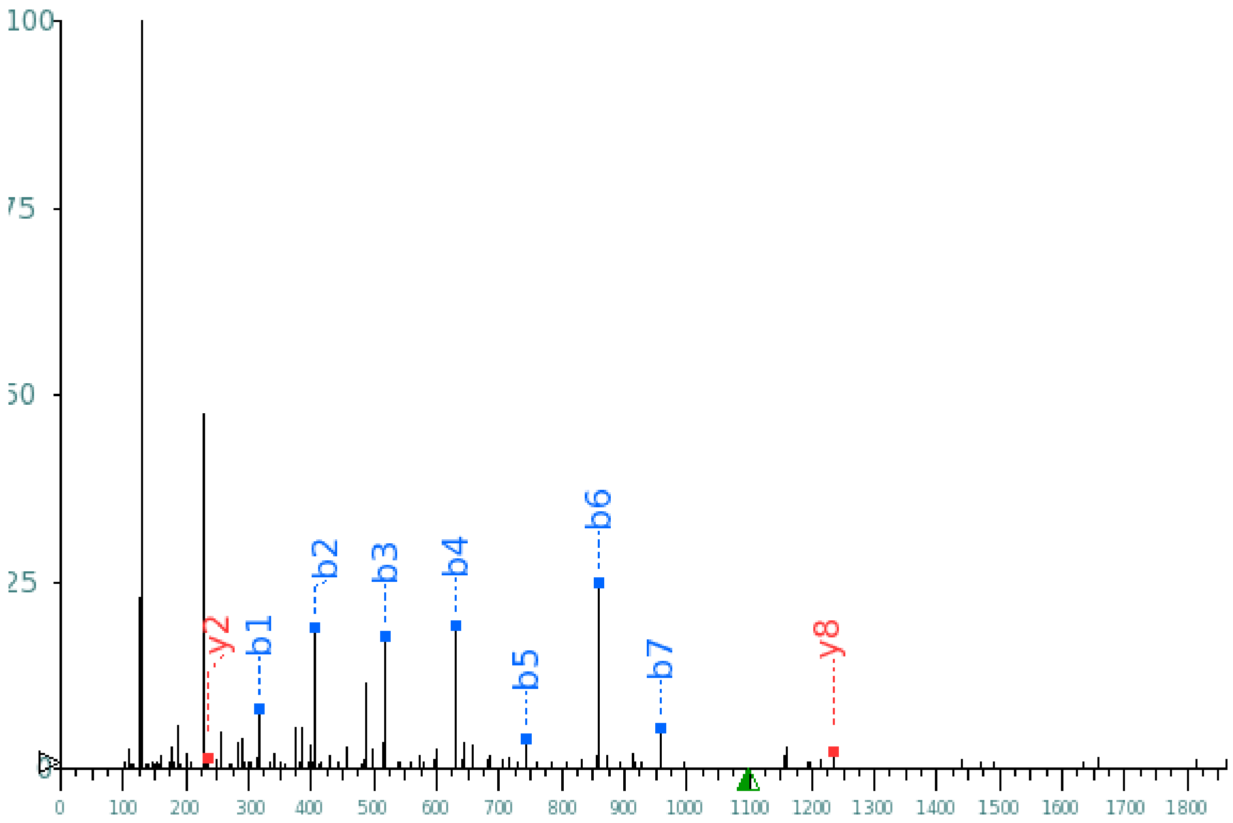

SSILLDVKPWDDETD (+2)

Primary Reference: EF1B_HUMAN
Search ID: 41760
Search Name: 20130328_ananiav_ERstress_TMT_TM_timecourse_trypsin_inj1_4mc_iaa
Scan#: 18741
Observed Mass: 1096.0827 (2.2 ppm)
PSM Score: 25.11

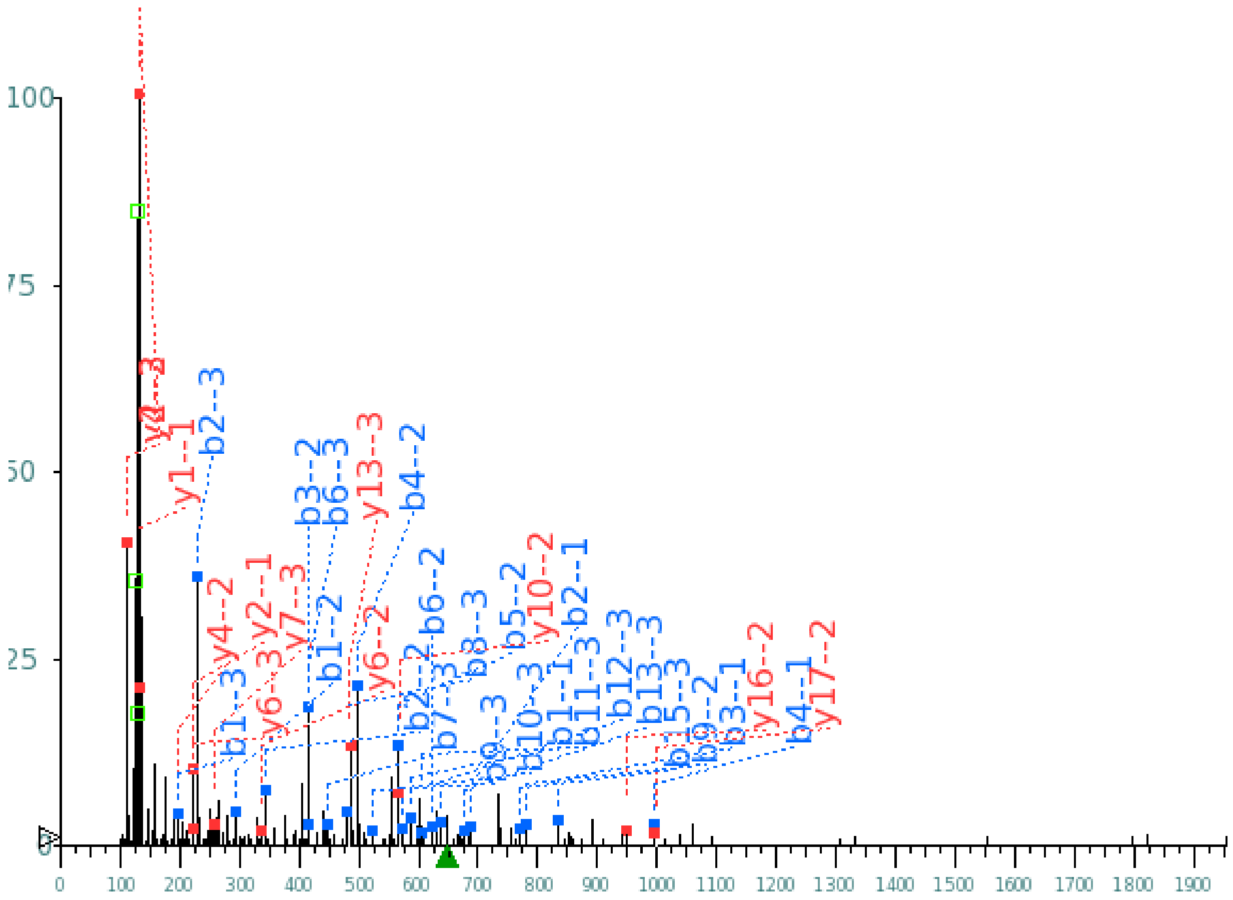

KVFYHLTVDRPVIYDGSD (+4)

Primary Reference: FYCO1_HUMAN
Search ID: 41760
Search Name: 20130328_ananiav_ERstress_TMT_TM_timecourse_trypsin_inj1_4mc_iaa
Scan#: 18743
Observed Mass: 646.3581 (3.4 ppm)
PSM Score: 11.87

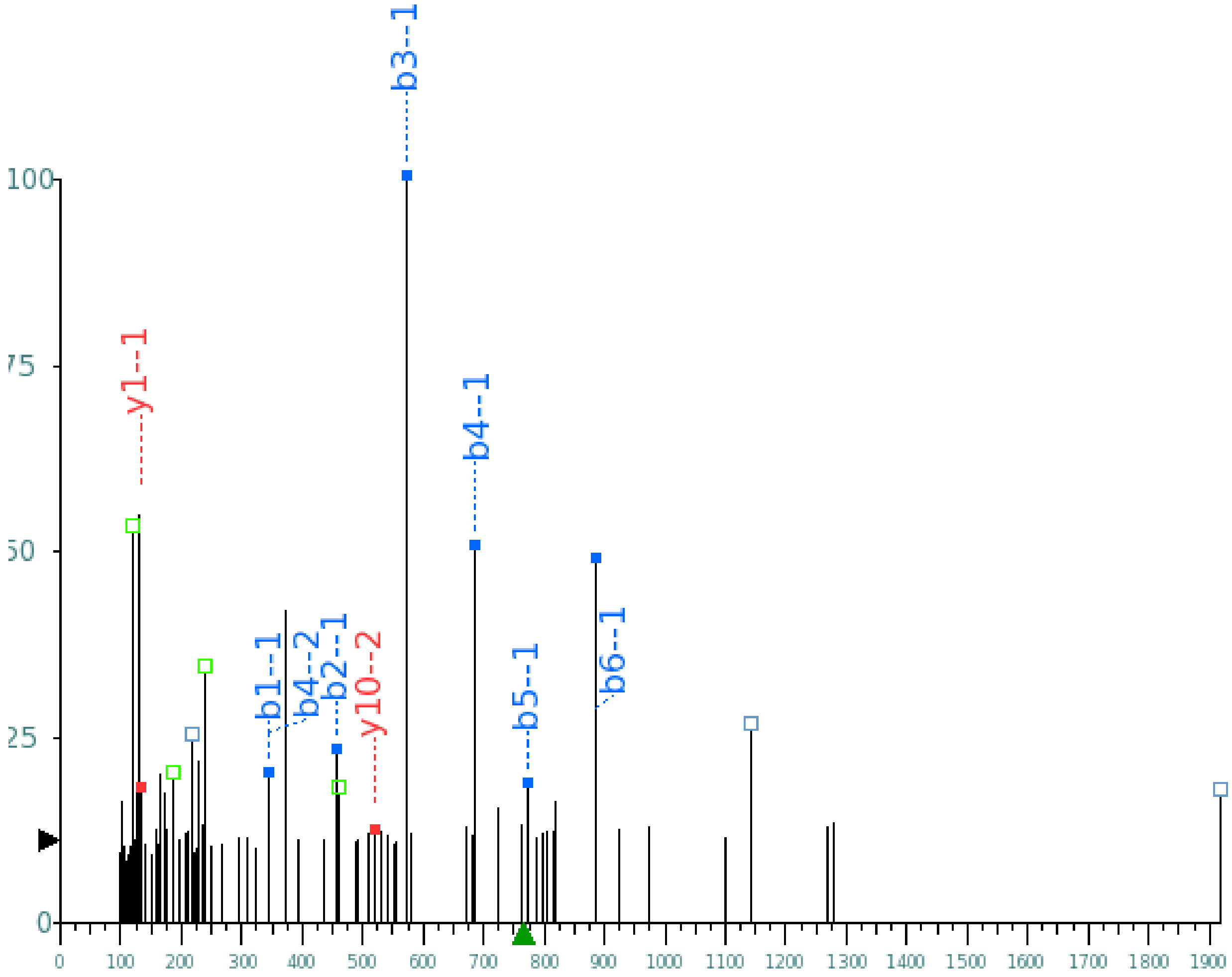

LLDLSDSTSVASFPSADETD (+3)

Primary Reference: ROCK1_HUMAN
Search ID: 41760
Search Name: 20130328_ananiav_ERstress_TMT_TM_timecourse_trypsin_inj1_4mc_iaa
Scan#: 18761
Observed Mass: 767.0416 (3.3 ppm)
PSM Score: 17.19

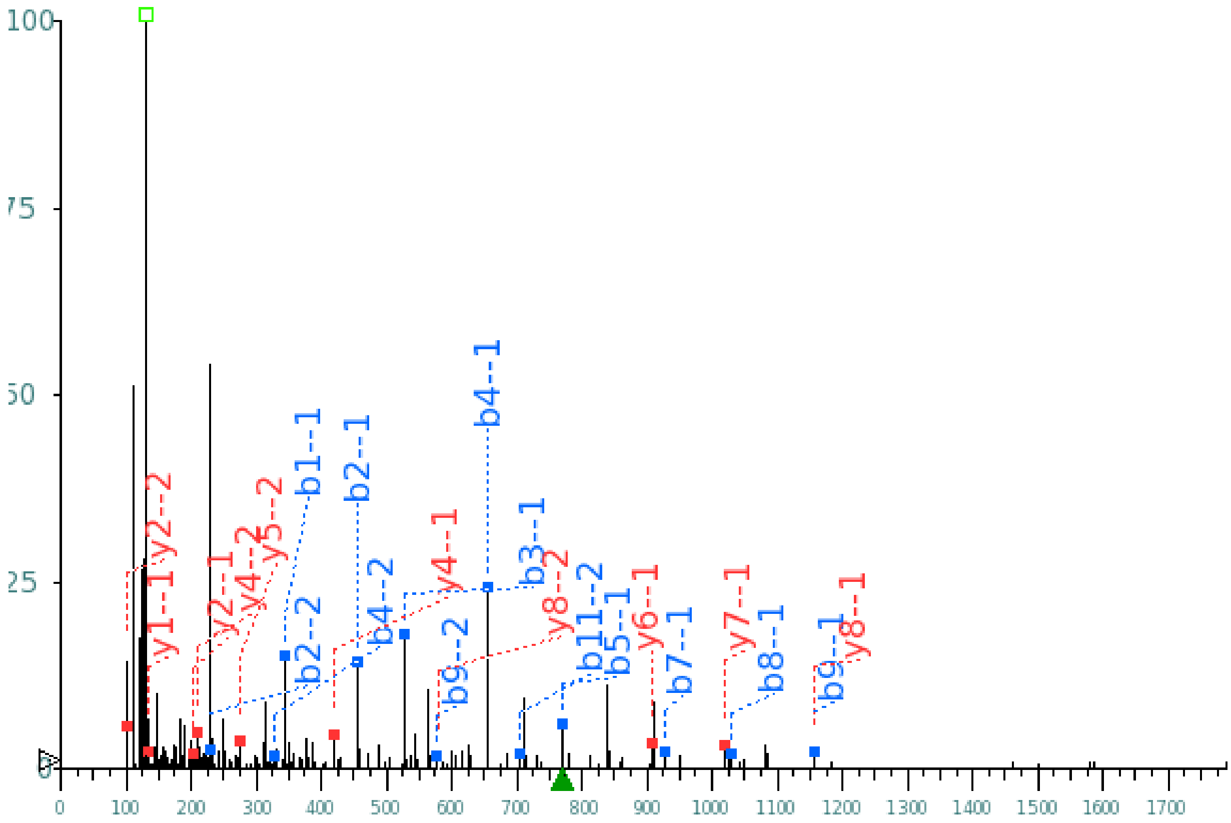

ILAQITGTEHLKESEAD (+3)

Primary Reference: PDLI5_HUMAN
Search ID: 41760
Search Name: 20130328_ananiav_ERstress_TMT_TM_timecourse_trypsin_inj1_4mc_iaa
Scan#: 18891
Observed Mass: 771.7636 (2.7 ppm)
PSM Score: 13.9

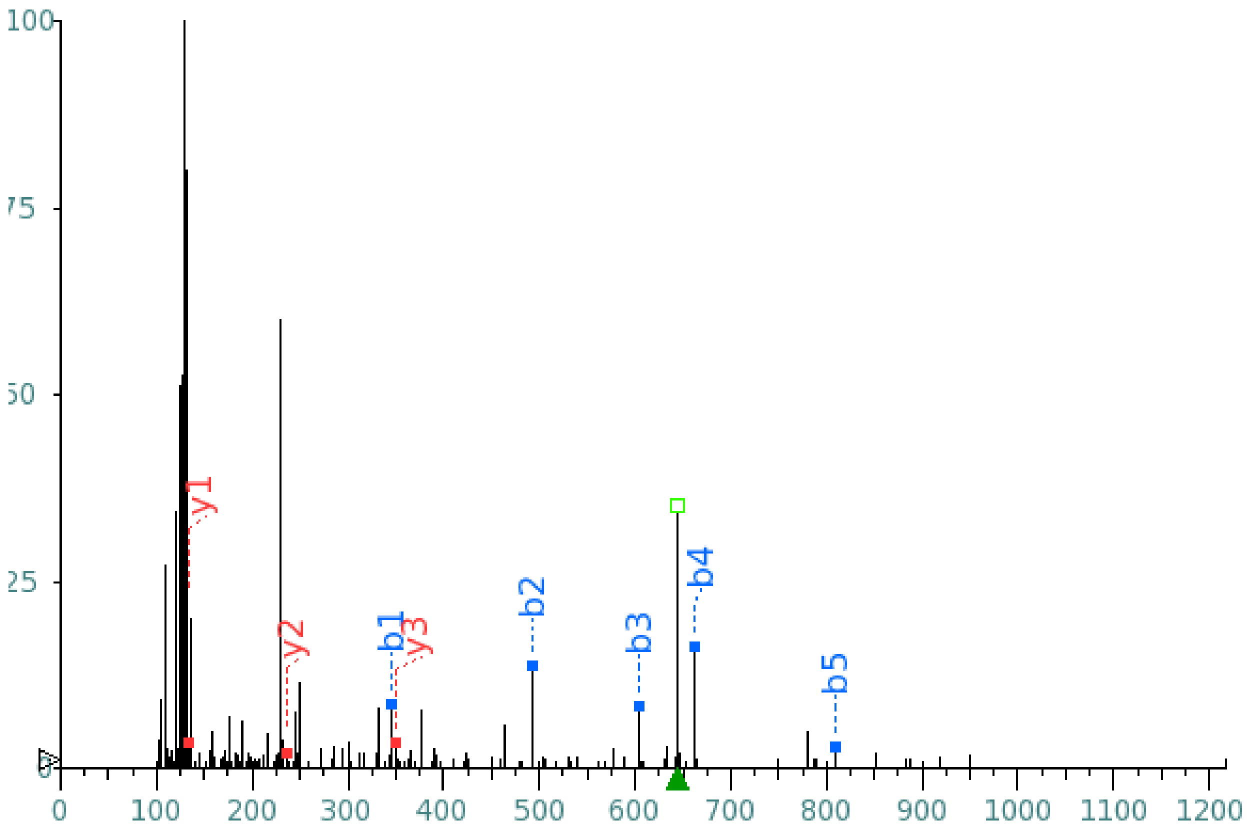

DFLGFMDTD (+2)

Primary Reference: MGAP_HUMAN
Search ID: 41760
Search Name: 20130328_ananiav_ERstress_TMT_TM_timecourse_trypsin_inj1_4mc_iaa
Scan#: 18893
Observed Mass: 645.3012 (2.3 ppm)
PSM Score: 8.82

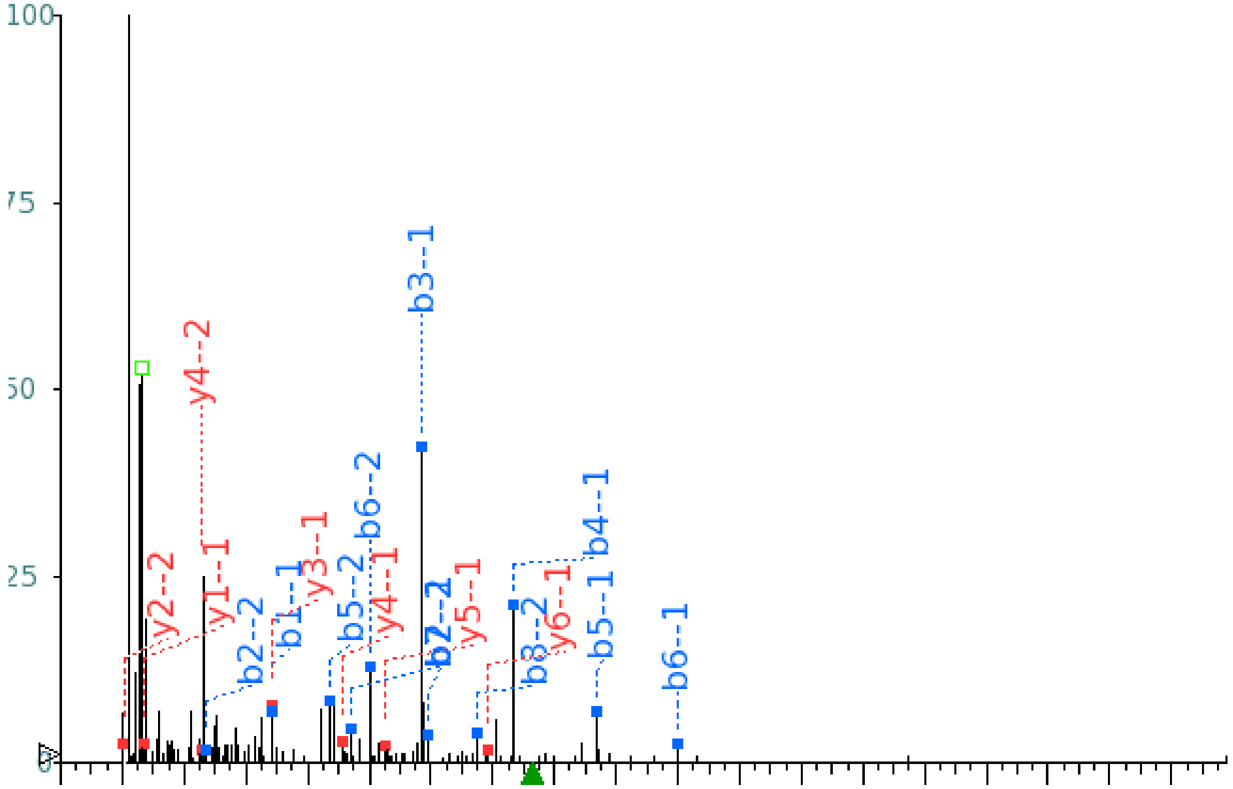

LQDFHQWYLAAYADHAD (+3)

Primary Reference: CNDH2_HUMAN
Search ID: 41760
Search Name: 20130328_ananiav_ERstress_TMT_TM_timecourse_trypsin_inj1_4mc_iaa
Scan#: 18912
Observed Mass: 765.038 (5.2 ppm)
PSM Score: 15.93

File name: 2015-02-04 04.01.53PM.pdf

Creation time: 2015-02-04 04:09PM

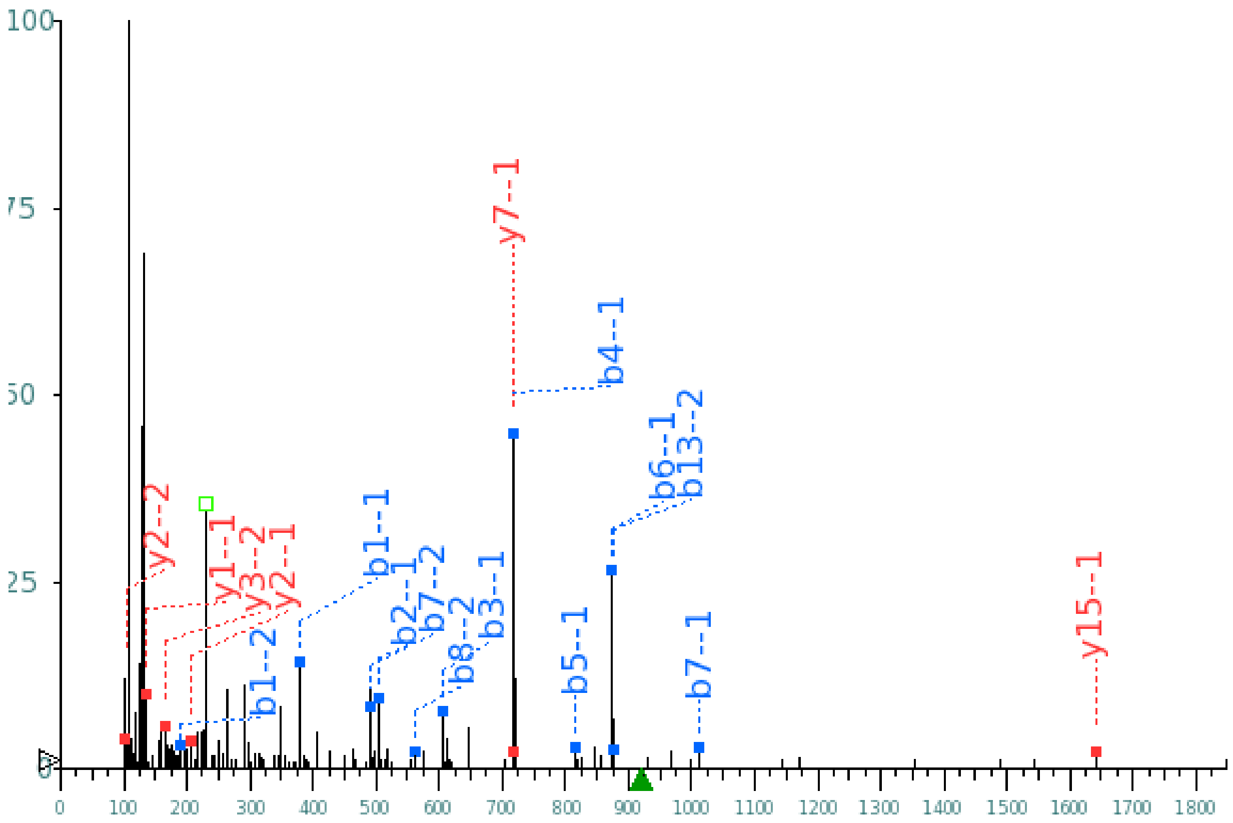

FLNDPGHLLWGDRPEAQLGSQAD (+3)

Primary Reference: RPP21_HUMAN
Search ID: 41760
Search Name: 20130328_ananiav_ERstress_TMT_TM_timecourse_trypsin_inj1_4mc_iaa
Scan#: 18943
Observed Mass: 922.4653 (-1.1 ppm)
PSM Score: 19.49

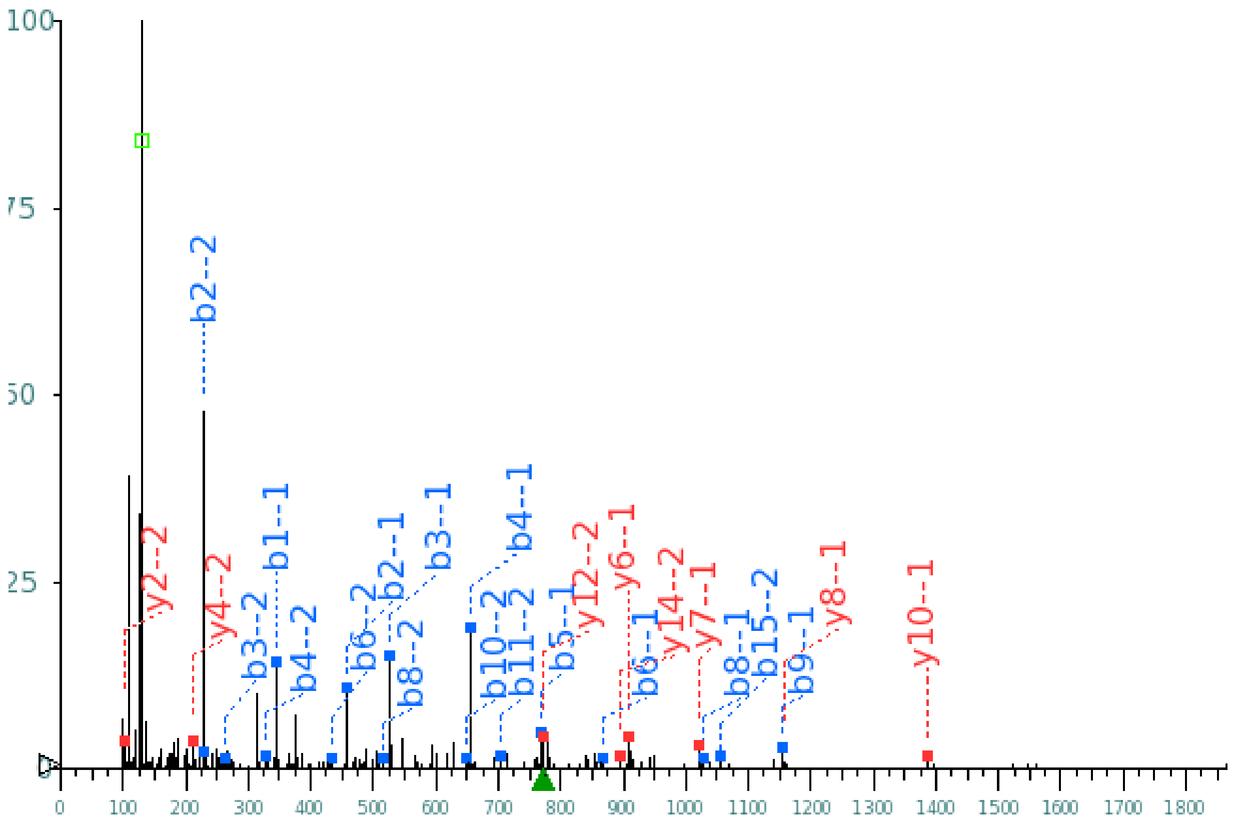

ILAQITGTEHLKESEAD (+3)

Primary Reference: PDLI5_HUMAN
Search ID: 41760
Search Name: 20130328_ananiav_ERstress_TMT_TM_timecourse_trypsin_inj1_4mc_iaa
Scan#: 19030
Observed Mass: 771.7644 (3.7 ppm)
PSM Score: 28.12

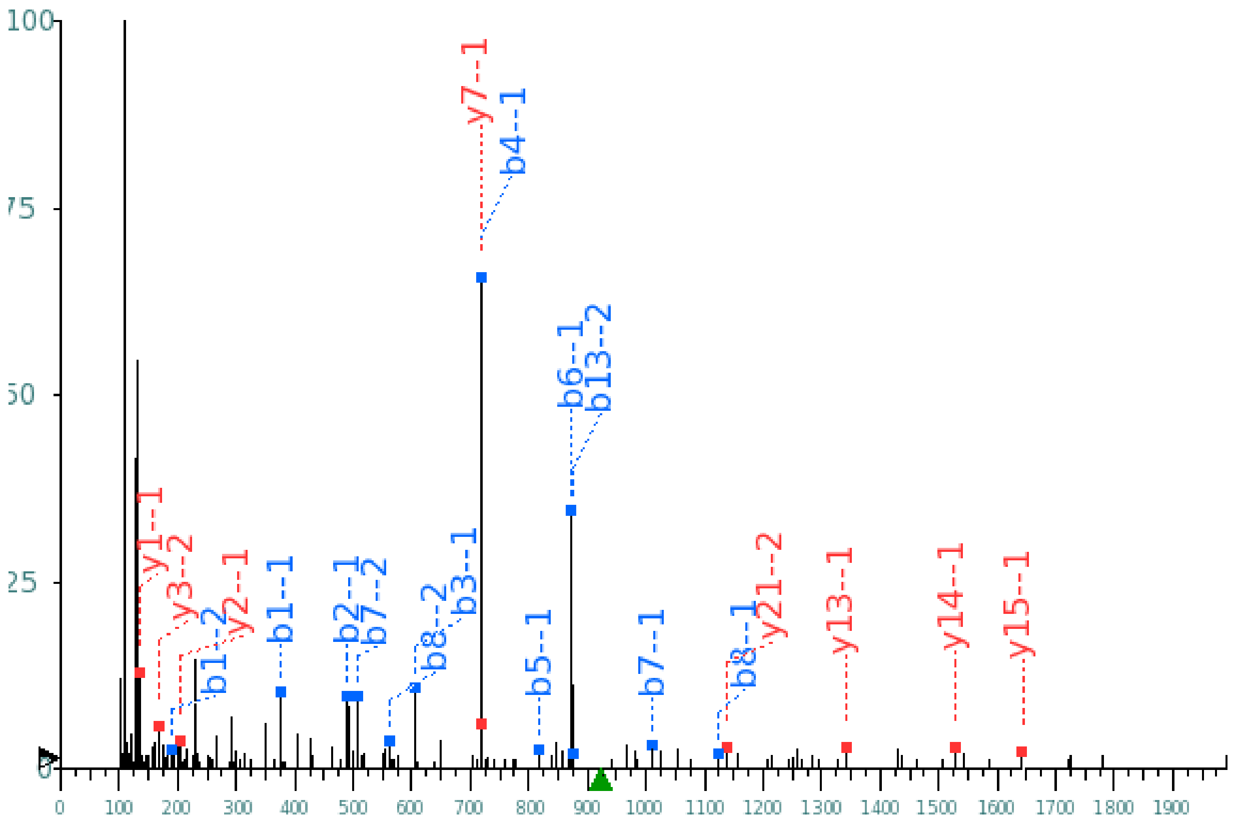

FLNDPGHLLWGDRPEAQLGSQAD (+3)

Primary Reference: RPP21_HUMAN
Search ID: 41760
Search Name: 20130328_ananiav_ERstress_TMT_TM_timecourse_trypsin_inj1_4mc_iaa
Scan#: 19070
Observed Mass: 922.4693 (3.2 ppm)
PSM Score: 29.26

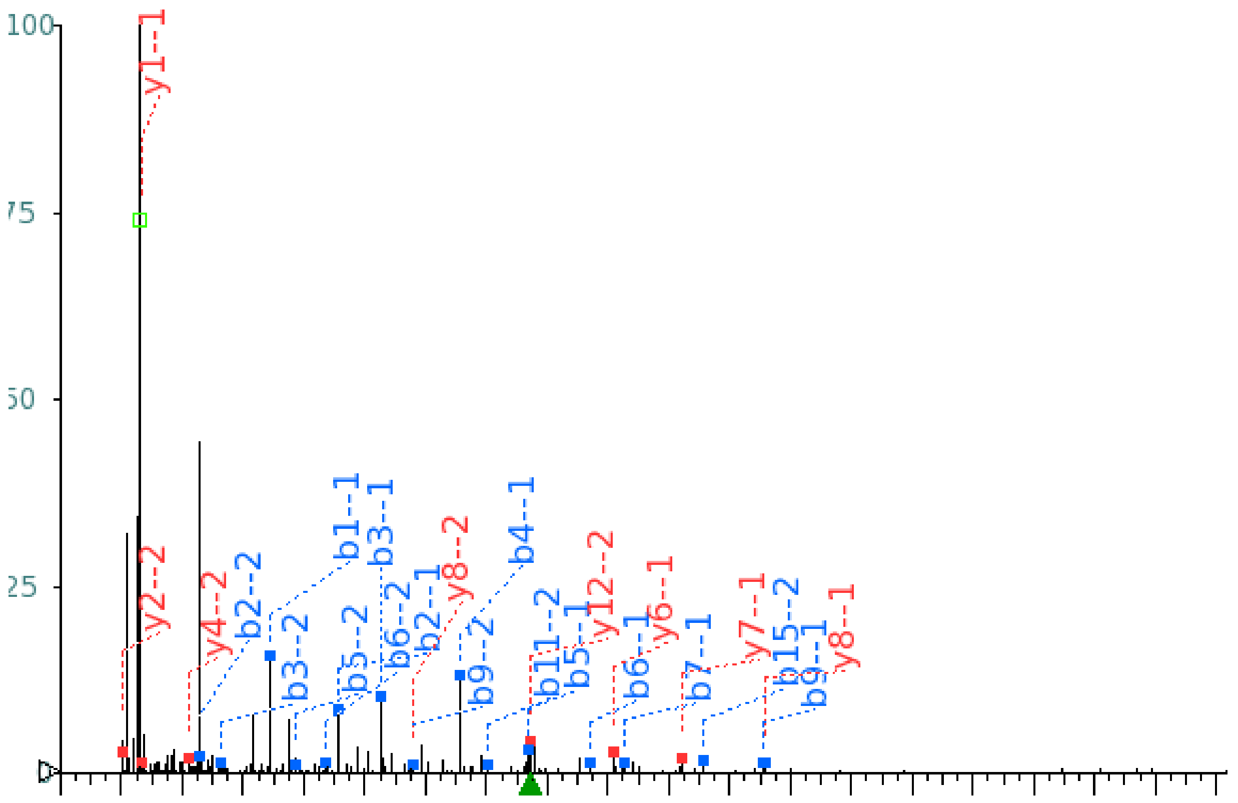

ILAQITGTEHLKESEAD (+3)

Primary Reference: PDLI5_HUMAN
Search ID: 41760
Search Name: 20130328_ananiav_ERstress_TMT_TM_timecourse_trypsin_inj1_4mc_iaa
Scan#: 19100
Observed Mass: 771.7636 (2.7 ppm)
PSM Score: 24.57

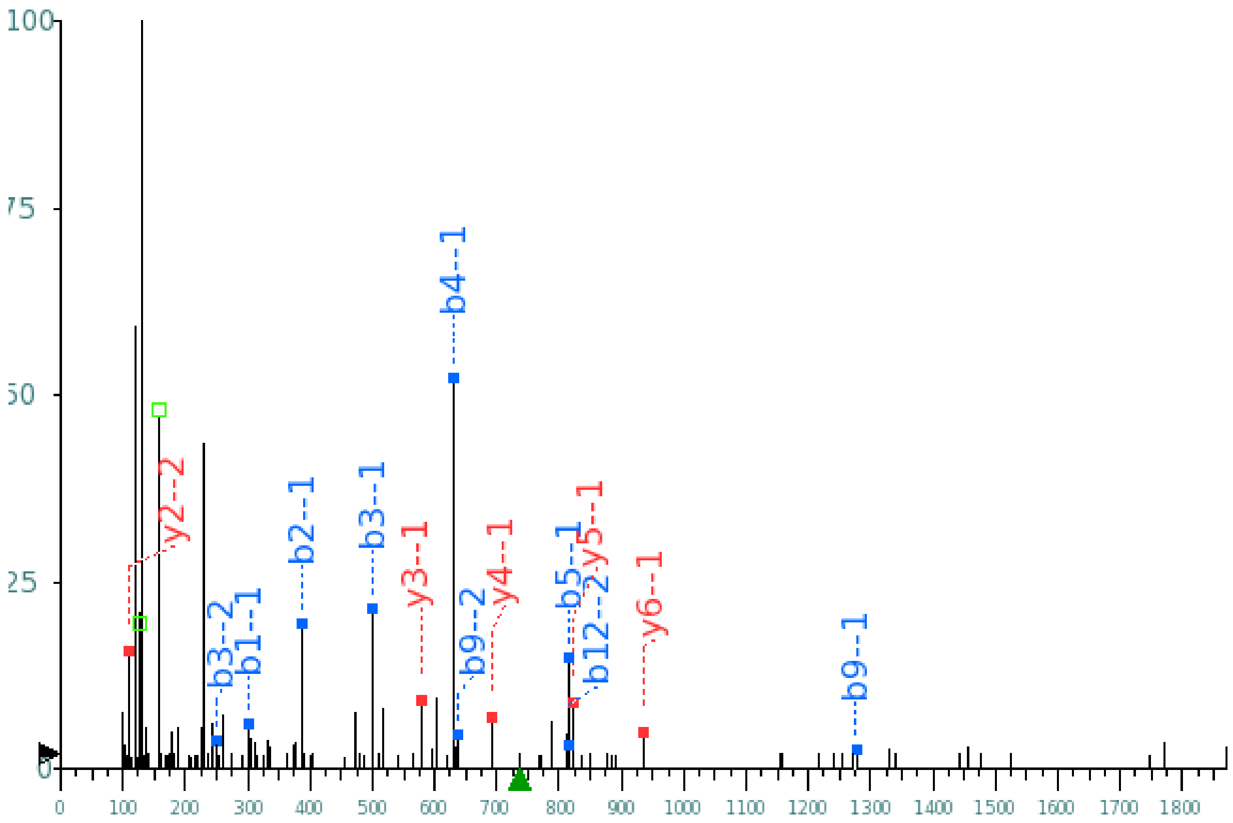

ASLQWFNQALEDKSD (+3)

Primary Reference: P121A_HUMAN
Search ID: 41760
Search Name: 20130328_ananiav_ERstress_TMT_TM_timecourse_trypsin_inj1_4mc_iaa
Scan#: 19149
Observed Mass: 737.3908 (3.9 ppm)
PSM Score: 13.89

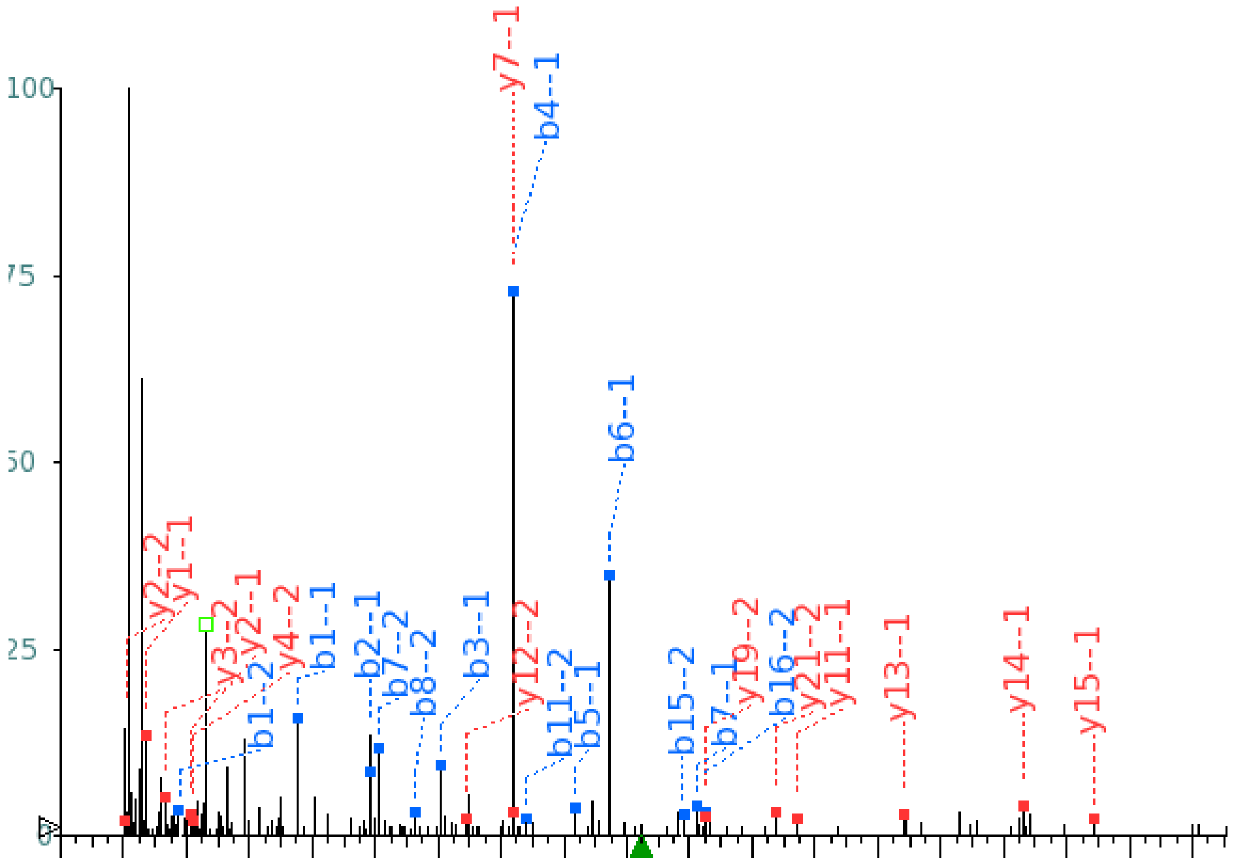

FLNDPGHLLWGDRPEAQLGSQAD (+3)

Primary Reference: RPP21_HUMAN
Search ID: 41760
Search Name: 20130328_ananiav_ERstress_TMT_TM_timecourse_trypsin_inj1_4mc_iaa
Scan#: 19152
Observed Mass: 922.4699 (3.9 ppm)
PSM Score: 22.24

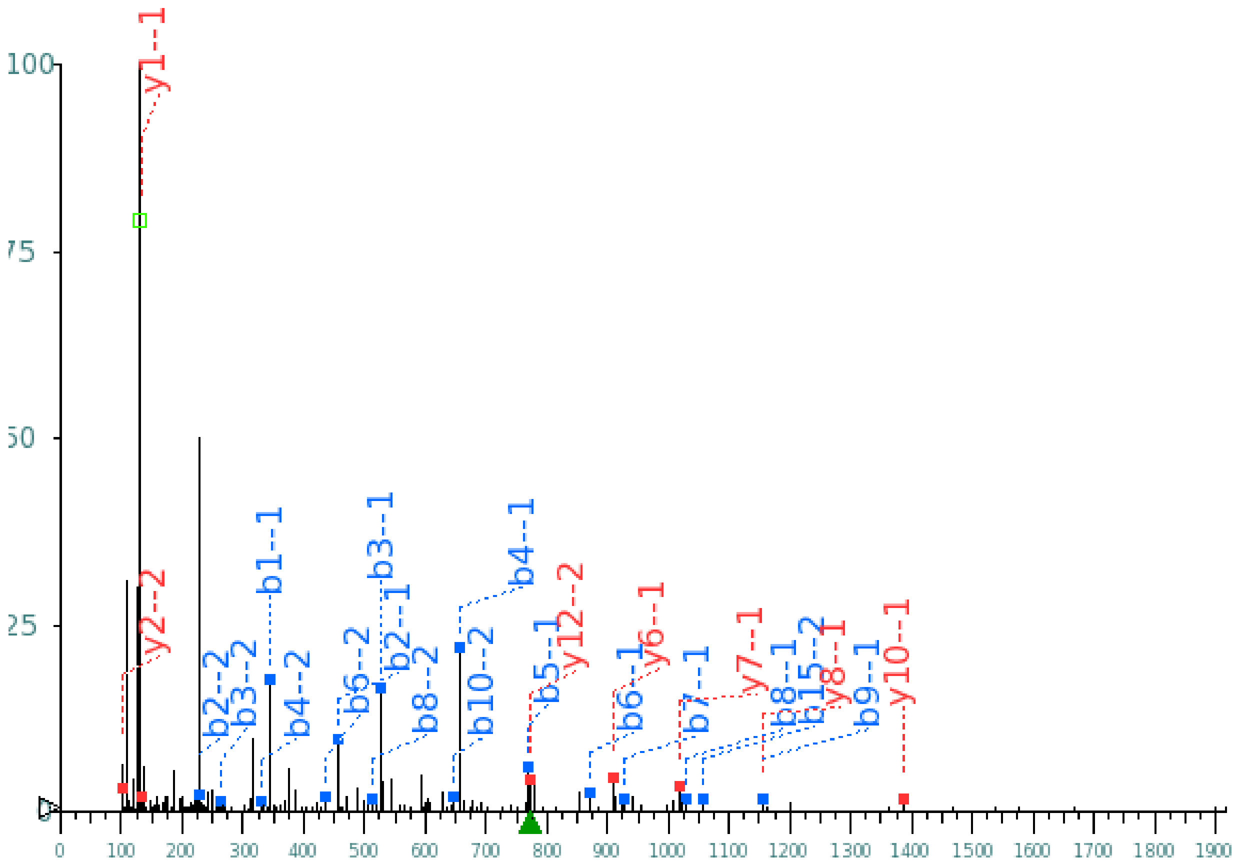

ILAQITGTEHLKESEAD (+3)

Primary Reference: PDLI5_HUMAN
Search ID: 41760
Search Name: 20130328_ananiav_ERstress_TMT_TM_timecourse_trypsin_inj1_4mc_iaa
Scan#: 19162
Observed Mass: 771.7621 (0.6 ppm)
PSM Score: 21.49

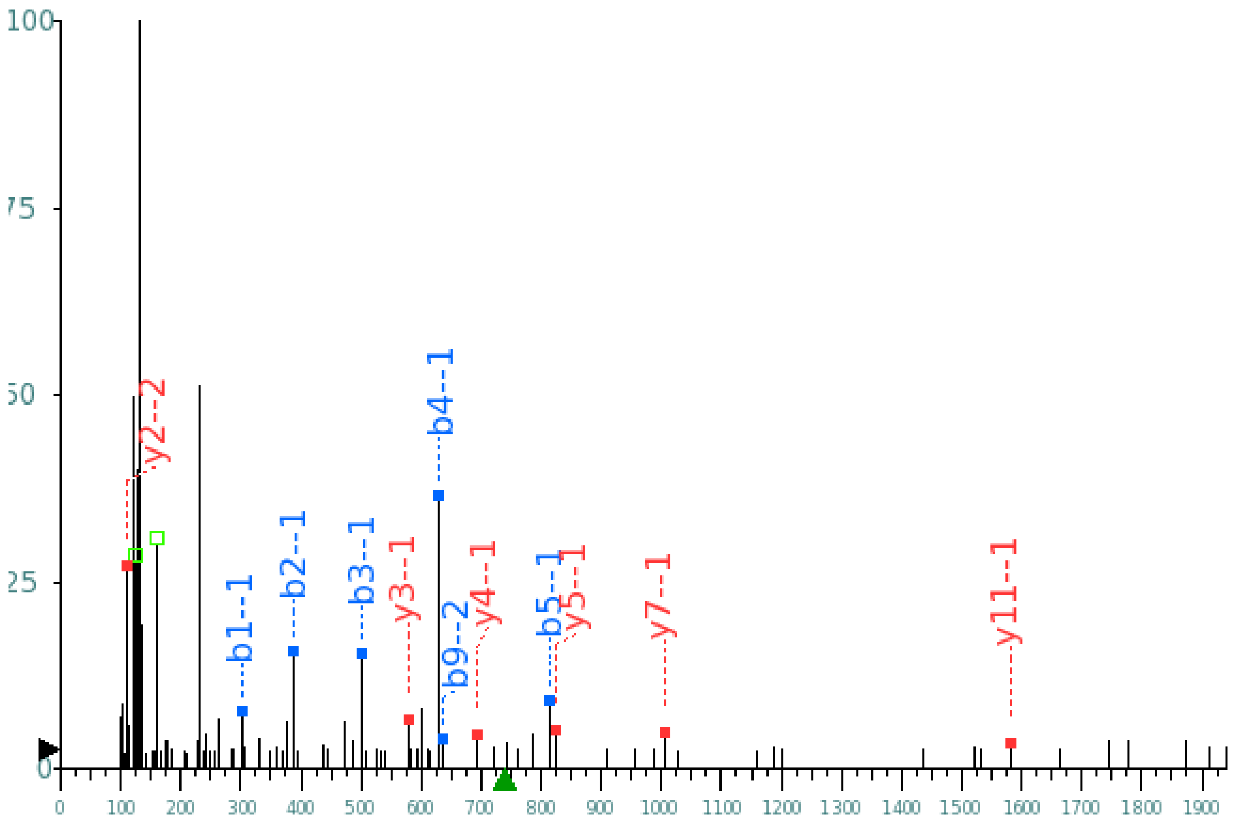

ASLQWFNQALEDKSD (+3)

Primary Reference: P121A_HUMAN
Search ID: 41760
Search Name: 20130328_ananiav_ERstress_TMT_TM_timecourse_trypsin_inj1_4mc_iaa
Scan#: 19214
Observed Mass: 737.3906 (3.6 ppm)
PSM Score: 11.63

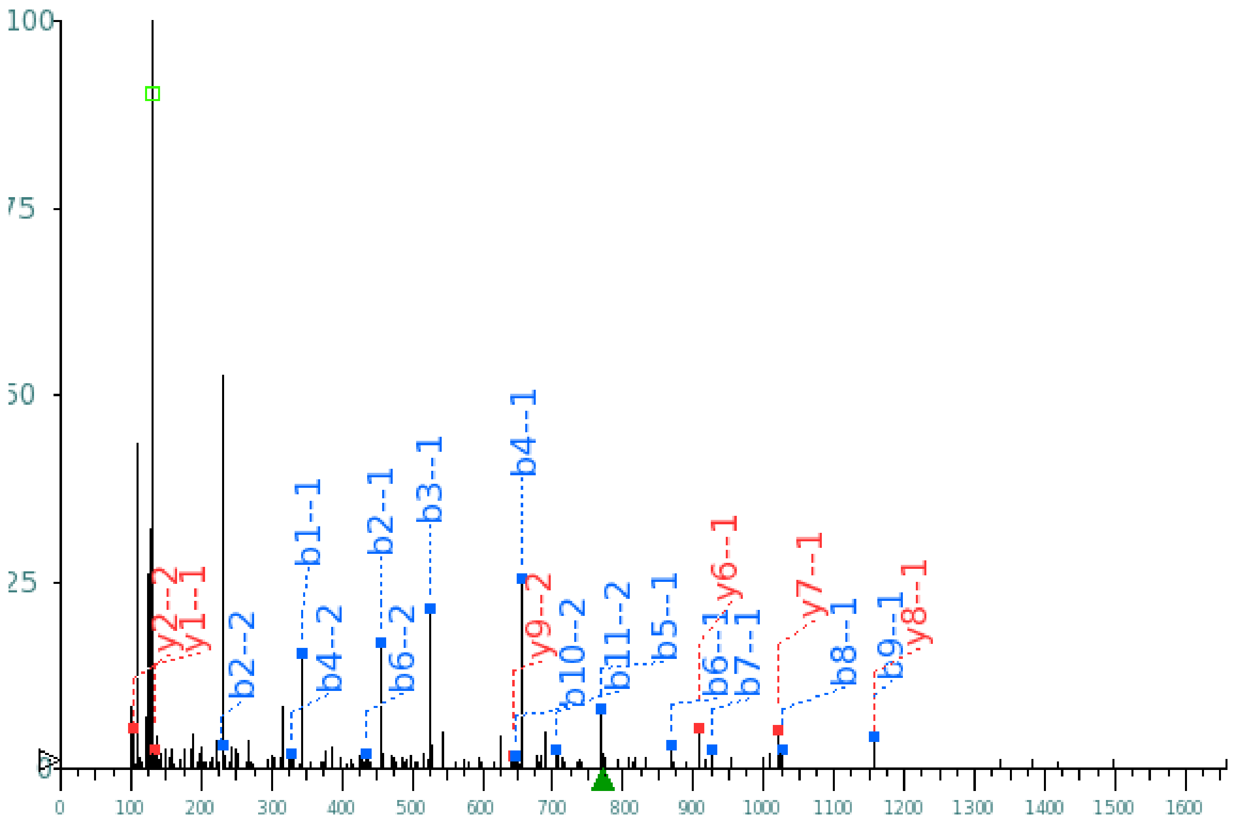

ILAQITGTEHLKESEAD (+3)

Primary Reference: PDLI5_HUMAN
Search ID: 41760
Search Name: 20130328_ananiav_ERstress_TMT_TM_timecourse_trypsin_inj1_4mc_iaa
Scan#: 19231
Observed Mass: 771.7647 (4.1 ppm)
PSM Score: 41.1

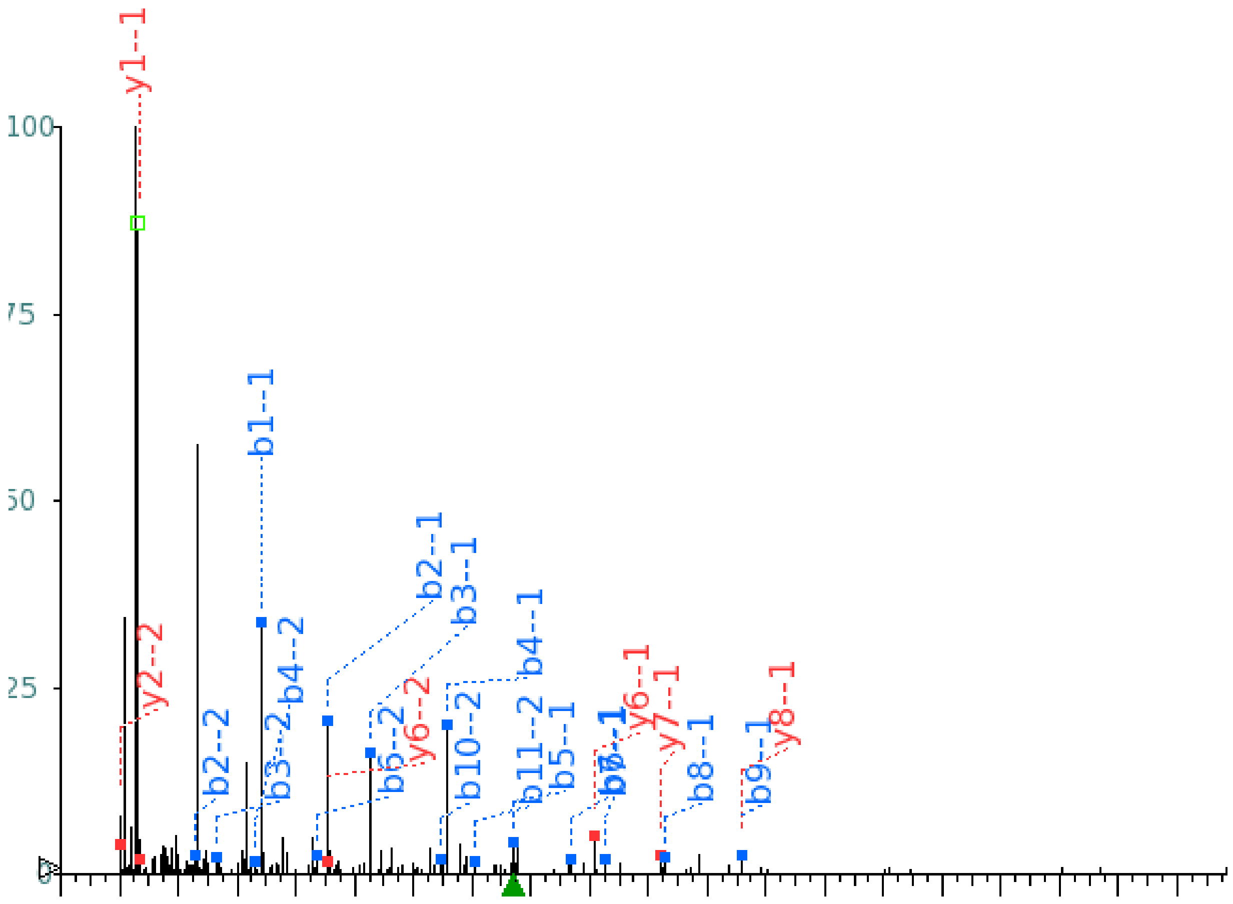

ILAQITGTEHLKESEAD (+3)

Primary Reference: PDLI5_HUMAN
Search ID: 41760
Search Name: 20130328_ananiav_ERstress_TMT_TM_timecourse_trypsin_inj1_4mc_iaa
Scan#: 19308
Observed Mass: 771.7647 (4 ppm)
PSM Score: 25.74

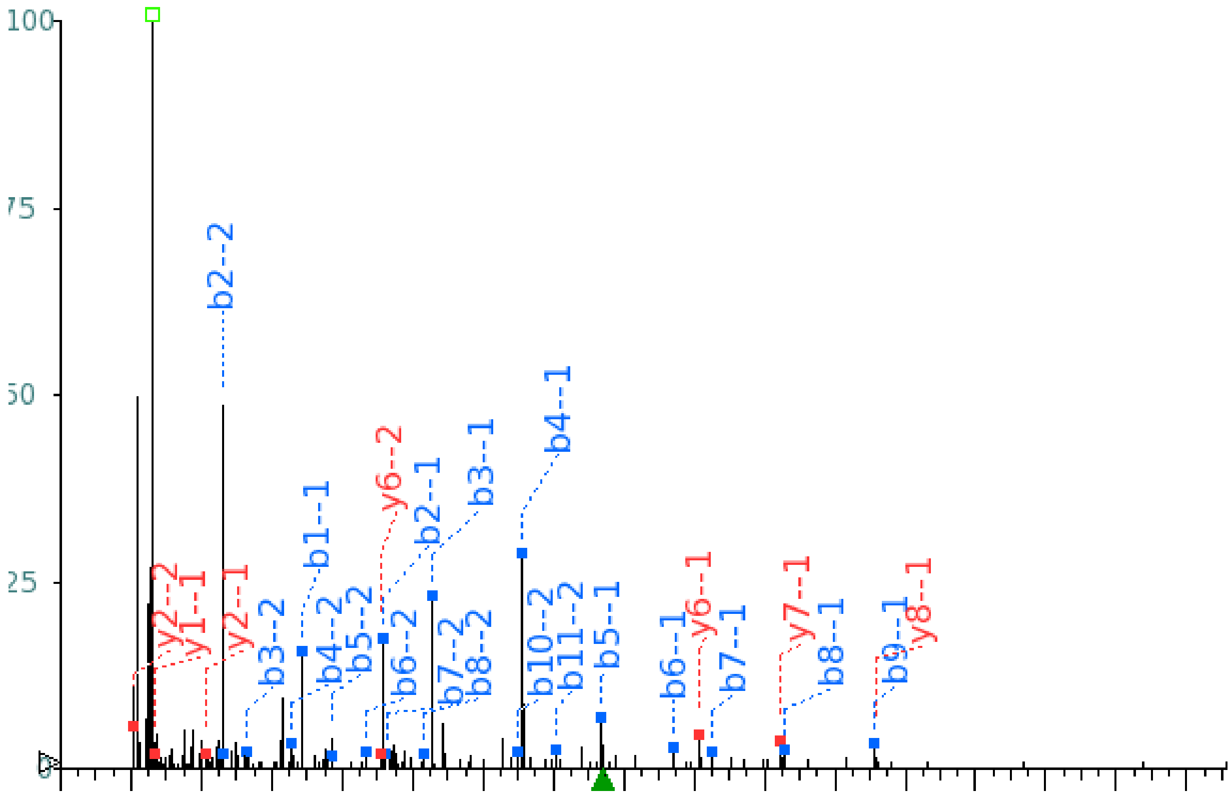

ILAQITGTEHLKESEAD (+3)

Primary Reference: PDLI5_HUMAN
Search ID: 41760
Search Name: 20130328_ananiav_ERstress_TMT_TM_timecourse_trypsin_inj1_4mc_iaa
Scan#: 19381
Observed Mass: 771.7635 (2.5 ppm)
PSM Score: 42.33

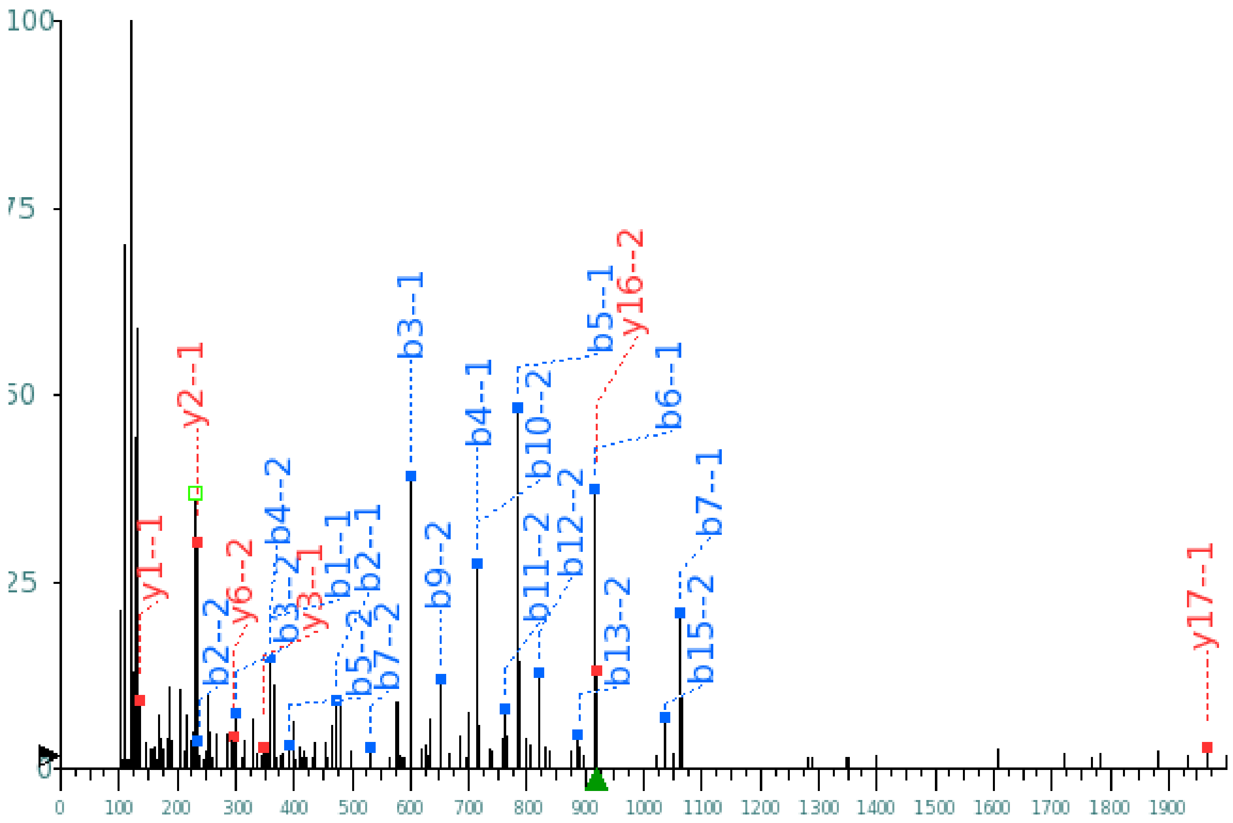

QNEIAMFPHEVNHFFSGSTITD (+3)

Primary Reference: OSBL3_HUMAN
Search ID: 41760
Search Name: 20130328_ananiav_ERstress_TMT_TM_timecourse_trypsin_inj1_4mc_iaa
Scan#: 19403
Observed Mass: 917.438 (-3.1 ppm)
PSM Score: 29.43

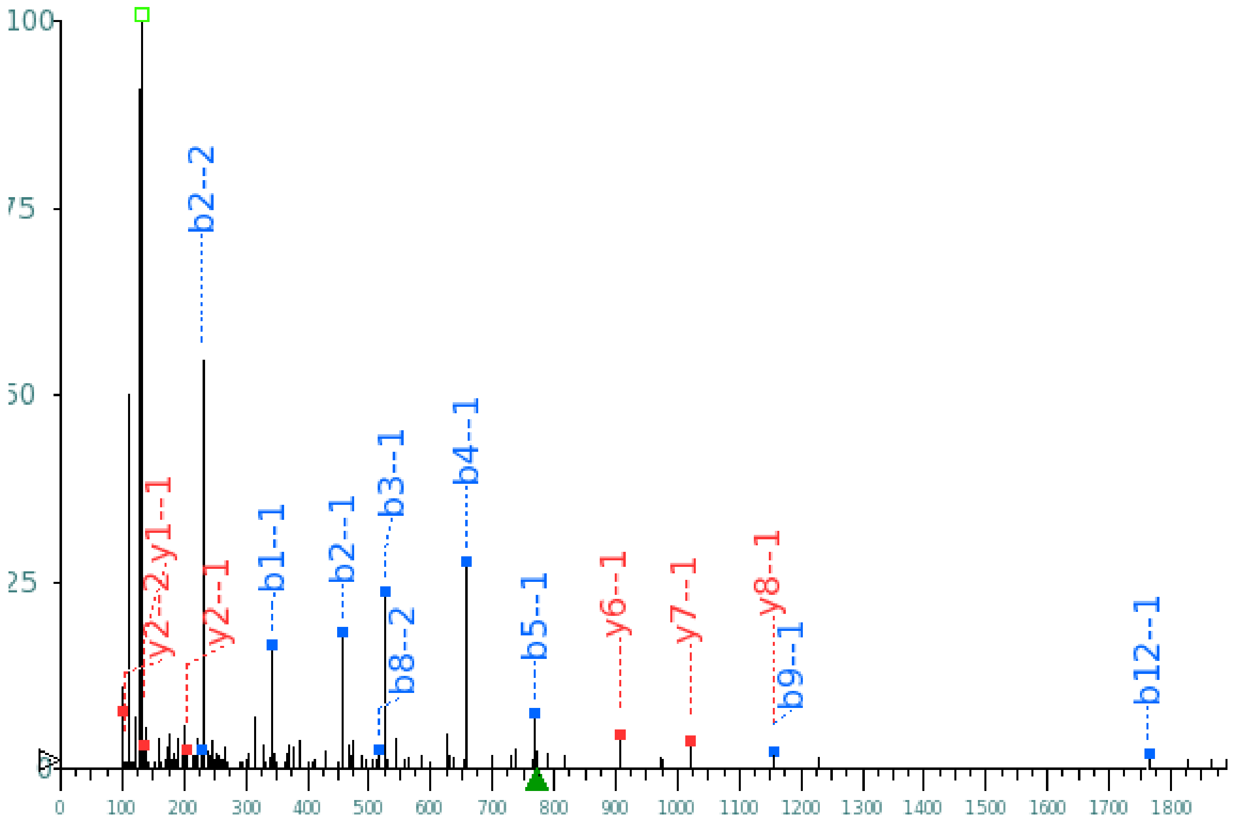

ILAQITGTEHLKESEAD (+3)

Primary Reference: PDLI5_HUMAN
Search ID: 41760
Search Name: 20130328_ananiav_ERstress_TMT_TM_timecourse_trypsin_inj1_4mc_iaa
Scan#: 19446
Observed Mass: 771.7639 (3 ppm)
PSM Score: 27.12

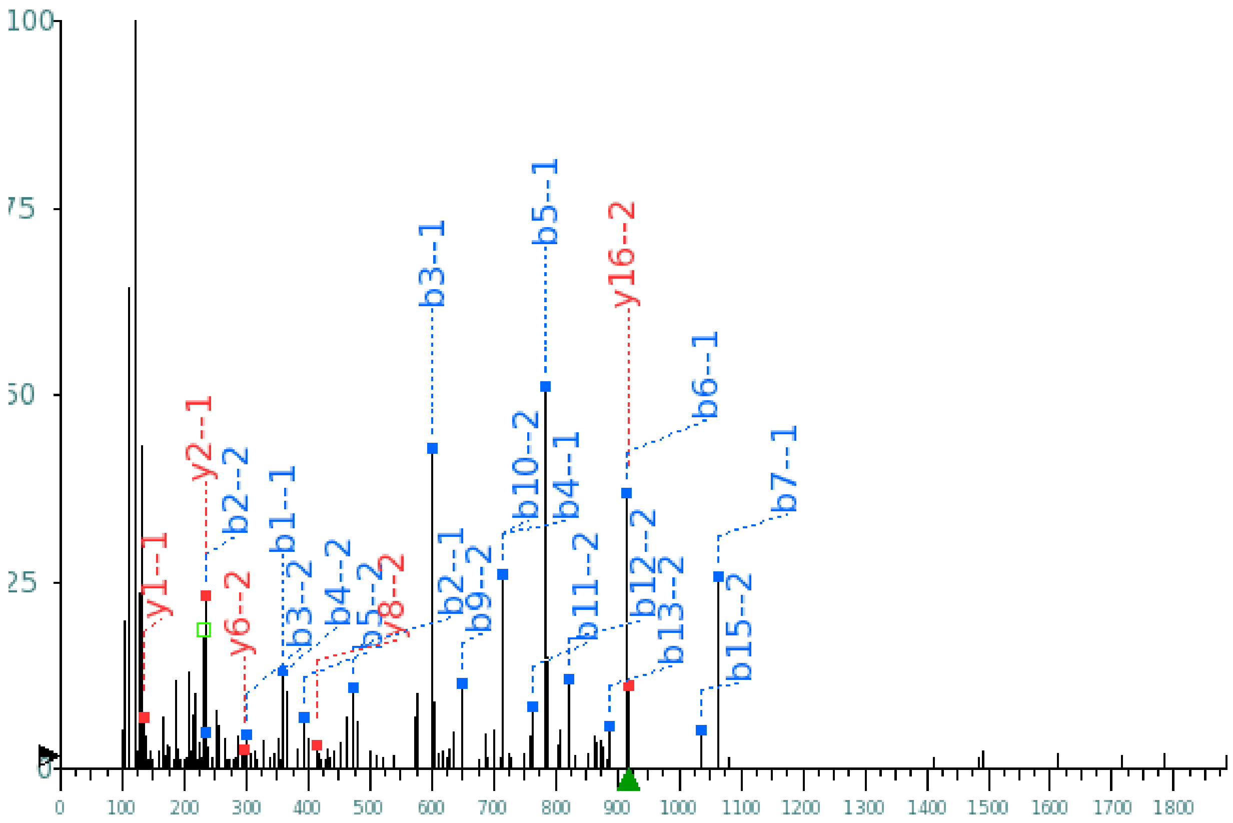

QNEIAMFPHEVNHFFSGSTITD (+3)

Primary Reference: OSBL3_HUMAN
Search ID: 41760
Search Name: 20130328_ananiav_ERstress_TMT_TM_timecourse_trypsin_inj1_4mc_iaa
Scan#: 19467
Observed Mass: 917.4421 (1.3 ppm)
PSM Score: 36.13

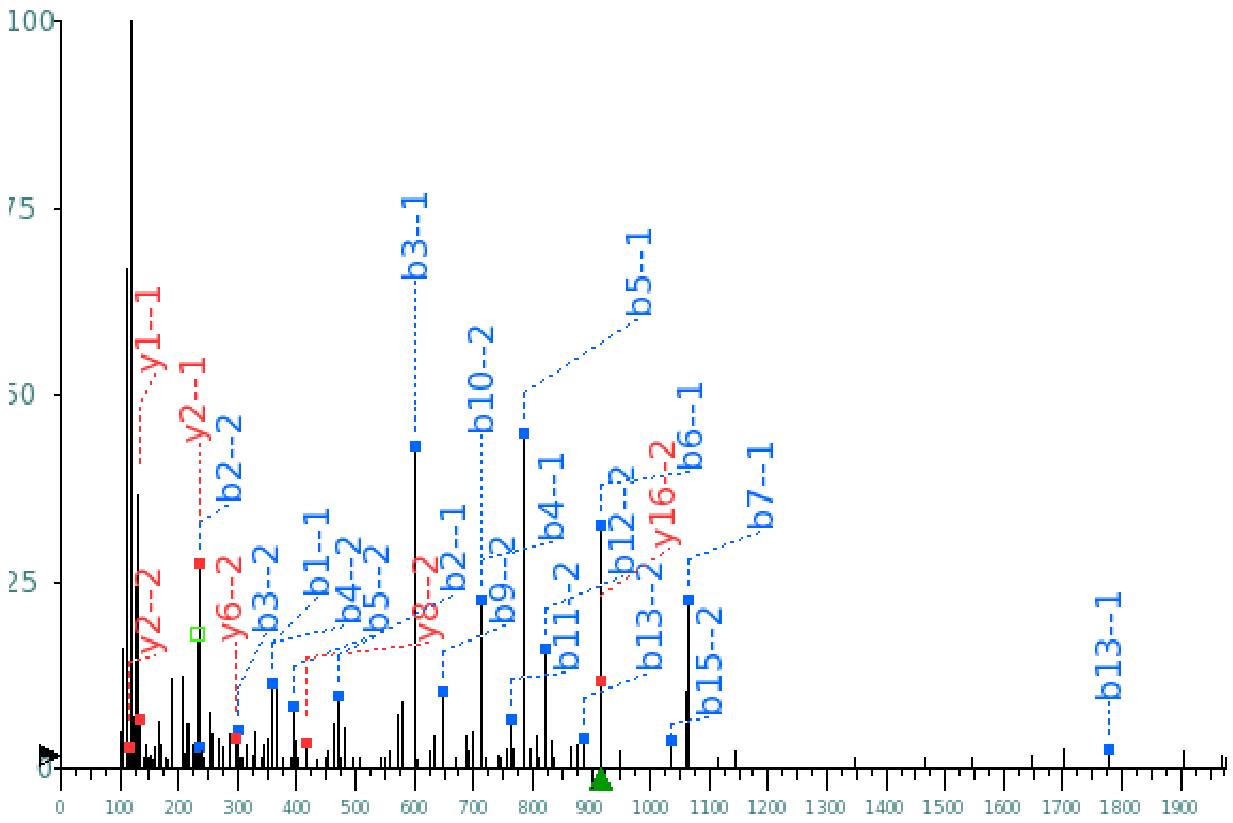

QNEIAMFPHEVNHFFSGSTITD (+3)

Primary Reference: OSBL3_HUMAN
Search ID: 41760
Search Name: 20130328_ananiav_ERstress_TMT_TM_timecourse_trypsin_inj1_4mc_iaa
Scan#: 19532
Observed Mass: 917.4429 (2.1 ppm)
PSM Score: 28.16

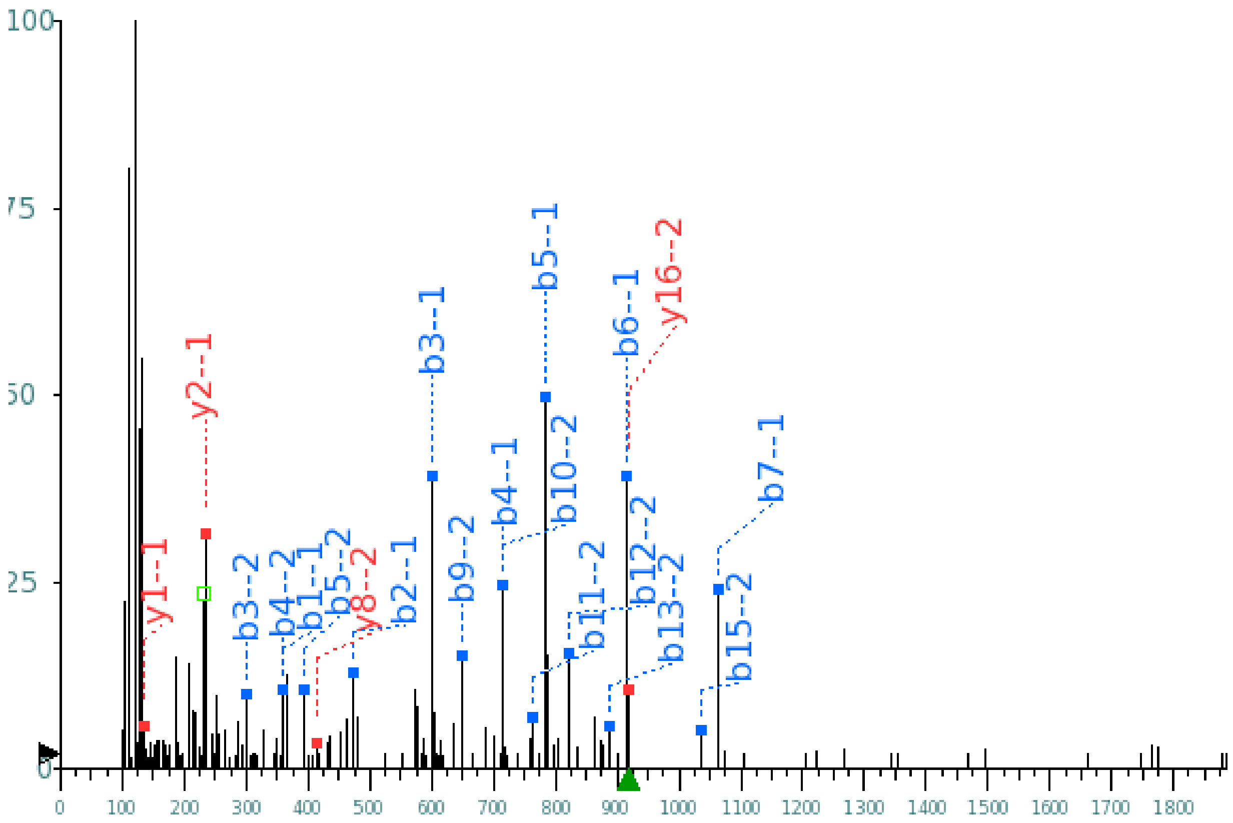

QNEIAMFPHEVNHFFSGSTITD (+3)

Primary Reference: OSBL3_HUMAN
Search ID: 41760
Search Name: 20130328_ananiav_ERstress_TMT_TM_timecourse_trypsin_inj1_4mc_iaa
Scan#: 19600
Observed Mass: 917.443 (2.2 ppm)
PSM Score: 23.84

File name: 2015-02-04 04.01.53PM.pdf

Creation time: 2015-02-04 04:09PM

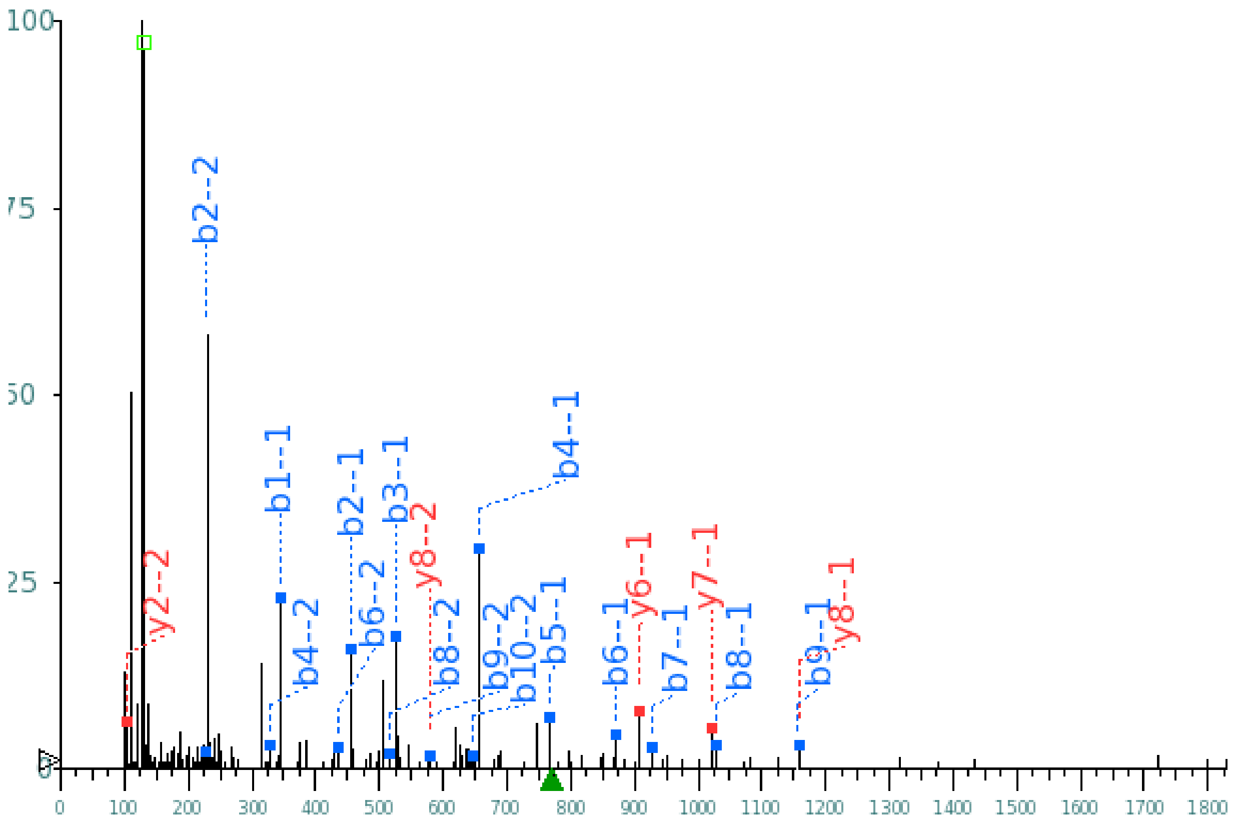

ILAQITGTEHLKESEAD (+3)

Primary Reference: PDLI5_HUMAN
Search ID: 41760
Search Name: 20130328_ananiav_ERstress_TMT_TM_timecourse_trypsin_inj1_4mc_iaa
Scan#: 20020
Observed Mass: 771.7674 (7.5 ppm)
PSM Score: 35.33

File name: 2015-02-04 04.01.53PM.pdf

Creation time: 2015-02-04 04:09PM

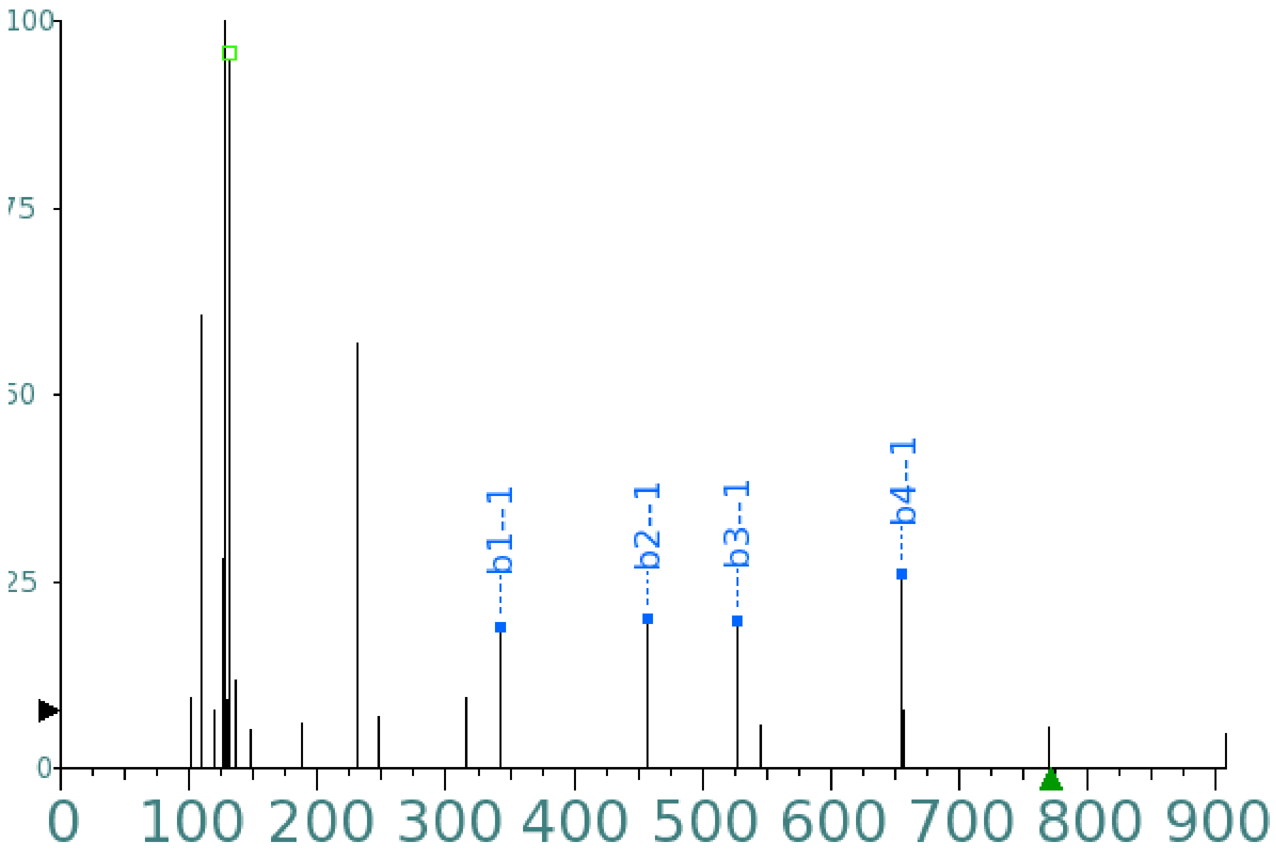

ILAQITGTEHLKESEAD (+3)

Primary Reference: PDLI5_HUMAN
Search ID: 41760
Search Name: 20130328_ananiav_ERstress_TMT_TM_timecourse_trypsin_inj1_4mc_iaa
Scan#: 20187
Observed Mass: 771.7652 (4.7 ppm)
PSM Score: 24.03

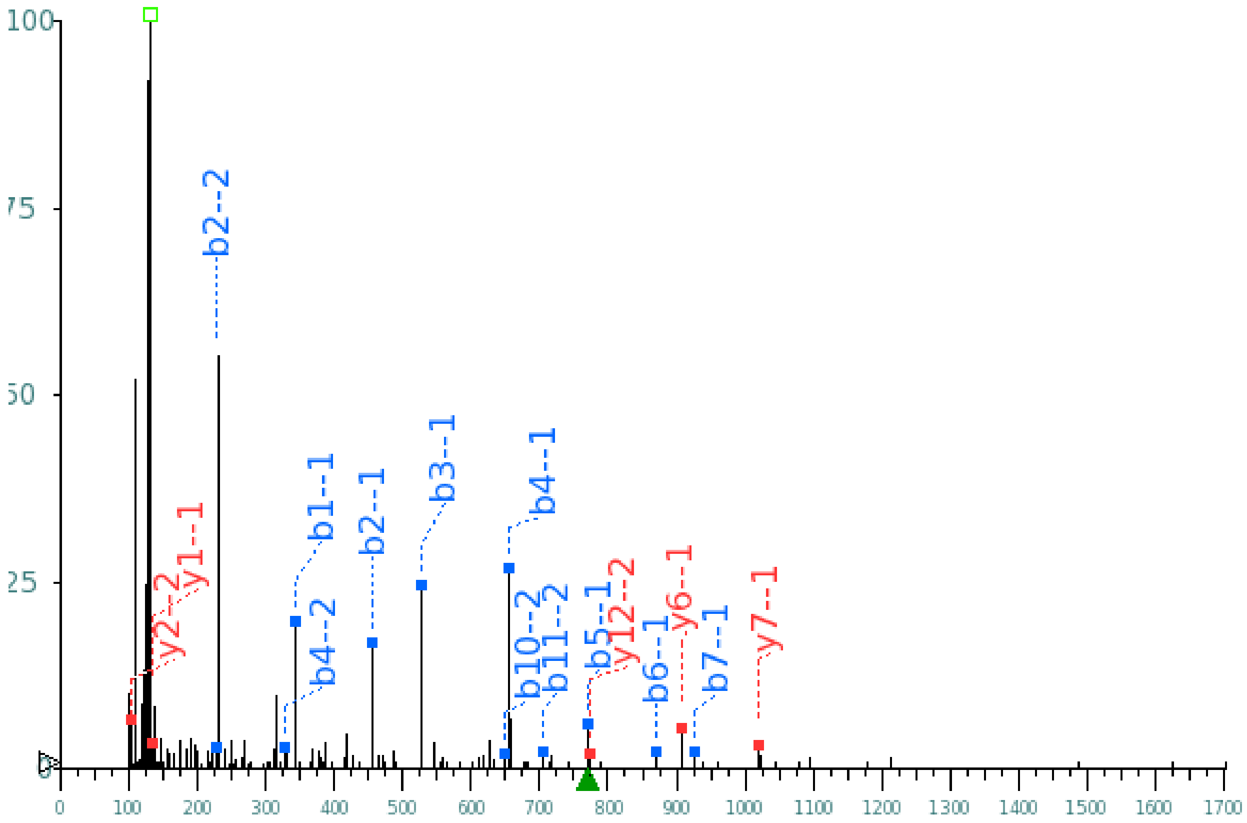

ILAQITGTEHLKESEAD (+3)

Primary Reference: PDLI5_HUMAN
Search ID: 41760
Search Name: 20130328_ananiav_ERstress_TMT_TM_timecourse_trypsin_inj1_4mc_iaa
Scan#: 20233
Observed Mass: 771.7654 (4.9 ppm)
PSM Score: 24.84

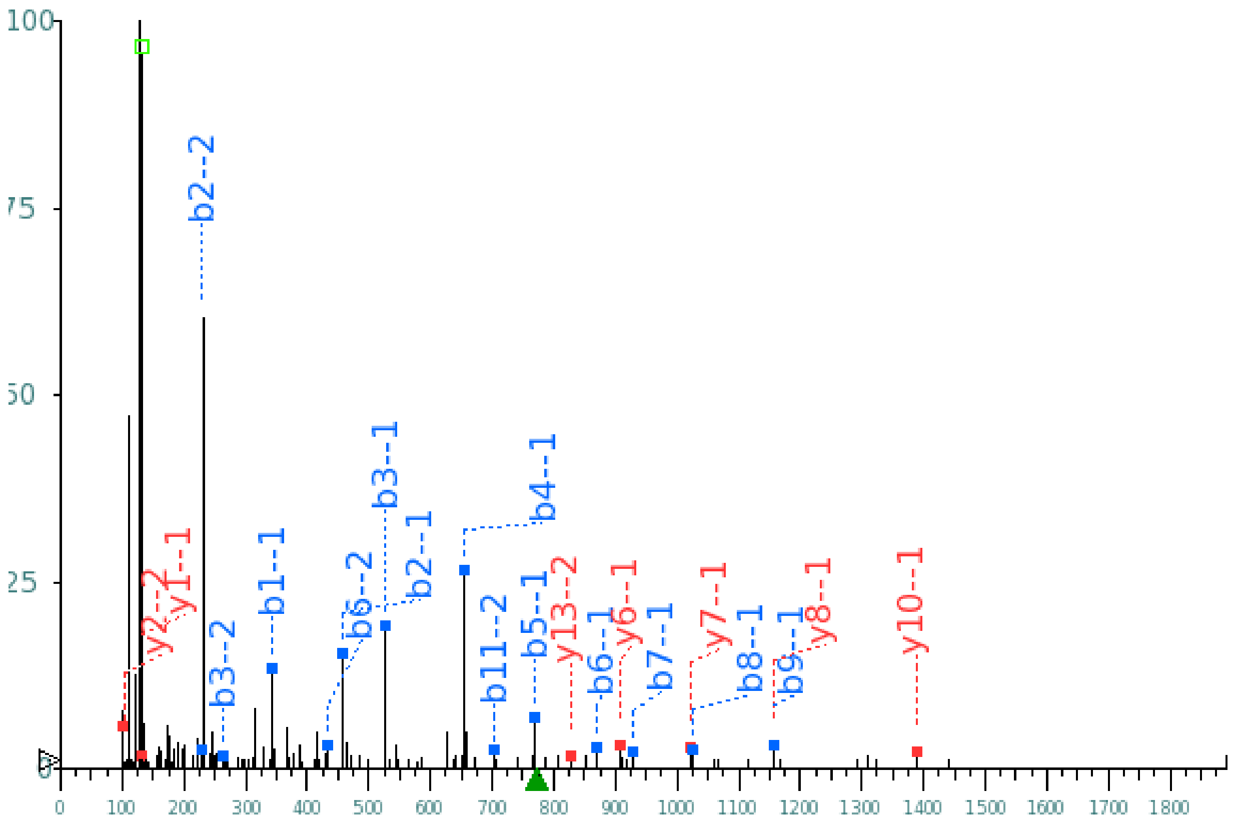

ILAQITGTEHLKESEAD (+3)

Primary Reference: PDLI5_HUMAN
Search ID: 41760
Search Name: 20130328_ananiav_ERstress_TMT_TM_timecourse_trypsin_inj1_4mc_iaa
Scan#: 20271
Observed Mass: 771.7668 (6.7 ppm)
PSM Score: 44.48

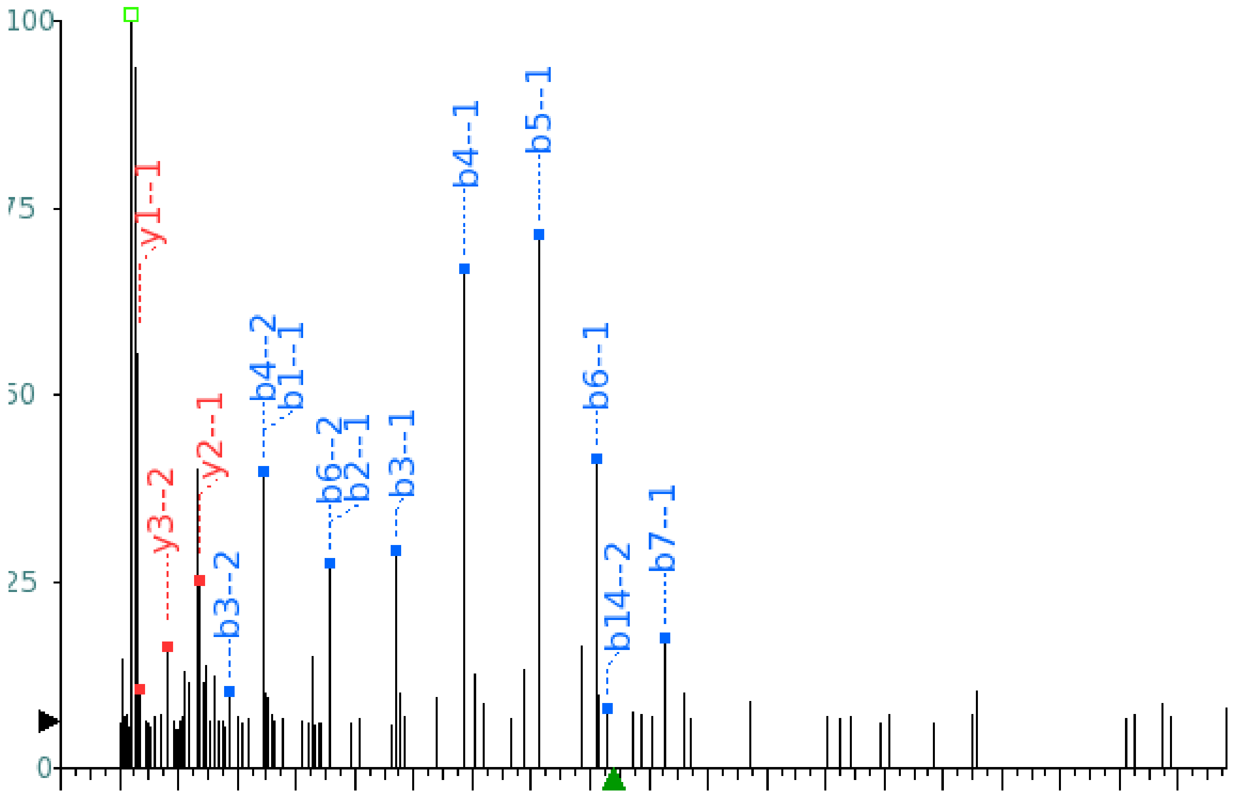

NLNDQVLFIDQGNRPLFEDMTD (+3)

Primary Reference: IL18_HUMAN
Search ID: 41760
Search Name: 20130328_ananiav_ERstress_TMT_TM_timecourse_trypsin_inj1_4mc_iaa
Scan#: 20303
Observed Mass: 941.8007 (1.9 ppm)
PSM Score: 28.07

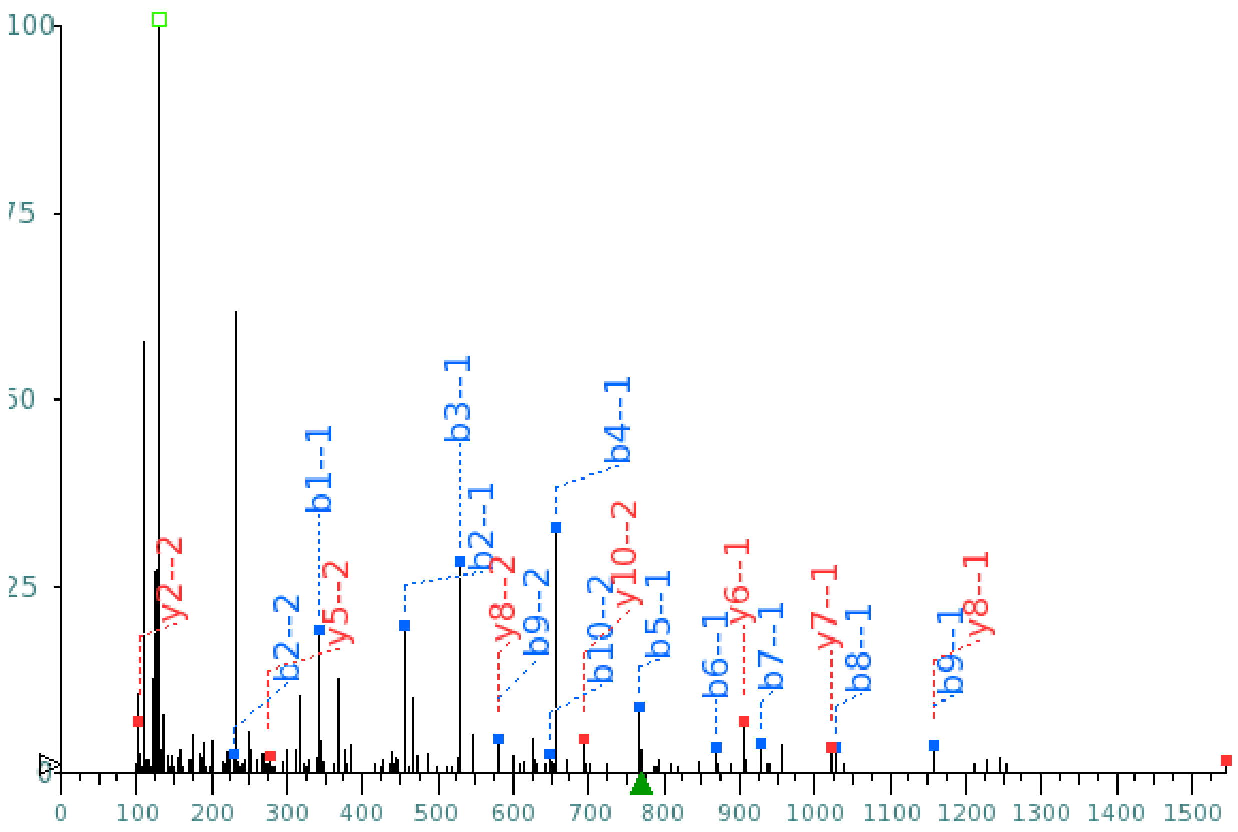

ILAQITGTEHLKESEAD (+3)

Primary Reference: PDLI5_HUMAN
Search ID: 41760
Search Name: 20130328_ananiav_ERstress_TMT_TM_timecourse_trypsin_inj1_4mc_iaa
Scan#: 20331
Observed Mass: 771.7645 (3.8 ppm)
PSM Score: 54.56

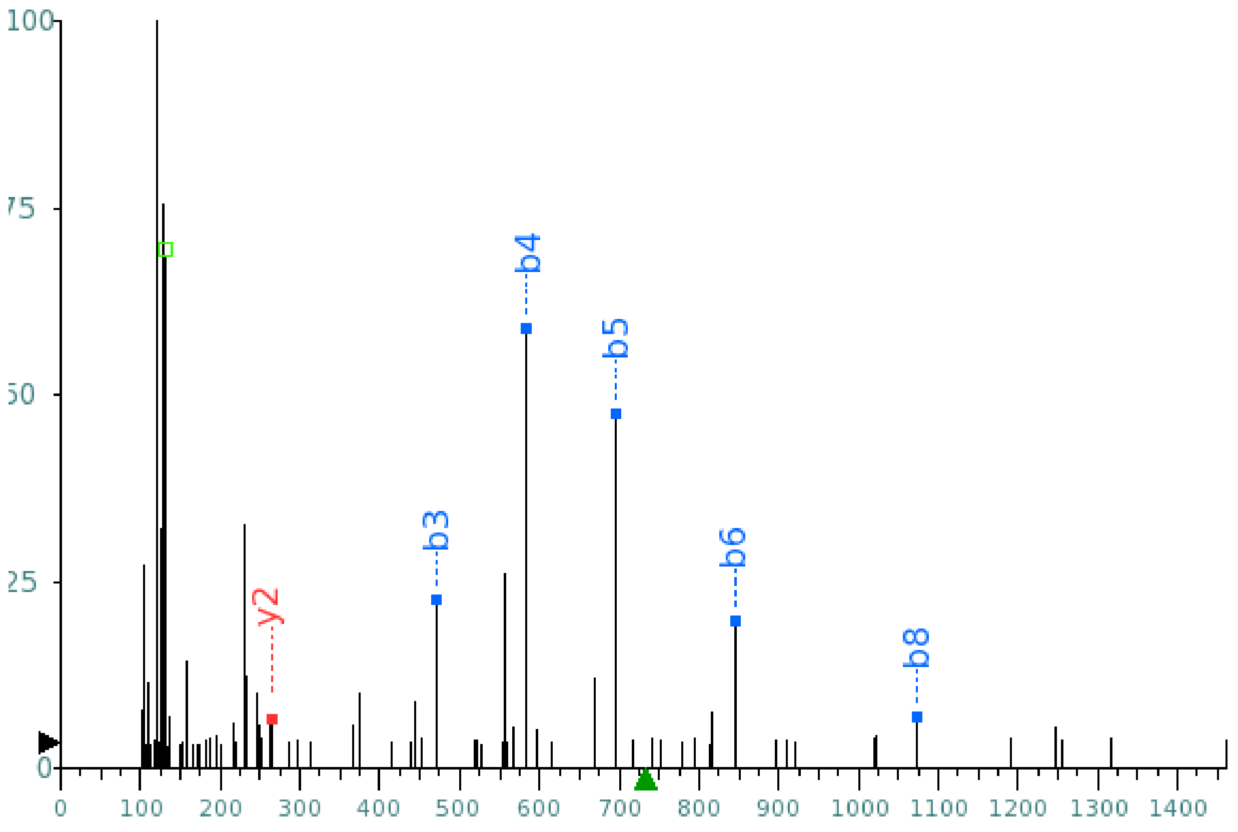

GPSLLFLDEMD (+2)

Primary Reference: SPA5L_HUMAN
Search ID: 41760
Search Name: 20130328_ananiav_ERstress_TMT_TM_timecourse_trypsin_inj1_4mc_iaa
Scan#: 20433
Observed Mass: 733.3783 (3.2 ppm)
PSM Score: 16.67

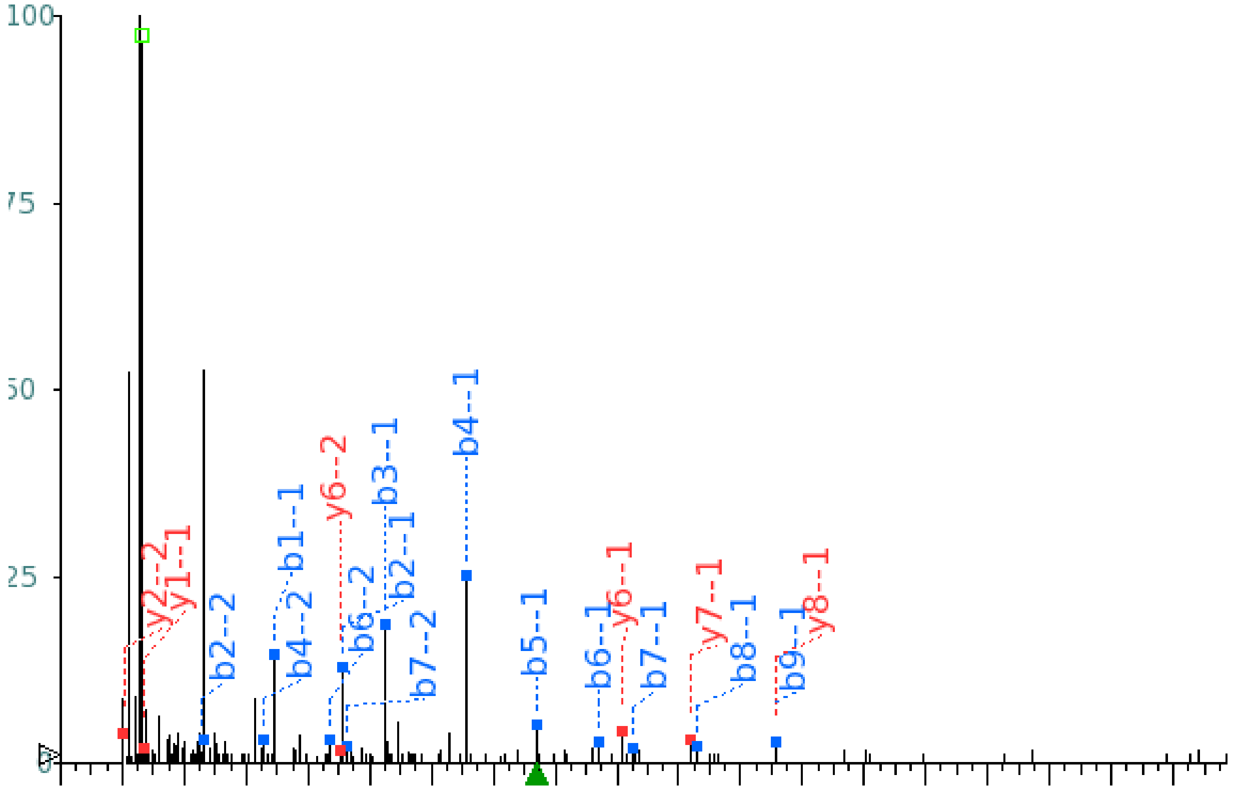

ILAQITGTEHLKESEAD (+3)

Primary Reference: PDLI5_HUMAN
Search ID: 41760
Search Name: 20130328_ananiav_ERstress_TMT_TM_timecourse_trypsin_inj1_4mc_iaa
Scan#: 20584
Observed Mass: 771.7628 (1.6 ppm)
PSM Score: 35.83

File name: 2015-02-04 04.01.53PM.pdf

Creation time: 2015-02-04 04:09PM

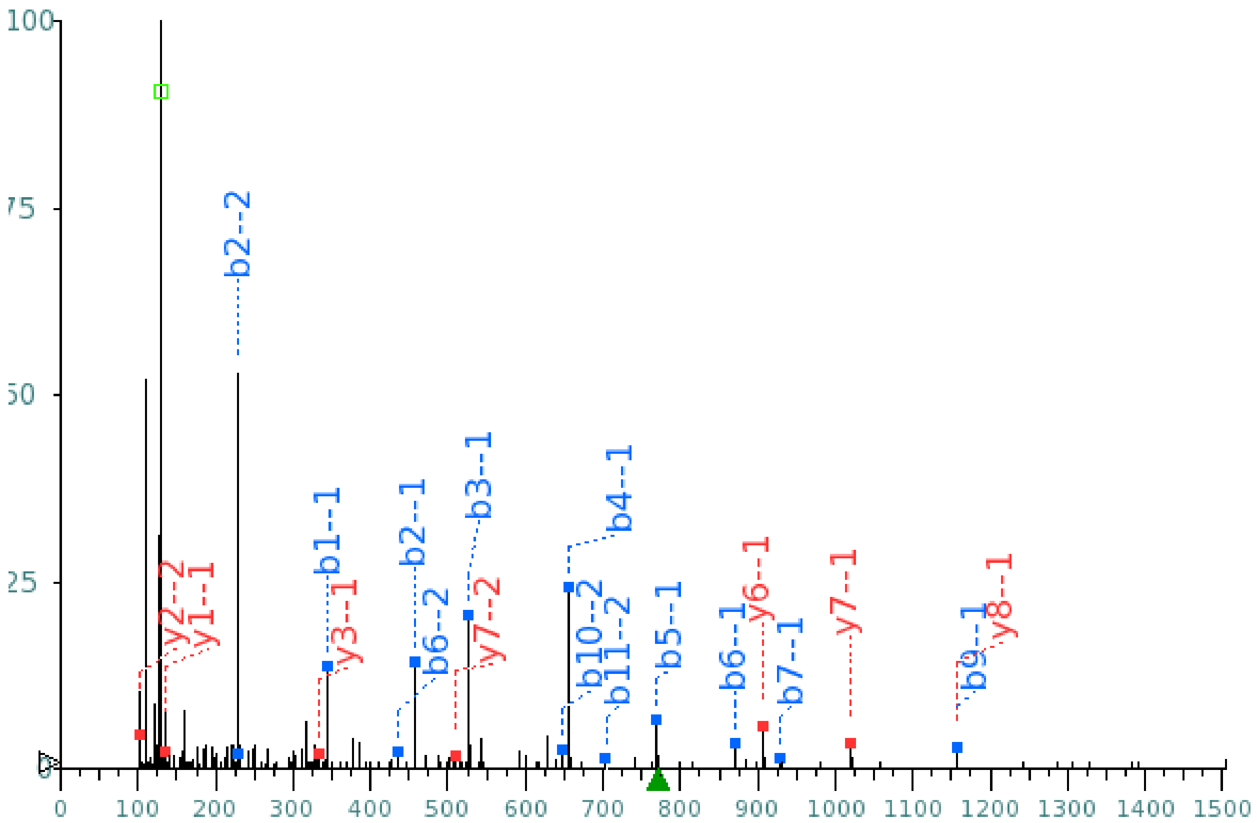

ILAQITGTEHLKESEAD (+3)

Primary Reference: PDLI5_HUMAN
Search ID: 41760
Search Name: 20130328_ananiav_ERstress_TMT_TM_timecourse_trypsin_inj1_4mc_iaa
Scan#: 20689
Observed Mass: 771.7623 (1 ppm)
PSM Score: 41.61

File name: 2015-02-04 04.01.53PM.pdf

Creation time: 2015-02-04 04:09PM

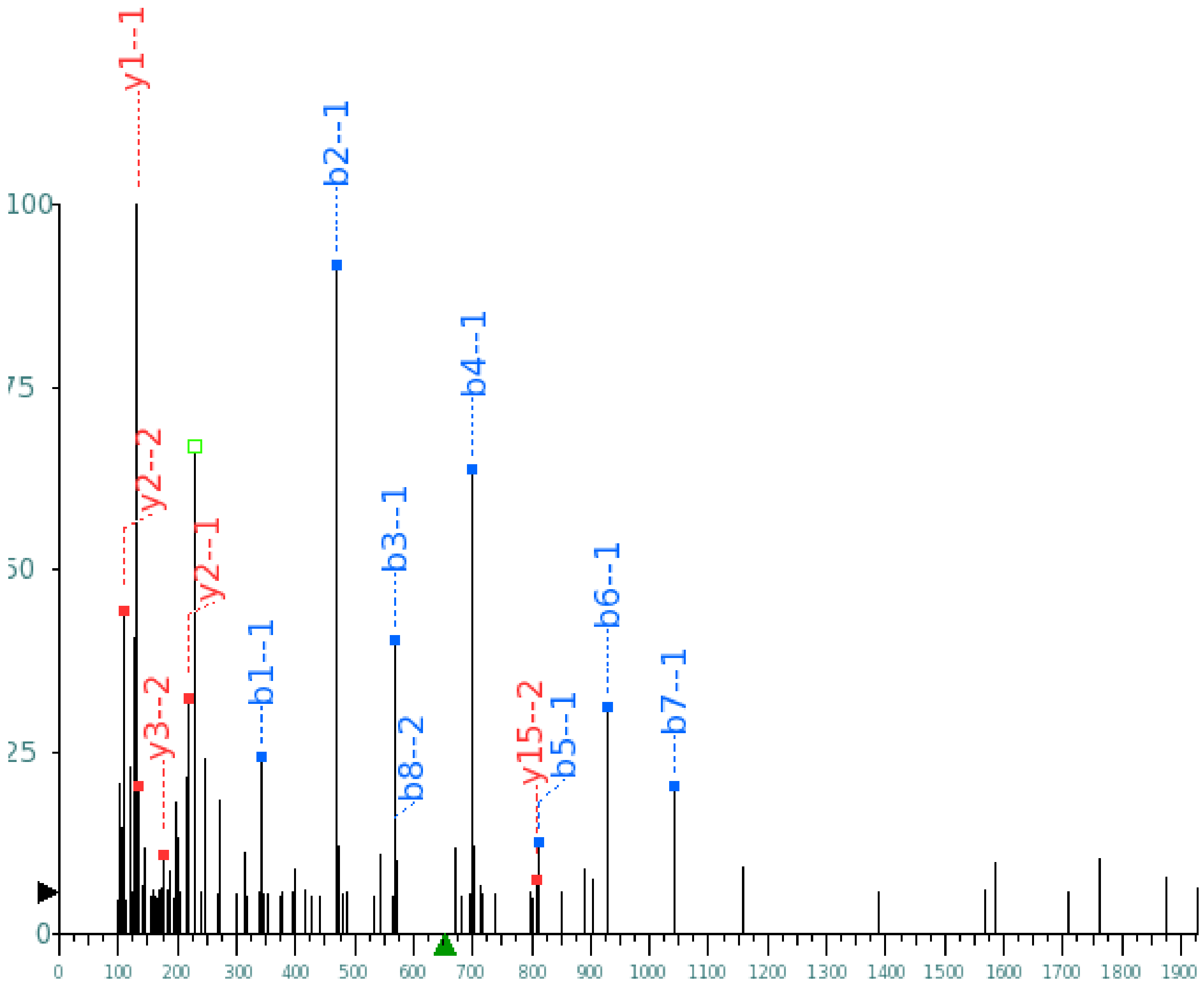

LQVELDNVTGLLSQSD (+3)

Primary Reference: MYH9_HUMAN
Search ID: 41760
Search Name: 20130328_ananiav_ERstress_TMT_TM_timecourse_trypsin_inj1_4mc_iaa
Scan#: 20753
Observed Mass: 654.0215 (3.3 ppm)
PSM Score: 32

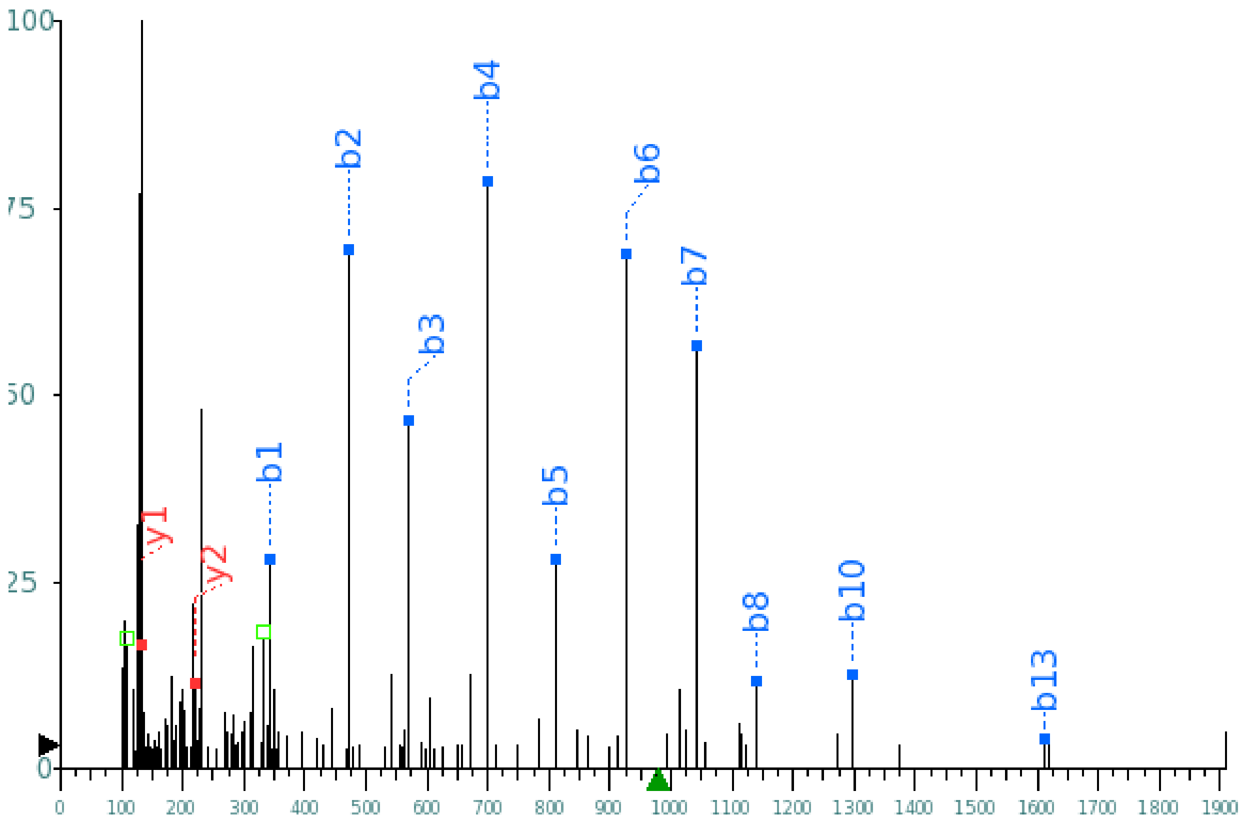

LQVELDNVTGLLSQSD (+2)

Primary Reference: MYH9_HUMAN
Search ID: 41760
Search Name: 20130328_ananiav_ERstress_TMT_TM_timecourse_trypsin_inj1_4mc_iaa
Scan#: 20756
Observed Mass: 980.5281 (2.7 ppm)
PSM Score: 61.76

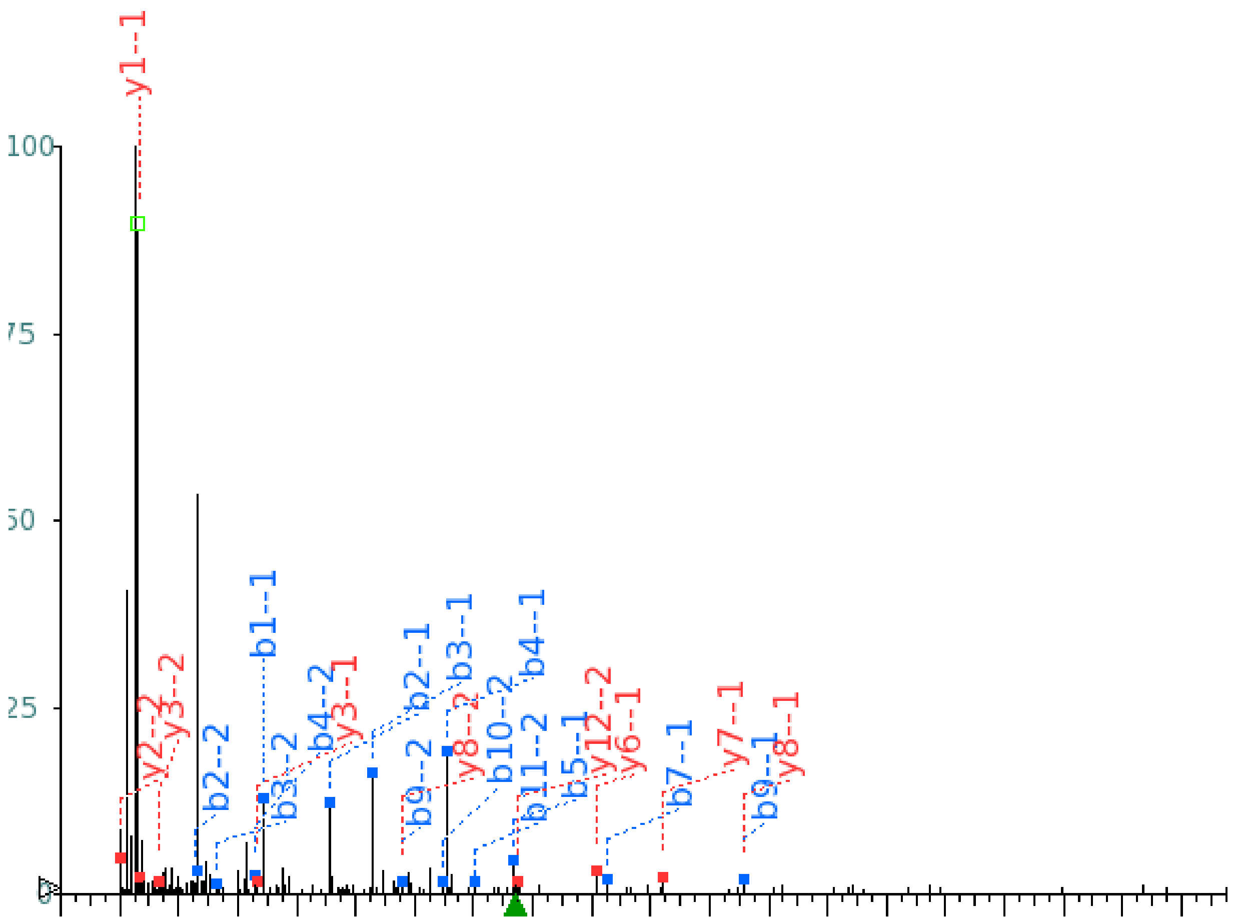

ILAQITGTEHLKESEAD (+3)

Primary Reference: PDLI5_HUMAN
Search ID: 41760
Search Name: 20130328_ananiav_ERstress_TMT_TM_timecourse_trypsin_inj1_4mc_iaa
Scan#: 20794
Observed Mass: 771.7646 (4 ppm)
PSM Score: 20.62

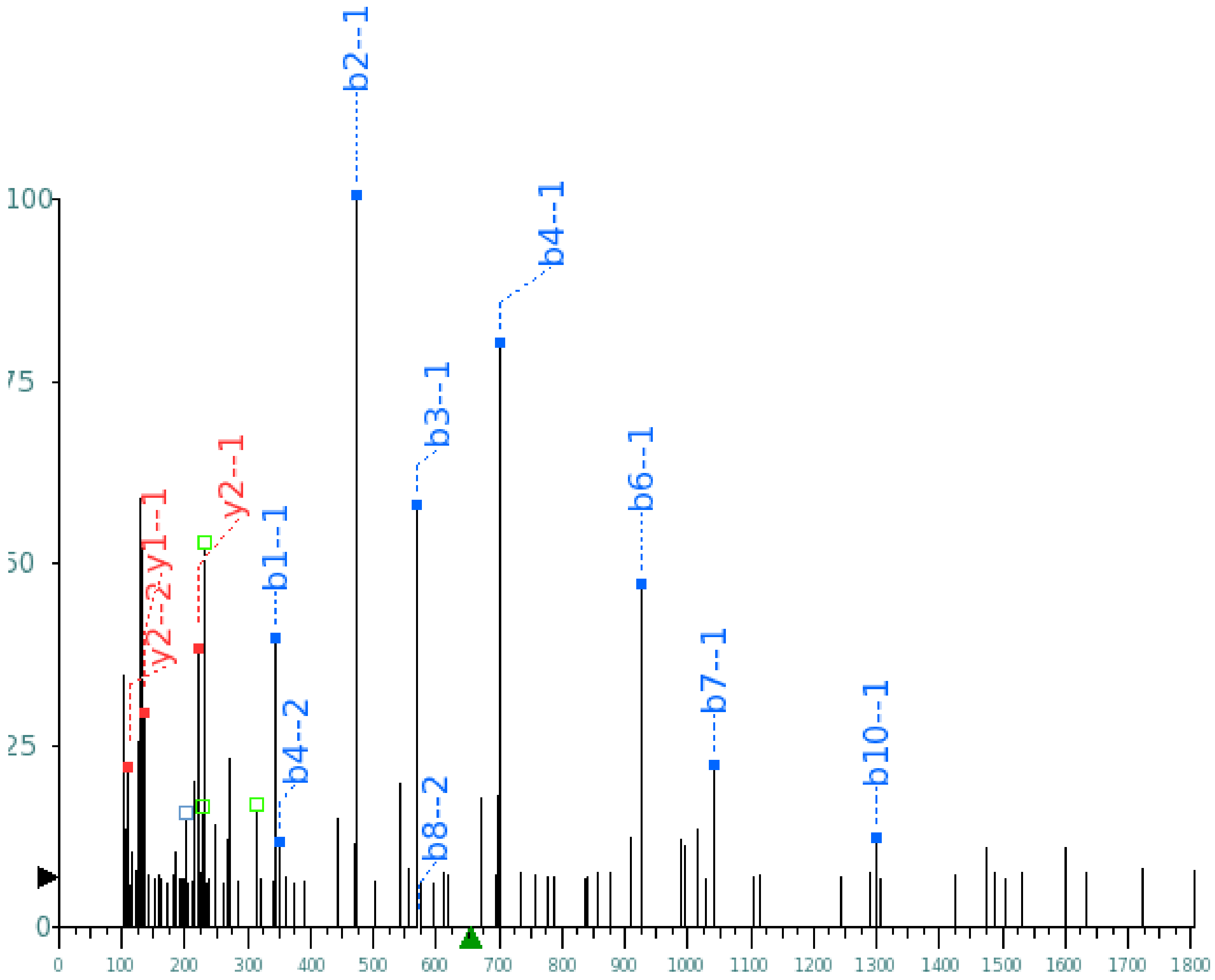

LQVELDNVTGLLSQSD (+3)

Primary Reference: MYH9_HUMAN
Search ID: 41760
Search Name: 20130328_ananiav_ERstress_TMT_TM_timecourse_trypsin_inj1_4mc_iaa
Scan#: 20818
Observed Mass: 654.0214 (3.1 ppm)
PSM Score: 29.68

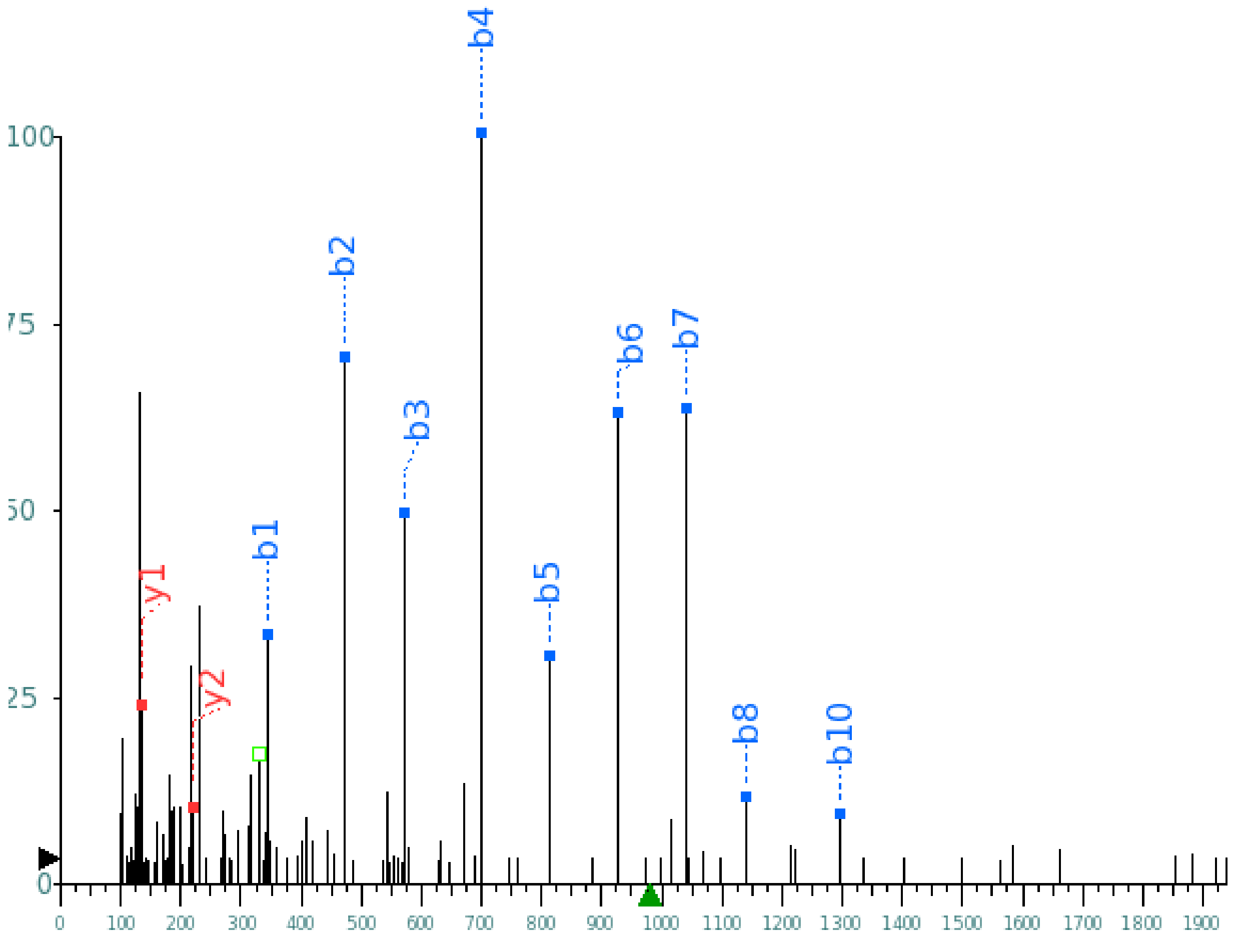

LQVELDNVTGLLSQSD (+2)

Primary Reference: MYH9_HUMAN
Search ID: 41760
Search Name: 20130328_ananiav_ERstress_TMT_TM_timecourse_trypsin_inj1_4mc_iaa
Scan#: 20819
Observed Mass: 980.5283 (2.9 ppm)
PSM Score: 54.71

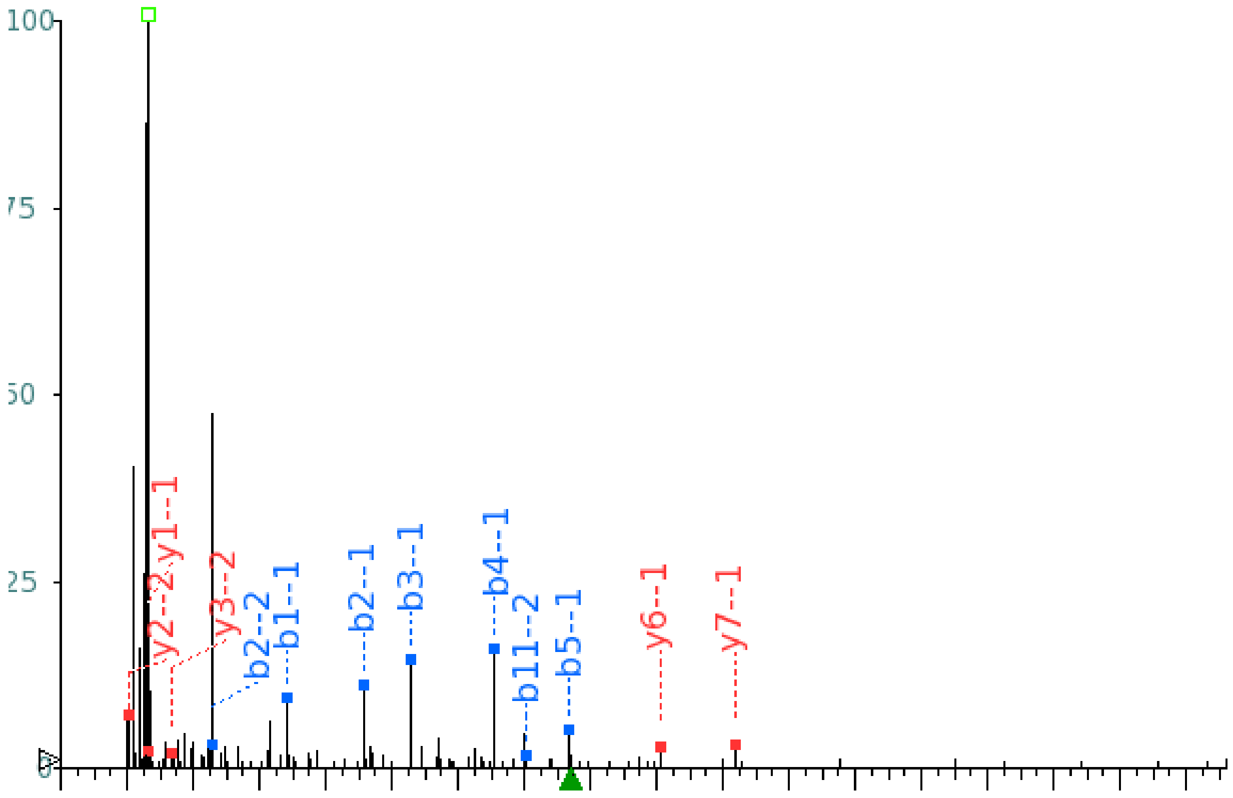

ILAQITGTEHLKESEAD (+3)

Primary Reference: PDLI5_HUMAN
Search ID: 41760
Search Name: 20130328_ananiav_ERstress_TMT_TM_timecourse_trypsin_inj1_4mc_iaa
Scan#: 20926
Observed Mass: 771.7617 (0.2 ppm)
PSM Score: 15.97

File name: 2015-02-04 04.01.53PM.pdf

Creation time: 2015-02-04 04:09PM

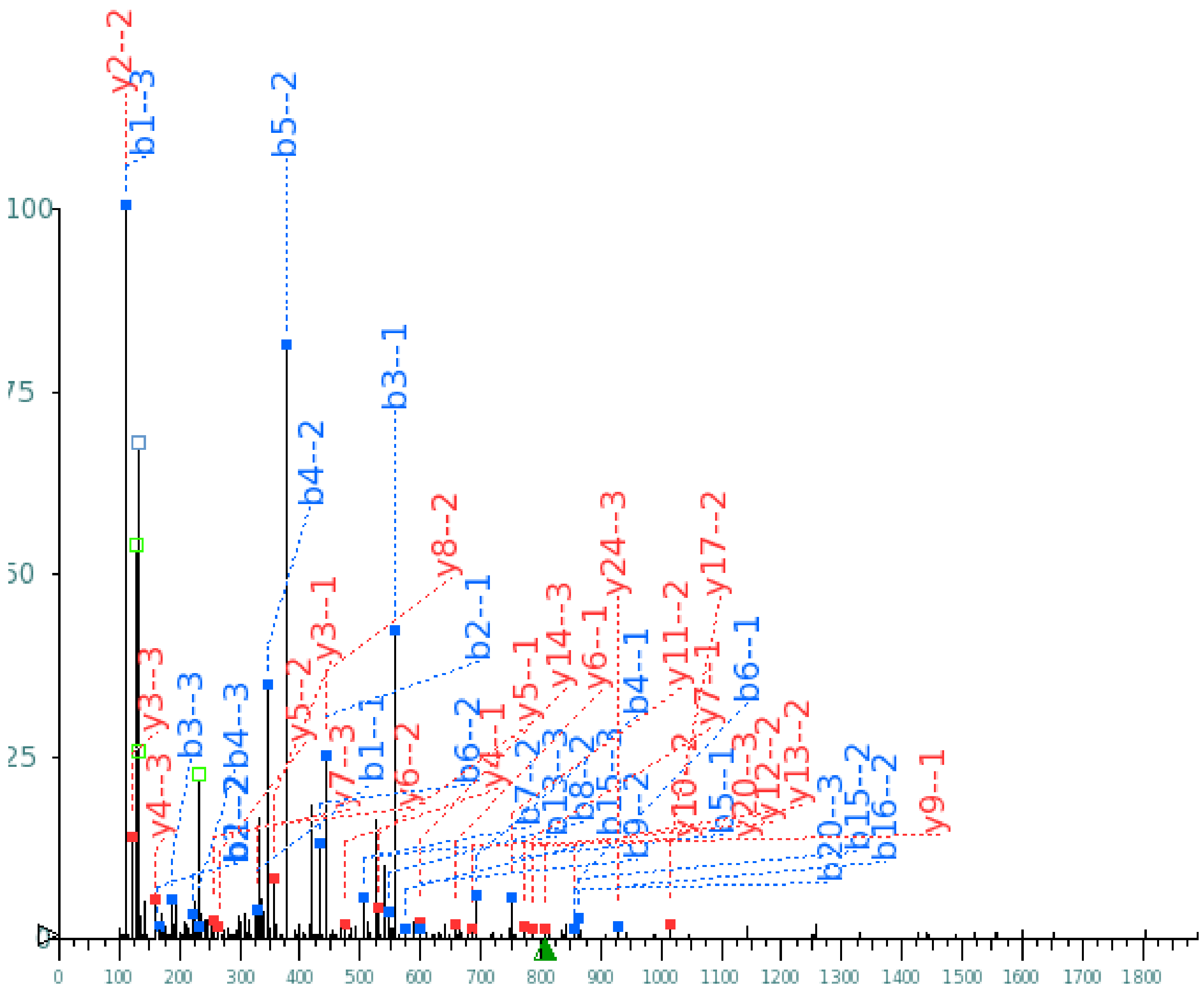

VNIHGIFSVASASVIEKQNLEGDHSD (+4)

Primary Reference: HS74L_HUMAN
Search ID: 41760
Search Name: 20130328_ananiav_ERstress_TMT_TM_timecourse_trypsin_inj1_4mc_iaa
Scan#: 20944
Observed Mass: 806.932 (3.4 ppm)
PSM Score: 18.1

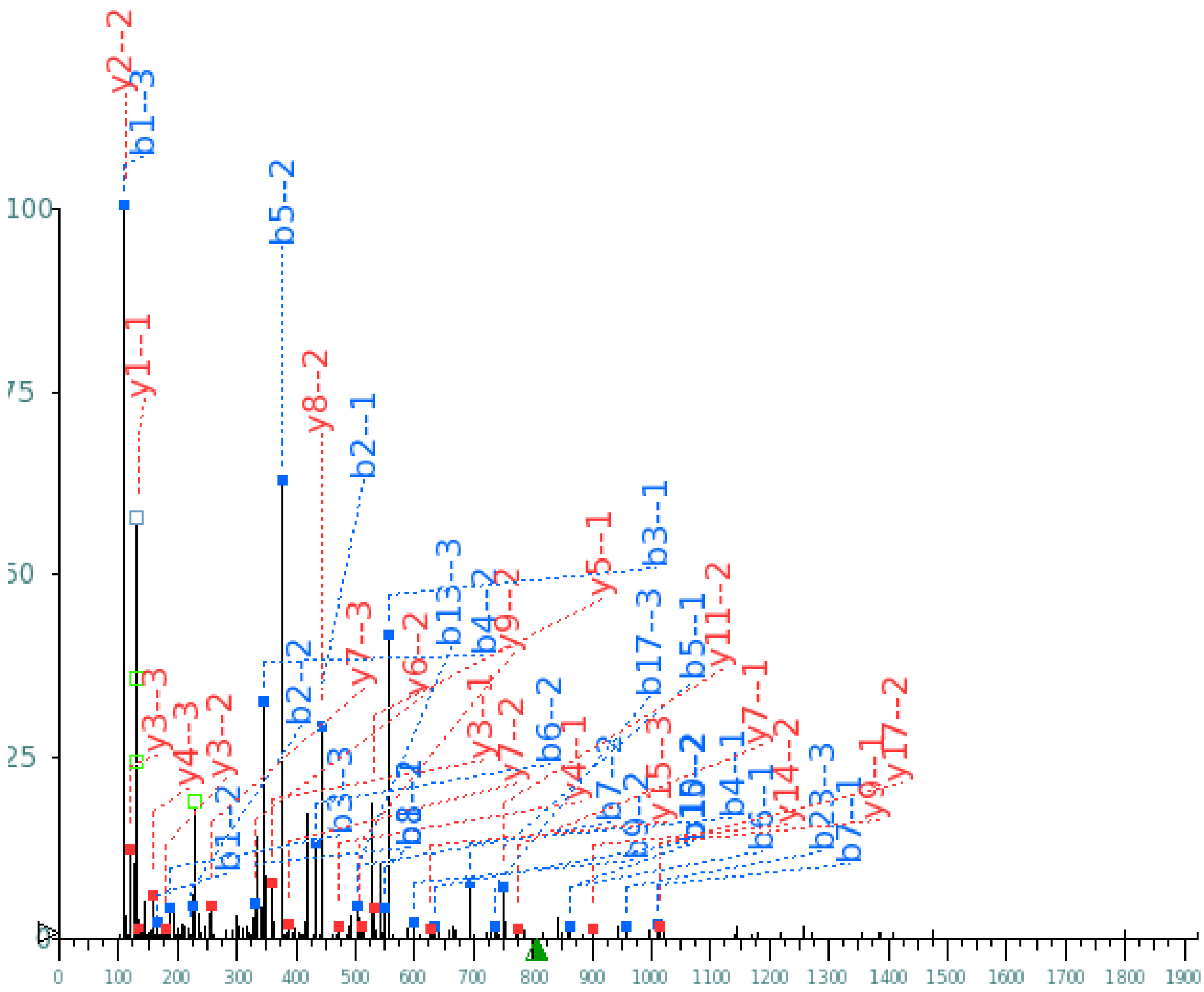

VNIHGIFSVASASVIEKQNLEGDHSD (+4)

Primary Reference: HS74L_HUMAN
Search ID: 41760
Search Name: 20130328_ananiav_ERstress_TMT_TM_timecourse_trypsin_inj1_4mc_iaa
Scan#: 20974
Observed Mass: 806.932 (3.4 ppm)
PSM Score: 18.77

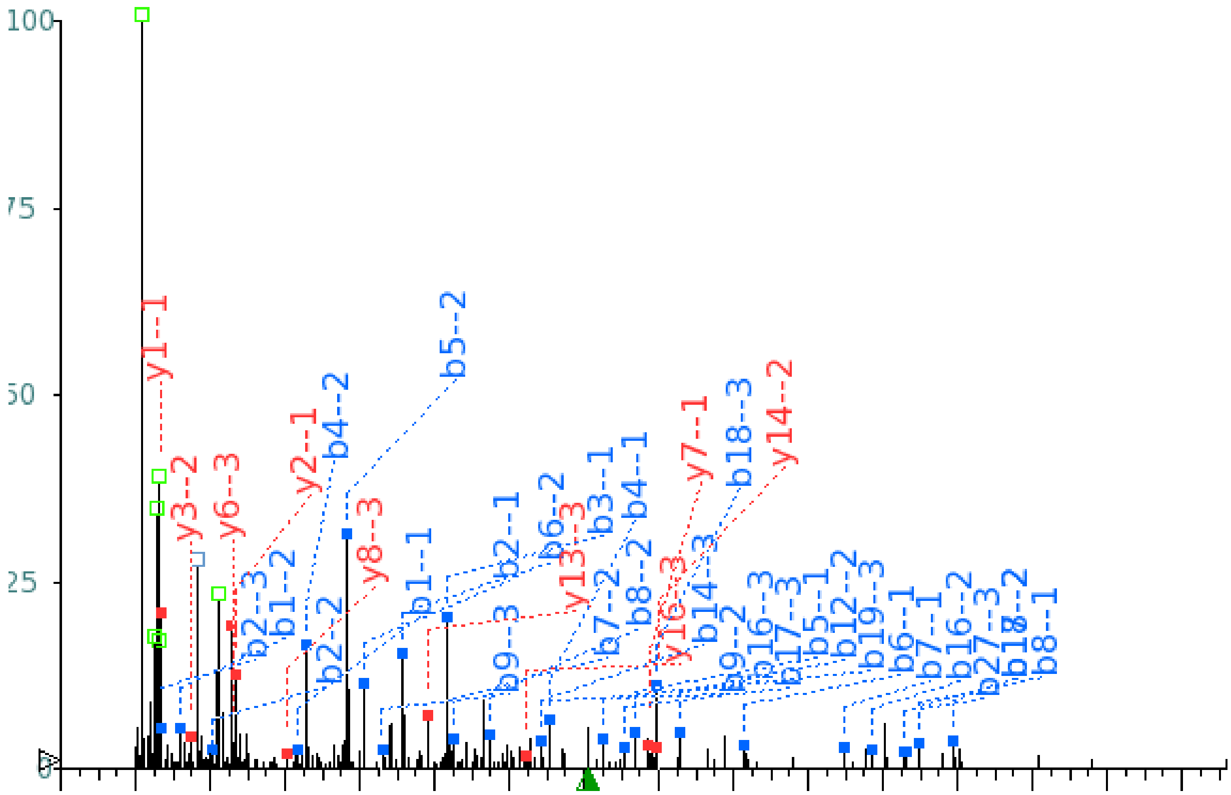

SSIHNFMTHPEFRIEDSEPHIPLIDDTD (+5)

Primary Reference: AT2B1_HUMAN
Search ID: 41760
Search Name: 20130328_ananiav_ERstress_TMT_TM_timecourse_trypsin_inj1_4mc_iaa
Scan#: 21131
Observed Mass: 705.1407 (-2.8 ppm)
PSM Score: 22.17

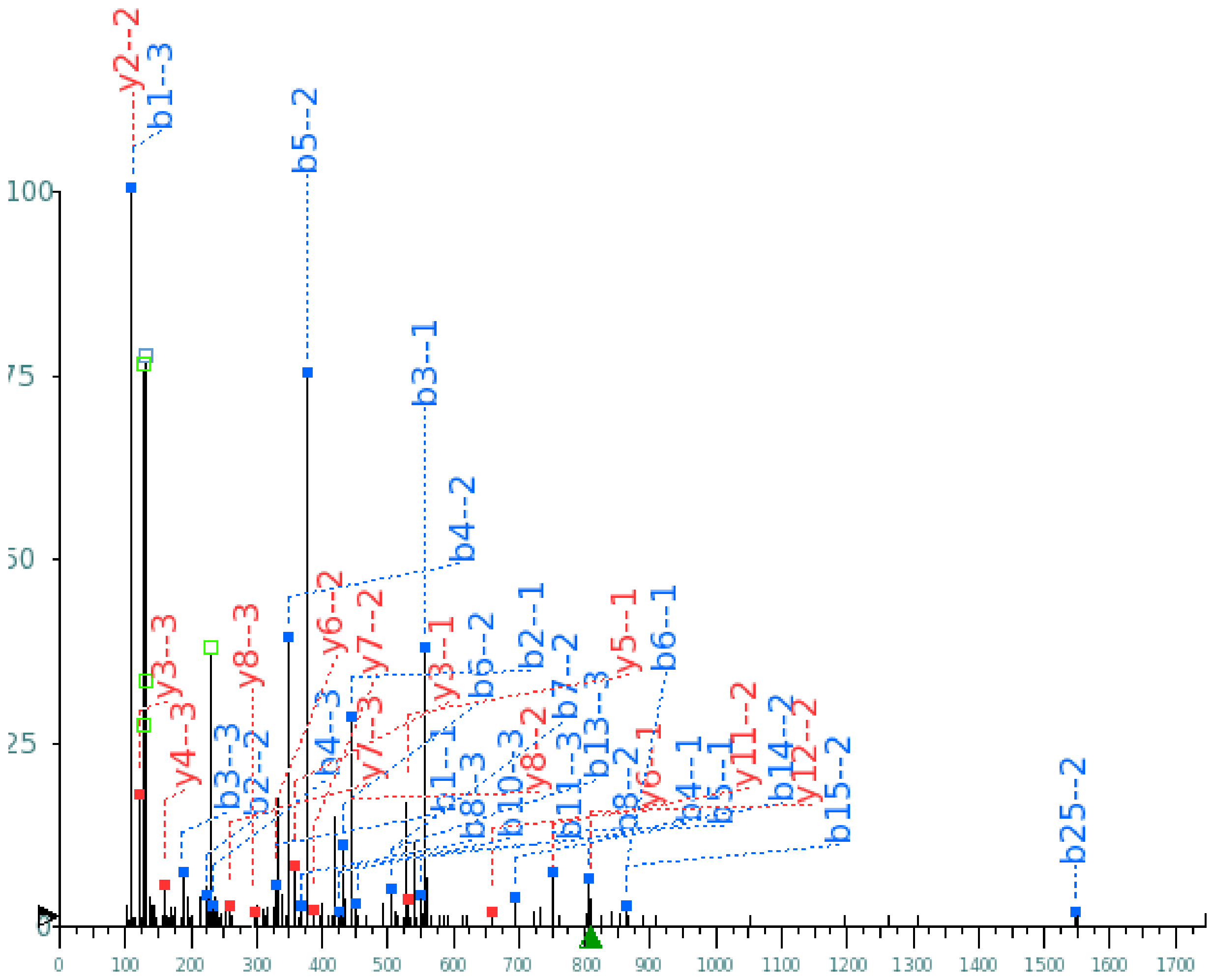

VNIHGIFSVASASVIEKQNLEGDHSD (+4)

Primary Reference: HS74L_HUMAN
Search ID: 41760
Search Name: 20130328_ananiav_ERstress_TMT_TM_timecourse_trypsin_inj1_4mc_iaa
Scan#: 21194
Observed Mass: 806.9358 (8.1 ppm)
PSM Score: 22.32

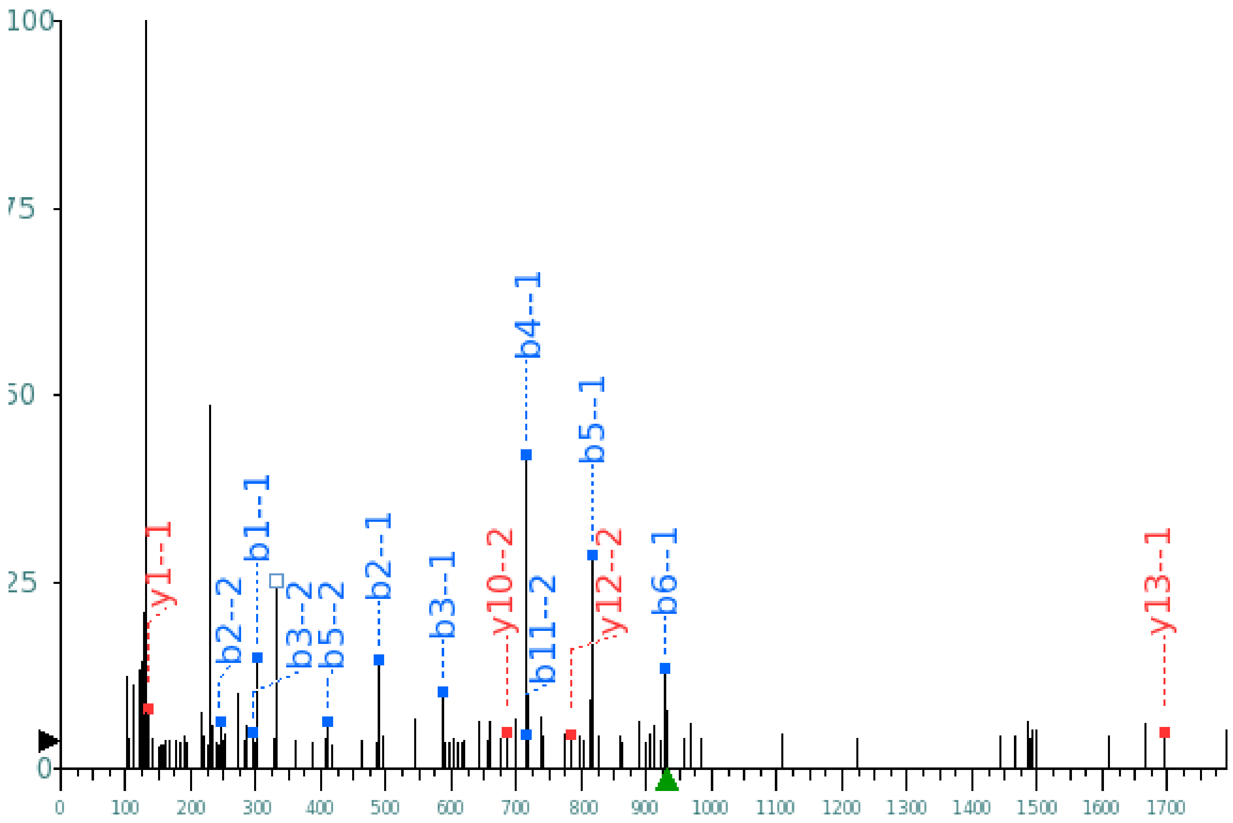

AWTQVLLGQAEDKAEVDMDTD (+3)

Primary Reference: DYHC1_HUMAN
Search ID: 41760
Search Name: 20130328_ananiav_ERstress_TMT_TM_timecourse_trypsin_inj1_4mc_iaa
Scan#: 21269
Observed Mass: 931.8072 (2 ppm)
PSM Score: 13.69

File name: 2015-02-04 04.01.53PM.pdf

Creation time: 2015-02-04 04:09PM

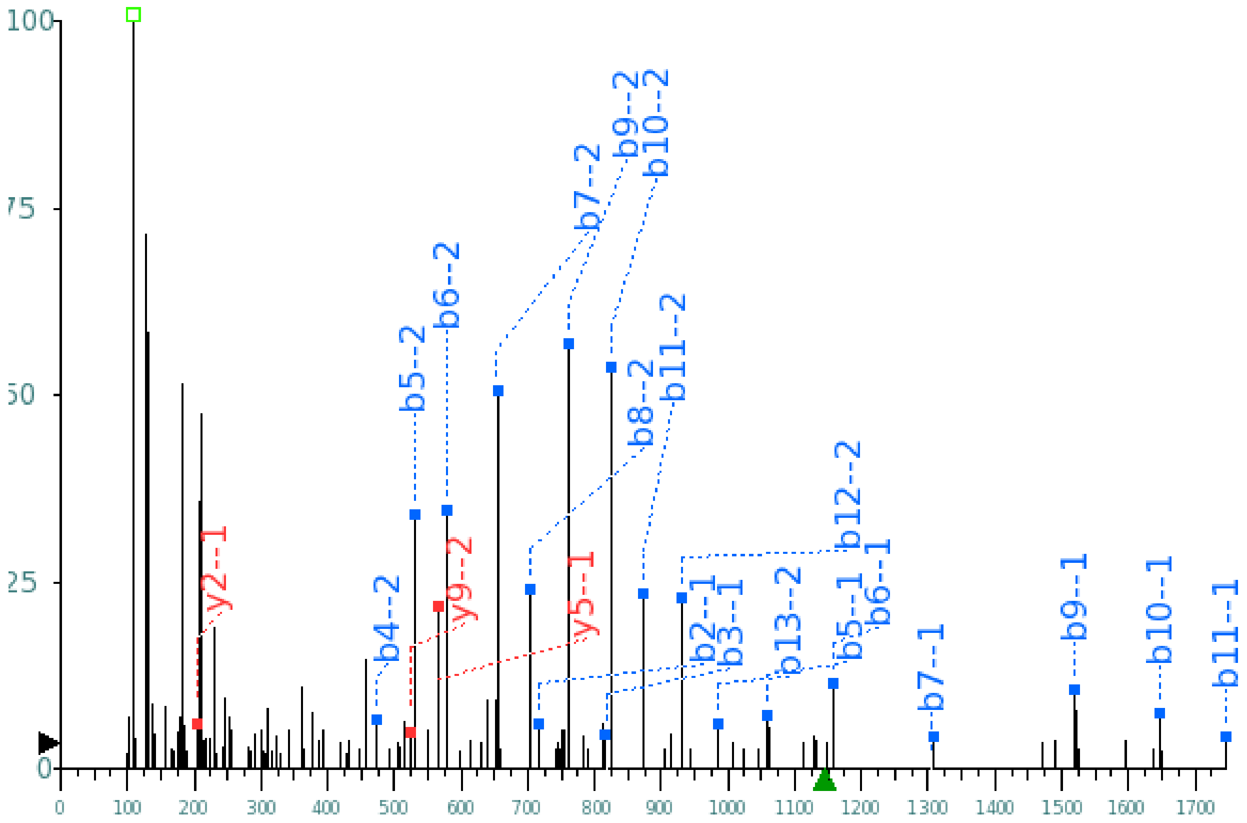

QKTENVFVDEPLIHATTYIPLMDNAD (+3)

Primary Reference: KTN1_HUMAN
Search ID: 41760
Search Name: 20130328_ananiav_ERstress_TMT_TM_timecourse_trypsin_inj1_4mc_iaa
Scan#: 21363
Observed Mass: 1144.9327 (2.2 ppm)
PSM Score: 43.37

File name: 2015-02-04 04.01.53PM.pdf

Creation time: 2015-02-04 04:09PM

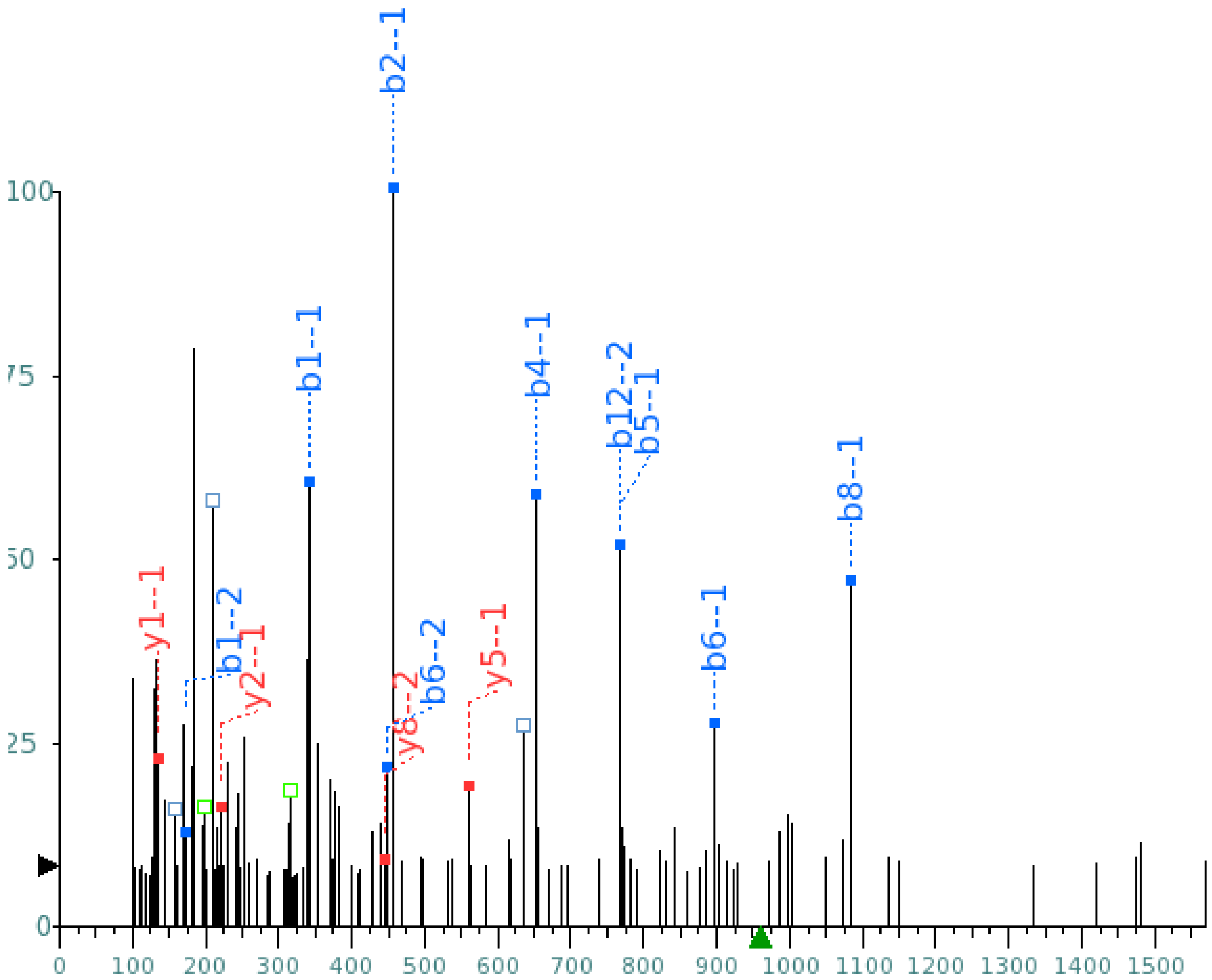

IIPTLEEGLQLPSPTATSQLPLESD (+3)

Primary Reference: HNRPK_HUMAN
Search ID: 41760
Search Name: 20130328_ananiav_ERstress_TMT_TM_timecourse_trypsin_inj1_4mc_iaa
Scan#: 21687
Observed Mass: 960.1907 (2.7 ppm)
PSM Score: 22.21

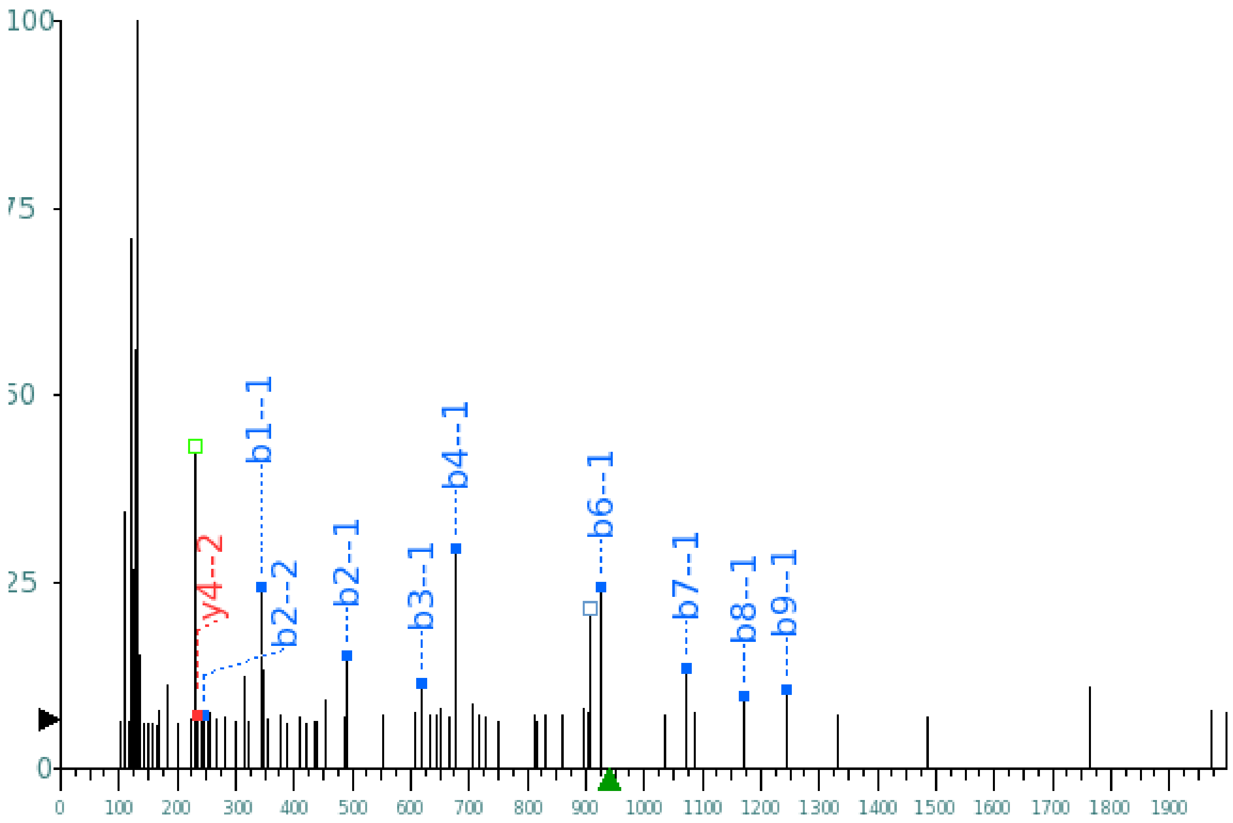

IFQGYSFVAPSILFDHNNAVMTD (+3)

Primary Reference: KS6A4_HUMAN
Search ID: 41760
Search Name: 20130328_ananiav_ERstress_TMT_TM_timecourse_trypsin_inj1_4mc_iaa
Scan#: 21732
Observed Mass: 939.1372 (0.3 ppm)
PSM Score: 19.85

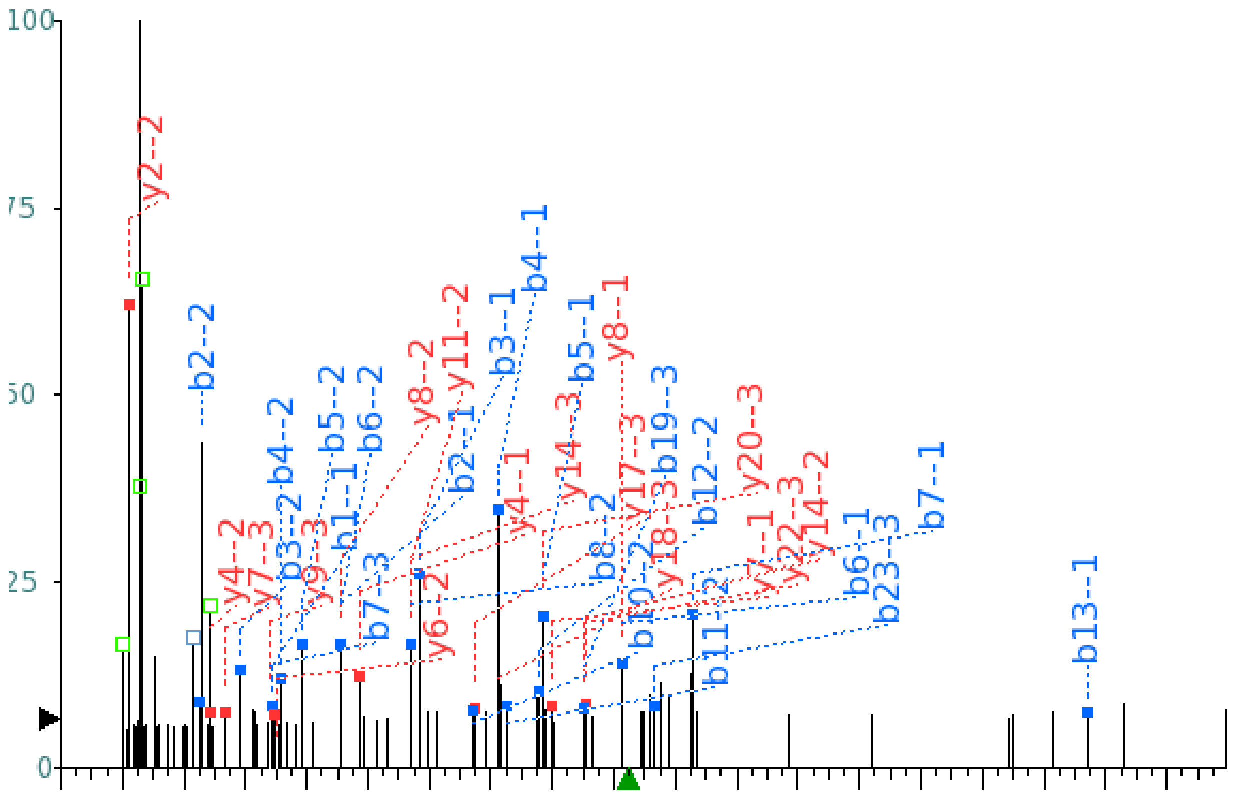

ILQEAQNLMALTNVDTPLKGGLNTPLHESD (+4)

Primary Reference: CDC5L_HUMAN
Search ID: 41760
Search Name: 20130328_ananiav_ERstress_TMT_TM_timecourse_trypsin_inj1_4mc_iaa
Scan#: 21864
Observed Mass: 923.5009 (1.1 ppm)
PSM Score: 19.77

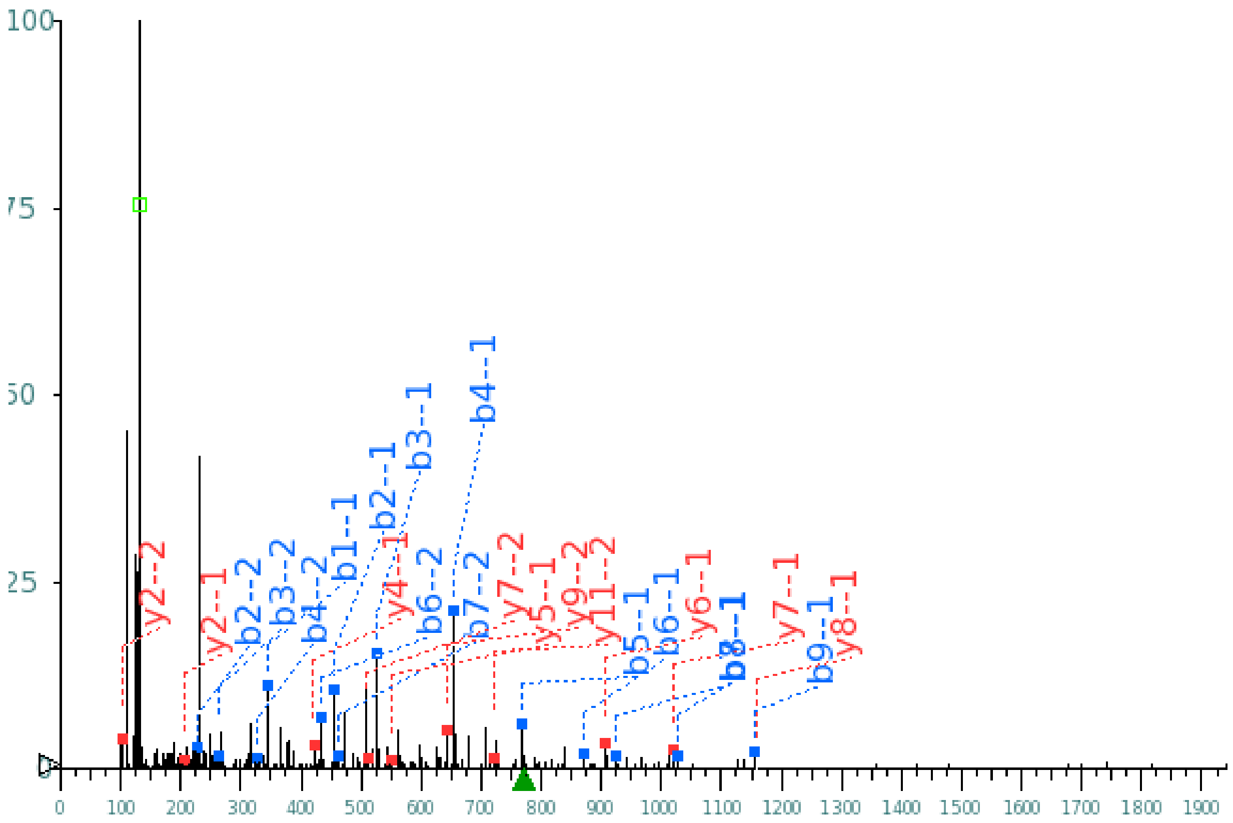

ILAQITGTEHLKESEAD (+3)

Primary Reference: PDLI5_HUMAN
Search ID: 41760
Search Name: 20130328_ananiav_ERstress_TMT_TM_timecourse_trypsin_inj1_4mc_iaa
Scan#: 21902
Observed Mass: 771.7632 (2.1 ppm)
PSM Score: 20

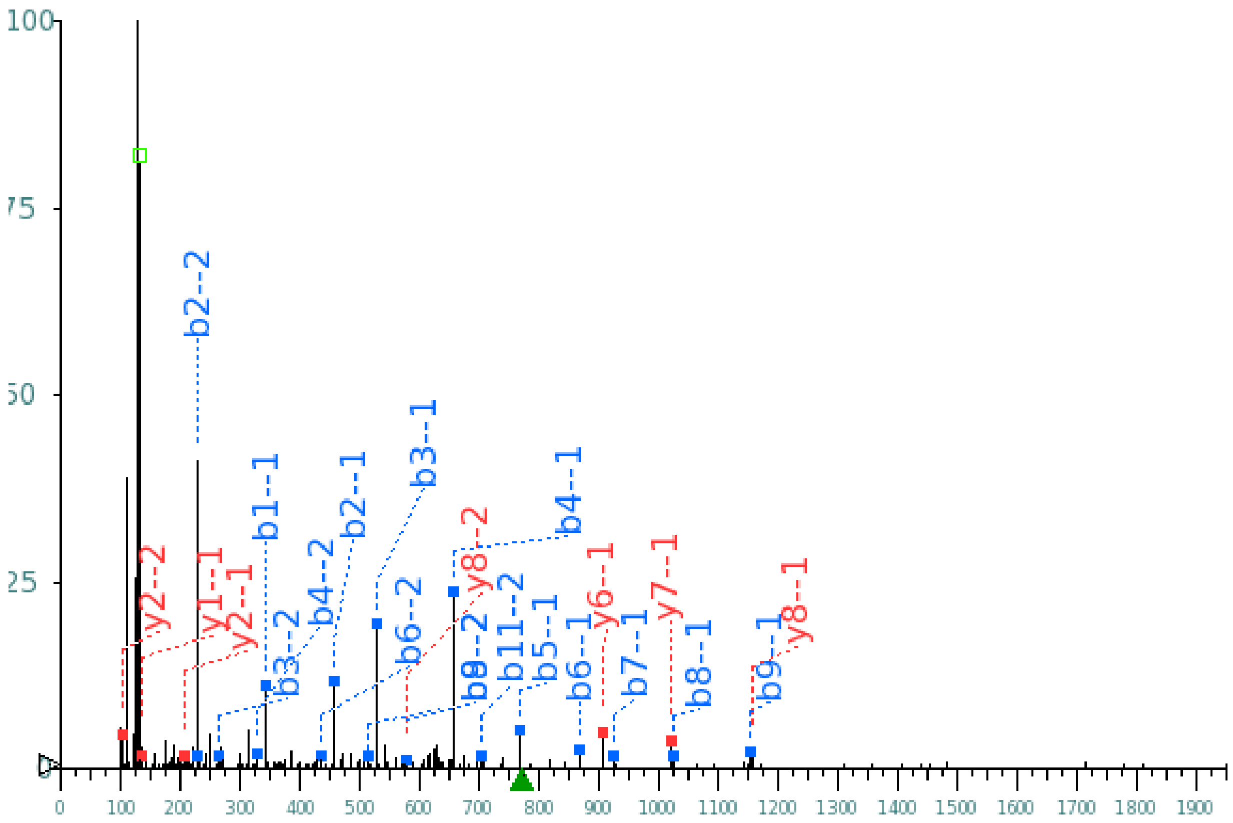

ILAQITGTEHLKESEAD (+3)

Primary Reference: PDLI5_HUMAN
Search ID: 41760
Search Name: 20130328_ananiav_ERstress_TMT_TM_timecourse_trypsin_inj1_4mc_iaa
Scan#: 22028
Observed Mass: 771.7619 (0.5 ppm)
PSM Score: 34.23

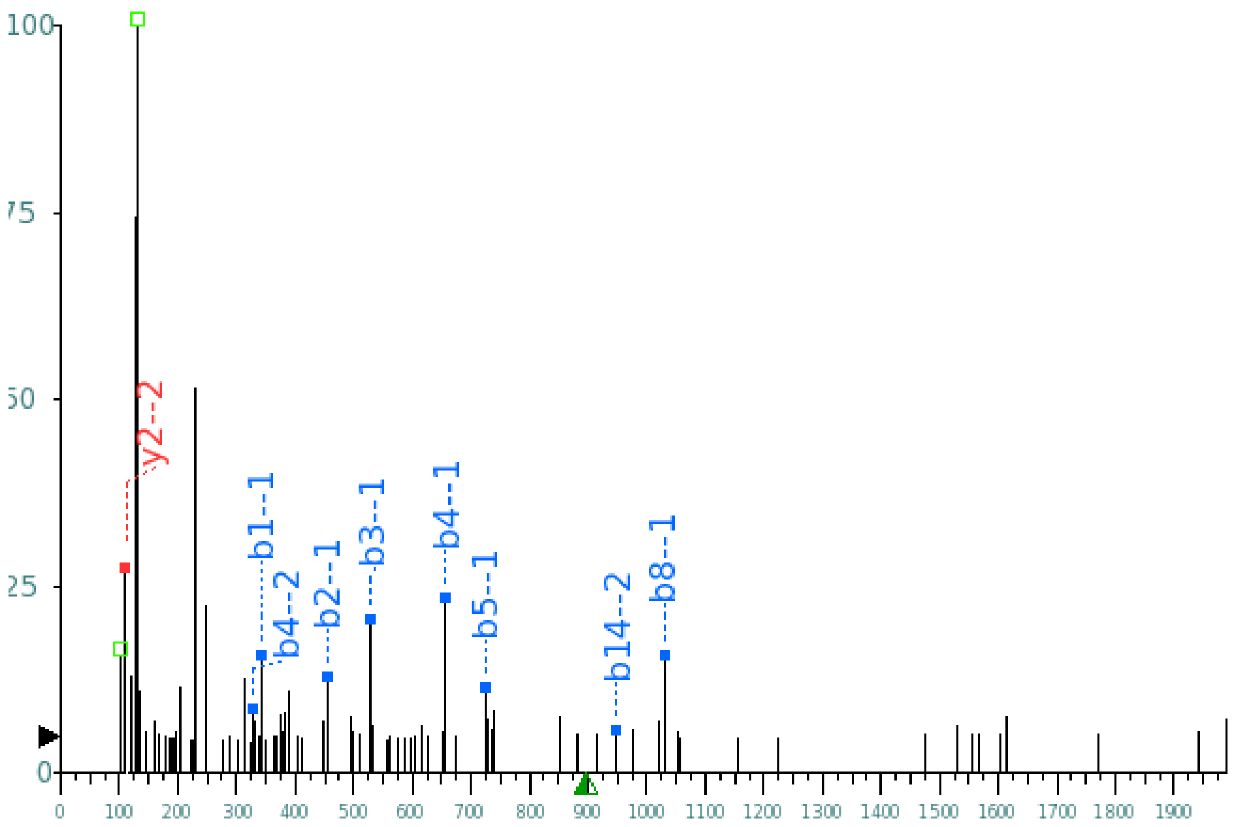

ILAQATSDLVNAIKADAEGESD (+3)

Primary Reference: TLN1_HUMAN
Search ID: 41760
Search Name: 20130328_ananiav_ERstress_TMT_TM_timecourse_trypsin_inj1_4mc_iaa
Scan#: 22039
Observed Mass: 897.1497 (1.9 ppm)
PSM Score: 16.81

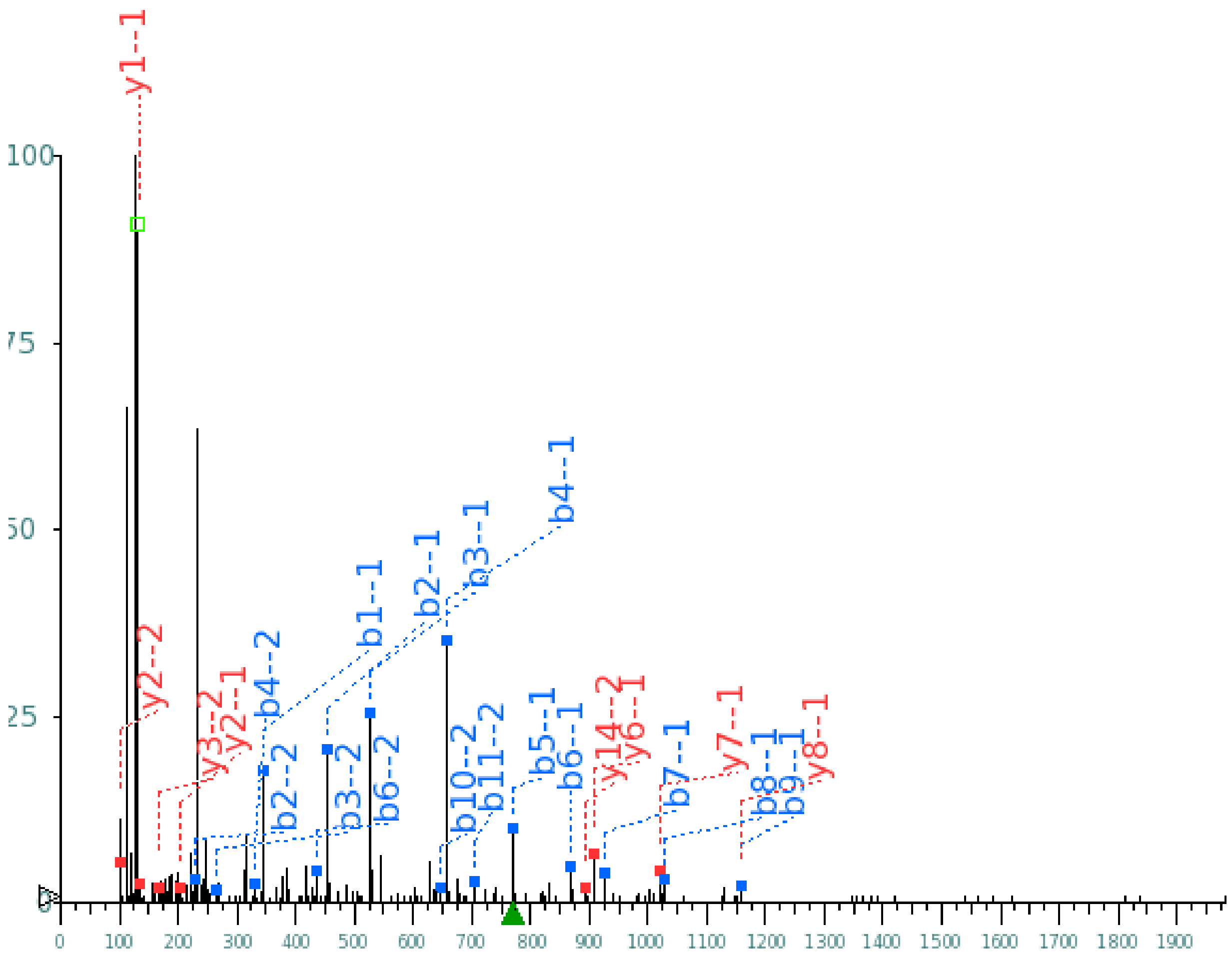

ILAQITGTEHLKESEAD (+3)

Primary Reference: PDLI5_HUMAN
Search ID: 41760
Search Name: 20130328_ananiav_ERstress_TMT_TM_timecourse_trypsin_inj1_4mc_iaa
Scan#: 22106
Observed Mass: 771.7624 (1.1 ppm)
PSM Score: 27.34

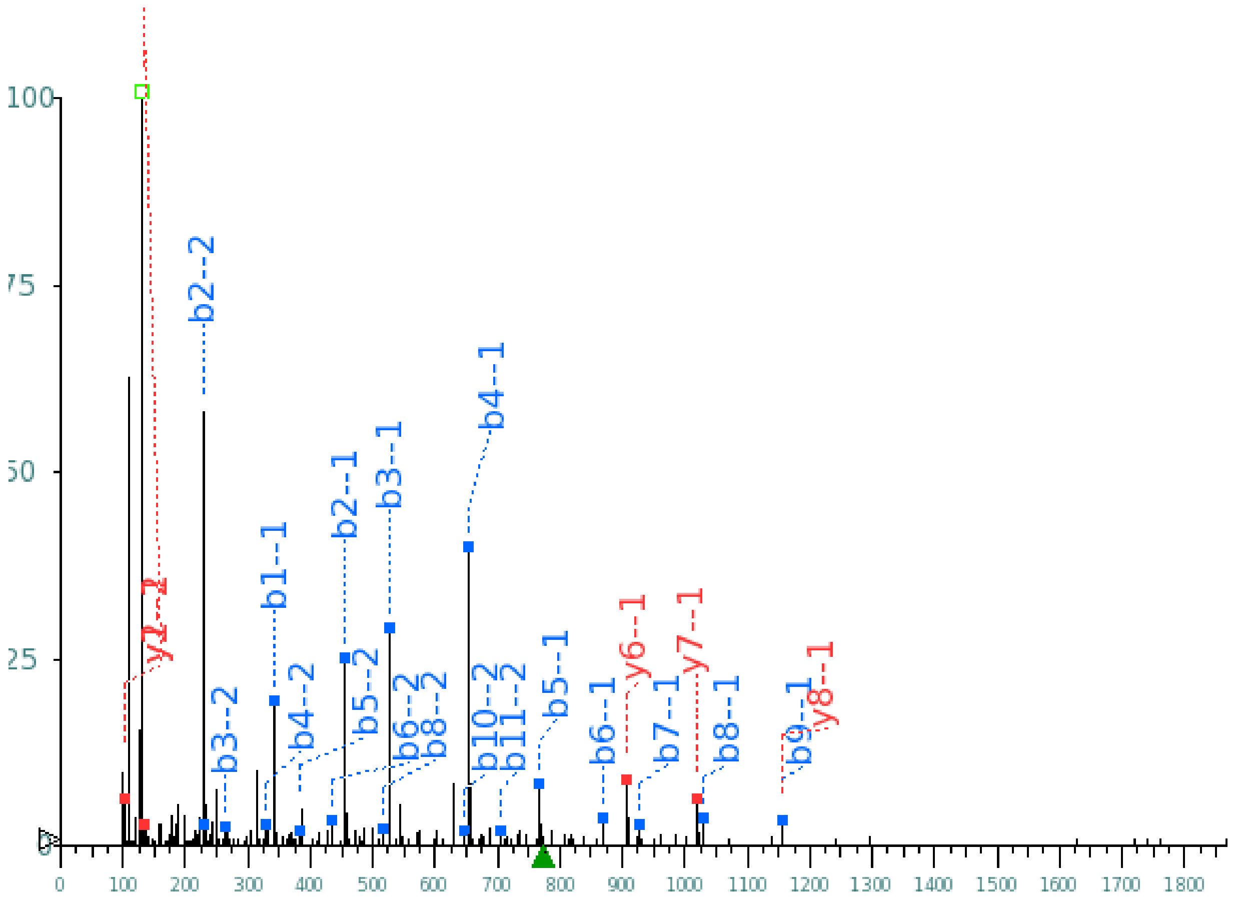

ILAQITGTEHLKESEAD (+3)

Primary Reference: PDLI5_HUMAN
Search ID: 41760
Search Name: 20130328_ananiav_ERstress_TMT_TM_timecourse_trypsin_inj1_4mc_iaa
Scan#: 22162
Observed Mass: 771.7624 (1 ppm)
PSM Score: 36.66

File name: 2015-02-04 04.01.53PM.pdf

Creation time: 2015-02-04 04:09PM

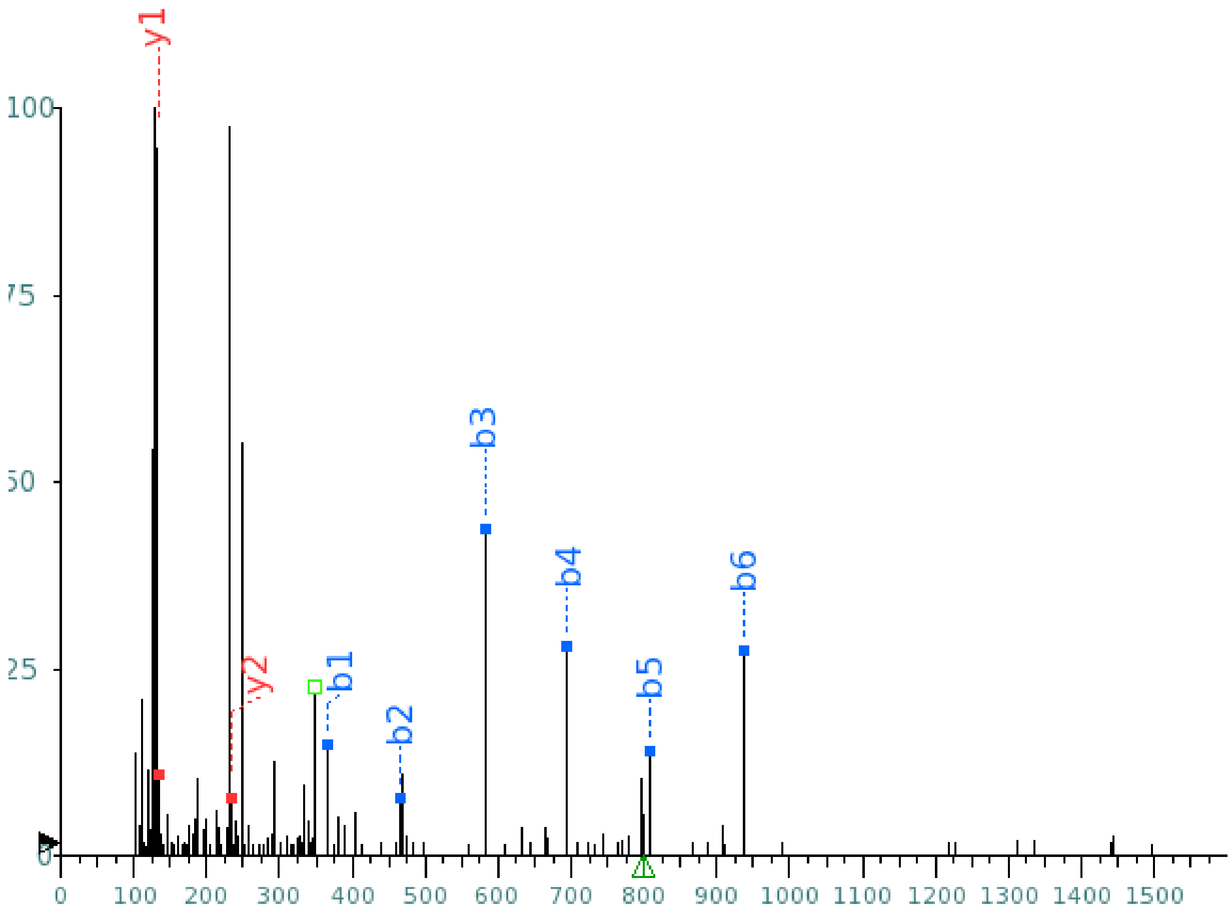

HVDLLEVAQETD (+2)

Primary Reference: ATAD1_HUMAN
Search ID: 41760
Search Name: 20130328_ananiav_ERstress_TMT_TM_timecourse_trypsin_inj1_4mc_iaa
Scan#: 22206
Observed Mass: 799.4164 (-1 ppm)
PSM Score: 17.26

File name: 2015-02-04 04.01.53PM.pdf

Creation time: 2015-02-04 04:09PM

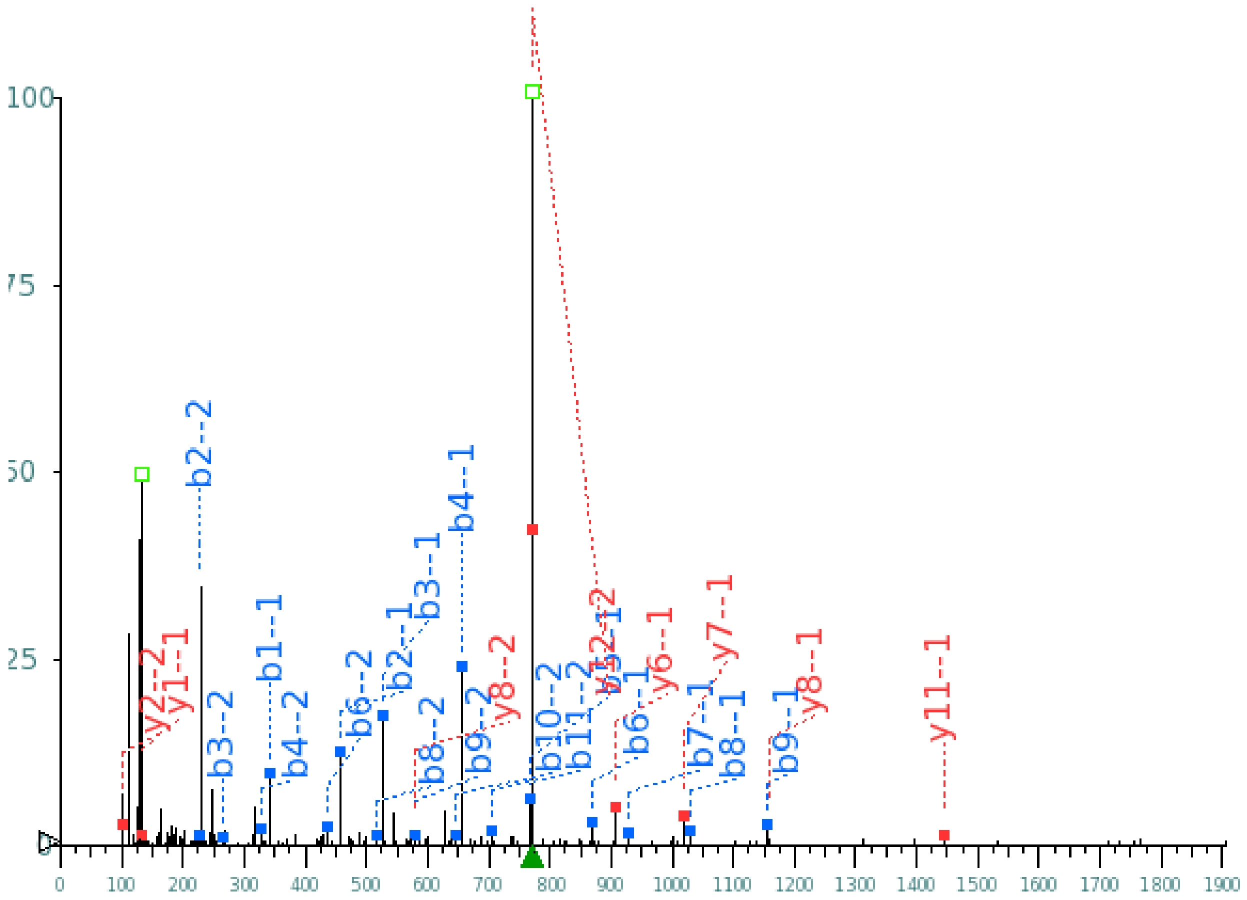

ILAQITGTEHLKESEAD (+3)

Primary Reference: PDLI5_HUMAN
Search ID: 41760
Search Name: 20130328_ananiav_ERstress_TMT_TM_timecourse_trypsin_inj1_4mc_iaa
Scan#: 22228
Observed Mass: 771.7627 (1.4 ppm)
PSM Score: 40.22

File name: 2015-02-04 04.01.53PM.pdf

Creation time: 2015-02-04 04:09PM

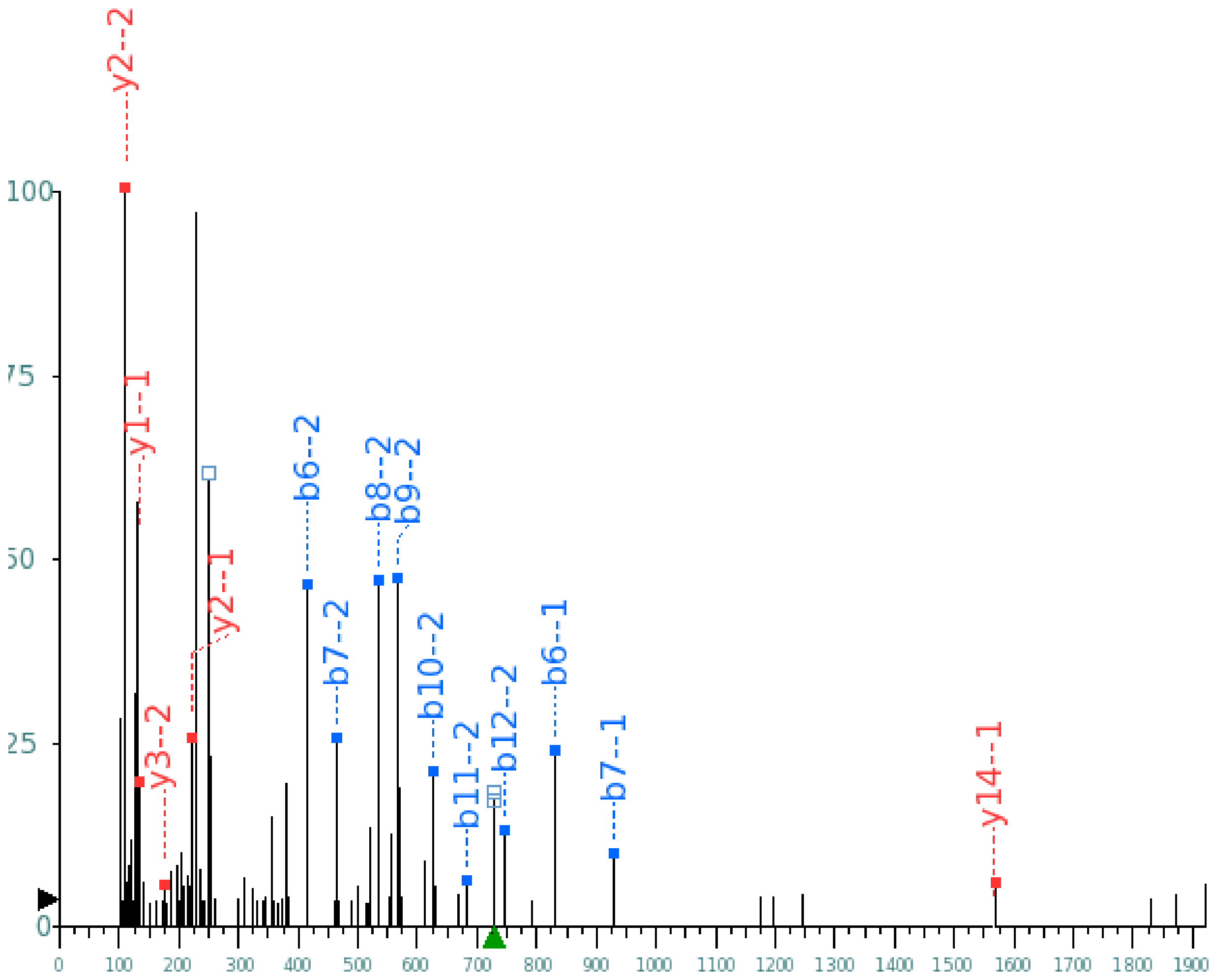

RGSSPDVHALLEITEESD (+3)

Primary Reference: PTRF_HUMAN
Search ID: 41760
Search Name: 20130328_ananiav_ERstress_TMT_TM_timecourse_trypsin_inj1_4mc_iaa
Scan#: 22246
Observed Mass: 728.7068 (3.5 ppm)
PSM Score: 17.04

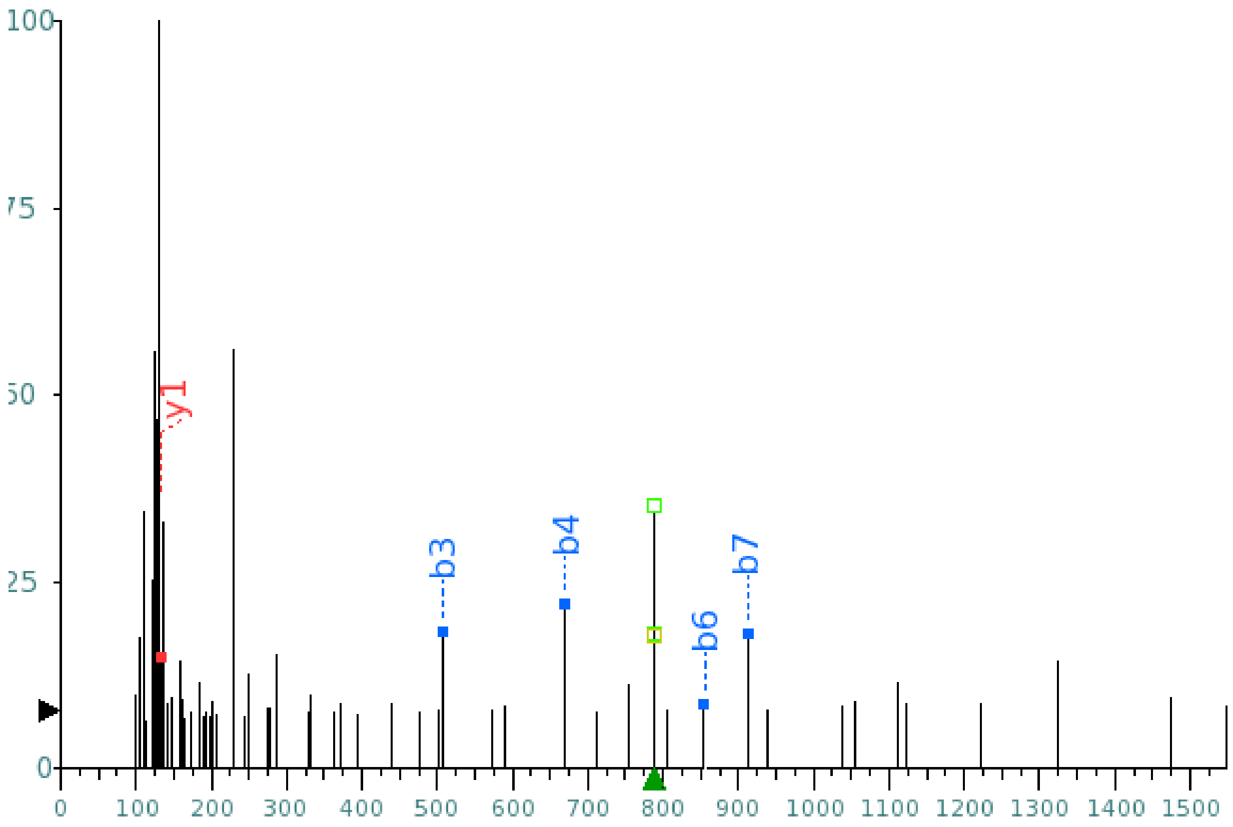

GYGYGGQGAGTLSTD (+2)

Primary Reference: CSRP1_HUMAN
Search ID: 41760
Search Name: 20130328_ananiav_ERstress_TMT_TM_timecourse_trypsin_inj1_4mc_iaa
Scan#: 22276
Observed Mass: 788.3794 (1.7 ppm)
PSM Score: 5.03

File name: 2015-02-04 04.01.53PM.pdf

Creation time: 2015-02-04 04:09PM

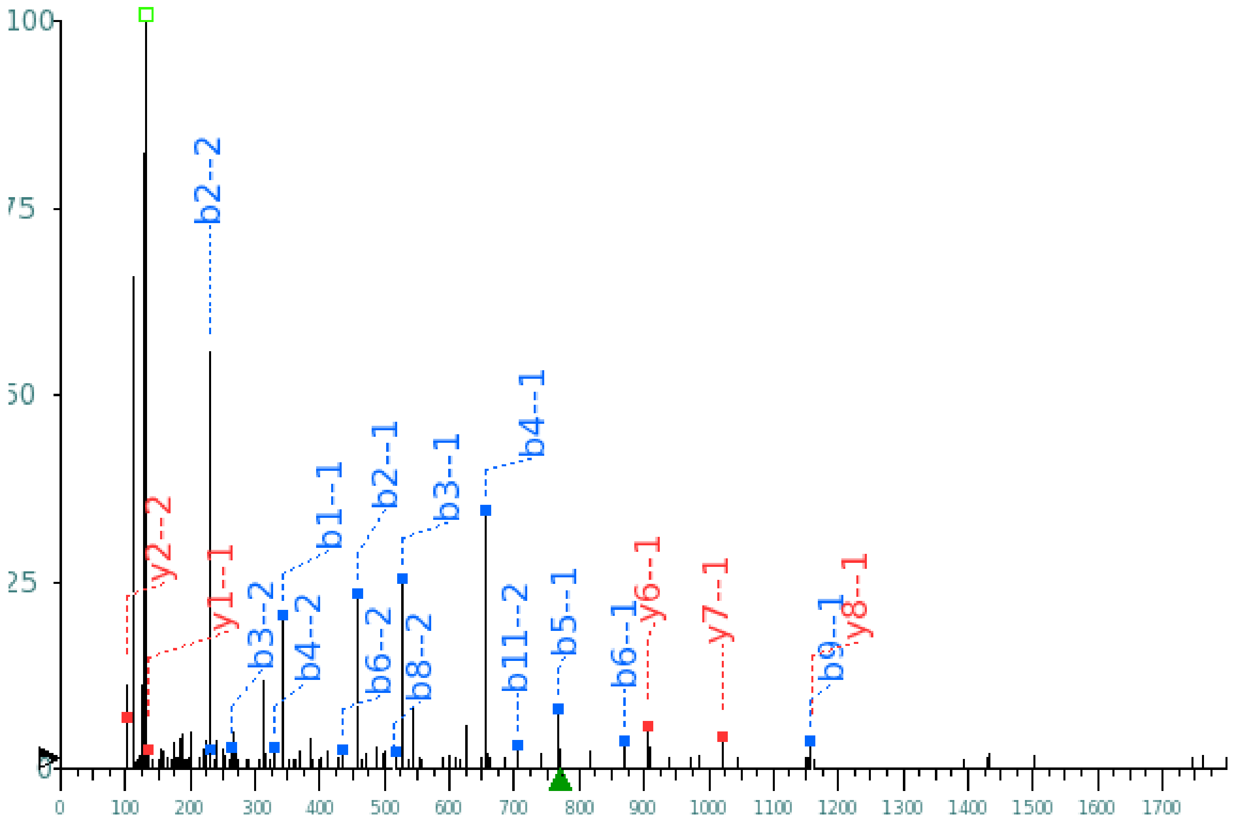

ILAQITGTEHLKESEAD (+3)

Primary Reference: PDLI5_HUMAN
Search ID: 41760
Search Name: 20130328_ananiav_ERstress_TMT_TM_timecourse_trypsin_inj1_4mc_iaa
Scan#: 22282
Observed Mass: 771.7625 (1.2 ppm)
PSM Score: 36.75

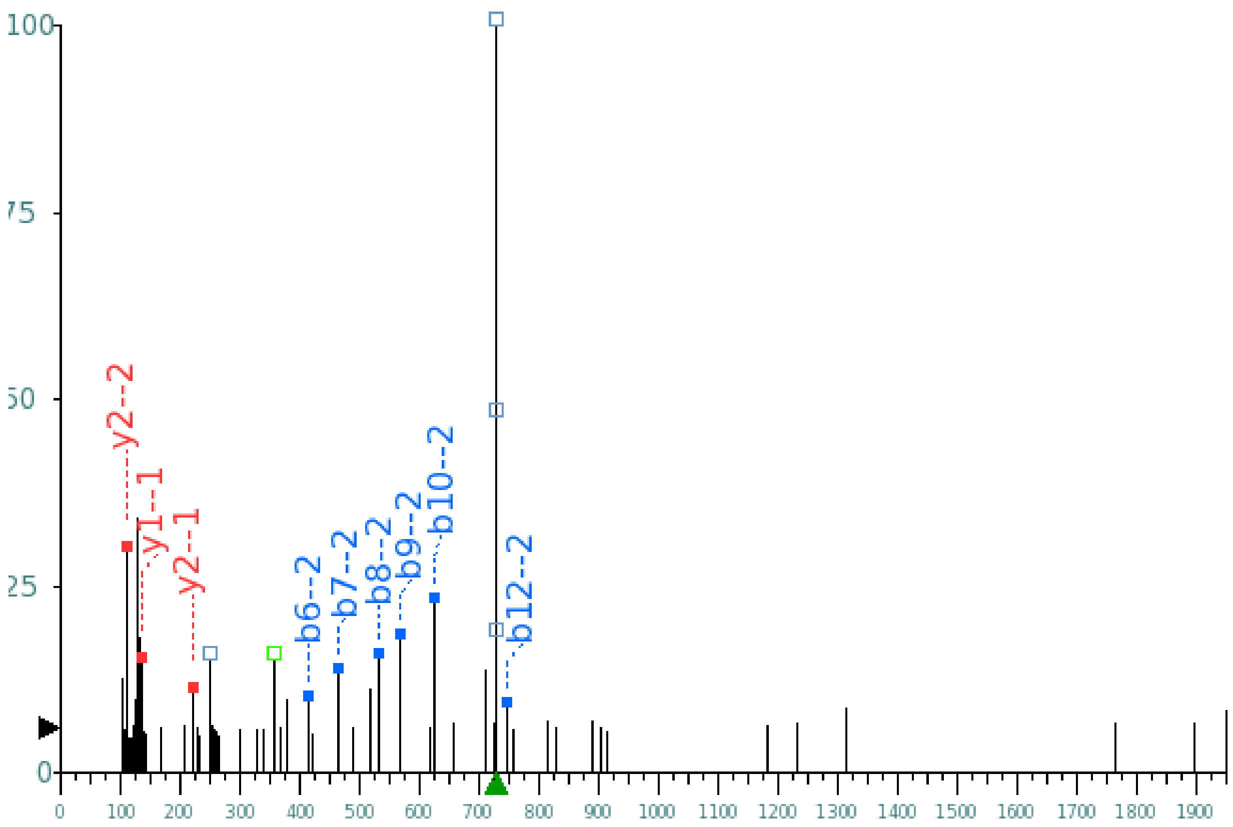

RGSSPDVHALLEITEESD (+3)

Primary Reference: PTRF_HUMAN
Search ID: 41760
Search Name: 20130328_ananiav_ERstress_TMT_TM_timecourse_trypsin_inj1_4mc_iaa
Scan#: 22320
Observed Mass: 728.7051 (1.1 ppm)
PSM Score: 12.54

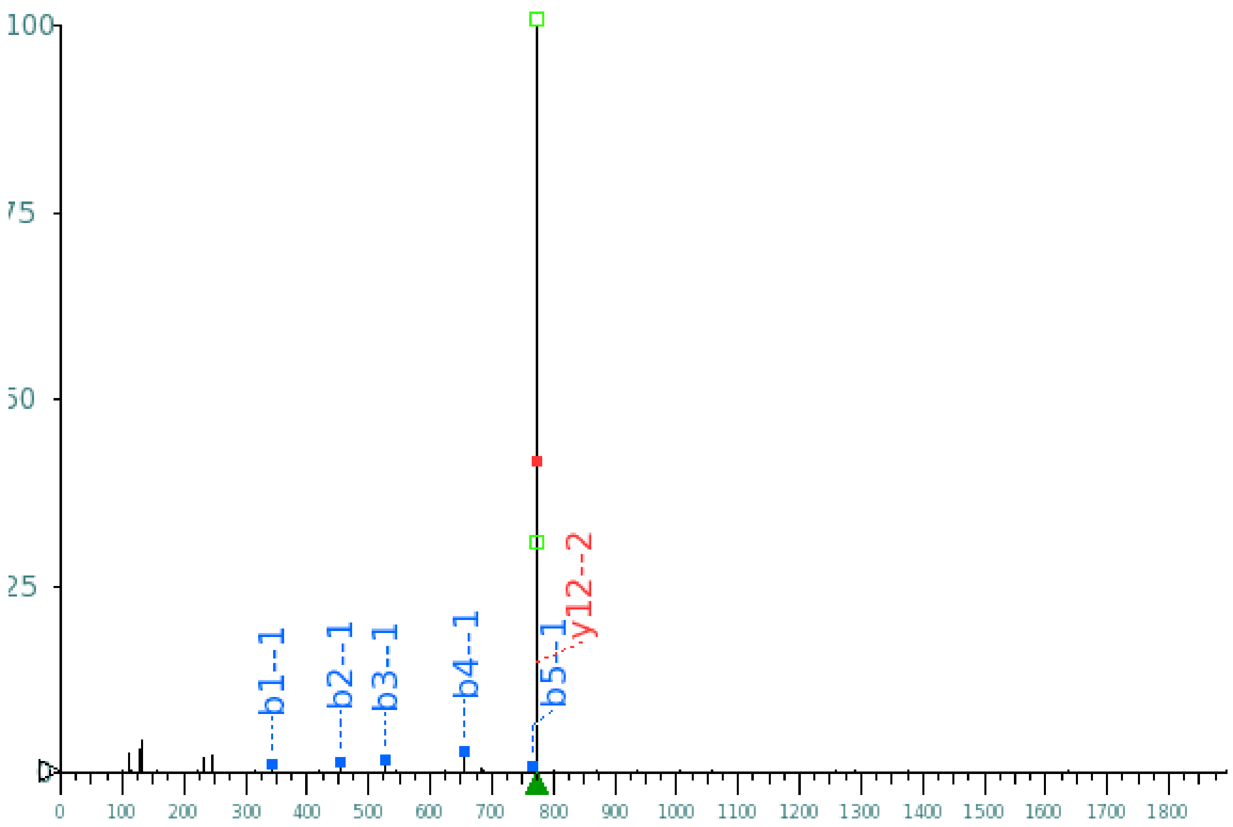

ILAQITGTEHLKESEAD (+3)

Primary Reference: PDLI5_HUMAN
Search ID: 41760
Search Name: 20130328_ananiav_ERstress_TMT_TM_timecourse_trypsin_inj1_4mc_iaa
Scan#: 22365
Observed Mass: 771.7626 (1.3 ppm)
PSM Score: 33.37

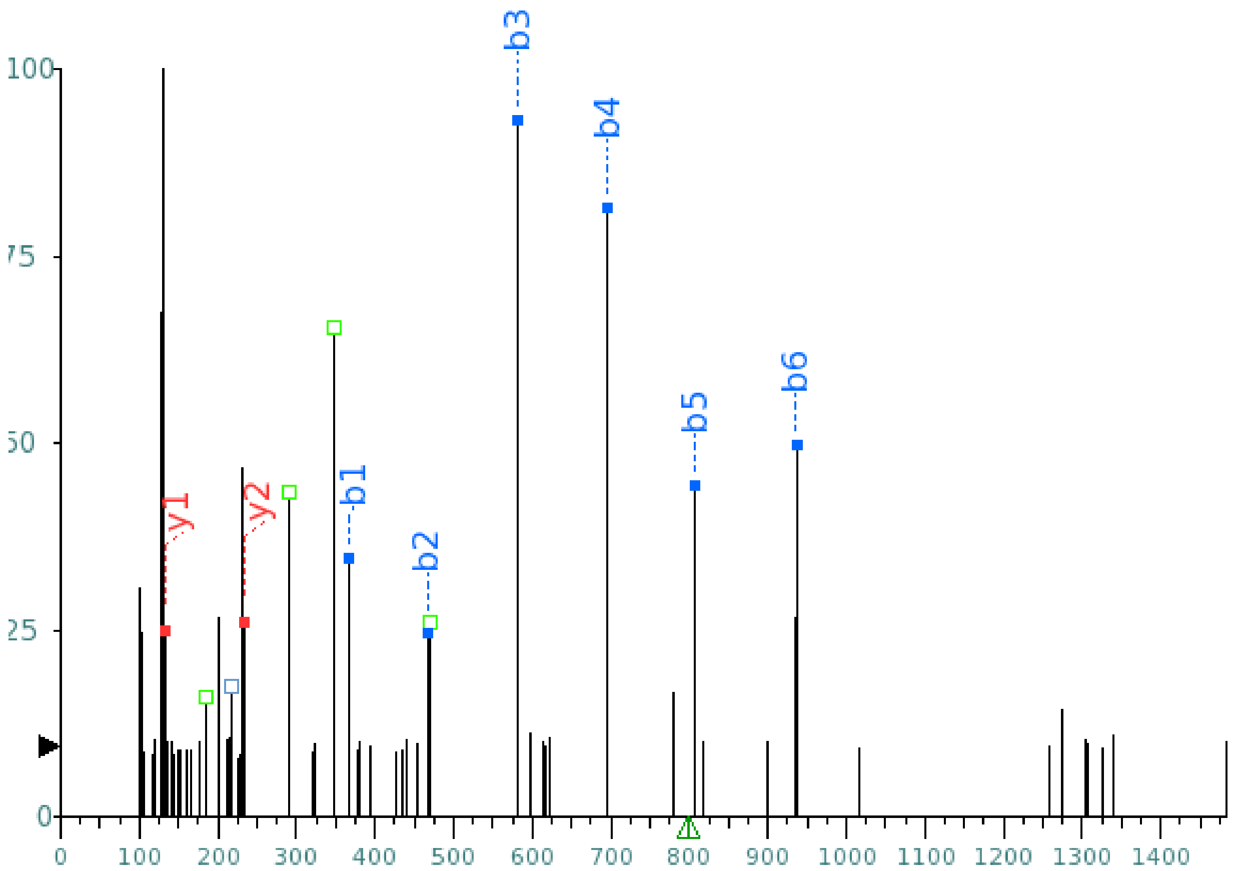

HVDLLEVAQETD (+2)

Primary Reference: ATAD1_HUMAN
Search ID: 41760
Search Name: 20130328_ananiav_ERstress_TMT_TM_timecourse_trypsin_inj1_4mc_iaa
Scan#: 23455
Observed Mass: 799.4174 (0.3 ppm)
PSM Score: 23.54

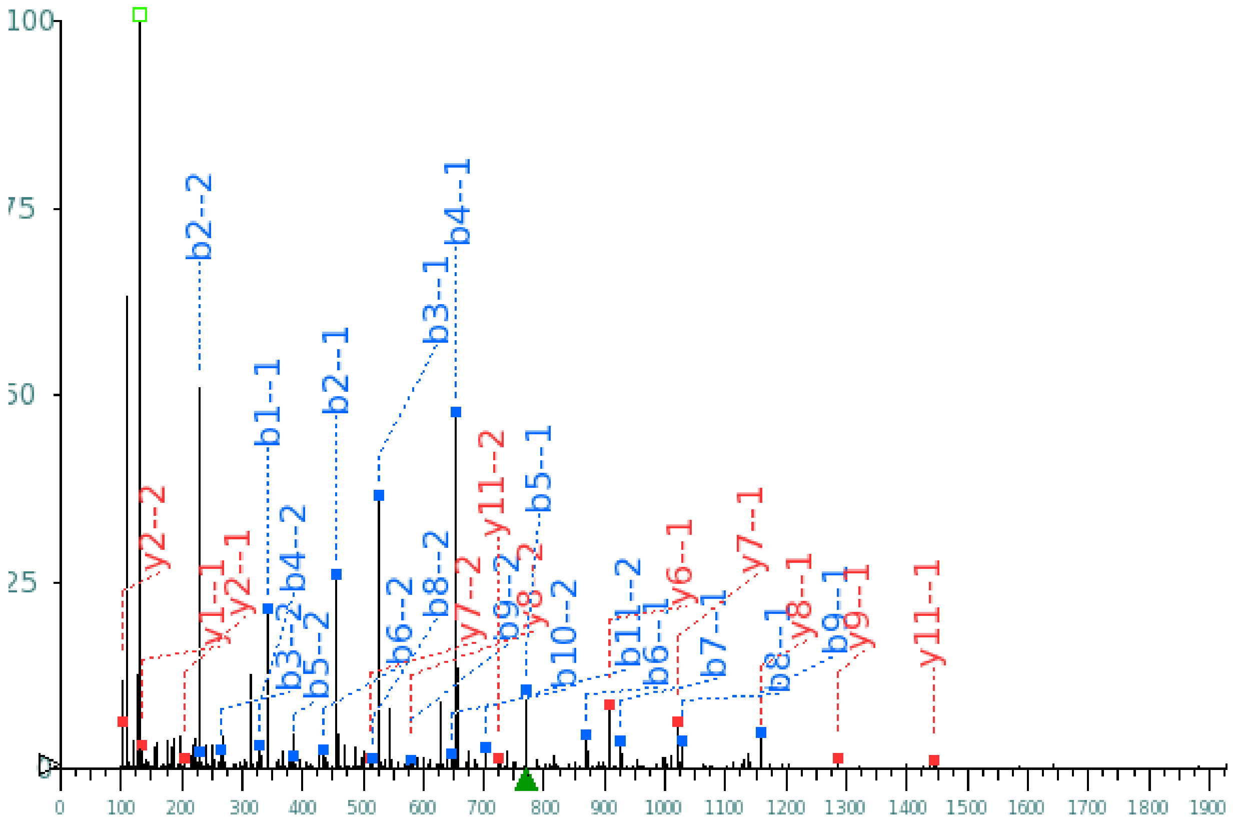

ILAQITGTEHLKESEAD (+3)

Primary Reference: PDLI5_HUMAN
Search ID: 41760
Search Name: 20130328_ananiav_ERstress_TMT_TM_timecourse_trypsin_inj1_4mc_iaa
Scan#: 23463
Observed Mass: 771.7637 (2.8 ppm)
PSM Score: 42.21

File name: 2015-02-04 04.01.53PM.pdf

Creation time: 2015-02-04 04:09PM

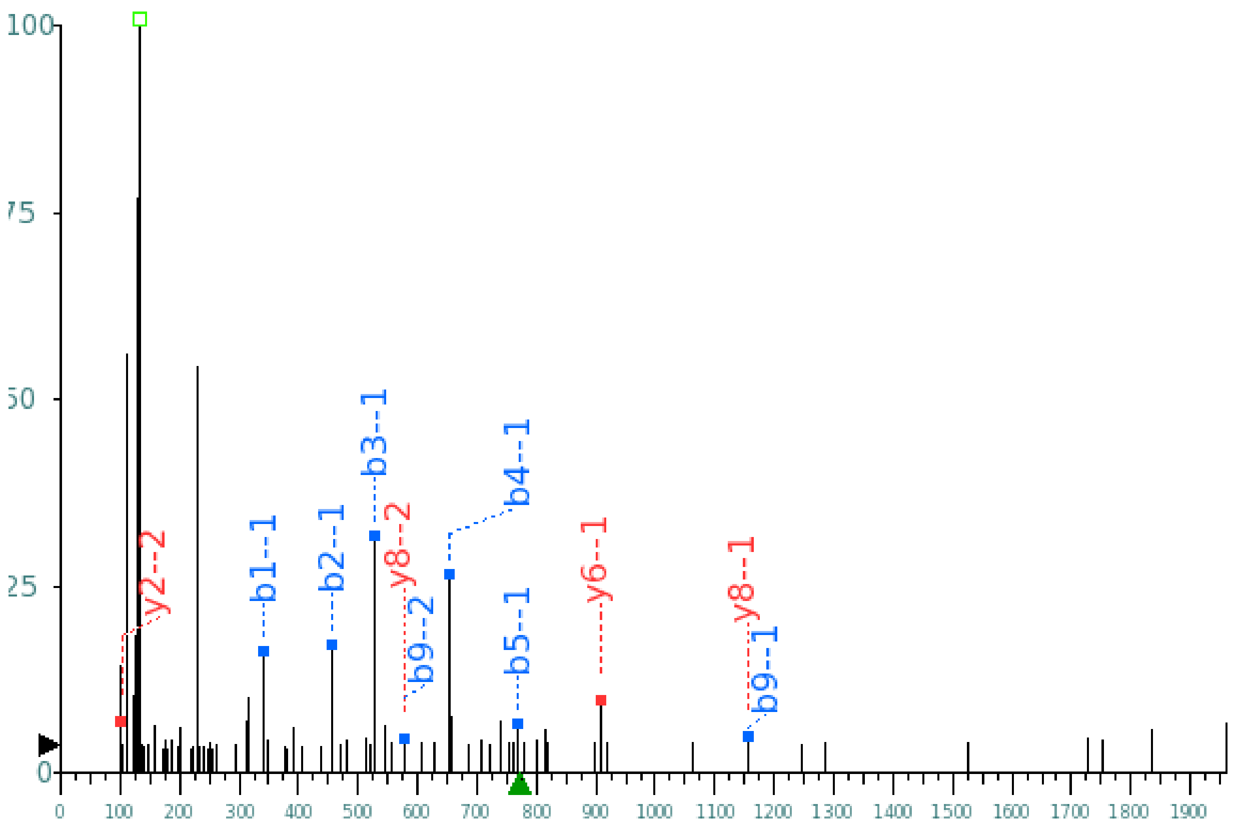

ILAQITGTEHLKESEAD (+3)

Primary Reference: PDLI5_HUMAN
Search ID: 41760
Search Name: 20130328_ananiav_ERstress_TMT_TM_timecourse_trypsin_inj1_4mc_iaa
Scan#: 23528
Observed Mass: 771.7625 (1.3 ppm)
PSM Score: 12.09

File name: 2015-02-04 04.01.53PM.pdf

Creation time: 2015-02-04 04:09PM

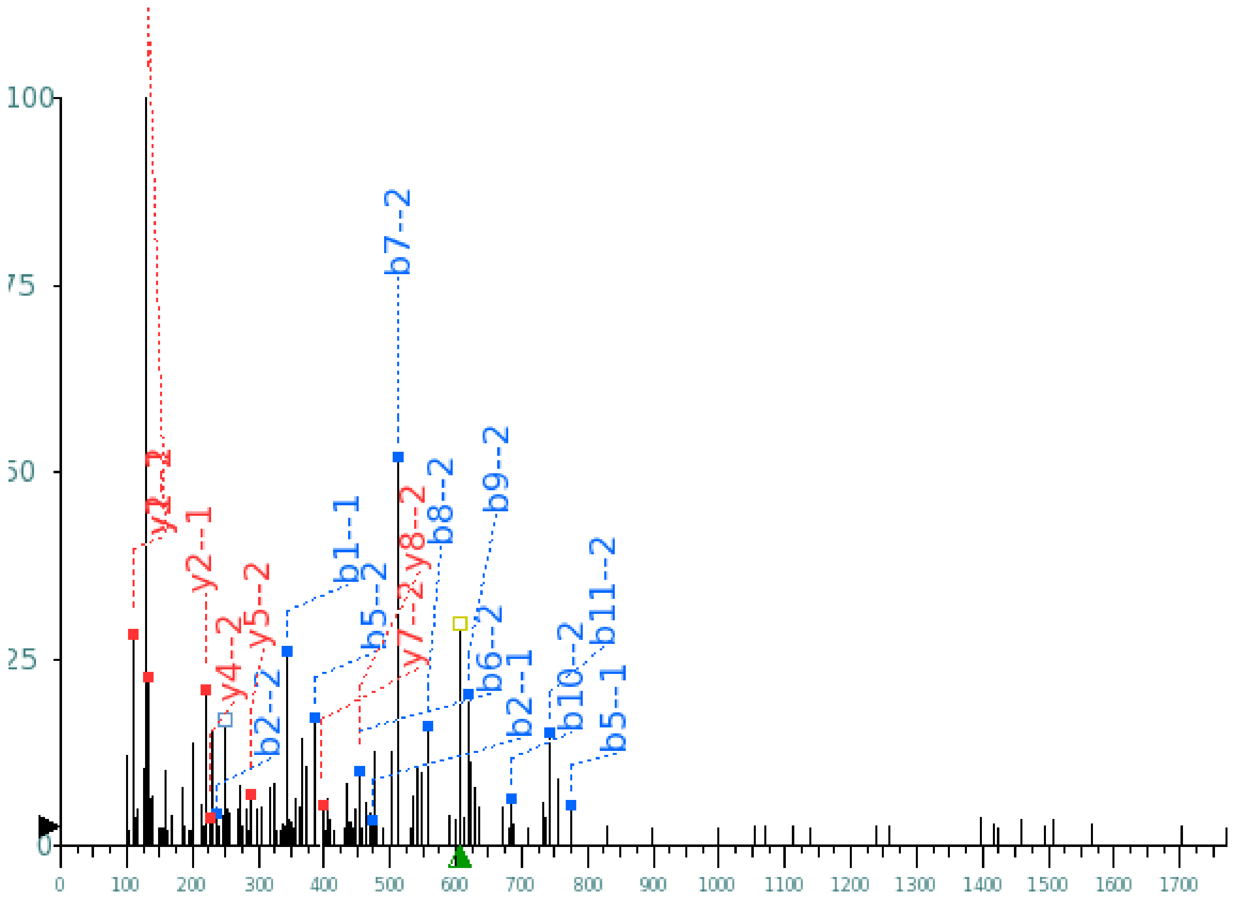

DQGSRHDSEQDNSD (+3)

Primary Reference: FUS_HUMAN
Search ID: 41761
Search Name: 20130328_ananiav_ERstress_TMT_TM_timecourse_trypsin_inj2_4mc_iaa
Scan#: 1750
Observed Mass: 606.9288 (1.8 ppm)
PSM Score: 10.96

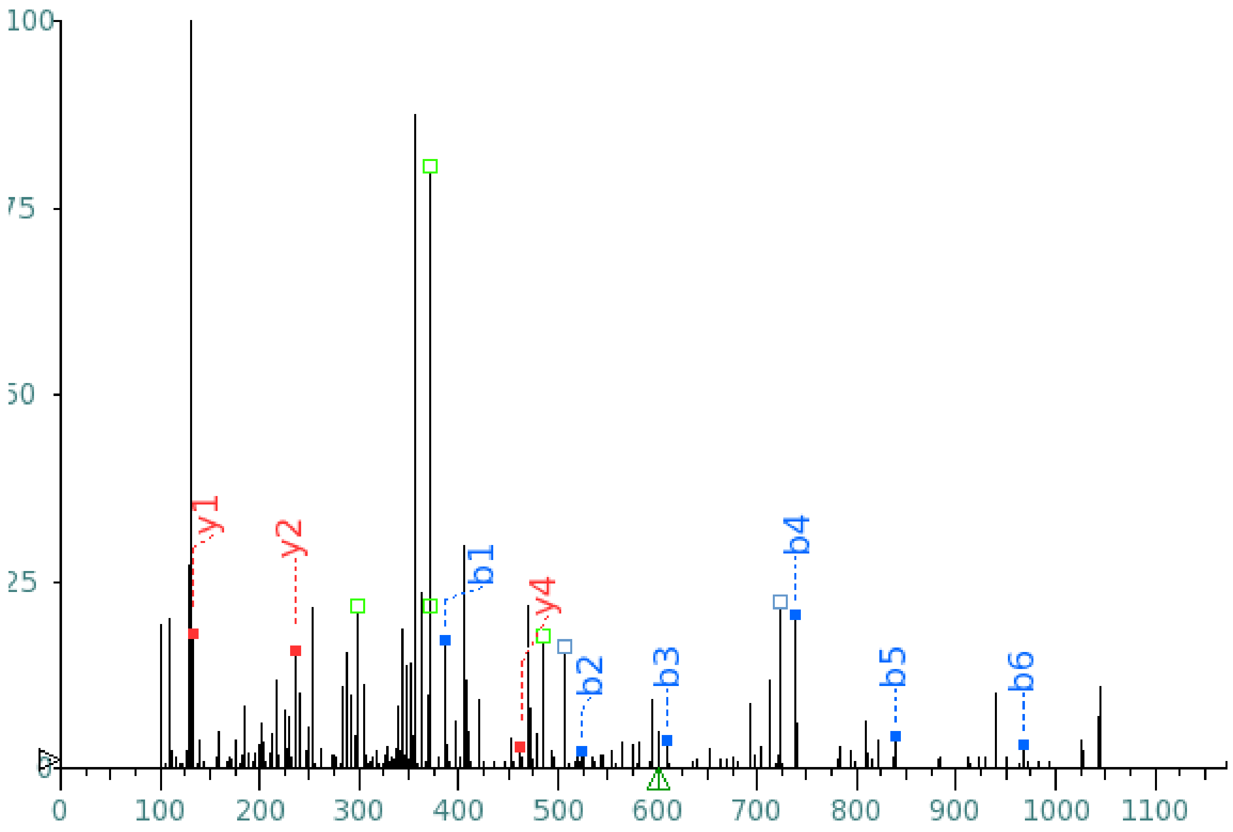

RHSEVETD (+2)

Primary Reference: LYAR_HUMAN
Search ID: 41761
Search Name: 20130328_ananiav_ERstress_TMT_TM_timecourse_trypsin_inj2_4mc_iaa
Scan#: 2051
Observed Mass: 601.3045 (0.6 ppm)
PSM Score: 10.97

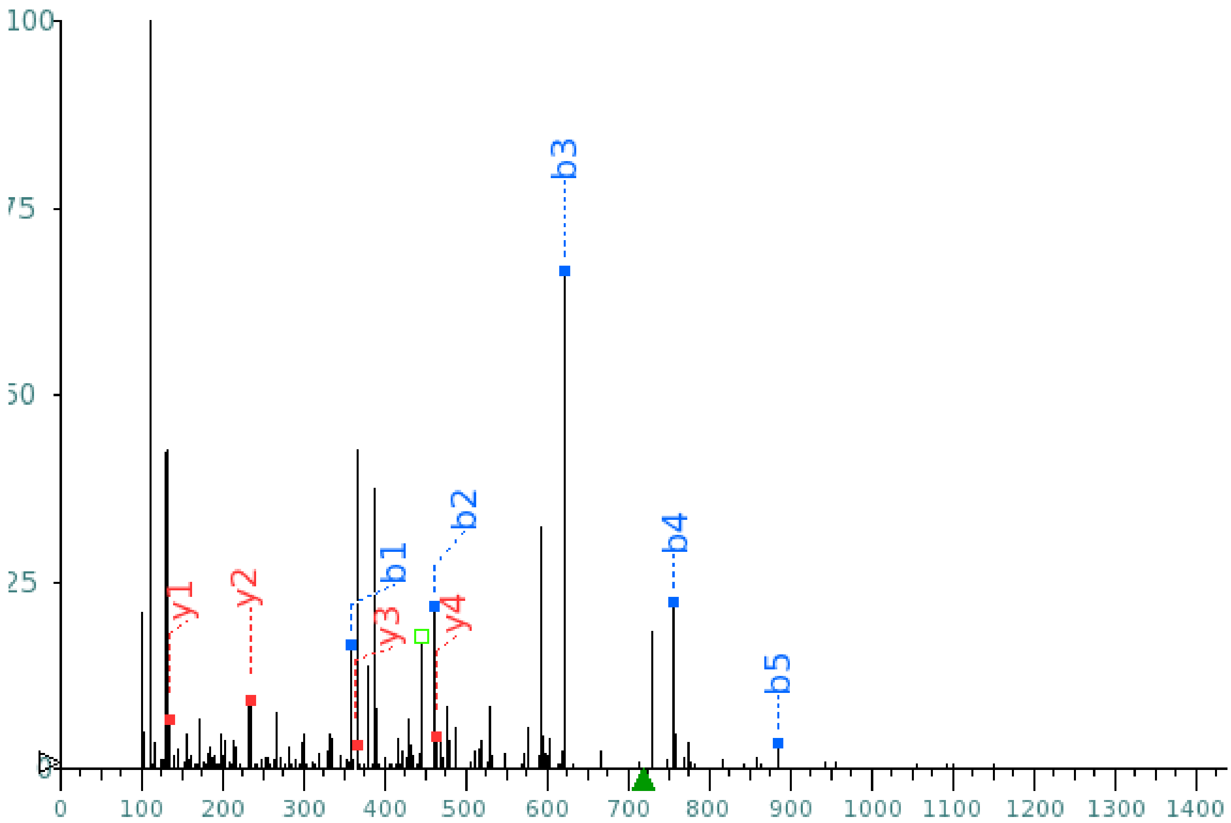

ETC*HQSTETD (+2)

Primary Reference: MORC3_HUMAN
Search ID: 41761
Search Name: 20130328_ananiav_ERstress_TMT_TM_timecourse_trypsin_inj2_4mc_iaa
Scan#: 2128
Observed Mass: 718.8125 (1.1 ppm)
PSM Score: 19.11

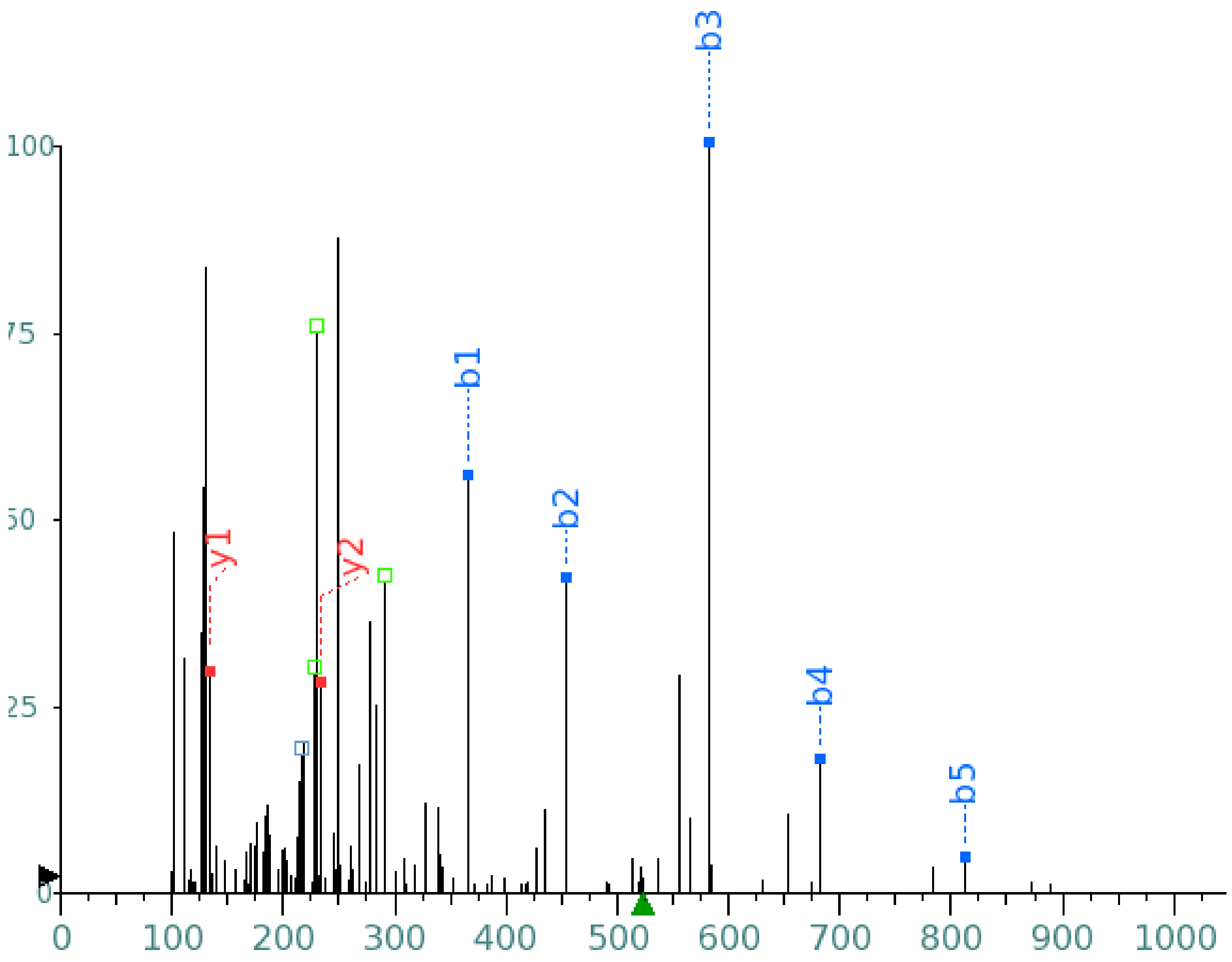

HSEVETD (+2)

Primary Reference: LYAR_HUMAN
Search ID: 41761
Search Name: 20130328_ananiav_ERstress_TMT_TM_timecourse_trypsin_inj2_4mc_iaa
Scan#: 2133
Observed Mass: 523.2542 (1.2 ppm)
PSM Score: 32.38

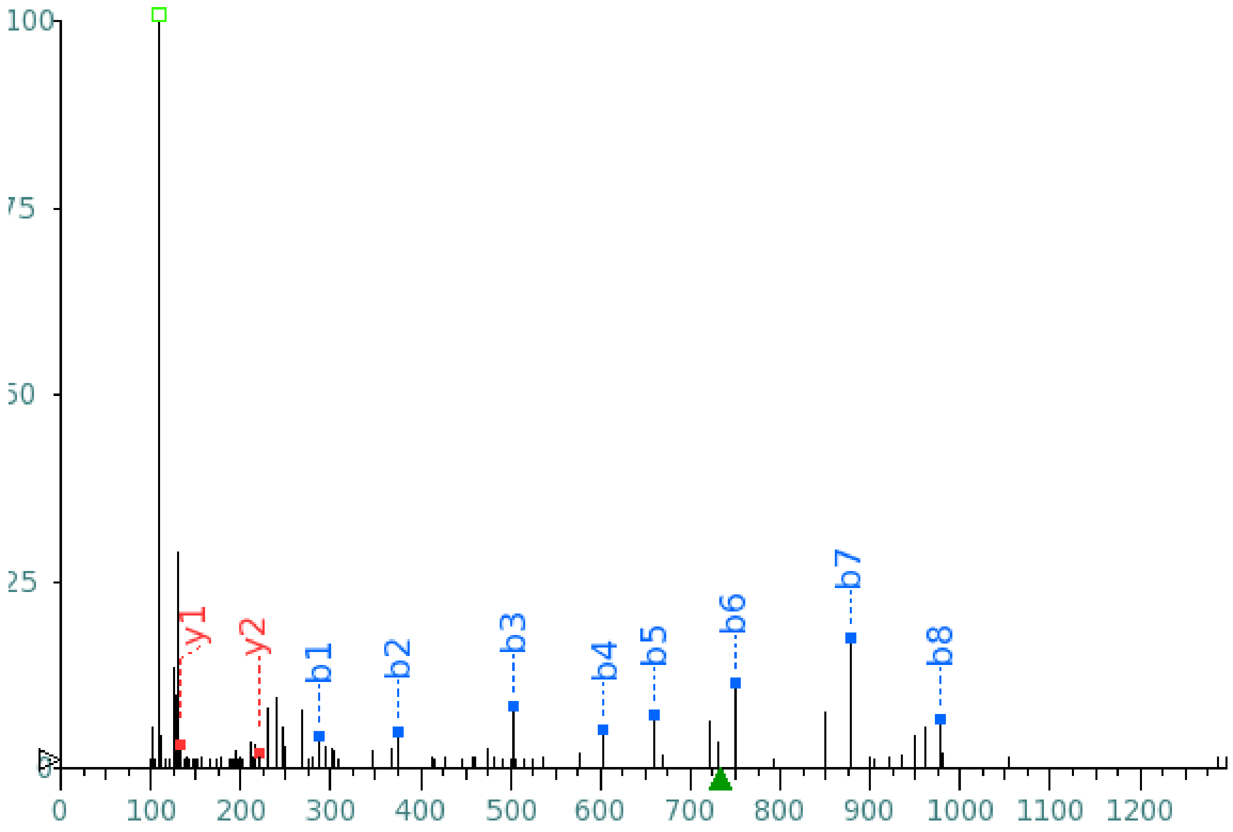

GSETGSETHESD (+2)

Primary Reference: PRC2A_HUMAN
Search ID: 41761
Search Name: 20130328_ananiav_ERstress_TMT_TM_timecourse_trypsin_inj2_4mc_iaa
Scan#: 2140
Observed Mass: 732.8195 (2 ppm)
PSM Score: 26.17

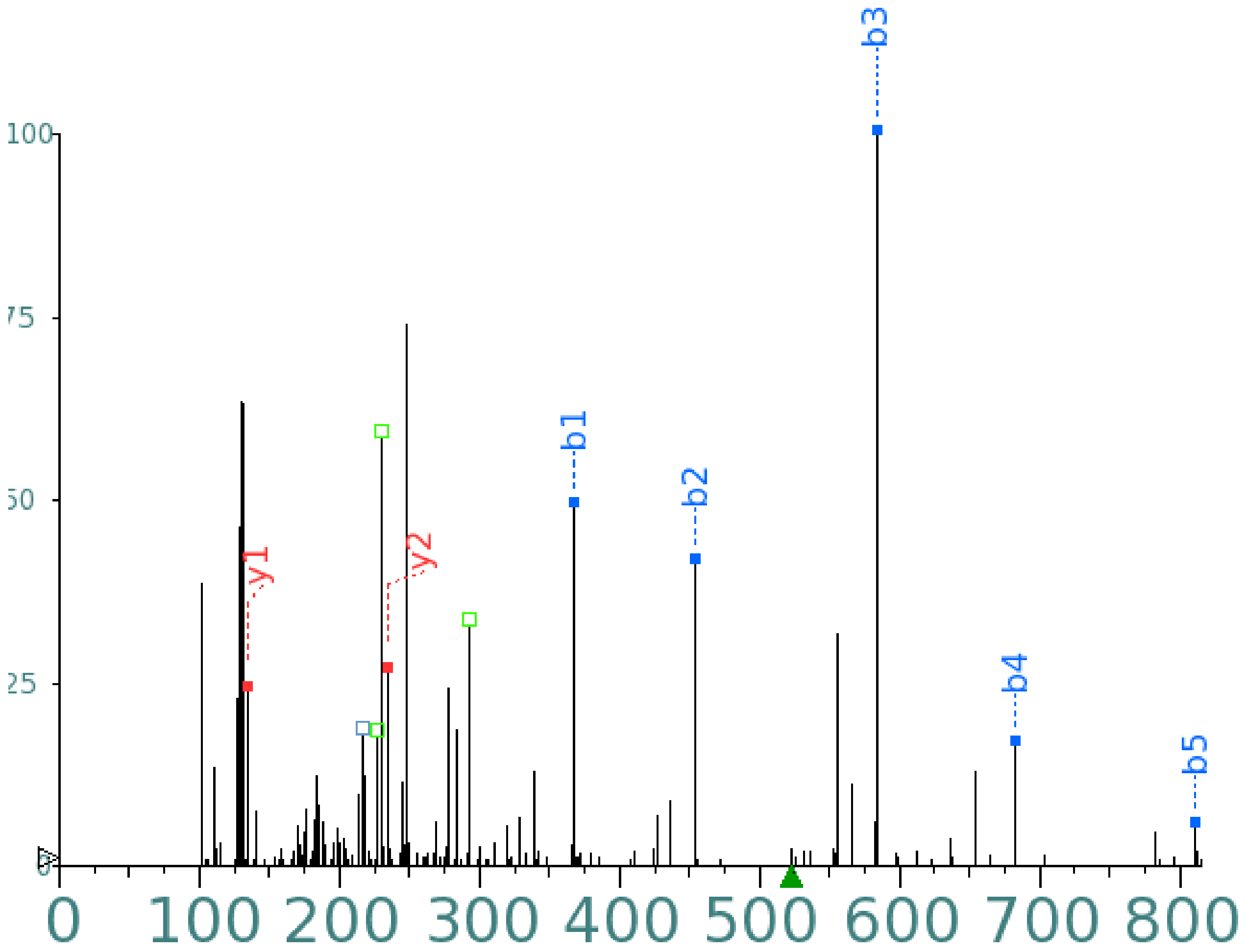

HSEVETD (+2)

Primary Reference: LYAR_HUMAN
Search ID: 41761
Search Name: 20130328_ananiav_ERstress_TMT_TM_timecourse_trypsin_inj2_4mc_iaa
Scan#: 2217
Observed Mass: 523.2551 (2.9 ppm)
PSM Score: 32.82

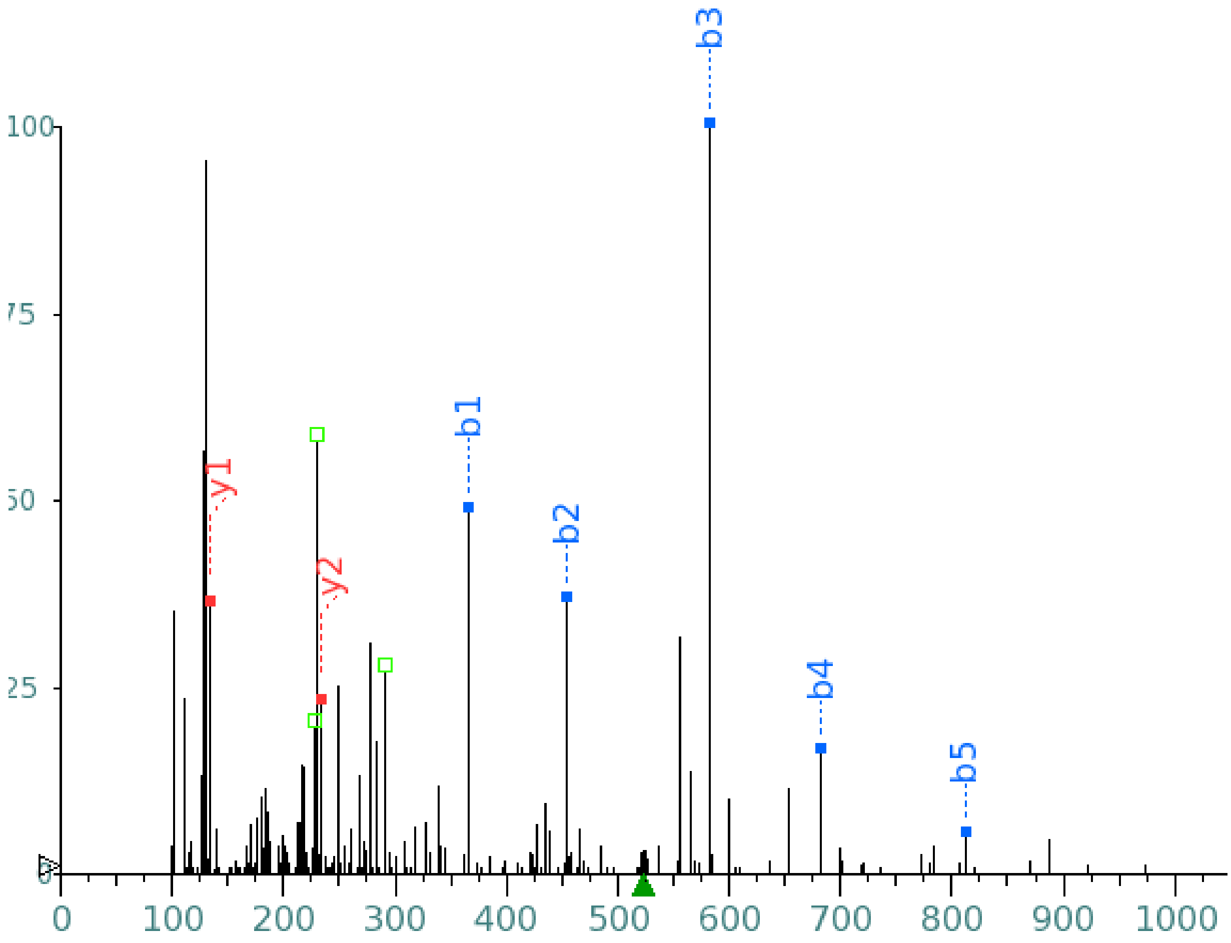

HSEVETD (+2)

Primary Reference: LYAR_HUMAN
Search ID: 41761
Search Name: 20130328_ananiav_ERstress_TMT_TM_timecourse_trypsin_inj2_4mc_iaa
Scan#: 2279
Observed Mass: 523.2552 (2.9 ppm)
PSM Score: 29.69

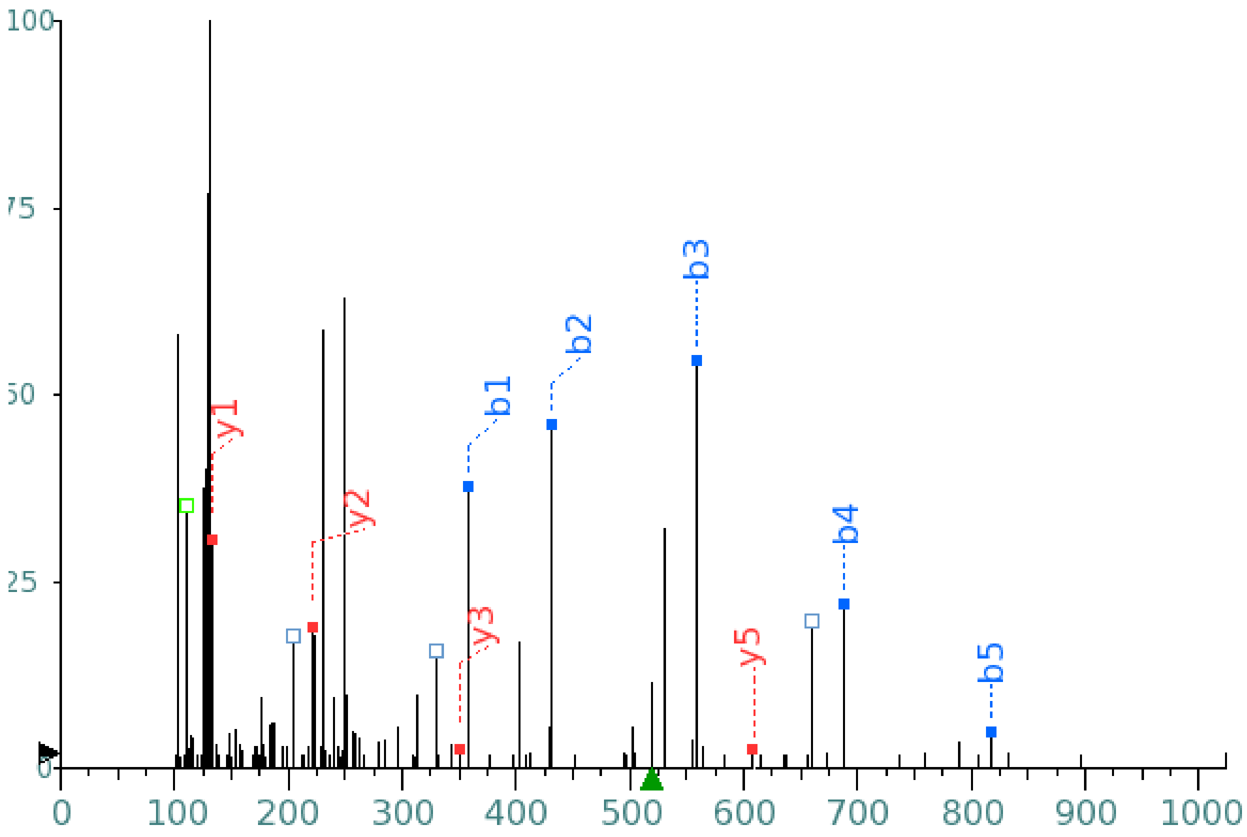

EAEEESD (+2)

Primary Reference: IF4G1_HUMAN
Search ID: 41761
Search Name: 20130328_ananiav_ERstress_TMT_TM_timecourse_trypsin_inj2_4mc_iaa
Scan#: 2326
Observed Mass: 519.2287 (2.8 ppm)
PSM Score: 28.88

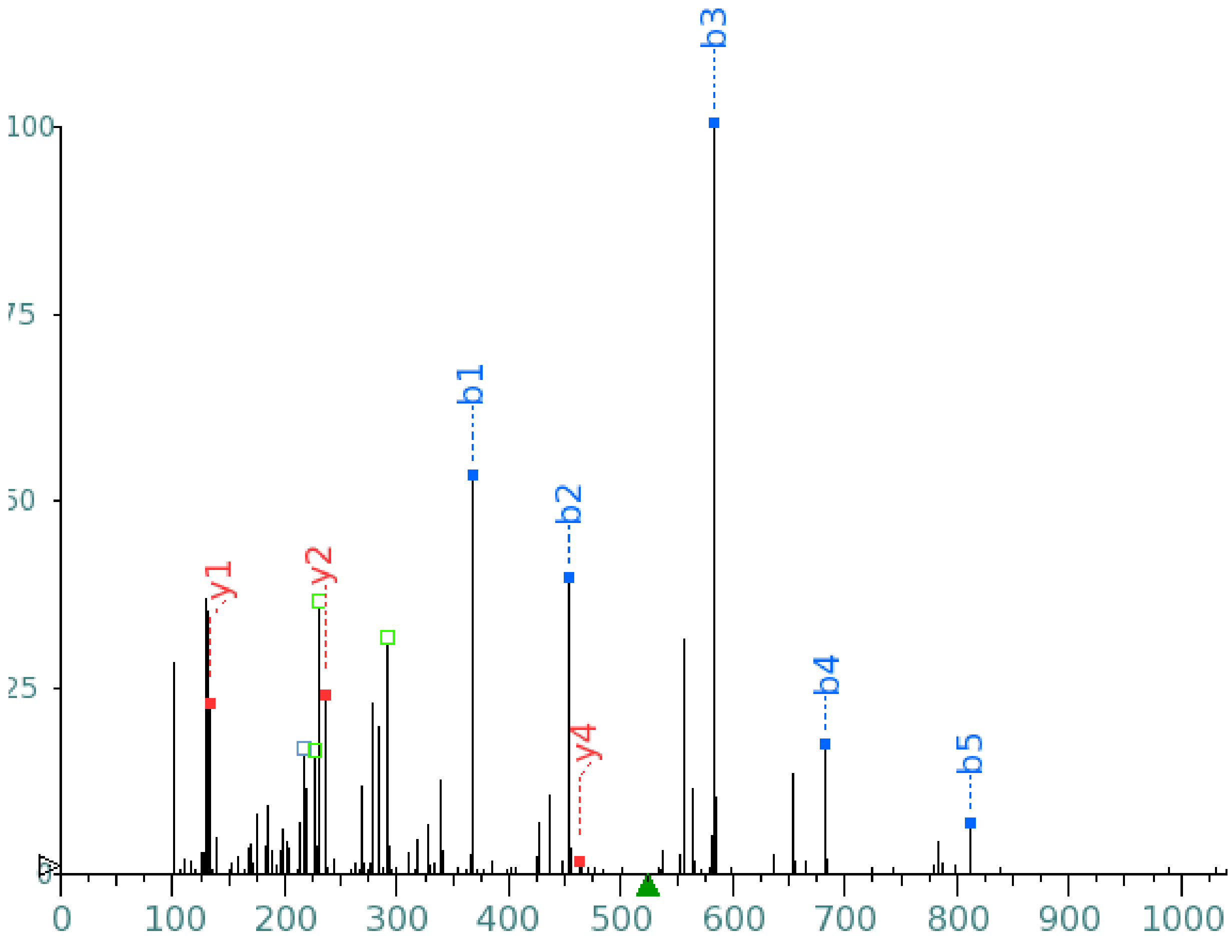

HSEVETD (+2)

Primary Reference: LYAR_HUMAN
Search ID: 41761
Search Name: 20130328_ananiav_ERstress_TMT_TM_timecourse_trypsin_inj2_4mc_iaa
Scan#: 2343
Observed Mass: 523.255 (2.6 ppm)
PSM Score: 28.74

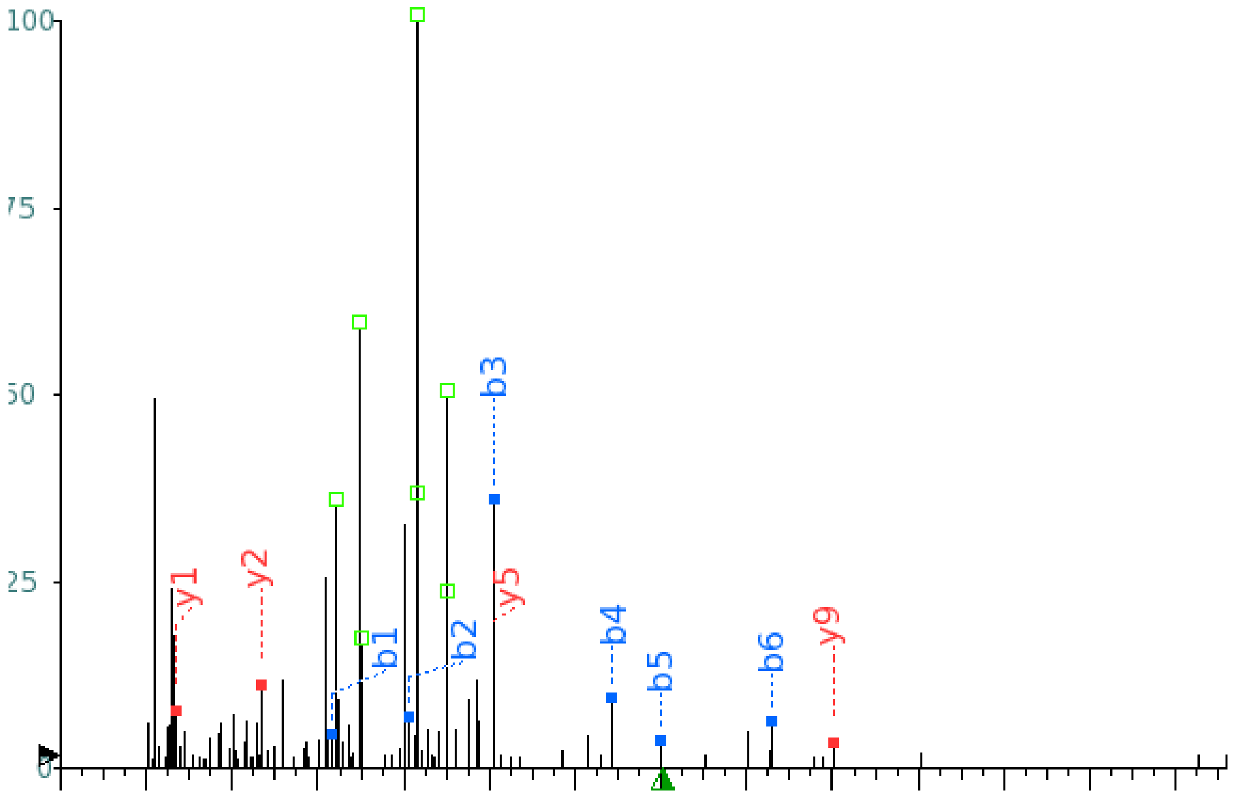

SSTHGEAAAETD (+2)

Primary Reference: UHRF1_HUMAN
Search ID: 41761
Search Name: 20130328_ananiav_ERstress_TMT_TM_timecourse_trypsin_inj2_4mc_iaa
Scan#: 2362
Observed Mass: 702.8275 (2.6 ppm)
PSM Score: 25.59

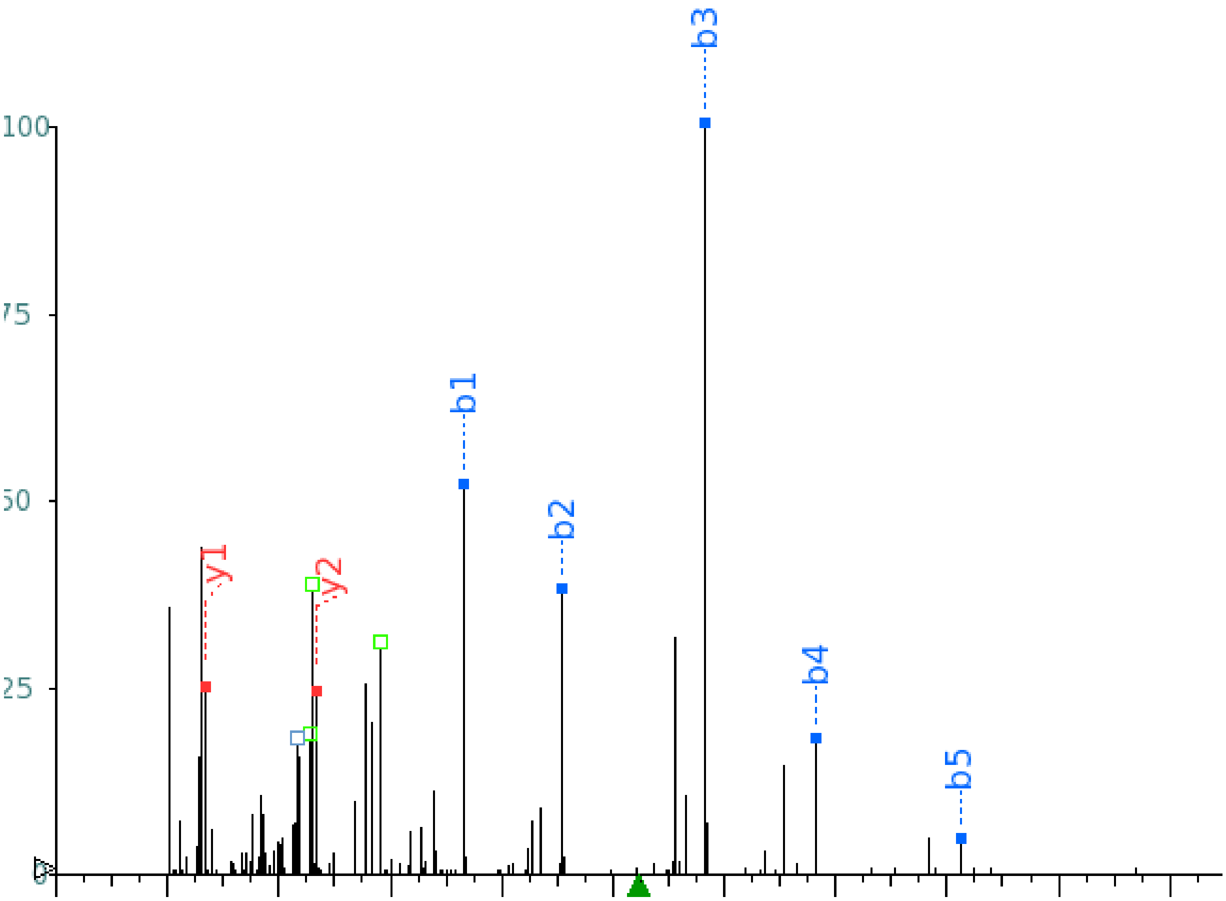

HSEVETD (+2)

Primary Reference: LYAR_HUMAN
Search ID: 41761
Search Name: 20130328_ananiav_ERstress_TMT_TM_timecourse_trypsin_inj2_4mc_iaa
Scan#: 2409
Observed Mass: 523.255 (2.6 ppm)
PSM Score: 31.08

File name: 2015-02-04 04.01.53PM.pdf

Creation time: 2015-02-04 04:09PM

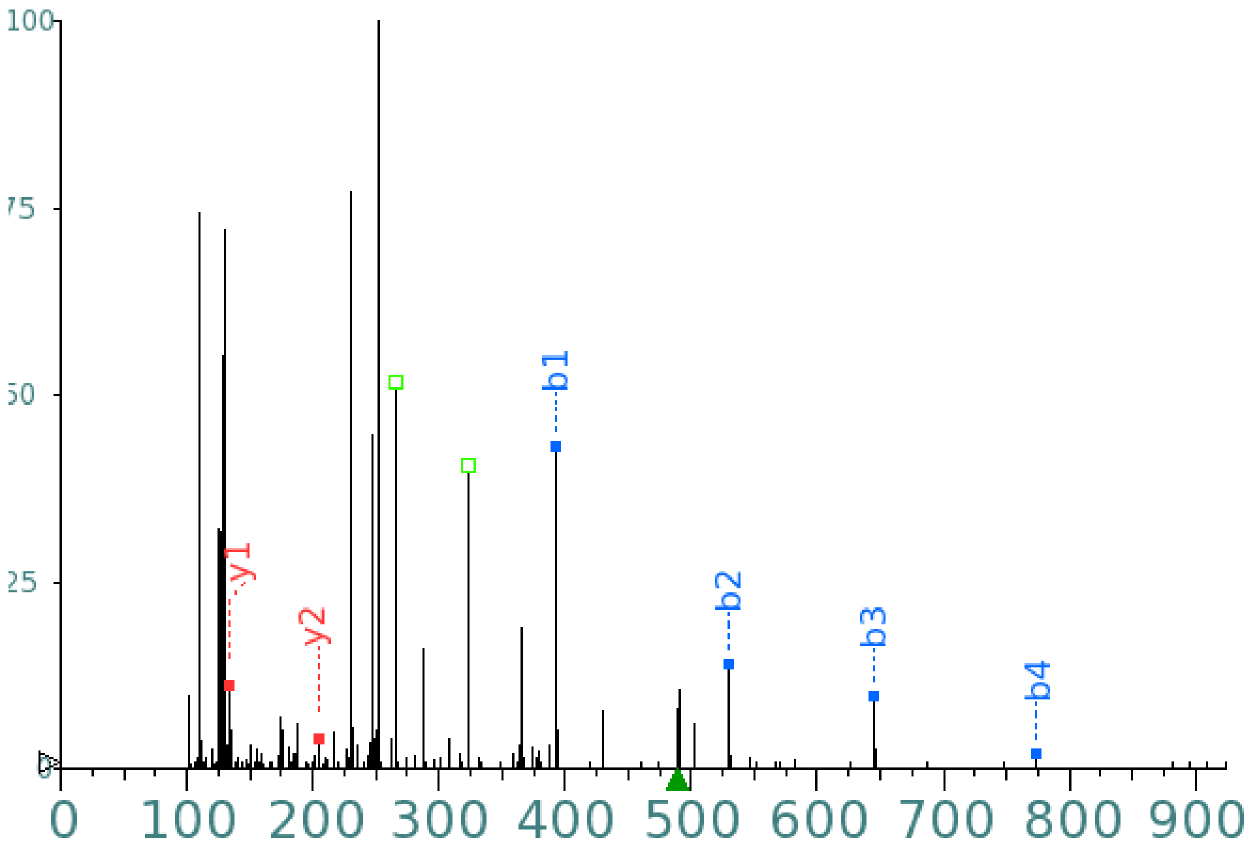

YHDEAD (+2)

Primary Reference: TF3C1_HUMAN
Search ID: 41761
Search Name: 20130328_ananiav_ERstress_TMT_TM_timecourse_trypsin_inj2_4mc_iaa
Scan#: 2435
Observed Mass: 489.7236 (3.4 ppm)
PSM Score: 13.78

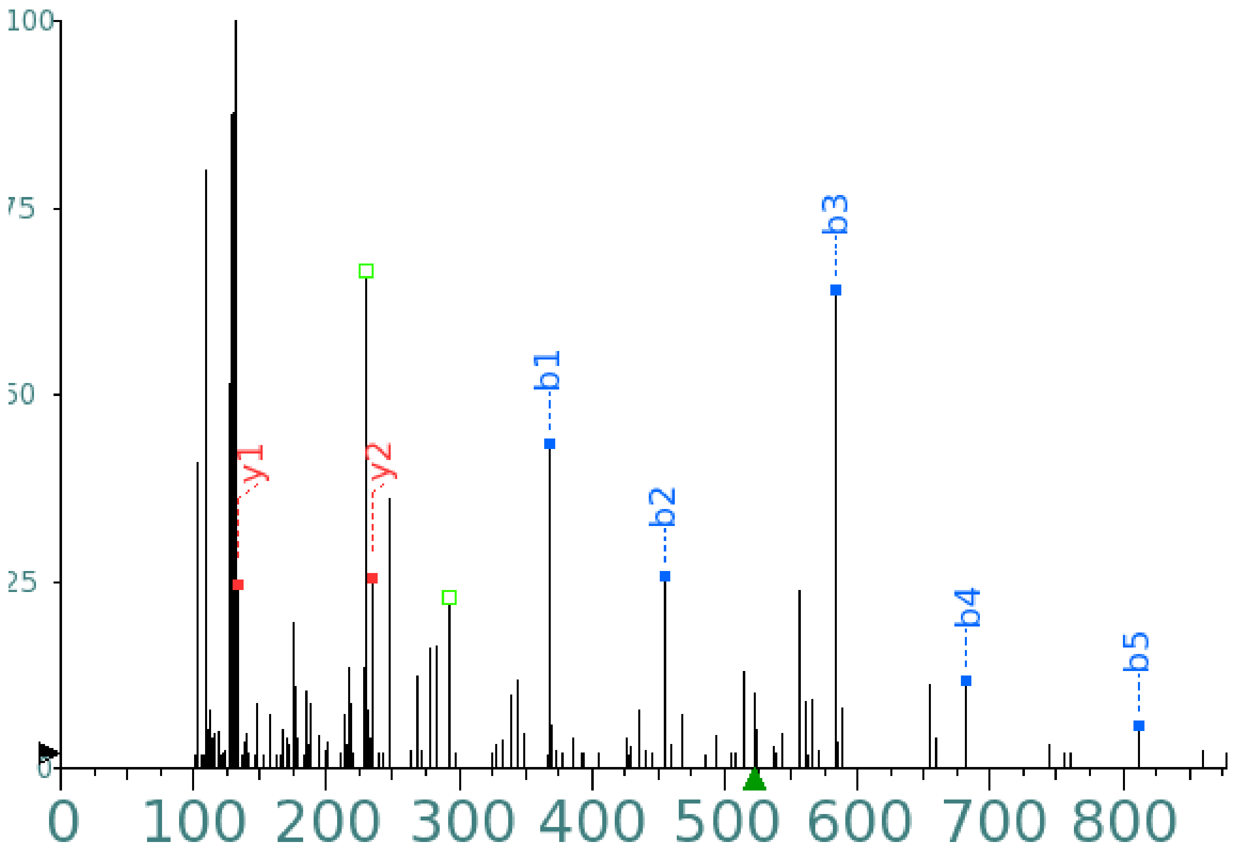

HSEVETD (+2)

Primary Reference: LYAR_HUMAN
Search ID: 41761
Search Name: 20130328_ananiav_ERstress_TMT_TM_timecourse_trypsin_inj2_4mc_iaa
Scan#: 2484
Observed Mass: 523.2549 (2.4 ppm)
PSM Score: 18.25

File name: 2015-02-04 04.01.53PM.pdf

Creation time: 2015-02-04 04:09PM

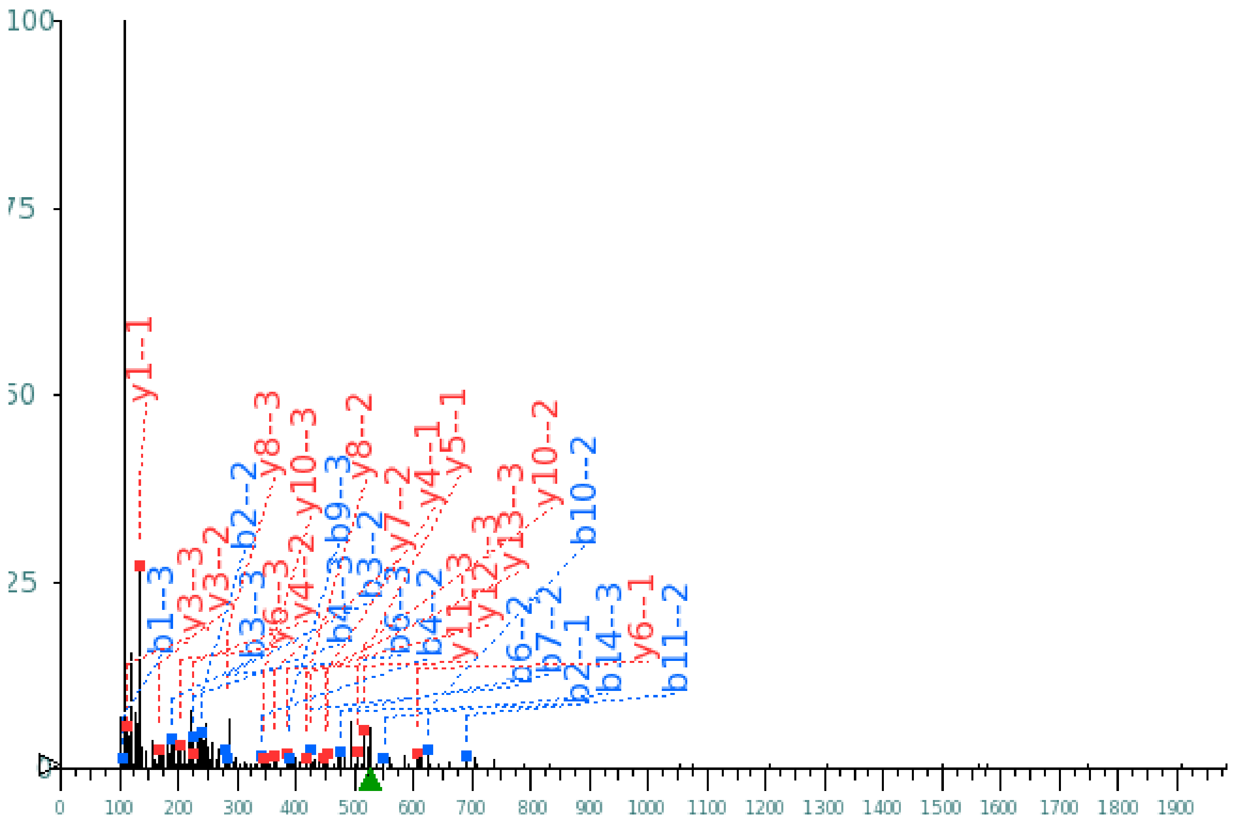

SC*SNGNCLPSEDTGNPCD (+4)

Primary Reference: ##LRP2_HUMAN
Search ID: 41761
Search Name: 20130328_ananiav_ERstress_TMT_TM_timecourse_trypsin_inj2_4mc_iaa
Scan#: 2512
Observed Mass: 525.4649 (3.1 ppm)
PSM Score: 6.7

File name: 2015-02-04 04.01.53PM.pdf

Creation time: 2015-02-04 04:09PM

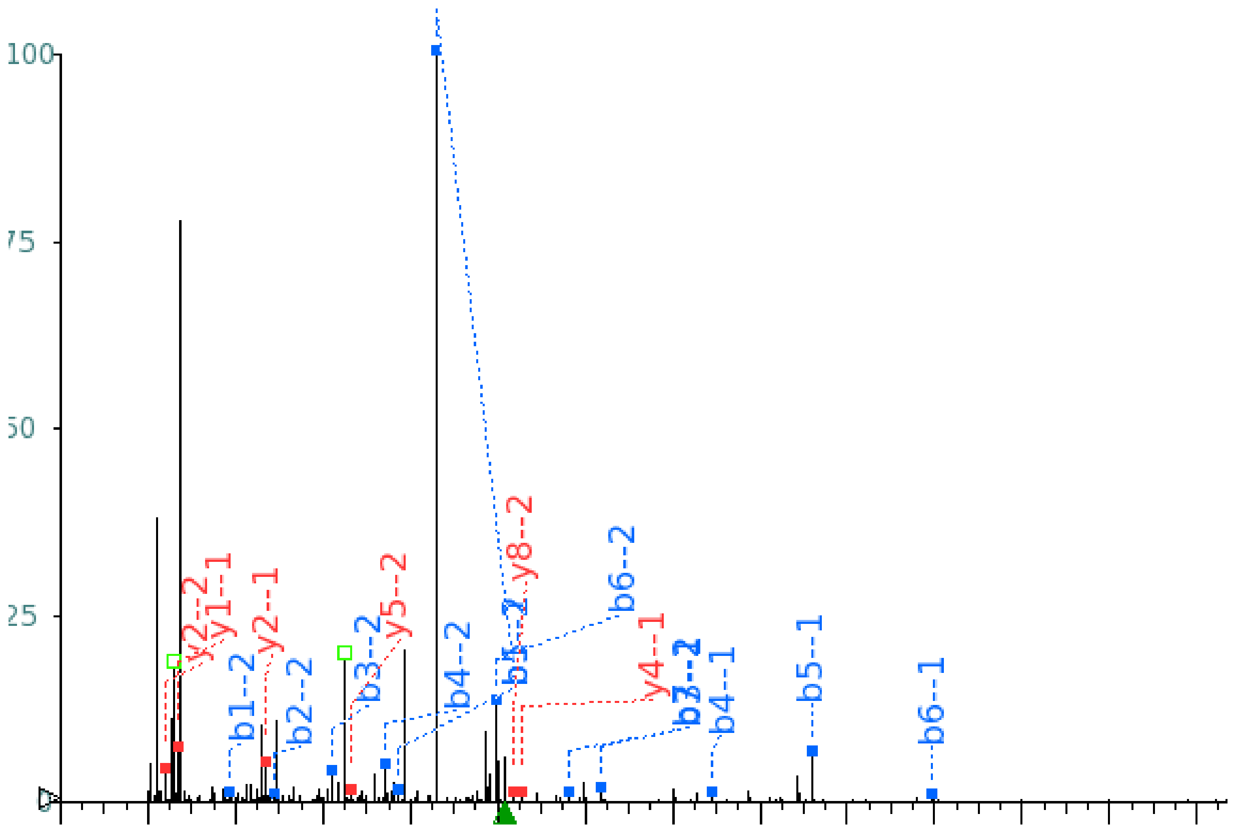

RTEQDHYETD (+3)

Primary Reference: OCLN_HUMAN
Search ID: 41761
Search Name: 20130328_ananiav_ERstress_TMT_TM_timecourse_trypsin_inj2_4mc_iaa
Scan#: 2547
Observed Mass: 508.2385 (2.4 ppm)
PSM Score: 8.91

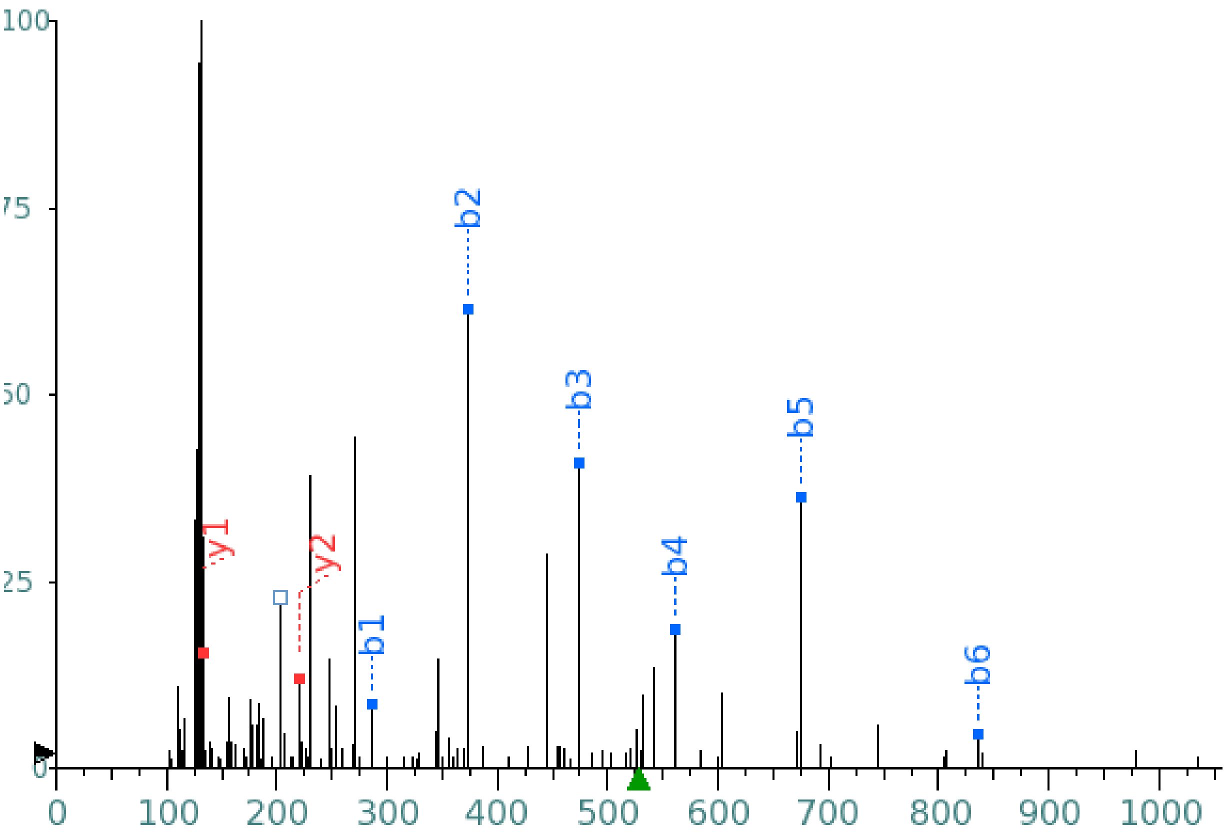

GSVSDC*SD (+2)

Primary Reference: ERF_HUMAN
Search ID: 41761
Search Name: 20130328_ananiav_ERstress_TMT_TM_timecourse_trypsin_inj2_4mc_iaa
Scan#: 2863
Observed Mass: 528.2305 (2.3 ppm)
PSM Score: 30.93

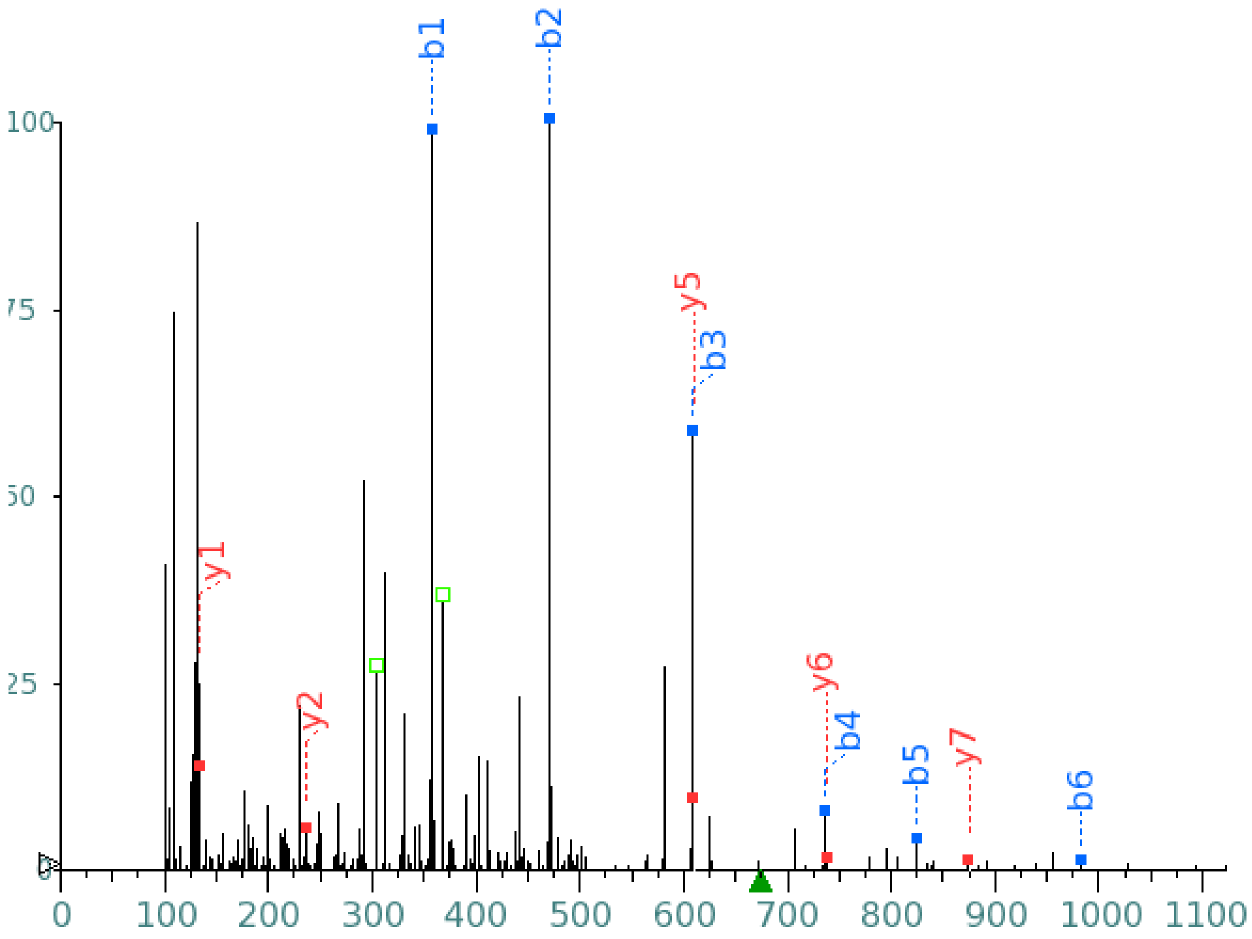

QLHQSC*QTD (+2)

Primary Reference: CSN2_HUMAN
Search ID: 41761
Search Name: 20130328_ananiav_ERstress_TMT_TM_timecourse_trypsin_inj2_4mc_iaa
Scan#: 2962
Observed Mass: 673.3237 (2.4 ppm)
PSM Score: 23.29

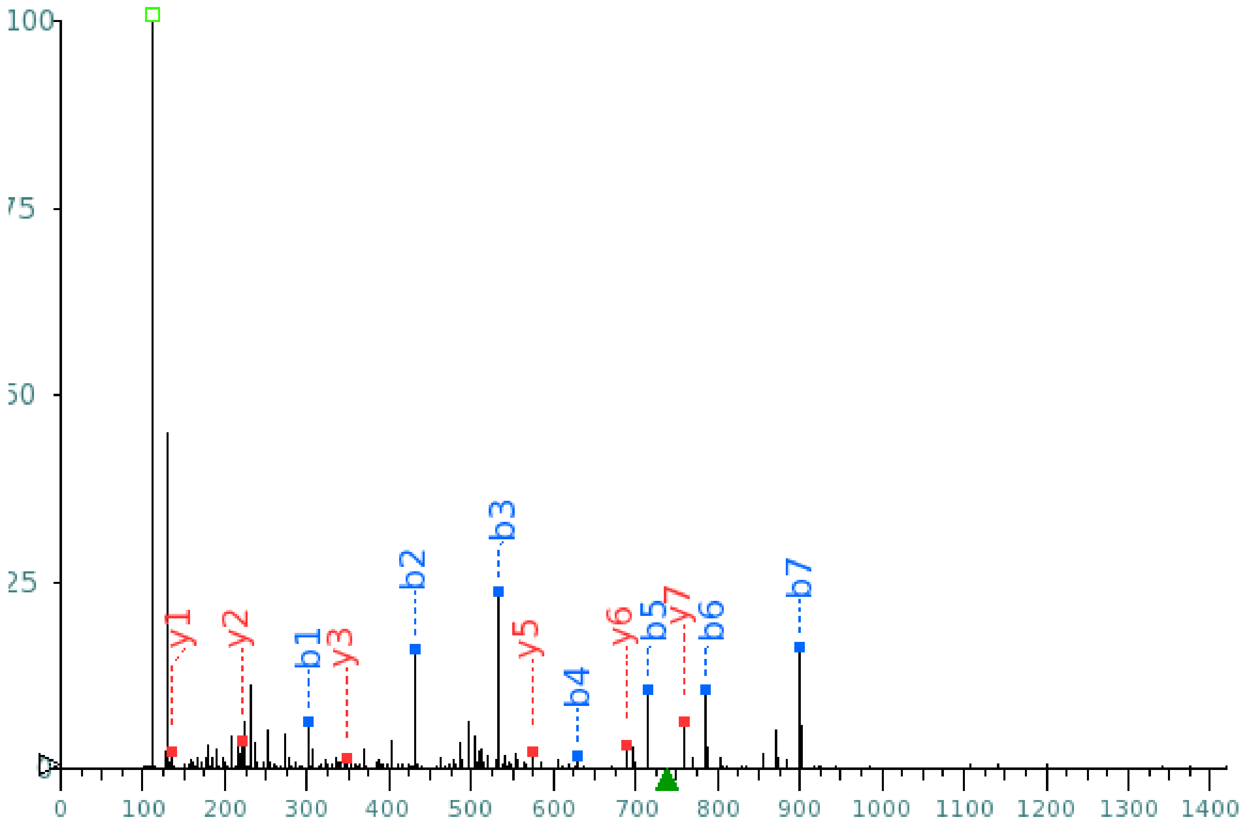

AETPSANHSESD (+2)

Primary Reference: YETS2_HUMAN
Search ID: 41761
Search Name: 20130328_ananiav_ERstress_TMT_TM_timecourse_trypsin_inj2_4mc_iaa
Scan#: 2987
Observed Mass: 737.3376 (1.6 ppm)
PSM Score: 32.48

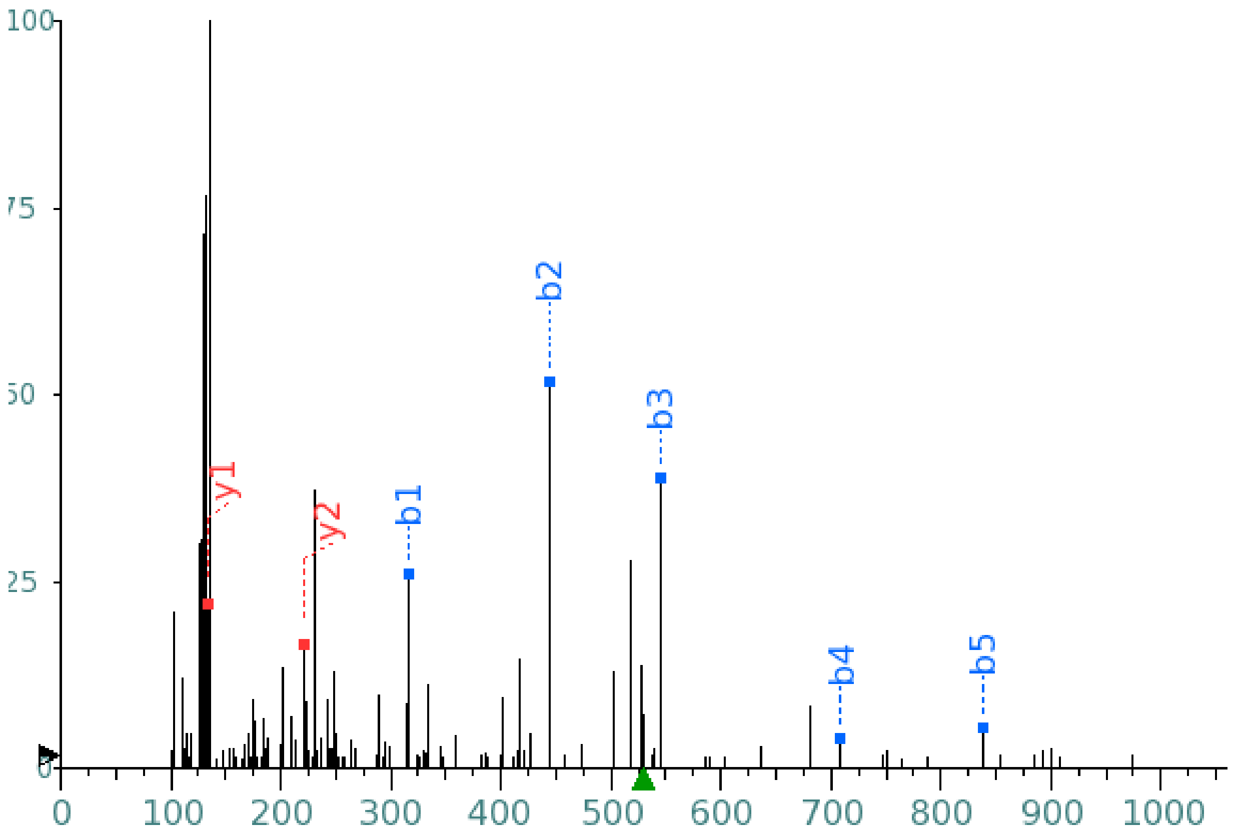

SQTYESD (+2)

Primary Reference: ADAM9_HUMAN
Search ID: 41761
Search Name: 20130328_ananiav_ERstress_TMT_TM_timecourse_trypsin_inj2_4mc_iaa
Scan#: 3012
Observed Mass: 529.7468 (2.2 ppm)
PSM Score: 28.42

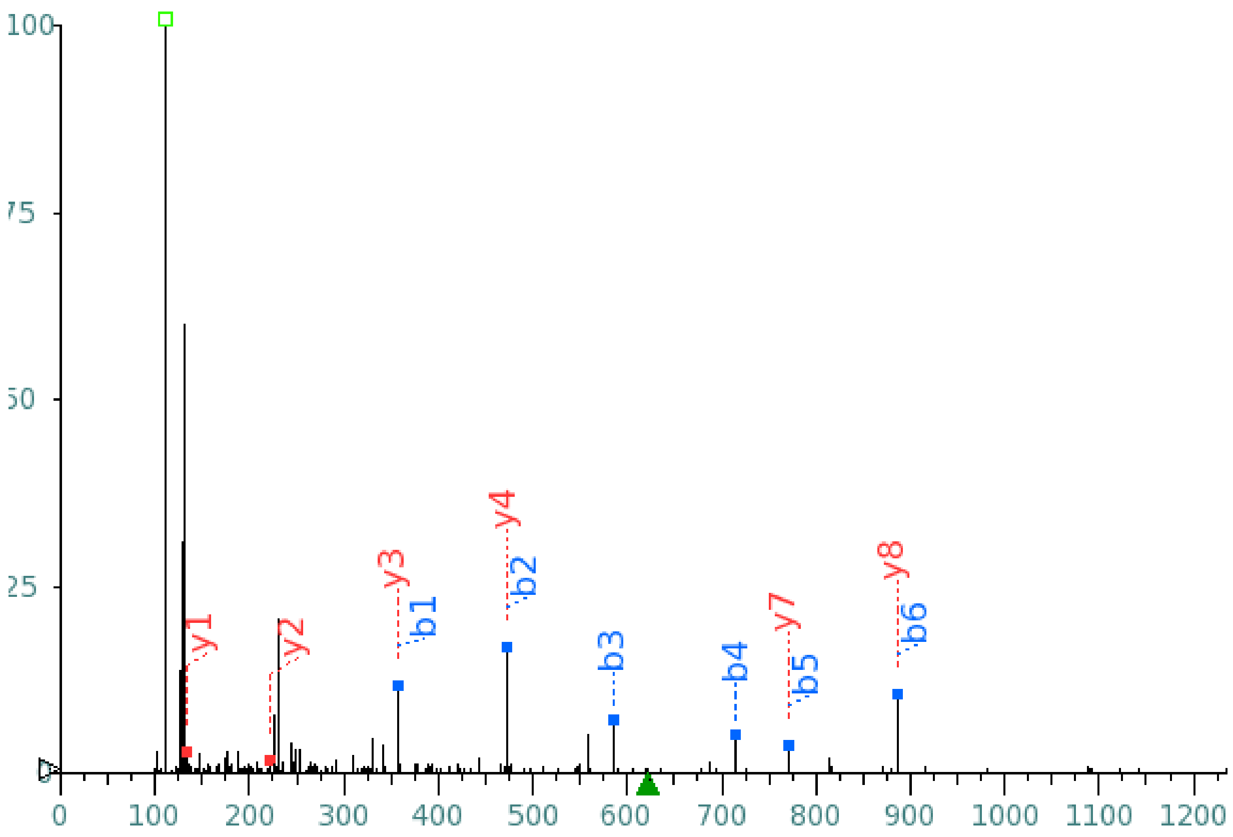

QNLEGDHSD (+2)

Primary Reference: HS74L_HUMAN
Search ID: 41761
Search Name: 20130328_ananiav_ERstress_TMT_TM_timecourse_trypsin_inj2_4mc_iaa
Scan#: 3107
Observed Mass: 622.2915 (0.4 ppm)
PSM Score: 22.04

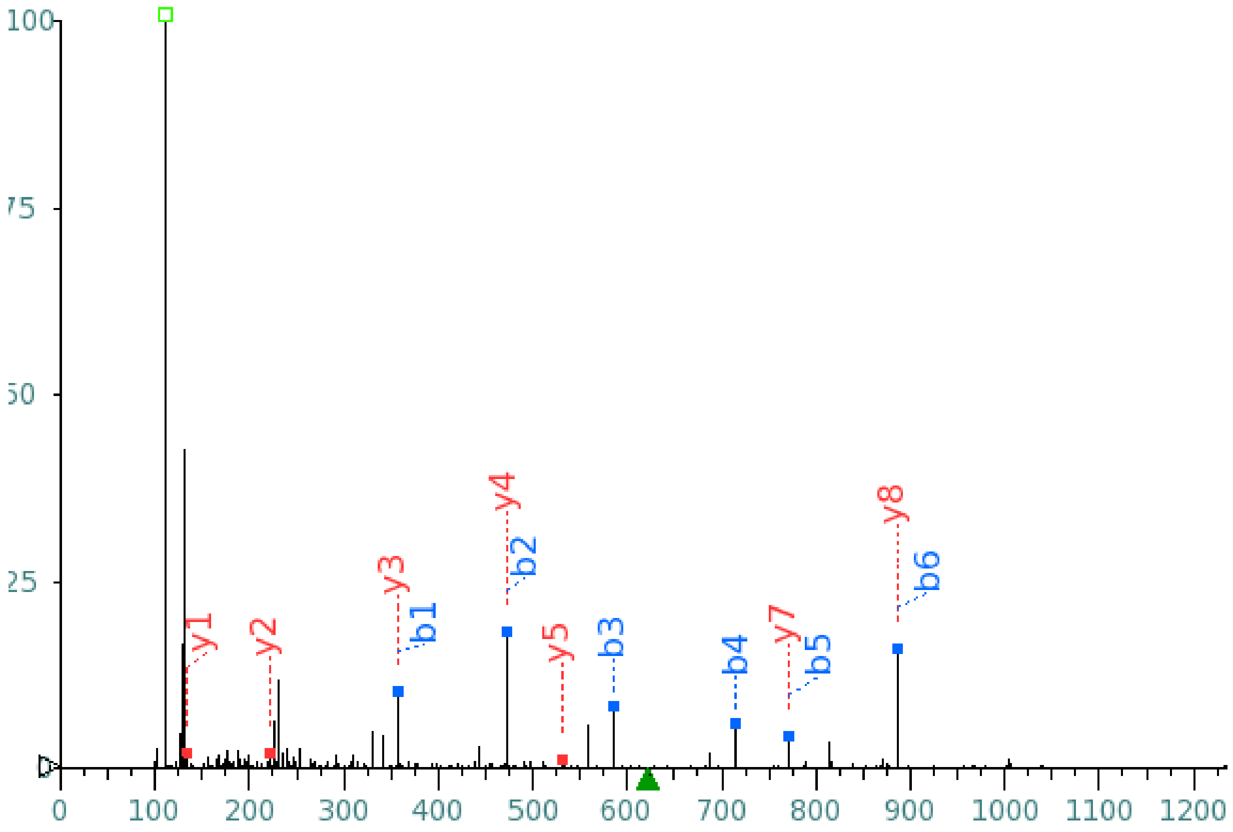

QNLEGDHSD (+2)

Primary Reference: HS74L_HUMAN
Search ID: 41761
Search Name: 20130328_ananiav_ERstress_TMT_TM_timecourse_trypsin_inj2_4mc_iaa
Scan#: 3170
Observed Mass: 622.2924 (1.9 ppm)
PSM Score: 24.44

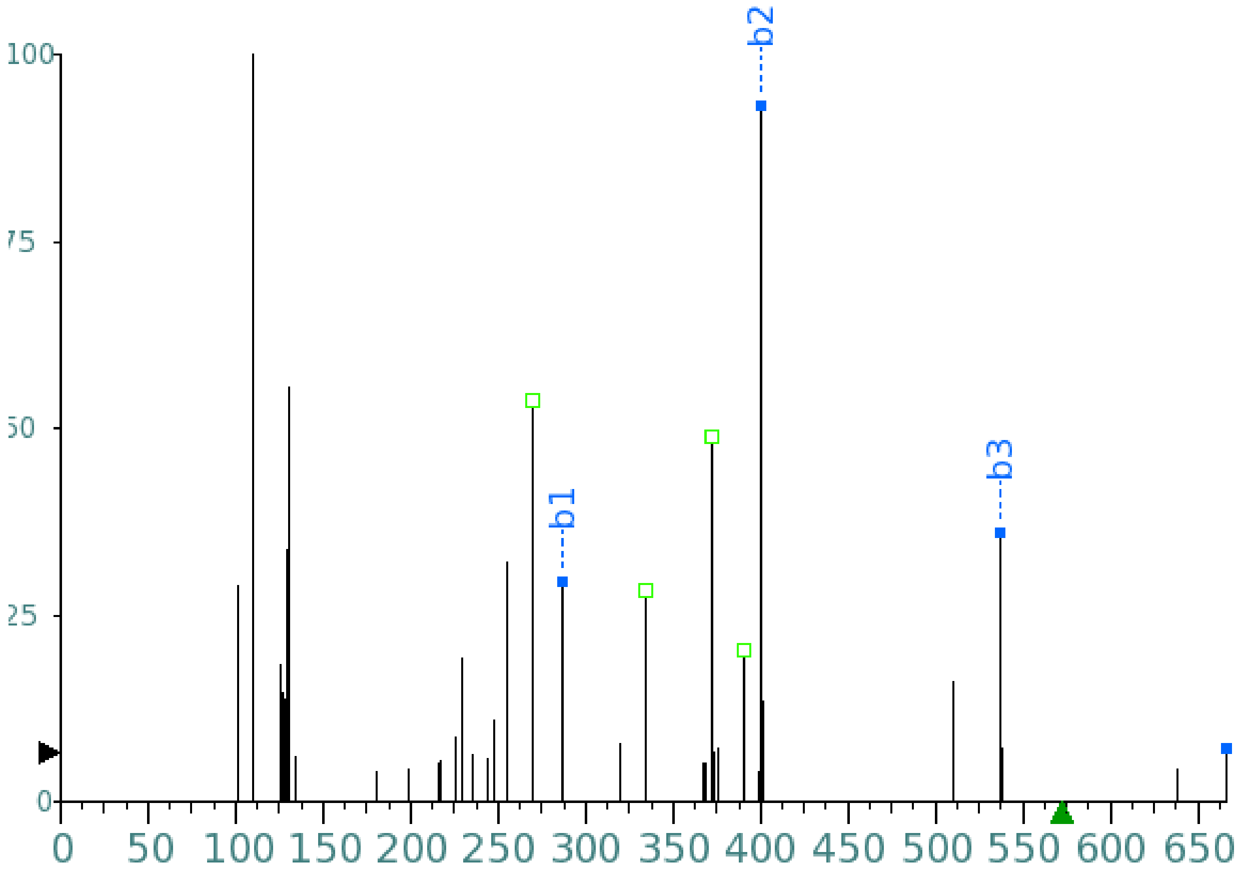

GIHENETD (+2)

Primary Reference: CC017_HUMAN
Search ID: 41761
Search Name: 20130328_ananiav_ERstress_TMT_TM_timecourse_trypsin_inj2_4mc_iaa
Scan#: 3205
Observed Mass: 572.2791 (2.6 ppm)
PSM Score: 16.46

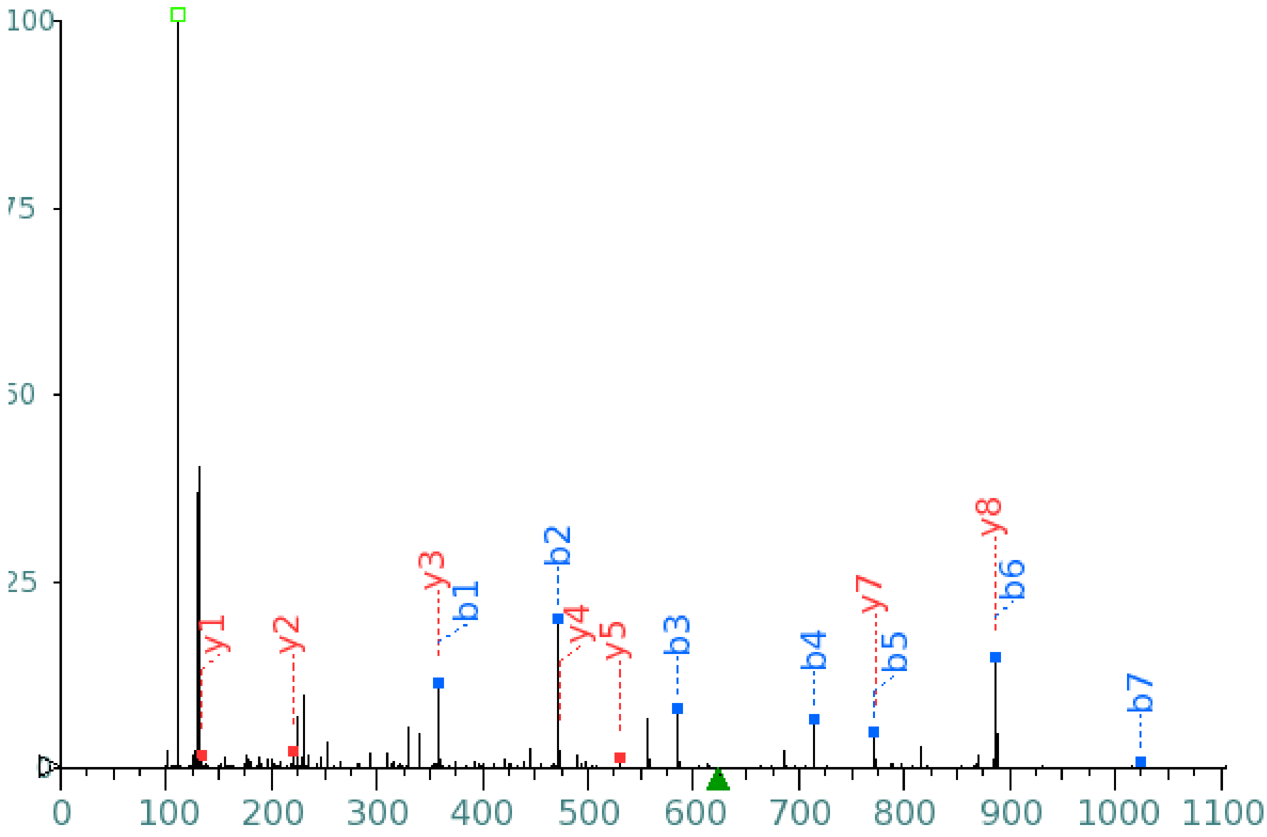

QNLEGDHSD (+2)

Primary Reference: HS74L_HUMAN
Search ID: 41761
Search Name: 20130328_ananiav_ERstress_TMT_TM_timecourse_trypsin_inj2_4mc_iaa
Scan#: 3231
Observed Mass: 622.2923 (1.6 ppm)
PSM Score: 41.2

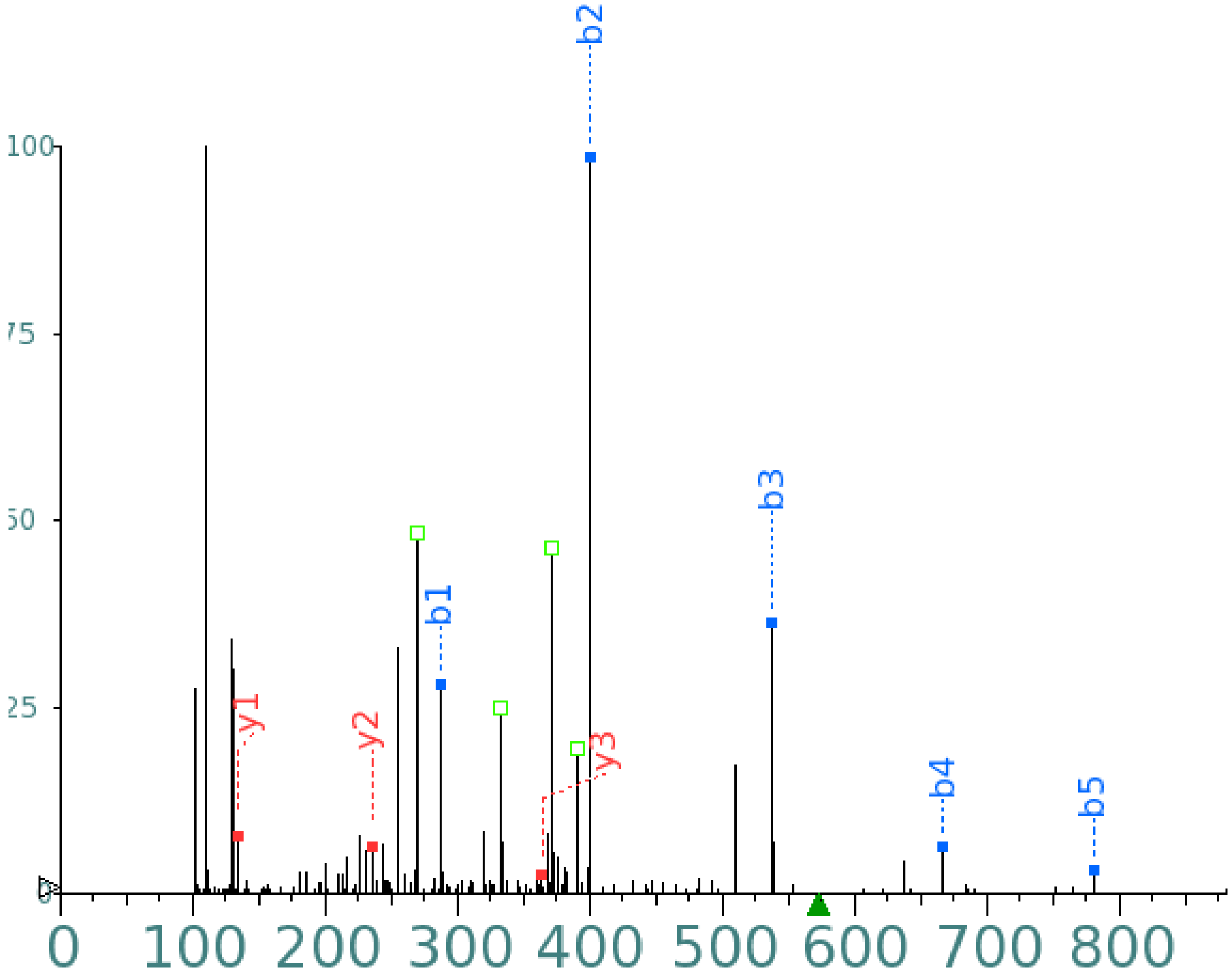

GIHENETD (+2)

Primary Reference: CC017_HUMAN
Search ID: 41761
Search Name: 20130328_ananiav_ERstress_TMT_TM_timecourse_trypsin_inj2_4mc_iaa
Scan#: 3264
Observed Mass: 572.2785 (1.6 ppm)
PSM Score: 19.83

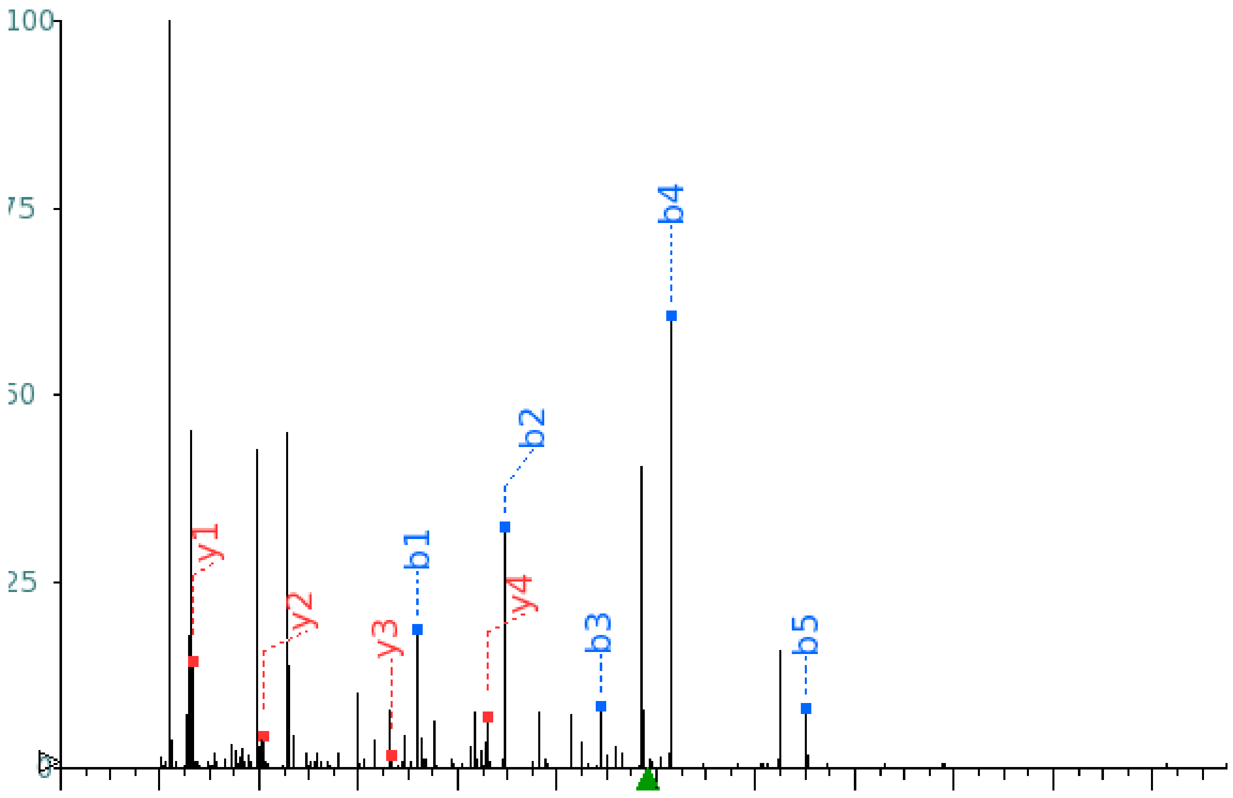

ESPAHPEAD (+2)

Primary Reference: AK17A_HUMAN
Search ID: 41761
Search Name: 20130328_ananiav_ERstress_TMT_TM_timecourse_trypsin_inj2_4mc_iaa
Scan#: 3266
Observed Mass: 591.2882 (4.7 ppm)
PSM Score: 12.12

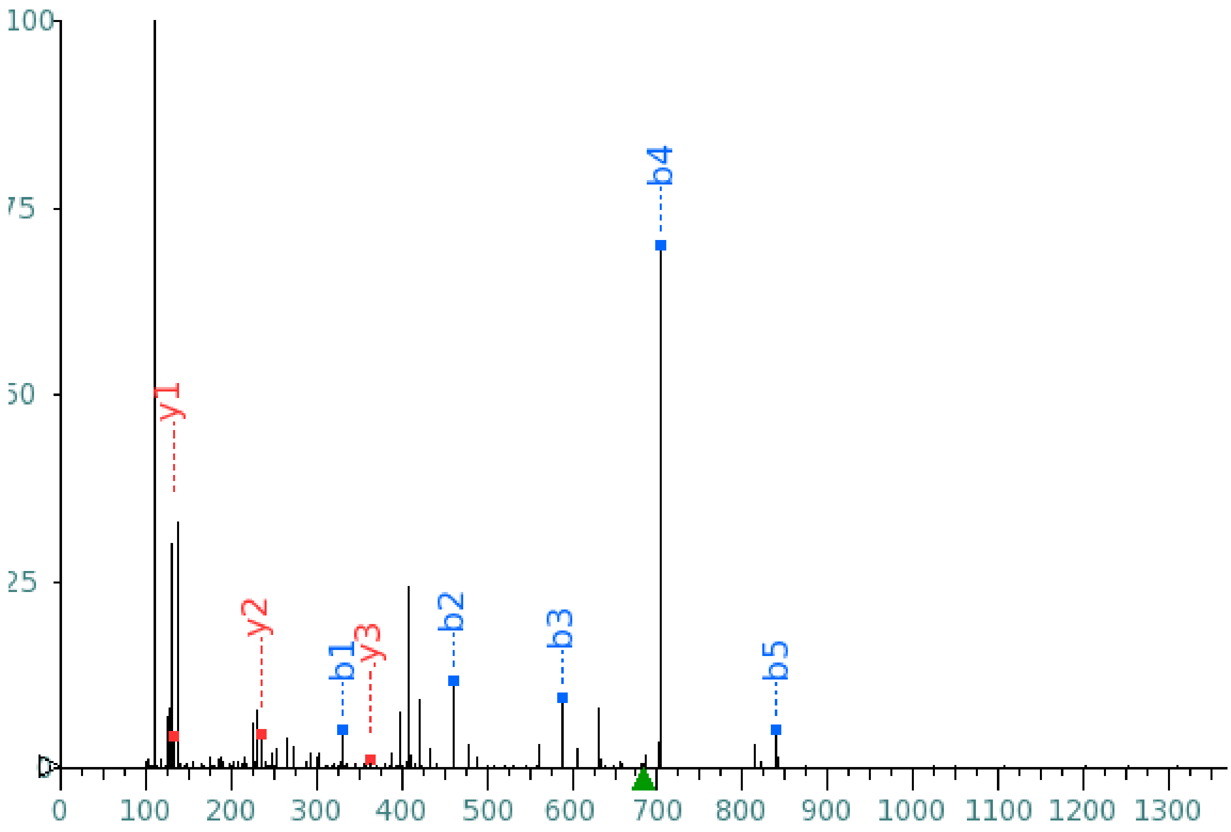

TEQDHYETD (+2)

Primary Reference: OCLN_HUMAN
Search ID: 41761
Search Name: 20130328_ananiav_ERstress_TMT_TM_timecourse_trypsin_inj2_4mc_iaa
Scan#: 3292
Observed Mass: 683.8024 (1.1 ppm)
PSM Score: 10.3

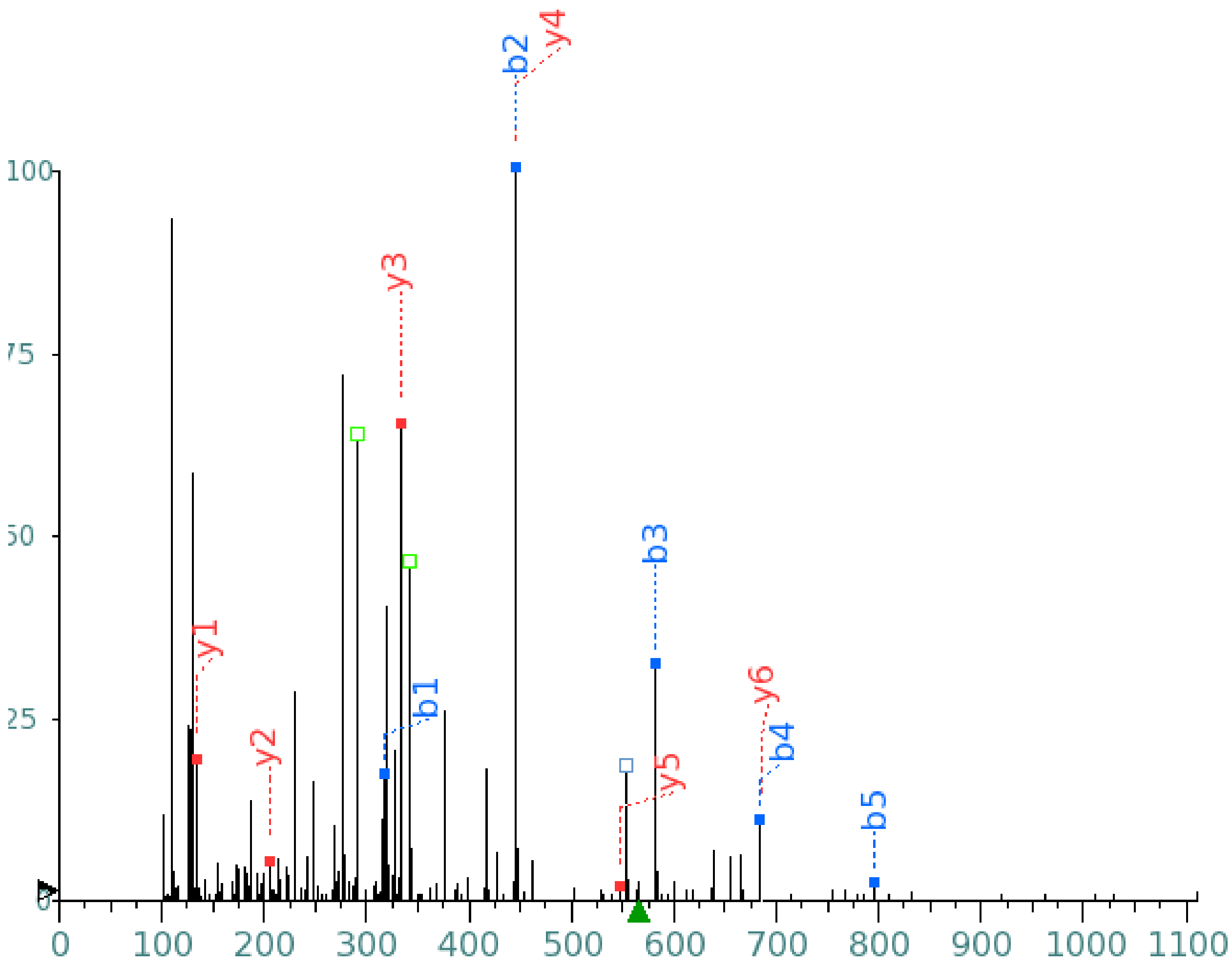

SQHTLQAD (+2)

Primary Reference: HD_HUMAN
Search ID: 41761
Search Name: 20130328_ananiav_ERstress_TMT_TM_timecourse_trypsin_inj2_4mc_iaa
Scan#: 3323
Observed Mass: 564.7958 (-0.3 ppm)
PSM Score: 17.7

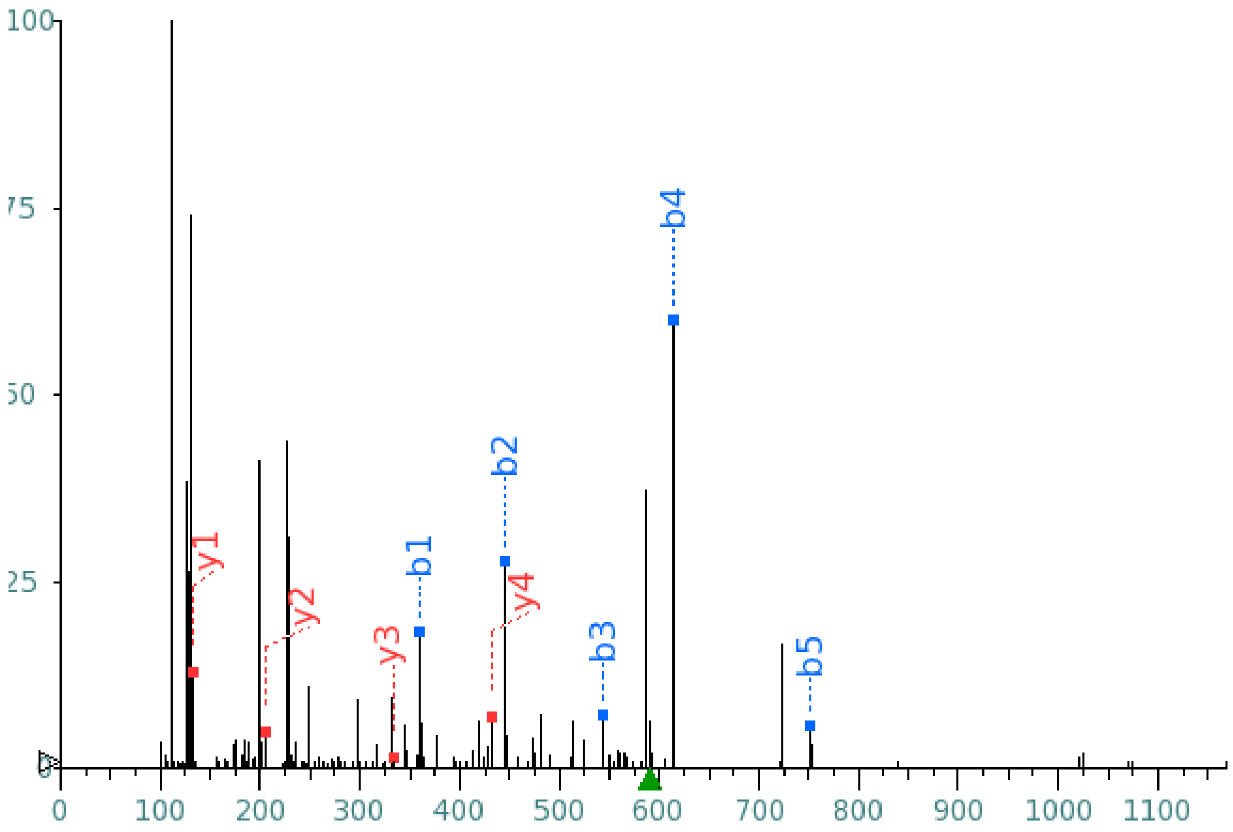

ESPAHPEAD (+2)

Primary Reference: AK17A_HUMAN
Search ID: 41761
Search Name: 20130328_ananiav_ERstress_TMT_TM_timecourse_trypsin_inj2_4mc_iaa
Scan#: 3343
Observed Mass: 591.286 (0.9 ppm)
PSM Score: 12.73

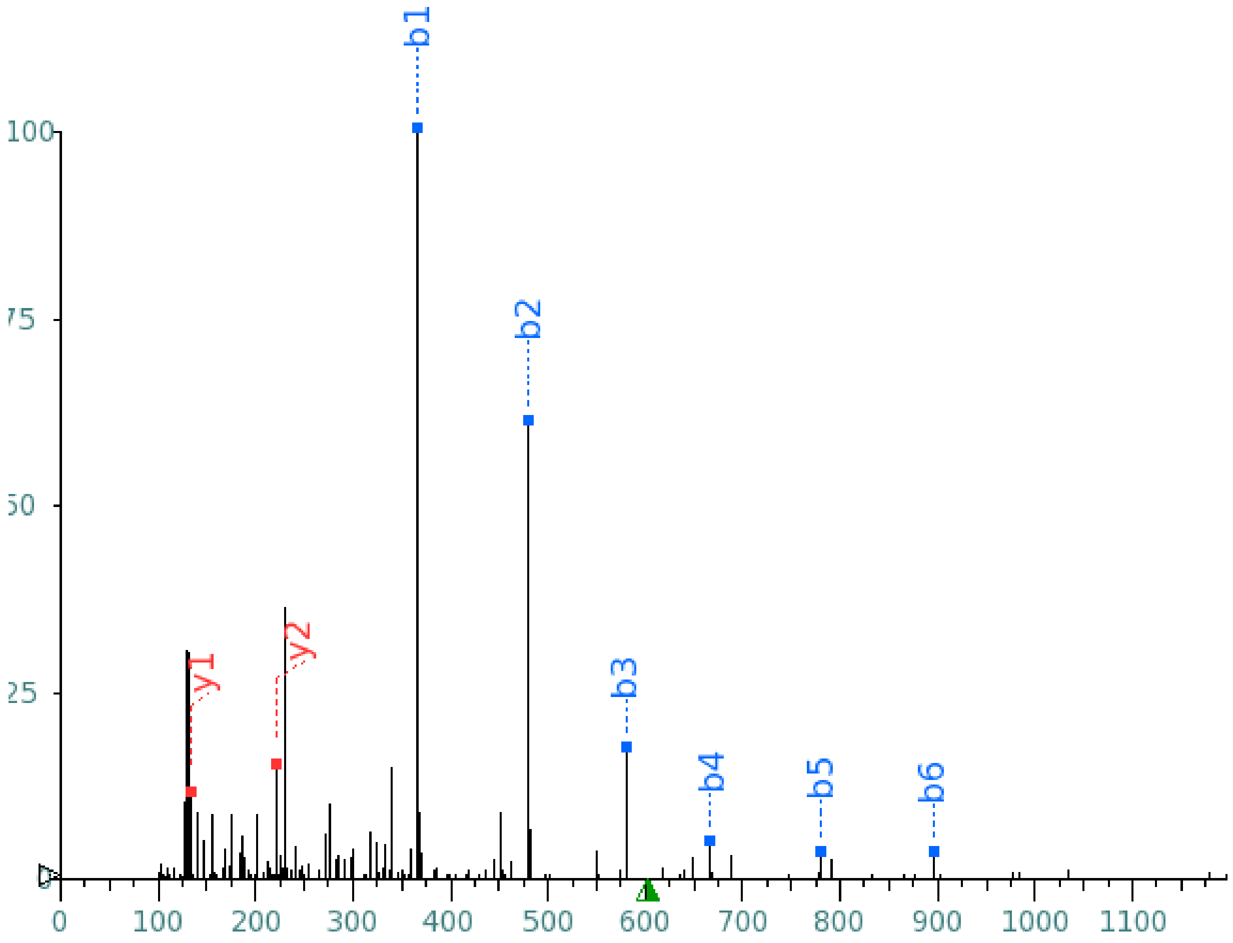

HIVSNDSSD (+2)

Primary Reference: PA24A_HUMAN
Search ID: 41761
Search Name: 20130328_ananiav_ERstress_TMT_TM_timecourse_trypsin_inj2_4mc_iaa
Scan#: 3475
Observed Mass: 601.7964 (0.4 ppm)
PSM Score: 39.34

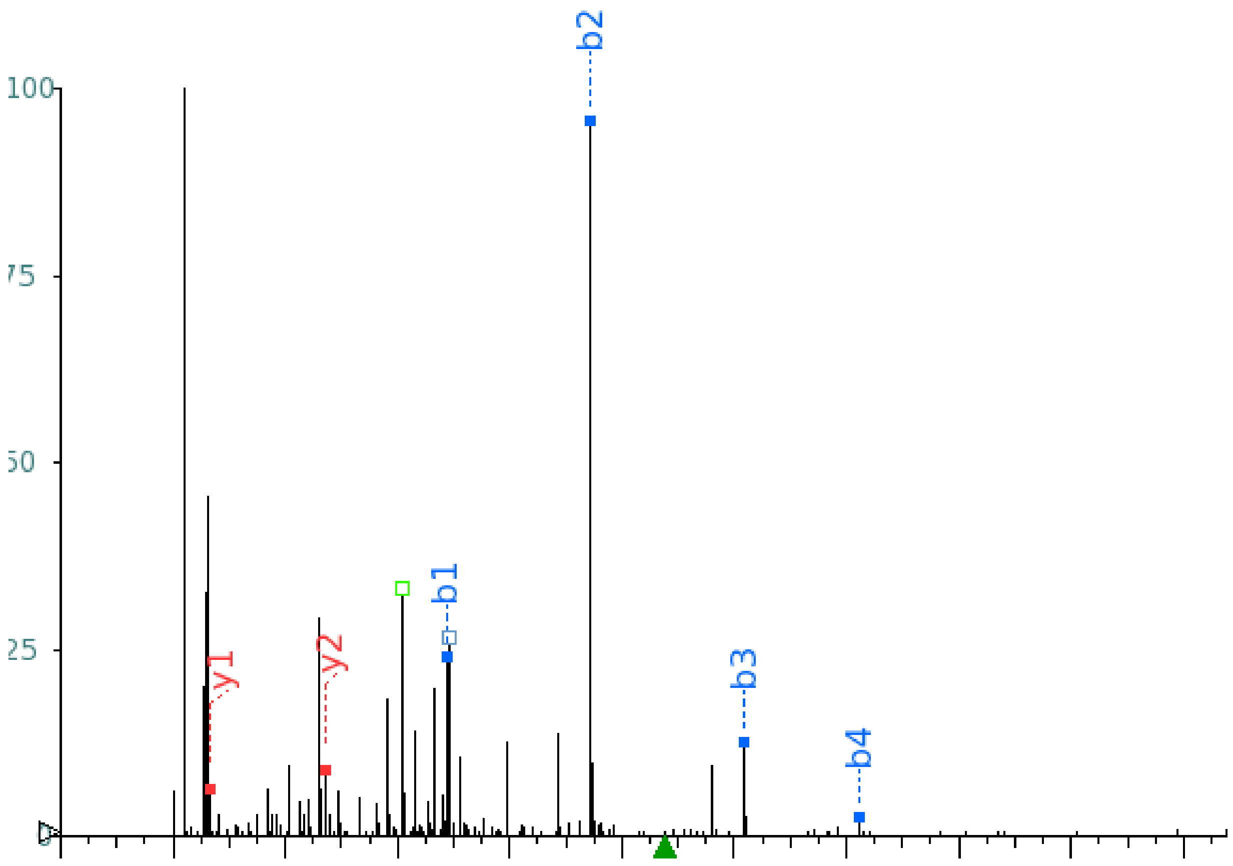

IEHTETD (+2)

Primary Reference: TM63B_HUMAN
Search ID: 41761
Search Name: 20130328_ananiav_ERstress_TMT_TM_timecourse_trypsin_inj2_4mc_iaa
Scan#: 3572
Observed Mass: 537.2701 (1.5 ppm)
PSM Score: 15.69

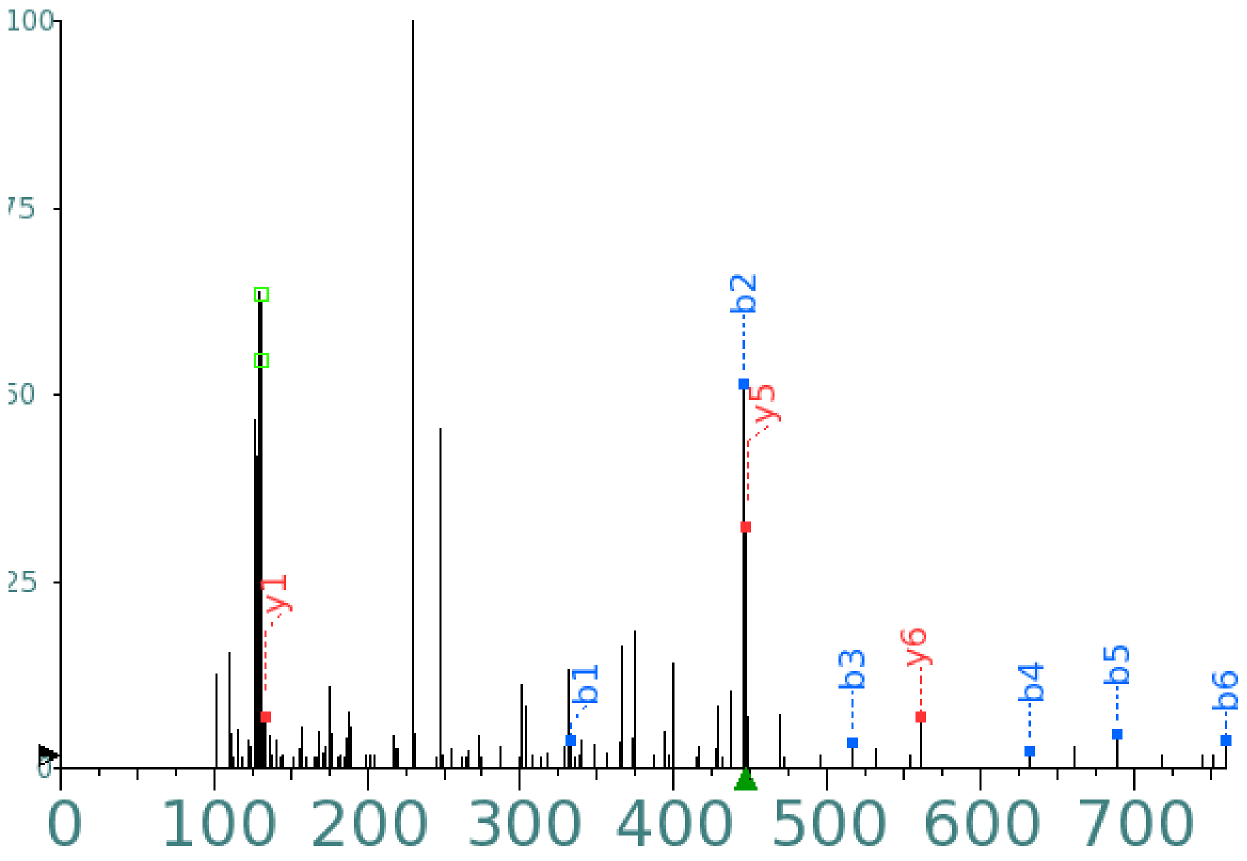

CLADGAD (+2)

Primary Reference: RFC4_HUMAN
Search ID: 41761
Search Name: 20130328_ananiav_ERstress_TMT_TM_timecourse_trypsin_inj2_4mc_iaa
Scan#: 3648
Observed Mass: 447.2165 (2.3 ppm)
PSM Score: 15.42

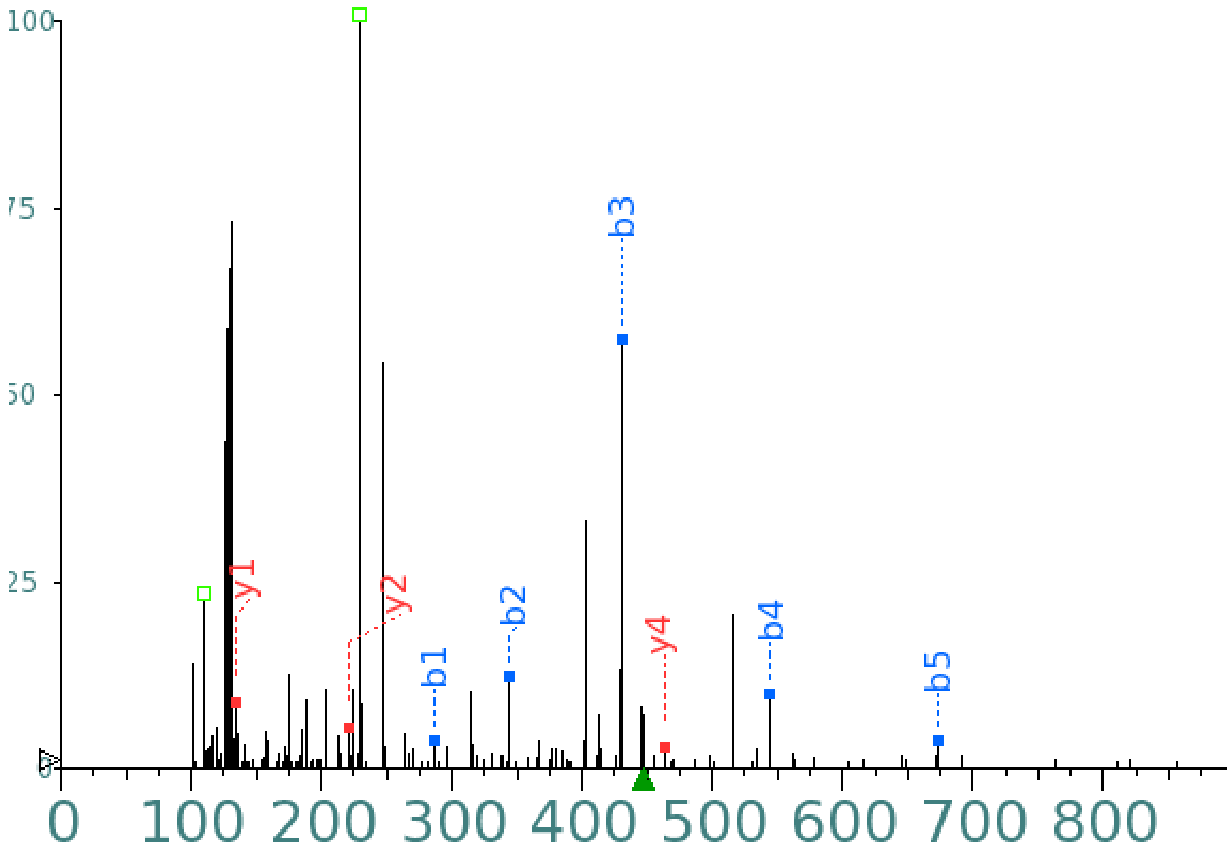

GGSLESD (+2)

Primary Reference: MECP2_HUMAN
Search ID: 41761
Search Name: 20130328_ananiav_ERstress_TMT_TM_timecourse_trypsin_inj2_4mc_iaa
Scan#: 3738
Observed Mass: 447.2252 (2.1 ppm)
PSM Score: 8.97

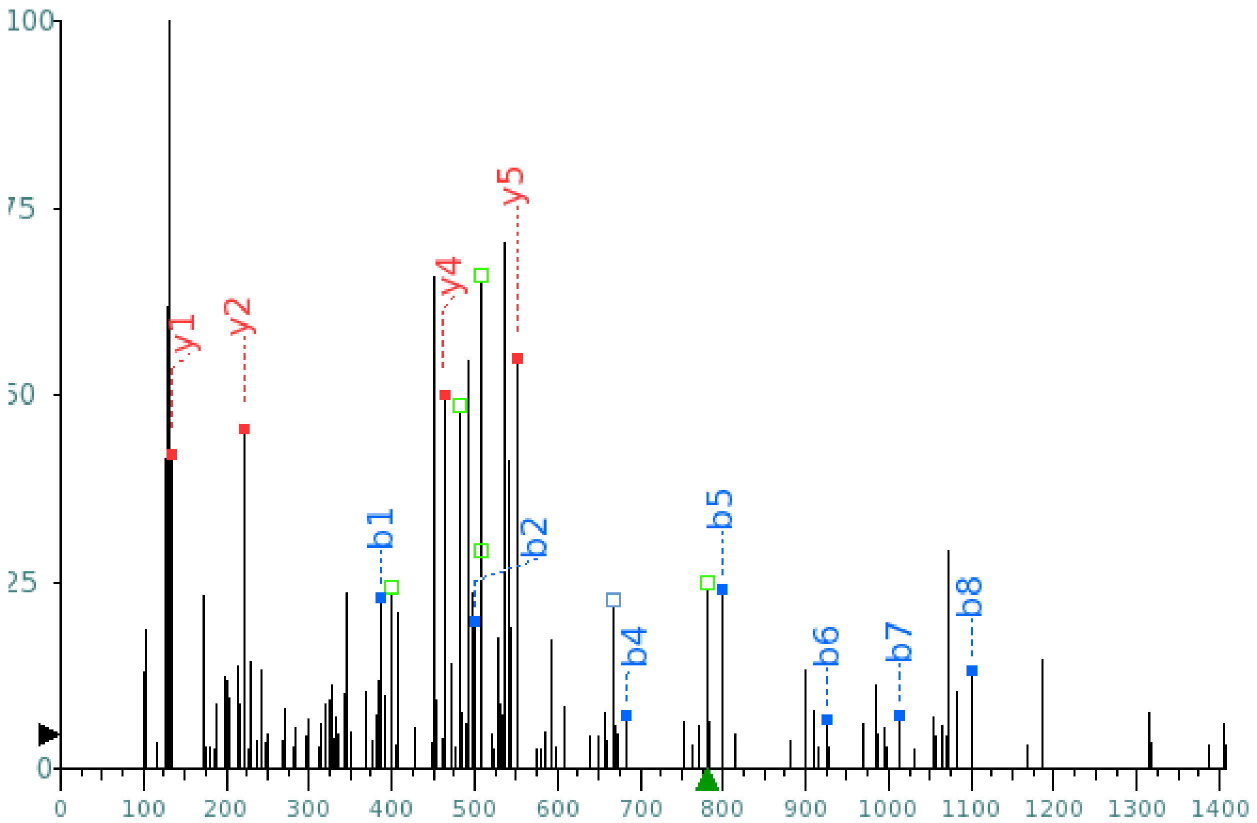

RNPSNQSSLESD (+2)

Primary Reference: MYO5B_HUMAN
Search ID: 41761
Search Name: 20130328_ananiav_ERstress_TMT_TM_timecourse_trypsin_inj2_4mc_iaa
Scan#: 3843
Observed Mass: 781.885 (1.3 ppm)
PSM Score: 27.39

File name: 2015-02-04 04.01.53PM.pdf

Creation time: 2015-02-04 04:09PM

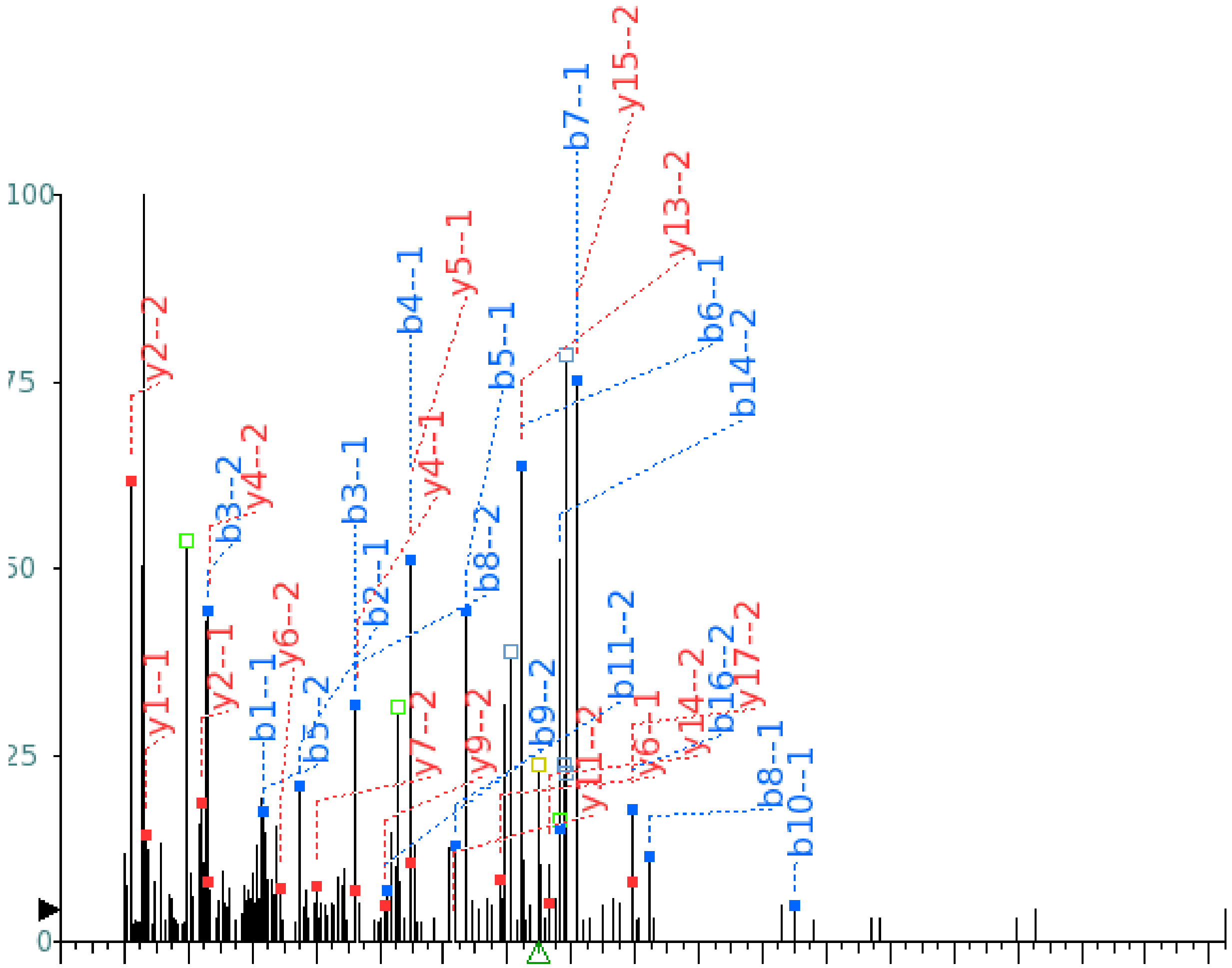

SGSSSSSSIPESQSNHSNQSD (+3)

Primary Reference: RAPH1_HUMAN
Search ID: 41761
Search Name: 20130328_ananiav_ERstress_TMT_TM_timecourse_trypsin_inj2_4mc_iaa
Scan#: 3862
Observed Mass: 751.0027 (1.3 ppm)
PSM Score: 33.82

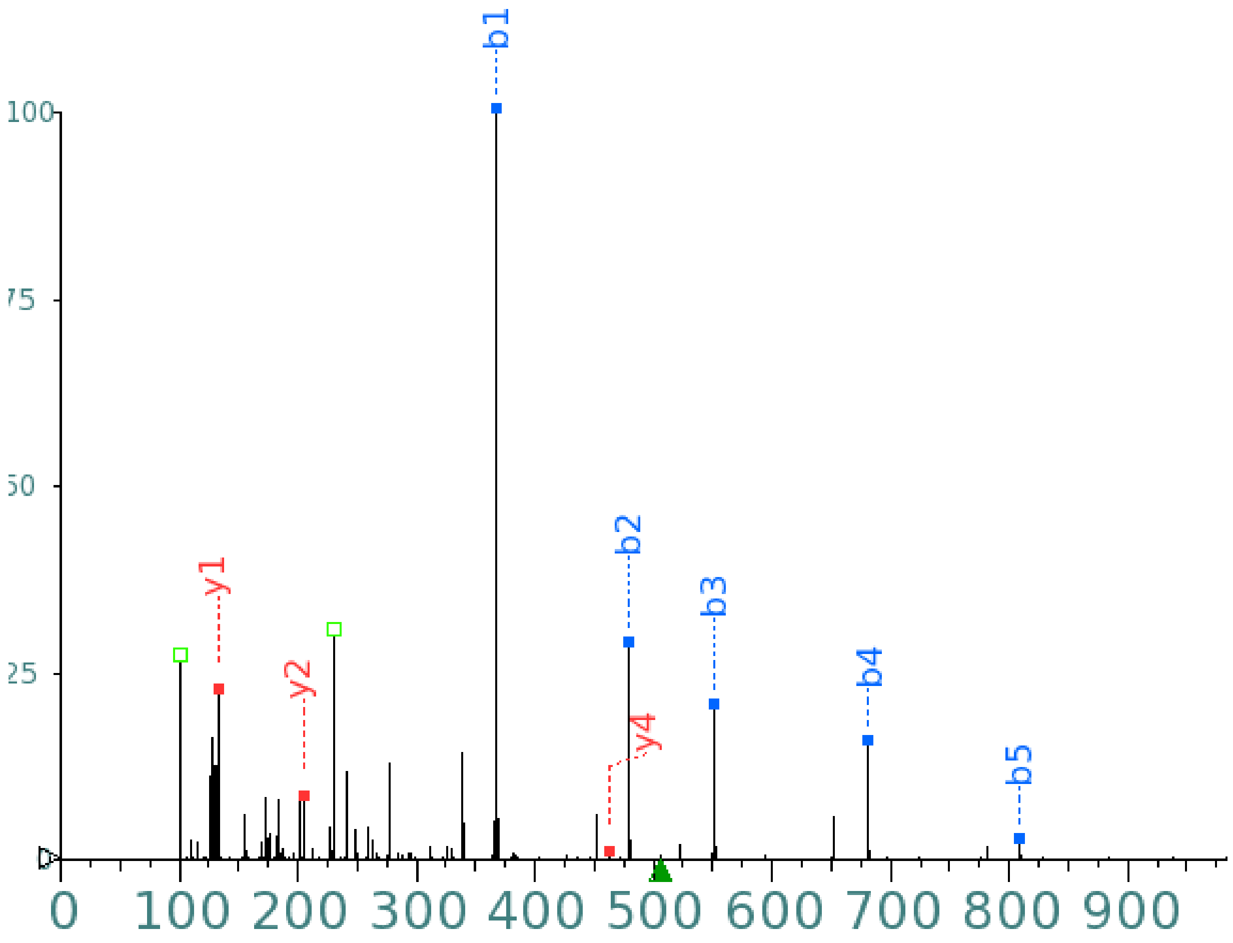

HIAEEAD (+2)

Primary Reference: TPM3L_HUMAN
Search ID: 41761
Search Name: 20130328_ananiav_ERstress_TMT_TM_timecourse_trypsin_inj2_4mc_iaa
Scan#: 3893
Observed Mass: 507.2595 (1.7 ppm)
PSM Score: 32.21

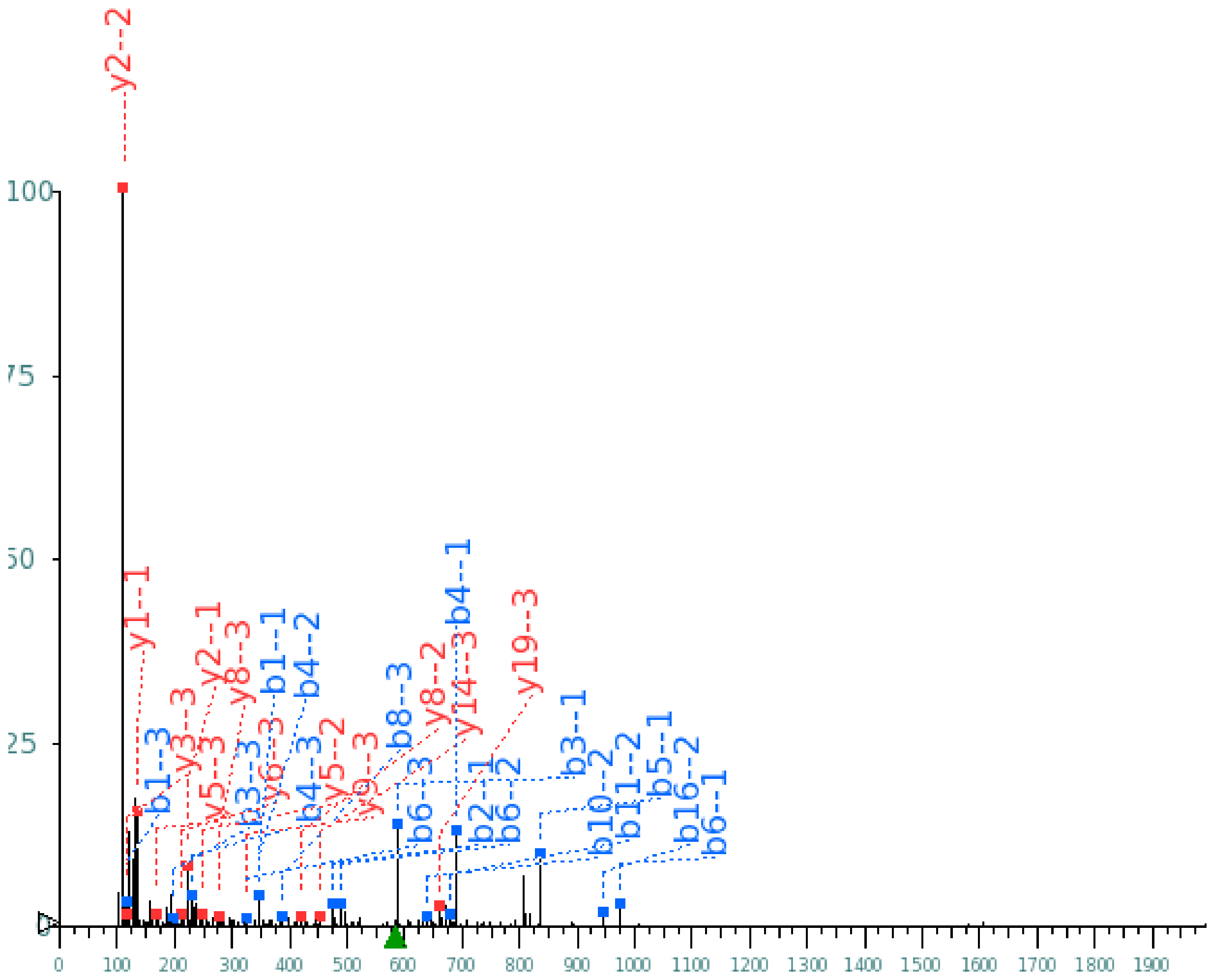

DQDTFHPSGGSHTTHGSESD (+4)

Primary Reference: A8K309_HUMAN
Search ID: 41761
Search Name: 20130328_ananiav_ERstress_TMT_TM_timecourse_trypsin_inj2_4mc_iaa
Scan#: 3930
Observed Mass: 582.7557 (2.1 ppm)
PSM Score: 17.56

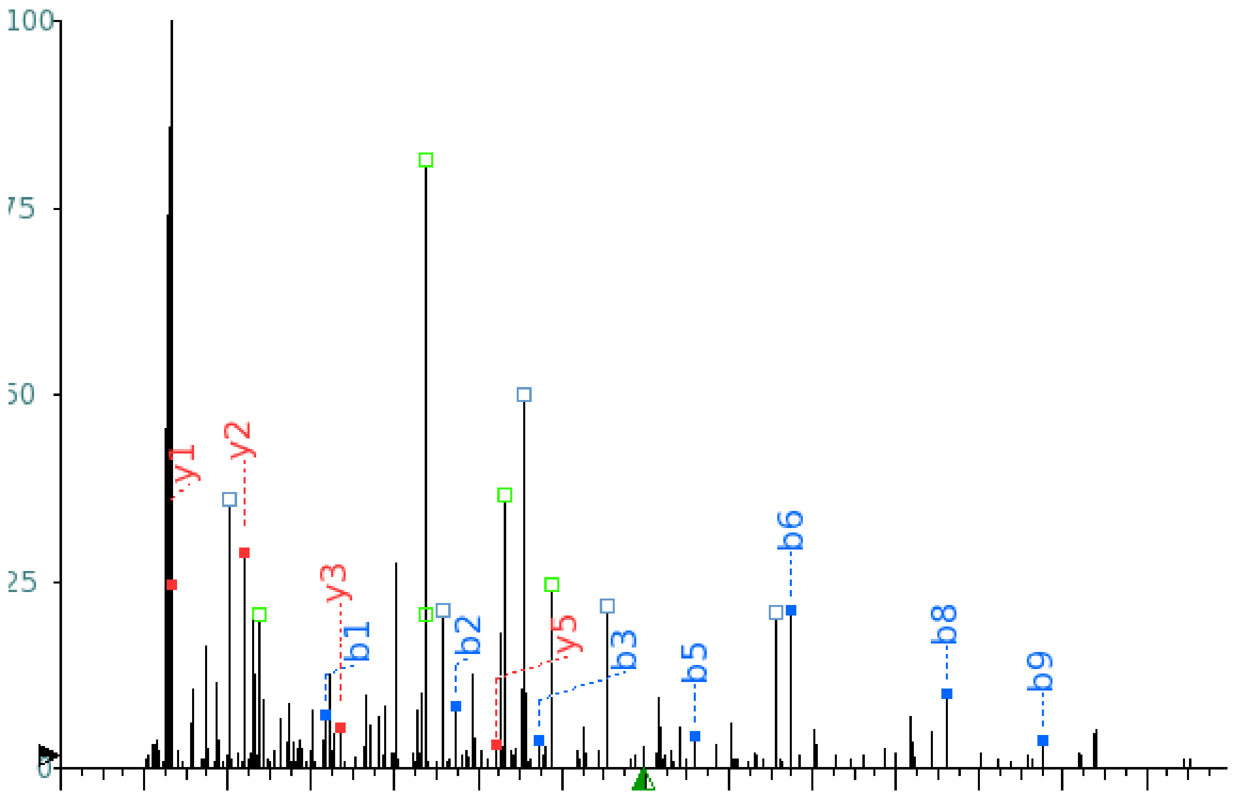

SRVVSDADDSD (+2)

Primary Reference: IWS1_HUMAN
Search ID: 41761
Search Name: 20130328_ananiav_ERstress_TMT_TM_timecourse_trypsin_inj2_4mc_iaa
Scan#: 3950
Observed Mass: 697.835 (2.2 ppm)
PSM Score: 16.76

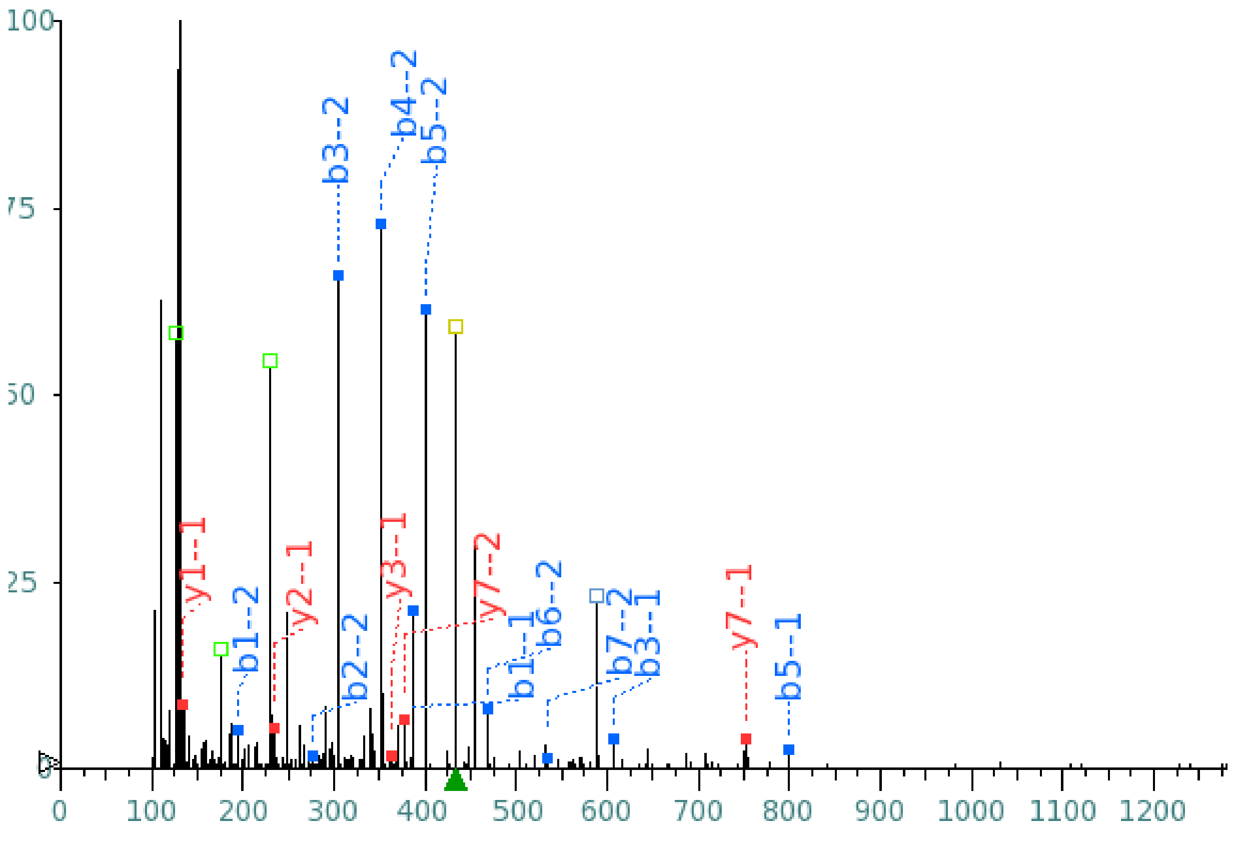

RYGPPHETD (+3)

Primary Reference: TCF20_HUMAN
Search ID: 41761
Search Name: 20130328_ananiav_ERstress_TMT_TM_timecourse_trypsin_inj2_4mc_iaa
Scan#: 3961
Observed Mass: 434.2217 (1.7 ppm)
PSM Score: 6.03

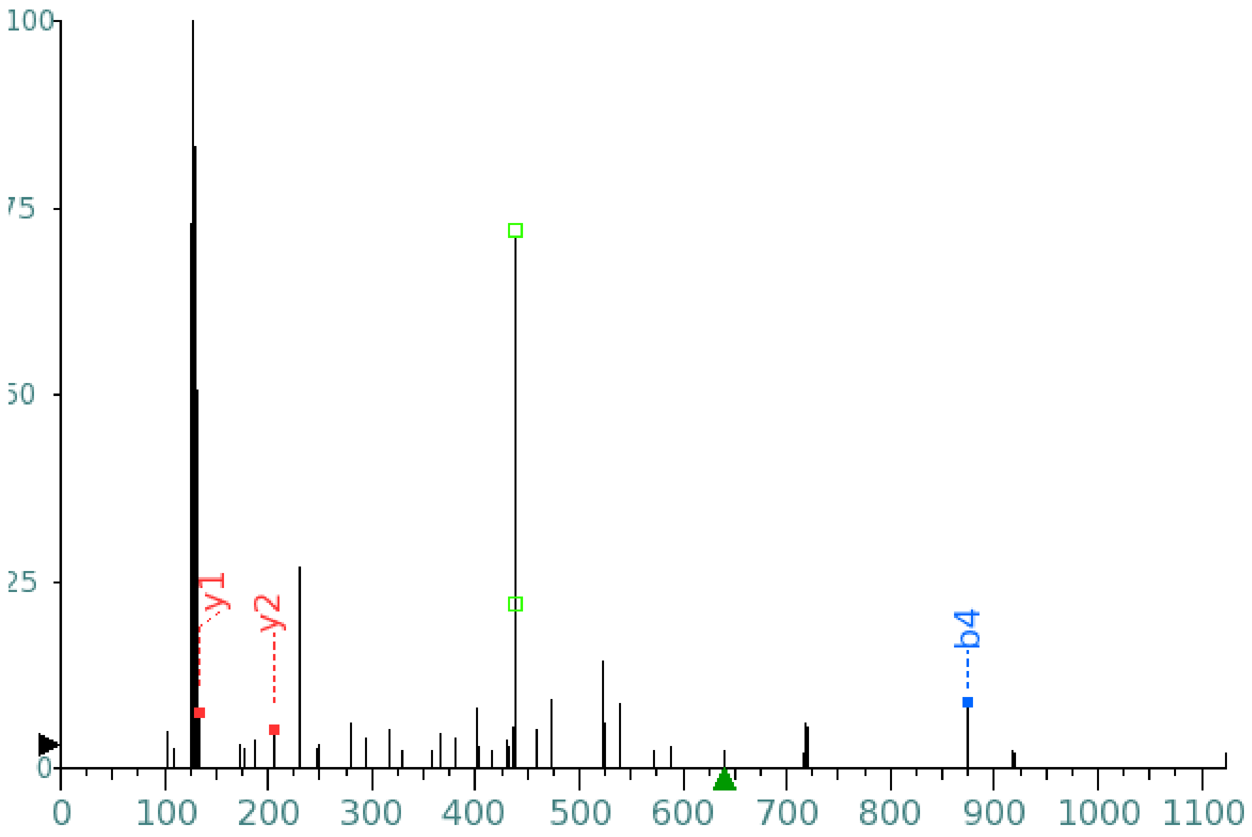

KATDAEAD (+2)

Primary Reference: TPM1_HUMAN
Search ID: 41761
Search Name: 20130328_ananiav_ERstress_TMT_TM_timecourse_trypsin_inj2_4mc_iaa
Scan#: 3996
Observed Mass: 639.8518 (1.8 ppm)
PSM Score: 19.2

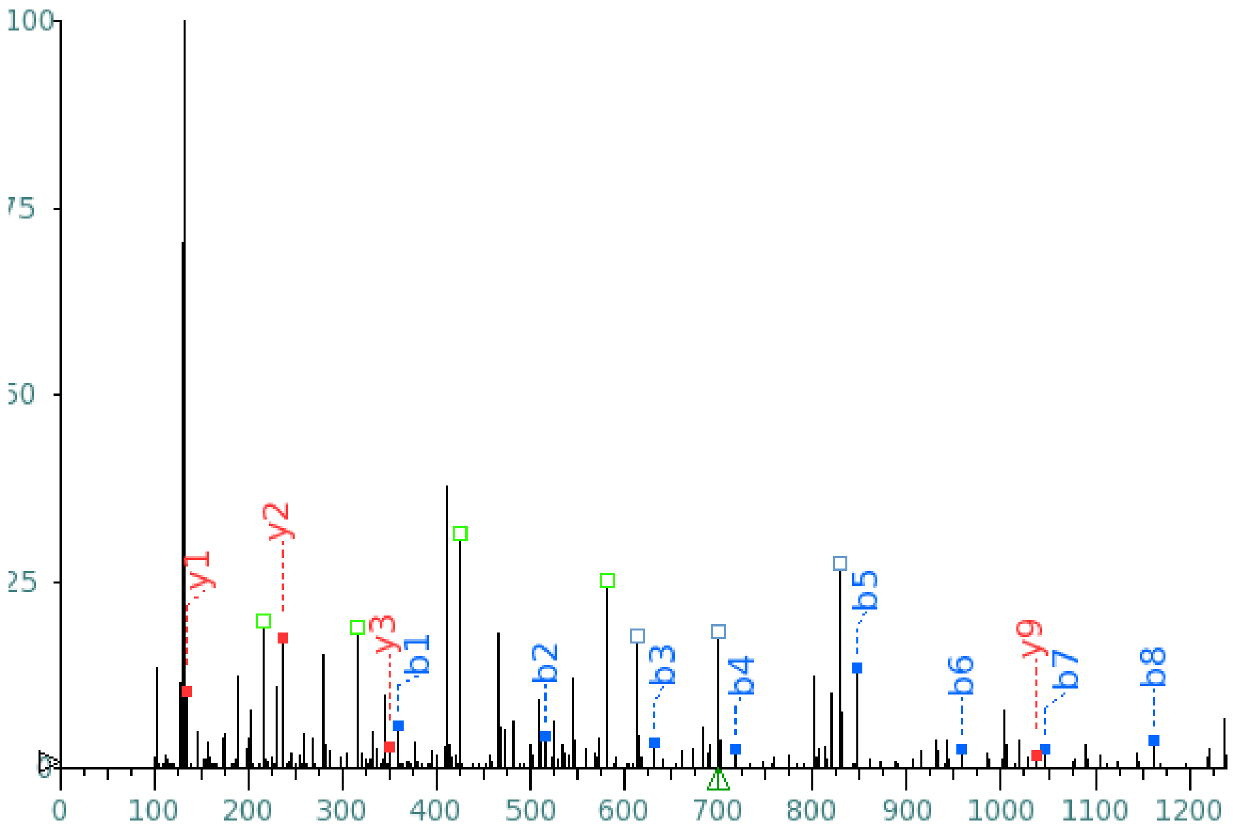

ERDSELSDTD (+2)

Primary Reference: UHRF1_HUMAN
Search ID: 41761
Search Name: 20130328_ananiav_ERstress_TMT_TM_timecourse_trypsin_inj2_4mc_iaa
Scan#: 4047
Observed Mass: 698.3276 (3 ppm)
PSM Score: 20.84

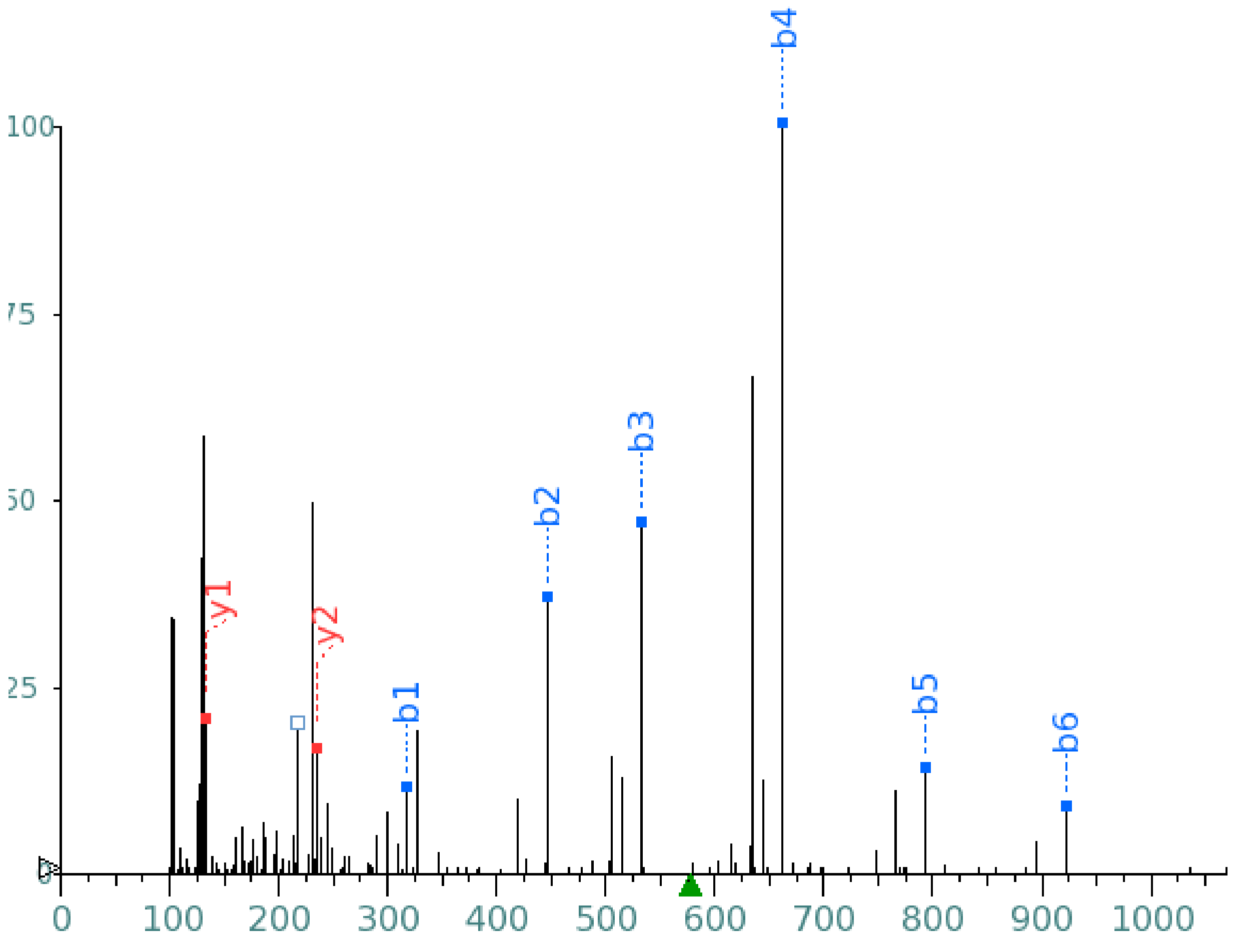

SESEMETD (+2)

Primary Reference: LARP7_HUMAN
Search ID: 41761
Search Name: 20130328_ananiav_ERstress_TMT_TM_timecourse_trypsin_inj2_4mc_iaa
Scan#: 4134
Observed Mass: 578.7487 (2.1 ppm)
PSM Score: 27.19

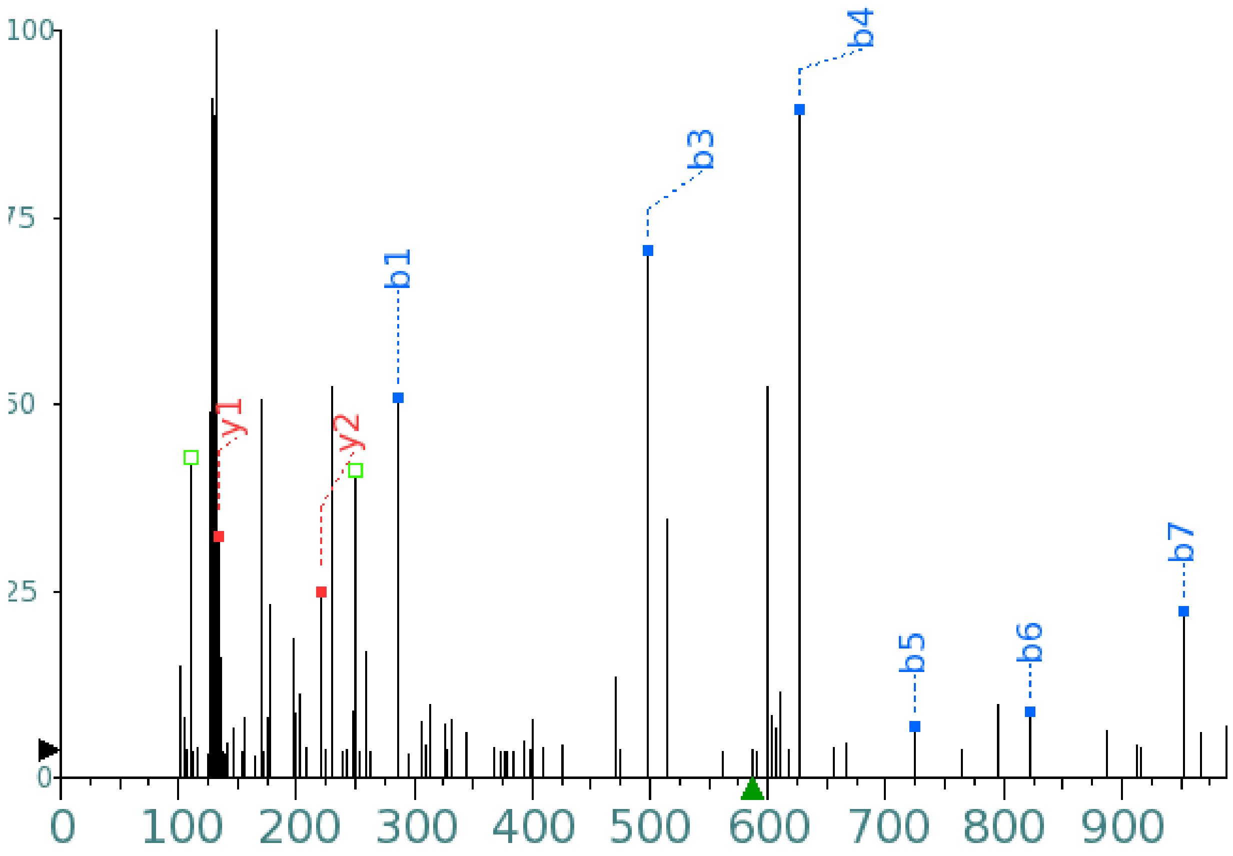

GPNQPVESD (+2)

Primary Reference: IRAK1_HUMAN
Search ID: 41761
Search Name: 20130328_ananiav_ERstress_TMT_TM_timecourse_trypsin_inj2_4mc_iaa
Scan#: 4182
Observed Mass: 586.2937 (0.8 ppm)
PSM Score: 27.28

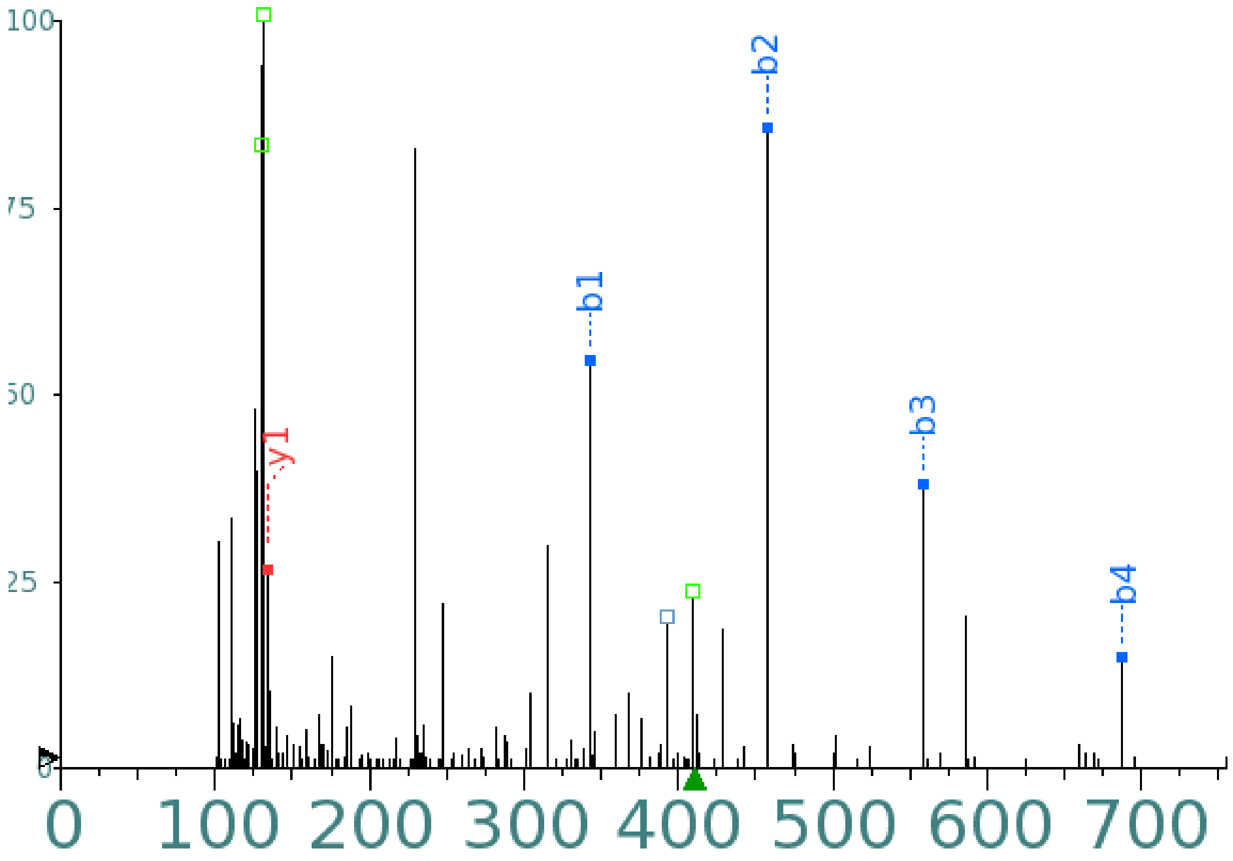

INTED (+2)

Primary Reference: CASQ2_HUMAN
Search ID: 41761
Search Name: 20130328_ananiav_ERstress_TMT_TM_timecourse_trypsin_inj2_4mc_iaa
Scan#: 4242
Observed Mass: 410.7161 (-0.1 ppm)
PSM Score: 23.19

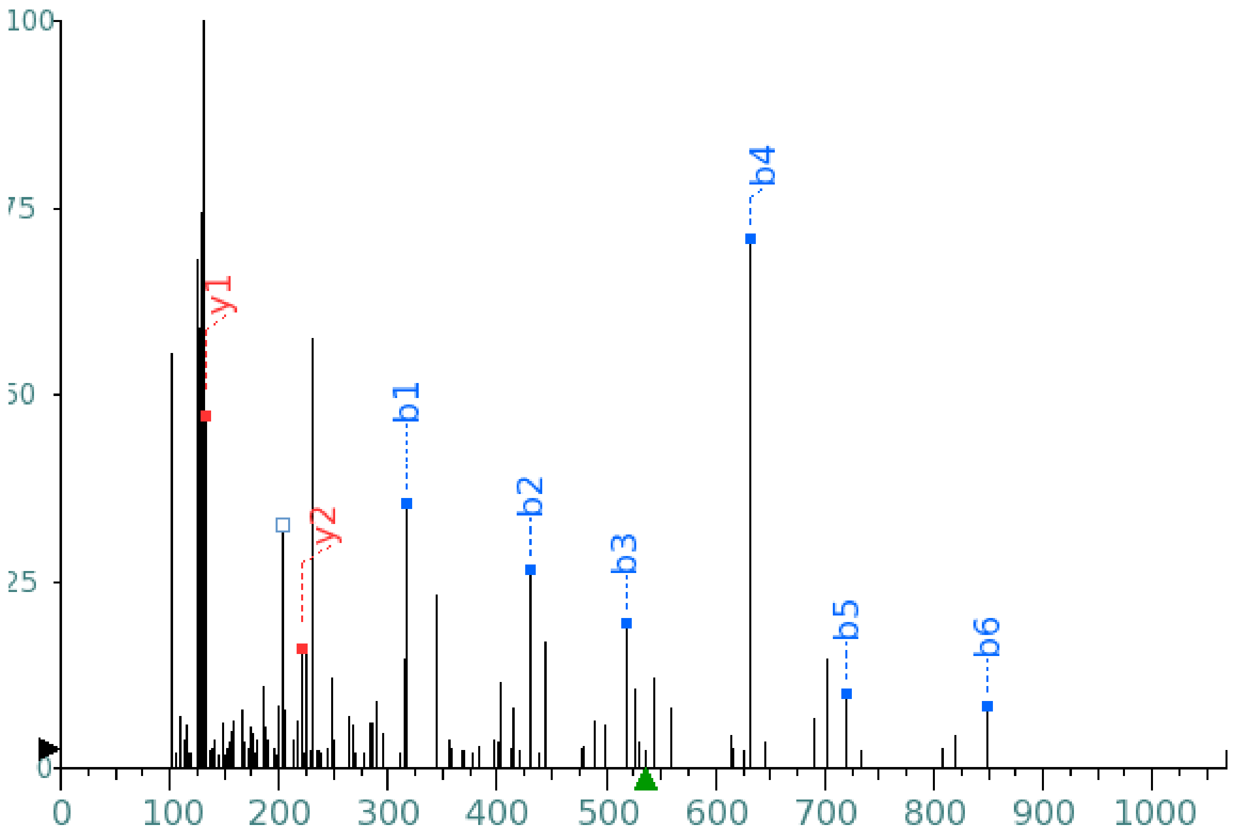

SLSDSESD (+2)

Primary Reference: CBX3_HUMAN
Search ID: 41761
Search Name: 20130328_ananiav_ERstress_TMT_TM_timecourse_trypsin_inj2_4mc_iaa
Scan#: 4319
Observed Mass: 534.7495 (2.1 ppm)
PSM Score: 35.72

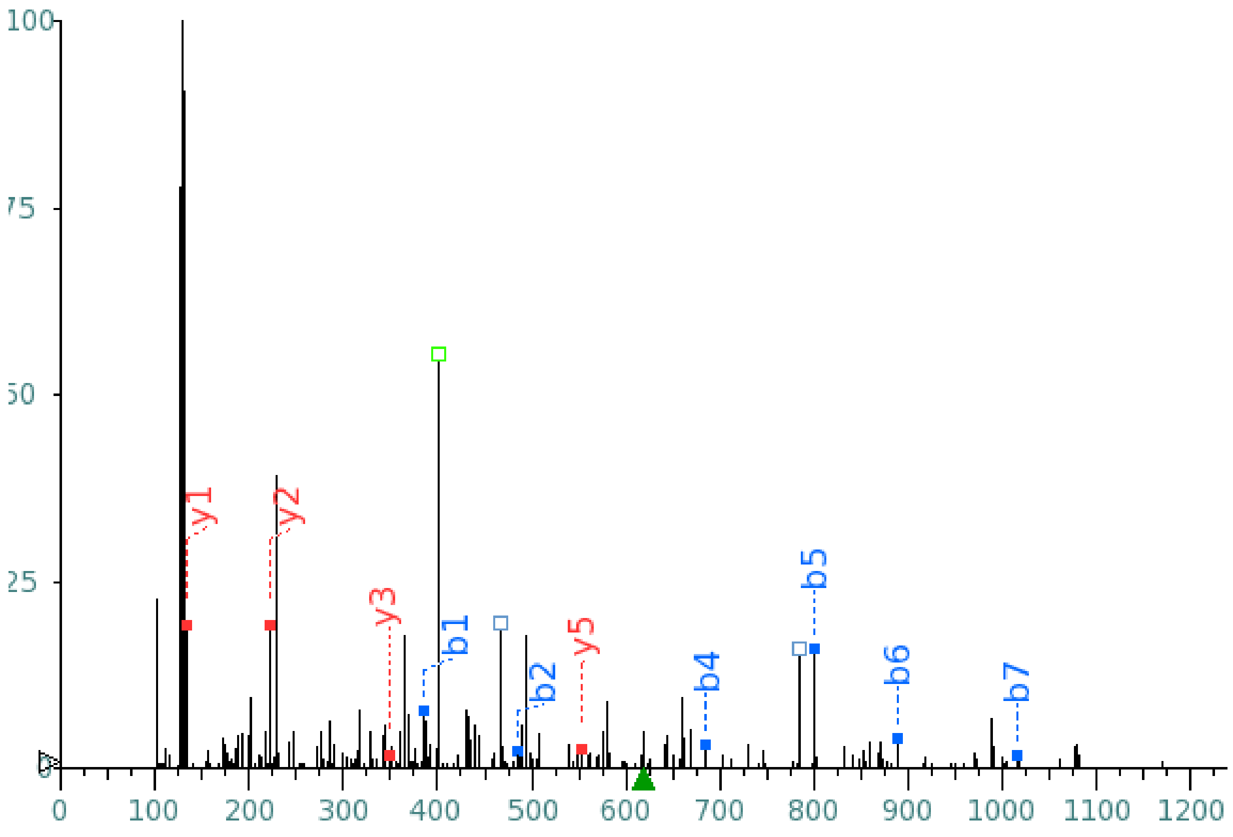

RVISDSESD (+2)

Primary Reference: MSH6_HUMAN
Search ID: 41761
Search Name: 20130328_ananiav_ERstress_TMT_TM_timecourse_trypsin_inj2_4mc_iaa
Scan#: 4389
Observed Mass: 618.8181 (1.6 ppm)
PSM Score: 10.1

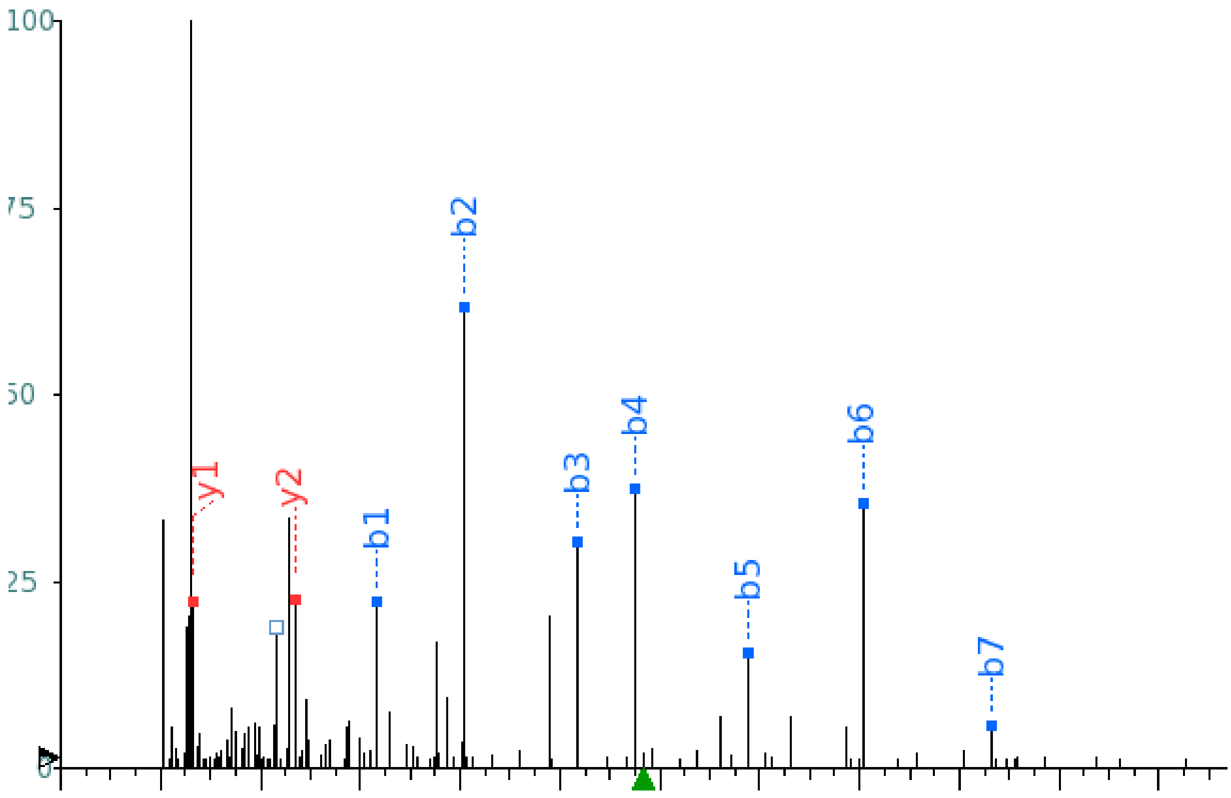

SSLGNDETD (+2)

Primary Reference: COBL_HUMAN
Search ID: 41761
Search Name: 20130328_ananiav_ERstress_TMT_TM_timecourse_trypsin_inj2_4mc_iaa
Scan#: 4410
Observed Mass: 583.7735 (1.9 ppm)
PSM Score: 37.65

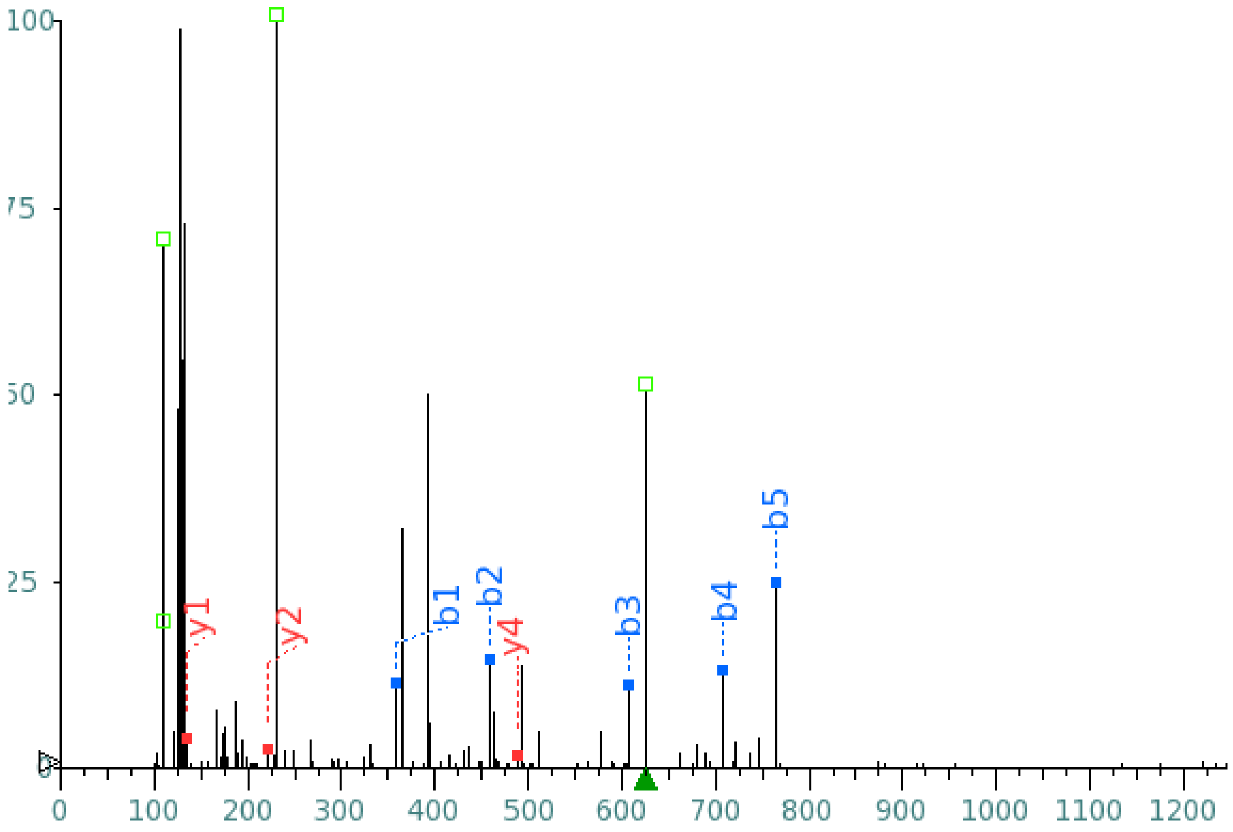

QTFTGHESD (+2)

Primary Reference: GBB1_HUMAN
Search ID: 41761
Search Name: 20130328_ananiav_ERstress_TMT_TM_timecourse_trypsin_inj2_4mc_iaa
Scan#: 4416
Observed Mass: 625.7972 (1.6 ppm)
PSM Score: 8.89

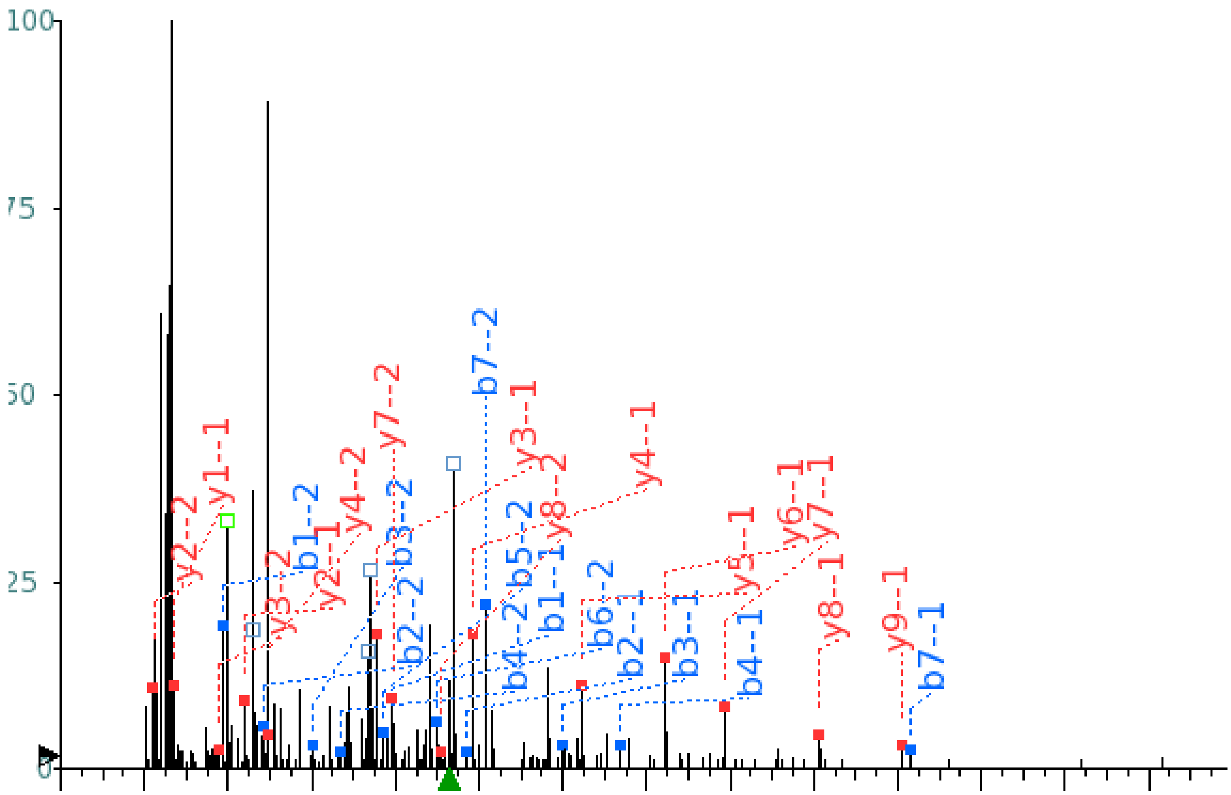

RVLATEDRSD (+3)

Primary Reference: RAB9A_HUMAN
Search ID: 41761
Search Name: 20130328_ananiav_ERstress_TMT_TM_timecourse_trypsin_inj2_4mc_iaa
Scan#: 4432
Observed Mass: 464.2546 (0.4 ppm)
PSM Score: 14.45

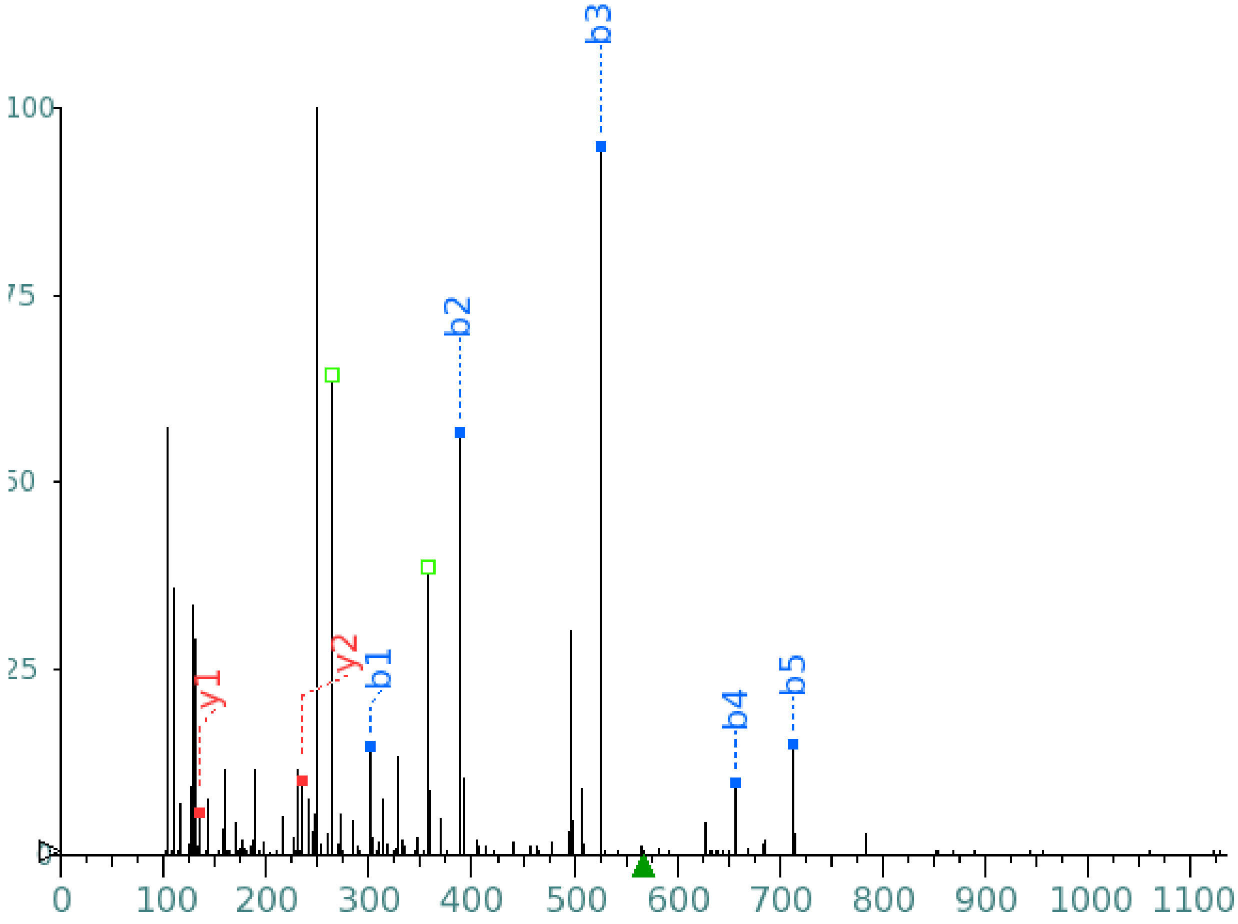

ASHMGVSTD (+2)

Primary Reference: LUZP1_HUMAN
Search ID: 41761
Search Name: 20130328_ananiav_ERstress_TMT_TM_timecourse_trypsin_inj2_4mc_iaa
Scan#: 4485
Observed Mass: 567.2765 (-0.1 ppm)
PSM Score: 13.5

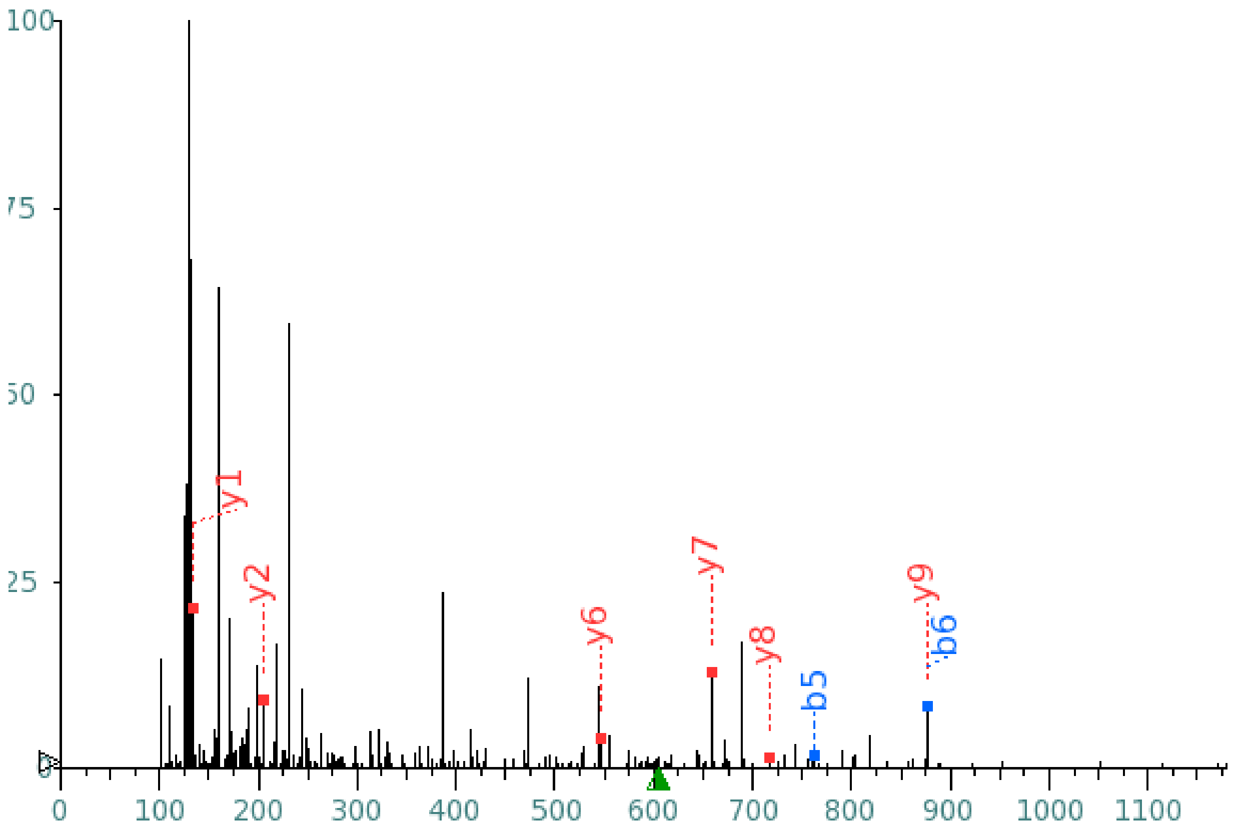

CC*GLVNGAAD (+2)

Primary Reference: TSN8_HUMAN
Search ID: 41761
Search Name: 20130328_ananiav_ERstress_TMT_TM_timecourse_trypsin_inj2_4mc_iaa
Scan#: 4512
Observed Mass: 604.785 (2.1 ppm)
PSM Score: 6.45

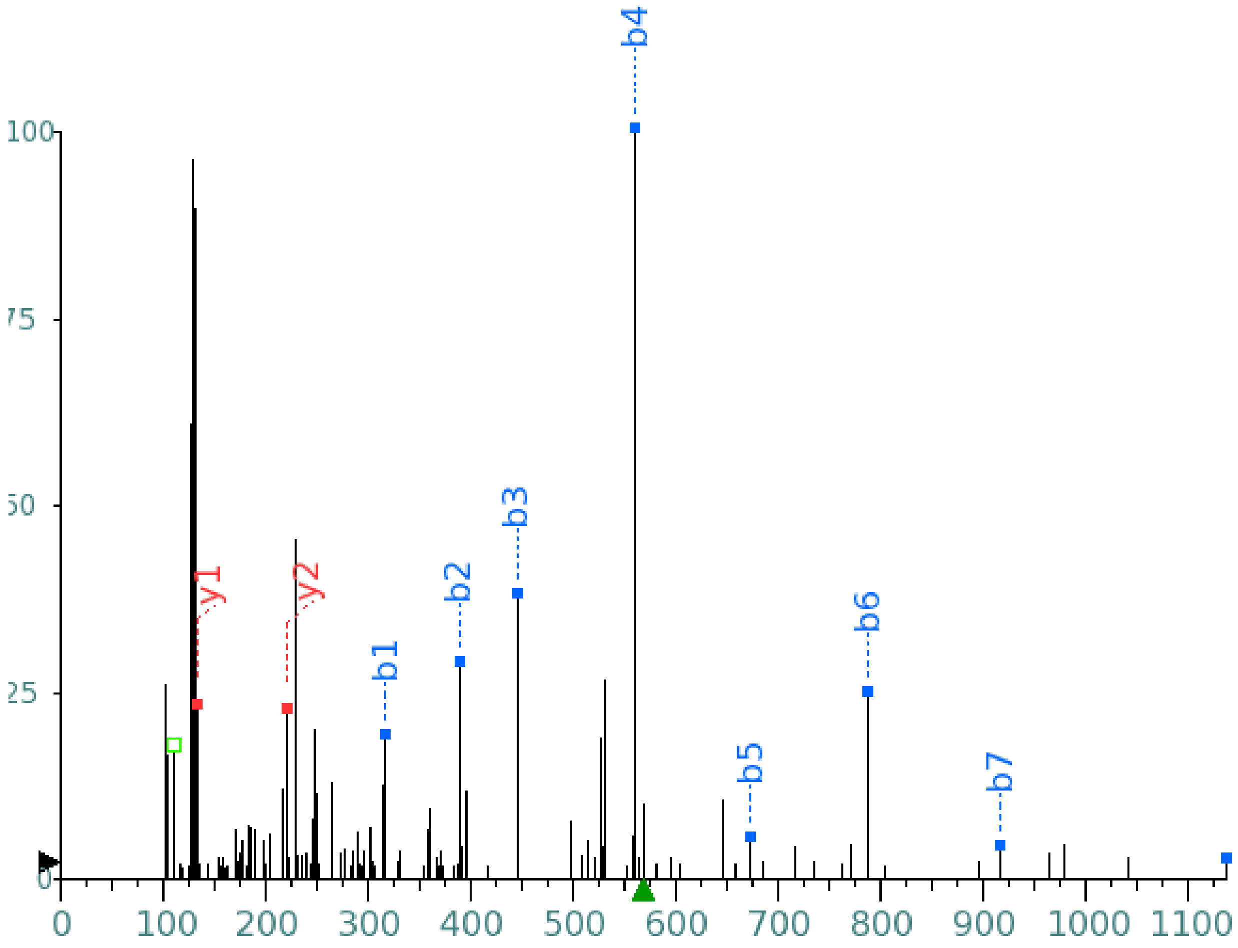

SAGNLDESD (+2)

Primary Reference: RABE1_HUMAN
Search ID: 41761
Search Name: 20130328_ananiav_ERstress_TMT_TM_timecourse_trypsin_inj2_4mc_iaa
Scan#: 4582
Observed Mass: 568.7678 (1.2 ppm)
PSM Score: 27.38

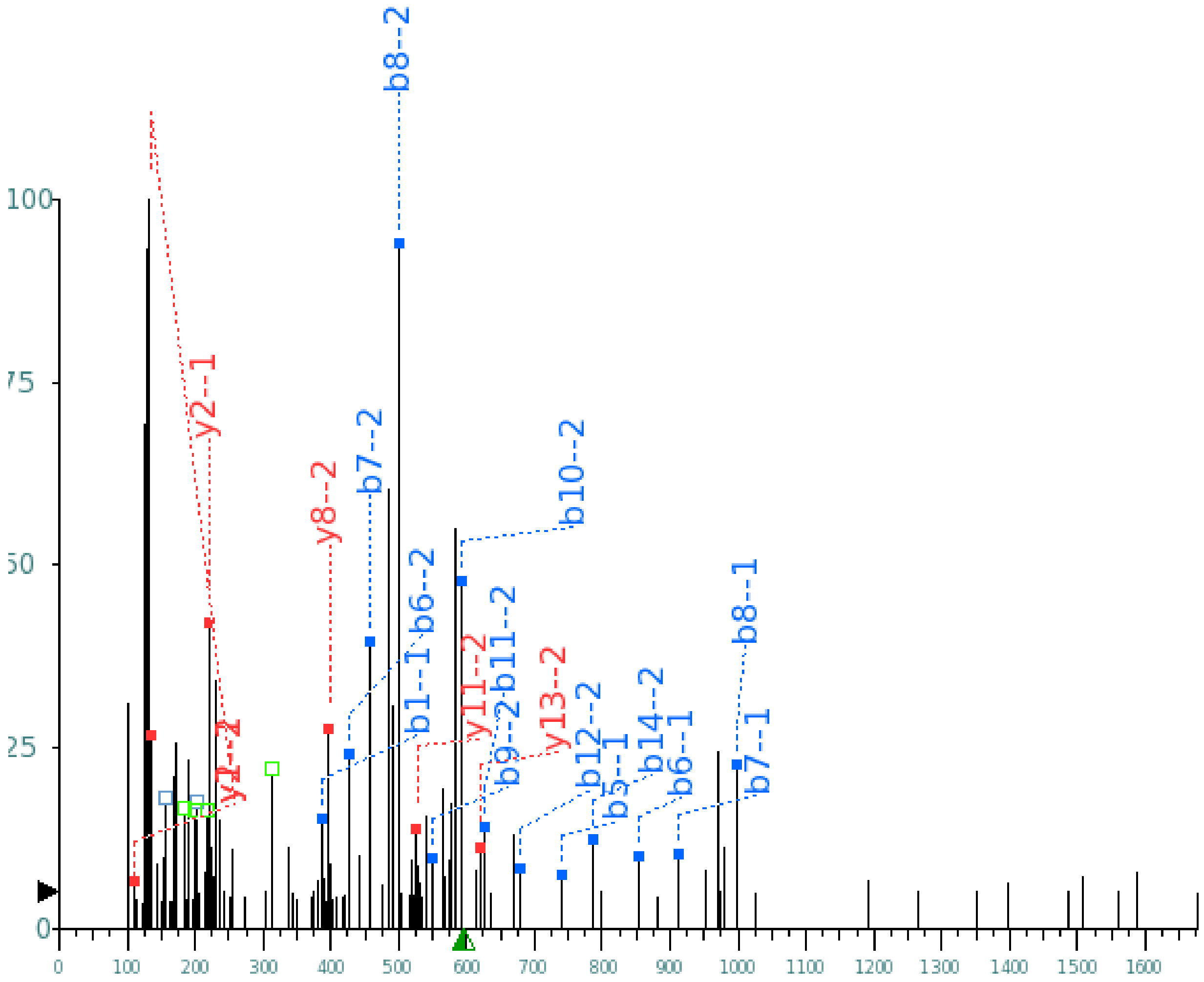

RPASVNGSPSATSESD (+3)

Primary Reference: ITCH_HUMAN
Search ID: 41761
Search Name: 20130328_ananiav_ERstress_TMT_TM_timecourse_trypsin_inj2_4mc_iaa
Scan#: 4594
Observed Mass: 597.6298 (1.7 ppm)
PSM Score: 21.03

File name: 2015-02-04 04.01.53PM.pdf

Creation time: 2015-02-04 04:09PM

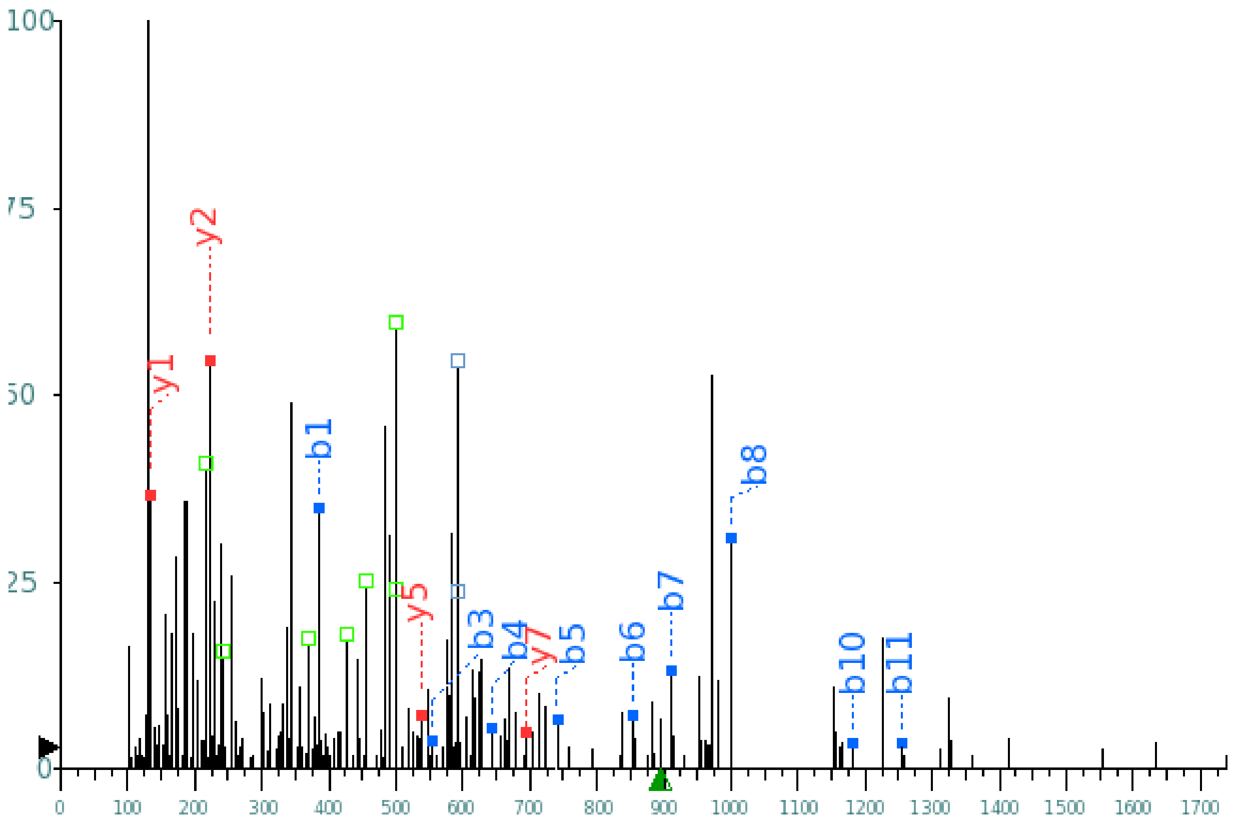

RPASVNGSPSATSESD (+2)

Primary Reference: ITCH_HUMAN
Search ID: 41761
Search Name: 20130328_ananiav_ERstress_TMT_TM_timecourse_trypsin_inj2_4mc_iaa
Scan#: 4595
Observed Mass: 895.9405 (1.1 ppm)
PSM Score: 29.28

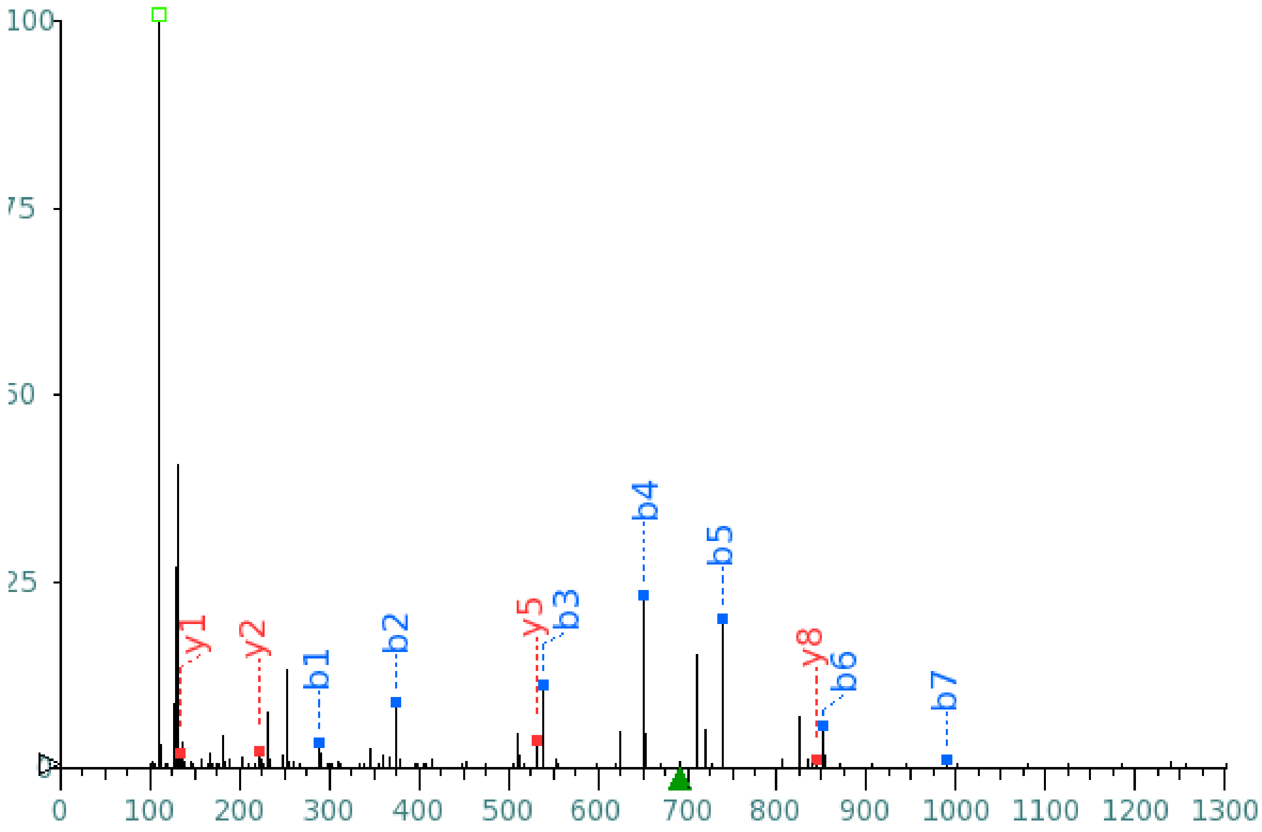

GSYNSLHDGSD (+2)

Primary Reference: CI150_HUMAN
Search ID: 41761
Search Name: 20130328_ananiav_ERstress_TMT_TM_timecourse_trypsin_inj2_4mc_iaa
Scan#: 4762
Observed Mass: 690.8156 (0.7 ppm)
PSM Score: 18.04

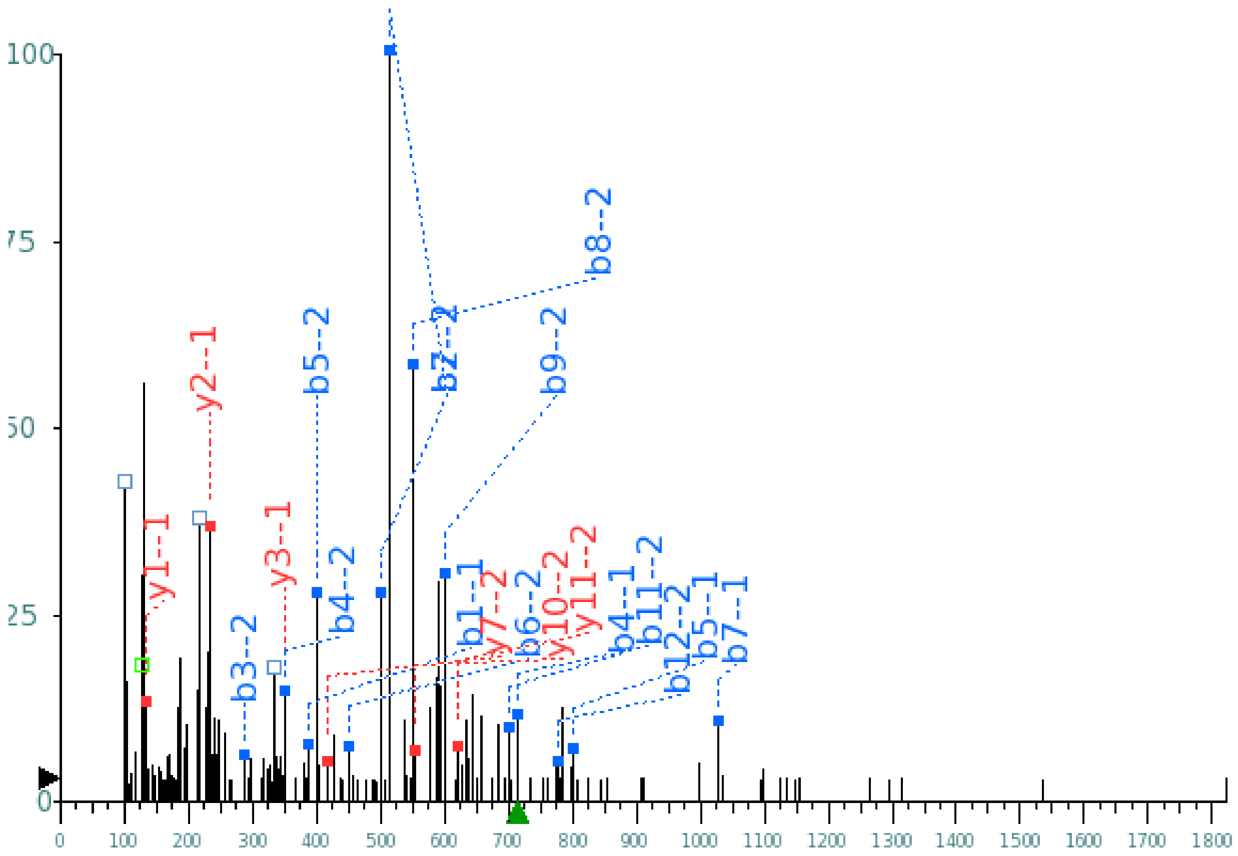

RNAETPEATTQQETDTD (+3)

Primary Reference: K1967_HUMAN
Search ID: 41761
Search Name: 20130328_ananiav_ERstress_TMT_TM_timecourse_trypsin_inj2_4mc_iaa
Scan#: 4767
Observed Mass: 712.6681 (0.4 ppm)
PSM Score: 23.48

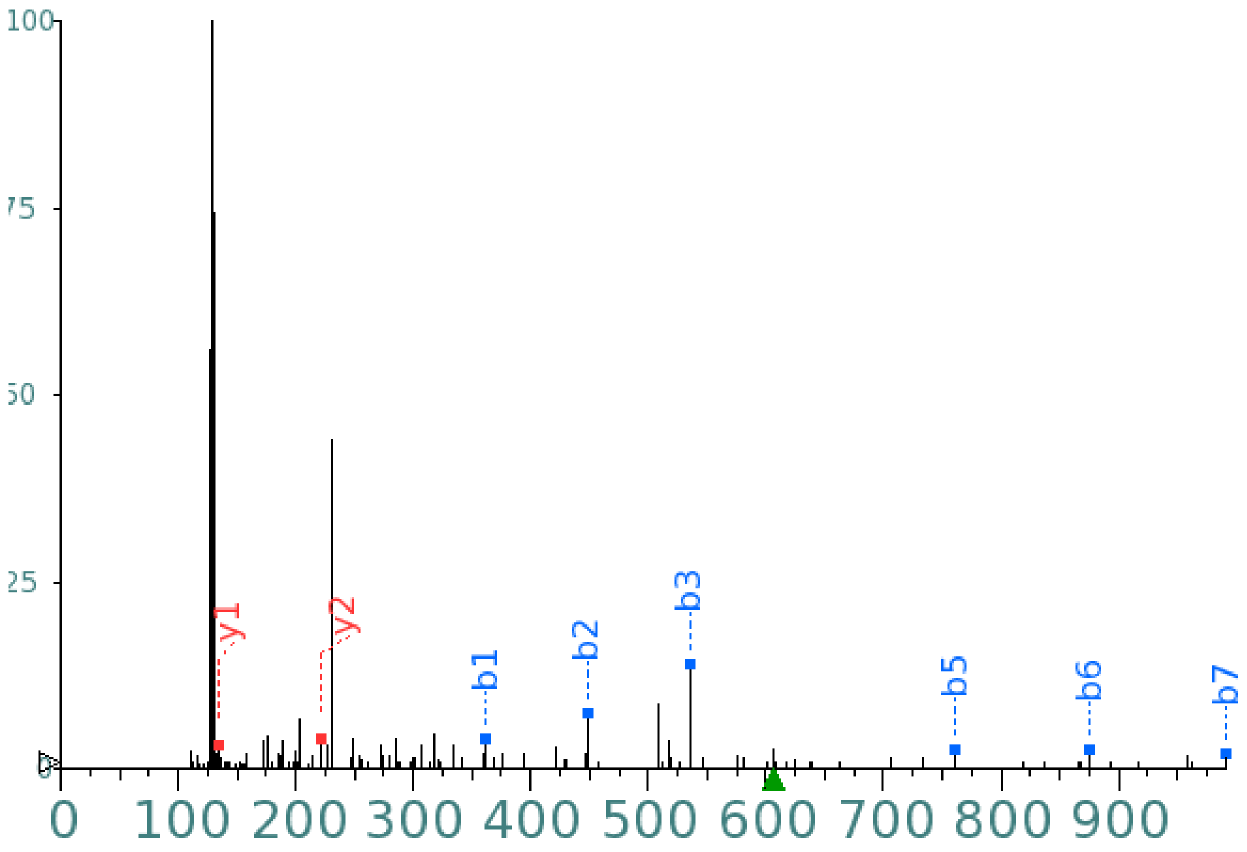

MSSPEDDSD (+2)

Primary Reference: SAFB1_HUMAN
Search ID: 41761
Search Name: 20130328_ananiav_ERstress_TMT_TM_timecourse_trypsin_inj2_4mc_iaa
Scan#: 4772
Observed Mass: 606.2513 (1.5 ppm)
PSM Score: 21.87

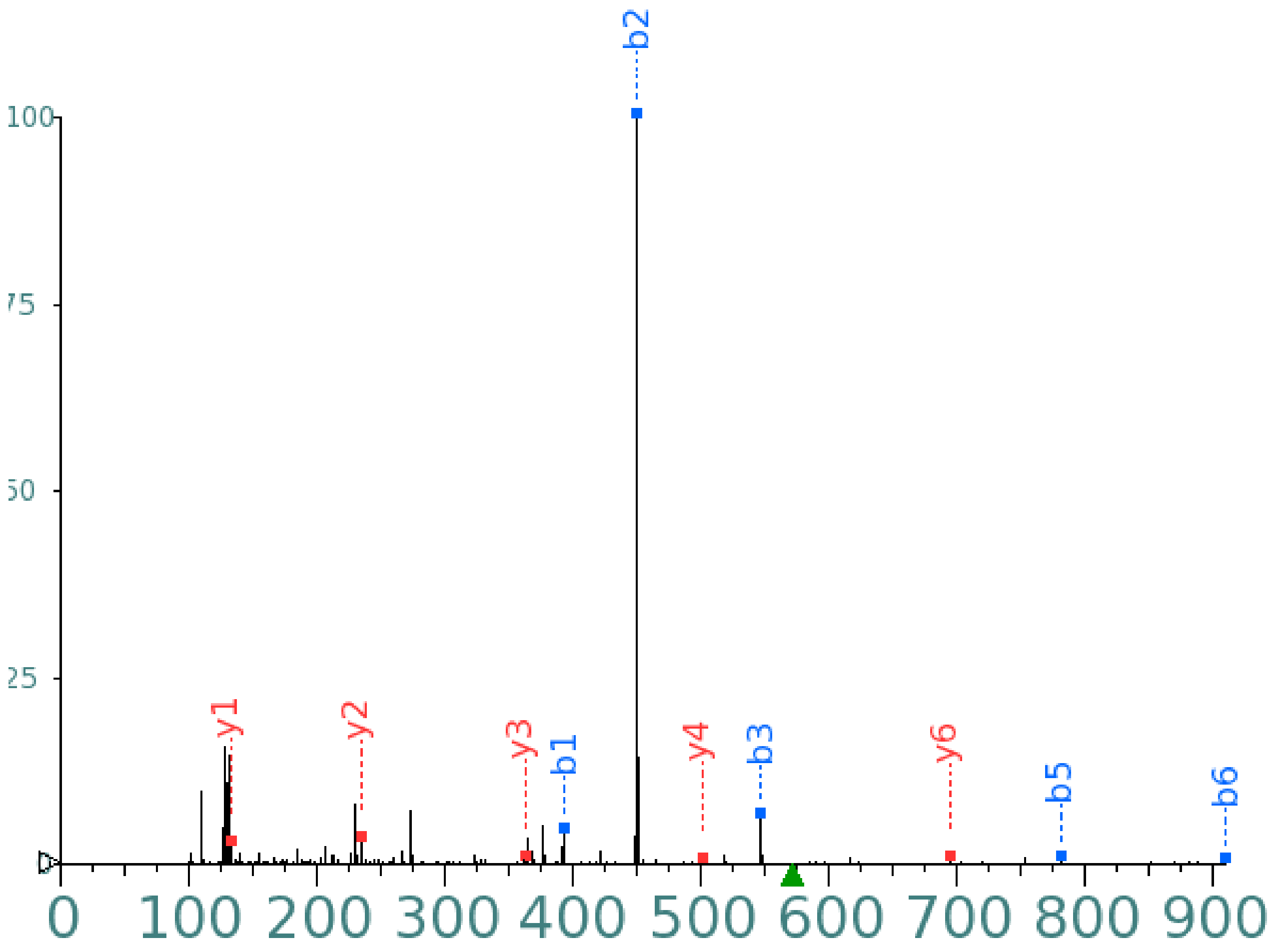

YGPPHETD (+2)

Primary Reference: TCF20_HUMAN
Search ID: 41761
Search Name: 20130328_ananiav_ERstress_TMT_TM_timecourse_trypsin_inj2_4mc_iaa
Scan#: 4846
Observed Mass: 572.7783 (1.8 ppm)
PSM Score: 19.44

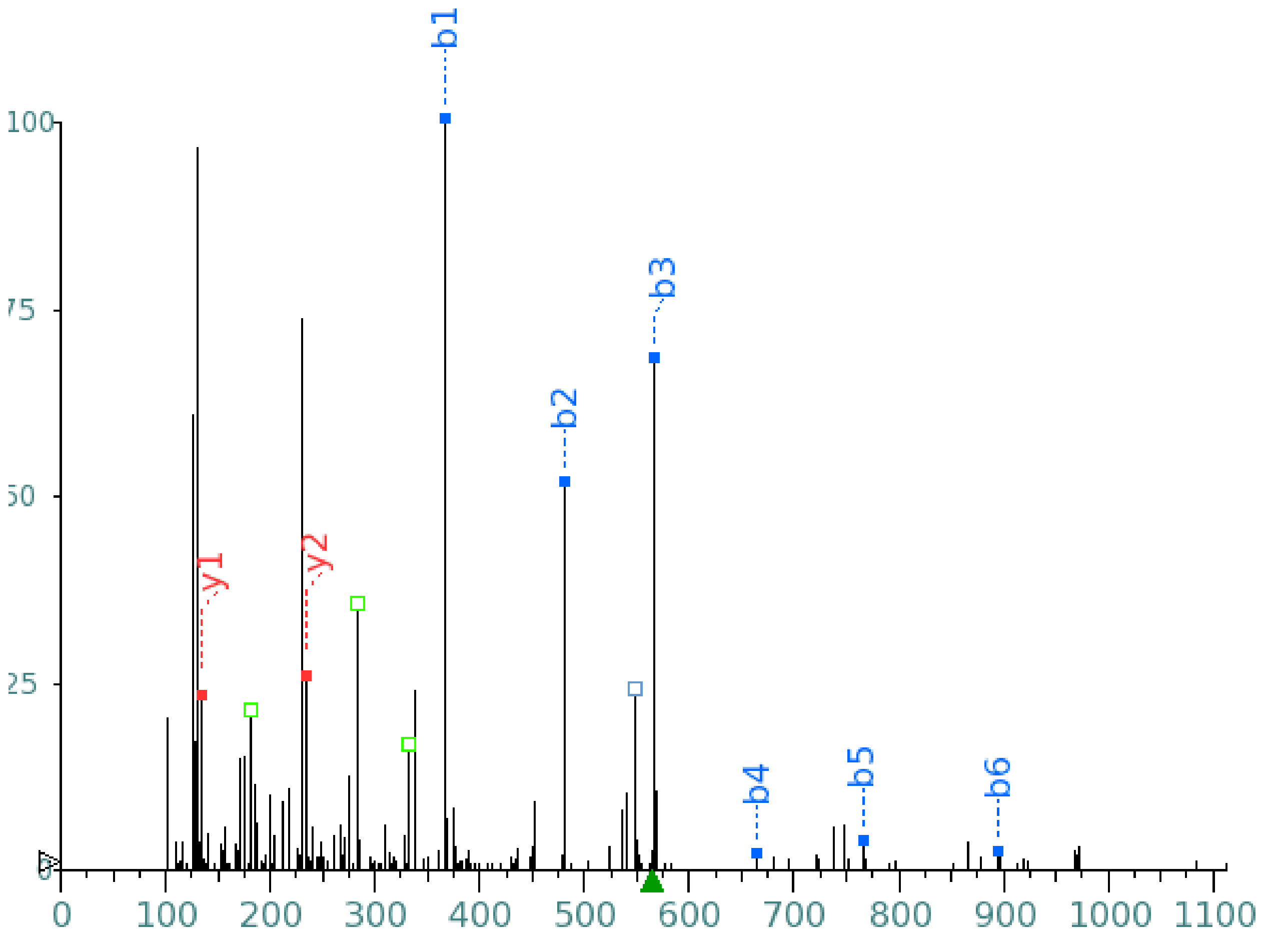

HLSPTETD (+2)

Primary Reference: KDIS_HUMAN
Search ID: 41761
Search Name: 20130328_ananiav_ERstress_TMT_TM_timecourse_trypsin_inj2_4mc_iaa
Scan#: 4857
Observed Mass: 564.7914 (1.8 ppm)
PSM Score: 18.94

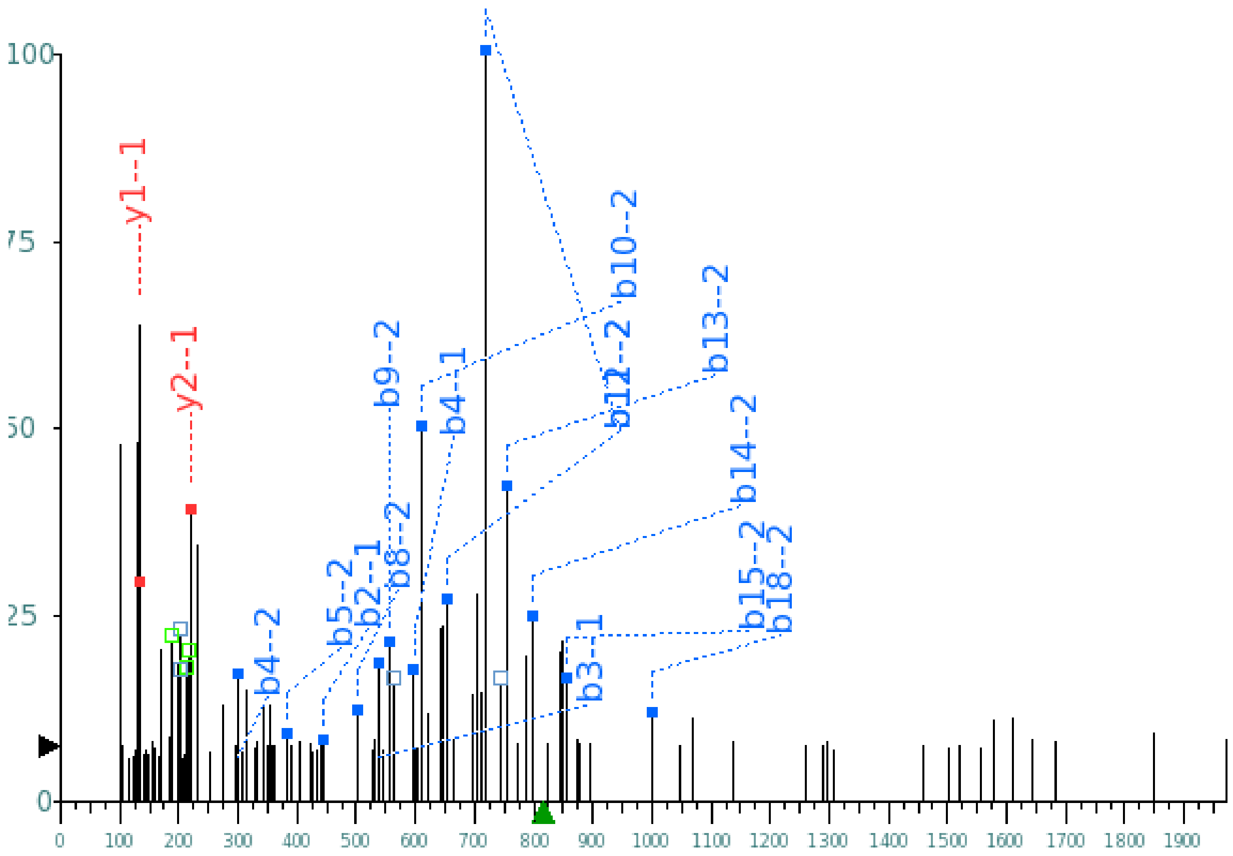

RGPGYTSGTNSEASNASETESD (+3)

Primary Reference: FMR1_HUMAN
Search ID: 41761
Search Name: 20130328_ananiav_ERstress_TMT_TM_timecourse_trypsin_inj2_4mc_iaa
Scan#: 4895
Observed Mass: 816.0331 (1.7 ppm)
PSM Score: 22.4

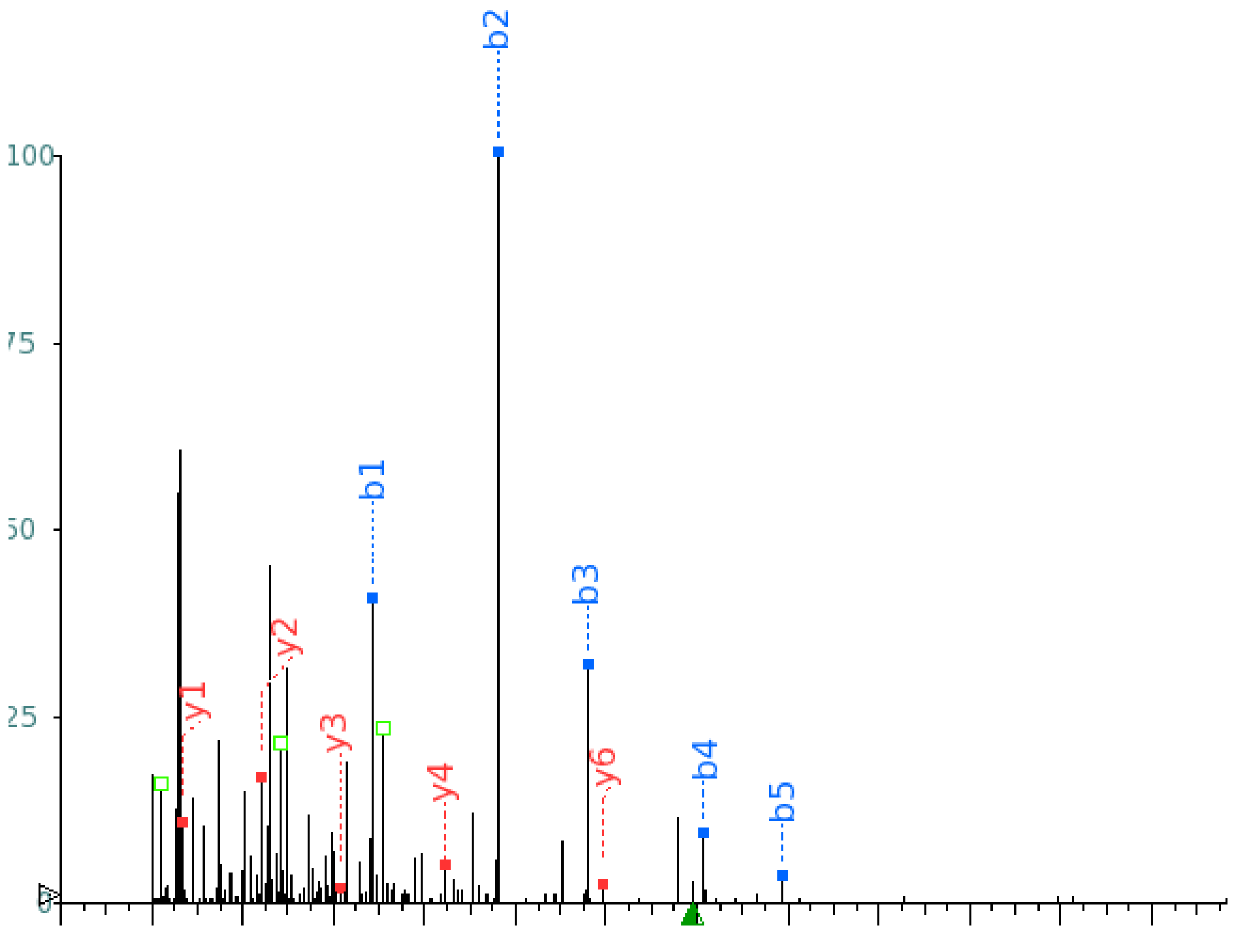

IHVQSSSDSSD (+2)

Primary Reference: F172A_HUMAN
Search ID: 41761
Search Name: 20130328_ananiav_ERstress_TMT_TM_timecourse_trypsin_inj2_4mc_iaa
Scan#: 4921
Observed Mass: 695.8371 (1.6 ppm)
PSM Score: 15.73

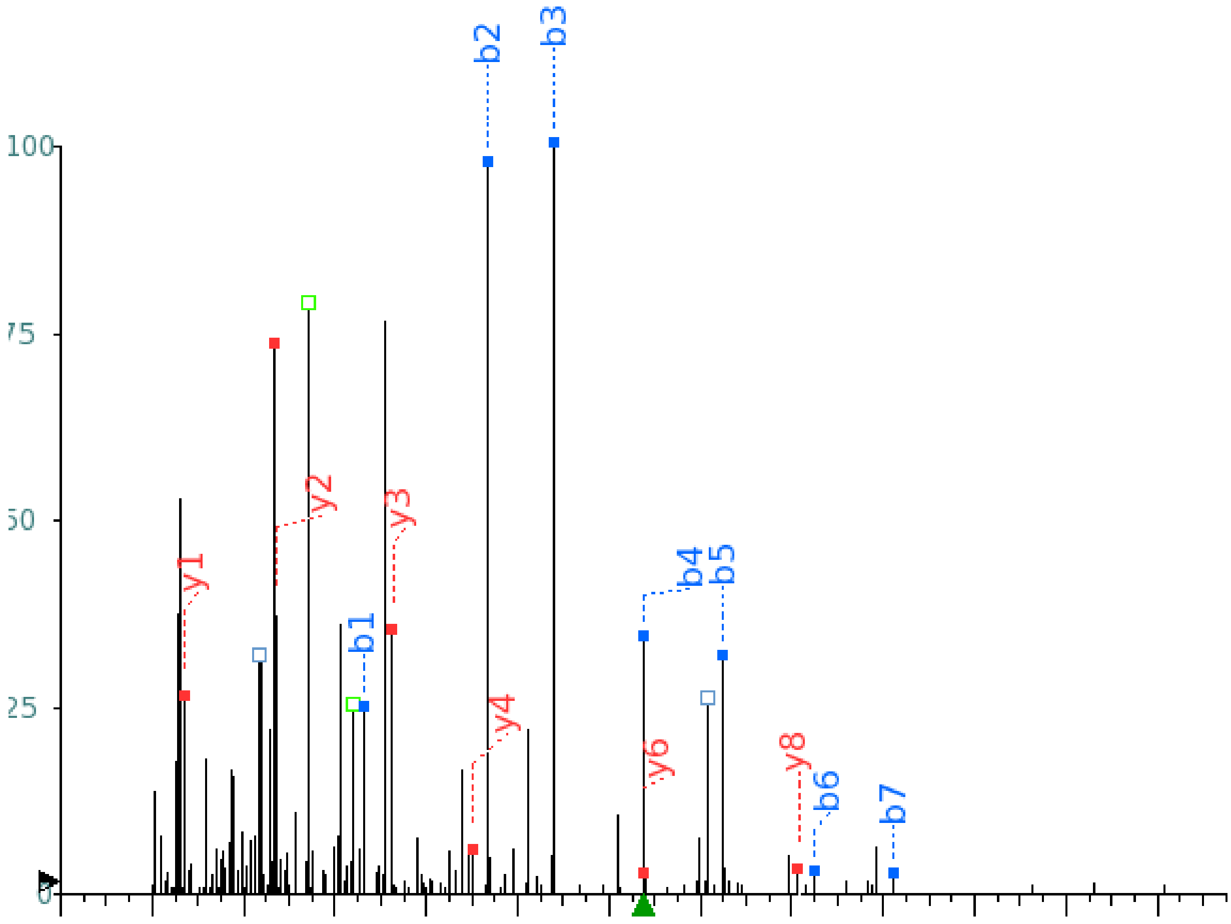

THAVSVSETD (+2)

Primary Reference: FAK1_HUMAN
Search ID: 41761
Search Name: 20130328_ananiav_ERstress_TMT_TM_timecourse_trypsin_inj2_4mc_iaa
Scan#: 4951
Observed Mass: 637.826 (1.7 ppm)
PSM Score: 23.42

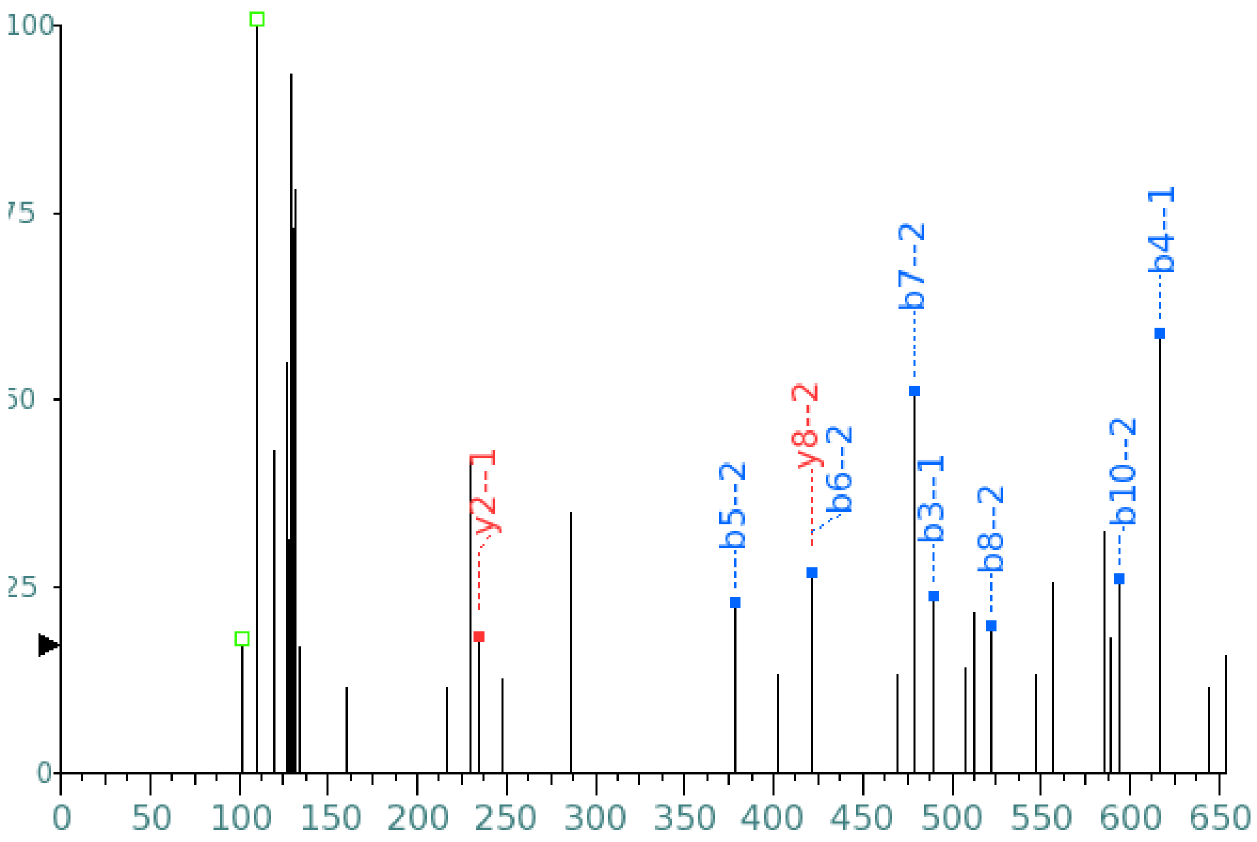

TASQHSDSSGFAEDSTD (+3)

Primary Reference: SSFA2_HUMAN
Search ID: 41761
Search Name: 20130328_ananiav_ERstress_TMT_TM_timecourse_trypsin_inj2_4mc_iaa
Scan#: 4985
Observed Mass: 657.6202 (2.4 ppm)
PSM Score: 19.29

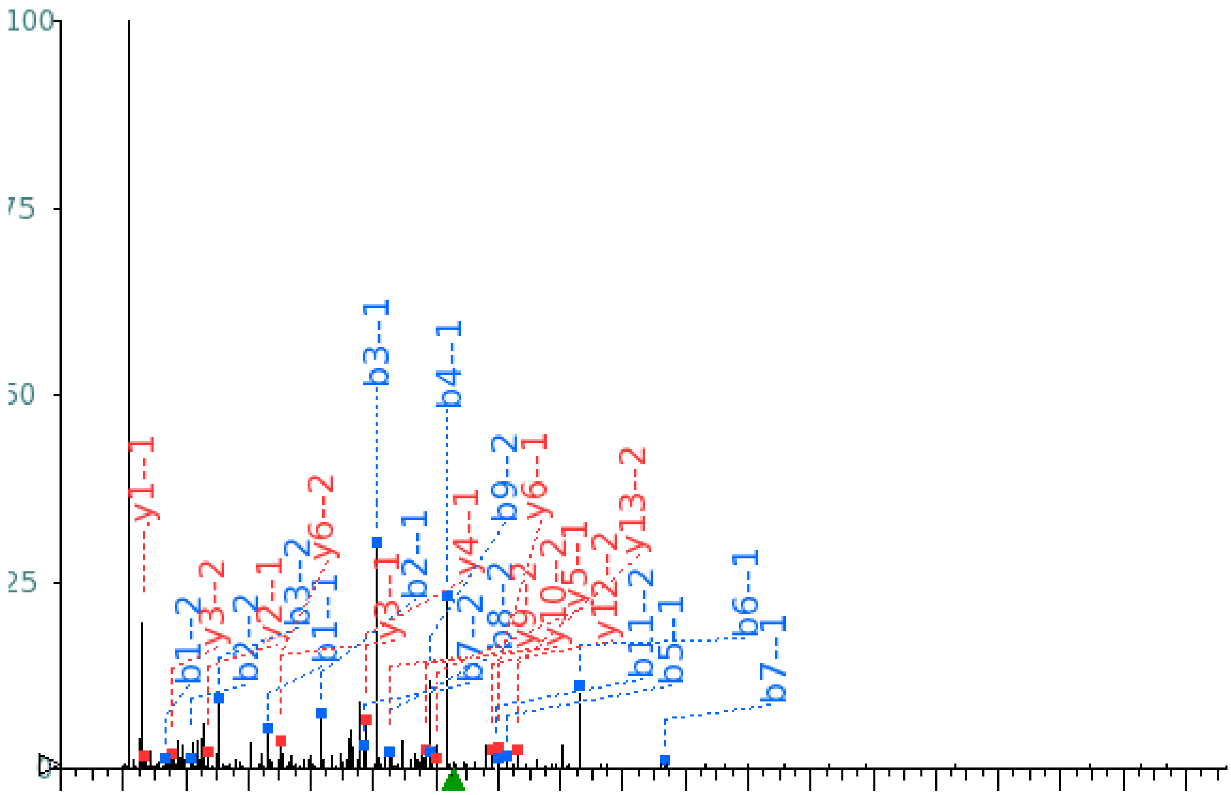

TSSLPNHSEPDHDTD (+3)

Primary Reference: VIR_HUMAN
Search ID: 41761
Search Name: 20130328_ananiav_ERstress_TMT_TM_timecourse_trypsin_inj2_4mc_iaa
Scan#: 4992
Observed Mass: 627.6211 (1.4 ppm)
PSM Score: 25.33

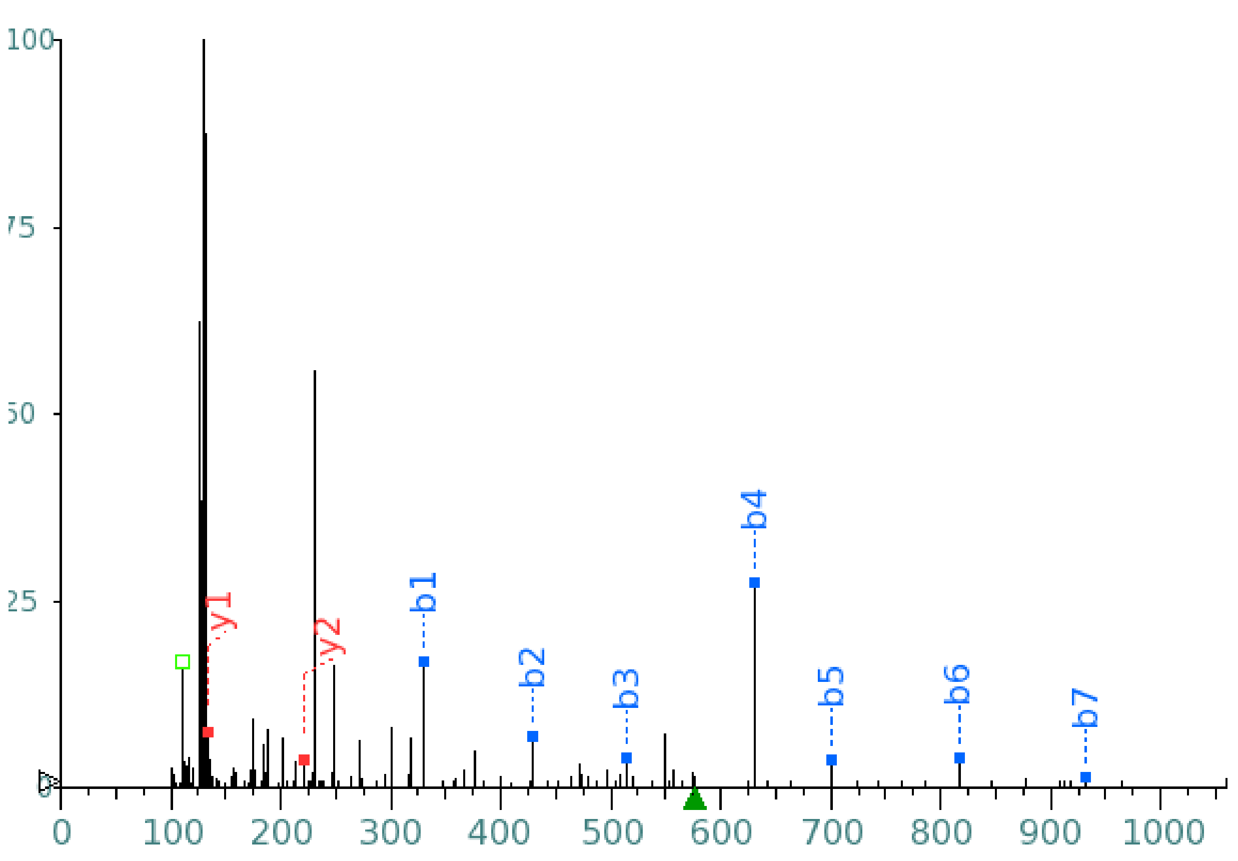

VVSDADDSD (+2)

Primary Reference: IWS1_HUMAN
Search ID: 41761
Search Name: 20130328_ananiav_ERstress_TMT_TM_timecourse_trypsin_inj2_4mc_iaa
Scan#: 5004
Observed Mass: 576.2683 (2.3 ppm)
PSM Score: 35.85

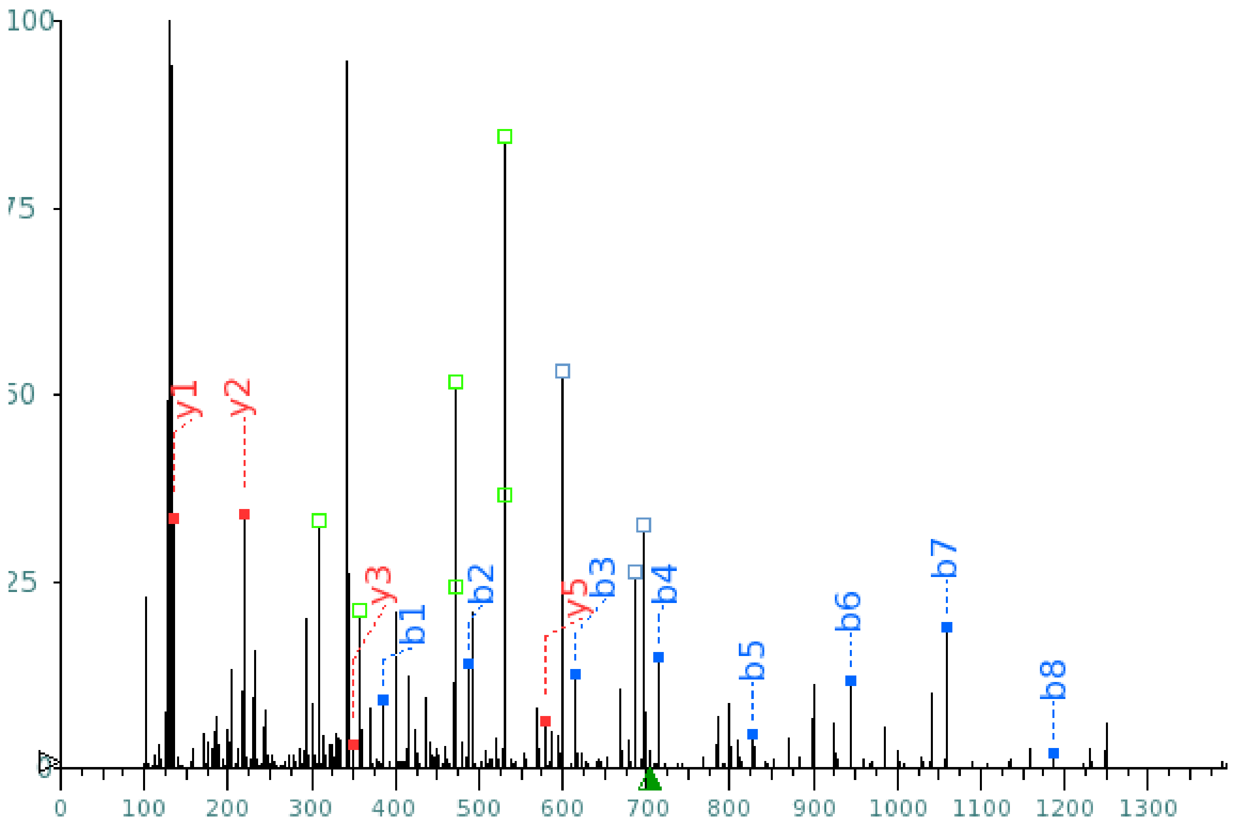

RTQVIDDESD (+2)

Primary Reference: TRIP4_HUMAN
Search ID: 41761
Search Name: 20130328_ananiav_ERstress_TMT_TM_timecourse_trypsin_inj2_4mc_iaa
Scan#: 5085
Observed Mass: 703.8528 (1.6 ppm)
PSM Score: 24.44

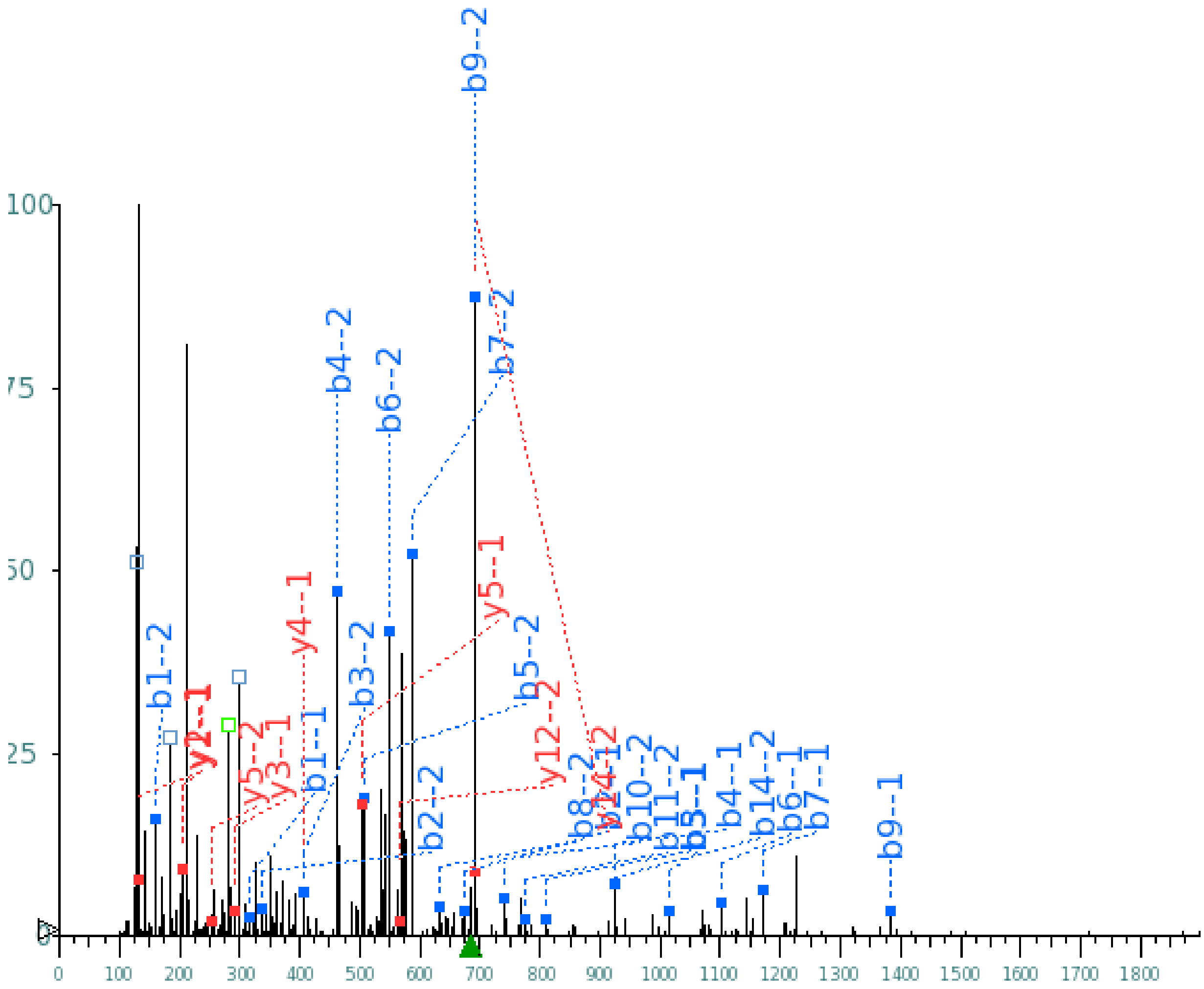

SKHDSSAPDPAPDSAD (+3)

Primary Reference: DIDO1_HUMAN
Search ID: 41761
Search Name: 20130328_ananiav_ERstress_TMT_TM_timecourse_trypsin_inj2_4mc_iaa
Scan#: 5151
Observed Mass: 685.6734 (1.2 ppm)
PSM Score: 33.84

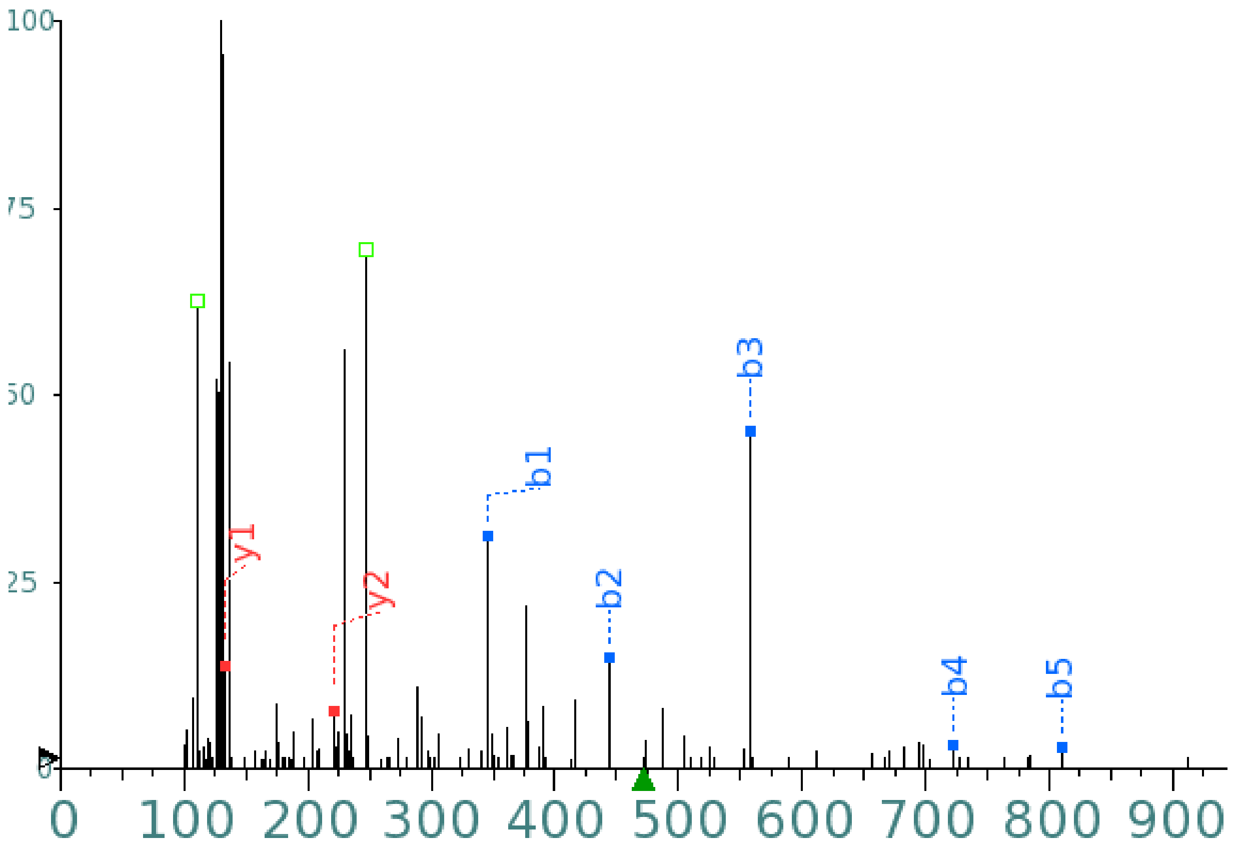

DVDYSD (+2)

Primary Reference: SMCA2_HUMAN
Search ID: 41761
Search Name: 20130328_ananiav_ERstress_TMT_TM_timecourse_trypsin_inj2_4mc_iaa
Scan#: 5155
Observed Mass: 471.717 (1.4 ppm)
PSM Score: 28.35

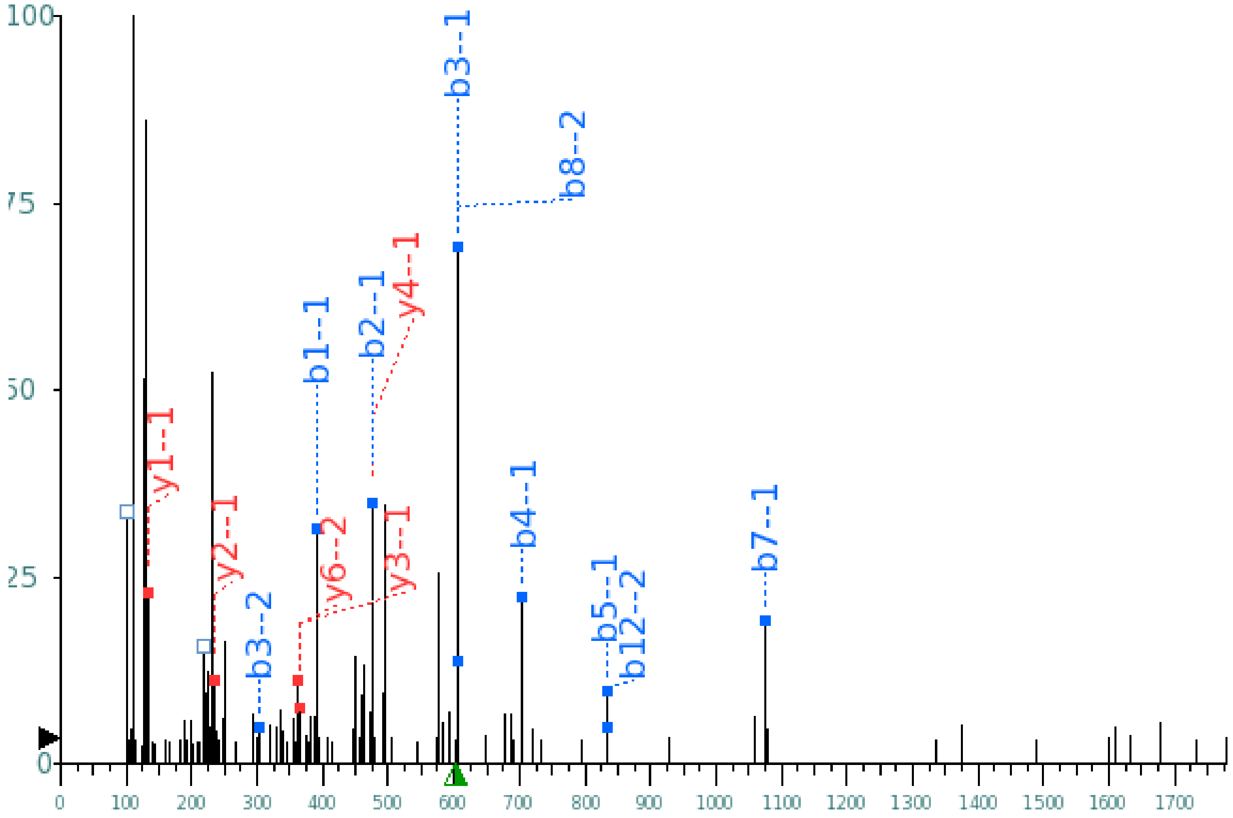

C*SQVQQDHLNQTD (+3)

Primary Reference: HOOK1_HUMAN
Search ID: 41761
Search Name: 20130328_ananiav_ERstress_TMT_TM_timecourse_trypsin_inj2_4mc_iaa
Scan#: 5214
Observed Mass: 601.283 (0.4 ppm)
PSM Score: 16.08

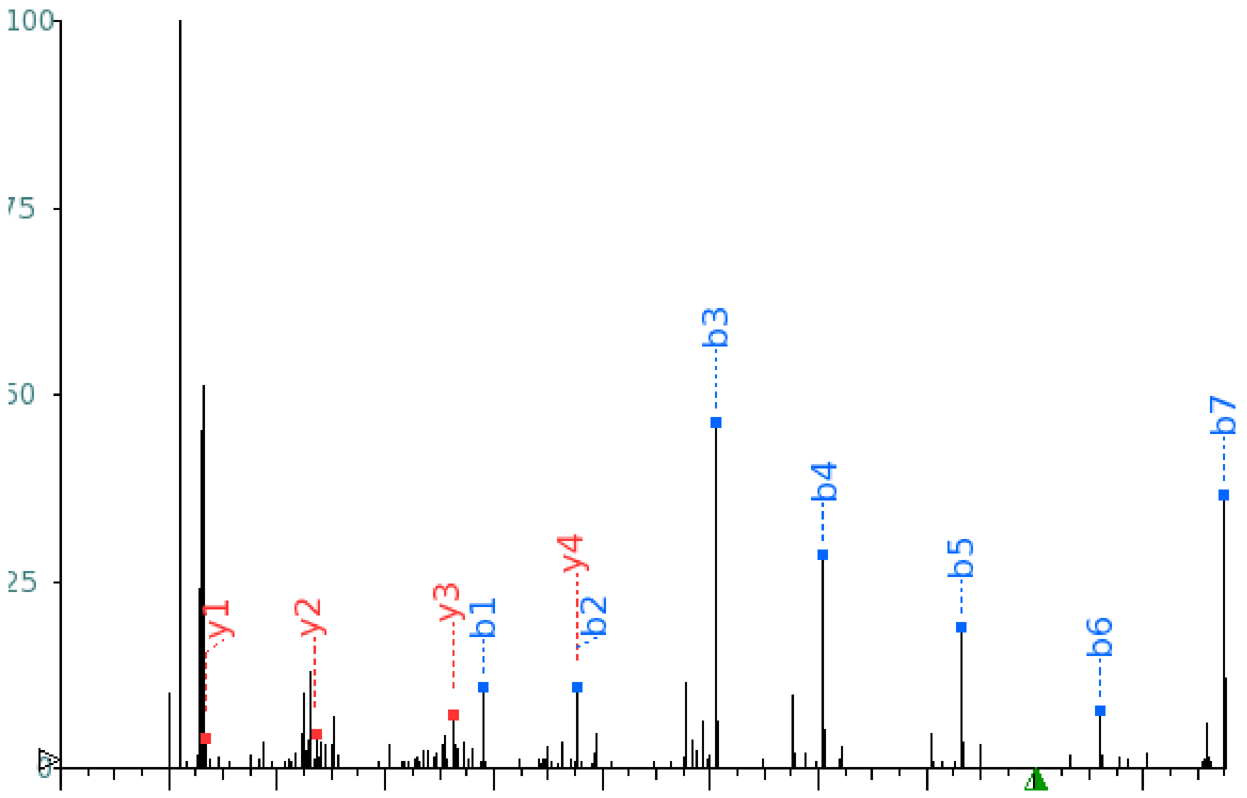

C*SQVQQDHLNQTD (+2)

Primary Reference: HOOK1_HUMAN
Search ID: 41761
Search Name: 20130328_ananiav_ERstress_TMT_TM_timecourse_trypsin_inj2_4mc_iaa
Scan#: 5217
Observed Mass: 901.4204 (-0.1 ppm)
PSM Score: 53.14

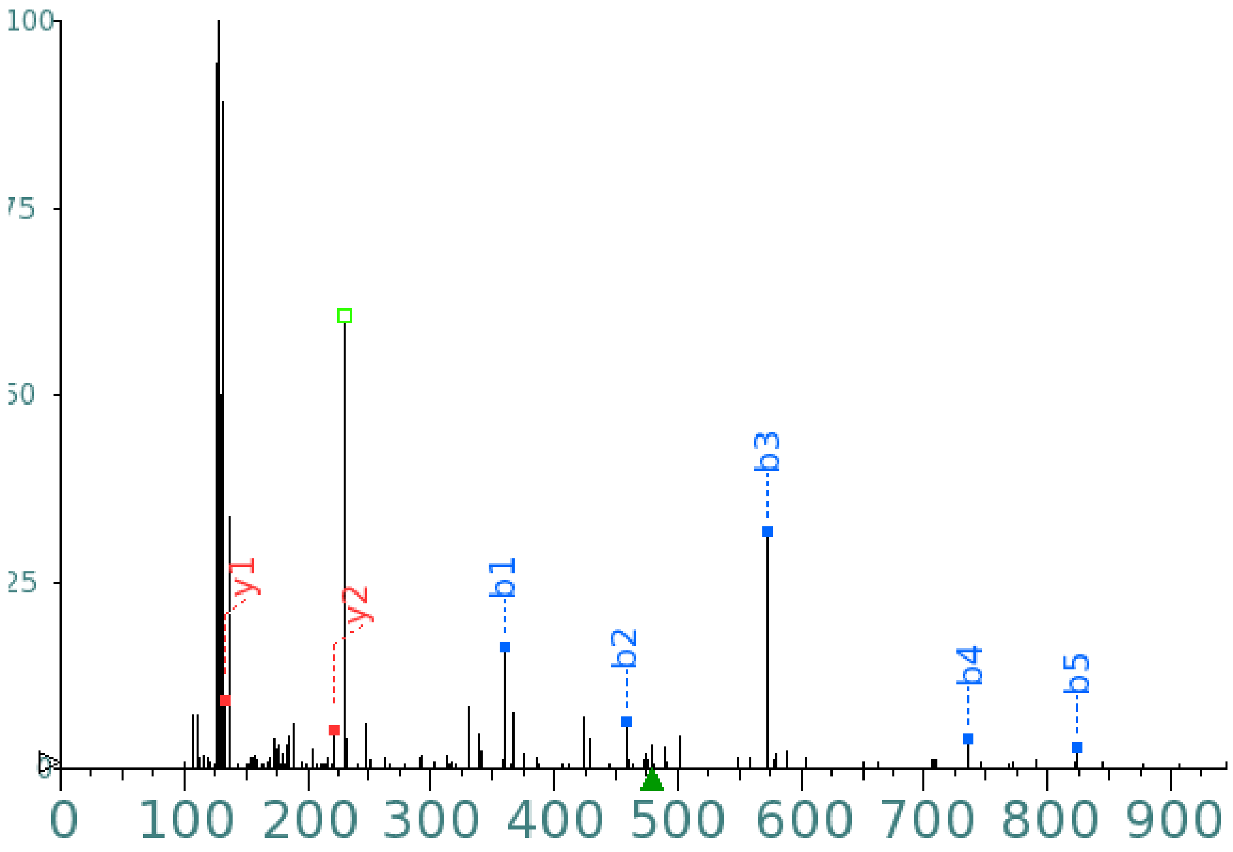

EVDYSD (+2)

Primary Reference: SMCA4_HUMAN
Search ID: 41761
Search Name: 20130328_ananiav_ERstress_TMT_TM_timecourse_trypsin_inj2_4mc_iaa
Scan#: 5290
Observed Mass: 478.7237 (-0.9 ppm)
PSM Score: 16.91

File name: 2015-02-04 04.01.53PM.pdf

Creation time: 2015-02-04 04:09PM

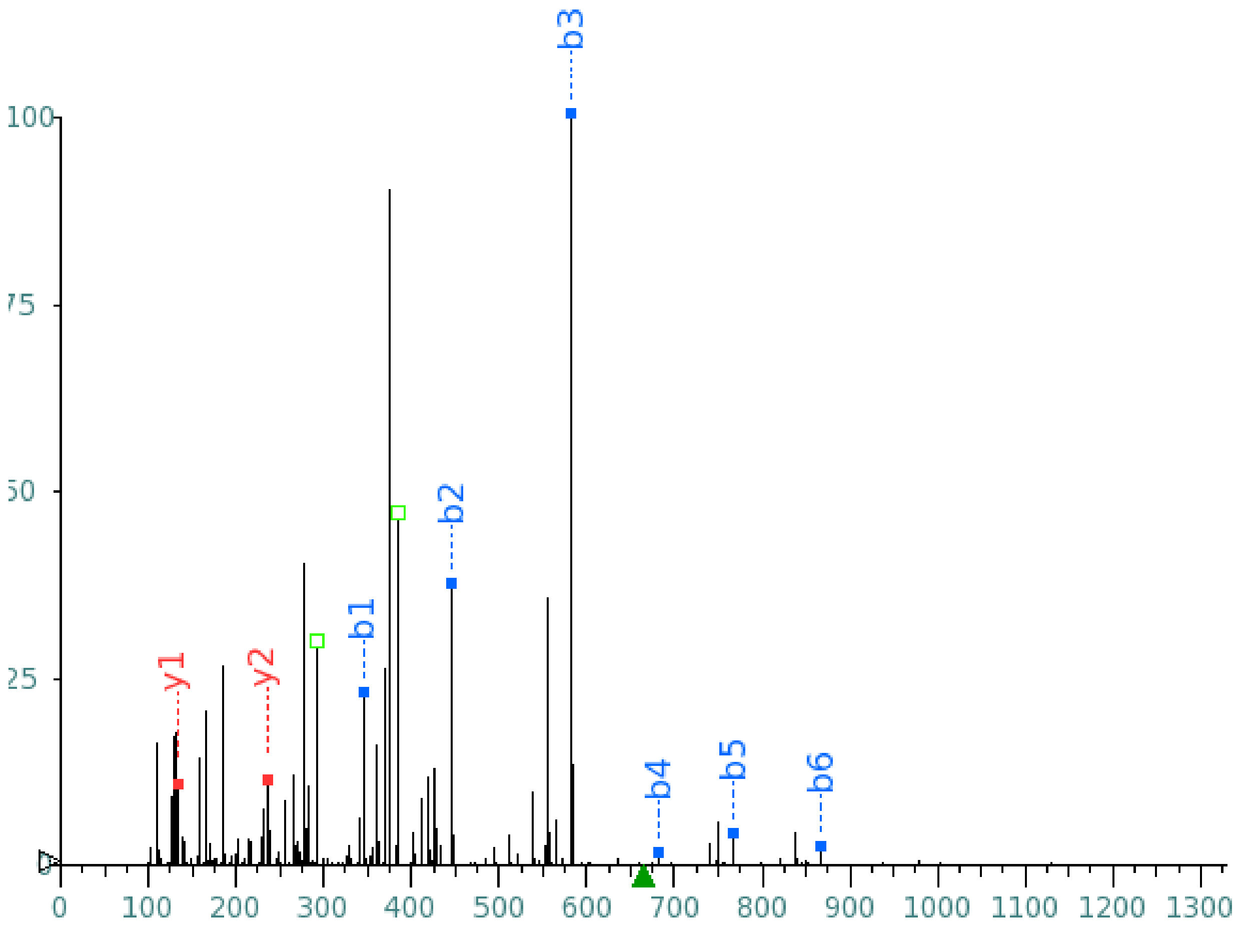

DTHPSVVETD (+2)

Primary Reference: ARHG5_HUMAN
Search ID: 41761
Search Name: 20130328_ananiav_ERstress_TMT_TM_timecourse_trypsin_inj2_4mc_iaa
Scan#: 5325
Observed Mass: 664.8307 (0.7 ppm)
PSM Score: 23.43

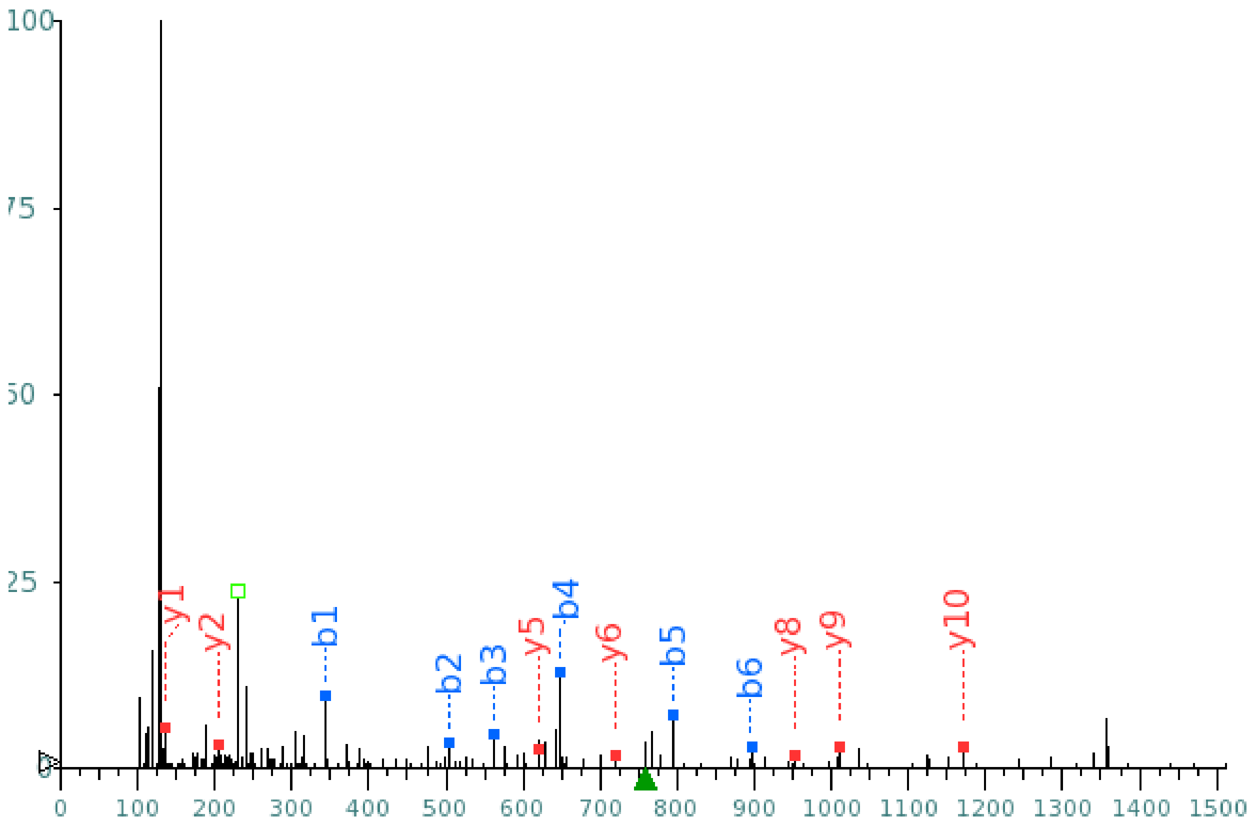

NC*GSFTREEAD (+2)

Primary Reference: ACLY_HUMAN
Search ID: 41761
Search Name: 20130328_ananiav_ERstress_TMT_TM_timecourse_trypsin_inj2_4mc_iaa
Scan#: 5339
Observed Mass: 757.8417 (1.2 ppm)
PSM Score: 13.63

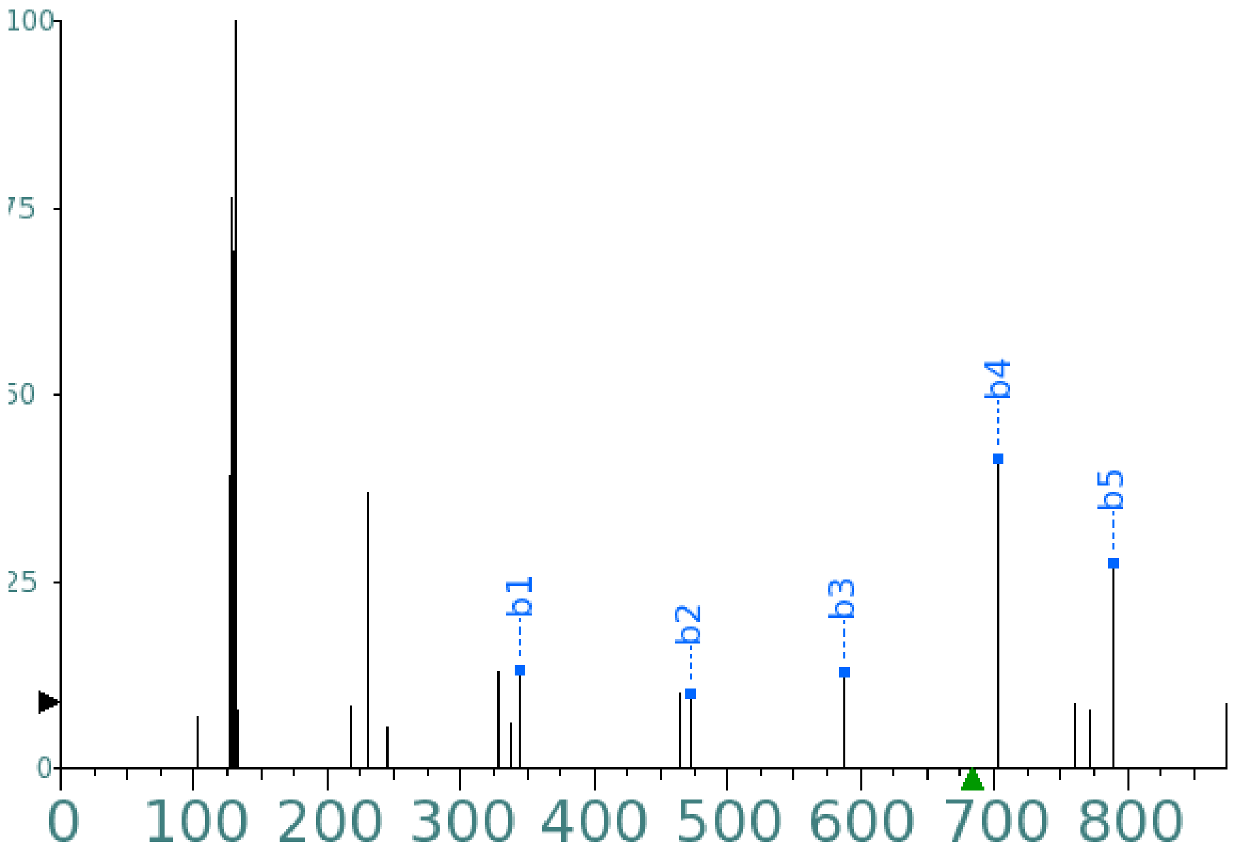

NQDDSLTETD (+2)

Primary Reference: PACS1_HUMAN
Search ID: 41761
Search Name: 20130328_ananiav_ERstress_TMT_TM_timecourse_trypsin_inj2_4mc_iaa
Scan#: 5392
Observed Mass: 683.8136 (2 ppm)
PSM Score: 20.35

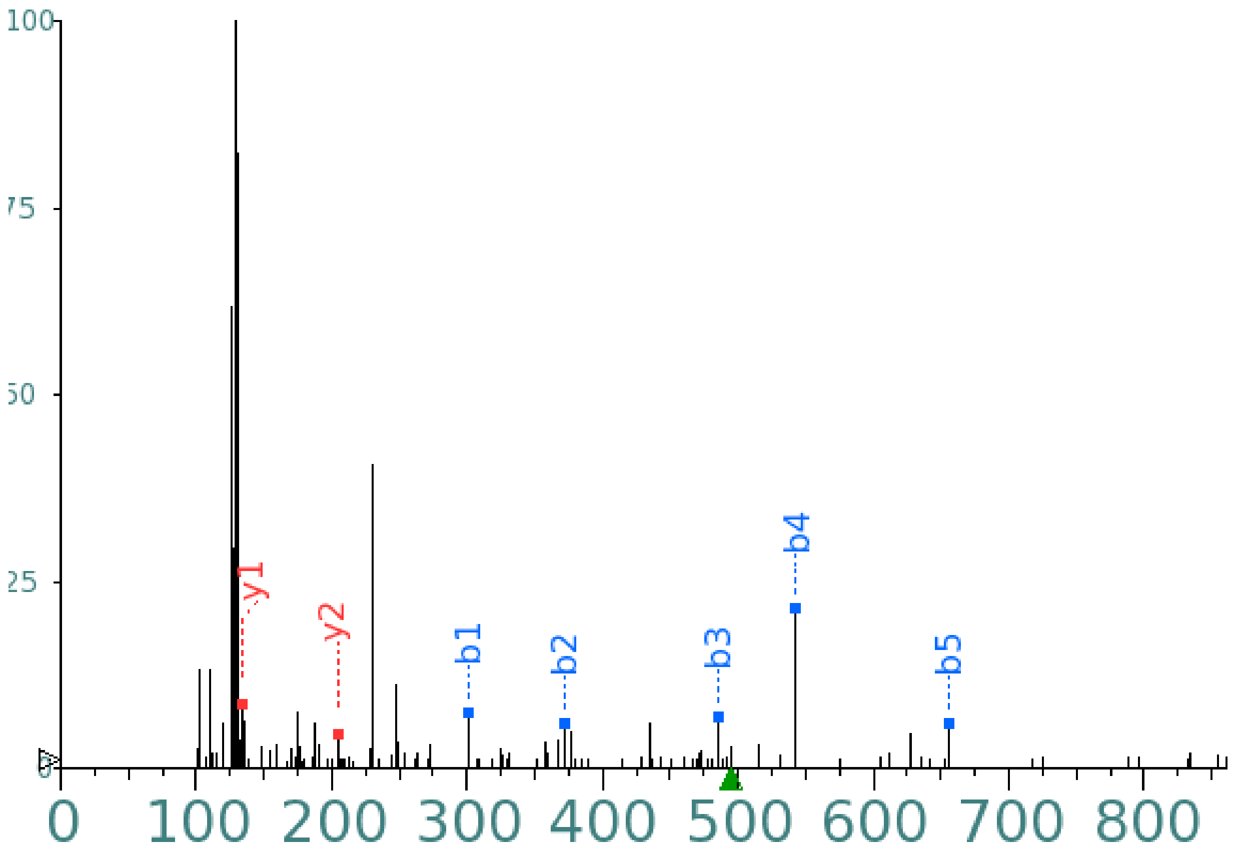

AANGLEAD (+2)

Primary Reference: LUZP1_HUMAN
Search ID: 41761
Search Name: 20130328_ananiav_ERstress_TMT_TM_timecourse_trypsin_inj2_4mc_iaa
Scan#: 5402
Observed Mass: 495.2589 (0.5 ppm)
PSM Score: 15.45

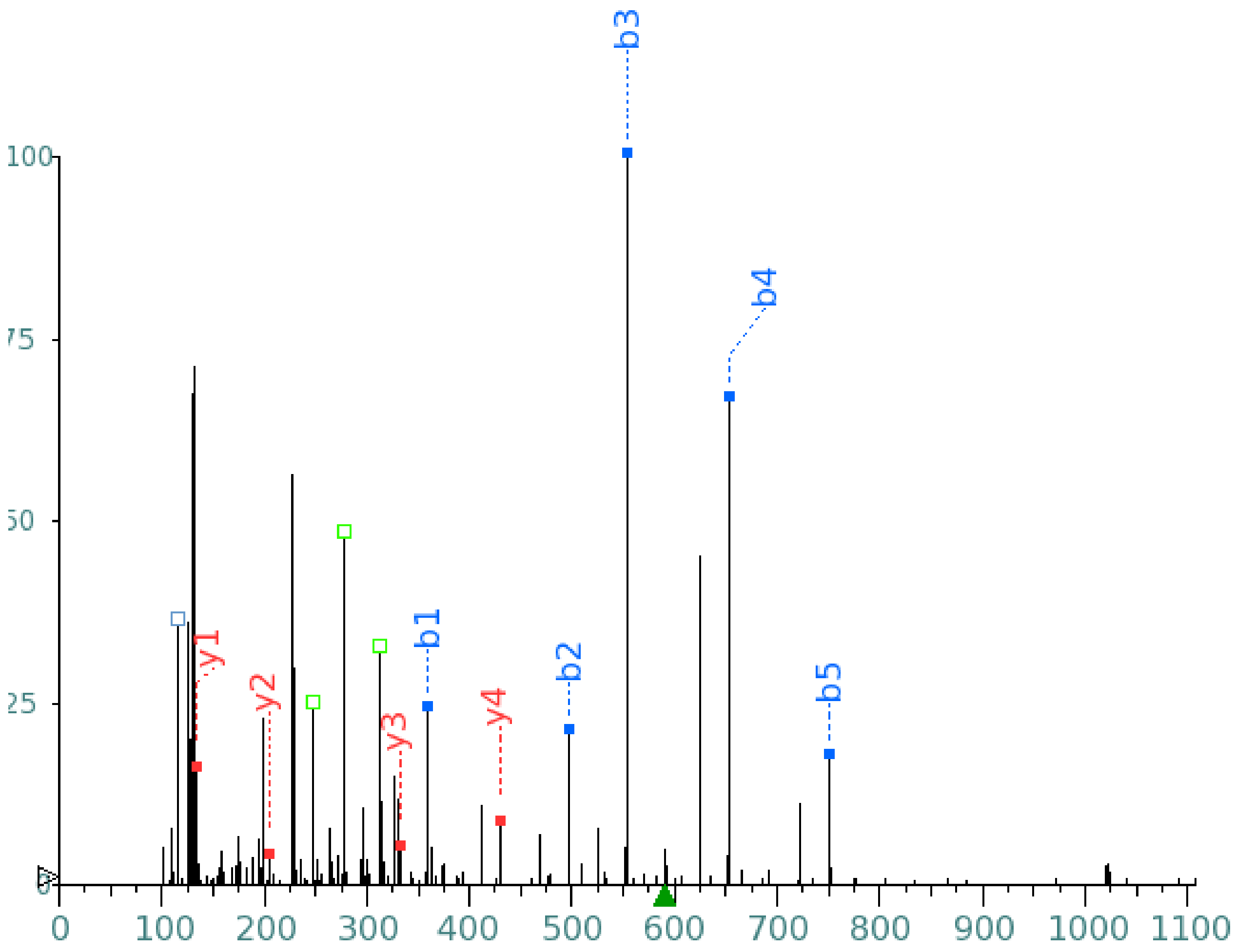

EHGVVPQAD (+2)

Primary Reference: DNJC2_HUMAN
Search ID: 41761
Search Name: 20130328_ananiav_ERstress_TMT_TM_timecourse_trypsin_inj2_4mc_iaa
Scan#: 5432
Observed Mass: 590.8127 (1.8 ppm)
PSM Score: 14.71

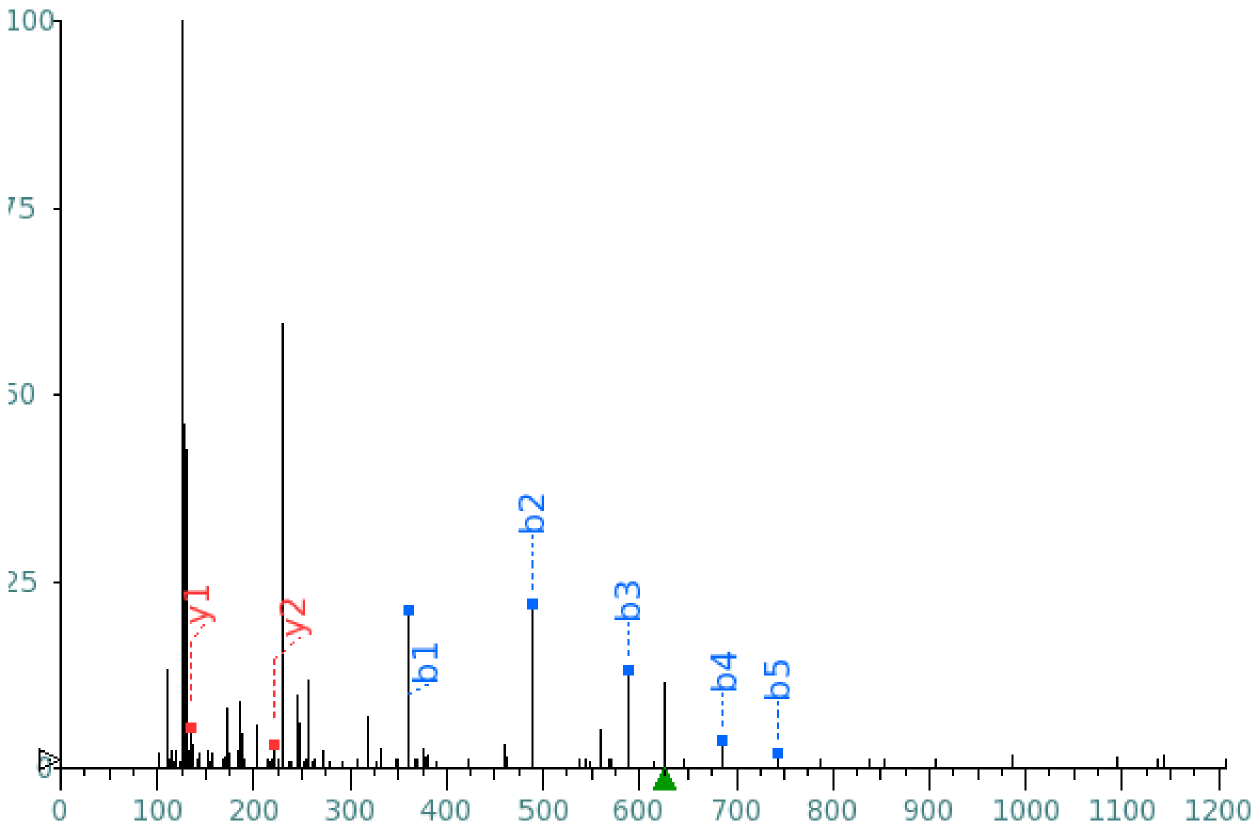

EEVVGGDDSD (+2)

Primary Reference: TYY1_HUMAN
Search ID: 41761
Search Name: 20130328_ananiav_ERstress_TMT_TM_timecourse_trypsin_inj2_4mc_iaa
Scan#: 5433
Observed Mass: 625.784 (1.7 ppm)
PSM Score: 7.28

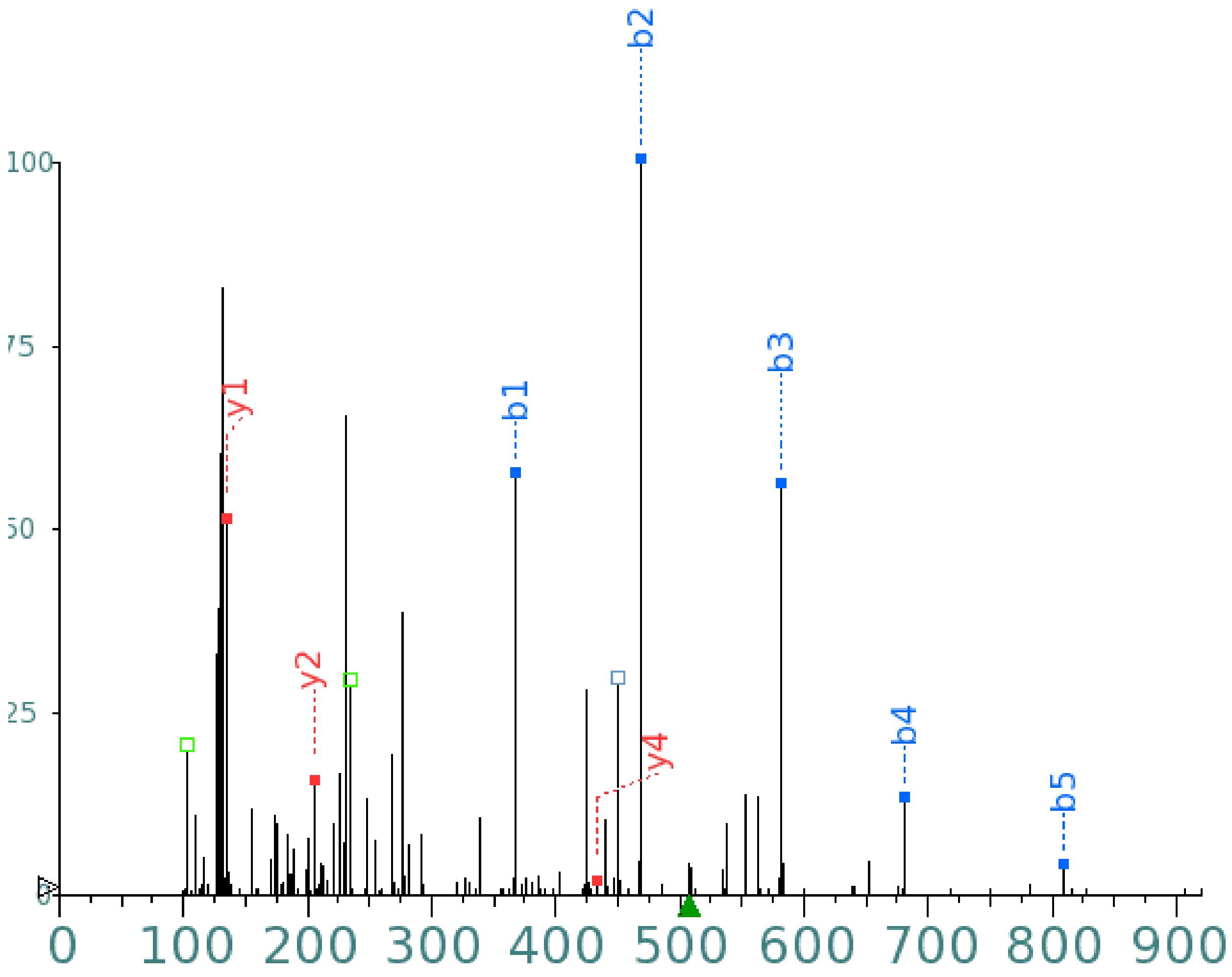

HTLVEAD (+2)

Primary Reference: SPTB2_HUMAN
Search ID: 41761
Search Name: 20130328_ananiav_ERstress_TMT_TM_timecourse_trypsin_inj2_4mc_iaa
Scan#: 5462
Observed Mass: 507.2779 (2.1 ppm)
PSM Score: 30.16

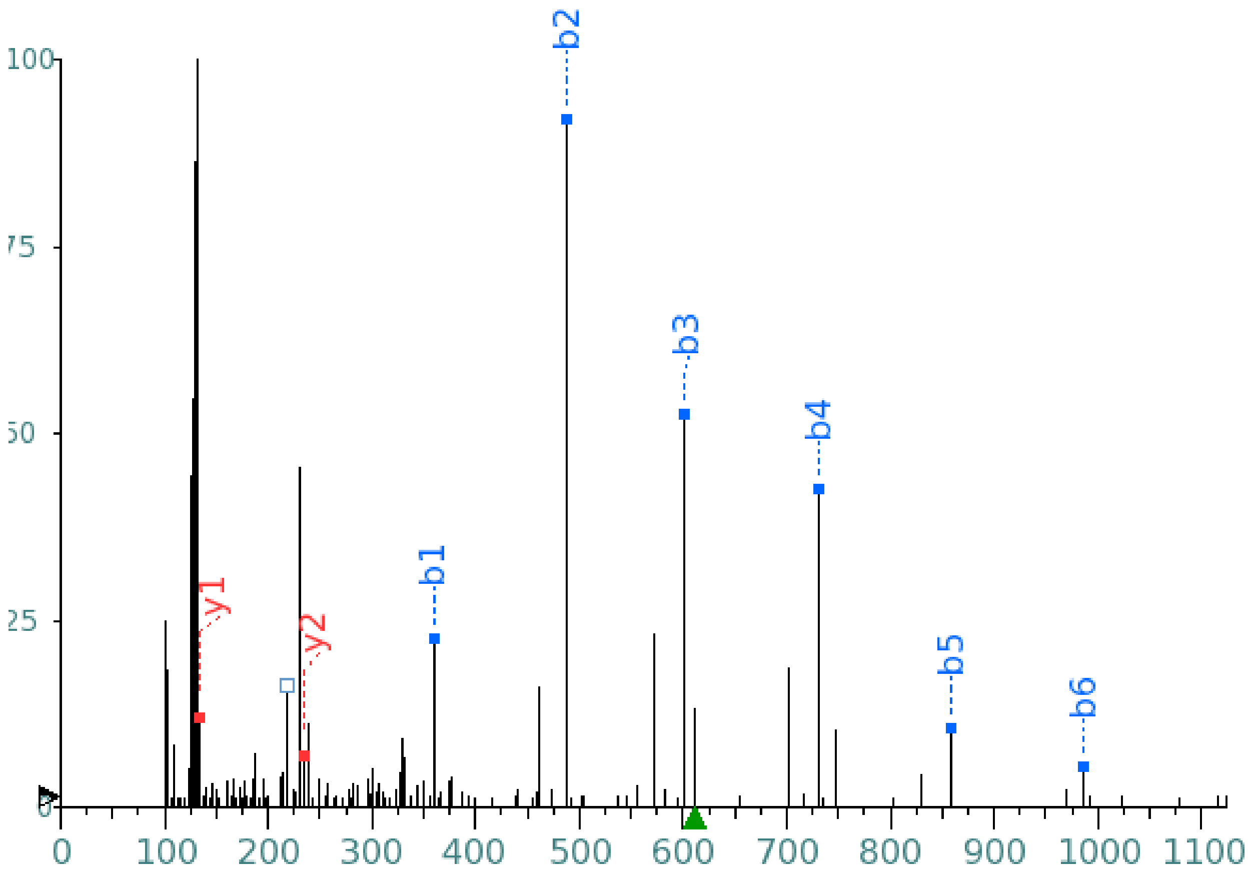

EELEQQTD (+2)

Primary Reference: SEC62_HUMAN
Search ID: 41761
Search Name: 20130328_ananiav_ERstress_TMT_TM_timecourse_trypsin_inj2_4mc_iaa
Scan#: 5463
Observed Mass: 610.7969 (1.8 ppm)
PSM Score: 37.65

File name: 2015-02-04 04.01.53PM.pdf

Creation time: 2015-02-04 04:09PM

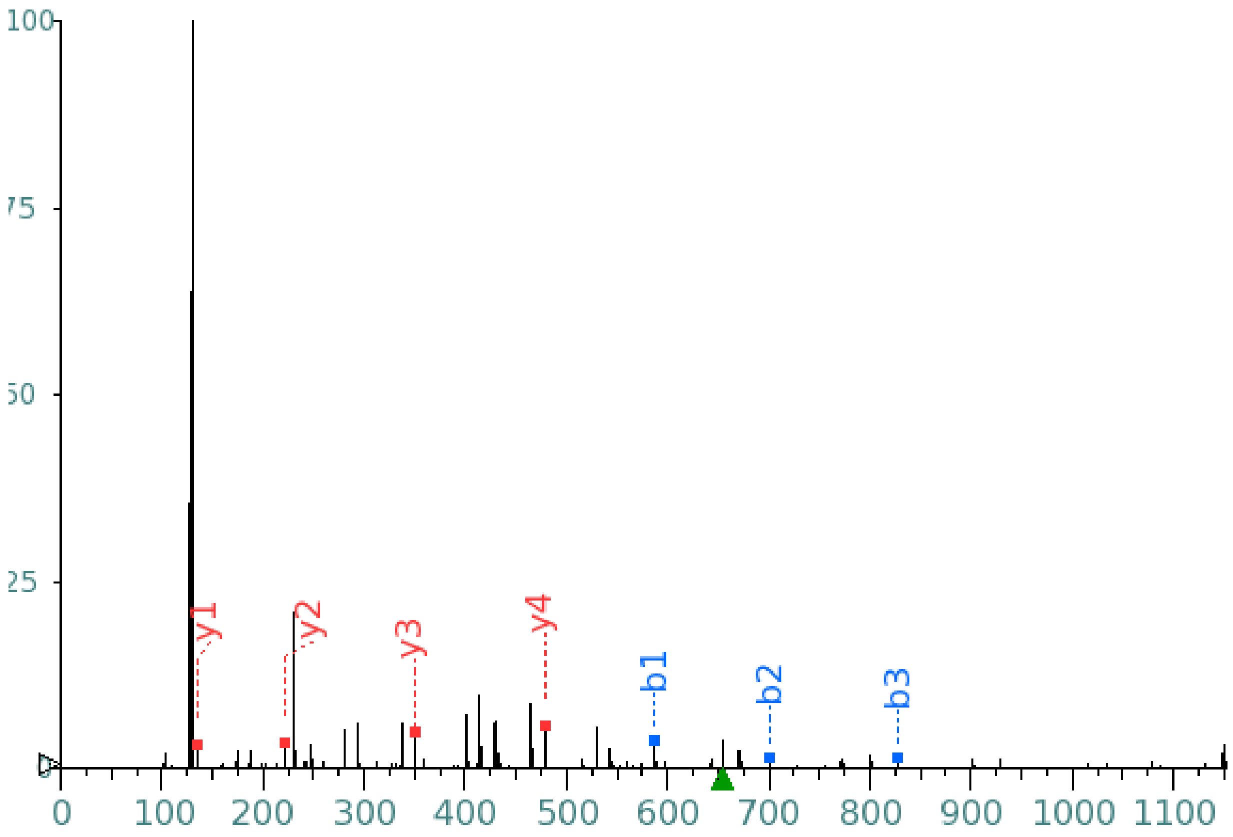

KLQEESD (+2)

Primary Reference: EIF3J_HUMAN
Search ID: 41761
Search Name: 20130328_ananiav_ERstress_TMT_TM_timecourse_trypsin_inj2_4mc_iaa
Scan#: 5491
Observed Mass: 653.8676 (1.9 ppm)
PSM Score: 9.63

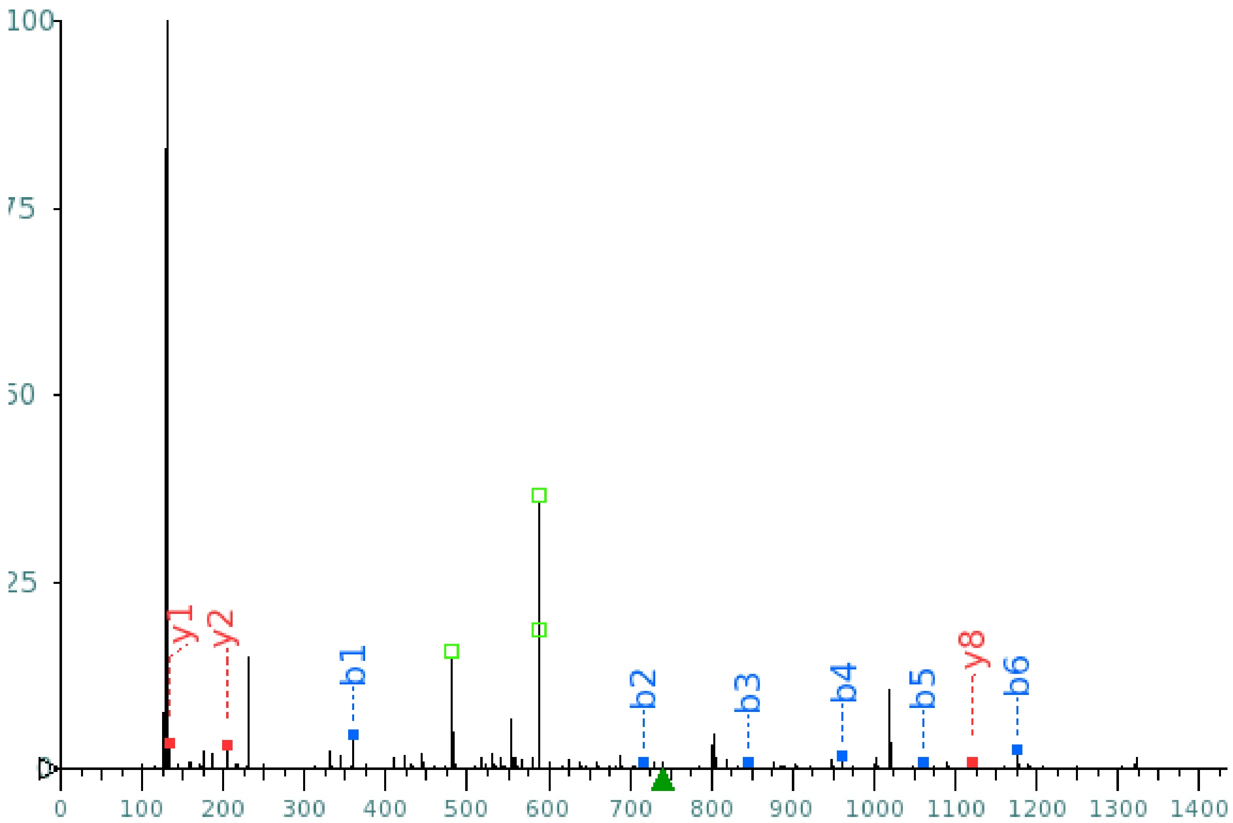

EKEDTDVAD (+2)

Primary Reference: AHNK2_HUMAN
Search ID: 41761
Search Name: 20130328_ananiav_ERstress_TMT_TM_timecourse_trypsin_inj2_4mc_iaa
Scan#: 5492
Observed Mass: 740.3838 (1.6 ppm)
PSM Score: 5.4

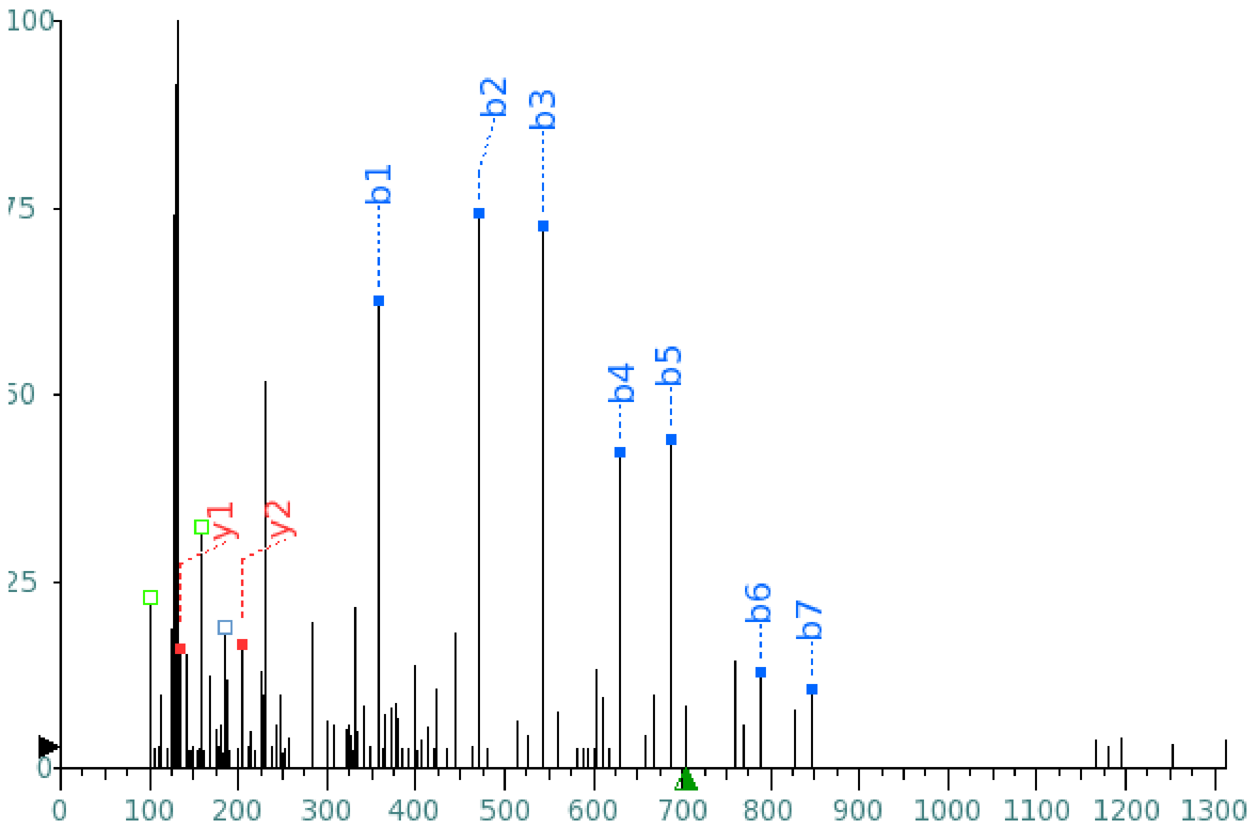

ELASGTGQDDAD (+2)

Primary Reference: E2QRJ1_HUMAN
Search ID: 41761
Search Name: 20130328_ananiav_ERstress_TMT_TM_timecourse_trypsin_inj2_4mc_iaa
Scan#: 5510
Observed Mass: 704.327 (2.2 ppm)
PSM Score: 33.88

File name: 2015-02-04 04.01.53PM.pdf

Creation time: 2015-02-04 04:09PM

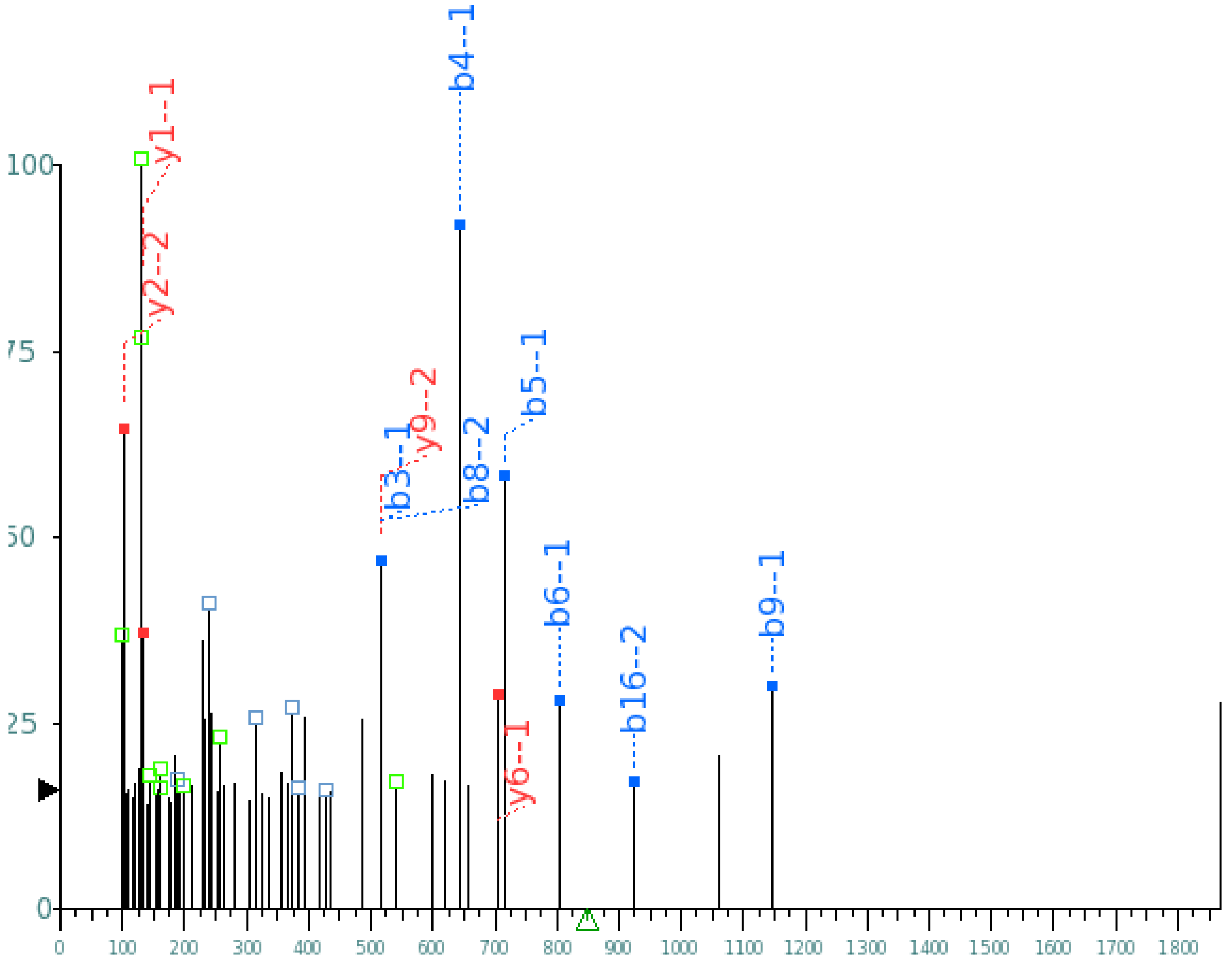

ASQEASTEDPSGEQAQEEDQAD (+3)

Primary Reference: PREX1_HUMAN
Search ID: 41761
Search Name: 20130328_ananiav_ERstress_TMT_TM_timecourse_trypsin_inj2_4mc_iaa
Scan#: 5534
Observed Mass: 851.032 (2.5 ppm)
PSM Score: 16.27

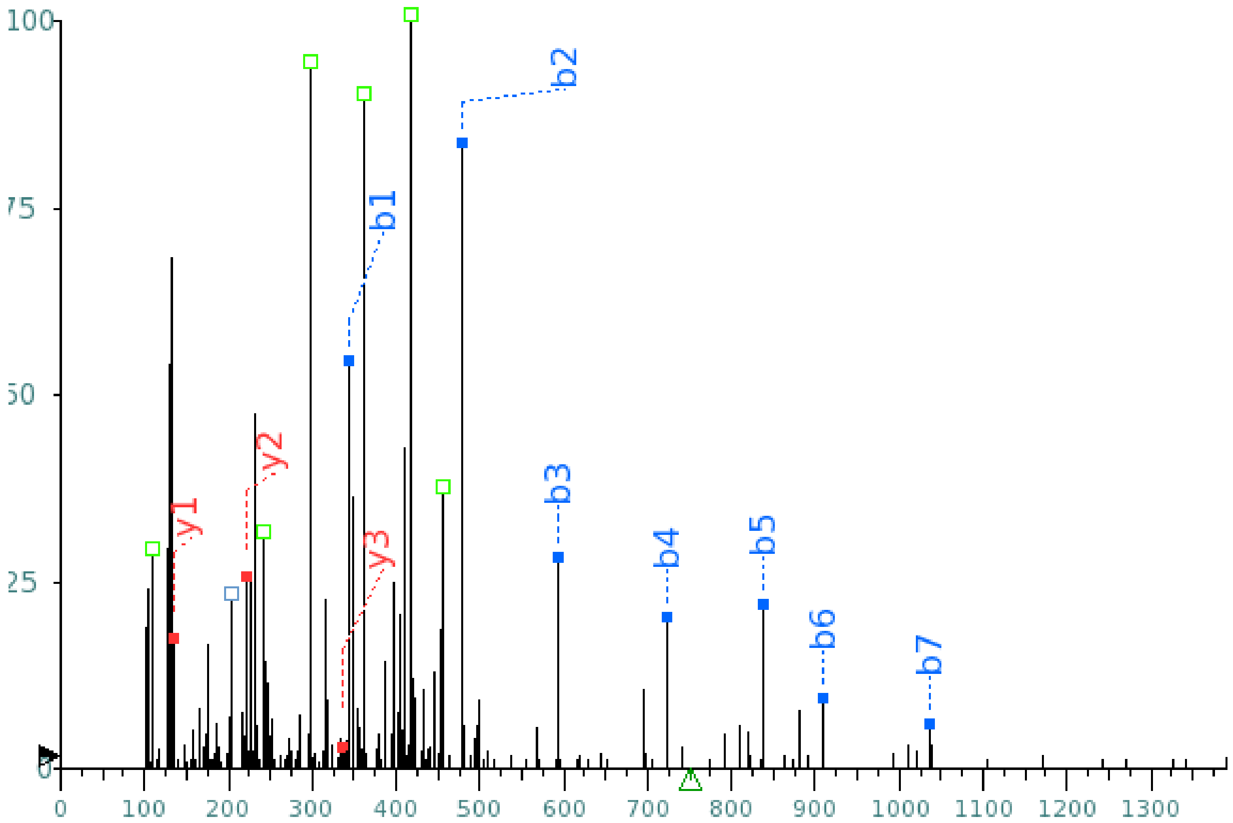

LHNENAEMDSD (+2)

Primary Reference: UBE2Z_HUMAN
Search ID: 41761
Search Name: 20130328_ananiav_ERstress_TMT_TM_timecourse_trypsin_inj2_4mc_iaa
Scan#: 5569
Observed Mass: 752.3349 (2.8 ppm)
PSM Score: 30.12

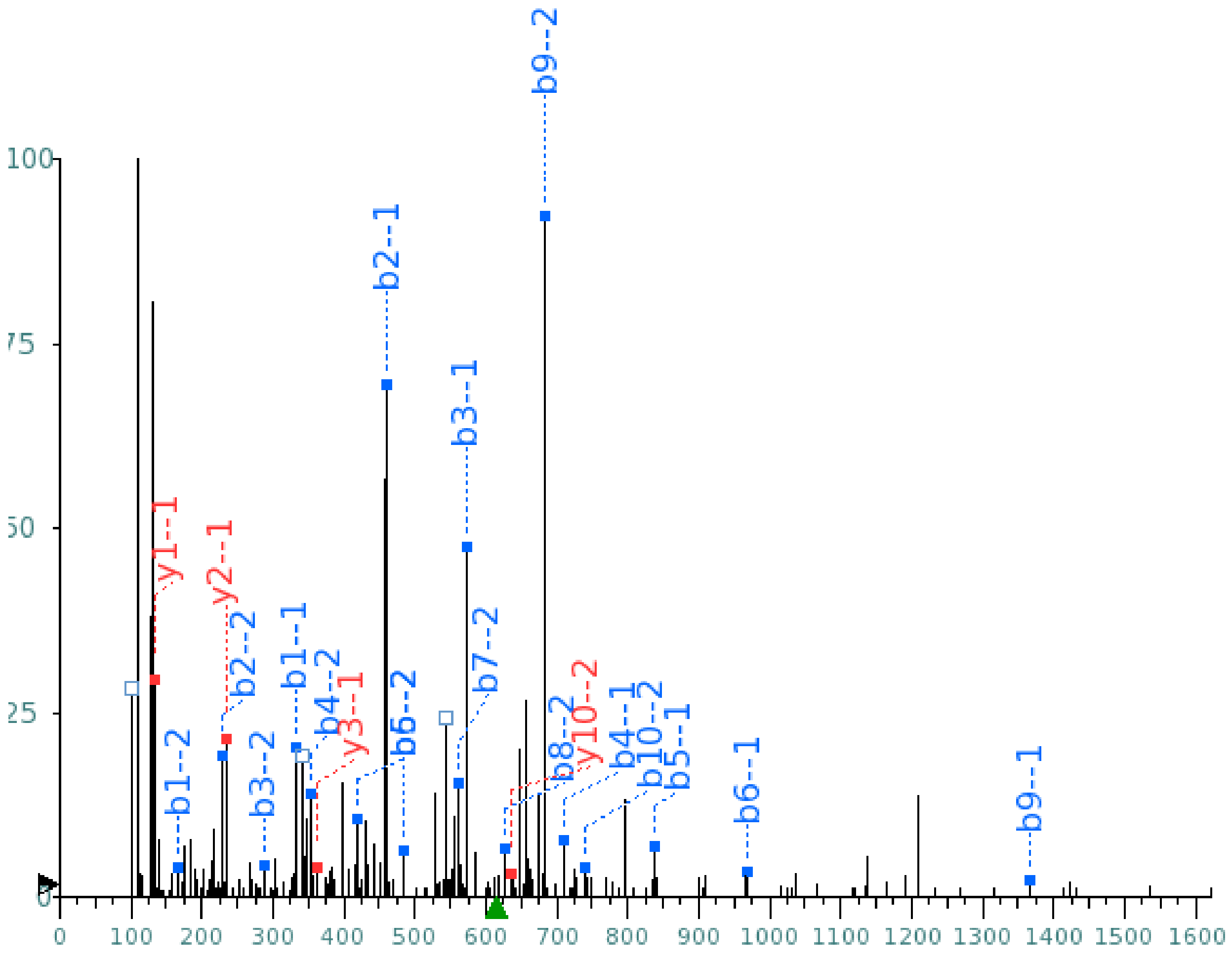

TQLHEERQDIQTD (+3)

Primary Reference: TRIPB_HUMAN
Search ID: 41761
Search Name: 20130328_ananiav_ERstress_TMT_TM_timecourse_trypsin_inj2_4mc_iaa
Scan#: 5574
Observed Mass: 614.6456 (1.8 ppm)
PSM Score: 29.22

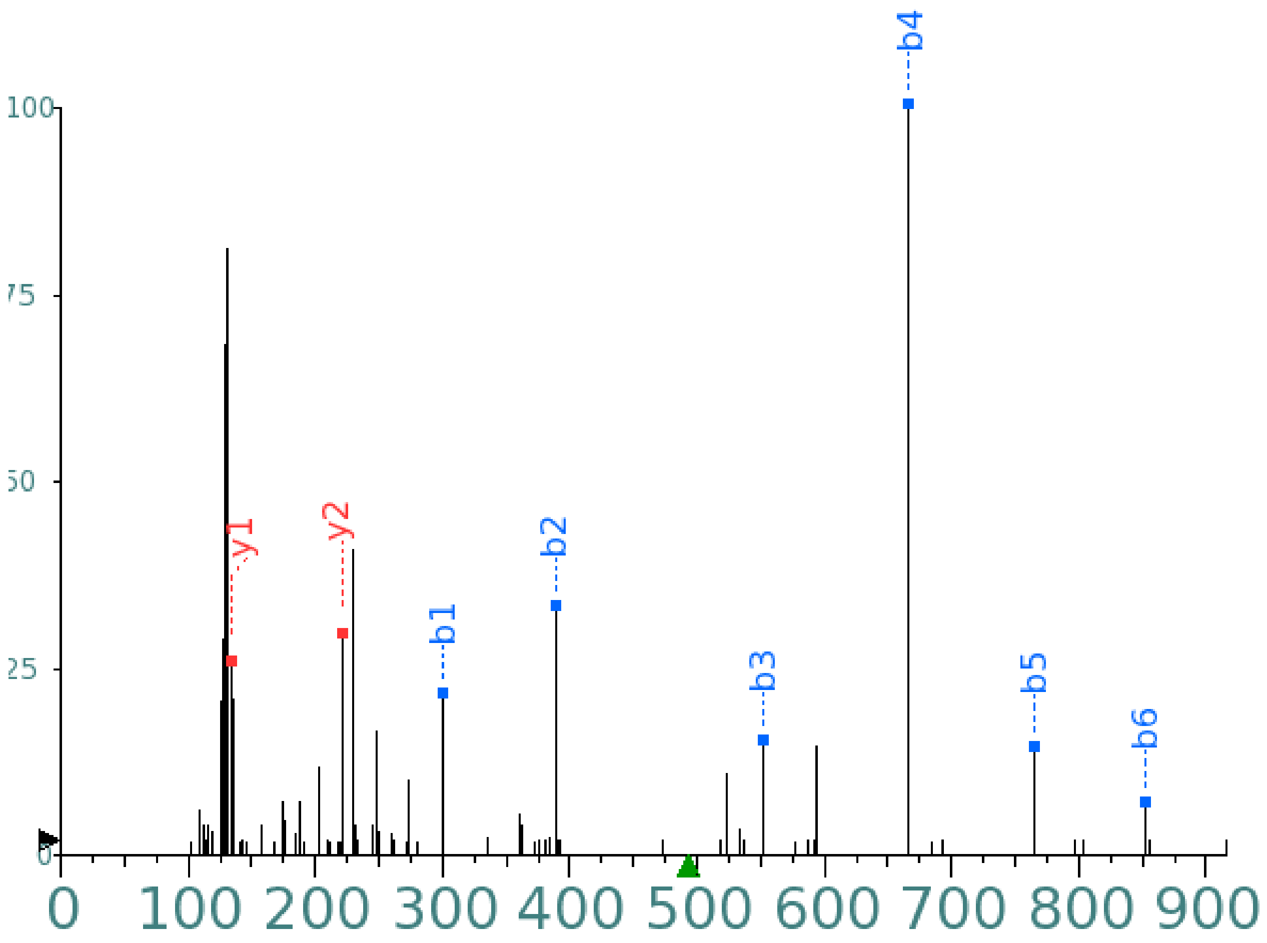

ASYDVSD (+2)

Primary Reference: K6PP_HUMAN
Search ID: 41761
Search Name: 20130328_ananiav_ERstress_TMT_TM_timecourse_trypsin_inj2_4mc_iaa
Scan#: 5627
Observed Mass: 493.2385 (2.2 ppm)
PSM Score: 31.94

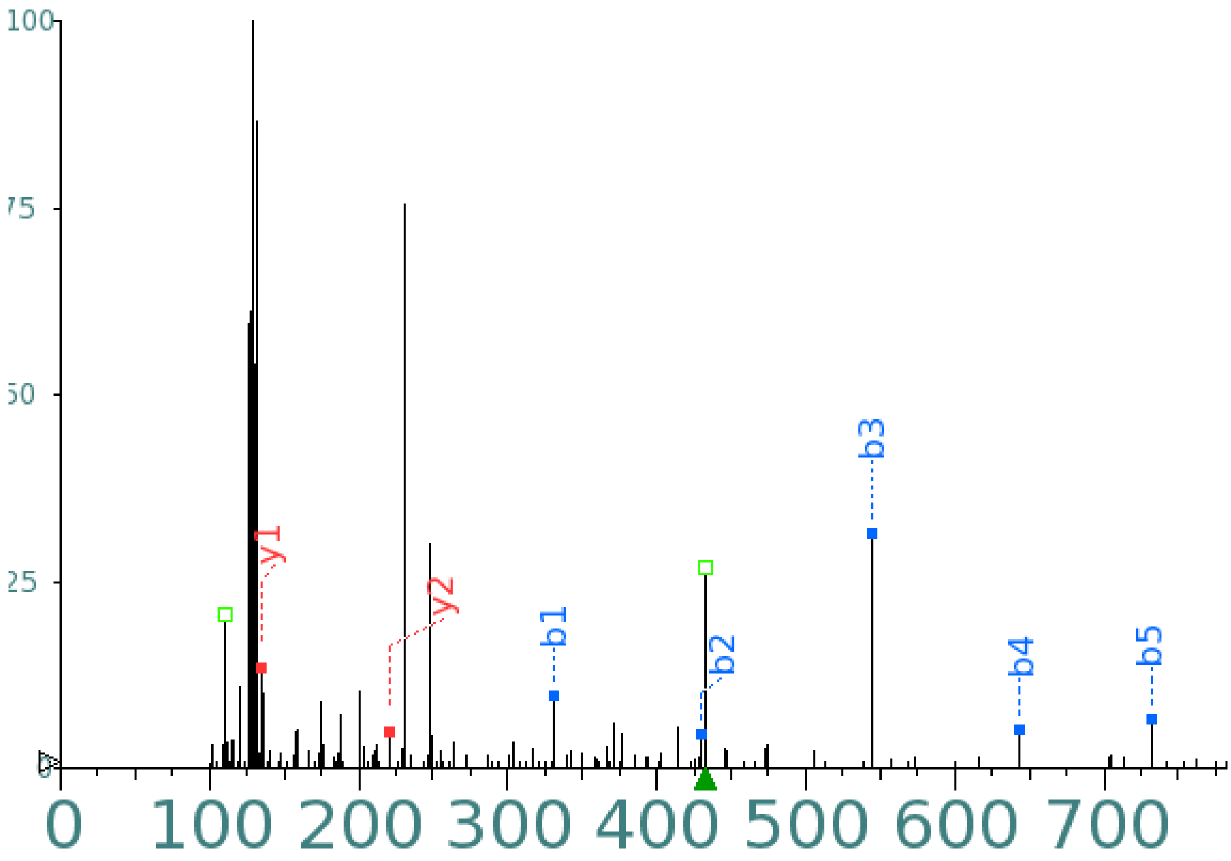

TVDVSD (+2)

Primary Reference: RPC4_HUMAN
Search ID: 41761
Search Name: 20130328_ananiav_ERstress_TMT_TM_timecourse_trypsin_inj2_4mc_iaa
Scan#: 5631
Observed Mass: 432.73 (1.8 ppm)
PSM Score: 18.79

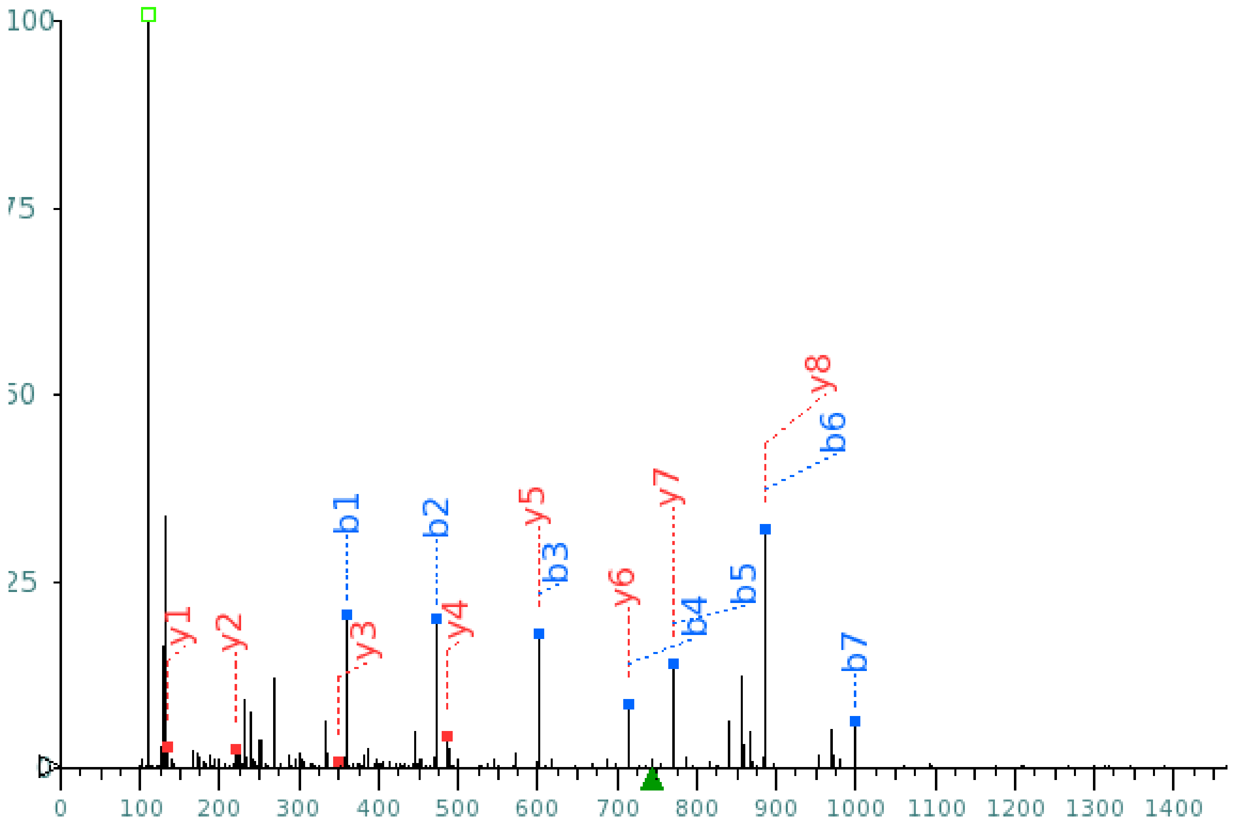

EIQNGNLHESD (+2)

Primary Reference: KTN1_HUMAN
Search ID: 41761
Search Name: 20130328_ananiav_ERstress_TMT_TM_timecourse_trypsin_inj2_4mc_iaa
Scan#: 5651
Observed Mass: 742.8638 (1.7 ppm)
PSM Score: 30.67

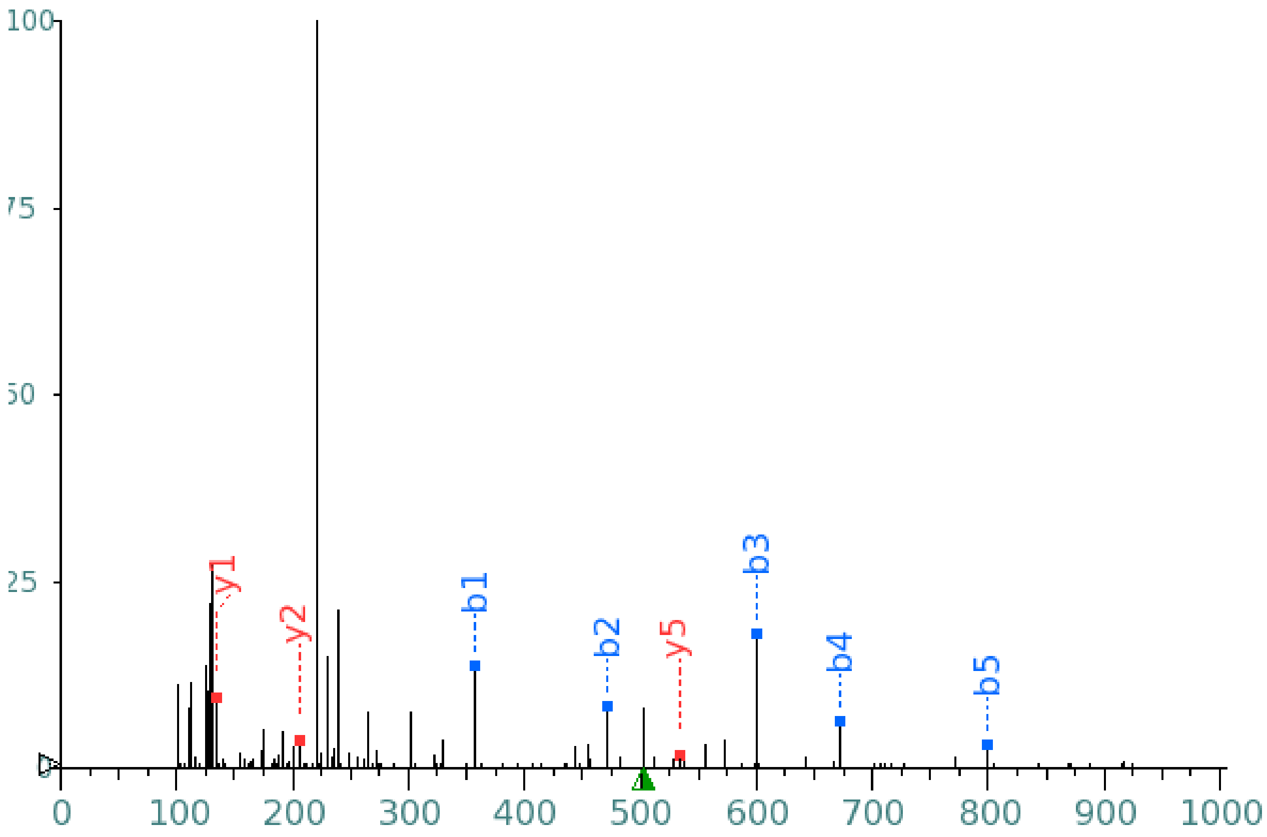

QLEAEAD (+2)

Primary Reference: HUWE1_HUMAN
Search ID: 41761
Search Name: 20130328_ananiav_ERstress_TMT_TM_timecourse_trypsin_inj2_4mc_iaa
Scan#: 5693
Observed Mass: 502.7593 (1.6 ppm)
PSM Score: 14.93

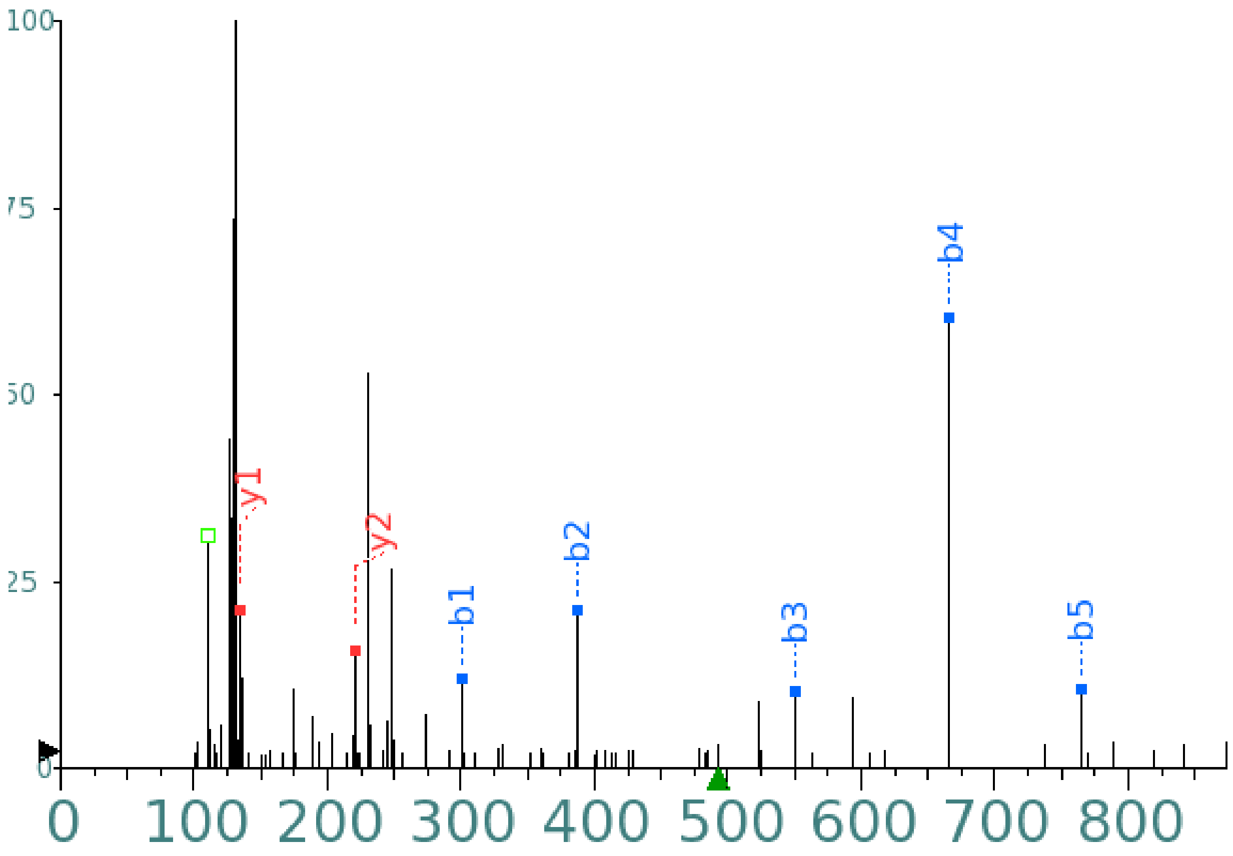

ASYDVSD (+2)

Primary Reference: K6PP_HUMAN
Search ID: 41761
Search Name: 20130328_ananiav_ERstress_TMT_TM_timecourse_trypsin_inj2_4mc_iaa
Scan#: 5717
Observed Mass: 493.2382 (1.6 ppm)
PSM Score: 18.66

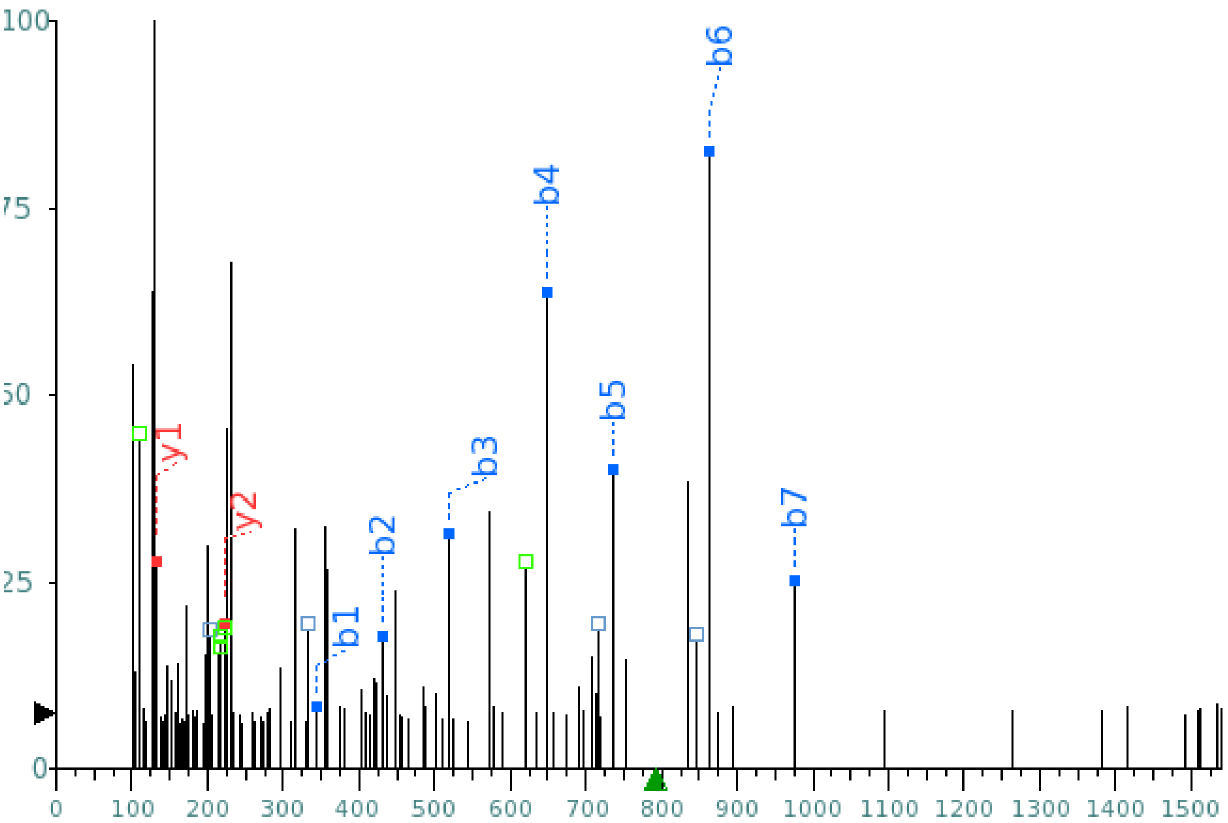

DSSESQLASTESD (+2)

Primary Reference: CR025_HUMAN
Search ID: 41761
Search Name: 20130328_ananiav_ERstress_TMT_TM_timecourse_trypsin_inj2_4mc_iaa
Scan#: 5732
Observed Mass: 792.8581 (1 ppm)
PSM Score: 12.98

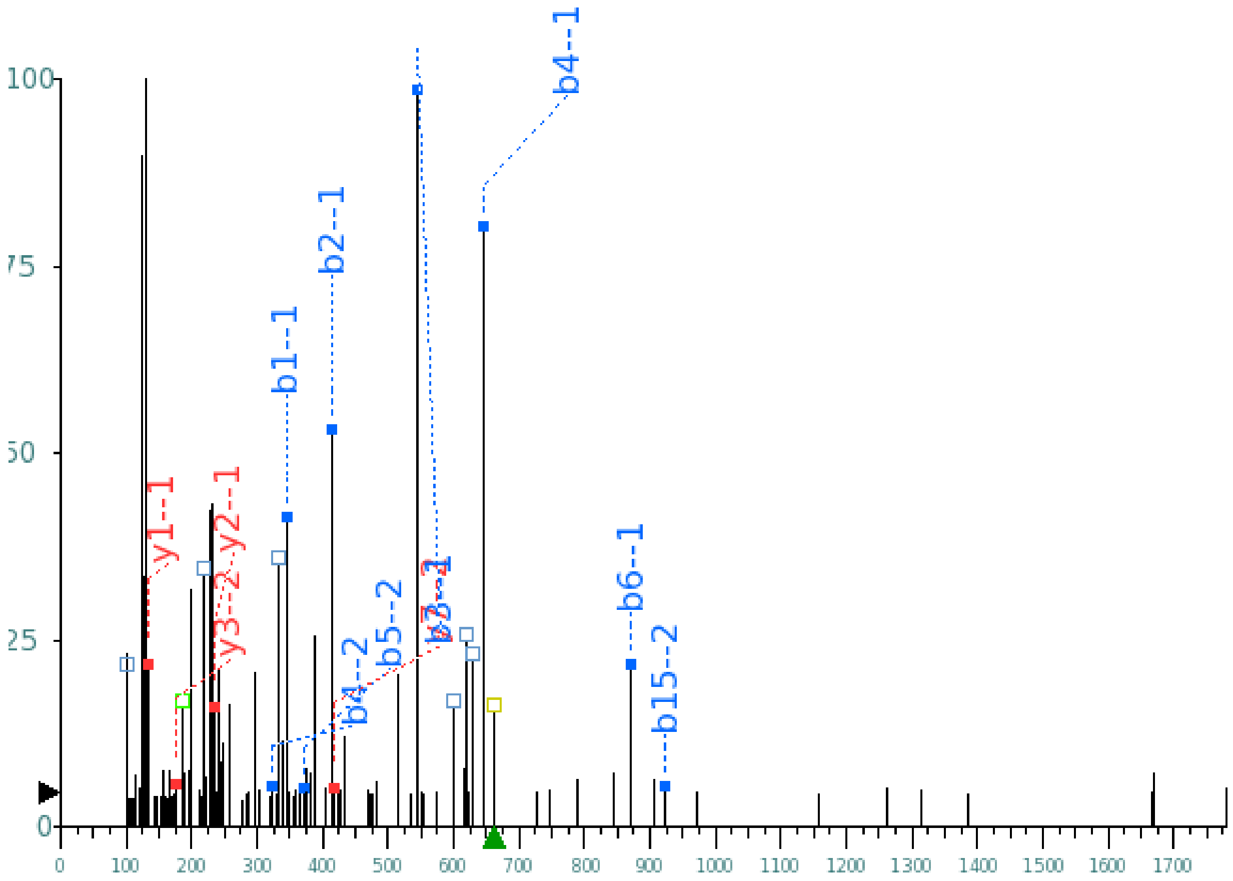

NAETPEATTQQETDTD (+3)

Primary Reference: K1967_HUMAN
Search ID: 41761
Search Name: 20130328_ananiav_ERstress_TMT_TM_timecourse_trypsin_inj2_4mc_iaa
Scan#: 5765
Observed Mass: 660.6345 (0.5 ppm)
PSM Score: 15.63

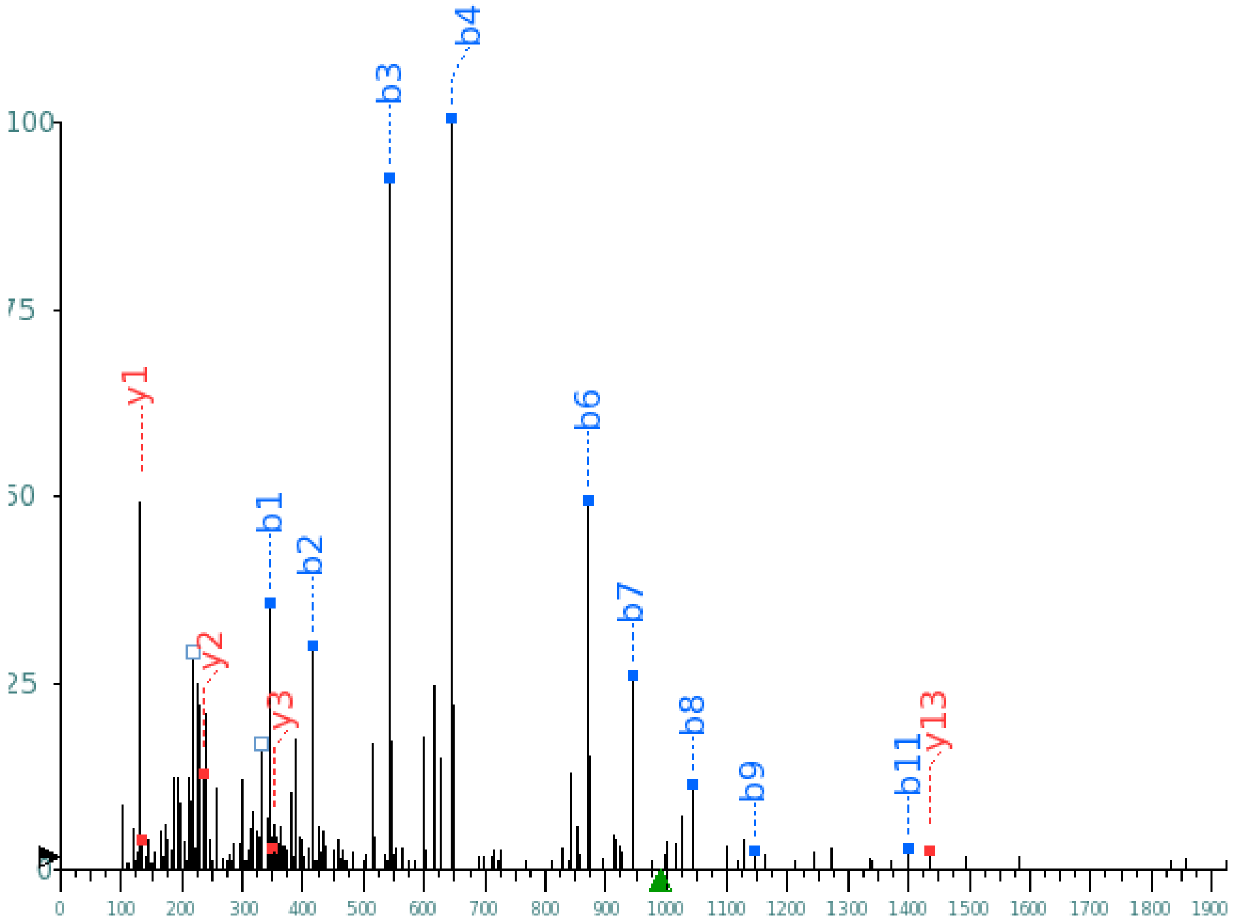

NAETPEATTQQETDTD (+2)

Primary Reference: K1967_HUMAN
Search ID: 41761
Search Name: 20130328_ananiav_ERstress_TMT_TM_timecourse_trypsin_inj2_4mc_iaa
Scan#: 5768
Observed Mass: 990.4475 (-0 ppm)
PSM Score: 48.8

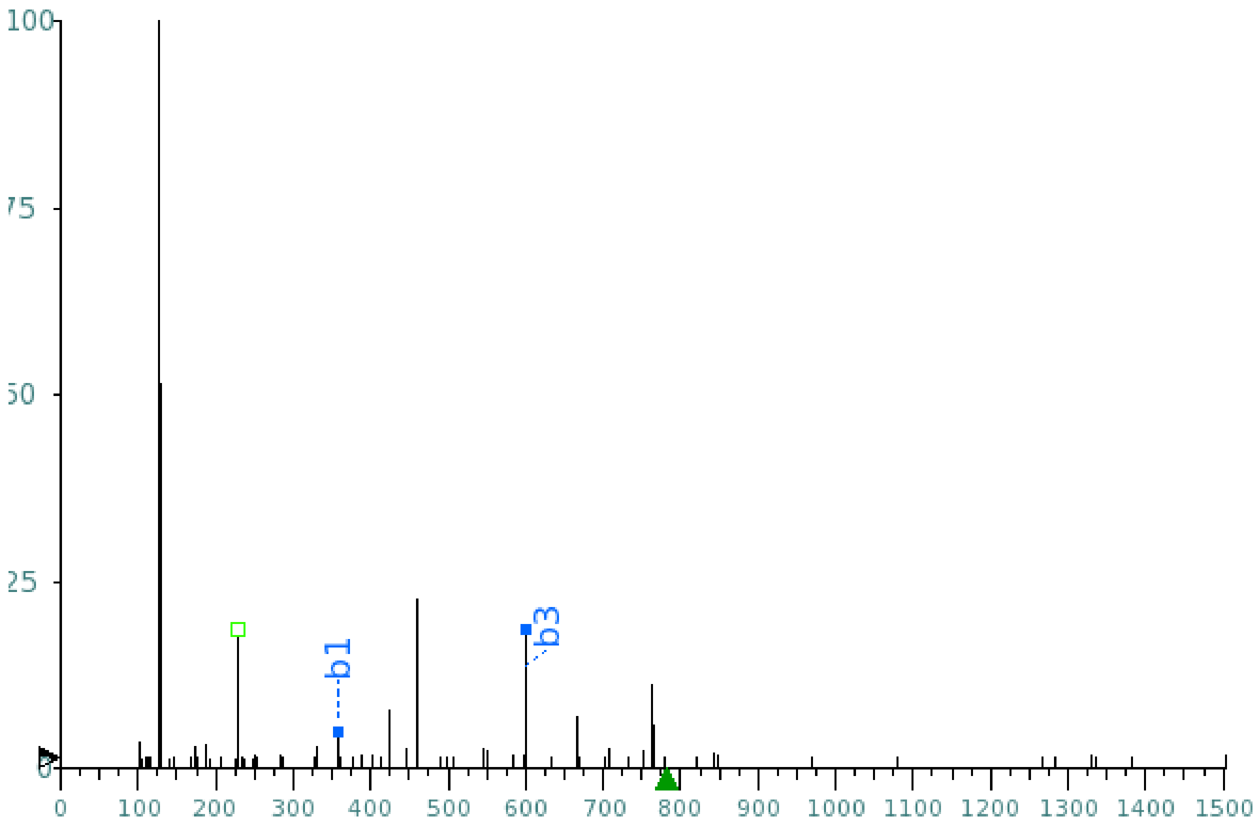

EDQLPSGFPNID (+2)

Primary Reference: PTPRS_HUMAN
Search ID: 41761
Search Name: 20130328_ananiav_ERstress_TMT_TM_timecourse_trypsin_inj2_4mc_iaa
Scan#: 5781
Observed Mass: 780.8933 (3.2 ppm)
PSM Score: 13.18

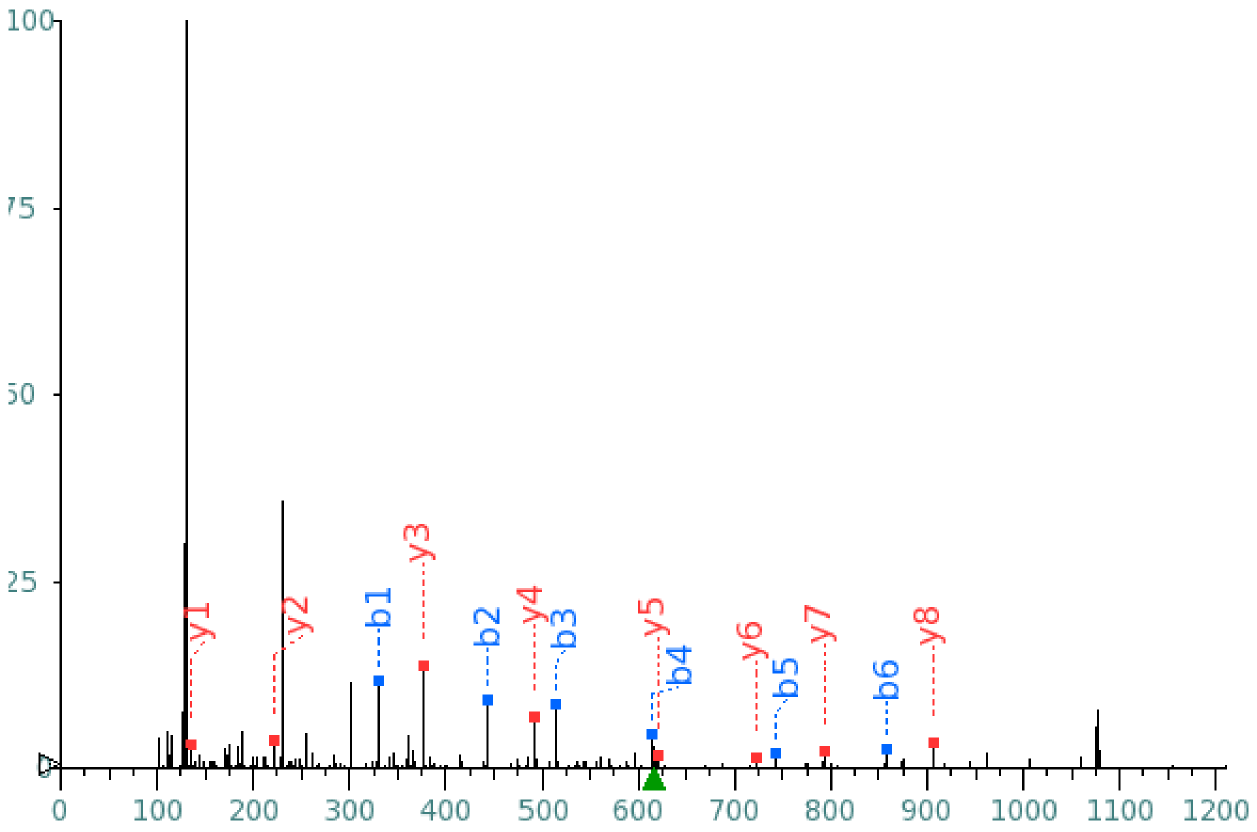

VLATEDRSD (+2)

Primary Reference: RAB9A_HUMAN
Search ID: 41761
Search Name: 20130328_ananiav_ERstress_TMT_TM_timecourse_trypsin_inj2_4mc_iaa
Scan#: 5847
Observed Mass: 617.8274 (-0.1 ppm)
PSM Score: 21.47

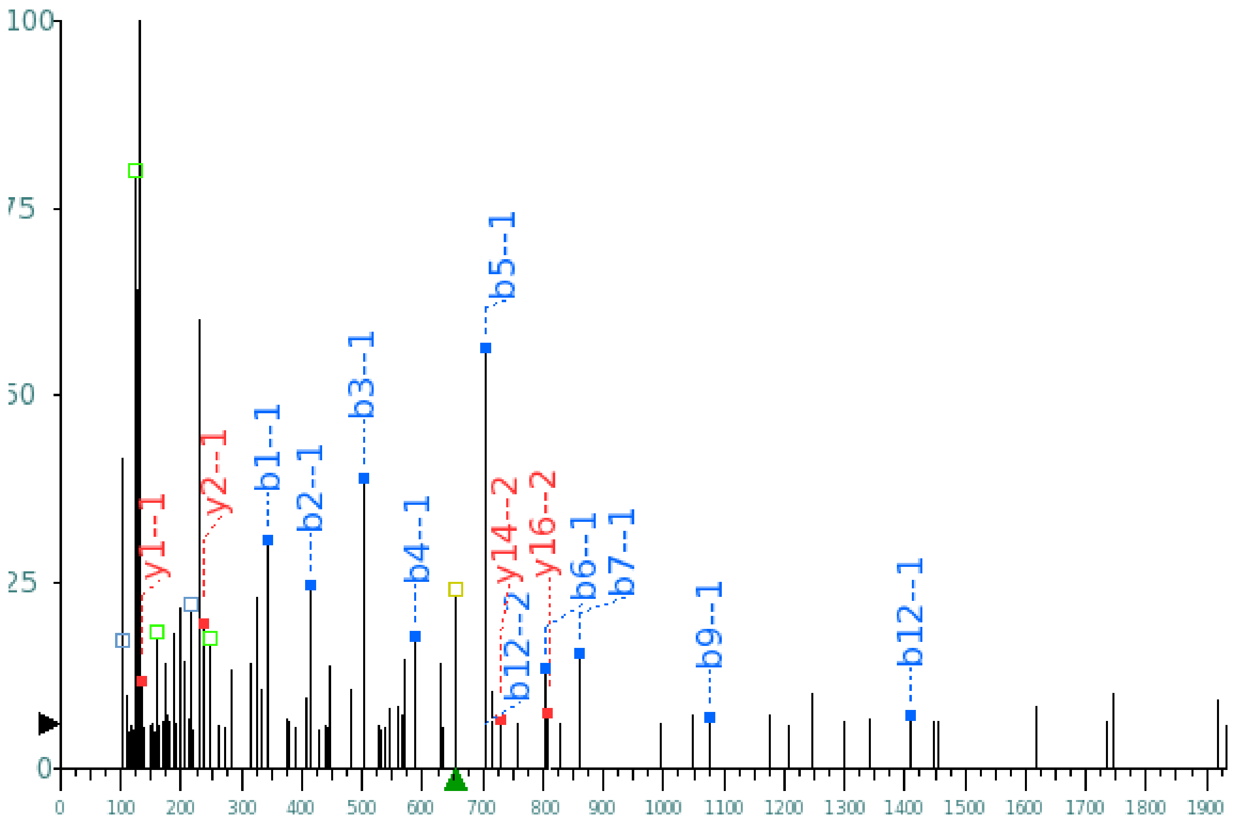

LASSDTGESDQSSTETD (+3)

Primary Reference: TTC28_HUMAN
Search ID: 41761
Search Name: 20130328_ananiav_ERstress_TMT_TM_timecourse_trypsin_inj2_4mc_iaa
Scan#: 5869
Observed Mass: 653.6218 (-0.2 ppm)
PSM Score: 32.41

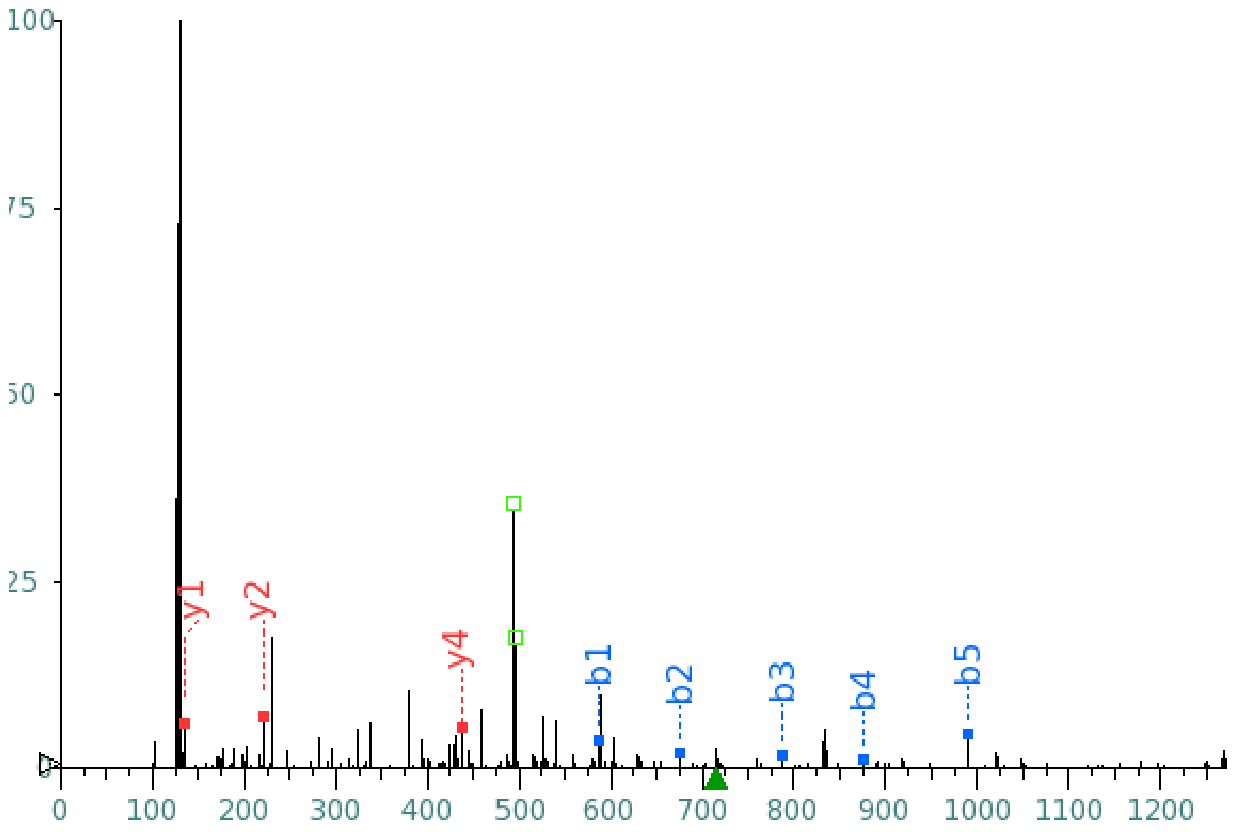

KSLSDSESD (+2)

Primary Reference: CBX3_HUMAN
Search ID: 41761
Search Name: 20130328_ananiav_ERstress_TMT_TM_timecourse_trypsin_inj2_4mc_iaa
Scan#: 5896
Observed Mass: 713.3775 (0.3 ppm)
PSM Score: 12.62

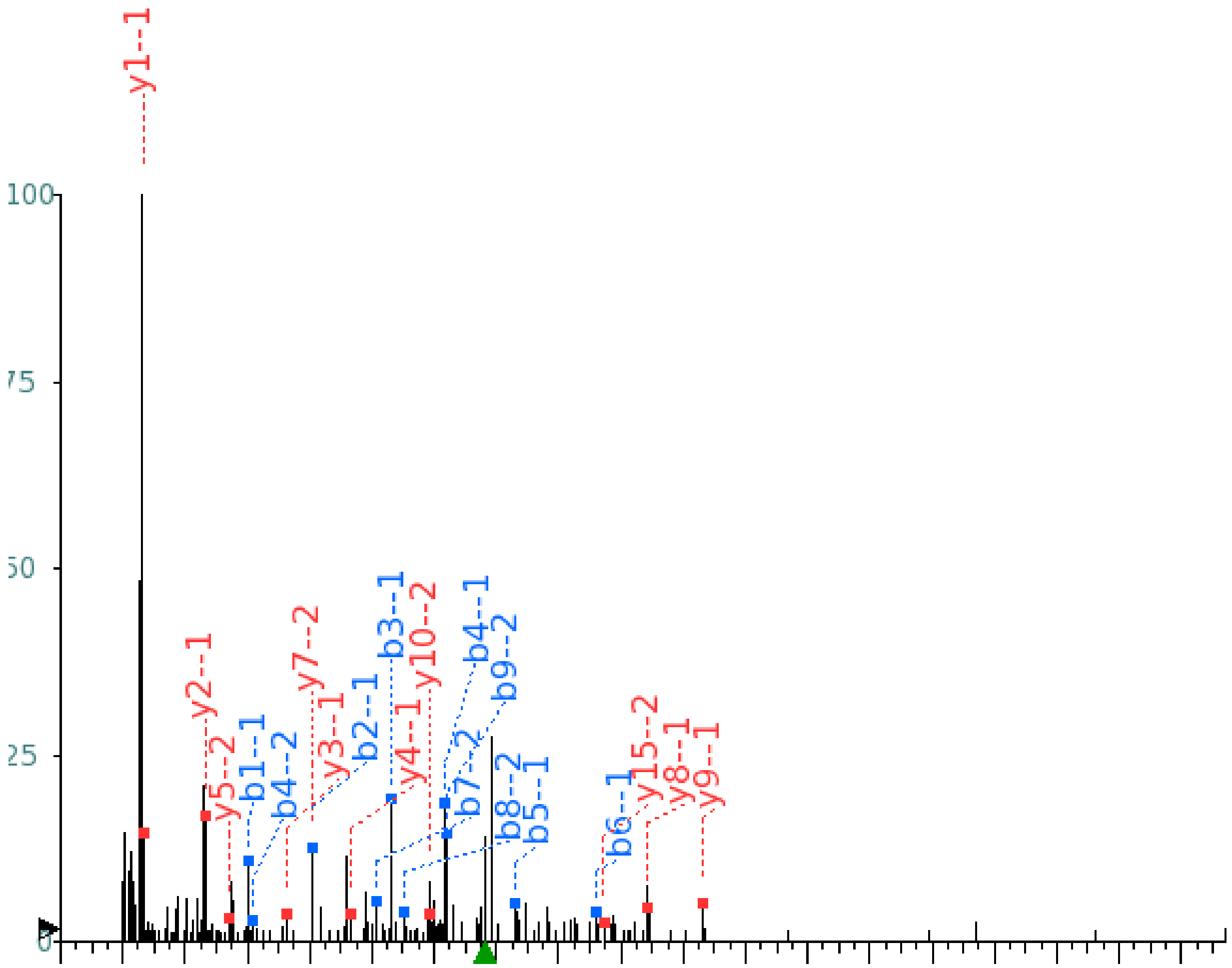

ATQSIERSHRIATETD (+3)

Primary Reference: VTI1B_HUMAN
Search ID: 41761
Search Name: 20130328_ananiav_ERstress_TMT_TM_timecourse_trypsin_inj2_4mc_iaa
Scan#: 5926
Observed Mass: 682.0259 (0.5 ppm)
PSM Score: 30.5

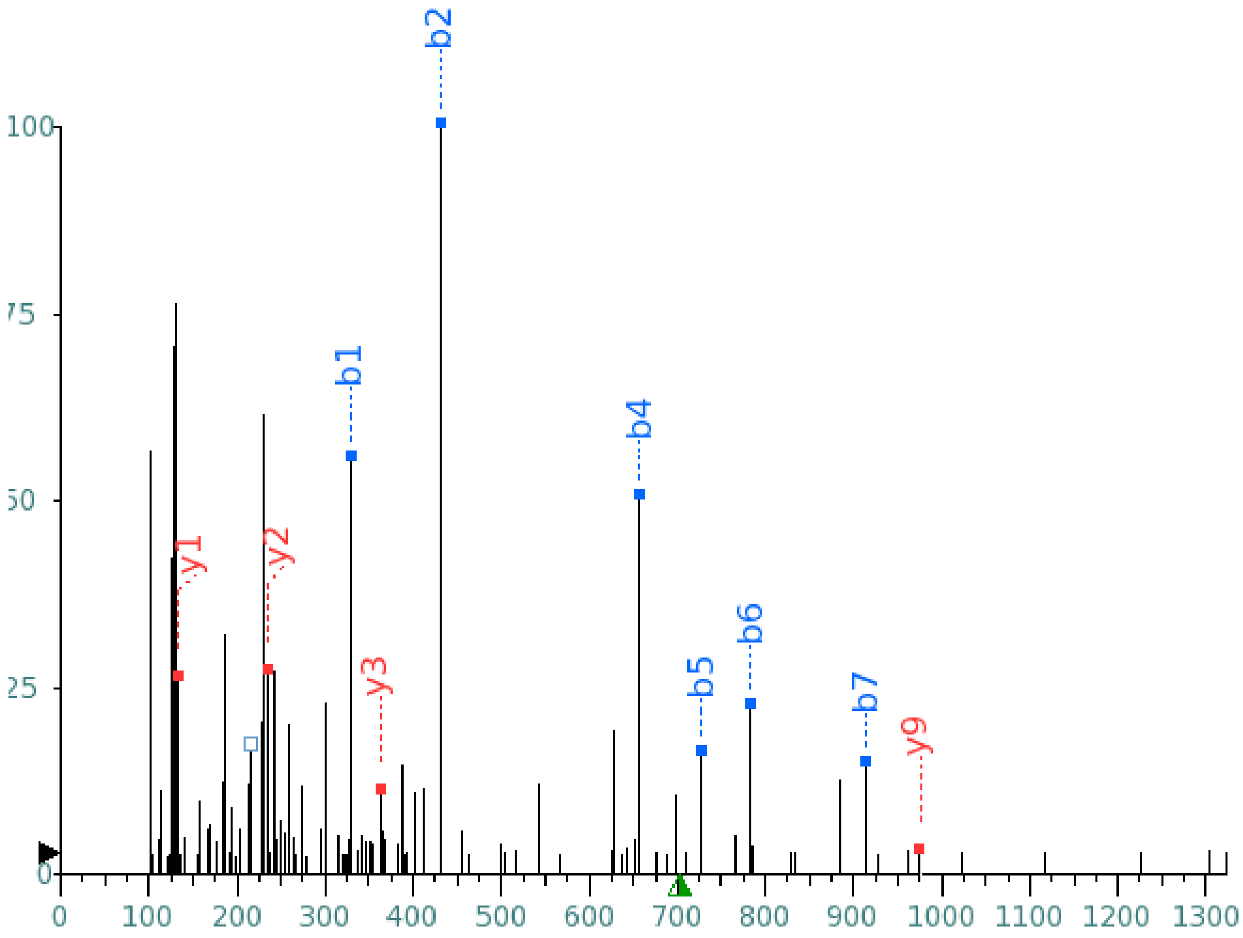

VTPQAGEEETD (+2)

Primary Reference: NACAD_HUMAN
Search ID: 41761
Search Name: 20130328_ananiav_ERstress_TMT_TM_timecourse_trypsin_inj2_4mc_iaa
Scan#: 5935
Observed Mass: 702.8386 (0.5 ppm)
PSM Score: 23.9

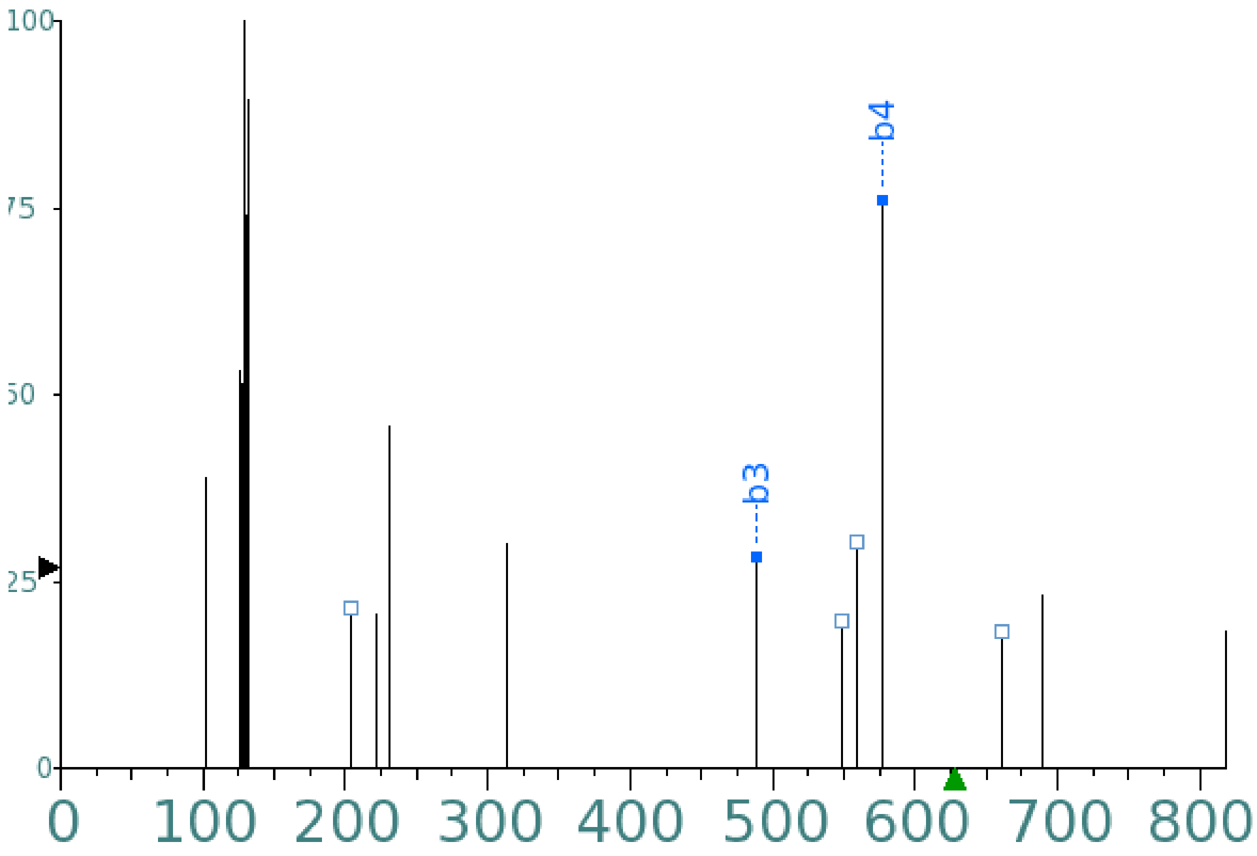

SATSLESESD (+2)

Primary Reference: RGNEF_HUMAN
Search ID: 41761
Search Name: 20130328_ananiav_ERstress_TMT_TM_timecourse_trypsin_inj2_4mc_iaa
Scan#: 5983
Observed Mass: 627.799 (0.7 ppm)
PSM Score: 20.04

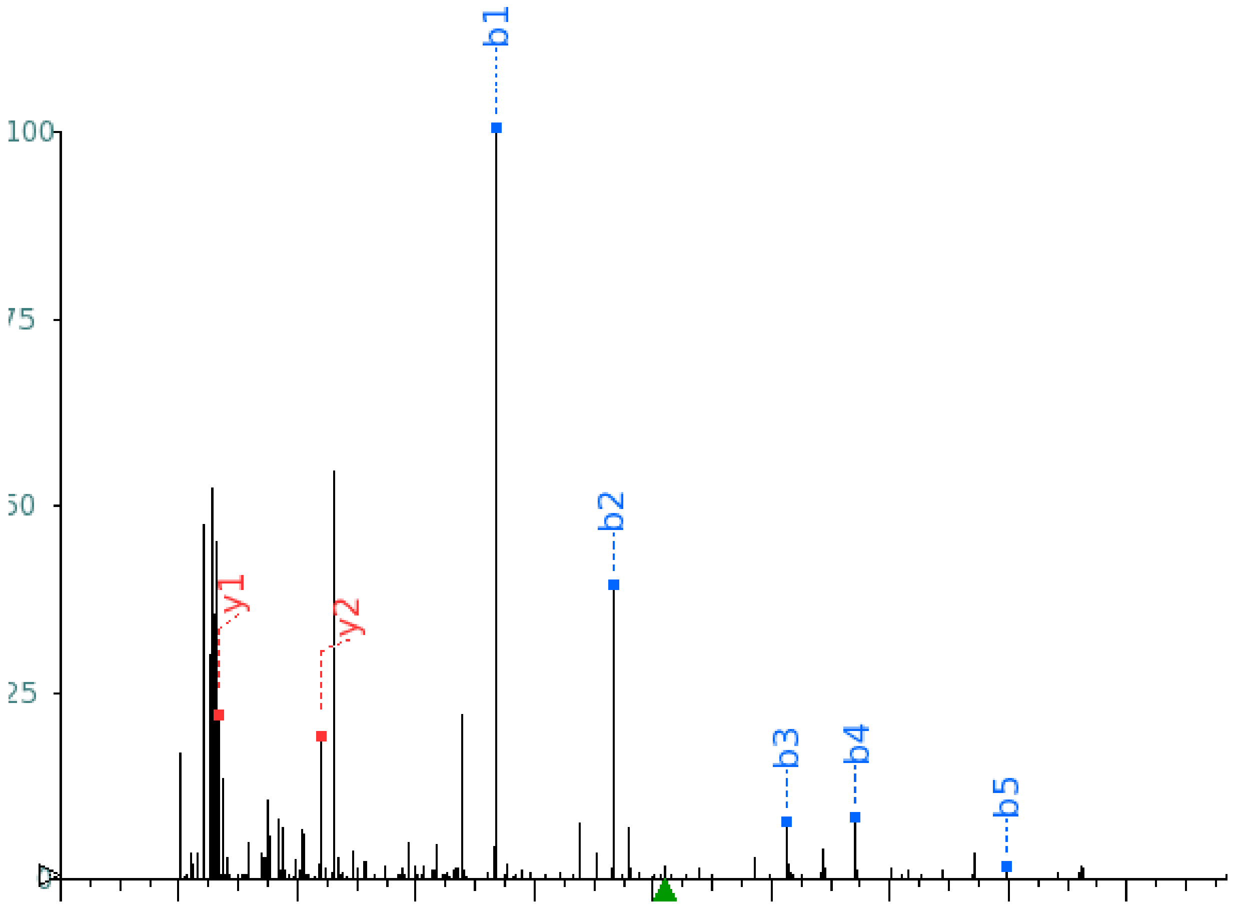

HVFGESD (+2)

Primary Reference: TPIS_HUMAN
Search ID: 41761
Search Name: 20130328_ananiav_ERstress_TMT_TM_timecourse_trypsin_inj2_4mc_iaa
Scan#: 6042
Observed Mass: 510.2543 (1.7 ppm)
PSM Score: 10.49

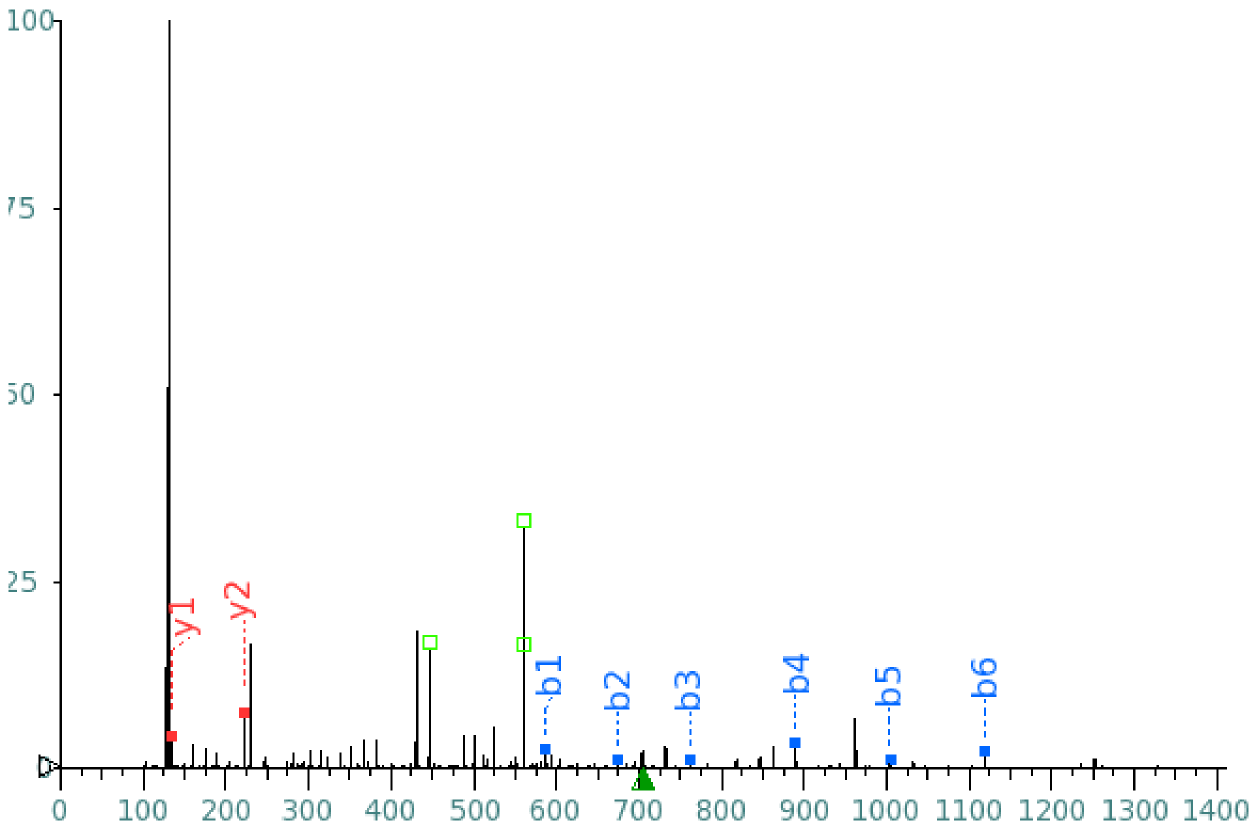

KSSELDASD (+2)

Primary Reference: MARK3_HUMAN
Search ID: 41761
Search Name: 20130328_ananiav_ERstress_TMT_TM_timecourse_trypsin_inj2_4mc_iaa
Scan#: 6051
Observed Mass: 705.3816 (2.5 ppm)
PSM Score: 13.14

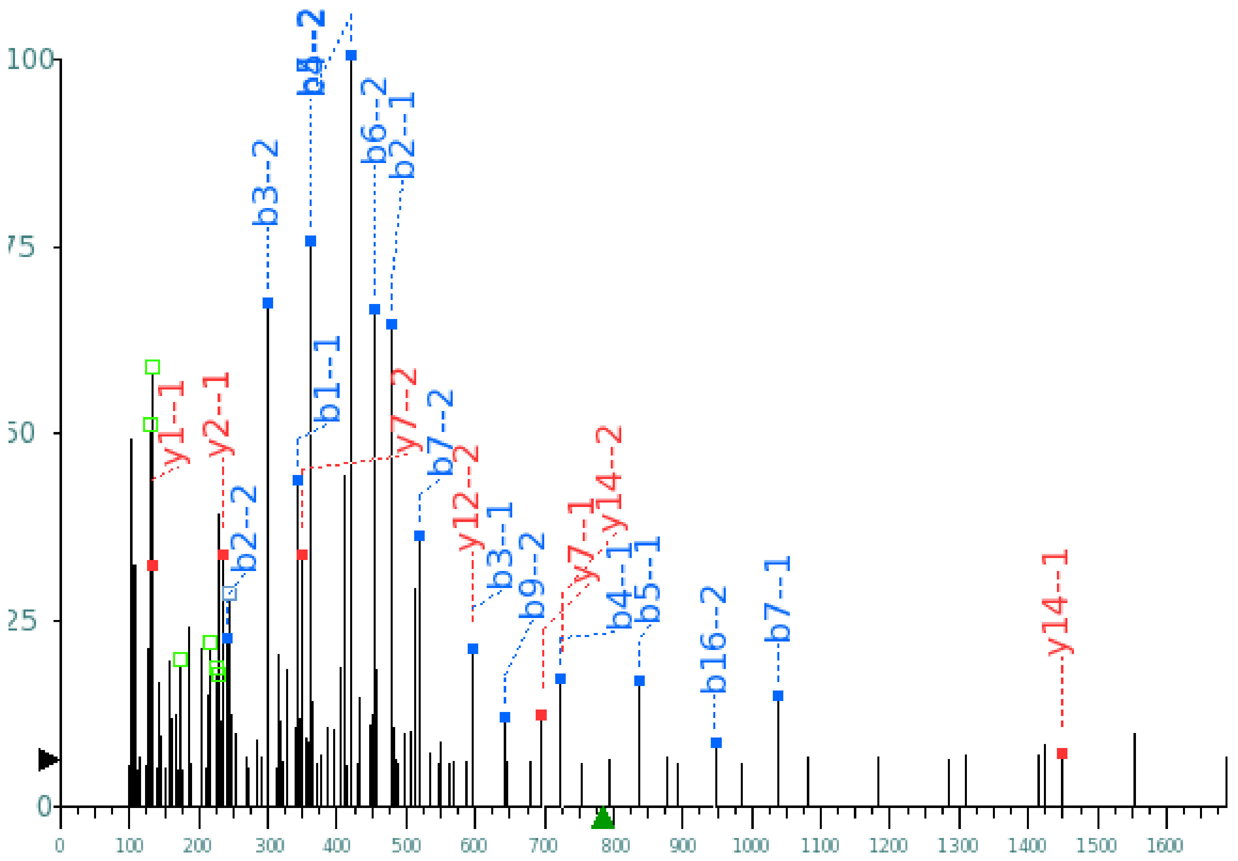

LHNENAEMDSDSSSSGTETD (+3)

Primary Reference: UBE2Z_HUMAN
Search ID: 41761
Search Name: 20130328_ananiav_ERstress_TMT_TM_timecourse_trypsin_inj2_4mc_iaa
Scan#: 6121
Observed Mass: 785.6629 (0.5 ppm)
PSM Score: 27.16

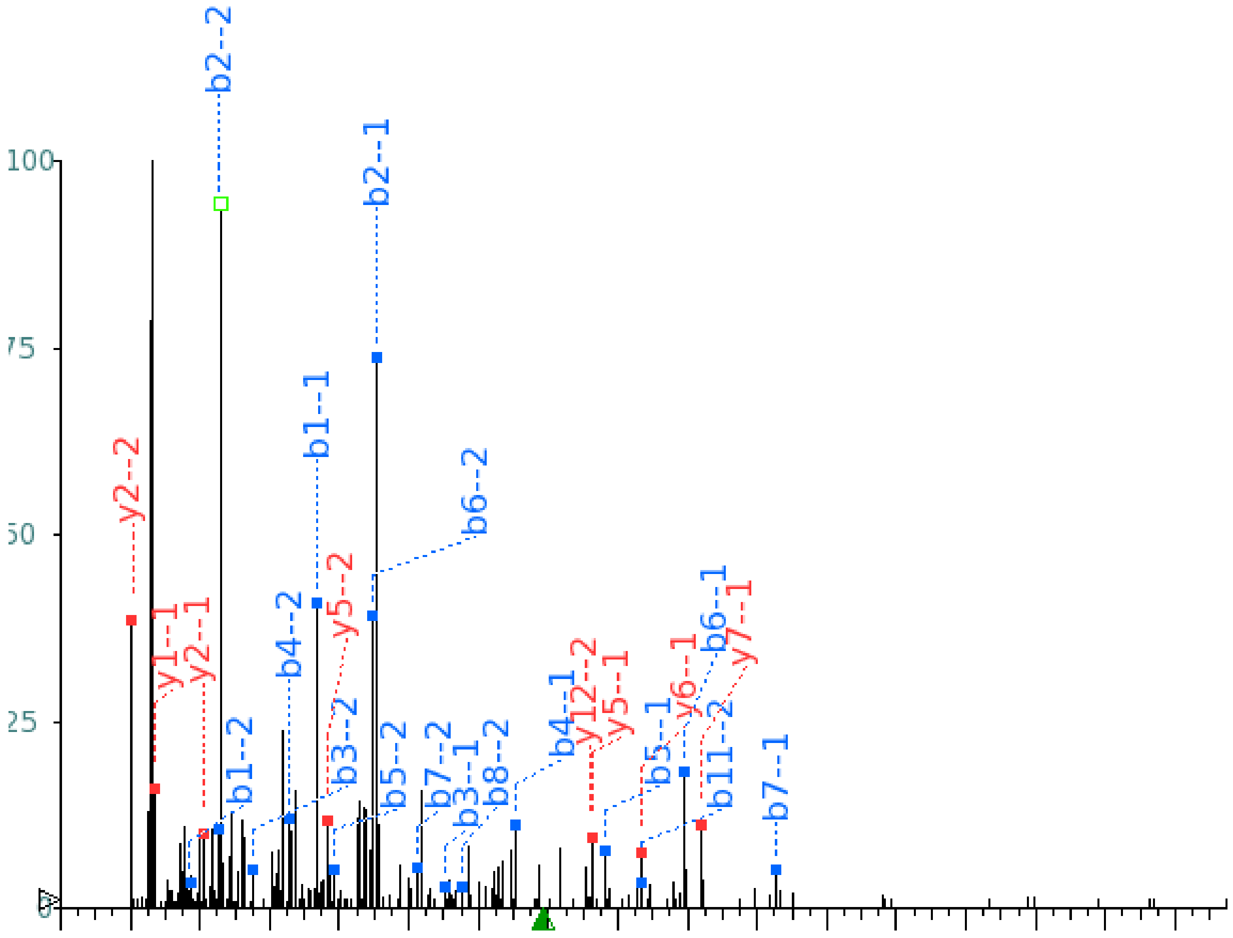

HSPTEDEESAKAEAD (+3)

Primary Reference: MAP1B_HUMAN
Search ID: 41761
Search Name: 20130328_ananiav_ERstress_TMT_TM_timecourse_trypsin_inj2_4mc_iaa
Scan#: 6132
Observed Mass: 692.0048 (1.1 ppm)
PSM Score: 18.55

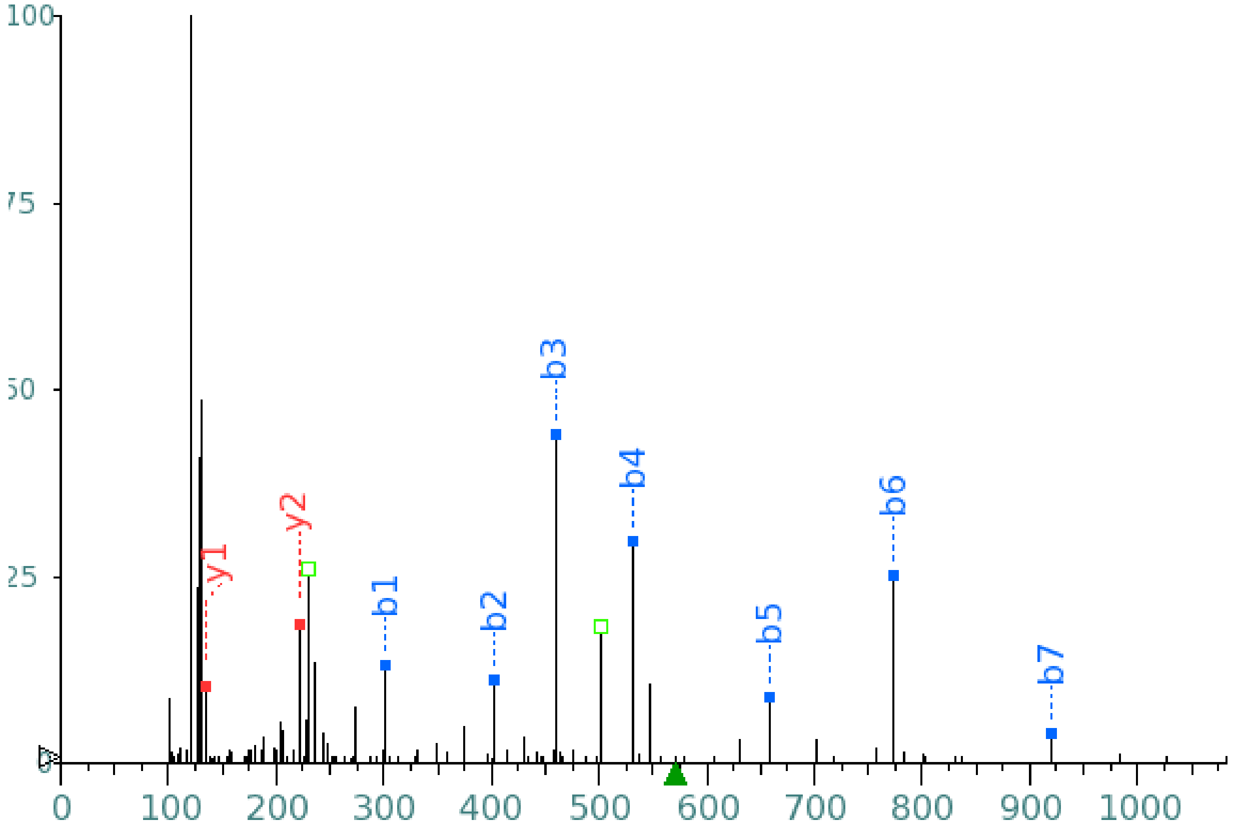

ATGAQDFSD (+2)

Primary Reference: GT2D1_HUMAN
Search ID: 41761
Search Name: 20130328_ananiav_ERstress_TMT_TM_timecourse_trypsin_inj2_4mc_iaa
Scan#: 6154
Observed Mass: 570.7731 (1.5 ppm)
PSM Score: 40.87

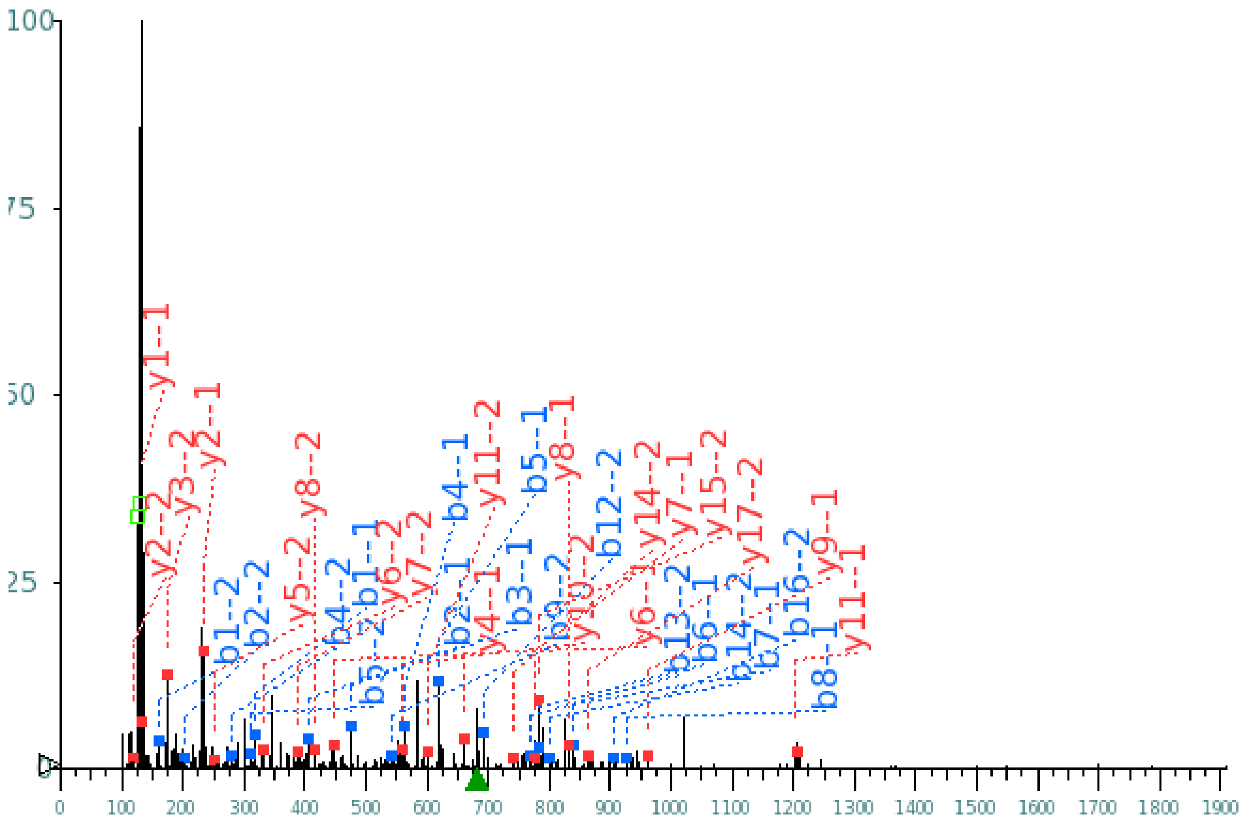

SSASGYGSREGDRGVITD (+3)

Primary Reference: SLTM_HUMAN
Search ID: 41761
Search Name: 20130328_ananiav_ERstress_TMT_TM_timecourse_trypsin_inj2_4mc_iaa
Scan#: 6161
Observed Mass: 681.6707 (1.8 ppm)
PSM Score: 33.39

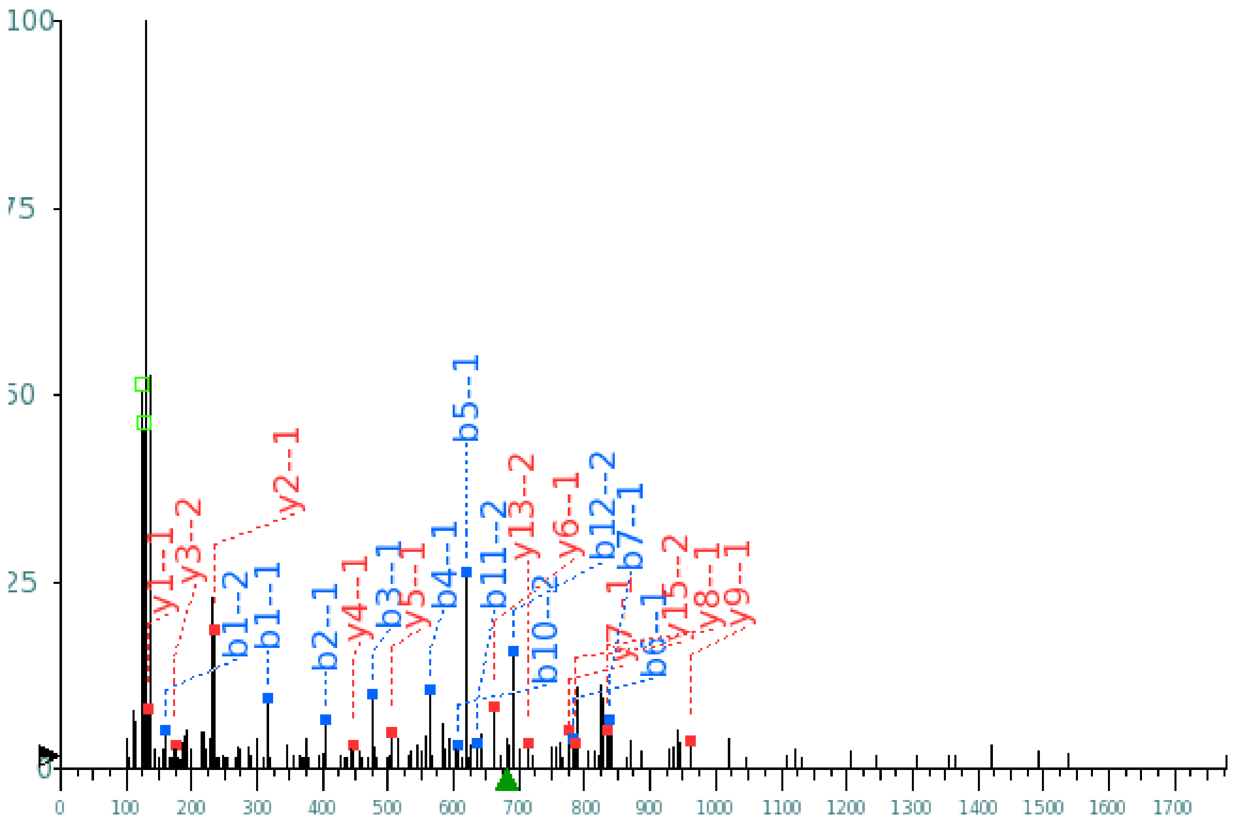

SSASGYGSREGDRGVITD (+3)

Primary Reference: SLTM_HUMAN
Search ID: 41761
Search Name: 20130328_ananiav_ERstress_TMT_TM_timecourse_trypsin_inj2_4mc_iaa
Scan#: 6187
Observed Mass: 681.6709 (2 ppm)
PSM Score: 33.21

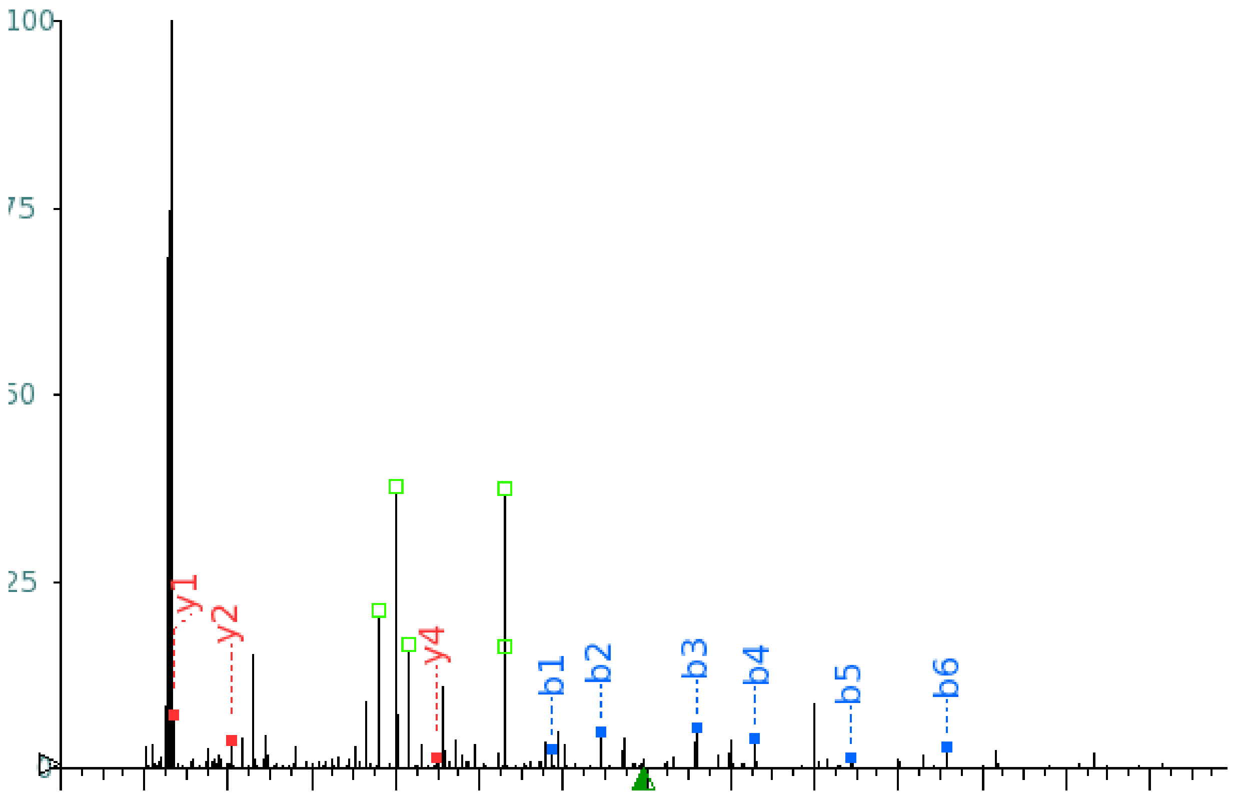

KGNAIDEAD (+2)

Primary Reference: C2D1A_HUMAN
Search ID: 41761
Search Name: 20130328_ananiav_ERstress_TMT_TM_timecourse_trypsin_inj2_4mc_iaa
Scan#: 6209
Observed Mass: 695.8837 (1.6 ppm)
PSM Score: 4.86

File name: 2015-02-04 04.01.53PM.pdf

Creation time: 2015-02-04 04:09PM

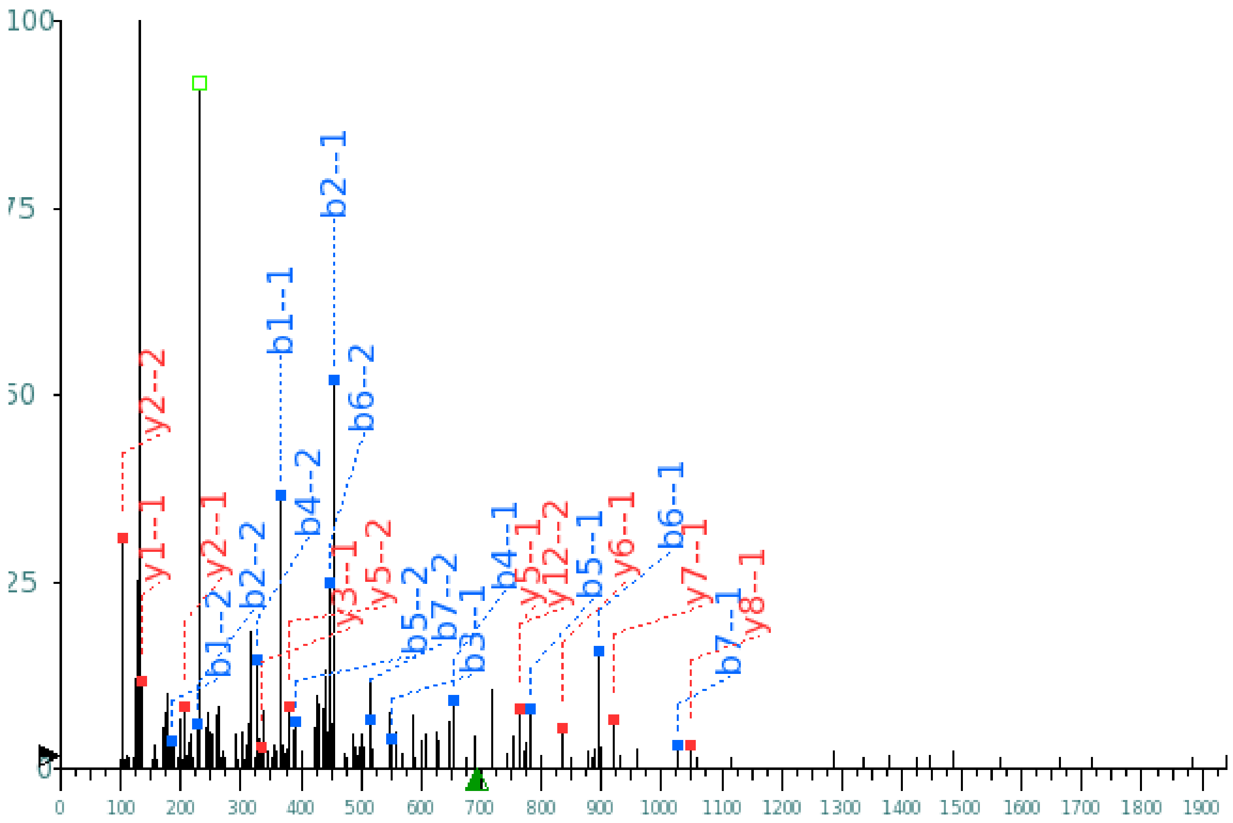

HSPTEDEESAKAEAD (+3)

Primary Reference: MAP1B_HUMAN
Search ID: 41761
Search Name: 20130328_ananiav_ERstress_TMT_TM_timecourse_trypsin_inj2_4mc_iaa
Scan#: 6221
Observed Mass: 692.0051 (1.4 ppm)
PSM Score: 22.68

File name: 2015-02-04 04.01.53PM.pdf

Creation time: 2015-02-04 04:09PM

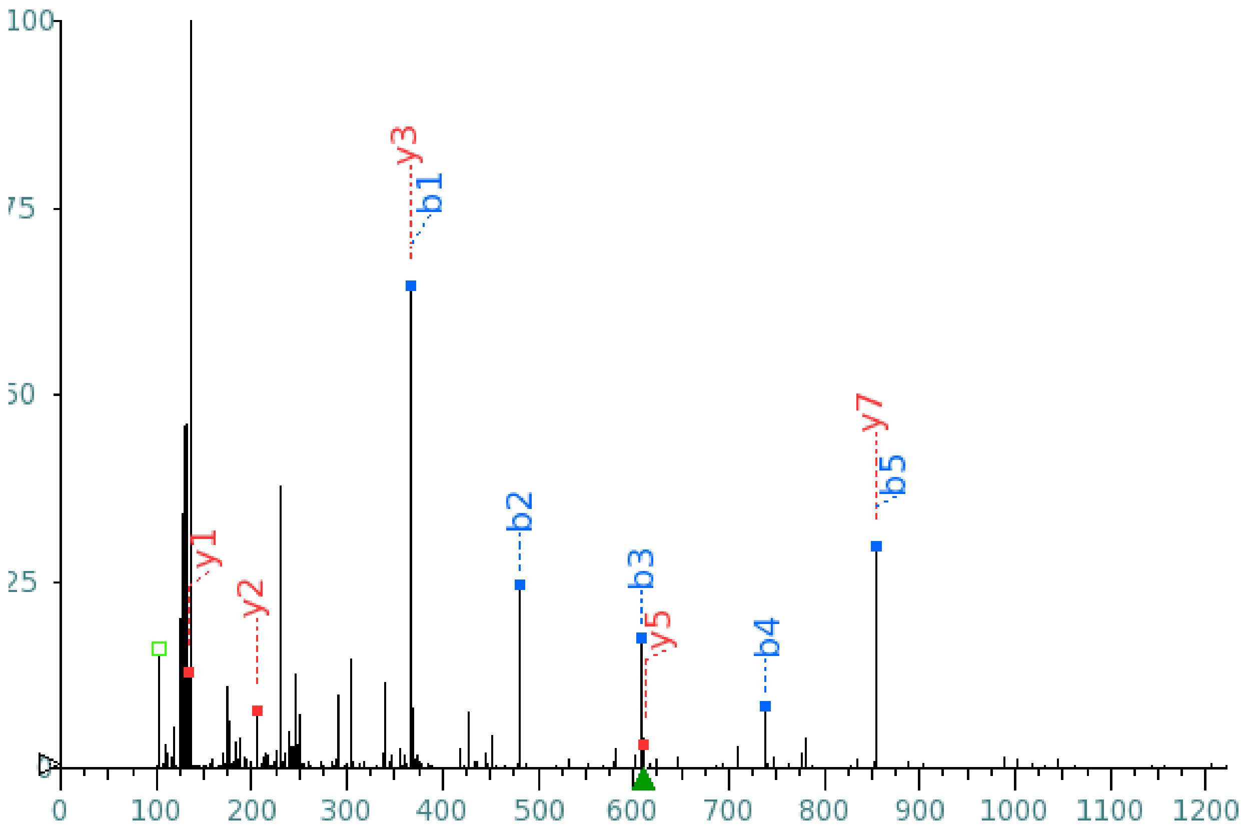

HLEEDYAD (+2)

Primary Reference: CACB3_HUMAN
Search ID: 41761
Search Name: 20130328_ananiav_ERstress_TMT_TM_timecourse_trypsin_inj2_4mc_iaa
Scan#: 6224
Observed Mass: 610.7862 (1.6 ppm)
PSM Score: 22.4

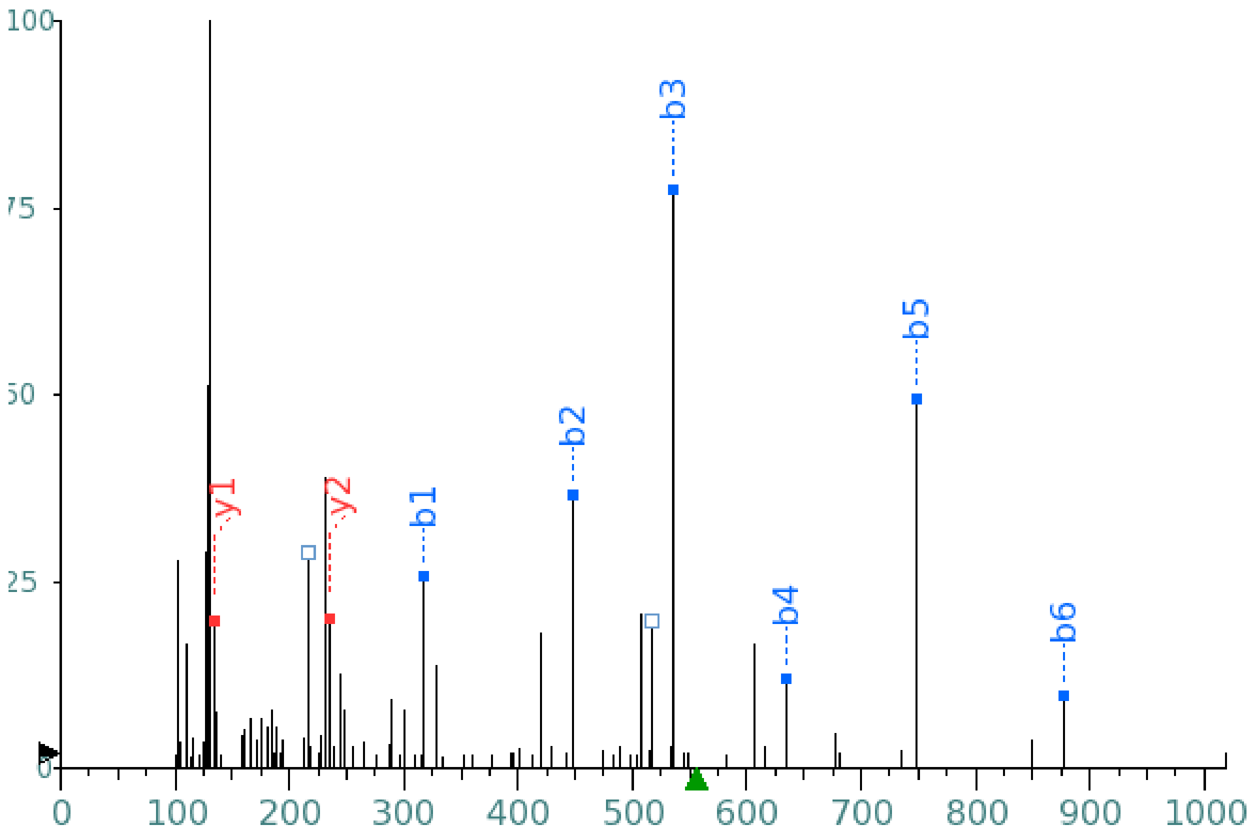

SMSVDETD (+2)

Primary Reference: COBL1_HUMAN
Search ID: 41761
Search Name: 20130328_ananiav_ERstress_TMT_TM_timecourse_trypsin_inj2_4mc_iaa
Scan#: 6230
Observed Mass: 556.7538 (2.2 ppm)
PSM Score: 29.61

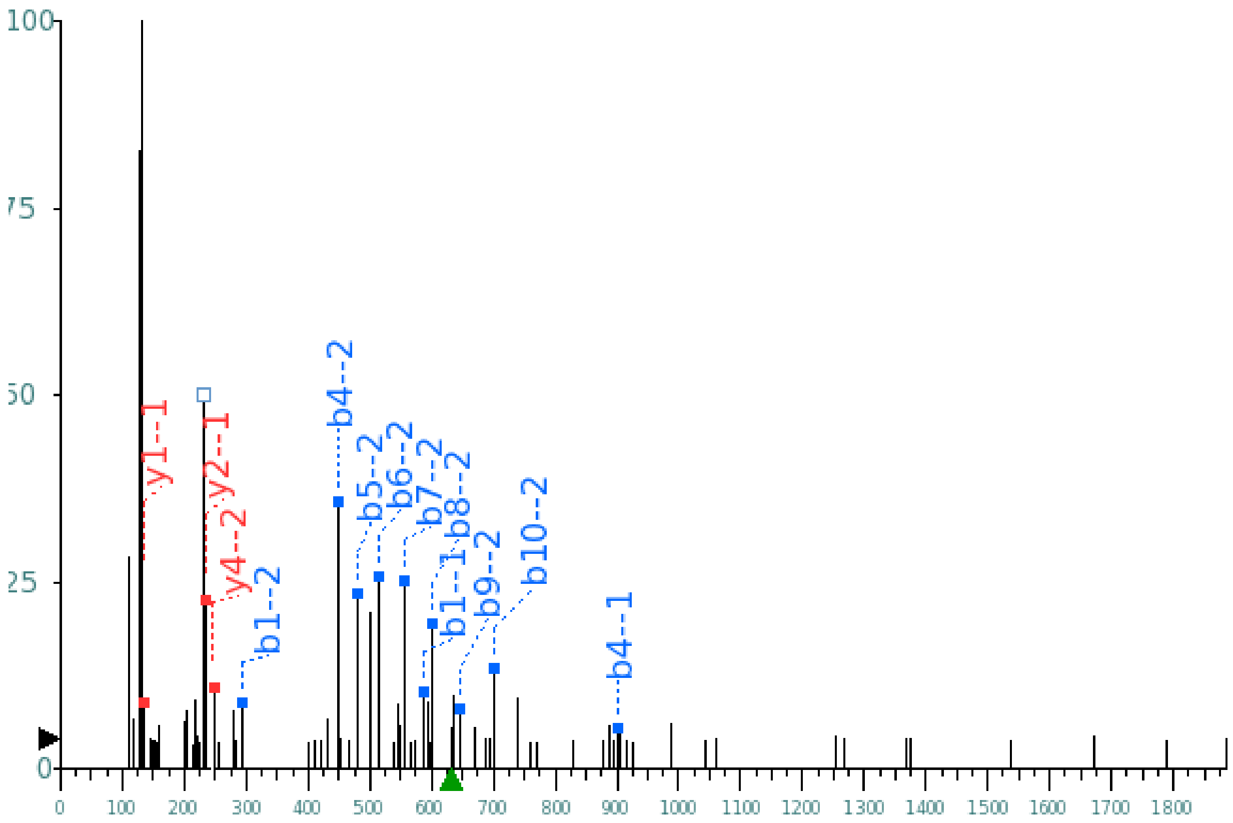

KPTDGASSSNC*VTD (+3)

Primary Reference: NASP_HUMAN
Search ID: 41761
Search Name: 20130328_ananiav_ERstress_TMT_TM_timecourse_trypsin_inj2_4mc_iaa
Scan#: 6298
Observed Mass: 632.9851 (1.8 ppm)
PSM Score: 13.01

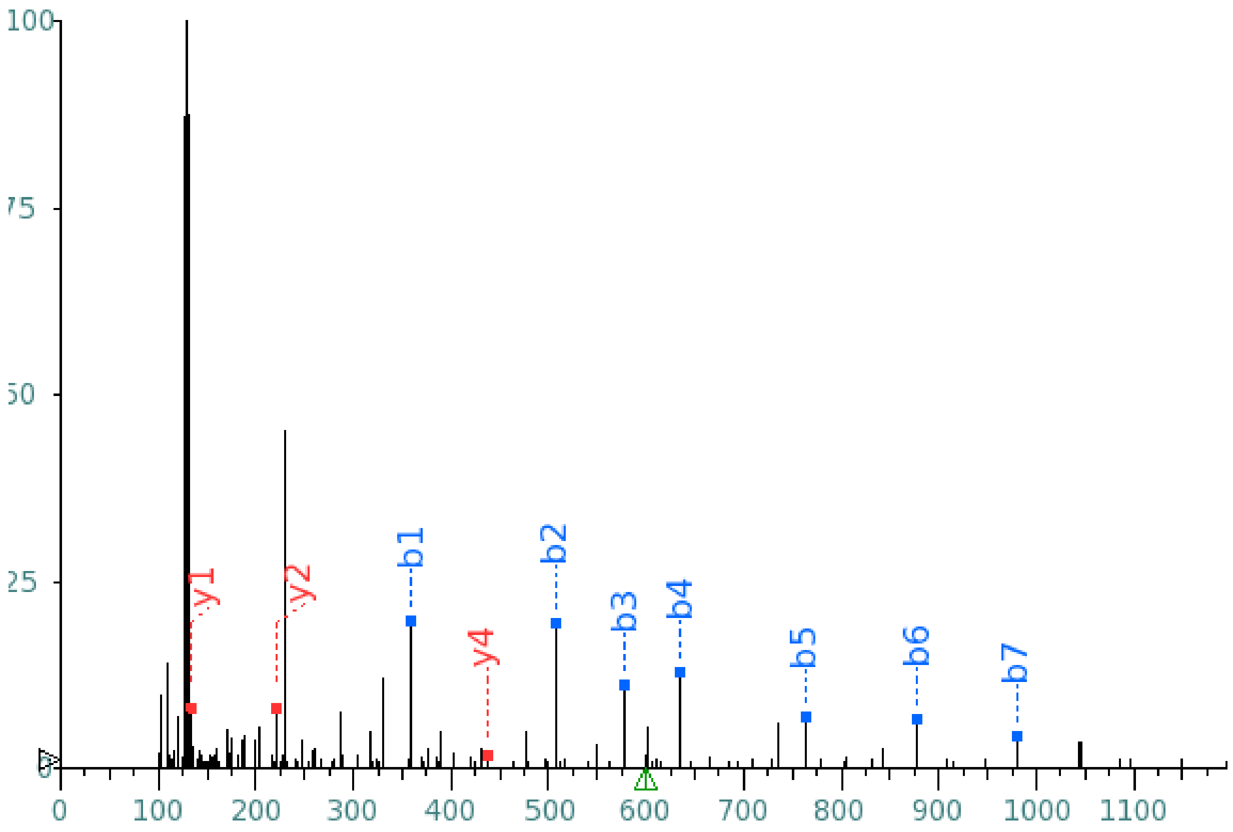

EFAGEDTSD (+2)

Primary Reference: XPO1_HUMAN
Search ID: 41761
Search Name: 20130328_ananiav_ERstress_TMT_TM_timecourse_trypsin_inj2_4mc_iaa
Scan#: 6300
Observed Mass: 600.2675 (1 ppm)
PSM Score: 30.6

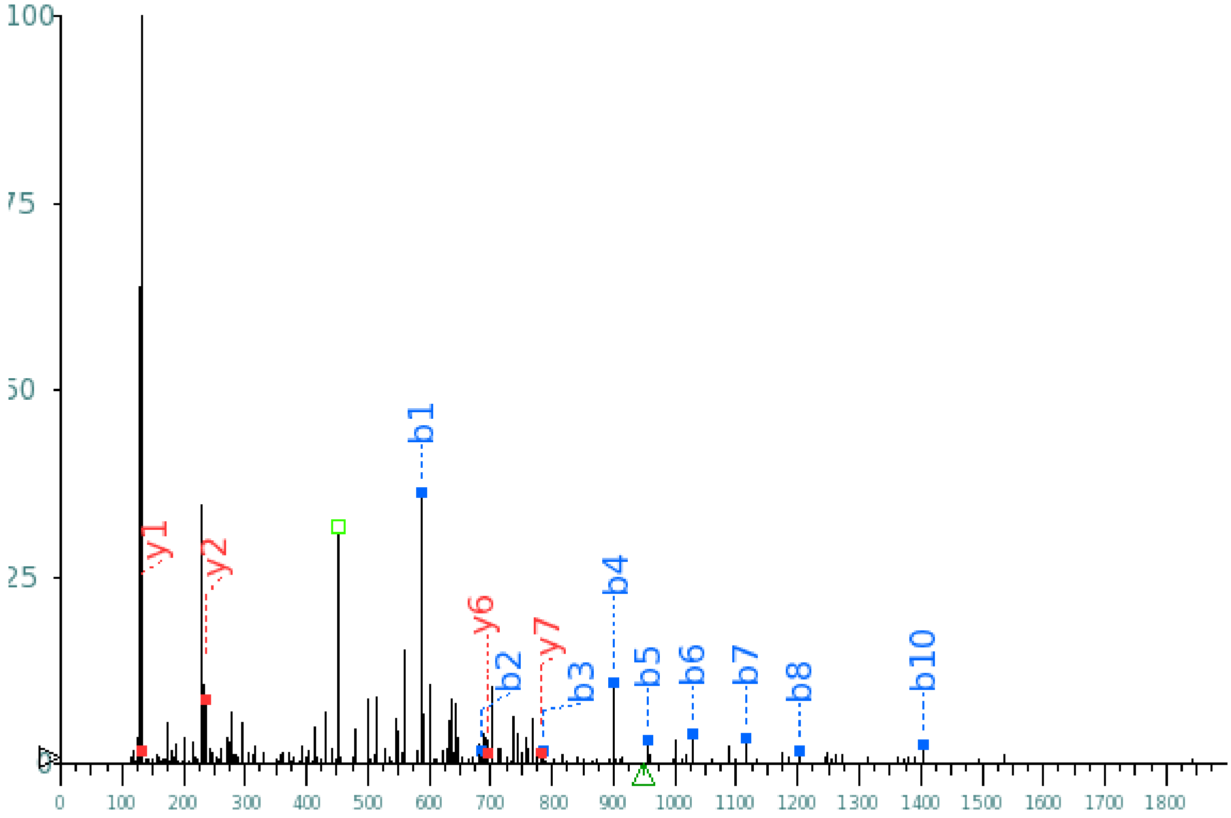

KPTDGASSSNC*VTD (+2)

Primary Reference: NASP_HUMAN
Search ID: 41761
Search Name: 20130328_ananiav_ERstress_TMT_TM_timecourse_trypsin_inj2_4mc_iaa
Scan#: 6303
Observed Mass: 948.9729 (0.6 ppm)
PSM Score: 21.67

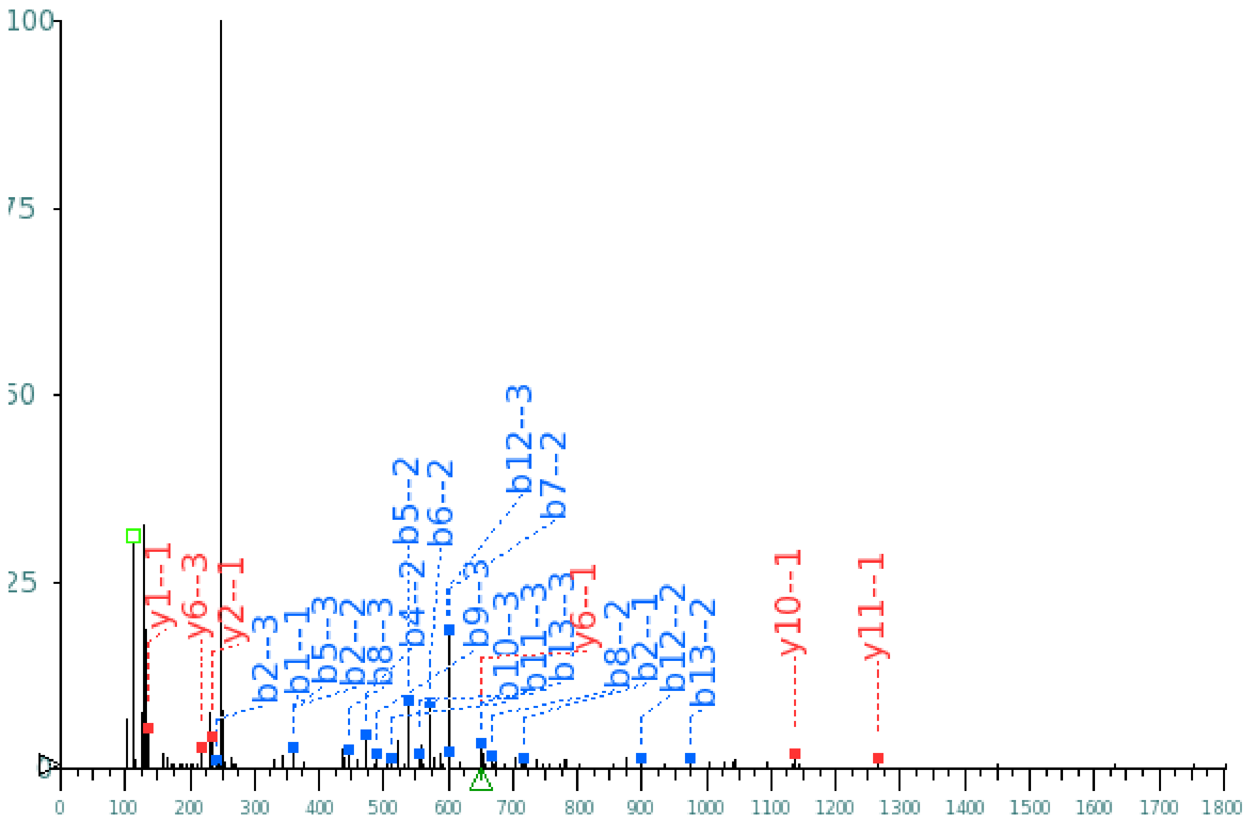

EKPEEAGHEAEERGETETD (+4)

Primary Reference: MDN1_HUMAN
Search ID: 41761
Search Name: 20130328_ananiav_ERstress_TMT_TM_timecourse_trypsin_inj2_4mc_iaa
Scan#: 6335
Observed Mass: 651.0646 (1.9 ppm)
PSM Score: 17.91

File name: 2015-02-04 04.01.53PM.pdf

Creation time: 2015-02-04 04:09PM

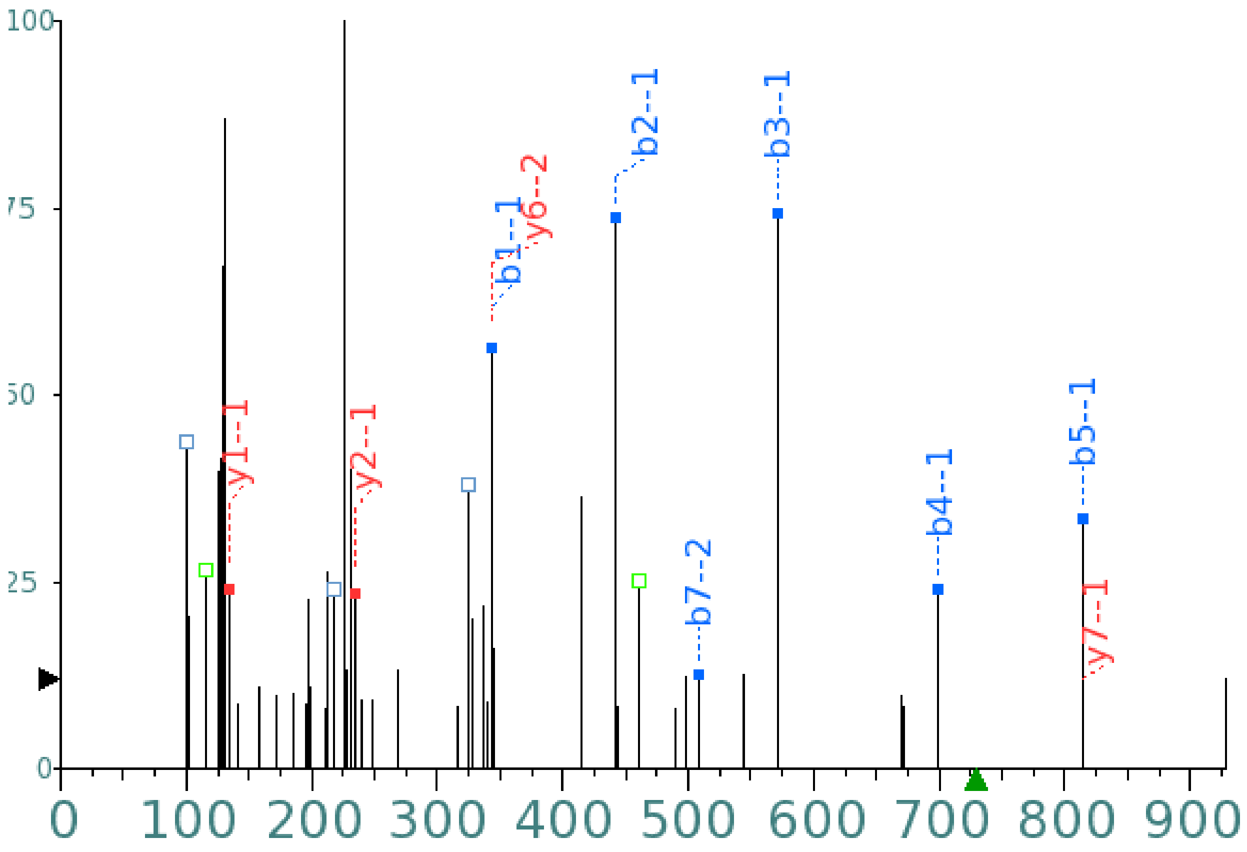

NVQQDNSEAGTQPQVQTD (+3)

Primary Reference: HS105_HUMAN
Search ID: 41761
Search Name: 20130328_ananiav_ERstress_TMT_TM_timecourse_trypsin_inj2_4mc_iaa
Scan#: 6346
Observed Mass: 730.0173 (2.7 ppm)
PSM Score: 39.11

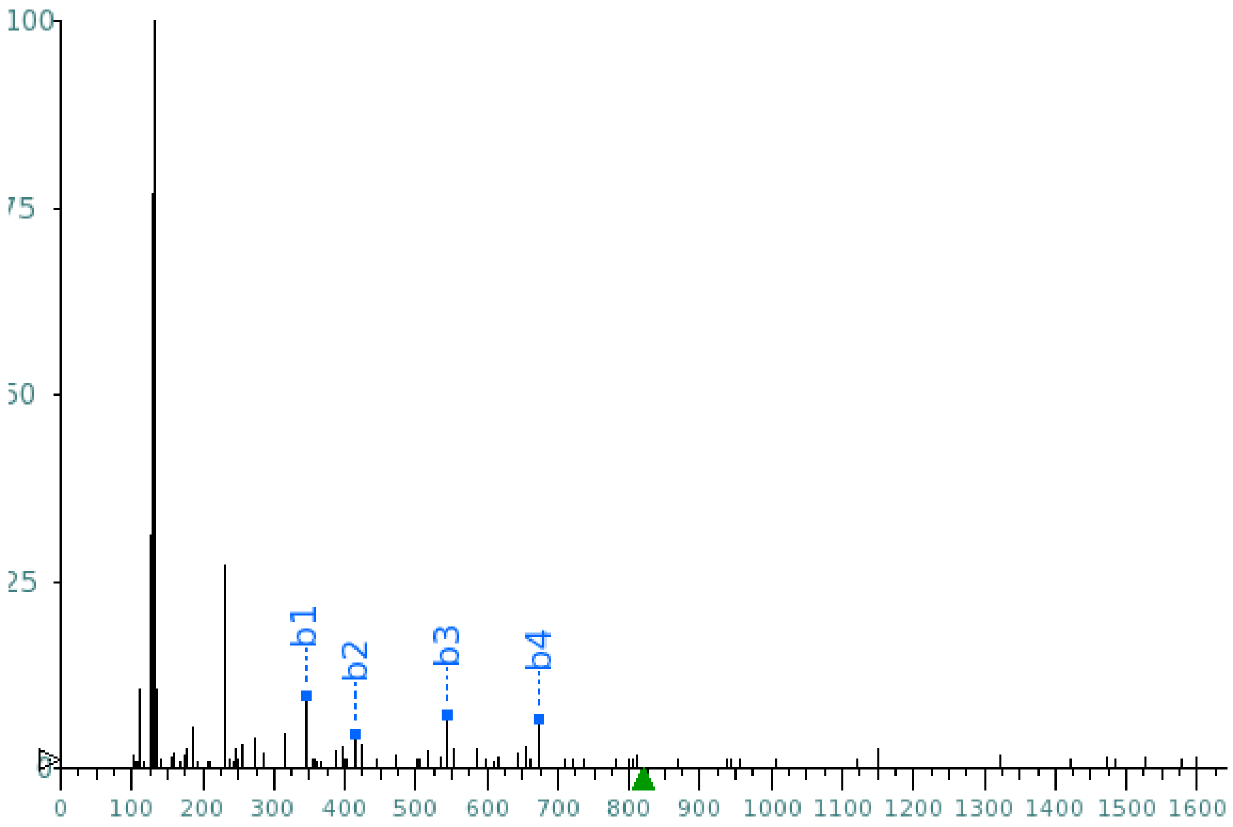

NAEQYKDQAD (+2)

Primary Reference: MYH9_HUMAN
Search ID: 41761
Search Name: 20130328_ananiav_ERstress_TMT_TM_timecourse_trypsin_inj2_4mc_iaa
Scan#: 6400
Observed Mass: 820.4218 (2.2 ppm)
PSM Score: 2.4

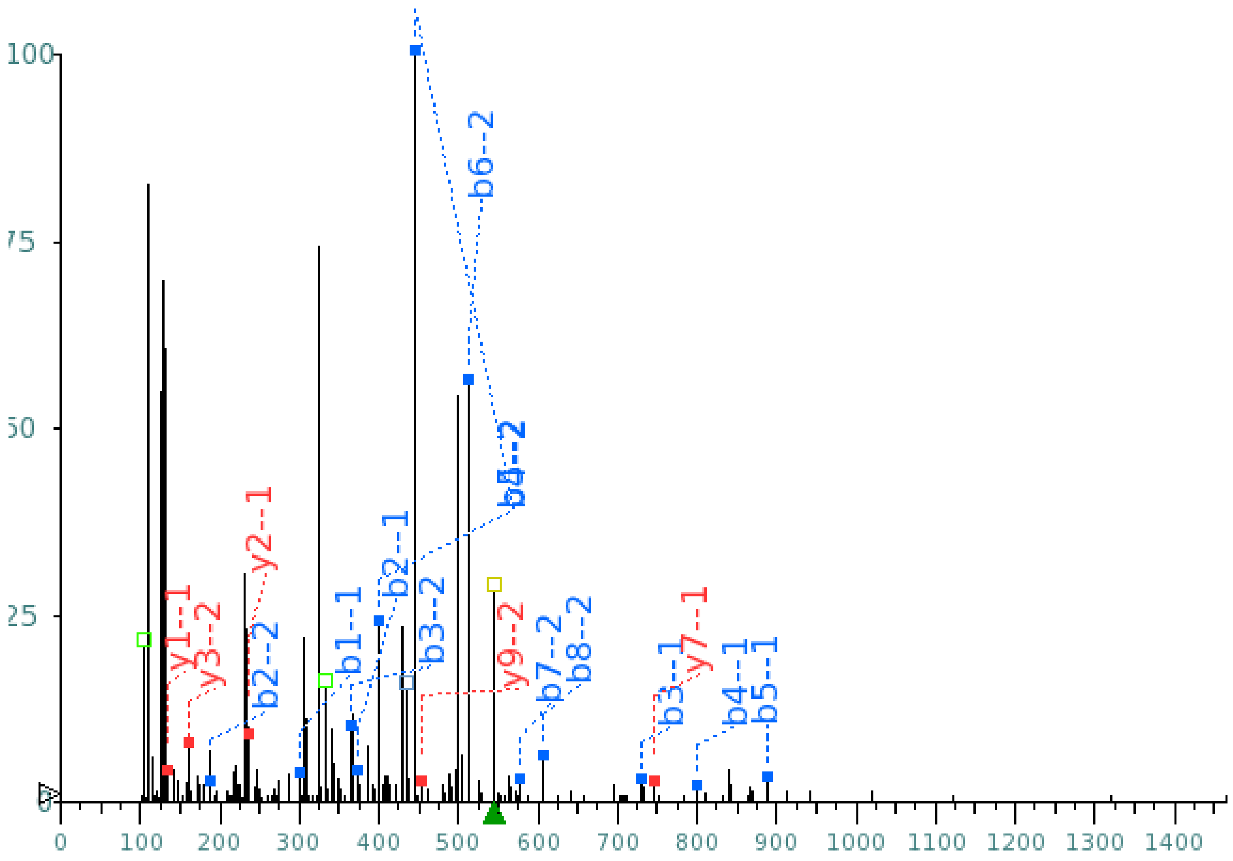

AAKASHMGVSTD (+3)

Primary Reference: LUZP1_HUMAN
Search ID: 41761
Search Name: 20130328_ananiav_ERstress_TMT_TM_timecourse_trypsin_inj2_4mc_iaa
Scan#: 6417
Observed Mass: 544.9649 (1.3 ppm)
PSM Score: 10.83

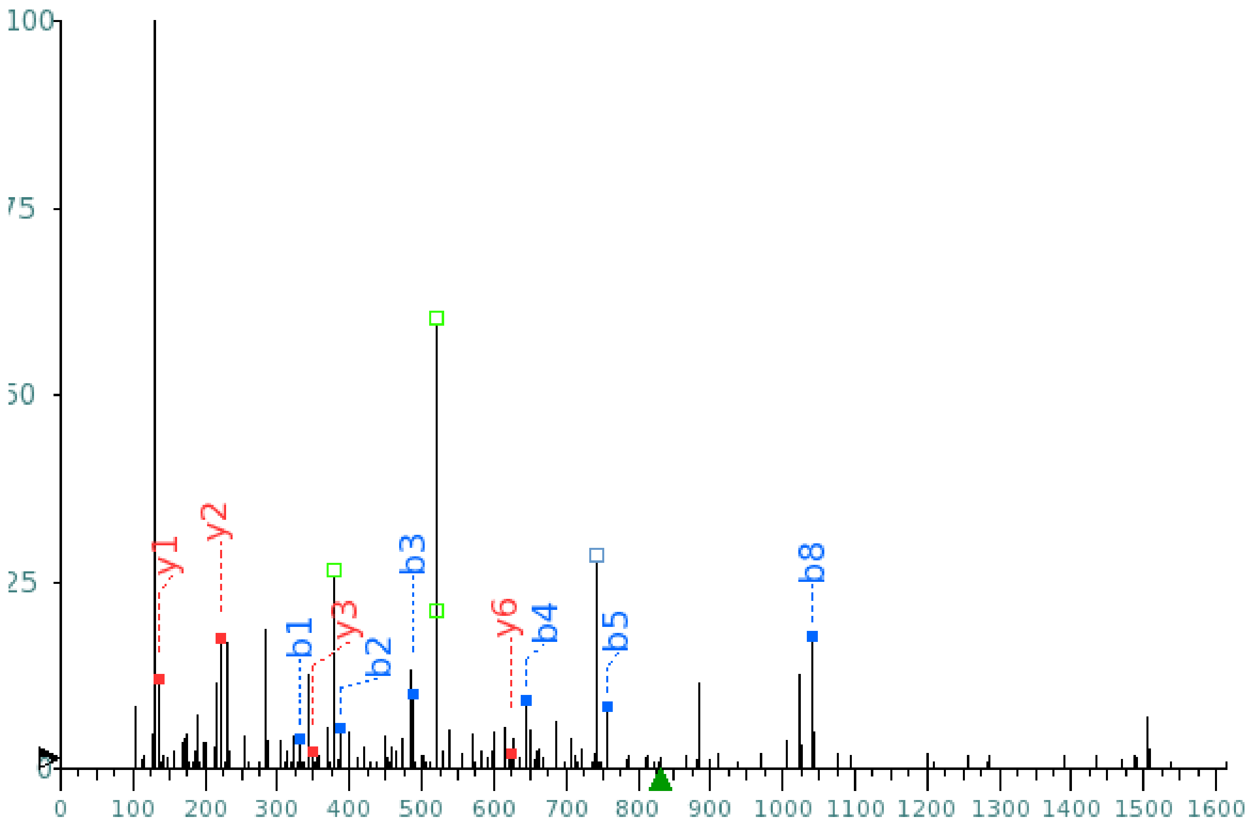

TGVRDPADVSSESD (+2)

Primary Reference: BAKOR_HUMAN
Search ID: 41761
Search Name: 20130328_ananiav_ERstress_TMT_TM_timecourse_trypsin_inj2_4mc_iaa
Scan#: 6497
Observed Mass: 832.4019 (-0.4 ppm)
PSM Score: 16.95

File name: 2015-02-04 04.01.53PM.pdf

Creation time: 2015-02-04 04:09PM

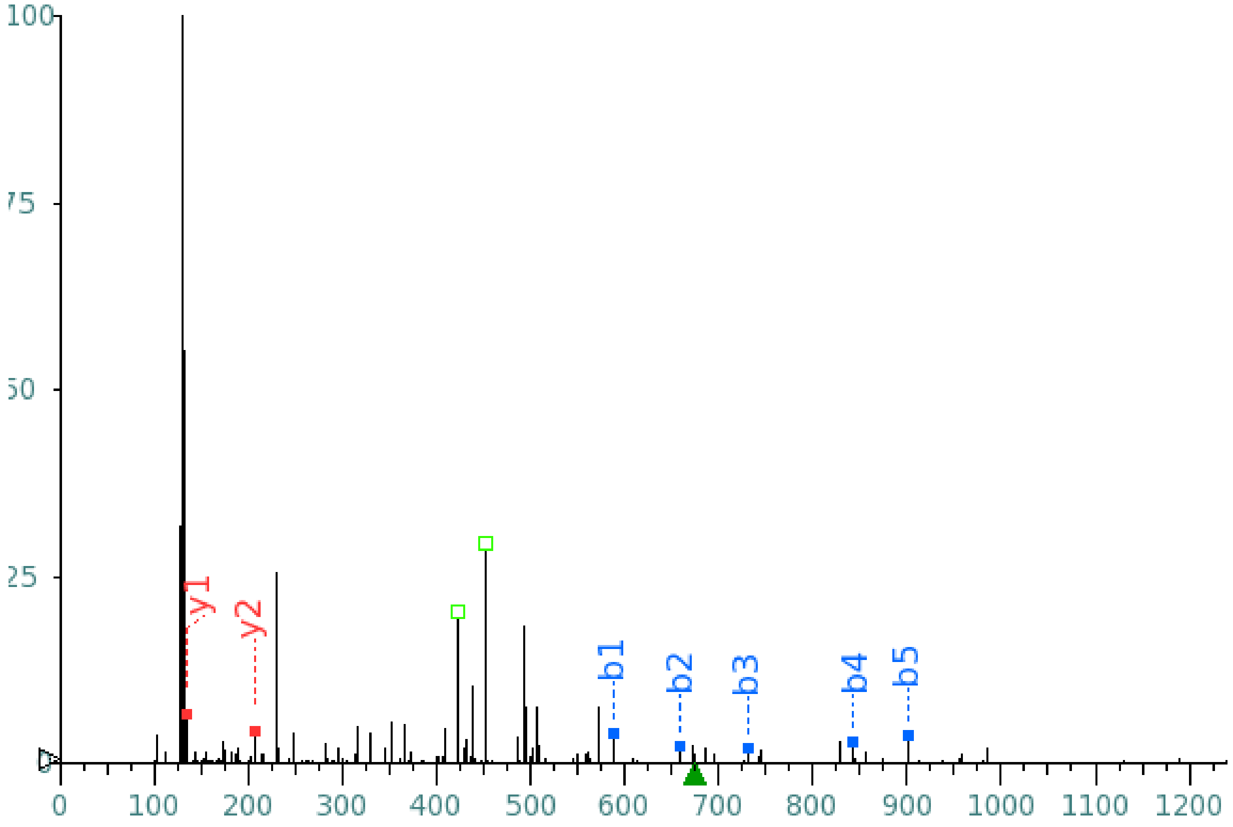

KAANGLEAD (+2)

Primary Reference: LUZP1_HUMAN
Search ID: 41761
Search Name: 20130328_ananiav_ERstress_TMT_TM_timecourse_trypsin_inj2_4mc_iaa
Scan#: 6513
Observed Mass: 673.8866 (-1.6 ppm)
PSM Score: 27.34

File name: 2015-02-04 04.01.53PM.pdf

Creation time: 2015-02-04 04:09PM

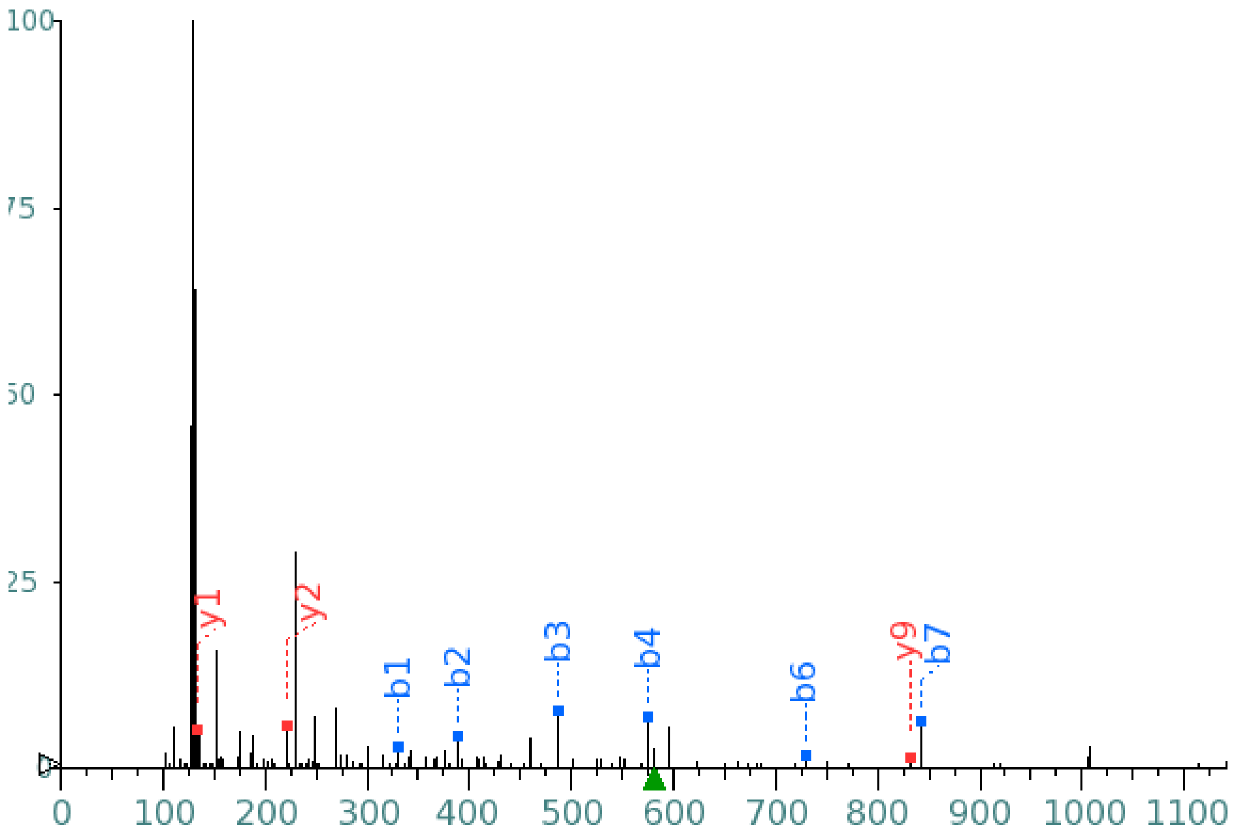

TGVSPGDVSD (+2)

Primary Reference: RHG21_HUMAN
Search ID: 41761
Search Name: 20130328_ananiav_ERstress_TMT_TM_timecourse_trypsin_inj2_4mc_iaa
Scan#: 6622
Observed Mass: 581.7934 (0.5 ppm)
PSM Score: 18.59

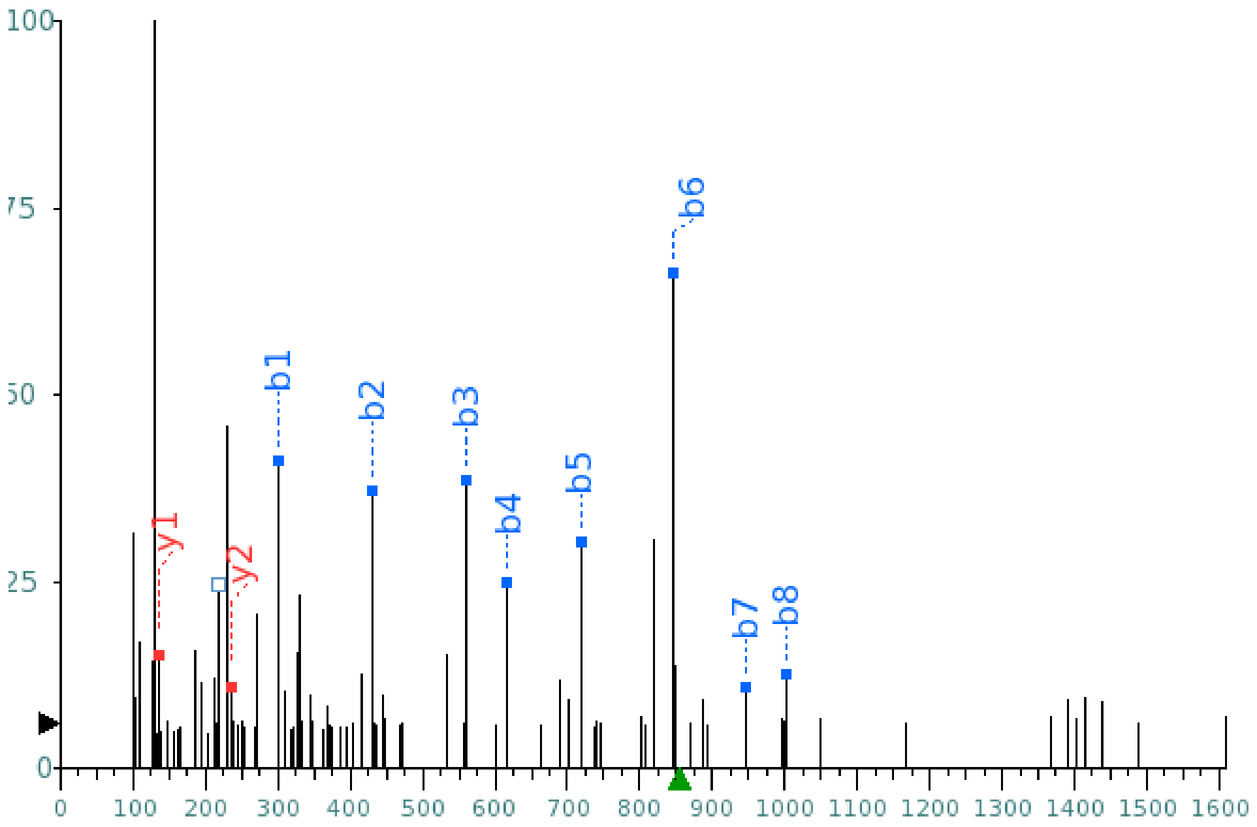

AMQGTEVGQTDQTD (+2)

Primary Reference: MIA3_HUMAN
Search ID: 41761
Search Name: 20130328_ananiav_ERstress_TMT_TM_timecourse_trypsin_inj2_4mc_iaa
Scan#: 6721
Observed Mass: 855.3958 (-0.4 ppm)
PSM Score: 51.18

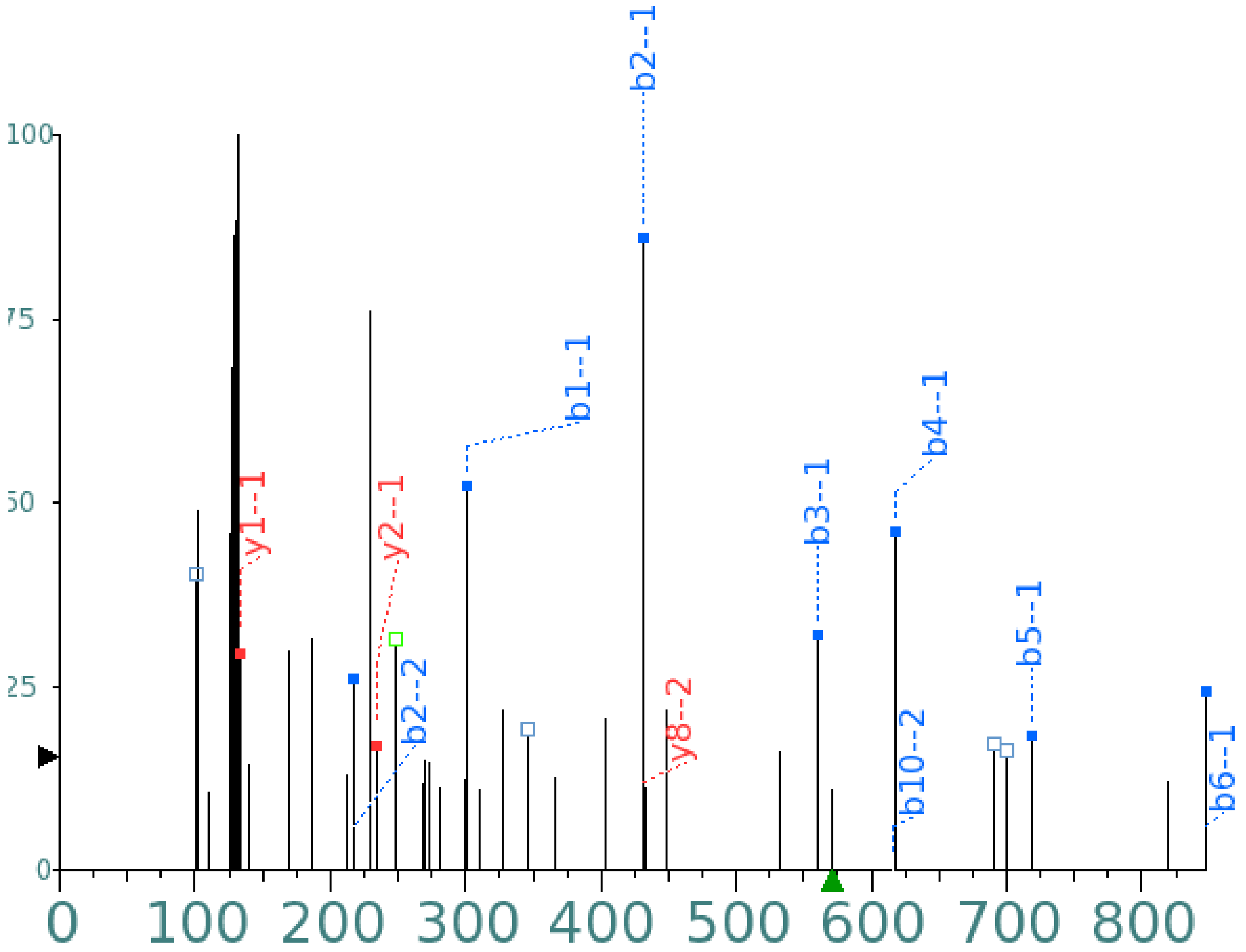

AMQGTEVGQTDQTD (+3)

Primary Reference: MIA3_HUMAN
Search ID: 41761
Search Name: 20130328_ananiav_ERstress_TMT_TM_timecourse_trypsin_inj2_4mc_iaa
Scan#: 6744
Observed Mass: 570.6006 (1.2 ppm)
PSM Score: 41.31

File name: 2015-02-04 04.01.53PM.pdf

Creation time: 2015-02-04 04:09PM

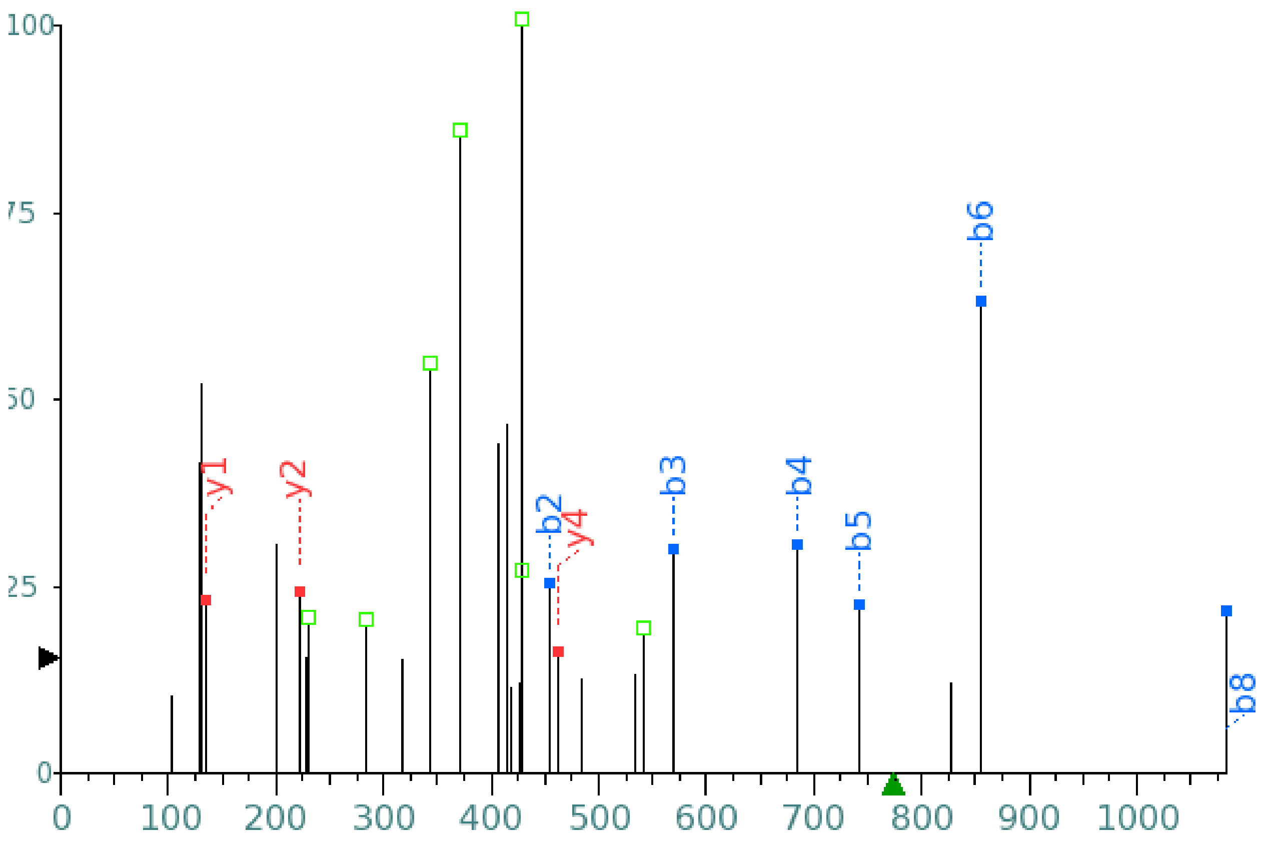

SHDDGNIDLESD (+2)

Primary Reference: SON_HUMAN
Search ID: 41761
Search Name: 20130328_ananiav_ERstress_TMT_TM_timecourse_trypsin_inj2_4mc_iaa
Scan#: 6753
Observed Mass: 773.347 (0 ppm)
PSM Score: 43.09

File name: 2015-02-04 04.01.53PM.pdf

Creation time: 2015-02-04 04:09PM

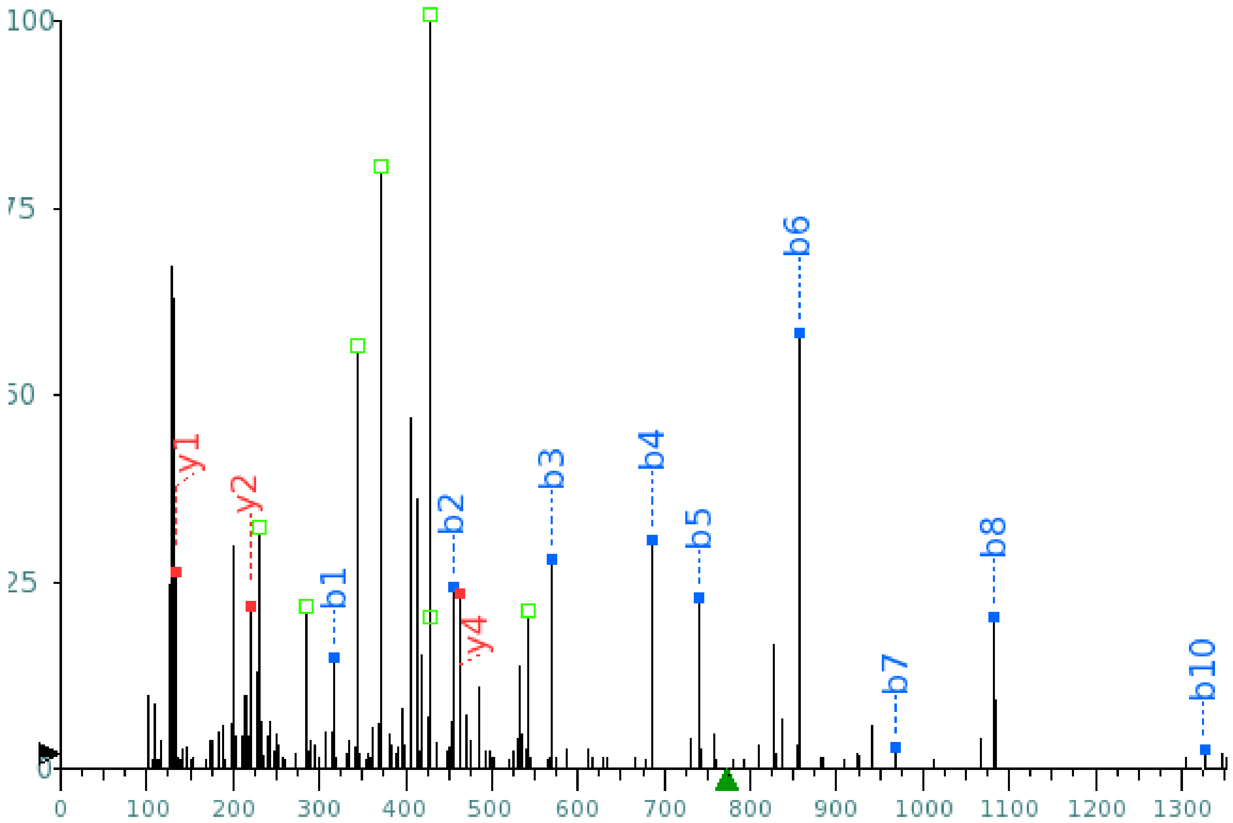

SHDDGNIDLESD (+2)

Primary Reference: SON_HUMAN
Search ID: 41761
Search Name: 20130328_ananiav_ERstress_TMT_TM_timecourse_trypsin_inj2_4mc_iaa
Scan#: 6838
Observed Mass: 773.3478 (1.1 ppm)
PSM Score: 35.57

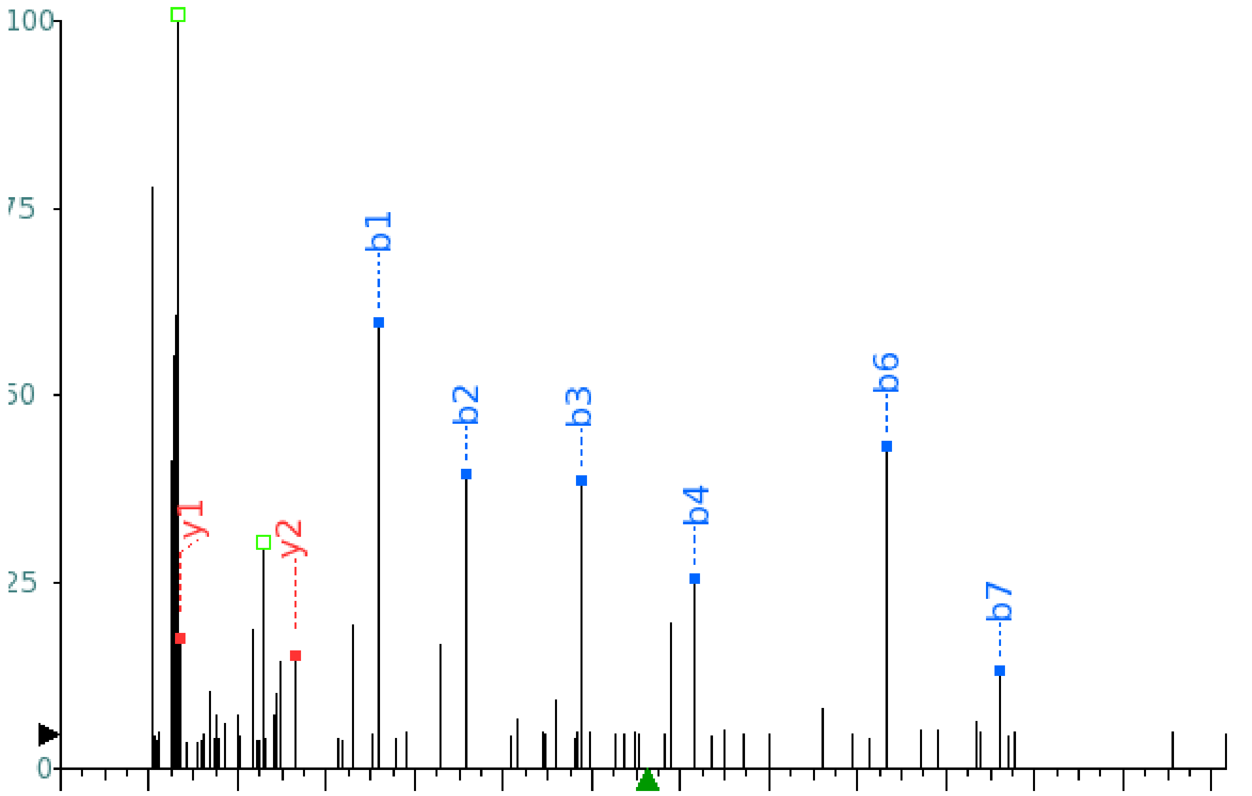

EVEETDEMD (+2)

Primary Reference: DNJA1_HUMAN
Search ID: 41761
Search Name: 20130328_ananiav_ERstress_TMT_TM_timecourse_trypsin_inj2_4mc_iaa
Scan#: 6859
Observed Mass: 663.2856 (1.8 ppm)
PSM Score: 37.7

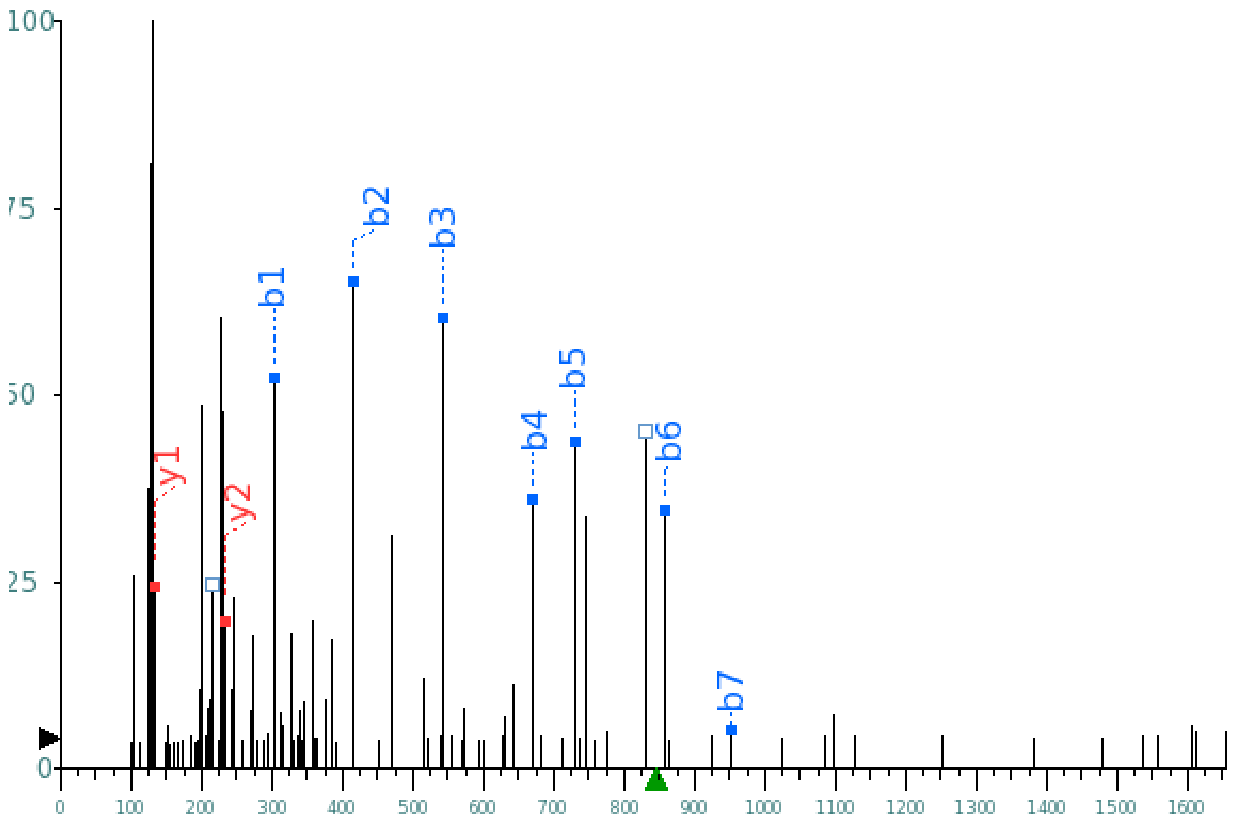

ALQEGQPEEDETD (+2)

Primary Reference: LENG1_HUMAN
Search ID: 41761
Search Name: 20130328_ananiav_ERstress_TMT_TM_timecourse_trypsin_inj2_4mc_iaa
Scan#: 6890
Observed Mass: 845.3876 (1.6 ppm)
PSM Score: 25.59

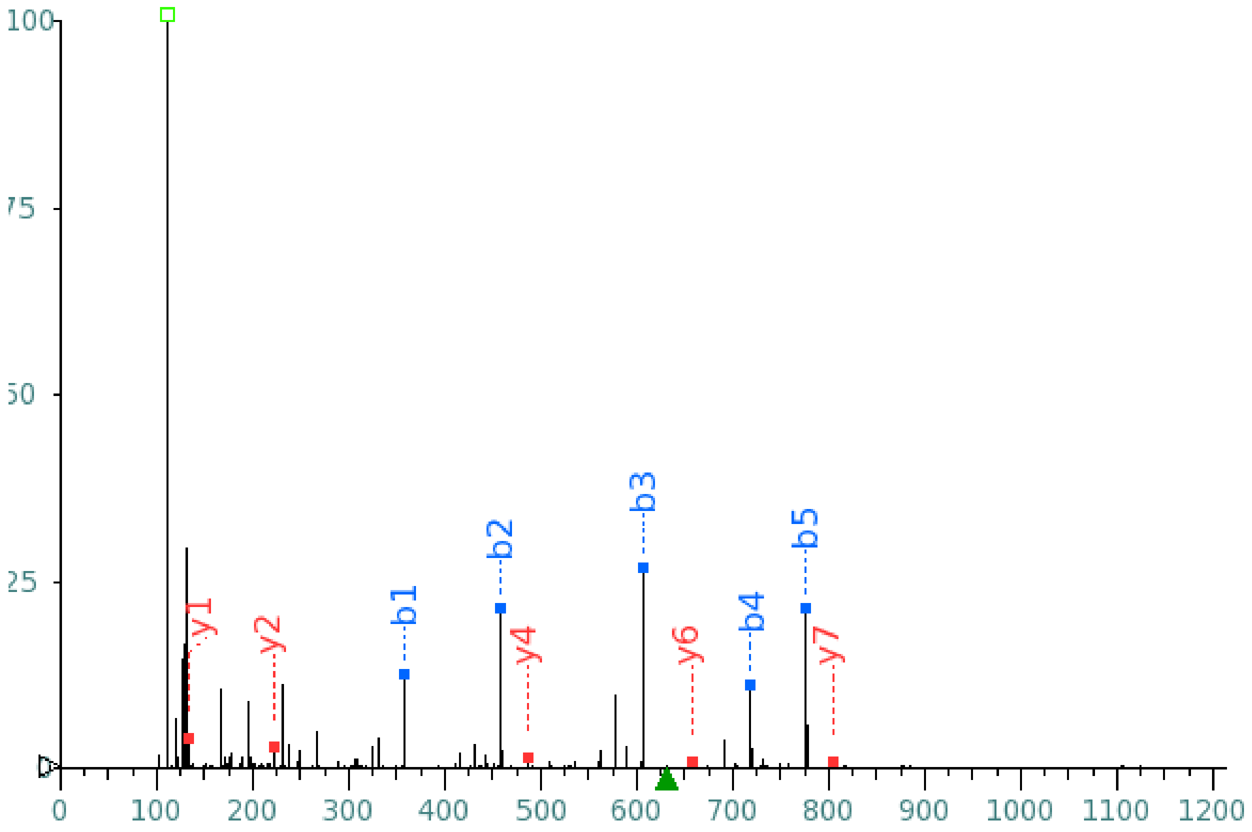

QTFIGHESD (+2)

Primary Reference: GBB2_HUMAN
Search ID: 41761
Search Name: 20130328_ananiav_ERstress_TMT_TM_timecourse_trypsin_inj2_4mc_iaa
Scan#: 6897
Observed Mass: 631.8158 (2.3 ppm)
PSM Score: 14.68

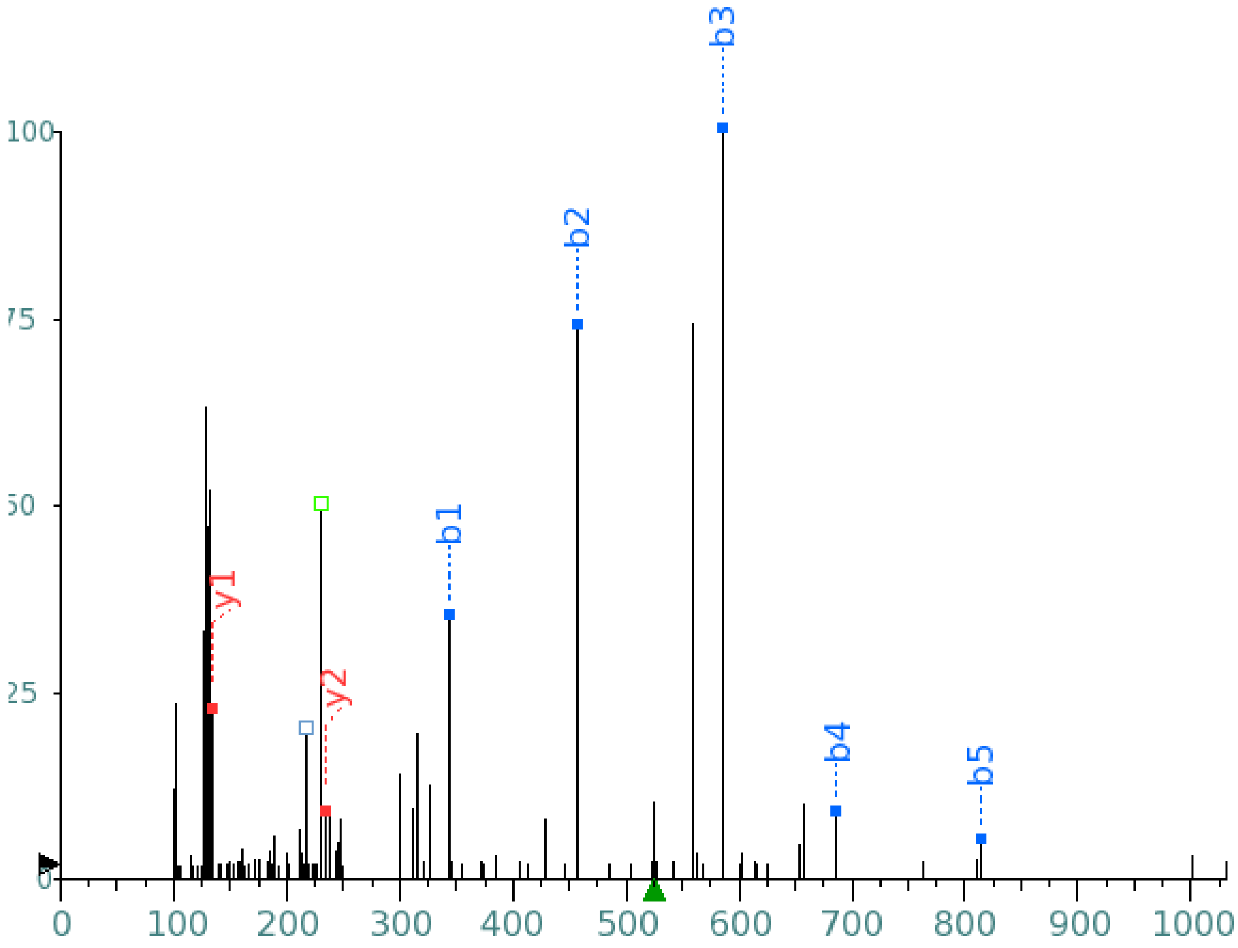

INEVQTD (+2)

Primary Reference: TWF1_HUMAN
Search ID: 41761
Search Name: 20130328_ananiav_ERstress_TMT_TM_timecourse_trypsin_inj2_4mc_iaa
Scan#: 6911
Observed Mass: 524.2807 (2.1 ppm)
PSM Score: 18.54

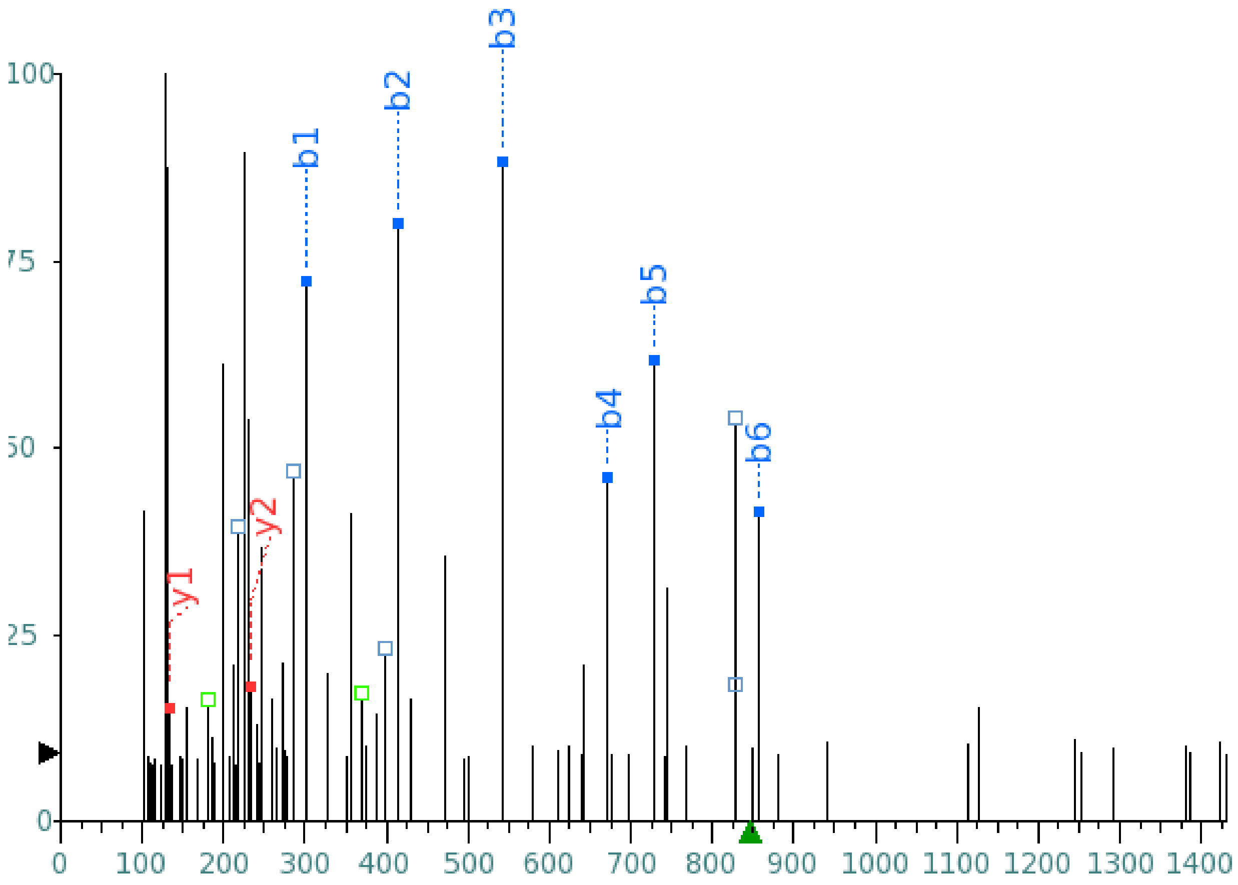

ALQEGGQPEEDETD (+2)

Primary Reference: LENG1_HUMAN
Search ID: 41761
Search Name: 20130328_ananiav_ERstress_TMT_TM_timecourse_trypsin_inj2_4mc_iaa
Scan#: 6981
Observed Mass: 845.3875 (1.5 ppm)
PSM Score: 18.19

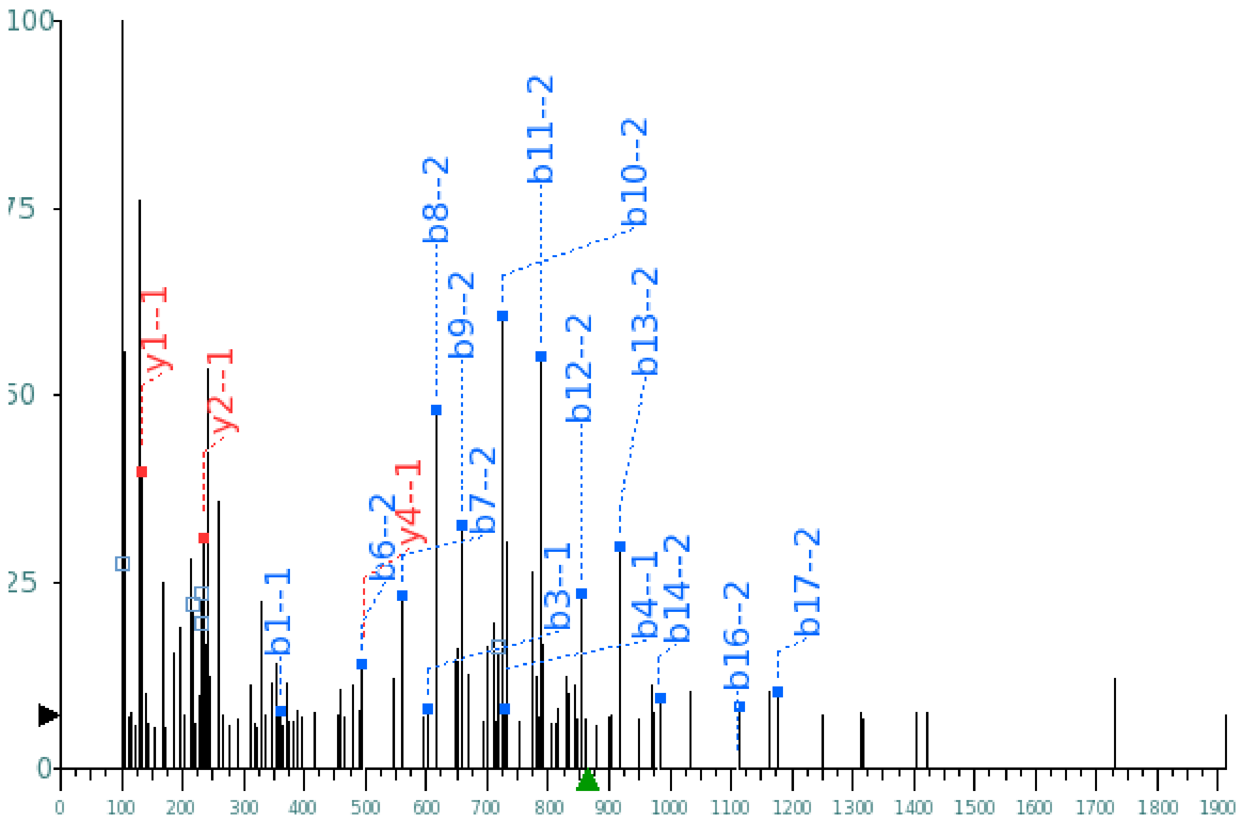

ESRQEEMNSQQEEEEMETD (+3)

Primary Reference: SF3B2_HUMAN
Search ID: 41761
Search Name: 20130328_ananiav_ERstress_TMT_TM_timecourse_trypsin_inj2_4mc_iaa
Scan#: 6991
Observed Mass: 863.0264 (1.5 ppm)
PSM Score: 29.26

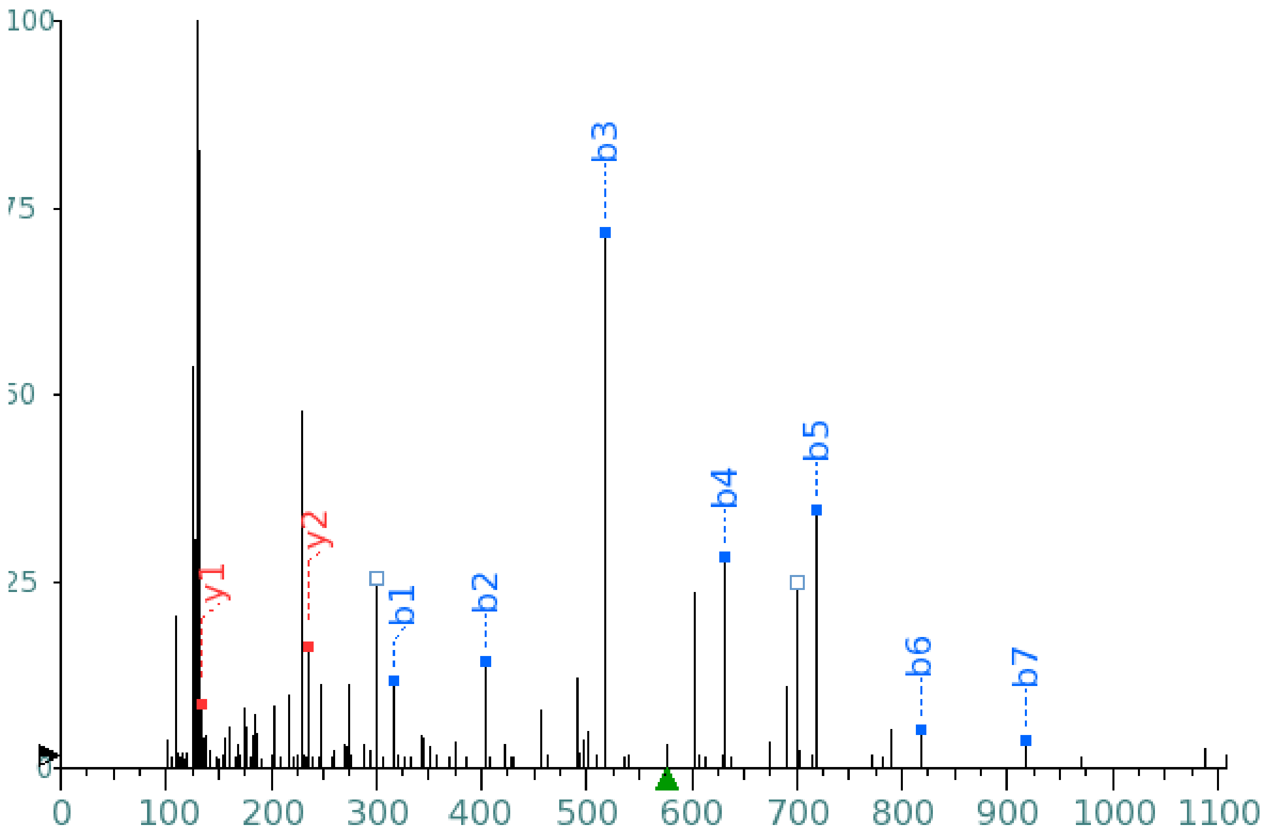

SSNLSVTTD (+2)

Primary Reference: LMO7_HUMAN
Search ID: 41761
Search Name: 20130328_ananiav_ERstress_TMT_TM_timecourse_trypsin_inj2_4mc_iaa
Scan#: 7047
Observed Mass: 576.8021 (2 ppm)
PSM Score: 51.74

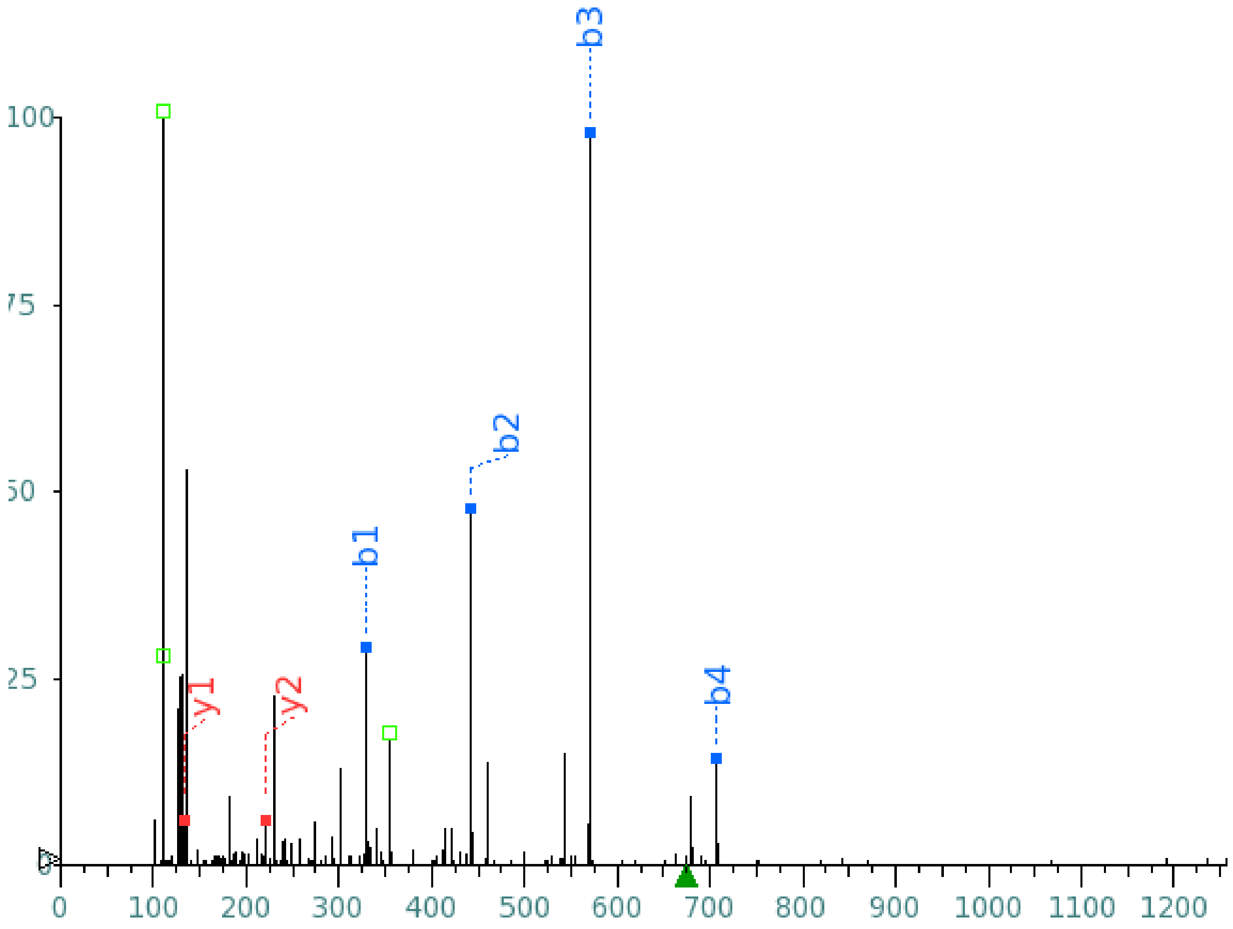

VLQHYQESD (+2)

Primary Reference: MTCH2_HUMAN
Search ID: 41761
Search Name: 20130328_ananiav_ERstress_TMT_TM_timecourse_trypsin_inj2_4mc_iaa
Scan#: 7052
Observed Mass: 674.3413 (0.8 ppm)
PSM Score: 13.29

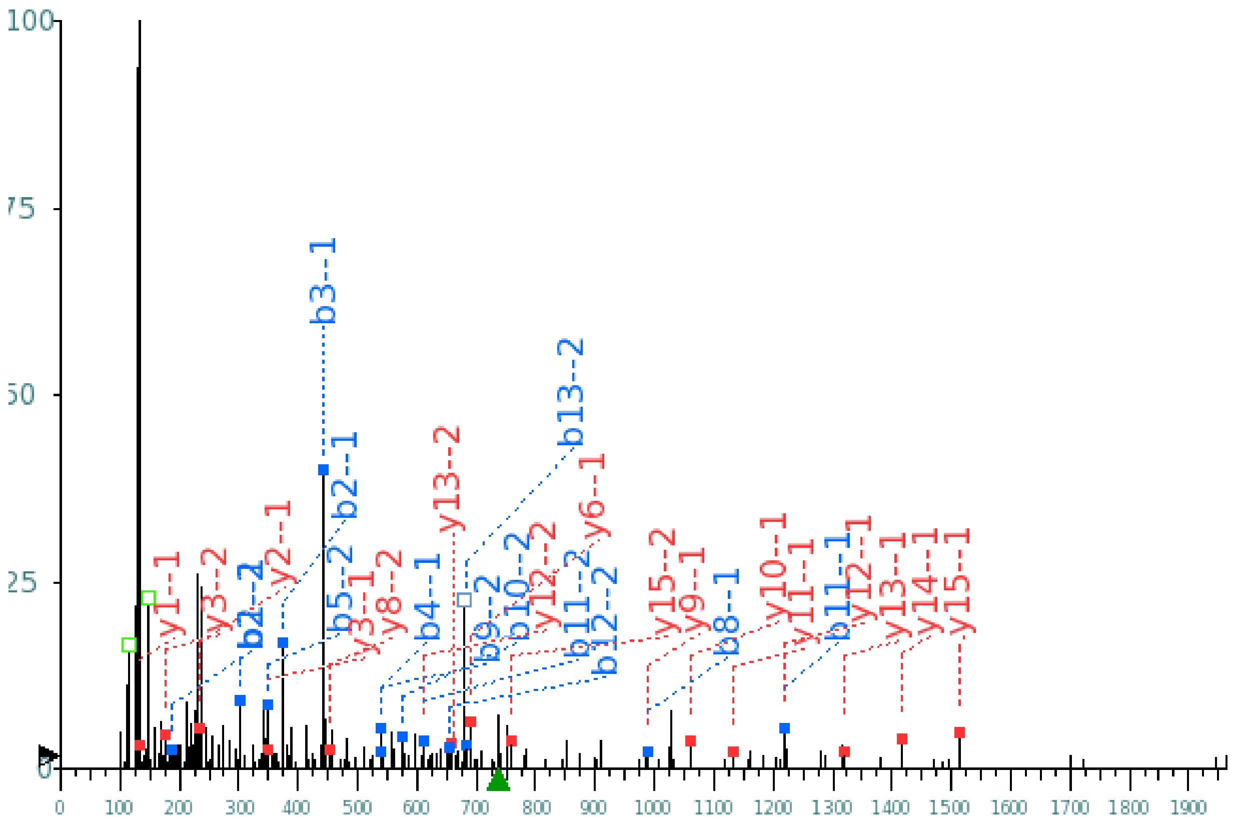

AAAPRPPVSAASGRPQDDTD (+3)

Primary Reference: WIPF2_HUMAN
Search ID: 41761
Search Name: 20130328_ananiav_ERstress_TMT_TM_timecourse_trypsin_inj2_4mc_iaa
Scan#: 7073
Observed Mass: 736.7136 (2.5 ppm)
PSM Score: 20.62

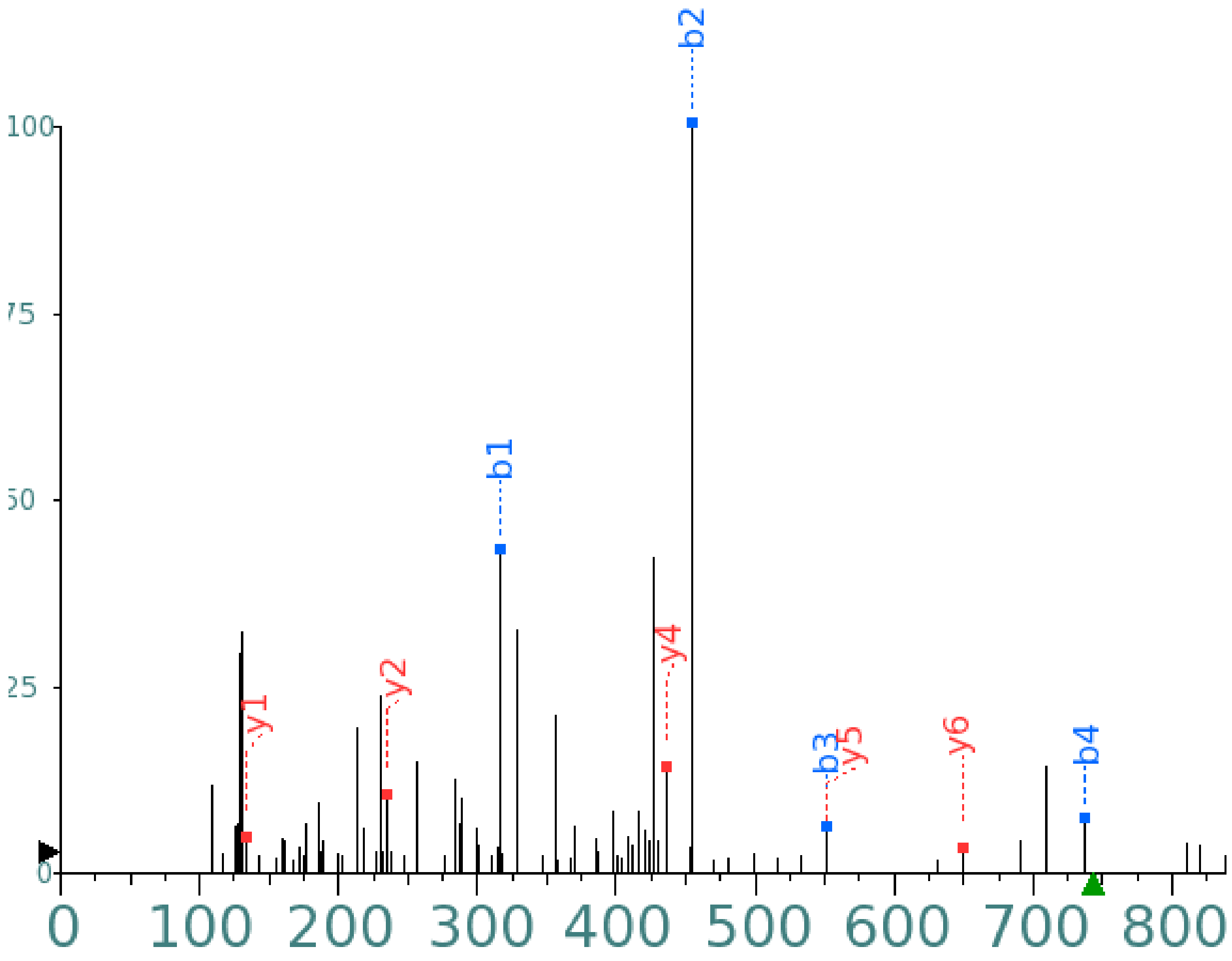

SHPWTPDDSTD (+2)

Primary Reference: M3K7_HUMAN
Search ID: 41761
Search Name: 20130328_ananiav_ERstress_TMT_TM_timecourse_trypsin_inj2_4mc_iaa
Scan#: 7105
Observed Mass: 743.8368 (1.1 ppm)
PSM Score: 17.51

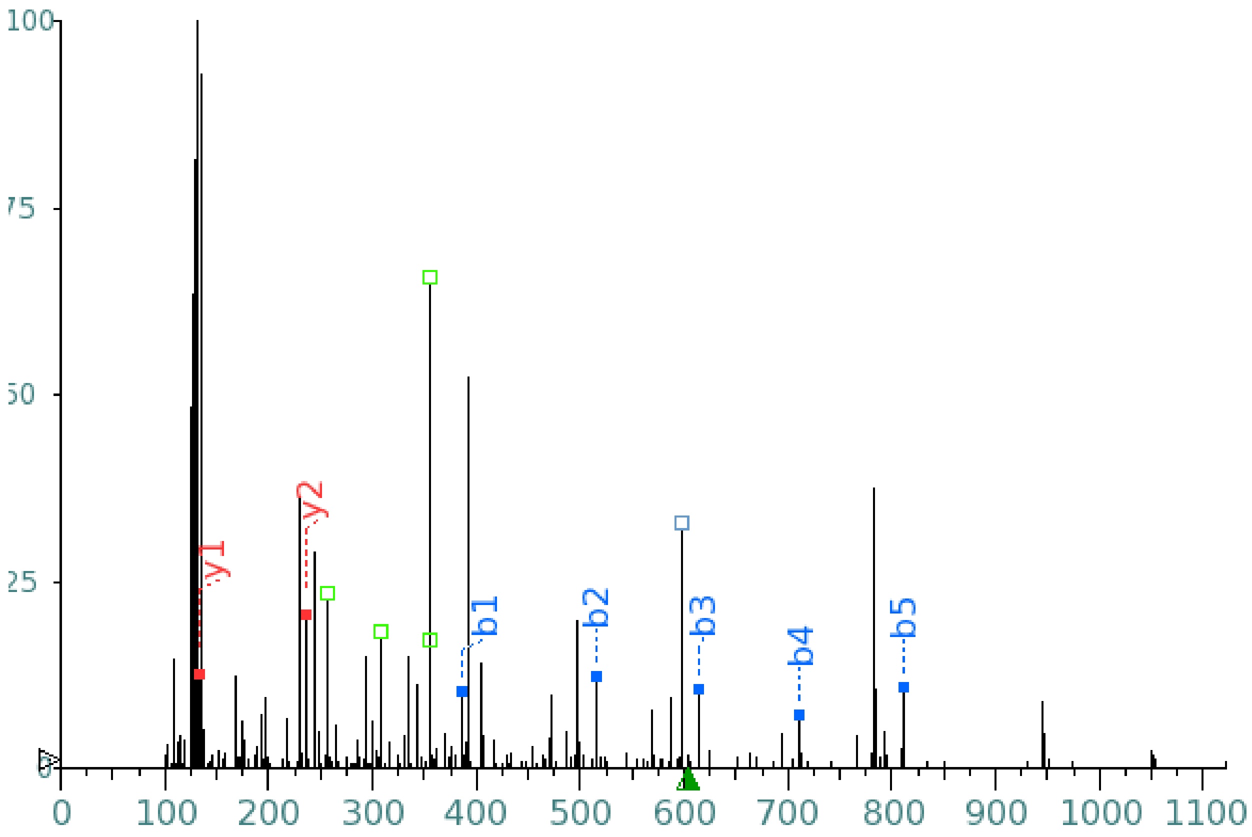

REVPVYTD (+2)

Primary Reference: F120B_HUMAN
Search ID: 41761
Search Name: 20130328_ananiav_ERstress_TMT_TM_timecourse_trypsin_inj2_4mc_iaa
Scan#: 7111
Observed Mass: 604.3311 (2.5 ppm)
PSM Score: 13.21

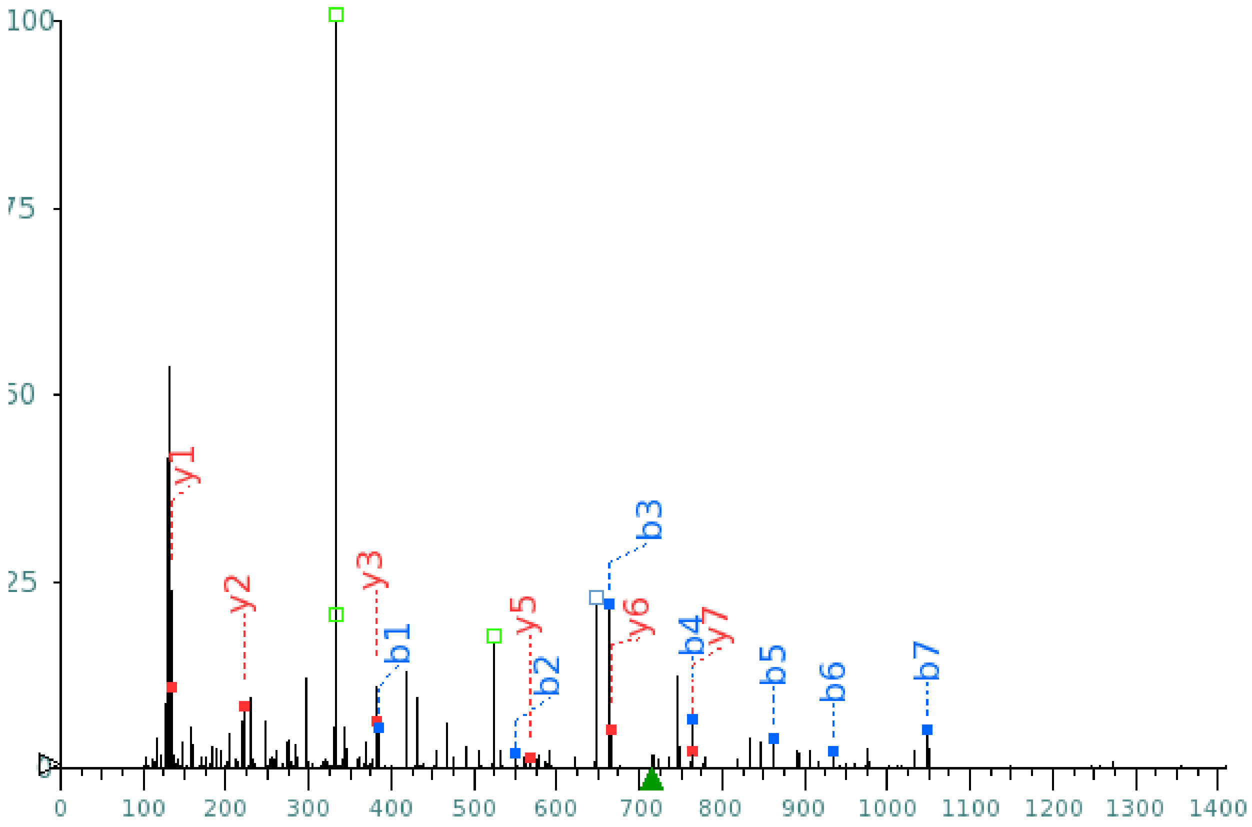

RYDVVADC*SD (+2)

Primary Reference: MOCS3_HUMAN
Search ID: 41761
Search Name: 20130328_ananiav_ERstress_TMT_TM_timecourse_trypsin_inj2_4mc_iaa
Scan#: 7129
Observed Mass: 714.8354 (0.6 ppm)
PSM Score: 25.08

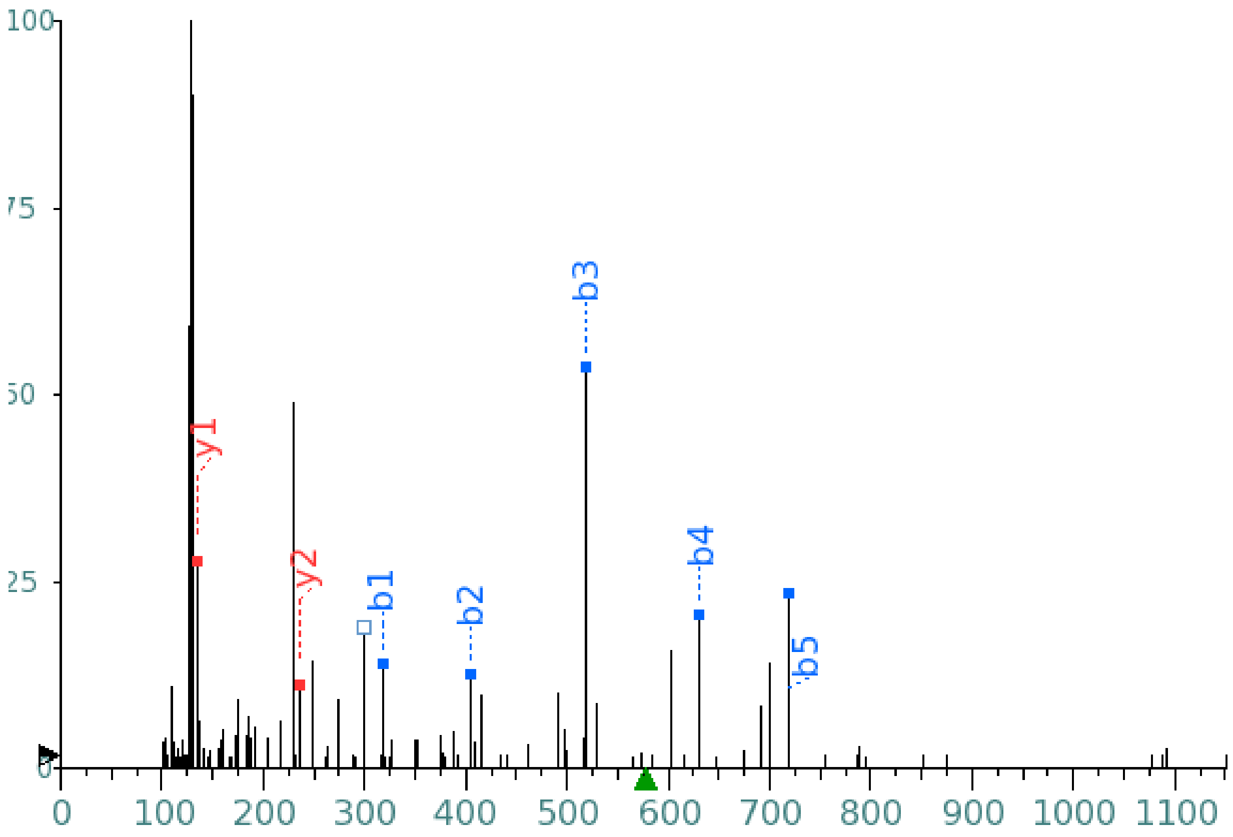

SSNLSVTTD (+2)

Primary Reference: LMO7_HUMAN
Search ID: 41761
Search Name: 20130328_ananiav_ERstress_TMT_TM_timecourse_trypsin_inj2_4mc_iaa
Scan#: 7135
Observed Mass: 576.8015 (1 ppm)
PSM Score: 25.06

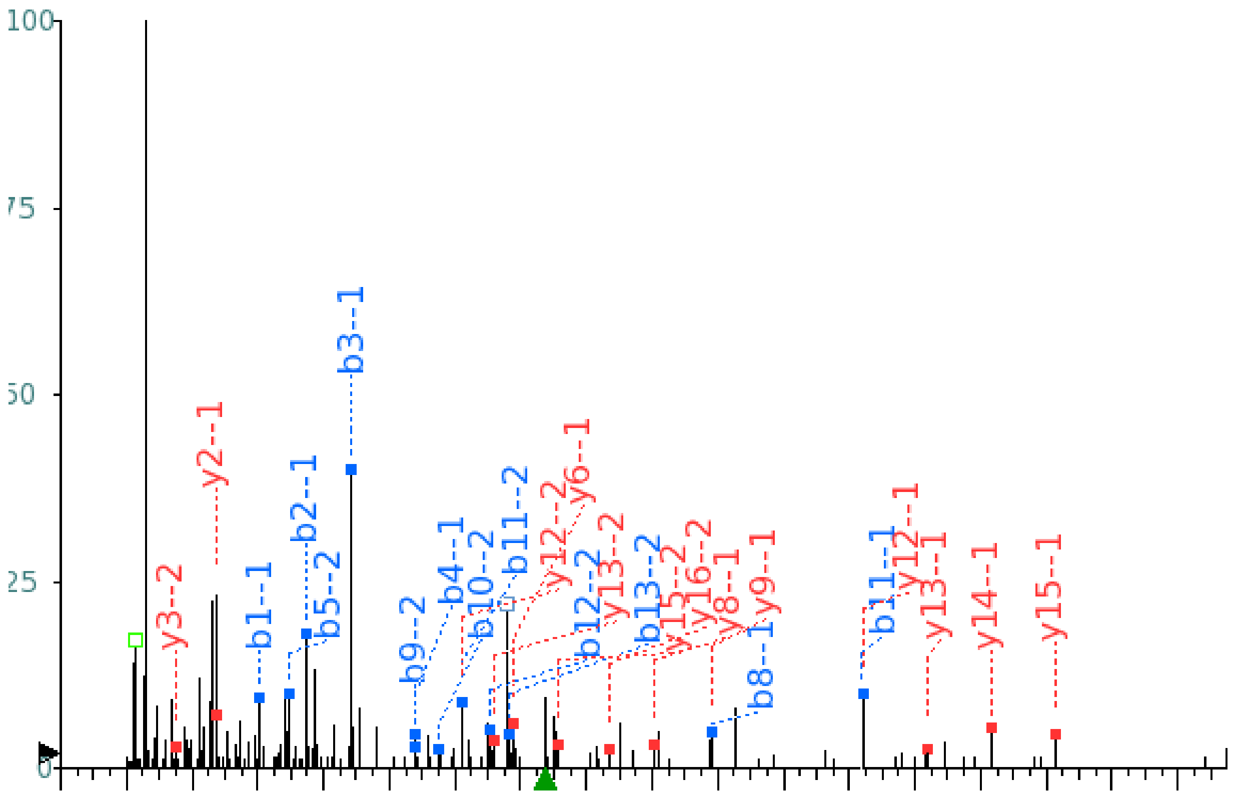

AAAPRPPVSAASGRPQDDTD (+3)

Primary Reference: WIPF2_HUMAN
Search ID: 41761
Search Name: 20130328_ananiav_ERstress_TMT_TM_timecourse_trypsin_inj2_4mc_iaa
Scan#: 7155
Observed Mass: 736.7128 (1.5 ppm)
PSM Score: 16.37

File name: 2015-02-04 04.01.53PM.pdf

Creation time: 2015-02-04 04:09PM

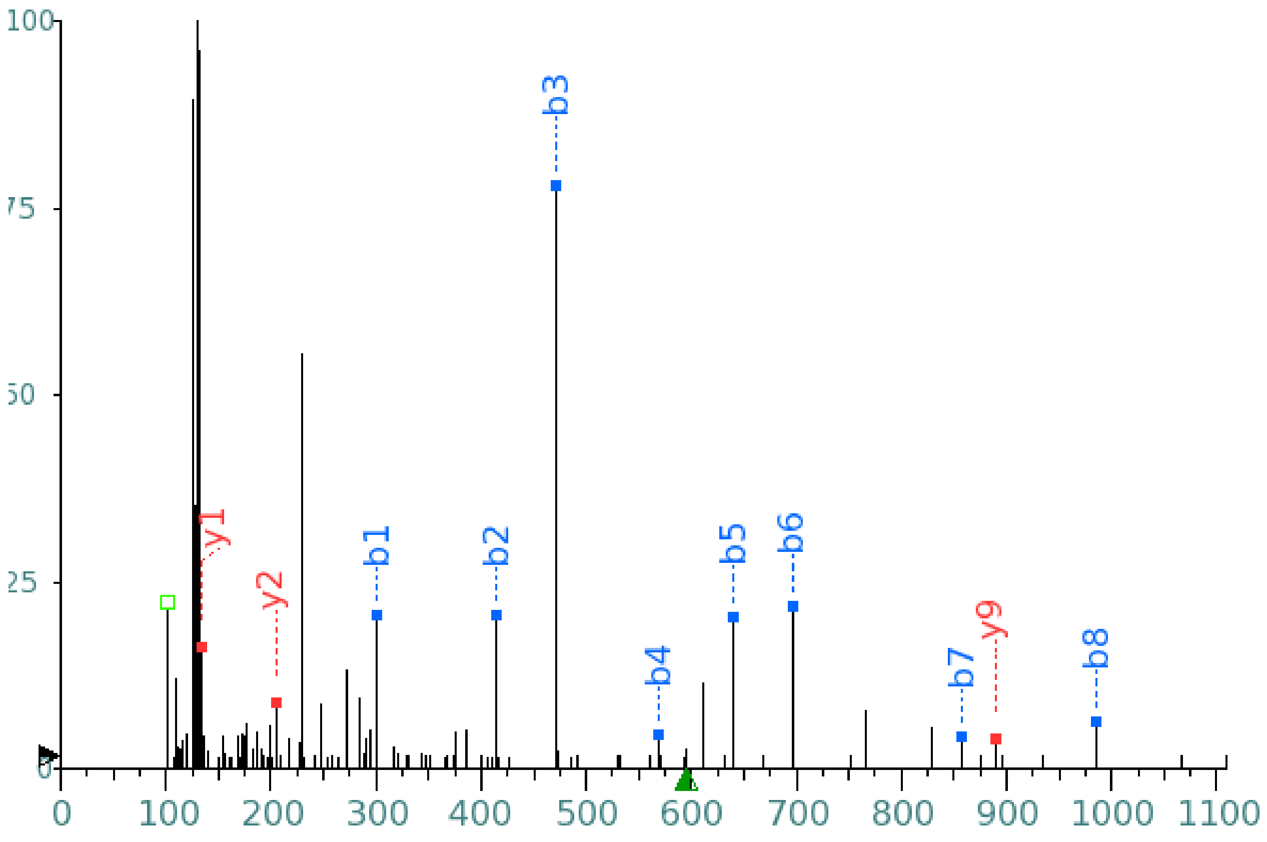

ALGPAGC*EAD (+2)

Primary Reference: AGRIN_HUMAN
Search ID: 41761
Search Name: 20130328_ananiav_ERstress_TMT_TM_timecourse_trypsin_inj2_4mc_iaa
Scan#: 7156
Observed Mass: 595.2904 (1.3 ppm)
PSM Score: 45.37

File name: 2015-02-04 04.01.53PM.pdf

Creation time: 2015-02-04 04:09PM

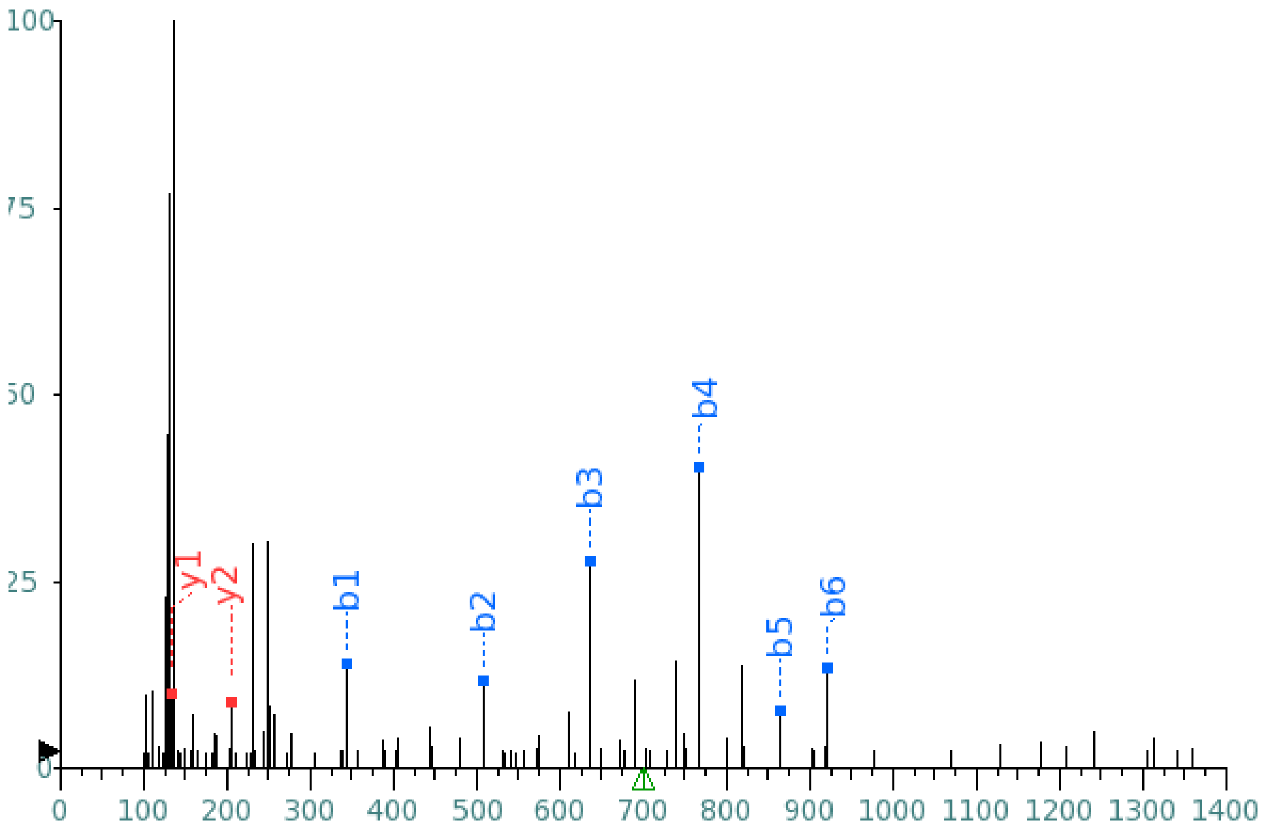

DYEEVGADSAD (+2)

Primary Reference: TBA1C_HUMAN
Search ID: 41761
Search Name: 20130328_ananiav_ERstress_TMT_TM_timecourse_trypsin_inj2_4mc_iaa
Scan#: 7166
Observed Mass: 700.3078 (1.5 ppm)
PSM Score: 20.55

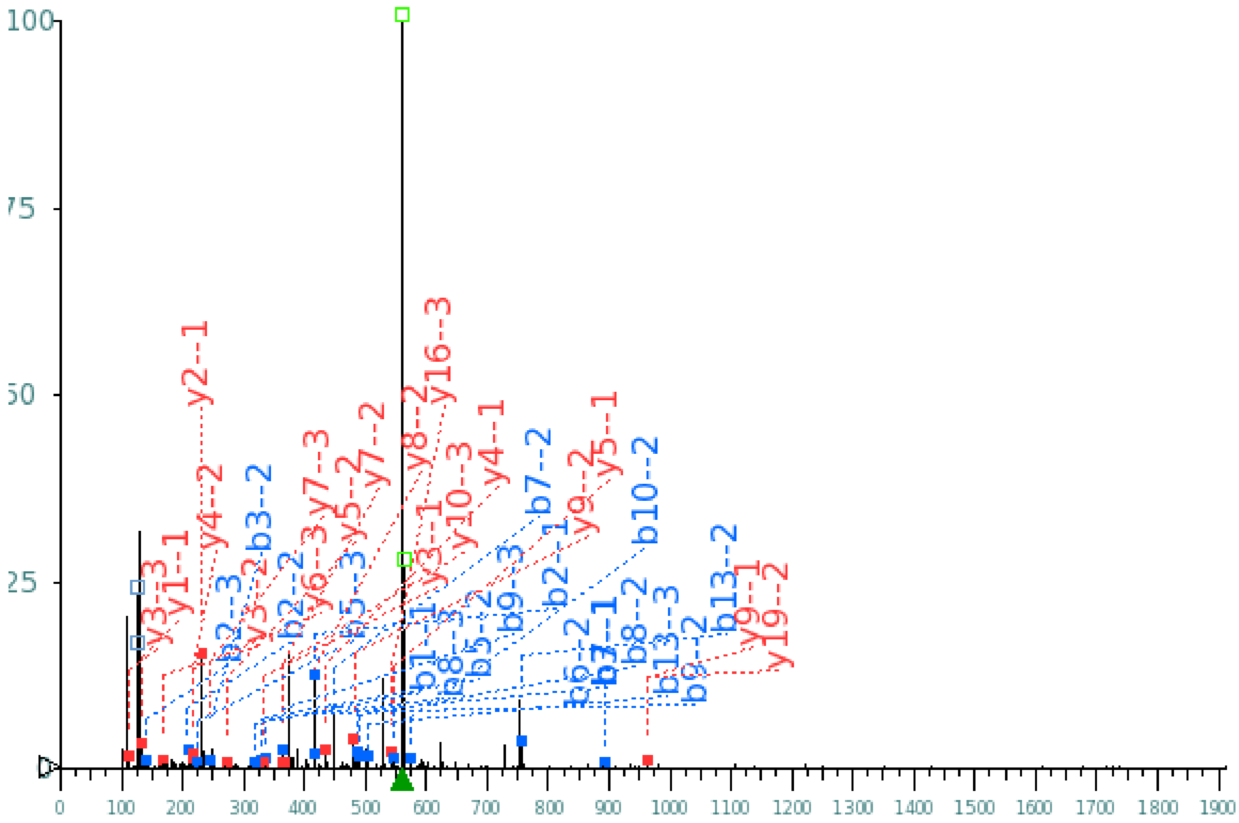

SVAQGYGSLGMMTSVLVCPD (+4)

Primary Reference: IDHC_HUMAN
Search ID: 41761
Search Name: 20130328_ananiav_ERstress_TMT_TM_timecourse_trypsin_inj2_4mc_iaa
Scan#: 7208
Observed Mass: 561.782 (6.6 ppm)
PSM Score: 17.98

File name: 2015-02-04 04.01.53PM.pdf

Creation time: 2015-02-04 04:09PM

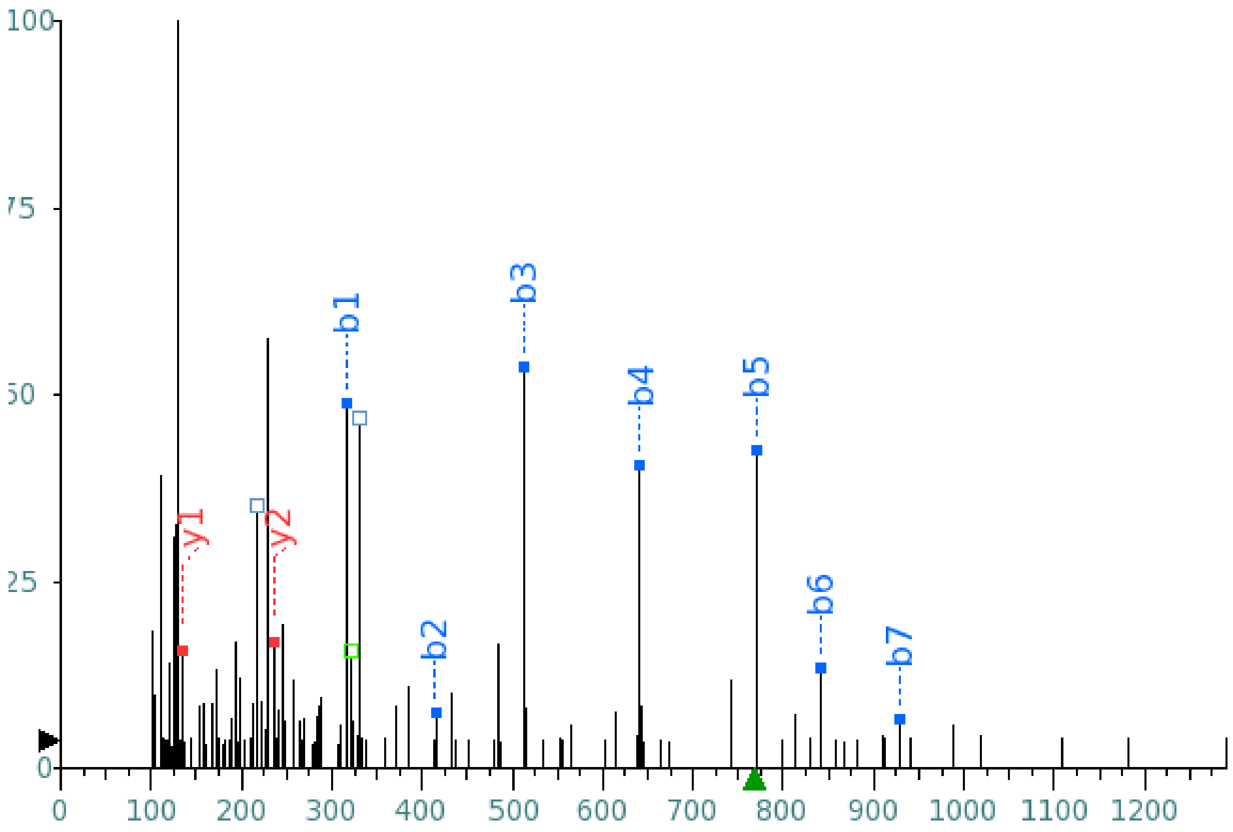

SPVQEASSATDTD (+2)

Primary Reference: F91A1_HUMAN
Search ID: 41761
Search Name: 20130328_ananiav_ERstress_TMT_TM_timecourse_trypsin_inj2_4mc_iaa
Scan#: 7382
Observed Mass: 768.8656 (0.8 ppm)
PSM Score: 18.72

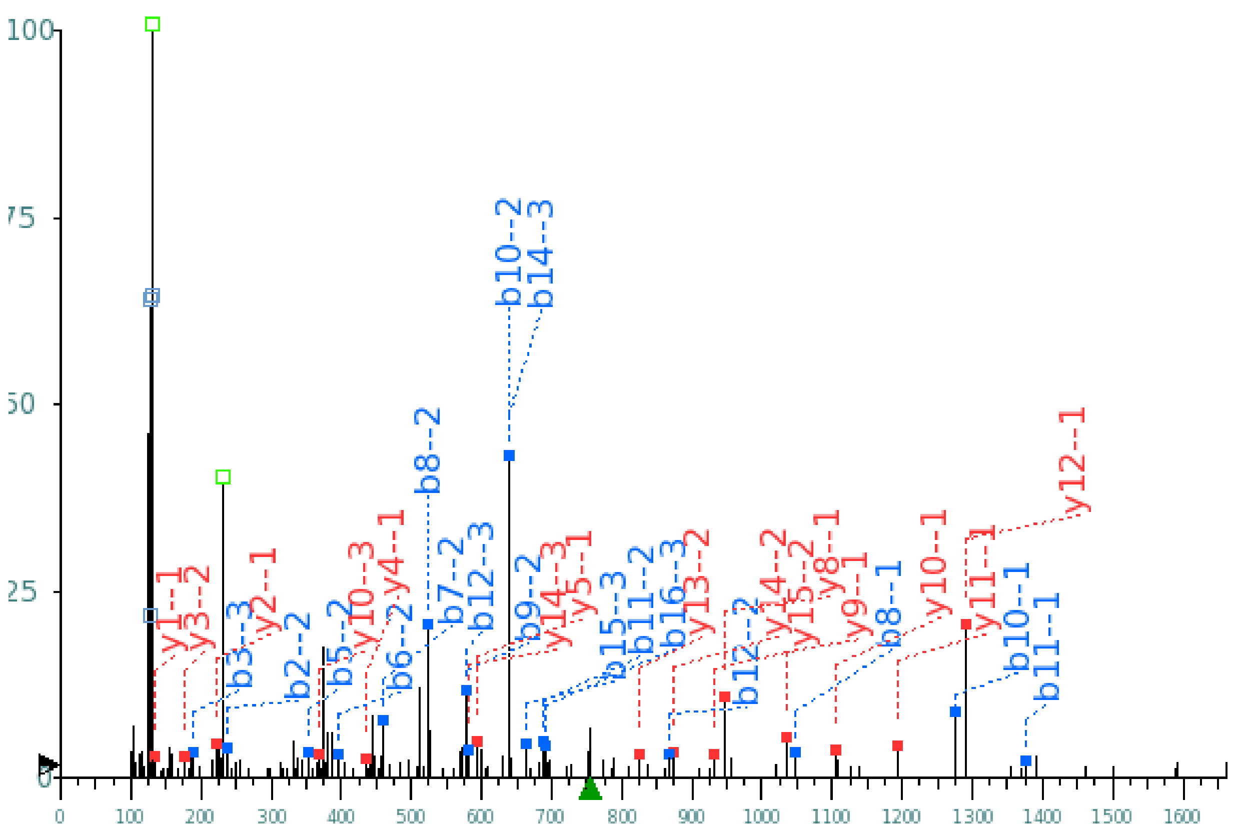

SRSGSSQELDVKPSASPQERSESD (+4)

Primary Reference: SRRM2_HUMAN
Search ID: 41761
Search Name: 20130328_ananiav_ERstress_TMT_TM_timecourse_trypsin_inj2_4mc_iaa
Scan#: 7399
Observed Mass: 756.1332 (-0.6 ppm)
PSM Score: 22.63

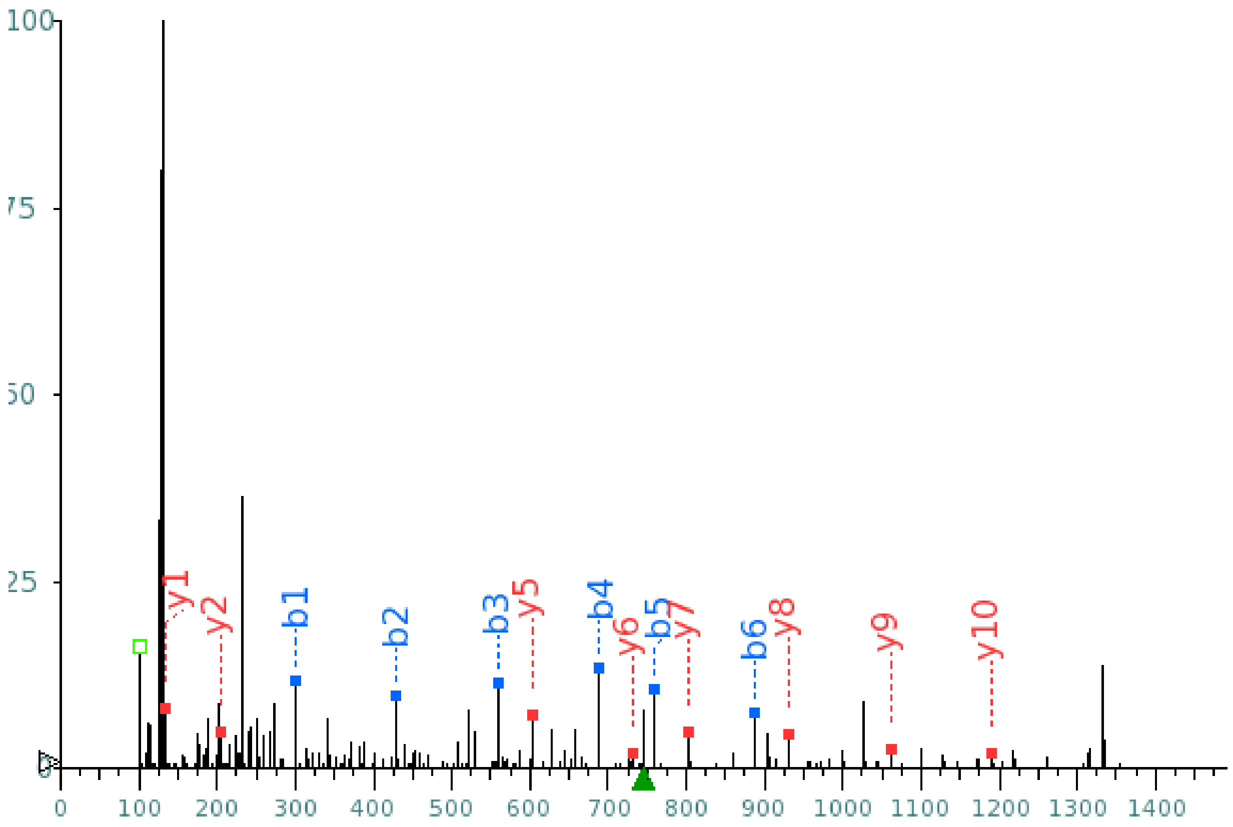

AQEEAERLEAD (+2)

Primary Reference: EZRI_HUMAN
Search ID: 41761
Search Name: 20130328_ananiav_ERstress_TMT_TM_timecourse_trypsin_inj2_4mc_iaa
Scan#: 7522
Observed Mass: 745.3703 (0.1 ppm)
PSM Score: 28.63

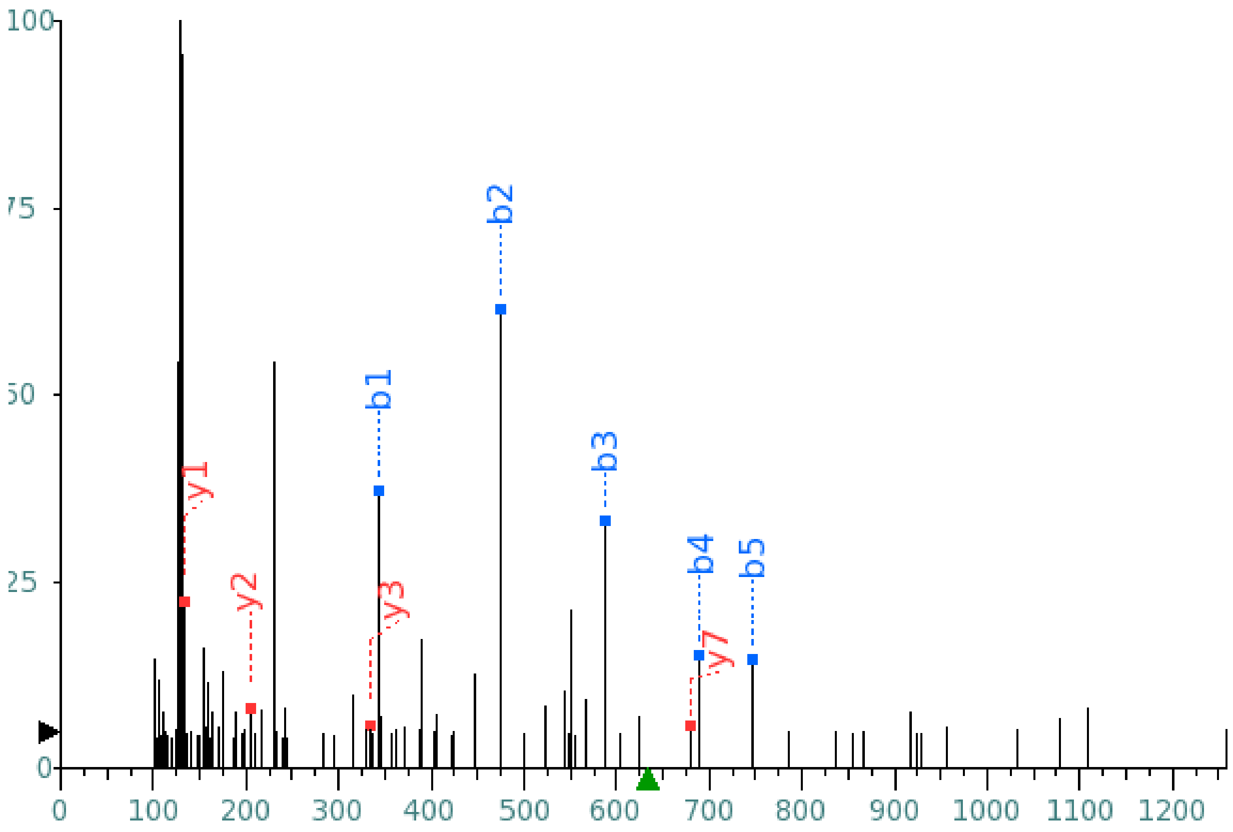

NMITGTSQAD (+2)

Primary Reference: EF1A1_HUMAN
Search ID: 41761
Search Name: 20130328_ananiav_ERstress_TMT_TM_timecourse_trypsin_inj2_4mc_iaa
Scan#: 7527
Observed Mass: 633.8146 (1.8 ppm)
PSM Score: 27.18

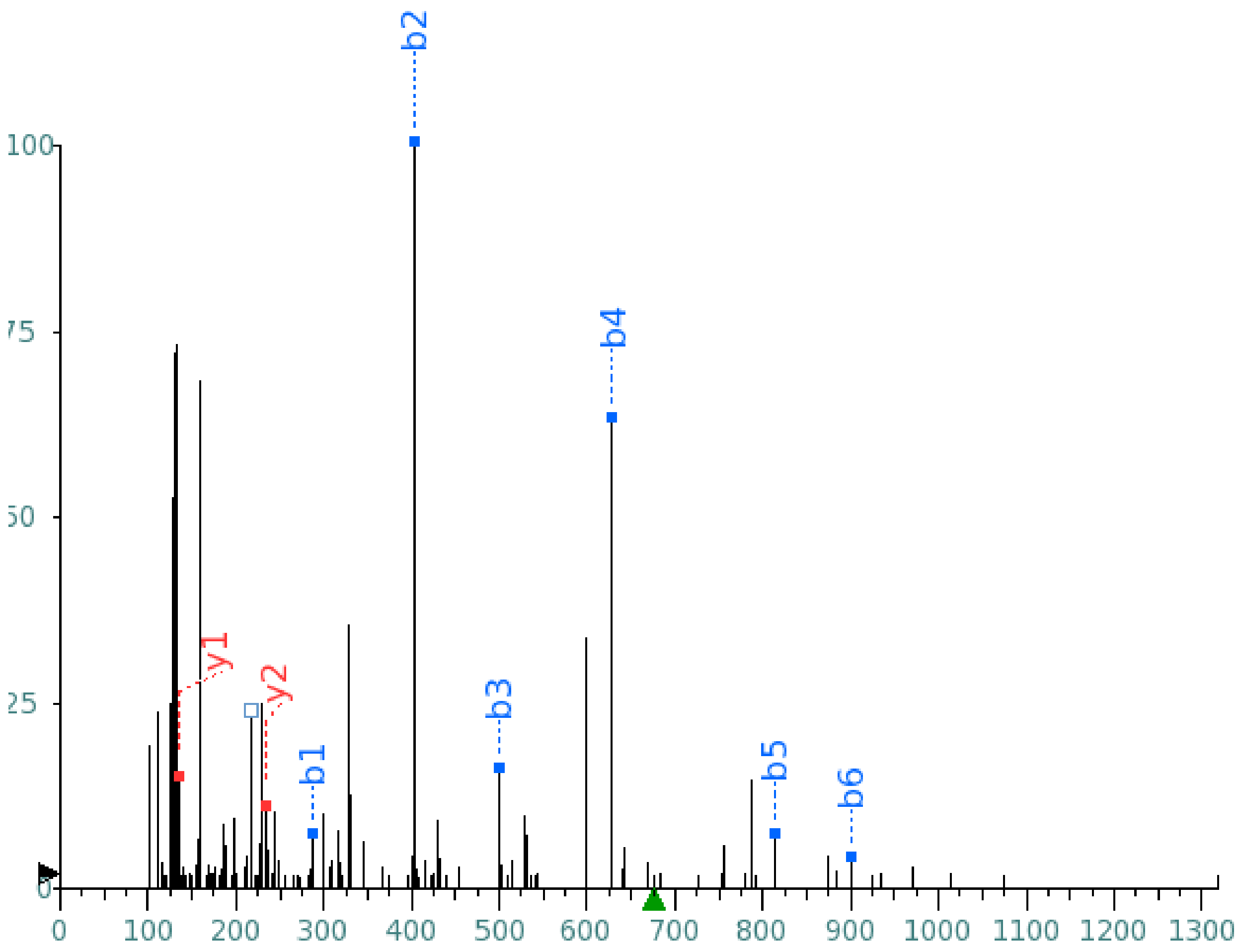

GDPEWSSETD (+2)

Primary Reference: T179B_HUMAN
Search ID: 41761
Search Name: 20130328_ananiav_ERstress_TMT_TM_timecourse_trypsin_inj2_4mc_iaa
Scan#: 7617
Observed Mass: 676.2973 (1.6 ppm)
PSM Score: 9.13

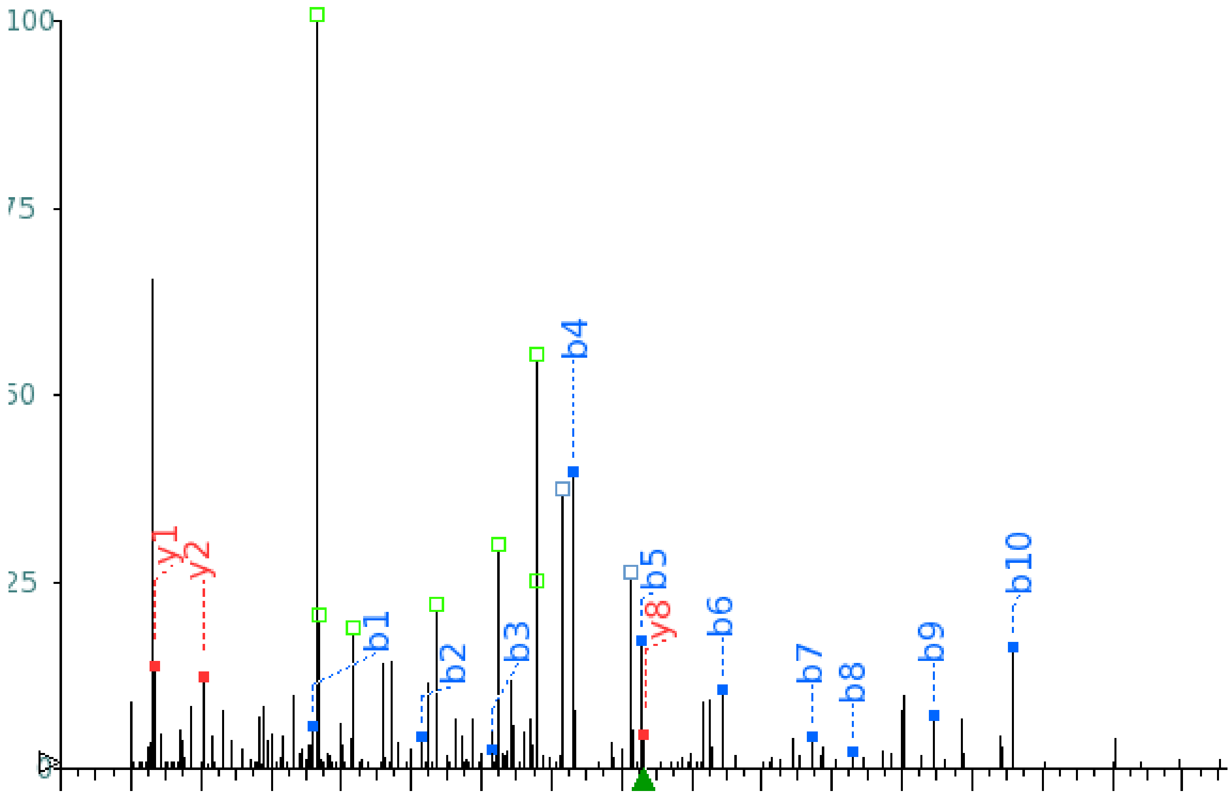

ERTDVIQGDDVAD (+2)

Primary Reference: RBP2_HUMAN
Search ID: 41761
Search Name: 20130328_ananiav_ERstress_TMT_TM_timecourse_trypsin_inj2_4mc_iaa
Scan#: 7747
Observed Mass: 831.413 (0.5 ppm)
PSM Score: 24.8

File name: 2015-02-04 04.01.53PM.pdf

Creation time: 2015-02-04 04:09PM

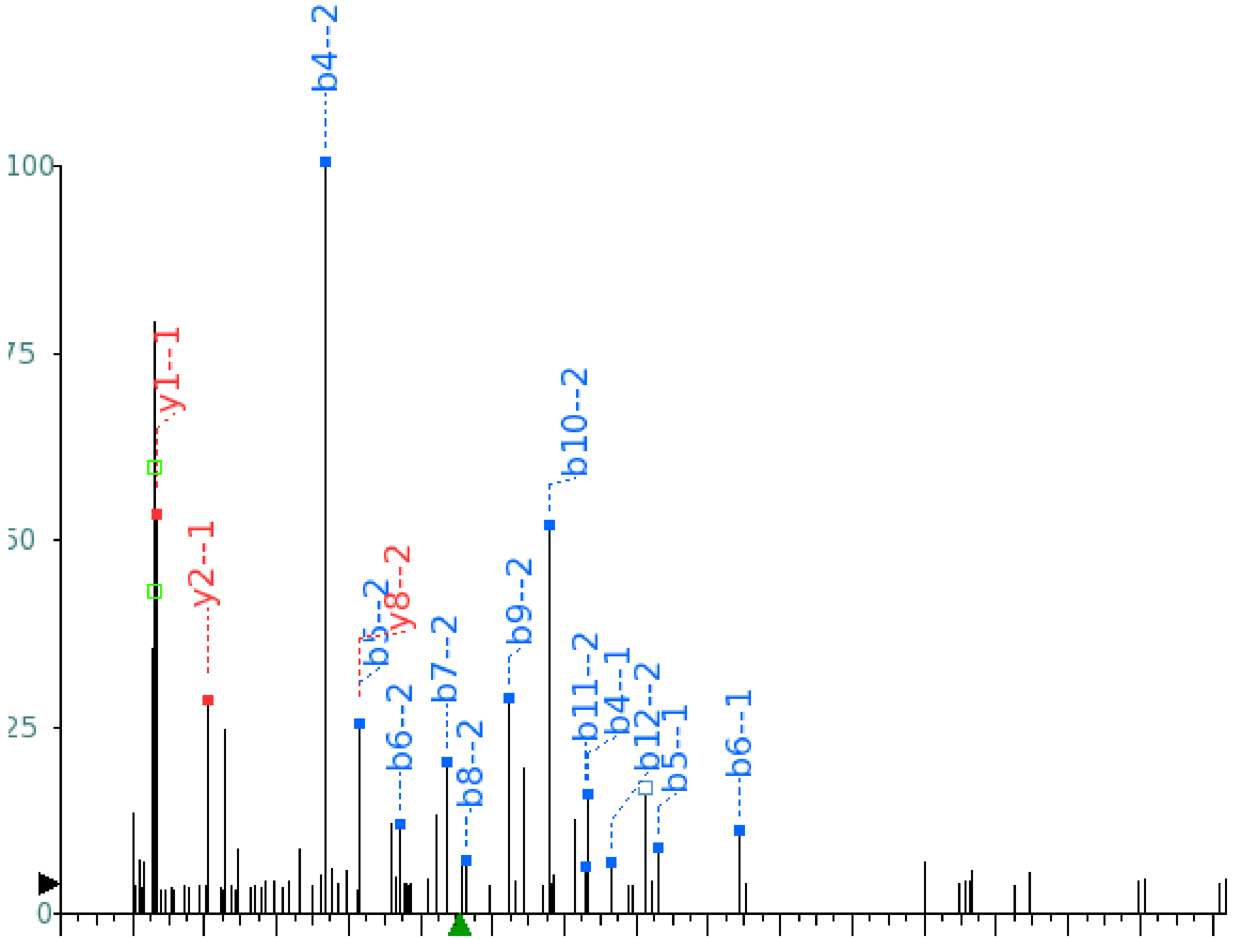

ERTDVIQGDDVAD (+3)

Primary Reference: RBP2_HUMAN
Search ID: 41761
Search Name: 20130328_ananiav_ERstress_TMT_TM_timecourse_trypsin_inj2_4mc_iaa
Scan#: 7769
Observed Mass: 554.6112 (0.6 ppm)
PSM Score: 36.64

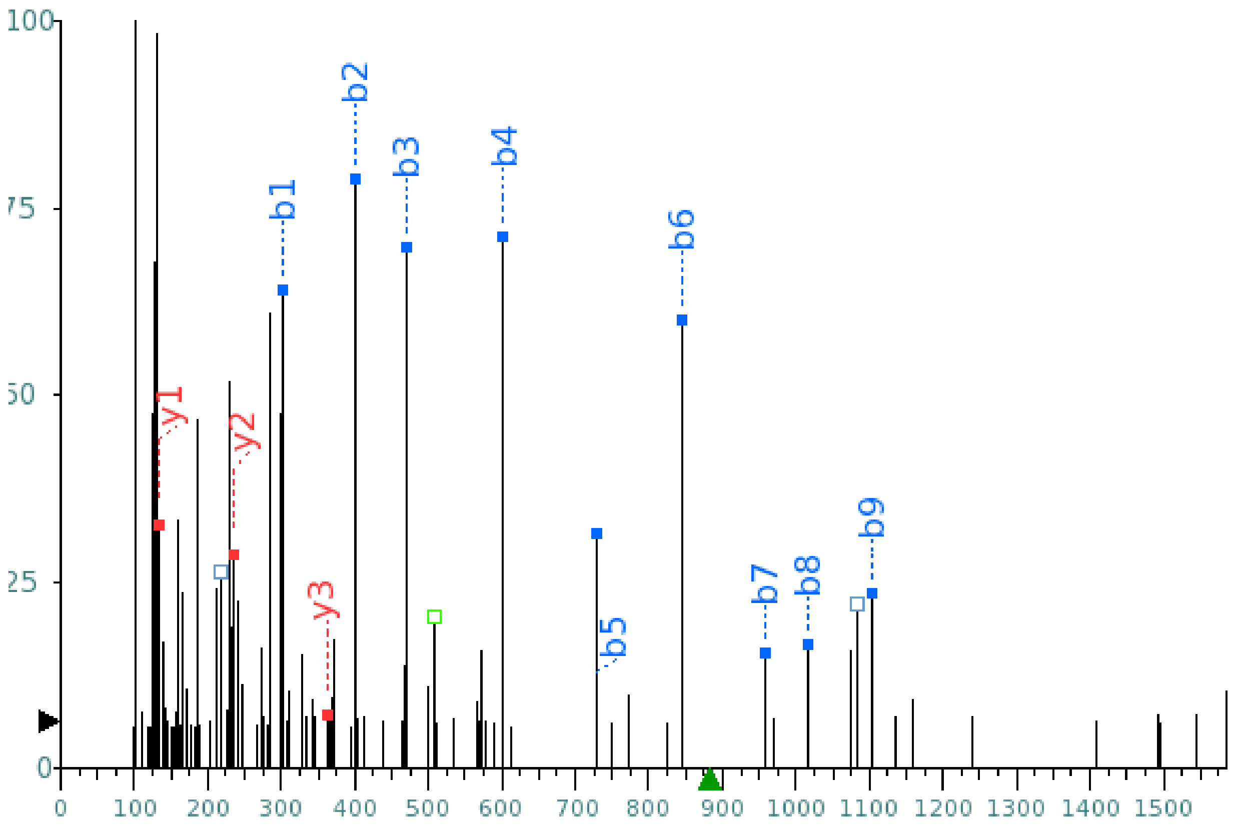

AVAEEDNGSIGEETD (+2)

Primary Reference: STIM1_HUMAN
Search ID: 41761
Search Name: 20130328_ananiav_ERstress_TMT_TM_timecourse_trypsin_inj2_4mc_iaa
Scan#: 7771
Observed Mass: 882.9028 (0.6 ppm)
PSM Score: 40.83

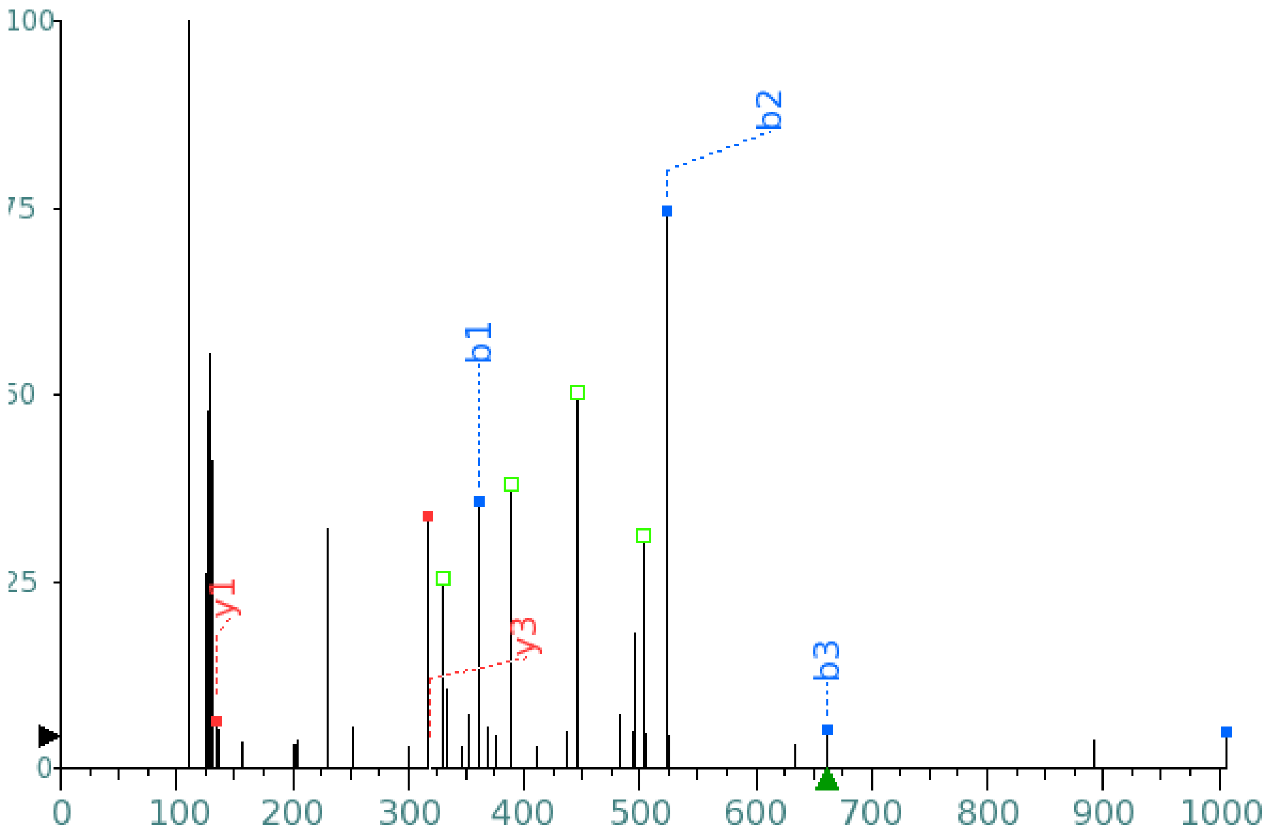

MYHDDDLAD (+2)

Primary Reference: MAP4_HUMAN
Search ID: 41761
Search Name: 20130328_ananiav_ERstress_TMT_TM_timecourse_trypsin_inj2_4mc_iaa
Scan#: 7787
Observed Mass: 662.2904 (0.8 ppm)
PSM Score: 27.28

File name: 2015-02-04 04.01.53PM.pdf

Creation time: 2015-02-04 04:09PM

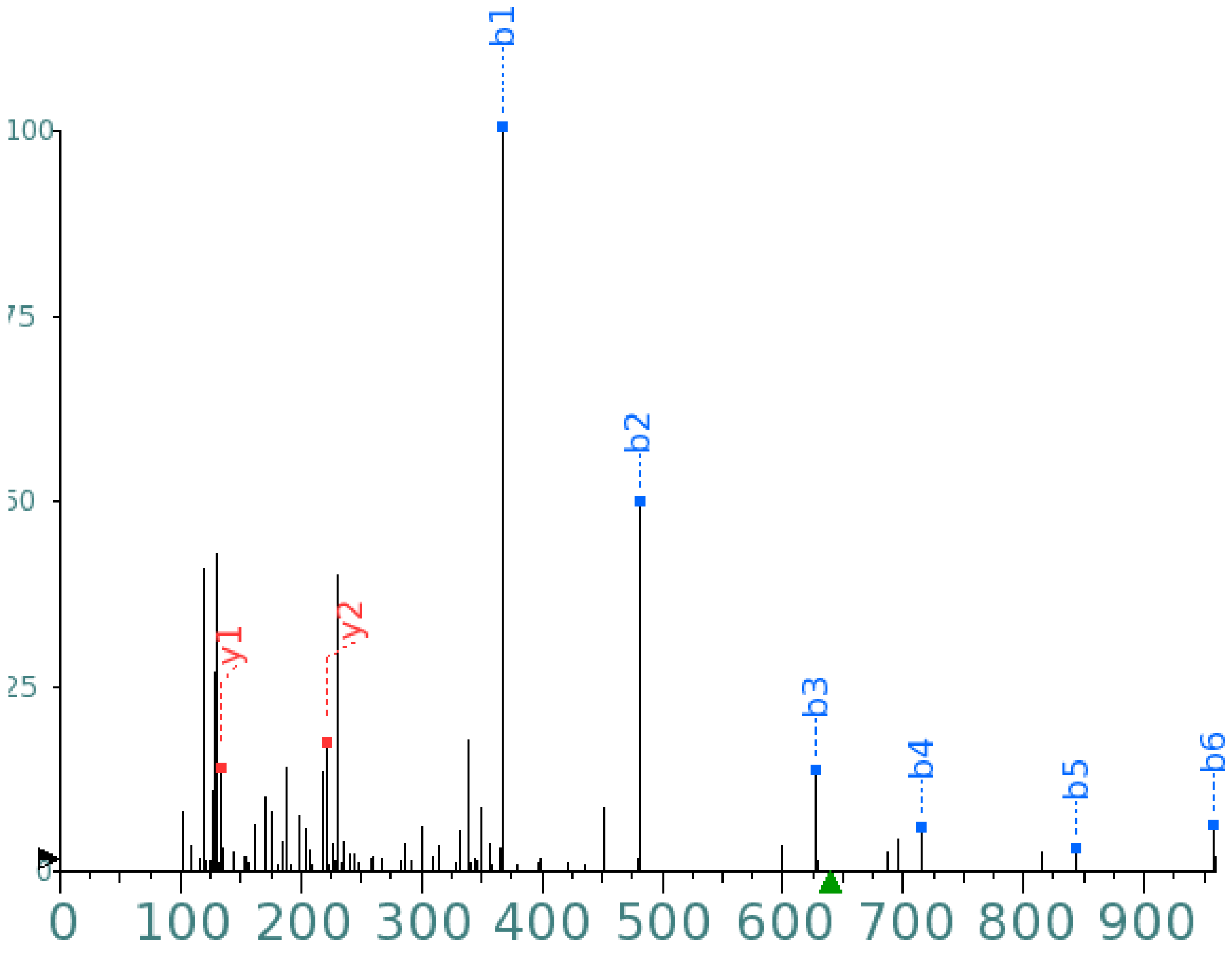

HIFSEDTSD (+2)

Primary Reference: SPB5_HUMAN
Search ID: 41761
Search Name: 20130328_ananiav_ERstress_TMT_TM_timecourse_trypsin_inj2_4mc_iaa
Scan#: 7809
Observed Mass: 640.3049 (1.7 ppm)
PSM Score: 29.42

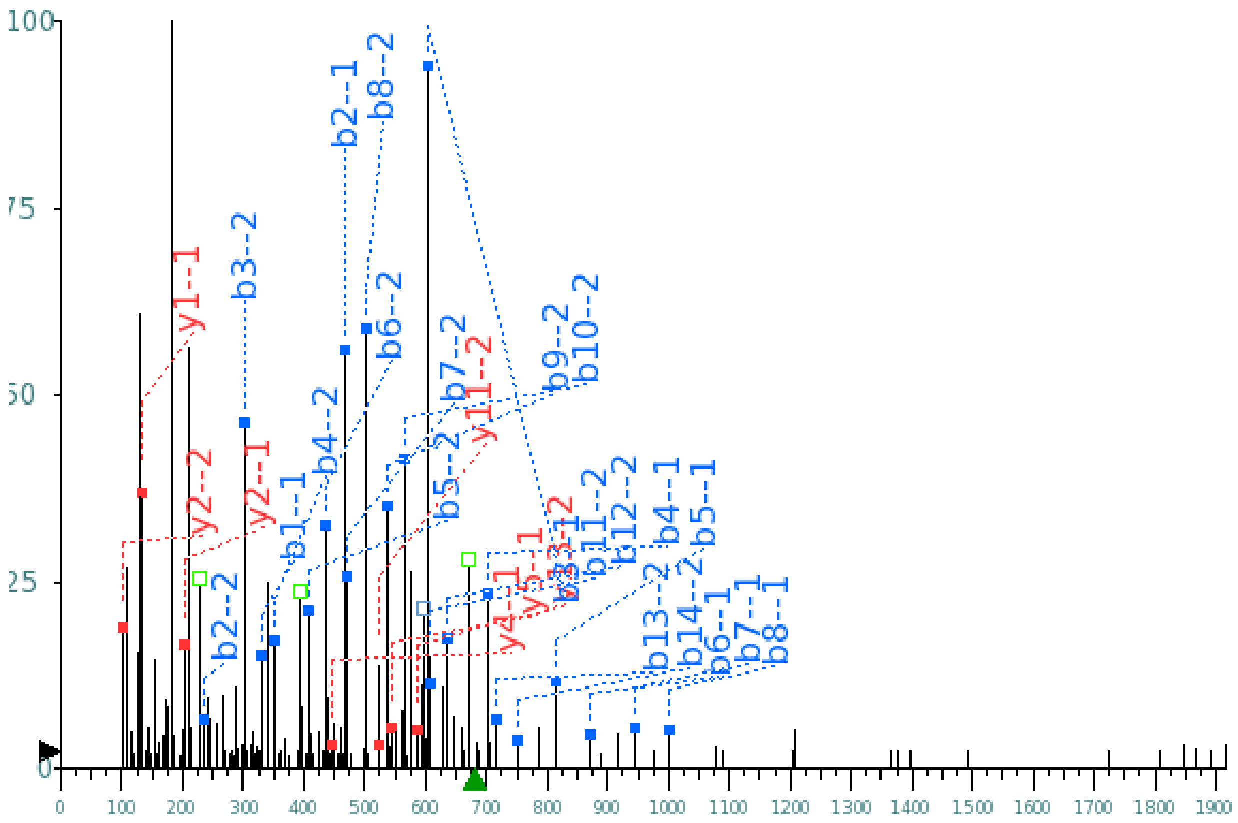

THHPLGAGAGSGC*APLEAD (+3)

Primary Reference: SC16A_HUMAN
Search ID: 41761
Search Name: 20130328_ananiav_ERstress_TMT_TM_timecourse_trypsin_inj2_4mc_iaa
Scan#: 7835
Observed Mass: 683.0014 (1.5 ppm)
PSM Score: 55.94

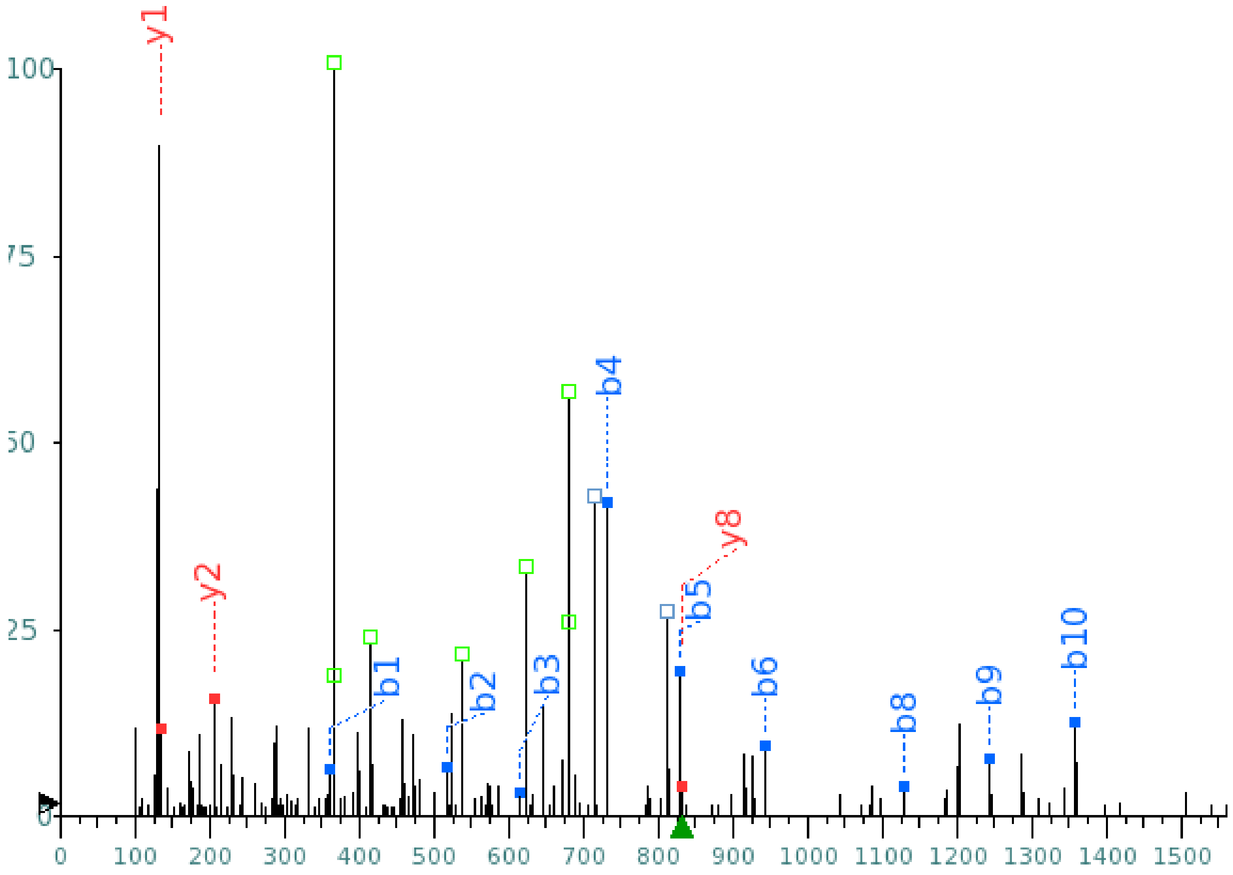

ERTDVIQGDDVAD (+2)

Primary Reference: RBP2_HUMAN
Search ID: 41761
Search Name: 20130328_ananiav_ERstress_TMT_TM_timecourse_trypsin_inj2_4mc_iaa
Scan#: 7841
Observed Mass: 831.4136 (1.1 ppm)
PSM Score: 23.32

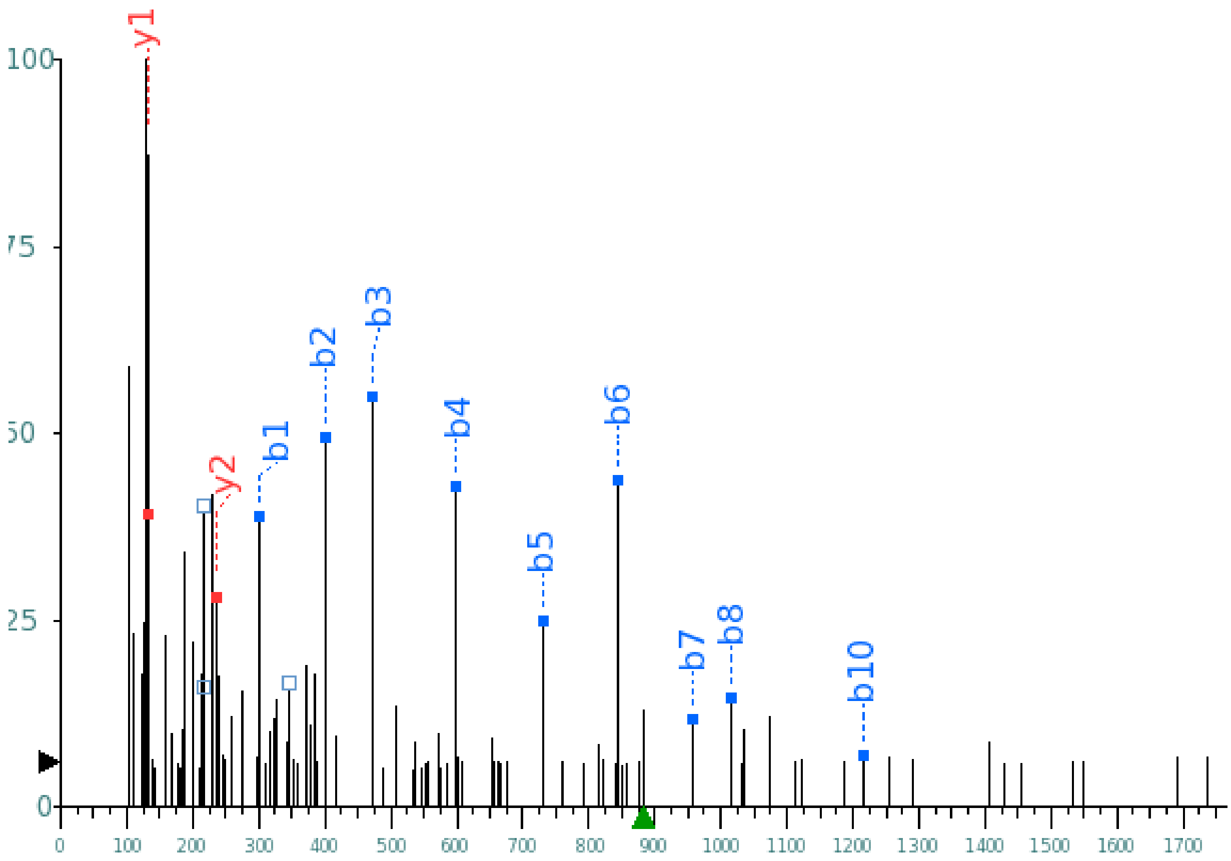

AVAEEDNGSIGEETD (+2)

Primary Reference: STIM1_HUMAN
Search ID: 41761
Search Name: 20130328_ananiav_ERstress_TMT_TM_timecourse_trypsin_inj2_4mc_iaa
Scan#: 7858
Observed Mass: 882.9036 (1.4 ppm)
PSM Score: 35.73

File name: 2015-02-04 04.01.53PM.pdf

Creation time: 2015-02-04 04:09PM

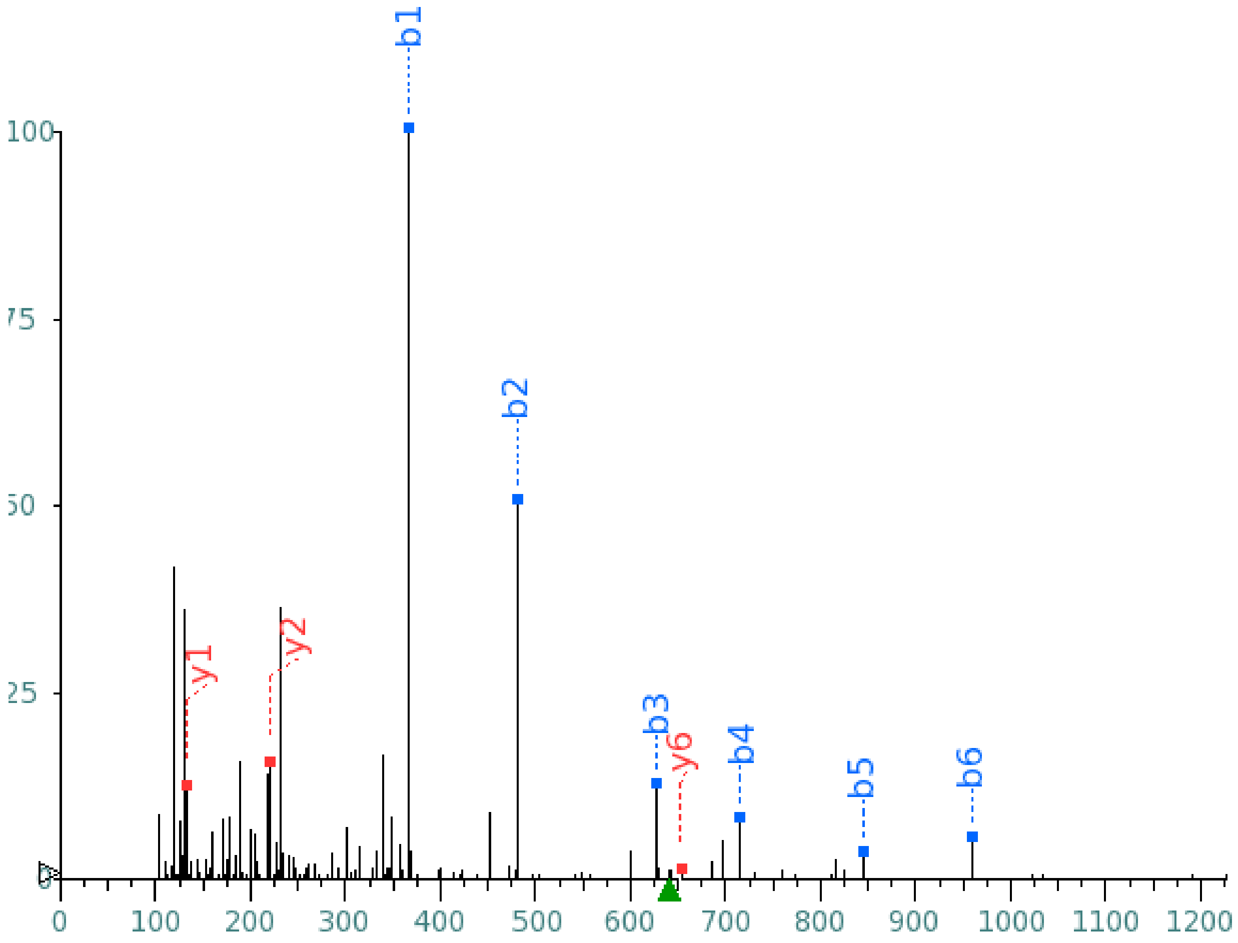

HIFSEDTSD (+2)

Primary Reference: SPB5_HUMAN
Search ID: 41761
Search Name: 20130328_ananiav_ERstress_TMT_TM_timecourse_trypsin_inj2_4mc_iaa
Scan#: 7890
Observed Mass: 640.3047 (1.4 ppm)
PSM Score: 35.35

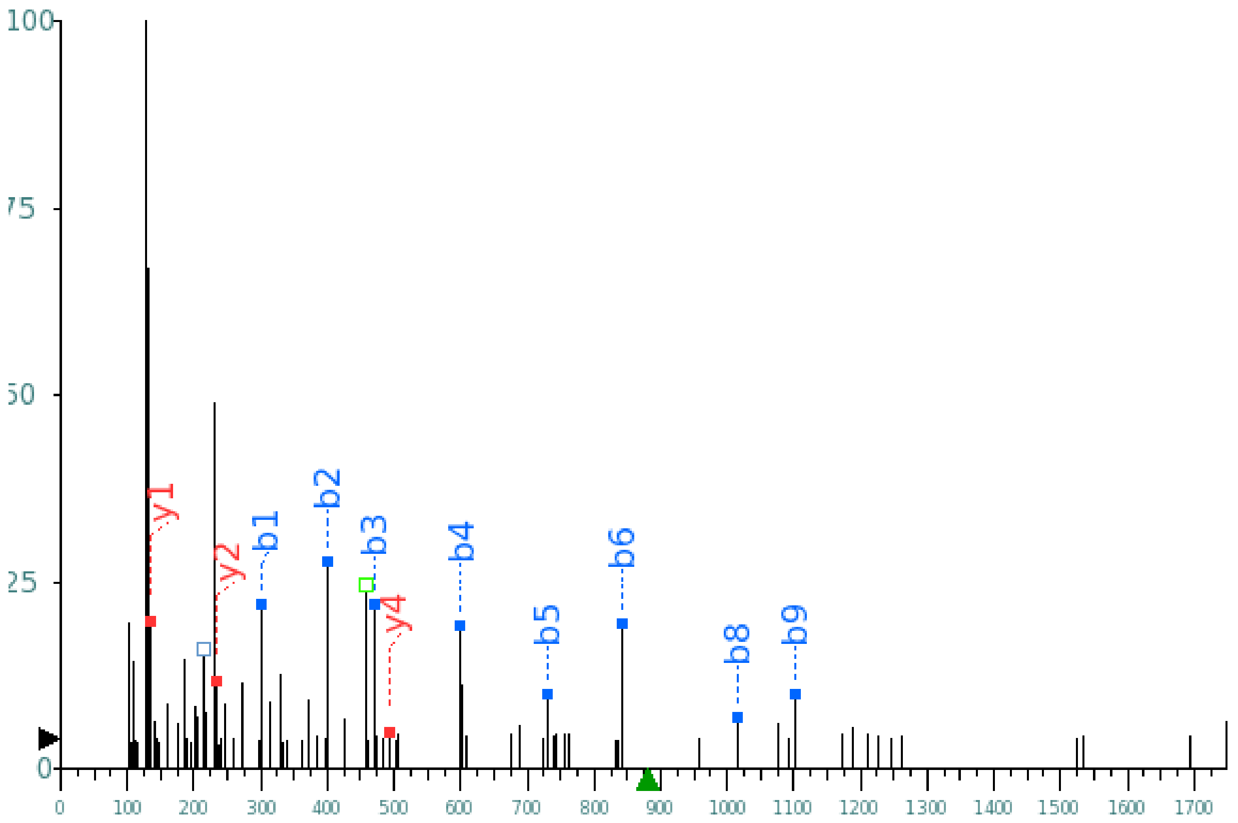

AVAEEDNGSIGEETD (+2)

Primary Reference: STIM1_HUMAN
Search ID: 41761
Search Name: 20130328_ananiav_ERstress_TMT_TM_timecourse_trypsin_inj2_4mc_iaa
Scan#: 7962
Observed Mass: 882.9024 (0.1 ppm)
PSM Score: 26.71

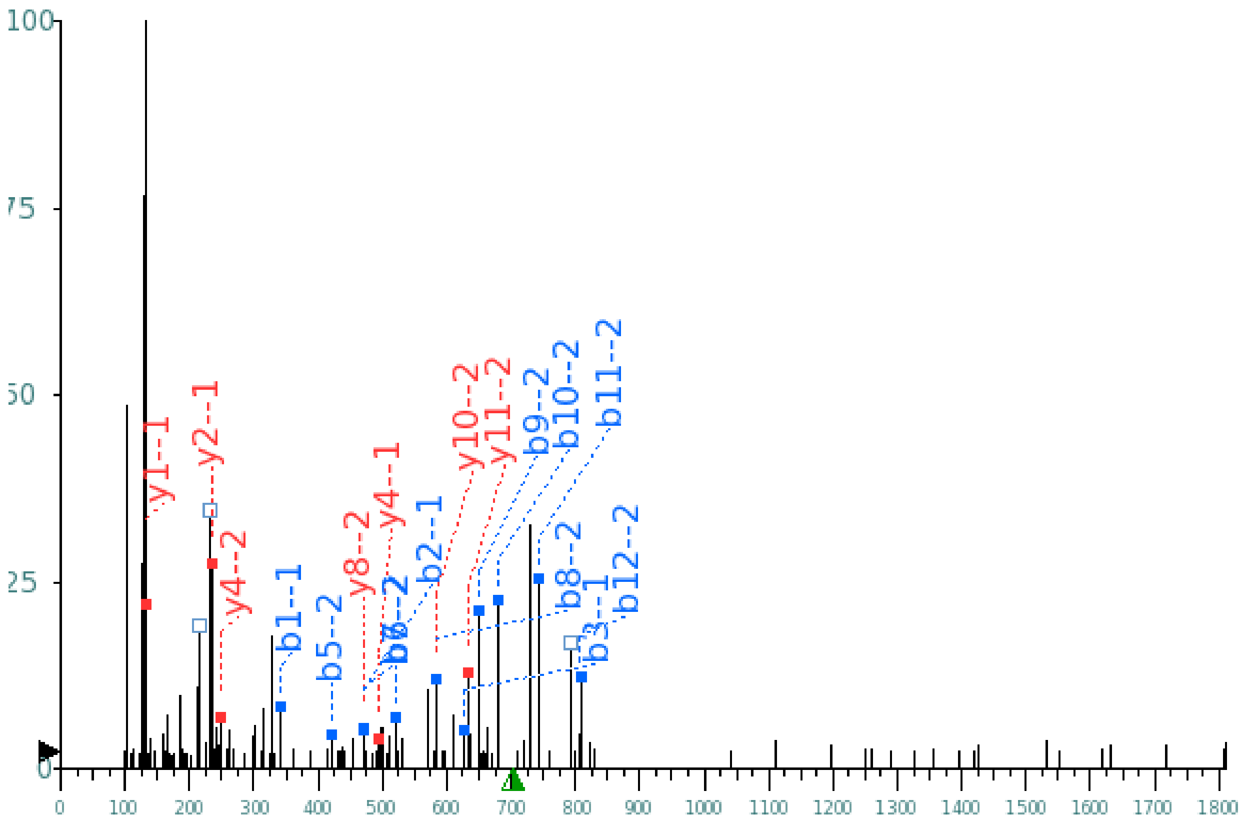

IQRPNTTQEGEEMETD (+3)

Primary Reference: HUWE1_HUMAN
Search ID: 41761
Search Name: 20130328_ananiav_ERstress_TMT_TM_timecourse_trypsin_inj2_4mc_iaa
Scan#: 7984
Observed Mass: 702.9986 (0 ppm)
PSM Score: 18.74

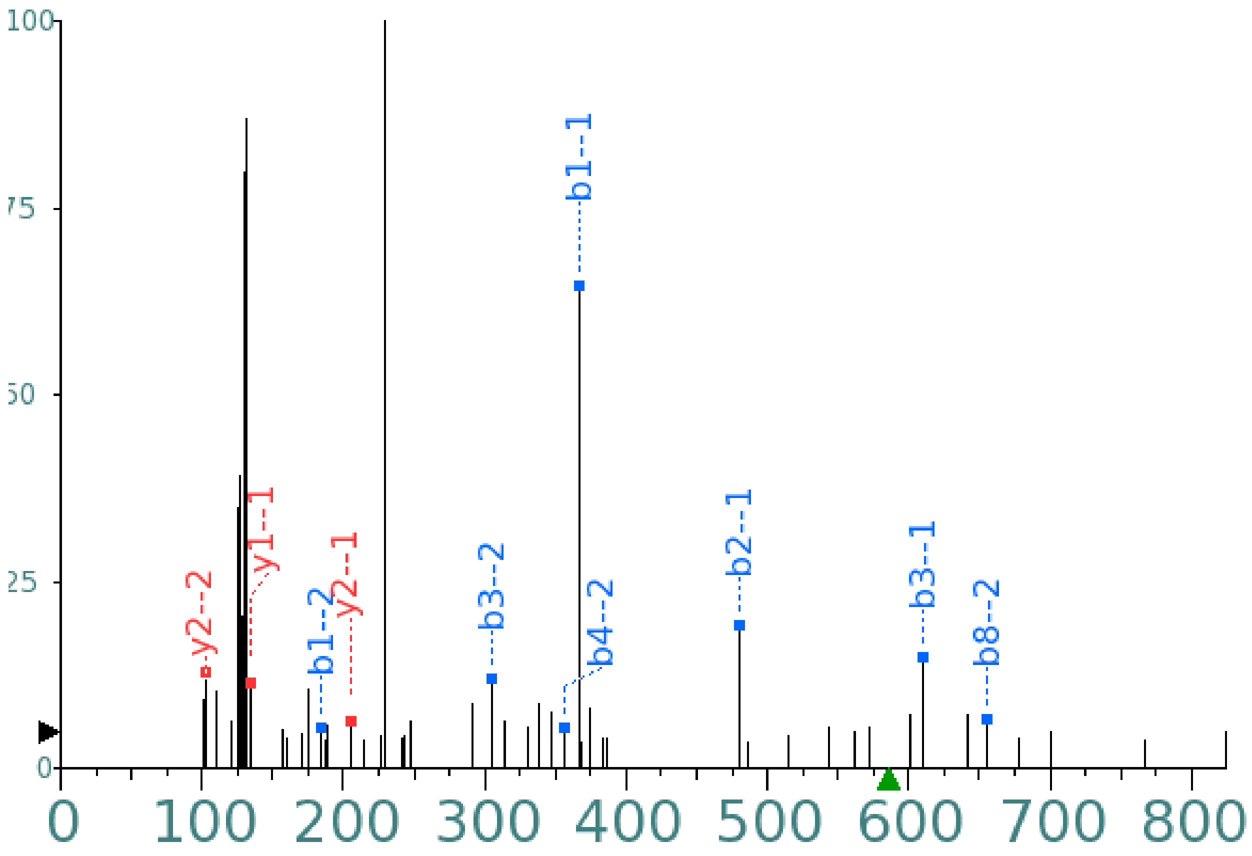

HLETGGQKIEAD (+3)

Primary Reference: GBF1_HUMAN
Search ID: 41761
Search Name: 20130328_ananiav_ERstress_TMT_TM_timecourse_trypsin_inj2_4mc_iaa
Scan#: 7985
Observed Mass: 585.9926 (-0.4 ppm)
PSM Score: 14.57

File name: 2015-02-04 04.01.53PM.pdf

Creation time: 2015-02-04 04:09PM

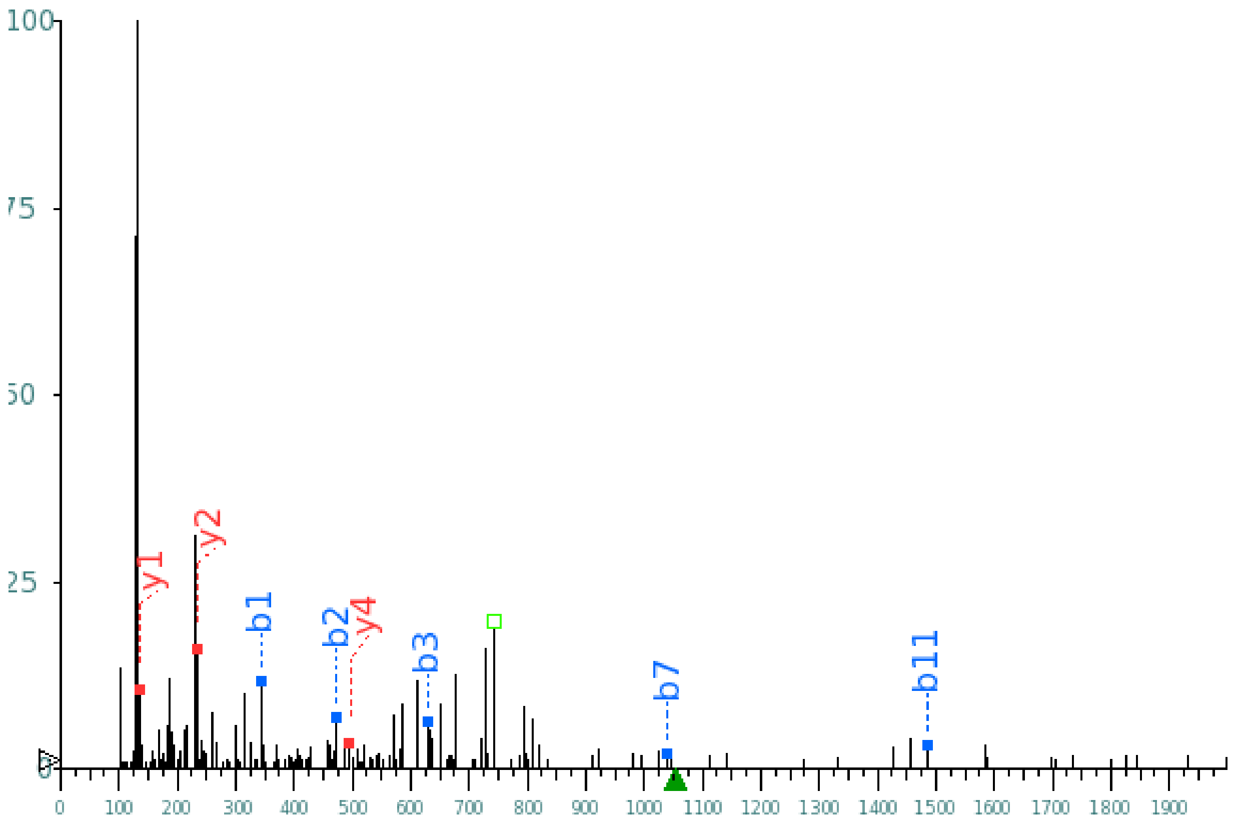

IQRPNTTQEGEEMETD (+2)

Primary Reference: HUWE1_HUMAN
Search ID: 41761
Search Name: 20130328_ananiav_ERstress_TMT_TM_timecourse_trypsin_inj2_4mc_iaa
Scan#: 8003
Observed Mass: 1053.9939 (-0.3 ppm)
PSM Score: 11.07

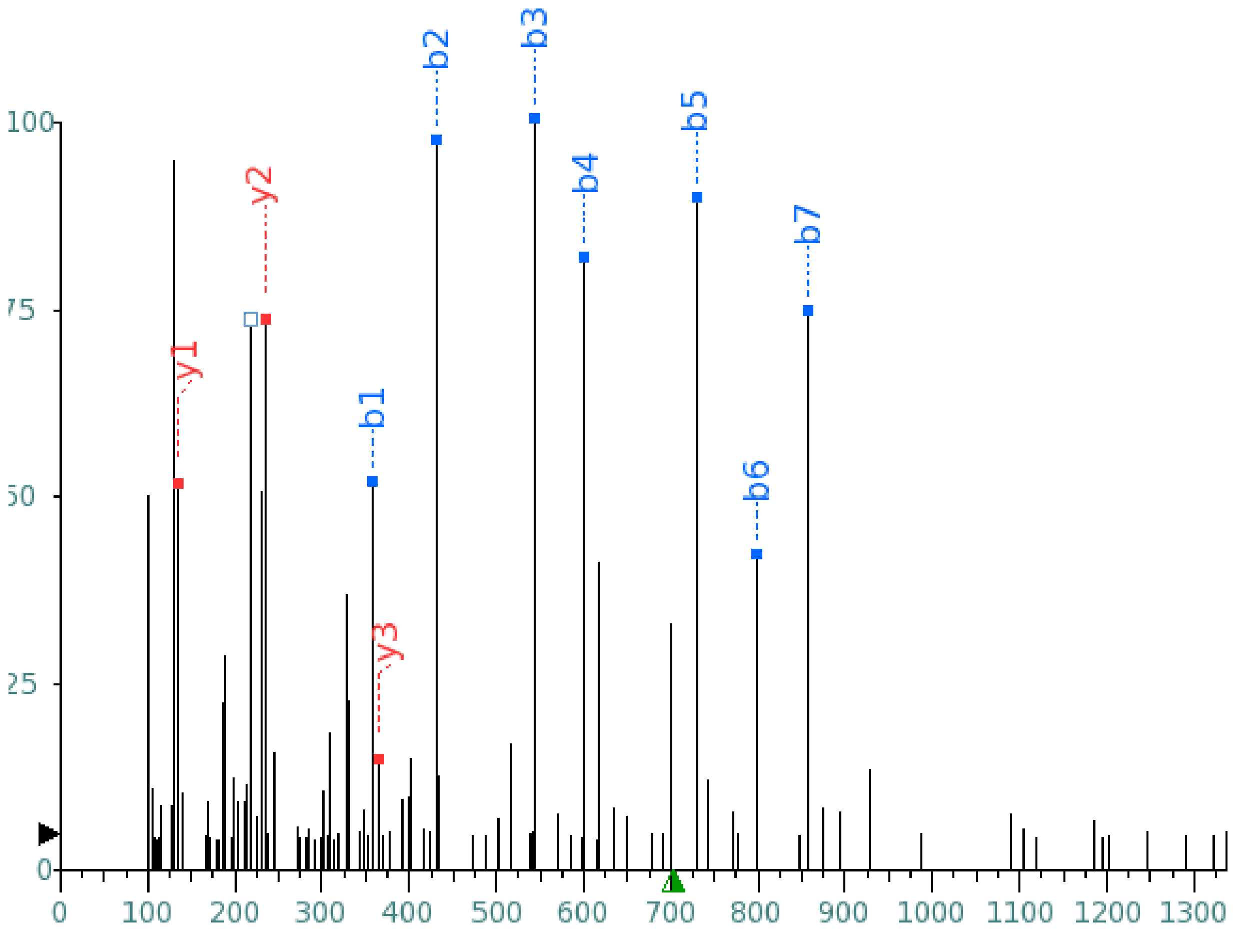

EALGQAGVSETD (+2)

Primary Reference: LIPA1_HUMAN
Search ID: 41761
Search Name: 20130328_ananiav_ERstress_TMT_TM_timecourse_trypsin_inj2_4mc_iaa
Scan#: 8044
Observed Mass: 703.3541 (0.1 ppm)
PSM Score: 26.76

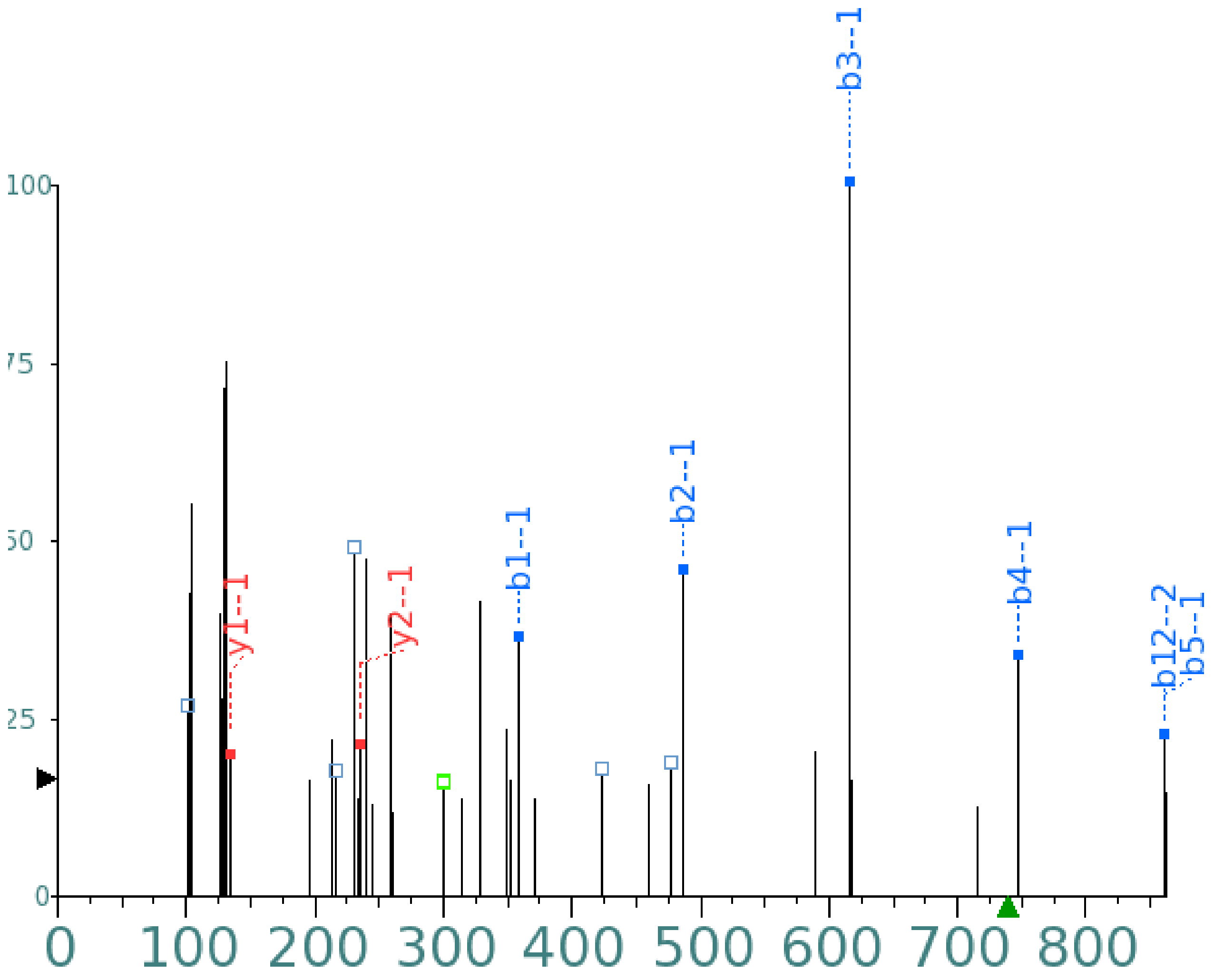

QEEMNSQQEEEEEMETD (+3)

Primary Reference: SF3B2_HUMAN
Search ID: 41761
Search Name: 20130328_ananiav_ERstress_TMT_TM_timecourse_trypsin_inj2_4mc_iaa
Scan#: 8106
Observed Mass: 738.9672 (0.9 ppm)
PSM Score: 26.04

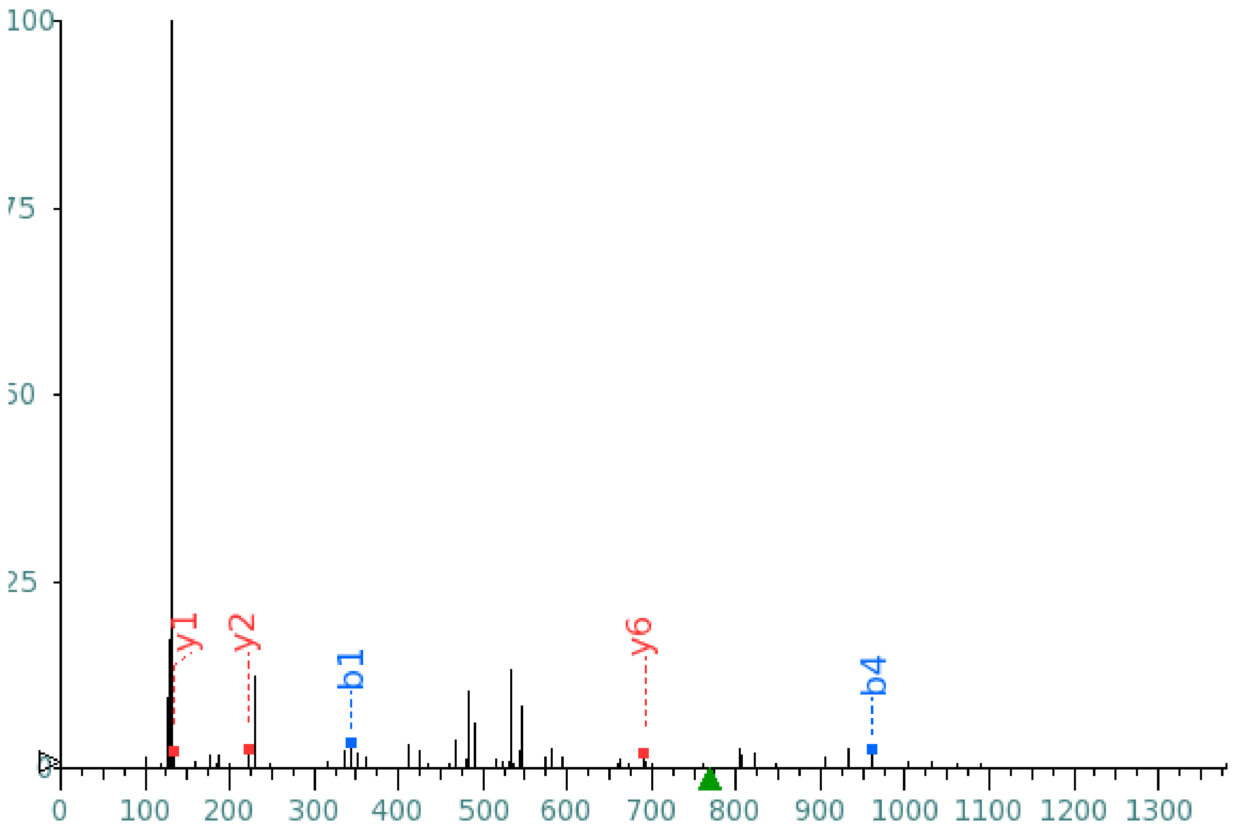

NKFNQVESD (+2)

Primary Reference: GALT2_HUMAN
Search ID: 41761
Search Name: 20130328_ananiav_ERstress_TMT_TM_timecourse_trypsin_inj2_4mc_iaa
Scan#: 8128
Observed Mass: 769.915 (0.9 ppm)
PSM Score: 11.64

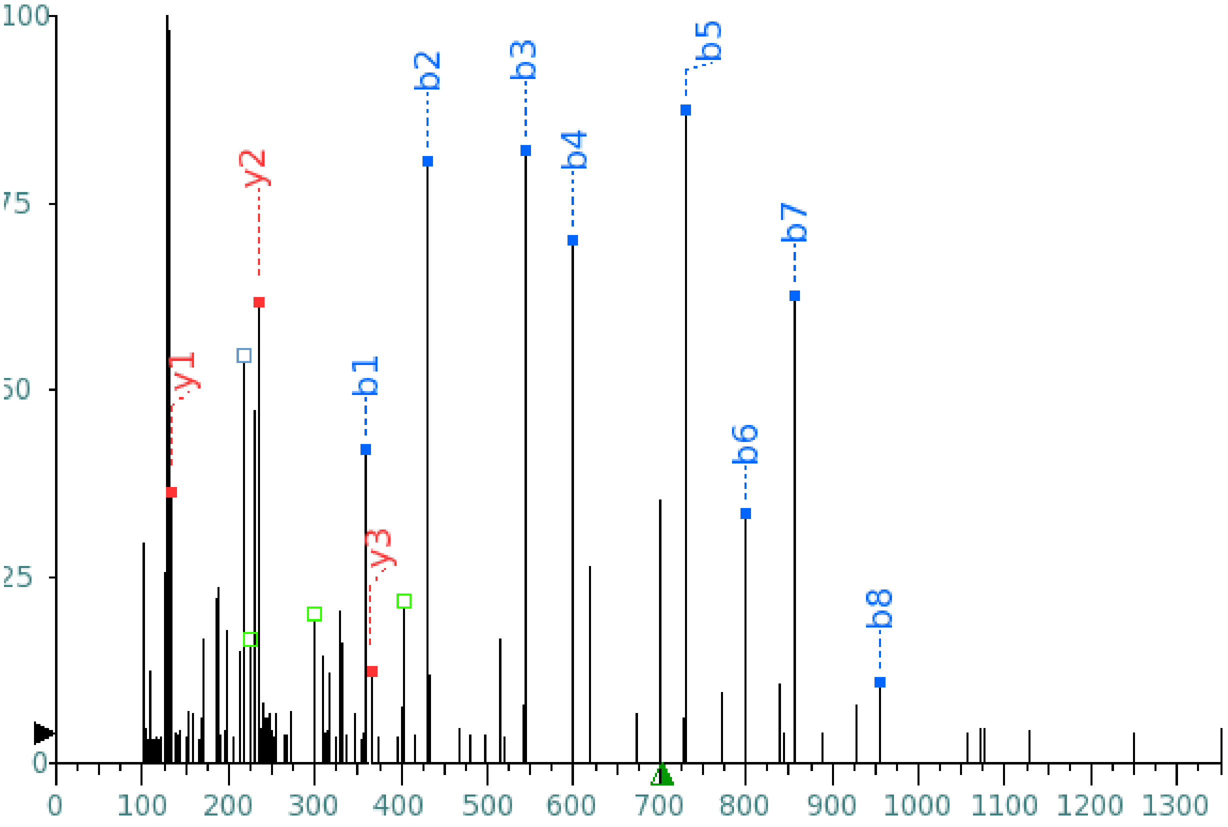

EALGQAGVSETD (+2)

Primary Reference: LIPA1_HUMAN
Search ID: 41761
Search Name: 20130328_ananiav_ERstress_TMT_TM_timecourse_trypsin_inj2_4mc_iaa
Scan#: 8132
Observed Mass: 703.3549 (1.2 ppm)
PSM Score: 42.47

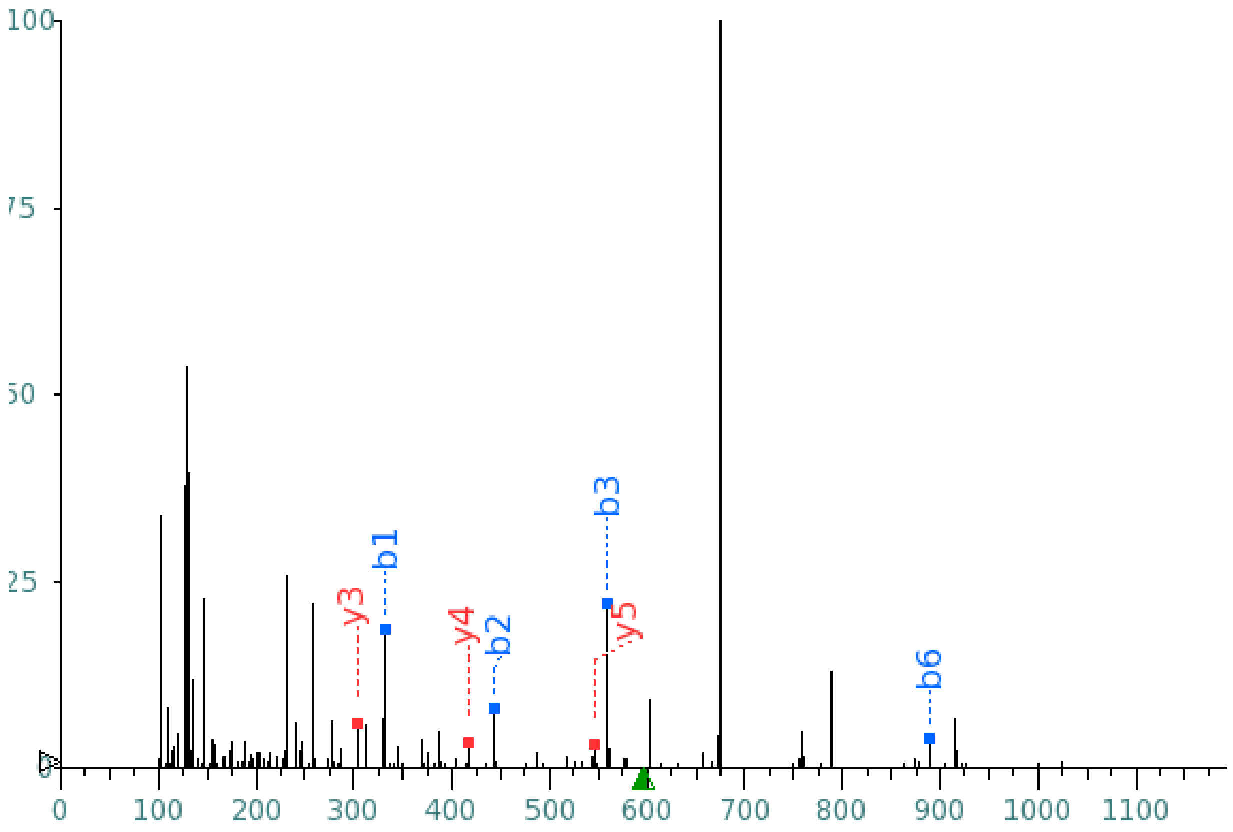

TLDSELLGD (+2)

Primary Reference: ##FXRD2_HUMAN
Search ID: 41761
Search Name: 20130328_ananiav_ERstress_TMT_TM_timecourse_trypsin_inj2_4mc_iaa
Scan#: 8144
Observed Mass: 596.3195 (0.9 ppm)
PSM Score: 10.23

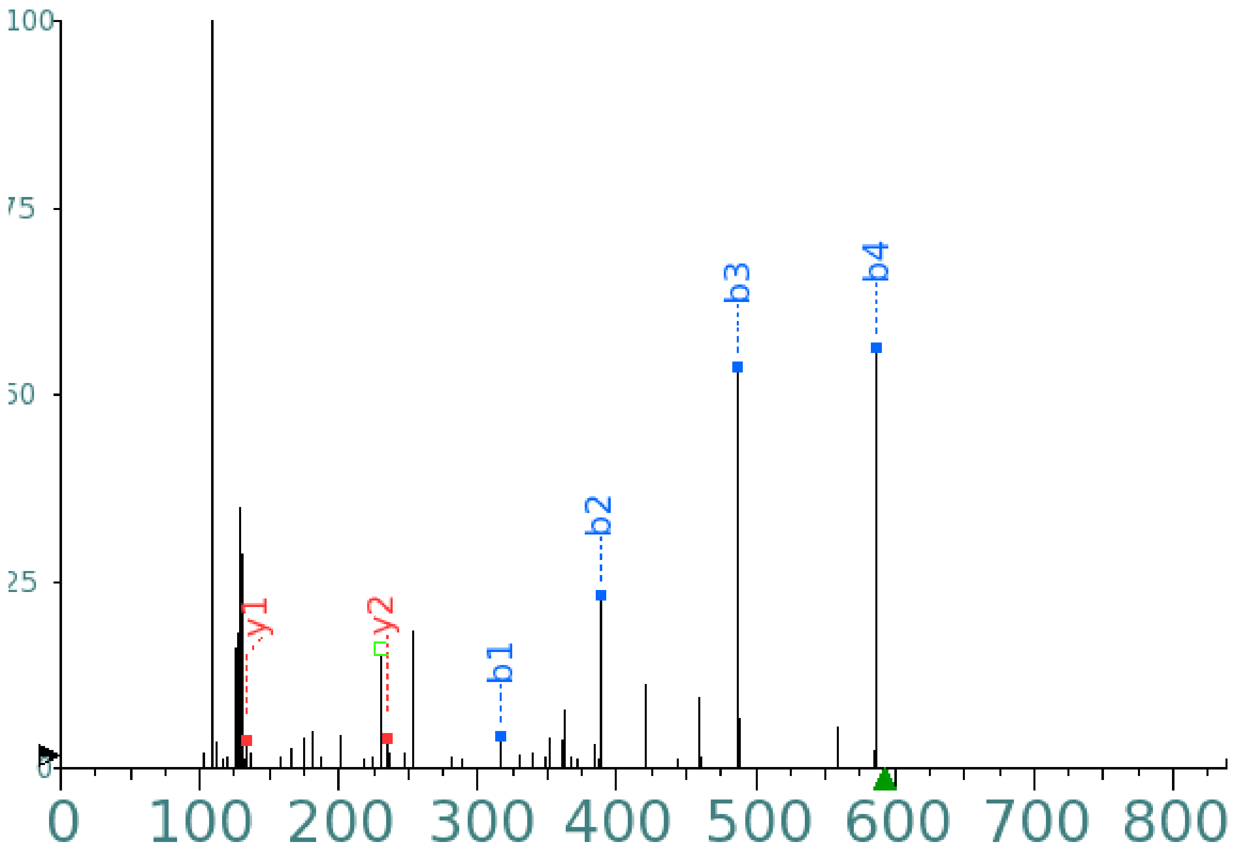

SAVVHDITD (+2)

Primary Reference: AKA12_HUMAN
Search ID: 41761
Search Name: 20130328_ananiav_ERstress_TMT_TM_timecourse_trypsin_inj2_4mc_iaa
Scan#: 8161
Observed Mass: 593.3199 (1.1 ppm)
PSM Score: 24.95

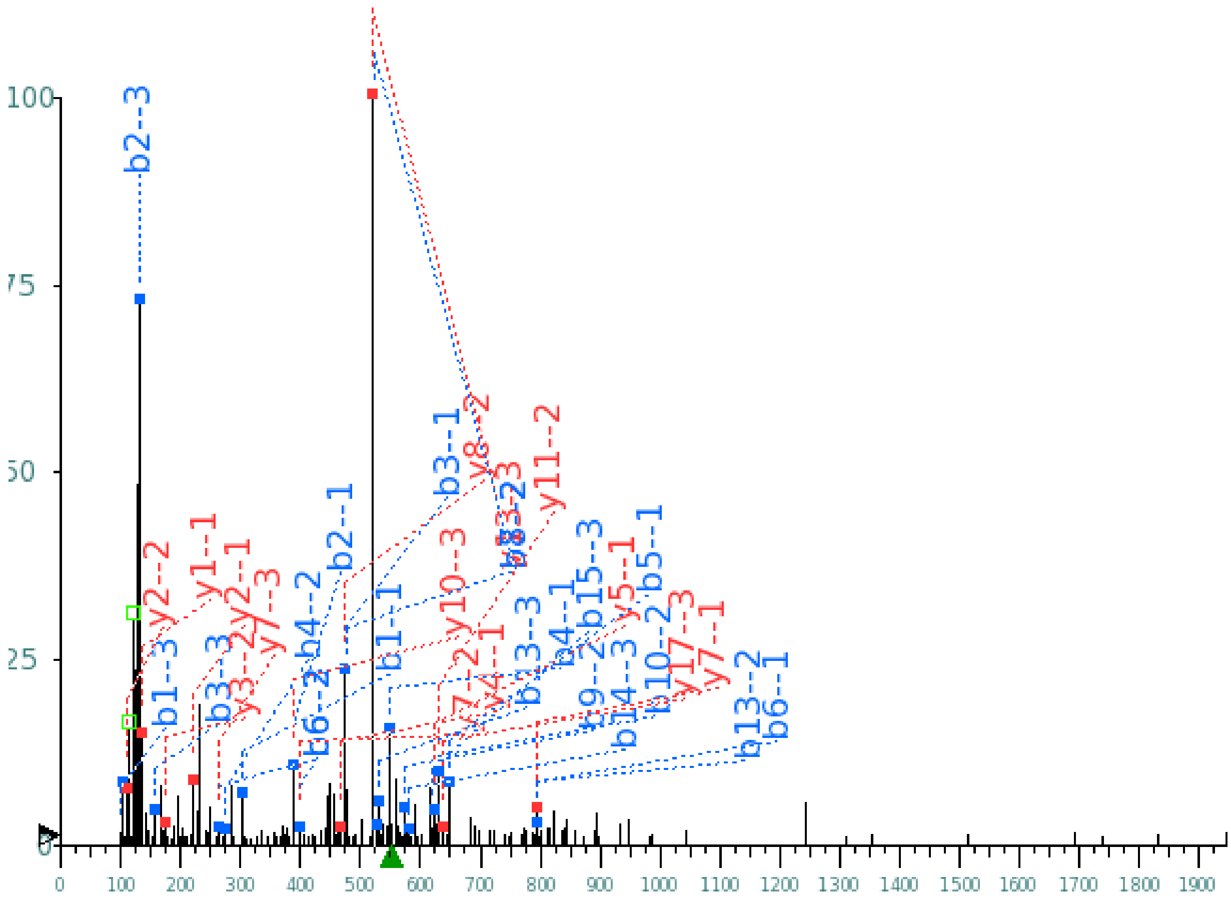

ASSATFRPVIRGDRDESD (+4)

Primary Reference: K0284_HUMAN
Search ID: 41761
Search Name: 20130328_ananiav_ERstress_TMT_TM_timecourse_trypsin_inj2_4mc_iaa
Scan#: 8188
Observed Mass: 552.7866 (1.8 ppm)
PSM Score: 19.24

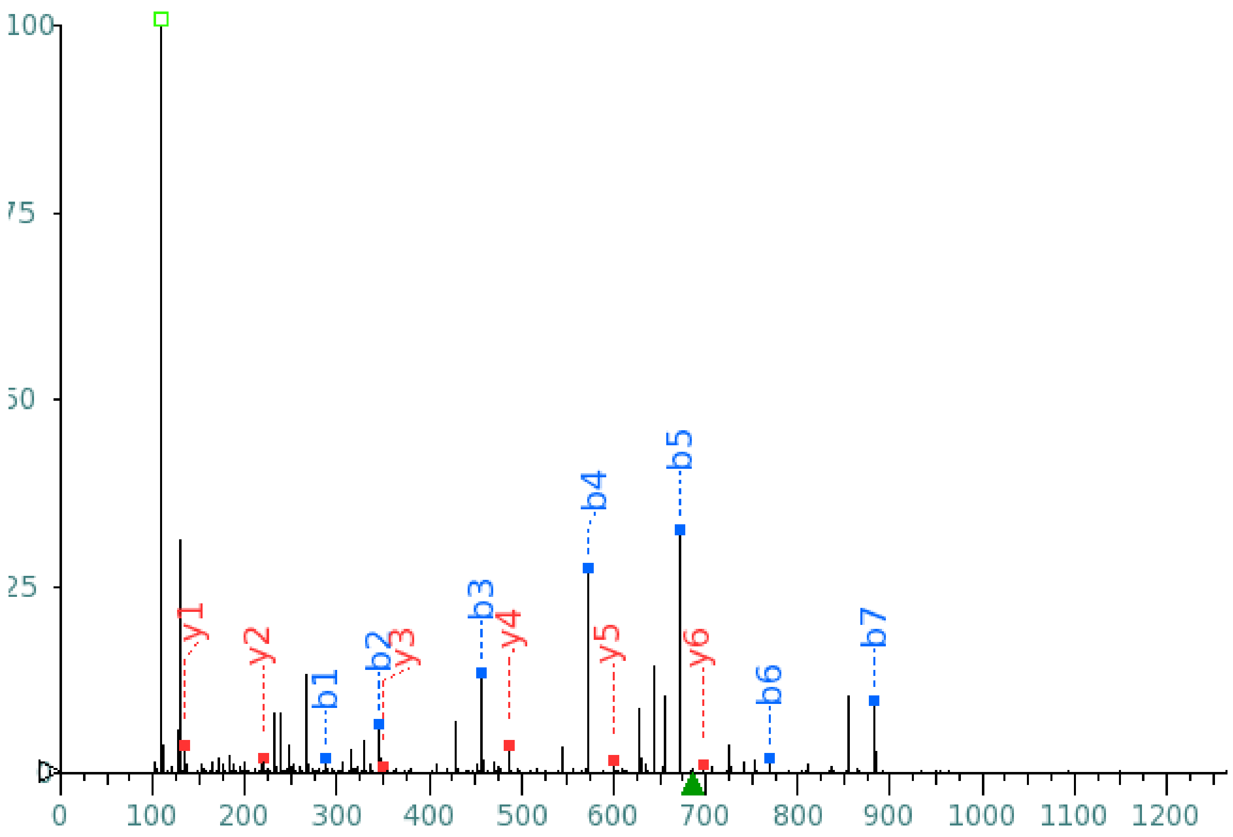

GGLNTPLHESD (+2)

Primary Reference: CDC5L_HUMAN
Search ID: 41761
Search Name: 20130328_ananiav_ERstress_TMT_TM_timecourse_trypsin_inj2_4mc_iaa
Scan#: 8226
Observed Mass: 684.8524 (1.4 ppm)
PSM Score: 13.96

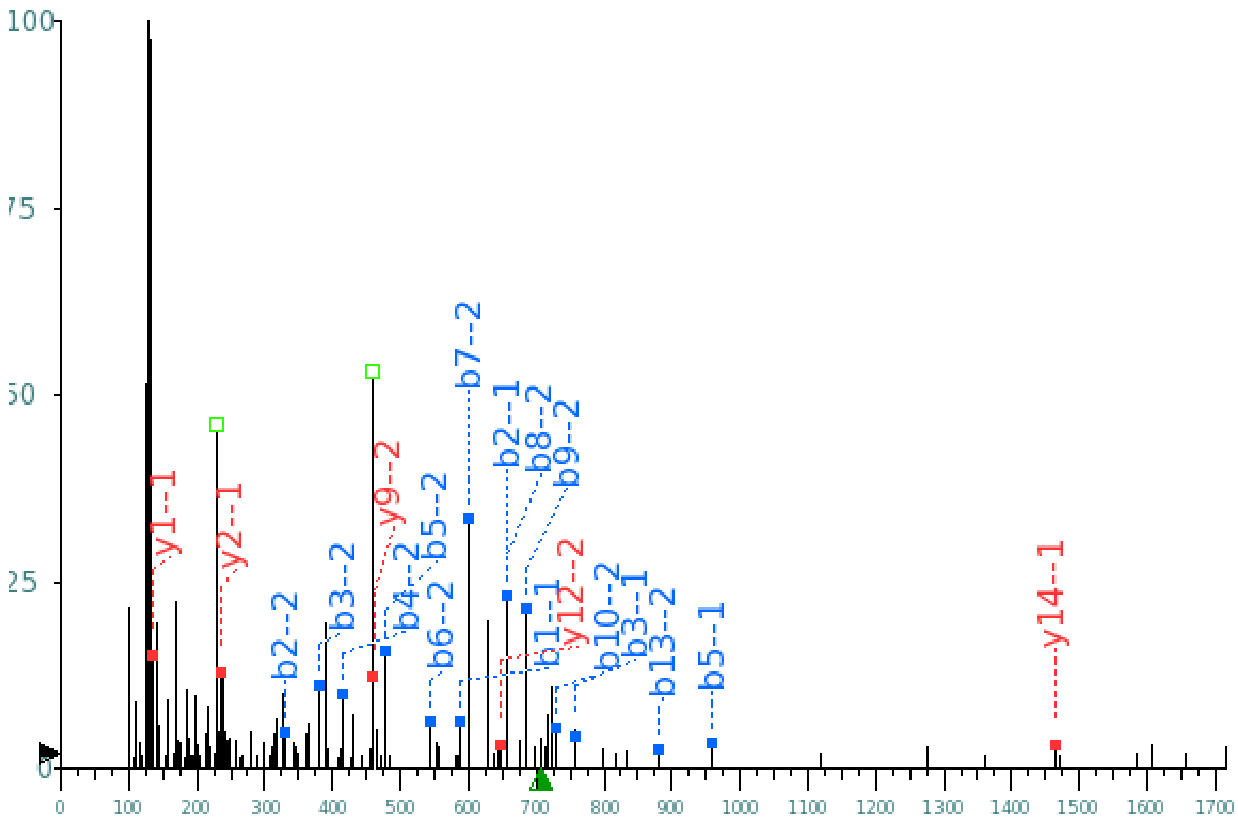

KAVAEEDNGSIGEETD (+3)

Primary Reference: STIM1_HUMAN
Search ID: 41761
Search Name: 20130328_ananiav_ERstress_TMT_TM_timecourse_trypsin_inj2_4mc_iaa
Scan#: 8279
Observed Mass: 708.0239 (0.9 ppm)
PSM Score: 26.91

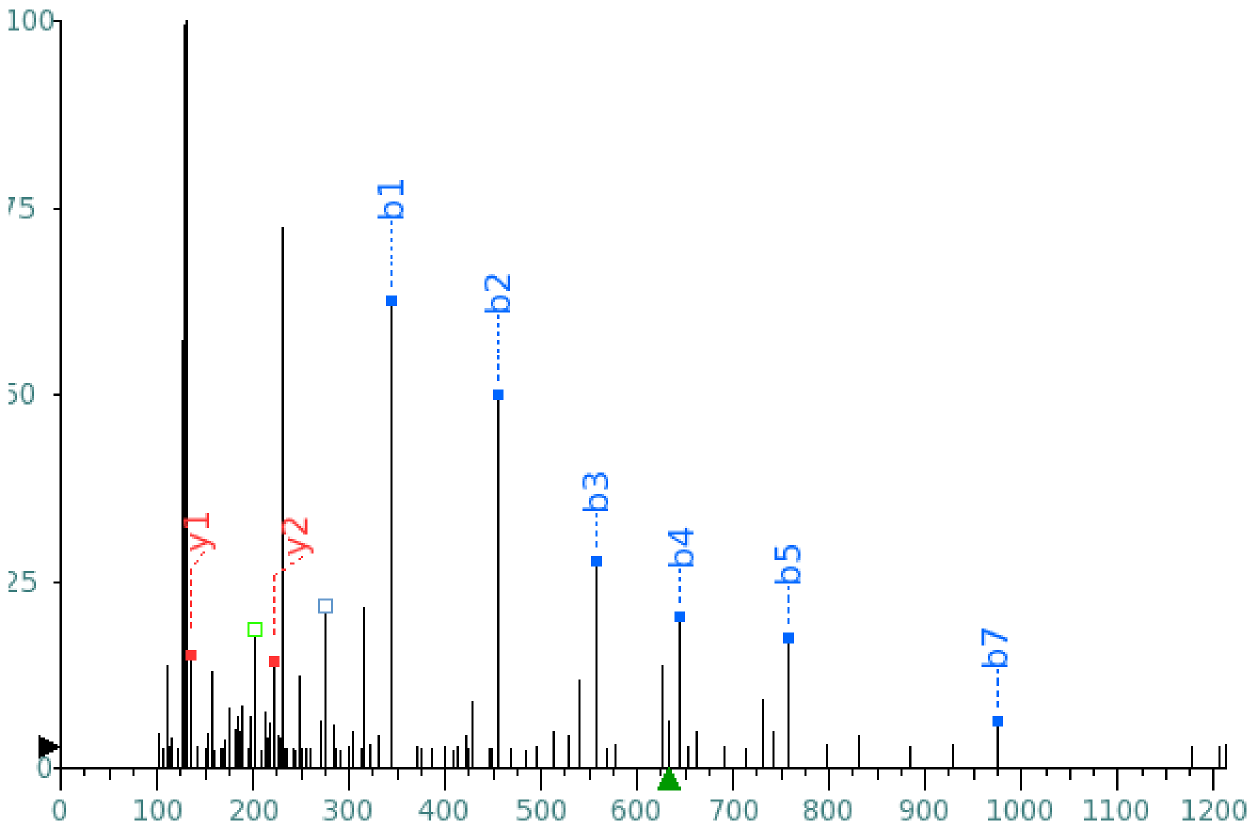

LITSNTDASD (+2)

Primary Reference: GOGB1_HUMAN
Search ID: 41761
Search Name: 20130328_ananiav_ERstress_TMT_TM_timecourse_trypsin_inj2_4mc_iaa
Scan#: 8311
Observed Mass: 633.326 (1.9 ppm)
PSM Score: 35.22

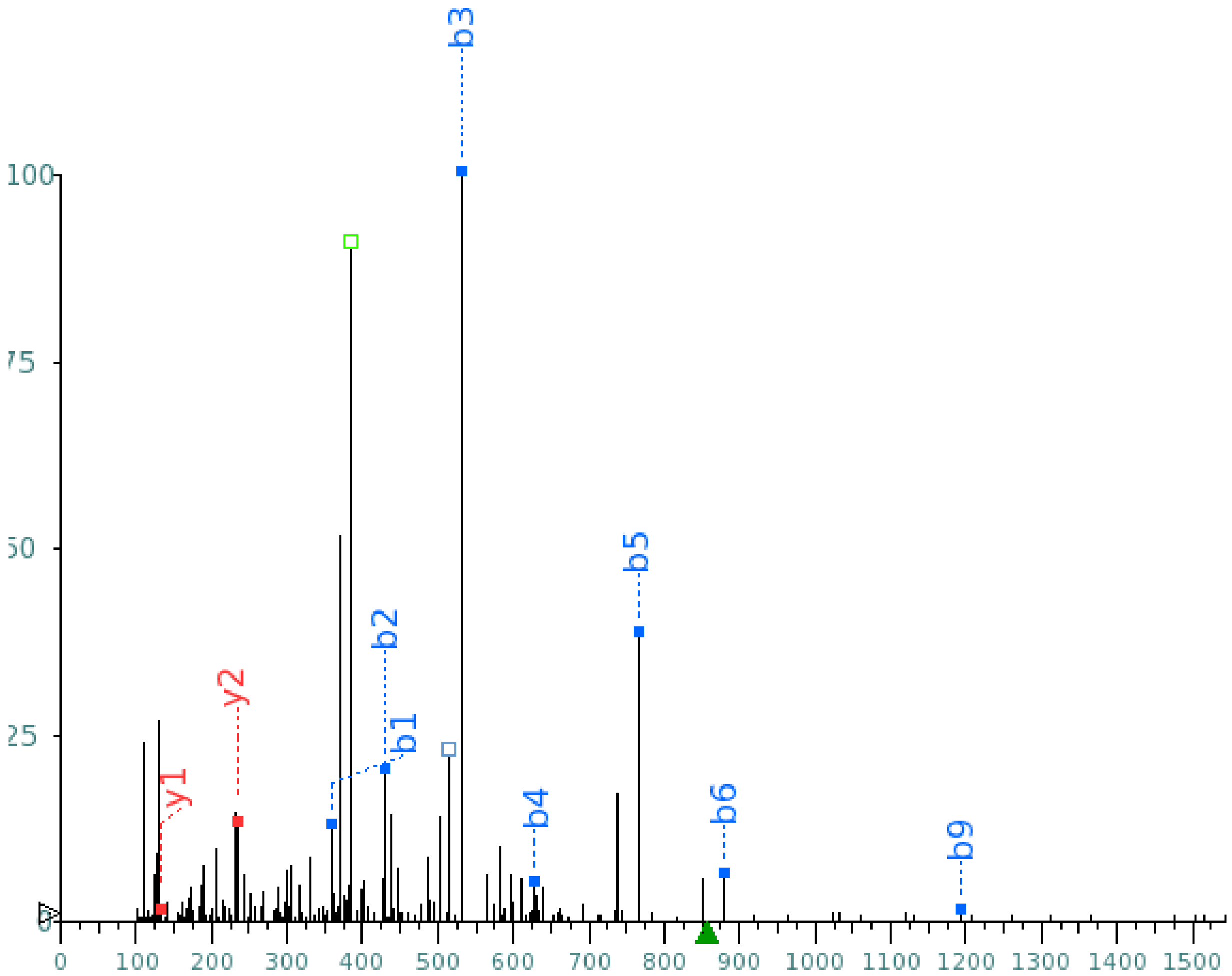

EATPHLQGETVSTD (+2)

Primary Reference: ZN646_HUMAN
Search ID: 41761
Search Name: 20130328_ananiav_ERstress_TMT_TM_timecourse_trypsin_inj2_4mc_iaa
Scan#: 8328
Observed Mass: 857.4295 (1.4 ppm)
PSM Score: 22.89

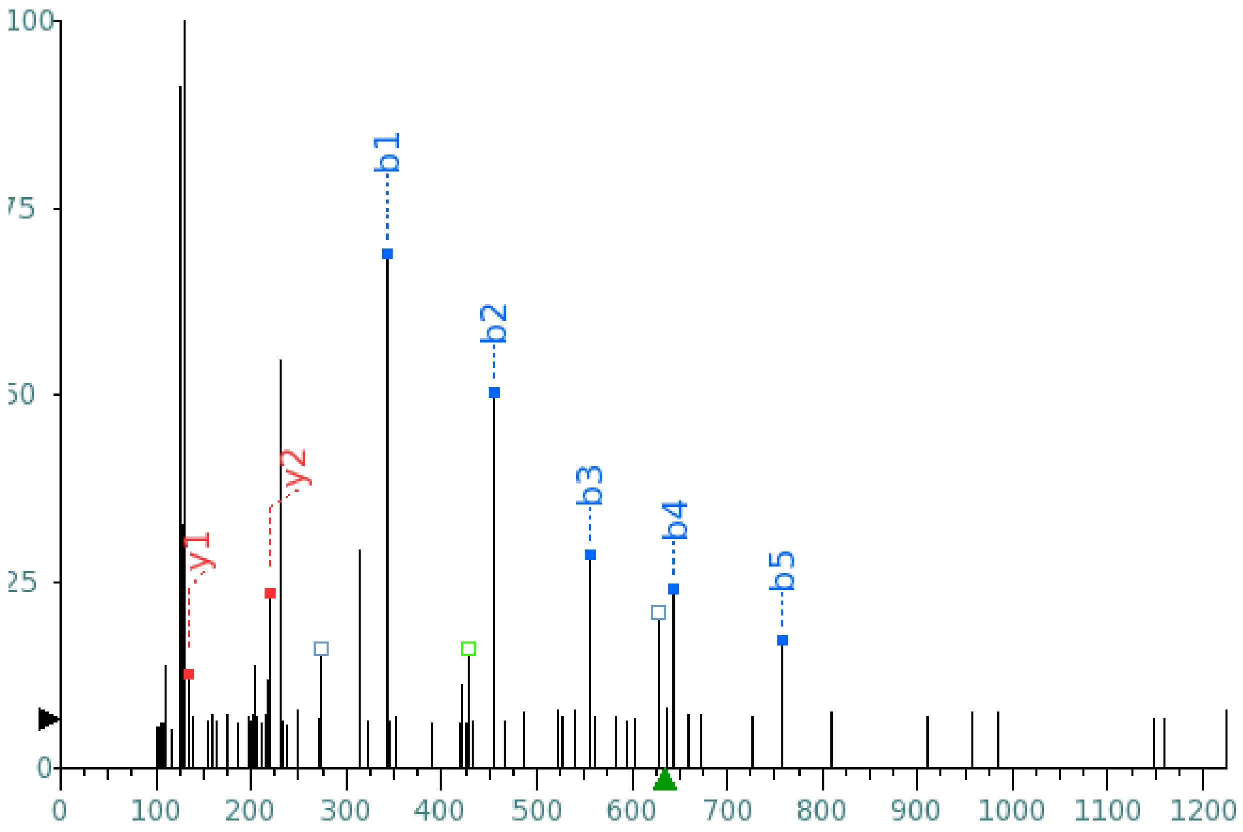

LITSNTDASD (+2)

Primary Reference: GOGB1_HUMAN
Search ID: 41761
Search Name: 20130328_ananiav_ERstress_TMT_TM_timecourse_trypsin_inj2_4mc_iaa
Scan#: 8398
Observed Mass: 633.3255 (1.2 ppm)
PSM Score: 22.38

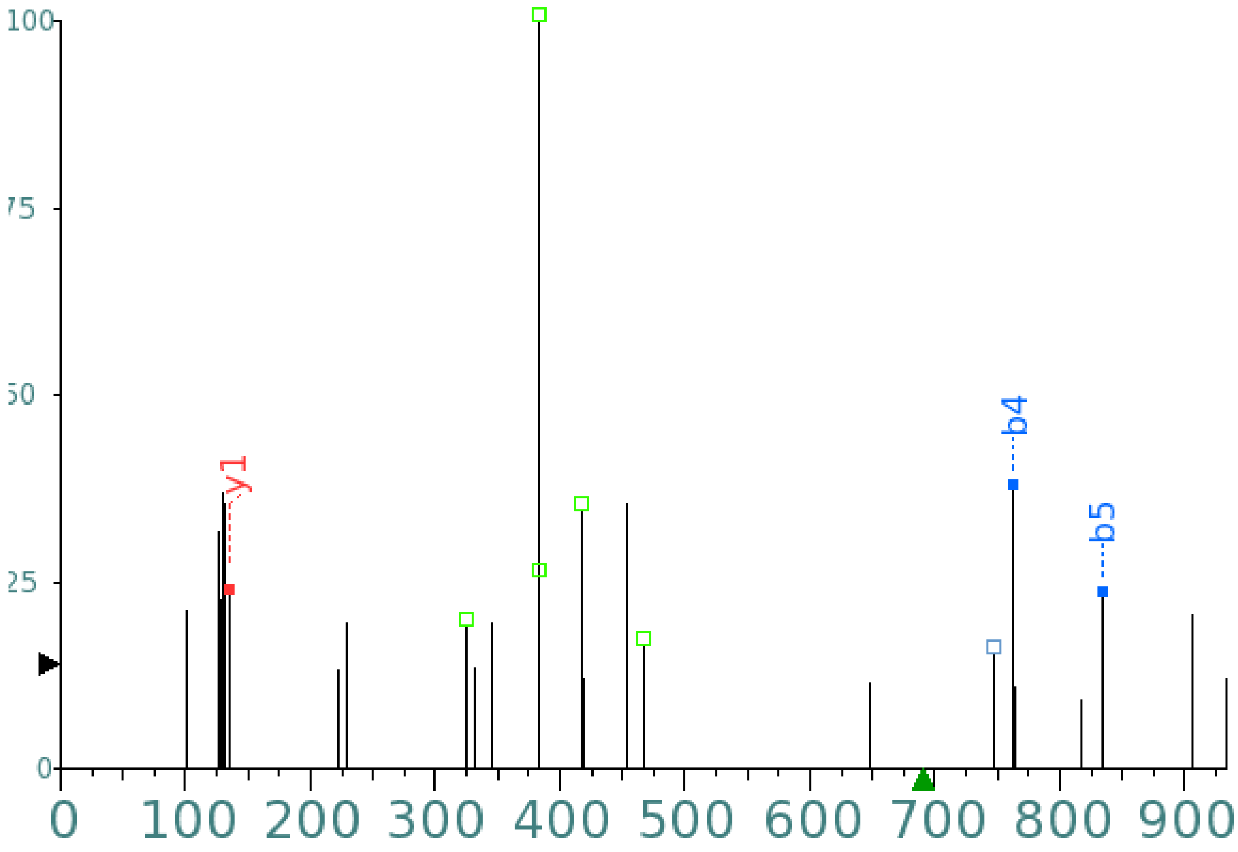

RFDDAVVQSD (+2)

Primary Reference: HSP7C_HUMAN
Search ID: 41761
Search Name: 20130328_ananiav_ERstress_TMT_TM_timecourse_trypsin_inj2_4mc_iaa
Scan#: 8414
Observed Mass: 690.8531 (2.3 ppm)
PSM Score: 19.64

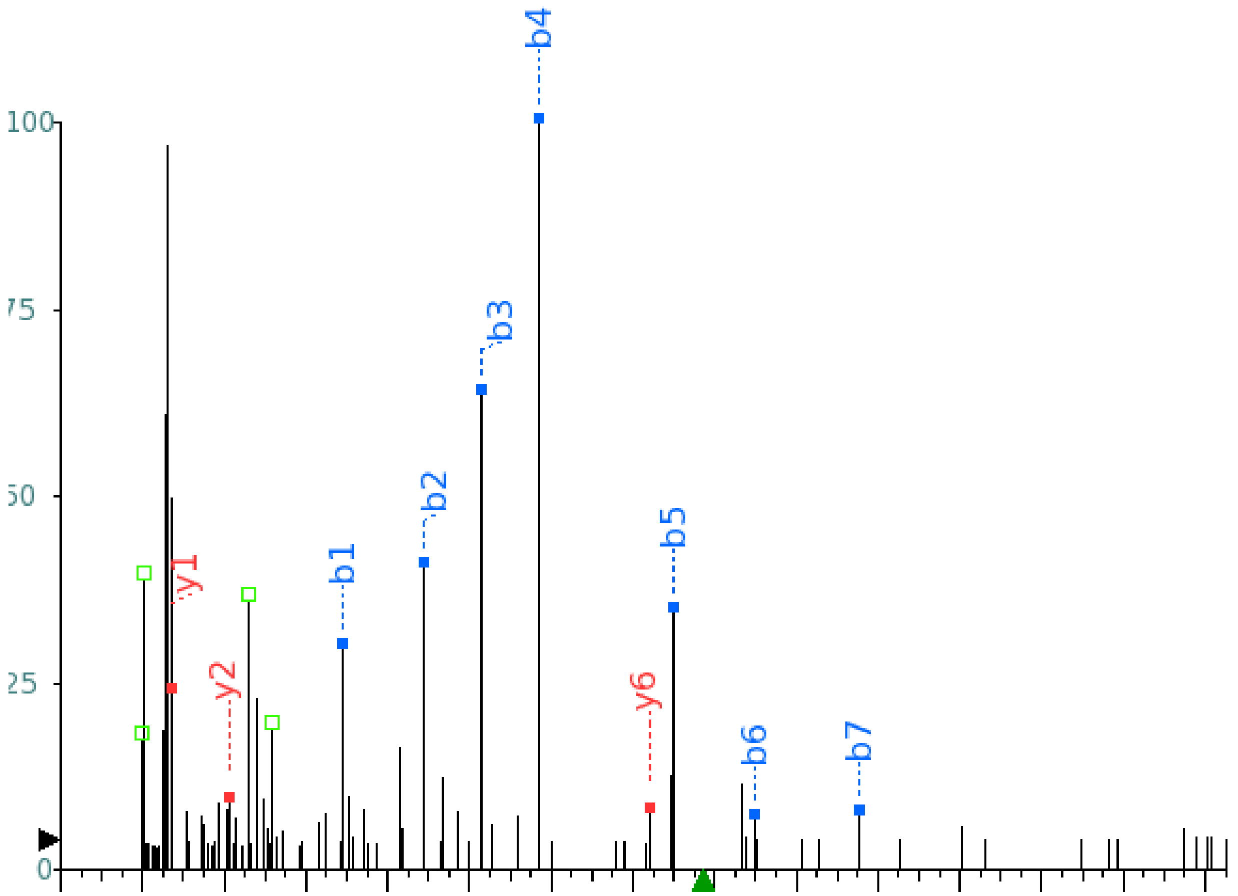

NVAAYTEQEEAD (+2)

Primary Reference: GOGA4_HUMAN
Search ID: 41761
Search Name: 20130328_ananiav_ERstress_TMT_TM_timecourse_trypsin_inj2_4mc_iaa
Scan#: 8444
Observed Mass: 784.867 (-0.7 ppm)
PSM Score: 23.54

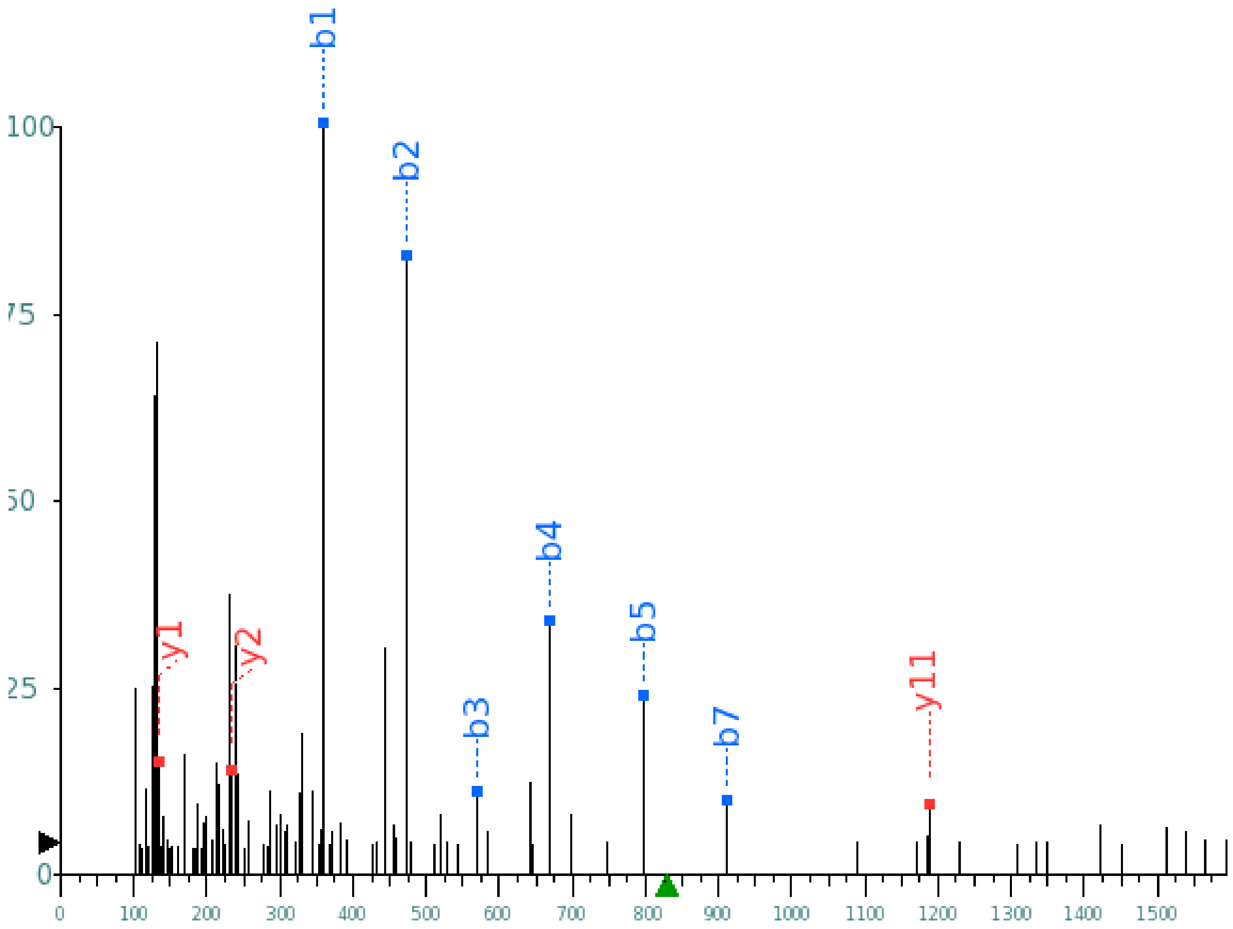

EIPVEGGQEQQTD (+2)

Primary Reference: B1AKC7_HUMAN
Search ID: 41761
Search Name: 20130328_ananiav_ERstress_TMT_TM_timecourse_trypsin_inj2_4mc_iaa
Scan#: 8466
Observed Mass: 829.9073 (0.2 ppm)
PSM Score: 27.76

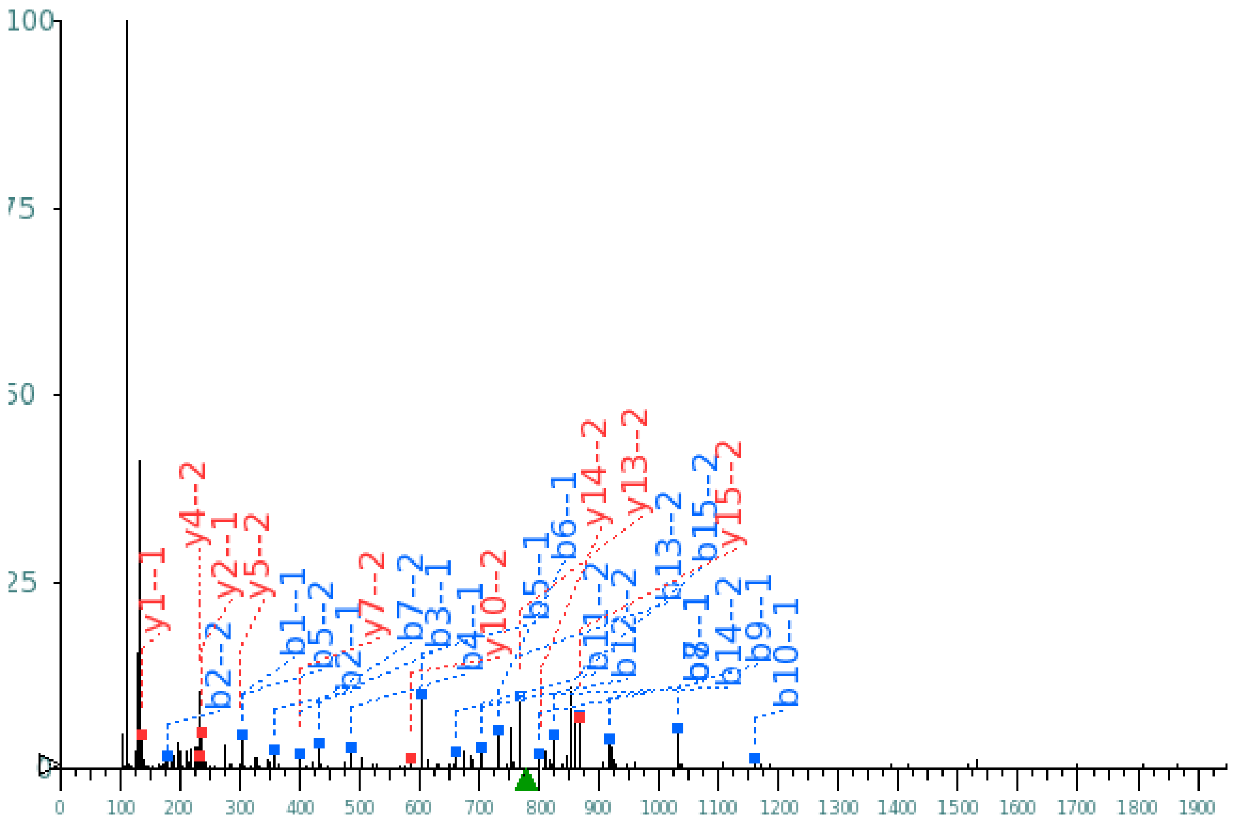

AGAGDEADDERSELSHVETD (+3)

Primary Reference: RHG23_HUMAN
Search ID: 41761
Search Name: 20130328_ananiav_ERstress_TMT_TM_timecourse_trypsin_inj2_4mc_iaa
Scan#: 8500
Observed Mass: 778.0178 (0.6 ppm)
PSM Score: 25.96

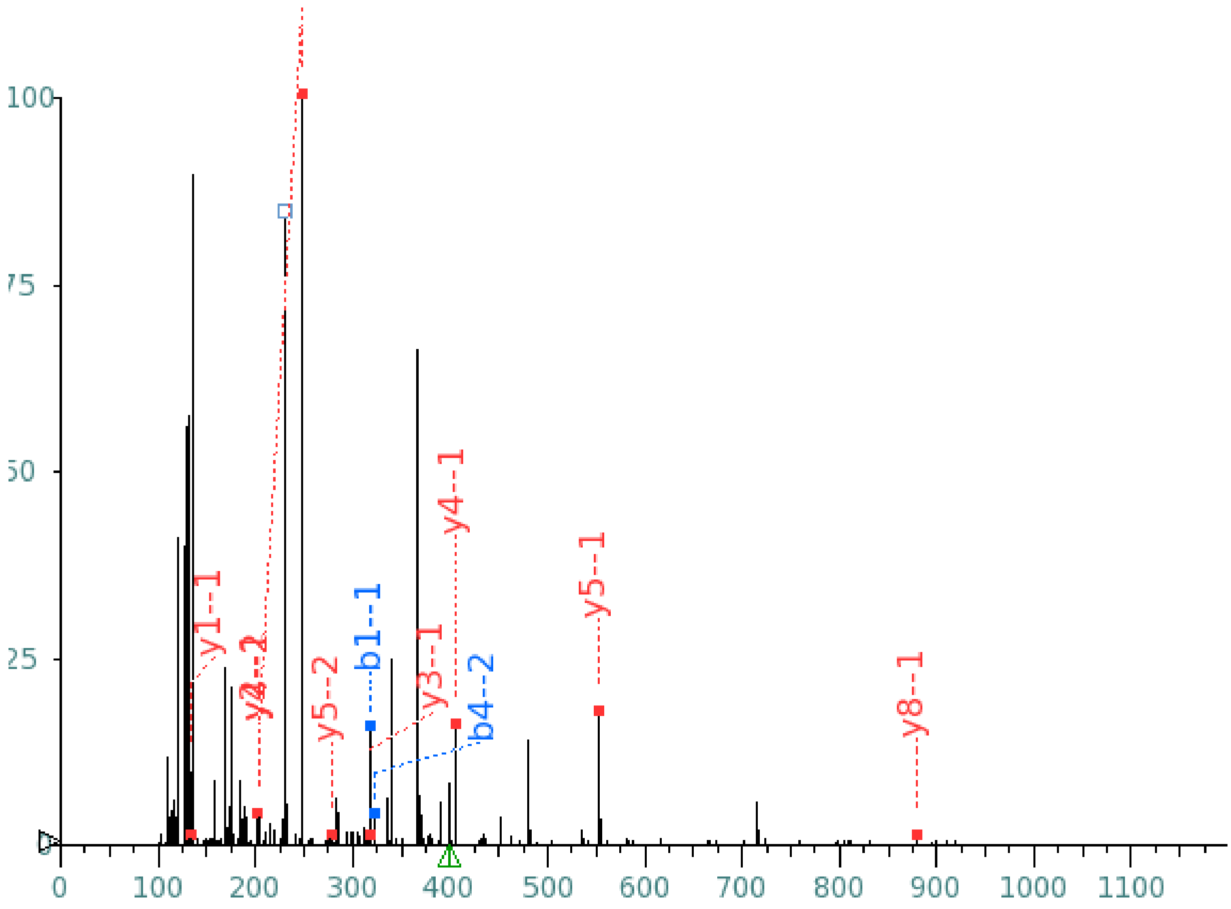

SRGLFSAND (+3)

Primary Reference: ZRAB2_HUMAN
Search ID: 41761
Search Name: 20130328_ananiav_ERstress_TMT_TM_timecourse_trypsin_inj2_4mc_iaa
Scan#: 8532
Observed Mass: 399.214 (0.6 ppm)
PSM Score: 15.36

File name: 2015-02-04 04.01.53PM.pdf

Creation time: 2015-02-04 04:09PM

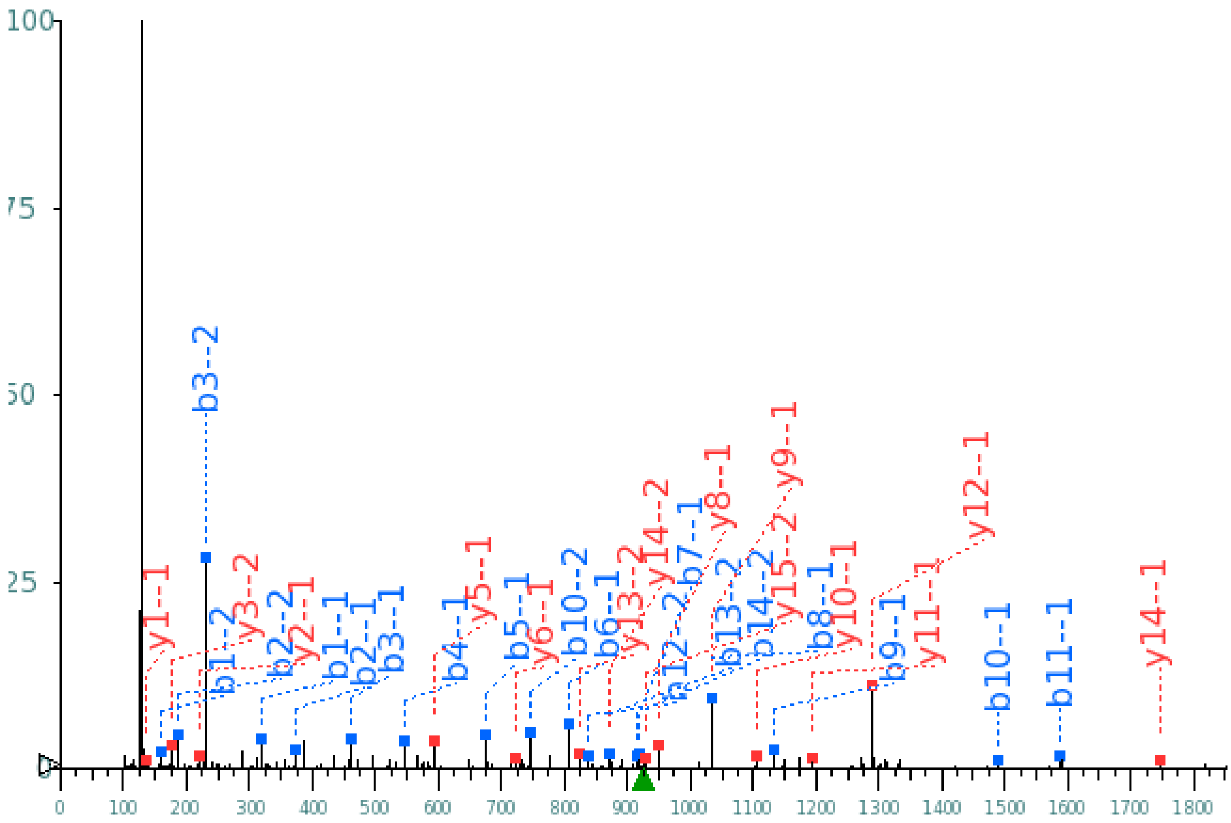

SGSSQELDVKPSASPQERSESD (+3)

Primary Reference: SRRM2_HUMAN
Search ID: 41761
Search Name: 20130328_ananiav_ERstress_TMT_TM_timecourse_trypsin_inj2_4mc_iaa
Scan#: 8534
Observed Mass: 926.799 (1.1 ppm)
PSM Score: 42.32

File name: 2015-02-04 04.01.53PM.pdf

Creation time: 2015-02-04 04:09PM

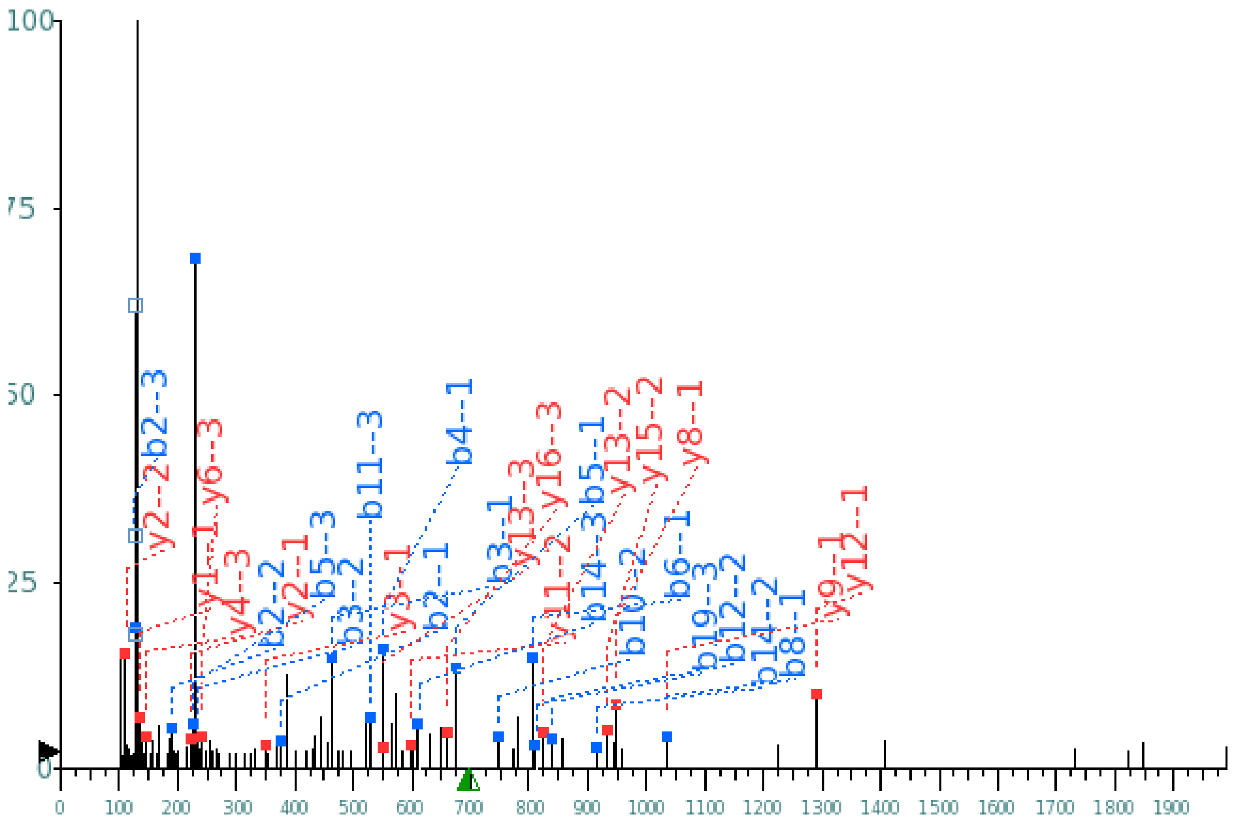

SGSSQELDVKPSASPQERSESD (+4)

Primary Reference: SRRM2_HUMAN
Search ID: 41761
Search Name: 20130328_ananiav_ERstress_TMT_TM_timecourse_trypsin_inj2_4mc_iaa
Scan#: 8541
Observed Mass: 695.3504 (0.2 ppm)
PSM Score: 13.53

File name: 2015-02-04 04.01.53PM.pdf

Creation time: 2015-02-04 04:09PM

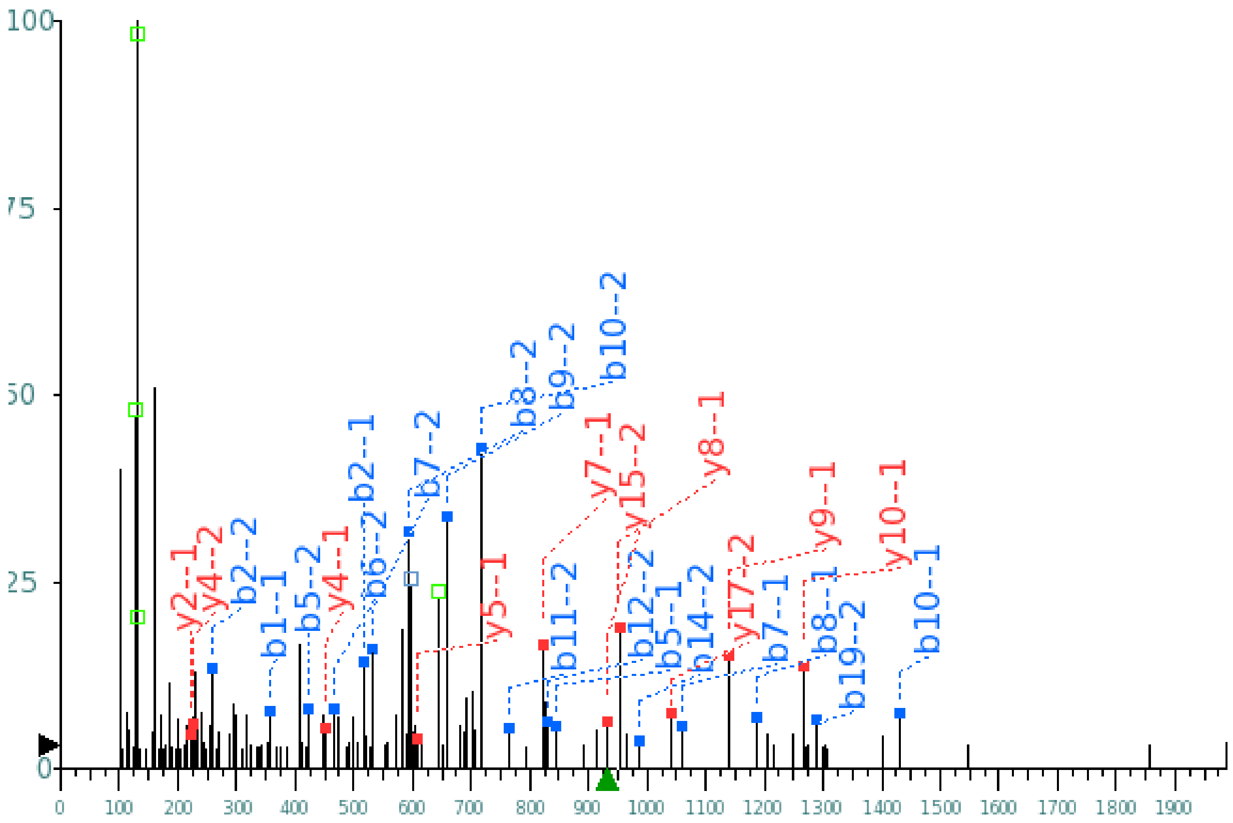

ERTPESEEEENVEWETNRDDSD (+3)

Primary Reference: ZC3HD_HUMAN
Search ID: 41761
Search Name: 20130328_ananiav_ERstress_TMT_TM_timecourse_trypsin_inj2_4mc_iaa
Scan#: 8554
Observed Mass: 932.4085 (1.2 ppm)
PSM Score: 42.37

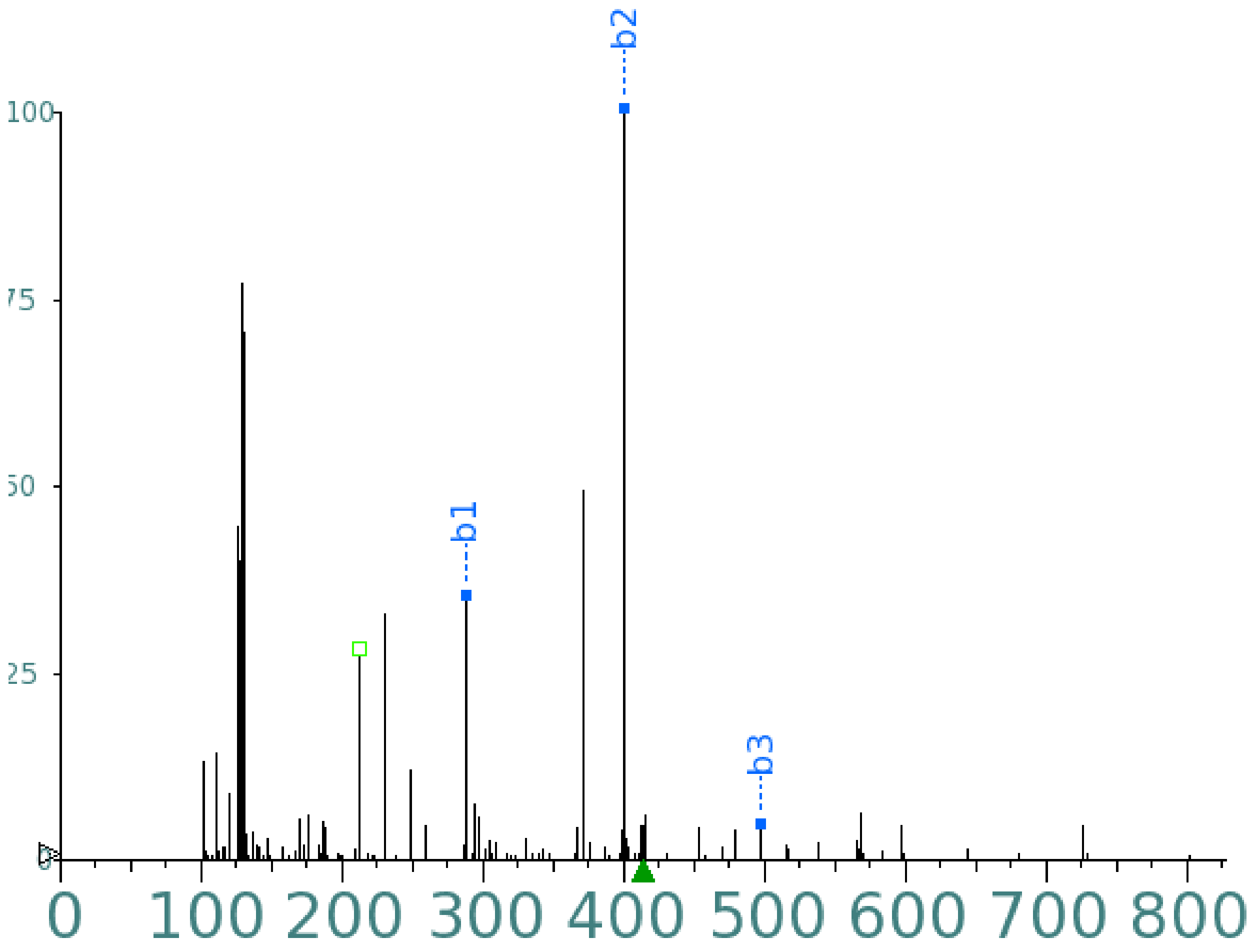

GLPPVD (+2)

Primary Reference: MA1A1_HUMAN
Search ID: 41761
Search Name: 20130328_ananiav_ERstress_TMT_TM_timecourse_trypsin_inj2_4mc_iaa
Scan#: 8562
Observed Mass: 413.7478 (1.5 ppm)
PSM Score: 11.12

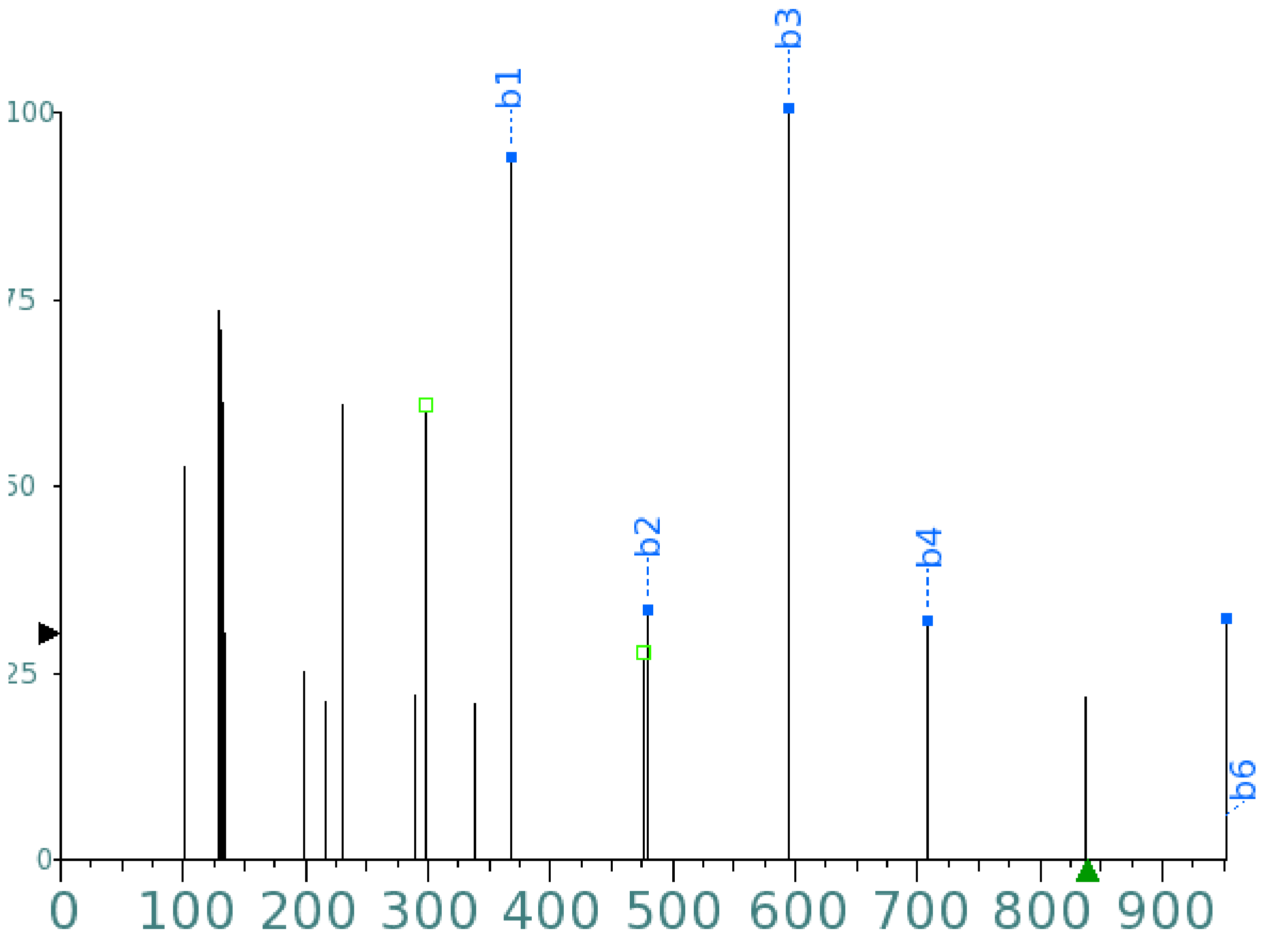

HIDLEDESC*ESD (+2)

Primary Reference: ZDBF2_HUMAN
Search ID: 41761
Search Name: 20130328_ananiav_ERstress_TMT_TM_timecourse_trypsin_inj2_4mc_iaa
Scan#: 8590
Observed Mass: 839.3607 (1.8 ppm)
PSM Score: 43.76

File name: 2015-02-04 04.01.53PM.pdf

Creation time: 2015-02-04 04:09PM

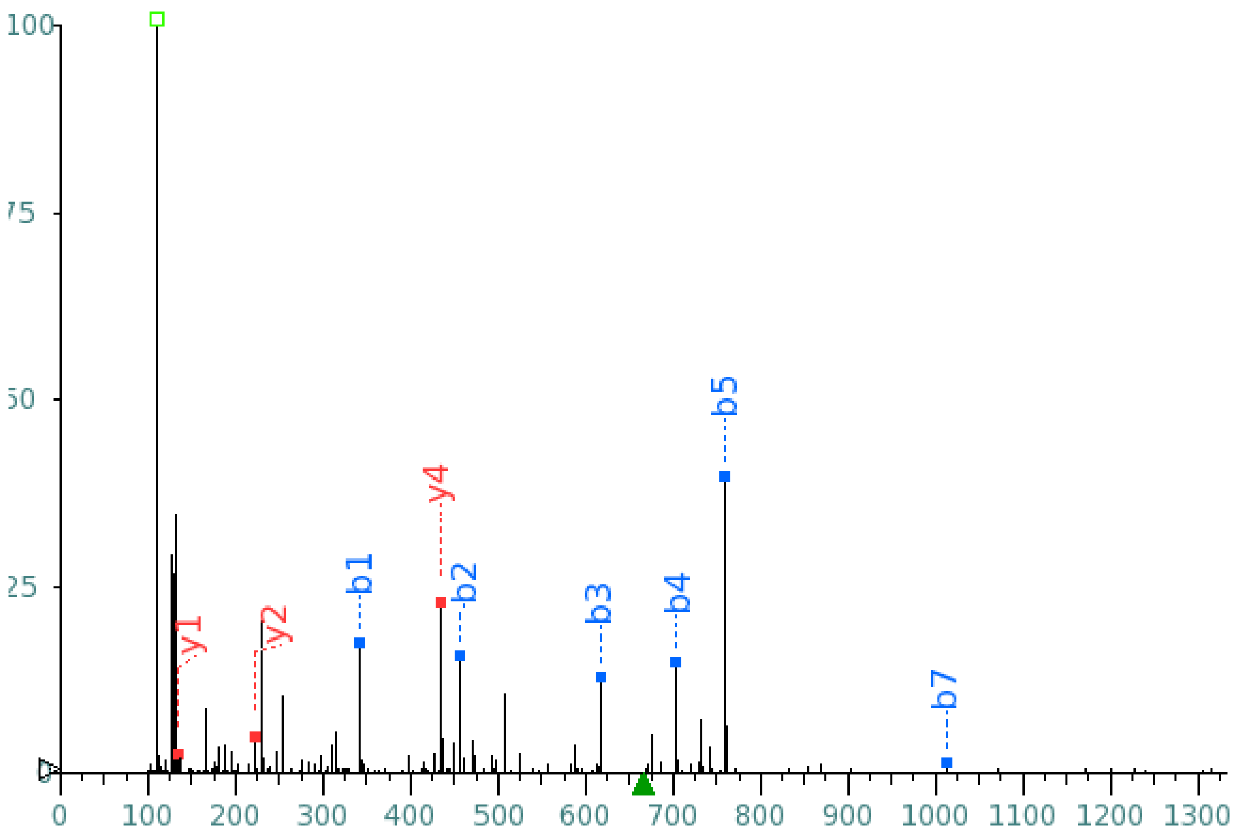

LLC*SGHDVSD (+2)

Primary Reference: PUR4_HUMAN
Search ID: 41761
Search Name: 20130328_ananiav_ERstress_TMT_TM_timecourse_trypsin_inj2_4mc_iaa
Scan#: 8599
Observed Mass: 666.3268 (-0 ppm)
PSM Score: 18.74

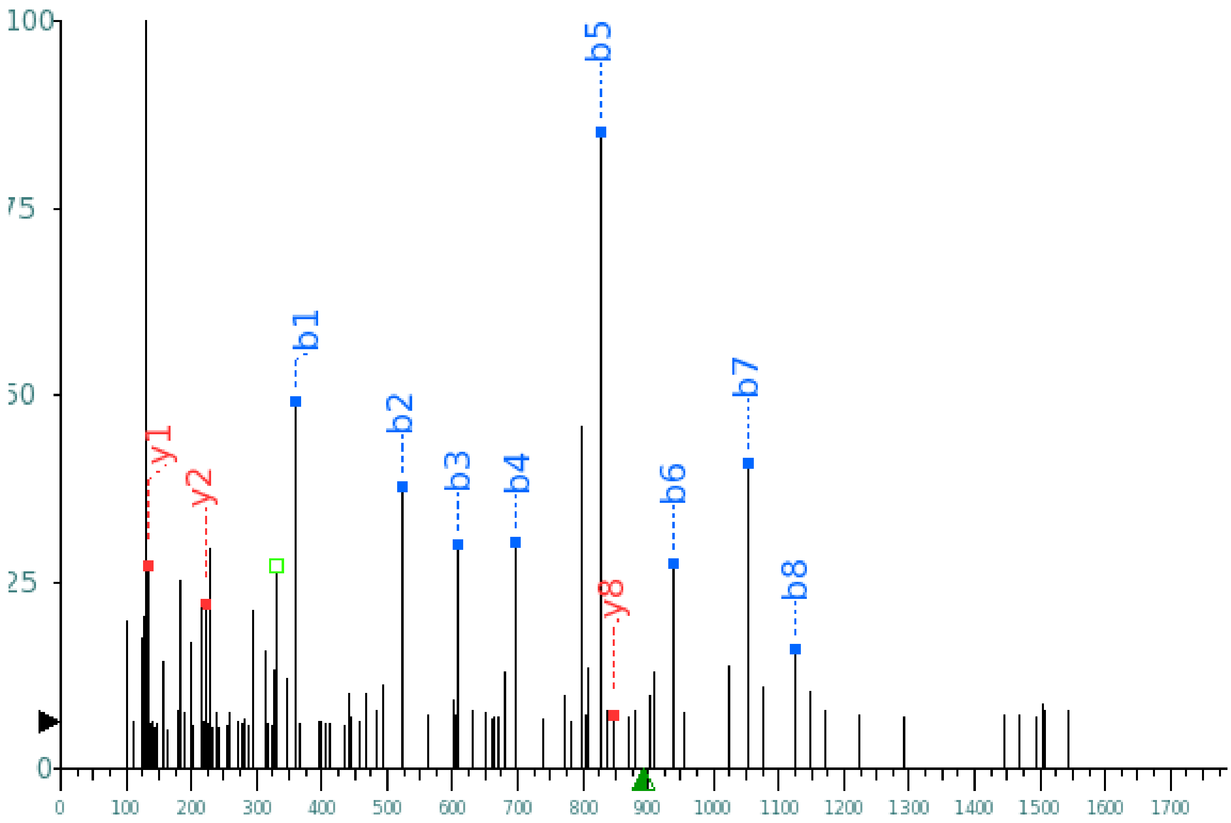

EYSSELNAPSQESD (+2)

Primary Reference: RBM25_HUMAN
Search ID: 41761
Search Name: 20130328_ananiav_ERstress_TMT_TM_timecourse_trypsin_inj2_4mc_iaa
Scan#: 8630
Observed Mass: 892.9057 (1.1 ppm)
PSM Score: 36.9

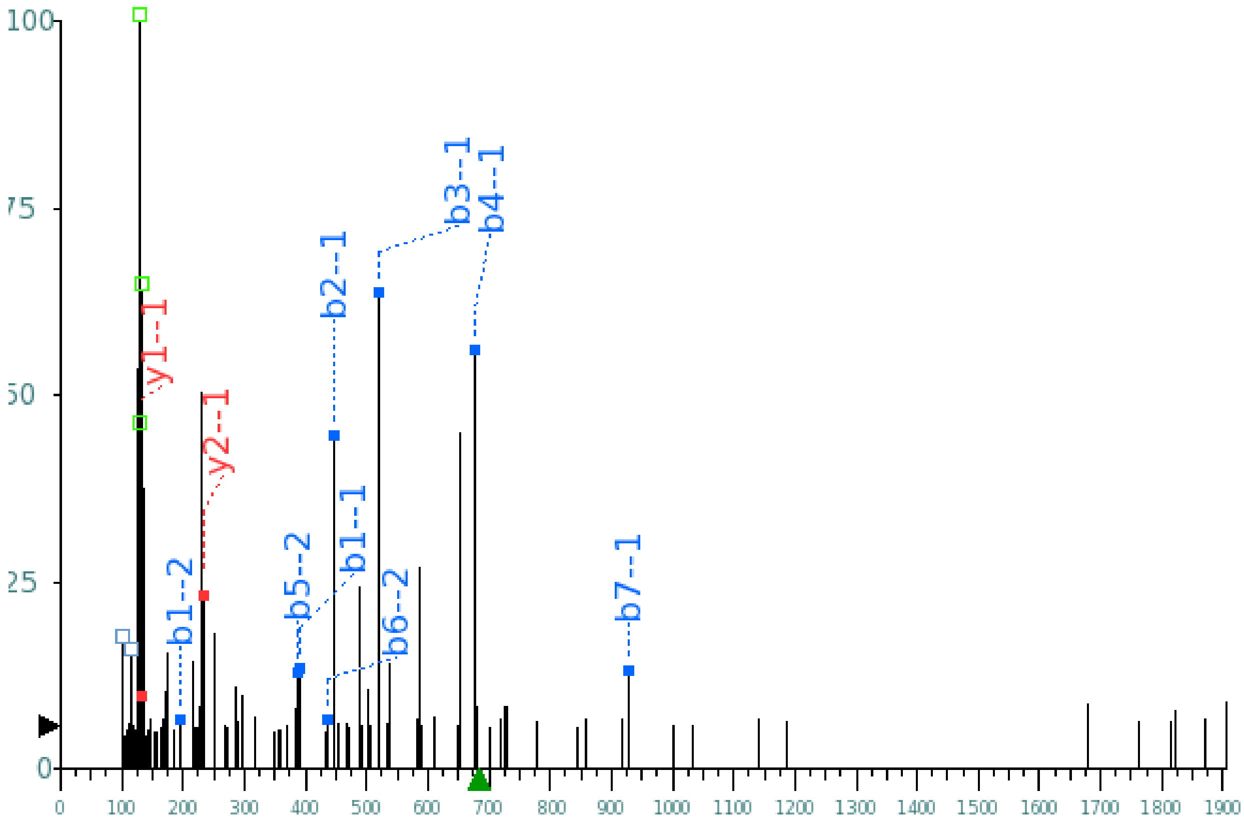

C*GAC*PPGYSGNGIQC*TD (+3)

Primary Reference: TSP1_HUMAN
Search ID: 41761
Search Name: 20130328_ananiav_ERstress_TMT_TM_timecourse_trypsin_inj2_4mc_iaa
Scan#: 8726
Observed Mass: 681.6246 (1.1 ppm)
PSM Score: 12.97

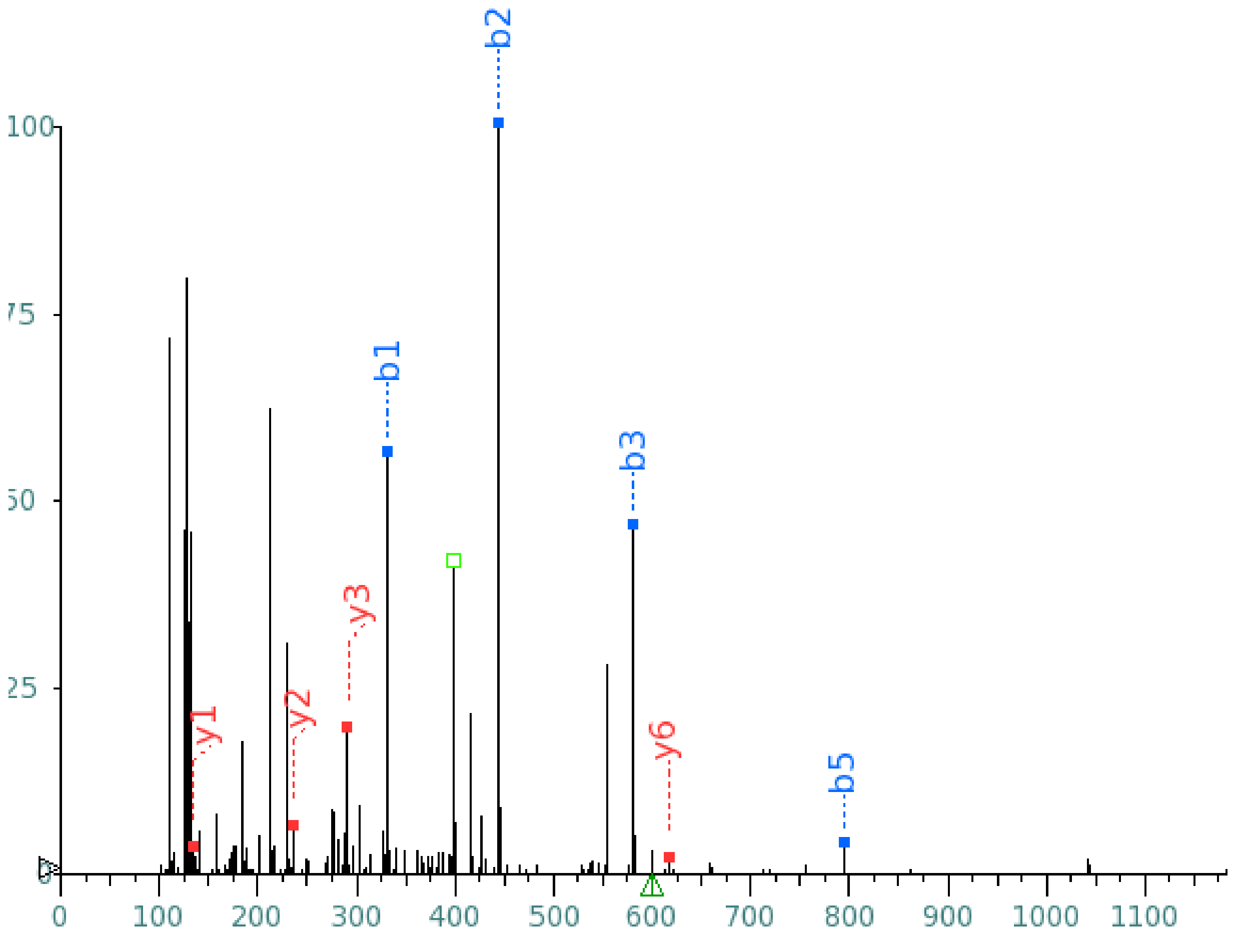

TLHPDLGTD (+2)

Primary Reference: LDHA_HUMAN
Search ID: 41761
Search Name: 20130328_ananiav_ERstress_TMT_TM_timecourse_trypsin_inj2_4mc_iaa
Scan#: 8728
Observed Mass: 599.32 (1.1 ppm)
PSM Score: 18.82

File name: 2015-02-04 04.01.53PM.pdf

Creation time: 2015-02-04 04:09PM

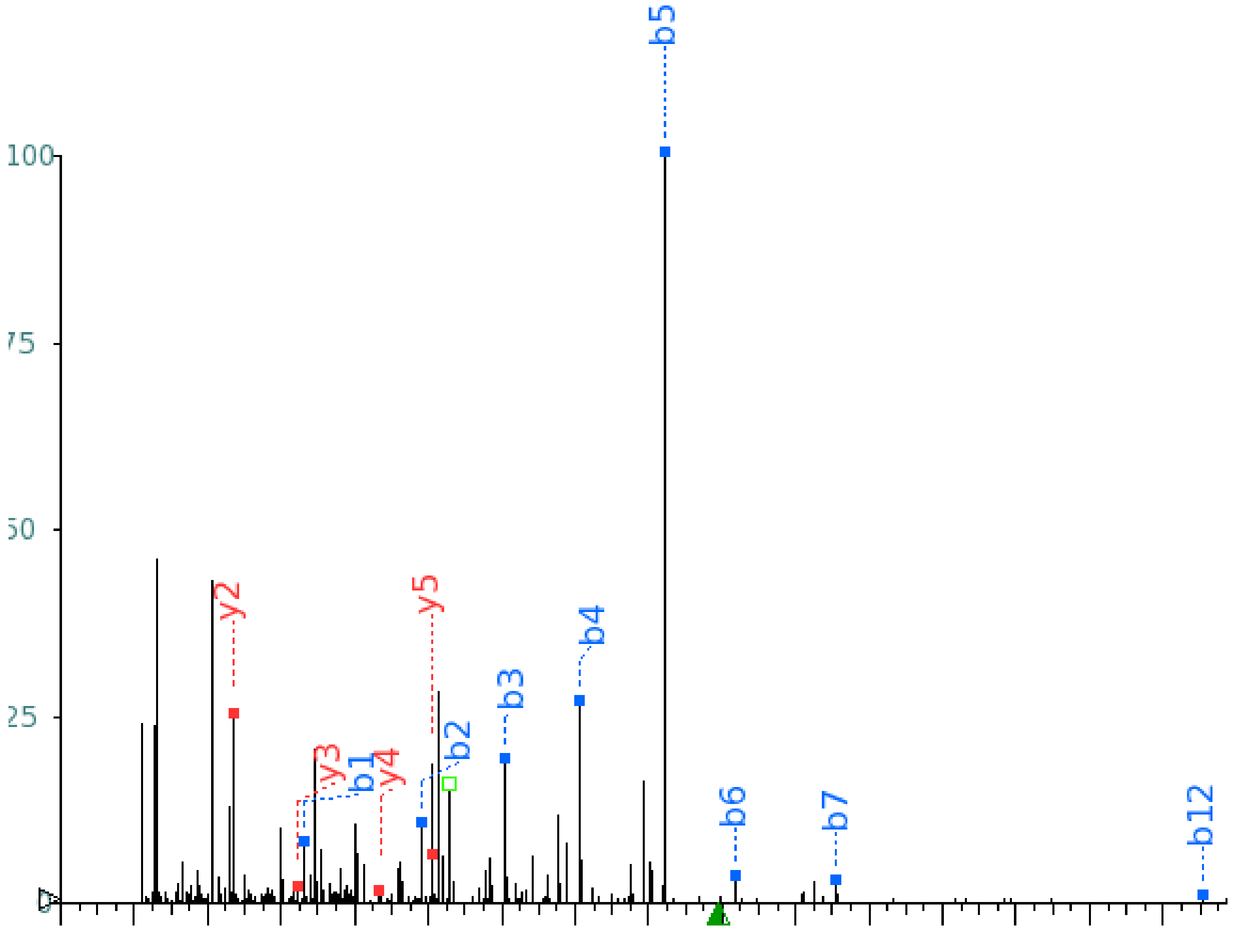

TC*NTNPHLNALSTD (+2)

Primary Reference: F120A_HUMAN
Search ID: 41761
Search Name: 20130328_ananiav_ERstress_TMT_TM_timecourse_trypsin_inj2_4mc_iaa
Scan#: 8743
Observed Mass: 893.9338 (0.7 ppm)
PSM Score: 32.28

File name: 2015-02-04 04.01.53PM.pdf

Creation time: 2015-02-04 04:09PM

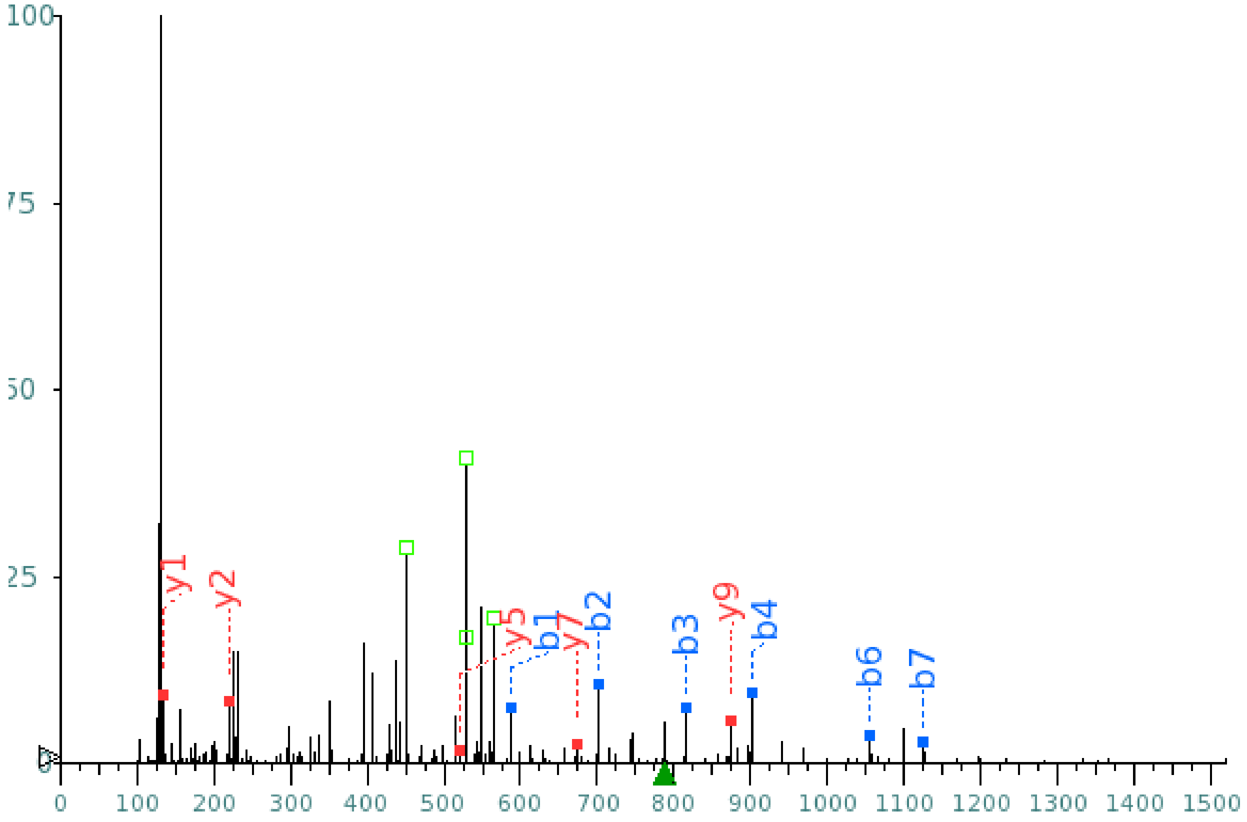

KNLSPGAVESD (+2)

Primary Reference: COPA_HUMAN
Search ID: 41761
Search Name: 20130328_ananiav_ERstress_TMT_TM_timecourse_trypsin_inj2_4mc_iaa
Scan#: 8787
Observed Mass: 787.9436 (0.6 ppm)
PSM Score: 16.16

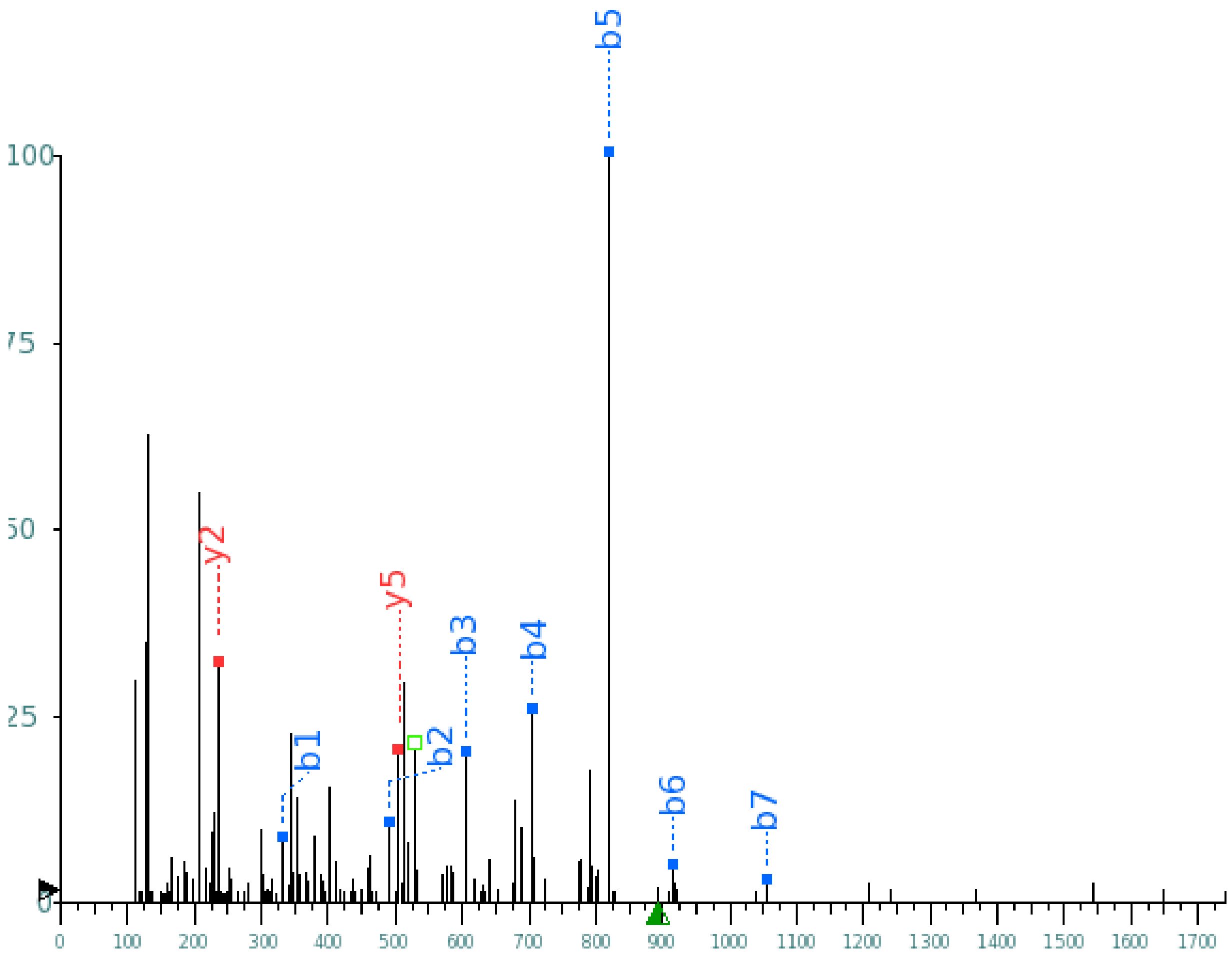

TC*NTNPHLNALSTD (+2)

Primary Reference: F120A_HUMAN
Search ID: 41761
Search Name: 20130328_ananiav_ERstress_TMT_TM_timecourse_trypsin_inj2_4mc_iaa
Scan#: 8830
Observed Mass: 893.934 (0.9 ppm)
PSM Score: 37.59

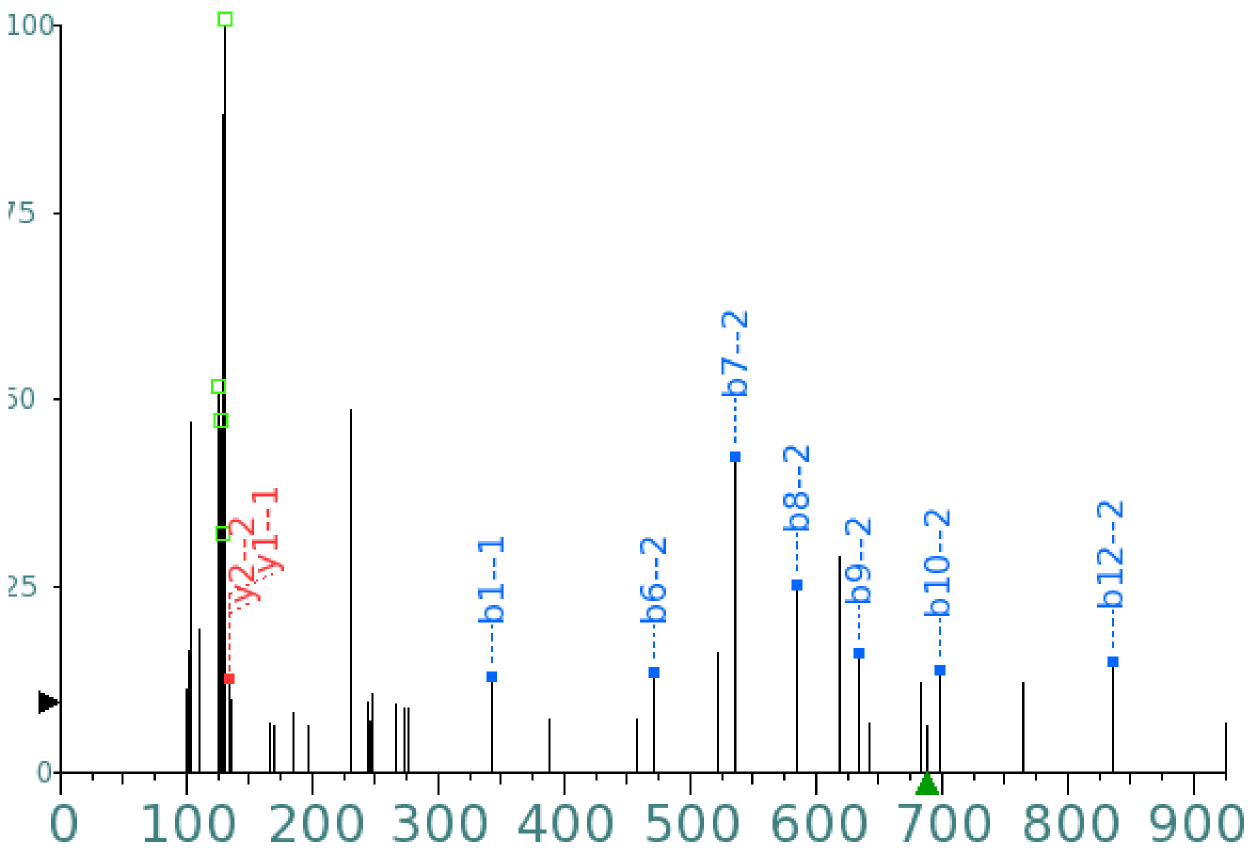

INAEREQPVQC*DEMD (+3)

Primary Reference: TUT4_HUMAN
Search ID: 41761
Search Name: 20130328_ananiav_ERstress_TMT_TM_timecourse_trypsin_inj2_4mc_iaa
Scan#: 8953
Observed Mass: 688.3168 (-0.6 ppm)
PSM Score: 28.15

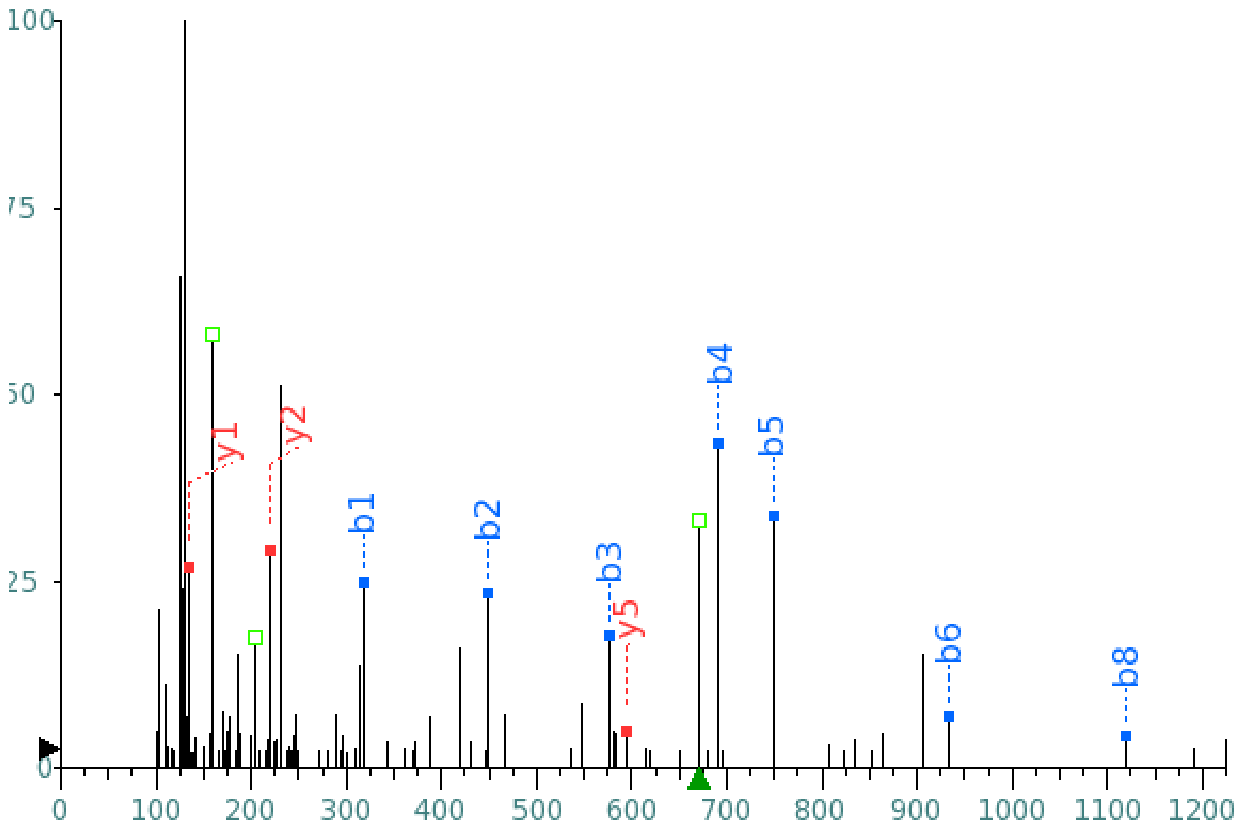

SMQDGWGESD (+2)

Primary Reference: TNR6B_HUMAN
Search ID: 41761
Search Name: 20130328_ananiav_ERstress_TMT_TM_timecourse_trypsin_inj2_4mc_iaa
Scan#: 8971
Observed Mass: 670.7859 (1.4 ppm)
PSM Score: 33.49

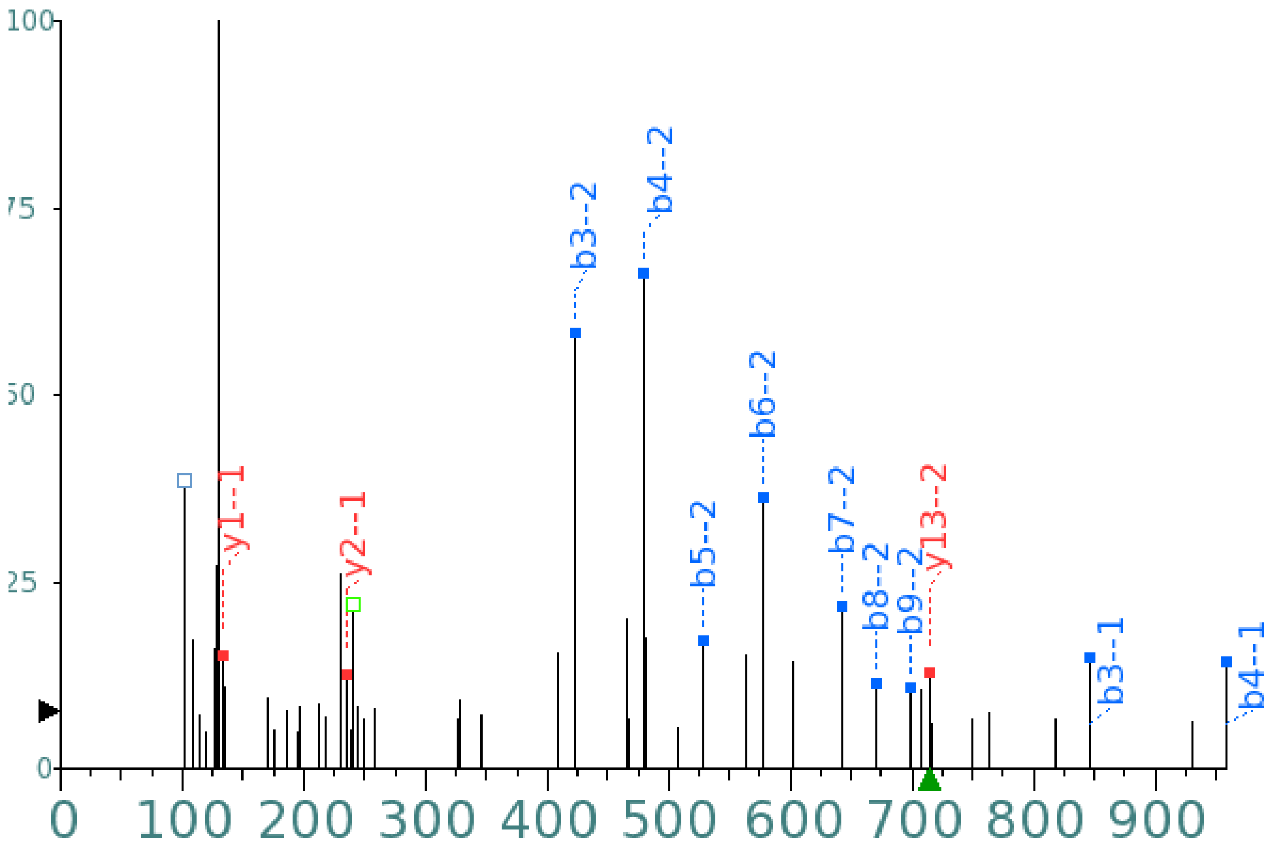

EKEIPVEGGGQEQQTD (+3)

Primary Reference: B1AKC7_HUMAN
Search ID: 41761
Search Name: 20130328_ananiav_ERstress_TMT_TM_timecourse_trypsin_inj2_4mc_iaa
Scan#: 8986
Observed Mass: 715.7084 (1.5 ppm)
PSM Score: 26.2

File name: 2015-02-04 04.01.53PM.pdf

Creation time: 2015-02-04 04:09PM

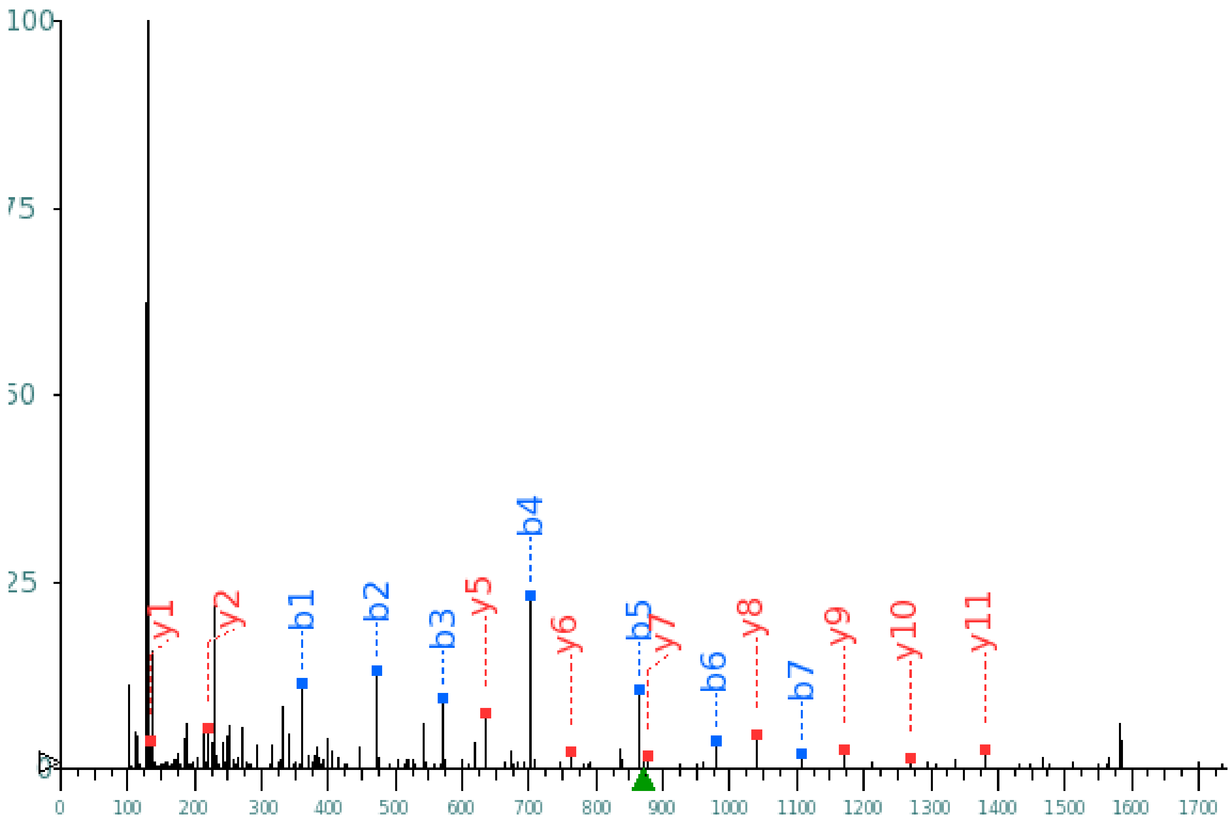

ENVEYIEREESD (+2)

Primary Reference: ZRAB2_HUMAN
Search ID: 41761
Search Name: 20130328_ananiav_ERstress_TMT_TM_timecourse_trypsin_inj2_4mc_iaa
Scan#: 9027
Observed Mass: 870.9112 (1.6 ppm)
PSM Score: 29.12

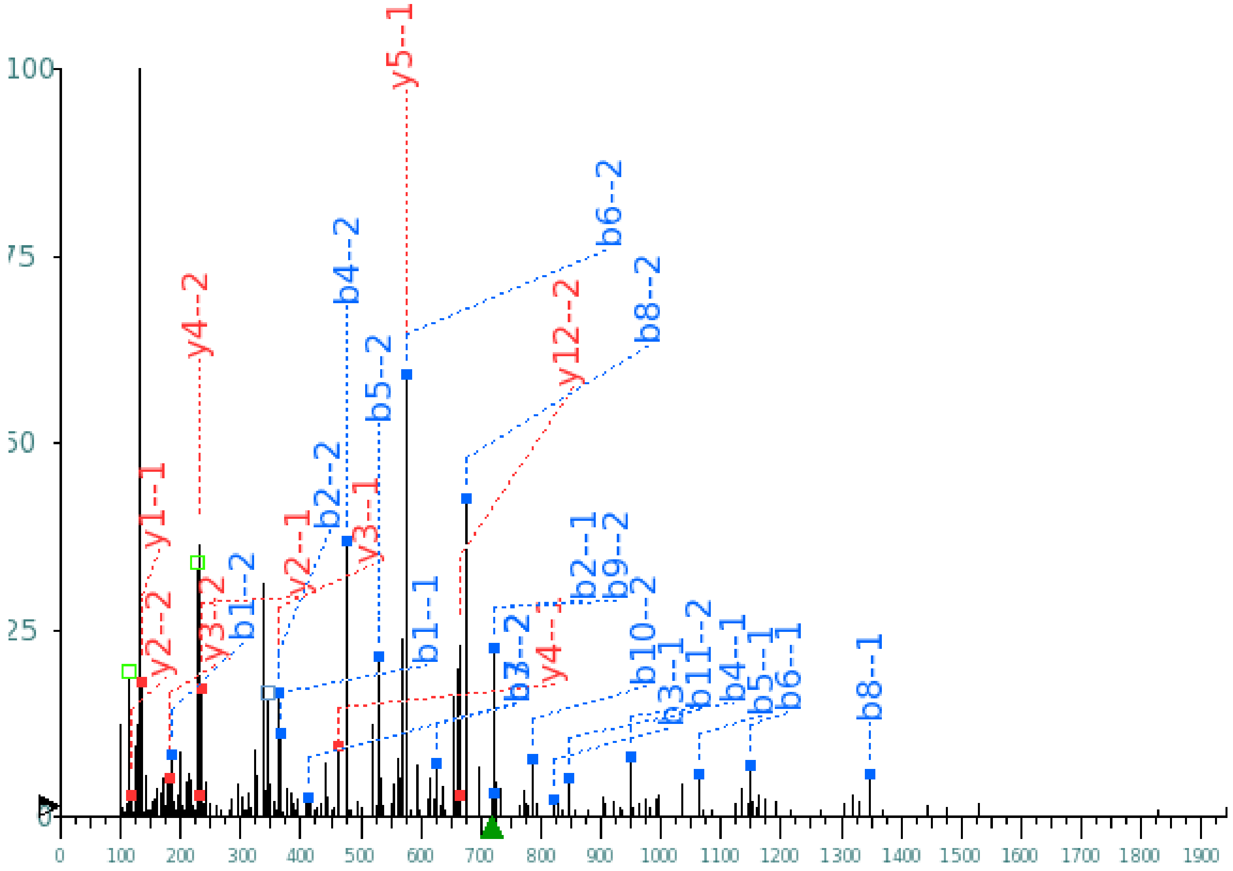

HKPELSPTPQNVQTD (+3)

Primary Reference: HAUS6_HUMAN
Search ID: 41761
Search Name: 20130328_ananiav_ERstress_TMT_TM_timecourse_trypsin_inj2_4mc_iaa
Scan#: 9084
Observed Mass: 717.0605 (0.8 ppm)
PSM Score: 31.19

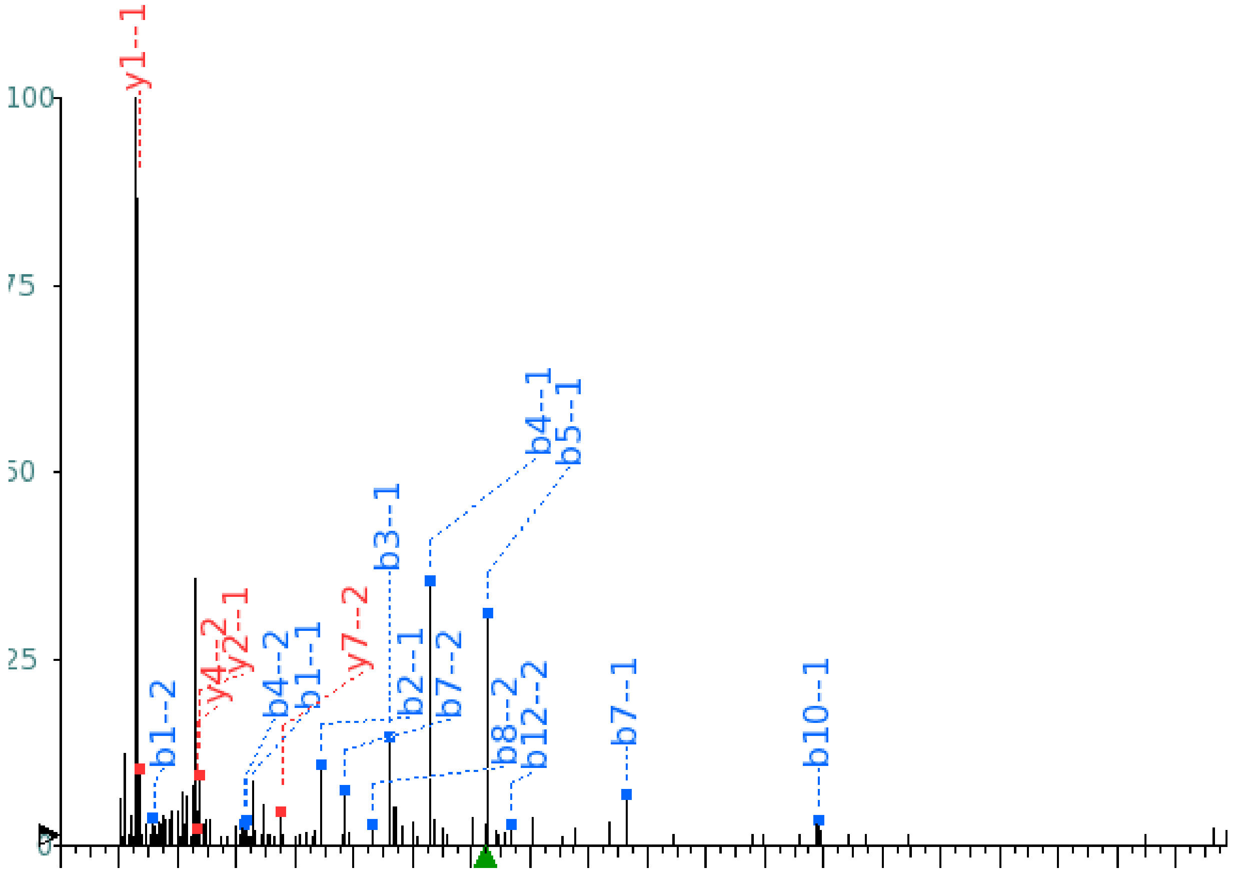

SQLAVPHPETQDSSTQTD (+3)

Primary Reference: SPAG5_HUMAN
Search ID: 41761
Search Name: 20130328_ananiav_ERstress_TMT_TM_timecourse_trypsin_inj2_4mc_iaa
Scan#: 9226
Observed Mass: 724.0225 (3.1 ppm)
PSM Score: 18.68

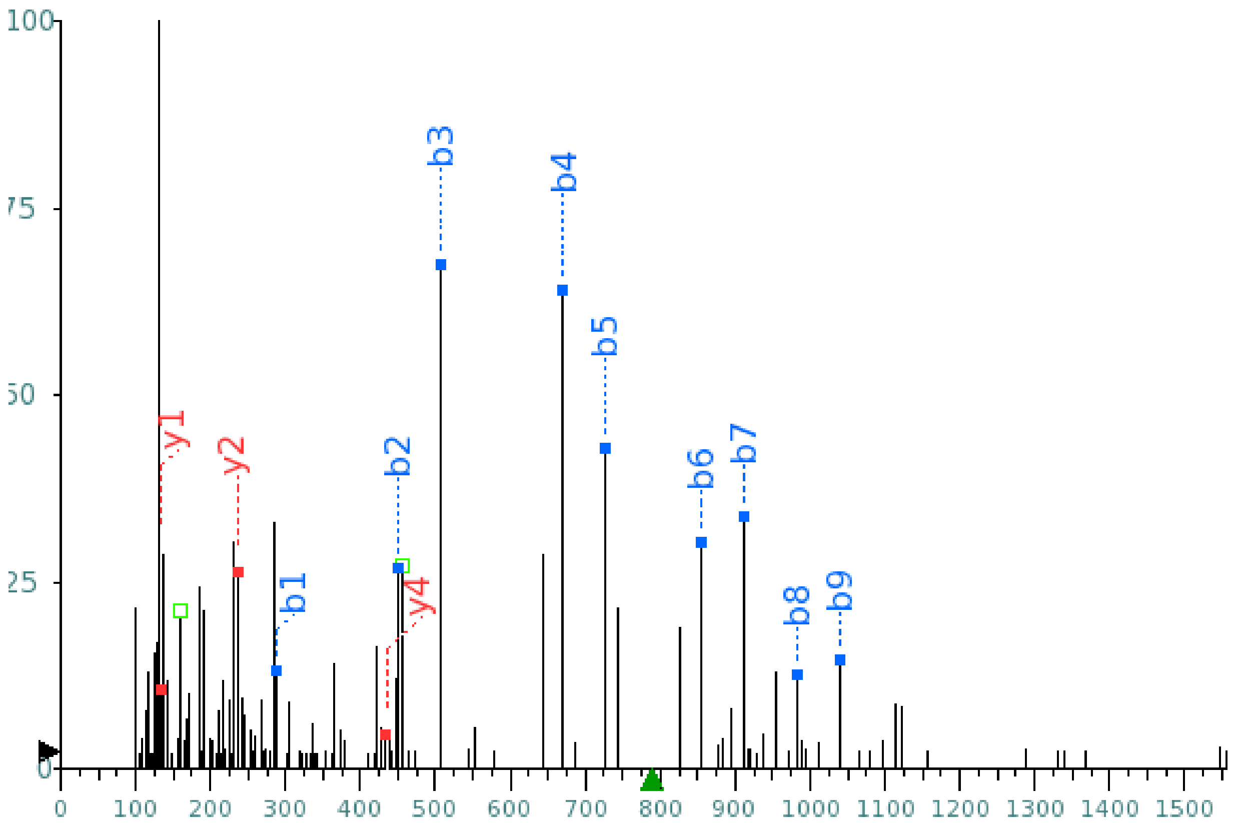

GYGYGQGAGTLSTD (+2)

Primary Reference: CSRP1_HUMAN
Search ID: 41761
Search Name: 20130328_ananiav_ERstress_TMT_TM_timecourse_trypsin_inj2_4mc_iaa
Scan#: 9266
Observed Mass: 788.379 (1.2 ppm)
PSM Score: 33.3

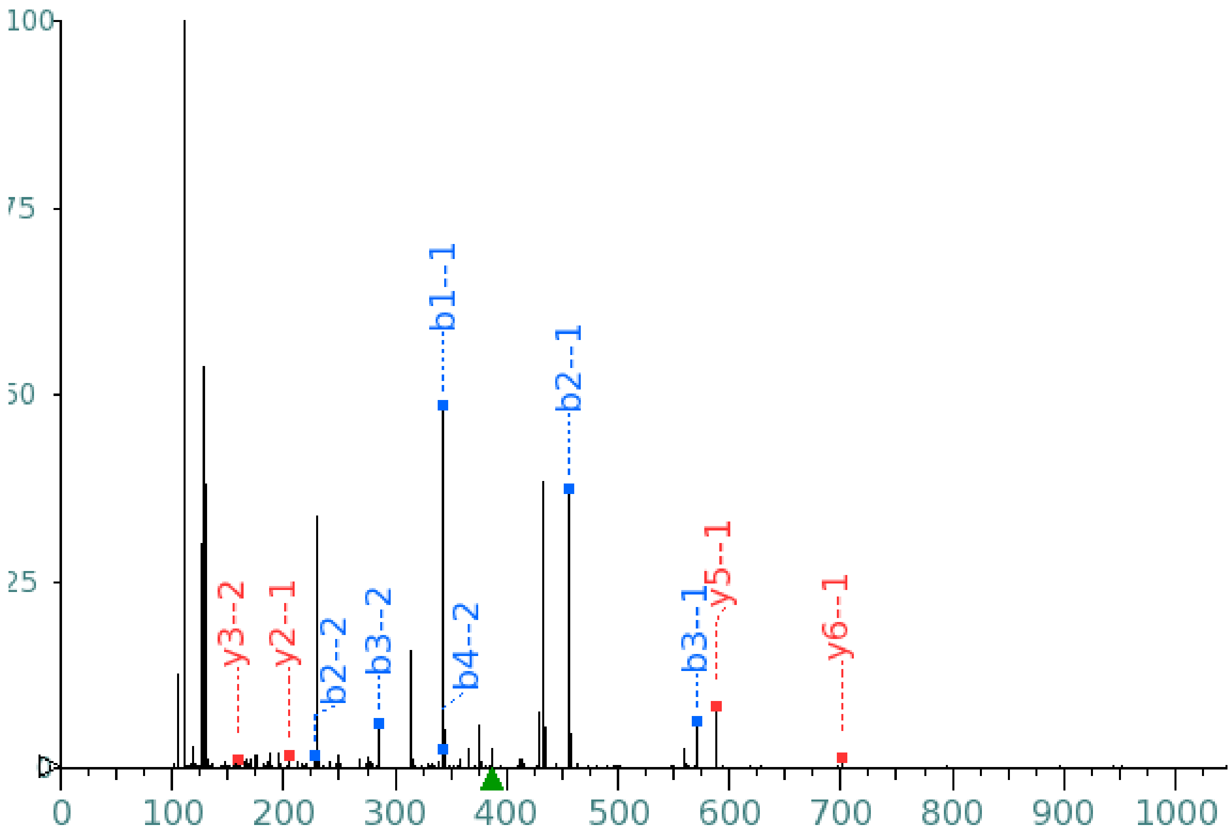

LLNLRLAD (+3)

Primary Reference: ##P66B_HUMAN
Search ID: 41761
Search Name: 20130328_ananiav_ERstress_TMT_TM_timecourse_trypsin_inj2_4mc_iaa
Scan#: 9268
Observed Mass: 386.2478 (3.4 ppm)
PSM Score: 10.92

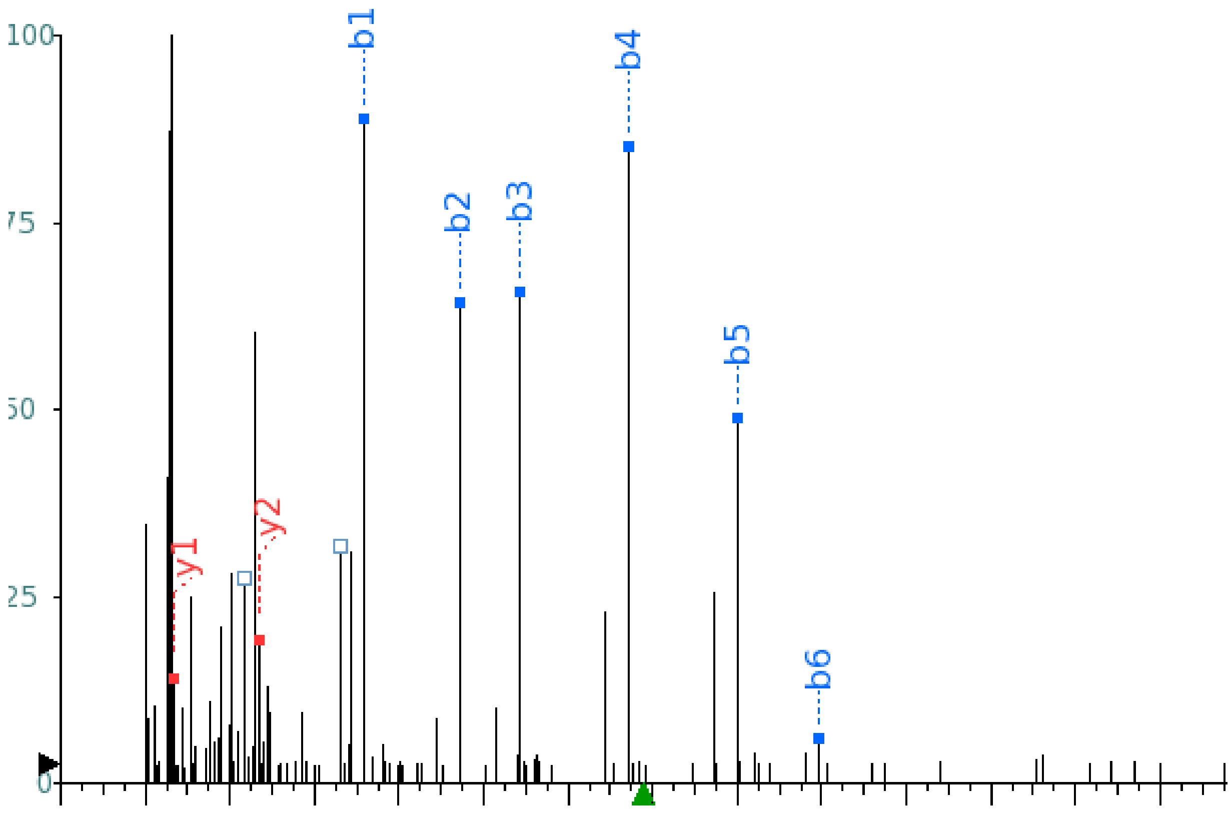

QLAEEPMDTD (+2)

Primary Reference: B4DKB3_HUMAN
Search ID: 41761
Search Name: 20130328_ananiav_ERstress_TMT_TM_timecourse_trypsin_inj2_4mc_iaa
Scan#: 9280
Observed Mass: 689.3246 (1 ppm)
PSM Score: 20.66

File name: 2015-02-04 04.01.53PM.pdf

Creation time: 2015-02-04 04:09PM

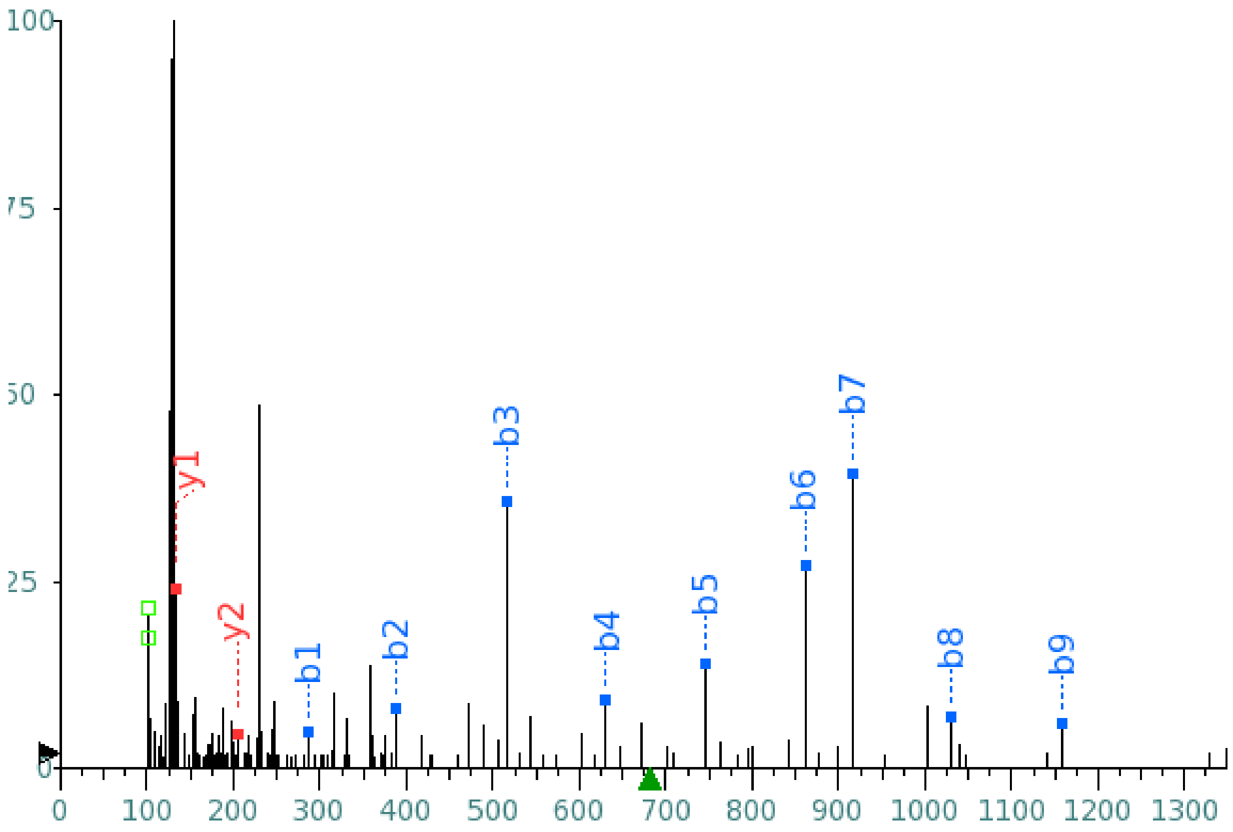

GTELDDGIQAD (+2)

Primary Reference: CASP7_HUMAN
Search ID: 41761
Search Name: 20130328_ananiav_ERstress_TMT_TM_timecourse_trypsin_inj2_4mc_iaa
Scan#: 9328
Observed Mass: 681.8335 (0.7 ppm)
PSM Score: 33.53

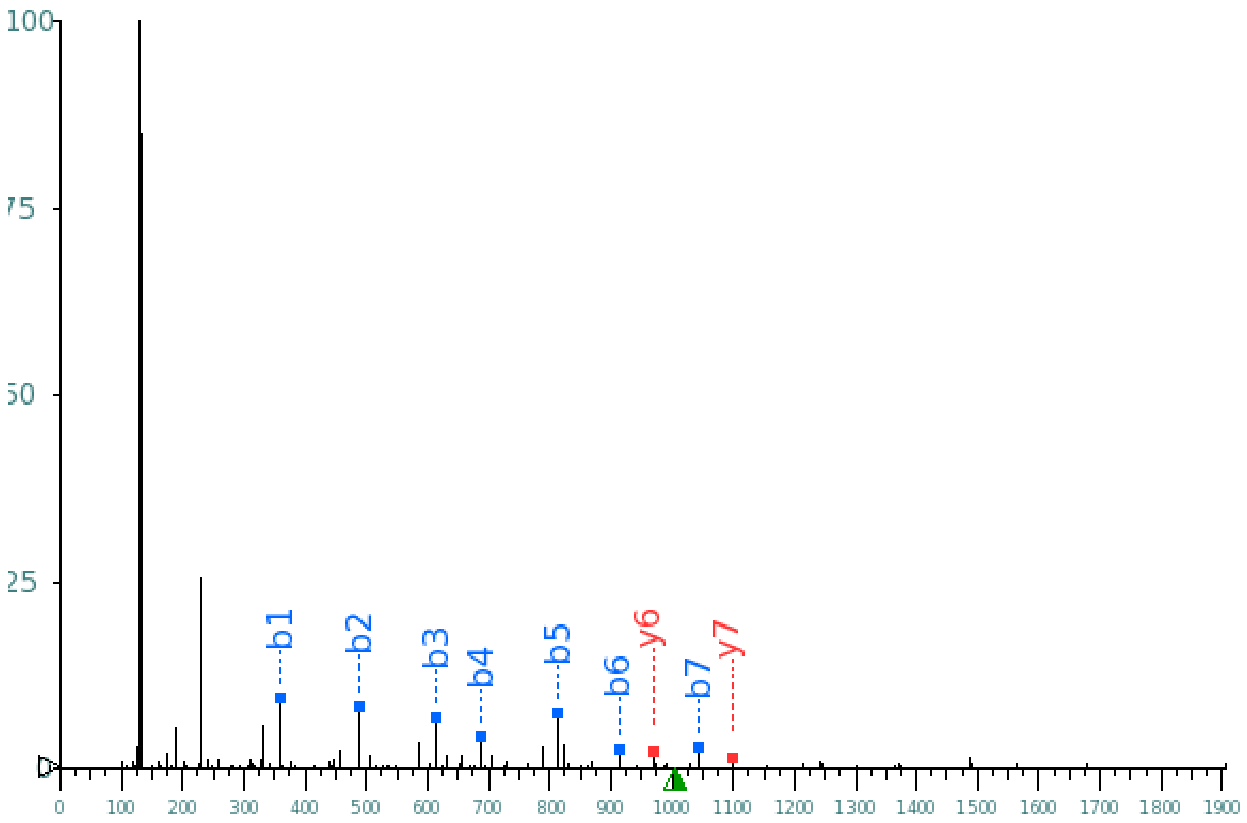

EQQAQVEKEDFSD (+2)

Primary Reference: SF3B2_HUMAN
Search ID: 41761
Search Name: 20130328_ananiav_ERstress_TMT_TM_timecourse_trypsin_inj2_4mc_iaa
Scan#: 9343
Observed Mass: 1006.0017 (-2.9 ppm)
PSM Score: 15.83

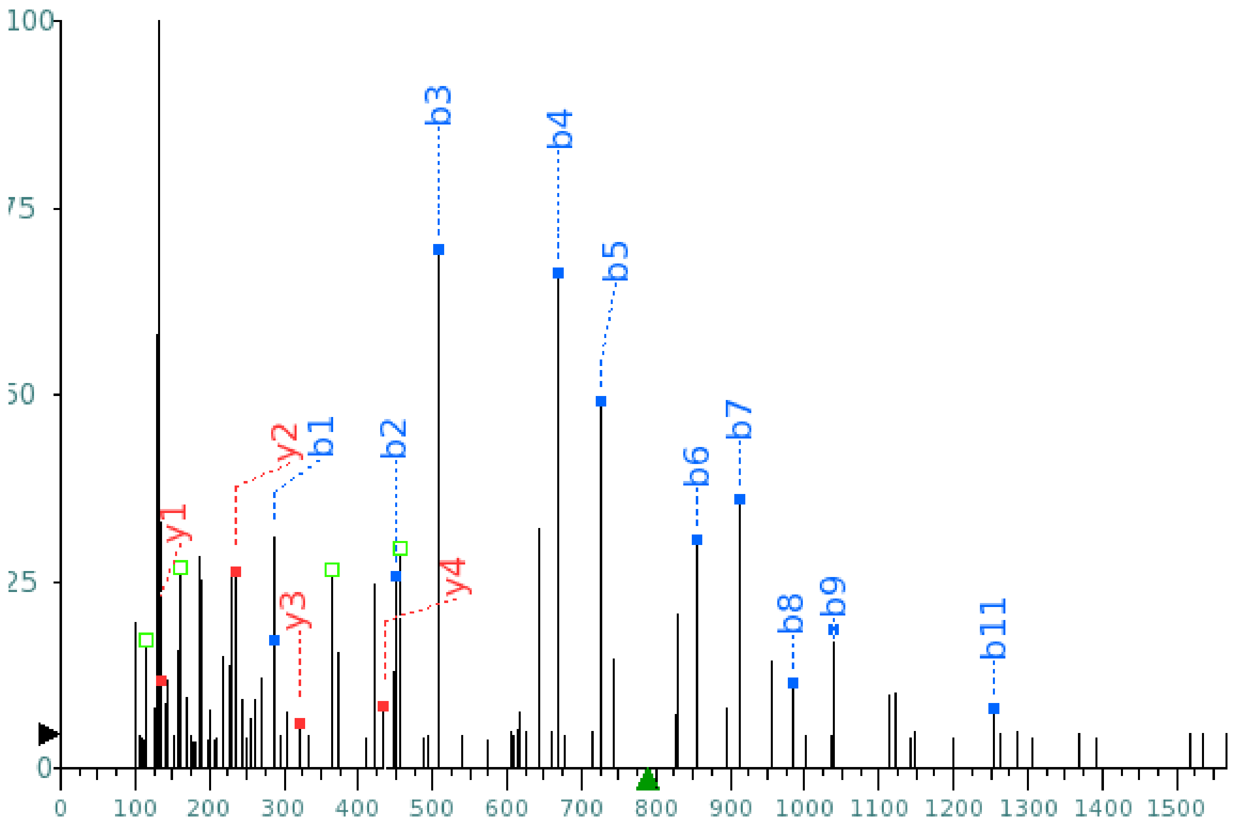

GYGYGQGAGTLSTD (+2)

Primary Reference: CSRP1_HUMAN
Search ID: 41761
Search Name: 20130328_ananiav_ERstress_TMT_TM_timecourse_trypsin_inj2_4mc_iaa
Scan#: 9364
Observed Mass: 788.3786 (0.7 ppm)
PSM Score: 49.54

File name: 2015-02-04 04.01.53PM.pdf

Creation time: 2015-02-04 04:09PM

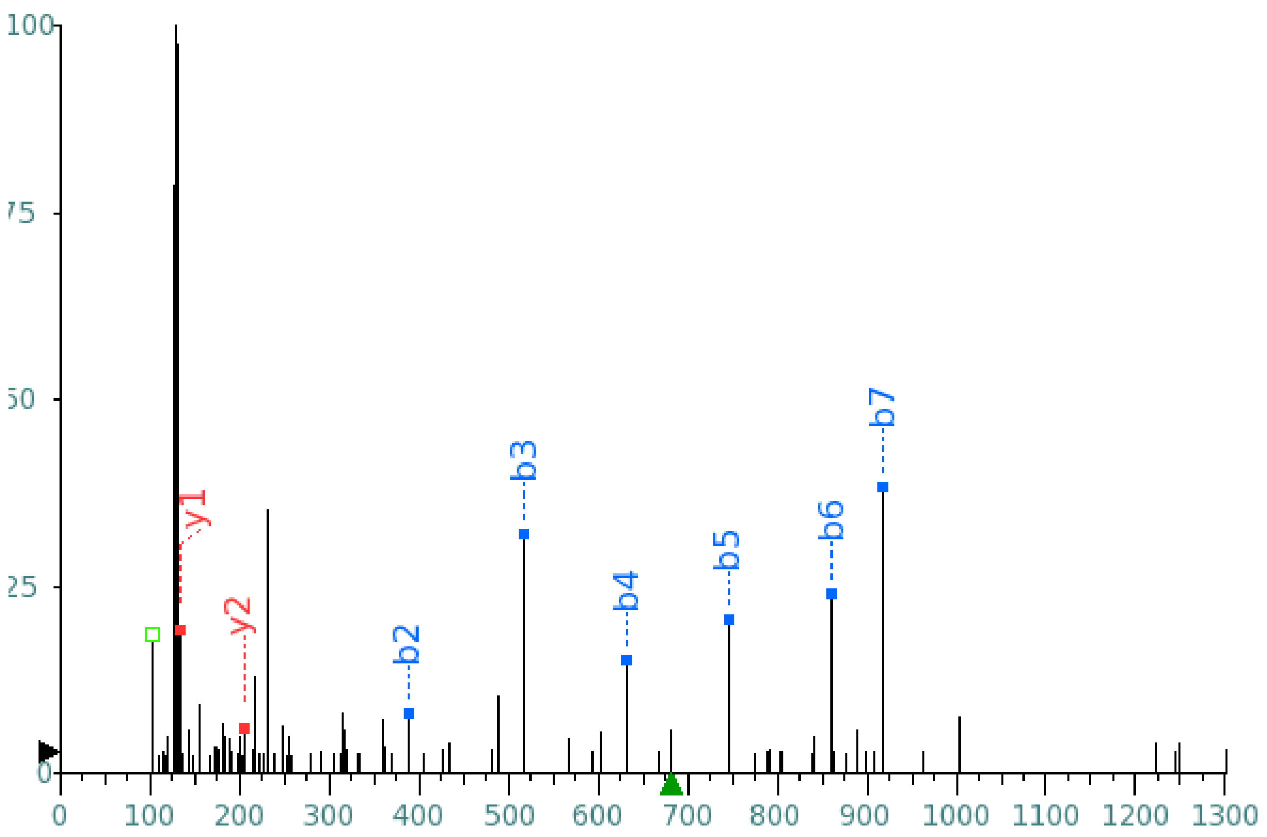

GTELDDGIQAD (+2)

Primary Reference: CASP7_HUMAN
Search ID: 41761
Search Name: 20130328_ananiav_ERstress_TMT_TM_timecourse_trypsin_inj2_4mc_iaa
Scan#: 9420
Observed Mass: 681.8338 (1.3 ppm)
PSM Score: 20.23

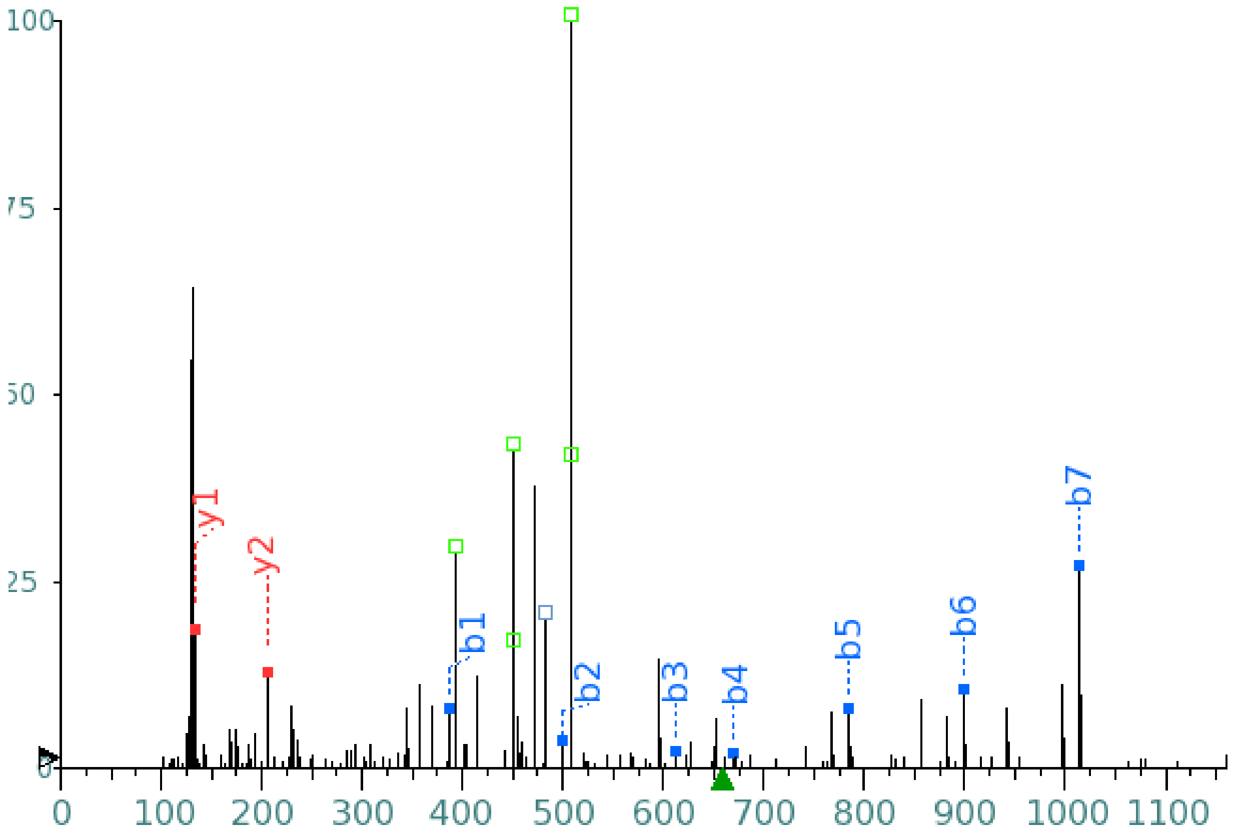

RLLGDDDVAD (+2)

Primary Reference: WDR62_HUMAN
Search ID: 41761
Search Name: 20130328_ananiav_ERstress_TMT_TM_timecourse_trypsin_inj2_4mc_iaa
Scan#: 9441
Observed Mass: 659.3467 (1 ppm)
PSM Score: 16.72

File name: 2015-02-04 04.01.53PM.pdf

Creation time: 2015-02-04 04:09PM

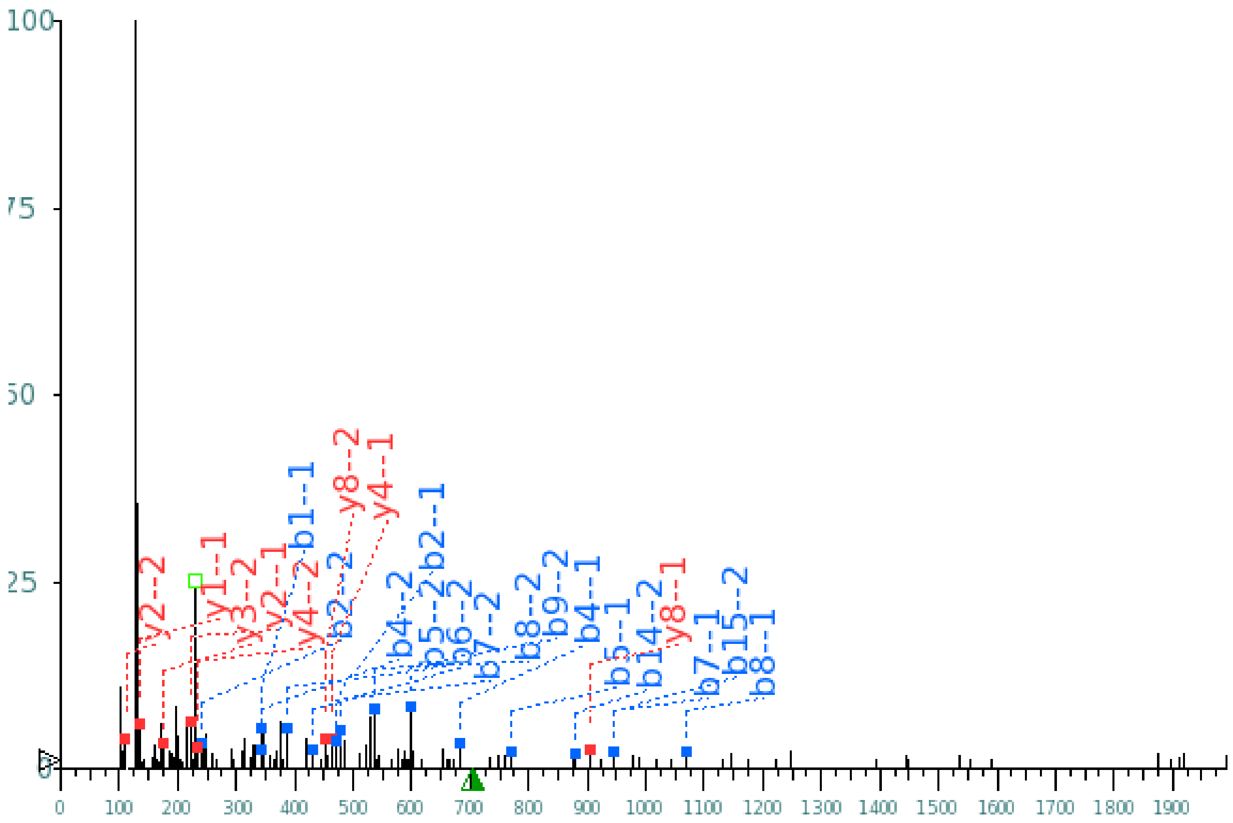

IHSNSSSEEVSQELESD (+3)

Primary Reference: PCLI1_HUMAN
Search ID: 41761
Search Name: 20130328_ananiav_ERstress_TMT_TM_timecourse_trypsin_inj2_4mc_iaa
Scan#: 9446
Observed Mass: 702.6608 (0.4 ppm)
PSM Score: 14.78

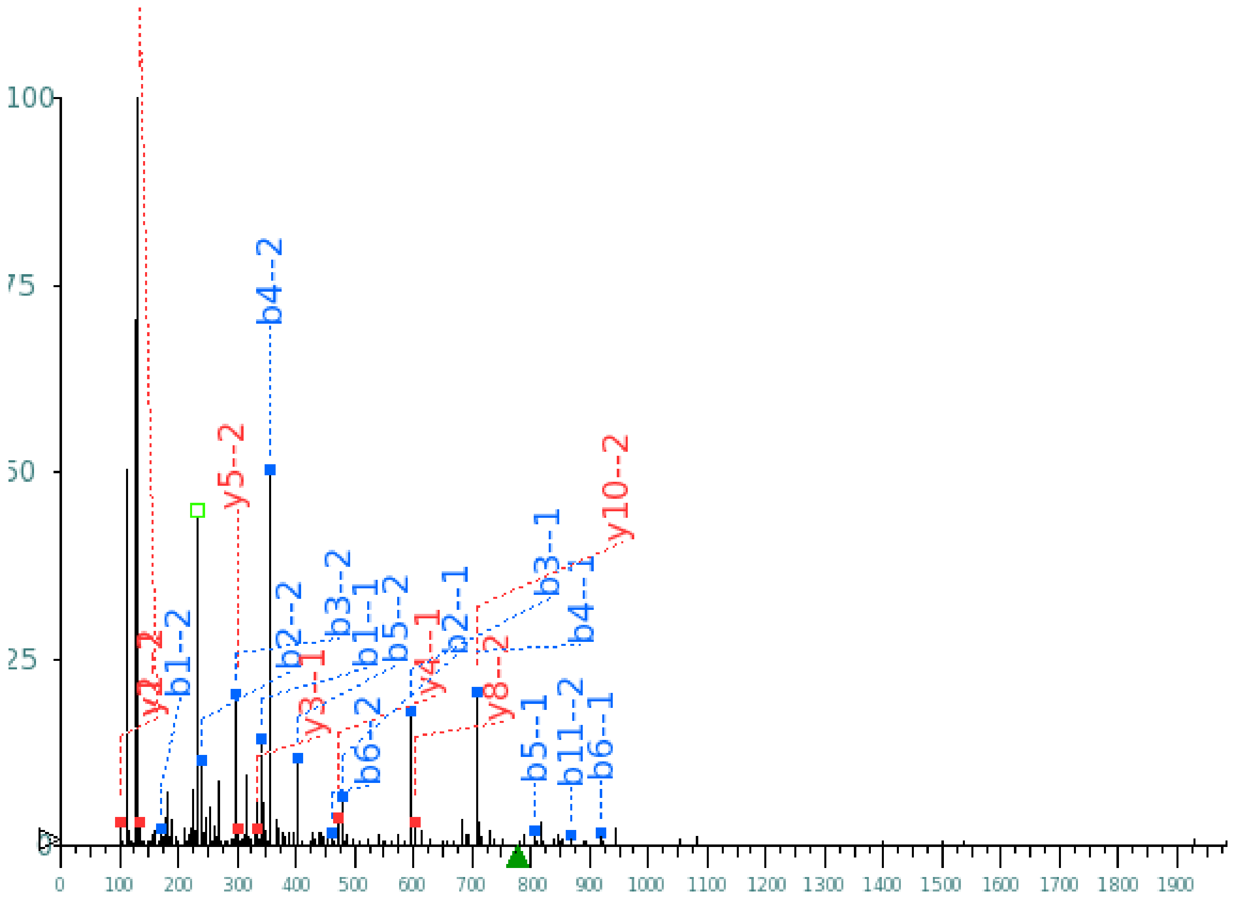

LHDNPLTDENKEHEAD (+3)

Primary Reference: NAA15_HUMAN
Search ID: 41761
Search Name: 20130328_ananiav_ERstress_TMT_TM_timecourse_trypsin_inj2_4mc_iaa
Scan#: 9461
Observed Mass: 779.0578 (1 ppm)
PSM Score: 15.5

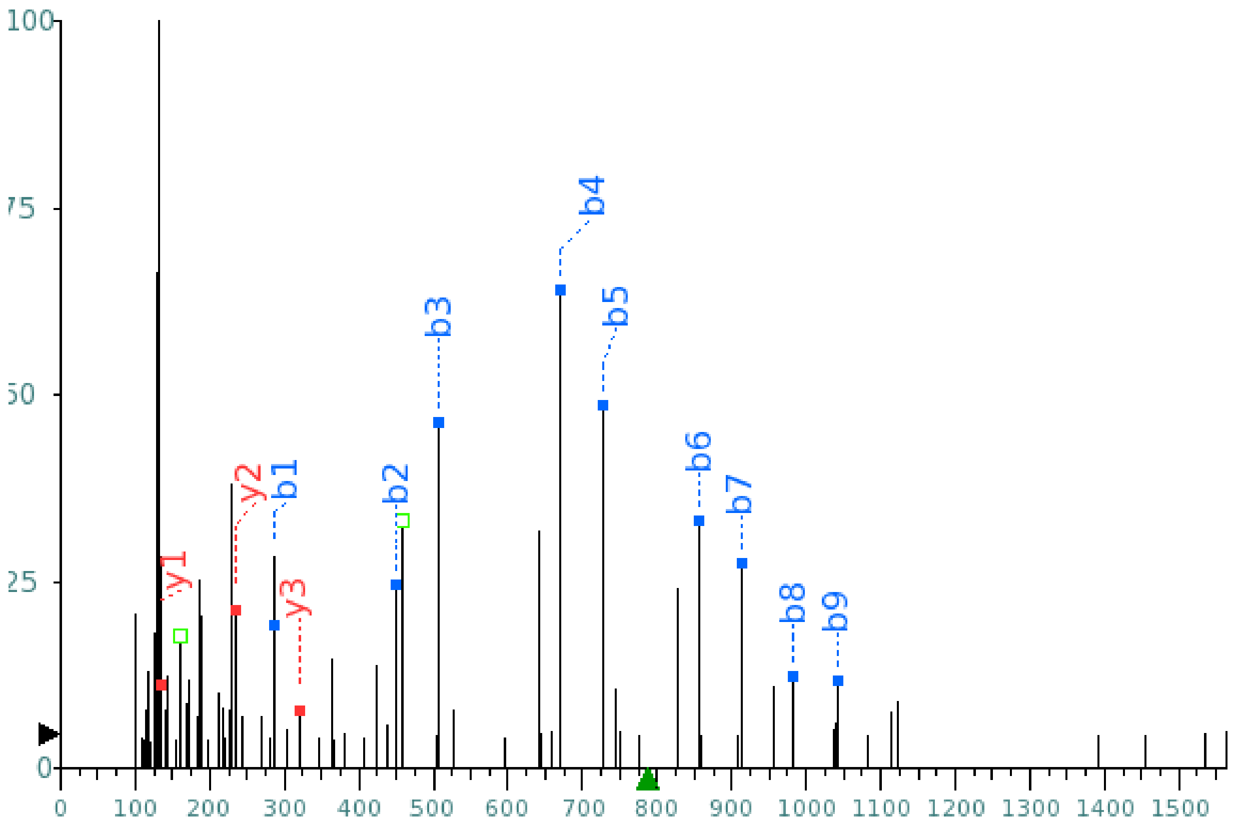

GYGYGGQGAGTLSTD (+2)

Primary Reference: CSRP1_HUMAN
Search ID: 41761
Search Name: 20130328_ananiav_ERstress_TMT_TM_timecourse_trypsin_inj2_4mc_iaa
Scan#: 9464
Observed Mass: 788.3792 (1.4 ppm)
PSM Score: 39.11

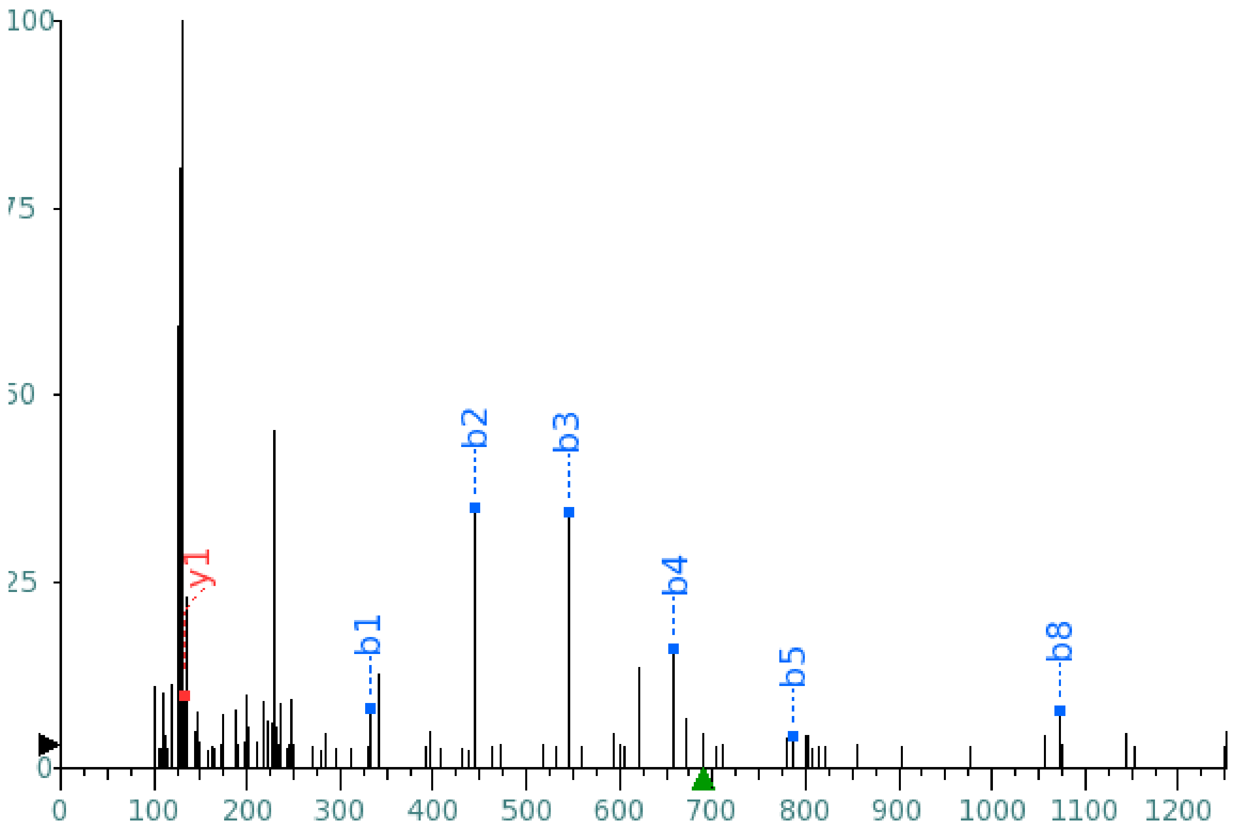

TDVIQGDDVAD (+2)

Primary Reference: RBP2_HUMAN
Search ID: 41761
Search Name: 20130328_ananiav_ERstress_TMT_TM_timecourse_trypsin_inj2_4mc_iaa
Scan#: 9466
Observed Mass: 688.8401 (-1 ppm)
PSM Score: 12.51

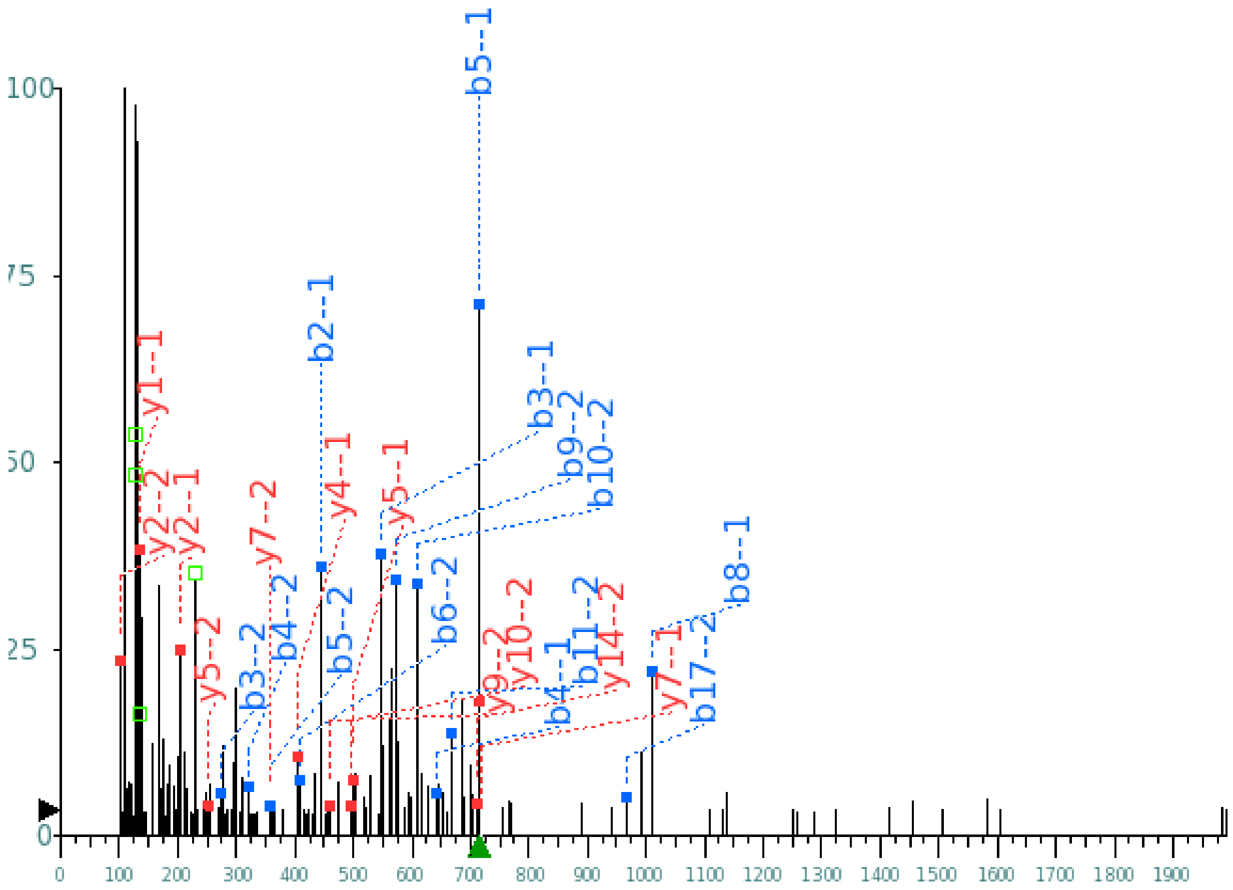

TDTPAPPTHALSQSPAEAD (+3)

Primary Reference: SNPC4_HUMAN
Search ID: 41761
Search Name: 20130328_ananiav_ERstress_TMT_TM_timecourse_trypsin_inj2_4mc_iaa
Scan#: 9468
Observed Mass: 712.3519 (-2 ppm)
PSM Score: 18.37

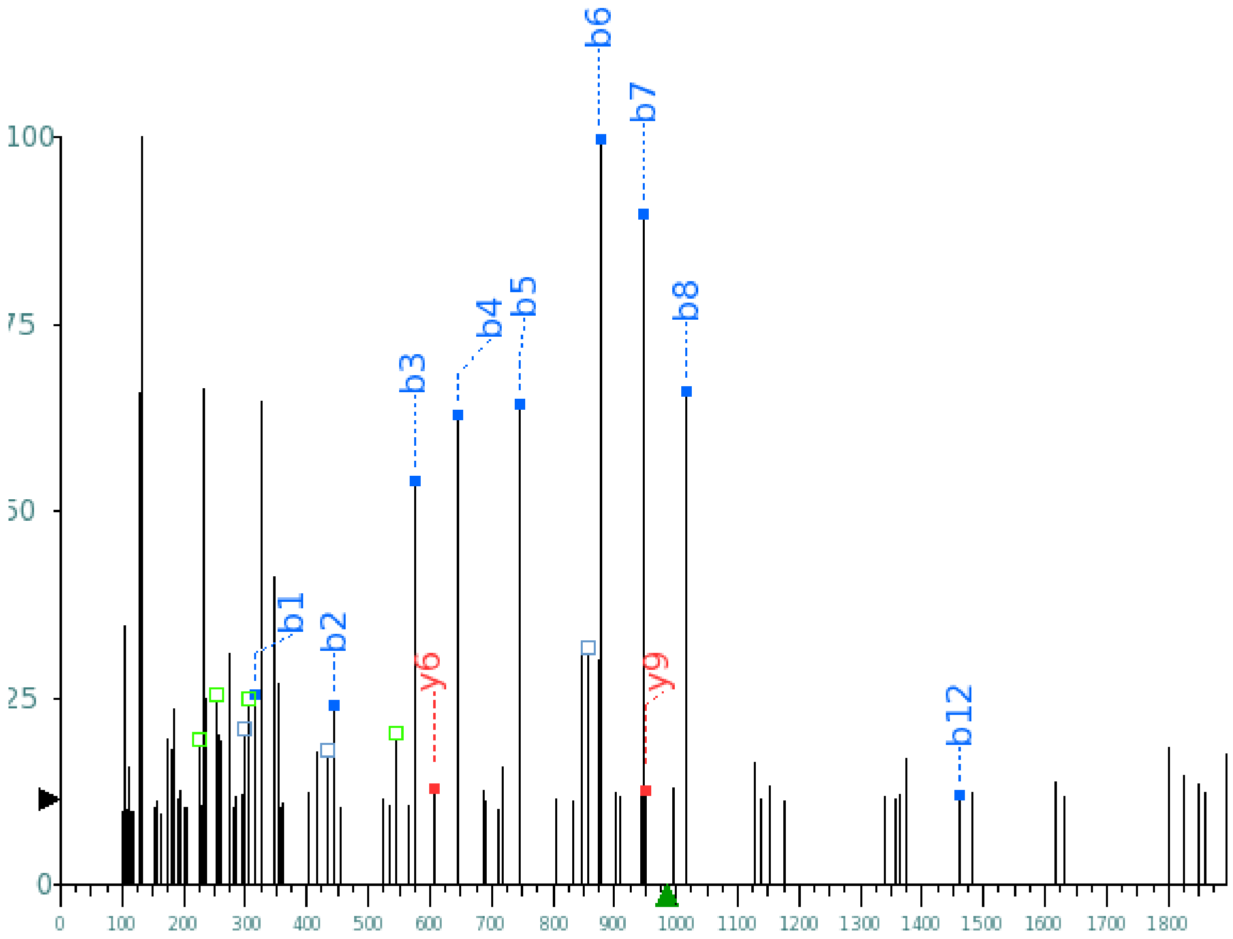

SQEATEAAPSC*VGDMAD (+2)

Primary Reference: SEPT9_HUMAN
Search ID: 41761
Search Name: 20130328_ananiav_ERstress_TMT_TM_timecourse_trypsin_inj2_4mc_iaa
Scan#: 9533
Observed Mass: 984.4309 (1.1 ppm)
PSM Score: 37.62

File name: 2015-02-04 04.01.53PM.pdf

Creation time: 2015-02-04 04:09PM

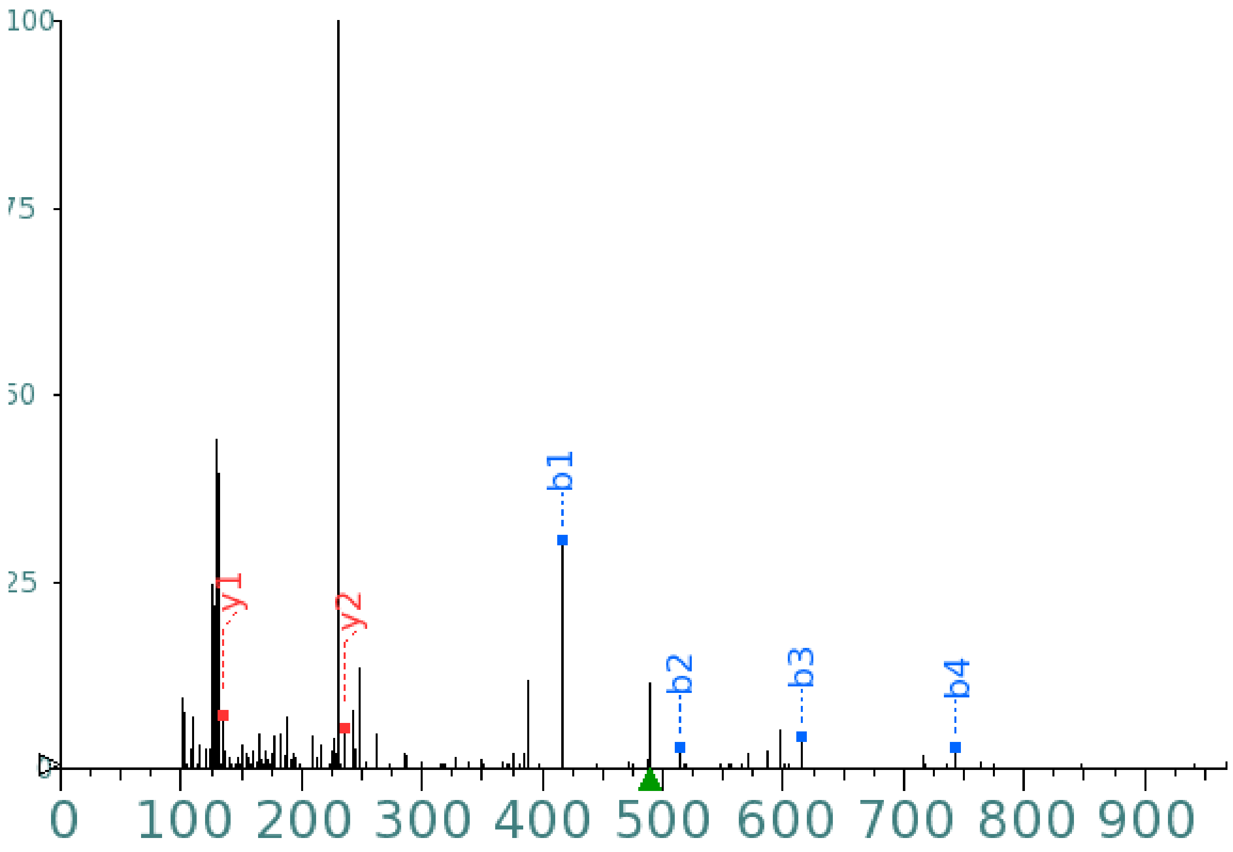

WPTETD (+2)

Primary Reference: MAP4_HUMAN
Search ID: 41761
Search Name: 20130328_ananiav_ERstress_TMT_TM_timecourse_trypsin_inj2_4mc_iaa
Scan#: 9541
Observed Mass: 489.2432 (1.4 ppm)
PSM Score: 8.89

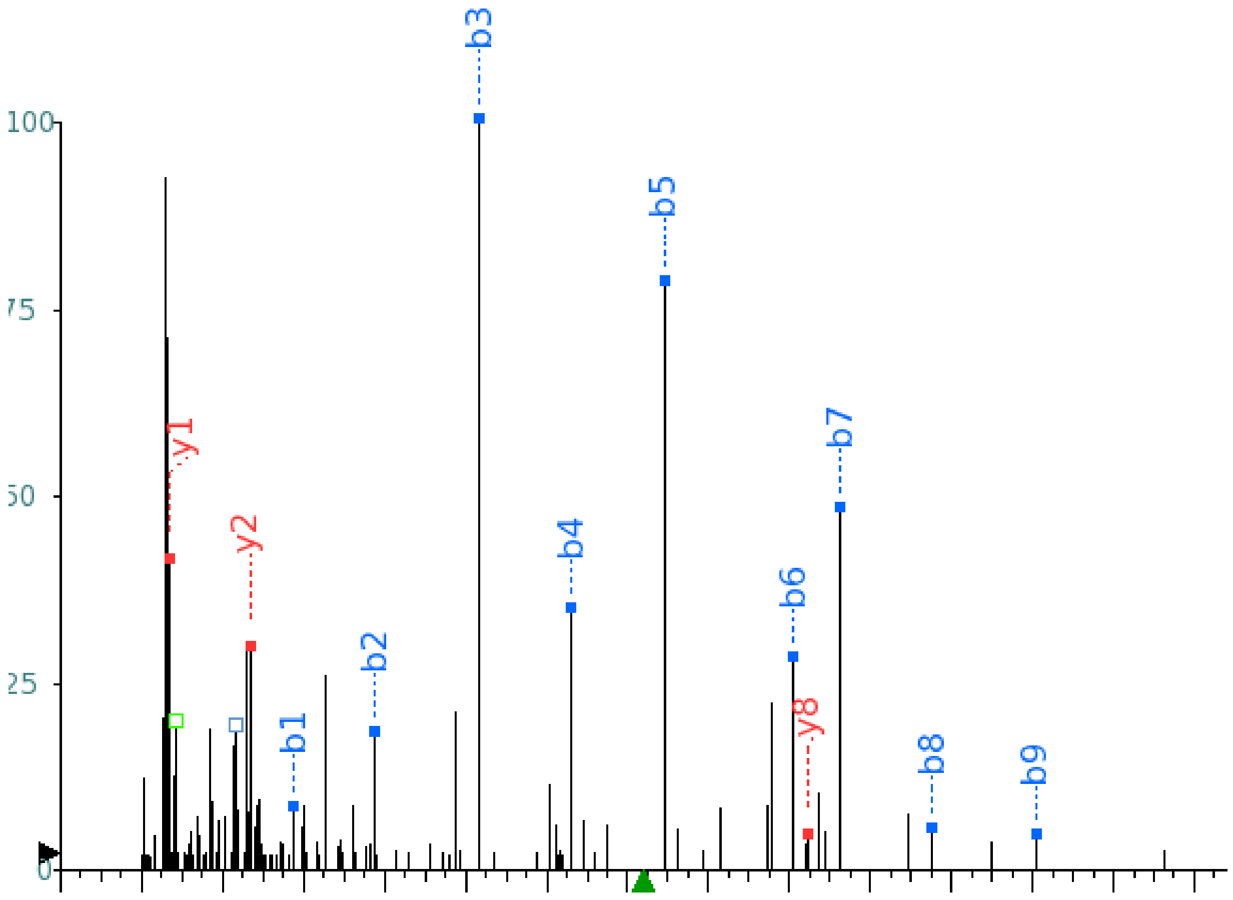

GTELDC*GIETD (+2)

Primary Reference: CASP3_HUMAN
Search ID: 41761
Search Name: 20130328_ananiav_ERstress_TMT_TM_timecourse_trypsin_inj2_4mc_iaa
Scan#: 9554
Observed Mass: 719.8327 (0.9 ppm)
PSM Score: 39.71

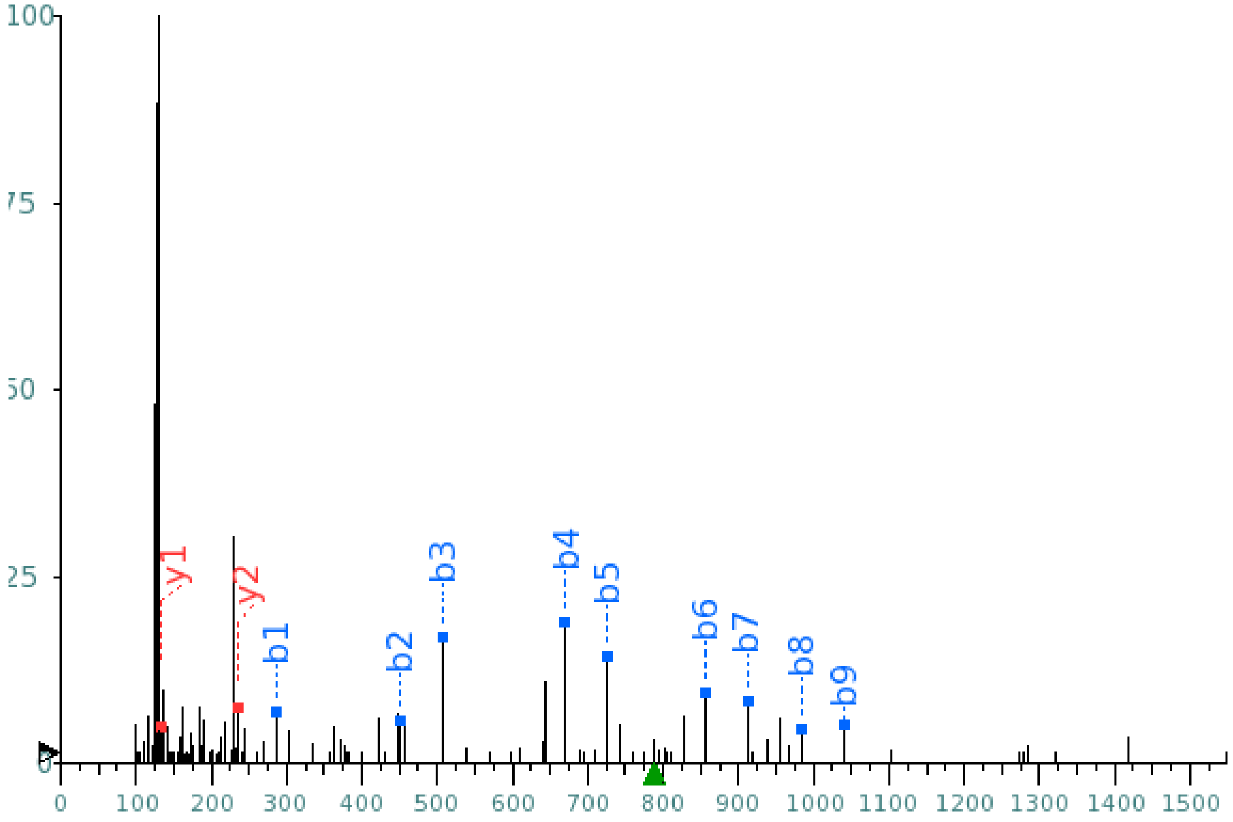

GYGYGQGAGTLSTD (+2)

Primary Reference: CSRP1_HUMAN
Search ID: 41761
Search Name: 20130328_ananiav_ERstress_TMT_TM_timecourse_trypsin_inj2_4mc_iaa
Scan#: 9567
Observed Mass: 788.3783 (0.3 ppm)
PSM Score: 29.73

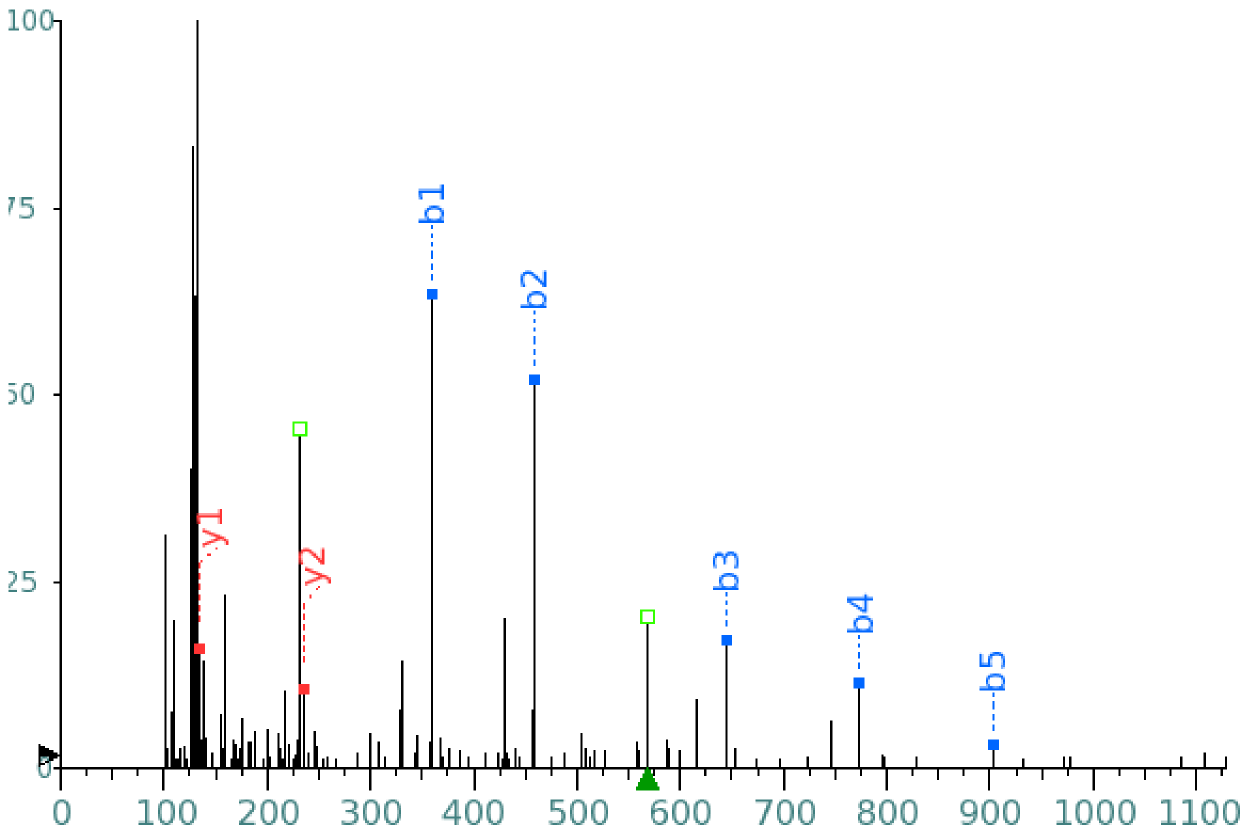

EVWEETD (+2)

Primary Reference: NUCB2_HUMAN
Search ID: 41761
Search Name: 20130328_ananiav_ERstress_TMT_TM_timecourse_trypsin_inj2_4mc_iaa
Scan#: 9574
Observed Mass: 568.7694 (0.5 ppm)
PSM Score: 24.5

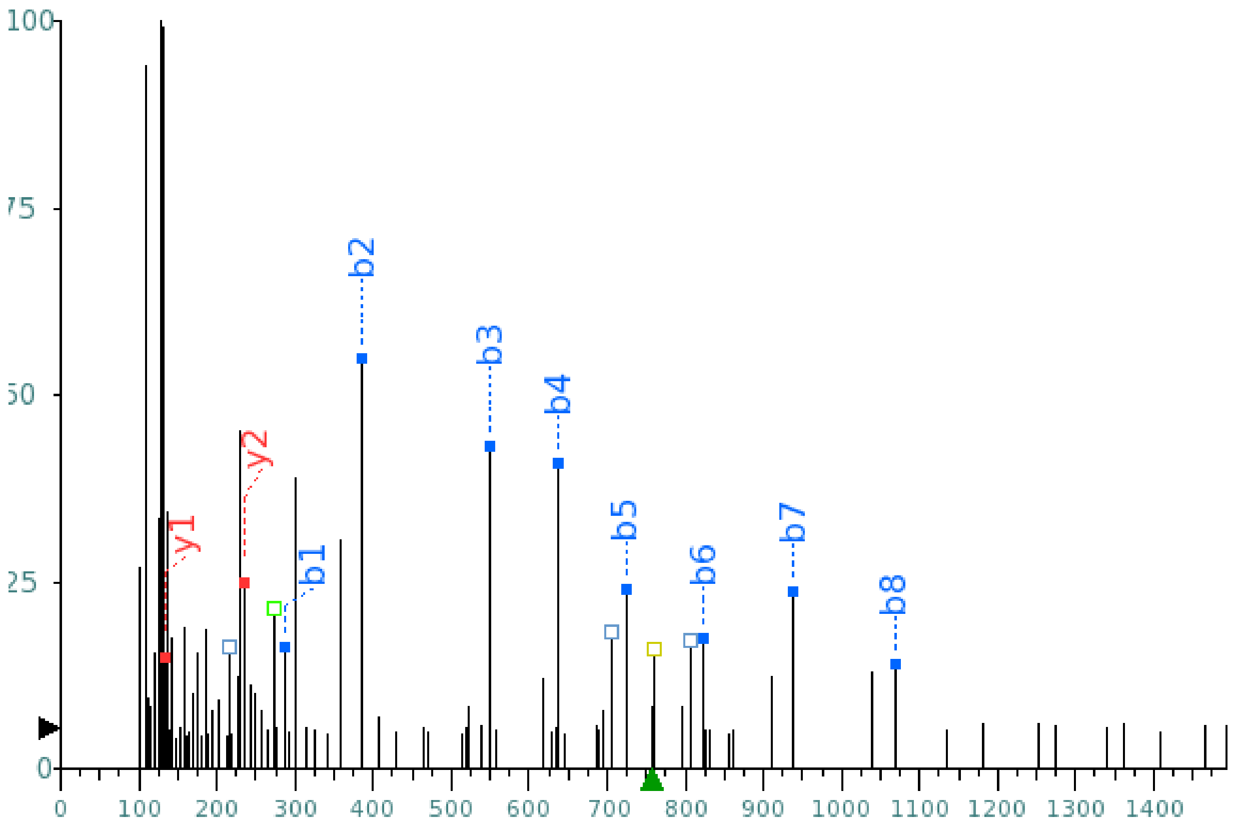

GVYSSTNELTTD (+2)

Primary Reference: N4BP1_HUMAN
Search ID: 41761
Search Name: 20130328_ananiav_ERstress_TMT_TM_timecourse_trypsin_inj2_4mc_iaa
Scan#: 9576
Observed Mass: 758.3727 (0.3 ppm)
PSM Score: 39.06

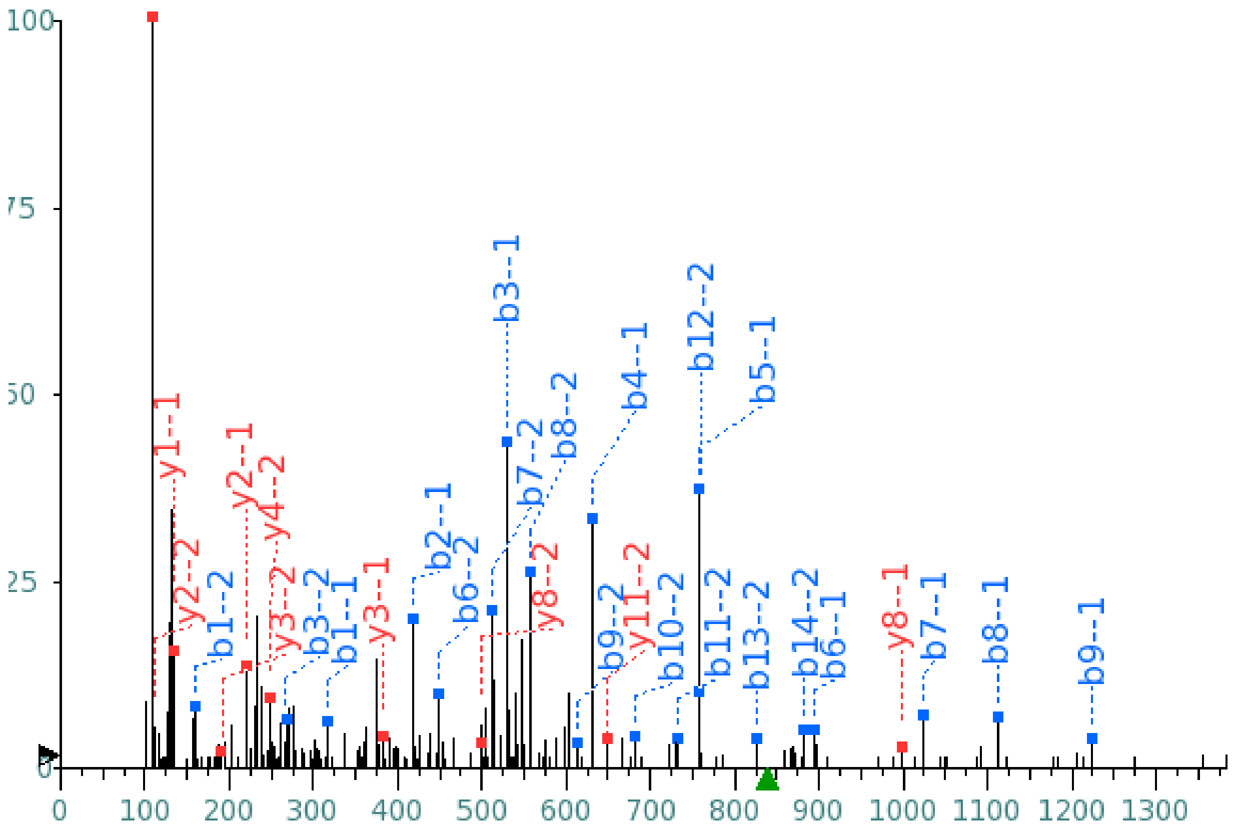

STLVQHESIHTGENPYDC*SD (+3)

Primary Reference: ZN134_HUMAN
Search ID: 41761
Search Name: 20130328_ananiav_ERstress_TMT_TM_timecourse_trypsin_inj2_4mc_iaa
Scan#: 9590
Observed Mass: 840.0501 (0.2 ppm)
PSM Score: 49.86

File name: 2015-02-04 04.01.53PM.pdf

Creation time: 2015-02-04 04:09PM

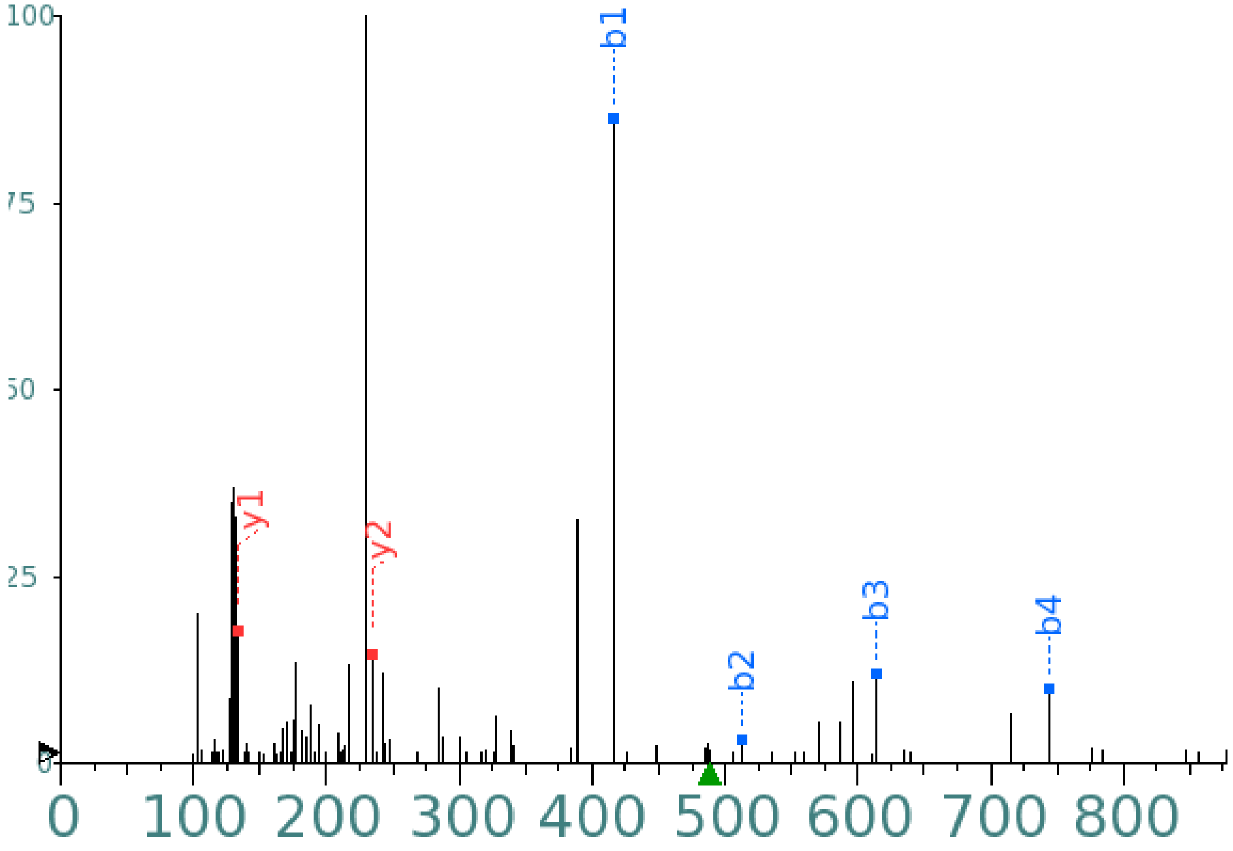

WPTETD (+2)

Primary Reference: MAP4_HUMAN
Search ID: 41761
Search Name: 20130328_ananiav_ERstress_TMT_TM_timecourse_trypsin_inj2_4mc_iaa
Scan#: 9628
Observed Mass: 489.2428 (0.6 ppm)
PSM Score: 11.62

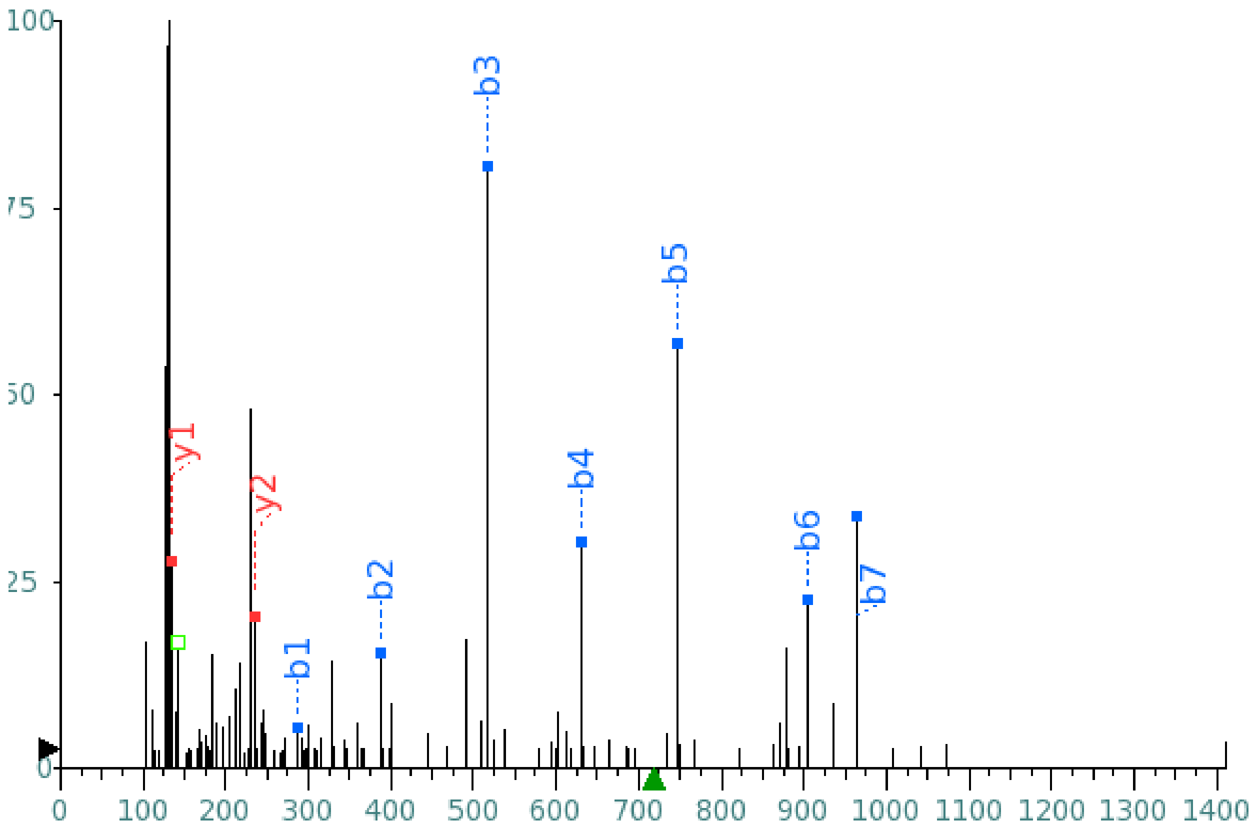

GTELDC*GIETD (+2)

Primary Reference: CASP3_HUMAN
Search ID: 41761
Search Name: 20130328_ananiav_ERstress_TMT_TM_timecourse_trypsin_inj2_4mc_iaa
Scan#: 9639
Observed Mass: 719.8325 (0.5 ppm)
PSM Score: 23.94

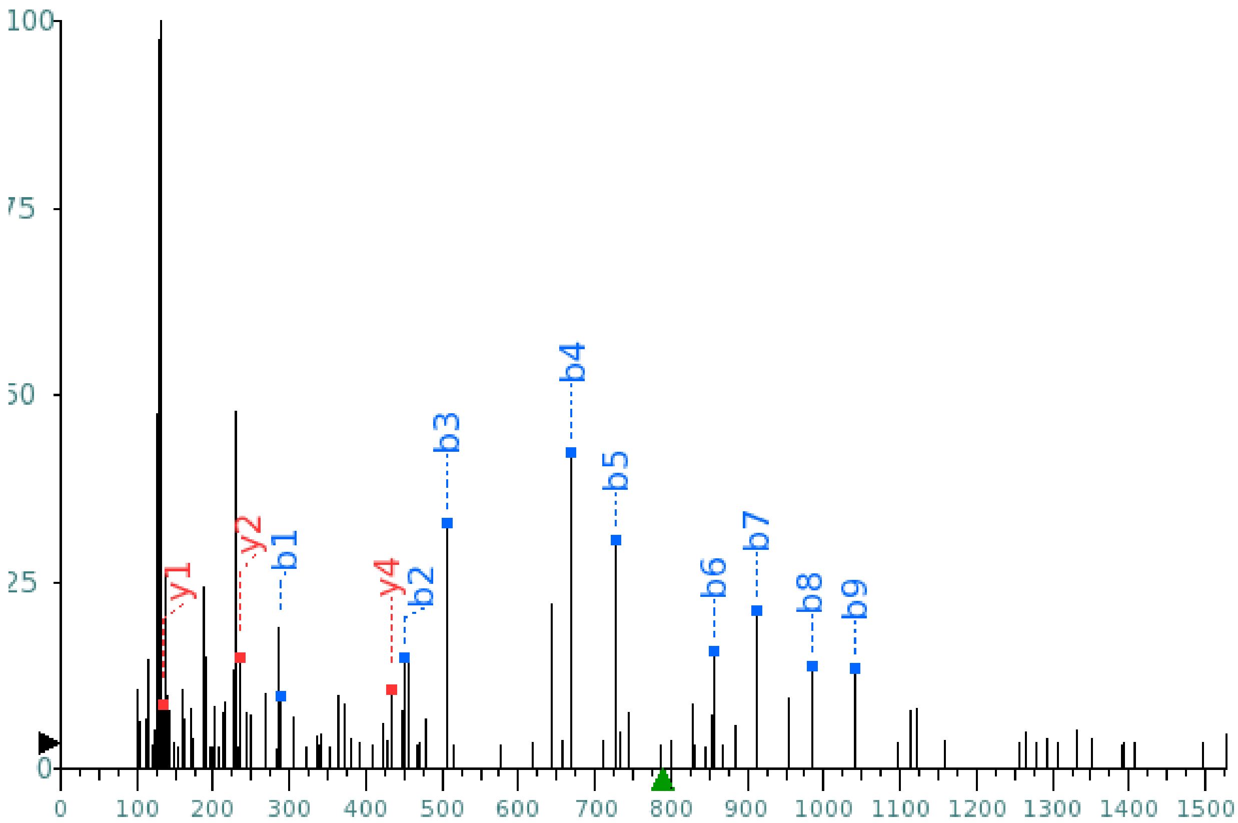

GYGYGGQGAGTLSTD (+2)

Primary Reference: CSRP1_HUMAN
Search ID: 41761
Search Name: 20130328_ananiav_ERstress_TMT_TM_timecourse_trypsin_inj2_4mc_iaa
Scan#: 9656
Observed Mass: 788.3781 (0.1 ppm)
PSM Score: 29.19

File name: 2015-02-04 04.01.53PM.pdf

Creation time: 2015-02-04 04:09PM

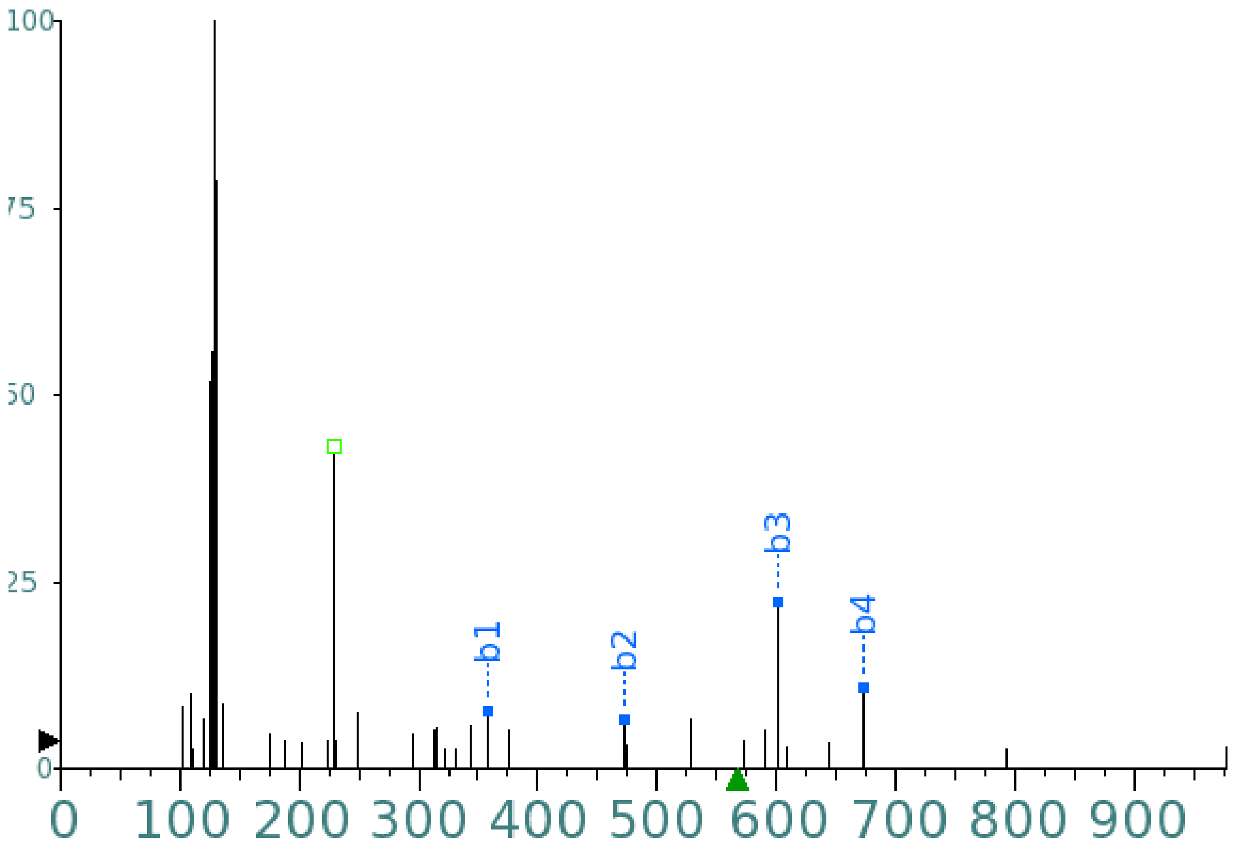

EIEAIESD (+2)

Primary Reference: RSRC1_HUMAN
Search ID: 41761
Search Name: 20130328_ananiav_ERstress_TMT_TM_timecourse_trypsin_inj2_4mc_iaa
Scan#: 9679
Observed Mass: 567.7909 (1.5 ppm)
PSM Score: 14.58

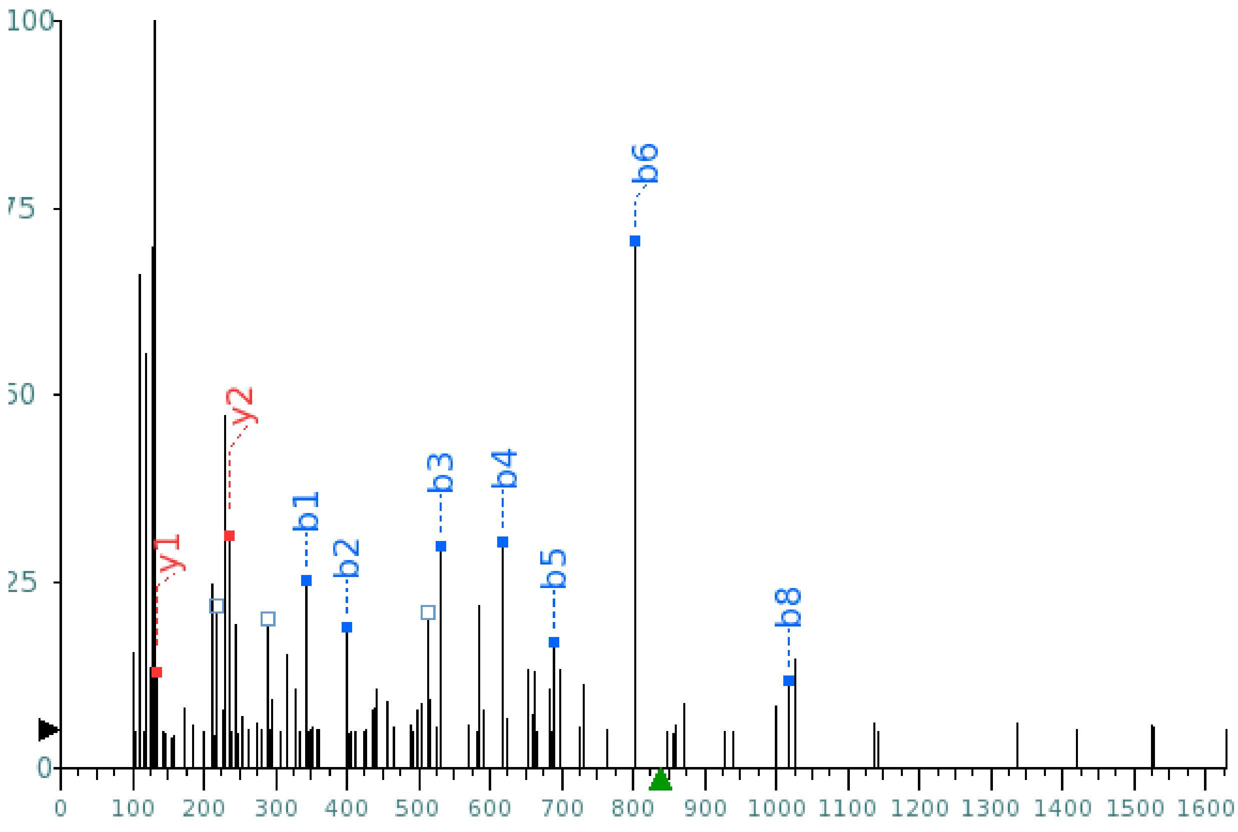

LGMSADPDNEDATD (+2)

Primary Reference: ZC11A_HUMAN
Search ID: 41761
Search Name: 20130328_ananiav_ERstress_TMT_TM_timecourse_trypsin_inj2_4mc_iaa
Scan#: 9772
Observed Mass: 840.3677 (0.8 ppm)
PSM Score: 20.96

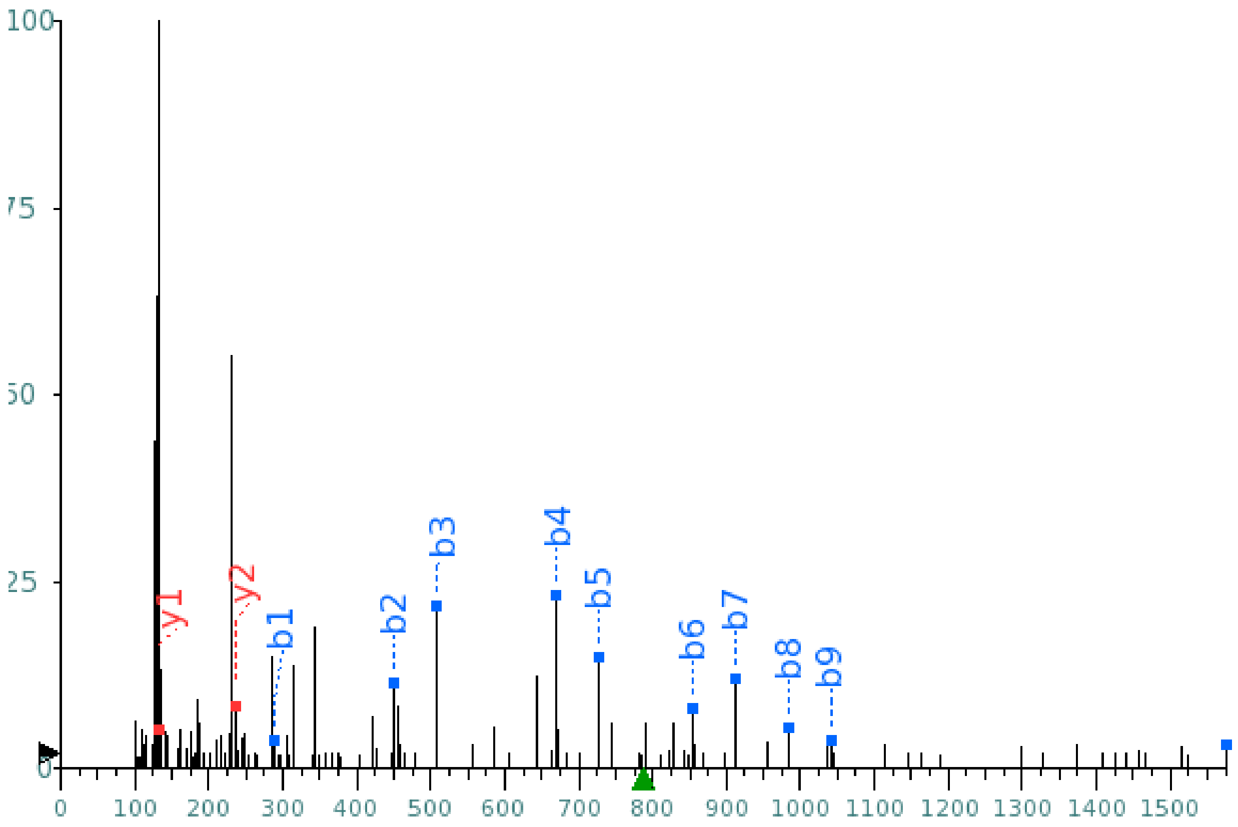

GYGYGQGAGTLSTD (+2)

Primary Reference: CSRP1_HUMAN
Search ID: 41761
Search Name: 20130328_ananiav_ERstress_TMT_TM_timecourse_trypsin_inj2_4mc_iaa
Scan#: 9791
Observed Mass: 788.3788 (0.9 ppm)
PSM Score: 25.19

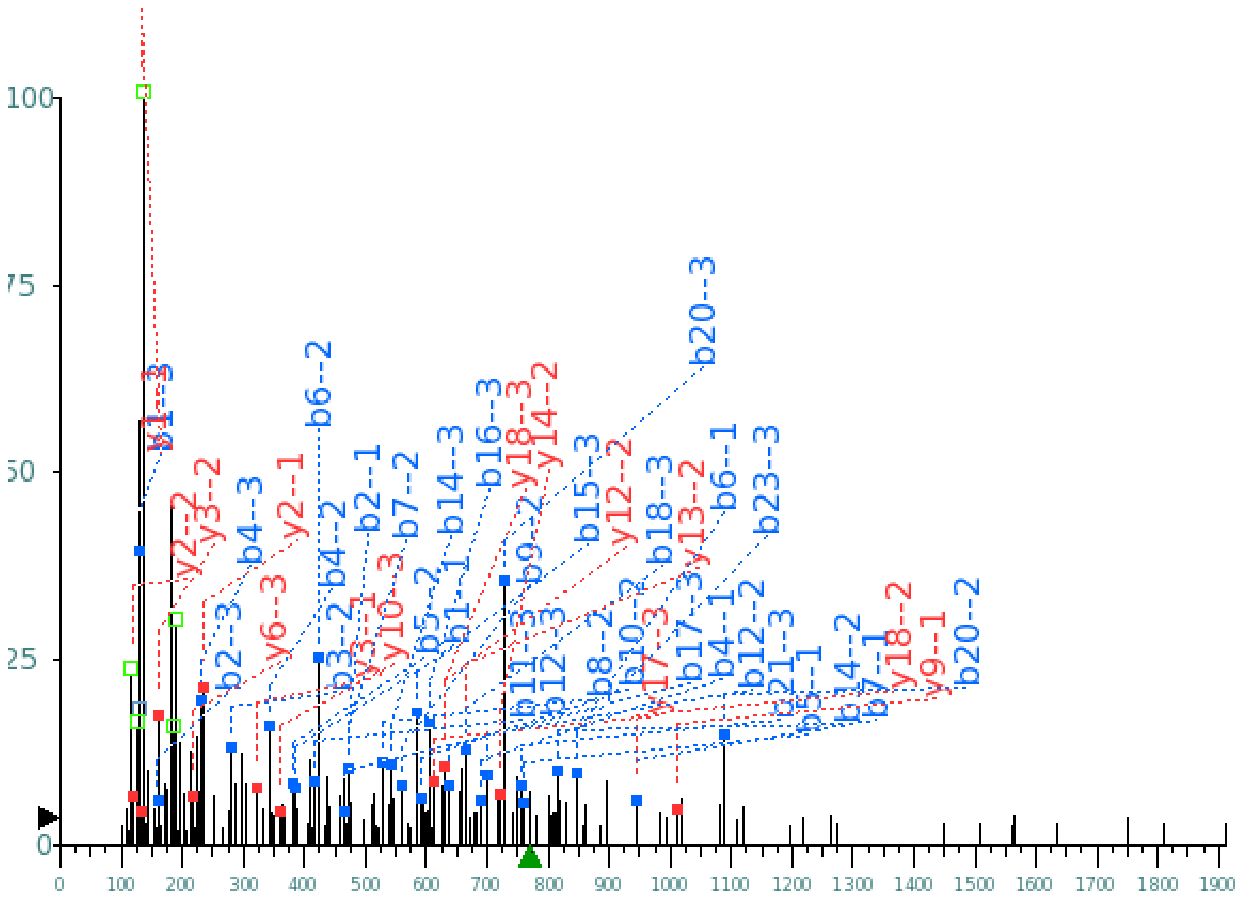

RSSEASPLGAGRPHNASSADSYDPISTD (+4)

Primary Reference: GLI2_HUMAN
Search ID: 41761
Search Name: 20130328_ananiav_ERstress_TMT_TM_timecourse_trypsin_inj2_4mc_iaa
Scan#: 9843
Observed Mass: 769.373 (2.9 ppm)
PSM Score: 24.81

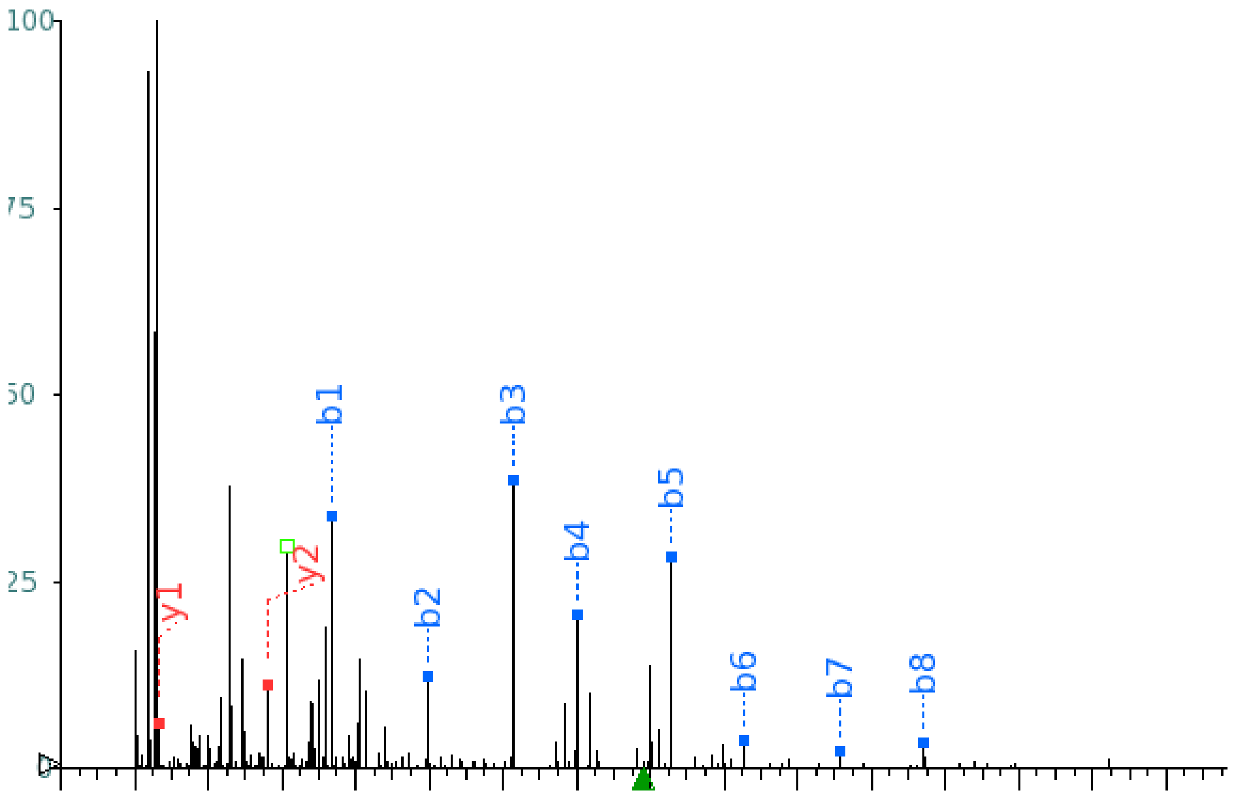

HMDSQVEDEFD (+2)

Primary Reference: SLBP_HUMAN
Search ID: 41761
Search Name: 20130328_ananiav_ERstress_TMT_TM_timecourse_trypsin_inj2_4mc_iaa
Scan#: 9883
Observed Mass: 790.8405 (0.1 ppm)
PSM Score: 37.21

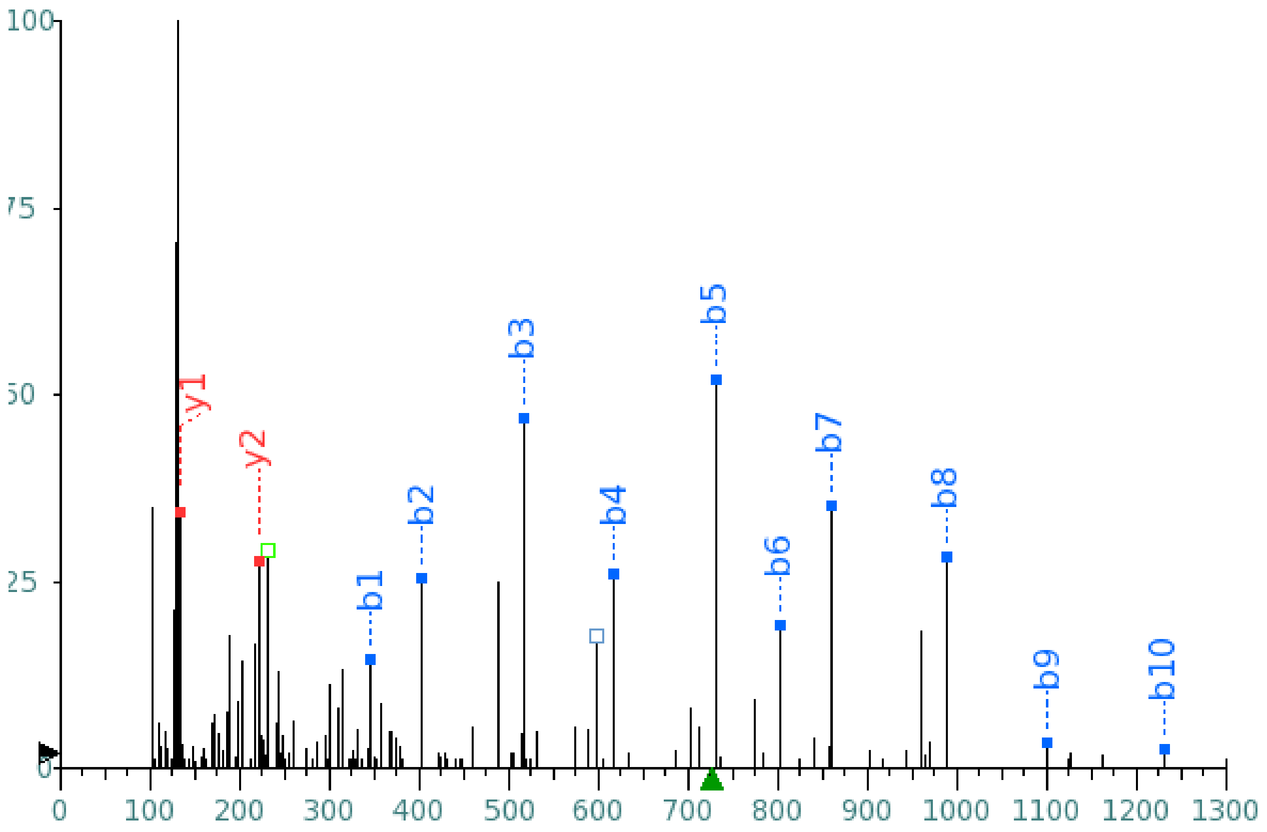

DGLTNAGELESD (+2)

Primary Reference: BMI1_HUMAN
Search ID: 41761
Search Name: 20130328_ananiav_ERstress_TMT_TM_timecourse_trypsin_inj2_4mc_iaa
Scan#: 9884
Observed Mass: 725.3493 (0.5 ppm)
PSM Score: 68.77

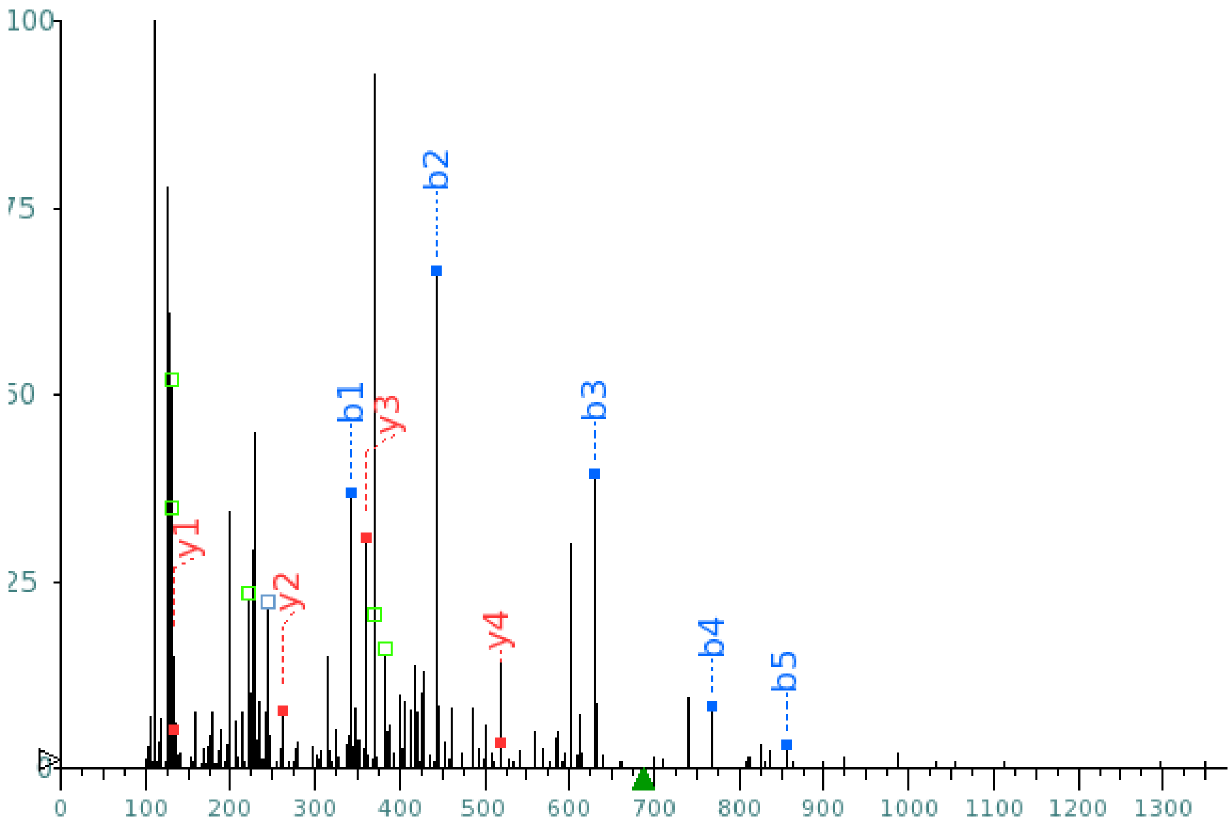

LTWHSC*PED (+2)

Primary Reference: CBX3_HUMAN
Search ID: 41761
Search Name: 20130328_ananiav_ERstress_TMT_TM_timecourse_trypsin_inj2_4mc_iaa
Scan#: 9896
Observed Mass: 687.3204 (-1.6 ppm)
PSM Score: 15.85

File name: 2015-02-04 04.01.53PM.pdf

Creation time: 2015-02-04 04:09PM

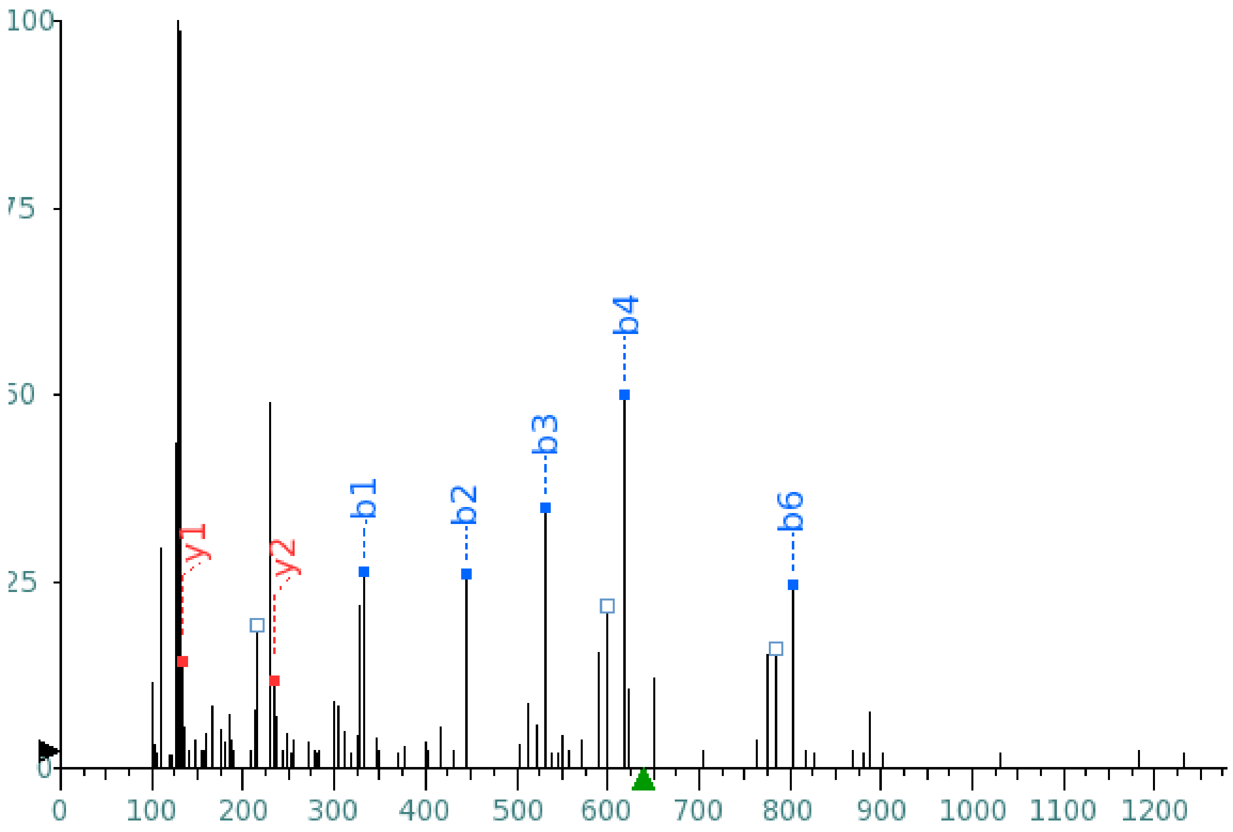

TLSSPSLQTD (+2)

Primary Reference: DOCK5_HUMAN
Search ID: 41761
Search Name: 20130328_ananiav_ERstress_TMT_TM_timecourse_trypsin_inj2_4mc_iaa
Scan#: 9960
Observed Mass: 639.3434 (0.8 ppm)
PSM Score: 24.05

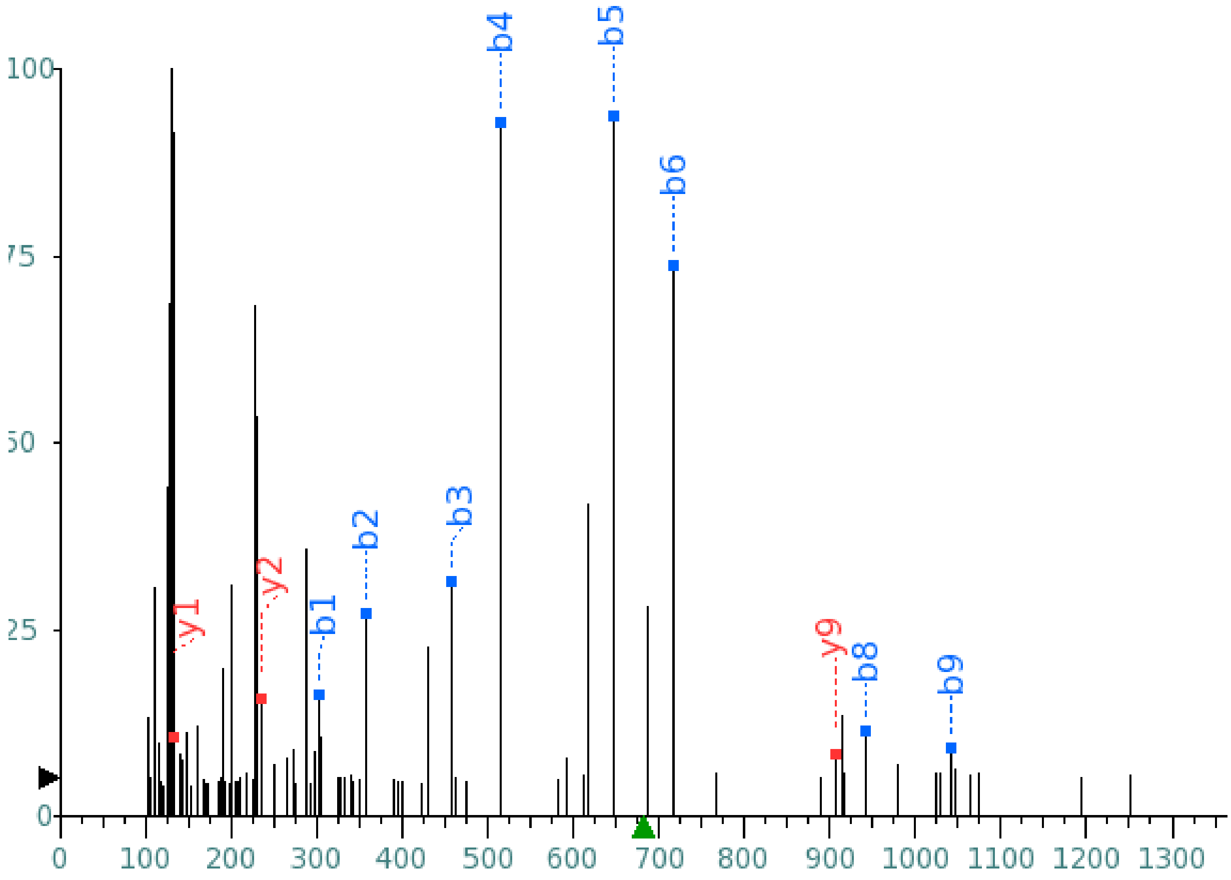

AGVGMAPEVSTD (+2)

Primary Reference: MA7D3_HUMAN
Search ID: 41761
Search Name: 20130328_ananiav_ERstress_TMT_TM_timecourse_trypsin_inj2_4mc_iaa
Scan#: 9995
Observed Mass: 681.8424 (0.1 ppm)
PSM Score: 33.55

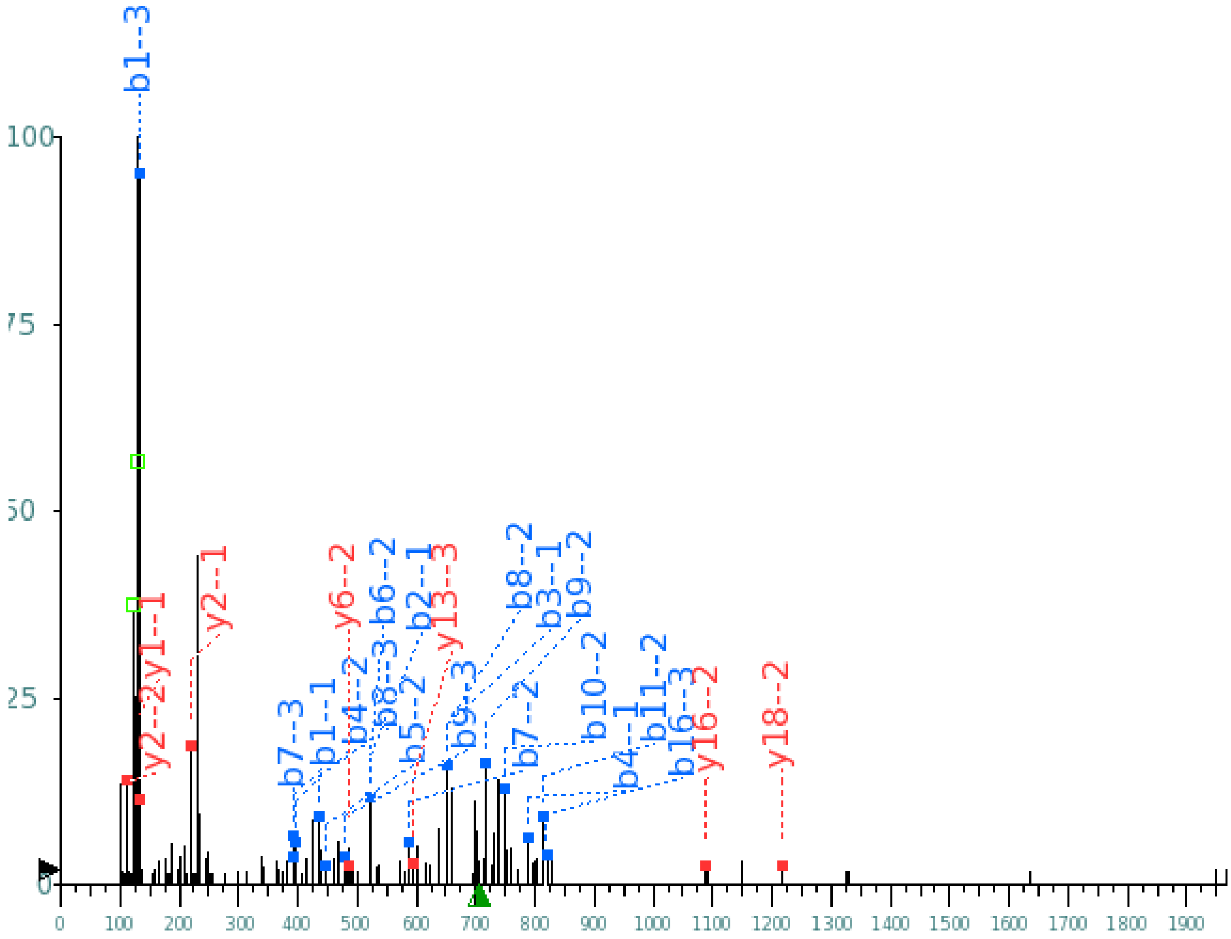

YEEHVREQQAQVEKEDFSD (+4)

Primary Reference: SF3B2_HUMAN
Search ID: 41761
Search Name: 20130328_ananiav_ERstress_TMT_TM_timecourse_trypsin_inj2_4mc_iaa
Scan#: 10019
Observed Mass: 706.852 (2.6 ppm)
PSM Score: 15.02

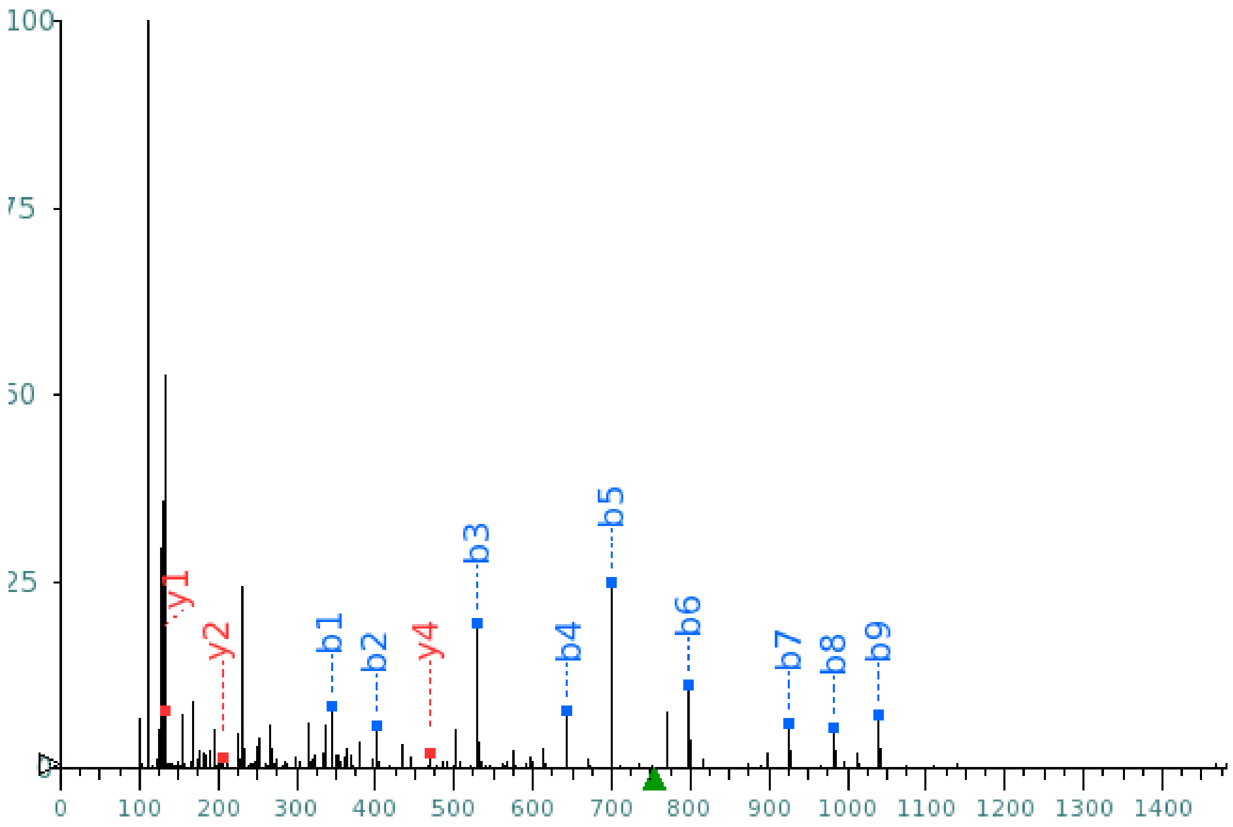

LGELGVQGGHQAD (+2)

Primary Reference: CC165_HUMAN
Search ID: 41761
Search Name: 20130328_ananiav_ERstress_TMT_TM_timecourse_trypsin_inj2_4mc_iaa
Scan#: 10088
Observed Mass: 755.3961 (-0.7 ppm)
PSM Score: 45.84

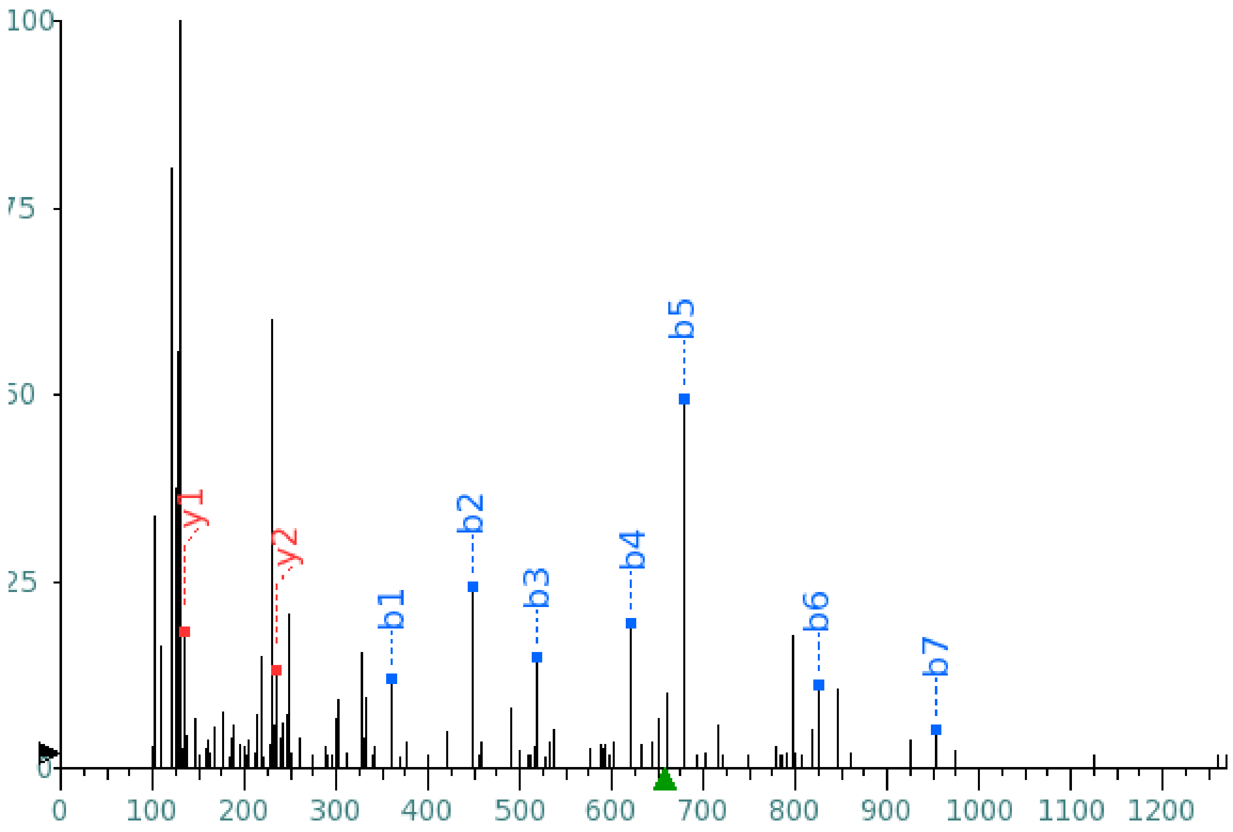

MSATGFEETD (+2)

Primary Reference: SRF_HUMAN
Search ID: 41761
Search Name: 20130328_ananiav_ERstress_TMT_TM_timecourse_trypsin_inj2_4mc_iaa
Scan#: 10140
Observed Mass: 658.7977 (0.3 ppm)
PSM Score: 28.29

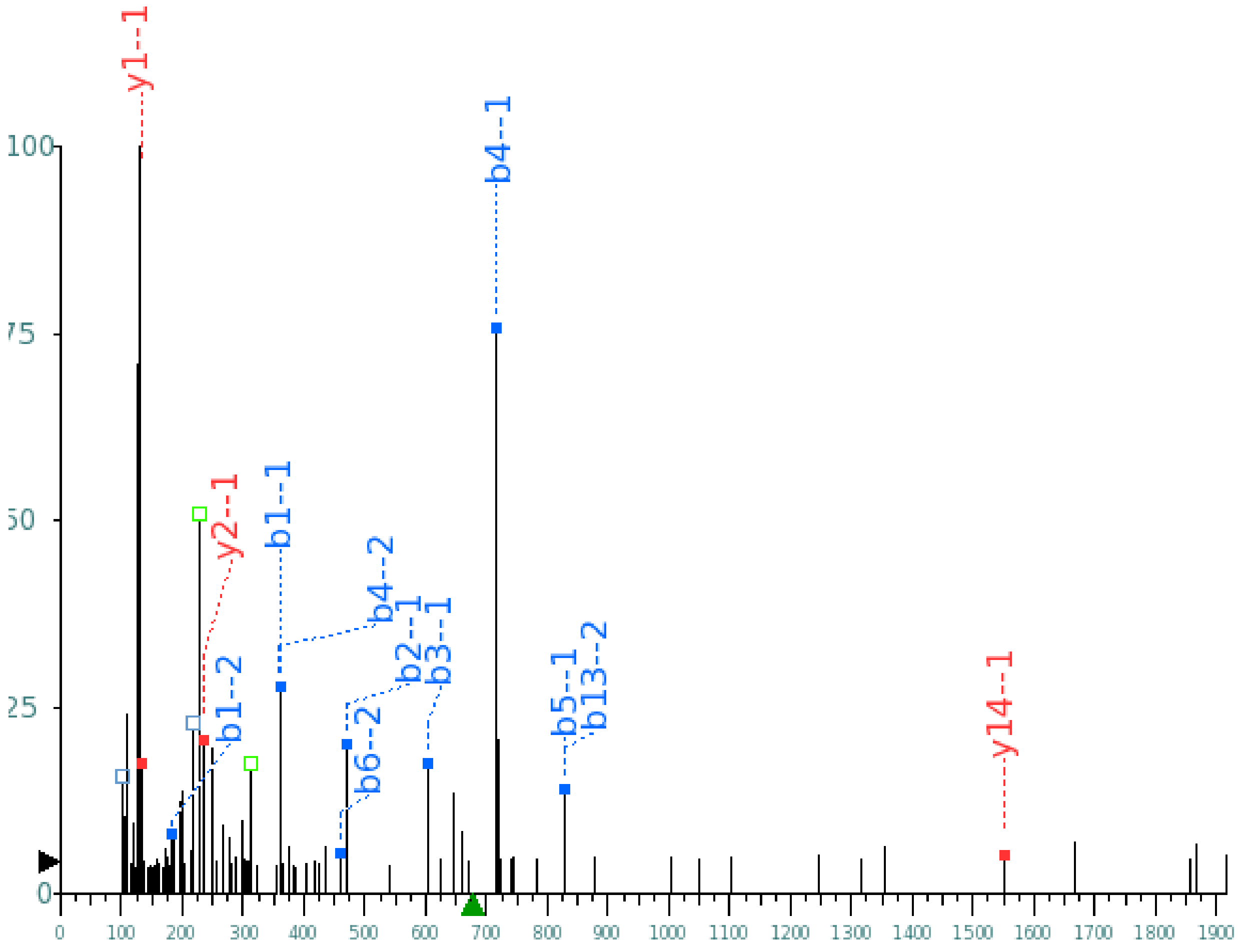

EIEDLSQSQSPESETD (+3)

Primary Reference: NBEA_HUMAN
Search ID: 41761
Search Name: 20130328_ananiav_ERstress_TMT_TM_timecourse_trypsin_inj2_4mc_iaa
Scan#: 10304
Observed Mass: 674.9782 (0.6 ppm)
PSM Score: 12.21

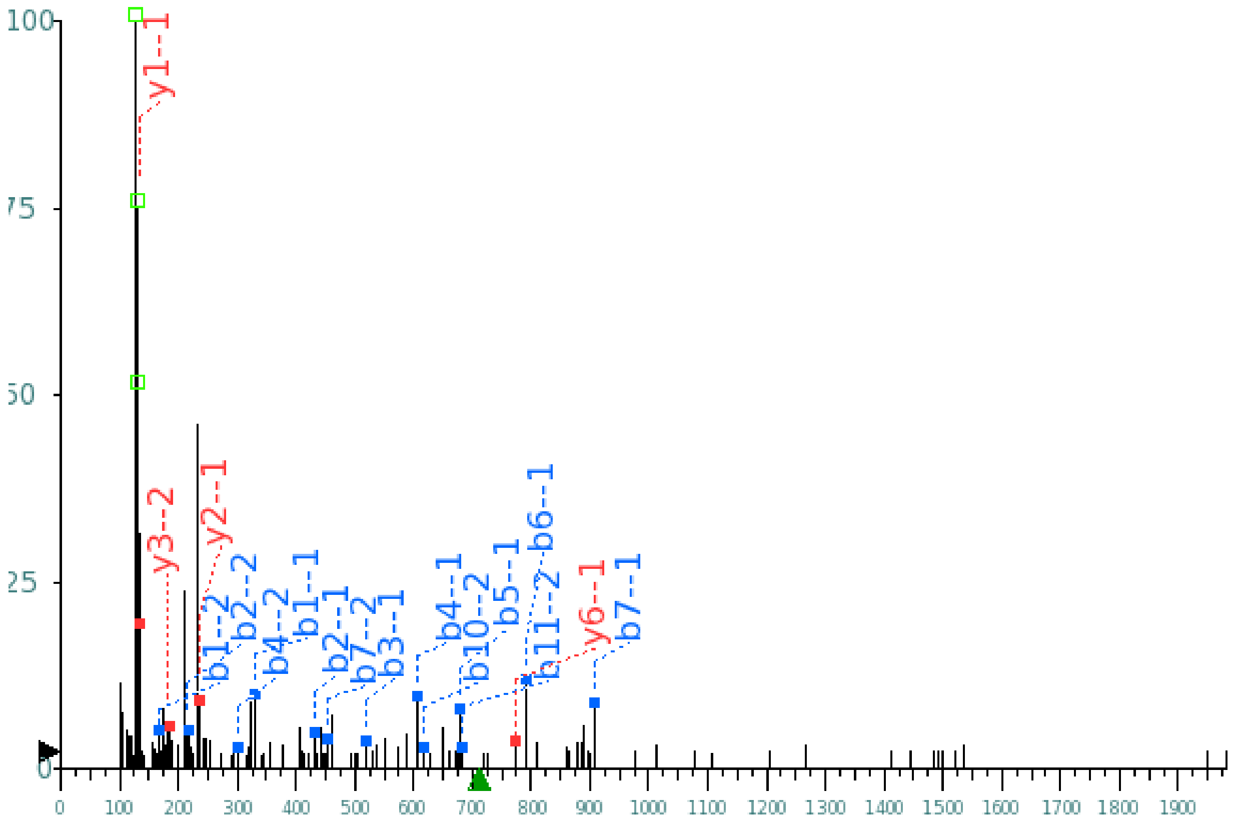

TTSSANNPNLMYQDECD (+3)

Primary Reference: DDX17_HUMAN
Search ID: 41761
Search Name: 20130328_ananiav_ERstress_TMT_TM_timecourse_trypsin_inj2_4mc_iaa
Scan#: 10490
Observed Mass: 711.3097 (1.7 ppm)
PSM Score: 10.31

File name: 2015-02-04 04.01.53PM.pdf

Creation time: 2015-02-04 04:09PM

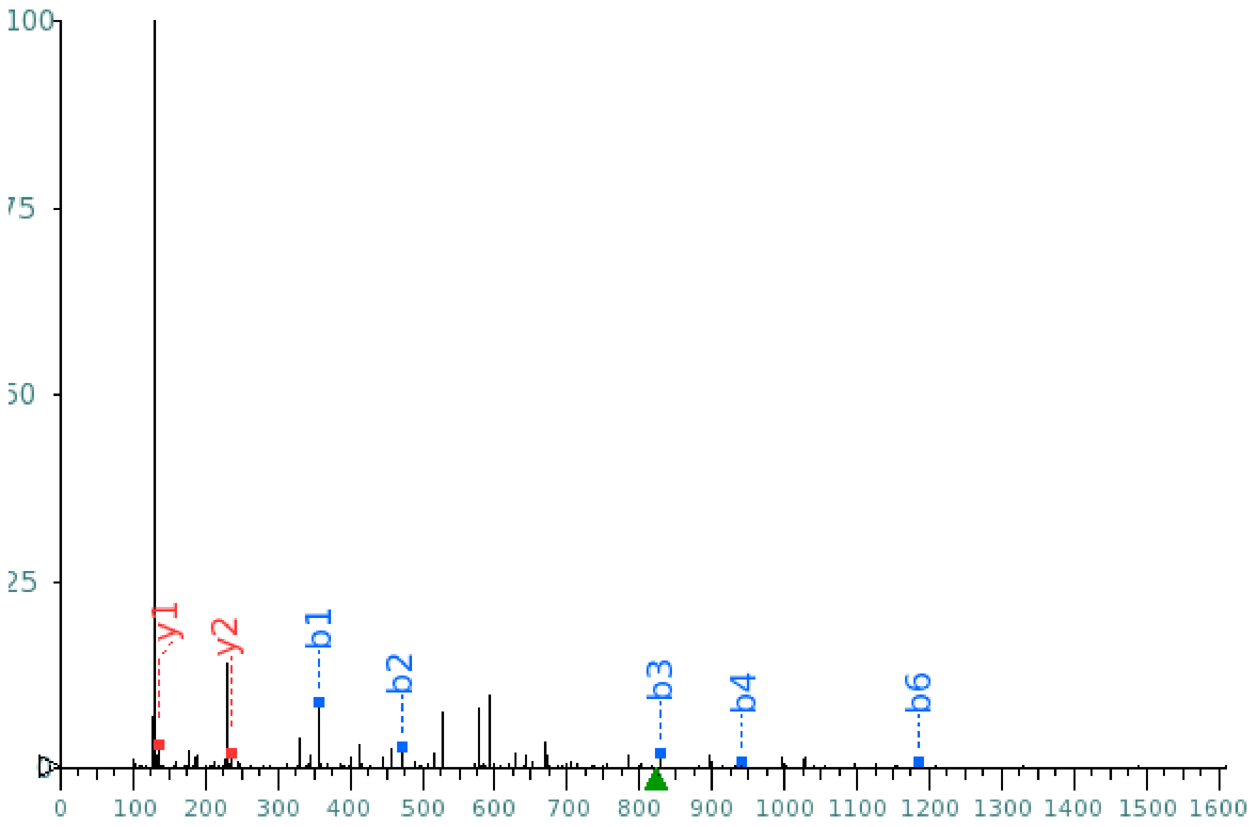

QIKINEVQTD (+2)

Primary Reference: TWF1_HUMAN
Search ID: 41761
Search Name: 20130328_ananiav_ERstress_TMT_TM_timecourse_trypsin_inj2_4mc_iaa
Scan#: 10576
Observed Mass: 823.48 (0.1 ppm)
PSM Score: 15.83

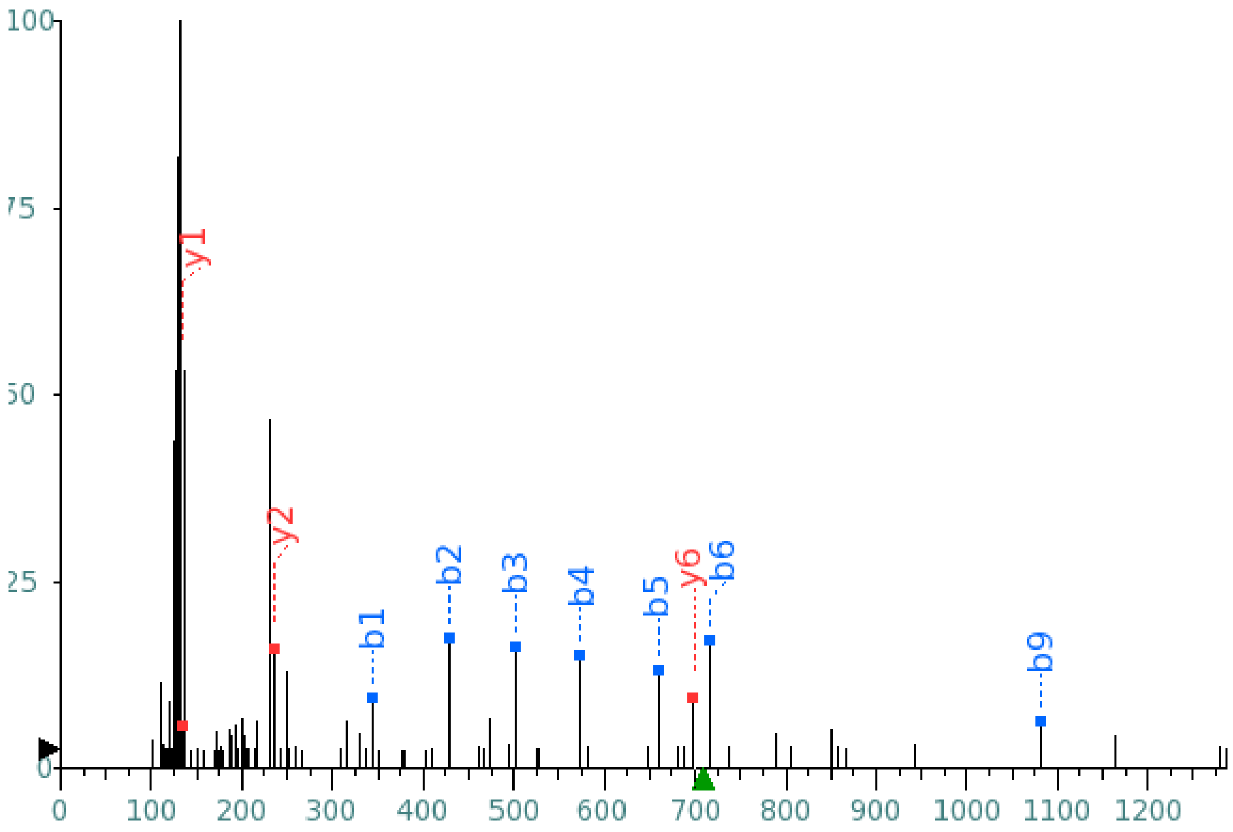

LSAASGYSDVTD (+2)

Primary Reference: PM2PB_HUMAN
Search ID: 41761
Search Name: 20130328_ananiav_ERstress_TMT_TM_timecourse_trypsin_inj2_4mc_iaa
Scan#: 10582
Observed Mass: 707.8494 (1.2 ppm)
PSM Score: 29.63

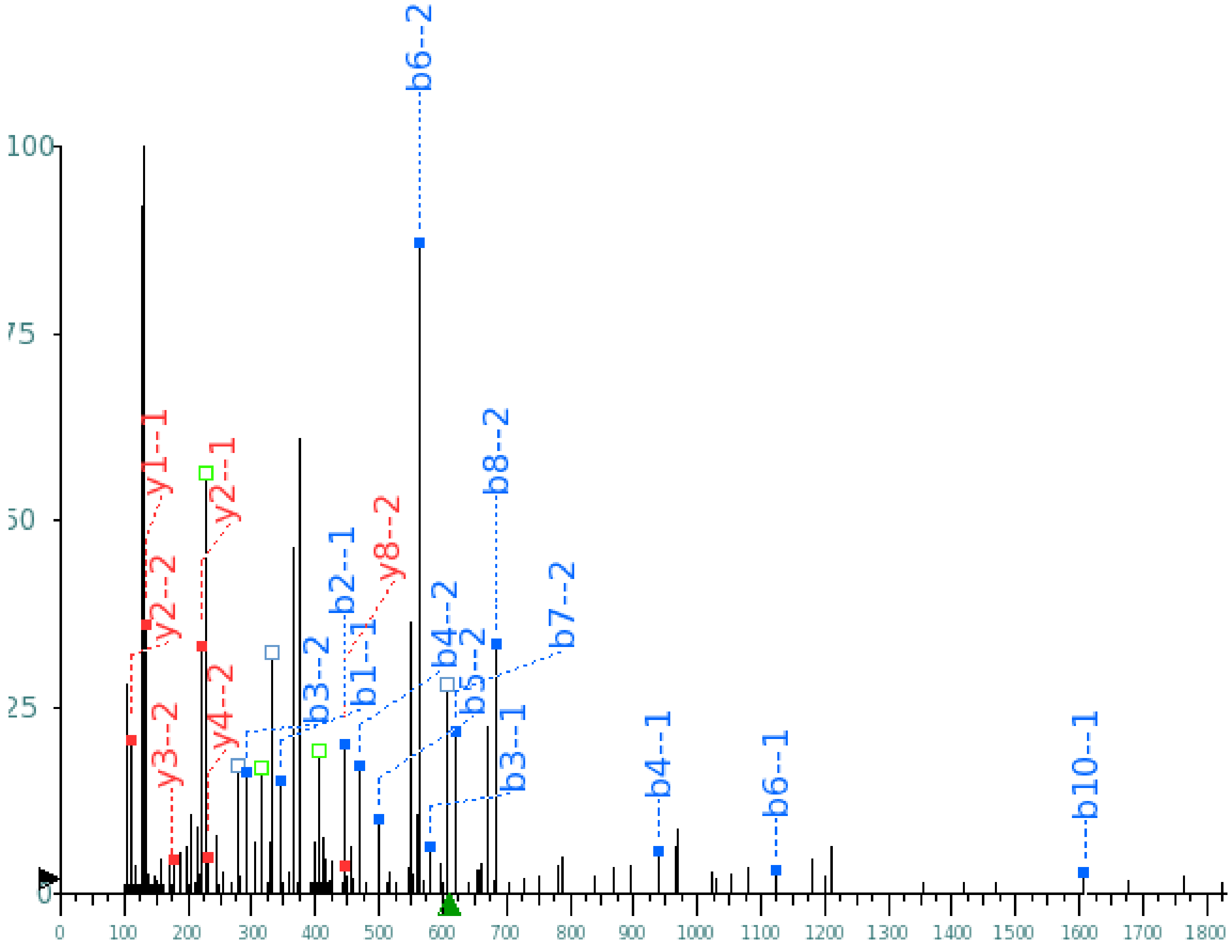

ITHKGEIEIESD (+3)

Primary Reference: PHF3_HUMAN
Search ID: 41761
Search Name: 20130328_ananiav_ERstress_TMT_TM_timecourse_trypsin_inj2_4mc_iaa
Scan#: 10762
Observed Mass: 610.3406 (0.9 ppm)
PSM Score: 10.18

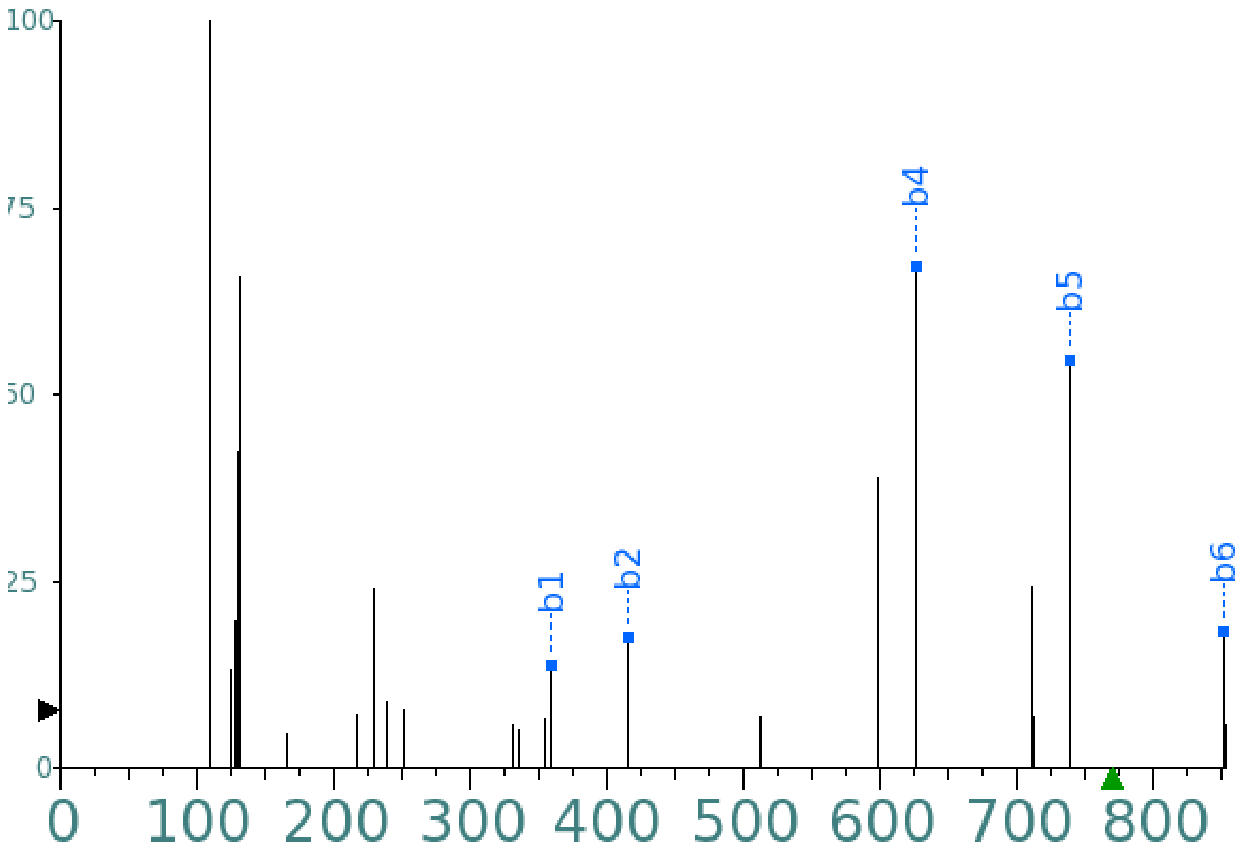

EGPIIIHTDEAD (+2)

Primary Reference: PPA6_HUMAN
Search ID: 41761
Search Name: 20130328_ananiav_ERstress_TMT_TM_timecourse_trypsin_inj2_4mc_iaa
Scan#: 10801
Observed Mass: 769.9008 (2.8 ppm)
PSM Score: 30.14

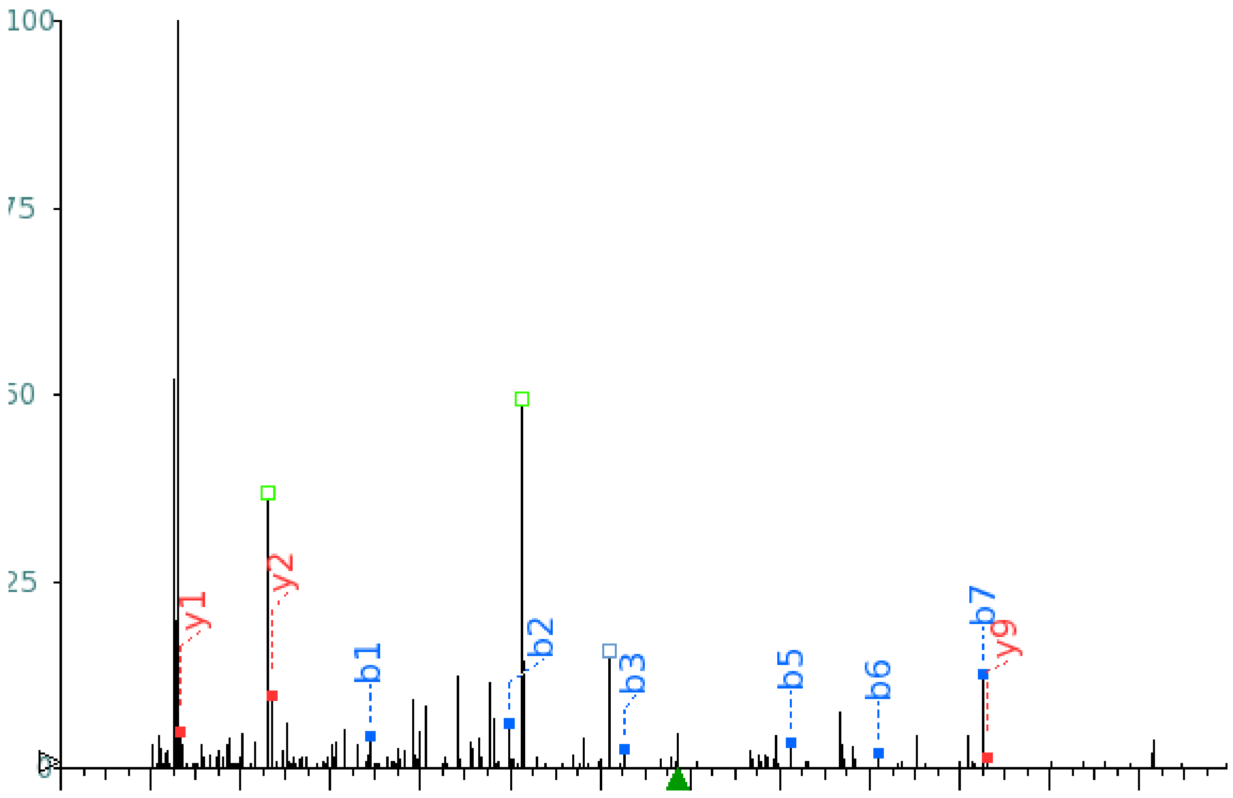

IRQPSVDLTD (+2)

Primary Reference: CE170_HUMAN
Search ID: 41761
Search Name: 20130328_ananiav_ERstress_TMT_TM_timecourse_trypsin_inj2_4mc_iaa
Scan#: 10846
Observed Mass: 686.8851 (-0.2 ppm)
PSM Score: 12.57

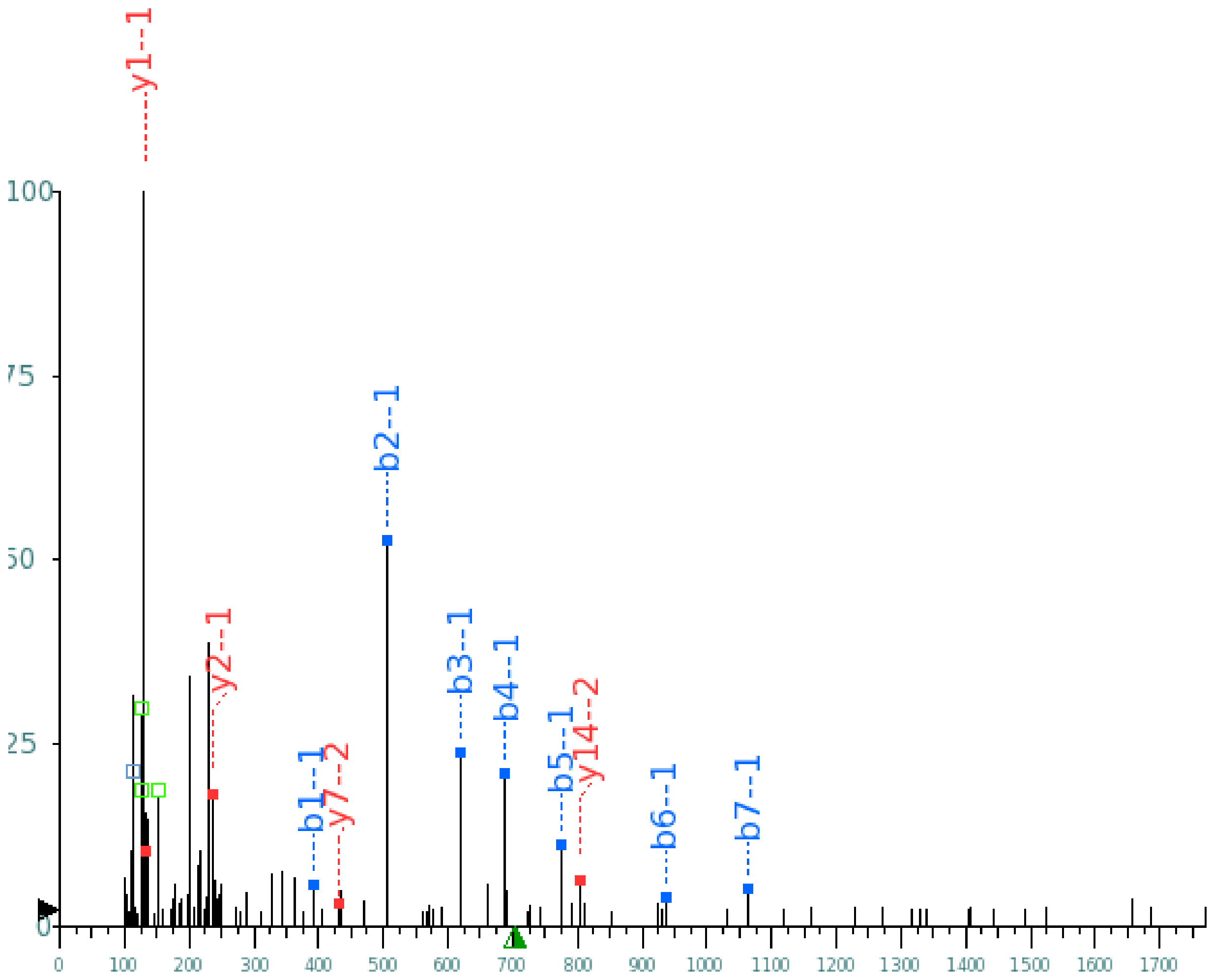

C*DLASC*QSTRPMLETD (+3)

Primary Reference: K0889_HUMAN
Search ID: 41761
Search Name: 20130328_ananiav_ERstress_TMT_TM_timecourse_trypsin_inj2_4mc_iaa
Scan#: 10875
Observed Mass: 704.9907 (0.7 ppm)
PSM Score: 9.81

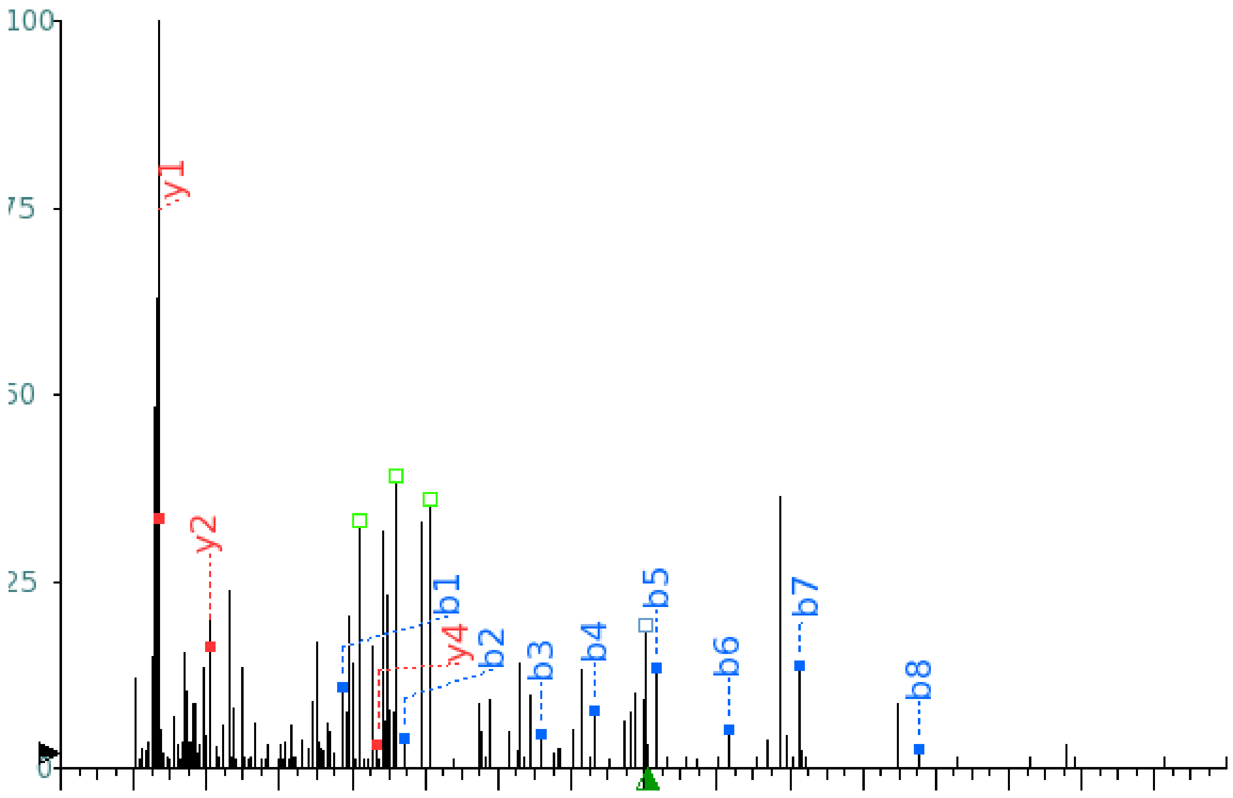

RSWASPVYTEAD (+2)

Primary Reference: LMO7_HUMAN
Search ID: 41761
Search Name: 20130328_ananiav_ERstress_TMT_TM_timecourse_trypsin_inj2_4mc_iaa
Scan#: 10921
Observed Mass: 805.9052 (1.2 ppm)
PSM Score: 17.45

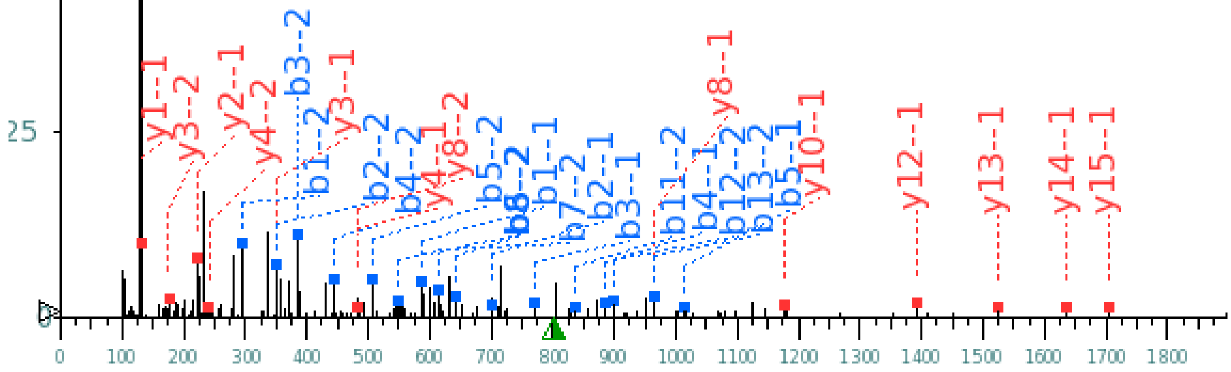

KIALESEGRPEEQMESD (+3)

Primary Reference: SQSTM_HUMAN
Search ID: 41761
Search Name: 20130328_ananiav_ERstress_TMT_TM_timecourse_trypsin_inj2_4mc_iaa
Scan#: 10928
Observed Mass: 802.7467 (1.4 ppm)
PSM Score: 36.27

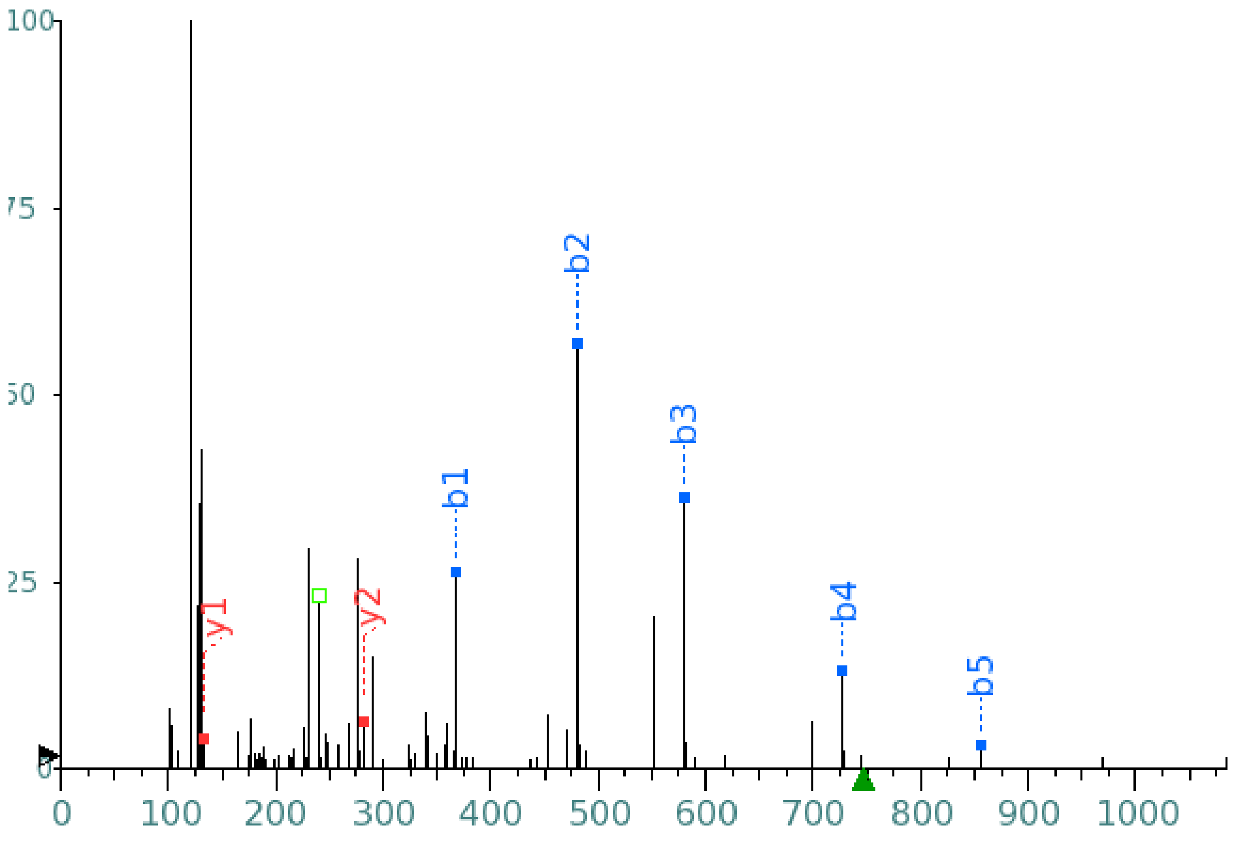

HNVFQNDEFD (+2)

Primary Reference: ASCC2_HUMAN
Search ID: 41761
Search Name: 20130328_ananiav_ERstress_TMT_TM_timecourse_trypsin_inj2_4mc_iaa
Scan#: 10933
Observed Mass: 747.3476 (1.4 ppm)
PSM Score: 51.02

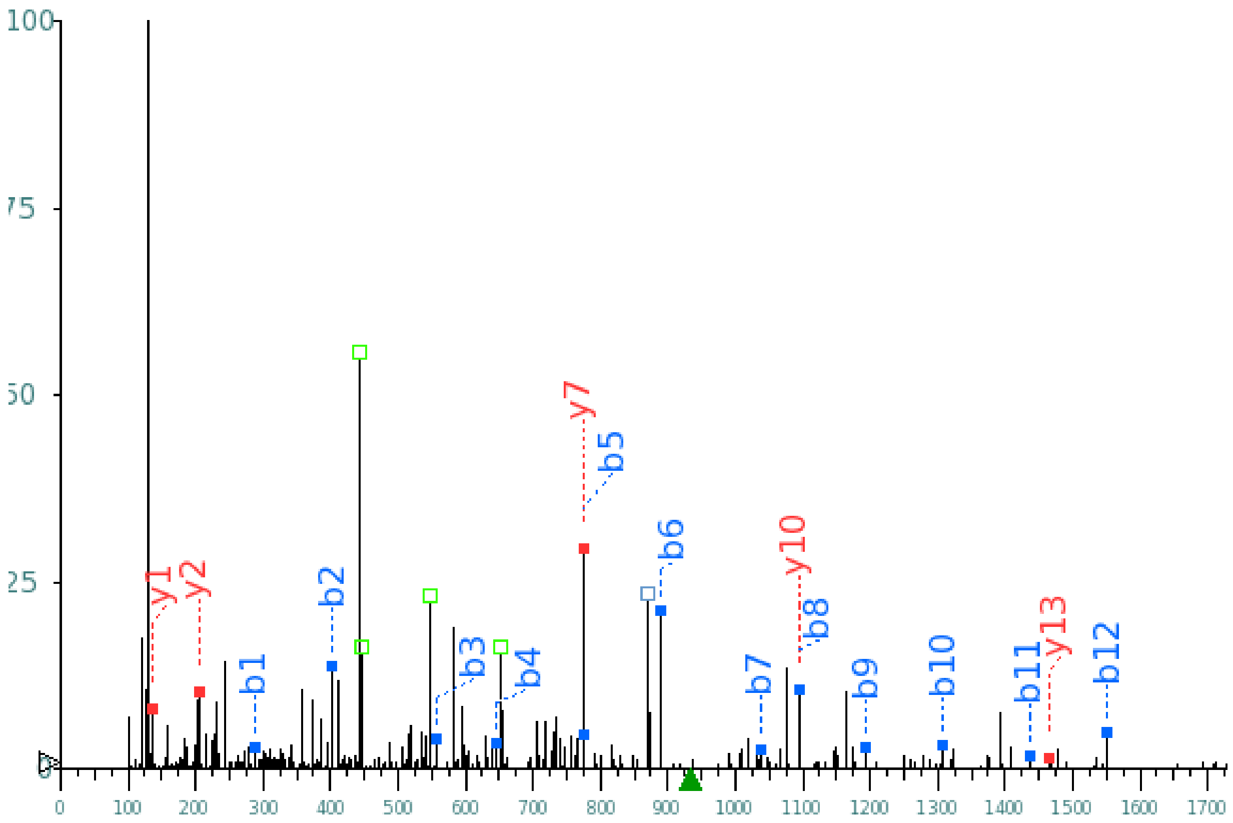

GDRSEDFGVNEDLAD (+2)

Primary Reference: ANXA1_HUMAN
Search ID: 41761
Search Name: 20130328_ananiav_ERstress_TMT_TM_timecourse_trypsin_inj2_4mc_iaa
Scan#: 10956
Observed Mass: 934.4288 (-0.3 ppm)
PSM Score: 27.93

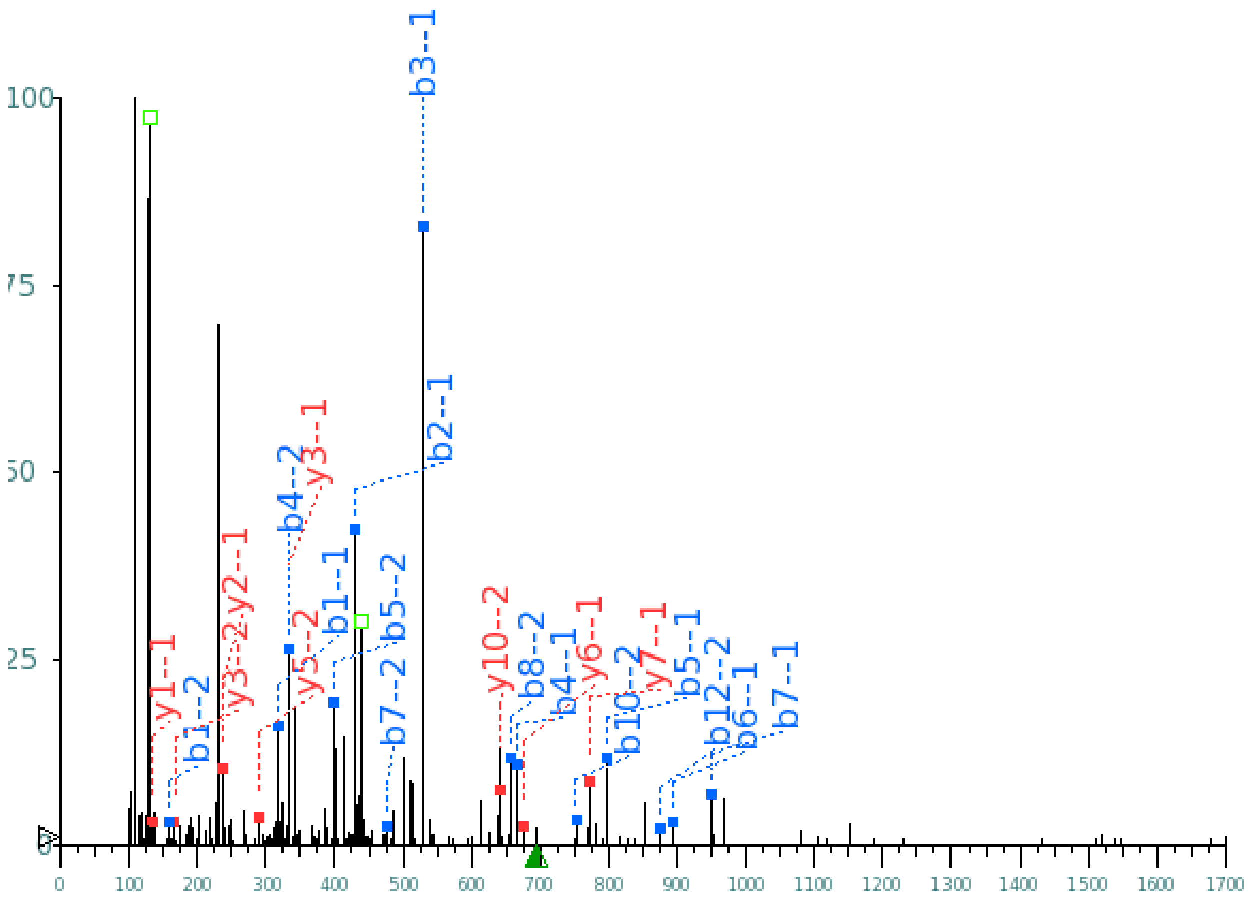

SLVHEVGKPPQDVTD (+3)

Primary Reference: MINT_HUMAN
Search ID: 41761
Search Name: 20130328_ananiav_ERstress_TMT_TM_timecourse_trypsin_inj2_4mc_iaa
Scan#: 10967
Observed Mass: 693.7227 (2.3 ppm)
PSM Score: 17.42

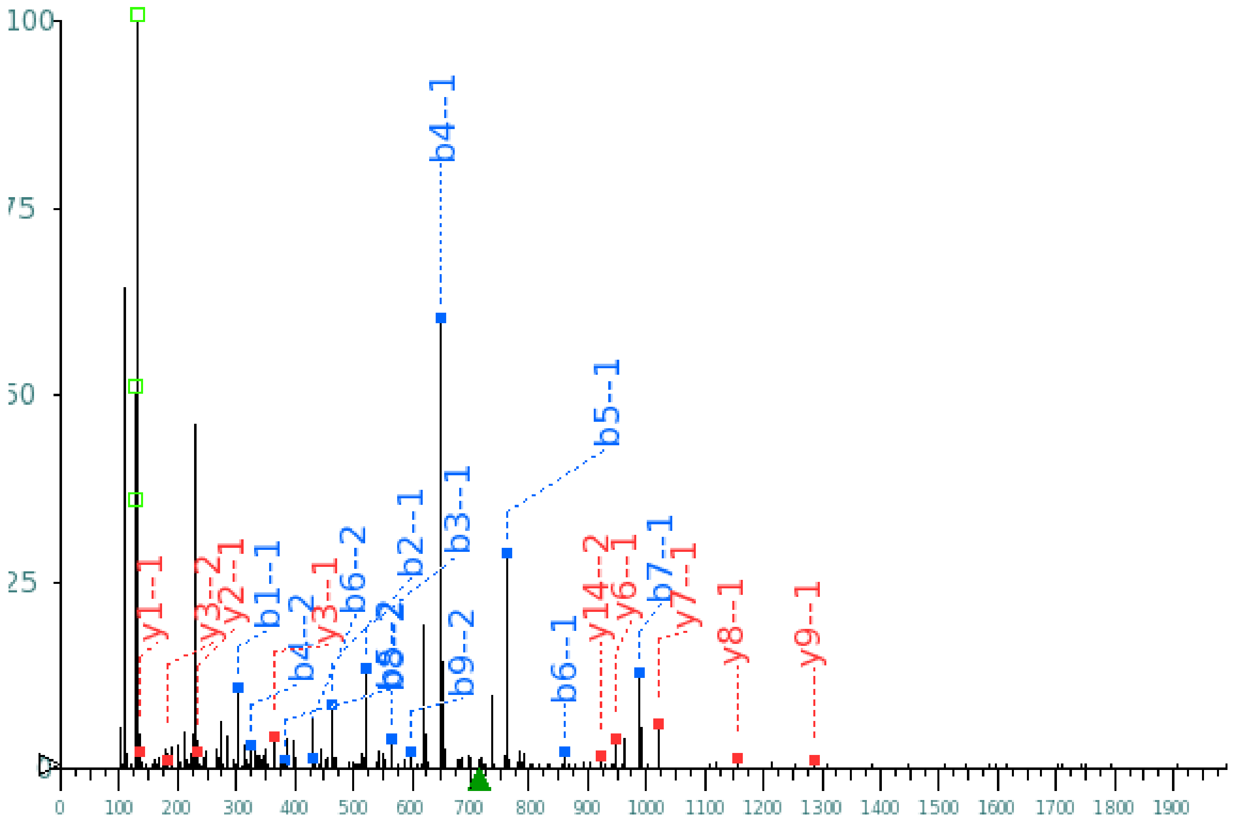

AYGELPEHAKINETD (+3)

Primary Reference: IF1AX_HUMAN
Search ID: 41761
Search Name: 20130328_ananiav_ERstress_TMT_TM_timecourse_trypsin_inj2_4mc_iaa
Scan#: 10972
Observed Mass: 715.7133 (1.2 ppm)
PSM Score: 35.4

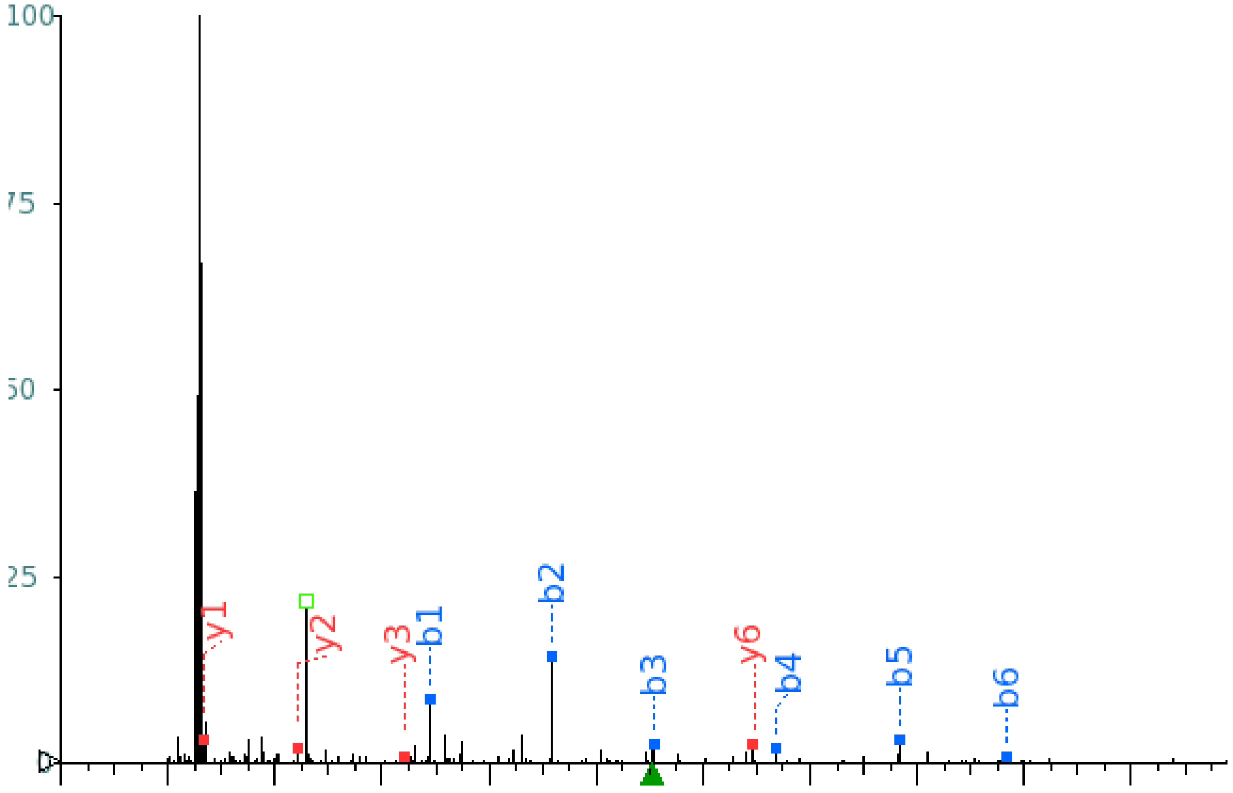

DLPLDTSD (+2)

Primary Reference: 3BP2_HUMAN
Search ID: 41761
Search Name: 20130328_ananiav_ERstress_TMT_TM_timecourse_trypsin_inj2_4mc_iaa
Scan#: 10985
Observed Mass: 552.7853 (1 ppm)
PSM Score: 13.08

File name: 2015-02-04 04.01.53PM.pdf

Creation time: 2015-02-04 04:09PM

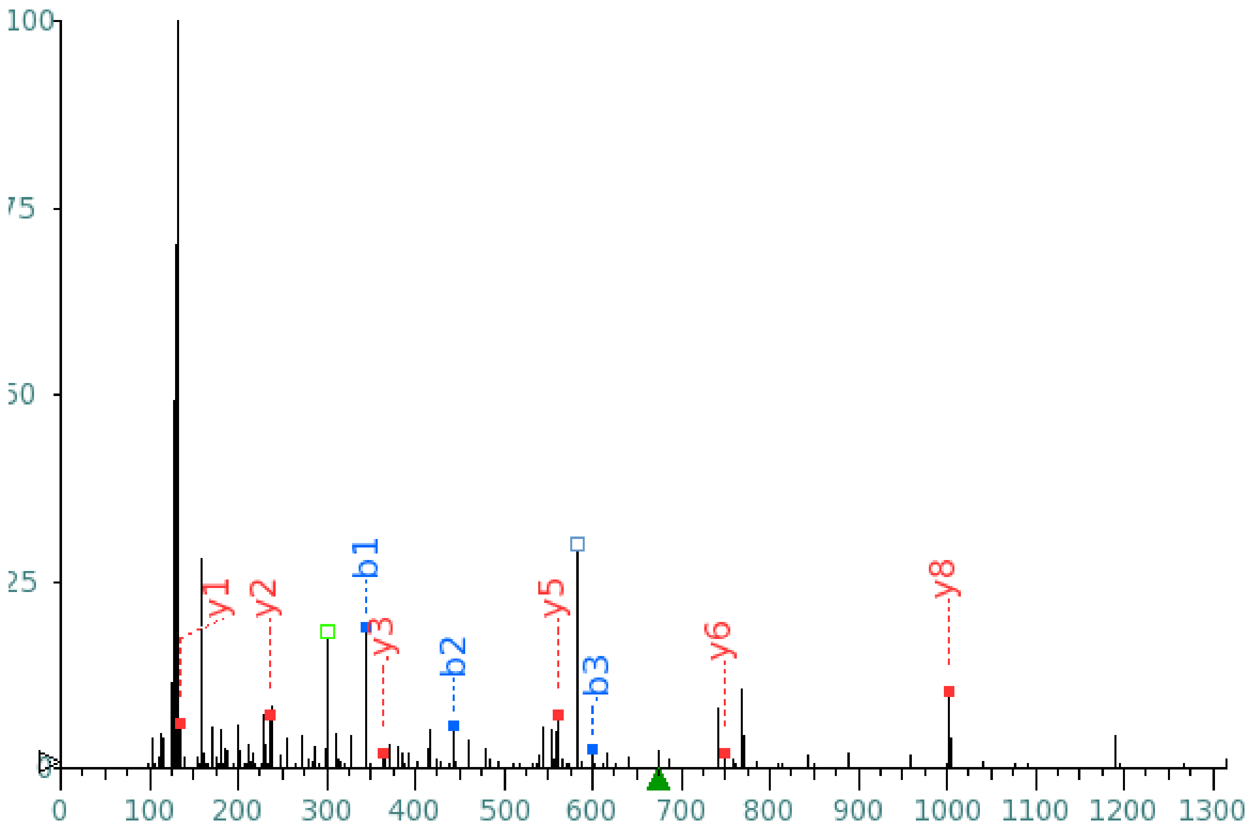

DVRWPTETD (+2)

Primary Reference: MAP4_HUMAN
Search ID: 41761
Search Name: 20130328_ananiav_ERstress_TMT_TM_timecourse_trypsin_inj2_4mc_iaa
Scan#: 11003
Observed Mass: 674.3413 (0.8 ppm)
PSM Score: 4.53

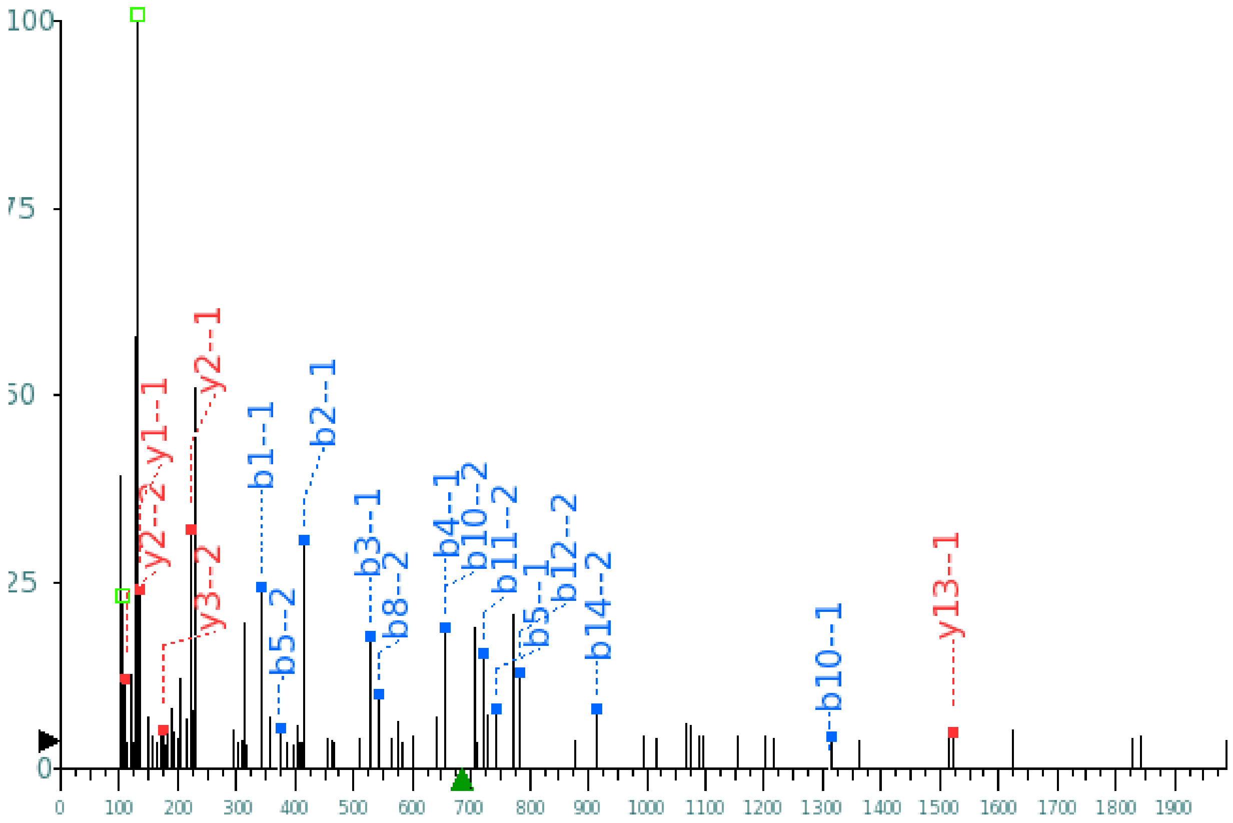

IALESEGRPEEQMESD (+3)

Primary Reference: SQSTM_HUMAN
Search ID: 41761
Search Name: 20130328_ananiav_ERstress_TMT_TM_timecourse_trypsin_inj2_4mc_iaa
Scan#: 11036
Observed Mass: 683.6609 (1.9 ppm)
PSM Score: 12.17

File name: 2015-02-04 04.01.53PM.pdf

Creation time: 2015-02-04 04:09PM

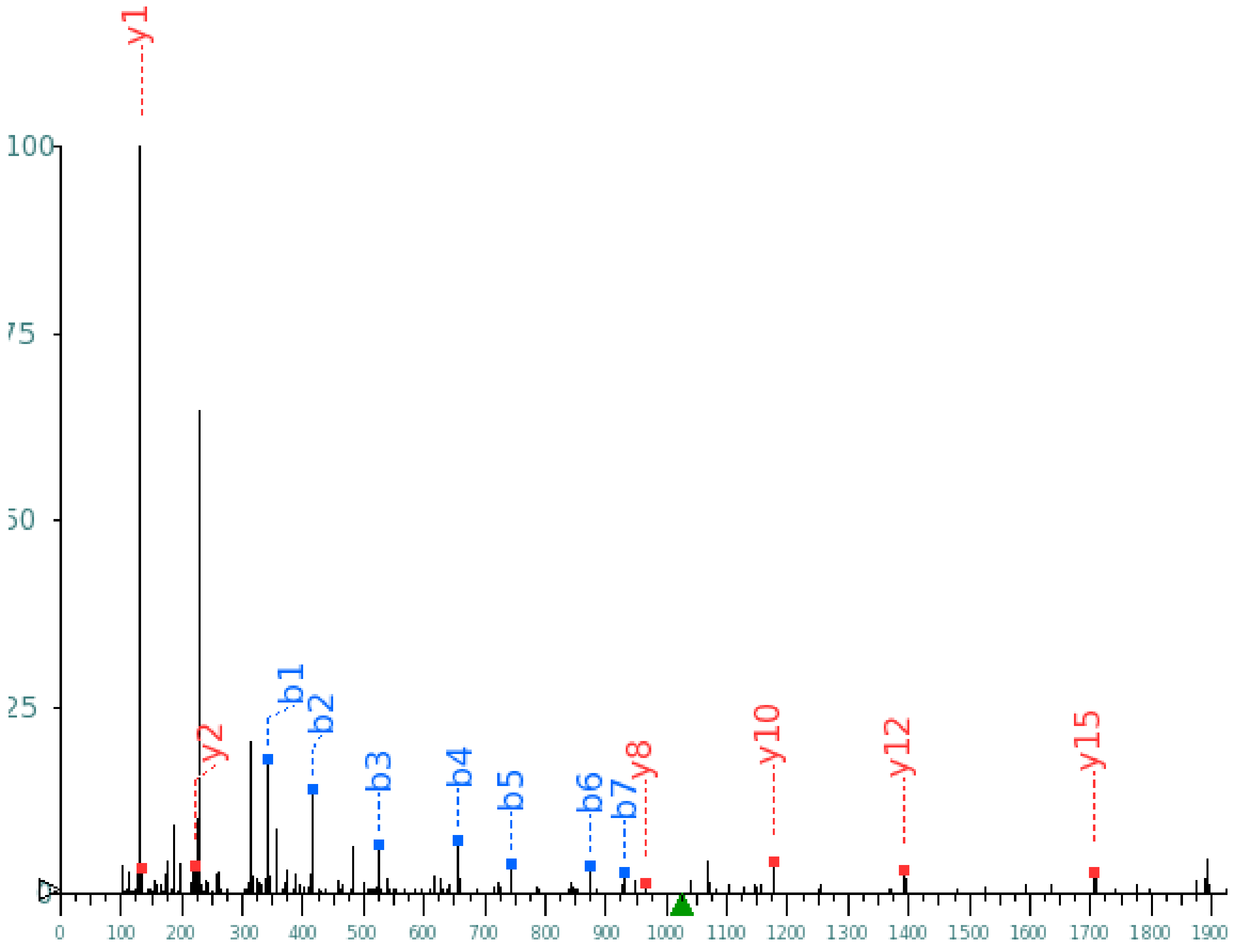

IALESEGRPEEQMESD (+2)

Primary Reference: SQSTM_HUMAN
Search ID: 41761
Search Name: 20130328_ananiav_ERstress_TMT_TM_timecourse_trypsin_inj2_4mc_iaa
Scan#: 11037
Observed Mass: 1024.9864 (0.6 ppm)
PSM Score: 19.28

File name: 2015-02-04 04.01.53PM.pdf

Creation time: 2015-02-04 04:09PM

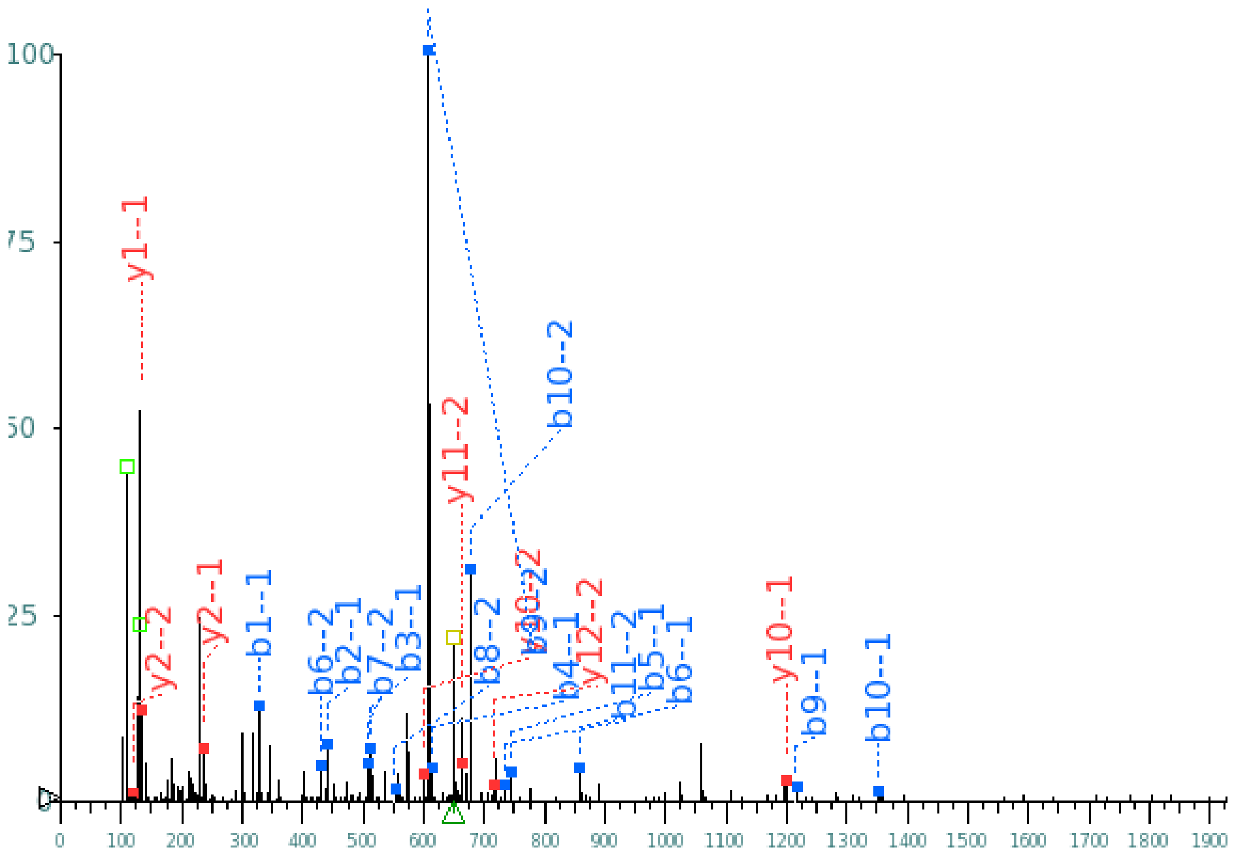

VLATEDRSDHLIQTD (+3)

Primary Reference: RAB9A_HUMAN
Search ID: 41761
Search Name: 20130328_ananiav_ERstress_TMT_TM_timecourse_trypsin_inj2_4mc_iaa
Scan#: 11039
Observed Mass: 648.0085 (1.6 ppm)
PSM Score: 10

File name: 2015-02-04 04.01.53PM.pdf

Creation time: 2015-02-04 04:09PM

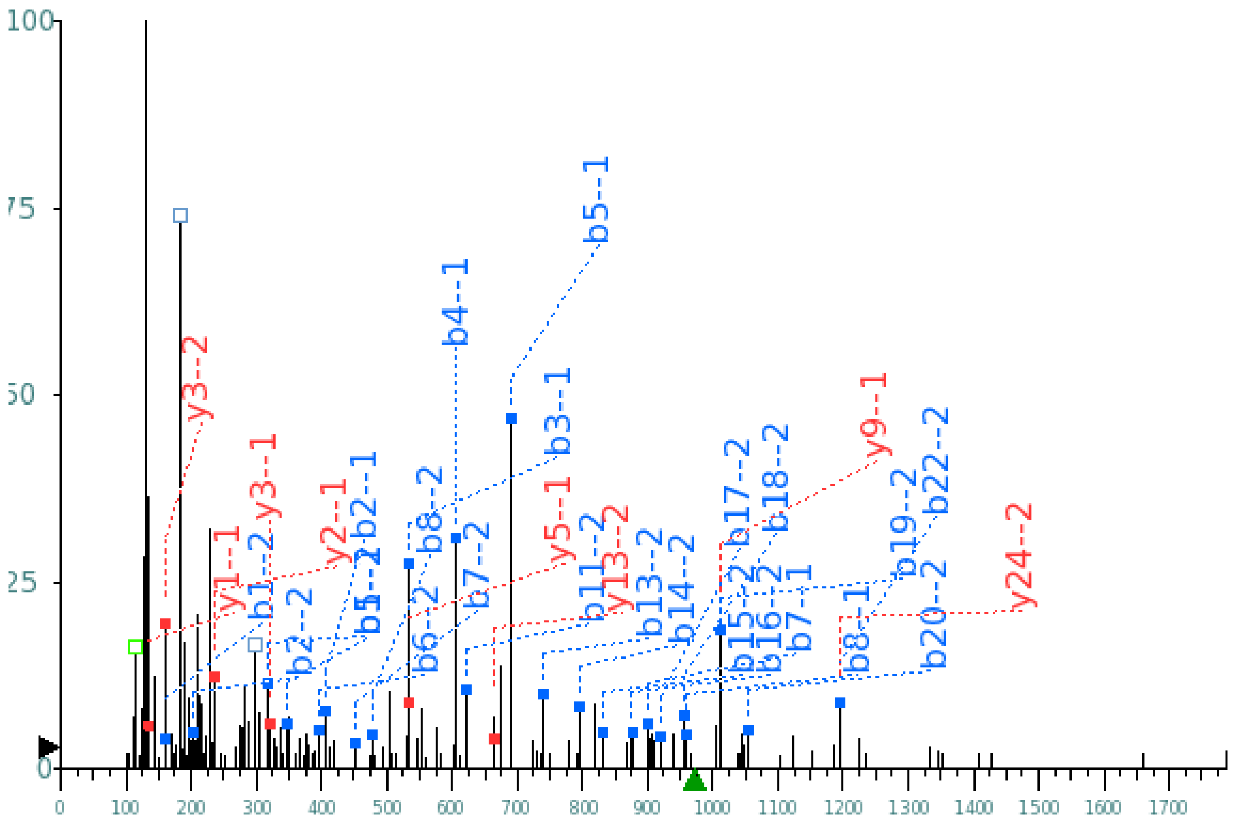

SSEASPLGAGRPHNASSADSYDPISTD (+3)

Primary Reference: GLI2_HUMAN
Search ID: 41761
Search Name: 20130328_ananiav_ERstress_TMT_TM_timecourse_trypsin_inj2_4mc_iaa
Scan#: 11053
Observed Mass: 973.4573 (-0.9 ppm)
PSM Score: 61.72

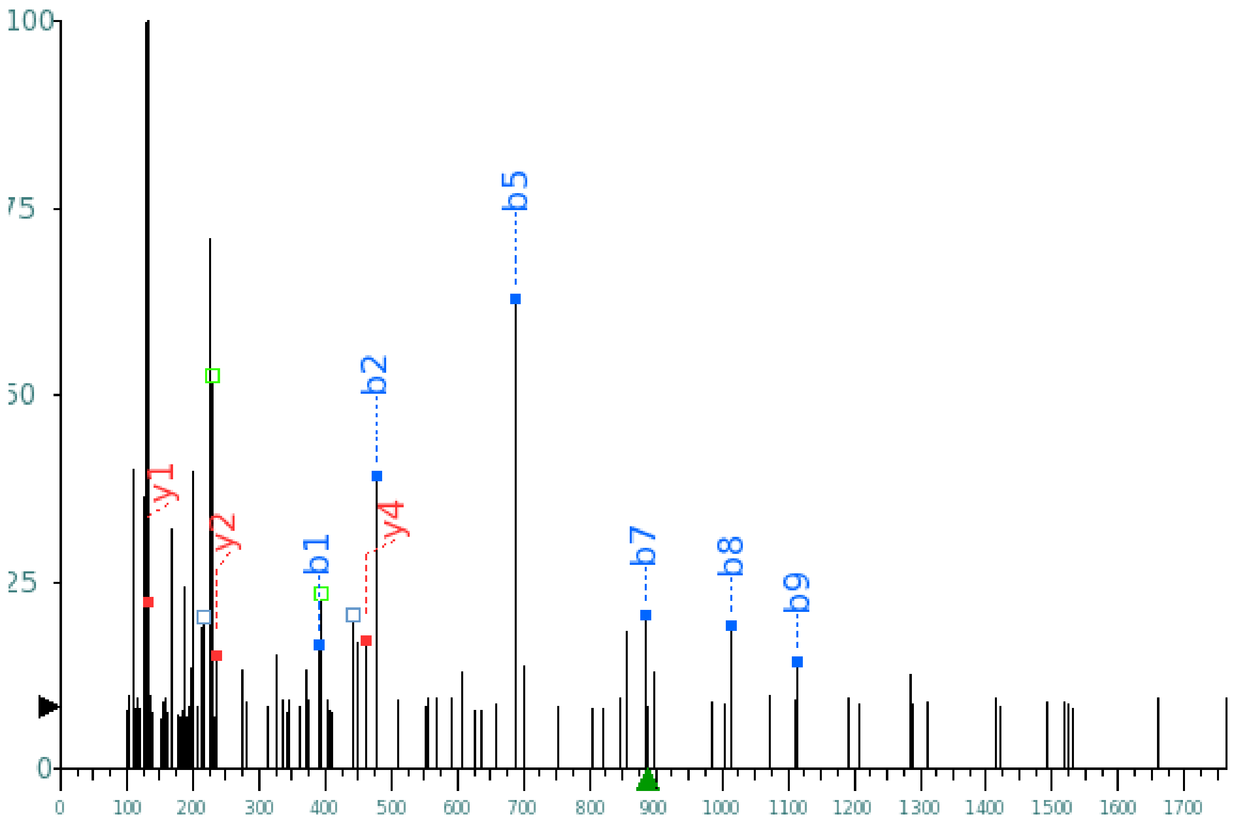

C*SPGGPVETLSPETD (+2)

Primary Reference: B3KR51_HUMAN
Search ID: 41761
Search Name: 20130328_ananiav_ERstress_TMT_TM_timecourse_trypsin_inj2_4mc_iaa
Scan#: 11066
Observed Mass: 887.9225 (0.5 ppm)
PSM Score: 10.54

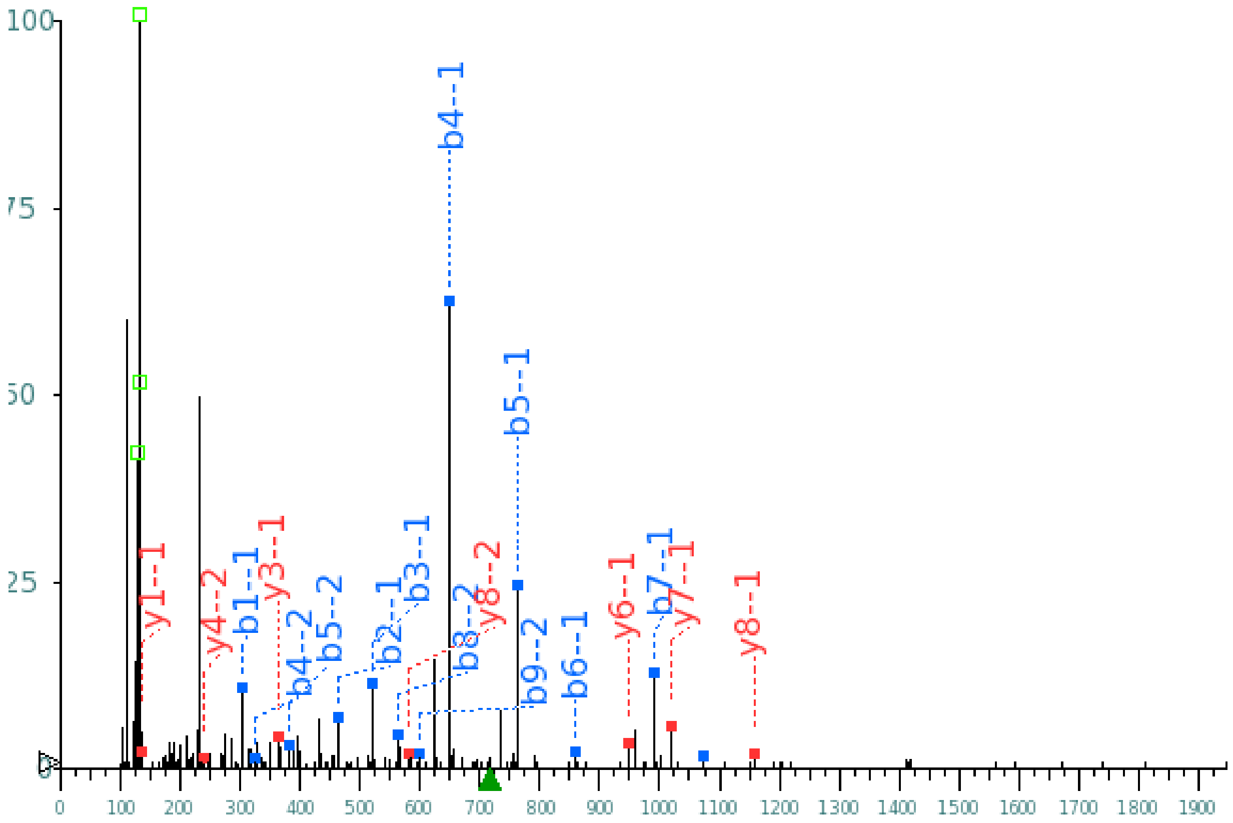

AYGELPEHAKINETD (+3)

Primary Reference: IF1AX_HUMAN
Search ID: 41761
Search Name: 20130328_ananiav_ERstress_TMT_TM_timecourse_trypsin_inj2_4mc_iaa
Scan#: 11071
Observed Mass: 715.7133 (1.3 ppm)
PSM Score: 24.96

File name: 2015-02-04 04.01.53PM.pdf

Creation time: 2015-02-04 04:09PM

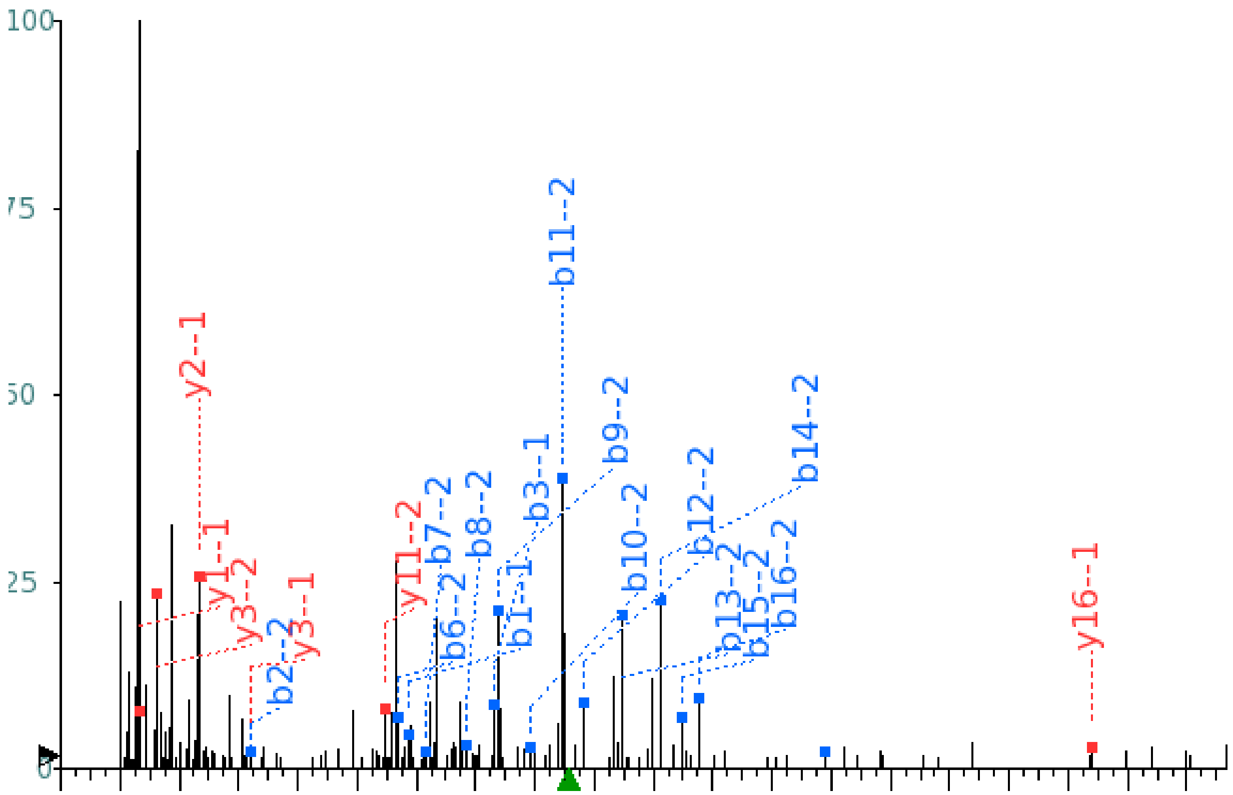

KGSTFRPMDTDAEEAGVSTD (+3)

Primary Reference: DCBD1_HUMAN
Search ID: 41761
Search Name: 20130328_ananiav_ERstress_TMT_TM_timecourse_trypsin_inj2_4mc_iaa
Scan#: 11083
Observed Mass: 858.0923 (0.8 ppm)
PSM Score: 19.62

File name: 2015-02-04 04.01.53PM.pdf

Creation time: 2015-02-04 04:09PM

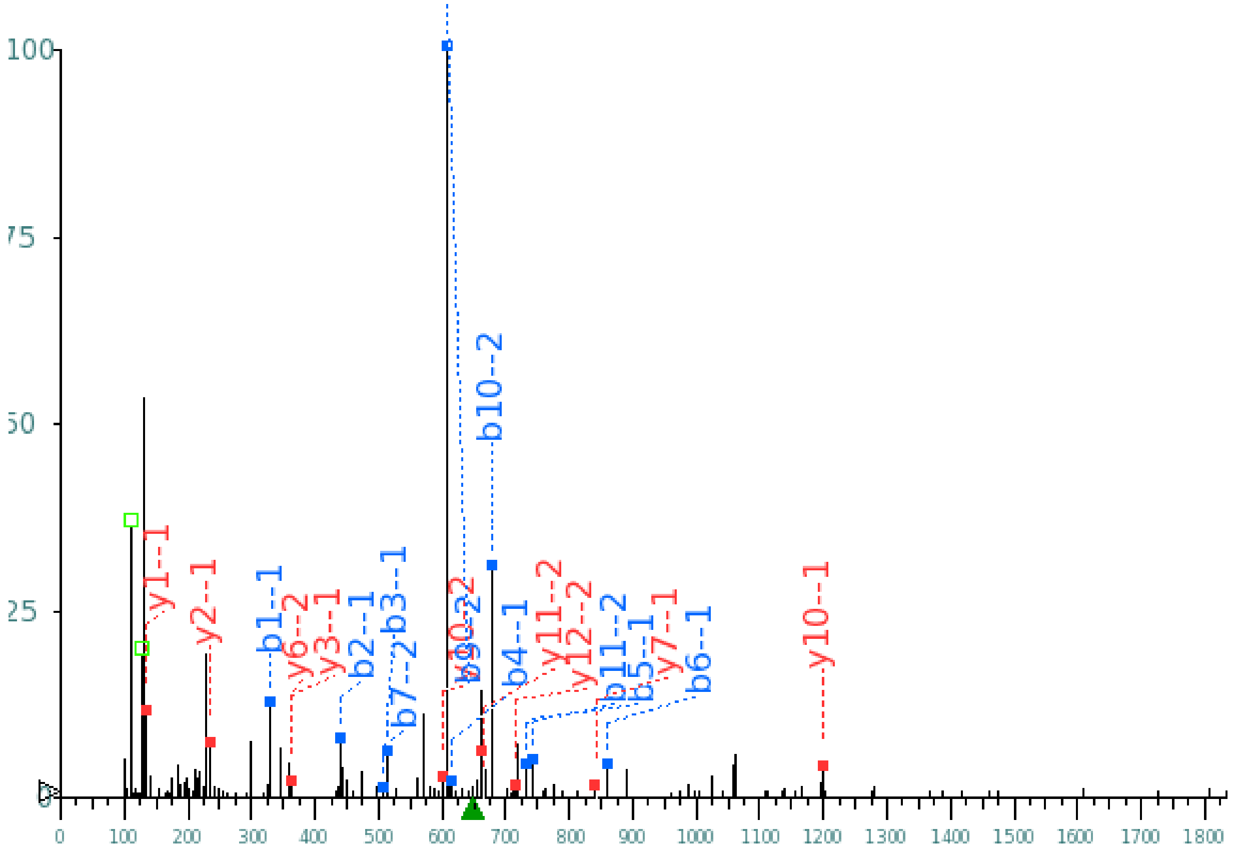

VLATEDRSDHLIQTD (+3)

Primary Reference: RAB9A_HUMAN
Search ID: 41761
Search Name: 20130328_ananiav_ERstress_TMT_TM_timecourse_trypsin_inj2_4mc_iaa
Scan#: 11124
Observed Mass: 648.009 (2.4 ppm)
PSM Score: 10.66

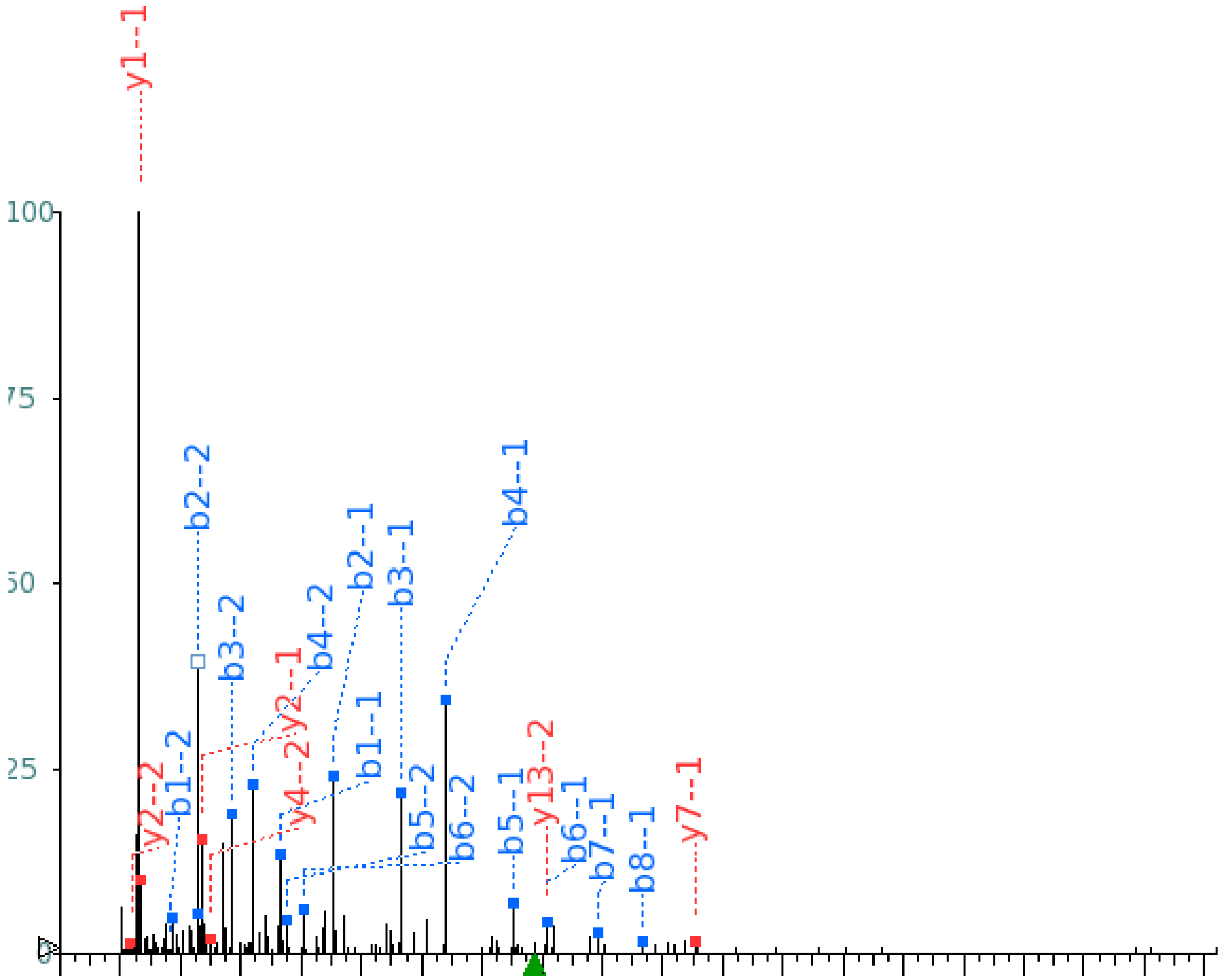

HSLALGSATEDKDSMETD (+3)

Primary Reference: NU188_HUMAN
Search ID: 41761
Search Name: 20130328_ananiav_ERstress_TMT_TM_timecourse_trypsin_inj2_4mc_iaa
Scan#: 11161
Observed Mass: 789.0591 (1.4 ppm)
PSM Score: 23.25

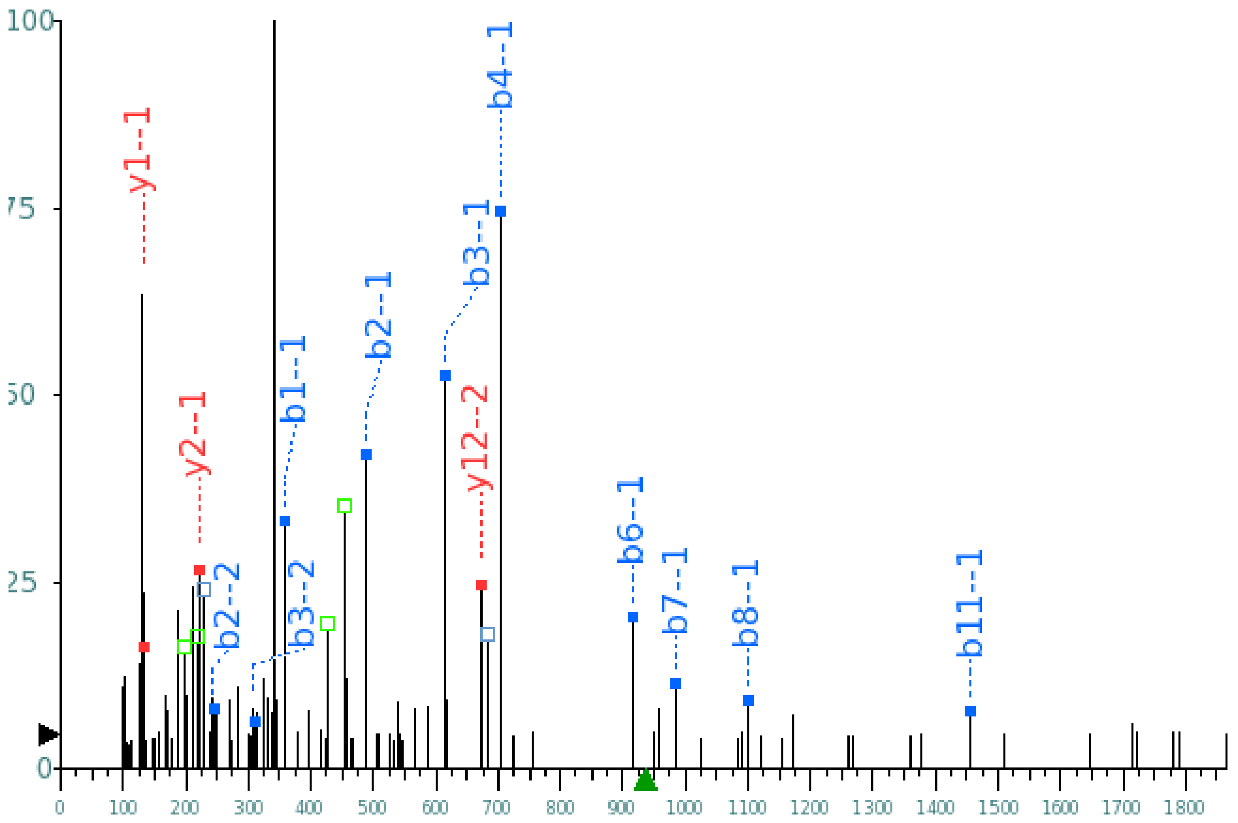

QEQSPNANNDQPEDLIQGC*SESD (+3)

Primary Reference: BPTF_HUMAN
Search ID: 41761
Search Name: 20130328_ananiav_ERstress_TMT_TM_timecourse_trypsin_inj2_4mc_iaa
Scan#: 11188
Observed Mass: 935.4106 (2 ppm)
PSM Score: 24.05

File name: 2015-02-04 04.01.53PM.pdf

Creation time: 2015-02-04 04:09PM

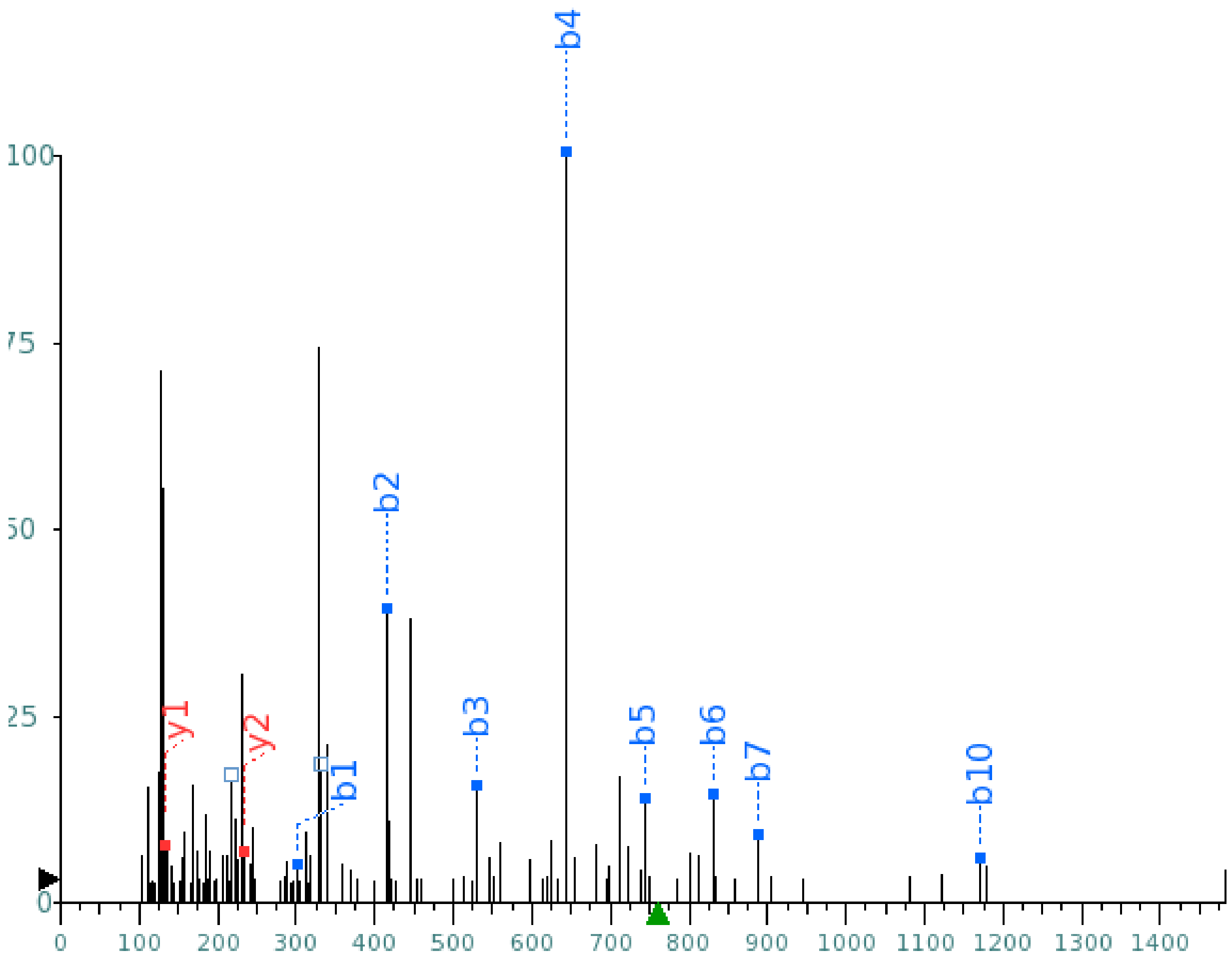

ADIDVSGPSVDTD (+2)

Primary Reference: AHNK_HUMAN
Search ID: 41761
Search Name: 20130328_ananiav_ERstress_TMT_TM_timecourse_trypsin_inj2_4mc_iaa
Scan#: 11202
Observed Mass: 760.3708 (1.2 ppm)
PSM Score: 31.29

File name: 2015-02-04 04.01.53PM.pdf

Creation time: 2015-02-04 04:09PM

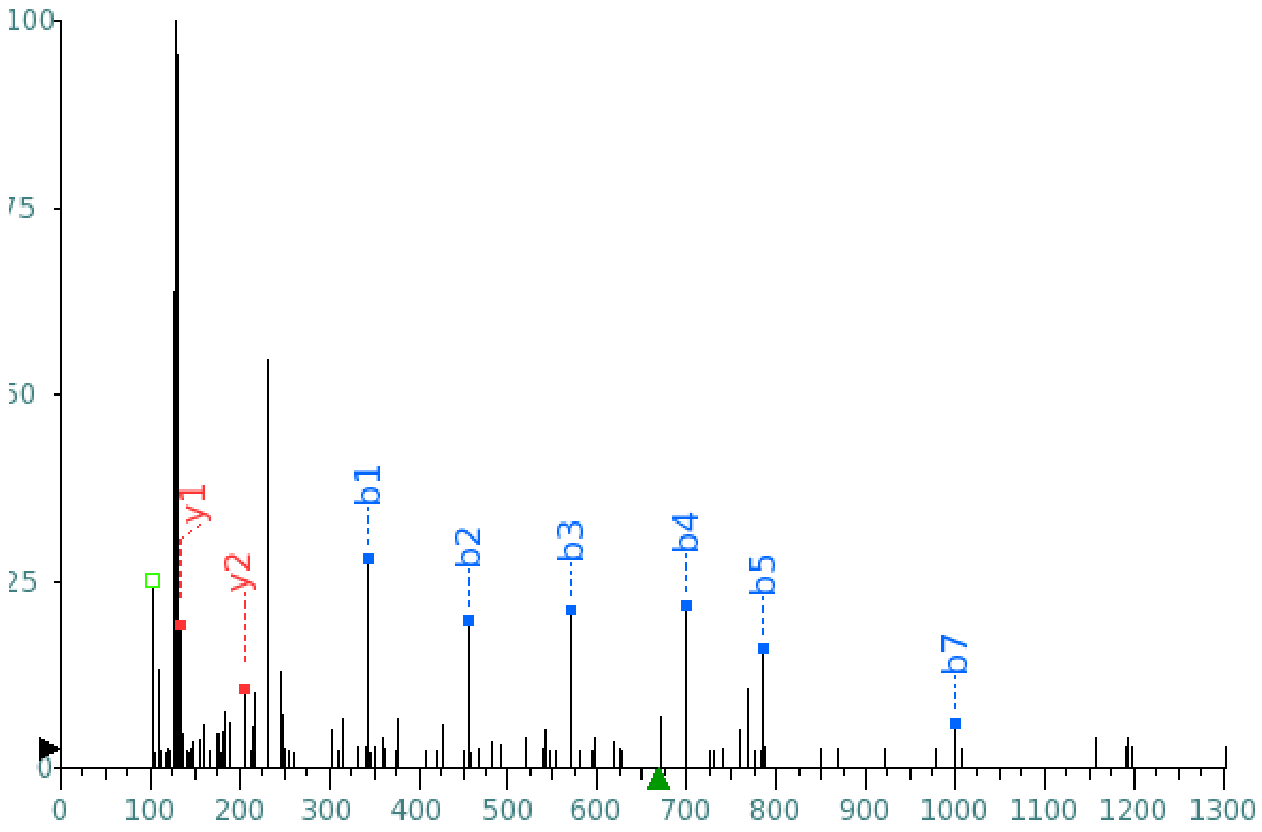

LINESVDEAD (+2)

Primary Reference: RANB3_HUMAN
Search ID: 41761
Search Name: 20130328_ananiav_ERstress_TMT_TM_timecourse_trypsin_inj2_4mc_iaa
Scan#: 11203
Observed Mass: 667.3389 (1.5 ppm)
PSM Score: 30.65

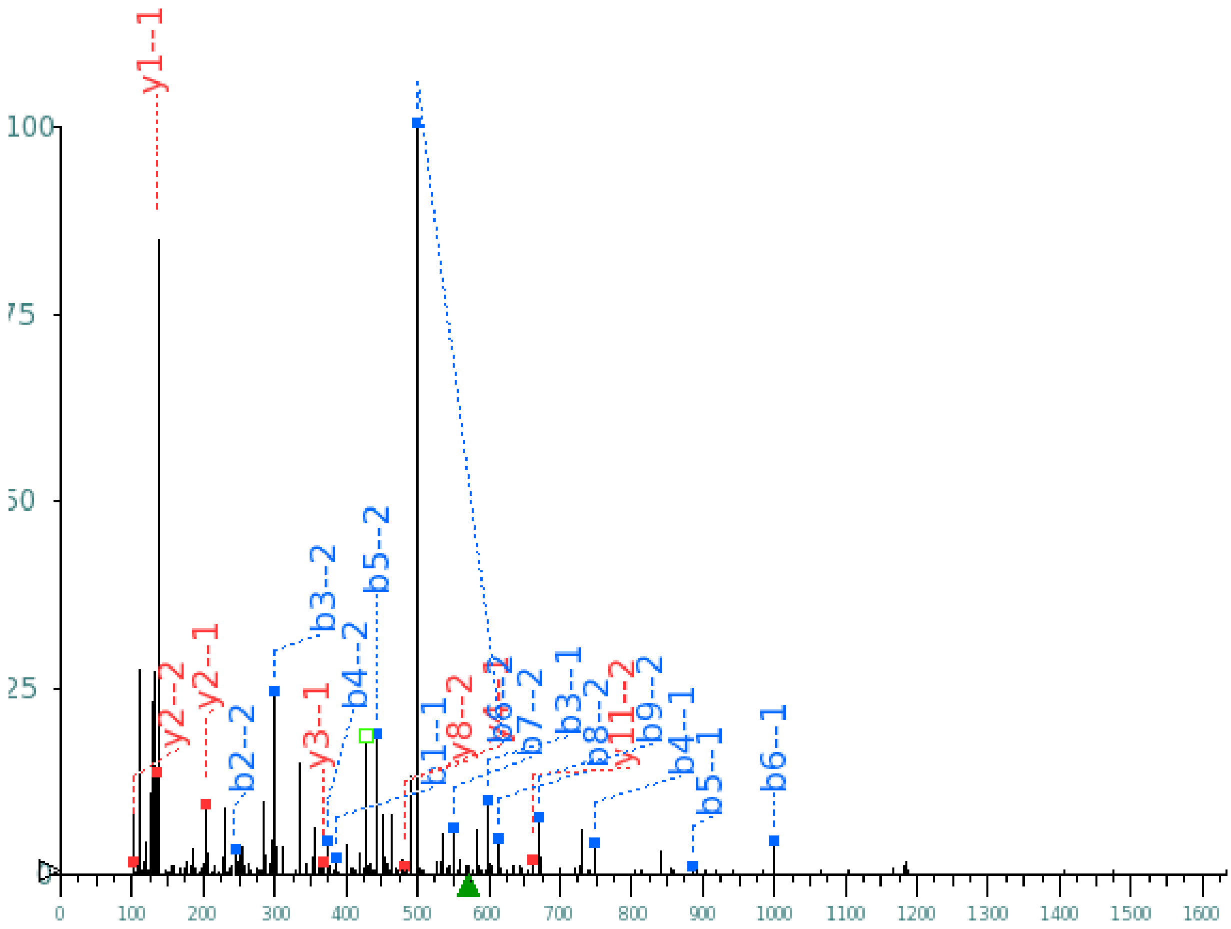

RVDFHDVQDYAD (+3)

Primary Reference: RAB24_HUMAN
Search ID: 41761
Search Name: 20130328_ananiav_ERstress_TMT_TM_timecourse_trypsin_inj2_4mc_iaa
Scan#: 11204
Observed Mass: 570.2768 (1.8 ppm)
PSM Score: 18.43

File name: 2015-02-04 04.01.53PM.pdf

Creation time: 2015-02-04 04:09PM

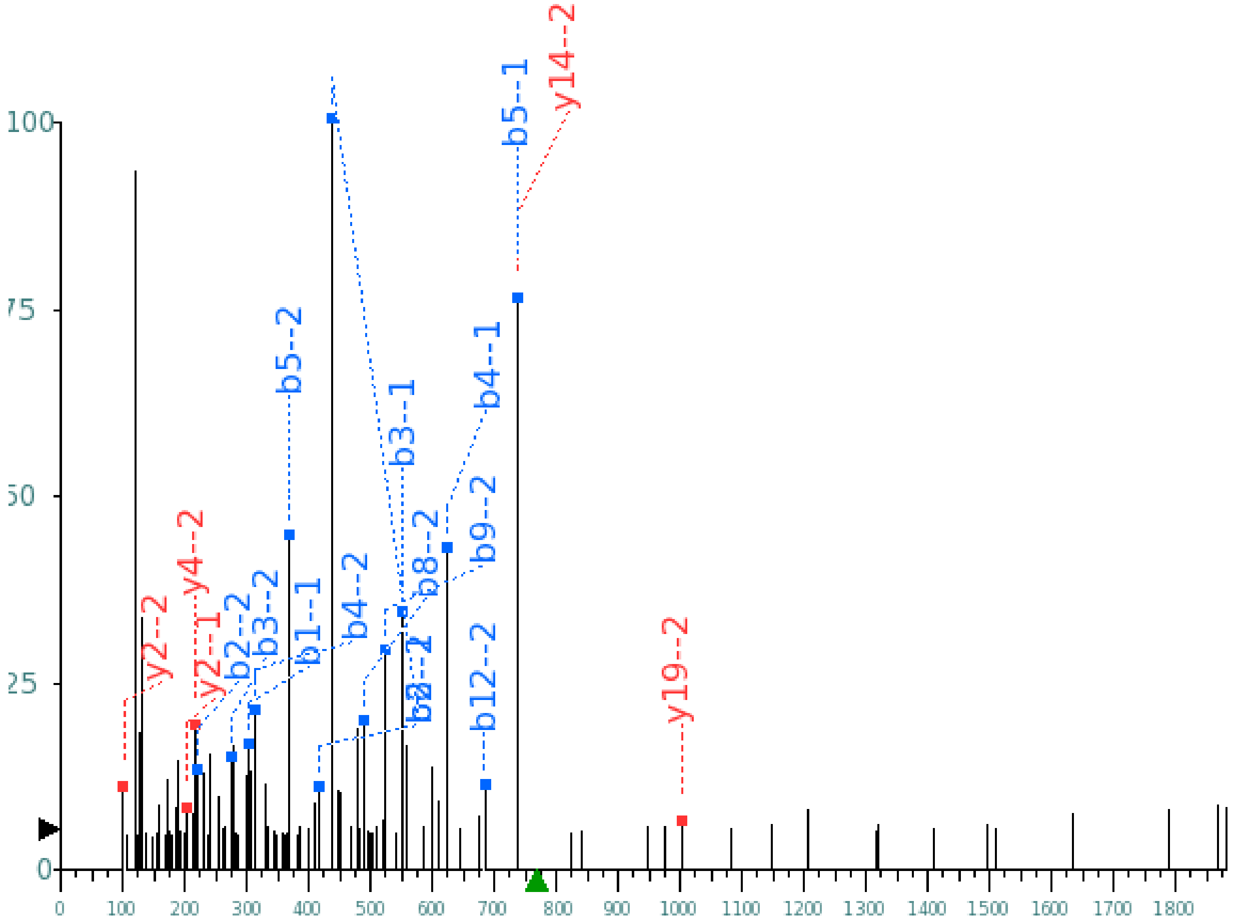

AHIANPSGASTEC*FVTDNAD (+3)

Primary Reference: FLNB_HUMAN
Search ID: 41761
Search Name: 20130328_ananiav_ERstress_TMT_TM_timecourse_trypsin_inj2_4mc_iaa
Scan#: 11234
Observed Mass: 769.355 (-2.3 ppm)
PSM Score: 23.12

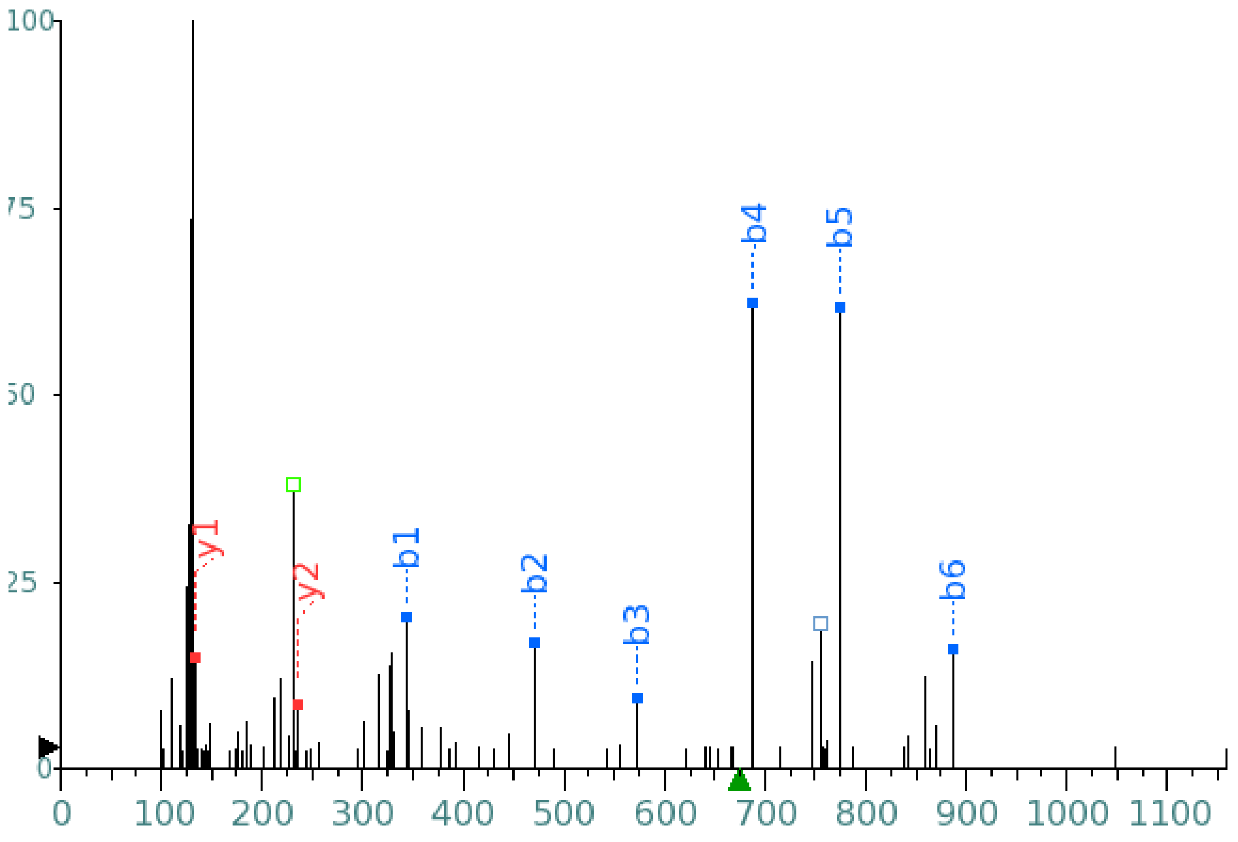

LQTDSIVQTD (+2)

Primary Reference: F1772_HUMAN
Search ID: 41761
Search Name: 20130328_ananiav_ERstress_TMT_TM_timecourse_trypsin_inj2_4mc_iaa
Scan#: 11268
Observed Mass: 674.8622 (1 ppm)
PSM Score: 38.16

File name: 2015-02-04 04.01.53PM.pdf

Creation time: 2015-02-04 04:09PM

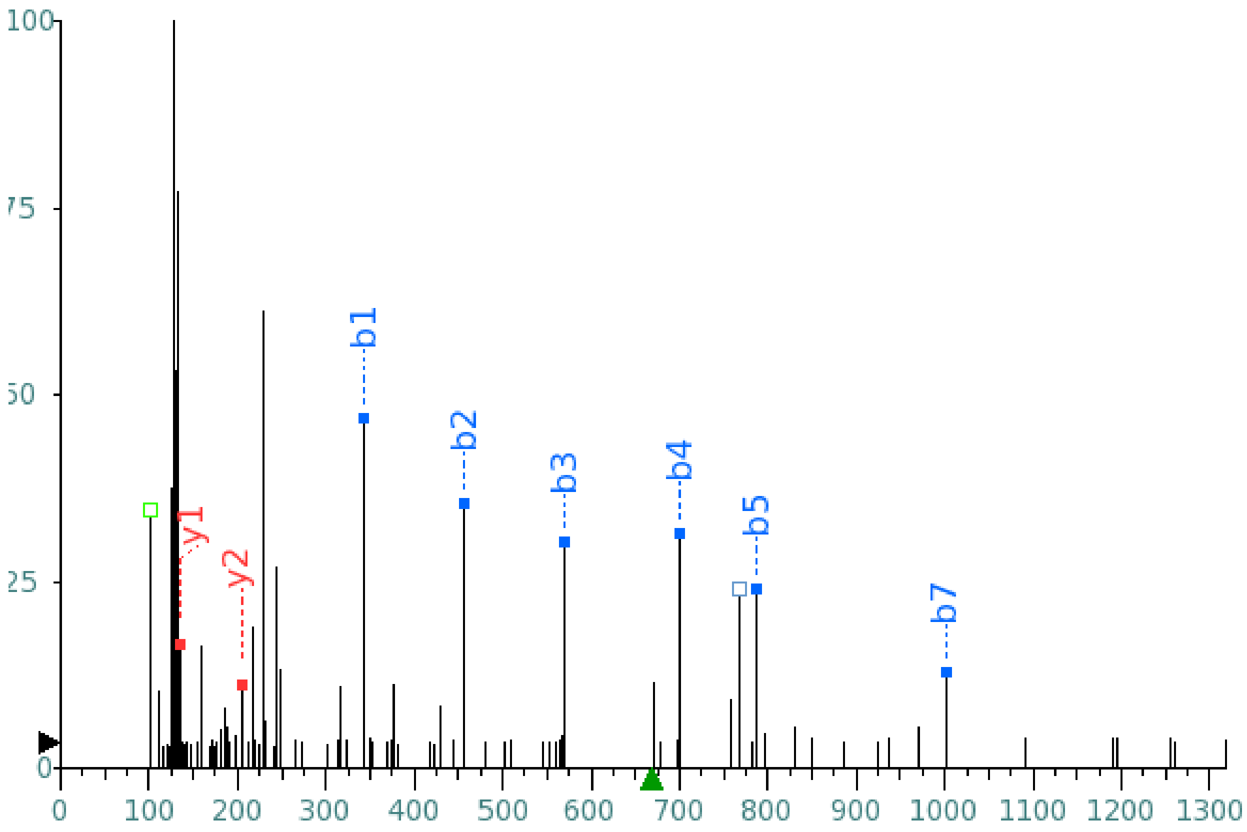

LINESVDEAD (+2)

Primary Reference: RANB3_HUMAN
Search ID: 41761
Search Name: 20130328_ananiav_ERstress_TMT_TM_timecourse_trypsin_inj2_4mc_iaa
Scan#: 11301
Observed Mass: 667.3387 (1.3 ppm)
PSM Score: 29.85

File name: 2015-02-04 04.01.53PM.pdf

Creation time: 2015-02-04 04:09PM

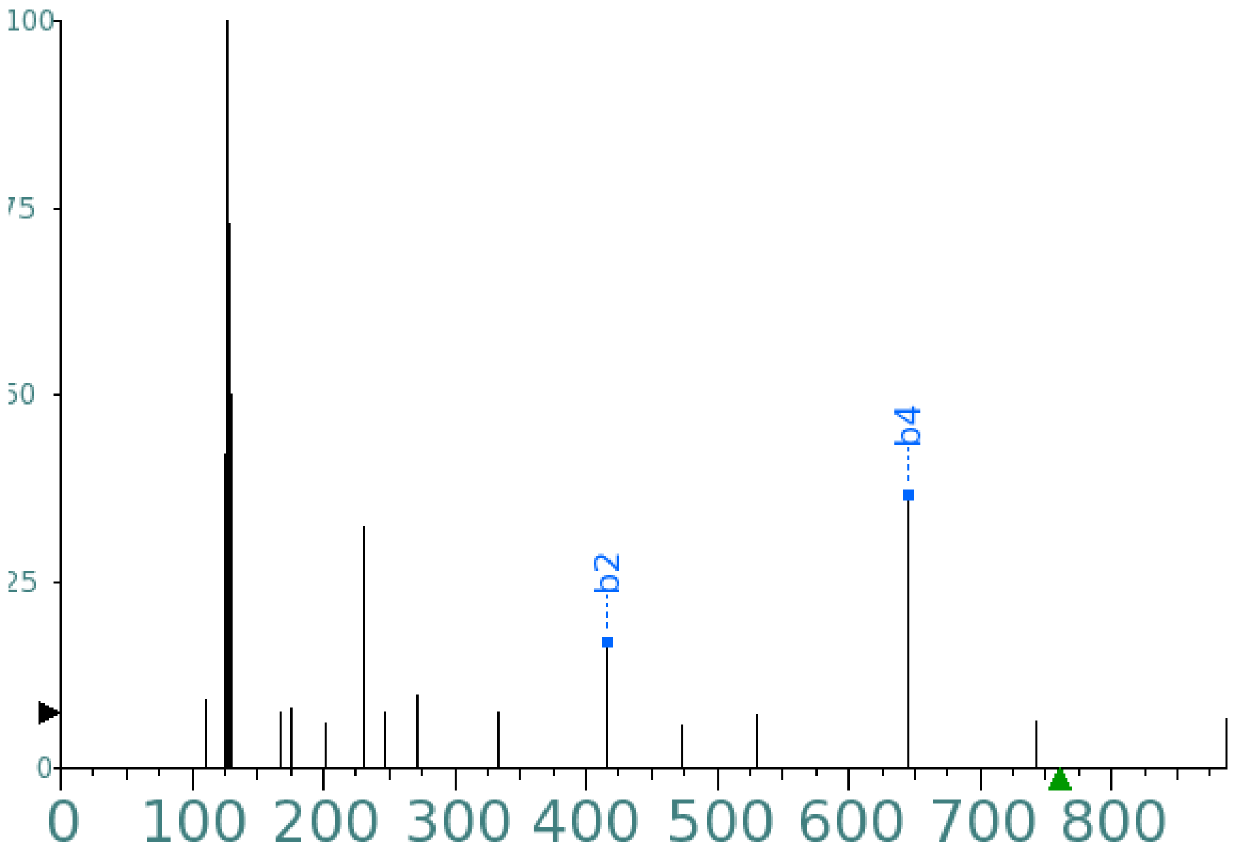

ADIDVSGPSVDTD (+2)

Primary Reference: AHNK_HUMAN
Search ID: 41761
Search Name: 20130328_ananiav_ERstress_TMT_TM_timecourse_trypsin_inj2_4mc_iaa
Scan#: 11307
Observed Mass: 760.3706 (1 ppm)
PSM Score: 27.53

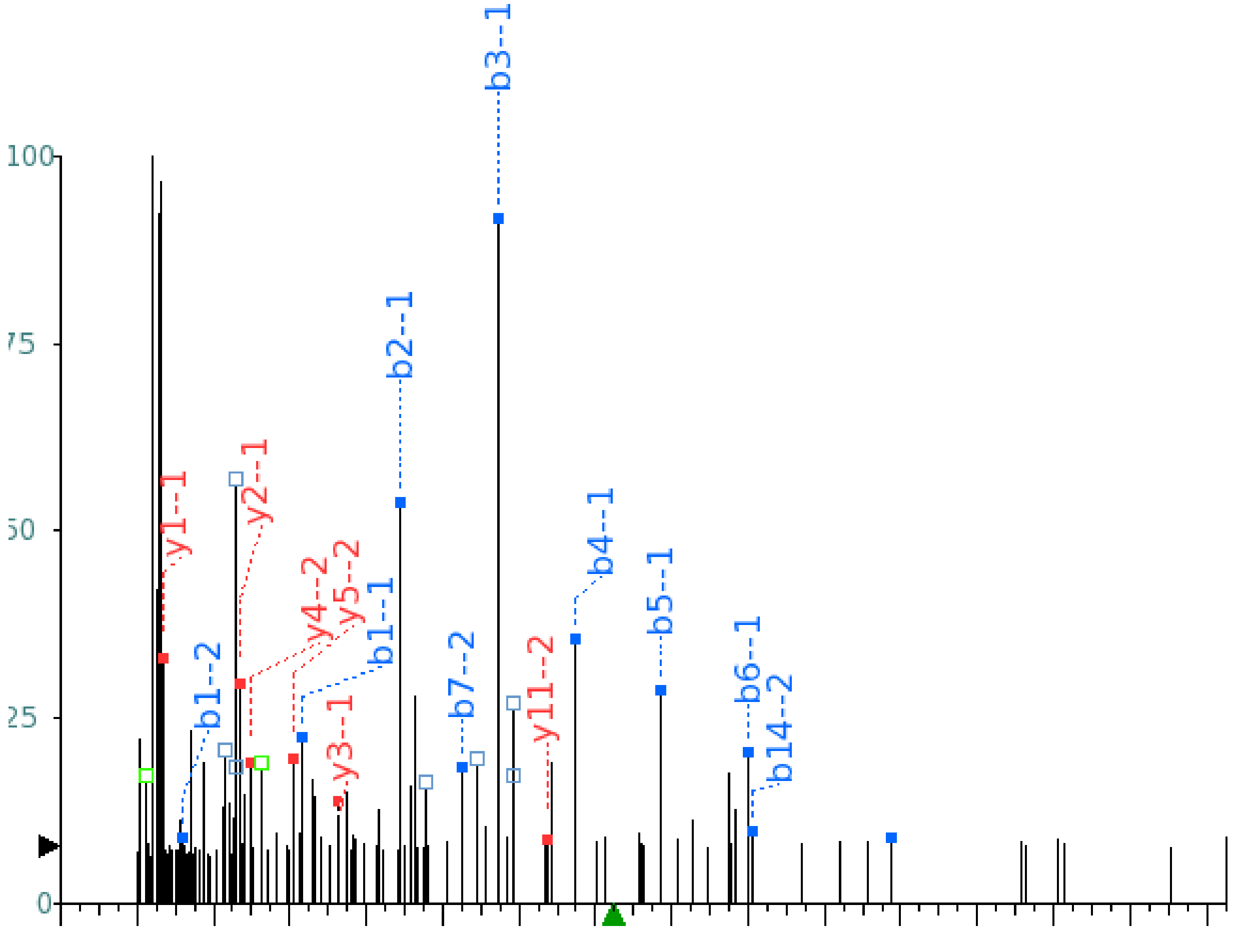

SQQVNNFTSNDVDMETD (+3)

Primary Reference: RANB9_HUMAN
Search ID: 41761
Search Name: 20130328_ananiav_ERstress_TMT_TM_timecourse_trypsin_inj2_4mc_iaa
Scan#: 11377
Observed Mass: 724.9911 (1.6 ppm)
PSM Score: 13.44

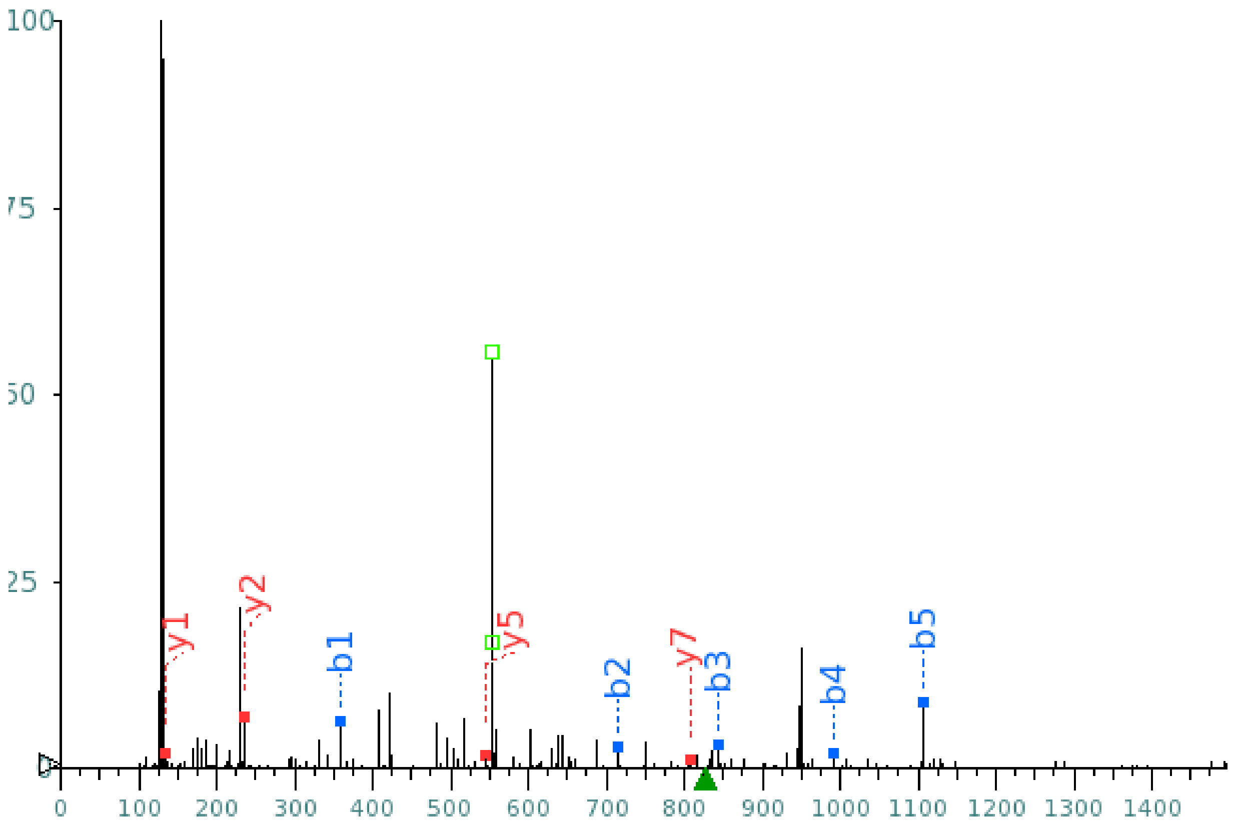

QKEFDPTITD (+2)

Primary Reference: CALD1_HUMAN
Search ID: 41761
Search Name: 20130328_ananiav_ERstress_TMT_TM_timecourse_trypsin_inj2_4mc_iaa
Scan#: 11380
Observed Mass: 826.4518 (1.2 ppm)
PSM Score: 10.38

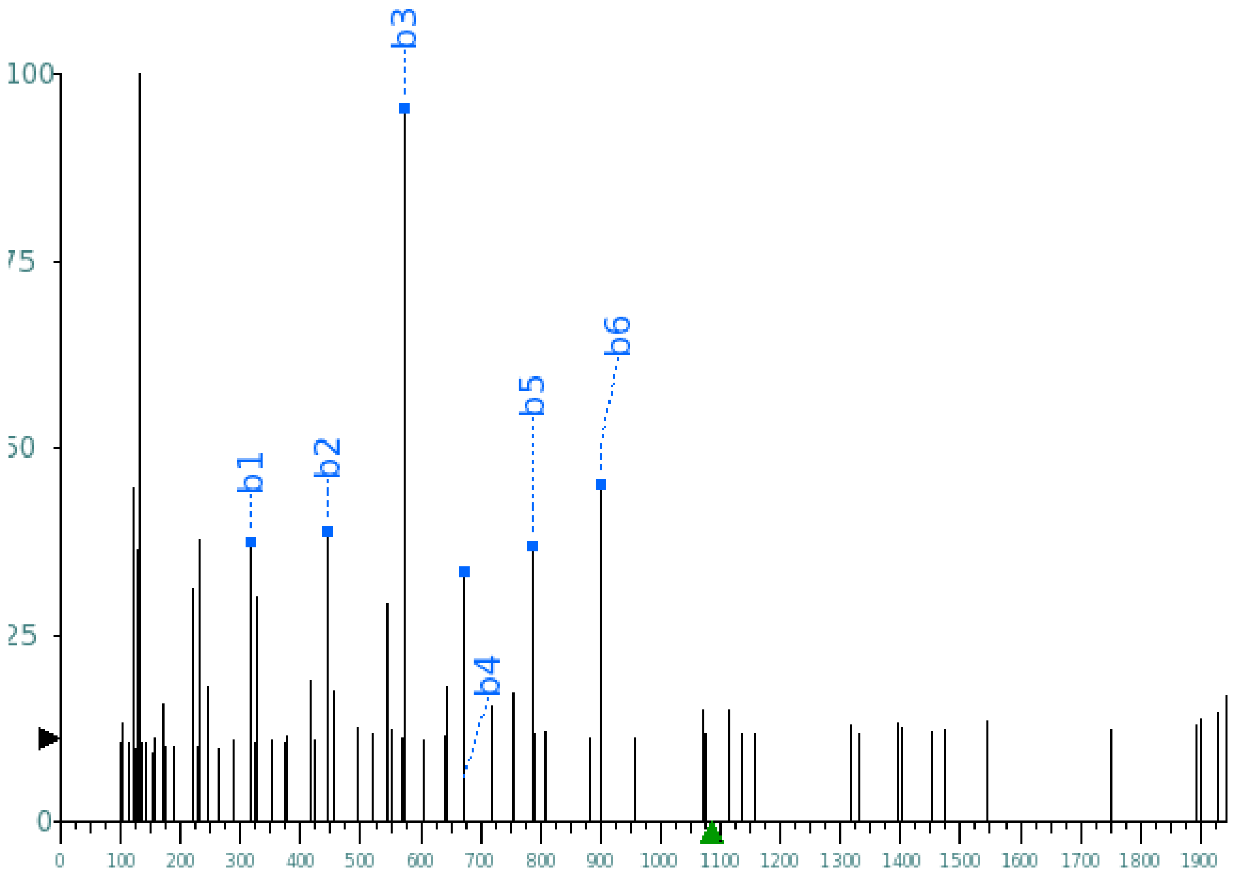

SQQVNNFTSNDVDMETD (+2)

Primary Reference: RANB9_HUMAN
Search ID: 41761
Search Name: 20130328_ananiav_ERstress_TMT_TM_timecourse_trypsin_inj2_4mc_iaa
Scan#: 11381
Observed Mass: 1086.9825 (1.1 ppm)
PSM Score: 19.93

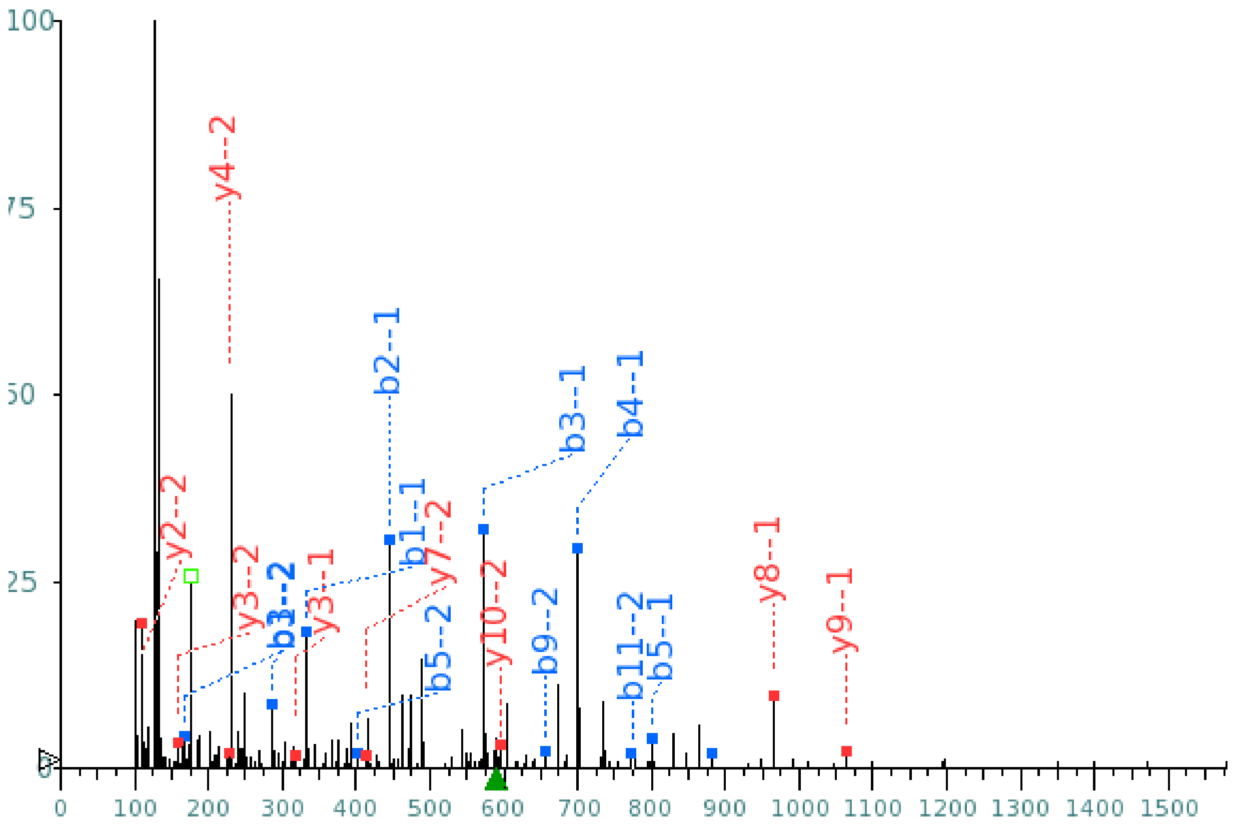

TLQEVHFLLHPSD (+3)

Primary Reference: PAR14_HUMAN
Search ID: 41761
Search Name: 20130328_ananiav_ERstress_TMT_TM_timecourse_trypsin_inj2_4mc_iaa
Scan#: 11396
Observed Mass: 588.9874 (-0.3 ppm)
PSM Score: 21.14

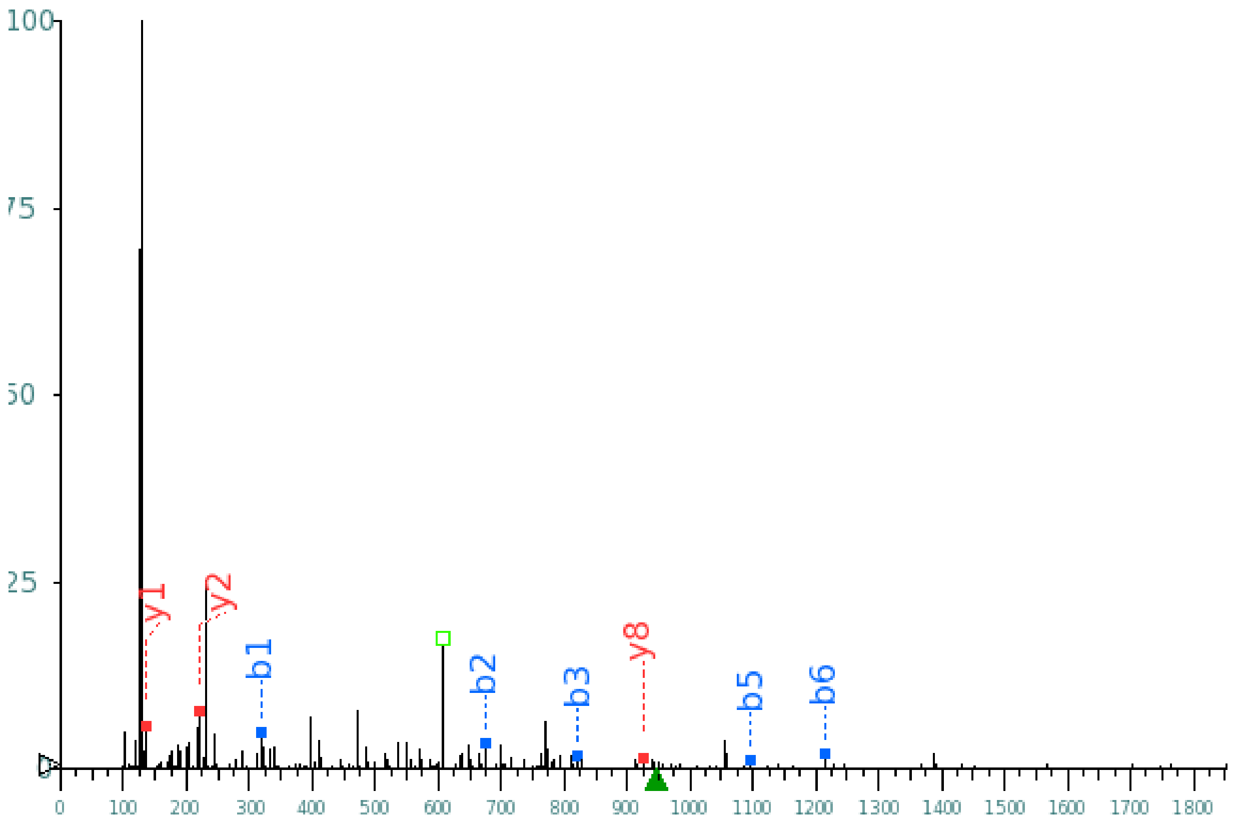

SKFFEDSEDESD (+2)

Primary Reference: PPM1G_HUMAN
Search ID: 41761
Search Name: 20130328_ananiav_ERstress_TMT_TM_timecourse_trypsin_inj2_4mc_iaa
Scan#: 11448
Observed Mass: 946.9454 (1.8 ppm)
PSM Score: 9.13

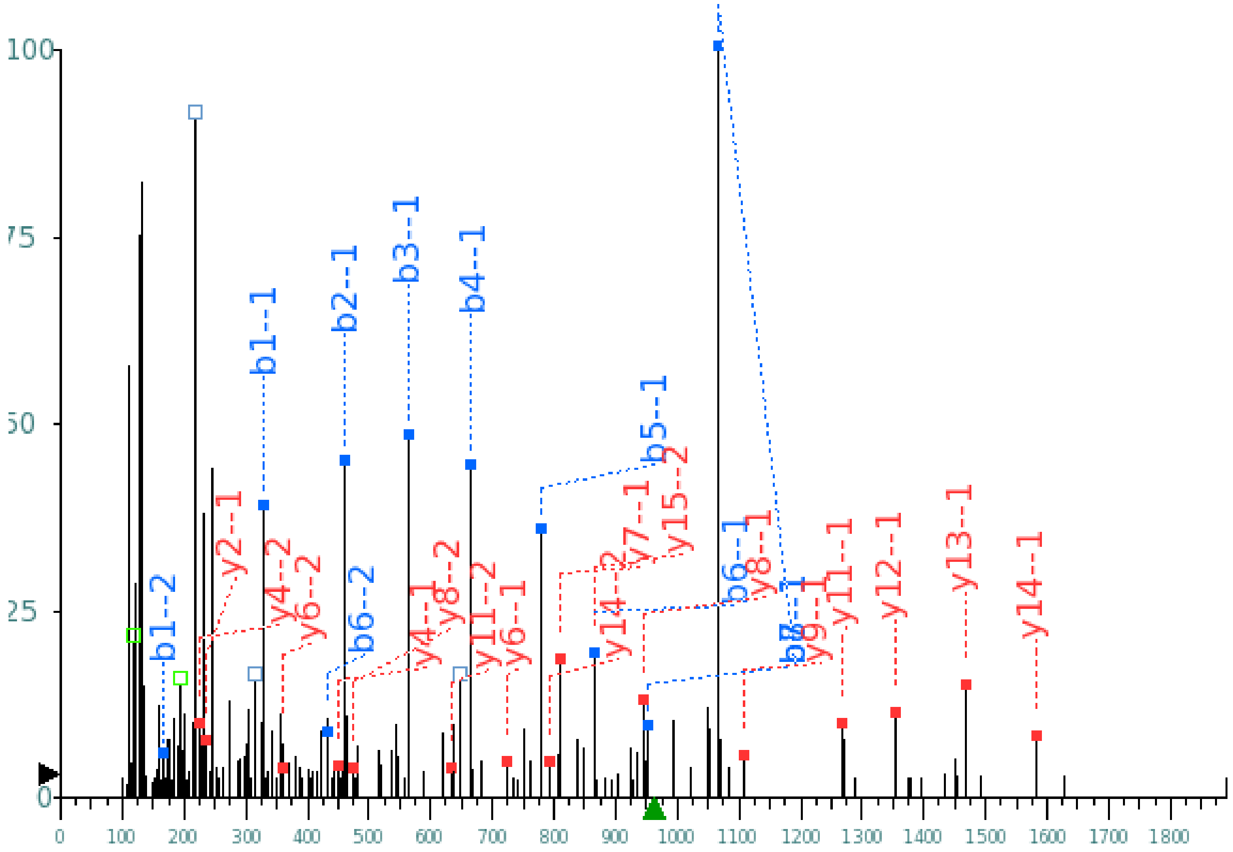

TMTTNSSDPFLNSGTYHSRDESTD (+3)

Primary Reference: YAP1_HUMAN
Search ID: 41761
Search Name: 20130328_ananiav_ERstress_TMT_TM_timecourse_trypsin_inj2_4mc_iaa
Scan#: 11459
Observed Mass: 964.7656 (1 ppm)
PSM Score: 63.3

File name: 2015-02-04 04.01.53PM.pdf

Creation time: 2015-02-04 04:09PM

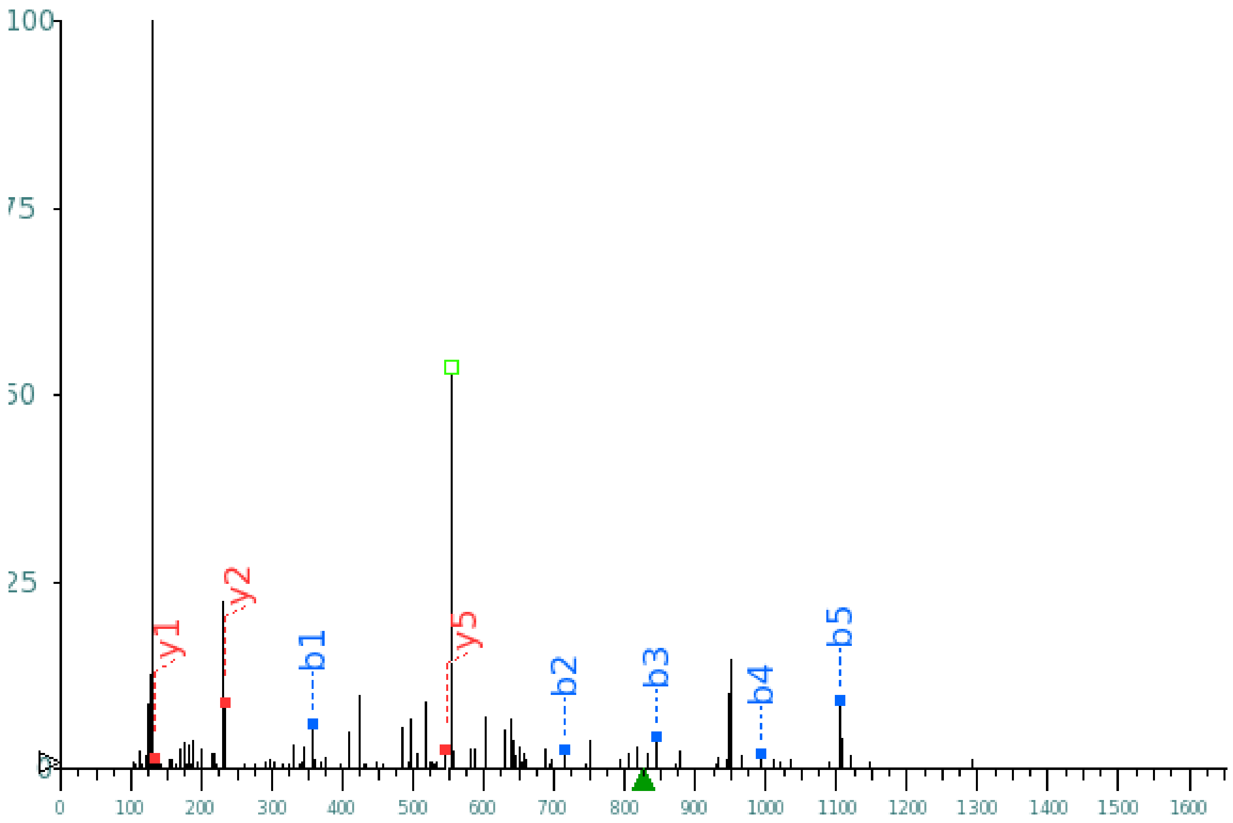

QKEFDPTITD (+2)

Primary Reference: CALD1_HUMAN
Search ID: 41761
Search Name: 20130328_ananiav_ERstress_TMT_TM_timecourse_trypsin_inj2_4mc_iaa
Scan#: 11469
Observed Mass: 826.4522 (1.7 ppm)
PSM Score: 15.49

File name: 2015-02-04 04.01.53PM.pdf

Creation time: 2015-02-04 04:09PM

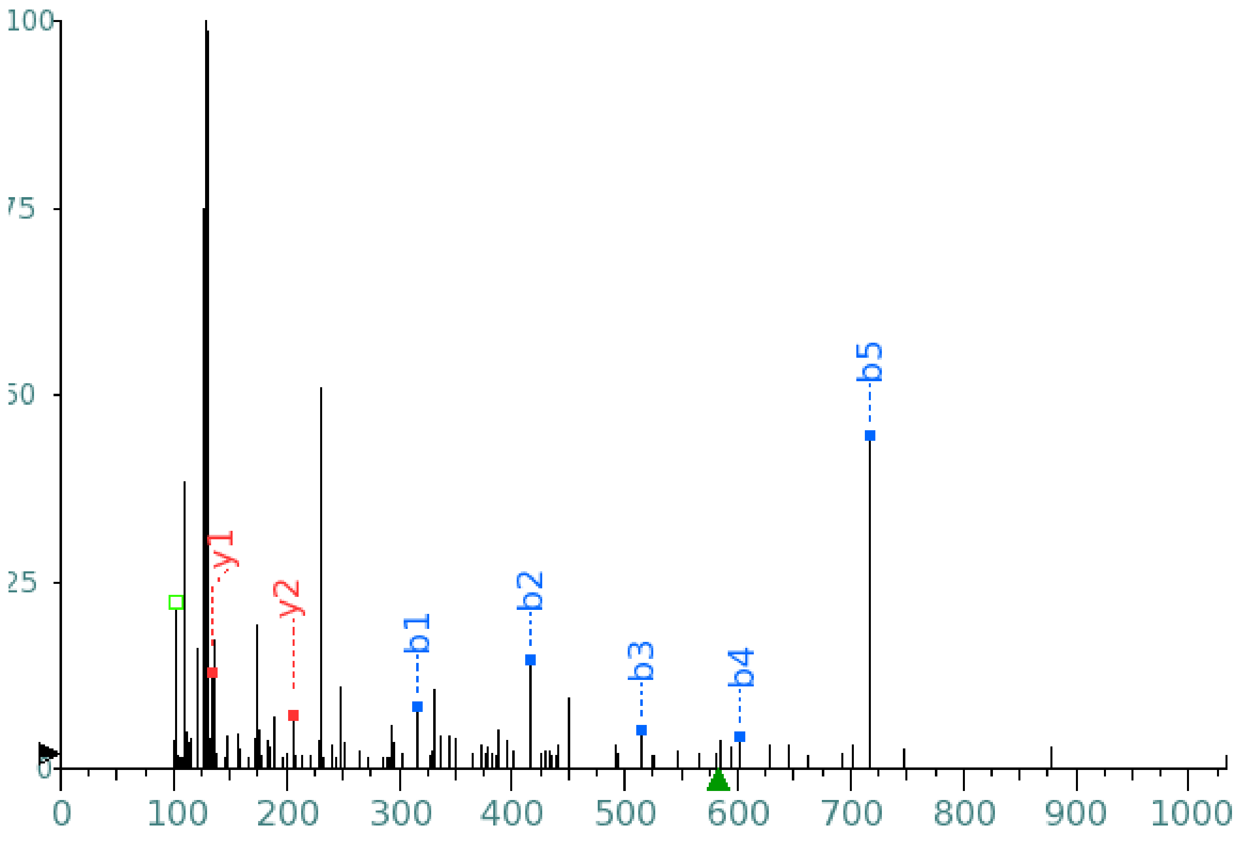

SVVSDLEAD (+2)

Primary Reference: TOP2A_HUMAN
Search ID: 41761
Search Name: 20130328_ananiav_ERstress_TMT_TM_timecourse_trypsin_inj2_4mc_iaa
Scan#: 11478
Observed Mass: 582.304 (1.2 ppm)
PSM Score: 13.34

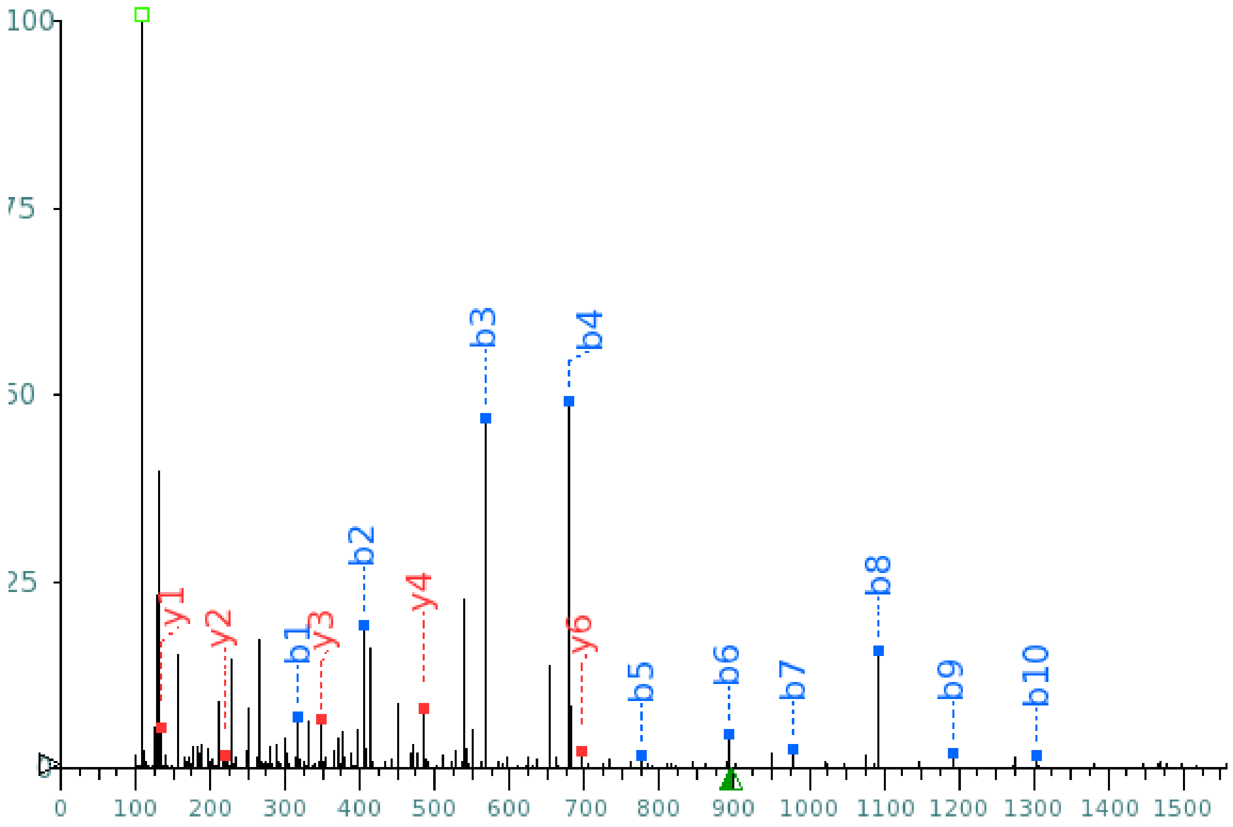

SSYLPNSDPLHQSD (+2)

Primary Reference: ANR17_HUMAN
Search ID: 41761
Search Name: 20130328_ananiav_ERstress_TMT_TM_timecourse_trypsin_inj2_4mc_iaa
Scan#: 11525
Observed Mass: 894.9338 (0.1 ppm)
PSM Score: 60.84

File name: 2015-02-04 04.01.53PM.pdf

Creation time: 2015-02-04 04:09PM

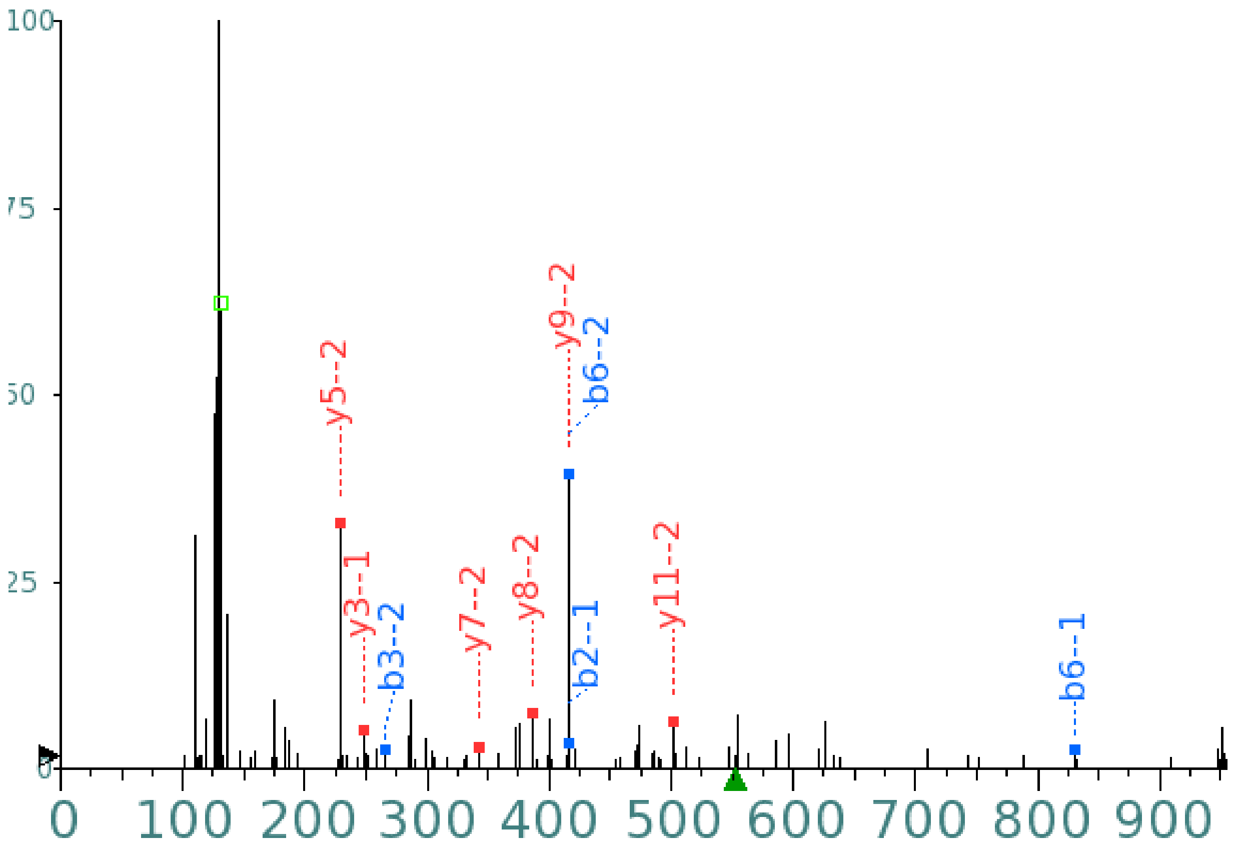

VSIQATGSRAPIGGD (+3)

Primary Reference: ##PCDC2_HUMAN
Search ID: 41761
Search Name: 20130328_ananiav_ERstress_TMT_TM_timecourse_trypsin_inj2_4mc_iaa
Scan#: 11549
Observed Mass: 553.3073 (0.2 ppm)
PSM Score: 12.76

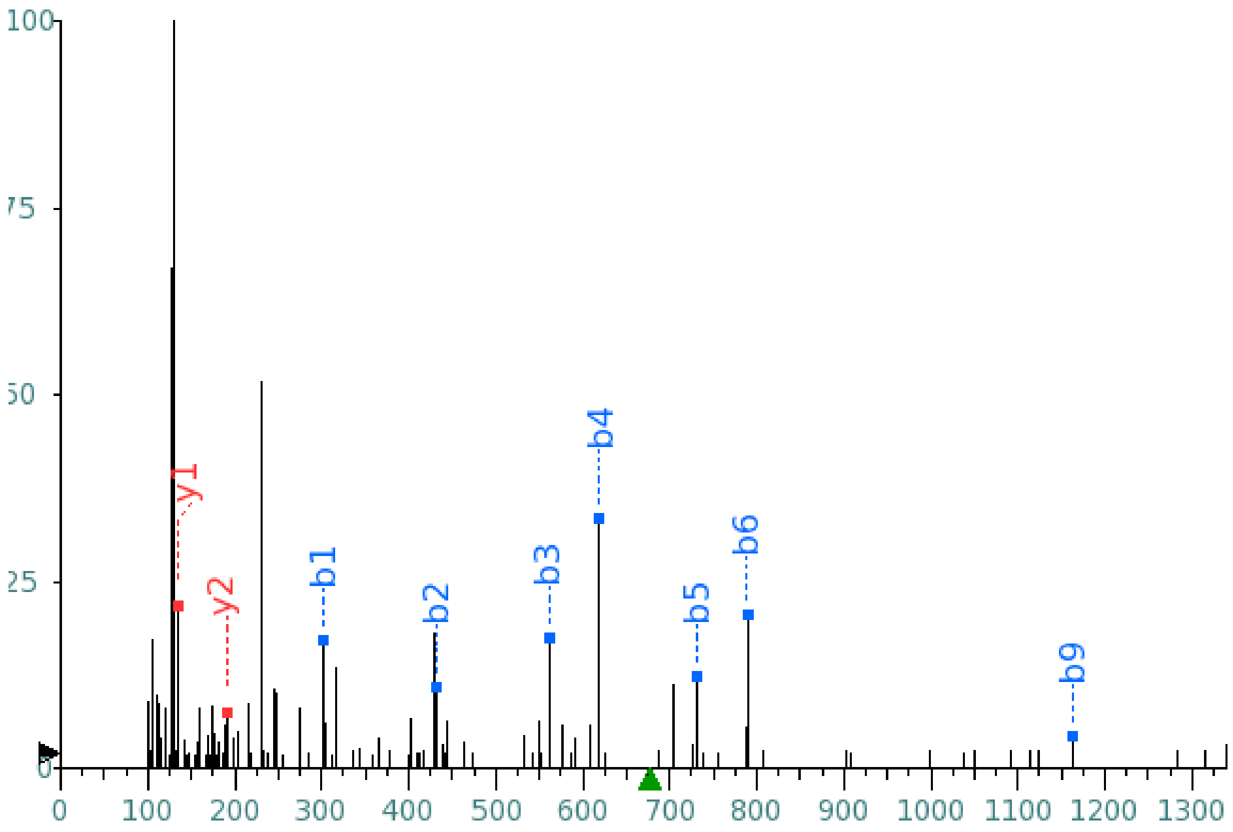

AMEGLGMDEGD (+2)

Primary Reference: PEX19_HUMAN
Search ID: 41761
Search Name: 20130328_ananiav_ERstress_TMT_TM_timecourse_trypsin_inj2_4mc_iaa
Scan#: 11554
Observed Mass: 677.2976 (1.1 ppm)
PSM Score: 22.88

File name: 2015-02-04 04.01.53PM.pdf

Creation time: 2015-02-04 04:09PM

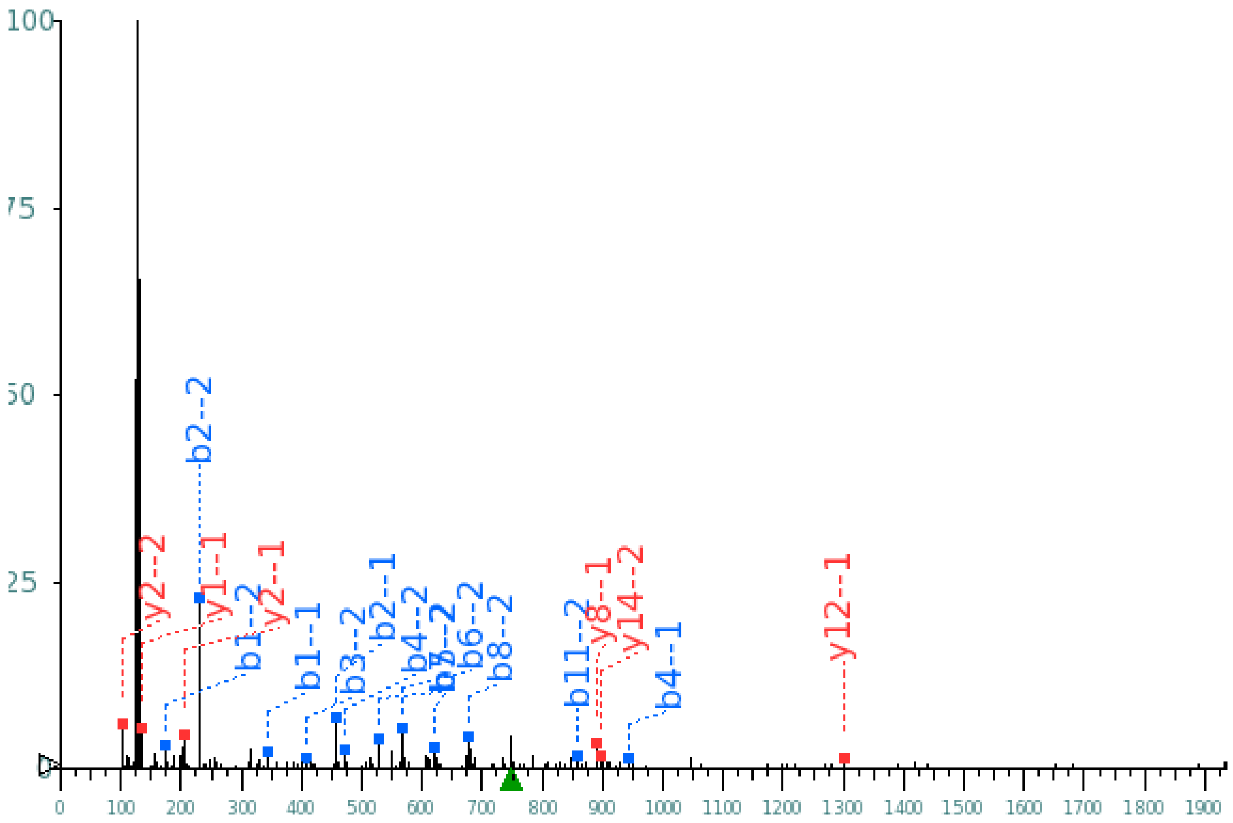

LDKENAIDRAEQAEAD (+3)

Primary Reference: TPM2_HUMAN
Search ID: 41761
Search Name: 20130328_ananiav_ERstress_TMT_TM_timecourse_trypsin_inj2_4mc_iaa
Scan#: 11646
Observed Mass: 749.3956 (2.6 ppm)
PSM Score: 16.6

File name: 2015-02-04 04.01.53PM.pdf

Creation time: 2015-02-04 04:09PM

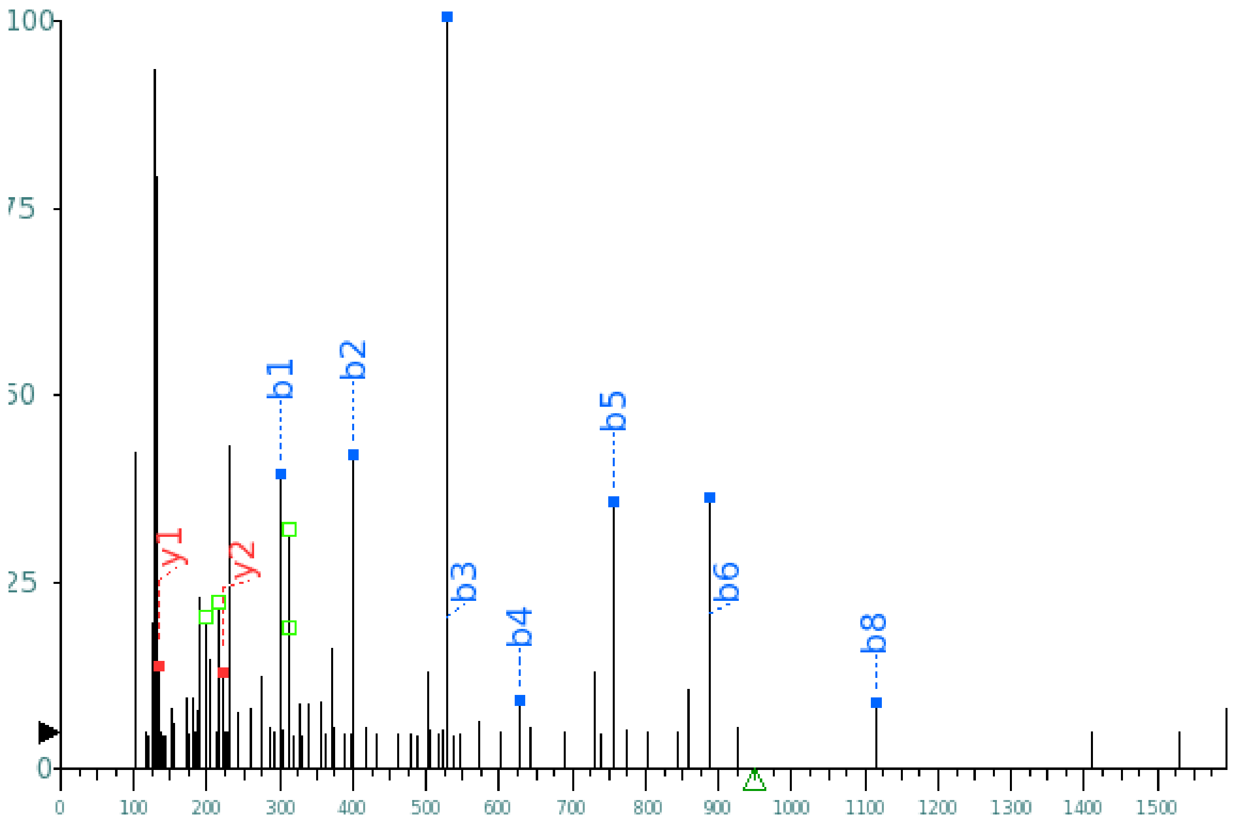

AVEPMEIDSEESESD (+2)

Primary Reference: ERCC5_HUMAN
Search ID: 41761
Search Name: 20130328_ananiav_ERstress_TMT_TM_timecourse_trypsin_inj2_4mc_iaa
Scan#: 11702
Observed Mass: 948.4176 (0.7 ppm)
PSM Score: 29.01

File name: 2015-02-04 04.01.53PM.pdf

Creation time: 2015-02-04 04:09PM

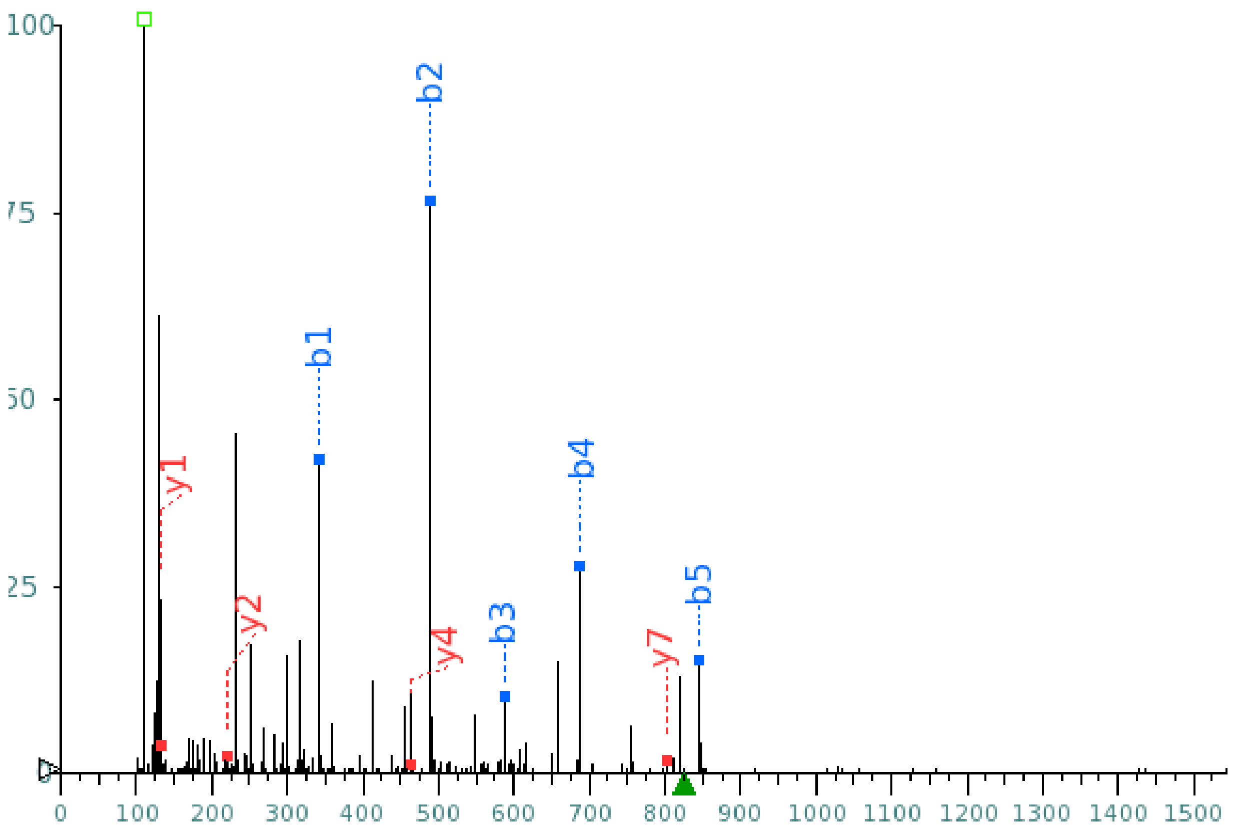

LFPVC*HDSDESD (+2)

Primary Reference: DNM3A_HUMAN
Search ID: 41761
Search Name: 20130328_ananiav_ERstress_TMT_TM_timecourse_trypsin_inj2_4mc_iaa
Scan#: 11733
Observed Mass: 825.3691 (-0.3 ppm)
PSM Score: 18.25

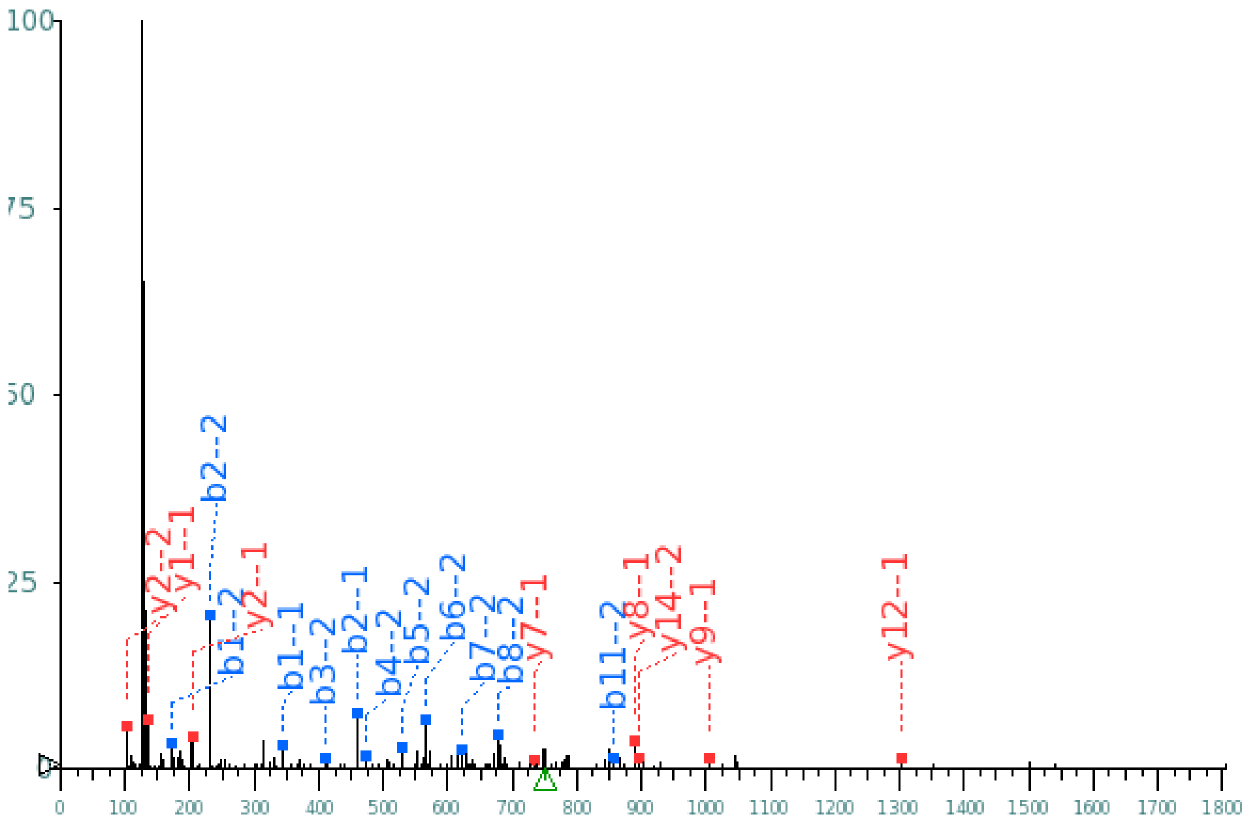

LDKENAIDRAEQAEAD (+3)

Primary Reference: TPM2_HUMAN
Search ID: 41761
Search Name: 20130328_ananiav_ERstress_TMT_TM_timecourse_trypsin_inj2_4mc_iaa
Scan#: 11757
Observed Mass: 749.3954 (2.4 ppm)
PSM Score: 19.69

File name: 2015-02-04 04.01.53PM.pdf

Creation time: 2015-02-04 04:09PM

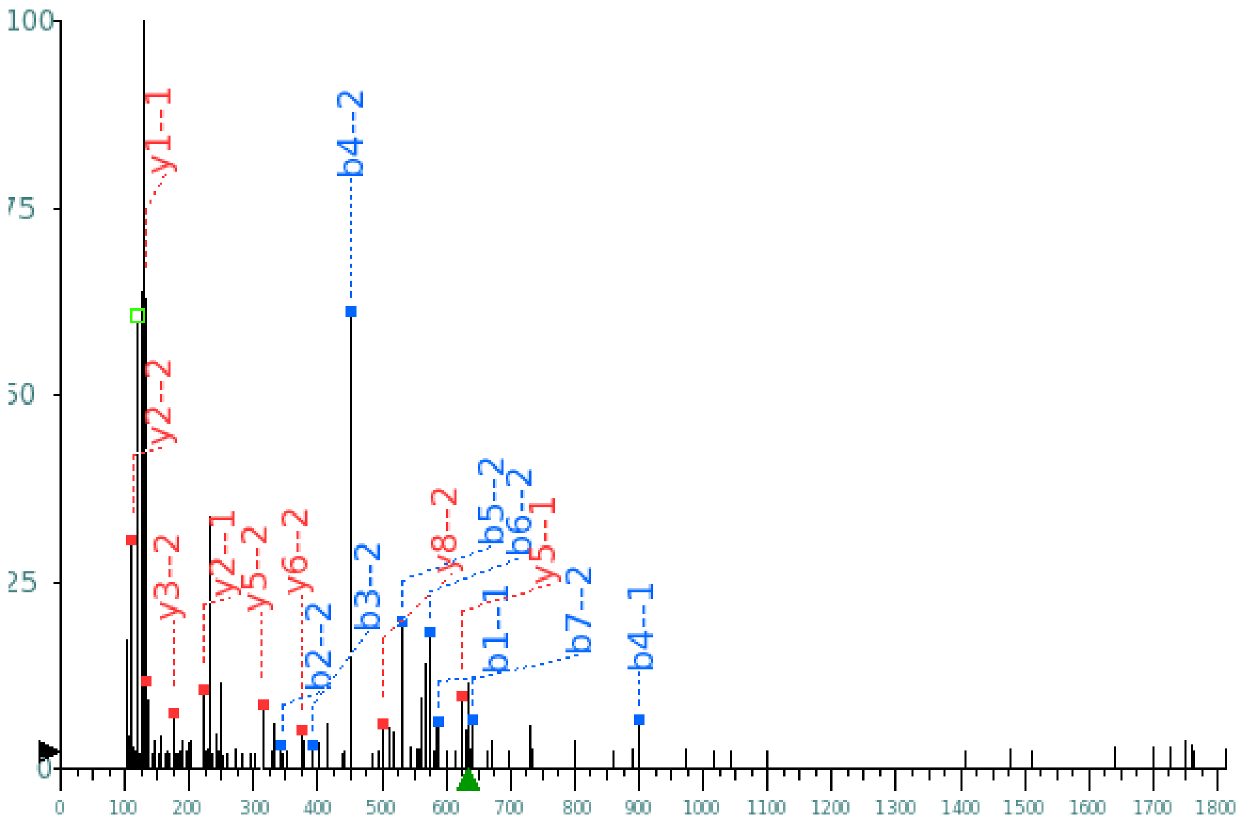

KVVDYSQFQESD (+3)

Primary Reference: NUCKS_HUMAN
Search ID: 41761
Search Name: 20130328_ananiav_ERstress_TMT_TM_timecourse_trypsin_inj2_4mc_iaa
Scan#: 11778
Observed Mass: 635.0011 (2 ppm)
PSM Score: 5.76

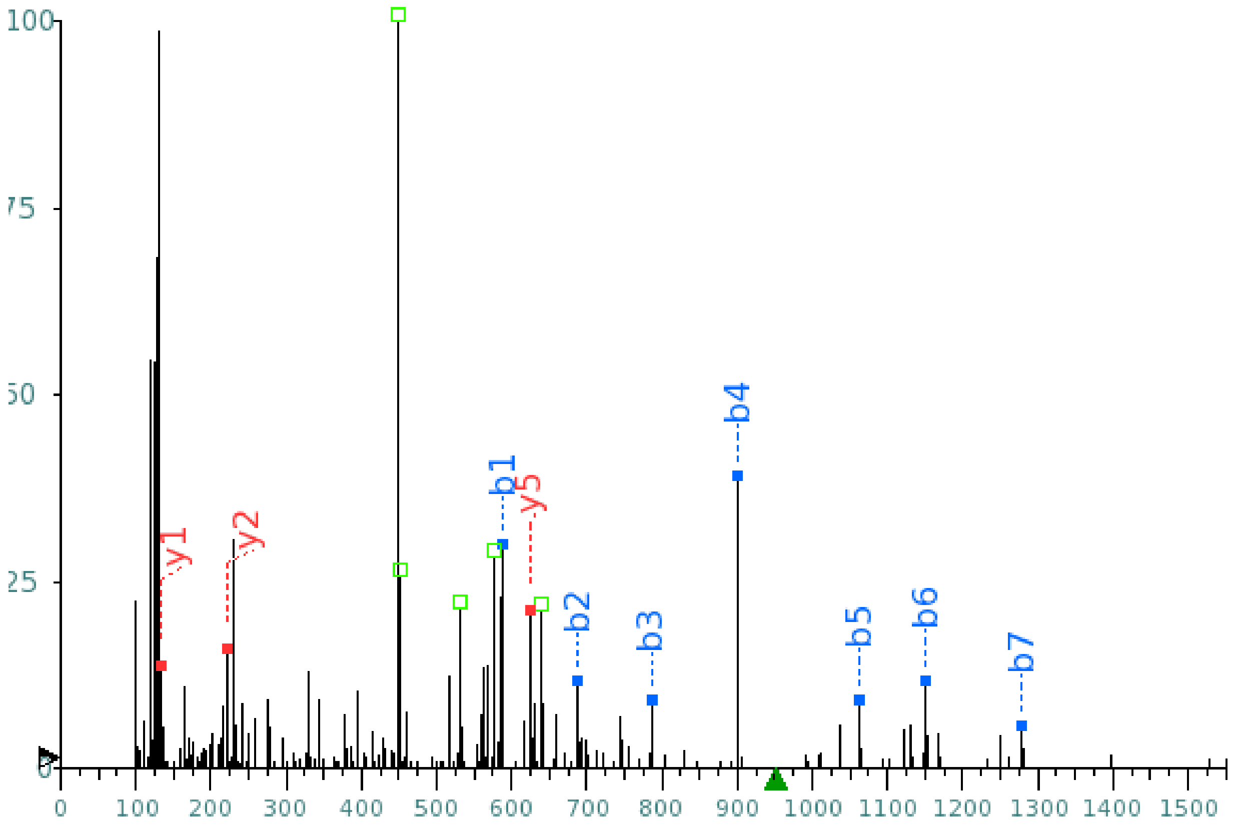

KVVDYSQFQESD (+2)

Primary Reference: NUCKS_HUMAN
Search ID: 41761
Search Name: 20130328_ananiav_ERstress_TMT_TM_timecourse_trypsin_inj2_4mc_iaa
Scan#: 11787
Observed Mass: 951.9968 (0.7 ppm)
PSM Score: 29.11

File name: 2015-02-04 04.01.53PM.pdf

Creation time: 2015-02-04 04:09PM

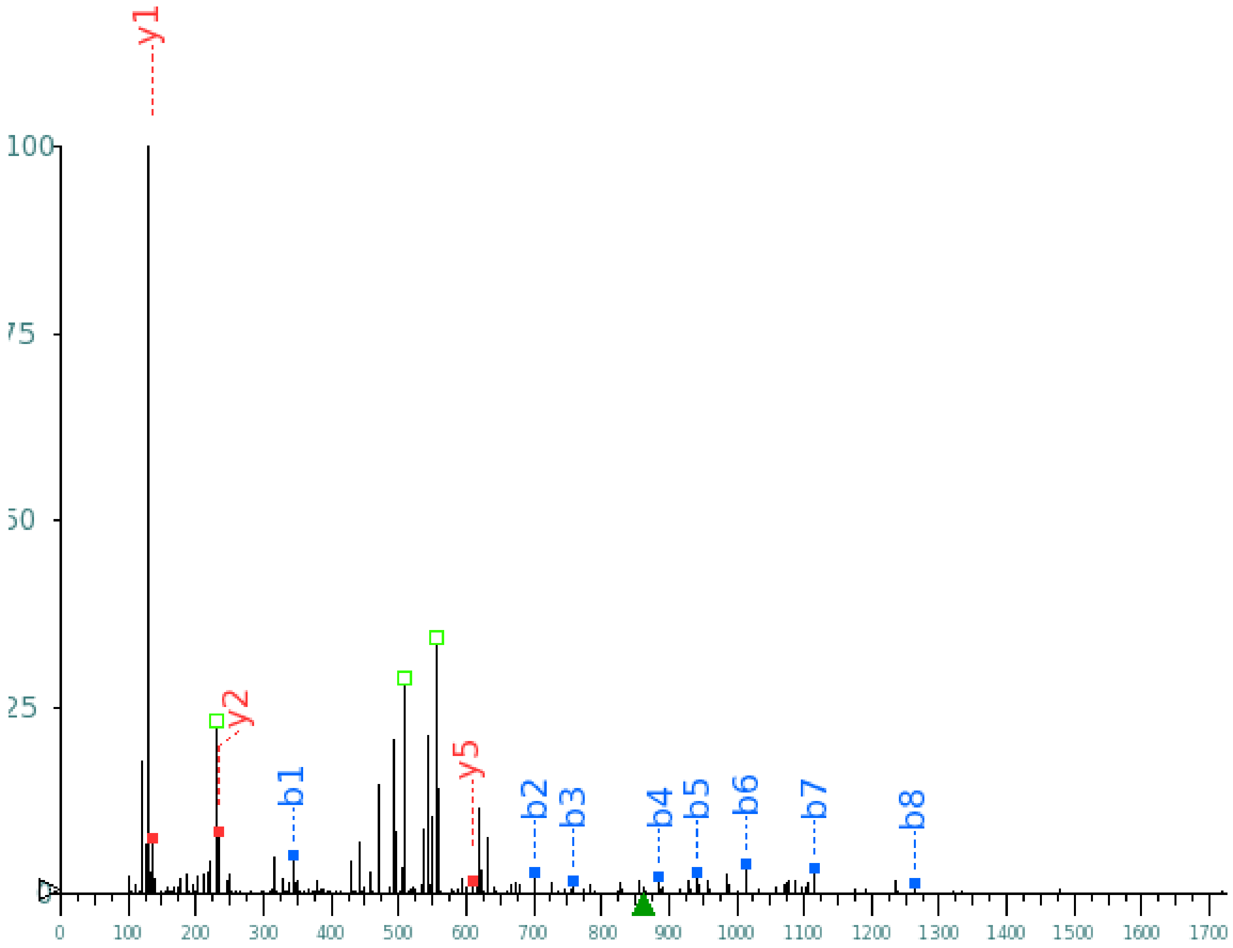

LKGQGATFVETD (+2)

Primary Reference: ZN407_HUMAN
Search ID: 41761
Search Name: 20130328_ananiav_ERstress_TMT_TM_timecourse_trypsin_inj2_4mc_iaa
Scan#: 11859
Observed Mass: 862.4862 (1.1 ppm)
PSM Score: 15.88

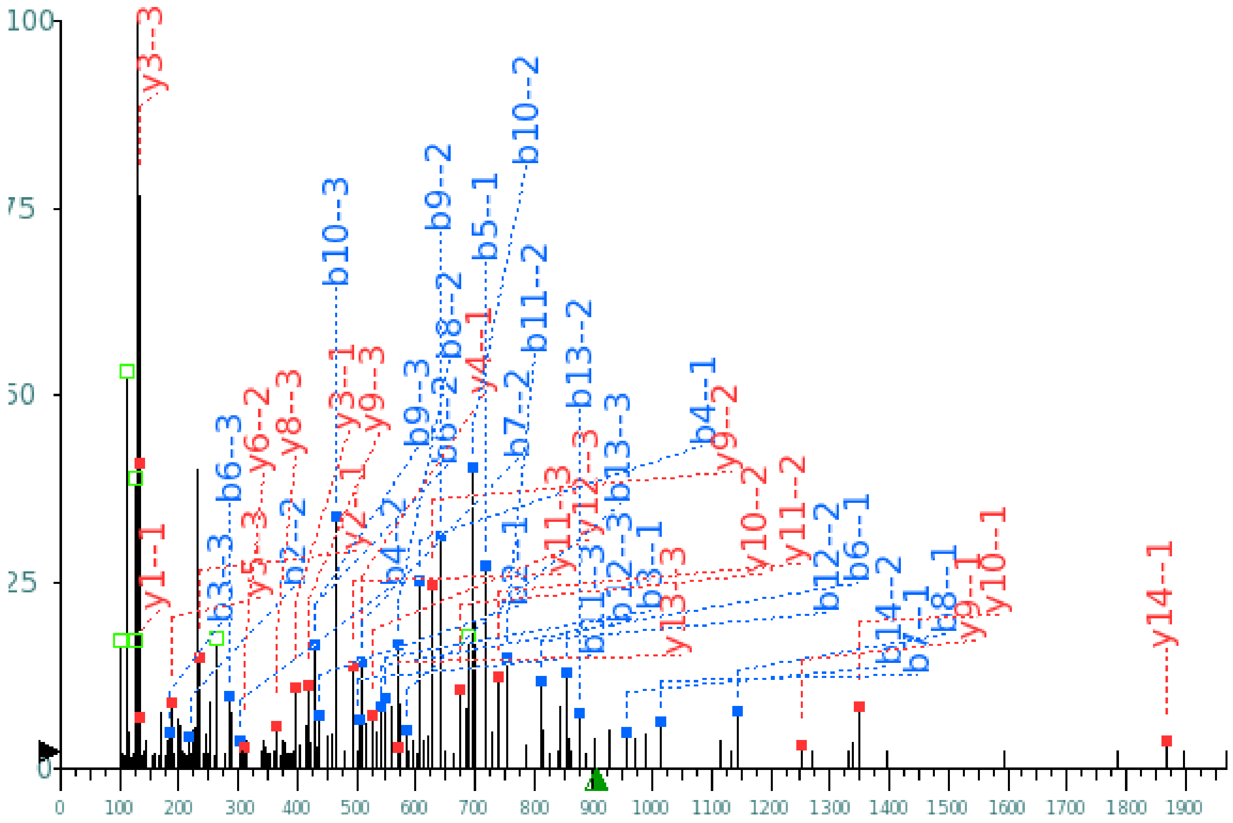

GFNGIHC*QHNINEC*EVEPC*KNGGIC*TD (+4)

Primary Reference: EDIL3_HUMAN
Search ID: 41761
Search Name: 20130328_ananiav_ERstress_TMT_TM_timecourse_trypsin_inj2_4mc_iaa
Scan#: 11886
Observed Mass: 905.1665 (1.3 ppm)
PSM Score: 34.68

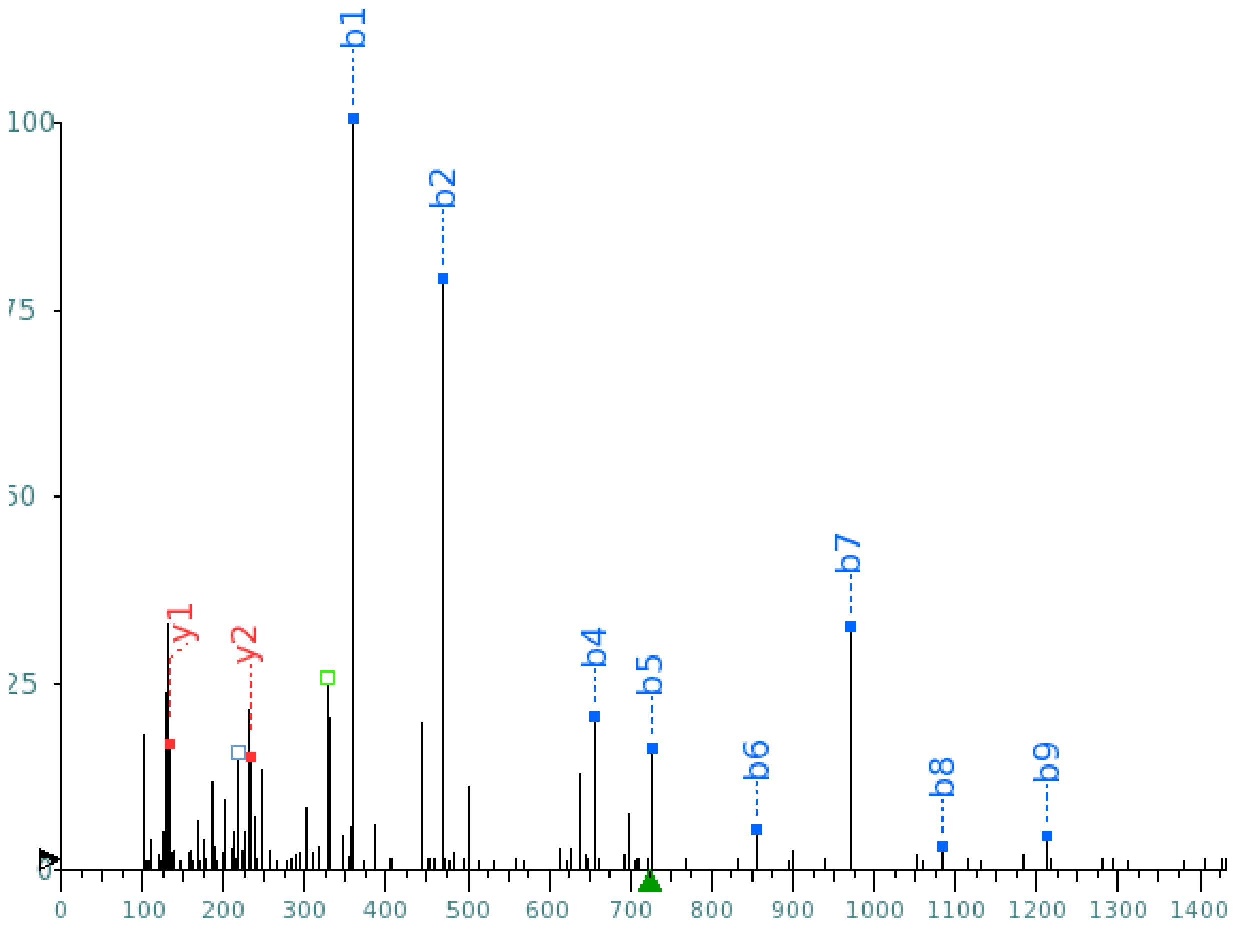

QIPSAEDLETD (+2)

Primary Reference: HDAC7_HUMAN
Search ID: 41761
Search Name: 20130328_ananiav_ERstress_TMT_TM_timecourse_trypsin_inj2_4mc_iaa
Scan#: 11938
Observed Mass: 723.8629 (1.7 ppm)
PSM Score: 59.5

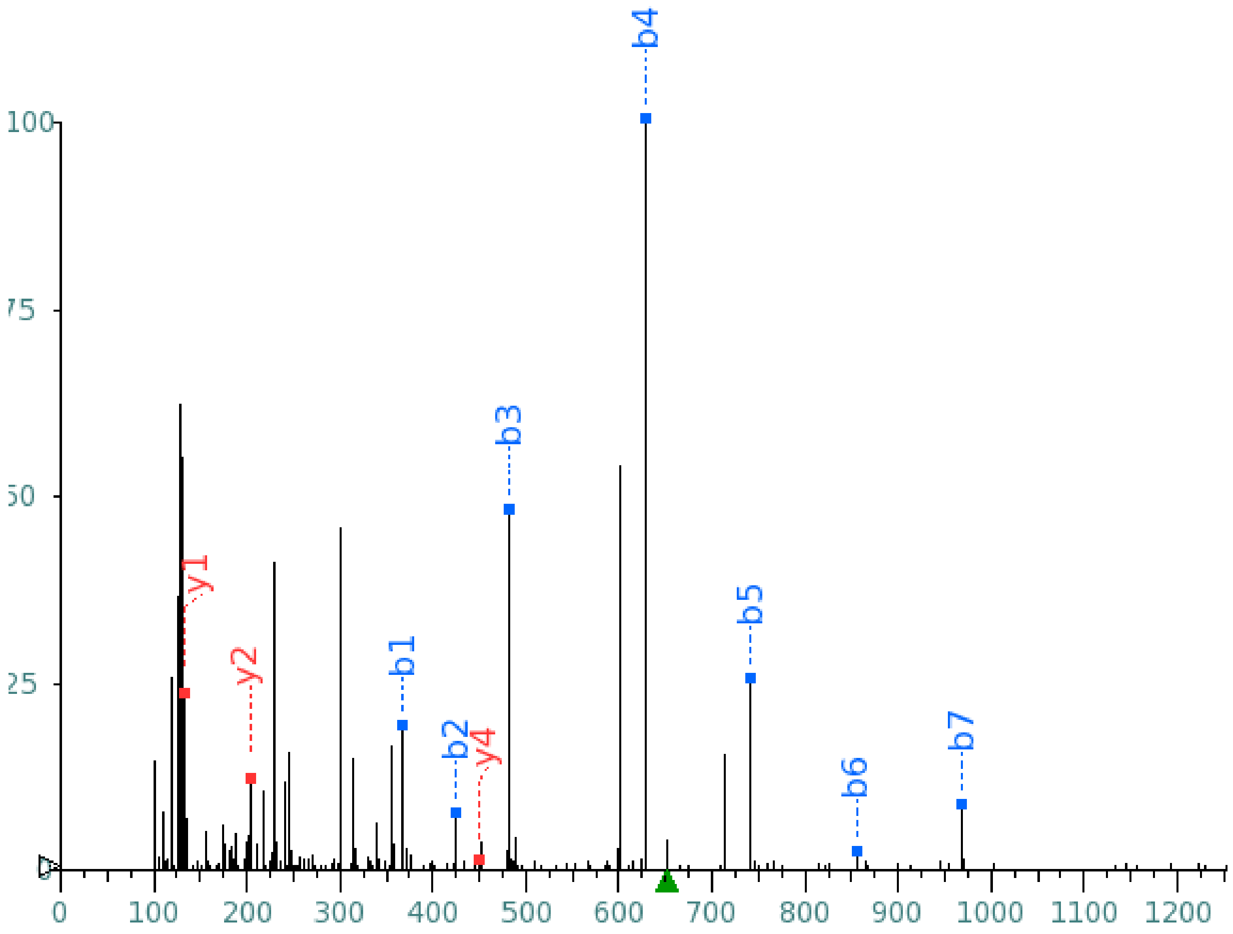

HGGFILDEAD (+2)

Primary Reference: SPT5H_HUMAN
Search ID: 41761
Search Name: 20130328_ananiav_ERstress_TMT_TM_timecourse_trypsin_inj2_4mc_iaa
Scan#: 11941
Observed Mass: 651.8303 (0.5 ppm)
PSM Score: 33.9

File name: 2015-02-04 04.01.53PM.pdf

Creation time: 2015-02-04 04:09PM

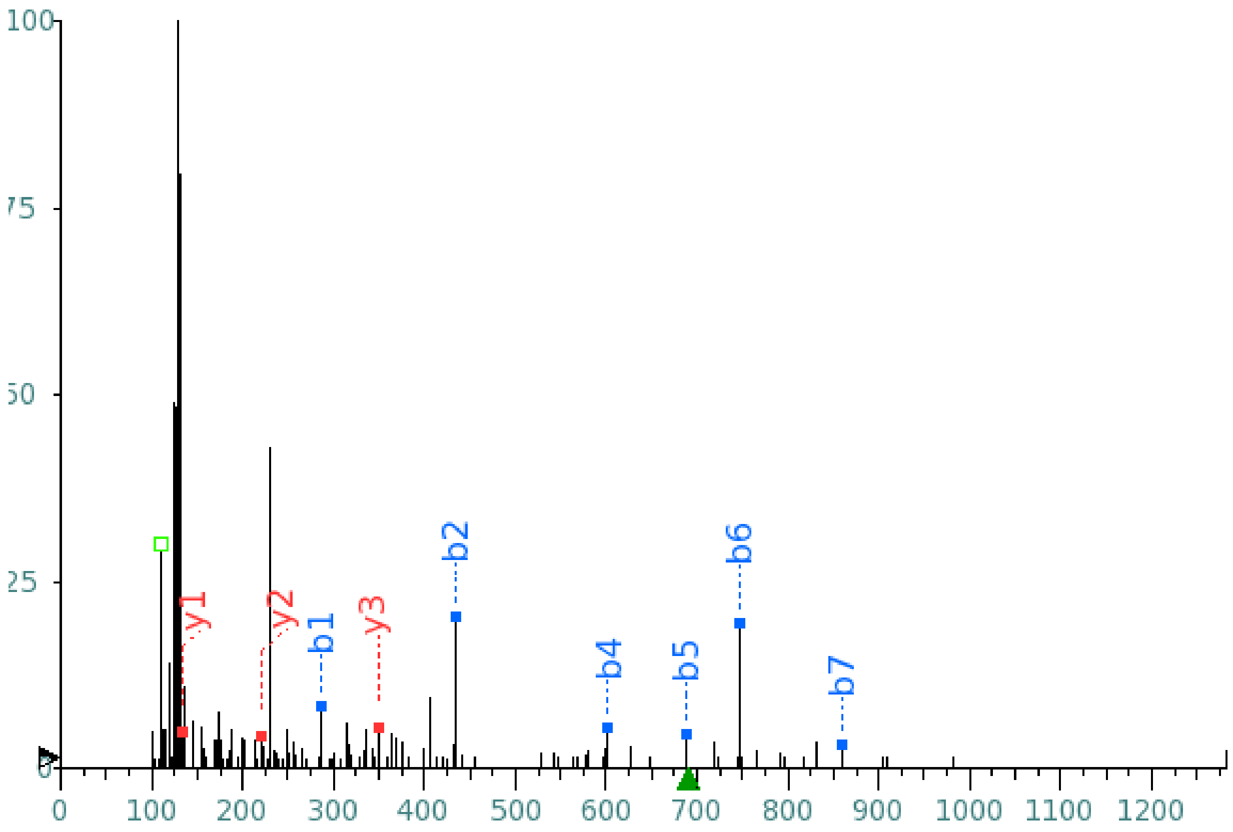

GFPASGLATESD (+2)

Primary Reference: AMRA1_HUMAN
Search ID: 41761
Search Name: 20130328_ananiav_ERstress_TMT_TM_timecourse_trypsin_inj2_4mc_iaa
Scan#: 11953
Observed Mass: 690.847 (1.7 ppm)
PSM Score: 19.97

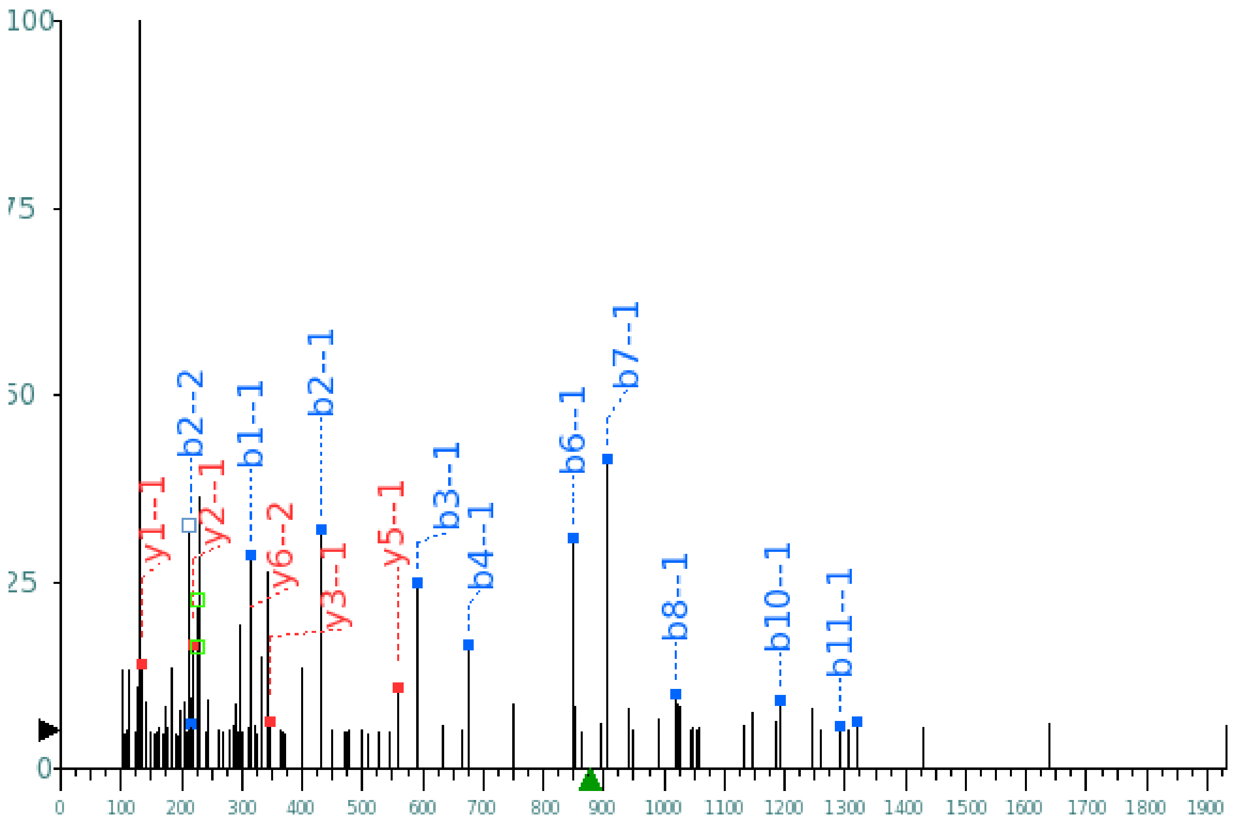

SLC*SGDGLGDTEISRPQAPDQSD (+3)

Primary Reference: HAUS5_HUMAN
Search ID: 41761
Search Name: 20130328_ananiav_ERstress_TMT_TM_timecourse_trypsin_inj2_4mc_iaa
Scan#: 11984
Observed Mass: 878.7442 (1.2 ppm)
PSM Score: 38.71

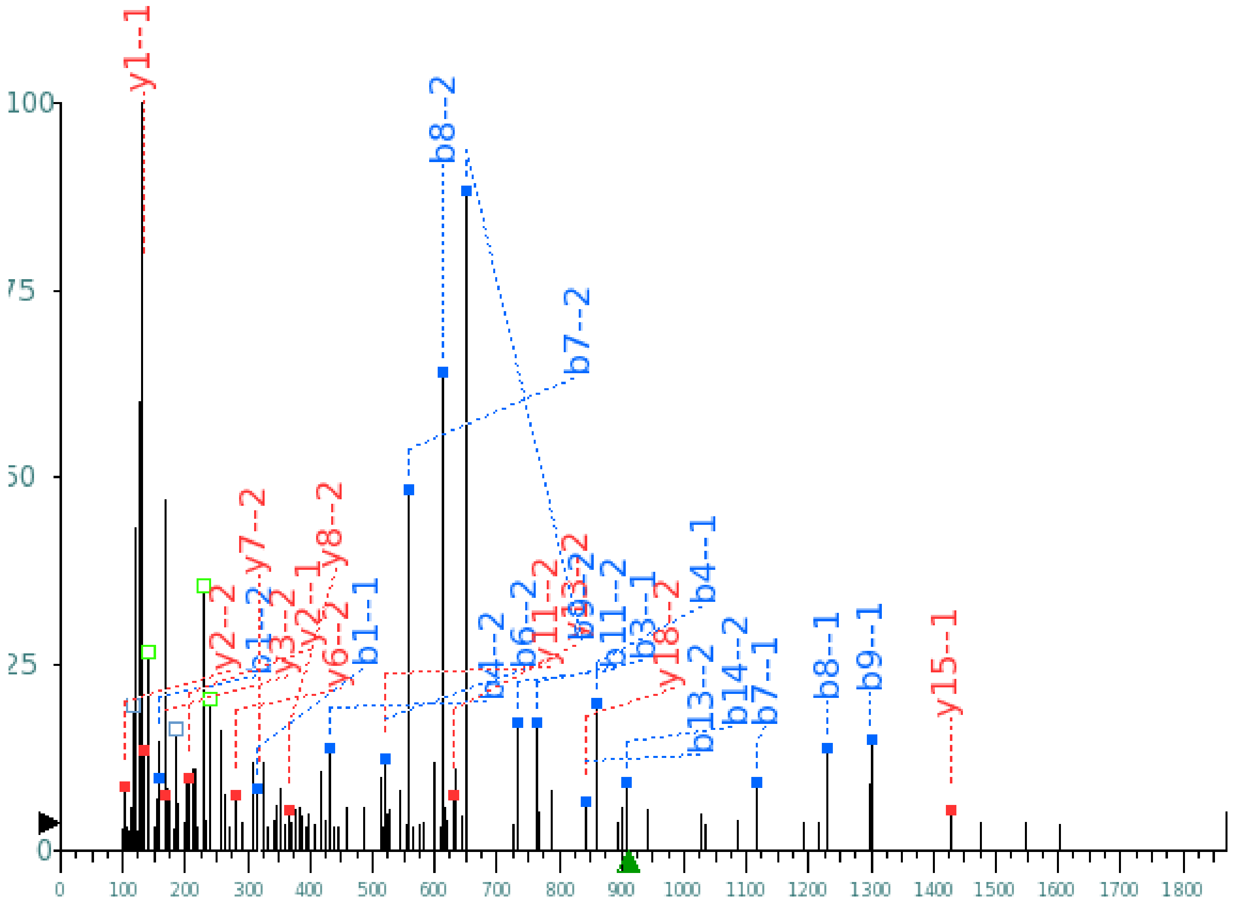

SSKVPSALAPASQEPSPAASAEAD (+3)

Primary Reference: DNM1L_HUMAN
Search ID: 41761
Search Name: 20130328_ananiav_ERstress_TMT_TM_timecourse_trypsin_inj2_4mc_iaa
Scan#: 11986
Observed Mass: 909.4807 (1 ppm)
PSM Score: 30.01

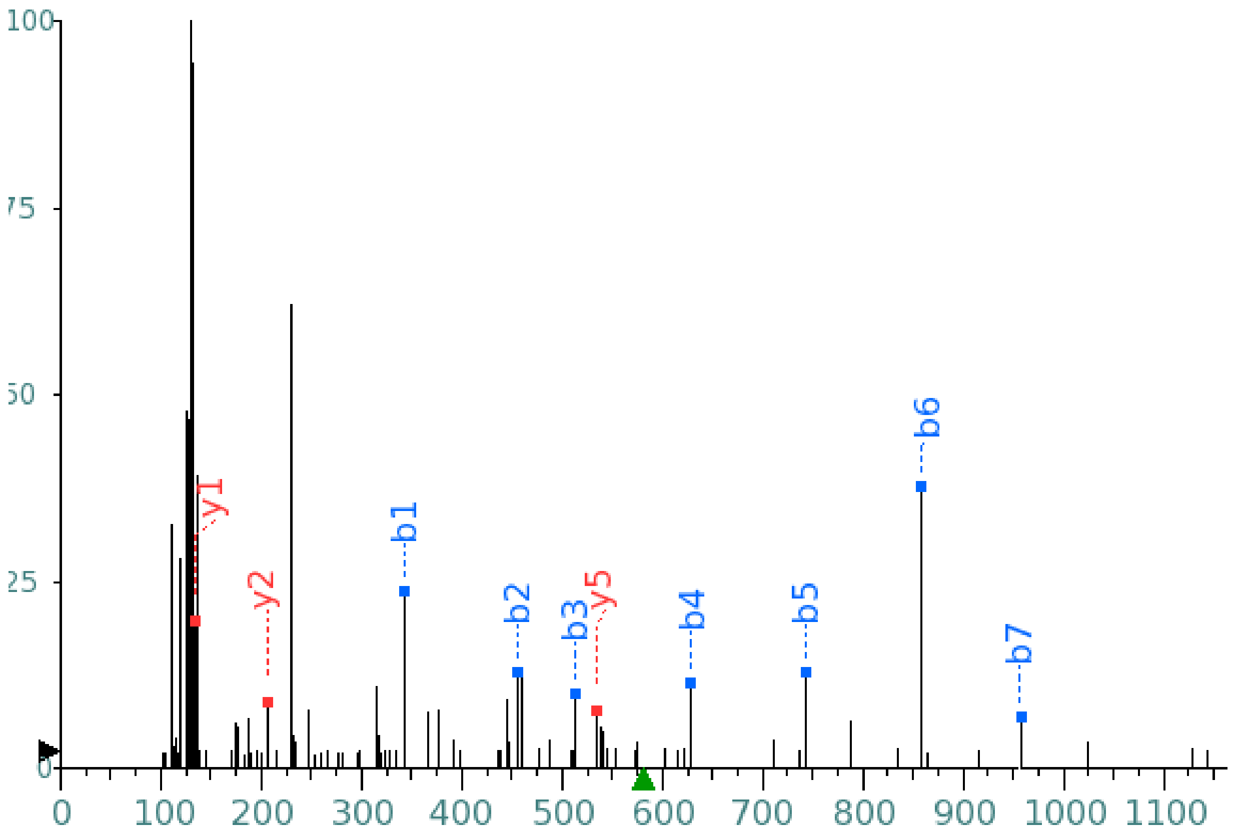

LLGDDDVAD (+2)

Primary Reference: WDR62_HUMAN
Search ID: 41761
Search Name: 20130328_ananiav_ERstress_TMT_TM_timecourse_trypsin_inj2_4mc_iaa
Scan#: 11994
Observed Mass: 581.2964 (1.5 ppm)
PSM Score: 38.09

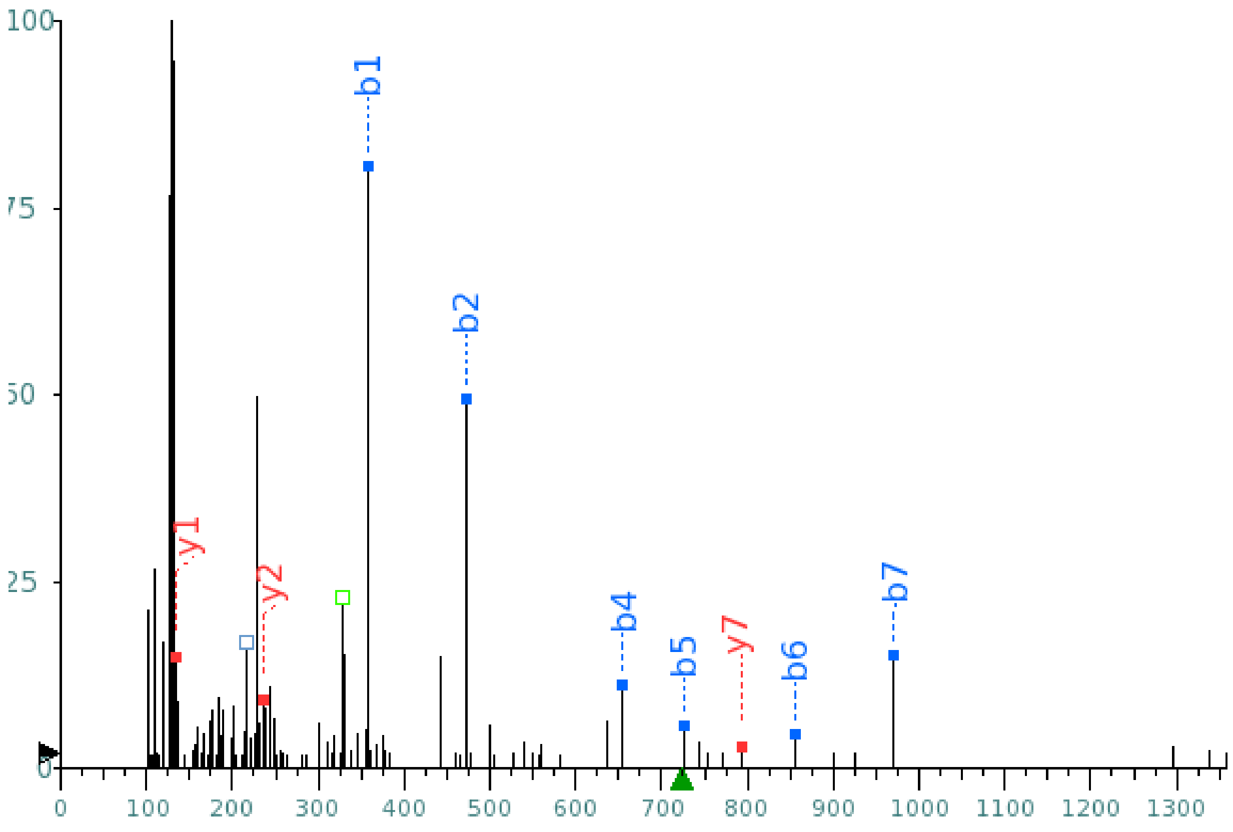

QIPSAEDLETD (+2)

Primary Reference: HDAC7_HUMAN
Search ID: 41761
Search Name: 20130328_ananiav_ERstress_TMT_TM_timecourse_trypsin_inj2_4mc_iaa
Scan#: 12028
Observed Mass: 723.863 (1.8 ppm)
PSM Score: 37.89

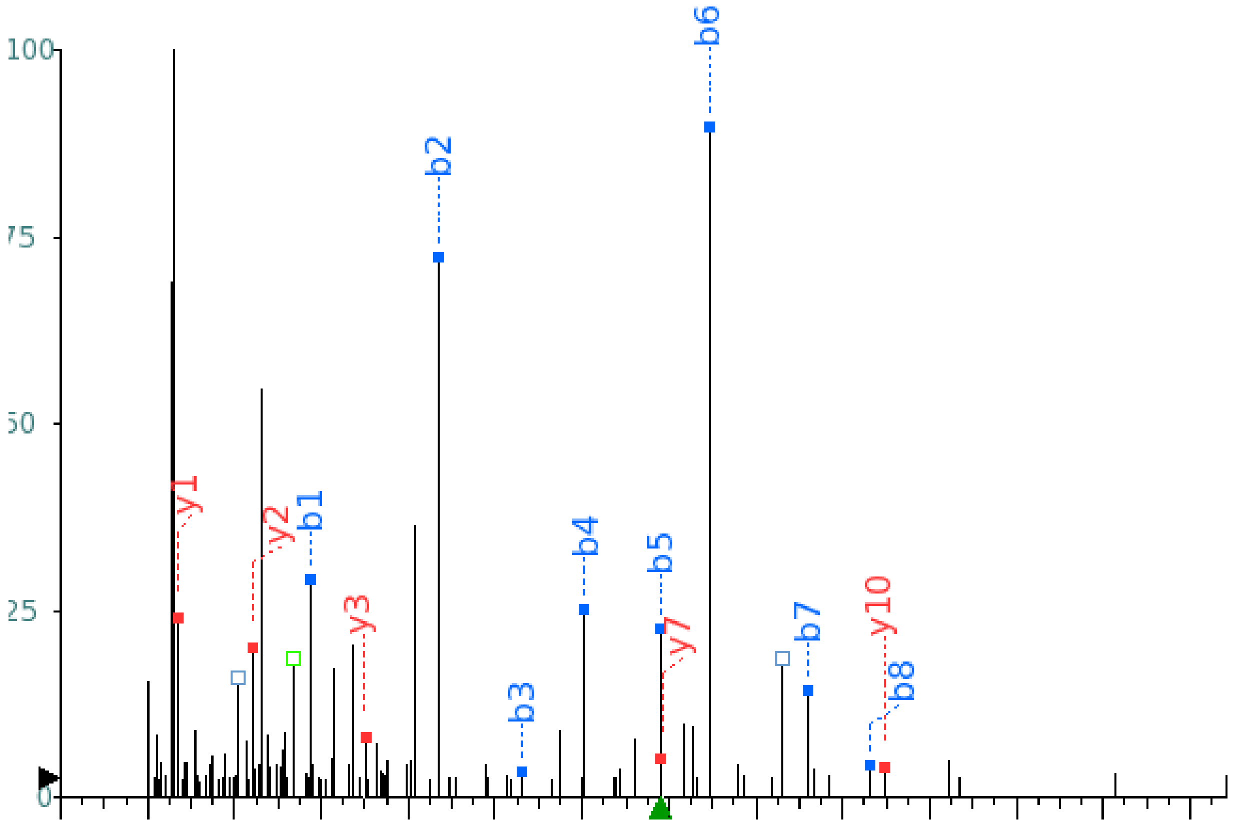

GFPASGLATESD (+2)

Primary Reference: AMRA1_HUMAN
Search ID: 41761
Search Name: 20130328_ananiav_ERstress_TMT_TM_timecourse_trypsin_inj2_4mc_iaa
Scan#: 12038
Observed Mass: 690.8469 (1.5 ppm)
PSM Score: 34.86

File name: 2015-02-04 04.01.53PM.pdf

Creation time: 2015-02-04 04:09PM

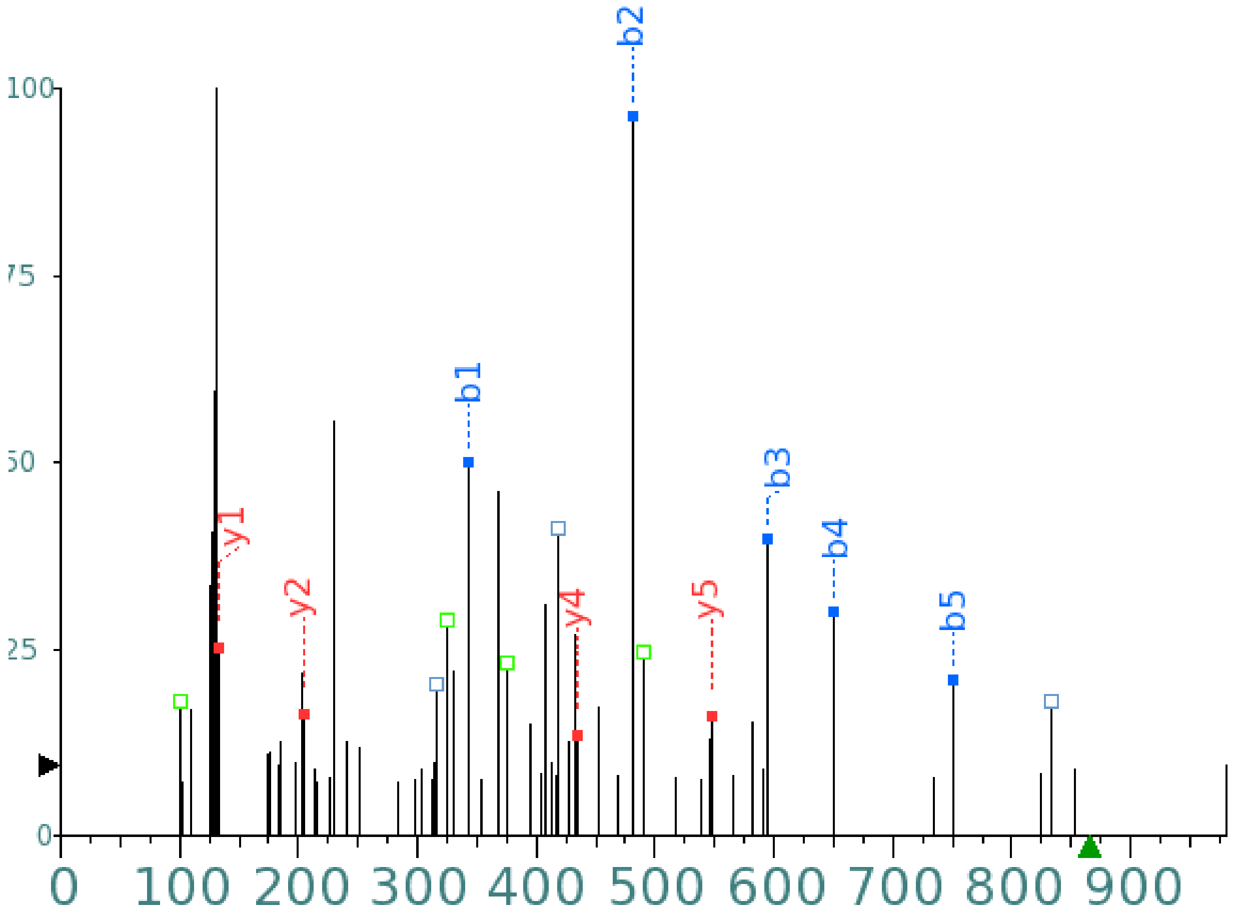

LHLGTTQNSLTEAD (+2)

Primary Reference: VPS4B_HUMAN
Search ID: 41761
Search Name: 20130328_ananiav_ERstress_TMT_TM_timecourse_trypsin_inj2_4mc_iaa
Scan#: 12045
Observed Mass: 864.9546 (3.1 ppm)
PSM Score: 45.99

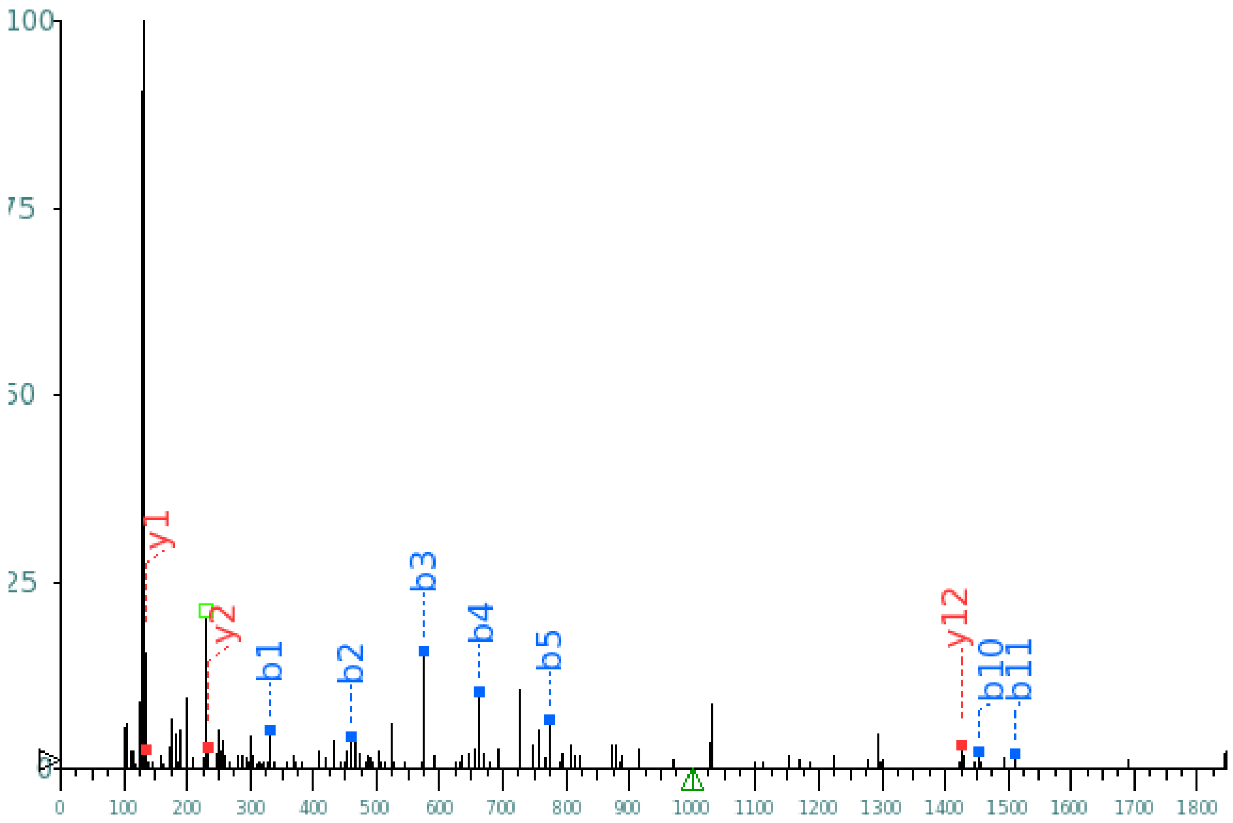

TEDSIRDYEDGMEVD (+2)

Primary Reference: ICLN_HUMAN
Search ID: 41761
Search Name: 20130328_ananiav_ERstress_TMT_TM_timecourse_trypsin_inj2_4mc_iaa
Scan#: 12048
Observed Mass: 1001.943 (2 ppm)
PSM Score: 22.13

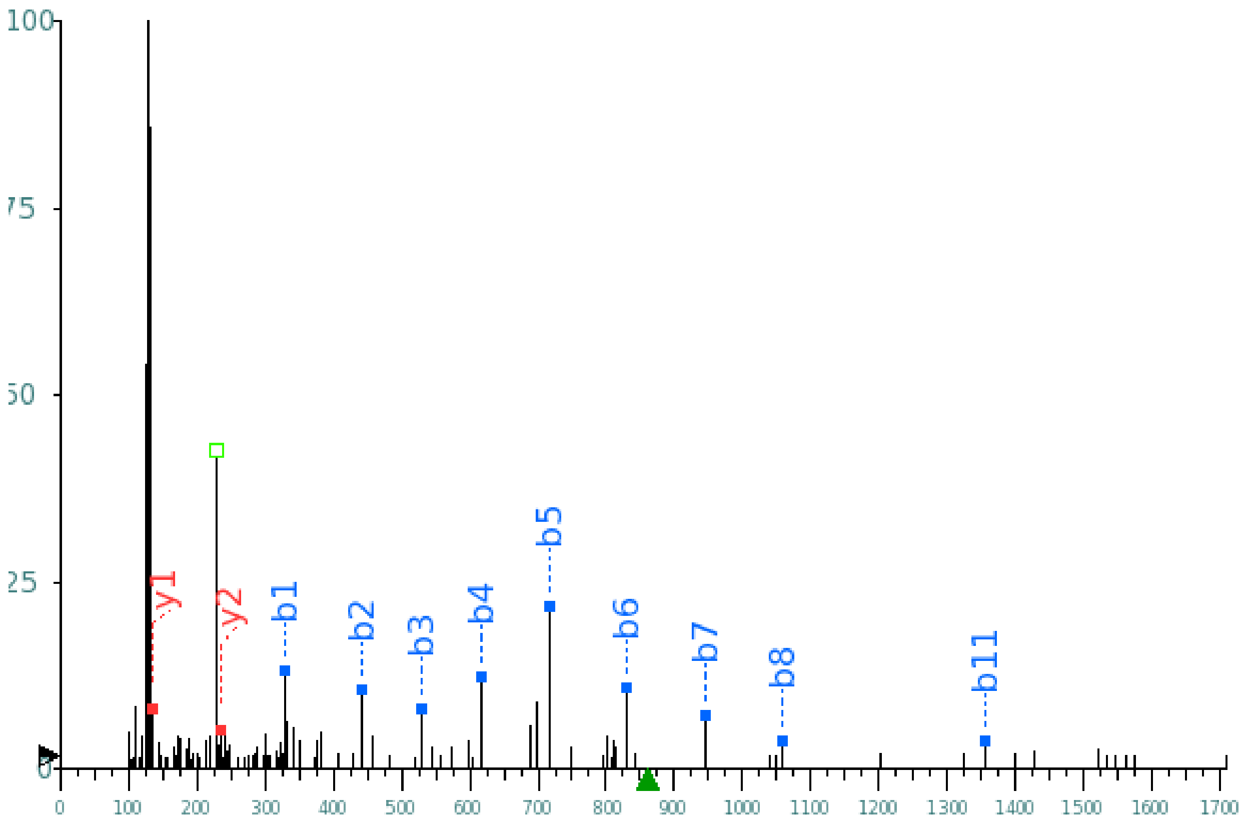

VLSSTINNQAVETD (+2)

Primary Reference: RHG12_HUMAN
Search ID: 41761
Search Name: 20130328_ananiav_ERstress_TMT_TM_timecourse_trypsin_inj2_4mc_iaa
Scan#: 12097
Observed Mass: 860.4532 (1.6 ppm)
PSM Score: 49.24

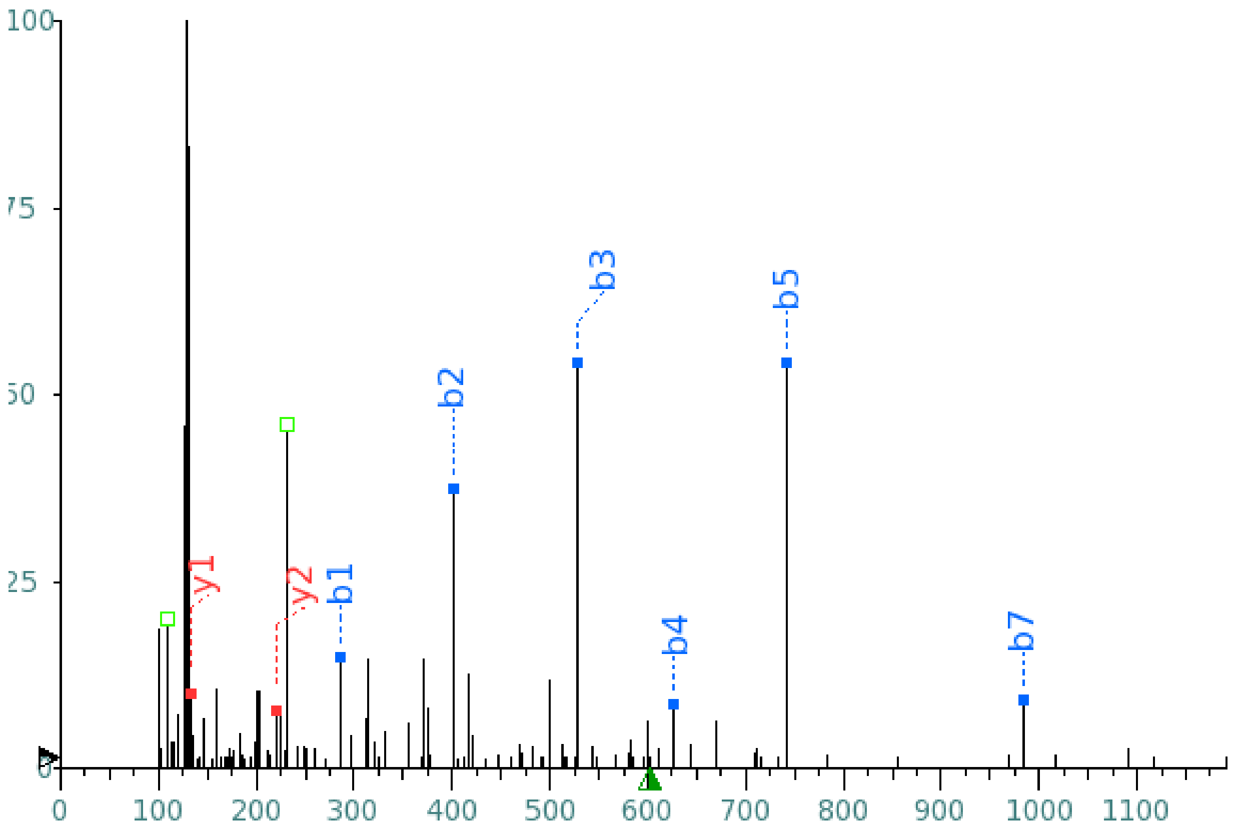

GLQVDLQSD (+2)

Primary Reference: MCM4_HUMAN
Search ID: 41761
Search Name: 20130328_ananiav_ERstress_TMT_TM_timecourse_trypsin_inj2_4mc_iaa
Scan#: 12114
Observed Mass: 602.3255 (1.6 ppm)
PSM Score: 24.49

File name: 2015-02-04 04.01.53PM.pdf

Creation time: 2015-02-04 04:09PM

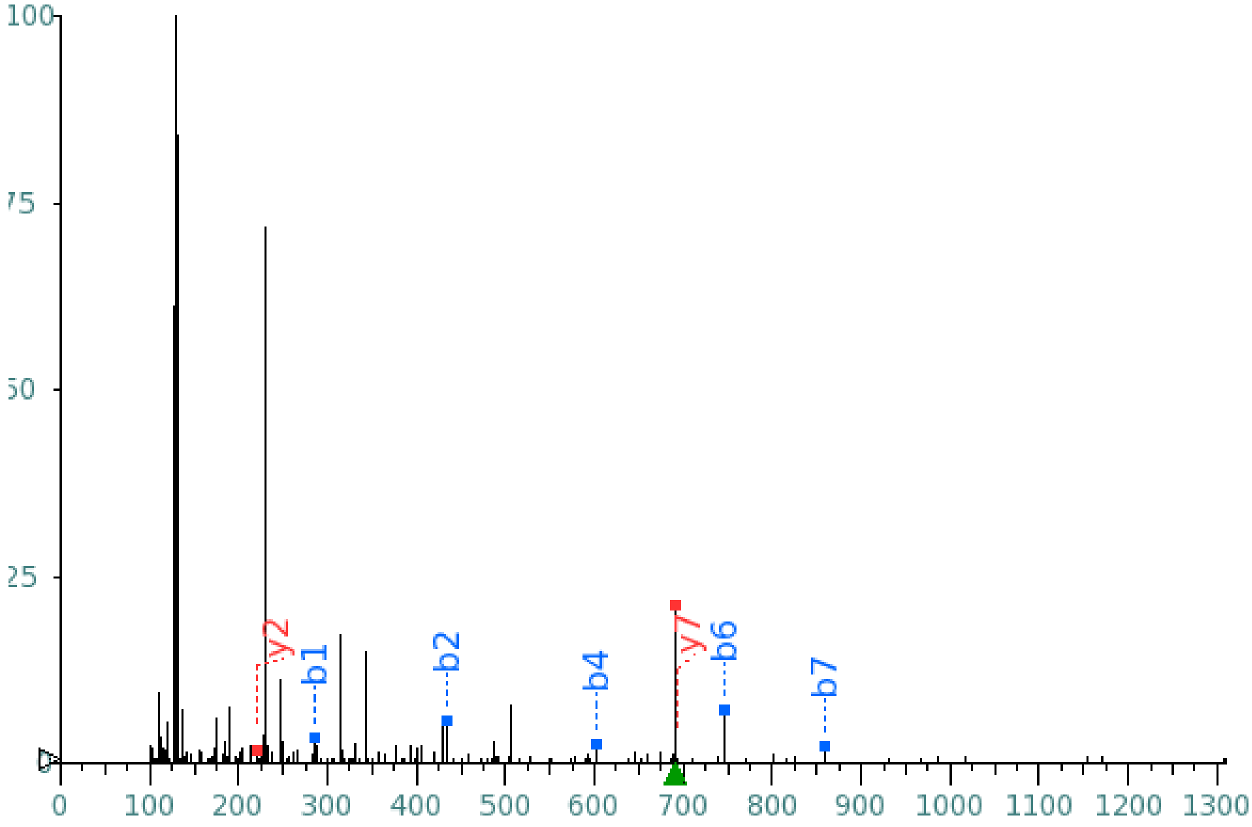

GFPASGLATESD (+2)

Primary Reference: AMRA1_HUMAN
Search ID: 41761
Search Name: 20130328_ananiav_ERstress_TMT_TM_timecourse_trypsin_inj2_4mc_iaa
Scan#: 12131
Observed Mass: 690.8478 (2.9 ppm)
PSM Score: 11.99

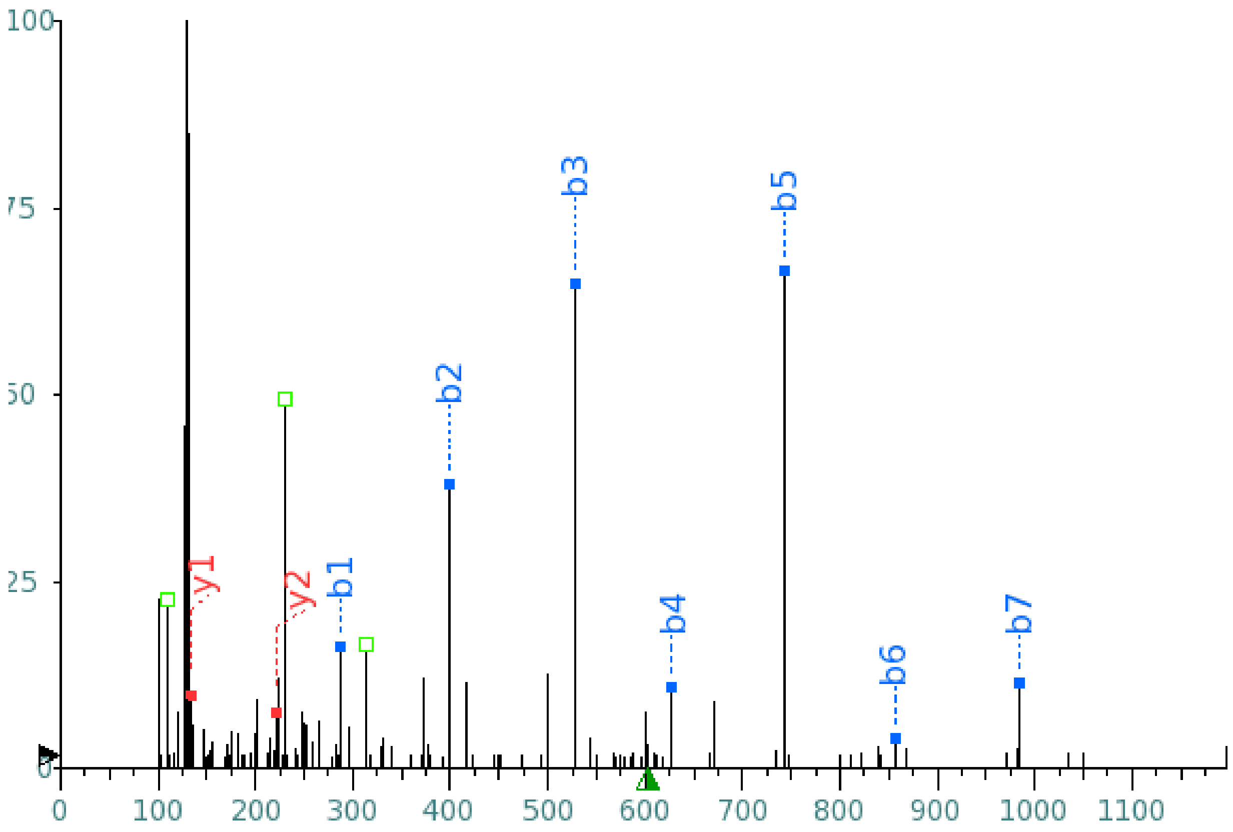

GLQVDLQSD (+2)

Primary Reference: MCM4_HUMAN
Search ID: 41761
Search Name: 20130328_ananiav_ERstress_TMT_TM_timecourse_trypsin_inj2_4mc_iaa
Scan#: 12192
Observed Mass: 602.3258 (2 ppm)
PSM Score: 38.01

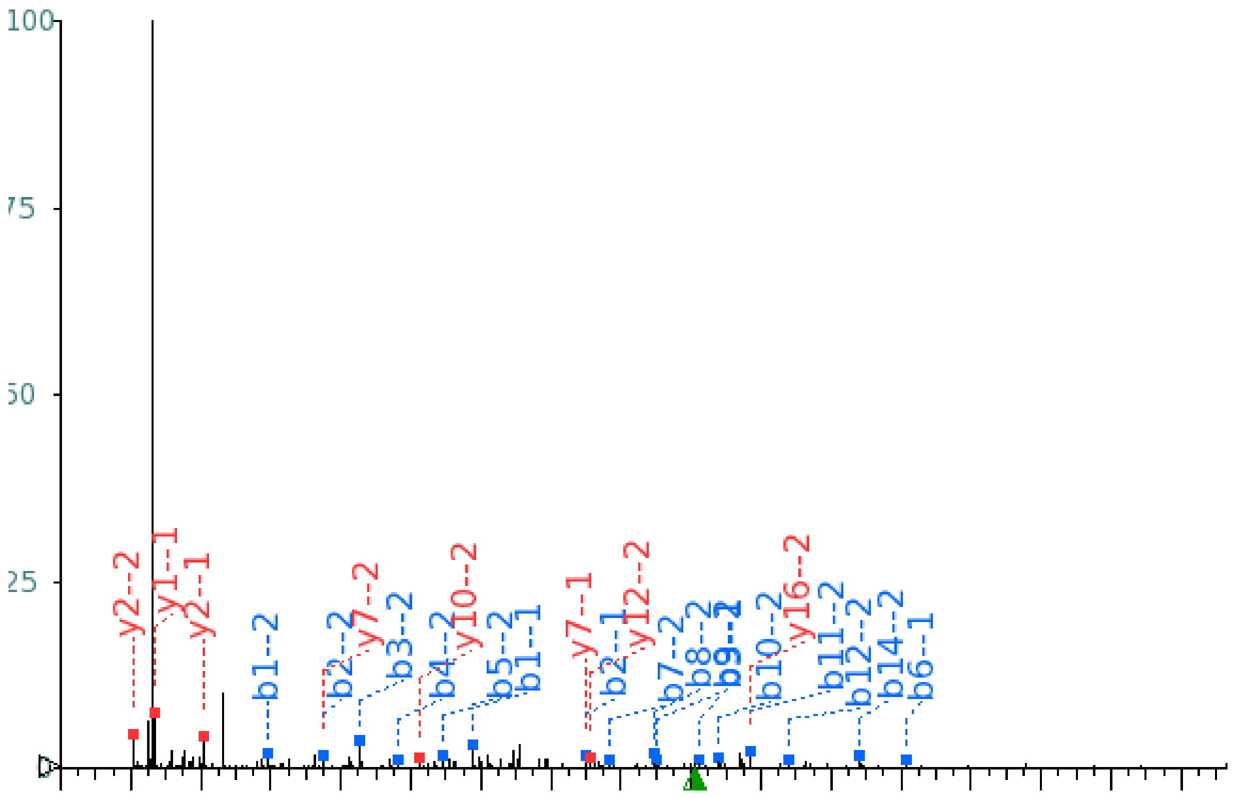

KYTLENKEEGSLSDTEAD (+3)

Primary Reference: LRC47_HUMAN
Search ID: 41761
Search Name: 20130328_ananiav_ERstress_TMT_TM_timecourse_trypsin_inj2_4mc_iaa
Scan#: 12207
Observed Mass: 906.1445 (2.1 ppm)
PSM Score: 16.19

File name: 2015-02-04 04.01.53PM.pdf

Creation time: 2015-02-04 04:09PM

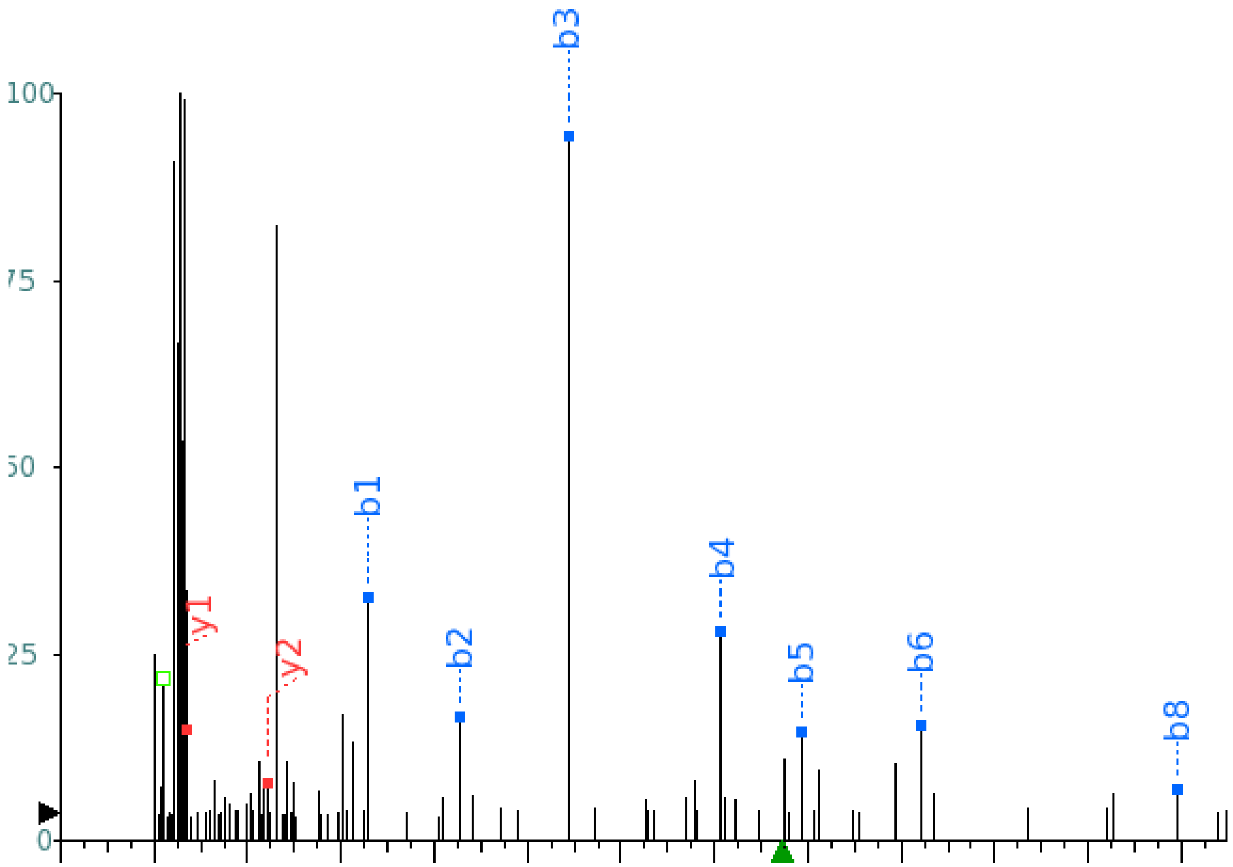

VVDYSQFQESD (+2)

Primary Reference: NUCKS_HUMAN
Search ID: 41761
Search Name: 20130328_ananiav_ERstress_TMT_TM_timecourse_trypsin_inj2_4mc_iaa
Scan#: 12230
Observed Mass: 773.3672 (0 ppm)
PSM Score: 24.37

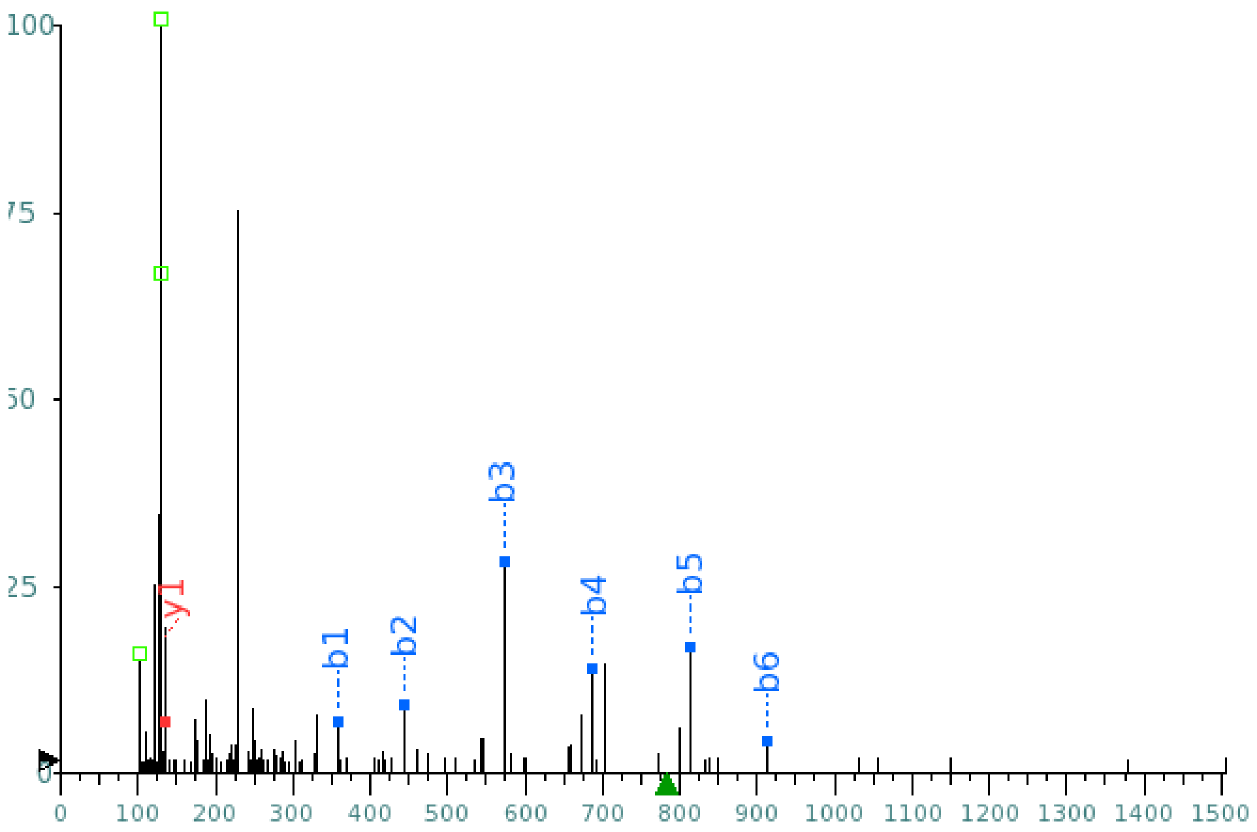

QSQIQVFEDGAD (+2)

Primary Reference: DCTN1_HUMAN
Search ID: 41761
Search Name: 20130328_ananiav_ERstress_TMT_TM_timecourse_trypsin_inj2_4mc_iaa
Scan#: 12259
Observed Mass: 783.3866 (0.9 ppm)
PSM Score: 19.23

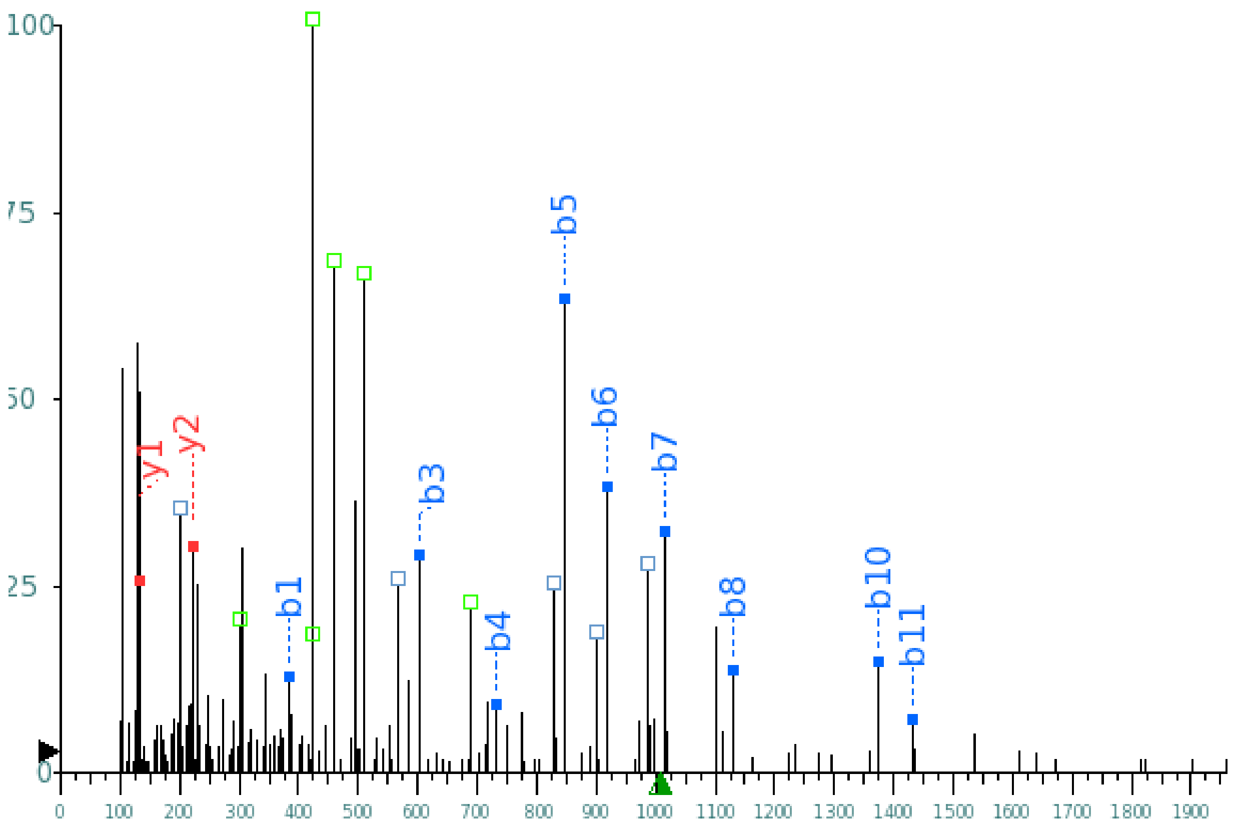

RTDEDAVLMDGMTESD (+2)

Primary Reference: FXR1_HUMAN
Search ID: 41761
Search Name: 20130328_ananiav_ERstress_TMT_TM_timecourse_trypsin_inj2_4mc_iaa
Scan#: 12294
Observed Mass: 1007.4524 (1.6 ppm)
PSM Score: 37.95

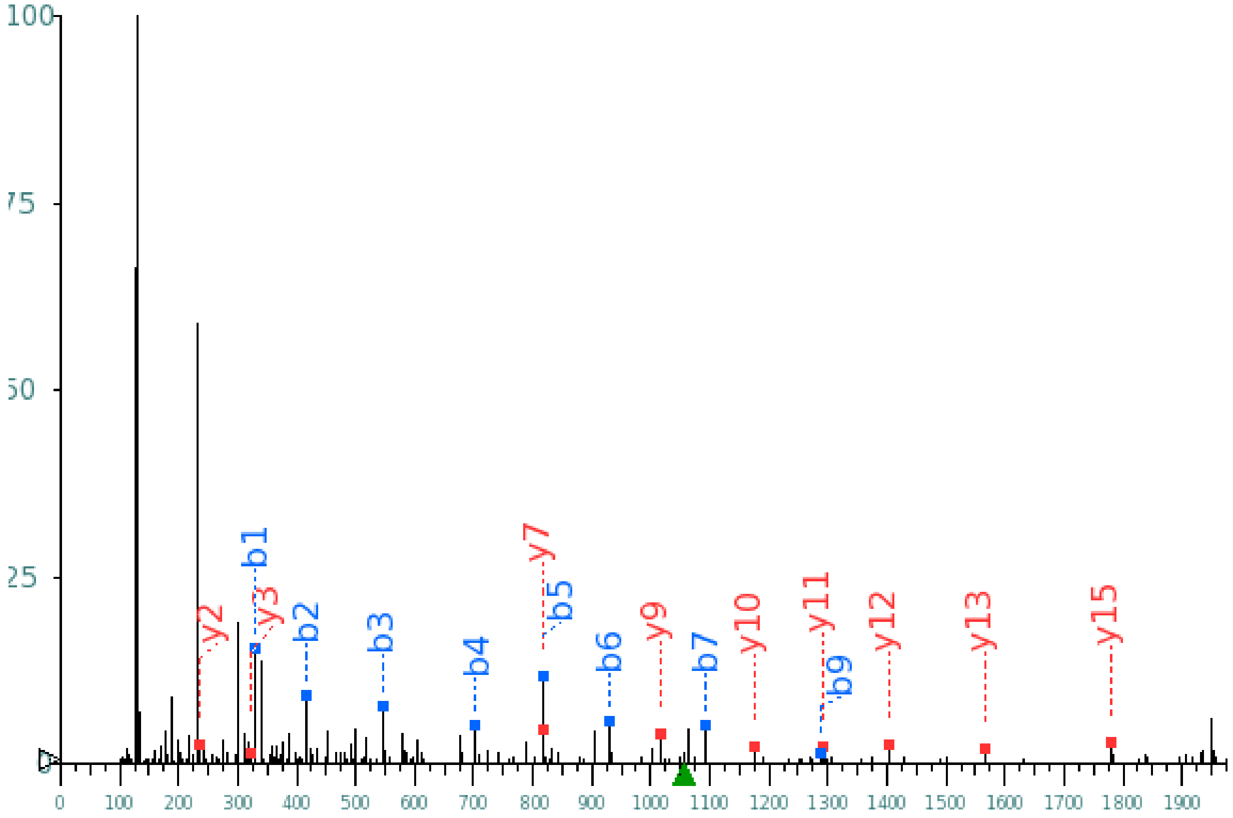

VSQC*NLC*PTRIEVSTD (+2)

Primary Reference: SPS2L_HUMAN
Search ID: 41761
Search Name: 20130328_ananiav_ERstress_TMT_TM_timecourse_trypsin_inj2_4mc_iaa
Scan#: 12313
Observed Mass: 1054.5196 (0.3 ppm)
PSM Score: 25.81

File name: 2015-02-04 04.01.53PM.pdf

Creation time: 2015-02-04 04:09PM

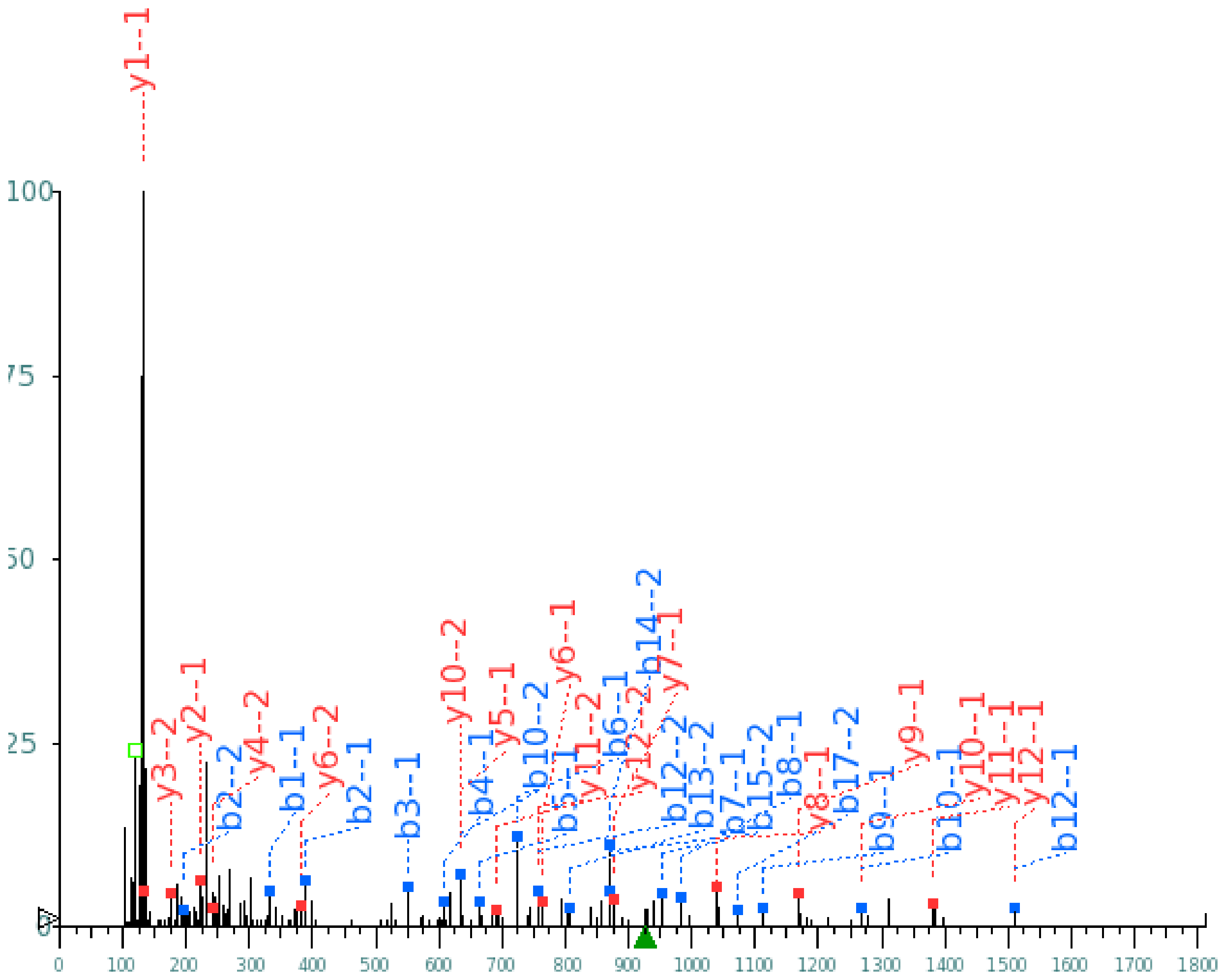

TGYGGGFNERENVEYIEREESD (+3)

Primary Reference: ZRAB2_HUMAN
Search ID: 41761
Search Name: 20130328_ananiav_ERstress_TMT_TM_timecourse_trypsin_inj2_4mc_iaa
Scan#: 12404
Observed Mass: 927.0927 (-0.4 ppm)
PSM Score: 54.82

File name: 2015-02-04 04.01.53PM.pdf

Creation time: 2015-02-04 04:09PM

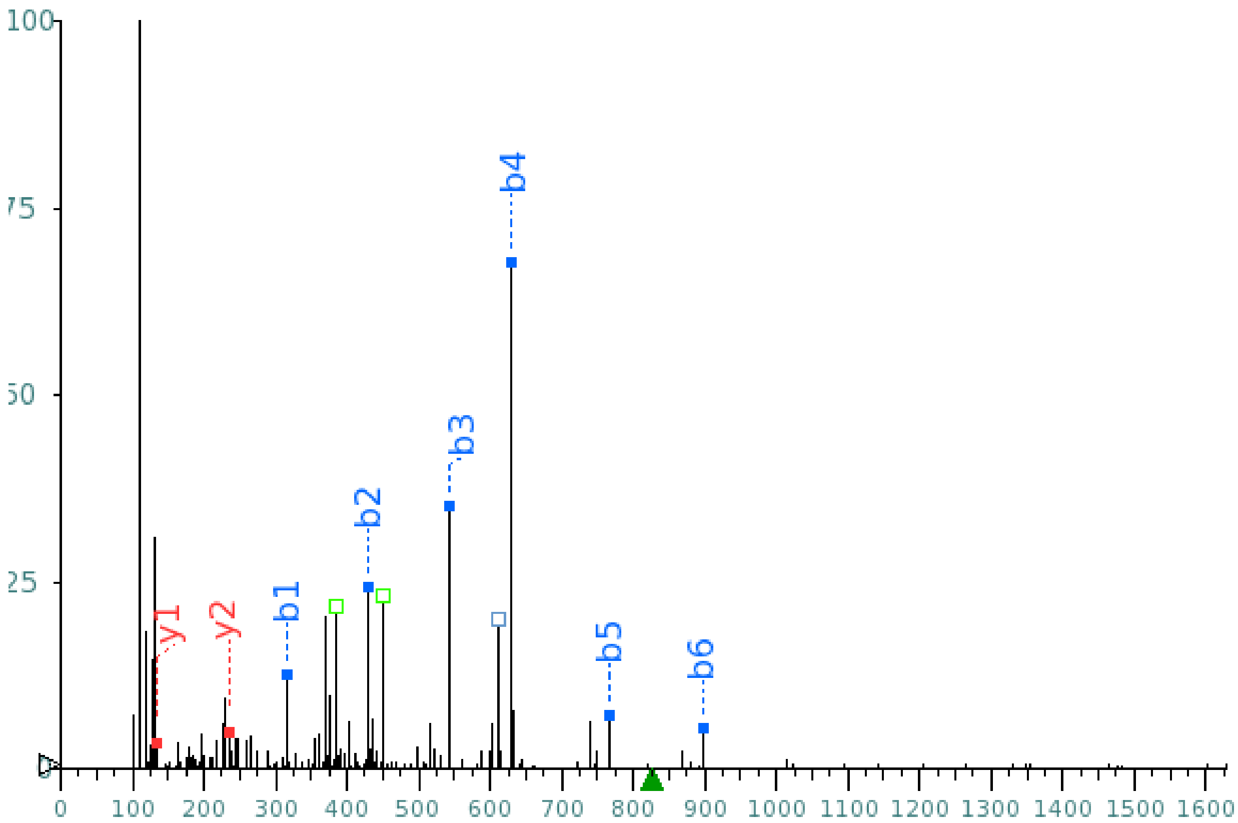

SLLSHEFQDETD (+2)

Primary Reference: CBPD_HUMAN
Search ID: 41761
Search Name: 20130328_ananiav_ERstress_TMT_TM_timecourse_trypsin_inj2_4mc_iaa
Scan#: 12436
Observed Mass: 825.3976 (1.4 ppm)
PSM Score: 13.5

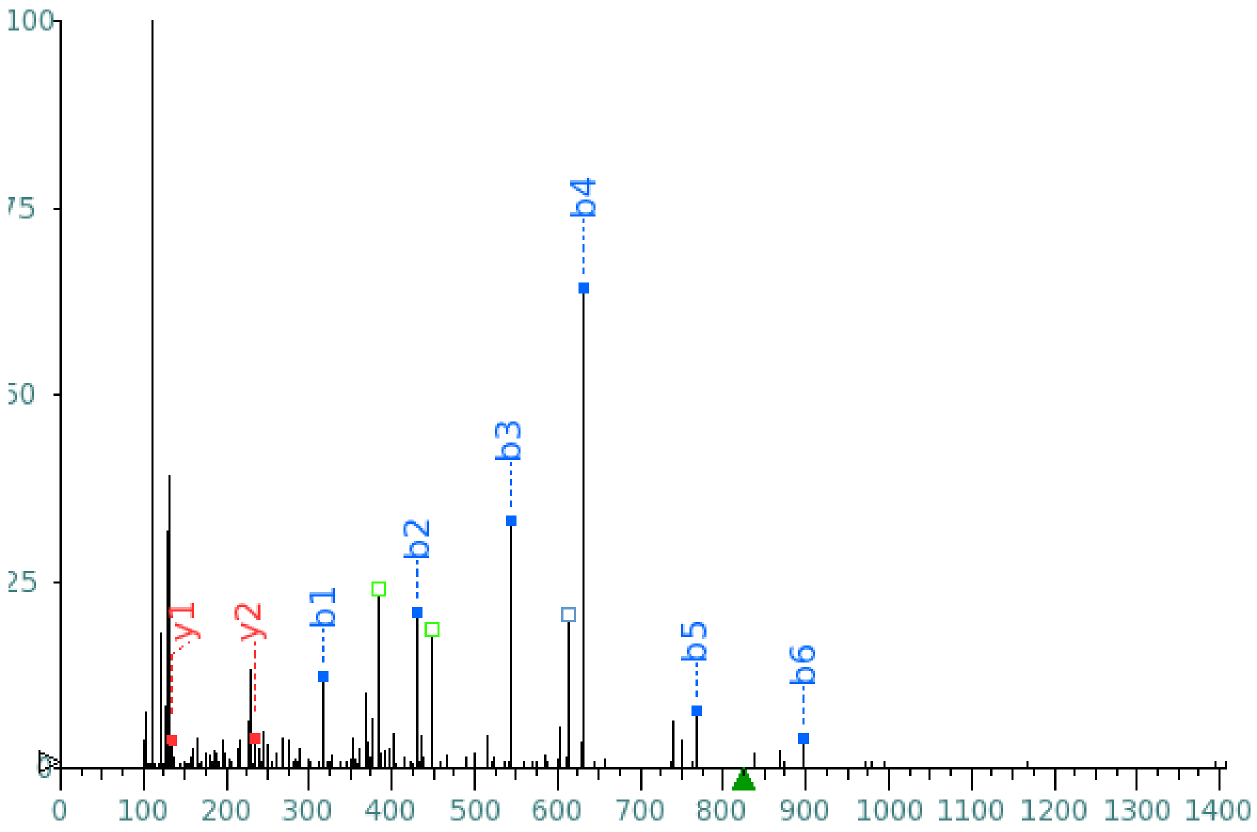

SLLSHEFQDETD (+2)

Primary Reference: CBPD_HUMAN
Search ID: 41761
Search Name: 20130328_ananiav_ERstress_TMT_TM_timecourse_trypsin_inj2_4mc_iaa
Scan#: 12522
Observed Mass: 825.398 (1.9 ppm)
PSM Score: 25.1

File name: 2015-02-04 04.01.53PM.pdf

Creation time: 2015-02-04 04:09PM

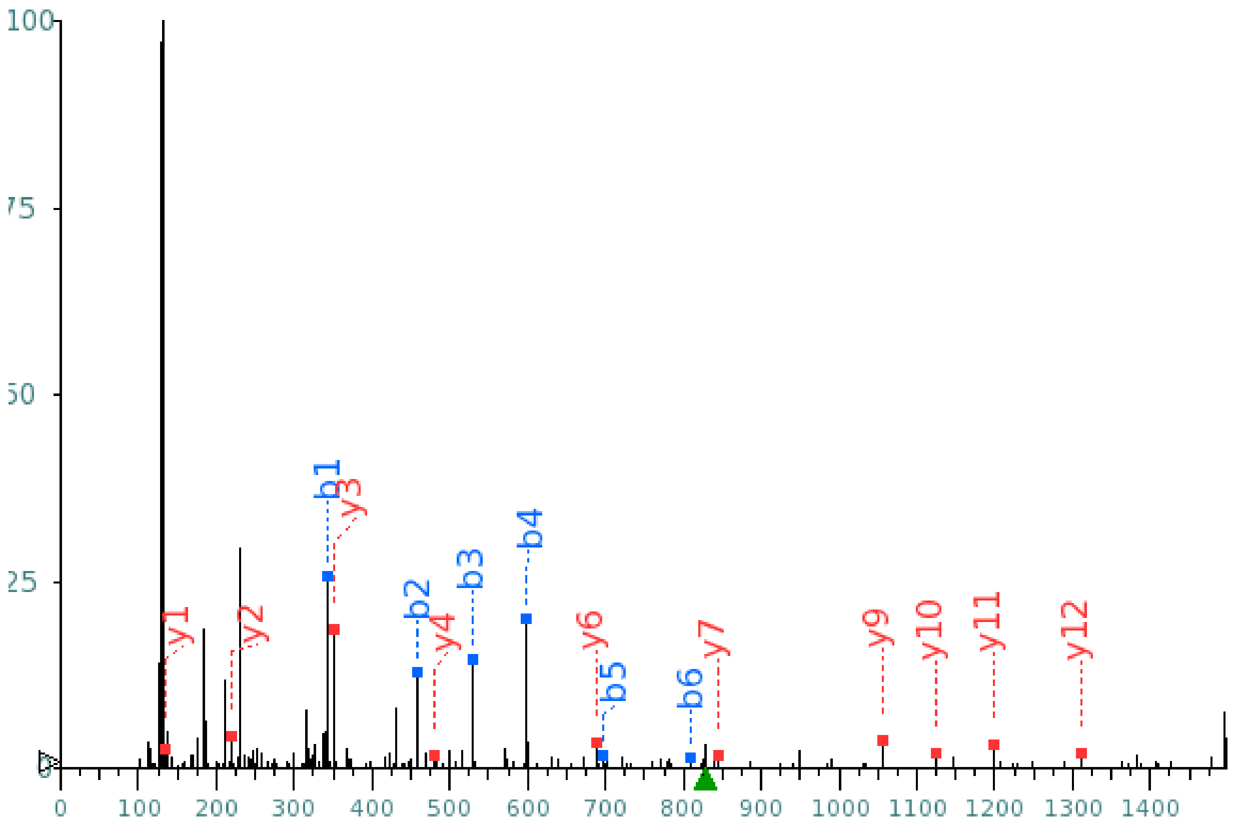

NLAAPIRPIEESD (+2)

Primary Reference: DCTN4_HUMAN
Search ID: 41761
Search Name: 20130328_ananiav_ERstress_TMT_TM_timecourse_trypsin_inj2_4mc_iaa
Scan#: 12565
Observed Mass: 827.4545 (0.5 ppm)
PSM Score: 29.34

File name: 2015-02-04 04.01.53PM.pdf

Creation time: 2015-02-04 04:09PM

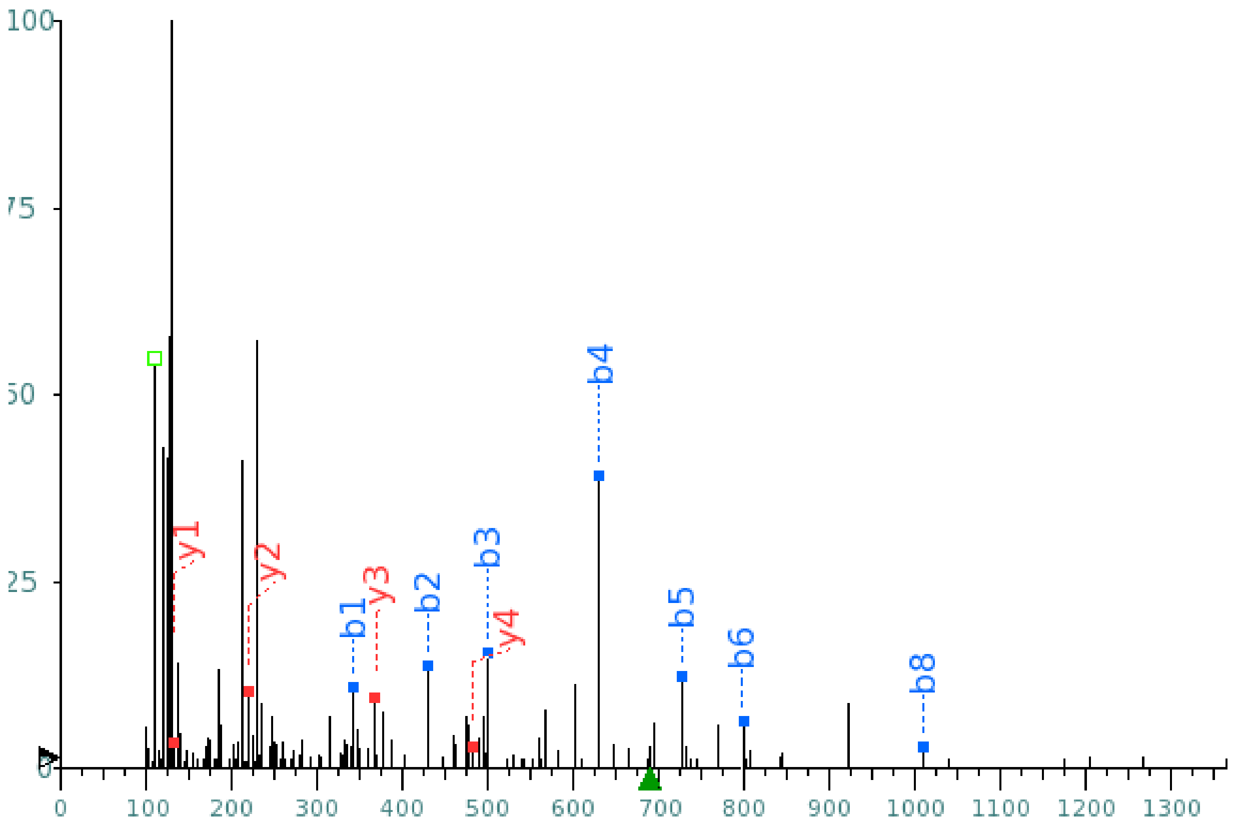

LSAEPAPDFSD (+2)

Primary Reference: RTN4_HUMAN
Search ID: 41761
Search Name: 20130328_ananiav_ERstress_TMT_TM_timecourse_trypsin_inj2_4mc_iaa
Scan#: 12566
Observed Mass: 689.341 (0.9 ppm)
PSM Score: 27.53

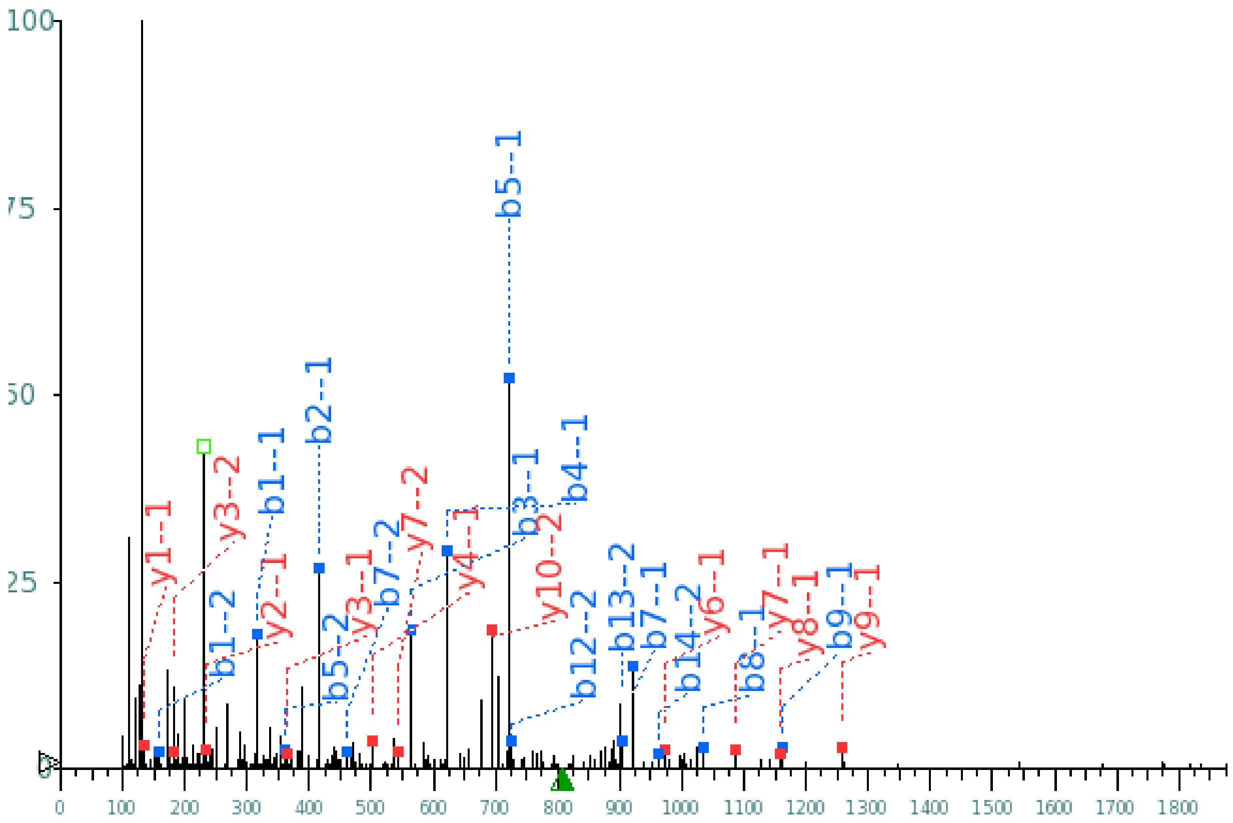

SVFGTPTLETANKNHETD (+3)

Primary Reference: RBP2_HUMAN
Search ID: 41761
Search Name: 20130328_ananiav_ERstress_TMT_TM_timecourse_trypsin_inj2_4mc_iaa
Scan#: 12569
Observed Mass: 807.0889 (0.6 ppm)
PSM Score: 27.21

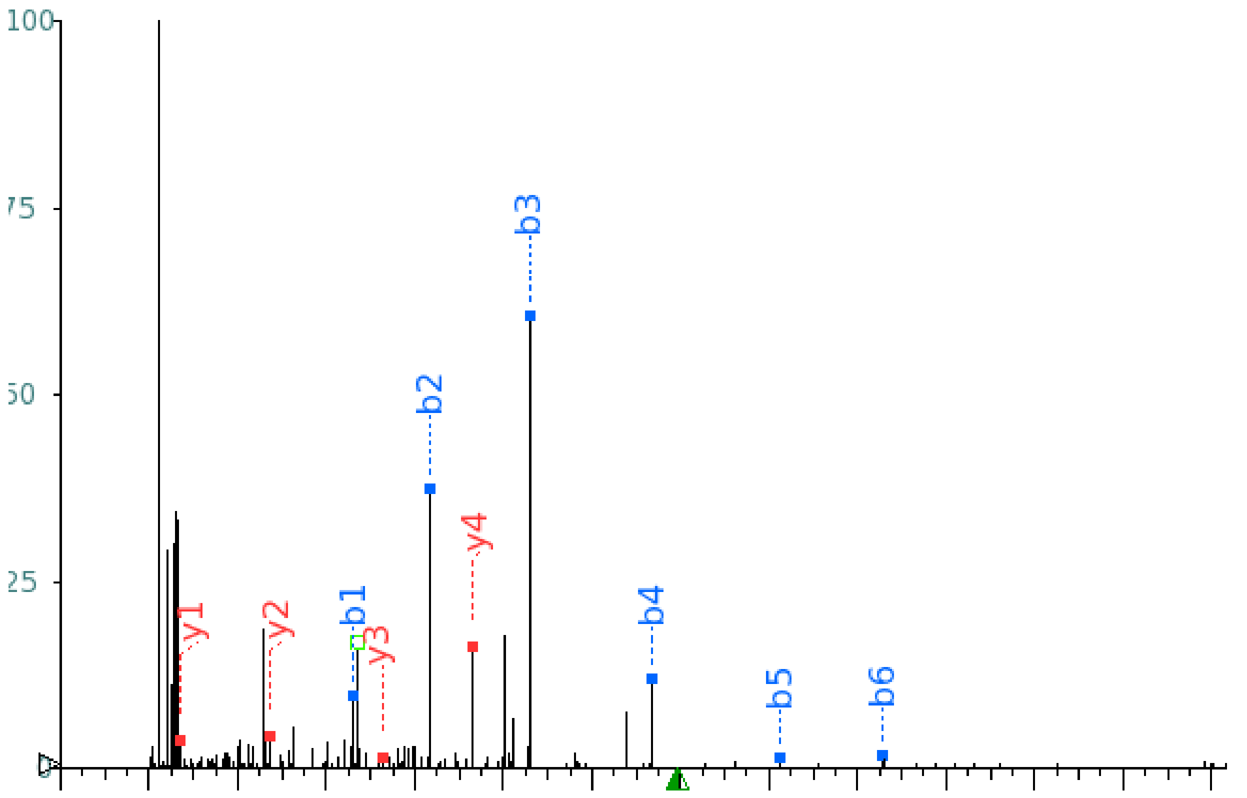

VSLHFDTETD (+2)

Primary Reference: JADE2_HUMAN
Search ID: 41761
Search Name: 20130328_ananiav_ERstress_TMT_TM_timecourse_trypsin_inj2_4mc_iaa
Scan#: 12644
Observed Mass: 696.8462 (0.5 ppm)
PSM Score: 23.39

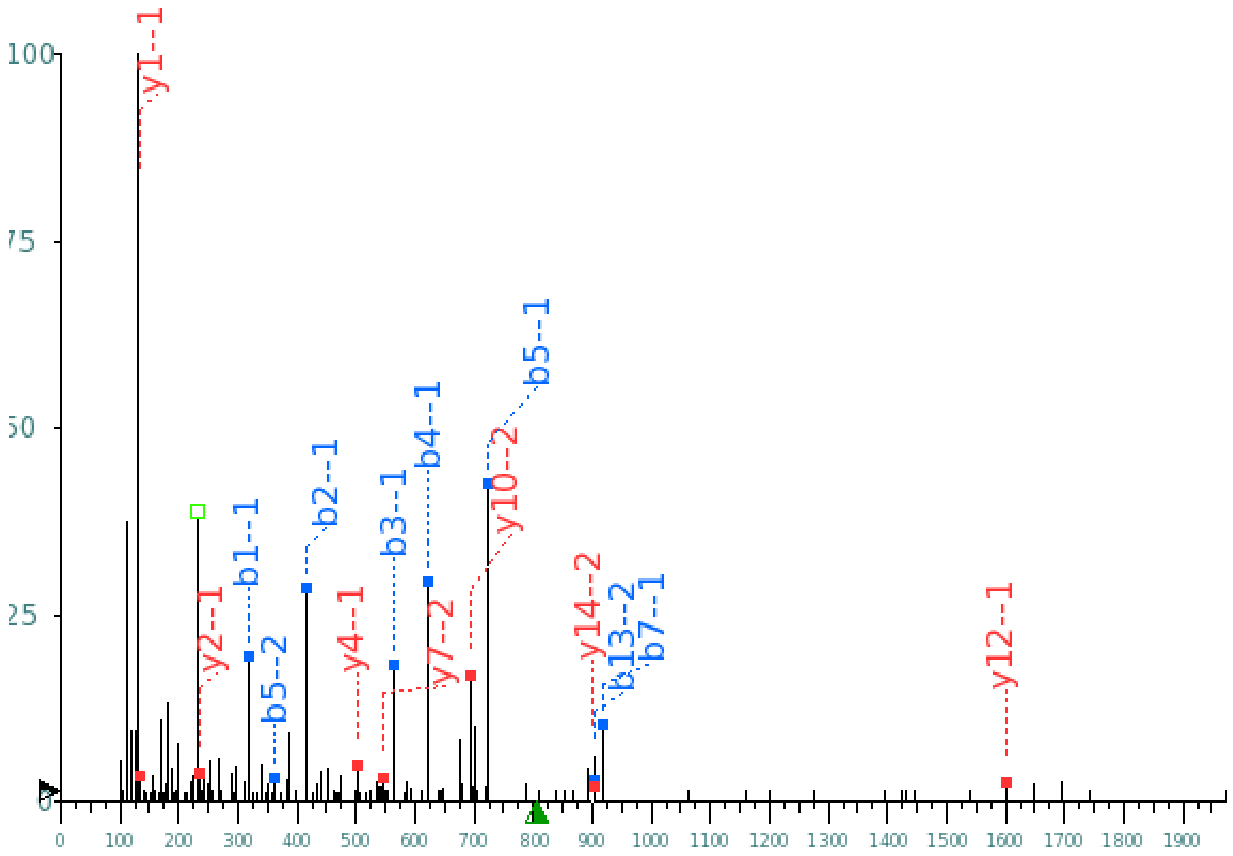

SVFGTPTLETANKNHETD (+3)

Primary Reference: RBP2_HUMAN
Search ID: 41761
Search Name: 20130328_ananiav_ERstress_TMT_TM_timecourse_trypsin_inj2_4mc_iaa
Scan#: 12664
Observed Mass: 807.0888 (0.5 ppm)
PSM Score: 19.06

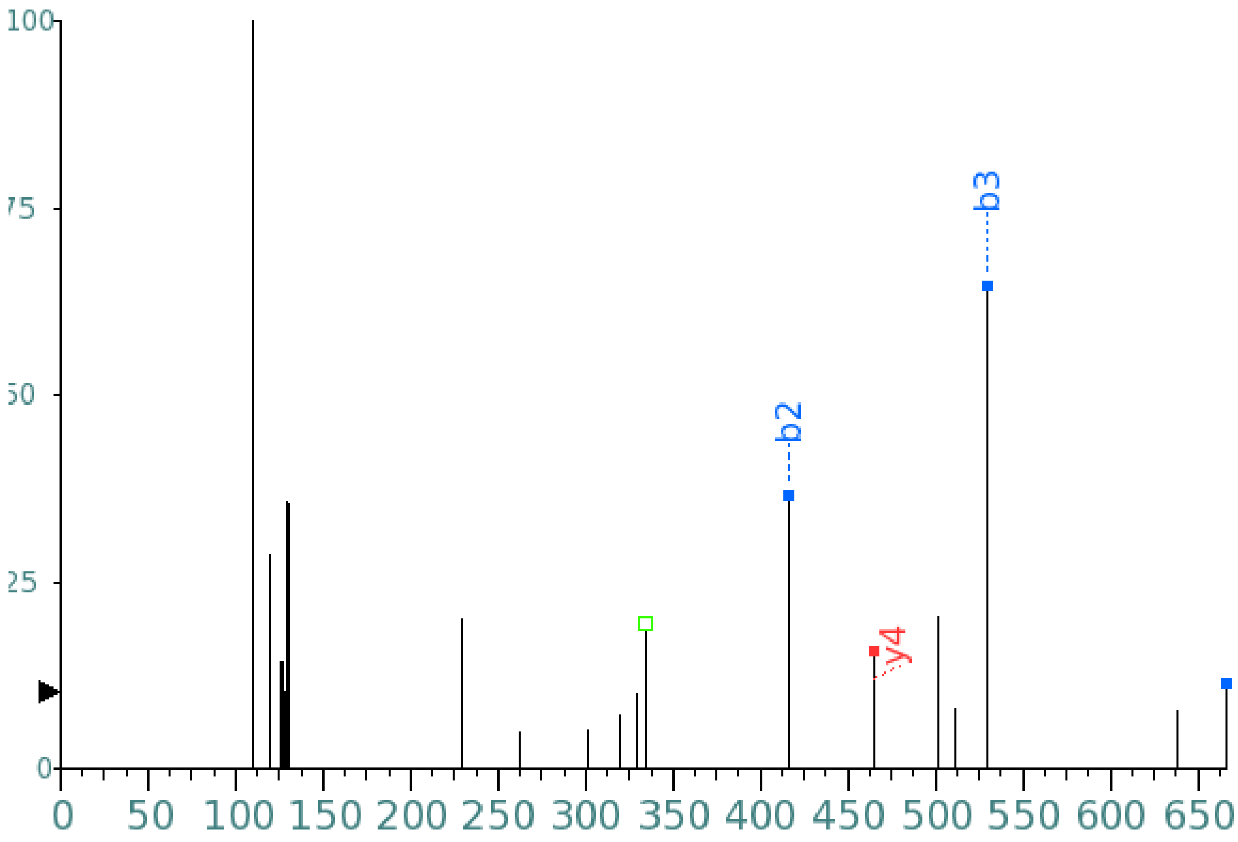

VSLHFDTETD (+2)

Primary Reference: JADE2_HUMAN
Search ID: 41761
Search Name: 20130328_ananiav_ERstress_TMT_TM_timecourse_trypsin_inj2_4mc_iaa
Scan#: 12737
Observed Mass: 696.8462 (0.5 ppm)
PSM Score: 14.94

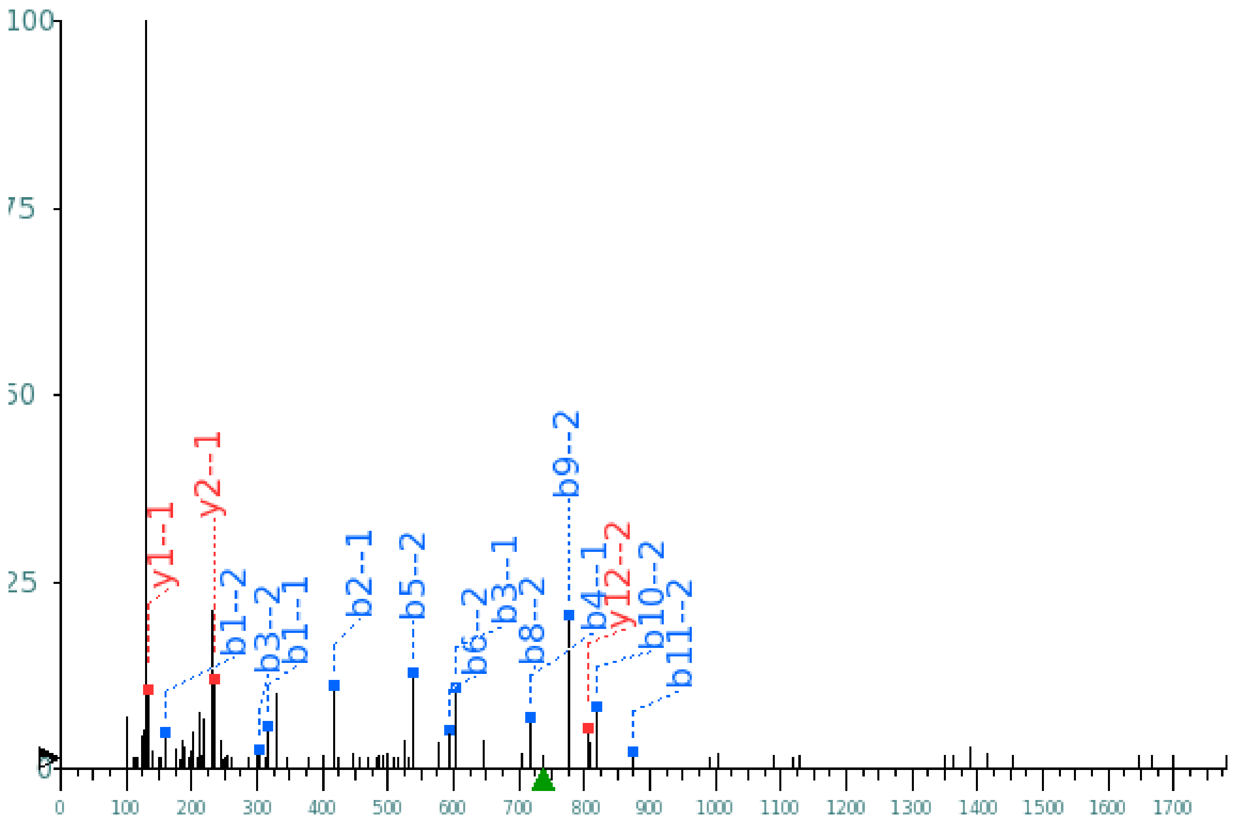

STWIKNQDDSLTETD (+3)

Primary Reference: PACS1_HUMAN
Search ID: 41761
Search Name: 20130328_ananiav_ERstress_TMT_TM_timecourse_trypsin_inj2_4mc_iaa
Scan#: 12786
Observed Mass: 737.7123 (1.9 ppm)
PSM Score: 22.07

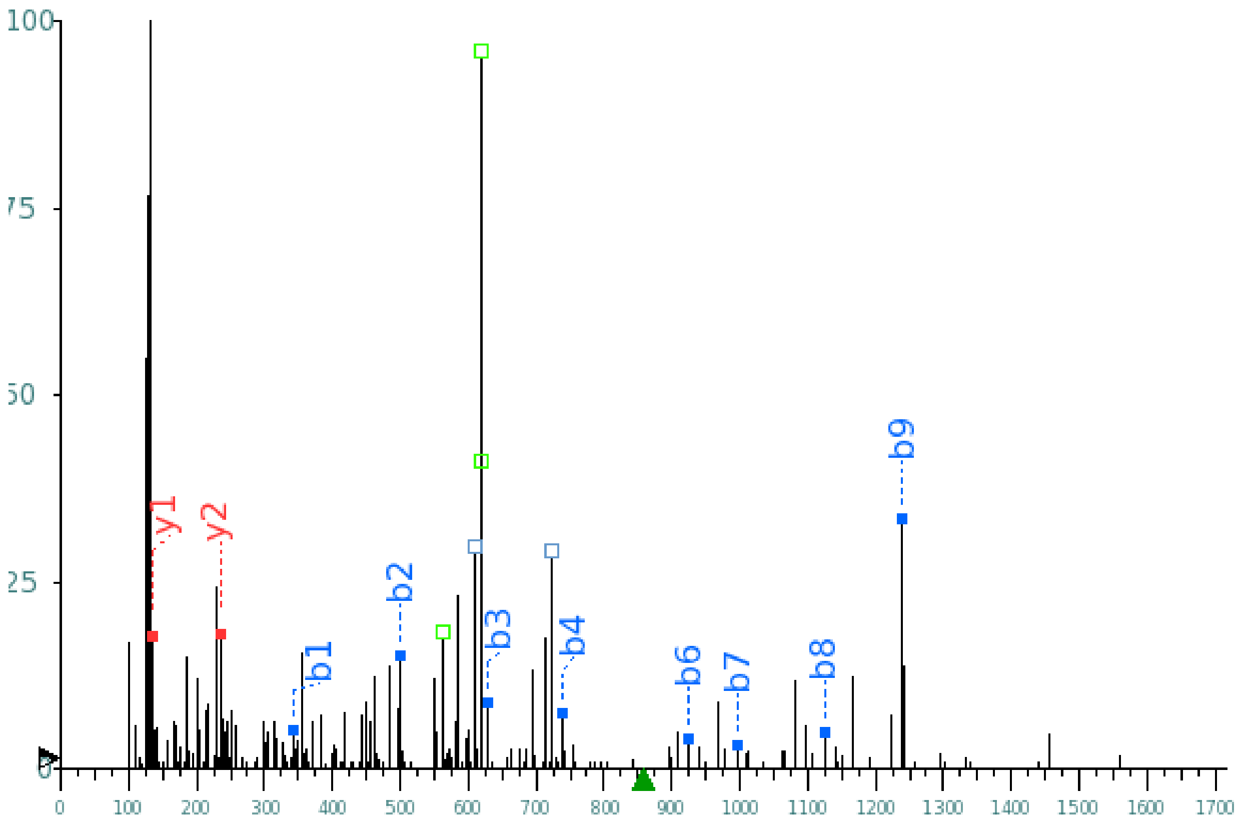

LRQIPSAEDLETD (+2)

Primary Reference: HDAC7_HUMAN
Search ID: 41761
Search Name: 20130328_ananiav_ERstress_TMT_TM_timecourse_trypsin_inj2_4mc_iaa
Scan#: 12798
Observed Mass: 858.4558 (1.8 ppm)
PSM Score: 21.18

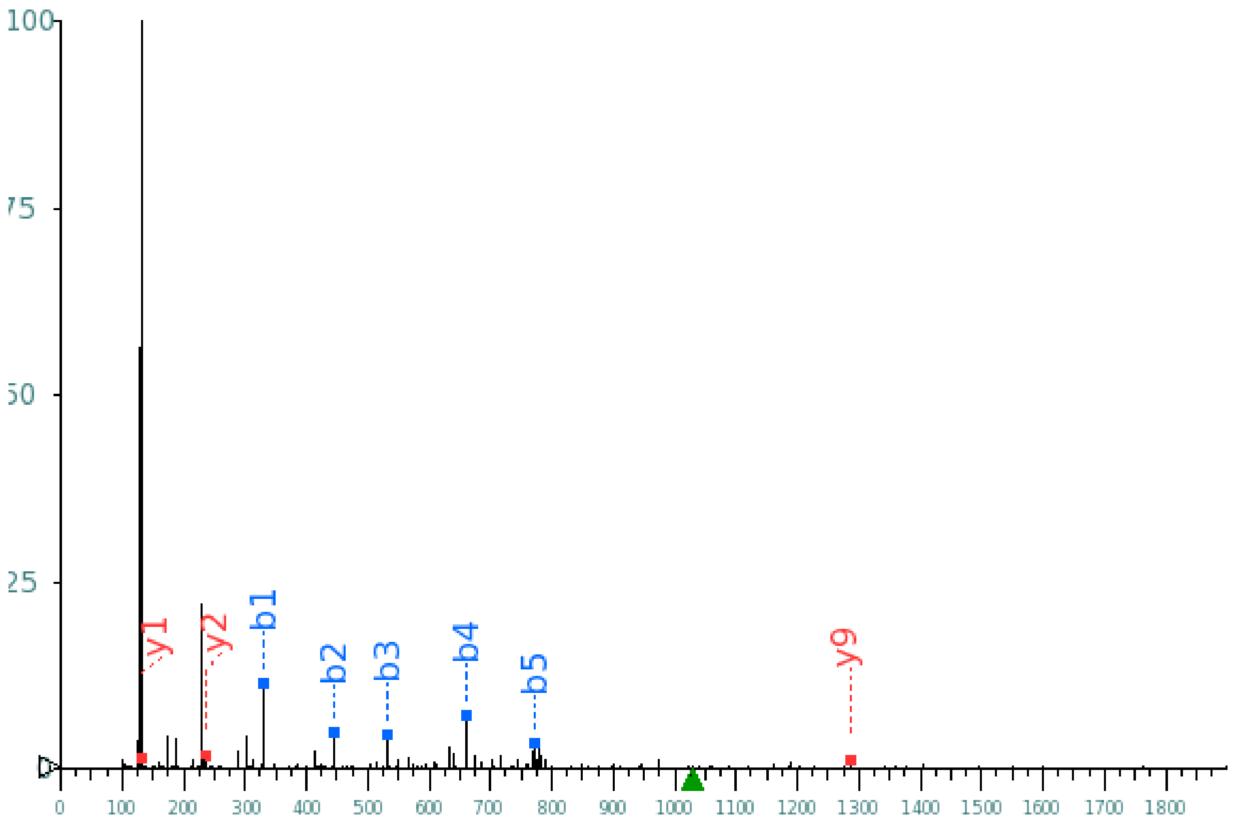

TISQIKSESEMETD (+2)

Primary Reference: LARP7_HUMAN
Search ID: 41761
Search Name: 20130328_ananiav_ERstress_TMT_TM_timecourse_trypsin_inj2_4mc_iaa
Scan#: 12818
Observed Mass: 1028.5312 (1.5 ppm)
PSM Score: 11.56

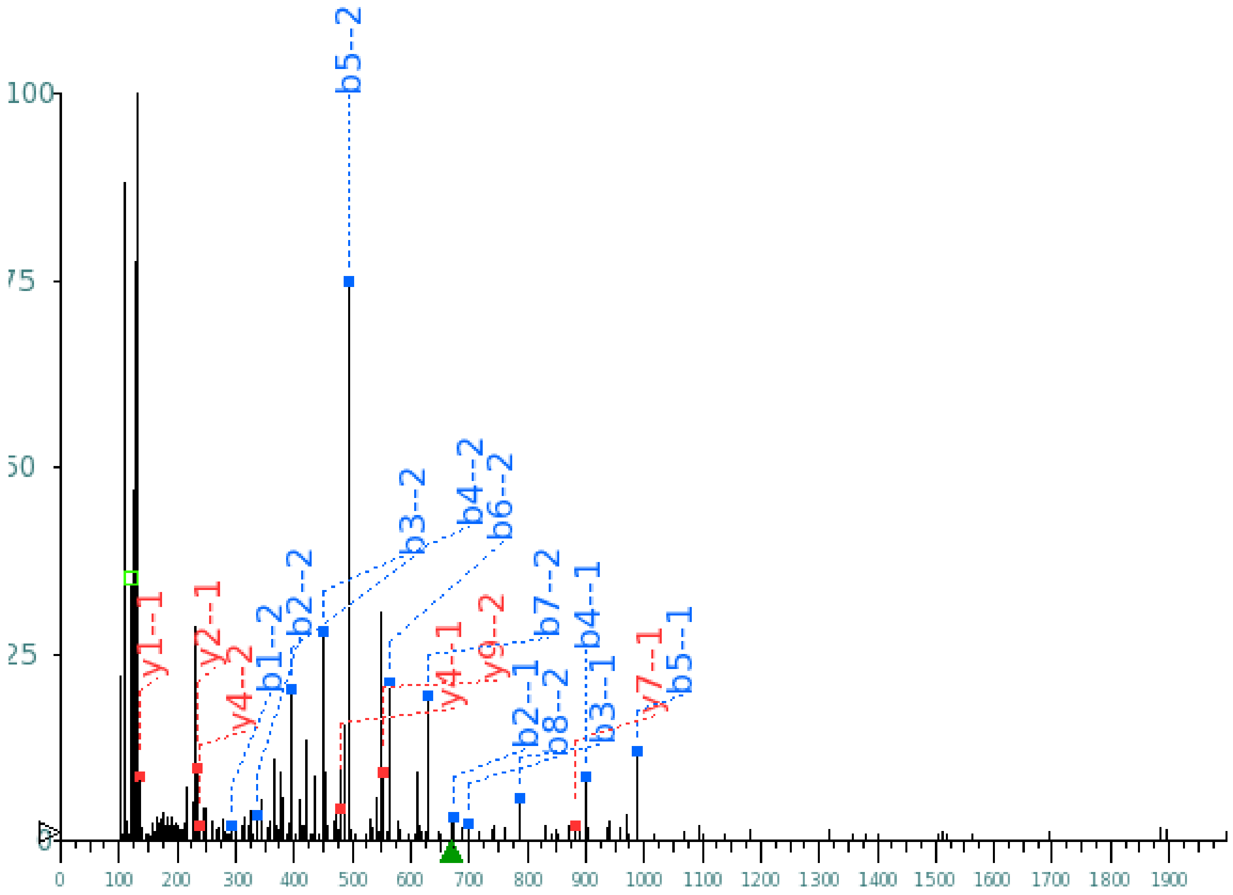

KSLLSHEFQDETD (+3)

Primary Reference: CBPD_HUMAN
Search ID: 41761
Search Name: 20130328_ananiav_ERstress_TMT_TM_timecourse_trypsin_inj2_4mc_iaa
Scan#: 12857
Observed Mass: 669.6871 (1.6 ppm)
PSM Score: 7.56

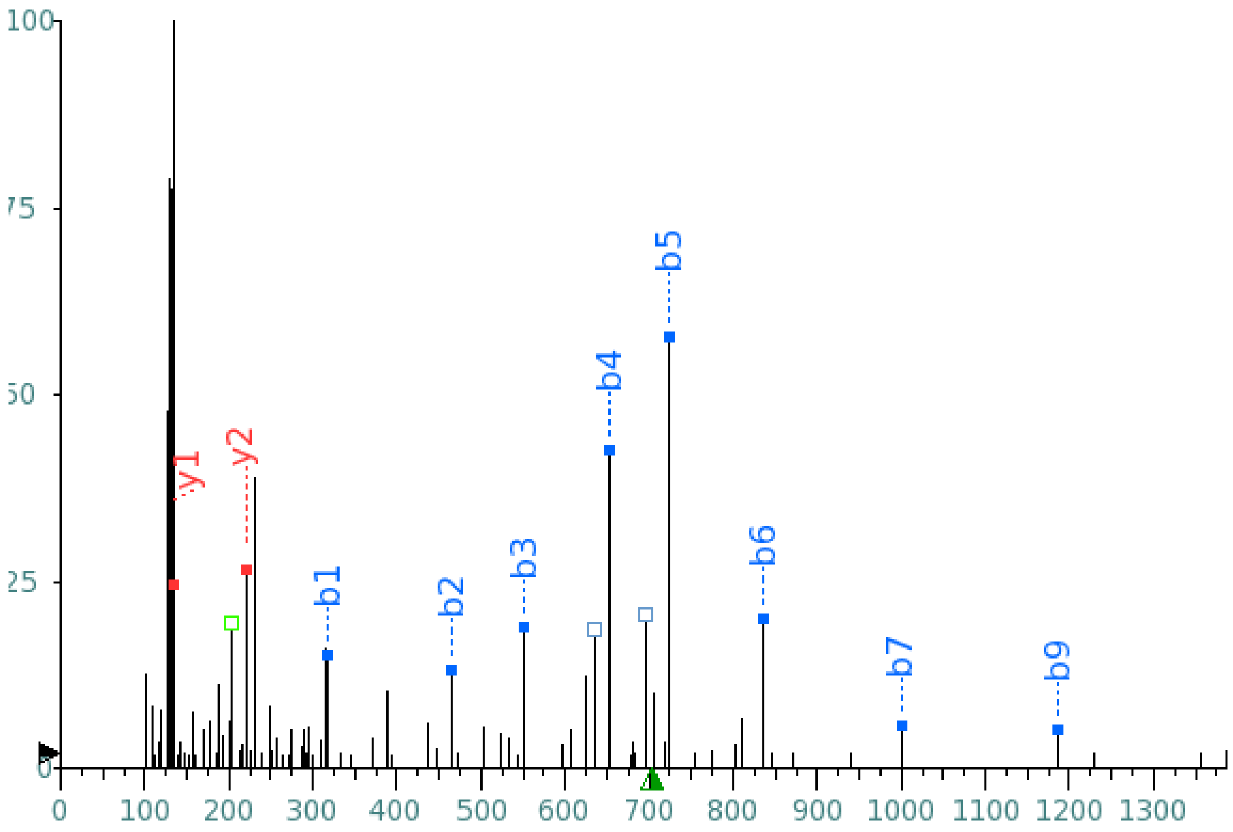

SFSTALYGESD (+2)

Primary Reference: ACTN4_HUMAN
Search ID: 41761
Search Name: 20130328_ananiav_ERstress_TMT_TM_timecourse_trypsin_inj2_4mc_iaa
Scan#: 12911
Observed Mass: 703.339 (1.7 ppm)
PSM Score: 45.7

File name: 2015-02-04 04.01.53PM.pdf

Creation time: 2015-02-04 04:09PM

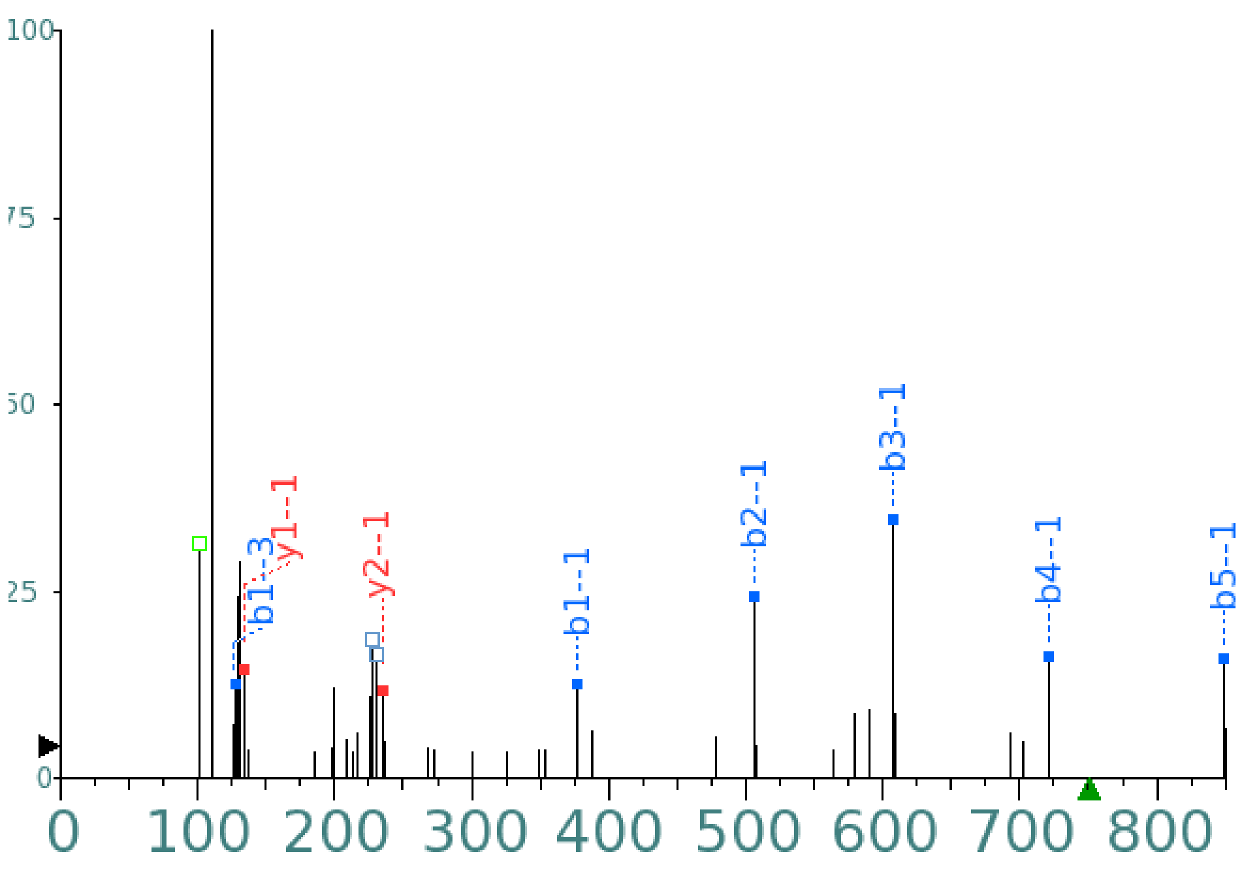

FETLEPEVSHEETEHSYHVEETD (+4)

Primary Reference: B4DQ07_HUMAN
Search ID: 41761
Search Name: 20130328_ananiav_ERstress_TMT_TM_timecourse_trypsin_inj2_4mc_iaa
Scan#: 12924
Observed Mass: 751.3409 (1.4 ppm)
PSM Score: 23.44

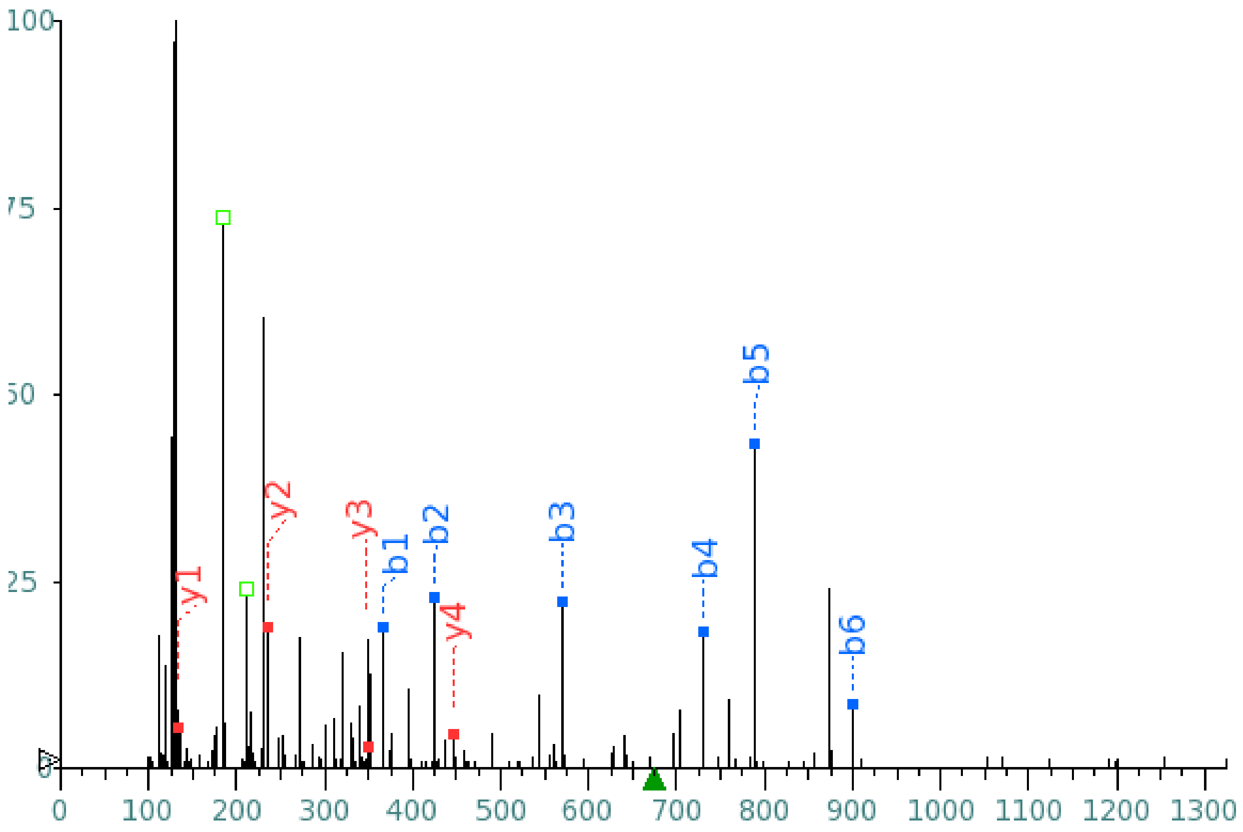

HGFC*GIPITD (+2)

Primary Reference: IMDH2_HUMAN
Search ID: 41761
Search Name: 20130328_ananiav_ERstress_TMT_TM_timecourse_trypsin_inj2_4mc_iaa
Scan#: 12931
Observed Mass: 673.3451 (4.2 ppm)
PSM Score: 16.83

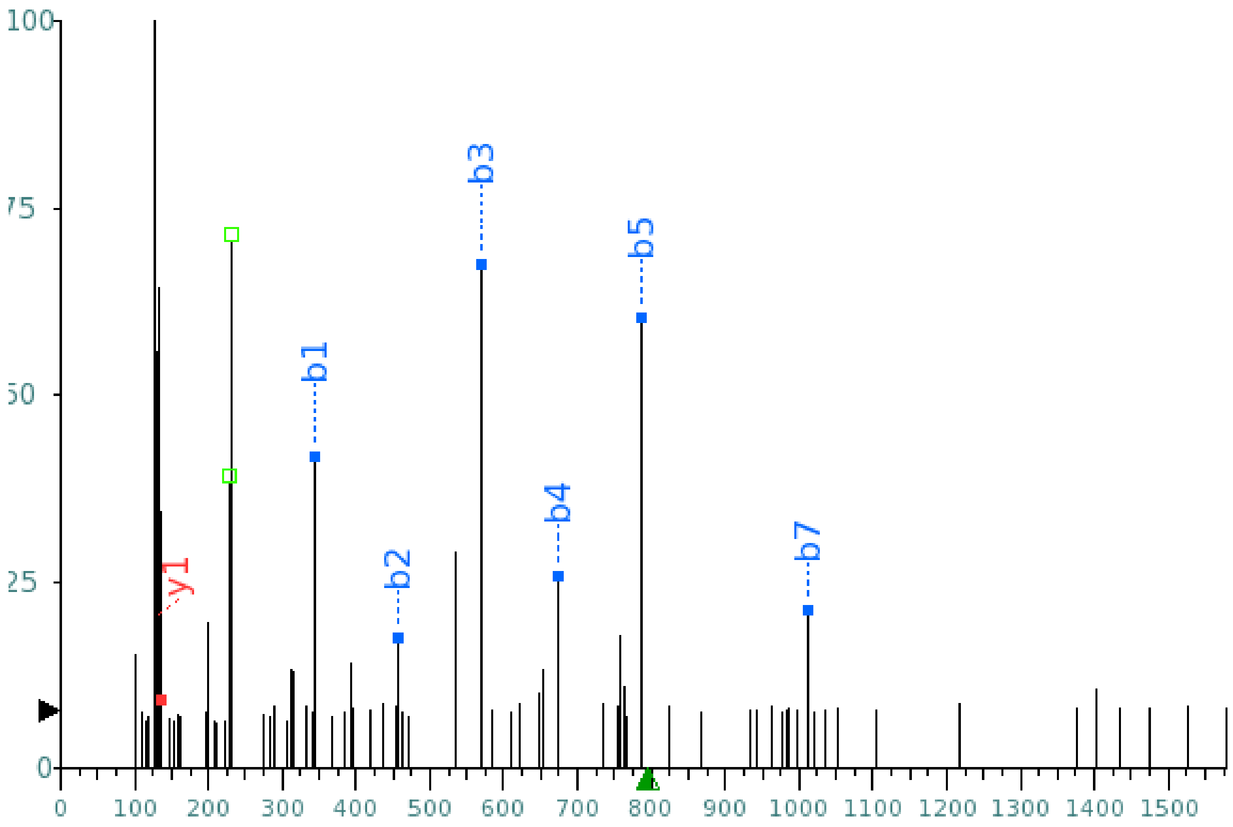

LLDTNPEINQSD (+2)

Primary Reference: TRIPC_HUMAN
Search ID: 41761
Search Name: 20130328_ananiav_ERstress_TMT_TM_timecourse_trypsin_inj2_4mc_iaa
Scan#: 12940
Observed Mass: 794.408 (1.5 ppm)
PSM Score: 24.49

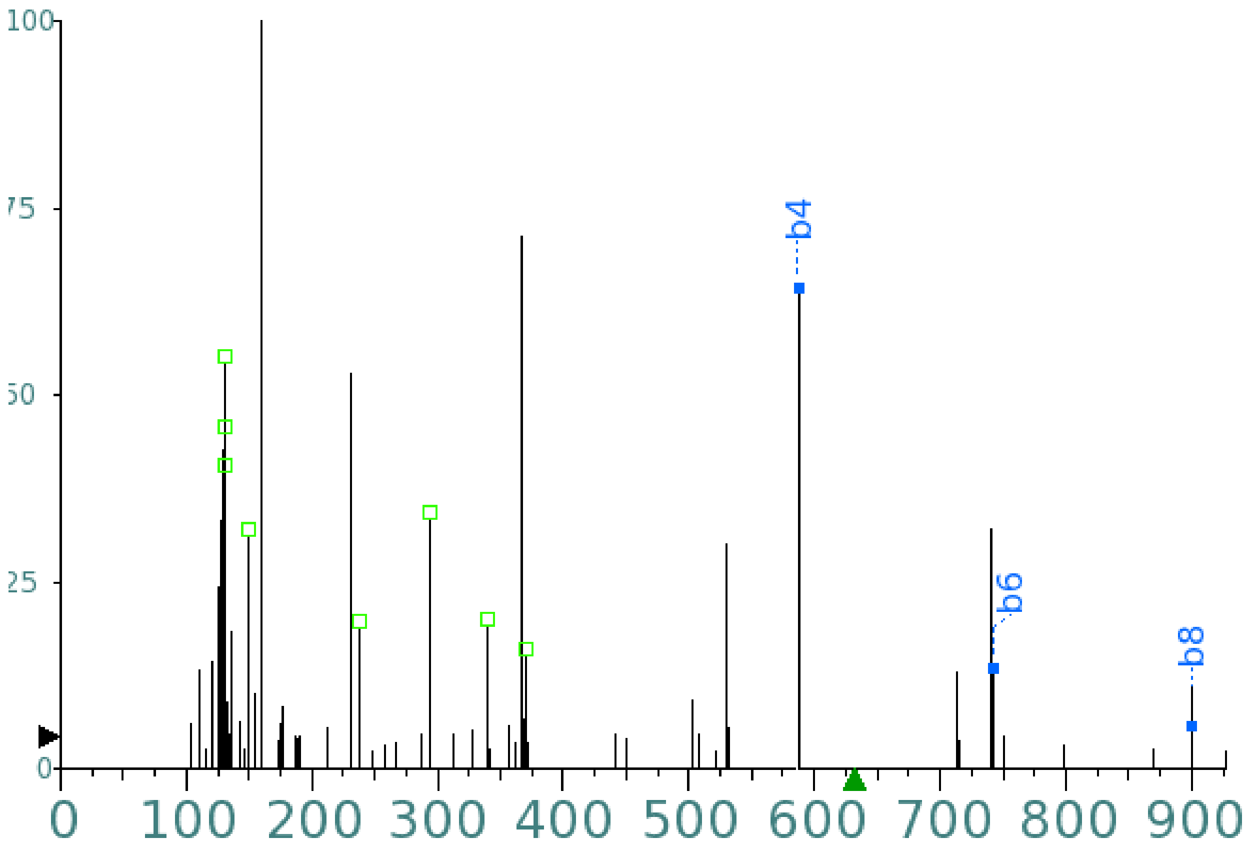

ANGNVGTGTQD (+2)

Primary Reference: F4MH31_HUMAN
Search ID: 41761
Search Name: 20130328_ananiav_ERstress_TMT_TM_timecourse_trypsin_inj2_4mc_iaa
Scan#: 12946
Observed Mass: 631.8135 (1.8 ppm)
PSM Score: 9.56

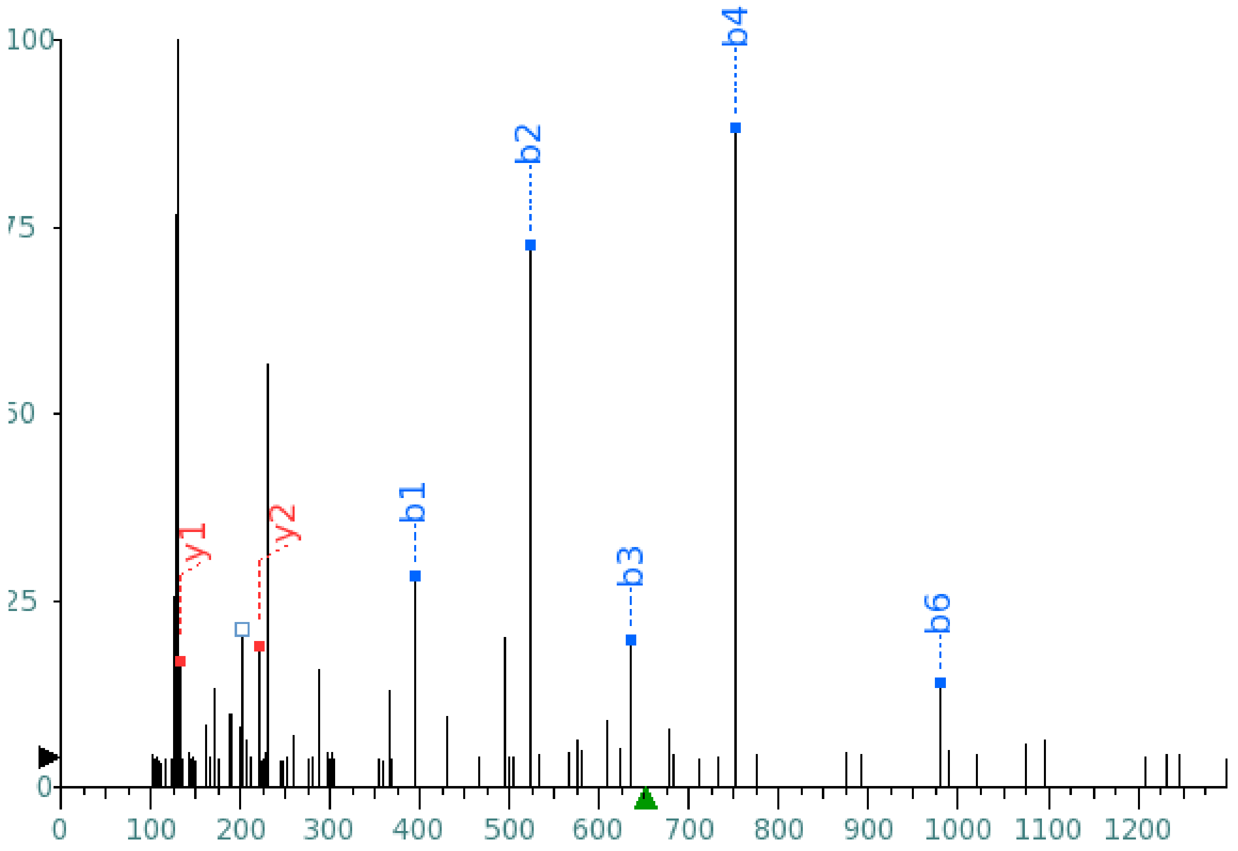

YEIDLDTSD (+2)

Primary Reference: K6PF_HUMAN
Search ID: 41761
Search Name: 20130328_ananiav_ERstress_TMT_TM_timecourse_trypsin_inj2_4mc_iaa
Scan#: 12973
Observed Mass: 650.3127 (2.2 ppm)
PSM Score: 24.62

File name: 2015-02-04 04.01.53PM.pdf

Creation time: 2015-02-04 04:09PM

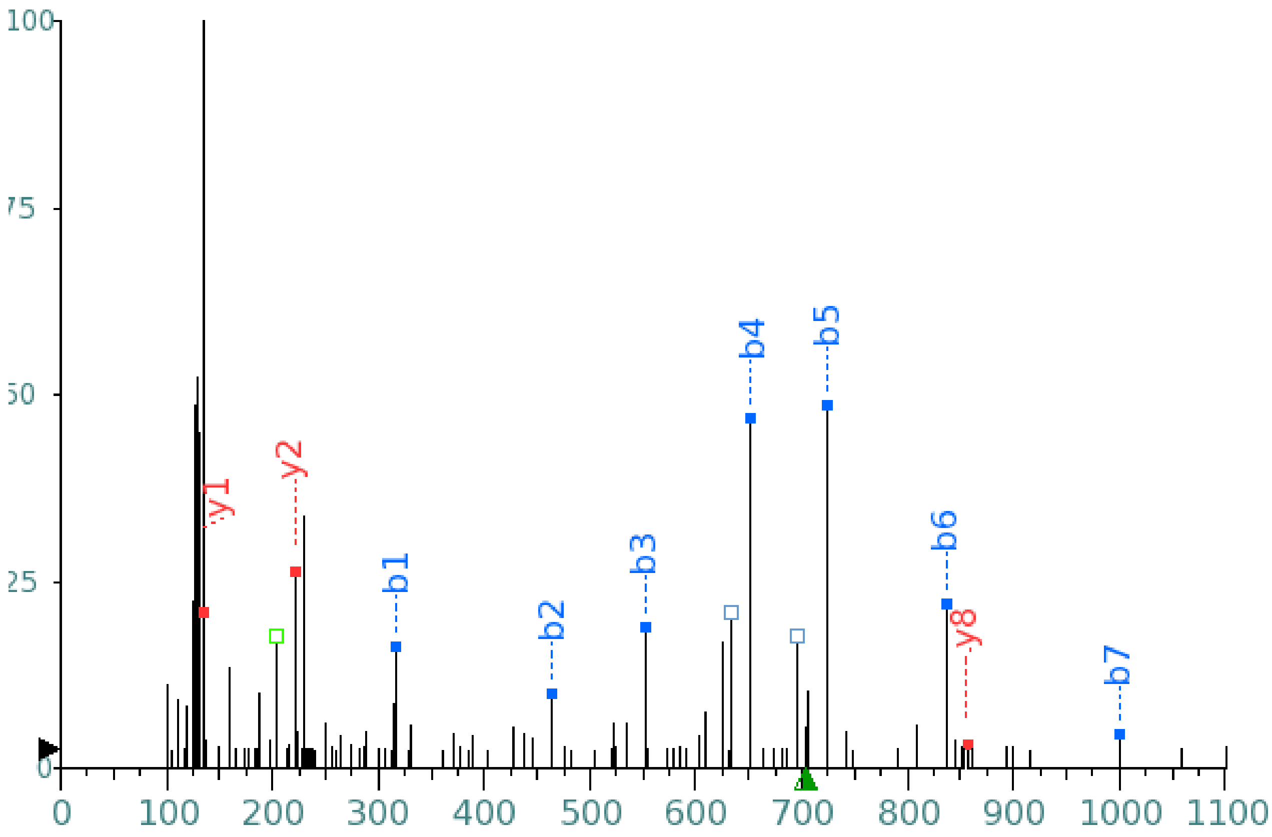

SFSTALYGESD (+2)

Primary Reference: ACTN4_HUMAN
Search ID: 41761
Search Name: 20130328_ananiav_ERstress_TMT_TM_timecourse_trypsin_inj2_4mc_iaa
Scan#: 12994
Observed Mass: 703.3394 (2.2 ppm)
PSM Score: 49.67

File name: 2015-02-04 04.01.53PM.pdf

Creation time: 2015-02-04 04:09PM

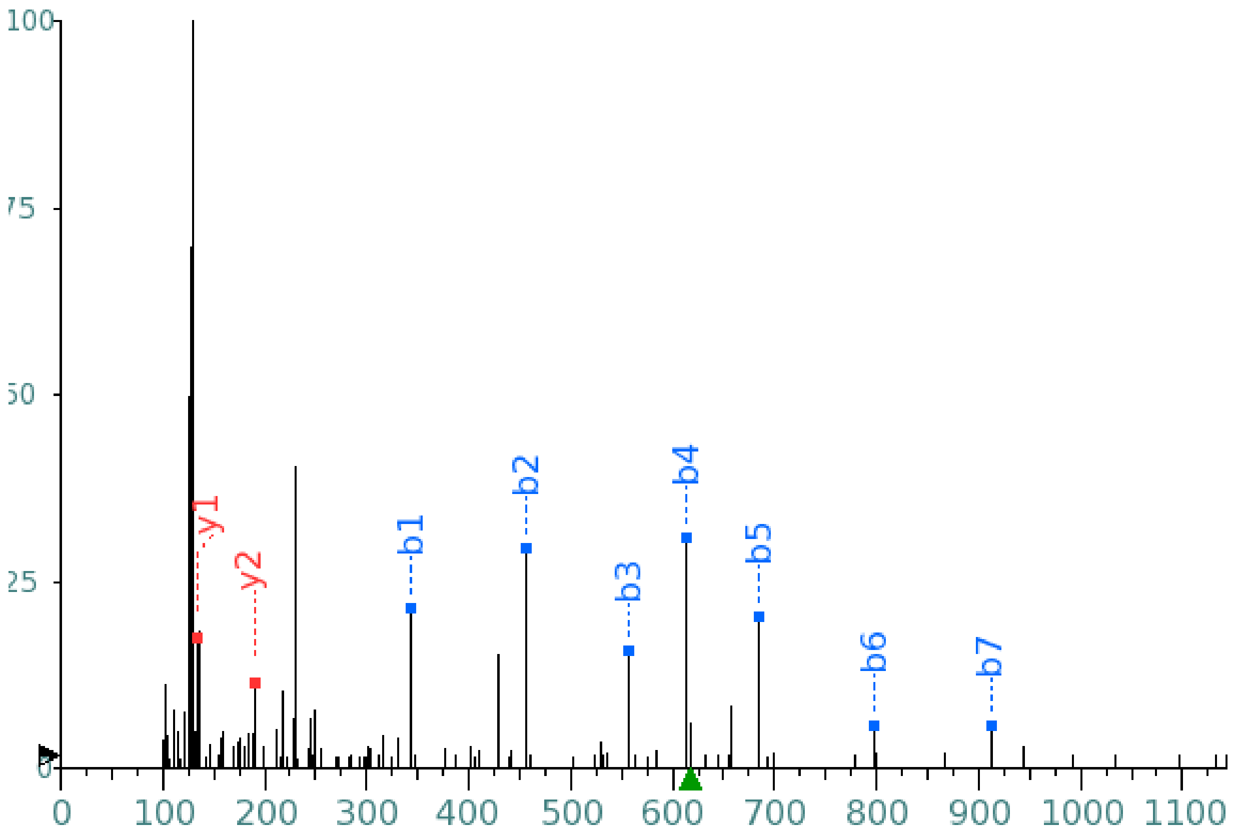

NLVGALDEGD (+2)

Primary Reference: ANR57_HUMAN
Search ID: 41761
Search Name: 20130328_ananiav_ERstress_TMT_TM_timecourse_trypsin_inj2_4mc_iaa
Scan#: 12996
Observed Mass: 616.3236 (2.5 ppm)
PSM Score: 34.18

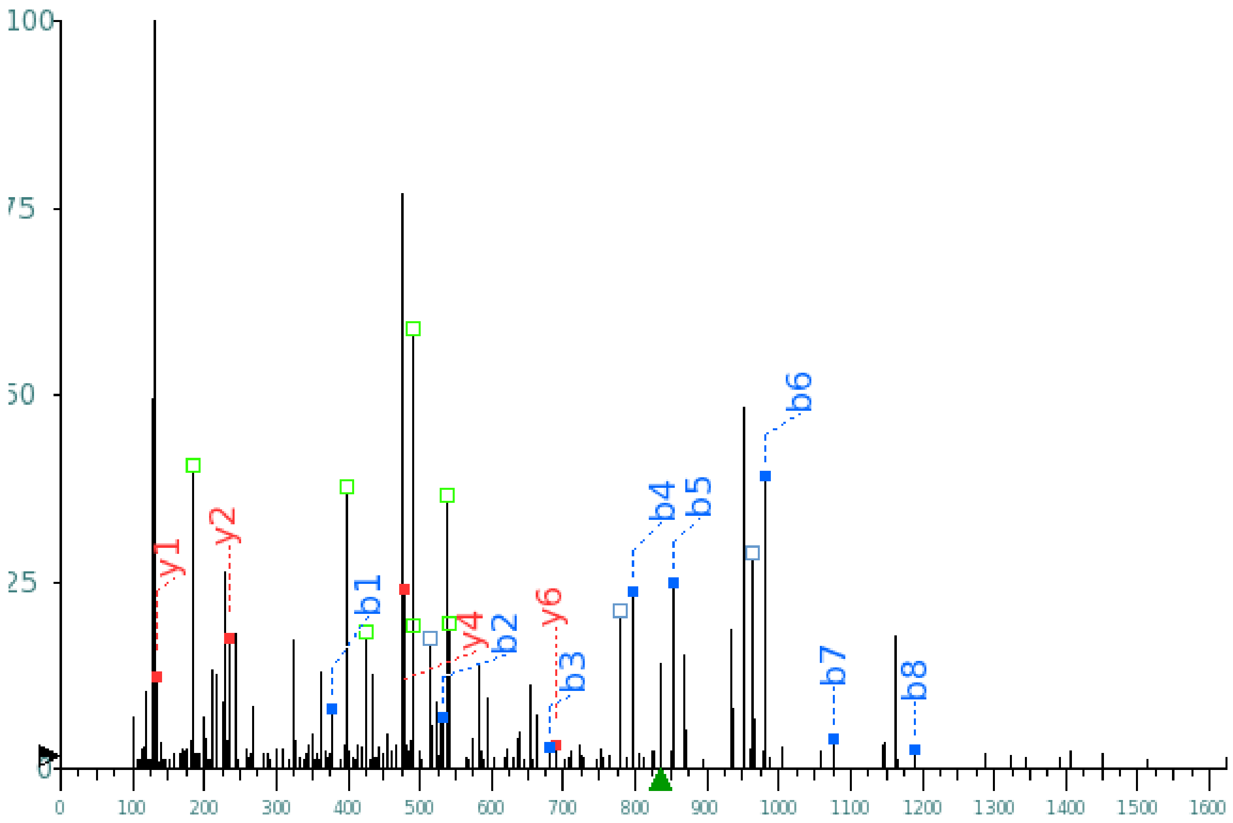

FRFDGQPINETD (+2)

Primary Reference: SUMO2_HUMAN
Search ID: 41761
Search Name: 20130328_ananiav_ERstress_TMT_TM_timecourse_trypsin_inj2_4mc_iaa
Scan#: 13005
Observed Mass: 834.4162 (1.4 ppm)
PSM Score: 14.21

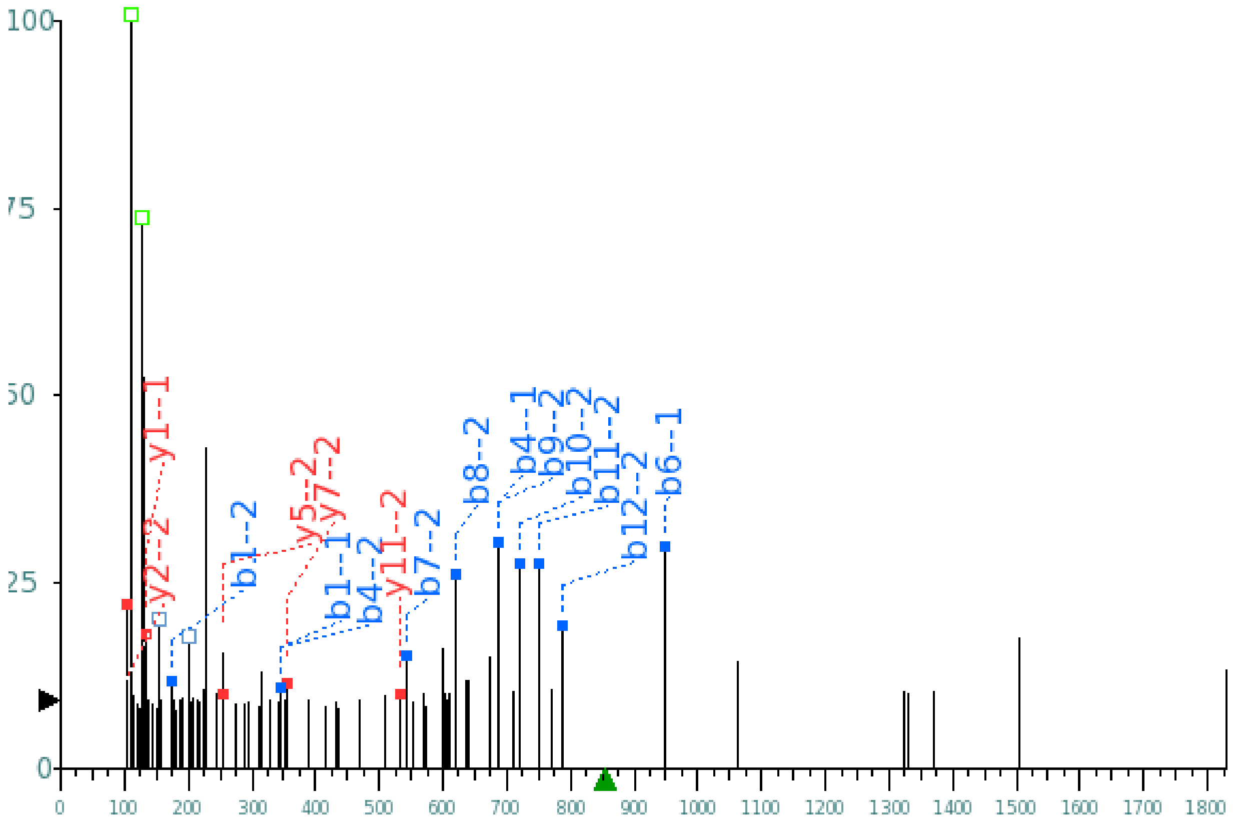

LLETPYHC*EAGAATDAEATEAD (+3)

Primary Reference: SELO_HUMAN
Search ID: 41761
Search Name: 20130328_ananiav_ERstress_TMT_TM_timecourse_trypsin_inj2_4mc_iaa
Scan#: 13017
Observed Mass: 855.3948 (1.5 ppm)
PSM Score: 17.72

File name: 2015-02-04 04.01.53PM.pdf

Creation time: 2015-02-04 04:09PM

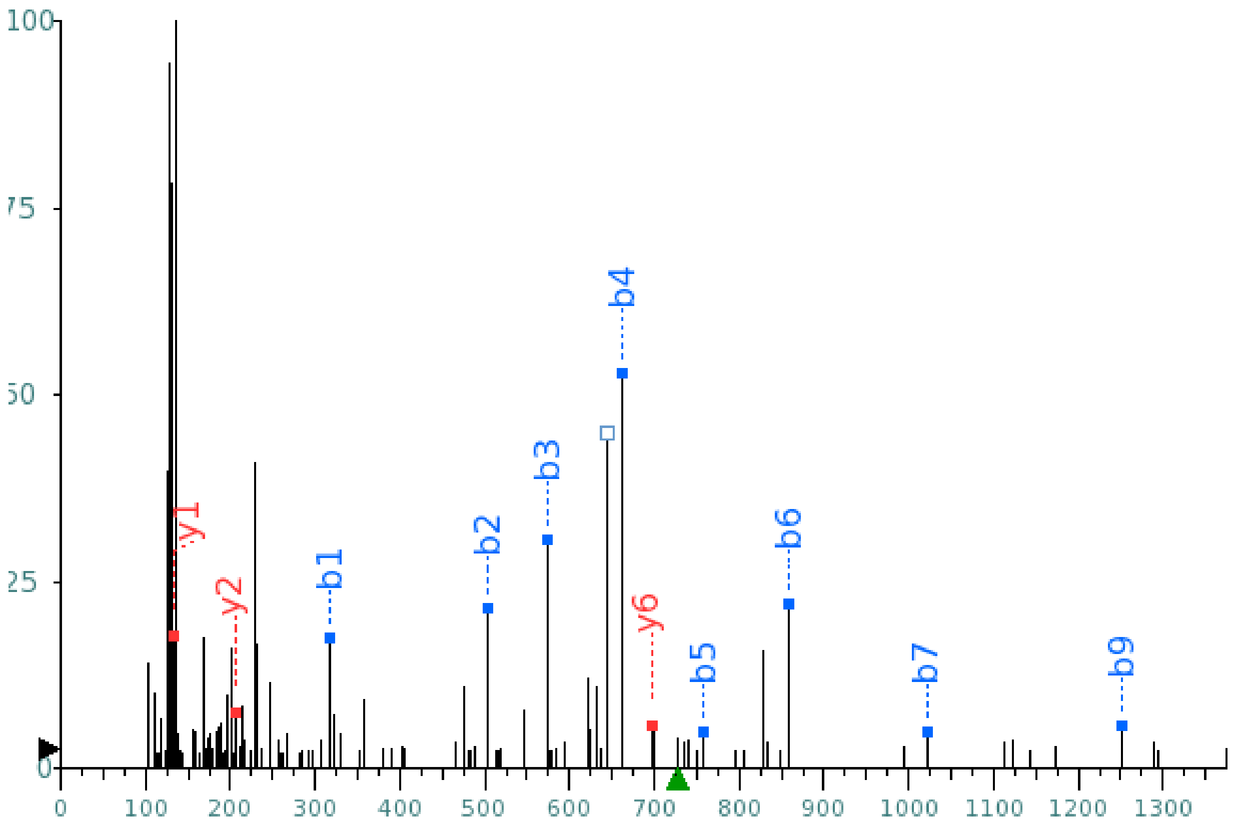

SWASPVYTEAD (+2)

Primary Reference: LMO7_HUMAN
Search ID: 41761
Search Name: 20130328_ananiav_ERstress_TMT_TM_timecourse_trypsin_inj2_4mc_iaa
Scan#: 13019
Observed Mass: 727.8551 (2 ppm)
PSM Score: 40.32

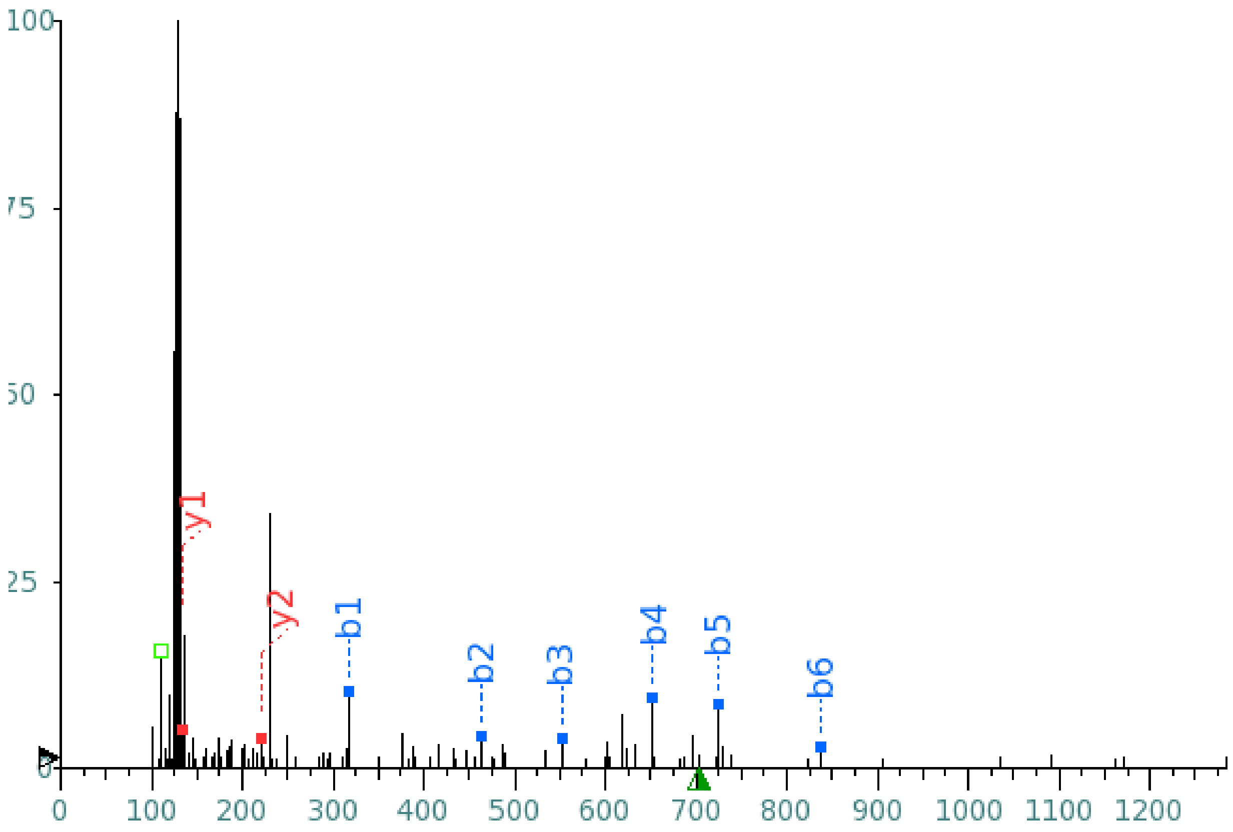

SFSTALYGESD (+2)

Primary Reference: ACTN4_HUMAN
Search ID: 41761
Search Name: 20130328_ananiav_ERstress_TMT_TM_timecourse_trypsin_inj2_4mc_iaa
Scan#: 13083
Observed Mass: 703.3397 (2.6 ppm)
PSM Score: 26.71

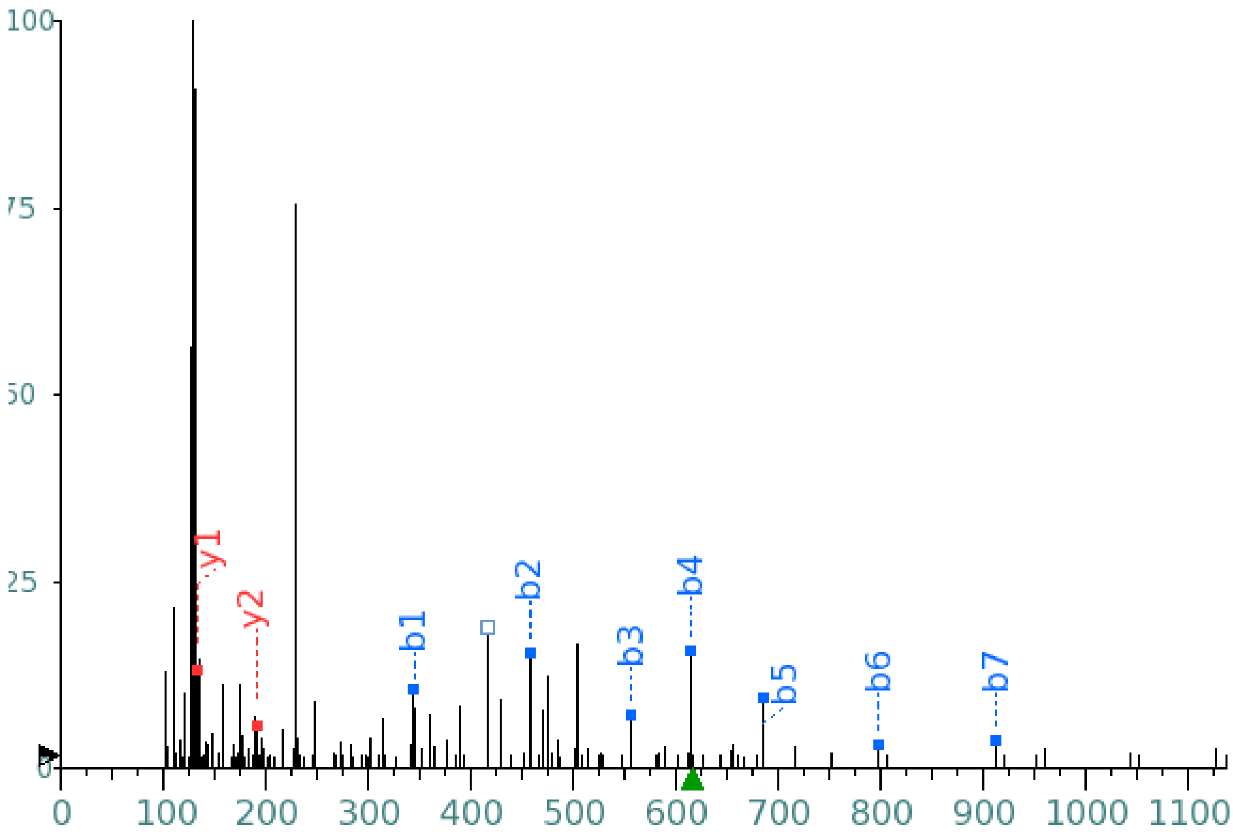

NLVGALDEGD (+2)

Primary Reference: ANR57_HUMAN
Search ID: 41761
Search Name: 20130328_ananiav_ERstress_TMT_TM_timecourse_trypsin_inj2_4mc_iaa
Scan#: 13084
Observed Mass: 616.3235 (2.4 ppm)
PSM Score: 18.04

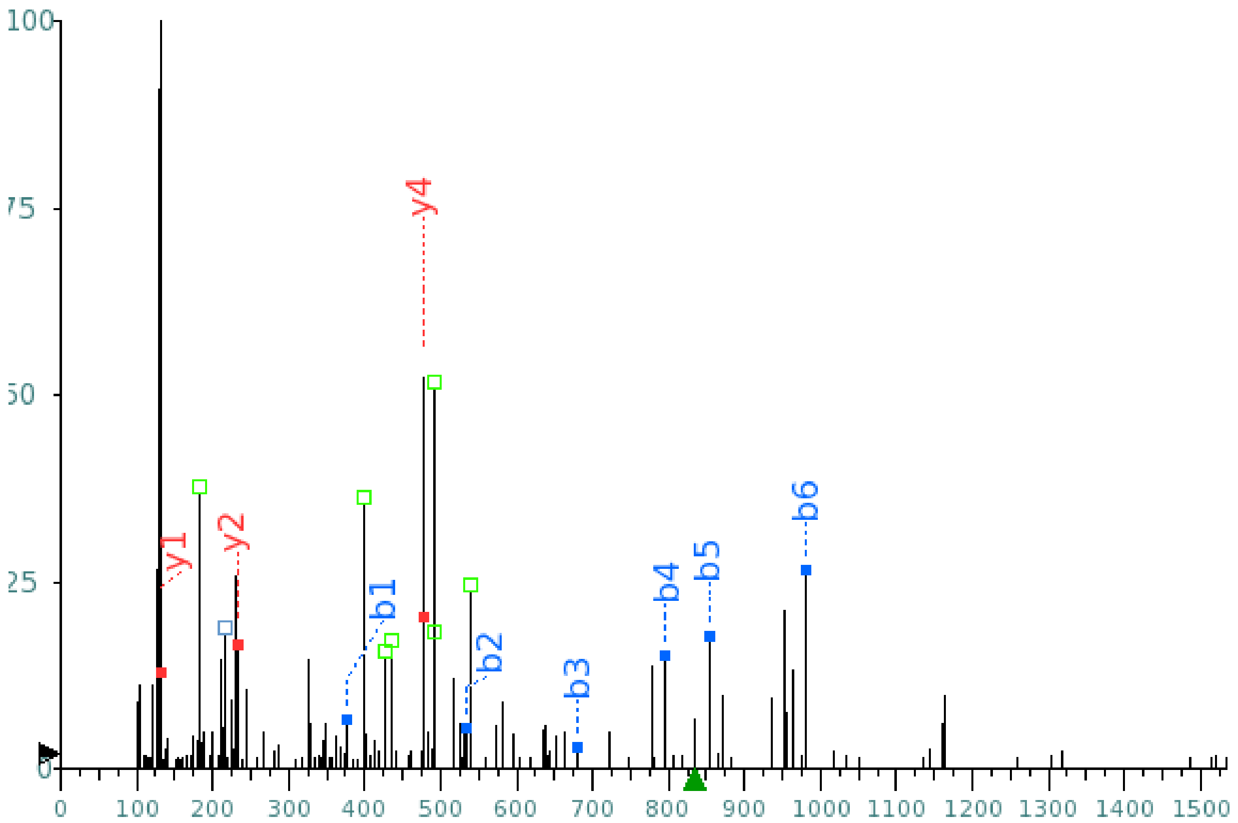

FRFDGQPINETD (+2)

Primary Reference: SUMO2_HUMAN
Search ID: 41761
Search Name: 20130328_ananiav_ERstress_TMT_TM_timecourse_trypsin_inj2_4mc_iaa
Scan#: 13096
Observed Mass: 834.4165 (1.8 ppm)
PSM Score: 16.95

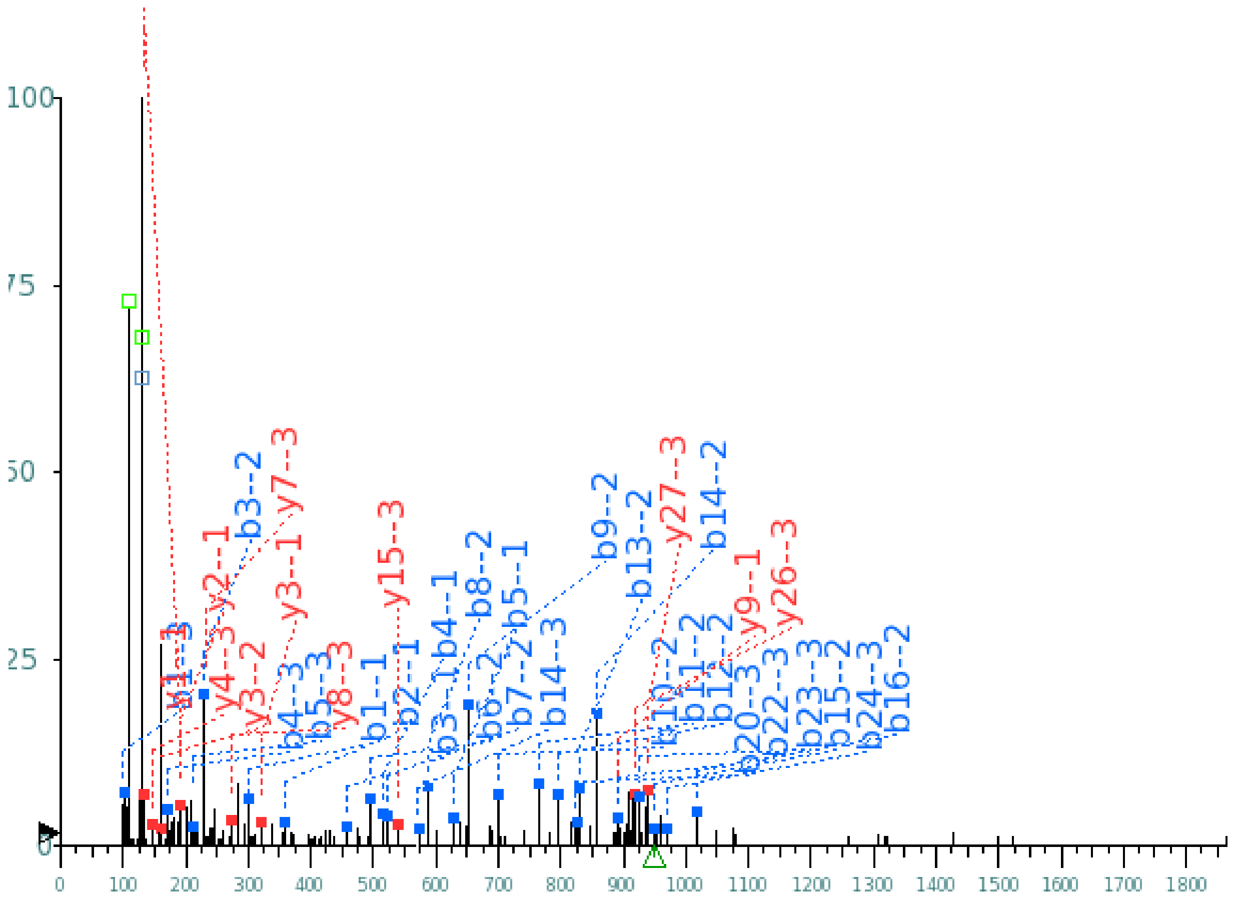

AGTGLKGEEVEGAGWMPGREPTTHAEAQGDEGD (+4)

Primary Reference: AHNK2_HUMAN
Search ID: 41761
Search Name: 20130328_ananiav_ERstress_TMT_TM_timecourse_trypsin_inj2_4mc_iaa
Scan#: 13131
Observed Mass: 950.2083 (1 ppm)
PSM Score: 34.67

File name: 2015-02-04 04.01.53PM.pdf

Creation time: 2015-02-04 04:09PM

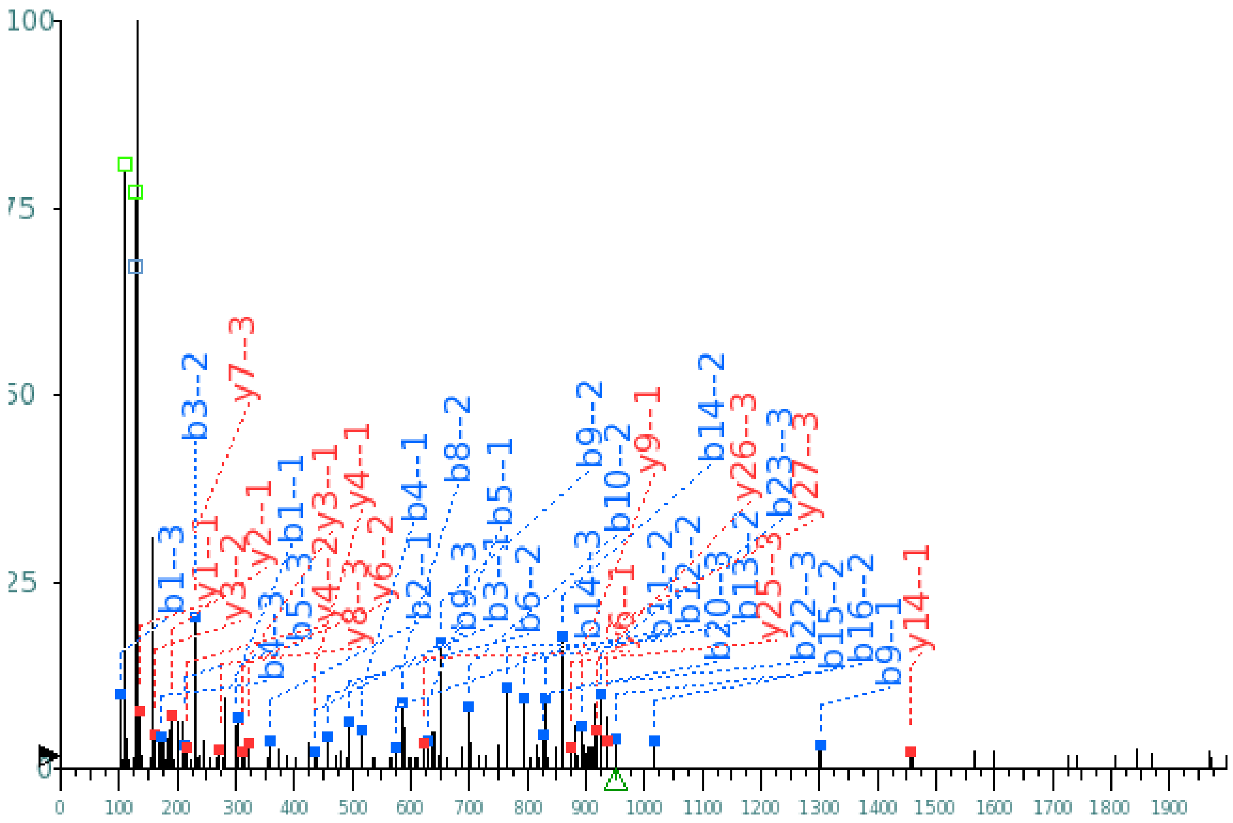

AGTGLKGEEVEGAGWMPGREPTTHAEAQGDEGD (+4)

Primary Reference: AHNK2_HUMAN

Search ID: 41761

Search Name: 20130328_ananiav_ERstress_TMT_TM_timecourse_trypsin_inj2_4mc_iaa

Scan#: 13138

Observed Mass: 950.2086 (1.3 ppm)

PSM Score: 31.68

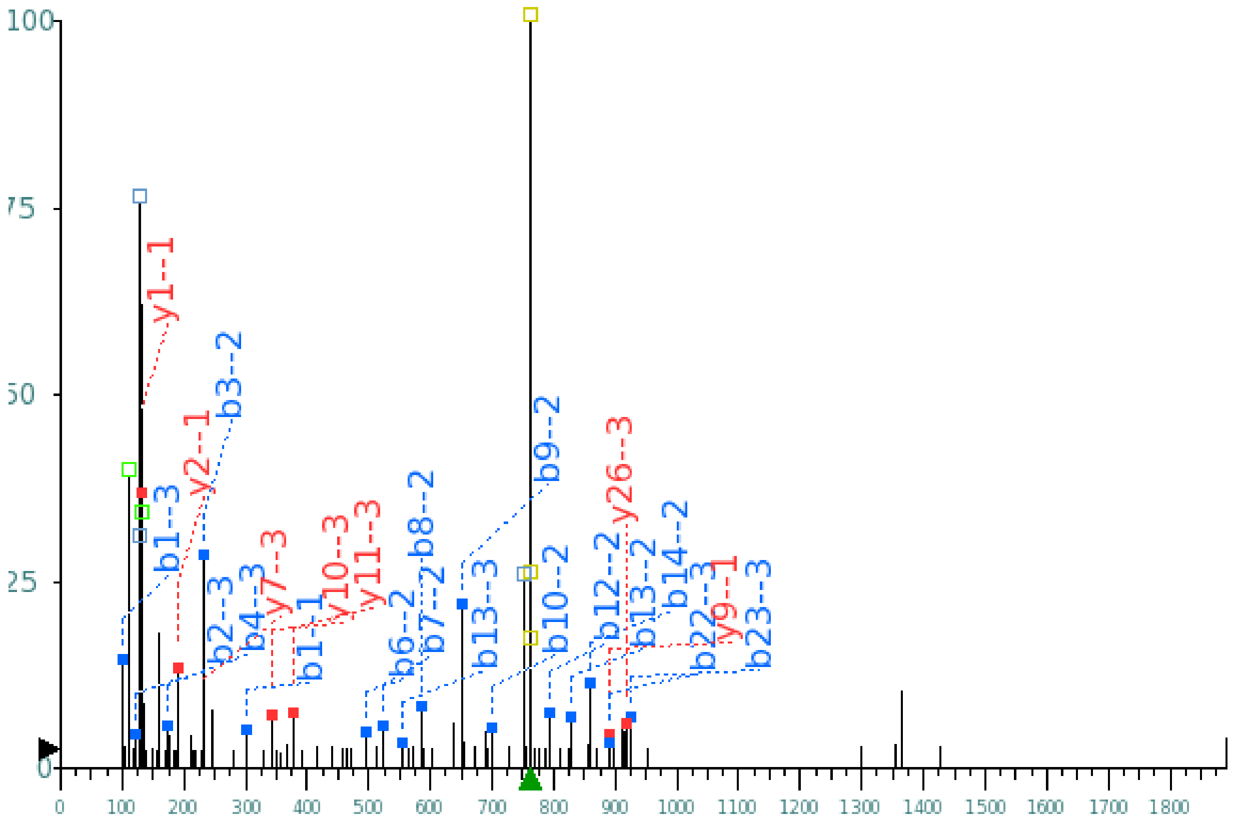

AGTGLKGEEVEGAGWMPGREPTTHAEAQGDEGD (+5)

Primary Reference: AHNK2_HUMAN
Search ID: 41761
Search Name: 20130328_ananiav_ERstress_TMT_TM_timecourse_trypsin_inj2_4mc_iaa
Scan#: 13157
Observed Mass: 760.3685 (1.6 ppm)
PSM Score: 17.64

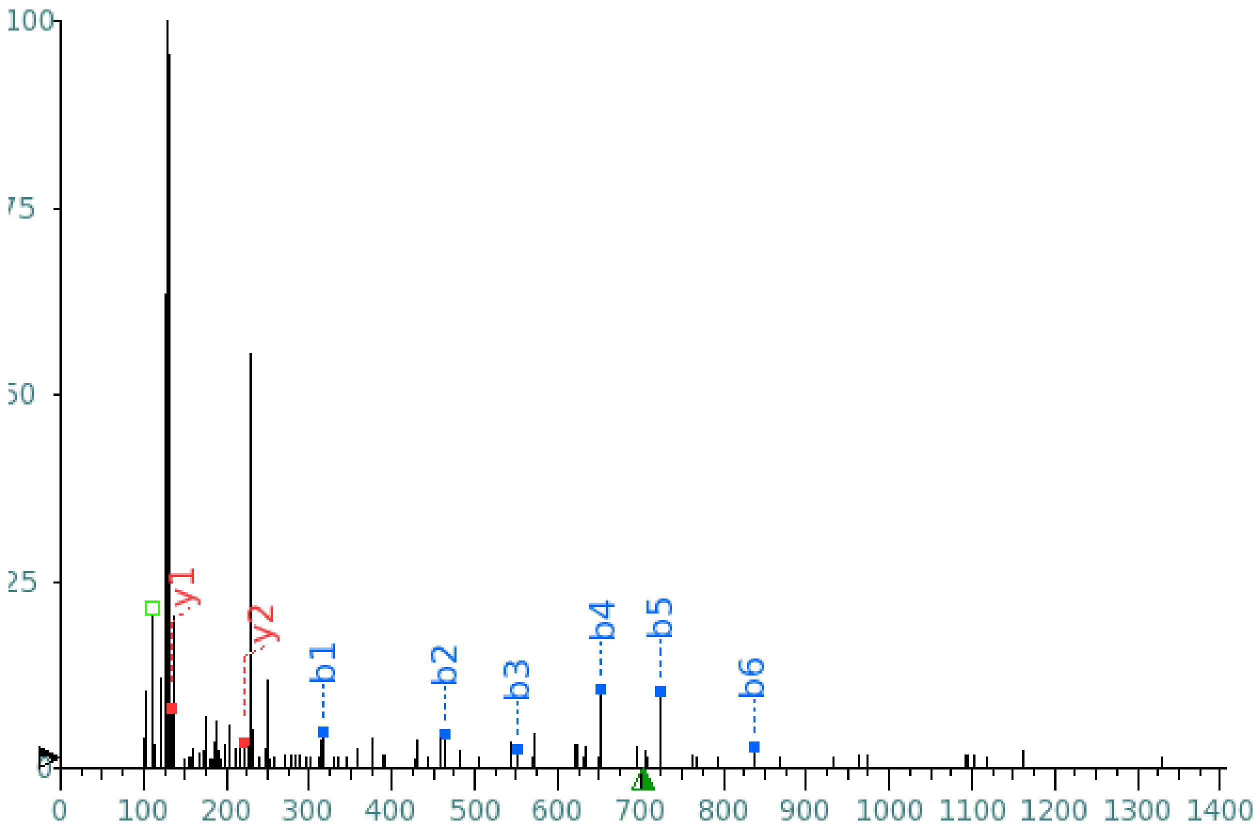

SFSTALYGESD (+2)

Primary Reference: ACTN4_HUMAN
Search ID: 41761
Search Name: 20130328_ananiav_ERstress_TMT_TM_timecourse_trypsin_inj2_4mc_iaa
Scan#: 13179
Observed Mass: 703.3398 (2.7 ppm)
PSM Score: 4.2

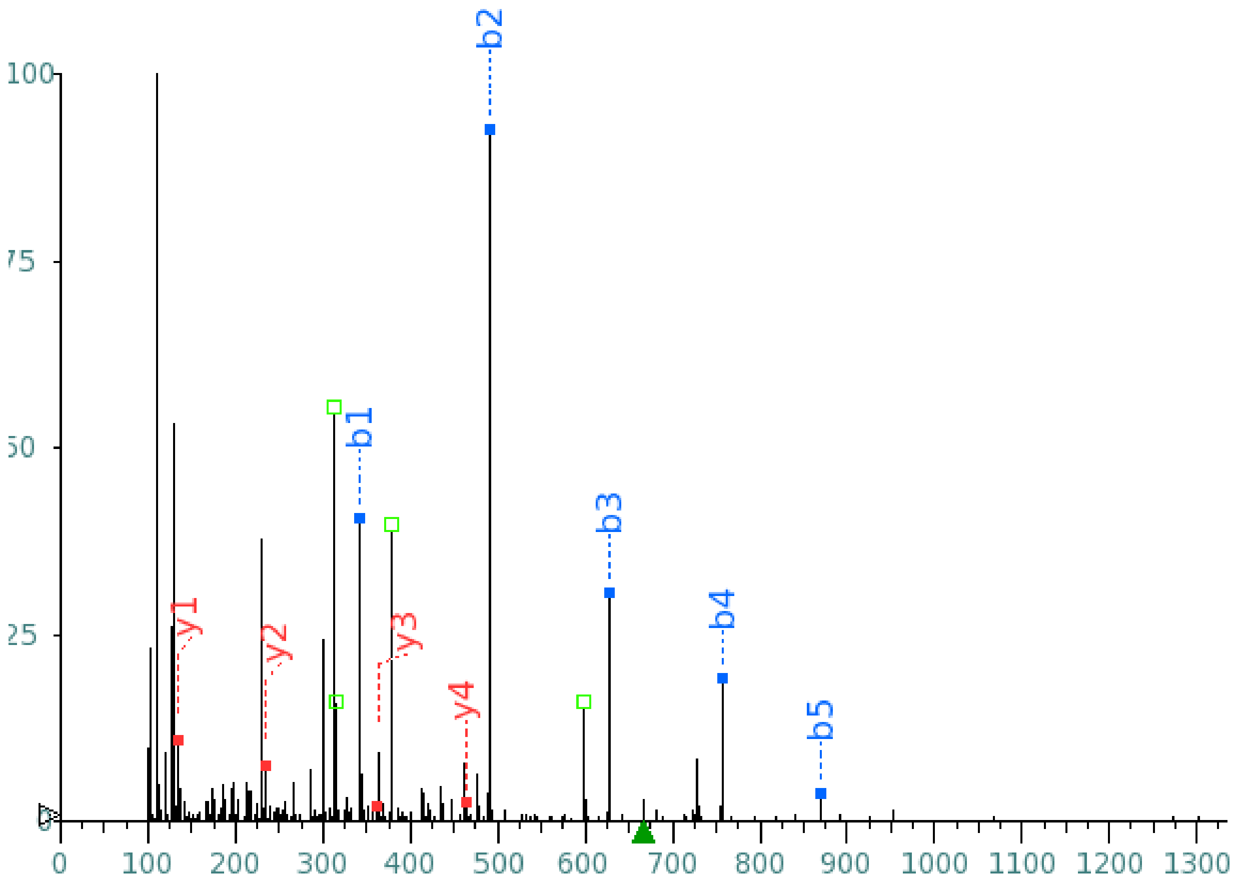

IFHELTQTD (+2)

Primary Reference: C1TC_HUMAN
Search ID: 41761
Search Name: 20130328_ananiav_ERstress_TMT_TM_timecourse_trypsin_inj2_4mc_iaa
Scan#: 13354
Observed Mass: 666.8524 (-1.7 ppm)
PSM Score: 17.46

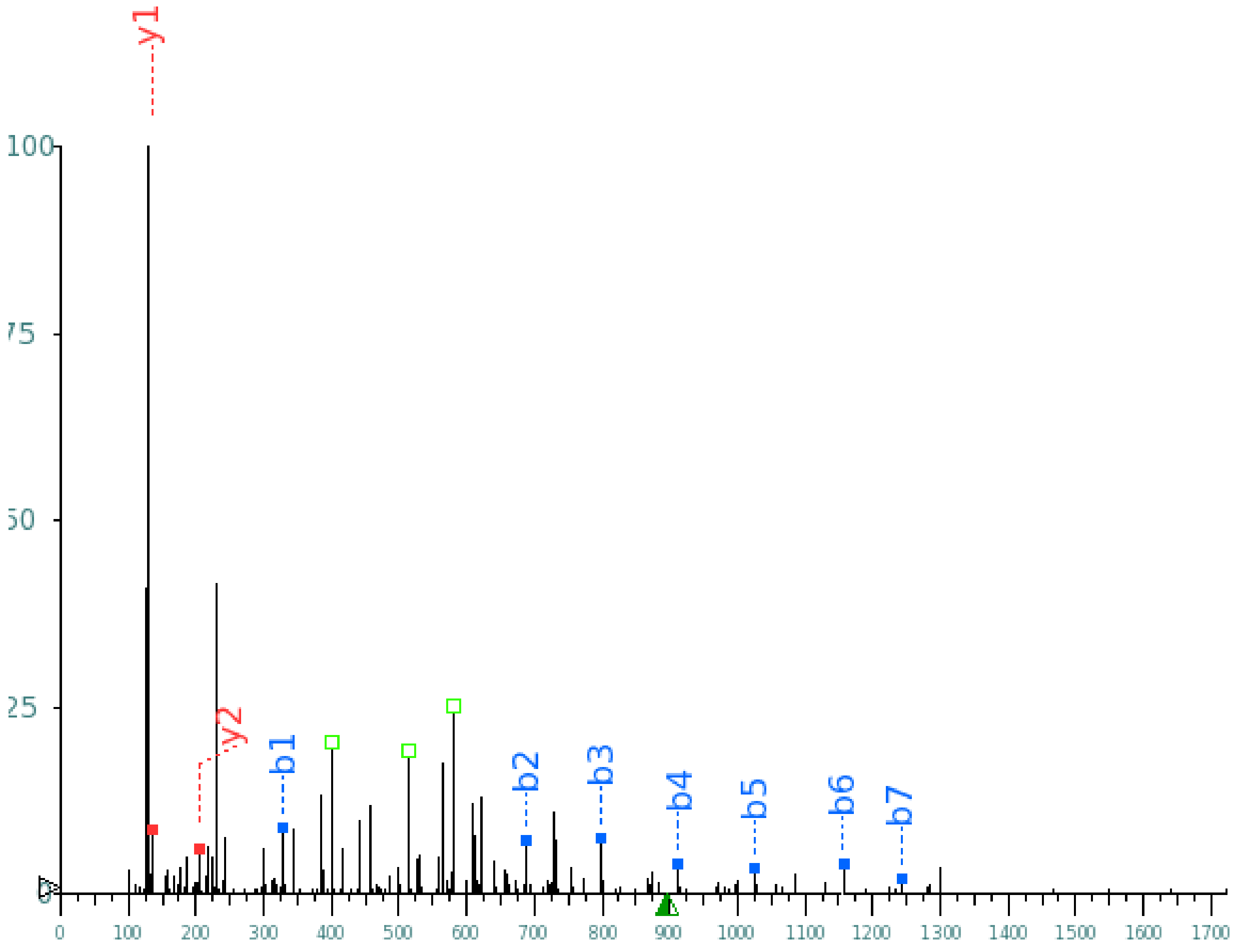

VKLINESVDEAD (+2)

Primary Reference: RANB3_HUMAN
Search ID: 41761
Search Name: 20130328_ananiav_ERstress_TMT_TM_timecourse_trypsin_inj2_4mc_iaa
Scan#: 13370
Observed Mass: 895.5031 (2.3 ppm)
PSM Score: 19.41

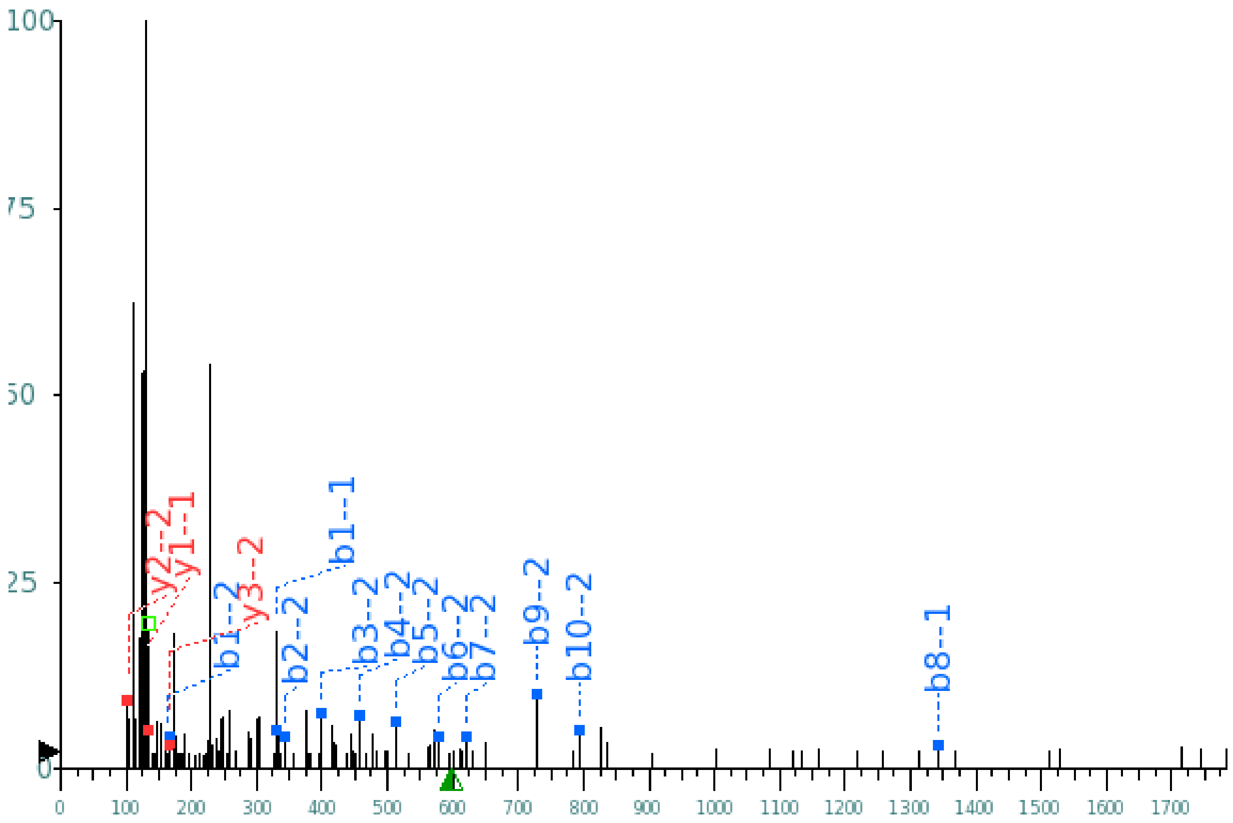

VKLINESVDEAD (+3)

Primary Reference: RANB3_HUMAN
Search ID: 41761
Search Name: 20130328_ananiav_ERstress_TMT_TM_timecourse_trypsin_inj2_4mc_iaa
Scan#: 13379
Observed Mass: 597.3371 (1.1 ppm)
PSM Score: 4.89

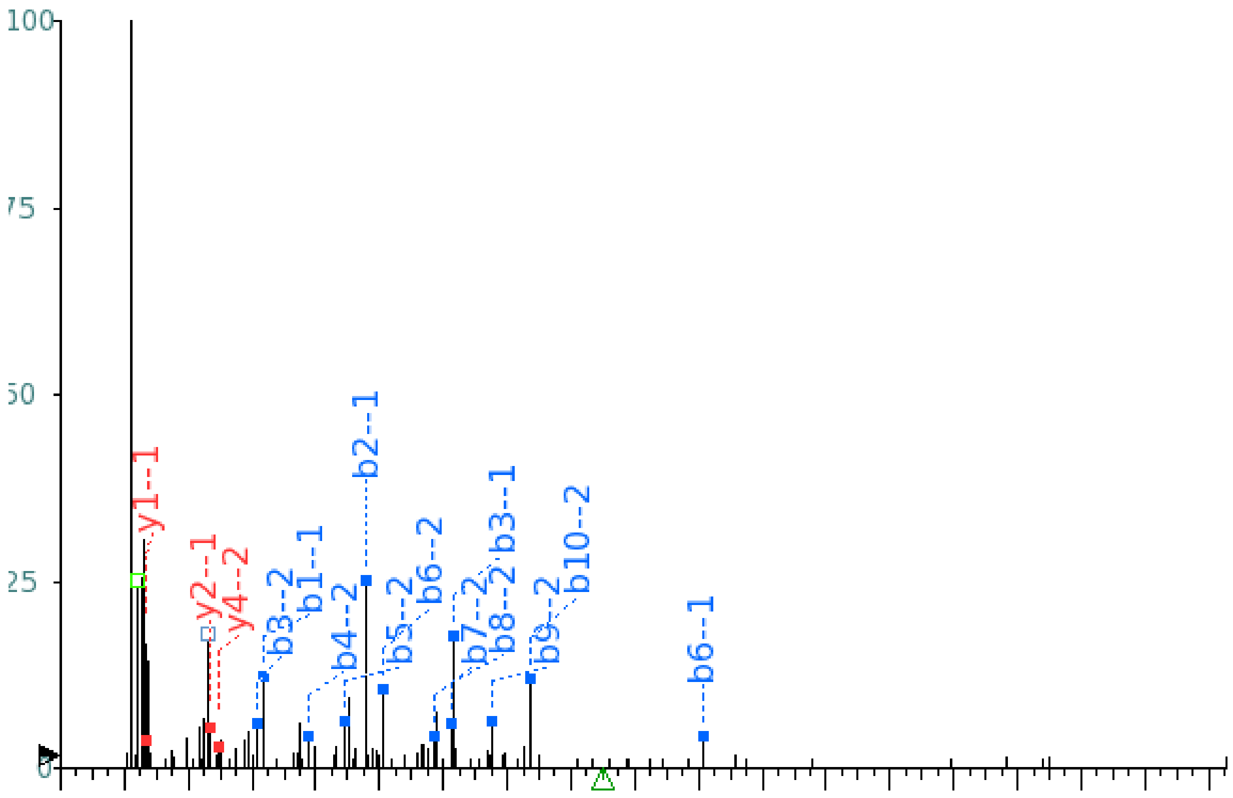

SYHC*NDC*GEDFSHITDFTD (+3)

Primary Reference: ZFP90_HUMAN
Search ID: 41761
Search Name: 20130328_ananiav_ERstress_TMT_TM_timecourse_trypsin_inj2_4mc_iaa
Scan#: 13431
Observed Mass: 850.3466 (2.7 ppm)
PSM Score: 23.74

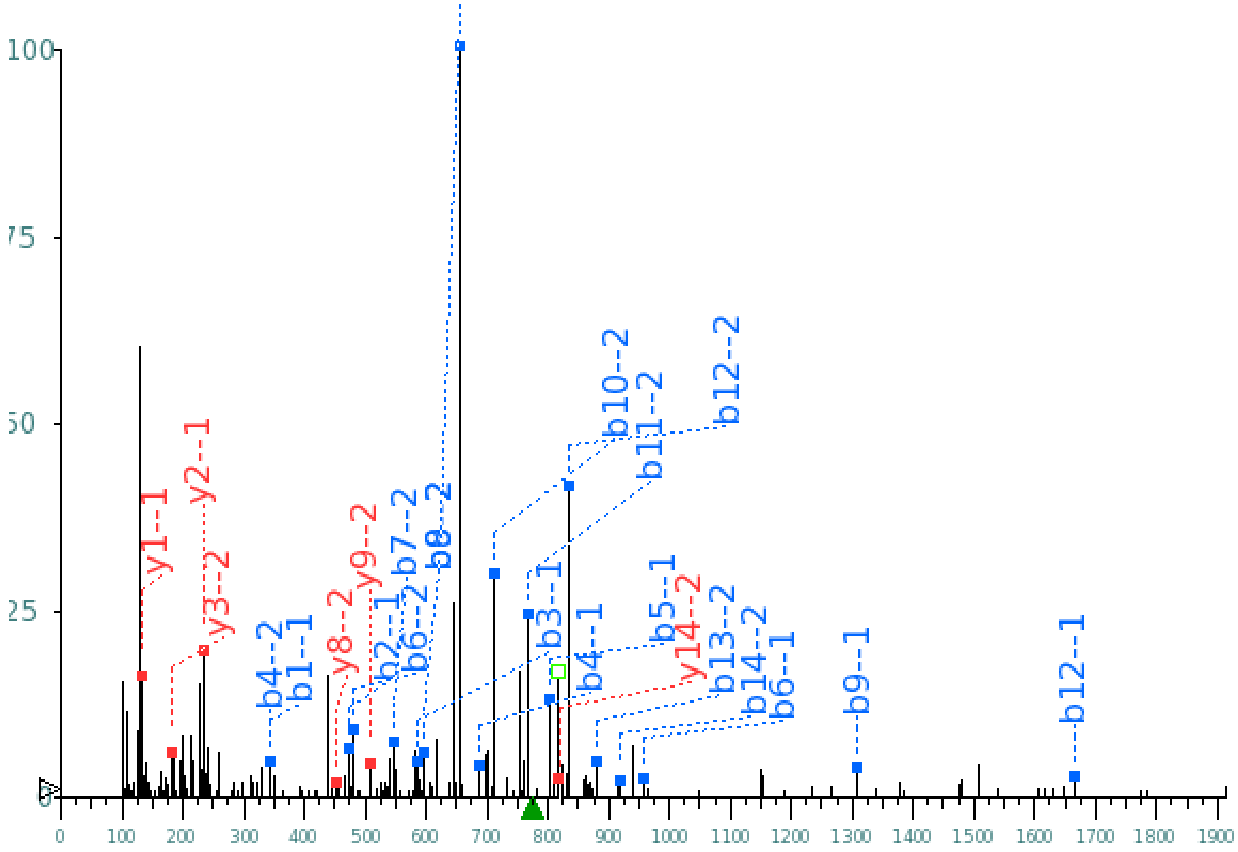

NENVDRHVDLLEVAQETD (+3)

Primary Reference: ATAD1_HUMAN
Search ID: 41761
Search Name: 20130328_ananiav_ERstress_TMT_TM_timecourse_trypsin_inj2_4mc_iaa
Scan#: 13467
Observed Mass: 775.7235 (1.7 ppm)
PSM Score: 27.54

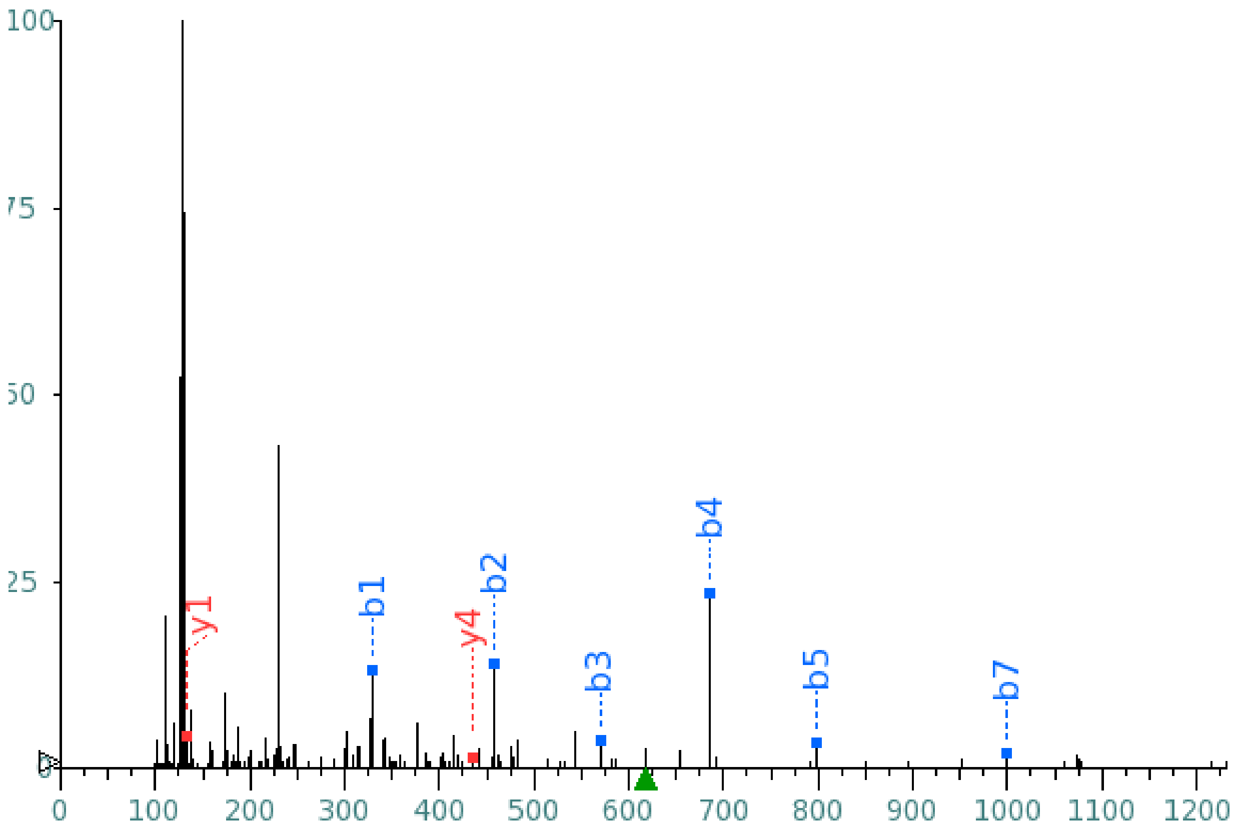

VQLDLAETD (+2)

Primary Reference: ESYT1_HUMAN
Search ID: 41761
Search Name: 20130328_ananiav_ERstress_TMT_TM_timecourse_trypsin_inj2_4mc_iaa
Scan#: 13503
Observed Mass: 616.8317 (-0.9 ppm)
PSM Score: 23.32

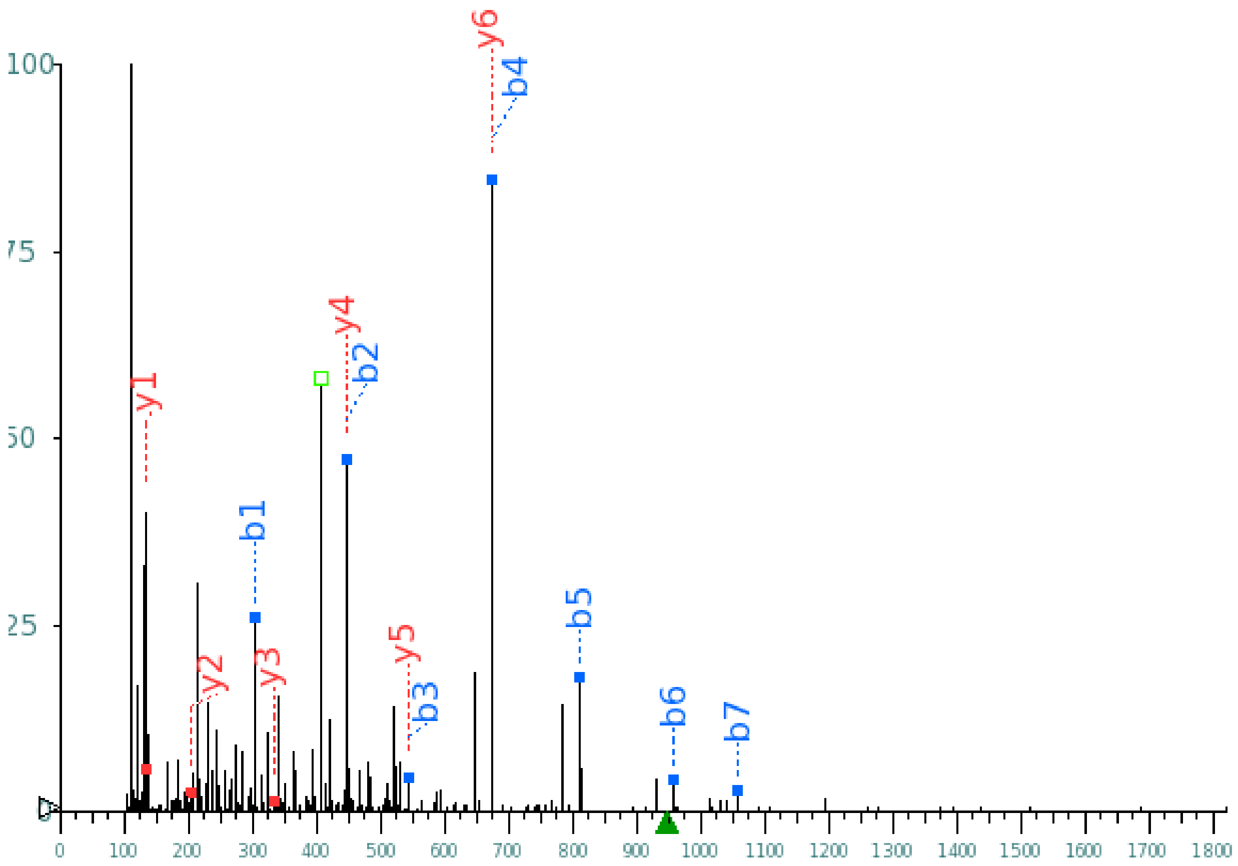

AFPEHFTYEPNEAD (+2)

Primary Reference: GYS1_HUMAN
Search ID: 41761
Search Name: 20130328_ananiav_ERstress_TMT_TM_timecourse_trypsin_inj2_4mc_iaa
Scan#: 13539
Observed Mass: 948.4374 (1.4 ppm)
PSM Score: 20.52

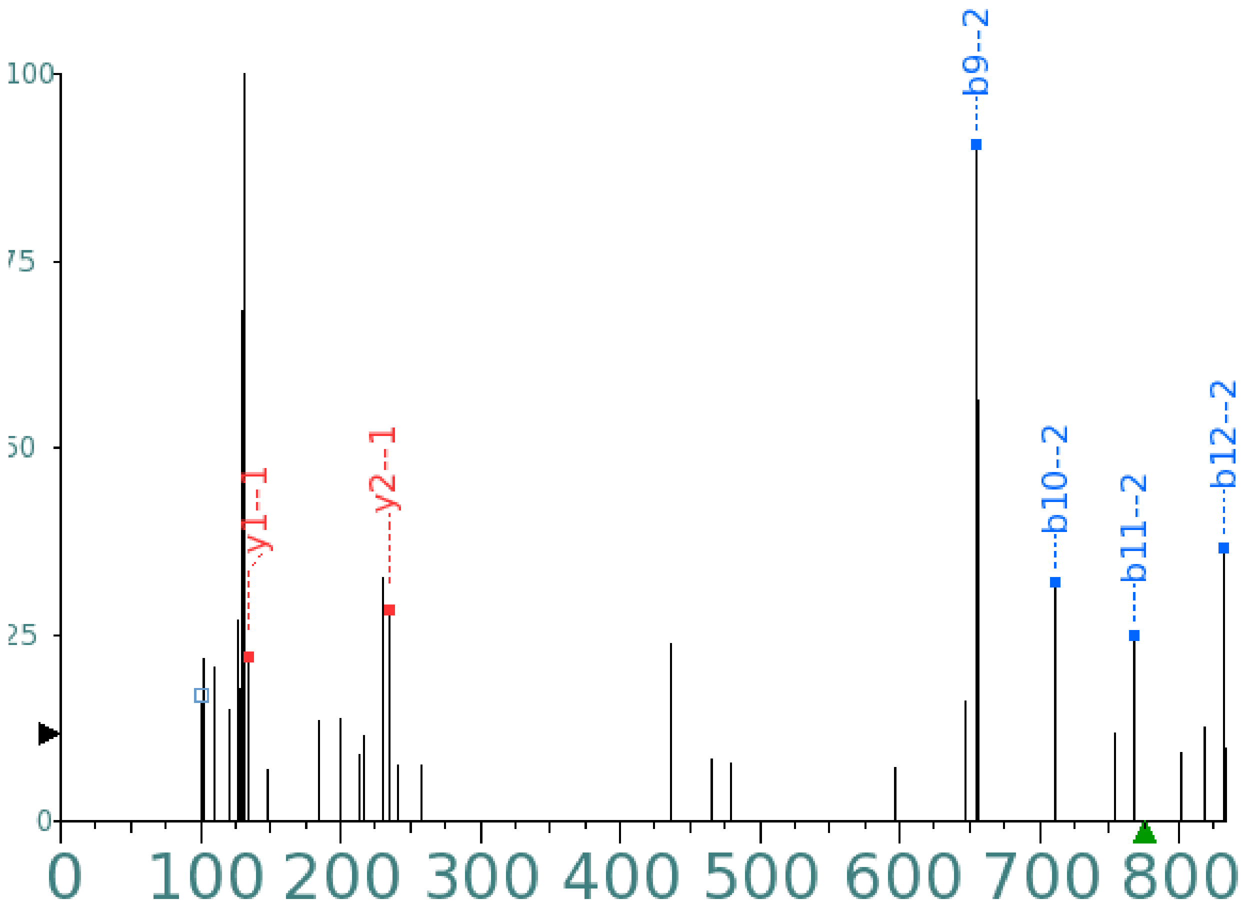

NENVDRHVDLLEVAQETD (+3)

Primary Reference: ATAD1_HUMAN
Search ID: 41761
Search Name: 20130328_ananiav_ERstress_TMT_TM_timecourse_trypsin_inj2_4mc_iaa
Scan#: 13551
Observed Mass: 775.7228 (0.8 ppm)
PSM Score: 25.17

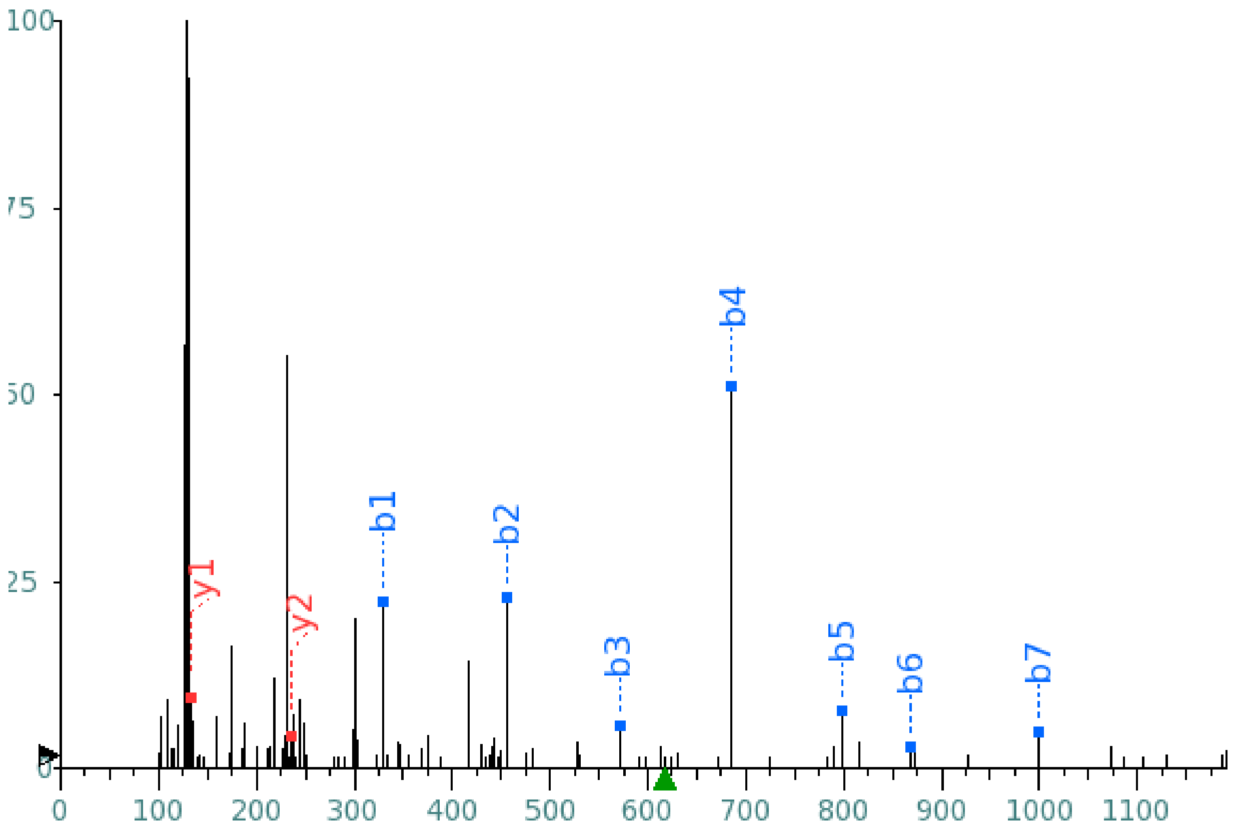

VQLDLAETD (+2)

Primary Reference: ESYT1_HUMAN
Search ID: 41761
Search Name: 20130328_ananiav_ERstress_TMT_TM_timecourse_trypsin_inj2_4mc_iaa
Scan#: 13590
Observed Mass: 616.8332 (1.6 ppm)
PSM Score: 34.69

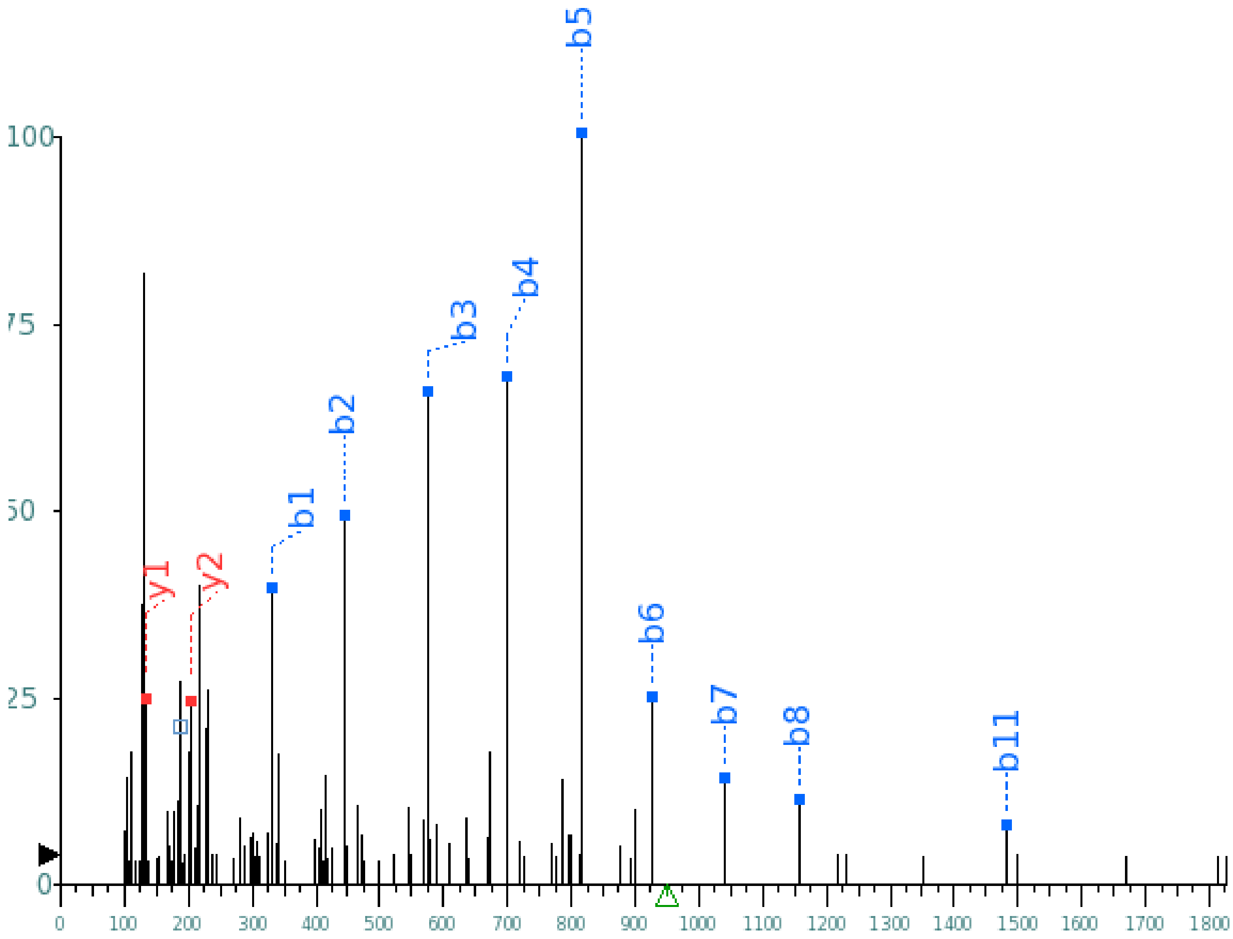

TIEQNINNLNVSEAD (+2)

Primary Reference: CND2_HUMAN
Search ID: 41761
Search Name: 20130328_ananiav_ERstress_TMT_TM_timecourse_trypsin_inj2_4mc_iaa
Scan#: 13601
Observed Mass: 951.9858 (2 ppm)
PSM Score: 58.64

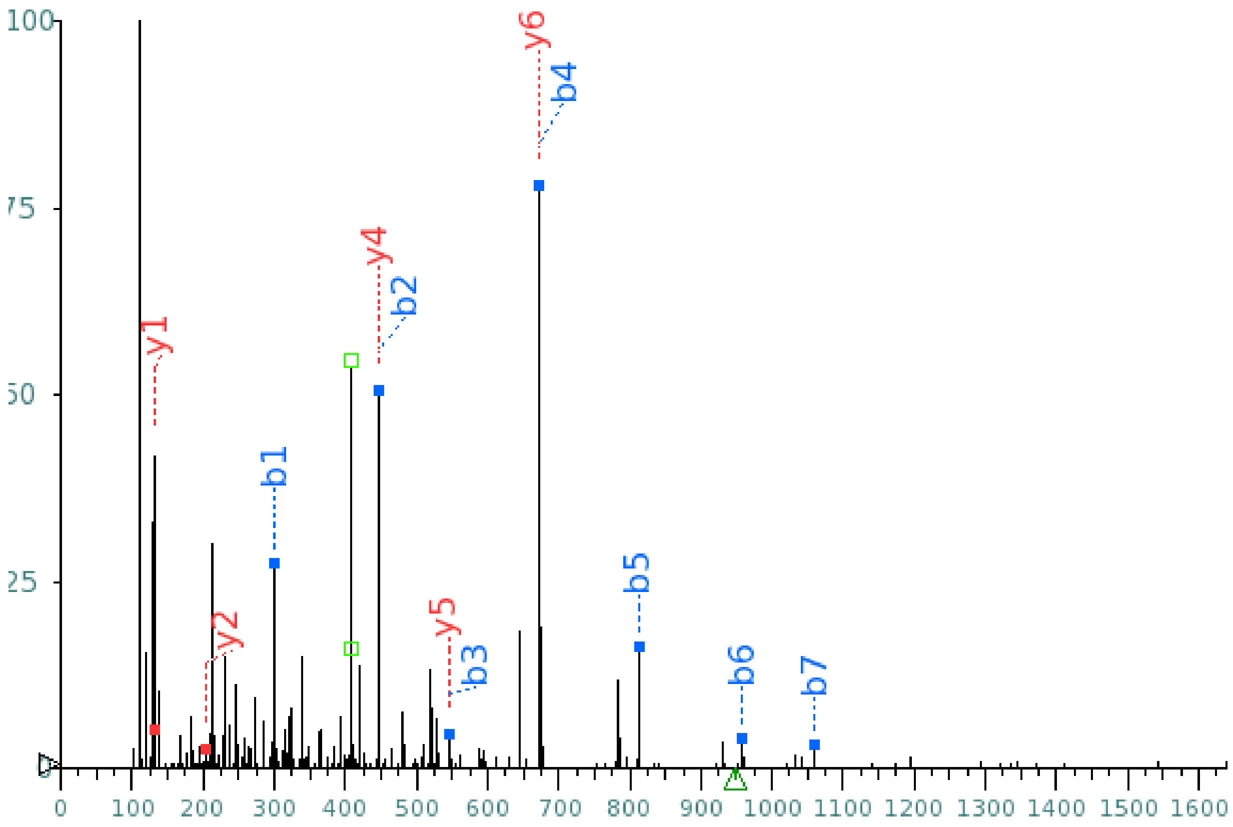

AFPEHFTYEPNEAD (+2)

Primary Reference: GYS1_HUMAN
Search ID: 41761
Search Name: 20130328_ananiav_ERstress_TMT_TM_timecourse_trypsin_inj2_4mc_iaa
Scan#: 13611
Observed Mass: 948.4383 (2.4 ppm)
PSM Score: 17.28

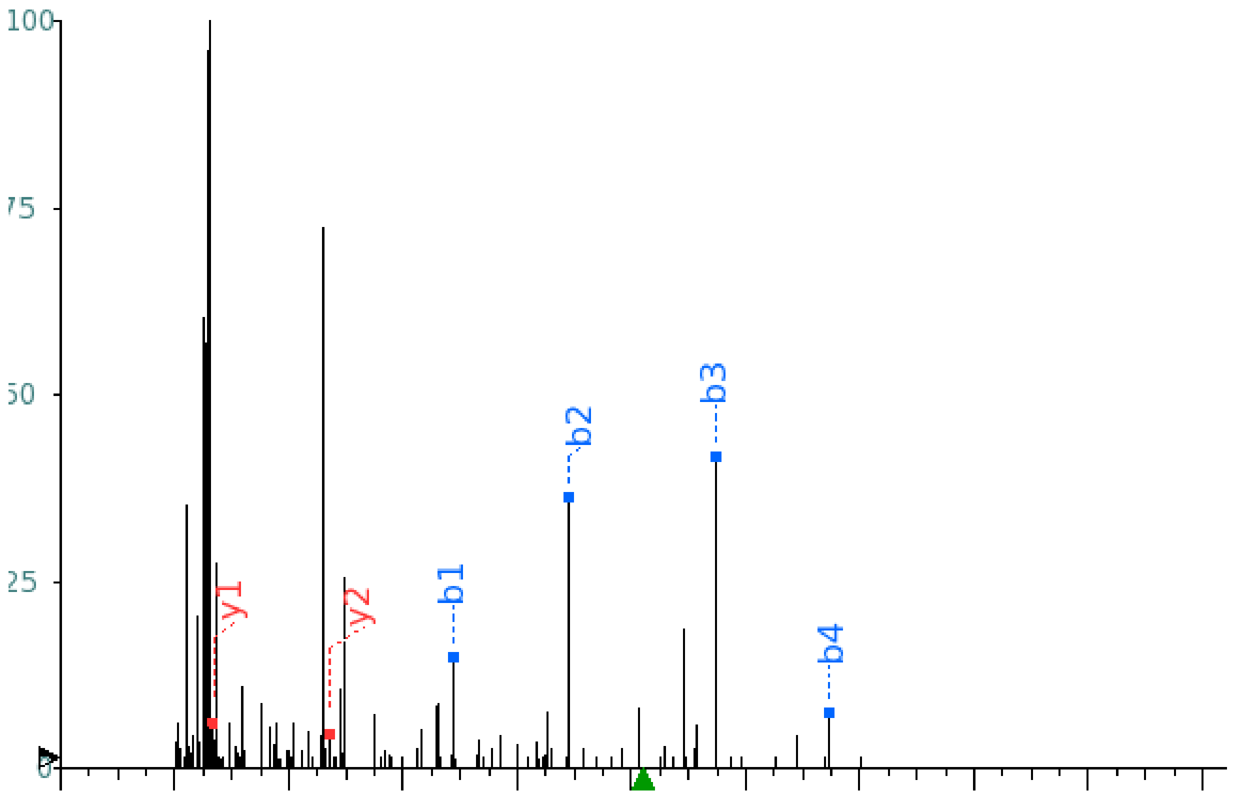

LTEVLTD (+2)

Primary Reference: GCN1L_HUMAN
Search ID: 41761
Search Name: 20130328_ananiav_ERstress_TMT_TM_timecourse_trypsin_inj2_4mc_iaa
Scan#: 13702
Observed Mass: 510.2957 (1.9 ppm)
PSM Score: 19.13

File name: 2015-02-04 04.01.53PM.pdf

Creation time: 2015-02-04 04:09PM

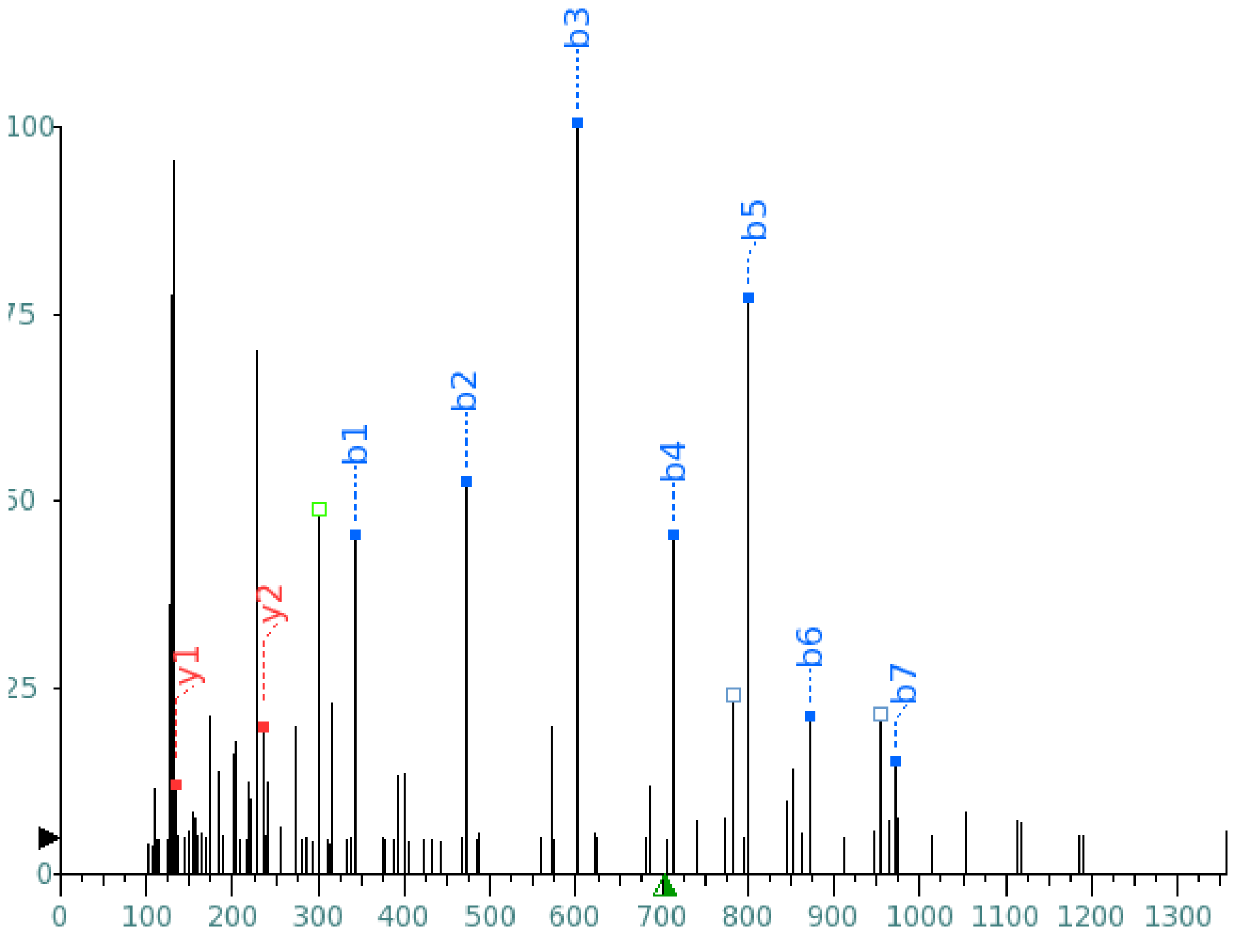

IQELSATVTTD (+2)

Primary Reference: TRIP6_HUMAN
Search ID: 41761
Search Name: 20130328_ananiav_ERstress_TMT_TM_timecourse_trypsin_inj2_4mc_iaa
Scan#: 13731
Observed Mass: 703.884 (2.2 ppm)
PSM Score: 37.38

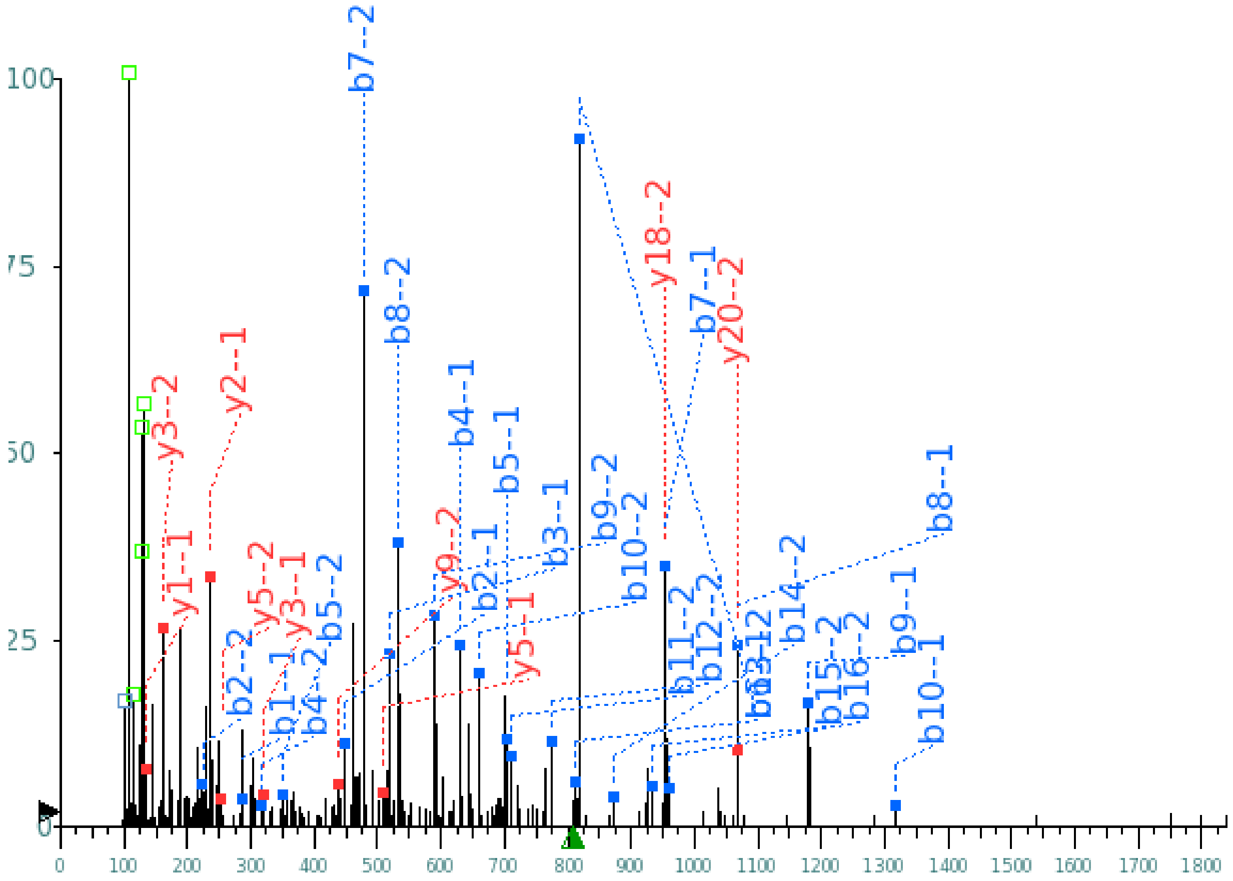

GC*ALADHLLHTQAQDGAISTD (+3)

Primary Reference: TPP2_HUMAN
Search ID: 41761
Search Name: 20130328_ananiav_ERstress_TMT_TM_timecourse_trypsin_inj2_4mc_iaa
Scan#: 13770
Observed Mass: 808.3997 (1 ppm)
PSM Score: 44.08

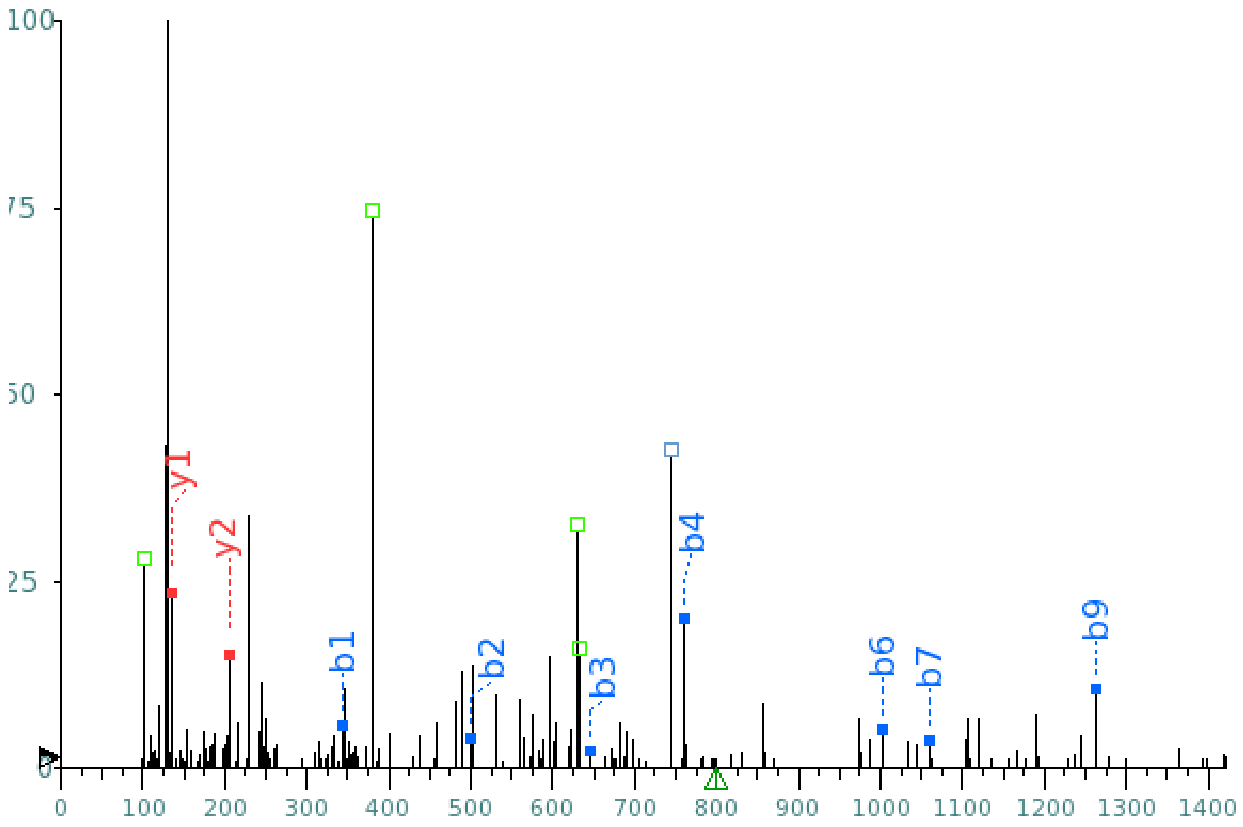

LRFDIEGSDEAD (+2)

Primary Reference: RB_HUMAN
Search ID: 41761
Search Name: 20130328_ananiav_ERstress_TMT_TM_timecourse_trypsin_inj2_4mc_iaa
Scan#: 13790
Observed Mass: 798.3917 (0.7 ppm)
PSM Score: 18.23

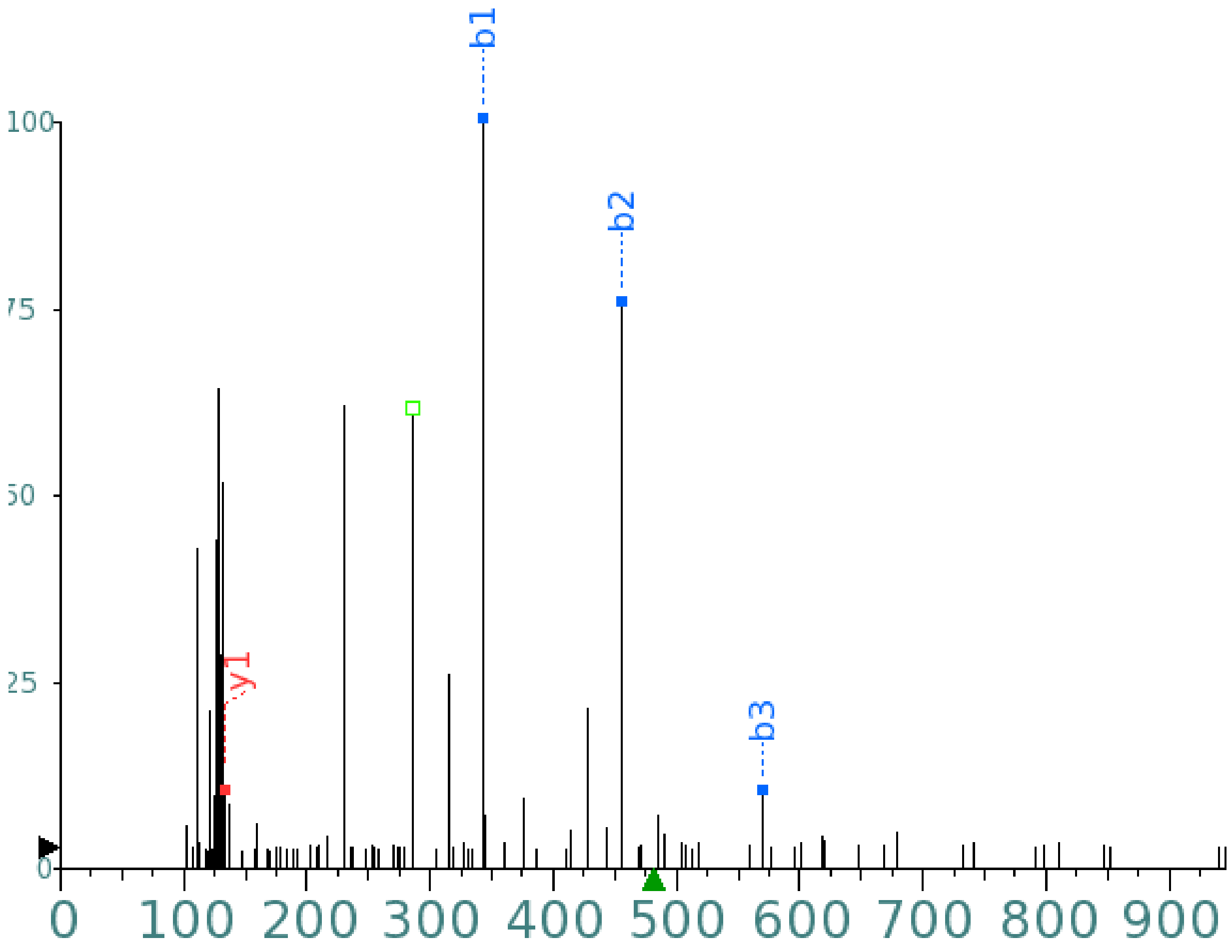

ILLC*TD (+2)

Primary Reference: DDX28_HUMAN
Search ID: 41761
Search Name: 20130328_ananiav_ERstress_TMT_TM_timecourse_trypsin_inj2_4mc_iaa
Scan#: 13808
Observed Mass: 482.2734 (1.3 ppm)
PSM Score: 10.33

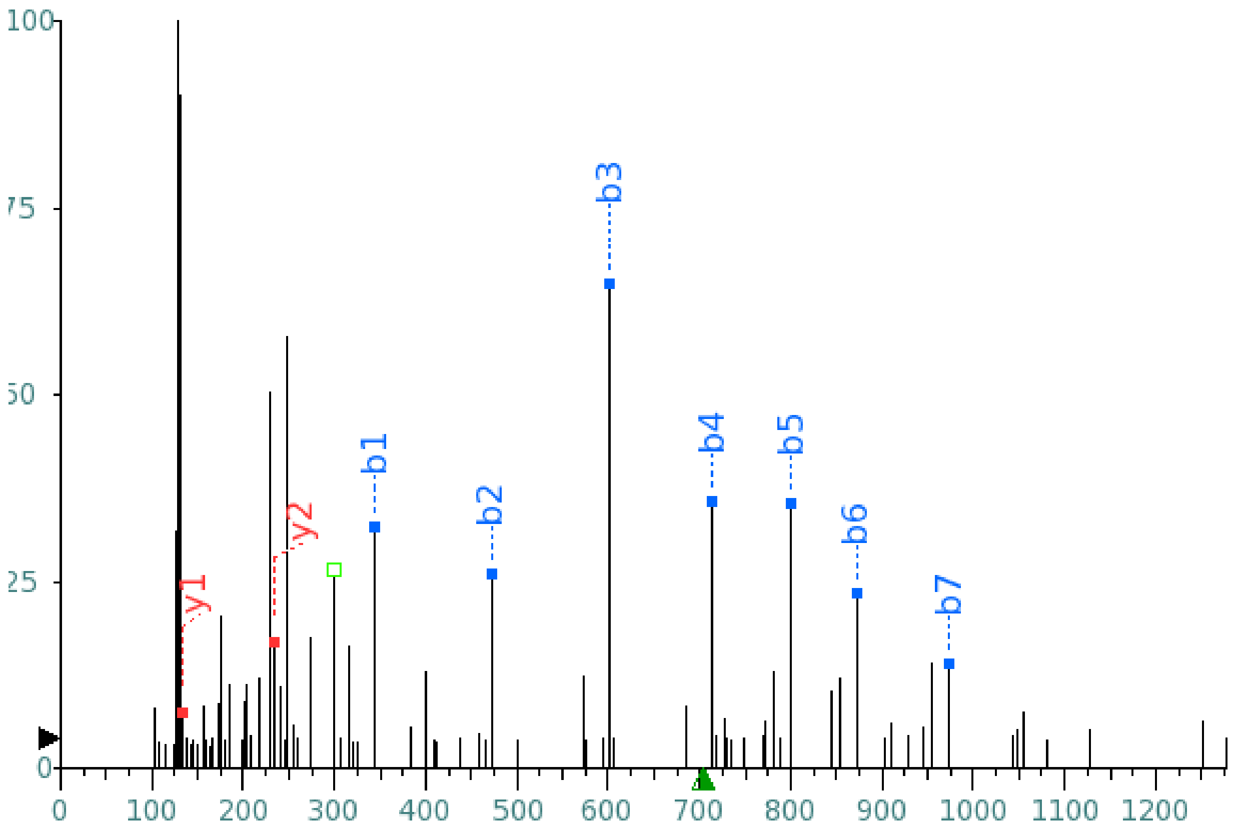

IQELSATVTTD (+2)

Primary Reference: TRIP6_HUMAN
Search ID: 41761
Search Name: 20130328_ananiav_ERstress_TMT_TM_timecourse_trypsin_inj2_4mc_iaa
Scan#: 13809
Observed Mass: 703.8834 (1.3 ppm)
PSM Score: 36

File name: 2015-02-04 04.01.53PM.pdf

Creation time: 2015-02-04 04:09PM

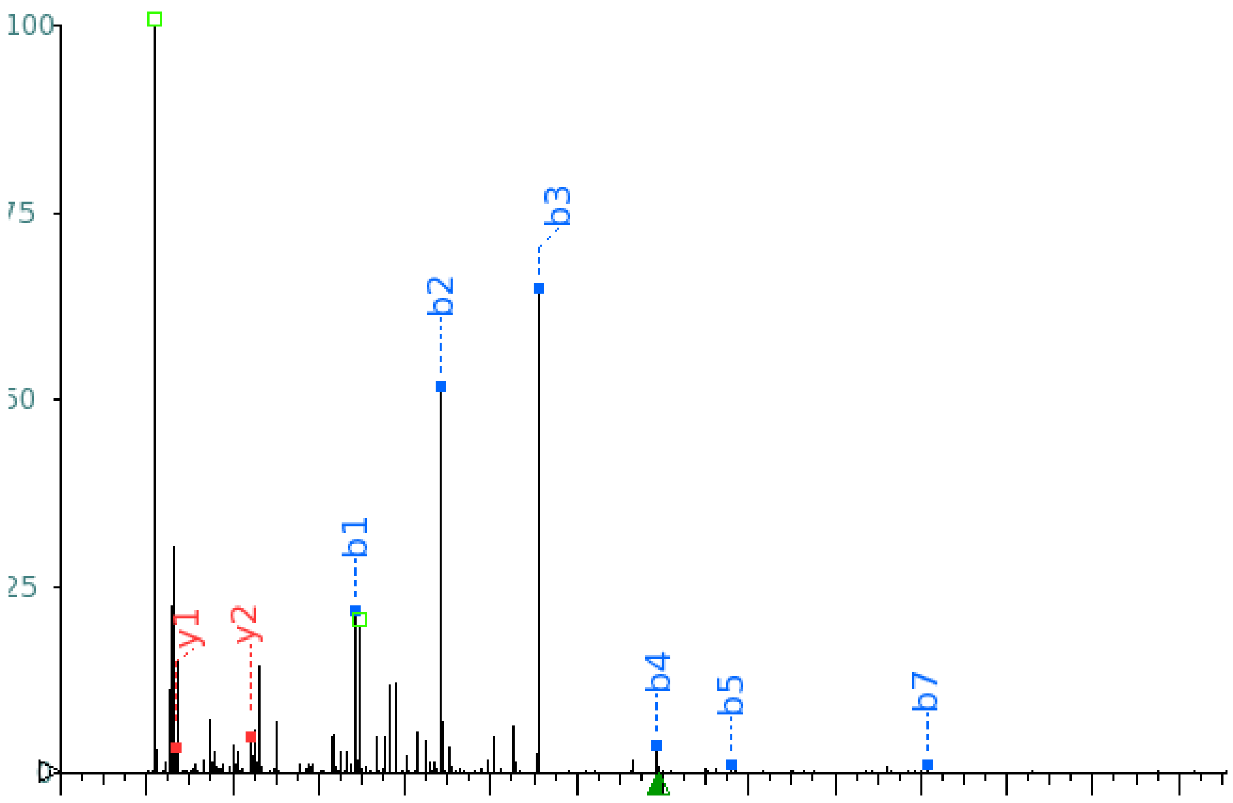

LVIHSLDYSD (+2)

Primary Reference: L1CAM_HUMAN
Search ID: 41761
Search Name: 20130328_ananiav_ERstress_TMT_TM_timecourse_trypsin_inj2_4mc_iaa
Scan#: 13858
Observed Mass: 695.8755 (1.6 ppm)
PSM Score: 31.21

File name: 2015-02-04 04.01.53PM.pdf

Creation time: 2015-02-04 04:09PM

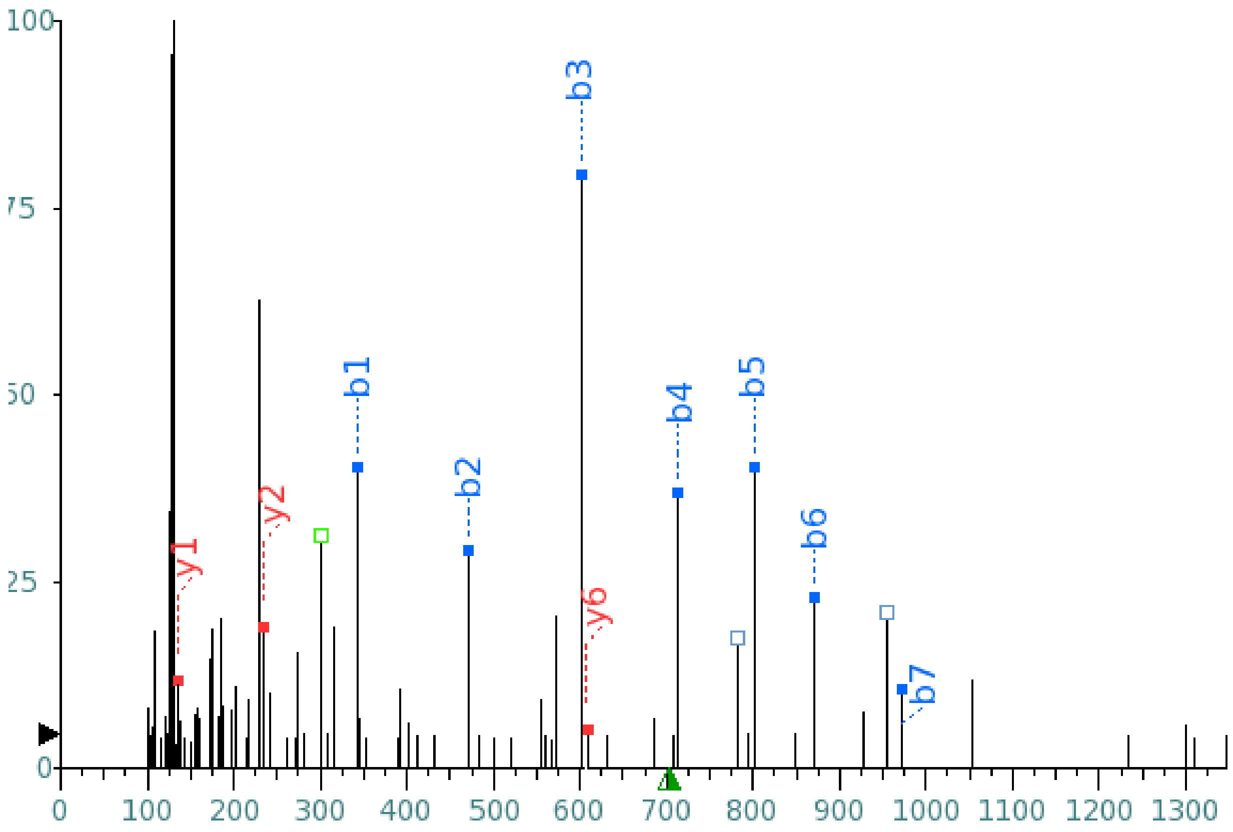

IQELSATVTTD (+2)

Primary Reference: TRIP6_HUMAN
Search ID: 41761
Search Name: 20130328_ananiav_ERstress_TMT_TM_timecourse_trypsin_inj2_4mc_iaa
Scan#: 13898
Observed Mass: 703.8839 (2.1 ppm)
PSM Score: 36.24

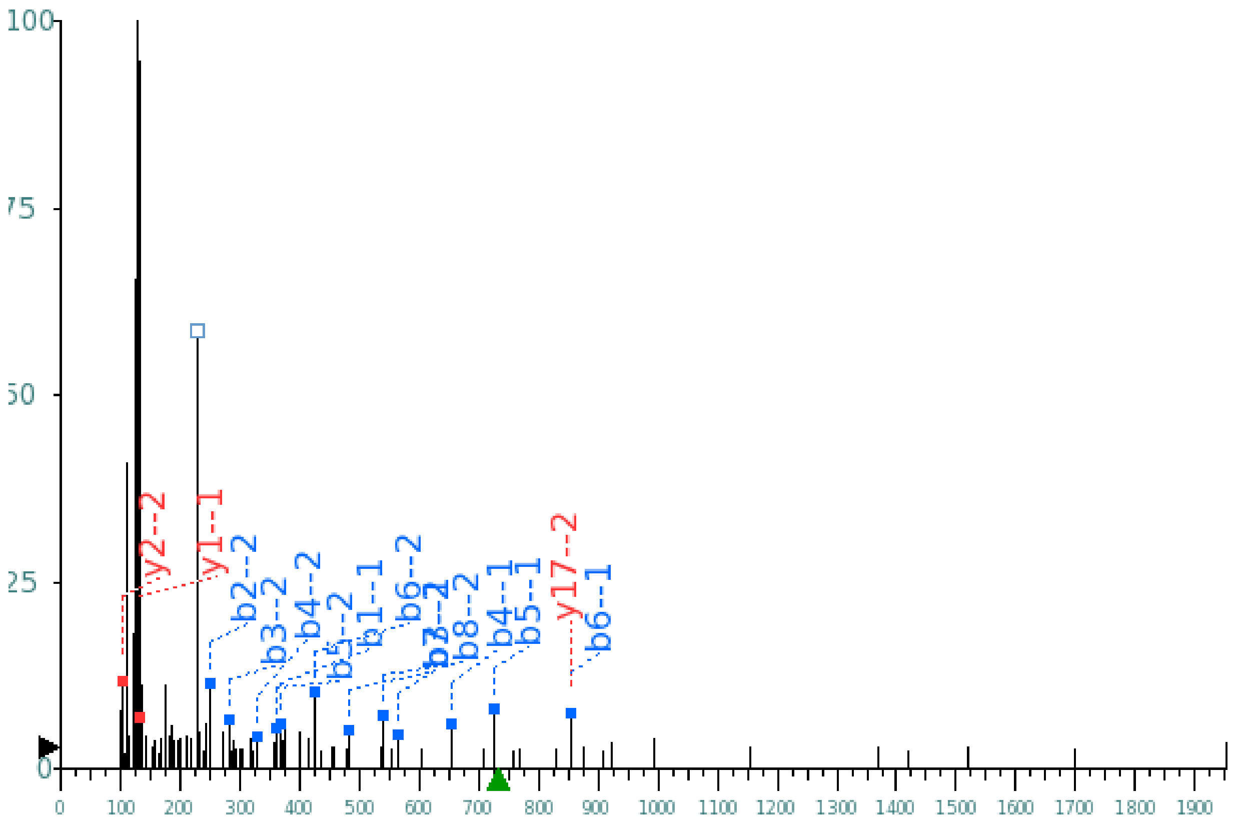

HEASAQIDEIVGETASEAD (+3)

Primary Reference: ASH1L_HUMAN
Search ID: 41761
Search Name: 20130328_ananiav_ERstress_TMT_TM_timecourse_trypsin_inj2_4mc_iaa
Scan#: 13923
Observed Mass: 734.352 (0.4 ppm)
PSM Score: 16.05

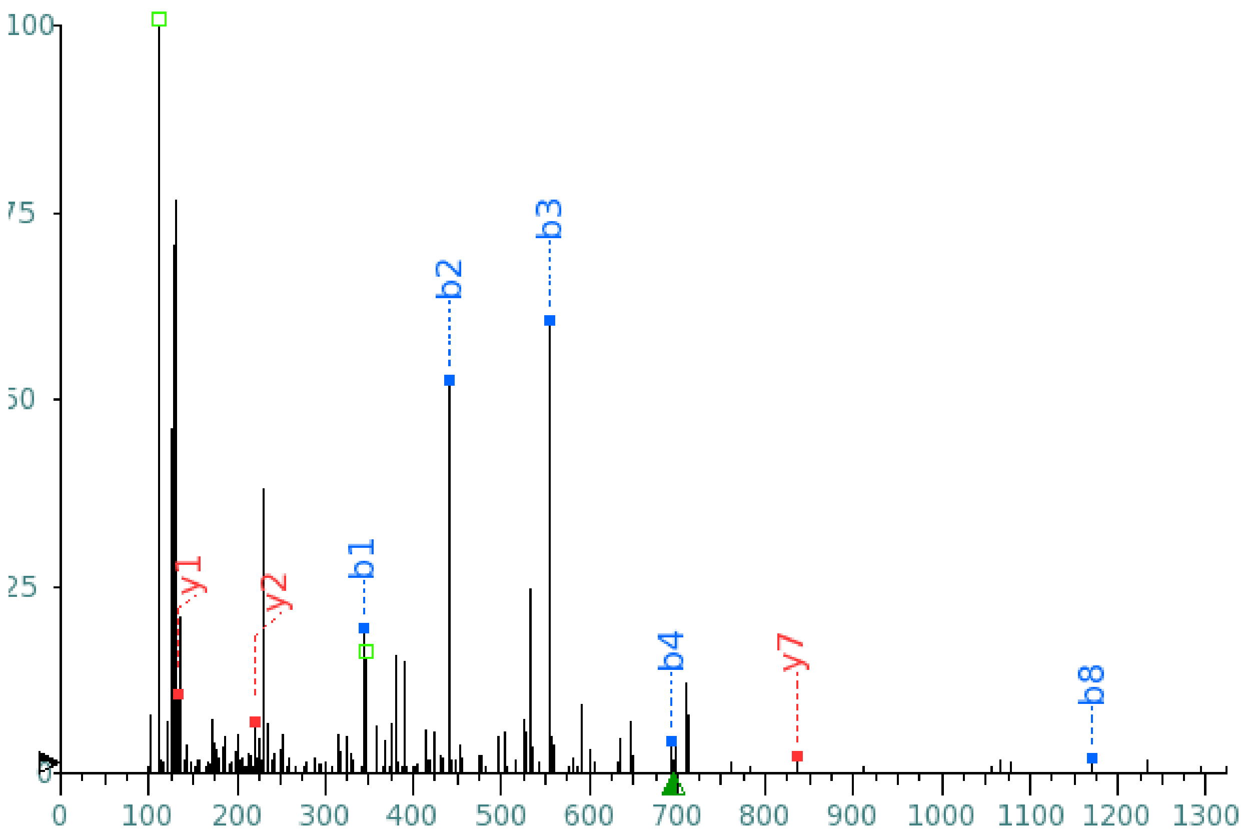

LVIHSLDYSD (+2)

Primary Reference: L1CAM_HUMAN
Search ID: 41761
Search Name: 20130328_ananiav_ERstress_TMT_TM_timecourse_trypsin_inj2_4mc_iaa
Scan#: 13937
Observed Mass: 695.8758 (1.9 ppm)
PSM Score: 8.83

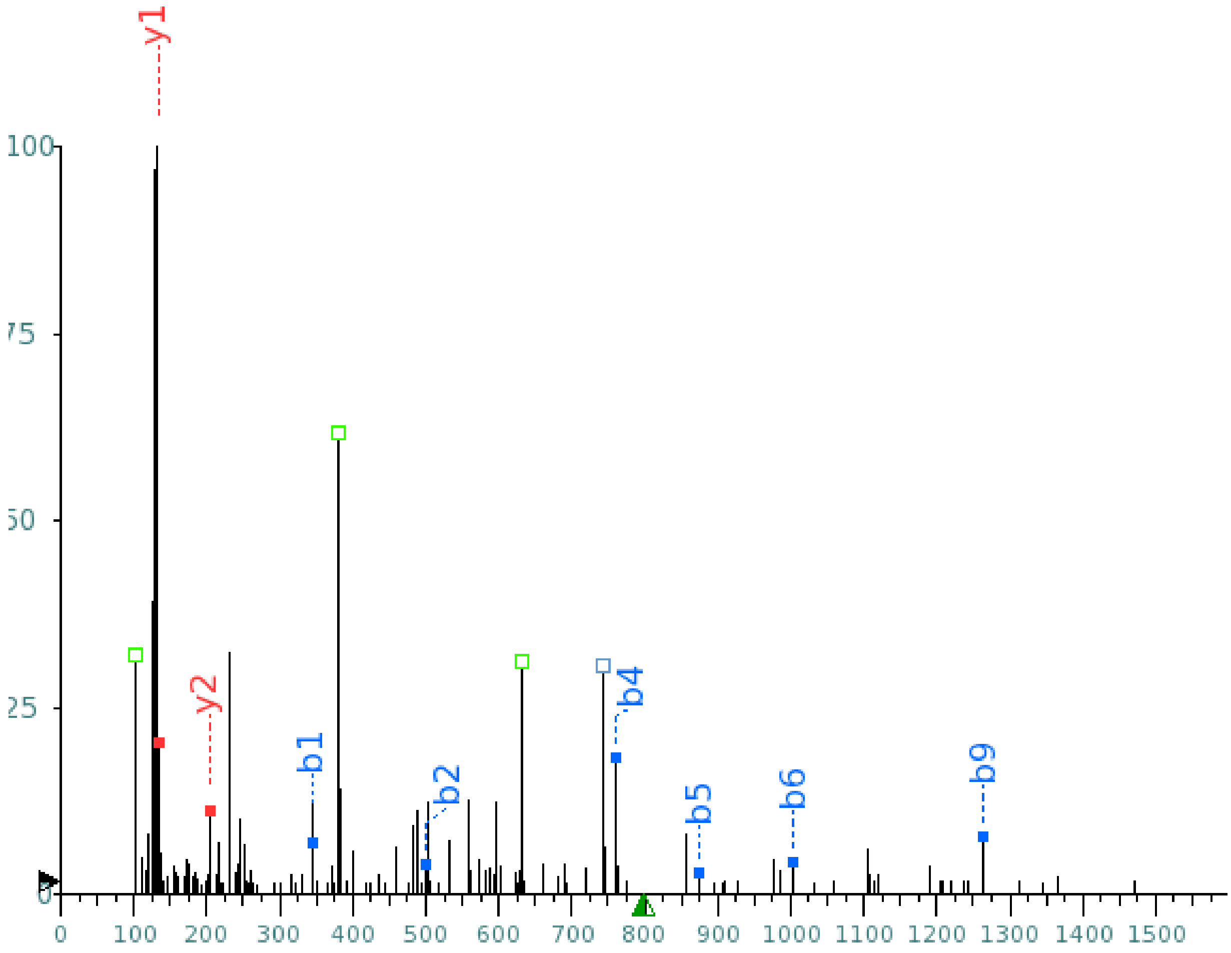

LRFDIEGSDEAD (+2)

Primary Reference: RB_HUMAN
Search ID: 41761
Search Name: 20130328_ananiav_ERstress_TMT_TM_timecourse_trypsin_inj2_4mc_iaa
Scan#: 13968
Observed Mass: 798.392 (1 ppm)
PSM Score: 17.09

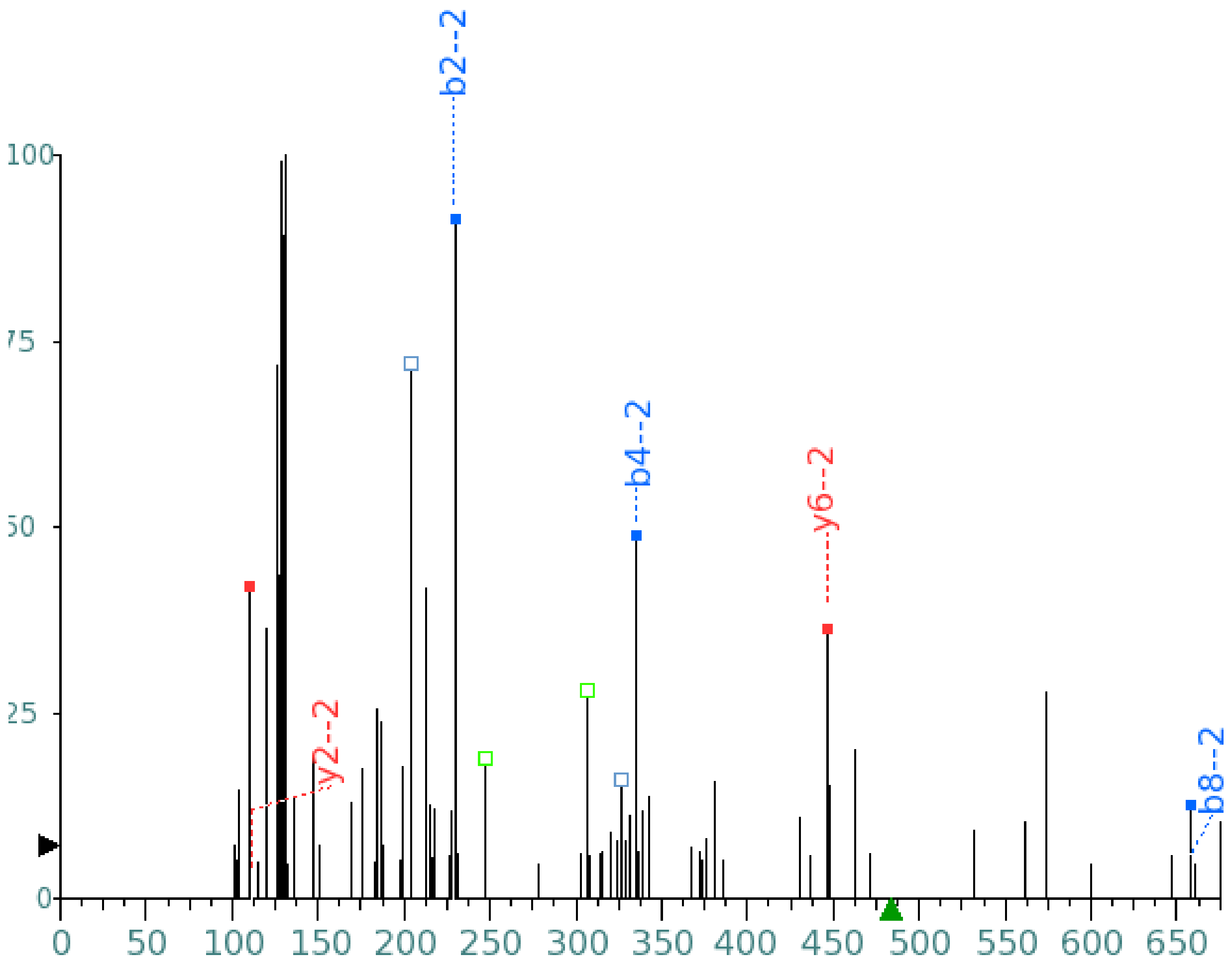

DLVLSLKSD (+3)

Primary Reference: ##AHNK2_HUMAN
Search ID: 41761
Search Name: 20130328_ananiav_ERstress_TMT_TM_timecourse_trypsin_inj2_4mc_iaa
Scan#: 13969
Observed Mass: 483.2988 (3.3 ppm)
PSM Score: 9.06

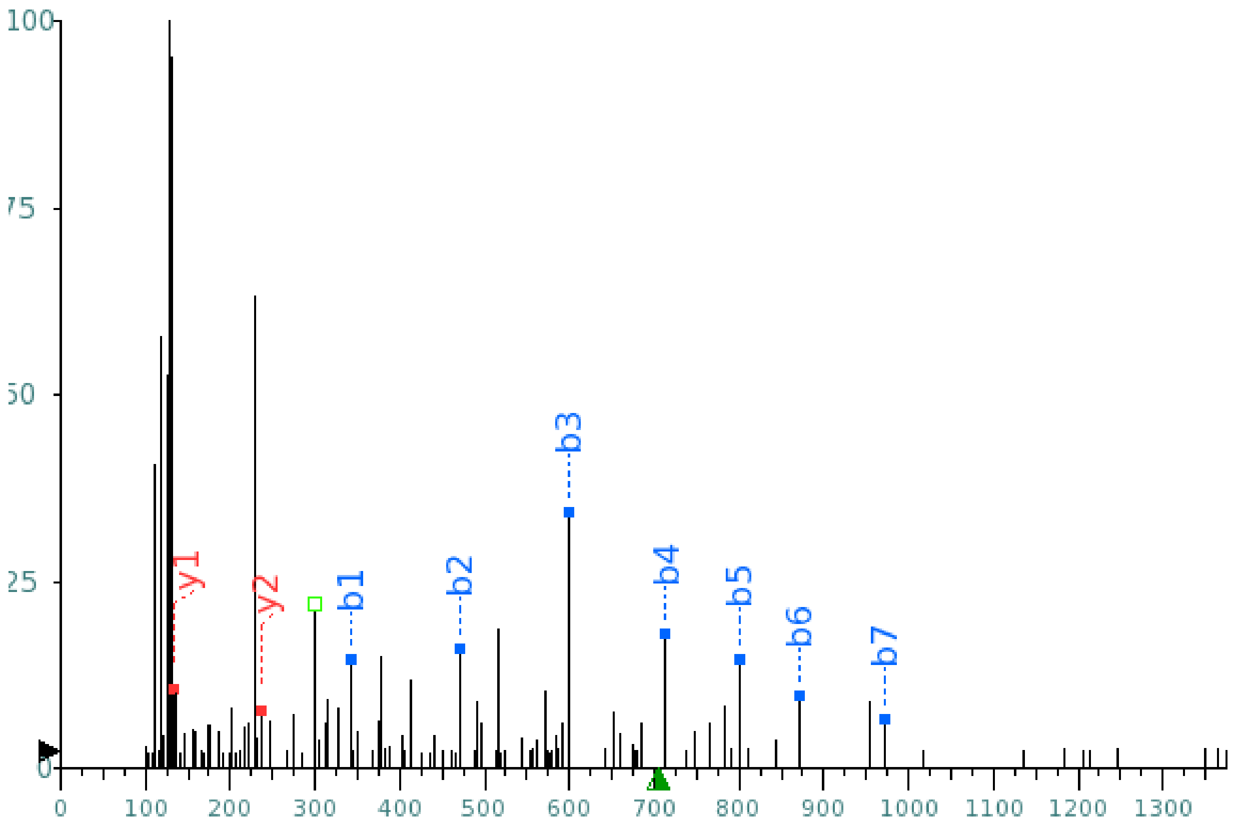

IQELSATVTTD (+2)

Primary Reference: TRIP6_HUMAN
Search ID: 41761
Search Name: 20130328_ananiav_ERstress_TMT_TM_timecourse_trypsin_inj2_4mc_iaa
Scan#: 13973
Observed Mass: 703.8853 (4 ppm)
PSM Score: 23.14

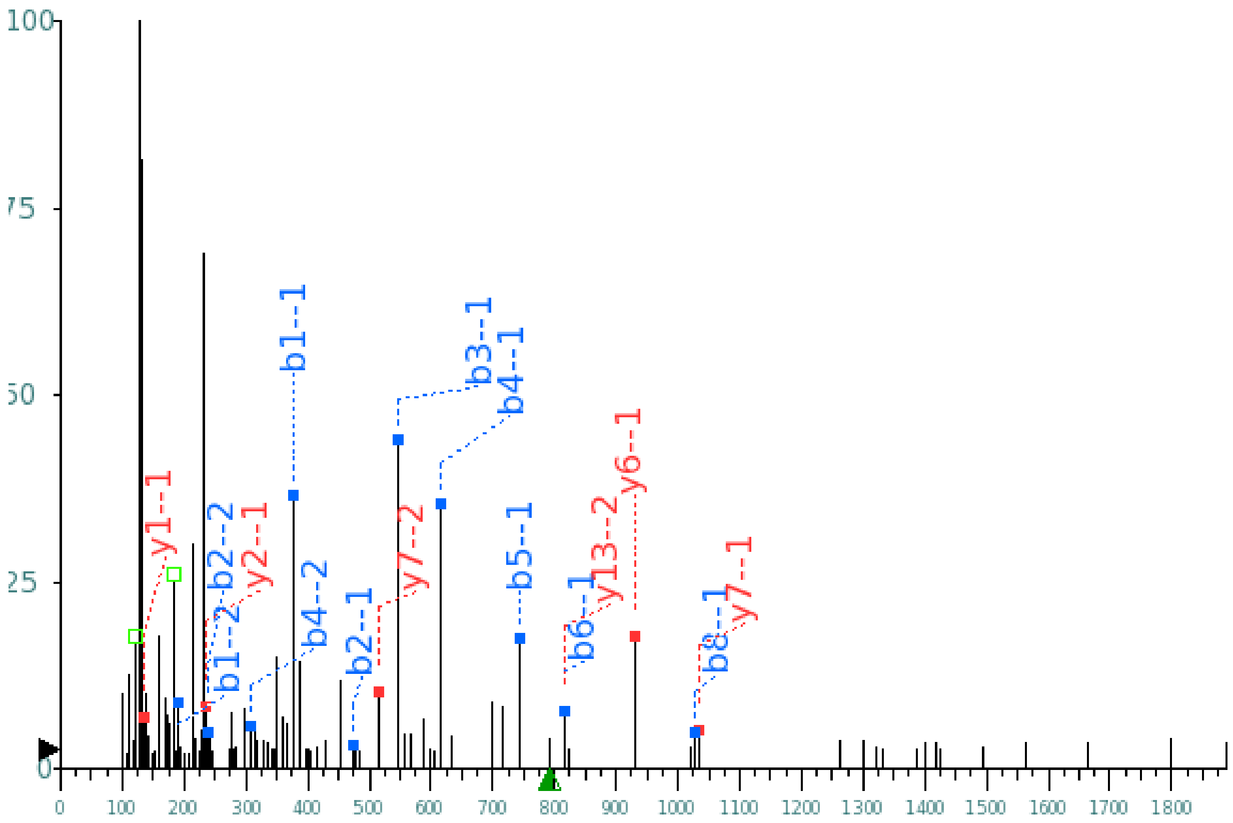

FPAAQAPDPSETPKIETD (+3)

Primary Reference: ABLM1_HUMAN
Search ID: 41761
Search Name: 20130328_ananiav_ERstress_TMT_TM_timecourse_trypsin_inj2_4mc_iaa
Scan#: 13977
Observed Mass: 791.4206 (3.6 ppm)
PSM Score: 17

File name: 2015-02-04 04.01.53PM.pdf

Creation time: 2015-02-04 04:09PM

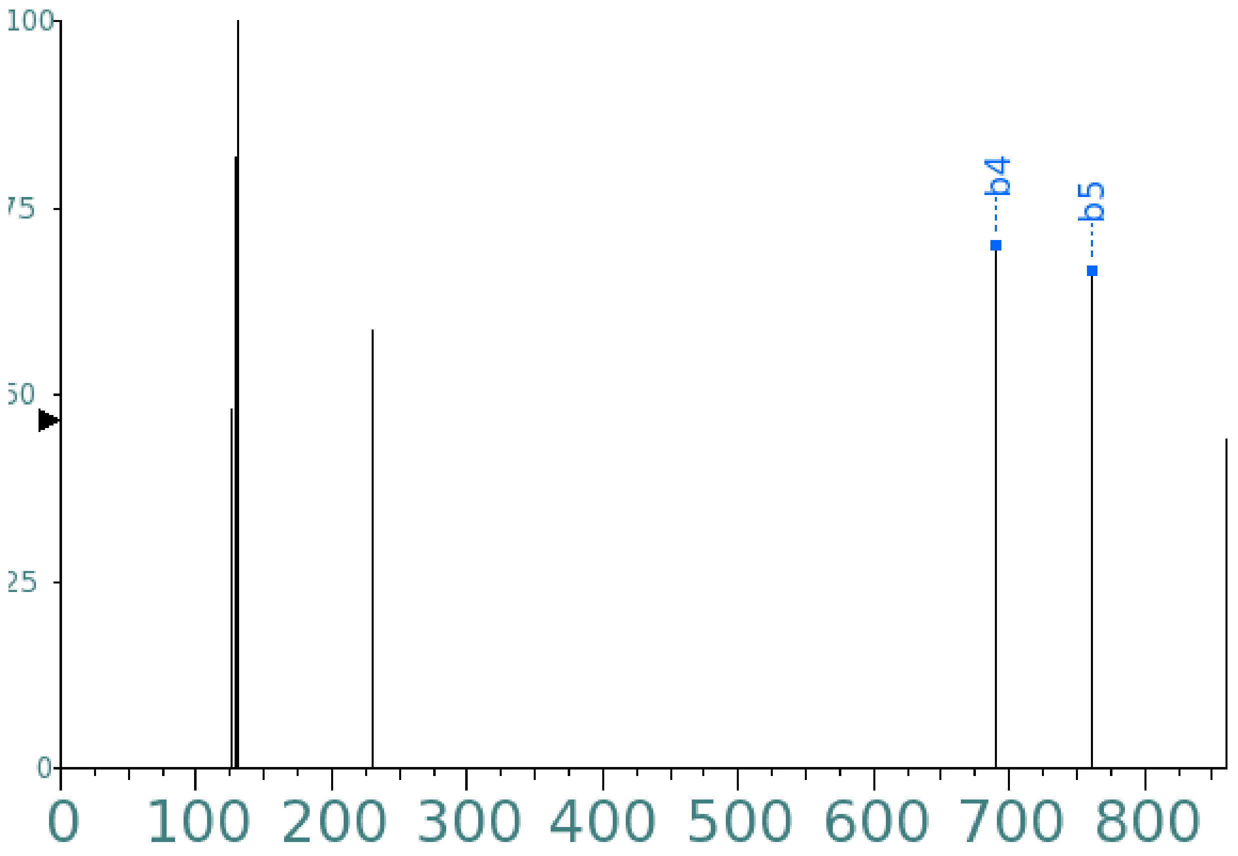

TDEDAVLMDGMTESD (+2)

Primary Reference: FXR1_HUMAN
Search ID: 41761
Search Name: 20130328_ananiav_ERstress_TMT_TM_timecourse_trypsin_inj2_4mc_iaa
Scan#: 14002
Observed Mass: 929.4016 (1.5 ppm)
PSM Score: 13.76

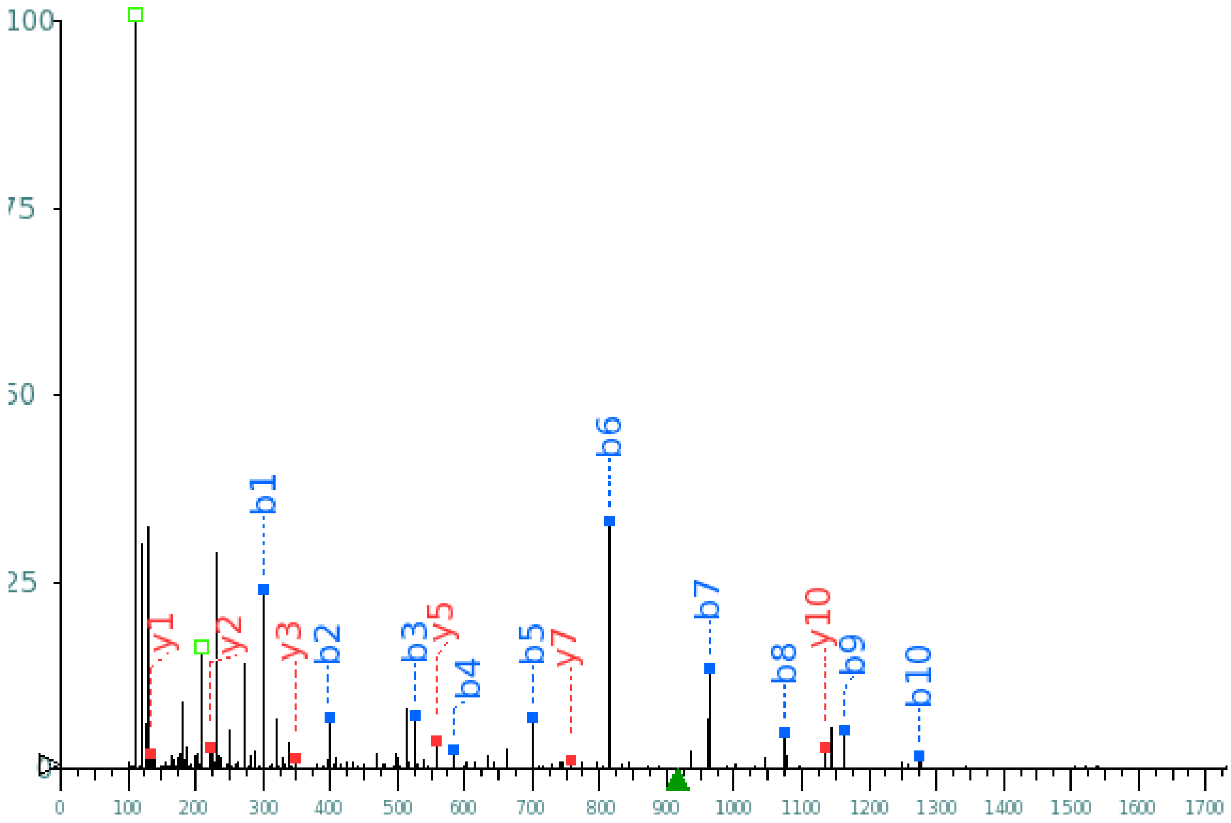

APEGDDFLSLHAESD (+2)

Primary Reference: SFR19_HUMAN
Search ID: 41761
Search Name: 20130328_ananiav_ERstress_TMT_TM_timecourse_trypsin_inj2_4mc_iaa
Scan#: 14004
Observed Mass: 916.4342 (3.5 ppm)
PSM Score: 28.62

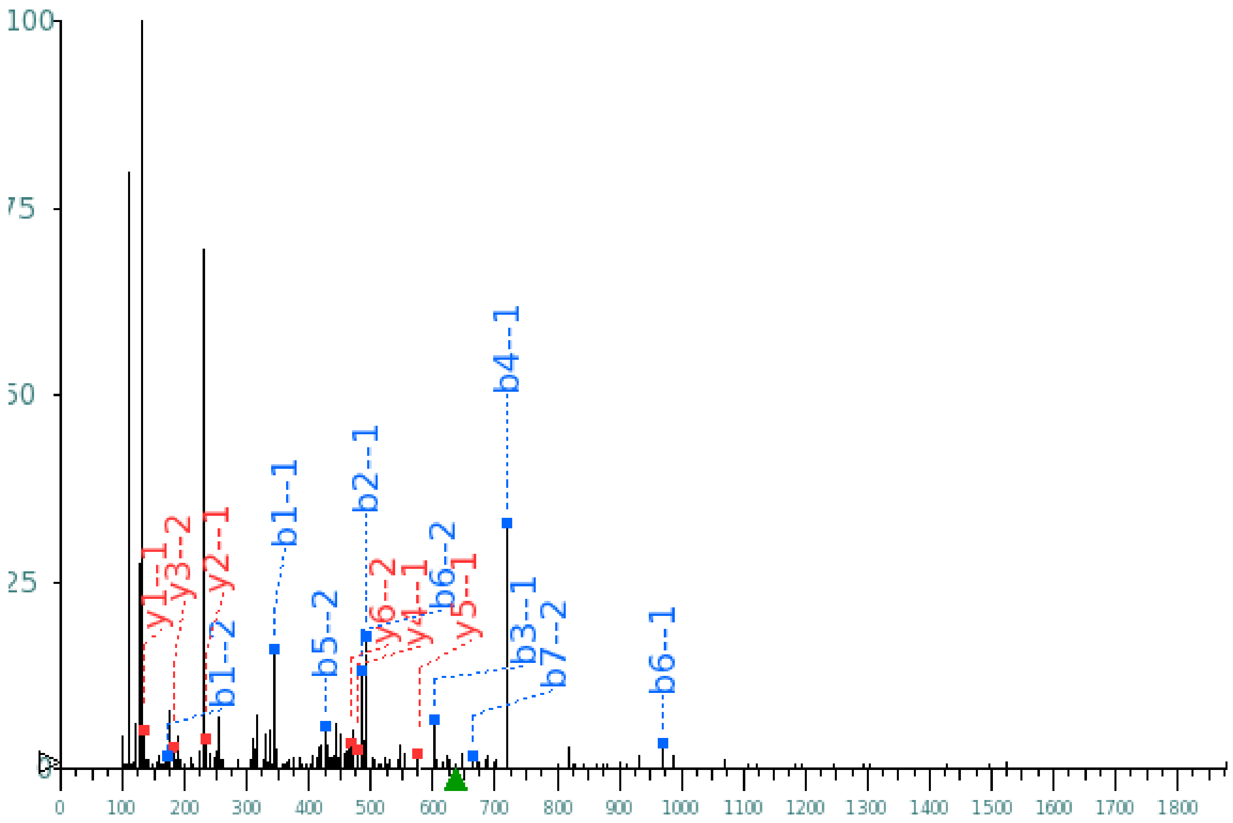

LFLDHDKTLQTD (+3)

Primary Reference: RB_HUMAN
Search ID: 41761
Search Name: 20130328_ananiav_ERstress_TMT_TM_timecourse_trypsin_inj2_4mc_iaa
Scan#: 14042
Observed Mass: 635.3582 (3.7 ppm)
PSM Score: 21.04

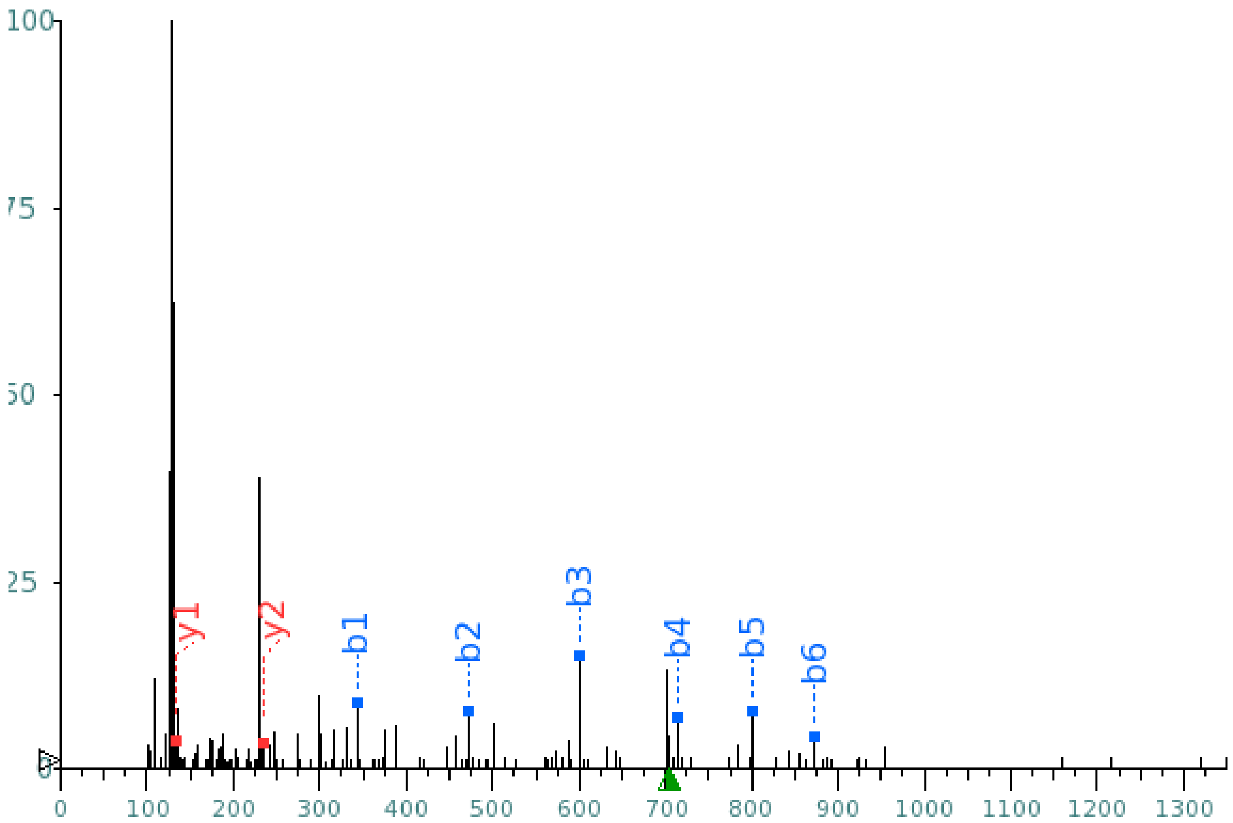

IQELSATVTTD (+2)

Primary Reference: TRIP6_HUMAN
Search ID: 41761
Search Name: 20130328_ananiav_ERstress_TMT_TM_timecourse_trypsin_inj2_4mc_iaa
Scan#: 14052
Observed Mass: 703.8844 (2.8 ppm)
PSM Score: 19.41

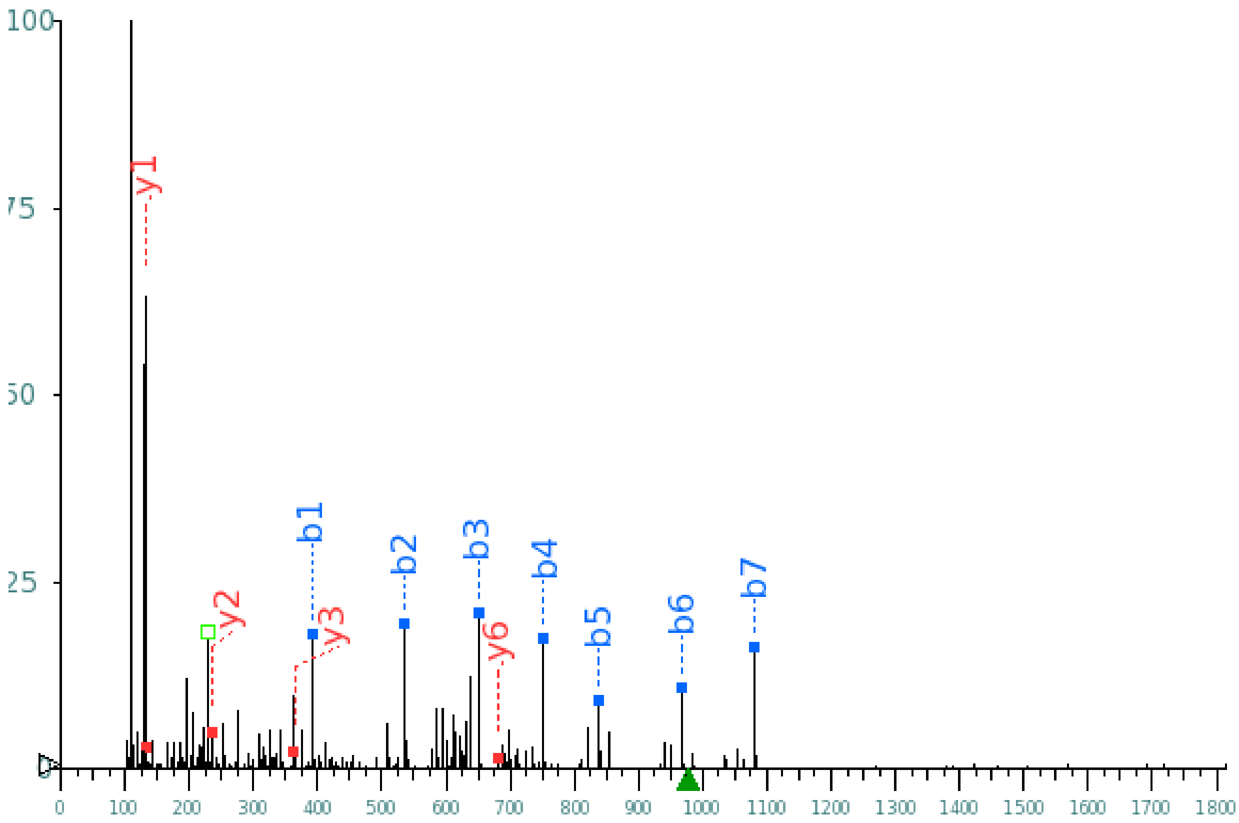

C*FLTSQNHGFAVETD (+2)

Primary Reference: PYR1_HUMAN
Search ID: 41761
Search Name: 20130328_ananiav_ERstress_TMT_TM_timecourse_trypsin_inj2_4mc_iaa
Scan#: 14075
Observed Mass: 977.9635 (1.6 ppm)
PSM Score: 29.47

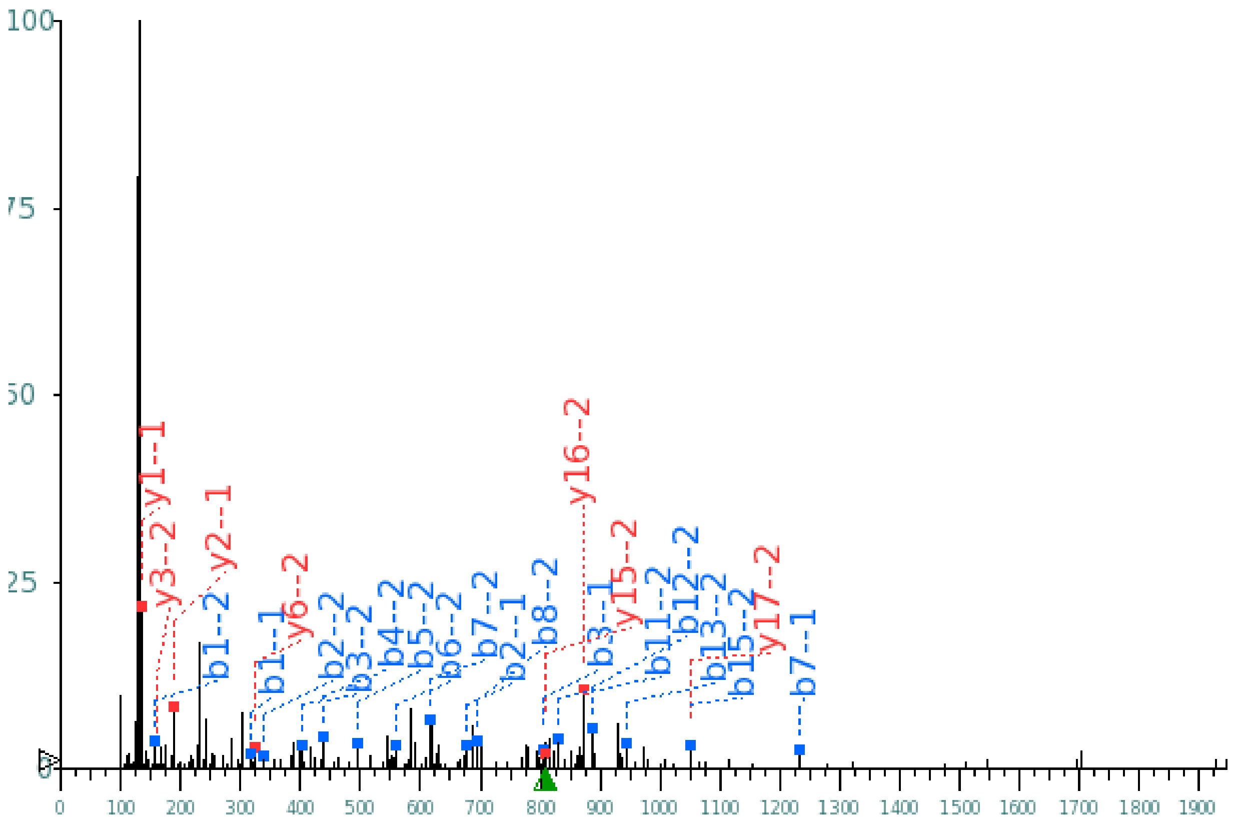

SKMALEDRPSSLLVDQGD (+3)

Primary Reference: TRIO_HUMAN
Search ID: 41761
Search Name: 20130328_ananiav_ERstress_TMT_TM_timecourse_trypsin_inj2_4mc_iaa
Scan#: 14100
Observed Mass: 807.1029 (1.6 ppm)
PSM Score: 14.91

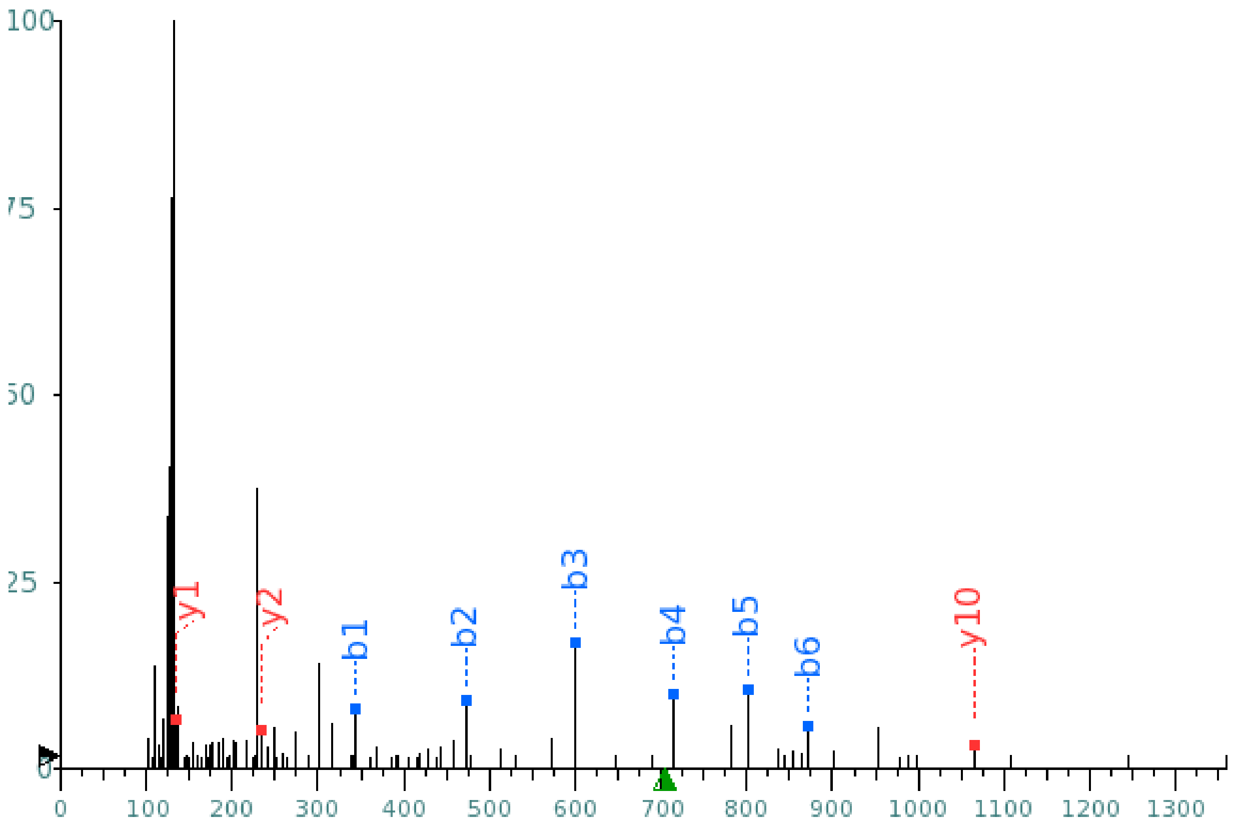

IQELSATVTTD (+2)

Primary Reference: TRIP6_HUMAN
Search ID: 41761
Search Name: 20130328_ananiav_ERstress_TMT_TM_timecourse_trypsin_inj2_4mc_iaa
Scan#: 14128
Observed Mass: 703.8841 (2.3 ppm)
PSM Score: 18.56

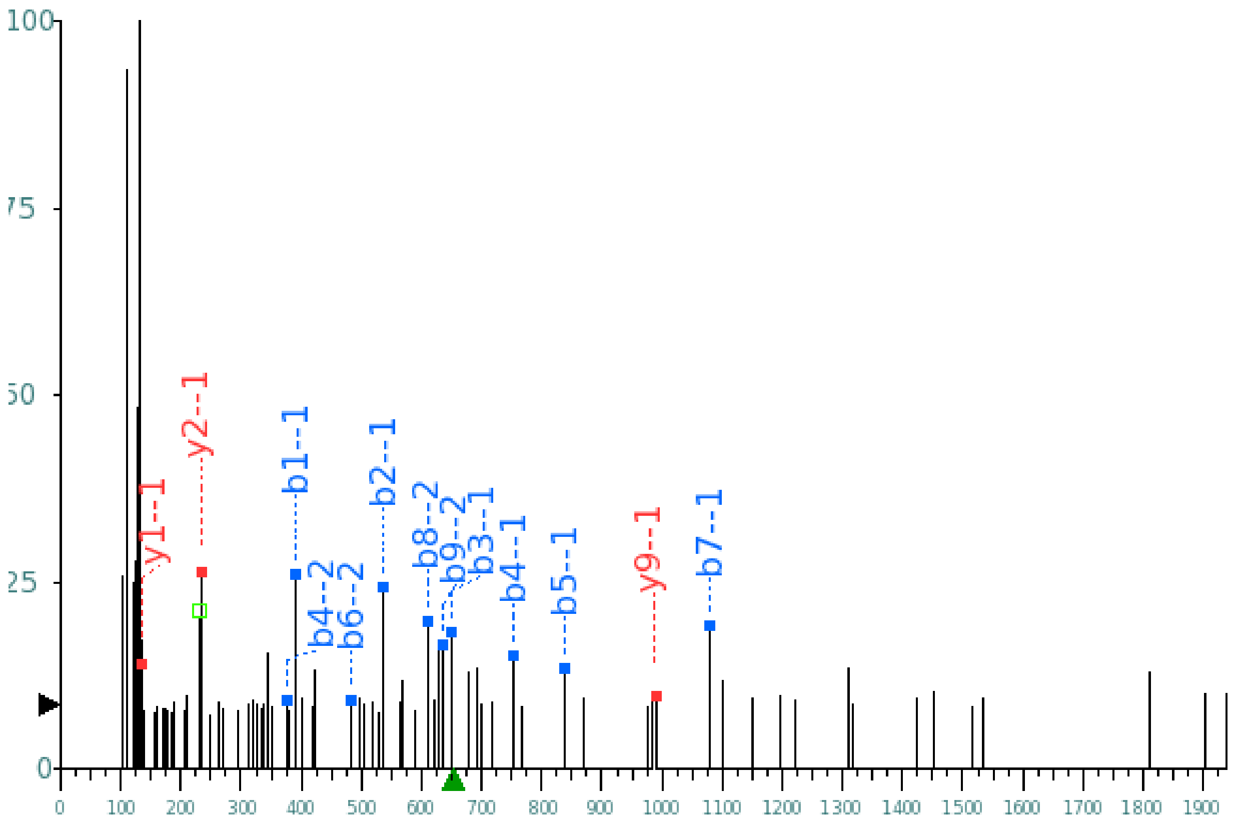

C*FLTSQNHGFAVETD (+3)

Primary Reference: PYR1_HUMAN
Search ID: 41761
Search Name: 20130328_ananiav_ERstress_TMT_TM_timecourse_trypsin_inj2_4mc_iaa
Scan#: 14138
Observed Mass: 652.3119 (2.2 ppm)
PSM Score: 9.29

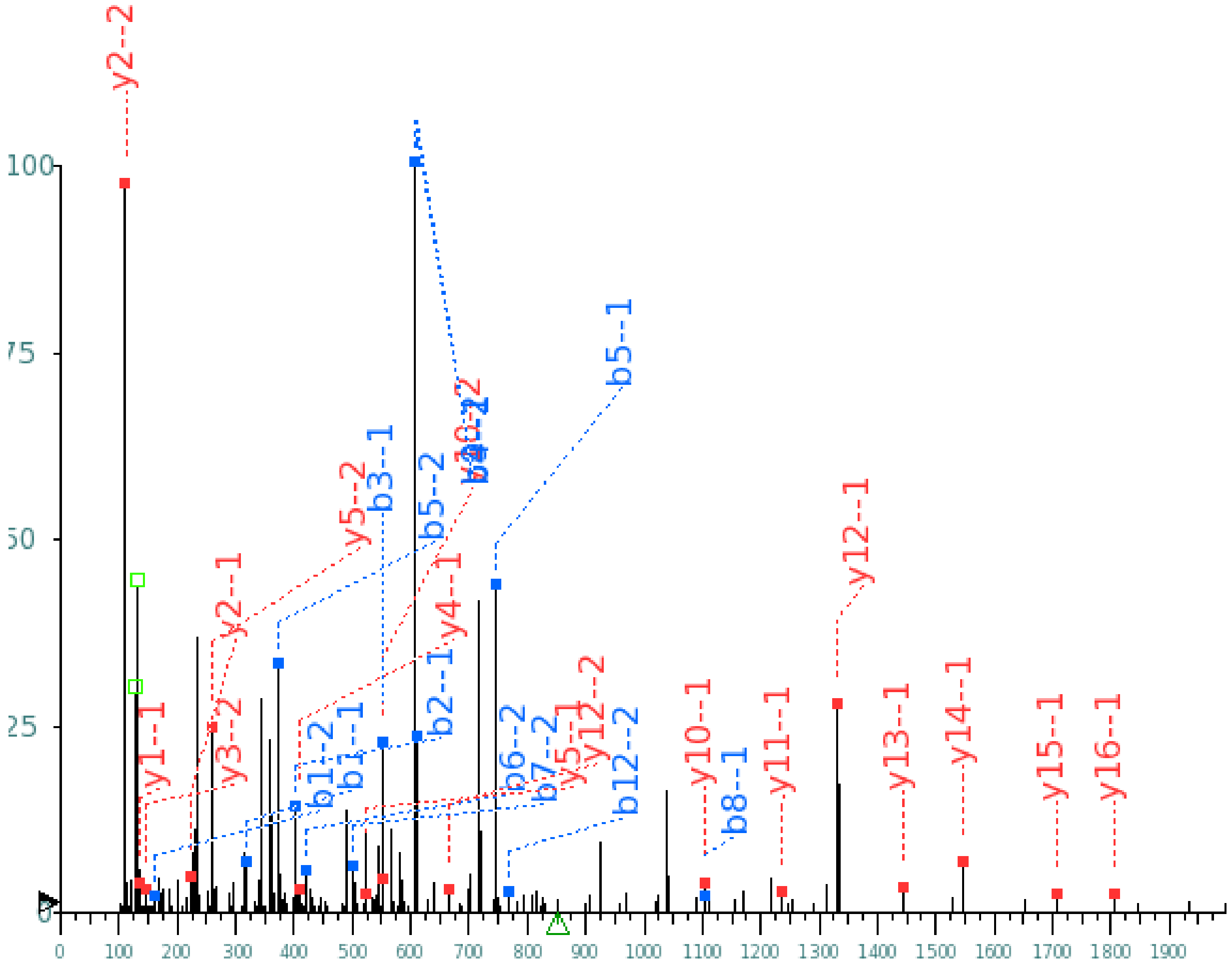

SSFGHPYTIPESLRVQDDASD (+3)

Primary Reference: TTC28_HUMAN
Search ID: 41761
Search Name: 20130328_ananiav_ERstress_TMT_TM_timecourse_trypsin_inj2_4mc_iaa
Scan#: 14140
Observed Mass: 850.7519 (4 ppm)
PSM Score: 25.69

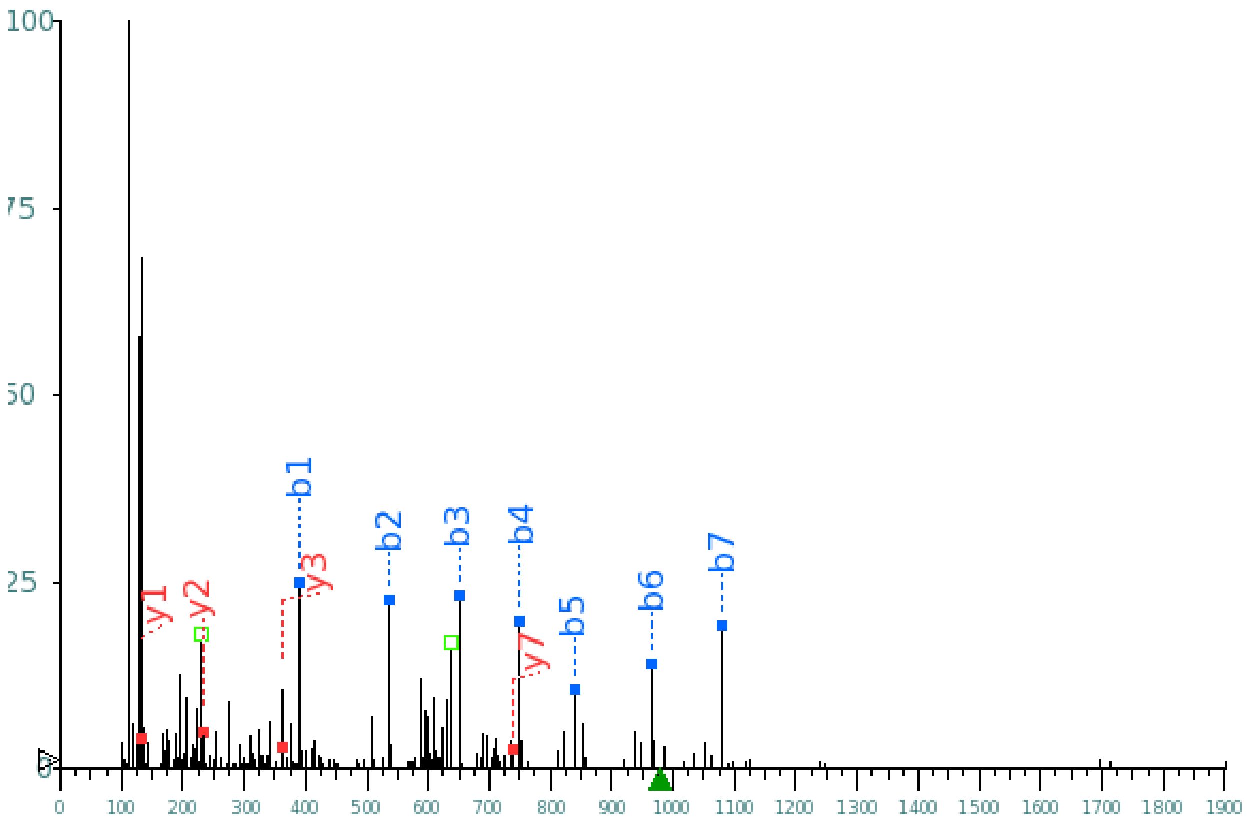

C*FLTSQNHGFAVETD (+2)

Primary Reference: PYR1_HUMAN
Search ID: 41761
Search Name: 20130328_ananiav_ERstress_TMT_TM_timecourse_trypsin_inj2_4mc_iaa
Scan#: 14151
Observed Mass: 977.9641 (2.1 ppm)
PSM Score: 33.35

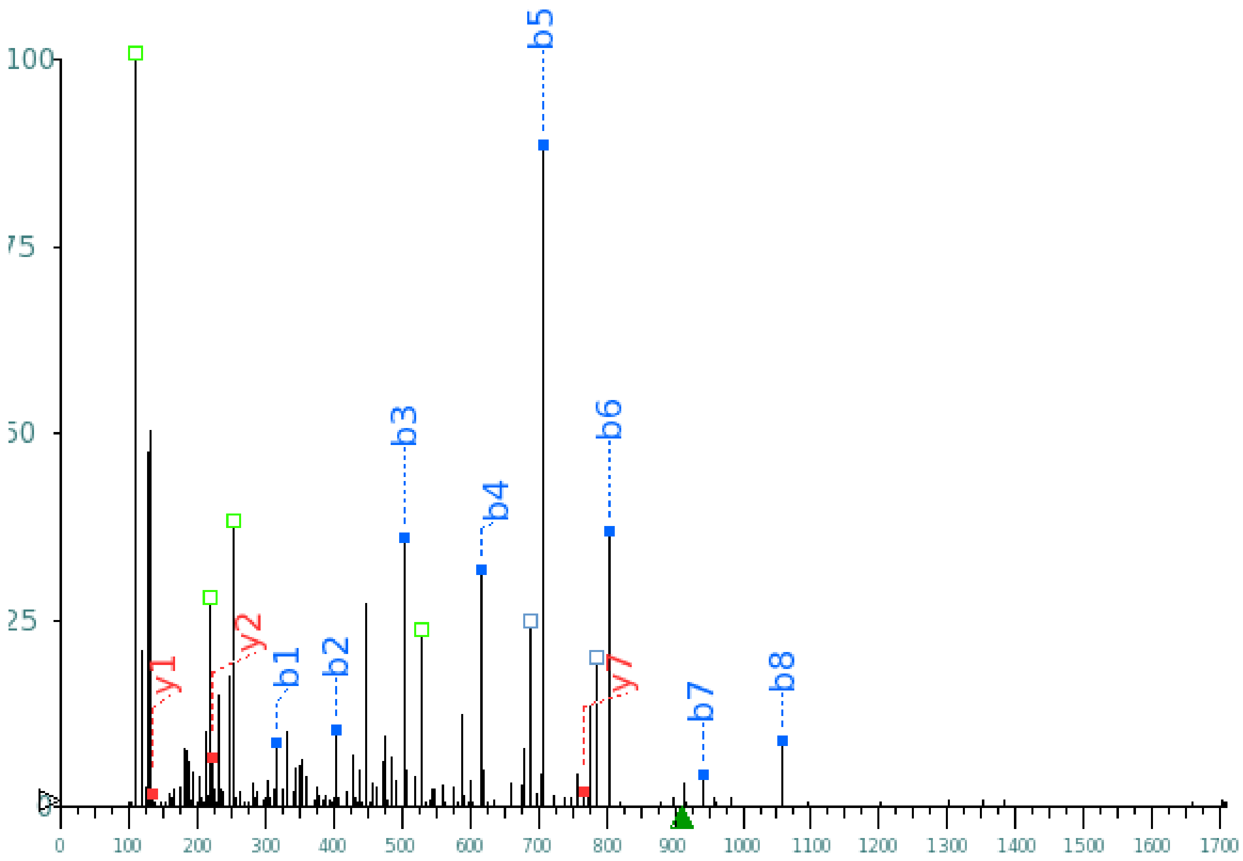

SSTISVHDPFSDVSD (+2)

Primary Reference: ARI1B_HUMAN
Search ID: 41761
Search Name: 20130328_ananiav_ERstress_TMT_TM_timecourse_trypsin_inj2_4mc_iaa
Scan#: 14183
Observed Mass: 911.4413 (2.7 ppm)
PSM Score: 39.07

File name: 2015-02-04 04.01.53PM.pdf

Creation time: 2015-02-04 04:09PM

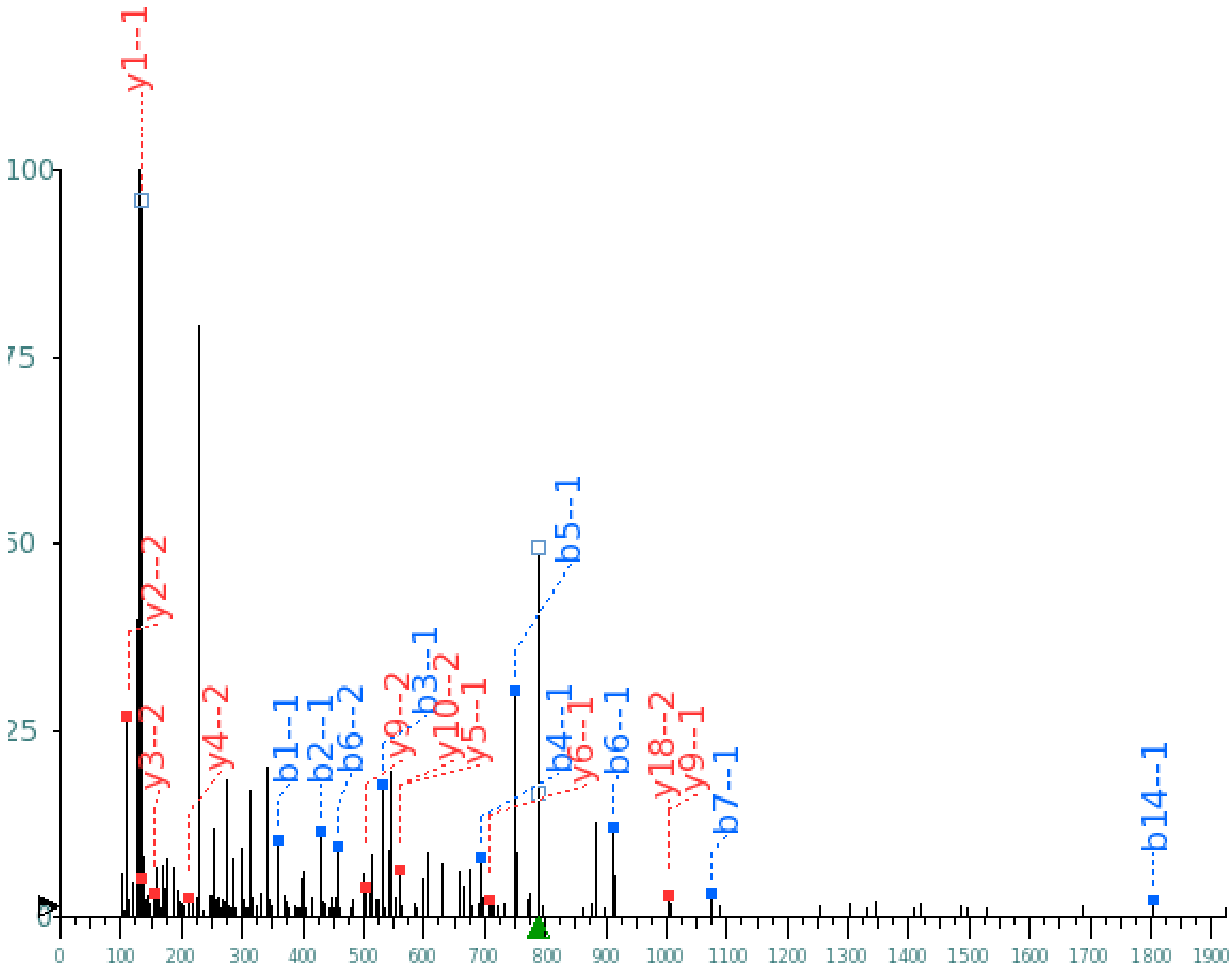

QATYGYYLGNPAEFHDSSD (+3)

Primary Reference: RCN1_HUMAN
Search ID: 41761
Search Name: 20130328_ananiav_ERstress_TMT_TM_timecourse_trypsin_inj2_4mc_iaa
Scan#: 14199
Observed Mass: 788.6948 (3.4 ppm)
PSM Score: 11.45

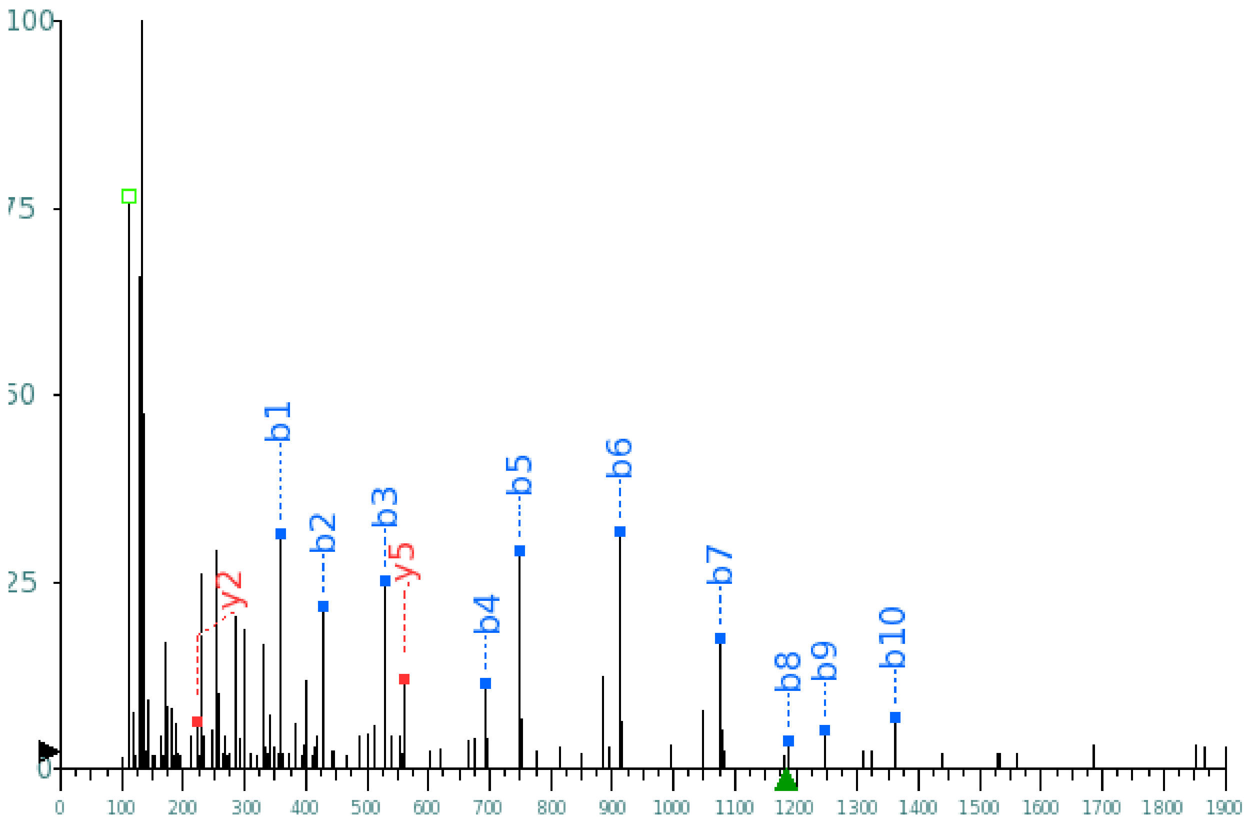

QATYGYYLGNPAEFHDSSD (+2)

Primary Reference: RCN1_HUMAN
Search ID: 41761
Search Name: 20130328_ananiav_ERstress_TMT_TM_timecourse_trypsin_inj2_4mc_iaa
Scan#: 14253
Observed Mass: 1182.5373 (2.3 ppm)
PSM Score: 66.58

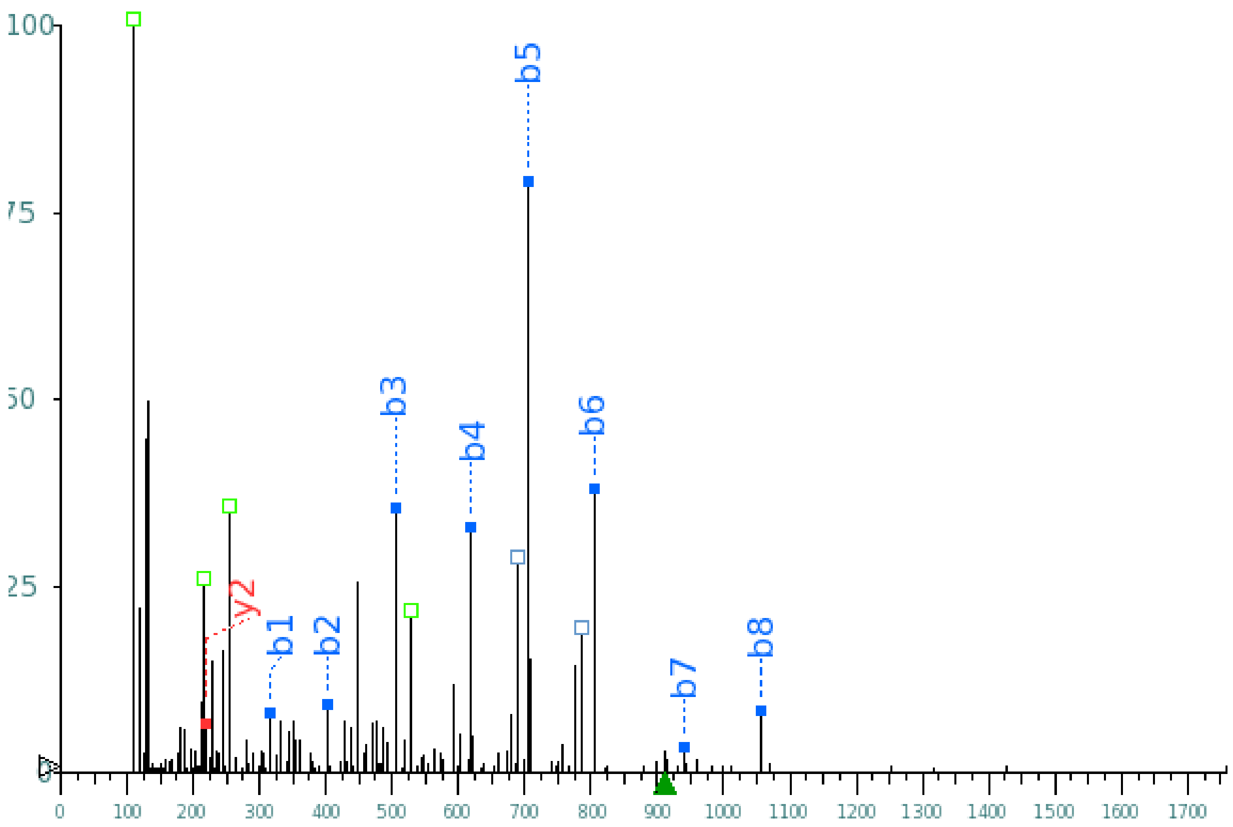

SSTISVHDPFSDVSD (+2)

Primary Reference: ARI1B_HUMAN
Search ID: 41761
Search Name: 20130328_ananiav_ERstress_TMT_TM_timecourse_trypsin_inj2_4mc_iaa
Scan#: 14261
Observed Mass: 911.4409 (2.2 ppm)
PSM Score: 27.9

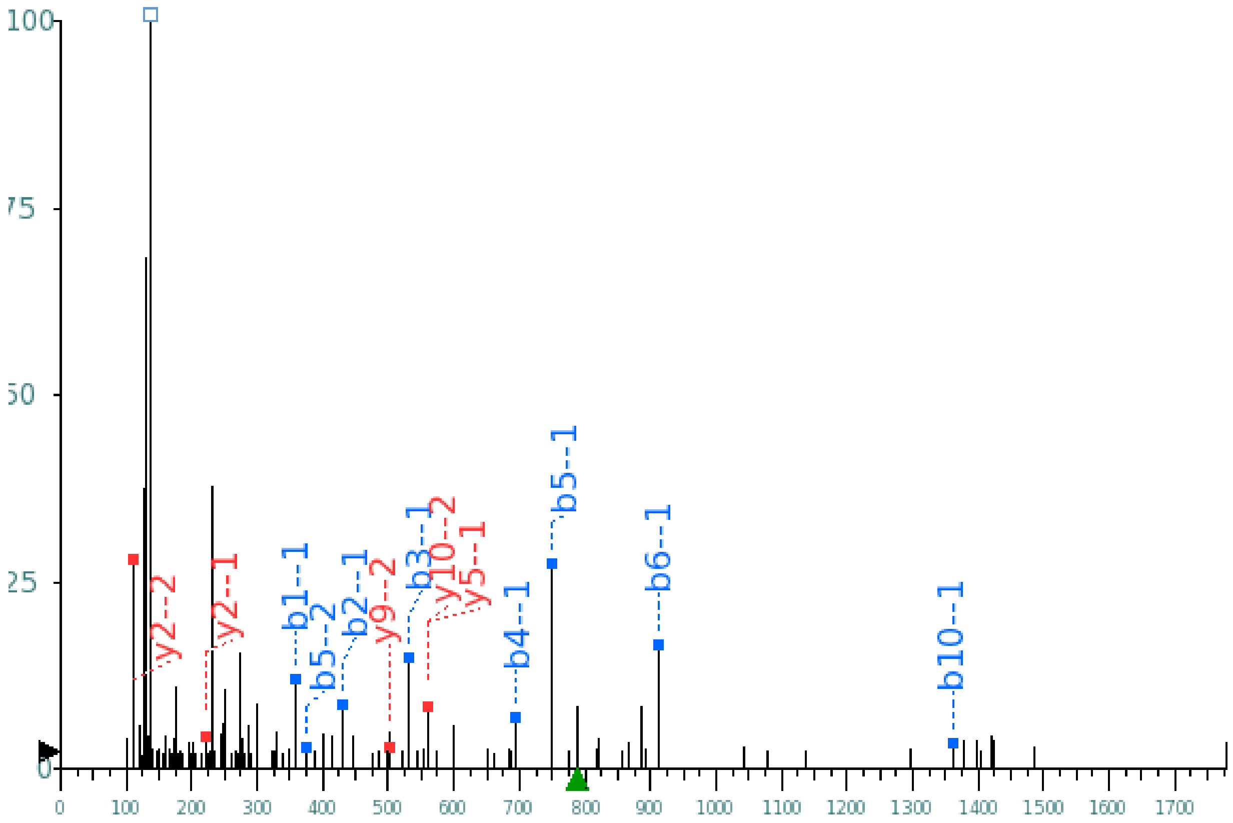

QATYGYYLGNPAEFHDSSD (+3)

Primary Reference: RCN1_HUMAN
Search ID: 41761
Search Name: 20130328_ananiav_ERstress_TMT_TM_timecourse_trypsin_inj2_4mc_iaa
Scan#: 14271
Observed Mass: 788.6944 (2.9 ppm)
PSM Score: 18.7

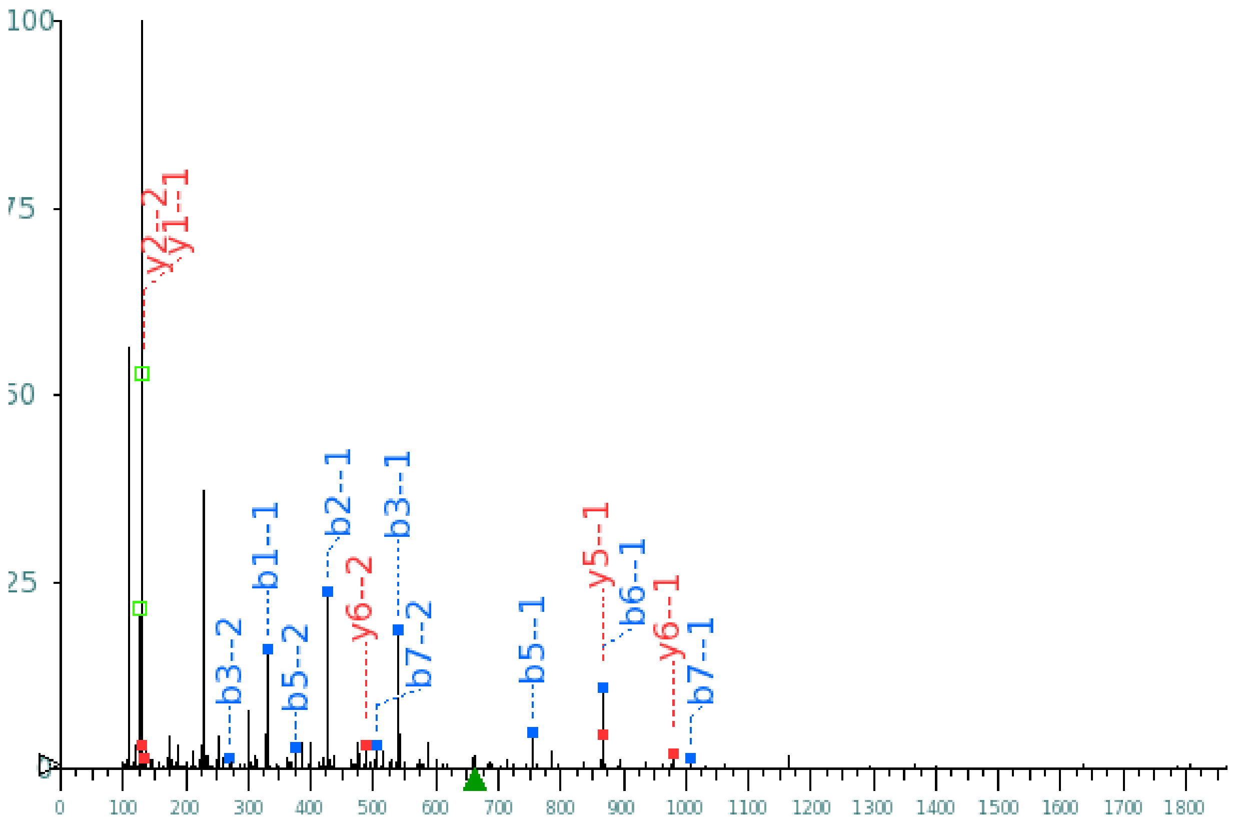

VVLPDDHLKDEMD (+3)

Primary Reference: RTN3_HUMAN
Search ID: 41761
Search Name: 20130328_ananiav_ERstress_TMT_TM_timecourse_trypsin_inj2_4mc_iaa
Scan#: 14316
Observed Mass: 662.0206 (0.5 ppm)
PSM Score: 19.7

File name: 2015-02-04 04.01.53PM.pdf

Creation time: 2015-02-04 04:09PM

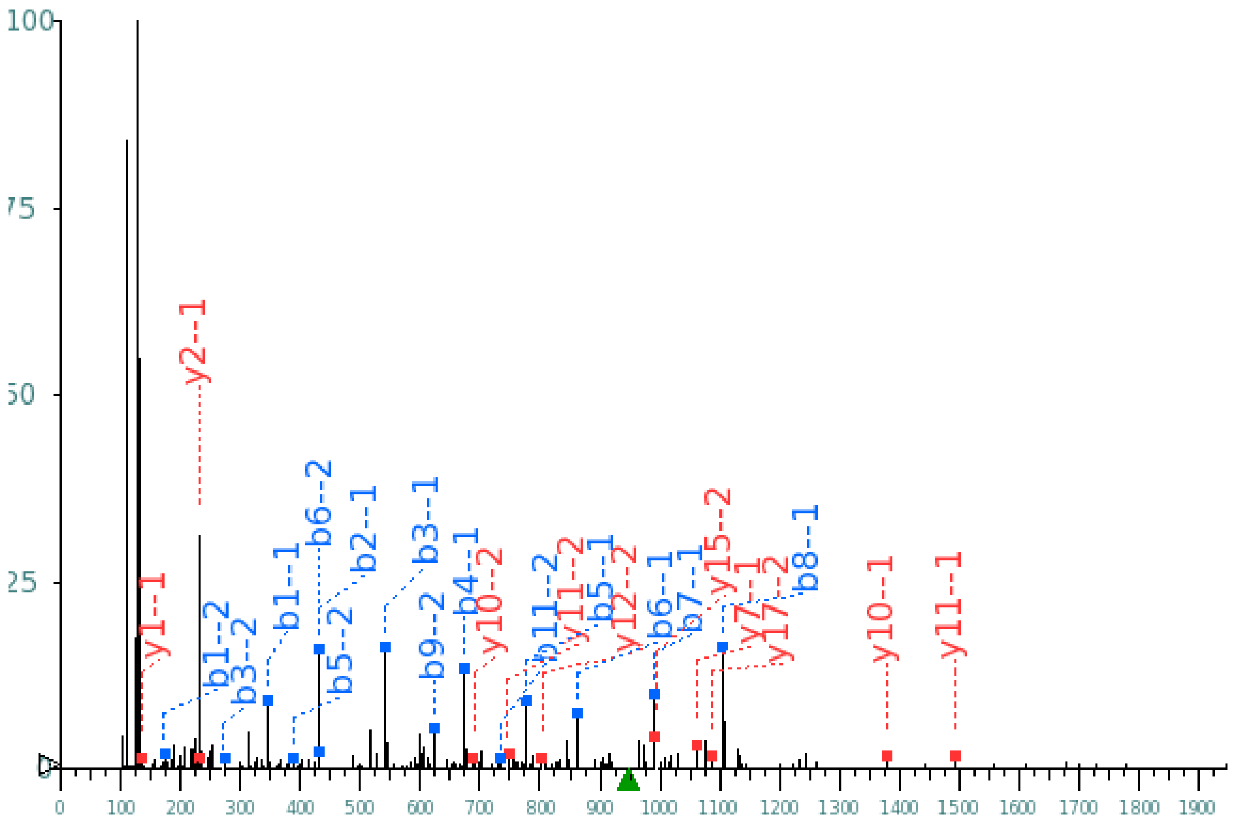

LSIMTSENHLNNSDKEVDEVD (+3)

Primary Reference: FERM2_HUMAN
Search ID: 41761
Search Name: 20130328_ananiav_ERstress_TMT_TM_timecourse_trypsin_inj2_4mc_iaa
Scan#: 14329
Observed Mass: 949.8092 (1.7 ppm)
PSM Score: 44.47

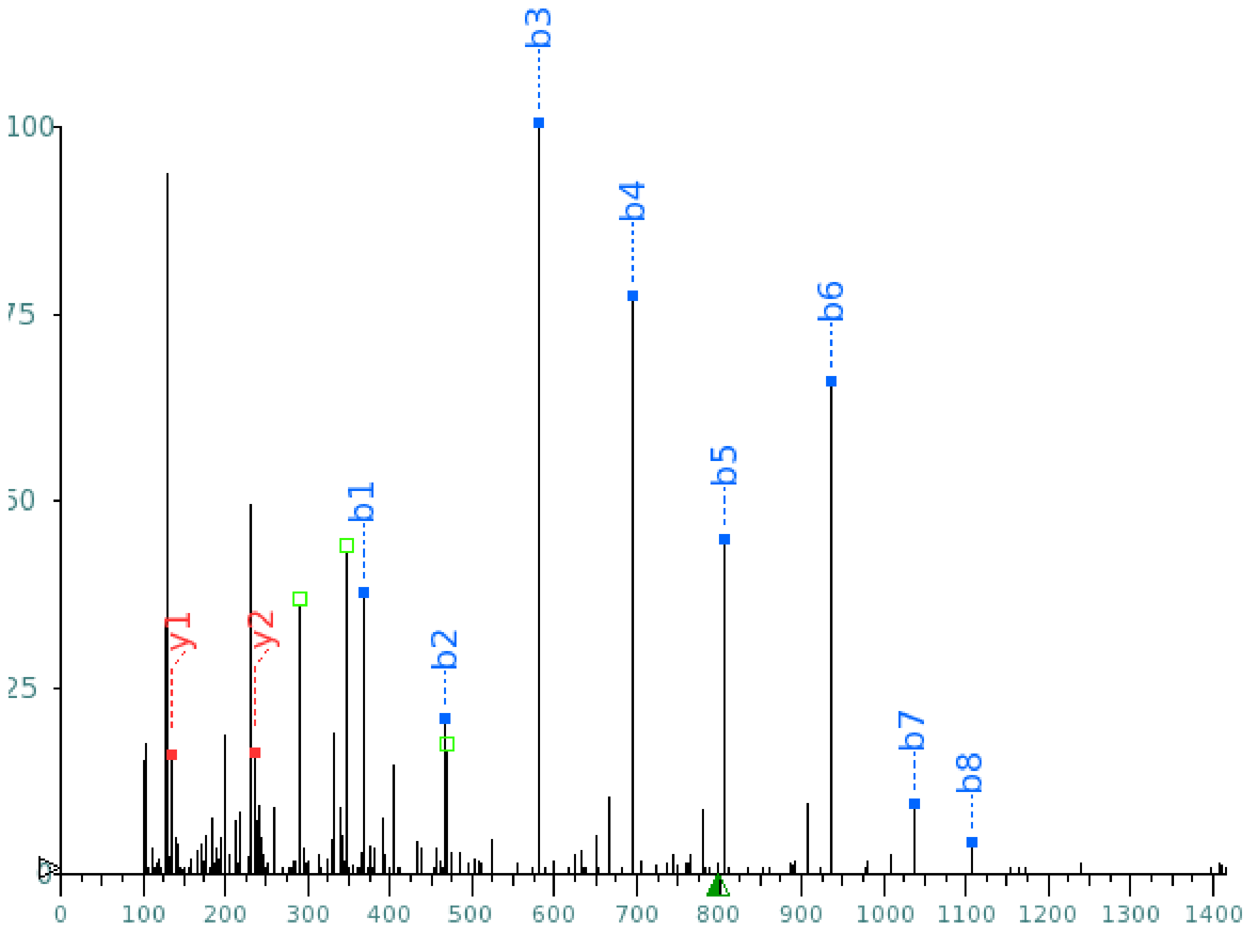

HVDLLEVAQETD (+2)

Primary Reference: ATAD1_HUMAN
Search ID: 41761
Search Name: 20130328_ananiav_ERstress_TMT_TM_timecourse_trypsin_inj2_4mc_iaa
Scan#: 14370
Observed Mass: 799.4187 (1.9 ppm)
PSM Score: 40.25

File name: 2015-02-04 04.01.53PM.pdf

Creation time: 2015-02-04 04:09PM

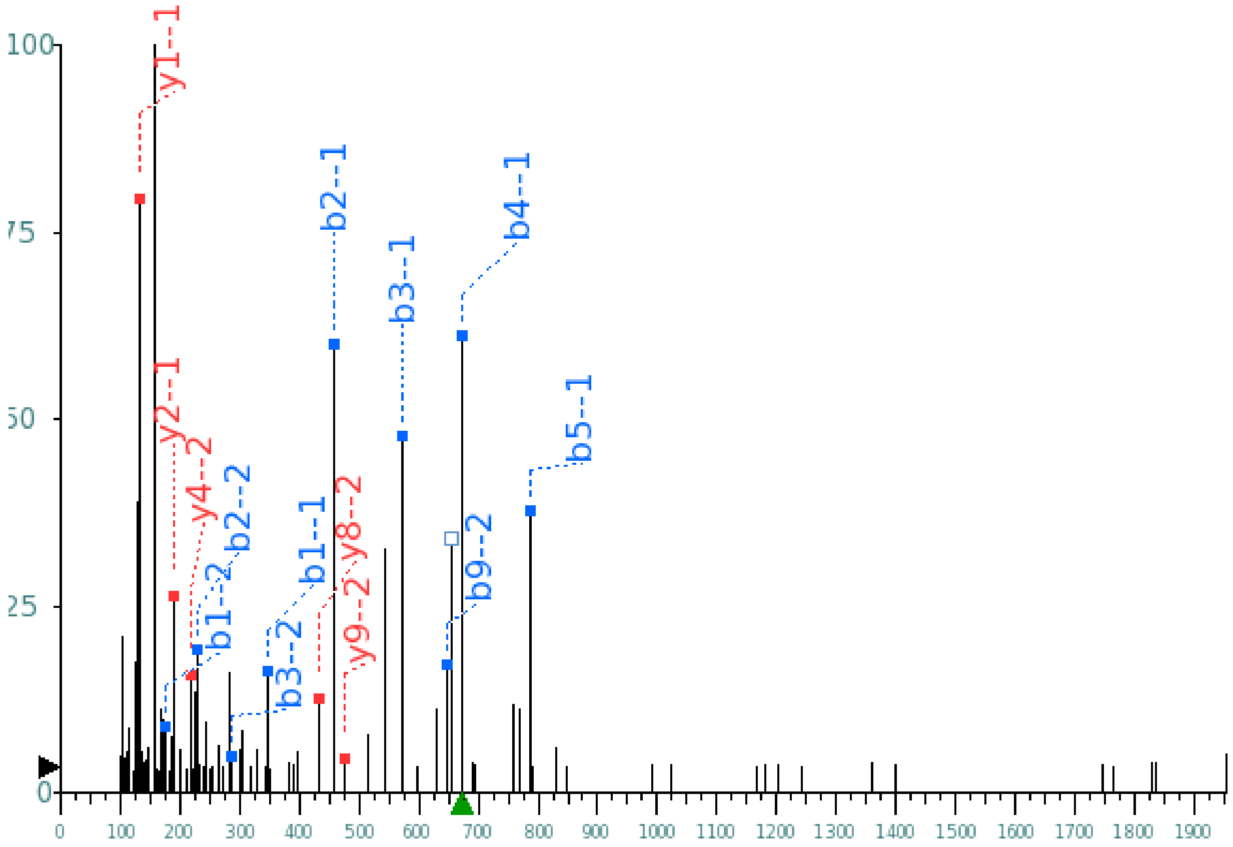

DNLTLWTSENQGDEGD (+3)

Primary Reference: 1433B_HUMAN
Search ID: 41761
Search Name: 20130328_ananiav_ERstress_TMT_TM_timecourse_trypsin_inj2_4mc_iaa
Scan#: 14392
Observed Mass: 674.9761 (2.3 ppm)
PSM Score: 13.75

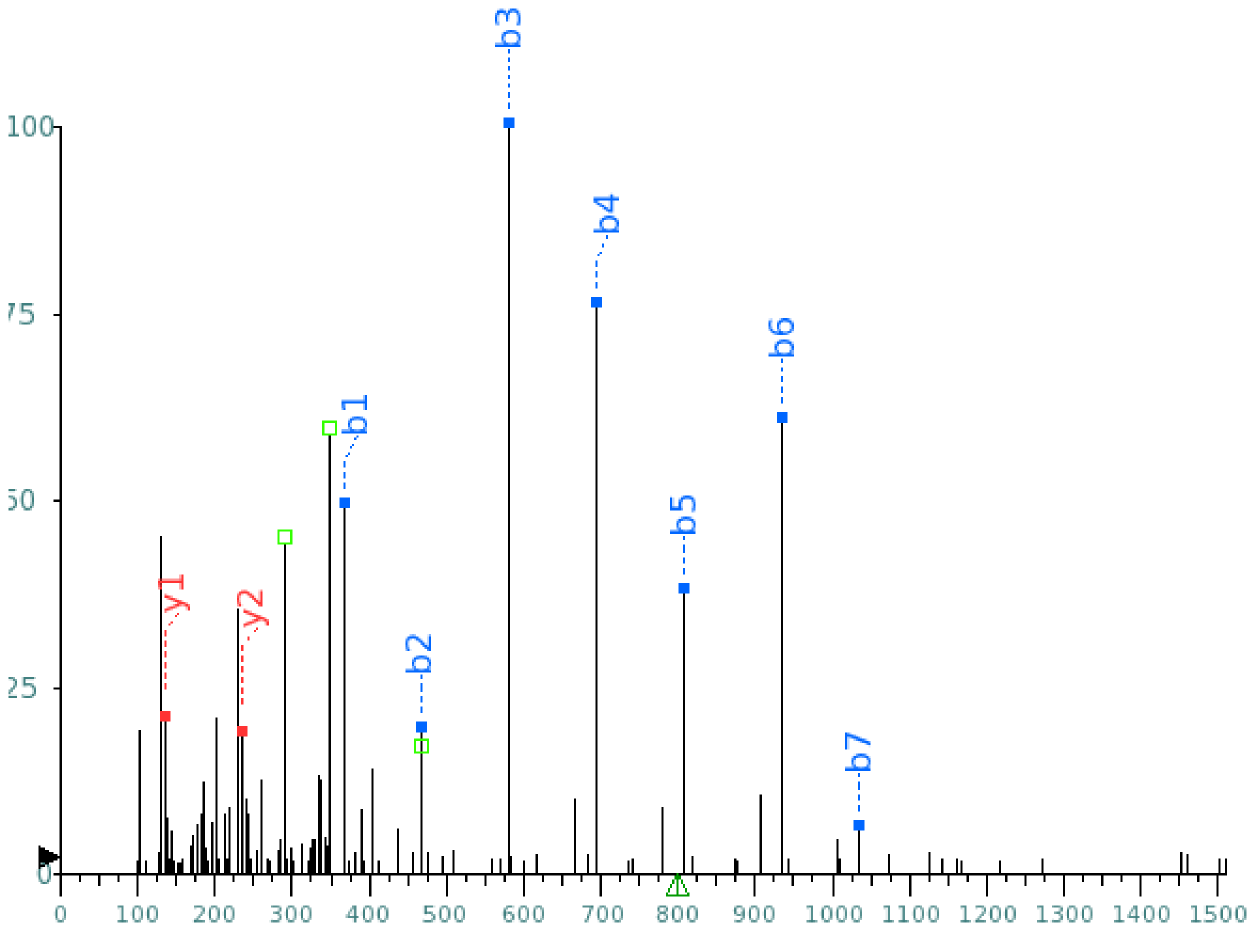

HVDLLEVAQETD (+2)

Primary Reference: ATAD1_HUMAN
Search ID: 41761
Search Name: 20130328_ananiav_ERstress_TMT_TM_timecourse_trypsin_inj2_4mc_iaa
Scan#: 14446
Observed Mass: 799.4178 (0.8 ppm)
PSM Score: 30.36

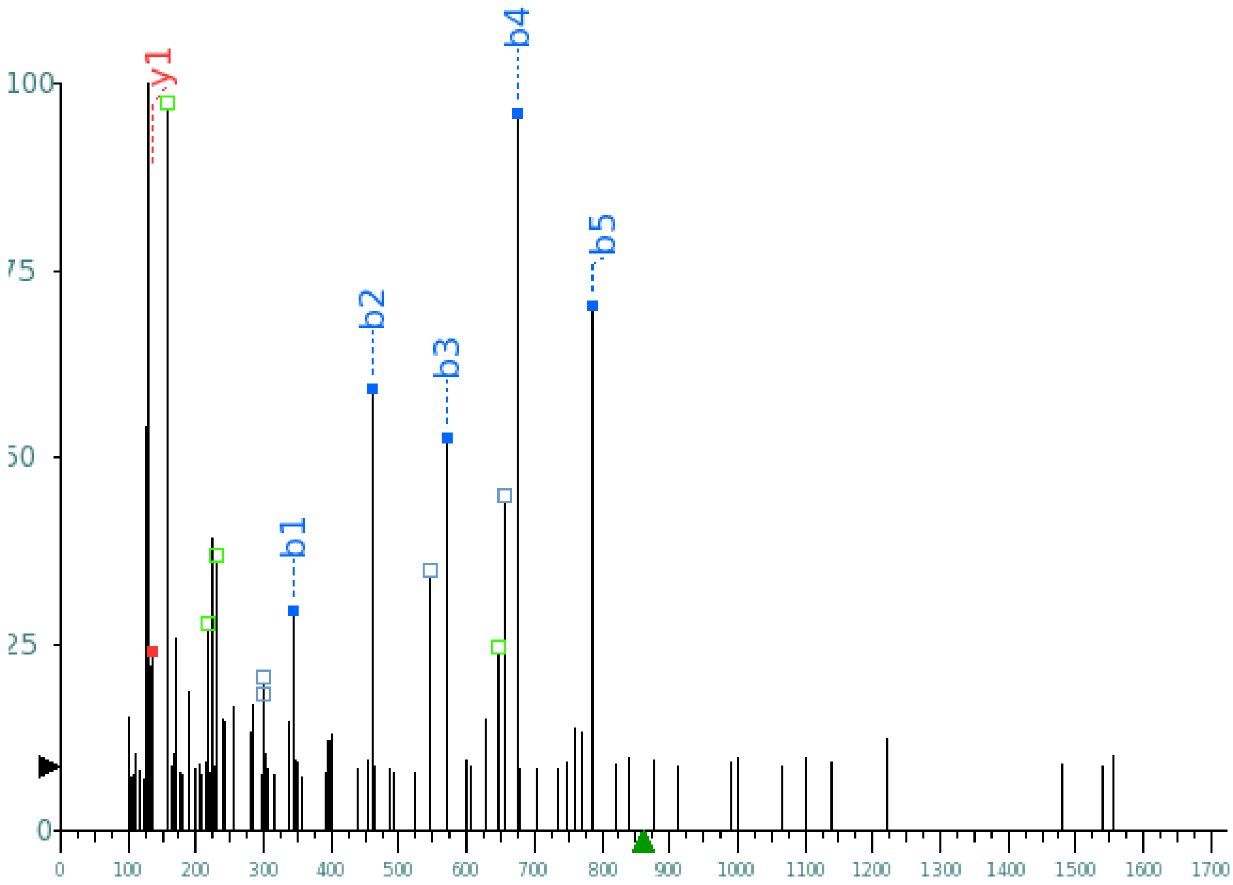

DNLTLWTSENQGD (+2)

Primary Reference: 1433B_HUMAN
Search ID: 41761
Search Name: 20130328_ananiav_ERstress_TMT_TM_timecourse_trypsin_inj2_4mc_iaa
Scan#: 14448
Observed Mass: 861.4132 (0.7 ppm)
PSM Score: 16.34

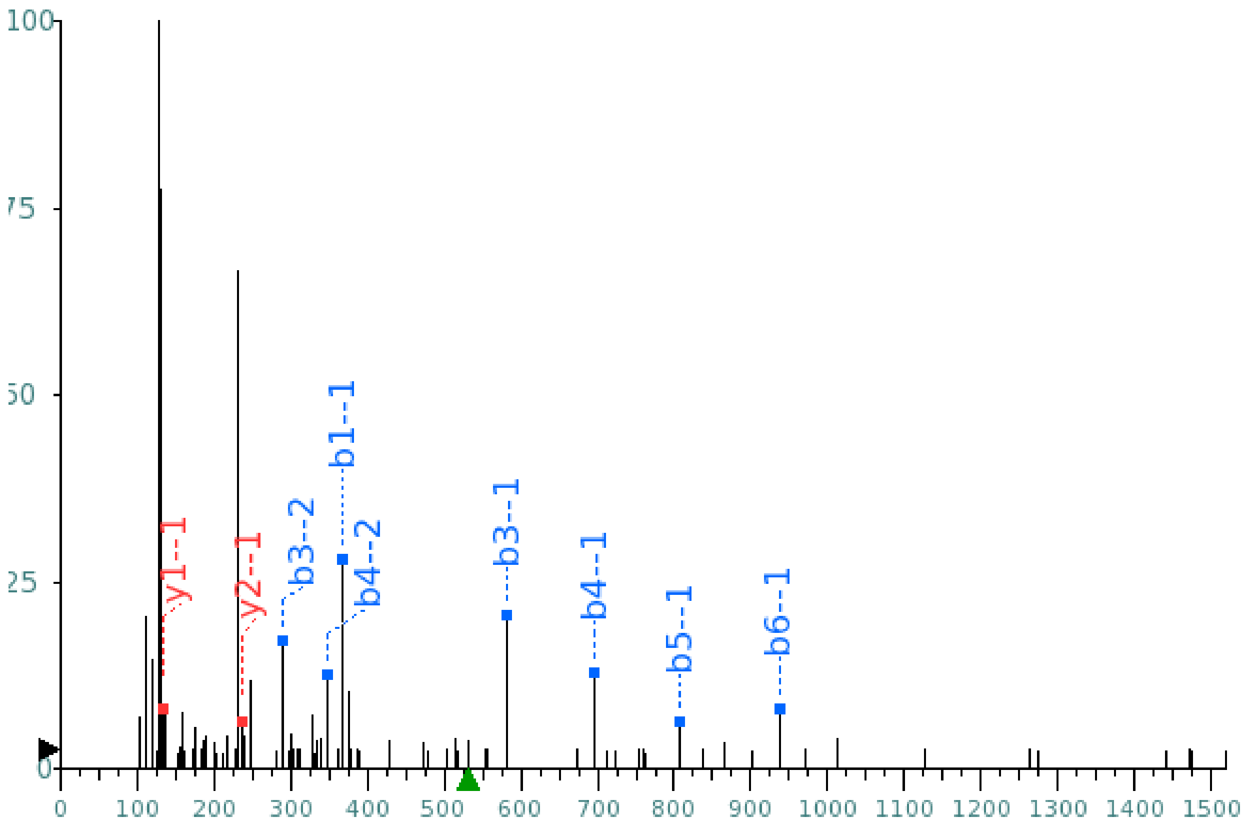

HVDLLEVAQETD (+3)

Primary Reference: ATAD1_HUMAN
Search ID: 41761
Search Name: 20130328_ananiav_ERstress_TMT_TM_timecourse_trypsin_inj2_4mc_iaa
Scan#: 14468
Observed Mass: 533.2808 (0.4 ppm)
PSM Score: 13.59

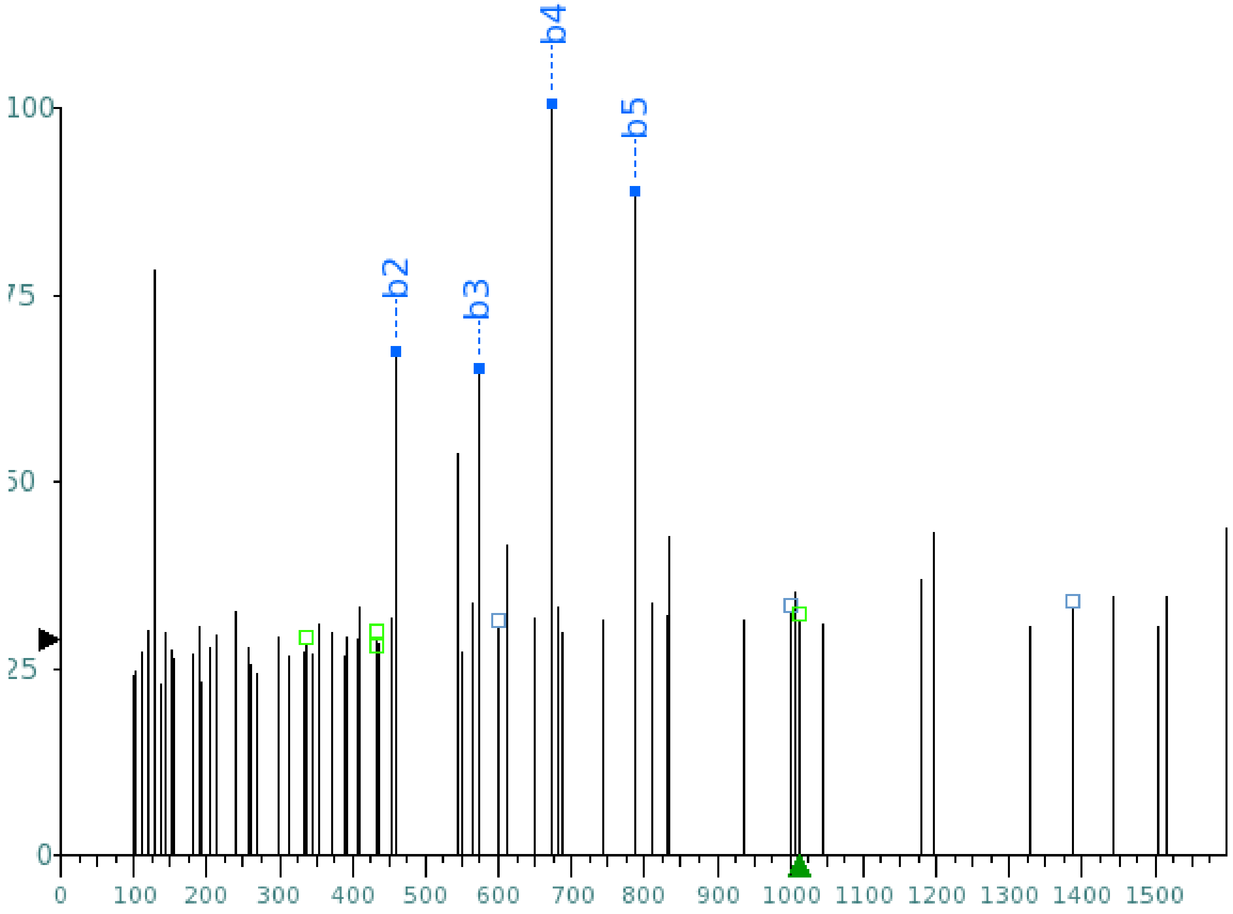

DNLTLWTSENQGDEGD (+2)

Primary Reference: 1433B_HUMAN
Search ID: 41761
Search Name: 20130328_ananiav_ERstress_TMT_TM_timecourse_trypsin_inj2_4mc_iaa
Scan#: 14479
Observed Mass: 1011.9594 (1.3 ppm)
PSM Score: 9.86

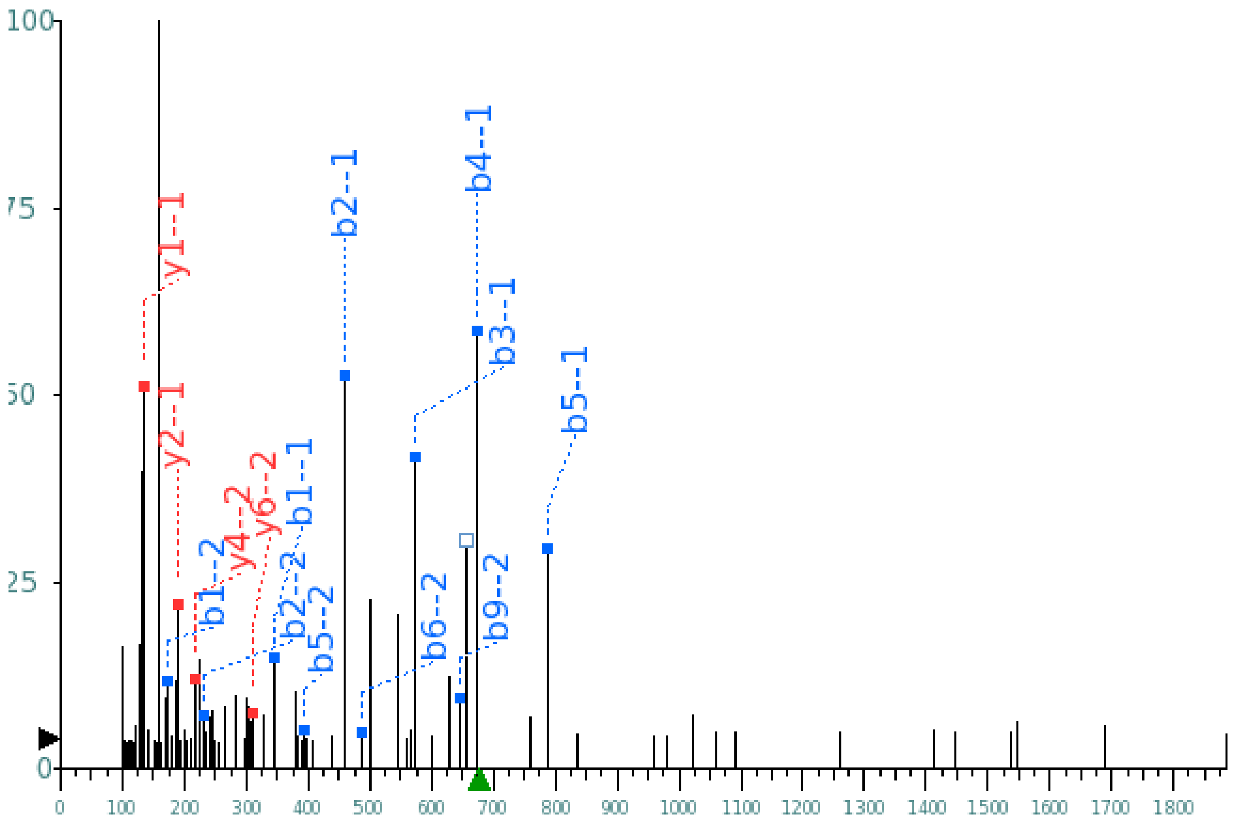

DNLTLWTSENQGDEGD (+3)

Primary Reference: 1433B_HUMAN

Search ID: 41761

Search Name: 20130328_ananiav_ERstress_TMT_TM_timecourse_trypsin_inj2_4mc_iaa

Scan#: 14480

Observed Mass: 674.9758 (2 ppm)

PSM Score: 14.47

File name: 2015-02-04 04.01.53PM.pdf

Creation time: 2015-02-04 04:09PM

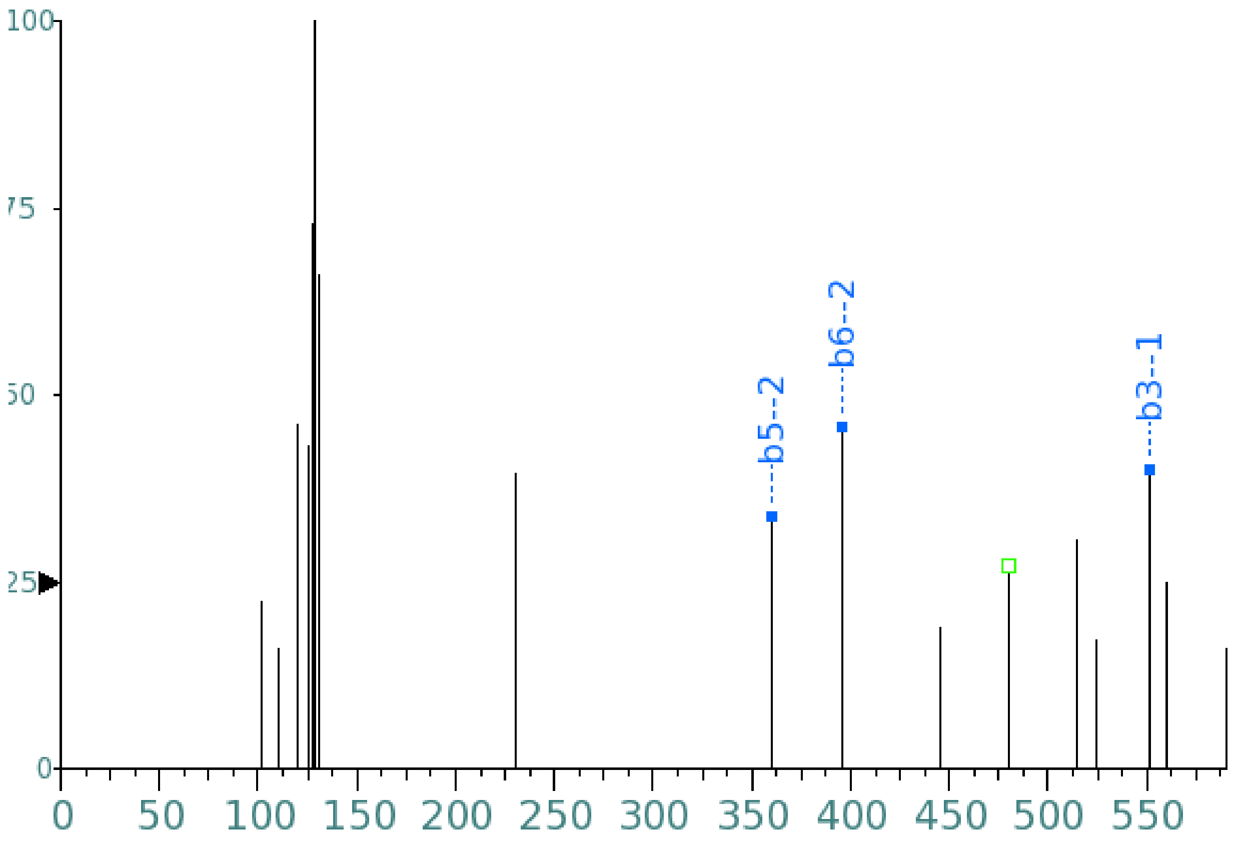

ALHPAAVSAAAEFENAESEAD (+3)

Primary Reference: C170L_HUMAN
Search ID: 41761
Search Name: 20130328_ananiav_ERstress_TMT_TM_timecourse_trypsin_inj2_4mc_iaa
Scan#: 14487
Observed Mass: 777.0445 (1.9 ppm)
PSM Score: 16.74

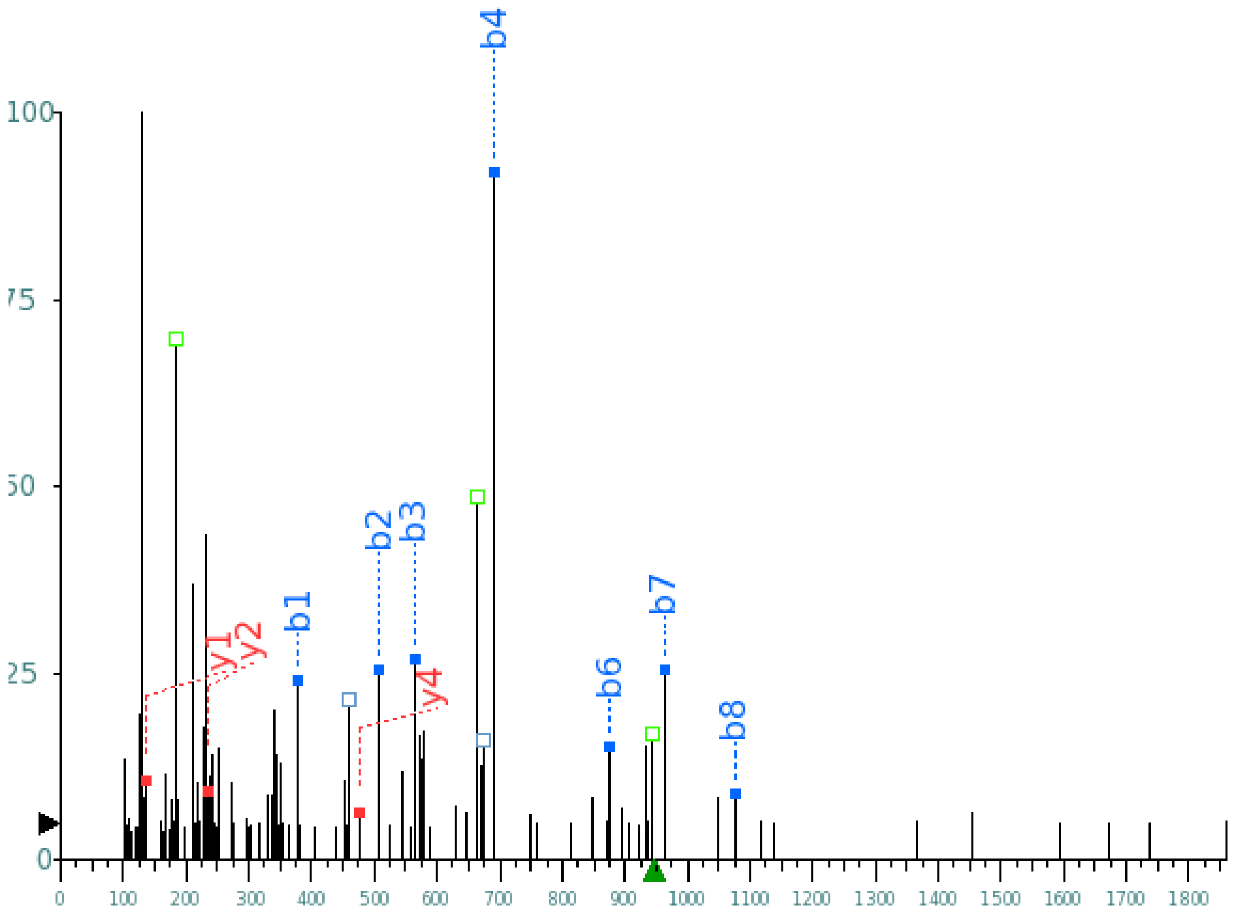

FEGQPSSLPLENETD (+2)

Primary Reference: ZN462_HUMAN
Search ID: 41761
Search Name: 20130328_ananiav_ERstress_TMT_TM_timecourse_trypsin_inj2_4mc_iaa
Scan#: 14492
Observed Mass: 946.4594 (-0.4 ppm)
PSM Score: 19.57

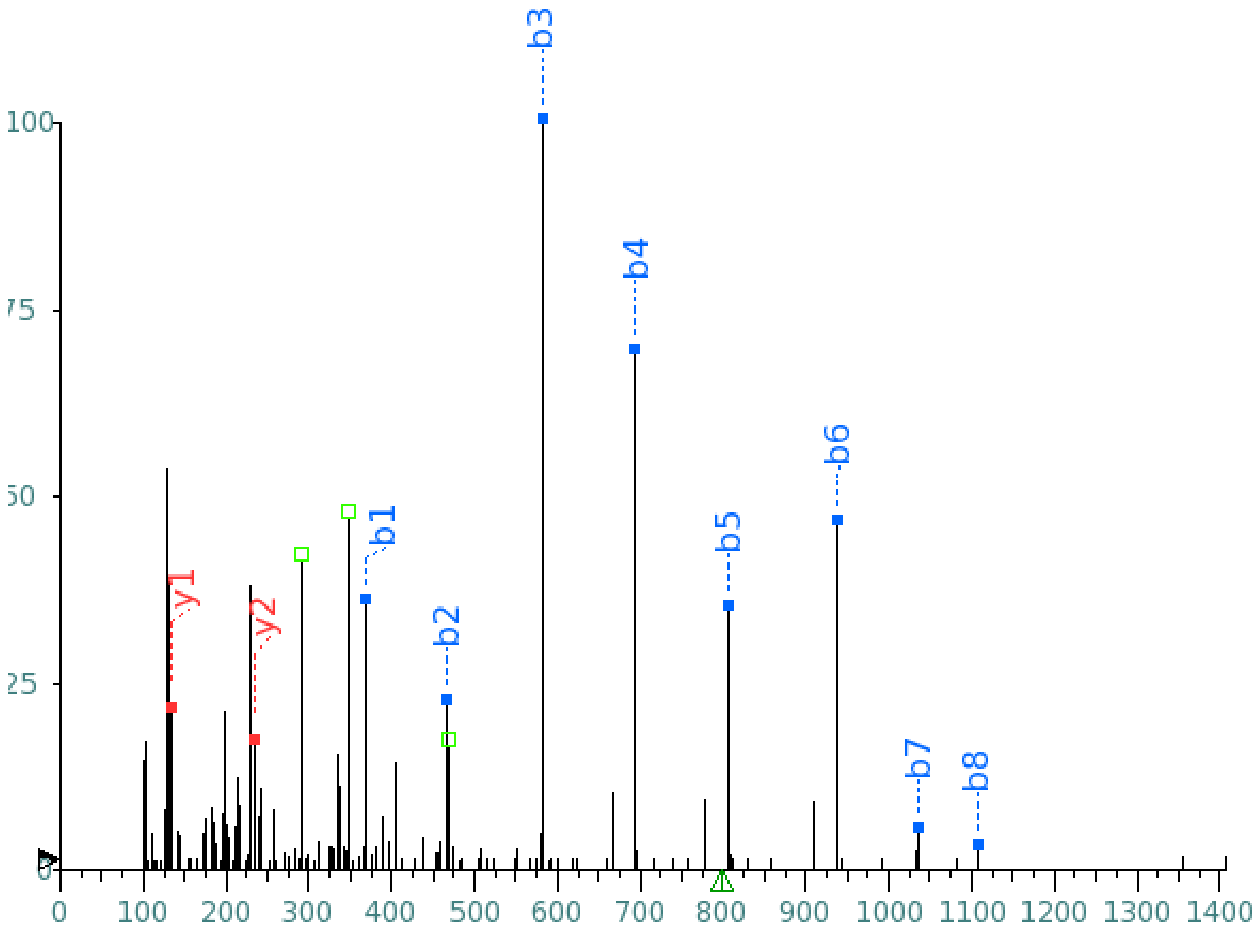

HVDLLEVAQETD (+2)

Primary Reference: ATAD1_HUMAN
Search ID: 41761
Search Name: 20130328_ananiav_ERstress_TMT_TM_timecourse_trypsin_inj2_4mc_iaa
Scan#: 14534
Observed Mass: 799.4188 (2.1 ppm)
PSM Score: 45.27

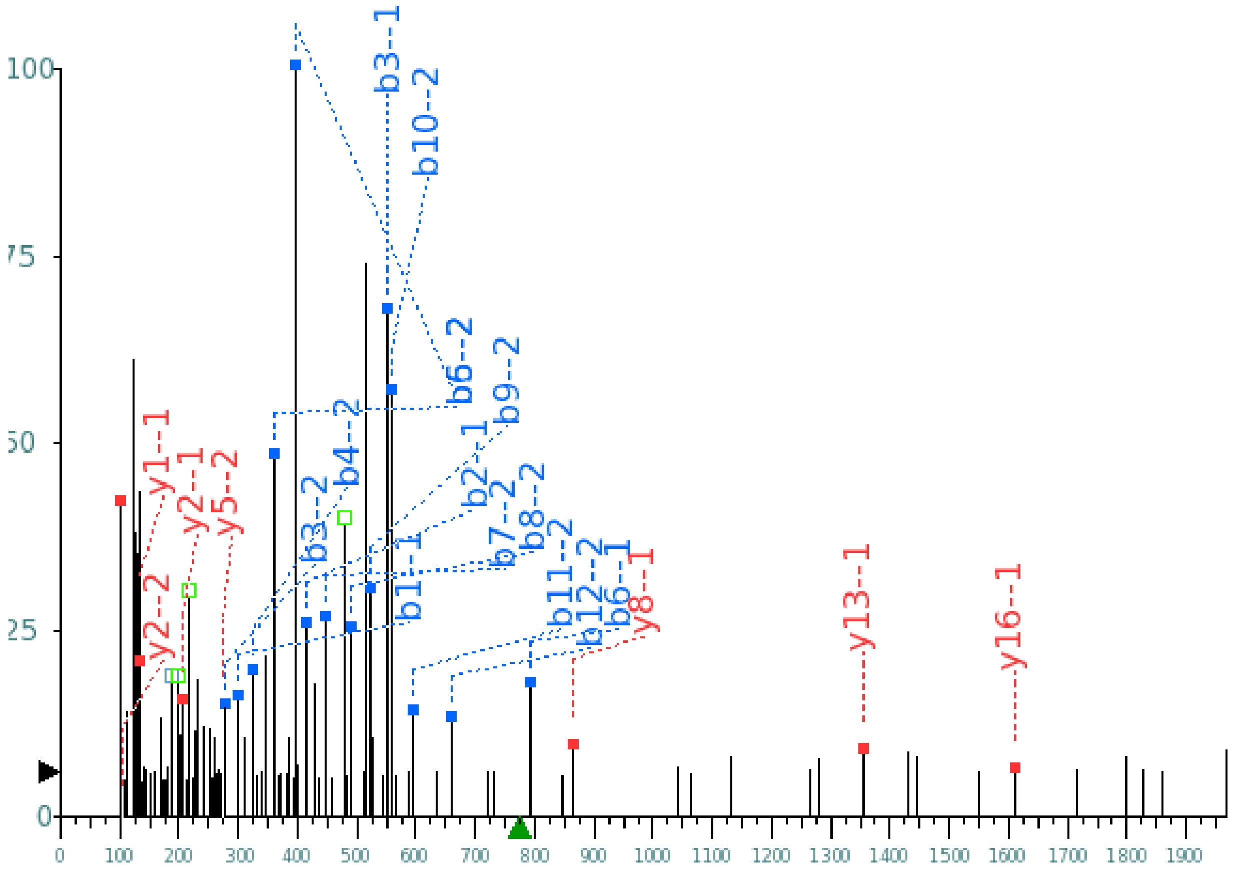

ALHPAAVSAAAEFENAESEAD (+3)

Primary Reference: C170L_HUMAN
Search ID: 41761
Search Name: 20130328_ananiav_ERstress_TMT_TM_timecourse_trypsin_inj2_4mc_iaa
Scan#: 14557
Observed Mass: 777.0454 (3.1 ppm)
PSM Score: 59.54

File name: 2015-02-04 04.01.53PM.pdf

Creation time: 2015-02-04 04:09PM

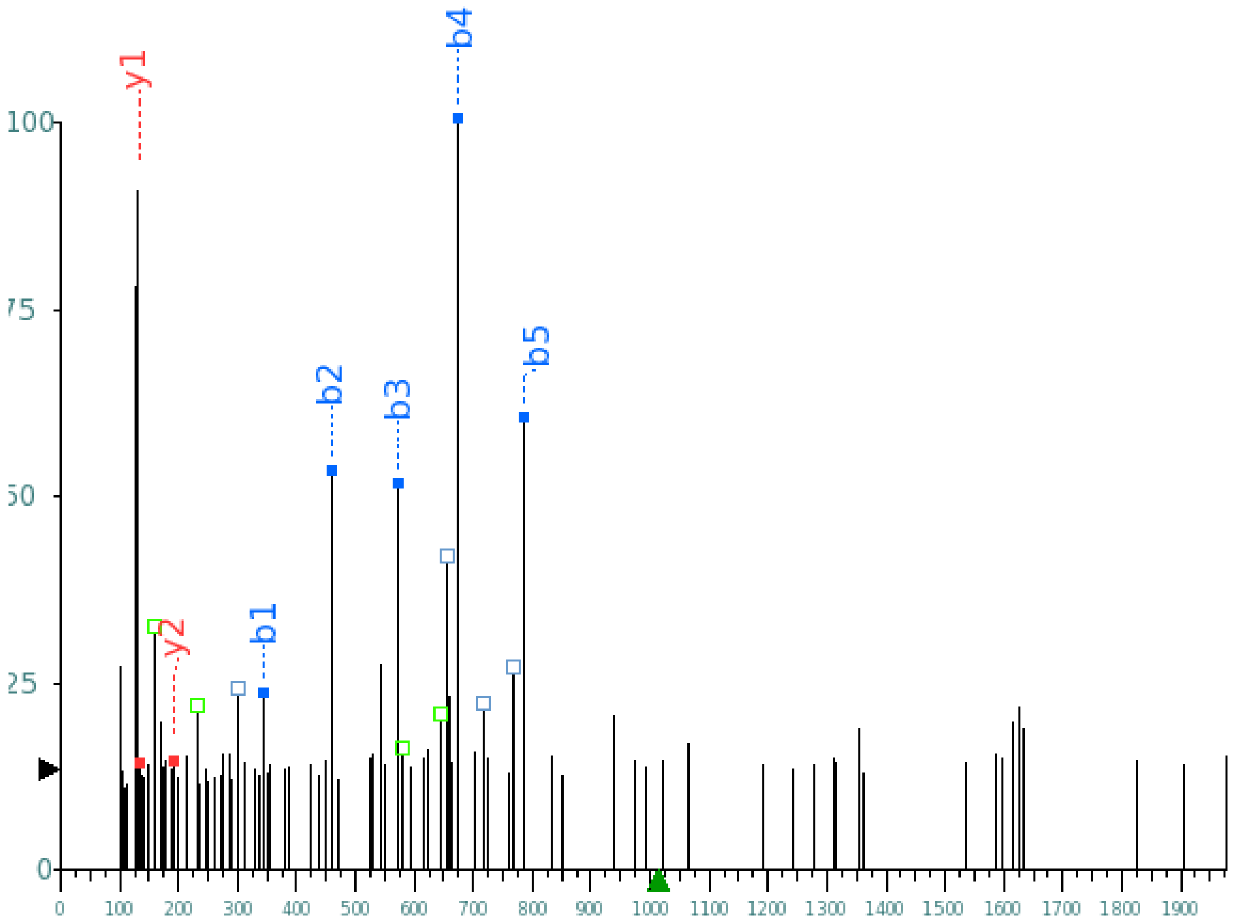

DNLTLWTSENQGDEGD (+2)

Primary Reference: 1433B_HUMAN
Search ID: 41761
Search Name: 20130328_ananiav_ERstress_TMT_TM_timecourse_trypsin_inj2_4mc_iaa
Scan#: 14558
Observed Mass: 1011.9597 (1.5 ppm)
PSM Score: 6.88

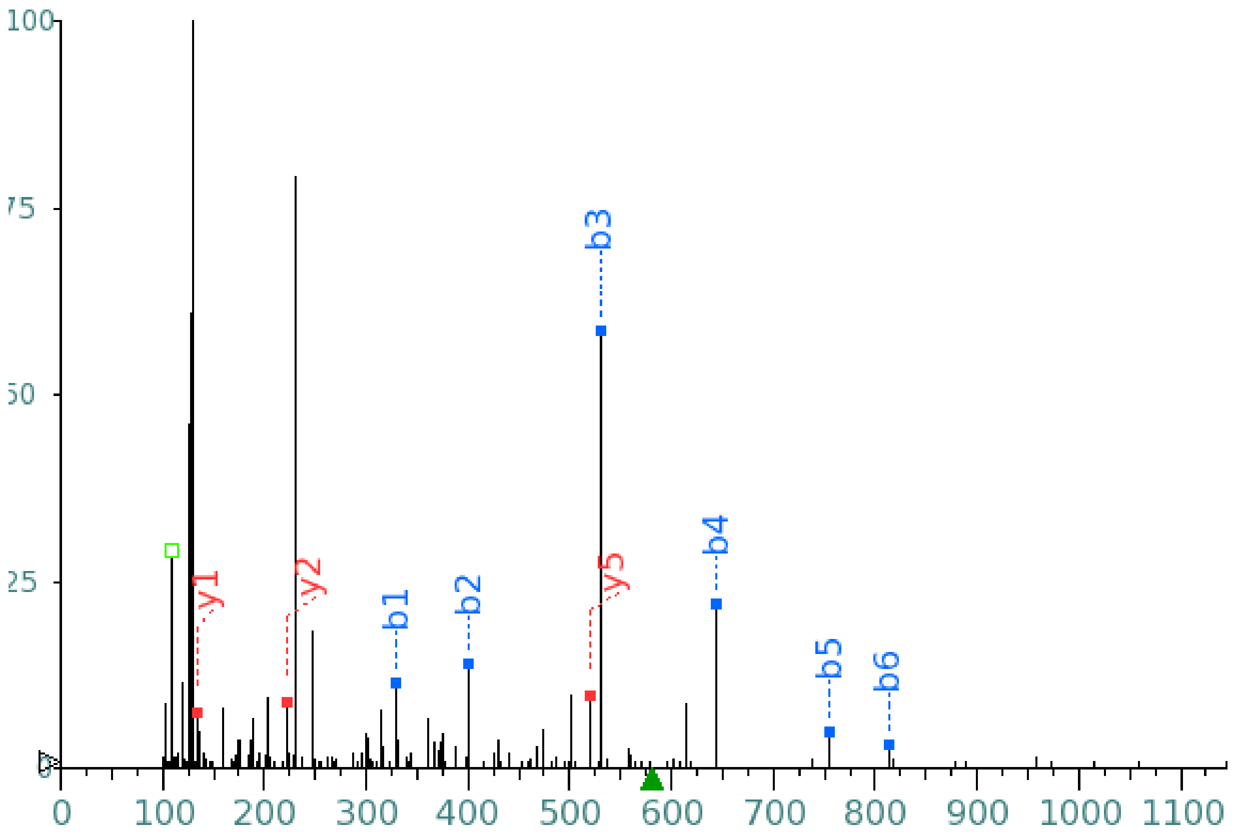

VAELLGESD (+2)

Primary Reference: KLC4_HUMAN
Search ID: 41761
Search Name: 20130328_ananiav_ERstress_TMT_TM_timecourse_trypsin_inj2_4mc_iaa
Scan#: 14560
Observed Mass: 581.3134 (-0.5 ppm)
PSM Score: 22.17

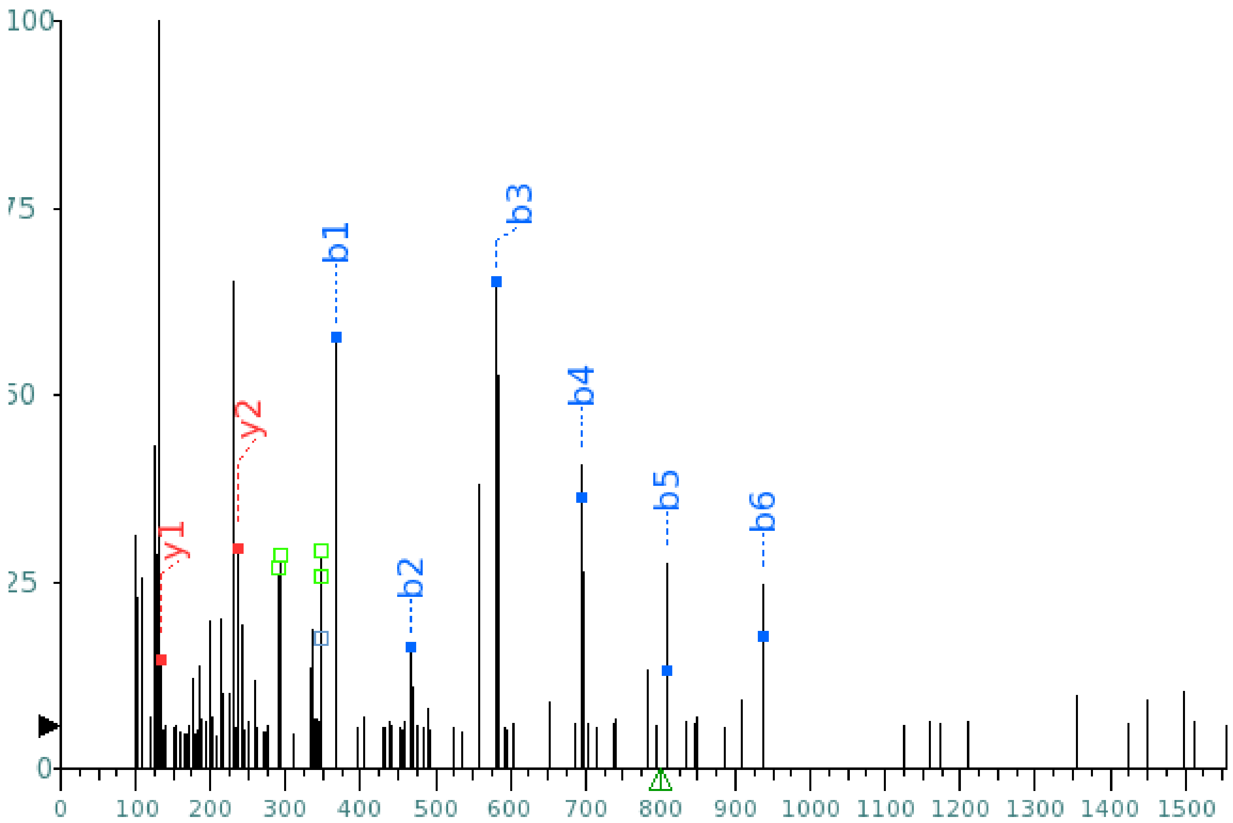

HVDLLEVAQETD (+2)

Primary Reference: ATAD1_HUMAN
Search ID: 41761
Search Name: 20130328_ananiav_ERstress_TMT_TM_timecourse_trypsin_inj2_4mc_iaa
Scan#: 14611
Observed Mass: 799.4189 (2.2 ppm)
PSM Score: 8.91

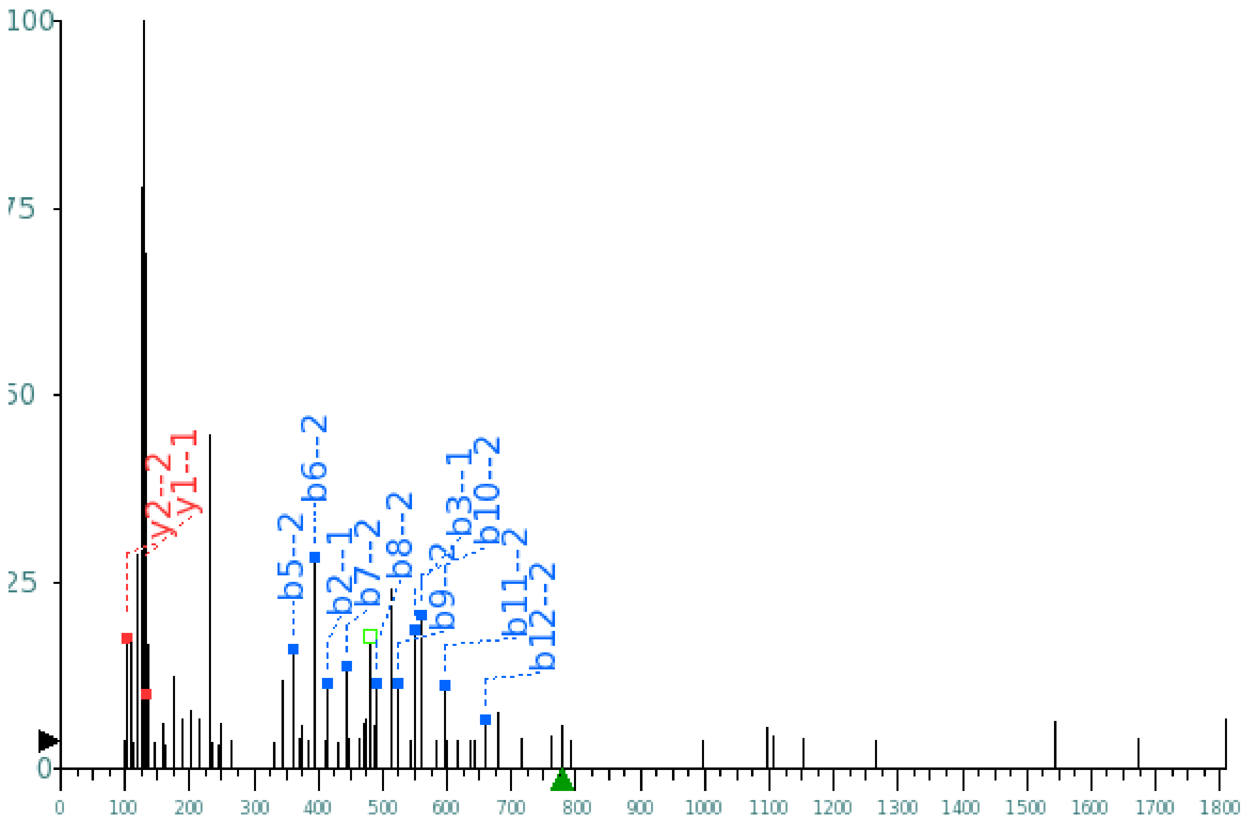

ALHPAAVSAAAEFENAESEAD (+3)

Primary Reference: C170L_HUMAN
Search ID: 41761
Search Name: 20130328_ananiav_ERstress_TMT_TM_timecourse_trypsin_inj2_4mc_iaa
Scan#: 14630
Observed Mass: 777.046 (3.8 ppm)
PSM Score: 18.79

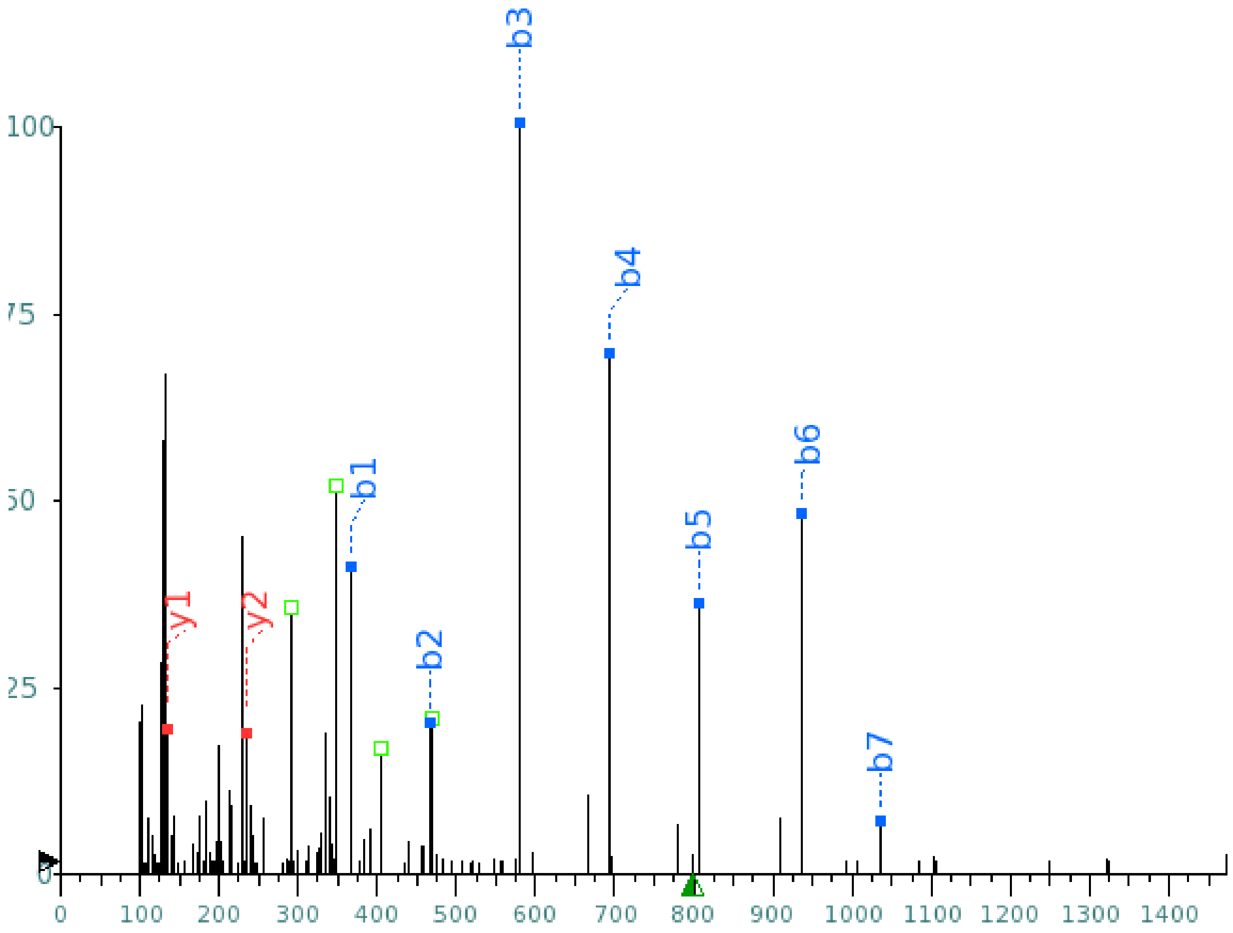

HVDLLEVAQETD (+2)

Primary Reference: ATAD1_HUMAN
Search ID: 41761
Search Name: 20130328_ananiav_ERstress_TMT_TM_timecourse_trypsin_inj2_4mc_iaa
Scan#: 14659
Observed Mass: 799.4192 (2.6 ppm)
PSM Score: 32.66

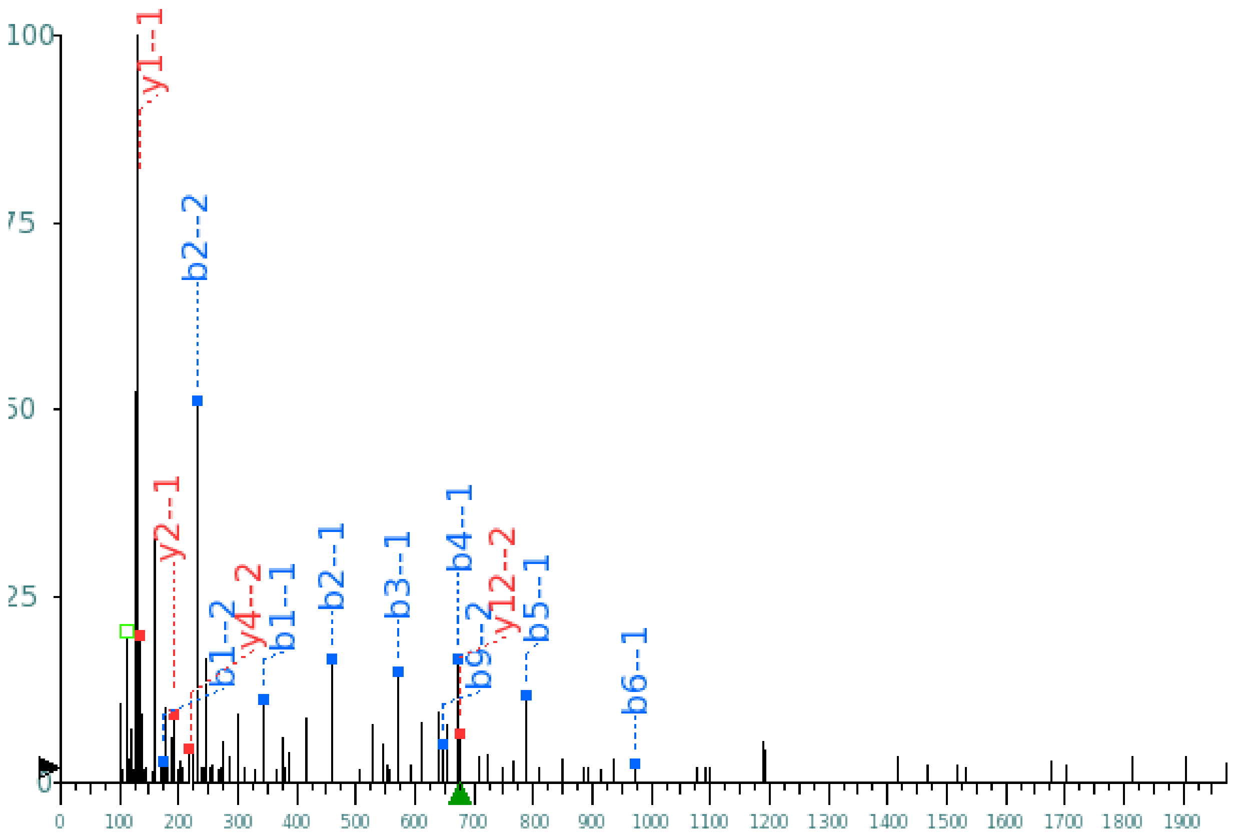

DNLTLWTSENQGDEGD (+3)

Primary Reference: 1433B_HUMAN
Search ID: 41761
Search Name: 20130328_ananiav_ERstress_TMT_TM_timecourse_trypsin_inj2_4mc_iaa
Scan#: 14704
Observed Mass: 674.9752 (1 ppm)
PSM Score: 10.46

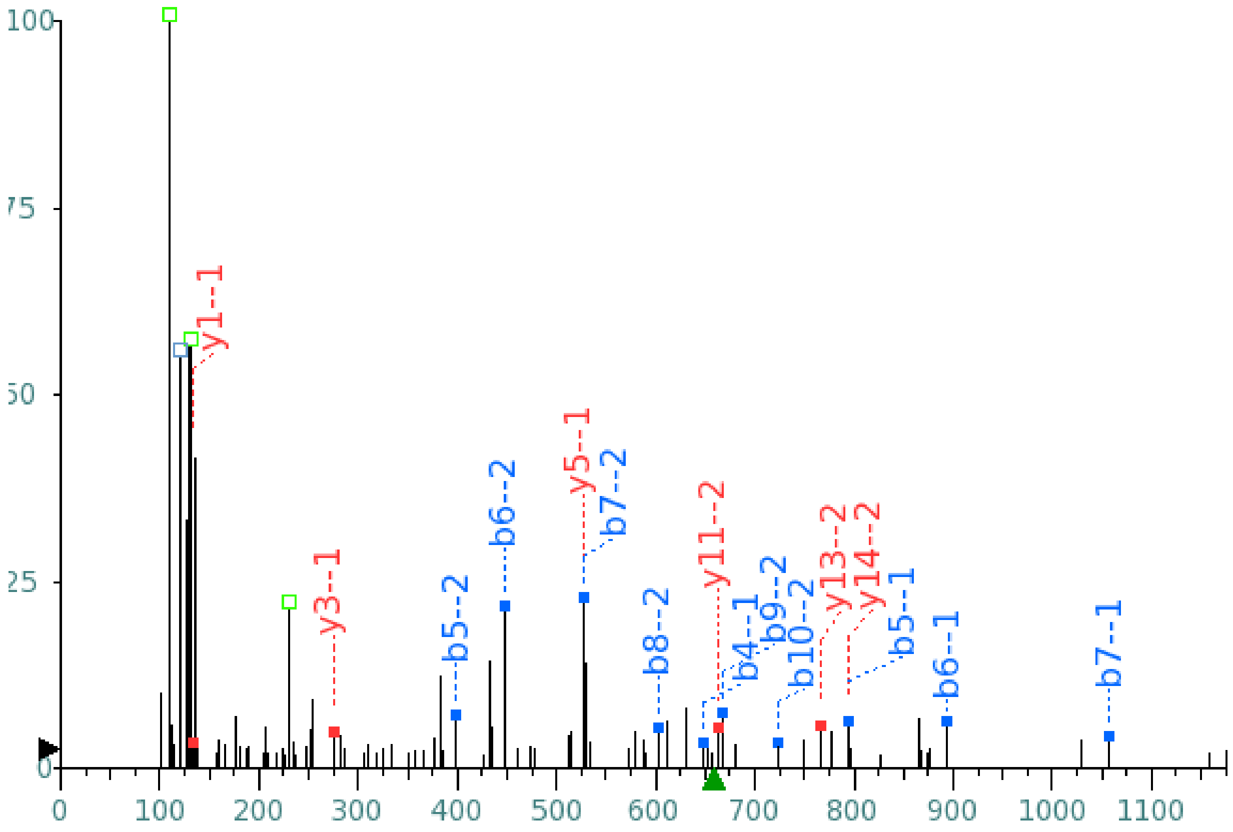

RGFGFVYFQNHDAAD (+3)

Primary Reference: ROA0_HUMAN
Search ID: 41761
Search Name: 20130328_ananiav_ERstress_TMT_TM_timecourse_trypsin_inj2_4mc_iaa
Scan#: 14717
Observed Mass: 658.3191 (-3.9 ppm)
PSM Score: 14.66

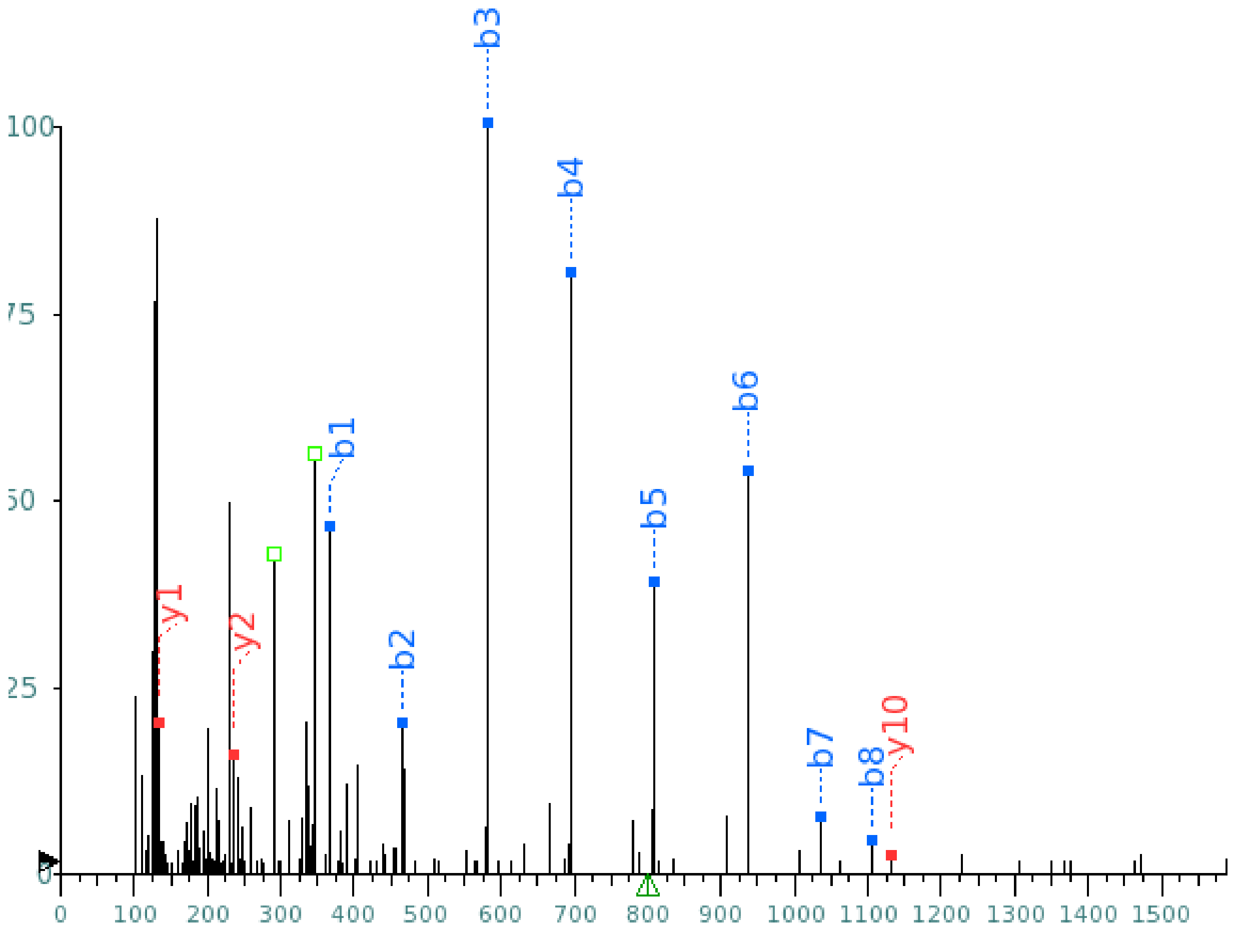

HVDLLEVAQETD (+2)

Primary Reference: ATAD1_HUMAN
Search ID: 41761
Search Name: 20130328_ananiav_ERstress_TMT_TM_timecourse_trypsin_inj2_4mc_iaa
Scan#: 14740
Observed Mass: 799.4184 (1.6 ppm)
PSM Score: 39.43

File name: 2015-02-04 04.01.53PM.pdf

Creation time: 2015-02-04 04:09PM

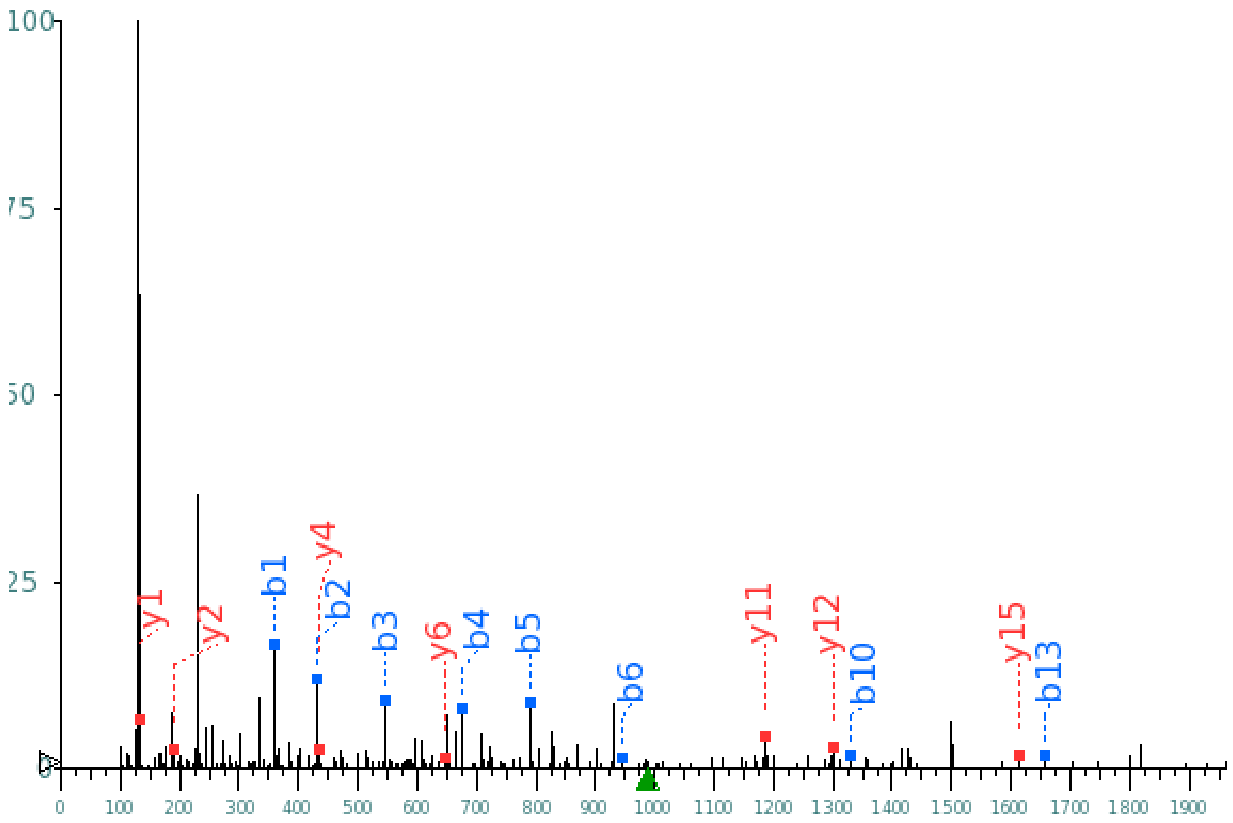

MALEDRPSSLLVDQGD (+2)

Primary Reference: TRIO_HUMAN
Search ID: 41761
Search Name: 20130328_ananiav_ERstress_TMT_TM_timecourse_trypsin_inj2_4mc_iaa
Scan#: 14776
Observed Mass: 988.0049 (1.1 ppm)
PSM Score: 15.69

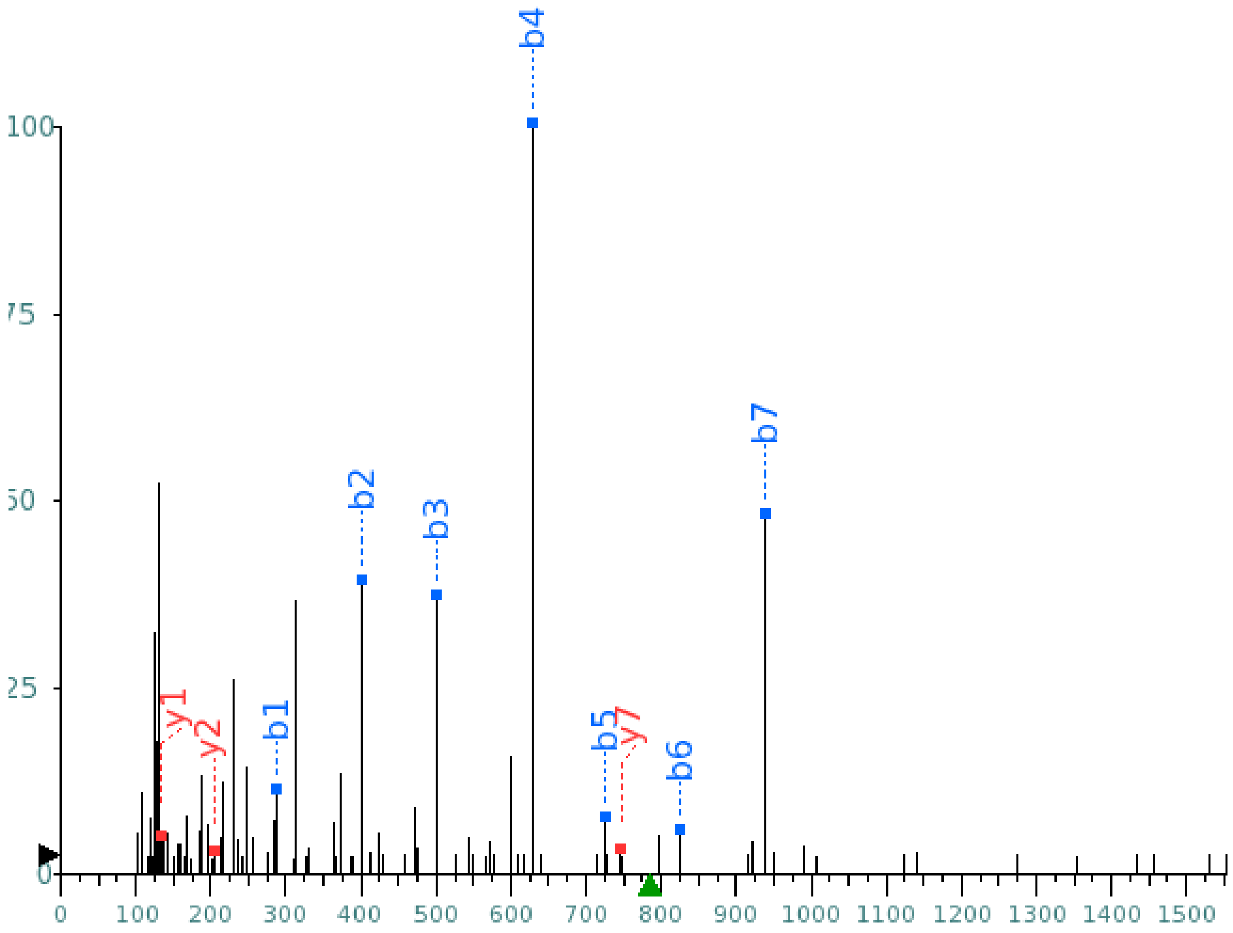

GLVEPVDVVDNAD (+2)

Primary Reference: FLNA_HUMAN
Search ID: 41761
Search Name: 20130328_ananiav_ERstress_TMT_TM_timecourse_trypsin_inj2_4mc_iaa
Scan#: 14799
Observed Mass: 785.9133 (2 ppm)
PSM Score: 29.47

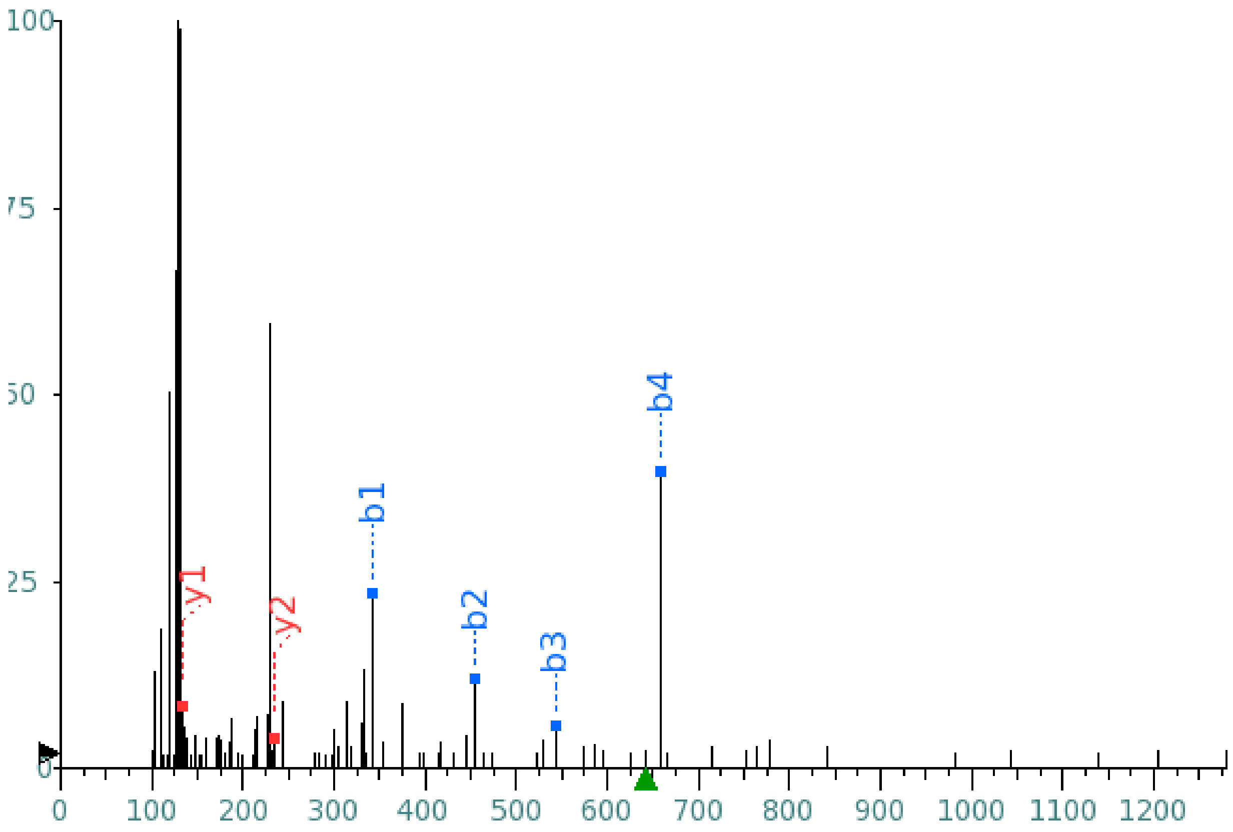

LISDFEDTD (+2)

Primary Reference: ZO2_HUMAN
Search ID: 41761
Search Name: 20130328_ananiav_ERstress_TMT_TM_timecourse_trypsin_inj2_4mc_iaa
Scan#: 14864
Observed Mass: 642.315 (1.8 ppm)
PSM Score: 8.89

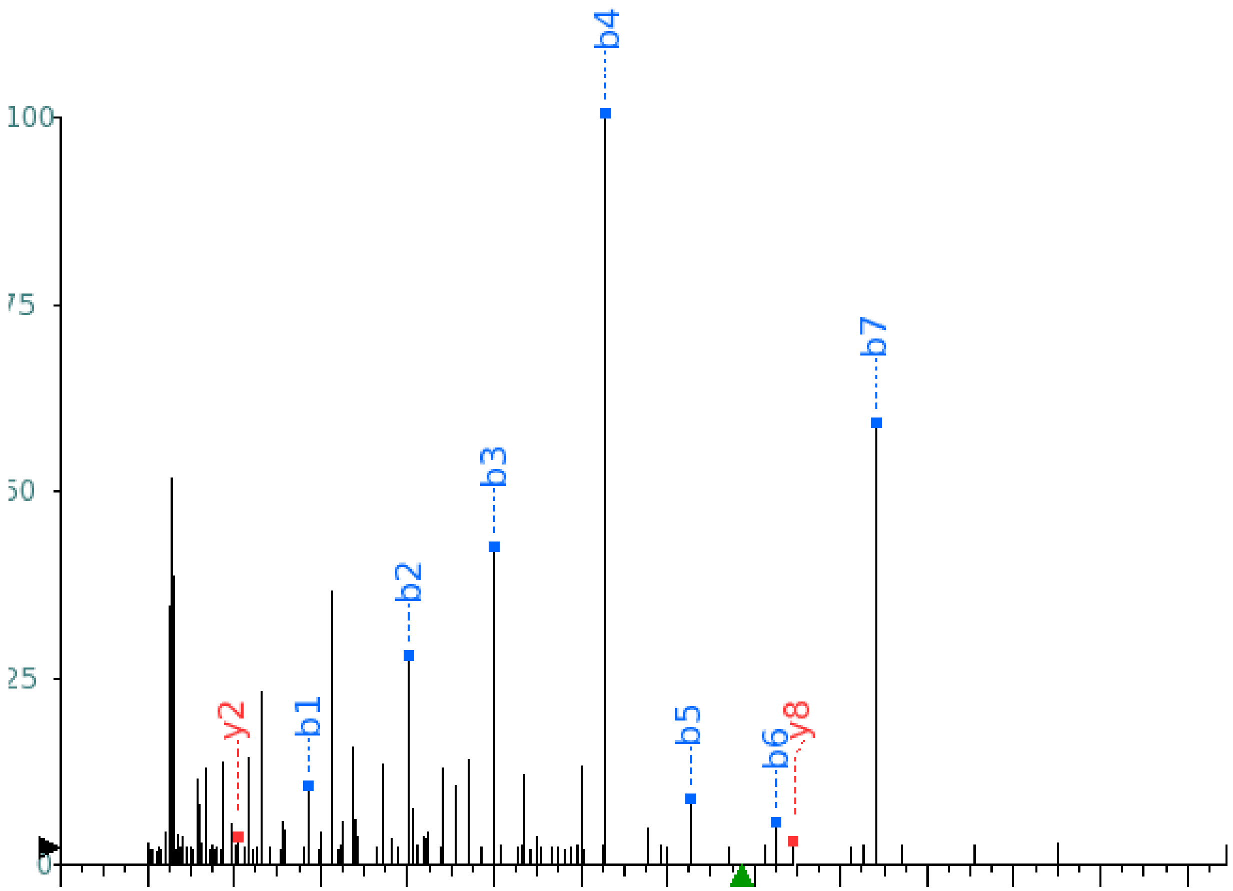

GLVEPVDVVDNAD (+2)

Primary Reference: FLNA_HUMAN
Search ID: 41761
Search Name: 20130328_ananiav_ERstress_TMT_TM_timecourse_trypsin_inj2_4mc_iaa
Scan#: 14877
Observed Mass: 785.9129 (1.5 ppm)
PSM Score: 20.93

File name: 2015-02-04 04.01.53PM.pdf

Creation time: 2015-02-04 04:09PM

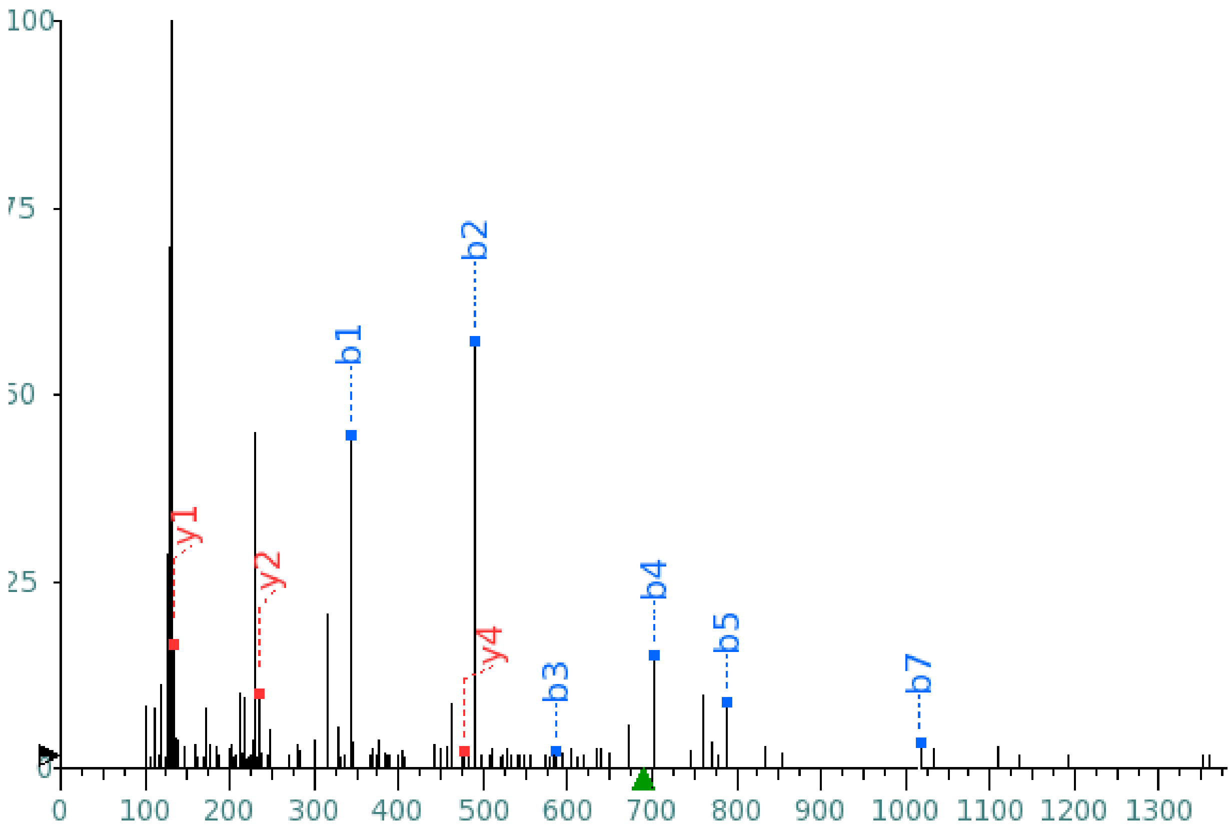

LFPNSLDQTD (+2)

Primary Reference: CALR_HUMAN
Search ID: 41761
Search Name: 20130328_ananiav_ERstress_TMT_TM_timecourse_trypsin_inj2_4mc_iaa
Scan#: 14897
Observed Mass: 689.857 (1.2 ppm)
PSM Score: 11.78

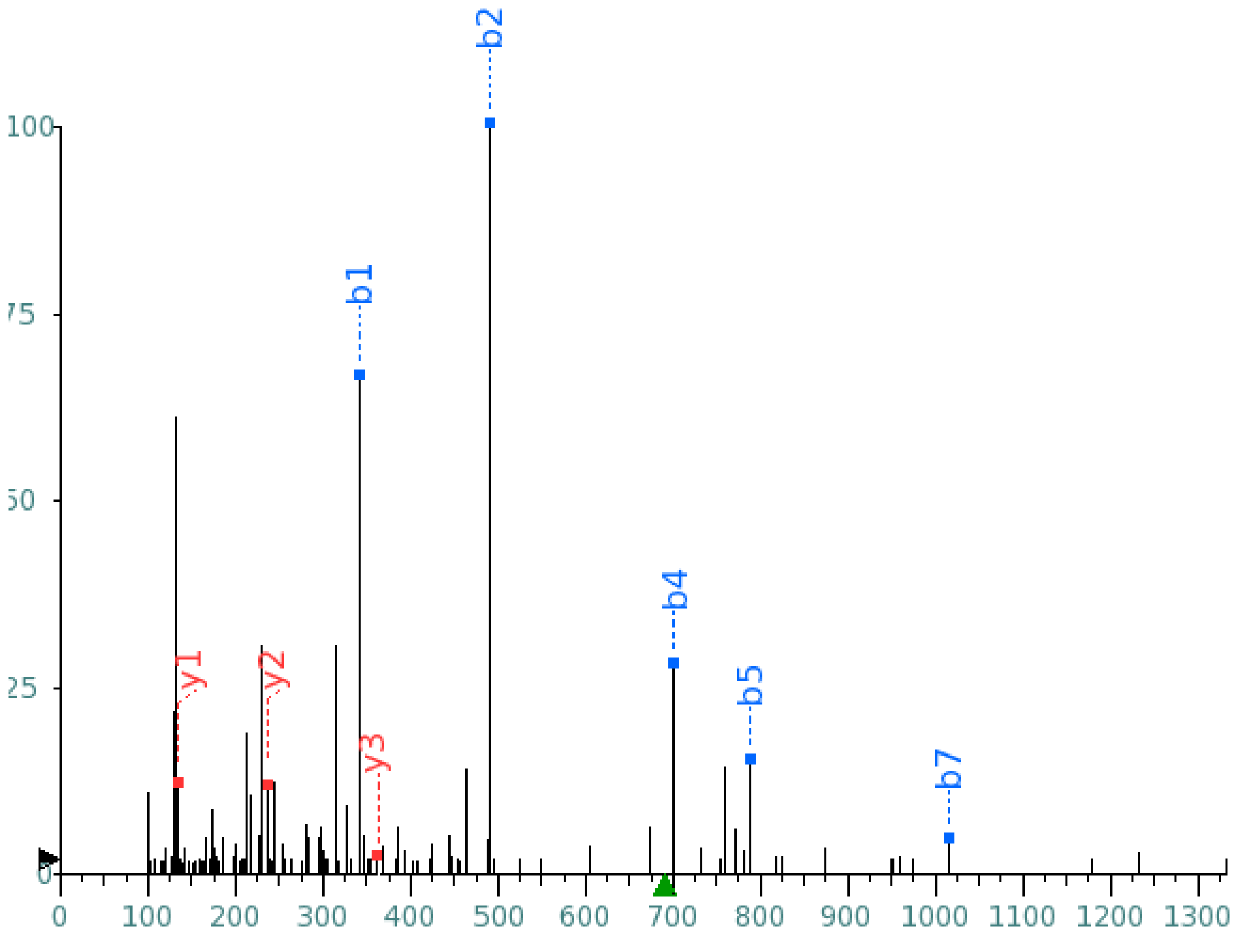

LFPNSLDQTD (+2)

Primary Reference: CALR_HUMAN
Search ID: 41761
Search Name: 20130328_ananiav_ERstress_TMT_TM_timecourse_trypsin_inj2_4mc_iaa
Scan#: 14974
Observed Mass: 689.8568 (0.8 ppm)
PSM Score: 12.3

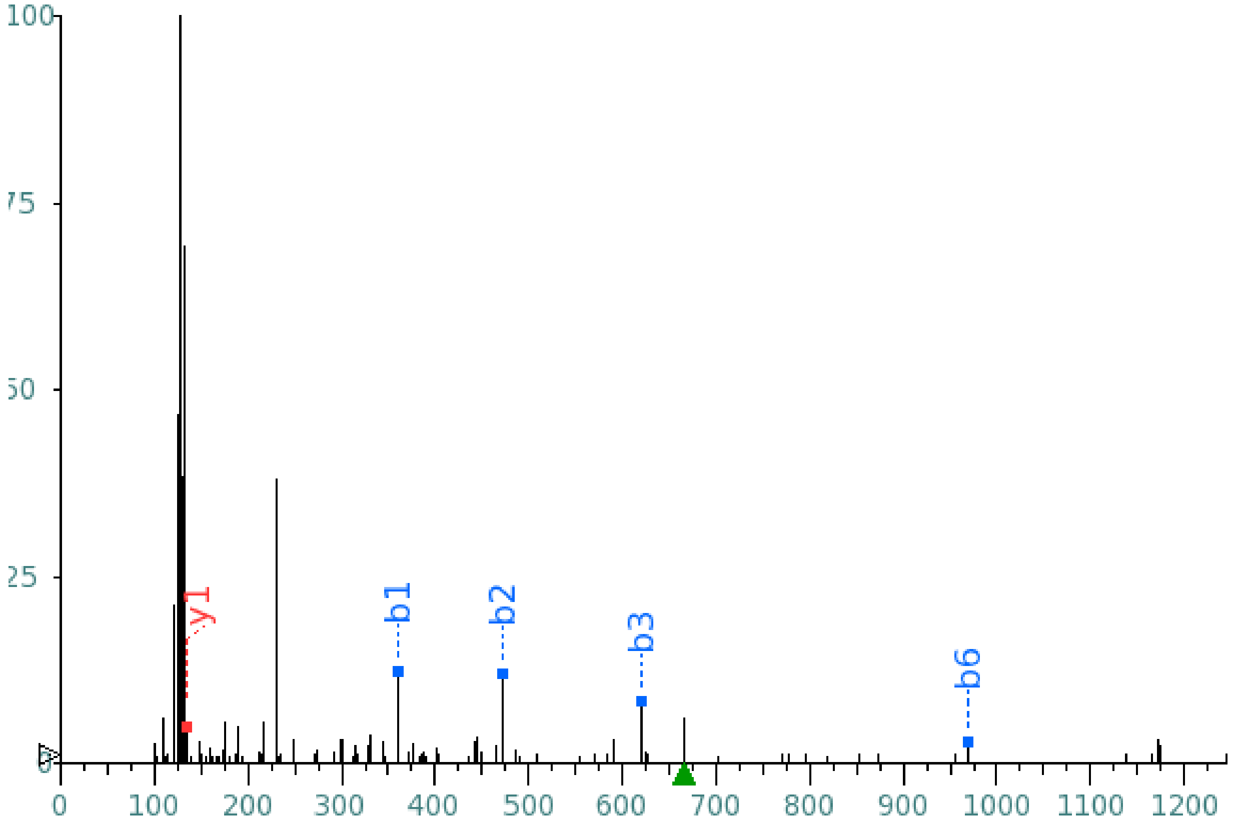

ELFFSDQTD (+2)

Primary Reference: PASK_HUMAN
Search ID: 41761
Search Name: 20130328_ananiav_ERstress_TMT_TM_timecourse_trypsin_inj2_4mc_iaa
Scan#: 14980
Observed Mass: 665.8229 (1.6 ppm)
PSM Score: 21.56

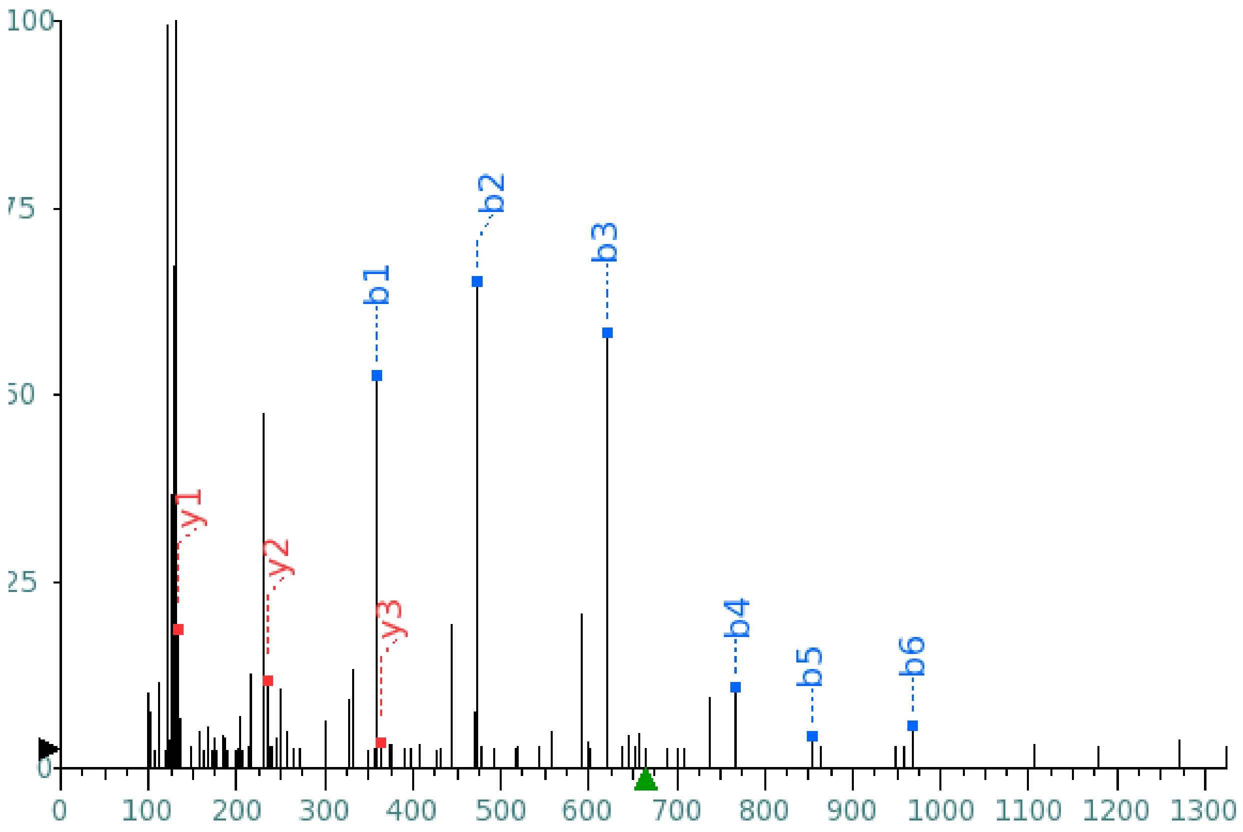

ELFFSDQTD (+2)

Primary Reference: PASK_HUMAN
Search ID: 41761
Search Name: 20130328_ananiav_ERstress_TMT_TM_timecourse_trypsin_inj2_4mc_iaa
Scan#: 15053
Observed Mass: 665.8231 (1.9 ppm)
PSM Score: 37.48

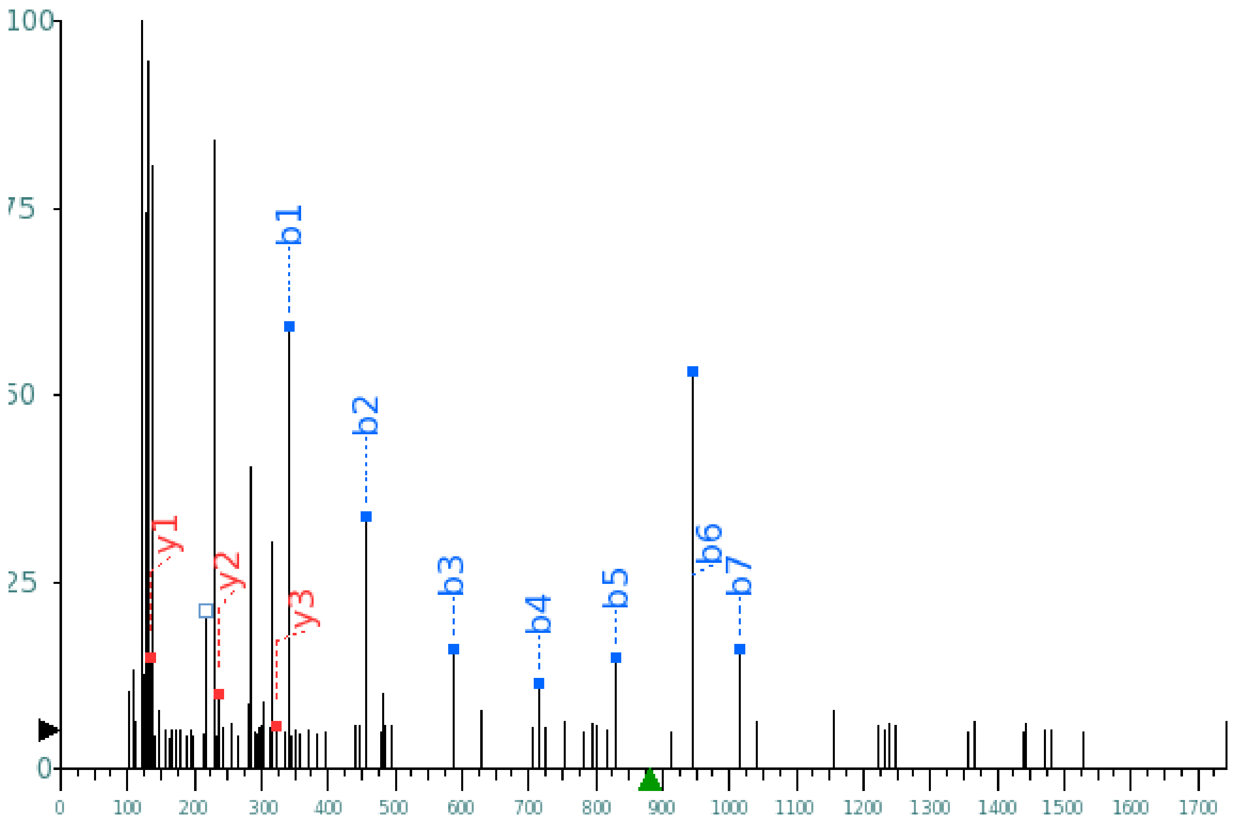

IIEEDDAYDFSTD (+2)

Primary Reference: GNL3_HUMAN
Search ID: 41761
Search Name: 20130328_ananiav_ERstress_TMT_TM_timecourse_trypsin_inj2_4mc_iaa
Scan#: 15056
Observed Mass: 881.4006 (2 ppm)
PSM Score: 32.86

File name: 2015-02-04 04.01.53PM.pdf

Creation time: 2015-02-04 04:09PM

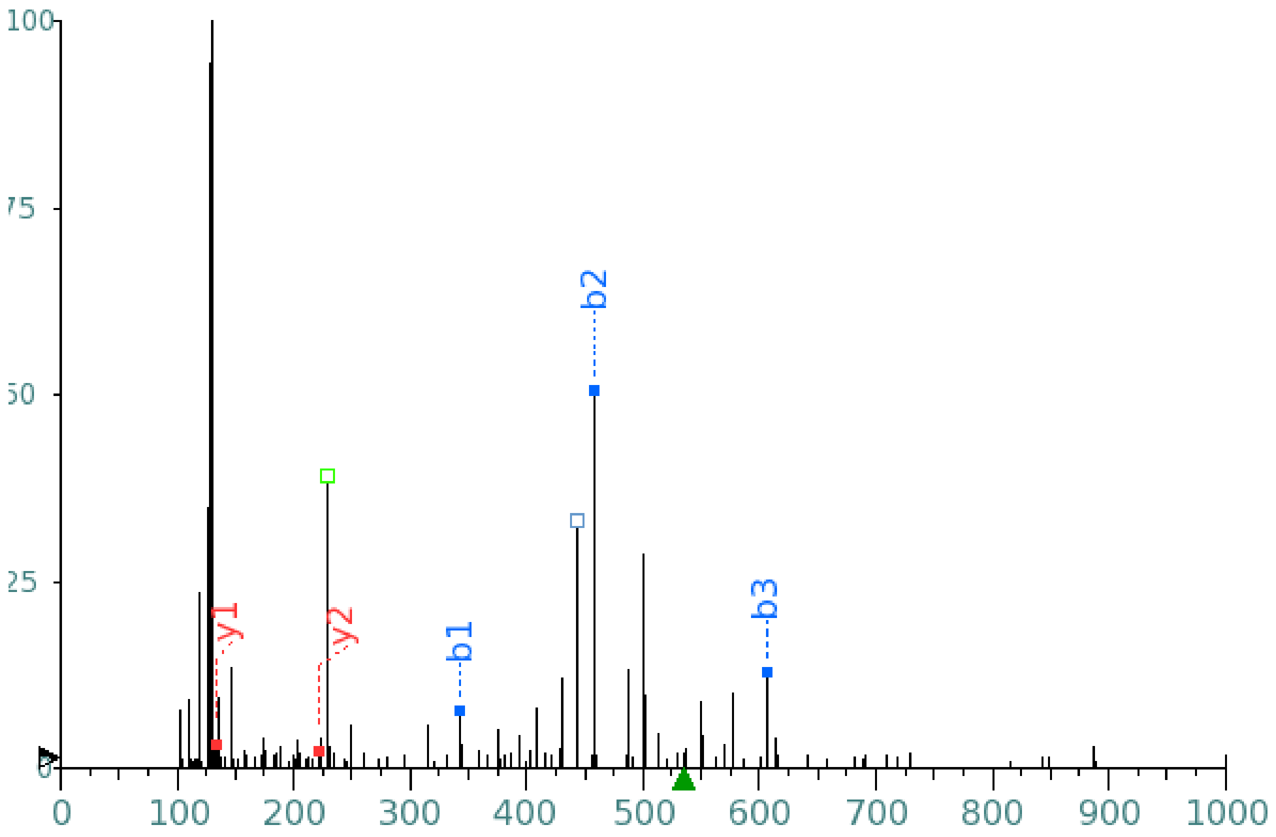

LDFIESD (+2)

Primary Reference: IF4G3_HUMAN
Search ID: 41761
Search Name: 20130328_ananiav_ERstress_TMT_TM_timecourse_trypsin_inj2_4mc_iaa
Scan#: 15059
Observed Mass: 534.2778 (2.3 ppm)
PSM Score: 6

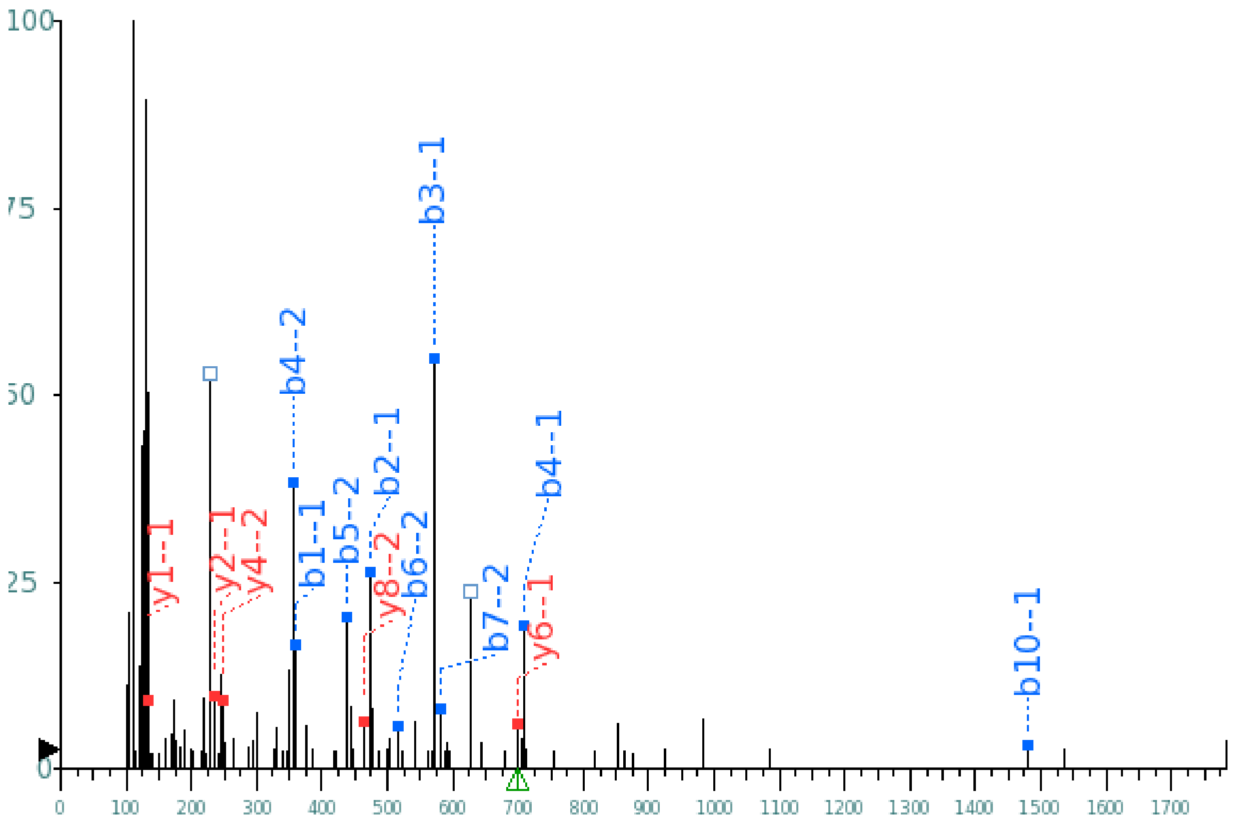

ELVHYYEQTSDMMTD (+3)

Primary Reference: PCM1_HUMAN
Search ID: 41761
Search Name: 20130328_ananiav_ERstress_TMT_TM_timecourse_trypsin_inj2_4mc_iaa
Scan#: 15076
Observed Mass: 697.6482 (2.5 ppm)
PSM Score: 14.03

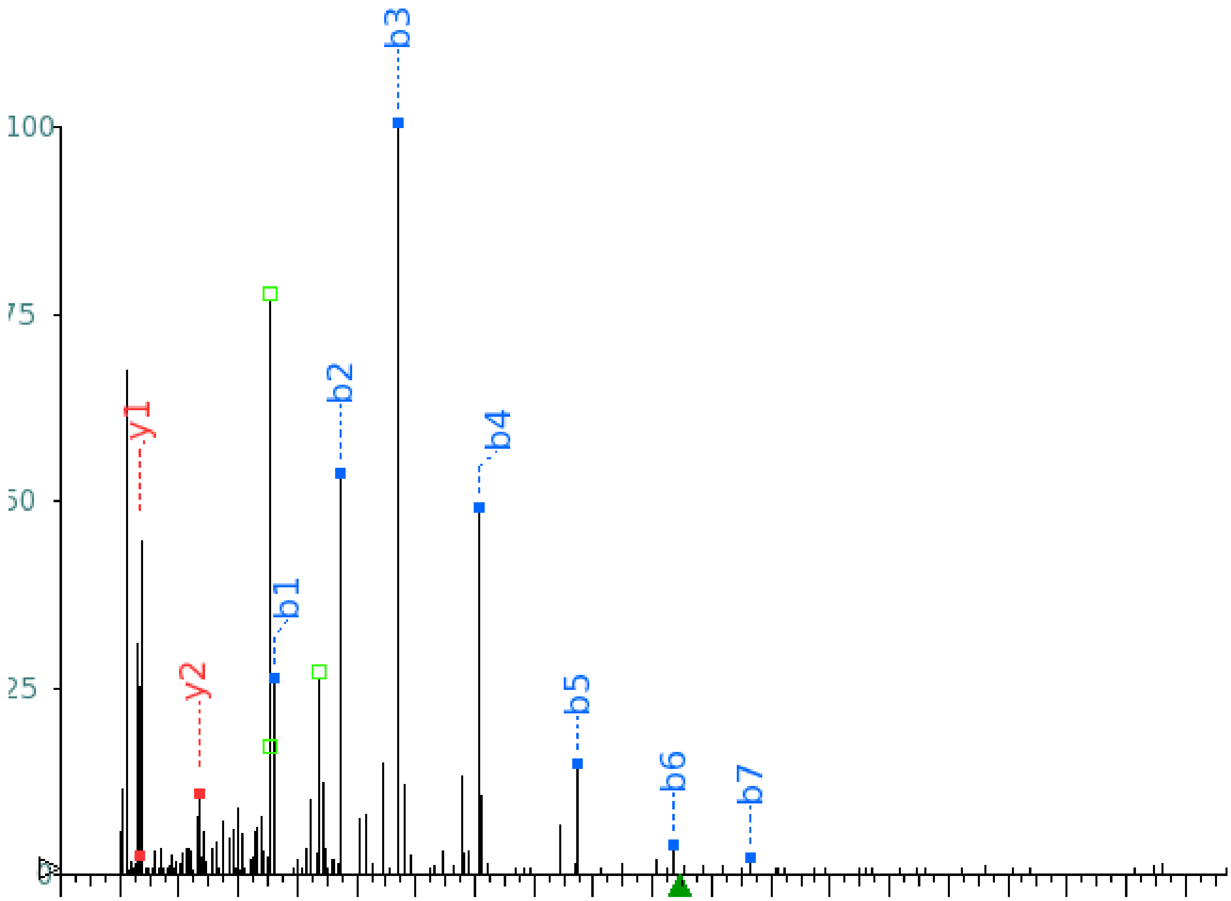

ELVHYYEQTSDMMTD (+2)

Primary Reference: PCM1_HUMAN
Search ID: 41761
Search Name: 20130328_ananiav_ERstress_TMT_TM_timecourse_trypsin_inj2_4mc_iaa
Scan#: 15087
Observed Mass: 1045.9645 (-1.5 ppm)
PSM Score: 27.92

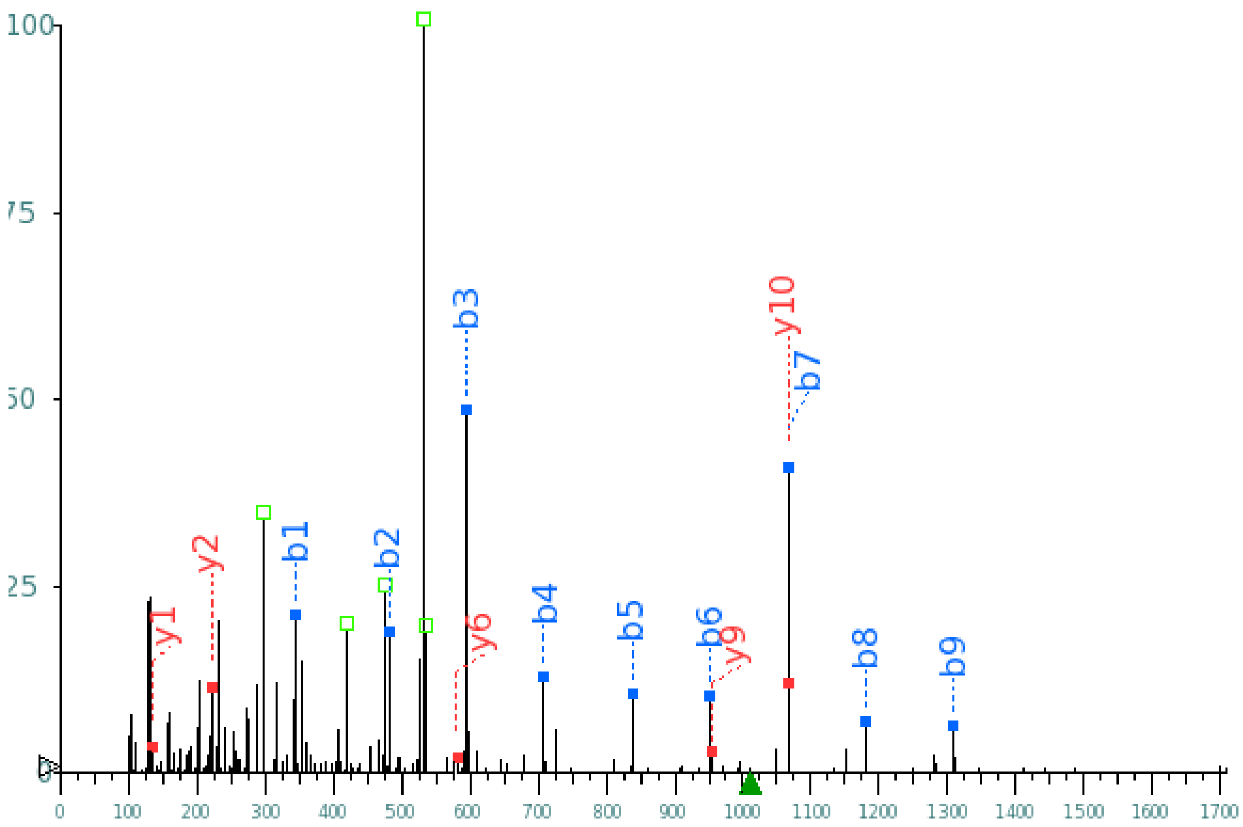

IHDLEDDLEMSSDASD (+2)

Primary Reference: T2FA_HUMAN
Search ID: 41761
Search Name: 20130328_ananiav_ERstress_TMT_TM_timecourse_trypsin_inj2_4mc_iaa
Scan#: 15100
Observed Mass: 1010.9478 (1.5 ppm)
PSM Score: 41.4

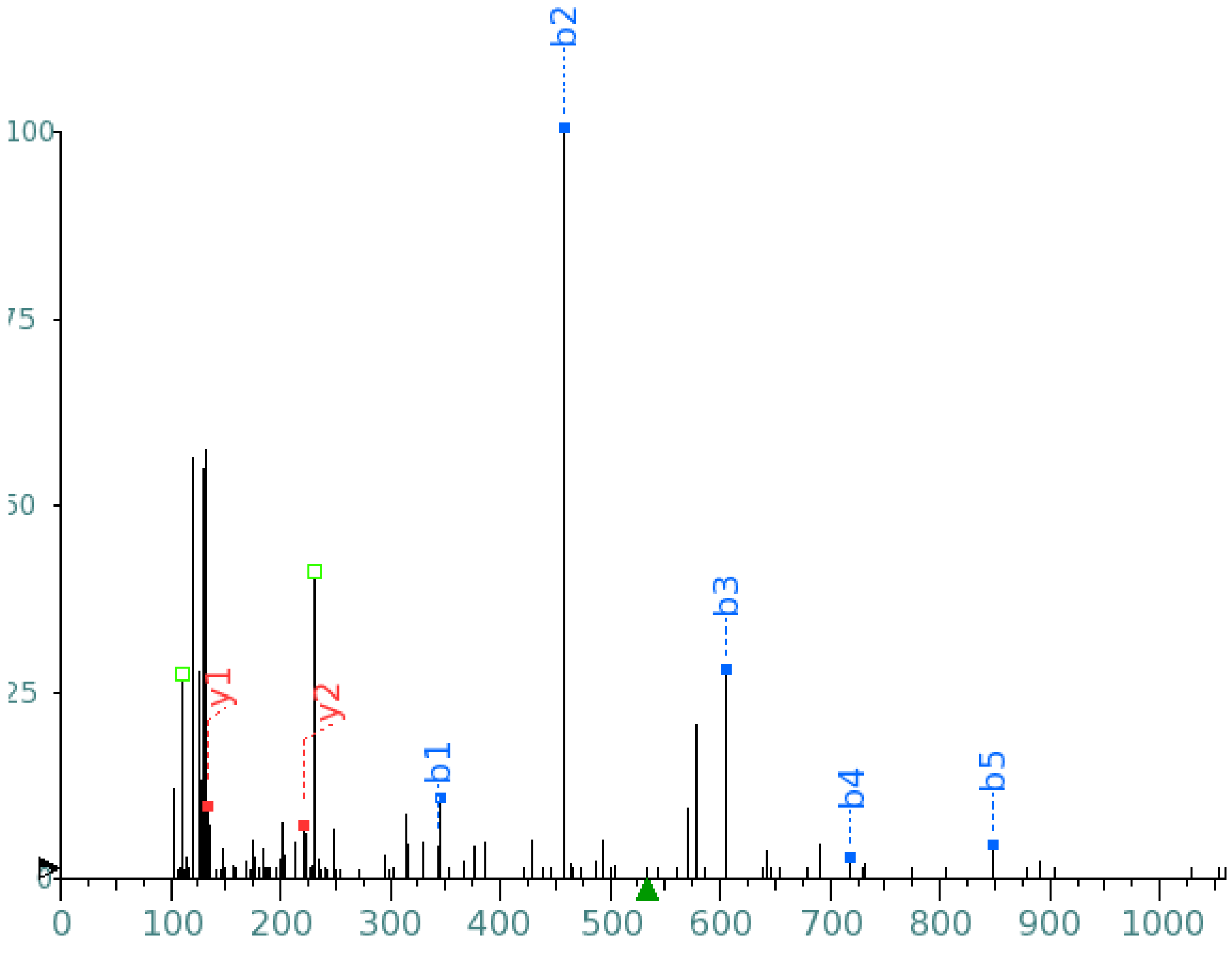

LDFIESD (+2)

Primary Reference: IF4G3_HUMAN
Search ID: 41761
Search Name: 20130328_ananiav_ERstress_TMT_TM_timecourse_trypsin_inj2_4mc_iaa
Scan#: 15129
Observed Mass: 534.2778 (2.2 ppm)
PSM Score: 16.58

File name: 2015-02-04 04.01.53PM.pdf

Creation time: 2015-02-04 04:09PM

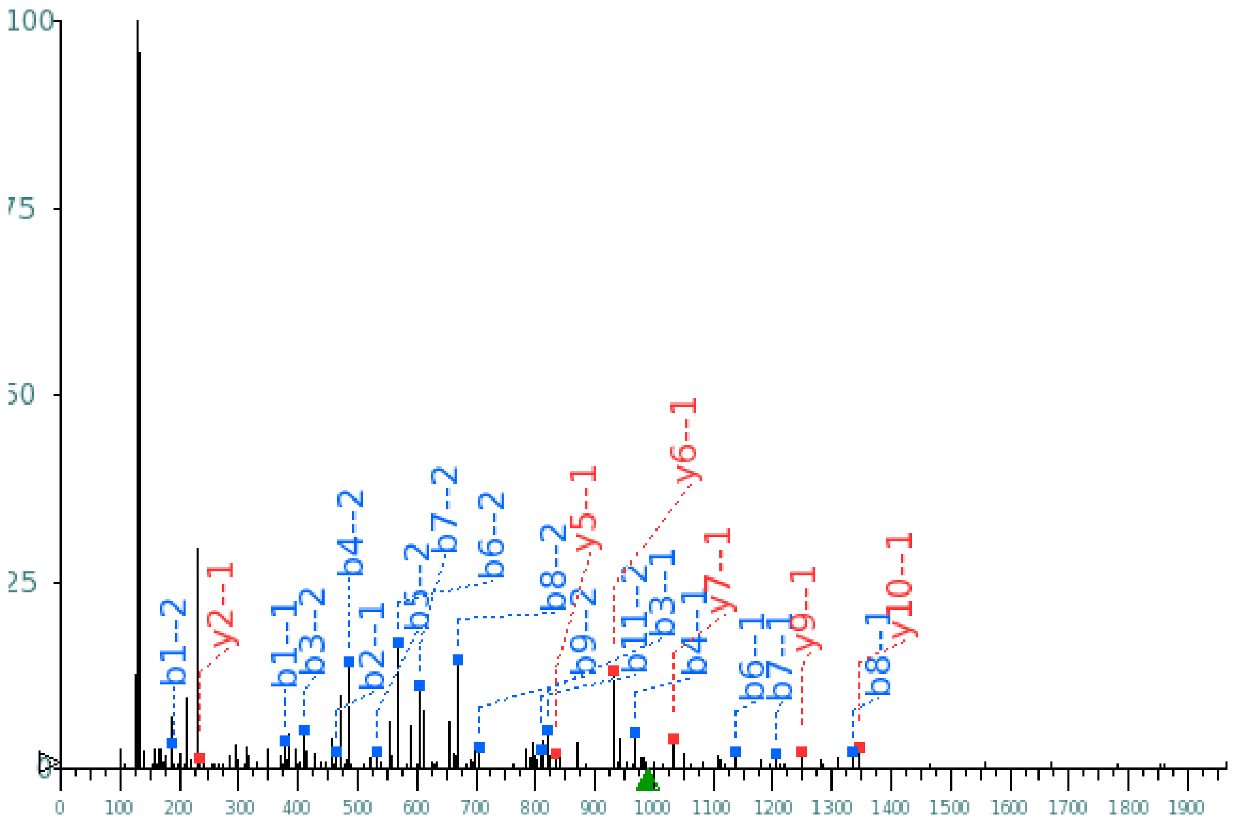

FSKFPAAQAPDPSETPKIETD (+3)

Primary Reference: ABLM1_HUMAN

Search ID: 41761

Search Name: 20130328_ananiav_ERstress_TMT_TM_timecourse_trypsin_inj2_4mc_iaa

Scan#: 15228

Observed Mass: 988.5394 (2.2 ppm)

PSM Score: 22.17

File name: 2015-02-04 04.01.53PM.pdf

Creation time: 2015-02-04 04:09PM

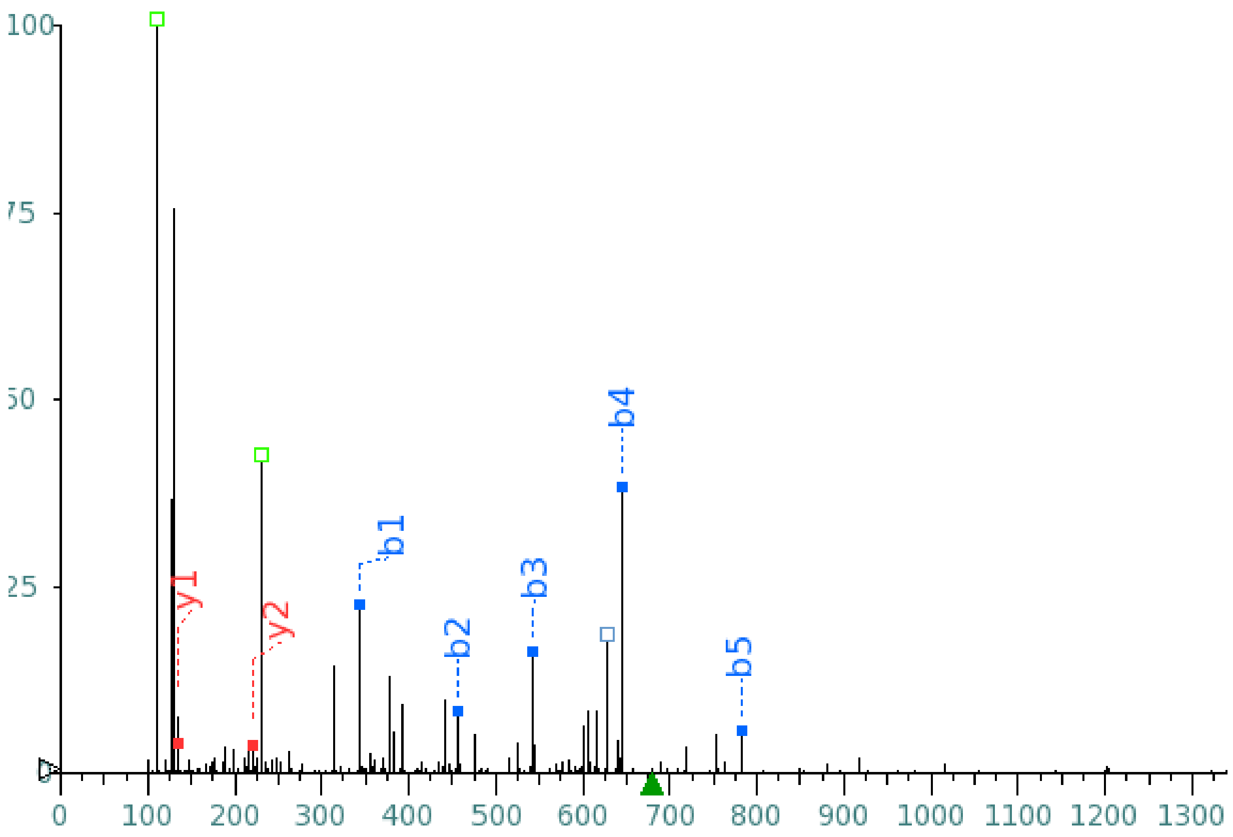

ILSTHLLESD (+2)

Primary Reference: CLOCK_HUMAN
Search ID: 41761
Search Name: 20130328_ananiav_ERstress_TMT_TM_timecourse_trypsin_inj2_4mc_iaa
Scan#: 15239
Observed Mass: 678.8844 (3.2 ppm)
PSM Score: 12.1

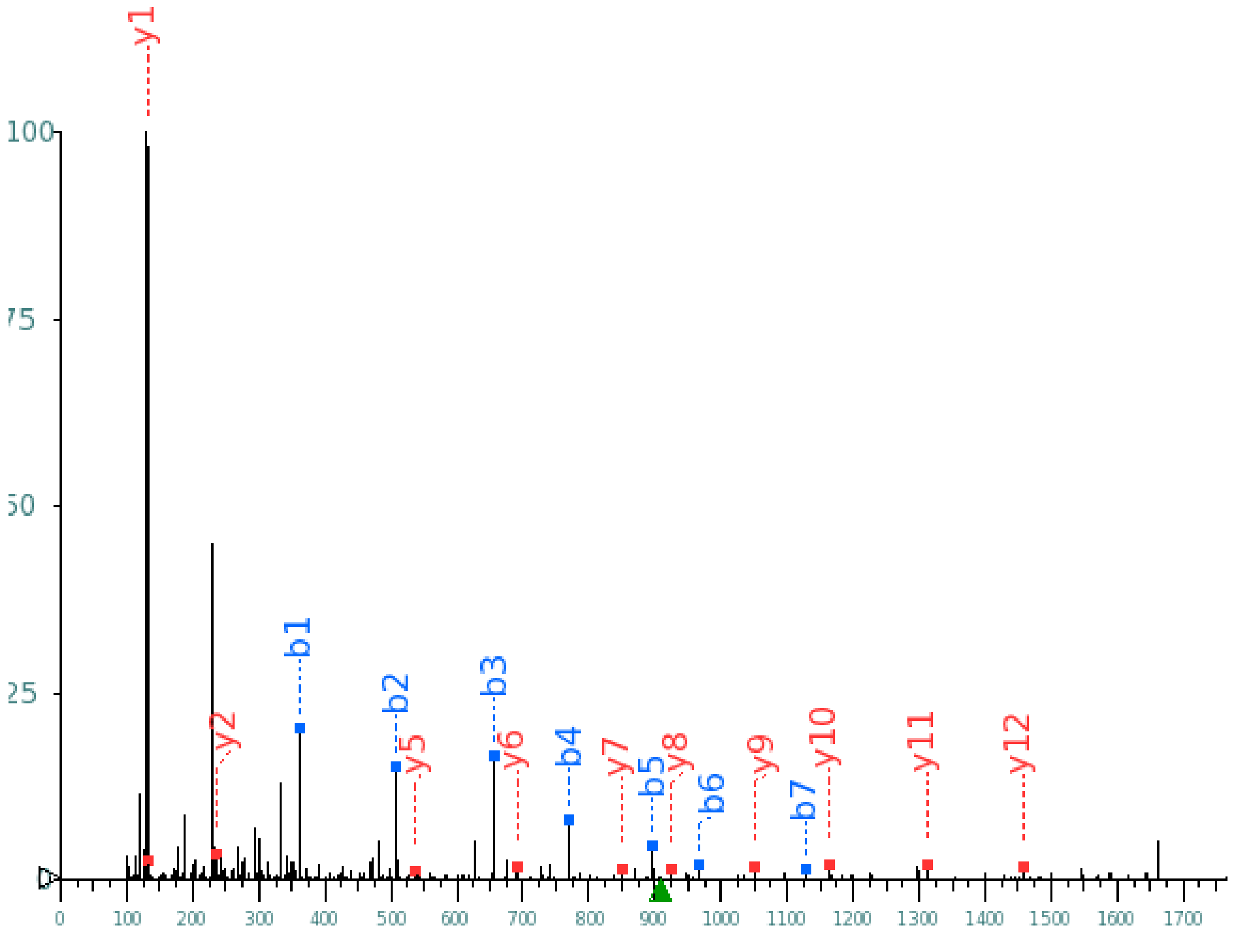

MFFIQAC*RGDETD (+2)

Primary Reference: CASP2_HUMAN
Search ID: 41761
Search Name: 20130328_ananiav_ERstress_TMT_TM_timecourse_trypsin_inj2_4mc_iaa
Scan#: 15266
Observed Mass: 909.9231 (2 ppm)
PSM Score: 24.87

File name: 2015-02-04 04.01.53PM.pdf

Creation time: 2015-02-04 04:09PM

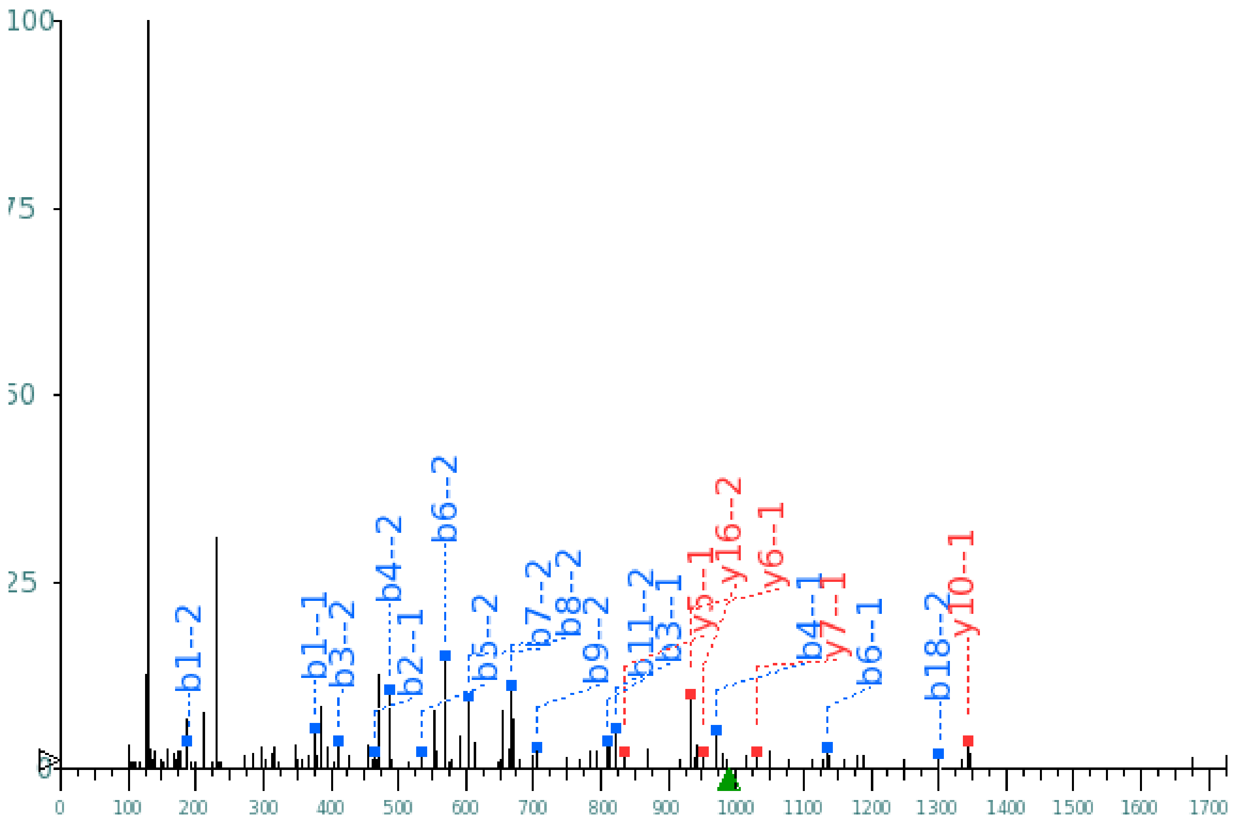

FSKFPAAQAPDPSETPKIETD (+3)

Primary Reference: ABLM1_HUMAN
Search ID: 41761
Search Name: 20130328_ananiav_ERstress_TMT_TM_timecourse_trypsin_inj2_4mc_iaa
Scan#: 15309
Observed Mass: 988.5397 (2.6 ppm)
PSM Score: 18.83

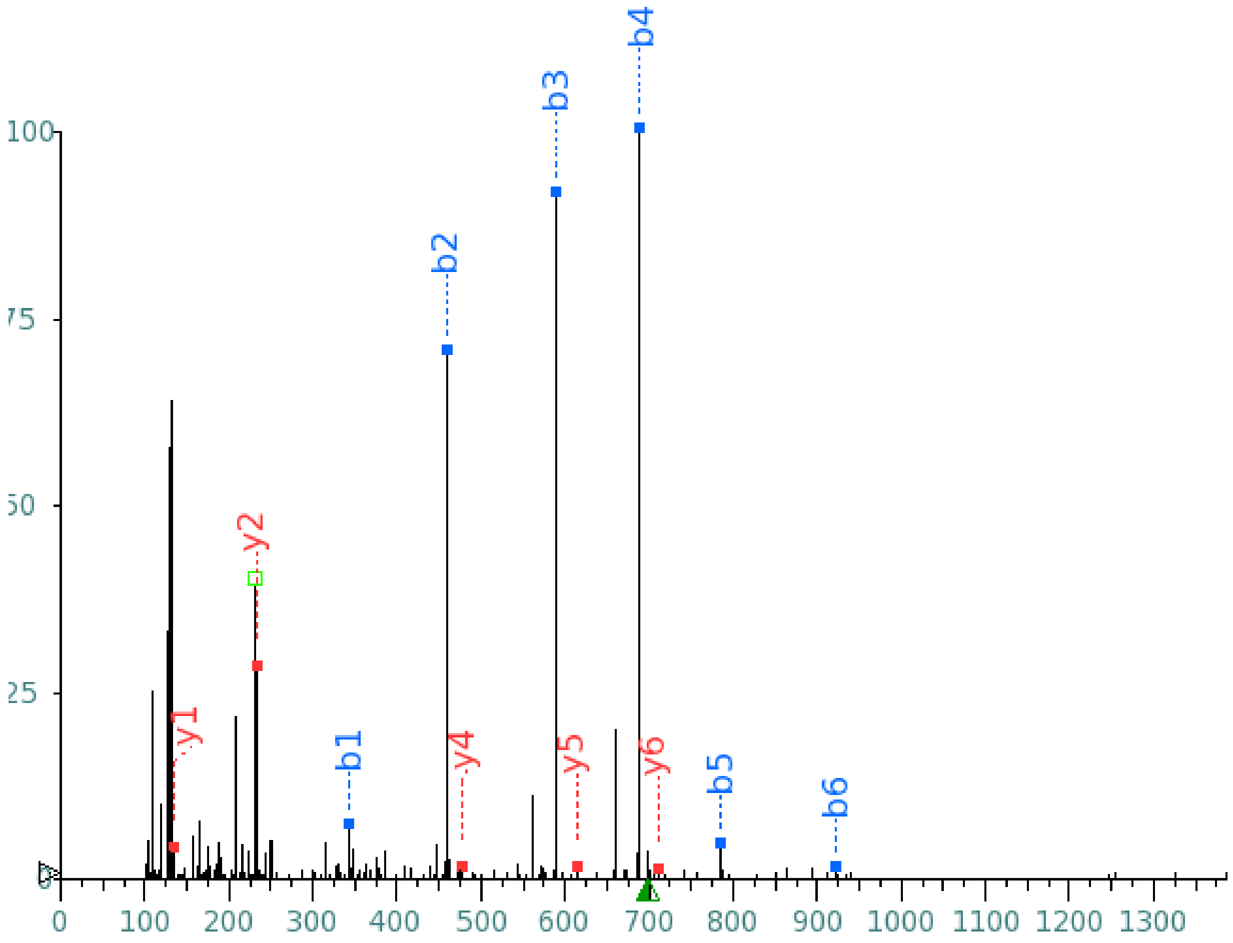

LDMVPHLETD (+2)

Primary Reference: AKP8L_HUMAN
Search ID: 41761
Search Name: 20130328_ananiav_ERstress_TMT_TM_timecourse_trypsin_inj2_4mc_iaa
Scan#: 15313
Observed Mass: 699.8622 (2.4 ppm)
PSM Score: 36.95

File name: 2015-02-04 04.01.53PM.pdf

Creation time: 2015-02-04 04:09PM

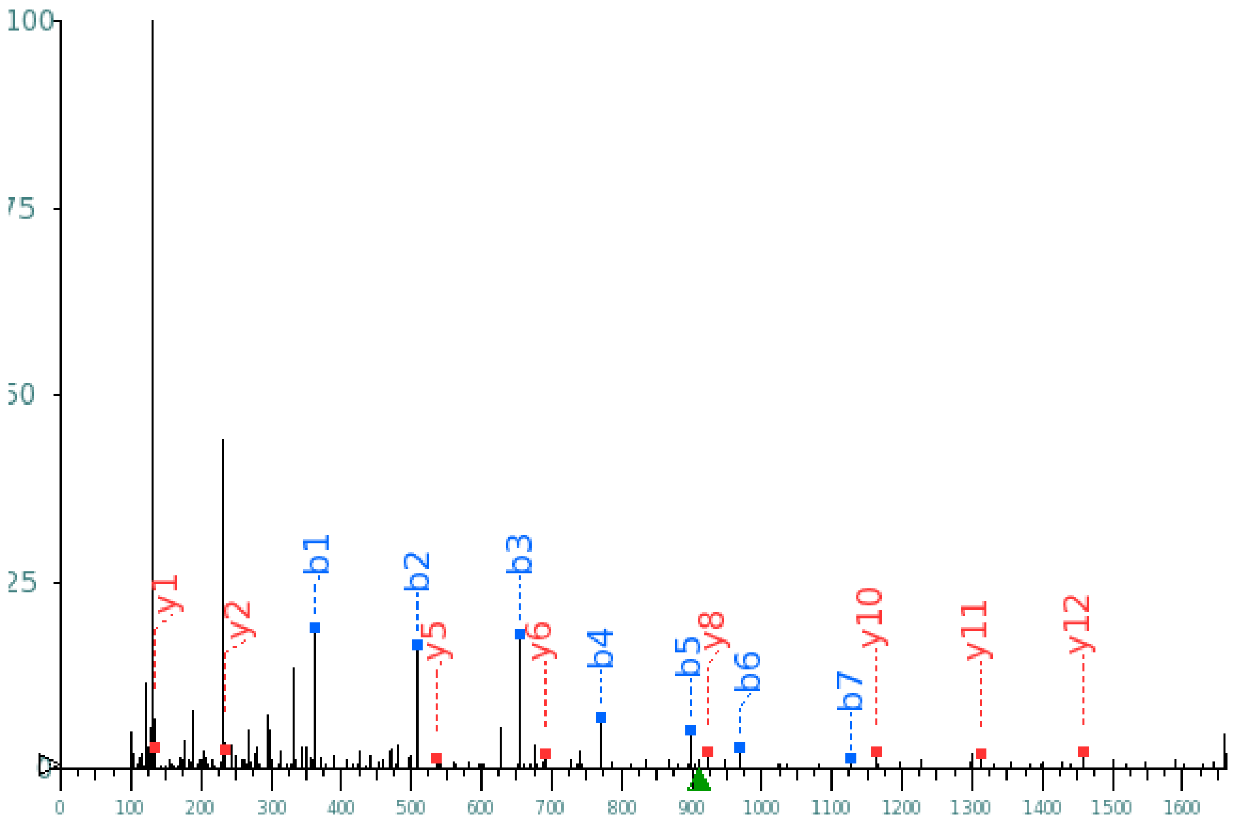

MFFIQAC*RGDETD (+2)

Primary Reference: CASP2_HUMAN
Search ID: 41761
Search Name: 20130328_ananiav_ERstress_TMT_TM_timecourse_trypsin_inj2_4mc_iaa
Scan#: 15338
Observed Mass: 909.9229 (1.8 ppm)
PSM Score: 29.29

File name: 2015-02-04 04.01.53PM.pdf

Creation time: 2015-02-04 04:09PM

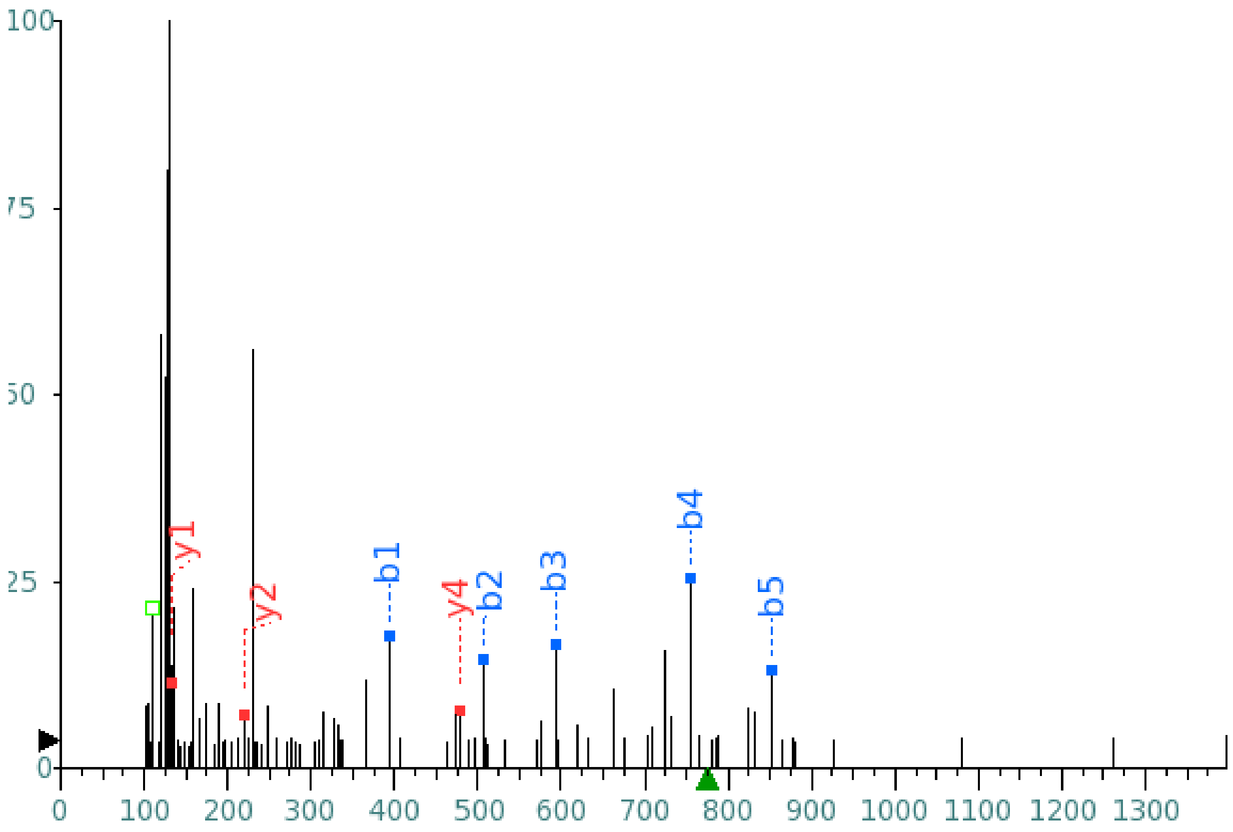

YISC*VFAEESD (+2)

Primary Reference: TF3C1_HUMAN
Search ID: 41761
Search Name: 20130328_ananiav_ERstress_TMT_TM_timecourse_trypsin_inj2_4mc_iaa
Scan#: 15405
Observed Mass: 774.8598 (2.2 ppm)
PSM Score: 9.62

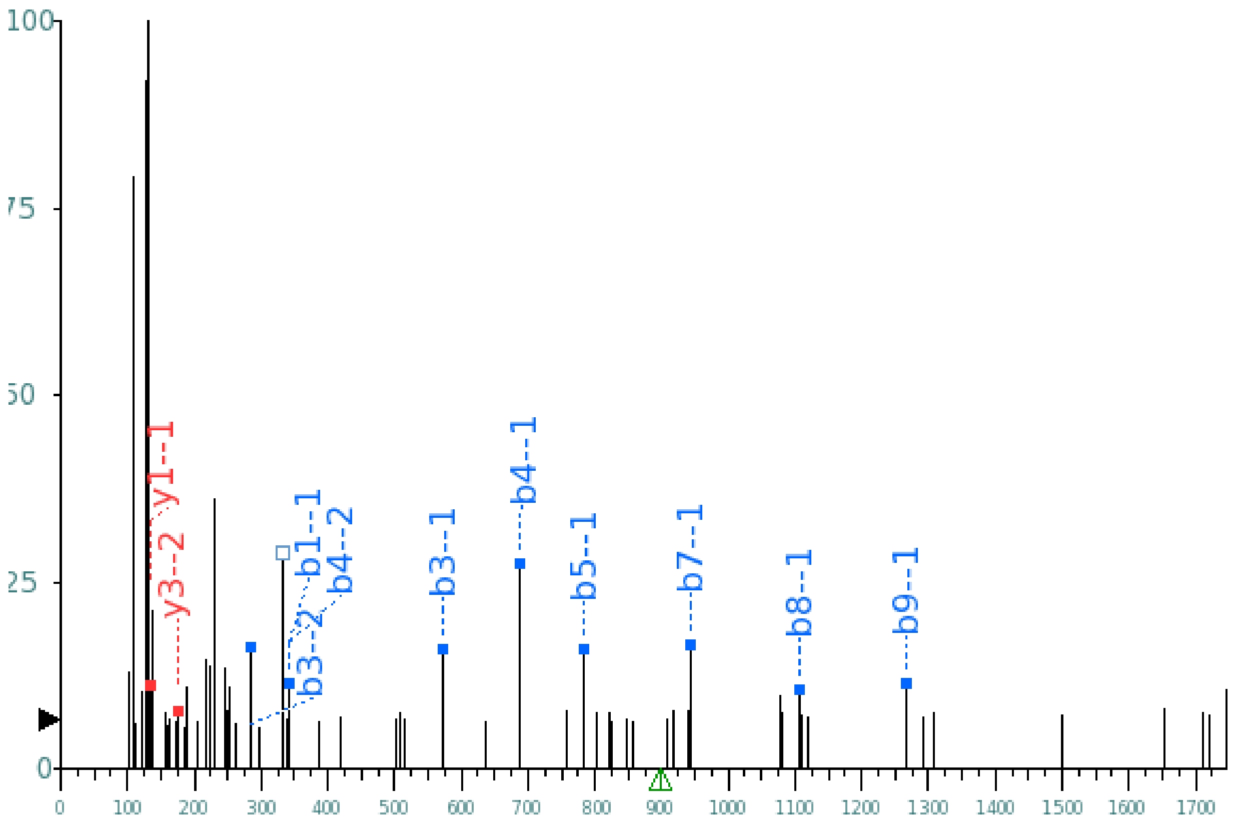

LNDNVSAYC*LHIAEDDGEVDTD (+3)

Primary Reference: SIN1_HUMAN
Search ID: 41761
Search Name: 20130328_ananiav_ERstress_TMT_TM_timecourse_trypsin_inj2_4mc_iaa
Scan#: 15462
Observed Mass: 898.7423 (3.2 ppm)
PSM Score: 18.01

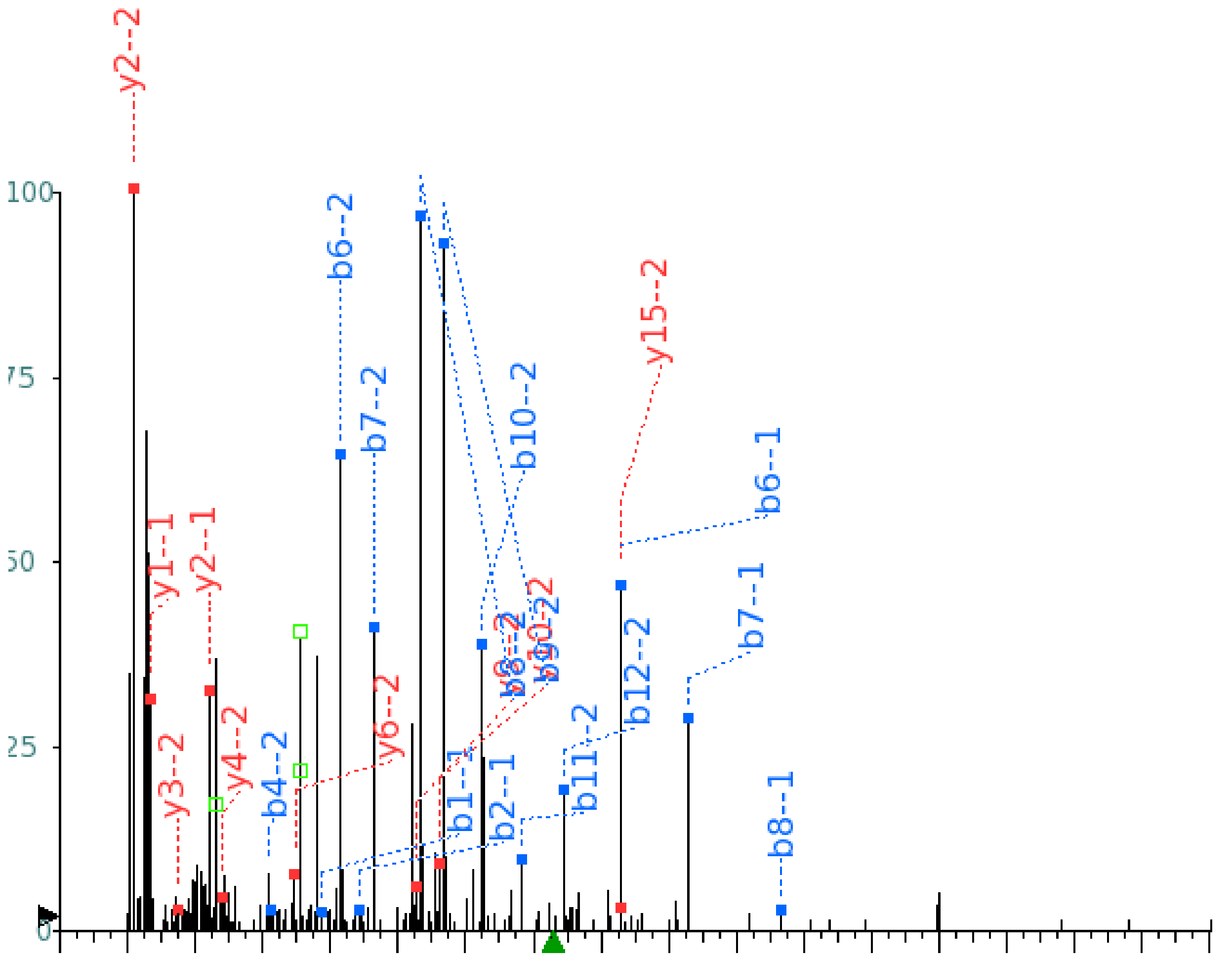

RGSSPDVHALLEITEESD (+3)

Primary Reference: PTRF_HUMAN
Search ID: 41761
Search Name: 20130328_ananiav_ERstress_TMT_TM_timecourse_trypsin_inj2_4mc_iaa
Scan#: 15660
Observed Mass: 728.7052 (1.3 ppm)
PSM Score: 23.24

File name: 2015-02-04 04.01.53PM.pdf

Creation time: 2015-02-04 04:09PM

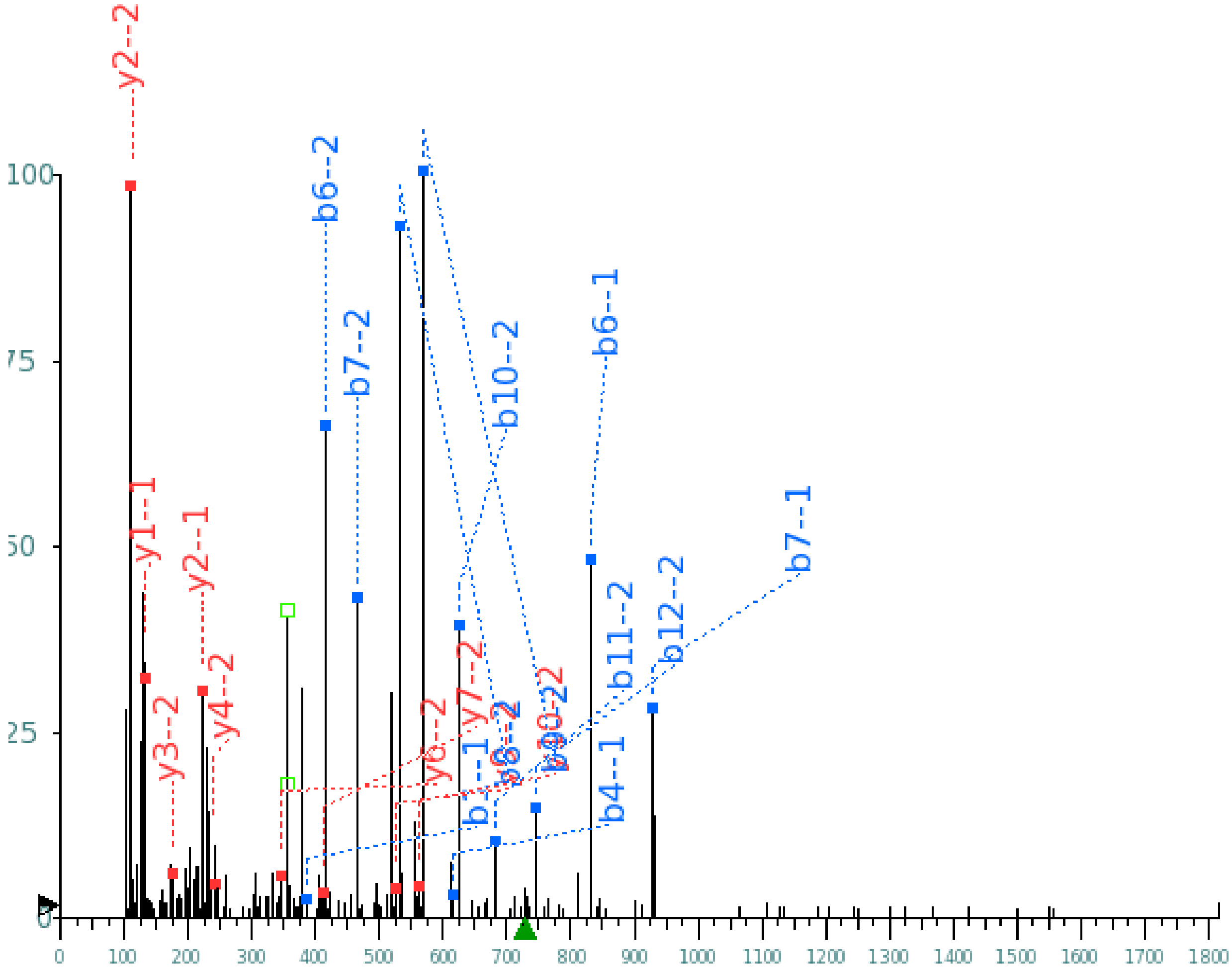

RGSSPDVHALLEITEESD (+3)

Primary Reference: PTRF_HUMAN
Search ID: 41761
Search Name: 20130328_ananiav_ERstress_TMT_TM_timecourse_trypsin_inj2_4mc_iaa
Scan#: 15738
Observed Mass: 728.7053 (1.4 ppm)
PSM Score: 21.35

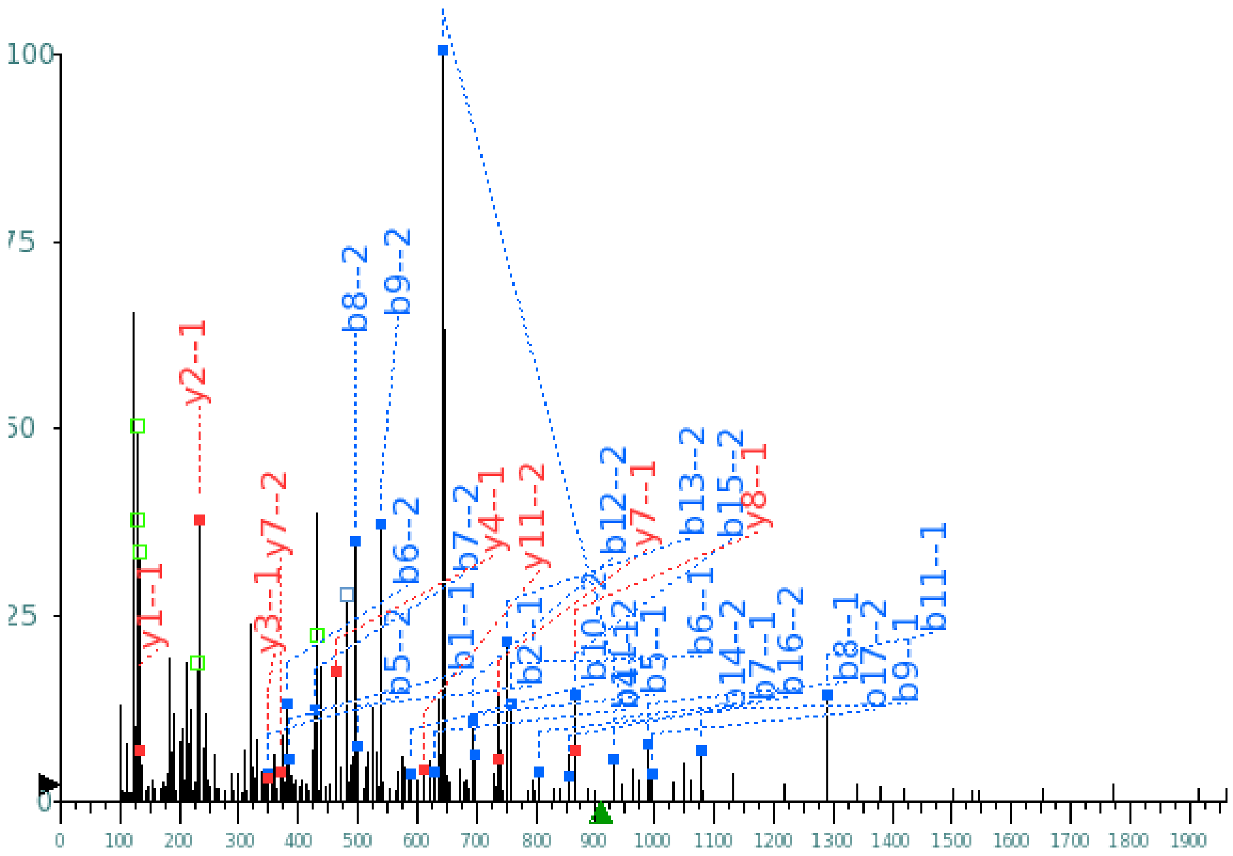

RNGAAGPHSPDPLLDEQAFGDLTD (+3)

Primary Reference: BORG4_HUMAN
Search ID: 41761
Search Name: 20130328_ananiav_ERstress_TMT_TM_timecourse_trypsin_inj2_4mc_iaa
Scan#: 15792
Observed Mass: 908.1147 (0.9 ppm)
PSM Score: 32.4

File name: 2015-02-04 04.01.53PM.pdf

Creation time: 2015-02-04 04:09PM

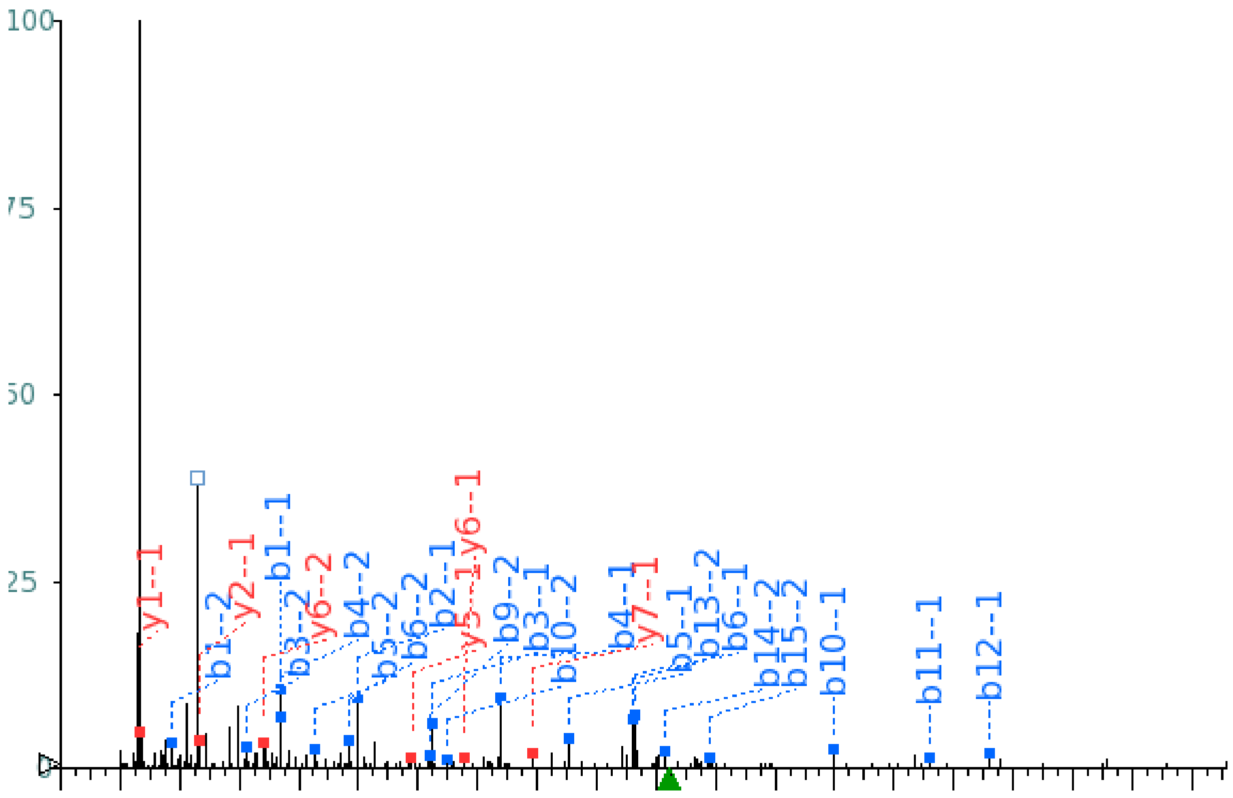

HEQNIDC*GGGYVKLFPNSLDQTD (+3)

Primary Reference: CALR_HUMAN
Search ID: 41761
Search Name: 20130328_ananiav_ERstress_TMT_TM_timecourse_trypsin_inj2_4mc_iaa
Scan#: 15803
Observed Mass: 1022.5095 (3.2 ppm)
PSM Score: 28.79

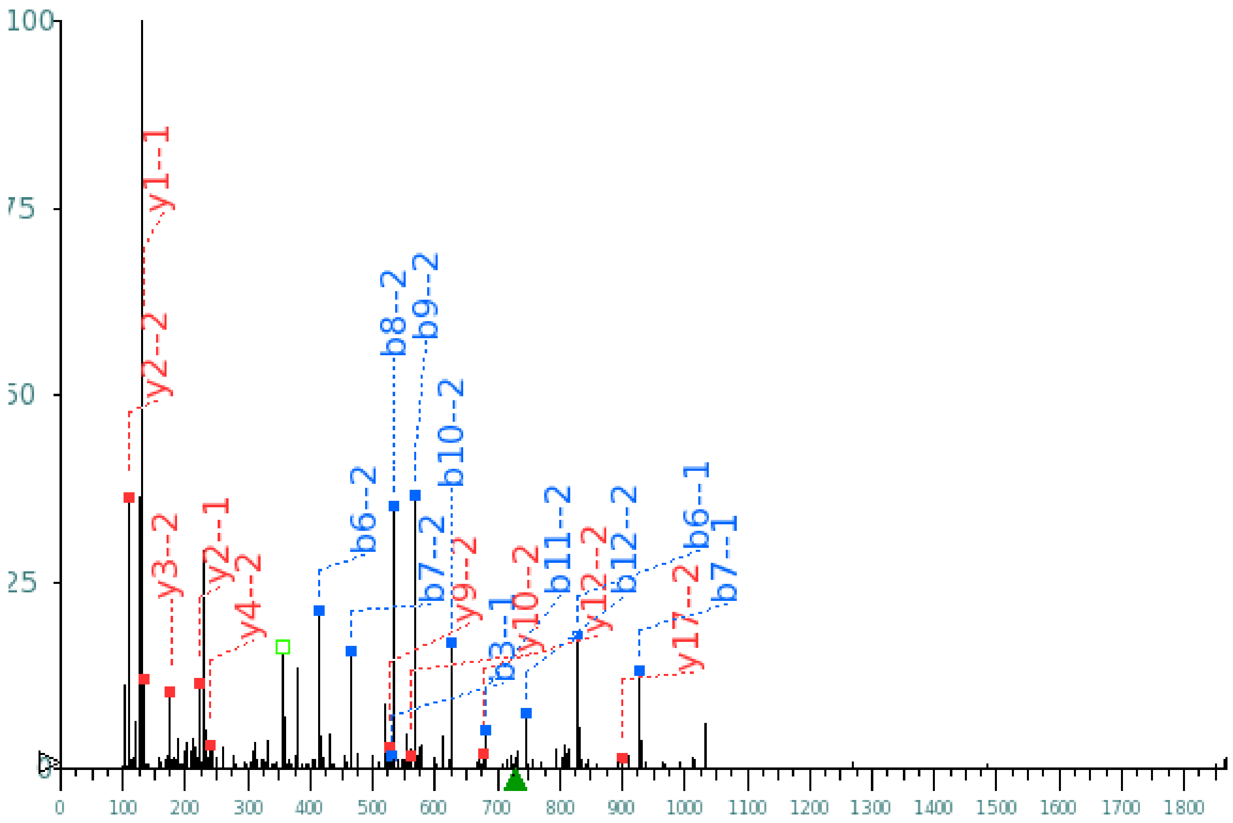

RGSSPDVHALLEITEESD (+3)

Primary Reference: PTRF_HUMAN
Search ID: 41761
Search Name: 20130328_ananiav_ERstress_TMT_TM_timecourse_trypsin_inj2_4mc_iaa
Scan#: 15814
Observed Mass: 728.7055 (1.7 ppm)
PSM Score: 18.79

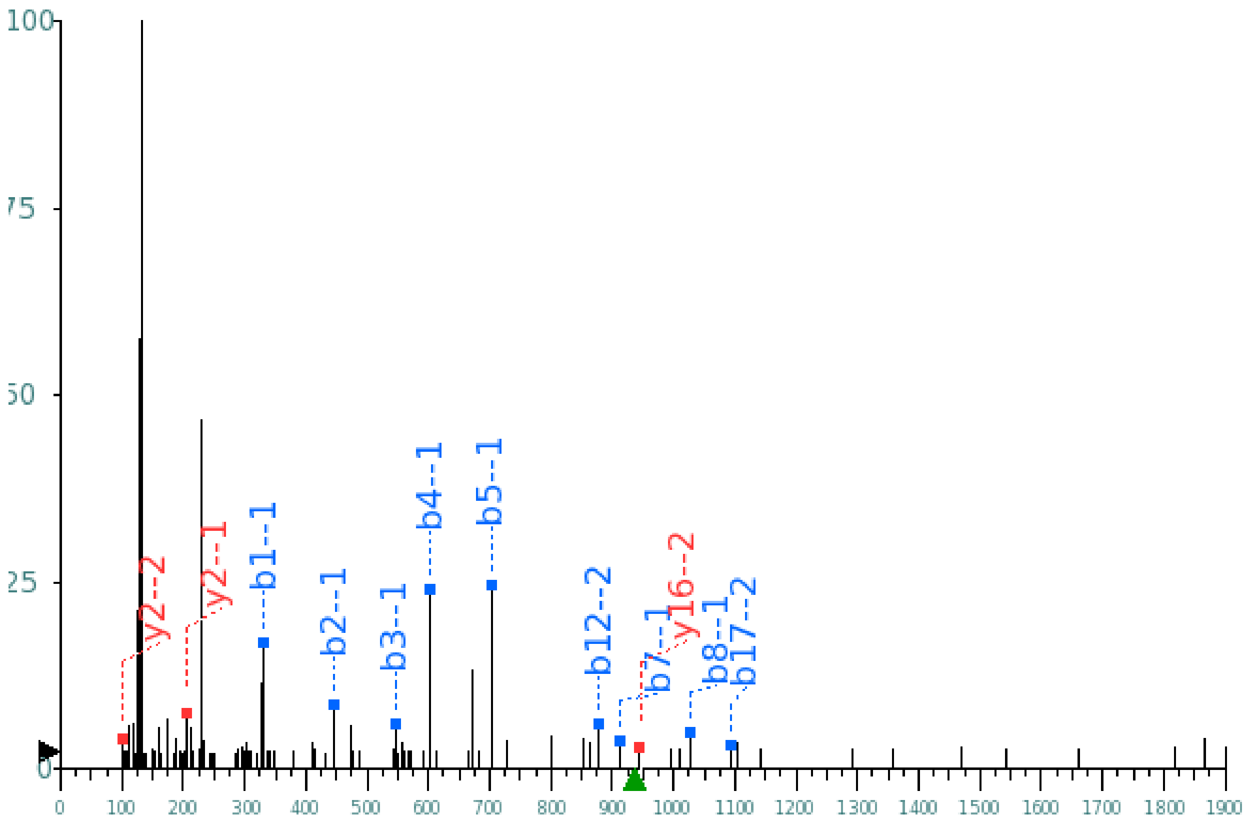

TITGVPDNIQKELASGTGQDDAD (+3)

Primary Reference: E2QRJ1_HUMAN
Search ID: 41761
Search Name: 20130328_ananiav_ERstress_TMT_TM_timecourse_trypsin_inj2_4mc_iaa
Scan#: 15867
Observed Mass: 935.1529 (2.8 ppm)
PSM Score: 22.92

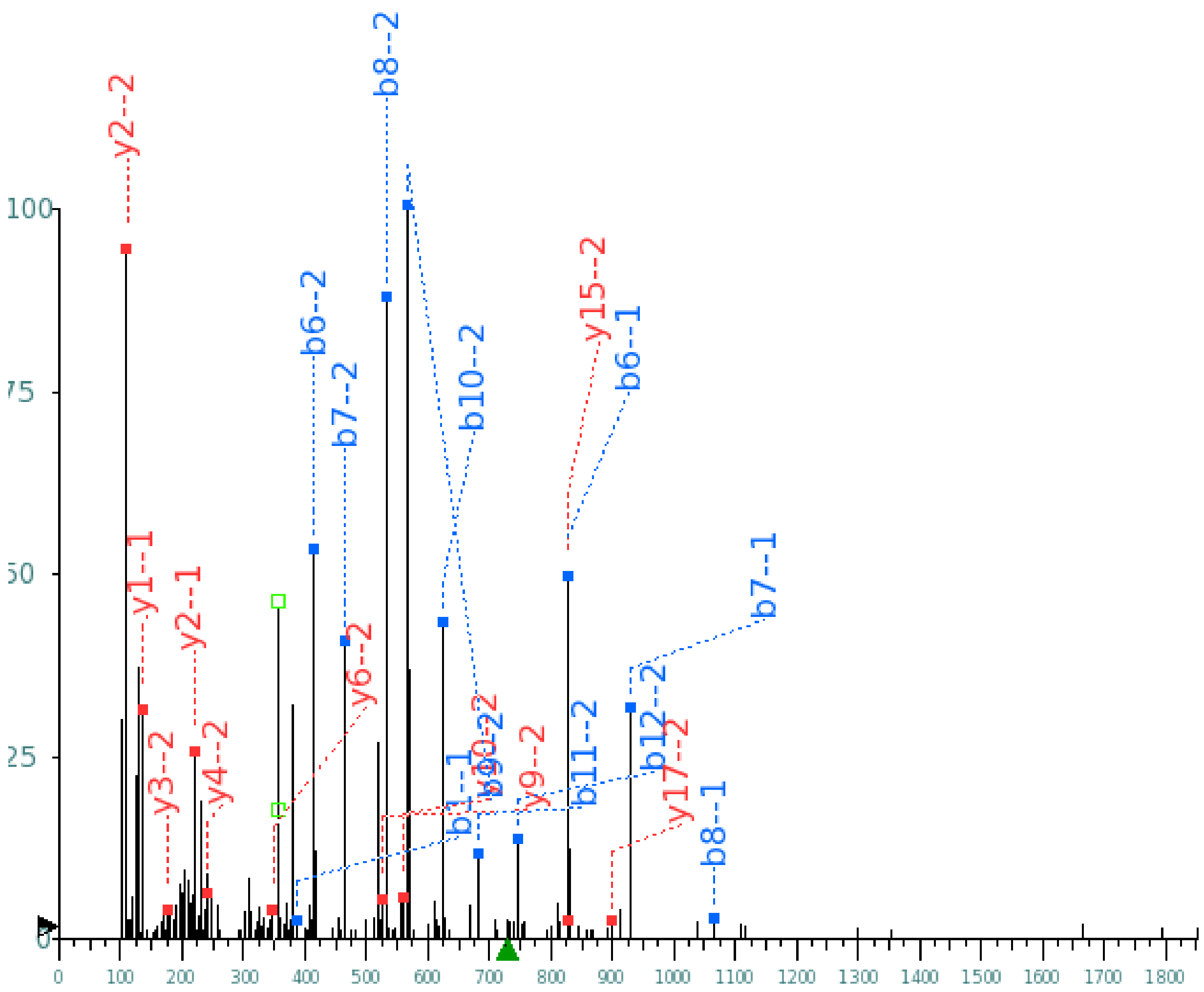

RGSSPDVHALLEITEESD (+3)

Primary Reference: PTRF_HUMAN
Search ID: 41761
Search Name: 20130328_ananiav_ERstress_TMT_TM_timecourse_trypsin_inj2_4mc_iaa
Scan#: 15879
Observed Mass: 728.7059 (2.2 ppm)
PSM Score: 25.28

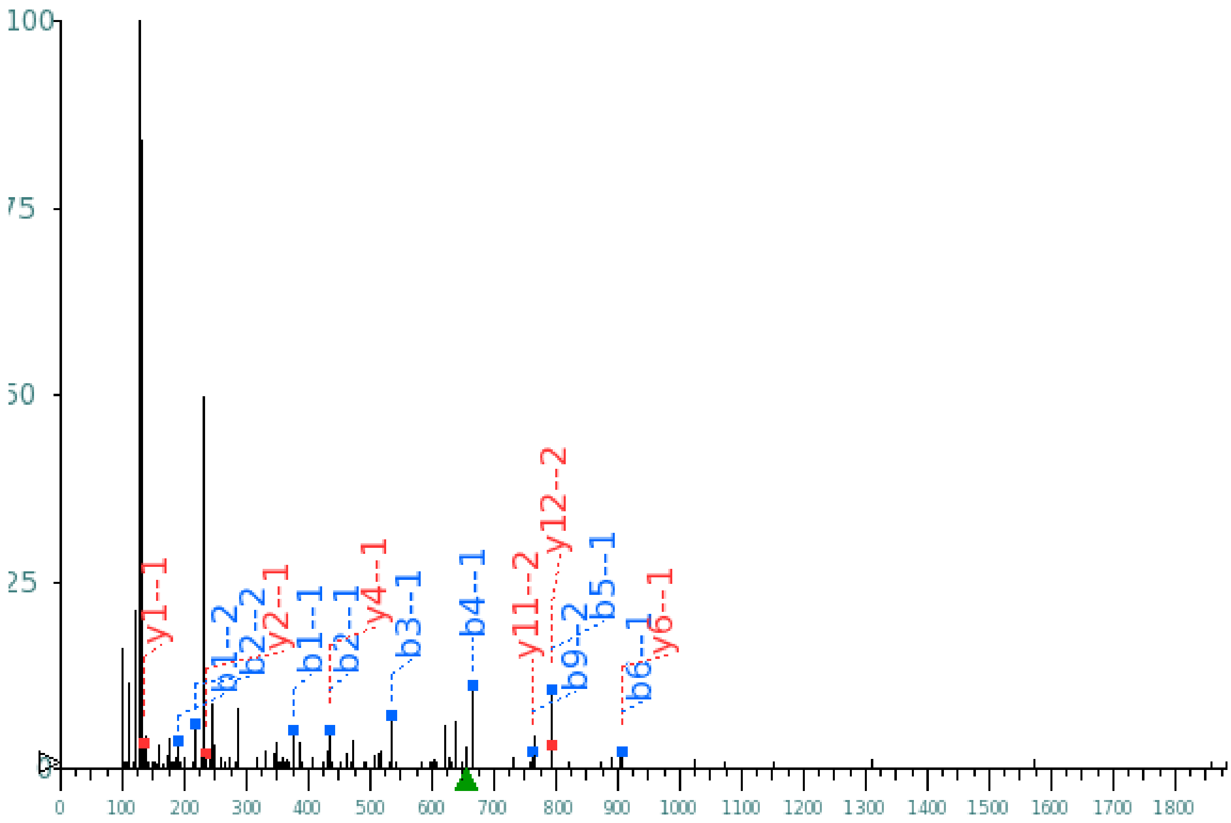

FGTEELFKDEATD (+3)

Primary Reference: CHD4_HUMAN
Search ID: 41761
Search Name: 20130328_ananiav_ERstress_TMT_TM_timecourse_trypsin_inj2_4mc_iaa
Scan#: 15893
Observed Mass: 654.0056 (3.5 ppm)
PSM Score: 10.12

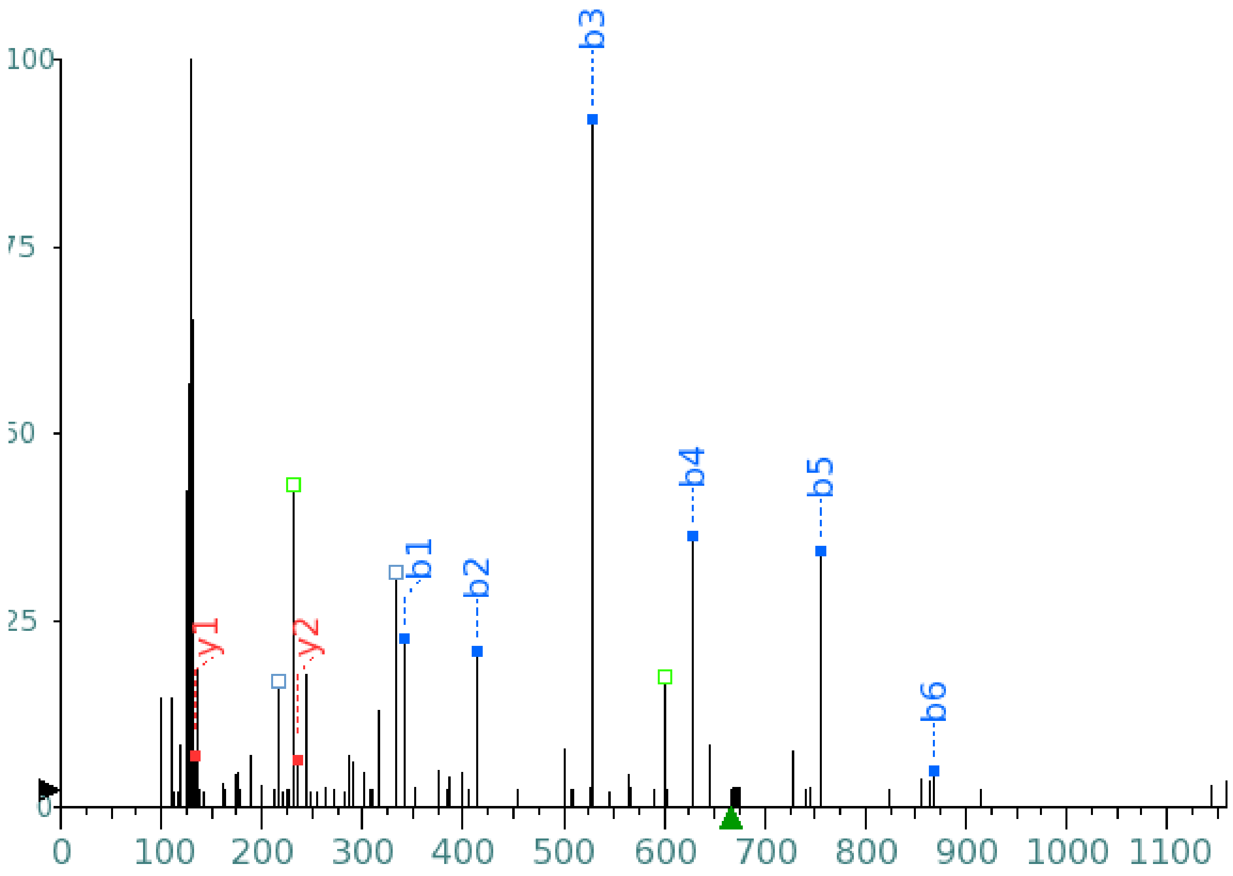

LANVQLLDTD (+2)

Primary Reference: PA1B2_HUMAN
Search ID: 41761
Search Name: 20130328_ananiav_ERstress_TMT_TM_timecourse_trypsin_inj2_4mc_iaa
Scan#: 15942
Observed Mass: 665.8759 (2.2 ppm)
PSM Score: 26.05

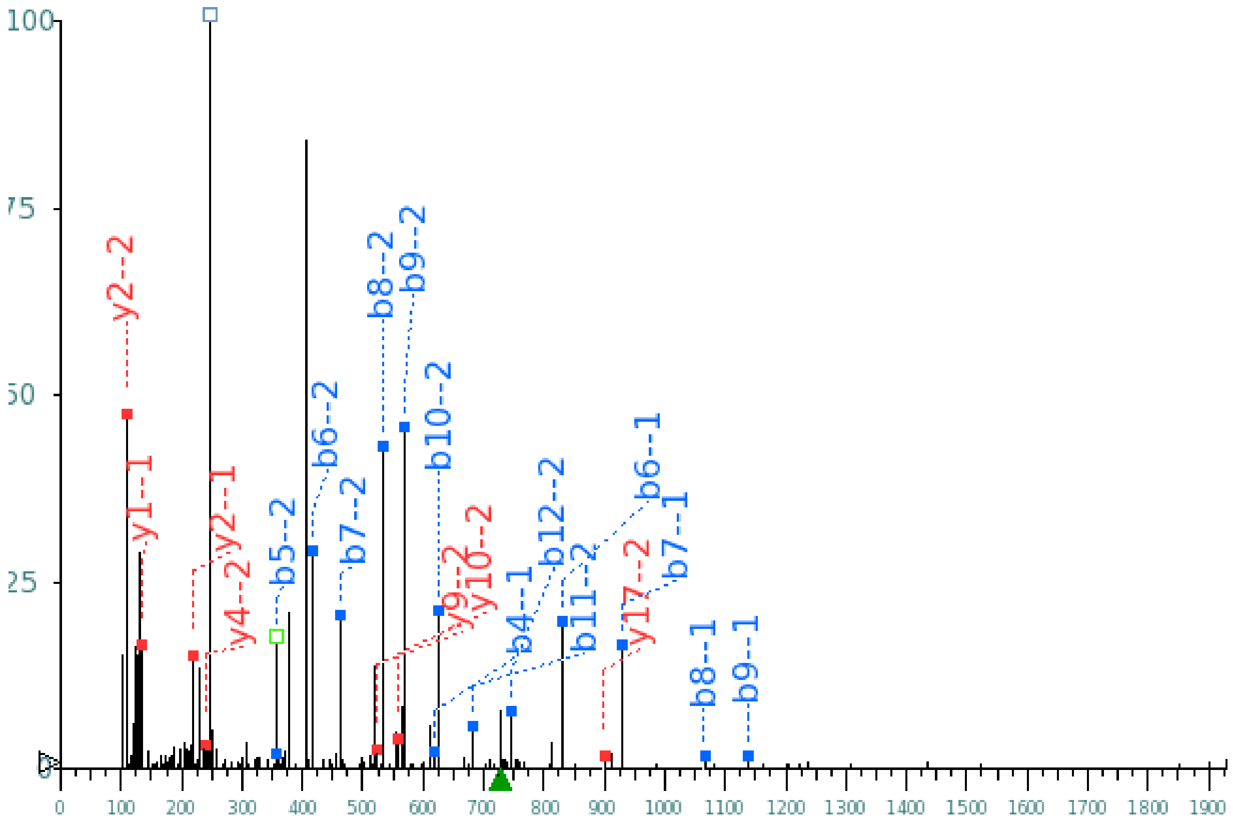

RGSSPDVHALLEITEESD (+3)

Primary Reference: PTRF_HUMAN
Search ID: 41761
Search Name: 20130328_ananiav_ERstress_TMT_TM_timecourse_trypsin_inj2_4mc_iaa
Scan#: 15958
Observed Mass: 728.7056 (1.9 ppm)
PSM Score: 16.36

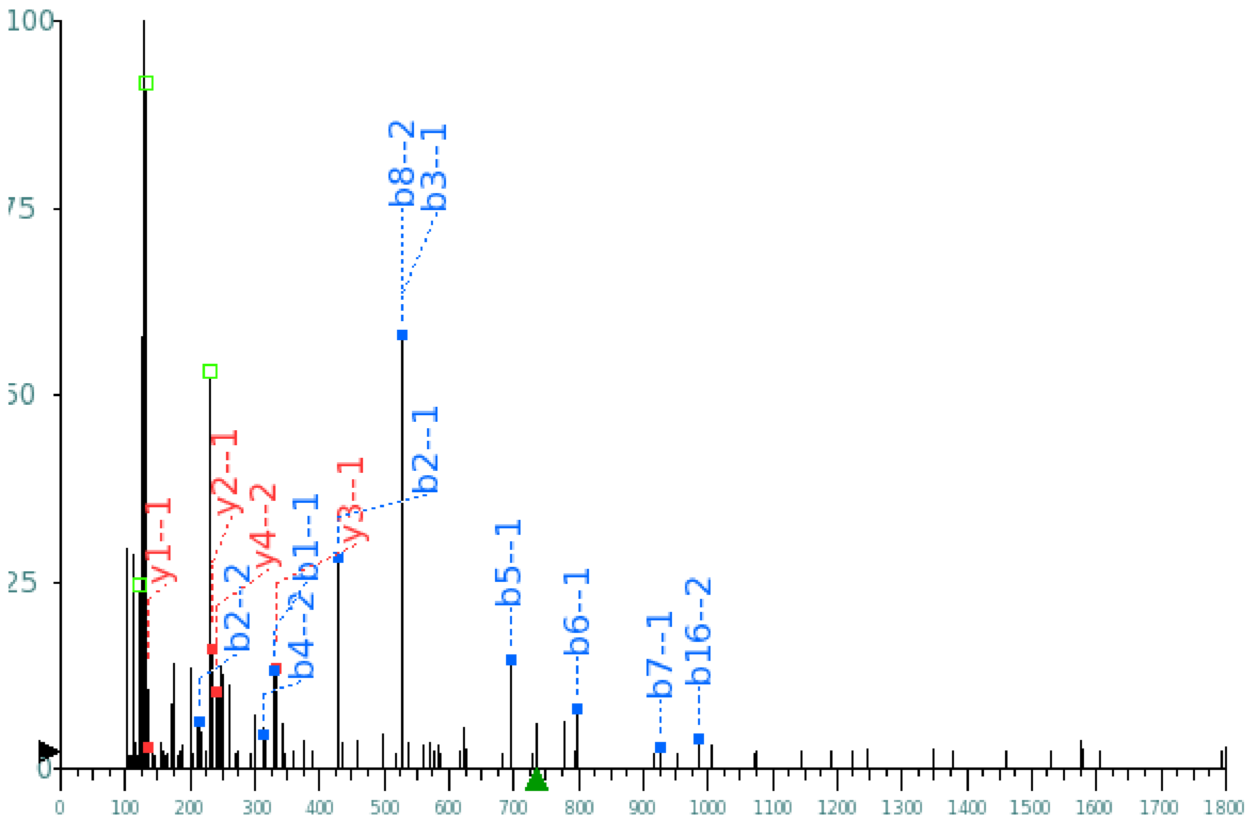

VVVPATEEEEAEVDEFPTD (+3)

Primary Reference: PESC_HUMAN
Search ID: 41761
Search Name: 20130328_ananiav_ERstress_TMT_TM_timecourse_trypsin_inj2_4mc_iaa
Scan#: 15968
Observed Mass: 735.6953 (2.9 ppm)
PSM Score: 10.34

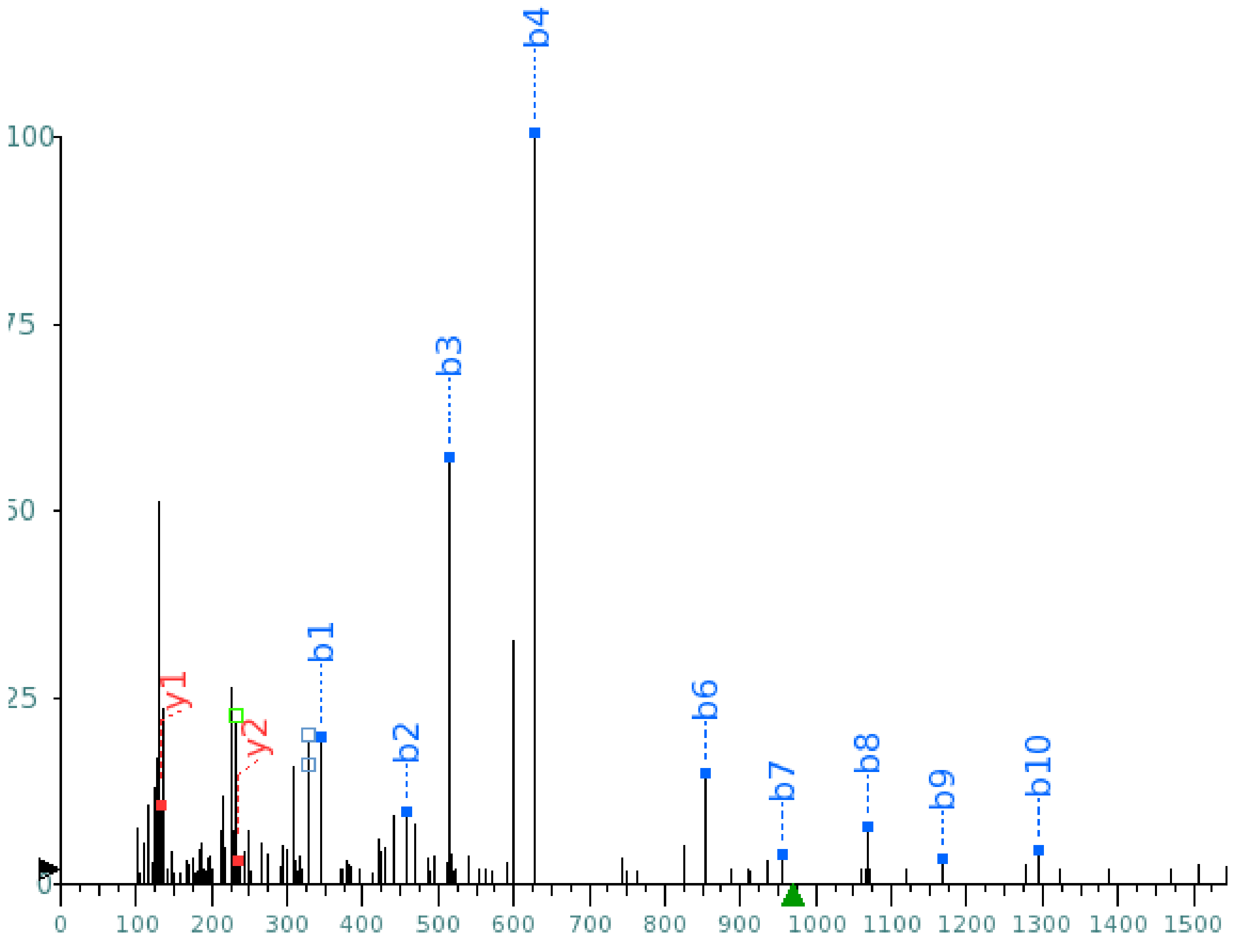

DLGLPQTNVEYIQTD (+2)

Primary Reference: HTRA2_HUMAN
Search ID: 41761
Search Name: 20130328_ananiav_ERstress_TMT_TM_timecourse_trypsin_inj2_4mc_iaa
Scan#: 16006
Observed Mass: 968 (0.9 ppm)
PSM Score: 61.05

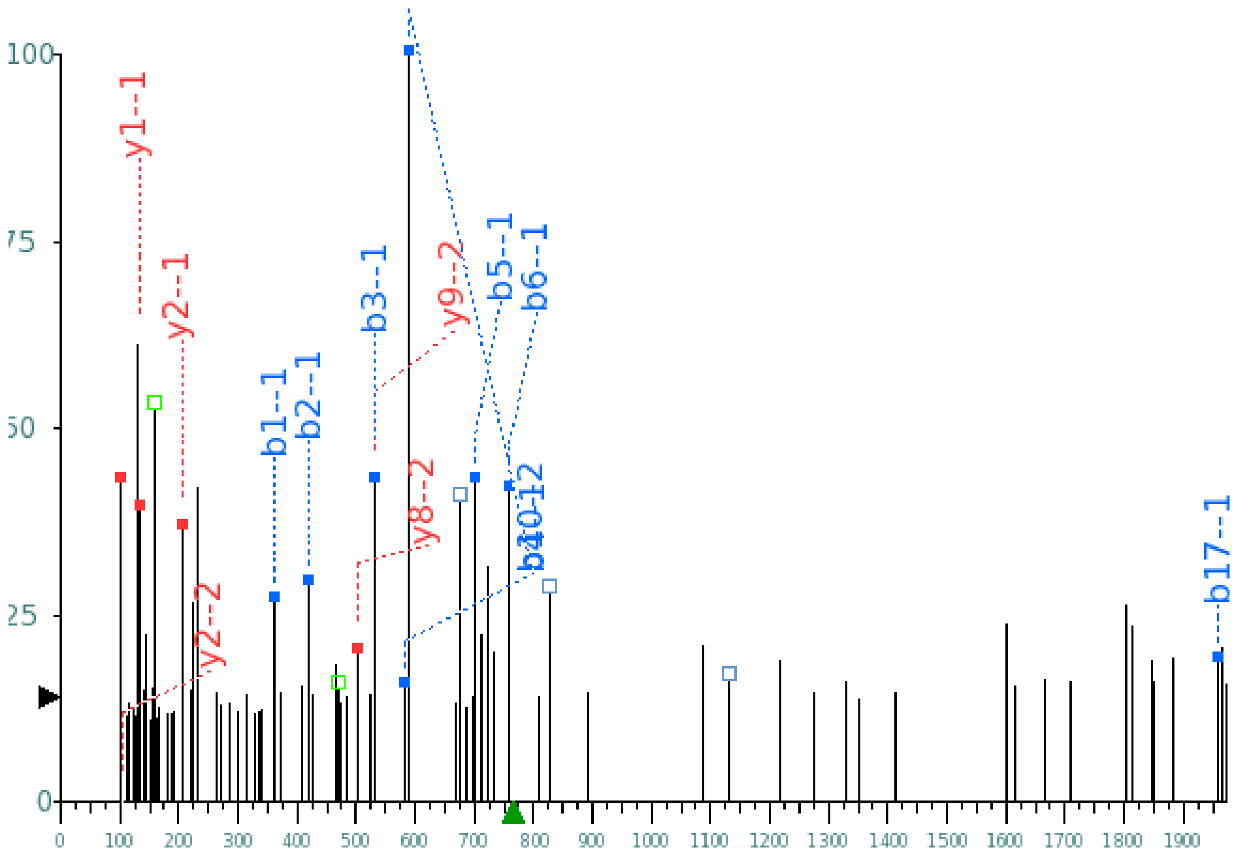

MGIGLGSENAAGPC*NWDEAD (+3)

Primary Reference: MAGD2_HUMAN
Search ID: 41761
Search Name: 20130328_ananiav_ERstress_TMT_TM_timecourse_trypsin_inj2_4mc_iaa
Scan#: 16030
Observed Mass: 765.0088 (2.4 ppm)
PSM Score: 16.05

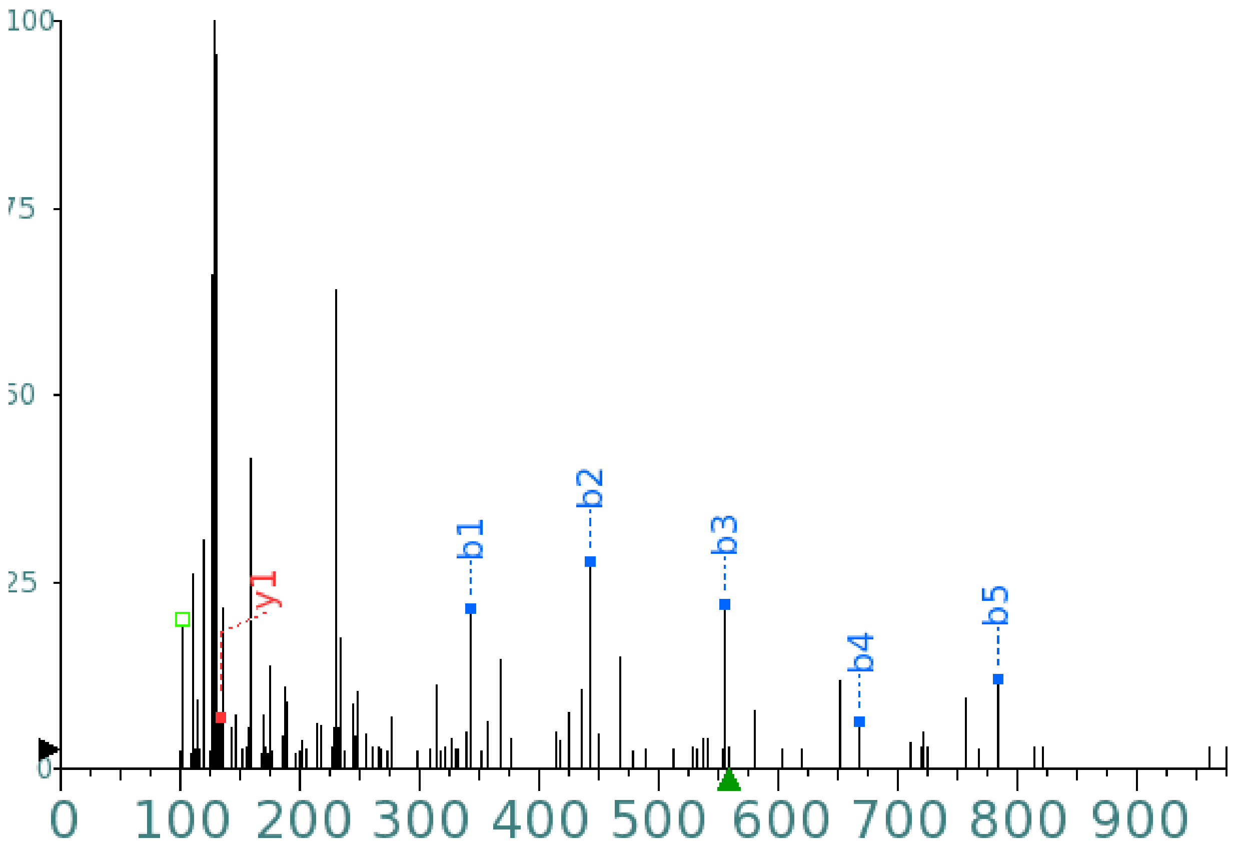

IVILDEAD (+2)

Primary Reference: RFC4_HUMAN
Search ID: 41761
Search Name: 20130328_ananiav_ERstress_TMT_TM_timecourse_trypsin_inj2_4mc_iaa
Scan#: 16031
Observed Mass: 558.8213 (0.4 ppm)
PSM Score: 12.84

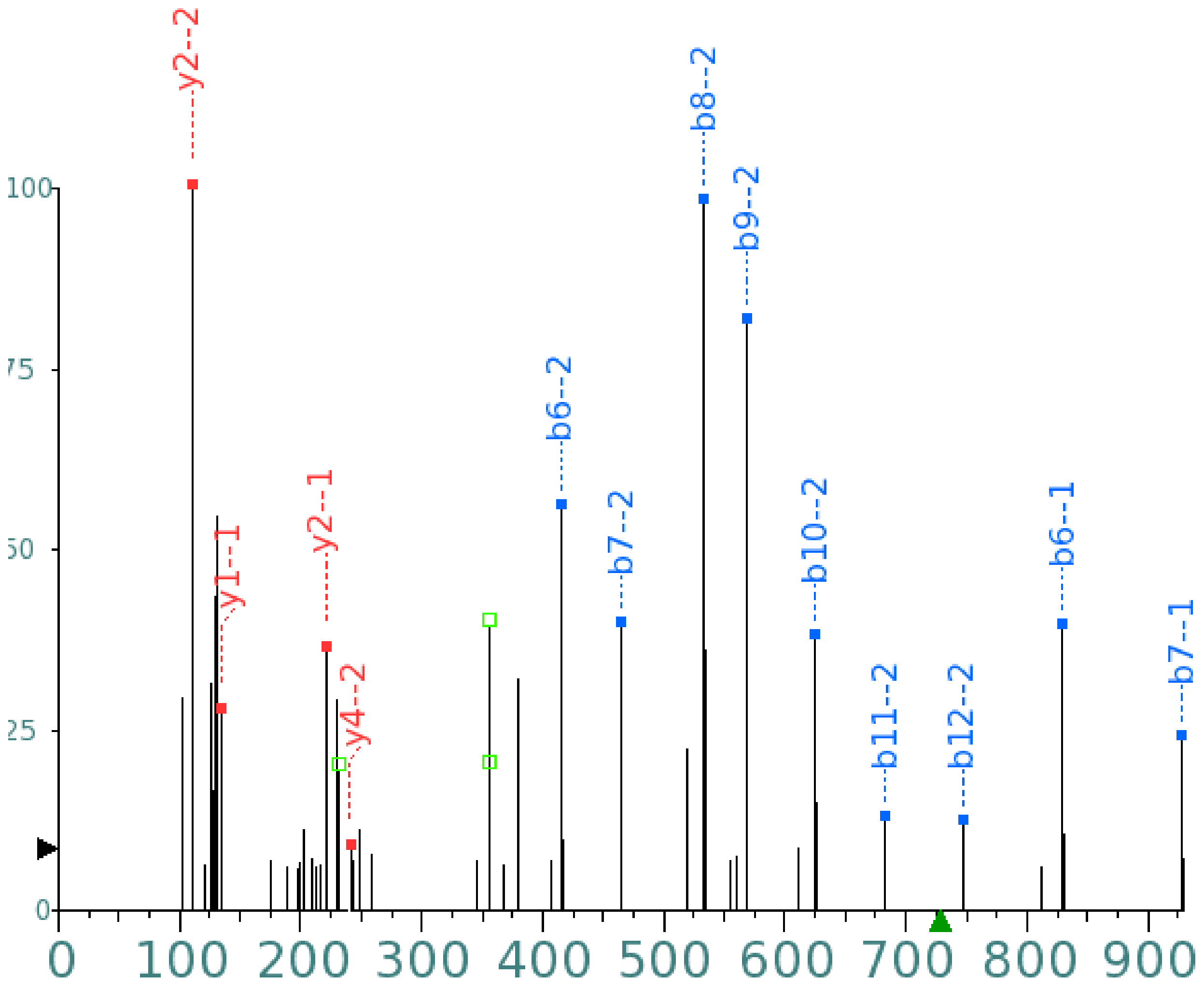

RGSSPDVHALLEITEESD (+3)

Primary Reference: PTRF_HUMAN
Search ID: 41761
Search Name: 20130328_ananiav_ERstress_TMT_TM_timecourse_trypsin_inj2_4mc_iaa
Scan#: 16037
Observed Mass: 728.7063 (2.9 ppm)
PSM Score: 33.91

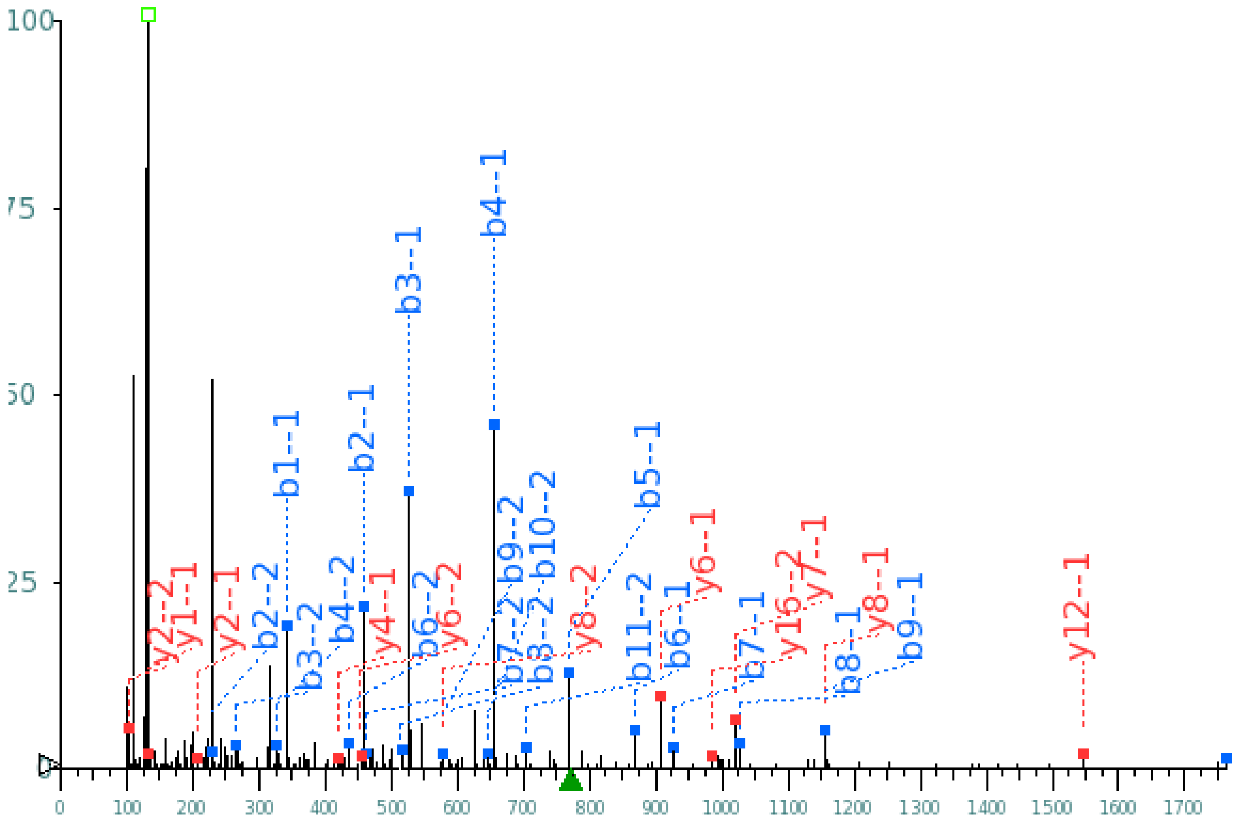

ILAQITGTEHLKESEAD (+3)

Primary Reference: PDLI5_HUMAN
Search ID: 41761
Search Name: 20130328_ananiav_ERstress_TMT_TM_timecourse_trypsin_inj2_4mc_iaa
Scan#: 16076
Observed Mass: 771.763 (1.8 ppm)
PSM Score: 37.69

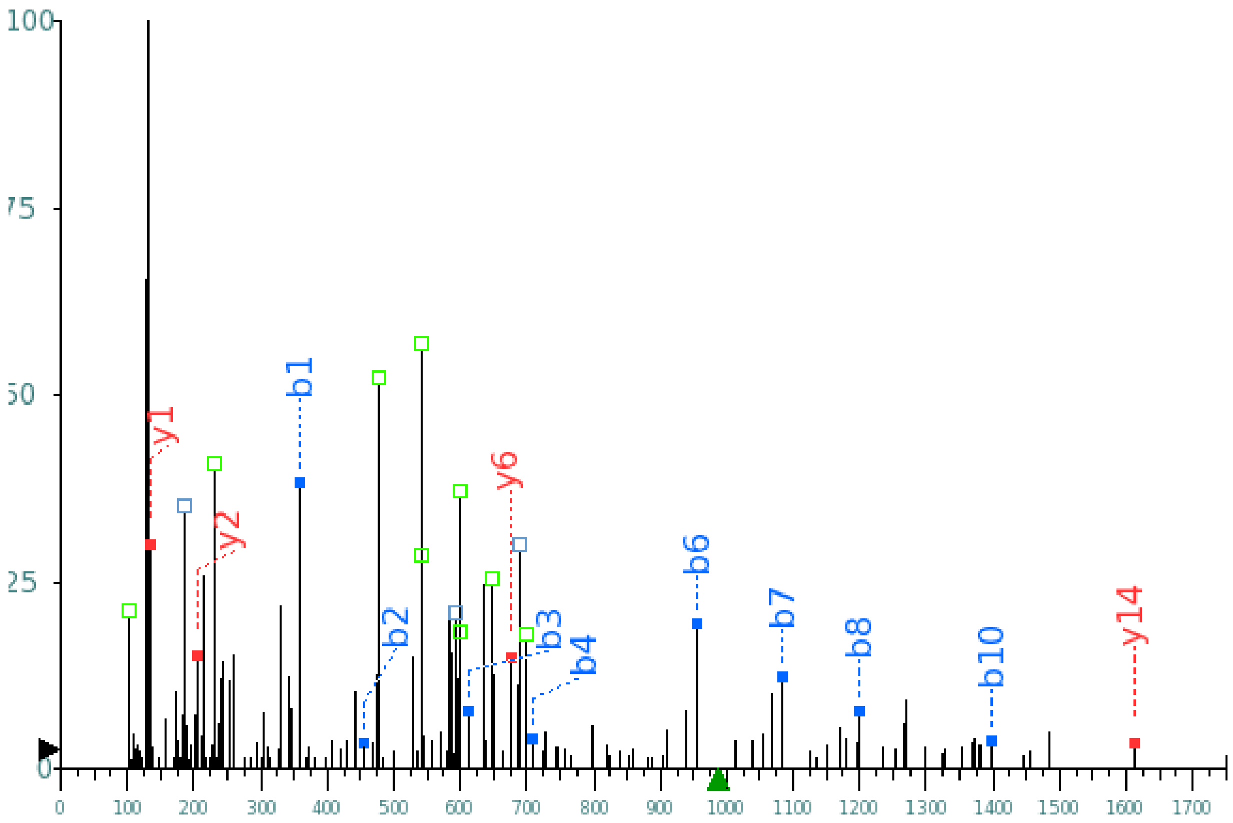

EPRPMDELVTLEEAD (+2)

Primary Reference: CACO1_HUMAN
Search ID: 41761
Search Name: 20130328_ananiav_ERstress_TMT_TM_timecourse_trypsin_inj2_4mc_iaa
Scan#: 16079
Observed Mass: 986.9923 (1.9 ppm)
PSM Score: 23.02

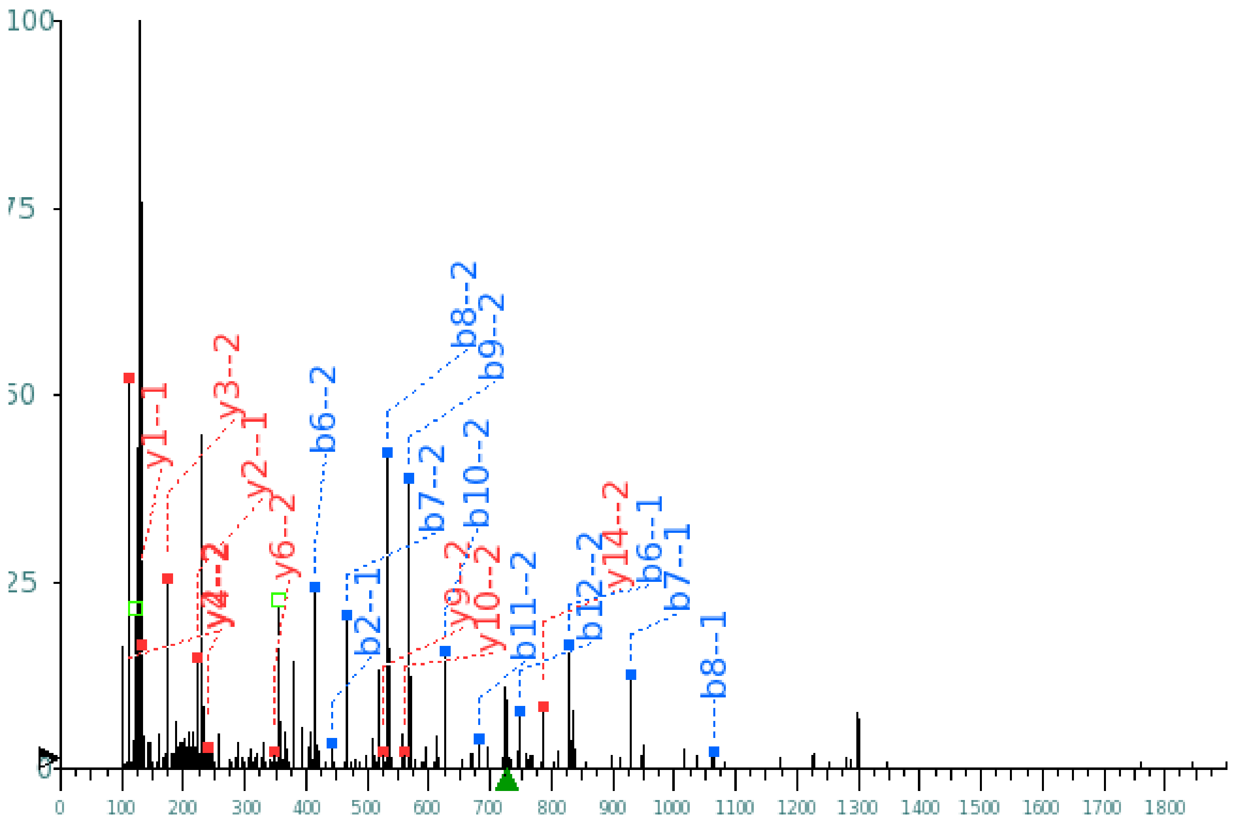

RGSSPDVHALLEITEESD (+3)

Primary Reference: PTRF_HUMAN
Search ID: 41761
Search Name: 20130328_ananiav_ERstress_TMT_TM_timecourse_trypsin_inj2_4mc_iaa
Scan#: 16101
Observed Mass: 728.7057 (2 ppm)
PSM Score: 13.72

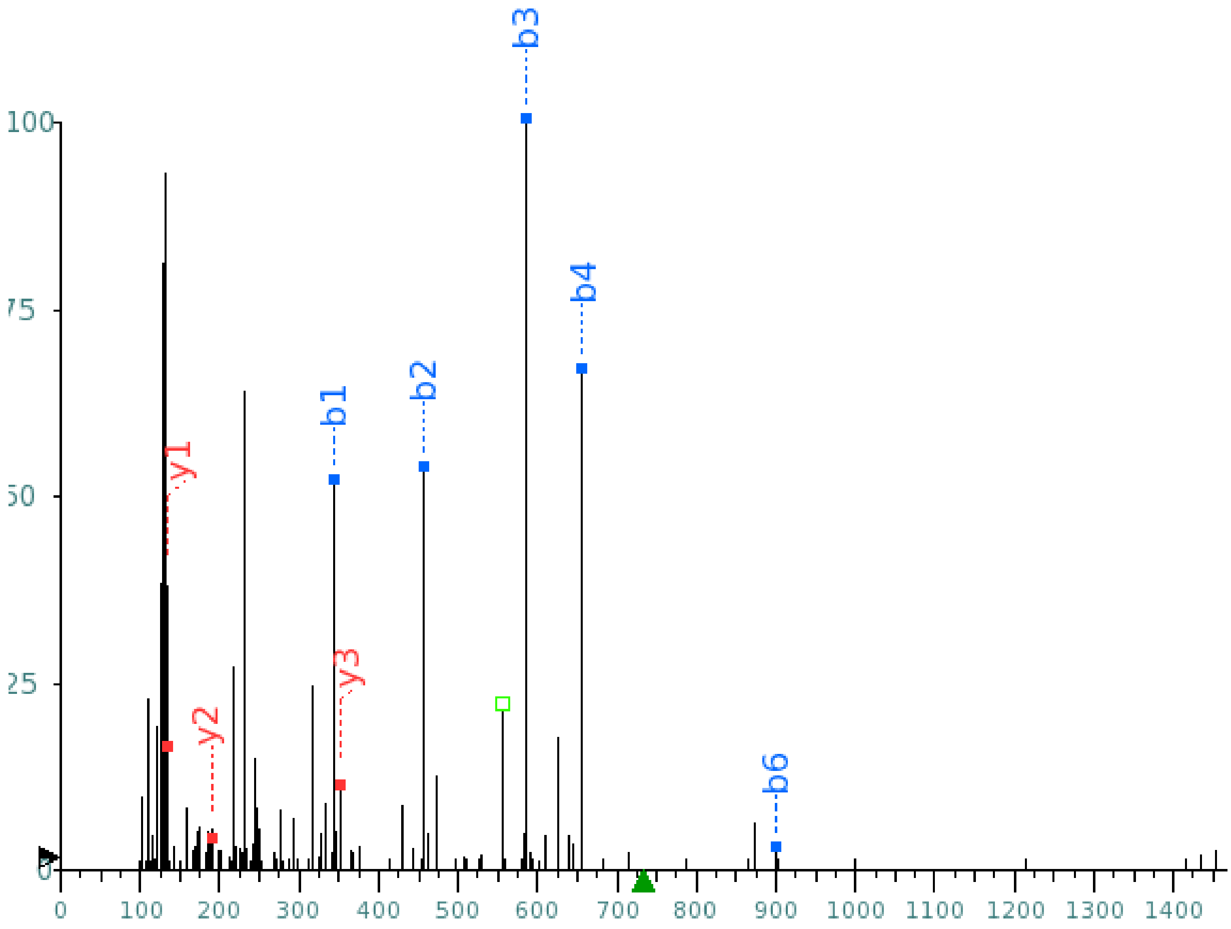

LLQAPFTDC*GD (+2)

Primary Reference: ITPR1_HUMAN
Search ID: 41761
Search Name: 20130328_ananiav_ERstress_TMT_TM_timecourse_trypsin_inj2_4mc_iaa
Scan#: 16122
Observed Mass: 733.3633 (-0 ppm)
PSM Score: 15.18

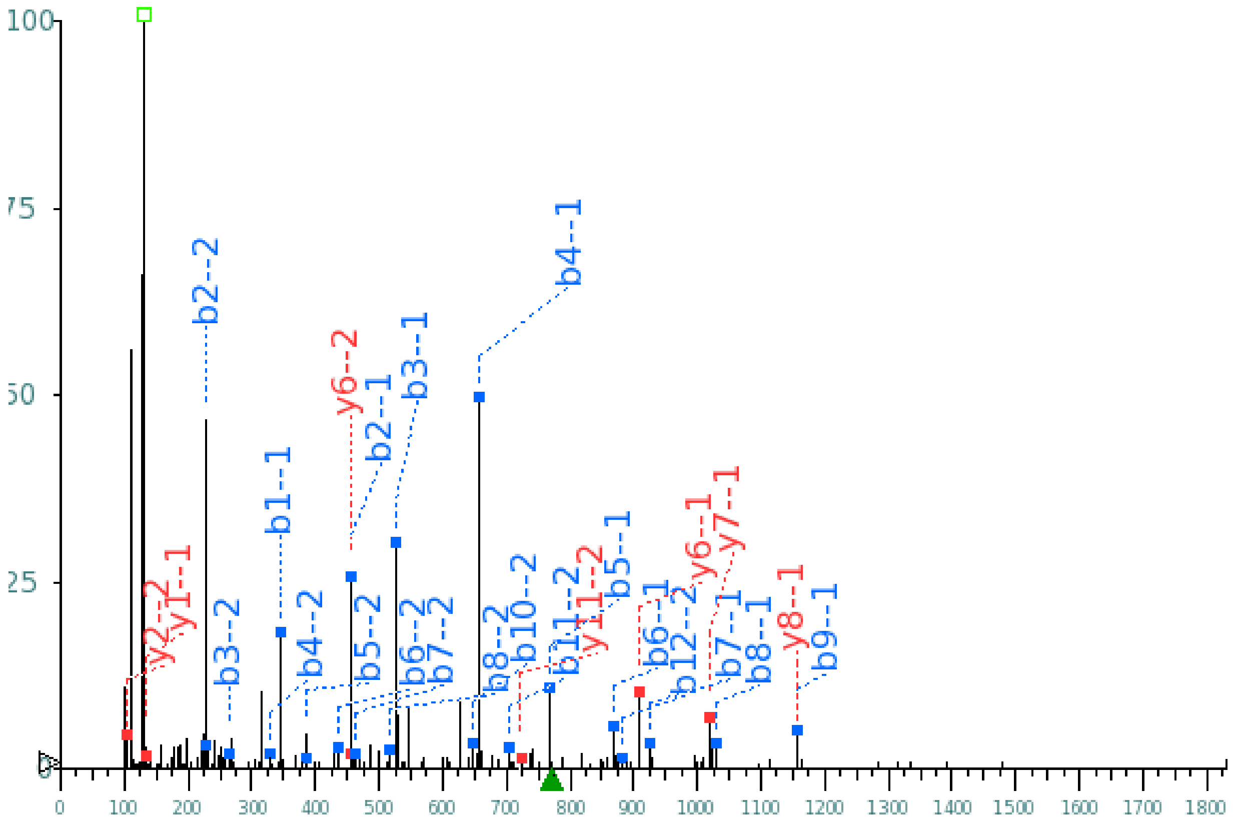

ILAQITGTEHLKESEAD (+3)

Primary Reference: PDLI5_HUMAN
Search ID: 41761
Search Name: 20130328_ananiav_ERstress_TMT_TM_timecourse_trypsin_inj2_4mc_iaa
Scan#: 16140
Observed Mass: 771.7631 (2 ppm)
PSM Score: 41.13

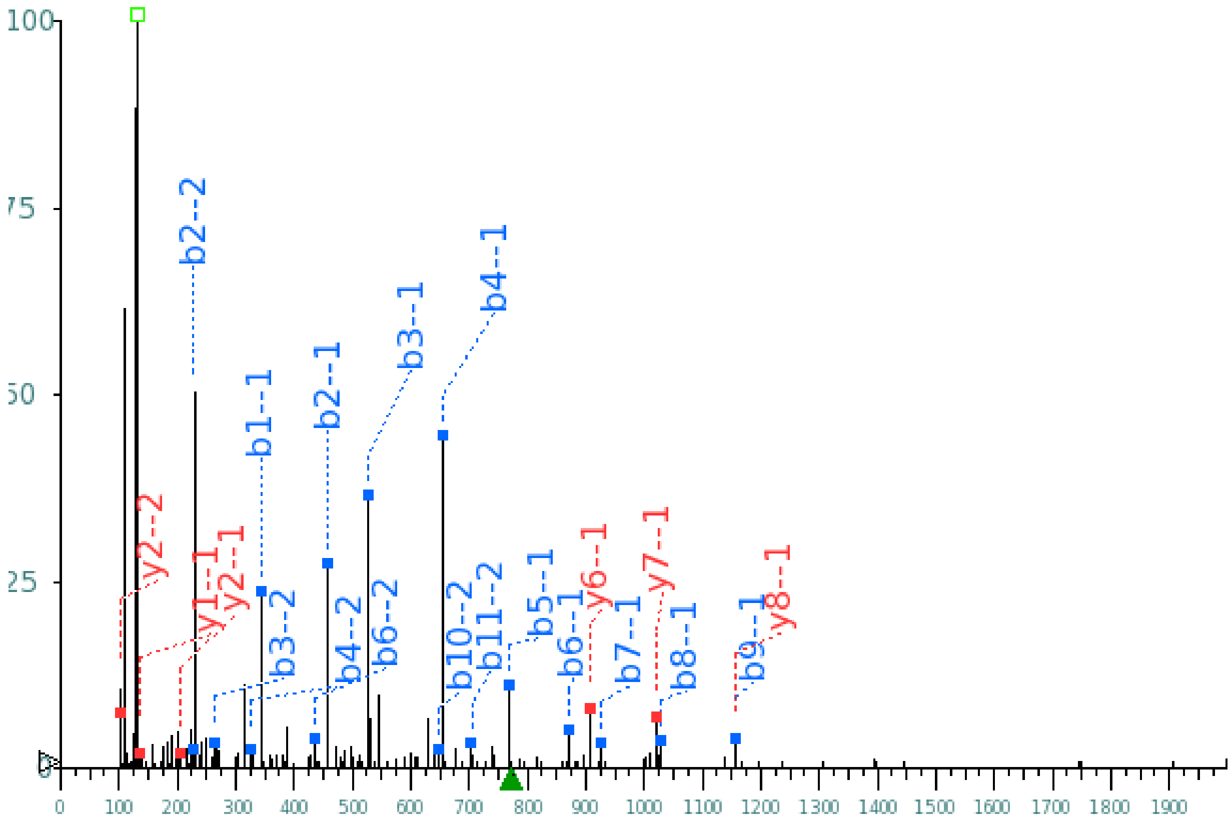

ILAQITGTEHLKESEAD (+3)

Primary Reference: PDLI5_HUMAN
Search ID: 41761
Search Name: 20130328_ananiav_ERstress_TMT_TM_timecourse_trypsin_inj2_4mc_iaa
Scan#: 16218
Observed Mass: 771.7632 (2.2 ppm)
PSM Score: 40.48

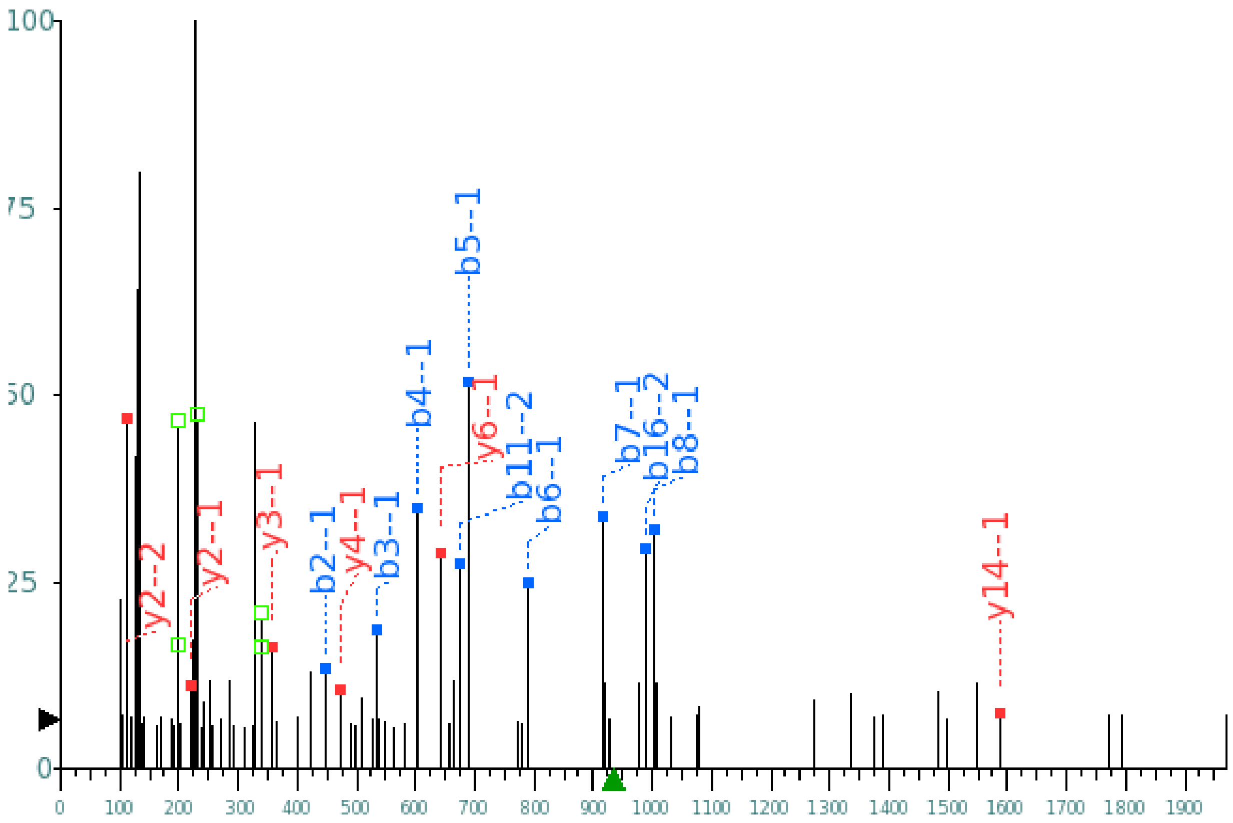

SQSASVESIPEVLEEC*TSPADHSD (+3)

Primary Reference: GBF1_HUMAN
Search ID: 41761
Search Name: 20130328_ananiav_ERstress_TMT_TM_timecourse_trypsin_inj2_4mc_iaa
Scan#: 16243
Observed Mass: 935.0993 (2 ppm)
PSM Score: 20.99

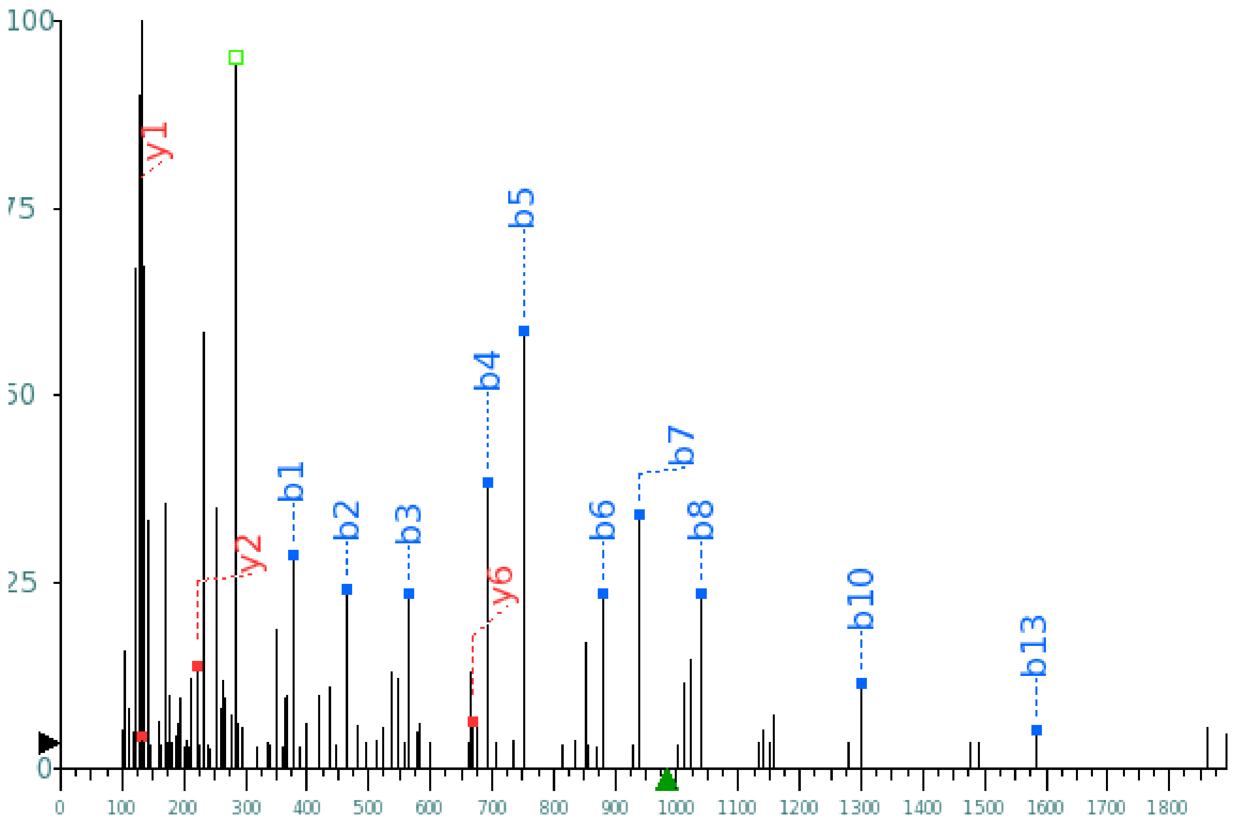

FSTQGMGTFNPADYSD (+2)

Primary Reference: UBAP2_HUMAN
Search ID: 41761
Search Name: 20130328_ananiav_ERstress_TMT_TM_timecourse_trypsin_inj2_4mc_iaa
Scan#: 16277
Observed Mass: 983.9394 (1.3 ppm)
PSM Score: 74.46

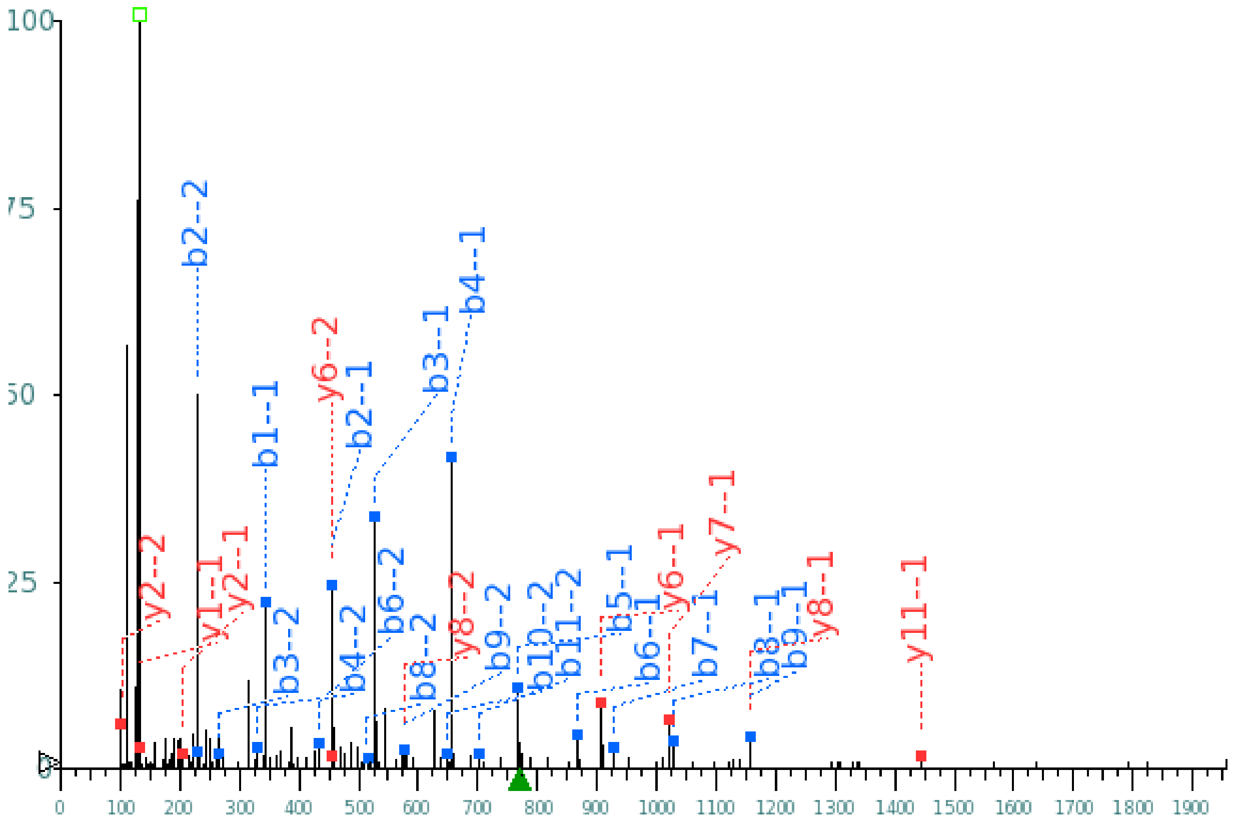

ILAQITGTEHLKESEAD (+3)

Primary Reference: PDLI5_HUMAN
Search ID: 41761
Search Name: 20130328_ananiav_ERstress_TMT_TM_timecourse_trypsin_inj2_4mc_iaa
Scan#: 16294
Observed Mass: 771.7632 (2.1 ppm)
PSM Score: 42.96

File name: 2015-02-04 04.01.53PM.pdf

Creation time: 2015-02-04 04:09PM

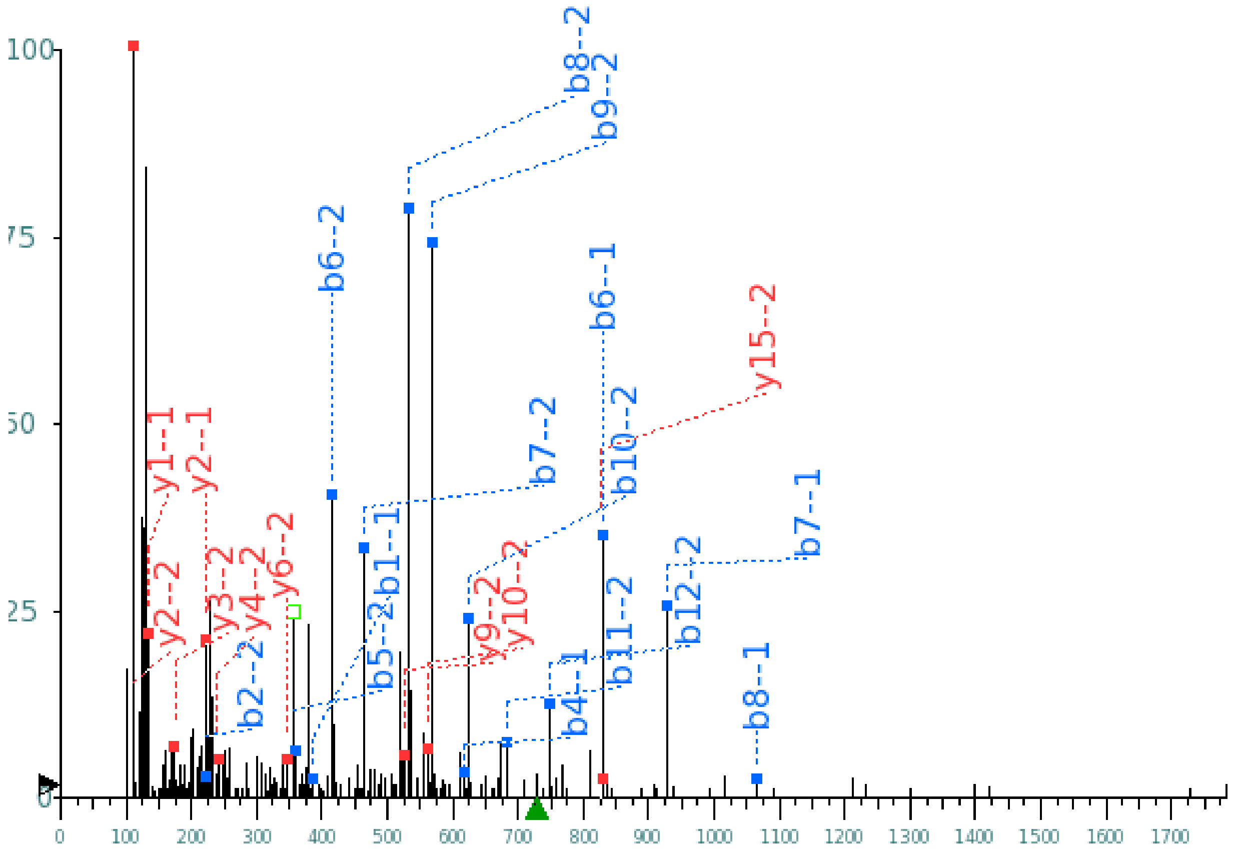

RGSSPDVHALLEITEESD (+3)

Primary Reference: PTRF_HUMAN
Search ID: 41761
Search Name: 20130328_ananiav_ERstress_TMT_TM_timecourse_trypsin_inj2_4mc_iaa
Scan#: 16314
Observed Mass: 728.7046 (0.4 ppm)
PSM Score: 20.11

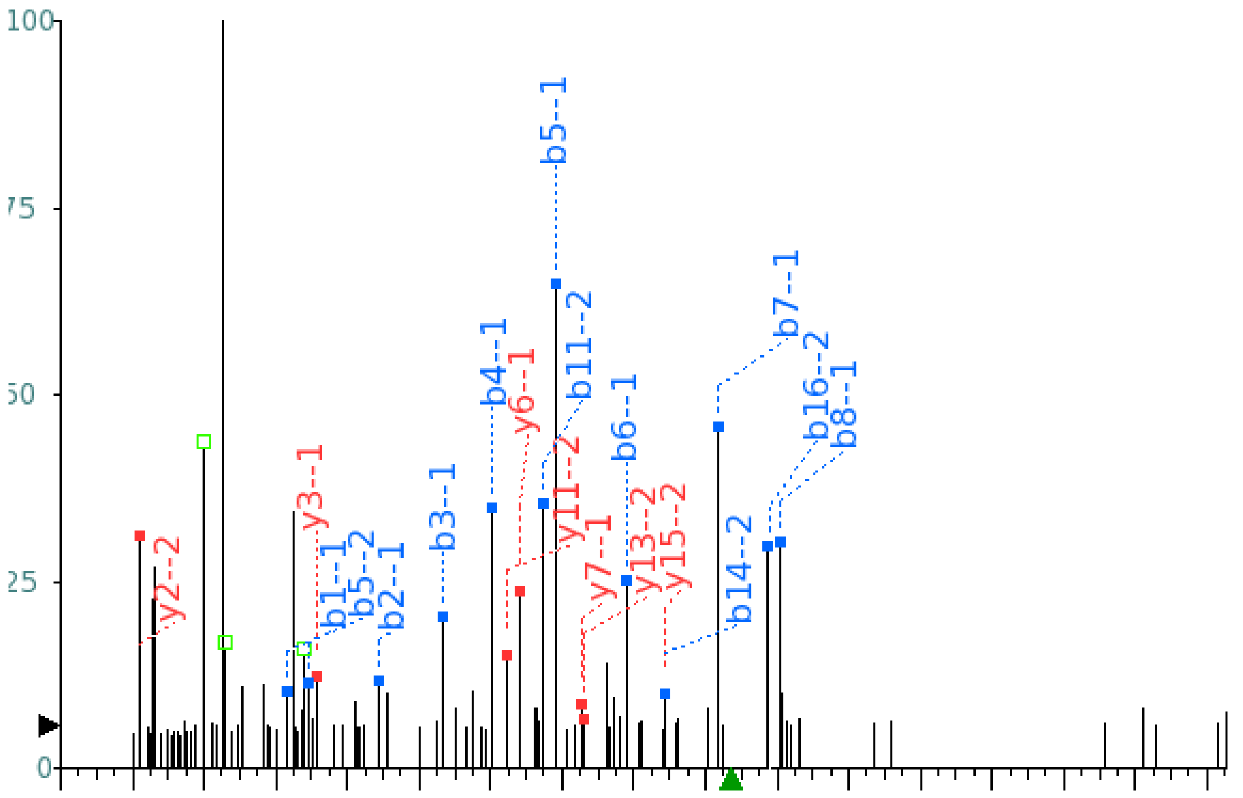

SQSASVESIPEVLEEC*TSPADHSD (+3)

Primary Reference: GBF1_HUMAN
Search ID: 41761
Search Name: 20130328_ananiav_ERstress_TMT_TM_timecourse_trypsin_inj2_4mc_iaa
Scan#: 16316
Observed Mass: 935.0994 (2.1 ppm)
PSM Score: 21.12

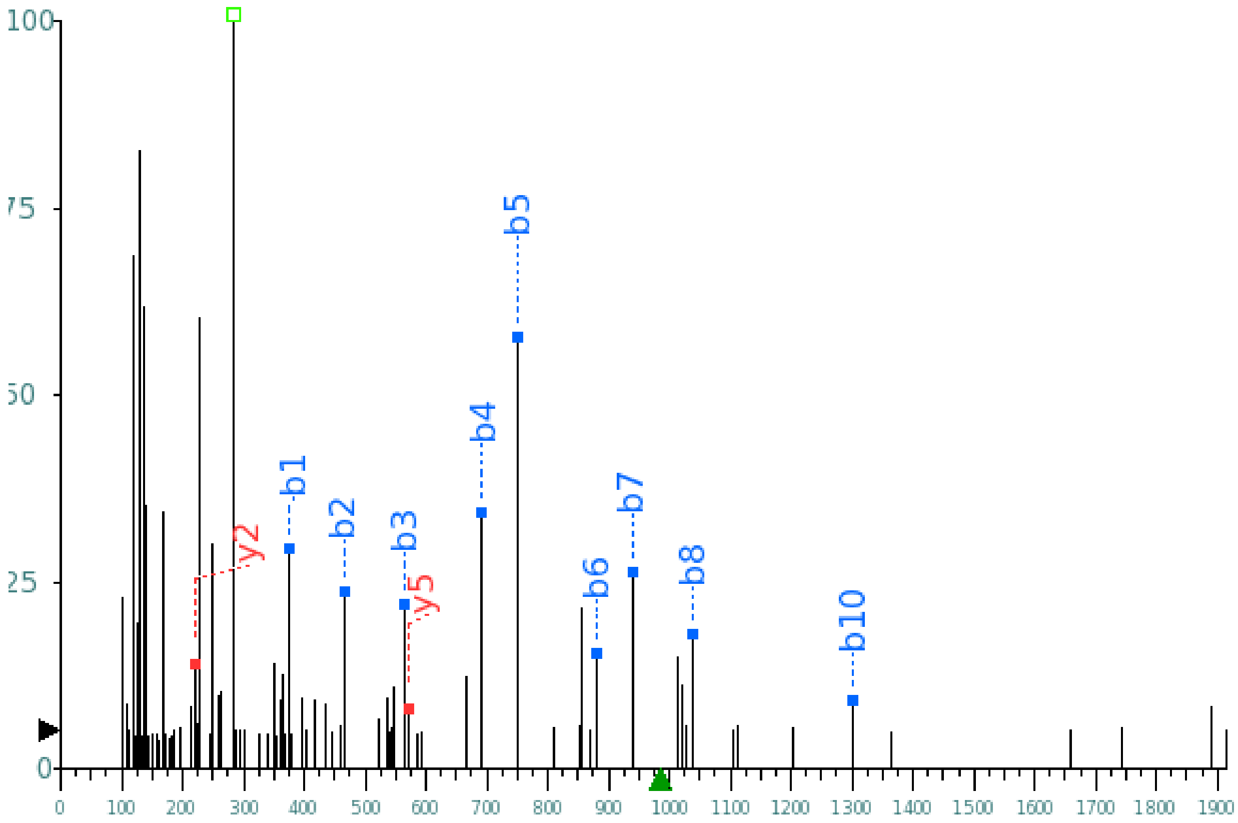

FSTQGMGTFNPADYSD (+2)

Primary Reference: UBAP2_HUMAN
Search ID: 41761
Search Name: 20130328_ananiav_ERstress_TMT_TM_timecourse_trypsin_inj2_4mc_iaa
Scan#: 16350
Observed Mass: 983.9398 (1.6 ppm)
PSM Score: 40.56

File name: 2015-02-04 04.01.53PM.pdf

Creation time: 2015-02-04 04:09PM

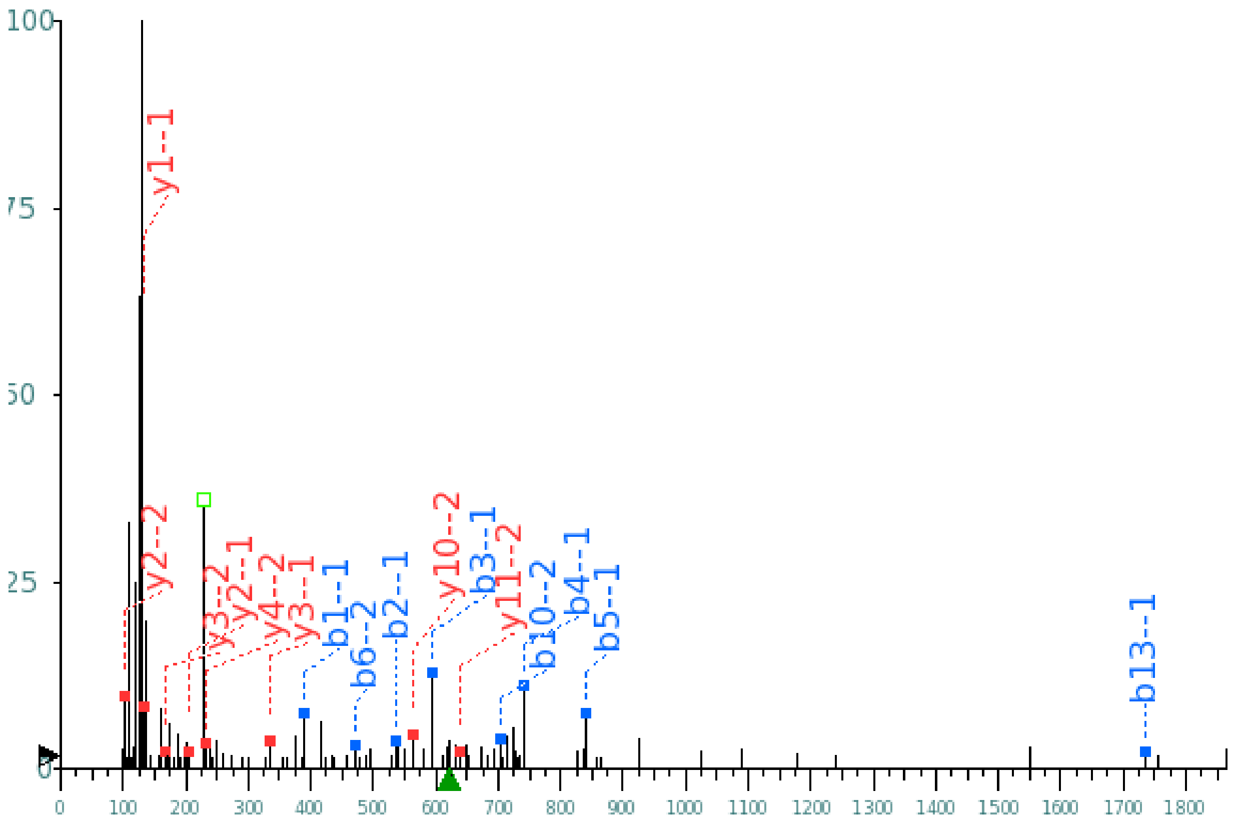

C*FGFVTYSNVEEAD (+3)

Primary Reference: ROA0_HUMAN
Search ID: 41761
Search Name: 20130328_ananiav_ERstress_TMT_TM_timecourse_trypsin_inj2_4mc_iaa
Scan#: 16352
Observed Mass: 622.9535 (2.2 ppm)
PSM Score: 12.04

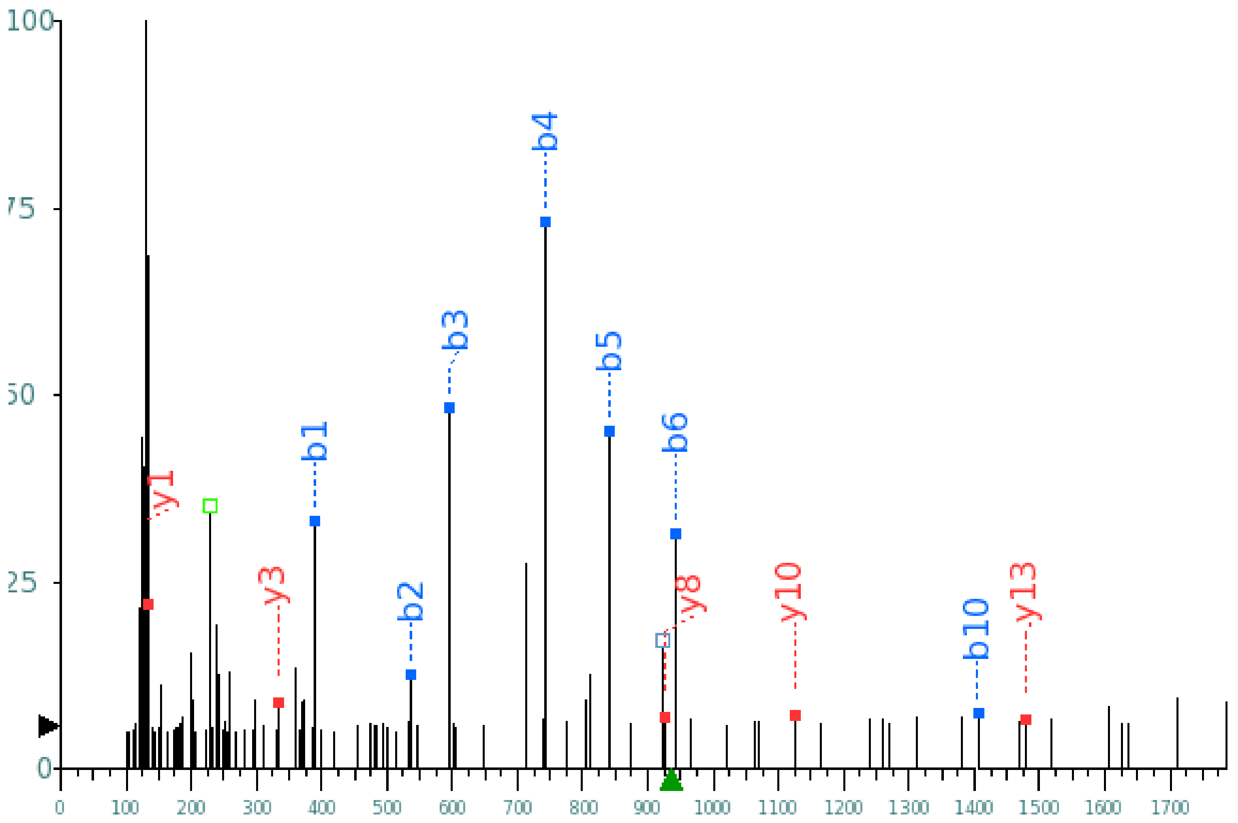

C*FGFVTYSNVEEAD (+2)

Primary Reference: ROA0_HUMAN
Search ID: 41761
Search Name: 20130328_ananiav_ERstress_TMT_TM_timecourse_trypsin_inj2_4mc_iaa
Scan#: 16371
Observed Mass: 933.9263 (1.9 ppm)
PSM Score: 21.24

File name: 2015-02-04 04.01.53PM.pdf

Creation time: 2015-02-04 04:09PM

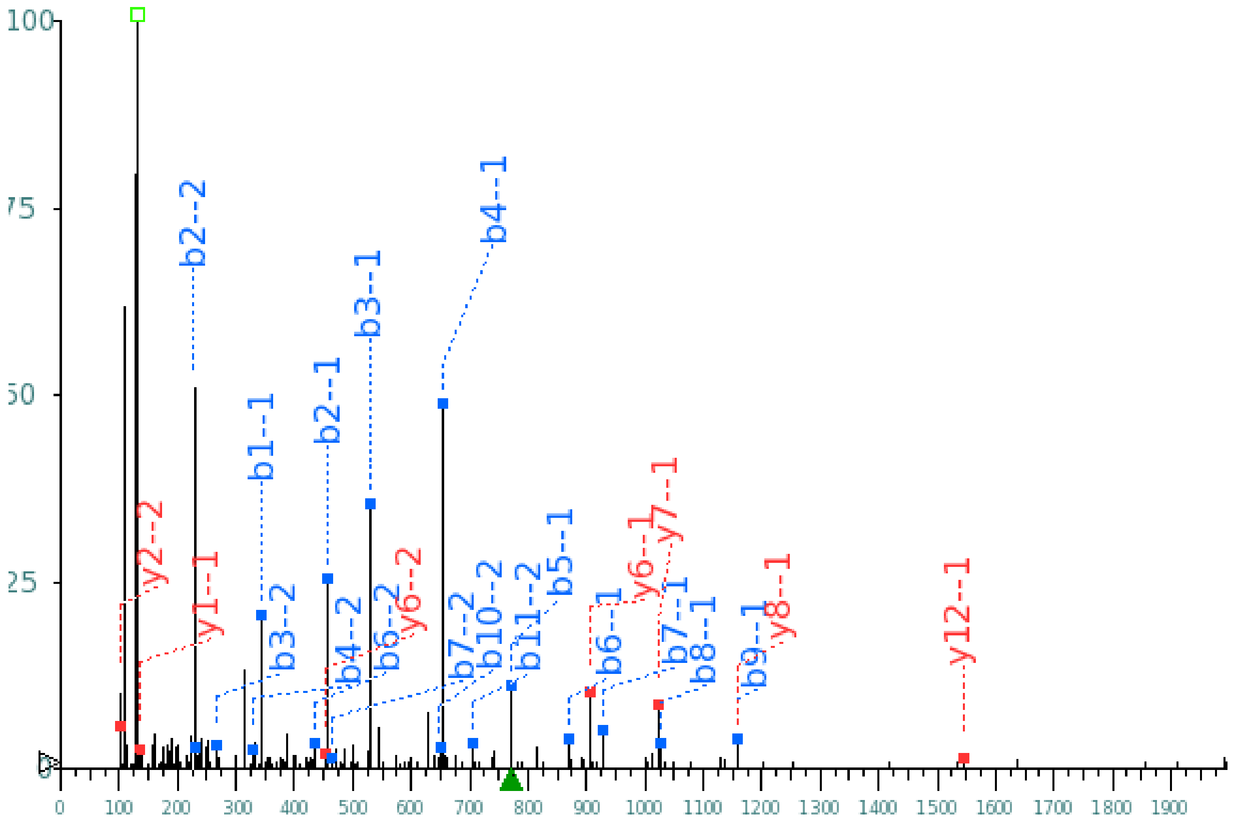

ILAQITGTEHLKESEAD (+3)

Primary Reference: PDLI5_HUMAN
Search ID: 41761
Search Name: 20130328_ananiav_ERstress_TMT_TM_timecourse_trypsin_inj2_4mc_iaa
Scan#: 16372
Observed Mass: 771.7633 (2.3 ppm)
PSM Score: 36.85

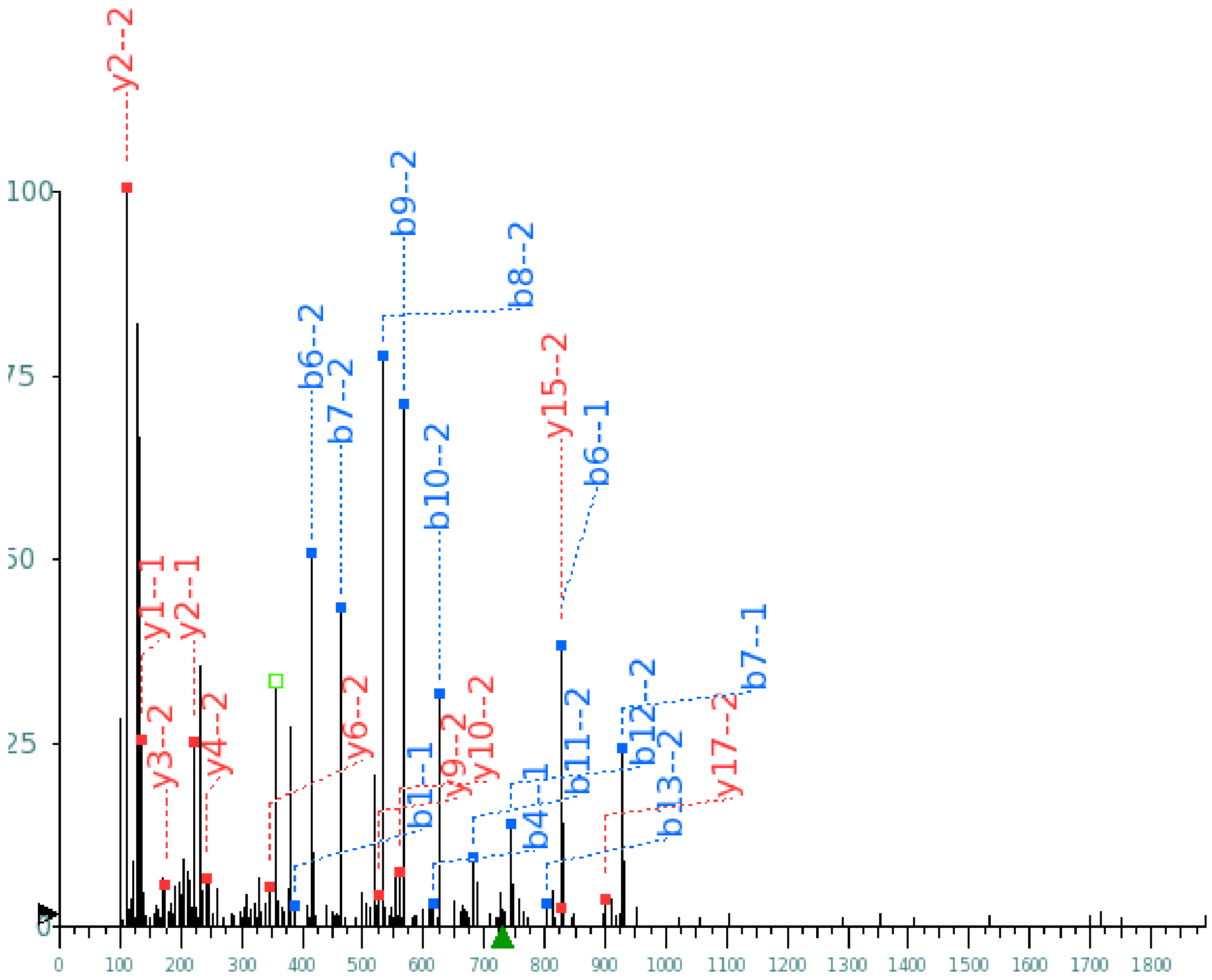

RGSSPDVHALLEITEESD (+3)

Primary Reference: PTRF_HUMAN
Search ID: 41761
Search Name: 20130328_ananiav_ERstress_TMT_TM_timecourse_trypsin_inj2_4mc_iaa
Scan#: 16385
Observed Mass: 728.7052 (1.3 ppm)
PSM Score: 18.87

File name: 2015-02-04 04.01.53PM.pdf

Creation time: 2015-02-04 04:09PM

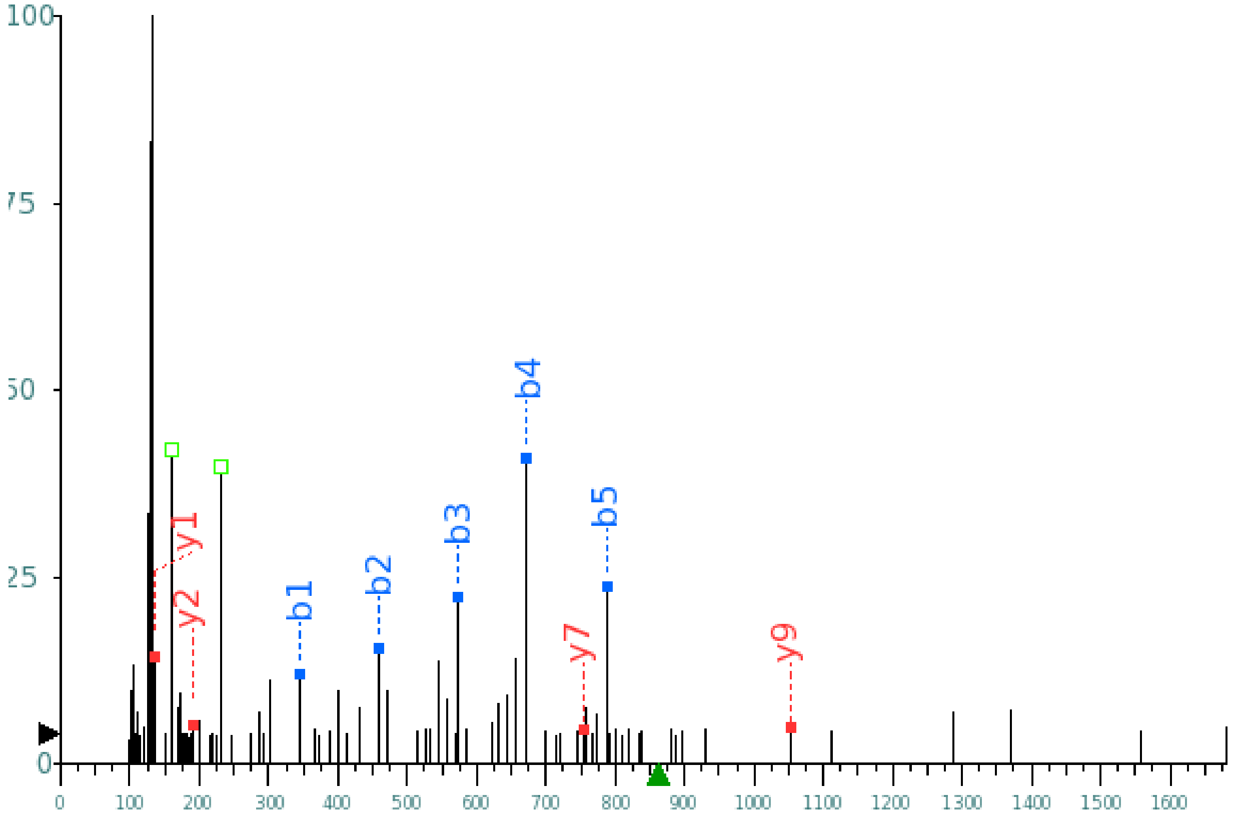

DNLTLWTSDMQGD (+2)

Primary Reference: 1433E_HUMAN
Search ID: 41761
Search Name: 20130328_ananiav_ERstress_TMT_TM_timecourse_trypsin_inj2_4mc_iaa
Scan#: 16415
Observed Mass: 862.906 (2.8 ppm)
PSM Score: 14.26

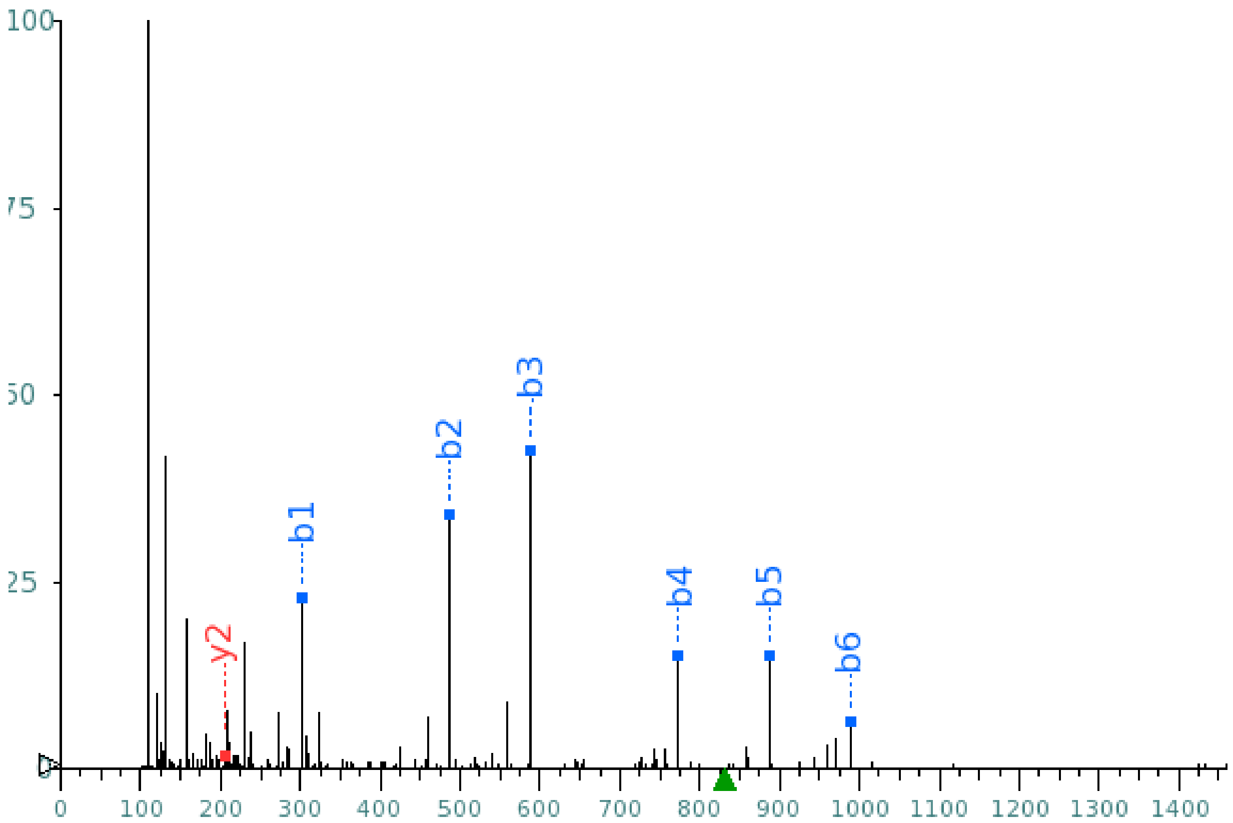

AWVWNTHADFAD (+2)

Primary Reference: RANG_HUMAN
Search ID: 41761
Search Name: 20130328_ananiav_ERstress_TMT_TM_timecourse_trypsin_inj2_4mc_iaa
Scan#: 16416
Observed Mass: 831.4016 (3 ppm)
PSM Score: 31.34

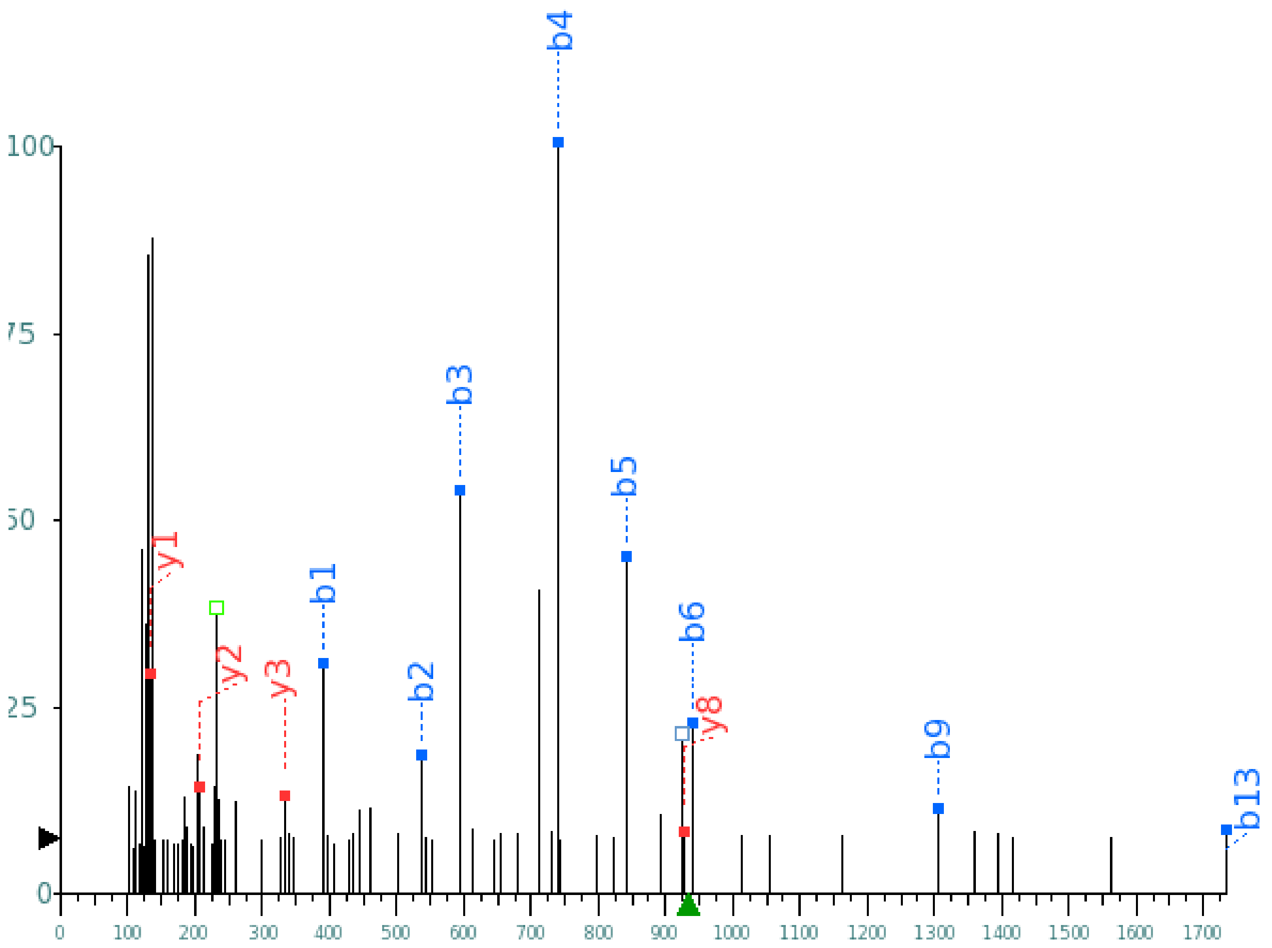

C*FGFVTYSNVEEAD (+2)

Primary Reference: ROA0_HUMAN
Search ID: 41761
Search Name: 20130328_ananiav_ERstress_TMT_TM_timecourse_trypsin_inj2_4mc_iaa
Scan#: 16437
Observed Mass: 933.9264 (2.1 ppm)
PSM Score: 34.97

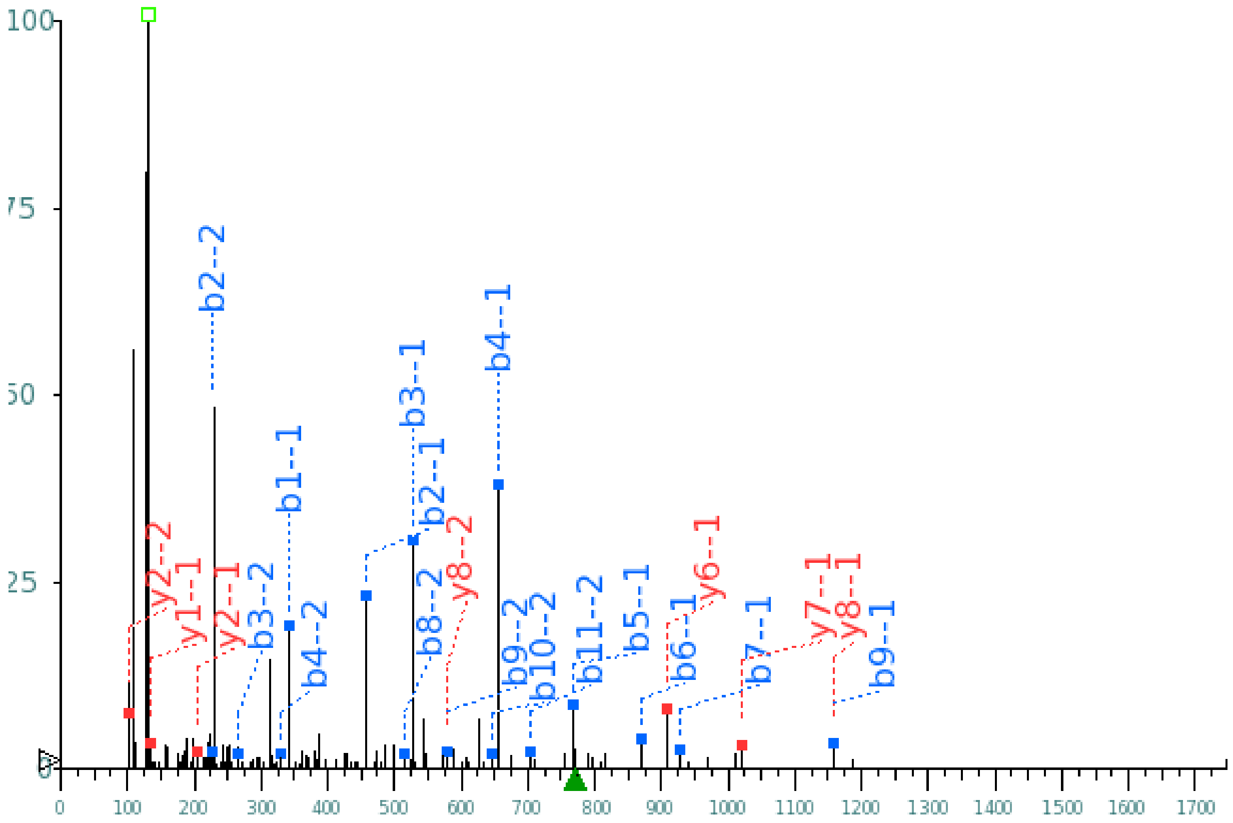

ILAQITGTEHLKESEAD (+3)

Primary Reference: PDLI5_HUMAN
Search ID: 41761
Search Name: 20130328_ananiav_ERstress_TMT_TM_timecourse_trypsin_inj2_4mc_iaa
Scan#: 16438
Observed Mass: 771.7636 (2.6 ppm)
PSM Score: 42.97

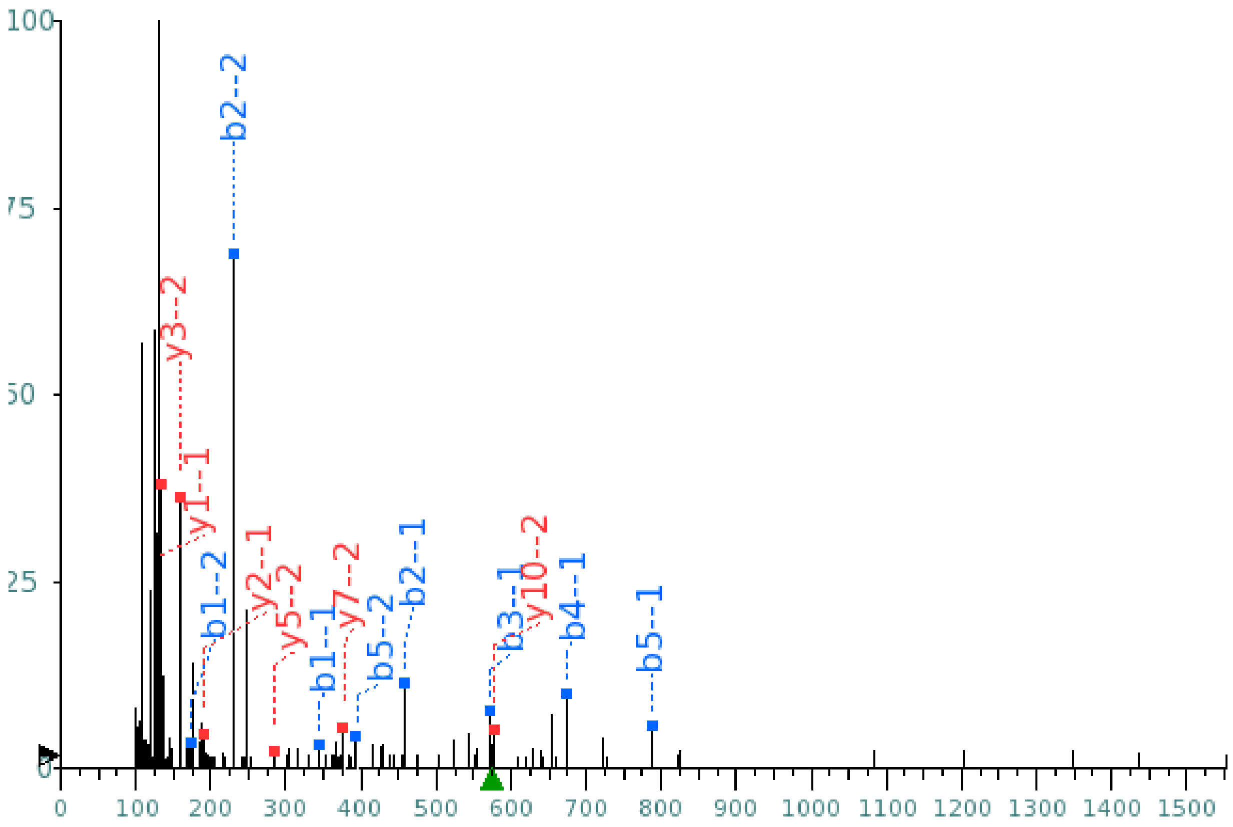

DNLTLWTSDMQGD (+3)

Primary Reference: 1433E_HUMAN
Search ID: 41761
Search Name: 20130328_ananiav_ERstress_TMT_TM_timecourse_trypsin_inj2_4mc_iaa
Scan#: 16442
Observed Mass: 575.6063 (2.6 ppm)
PSM Score: 7.4

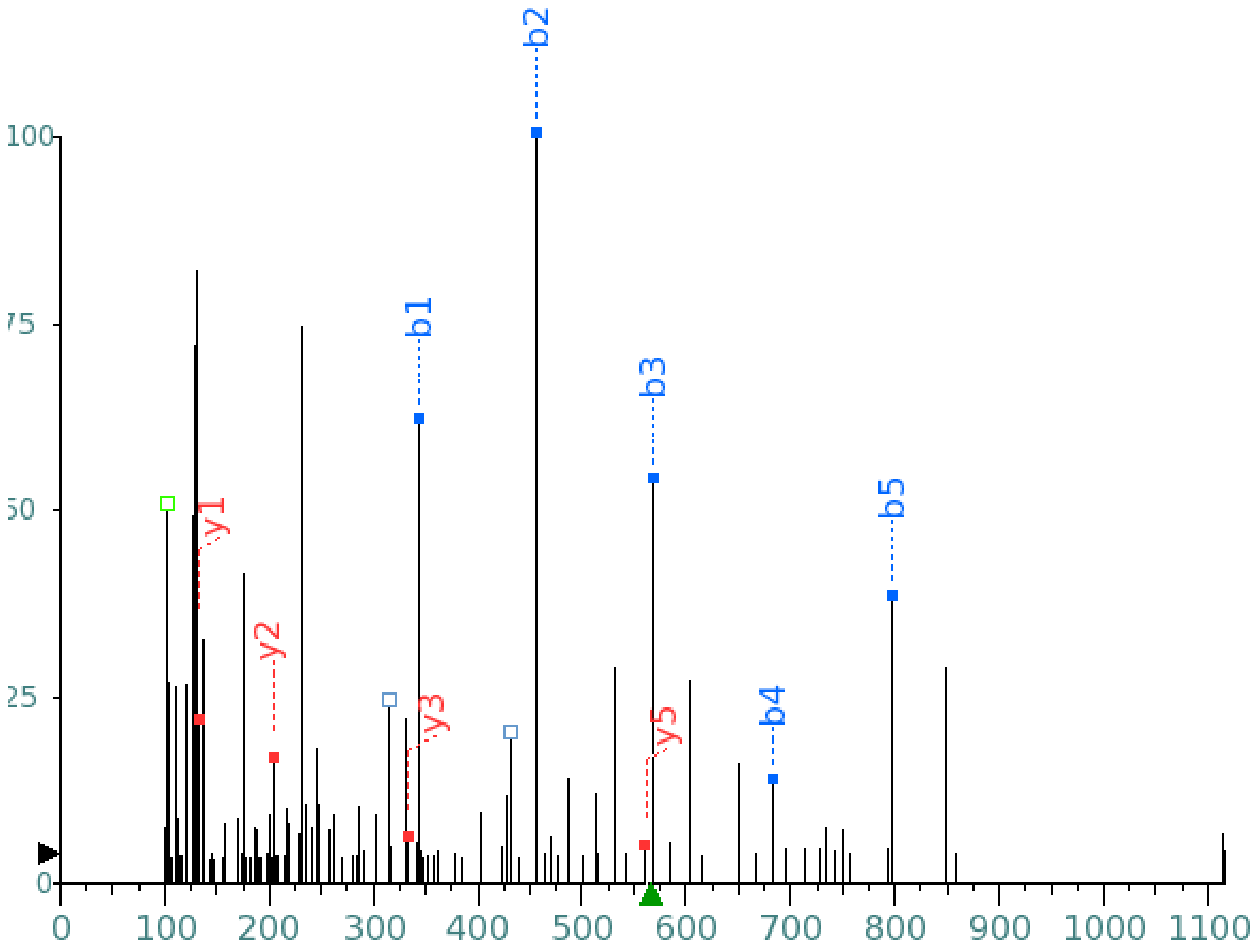

IIILDEAD (+2)

Primary Reference: RFC2_HUMAN
Search ID: 41761
Search Name: 20130328_ananiav_ERstress_TMT_TM_timecourse_trypsin_inj2_4mc_iaa
Scan#: 16459
Observed Mass: 565.8302 (2.2 ppm)
PSM Score: 16.7

File name: 2015-02-04 04.01.53PM.pdf

Creation time: 2015-02-04 04:09PM

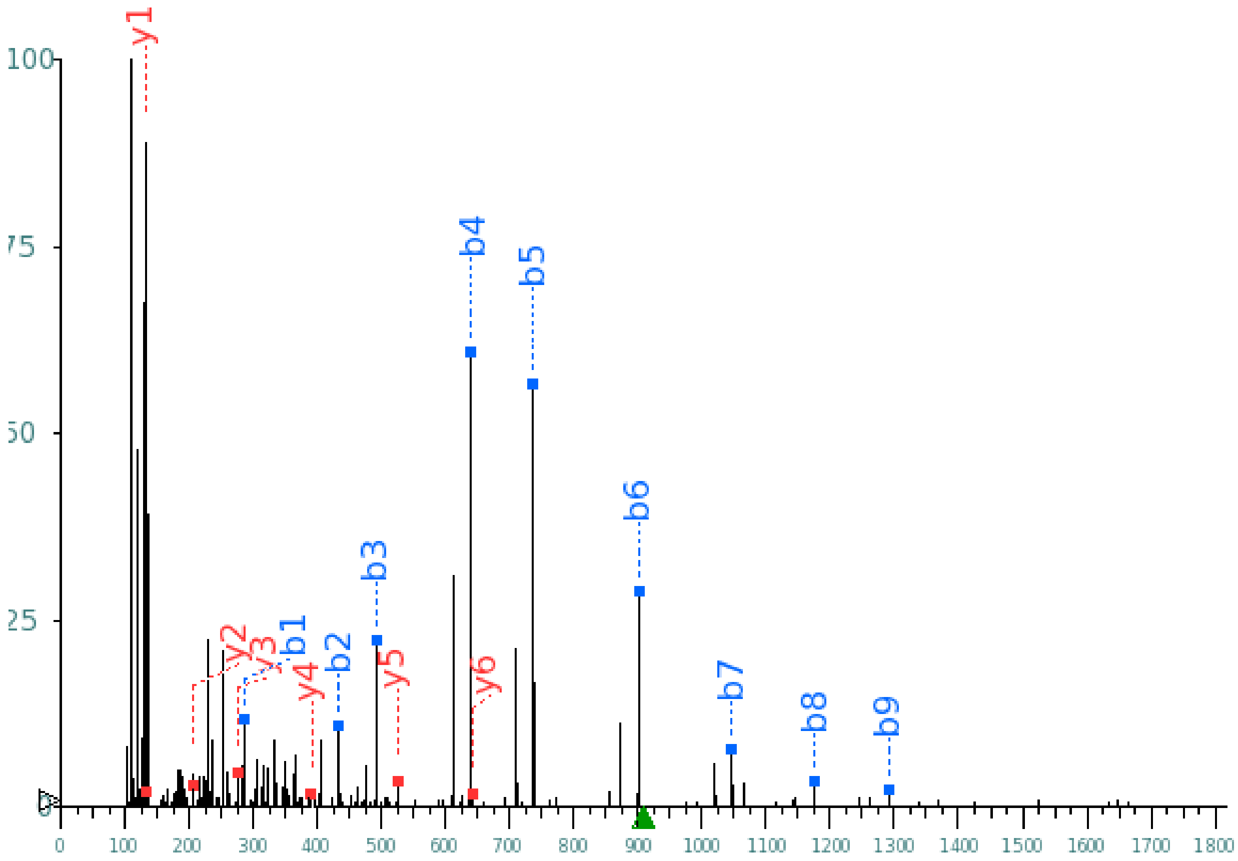

GFGFVYFQNHDAAD (+2)

Primary Reference: ROA0_HUMAN
Search ID: 41761
Search Name: 20130328_ananiav_ERstress_TMT_TM_timecourse_trypsin_inj2_4mc_iaa
Scan#: 16475
Observed Mass: 908.9296 (1.5 ppm)
PSM Score: 34.87

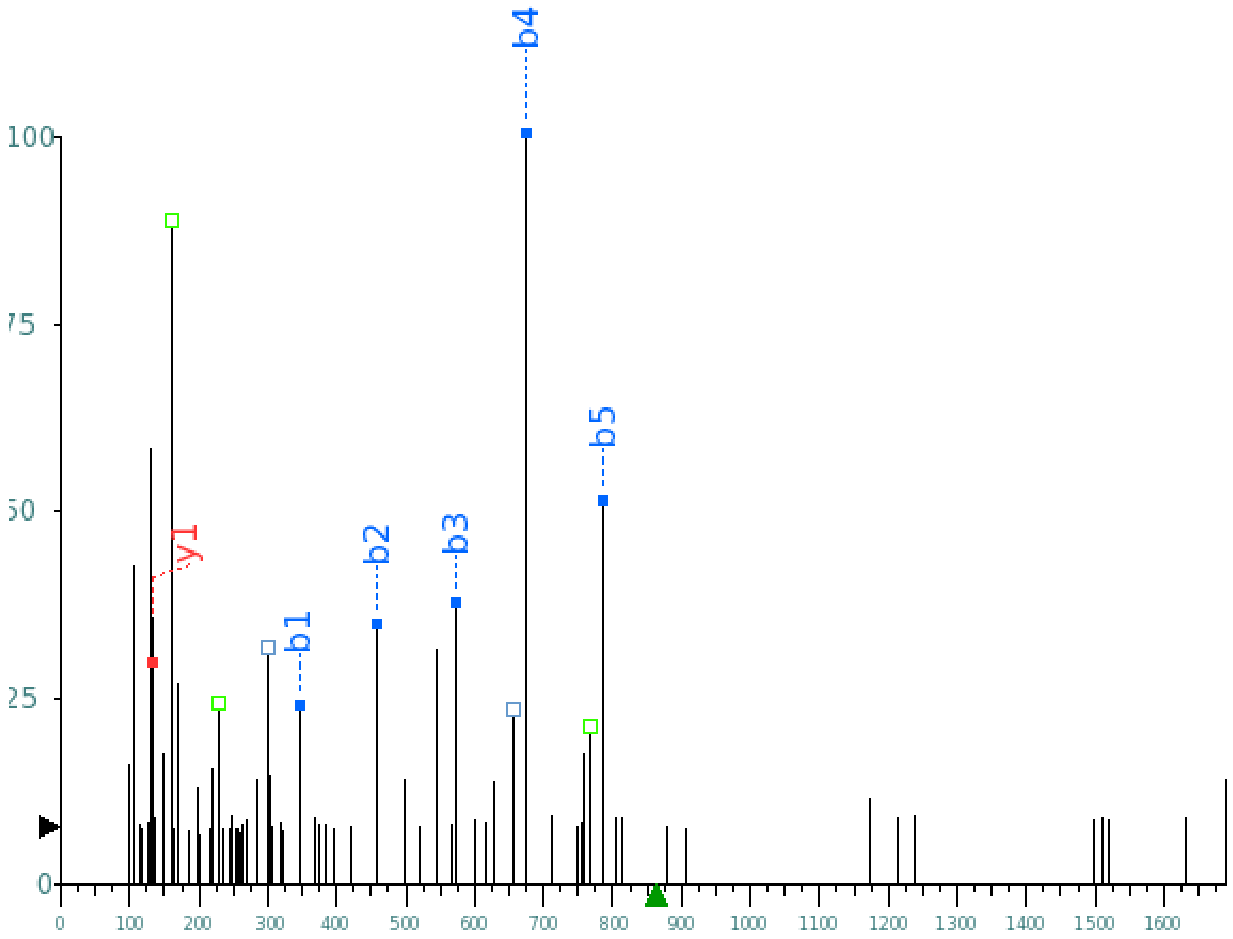

DNLTLWTSDMQGD (+2)

Primary Reference: 1433E_HUMAN
Search ID: 41761
Search Name: 20130328_ananiav_ERstress_TMT_TM_timecourse_trypsin_inj2_4mc_iaa
Scan#: 16481
Observed Mass: 862.9057 (2.4 ppm)
PSM Score: 13.43

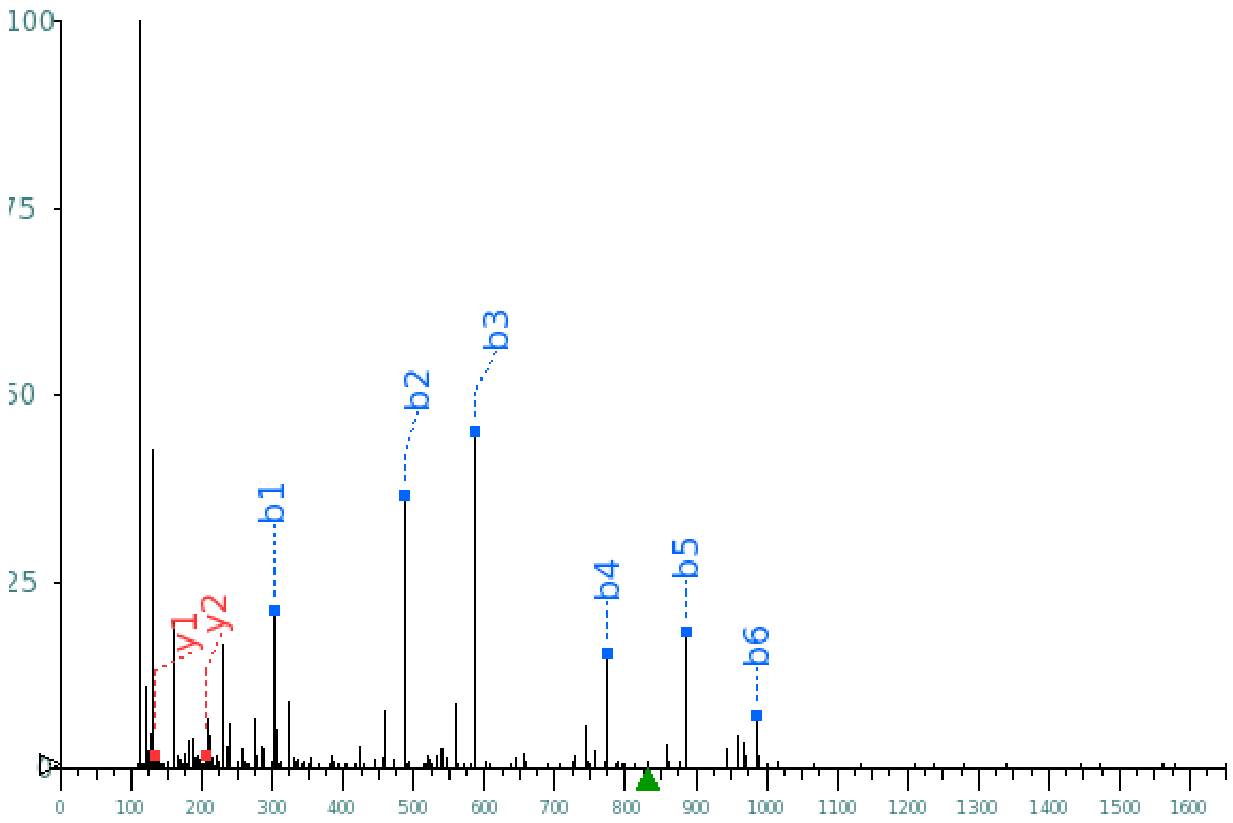

AWVWNTHADFAD (+2)

Primary Reference: RANG_HUMAN
Search ID: 41761
Search Name: 20130328_ananiav_ERstress_TMT_TM_timecourse_trypsin_inj2_4mc_iaa
Scan#: 16482
Observed Mass: 831.4012 (2.5 ppm)
PSM Score: 24.25

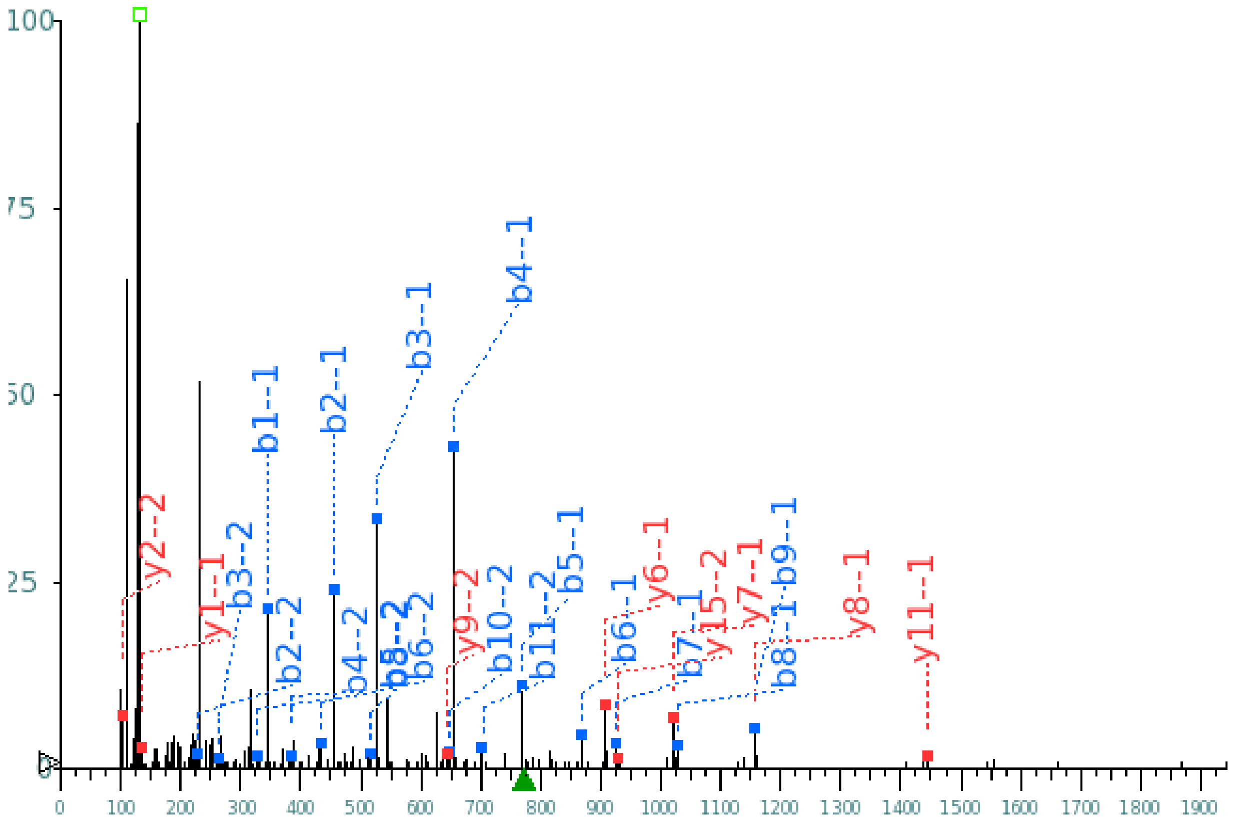

ILAQITGTEHLKESEAD (+3)

Primary Reference: PDLI5_HUMAN
Search ID: 41761
Search Name: 20130328_ananiav_ERstress_TMT_TM_timecourse_trypsin_inj2_4mc_iaa
Scan#: 16515
Observed Mass: 771.7635 (2.5 ppm)
PSM Score: 41.49

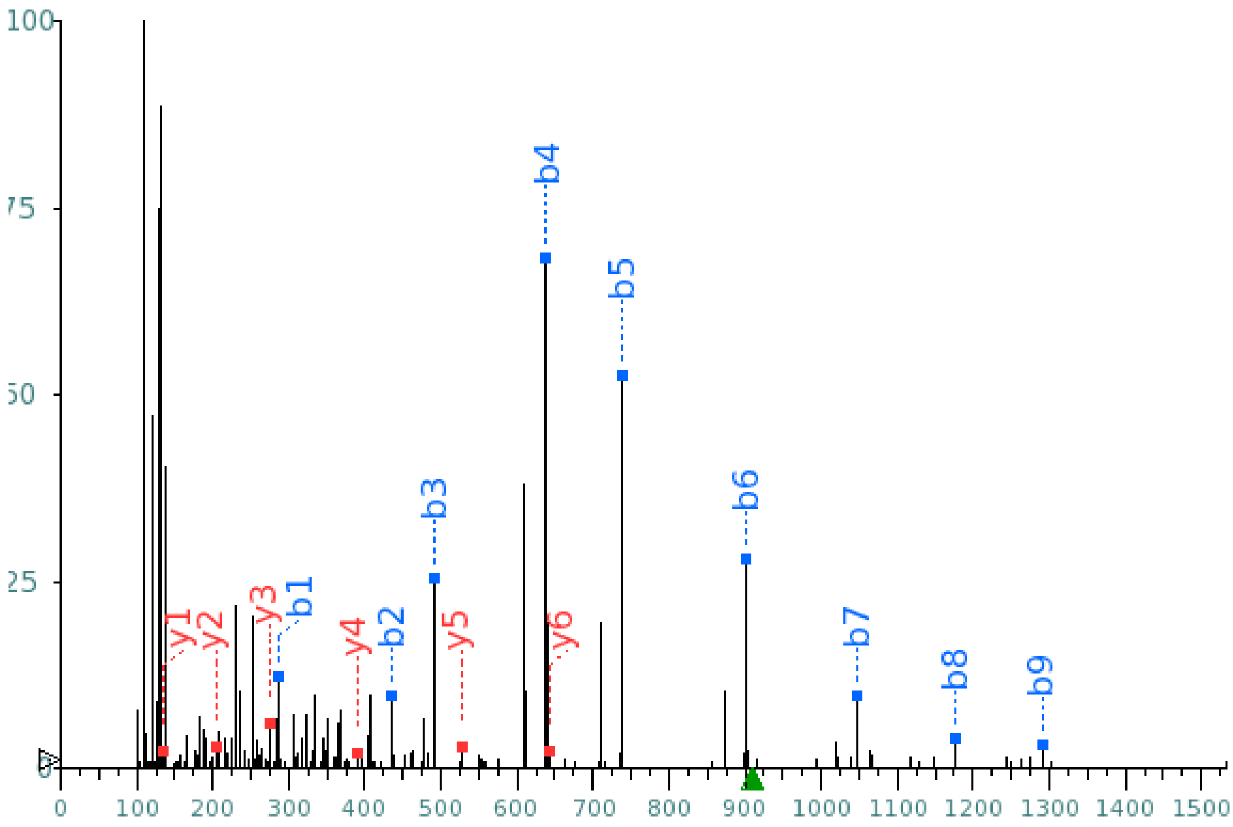

GFGFVYFQNHDAAD (+2)

Primary Reference: ROA0_HUMAN
Search ID: 41761
Search Name: 20130328_ananiav_ERstress_TMT_TM_timecourse_trypsin_inj2_4mc_iaa
Scan#: 16538
Observed Mass: 908.9299 (1.8 ppm)
PSM Score: 36.1

File name: 2015-02-04 04.01.53PM.pdf

Creation time: 2015-02-04 04:09PM

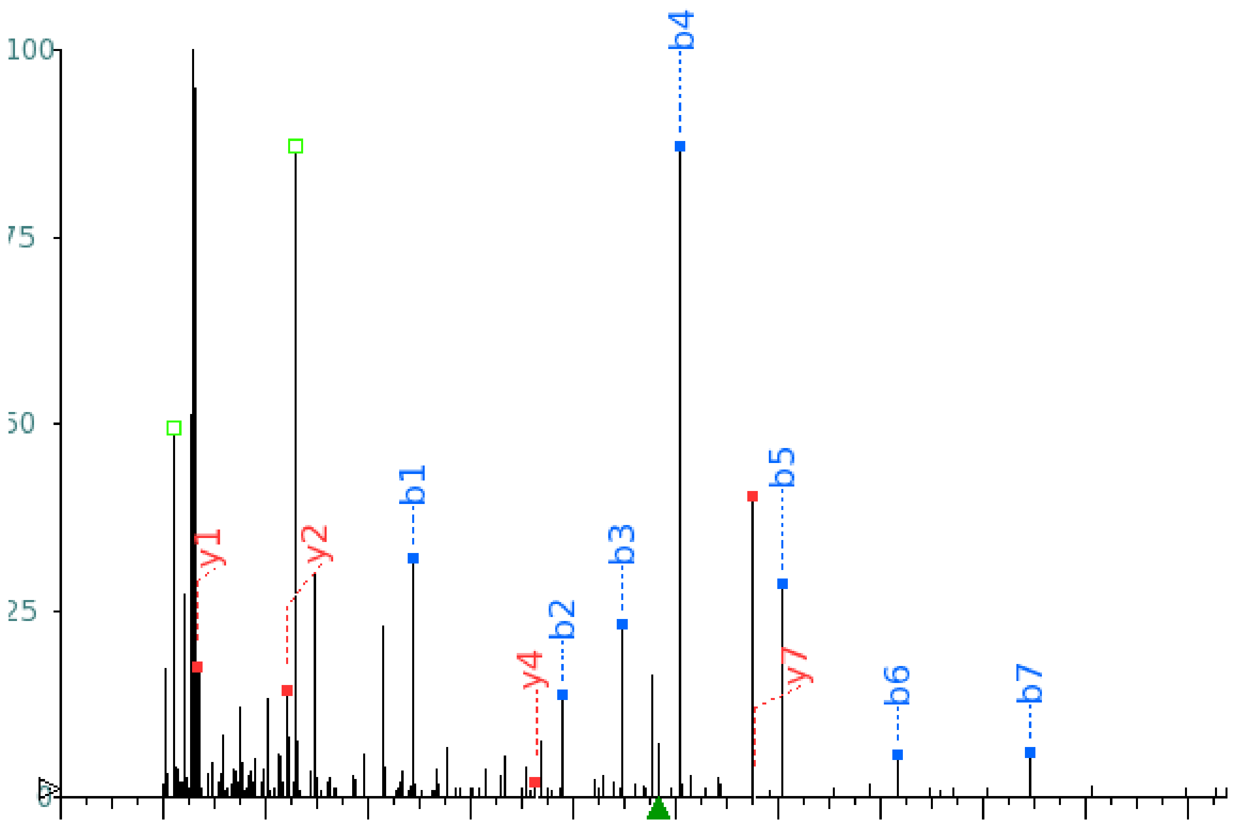

IFGGVLESD (+2)

Primary Reference: HAX1_HUMAN
Search ID: 41761
Search Name: 20130328_ananiav_ERstress_TMT_TM_timecourse_trypsin_inj2_4mc_iaa
Scan#: 16544
Observed Mass: 583.3199 (1.9 ppm)
PSM Score: 51.38

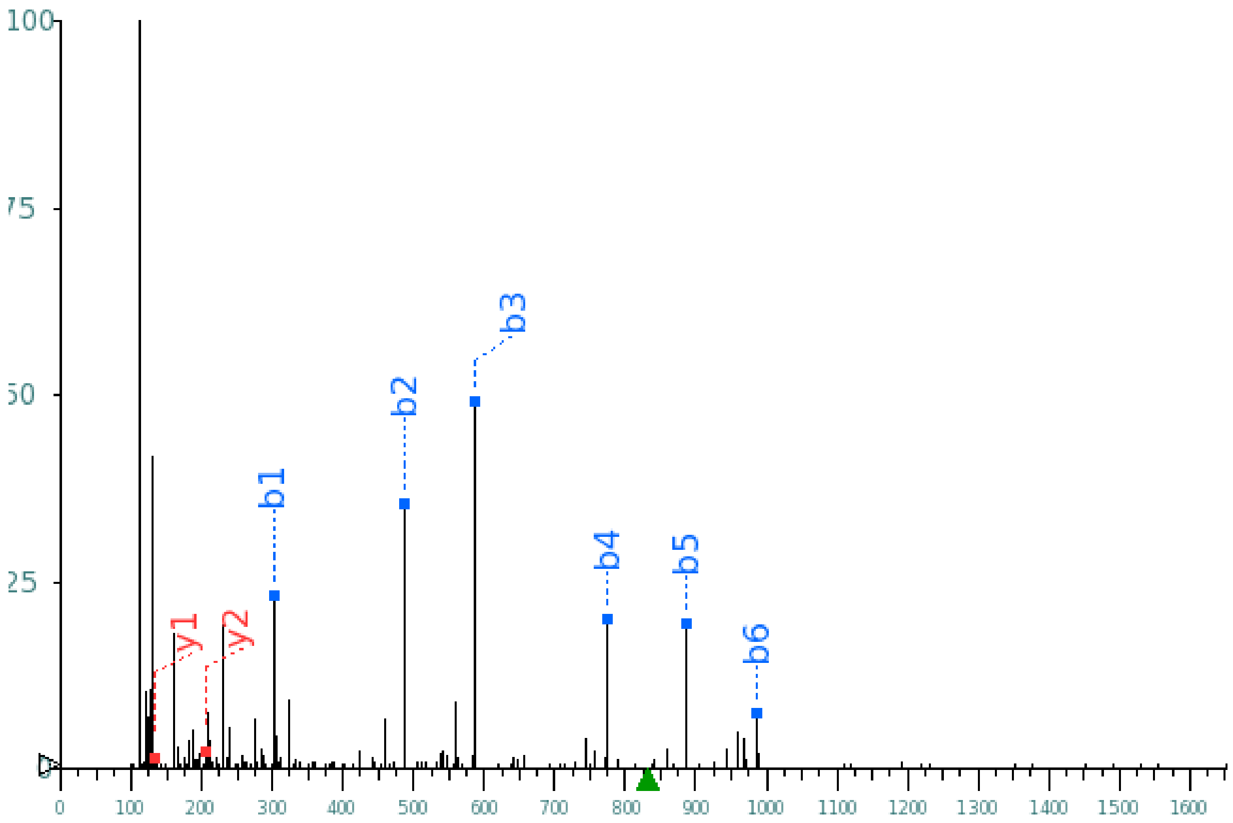

AWVWNTHADFAD (+2)

Primary Reference: RANG_HUMAN
Search ID: 41761
Search Name: 20130328_ananiav_ERstress_TMT_TM_timecourse_trypsin_inj2_4mc_iaa
Scan#: 16547
Observed Mass: 831.4003 (1.4 ppm)
PSM Score: 26

File name: 2015-02-04 04.01.53PM.pdf

Creation time: 2015-02-04 04:09PM

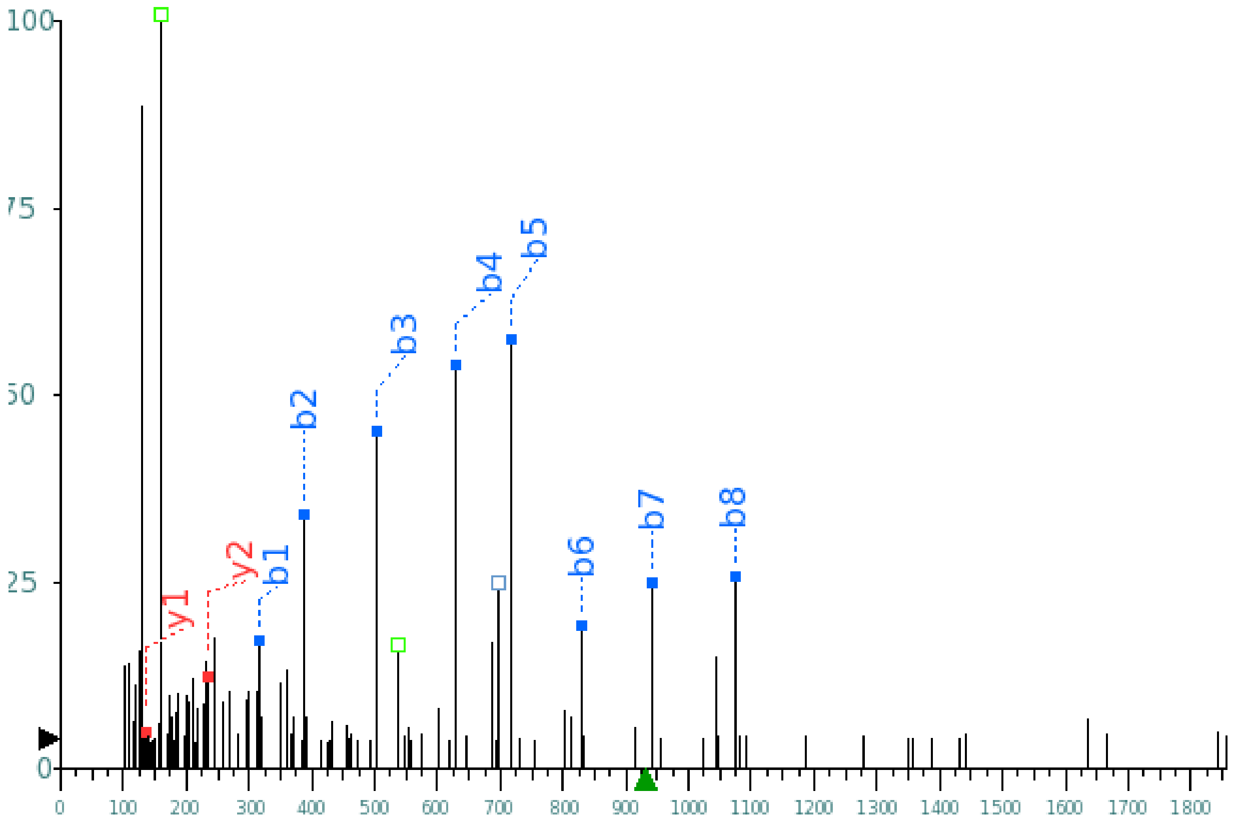

SALQSINEWAAQTTD (+2)

Primary Reference: SERPH_HUMAN
Search ID: 41761
Search Name: 20130328_ananiav_ERstress_TMT_TM_timecourse_trypsin_inj2_4mc_iaa
Scan#: 16571
Observed Mass: 932.4706 (2.9 ppm)
PSM Score: 37.49

File name: 2015-02-04 04.01.53PM.pdf

Creation time: 2015-02-04 04:09PM

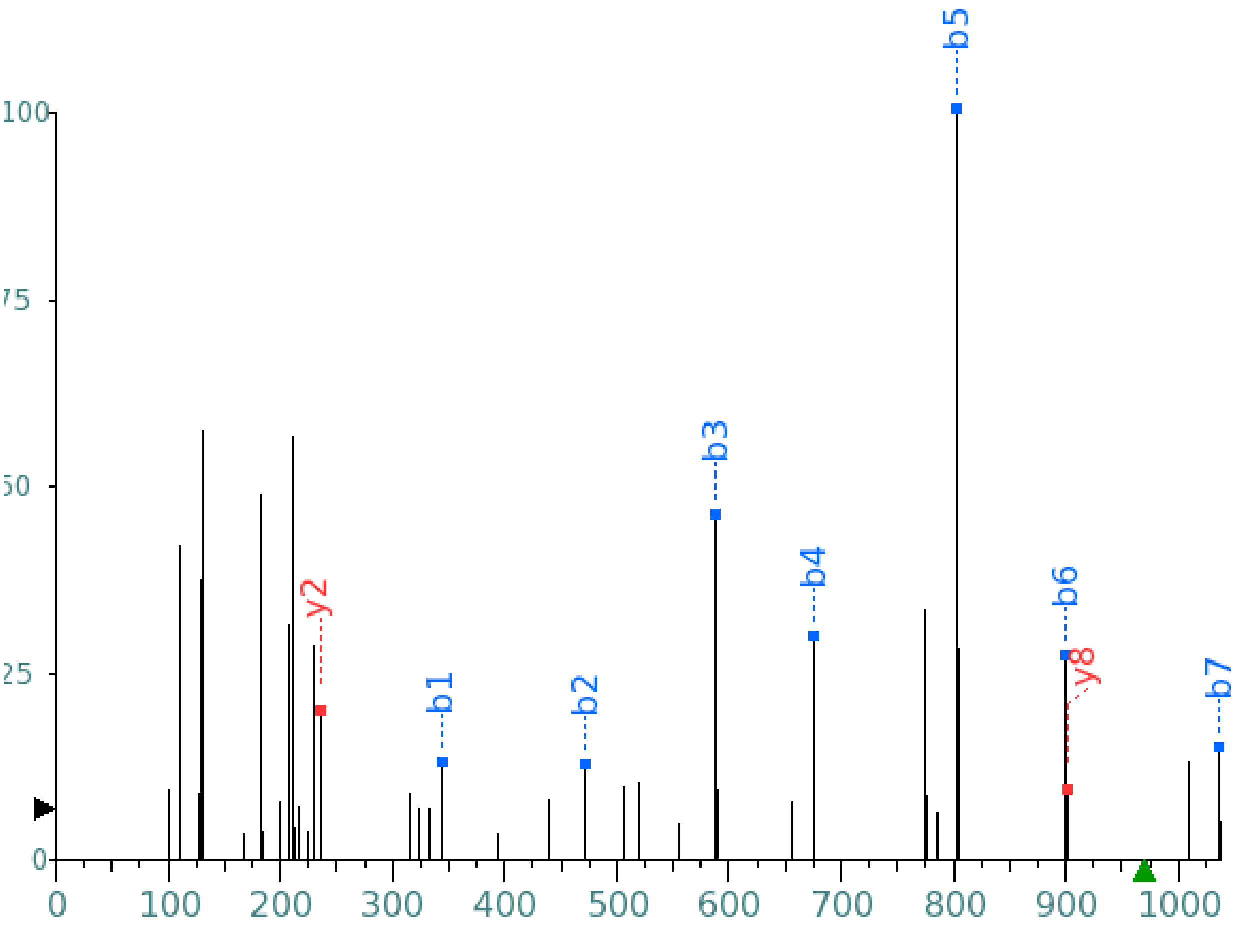

IEDSEPHIPLIDDTD (+2)

Primary Reference: AT2B1_HUMAN
Search ID: 41761
Search Name: 20130328_ananiav_ERstress_TMT_TM_timecourse_trypsin_inj2_4mc_iaa
Scan#: 16587
Observed Mass: 969.4826 (1.9 ppm)
PSM Score: 37.16

File name: 2015-02-04 04.01.53PM.pdf

Creation time: 2015-02-04 04:09PM

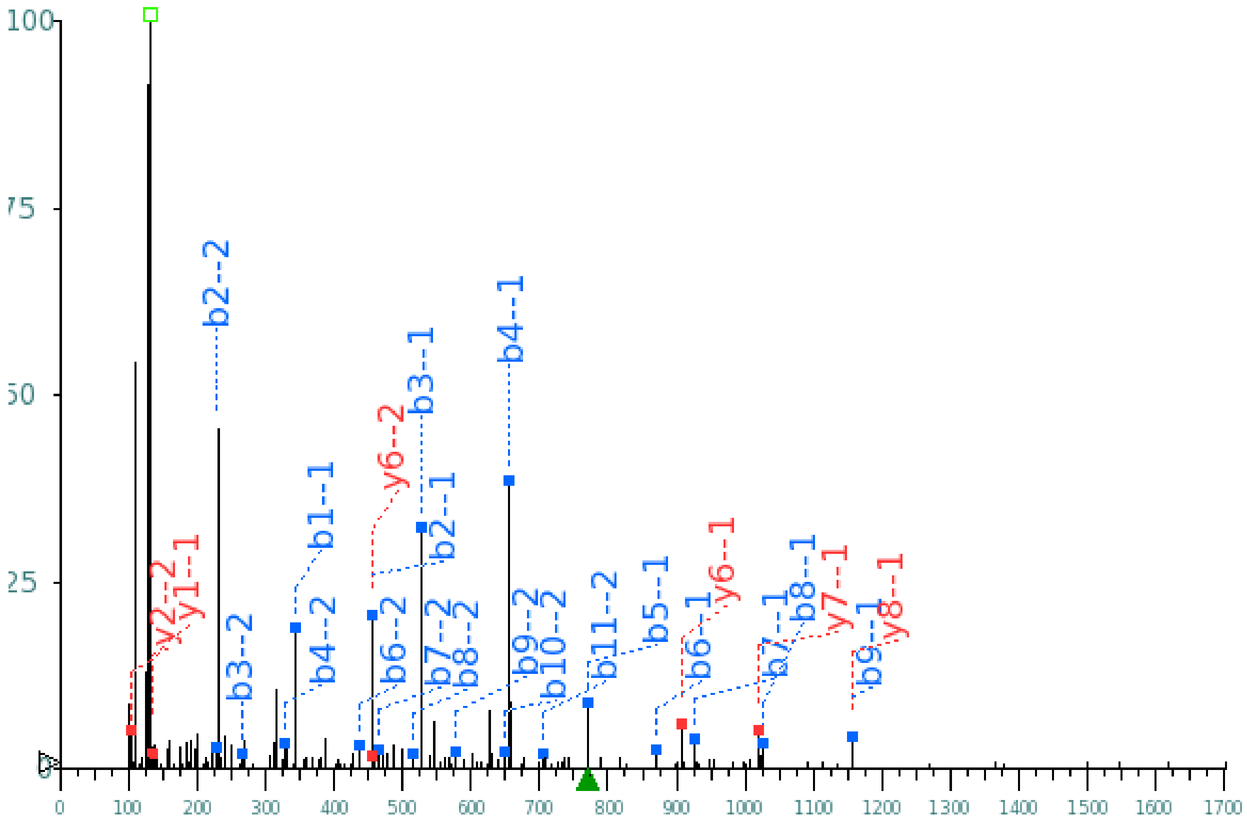

ILAQITGTEHLKESEAD (+3)

Primary Reference: PDLI5_HUMAN
Search ID: 41761
Search Name: 20130328_ananiav_ERstress_TMT_TM_timecourse_trypsin_inj2_4mc_iaa
Scan#: 16593
Observed Mass: 771.7638 (2.9 ppm)
PSM Score: 40.97

File name: 2015-02-04 04.01.53PM.pdf

Creation time: 2015-02-04 04:09PM

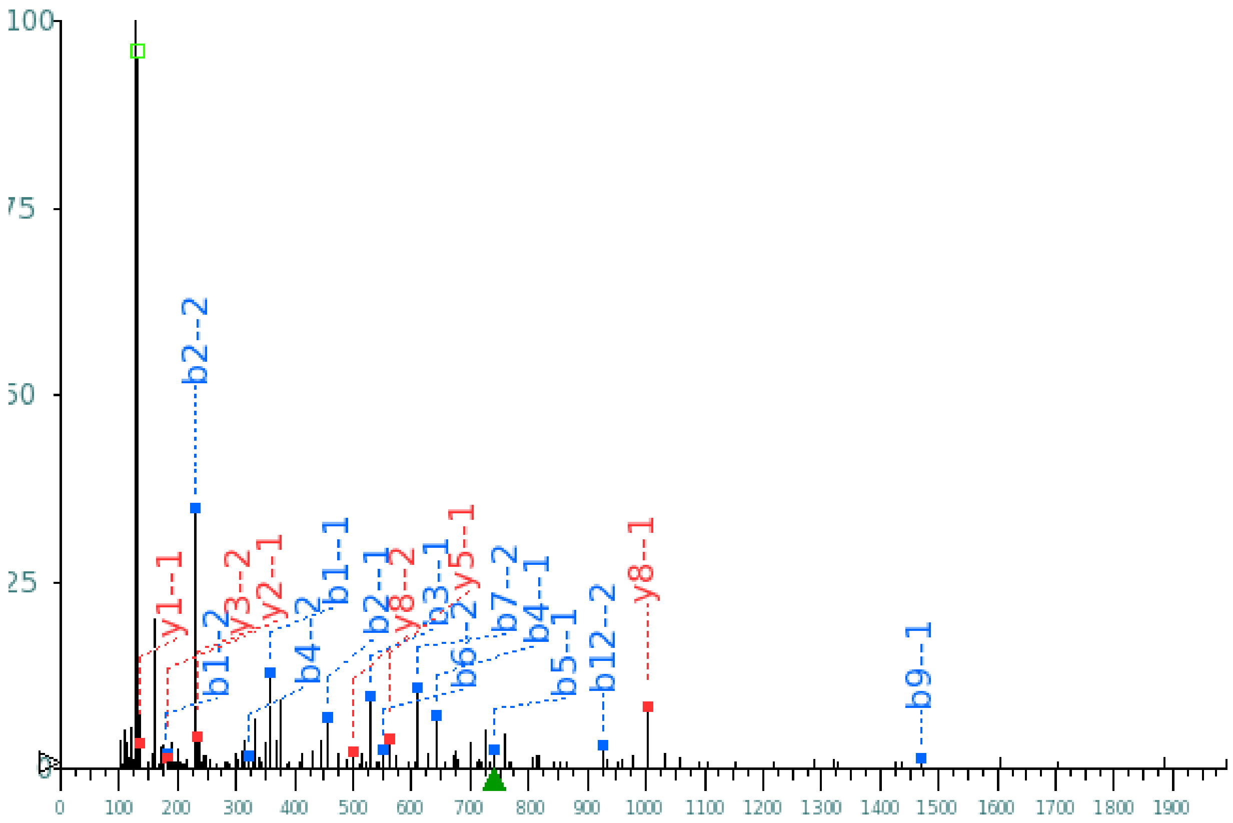

EVALVKDVRWPTETD (+3)

Primary Reference: MAP4_HUMAN
Search ID: 41761
Search Name: 20130328_ananiav_ERstress_TMT_TM_timecourse_trypsin_inj2_4mc_iaa
Scan#: 16595
Observed Mass: 739.4137 (-2.7 ppm)
PSM Score: 8.43

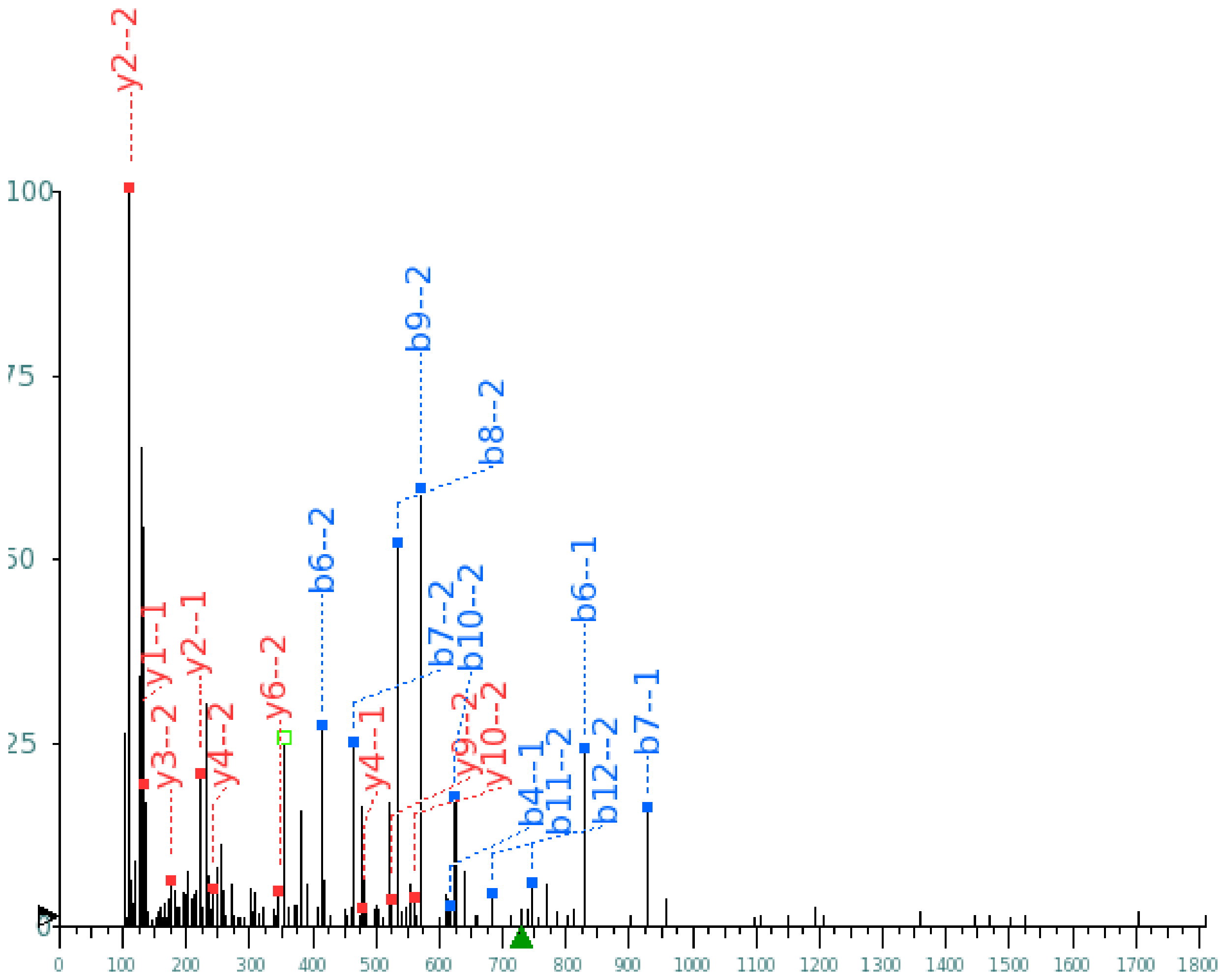

RGSSPDVHALLEITEESD (+3)

Primary Reference: PTRF_HUMAN
Search ID: 41761
Search Name: 20130328_ananiav_ERstress_TMT_TM_timecourse_trypsin_inj2_4mc_iaa
Scan#: 16608
Observed Mass: 728.7054 (1.6 ppm)
PSM Score: 12.81

File name: 2015-02-04 04.01.53PM.pdf

Creation time: 2015-02-04 04:09PM

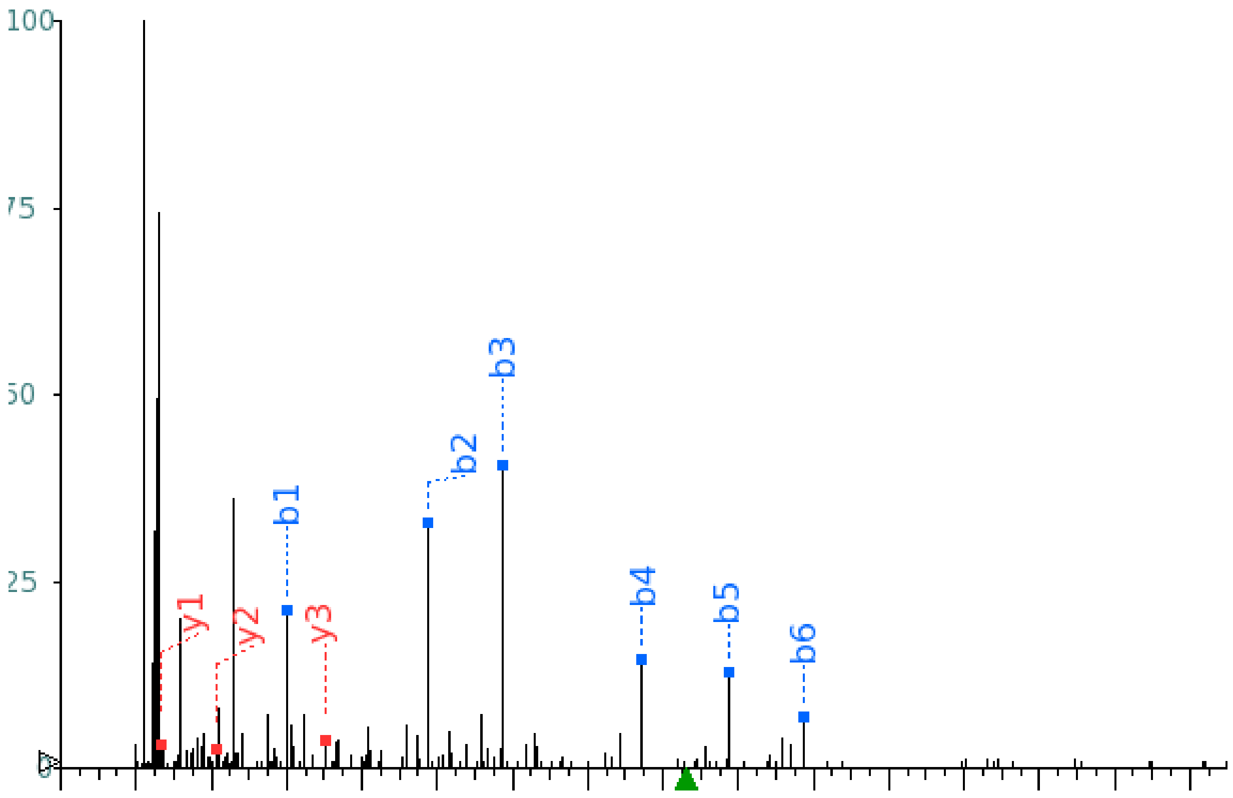

AWVWNTHADFAD (+2)

Primary Reference: RANG_HUMAN
Search ID: 41761
Search Name: 20130328_ananiav_ERstress_TMT_TM_timecourse_trypsin_inj2_4mc_iaa
Scan#: 16637
Observed Mass: 831.399 (-0.1 ppm)
PSM Score: 24.32

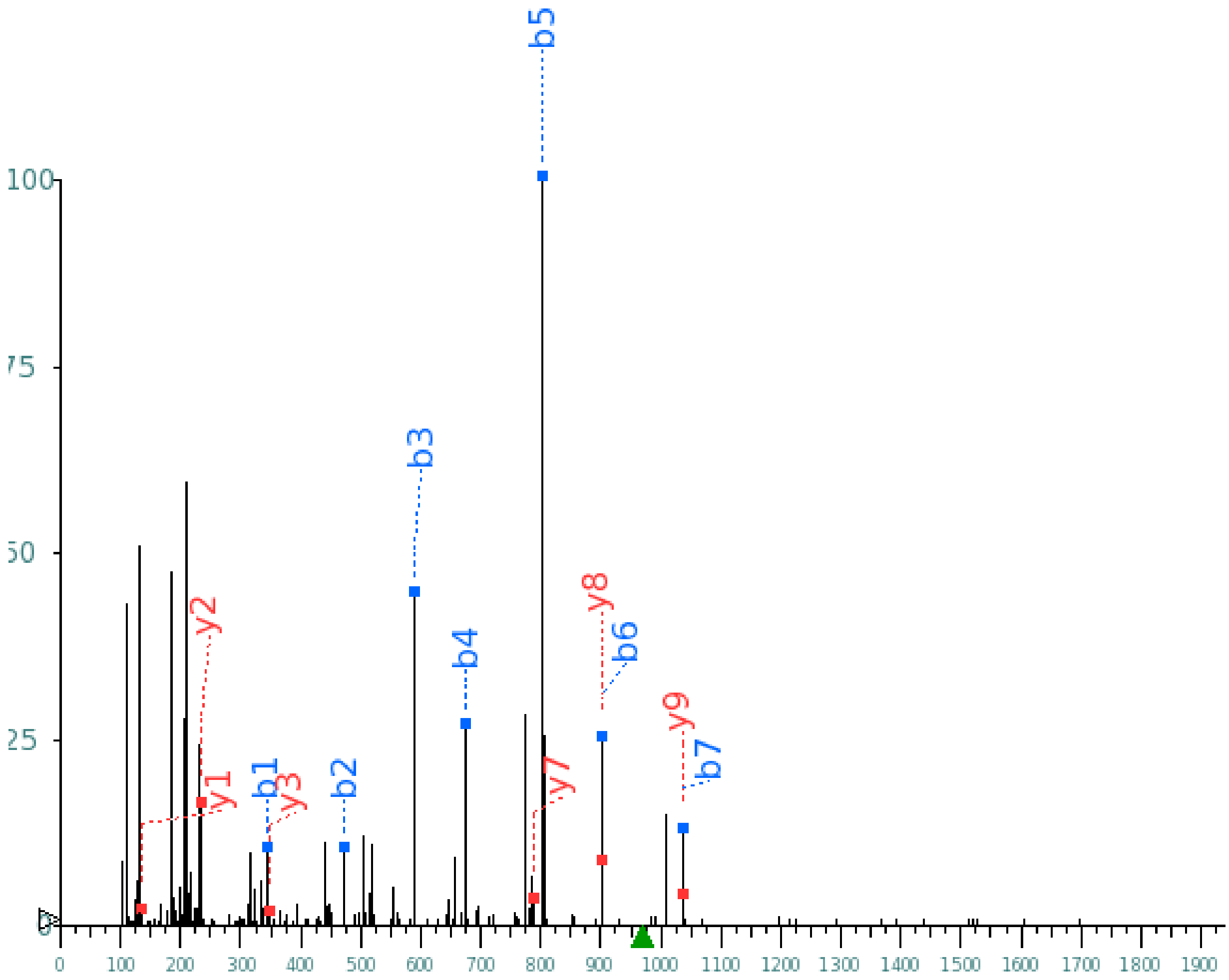

IEDSEPHIPLIDDTD (+2)

Primary Reference: AT2B1_HUMAN
Search ID: 41761
Search Name: 20130328_ananiav_ERstress_TMT_TM_timecourse_trypsin_inj2_4mc_iaa
Scan#: 16648
Observed Mass: 969.4845 (3.9 ppm)
PSM Score: 15.21

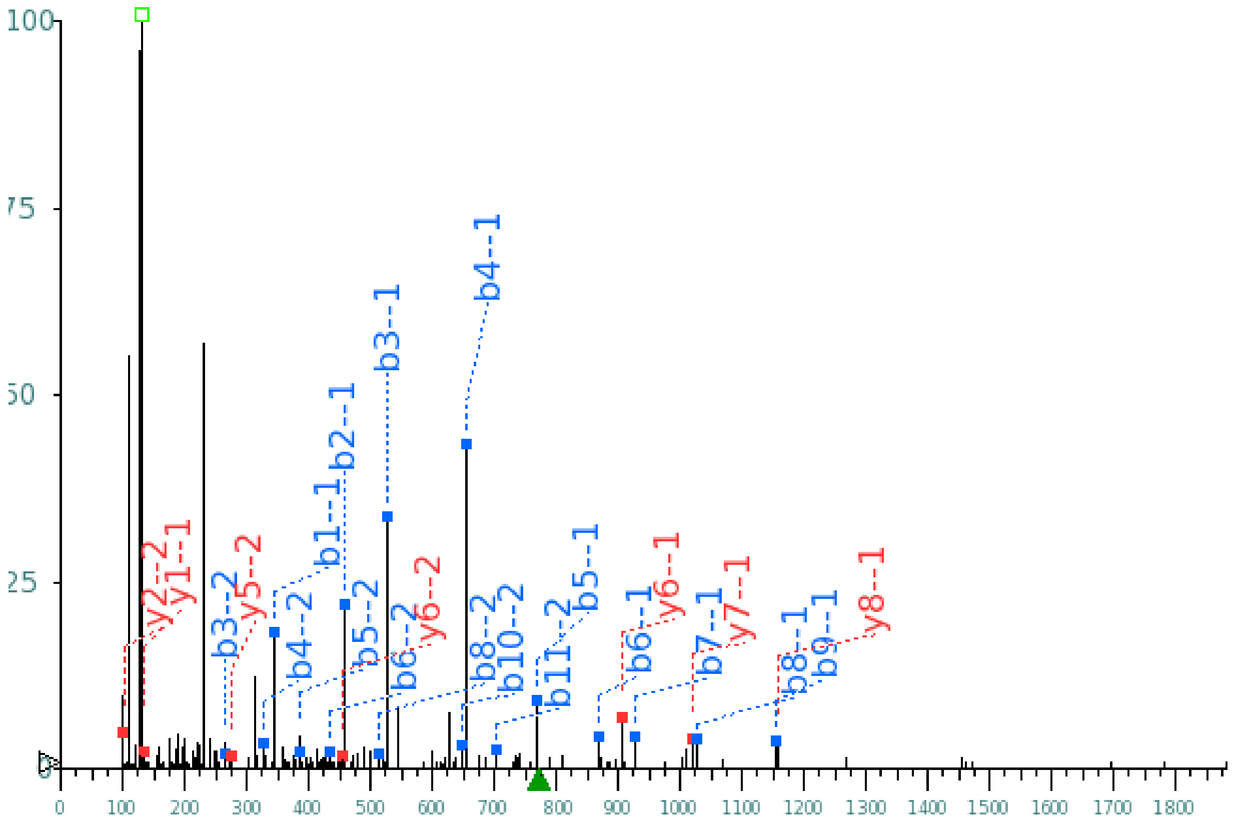

ILAQITGTEHLKESEAD (+3)

Primary Reference: PDLI5_HUMAN
Search ID: 41761
Search Name: 20130328_ananiav_ERstress_TMT_TM_timecourse_trypsin_inj2_4mc_iaa
Scan#: 16668
Observed Mass: 771.7639 (3 ppm)
PSM Score: 40.34

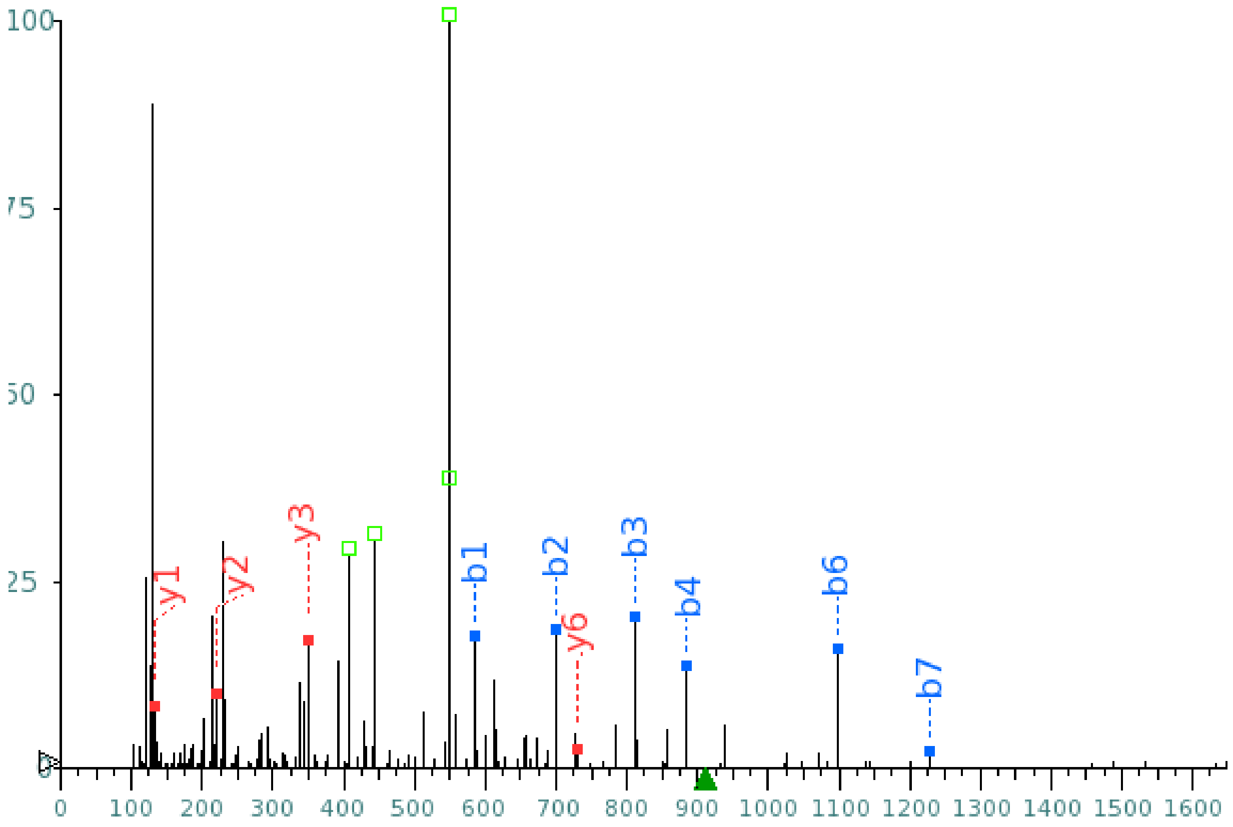

KLLAPDMFTESD (+2)

Primary Reference: PRP4B_HUMAN
Search ID: 41761
Search Name: 20130328_ananiav_ERstress_TMT_TM_timecourse_trypsin_inj2_4mc_iaa
Scan#: 16676
Observed Mass: 912.9979 (3.7 ppm)
PSM Score: 15.78

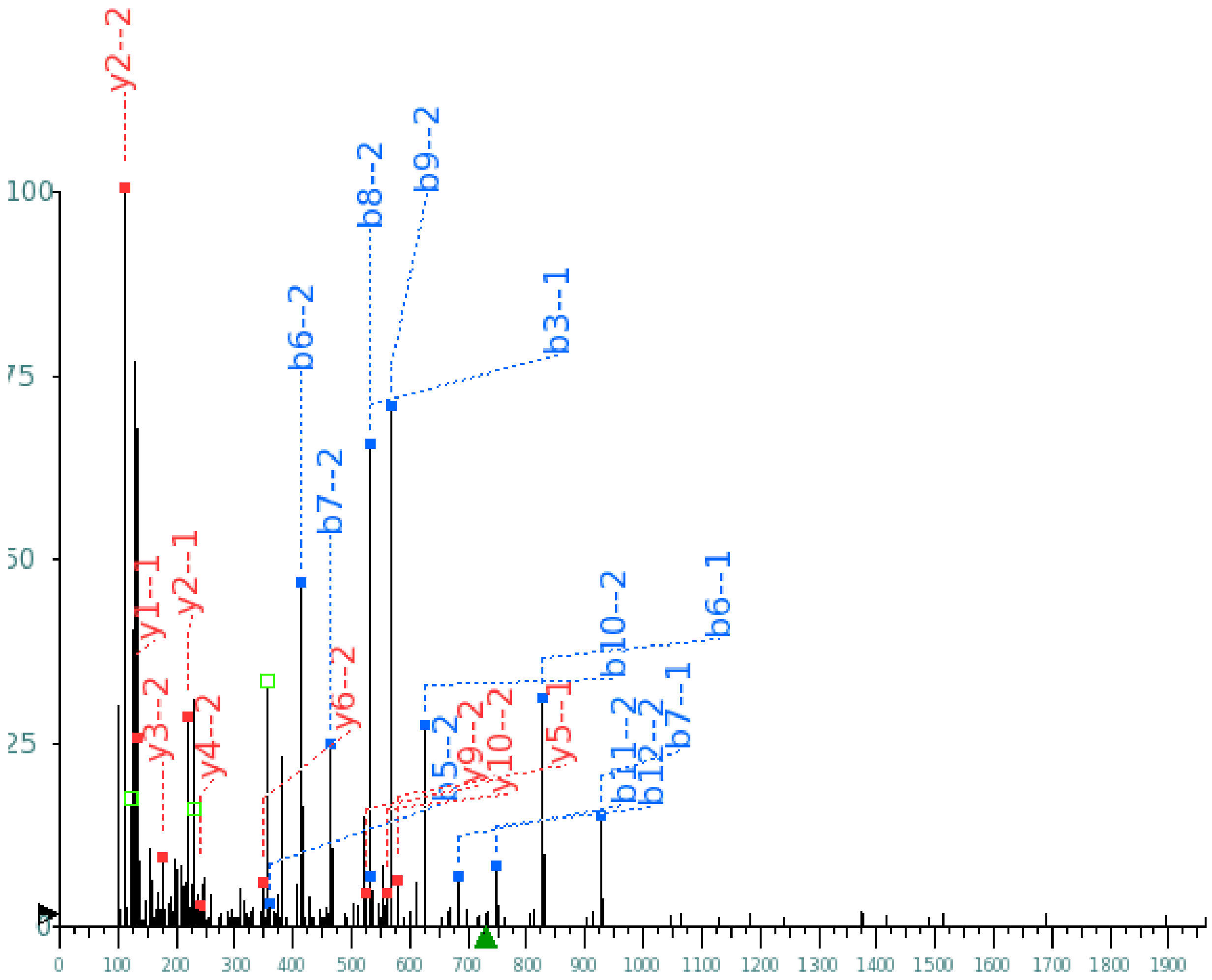

RGSSPDVHALLEITEESD (+3)

Primary Reference: PTRF_HUMAN
Search ID: 41761
Search Name: 20130328_ananiav_ERstress_TMT_TM_timecourse_trypsin_inj2_4mc_iaa
Scan#: 16688
Observed Mass: 728.7042 (-0.1 ppm)
PSM Score: 21.41

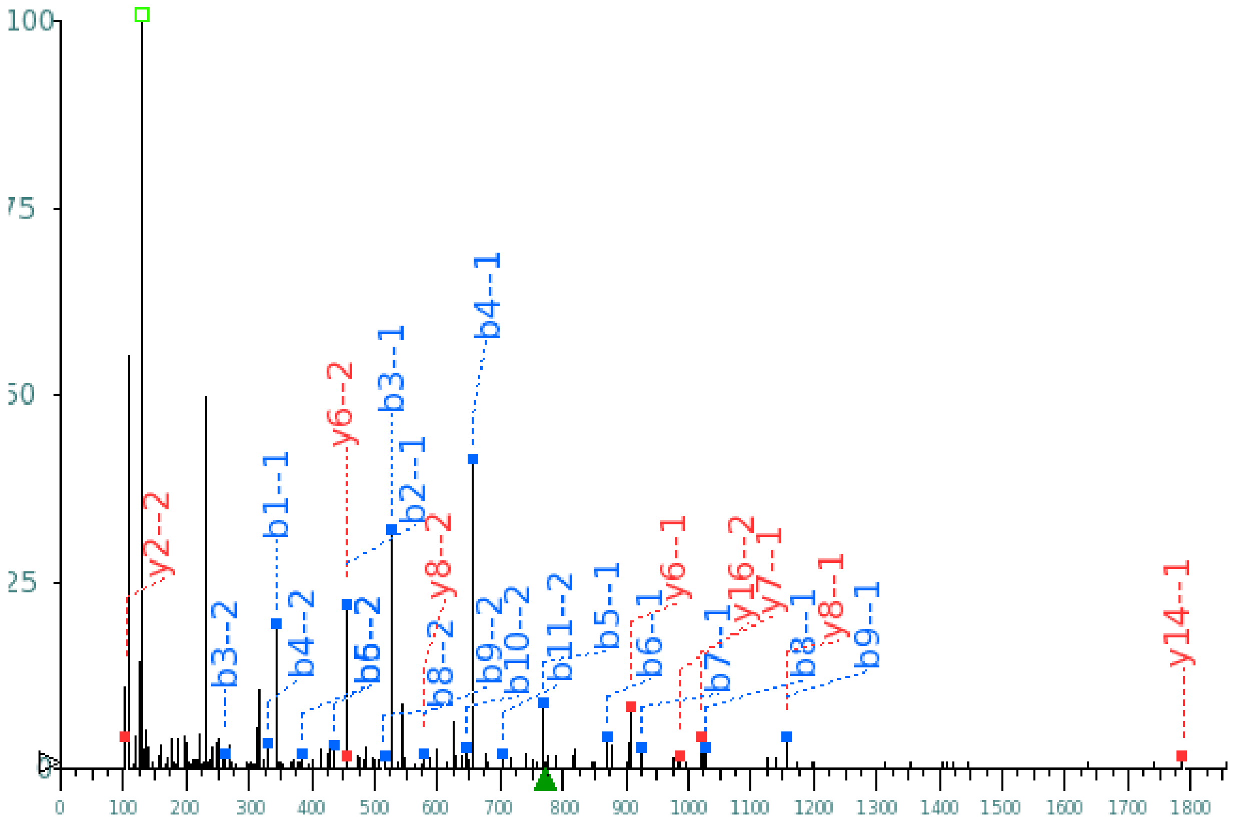

ILAQITGTEHLKESEAD (+3)

Primary Reference: PDLI5_HUMAN
Search ID: 41761
Search Name: 20130328_ananiav_ERstress_TMT_TM_timecourse_trypsin_inj2_4mc_iaa
Scan#: 16736
Observed Mass: 771.7632 (2.1 ppm)
PSM Score: 34.49

File name: 2015-02-04 04.01.53PM.pdf

Creation time: 2015-02-04 04:09PM

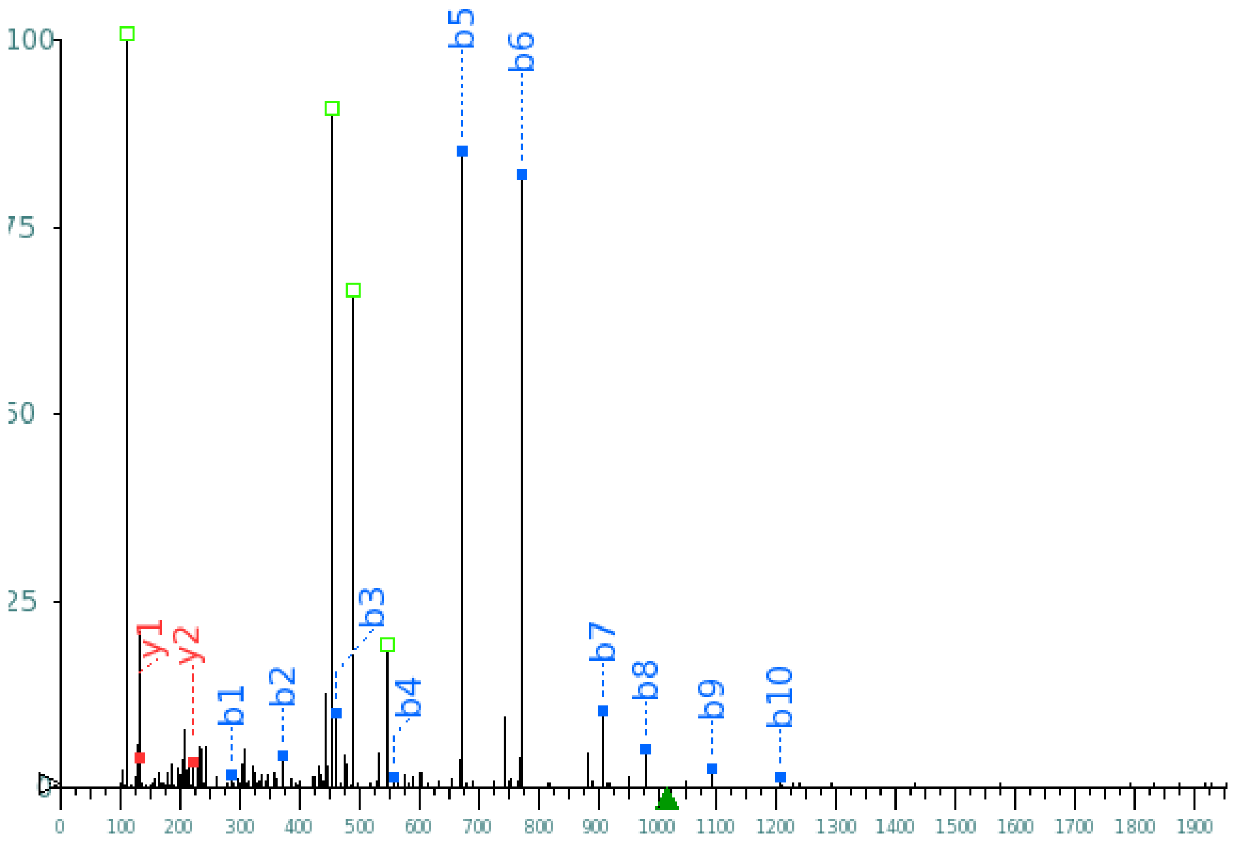

GSSPDVHALLEITEESD (+2)

Primary Reference: PTRF_HUMAN
Search ID: 41761
Search Name: 20130328_ananiav_ERstress_TMT_TM_timecourse_trypsin_inj2_4mc_iaa
Scan#: 16759
Observed Mass: 1014.5047 (2.5 ppm)
PSM Score: 24.38

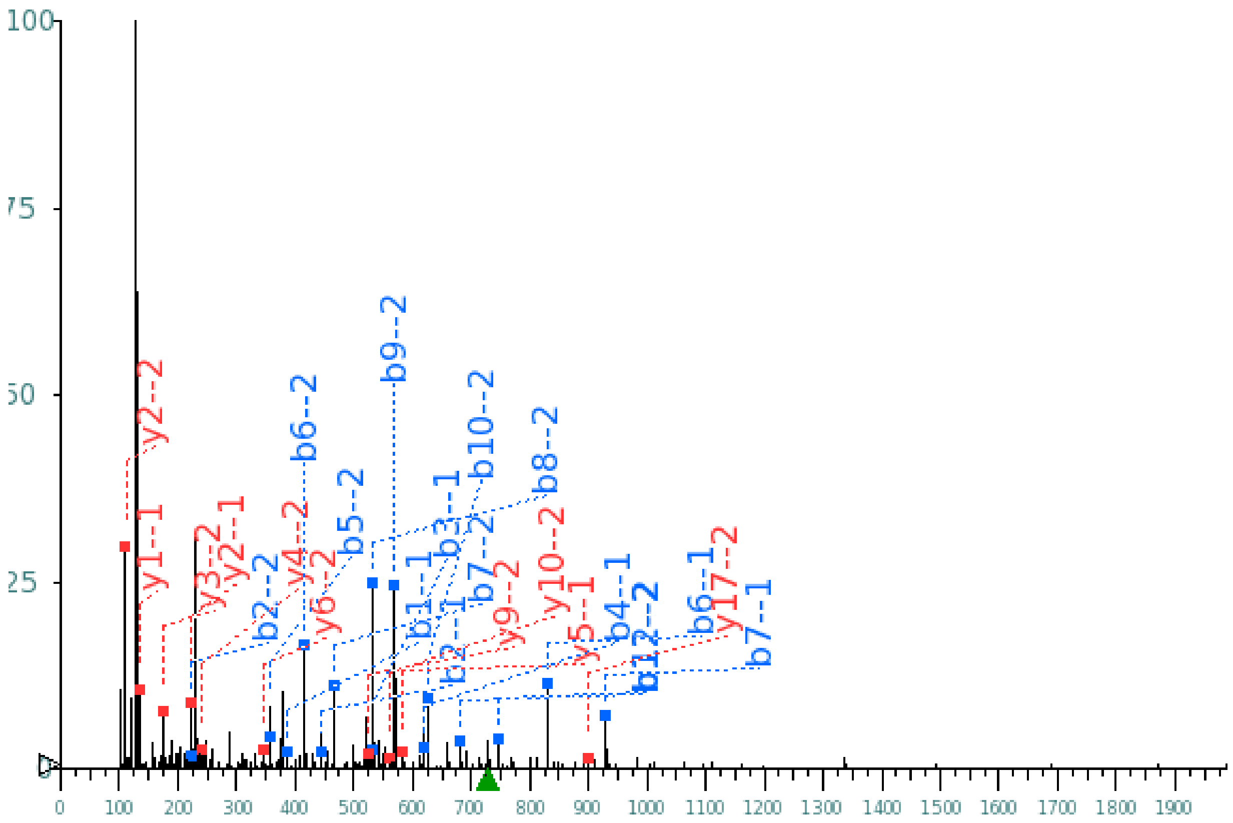

RGSSPDVHALLEITEESD (+3)

Primary Reference: PTRF_HUMAN
Search ID: 41761
Search Name: 20130328_ananiav_ERstress_TMT_TM_timecourse_trypsin_inj2_4mc_iaa
Scan#: 16764
Observed Mass: 728.7022 (-2.7 ppm)
PSM Score: 14.2

File name: 2015-02-04 04.01.53PM.pdf

Creation time: 2015-02-04 04:09PM

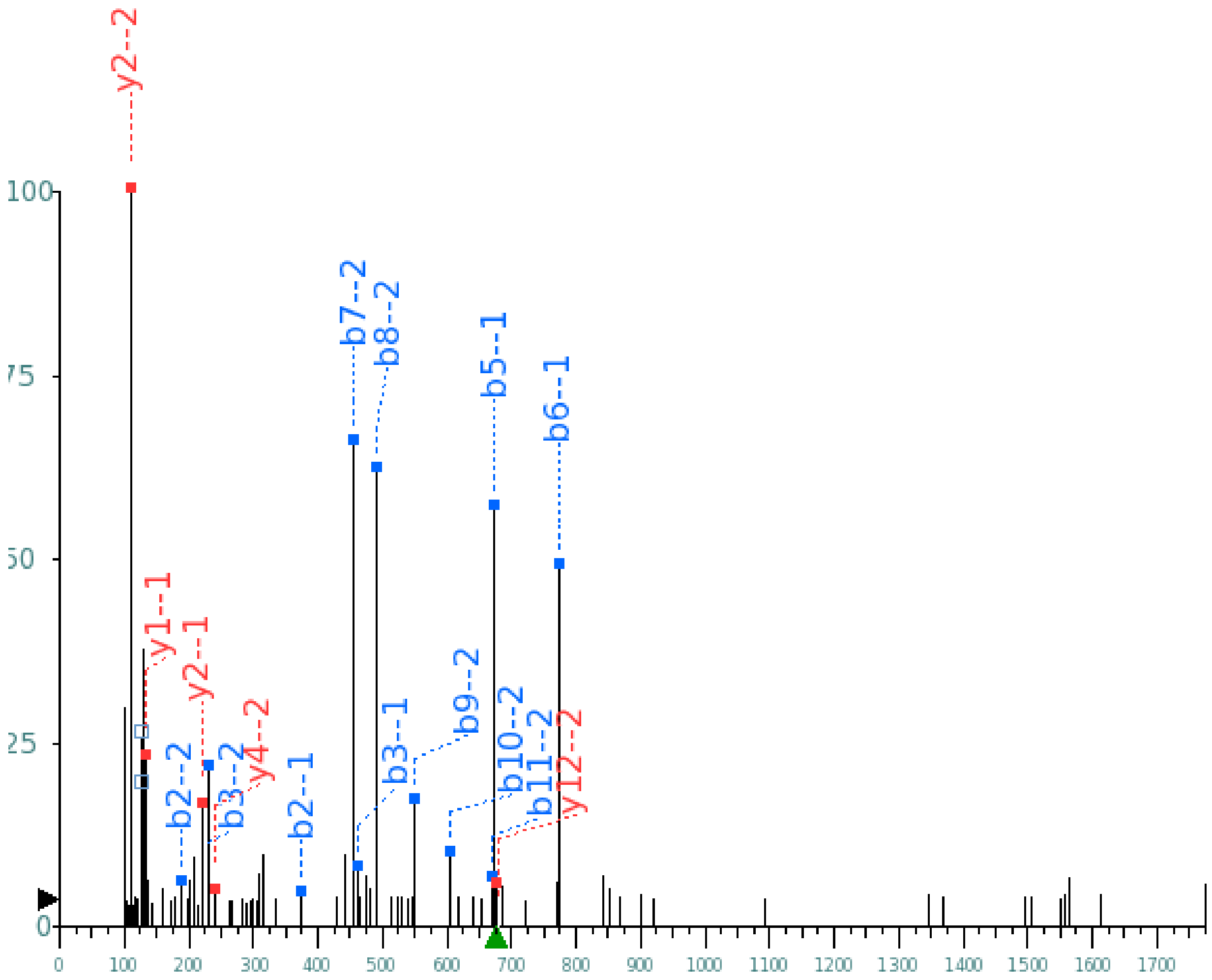

GSSPDVHALLEITEESD (+3)

Primary Reference: PTRF_HUMAN
Search ID: 41761
Search Name: 20130328_ananiav_ERstress_TMT_TM_timecourse_trypsin_inj2_4mc_iaa
Scan#: 16811
Observed Mass: 676.6723 (2.6 ppm)
PSM Score: 10.04

File name: 2015-02-04 04.01.53PM.pdf

Creation time: 2015-02-04 04:09PM

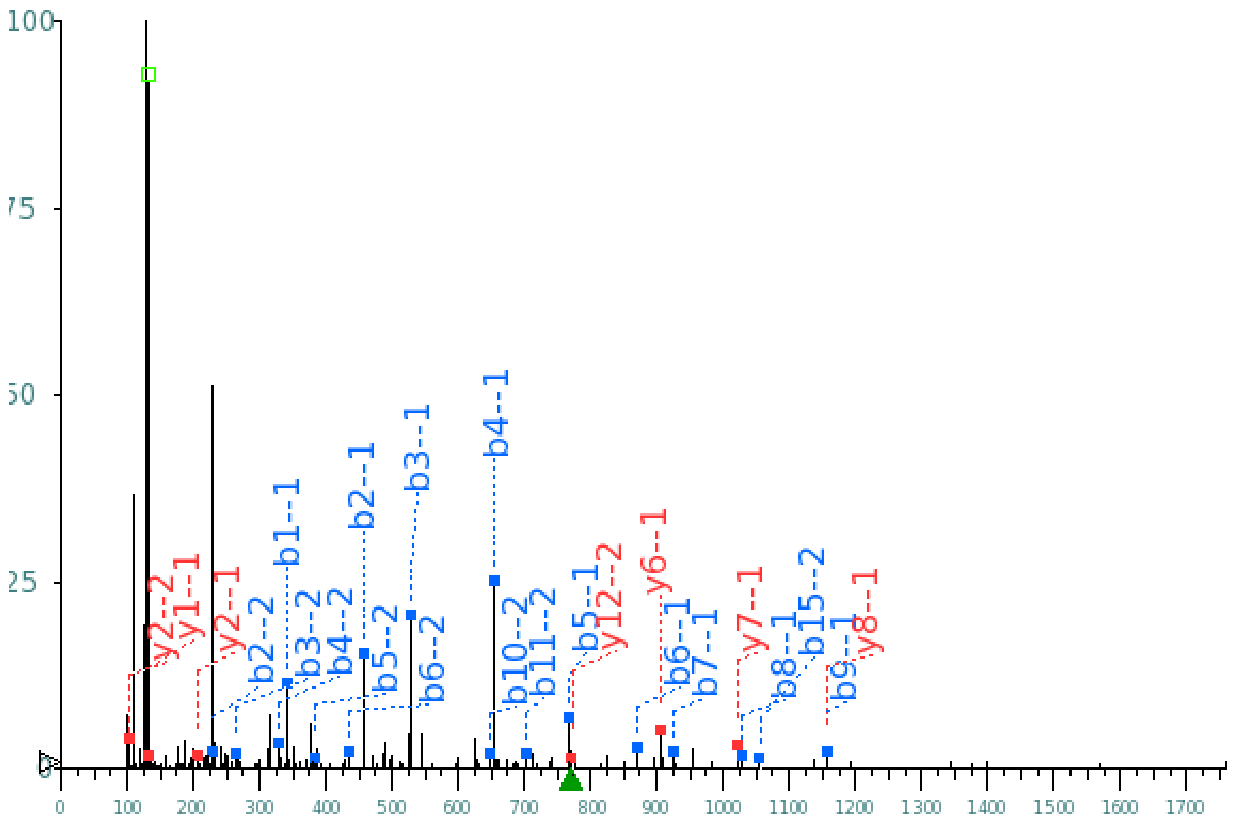

ILAQITGTEHLKESEAD (+3)

Primary Reference: PDLI5_HUMAN
Search ID: 41761
Search Name: 20130328_ananiav_ERstress_TMT_TM_timecourse_trypsin_inj2_4mc_iaa
Scan#: 16814
Observed Mass: 771.7639 (3 ppm)
PSM Score: 37.92

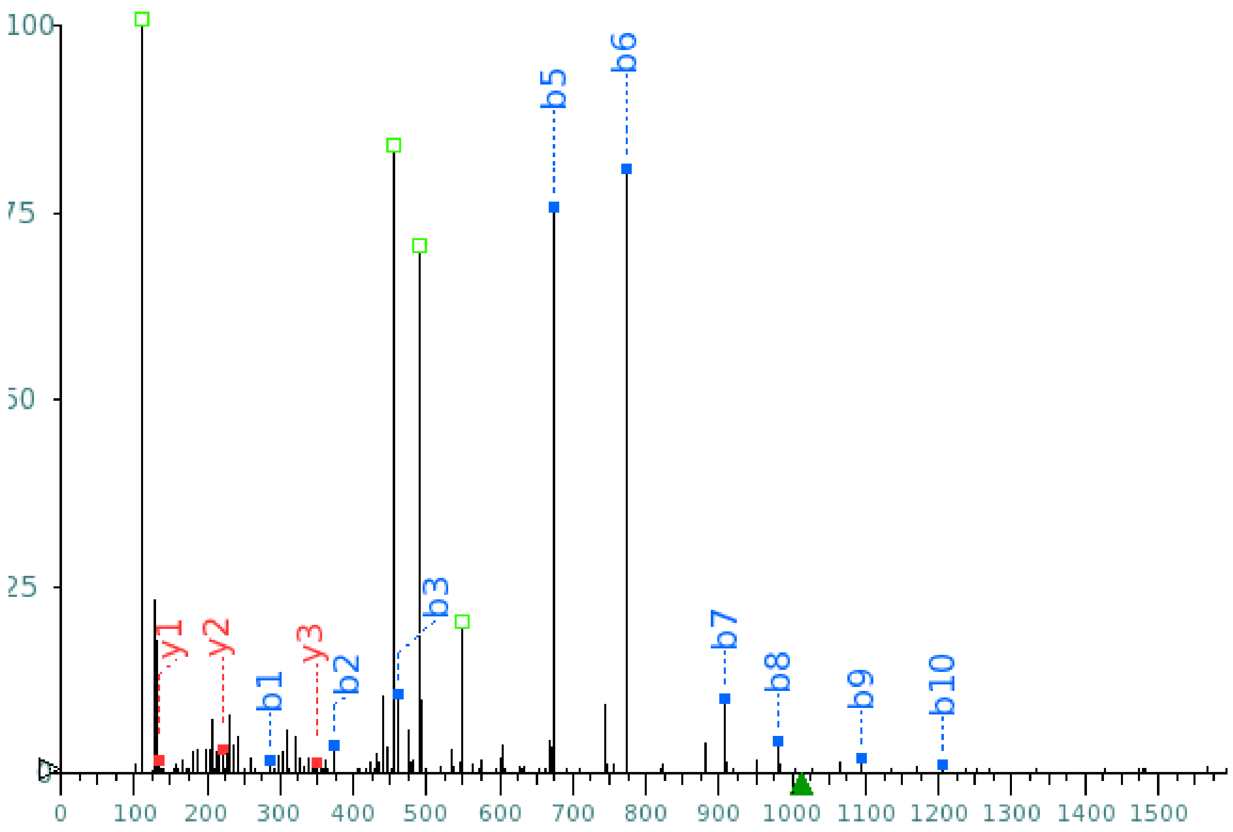

GSSPDVHALLEITEESD (+2)

Primary Reference: PTRF_HUMAN
Search ID: 41761
Search Name: 20130328_ananiav_ERstress_TMT_TM_timecourse_trypsin_inj2_4mc_iaa
Scan#: 16822
Observed Mass: 1014.5043 (2.1 ppm)
PSM Score: 22.85

File name: 2015-02-04 04.01.53PM.pdf

Creation time: 2015-02-04 04:09PM

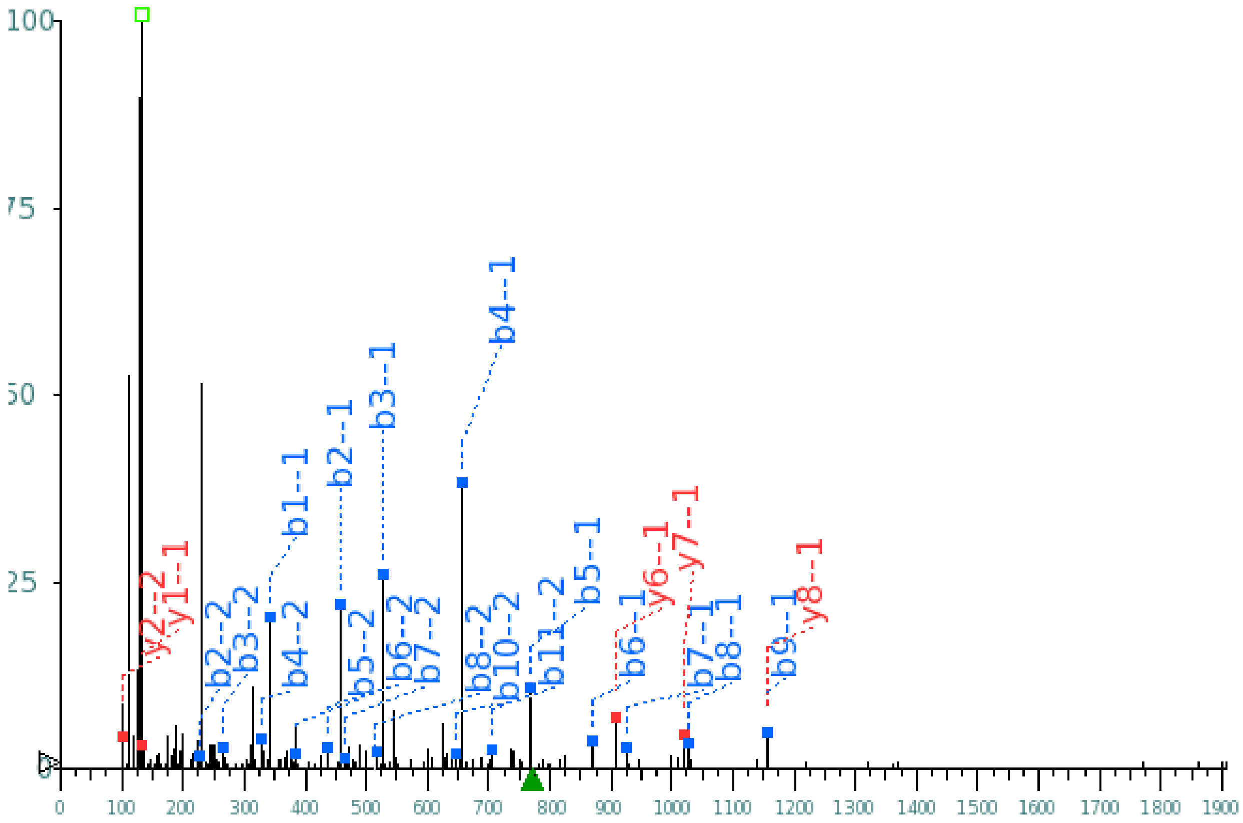

ILAQITGTEHLKESEAD (+3)

Primary Reference: PDLI5_HUMAN
Search ID: 41761
Search Name: 20130328_ananiav_ERstress_TMT_TM_timecourse_trypsin_inj2_4mc_iaa
Scan#: 16890
Observed Mass: 771.7642 (3.4 ppm)
PSM Score: 37.21

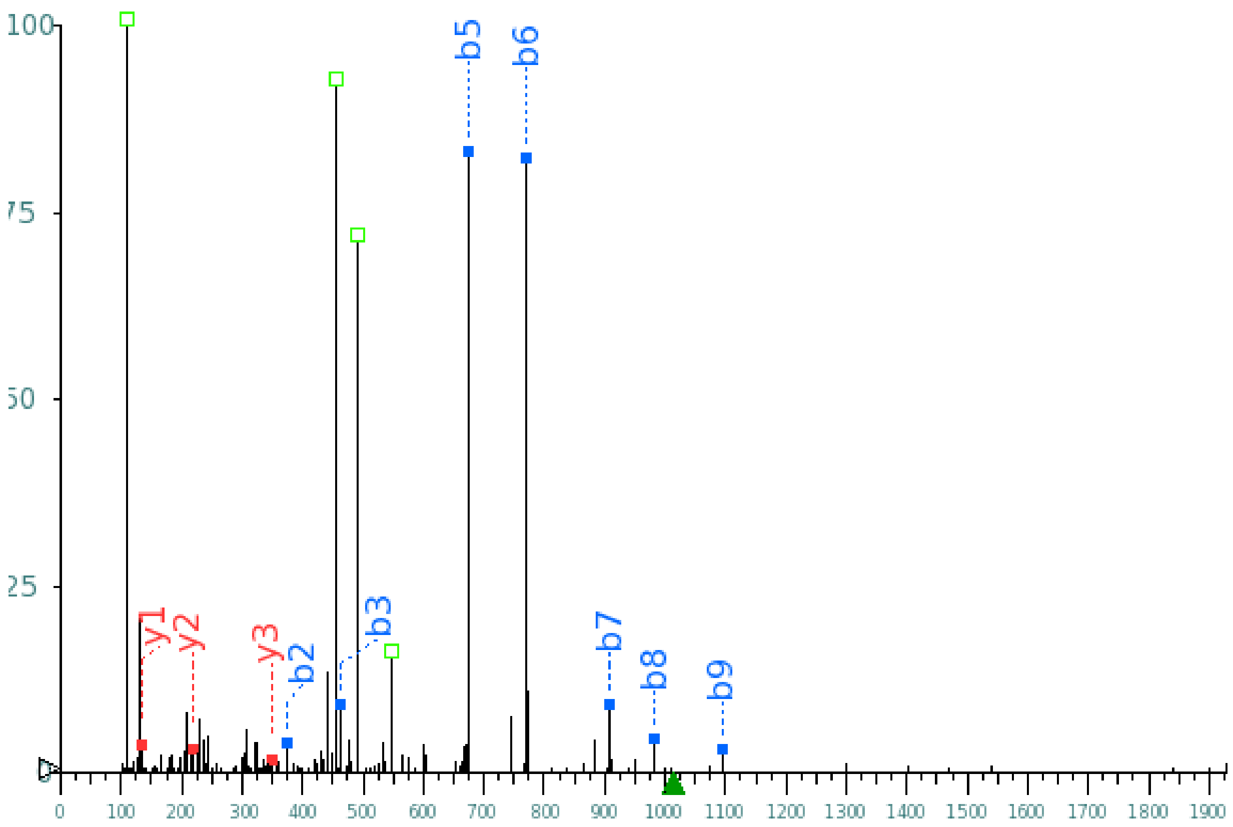

GSSPDVHALLEITEESD (+2)

Primary Reference: PTRF_HUMAN
Search ID: 41761
Search Name: 20130328_ananiav_ERstress_TMT_TM_timecourse_trypsin_inj2_4mc_iaa
Scan#: 16902
Observed Mass: 1014.5041 (1.9 ppm)
PSM Score: 23.45

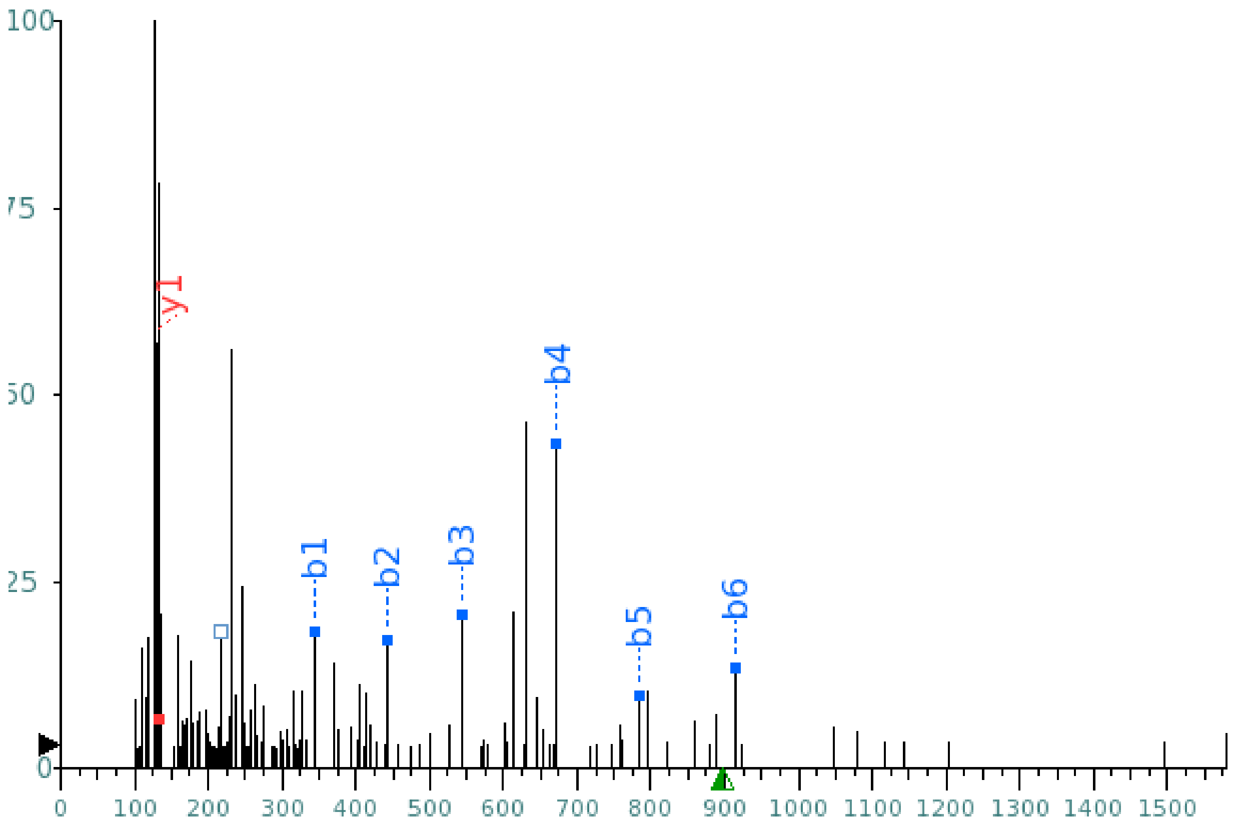

NVTQIEPFC*LETD (+2)

Primary Reference: TPX2_HUMAN
Search ID: 41761
Search Name: 20130328_ananiav_ERstress_TMT_TM_timecourse_trypsin_inj2_4mc_iaa
Scan#: 16947
Observed Mass: 897.9454 (3 ppm)
PSM Score: 10.2

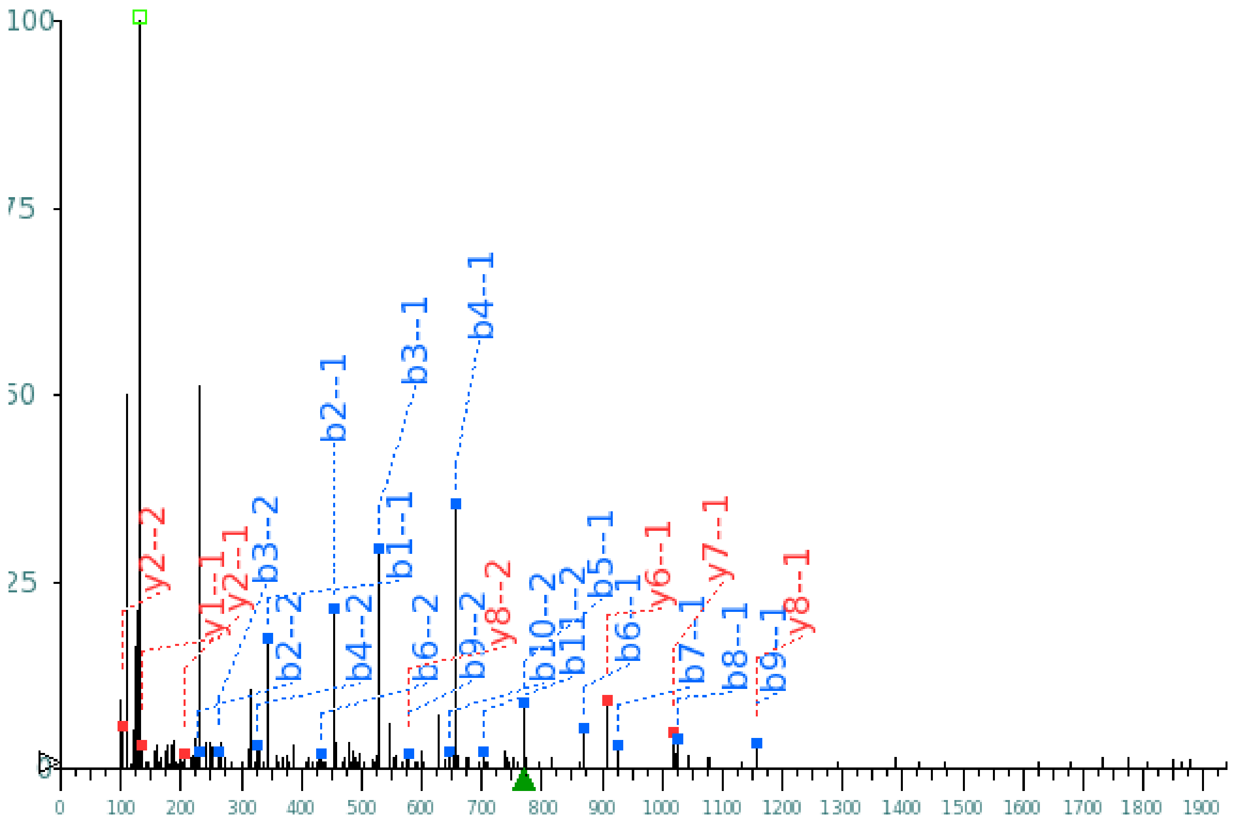

ILAQITGTEHLKESEAD (+3)

Primary Reference: PDLI5_HUMAN
Search ID: 41761
Search Name: 20130328_ananiav_ERstress_TMT_TM_timecourse_trypsin_inj2_4mc_iaa
Scan#: 16957
Observed Mass: 771.7644 (3.6 ppm)
PSM Score: 32.91

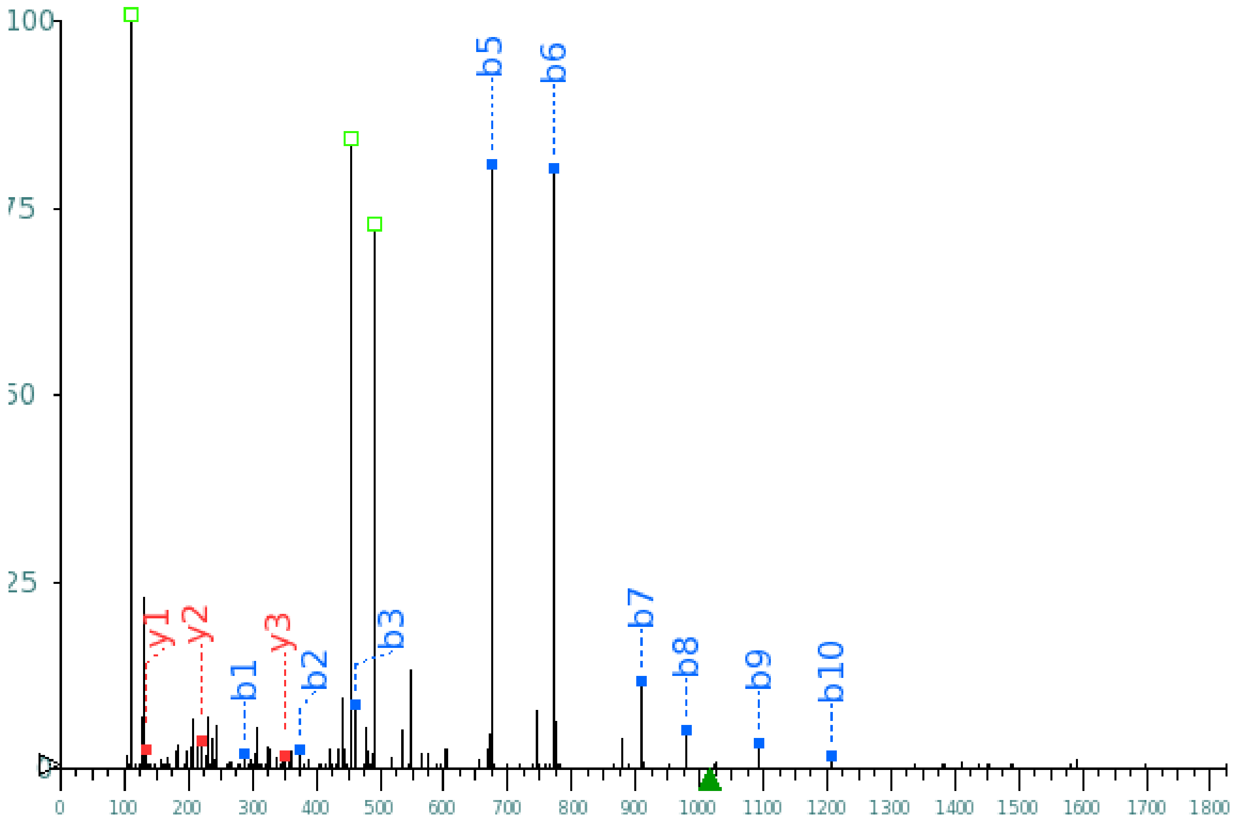

GSSPDVHALLEITEESD (+2)

Primary Reference: PTRF_HUMAN
Search ID: 41761
Search Name: 20130328_ananiav_ERstress_TMT_TM_timecourse_trypsin_inj2_4mc_iaa
Scan#: 16967
Observed Mass: 1014.5052 (3 ppm)
PSM Score: 18.98

File name: 2015-02-04 04.01.53PM.pdf

Creation time: 2015-02-04 04:09PM

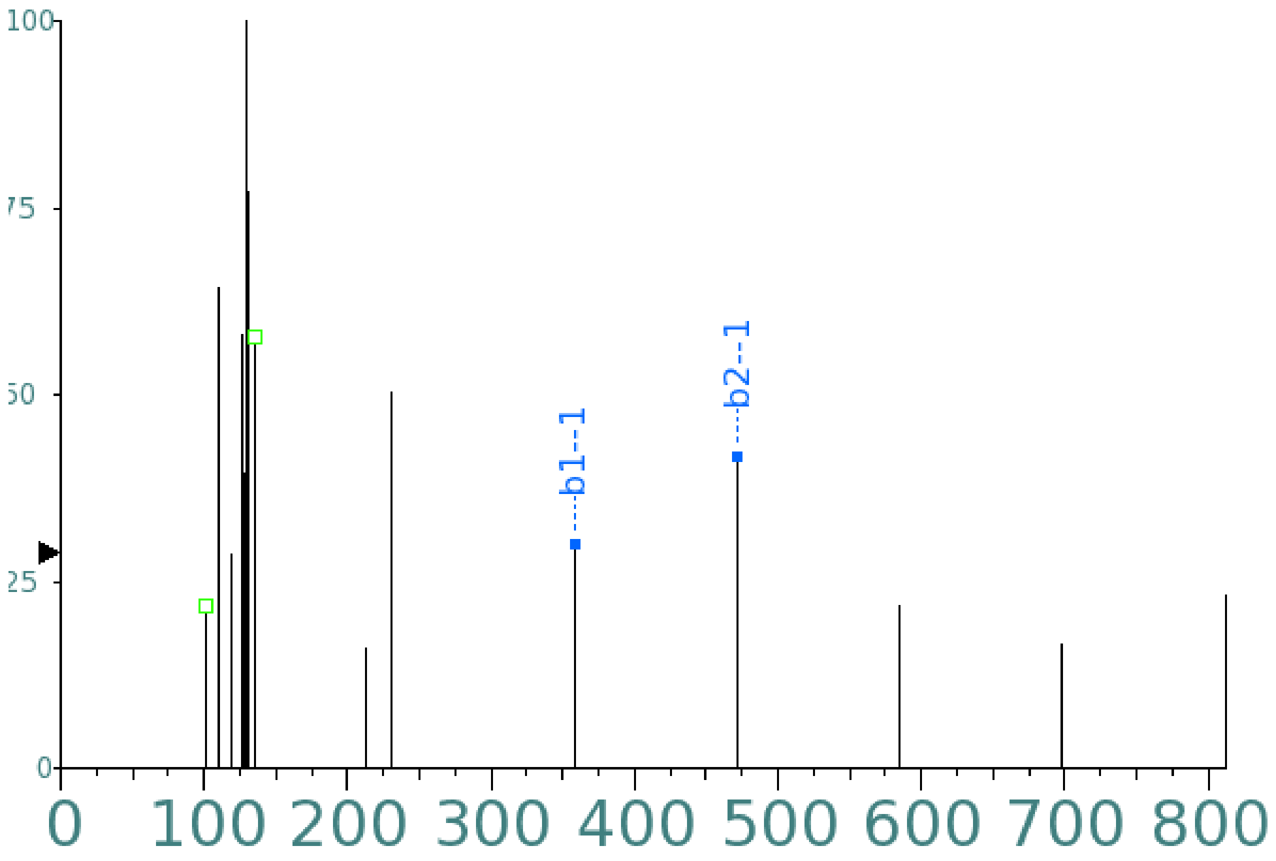

QILNNYSAQFSLEHMSPDQAD (+3)

Primary Reference: SYNE2_HUMAN
Search ID: 41761
Search Name: 20130328_ananiav_ERstress_TMT_TM_timecourse_trypsin_inj2_4mc_iaa
Scan#: 16983
Observed Mass: 879.7542 (1 ppm)
PSM Score: 26.55

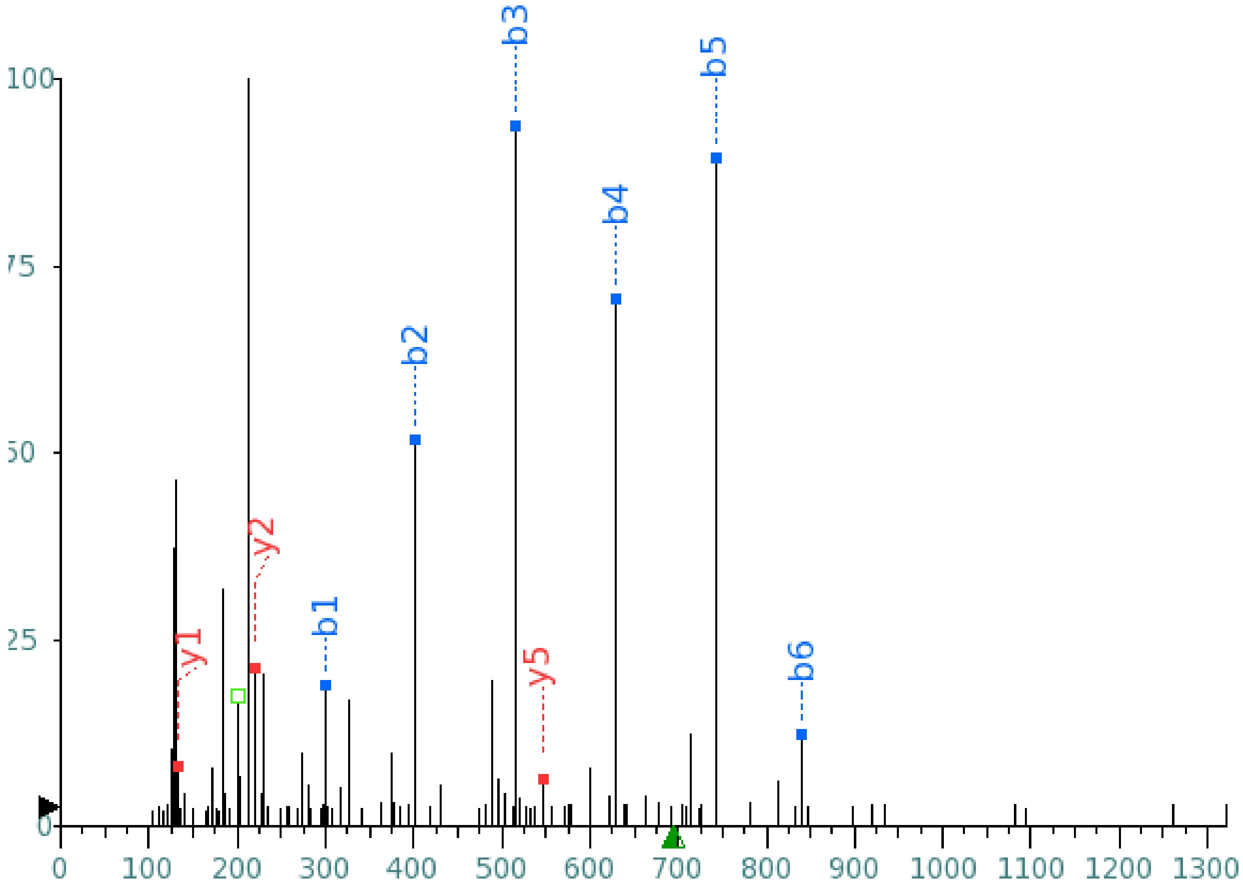

ATLLNVPDLSD (+2)

Primary Reference: ZO1_HUMAN
Search ID: 41761
Search Name: 20130328_ananiav_ERstress_TMT_TM_timecourse_trypsin_inj2_4mc_iaa
Scan#: 17009
Observed Mass: 693.8894 (2.7 ppm)
PSM Score: 23.48

File name: 2015-02-04 04.01.53PM.pdf

Creation time: 2015-02-04 04:09PM

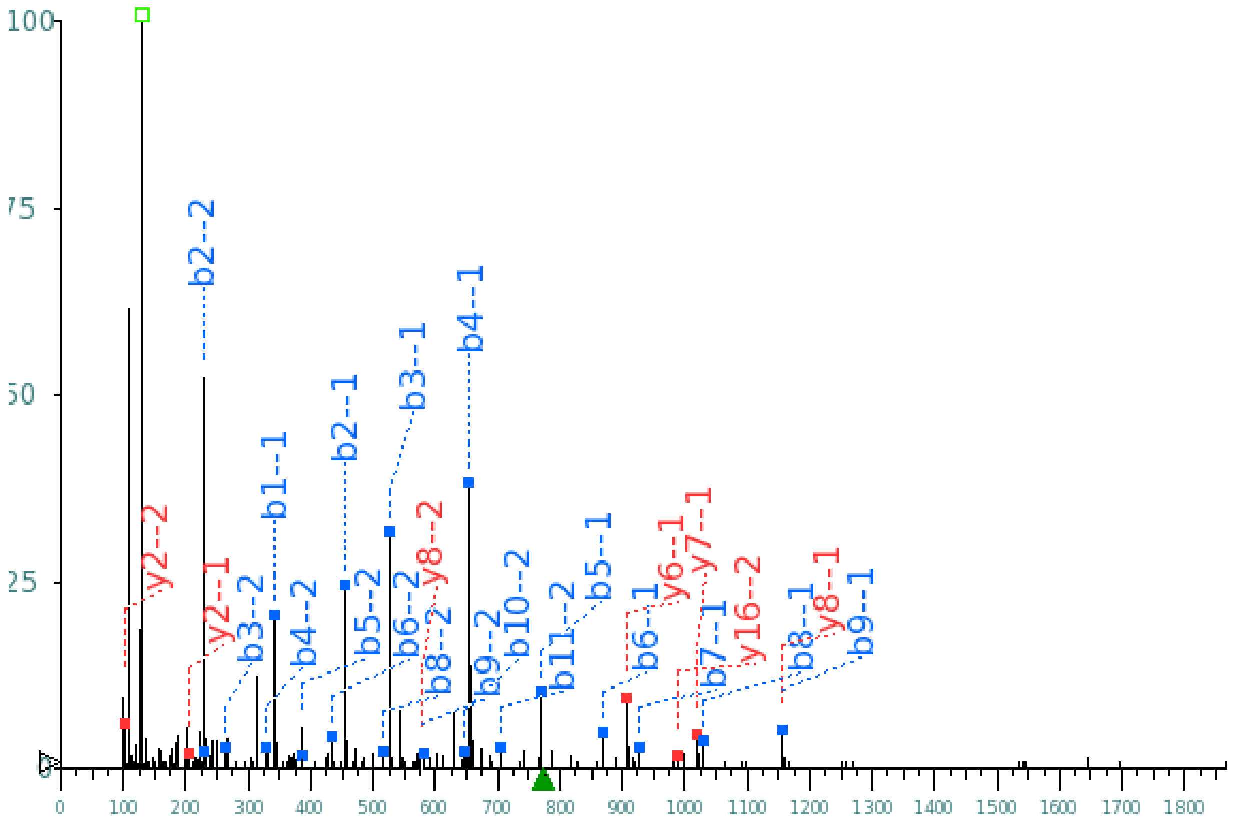

ILAQITGTEHLKESEAD (+3)

Primary Reference: PDLI5_HUMAN

Search ID: 41761

Search Name: 20130328_ananiav_ERstress_TMT_TM_timecourse_trypsin_inj2_4mc_iaa

Scan#: 17035

Observed Mass: 771.7638 (2.8 ppm)

PSM Score: 39.64

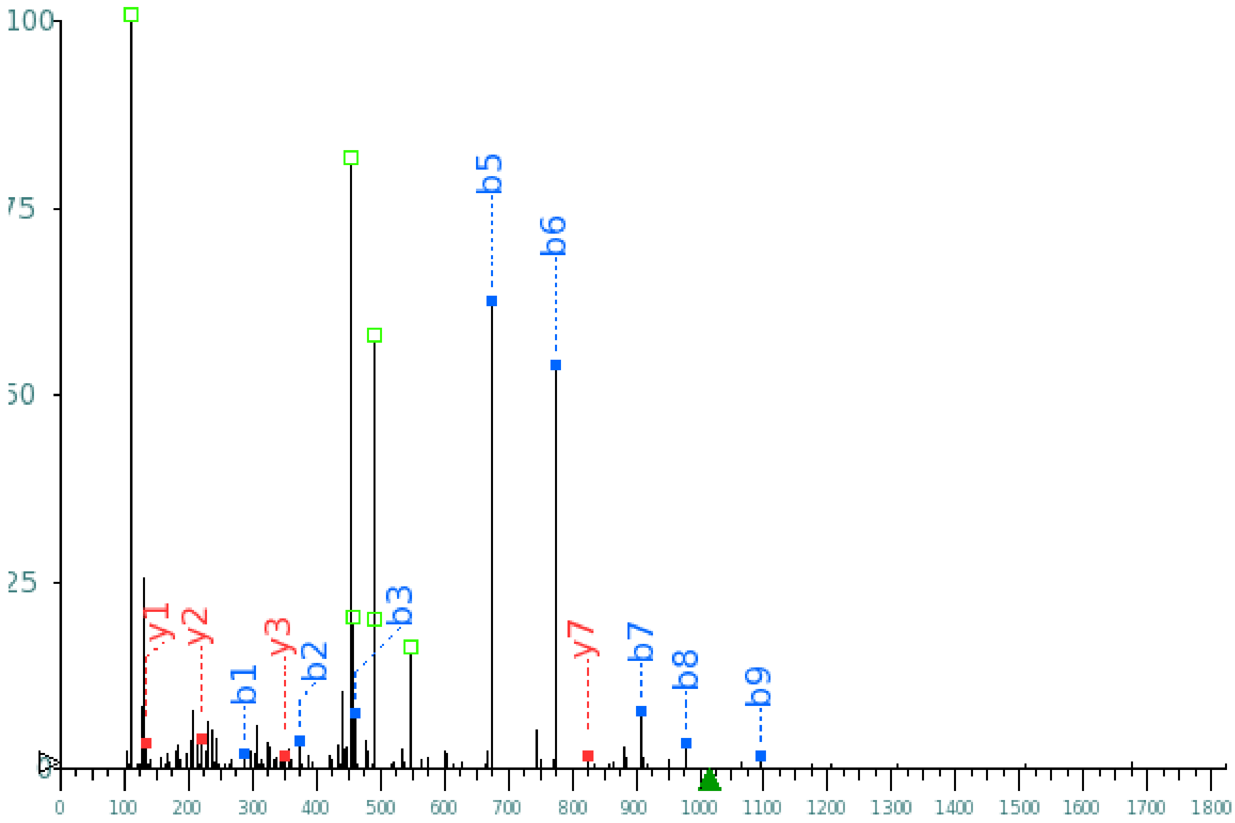

GSSPDVHALLEITEESD (+2)

Primary Reference: PTRF_HUMAN
Search ID: 41761
Search Name: 20130328_ananiav_ERstress_TMT_TM_timecourse_trypsin_inj2_4mc_iaa
Scan#: 17040
Observed Mass: 1014.5043 (2.1 ppm)
PSM Score: 22.08

File name: 2015-02-04 04.01.53PM.pdf

Creation time: 2015-02-04 04:09PM

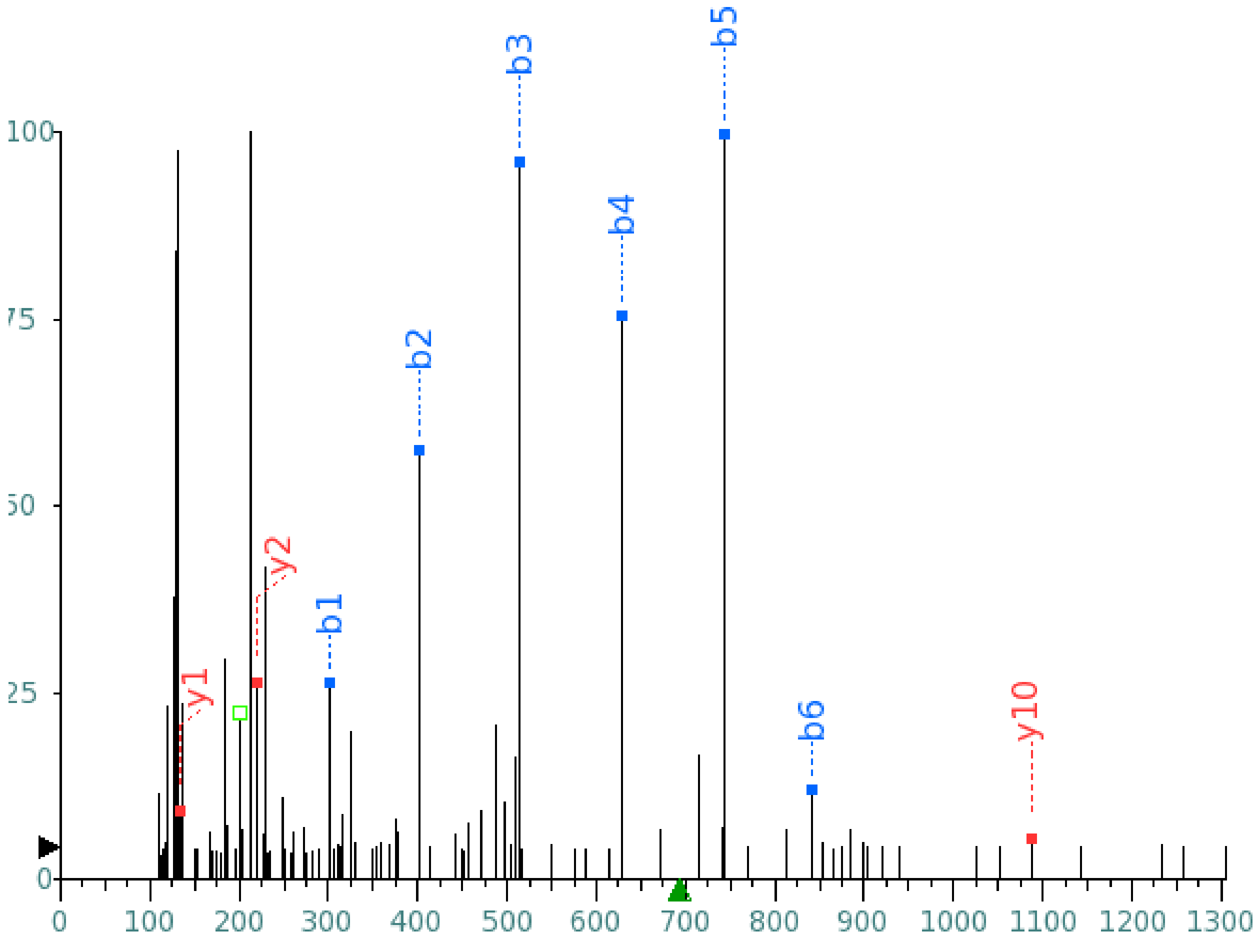

ATLLNVPDLSD (+2)

Primary Reference: ZO1_HUMAN
Search ID: 41761
Search Name: 20130328_ananiav_ERstress_TMT_TM_timecourse_trypsin_inj2_4mc_iaa
Scan#: 17075
Observed Mass: 693.8889 (1.9 ppm)
PSM Score: 22.37

File name: 2015-02-04 04.01.53PM.pdf

Creation time: 2015-02-04 04:09PM

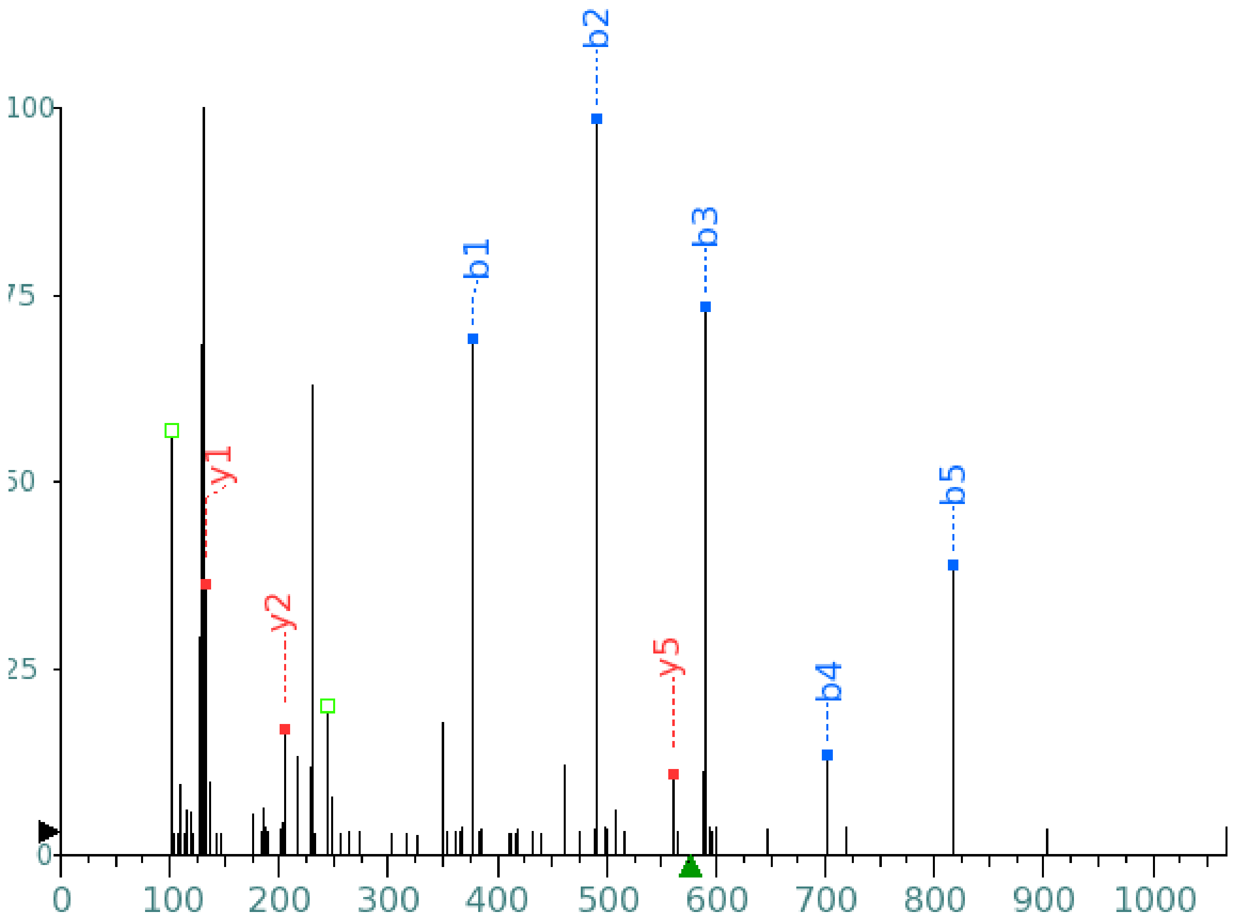

FLVLDEAD (+2)

Primary Reference: DDX1_HUMAN
Search ID: 41761
Search Name: 20130328_ananiav_ERstress_TMT_TM_timecourse_trypsin_inj2_4mc_iaa
Scan#: 17097
Observed Mass: 575.8147 (2.4 ppm)
PSM Score: 28.38

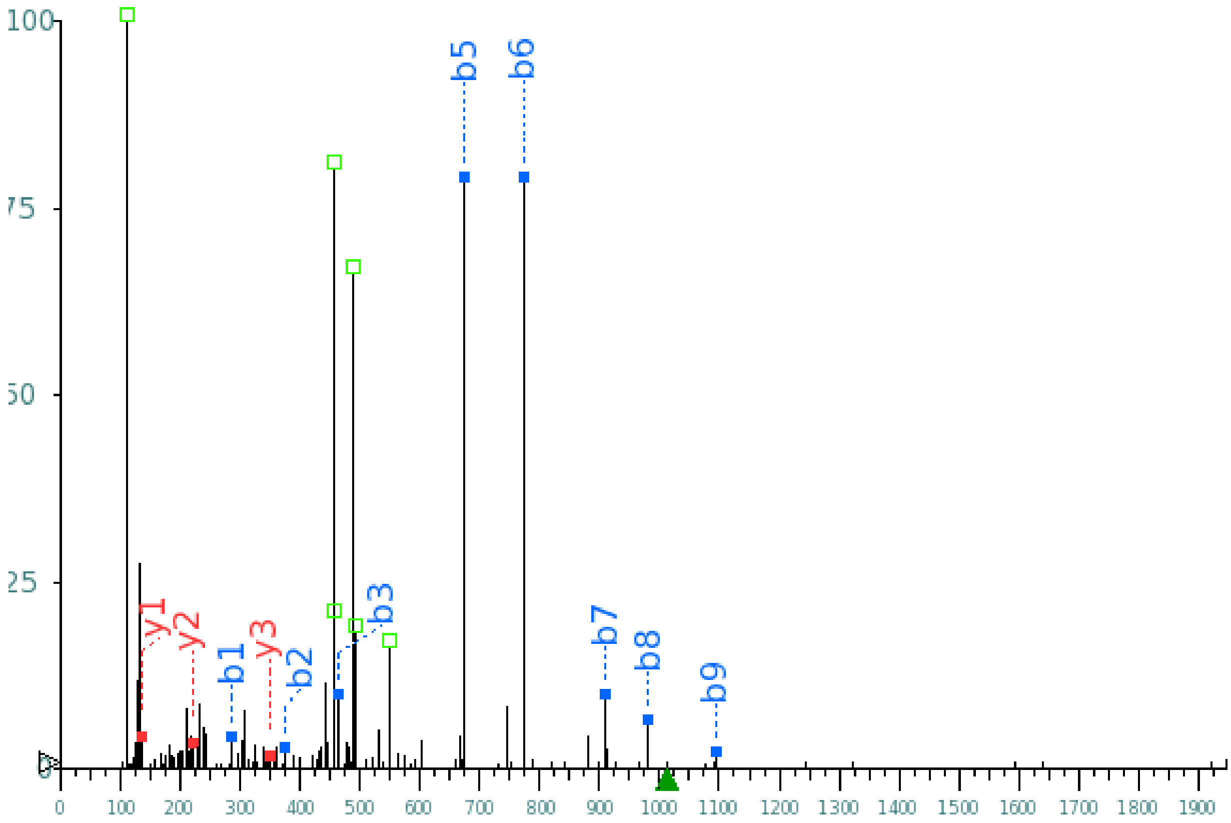

GSSPDVHALLEITEESD (+2)

Primary Reference: PTRF_HUMAN
Search ID: 41761
Search Name: 20130328_ananiav_ERstress_TMT_TM_timecourse_trypsin_inj2_4mc_iaa
Scan#: 17103
Observed Mass: 1014.5045 (2.3 ppm)
PSM Score: 25.78

File name: 2015-02-04 04.01.53PM.pdf

Creation time: 2015-02-04 04:09PM

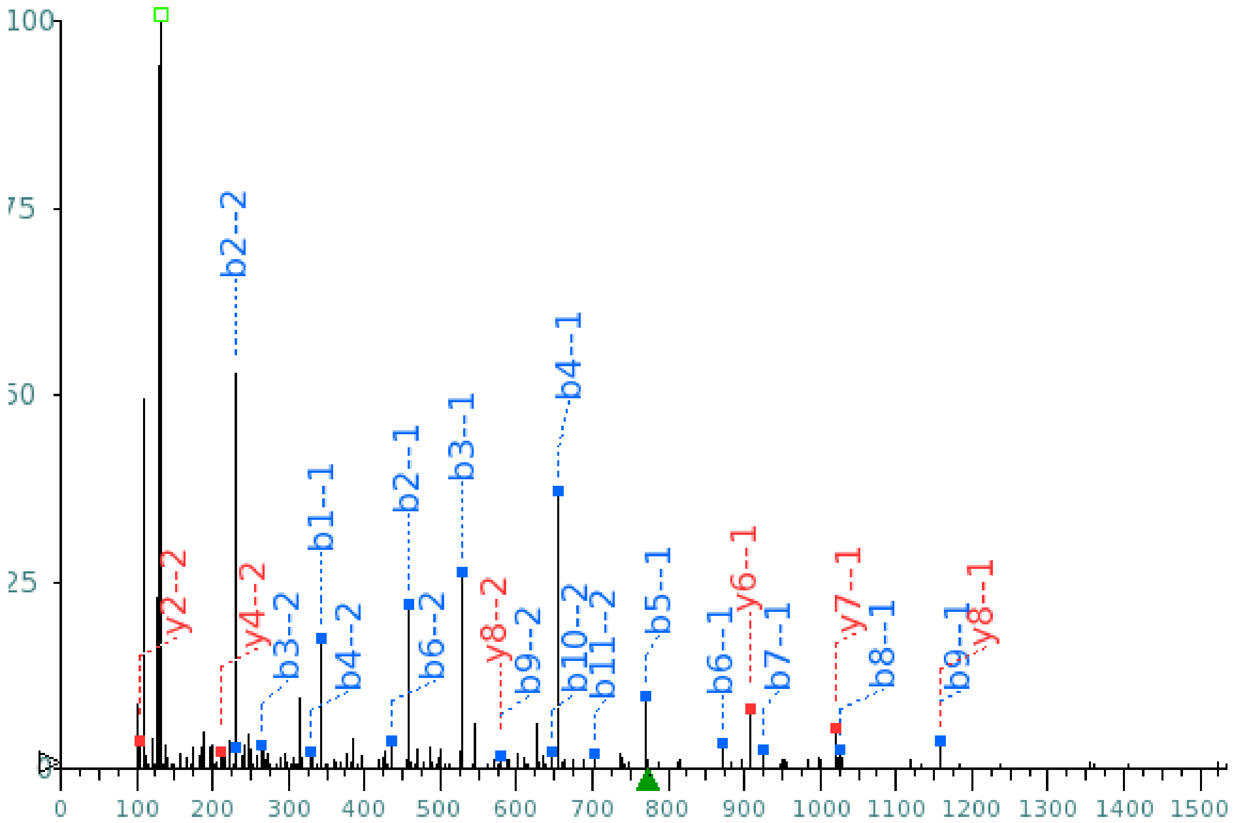

ILAQITGTEHLKESEAD (+3)

Primary Reference: PDLI5_HUMAN
Search ID: 41761
Search Name: 20130328_ananiav_ERstress_TMT_TM_timecourse_trypsin_inj2_4mc_iaa
Scan#: 17132
Observed Mass: 771.7643 (3.5 ppm)
PSM Score: 48.93

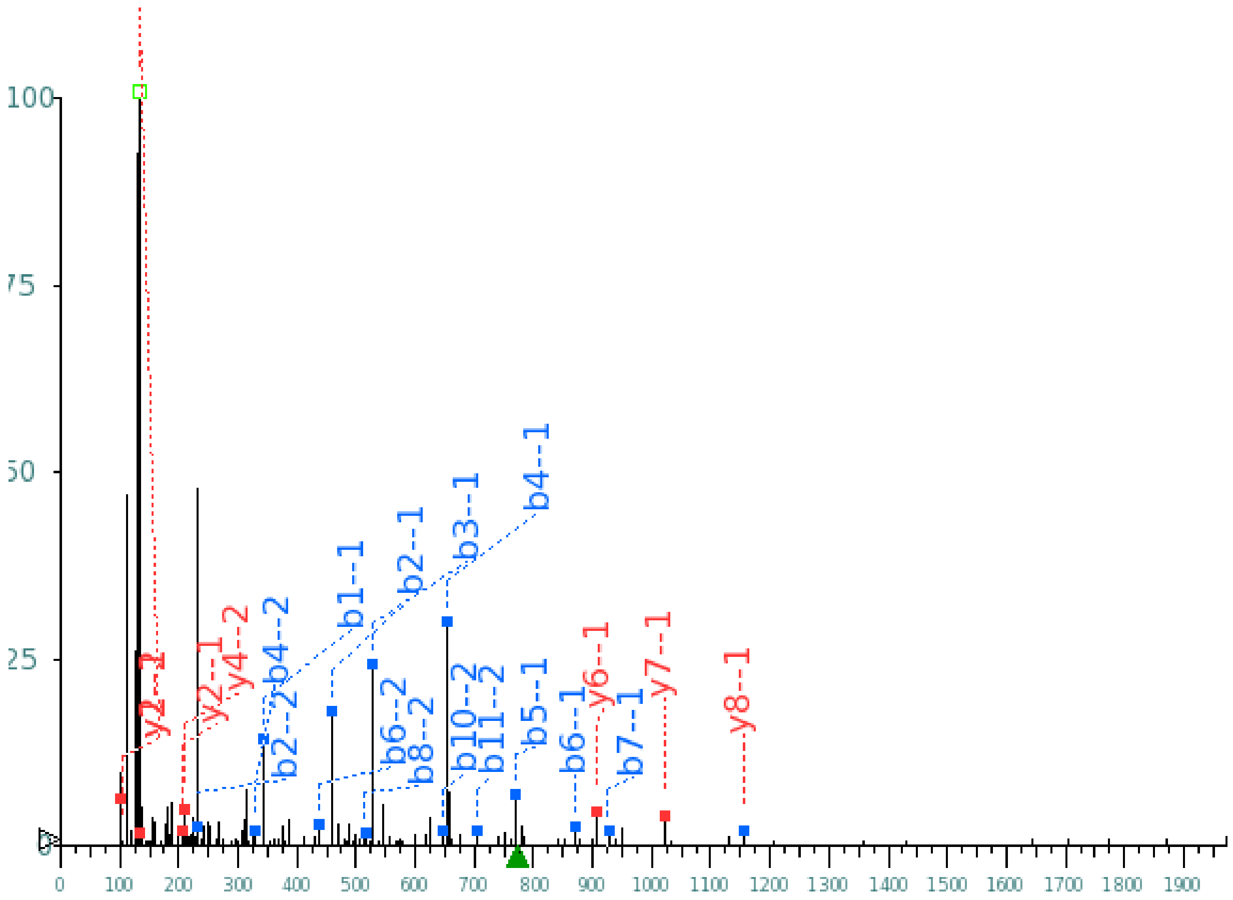

ILAQITGTEHLKESEAD (+3)

Primary Reference: PDLI5_HUMAN
Search ID: 41761
Search Name: 20130328_ananiav_ERstress_TMT_TM_timecourse_trypsin_inj2_4mc_iaa
Scan#: 17205
Observed Mass: 771.7642 (3.4 ppm)
PSM Score: 25.21

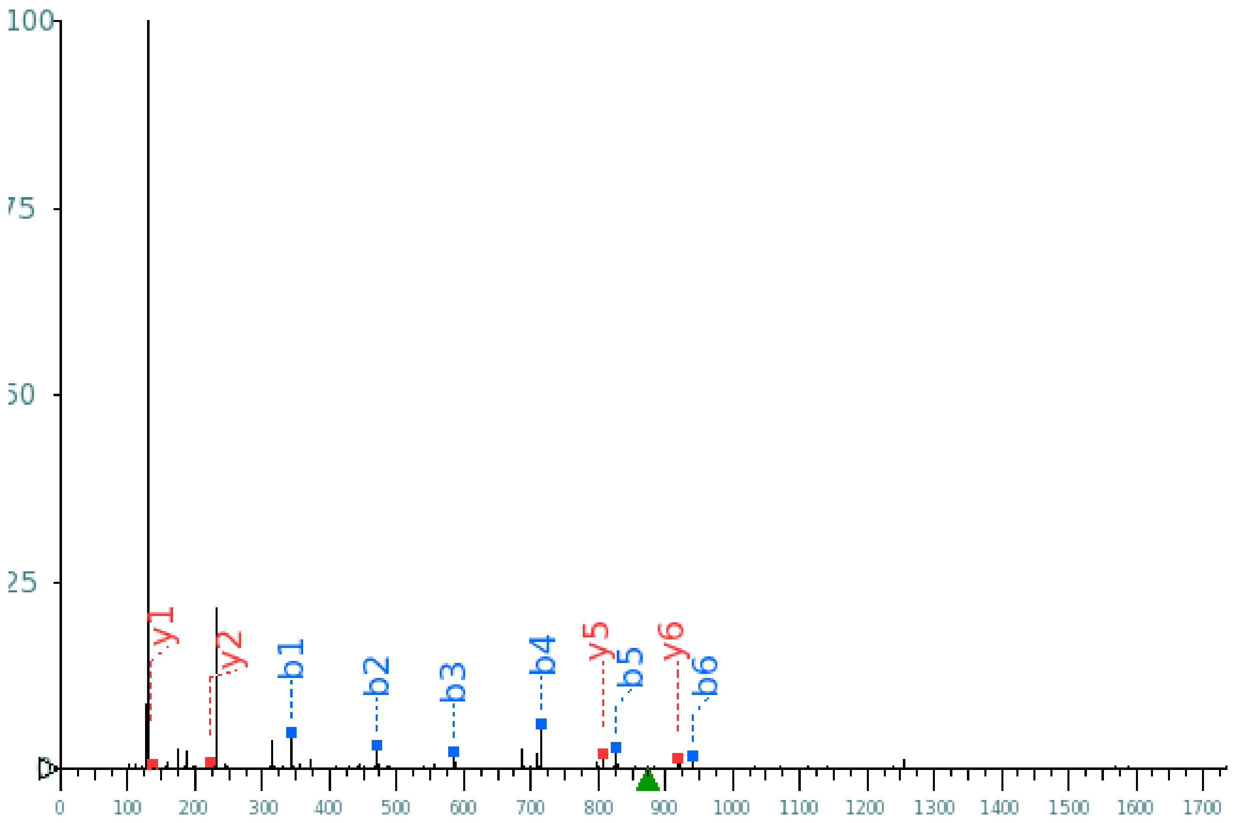

LQNEILKDLSD (+2)

Primary Reference: CKAP4_HUMAN
Search ID: 41761
Search Name: 20130328_ananiav_ERstress_TMT_TM_timecourse_trypsin_inj2_4mc_iaa
Scan#: 17234
Observed Mass: 873.5081 (2.3 ppm)
PSM Score: 18.16

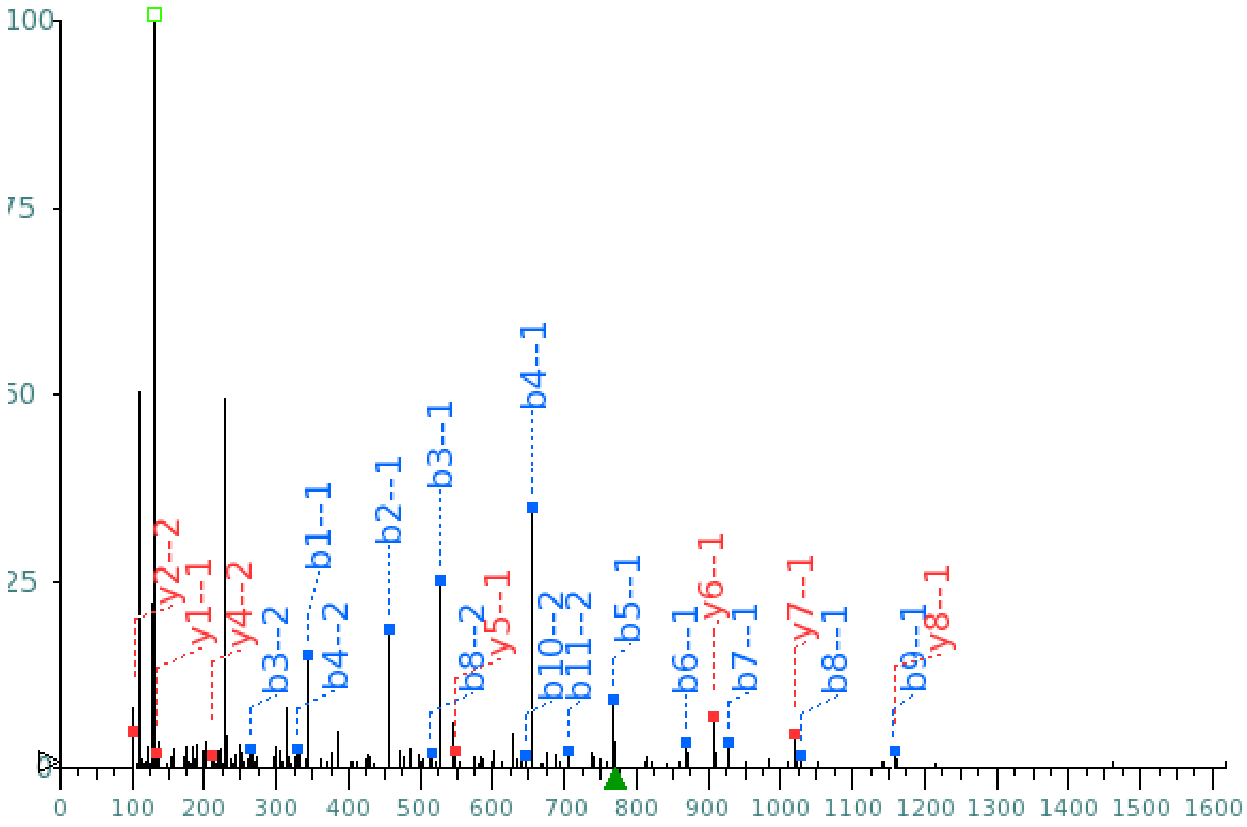

ILAQITGTEHLKESEAD (+3)

Primary Reference: PDLI5_HUMAN
Search ID: 41761
Search Name: 20130328_ananiav_ERstress_TMT_TM_timecourse_trypsin_inj2_4mc_iaa
Scan#: 17270
Observed Mass: 771.7635 (2.5 ppm)
PSM Score: 38.7

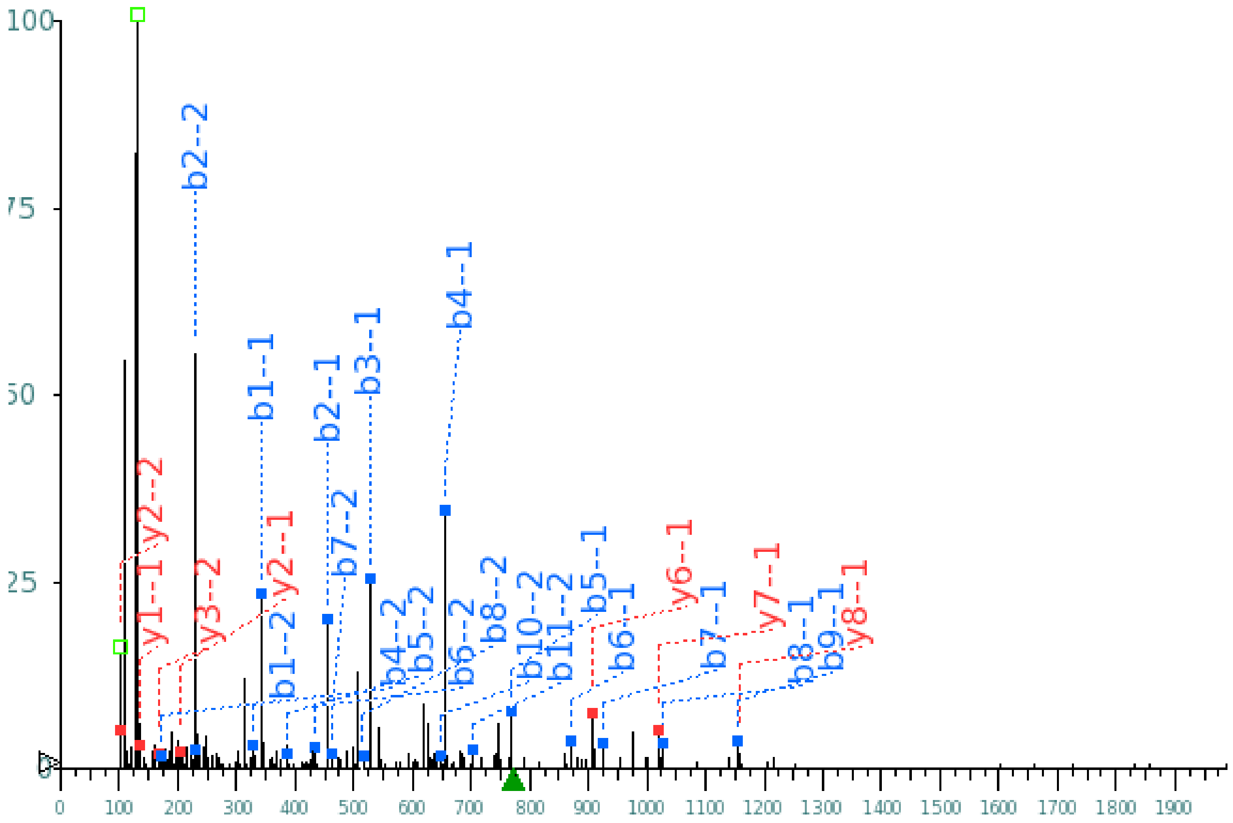

ILAQITGTEHLKESEAD (+3)

Primary Reference: PDLI5_HUMAN
Search ID: 41761
Search Name: 20130328_ananiav_ERstress_TMT_TM_timecourse_trypsin_inj2_4mc_iaa
Scan#: 17333
Observed Mass: 771.7671 (7.1 ppm)
PSM Score: 31.89

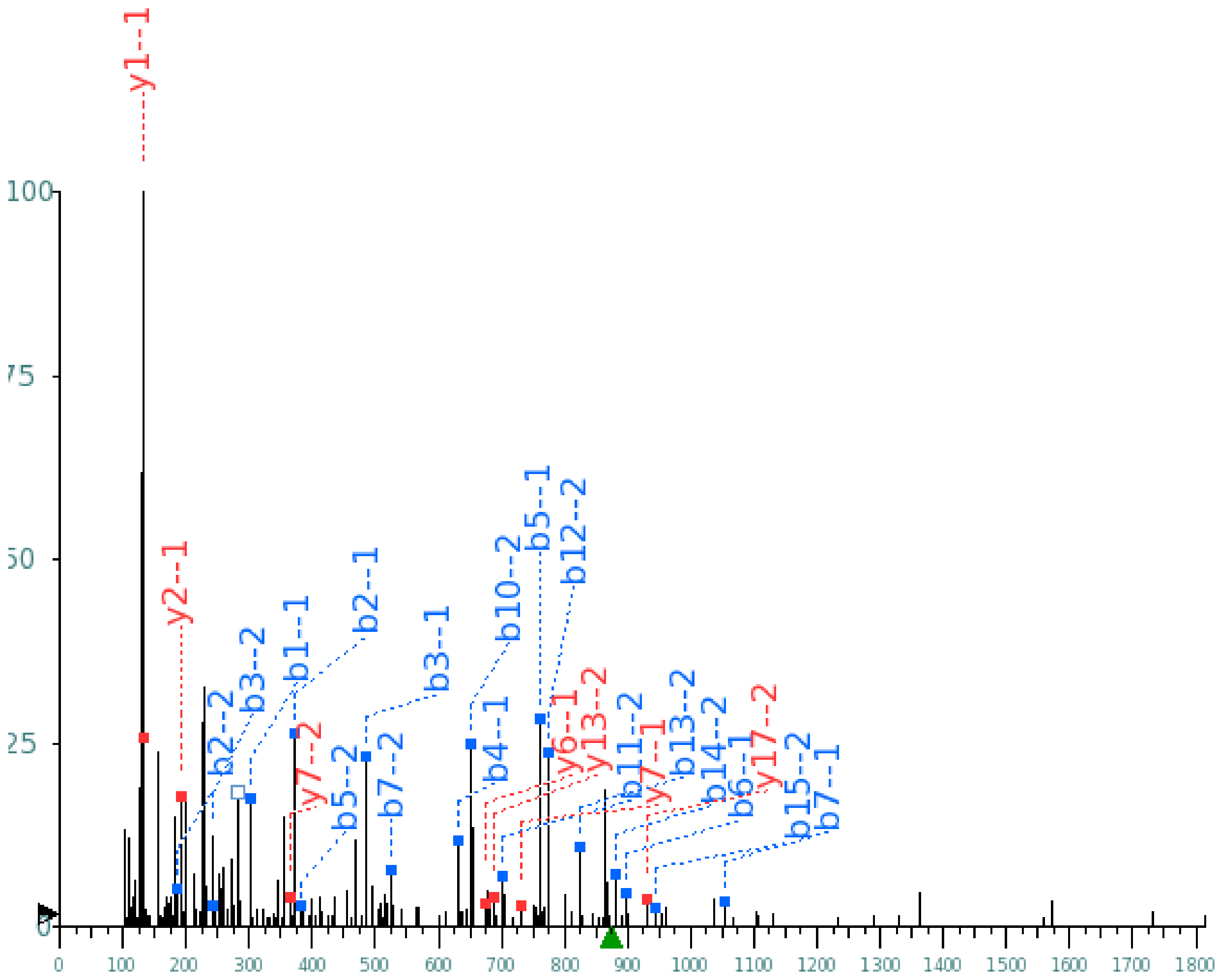

AALFQHRPPGPFPIMGPEEEGD (+3)

Primary Reference: PHKG2_HUMAN
Search ID: 41761
Search Name: 20130328_ananiav_ERstress_TMT_TM_timecourse_trypsin_inj2_4mc_iaa
Scan#: 17334
Observed Mass: 874.4407 (2.2 ppm)
PSM Score: 16.56

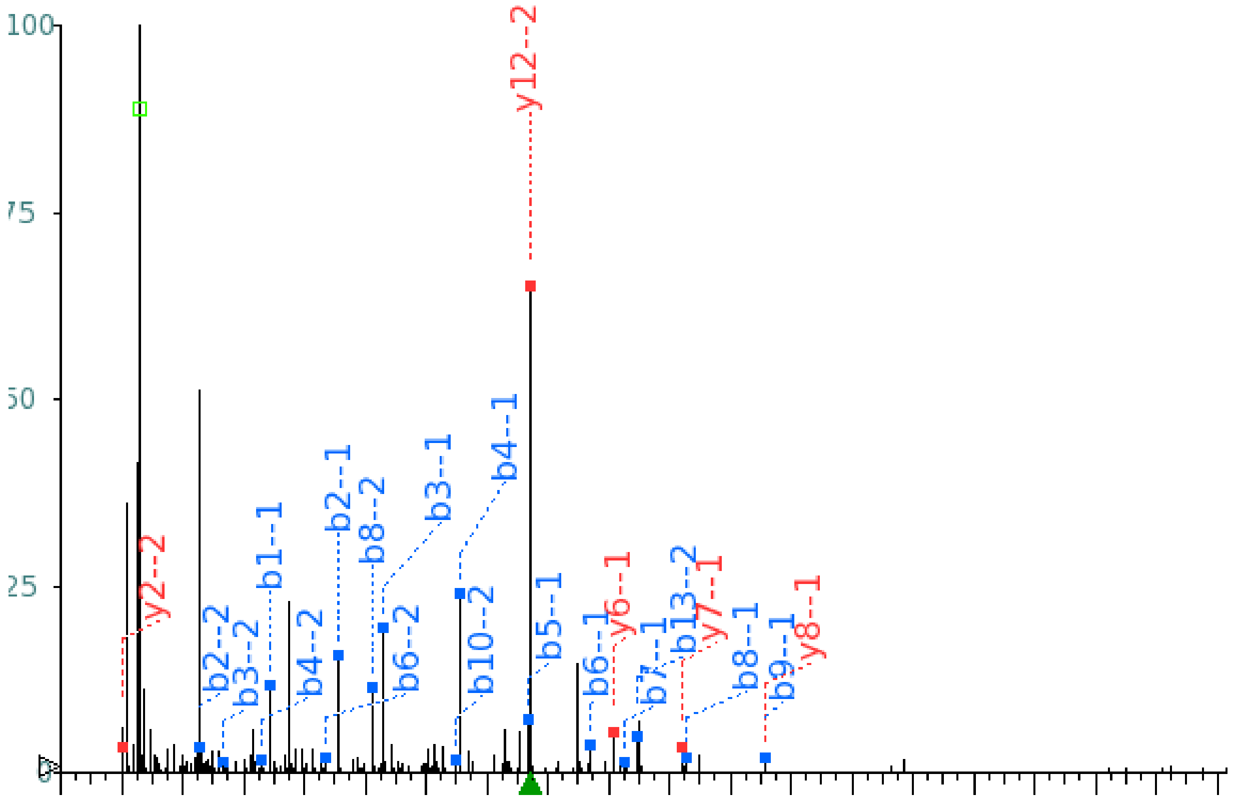

ILAQITGTEHLKESEAD (+3)

Primary Reference: PDLI5_HUMAN
Search ID: 41761
Search Name: 20130328_ananiav_ERstress_TMT_TM_timecourse_trypsin_inj2_4mc_iaa
Scan#: 17543
Observed Mass: 771.7641 (3.3 ppm)
PSM Score: 12.65

File name: 2015-02-04 04.01.53PM.pdf

Creation time: 2015-02-04 04:09PM

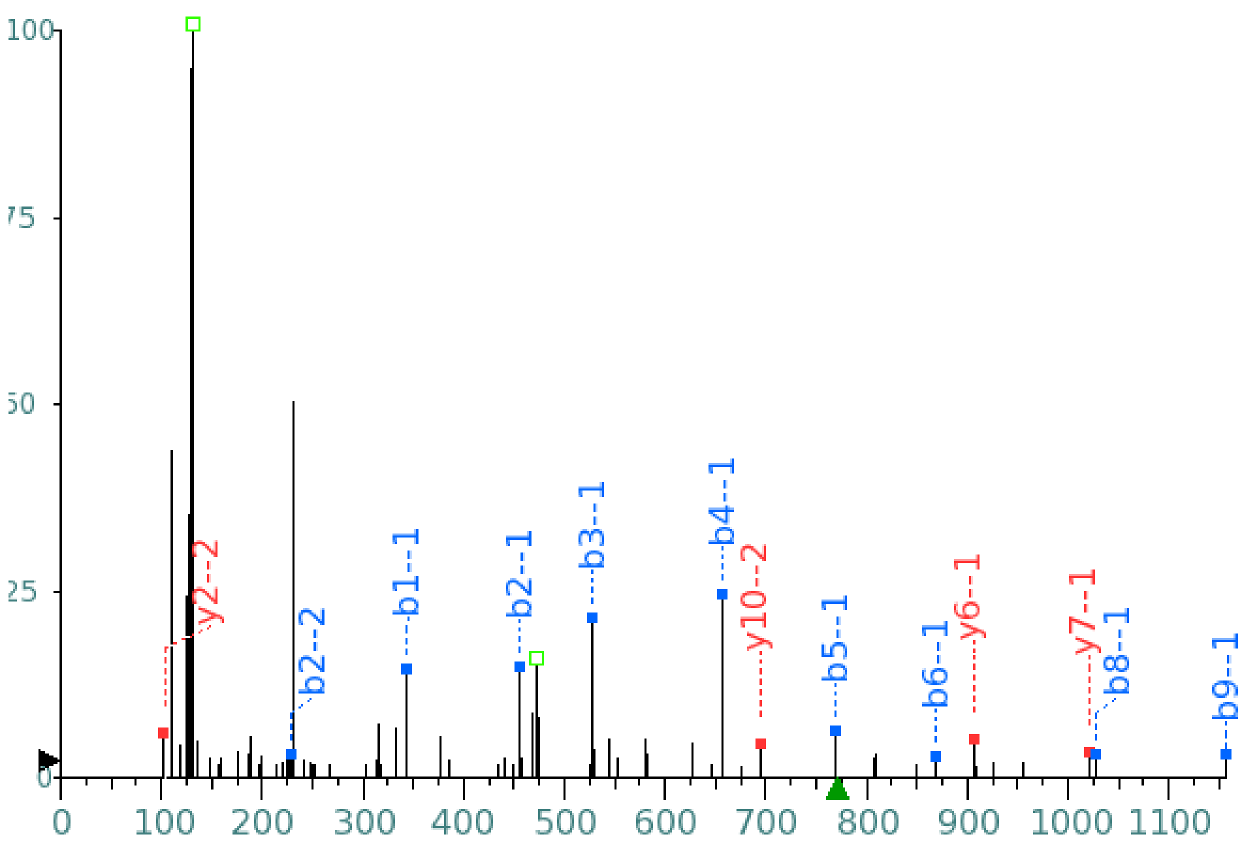

ILAQITGTEHLKESEAD (+3)

Primary Reference: PDLI5_HUMAN
Search ID: 41761
Search Name: 20130328_ananiav_ERstress_TMT_TM_timecourse_trypsin_inj2_4mc_iaa
Scan#: 17666
Observed Mass: 771.7624 (1.1 ppm)
PSM Score: 28.82

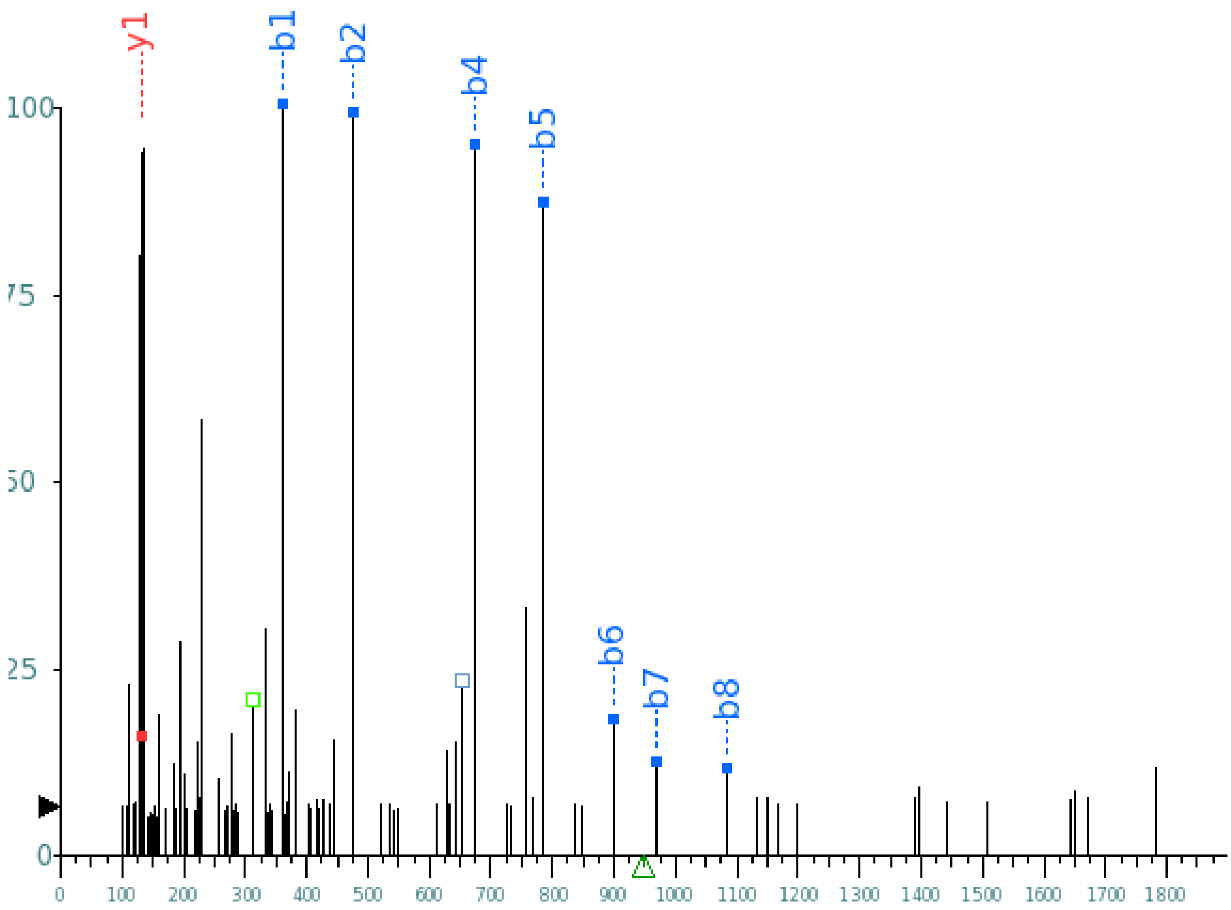

MLPTIIADNAGYDSAD (+2)

Primary Reference: TCPB_HUMAN
Search ID: 41761
Search Name: 20130328_ananiav_ERstress_TMT_TM_timecourse_trypsin_inj2_4mc_iaa
Scan#: 17715
Observed Mass: 948.4689 (2.5 ppm)
PSM Score: 33.07

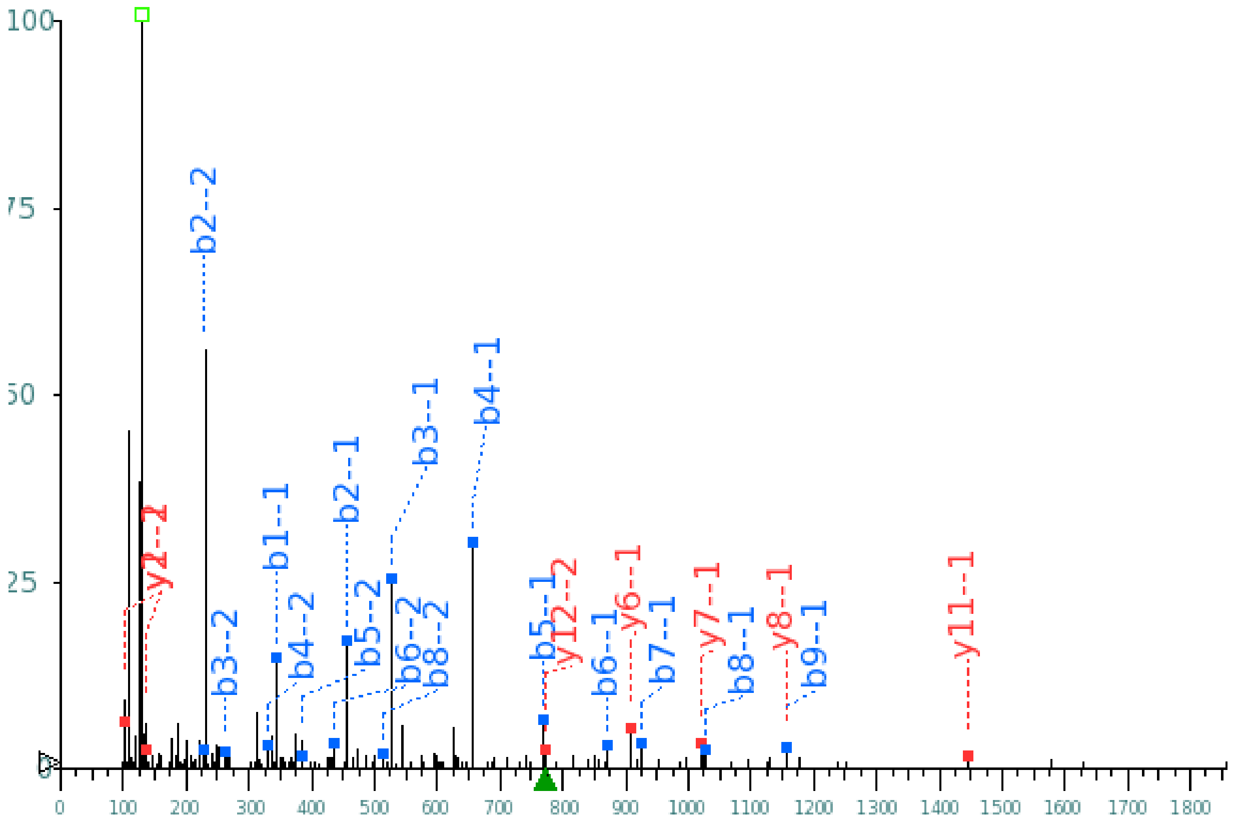

ILAQITGTEHLKESEAD (+3)

Primary Reference: PDLI5_HUMAN
Search ID: 41761
Search Name: 20130328_ananiav_ERstress_TMT_TM_timecourse_trypsin_inj2_4mc_iaa
Scan#: 17729
Observed Mass: 771.7637 (2.8 ppm)
PSM Score: 35.91

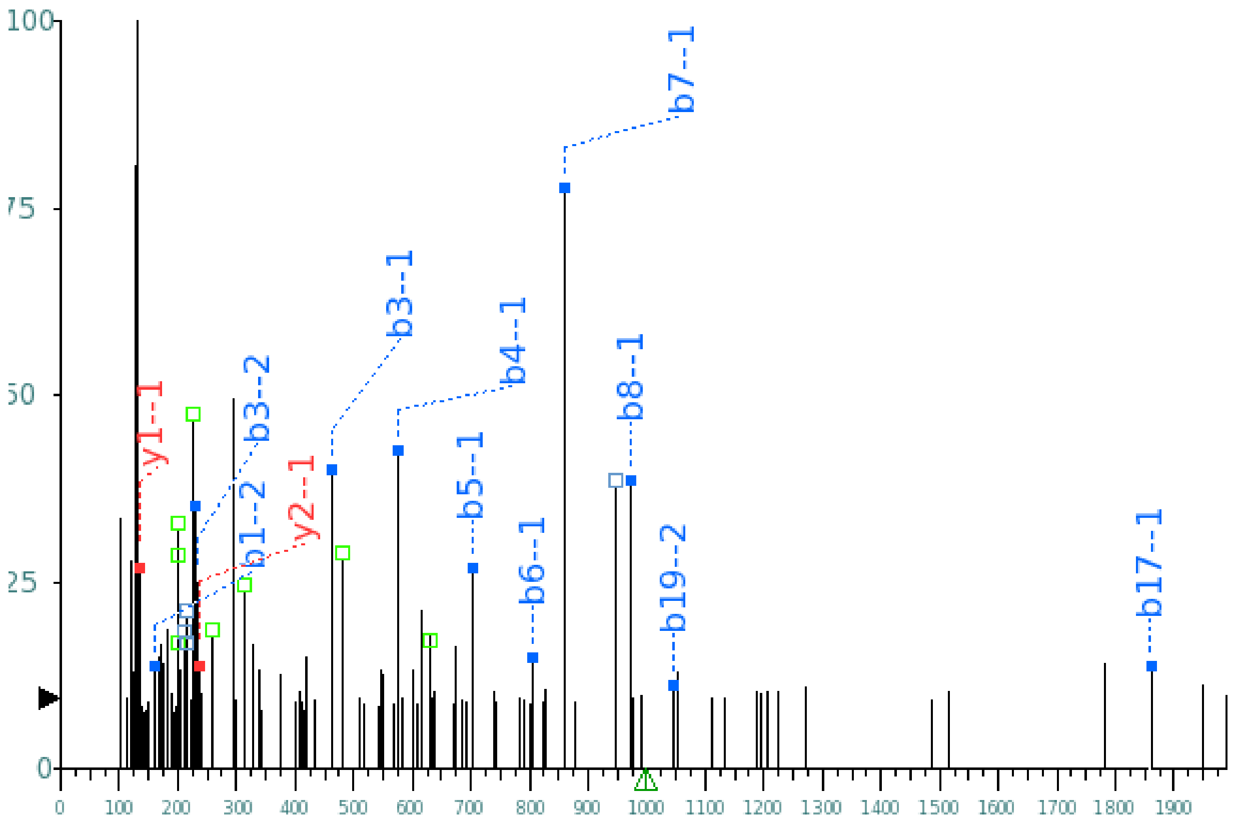

SGSLQTGLPESFPLTGGTENLNTETTD (+3)

Primary Reference: VCIP1_HUMAN
Search ID: 41761
Search Name: 20130328_ananiav_ERstress_TMT_TM_timecourse_trypsin_inj2_4mc_iaa
Scan#: 17779
Observed Mass: 999.1582 (0.6 ppm)
PSM Score: 20.02

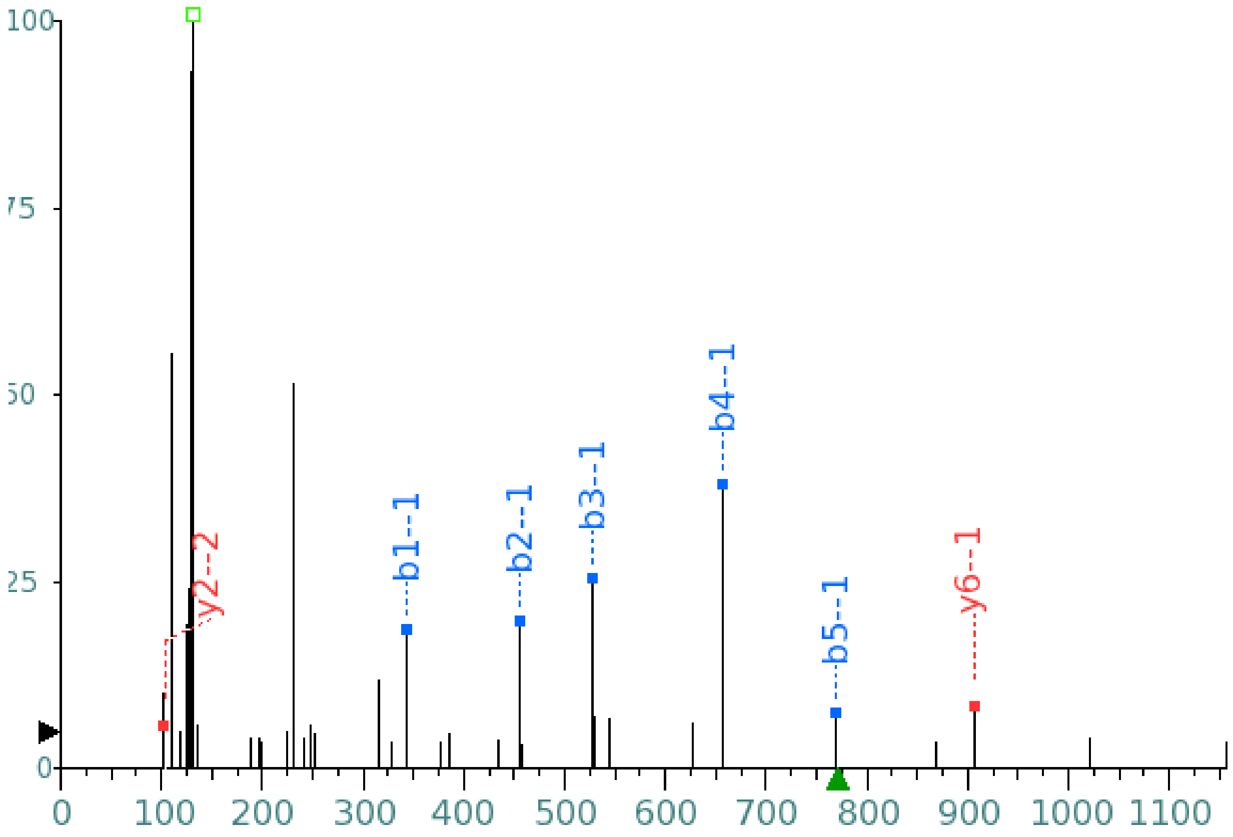

ILAQITGTEHLKESEAD (+3)

Primary Reference: PDLI5_HUMAN
Search ID: 41761
Search Name: 20130328_ananiav_ERstress_TMT_TM_timecourse_trypsin_inj2_4mc_iaa
Scan#: 17797
Observed Mass: 771.7642 (3.4 ppm)
PSM Score: 48.78

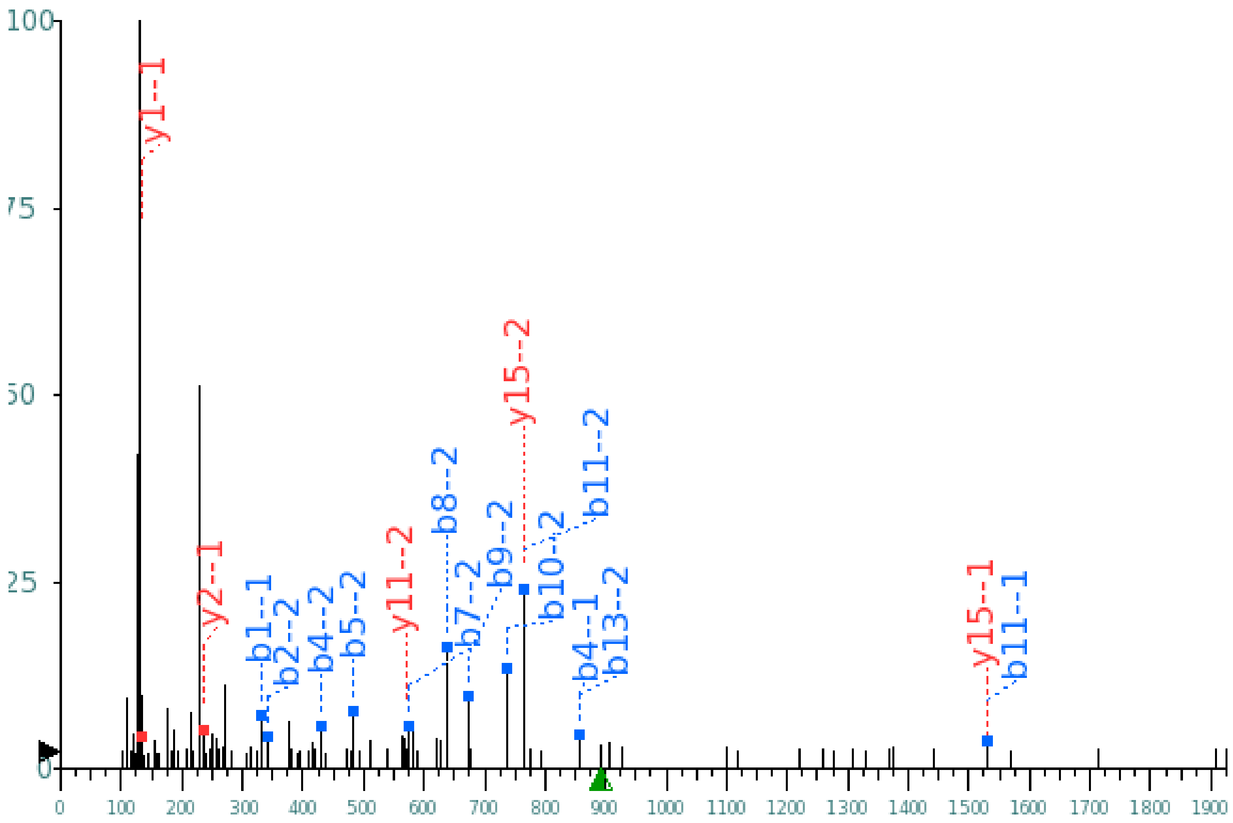

VKGILSSQAEGLANGPDVLETD (+3)

Primary Reference: EHMT1_HUMAN
Search ID: 41761
Search Name: 20130328_ananiav_ERstress_TMT_TM_timecourse_trypsin_inj2_4mc_iaa
Scan#: 17846
Observed Mass: 891.1593 (2.9 ppm)
PSM Score: 27.77

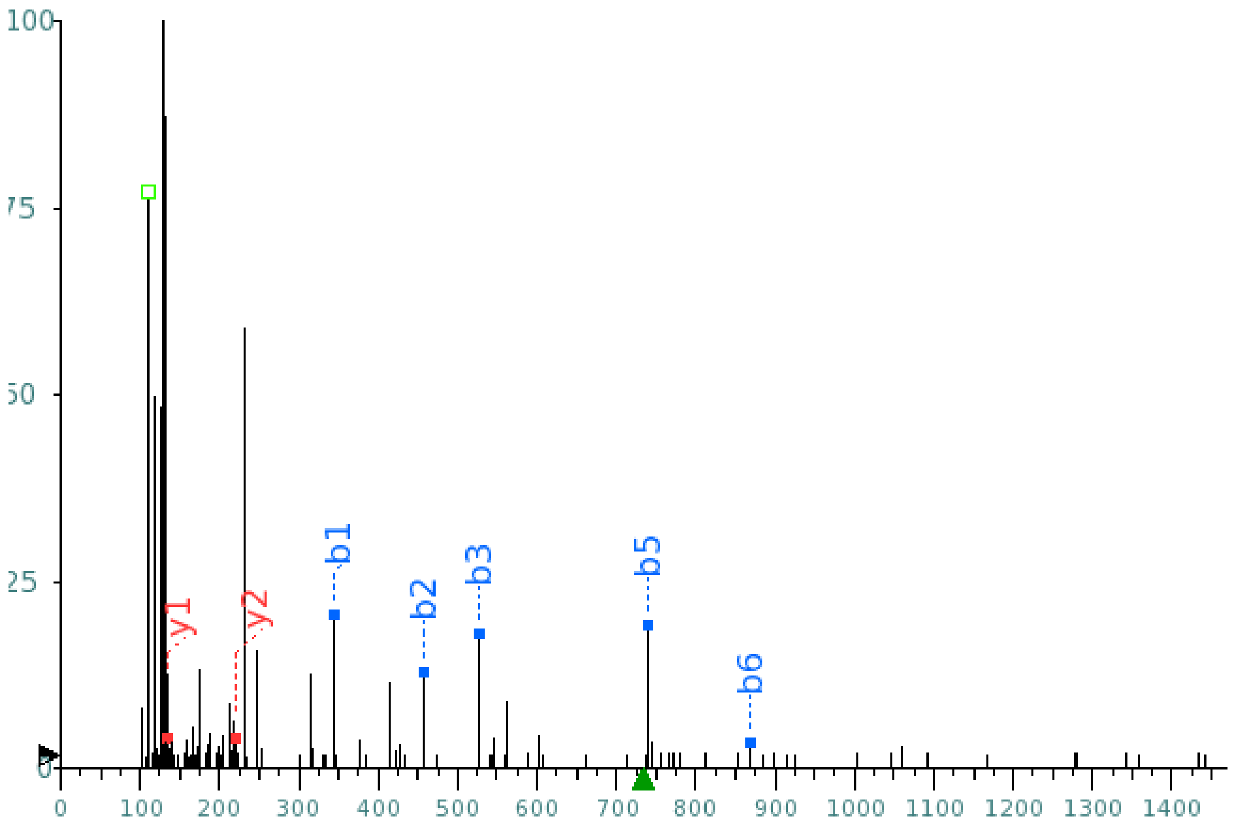

LLAPDMFTESD (+2)

Primary Reference: PRP4B_HUMAN
Search ID: 41761
Search Name: 20130328_ananiav_ERstress_TMT_TM_timecourse_trypsin_inj2_4mc_iaa
Scan#: 17849
Observed Mass: 734.3663 (1 ppm)
PSM Score: 13.57

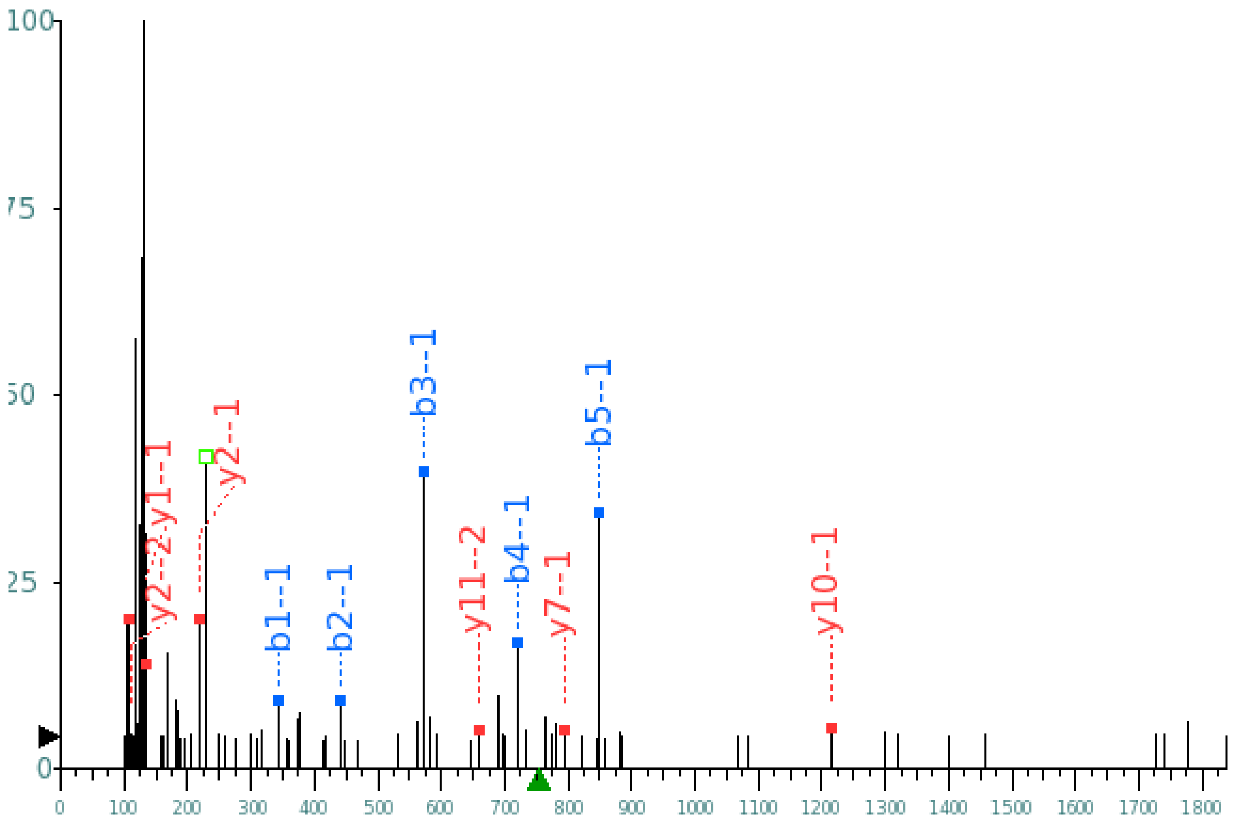

NVEFEPTYEEFPSLESD (+3)

Primary Reference: VPP2_HUMAN
Search ID: 41761
Search Name: 20130328_ananiav_ERstress_TMT_TM_timecourse_trypsin_inj2_4mc_iaa
Scan#: 17867
Observed Mass: 754.3499 (0.7 ppm)
PSM Score: 14.91

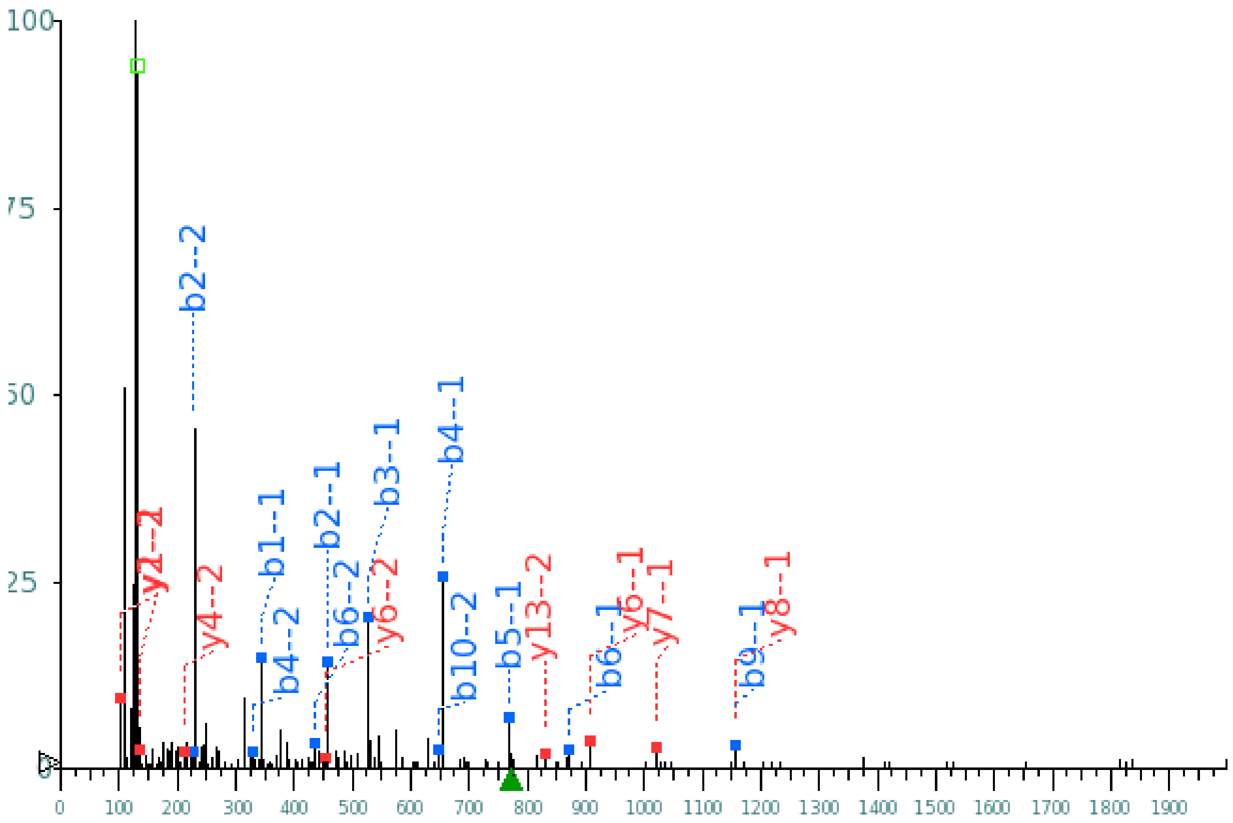

ILAQITGTEHLKESEAD (+3)

Primary Reference: PDLI5_HUMAN
Search ID: 41761
Search Name: 20130328_ananiav_ERstress_TMT_TM_timecourse_trypsin_inj2_4mc_iaa
Scan#: 17869
Observed Mass: 771.7633 (2.2 ppm)
PSM Score: 31.97

File name: 2015-02-04 04.01.53PM.pdf

Creation time: 2015-02-04 04:09PM

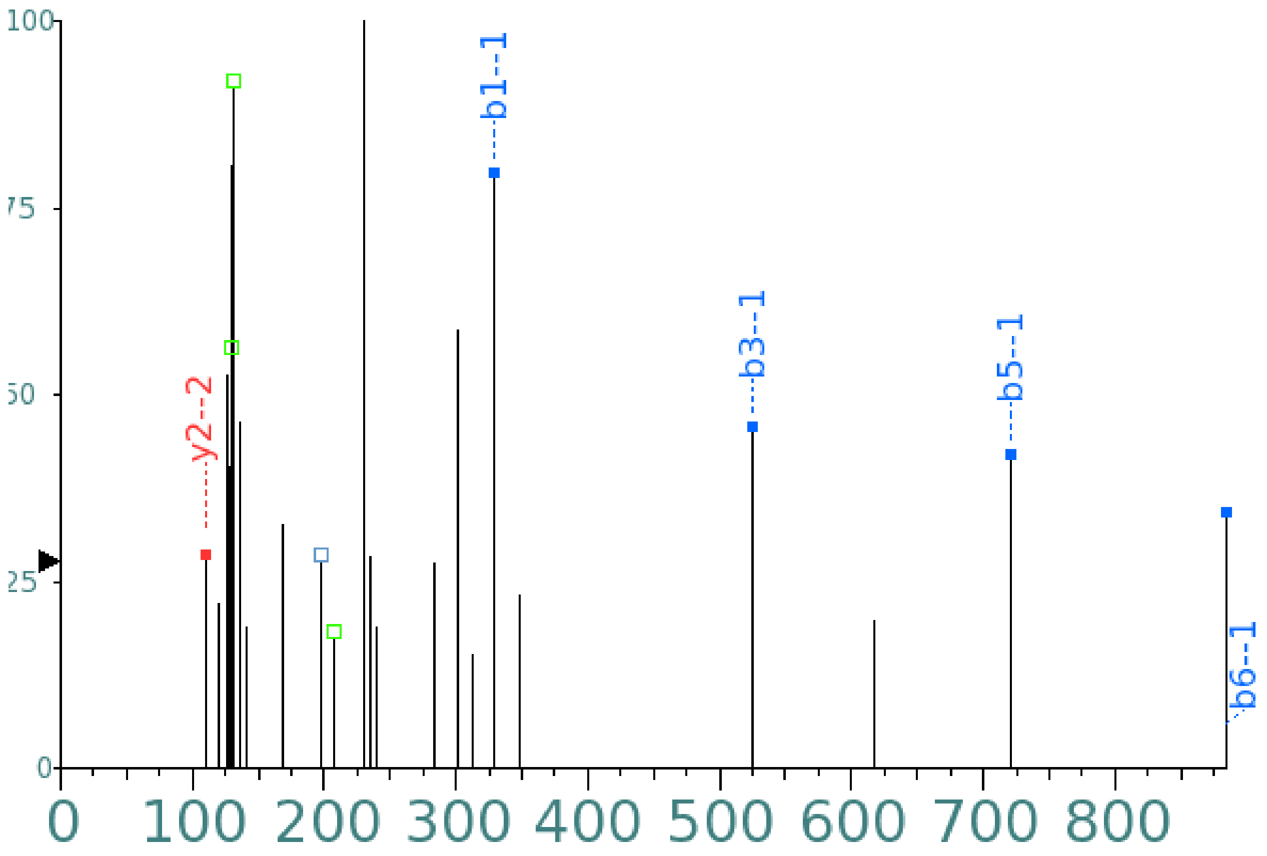

VPVPVYPHNPWFQEAPAAQNESD (+3)

Primary Reference: OTUD4_HUMAN
Search ID: 41761
Search Name: 20130328_ananiav_ERstress_TMT_TM_timecourse_trypsin_inj2_4mc_iaa
Scan#: 17896
Observed Mass: 941.1312 (0.3 ppm)
PSM Score: 16.33

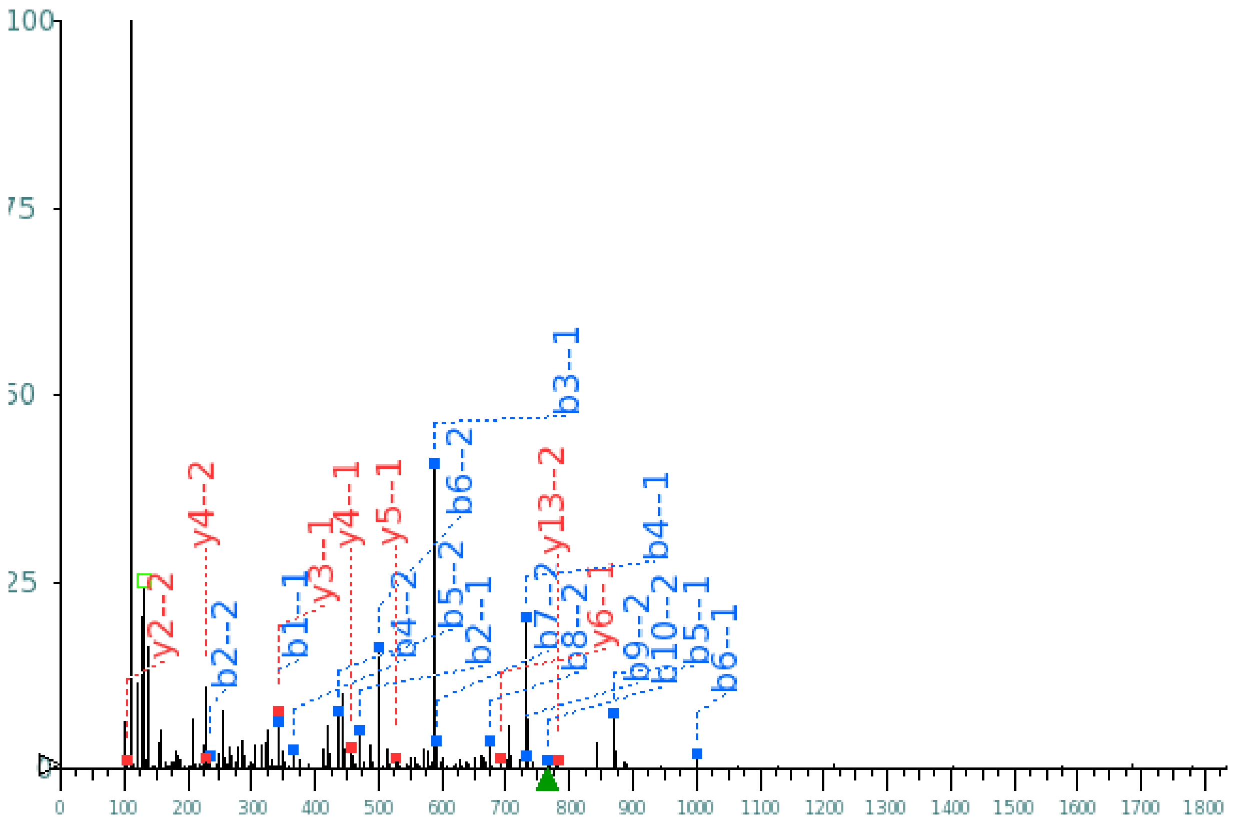

LQDFHQWYLAAYADHAD (+3)

Primary Reference: CNDH2_HUMAN
Search ID: 41761
Search Name: 20130328_ananiav_ERstress_TMT_TM_timecourse_trypsin_inj2_4mc_iaa
Scan#: 17897
Observed Mass: 765.0349 (1.2 ppm)
PSM Score: 11.67

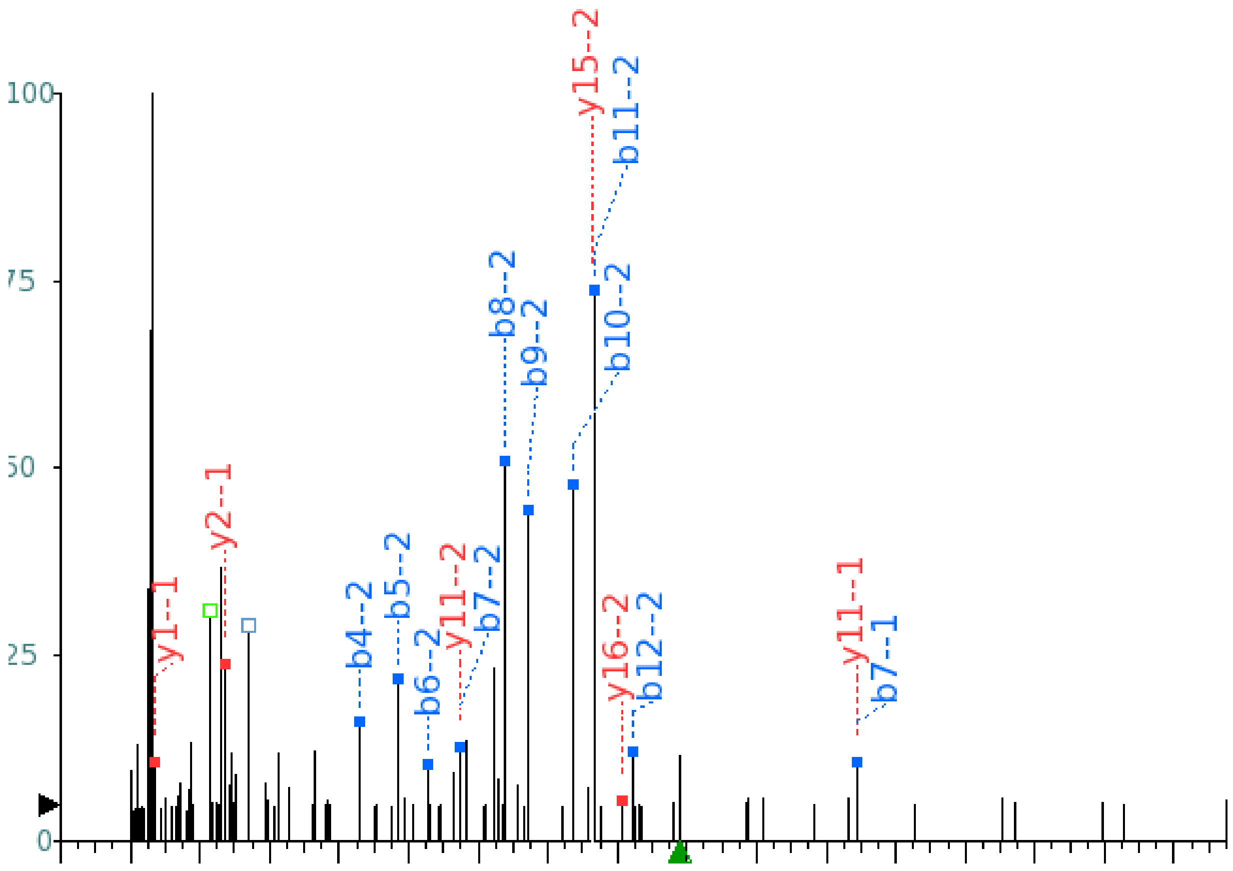

VKGILSSQAEGLANGPDVLETD (+3)

Primary Reference: EHMT1_HUMAN
Search ID: 41761
Search Name: 20130328_ananiav_ERstress_TMT_TM_timecourse_trypsin_inj2_4mc_iaa
Scan#: 17912
Observed Mass: 891.1572 (0.6 ppm)
PSM Score: 25.51

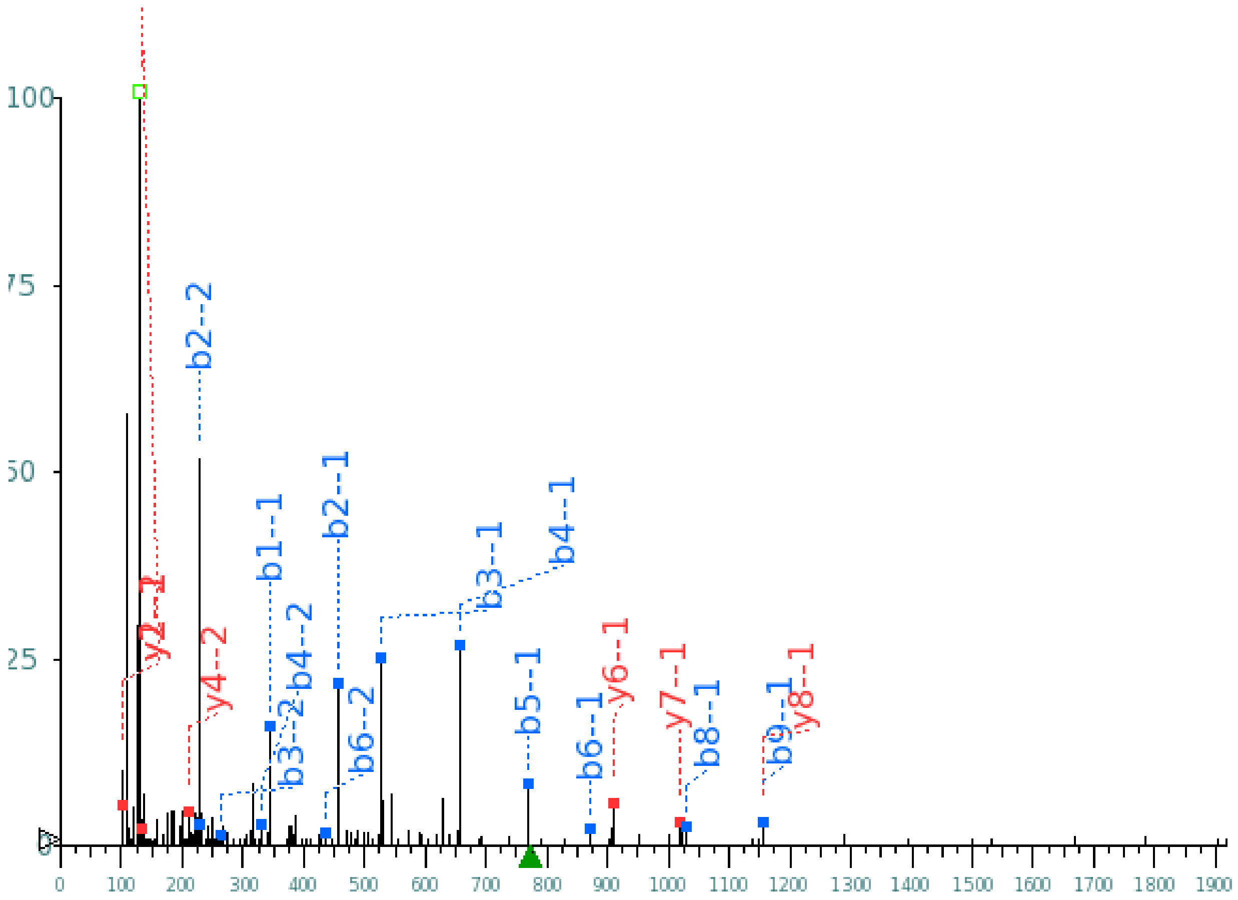

ILAQITGTEHLKESEAD (+3)

Primary Reference: PDLI5_HUMAN
Search ID: 41761
Search Name: 20130328_ananiav_ERstress_TMT_TM_timecourse_trypsin_inj2_4mc_iaa
Scan#: 17986
Observed Mass: 771.7611 (-0.7 ppm)
PSM Score: 30.2

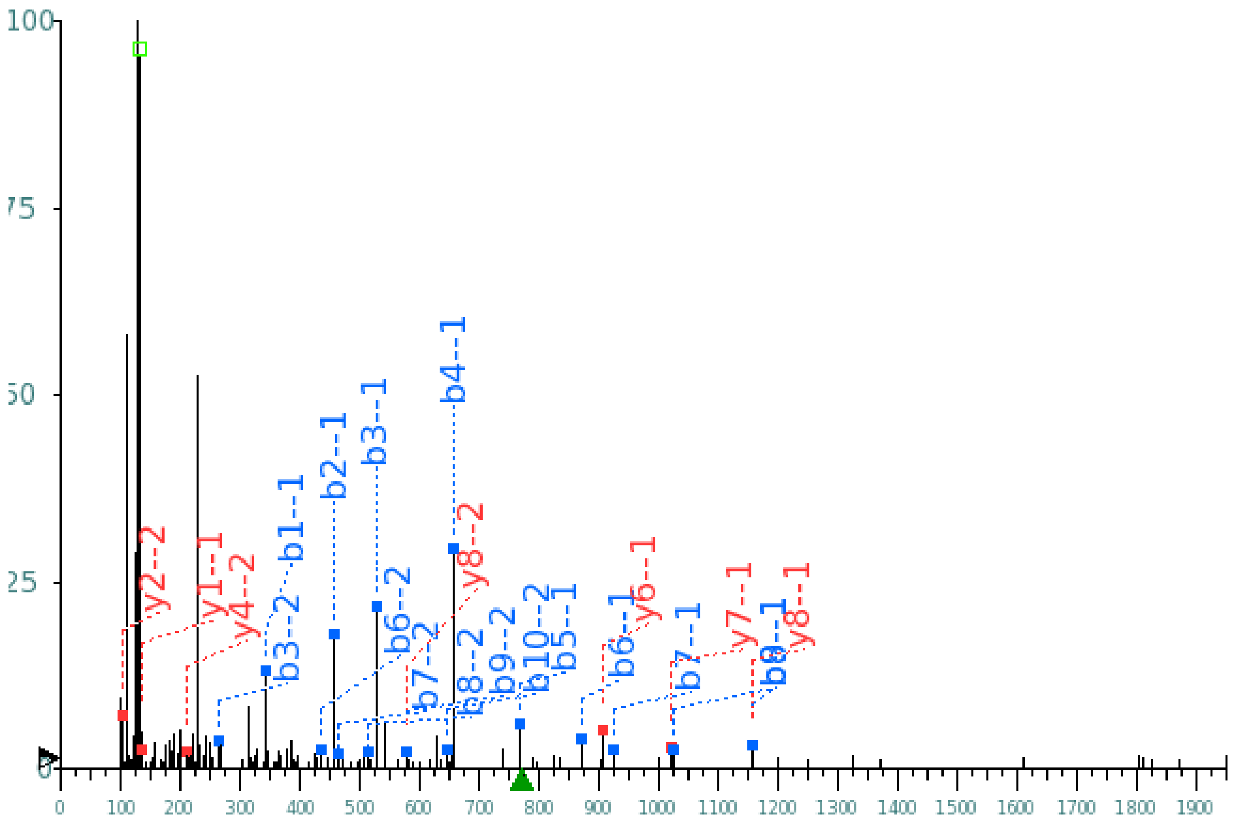

ILAQITGTEHLKESEAD (+3)

Primary Reference: PDLI5_HUMAN
Search ID: 41761
Search Name: 20130328_ananiav_ERstress_TMT_TM_timecourse_trypsin_inj2_4mc_iaa
Scan#: 17994
Observed Mass: 771.7613 (-0.4 ppm)
PSM Score: 33.74

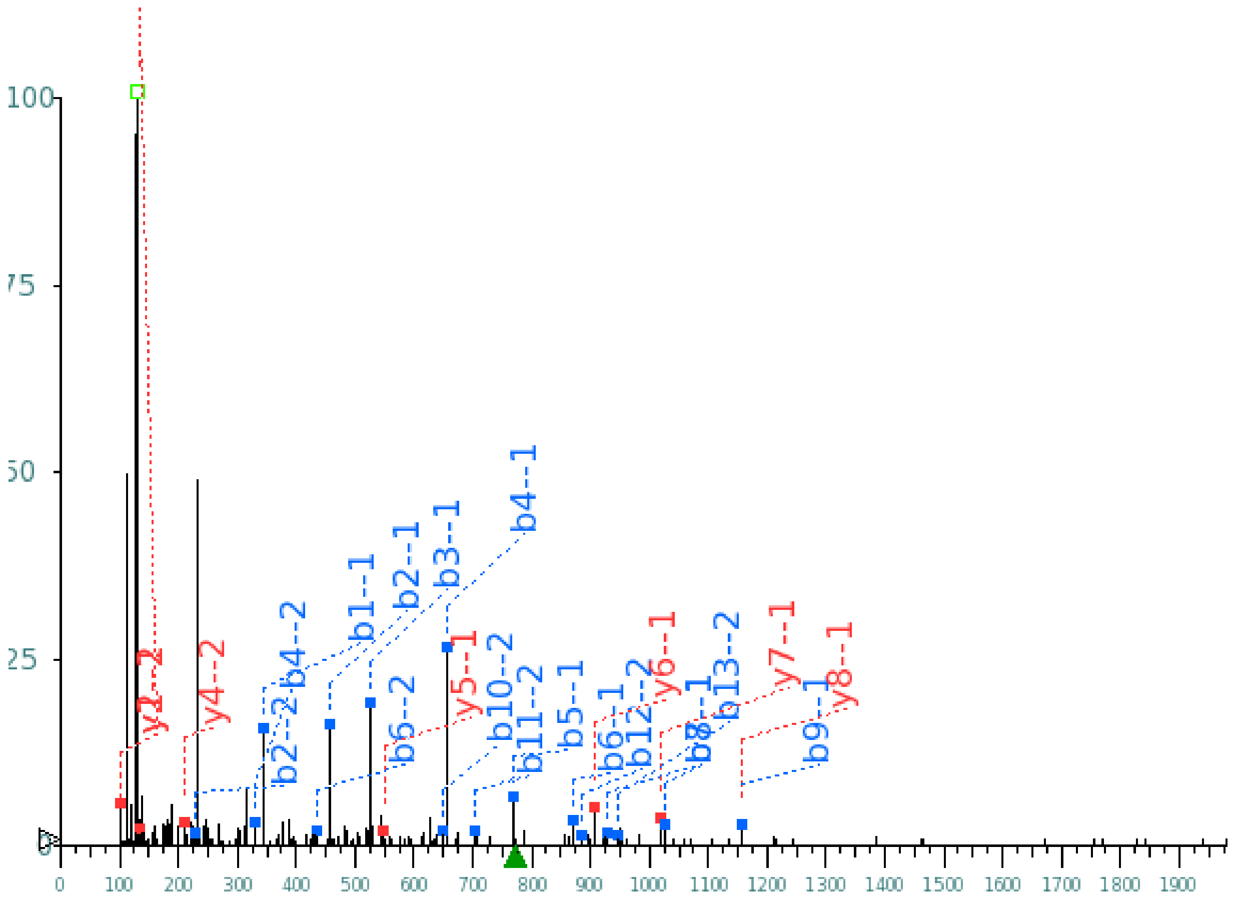

ILAQITGTEHLKESEAD (+3)

Primary Reference: PDLI5_HUMAN
Search ID: 41761
Search Name: 20130328_ananiav_ERstress_TMT_TM_timecourse_trypsin_inj2_4mc_iaa
Scan#: 18058
Observed Mass: 771.7629 (1.8 ppm)
PSM Score: 33.29

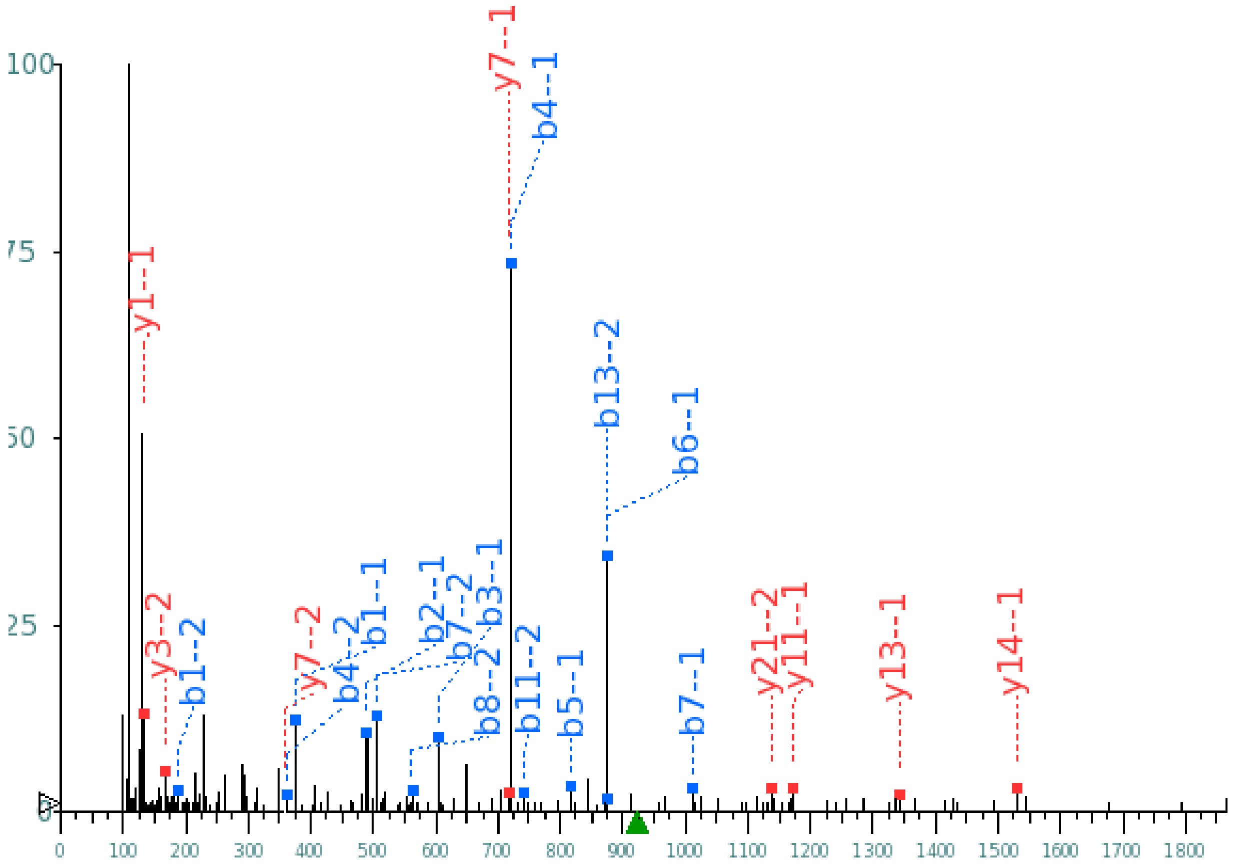

FLNDPGHLLWGDRPEAQLGSQAD (+3)

Primary Reference: RPP21_HUMAN
Search ID: 41761
Search Name: 20130328_ananiav_ERstress_TMT_TM_timecourse_trypsin_inj2_4mc_iaa
Scan#: 18109
Observed Mass: 922.4676 (1.4 ppm)
PSM Score: 18.2

File name: 2015-02-04 04.01.53PM.pdf

Creation time: 2015-02-04 04:09PM

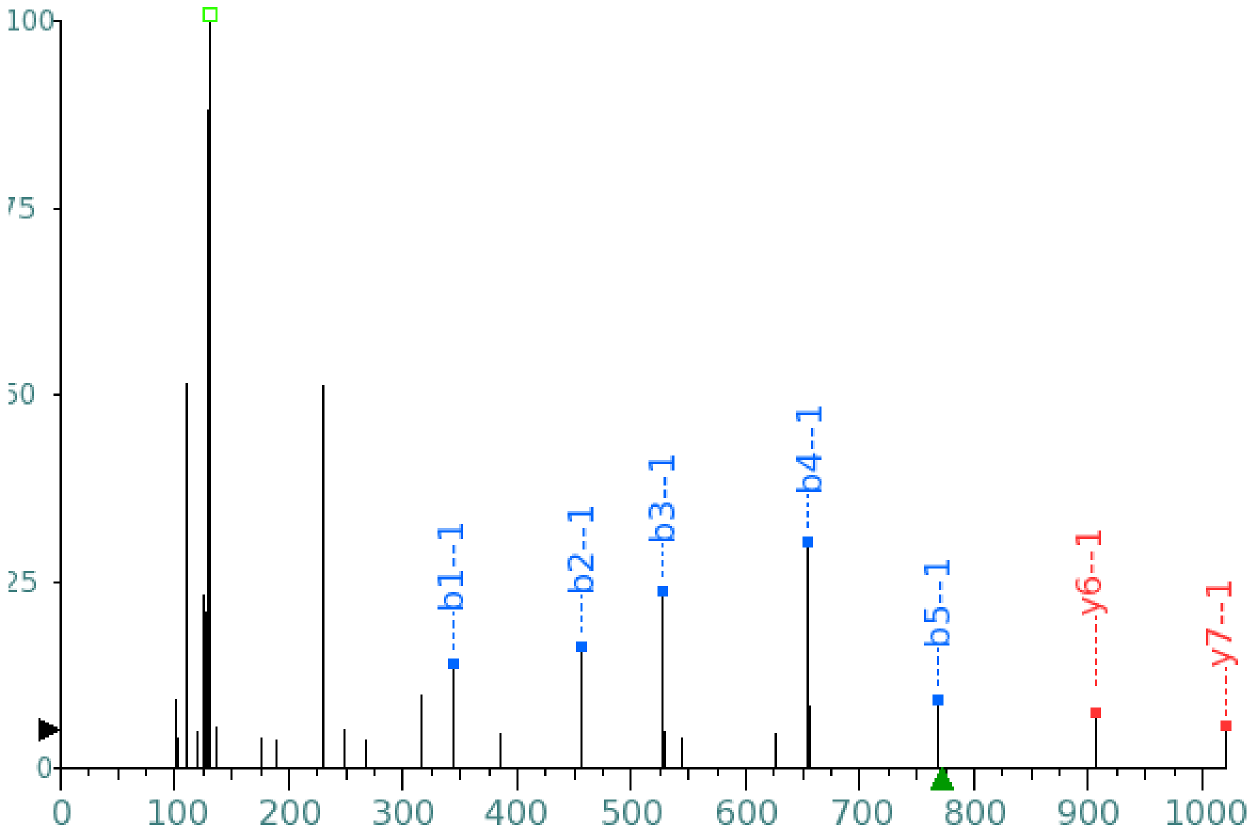

ILAQITGTEHLKESEAD (+3)

Primary Reference: PDLI5_HUMAN
Search ID: 41761
Search Name: 20130328_ananiav_ERstress_TMT_TM_timecourse_trypsin_inj2_4mc_iaa
Scan#: 18149
Observed Mass: 771.7624 (1 ppm)
PSM Score: 28.4

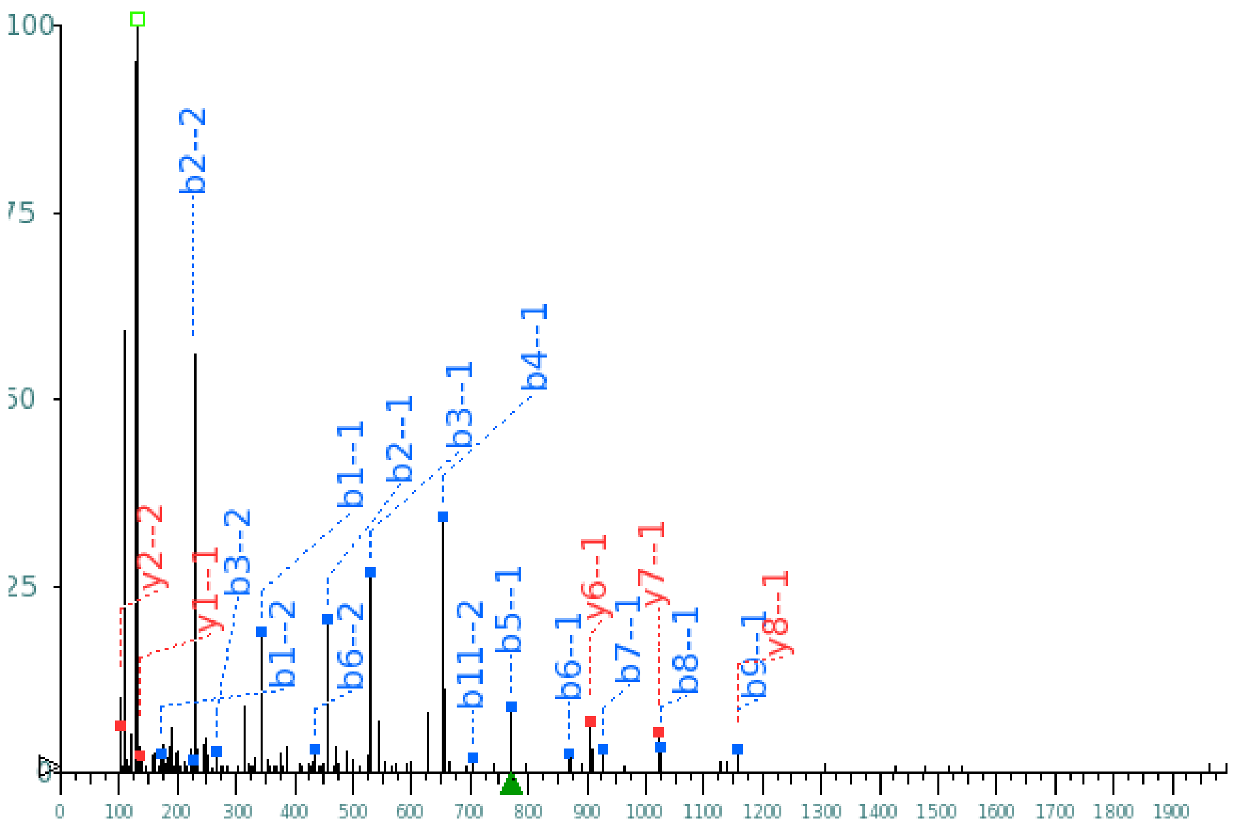

ILAQITGTEHLKESEAD (+3)

Primary Reference: PDLI5_HUMAN
Search ID: 41761
Search Name: 20130328_ananiav_ERstress_TMT_TM_timecourse_trypsin_inj2_4mc_iaa
Scan#: 18225
Observed Mass: 771.7645 (3.8 ppm)
PSM Score: 42.12

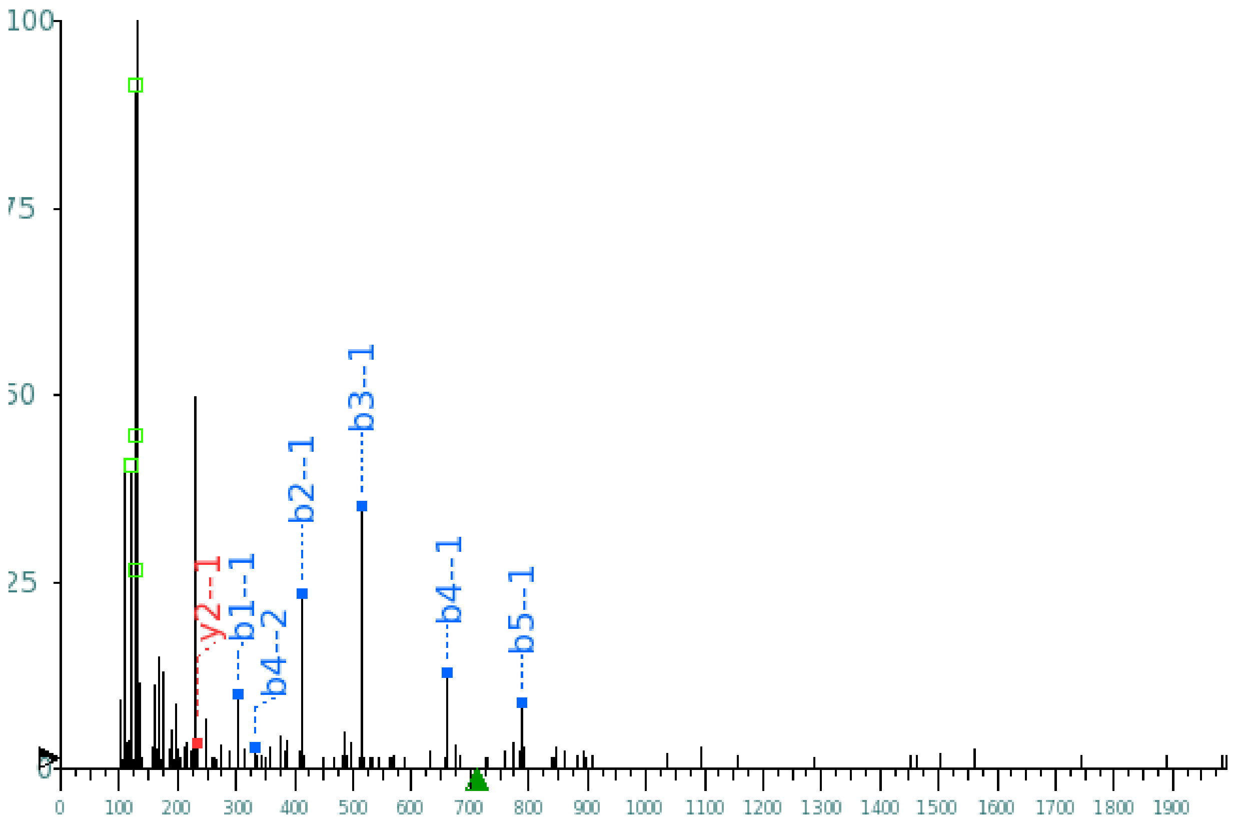

ALVFQPVAELKDQTD (+3)

Primary Reference: K6PF_HUMAN
Search ID: 41761
Search Name: 20130328_ananiav_ERstress_TMT_TM_timecourse_trypsin_inj2_4mc_iaa
Scan#: 18246
Observed Mass: 711.4074 (3.4 ppm)
PSM Score: 9.15

File name: 2015-02-04 04.01.53PM.pdf

Creation time: 2015-02-04 04:09PM

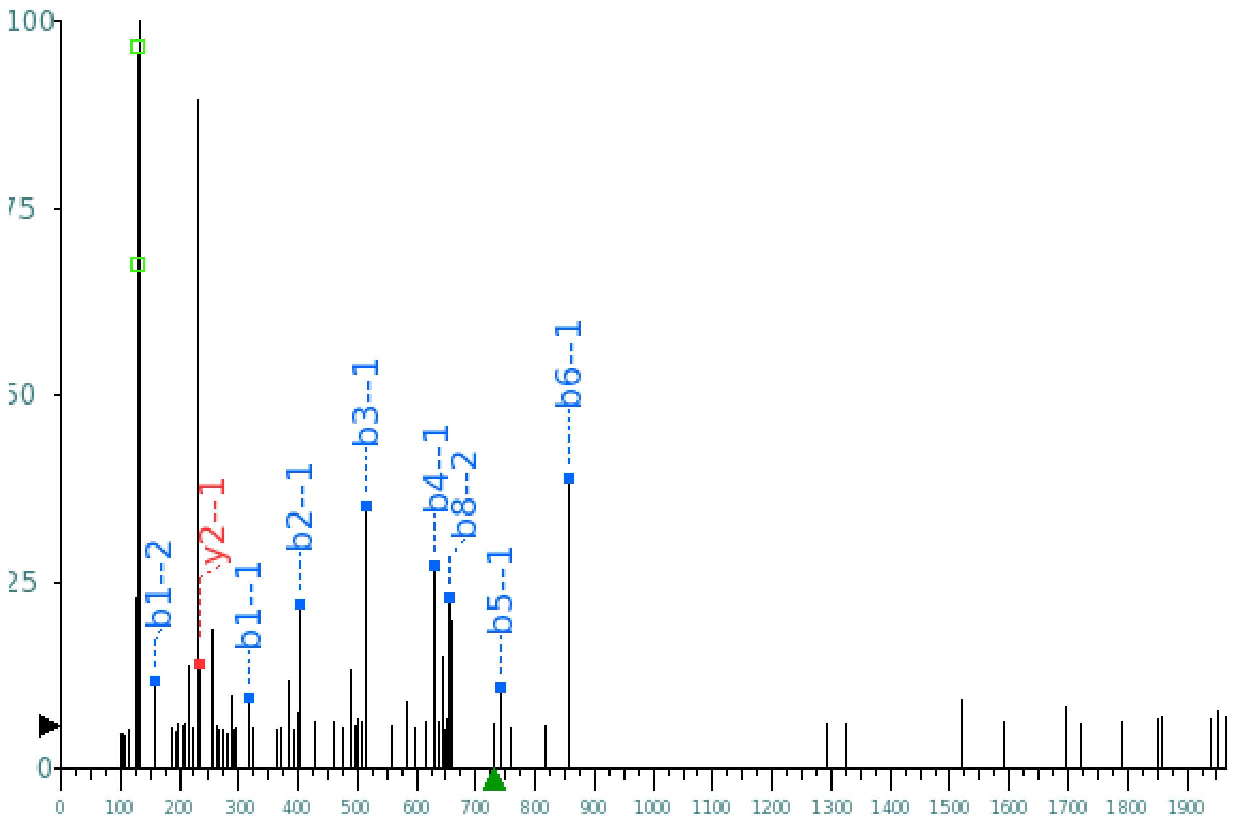

SSILLDVKPWDDETD (+3)

Primary Reference: EF1B_HUMAN
Search ID: 41761
Search Name: 20130328_ananiav_ERstress_TMT_TM_timecourse_trypsin_inj2_4mc_iaa
Scan#: 18307
Observed Mass: 731.0579 (2.6 ppm)
PSM Score: 16.47

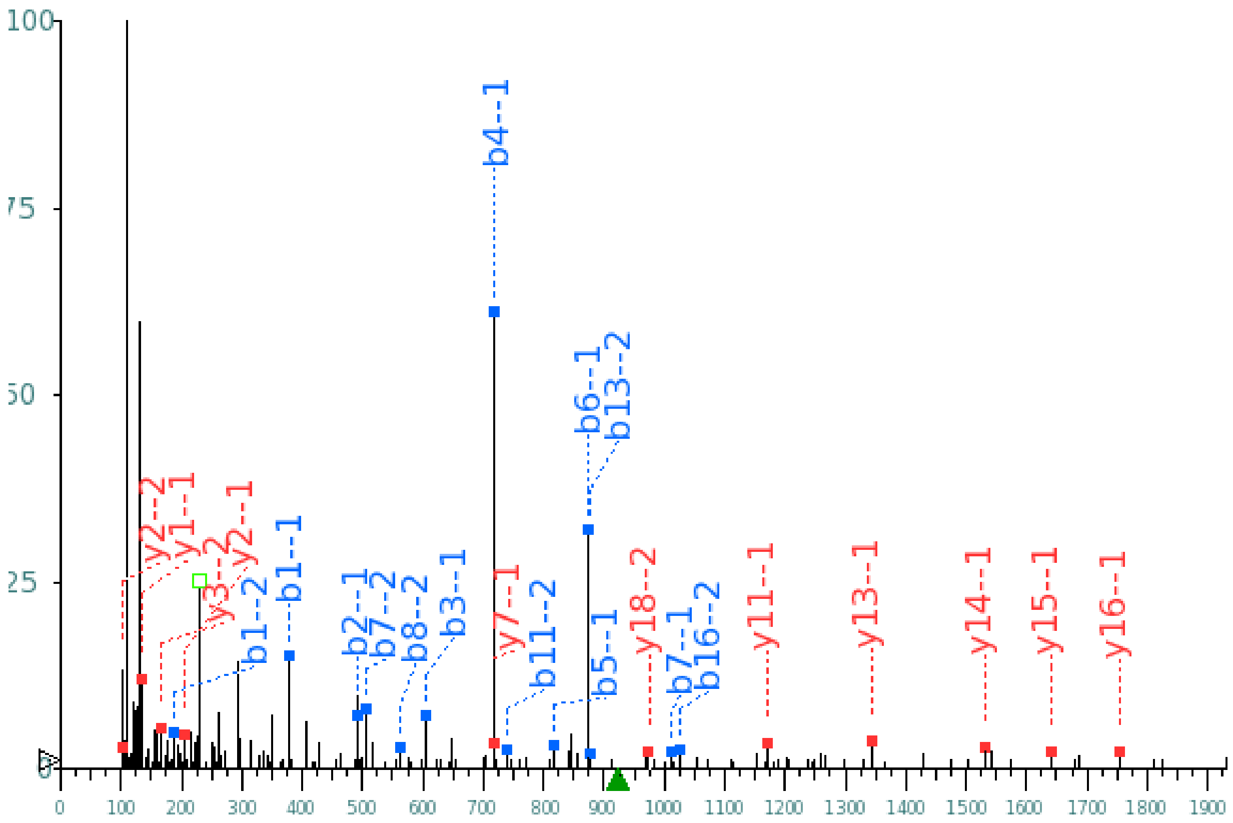

FLNDPGHLLWGDRPEAQLGSQAD (+3)

Primary Reference: RPP21_HUMAN
Search ID: 41761
Search Name: 20130328_ananiav_ERstress_TMT_TM_timecourse_trypsin_inj2_4mc_iaa
Scan#: 18332
Observed Mass: 922.4666 (0.3 ppm)
PSM Score: 23.22

File name: 2015-02-04 04.01.53PM.pdf

Creation time: 2015-02-04 04:09PM

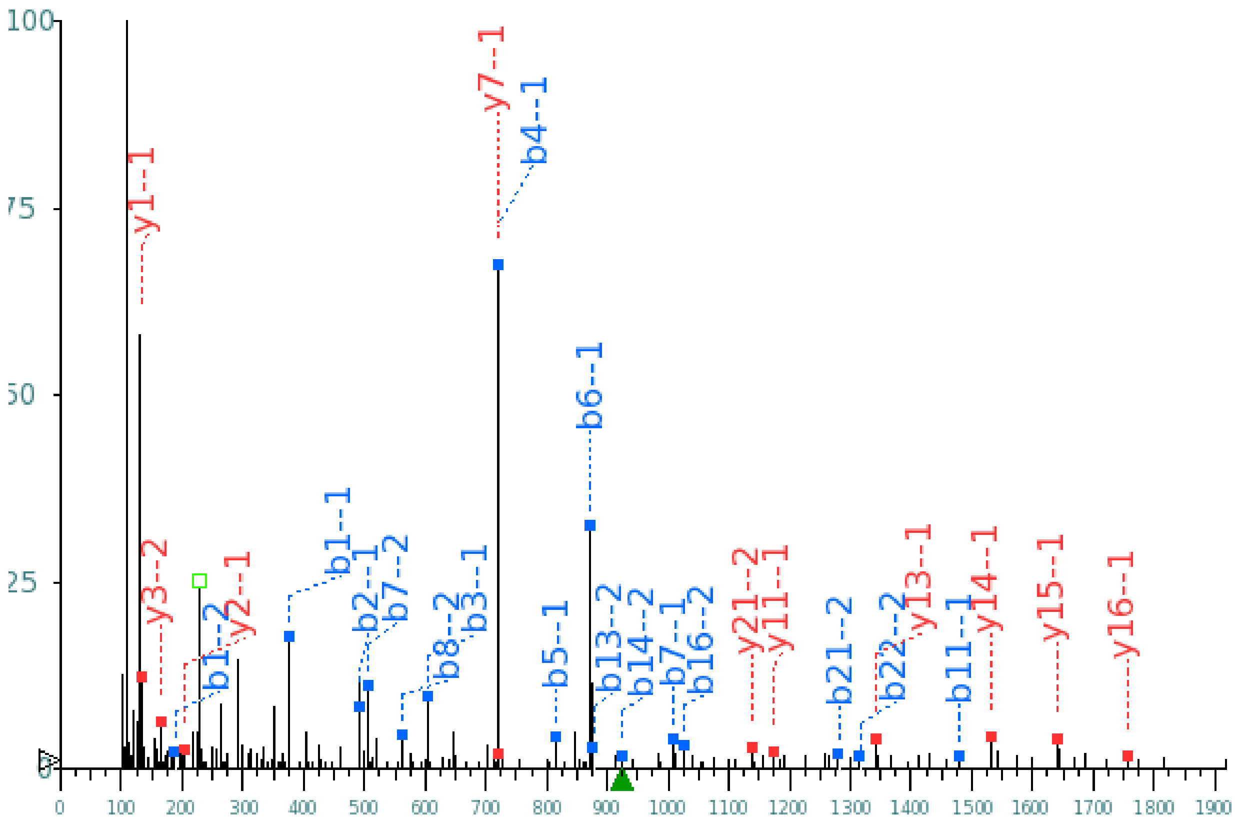

FLNDPGHLLWGDRPEAQLGSQAD (+3)

Primary Reference: RPP21_HUMAN
Search ID: 41761
Search Name: 20130328_ananiav_ERstress_TMT_TM_timecourse_trypsin_inj2_4mc_iaa
Scan#: 18400
Observed Mass: 922.4693 (3.2 ppm)
PSM Score: 17.99

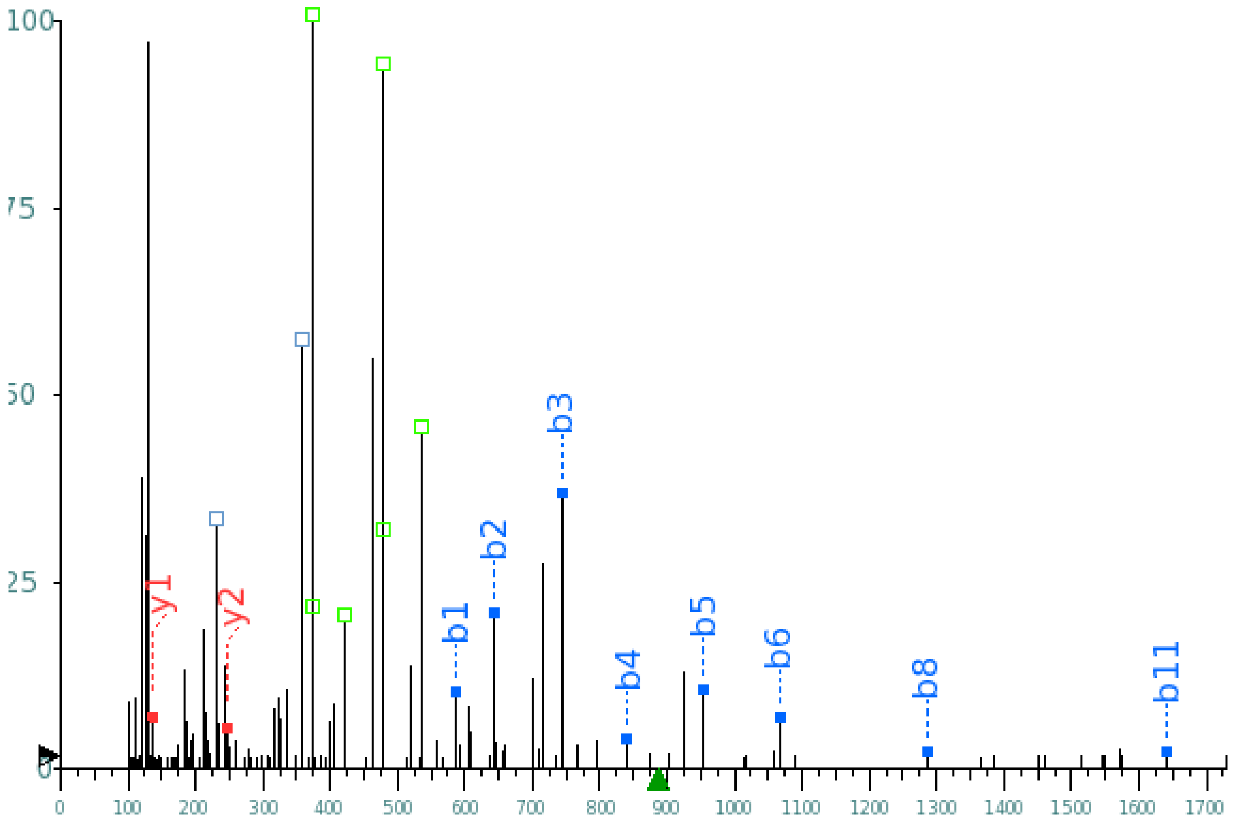

KGVPIIFADELD (+2)

Primary Reference: DAG1_HUMAN
Search ID: 41761
Search Name: 20130328_ananiav_ERstress_TMT_TM_timecourse_trypsin_inj2_4mc_iaa
Scan#: 18412
Observed Mass: 888.0221 (0.8 ppm)
PSM Score: 32.85

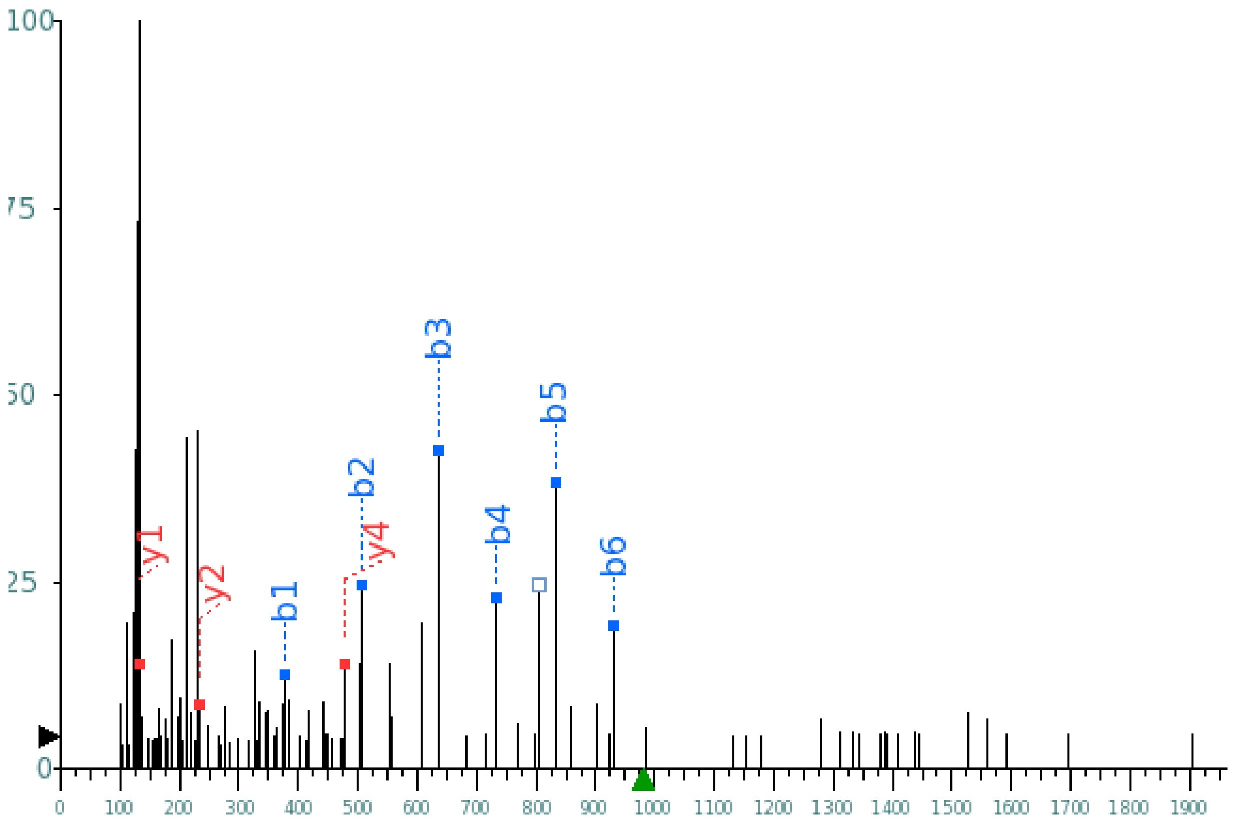

FEEPVVLPDLDDQTD (+2)

Primary Reference: NOTC1_HUMAN
Search ID: 41761
Search Name: 20130328_ananiav_ERstress_TMT_TM_timecourse_trypsin_inj2_4mc_iaa
Scan#: 18420
Observed Mass: 980.9847 (1.7 ppm)
PSM Score: 12.15

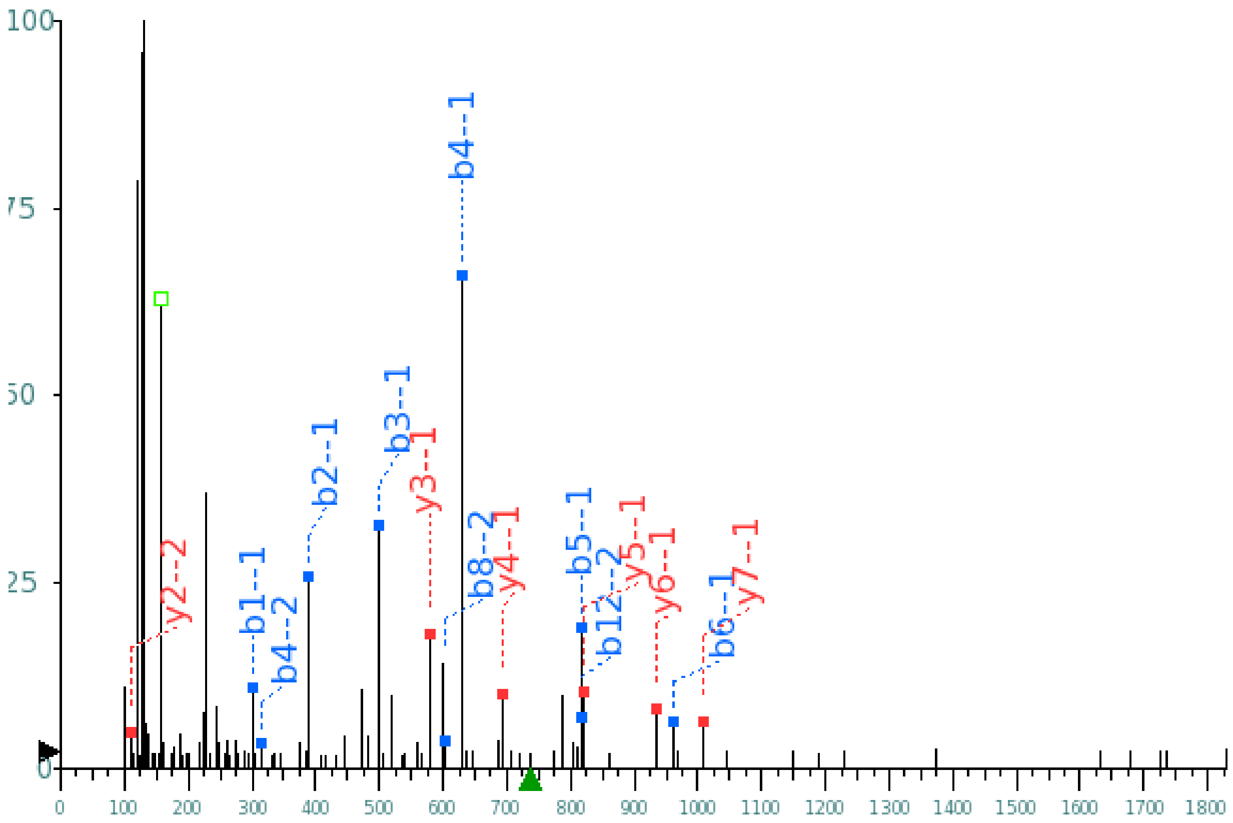

ASLQWFNQALEDKSD (+3)

Primary Reference: P121A_HUMAN

Search ID: 41761

Search Name: 20130328_ananiav_ERstress_TMT_TM_timecourse_trypsin_inj2_4mc_iaa

Scan#: 18703

Observed Mass: 737.39 (2.7 ppm)

PSM Score: 24.5

File name: 2015-02-04 04.01.53PM.pdf

Creation time: 2015-02-04 04:09PM

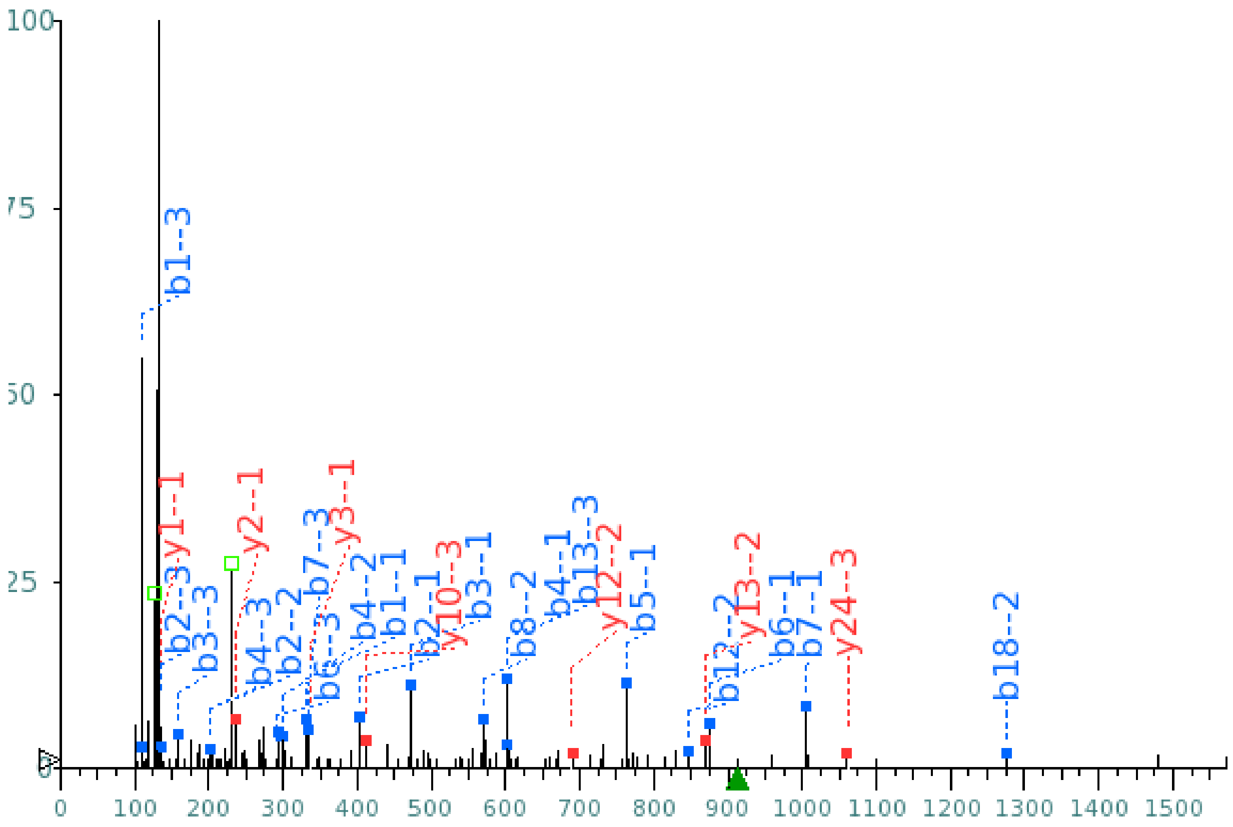

TAAQC*LEHYEFLLDKAAQRDNEEETTD (+4)

Primary Reference: CDC5L_HUMAN
Search ID: 41761
Search Name: 20130328_ananiav_ERstress_TMT_TM_timecourse_trypsin_inj2_4mc_iaa
Scan#: 18723
Observed Mass: 914.6943 (-0.9 ppm)
PSM Score: 21.61

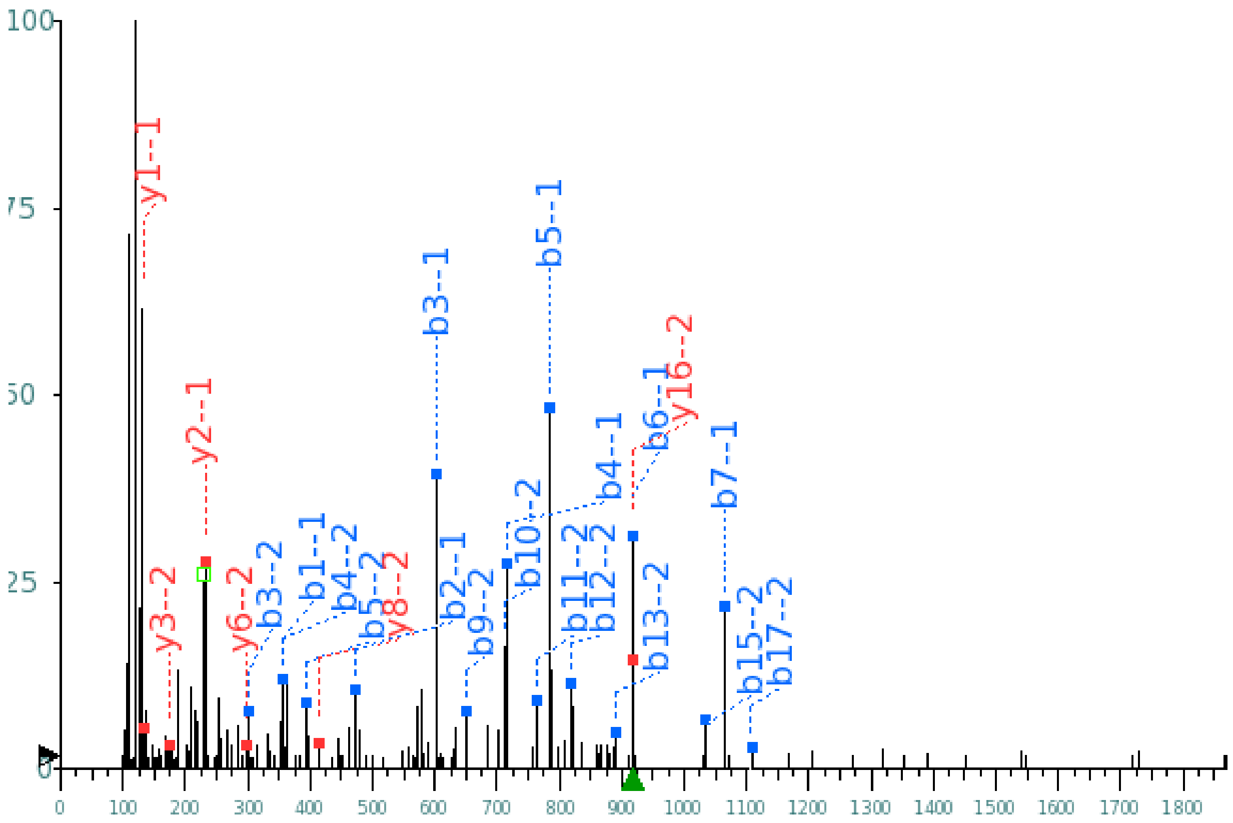

QNEIAMFPHEVNHFFSGSTITD (+3)

Primary Reference: OSBL3_HUMAN
Search ID: 41761
Search Name: 20130328_ananiav_ERstress_TMT_TM_timecourse_trypsin_inj2_4mc_iaa
Scan#: 18737
Observed Mass: 917.4427 (1.9 ppm)
PSM Score: 30.6

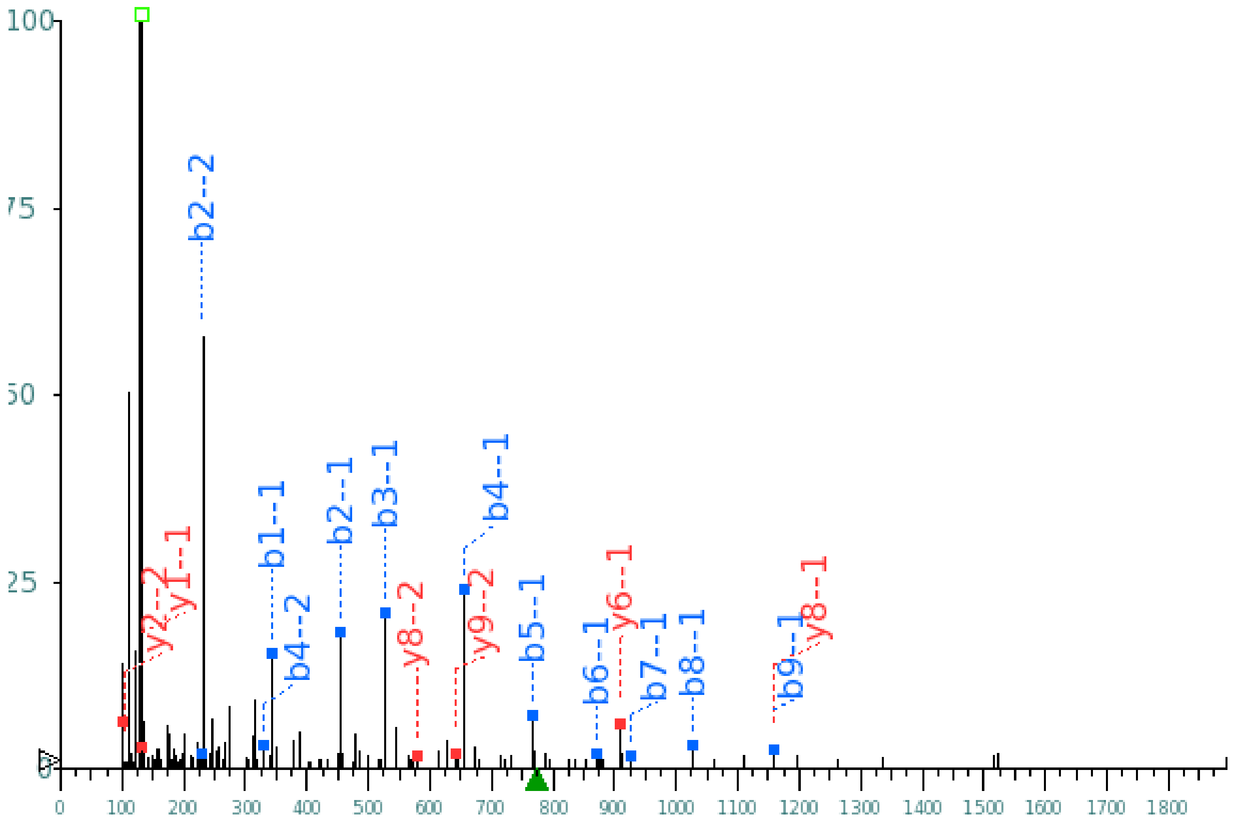

ILAQITGTEHLKESEAD (+3)

Primary Reference: PDLI5_HUMAN
Search ID: 41761
Search Name: 20130328_ananiav_ERstress_TMT_TM_timecourse_trypsin_inj2_4mc_iaa
Scan#: 18745
Observed Mass: 771.7625 (1.2 ppm)
PSM Score: 32.5

File name: 2015-02-04 04.01.53PM.pdf

Creation time: 2015-02-04 04:09PM

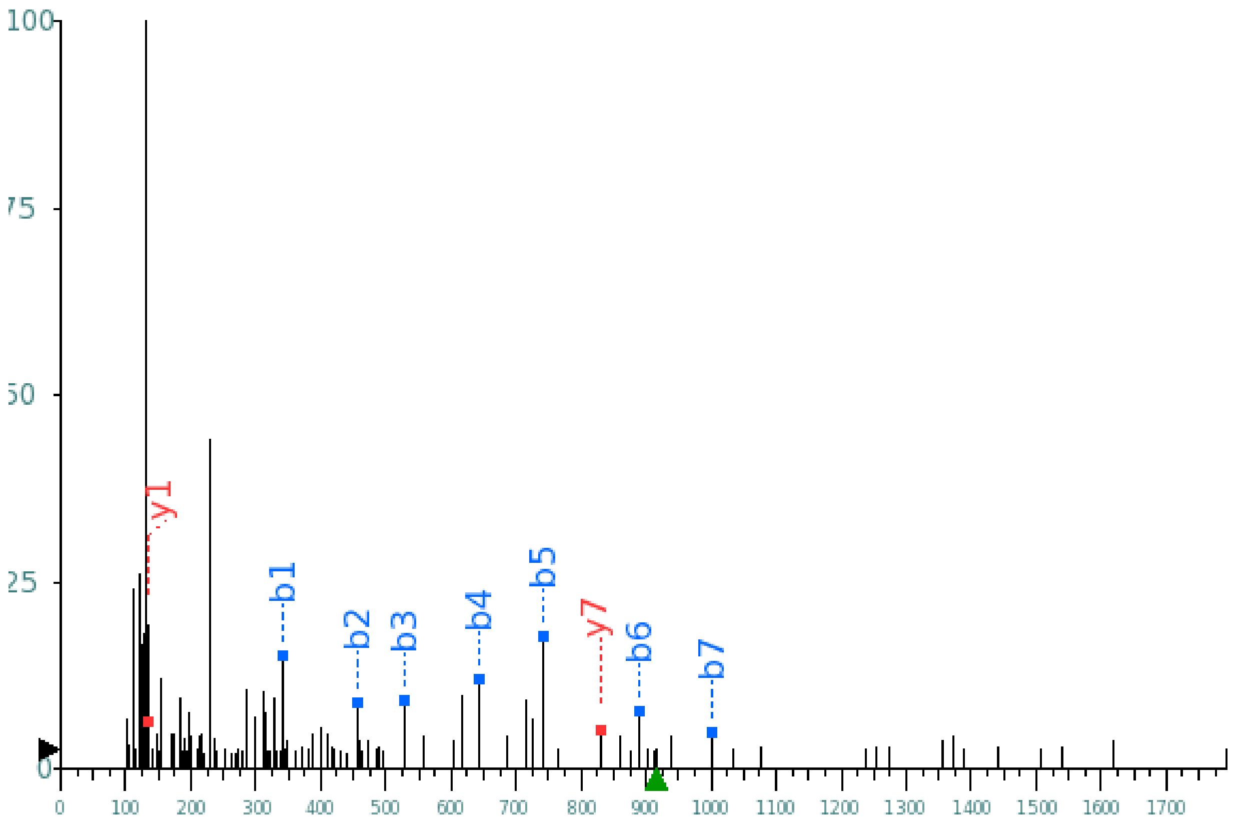

ILANTFITYTTQTD (+2)

Primary Reference: NFXL1_HUMAN
Search ID: 41761
Search Name: 20130328_ananiav_ERstress_TMT_TM_timecourse_trypsin_inj2_4mc_iaa
Scan#: 18792
Observed Mass: 915.9902 (2.4 ppm)
PSM Score: 29.6

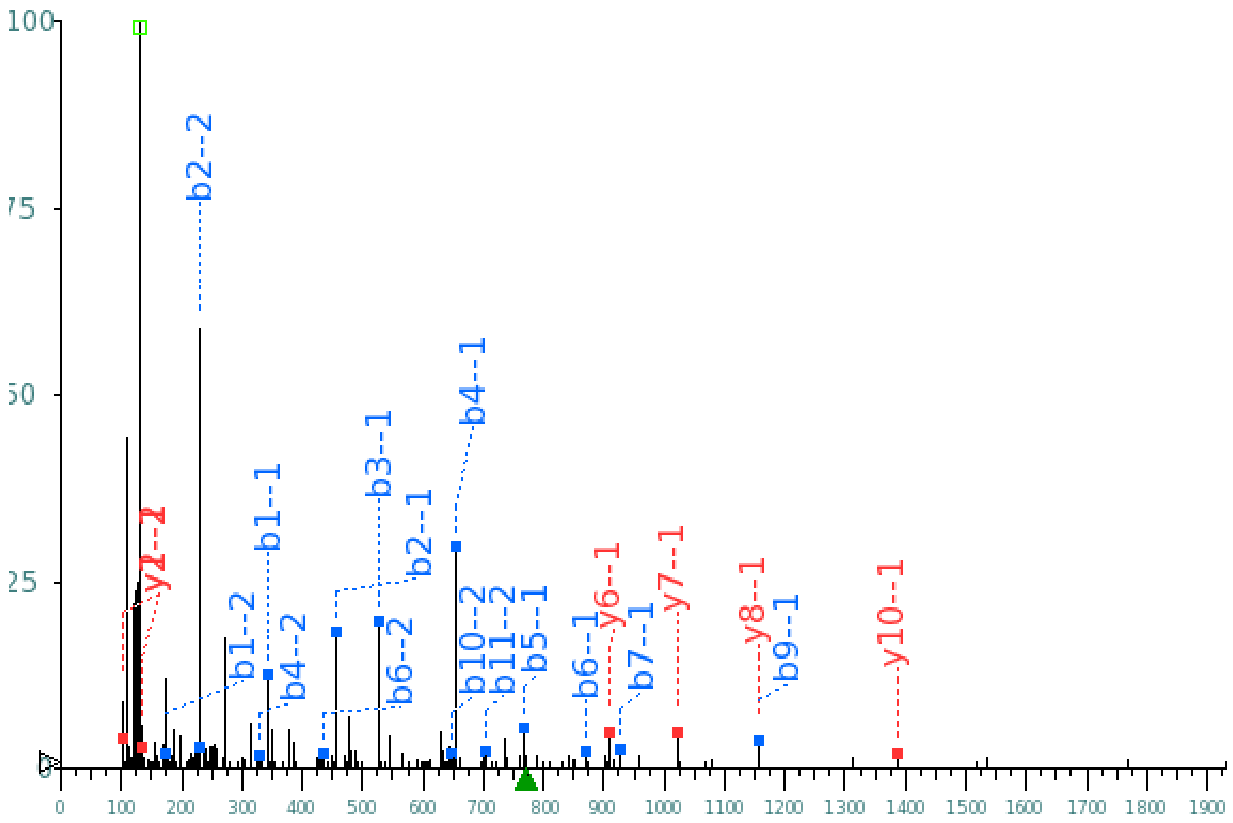

ILAQITGTEHLKESEAD (+3)

Primary Reference: PDLI5_HUMAN
Search ID: 41761
Search Name: 20130328_ananiav_ERstress_TMT_TM_timecourse_trypsin_inj2_4mc_iaa
Scan#: 18832
Observed Mass: 771.7629 (1.8 ppm)
PSM Score: 28.72

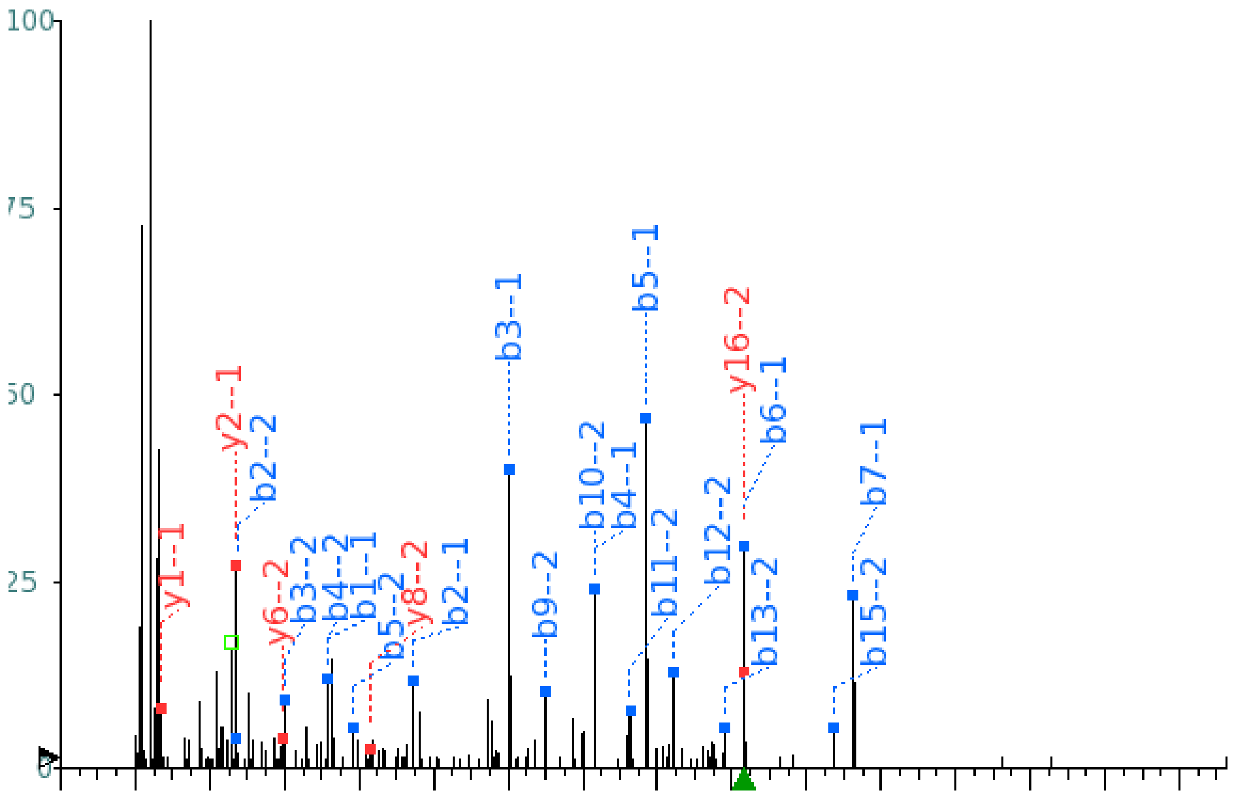

QNEIAMFPHEVNHFFSGSTITD (+3)

Primary Reference: OSBL3_HUMAN
Search ID: 41761
Search Name: 20130328_ananiav_ERstress_TMT_TM_timecourse_trypsin_inj2_4mc_iaa
Scan#: 18837
Observed Mass: 917.4393 (-1.7 ppm)
PSM Score: 38.25

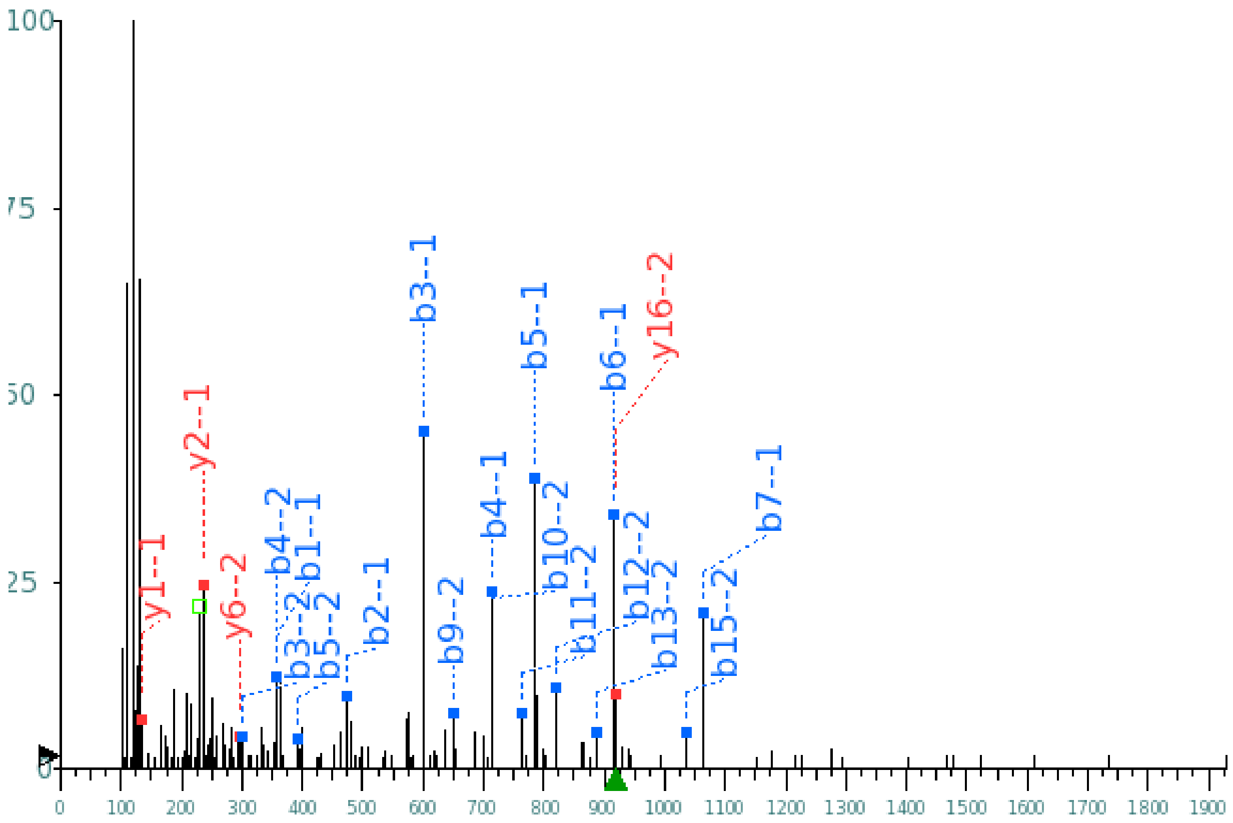

QNEIAMFPHEVNHFFSGSTITD (+3)

Primary Reference: OSBL3_HUMAN
Search ID: 41761
Search Name: 20130328_ananiav_ERstress_TMT_TM_timecourse_trypsin_inj2_4mc_iaa
Scan#: 18901
Observed Mass: 917.4418 (0.9 ppm)
PSM Score: 19.98

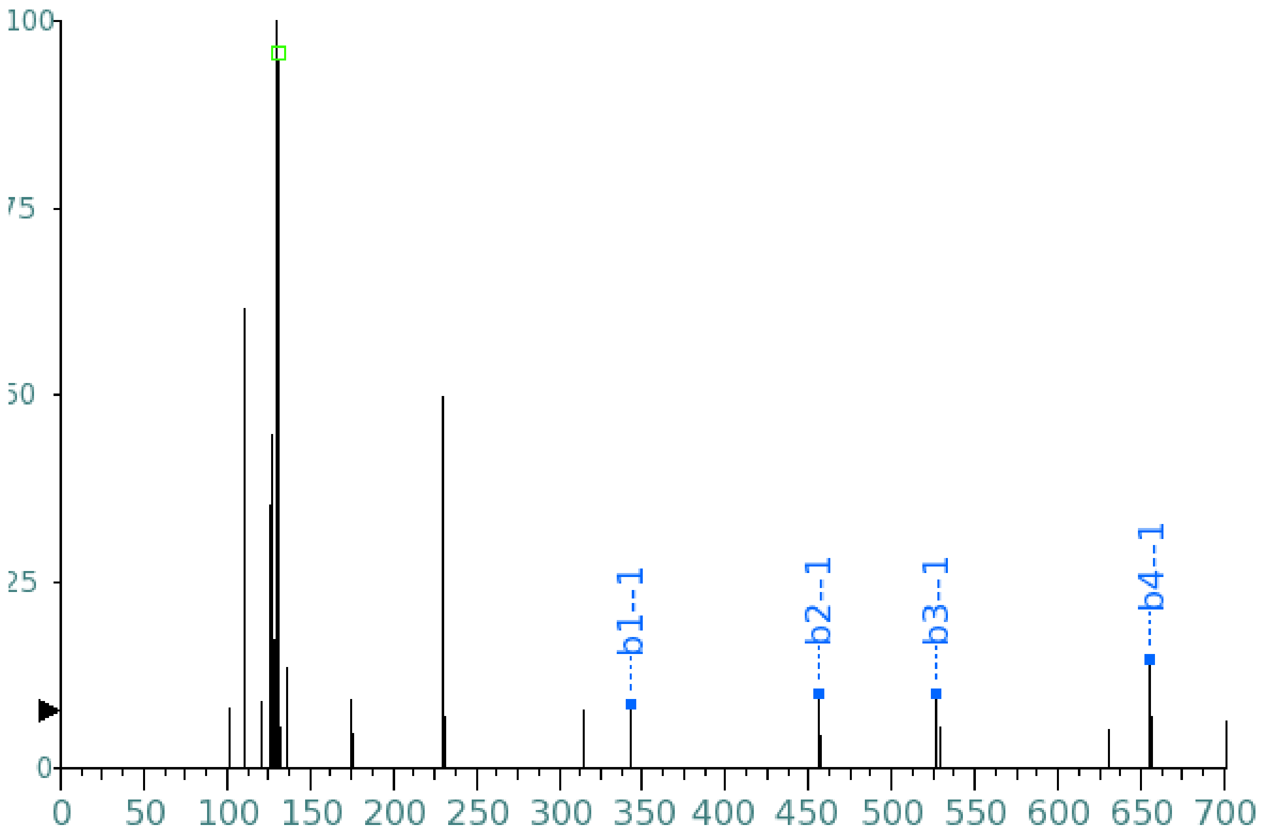

ILAQITGTEHLKESEAD (+3)

Primary Reference: PDLI5_HUMAN
Search ID: 41761
Search Name: 20130328_ananiav_ERstress_TMT_TM_timecourse_trypsin_inj2_4mc_iaa
Scan#: 19381
Observed Mass: 771.7628 (1.5 ppm)
PSM Score: 12.24

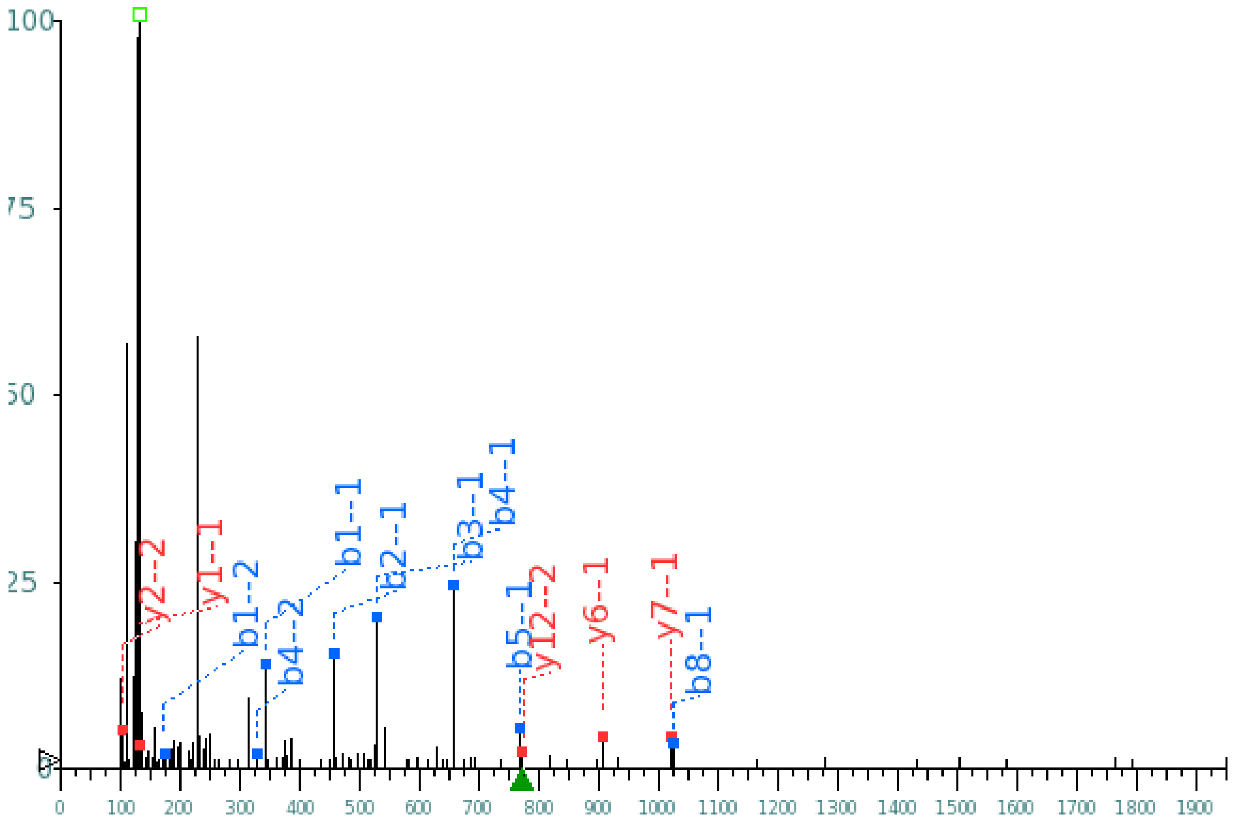

ILAQITGTEHLKESEAD (+3)

Primary Reference: PDLI5_HUMAN
Search ID: 41761
Search Name: 20130328_ananiav_ERstress_TMT_TM_timecourse_trypsin_inj2_4mc_iaa
Scan#: 19613
Observed Mass: 771.7638 (2.8 ppm)
PSM Score: 13.66

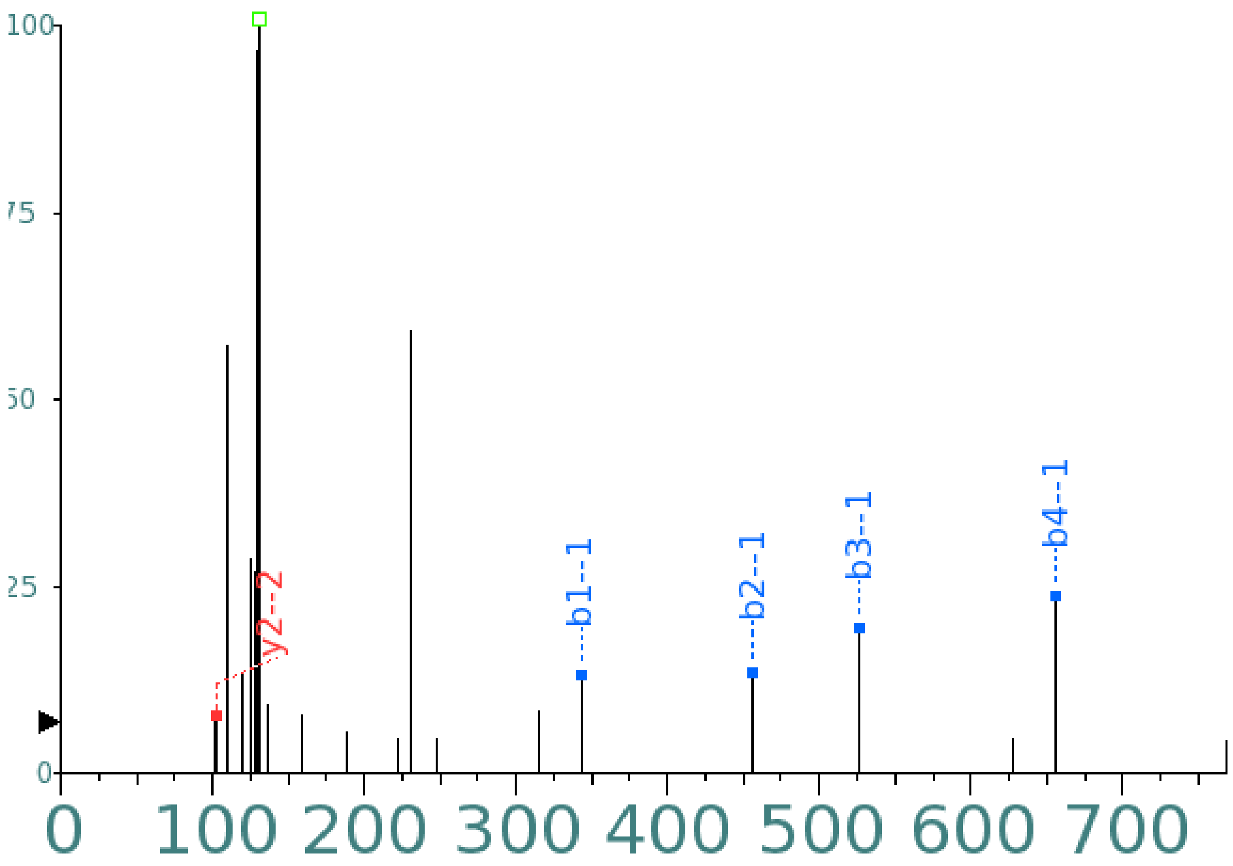

ILAQITGTEHLKESEAD (+3)

Primary Reference: PDLI5_HUMAN
Search ID: 41761
Search Name: 20130328_ananiav_ERstress_TMT_TM_timecourse_trypsin_inj2_4mc_iaa
Scan#: 19634
Observed Mass: 771.7632 (2.2 ppm)
PSM Score: 26.26

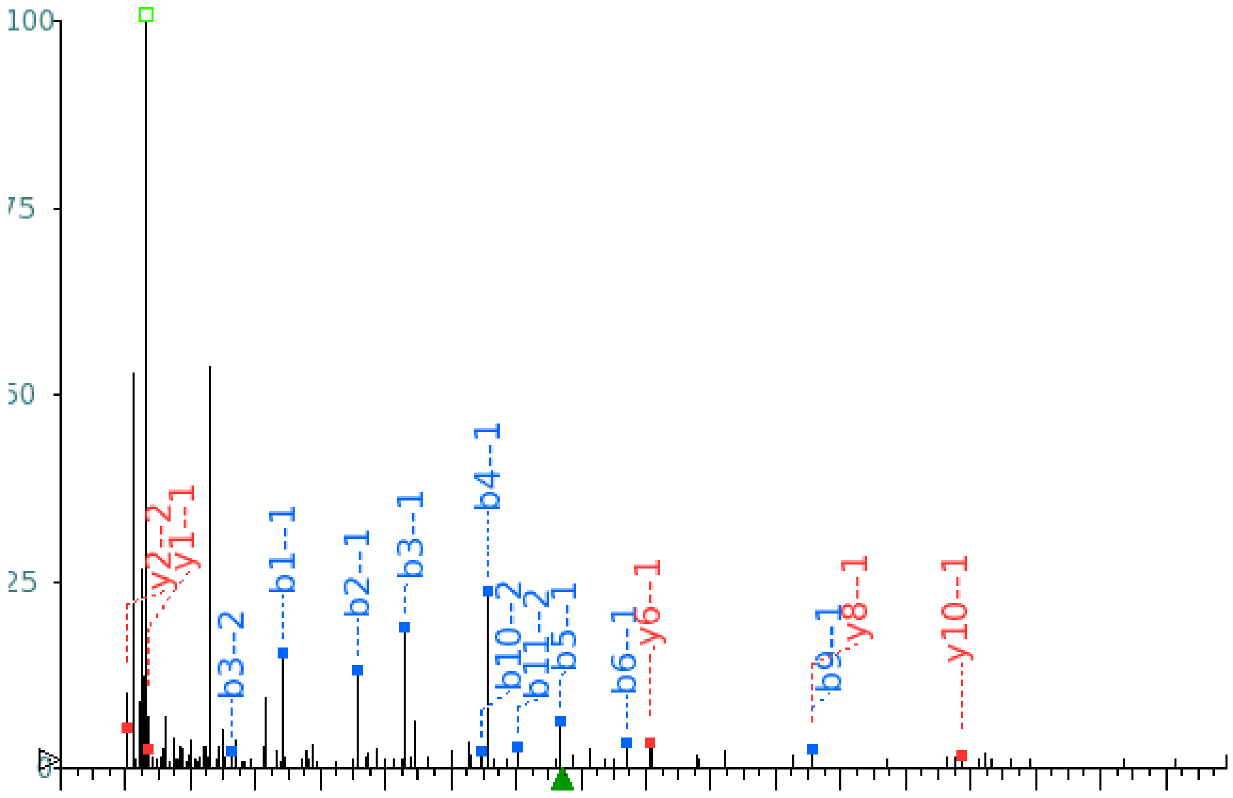

ILAQITGTEHLKESEAD (+3)

Primary Reference: PDLI5_HUMAN
Search ID: 41761
Search Name: 20130328_ananiav_ERstress_TMT_TM_timecourse_trypsin_inj2_4mc_iaa
Scan#: 19712
Observed Mass: 771.7636 (2.6 ppm)
PSM Score: 13.38

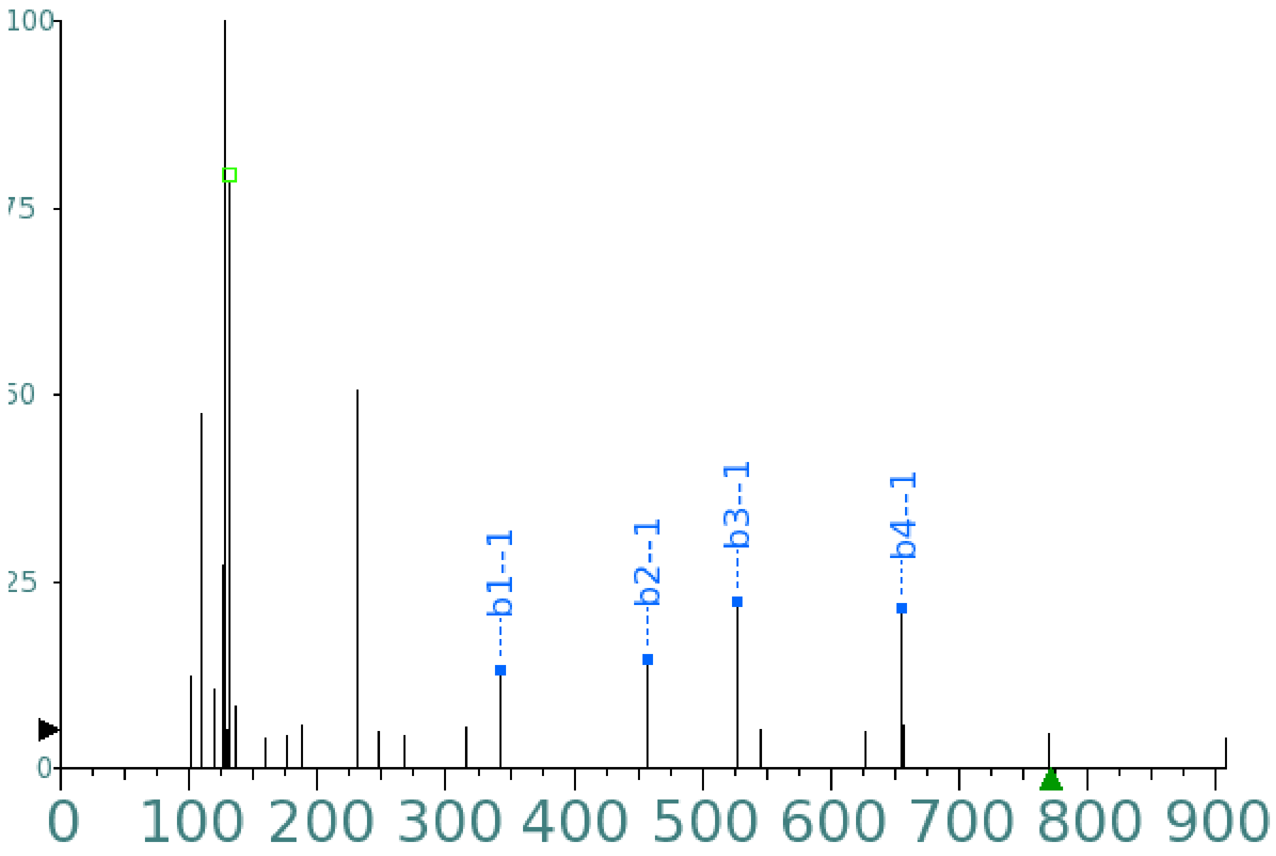

ILAQITGTEHLKESEAD (+3)

Primary Reference: PDLI5_HUMAN
Search ID: 41761
Search Name: 20130328_ananiav_ERstress_TMT_TM_timecourse_trypsin_inj2_4mc_iaa
Scan#: 19777
Observed Mass: 771.7638 (2.9 ppm)
PSM Score: 23.85

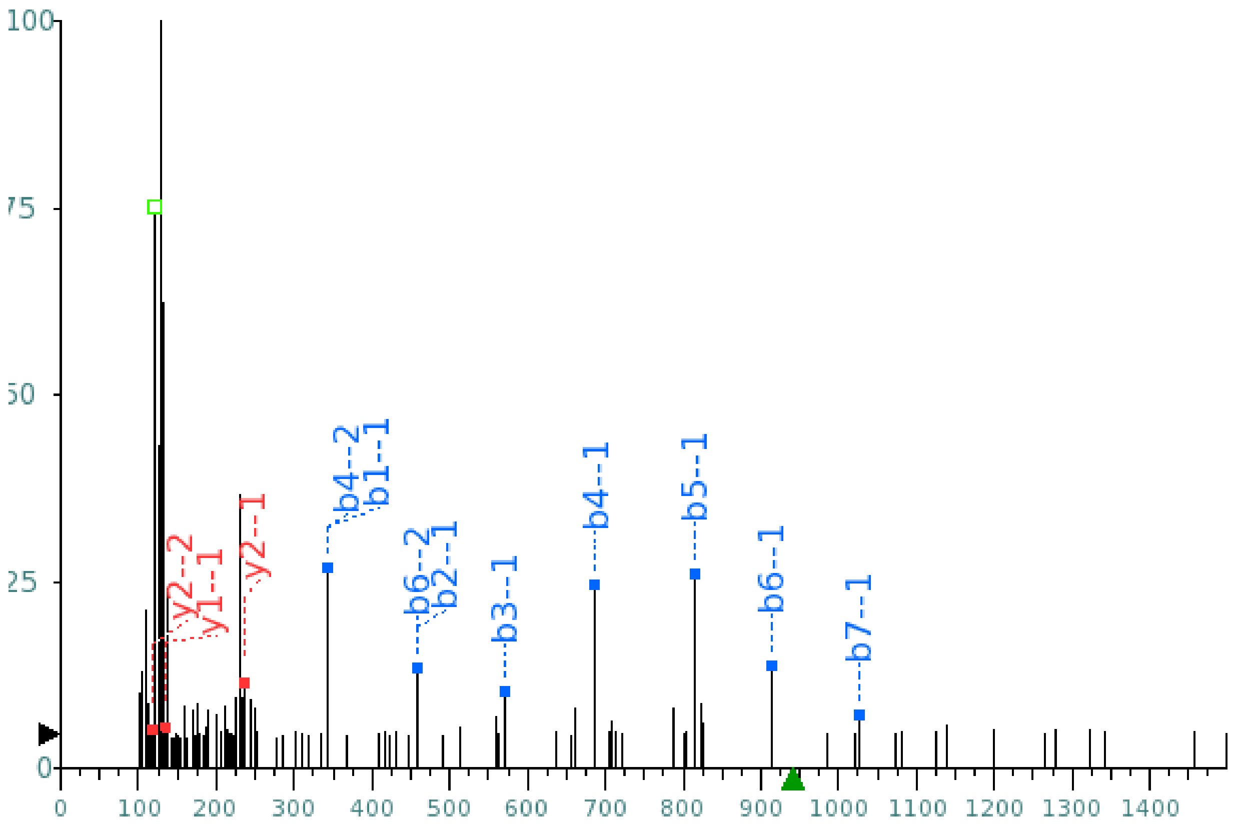

NLNDQVLFIDQGNRPLFEDMTD (+3)

Primary Reference: IL18_HUMAN
Search ID: 41761
Search Name: 20130328_ananiav_ERstress_TMT_TM_timecourse_trypsin_inj2_4mc_iaa
Scan#: 19796
Observed Mass: 941.8006 (1.9 ppm)
PSM Score: 19.43

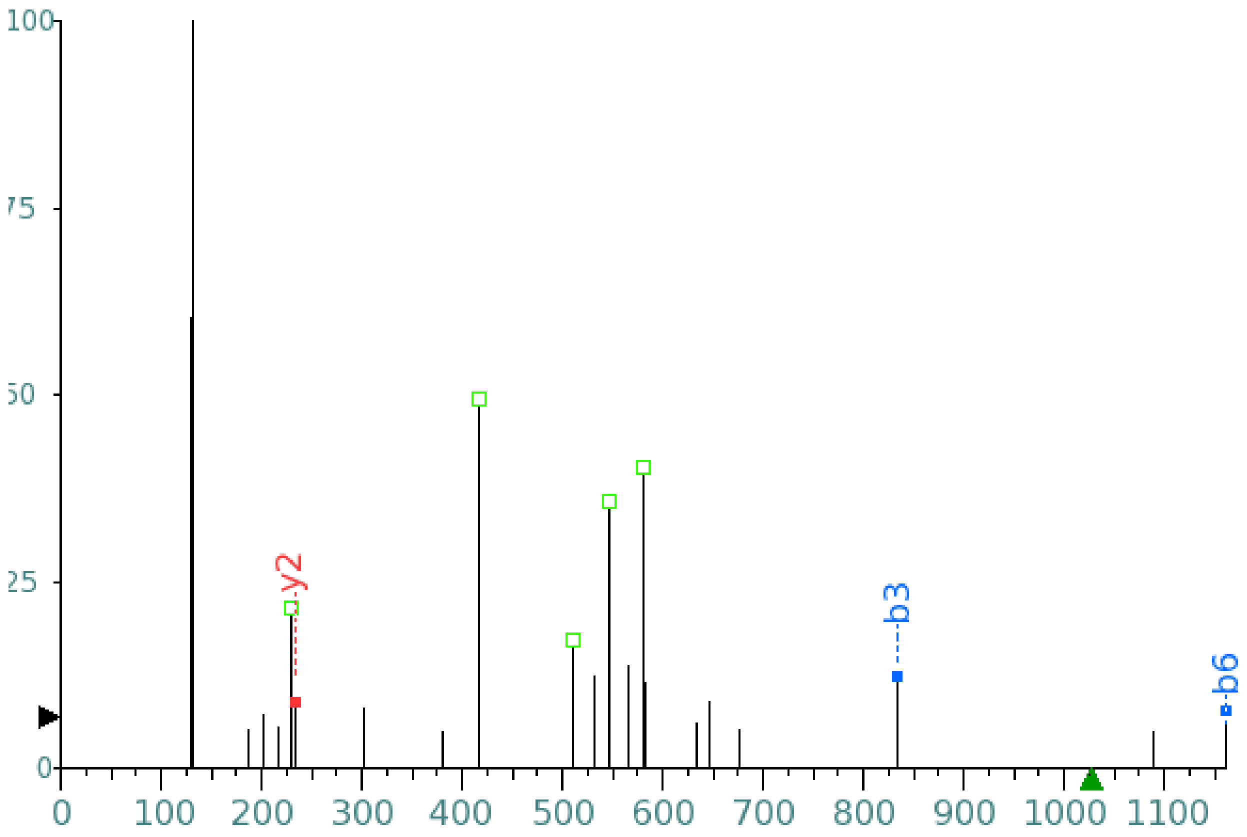

MKDWAAMLGEDITD (+2)

Primary Reference: CTGE5_HUMAN

Search ID: 41761

Search Name: 20130328_ananiav_ERstress_TMT_TM_timecourse_trypsin_inj2_4mc_iaa

Scan#: 19810

Observed Mass: 1027.5237 (3 ppm)

PSM Score: 26.04

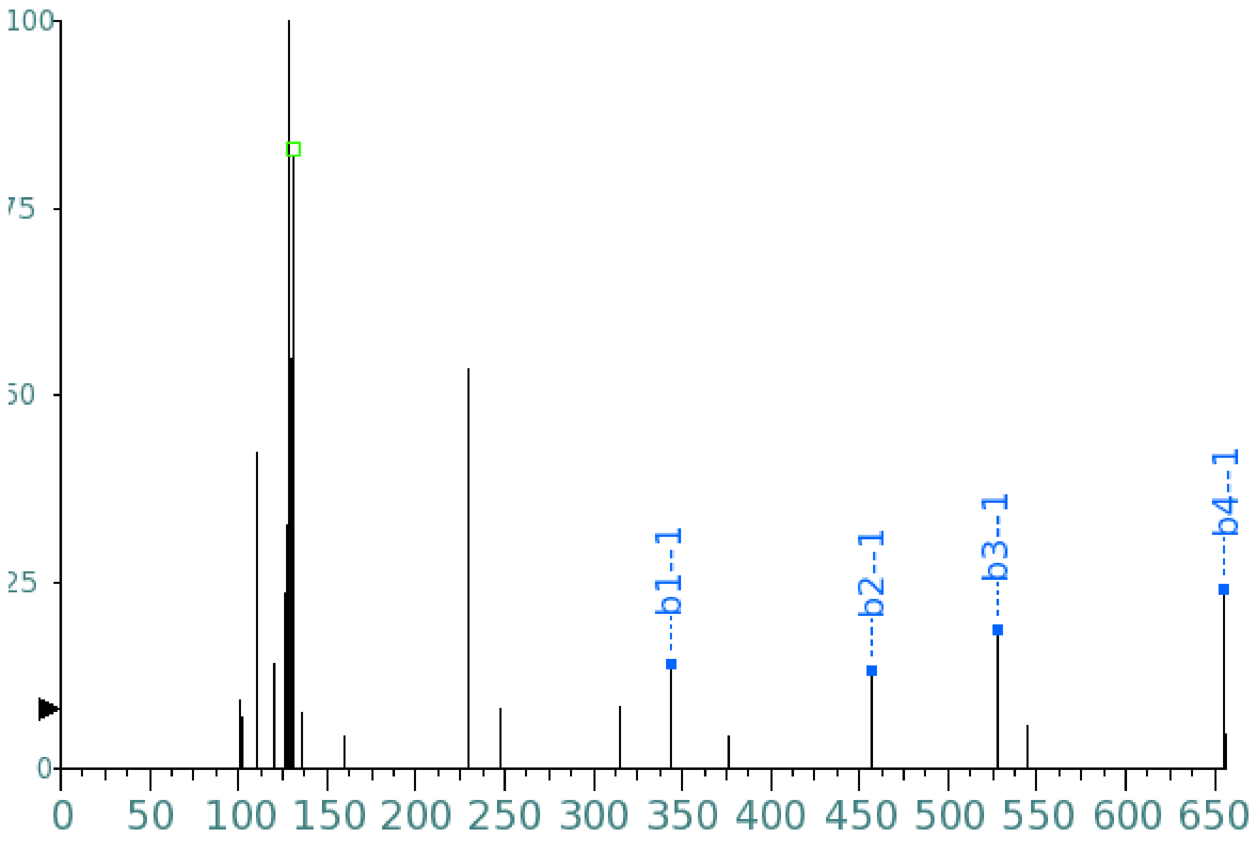

ILAQITGTEHLKESEAD (+3)

Primary Reference: PDLI5_HUMAN
Search ID: 41761
Search Name: 20130328_ananiav_ERstress_TMT_TM_timecourse_trypsin_inj2_4mc_iaa
Scan#: 19854
Observed Mass: 771.7613 (-0.3 ppm)
PSM Score: 17.47

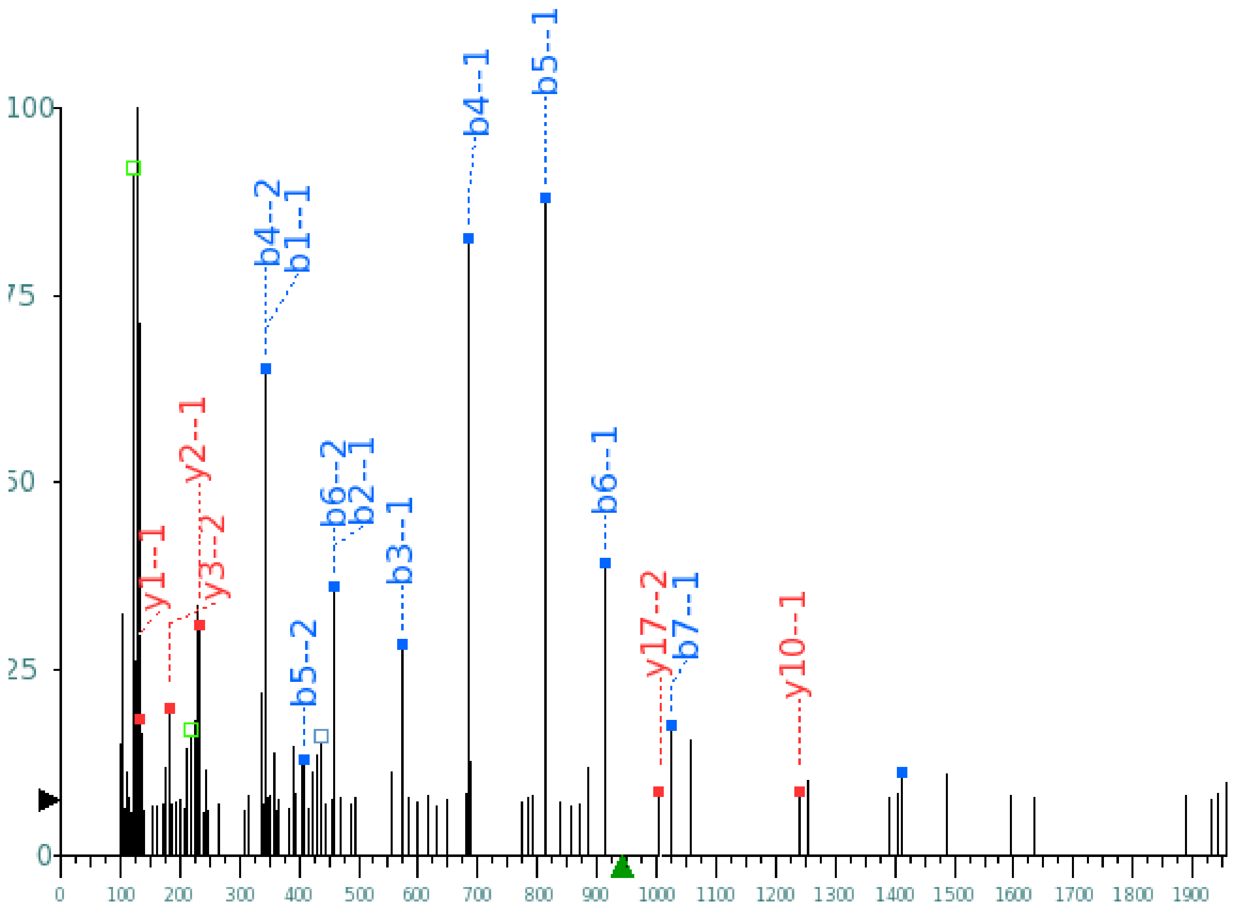

NLNDQVLFIDQGNRPLFEDMTD (+3)

Primary Reference: IL18_HUMAN
Search ID: 41761
Search Name: 20130328_ananiav_ERstress_TMT_TM_timecourse_trypsin_inj2_4mc_iaa
Scan#: 19859
Observed Mass: 941.8012 (2.4 ppm)
PSM Score: 26.56

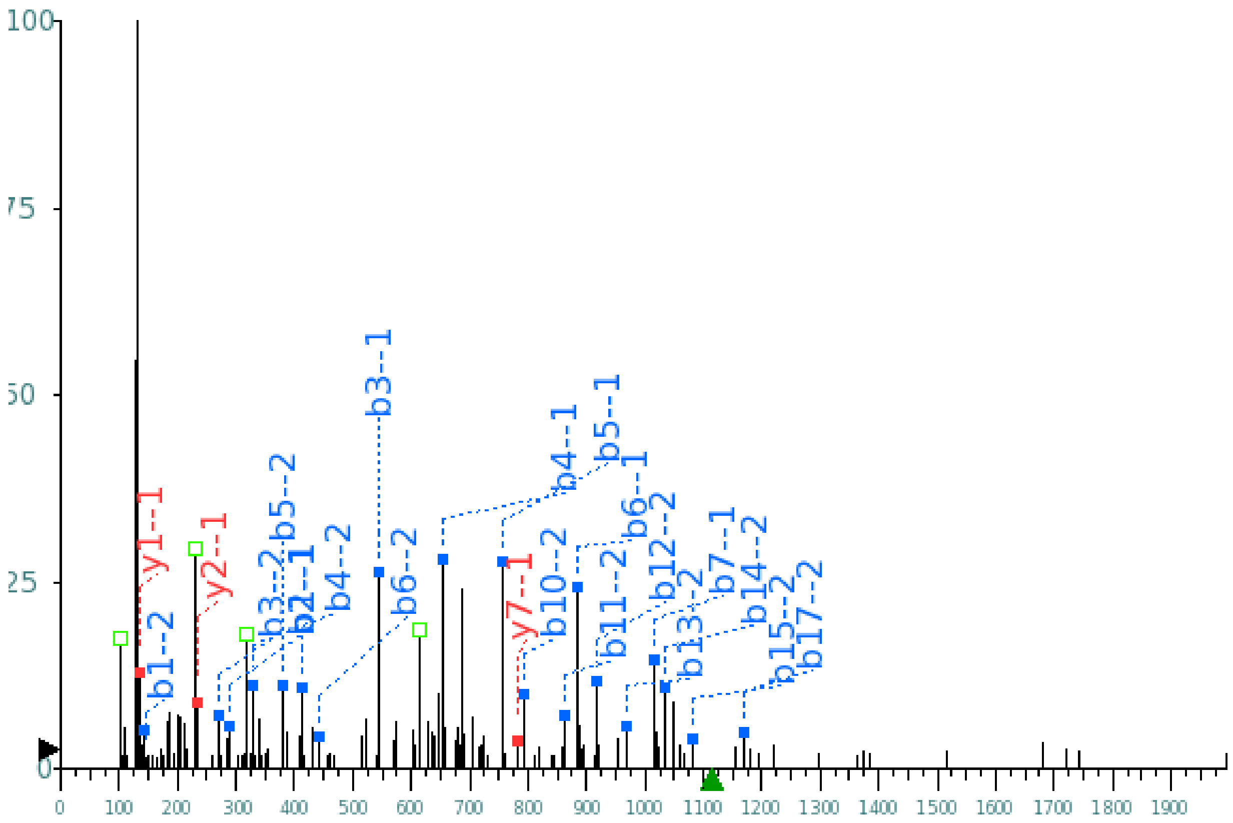

GQQLVMMPNKHNVEVLGILSDDVETD (+3)

Primary Reference: ERF3A_HUMAN
Search ID: 41761
Search Name: 20130328_ananiav_ERstress_TMT_TM_timecourse_trypsin_inj2_4mc_iaa
Scan#: 19897
Observed Mass: 1113.9176 (1.6 ppm)
PSM Score: 26.54

File name: 2015-02-04 04.01.53PM.pdf

Creation time: 2015-02-04 04:09PM

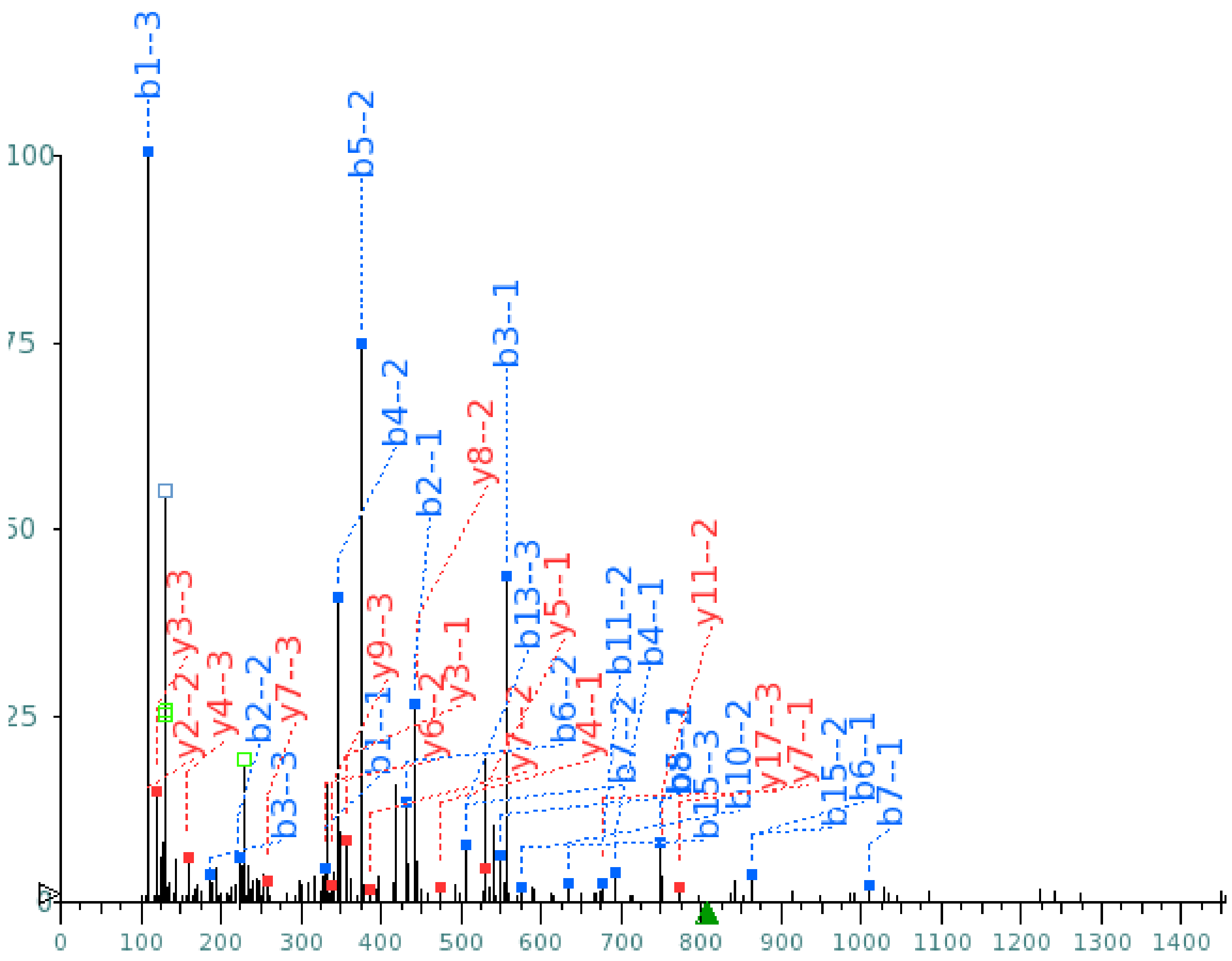

VNIHGIFSVASASVIEKQNLEGDHSD (+4)

Primary Reference: HS74L_HUMAN
Search ID: 41761
Search Name: 20130328_ananiav_ERstress_TMT_TM_timecourse_trypsin_inj2_4mc_iaa
Scan#: 19930
Observed Mass: 806.9318 (3.1 ppm)
PSM Score: 19.22

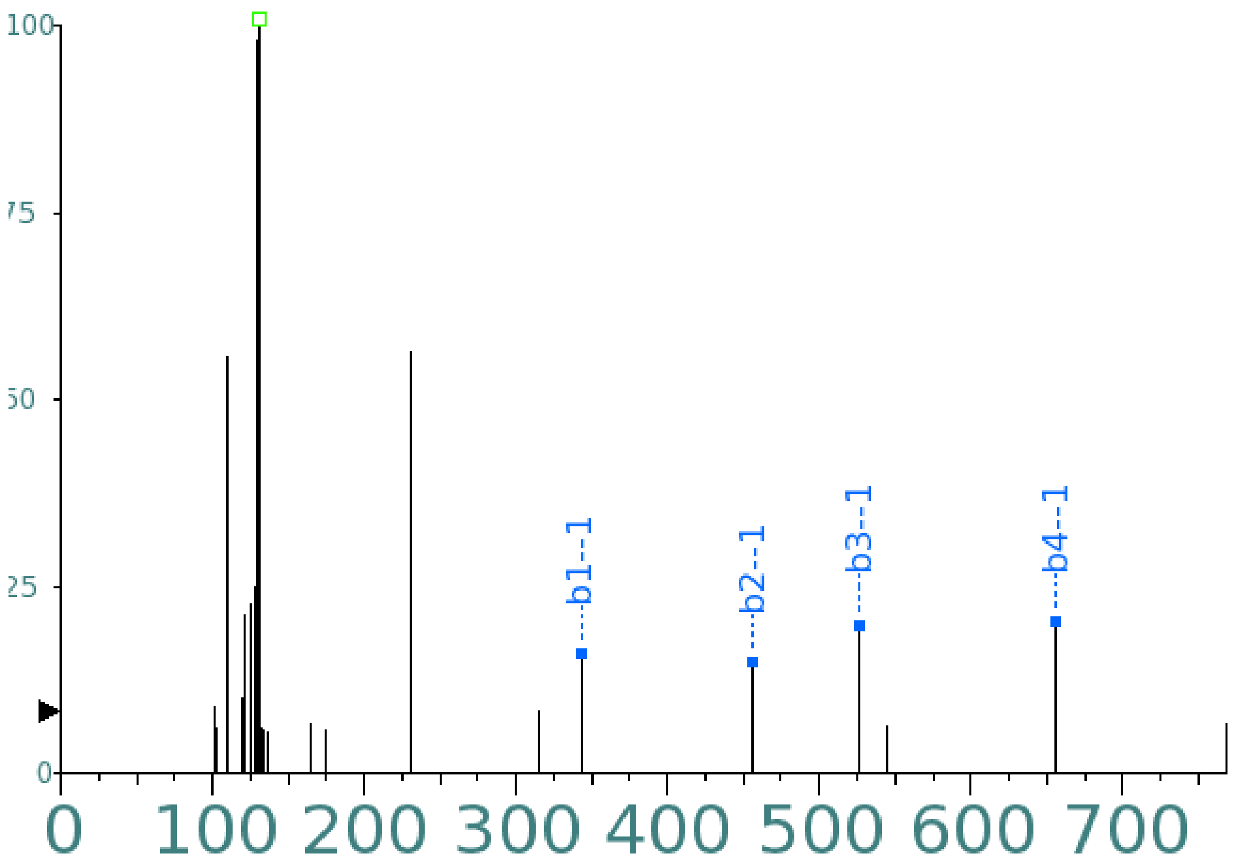

ILAQITGTEHLKESEAD (+3)

Primary Reference: PDLI5_HUMAN
Search ID: 41761
Search Name: 20130328_ananiav_ERstress_TMT_TM_timecourse_trypsin_inj2_4mc_iaa
Scan#: 19942
Observed Mass: 771.7626 (1.3 ppm)
PSM Score: 26.34

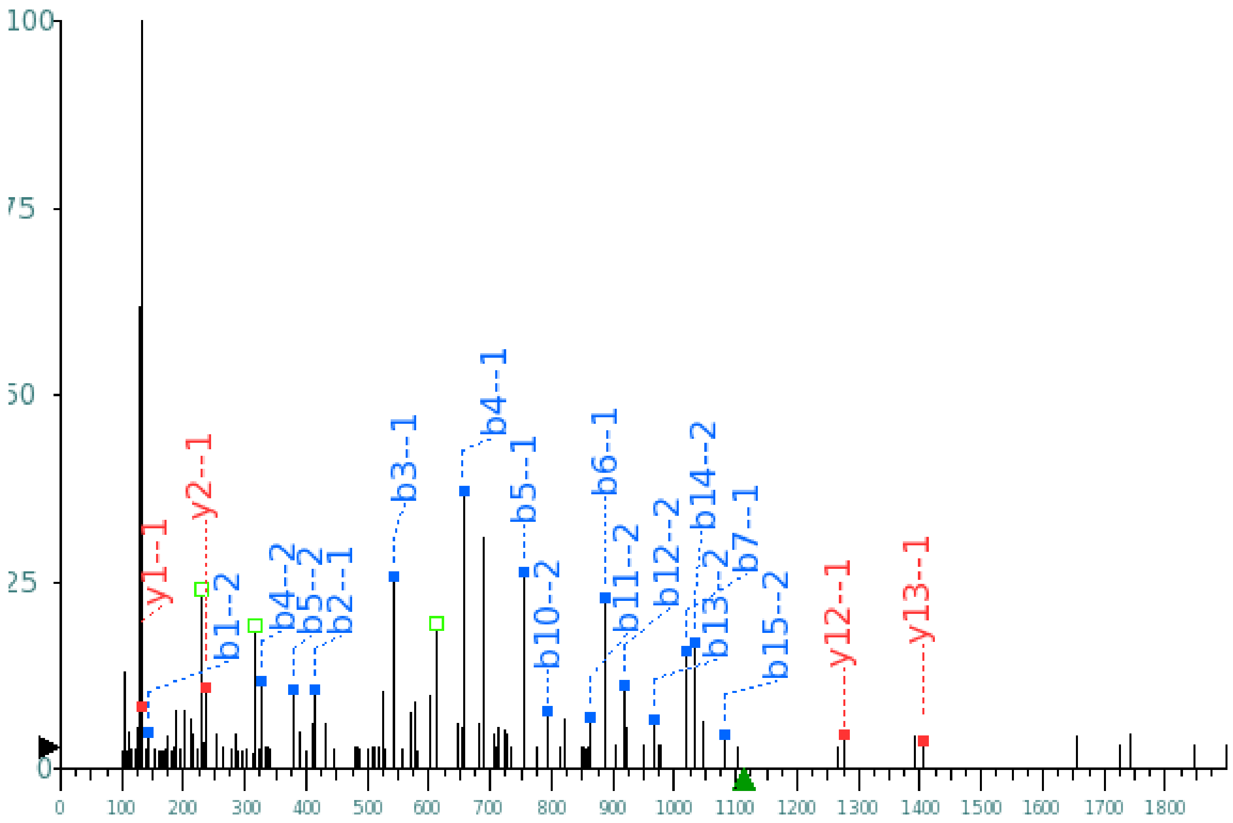

GQQLVMMPNKHNVEVLGILSDDVETD (+3)

Primary Reference: ERF3A_HUMAN
Search ID: 41761
Search Name: 20130328_ananiav_ERstress_TMT_TM_timecourse_trypsin_inj2_4mc_iaa
Scan#: 19960
Observed Mass: 1113.9168 (0.8 ppm)
PSM Score: 18.46

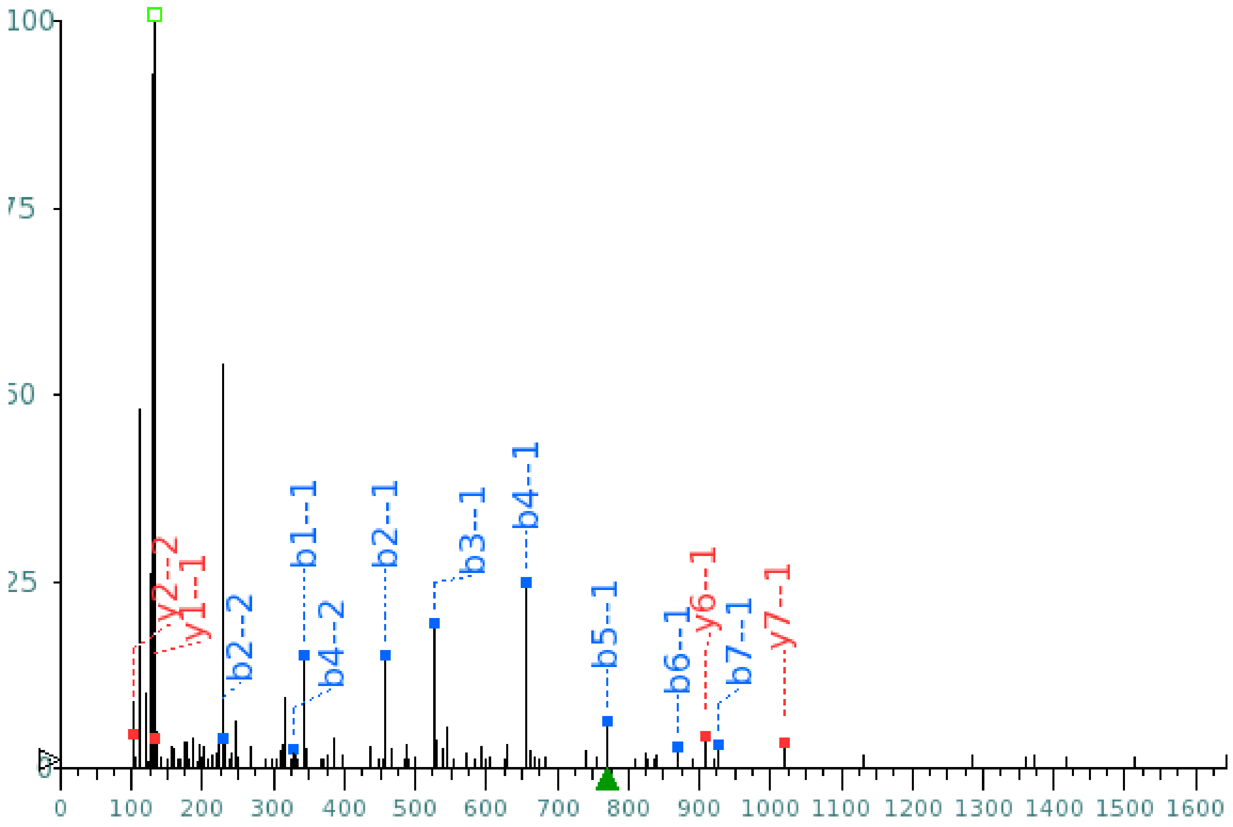

ILAQITGTEHLKESEAD (+3)

Primary Reference: PDLI5_HUMAN
Search ID: 41761
Search Name: 20130328_ananiav_ERstress_TMT_TM_timecourse_trypsin_inj2_4mc_iaa
Scan#: 20043
Observed Mass: 771.7639 (3 ppm)
PSM Score: 22.68

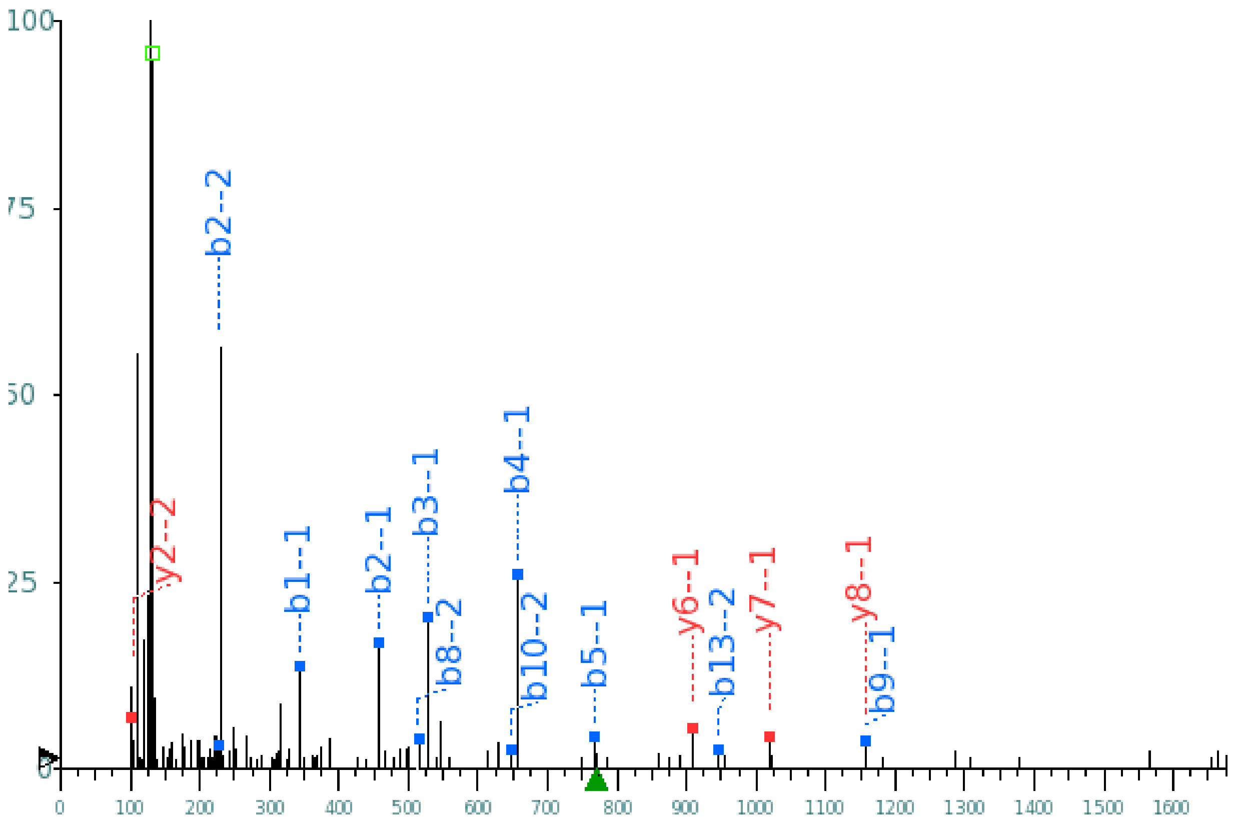

ILAQITGTEHLKESEAD (+3)

Primary Reference: PDLI5_HUMAN
Search ID: 41761
Search Name: 20130328_ananiav_ERstress_TMT_TM_timecourse_trypsin_inj2_4mc_iaa
Scan#: 20109
Observed Mass: 771.7643 (3.5 ppm)
PSM Score: 24.42

File name: 2015-02-04 04.01.53PM.pdf

Creation time: 2015-02-04 04:09PM

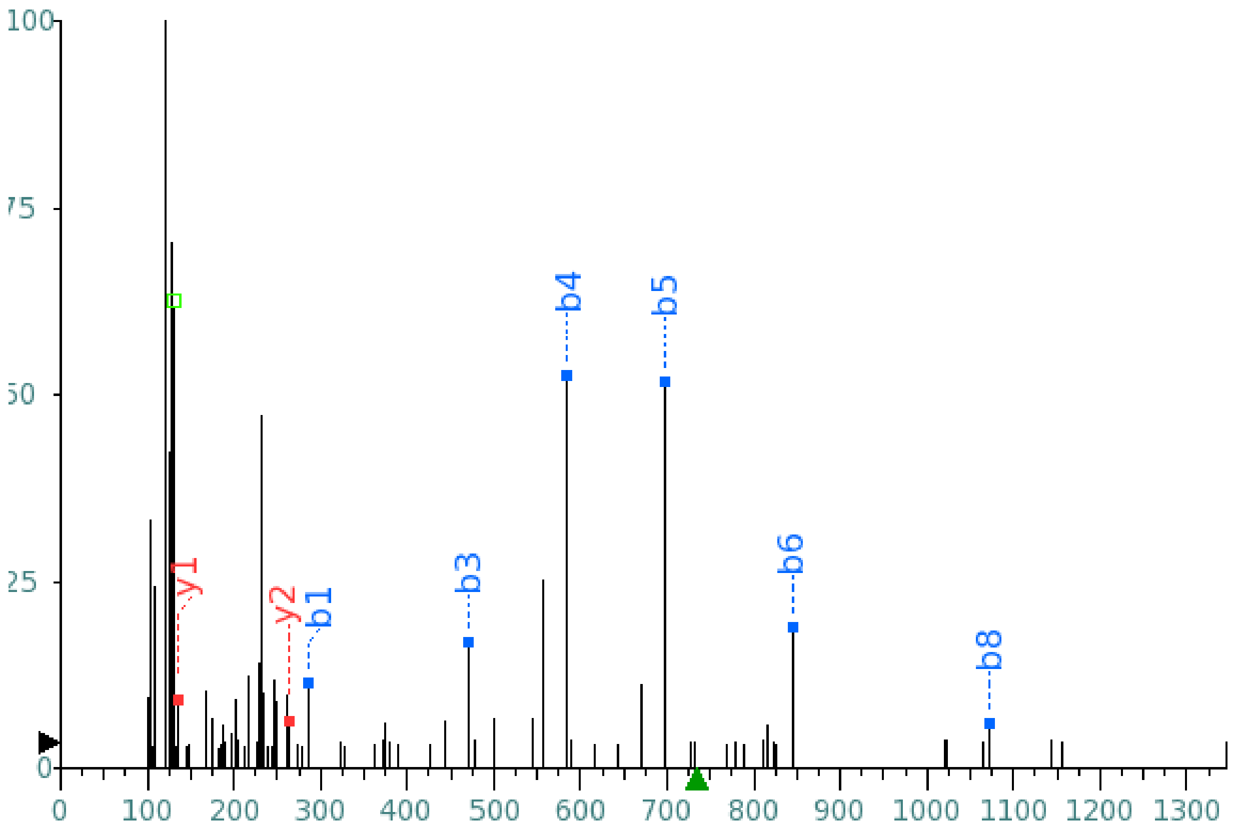

GPSLLFLDEMD (+2)

Primary Reference: SPA5L_HUMAN
Search ID: 41761
Search Name: 20130328_ananiav_ERstress_TMT_TM_timecourse_trypsin_inj2_4mc_iaa
Scan#: 20111
Observed Mass: 733.3776 (2.3 ppm)
PSM Score: 21.42

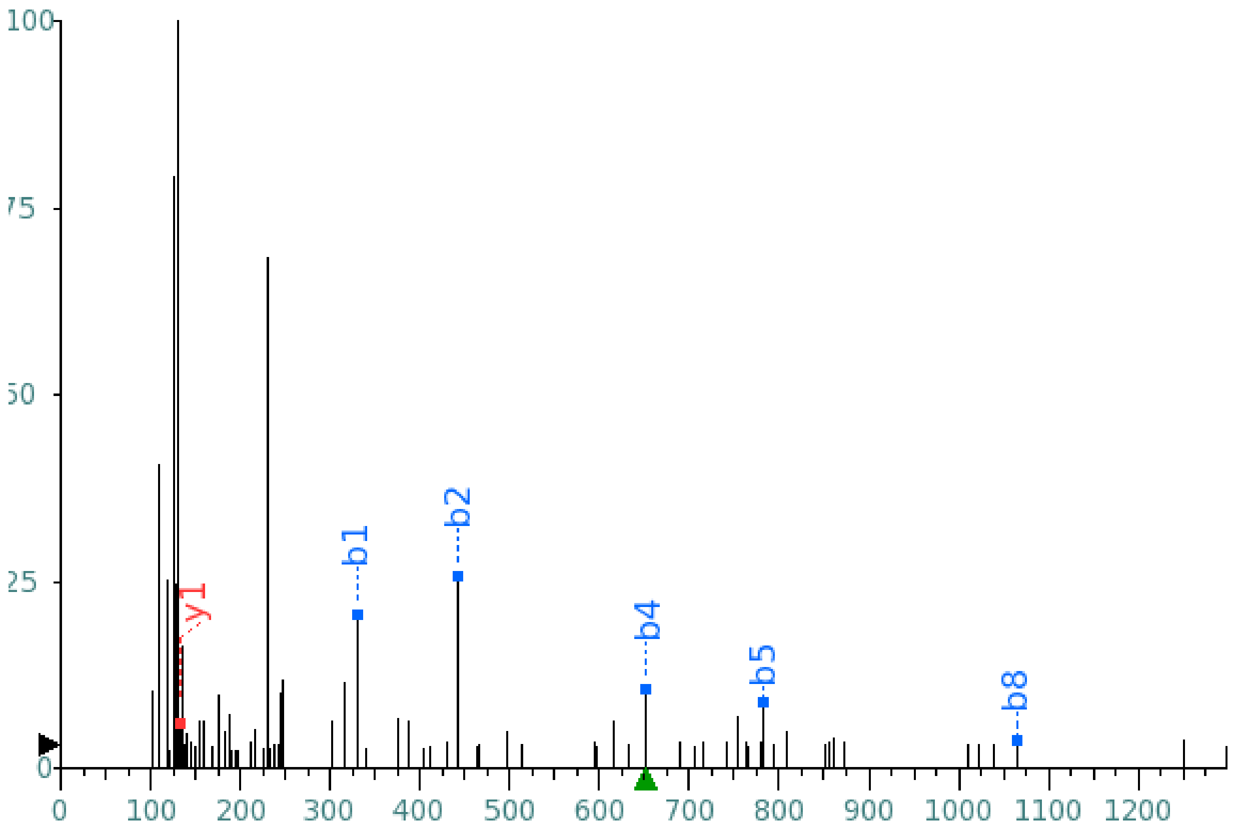

VLPLEALVTD (+2)

Primary Reference: GCN1L_HUMAN
Search ID: 41761
Search Name: 20130328_ananiav_ERstress_TMT_TM_timecourse_trypsin_inj2_4mc_iaa
Scan#: 20145
Observed Mass: 649.8937 (2.5 ppm)
PSM Score: 10.36

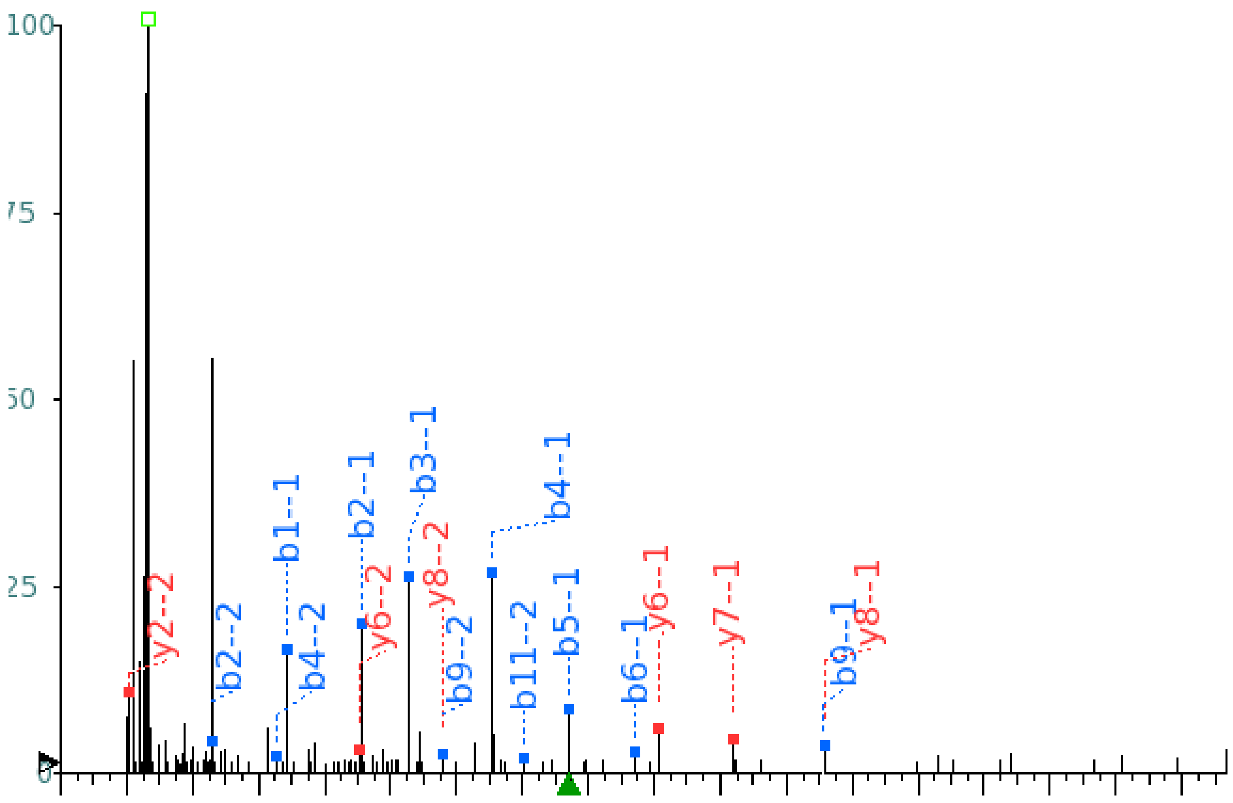

ILAQITGTEHLKESEAD (+3)

Primary Reference: PDLI5_HUMAN
Search ID: 41761
Search Name: 20130328_ananiav_ERstress_TMT_TM_timecourse_trypsin_inj2_4mc_iaa
Scan#: 20193
Observed Mass: 771.7639 (3.1 ppm)
PSM Score: 31.83

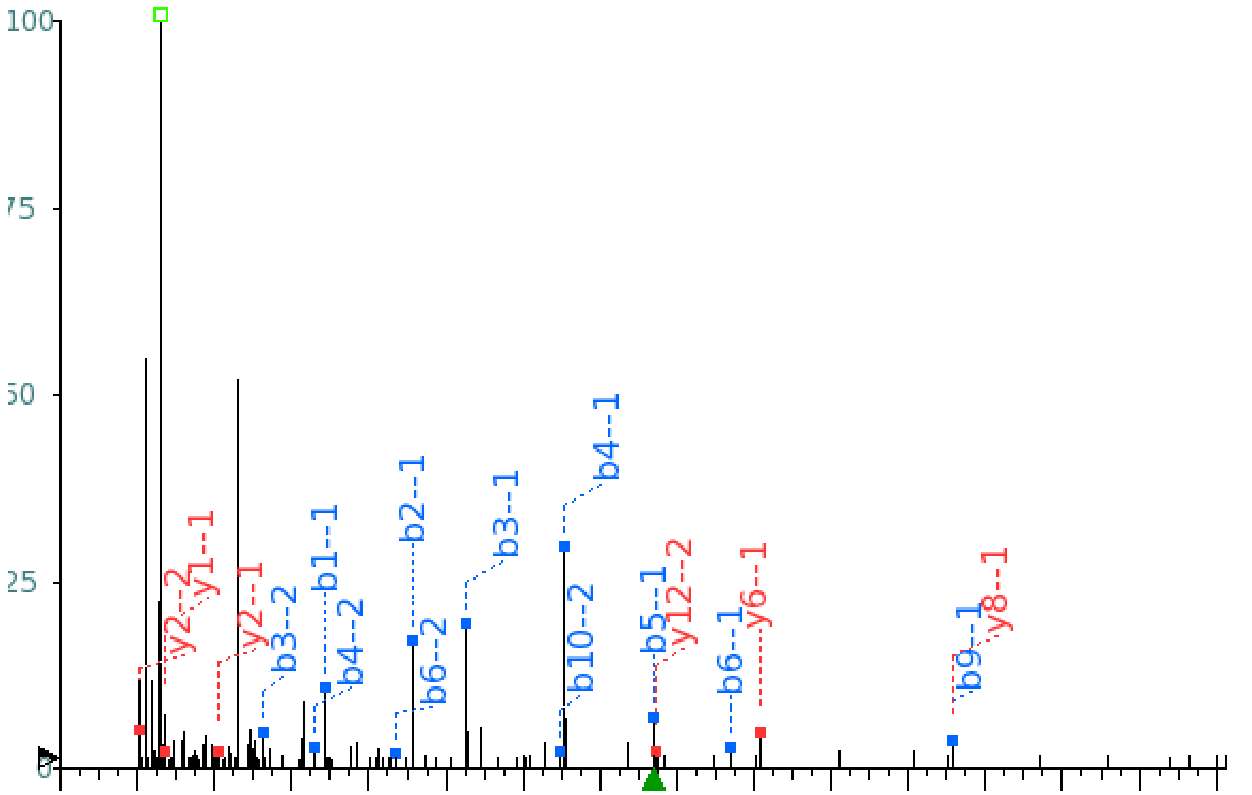

ILAQITGTEHLKESEAD (+3)

Primary Reference: PDLI5_HUMAN
Search ID: 41761
Search Name: 20130328_ananiav_ERstress_TMT_TM_timecourse_trypsin_inj2_4mc_iaa
Scan#: 20259
Observed Mass: 771.7654 (4.9 ppm)
PSM Score: 32.59

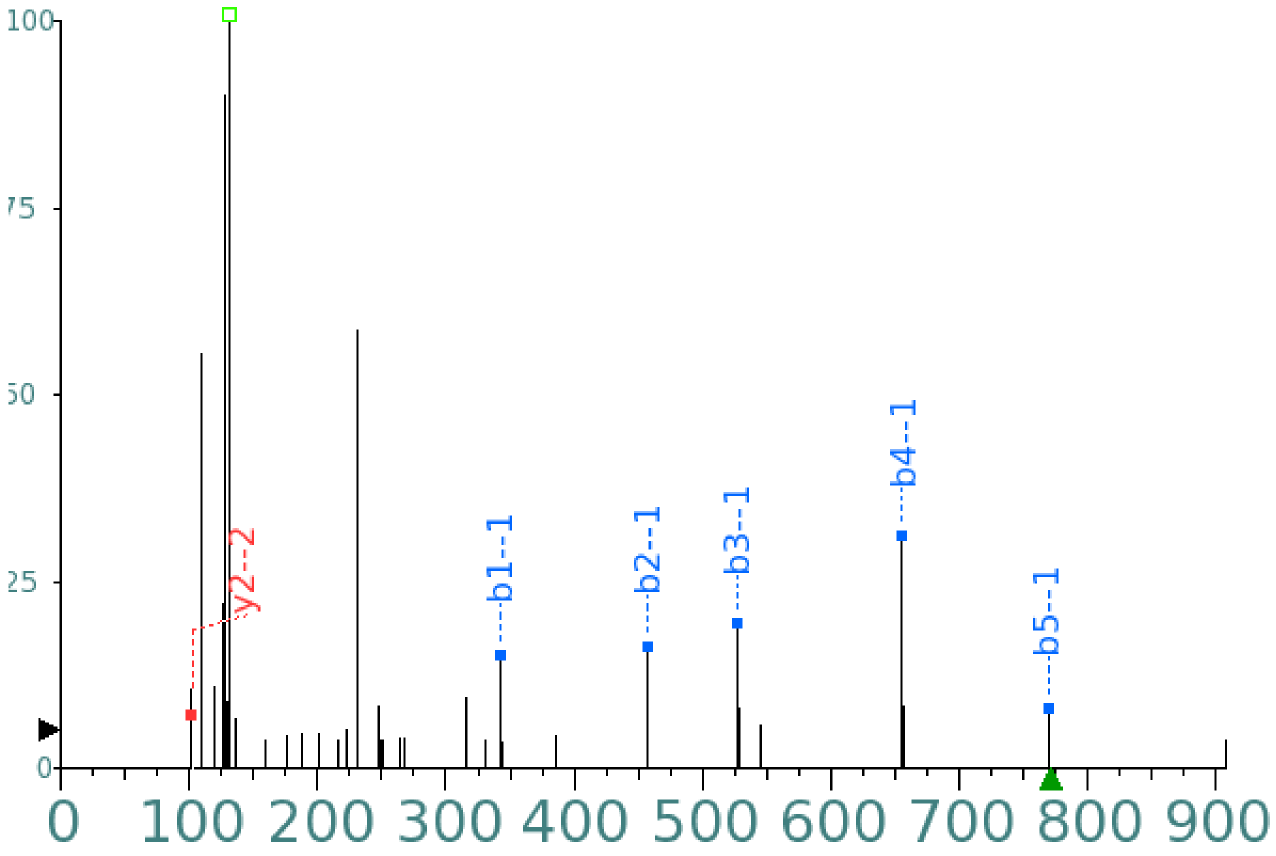

ILAQITGTEHLKESEAD (+3)

Primary Reference: PDLI5_HUMAN
Search ID: 41761
Search Name: 20130328_ananiav_ERstress_TMT_TM_timecourse_trypsin_inj2_4mc_iaa
Scan#: 20283
Observed Mass: 771.7651 (4.6 ppm)
PSM Score: 24

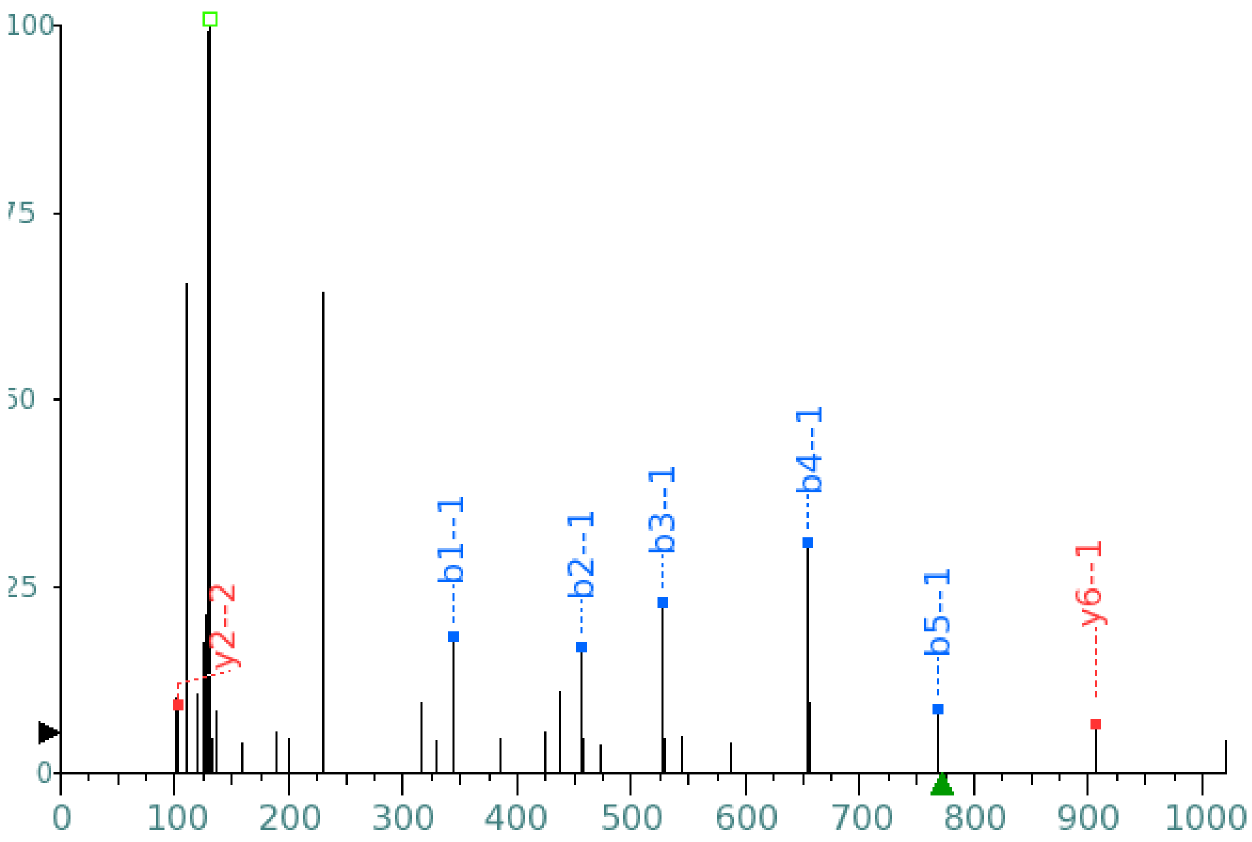

ILAQITGTEHLKESEAD (+3)

Primary Reference: PDLI5_HUMAN
Search ID: 41761
Search Name: 20130328_ananiav_ERstress_TMT_TM_timecourse_trypsin_inj2_4mc_iaa
Scan#: 20294
Observed Mass: 771.764 (3.1 ppm)
PSM Score: 28.18

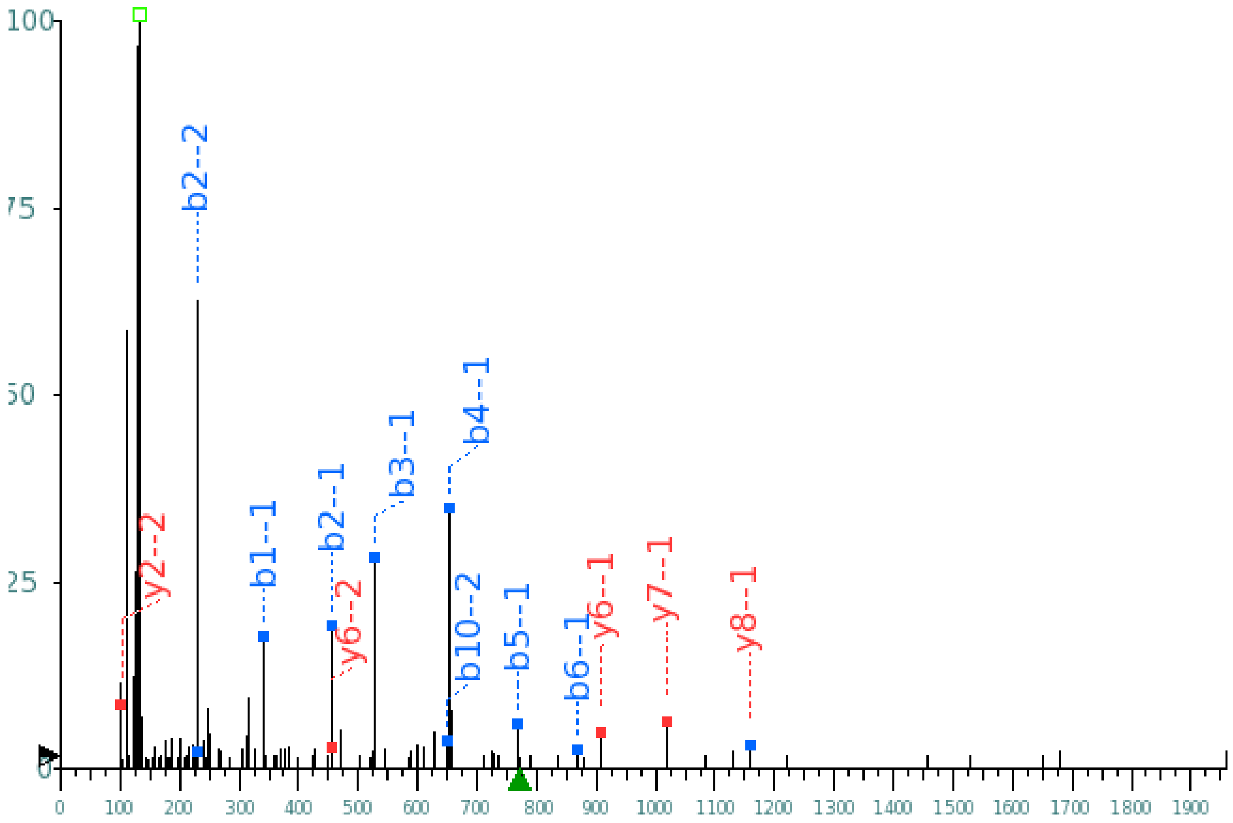

ILAQITGTEHLKESEAD (+3)

Primary Reference: PDLI5_HUMAN
Search ID: 41761
Search Name: 20130328_ananiav_ERstress_TMT_TM_timecourse_trypsin_inj2_4mc_iaa
Scan#: 20312
Observed Mass: 771.7636 (2.6 ppm)
PSM Score: 31.15

File name: 2015-02-04 04.01.53PM.pdf

Creation time: 2015-02-04 04:09PM

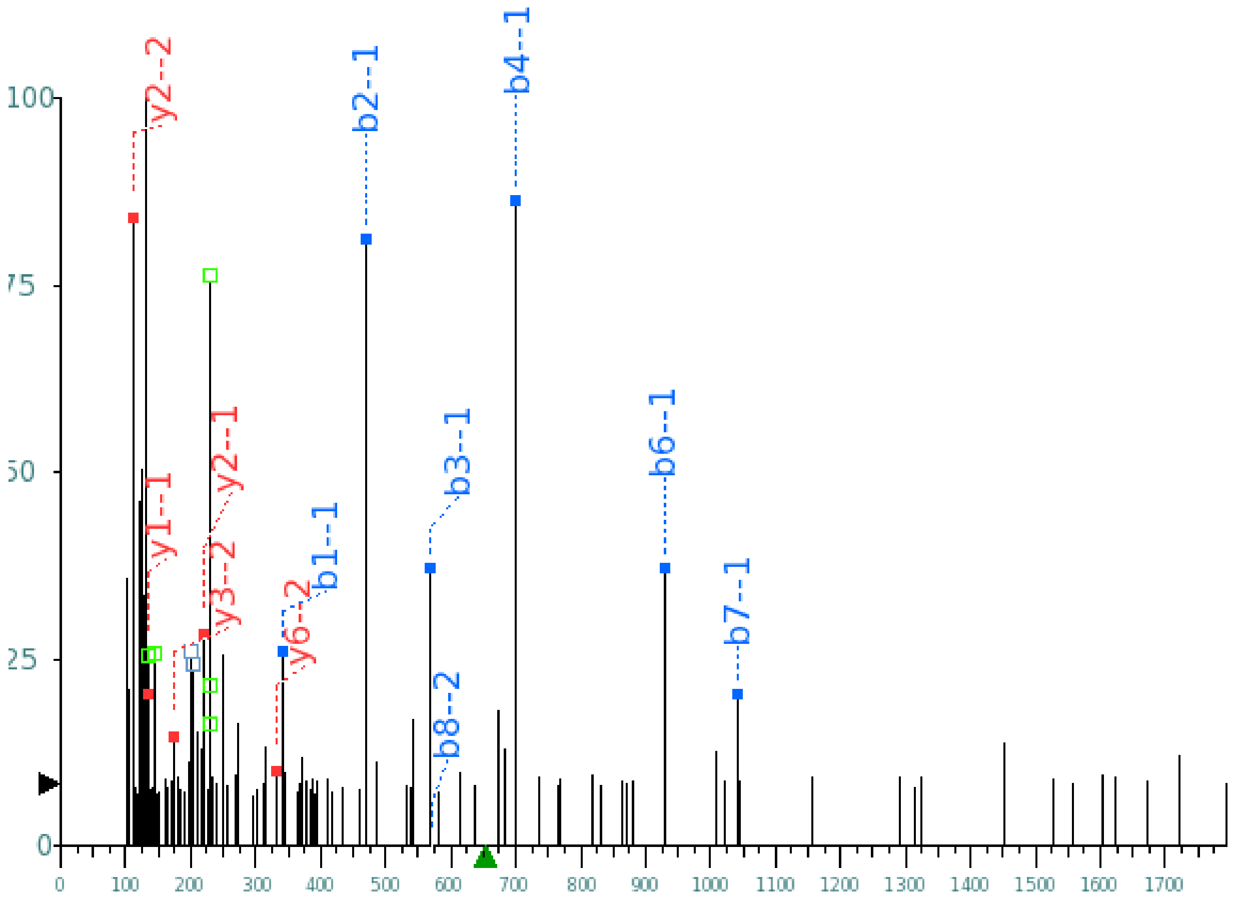

LQVELDNVTGLLSQSD (+3)

Primary Reference: MYH9_HUMAN
Search ID: 41761
Search Name: 20130328_ananiav_ERstress_TMT_TM_timecourse_trypsin_inj2_4mc_iaa
Scan#: 20343
Observed Mass: 654.0212 (2.8 ppm)
PSM Score: 22.26

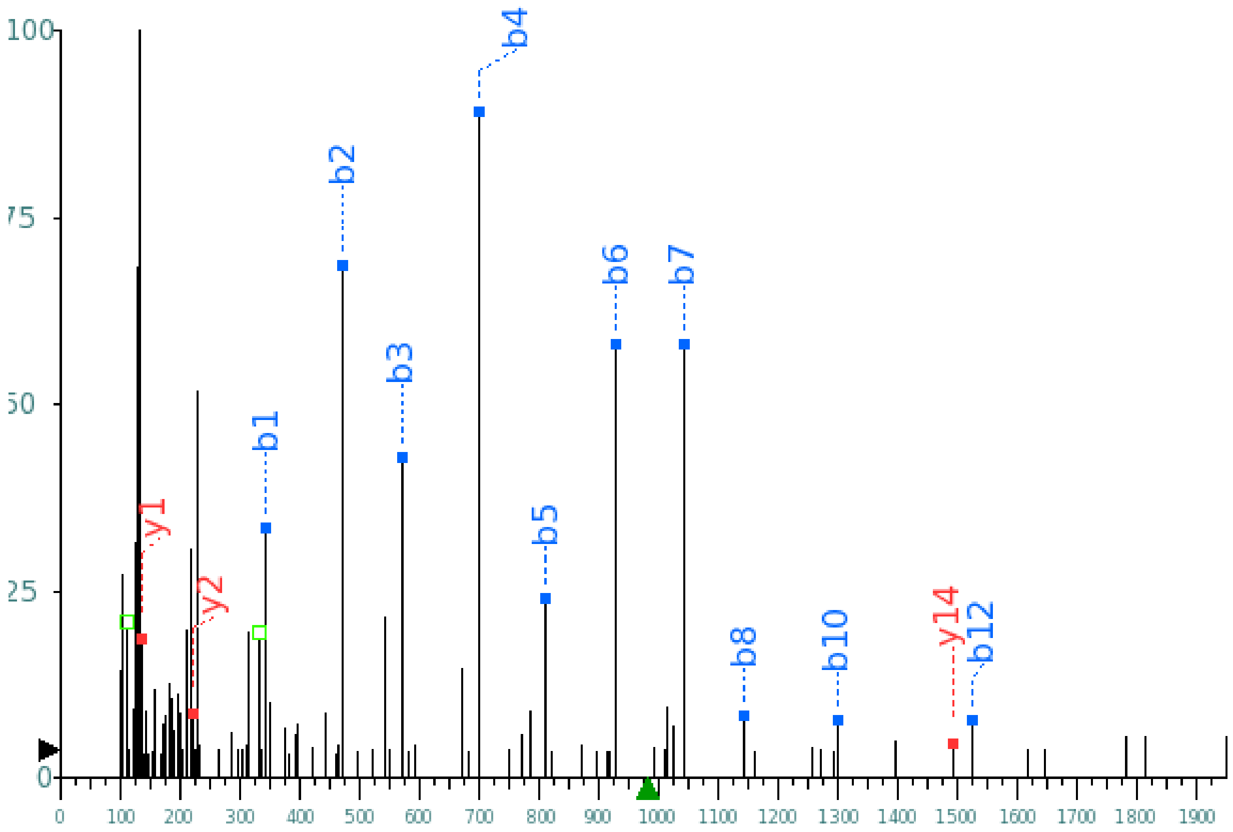

LQVELDNVTGLLSQSD (+2)

Primary Reference: MYH9_HUMAN
Search ID: 41761
Search Name: 20130328_ananiav_ERstress_TMT_TM_timecourse_trypsin_inj2_4mc_iaa
Scan#: 20347
Observed Mass: 980.5283 (2.9 ppm)
PSM Score: 64.67

File name: 2015-02-04 04.01.53PM.pdf

Creation time: 2015-02-04 04:09PM

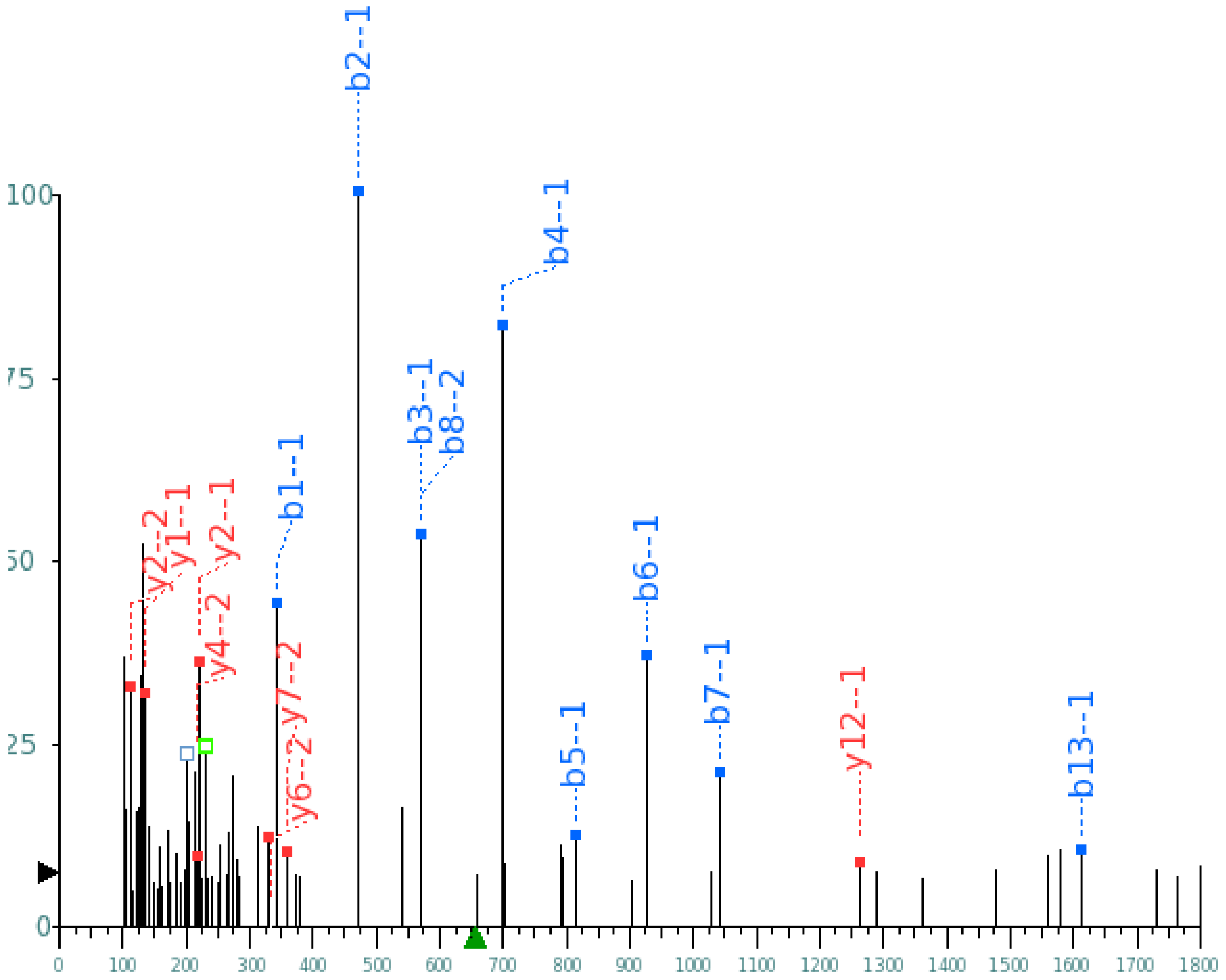

LQVELDNVTGLLSQSD (+3)

Primary Reference: MYH9_HUMAN
Search ID: 41761
Search Name: 20130328_ananiav_ERstress_TMT_TM_timecourse_trypsin_inj2_4mc_iaa
Scan#: 20408
Observed Mass: 654.0213 (2.9 ppm)
PSM Score: 52.37

File name: 2015-02-04 04.01.53PM.pdf

Creation time: 2015-02-04 04:09PM

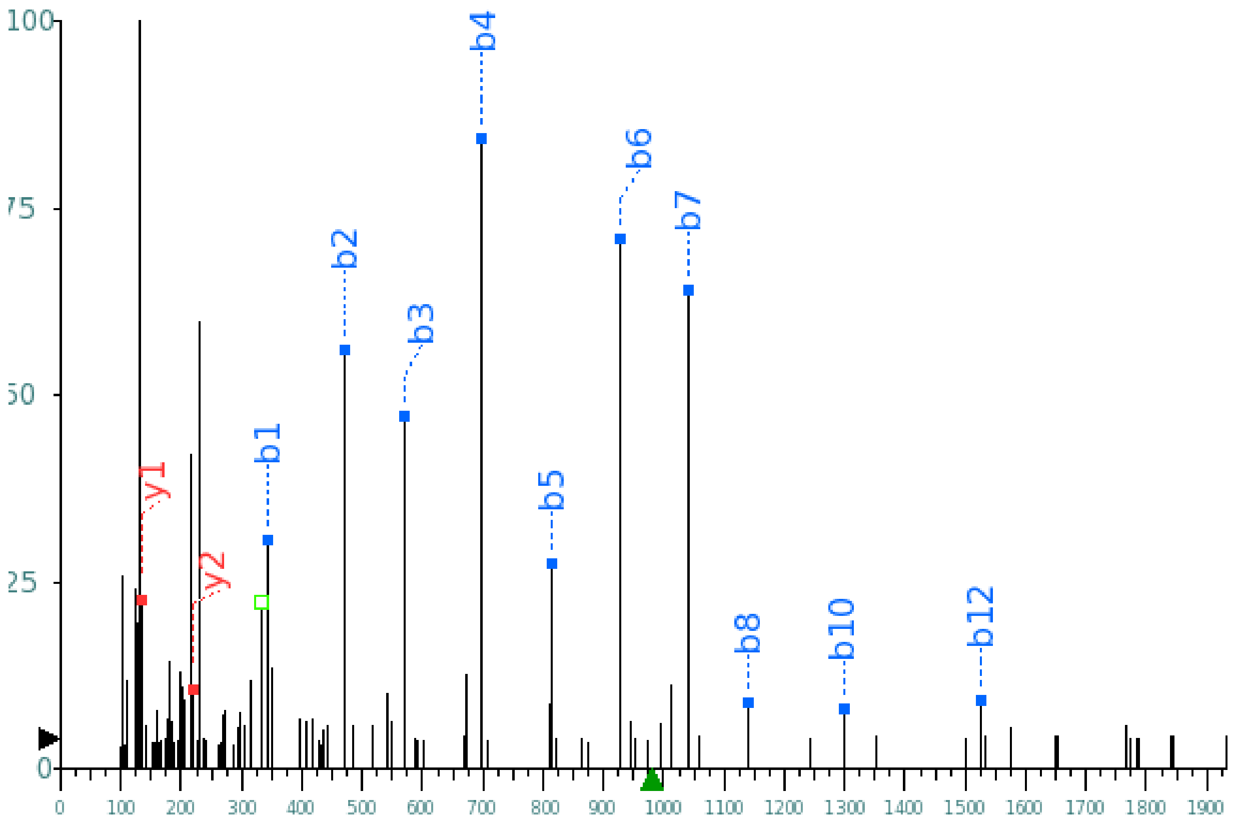

LQVELDNVTGLLSQSD (+2)

Primary Reference: MYH9_HUMAN
Search ID: 41761
Search Name: 20130328_ananiav_ERstress_TMT_TM_timecourse_trypsin_inj2_4mc_iaa
Scan#: 20409
Observed Mass: 980.5281 (2.7 ppm)
PSM Score: 70.98

File name: 2015-02-04 04.01.53PM.pdf

Creation time: 2015-02-04 04:09PM

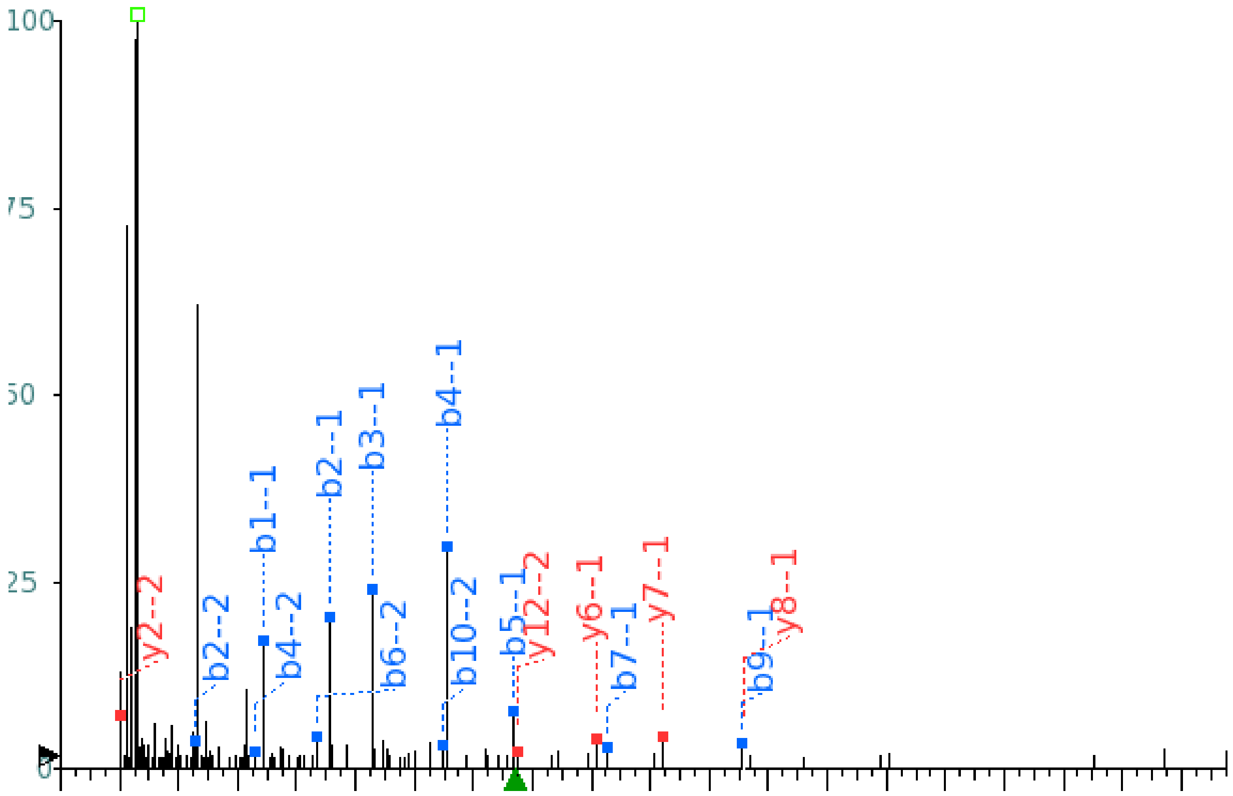

ILAQITGTEHLKESEAD (+3)

Primary Reference: PDLI5_HUMAN
Search ID: 41761
Search Name: 20130328_ananiav_ERstress_TMT_TM_timecourse_trypsin_inj2_4mc_iaa
Scan#: 20427
Observed Mass: 771.7634 (2.4 ppm)
PSM Score: 23.67

File name: 2015-02-04 04.01.53PM.pdf

Creation time: 2015-02-04 04:09PM

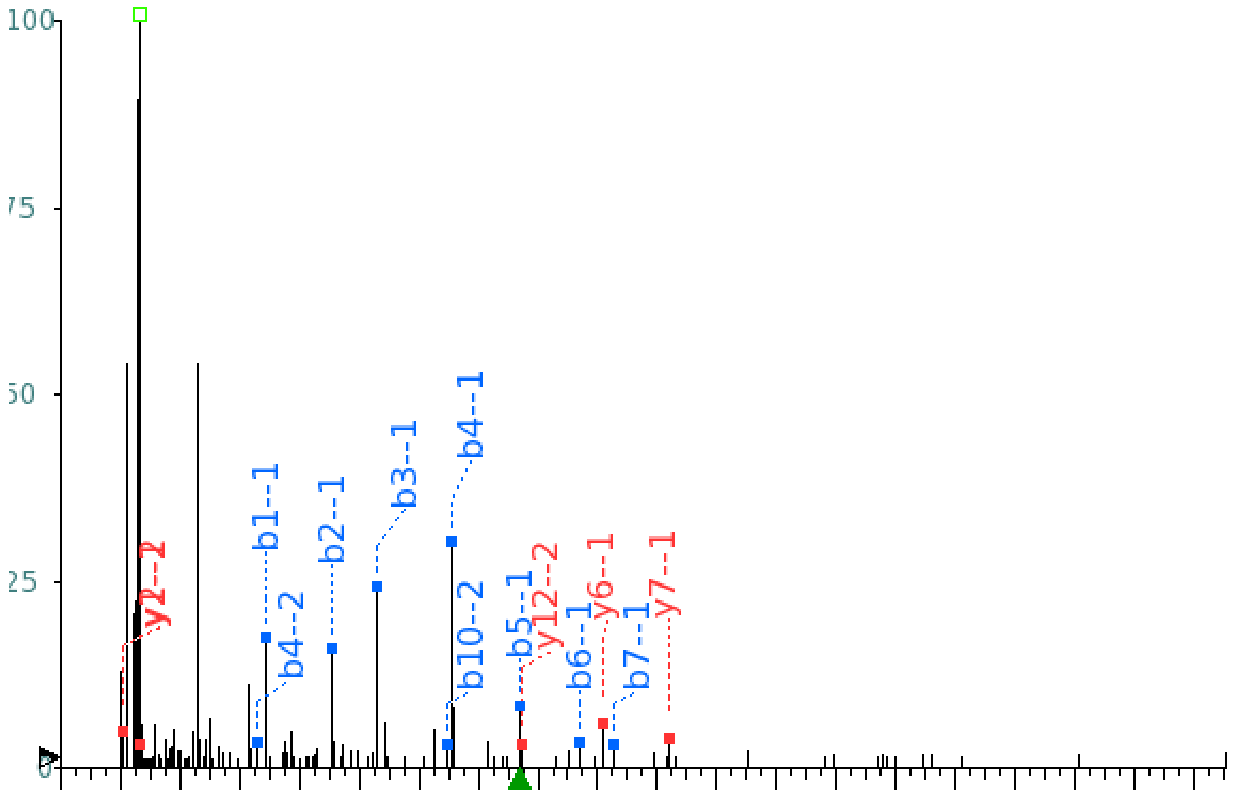

ILAQITGTEHLKESEAD (+3)

Primary Reference: PDLI5_HUMAN
Search ID: 41761
Search Name: 20130328_ananiav_ERstress_TMT_TM_timecourse_trypsin_inj2_4mc_iaa
Scan#: 20438
Observed Mass: 771.7618 (0.3 ppm)
PSM Score: 23.67

File name: 2015-02-04 04.01.53PM.pdf

Creation time: 2015-02-04 04:09PM

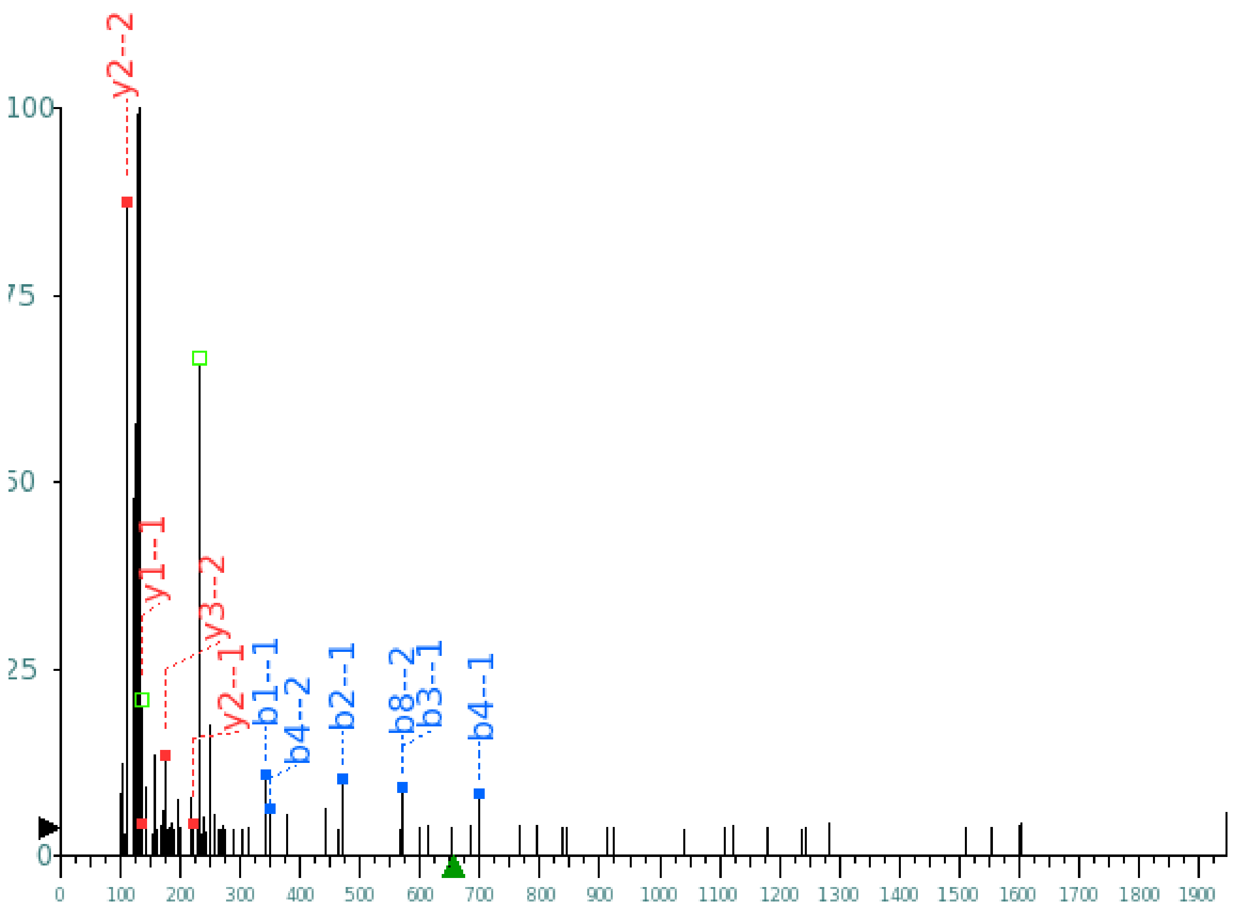

LQVELDNVTGLLSQSD (+3)

Primary Reference: MYH9_HUMAN
Search ID: 41761
Search Name: 20130328_ananiav_ERstress_TMT_TM_timecourse_trypsin_inj2_4mc_iaa
Scan#: 20501
Observed Mass: 654.021 (2.5 ppm)
PSM Score: 3.9

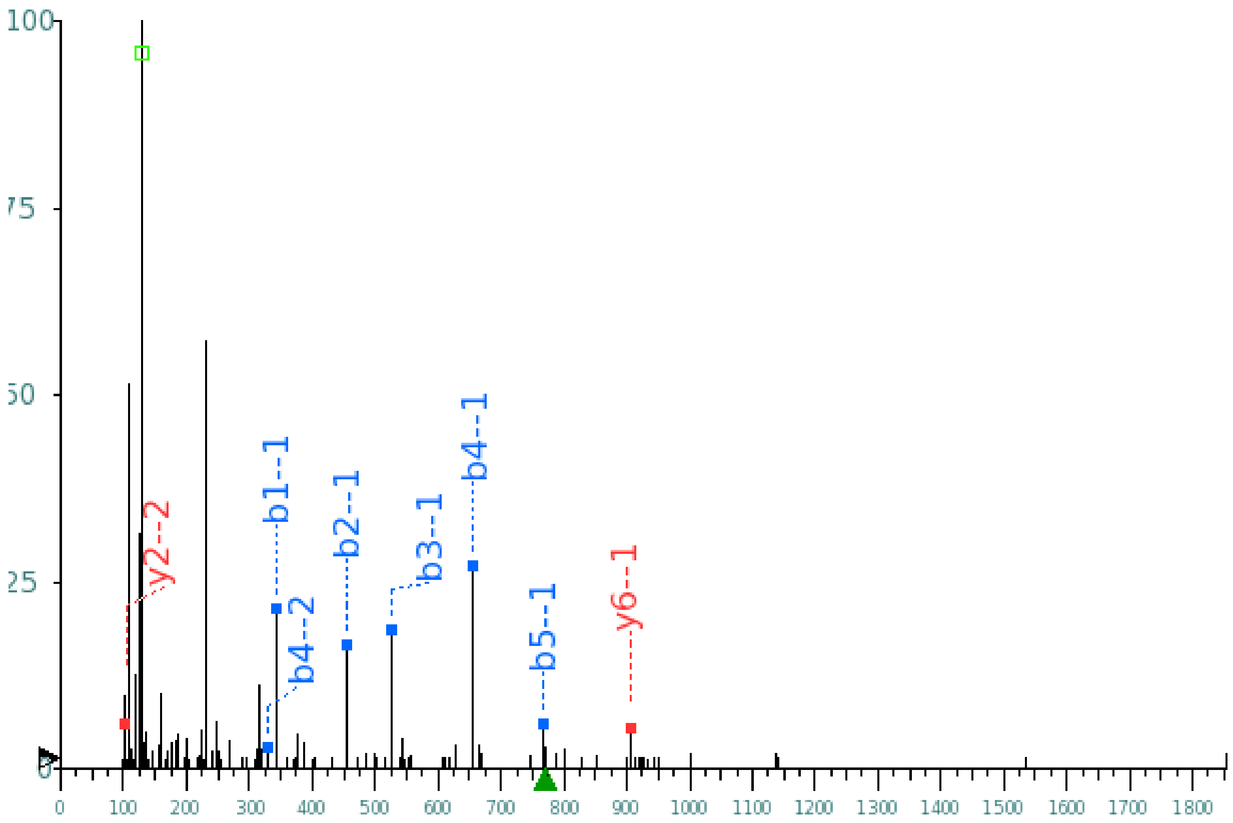

ILAQITGTEHLKESEAD (+3)

Primary Reference: PDLI5_HUMAN
Search ID: 41761
Search Name: 20130328_ananiav_ERstress_TMT_TM_timecourse_trypsin_inj2_4mc_iaa
Scan#: 20548
Observed Mass: 771.7627 (1.4 ppm)
PSM Score: 15.92

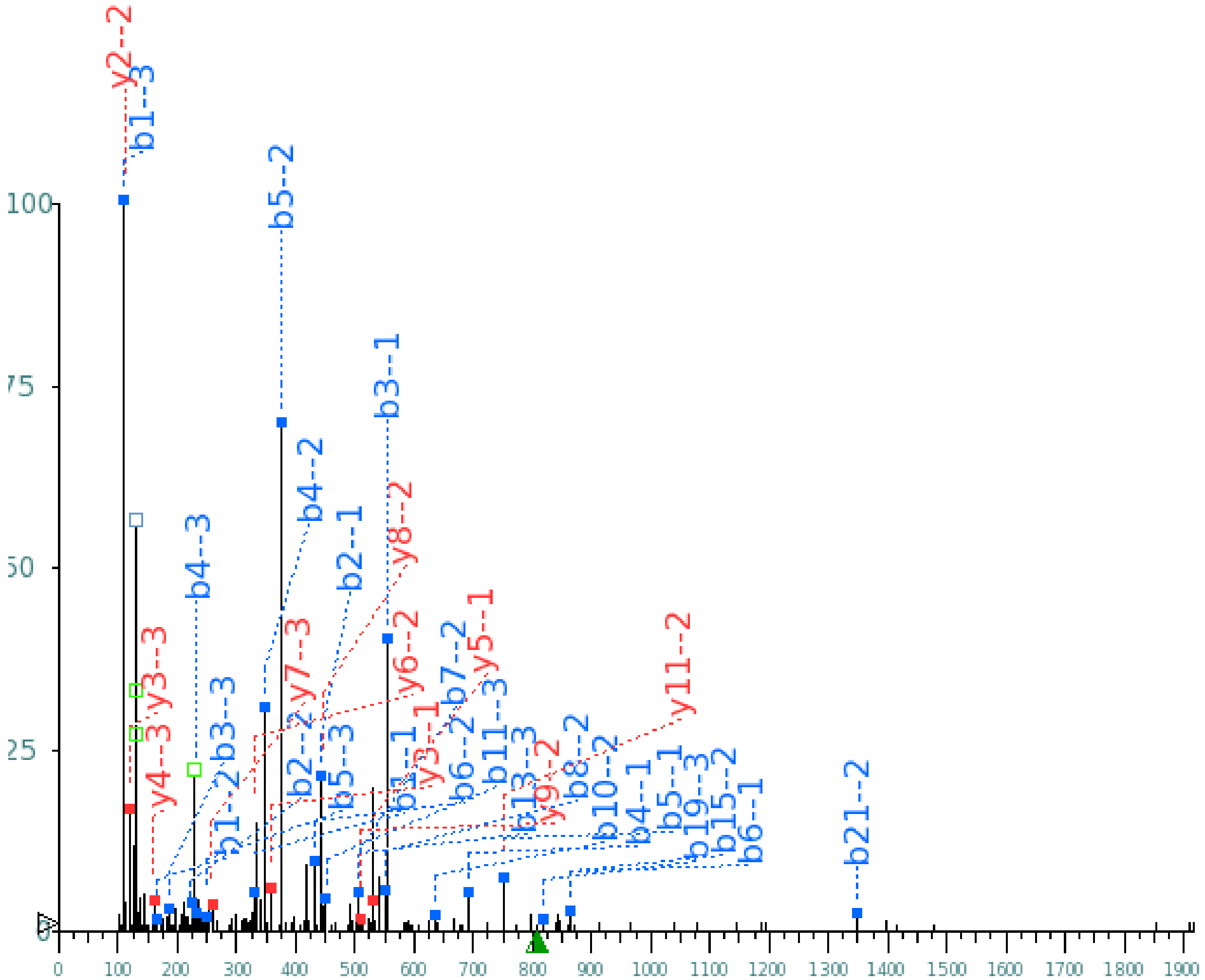

VNIHGIFSVASASVIEKQNLEGDHSD (+4)

Primary Reference: HS74L_HUMAN
Search ID: 41761
Search Name: 20130328_ananiav_ERstress_TMT_TM_timecourse_trypsin_inj2_4mc_iaa
Scan#: 20565
Observed Mass: 806.9324 (3.9 ppm)
PSM Score: 25.17

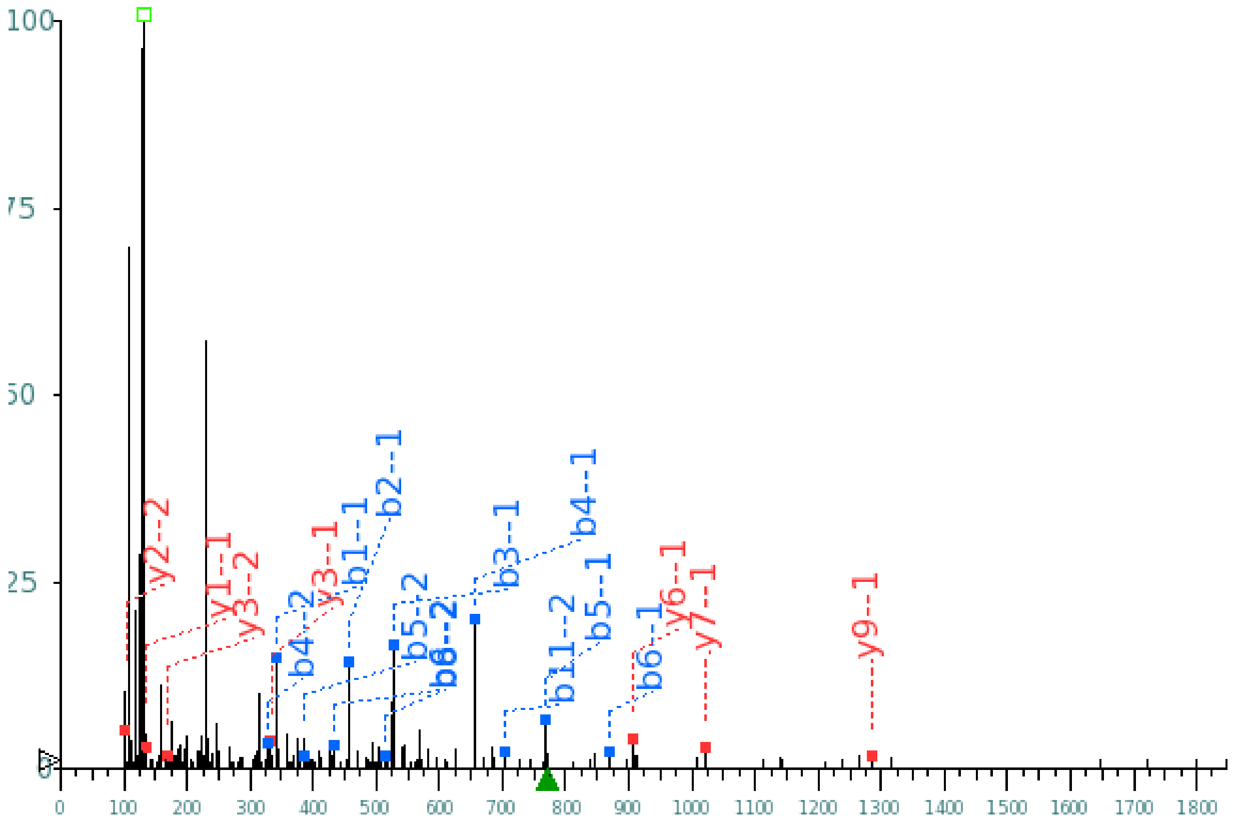

ILAQITGTEHLKESEAD (+3)

Primary Reference: PDLI5_HUMAN
Search ID: 41761
Search Name: 20130328_ananiav_ERstress_TMT_TM_timecourse_trypsin_inj2_4mc_iaa
Scan#: 20647
Observed Mass: 771.7604 (-1.6 ppm)
PSM Score: 14.67

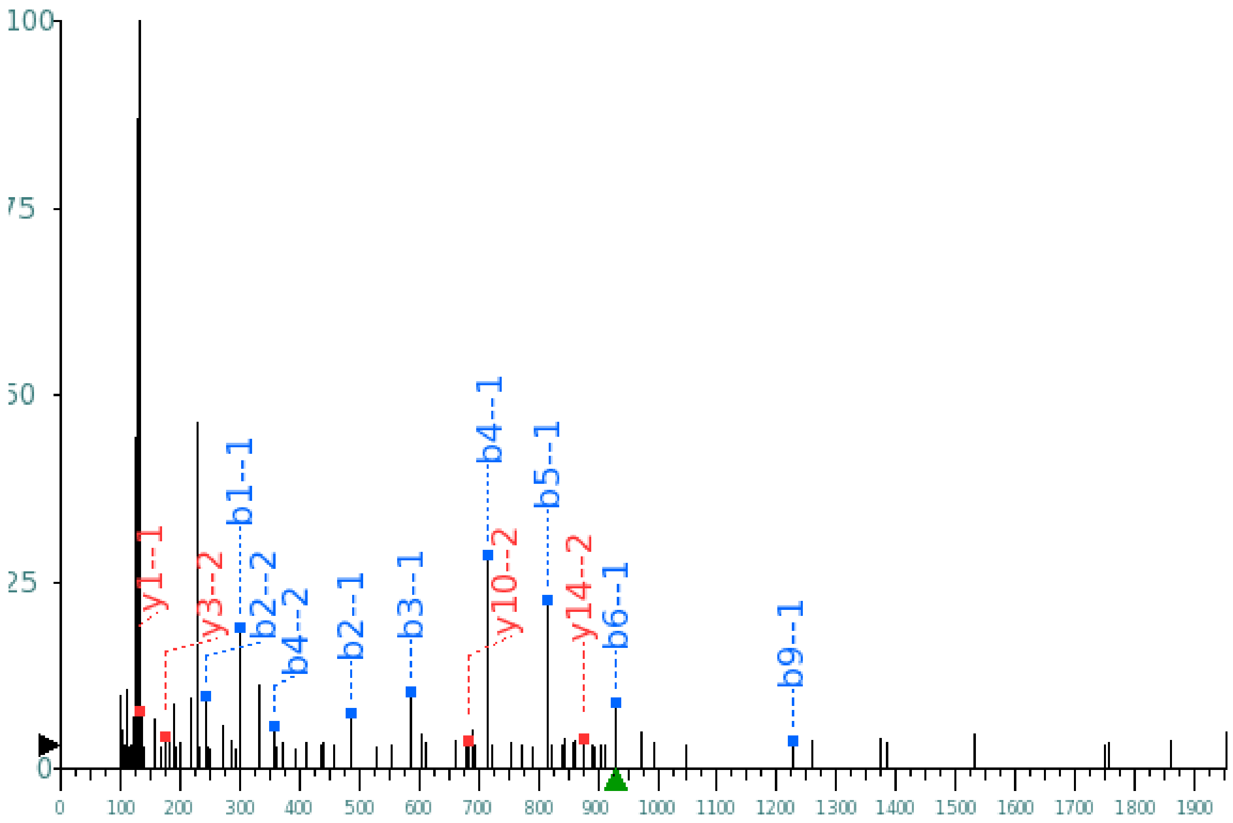

AWTQVLLGQAEDKAEVDMDTD (+3)

Primary Reference: DYHC1_HUMAN
Search ID: 41761
Search Name: 20130328_ananiav_ERstress_TMT_TM_timecourse_trypsin_inj2_4mc_iaa
Scan#: 20728
Observed Mass: 931.8067 (1.4 ppm)
PSM Score: 19.09

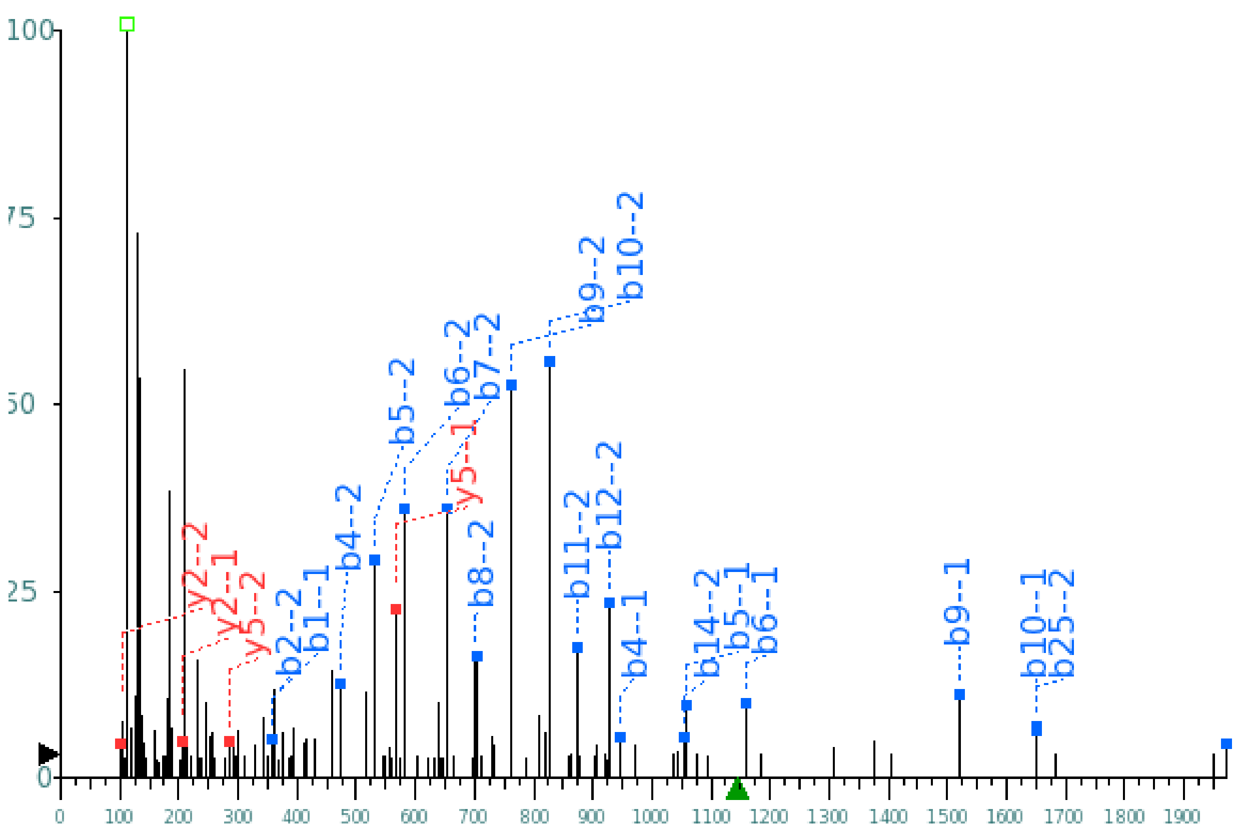

QKTENVFVDEPLIHATTYIPLMDNAD (+3)

Primary Reference: KTN1_HUMAN
Search ID: 41761
Search Name: 20130328_ananiav_ERstress_TMT_TM_timecourse_trypsin_inj2_4mc_iaa
Scan#: 20849
Observed Mass: 1144.9314 (1 ppm)
PSM Score: 49.11

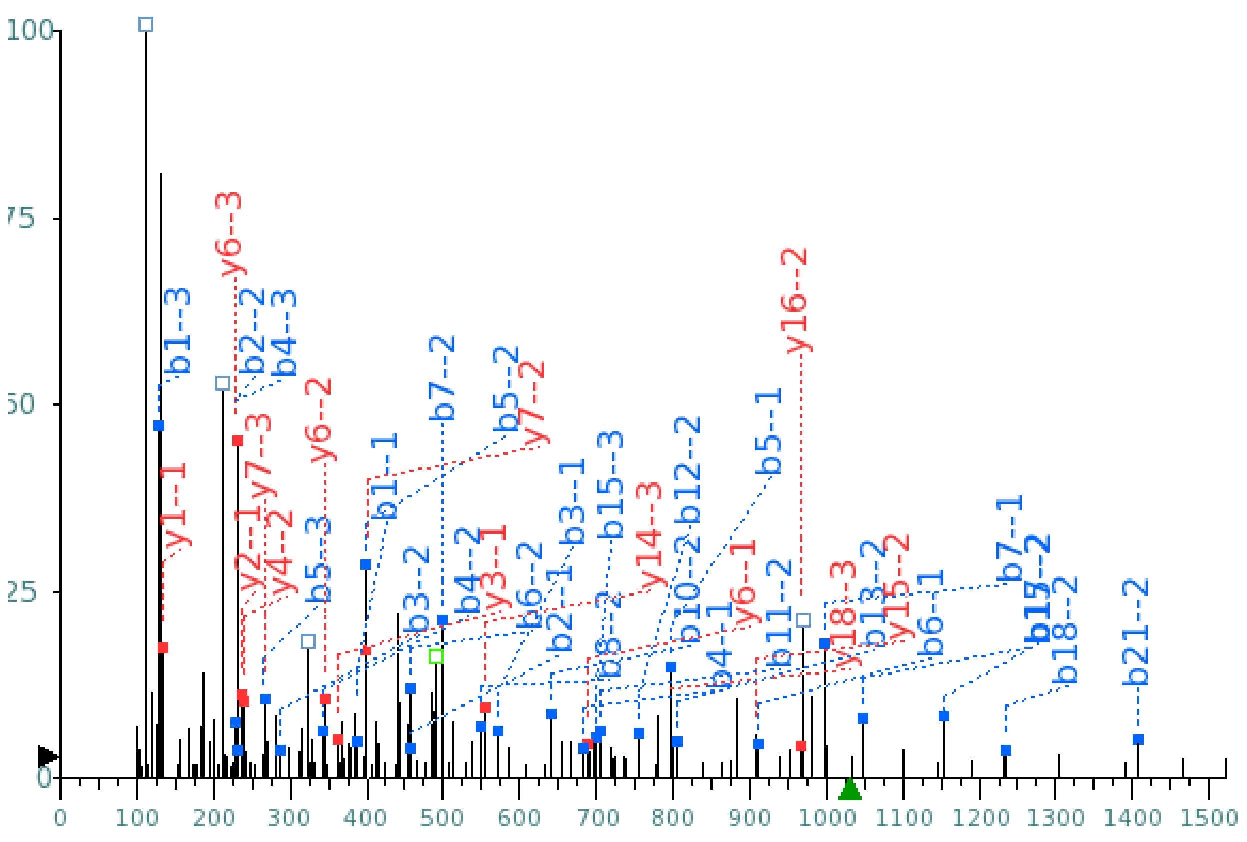

RALINLSPPSMVVQKPDYLNDFTHEIPNIQTD (+4)

Primary Reference: IKZF5_HUMAN
Search ID: 41761
Search Name: 20130328_ananiav_ERstress_TMT_TM_timecourse_trypsin_inj2_4mc_iaa
Scan#: 20920
Observed Mass: 1031.8024 (-0.3 ppm)
PSM Score: 52.11

File name: 2015-02-04 04.01.53PM.pdf

Creation time: 2015-02-04 04:09PM

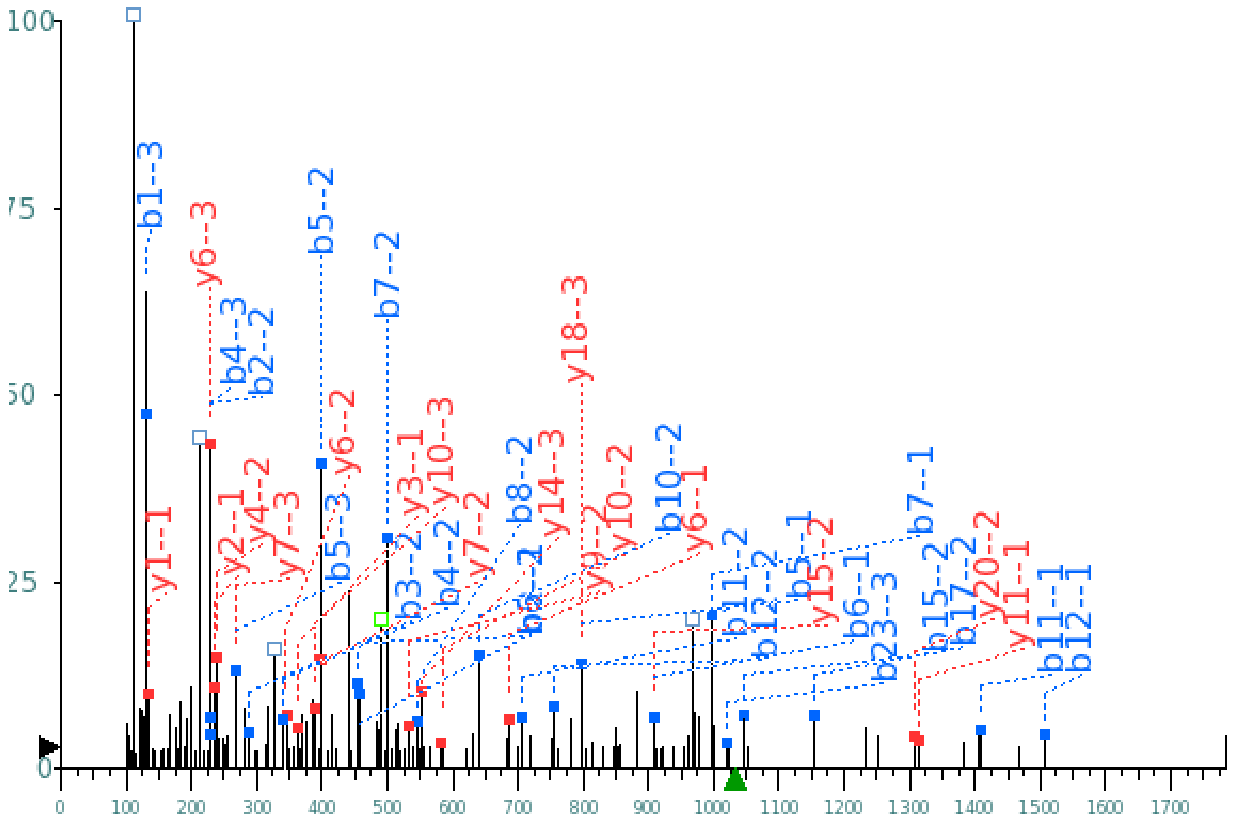

RALINLSPPSMVVQKPDYLNDFTHEIPNIQTD (+4)

Primary Reference: IKZF5_HUMAN
Search ID: 41761
Search Name: 20130328_ananiav_ERstress_TMT_TM_timecourse_trypsin_inj2_4mc_iaa
Scan#: 20928
Observed Mass: 1031.8014 (-1.3 ppm)
PSM Score: 23.04

File name: 2015-02-04 04.01.53PM.pdf

Creation time: 2015-02-04 04:09PM

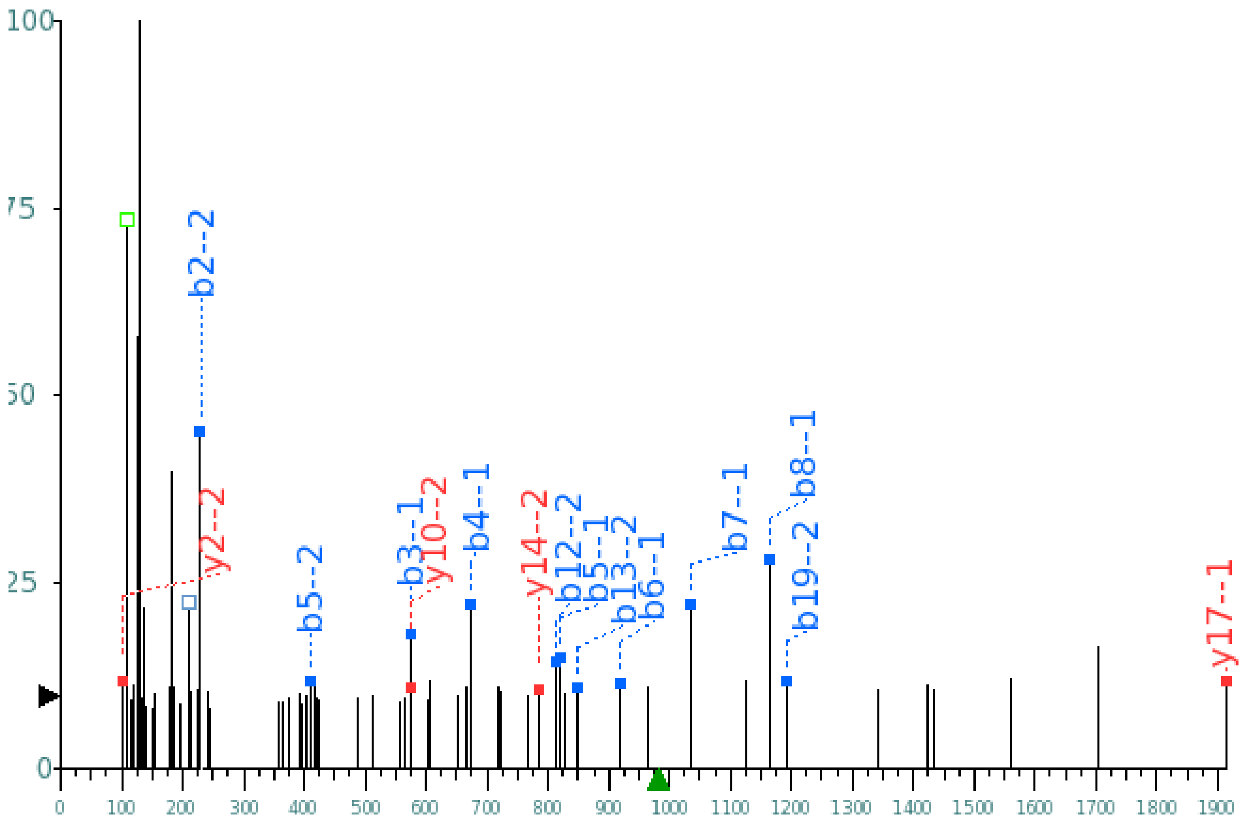

TENVFVDEPLIHATTYIPLMDNAD (+3)

Primary Reference: KTN1_HUMAN
Search ID: 41761
Search Name: 20130328_ananiav_ERstress_TMT_TM_timecourse_trypsin_inj2_4mc_iaa
Scan#: 21025
Observed Mass: 983.1586 (0.5 ppm)
PSM Score: 15.83

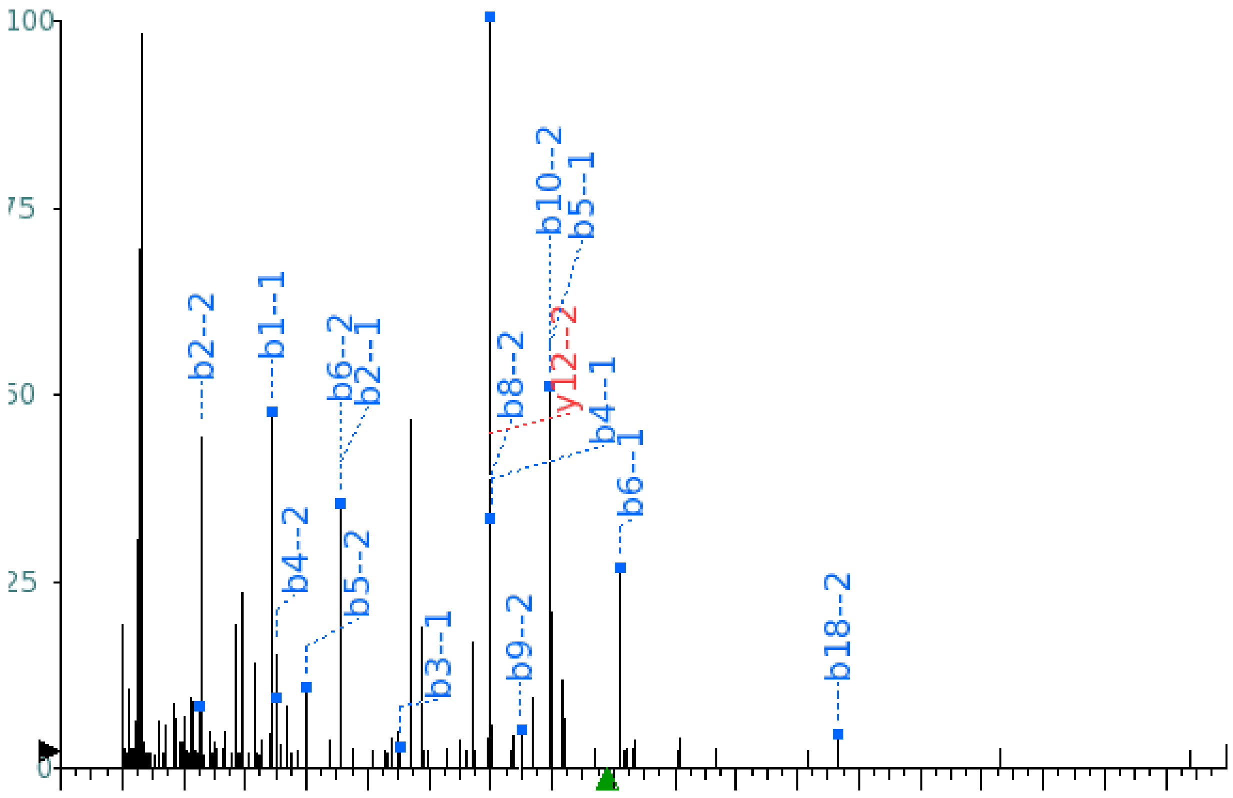

IIPFPLKETPEECNYANID (+3)

Primary Reference: ##PRDX6_HUMAN
Search ID: 41761
Search Name: 20130328_ananiav_ERstress_TMT_TM_timecourse_trypsin_inj2_4mc_iaa
Scan#: 21212
Observed Mass: 888.8037 (-1.1 ppm)
PSM Score: 17.51

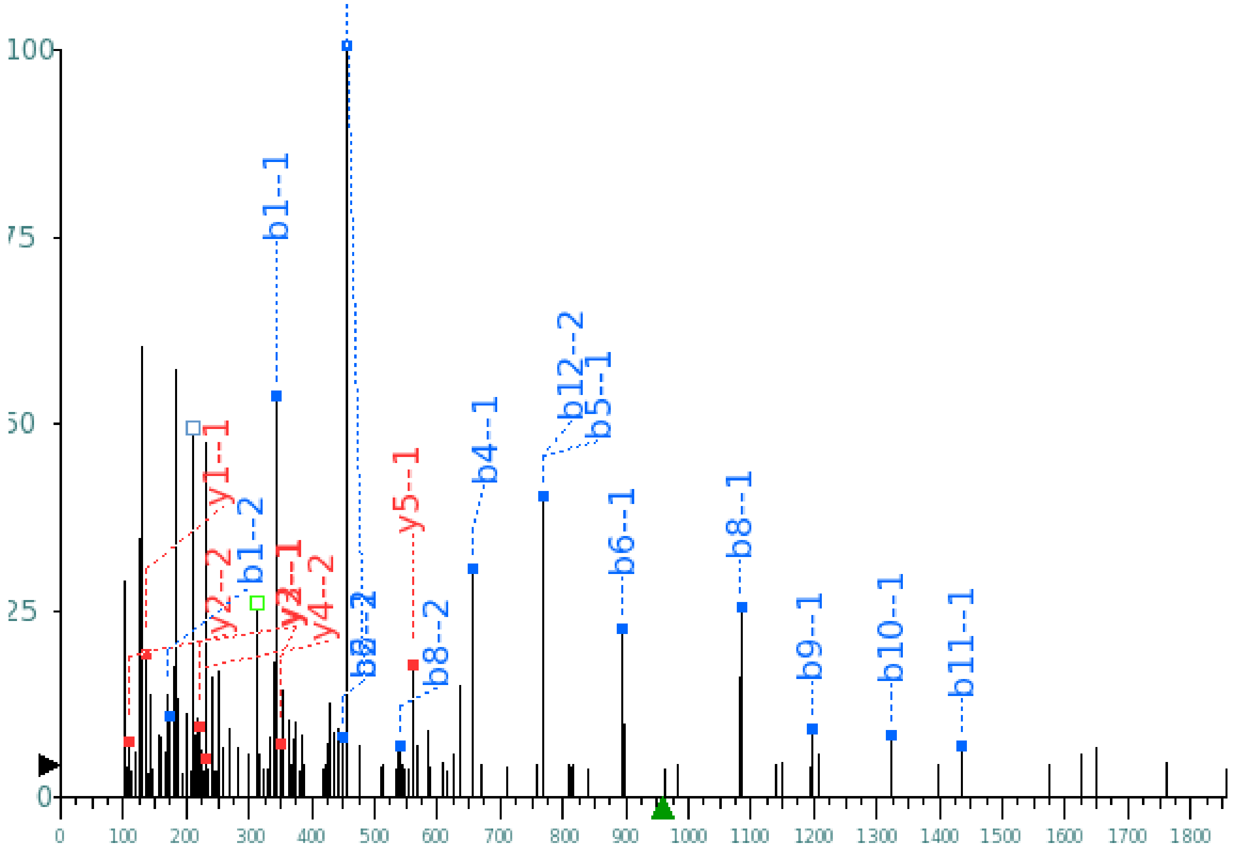

IIPTLEEGLQLPSPTATSQLPLESD (+3)

Primary Reference: HNRPK_HUMAN
Search ID: 41761
Search Name: 20130328_ananiav_ERstress_TMT_TM_timecourse_trypsin_inj2_4mc_iaa
Scan#: 21224
Observed Mass: 960.1905 (2.4 ppm)
PSM Score: 45.67

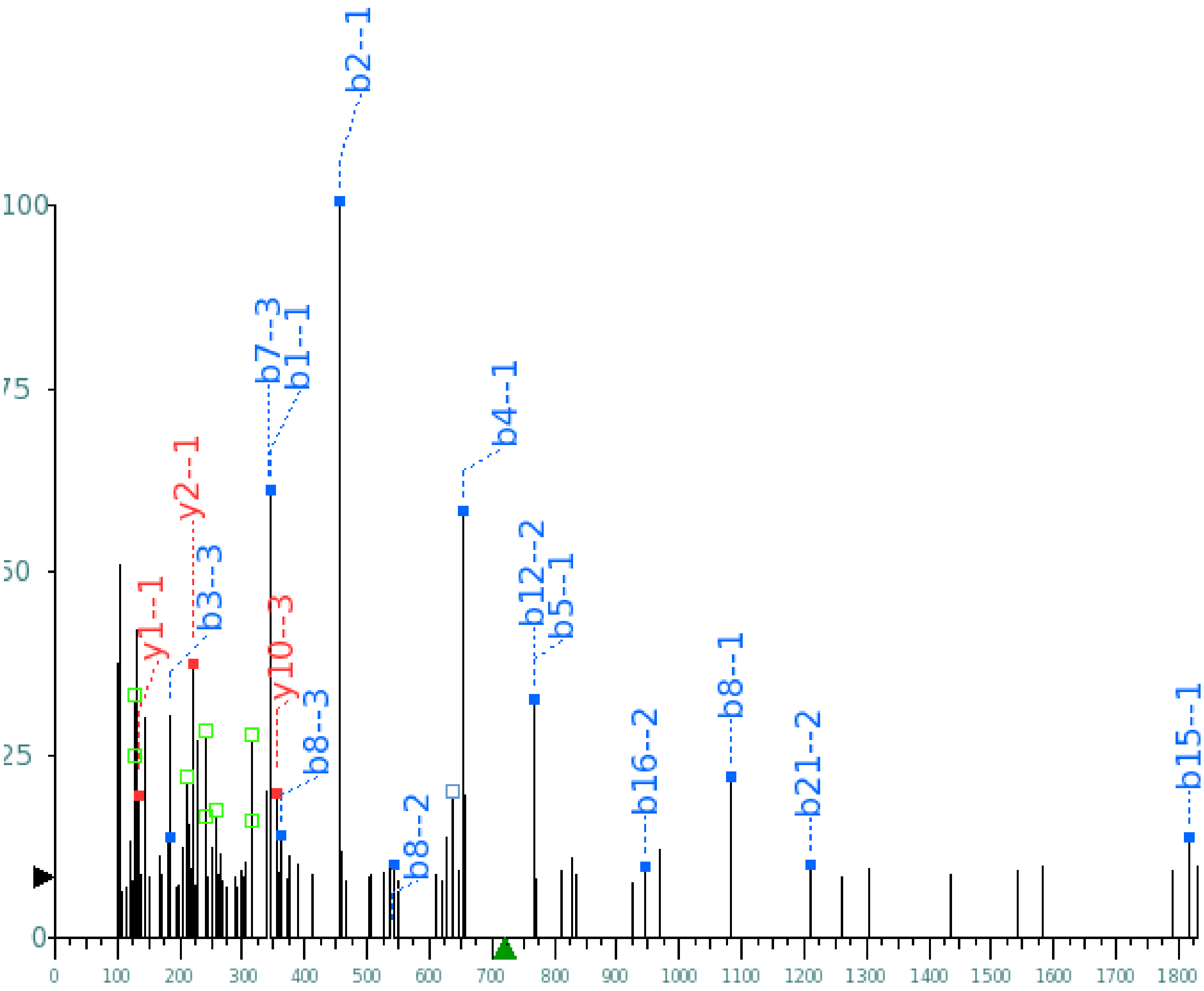

IIPTLEEGLQLPSPTATSQLPLESD (+4)

Primary Reference: HNRPK_HUMAN
Search ID: 41761
Search Name: 20130328_ananiav_ERstress_TMT_TM_timecourse_trypsin_inj2_4mc_iaa
Scan#: 21234
Observed Mass: 720.394 (1.4 ppm)
PSM Score: 23.77

File name: 2015-02-04 04.01.53PM.pdf

Creation time: 2015-02-04 04:09PM

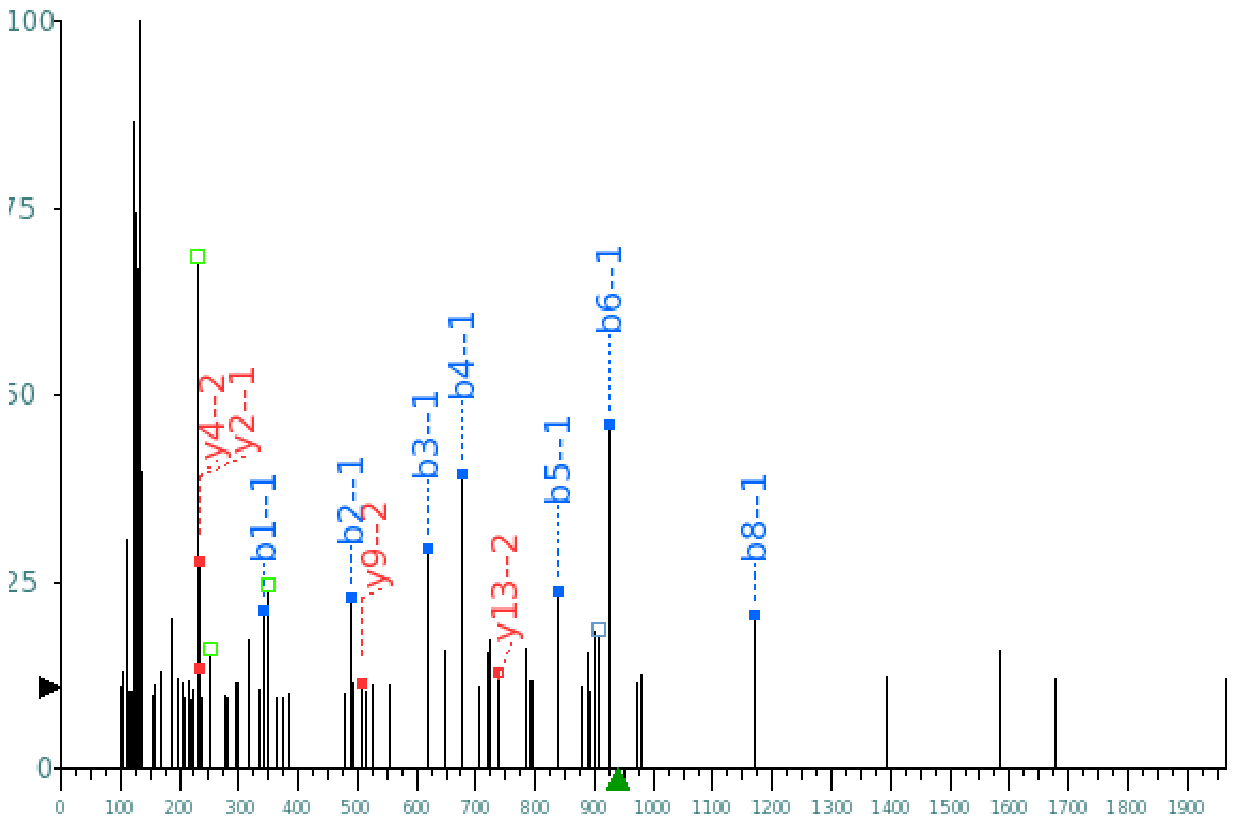

IFQGYSFVAPSILFDHNNAVMTD (+3)

Primary Reference: KS6A4_HUMAN
Search ID: 41761
Search Name: 20130328_ananiav_ERstress_TMT_TM_timecourse_trypsin_inj2_4mc_iaa
Scan#: 21244
Observed Mass: 939.1394 (2.6 ppm)
PSM Score: 19.42

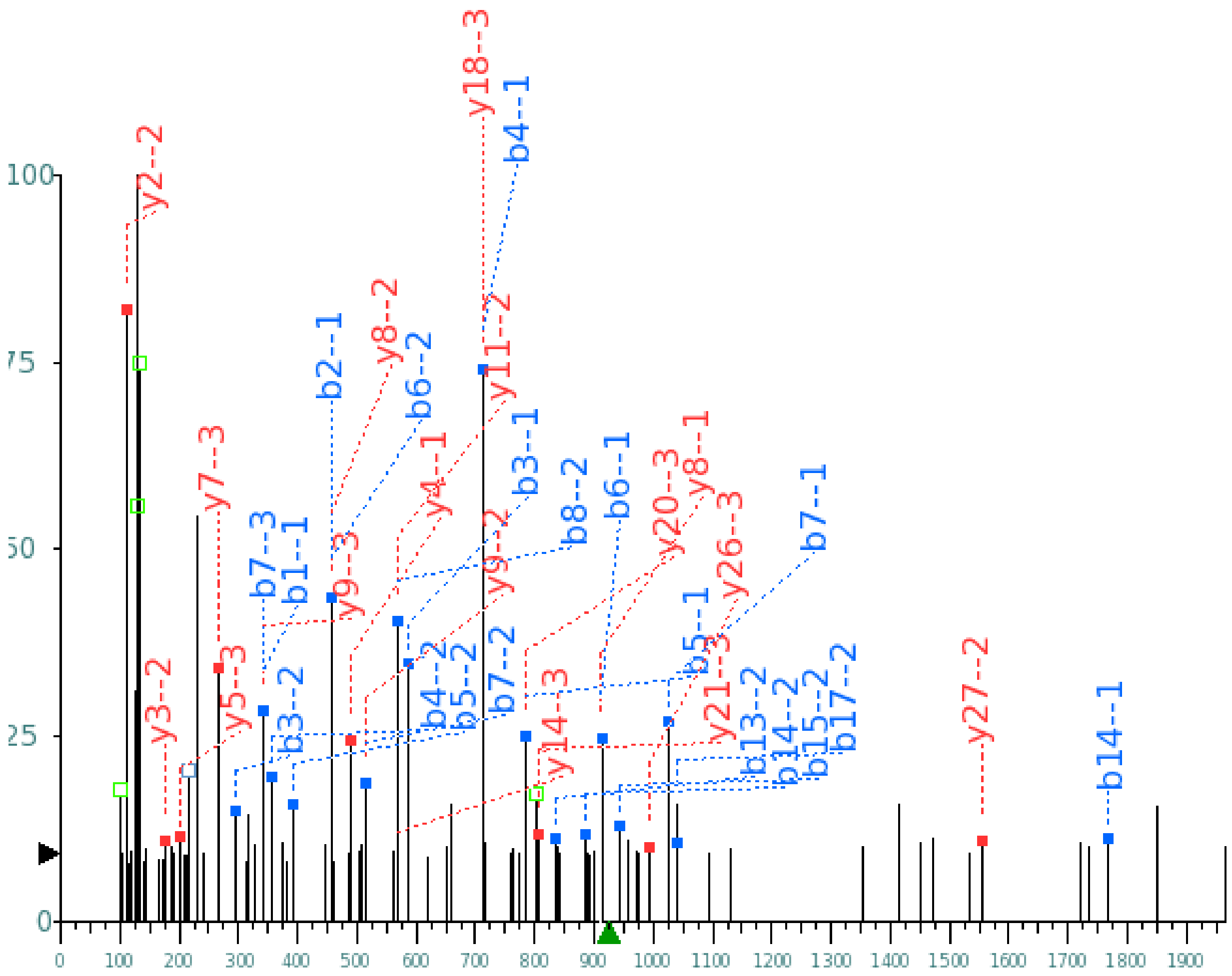

ILQEAQNLMALTNVDTPLKGGLNTPLHESD (+4)

Primary Reference: CDC5L_HUMAN
Search ID: 41761
Search Name: 20130328_ananiav_ERstress_TMT_TM_timecourse_trypsin_inj2_4mc_iaa
Scan#: 21344
Observed Mass: 923.5019 (2.2 ppm)
PSM Score: 26.32

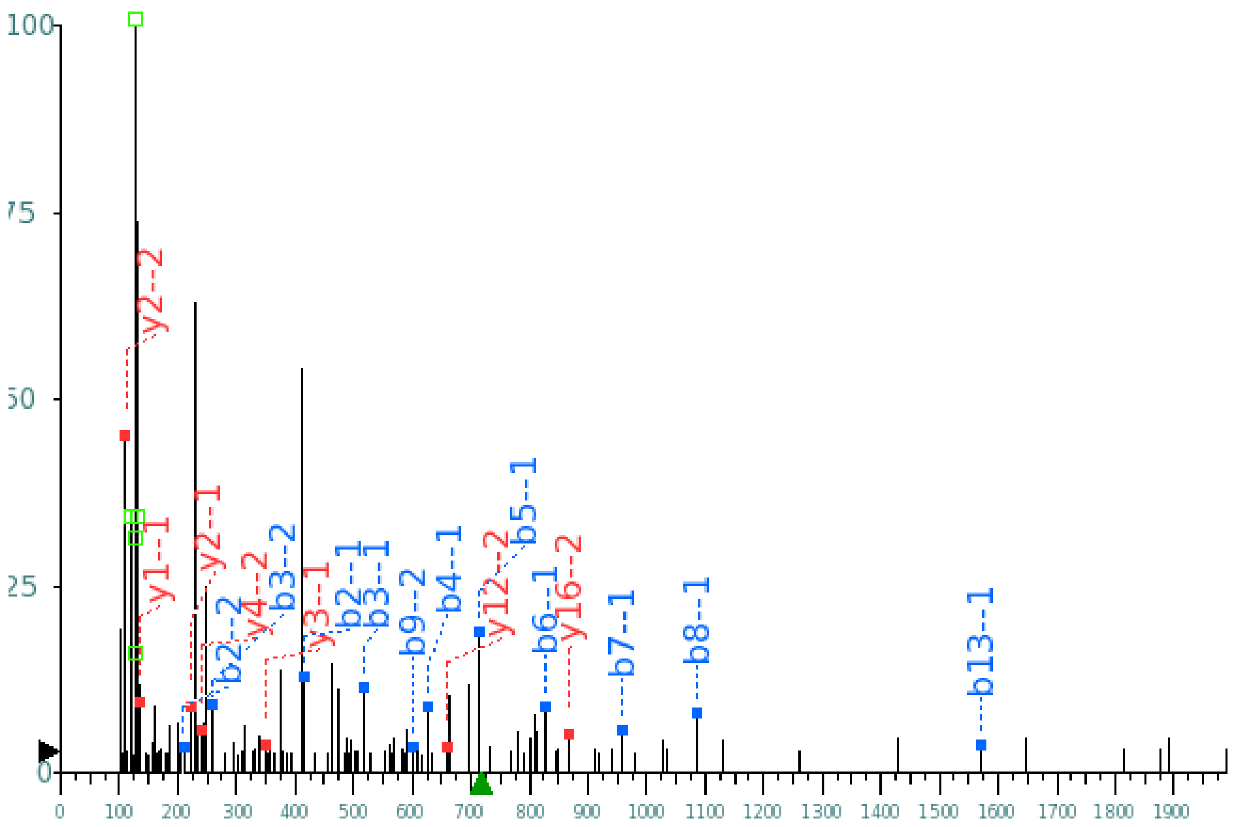

ADVLSLEEILAEGVEESD (+3)

Primary Reference: ABTB2_HUMAN

Search ID: 41761

Search Name: 20130328_ananiav_ERstress_TMT_TM_timecourse_trypsin_inj2_4mc_iaa

Scan#: 21363

Observed Mass: 716.3673 (3.2 ppm)

PSM Score: 16.29

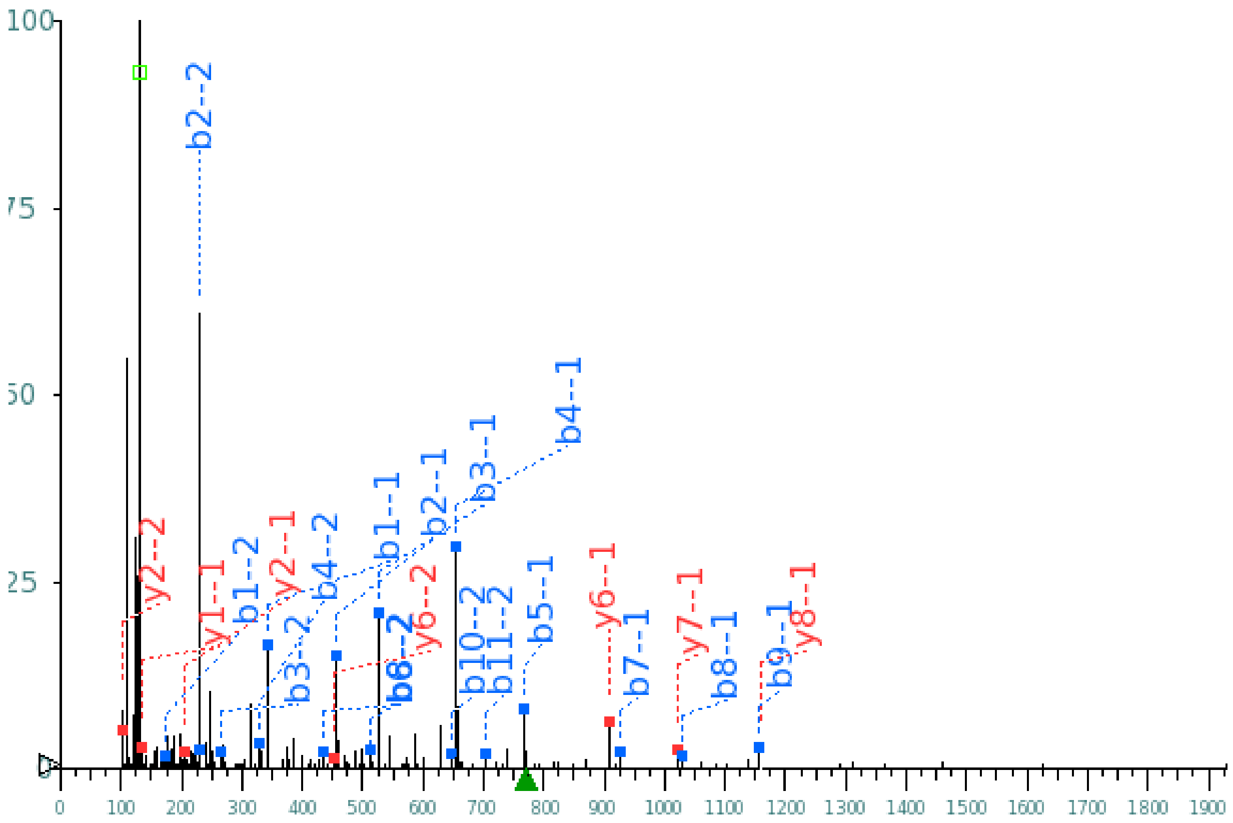

ILAQITGTEHLKESEAD (+3)

Primary Reference: PDLI5_HUMAN
Search ID: 41761
Search Name: 20130328_ananiav_ERstress_TMT_TM_timecourse_trypsin_inj2_4mc_iaa
Scan#: 21407
Observed Mass: 771.7615 (-0.1 ppm)
PSM Score: 23.53

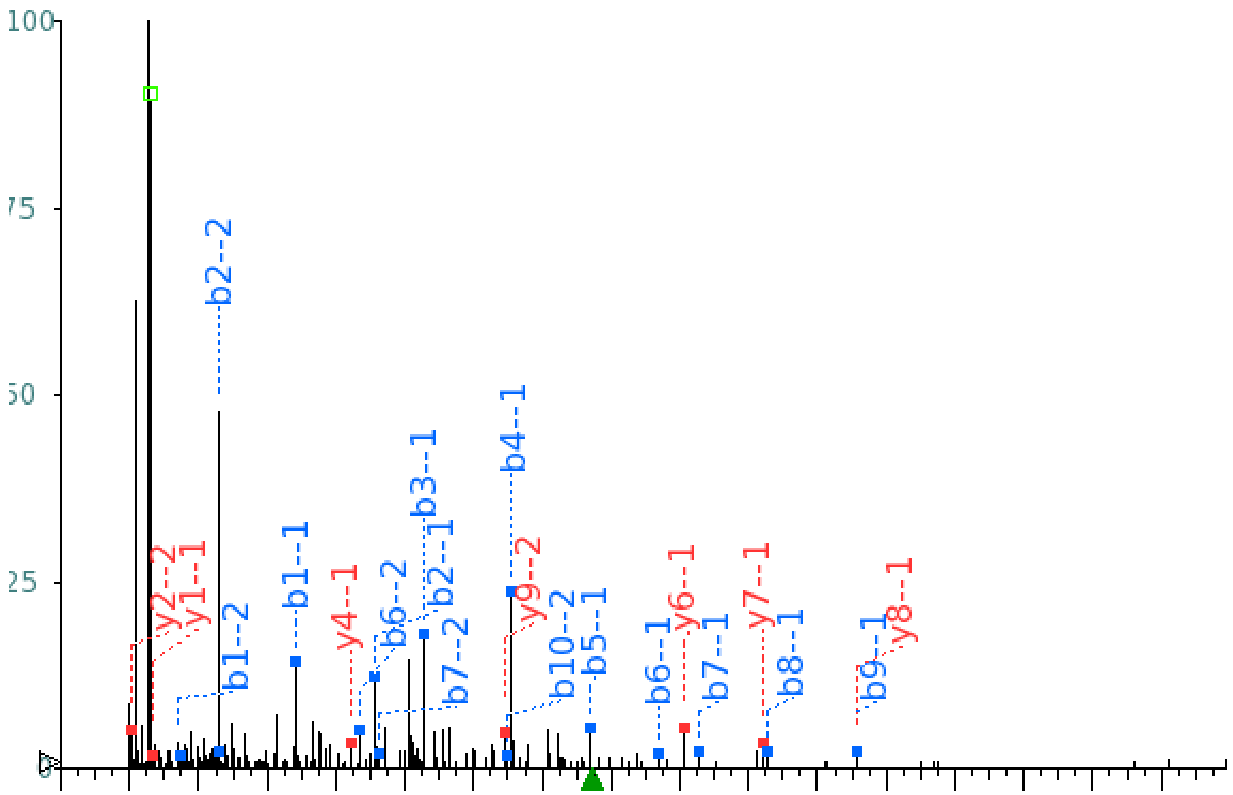

ILAQITGTEHLKESEAD (+3)

Primary Reference: PDLI5_HUMAN
Search ID: 41761
Search Name: 20130328_ananiav_ERstress_TMT_TM_timecourse_trypsin_inj2_4mc_iaa
Scan#: 21436
Observed Mass: 771.763 (1.9 ppm)
PSM Score: 27.61

File name: 2015-02-04 04.01.53PM.pdf

Creation time: 2015-02-04 04:09PM

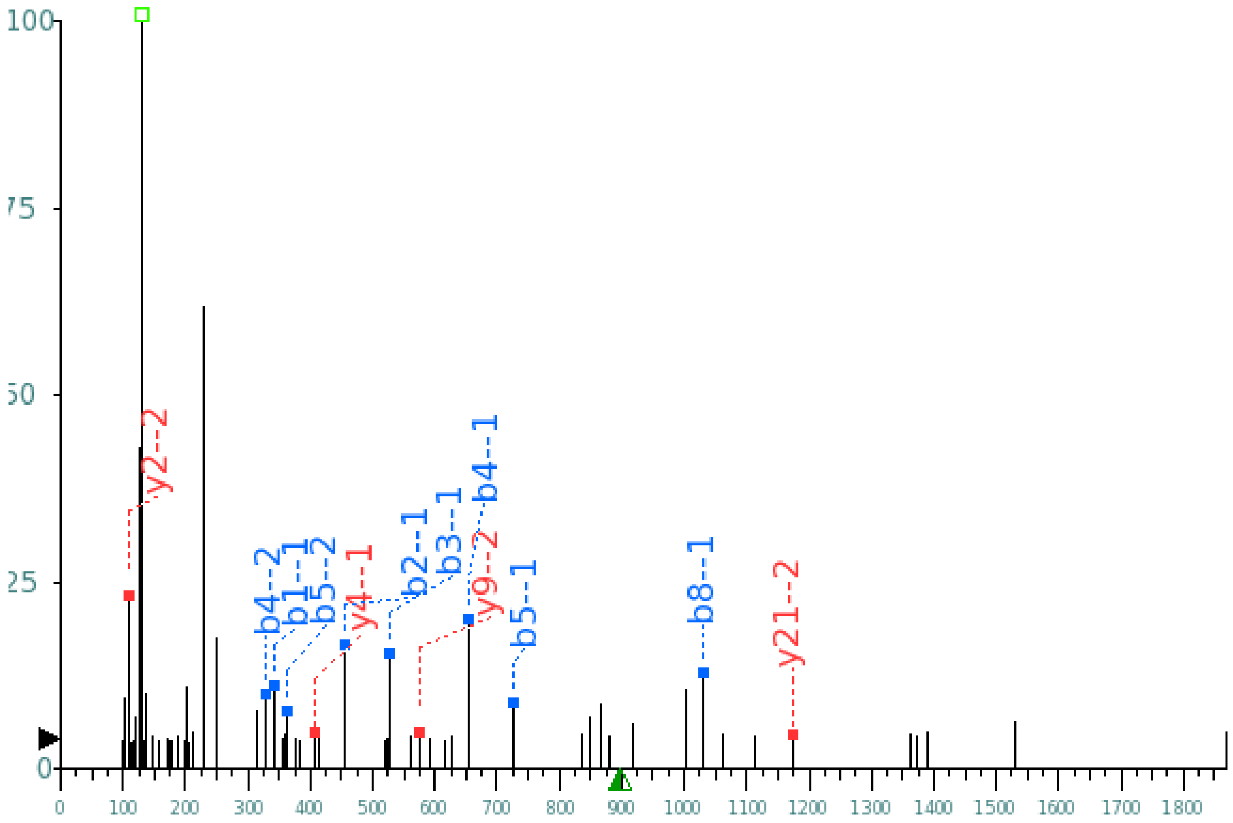

ILAQATSDLVNAIKADAEGESD (+3)

Primary Reference: TLN1_HUMAN
Search ID: 41761
Search Name: 20130328_ananiav_ERstress_TMT_TM_timecourse_trypsin_inj2_4mc_iaa
Scan#: 21552
Observed Mass: 897.1507 (3 ppm)
PSM Score: 18.99

File name: 2015-02-04 04.01.53PM.pdf

Creation time: 2015-02-04 04:09PM

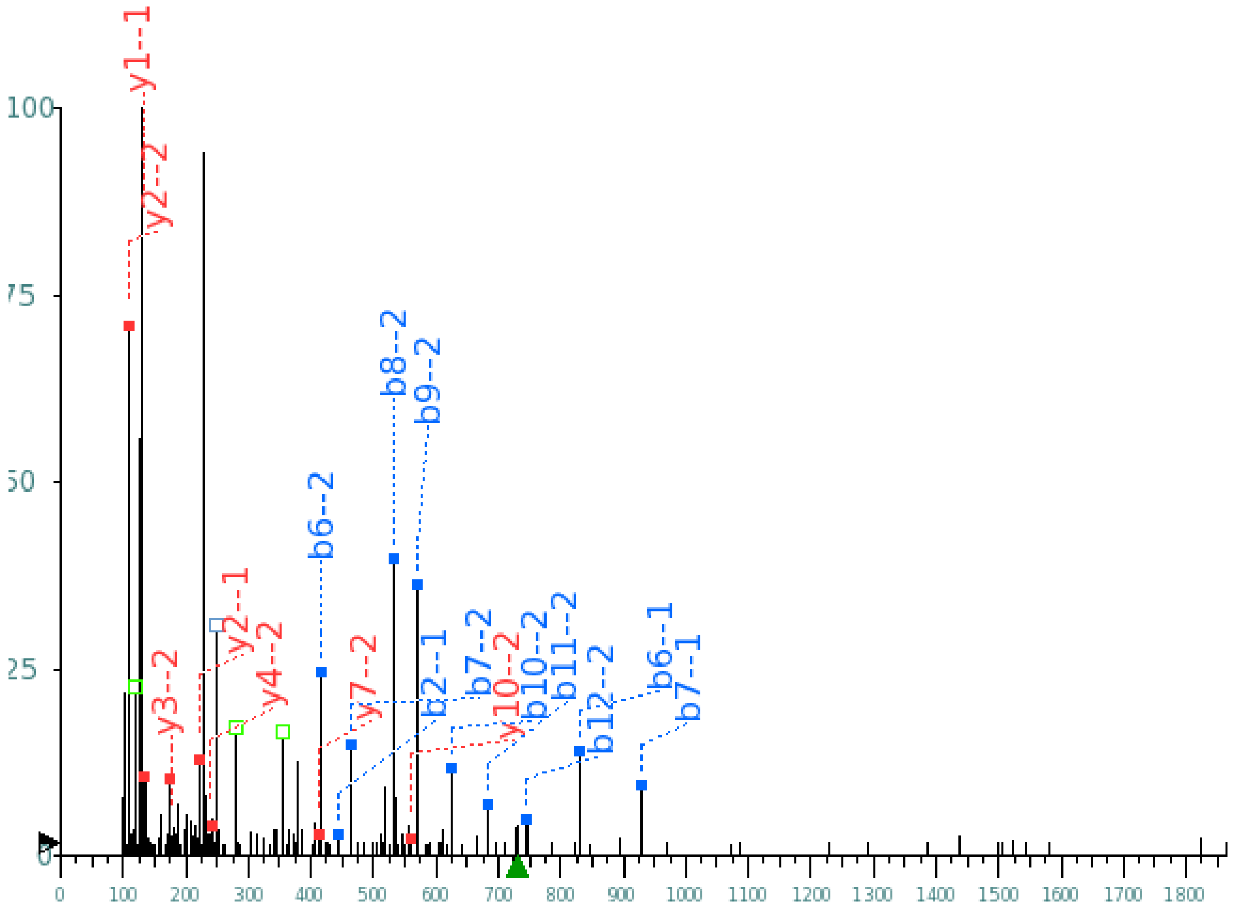

RGSSPDVHALLEITEESD (+3)

Primary Reference: PTRF_HUMAN
Search ID: 41761
Search Name: 20130328_ananiav_ERstress_TMT_TM_timecourse_trypsin_inj2_4mc_iaa
Scan#: 21572
Observed Mass: 728.7031 (-1.6 ppm)
PSM Score: 16.06

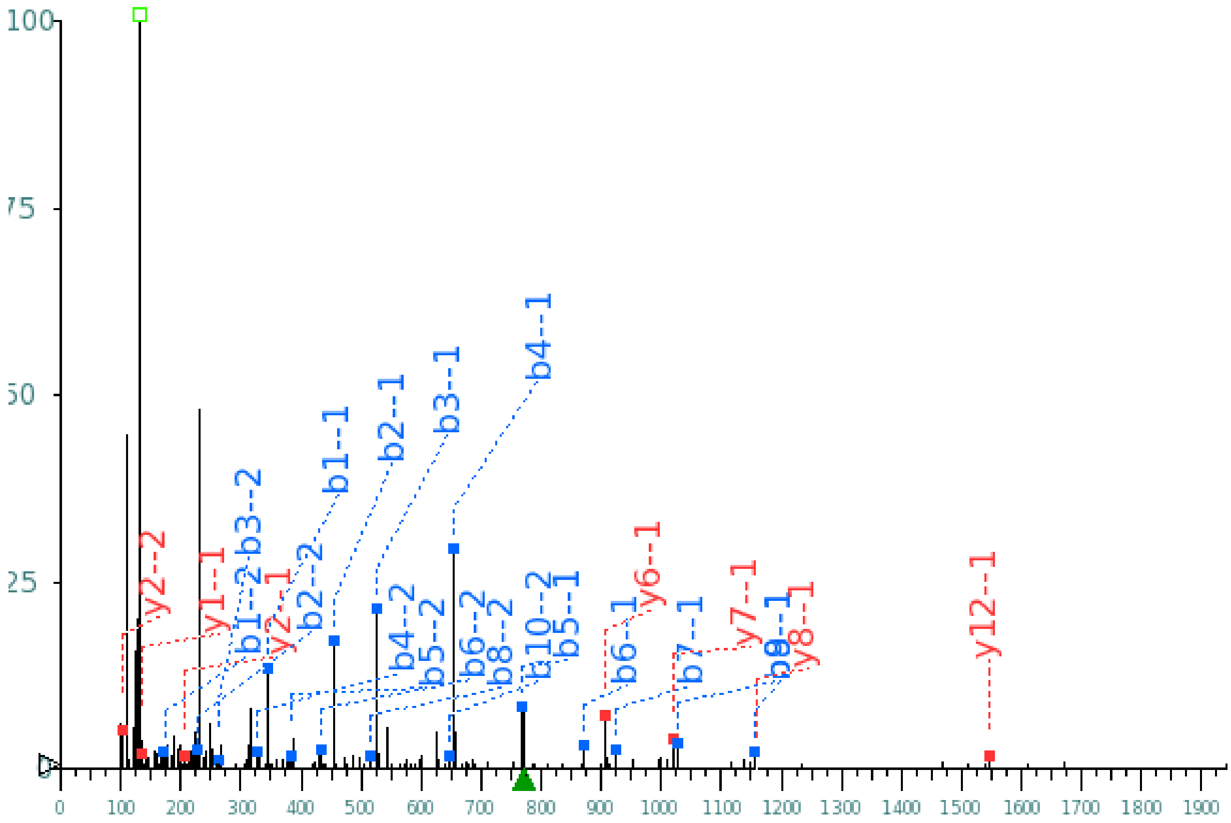

ILAQITGTEHLKESEAD (+3)

Primary Reference: PDLI5_HUMAN
Search ID: 41761
Search Name: 20130328_ananiav_ERstress_TMT_TM_timecourse_trypsin_inj2_4mc_iaa
Scan#: 21587
Observed Mass: 771.7623 (0.9 ppm)
PSM Score: 38.14

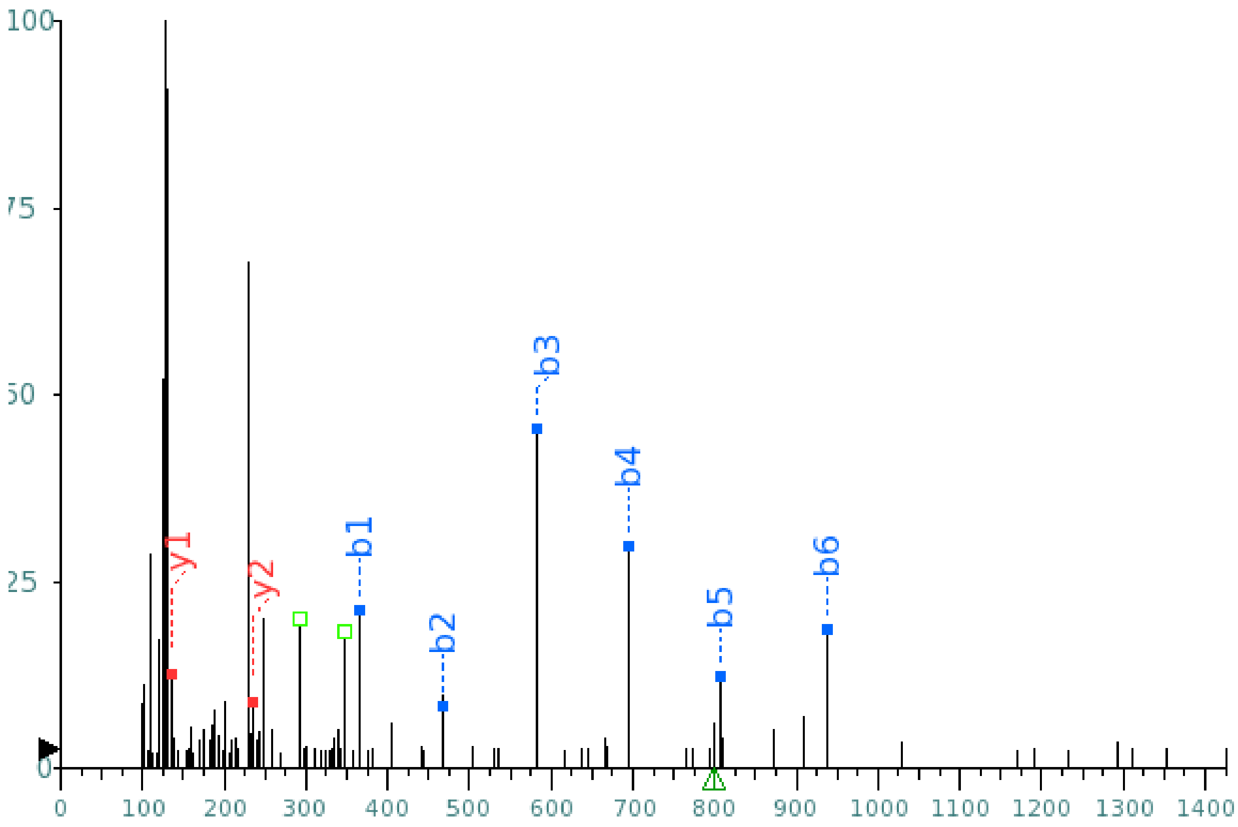

HVDLLEVAQETD (+2)

Primary Reference: ATAD1_HUMAN
Search ID: 41761
Search Name: 20130328_ananiav_ERstress_TMT_TM_timecourse_trypsin_inj2_4mc_iaa
Scan#: 21635
Observed Mass: 799.4182 (1.3 ppm)
PSM Score: 24.49

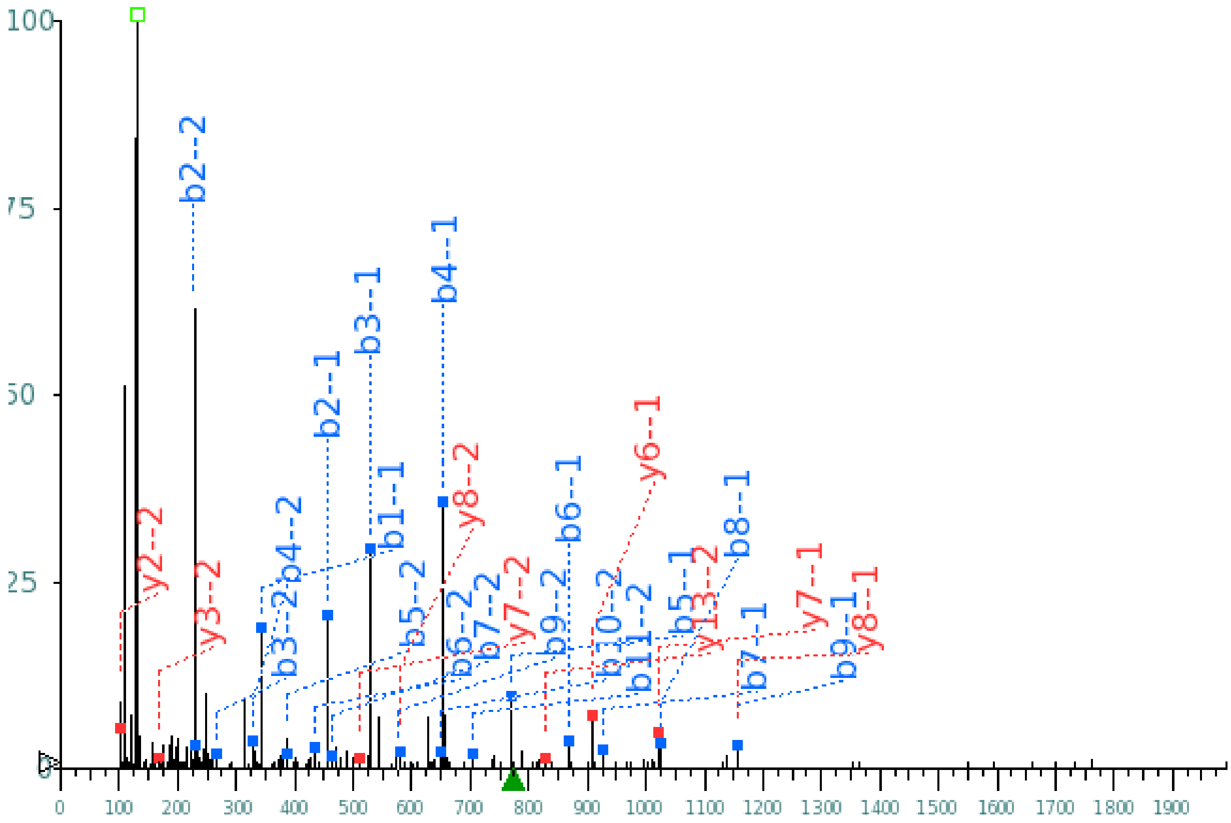

ILAQITGTEHLKESEAD (+3)

Primary Reference: PDLI5_HUMAN
Search ID: 41761
Search Name: 20130328_ananiav_ERstress_TMT_TM_timecourse_trypsin_inj2_4mc_iaa
Scan#: 21653
Observed Mass: 771.7634 (2.4 ppm)
PSM Score: 34.17

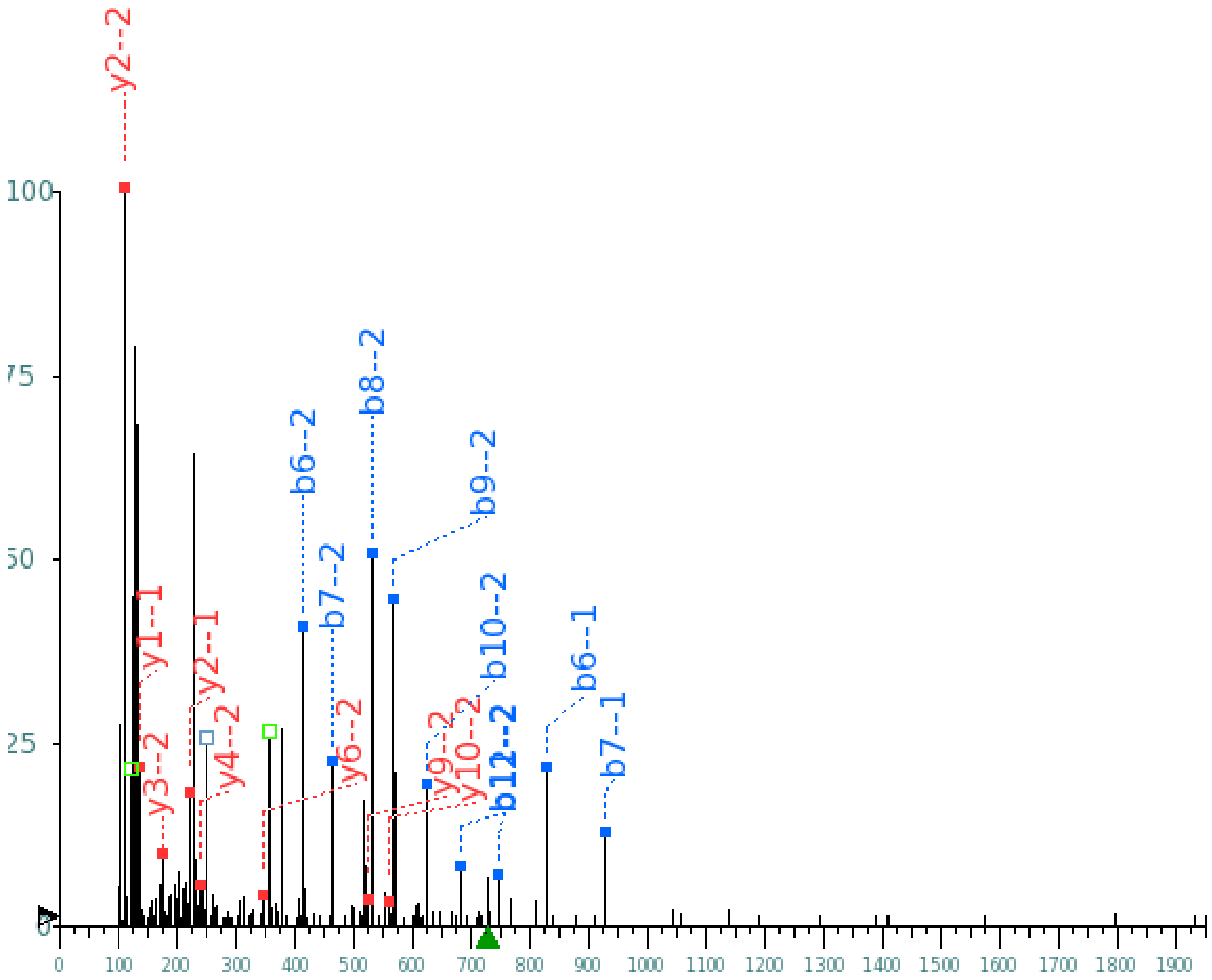

RGSSPDVHALLEITEESD (+3)

Primary Reference: PTRF_HUMAN
Search ID: 41761
Search Name: 20130328_ananiav_ERstress_TMT_TM_timecourse_trypsin_inj2_4mc_iaa
Scan#: 21690
Observed Mass: 728.7055 (1.8 ppm)
PSM Score: 16.24

File name: 2015-02-04 04.01.53PM.pdf

Creation time: 2015-02-04 04:09PM

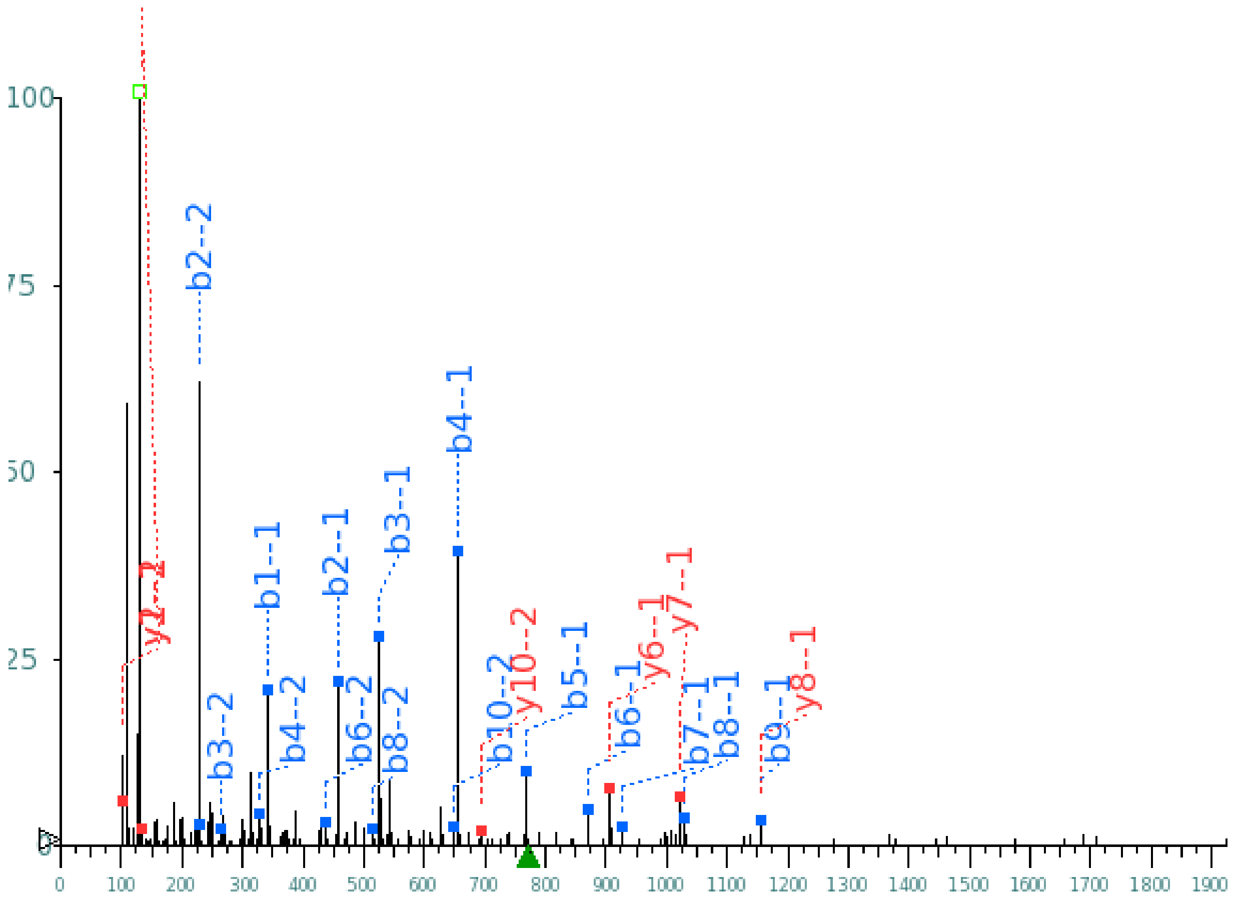

ILAQITGTEHLKESEAD (+3)

Primary Reference: PDLI5_HUMAN
Search ID: 41761
Search Name: 20130328_ananiav_ERstress_TMT_TM_timecourse_trypsin_inj2_4mc_iaa
Scan#: 21707
Observed Mass: 771.7626 (1.3 ppm)
PSM Score: 30.58

File name: 2015-02-04 04.01.53PM.pdf

Creation time: 2015-02-04 04:09PM

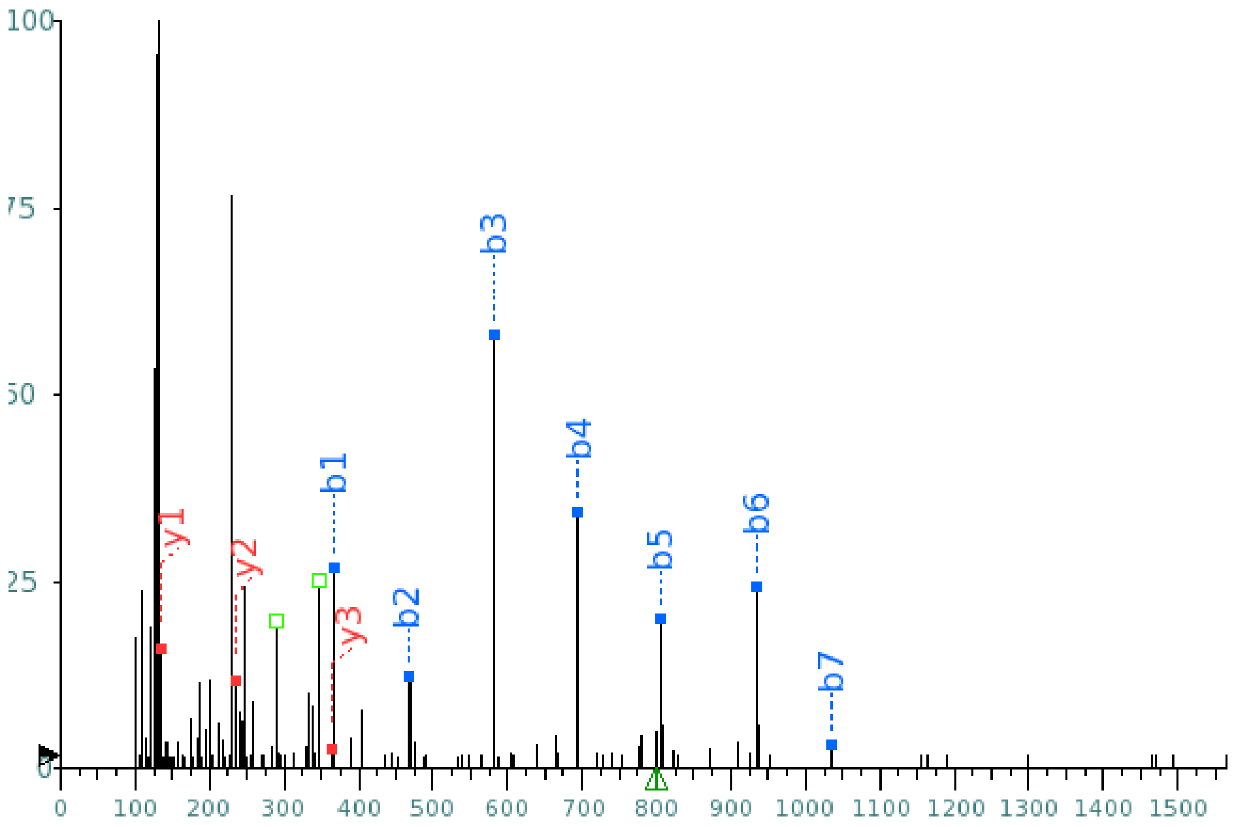

HVDLLEVAQETD (+2)

Primary Reference: ATAD1_HUMAN
Search ID: 41761
Search Name: 20130328_ananiav_ERstress_TMT_TM_timecourse_trypsin_inj2_4mc_iaa
Scan#: 21711
Observed Mass: 799.4181 (1.2 ppm)
PSM Score: 38.21

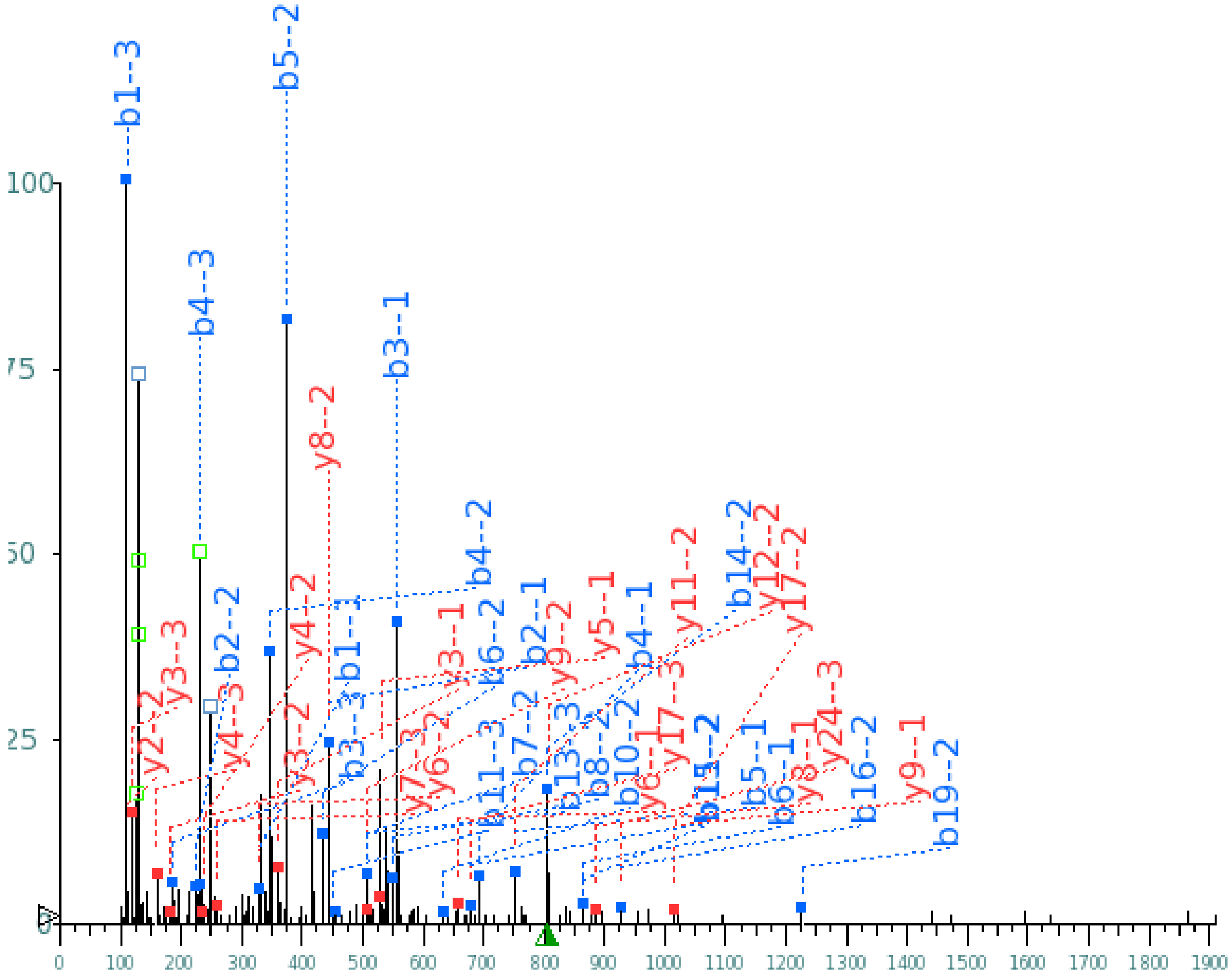

VNIHGIFSVASASVIEKQNLEGDHSD (+4)

Primary Reference: HS74L_HUMAN
Search ID: 41761
Search Name: 20130328_ananiav_ERstress_TMT_TM_timecourse_trypsin_inj2_4mc_iaa
Scan#: 21729
Observed Mass: 806.9305 (1.5 ppm)
PSM Score: 29.19

File name: 2015-02-04 04.01.53PM.pdf

Creation time: 2015-02-04 04:09PM

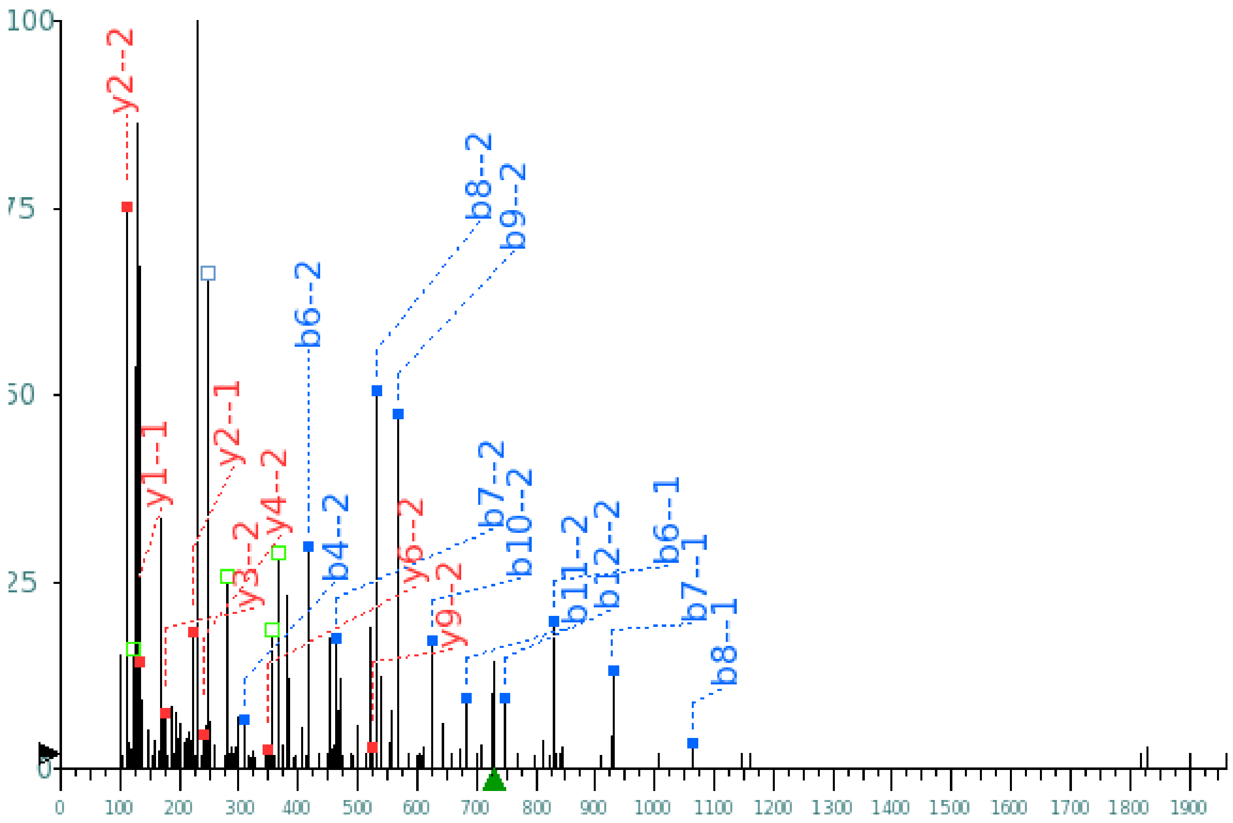

RGSSPDVHALLEITEESD (+3)

Primary Reference: PTRF_HUMAN
Search ID: 41761
Search Name: 20130328_ananiav_ERstress_TMT_TM_timecourse_trypsin_inj2_4mc_iaa
Scan#: 21747
Observed Mass: 728.7056 (1.9 ppm)
PSM Score: 16.74

File name: 2015-02-04 04.01.53PM.pdf

Creation time: 2015-02-04 04:09PM

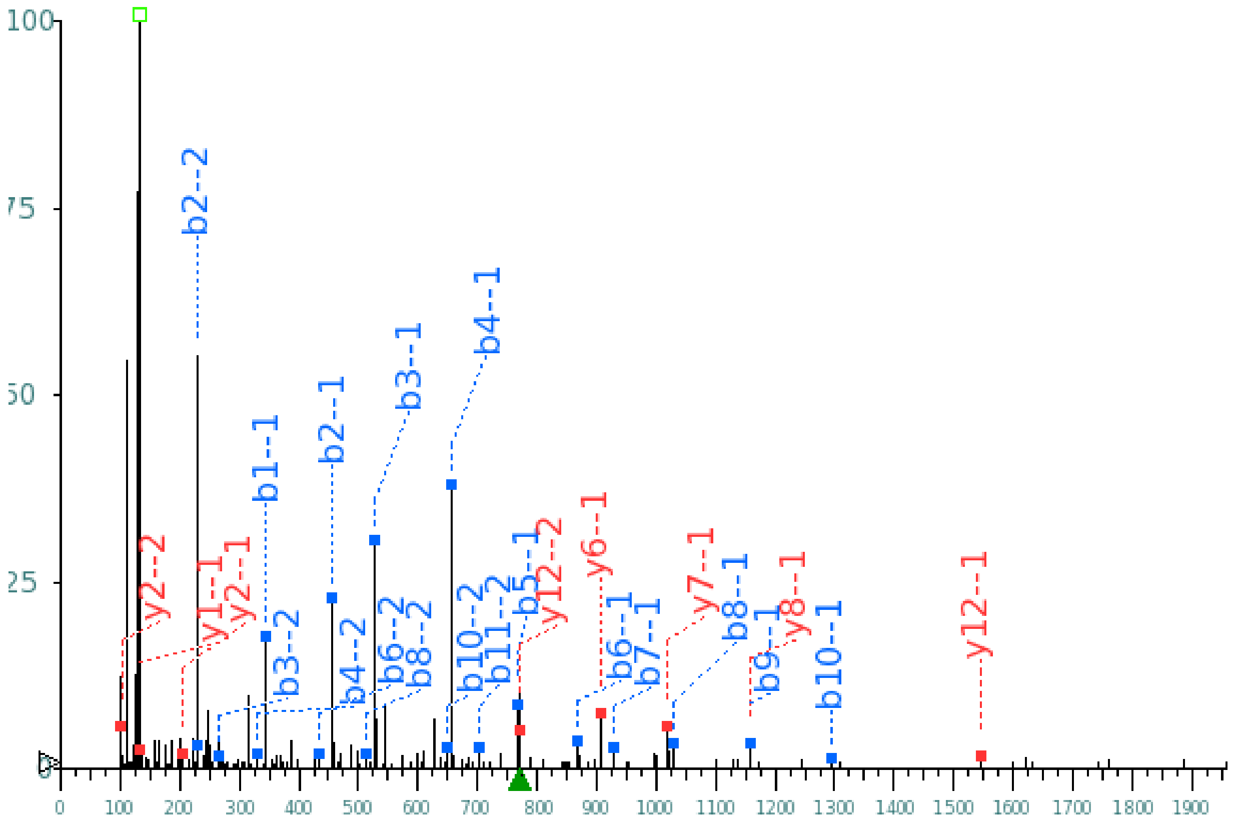

ILAQITGTEHLKESEAD (+3)

Primary Reference: PDLI5_HUMAN
Search ID: 41761
Search Name: 20130328_ananiav_ERstress_TMT_TM_timecourse_trypsin_inj2_4mc_iaa
Scan#: 21763
Observed Mass: 771.7627 (1.5 ppm)
PSM Score: 43.67

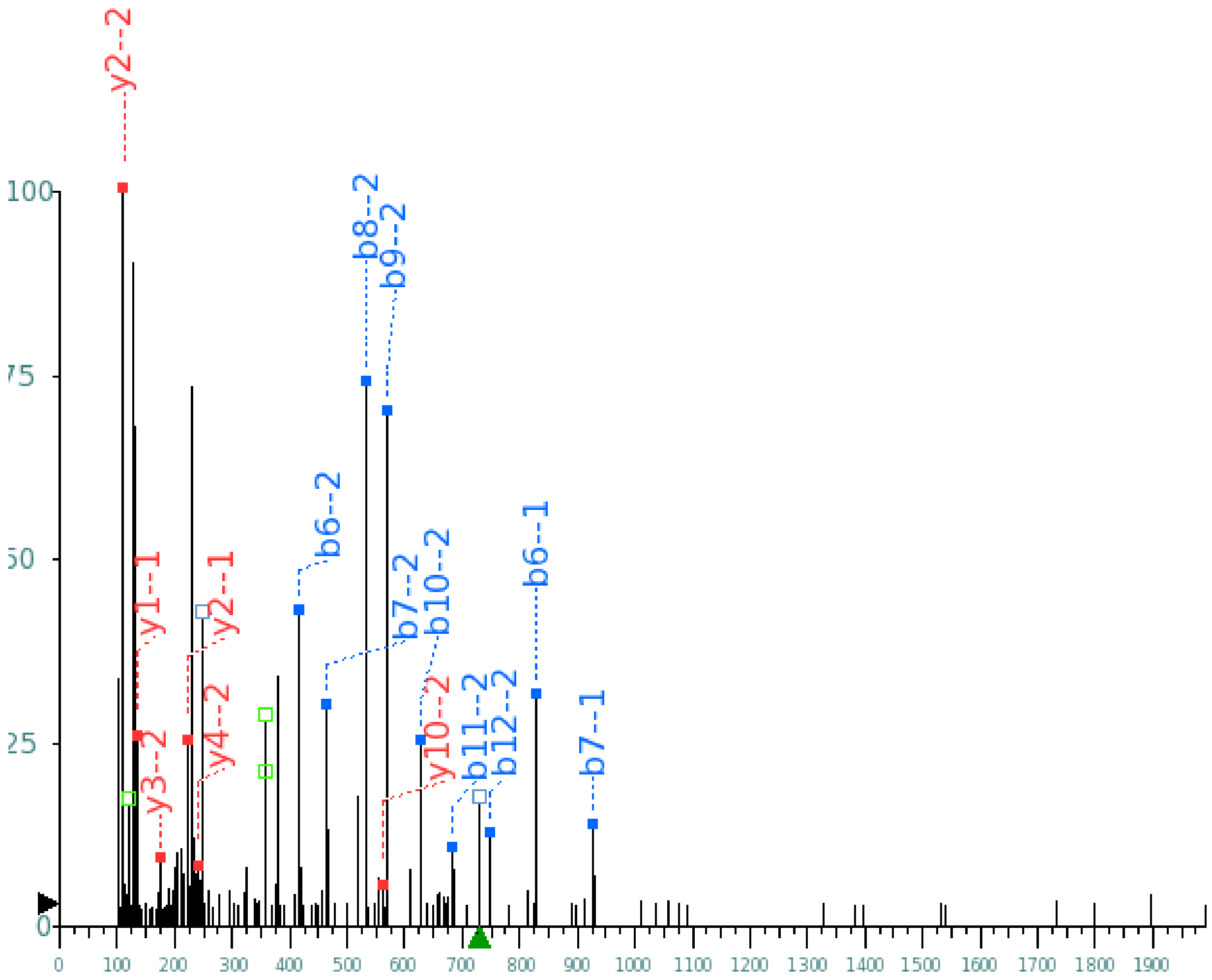

RGSSPDVHALLEITEESD (+3)

Primary Reference: PTRF_HUMAN
Search ID: 41761
Search Name: 20130328_ananiav_ERstress_TMT_TM_timecourse_trypsin_inj2_4mc_iaa
Scan#: 21767
Observed Mass: 728.7054 (1.5 ppm)
PSM Score: 13.11

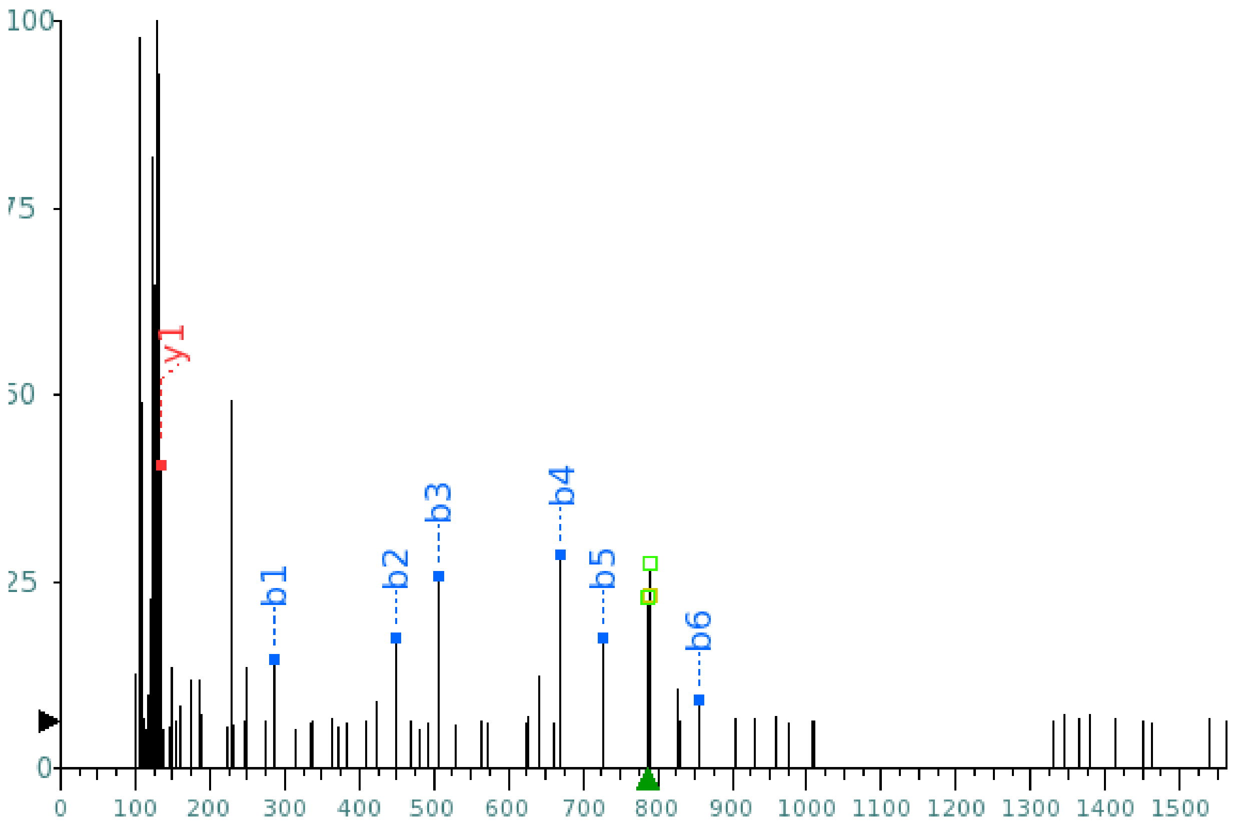

GYGYGQGAGTLSTD (+2)

Primary Reference: CSRP1_HUMAN
Search ID: 41761
Search Name: 20130328_ananiav_ERstress_TMT_TM_timecourse_trypsin_inj2_4mc_iaa
Scan#: 21795
Observed Mass: 788.3771 (-1.2 ppm)
PSM Score: 14.64

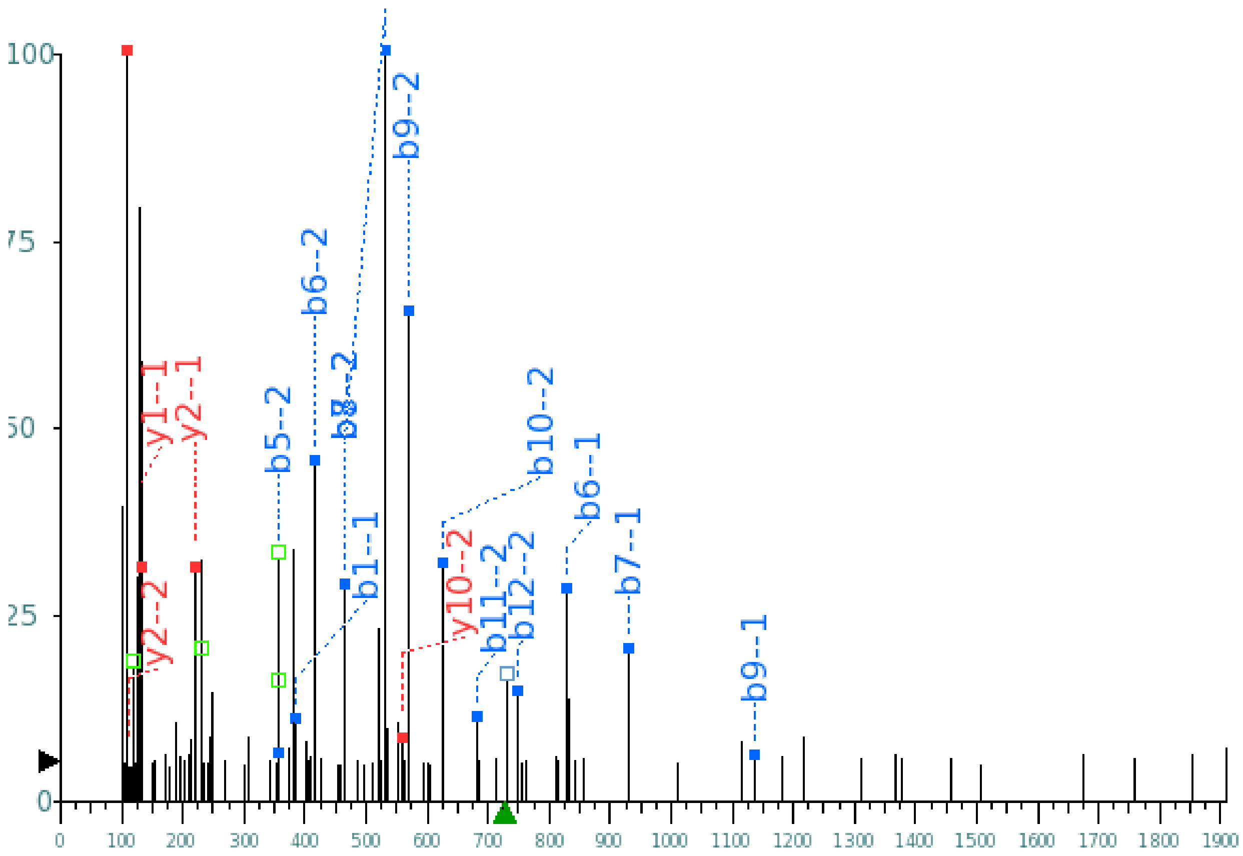

RGSSPDVHALLEITEESD (+3)

Primary Reference: PTRF_HUMAN
Search ID: 41761
Search Name: 20130328_ananiav_ERstress_TMT_TM_timecourse_trypsin_inj2_4mc_iaa
Scan#: 21802
Observed Mass: 728.706 (2.4 ppm)
PSM Score: 19.13

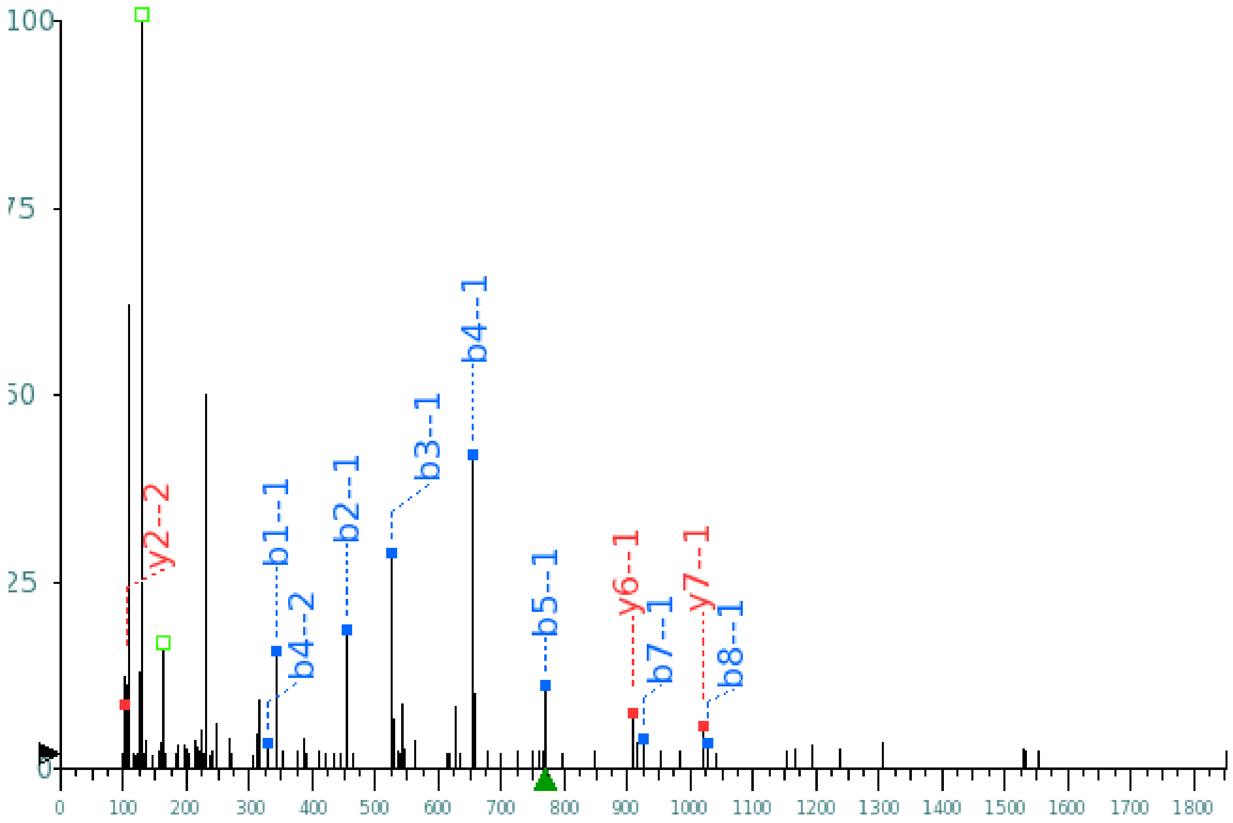

ILAQITGTEHLKESEAD (+3)

Primary Reference: PDLI5_HUMAN
Search ID: 41761
Search Name: 20130328_ananiav_ERstress_TMT_TM_timecourse_trypsin_inj2_4mc_iaa
Scan#: 21827
Observed Mass: 771.7633 (2.3 ppm)
PSM Score: 18.15

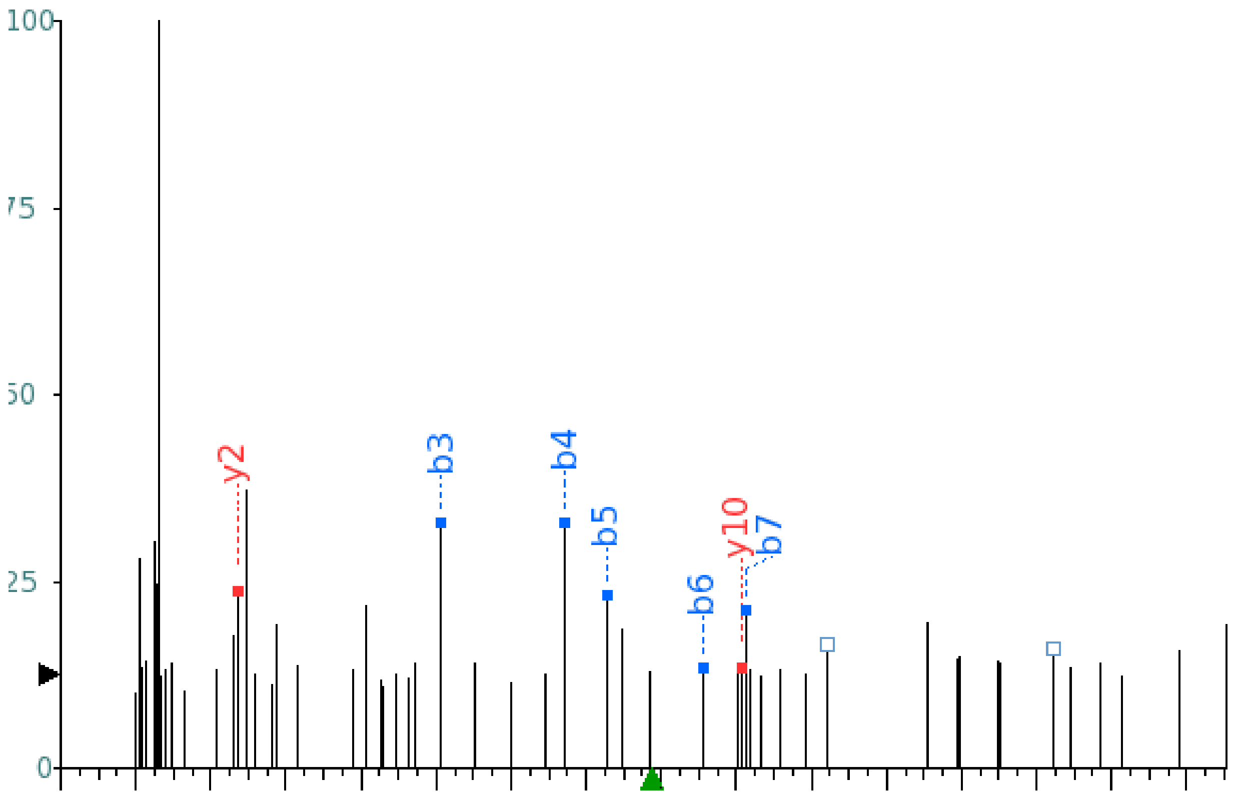

GYGYGQGAGTLSTD (+2)

Primary Reference: CSRP1_HUMAN
Search ID: 41761
Search Name: 20130328_ananiav_ERstress_TMT_TM_timecourse_trypsin_inj2_4mc_iaa
Scan#: 21864
Observed Mass: 788.3788 (0.9 ppm)
PSM Score: 15.25

File name: 2015-02-04 04.01.53PM.pdf

Creation time: 2015-02-04 04:09PM

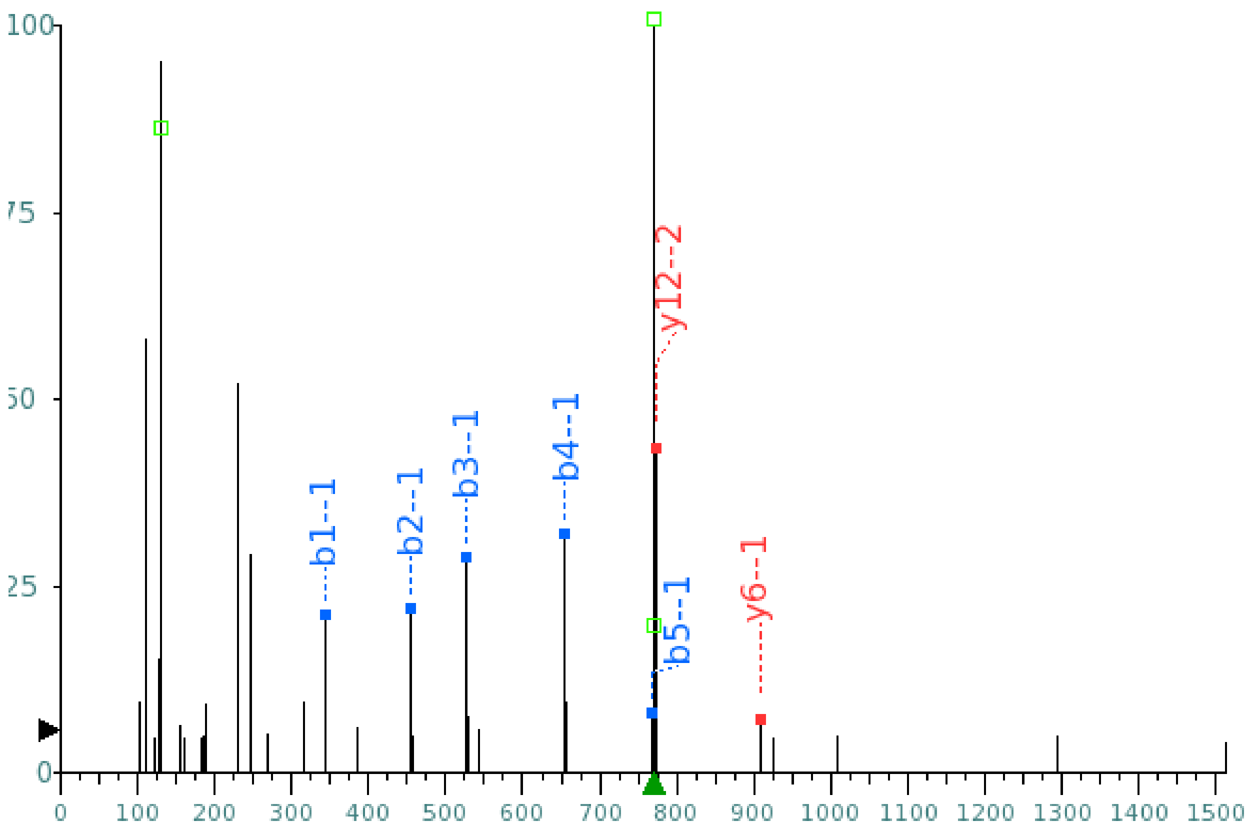

ILAQITGTEHLKESEAD (+3)

Primary Reference: PDLI5_HUMAN
Search ID: 41761
Search Name: 20130328_ananiav_ERstress_TMT_TM_timecourse_trypsin_inj2_4mc_iaa
Scan#: 21884
Observed Mass: 771.7629 (1.7 ppm)
PSM Score: 28.8

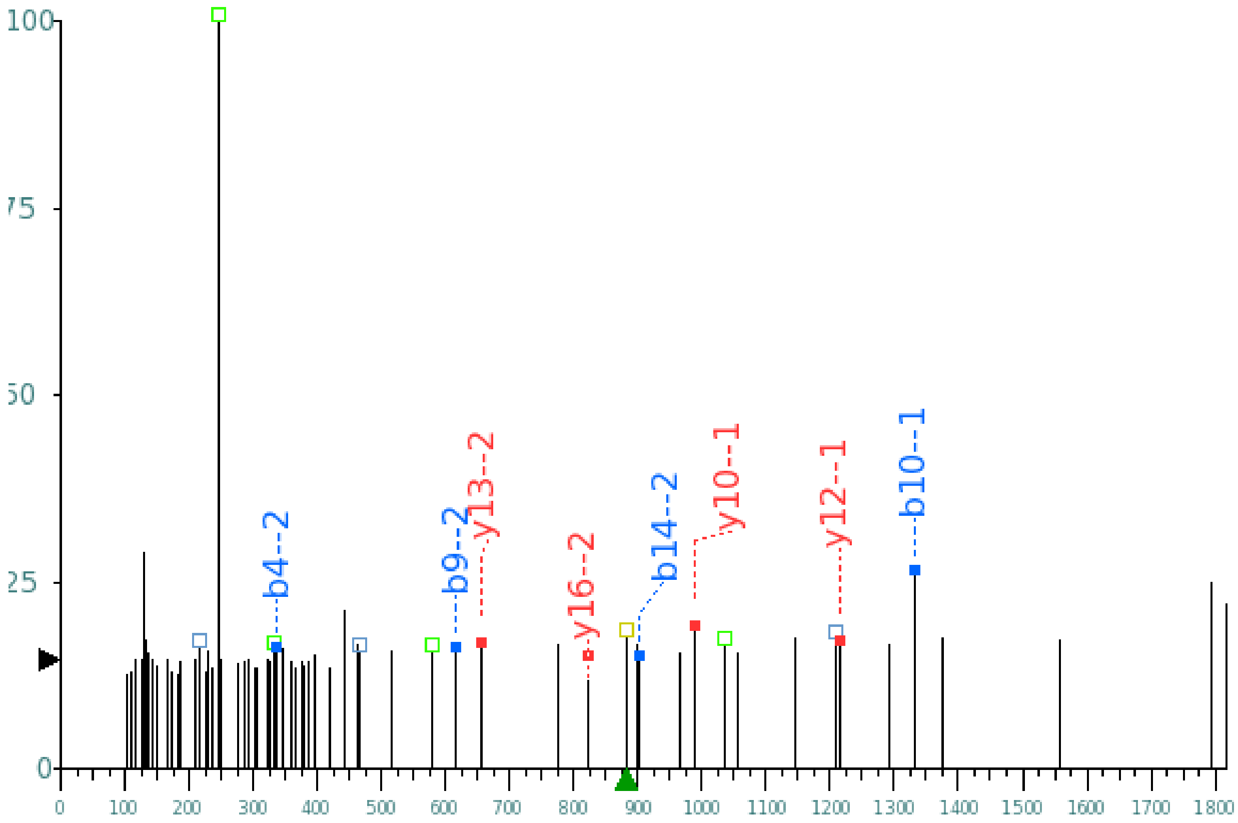

VRSPDC*GETVPVQFPSSPVPSGD (+3)

Primary Reference: ##FLNA_HUMAN
Search ID: 41761
Search Name: 20130328_ananiav_ERstress_TMT_TM_timecourse_trypsin_inj2_4mc_iaa
Scan#: 22048
Observed Mass: 881.7695 (0.7 ppm)
PSM Score: 6.02

File name: 2015-02-04 04.01.53PM.pdf

Creation time: 2015-02-04 04:09PM

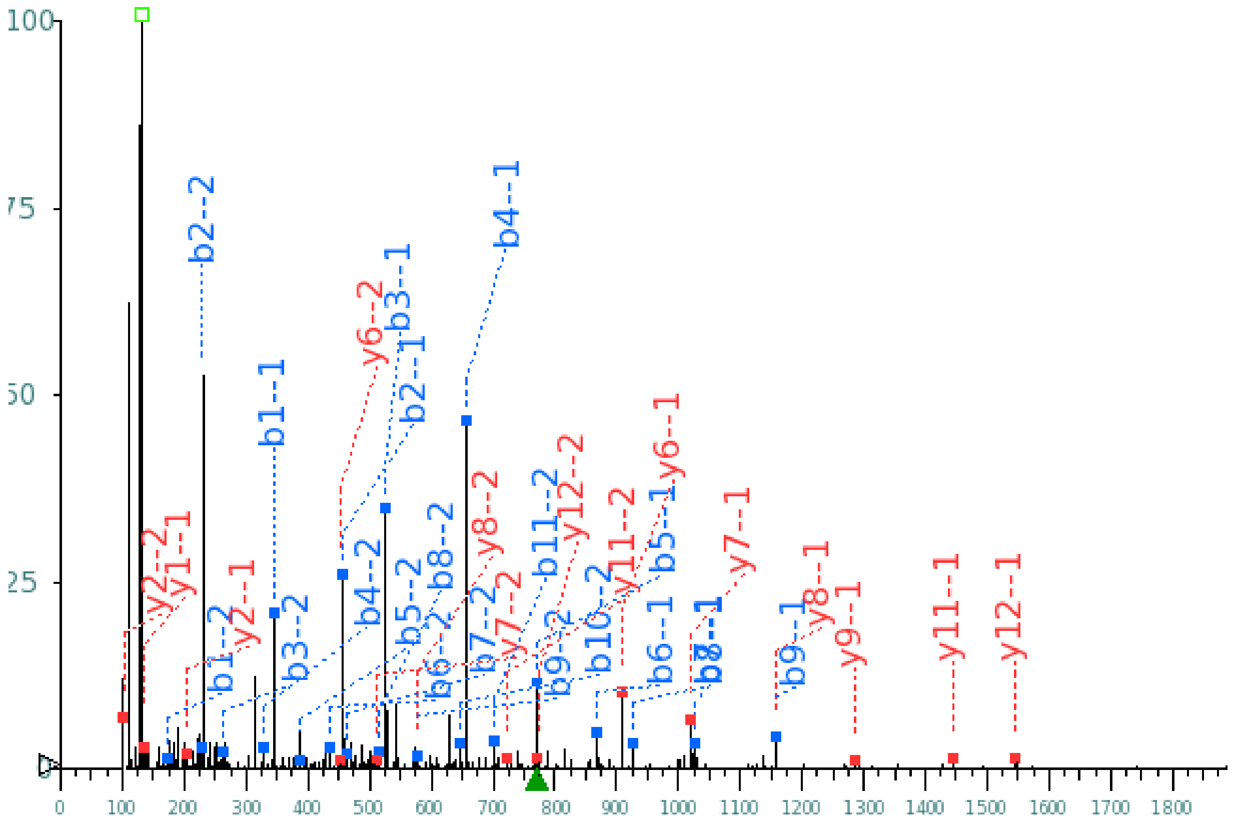

ILAQITGTEHLKESEAD (+3)

Primary Reference: PDLI5_HUMAN
Search ID: 41761
Search Name: 20130328_ananiav_ERstress_TMT_TM_timecourse_trypsin_inj2_4mc_iaa
Scan#: 22930
Observed Mass: 771.7646 (3.9 ppm)
PSM Score: 47.88

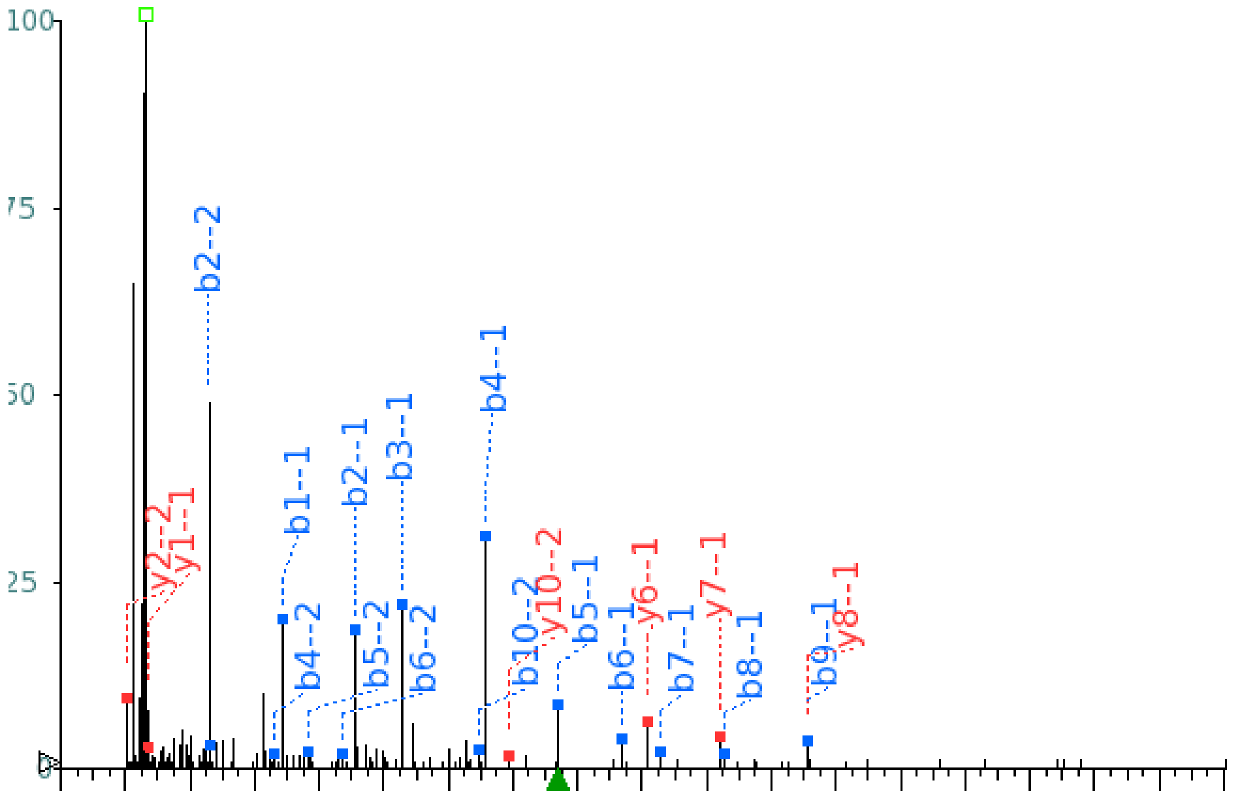

ILAQITGTEHLKESEAD (+3)

Primary Reference: PDLI5_HUMAN
Search ID: 41761
Search Name: 20130328_ananiav_ERstress_TMT_TM_timecourse_trypsin_inj2_4mc_iaa
Scan#: 22961
Observed Mass: 771.7643 (3.5 ppm)
PSM Score: 34.18

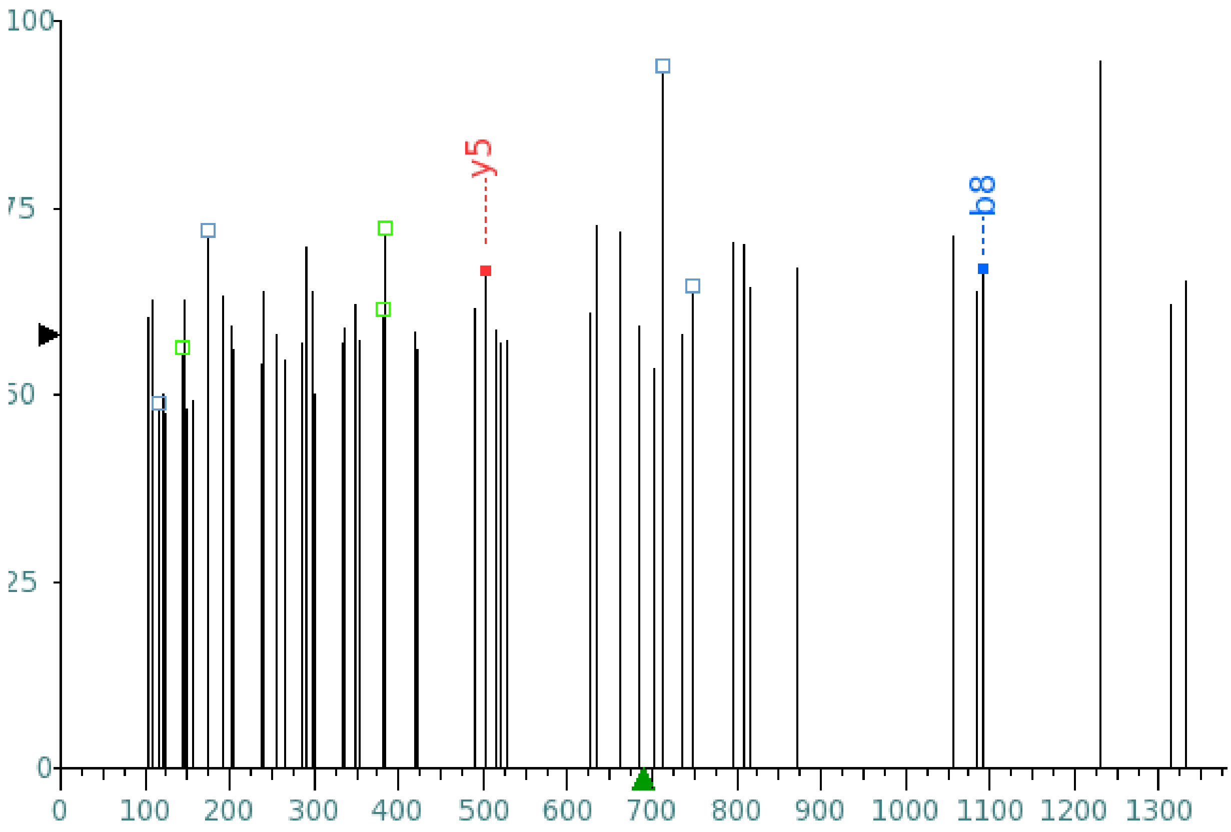

DFSALLQSPGD (+2)

Primary Reference: KHNYN_HUMAN
Search ID: 41761
Search Name: 20130328_ananiav_ERstress_TMT_TM_timecourse_trypsin_inj2_4mc_iaa
Scan#: 23561
Observed Mass: 689.8576 (2 ppm)
PSM Score: 8.69

File name: 2015-02-04 04.01.53PM.pdf

Creation time: 2015-02-04 04:09PM
